# Supplementary material for: Research on the development of an automated system for psychology questionnaire generation based on large language models
Source: PLoS One. 2026 Apr 24;21(4):e0345117. doi: 10.1371/journal.pone.0345117 (PMC13108753; doi:10.1371/journal.pone.0345117)
Supplement: S4 Data — (ZIP) [file pone.0345117.s004.zip › S5_Code (Model & Training Configuration)/psychological-questionnaire-label.en_only.docx]

{"questionnaire_title": "General sense of self-efficiency", "questionnaire_category": "Self-cognition", "item_count": 10, "emotion_shift_overall": "No obvious trend", "language_style_overall": "Neutral", "items": [{"content": "I can always solve the problem if I do my best.", "word_count": 19, "label": "", "sentence_structure": "Subject鈥損redicate", "subjective_objective": {"answer": "Subjective", "rationale": "Because \"do your best\" and \" always solve your problems\" are patients' subjective attitudes and beliefs."}, "sentence_sentiment": {"answer": "Positive", "rationale": "Because it expresses confidence and a positive attitude towards its abilities."}, "language_style": {"answer": "Tactful and warm", "rationale": "The language style of this questionnaire entry is relatively moderate, with dialogue taking place in an invitation tone, rather than direct statements or orders, which are more personal and interactive. At the same time, the entry also uses positive language, such as \"if I try to do it,\" \"it always solves the problem\", which gives positive psychological hints and encouragement, encouraging and encouraging."}, "time_dimension": {"answer": "Present", "rationale": "The keyword \"if I try to do it\" suggests that this is a hypothesis, and the behaviour (do as much as I can) and the result (can solve the problem) in that scenario are in their present state. The question is therefore about the current attitude or conviction of the testee, which can be attributed to the time dimension of the present."}, "sentence_type": "Affirmative declarative sentence", "pos": {"nouns": {"Problem": 2}, "verbs": {"Do it": 1, "be": 1, "Resolve": 1}, "adjectives": {"Always": 1}, "adverbs": {"If": 1, "Try": 1}, "pronouns": {"I": 2}}}, {"content": "I still have a way to get what I want, even if I'm opposed.", "word_count": 18, "label": "", "sentence_structure": "Subject鈥損redicate", "subjective_objective": {"answer": "Subjective", "rationale": "Because \"even if someone objects to me, I still have the means to get what I want\" is the belief and attitude of the patient in the face of difficulties and is therefore subjective."}, "sentence_sentiment": {"answer": "Positive", "rationale": "Because the behavior in the entry \"even if someone objects to me, I still have the way to get what I want\" expresses confidence and resolve."}, "language_style": {"answer": "Tactful and warm", "rationale": "The entry is more adept and interactive when it is spoken in a gentle and inviting manner, rather than by direct statements or orders. At the same time, the content of this entry relates to the ability of individuals to make decisions and to think independently, so that the euphemism of language is more appropriate to the context and context of the questionnaire."}, "time_dimension": {"answer": "Present", "rationale": "According to the phrases \"even if\" and \"even if there is still a way to get it\" in the sentence, it can be judged that the entry in the questionnaire is a question of the manner or attitude of the respondent in the face of an objection and is therefore of the current temporal dimension."}, "sentence_type": "Affirmative declarative sentence", "pos": {"nouns": {"person": 1, "Methodology": 1, "Something": 1}, "verbs": {"have": 1, "Access": 1, "Objection": 1}, "adjectives": {"others": 1}, "adverbs": {"Still": 1, "All I want": 1}, "pronouns": {"I": 1}}}, {"content": "It's easy for me to stick to ideals and achieve goals.", "word_count": 19, "label": "", "sentence_structure": "Subject鈥揷opula鈥損redicative", "subjective_objective": {"answer": "Subjective", "rationale": "Because \"easy\" is an individual's feeling and attitude and is subjective."}, "sentence_sentiment": {"answer": "Positive", "rationale": "Because the expression \"maintenance of ideals and achievement of goals is easy\" in the entry has a positive emotional tone."}, "language_style": {"answer": "Calm and direct", "rationale": "The language style of the entry is more direct, it is not over-modified or emotionalized, and it is balanced and objective, expressing the researcher ' s views on the attitude of the person under investigation towards the ideals and objectives."}, "time_dimension": {"answer": "Present", "rationale": "The entry describes the current feelings or attitudes of the testees, indicating that they believe it is easy to adhere to ideals and achieve goals. The keyword \"to say,\" \"to be easy\" is a description of the current state, so the temporal dimension is now."}, "sentence_type": "Affirmative declarative sentence", "pos": {"nouns": {"I": 1, "Ideal": 1, "Objective": 1}, "verbs": {"Hold on": 1, "Achieved": 1, "be": 1}, "adjectives": {"Easy": 1}}}, {"content": "4. I'm confident that I can deal effectively with anything unexpected.", "word_count": 18, "label": "", "sentence_structure": "Subject鈥搗erb鈥搊bject", "subjective_objective": {"answer": "Subjective", "rationale": "Because self-confidence is the subjective feeling and attitude of a person."}, "sentence_sentiment": {"answer": "Positive", "rationale": "Because the entry \"confidently able to deal effectively with anything unexpected\" expresses an optimistic, positive mentality."}, "language_style": {"answer": "Tactful and warm", "rationale": "The entry is more personal and interactive when it is spoken in a question-and-answer manner rather than by direct statements or orders. At the same time, positive words of \"confidence\" and \"act effectively\" are used to convey a message of encouragement and support, and the language style is more euphemistic."}, "time_dimension": {"answer": "Present", "rationale": "The key word is \"now can\" and it means the power and the state."}, "sentence_type": "Affirmative declarative sentence", "pos": {"nouns": {"Problem": 1, "Questionnaire": 1, "Entry": 1, "Things": 1}, "verbs": {"Analysis": 1, "Confidence": 1, "Yes": 2, "Payable": 1, "Break": 1, "Come on": 1}, "adjectives": {"Any": 1, "It works": 1, "Suddenly": 1}, "numerals": {"4": 1}, "classifiers": {"individual": 1}, "pronouns": {"I": 1}}}, {"content": "In my wisdom, I'll be able to cope with unexpected circumstances.", "word_count": 17, "label": "", "sentence_structure": "Subject鈥搗erb鈥搊bject", "subjective_objective": {"answer": "Subjective", "rationale": "Because this is the patient ' s subjective assessment and expectation of self-capacity."}, "sentence_sentiment": {"answer": "Positive", "rationale": "Because the expression in the entry, \"With my talents, I will be able to cope with unexpected circumstances,\" expresses a positive confidence and determination."}, "language_style": {"answer": "Calm and direct", "rationale": "Because the entry is a direct expression of the interviewee ' s confidence in his or her abilities, he or she does not have too many rhetorical or emotional expressions, and is speaking fairly and objectively."}, "time_dimension": {"answer": "Future", "rationale": "It's a vision of the future. The entry is therefore judged as the future in terms of time dimension, inquiring about the testee ' s confidence in the future and his ability to respond to contingencies."}, "sentence_type": "Affirmative declarative sentence", "pos": {"verbs": {"Payable": 1, "be": 1, "Yes": 1}, "adjectives": {"- Unexpected": 1}, "adverbs": {"Freeze": 1}, "pronouns": {"Mine": 1}}}, {"content": "If I make the necessary effort, I can solve most of the problems.", "word_count": 22, "label": "", "sentence_structure": "Subject鈥損redicate", "subjective_objective": {"answer": "Subjective", "rationale": "This is a self-evaluation and conviction of patients and a subjective attitude and view."}, "sentence_sentiment": {"answer": "Positive", "rationale": "Because it expresses confidence and determination that it is capable of solving the problem, it is a positive attitude and emotion."}, "language_style": {"answer": "Calm and direct", "rationale": "In a direct presentation, the entry expressed the view that most difficulties could be solved with sufficient effort. This tone is more objective and firm, does not have too many emotional or rhetorical features and is consistent with a calm and direct linguistic style."}, "time_dimension": {"answer": "Present", "rationale": "The temporal dimension of the entry in the questionnaire does not clearly indicate past, present or future behaviour or circumstances, so it is judged to be \"now\". The keyword \"must solve most of the difficulties\" suggests that it is a belief or attitude, possibly the current mindset or idea of the testee."}, "sentence_type": "Affirmative declarative sentence", "pos": {"nouns": {"I": 1, "Difficulty": 1}, "verbs": {"Give": 1, "Try": 1, "It'll work out": 1, "I'm sure": 1}, "adjectives": {"Required": 1, "Most of them": 1}, "numerals": {"6": 1}, "classifiers": {"individual": 1}}}, {"content": "I can calm myself to difficulties because I trust my ability to handle them.", "word_count": 23, "label": "", "sentence_structure": "Subject鈥搗erb鈥搊bject", "subjective_objective": {"answer": "Subjective", "rationale": "Because \"I trust my own ability to deal with problems\" is a subjective attitude and belief of a human being. While this attitude may affect the way in which a person deals with difficulties, the entry is primarily concerned with the subjective views of the individual rather than with objective results."}, "sentence_sentiment": {"answer": "Positive", "rationale": "It is positive because the entries express confidence in their abilities and a calm response to difficulties."}, "language_style": {"answer": "Tactful and warm", "rationale": ""}, "time_dimension": {"answer": "Present", "rationale": ""}, "sentence_type": "Affirmative declarative sentence", "pos": {"nouns": {"Difficulties": 1, "Problem": 1}, "verbs": {"Face it": 1, "Trust": 1, "Processing": 1, "Yes": 1}, "adjectives": {"Calm down": 1}, "adverbs": {"Because": 1}, "pronouns": {"self": 1}}}, {"content": "8. I usually find solutions to a problem.", "word_count": 19, "label": "", "sentence_structure": "Subject鈥搗erb鈥搊bject", "subjective_objective": {"answer": "Subjective", "rationale": "Because this issue is primarily concerned with individual thinking and habits, which are subjective. For example, one person may be born better at finding multiple solutions, while another may prefer a single solution. So, \"In the face of a problem, I usually find a few solutions\" is subjective."}, "sentence_sentiment": {"answer": "Neutral", "rationale": ""}, "language_style": {"answer": "Calm and direct", "rationale": "The entry uses a direct question-and-answer approach, with no excessive qualifier or emotional colour, tone and consistency with a calm and direct language style."}, "time_dimension": {"answer": "Present", "rationale": "The entry asks about the testee's customary behaviour in the face of the current difficulties, namely, \"some solutions\". Such descriptive behaviour is directed at the present situation and does not involve past or future scenarios. \""}, "sentence_type": "Affirmative declarative sentence", "pos": {"nouns": {"Difficulty": 1, "Solutions": 1}, "verbs": {"Face it": 1, "Found it": 1}, "numerals": {"How many": 1}, "pronouns": {"I": 1}}}, {"content": "When there's trouble, I usually think of some way to deal with it.", "word_count": 19, "label": "", "sentence_structure": "Subject鈥揷opula鈥損redicative", "subjective_objective": {"answer": "Subjective", "rationale": "Because finding a way to respond is a patient's subjective thinking and action."}, "sentence_sentiment": {"answer": "Positive", "rationale": "This entry is an expression of the positive attitude and approach that individuals can take when they encounter difficulties."}, "language_style": {"answer": "Calm and direct", "rationale": "Basis for the determination: The entry expresses a direct question in a concise and clear manner, asking the respondents if there are ways to respond to the difficulties encountered. The tone is more balanced and objective, and there is no excessive rhetoric or emotional rendering, which is consistent with the calm and direct language style."}, "time_dimension": {"answer": "Present", "rationale": ""}, "sentence_type": "Affirmative declarative sentence", "pos": {"nouns": {"Trouble": 1, "Methodology": 1}, "verbs": {"Yes": 2, "Payable": 1}, "adverbs": {"Usually": 1}, "numerals": {"9": 1}, "pronouns": {"I": 1}}}, {"content": "I can handle whatever happens to me.", "word_count": 18, "label": "", "sentence_structure": "Subject鈥揷opula鈥損redicative", "subjective_objective": {"answer": "Subjective", "rationale": "Because this is the patient ' s subjective attitude and conviction."}, "sentence_sentiment": {"answer": "Positive", "rationale": "Because behavior in the entries is positive."}, "language_style": {"answer": "Calm and direct", "rationale": "Basis of judgement: The entry is a direct expression of the question of whether the counterparty can respond to the subject when it encounters anything, without too much rhetoric or emotional rendering, and with a more balanced and objective tone. Nor does it use euphemistic and warm expressions to increase kinship and interaction, so it can be judged as \"cool and direct\"."}, "time_dimension": {"answer": "Present", "rationale": "The keywords \"incidents\" and \"I can handle myself\" indicate that this is a description of the person's present state or ability. The time dimension of the article is therefore the present."}, "sentence_type": "Affirmative declarative sentence", "pos": {"nouns": {"Yes": 1, "I": 2}, "verbs": {"Come on": 1, "Pay for it": 1}, "adverbs": {"Whatever": 1}, "numerals": {"10": 1}}}]}

{"questionnaire_title": "Response questionnaire", "questionnaire_category": "Behavioral questionnaire", "item_count": 62, "emotion_shift_overall": "From positive to negative", "language_style_overall": "Tactful and warm", "items": [{"content": "1. Reasonable response to dilemmas", "word_count": 8, "label": "", "sentence_structure": "Subject鈥搗erb鈥揷omplement", "subjective_objective": {"answer": "Subjective", "rationale": "Because \"sure to deal with dilemmas\" is a subjective assessment of a person's ability, not an objective reality or event. It is the subjective aspect of psychological assessment to examine how a person responds to a difficult situation."}, "sentence_sentiment": {"answer": "Positive", "rationale": "It is a positive attitude and behaviour that the \"sure to deal with the dilemma\" shows that the respondents are able to remain calm and take effective measures in the face of difficulties."}, "language_style": {"answer": "Calm and direct", "rationale": ""}, "time_dimension": {"answer": "Future", "rationale": "The key word, \"sensibly able to cope with a dilemma\", shows that it is an expectation or a goal for the future and therefore belongs to the behaviour or mentality of the future."}, "sentence_type": "Imperative sentence", "pos": {"nouns": {"The dilemma": 1}, "verbs": {"Payable": 1, "- I'm not": 1}, "adverbs": {"- I'm not": 1}}}, {"content": "2. Learning from failures", "word_count": 10, "label": "", "sentence_structure": "Subject鈥搗erb鈥揷omplement", "subjective_objective": {"answer": "Subjective", "rationale": "Because learning from a failure is an attitude or a capacity that needs to be realized through self-reflection and subjectivity."}, "sentence_sentiment": {"answer": "Positive", "rationale": "Because the action of learning from failures in the entries is positive."}, "language_style": {"answer": "Tactful and warm", "rationale": "The language style of the entry is more natural, and it does not use too direct or hard language, but rather, in a way that encourages and supports respondents to answer questions and to feel respected and understood. At the same time, the wording of the entry is more positive and helps to mobilize the motivation and self-confidence of the interviewee, thereby providing a more accurate picture of his or her psychological state."}, "time_dimension": {"answer": "Present", "rationale": "The entry describes a habit or attitude of the testee to learn from experience in the face of failure. Such behaviour is a state of affairs or a tendency that is currently under way or has developed and is therefore judged to be \"now\"."}, "sentence_type": "Affirmative declarative sentence", "pos": {"nouns": {"Experience": 1, "Failed": 1}, "verbs": {"Good at it": 1, "Draw": 1}, "adverbs": {"Medium": 1}, "classifiers": {"2": 1}}}, {"content": "3. Develop and implement plans to overcome difficulties", "word_count": 17, "label": "", "sentence_structure": "Subject鈥搗erb鈥搊bject", "subjective_objective": {"answer": "Subjective", "rationale": "Because \"programs to overcome difficulties and do so as planned\" are subjective actions by patients to change their attitudes and behaviour. While these plans may be influenced by objective circumstances, their formulation and implementation are themselves based on the patient ' s subjective will and determination."}, "sentence_sentiment": {"answer": "Positive", "rationale": "Because the \"plan and do as planned\" in the entry is positive."}, "language_style": {"answer": "Calm and direct", "rationale": "The entry is balanced and objective, stating in a direct manner that the plan is to be formulated and is to be acted upon in order to overcome difficulties, that there is no excessive emotional rendering or rhetoric, and that the language style is straightforward."}, "time_dimension": {"answer": "Present", "rationale": "The entry asks the testee about his future behaviour and mentality, and the time dimension is judged to be the future. The keywords \"formulation\" and \"do as planned\" suggest that the act has not yet taken place and needs to be implemented in the future. Thus, the entry describes future behaviour or attitudes."}, "sentence_type": "Imperative sentence", "pos": {"nouns": {"Difficulties": 1, "Planned": 2, "Do it": 1}, "verbs": {"Development": 1, "Overcoming": 1, "Press": 1}, "adverbs": {"Some": 1}, "numerals": {"3": 1}, "classifiers": {"Some": 1, "individual": 1}}}, {"content": "4. It is often hoped that they have resolved the difficulties they face", "word_count": 15, "label": "", "sentence_structure": "Subject鈥搗erb鈥揷omplement", "subjective_objective": {"answer": "Subjective", "rationale": "Because hope is the patient's subjective attitude."}, "sentence_sentiment": {"answer": "Positive", "rationale": "Because the entry \"I hope I've solved the difficulties\" expresses a positive mindset and willingness to solve the problems."}, "language_style": {"answer": "Tactful and warm", "rationale": "The language style of the entry is more euphemistic and encouraging, using the word \"hope\" rather than directly ordering or compelling the other party to solve the problem. This expression is more comfortable and respected for the interviewees, thus making it easier to open the door to questions."}, "time_dimension": {"answer": "Future", "rationale": "The future."}, "sentence_type": "Affirmative declarative sentence", "pos": {"nouns": {"Problem": 1, "Investigations": 1, "Answer": 1}, "verbs": {"Resolve": 1, "Faced": 1}, "adverbs": {"self": 1, "Regular": 1}, "numerals": {"4": 1}, "pronouns": {"Already": 1}}}, {"content": "5. Confidence in their ability to succeed", "word_count": 14, "label": "", "sentence_structure": "Subject鈥搗erb鈥揷omplement", "subjective_objective": {"answer": "Subjective", "rationale": "Because \"confident\" is a subjective assessment or attitude."}, "sentence_sentiment": {"answer": "Positive", "rationale": "Because the \"confidence in your ability to succeed\" approach is positive."}, "language_style": {"answer": "Tactful and warm", "rationale": ""}, "time_dimension": {"answer": "Present", "rationale": "According to the above-mentioned example, the word \"confident\" in \"confident in their ability to succeed\" indicates the current mindset of the testee and the entry should therefore be judged now."}, "sentence_type": "Affirmative declarative sentence", "pos": {"nouns": {"self": 1, "Access": 1, "Success": 1, "Capacity": 1, "Confidence": 1}, "verbs": {"Fill": 1}, "numerals": {"5": 1}}}, {"content": "6. Considering that \"life experience is suffering\"", "word_count": 10, "label": "", "sentence_structure": "Subject鈥搗erb鈥搊bject", "subjective_objective": {"answer": "Subjective", "rationale": "Because this sentence relates to patients ' perception and evaluation of their life experience, it is an expression of a subjective attitude."}, "sentence_sentiment": {"answer": "Negative", "rationale": "Because the expression \"life experience is bitter\" in the entries has a pessimistic, negative emotional connotation."}, "language_style": {"answer": "Calm and direct", "rationale": "The basis for the determination is that the entry, in a direct statement, asks the respondents about \"life experience is bitterness\" and that there are no too many rhetorical techniques or emotional renderings, and that the tone is more objective, so that its language style can be considered as calm and straightforward."}, "time_dimension": {"answer": "Present", "rationale": "The keyword \"considers\" indicates that it is a subjective view or view, which is not clearly time-directed, so the temporal dimension is now judged."}, "sentence_type": "Affirmative declarative sentence", "pos": {"nouns": {"Life experience": 1, "The trials": 1}, "verbs": {"I think": 1}}}, {"content": "7 . . . . . . . . . . . . . . . . . . . . . . . . . . . . . . . . . . . . . . . . . . . . . . . . . . . . . . . . . . . . . . . . . . . . . . . . . . . .", "word_count": 8, "label": "", "sentence_structure": "Subject鈥搗erb鈥揷omplement", "subjective_objective": {"answer": "Subjective", "rationale": "Because the \"distress of living\" is the feeling and emotional reaction of the patient, which is subjective."}, "sentence_sentiment": {"answer": "Negative", "rationale": "Because the description of \"hard life\" in the entry is negative."}, "language_style": {"answer": "Tactful and warm", "rationale": "The entry is more adept and interactive when it is spoken in a gentle and inviting manner, rather than by direct statements or orders. At the same time, the use of the word \"satisfaction\" conveys an emotional colour that brings the questionnaire closer to the emotional experience of the respondents, thus making it easier to obtain real feedback."}, "time_dimension": {"answer": "Past", "rationale": ""}, "sentence_type": "Exclamatory sentence", "pos": {"nouns": {"Life": 1, "Hard": 1}, "verbs": {"I'm sorry": 1}, "adverbs": {"Regular": 1}}}, {"content": "8. Focusing on work or learning to forget", "word_count": 13, "label": "", "sentence_structure": "Subject鈥損redicate", "subjective_objective": {"answer": "Subjective", "rationale": "Focusing on work or learning requires subjective efforts and decisions, not objective circumstances or results."}, "sentence_sentiment": {"answer": "Positive", "rationale": "Because the purpose of the entry is to focus on work or learning."}, "language_style": {"answer": "Tactful and warm", "rationale": "The entry uses a moderate and encouraging tone and proposes a response that is not directed at accusing or ordering, which is more readily acceptable to respondents and willing to try. At the same time, the expression \"forget it\" is euphemism and avoids the sensitive points that directly touch the interviewees."}, "time_dimension": {"answer": "Present", "rationale": "The keywords \"focusing on work or learning\" and \"failure to forget\" indicate that this is a description of current behaviour, and therefore judgement is now."}, "sentence_type": "Imperative sentence", "pos": {"nouns": {"Work": 1, "Learning": 1, "Not fast": 1}, "verbs": {"Focus": 1, "Forget it": 1}}}, {"content": "It is often thought that 鈥渓ife and death are in heaven rich鈥?, "word_count": 11, "label": "", "sentence_structure": "Subject鈥搗erb鈥搊bject", "subjective_objective": {"answer": "Objective", "rationale": "Because the word \"live or die, rich or rich\" is the patient's personal opinion and conviction and is subjective."}, "sentence_sentiment": {"answer": "Negative", "rationale": "Because the attitudes in the entries are negative."}, "language_style": {"answer": "Tactful and warm", "rationale": "The entry uses a more euphemistic tone, by asking the interviewees whether they often hold such a perception, rather than making direct statements or orders. This approach is more resonant and thought-provoking, as well as more personal and interactive."}, "time_dimension": {"answer": "Past", "rationale": "The keyword \"often believes\" indicates that the entry is questioned by the testee ' s past ideas and thoughts and is therefore judged to be past."}, "sentence_type": "Affirmative declarative sentence", "pos": {"nouns": {"Live or die": 2, "Life": 1, "Rich": 2, "Oh, my God": 1}, "verbs": {"I think it's a good idea": 1}, "numerals": {"9": 1}}}, {"content": "Ten. Always likes to talk to people to make things easier.", "word_count": 13, "label": "", "sentence_structure": "Subject鈥搗erb鈥揷omplement", "subjective_objective": {"answer": "Subjective", "rationale": "Because \"often like\" is the patient's own feelings and thoughts, and it's subjective."}, "sentence_sentiment": {"answer": "Positive", "rationale": "Because the purpose of the entry is to find someone to talk to to ease trouble."}, "language_style": {"answer": "Tactful and warm", "rationale": ""}, "time_dimension": {"answer": "Present", "rationale": ""}, "sentence_type": "Affirmative declarative sentence", "pos": {"nouns": {"Questionnaire Entry": 1, "People chatting": 1, "Trouble": 1}, "verbs": {"Yeah": 1, "Find": 1, "Mitigation": 1}, "adverbs": {"Often": 1}, "numerals": {"10": 1}}}, {"content": "11. Requesting help to overcome difficulties", "word_count": 12, "label": "", "sentence_structure": "Subject鈥搗erb鈥搊bject", "subjective_objective": {"answer": "Subjective", "rationale": "Because asking someone to help themselves out is the patient's subjective idea and need."}, "sentence_sentiment": {"answer": "Positive", "rationale": "Because asking someone to help themselves overcome difficulties is a positive act, demonstrating the willingness of those seeking help to work with others to resolve problems."}, "language_style": {"answer": "Tactful and warm", "rationale": "The entry opens a conversation in a gentle and inviting tone and asks the interviewees whether they need help to cope with the difficulties and challenges of life. This expression is more personal and interactive and better attracts the participation and feedback of interviewees."}, "time_dimension": {"answer": "Present", "rationale": "The entry is for the testee to describe the current behaviour or situation, and the \"request for help to overcome the difficulties\" is an ongoing exercise, so the time dimension is now judged."}, "sentence_type": "Imperative sentence", "pos": {"nouns": {"Request": 1, "others": 1, "Help": 1, "self": 1, "Difficulties": 1}, "verbs": {"Overcoming": 1}, "numerals": {"11": 1}}}, {"content": "12. Always do what you want, without consequences", "word_count": 14, "label": "", "sentence_structure": "Subject鈥損redicate", "subjective_objective": {"answer": "Subjective", "rationale": "Because it is only what it wants, it is the patient ' s subjective attitude and behaviour to ignore the consequences."}, "sentence_sentiment": {"answer": "Negative", "rationale": "Because the act of \"always doing what you want without taking into account the consequences\" in the entry is negative."}, "language_style": {"answer": "Calm and direct", "rationale": "The entry was asked in a straightforward manner, without any rhetorical or emotional presentation, and in a more objective manner. The question was simply and directly asked whether respondents often acted on their own ideas without considering the outcome. This language style makes the questionnaire more relevant and operational."}, "time_dimension": {"answer": "Present", "rationale": ""}, "sentence_type": "Negative declarative sentence", "pos": {"nouns": {"Regular": 1, "self": 1, "Consequences": 1}, "verbs": {"Press": 1, "Do it": 1, "Consider": 1}, "adverbs": {"Only": 1}, "numerals": {"12": 1}}}, {"content": "I don't want to think too much about what affects my emotions.", "word_count": 15, "label": "", "sentence_structure": "Subject鈥搗erb鈥揷omplement", "subjective_objective": {"answer": "Subjective", "rationale": "Because it's the patient's own subjective attitude that doesn't want to think."}, "sentence_sentiment": {"answer": "Negative", "rationale": "Because the act of \"not thinking too much\" in the entry is negative."}, "language_style": {"answer": "Calm and direct", "rationale": "The language style of the entry is calm and straightforward. In a brief and clear query, it asked the interviewees whether they were unwilling to reflect on issues that might affect their emotions, without any aesthetic or emotional colour. This language style is more neutral and objective."}, "time_dimension": {"answer": "Present", "rationale": "The entry of the questionnaire asks about the current behaviour or state of the subject, and the keywords \"unwilling\" and \"too much thinking\" indicate that this is a present behaviour or attitude."}, "sentence_type": "Negative declarative sentence", "pos": {"nouns": {"Problem": 1, "Emotion": 1}, "verbs": {"I don't want to": 1, "Think": 1, "Impact": 1}, "adverbs": {"Too much": 1}, "numerals": {"13": 1}}}, {"content": "14. To engage in other social activities and find new ones", "word_count": 13, "label": "", "sentence_structure": "Subject鈥搗erb鈥搊bject", "subjective_objective": {"answer": "Subjective", "rationale": "The search for new care is a subjective choice and action in the face of psychological distress."}, "sentence_sentiment": {"answer": "Positive", "rationale": "Since the behaviour in the entry \"involved in other social activities\" can bring new experiences and opportunities, it can also help individuals find new endowments and support and is a positive attitude and behaviour."}, "language_style": {"answer": "Calm and direct", "rationale": "The basis for the determination is that the entry, in a simple and clear language, is a direct expression of the desire to ask the other party whether or not it is regularly involved in other social activities in search of a new subject, and that it does not use too many rhetorical techniques or emotional renderings, and is speaking in a more objective manner."}, "time_dimension": {"answer": "Present", "rationale": "The entry describes the behaviour or state of the person currently being tested, and the time dimension is now judged. The verbs \"into the body\" and \"in the search\" show that the testees are actively participating in and exploring new social events to find new ones."}, "sentence_type": "Imperative sentence", "pos": {"nouns": {"Activities": 2, "Mailing": 1}, "verbs": {"- Get in": 1, "Search": 1}, "adverbs": {"Other": 1}, "numerals": {"14": 1}}}, {"content": "15, frequent self-absorption.", "word_count": 5, "label": "", "sentence_structure": "Subject鈥搗erb鈥揷omplement", "subjective_objective": {"answer": "Subjective", "rationale": "Because self-absorption is a subjective attitude and emotional experience that reflects an individual's negative assessment of his or her abilities and values."}, "sentence_sentiment": {"answer": "Negative", "rationale": "Because self-absorption in the entry is a negative act or attitude."}, "language_style": {"answer": "Tactful and warm", "rationale": "The language style of the entry is more euphemistic, using the word \"normal\". This term is used to express frequent self-negative behaviour, rather than directly accusing or ordering the other. In addition, the entries do not contain too many emotional colours and rhetorical techniques, are more balanced and objective, are more friendly and interactive, and help to increase the confidence and participation of respondents."}, "time_dimension": {"answer": "Past", "rationale": "The key words in the entry are \"always\" and \"responsible.\" In it, \"often\" is a constant, customary act, while \"smuggling\" is a state of emotion. The entry therefore asks whether the testee has often experienced a self-absorbed emotional state in the past."}, "sentence_type": "Negative declarative sentence", "pos": {"nouns": {"Questionnaire": 1, "Entry": 1, "You're a self-absorbed": 1}, "verbs": {"Analysis": 1}, "numerals": {"15": 1}}}, {"content": "16, often to hide what's inside.", "word_count": 16, "label": "", "sentence_structure": "Subject鈥搗erb鈥揷omplement", "subjective_objective": {"answer": "Subjective", "rationale": "Because \"always indifferent to the feeling inside\" describes the patient's subjective mentality and behaviour."}, "sentence_sentiment": {"answer": "Negative", "rationale": "Because it's a negative act in the entry, \"I don't care to hide my feelings.\""}, "language_style": {"answer": "Tactful and warm", "rationale": "The language style of the entry is more euphemistic and triggers reflection and reflection by asking the respondents whether they have done so, rather than making a direct statement or order. At the same time, words such as \"disguised\" and \"inspiration\" were used to express an attitude of understanding, care and concern, making it easier for respondents to answer questions."}, "time_dimension": {"answer": "Present", "rationale": ""}, "sentence_type": "Negative declarative sentence", "pos": {"nouns": {"Attitude": 1, "Inside": 1, "Feel": 1}, "verbs": {"Always": 1, "Cover it up": 1}, "adjectives": {"It doesn't matter": 1}, "numerals": {"16": 1}}}, {"content": "\"That's not true.\"", "word_count": 10, "label": "", "sentence_structure": "Subject鈥搗erb鈥揷omplement", "subjective_objective": {"answer": "Subjective", "rationale": "Because it is often the patient ' s subjective attitude."}, "sentence_sentiment": {"answer": "Negative", "rationale": "For the purpose of the article is expressed with negative emotions and attitudes, i.e. dissatisfaction with the reality and a sense of flight."}, "language_style": {"answer": "Tactful and warm", "rationale": "The entry is more adept and interactive when it is spoken in a gentle and inviting manner, rather than by direct statements or orders. At the same time, \"if not true\" is an expression of a sense of evasiveness to reality, so the language of this purpose is more euphemism, and it is an attempt to guide the interviewee to a more realistic view of his thoughts and emotions."}, "time_dimension": {"answer": "Past", "rationale": "According to the keyword \"always\", the entry asks about the frequent reflection of the subject on a particular content, i.e., past, present or future, and therefore is judged to be \"now\"."}, "sentence_type": "Exclamatory sentence", "pos": {"nouns": {"Here": 2, "True": 1, "Okay": 1, "Yes": 1}, "verbs": {"Regular": 1, "Yes": 1, "Okay": 1}, "adjectives": {"No, it's not": 1, "Really": 1}, "adverbs": {"Just": 1}, "numerals": {"17": 1}}}, {"content": "18. Considering that their failure is due to external causes", "word_count": 13, "label": "", "sentence_structure": "Subject鈥損redicate", "subjective_objective": {"answer": "Subjective", "rationale": "Because it's the patient's subjective attitude, it comes from his own thoughts and perceptions."}, "sentence_sentiment": {"answer": "Negative", "rationale": "Because the attitude of \"failure is due mostly to external causes\" in the entry is negative."}, "language_style": {"answer": "Calm and direct", "rationale": ""}, "time_dimension": {"answer": "Present", "rationale": ""}, "sentence_type": "Negative declarative sentence", "pos": {"nouns": {"self": 1, "Failed": 1, "External factors": 1}, "verbs": {"I think": 1, "Result": 1}}}, {"content": "19. A wait-and-see attitude to difficulties", "word_count": 16, "label": "", "sentence_structure": "Subject鈥搗erb鈥搊bject", "subjective_objective": {"answer": "Subjective", "rationale": "Because the words \"take\" and \"attitude\" indicate that this is a subjective perception of the patient's treatment of difficulties and a tendency to behave."}, "sentence_sentiment": {"answer": "Negative", "rationale": "Because the attitude in the entry of \"waiting for its development\" is negative."}, "language_style": {"answer": "Calm and direct", "rationale": "The language style of the entry is relatively calm and objective, and there is no excessive emotional qualifier or subjective judgement to ask directly whether the other party prefers to wait and see in the face of difficulties. This language style helps to maintain the neutrality and accuracy of the questionnaire and avoids introducing personal bias or emotional colours."}, "time_dimension": {"answer": "Past", "rationale": "The entry describes the attitude and manner in which the testee has dealt with difficulties, a past behaviour or mentality."}, "sentence_type": "Negative declarative sentence", "pos": {"nouns": {"Difficulties": 1, "Attitude": 1}, "verbs": {"Adoption": 1, "Wait": 1, "Watch": 1, "Development": 1}, "numerals": {"19": 1}}}, {"content": "20, conflict with people, often caused by the weirdness of the other.", "word_count": 14, "label": "", "sentence_structure": "Subject鈥搗erb鈥搊bject", "subjective_objective": {"answer": "Objective", "rationale": "Because \"the weirdness of the other side\" is a description of the facts, the objective information that can be derived from observation or investigation. The situation of \"conflicts with people\" may be caused by factors such as differences in character or poor communication between the parties, which are objective. So, \"20, conflict with people, often caused by the weirdness of each other.\" This questionnaire entry was examined from an objective perspective."}, "sentence_sentiment": {"answer": "Negative", "rationale": "Because the words \"conflict\" and \"cause of personality\" in the entries are negative terms, expressing a disharmony and dissatisfaction."}, "language_style": {"answer": "Tactful and warm", "rationale": "The entry uses a more euphemistic formulation, without directly accusing or attacking the character of the other party, but rather describing the causes of the conflict in an objective manner. This expression is more acceptable and understandable and allows for good interpersonal relations."}, "time_dimension": {"answer": "Present", "rationale": "The words \"conflicting with people\" and \"often caused by the strangeness of each other\" in that sentence describe current or recent experiences and behaviour, and are therefore judged as \"now\"."}, "sentence_type": "Affirmative declarative sentence", "pos": {"nouns": {"Conflict": 1, "person": 1, "Character": 1}, "verbs": {"be": 1, "Cause": 1}, "adjectives": {"Regular": 1, "Weird": 1}, "numerals": {"20": 1}}}, {"content": "21. Too often angry at people and things that cause problems", "word_count": 13, "label": "", "sentence_structure": "Subject鈥搗erb鈥搊bject", "subjective_objective": {"answer": "Subjective", "rationale": "Because \"the person who often causes the problem and the incident\" is a person's behaviour, a patient's subjective attitude."}, "sentence_sentiment": {"answer": "Negative", "rationale": "Because it's negative in the entry."}, "language_style": {"answer": "Calm and direct", "rationale": "The entry describes a behavioral phenomenon in a concise manner and does not use excessive qualifiers or emotional colours, so that the language style is straightforward."}, "time_dimension": {"answer": "Present", "rationale": "The word \"always\" denotes a regular, customary act, and the expression \"to the person who caused the problem and to the incident\" describes an act that is now in progress, so the temporal dimension of the article is now."}, "sentence_type": "Imperative sentence", "pos": {"nouns": {"Problem": 1, "person": 1}, "verbs": {"Cause": 1, "Let's go": 1, "- Grumpy": 1}, "adverbs": {"Regular": 1}, "numerals": {"21": 1}}}, {"content": "Twenty-two. I've always imagined that I have a superhuman skill to overcome difficulties.", "word_count": 15, "label": "", "sentence_structure": "Subject鈥搗erb鈥搊bject", "subjective_objective": {"answer": "Subjective", "rationale": "Because fantasies are subjective ideas or emotions."}, "sentence_sentiment": {"answer": "Positive", "rationale": "Because in the entry, \"The Overcoming Hard Superman.\" This image is positive and expresses confidence in its capabilities and a positive attitude towards challenges."}, "language_style": {"answer": "Tactful and warm", "rationale": "The entry is more adept and interactive when it is spoken in a gentle and inviting manner, rather than by direct statements or orders. At the same time, this expression is better able to resonate and engage the respondents and make them more willing to share their thoughts and feelings."}, "time_dimension": {"answer": "Past", "rationale": "The entry is a description of the testee ' s customary thinking or ideas and does not specify the past, present or future circumstances and can therefore be judged neutral."}, "sentence_type": "Affirmative declarative sentence", "pos": {"nouns": {"Difficulties": 1, "Superman": 1}, "verbs": {"Fantasy": 1, "have": 1, "Overcoming": 1}, "numerals": {"22": 1}, "classifiers": {"Regular": 1}}}, {"content": "Regular self-criticism", "word_count": 5, "label": "", "sentence_structure": "Subject鈥搗erb鈥揷omplement", "subjective_objective": {"answer": "Subjective", "rationale": "Self-pity is a manifestation of subjective feelings and attitudes."}, "sentence_sentiment": {"answer": "Negative", "rationale": "Because the act of \"self-criticism\" is usually considered negative and has a negative emotional connotation."}, "language_style": {"answer": "Tactful and warm", "rationale": ""}, "time_dimension": {"answer": "Present", "rationale": ""}, "sentence_type": "Negative declarative sentence", "pos": {"nouns": {"You blame yourself": 1, "Regular": 1}, "verbs": {"Blame": 1}, "adverbs": {"Regular": 1}, "numerals": {"23": 1}}}, {"content": "24, often sleeps to escape pain.", "word_count": 11, "label": "", "sentence_structure": "Subject鈥搗erb鈥搊bject", "subjective_objective": {"answer": "Subjective", "rationale": "Because of the subjective fact that it's often the way you sleep to escape pain."}, "sentence_sentiment": {"answer": "Negative", "rationale": "Because the act of \"evading pain\" in the entry is negative."}, "language_style": {"answer": "Tactful and warm", "rationale": "The entry uses a gentle and inviting tone for dialogue rather than a direct statement or order, which is more personal and interactive. At the same time, the purpose of the article is more euphemistic, not overly emotional, and is easier for respondents to accept and to answer questions."}, "time_dimension": {"answer": "Past", "rationale": "The terms \"commonly used\" and \"evading\" imply a customary act of using sleep to avoid treatment in the face of pain. At the same time, the \"usual\" of \"the past\" also shows that such behaviour has existed for some time. The entry therefore asks about the testee ' s past experience."}, "sentence_type": "Affirmative declarative sentence", "pos": {"nouns": {"Modalities": 1, "Pain": 1, "Sleep": 1}, "verbs": {"Use": 1, "Common": 1, "Run away": 1}, "numerals": {"24": 1}}}, {"content": "25: Recreational activities are often used to ease trouble.", "word_count": 11, "label": "", "sentence_structure": "Subject鈥搗erb鈥揷omplement", "subjective_objective": {"answer": "Subjective", "rationale": "Because \"receiving troubles through recreational activities\" is a personal choice and preference and a patient's subjective attitude."}, "sentence_sentiment": {"answer": "Positive", "rationale": "Because the purpose of the act \"to remove troubles through recreational activities\" is positive and can be seen as a positive attitude or quality."}, "language_style": {"answer": "Tactful and warm", "rationale": ""}, "time_dimension": {"answer": "Present", "rationale": "The entry indicates that the testees often resolve their problems through recreational activities and describes current behaviour."}, "sentence_type": "Affirmative declarative sentence", "pos": {"nouns": {"Recreational activities": 1, "Trouble": 1}, "verbs": {"Regular borrowing": 1, "Elimination": 1}, "adverbs": {"Regular": 1}, "numerals": {"25": 1}, "classifiers": {"individual": 1}}}, {"content": "Twenty-six. Always thinking about something happy.", "word_count": 12, "label": "", "sentence_structure": "Subject鈥搗erb鈥搊bject", "subjective_objective": {"answer": "Subjective", "rationale": "Because it's the patient's subjective attitude."}, "sentence_sentiment": {"answer": "Positive", "rationale": "Because it's positive to think of something happy and comfort yourself."}, "language_style": {"answer": "Tactful and warm", "rationale": "The language style of the entry is euphemistic, as it uses invitations to guide respondents to answer questions, and the expression is relatively gentle. This expression is more personal and interactive and can help the respondents to feel more comfortable and relaxed and thus be more willing to share real ideas and feelings."}, "time_dimension": {"answer": "Present", "rationale": "The word \"always\" in \"always\" denotes regular, customary behaviour, while the word \"want\" adopts the normal current state of affairs, which means that this behaviour has not only occurred in the past, but may still persist. \"26\" is a serial number, not a word of time. The entry therefore describes the current situation of the person tested."}, "sentence_type": "Affirmative declarative sentence", "pos": {"nouns": {"26": 1, "Always": 1, "Happy things": 1, "Self-pity": 1}, "numerals": {"26": 1}}}, {"content": "27: Avoiding difficulties to find peace in your heart", "word_count": 10, "label": "", "sentence_structure": "Subject鈥搗erb鈥揷omplement", "subjective_objective": {"answer": "Subjective", "rationale": "Since avoiding difficulties is the patient ' s subjective choice and behaviour, it is his psychological state and attitude that are examined."}, "sentence_sentiment": {"answer": "Negative", "rationale": "Because the action of avoiding difficulties in the entry is negative."}, "language_style": {"answer": "Tactful and warm", "rationale": "The entry uses a more euphemistic wording, and \"avoiding difficulties\" does not directly require the other party to overcome them, but rather encourages them to look for inner peace in the face of problems. This expression is more cordial and encouraging and better guides respondents to express their true feelings."}, "time_dimension": {"answer": "Present", "rationale": ""}, "sentence_type": "Imperative sentence", "pos": {"nouns": {"Difficulties": 1, "鈾?In the heart 鈾?: 1, "Peace": 1}, "verbs": {"Avoid": 1, "Please": 1}, "numerals": {"27": 1}}}, {"content": "28, upset over not avoiding difficulties.", "word_count": 10, "label": "", "sentence_structure": "Subject鈥搗erb鈥揷omplement", "subjective_objective": {"answer": "Subjective", "rationale": "Because pain is the patient's subjective reaction to the difficulty."}, "sentence_sentiment": {"answer": "Negative", "rationale": "Because the attitude in the entry is negative."}, "language_style": {"answer": "Calm and direct", "rationale": "The entry asks the interviewees, in a straightforward and clear manner, whether they would be upset at the difficulties they face and whether the language style is straightforward."}, "time_dimension": {"answer": "Past", "rationale": "According to the time dimension test, \"28\" is the respondent's answer to a particular question and does not address past or future behavioural or mental changes, so the temporal dimension of the article cannot be determined."}, "sentence_type": "Negative declarative sentence", "pos": {"nouns": {"Difficulties": 1, "I'm upset": 1}, "verbs": {"Avoidance": 1, "be": 1}, "adverbs": {"No, I can't": 1}, "numerals": {"28": 1}, "classifiers": {"individual": 1}, "pronouns": {"Here": 1}}}, {"content": "29. More than two common solutions to difficulties", "word_count": 13, "label": "", "sentence_structure": "Subject鈥搗erb鈥搊bject", "subjective_objective": {"answer": "Subjective", "rationale": "Because the solution is chosen and used by the patient himself, it is a test of subjective behaviour and attitudes."}, "sentence_sentiment": {"answer": "Positive", "rationale": "Because the act of \"always two or more solutions to difficulties\" in the entry is positive."}, "language_style": {"answer": "Tactful and warm", "rationale": "The entry uses a more moderate approach to ask respondents whether they often use a variety of methods to resolve difficulties, without direct orders or strong demands, which makes it easier for respondents to accept and answer questions. At the same time, this euphemism allows respondents to express their ideas and experiences in a more comfortable manner, facilitating the quality of the questionnaire and the effectiveness of its findings."}, "time_dimension": {"answer": "Present", "rationale": "The entry now asks whether the testees are now using a variety of methods to solve problems, so the time dimension is now judged. The qualifier of the keyword \"usual\" means that it is a customary act, and the qualifier of \"two or more\" indicates that it occurs frequently, not occasionally."}, "sentence_type": "Affirmative declarative sentence", "pos": {"nouns": {"Approach": 2, "Difficulties": 1}, "verbs": {"Resolve": 1, "Common": 1}, "adverbs": {"Regular": 1, "Two": 1, "More than one type": 1}, "numerals": {"Two": 1, "More than one type": 1}}}, {"content": "I've always thought there's no need to fight for success.", "word_count": 15, "label": "", "sentence_structure": "Subject鈥搗erb鈥搊bject", "subjective_objective": {"answer": "Subjective", "rationale": "Because it is the patient's subjective attitude and thinking that sees no need for success or failure."}, "sentence_sentiment": {"answer": "Negative", "rationale": "Because the entry \"there's no need to struggle to win or lose\" expresses a negative attitude and behaviour."}, "language_style": {"answer": "Tactful and warm", "rationale": ""}, "time_dimension": {"answer": "Present", "rationale": "The word \"normal\" is often considered to indicate a regular, customary act, and the \"conception\" adopts a general current, which means that the act has not only occurred in the past, but may still continue. Therefore, the entry primarily asks the testee about his current thoughts and attitudes, and the dimension of judgement is now."}, "sentence_type": "Negative declarative sentence", "pos": {"nouns": {"Success or failure": 1, "Required": 1, "- It's hard": 1, "Fight": 1}, "verbs": {"I think": 1, "Yes": 1, "Go on": 1, "Fight": 1, "Regular": 1}, "adjectives": {"Well": 1}, "adverbs": {"None": 1, "Required": 1}, "numerals": {"30": 1}}}, {"content": "31, trying to change the status quo and make it work for the better", "word_count": 17, "label": "", "sentence_structure": "Subject鈥損redicate", "subjective_objective": {"answer": "Subjective", "rationale": "Because \"to try to change the status quo\" and \"to turn the situation to the good side\" are personal will or attitude of the patient and subjective."}, "sentence_sentiment": {"answer": "Positive", "rationale": "Because \"working to change the status quo\" is a positive attitude and behavior, and \"transforming the situation to the better\" is the purpose and is positive."}, "language_style": {"answer": "Tactful and warm", "rationale": "The language style of the entry is more euphemistic and encouraging, using positive terms such as \"temporarily\" and \"transforming the situation to the better\", without directly criticizing or accusing each other of failing. This expression is more likely to stimulate the motivation and participation of the respondents."}, "time_dimension": {"answer": "Present", "rationale": "The keywords \"work,\" \"change\" and \"transformation\" point to the actions and goals of the moment."}, "sentence_type": "Imperative sentence", "pos": {"nouns": {"Status": 1, "Situation": 1}, "verbs": {"Try": 1, "Change": 1, "Conversion": 1}, "adjectives": {"Okay": 1}, "numerals": {"31": 1}}}, {"content": "32 The use of tobacco or alcohol as a source of grief", "word_count": 6, "label": "", "sentence_structure": "Subject鈥搗erb鈥搊bject", "subjective_objective": {"answer": "Subjective", "rationale": "Because it is the patient ' s subjective choice and behaviour, rather than the objective fact, to smoke or drink. This reflects the emotional state and coping strategies of the patients and is therefore viewed from a subjective perspective."}, "sentence_sentiment": {"answer": "Negative", "rationale": "Because smoking or alcohol is a negative behaviour, it can easily lead to negative effects such as physical health problems and addiction."}, "language_style": {"answer": "Tactful and warm", "rationale": "The entry is more adept and interactive when it is spoken in a gentle and inviting manner, rather than by direct statements or orders. At the same time, it does not use excessive rhetoric or emotional rendering, which is more objective in tone."}, "time_dimension": {"answer": "Present", "rationale": "Past"}, "sentence_type": "Imperative sentence", "pos": {"nouns": {"Smoke": 1, "Wine": 1}, "verbs": {"Borrow": 1, "鈾?To be sad 鈾?: 1}, "numerals": {"32": 1}}}, {"content": "33 - Too often blaming others", "word_count": 5, "label": "", "sentence_structure": "Subject鈥搗erb鈥揷omplement", "subjective_objective": {"answer": "Subjective", "rationale": "Because \"always blaming others\" is a subjective attitude or behavioural tendency that reflects the patient's own inner feelings and emotional reactions, not objective facts or outcomes."}, "sentence_sentiment": {"answer": "Negative", "rationale": "Because the act of blaming others is negative."}, "language_style": {"answer": "Calm and direct", "rationale": "The entry is a direct expression of the subject matter on which the other party is often asked to blame others, without using excessive rhetorical techniques or emotional rendering, and with a more balanced and objective tone."}, "time_dimension": {"answer": "Present", "rationale": "Now the word \"normal\" in the entry denotes a regular, customary act, and the word \"critic\" and \"other\" is an act of the present, indicating that the act is taking place. The entry therefore describes the person ' s current state of behaviour."}, "sentence_type": "Imperative sentence", "pos": {"verbs": {"Blame": 1, "Others": 1}, "adjectives": {"Regular": 1}, "numerals": {"33": 1}}}, {"content": "34. A frequent evasive attitude towards difficulties", "word_count": 11, "label": "", "sentence_structure": "Subject鈥搗erb鈥揷omplement", "subjective_objective": {"answer": "Subjective", "rationale": "Because \"often evasive\" is the patient's subjective attitude."}, "sentence_sentiment": {"answer": "Negative", "rationale": "Because the behavior of evasive attitude in the entries is negative."}, "language_style": {"answer": "Calm and direct", "rationale": "The entry expresses in a more direct manner the attitude of the party to the challenge, is not overly emotional or rhetorical, and is more balanced and objective."}, "time_dimension": {"answer": "Present", "rationale": "In this entry, the \"attitude of evasiveness\" is an expression of behaviour that uses the general current state of affairs, so it can be judged to be a description of the person's current attitude and behaviour."}, "sentence_type": "Negative declarative sentence", "pos": {"nouns": {"Difficulties": 1, "Attitude": 1}, "verbs": {"Adopt": 1, "Avoidance": 1}, "numerals": {"34": 1}}}, {"content": "35 - Considering that 鈥渁 step back is natural鈥?, "word_count": 9, "label": "", "sentence_structure": "Subject鈥搗erb鈥揷omplement", "subjective_objective": {"answer": "Objective", "rationale": "Because it is a widespread psychological phenomenon, it is not the patient ' s own subjective attitude or feelings."}, "sentence_sentiment": {"answer": "Neutral", "rationale": "Because the entry does not have any emotional color."}, "language_style": {"answer": "Tactful and warm", "rationale": "The entry leads the interviewer to think by quoting a proverb, without direct questioning or ordering of the interviewee, and with a more euphemism and moderation of language style. At the same time, the questionnaire was used to increase the willingness of respondents to respond, to increase the interaction of the questionnaire and to increase the participation of respondents."}, "time_dimension": {"answer": "Present", "rationale": "The entry is a description of a view or belief by the testee and does not have a clear temporal dimension towards the past, present or future. So it should be judged \"now\"."}, "sentence_type": "Affirmative declarative sentence", "pos": {"nouns": {"Step": 1, "Kwang": 1}, "verbs": {"Back off": 1, "I think": 1}, "adjectives": {"Naturally": 1}, "numerals": {"35": 1}}}, {"content": "36: Bury unpleasant things in your heart.", "word_count": 10, "label": "", "sentence_structure": "Subject鈥搗erb鈥揷omplement", "subjective_objective": {"answer": "Subjective", "rationale": "Because this is the psychological activity and treatment of patients. \""}, "sentence_sentiment": {"answer": "Negative", "rationale": "Because the entry \"suck in the heart\" expresses a negative attitude."}, "language_style": {"answer": "Calm and direct", "rationale": "The language style of the entry is more straightforward and concise, without too many rhetorical or emotional colours, and the other person is asked whether he or she likes to bury unpleasant things in his or her heart. Such language styles are objective and calm and facilitate access to accurate information."}, "time_dimension": {"answer": "Past", "rationale": "But if we assume that this is a question of the present situation of the subject, then it can be interpreted as \"Do you often take unpleasant things to heart and not say them? \" In this case, the entry is a question of the current state of affairs and is used to understand the daily habits and behaviour of the person being tested."}, "sentence_type": "Imperative sentence", "pos": {"nouns": {"Yes": 1, "In my heart": 1}, "verbs": {"- Put it on": 1, "Buried": 1}, "adverbs": {"It's not good": 1}, "numerals": {"36": 1}}}, {"content": "37. Always humbled and humbled.", "word_count": 5, "label": "", "sentence_structure": "Subject鈥搗erb鈥揷omplement", "subjective_objective": {"answer": "Subjective", "rationale": "Because self-pity is a subjective feeling and emotional experience."}, "sentence_sentiment": {"answer": "Negative", "rationale": "Because the act of \"always ashamed\" in the entry is negative."}, "language_style": {"answer": "Tactful and warm", "rationale": ""}, "time_dimension": {"answer": "Present", "rationale": ""}, "sentence_type": "Negative declarative sentence", "pos": {"nouns": {"You're a low man": 1, "Self-pity": 1}, "verbs": {"Regular": 1}, "numerals": {"37": 1}}}, {"content": "It's often seen as an expression of the unfairness of life.", "word_count": 16, "label": "", "sentence_structure": "Subject鈥搗erb鈥揷omplement", "subjective_objective": {"answer": "Subjective", "rationale": ""}, "sentence_sentiment": {"answer": "Negative", "rationale": "Because the idea that life is not fair to yourself in the entry is negative."}, "language_style": {"answer": "Calm and direct", "rationale": ""}, "time_dimension": {"answer": "Past", "rationale": "The keyword \"often considers\" to be an expression of past habitual behaviour, and the entry is therefore an inquiry as to whether the testees often have such an idea in the past and the temporal dimension is judged to be the past."}, "sentence_type": "Negative declarative sentence", "pos": {"nouns": {"38": 1, "Performance": 1}, "verbs": {"I think it's a good idea": 1, "be": 1}, "adjectives": {"It's not fair": 1}, "adverbs": {"to": 1, "self": 1}, "numerals": {"Here": 1}}}, {"content": "39: Repression of anger and discontent", "word_count": 11, "label": "", "sentence_structure": "Subject鈥搗erb鈥揷omplement", "subjective_objective": {"answer": "Subjective", "rationale": "Because the suppression of internal anger and discontent is a feeling and attitude within a person, it is a subjective approach."}, "sentence_sentiment": {"answer": "Negative", "rationale": "Because the act of \"pressing inner anger and discontent\" in the entries is negative."}, "language_style": {"answer": "Tactful and warm", "rationale": ""}, "time_dimension": {"answer": "Present", "rationale": "The keyword \"always\" indicates that it is a constant act, that \"pressing internal anger and dissatisfaction\" is an inherent state of feeling or mental state, so it can be judged that the entry describes the current behavior or mentality."}, "sentence_type": "Negative declarative sentence", "pos": {"nouns": {"Anger": 1, "Dissatisfied": 1}, "verbs": {"It's always depressing": 1}, "adjectives": {"Inside": 1}, "numerals": {"39": 1}}}, {"content": "To learn from your own experience or that of others to cope with difficulties", "word_count": 15, "label": "", "sentence_structure": "Subject鈥搗erb鈥搊bject", "subjective_objective": {"answer": "Objective", "rationale": "Since the entry mainly describes an approach taken in the face of difficulties, which can be achieved by learning and learning from the experience of others, it does not involve emotional attitudes or subjectivity of individuals. The perspective of the entry is therefore objective."}, "sentence_sentiment": {"answer": "Positive", "rationale": "Because \"learning from your own experience or that of others\" and \"dealing with difficulties\" are positive behaviours and attitudes."}, "language_style": {"answer": "Tactful and warm", "rationale": "The language style of the entry is more moderate, encouraging respondents to learn from their own or others ' experiences how to cope with difficulties in an invitation-style tone, and this expression is more personal and interactive."}, "time_dimension": {"answer": "Future", "rationale": "\"Take your own experience and others' experience to deal with it.\" It is expressed as a future goal or plan and is therefore judged as the future."}, "sentence_type": "Imperative sentence", "pos": {"nouns": {"Experience": 1, "self": 1, "Others": 1, "Difficulties": 1}, "verbs": {"Draw": 1, "Payable": 1}, "numerals": {"40": 1}}}, {"content": "I don't believe in things that hurt me.", "word_count": 13, "label": "", "sentence_structure": "Subject鈥搗erb鈥搊bject", "subjective_objective": {"answer": "Subjective", "rationale": "This is viewed from a subjective point of view because of the psychological attitude and conviction of a person to believe in something harmful to the individual."}, "sentence_sentiment": {"answer": "Negative", "rationale": "Because \"no\" is negative."}, "language_style": {"answer": "Calm and direct", "rationale": "The language style of the entry is relatively concise, with no excessive emotional accommodations and euphemisms, with a calmer tone and directness, which directly conveys the core meaning of the problem."}, "time_dimension": {"answer": "Future", "rationale": "The keyword is \"often unconvinced\", indicating that it is a vision and a expectation of what may happen in the future, and therefore judgement is the future."}, "sentence_type": "Negative declarative sentence", "pos": {"nouns": {"Yes": 1, "Volume": 1}, "verbs": {"Regular": 1, "not": 1, "Marriage": 1, "Letter": 1, "to": 1, "Well": 1, "have": 1}, "adjectives": {"Lee": 1}, "adverbs": {"From": 1}, "numerals": {"41": 1}, "pronouns": {"Right": 1}}}, {"content": "42. For self-esteem, people are often reluctant to know what happened to them.", "word_count": 16, "label": "", "sentence_structure": "Subject鈥搗erb鈥搊bject", "subjective_objective": {"answer": "Subjective", "rationale": "Because it's not the patient's subjective attitude."}, "sentence_sentiment": {"answer": "Negative", "rationale": "Because the \"unwilling to let people know what happened to them\" in the entry is negative."}, "language_style": {"answer": "Tactful and warm", "rationale": "The language style of the entry is more euphemistic and sensitive to each other ' s feelings, leading the interviewee to reflect on and express his/her situation in the form of questions, and reflecting respect for and interest in the interviewee."}, "time_dimension": {"answer": "Present", "rationale": "In the words \"for self-esteem\", \"for purposes and motivations, \"self-esteem\" means respect for one's own values and abilities; and in the words \"always unwilling to let anyone know\" it means that the act is recurrent. In conclusion, the entry is an inquiry into the present state of behaviour and mental state of the person being tested."}, "sentence_type": "Negative declarative sentence", "pos": {"nouns": {"Dignity": 1, "person": 1, "Problems": 1}, "verbs": {"Yes": 2, "Jean": 1}, "adjectives": {"Self-esteem": 1}, "adverbs": {"Regular": 1}, "numerals": {"42": 1}}}, {"content": "43. Often discussing solutions with colleagues and friends", "word_count": 17, "label": "", "sentence_structure": "Subject鈥搗erb鈥搊bject", "subjective_objective": {"answer": "Objective", "rationale": "This is an objective fact because of the living conditions and social behaviour of the patient."}, "sentence_sentiment": {"answer": "Positive", "rationale": "Because the purpose of the entry is positive."}, "language_style": {"answer": "Tactful and warm", "rationale": "The entry uses euphemisms and expressions like \"always\" to make people feel more friendly and natural, while avoiding direct command tone. This would better stimulate the willingness and involvement of respondents and produce more accurate results."}, "time_dimension": {"answer": "Past", "rationale": "So the time dimension is now judged."}, "sentence_type": "Affirmative declarative sentence", "pos": {"nouns": {"Co-workers": 1, "Problem": 1, "Approach": 1}, "verbs": {"Discussion": 1, "Resolve": 1, "Give": 1}, "numerals": {"43": 1}}}, {"content": "* The present document is being issued without formal editing.", "word_count": 10, "label": "", "sentence_structure": "Subject鈥搗erb鈥揷omplement", "subjective_objective": {"answer": "Subjective", "rationale": "Because it's the subjective attitude of the patient."}, "sentence_sentiment": {"answer": "Positive", "rationale": "Because the act in the entry, \"Assisting the Ninja,\" expresses positive qualities and attitudes."}, "language_style": {"answer": "Tactful and warm", "rationale": "The language style of the entry is soft, encouraging and self-consoling, friendly and positive. The importance of self-alarming and self-adaptation was emphasized through the use of the expression of \"common caution\" and greater emphasis on emotional communication and interaction."}, "time_dimension": {"answer": "Present", "rationale": "The act of \"caution\" takes place in the current state of affairs, and the act of \"conserve the Ninja\" is a current fact or belief, so the entire sentence describes the present behaviour or attitude of the subject."}, "sentence_type": "Affirmative declarative sentence", "pos": {"nouns": {"self": 1, "ninjas": 1}, "verbs": {"Always warn": 1, "Shino-kun": 1}, "adjectives": {"Ann": 1}, "adverbs": {"From": 1}, "numerals": {"44": 1}}}, {"content": "\"45, I pray to the gods", "word_count": 7, "label": "", "sentence_structure": "Subject鈥搗erb鈥揷omplement", "subjective_objective": {"answer": "Subjective", "rationale": "Because \"often\" means it's a constant practice of patients, and \"pity and blessings\" is also their personal faith and attitude. These are subjective information."}, "sentence_sentiment": {"answer": "Positive", "rationale": "Since prayer is considered a positive emotional expression and belief act, it is generally considered to have a positive meaning."}, "language_style": {"answer": "Tactful and warm", "rationale": "The entry, in a gentle and respectful manner, asked the respondents whether they had a habit of praying for God ' s blessing, were not taking orders or making accusations, and were more kind and considerate. Such an expression would make it easier for interviewees to share their real ideas and would help create a good research climate."}, "time_dimension": {"answer": "Present", "rationale": "The keyword is \"a common prayer\", which means that it is a regular act and that it happens in the past and now, so it's a time dimension."}, "sentence_type": "Imperative sentence", "pos": {"nouns": {"45": 1, "Spirit": 1, "Bless you": 1}, "verbs": {"Pray": 1}, "adverbs": {"Regular": 1}, "numerals": {"45": 1}}}, {"content": "46. Common humor or jokes to mitigate conflict or delay", "word_count": 17, "label": "", "sentence_structure": "Subject鈥搗erb鈥搊bject", "subjective_objective": {"answer": "Subjective", "rationale": "This approach is determined subjectively by the patient and reflects his psychological state."}, "sentence_sentiment": {"answer": "Positive", "rationale": "Because the purpose of the entry is to \"mitigate conflict or delay in a way that is funny or funny\"."}, "language_style": {"answer": "Tactful and warm", "rationale": "The language style of the entry is more euphemistic, allowing interviewees to share their behaviour in an invitation-style manner, while using terms such as \"usual\" and \"mitigation\", implying the universality and positive effects of the act and creating a positive and friendly atmosphere."}, "time_dimension": {"answer": "Present", "rationale": "The subject of the questionnaire was asked about the current behaviour and attitudes of the subjects, and thus judged as \"now\"."}, "sentence_type": "Imperative sentence", "pos": {"nouns": {"Questionnaire": 1, "Entry": 1, "Humor": 1, "A joke": 1, "Modalities": 2, "Conflict": 1, "Not fast": 1}, "verbs": {"Common": 1, "Mitigation": 1}, "numerals": {"46": 1}}}, {"content": "47. Limited capacity and patience", "word_count": 10, "label": "", "sentence_structure": "Subject鈥搗erb鈥揷omplement", "subjective_objective": {"answer": "Subjective", "rationale": "This is because patients perceive and evaluate their abilities and are subject to subjective perceptions."}, "sentence_sentiment": {"answer": "Negative", "rationale": "Because \"capacity\" and \"resistance\" in the entries are both negative emotional tendencies."}, "language_style": {"answer": "Calm and direct", "rationale": ""}, "time_dimension": {"answer": "Present", "rationale": "In the future, the words \"self-limited capacity\" and \"resistence only\" in the entry imply a vision or expectation for the future, and are therefore judged to be the future. At the same time, the word \"only\" indicates that the idea is a possible solution or response strategy, which also supports a decision to classify it as a future."}, "sentence_type": "Negative declarative sentence", "pos": {"nouns": {"Capacity": 1, "Hold on": 1}, "verbs": {"Limited": 1, "Only": 1, "Hold on": 1}, "numerals": {"47": 1}, "pronouns": {"self": 1}}}, {"content": "48, I blame myself for being useless.", "word_count": 7, "label": "", "sentence_structure": "Subject鈥搗erb鈥揷omplement", "subjective_objective": {"answer": "Subjective", "rationale": "Because \"unusual\" is a subjective assessment and attitude that reflects patients' perception of their negative emotions."}, "sentence_sentiment": {"answer": "Negative", "rationale": "Because the act of \"always blaming itself for being useless\" means denial and self-demeaning, which is a negative sentiment."}, "language_style": {"answer": "Calm and direct", "rationale": "The entry is a direct expression of the question about whether the other party often feels that it has no interest in the subject, does not use excessive rhetorical techniques or emotional rendering, and is more objective in tone."}, "time_dimension": {"answer": "Past", "rationale": ""}, "sentence_type": "Negative declarative sentence", "pos": {"nouns": {"48": 1, "It's weird": 1, "self": 1, "- It's good": 1}, "adjectives": {"Regular": 1}, "adverbs": {"Nope": 1}, "numerals": {"48": 1}, "pronouns": {"self": 1}}}, {"content": "I always fantasize about things that aren't realistic to get rid of.", "word_count": 16, "label": "", "sentence_structure": "Subject鈥搗erb鈥搊bject", "subjective_objective": {"answer": "Subjective", "rationale": "Because the fantasy of love is a subjective attitude or psychological state."}, "sentence_sentiment": {"answer": "Negative", "rationale": "It's a negative act because the entry says, \"Imagine something unrealistic to remove trouble.\""}, "language_style": {"answer": "Tactful and warm", "rationale": "The entry is more adept and interactive when it is spoken in a gentle and inviting manner, rather than by direct statements or orders. At the same time, it does not use excessive rhetorical techniques or emotional rendering, which is more balanced and objective."}, "time_dimension": {"answer": "Present", "rationale": "Now. Because \"Always Love\" means constant behavior, \"Imagine something unrealistic to remove troubles\" is a description of the current behavior, so the sentence describes the current situation."}, "sentence_type": "Affirmative declarative sentence", "pos": {"nouns": {"Fantasy": 1, "Yes": 1, "Trouble": 1}, "verbs": {"Always": 1, "Elimination": 1}, "adverbs": {"Some": 1, "It's not realistic": 1}, "numerals": {"49": 1}}}, {"content": "50, often complaining about incompetence.", "word_count": 7, "label": "", "sentence_structure": "Subject鈥搗erb鈥揷omplement", "subjective_objective": {"answer": "Subjective", "rationale": "Because \"often complaining about incompetence\" is the patient's subjective attitude and thoughts."}, "sentence_sentiment": {"answer": "Negative", "rationale": "Because the action of complaining about incompetence in the entry is negative."}, "language_style": {"answer": "Tactful and warm", "rationale": ""}, "time_dimension": {"answer": "Present", "rationale": "The entry asks the testee about his current behaviour or mental state, because \"often\" means a regular act, and \"complaining for his incompetence\" is a description of the current state of affairs, so it is judged now."}, "sentence_type": "Negative declarative sentence", "pos": {"nouns": {"Entry": 1, "Questionnaire": 1}, "verbs": {"Analysis": 1, "- He's complaining": 1, "Incompetent": 1}, "numerals": {"50": 1}}}, {"content": "There's a good side to seeing bad things.", "word_count": 12, "label": "", "sentence_structure": "Subject鈥揷opula鈥損redicative", "subjective_objective": {"answer": "Subjective", "rationale": "Because perceptions of good and bad are determined by the thoughts of individuals and are subjective."}, "sentence_sentiment": {"answer": "Positive", "rationale": "For the expression \"a good side of bad things is often visible\" emphasizes a positive attitude, namely, finding positive aspects in difficulties and setbacks, as an expression of optimism."}, "language_style": {"answer": "Tactful and warm", "rationale": "The entry, which guides the interviewees in a spirit of encouragement and certainty, does not directly raise questions or orders, and the language style is relatively gentle and friendly and acceptable."}, "time_dimension": {"answer": "Future", "rationale": "The \"often visible\" expression of customary behaviour in the entry, while \"seeing the good side of bad things\" takes place in the future to reflect it, and the temporal dimension of the article can be judged to be future."}, "sentence_type": "Affirmative declarative sentence", "pos": {"nouns": {"Bad things": 1, "Okay": 1, "Noodles": 1}, "verbs": {"Look": 1, "have": 1}, "adverbs": {"- I'm not sure": 1}, "numerals": {"51": 1}}}, {"content": "52: Self-frustration is a test.", "word_count": 11, "label": "", "sentence_structure": "Subject鈥損redicate", "subjective_objective": {"answer": "Subjective", "rationale": "Because \"frustration is a test of self\" is a personal view or feeling that belongs to the patient's subjective perspective."}, "sentence_sentiment": {"answer": "Negative", "rationale": "Because frustration is negative, and the entry uses test. This term describes frustration and gives people a sense of challenge and pressure, and thus has a negative emotional colour as a whole."}, "language_style": {"answer": "Calm and direct", "rationale": "The entry is a direct illustration of the value of self-defeating, i.e., a more straightforward tone for exercise and testing, and does not use excessive rhetorical or emotional rendering."}, "time_dimension": {"answer": "Past", "rationale": "The entry contains a time reference term for \"self-depression\", which points to past experiences and feelings, so it is judged to be past."}, "sentence_type": "Affirmative declarative sentence", "pos": {"nouns": {"Frustration": 1, "Test": 1}, "verbs": {"Self-conscious": 1}, "adverbs": {"to": 1, "self": 1}, "numerals": {"52": 1}}}, {"content": "53. Ways to solve problems with experienced relatives and teachers", "word_count": 18, "label": "", "sentence_structure": "Subject鈥搗erb鈥搊bject", "subjective_objective": {"answer": "Subjective", "rationale": "The issue involves the patient ' s behaviour and attitude in seeking help on his own initiative and is therefore examined from a subjective perspective."}, "sentence_sentiment": {"answer": "Positive", "rationale": "Because the entry \"Teaching experienced friends and friends and teachers\" is a positive act and attitude that helps solve problems."}, "language_style": {"answer": "Tactful and warm", "rationale": "The entry raises questions in a question-and-answer manner, respects each other ' s knowledge and experience and wishes to receive their help and support. This expression is moderate, friendly and interactive, and it is easy for respondents to feel valued and focused."}, "time_dimension": {"answer": "Past", "rationale": "Present"}, "sentence_type": "Imperative sentence", "pos": {"nouns": {"Experience": 1, "Friends": 1, "Sir": 1, "Problem": 1, "Methodology": 1}, "verbs": {"In": 1, "- You're a teacher": 1, "Resolve": 1}, "numerals": {"53": 1}}}, {"content": "54, calm down, calm down.", "word_count": 8, "label": "", "sentence_structure": "Subject鈥搗erb鈥揷omplement", "subjective_objective": {"answer": "Subjective", "rationale": "Because \"silent, lightened\" is an individual psychological feeling and attitude, a patient's subjective idea."}, "sentence_sentiment": {"answer": "Positive", "rationale": "Because \"silent\" and \"silent\" are both positive emotional adjustments."}, "language_style": {"answer": "Tactful and warm", "rationale": "The entry uses a more moderate tone, encouraging respondents to maintain a calm mentality and to try to dilute their distress, which is more friendly and friendly."}, "time_dimension": {"answer": "Present", "rationale": "The words \"silentness\" and \"reduced distress\" in this entry are suggestions for the current emotional state of the subjects and do not relate to past or future situations and are therefore judged to be the present."}, "sentence_type": "Imperative sentence", "pos": {"nouns": {"Calm down": 1, "Dilution": 1, "Trouble": 1}, "verbs": {"54": 1}, "numerals": {"54": 1}}}, {"content": "55 - Towards solutions", "word_count": 11, "label": "", "sentence_structure": "Subject鈥搗erb鈥搊bject", "subjective_objective": {"answer": "Subjective", "rationale": "Because finding solutions is an act of initiative that requires the patient's subjective will and action. This is the subjective attitude and behaviour of the patient and is therefore the subject of a subjective examination."}, "sentence_sentiment": {"answer": "Positive", "rationale": "Because the purpose of the action of finding solutions in the entry is positive."}, "language_style": {"answer": "Calm and direct", "rationale": ""}, "time_dimension": {"answer": "Past", "rationale": "The words \"to try to find\" and \"to solve problems\" in this entry show that the testees have tried to solve problems in the past, so the dimension of time is the past."}, "sentence_type": "Imperative sentence", "pos": {"nouns": {"Try": 1, "Search": 1, "Problem": 1, "Methodology": 1}, "verbs": {"Resolve": 1}, "numerals": {"55": 1}}}, {"content": "56. Improper career choices are the main cause of frequent setbacks.", "word_count": 18, "label": "", "sentence_structure": "Subject鈥搗erb鈥揷omplement", "subjective_objective": {"answer": "Objective", "rationale": "Since the choice of occupation is in itself an objective situation, whether the frustration is related to the choice of occupation is to be judged by data analysis. Thus, the entry examines objectively whether the patient is aware of the impact of the choice of occupation on his or her life."}, "sentence_sentiment": {"answer": "Negative", "rationale": "'Cause \"Improper choice of profession\" and \"unusual frustration\" in the entries are both negative."}, "language_style": {"answer": "Calm and direct", "rationale": "The language style of the entry is more straightforward and clearly identifies \"improper choice of profession\" as the main reason for the frequent frustration. Such a presentation is concise, does not have too many embellishment or emotional colours and is more in keeping with the nature and tone of the questions surveyed."}, "time_dimension": {"answer": "Past", "rationale": "The entry describes a past experience or fact, and the keywords \"mischored career\" and \"principal cause of frequent frustration\" point to what has happened. Thus, the temporal dimension of the purpose of the article is a judgement of the past."}, "sentence_type": "Negative declarative sentence", "pos": {"nouns": {"Selection": 1, "Occupation": 2, "Main reasons": 1, "Frustration": 1}, "verbs": {"Choose": 1, "Yes": 1, "When": 1, "be": 1, "self": 1}, "adverbs": {"Not right": 1}, "numerals": {"56": 1, "2": 1}, "classifiers": {"individual": 1}}}, {"content": "It's your fault.", "word_count": 6, "label": "", "sentence_structure": "Subject鈥搗erb鈥揷omplement", "subjective_objective": {"answer": "Subjective", "rationale": "Because blame yourself is a subjective view and evaluation."}, "sentence_sentiment": {"answer": "Negative", "rationale": "Because in the entry, \"I'm sorry\" expresses a self-criticism and has a negative emotional tone."}, "language_style": {"answer": "Tactful and warm", "rationale": "The language style of the entry is gentle and friendly, expressing a sense of concern and understanding by asking if the other side often blames itself, and attempting to make the other side feel its support."}, "time_dimension": {"answer": "Present", "rationale": "The entry asks the testee about his current behavior or mental state, and it's \"bad\" that the testee regularly blames himself, which is a description of his current behaviour. The time dimension of the article could therefore be judged as now."}, "sentence_type": "Negative declarative sentence", "pos": {"nouns": {"self": 1}, "verbs": {"Weird": 1, "Okay": 1}, "adverbs": {"Total": 1}, "numerals": {"57": 1}}}, {"content": "58, often to see the dust, not to worry about what happened to you.", "word_count": 17, "label": "", "sentence_structure": "Subject鈥搗erb鈥搊bject", "subjective_objective": {"answer": "Subjective", "rationale": "Because the words \"seeing the dust\" and \"don't care about your own misfortune\" express the patient's subjective attitude."}, "sentence_sentiment": {"answer": "Negative", "rationale": "Because \"seeing red dust\" and \"don't care about your own misfortune\" express a negative emotional attitude."}, "language_style": {"answer": "Calm and direct", "rationale": "The language style of the entry is more concise, with no excessive use of emotional qualifiers or rhetorical techniques, and more objective and direct. In a direct question, it asked whether respondents often felt that they were secular and did not care about their plight, which was a calm and straightforward expression."}, "time_dimension": {"answer": "Past", "rationale": ""}, "sentence_type": "Negative declarative sentence", "pos": {"nouns": {"Reddust": 1, "Unfortunate encounters": 1}, "verbs": {"- Look at that": 1, "I care": 1}, "adjectives": {"Regular": 1}}}, {"content": "59. I'm always feeling bad luck.", "word_count": 7, "label": "", "sentence_structure": "Subject鈥揷opula鈥損redicative", "subjective_objective": {"answer": "Subjective", "rationale": "Because luck is an abstract concept, a subjective assessment of the patient's own feelings."}, "sentence_sentiment": {"answer": "Negative", "rationale": "Because the expression \"bad luck\" in the entry is negative."}, "language_style": {"answer": "Calm and direct", "rationale": "Because the entry is a direct expression of the subject of asking the other party whether he or she often feels bad luck, it does not use excessive rhetorical techniques or emotional rendering, which is more objective in tone."}, "time_dimension": {"answer": "Past", "rationale": "The keyword \"common sense of self\" indicates a customary feeling or perception that is usually a response to past experiences and events. The entry therefore asks whether the person tested had had any experience with bad luck in the past."}, "sentence_type": "Negative declarative sentence", "pos": {"nouns": {"Lucky": 1}, "verbs": {"Sensor": 1, "Regular": 1, "not": 1}, "adverbs": {"Okay": 1}, "numerals": {"59": 1}}}, {"content": "60, talking to others about your problems.", "word_count": 10, "label": "", "sentence_structure": "Subject鈥搗erb鈥搊bject", "subjective_objective": {"answer": "Subjective", "rationale": "Because \"talking to others about your worries\" is a personal feeling and thought, it is a subjective examination."}, "sentence_sentiment": {"answer": "Positive", "rationale": "Because the behavior in the entry \"to tell others what's in your heart\" is an act that seeks support and help and is positive."}, "language_style": {"answer": "Tactful and warm", "rationale": "The entry uses a moderate approach to questioning, attempts to establish contact with the interviewees and encourages them to share their inner distress and to speak more with them. At the same time, the issue was not directed or criticized by interviewees, but rather provided a safe and supportive environment for them to express their feelings."}, "time_dimension": {"answer": "Past", "rationale": "Based on the key words in the title, \"to say to others\" and \"to the mind\" it can be deduced that the entry is a judgement of the past by asking whether the person who was tested has spoken to others about their concerns at some point in the past."}, "sentence_type": "Imperative sentence", "pos": {"nouns": {"鈾?In the heart 鈾?: 1, "Trouble": 1}, "verbs": {"Tell me": 1, "To others": 1}, "numerals": {"60": 1}, "classifiers": {"individual": 1}}}, {"content": "61, I've always felt like I'm not doing anything.", "word_count": 12, "label": "", "sentence_structure": "Subject鈥搗erb鈥搊bject", "subjective_objective": {"answer": "Subjective", "rationale": "Because \"inaction is left to nature\" is an individual feeling and attitude that falls within the realm of subjective experience."}, "sentence_sentiment": {"answer": "Negative", "rationale": "Because \"inaction\" and \"intentional nature\" in the entries are negative emotional attitudes."}, "language_style": {"answer": "Calm and direct", "rationale": "The entry is a direct expression of a question about whether the other party often feels that it has not done anything, that the language style is simpler, that there are no excessive modifications or emotional renderings, and that the tone is more objective and neutral."}, "time_dimension": {"answer": "Present", "rationale": "The \"normal\" expression of a regular, customary act, the \"reception\" of the general current, means that the act has not only occurred in the past, but may still continue. The entry is therefore a description of current behaviour or mentality."}, "sentence_type": "Negative declarative sentence", "pos": {"nouns": {"Entry": 1, "Questionnaire": 1, "I'm not doing anything": 1}, "verbs": {"I feel like I'm": 1, "Ren": 1, "Yes": 2}, "adjectives": {"Naturally": 1}, "numerals": {"61銆?: 1}}}, {"content": "Seeking understanding and sympathy from others", "word_count": 10, "label": "", "sentence_structure": "Subject鈥搗erb鈥搊bject", "subjective_objective": {"answer": "Subjective", "rationale": "The search for understanding and compassion is the subjective will and emotional need of a person, and it is an inner feeling and expectation of a patient in the face of problems or difficulties. This questionnaire entry is therefore viewed from the patient ' s subjective perspective."}, "sentence_sentiment": {"answer": "Positive", "rationale": "Because the act of \"seeking understanding and compassion from others\" in the entry expresses a desire for help and support from others and is positive."}, "language_style": {"answer": "Tactful and warm", "rationale": "The language style of the entry is more euphemistic and caring, using the term \"understanding and compassion\" and giving people a soft feeling, less direct and tough. At the same time, attention to and respect for the emotional needs of the interviewees are reflected."}, "time_dimension": {"answer": "Present", "rationale": ""}, "sentence_type": "Imperative sentence", "pos": {"nouns": {"Understood": 1, "Compassion": 1}, "verbs": {"Seek": 1}, "numerals": {"62": 1}}}]}

{"questionnaire_title": "Rasch-type solitude table", "questionnaire_category": "Behavioral questionnaire", "item_count": 28, "emotion_shift_overall": "From negative to positive", "language_style_overall": "Tactful and warm", "items": [{"content": "I really want to have a good friend.", "word_count": 7, "label": "", "sentence_structure": "Subject鈥損redicate", "subjective_objective": {"answer": "Subjective", "rationale": "Because \"real\" is the personal feeling and desire of the patient. It's a subjective attitude."}, "sentence_sentiment": {"answer": "Positive", "rationale": "It is a positive emotional attitude because it expresses the desire of a person to have a friend."}, "language_style": {"answer": "Tactful and warm", "rationale": "The entry uses a more euphemistic expression, \"I really want to have a good friend.\" Rather than directly showing whether the other side really needs or desires friends, this expression is more personal and interactive in a more euphemistic way to ask or invite the other side to share their thoughts and feelings."}, "time_dimension": {"answer": "Present", "rationale": "In the future, the entry expresses one of the expectations and visions of the testee for the future, i.e. for having a good friend. The keyword \"I really want\" indicates that this expectation is very strong and may be realized in the future."}, "sentence_type": "Exclamatory sentence", "pos": {"nouns": {"Friend": 1}, "verbs": {"I wish I could": 1}, "adverbs": {"True": 1}}}, {"content": "I feel a void around me.", "word_count": 9, "label": "", "sentence_structure": "Subject鈥揷opula鈥損redicative", "subjective_objective": {"answer": "Subjective", "rationale": "Because it's a subjective feeling."}, "sentence_sentiment": {"answer": "Negative", "rationale": "Because the feeling in the entry is negative."}, "language_style": {"answer": "Calm and direct", "rationale": "In a direct way, the entry asks the interviewee whether he or she feels emptiness in his or her surroundings, does not use too much rhetoric or emotional rendering, and is relatively objective and calm."}, "time_dimension": {"answer": "Present", "rationale": ""}, "sentence_type": "Affirmative declarative sentence", "pos": {"nouns": {"Around": 1, "Snippets": 1, "Empty": 1}, "verbs": {"I feel": 1}}}, {"content": "There is no one who can truly join me in honour.", "word_count": 11, "label": "", "sentence_structure": "Subject鈥搗erb鈥揷omplement", "subjective_objective": {"answer": "Subjective", "rationale": "Because \"honor and communion\" is a subjective feeling of emotional connection and shared experience."}, "sentence_sentiment": {"answer": "Negative", "rationale": "Because the expression \"no one can really shame and be with me\" in the entry is negative."}, "language_style": {"answer": "Calm and direct", "rationale": "The language style of the entry is more direct and concise, there is no excessive qualifier or emotional colour, and the tone is more calm and objective. It expresses the theme through a simple and direct statement that very few people in human relations truly share their own suffering, a statement that neither praises nor demeans others, but presents itself in a calm manner."}, "time_dimension": {"answer": "Past", "rationale": "The entry expresses the past feelings and experiences of the testees, and the keyword \"no one\" indicates that these feelings and experiences are related to past events or events."}, "sentence_type": "Negative declarative sentence", "pos": {"nouns": {"person": 1, "Honor": 1, "Total": 1}, "verbs": {"True": 1, "Yes": 1, "Call": 1, "Together": 1}, "adverbs": {"No, I'm not": 1}, "pronouns": {"I": 1}}}, {"content": "I really have no friends.", "word_count": 8, "label": "", "sentence_structure": "Subject鈥搗erb鈥揷omplement", "subjective_objective": {"answer": "Subjective", "rationale": "Because having friends is a subjective feeling and an assessment."}, "sentence_sentiment": {"answer": "Negative", "rationale": "Because the expression \"no friends\" in the entry is negative, which is a sign of feelings of loneliness, helplessness, etc."}, "language_style": {"answer": "Calm and direct", "rationale": "Instead of using excessive rhetorical techniques or emotional renderings, the entry is more concise in its linguistic style and raises questions in a direct and objective manner, expressing concern about whether the person under investigation has friends. So I think the language style of this questionnaire entry is cool and straightforward."}, "time_dimension": {"answer": "Past", "rationale": "The entry is about the state and feeling of the past. This phrase shows that speakers have had no friends at some point in the past."}, "sentence_type": "Negative declarative sentence", "pos": {"nouns": {"friends": 1}, "verbs": {"have": 1, "True": 1, "Any": 1, "No, I'm not": 1, "be": 1}, "adjectives": {"Really": 1}, "pronouns": {"I": 1}}}, {"content": "No one cares about you in particular.", "word_count": 8, "label": "", "sentence_structure": "Subject鈥損redicate", "subjective_objective": {"answer": "Subjective", "rationale": "Because \"special care\" is a subjective feeling and assessment, it is not entirely possible to describe the fact that an individual is cared for by others."}, "sentence_sentiment": {"answer": "Negative", "rationale": "Because the expression \"no one cares about you\" in the entry is a negative experience or state, expressing a feeling of lack of love and support."}, "language_style": {"answer": "Tactful and warm", "rationale": "The language style of the entry belongs to the euphemism and warmth category. It expresses the concerns of the interviewees in a more moderate and cordial manner, without being too direct or inflexible to attract and maintain the participation of the interviewees. In addition, the entry has the metaphor of suggesting that the interviewee may feel neglected or alone, a formulation that is more likely to generate resonance among the interviewee and enhance the effectiveness of the questionnaire."}, "time_dimension": {"answer": "Past", "rationale": "The entry describes the emotional state of the person being tested in the present social context, with no clear time-indicators pointing to the past, present or future, and is therefore an emotional assessment of the current state of affairs."}, "sentence_type": "Negative declarative sentence", "pos": {"nouns": {"person": 1, "Special": 1, "Care": 1, "you": 1}, "verbs": {"No, I'm not": 1, "Of special interest": 1}}}, {"content": "6. I often feel rejected.", "word_count": 7, "label": "", "sentence_structure": "Subject鈥揷opula鈥損redicative", "subjective_objective": {"answer": "Subjective", "rationale": "Because \"always feeling rejected\" is the patient's subjective feeling and experience and belongs to the emotional state of the patient. This perception may be influenced by a variety of factors, including individual emotions, perceptions and past experiences. This questionnaire entry is therefore viewed from the patient ' s subjective perspective."}, "sentence_sentiment": {"answer": "Negative", "rationale": "Because \"rejected\" is usually considered negative."}, "language_style": {"answer": "Tactful and warm", "rationale": ""}, "time_dimension": {"answer": "Present", "rationale": "I often feel that the word \"always\" denotes a regular, customary act, and that it uses the general current, which means that it has not only happened in the past, but may still continue. The entry is therefore to ask whether the person who was tested is now often feeling rejected."}, "sentence_type": "Negative declarative sentence", "pos": {"nouns": {"Questionnaire Entry": 1, "Reject": 1}, "verbs": {"I feel": 1, "Rejected": 1}, "adverbs": {"Regular": 1}, "pronouns": {"I": 1}}}, {"content": "7. Even your family is no longer interested in you.", "word_count": 14, "label": "", "sentence_structure": "Subject鈥搗erb鈥揷omplement", "subjective_objective": {"answer": "Subjective", "rationale": "Because \"interest\" is a subjective criterion, different people may have different levels of interest in the same thing. Therefore, \"interest in someone or something\" is usually a question of the subjective attitude and perception of the person."}, "sentence_sentiment": {"answer": "Negative", "rationale": "Because the \"I'm not interested\" act in the entry is negative."}, "language_style": {"answer": "Calm and direct", "rationale": "The language style of the entry is relatively straightforward and concise, and there is no excessive use of rhetorical techniques or emotional rendering to allow respondents to understand the meaning and intent of the issue directly. At the same time, this condensed form of expression can avoid plaguing or maladapting the respondents."}, "time_dimension": {"answer": "Present", "rationale": "The keywords are \"even\" and \"also\", which indicate that this is not only true, but very serious. At the same time, the description of \"no interest\" also shows that the current situation is more serious than in the past, so it can be judged that the entry is an inquiry into the present behaviour or mental state of the subject."}, "sentence_type": "Negative declarative sentence", "pos": {"nouns": {"Family": 1, "you": 2, "Interest": 1}, "verbs": {"not": 1, "to": 1, "Interested": 1, "Yes": 1}, "adjectives": {"Even": 1}, "numerals": {"7": 1}}}, {"content": "Only a few people have spoken to you.", "word_count": 10, "label": "", "sentence_structure": "Subject鈥損redicate", "subjective_objective": {"answer": "Objective", "rationale": "Because \"only a few people can talk to you\" describes the number and extent of human relationships, an objective fact that can be observed and quantified. While this may reflect some subjective feelings or emotional state of the patient (e.g. loneliness or social anxiety), the subject itself does not directly question these subjective experiences. Therefore, the entry is primarily for objective purposes."}, "sentence_sentiment": {"answer": "Neutral", "rationale": "Because the entry does not have any emotional color."}, "language_style": {"answer": "Tactful and warm", "rationale": "The entry is more adept and interactive when it is spoken in a gentle and inviting manner, rather than by direct statements or orders."}, "time_dimension": {"answer": "Past", "rationale": "The entry asks the testee about the past, and the time dimension is the past."}, "sentence_type": "Affirmative declarative sentence", "pos": {"nouns": {"person": 1, "Talk": 1}, "verbs": {"and": 1, "It's working": 1}, "numerals": {"How many": 1}, "classifiers": {"individual": 1}, "pronouns": {"you": 1}}}, {"content": "9. Only a few people are tired of listening to you.", "word_count": 14, "label": "", "sentence_structure": "Subject鈥搗erb鈥搊bject", "subjective_objective": {"answer": "Subjective", "rationale": "Because it's a subjective judgment to listen to you, and it needs to be assessed according to how the patient feels."}, "sentence_sentiment": {"answer": "Neutral", "rationale": "Because the entry only describes a phenomenon without any emotional colour."}, "language_style": {"answer": "Tactful and warm", "rationale": "The language style of the entry is more euphemistic. First of all, the phrase \"to hear you speak\" is more positive and positive and does not give rise to accusations or criticisms of the other side. Secondly, the tone of the entire sentence is relatively mild, giving people a sense of affection and concern, and thus of comfort and acceptance."}, "time_dimension": {"answer": "Present", "rationale": "The key words \"hearing\" and \"spoken\" all happen now, so the entry describes the person's current behaviour or state."}, "sentence_type": "Negative declarative sentence", "pos": {"nouns": {"person": 2, "Tell me": 1}, "verbs": {"I'm not tired of listening": 1}, "adjectives": {"How many": 1, "Boring": 1}, "adverbs": {"Only": 1, "Land": 1}, "numerals": {"9": 1}}}, {"content": "10. When you feel good, you are welcome, but when you are depressed, you are completely different.", "word_count": 24, "label": "", "sentence_structure": "Subject鈥搗erb鈥揷omplement", "subjective_objective": {"answer": "Subjective", "rationale": "Because emotional state is described on the basis of the patient ' s own feelings and experiences, it is subjective."}, "sentence_sentiment": {"answer": "Negative", "rationale": "Because \"when you feel good, you'll be welcomed\" is positive, but when you're depressed, it's completely different.\" It's a negative emotional expression, suggesting that people may be excluded or left alone when they're depressed."}, "language_style": {"answer": "Tactful and warm", "rationale": "Basis of judgement: In describing people ' s feelings and behaviour, the entry does not use too direct or hard language, but rather is expressed in gentle and emotional language. For example, the use of words such as \"good feeling\", \"popularness\" and \"depressiveness\" conveys an emotional atmosphere of care, understanding and support that makes people feel comfortable and warm. At the same time, the entry does not place too much emphasis on its own views or positions, but rather engages in a dialogue based on building a resonance with the other party, a softer expression that attracts more attention and participation."}, "time_dimension": {"answer": "Present", "rationale": ""}, "sentence_type": "Affirmative declarative sentence", "pos": {"nouns": {"you": 2, "Time": 2, "Emotion": 1, "Good": 1, "Welcome": 1, "I'm depressed": 1, "Different": 1, "Questionnaire": 1, "Entry": 1}, "verbs": {"Feel": 1, "Yes": 3, "Just": 1, "It's completely different": 1}, "adjectives": {"Good": 1, "I'm depressed": 1, "Different": 1}, "adverbs": {"When": 2, "Time": 2, "Just": 1}, "numerals": {"10": 1}, "pronouns": {"you": 2}}}, {"content": "11. Recently I felt that even the closest family had misunderstood me.", "word_count": 18, "label": "", "sentence_structure": "Subject鈥揷opula鈥損redicative", "subjective_objective": {"answer": "Subjective", "rationale": "Because \"fear\" and \"misunderstanding\" are patients' own subjective feelings and understandings."}, "sentence_sentiment": {"answer": "Negative", "rationale": "Because the entry \"understood by the closest family\" is negative."}, "language_style": {"answer": "Tactful and warm", "rationale": "The language style of the entry tends to be gentle and euphemistic, expressing the feelings and experiences of the author in a relatively soft way. In this sentence, \"even the closest family misconstrued me\" indicates that the author may feel lonely and frustrated, but rather than directly accusing or complaining about the other, he uses a more relaxed way of describing his feelings, which makes it easy to generate compassion and understanding. This language style helps to create a good climate of interaction and gives respondents a greater willingness to share their real ideas and experiences."}, "time_dimension": {"answer": "Present", "rationale": ""}, "sentence_type": "Negative declarative sentence", "pos": {"nouns": {"Questionnaire": 1, "Entry": 1, "person": 1, "Family": 1}, "verbs": {"I think": 1, "Misunderstanding": 1, "Yes": 2}, "adjectives": {"Most recently": 1, "The closest": 1}, "adverbs": {"Yeah": 1}, "numerals": {"11": 1}, "pronouns": {"I": 1}}}, {"content": "I'm in love with people around me.", "word_count": 10, "label": "", "sentence_structure": "Subject鈥揷opula鈥損redicative", "subjective_objective": {"answer": "Subjective", "rationale": "Because \"relationship\" and \"persons around\" are the feelings and subjective experiences of patients, this is a subjective approach."}, "sentence_sentiment": {"answer": "Positive", "rationale": "Because the words \"relationship\" and \"when there are people around you\" are both positive emotional."}, "language_style": {"answer": "Tactful and warm", "rationale": "The entry uses a gentle and inviting tone to guide respondents to think and share their feelings, which is more personal and interactive. At the same time, the word \"relationship\" also implies a deep emotional connection, further highlighting the gentleness of the questionnaire."}, "time_dimension": {"answer": "Present", "rationale": ""}, "sentence_type": "Affirmative declarative sentence", "pos": {"nouns": {"Time": 1}, "verbs": {"I'm in love": 1, "have": 1}, "adverbs": {"Around": 1}, "pronouns": {"I": 1}}}, {"content": "I have lost all my past friends.", "word_count": 12, "label": "", "sentence_structure": "Subject鈥搗erb鈥搊bject", "subjective_objective": {"answer": "Subjective", "rationale": "Because \"all friends\" is a subjective expression that reflects what patients think and feel about their social status. While this may occur in reality, it is itself an internal experience and assessment of the patient and therefore falls within the realm of subjective examination."}, "sentence_sentiment": {"answer": "Negative", "rationale": "Because \"lost all friends\" in the entry is negative."}, "language_style": {"answer": "Tactful and warm", "rationale": "The entry is more adept and interactive when it is spoken in a gentle and inviting manner, rather than by direct statements or orders."}, "time_dimension": {"answer": "Past", "rationale": "The keyword \"has\" indicates that this occurred at some point in the past and still exists. The expression \"lost everything\" emphasizes the distress and loneliness of the subjects in their interpersonal contacts. Thus, the main message of this entry is a description of the current state and emotional experience of the person being tested, which is the current state of affairs."}, "sentence_type": "Negative declarative sentence", "pos": {"nouns": {"Past": 1, "All": 1, "friends": 1}, "verbs": {"Lost": 1}, "numerals": {"13": 1}, "pronouns": {"I": 1}}}, {"content": "14. I feel that I am a prisoner in my own room.", "word_count": 12, "label": "", "sentence_structure": "Subject鈥揷opula鈥損redicative", "subjective_objective": {"answer": "Subjective", "rationale": "Because \"I think\" expresses patients' subjective feelings and views."}, "sentence_sentiment": {"answer": "Negative", "rationale": "Because the expression \"a prisoner in his own room\" in the entry conveys a negative emotion and attitude."}, "language_style": {"answer": "Tactful and warm", "rationale": "The entry expresses a sense of the interviewee in the first person ' s name, using a moderate and self-reflective language style, without direct assessment or criticism, which is more euphemism and goodwill. At the same time, it implies that there may be some problem or dilemma that needs to be addressed. This language style is therefore more suitable for use in psychological questionnaire studies in order to better understand and accept the feelings and circumstances of the interviewees."}, "time_dimension": {"answer": "Present", "rationale": "Now it's the testee's feelings and cognitive description of his current state, and \"I think\" in it means that it's subjective, while \"the prisoner in his cell\" depicts the living conditions of the testee, so it can be judged now."}, "sentence_type": "Affirmative declarative sentence", "pos": {"nouns": {"I": 1, "Prisoners": 1, "Room": 1}, "verbs": {"I think": 1}, "adjectives": {"Your own": 1}, "numerals": {"14": 1}}}, {"content": "15. I am saddened by the absence of a companion.", "word_count": 12, "label": "", "sentence_structure": "Subject鈥揷opula鈥損redicative", "subjective_objective": {"answer": "Subjective", "rationale": "Because making me sad is a subjective feeling or emotion, so it's a subjective angle."}, "sentence_sentiment": {"answer": "Negative", "rationale": "Because in the entry, \"I'm sad because no one's accompanying me\" expresses negative emotions."}, "language_style": {"answer": "Tactful and warm", "rationale": "The entry uses a gentle and caring tone for each other ' s feelings, using more euphemistic language and expressing sympathy and understanding for the interviewees. This language style, which is more common in psychological questionnaires, is better able to resonate and improve the collaboration of respondents."}, "time_dimension": {"answer": "Past", "rationale": "\"No one is with me\" is a statement describing the experience or state of the past, and \"depressing me\" is also the feeling and emotional reaction of the past point of time. This entry is therefore a question of past behaviour or mentality."}, "sentence_type": "Negative declarative sentence", "pos": {"nouns": {"person": 1, "Stay with me": 1, "Sorry": 1}, "verbs": {"Nobody": 1, "鈾?And I'm gonna be 鈾?: 1}, "adjectives": {"No, I'm not": 1, "I": 1}, "numerals": {"15": 1}}}, {"content": "I feel neglected by my neighbours.", "word_count": 9, "label": "", "sentence_structure": "Subject鈥揷opula鈥損redicative", "subjective_objective": {"answer": "Subjective", "rationale": "Because feeling ignored by neighbours is the patient's own feeling and subjective judgment."}, "sentence_sentiment": {"answer": "Negative", "rationale": "Because the feeling of being ignored by the neighbors is negative."}, "language_style": {"answer": "Tactful and warm", "rationale": "By describing the emotional feelings of the interviewee, the entry leads the other party to answer the questions rather than directly questioning or blaming the neighbour for his behaviour. This expression is more moderate and understanding and can create a better climate for interaction."}, "time_dimension": {"answer": "Present", "rationale": "The key word in the sentence is \"I feel\", which means the subjective feeling of the speaker, and the temporal dimension is now judged."}, "sentence_type": "Negative declarative sentence", "pos": {"nouns": {"I": 1, "Neighbors": 1, "Neglect": 1}, "verbs": {"I feel": 1, "Neglect": 1}, "numerals": {"16": 1}}}, {"content": "I really want to have a relationship with my neighbours.", "word_count": 9, "label": "", "sentence_structure": "Subject鈥搗erb鈥揷omplement", "subjective_objective": {"answer": "Subjective", "rationale": "Because \"I really want\" expresses a patient's desire or emotional attitude, which is considered subjective."}, "sentence_sentiment": {"answer": "Positive", "rationale": "Because the entry expresses the author's desire to interact with his neighbours, it is positive and emotional."}, "language_style": {"answer": "Tactful and warm", "rationale": "The entry is more adept and interactive when it is spoken in a gentle and inviting manner, rather than by direct statements or orders."}, "time_dimension": {"answer": "Present", "rationale": ""}, "sentence_type": "Exclamatory sentence", "pos": {"nouns": {"I": 1, "Neighbors": 1, "Travel": 1}, "verbs": {"Yes": 1, "have": 1}, "adverbs": {"True": 1}}}, {"content": "I think the circle of friends and acquaintances is too small.", "word_count": 14, "label": "", "sentence_structure": "Subject鈥揷opula鈥損redicative", "subjective_objective": {"answer": "Subjective", "rationale": "Because it's the patient's subjective feeling."}, "sentence_sentiment": {"answer": "Negative", "rationale": "Because the description \"too small\" is negative."}, "language_style": {"answer": "Tactful and warm", "rationale": "The entry uses a mild and inviting tone, which attempts to provoke the other side to think and reflect on its own social circle, without directly rejecting or blaming the other side. Such expressions are more personal and interactive."}, "time_dimension": {"answer": "Present", "rationale": "The entry describes the current feeling of the testees that they feel that their social circle is \"too small\" and is therefore judged to be current behavior or mentality."}, "sentence_type": "Affirmative declarative sentence", "pos": {"nouns": {"friends": 1, "I know someone": 1, "Circle": 1}, "verbs": {"I think": 1, "Too small": 1, "be": 1}, "adjectives": {"Small": 1}, "numerals": {"18": 1}, "pronouns": {"I": 1}}}, {"content": "Neighbors are too cold.", "word_count": 6, "label": "", "sentence_structure": "Subject鈥揷opula鈥損redicative", "subjective_objective": {"answer": "Subjective", "rationale": "Because \"neighbors are too indifferent\" is a subjective judgement and feeling that reflects the patient's attitude towards neighbours rather than an objective fact."}, "sentence_sentiment": {"answer": "Negative", "rationale": "Because \"apathy\" is usually considered a negative emotion or attitude."}, "language_style": {"answer": "Calm and direct", "rationale": "The language of the entry is a direct and concise expression of dissatisfaction or criticism of the apathy of the neighbour, without the use of euphemism, encouragement, etc., and is more direct and cold."}, "time_dimension": {"answer": "Past", "rationale": "The entry describes the interviewee's account of his past experience and feelings, and the key word is \"neighbors are too cold\", which indicates that the testee has had experience with his neighbour at some point in the past and has a negative feeling about it."}, "sentence_type": "Negative declarative sentence", "pos": {"nouns": {"Neighbors": 1, "Coldness": 1}, "verbs": {"Too much": 1}, "adverbs": {"Yes": 1}}}, {"content": "20. There are a number of people I would like to pay.", "word_count": 10, "label": "", "sentence_structure": "Subject鈥搗erb鈥搊bject", "subjective_objective": {"answer": "Subjective", "rationale": "For being willing to interact is an expression of subjective attitudes and will."}, "sentence_sentiment": {"answer": "Neutral", "rationale": "Because the entry does not have any emotional color."}, "language_style": {"answer": "Tactful and warm", "rationale": "The language style of the entry is more euphemistic, using an invitation-style tone and expressing queries about the willingness of the interviewee to establish relationships with others. This expression makes it easier to elicit positive responses and interactive participation from interviewees."}, "time_dimension": {"answer": "Present", "rationale": "The keywords \"a few\" and \"willing to have sex\" are all expressions of the current state and feeling."}, "sentence_type": "Affirmative declarative sentence", "pos": {"nouns": {"person": 1, "Hand it over": 1}, "verbs": {"May I": 1}, "adverbs": {"How many": 1, "One": 1}, "numerals": {"individual": 1, "20": 1}, "pronouns": {"I": 1}}}, {"content": "21. Several of the neighbours can talk to me.", "word_count": 13, "label": "", "sentence_structure": "Subject鈥搗erb鈥搊bject", "subjective_objective": {"answer": "Subjective", "rationale": "Because \"can talk to me\" is the patient's subjective feeling and expectation, and \"number of neighbors\" is objective statistical information."}, "sentence_sentiment": {"answer": "Neutral", "rationale": "Because the entry is a factual question and does not contain any positive or negative emotional aspects."}, "language_style": {"answer": "Tactful and warm", "rationale": "The language style of the entry is more euphemistic, the language is more friendly and open, there is no direct order or pressure to answer, and there is a feeling of affection and comfort."}, "time_dimension": {"answer": "Present", "rationale": "In the past, the entry enquired about the social interaction of the testee with his neighbour and the time frame involved was the past and therefore belonged to the past behaviour or mentality."}, "sentence_type": "Interrogative sentence", "pos": {"nouns": {"Neighbors": 1, "person": 1, "Chat": 1}, "verbs": {"have": 1, "Talk": 1}, "numerals": {"How many": 1}, "classifiers": {"individual": 1}, "pronouns": {"I": 1}}}, {"content": "22. I can always find someone to talk about my daily problems.", "word_count": 15, "label": "", "sentence_structure": "Subject鈥搗erb鈥搊bject", "subjective_objective": {"answer": "Subjective", "rationale": "Because finding someone to talk about my daily problems is the patient's subjective feeling and need."}, "sentence_sentiment": {"answer": "Positive", "rationale": "Because the purpose of the entry is to find someone to talk about my everyday problems."}, "language_style": {"answer": "Tactful and warm", "rationale": "The entry expresses a positive attitude through the use of the positive word \"always\" and asks the other side, in an invitation manner, whether it agrees with this view. This expression is more personal and interactive than direct statements."}, "time_dimension": {"answer": "Present", "rationale": ""}, "sentence_type": "Affirmative declarative sentence", "pos": {"nouns": {"person": 1, "Problem": 1}, "verbs": {"Found it": 1, "Talk": 1}, "adverbs": {"Total": 1, "Mine": 1}, "numerals": {"22": 1}, "pronouns": {"I": 1}}}, {"content": "When there is trouble, I have many people to trust.", "word_count": 13, "label": "", "sentence_structure": "Subject鈥搗erb鈥搊bject", "subjective_objective": {"answer": "Subjective", "rationale": "Because \"when there's trouble, I have a lot of people who can trust\" is the patient's subjective assessment of his social relationships."}, "sentence_sentiment": {"answer": "Neutral", "rationale": "Because the entry does not have any emotional color."}, "language_style": {"answer": "Tactful and warm", "rationale": "The language style of the entry is more euphemistic and avoids the sensitive question of whether the person surveyed has a person to rely on by using the expression \"I have many people to trust\". At the same time, this expression gives people a warm and warm feeling, which helps to increase the participation and cooperation of the respondents."}, "time_dimension": {"answer": "Present", "rationale": ""}, "sentence_type": "Affirmative declarative sentence", "pos": {"nouns": {"Trouble": 1, "Many people": 1, "Trust": 1}, "verbs": {"have": 1, "Yeah": 1, "Trust": 1}, "adverbs": {"Many": 1, "Both": 1}, "numerals": {"23": 1, "person": 1}, "pronouns": {"I": 1}}}, {"content": "24. I have enough friends.", "word_count": 9, "label": "", "sentence_structure": "Subject鈥搗erb鈥揷omplement", "subjective_objective": {"answer": "Subjective", "rationale": "It is not an objective fact because it is not the subjective feeling and perception of a person to think that they have enough friends."}, "sentence_sentiment": {"answer": "Positive", "rationale": "Because the expression \"have enough good friends\" in the entry expresses a positive emotional attitude."}, "language_style": {"answer": "Tactful and warm", "rationale": "The entry uses a more moderate tone, \"a good enough friend.\" This expression is used to stimulate positive emotions and memories of the other party and is not a direct question or request. The language style of the article is therefore euphemistic."}, "time_dimension": {"answer": "Present", "rationale": "The entry asks the testee about his/her personal relationship and the temporal dimension is now judged."}, "sentence_type": "Affirmative declarative sentence", "pos": {"nouns": {"Friends": 1}, "verbs": {"have": 1, "That's enough": 1}, "adjectives": {"More": 1}, "adverbs": {"That's enough": 1}, "numerals": {"24": 1}}}, {"content": "25. I can rely on friends when needed.", "word_count": 10, "label": "", "sentence_structure": "Subject鈥搗erb鈥揷omplement", "subjective_objective": {"answer": "Subjective", "rationale": "Because \"I can trust friends when I need them\" is a patient's sense of trust in friends, a subjective assessment and attitude."}, "sentence_sentiment": {"answer": "Positive", "rationale": "Because \"friend\" is a positive word and \"trust\" is a positive emotional attitude."}, "language_style": {"answer": "Tactful and warm", "rationale": "The entry uses more euphemistic language and does not directly ask the other party whether it can trust a friend, but rather allows the other party to think about the issue when it is needed. This expression is relatively soft, respectful and encourages self-reflection and evaluation."}, "time_dimension": {"answer": "Present", "rationale": "\"I can count on my friends when I need them\" and \"trust\" means the state and capacity of the moment, so time dimensions are now judged."}, "sentence_type": "Affirmative declarative sentence", "pos": {"nouns": {"Problem": 1, "friends": 1}, "verbs": {"Yes": 1, "Yeah": 1, "Trust": 1}, "adverbs": {"Time": 1}, "numerals": {"25": 1}, "pronouns": {"I": 1}}}, {"content": "26. I have some trusted friends.", "word_count": 10, "label": "", "sentence_structure": "Subject鈥搗erb鈥揷omplement", "subjective_objective": {"answer": "Subjective", "rationale": "Because \"reliable\" is the patient's subjective perception and evaluation, and the patient's subjective judgement of his social relationship."}, "sentence_sentiment": {"answer": "Positive", "rationale": "Because the expression \"trustable friends\" in the entry is positive."}, "language_style": {"answer": "Tactful and warm", "rationale": "The entry is more adept and interactive when it is spoken in a gentle and inviting manner, rather than by direct statements or orders. Instead of using strong tone or negative terminology, it asked the interviewee, in a calm tone, whether there were any trusted friends, a language that would make it easier for the interviewee to relax and answer the questions."}, "time_dimension": {"answer": "Present", "rationale": "The keyword is \"some,\" which means that there are still friends, and the description of \"trustable\" indicates that the act continues in its present state, so the temporal dimension is now."}, "sentence_type": "Affirmative declarative sentence", "pos": {"nouns": {"friends": 1, "Trust": 1}, "verbs": {"have": 1, "be": 1}, "adjectives": {"Some": 1}, "numerals": {"26": 1}, "pronouns": {"I": 1}}}, {"content": "There are many who deserve my full credit.", "word_count": 11, "label": "", "sentence_structure": "Subject鈥搗erb鈥搊bject", "subjective_objective": {"answer": "Subjective", "rationale": "Because it is a subjective judgement and feeling of one who is trustworthy."}, "sentence_sentiment": {"answer": "Positive", "rationale": "Because the phrase \"many people deserve my trust\" is positive."}, "language_style": {"answer": "Tactful and warm", "rationale": "The entry uses a more euphemistic formulation, using the vague concept of \"many\" rather than directly identifying specific persons. This expression avoids possible affronts or misunderstandings and encourages interviewees to share those they believe to be trustworthy. In addition, the expression \"fully worthy of my trust\" gives positive and positive impressions and helps to attract respondents to the questionnaire."}, "time_dimension": {"answer": "Present", "rationale": ""}, "sentence_type": "Affirmative declarative sentence", "pos": {"nouns": {"person": 1, "Trust": 1}, "verbs": {"have": 1, "It's worth it": 1}, "adverbs": {"Total": 1}, "numerals": {"27": 1}}}, {"content": "There are enough people to accept me.", "word_count": 14, "label": "", "sentence_structure": "Subject鈥搗erb鈥揷omplement", "subjective_objective": {"answer": "Subjective", "rationale": "Because \"there are enough people to accept me\" is a person's self-perception and expectations, a patient's subjective attitude."}, "sentence_sentiment": {"answer": "Neutral", "rationale": "Since the entry does not address specific aspects of emotion, attitude, behaviour, purpose or quality, it merely describes a factual situation."}, "language_style": {"answer": "Tactful and warm", "rationale": "The entry reflects respect for and concern for individual differences by asking the question of whether there are people around the other party who can accommodate their unique personality. This expression, which is relatively moderate, inclusive and encouraging, is more likely to generate resonance and responsiveness among the respondents."}, "time_dimension": {"answer": "Present", "rationale": "The entry asks about the testee's current self-perception and other people's perception of him, and the keyword \"there are enough people to accept me\" suggests that this is a subjective feeling and an assessment that can be judged now."}, "sentence_type": "Affirmative declarative sentence", "pos": {"nouns": {"person": 2, "Accept": 1}, "verbs": {"have": 1, "Yes": 1, "Accept": 1}, "adjectives": {"Enough": 1, "Here's the thing": 1}, "numerals": {"28": 1, "One": 2}, "pronouns": {"I": 1}}}]}

{"questionnaire_title": "AUDIT", "questionnaire_category": "Behavioral questionnaire", "item_count": 8, "emotion_shift_overall": "No obvious trend", "language_style_overall": "Calm and direct", "items": [{"content": "Have you had any head injuries since you were 18?", "word_count": 14, "label": "", "sentence_structure": "Subject鈥搗erb鈥搊bject", "subjective_objective": {"answer": "Subjective", "rationale": "It's from the patient's subjective perspective. Although the injury was an objective fact, it was a subjective question to ask whether the patient had been injured."}, "sentence_sentiment": {"answer": "Neutral", "rationale": "Because the entry merely asks a fact and does not express any feelings or attitudes."}, "language_style": {"answer": "Calm and direct", "rationale": "The entry asks questions in a direct and clear manner, without adding too much emotional colour or rhetoric, and the language style is straightforward."}, "time_dimension": {"answer": "Past", "rationale": "Based on the temporal expression \"since you were 18\" it is the testee's past that can be judged, so the answer is \"the past\"."}, "sentence_type": "Interrogative sentence", "pos": {"nouns": {"Year": 2, "Head": 1, "Wounded": 1}, "verbs": {"Yes": 1, "have": 1, "Come on": 1}, "adverbs": {"Since": 1}, "numerals": {"18": 1, "One": 1}, "classifiers": {"individual": 1}, "pronouns": {"you": 1}}}, {"content": "Have you had any fractures since you were 18?", "word_count": 11, "label": "", "sentence_structure": "Subject鈥搗erb鈥搊bject", "subjective_objective": {"answer": "Objective", "rationale": "Because the fracture is a physical phenomenon that can be measured, an objective fact that patients have experienced."}, "sentence_sentiment": {"answer": "Neutral", "rationale": "Because the entry does not have any emotional color."}, "language_style": {"answer": "Calm and direct", "rationale": "As the question of this entry is clear and concise, it does not use excessive emotional colours or rhetorical techniques, but rather asks the counterparty in a direct and objective manner whether there has been a fracture. This language style applies to questionnaires requiring specific information or data."}, "time_dimension": {"answer": "Past", "rationale": "The entry asks if the testee has experienced a fracture since the age of 18 and therefore the temporal dimension is judged to be past."}, "sentence_type": "Interrogative sentence", "pos": {"nouns": {"Questionnaire": 1, "Entry": 1, "Eighteen": 1, "Broken bones": 1}, "verbs": {"have": 1, "Pass": 1, "Since": 1}, "adverbs": {"Since then": 1, "- You're not": 1}, "numerals": {"1": 2, "18": 1}, "classifiers": {"individual": 1}, "pronouns": {"you": 1}}}, {"content": "Osteoporosis", "word_count": 5, "label": "", "sentence_structure": "Subject鈥搗erb鈥揷omplement", "subjective_objective": {"answer": "Objective", "rationale": "Because \"eye coronary infection\" is a specific symptom or disease, it is not the patient's subjective attitude or feelings, but the objective facts that can be determined by medical examination."}, "sentence_sentiment": {"answer": "Negative", "rationale": "Because \"eye complication\" is negative."}, "language_style": {"answer": "Calm and direct", "rationale": "The language style of the entry is calm and straightforward. It simply raises the issue of the health of the eyes, without too much embellishment or emotional rendering, and more focused on the transmission of facts and information."}, "time_dimension": {"answer": "Past", "rationale": "The entry does not provide a time trail, so it is not possible to determine whether it is past, present or future behaviour or mentality."}, "sentence_type": "Declarative sentence", "pos": {"nouns": {"Eye amplitude": 1, "Infection": 1}}}, {"content": "4. Unusual skin-forming small vein expansion", "word_count": 10, "label": "", "sentence_structure": "Subject鈥搗erb鈥揷omplement", "subjective_objective": {"answer": "Objective", "rationale": "Because abnormally small vascular expansion is a symptom or a sign of the patient ' s body, it is an objective fact."}, "sentence_sentiment": {"answer": "Negative", "rationale": "Because \"unusual skin-skinned small-vascular expansion\" is a pathological phenomenon, expressing a negative picture."}, "language_style": {"answer": "Calm and direct", "rationale": "The language style of the entry is straightforward and calm. It is a clear question, which does not have too many emotional renderings or rhetorical techniques, and which is simple and directly expresses the subject of asking the other party whether there is an abnormal expansion of skin vessels."}, "time_dimension": {"answer": "Past", "rationale": "In this entry, \"unusual\" and \"skinned small angiogenesis\" describe a condition or symptom that exists in the testee. The entry is therefore an inquiry into the testee ' s past status."}, "sentence_type": "Declarative sentence", "pos": {"nouns": {"Unusual": 1, "Skinny veins": 1, "Expansion": 1}, "numerals": {"4": 1}}}, {"content": "Handouts", "word_count": 3, "label": "", "sentence_structure": "Subject鈥損redicate", "subjective_objective": {"answer": "Objective", "rationale": "Because hand show is a physical act or condition, it is not a subjective idea or feeling inside the patient, and it is therefore objective."}, "sentence_sentiment": {"answer": "Neutral", "rationale": "Basis of judgement: The term \"showing\" in the entry lacks specific context information, does not accurately understand its meaning and emotional colour and is therefore judged neutral."}, "language_style": {"answer": "Tactful and warm", "rationale": "Please provide the full text of the entry and I will try to analyse it for you."}, "time_dimension": {"answer": "Present", "rationale": "The key word is \"handshow\", which does not have a clear point of time or a clear past or future event, so it is now judged."}, "sentence_type": "Interrogative sentence", "pos": {"verbs": {"Exhibition": 1, "Pong": 1}, "adverbs": {"very": 1}}}, {"content": "6. Use of the tongue", "word_count": 3, "label": "", "sentence_structure": "Subject鈥損redicate", "subjective_objective": {"answer": "Subjective", "rationale": "Because \"tip-drive\" is a subjective act that requires the patient's own description and assessment."}, "sentence_sentiment": {"answer": "Neutral", "rationale": "Basis of judgement: The entry describes a biological act that does not involve emotional, attitudinal or behavioural tendencies and is therefore neutral."}, "language_style": {"answer": "Calm and direct", "rationale": "The language style of this questionnaire entry is \"cool straight\". It uses a simple and straightforward question, without too much emotional colour or rhetoric, to ask directly whether the person under investigation was able to spread his tongue. This expression is more appropriate for objective, scientific data collection and analysis."}, "time_dimension": {"answer": "Future", "rationale": "The entry asks if the subject is willing to engage in a tongue-sharp in the future. Such behaviour or activity is judged by the temporal dimension as the future."}, "sentence_type": "Imperative sentence", "pos": {"nouns": {"- Put your tongue in the air": 1}}}, {"content": "7. Hepatic swelling", "word_count": 4, "label": "", "sentence_structure": "Subject鈥揷opula鈥損redicative", "subjective_objective": {"answer": "Objective", "rationale": "Because \"hepatic swelling\" is an observed sign or symptom and is an objective fact."}, "sentence_sentiment": {"answer": "Negative", "rationale": "Because the symptoms of \"hepatic swelling\" usually indicate a disease or pathological condition of the body and have a negative impact."}, "language_style": {"answer": "Calm and direct", "rationale": "The language style of the entry is \"cool straight\". It did not use any euphemism or rhetorical techniques to directly present the subject of the investigation, i.e. whether the person under investigation had any liver swelling. This style is more objective and accurate and helps to obtain real data results."}, "time_dimension": {"answer": "Past", "rationale": "The entry describes the physical condition of the person being tested and relates to past medical or medical examinations."}, "sentence_type": "Affirmative declarative sentence", "pos": {"nouns": {"Liver": 1, "Big": 1}}}, {"content": "8. GPT", "word_count": 1, "label": "", "sentence_structure": "Subject鈥搗erb鈥揷omplement", "subjective_objective": {"answer": "Objective", "rationale": "Because GPT values are an objective assessment criterion."}, "sentence_sentiment": {"answer": "Neutral", "rationale": "As the entry simply presents a value, it does not express any positive or negative feelings."}, "language_style": {"answer": "Calm and direct", "rationale": "It asked whether respondents were aware of the concept of \"GPT values\", which was expressed more objectively and succinctly. So I think the language style of this purpose is straightforward."}, "time_dimension": {"answer": "Present", "rationale": ""}, "sentence_type": "Interrogative sentence", "pos": {"nouns": {"GPT": 1}}}]}

{"questionnaire_title": "Social support scale", "questionnaire_category": "Emotion and affect", "item_count": 23, "emotion_shift_overall": "From negative to positive", "language_style_overall": "Tactful and warm", "items": [{"content": "1. How many friends do you have close ties of support and help?", "word_count": 20, "label": "", "sentence_structure": "Subject鈥搗erb鈥搊bject", "subjective_objective": {"answer": "Subjective", "rationale": "Basis of judgement: The questionnaire entry asks how many \"close friends\" the respondent has, which is a description of the individual circumstances and feelings of the respondent and is therefore subjective."}, "sentence_sentiment": {"answer": "Neutral", "rationale": "Since the entry is about personal social networks, it does not involve positive or negative emotions, attitudes or behaviours."}, "language_style": {"answer": "Calm and direct", "rationale": "The entry is of a more direct language style, does not have too many rhetorical techniques or emotional renderings, and is more balanced and objective by directly asking the number of close friends the interviewee has."}, "time_dimension": {"answer": "Present", "rationale": "The entry asks about the current situation of the subjects, i.e. how many close friends they have who can provide support and help. So the time dimension is now judged."}, "sentence_type": "Interrogative sentence", "pos": {"nouns": {"you": 1, "Relations": 1, "friends": 2}, "verbs": {"have": 1, "Got it": 1, "Support": 1, "Help": 1}, "adjectives": {"Close": 1}, "numerals": {"How much": 1}}}, {"content": "For almost a year you have been far from your family and living alone.", "word_count": 13, "label": "", "sentence_structure": "Subject鈥搗erb鈥揷omplement", "subjective_objective": {"answer": "Objective", "rationale": "Because being away from family members and living alone is an objective condition for patients."}, "sentence_sentiment": {"answer": "Negative", "rationale": "This is because being alone can lead to negative feelings of loneliness and helplessness."}, "language_style": {"answer": "Calm and direct", "rationale": "The language style of the entry is more concise, with no excessive accoutrements and emotional colours, but rather directly raises questions and is more calm and objective. \" ."}, "time_dimension": {"answer": "Past", "rationale": ""}, "sentence_type": "Affirmative declarative sentence", "pos": {"nouns": {"Year": 1, "Family": 1, "One": 1, "Room": 1}, "verbs": {"Stay away": 1, "and": 1, "Living alone": 1}, "adverbs": {"Near": 1}, "numerals": {"One year": 1}, "classifiers": {"One": 1}}}, {"content": "3. You and your neighbours never cared about each other, but only noded.", "word_count": 18, "label": "", "sentence_structure": "Subject鈥搗erb鈥揷omplement", "subjective_objective": {"answer": "Objective", "rationale": "Because \"you and your neighbors never care about each other, just nods\" is an objective fact that does not need to be assessed by the patient's subjective attitude."}, "sentence_sentiment": {"answer": "Neutral", "rationale": "Because the entry describes objective facts, no positive or negative assessment of the phenomenon is expressed."}, "language_style": {"answer": "Tactful and warm", "rationale": "The language style of the entry is more euphemistic, using more objective and neutral expressions such as \"never care\" or \"noding\" and not too emotional or strong. At the same time, it did not directly accuse the other party of his behaviour, but instead guided the respondents in a moderate manner to answer questions. This makes the questionnaire more acceptable and responsive."}, "time_dimension": {"answer": "Present", "rationale": ""}, "sentence_type": "Affirmative declarative sentence", "pos": {"nouns": {"you": 1, "Neighbors": 2, "Care": 1, "Nod your head": 1}, "verbs": {"and": 1, "To each other": 1, "Never": 1, "Just": 1, "Care": 1, "Nod your head": 1, "Hand it over": 1}, "adverbs": {"Never": 1, "Only": 1}, "numerals": {"3": 1}, "classifiers": {"individual": 1}, "pronouns": {"you": 1}}}, {"content": "4. You and your colleagues have never cared about each other, but have nodes.", "word_count": 18, "label": "", "sentence_structure": "Subject鈥搗erb鈥揷omplement", "subjective_objective": {"answer": "Objective", "rationale": "Because \"never care, just nod\" is a description of the relationship, not the patient's own subjective feelings or attitudes. The existence, depth and nature of such a relationship is an objective fact and therefore an objective question."}, "sentence_sentiment": {"answer": "Negative", "rationale": "Because the \"never care\" act in the entry is negative."}, "language_style": {"answer": "Calm and direct", "rationale": "The entry expresses, in a straightforward and concise tone, the question of whether respondents have good interaction with their colleagues in the working environment, and the language style is more calm and objective."}, "time_dimension": {"answer": "Present", "rationale": ""}, "sentence_type": "Negative declarative sentence", "pos": {"nouns": {"Co-workers": 1, "Care": 1, "Nod your head": 1}, "verbs": {"Never": 1, "To each other": 1}, "adverbs": {"Between them": 1}, "numerals": {"4": 1}}}, {"content": "5. Support and care from husband and wife (lovers)", "word_count": 13, "label": "", "sentence_structure": "Subject鈥搗erb鈥搊bject", "subjective_objective": {"answer": "Subjective", "rationale": "Since this questionnaire entry asks about the patient ' s own feelings and experiences, it examines the patient ' s subjective views."}, "sentence_sentiment": {"answer": "Positive", "rationale": "Because \"support and care\" in the entry expresses positive emotional attitudes and behaviour, meaning mutual assistance and care between spouses or lovers."}, "language_style": {"answer": "Tactful and warm", "rationale": "The language style of the entry appears to be mild and caring about each other's feelings. The term \"spousal (lover)\" has been used to make it more humane, and the term \"support and care\" has been used, emphasizing mutual care and support in a partner's relationship, which is more intimate and warm."}, "time_dimension": {"answer": "Present", "rationale": ""}, "sentence_type": "Interrogative sentence", "pos": {"nouns": {"Couples": 1, "Lovers": 1, "Support": 1, "Care": 1}, "numerals": {"5": 1}}}, {"content": "Support and care from parents.", "word_count": 11, "label": "", "sentence_structure": "Subject鈥搗erb鈥揷omplement", "subjective_objective": {"answer": "Subjective", "rationale": "Because \"support and care\" is the emotional experience of the parents that the patient feels, it is viewed from the patient's subjective perspective."}, "sentence_sentiment": {"answer": "Positive", "rationale": "Because it expresses a positive feeling and attitude of support and care."}, "language_style": {"answer": "Tactful and warm", "rationale": "The entry is more personal and interactive when it is spoken in a question-and-answer manner rather than by direct statements or orders. At the same time, the terms \"support\" and \"care\" have been used to emphasize the mutual interest and support of family members, which are more moderate and caring."}, "time_dimension": {"answer": "Past", "rationale": "So the answer is \"the past\"."}, "sentence_type": "Affirmative declarative sentence", "pos": {"nouns": {"parents": 1, "Support": 1, "Care": 1}, "numerals": {"6": 1}}}, {"content": "7. Support and care from children", "word_count": 11, "label": "", "sentence_structure": "Subject鈥搗erb鈥揷omplement", "subjective_objective": {"answer": "Subjective", "rationale": "Since support and care is a subjective feeling or evaluation, it is seen from the perspective of the patient himself and not as an objective fact."}, "sentence_sentiment": {"answer": "Positive", "rationale": "Because both \"support and care\" are positive words that express the concern and support of children for their parents."}, "language_style": {"answer": "Tactful and warm", "rationale": ""}, "time_dimension": {"answer": "Present", "rationale": "The action of \"getting\" occurred on the subject, indicating a state of experience now under way or in the recent past, and therefore a time dimension of the present."}, "sentence_type": "Interrogative sentence", "pos": {"nouns": {"Children": 1, "Support": 1, "Care": 1}, "verbs": {"Got it": 1}, "numerals": {"7": 1}}}, {"content": "8. Support and care from siblings", "word_count": 13, "label": "", "sentence_structure": "Subject鈥搗erb鈥揷omplement", "subjective_objective": {"answer": "Objective", "rationale": "The objective fact of support and care from siblings, rather than subjective feelings or attitudes of patients, is examined in the title."}, "sentence_sentiment": {"answer": "Positive", "rationale": "Because it expresses mutual assistance and support within the family, it is a positive emotional and behavioural description."}, "language_style": {"answer": "Tactful and warm", "rationale": "The entry uses a euphemism and affection, asking the interviewees whether they are supported and cared for by their brothers and sisters as they grow up, without direct requests or orders. This expression is more cozy and human, and it is better able to resonate and think."}, "time_dimension": {"answer": "Present", "rationale": "The entry describes what is now felt by the testee, i.e. whether he/she is supported and cared for by his/her siblings."}, "sentence_type": "Affirmative declarative sentence", "pos": {"nouns": {"Brothers and sisters": 1, "Support": 1, "Care": 1}, "verbs": {"Got it": 1, "Give": 0}, "numerals": {"8": 1, "Brothers and sisters": 1, "Support": 1, "Care": 1}}}, {"content": "Support and care from other members of the family (e.g. sister-in-law)", "word_count": 18, "label": "", "sentence_structure": "Subject鈥搗erb鈥揷omplement", "subjective_objective": {"answer": "Subjective", "rationale": "Basis of judgement:\nThe subject asked whether the patient had received support and care from other members of the family, which was a subjective feeling and experience of the patient and was therefore considered subjectively."}, "sentence_sentiment": {"answer": "Neutral", "rationale": "Because the entry does not have any emotional color."}, "language_style": {"answer": "Tactful and warm", "rationale": "The language style of the entry is euphemistic, as it uses a gentle and inviting tone to ask if the other side has received support and care from other members of the family. This expression is more friendly and interactive and does not put pressure or discomfort on the interviewees."}, "time_dimension": {"answer": "Present", "rationale": ""}, "sentence_type": "Interrogative sentence", "pos": {"nouns": {"Family": 1, "Members": 2, "Sister-in-law": 1, "Support": 1, "Care": 1}, "verbs": {"Got it": 1, "be": 1}, "adverbs": {"From": 1, "Other": 1, "Like": 1}, "numerals": {"9锛?: 1}, "pronouns": {"you": 1}}}, {"content": "10. In the past, when you were in an emergency situation, the sources of financial support and help to solve the real problems were:", "word_count": 34, "label": "", "sentence_structure": "Subject鈥搗erb鈥搊bject", "subjective_objective": {"answer": "Objective", "rationale": "Objective\nBecause \"the source of financial support and help to solve practical problems\" is the objective fact of the patient's experience and does not involve subjective attitudes or feelings of the patient."}, "sentence_sentiment": {"answer": "Neutral", "rationale": "Since the entry describes an objective fact, it does not have any emotional colour."}, "language_style": {"answer": "Calm and direct", "rationale": ""}, "time_dimension": {"answer": "Past", "rationale": "The entry asks what financial support and problem-solving assistance the person tested has received in the past in the event of an emergency. The time dimension is the past."}, "sentence_type": "Interrogative sentence", "pos": {"nouns": {"Past": 1, "you": 1, "Emergency situations": 1, "Economic support": 1, "Addressing practical issues": 1, "Help": 1, "Source": 1}, "verbs": {"Got it": 1, "have": 1, "Provision": 1}, "adverbs": {"Once": 1, "of": 2}, "numerals": {"10锛?: 1}, "pronouns": {"you": 1}}}, {"content": "In the past, the sources of consolation and concern you have received in times of acute distress include:", "word_count": 25, "label": "", "sentence_structure": "Subject鈥搗erb鈥搊bject", "subjective_objective": {"answer": "Subjective", "rationale": "Because the answer to this topic is mainly from the personal experiences and feelings of the interviewees, it looks at their subjective thoughts and experiences and is therefore subjective."}, "sentence_sentiment": {"answer": "Neutral", "rationale": "Because the entry does not have any emotional color."}, "language_style": {"answer": "Calm and direct", "rationale": ""}, "time_dimension": {"answer": "Past", "rationale": ""}, "sentence_type": "Interrogative sentence", "pos": {"nouns": {"Past": 1, "Emergency situations": 1, "Consolation": 1, "Care": 1, "Source": 1}, "verbs": {"Got it": 1, "be": 1}, "adverbs": {"Once": 1}, "numerals": {"11锛?: 1, "Yes": 1}}}, {"content": "12. The way you talk when you encounter trouble:", "word_count": 11, "label": "", "sentence_structure": "Subject鈥搗erb鈥揷omplement", "subjective_objective": {"answer": "Subjective", "rationale": "Because it is an inner thought and choice of a person, it is a subjective examination."}, "sentence_sentiment": {"answer": "Neutral", "rationale": "Because the entry simply asked the interviewee what kind of talk he would have in case of trouble, it did not express any emotion."}, "language_style": {"answer": "Calm and direct", "rationale": "This is a question of directly asking the interviewee how to talk when you're upset."}, "time_dimension": {"answer": "Present", "rationale": "The entry is the type of representation that is usually used when asking the testee what he or she is now facing, and therefore the judgement is now."}, "sentence_type": "Interrogative sentence", "pos": {"nouns": {"you": 1, "Trouble": 1, "Rule 39 Communication": 1}, "verbs": {"I met him": 1, "Speech": 1}, "adverbs": {"Time": 1}, "numerals": {"12锛?: 1}}}, {"content": "13. The means of recourse in case of distress:", "word_count": 11, "label": "", "sentence_structure": "Subject鈥搗erb鈥搊bject", "subjective_objective": {"answer": "Subjective", "rationale": "It is a highly subjective question because the subject is the behaviour and attitude of the patient to choose how to seek help in the face of a problem. Although this issue is based on objective facts (i.e. whether the patient is troubled and whether they seek help), its examination focuses on the patient ' s subjective feelings, thoughts and behavioural decisions. The entry is therefore subjective."}, "sentence_sentiment": {"answer": "Neutral", "rationale": "Because the entry does not have any emotional colour, it only asks for help."}, "language_style": {"answer": "Tactful and warm", "rationale": "The language style of the entry is more moderate and respects the choices and privacy of the interviewee, rather than using language that is too direct or intrusive, it is presented in a way that gives the interviewee more autonomy and freedom. At the same time, this expression is more in line with the professional image of the psychologist and better enables good interaction with the interviewees."}, "time_dimension": {"answer": "Present", "rationale": "The entry asks the testee about the means of recourse in the current situation of distress, so the time dimension is now judged."}, "sentence_type": "Interrogative sentence", "pos": {"nouns": {"Trouble": 1, "Help": 2}, "verbs": {"I met him": 1, "of": 1, "Modalities": 1}, "numerals": {"13锛?: 1}}}, {"content": "With regard to the organization of activities by groups (such as party organizations, religious organizations, trade unions, student associations, etc.), you:", "word_count": 24, "label": "", "sentence_structure": "Subject鈥搗erb鈥搊bject", "subjective_objective": {"answer": "Objective", "rationale": "Since participation in group organization is the objective behaviour and experience of patients, their objectivity and ideas are examined."}, "sentence_sentiment": {"answer": "Neutral", "rationale": "Because the entry does not have any emotional color."}, "language_style": {"answer": "Calm and direct", "rationale": "The entry uses a more neutral and objective language style, in which the other party is asked directly about the attitude or involvement of the group in the organization of its activities, without excessive rhetoric or emotional rendering."}, "time_dimension": {"answer": "Present", "rationale": "The questionnaire entry asked the testee whether he was currently involved in the organization of the group and judged the time dimension to be the present."}, "sentence_type": "Interrogative sentence", "pos": {"nouns": {"Groups": 1, "Organizations": 2, "Party Group": 1, "Religious organizations": 1, "Trade unions": 1, "Students ' Association": 1, "Activities": 1}, "verbs": {"Organizations": 2, "Activities": 1}, "numerals": {"14": 1}, "classifiers": {"individual": 1}, "pronouns": {"you": 1}}}, {"content": "Your place of residence has changed regularly for the last year, mostly with strangers.", "word_count": 23, "label": "", "sentence_structure": "Subject鈥搗erb鈥搊bject", "subjective_objective": {"answer": "Objective", "rationale": "This is an objective fact of the patient ' s living environment and does not involve subjective attitudes or feelings."}, "sentence_sentiment": {"answer": "Negative", "rationale": "Because the entry describes precarious living conditions and dealing with strangers, it may create negative feelings of anxiety and loneliness."}, "language_style": {"answer": "Tactful and warm", "rationale": ""}, "time_dimension": {"answer": "Past", "rationale": ""}, "sentence_type": "Affirmative declarative sentence", "pos": {"nouns": {"Year": 1, "Accommodation": 1, "Time": 1, "person": 1}, "verbs": {"Change": 1, "Stay": 1, "Together": 1}, "adjectives": {"Most": 1, "Strange": 1}, "adverbs": {"Regular": 1, "Near": 1}, "numerals": {"One": 2, "How many": 1}, "pronouns": {"you": 1}}}, {"content": "You've lived with classmates, colleagues or friends for almost a year.", "word_count": 17, "label": "", "sentence_structure": "Subject鈥搗erb鈥搊bject", "subjective_objective": {"answer": "Objective", "rationale": "Because living with classmates, colleagues or friends is an objective fact."}, "sentence_sentiment": {"answer": "Neutral", "rationale": "Because the entry does not have any emotional color."}, "language_style": {"answer": "Tactful and warm", "rationale": "The language style of the entry is more euphemistic, leading the respondents to answer questions by asking questions and avoiding direct orders or mandatory requirements. At the same time, the time frame of the \"near year\" also provides the respondent with space for reflection and memory, making the responses to the questionnaire more natural."}, "time_dimension": {"answer": "Present", "rationale": "The entry is now asked about the testee ' s behaviour over the past year, and the temporal dimension is now judged. The keyword \"for almost a year\" and \"for living together\" point to recent events and do not clearly point to past or future time lines."}, "sentence_type": "Affirmative declarative sentence", "pos": {"nouns": {"Year": 1, "Students": 1, "Co-workers": 1, "friends": 1, "Stay": 1}, "verbs": {"and": 1, "Together": 1}, "adverbs": {"Near": 1}, "numerals": {"One": 1}, "pronouns": {"you": 1}}}, {"content": "You lived with your family for almost a year.", "word_count": 12, "label": "", "sentence_structure": "Subject鈥搗erb鈥揷omplement", "subjective_objective": {"answer": "Objective", "rationale": "Since it is an objective fact to live with family members in the last year, it does not involve subjective attitudes or feelings of the patient."}, "sentence_sentiment": {"answer": "Neutral", "rationale": "Because the entry does not have any emotional color."}, "language_style": {"answer": "Tactful and warm", "rationale": "Warm and warm."}, "time_dimension": {"answer": "Present", "rationale": ""}, "sentence_type": "Affirmative declarative sentence", "pos": {"nouns": {"About a year ago": 1, "you": 1, "Family": 1, "Live together": 1}, "numerals": {"One": 1}, "classifiers": {"Year": 1}, "pronouns": {"and": 1}}}, {"content": "You and your neighbors may have a little trouble with each other.", "word_count": 18, "label": "", "sentence_structure": "Subject鈥搗erb鈥揷omplement", "subjective_objective": {"answer": "Subjective", "rationale": "Because \"a little care\" is a description of a subjective attitude that reflects the patient's perception of his relationship with his neighbour."}, "sentence_sentiment": {"answer": "Neutral", "rationale": "Since the entry was not of any emotional colour, it merely presented an objective fact."}, "language_style": {"answer": "Tactful and warm", "rationale": "The entry uses a euphemistic and polite language style, expressing positive encouragement and affirmation of the interviewee ' s concern for the neighbour, without direct orders or criticism. This language style is more relevant and more accessible to respondents."}, "time_dimension": {"answer": "Present", "rationale": "Keywords include \"to each other\" , \"to have difficulties\" and \"to have a little concern\", which point to current behaviour or circumstances. Therefore, the time dimension of this questionnaire entry is now."}, "sentence_type": "Interrogative sentence", "pos": {"nouns": {"you": 1, "Neighbors": 2, "Difficulties": 1, "Maybe": 1, "A little": 1, "Care": 1}, "verbs": {"I met him": 1, "Care": 1}, "adjectives": {"Between them": 1, "of": 3}, "adverbs": {"Maybe": 1, "A little": 1}, "pronouns": {"you": 1}}}, {"content": "You and your neighbors have some neighbors who care about you.", "word_count": 16, "label": "", "sentence_structure": "Subject鈥搗erb鈥搊bject", "subjective_objective": {"answer": "Subjective", "rationale": "Because \"care\" is a subjective feeling or attitude that reflects the attitude and perception of the person questioned towards the neighbour."}, "sentence_sentiment": {"answer": "Neutral", "rationale": "As the entry merely describes a fact, it does not contain any apparent emotional or tendentious colour and is therefore neutral."}, "language_style": {"answer": "Tactful and warm", "rationale": "The entry leads the interviewee to remember his/her relationship with his/her neighbourhood by asking a question and using \"care.\" This expression of positive emotion, with a moderate and inviting tone, is more acrimonym and interactive."}, "time_dimension": {"answer": "Present", "rationale": "The words \"some\" in the sentence indicate that some people, \"concerning\" is an ongoing move, so the act is taking place, and it is in the context of the present."}, "sentence_type": "Affirmative declarative sentence", "pos": {"nouns": {"you": 1, "Neighbors": 2, "Care": 1}, "verbs": {"Between them": 1, "Some": 1}, "adjectives": {"very": 1}, "adverbs": {"and": 1, "Between them": 1, "Both": 1}, "classifiers": {"individual": 1}}}, {"content": "Most of your neighbors care about you.", "word_count": 18, "label": "", "sentence_structure": "Subject鈥搗erb鈥搊bject", "subjective_objective": {"answer": "Subjective", "rationale": "Because the entry is asking patients about their subjective feelings and perceptions about whether they feel that neighbours care about themselves. Although the behaviour of a neighbour can be considered objective, the patient ' s feelings about these acts are subjective. The perspective of this questionnaire entry is therefore viewed from the patient ' s subjective perspective."}, "sentence_sentiment": {"answer": "Positive", "rationale": "Because it expresses a relationship of love and mutual assistance between neighbours, it is a positive emotional attitude."}, "language_style": {"answer": "Tactful and warm", "rationale": "The entry, in an interview and care, expresses concern about the interpersonal relationship of the interviewee rather than a direct statement or criticism. Such expressions are more intimate, respectful and encouraging the sharing of real ideas and feelings. \" ."}, "time_dimension": {"answer": "Present", "rationale": "The entry asks about the current situation of the subject, i.e. how he/she is related to most neighbours. The keyword \"most neighbors care about you\" shows that it's a constant behavior or state, so it's now."}, "sentence_type": "Affirmative declarative sentence", "pos": {"nouns": {"you": 1, "Neighbors": 2, "Relations": 1}, "verbs": {"To each other": 1, "Care": 1}, "adjectives": {"very": 1, "Both": 1, "of": 2}, "numerals": {"Most of them": 1}}}, {"content": "You and your colleagues may have a little problem with each other.", "word_count": 18, "label": "", "sentence_structure": "Subject鈥搗erb鈥揷omplement", "subjective_objective": {"answer": "Objective", "rationale": "Because \"difficult\" and \"a little care\" are objective factual descriptions, and do not involve subjective attitudes or feelings of patients."}, "sentence_sentiment": {"answer": "Neutral", "rationale": "Since the entry was not of any emotional colour, it merely stated a fact."}, "language_style": {"answer": "Tactful and warm", "rationale": "The entry uses a more euphemistic tone and raises the issue in a more moderate manner rather than directly accusing or ordering the other party to care more about colleagues. At the same time, the word \"a little care\" reduces the intensity of the expression and makes the whole question more acceptable and answerable. The language style of the article ' s purpose could therefore be described as euphemism."}, "time_dimension": {"answer": "Past", "rationale": "The keywords are \"to each other\" and \"slightly\" , which all indicate that the act is a normal, general act with no clear past or future direction."}, "sentence_type": "Interrogative sentence", "pos": {"nouns": {"Co-workers": 1, "Difficulties": 1}, "verbs": {"I met him": 1, "Care": 1}, "adjectives": {"A little": 1}, "adverbs": {"Between them": 1}}}, {"content": "You and your colleagues have some neighbors who care about you.", "word_count": 16, "label": "", "sentence_structure": "Subject鈥搗erb鈥揷omplement", "subjective_objective": {"answer": "Objective", "rationale": "Since the behaviour of colleagues and neighbours affects patients, these effects are objective."}, "sentence_sentiment": {"answer": "Positive", "rationale": "Because the description \"some of our colleagues are concerned about you\" in the entry expresses a positive emotional attitude."}, "language_style": {"answer": "Tactful and warm", "rationale": "The entry uses a more euphemistic and moderate expression, such as the use of terms such as \"some\" and \"very concerned\", and does not ask the other side too directly or forcefully whether they feel that they are concerned or not. Such language styles are more likely to create an atmosphere of friendly and comfortable dialogue and make interviewees more willing to share their real feelings and views."}, "time_dimension": {"answer": "Present", "rationale": "The entry describes the current situation, in which \"some neighbours are concerned about you\" refers to what is happening at the present time and does not refer to past or future situations. So the time dimension is now judged."}, "sentence_type": "Affirmative declarative sentence", "pos": {"nouns": {"you": 1, "Co-workers": 1, "Between them": 1, "Neighbors": 1, "Care": 1}, "verbs": {"have": 1, "Care": 1}, "adjectives": {"very": 1}, "classifiers": {"Some": 1}}}, {"content": "Most of your neighbors and colleagues care about you.", "word_count": 18, "label": "", "sentence_structure": "Subject鈥搗erb鈥揷omplement", "subjective_objective": {"answer": "Objective", "rationale": "Because \"most of our neighbors care about you\" is an observed fact or situation, not a patient's personal feeling or attitude. It is therefore an objective description."}, "sentence_sentiment": {"answer": "Neutral", "rationale": "Because the entry does not have any emotional color."}, "language_style": {"answer": "Tactful and warm", "rationale": "The entry uses a more euphemistic presentation, in which it is asked whether the relationship between the interviewees and their colleagues is harmonious and caring for each other. At the same time, this expression has become more personal and interactive and more likely to trigger positive responses from interviewees."}, "time_dimension": {"answer": "Past", "rationale": "The keyword \"most\" and \"very concerned\" indicates that it is a description of the current state, not the past or the future. The time dimension of the article is therefore now."}, "sentence_type": "Affirmative declarative sentence", "pos": {"nouns": {"Co-workers": 1, "Neighbors": 2, "Care": 1, "you": 2}, "verbs": {"To each other": 1, "Both": 1, "very": 1, "Care": 1}, "adverbs": {"Most of them": 1}, "numerals": {"One": 1, "Two": 1}, "classifiers": {"individual": 2}, "pronouns": {"you": 2}}}]}

{"questionnaire_title": "Old-age depression table", "questionnaire_category": "Mental health", "item_count": 30, "emotion_shift_overall": "From positive to negative", "language_style_overall": "Neutral", "items": [{"content": "1. Are you basically satisfied with life?", "word_count": 10, "label": "", "sentence_structure": "Subject鈥損redicate", "subjective_objective": {"answer": "Subjective", "rationale": "Because satisfaction with life is a subjective feeling and evaluation."}, "sentence_sentiment": {"answer": "Positive", "rationale": "Because the entry describes personal satisfaction with life and is a positive emotional attitude."}, "language_style": {"answer": "Calm and direct", "rationale": "The entry is based on a simple and direct question, with no excessive embellishment or emotional rendering, and a more calm and straightforward language style."}, "time_dimension": {"answer": "Present", "rationale": "The entry now asks the testee about his current life satisfaction and the time dimension is now judged."}, "sentence_type": "Interrogative sentence", "pos": {"nouns": {"Life": 1, "Basic": 1, "Satisfied": 1}, "verbs": {"to": 1, "have": 1}, "adverbs": {"Basically": 1}}}, {"content": "Have you given up many activities and interests?", "word_count": 14, "label": "", "sentence_structure": "Subject鈥搗erb鈥搊bject", "subjective_objective": {"answer": "Subjective", "rationale": "For giving up activities and interests is a personal choice and an expression of patients ' subjective attitudes."}, "sentence_sentiment": {"answer": "Negative", "rationale": "Because the act of \"abandoning many activities and interests\" in the entry is negative."}, "language_style": {"answer": "Tactful and warm", "rationale": "The entry is more adept and interactive when it is spoken in a gentle and inviting manner, rather than by direct statements or orders."}, "time_dimension": {"answer": "Present", "rationale": "The entry asks whether the testee ' s state of conduct at the current time, i.e. whether the abandonment of many activities and interests has occurred, is now judged in time dimension. \""}, "sentence_type": "Interrogative sentence", "pos": {"nouns": {"you": 1, "Many": 1, "Activities": 1, "Interest": 1}, "verbs": {"Abandon": 1, "have": 1}, "adverbs": {"Already": 1}, "numerals": {"2": 1}}}, {"content": "Do you feel that life is empty?", "word_count": 9, "label": "", "sentence_structure": "Subject鈥損redicate", "subjective_objective": {"answer": "Subjective", "rationale": "Because feeling empty is a subjective feeling or experience."}, "sentence_sentiment": {"answer": "Negative", "rationale": "Because the emotional description of \"living empty\" in the entry is negative."}, "language_style": {"answer": "Calm and direct", "rationale": "The entry directly asks the respondents whether they feel that life is empty and that their tone is more direct and that they are not over-modified or emotional. This approach is more objective and practical and suitable for conducting psychological questionnaires."}, "time_dimension": {"answer": "Present", "rationale": "The entry asks the testee about his current feelings and experiences, and the time dimension is now judged."}, "sentence_type": "Interrogative sentence", "pos": {"nouns": {"Life": 1, "Empty": 1}, "verbs": {"I think": 1}}}, {"content": "Do you often get bored?", "word_count": 8, "label": "", "sentence_structure": "Subject鈥搗erb鈥搊bject", "subjective_objective": {"answer": "Subjective", "rationale": "Because \"often feeling\" is an individual subjective feeling that requires patients to answer and judge themselves, it is subjective."}, "sentence_sentiment": {"answer": "Negative", "rationale": "Because \"weary\" in the entry is a negative emotional attitude."}, "language_style": {"answer": "Tactful and warm", "rationale": "The language style of the entry is more euphemistic, with a gentle and inviting tone to ask whether the other side is often tired. Such expressions are more resonant and thought-provoking, as well as more personal and interactive."}, "time_dimension": {"answer": "Present", "rationale": "The word \"always\" in the word \"often\" denotes a regular, customary act, and the fact that the expression \"likes\" uses the general current means that the act has not only occurred in the past, but may still continue. The entry is therefore an inquiry into the present emotional state of the subject."}, "sentence_type": "Interrogative sentence", "pos": {"nouns": {"Questionnaire": 1, "Entry": 1, "Tired": 1}, "verbs": {"Common sense": 1, "I feel": 1}, "numerals": {"4": 1}}}, {"content": "Do you think there's hope for the future?", "word_count": 9, "label": "", "sentence_structure": "Subject鈥損redicate", "subjective_objective": {"answer": "Subjective", "rationale": "Because \"if there is hope for the future\" is a subjective question, different people may have different views and answers. For example, some may be hopeful about the future, while others may be pessimistic about the future. So, \"Do you think there's hope for the future? \"The subject of examining the subjective attitudes or beliefs of the interviewees."}, "sentence_sentiment": {"answer": "Positive", "rationale": "Because \"promising the future\" is a positive expression, expressing optimism."}, "language_style": {"answer": "Tactful and warm", "rationale": "Instead of directly asking the other party whether they had confidence or pessimism in the future, the wording of the entry gave the interviewees a more natural expression of their thoughts in an encouraging and positive tone. At the same time, this euphemism is more likely to resonate with the interviewees and make them more willing to share their feelings."}, "time_dimension": {"answer": "Future", "rationale": "The entry contains visions and expectations for the future, and the keywords are \"the future\" and \"if there is hope\", which point to situations that have not yet occurred and that may arise in the future."}, "sentence_type": "Interrogative sentence", "pos": {"nouns": {"Hope": 1, "The future": 1}, "verbs": {"I think": 1}}}, {"content": "Do you have any trouble getting rid of some ideas in your head?", "word_count": 19, "label": "", "sentence_structure": "Subject鈥搗erb鈥搊bject", "subjective_objective": {"answer": "Subjective", "rationale": "Because worry is usually the patient's subjective feeling."}, "sentence_sentiment": {"answer": "Negative", "rationale": "Because the expression \"some thoughts in the brain can't get out of trouble\" expresses negative sentiment."}, "language_style": {"answer": "Tactful and warm", "rationale": "This entry is more personal and interactive when it is spoken in a question-and-answer tone, rather than in direct statements or orders. At the same time, words such as \"because\" are used to guide each other's thinking, to avoid exerting pressure or judgement on the other and to make the questionnaire more objective and neutral."}, "time_dimension": {"answer": "Present", "rationale": "The entry asks about the current status and feelings of the subject, and the keyword \"if\" and \"because\" point to the current situation. Therefore, it can be judged that the entry is a survey of current behaviour or mentality."}, "sentence_type": "Interrogative sentence", "pos": {"nouns": {"Questionnaire": 1, "Entry": 1, "In my head": 1, "Thoughts": 2, "Get rid of it": 1, "Trouble": 1}, "verbs": {"be": 1, "Because": 1, "Yes": 1}, "adjectives": {"Some": 1}, "numerals": {"6": 1, "Two": 1}, "classifiers": {"individual": 1}, "pronouns": {"you": 1}}}, {"content": "Do you have much of your energy?", "word_count": 12, "label": "", "sentence_structure": "Subject鈥損redicate", "subjective_objective": {"answer": "Subjective", "rationale": "Because \"most of the time is energetic\" is the patient's subjective feeling and experience, not the objective fact or result."}, "sentence_sentiment": {"answer": "Positive", "rationale": "Because the expression \"most of the time is energetic\" is positive, it indicates that the person surveyed has a high level of energy."}, "language_style": {"answer": "Tactful and warm", "rationale": "The entry is more adept and interactive when it is spoken in a gentle and inviting manner, rather than by direct statements or orders."}, "time_dimension": {"answer": "Present", "rationale": "The entry asks about the current state of the subject, and the keyword is \"are most of the time energetic,\" which is linked to the current time dimension."}, "sentence_type": "Interrogative sentence", "pos": {"nouns": {"- The energy": 1, "鈾?Full of 鈾?: 1}, "verbs": {"Is it possible": 2, "have": 1}, "adjectives": {"Most of them": 1}, "numerals": {"7": 1}}}, {"content": "8. Are you afraid something unfortunate will happen to you?", "word_count": 16, "label": "", "sentence_structure": "Subject鈥搗erb鈥搊bject", "subjective_objective": {"answer": "Subjective", "rationale": "Because fear is a subjective feeling or emotional reaction."}, "sentence_sentiment": {"answer": "Negative", "rationale": "Because the mental state of \"fearing that something bad will fall on your head\" in the entry is negative."}, "language_style": {"answer": "Calm and direct", "rationale": "The entry directly asked the respondents whether they feared that they would experience misfortune and did not use any euphemism or tender language. This language style is usually more common in psychological questionnaires and allows for more accurate collection of actual ideas and emotional reactions of the respondents."}, "time_dimension": {"answer": "Present", "rationale": ""}, "sentence_type": "Interrogative sentence", "pos": {"nouns": {"Unfortunately": 1, "Yes": 1, "Head": 1}, "verbs": {"Fall": 1, "Present": 1, "Scared": 1}, "adverbs": {"Did you": 1, "Is it possible": 1}, "numerals": {"8": 1}, "classifiers": {"individual": 1}}}, {"content": "Are you happy most of the time?", "word_count": 12, "label": "", "sentence_structure": "Subject鈥搗erb鈥搊bject", "subjective_objective": {"answer": "Subjective", "rationale": "Because \"happiness\" is a subjective feeling and assessment, different people may have different understandings and standards of happiness, the answer to this questionnaire is subjective."}, "sentence_sentiment": {"answer": "Positive", "rationale": "Because the words \"mostly happy\" express a positive emotional attitude and a positive description."}, "language_style": {"answer": "Tactful and warm", "rationale": "The language style of the entry is more euphemistic and it uses a statement of inquiry and is expressed in a kind and gentle manner, without direct orders or judgment. Such a presentation could be more conducive to creating a good climate for dialogue and enhancing the participation and collaboration of respondents."}, "time_dimension": {"answer": "Present", "rationale": "The entry asks about the emotional state of the subject during the current period, the keywords being \"most of the time\" and \"happiness\", which point to what is now happening. Thus, the temporal dimension of the purpose of the article is now judged."}, "sentence_type": "Interrogative sentence", "pos": {"nouns": {"you": 1, "Time": 1, "Happiness": 1}, "verbs": {"Is it possible": 1, "I feel": 1, "Most of them": 1}, "adverbs": {"Most of them": 1}, "numerals": {"9": 1}}}, {"content": "Do you often feel isolated?", "word_count": 10, "label": "", "sentence_structure": "Subject鈥損redicate", "subjective_objective": {"answer": "Subjective", "rationale": "Because the feeling of being alone is usually the emotional experience inside the patient and is subjective."}, "sentence_sentiment": {"answer": "Negative", "rationale": "Because the description of isolation is negative."}, "language_style": {"answer": "Calm and direct", "rationale": ""}, "time_dimension": {"answer": "Present", "rationale": "The keyword \"Do you often feel alone\" refers to the current feeling or state of the person being tested, so it's now."}, "sentence_type": "Interrogative sentence", "pos": {"nouns": {"you": 1, "I'm alone": 1}, "verbs": {"Regular": 1, "I feel": 1, "Is it possible": 1, "No help": 1}, "adverbs": {"Regular": 1}, "numerals": {"10": 1}}}, {"content": "Do you often sit up and be upset?", "word_count": 13, "label": "", "sentence_structure": "Subject鈥搗erb鈥搊bject", "subjective_objective": {"answer": "Subjective", "rationale": "Because \"sitting\" and \"disturbing\" are the feelings and experiences inside the patient and are a reflection of subjective emotions."}, "sentence_sentiment": {"answer": "Negative", "rationale": "Because the entry \"Sitting, Disturbing\" describes a negative emotional state."}, "language_style": {"answer": "Calm and direct", "rationale": "The language style of the entry is relatively calm and straightforward, using simple vocabulary and grammar structures, and there is no excessive rhetorical or emotional rendering. This expression is more objective, concise and accessible to respondents."}, "time_dimension": {"answer": "Present", "rationale": "\"Are you \" adopts the general current, indicating that it is a general act and that it may continue now. The entry therefore asks about the situation of the person being tested."}, "sentence_type": "Interrogative sentence", "pos": {"nouns": {"Questionnaire": 1, "Entry": 1, "Heart": 1, "Frenzied": 1}, "verbs": {"Sit tight": 1, "Boring": 1}, "adverbs": {"Regular": 1}, "numerals": {"11": 1}}}, {"content": "Do you want to stay home rather than do something new?", "word_count": 18, "label": "", "sentence_structure": "Subject鈥搗erb鈥搊bject", "subjective_objective": {"answer": "Subjective", "rationale": "Because hope is the patient's subjective attitude and involves personal will and choice."}, "sentence_sentiment": {"answer": "Negative", "rationale": "Because the \"not wanting to do something new\" in the entry is negative."}, "language_style": {"answer": "Calm and direct", "rationale": "The language style of the entry is relatively straightforward and does not use much rhetorical or emotional colours, but simply asks the respondents whether they are reluctant to do new things and prefer to stay at home. This direct way of asking questions effectively captures the reality and needs of the interviewees."}, "time_dimension": {"answer": "Past", "rationale": "The entry uses the expression \"if you want\" to describe the hopes and expectations of the past. At the same time, the words \"stay at home\" and \"resistance to do something new\" indicate that this is a question of past behaviour or mentality of the subjects. The time dimension of the questionnaire entry is therefore judged to be past."}, "sentence_type": "Interrogative sentence", "pos": {"nouns": {"Questionnaire": 1, "Entry": 1, "Home": 1, "Something new": 1}, "verbs": {"Hope": 1, "Stay": 1, "Do it": 2, "Go on": 1}, "adverbs": {"not": 1}, "numerals": {"12": 1}}}, {"content": "Do you often worry about the future?", "word_count": 9, "label": "", "sentence_structure": "Subject鈥搗erb鈥搊bject", "subjective_objective": {"answer": "Subjective", "rationale": "Because fear is a subjective emotional experience."}, "sentence_sentiment": {"answer": "Negative", "rationale": "For fear of the future is a negative sentiment and attitude."}, "language_style": {"answer": "Calm and direct", "rationale": "The entry directly asked the respondents whether they were often concerned about the future, the language was concise and there was no excessive emotional or speech modification. This expression makes the question more objective, direct and easily understandable and accurate."}, "time_dimension": {"answer": "Future", "rationale": "The entry contains visions and expectations for the future, and the key words are \" worry\" and \"the future\", all of which point to situations that have not yet occurred and that may arise in the future."}, "sentence_type": "Interrogative sentence", "pos": {"nouns": {"Future": 1}, "verbs": {"I'm worried": 1}, "adverbs": {"Often": 1}}}, {"content": "Do you feel worse than before?", "word_count": 12, "label": "", "sentence_structure": "Subject鈥搗erb鈥搊bject", "subjective_objective": {"answer": "Subjective", "rationale": "Because the change in memory is the patient's own subjective feeling and perception."}, "sentence_sentiment": {"answer": "Negative", "rationale": "Because the expression \"less memory\" in the entry is negative and represents a negative assessment of its own situation."}, "language_style": {"answer": "Tactful and warm", "rationale": "The language style of the entry is more euphemistic, using the form of questions to guide respondents to answer questions, without direct orders or criticism, which makes people feel more friendly and comfortable. At the same time, the term \"failer\" is more implicit, avoiding the use of strong language to stimulate the self-esteem of the respondents or to provoke their defence."}, "time_dimension": {"answer": "Past", "rationale": "The entry asks the testee about his or her past, and the keywords are \"the memory is worse\" and \"the past\", all pointing to what has happened. Thus, the temporal dimension of the purpose of the article can be judged to be past."}, "sentence_type": "Interrogative sentence", "pos": {"nouns": {"Questionnaire": 1, "Entry": 1, "Memory": 1, "Before": 1}, "verbs": {"I think": 1, "- Yeah": 1, "Bad": 1}}}, {"content": "You think it's easy to live now?", "word_count": 11, "label": "", "sentence_structure": "Subject鈥搗erb鈥揷omplement", "subjective_objective": {"answer": "Subjective", "rationale": "Because feeling is a subjective verb, which means it's the patient's personal opinion and feelings."}, "sentence_sentiment": {"answer": "Neutral", "rationale": "Basis of judgement.\n\n\"Do you feel comfortable living now? \"This question may be answered differently by different people. Some people may find their lives very pleasant, and they may have a lot of things to do and a lot of friends and family to accompany them. Their response may therefore be positive.\n\nHowever, there may also be people who do not think that their lives are comfortable. They may feel that their lives are full of stress and challenges, or they may feel that they lack something important (such as money, love or success). Therefore, their answers may be negative.\n\nFinally, others may feel that their lives are neither good nor bad, and that there are no obvious positive or negative factors. They may feel that their lives are like that and that there is nothing to be particularly happy about and nothing to be particularly worried about. Their answers may therefore be neutral."}, "language_style": {"answer": "Tactful and warm", "rationale": "The language style of the entry is euphemism and warmness, as it asks each other ' s feelings in a gentle and inviting tone, rather than directly speaking or ordering. This expression is more personal and interactive, making interviewees more willing to share their thoughts and feelings. At the same time, the word \"pity\" itself has a certain subjectivity and emotion, and is in keeping with the euphemism of language."}, "time_dimension": {"answer": "Present", "rationale": "The entry asks about the testee's current feelings and experience, \"Is it comfortable to live now? The \"now\" indicates that the entry is being investigated for the current state."}, "sentence_type": "Interrogative sentence", "pos": {"nouns": {"Questionnaire Entry": 1, "you": 1, "Present": 1, "Alive": 1, "Nice": 1}, "verbs": {"I think": 1}, "adverbs": {"very": 1}, "numerals": {"15": 1}}}, {"content": "Do you often feel heavy and depressed?", "word_count": 12, "label": "", "sentence_structure": "Subject鈥搗erb鈥搊bject", "subjective_objective": {"answer": "Subjective", "rationale": "Because it is a subjective feeling, it is often the patient who feels heavy and depressed."}, "sentence_sentiment": {"answer": "Negative", "rationale": "Because \"heavy, depressed\" is a negative emotional experience."}, "language_style": {"answer": "Tactful and warm", "rationale": "The question was asked, in a mild and inviting tone, whether the other party often felt heavy and depressed rather than directly ordering or making statements. Such expressions are more personal and interactive."}, "time_dimension": {"answer": "Present", "rationale": ""}, "sentence_type": "Interrogative sentence", "pos": {"nouns": {"you": 1, "The mood": 1, "Heavy": 1, "I'm depressed": 1}, "verbs": {"I always feel": 1}, "adverbs": {"Regular": 1}}}, {"content": "Do you find it pointless to live like this?", "word_count": 16, "label": "", "sentence_structure": "Subject鈥搗erb鈥搊bject", "subjective_objective": {"answer": "Subjective", "rationale": "Because the problem relates to the patient ' s subjective perception and perception of life, it is a subjective examination."}, "sentence_sentiment": {"answer": "Negative", "rationale": "Because the phrase in the entry, \"I feel like living like this doesn't make sense\" is negative."}, "language_style": {"answer": "Calm and direct", "rationale": "The entry is open to dialogue in a direct question-and-answer manner, with no excessive rhetorical or emotional rendering, and is more objective in tone. \" ."}, "time_dimension": {"answer": "Present", "rationale": "In this entry, \"you're like this\" refers to the state or situation of the moment, so it's the past."}, "sentence_type": "Interrogative sentence", "pos": {"nouns": {"17": 1, "you": 1, "Elephant": 1, "Here": 1, "Life": 1, "It's pointless": 1}, "verbs": {"I think": 1, "Elephant": 1, "Present": 1, "Alive": 1}, "adjectives": {"It's pointless": 1}, "numerals": {"17": 1}, "classifiers": {"Elephant": 1}, "pronouns": {"you": 1}}}, {"content": "18. Do you always grieve about the past?", "word_count": 11, "label": "", "sentence_structure": "Subject鈥搗erb鈥搊bject", "subjective_objective": {"answer": "Subjective", "rationale": "Because sorrow is a subjective feeling and emotional experience."}, "sentence_sentiment": {"answer": "Negative", "rationale": "Because \"pity\" is a negative emotional state."}, "language_style": {"answer": "Calm and direct", "rationale": "\"Calm down straight.\""}, "time_dimension": {"answer": "Present", "rationale": "The entry asks the testee whether he is currently worried about the past and the time dimension is now judged. The keywords \"whether\" and \"total\" and \"torture for the past\" point to the current emotional state and experience of the subject, so it can be established that this is the description of the present."}, "sentence_type": "Interrogative sentence", "pos": {"nouns": {"Yes": 1, "Sad": 1}, "verbs": {"Total": 1, "Yes": 1, "Sad": 1}, "adverbs": {"Total": 1}, "numerals": {"18": 1}, "classifiers": {"individual": 1}}}, {"content": "You think life is exciting?", "word_count": 11, "label": "", "sentence_structure": "Subject鈥損redicate", "subjective_objective": {"answer": "Subjective", "rationale": "Because \"the feeling\" reflects the patient's personal feelings and perceptions, it's a subjective examination."}, "sentence_sentiment": {"answer": "Positive", "rationale": "Because \"life is exciting\" in the entries expresses positive attitudes and feelings."}, "language_style": {"answer": "Tactful and warm", "rationale": "The language style of the entry is more euphemistic, asking each other how they feel about life in a more moderate way, and using the word \"like\" to make each other more free to express their views. This expression is more friendly, friendly and acceptable."}, "time_dimension": {"answer": "Present", "rationale": "The entry asks the testee ' s current feelings and attitudes, so the time dimension is now judged. Is life exciting? It shows that this is a subject of subjective evaluation of the current emotional state."}, "sentence_type": "Interrogative sentence", "pos": {"nouns": {"Life": 1}, "verbs": {"I think": 1, "It's exciting": 1}, "adjectives": {"very": 1, "Excited": 1}, "pronouns": {"you": 1}}}, {"content": "Is it hard for you to start a new job?", "word_count": 13, "label": "", "sentence_structure": "Subject鈥搗erb鈥搊bject", "subjective_objective": {"answer": "Subjective", "rationale": "The difficulty of starting a new job is the subjective feeling and judgement of the patient."}, "sentence_sentiment": {"answer": "Negative", "rationale": "Because it's a negative act in the entry, \"It's difficult to start a new job.\""}, "language_style": {"answer": "Calm and direct", "rationale": ""}, "time_dimension": {"answer": "Present", "rationale": "The keywords are \"start\" and \"hard\" and they represent the feelings and experiences of the testees in their current state, so they are judged now."}, "sentence_type": "Interrogative sentence", "pos": {"nouns": {"you": 1, "New job": 1}, "verbs": {"Start": 1, "It's hard": 1}, "adjectives": {"Hard": 1}, "adverbs": {"very": 1}, "numerals": {"20": 1}}}, {"content": "21. Do you feel alive?", "word_count": 10, "label": "", "sentence_structure": "Subject鈥損redicate", "subjective_objective": {"answer": "Subjective", "rationale": "Because \"I feel\" is a subjective term that relates to patients' perceptions and feelings of life, the entry to the questionnaire is subjective."}, "sentence_sentiment": {"answer": "Positive", "rationale": "Because dynamic is a positive description."}, "language_style": {"answer": "Tactful and warm", "rationale": "The language style of the entry is more euphemistic, leading the respondents to reflect on their feelings in a question-and-answer manner, and is not expressed too directly or strongly. Besides, the phrase \"you feel\" is more friendly and friendly, which enhances the interaction between the questionnaire and the respondents."}, "time_dimension": {"answer": "Present", "rationale": "The entry enquires about the current feelings or state of the subject, and the keywords are \"fear\" and \"viable\", which point to what is happening, and the temporal dimension is now."}, "sentence_type": "Interrogative sentence", "pos": {"nouns": {"Life": 1, "Dynamic": 1}, "verbs": {"I think": 1, "Fill": 1}, "adverbs": {"Very": 1}}}, {"content": "22. Do you find your situation hopeless?", "word_count": 14, "label": "", "sentence_structure": "Subject鈥搗erb鈥搊bject", "subjective_objective": {"answer": "Subjective", "rationale": "Because \"you feel\" is a subjective judgment and feeling."}, "sentence_sentiment": {"answer": "Negative", "rationale": "Because the expression \"situation is hopeless\" in the entry is negative and reflects negative sentiment."}, "language_style": {"answer": "Calm and direct", "rationale": ""}, "time_dimension": {"answer": "Past", "rationale": "The entry asks whether the person tested had experienced despair in the past."}, "sentence_type": "Interrogative sentence", "pos": {"nouns": {"Situation": 1, "Hope": 1}, "verbs": {"I think": 1, "Nothing": 1, "have": 1}, "adjectives": {"Already": 1}, "numerals": {"22": 1}}}, {"content": "Do you think most people are much better than you?", "word_count": 14, "label": "", "sentence_structure": "Subject鈥損redicate", "subjective_objective": {"answer": "Subjective", "rationale": "Because this is the patient ' s subjective assessment of most people ' s abilities, it is the patient ' s comparison and perception of himself and others."}, "sentence_sentiment": {"answer": "Negative", "rationale": "Because the entry expresses a negative assessment of itself that most people are stronger than themselves. This may lead to negative feelings and attitudes of self-doubt, inferiority and so on."}, "language_style": {"answer": "Calm and direct", "rationale": "The entry uses a direct question to ask if the counterparty feels that others are stronger than themselves, is not over-modified or emotional, and is more objective and quiet."}, "time_dimension": {"answer": "Present", "rationale": "The key words are \"Does it feel\" and \"Most people are much better than you\" and all point to the current mindset or perception of the subject, so the time dimension of this entry should be now."}, "sentence_type": "Interrogative sentence", "pos": {"nouns": {"Questionnaire": 1, "Entry": 1, "person": 1}, "verbs": {"I think": 1, "Strong": 1, "More": 1}, "adjectives": {"Large": 1}, "adverbs": {"- Yeah": 1, "Both": 1}, "numerals": {"23": 1}, "pronouns": {"you": 1}}}, {"content": "Are you often sad about small things?", "word_count": 10, "label": "", "sentence_structure": "Subject鈥搗erb鈥搊bject", "subjective_objective": {"answer": "Subjective", "rationale": "Because the subject is asking if the patient has such a subjective feeling."}, "sentence_sentiment": {"answer": "Negative", "rationale": "Because in the entry, the act of \"sorting for little things\" is negative."}, "language_style": {"answer": "Tactful and warm", "rationale": "The entry uses a gentle and caring tone of the other ' s feelings, asking the interviewee whether he or she is often sad about small things and expressing himself in a more caring and caring manner."}, "time_dimension": {"answer": "Present", "rationale": "In the entry, \"Whether or not\" indicates that this is a selective question, asking the testee's current situation or feelings, and therefore judging now."}, "sentence_type": "Interrogative sentence", "pos": {"nouns": {"Things": 1, "Sad": 1}, "verbs": {"Always": 1}, "adverbs": {"Some": 1}, "numerals": {"24": 1}}}, {"content": "Do you often feel like crying?", "word_count": 8, "label": "", "sentence_structure": "Subject鈥搗erb鈥搊bject", "subjective_objective": {"answer": "Subjective", "rationale": "Because it's a personal feeling and emotional experience, and it's the patient's subjective perspective."}, "sentence_sentiment": {"answer": "Negative", "rationale": "Because the \"want to cry\" mood in the entry is negative."}, "language_style": {"answer": "Calm and direct", "rationale": ""}, "time_dimension": {"answer": "Present", "rationale": "\"Do you often feel like crying? The word \"always\" denotes a regular, customary act, and \"like to cry\" is a past act, but whether the person under investigation currently feels this feeling is the focus of attention, so the entry describes the current state."}, "sentence_type": "Interrogative sentence", "pos": {"nouns": {"Questionnaire": 1, "Entry": 1, "Cry": 1}, "verbs": {"Regular": 1, "I think": 1, "Yes": 1, "Cry": 1}, "adverbs": {"very": 1}, "numerals": {"25": 1}}}, {"content": "26. Is it difficult to concentrate?", "word_count": 9, "label": "", "sentence_structure": "Subject鈥損redicate", "subjective_objective": {"answer": "Subjective", "rationale": "Because the question is about the patient ' s own feelings and experiences, it is a subjective examination."}, "sentence_sentiment": {"answer": "Negative", "rationale": "Because the action of focusing on difficulties in the entries is negative."}, "language_style": {"answer": "Calm and direct", "rationale": "The language style of the entry is calm and straightforward. It simply raises the question of not using excessive rhetorical techniques or emotional rendering, which is more balanced and objective. \""}, "time_dimension": {"answer": "Present", "rationale": ""}, "sentence_type": "Interrogative sentence", "pos": {"nouns": {"Questionnaire": 1, "Entry": 1, "- The energy": 1, "Difficulties": 1}, "verbs": {"Focus": 1, "have": 1}, "numerals": {"26": 1}}}, {"content": "Did you get up in the morning?", "word_count": 9, "label": "", "sentence_structure": "Subject鈥損redicate", "subjective_objective": {"answer": "Subjective", "rationale": "Because \"fast living\" is the patient's subjective feeling and emotional state."}, "sentence_sentiment": {"answer": "Positive", "rationale": "Because the description \"fast live\" in the entry is positive."}, "language_style": {"answer": "Tactful and warm", "rationale": "The language style of the entry is more euphemistic, asking questions in a way that is sensitive to each other ' s feelings, rather than directly asking or ordering, which is more personal and interactive."}, "time_dimension": {"answer": "Present", "rationale": "The entry asks about the person's current state or feelings, and \"get up in the morning\" indicates that this is an act or state that occurs now."}, "sentence_type": "Interrogative sentence", "pos": {"nouns": {"Morning": 1, "Quickie": 1}, "verbs": {"Get up": 1, "very": 1, "- You're not": 1}, "adverbs": {"very": 1}}}, {"content": "Do you wish to avoid the party?", "word_count": 8, "label": "", "sentence_structure": "Subject鈥搗erb鈥搊bject", "subjective_objective": {"answer": "Subjective", "rationale": "Because hope is the expression of a subjective attitude that reflects patients' psychological preference and willingness to avoid a party."}, "sentence_sentiment": {"answer": "Negative", "rationale": "Because avoiding parties is negative."}, "language_style": {"answer": "Calm and direct", "rationale": "In a direct manner, the entry asks the respondents whether they wish to avoid the party, without using excessive rhetorical techniques or emotional rendering, and with a more calm and direct language style."}, "time_dimension": {"answer": "Future", "rationale": "The entry asks the testee about future behavioural tendencies, i.e. whether there is any hope of avoiding the event, and the temporal dimension is judged to be future. \""}, "sentence_type": "Interrogative sentence", "pos": {"nouns": {"Party": 1}, "verbs": {"Hope": 1, "Avoid": 1}, "numerals": {"28": 1}}}, {"content": "Is it easy for you to make decisions?", "word_count": 8, "label": "", "sentence_structure": "Subject鈥損redicate", "subjective_objective": {"answer": "Subjective", "rationale": "Because ease is the patient's own perception and feelings, subjective."}, "sentence_sentiment": {"answer": "Neutral", "rationale": "Because the entry is merely an inquiry into the ease of the interviewee ' s decision, it does not express positive or negative emotional tendencies."}, "language_style": {"answer": "Calm and direct", "rationale": "Calm down straight."}, "time_dimension": {"answer": "Present", "rationale": "The entry asks the testee ' s decision-making ability in the current state, so the time dimension is now judged."}, "sentence_type": "Interrogative sentence", "pos": {"nouns": {"you": 1, "Decision": 1}, "verbs": {"Do it": 1, "Easy": 1}, "adjectives": {"very": 1}, "adverbs": {"- You're not": 1}, "numerals": {"29": 1}}}, {"content": "Is your mind as clear as usual?", "word_count": 12, "label": "", "sentence_structure": "Subject鈥揷opula鈥損redicative", "subjective_objective": {"answer": "Subjective", "rationale": "Because this is the patient ' s perception and assessment of his current state, it is a subjective observation."}, "sentence_sentiment": {"answer": "Neutral", "rationale": "Because the entry does not have any emotional color."}, "language_style": {"answer": "Calm and direct", "rationale": "The entry is more objective and concise in its tone by asking the respondents in a straightforward language style about their current state of mind, without adding too much emotional colour or rhetoric."}, "time_dimension": {"answer": "Present", "rationale": ""}, "sentence_type": "Interrogative sentence", "pos": {"nouns": {"Brain": 1, "Elephant": 1, "As usual": 1, "Same thing": 1, "Clear": 1}, "verbs": {"Clear": 2, "Elephant": 1, "As usual": 1, "Same thing": 1}, "adjectives": {"Brain": 1, "Elephant": 1, "As usual": 1, "Same thing": 1, "Clear": 1}, "numerals": {"30": 1}}}]}

{"questionnaire_title": "SAS Axis Self-Measuring Table", "questionnaire_category": "Mental health", "item_count": 20, "emotion_shift_overall": "From negative to positive", "language_style_overall": "Neutral", "items": [{"content": "One, feeling more nervous and anxious than usual.", "word_count": 12, "label": "", "sentence_structure": "Subject鈥搗erb鈥揷omplement", "subjective_objective": {"answer": "Subjective", "rationale": "Because the word \"thinks\" indicates that it's the patient's own feelings and experiences, which are described subjectively."}, "sentence_sentiment": {"answer": "Negative", "rationale": "Because \"Easy and Excited\" in the entry is a negative emotional experience."}, "language_style": {"answer": "Calm and direct", "rationale": "The language style of the entry is \"cool straight\". It directly asked whether the interviewee ' s feelings had changed under normal circumstances, without using excessive emotional modifications or metaphors, and in a more straightforward and concise manner."}, "time_dimension": {"answer": "Present", "rationale": "Now the entry describes the current feeling of the testee, which is, \"I feel more nervous and anxious than usual,\" so the temporal dimension is now."}, "sentence_type": "Affirmative declarative sentence", "pos": {"nouns": {"Normal": 1, "Nervous": 1, "I'm in a hurry": 1}, "verbs": {"I think": 1, "- Yeah": 1, "Easy": 1}, "adjectives": {"Nervous": 1, "I'm in a hurry": 1}, "numerals": {"1": 2}, "pronouns": {"I": 1}}}, {"content": "2. Fear without cause", "word_count": 9, "label": "", "sentence_structure": "Subject鈥搗erb鈥揷omplement", "subjective_objective": {"answer": "Subjective", "rationale": "Because fear is the patient's subjective feeling."}, "sentence_sentiment": {"answer": "Negative", "rationale": "Because the feeling of fear in the entries is negative."}, "language_style": {"answer": "Calm and direct", "rationale": "The language style of the entry is relatively calm and straightforward, and there is no use of excessive rhetorical or emotional colours to ask respondents in a simple and straightforward manner whether they are afraid for no reason."}, "time_dimension": {"answer": "Present", "rationale": "The entry describes a subjective feeling or emotional state that usually occurs at a certain point in time and lasts for some time. As there is no specific time frame or past events for reference, the judgement is \"now\"."}, "sentence_type": "Negative declarative sentence", "pos": {"nouns": {"For no reason": 1, "Scared": 1}, "adverbs": {"Land": 1}}}, {"content": "Three, easy to upset or panic.", "word_count": 11, "label": "", "sentence_structure": "Subject鈥搗erb鈥揷omplement", "subjective_objective": {"answer": "Subjective", "rationale": "Subjectivity: Because psychological feelings are usually subjective experiences and judgments, \"easy or frightened\" is the patient's own psychological state."}, "sentence_sentiment": {"answer": "Negative", "rationale": "Because \"psychotic\" and \"frightful\" are negative emotional expressions."}, "language_style": {"answer": "Calm and direct", "rationale": "The entry is a straightforward question of whether the interviewee is prone to irritation or panic, is free of excessive qualifiers or emotional colours and is more calm and objective."}, "time_dimension": {"answer": "Present", "rationale": "The entry describes the current psychological state of the person being tested, involving emotional reactions (irritation or panic) that are occurring or often occurring, so it is now judged."}, "sentence_type": "Negative declarative sentence", "pos": {"nouns": {"Heart": 1, "Disturbing": 1, "Scared": 1}, "verbs": {"Easy": 1, "I think": 1}, "numerals": {"3": 1}}}, {"content": "Four, I think it might go crazy.", "word_count": 7, "label": "", "sentence_structure": "Subject鈥搗erb鈥揷omplement", "subjective_objective": {"answer": "Subjective", "rationale": "Because \"the feeling of possible madness\" is the patient's subjective feelings and thoughts."}, "sentence_sentiment": {"answer": "Negative", "rationale": "Because the idea of \"like to go crazy\" is negative, expressing concern and anxiety about the future."}, "language_style": {"answer": "Tactful and warm", "rationale": "The entry is more adept and interactive when it is spoken in a gentle and inviting manner, rather than by direct statements or orders. At the same time, the expression \"likely to go crazy\" is more euphemistic and avoids too direct or offensive tone."}, "time_dimension": {"answer": "Present", "rationale": "The entry does not contain clear time indicators or references to points of time, nor does it describe past conduct or expectations for the future, so it is judged to be present conduct or state. At the same time, \"the feeling\" shows that it is a subjective feeling, not an objective fact, which further confirms the present situation."}, "sentence_type": "Interrogative sentence", "pos": {"nouns": {"Crazy": 1}, "verbs": {"Let's go": 1, "I think": 1, "Maybe": 1}, "numerals": {"4": 1}}}, {"content": "Five, I think everything's fine. Nothing bad happens.", "word_count": 16, "label": "", "sentence_structure": "Subject鈥搗erb鈥揷omplement", "subjective_objective": {"answer": "Subjective", "rationale": "Because it is a subjective view and expectation of what may happen in the future."}, "sentence_sentiment": {"answer": "Negative", "rationale": "Because \"there won't be any misfortune\" expresses a negative belief that expectations for the future are negative."}, "language_style": {"answer": "Tactful and warm", "rationale": "The language style of the entry is euphemistic and warm. It is not a direct statement or an order, but rather an invitation to engage in dialogue in a moderate manner, giving people a sense of affection and comfort. Moreover, the expression of the purpose of the article avoids negative emotions and pressures and is reassuring and pleasant."}, "time_dimension": {"answer": "Present", "rationale": "The keyword \"thinks it's all very good\" and \"doesn't have any misfortune\" shows that the testees think that life is going well and are optimistic about the future."}, "sentence_type": "Affirmative declarative sentence", "pos": {"nouns": {"Everything": 1, "Unfortunately": 1}, "verbs": {"I think": 1, "Come on": 1}, "adjectives": {"Okay": 1}}}, {"content": "Six, hands and feet shaking.", "word_count": 6, "label": "", "sentence_structure": "Subject鈥搗erb鈥揷omplement", "subjective_objective": {"answer": "Objective", "rationale": "As a result of tremors on hands and feet, the patient's condition is objectively manifested."}, "sentence_sentiment": {"answer": "Negative", "rationale": "Because the physical reaction of tremors is usually associated with negative emotions such as fear, stress or anxiety."}, "language_style": {"answer": "Calm and direct", "rationale": "The entry directly asks the interviewee whether he has symptoms of tremors on his hands and feet, does not use excessive qualifiers or emotional colours, and the language style is more objective and calm."}, "time_dimension": {"answer": "Past", "rationale": "According to the keyword \"hands and feet shaking\", it can be seen that the entry is a question of the testee's present or past symptoms or behaviour and therefore the temporal dimension is judged to be past or present."}, "sentence_type": "Affirmative declarative sentence", "pos": {"nouns": {"Hands and feet": 1, "Wreck": 1}, "verbs": {"# Wresh #": 1}}}, {"content": "7 - A headache, a headache and a back.", "word_count": 13, "label": "", "sentence_structure": "Subject鈥搗erb鈥揷omplement", "subjective_objective": {"answer": "Objective", "rationale": "This is an objective problem, not a subjective attitude or idea."}, "sentence_sentiment": {"answer": "Negative", "rationale": "Because \"headaches, neck pains and back pains\" in the entry are negative physical symptoms and are described as causes of \"suffering\"."}, "language_style": {"answer": "Calm and direct", "rationale": "For the following reasons: The entry is in plain and clear language, asking directly whether the person under investigation is suffering from headaches, headaches and back pains and does not use excessive qualifiers or strong emotional expressions. This calm and direct inquiry allows for more accurate access to the actual situation of the person under investigation."}, "time_dimension": {"answer": "Present", "rationale": "The entry describes the current situation of the subjects, who are suffering from headaches, headaches and back pains. This feeling is present, so it is judged now."}, "sentence_type": "Affirmative declarative sentence", "pos": {"nouns": {"Headache": 2, "Backache": 1, "Trouble": 1}, "verbs": {"Because": 1, "Trouble": 1}}}, {"content": "8. Feeling vulnerable to weakness and fatigue", "word_count": 9, "label": "", "sentence_structure": "Subject鈥搗erb鈥揷omplement", "subjective_objective": {"answer": "Subjective", "rationale": "Because it's a subjective experience."}, "sentence_sentiment": {"answer": "Negative", "rationale": "Because \"fear vulnerability and fatigue\" is a physical discomfort that is usually considered negative."}, "language_style": {"answer": "Tactful and warm", "rationale": ""}, "time_dimension": {"answer": "Past", "rationale": "The entry uses the word \"feel\" and describes the subjective feelings of the testee, judging by time."}, "sentence_type": "Negative declarative sentence", "pos": {"nouns": {"Feel": 1, "Weakness": 1, "Weary": 1}, "verbs": {"Easy": 1, "Here": 1}}}, {"content": "Nine. Feel calm and sit quietly.", "word_count": 15, "label": "", "sentence_structure": "Subject鈥揷opula鈥損redicative", "subjective_objective": {"answer": "Subjective", "rationale": "Because words like \"felt\" and \"easy\" are the expression of patients' subjective feelings and attitudes."}, "sentence_sentiment": {"answer": "Neutral", "rationale": "Since the entry describes an emotional state, there is no apparent positive or negative bias."}, "language_style": {"answer": "Tactful and warm", "rationale": "The entry is more adept and interactive when it is spoken in a gentle and inviting manner, rather than by direct statements or orders. At the same time, the terms \"like,\" \"and\" were used to guide respondents to self-assessment, making the questionnaire more humane and acceptable."}, "time_dimension": {"answer": "Present", "rationale": "According to the entry sentence, \"Feel\" and \"sit quietly\" it can be judged as a sentence describing the current feelings and state of the interviewee, so the temporal dimension is now."}, "sentence_type": "Affirmative declarative sentence", "pos": {"nouns": {"Calm down": 1, "Quiet": 1, "Sit down": 1}, "verbs": {"I think": 1, "Easy": 1}, "adverbs": {"And": 1}, "numerals": {"9": 1}}}, {"content": "I feel like my heart is beating fast.", "word_count": 7, "label": "", "sentence_structure": "Subject鈥搗erb鈥揷omplement", "subjective_objective": {"answer": "Subjective", "rationale": "Because \"heart beats fast\" is a feeling, usually subjective experience and feeling. This feeling may be due to physical, emotional or other reasons and is therefore subjective.\n\nSo the answer is: subjective."}, "sentence_sentiment": {"answer": "Negative", "rationale": "As ulterior heart rate is often considered physical or emotional discomfort, it is a negative situation."}, "language_style": {"answer": "Tactful and warm", "rationale": "Basis for judgement: the language style of the article is relatively euphemistic and does not involve too many negative evaluations or direct expressions of sentiment. It uses the term \"like\" to guide the respondents to express their feelings and avoids too strong a tone or language. So, I think the language style of the questionnaire entry belongs to the \"precipitous warmth\"."}, "time_dimension": {"answer": "Present", "rationale": "The entry asks the testee about his current feelings or state, so the basis of the judgement is \"now\"."}, "sentence_type": "Affirmative declarative sentence", "pos": {"nouns": {"Heart rate": 1}, "verbs": {"I think": 1, "Come on": 1}, "numerals": {"10": 1}}}, {"content": "Eleven. A moment of dizziness.", "word_count": 10, "label": "", "sentence_structure": "Subject鈥搗erb鈥揷omplement", "subjective_objective": {"answer": "Objective", "rationale": "Since dizziness is a symptom of the patient, it is viewed from the patient ' s subjective perspective."}, "sentence_sentiment": {"answer": "Negative", "rationale": "Because \"difficult\" and \"tort\" are both negative terms."}, "language_style": {"answer": "Calm and direct", "rationale": "The language style of the entry is more direct and objective and does not use excessive emotional colours or rhetorical techniques, mainly to direct respondents to questions. At the same time, the purpose of the article was neutral and did not manifest a clear bias or attitude. The language style of this questionnaire entry is therefore clear and straightforward."}, "time_dimension": {"answer": "Past", "rationale": "According to the key words in the sentence, \"for a while\" and \"for a while\", it can be judged that the entry asks whether the person who was tested had experienced vertigo in the past and suffered from it. The time dimension is the past."}, "sentence_type": "Affirmative declarative sentence", "pos": {"nouns": {"Dizziness": 1, "Trouble": 1}, "verbs": {"Because": 1, "For a while": 1, "And": 1, "Trouble": 1}, "adverbs": {"For a while": 1}, "numerals": {"11": 1}}}, {"content": "12, fainting, or feeling fainting.", "word_count": 13, "label": "", "sentence_structure": "Subject鈥搗erb鈥揷omplement", "subjective_objective": {"answer": "Objective", "rationale": "Because it's an objective fact of what patients experience."}, "sentence_sentiment": {"answer": "Negative", "rationale": "Since the entry describes symptoms of physical discomfort, it may cause panic and anxiety."}, "language_style": {"answer": "Calm and direct", "rationale": "The language style of the entry is relatively direct and objective, there is no excessive rhetorical or emotional rendering, and direct inquiries are used to express concern and concern about whether the person under investigation has fainted."}, "time_dimension": {"answer": "Past", "rationale": "In the entry, the words \"with fainting\" and \"with feeling fainting\" describe the testee's experience and therefore judge the past."}, "sentence_type": "Interrogative sentence", "pos": {"nouns": {"鈾?And the attacks 鈾?: 1, "I'm fainted": 1}, "verbs": {"have": 1, "I think": 1, "Like": 1, "of": 1}, "numerals": {"12": 1}}}, {"content": "It's easy to breathe.", "word_count": 10, "label": "", "sentence_structure": "Subject鈥揷opula鈥損redicative", "subjective_objective": {"answer": "Objective", "rationale": "Because the feelings of inhaling and exhale are physical experiences, they are objective."}, "sentence_sentiment": {"answer": "Positive", "rationale": "Because it's an act of physical comfort, of positive emotion."}, "language_style": {"answer": "Tactful and warm", "rationale": "The entry is more adept and interactive when it is spoken in a gentle and inviting manner, rather than by direct statements or orders. At the same time, the use of the term \"easy\" also conveys a positive tone and gives people a sense of comfort."}, "time_dimension": {"answer": "Present", "rationale": "The entry describes the current feeling or experience of the respondent in replying to the questionnaire, and thus judged \"now\"."}, "sentence_type": "Affirmative declarative sentence", "pos": {"verbs": {"I feel": 1, "Breathe in": 1, "Breathe out": 1}, "adjectives": {"Both": 1, "very": 1, "Easy": 1}, "numerals": {"13": 1}}}, {"content": "14: A numbness and sting.", "word_count": 7, "label": "", "sentence_structure": "Subject鈥損redicate", "subjective_objective": {"answer": "Objective", "rationale": "Since numbing and stinging are symptoms of the patient, it is a description of objective facts."}, "sentence_sentiment": {"answer": "Neutral", "rationale": "Because the entry only describes a symptom and does not contain any emotional colour or evaluation."}, "language_style": {"answer": "Calm and direct", "rationale": ""}, "time_dimension": {"answer": "Present", "rationale": ""}, "sentence_type": "Interrogative sentence", "pos": {"nouns": {"Hands and feet": 2, "I'm numb": 1, "Stings": 1}, "numerals": {"14": 1}}}, {"content": "15: Worried by stomach pain and indigestion", "word_count": 12, "label": "", "sentence_structure": "Subject鈥搗erb鈥揷omplement", "subjective_objective": {"answer": "Subjective", "rationale": "The pain caused by stomach pain and indigestion is the patient ' s subjective feeling and emotional experience."}, "sentence_sentiment": {"answer": "Negative", "rationale": "Because in the entry, stomach pains and indigestion are described as physical discomfort, with negative emotions."}, "language_style": {"answer": "Tactful and warm", "rationale": "The entry is more personal and interactive when it is spoken in an expression of concern and understanding, rather than in direct statements or orders. At the same time, the issue implies concern for the health of the respondents and reflects the care and kindness of the surveyer."}, "time_dimension": {"answer": "Past", "rationale": ""}, "sentence_type": "Affirmative declarative sentence", "pos": {"nouns": {"Stomach pain": 1, "Indigestion": 1, "Trouble": 1}, "verbs": {"Because": 1, "And": 1, "Trouble": 1}, "numerals": {"15": 1, "1": 1}}}, {"content": "16, often pee.", "word_count": 5, "label": "", "sentence_structure": "Subject鈥搗erb鈥揷omplement", "subjective_objective": {"answer": "Objective", "rationale": "Because \"often pee\" is a physical need and experience of a patient, which is an objective fact and is not controlled by the patient's subjective consciousness."}, "sentence_sentiment": {"answer": "Negative", "rationale": "Because \"often to urinate\" is usually an expression of physical discomfort, implying that there may be some health problems and therefore negative."}, "language_style": {"answer": "Calm and direct", "rationale": "The entry asked about the other ' s physical condition in a directly explicit language, and there was no excessive emotional rendering or rhetorical technique, so the language style was more calm and straightforward."}, "time_dimension": {"answer": "Present", "rationale": "The term \"often to urinate\" in this entry indicates a physical reaction of a regular or customary nature, which does not relate to the temporal dimension of the past or the future, and therefore is now judged."}, "sentence_type": "Imperative sentence", "pos": {"nouns": {"Often": 1, "Pee": 1}, "adverbs": {"Yes": 1}, "numerals": {"16": 1}}}, {"content": "17, hands are often dry and warm.", "word_count": 9, "label": "", "sentence_structure": "Subject鈥揷opula鈥損redicative", "subjective_objective": {"answer": "Objective", "rationale": "Because \"the hand is often dry and warm\" is an objective fact of the patient's condition."}, "sentence_sentiment": {"answer": "Neutral", "rationale": "Since the entry merely describes a fact, it does not contain any emotion or attitude."}, "language_style": {"answer": "Tactful and warm", "rationale": "The entry uses a mild and descriptive language style, which does not directly question or order the behaviour of the other party, but rather resonates by depicting a situation. Such expressions are more friendly and sweet and contribute to a good atmosphere of interaction."}, "time_dimension": {"answer": "Present", "rationale": ""}, "sentence_type": "Affirmative declarative sentence", "pos": {"nouns": {"Hands": 1, "Dry": 1, "Warm": 1}, "verbs": {"Always": 1}, "adjectives": {"Fuck": 1, "It's warm": 1}, "adverbs": {"very": 1, "of": 2}, "numerals": {"17": 1}}}, {"content": "It's red.", "word_count": 4, "label": "", "sentence_structure": "Subject鈥搗erb鈥揷omplement", "subjective_objective": {"answer": "Objective", "rationale": "Because of the red fever that patients experience."}, "sentence_sentiment": {"answer": "Neutral", "rationale": "Because the entry has no obvious emotional colour, it merely describes a physical reaction."}, "language_style": {"answer": "Tactful and warm", "rationale": "The entry is more adept and interactive when it is spoken in a gentle and inviting manner, rather than by direct statements or orders. In addition, the description of \"face red fever\" tends more to be depicted as subjective than as an objective fact, which is consistent with the euphemism of the language."}, "time_dimension": {"answer": "Past", "rationale": ""}, "sentence_type": "Affirmative declarative sentence", "pos": {"nouns": {"Red": 1, "Heat": 1}}}, {"content": "It's easy to sleep and it's good.", "word_count": 10, "label": "", "sentence_structure": "Subject鈥揷opula鈥損redicative", "subjective_objective": {"answer": "Subjective", "rationale": "Because it is easy to sleep and sleep well is a patient ' s subjective feeling and experience, which involves the patient ' s psychological state and emotional response. While the quality of sleep may be influenced by objective factors (such as environmental noise or uncomfortable beds), the sense of \"easy and well-sleeping\" is often subjective and reflects individual comfort and satisfaction."}, "sentence_sentiment": {"answer": "Positive", "rationale": "Because \"sleep easy and well\" is positive."}, "language_style": {"answer": "Tactful and warm", "rationale": "The entry uses a milder, intimate tone to describe people ' s sleep, without too direct or hard language, and gives people a more intimate and natural feeling. At the same time, the active upward wording of \"sleep easy and well\" has made it easier for interviewees to accept and be willing to answer questions."}, "time_dimension": {"answer": "Present", "rationale": "The entry describes the general habits or state of life, using the general current, indicating that the situation still exists. So the time dimension is now judged."}, "sentence_type": "Affirmative declarative sentence", "pos": {"nouns": {"19": 1, "It's easy to sleep": 1, "Sleep well": 1}, "adjectives": {"Okay": 1}, "adverbs": {"Easy": 1}, "numerals": {"19": 1}, "classifiers": {"individual": 1}}}, {"content": "20, nightmares.", "word_count": 3, "label": "", "sentence_structure": "Subject鈥搗erb鈥揷omplement", "subjective_objective": {"answer": "Subjective", "rationale": "The question is whether the patient has had nightmare experiences and is the subject of subjective feelings and experiences."}, "sentence_sentiment": {"answer": "Negative", "rationale": "Because the \"Nightmare\" act is negative."}, "language_style": {"answer": "Calm and direct", "rationale": "The language style of the entry is more direct, with no excessive qualifiers or emotional colours, and more of a discussion of the question itself, allowing the respondents to answer the questions clearly and clearly."}, "time_dimension": {"answer": "Past", "rationale": ""}, "sentence_type": "Imperative sentence", "pos": {"nouns": {"Nightmare": 1}, "verbs": {"Do it": 1}, "numerals": {"20": 1}}}]}

{"questionnaire_title": "Revised adult attachment scale (AAS)", "questionnaire_category": "Emotion and affect", "item_count": 18, "emotion_shift_overall": "From positive to negative", "language_style_overall": "Tactful and warm", "items": [{"content": "1. I find it easier to be close.", "word_count": 11, "label": "", "sentence_structure": "Subject鈥搗erb鈥搊bject", "subjective_objective": {"answer": "Subjective", "rationale": "Because \"discovery\" is the patient's subjective feeling and perception."}, "sentence_sentiment": {"answer": "Positive", "rationale": "Because the expression \"discovery of proximity\" in the entry is positive."}, "language_style": {"answer": "Tactful and warm", "rationale": "Warm and warm."}, "time_dimension": {"answer": "Present", "rationale": "The entry is for self-evaluation of the human ability of the testee and the time dimension is now judged. In it, the word \"I\" indicates that it is a statement of a personal subjective feeling, and \"discovery\" and \"easy\" also indicates that it is a recent experience or observation. This questionnaire entry is therefore the subject of current behaviour or mentality."}, "sentence_type": "Affirmative declarative sentence", "pos": {"nouns": {"Found": 1, "person": 1}, "verbs": {"Close": 1, "Easy": 1}, "adjectives": {"Comparison": 1}, "numerals": {"1": 2}, "pronouns": {"I": 1}}}, {"content": "I found it difficult to rely on someone else.", "word_count": 13, "label": "", "sentence_structure": "Subject鈥搗erb鈥揷omplement", "subjective_objective": {"answer": "Subjective", "rationale": "Because it is \"difficult to find one's way of relying on another person\" is a personal perception and perception that needs to be shaped by one's own subjective experience, beliefs and attitudes, it is an entry from a subjective perspective."}, "sentence_sentiment": {"answer": "Negative", "rationale": "Because it is difficult to rely on someone else in the entry, it expresses a negative attitude or feeling that an individual may feel uncomfortable or resistant when facing a situation that requires help from others."}, "language_style": {"answer": "Tactful and warm", "rationale": "The language style of the entry is more euphemistic, gentle, without direct command or criticism of the other, but rather as an understanding and support to guide the dialogue, making the interviewees feel more comfortable and free to express their thoughts and feelings."}, "time_dimension": {"answer": "Present", "rationale": "The keyword 鈥渄iscovery鈥?means that the dominant language is aware of this fact at some point or time, so that it can be judged to be describing the current mindset or state of the subject."}, "sentence_type": "Negative declarative sentence", "pos": {"nouns": {"Found": 1, "I": 1, "others": 1, "Dependency": 1}, "verbs": {"I'm going": 1, "It's hard": 1}, "adjectives": {"Difficulties": 2}, "numerals": {"2": 1}}}, {"content": "3. I often worry that couples do not really love me.", "word_count": 13, "label": "", "sentence_structure": "Subject鈥搗erb鈥搊bject", "subjective_objective": {"answer": "Subjective", "rationale": "Fear is the patient's subjective feeling and attitude."}, "sentence_sentiment": {"answer": "Negative", "rationale": "Because the attitude in the entry that \"fears that couples don't really love me\" is negative."}, "language_style": {"answer": "Calm and direct", "rationale": "The entry explicitly asks the respondents whether they often have concerns about the true love of the couple, and does not use excessive rhetorical or emotional accents to be more objective."}, "time_dimension": {"answer": "Present", "rationale": ""}, "sentence_type": "Negative declarative sentence", "pos": {"nouns": {"Questionnaire": 1, "Entry": 1, "Couple": 1, "Really": 1, "Love": 1}, "verbs": {"I'm worried": 1, "not": 1, "Love": 1}, "adverbs": {"Often": 1}, "numerals": {"3": 1}, "pronouns": {"I": 1}}}, {"content": "I found out people didn't want to be as close to me as I wanted.", "word_count": 18, "label": "", "sentence_structure": "Subject鈥搗erb鈥揷omplement", "subjective_objective": {"answer": "Subjective", "rationale": "Because this is the patient ' s personal feeling and perception, it is a subjective observation."}, "sentence_sentiment": {"answer": "Negative", "rationale": "Because the expression \"no one else wants to be as close to me as I want\" in the entry expresses negative emotions and attitudes."}, "language_style": {"answer": "Calm and direct", "rationale": "The entry presents in a more straightforward manner the problems experienced by the respondents in their interpersonal contacts, without excessive rhetoric and emotional rendering, and with a more objective and calm tone."}, "time_dimension": {"answer": "Present", "rationale": "The keyword \"I find\" and \"I don't want\" to indicate that the question describes what the subjects feel or observe at the moment, so the judgment is now."}, "sentence_type": "Negative declarative sentence", "pos": {"nouns": {"others": 1, "Hope": 1, "Close": 1}, "verbs": {"Found": 1, "Yes": 1}, "adverbs": {"not": 1, "That way": 1}, "numerals": {"4": 1}, "pronouns": {"I": 1}}}, {"content": "I'm comfortable with being dependent.", "word_count": 12, "label": "", "sentence_structure": "Subject鈥揷opula鈥損redicative", "subjective_objective": {"answer": "Subjective", "rationale": "Because relying on the ideas and feelings of others is a subjective attitude of one."}, "sentence_sentiment": {"answer": "Positive", "rationale": "The entry is a positive emotional attitude because it expresses a trust and dependence on others."}, "language_style": {"answer": "Tactful and warm", "rationale": "The entry is more adept and interactive when it is spoken in a gentle and inviting manner, rather than by direct statements or orders. At the same time, the language style of the article is more nuanced and the term \"comfort\" is used to describe feelings and convey a warm, comfortable emotional atmosphere."}, "time_dimension": {"answer": "Present", "rationale": "At the same time, \"sweet\" is an indication of the feelings and emotions of the subjects and supports this conclusion."}, "sentence_type": "Affirmative declarative sentence", "pos": {"nouns": {"others": 1, "I": 1}, "verbs": {"Jean": 1, "I feel": 1, "Dependency": 1}, "adjectives": {"- Yeah": 1}}}, {"content": "I don't care about being too close to me.", "word_count": 10, "label": "", "sentence_structure": "Subject鈥搗erb鈥揷omplement", "subjective_objective": {"answer": "Subjective", "rationale": "Because it is a subjective attitude and emotional experience to care about the proximity of others."}, "sentence_sentiment": {"answer": "Negative", "rationale": "Because the attitude in the entry, \"Don't care about being too close to me,\" is negative."}, "language_style": {"answer": "Calm and direct", "rationale": "The entry expresses, in a negative form, the attitude of the other party to being close to the other person, without too much ado or emotion, and with a relatively straightforward and calm language style."}, "time_dimension": {"answer": "Present", "rationale": "The sentence contains a description of the present, where \"no care\" and \"too close to me\" may be occurring or present, and expresses the attitude or behaviour of the subject."}, "sentence_type": "Negative declarative sentence", "pos": {"nouns": {"Questionnaire": 1, "Entry": 1}, "verbs": {"I don't care": 1, "Close": 1}, "adjectives": {"Too much": 1}, "pronouns": {"I": 1}}}, {"content": "7. I find no one will help me when I need help.", "word_count": 17, "label": "", "sentence_structure": "Subject鈥搗erb鈥揷omplement", "subjective_objective": {"answer": "Subjective", "rationale": "Because \"discover\" and \"need\" and \"negative\" are patients' subjective feelings and perceptions, it's not objective."}, "sentence_sentiment": {"answer": "Negative", "rationale": "Because \"no one will help me\" in the entry expresses negative feelings and attitudes."}, "language_style": {"answer": "Calm and direct", "rationale": "\"Calm down straight.\" It's a direct statement. It's a calm and objective expression.\" Nobody's willing to help when they need help.\" There's not too much rhetorical or emotional."}, "time_dimension": {"answer": "Present", "rationale": "The keyword \"discovery\" represents a perception of the state of the moment, and \"when needing help from someone else\" indicates that it is the feeling or experience of the subject during a certain period of time, so the entry describes the current feeling of the testee, and the time dimension is now judged."}, "sentence_type": "Negative declarative sentence", "pos": {"nouns": {"I": 1, "Found": 1, "person": 1, "Help": 1}, "verbs": {"Yes": 2, "Help": 1}, "adverbs": {"When": 1, "Time": 1}, "numerals": {"7": 1}, "classifiers": {"individual": 1}, "pronouns": {"Nope": 1}}}, {"content": "8. I feel a little uncomfortable being close to others.", "word_count": 14, "label": "", "sentence_structure": "Subject鈥揷opula鈥損redicative", "subjective_objective": {"answer": "Subjective", "rationale": "It is a subjective experience to examine how the patient feels close to others."}, "sentence_sentiment": {"answer": "Negative", "rationale": "Because the \"uncomfortable\" emotion in the entry is negative."}, "language_style": {"answer": "Calm and direct", "rationale": "The language style of the entry is relatively straightforward, and it does not use excessive rhetorical techniques or emotional rendering, but rather presents the matter in an objective and concise manner, which is consistent with the immediate and calm character of the entry."}, "time_dimension": {"answer": "Present", "rationale": "The entry describes the current feeling of the testee, i.e., that he/she would feel uncomfortable when he/she was close to another person, and therefore falls within the current temporal dimension."}, "sentence_type": "Negative declarative sentence", "pos": {"nouns": {"person": 1, "Close": 1, "I'm not feeling well": 1}, "verbs": {"鈾?And I'm gonna be 鈾?: 1, "I feel": 1, "Some": 1, "I'm not feeling well": 1}, "adjectives": {"- Yeah": 1}, "adverbs": {"Some": 1}, "pronouns": {"I": 1}}}, {"content": "I'm always worried that couples don't want to be with me.", "word_count": 14, "label": "", "sentence_structure": "Subject鈥搗erb鈥搊bject", "subjective_objective": {"answer": "Subjective", "rationale": "Because \"fear\" is a subjective feeling and a fear that means the patient wants to be with herself."}, "sentence_sentiment": {"answer": "Negative", "rationale": "Because the words \"fear\" and \"don't want to be with me\" in the entries express negative emotional attitudes."}, "language_style": {"answer": "Tactful and warm", "rationale": "The entry uses the first person's expression and the phrase 鈥渙ften feared鈥?expresses concern about his emotional situation, but does not directly accuse or complain about the other party. Besides, the expression \"with me\" is even more mild, without mandatory language or command. Thus, the language style of this questionnaire entry is generally euphemistic."}, "time_dimension": {"answer": "Present", "rationale": "The phrase \"I'm often worried\" in the entry indicates a situation that is now worrying, and the word \"want\" uses the usual current state of affairs, which means that this situation may continue. Thus, the temporal dimension of the purpose of the article is now judged."}, "sentence_type": "Negative declarative sentence", "pos": {"nouns": {"Questionnaire Entry": 1, "Couple": 1, "I": 2}, "verbs": {"I'm worried": 1, "Yes": 1, "in": 1, "Together": 1}, "adverbs": {"Often": 1}, "numerals": {"9": 1}}}, {"content": "When I express my feelings to others, I fear that they will feel different from me.", "word_count": 26, "label": "", "sentence_structure": "Subject鈥揷opula鈥損redicative", "subjective_objective": {"answer": "Subjective", "rationale": "Because fearing that they will feel different from me is a subjective feeling and emotion."}, "sentence_sentiment": {"answer": "Negative", "rationale": "Because the attitude in the entry \"to fear that they will feel different from me\" is negative."}, "language_style": {"answer": "Tactful and warm", "rationale": "The entry opens a conversation in a moderate, caring tone about what the other person feels and asks whether the other is afraid and worried when expressing feelings. This expression is more caring and understanding."}, "time_dimension": {"answer": "Past", "rationale": "And \"I'm expressing my feelings\" and \"I'm afraid they'll feel different from me\" describe the situation, so the time dimension of this questionnaire is now."}, "sentence_type": "Negative declarative sentence", "pos": {"nouns": {"I": 2, "others": 1, "Emotion": 1, "Scared": 1}, "verbs": {"Expression": 1, "be": 1, "Yes": 1, "It's different": 1}, "adverbs": {"to": 1, "When": 1}, "numerals": {"10": 1}, "pronouns": {"they": 1}}}, {"content": "I often wonder if couples really care about me.", "word_count": 14, "label": "", "sentence_structure": "Subject鈥搗erb鈥搊bject", "subjective_objective": {"answer": "Subjective", "rationale": "Because \"suspicion\" is a subjective emotion and perception, not a description of objective facts."}, "sentence_sentiment": {"answer": "Negative", "rationale": "Because the contrast between \"suspicion\" and \"real concern\" in the entries expresses a negative sentiment."}, "language_style": {"answer": "Tactful and warm", "rationale": ""}, "time_dimension": {"answer": "Present", "rationale": "Now the entry describes a subjective feeling and way of thinking of the subject, \"I'm often\" indicating that it's a constant act or state, and \"suspecting that couples really care about me\" is a reflection and evaluation of current relationships. The time dimension of this entry should therefore be the present."}, "sentence_type": "Interrogative sentence", "pos": {"nouns": {"Couple": 1, "I": 2, "Care": 1}, "verbs": {"Doubt": 1, "be": 1}, "adjectives": {"True": 1}, "adverbs": {"Often": 1}, "numerals": {"11": 1, "One": 2}}}, {"content": "12. I am comfortable with the establishment of close relationships.", "word_count": 16, "label": "", "sentence_structure": "Subject鈥揷opula鈥損redicative", "subjective_objective": {"answer": "Subjective", "rationale": "Because feeling comfortable is the subjective feeling and attitude of the patient."}, "sentence_sentiment": {"answer": "Positive", "rationale": "Because it's a positive attitude in the entry to be comfortable with being intimate."}, "language_style": {"answer": "Tactful and warm", "rationale": "The language style of the entry is more euphemistic, using the expression \"I'm comfy\" and not directly stating or ordering the other person to be comfortable in establishing a close relationship. This gentle and inviting tone makes it easier for interviewees to share their views and feelings."}, "time_dimension": {"answer": "Present", "rationale": ""}, "sentence_type": "Affirmative declarative sentence", "pos": {"nouns": {"Questionnaire": 1, "Entry": 1, "Relations": 1, "others": 1}, "verbs": {"Create": 1, "I feel": 1, "- Yeah": 1}, "adjectives": {"very": 1, "Intimacy": 1}, "numerals": {"12": 1}, "pronouns": {"I": 1}}}, {"content": "I feel uncomfortable when someone is too emotionally close to me.", "word_count": 18, "label": "", "sentence_structure": "Subject鈥揷opula鈥損redicative", "subjective_objective": {"answer": "Subjective", "rationale": "Because feeling uncomfortable is a subjective experience and feeling."}, "sentence_sentiment": {"answer": "Negative", "rationale": "Because \"uncomfortable\" expresses a negative mood and attitude."}, "language_style": {"answer": "Calm and direct", "rationale": "The entry is of a more direct language style, with no too many qualifiers or euphemisms, opens the door to questions and conveys a clearer negative sentiment through the use of the words \"uncomfort\". Such language styles apply to situations in which it is necessary to obtain the true ideas of the other party quickly."}, "time_dimension": {"answer": "Present", "rationale": ""}, "sentence_type": "Negative declarative sentence", "pos": {"nouns": {"person": 1, "Emotion": 1, "Close": 1, "I'm not feeling well": 1}, "verbs": {"have": 1, "I feel": 1}, "adverbs": {"Too much": 1}, "numerals": {"13": 1}, "pronouns": {"I": 1}}}, {"content": "I know that when I need help, someone will help me.", "word_count": 18, "label": "", "sentence_structure": "Subject鈥搗erb鈥搊bject", "subjective_objective": {"answer": "Subjective", "rationale": "Output: Subjective\nIt is subjective to examine whether he is aware of the personal perception and conviction of being a patient."}, "sentence_sentiment": {"answer": "Positive", "rationale": "Because the idea of knowing that someone will help me when I need help is positive and can give people a sense of security and trust."}, "language_style": {"answer": "Tactful and warm", "rationale": "The survey questioned the use of a more euphemistic and encouraging language style, which did not directly indicate the seriousness or urgency of the problem but rather raised it in a more moderate manner. This expression is more acceptable and responsive and may encourage respondents to express their thoughts and feelings more honestly and frankly."}, "time_dimension": {"answer": "Present", "rationale": "The entry is a question of the testee ' s subjective perception of his/her abilities and relationships, and the temporal dimension is now judged."}, "sentence_type": "Affirmative declarative sentence", "pos": {"nouns": {"I know": 1, "Time": 1, "person": 1, "Help": 1}, "verbs": {"Yes": 2, "Help": 1}, "adverbs": {"Total": 1}, "numerals": {"14": 1}, "pronouns": {"I": 1}}}, {"content": "I wanted to be close, but I was afraid I might get hurt.", "word_count": 16, "label": "", "sentence_structure": "Subject鈥搗erb鈥揷omplement", "subjective_objective": {"answer": "Subjective", "rationale": "The content of this questionnaire, \"I\" wanted to be close to people, \"I\" feared that I would get hurt, and these statements were the patient's own feelings and thoughts, so it looked at the patient's subjective perspective. So the answer to this questionnaire is \"subjective.\""}, "sentence_sentiment": {"answer": "Negative", "rationale": "Because the emotional expression in the entry is fear of harm, which is a negative emotion and attitude."}, "language_style": {"answer": "Tactful and warm", "rationale": ""}, "time_dimension": {"answer": "Present", "rationale": ""}, "sentence_type": "Affirmative declarative sentence", "pos": {"nouns": {"Questionnaire": 1, "Entry": 1, "person": 1, "Close": 1, "Hurt": 1}, "verbs": {"Yes": 3, "I'm worried": 1, "Received": 1, "Close": 1}, "adverbs": {"But": 1}, "numerals": {"15": 1}, "pronouns": {"self": 1}}}, {"content": "16. I find it difficult to rely entirely on others.", "word_count": 12, "label": "", "sentence_structure": "Subject鈥搗erb鈥揷omplement", "subjective_objective": {"answer": "Subjective", "rationale": "Because it is the patient's feeling or perception that \"it is difficult to rely fully on\" and the patient's subjective attitude."}, "sentence_sentiment": {"answer": "Negative", "rationale": "Because the entry \"It's hard to trust people completely\" expresses a negative emotional attitude."}, "language_style": {"answer": "Tactful and warm", "rationale": "The entry is more adept and interactive when it is spoken in a gentle and inviting manner, rather than by direct statements or orders."}, "time_dimension": {"answer": "Past", "rationale": "The entry describes the experience and state of the person being tested, i.e., \"I can't trust anyone completely\" and therefore judges the past."}, "sentence_type": "Negative declarative sentence", "pos": {"nouns": {"Found": 1, "I": 1, "others": 1}, "verbs": {"It's hard": 1, "Total": 1, "Trust": 1}, "adverbs": {"very": 1, "Total": 1}, "numerals": {"16": 1}}}, {"content": "17. Couples want me to be more emotional, which often makes me uncomfortable.", "word_count": 23, "label": "", "sentence_structure": "Subject鈥揷opula鈥損redicative", "subjective_objective": {"answer": "Subjective", "rationale": "Because \"want\" and \"discomfort\" are the personal feelings and attitudes of the patient, it is seen from a subjective perspective."}, "sentence_sentiment": {"answer": "Negative", "rationale": "Because the emotional reaction in the entry is negative."}, "language_style": {"answer": "Tactful and warm", "rationale": "The entry is more adept and interactive when it is spoken in a gentle and inviting manner, rather than by direct statements or orders. At the same time, descriptive terms such as \"want\" and \"relationship\" were used, and it was emphasized that such demands were uncomfortable, euphemistic and sensitive to each other's feelings, and reflected a humanistic and understanding attitude."}, "time_dimension": {"answer": "Present", "rationale": "The entry describes the current situation and feelings, in which \"wanted me to be more emotional\" and \"often uncomfortable\" are expressions of the current situation. So the time dimension is now judged."}, "sentence_type": "Negative declarative sentence", "pos": {"nouns": {"Couple": 1, "Emotion": 1, "Close": 1, "I'm not feeling well": 1}, "verbs": {"Yes": 1, "鈾?Make you feel 鈾?: 1}, "adverbs": {"More": 1, "Regular": 1}, "numerals": {"17": 1}, "pronouns": {"I": 2}}}, {"content": "18. I am not sure that I can always find someone I can rely on when I need them.", "word_count": 20, "label": "", "sentence_structure": "Subject鈥搗erb鈥搊bject", "subjective_objective": {"answer": "Subjective", "rationale": "Because \"persons who can be relied upon\" is an individual subjective feeling and evaluation."}, "sentence_sentiment": {"answer": "Positive", "rationale": "Because it's positive that \"you can always find someone you can rely on.\""}, "language_style": {"answer": "Calm and direct", "rationale": "The language style of the entry is more straightforward, with no excessive rhetorical or emotional colours, but rather a simple and straightforward query as to whether the interviewee can find reliable support when he or she needs help. This expression is more in line with the principles of objectivity and neutrality in the questionnaire survey and is better able to obtain real feedback from respondents."}, "time_dimension": {"answer": "Present", "rationale": "\"I'm not sure\" in \"I'm not sure\" is an expression of current attitude and conviction, and \"when I need it\" means that, at some point in the present or in the future, \"you can always find someone you can rely on\" is a future act or state, so the entry is mainly concerned with the current and future dimensions of time."}, "sentence_type": "Negative declarative sentence", "pos": {"nouns": {"Questionnaire Entry": 1, "person": 1}, "verbs": {"Found it": 1, "Dependency": 1}, "adverbs": {"Sure": 1, "Total": 1}, "numerals": {"18": 1}, "pronouns": {"I": 1}}}]}

{"questionnaire_title": "Suicide attitude questionnaire (QSA)", "questionnaire_category": "Behavioral questionnaire", "item_count": 29, "emotion_shift_overall": "From positive to negative", "language_style_overall": "Calm and direct", "items": [{"content": "1. Suicide is a madness...", "word_count": 10, "label": "", "sentence_structure": "Subject鈥揷opula鈥損redicative", "subjective_objective": {"answer": "Subjective", "rationale": "This expression is an expression of the subjective perception or values of suicide on the part of researchers, rather than a description of an objective fact or situation."}, "sentence_sentiment": {"answer": "Negative", "rationale": "Because in the entry, \"suicide is a madness.\" This expression is negative and expresses a negative and negative attitude towards suicide."}, "language_style": {"answer": "Calm and direct", "rationale": "The language style of the entry is more dispassionate and straightforward, without too much emotional or acrimony, but rather expresses the serious consequences and negative effects of suicide in an objective and factual manner. This formulation is clearer and can help the respondents to understand the problem in greater depth and to think about themselves."}, "time_dimension": {"answer": "Past", "rationale": ""}, "sentence_type": "Affirmative declarative sentence", "pos": {"nouns": {"Suicide": 1, "Behaviour": 1}, "verbs": {"be": 1}, "adjectives": {"One": 1, "Crazy": 1}, "numerals": {"One": 2}}}, {"content": "2. Deaths due to suicide shall be treated in the same manner as natural deaths.", "word_count": 19, "label": "", "sentence_structure": "Subject鈥搗erb鈥搊bject", "subjective_objective": {"answer": "Subjective", "rationale": "Because this phrase reflects the subjective view of researchers that \"suicide deaths\" and \"natural deaths\" deserve equal rights."}, "sentence_sentiment": {"answer": "Neutral", "rationale": "Since the entry merely stated one view or proposition, it did not contain any emotional colour."}, "language_style": {"answer": "Calm and direct", "rationale": "The language style of the entry is straight and cool. It clearly raised a question and was expressed in direct language. There is no qualifier or rhetoric to glorify or emphasize the importance of the issue. This language style is therefore more suitable for use in official settings or in academic research."}, "time_dimension": {"answer": "Present", "rationale": "The entry does not have a clear indication or phrase of past or future time, but rather presents a current view or proposition. Therefore, it should be classified as \"now\"."}, "sentence_type": "Affirmative declarative sentence", "pos": {"nouns": {"Suicide": 1, "Dead": 2, "Naturally": 1, "Treatment": 1}, "verbs": {"Reactions": 1, "Enjoy": 1, "Same thing": 1}, "numerals": {"2": 1}}}, {"content": "In general, I do not want to be deeply involved with those who have committed suicide.", "word_count": 20, "label": "", "sentence_structure": "Subject鈥搗erb鈥搊bject", "subjective_objective": {"answer": "Subjective", "rationale": "Because \"involved in suicide\" is a personal attitude or emotional expression and a patient's subjective idea."}, "sentence_sentiment": {"answer": "Negative", "rationale": "Because the attitude in the entry of \"unwillingness to engage in suicide\" is negative."}, "language_style": {"answer": "Calm and direct", "rationale": "The language style of the entry is relatively calm and straightforward, with no excessive rhetoric or emotional colour. It clearly expresses the author ' s view and position that he is not willing to engage with those who have committed suicide and that his tone is more decisive and resolute."}, "time_dimension": {"answer": "Present", "rationale": "The entry asks the testee about his or her attitude or behaviour in general and does not relate to past or future time dimensions, so it is now judged."}, "sentence_type": "Negative declarative sentence", "pos": {"nouns": {"Situation": 1, "person": 2, "Suicide": 1}, "verbs": {"Yes": 1, "Hand it over": 1, "Deep": 1}, "adjectives": {"General": 1, "Pass": 1}, "adverbs": {"not": 1, "and": 1, "Yes": 2}, "classifiers": {"One": 2, "individual": 1}, "pronouns": {"I": 1}}}, {"content": "4. Throughout the suicides, the most painful were the families of the suicide victims.", "word_count": 19, "label": "", "sentence_structure": "Subject鈥搗erb鈥搊bject", "subjective_objective": {"answer": "Subjective", "rationale": "Because the most painful thoughts and feelings are usually based on individual subjective experience, the \"most painful\" is a subjective assessment."}, "sentence_sentiment": {"answer": "Negative", "rationale": "Because the entries \"suicide\" and \"most painful is the family of the suicide victim\" are described with negative emotions."}, "language_style": {"answer": "Calm and direct", "rationale": "The language style of the entry is direct and calm. It succinctly expresses the subject of the study 鈥?the effects of suicide on families 鈥?without too much rhetoric or emotional colour and with a more objective and straightforward tone."}, "time_dimension": {"answer": "Past", "rationale": "The entry describes the effects of suicide on the family, and the temporal dimension is the past."}, "sentence_type": "Affirmative declarative sentence", "pos": {"nouns": {"Suicide": 2, "Pain": 1, "Family": 1}, "verbs": {"be": 1}, "numerals": {"The whole thing": 1, "Most": 1}}}, {"content": "5. In cases of terminally ill and extremely painful patients, medical personnel, with the support of the law, can help them to end their lives (active euthanasia).", "word_count": 40, "label": "", "sentence_structure": "Subject鈥搗erb鈥搊bject", "subjective_objective": {"answer": "Subjective", "rationale": "This is the patient ' s personal view and opinion and is a subjective one."}, "sentence_sentiment": {"answer": "Neutral", "rationale": "Because the entry does not express any emotion. It offers support for a possible choice and legitimacy, but it does not itself evaluate the merits or validity of that choice."}, "language_style": {"answer": "Calm and direct", "rationale": "The language style of the entry is relatively calm and straightforward, and it clearly raises a question and describes the situation. Moreover, the issue itself involved several dimensions, including moral, ethical and legal provisions, and therefore needed to be objectively expressed and explored."}, "time_dimension": {"answer": "Present", "rationale": ""}, "sentence_type": "Affirmative declarative sentence", "pos": {"nouns": {"Patients": 2, "Pain": 1, "Insane": 1, "Medical personnel": 1, "Law": 1, "Support": 1, "Life": 2, "I'm gonna get a little euthanic": 1}, "verbs": {"I'm sick": 1, "Extreme": 1, "Help": 1, "End": 1, "(Present euthanasia)": 1}, "adverbs": {"And": 1}, "numerals": {"5": 1}}}, {"content": "6. In dealing with suicides, sympathy and concern should be extended to their families and, to the extent possible, assistance provided to them.", "word_count": 34, "label": "", "sentence_structure": "Subject鈥搗erb鈥搊bject", "subjective_objective": {"answer": "Subjective", "rationale": "The expression of sympathy, concern and assistance is subjective because of the investigator ' s attitude and behaviour towards the family members of the suicide."}, "sentence_sentiment": {"answer": "Positive", "rationale": "Basis: The content of the entry is to encourage care and assistance to others and is a positive emotional expression."}, "language_style": {"answer": "Tactful and warm", "rationale": "The language style of the entry is relatively gentle, thoughtful and caring. It does not directly order or strongly accuse the person concerned of acting, but rather conveys the message by expressing sympathy, concern and offering help, a tone that is more acceptable to the reader and willing to act on the recommendation. At the same time, the word \"as far as possible\" also shows understanding and support for the family, which is warm and comforting."}, "time_dimension": {"answer": "Present", "rationale": ""}, "sentence_type": "Affirmative declarative sentence", "pos": {"nouns": {"Process": 1, "Events": 1, "Family": 1, "Suicide": 1, "Compassion": 1, "Care": 1, "Help": 1}, "verbs": {"Processing": 1, "Organisation": 1, "As far as possible": 1, "Yes": 1, "Provision": 1}, "adverbs": {"in": 1, "Process": 1, "Suicide": 1, "and": 1, "As far as possible": 1, "they": 1}, "numerals": {"6": 1}, "pronouns": {"Other": 1, "they": 1}}}, {"content": "Suicide was an affront to the dignity of human life.", "word_count": 12, "label": "", "sentence_structure": "Subject鈥揷opula鈥損redicative", "subjective_objective": {"answer": "Objective", "rationale": "Because \"suicide is an affront to the dignity of human life\" is a statement of fact or a general view and does not involve personal feelings or attitudes. Therefore, it is examined from an objective perspective."}, "sentence_sentiment": {"answer": "Negative", "rationale": "Because \"suicide\" in the entry is negative."}, "language_style": {"answer": "Calm and direct", "rationale": "The entry uses direct and clear language to describe the effects of suicide on the dignity of human life, does not use excessive rhetoric or emotional rendering, and is more objective in tone. Such language styles help to convey serious messages and perspectives and enhance persuasiveness."}, "time_dimension": {"answer": "Past", "rationale": "The entry expresses a widely shared view or belief that does not relate to the individual ' s specific experience or future expectations, and therefore determines the past."}, "sentence_type": "Affirmative declarative sentence", "pos": {"nouns": {"Suicide": 1, "person": 1, "Life": 1, "Dignity": 1}, "verbs": {"to": 1, "be": 1, "Scrubs": 1}, "adjectives": {"person": 1, "Life": 1, "Yours": 1, "Heavy": 2}, "numerals": {"7": 1}}}, {"content": "There should be no memorial service for suicide victims.", "word_count": 12, "label": "", "sentence_structure": "Subject鈥損redicate", "subjective_objective": {"answer": "Subjective", "rationale": "It is subjective to consider whether to hold a memorial service for a suicide victim is a decision of personal opinion and value."}, "sentence_sentiment": {"answer": "Negative", "rationale": "Because the act in the entry \"a memorial service for a suicide victim\" may be seen as a rationalization or encouragement of suicide and therefore considered negative."}, "language_style": {"answer": "Calm and direct", "rationale": "The language style of the entry is more straightforward, it does not use excessive rhetorical techniques or emotional rendering, it is more balanced and objective, and it expresses a clear view."}, "time_dimension": {"answer": "Past", "rationale": "The entry does not contain any particular time indicator or time-related information, so it is not possible to determine whether it describes past, present or future behaviour or mindset."}, "sentence_type": "Negative declarative sentence", "pos": {"nouns": {"Suicide Deaths": 1, "The memorial service": 1}, "verbs": {"No, I shouldn't": 1, "Open": 1}}}, {"content": "9. If my friend attempted suicide, I would care more about him than ever before.", "word_count": 19, "label": "", "sentence_structure": "Subject鈥搗erb鈥搊bject", "subjective_objective": {"answer": "Subjective", "rationale": "Since the entry relates to patients ' subjective attitudes and emotional reactions, they are asked whether they will change the level of interest in friends. It is therefore viewed from a subjective perspective."}, "sentence_sentiment": {"answer": "Positive", "rationale": "Because the purpose of the action in the entry is positive."}, "language_style": {"answer": "Tactful and warm", "rationale": "The language style of the entry is more euphemistic. In a concerned manner, it enquired about the responses of the interviewees in the face of an attempted suicide of a friend, expressed its understanding and concern at the interviewees and did not directly convey any accusations or pressure, and was more cordial and gentle."}, "time_dimension": {"answer": "Present", "rationale": "The word \"if\" indicates that this is a hypothetical situation, and the word \"will\" indicates actions that may occur in the future. Thus, the sentence describes the current attitude and the idea that when a friend attempts to commit suicide, the testee will pay more attention to him."}, "sentence_type": "Affirmative declarative sentence", "pos": {"nouns": {"friends": 1, "Suicide": 1, "Attempt": 1, "I": 1}, "verbs": {"Yes": 1, "Care": 1, "- Yeah": 1}, "adjectives": {"Before": 1}, "adverbs": {"More": 1}, "numerals": {"9": 1}}}, {"content": "10. If my neighbours had committed suicide in their homes, I would have become alienated from them.", "word_count": 24, "label": "", "sentence_structure": "Subject鈥搗erb鈥搊bject", "subjective_objective": {"answer": "Subjective", "rationale": "Since the entry expresses the action or attitude that the patient may take in certain circumstances, it is subjective or emotional."}, "sentence_sentiment": {"answer": "Negative", "rationale": "Because in the entry, the act of \"disconnection and their relationship\" is negative."}, "language_style": {"answer": "Calm and direct", "rationale": "The language style of the entry is straightforward, as it clearly expresses a hypothetical result, i.e., what would you do if that happened. There are no too many words or emotions, and the tone is more objective."}, "time_dimension": {"answer": "Past", "rationale": "The term \"will\" in the entry represents a future-modern behavioural assumption, but it describes the effect or result of an impending event on the present. Thus, the time dimension is judged to be the past."}, "sentence_type": "Affirmative declarative sentence", "pos": {"nouns": {"Questionnaire": 1, "Entry": 1, "person": 2, "Neighbors": 2, "Home": 1, "Relations": 1, "Suicide": 1}, "verbs": {"Yes": 3, "Distant": 1, "Progressive": 1, "and": 1, "of": 5, "be": 1}, "adverbs": {"If": 1, "Mine": 2, "they": 1, "of": 2, "Progressive": 1}, "numerals": {"10": 1}, "classifiers": {"individual": 1}, "pronouns": {"I": 1}}}, {"content": "Euthanasia is an affront to the dignity of human life.", "word_count": 13, "label": "", "sentence_structure": "Subject鈥揷opula鈥損redicative", "subjective_objective": {"answer": "Subjective", "rationale": "Because euthanasia is an affront to the dignity of human life, it is the opinion or opinion of researchers or interviewees that euthanasia is subjective."}, "sentence_sentiment": {"answer": "Negative", "rationale": "Because the idea that euthanasia is a violation of human dignity is negative."}, "language_style": {"answer": "Calm and direct", "rationale": "The entry expresses the author ' s view of euthanasia in simple and clear language, without using any rhetorical or emotional expressions and with a calm and straightforward language style."}, "time_dimension": {"answer": "Present", "rationale": "The sentence describes views and attitudes towards euthanasia, and does not indicate clearly whether it is past, present or future in time, so it is now."}, "sentence_type": "Negative declarative sentence", "pos": {"nouns": {"I'm glad to see you": 1, "Life": 1, "Dignity": 1}, "verbs": {"be": 1, "Scrubs": 1}, "adverbs": {"to": 1}, "numerals": {"11": 1}}}, {"content": "Suicide is an act of impotence on the part of the family and society.", "word_count": 18, "label": "", "sentence_structure": "Subject鈥揷opula鈥損redicative", "subjective_objective": {"answer": "Objective", "rationale": "Because \"irresponsibility\" and \"suicide\" are objective facts and descriptions of family and society."}, "sentence_sentiment": {"answer": "Negative", "rationale": "Because the expression \"suicide\" and \"irresponsibility\" in this entry have negative feelings."}, "language_style": {"answer": "Calm and direct", "rationale": "The entry uses a strong language style, characterizing suicide as \"irresponsible\", which is straightforward and does not use euphemism or consolation. This language style is usually used as a warning or exhortation to draw attention and vigilance from the other side."}, "time_dimension": {"answer": "Past", "rationale": "The entry now expresses a point of view, does not involve a specific period of time and is neutral in tone."}, "sentence_type": "Negative declarative sentence", "pos": {"nouns": {"Suicide": 1, "Family": 1, "Social": 1, "Behaviour": 1}, "verbs": {"be": 1, "to": 1, "Irresponsible": 1, "Behaviour": 1}, "adverbs": {"One": 2}, "numerals": {"12": 1, "Plant": 1}}}, {"content": "People shouldn't comment on suicides.", "word_count": 15, "label": "", "sentence_structure": "Subject鈥搗erb鈥搊bject", "subjective_objective": {"answer": "Subjective", "rationale": "Subjective. Because it is an attitude or opinion, it is the patient ' s subjective idea."}, "sentence_sentiment": {"answer": "Neutral", "rationale": "Because the entry expresses a moral norm and there is no apparent emotional tendency."}, "language_style": {"answer": "Tactful and warm", "rationale": "The entry, in a respectful and understandable sense, recommends that the subject of the investigation be careful in the face of the statement of the suicide victim and that it not be unduly evaluated. This expression is more humane, friendly and resonant with those surveyed."}, "time_dimension": {"answer": "Present", "rationale": ""}, "sentence_type": "Negative declarative sentence", "pos": {"nouns": {"People": 1, "Suicide": 1, "Dead": 1, "- The headline": 1}, "verbs": {"I think so": 1, "to": 1, "Say it": 1}, "numerals": {"13": 1}}}, {"content": "14. I am repulsed by those who repeatedly commit suicide because they often use suicide as a means of controlling others.", "word_count": 32, "label": "", "sentence_structure": "Subject鈥搗erb鈥搊bject", "subjective_objective": {"answer": "Objective", "rationale": "Because it describes a view or view, it is the subjective feeling of the observer. The existence and behaviour of repeated suicides are objective facts."}, "sentence_sentiment": {"answer": "Negative", "rationale": "Because the entry contains the word \"very distasteful\" and expresses a negative attitude. At the same time, \"they often use suicide as a means of controlling others\" is also a negative description, expressing resentment and criticism of suicides, and thus a negative emotional aspect in general."}, "language_style": {"answer": "Calm and direct", "rationale": "The entry is presented in a direct manner, without too much qualifying or euphemism. It clearly expressed the author ' s attitude towards repeated suicides and indicated that they might use suicide to control the behaviour of others. This approach is more objective and accurate."}, "time_dimension": {"answer": "Present", "rationale": "The entry describes a current attitude or view, \"I'm sick of it,\" which suggests that this is an assessment of the current situation. At the same time, they often describe a common phenomenon or behaviour, namely, \"successful suicides are often used as a means of controlling others\". The time dimension of the article is therefore now."}, "sentence_type": "Affirmative declarative sentence", "pos": {"nouns": {"Repeatedly": 1, "Suicide": 1, "they": 1, "Means": 1}, "verbs": {"Disgusting": 1, "Will": 1, "As": 1, "Suicide": 1, "Control": 1, "others": 1}, "adjectives": {"very": 1}, "numerals": {"14": 1}, "pronouns": {"I": 1}}}, {"content": "15. In the case of suicide, the families of the suicide victims should, to varying degrees, be given a certain responsibility.", "word_count": 25, "label": "", "sentence_structure": "Subject鈥搗erb鈥搊bject", "subjective_objective": {"answer": "Subjective", "rationale": "Because \"whether or not the family is responsible\" is the subjective view of the family of the suicide victim and belongs to the values and attitudes of the individual. It's a question of the patient's subjective perspective."}, "sentence_sentiment": {"answer": "Negative", "rationale": "Because in the entry, \"the family of a suicide victim should be responsible to some extent.\" This expression is somewhat remiss and negative."}, "language_style": {"answer": "Tactful and warm", "rationale": "The language style of the entry is more euphemistic and moderate, using the expression \"to some extent\" to suggest the size of the responsibility and without directly blaming or criticizing the suicide victim's family. This expression is more acceptable and encourages respondents to reflect and participate actively in the discussions."}, "time_dimension": {"answer": "Present", "rationale": "The entry enquires about the views and attitudes of the subjects on current social phenomena, and the keywords \"for suicide\", \"for the family of the suicide victim\" and \"to some extent for the family of the suicide victim\" describe the current situation."}, "sentence_type": "Affirmative declarative sentence", "pos": {"nouns": {"Suicide": 1, "Family": 1, "Responsibility": 1}, "verbs": {"in": 1, "Responsible": 1, "I'm sure": 1, "Level": 1, "Reactions": 1}, "adjectives": {"You're a good man": 1}, "numerals": {"15": 1}}}, {"content": "16. If I am suffering from terminal illness and suffering, I hope the medical staff will help me end my life.", "word_count": 36, "label": "", "sentence_structure": "Subject鈥搗erb鈥搊bject", "subjective_objective": {"answer": "Subjective", "rationale": "Because it is a patient's personal thought and hope about the situation he may face, it is subjective."}, "sentence_sentiment": {"answer": "Negative", "rationale": "Because it relates to life and death and expresses a negative desire or attitude."}, "language_style": {"answer": "Calm and direct", "rationale": "The entry is asked in a straightforward manner without any modification or emotional rendering. It sought to understand the views of the interviewees in the face of terminal illness and extreme suffering regarding the assistance of medical personnel in ending life. This language style seems objective and calm."}, "time_dimension": {"answer": "Present", "rationale": "Keywords: \"I hope,\" \"in extreme pain\" and \"help me end my life.\" It shows that the entry is exploring the current thoughts, emotions or attitudes of the subjects. In addition, \"if I am suffering from terminal illness\" is a hypothetical scenario that suggests that this is a possible expectation or scenario for the present. Thus, in sum, the time dimension of this questionnaire entry should be now."}, "sentence_type": "Affirmative declarative sentence", "pos": {"nouns": {"self": 2, "Insane": 1, "Extreme pain": 1, "Medical personnel": 1, "Life": 1}, "verbs": {"Hope": 1, "Help": 1, "End": 1}, "adjectives": {"I'm sick": 1, "Extreme": 1}, "numerals": {"16": 1}, "pronouns": {"I": 1}}}, {"content": "17. It was commendable that individuals had committed suicide for something great that exceeded the value of human life.", "word_count": 27, "label": "", "sentence_structure": "Subject鈥揷opula鈥損redicative", "subjective_objective": {"answer": "Subjective", "rationale": "Because \"if it's commendable\" is a subjective value judgement."}, "sentence_sentiment": {"answer": "Negative", "rationale": "Because the suicide act in the entry is negative."}, "language_style": {"answer": "Calm and direct", "rationale": "The language style of the entry is direct and calm. It presents its views in the form of a statement of the facts, without using euphemisms of euphemism or emotionality, which are more direct and objective."}, "time_dimension": {"answer": "Present", "rationale": "The key words \"individual,\" \"great,\" \"over\" and the verb \"suicide\" can be judged to describe a current phenomenon or view. Therefore, the time dimension judges the entry to be now."}, "sentence_type": "Affirmative declarative sentence", "pos": {"nouns": {"Individual": 1, "Purpose": 1, "Value": 1, "Suicide": 1}, "verbs": {"Yes": 1, "Praise": 1}, "adjectives": {"Great": 1, "More than": 1, "Life": 1}, "numerals": {"17": 1}}}, {"content": "18. In general, I do not wish to visit people who have attempted suicide, even when they are relatives or good friends.", "word_count": 29, "label": "", "sentence_structure": "Subject鈥搗erb鈥搊bject", "subjective_objective": {"answer": "Subjective", "rationale": "The issue is based on the attitude and opinion of the individual, i.e. whether he or she is willing to visit the person who attempted to commit suicide and is therefore subjective. While this attitude may be influenced by objective factors (e.g. mental health awareness, compassion for suicides, etc.), in this case these are all references to the assessment of subjective attitudes rather than the problem itself."}, "sentence_sentiment": {"answer": "Negative", "rationale": "Because the entry expresses a reluctance to help others, it is negative."}, "language_style": {"answer": "Calm and direct", "rationale": "The entry answered a question in a straightforward tone and did not use too many emotional language or rhetorical techniques to promote the atmosphere, giving people a sense of objectivity and calmness. At the same time, this response can more accurately reflect the real thinking and attitude of the respondents."}, "time_dimension": {"answer": "Past", "rationale": "The entry describes a habit or attitude of the person being tested, i.e., a general reluctance to visit an attempted suicide. Such conduct or attitude is directed at a certain period of time in the past (not specified) and is therefore judged to be \"the past\"."}, "sentence_type": "Affirmative declarative sentence", "pos": {"nouns": {"Situation": 1, "General": 1, "Time": 1, "Wait": 1, "I": 1, "Guys": 1, "Kiss": 1, "person": 1, "Pun": 1, "Okay": 1, "friends": 1, "Organisation": 1, "Suicide": 1, "Attempted": 1}, "verbs": {"May I": 1, "Look": 1, "Go on": 1, "Visits": 1, "be": 1, "not": 1, "Marriage": 1, "None": 1, "Yeah": 1, "Example": 1, "External": 1, "Divide": 1}, "adverbs": {"One": 1, "General": 1}, "numerals": {"18": 1, "1": 9}, "classifiers": {"individual": 2}, "pronouns": {"Here": 1, "Well": 1, "Other": 1, "he": 1}}}, {"content": "19. Suicide was only a human phenomenon, regardless of moral good or bad.", "word_count": 20, "label": "", "sentence_structure": "Subject鈥損redicate", "subjective_objective": {"answer": "Objective", "rationale": "Because \"suicide is only a life phenomenon\" is a statement of fact, and \"moral good and bad\" is based on subjective values. The view of this questionnaire entry is therefore considered from an objective perspective."}, "sentence_sentiment": {"answer": "Negative", "rationale": "Because the act of suicide in the entry is negative."}, "language_style": {"answer": "Calm and direct", "rationale": "The language style of the entry is calm and straightforward. It clearly expressed the view that suicide was a life phenomenon and that there was no moral appreciation. This expression is not overly emotional or rhetorical and focuses more on the accuracy of the message itself."}, "time_dimension": {"answer": "Present", "rationale": "In the past, the entry asked the testee's views on \"suicide\", and the temporal dimension judged the past."}, "sentence_type": "Affirmative declarative sentence", "pos": {"nouns": {"Suicide": 1, "Life phenomena": 1, "Ethics": 1, "Okay": 1, "Bad": 1}, "verbs": {"be": 1, "have": 1, "I care": 1}, "adverbs": {"EMPTY": 4}, "numerals": {"One": 2}}}, {"content": "Attempts to commit suicide do not deserve sympathy.", "word_count": 10, "label": "", "sentence_structure": "Subject鈥搗erb鈥搊bject", "subjective_objective": {"answer": "Subjective", "rationale": "Because \"are compassionate\" is a subjective value judgement, depending on the individual's feelings and values, it is a subjective examination."}, "sentence_sentiment": {"answer": "Negative", "rationale": "Because this entry expresses a negative attitude towards suicide attempts."}, "language_style": {"answer": "Calm and direct", "rationale": "The entry expresses a clear view that there is no excessive accommodative or emotional colour and that the language style is more straightforward and concise."}, "time_dimension": {"answer": "Present", "rationale": ""}, "sentence_type": "Negative declarative sentence", "pos": {"nouns": {"Attempted suicide": 1, "Compassion": 1}, "verbs": {"It's not worth it": 1}}}, {"content": "21. Life-support treatment (passive euthanasia) is no longer available to patients suffering from terminal illness and extreme pain.", "word_count": 33, "label": "", "sentence_structure": "Subject鈥搗erb鈥搊bject", "subjective_objective": {"answer": "Subjective", "rationale": "Because this is a doctor ' s judgement and decision on the patient, it involves values, ethics, etc., and it is the patient ' s subjective attitude."}, "sentence_sentiment": {"answer": "Negative", "rationale": "Because in the entry, \"No more life-support therapy.\" This may lead to an increase in the suffering of the patient and is negative."}, "language_style": {"answer": "Calm and direct", "rationale": "The entry for the questionnaire is in plain and simple language and does not contain excessive emotional colours or rhetorical techniques. It directly presented a view of passive euthanasia, leaving the interviewee to judge and express his/her own views, so that the language style was relatively calm and straightforward."}, "time_dimension": {"answer": "Future", "rationale": "The entry discusses a possible future scenario where, in some cases, patients may choose to cease life-support treatment. The keywords \"no longer for them\" and \"passive euthanasia\" point to medical ethics and decision-making that may arise in the future."}, "sentence_type": "Affirmative declarative sentence", "pos": {"nouns": {"Patients": 1, "I'm sick": 1, "Insane": 1, "Extreme": 1, "Pain": 1, "Treatment": 2, "Passive": 1, "I'm glad to see you": 1}, "verbs": {"Yeah": 1, "Not anymore": 1, "Conduct": 1, "Maintenance": 1, "Life": 1}, "adjectives": {"I'm sick": 1, "Insane": 1, "Extreme": 1, "Pain": 1, "Passive": 1}, "numerals": {"21": 1, "1": 5}, "classifiers": {"individual": 1}, "pronouns": {"Other": 1}}}, {"content": "Suicide was a betrayal of loved ones and friends.", "word_count": 11, "label": "", "sentence_structure": "Subject鈥搗erb鈥搊bject", "subjective_objective": {"answer": "Subjective", "rationale": "This is a perception and evaluation of suicide and is subjective."}, "sentence_sentiment": {"answer": "Negative", "rationale": "Because the word \"treachery\" in the entry is negative emotional."}, "language_style": {"answer": "Tactful and warm", "rationale": "The more moderate and friendly language of the entry, which emphasizes the harm that suicide can cause to relatives and friends rather than direct accusations or warnings, is more acceptable and understandable. At the same time, the term \"treachery\" has been used to give the title some warning, reminding those under investigation to cherish life and respect those around them."}, "time_dimension": {"answer": "Present", "rationale": "The entry indicates a point of view or opinion of the speaker and does not indicate a clear point of time, so it is now judged."}, "sentence_type": "Affirmative declarative sentence", "pos": {"nouns": {"Suicide": 1, "Family": 1, "friends": 1, "Betrayal": 1}, "numerals": {"22": 1}}}, {"content": "23. People sometimes have to commit suicide for their dignity and honour.", "word_count": 16, "label": "", "sentence_structure": "Subject鈥搗erb鈥搊bject", "subjective_objective": {"answer": "Subjective", "rationale": "Since suicide is an individual decision, involving factors such as individual values, emotional state and so forth, the problem is examined more subjectively from the perspective of the patient."}, "sentence_sentiment": {"answer": "Negative", "rationale": "Because suicide in the entry is a negative behavior."}, "language_style": {"answer": "Calm and direct", "rationale": "The entry uses a more direct and clear language style, does not use euphemism and subtle expression and is more objective and neutral in tone. At the same time, the content of this article is concerned with serious social issues and therefore needs to be expressed in more formal language."}, "time_dimension": {"answer": "Present", "rationale": "The entry describes the reactions or behaviour (suicides) that people may have in certain circumstances, which is a possible current situation."}, "sentence_type": "Affirmative declarative sentence", "pos": {"nouns": {"person": 1, "Dignity": 1, "Honours": 1, "Suicide": 1}, "verbs": {"For": 1, "Okay": 1, "Suicide": 1}, "adjectives": {"Sometimes": 1}, "numerals": {"23": 1}, "pronouns": {"And": 1}}}, {"content": "24. When making friends, I do not mind whether the other side has committed suicide.", "word_count": 19, "label": "", "sentence_structure": "Subject鈥搗erb鈥搊bject", "subjective_objective": {"answer": "Subjective", "rationale": "Because \"not minded\" is the patient's attitude to whether or not he or she has committed suicide."}, "sentence_sentiment": {"answer": "Neutral", "rationale": "Because the entry simply stated a fact and did not express any emotional tendencies."}, "language_style": {"answer": "Calm and direct", "rationale": "The language style of the entry is more direct and there are no too many embellishments or euphemisms, so that it can be judged as straightforward."}, "time_dimension": {"answer": "Present", "rationale": "The keyword \"when we're friends\" means that it's an exercise that's happening, and the state of \"not minding\" means that it's the attitude and the idea. So the time dimension of this entry is judged now."}, "sentence_type": "Affirmative declarative sentence", "pos": {"nouns": {"Friends": 1, "The other side": 1, "Suicide": 1}, "verbs": {"Yes": 1, "have": 1}, "adverbs": {"Not really": 1}, "numerals": {"24": 1}}}, {"content": "25. More attention and assistance should be given to those who attempted suicide.", "word_count": 17, "label": "", "sentence_structure": "Subject鈥搗erb鈥搊bject", "subjective_objective": {"answer": "Subjective", "rationale": "Since recommendations or points of view are given, this is usually a subjective perspective."}, "sentence_sentiment": {"answer": "Positive", "rationale": "Because it expresses support and encouragement for suicide attempts, it is positive emotional."}, "language_style": {"answer": "Tactful and warm", "rationale": "The language style of the entry is more euphemistic and moderate. It highlighted the need to give more attention and assistance to those who attempted suicide, a expression that was relatively polite and respectful of each other and more resonating and identifying than direct orders or criticism."}, "time_dimension": {"answer": "Future", "rationale": "The entry is a description of an act or measure that should be taken, the temporal dimension of which is judged to be the future because it is an expectation and a requirement for the future."}, "sentence_type": "Affirmative declarative sentence", "pos": {"nouns": {"Suicide": 1, "Attempt": 1, "Who": 1, "Care": 1, "Help": 1}, "verbs": {"Give it to me": 1}, "numerals": {"25": 1}}}, {"content": "26. Suicide is understandable when life is over.", "word_count": 18, "label": "", "sentence_structure": "Subject鈥搗erb鈥揷omplement", "subjective_objective": {"answer": "Subjective", "rationale": "Because the \"understandable\" view is the result of subjective judgement by researchers, not on objective facts."}, "sentence_sentiment": {"answer": "Negative", "rationale": "Because the act of suicide in the entry is negative."}, "language_style": {"answer": "Calm and direct", "rationale": "The entry uses direct and concise language to express an understanding of suicide, with no excessive emotional colours and rhetoric. This expression is more objective and clearly conveys information and is more in line with the professional requirements of the psychological questionnaire study."}, "time_dimension": {"answer": "Future", "rationale": "The entry contains ideas and expectations for the future, and the key words are \"when life is over\" and \"understandable\" and all point to situations that have not yet occurred and that may occur in the future."}, "sentence_type": "Negative declarative sentence", "pos": {"nouns": {"Life": 1, "Happy": 1, "Suicide": 1}, "verbs": {"have": 1, "be": 1, "Yeah": 2, "Understood": 1}, "adjectives": {"None": 1, "- I'm sorry": 1}, "adverbs": {"Already": 1, "When": 1}, "numerals": {"26": 1}}}, {"content": "27. If I am suffering from terminal illness and suffering, I will no longer receive life-sustaining treatment.", "word_count": 31, "label": "", "sentence_structure": "Subject鈥損redicate", "subjective_objective": {"answer": "Subjective", "rationale": "This is because it is a manifestation of patients ' subjective attitudes and values. At a critical juncture in life or death, patients choose to give up treatment as an expression of their individual will, reflecting their awareness and attitude to life, death and suffering. This questionnaire entry is therefore examined from a subjective perspective."}, "sentence_sentiment": {"answer": "Negative", "rationale": "For the purpose of this article, the act of \"resistance to life-sustaining treatment\" is negative."}, "language_style": {"answer": "Tactful and warm", "rationale": "The language style of the entry is more euphemistic, using virtual and descriptive expressions such as \"if\" and \"infection\" to guide respondents to express their thoughts and feelings in an understanding and respectable manner. At the same time, this description avoids direct statements or orders and increases the comfort and participation of interviewees."}, "time_dimension": {"answer": "Present", "rationale": "The entry asks about the current attitude or perception of the testees, i.e. whether they are willing to receive life-sustaining treatment, assuming they are terminally ill and in extreme pain. So time dimensions are judged \"now\"."}, "sentence_type": "Affirmative declarative sentence", "pos": {"nouns": {"Insane": 1, "Pain": 1, "Treatment": 1}, "verbs": {"I'm sick": 1, "Yes": 1, "Accept": 1, "Maintenance": 1}, "adjectives": {"Extreme": 1}, "numerals": {"27": 1}, "pronouns": {"I": 1}}}, {"content": "28. Generally speaking, I will not marry a person who has committed suicide at home.", "word_count": 20, "label": "", "sentence_structure": "Subject鈥搗erb鈥搊bject", "subjective_objective": {"answer": "Subjective", "rationale": "It is a subjective judgement that it is personal will and attitude to marry a person who has committed suicide at home."}, "sentence_sentiment": {"answer": "Negative", "rationale": "Because the presence of \"suicides in the family\" in the entry may create negative feelings and perceptions that affect marriage decisions."}, "language_style": {"answer": "Calm and direct", "rationale": "The language style of the entry is of a calm and straightforward type, asking in a straightforward manner whether the other party is unwilling to marry a person who has committed suicide at home. Too many rhetorical techniques or emotional renderings are not used, the tone is more objective, the emphasis on facts and logic is more practical."}, "time_dimension": {"answer": "Present", "rationale": "The expression \"generally\" suggests that this is a regular situation, that \"I will not marry a person who has committed suicide in the family\" is an attitude or belief of the individual and that such an attitude or belief may influence the decision-making process. Therefore, this questionnaire entry relates to current behaviour or mentality."}, "sentence_type": "Negative declarative sentence", "pos": {"nouns": {"Situation": 1, "Family": 1, "person": 1, "Suicide": 1, "Marriage": 1}, "verbs": {"Yes": 1, "Conclusion": 1, "Marriage": 2}, "adjectives": {"General": 1}, "adverbs": {"In general": 1}, "numerals": {"28": 1}}}, {"content": "29. A person should have the right to choose suicide.", "word_count": 11, "label": "", "sentence_structure": "Subject鈥搗erb鈥揷omplement", "subjective_objective": {"answer": "Subjective", "rationale": "Because \"the right to choose suicide\" is a subjective view and proposition of the interviewees, not an objective situation or fact."}, "sentence_sentiment": {"answer": "Negative", "rationale": "Because the choice of suicide in the entry is negative."}, "language_style": {"answer": "Calm and direct", "rationale": "The language style of the entry is relatively calm and straightforward, and there are no excessive emotional modifications or rhetorical techniques. It clearly sets out a point of view and asks respondents to comment on it in a more straightforward manner."}, "time_dimension": {"answer": "Past", "rationale": "It is more like a statement or position than a description of behaviour or mentality within a given period of time. So I classified it as \"other.\""}, "sentence_type": "Interrogative sentence", "pos": {"nouns": {"person": 1, "Suicide": 1, "Powers": 1}, "verbs": {"I think so": 1, "have": 1, "Selection": 1}, "numerals": {"29": 1}}}]}

{"questionnaire_title": "Olson Marriage Questionnaire (ENRICH)", "questionnaire_category": "Emotion and affect", "item_count": 124, "emotion_shift_overall": "From negative to positive", "language_style_overall": "Tactful and warm", "items": [{"content": "1. Both spouses love the same type of social activity.", "word_count": 15, "label": "", "sentence_structure": "Subject鈥搗erb鈥搊bject", "subjective_objective": {"answer": "Subjective", "rationale": "Because \"both husband and wife love the same type of social activity\" is the patient's subjective view and attitude, which is the expression of individual subjective experience."}, "sentence_sentiment": {"answer": "Positive", "rationale": "Because the entry \"Spouses love the same type of social activity\" expresses a positive feature or state, indicating that the couple has a common hobby, which enhances their feelings and interactions."}, "language_style": {"answer": "Tactful and warm", "rationale": "The entry uses an invitation language, implying that the subjects are willing to share their own social behaviour and avoiding direct orders or evaluations. This approach makes it easier to obtain positive responses and cooperation from interviewees."}, "time_dimension": {"answer": "Present", "rationale": "The word \"both\" in the entry indicates that the conduct of the spouses is consistent and that such conduct is an ongoing one and is therefore judged to be current."}, "sentence_type": "Affirmative declarative sentence", "pos": {"nouns": {"Couple": 2, "Same category": 1, "Social activities": 1}, "adjectives": {"Both": 1}, "numerals": {"1": 1}}}, {"content": "It is very easy to express my true feelings to a spouse.", "word_count": 17, "label": "", "sentence_structure": "Subject鈥揷opula鈥損redicative", "subjective_objective": {"answer": "Subjective", "rationale": "Because this statement relates to the feelings and views of the patient, it is a subjective judgement."}, "sentence_sentiment": {"answer": "Positive", "rationale": "Because it's very easy to express my true feelings to a spouse in the entry."}, "language_style": {"answer": "Tactful and warm", "rationale": "The entry is more adept and interactive when it is spoken in a gentle and inviting manner, rather than by direct statements or orders."}, "time_dimension": {"answer": "Present", "rationale": "The term \"Yes\" in this entry uses the general current, indicating that the act is currently being performed or is occurring on a regular basis, so the temporal dimension is now judged. At the same time, the words \"expressing my true feelings\" and \"very easy\" also indicate that this is the current attitude or state of the subject, not the past or the future."}, "sentence_type": "Affirmative declarative sentence", "pos": {"nouns": {"Spouse": 1, "Feel": 1, "Real": 1}, "verbs": {"Expression": 1, "be": 1, "Easy": 1}, "adjectives": {"Very": 1}, "numerals": {"2": 1}, "pronouns": {"I": 1}}}, {"content": "3. It is difficult for me to receive the education I have received on religious beliefs.", "word_count": 22, "label": "", "sentence_structure": "Subject鈥搗erb鈥搊bject", "subjective_objective": {"answer": "Subjective", "rationale": "Because \"hard to accept\" is the patient's personal opinion and feelings."}, "sentence_sentiment": {"answer": "Negative", "rationale": "Because the \"difficult to accept\" attitude in the entry is negative."}, "language_style": {"answer": "Calm and direct", "rationale": "The entry expresses in a straightforward manner the attitudes and views of the interviewees regarding their religious education. There is no excessive emotional qualifier or rhetoric, but rather a straightforward description of one ' s views, objectivity and authenticity."}, "time_dimension": {"answer": "Present", "rationale": "The use of the usual current \"I'm Hard\" in the entry indicates that the testee's attitude towards religious education at the current point in time is not entirely acceptable. At the same time, \"get\" and \"take it all\" means it's an experience or state that has happened, so time dimension is now."}, "sentence_type": "Negative declarative sentence", "pos": {"nouns": {"Religious beliefs": 1, "Education": 1}, "verbs": {"Yes": 1, "Accept": 1}, "adjectives": {"Hard": 1, "Overall": 1}, "adverbs": {"very": 1}}}, {"content": "4. In order to put an end to the dispute as soon as possible, I have often given in.", "word_count": 14, "label": "", "sentence_structure": "Subject鈥搗erb鈥搊bject", "subjective_objective": {"answer": "Subjective", "rationale": "Because concessions are a subjective choice and action. \""}, "sentence_sentiment": {"answer": "Negative", "rationale": "Because the act of \"concession\" in the entry is usually considered to be negative, as it implies giving up one's views or positions."}, "language_style": {"answer": "Calm and direct", "rationale": "The language style of the entry, which is calm and straightforward, expresses in a straightforward manner the attitude often taken by the subjects of the survey in dealing with the arguments, without too much emotional or tautological language."}, "time_dimension": {"answer": "Present", "rationale": "The entry can be judged by the verb \"I often\" in the sentence and by the general verb \"concession\" in the current state of affairs."}, "sentence_type": "Affirmative declarative sentence", "pos": {"verbs": {"End": 1, "Argument": 1, "Compliment": 1}, "adjectives": {"As soon as possible": 1}, "pronouns": {"I": 1}}}, {"content": "5. In our home, fathers spend not enough time with their children.", "word_count": 21, "label": "", "sentence_structure": "Subject鈥搗erb鈥搊bject", "subjective_objective": {"answer": "Objective", "rationale": "Because the subject is a description of an objective situation where fathers and children are not spent enough time together. While this situation may affect the mood or feelings of the patient, it is itself an observable fact and therefore objective."}, "sentence_sentiment": {"answer": "Negative", "rationale": "Since the entry describes the lack of time for fathers to accompany their children, it is a negative issue, expressing concern and dissatisfaction with family relations."}, "language_style": {"answer": "Tactful and warm", "rationale": "The language style of the entry is euphemistic and warm. Rather than directly blaming or criticizing fathers, it raises questions in an attitude of concern and understanding, which is more acceptable and encourages each other to participate in discussions and improvements."}, "time_dimension": {"answer": "Present", "rationale": ""}, "sentence_type": "Negative declarative sentence", "pos": {"nouns": {"Home": 1, "Father": 1, "Son": 1, "Time": 2}, "verbs": {"Cost": 1, "Stay together": 1}, "adjectives": {"Enough": 1}, "numerals": {"5": 1}}}, {"content": "6. When a couple is in conflict, my spouse is often silent.", "word_count": 18, "label": "", "sentence_structure": "Subject鈥搗erb鈥揷omplement", "subjective_objective": {"answer": "Objective", "rationale": "Since silence is an objective expression of behaviour, it is not the patient ' s own subjective feelings or attitudes."}, "sentence_sentiment": {"answer": "Negative", "rationale": "Because the act of silence in the entry is negative."}, "language_style": {"answer": "Calm and direct", "rationale": "Basis of judgement: The description of the article is objective and does not involve too much emotional or subjective evaluation. It refers directly to the fact that when there is a conflict between spouses, the spouse often chooses to remain silent, and that the language is simple and clear, without using euphemism or indirect expression, so that his or her language style can be considered calm and straightforward."}, "time_dimension": {"answer": "Past", "rationale": "\"In case of contradiction\" and \"always silent\" are descriptions or observations of the past, so the entry asks about the conduct of the subject in the past."}, "sentence_type": "Affirmative declarative sentence", "pos": {"nouns": {"Couples": 1, "Paradoxious": 1, "Spouse": 1, "Words": 1}, "verbs": {"Come on": 1, "Regular": 1, "Silence": 1, "No words": 1}, "numerals": {"6": 1}, "pronouns": {"Mine": 1}}}, {"content": "7. Some of our relatives and friends have made our marriage tense.", "word_count": 16, "label": "", "sentence_structure": "Subject鈥搗erb鈥搊bject", "subjective_objective": {"answer": "Subjective", "rationale": "It is the patient ' s subjective view, since the behaviour of some of the relatives affects the relationship between the patient and the spouse."}, "sentence_sentiment": {"answer": "Negative", "rationale": "Because the result of \"marriage gets nervous\" in the entry is negative."}, "language_style": {"answer": "Calm and direct", "rationale": "The entry describes the core of the problem in a direct and concise language, i.e. that certain acts of relatives and friends result in marital tension. Language styles are calm and objective, with no too much emotional or euphemism, with greater emphasis on the communication of information and clarity of issues."}, "time_dimension": {"answer": "Present", "rationale": ""}, "sentence_type": "Affirmative declarative sentence", "pos": {"nouns": {"Friends": 1, "Marriage": 1}, "verbs": {"Change": 1, "鈾?And I'm gonna be 鈾?: 1}, "adjectives": {"Some": 1, "Nervous": 1}, "pronouns": {"we": 1}}}, {"content": "8. My spouse is too picky or often negative.", "word_count": 17, "label": "", "sentence_structure": "Subject鈥揷opula鈥損redicative", "subjective_objective": {"answer": "Subjective", "rationale": "Since the entry in the questionnaire describes the attitude and perception of patients towards their spouses, it is subjective."}, "sentence_sentiment": {"answer": "Negative", "rationale": "Because the act of \"too picky or often negative\" in the entry is negative."}, "language_style": {"answer": "Tactful and warm", "rationale": "The entry is more adept and interactive when it is spoken in a gentle and inviting manner, rather than by direct statements or orders."}, "time_dimension": {"answer": "Present", "rationale": "The entry describes the way in which the testee interacts with his spouse and relates to the present situation, so it can be judged as \"now\"."}, "sentence_type": "Negative declarative sentence", "pos": {"nouns": {"Spouse": 1, "Views": 1}, "verbs": {"Picky": 1, "Hold": 1, "Negative": 1}, "adjectives": {"Too much": 1}, "numerals": {"8": 1}, "pronouns": {"I": 1}}}, {"content": "I am fully satisfied with my spouse's feelings for me.", "word_count": 12, "label": "", "sentence_structure": "Subject鈥揷opula鈥損redicative", "subjective_objective": {"answer": "Subjective", "rationale": "Because \"satisfaction\" is a subjective feeling and attitude based on the patient's personal views and experience, rather than a fact that can be objectively measured."}, "sentence_sentiment": {"answer": "Positive", "rationale": "Because the entry expresses emotional satisfaction with the spouse and is a positive emotional attitude."}, "language_style": {"answer": "Tactful and warm", "rationale": "The expression \"fully satisfied\" in the entry is relatively positive, but it is not too direct or strong, but rather expresses the respondent's attitude towards the emotional aspects of the spouses in a moderate and understandable way. Moreover, the use of a stop rather than a question mark at the end of the sentence implied that it was a narrative rather than a question, making language style more euphemismal and sweeter."}, "time_dimension": {"answer": "Present", "rationale": "The entry enquired about the satisfaction of the testee with the emotional relationship of the spouse, and the time dimension is now judged."}, "sentence_type": "Affirmative declarative sentence", "pos": {"nouns": {"Questionnaire": 1, "Entry": 1, "Spouse": 1, "Emotion": 1}, "verbs": {"Satisfied": 1}, "adjectives": {"Total": 1}, "pronouns": {"I": 1}}}, {"content": "10. My spouse and I often do not agree on how best to resolve conflicts.", "word_count": 23, "label": "", "sentence_structure": "Subject鈥搗erb鈥揷omplement", "subjective_objective": {"answer": "Subjective", "rationale": "Because the questionnaire entry asks about patients ' subjective feelings and attitudes, i.e. whether there are differences between the patient and his/her spouse in resolving the conflict and the impact of such differences on the patient. This is an examination of the patient ' s subjective perception and emotional experience and is therefore a subjective one."}, "sentence_sentiment": {"answer": "Negative", "rationale": "For the entry, the act of \"how best to resolve a contradiction is often unopposed\" is negative."}, "language_style": {"answer": "Tactful and warm", "rationale": "The entry is more adept and interactive when it is spoken in a gentle and inviting manner, rather than by direct statements or orders. At the same time, the language style of the article, which is objective and neutral, is not overly emotional or subjective and meets the requirements of the questionnaire study."}, "time_dimension": {"answer": "Present", "rationale": "The entry describes the current marital status of the person tested, and the keyword \"often\" indicates that this is a frequent occurrence. So the time dimension of this entry is now."}, "sentence_type": "Negative declarative sentence", "pos": {"nouns": {"I": 1, "Spouse": 1, "Best Practices": 1, "Paradoxious": 1}, "verbs": {"and": 1, "Adoption": 1, "Resolve": 1, "Often": 1, "Observations": 1, "No, I'm fine": 1}, "adverbs": {"Just": 1, "How's that": 1}, "numerals": {"10": 1}}}, {"content": "I believe that both spouses should have the same understanding of religion.", "word_count": 17, "label": "", "sentence_structure": "Subject鈥搗erb鈥搊bject", "subjective_objective": {"answer": "Subjective", "rationale": "Because \"think\" expresses patients' subjective attitudes and opinions."}, "sentence_sentiment": {"answer": "Neutral", "rationale": "As the entry does not express a positive or negative emotional tendency, it is presenting an opinion or a request."}, "language_style": {"answer": "Tactful and warm", "rationale": "The entry, in a more euphemistic tone, did not directly express an individual position or view, but rather invited respondents to express their views. This expression makes it easier for interviewees to open their minds and participate actively in the discussions."}, "time_dimension": {"answer": "Present", "rationale": "The keyword \"considers\" and \"shall\" both represent current thinking and expectations, while the words \"both husband and wife\" and \"religious\" refer to situations and themes that currently exist, so the temporal dimension of the article is now."}, "sentence_type": "Affirmative declarative sentence", "pos": {"nouns": {"I": 1, "Couple": 1, "Religion": 1, "Same": 1, "Understood": 1}, "verbs": {"I think": 1}, "numerals": {"11": 1}}}, {"content": "12. I think women should stay mainly at home.", "word_count": 12, "label": "", "sentence_structure": "Subject鈥搗erb鈥搊bject", "subjective_objective": {"answer": "Subjective", "rationale": "Because it's personal or personal, it's patient's subjective attitude."}, "sentence_sentiment": {"answer": "Negative", "rationale": "Because the view in the entry is demeaning and discriminatory towards women, it is incompatible with the principles of equality, respect and equality."}, "language_style": {"answer": "Calm and direct", "rationale": "This is based on the following criteria: 1. The entry clearly expresses the traditional stereotypes and limitations of the role of women, considering that their primary responsibility is in the family, while neglecting their abilities and contributions in other areas, such as society and the workplace; 2. This view is not only incompatible with the values of equality and pluralism in modern society, but may also lead to the exacerbation and spread of gender discrimination, which has a negative impact on the promotion of gender equality and the emancipation of women."}, "time_dimension": {"answer": "Past", "rationale": "The \"concept\" in the entry indicates that this was the view and attitude of the interviewees in the past and is therefore a judgement of the past."}, "sentence_type": "Affirmative declarative sentence", "pos": {"nouns": {"Women": 1, "Home": 1}, "verbs": {"I think": 1, "- You should stay": 1}, "adjectives": {"Main": 1, "Home": 1}, "numerals": {"12": 1}, "pronouns": {"I": 1}}}, {"content": "Sometimes, I care about my spouse's temper.", "word_count": 12, "label": "", "sentence_structure": "Subject鈥揷opula鈥損redicative", "subjective_objective": {"answer": "Subjective", "rationale": "Because \"care\" is the patient's subjective feeling and attitude."}, "sentence_sentiment": {"answer": "Negative", "rationale": "It's because the entry \"takes the temper of the spouse\" expresses a negative mood and attitude."}, "language_style": {"answer": "Calm and direct", "rationale": "The entry directly asked the respondents about the level of interest in the temperament of the spouses, without using any means of rhetoric or euphemism, and the more direct and objective language style."}, "time_dimension": {"answer": "Present", "rationale": "The entry \"sometimes\" indicates a situation that has occurred in the past and may happen again in the future. At the same time, my subjective feelings are now. The time dimension of the article is therefore now."}, "sentence_type": "Affirmative declarative sentence", "pos": {"nouns": {"Spouse": 1, "- Grumpy": 1}, "verbs": {"to": 1, "in": 1, "Meaning": 1}, "adjectives": {"very": 1}, "adverbs": {"Time": 1, "Sometimes": 1}, "numerals": {"13": 1}, "pronouns": {"I": 1}}}, {"content": "I don't like the personality and personal habits of spouses.", "word_count": 14, "label": "", "sentence_structure": "Subject鈥搗erb鈥揷omplement", "subjective_objective": {"answer": "Subjective", "rationale": "Because \"not like\" is a subjective feeling and attitude."}, "sentence_sentiment": {"answer": "Negative", "rationale": "Because \"not like\" in the entry expresses a negative attitude."}, "language_style": {"answer": "Calm and direct", "rationale": "The entry uses plain, simple language to describe the problem, with no excessive modifications or emotional rendering, and is more objective and calm."}, "time_dimension": {"answer": "Present", "rationale": "The entry expresses a subjective feeling and perception that the words \"not like\" , \"personality\" and \"personal habits\" point to the current state and can therefore be judged to be the present."}, "sentence_type": "Negative declarative sentence", "pos": {"nouns": {"Questionnaire": 1, "Entry": 1, "Character": 1, "Personal habits": 1, "Spouse": 1}, "verbs": {"Yeah": 1, "not": 1, "have": 1}, "adjectives": {"I don't like it": 2}, "numerals": {"14": 1}, "pronouns": {"I": 1}}}, {"content": "15. In order to keep sex fun, we have tried to find new ways.", "word_count": 21, "label": "", "sentence_structure": "Subject鈥搗erb鈥搊bject", "subjective_objective": {"answer": "Subjective", "rationale": "The search for new methods is an act of the patient ' s own initiative and is a subjective act of the patient."}, "sentence_sentiment": {"answer": "Positive", "rationale": "Because the purpose of the act of trying to find some new way is to be positive, to keep sex fun."}, "language_style": {"answer": "Tactful and warm", "rationale": "The entry is more adept and interactive when it is spoken in a gentle and inviting manner, rather than by direct statements or orders. It does not explicitly ask the other side whether it has had experience of finding new ways of maintaining the pleasure of sexual relations, but offers a possibility and invites the other side to share its views or experience. So its language style can be described as euphemism."}, "time_dimension": {"answer": "Future", "rationale": ""}, "sentence_type": "Affirmative declarative sentence", "pos": {"nouns": {"Sexual relations": 1, "Fun": 1, "Approach": 1}, "verbs": {"鈾?And I'm gonna be 鈾?: 1, "Hold on": 1, "Try": 1, "Find": 1}, "adverbs": {"For": 1, "Some": 1, "New": 1}, "numerals": {"15": 1}}}, {"content": "16. Sometimes, I hope that spouses will not spend money.", "word_count": 11, "label": "", "sentence_structure": "Subject鈥搗erb鈥搊bject", "subjective_objective": {"answer": "Subjective", "rationale": "Based on the judgement: This entry expresses the subjective attitude of the subject of the investigation, who has expectations and hopes for the conduct of the spouse."}, "sentence_sentiment": {"answer": "Negative", "rationale": "Because the expression \"I hope that the spouse will not spend money\" in the entry implies a negative attitude, i.e. dissatisfaction with and stigmatization of the spouse."}, "language_style": {"answer": "Tactful and warm", "rationale": "The language style of the entry is more euphemistic, using a form of expression of hope rather than direct criticism or stigmatization, which is more polite and respectful of each other ' s feelings. At the same time, it has also increased the sensitivity and interaction of dialogue through the use of the pronoun \"spouse\"."}, "time_dimension": {"answer": "Present", "rationale": "Keywords include \"sometimes\", \"hope,\" all of which represent a description and a sense of the current situation, so the time dimension of this entry is now."}, "sentence_type": "Negative declarative sentence", "pos": {"nouns": {"Spouse": 1, "Money": 1}, "verbs": {"Hope": 1, "鈾?And flowers 鈾?: 1}, "adverbs": {"Sometimes": 1}, "pronouns": {"I": 1}}}, {"content": "My spouse seems to lack the time and energy to entertain with me.", "word_count": 19, "label": "", "sentence_structure": "Subject鈥揷opula鈥損redicative", "subjective_objective": {"answer": "Subjective", "rationale": "Because \"lack of time and energy\" is a subjective assessment that reflects patients' perceptions and feelings about the behaviour of their spouses."}, "sentence_sentiment": {"answer": "Negative", "rationale": "Because \"lack of time and energy\" in the entries is a negative description."}, "language_style": {"answer": "Tactful and warm", "rationale": ""}, "time_dimension": {"answer": "Present", "rationale": ""}, "sentence_type": "Affirmative declarative sentence", "pos": {"nouns": {"Spouse": 1, "Time": 1, "- The energy": 1, "Recreation": 1}, "verbs": {"Missing": 1, "With me": 1}, "adverbs": {"Seems like": 1}, "numerals": {"17": 1}, "pronouns": {"Mine": 1}}}, {"content": "I would rather do anything else than spend one night alone.", "word_count": 20, "label": "", "sentence_structure": "Subject鈥搗erb鈥搊bject", "subjective_objective": {"answer": "Subjective", "rationale": "Because \"I'd rather do anything else\" and \"I don't want to spend one night alone\" are patients' subjective attitudes and thoughts."}, "sentence_sentiment": {"answer": "Negative", "rationale": "Because in the entry, \"I'd rather do anything else than spend the night alone.\" This expression, which expresses a fear of loneliness and avoidance, is a negative emotional attitude."}, "language_style": {"answer": "Tactful and warm", "rationale": "In a euphemism, the entry expresses the idea that the other party might prefer to spend time with others rather than directly questioning or challenging the other party ' s ability to be alone. This language style is more conducive to creating a friendly atmosphere of communication and enhancing the participation of interviewees."}, "time_dimension": {"answer": "Present", "rationale": "\"Better\" and \"not\" express the testee's attitude towards being alone for one night, which is for the current state, so the temporal dimension is now."}, "sentence_type": "Affirmative declarative sentence", "pos": {"verbs": {"Do it": 1, "I'd rather": 1, "Stay": 1}, "adjectives": {"Don't": 1, "Any": 1, "Single": 1, "One": 2}, "adverbs": {"Yeah": 1, "not": 1, "I'd rather not": 1, "Alone": 1, "One": 1}, "numerals": {"8": 1}, "pronouns": {"I": 1}}}, {"content": "19. I am very satisfied with the responsibilities of both spouses in marriage.", "word_count": 18, "label": "", "sentence_structure": "Subject鈥搗erb鈥揷omplement", "subjective_objective": {"answer": "Subjective", "rationale": "Because \"very satisfied\" is the patient's subjective attitude, reflecting the degree to which they are satisfied with their marital responsibilities."}, "sentence_sentiment": {"answer": "Positive", "rationale": "Because the \"very satisfied\" attitude in the entries is positive."}, "language_style": {"answer": "Calm and direct", "rationale": "The language style of the entry is calm and straightforward, and it clearly sets out a point of view and gives positive expression, without too much emotional qualification or euphemism. Such language styles are more objective and concise and apply where a clear message is needed."}, "time_dimension": {"answer": "Present", "rationale": ""}, "sentence_type": "Affirmative declarative sentence", "pos": {"nouns": {"Questionnaire": 1, "Entry": 1, "Responsibility": 1}, "verbs": {"Undertaking": 1, "Satisfied": 1}, "adjectives": {"Very": 1}, "numerals": {"19": 1}, "pronouns": {"I": 1, "Couple": 1}}}, {"content": "20. My spouse and I always agree on how to spend money.", "word_count": 15, "label": "", "sentence_structure": "Subject鈥揷opula鈥損redicative", "subjective_objective": {"answer": "Subjective", "rationale": "Since it is the subjective perception and perception that the agreement on spending is made between the spouses, which is based on their communication and consultation, the entry looks at the subjective perspective."}, "sentence_sentiment": {"answer": "Neutral", "rationale": "As this entry does not express positive or negative emotions."}, "language_style": {"answer": "Tactful and warm", "rationale": "This topic uses more euphemistic language to ask the respondents and their spouses whether they agree on spending money, rather than asking directly or rudely. Such a gentle and polite language would make it easier for respondents to respond positively and create a good atmosphere of communication."}, "time_dimension": {"answer": "Present", "rationale": "The entry describes a situation where \"me\" and a spouse are always the same in terms of spending money. No past or future acts or events are involved."}, "sentence_type": "Affirmative declarative sentence", "pos": {"nouns": {"I": 1, "Spouse": 1, "Money": 1, "Observations": 1}, "verbs": {"Flowers": 1, "Always": 1, "Unanimously": 1}, "adverbs": {"What": 1}, "classifiers": {"individual": 1}}}, {"content": "21. I am pleased with the division of responsibilities for the upbringing of children.", "word_count": 16, "label": "", "sentence_structure": "Subject鈥揷opula鈥損redicative", "subjective_objective": {"answer": "Subjective", "rationale": "Because \"satisfaction\" is the patient's subjective assessment and perception of the division of responsibilities."}, "sentence_sentiment": {"answer": "Positive", "rationale": "Because \"satisfaction\" in the entry is a positive emotional attitude."}, "language_style": {"answer": "Tactful and warm", "rationale": "The fact that the entry expresses satisfaction with the division of family responsibilities in a moderate and affirmative manner, without the use of language that is directly negative or critical, makes the issue more friendly and understandable. At the same time, it encouraged interviewees to share their thoughts and feelings, which contributed to a good climate of communication."}, "time_dimension": {"answer": "Present", "rationale": "The keywords are \"we are satisfied\" and \"division of responsibility for the upbringing of children\", which describe the state and behaviour of the present, and therefore judge the present."}, "sentence_type": "Affirmative declarative sentence", "pos": {"nouns": {"we": 1, "Division of responsibilities": 1, "Children": 1}, "verbs": {"very": 1, "Satisfied": 1, "Maintenance": 1, "Division of labour": 1}, "adverbs": {"very": 1}, "numerals": {"21": 1}, "pronouns": {"I": 1}}}, {"content": "22. Common faith contributes to the development of our relationship.", "word_count": 15, "label": "", "sentence_structure": "Subject鈥搗erb鈥揷omplement", "subjective_objective": {"answer": "Subjective", "rationale": "This reflects patients ' subjective views and convictions. \""}, "sentence_sentiment": {"answer": "Positive", "rationale": "Because in the entry, \"Community of Faith helps our relationship develop.\" This expression is positive and indicates that shared faith can foster the development of human relations."}, "language_style": {"answer": "Tactful and warm", "rationale": "The entry is more adept and interactive when it is spoken in a gentle and inviting manner, rather than by direct statements or orders. At the same time, the term \"helping\" does not express in an overly positive or absolute way the role of common beliefs in the development of relations, but rather leaves some room for openness and possibility, which reflects a euphemism of language."}, "time_dimension": {"answer": "Present", "rationale": "The entry describes the current situation or view, does not have a clear time frame and can therefore be judged as \"now\"."}, "sentence_type": "Affirmative declarative sentence", "pos": {"nouns": {"Faith": 1, "we": 1, "Relations": 1, "Development": 1}, "verbs": {"have": 1, "Helping": 1}, "adjectives": {"Common": 1}, "numerals": {"22": 1}}}, {"content": "23. If both husband and wife have a job, the husband should have as much domestic work as his wife.", "word_count": 27, "label": "", "sentence_structure": "Subject鈥損redicate", "subjective_objective": {"answer": "Subjective", "rationale": "This is because it raises a hypothetical situation and examines attitudes and perceptions of the situation and is therefore subjective."}, "sentence_sentiment": {"answer": "Positive", "rationale": "Since the article is intended to promote equality between men and women, the emphasis on shared responsibilities and responsibilities of both spouses is positive and emotional."}, "language_style": {"answer": "Calm and direct", "rationale": "The entry expresses the view of the division of household tasks in a descriptive manner, with no excessive emotional or rhetorical colours, and is more direct and objective."}, "time_dimension": {"answer": "Present", "rationale": "In conclusion, I think the time dimension of this questionnaire entry is \"now\" and \"for the future.\""}, "sentence_type": "Affirmative declarative sentence", "pos": {"nouns": {"Couples": 1, "Both sides": 1, "Work": 1, "Husband": 1, "Wife": 1, "Domestic work": 1}, "verbs": {"have": 1, "Undertaking": 1, "I think so": 1, "and": 1}, "adverbs": {"The same": 1}, "numerals": {"23": 1}}}, {"content": "On occasion, I am concerned about the unhappiness and isolation of spouses.", "word_count": 18, "label": "", "sentence_structure": "Subject鈥揷opula鈥損redicative", "subjective_objective": {"answer": "Subjective", "rationale": "Because the descriptions of \"to my spouse\" and \"silent\" are patients' subjective feelings and judgment of their own situation."}, "sentence_sentiment": {"answer": "Negative", "rationale": "Because the entry \"disturbed and isolated\" expresses a negative emotional attitude."}, "language_style": {"answer": "Calm and direct", "rationale": "Calm down straight."}, "time_dimension": {"answer": "Past", "rationale": "The entry describes the concerns that have arisen in the testee and concerns the experiences and feelings of the past. \""}, "sentence_type": "Negative declarative sentence", "pos": {"nouns": {"Time": 1, "I": 1, "Spouse": 1, "I'm alone": 1, "I'm worried": 1}, "verbs": {"It seems": 1, "I feel": 1}, "adjectives": {"It's not good": 1}, "numerals": {"24": 1}}}, {"content": "25. I am concerned that spouses may not be interested in sex.", "word_count": 17, "label": "", "sentence_structure": "Subject鈥搗erb鈥搊bject", "subjective_objective": {"answer": "Subjective", "rationale": "Because the fear that a spouse may not be interested in sex is a personal subjectivity and concern. This relates to the feelings, perceptions and thoughts inside the patient, rather than the results of objective observations or measurements."}, "sentence_sentiment": {"answer": "Negative", "rationale": "Because the concerns in the entries express a negative emotional attitude."}, "language_style": {"answer": "Tactful and warm", "rationale": "The language of the entry is more euphemistic and uses \"fear.\" This term expresses the inner feelings of the master and raises questions in a more polite and understanding manner, avoiding direct accusations or attacks. Such a formulation would be more acceptable and willing to answer."}, "time_dimension": {"answer": "Present", "rationale": ""}, "sentence_type": "Negative declarative sentence", "pos": {"nouns": {"Questionnaire": 1, "Entry": 1, "Spouse": 1, "Sex": 1, "In terms of": 1, "Interest": 1}, "verbs": {"I'm worried": 1, "Maybe": 1, "Not interested in": 1}, "adjectives": {"Sex": 1, "Not interested": 1}, "numerals": {"25": 1}, "pronouns": {"I": 1}}}, {"content": "26. It is difficult for us to decide on economic arrangements.", "word_count": 14, "label": "", "sentence_structure": "Subject鈥搗erb鈥搊bject", "subjective_objective": {"answer": "Subjective", "rationale": "This issue relates to individual decision-making processes and perceptions of economic arrangements and is therefore more subjective. So the answer is \"subjective.\""}, "sentence_sentiment": {"answer": "Negative", "rationale": "Because the act of \"hard to make a decision\" in the entry is negative."}, "language_style": {"answer": "Calm and direct", "rationale": "The language style of the entry is calm and straightforward. It is a simple and clear statement of the fact that economic arrangements are difficult to make and that too many emotional modifications or euphemisms are not used. This direct language style allows for rapid understanding of the subject and avoids misunderstanding."}, "time_dimension": {"answer": "Present", "rationale": "The entry describes the current situation and difficulties and does not address past or future time dimensions, so it is now judged."}, "sentence_type": "Negative declarative sentence", "pos": {"nouns": {"we": 1, "Economic arrangements": 1, "Decision": 1}, "verbs": {"Do it": 1, "Out": 1, "have": 1}, "adjectives": {"Hard": 1}, "numerals": {"26": 1}, "pronouns": {"Here": 1}}}, {"content": "27. The time we spend on relatives and friends is appropriate.", "word_count": 13, "label": "", "sentence_structure": "Subject鈥揷opula鈥損redicative", "subjective_objective": {"answer": "Subjective", "rationale": "Because \"we spend the right amount of time for our family and friends\" is a subjective assessment of the patients, reflecting their satisfaction with their commitment to the relationship."}, "sentence_sentiment": {"answer": "Positive", "rationale": "Because the \"time is right\" behavior and attitude are positive."}, "language_style": {"answer": "Tactful and warm", "rationale": "\"The language of the entry is more euphemistic and warm. It uses a positive word.\" Rather than directly indicating whether there is a problem or need for improvement, it is a positive way to guide respondents to reflect on the way they spend time dealing with their relatives and friends. This expression is more compassionate and encouraging."}, "time_dimension": {"answer": "Present", "rationale": "Now the entry describes the current state or habit of the testee, which is that \"the time we spend for our relatives and friends is appropriate\" and therefore the judgment is now."}, "sentence_type": "Affirmative declarative sentence", "pos": {"nouns": {"we": 1, "Friends": 1, "Time": 1}, "verbs": {"Cost": 1, "Appropriate": 1}, "adjectives": {"very": 1}, "numerals": {"27": 1}}}, {"content": "28. I care that there is too little interest or hobby in spouses.", "word_count": 14, "label": "", "sentence_structure": "Subject鈥揷opula鈥損redicative", "subjective_objective": {"answer": "Subjective", "rationale": "Because the title says \"I care,\" it's a subjective feeling or attitude. It looks at the patient ' s own feelings and perceptions and is therefore a subjective one."}, "sentence_sentiment": {"answer": "Negative", "rationale": "Because it's \"heavy\" in the entries, it's a negative emotional expression."}, "language_style": {"answer": "Calm and direct", "rationale": "Basis of judgement: - The entry expresses its views directly and does not use excessive rhetoric or emotional colours. - The sentence is simple and clear-minded and does not deliberately create an emotional atmosphere. - The term \"careful\" has been used to highlight issues of interest to, or preference for, spouses and to demonstrate a more explicit attitude and position."}, "time_dimension": {"answer": "Present", "rationale": "The word \"very\" indicates a degree of intensity, while the word \"care\" uses the general current, which means that the act has not only happened in the past, but may still continue. Thus, the entry describes the current attitude and feelings of the testee with regard to his/her spouse ' s interests or preferences, and the temporal dimension is now judged."}, "sentence_type": "Negative declarative sentence", "pos": {"nouns": {"Spouse": 1, "Interest": 1, "Hobbies": 1, "Yes": 1}, "verbs": {"I care": 1, "Too little": 1}, "numerals": {"28": 1}, "pronouns": {"I": 1}}}, {"content": "29. My wife shall not go out to work unless it is economically necessary.", "word_count": 17, "label": "", "sentence_structure": "Subject鈥搗erb鈥搊bject", "subjective_objective": {"answer": "Objective", "rationale": "Since `exit or not' is an objective fact and circumstance, and `economic necessity' is an objective criterion, the sentence is primarily concerned with the subjective view of whether the wife should be out of work. \""}, "sentence_sentiment": {"answer": "Negative", "rationale": "This is because it expresses the idea of inequality towards women, who should be considered caregivers in the family. This reflects a sexist attitude and perception and is negative."}, "language_style": {"answer": "Calm and direct", "rationale": "The language style of the entry is relatively straightforward, not using too many euphemisms or implicit expressions, but rather communicating through the direct presentation of personal views. This style is usually more concise and clear, but may cause some discomfort or resentment."}, "time_dimension": {"answer": "Past", "rationale": ""}, "sentence_type": "Negative declarative sentence", "pos": {"nouns": {"Economy": 1, "Yes": 1, "My wife": 1, "Work": 1}, "verbs": {"Reactions": 1, "Out": 1}, "adverbs": {"Unless": 1, "Go, go, go": 1}, "numerals": {"29": 1}, "pronouns": {"I": 1}}}, {"content": "My spouse has problems with smoking and/or drinking.", "word_count": 12, "label": "", "sentence_structure": "Subject鈥搗erb鈥搊bject", "subjective_objective": {"answer": "Objective", "rationale": "The question of whether the spouse smokes and/or drinks is an objective fact observed by the patient."}, "sentence_sentiment": {"answer": "Negative", "rationale": "Because \"smoking and/or drinking is a problem\" is a negative description."}, "language_style": {"answer": "Tactful and warm", "rationale": "The language style of the entry is euphemistic, because it is more respectful and polite to use the term \"my spouse\" rather than to speak directly to each other (such as \"he/her\". At the same time, the expression \"smoking and/or drinking is problematic\" is more objective, neutral and does not imply too much emotion and blame."}, "time_dimension": {"answer": "Present", "rationale": "The reference in the entry to \"smoking\" and \"drinking is a problem\" indicates the current situation and therefore the judgement is now."}, "sentence_type": "Affirmative declarative sentence", "pos": {"nouns": {"I": 1, "Spouse": 1, "Smoke": 1, "Wine": 1, "Problem": 1}, "verbs": {"Smoke": 1, "Drink": 1, "Done": 1}, "adjectives": {"and/or": 1}, "numerals": {"30": 1}}}, {"content": "31. I have little pressure to engage in social activities with my spouse.", "word_count": 16, "label": "", "sentence_structure": "Subject鈥搗erb鈥揷omplement", "subjective_objective": {"answer": "Subjective", "rationale": "Because feeling pressure is a subjective feeling, different people may react differently to the same thing. The answer to this questionnaire is therefore subjective."}, "sentence_sentiment": {"answer": "Positive", "rationale": "Because the entry describes positive behaviour and feelings."}, "language_style": {"answer": "Tactful and warm", "rationale": "The language style of the entry is more euphemistic, using softer terminology and presentation, such as \"very little pressure\", and not too direct or strong. At the same time, the sentence is carried out in a question-and-answer manner and is more interactive and personal."}, "time_dimension": {"answer": "Present", "rationale": ""}, "sentence_type": "Affirmative declarative sentence", "pos": {"nouns": {"Spouse": 1, "Social events": 1, "Pressure": 1}, "verbs": {"Participation": 1, "I feel": 1}, "adjectives": {"Very few": 1}, "numerals": {"31": 1}, "pronouns": {"I": 1}}}, {"content": "I am not satisfied with the communication between husband and wife, and my spouse does not understand me.", "word_count": 18, "label": "", "sentence_structure": "Subject鈥搗erb鈥揷omplement", "subjective_objective": {"answer": "Subjective", "rationale": "Because \"unsatisfactory\" and \"not understanding\" are subjective feelings and evaluations of patients."}, "sentence_sentiment": {"answer": "Negative", "rationale": "This is because the entries express dissatisfaction with the communication between husband and wife and the negative feelings that the other party does not understand."}, "language_style": {"answer": "Tactful and warm", "rationale": "The entry expresses the displeasure of the speaker with regard to communication between husband and wife in a first person and points out that the spouse does not understand himself or herself. However, the overall language style is more moderate and euphemistic, rather than directly accusing or attacking the other, preferring to express one ' s feelings and needs, unlike the calm and direct language style."}, "time_dimension": {"answer": "Present", "rationale": "The words \"unsatisfactory\" and \"unsense\" in the entry represent the current situation and feelings, so it can be judged now. Moreover, the word \"me\" indicates that the entry is described from the perspective of the person being tested and supports this judgement."}, "sentence_type": "Negative declarative sentence", "pos": {"nouns": {"Couples": 1, "Communications": 1, "Spouse": 1, "Understood": 1}, "verbs": {"Dissatisfied": 1, "be": 1, "Understood": 1}, "adjectives": {"of": 2, "not": 1, "Understood": 1}, "numerals": {"1": 2}, "classifiers": {"individual": 1}, "pronouns": {"I": 1}}}, {"content": "I'm always happy about how and where our family is on vacation.", "word_count": 20, "label": "", "sentence_structure": "Subject鈥揷opula鈥損redicative", "subjective_objective": {"answer": "Subjective", "rationale": "Because I'm always satisfied with a description of my subjective feelings."}, "sentence_sentiment": {"answer": "Positive", "rationale": "Because it expresses satisfaction with family holidays, it is a positive emotional colour."}, "language_style": {"answer": "Tactful and warm", "rationale": "The entry is more euphemistic and warm, using the first person's pronoun, \"I,\" to express personal views, softer and more friendly. In addition, the end of the sentence shows a level of calm and stable emotional state."}, "time_dimension": {"answer": "Present", "rationale": "The keyword \"always satisfied\" indicates a positive attitude and assessment of the current situation, and the term \"sent\" also indicates a state of continuity, so the temporal dimension is now judged."}, "sentence_type": "Affirmative declarative sentence", "pos": {"nouns": {"Home": 1, "Holiday": 1}, "verbs": {"I always thought": 1, "Satisfied": 1}, "adjectives": {"What": 1, "Where": 1}, "pronouns": {"I": 1}}}, {"content": "3. We have a complete understanding of each other.", "word_count": 11, "label": "", "sentence_structure": "Subject鈥揷opula鈥損redicative", "subjective_objective": {"answer": "Subjective", "rationale": "Because \"complete understanding\" is a subjective view or feeling, depending on the interaction and communication between individuals."}, "sentence_sentiment": {"answer": "Positive", "rationale": "Because the expression \"mutual understanding\" reflects good communication and trust between the two sides, it is a positive emotional attitude."}, "language_style": {"answer": "Tactful and warm", "rationale": "The entry uses the more positive and positive expression of \"full mutual understanding\" and does not have too many emotional or direct judgement terms to give people a feeling of calmer and friendly, and therefore can be judged as a euphemism of language style."}, "time_dimension": {"answer": "Present", "rationale": "The entry now expresses a state of affairs or experience in the couple's relationship between the interviewee and their spouse, and it points to the current reality. At the same time, the description of \"complete understanding\" also highlights the importance of this state of affairs, which shows that the couple has a high level of understanding and acceptance of each other, which is an ongoing and ongoing state of affairs."}, "sentence_type": "Affirmative declarative sentence", "pos": {"nouns": {"we": 1, "Couples": 1, "Understood": 1}, "verbs": {"STAGE": 1, "Total": 1, "To each other": 1, "be": 1}, "adverbs": {"Land": 1}, "numerals": {"3": 1}, "pronouns": {"we": 1}}}, {"content": "4. The spouses are in agreement in matters of child-rearing.", "word_count": 13, "label": "", "sentence_structure": "Subject鈥揷opula鈥損redicative", "subjective_objective": {"answer": "Subjective", "rationale": "The consensus on child control is a reflection of the individual subjective view and is examined from a subjective perspective."}, "sentence_sentiment": {"answer": "Neutral", "rationale": "Because the entry does not have any emotional color."}, "language_style": {"answer": "Tactful and warm", "rationale": "The language style of the entry is more euphemistic, as it does not directly ask the couple about the specific circumstances and conflicts in the discipline of their children, but rather in a more moderate way, making the respondents more comfortable to answer. At the same time, the phrase suggests that there may be disagreement between the spouses, but does not make it clear that this is more polite and respects the privacy of the respondents."}, "time_dimension": {"answer": "Present", "rationale": "In the sentence, \"in respect of\" means an act or a state, while \"consistent\" is a description of that act or state, so the entry relates to the current state and behaviour of the couple."}, "sentence_type": "Affirmative declarative sentence", "pos": {"nouns": {"Child control": 1, "Couples": 1, "Observations": 1}, "verbs": {"Unanimously": 1}, "numerals": {"4": 1}}}, {"content": "I am very pleased with the way in which we have taken decisions and resolved conflicts.", "word_count": 18, "label": "", "sentence_structure": "Subject鈥搗erb鈥搊bject", "subjective_objective": {"answer": "Subjective", "rationale": "Because satisfaction is a subjective emotional response, it is evaluated on the basis of individual values and expectations. The sentence looks at patients ' subjective feelings and satisfaction with decision-making and conflict resolution."}, "sentence_sentiment": {"answer": "Positive", "rationale": "Because the \"very satisfied\" assessment in the entry is positive and expresses a positive attitude towards decision-making and conflict resolution."}, "language_style": {"answer": "Tactful and warm", "rationale": "The entry uses a positive expression to ask each other about how they feel about decision-making and conflict resolution, and uses the adverb \"very\" to emphasize satisfaction. This way makes language style more friendly and inspiring."}, "time_dimension": {"answer": "Present", "rationale": "The entry describes the respondent ' s satisfaction with the current decision-making and conflict resolution methods, so the time dimension is now judged."}, "sentence_type": "Affirmative declarative sentence", "pos": {"nouns": {"Questionnaire": 1, "Entry": 1, "Modalities": 1}, "verbs": {"I'm very satisfied": 1, "Make a decision": 1, "Resolve": 1, "Conflict": 1}, "adjectives": {"Satisfied": 1}, "adverbs": {"Very": 1}, "numerals": {"5": 1}}}, {"content": "At times, my spouse does not depend on me, and not always has a cloud of the sky.", "word_count": 17, "label": "", "sentence_structure": "Subject鈥搗erb鈥揷omplement", "subjective_objective": {"answer": "Subjective", "rationale": "Because the description of \"sometimes\" and \"not always\" involves the patient's personal feelings and perceptions, it looks at subjective attitudes."}, "sentence_sentiment": {"answer": "Negative", "rationale": "Because both \"no reliance on me\" and \"not always \"no support\" describe the lack of support of the spouses, they have a negative emotional tone."}, "language_style": {"answer": "Calm and direct", "rationale": "The entry uses a direct language style, with no excessive rhetorical or emotional rendering, and presents the question in an objective, concise manner. This way allows the interviewee to understand and answer the question with precision and without personal emotions."}, "time_dimension": {"answer": "Past", "rationale": "It is therefore not possible to judge its temporal dimension."}, "sentence_type": "Affirmative declarative sentence", "pos": {"nouns": {"Spouse": 1, "person": 1, "Clouds": 1}, "verbs": {"No dependent": 1, "Not always": 1, "Man and woman": 1}, "adverbs": {"Sometimes": 1}, "classifiers": {"individual": 1}, "pronouns": {"I": 1}}}, {"content": "7. I am satisfied with the decision on how much money families should save.", "word_count": 18, "label": "", "sentence_structure": "Subject鈥揷opula鈥損redicative", "subjective_objective": {"answer": "Subjective", "rationale": "Because \"satisfaction\" is a subjective feeling and attitude."}, "sentence_sentiment": {"answer": "Positive", "rationale": "Since the purpose of the article is to express satisfaction, this indicates a positive attitude and emotion towards the decision of the author as to how much the family should save."}, "language_style": {"answer": "Calm and direct", "rationale": ""}, "time_dimension": {"answer": "Past", "rationale": "The entry describes the feelings of the testee regarding the decision-making of household savings at a certain point in the past (i.e., when the answer is given), the temporal dimension of which is the past."}, "sentence_type": "Affirmative declarative sentence", "pos": {"nouns": {"Family": 1, "Money": 1, "Decision": 1}, "verbs": {"I think so": 1, "Savings": 1, "I feel": 1, "Satisfied": 1}, "numerals": {"How many": 1}, "pronouns": {"I": 1}}}, {"content": "When a matter is discussed, I usually feel that the spouse understands me.", "word_count": 20, "label": "", "sentence_structure": "Subject鈥揷opula鈥損redicative", "subjective_objective": {"answer": "Subjective", "rationale": "Because the word \"fear\" expresses patients' subjective attitudes and feelings."}, "sentence_sentiment": {"answer": "Positive", "rationale": "Because the entry is expressed as a positive attitude or quality, i.e. a sense of the spouse ' s understanding in the discussion of a particular issue, it is a positive experience and emotional tendency."}, "language_style": {"answer": "Tactful and warm", "rationale": "The entry expresses concern and respect for the feelings of the interviewees in family relations in positive language, is gentle and friendly, is not directly evaluated or criticized and can therefore be judged as \" euphemismal \" ."}, "time_dimension": {"answer": "Present", "rationale": "The key words \"normally\" and \"influence\" make it possible to judge that the entry describes the current feelings and attitudes of the testee, and therefore the temporal dimension is now."}, "sentence_type": "Affirmative declarative sentence", "pos": {"nouns": {"Questionnaire": 1, "Entry": 1, "Problem": 2, "Spouse": 1}, "verbs": {"Discussion": 1, "I feel": 1, "It's understanding": 1, "of": 1}, "numerals": {"8": 1}, "pronouns": {"I": 1}}}, {"content": "My spouse sometimes makes some disparaging remarks.", "word_count": 16, "label": "", "sentence_structure": "Subject鈥搗erb鈥搊bject", "subjective_objective": {"answer": "Subjective", "rationale": "This is because of the patient ' s subjective perception and understanding of his/her spouse ' s views."}, "sentence_sentiment": {"answer": "Negative", "rationale": "Because the \"demeaning\" act in the entry is negative."}, "language_style": {"answer": "Tactful and warm", "rationale": "The language style of the entry is euphemism. It implies that problems are not always present by using the term \"sometimes\" and tries to express the problems that the other side may suffer in a moderate way, rather than directly accusing or attacking. Such expression can only trigger the resonance and willingness of the person under investigation to cooperate, and help to better gather valuable information."}, "time_dimension": {"answer": "Present", "rationale": "The word \"time\" in \"sometimes\" denotes conduct in the past at a time or in a period of time, while the word \"publication\" adopts the general current, meaning that the act not only has occurred in the past but may continue to exist. Thus, \"sometimes published\" is a description of the situation that is present."}, "sentence_type": "Negative declarative sentence", "pos": {"nouns": {"Spouse": 1, "Observations": 1}, "verbs": {"Publication": 1, "Demeaning": 1}, "adverbs": {"Sometimes": 1}, "pronouns": {"I": 1}}}, {"content": "10. It was easy and easy for me to talk about sex with a spouse.", "word_count": 20, "label": "", "sentence_structure": "Subject鈥揷opula鈥損redicative", "subjective_objective": {"answer": "Subjective", "rationale": "Because \"easy\" and \"easy\" are subjective descriptions of how patients feel about themselves."}, "sentence_sentiment": {"answer": "Positive", "rationale": "Because the \"talking about sex\" act is described in the sentence as \"easy and easy\" and has a positive emotional color."}, "language_style": {"answer": "Tactful and warm", "rationale": "The language style of the entry is a euphemism, because it does not directly ask the respondents whether they are willing or able to talk about sex with their spouses, but rather conveys a positive, open attitude by expressing \"it is easy and easy for me\". At the same time, this expression is more respectful of the privacy and personal feelings of the respondents."}, "time_dimension": {"answer": "Present", "rationale": "\"It is easy and easy for me\" indicates that it is the current feeling and experience of the speaker, so the time dimension of the article is now."}, "sentence_type": "Affirmative declarative sentence", "pos": {"nouns": {"Spouse": 1, "Sexual issues": 1, "I": 1}, "verbs": {"Talk": 1, "be": 2, "Easy": 2}, "adjectives": {"very": 1}, "adverbs": {"and": 1, "of": 2}, "numerals": {"10": 1}, "pronouns": {"This": 1}}}, {"content": "11. My spouse fully understands and shares every emotional change that has occurred to me.", "word_count": 27, "label": "", "sentence_structure": "Subject鈥揷opula鈥損redicative", "subjective_objective": {"answer": "Subjective", "rationale": "Because the spouses understand and feel the emotional state of the patient is reflected in their subjective perception."}, "sentence_sentiment": {"answer": "Positive", "rationale": "Because the entry describes the ability of the spouse to understand and share his or her emotions, it is a positive emotional attitude and behaviour."}, "language_style": {"answer": "Tactful and warm", "rationale": "The language style of the entry is more euphemistic and sweet, emphasizing personal feelings and subjective experiences rather than objective facts, by using the word \"my spouse\". At the same time, the description \"who understands and feels the same\" is more general and subjective, without giving specific criteria or a basis for judgement, and is more personal and emotional, and more able to guide respondents to share their real thoughts and feelings."}, "time_dimension": {"answer": "Present", "rationale": ""}, "sentence_type": "Affirmative declarative sentence", "pos": {"nouns": {"Spouse": 1, "Emotional change": 1, "Feel": 1}, "verbs": {"Understood": 1, "have": 2}, "adverbs": {"Total": 1}, "numerals": {"Every time": 1}, "pronouns": {"I": 1}}}, {"content": "12. In our marriage, the wife should be more obedient to the wishes of her husband.", "word_count": 19, "label": "", "sentence_structure": "Subject鈥搗erb鈥搊bject", "subjective_objective": {"answer": "Objective", "rationale": "Because this is a description of what is expected of a social role, it is an objective perspective."}, "sentence_sentiment": {"answer": "Negative", "rationale": "Because the entry implies that women should obey the will of men, it is a sexist and unfair attitude."}, "language_style": {"answer": "Calm and direct", "rationale": "The language style of the entry is more straightforward, and it is not expressed in a euphemistic fashion, which is relatively calm and objective. At the same time, the article is gender-discriminatory in its content, implying that women should obey men."}, "time_dimension": {"answer": "Past", "rationale": "The entry describes a past situation or phenomenon in which \"in our marriage\" and \"more complying with the wishes of the husband\" have happened or exist."}, "sentence_type": "Affirmative declarative sentence", "pos": {"nouns": {"Questionnaire": 1, "Entry": 1, "Marriage": 1, "Wife": 1, "Husband": 1, "Wish": 1}, "verbs": {"Reactions": 1, "More": 1, "Submissive": 1}, "adverbs": {"in": 1, "Ours": 1}, "classifiers": {"individual": 1}}}, {"content": "13. When we are with others, I sometimes feel uncomfortable about the behaviour of my spouse.", "word_count": 22, "label": "", "sentence_structure": "Subject鈥揷opula鈥損redicative", "subjective_objective": {"answer": "Subjective", "rationale": "Because \"uneasy\" is a subjective feeling, a personal reaction and emotional experience of the patient with the behaviour of the spouse."}, "sentence_sentiment": {"answer": "Negative", "rationale": "Because the mood of \"uneasy\" in the entry is negative."}, "language_style": {"answer": "Tactful and warm", "rationale": ""}, "time_dimension": {"answer": "Present", "rationale": "The entry describes the feelings and experiences of the person being tested in dealings with others, which are ongoing and present, and therefore judged to be the present."}, "sentence_type": "Affirmative declarative sentence", "pos": {"nouns": {"Questionnaire Entry": 1, "we": 1, "others": 1, "Together": 1, "Spouse": 1, "Behaviour": 1, "Unsettled": 1}, "verbs": {"Sometimes": 1, "I feel": 1}, "adverbs": {"When": 1, "and": 1, "Time": 1}, "numerals": {"13": 1}, "pronouns": {"I": 1}}}, {"content": "14. We all know that we owe debt and that it is not a problem.", "word_count": 19, "label": "", "sentence_structure": "Subject鈥搗erb鈥搊bject", "subjective_objective": {"answer": "Subjective", "rationale": "Because \"know\" and \"not a problem\" are subjective views and judgments of patients."}, "sentence_sentiment": {"answer": "Neutral", "rationale": "The entry does not involve any emotional or attitudinal tendencies."}, "language_style": {"answer": "Tactful and warm", "rationale": "The entry expresses the view in a less intrusive and encouraging way that we can recognize our debt and have confidence in solving the problem. This language style is more acceptable and resonant."}, "time_dimension": {"answer": "Present", "rationale": "The \"both\" and \"it's not a problem\" in the entry indicate that it is a perception and assessment of the current situation, and therefore the temporal dimension is now judged."}, "sentence_type": "Affirmative declarative sentence", "pos": {"nouns": {"Debt": 1}, "verbs": {"Yes": 1, "Own": 1, "Done": 1, "Problem": 1}, "adverbs": {"Both": 1, "we": 1}, "numerals": {"14": 1}}}, {"content": "15. My religious beliefs are an important part of the influence of our marriage.", "word_count": 20, "label": "", "sentence_structure": "Subject鈥揷opula鈥損redicative", "subjective_objective": {"answer": "Subjective", "rationale": "Because it is an individual religious belief and a subjective attitude of the patient."}, "sentence_sentiment": {"answer": "Negative", "rationale": "Because the expression \"religious belief is an important part of our marriage\" implies that people may face marriage problems because of their religious differences, which is a negative emotional tendency."}, "language_style": {"answer": "Tactful and warm", "rationale": "The entry is more personal and interactive when it is spoken in a gentle and inviting manner, rather than in direct statements or orders. At the same time, it does not use excessive rhetoric or emotional rendering, but rather raises questions in a relatively objective way that makes it easier for the respondents to answer."}, "time_dimension": {"answer": "Present", "rationale": "The entry now asks about the current status and beliefs of the person tested, i.e. whether religious beliefs influence their marriage."}, "sentence_type": "Affirmative declarative sentence", "pos": {"nouns": {"Faith": 1, "Religion": 1, "Marriage": 1, "Part": 1}, "verbs": {"be": 1, "Impact": 1}, "adjectives": {"Important": 1}, "numerals": {"15": 1}, "pronouns": {"I": 1}}}, {"content": "16. Sometimes I fear that spouses may have the idea of seeking extramarital sexual relations.", "word_count": 19, "label": "", "sentence_structure": "Subject鈥搗erb鈥搊bject", "subjective_objective": {"answer": "Subjective", "rationale": "Because the fear is the patient's subjective attitude."}, "sentence_sentiment": {"answer": "Negative", "rationale": "Because the mood of \"fear\" in the entry and the assumption that \"the spouses will have the idea of seeking extramarital sexual relations\" have a negative emotional effect."}, "language_style": {"answer": "Calm and direct", "rationale": "The entry is more calm and straightforward, asking if the other spouse has any concerns about possible extramarital sex, without too much embellishment or emotional rendering. This is more objective and clear, and helps to obtain accurate information.\""}, "time_dimension": {"answer": "Present", "rationale": "The entry \"sometimes\" indicates occasional behaviour or thought, while \"fear\" uses the usual current state of affairs, indicating that the fear is a current feeling. The entry therefore describes the person's current emotional and psychological state."}, "sentence_type": "Negative declarative sentence", "pos": {"nouns": {"Time": 1, "Spouse": 1, "Thoughts": 1}, "verbs": {"I'm worried": 1, "have": 1, "Seek": 1, "Relations": 1}, "adverbs": {"Sometimes": 1}, "numerals": {"16": 1}, "pronouns": {"I": 1}}}, {"content": "17. I believe that the spouse is too close to his/her home or is disproportionately affected by it.", "word_count": 22, "label": "", "sentence_structure": "Subject鈥搗erb鈥搊bject", "subjective_objective": {"answer": "Subjective", "rationale": "Because \"conception\" expresses patients' subjective view of the relationship between the spouses and their families."}, "sentence_sentiment": {"answer": "Negative", "rationale": "Because the expression \"too close or too affected\" in the entry is negative."}, "language_style": {"answer": "Tactful and warm", "rationale": ""}, "time_dimension": {"answer": "Present", "rationale": ""}, "sentence_type": "Affirmative declarative sentence", "pos": {"nouns": {"Spouse": 1, "he": 1, "she": 1, "Home": 1, "Impact": 1, "Too big": 1}, "verbs": {"I think": 1, "Too much": 2, "Close": 1, "To hell with it": 1}, "adverbs": {"and": 1}, "numerals": {"17": 1}, "pronouns": {"he": 1, "she": 1}}}, {"content": "18. Children appear to be a major source of conflict in our marriage.", "word_count": 19, "label": "", "sentence_structure": "Subject鈥揷opula鈥損redicative", "subjective_objective": {"answer": "Subjective", "rationale": "Because \"primary sources\" are subjective judgements, they need to be assessed on the basis of the individual experience and perceptions of the respondent. While children may have contradictory factors, the specific extent and impact factors require self-assessment and reflection by the respondent."}, "sentence_sentiment": {"answer": "Negative", "rationale": "Because the \"continuation\" and \"main source\" in the entry are all negative emotional."}, "language_style": {"answer": "Calm and direct", "rationale": "The entry raises the question in a direct and objective manner, without too much emotional rendering or rhetorical techniques, and with a more calm and straightforward language style."}, "time_dimension": {"answer": "Present", "rationale": "The entry now describes the current marital status of the person tested, i.e., the child appears to be a source of problems in marriage. While it does not explicitly refer to the past or the future, it is about the current situation and feelings and is therefore the current state of affairs."}, "sentence_type": "Affirmative declarative sentence", "pos": {"nouns": {"Children": 1, "Marriage": 1, "Paradoxious": 1, "One": 1, "Main sources": 1}, "verbs": {"It seems so": 1}, "adjectives": {"we": 1}, "numerals": {"18": 1}, "pronouns": {"EMPTY": 1, "Here": 1}}}, {"content": "19. We agree on the number of children needed.", "word_count": 14, "label": "", "sentence_structure": "Subject鈥搗erb鈥搊bject", "subjective_objective": {"answer": "Subjective", "rationale": "This is a subjective examination of the patient ' s personal perception and attitude towards the number of children."}, "sentence_sentiment": {"answer": "Neutral", "rationale": "The entry is merely an inquiry into whether the respondents agree on the number of children required, and does not contain any emotional or value judgement."}, "language_style": {"answer": "Tactful and warm", "rationale": "The language style of the entry is more euphemistic, gentle, and the questioning is conducted in a polite and respectful manner, without directly expressing one ' s views or position, and is more in line with the psychological questionnaire."}, "time_dimension": {"answer": "Present", "rationale": "The entry indicates the current situation or state of affairs, i.e. whether the person interviewed and his/her group have agreed on the number of children. It does not address the time dimension of the past or the future."}, "sentence_type": "Affirmative declarative sentence", "pos": {"nouns": {"Children": 1, "Number": 1, "Observations": 1}, "verbs": {"to": 1, "Unanimously": 1}, "numerals": {"19": 1}}}, {"content": "20. We spend our money in a regular manner, according to our economic strength.", "word_count": 16, "label": "", "sentence_structure": "Subject鈥搗erb鈥揷omplement", "subjective_objective": {"answer": "Subjective", "rationale": "According to the sentence that was entered, \"We pay regularly according to our economic power\" is the subjective view and description of the patient's own consumption behaviour. The purpose of the questionnaire is therefore based on \"subjective\"."}, "sentence_sentiment": {"answer": "Positive", "rationale": "Because the purpose of the article is positive, the regular spending of money in accordance with economic strength is a rational and responsible consumption."}, "language_style": {"answer": "Calm and direct", "rationale": "The entry is written in a calm and straightforward manner, asking the interviewee whether he or she spends according to his or her economic power."}, "time_dimension": {"answer": "Present", "rationale": "The keyword \"sequenced spending\" indicates that it is a customary act, and that it is done according to their economic power, so the time dimension of this entry should be now."}, "sentence_type": "Affirmative declarative sentence", "pos": {"nouns": {"we": 1, "Economic power": 1, "Pattern": 1, "Money": 1}, "verbs": {"Press": 1, "Flowers": 1}, "adverbs": {"There's a regular pattern": 1}, "numerals": {"20": 1}}}, {"content": "21. I am not satisfied with our economic status and the way in which economic affairs are determined.", "word_count": 21, "label": "", "sentence_structure": "Subject鈥搗erb鈥揷omplement", "subjective_objective": {"answer": "Subjective", "rationale": "Because the terms \"unsatisfactory\" and \"methodical methods of economic decision-making\" are subjectively perceived and evaluated by patients, the entry on the questionnaire is viewed from the subjective point of view of the patients."}, "sentence_sentiment": {"answer": "Negative", "rationale": "Because the words \"unsatisfied\" and \"unsatisfaction\" in the entries all have a negative emotional color."}, "language_style": {"answer": "Calm and direct", "rationale": "The entry expresses the respondent ' s dissatisfaction with the current economic status and decision-making methods in an objective and direct manner, with a more calm and straightforward language style."}, "time_dimension": {"answer": "Past", "rationale": "\"Unsatisfactory\" indicates that the testee has a negative view of a certain state of the past, and that \"methods of economic decision\" suggests that such dissatisfaction may have been caused by a previous decision or situation. Thus, the entry is a judgement of past behaviour and mentality."}, "sentence_type": "Negative declarative sentence", "pos": {"nouns": {"we": 1, "Economic status": 1, "Economic affairs": 1}, "verbs": {"Unsatisfactory": 1, "Decision": 1}, "adverbs": {"not": 1, "Land": 1}, "numerals": {"21": 1}, "pronouns": {"I": 1}}}, {"content": "22. I am very pleased with our amateur activities and the time spent together with our spouses.", "word_count": 22, "label": "", "sentence_structure": "Subject鈥搗erb鈥揷omplement", "subjective_objective": {"answer": "Subjective", "rationale": "Because satisfaction is an expression of subjective emotion."}, "sentence_sentiment": {"answer": "Positive", "rationale": "Because this entry expresses satisfaction with amateur activity and the time of the couple, it is a positive emotional trend."}, "language_style": {"answer": "Tactful and warm", "rationale": ""}, "time_dimension": {"answer": "Present", "rationale": ""}, "sentence_type": "Affirmative declarative sentence", "pos": {"nouns": {"Amateur activities": 1, "Time": 1, "we": 1}, "verbs": {"Very": 1, "Satisfied": 1, "Pass": 1}, "adjectives": {"of": 2, "Together": 1}, "adverbs": {"Very": 1}, "numerals": {"22": 1}, "classifiers": {"individual": 1}, "pronouns": {"I": 1, "we": 1}}}, {"content": "23. Sometimes I do not dare to look for what my spouse wants.", "word_count": 15, "label": "", "sentence_structure": "Subject鈥搗erb鈥搊bject", "subjective_objective": {"answer": "Subjective", "rationale": "Because \"never dared\" is a subjective feeling and attitude, which reflects the patient鈥檚 fear or anxiety about seeking help."}, "sentence_sentiment": {"answer": "Negative", "rationale": "Because the \"never dared to find what my spouse wanted\" behavior is negative."}, "language_style": {"answer": "Calm and direct", "rationale": "The language style of the entry is more calm and straightforward, and there is no excessive amount of rhetoric or emotional rendering, which is the subject of questioning whether the other party is confused or insecure in a given situation. This expression is more in keeping with the linguistic characteristics of the psychological questionnaire, which enables respondents to express their thoughts and feelings more honestly and objectively."}, "time_dimension": {"answer": "Present", "rationale": "\"Sometimes\" means that the act is not always being performed, but is of a certain frequency; the verbs \"never\" and \"spouse\" indicate that the act is taking place in the present situation. Thus, the entry is mainly concerned with the present behaviour and attitude of the person being tested."}, "sentence_type": "Negative declarative sentence", "pos": {"nouns": {"Time": 1, "I": 1, "Spouse": 1, "Something": 1}, "verbs": {"I dare you": 1, "Find": 1, "Yes": 2, "Okay": 1}, "adjectives": {"not": 2}, "adverbs": {"Sometimes": 1}, "numerals": {"23": 1}}}, {"content": "24. Even when the wife works outside the home, she should be responsible for managing the household.", "word_count": 20, "label": "", "sentence_structure": "Subject鈥搗erb鈥搊bject", "subjective_objective": {"answer": "Subjective", "rationale": "Because it expresses a view and attitude towards family responsibilities, it is the subjective view of the interviewees."}, "sentence_sentiment": {"answer": "Negative", "rationale": "The phrase \"shall be responsible for managing the household\" in the entry is clearly patriarchal and sexist and unfair to women."}, "language_style": {"answer": "Calm and direct", "rationale": "The language style of the entry is more straightforward, and it does not use excessive rhetorical or emotional colours, but rather presents the idea in a simple language that men should assume family responsibilities."}, "time_dimension": {"answer": "Present", "rationale": ""}, "sentence_type": "Affirmative declarative sentence", "pos": {"nouns": {"Wife": 1, "Domestic work": 1}, "verbs": {"Work": 1, "I think so": 1, "Burden": 1, "Management": 1}, "adverbs": {"Even if": 1}, "numerals": {"24": 1}, "pronouns": {"Wife": 1}}}, {"content": "25. The spouses disagree in their religious activities.", "word_count": 20, "label": "", "sentence_structure": "Subject鈥搗erb鈥揷omplement", "subjective_objective": {"answer": "Subjective", "rationale": "The basis for the judgement: \"The difference between the spouses in their religious activities\" describes the different attitudes and perceptions of the patient and the partner regarding religious beliefs, which are subjective."}, "sentence_sentiment": {"answer": "Negative", "rationale": "Since \"dissidents\" usually indicate conflict and conflict, this may have a negative impact in the context of a couple's relationship."}, "language_style": {"answer": "Calm and direct", "rationale": "The entry presents the survey in a straightforward and concise manner, without adding too many rhetorical or emotional features. At the same time, as the issue involves sensitive or private topics, this calm and straightforward expression helps to avoid unnecessary misunderstandings and controversy and allows respondents to answer questions more honestly and objectively."}, "time_dimension": {"answer": "Present", "rationale": ""}, "sentence_type": "Affirmative declarative sentence", "pos": {"nouns": {"Couple": 1, "Religious beliefs": 1, "Activities": 1, "Observations": 1}, "verbs": {"in": 1, "Related": 1, "No, no, no": 1}, "numerals": {"25": 1}}}, {"content": "I am unhappy with my relatives or those of my spouse.", "word_count": 19, "label": "", "sentence_structure": "Subject鈥揷opula鈥損redicative", "subjective_objective": {"answer": "Subjective", "rationale": "Because it is a subjective feeling, it is the emotional reaction and psychological state of the patient that is examined."}, "sentence_sentiment": {"answer": "Negative", "rationale": "Because the \"unhappy\" attitude in the entry is negative."}, "language_style": {"answer": "Calm and direct", "rationale": "The language style of the entry is calm and straightforward, as it is a simple and straightforward expression of the question of whether the person interviewed is uncomfortable with his relatives, and does not use any euphemism, warm language or emotional publicity. At the same time, the language temperature and objectivity do not show too much emotional colour."}, "time_dimension": {"answer": "Past", "rationale": ""}, "sentence_type": "Negative declarative sentence", "pos": {"nouns": {"Relatives": 1, "Partner": 1, "Family": 1}, "verbs": {"in": 1, "I feel": 1, "It's not good": 1}, "adjectives": {"Mine": 1, "Spouse": 1}, "numerals": {"26": 1}, "pronouns": {"I": 1}}}, {"content": "When I had difficulties, I always told my spouse.", "word_count": 14, "label": "", "sentence_structure": "Subject鈥搗erb鈥搊bject", "subjective_objective": {"answer": "Subjective", "rationale": "It is a subjective decision-making and action, and reflects the patient鈥檚 attitude and behaviour in the face of difficulties."}, "sentence_sentiment": {"answer": "Negative", "rationale": "Because \"when you're having trouble\" and \"when you're always telling your spouse\" these acts and attitudes have a negative emotional effect."}, "language_style": {"answer": "Tactful and warm", "rationale": "The entry is in a gentle manner asking if the other spouse will be told when the difficulty is encountered that this is more friendly and acceptable and helps to create a good atmosphere of communication."}, "time_dimension": {"answer": "Present", "rationale": "\"Now in the entry, \"when\" means \"when\" is an act occurring in a particular situation, \" always telling the spouse\" is the act of the present, customary, and therefore is judged to be \"now\"."}, "sentence_type": "Affirmative declarative sentence", "pos": {"nouns": {"Difficulties": 1, "Spouse": 1}, "verbs": {"I met him": 1, "Tell": 1}, "adverbs": {"Always": 1}, "pronouns": {"I": 1}}}, {"content": "28. It makes me uncomfortable that my spouse has given more attention to our children than to our marriage.", "word_count": 24, "label": "", "sentence_structure": "Subject鈥揷opula鈥損redicative", "subjective_objective": {"answer": "Subjective", "rationale": "This is a subjective attitude of the patient, since the sentence contains the patient ' s feelings and perceptions of the spouse ' s concerns."}, "sentence_sentiment": {"answer": "Negative", "rationale": "Because the \"I'm not feeling well\" act is negative."}, "language_style": {"answer": "Calm and direct", "rationale": "This entry is a direct expression of the core of the problem - the spouse is uncomfortable with the questioner because of the excessive attention given to the child, and does not use too many rhetorical techniques or emotional rendering."}, "time_dimension": {"answer": "Present", "rationale": "The entry on the questionnaire relates to the feelings and mindset of the person tested and therefore is judged as \"now\"."}, "sentence_type": "Negative declarative sentence", "pos": {"nouns": {"Spouse": 1, "Children": 1, "Marriage": 1, "Attention": 1, "I'm not feeling well": 1}, "verbs": {"鈾?And I'm gonna be 鈾?: 1}, "adverbs": {"More than": 1}, "numerals": {"28": 1}, "pronouns": {"we": 1}}}, {"content": "29. I think our holidays and trips have been very well.", "word_count": 15, "label": "", "sentence_structure": "Subject鈥揷opula鈥損redicative", "subjective_objective": {"answer": "Subjective", "rationale": "As it is perceived as a subjective feeling, the patient ' s satisfaction with the holidays and travel is examined from the patient ' s subjective point of view."}, "sentence_sentiment": {"answer": "Positive", "rationale": "Because the expression \"I think the holidays and the trips are going well\" is positive."}, "language_style": {"answer": "Tactful and warm", "rationale": "The entry is a first-person expression of personal feelings, is gentle and subjective and does not use strong adjectives or adverbs to qualify and emphasize, and reflects a soft and warm language style."}, "time_dimension": {"answer": "Present", "rationale": "The entry describes the subjective assessment of the present perception of the testees, that is, their review of their past experience (in this case, \"leave and travel\") and draws positive conclusions. Thus, the time dimension is now judged."}, "sentence_type": "Affirmative declarative sentence", "pos": {"nouns": {"we": 1, "Holiday": 1, "Tourism": 1}, "verbs": {"I think": 1, "Pass": 1}, "adjectives": {"Okay": 1}, "numerals": {"29": 1}}}, {"content": "Our husband is the head of the family.", "word_count": 10, "label": "", "sentence_structure": "Subject鈥揷opula鈥損redicative", "subjective_objective": {"answer": "Subjective", "rationale": "Because \"household\" is a subjective evaluation or role-positioning, not an objective reality. This view is derived from the subjective perception and perception of family members and may differ from one culture to another. Therefore, \"We think that our husband is the head of the family\" is a subjective view and attitude."}, "sentence_sentiment": {"answer": "Neutral", "rationale": "Because the entry does not have any emotional color."}, "language_style": {"answer": "Calm and direct", "rationale": "The entry uses a plain and simple language style to describe the problem, which does not add too many rhetorical or emotional colours, but rather directly presents a view or fact."}, "time_dimension": {"answer": "Present", "rationale": ""}, "sentence_type": "Affirmative declarative sentence", "pos": {"nouns": {"Home": 1, "Husband": 1, "Family": 1, "Lord": 1}, "verbs": {"be": 1}, "numerals": {"30": 1}}}, {"content": "31. For me, our sexual relations are satisfactory and perfect.", "word_count": 17, "label": "", "sentence_structure": "Subject鈥揷opula鈥損redicative", "subjective_objective": {"answer": "Subjective", "rationale": "Because \"satisfactory\" and \"perfect\" are subjective feelings and evaluations of patients."}, "sentence_sentiment": {"answer": "Neutral", "rationale": "Because the entry does not have a clear emotional colour and behavioural orientation."}, "language_style": {"answer": "Tactful and warm", "rationale": "The entry is more euphemistic, using the more subjective term \"satisfaction and perfection\" to describe the other side's feelings of sexual relations, rather than directly evaluating or criticizing them. This expression is more moderate and easy to accept and understand."}, "time_dimension": {"answer": "Present", "rationale": "The entry is an expression of the person currently tested on his or her sexual relationship, and the keyword \"to me\", \"satisfaction and perfection\" is all emphasizing the state of the moment. So the time dimension of the article is now."}, "sentence_type": "Affirmative declarative sentence", "pos": {"nouns": {"I": 1, "Sexual relations": 1}, "verbs": {"be": 1, "Satisfied": 1, "Perfect": 1}, "adjectives": {"Satisfied": 1, "Perfect": 1}, "numerals": {"31": 1}}}, {"content": "32. Sometimes my spouse is too stubborn.", "word_count": 9, "label": "", "sentence_structure": "Subject鈥揷opula鈥損redicative", "subjective_objective": {"answer": "Subjective", "rationale": "Because of its persistence, which is a subjective evaluation term, it shows the attitude and perception of the respondents to the questionnaire towards their spouses."}, "sentence_sentiment": {"answer": "Negative", "rationale": "Because \"obstinate\" is a negative adjective in the entry."}, "language_style": {"answer": "Calm and direct", "rationale": "The entry is more directly linguistically, is not subject to too much rhetoric or emotional overture, and is a concise expression of the subject matter. Moreover, the entry uses neutral vocabulary and speech structures, and does not show any apparent emotional or attitudinal orientation. It is therefore judged that the language style of the entry is \"cool and direct\"."}, "time_dimension": {"answer": "Present", "rationale": "The entry is a description of the person's current behaviour or state, and the keyword is \"sometimes\", implying that this situation does not always exist, but may happen occasionally. It is therefore judged to be in the present category."}, "sentence_type": "Negative declarative sentence", "pos": {"nouns": {"Time": 1, "Spouse": 1, "Obstinacy": 1}, "verbs": {"be": 1}, "adjectives": {"Too much": 1}, "numerals": {"32": 1}}}, {"content": "33. Our marriage has been very successful.", "word_count": 11, "label": "", "sentence_structure": "Subject鈥揷opula鈥損redicative", "subjective_objective": {"answer": "Subjective", "rationale": "The question of whether a successful marriage is considered a subjective view and attitude of the patient."}, "sentence_sentiment": {"answer": "Positive", "rationale": "Because this entry is a positive assessment of the success of the marriage."}, "language_style": {"answer": "Calm and direct", "rationale": "The entry directly describes the status of marriage, is not expressed with a qualifier or acrimonym, and is more objective and clear. It is therefore considered that its language style is \"cool and direct\"."}, "time_dimension": {"answer": "Present", "rationale": "The entry is a description of the current marital status of the interviewee, and therefore the time dimension is now judged."}, "sentence_type": "Affirmative declarative sentence", "pos": {"nouns": {"Marriage": 1, "Success": 1}, "verbs": {"be": 1, "Very": 1}, "adverbs": {"Very": 1}}}, {"content": "34. It is important to me to pray with my spouse.", "word_count": 12, "label": "", "sentence_structure": "Subject鈥揷opula鈥損redicative", "subjective_objective": {"answer": "Subjective", "rationale": "Because \"important\" is a subjective feeling or evaluation that represents the attitude and values of patients to pray with their spouses."}, "sentence_sentiment": {"answer": "Positive", "rationale": "Because the behavior and attitude of \"Pray with the spouses\" in the entry is positive."}, "language_style": {"answer": "Tactful and warm", "rationale": "The language style of the entry is a euphemism and warmth type. It uses polite and invitee language, displays respect for the individual beliefs and values of the interviewee and encourages the interviewer to share his/her views through questioning."}, "time_dimension": {"answer": "Present", "rationale": "Both the expressions \"pray with spouses\" and \"important to me\" follow the general current, indicating that this is a current act or attitude and can therefore be judged as \"now\"."}, "sentence_type": "Affirmative declarative sentence", "pos": {"nouns": {"Spouse": 1, "Pray": 1}, "verbs": {"be": 1, "Important": 1}, "adjectives": {"very": 1}, "numerals": {"34": 1}, "pronouns": {"I": 1}}}, {"content": "35. I hope that the spouse will be more willing to share his/her feelings with me.", "word_count": 17, "label": "", "sentence_structure": "Subject鈥搗erb鈥揷omplement", "subjective_objective": {"answer": "Subjective", "rationale": "Because the word \"hope\" indicates that it is the patient's wish and desire, and that it is the patient's subjective attitude."}, "sentence_sentiment": {"answer": "Positive", "rationale": "Because the behaviour and attitude of \"I want my spouse to be more willing to share his/her feelings\" in the entry is positive and expresses the expectation of better communication and relationships."}, "language_style": {"answer": "Tactful and warm", "rationale": "The language style of the entry is more euphemistic and emphasizes the desire to be more active in expressing one鈥檚 feelings than to direct orders or demands. It is more friendly and friendly, and it is easily resonant and willing to cooperate with the investigation."}, "time_dimension": {"answer": "Present", "rationale": "The keywords \"hope\" and \"better\" both indicate that this is an expectation or desire for the act in hand and that it is directed at relations between oneself and the spouse. Thus, the purpose of the article is now time-bound."}, "sentence_type": "Imperative sentence", "pos": {"nouns": {"Questionnaire Entry": 1, "Spouse": 1, "Feel": 1}, "verbs": {"Hope": 1, "Yes": 1, "Share": 1, "he": 1, "she": 1}, "adverbs": {"More": 1}, "numerals": {"35": 1}, "pronouns": {"I": 1}}}, {"content": "36. With children, we are closer to marriage.", "word_count": 15, "label": "", "sentence_structure": "Subject鈥搗erb鈥揷omplement", "subjective_objective": {"answer": "Subjective", "rationale": "Because \"closer\" is a subjective feeling and assessment, it reflects the subjective view of the respondent of marriage."}, "sentence_sentiment": {"answer": "Positive", "rationale": "Because the result of the entry \"the closer marriage\" is positive."}, "language_style": {"answer": "Tactful and warm", "rationale": "The entry uses a more subjective description, which makes it easier for the interviewee to resonate and identify by using the language \"let's get closer\" and, as a precondition, to direct the interviewer to answer with the objective fact that \"have a child.\" This soft, invitations expression is more personal and interactive and is more suitable for psychological surveying."}, "time_dimension": {"answer": "Present", "rationale": "The entry now describes the marital status of the child and does not address information such as past or future behaviour or mental state."}, "sentence_type": "Affirmative declarative sentence", "pos": {"nouns": {"Son": 1, "Marriage": 1}, "verbs": {"have": 1, "鈾?And I'm gonna be 鈾?: 1, "Closer": 1}, "numerals": {"36": 1}}}, {"content": "My spouse likes all my friends.", "word_count": 12, "label": "", "sentence_structure": "Subject鈥搗erb鈥搊bject", "subjective_objective": {"answer": "Subjective", "rationale": "Because \"like\" is a description of the patient's emotional attitude towards his spouse, it is a subjective examination."}, "sentence_sentiment": {"answer": "Positive", "rationale": "Because the expression in the entry is positive, it expresses the acceptance and affection of the spouse to all friends of the interviewee."}, "language_style": {"answer": "Tactful and warm", "rationale": ""}, "time_dimension": {"answer": "Present", "rationale": ""}, "sentence_type": "Affirmative declarative sentence", "pos": {"nouns": {"Spouse": 1, "friends": 1}, "verbs": {"Yeah": 1}, "adverbs": {"All": 1}, "numerals": {"37": 1}, "pronouns": {"I": 1}}}, {"content": "38. I do not want to show the same tenderness to a spouse, as it is often misconstrued as a sexual expression.", "word_count": 28, "label": "", "sentence_structure": "Subject鈥損redicate", "subjective_objective": {"answer": "Subjective", "rationale": "Subjective. The reluctance to display a very gentle attitude towards the spouse is one person ' s subjective idea and attitude, while the expression of falsely perceived sexuality is a possible objective result."}, "sentence_sentiment": {"answer": "Negative", "rationale": "Because the expression \"unwilling\" and \"often misconstrued as \"is\" in the entry is negative and indicates that the author is dissatisfied or contradicted."}, "language_style": {"answer": "Tactful and warm", "rationale": "The language style of the entry is euphemism. First, it uses a more polite tone, without direct orders or accusations. Second, it attempts to avoid possible misunderstandings by moderate language, showing a sense of concern and understanding. Such expression is more acceptable and willing to cooperate in answering questions."}, "time_dimension": {"answer": "Present", "rationale": "The entry describes the current feelings and behaviour, \"not willing\" and \"because\" all indicate that this is the current situation and may be ongoing. Moreover, \"often misperception of a sexual expression\" also highlights the impact and importance of this situation on the voice. So the temporal dimension is now."}, "sentence_type": "Negative declarative sentence", "pos": {"nouns": {"Questionnaire": 1, "Entry": 1, "Spouse": 1, "Sex": 2}, "verbs": {"Performance": 1, "鈾?Mistaked 鈾?: 1, "be": 3, "Give": 1}, "adjectives": {"Be gentle": 1, "Regular": 1, "Organisation": 1}, "numerals": {"38": 1, "One": 4}, "classifiers": {"Plant": 1}, "pronouns": {"it": 1}}}, {"content": "39. I feel that our marriage lacks something.", "word_count": 16, "label": "", "sentence_structure": "Subject鈥揷opula鈥損redicative", "subjective_objective": {"answer": "Subjective", "rationale": "Because \"I think\" is the patient's subjective feeling and thoughts."}, "sentence_sentiment": {"answer": "Negative", "rationale": "Because the expression \"lack of something\" in the entry is negative."}, "language_style": {"answer": "Tactful and warm", "rationale": "The entry expresses, in a more euphemistic way, the dissatisfaction or lack of a marriage relationship, without using too direct or harsh language. At the same time, it leaves the respondent a space for self-reflection and answers, avoiding possible embarrassment or conflict."}, "time_dimension": {"answer": "Present", "rationale": ""}, "sentence_type": "Affirmative declarative sentence", "pos": {"nouns": {"Marriage": 1, "Something": 1}, "verbs": {"I think": 1, "Missing": 1}, "adjectives": {"Some": 1}, "numerals": {"39": 1}, "pronouns": {"we": 1}}}, {"content": "40. We have sometimes had serious disputes over issues of little importance.", "word_count": 22, "label": "", "sentence_structure": "Subject鈥揷opula鈥損redicative", "subjective_objective": {"answer": "Objective", "rationale": "Because the dispute is an objective result, it is not controlled by subjective will. Although it may affect the mood and relationship of the patient, it is itself occurring objectively."}, "sentence_sentiment": {"answer": "Negative", "rationale": "Because the entry \"sometimes we get serious disputes about something that is not important\" is negative."}, "language_style": {"answer": "Tactful and warm", "rationale": "The entry uses a more moderate, inviting language style, which does not directly convey the meaning of blame or criticism, but rather directs the respondents to share their views and experiences in an objective and neutral manner. This expression makes the interviewee comfortable and receptive, more willing to participate and answer questions honestly."}, "time_dimension": {"answer": "Past", "rationale": "The entry describes \"sometimes\" and \"frequently\" behaviour, which indicates that it is a regular, customary act and therefore a judgement of the past."}, "sentence_type": "Affirmative declarative sentence", "pos": {"nouns": {"Problem": 2, "Arguments": 1}, "verbs": {"Generate": 1, "in": 1, "Sometimes": 1}, "adjectives": {"Serious": 1, "Some": 1, "It doesn't matter": 1}, "numerals": {"40": 1}}}, {"content": "41. I feel that the couple have not spent enough time together to spend their leisure time.", "word_count": 24, "label": "", "sentence_structure": "Subject鈥搗erb鈥搊bject", "subjective_objective": {"answer": "Subjective", "rationale": "This view is based on the patient ' s own subjective feelings and perceptions of the relationship."}, "sentence_sentiment": {"answer": "Negative", "rationale": "Because the expression \"not spending enough time to spend leisure time together\" in the entry is negative emotional."}, "language_style": {"answer": "Tactful and warm", "rationale": ""}, "time_dimension": {"answer": "Present", "rationale": "\"I feel\" means the subjective feeling or experience of the testee, and \"I feel that the couple has not spent enough time together to spend their leisure time\" describes the current situation and therefore the present."}, "sentence_type": "Negative declarative sentence", "pos": {"nouns": {"Time": 1, "Both sides": 1, "Amateur": 1, "- Free": 1}, "verbs": {"I feel": 1, "Cost": 1, "Pass": 1}, "adverbs": {"Enough": 1, "Together": 1}, "numerals": {"41": 1}, "pronouns": {"I": 1}}}, {"content": "42. Sometimes, I find it difficult to believe everything that my spouse tells me.", "word_count": 17, "label": "", "sentence_structure": "Subject鈥揷opula鈥損redicative", "subjective_objective": {"answer": "Subjective", "rationale": "Because \"It's hard to believe that everything a spouse tells me\" is the patient's own feelings and perceptions."}, "sentence_sentiment": {"answer": "Negative", "rationale": "Because the expression \"it's hard to believe everything a spouse tells me\" in the entry is negative."}, "language_style": {"answer": "Calm and direct", "rationale": "The language style of the entry is calm and straightforward. It asks the respondents whether they are sometimes suspicious of the truth of what the spouse says, and do not have too many emotional renderings or qualifiers. This expression is more objective, neutral and more accurate in reflecting the views and feelings of the respondents."}, "time_dimension": {"answer": "Present", "rationale": "The entry asks the testee about his current thoughts and feelings, and the temporal dimension is now. \"Sometimes\" means that the act may have occurred in the past, but here more emphasis is placed on the current situation."}, "sentence_type": "Negative declarative sentence", "pos": {"nouns": {"Time": 1, "I": 1, "Spouse": 1, "Something": 1}, "verbs": {"Yes": 1, "Tell": 1, "Hard": 1}, "adjectives": {"It's hard": 1}, "adverbs": {"Sometimes": 1}, "numerals": {"42": 1}, "pronouns": {"Every piece": 1}}}, {"content": "43. I have tried to avoid conflict with spouses.", "word_count": 12, "label": "", "sentence_structure": "Subject鈥搗erb鈥搊bject", "subjective_objective": {"answer": "Subjective", "rationale": "Because \"do your best to avoid\" means that it's subjective and hard work."}, "sentence_sentiment": {"answer": "Negative", "rationale": "Because the action \"avoid conflict with spouse\" in the entry is negative."}, "language_style": {"answer": "Tactful and warm", "rationale": "The entry is more personal and interactive when it is spoken in a gentle and inviting tone, rather than in direct statements or orders. The phrase \"as far as possible avoids\" also shows that the questioner does not ask the other party to do absolutely nothing against the spouse, but rather that they try to reduce the occurrence of the conflict. This expression is more euphemistic and does not put undue pressure on people."}, "time_dimension": {"answer": "Present", "rationale": "The words \"as far as possible avoid\" and \"conflict\" in this entry indicate that this is a present act or attitude, that is, that the testees are currently trying to avoid disputes in their marriage. Therefore, the temporal dimension is now judged."}, "sentence_type": "Affirmative declarative sentence", "pos": {"nouns": {"Conflict": 1, "Spouse": 1}, "verbs": {"Avoid": 1, "Come on": 1}, "adjectives": {"Try": 1}, "numerals": {"43": 1}}}, {"content": "44. For us, the husband ' s profession is more important than the wife ' s occupation.", "word_count": 20, "label": "", "sentence_structure": "Subject鈥揷opula鈥損redicative", "subjective_objective": {"answer": "Subjective", "rationale": "This view reflects the subjective view of the respondent on the importance of the husband ' s profession and that of the wife."}, "sentence_sentiment": {"answer": "Negative", "rationale": "Because it implies that men are more important than women and that there is a tendency to discriminate on the basis of sex."}, "language_style": {"answer": "Tactful and warm", "rationale": "The entry is more personal and interactive when it is spoken in a gentle and inviting tone, rather than in direct statements or orders. At the same time, it is not overly emotional or value-judged, but rather presents a point of view for the interviewees to reflect on."}, "time_dimension": {"answer": "Present", "rationale": "The term \"for the \" and \" important\" in the sentence shows that this is an assessment or view of the current situation, so that the temporal dimension of the article can be judged to be the present. At the same time, since the sentence does not relate to past or future situations, the entry is not about past experiences or future expectations."}, "sentence_type": "Affirmative declarative sentence", "pos": {"nouns": {"we": 1, "Husband": 1, "Occupation": 2, "Wife": 1}, "verbs": {"Compare": 1, "Important": 1}, "numerals": {"44": 1}, "pronouns": {"he": 1}}}, {"content": "45. I feel that our marriage is influenced by religious beliefs.", "word_count": 16, "label": "", "sentence_structure": "Subject鈥揷opula鈥損redicative", "subjective_objective": {"answer": "Subjective", "rationale": "Because \"think\" expresses the patient's perception or perception of the religious influence of marriage, it is the patient's subjective attitude."}, "sentence_sentiment": {"answer": "Negative", "rationale": "Because the description of \"affected by religious beliefs\" in the entries can give rise to negative emotional reactions, such as anxiety, distress, etc."}, "language_style": {"answer": "Tactful and warm", "rationale": "The entry uses a first person鈥檚 perspective, which increases the willingness of the interviewee to express his/her views and ideas, while avoiding direct evaluation or criticism of the other person鈥檚 religious beliefs."}, "time_dimension": {"answer": "Present", "rationale": "So the time dimension is now judged."}, "sentence_type": "Affirmative declarative sentence", "pos": {"nouns": {"Marriage": 1, "Religion": 1, "Perceptions": 1}, "verbs": {"I think": 1, "Impact": 1}, "numerals": {"45": 1}, "pronouns": {"we": 1}}}, {"content": "46. Our economy has become tense, as in the case of excessive credit.", "word_count": 15, "label": "", "sentence_structure": "Subject鈥搗erb鈥揷omplement", "subjective_objective": {"answer": "Objective", "rationale": "This is a description of the current situation, a reflection of the current economic situation and an objective perspective."}, "sentence_sentiment": {"answer": "Negative", "rationale": "Because the entries \"the economy has become tense\" and \"over credit\" are negative expressions."}, "language_style": {"answer": "Calm and direct", "rationale": "The entry presents a fact in a direct and objective tone 鈥?economic conditions have become tense, and a specific example is given. This is more concise and clear, and it conveys the message accurately."}, "time_dimension": {"answer": "Present", "rationale": ""}, "sentence_type": "Affirmative declarative sentence", "pos": {"nouns": {"Economy": 1, "Nervous": 1, "Credit": 1}, "verbs": {"Has become": 1}, "adjectives": {"Too much": 1}, "numerals": {"46": 1}}}, {"content": "47. I am troubled by the frequent delays in the marriage of spouses.", "word_count": 13, "label": "", "sentence_structure": "Subject鈥搗erb鈥揷omplement", "subjective_objective": {"answer": "Subjective", "rationale": "Because \"slowing and dragging\" and \"fearing\" are subjective feelings and evaluations of patients."}, "sentence_sentiment": {"answer": "Negative", "rationale": "Because the behavior in the entry \"spouses often drag and pull\" is negative and it leads to \"I'm upset\" and further reinforces negative emotions."}, "language_style": {"answer": "Tactful and warm", "rationale": "The language style of this questionnaire entry is more euphemistic, using descriptive terms of \"treasure\" and being expressed in a first person way, which allows respondents to share their feelings and experiences more easily than to be asked directly or blamed. Moreover, the word \"often\" implies that it is a long-standing problem, not a one-off event, which gives the interviewee sufficient time to think and answer."}, "time_dimension": {"answer": "Past", "rationale": ""}, "sentence_type": "Affirmative declarative sentence", "pos": {"nouns": {"Spouse": 1, "- I'm sorry": 1, "Trouble": 1}, "verbs": {"鈾?And I'm gonna be 鈾?: 1, "Regular": 1, "Trouble": 1}, "adjectives": {"Regular": 1, "very": 1}, "numerals": {"47": 1}}}, {"content": "48. Sometimes, I feel that the dispute between husband and wife is endless and never resolved.", "word_count": 22, "label": "", "sentence_structure": "Subject鈥搗erb鈥揷omplement", "subjective_objective": {"answer": "Subjective", "rationale": "Because the sentence expresses the patient ' s personal feelings and thoughts that the dispute between the spouses is not resolved, and that feeling is subjective."}, "sentence_sentiment": {"answer": "Negative", "rationale": "Because \"continues\" and \"never solved\" in the entries are negative descriptions."}, "language_style": {"answer": "Tactful and warm", "rationale": "The language style of the entry is more euphemistic and caring, and it does not directly blame or criticize the dispute between the spouses, but rather expresses the problem in a way that is understood and cared for. This expression is more acceptable and may lead the respondents to more willing to share their thoughts and feelings."}, "time_dimension": {"answer": "Present", "rationale": "The keywords \"thinking\" and \" never ending\" and \" never solving\" point to the current state and feeling."}, "sentence_type": "Negative declarative sentence", "pos": {"nouns": {"Couples": 2, "Arguments": 2, "Resolve": 2}, "verbs": {"I think": 1, "Okay": 1, "Never": 1, "Present": 1}, "adjectives": {"It's not over": 1}, "adverbs": {"Sometimes": 1}, "numerals": {"48": 1}}}, {"content": "49. A wife should not be absent from work if there were a small child in the family.", "word_count": 18, "label": "", "sentence_structure": "Subject鈥搗erb鈥搊bject", "subjective_objective": {"answer": "Subjective", "rationale": "Because the content of the entry relates to subjective perceptions and values of family and gender roles, it is viewed from a subjective perspective."}, "sentence_sentiment": {"answer": "Negative", "rationale": "Because the \"not to go out to work\" view in the entry is gender-discriminatory and unfair, it is a negative emotional colour."}, "language_style": {"answer": "Calm and direct", "rationale": "This entry uses a direct and explicit language style, which presents a view or proposal, without using euphemism or other emotional language."}, "time_dimension": {"answer": "Past", "rationale": ""}, "sentence_type": "Negative declarative sentence", "pos": {"nouns": {"Home": 1, "Children": 1, "Wife": 1, "Work": 1}, "verbs": {"have": 1, "Reactions": 1, "Out": 1, "Work": 1}, "adjectives": {"Small": 1}, "numerals": {"49": 1}, "pronouns": {"Home": 1, "Wife": 1}}}, {"content": "I often do not tell my spouse what I feel, because he/she should know.", "word_count": 23, "label": "", "sentence_structure": "Subject鈥搗erb鈥搊bject", "subjective_objective": {"answer": "Subjective", "rationale": "Because \"he/she should have known\" is the patient's subjective idea."}, "sentence_sentiment": {"answer": "Negative", "rationale": "Because the words \"not to tell the spouse\" and \"he/she should know\" in the entries have a certain negative emotional connotation."}, "language_style": {"answer": "Tactful and warm", "rationale": "The language style of the entry is more euphemistic and warm, using the word \"should be felt\" to express the author鈥檚 desire to understand his/her feelings, while also showing some expectation and trust. This expression is more acclaimable and more likely to trigger resonance and reaction."}, "time_dimension": {"answer": "Present", "rationale": "The time dimension of the entry is now, as it describes a current act or state."}, "sentence_type": "Negative declarative sentence", "pos": {"nouns": {"Spouse": 1, "Feel": 1}, "verbs": {"Tell": 1, "Feel it": 1}, "adjectives": {"Regular": 1}, "numerals": {"50": 1}, "pronouns": {"he": 1, "she": 1}}}, {"content": "51. I'm happy about how we express our feelings and our sexuality.", "word_count": 24, "label": "", "sentence_structure": "Subject鈥揷opula鈥損redicative", "subjective_objective": {"answer": "Subjective", "rationale": "The sentence is an expression of patients' attitudes and feelings regarding the expression of emotional and sexual relations between spouses."}, "sentence_sentiment": {"answer": "Positive", "rationale": "Because the word \"satisfactory\" conveys positive emotions."}, "language_style": {"answer": "Tactful and warm", "rationale": "The language style used in this entry is more euphemistic and moderate, and conveys the satisfaction of the interviewee with the expression of emotional and sexual relations between the couple, rather than using direct or hard language. This expression is more comfortable and acceptable."}, "time_dimension": {"answer": "Present", "rationale": "\"Fully satisfied\" is the description of the past, but the main words of the sentence \"I\" and \"satisfaction\" are both present. So the entry is mainly about the attitude and satisfaction of the interviewees with the current communication between the spouses, and does not address past or future behaviour or mindset. So it is \"now.\""}, "sentence_type": "Affirmative declarative sentence", "pos": {"nouns": {"we": 2, "Couples": 1, "Emotion": 1, "Sex": 1, "Yes": 1}, "verbs": {"What": 1, "Expression": 1, "have": 1, "Satisfied": 1}, "adjectives": {"very": 1}, "numerals": {"51": 1}, "pronouns": {"I": 1}}}, {"content": "52. When there is disagreement between spouses, we communicate our feelings openly and decide how to deal with it.", "word_count": 31, "label": "", "sentence_structure": "Subject鈥搗erb鈥搊bject", "subjective_objective": {"answer": "Subjective", "rationale": "According to the sentence, \"the situation of different views between spouses\" is an objective fact, and \"the open exchange of feelings and the determination of how to solve it\" is a subjective attitude or strategy. The questionnaire entry is therefore viewed from the patient's subjective perspective."}, "sentence_sentiment": {"answer": "Positive", "rationale": "Because the entry \"to communicate with one another openly and to decide how to deal with it\" is positive."}, "language_style": {"answer": "Tactful and warm", "rationale": "Starting with the description of \"when there is a disagreement between husband and wife\" , there is neither direct accusation nor order nor too negative emotional expression. And, in this part of the phrase, \"We communicate our feelings and decide how to solve it in an open and honest manner,\" the more positive language is used, emphasizing the need to solve problems through communication and cooperation, with a more moderate and friendly tone."}, "time_dimension": {"answer": "Present", "rationale": ""}, "sentence_type": "Affirmative declarative sentence", "pos": {"nouns": {"Couples": 1, "Observations": 1, "Decision": 1, "Problem": 1, "Communications": 1, "Feel": 1}, "verbs": {"Come on": 1, "Open and open": 1, "Resolve": 1}, "adverbs": {"What": 1}, "numerals": {"52": 1}, "pronouns": {"we": 1}}}, {"content": "53. I rarely joke unless I am with my spouse.", "word_count": 16, "label": "", "sentence_structure": "Subject鈥搗erb鈥搊bject", "subjective_objective": {"answer": "Subjective", "rationale": "Because \"I'm rarely joking\" is the patient's subjective feeling and attitude, and it's the individual's psychological state and emotional experience."}, "sentence_sentiment": {"answer": "Negative", "rationale": "Because the \"I don't joke\" in the entry is negative."}, "language_style": {"answer": "Calm and direct", "rationale": "The language style of the entry is relatively calm and straightforward, without excessive rhetoric or emotional expressions, but rather a simple and straightforward presentation of a fact or view."}, "time_dimension": {"answer": "Present", "rationale": "The keywords \"less\" and \"unless...\" express a normal, customary situation, where the testee is less joking when he or she is not with his or her spouse. This is the time dimension of the present."}, "sentence_type": "Affirmative declarative sentence", "pos": {"nouns": {"Questionnaire": 1, "Entry": 1, "Spouse": 1}, "verbs": {"Analysis": 1, "in": 1, "Together": 1, "Open": 1}, "adjectives": {"Very few": 1}, "adverbs": {"Unless": 1, "Otherwise": 1}, "numerals": {"53": 1}, "pronouns": {"I": 1}}}, {"content": "54. We are very focused on deciding how to spend our money on the most important things.", "word_count": 20, "label": "", "sentence_structure": "Subject鈥搗erb鈥搊bject", "subjective_objective": {"answer": "Subjective", "rationale": "Because the words \"are very focused\" and \"most important things\" are the personal views and attitudes of the patients."}, "sentence_sentiment": {"answer": "Positive", "rationale": "Because the entry is a reflection of the importance of the matter and a wise financial management attitude, it is a positive emotional tendency."}, "language_style": {"answer": "Tactful and warm", "rationale": ""}, "time_dimension": {"answer": "Present", "rationale": "The entry describes the current behaviour and attitude of the interviewees, who are concerned about how funds are being allocated in a rational manner and are therefore in the current state of affairs."}, "sentence_type": "Affirmative declarative sentence", "pos": {"nouns": {"Decision": 1, "Money": 1, "Things": 1}, "verbs": {"Flowers": 1, "Focus": 1, "- Put it on": 1, "in": 1, "Go, go, go": 1}, "adjectives": {"Most important": 1}, "numerals": {"54": 1}}}, {"content": "55. Sometimes my spouse spends too much time with friends.", "word_count": 17, "label": "", "sentence_structure": "Subject鈥搗erb鈥搊bject", "subjective_objective": {"answer": "Subjective", "rationale": "Because \"too much\" is the result of subjective perceptions and evaluation of patients, it is subjective."}, "sentence_sentiment": {"answer": "Negative", "rationale": "Because \"too much time\" in the entries implies negative emotions and attitudes. This may mean that respondents think their spouses have ignored them or given more time to their friends, which may cause resentment or jealousy."}, "language_style": {"answer": "Calm and direct", "rationale": "The entry expresses concerns in a straightforward manner about possible problems in the marital relationship of the person interviewed, with no excessive adoration or emotional colour, and with a more calm and straightforward language style."}, "time_dimension": {"answer": "Present", "rationale": "The entry describes a status quo or state, which is that \"sometimes\" spouses spend too much time with friends, and therefore judges now.\""}, "sentence_type": "Negative declarative sentence", "pos": {"nouns": {"Spouse": 1, "friends": 1, "Time": 1}, "verbs": {"and": 1, "Together": 1, "be": 1}, "adverbs": {"Sometimes": 1}, "numerals": {"55": 1}, "pronouns": {"I": 1}}}, {"content": "56. My spouse and I have different views on the religious education of our children.", "word_count": 21, "label": "", "sentence_structure": "Subject鈥搗erb鈥搊bject", "subjective_objective": {"answer": "Subjective", "rationale": "Because \"different views\" are subjective perceptions or feelings, which indicate differences between the respondents and their spouses in how to educate their children about religion."}, "sentence_sentiment": {"answer": "Negative", "rationale": "Because the act of \"different opinion\" in the entry indicates a disagreement between the parties, it may lead to contradictions and conflicts."}, "language_style": {"answer": "Tactful and warm", "rationale": "This questionnaire entry is presented in a more euphemistic way, avoiding direct accusations or conflicting language, and instead directing respondents to express their views and positions in the form of inquiries."}, "time_dimension": {"answer": "Present", "rationale": "The questionnaire entry asks about the current situation of the respondents, i.e. they have different views with their spouses on the religious education of their children."}, "sentence_type": "Affirmative declarative sentence", "pos": {"nouns": {"I": 1, "Spouse": 1, "Children": 2, "Religious education": 2, "Observations": 1}, "verbs": {"in": 1, "have": 1, "Different": 1, "Here": 1}, "adverbs": {"very": 1}, "numerals": {"56": 1, "Two": 1}}}, {"content": "57. I am not satisfied with the division of responsibilities for parenthood.", "word_count": 17, "label": "", "sentence_structure": "Subject鈥搗erb鈥搊bject", "subjective_objective": {"answer": "Subjective", "rationale": "Because \"unsatisfied\" is the patient's subjective attitude and feelings about this."}, "sentence_sentiment": {"answer": "Negative", "rationale": "Because the attitude of \"unsatisfactory\" in the entries is negative."}, "language_style": {"answer": "Calm and direct", "rationale": "The entry is in plain and clear language, expressing the respondent ' s dissatisfaction with the division of parental responsibilities, without too many rhetorical or emotional expressions, and with a more calm and straightforward language style."}, "time_dimension": {"answer": "Present", "rationale": "The \"division of parental responsibility\" in \"the division of parental responsibility\" indicates that it is a present state of behaviour or state, and the expression \"unsatisfactory\" also implies the continuity of the situation, so the entry describes the current situation."}, "sentence_type": "Negative declarative sentence", "pos": {"nouns": {"Division of responsibilities": 1, "parents": 2}, "verbs": {"Undertaking": 1, "Unsatisfactory": 1, "Division of labour": 1}, "numerals": {"57": 1}}}, {"content": "Love for a spouse makes me feel even more deeply: God is kind.", "word_count": 17, "label": "", "sentence_structure": "Subject鈥揷opula鈥損redicative", "subjective_objective": {"answer": "Subjective", "rationale": "Because \"appear\" is a subjective feeling, the subject is considered from the subjective point of view of the patient."}, "sentence_sentiment": {"answer": "Positive", "rationale": "Because the words \"love\" and \"know God is kind\" in the entry are positive emotions and attitudes."}, "language_style": {"answer": "Tactful and warm", "rationale": "The language style of the entry is more euphemistic and contagious. It uses a positive emotional vocabulary, \"love\" and \"more experienced\", to convey love for a spouse and to impress the whole phrase by introducing religious belief (God is kind) with a quiet, far-reaching emotional color."}, "time_dimension": {"answer": "Present", "rationale": "The entry describes the current feelings and experiences of the testee, and the keywords \"love\" and \"appear\" all indicate that this is the state or feeling in the moment, so it is judged to be the present. And, \"make\" is also the current description, highlighting the impact of the existence of a spouse on the testee's perception of God's love."}, "sentence_type": "Affirmative declarative sentence", "pos": {"nouns": {"Spouse": 1, "Jesus": 1}, "verbs": {"鈾?And I'm gonna be 鈾?: 1, "Feel it": 1, "Love": 1}, "adjectives": {"Deep": 1, "\"Careful.\"": 1}, "numerals": {"58": 1}, "pronouns": {"I": 1}}}, {"content": "59. I feel that the high expectations of parents on both sides are receiving our attention and help.", "word_count": 23, "label": "", "sentence_structure": "Subject鈥搗erb鈥揷omplement", "subjective_objective": {"answer": "Subjective", "rationale": "Because \"the feeling\" shows how the patient feels about his relationship with his parents, and is subjective."}, "sentence_sentiment": {"answer": "Negative", "rationale": "Because the \"high expectations\" in the entries express a negative attitude or emotion."}, "language_style": {"answer": "Calm and direct", "rationale": "The entry uses more objective and neutral language to express the views of the investigators, without excessive emotional or rhetorical styles, and is more intuitive and direct."}, "time_dimension": {"answer": "Past", "rationale": ""}, "sentence_type": "Affirmative declarative sentence", "pos": {"nouns": {"Parents of both parties": 1, "Expectations": 1, "Care": 1, "Help": 1}, "verbs": {"I think": 1, "Got it": 1}, "numerals": {"59": 1}}}, {"content": "60. I am very pleased with the way in which couples talk to each other.", "word_count": 16, "label": "", "sentence_structure": "Subject鈥搗erb鈥揷omplement", "subjective_objective": {"answer": "Subjective", "rationale": "Because \"very satisfied\" is the patient's subjective perception and evaluation of the way in which the couple talks."}, "sentence_sentiment": {"answer": "Positive", "rationale": "This is a positive emotional attitude, since it expresses satisfaction with and acceptance of the way in which couples talk to each other."}, "language_style": {"answer": "Tactful and warm", "rationale": "Warm and warm."}, "time_dimension": {"answer": "Present", "rationale": "The entry describes the satisfaction of the interviewees with the current way in which couples talk to each other, and therefore the time dimension is now judged."}, "sentence_type": "Affirmative declarative sentence", "pos": {"nouns": {"Couples": 1, "Talk": 1, "Modalities": 1}, "verbs": {"I'm very satisfied": 1}, "adverbs": {"Between": 1, "To each other": 1, "Very": 1}, "numerals": {"60": 1}}}, {"content": "61. I think our parents have caused problems in our marriage.", "word_count": 18, "label": "", "sentence_structure": "Subject鈥揷opula鈥損redicative", "subjective_objective": {"answer": "Subjective", "rationale": "Because it expresses the patient ' s subjective feelings and views of a certain view."}, "sentence_sentiment": {"answer": "Negative", "rationale": "Because the act or result of \"problems our marriage\" in the entry is negative."}, "language_style": {"answer": "Tactful and warm", "rationale": "The entry, which expresses personal feelings in the first person ' s name, does not directly accuse or deny parents, but uses a more euphemism, which can reduce unnecessary conflicts and contradictions."}, "time_dimension": {"answer": "Present", "rationale": ""}, "sentence_type": "Negative declarative sentence", "pos": {"nouns": {"Questionnaire Entry": 1, "parents": 1, "Marriage": 1, "Problem": 1}, "verbs": {"I think": 1, "Here": 1, "Cause": 1}, "adjectives": {"we": 2}, "numerals": {"61": 1}, "pronouns": {"I": 1}}}, {"content": "62. I am troubled that I cannot spend without the permission of a spouse.", "word_count": 16, "label": "", "sentence_structure": "Subject鈥搗erb鈥搊bject", "subjective_objective": {"answer": "Subjective", "rationale": "This is because of the patient ' s personal feelings and attitudes, reflecting his or her perception of the desire of the spouse to control it, and is a subjective view."}, "sentence_sentiment": {"answer": "Negative", "rationale": "Because the \"I'm upset\" in the entry expresses negative emotions and attitudes, and the \"I can't spend without a spouse\" is negative."}, "language_style": {"answer": "Tactful and warm", "rationale": "The language style of the entry is euphemism. It uses the expression \"I'm upset\" to convey the pain and impatience of the voice, and to show respect and understanding for the listener. Moreover, the condition that I cannot spend without the permission of a spouse implies the place and limitations of the voice in family life, which is more moderate and euphemistic."}, "time_dimension": {"answer": "Present", "rationale": ""}, "sentence_type": "Negative declarative sentence", "pos": {"nouns": {"Questionnaire": 1, "Entry": 1, "I": 1, "Spouse": 1, "Allow": 1, "Money": 1}, "verbs": {"very": 1, "Trouble": 1, "No, I'm not": 1, "Yes": 1, "Flowers": 1}, "adverbs": {"No, I can't": 1}, "numerals": {"62": 1}}}, {"content": "63. Since having children, couples have had little time alone.", "word_count": 19, "label": "", "sentence_structure": "Subject鈥搗erb鈥揷omplement", "subjective_objective": {"answer": "Subjective", "rationale": "Because there is a great deal of time alone for a subjective feeling and experience of a person, it is a subjective perspective."}, "sentence_sentiment": {"answer": "Negative", "rationale": "The entry describes the negative effects of the couple ' s relationship due to the fact that a child is born."}, "language_style": {"answer": "Calm and direct", "rationale": "The entry was in a simple and clear language, asking the respondents about the decrease in the time spent alone with the partner after becoming a parent, and the language style was more calm and objective."}, "time_dimension": {"answer": "Present", "rationale": ""}, "sentence_type": "Affirmative declarative sentence", "pos": {"nouns": {"Son": 1, "Couples": 1, "Time": 1, "Alone": 1}, "verbs": {"Got it": 1, "Very few": 1, "Together": 1}, "adjectives": {"Since": 1}, "numerals": {"63": 1}}}, {"content": "64. I sometimes feel helpless about the mood of the spouses.", "word_count": 18, "label": "", "sentence_structure": "Subject鈥搗erb鈥揷omplement", "subjective_objective": {"answer": "Subjective", "rationale": "Because \"frightless\" is a subjective feeling and attitude. While it describes an objective situation (i.e. the grumpyness of the spouse), it looks primarily at the patient's subjective reaction to and feelings of the situation."}, "sentence_sentiment": {"answer": "Negative", "rationale": "Because the \"frightful\" attitude in the entry is negative."}, "language_style": {"answer": "Tactful and warm", "rationale": "The entry uses moderate language, without directly accusing or attacking the other, but rather expressing their feelings and confusion. This expression avoids unnecessary contradictions and conflicts, and makes it easier for the other to accept and understand their views."}, "time_dimension": {"answer": "Present", "rationale": "The entry describes the feelings and experiences of the testees in the current situation, and the keyword \"Established\" indicates that this is the current state.\""}, "sentence_type": "Negative declarative sentence", "pos": {"nouns": {"Spouse": 1, "鈾?The mood is unchanging 鈾?: 1, "Nothing": 1}, "verbs": {"to": 1, "I feel": 1, "Nothing": 1}, "adjectives": {"Unusual": 1}, "adverbs": {"Sometimes": 1}, "numerals": {"64": 1}, "pronouns": {"I": 1}}}, {"content": "65. I often feel that spouses do not take our differences seriously.", "word_count": 18, "label": "", "sentence_structure": "Subject鈥搗erb鈥揷omplement", "subjective_objective": {"answer": "Subjective", "rationale": "Because \"feel\" is a subjective experience and evaluation that reflects patients' attitudes and perceptions about the way spouses deal with differences."}, "sentence_sentiment": {"answer": "Negative", "rationale": "Because the \"not taking our differences seriously\" act in the entry is negative."}, "language_style": {"answer": "Calm and direct", "rationale": "The language style of the entry is more direct and objective, and it does not use too many emotional or euphemisms, but rather expresses what the investigator wants to understand in a straightforward way."}, "time_dimension": {"answer": "Present", "rationale": "The entry describes the current feelings and experiences of the testees, the keyword being \"often feeling\" and pointing to the state of the situation that is taking place."}, "sentence_type": "Negative declarative sentence", "pos": {"nouns": {"Spouse": 1, "Difference": 1}, "verbs": {"I feel": 1, "Seriously": 1}, "adverbs": {"Regular": 1}, "pronouns": {"I": 1, "Ours": 1}}}, {"content": "66. In our home, the husband has the final say on most important matters.", "word_count": 25, "label": "", "sentence_structure": "Subject鈥揷opula鈥損redicative", "subjective_objective": {"answer": "Subjective", "rationale": "Since the entry addresses patients ' perceptions and attitudes to family decision-making power, it is subjective."}, "sentence_sentiment": {"answer": "Positive", "rationale": "Since the entry suggests that the husband should have final decision-making power on most important issues, which is a traditional gender role concept, it also emphasizes the position and responsibility of men as family leaders and can therefore be considered a positive attitude."}, "language_style": {"answer": "Calm and direct", "rationale": "The entry expresses a clear and straightforward view of family decision-making power, without excessive rhetoric or emotional rendering. At the same time, this expression is more persuasive and objective and does not attract resentment."}, "time_dimension": {"answer": "Present", "rationale": "The entry describes a general phenomenon or view and does not address past or future acts or events and is therefore current."}, "sentence_type": "Affirmative declarative sentence", "pos": {"nouns": {"Our house": 1, "Husband": 1, "Things": 1, "Finally": 1, "Decision-making authority": 1}, "verbs": {"You should": 1, "in": 1, "Go, go, go": 1}, "adverbs": {"Most of them": 1}, "numerals": {"66": 1}}}, {"content": "67. Fearing that the spouse is angry, I do not always tell him/her some of the worries that are in his/her heart.", "word_count": 27, "label": "", "sentence_structure": "Subject鈥搗erb鈥搊bject", "subjective_objective": {"answer": "Subjective", "rationale": "Because \"I\" fear that a spouse's temper is a patient's subjective feeling and thoughts."}, "sentence_sentiment": {"answer": "Negative", "rationale": "Because it expresses fear and fear, it is a negative attitude."}, "language_style": {"answer": "Tactful and warm", "rationale": "The entry uses a euphemism, expressing a more implicit emotional attitude through the words \"rememble\" and \"not always\" and does not directly condemn or criticize each other's words and actions, a language that is more comfortable and acceptable to the interviewees."}, "time_dimension": {"answer": "Past", "rationale": "The entry describes the past behaviour of the person tested, i.e., the failure to tell them about the anguish of the person concerned because of fear of the spouse ' s temper."}, "sentence_type": "Affirmative declarative sentence", "pos": {"nouns": {"Problem": 1, "Questionnaire": 1, "Entry": 1, "Spouse": 1, "Trouble": 1}, "verbs": {"I'm worried": 1, "- You're losing your temper": 1, "Always": 1, "- Put it on": 1, "Tell": 1, "he": 1, "she": 1}, "adjectives": {"Because": 1, "Some": 1}, "adverbs": {"So": 1, "Not always": 1}, "numerals": {"67": 1}, "classifiers": {"individual": 1}, "pronouns": {"I": 1}}}, {"content": "68. I am not satisfied with our relationship with both parents and friends.", "word_count": 16, "label": "", "sentence_structure": "Subject鈥搗erb鈥揷omplement", "subjective_objective": {"answer": "Subjective", "rationale": "Because dissatisfaction is our personal feeling and perception, it is subjective."}, "sentence_sentiment": {"answer": "Negative", "rationale": "Because the word \"unsatisfactory\" in the entry is an expression of a negative emotion."}, "language_style": {"answer": "Calm and direct", "rationale": "The language style of the entry is calm and straightforward, as it clearly expresses the dissatisfaction of the respondents with their relationship with their parents and friends."}, "time_dimension": {"answer": "Present", "rationale": "The entry is for the testee ' s satisfaction with the current relationship, and does not address past or future time dimensions."}, "sentence_type": "Negative declarative sentence", "pos": {"nouns": {"Relations": 1, "Unsatisfactory": 1, "we": 1, "Both sides": 1, "parents": 1, "friends": 1}, "verbs": {"have": 1, "Dissatisfied": 1, "be": 1}, "adverbs": {"not": 2}, "numerals": {"68": 1}, "pronouns": {"I": 1}}}, {"content": "69. My husband and I differ in the education of my religion.", "word_count": 20, "label": "", "sentence_structure": "Subject鈥搗erb鈥揷omplement", "subjective_objective": {"answer": "Subjective", "rationale": "This is because the patient ' s perception of his/her spouse ' s religious education is subjective."}, "sentence_sentiment": {"answer": "Negative", "rationale": "Because the term \"dissident\" in the entry implies differences and conflicts between the two sides, and is a reflection of negative sentiments."}, "language_style": {"answer": "Tactful and warm", "rationale": ""}, "time_dimension": {"answer": "Past", "rationale": "The entry is therefore a question about the current situation of the person being tested and is judged on the basis of the distinction between past, present and future verbs in the time dimension and the verb pattern."}, "sentence_type": "Negative declarative sentence", "pos": {"nouns": {"Religious education": 1, "Me and my husband": 2}, "verbs": {"to": 2, "Yes": 1, "Dissenting": 1}, "numerals": {"69": 1}, "pronouns": {"I": 1, "Spouse": 1}}}, {"content": "70. I never regret my parents ' relationship, even for a moment.", "word_count": 18, "label": "", "sentence_structure": "Subject鈥搗erb鈥揷omplement", "subjective_objective": {"answer": "Subjective", "rationale": "Because \"I never regret\" is the patient's subjective attitude."}, "sentence_sentiment": {"answer": "Positive", "rationale": "This item is a positive attitude and emotion towards parental relations."}, "language_style": {"answer": "Calm and direct", "rationale": "The entry expresses, in a direct presentation, the attitude and views of the subject in relation to his or her parentage. Language style is more direct, with no excessive adoration or emotional colour, and more focused on the presentation of the facts themselves."}, "time_dimension": {"answer": "Past", "rationale": "The entry is for the testee ' s past behaviour or state, and the time dimension is judged to be past."}, "sentence_type": "Affirmative declarative sentence", "pos": {"nouns": {"Questionnaire": 1, "Entry": 1, "Relations": 1, "parents": 1, "A moment": 1}, "verbs": {"No regrets": 1, "be": 1}, "adjectives": {"Never": 1}, "adverbs": {"Even": 1}, "numerals": {"70": 1}, "pronouns": {"I": 2}}}, {"content": "71. The amount of work that should be done for our children is one of the reasons for our conflict.", "word_count": 21, "label": "", "sentence_structure": "Subject鈥揷opula鈥損redicative", "subjective_objective": {"answer": "Objective", "rationale": "Because \"doing or not doing for the children\" is an objective fact, and \"does it cause conflict\" is an observable and quantifiable result. Although there may be an impact of individual subjective views, there is no apparent subjective tendency or attitude in the description of the issue, so we can judge it as an objective entry."}, "sentence_sentiment": {"answer": "Neutral", "rationale": "Because the entry presents a fact, there is no apparent emotional tendency."}, "language_style": {"answer": "Tactful and warm", "rationale": "Although the entry addresses the potential conflict between parents and children, it does not directly criticize or dictate what anyone should do. Rather, it uses a more moderate way of expressing itself, which is more respectful of and understanding the feelings and positions of the parties."}, "time_dimension": {"answer": "Present", "rationale": "The entry asks the testee about the perception and attitude of the child ' s responsibility in dealing with family conflicts, and the temporal dimension is now judged. The keyword \"How much should be done for the child\" suggests that this is a problem at hand, and \"a cause of our conflict\" suggests that the existence of this problem has an impact on the current family relationship."}, "sentence_type": "Interrogative sentence", "pos": {"nouns": {"Children": 1, "Things": 2, "Reason": 1, "Conflict": 1}, "verbs": {"I think so": 1, "Do it": 1, "be": 1, "Come on": 1}, "numerals": {"71": 1}, "pronouns": {"we": 1}}}, {"content": "72. I am indeed pleased to be with all my friends.", "word_count": 16, "label": "", "sentence_structure": "Subject鈥揷opula鈥損redicative", "subjective_objective": {"answer": "Subjective", "rationale": "Because happiness is an emotional experience, it belongs to the patient ' s own subjective feelings and attitudes."}, "sentence_sentiment": {"answer": "Positive", "rationale": "Because the entry \"Enjoyed to meet all friends of the spouses\" expresses positive attitudes and emotions."}, "language_style": {"answer": "Tactful and warm", "rationale": "The entry is a soft, inviting conversation, emphasizing personal pleasure rather than direct statements or orders. At the same time, the positive attitude of the other to the relationship between the spouse's friends is expressed by using the words \"actually\" and \"all\"."}, "time_dimension": {"answer": "Present", "rationale": "The entry for the questionnaire is a description of the current status and feelings of the testee, the keyword being \"surely happy\" and using the general current, which means that this feeling is continuing. It is therefore possible to judge the time dimension of the entry as it is."}, "sentence_type": "Affirmative declarative sentence", "pos": {"nouns": {"I": 1, "Spouse": 1, "friends": 1, "Travel": 1}, "verbs": {"Indeed": 1, "Happy": 1, "and": 1}, "adjectives": {"All of it": 1}, "numerals": {"72": 1}}}, {"content": "73. Because of our religious beliefs, I feel very close to my spouse.", "word_count": 18, "label": "", "sentence_structure": "Subject鈥揷opula鈥損redicative", "subjective_objective": {"answer": "Subjective", "rationale": "Because this entry describes the feelings of the patient and the spouse, which are their subjective experiences."}, "sentence_sentiment": {"answer": "Positive", "rationale": "Because the description of \"fearing intimacy\" in the entry is positive."}, "language_style": {"answer": "Tactful and warm", "rationale": "This is a more intimate and acceptable expression, which has a better communication effect."}, "time_dimension": {"answer": "Past", "rationale": "The keyword \"because\" and \"like\" all indicate that it is a description and a sense of past events, and therefore the temporal dimension of the article can be judged to be the past."}, "sentence_type": "Affirmative declarative sentence", "pos": {"nouns": {"Faith": 1, "Spouse": 1, "Intimacy": 1}, "verbs": {"I think": 1, "very": 1}, "adjectives": {"Because": 1, "of": 2, "Ours": 1, "Religion": 1, "Close": 1}, "numerals": {"73": 1}, "pronouns": {"I": 1}}}, {"content": "74. The wife should have faith in and acceptance of her husband ' s judgement on important issues.", "word_count": 20, "label": "", "sentence_structure": "Subject鈥搗erb鈥搊bject", "subjective_objective": {"answer": "Subjective", "rationale": "Since the content of the questionnaire relates to the subjective attitude and level of trust of the \"wife\", it is examined whether she is willing to believe and accept the judgement of her husband and is therefore subjective in nature."}, "sentence_sentiment": {"answer": "Positive", "rationale": "The view is positive, that the wife should trust and accept the husband ' s judgment on important issues."}, "language_style": {"answer": "Calm and direct", "rationale": "Calm down straight."}, "time_dimension": {"answer": "Present", "rationale": "In the past, the entry describes a traditional gender role and expectation that men are expected to be decision makers and supporters in the family. This view has been more prevalent in the past and may still affect the behaviour and perceptions of some people. The content of this questionnaire may therefore be considered a historical and past perspective."}, "sentence_type": "Affirmative declarative sentence", "pos": {"nouns": {"Wife": 1, "Significant issues": 1, "Judgement": 1}, "verbs": {"I think so": 1, "Yes": 1, "Accept": 1}, "adverbs": {"in": 1, "Go, go, go": 1}, "numerals": {"74": 1}, "pronouns": {"Husband": 1}}}, {"content": "75. Sometimes I am concerned that the sexual interests of the spouses are not in line with me.", "word_count": 17, "label": "", "sentence_structure": "Subject鈥搗erb鈥揷omplement", "subjective_objective": {"answer": "Subjective", "rationale": "Because \"care\" is the patient's subjective feeling and attitude. Although sexual interest may be an objective fact (i.e. whether the spouse is really interested in a sexual act), patients' feelings and reactions to this situation are subjective."}, "sentence_sentiment": {"answer": "Negative", "rationale": "Because the \"scareful\" in the entry is a negative attitude."}, "language_style": {"answer": "Tactful and warm", "rationale": "The entry uses a more euphemistic and moderate language style, which does not directly indicate whether the other party has a different sexual interest, but rather leads the respondents to express their thoughts and feelings by asking questions."}, "time_dimension": {"answer": "Present", "rationale": "\"Sometimes\" indicates that this is a regular act or condition, while \"very\" shows the emotional state and attitude of the testee. So, \"sometimes, I care that the sexual interest of the spouse is not in line with me.\" The description is the emotional experience and feelings of the testee, which are now time-dimensional."}, "sentence_type": "Affirmative declarative sentence", "pos": {"nouns": {"75": 1, "Time": 1, "I": 1, "Spouse": 1, "Sexual interest": 1, "Inconsistencies": 1}, "verbs": {"very": 1, "I care": 1, "and": 1}, "adverbs": {"Sometimes": 1}, "numerals": {"75": 1}, "pronouns": {"I": 1, "Spouse": 1}}}, {"content": "76. I am pleased with the decision on family planning and the number of children to be born.", "word_count": 19, "label": "", "sentence_structure": "Subject鈥搗erb鈥揷omplement", "subjective_objective": {"answer": "Subjective", "rationale": "This item examines patients鈥?subjective attitudes, i.e., their satisfaction with their decisions regarding family planning and the number of children they have."}, "sentence_sentiment": {"answer": "Positive", "rationale": "Because the description of \"satisfaction\" in the entry is a positive emotional attitude."}, "language_style": {"answer": "Tactful and warm", "rationale": "The language style of the entry is more euphemistic and warm, and the tone is more balanced and concerned and understanding by asking the other party whether it is satisfied with the family plan and the number of children to be born."}, "time_dimension": {"answer": "Present", "rationale": ""}, "sentence_type": "Affirmative declarative sentence", "pos": {"nouns": {"I": 1, "Family programme": 1, "Number of children born": 1, "Decision": 1}, "verbs": {"very": 1, "Satisfied": 1}, "adverbs": {"About": 1}, "numerals": {"76": 1}}}, {"content": "77. I do not care that a spouse is with a heterosexual friend.", "word_count": 14, "label": "", "sentence_structure": "Subject鈥損redicate", "subjective_objective": {"answer": "Subjective", "rationale": "Because \"not to care\" is a personal feeling and attitude that reflects the patient's attitude towards the relationship between his spouse and a heterosexual friend, and is a subjective examination."}, "sentence_sentiment": {"answer": "Negative", "rationale": "Because the \"insensitive\" attitude could have a negative impact on marriage."}, "language_style": {"answer": "Tactful and warm", "rationale": "The entry is based on the question of whether the other spouse is satisfied with their dealings with heterosexual friends and is expressed in a more euphemistic manner."}, "time_dimension": {"answer": "Present", "rationale": ""}, "sentence_type": "Negative declarative sentence", "pos": {"nouns": {"Spouse": 1, "Fae friend": 1, "Together": 1}, "verbs": {"I don't care": 1}, "numerals": {"77": 1}}}, {"content": "78. When I speak, the spouse listens carefully.", "word_count": 12, "label": "", "sentence_structure": "Subject鈥搗erb鈥搊bject", "subjective_objective": {"answer": "Subjective", "rationale": "Because \"I always listen carefully when I speak\" is a subjective feeling and an assessment, not an objective fact."}, "sentence_sentiment": {"answer": "Positive", "rationale": "Because the \"listen carefully\" act in the entry is positive, it means that the spouse has enough respect and attention to the person who speaks."}, "language_style": {"answer": "Tactful and warm", "rationale": "The entry uses a more euphemistic formulation, expresses a positive attitude by \"always\" and uses the positive behavioural verb \"listen\" to give a positive impression. Overall, the entry is more euphemistic, moderate and encouraging."}, "time_dimension": {"answer": "Present", "rationale": ""}, "sentence_type": "Affirmative declarative sentence", "pos": {"nouns": {"I": 1, "Talk": 1, "Spouse": 1, "Always": 1, "Seriously": 1, "Listen": 1}, "verbs": {"Say it": 1, "Listen": 1}, "adjectives": {"Seriously": 1}, "adverbs": {"Always": 1}, "numerals": {"78": 1}}}, {"content": "I care who cares about the money.", "word_count": 7, "label": "", "sentence_structure": "Subject鈥揷opula鈥損redicative", "subjective_objective": {"answer": "Subjective", "rationale": "Because \"care\" is a manifestation of subjective emotions and attitudes."}, "sentence_sentiment": {"answer": "Negative", "rationale": "Because the act of \"careful who cares about money\" may involve control and mistrust, with some negative emotional connotations."}, "language_style": {"answer": "Calm and direct", "rationale": "The entry is in a concise language asking respondents about financial management, without adding too many adorations or emotional colours, and with a more direct and objective language style."}, "time_dimension": {"answer": "Present", "rationale": "The keyword \"satisfaction\" indicates that it is a current feeling or attitude, so the entry describes the current state and the thoughts of the testee and falls within the scope of the present."}, "sentence_type": "Affirmative declarative sentence", "pos": {"nouns": {"Questionnaire": 1, "Entry": 1, "person": 1, "Money": 1}, "verbs": {"I care": 1, "Pipe": 1}, "adverbs": {"79": 1}, "numerals": {"79": 1}, "classifiers": {"individual": 1}}}, {"content": "80. I am troubled by the unfair manner in which spouses agree or refuse to have sex.", "word_count": 23, "label": "", "sentence_structure": "Subject鈥搗erb鈥搊bject", "subjective_objective": {"answer": "Objective", "rationale": "The observation of \"I'm upset\" in the sentence shows that it is a subjective feeling of dissatisfaction and distress among patients about the behaviour of their partners. So the questionnaire is subjective in its approach. The basis for the judgement is: - Subjectivity: the sentence contains words that describe the patient's emotions, feelings, or wishes, such as \"problem.\" Objective: the sentence describes objective facts or behaviour, such as \"the spouse's unfair way of agreeing or refusing to have sex.\""}, "sentence_sentiment": {"answer": "Negative", "rationale": "Because the \"unfair way of agreeing or refusing to have sex\" in the entry is negative."}, "language_style": {"answer": "Tactful and warm", "rationale": ""}, "time_dimension": {"answer": "Present", "rationale": "The entry describes the current feeling and state of the testee, and the keyword \"suffering\" suggests that it is an emotional reaction that is going on. So, judging by the dimensions of time, the entry is now."}, "sentence_type": "Affirmative declarative sentence", "pos": {"nouns": {"Spouse": 1, "Modalities": 1, "Sex": 1, "Trouble": 1}, "verbs": {"Apply": 1, "Agreed": 1, "Reject": 1}, "adjectives": {"It's not fair": 1}, "numerals": {"80": 1}, "pronouns": {"I": 1}}}, {"content": "81. When we argue, I usually do not think it was my fault.", "word_count": 18, "label": "", "sentence_structure": "Subject鈥搗erb鈥揷omplement", "subjective_objective": {"answer": "Subjective", "rationale": "Because it involves the views and opinions of the patient himself, it is a subjective attitude.\""}, "sentence_sentiment": {"answer": "Negative", "rationale": "Because the entry \"not to think it was my fault\" shows a reluctance to take responsibility in a fight, and this attitude is negative."}, "language_style": {"answer": "Tactful and warm", "rationale": "The entry is more personal and interactive, by means of a gentle and inviting tone of speech rather than by direct statement or order. At the same time, the entry does not use too strong language to guide respondents to answer questions, but rather uses a more euphemism that makes them more willing to share their real ideas."}, "time_dimension": {"answer": "Present", "rationale": "The entry describes \"we\" when we argue, \"I\" is usually not thinking about \"my fault\" and means it's a customary, regular act, so it's judged as \"now\"."}, "sentence_type": "Negative declarative sentence", "pos": {"nouns": {"Argument": 1, "Wrong": 1}, "verbs": {"Go on": 1, "Yes": 1}, "adverbs": {"Usually": 1}, "numerals": {"81": 1}, "pronouns": {"I": 1}}}, {"content": "82. I feel good about our religious beliefs and values.", "word_count": 18, "label": "", "sentence_structure": "Subject鈥揷opula鈥損redicative", "subjective_objective": {"answer": "Subjective", "rationale": "Because it is the patient鈥檚 subjective attitude and feelings. Although religious beliefs and values are objective things, patients鈥?feelings and perceptions of them are subjective."}, "sentence_sentiment": {"answer": "Positive", "rationale": "Because the expression \"well felt good\" in the entry is a positive expression."}, "language_style": {"answer": "Tactful and warm", "rationale": "The language style of the entry is euphemism. It expresses a positive view of religious beliefs and values, but does not directly emphasize this or criticize the different views that others may have, but expresses itself in a more moderate, respectful and understanding way. This language style is more likely to create a good atmosphere of communication and mutual respect."}, "time_dimension": {"answer": "Present", "rationale": "The keyword \"well felt\" is the person's perception of the current situation, so the time dimension of the entry is now."}, "sentence_type": "Affirmative declarative sentence", "pos": {"verbs": {"I think": 1}, "adjectives": {"Okay": 1}, "adverbs": {"very": 1}, "numerals": {"82": 1}, "pronouns": {"Ours": 1, "I": 1}}}, {"content": "83. The time spent on the business with my spouse and in separate travels was fairly distributed.", "word_count": 22, "label": "", "sentence_structure": "Subject鈥搗erb鈥搊bject", "subjective_objective": {"answer": "Subjective", "rationale": "Because \"I have a fair distribution of time between my spouse and my spouse's work\" is a patient's self-evaluation and subjective view."}, "sentence_sentiment": {"answer": "Positive", "rationale": "Because the \"fair distribution of time for the journey\" is positive."}, "language_style": {"answer": "Tactful and warm", "rationale": "The entry is more adept and interactive when it is spoken in a gentle and inviting manner, rather than by direct statements or orders."}, "time_dimension": {"answer": "Present", "rationale": ""}, "sentence_type": "Affirmative declarative sentence", "pos": {"nouns": {"I": 1, "Spouse": 1, "Business time": 1}, "verbs": {"Allocation": 1, "in": 1, "Pass": 1}, "adjectives": {"Fair": 1}, "numerals": {"83": 1}}}, {"content": "84. Sometimes I think that spouses are too aggressive.", "word_count": 13, "label": "", "sentence_structure": "Subject鈥揷opula鈥損redicative", "subjective_objective": {"answer": "Subjective", "rationale": "Because \"too much to be aggressive\" is a subjective assessment and feeling."}, "sentence_sentiment": {"answer": "Negative", "rationale": "Because the description of \"spouses too much to be a man\" in the entry is negative."}, "language_style": {"answer": "Tactful and warm", "rationale": "The entry implies, by using the term \"conceptive\" to suggest the attitude and feelings of the respondent towards his spouse, rather than directly accusing or criticizing him. Moreover, the term \"insolent\" is more subtle, without using overly sharp or harsh language, thus reflecting a euphemism."}, "time_dimension": {"answer": "Present", "rationale": ""}, "sentence_type": "Negative declarative sentence", "pos": {"nouns": {"Questionnaire Entry": 1, "Time": 1, "I think": 1, "Spouse": 1, "You're a badass": 1}, "verbs": {"have": 1, "I think": 2}, "adjectives": {"Too much": 1}, "numerals": {"84": 1}, "pronouns": {"I": 1}}}, {"content": "85. I do not think that any spouse living together has any harmony between us.", "word_count": 22, "label": "", "sentence_structure": "Subject鈥揷opula鈥損redicative", "subjective_objective": {"answer": "Subjective", "rationale": "Because \"I think\" shows that it's a patient's personal view and conviction, and it's a subjective perspective."}, "sentence_sentiment": {"answer": "Negative", "rationale": "This comparative language is negative in emotional terms because it expresses dissatisfaction with one ' s marital life and the affirmation of another ' s marital life."}, "language_style": {"answer": "Tactful and warm", "rationale": "The entry expresses a high rating of the status of the marriage in a positive and optimistic manner, without directly denigrating or criticizing the marriage of others, which is more moderate and friendly."}, "time_dimension": {"answer": "Present", "rationale": ""}, "sentence_type": "Negative declarative sentence", "pos": {"nouns": {"Spouse": 2, "Life": 1, "Couples": 2}, "verbs": {"I think": 1, "Harmony": 1}, "adjectives": {"No, I'm not": 1, "Any": 1, "Together": 1}, "adverbs": {"Both": 1, "we": 1}, "numerals": {"85": 1}, "pronouns": {"I": 1}}}, {"content": "86. Sometimes I feel like I have no love or affection for a spouse.", "word_count": 16, "label": "", "sentence_structure": "Subject鈥揷opula鈥損redicative", "subjective_objective": {"answer": "Subjective", "rationale": "Because \"I feel like I can't feel love and feelings for my spouse\" is a subjective feeling for the patient."}, "sentence_sentiment": {"answer": "Negative", "rationale": "Because the description \"I can't feel love and feelings\" in the entry is negative."}, "language_style": {"answer": "Tactful and warm", "rationale": "The language style of the entry is a soft and warmer type. It asks the interviewee in a more soft, indirect way whether he or she has ever had a feeling of love and affection."}, "time_dimension": {"answer": "Present", "rationale": "Now the entry uses the usual current \"fright\" to describe the feelings of the testee, implying that they are present, ongoing, not past or future. At the same time, \"sometimes\" also means that it is an occasional feeling, not always. So, the entry focuses more on the current emotional state of the testee, which should be \"now\"."}, "sentence_type": "Negative declarative sentence", "pos": {"nouns": {"Questionnaire": 1, "Entry": 1, "Spouse": 1, "Love": 1, "Emotion": 2}, "verbs": {"I think": 1}, "adverbs": {"Sometimes": 1}, "numerals": {"86": 1}}}, {"content": "87. Sometimes, spouses do things that are not pleasant to them.", "word_count": 14, "label": "", "sentence_structure": "Subject鈥搗erb鈥搊bject", "subjective_objective": {"answer": "Subjective", "rationale": "This is because of patients ' subjective feelings and perceptions of the behaviour of their spouses."}, "sentence_sentiment": {"answer": "Negative", "rationale": "Because the \"spouse does something that makes me unhappy\" in the entry is negative."}, "language_style": {"answer": "Tactful and warm", "rationale": "The language style of the entry is more euphemistic. It does not directly criticize or accuse the other spouse of doing something wrong, but rather expresses its feelings about certain behaviours in a relatively objective way. Such expression helps to reduce conflict and enhance the effectiveness of dialogue."}, "time_dimension": {"answer": "Present", "rationale": "The entry describes the current situation of the person being tested, and the keywords \"sometimes\" and \"do something\" indicate that the act is being or exists, so it is now judged."}, "sentence_type": "Affirmative declarative sentence", "pos": {"nouns": {"Spouse": 1, "Yes": 1}, "verbs": {"Do it": 1, "鈾?And I'm gonna be 鈾?: 1, "It's not good": 1}, "adjectives": {"Have a nice day": 1}, "adverbs": {"Sometimes": 1}, "numerals": {"87": 1}}}, {"content": "88. I am not aware of any faults of the spouses.", "word_count": 14, "label": "", "sentence_structure": "Subject鈥搗erb鈥搊bject", "subjective_objective": {"answer": "Subjective", "rationale": "Because \"not knowing\" is a description of the patient's subjective attitude and state of consciousness."}, "sentence_sentiment": {"answer": "Negative", "rationale": "Because the attitude of \"not knowing the fault of the spouse\" in the entry is negative."}, "language_style": {"answer": "Tactful and warm", "rationale": "This is a more euphemistic and implicit way of expressing one鈥檚 own spouse鈥檚 awareness and understanding in a moderate way."}, "time_dimension": {"answer": "Present", "rationale": "In the entry, the words \"if there is\" and \"if not aware\" indicate that this is a description and perception of current marital status, so the temporal dimension is now judged."}, "sentence_type": "Negative declarative sentence", "pos": {"nouns": {"Spouse": 1, "Wrong": 1, "Awareness": 1}, "verbs": {"have": 1, "I didn't realize": 2}, "numerals": {"88": 1}, "pronouns": {"I": 1}}}, {"content": "120. Even if every person of the opposite sex in the world wishes to marry me, I cannot make a better choice than the marriage that is now.", "word_count": 32, "label": "", "sentence_structure": "Subject鈥損redicate", "subjective_objective": {"answer": "Subjective", "rationale": "This is because patients are subjectively evaluating and expressing their current marital status."}, "sentence_sentiment": {"answer": "Negative", "rationale": "The entry is somewhat negative because of the firm and unchanging attitude expressed in the entries towards existing marriages."}, "language_style": {"answer": "Tactful and warm", "rationale": "The language style of the entry is more euphemistic and soft, raising questions in a way that is invited and explored, rather than direct orders or statements. At the same time, the phrase \"even if\" is used in the language, which expresses a relatively gentle attitude and encourages respondents to share their ideas. The language style of the questionnaire entry is therefore considered to be euphemistic and warm."}, "time_dimension": {"answer": "Present", "rationale": "The word \"also\" in the title of the topic indicates that even in extreme cases (i.e. \"every person in the world who is different wants to marry me\") the decision cannot be changed. This indicates the degree of satisfaction of the person tested with the current marital status and that this behaviour or mentality is taking place now."}, "sentence_type": "Affirmative declarative sentence", "pos": {"nouns": {"World": 1, "Heterosexual": 1, "Marriage": 1}, "verbs": {"May I": 1, "and": 1, "Conclusion": 1, "Do it": 1, "Out": 1, "- Yeah": 1, "Better": 1, "Selection": 1}, "adverbs": {"Both": 1, "More": 1}, "numerals": {"120": 1, "Every one of them": 1}, "pronouns": {"I": 1}}}, {"content": "121. Our husband and wife are better suited to each other than anyone else in the world.", "word_count": 18, "label": "", "sentence_structure": "Subject鈥搗erb鈥揷omplement", "subjective_objective": {"answer": "Subjective", "rationale": "Because \"good-to-good\" is a subjective assessment based on the patient's perception and feelings about their marriage."}, "sentence_sentiment": {"answer": "Positive", "rationale": "Because the words in the entry \"the couple is better suited to each other than anyone else in the world\" are positive."}, "language_style": {"answer": "Tactful and warm", "rationale": "The entry uses a more euphemistic language style, which is a positive expression of the excellent relationship between the spouses, the compassionate and positive emotional colours. It is also used to reinforce the tone and the emotional colour of expression by using the relatively exaggerated term \"the world's most diverse\"."}, "time_dimension": {"answer": "Present", "rationale": "The entry describes the current state and feeling, and therefore judges \"now\"."}, "sentence_type": "Affirmative declarative sentence", "pos": {"nouns": {"We're married": 1, "World": 1, "person": 1, "Adaptation": 1}, "verbs": {"- Yeah": 1, "To each other": 1, "Okay": 1}, "numerals": {"121": 1}}}, {"content": "122. I am pleased with every new thing about a spouse.", "word_count": 16, "label": "", "sentence_structure": "Subject鈥搗erb鈥揷omplement", "subjective_objective": {"answer": "Subjective", "rationale": "Because making me happy is the patient's subjective feeling and attitude."}, "sentence_sentiment": {"answer": "Positive", "rationale": "Because the \"I'm happy\" act in the entry is positive."}, "language_style": {"answer": "Tactful and warm", "rationale": "And by using the first person's name, I am the person who has been the direct and personal friend of the language. So the language style of this questionnaire entry is euphemism."}, "time_dimension": {"answer": "Present", "rationale": ""}, "sentence_type": "Affirmative declarative sentence", "pos": {"nouns": {"Annex": 1, "Something new": 1, "Happy": 1}, "verbs": {"鈾?And I'm gonna be 鈾?: 1}, "adjectives": {"Every piece": 1}, "numerals": {"122": 1}}}, {"content": "123. Our relationship is better than it should be.", "word_count": 14, "label": "", "sentence_structure": "Subject鈥揷opula鈥損redicative", "subjective_objective": {"answer": "Subjective", "rationale": "Because \"the relationship between us\" is a subjective concept, with different people having different views and feelings, it is viewed from the subjective perspective of the patient."}, "sentence_sentiment": {"answer": "Positive", "rationale": "Because the expression \"better than it should\" in the entry expresses a positive emotional attitude."}, "language_style": {"answer": "Tactful and warm", "rationale": "The entry is more adept and interactive when it is spoken in a gentle and inviting manner, rather than by direct statements or orders."}, "time_dimension": {"answer": "Present", "rationale": "By the way, \"our relationship\" and \"better than it should be\" describe the situation, so the temporal dimension of the article is now."}, "sentence_type": "Affirmative declarative sentence", "pos": {"verbs": {"be": 1, "have": 1, "- Yeah": 1, "Better": 1}, "adjectives": {"More": 1}, "numerals": {"123": 1, "It's in the right place": 1}, "pronouns": {"Our relationship": 1}}}, {"content": "124. When I am with my spouse, I do not think anyone can be happier than us.", "word_count": 24, "label": "", "sentence_structure": "Subject鈥揷opula鈥損redicative", "subjective_objective": {"answer": "Subjective", "rationale": "Because it is not possible for anyone to feel and evaluate happiness more than he is."}, "sentence_sentiment": {"answer": "Positive", "rationale": "The entries are written in the form of positive emotional attitudes, which are expressed in terms of recognition of and confidence in their relationship with their spouses."}, "language_style": {"answer": "Tactful and warm", "rationale": "The entry describes the individual鈥檚 feelings of being with his or her spouse in a softer way and emphasizes his or her happiness in a more comparable and advanced way. This language style has a certain degree of affection and emotional impact, which is better able to guide the person surveyed to open the box and share more real voices."}, "time_dimension": {"answer": "Present", "rationale": "The entry describes the person who was tested now in his senses and thoughts, and \"when I'm with my spouse\" is a time-style expression of what is happening now. So the basis of the judgement is now."}, "sentence_type": "Affirmative declarative sentence", "pos": {"nouns": {"Spouse": 1, "person": 1, "Happiness": 1}, "verbs": {"I think": 1, "in": 1, "- Yeah": 1}, "adverbs": {"When": 1, "Any": 1, "Impossible": 1}, "numerals": {"124": 1}, "classifiers": {"individual": 1}, "pronouns": {"I": 1, "we": 1}}}]}

{"questionnaire_title": "Big 5 personality questionnaire", "questionnaire_category": "Self-cognition", "item_count": 60, "emotion_shift_overall": "From positive to negative", "language_style_overall": "Neutral", "items": [{"content": "I am not a man of trouble.", "word_count": 11, "label": "", "sentence_structure": "Subject鈥揷opula鈥損redicative", "subjective_objective": {"answer": "Subjective", "rationale": "Because \"I'm not a person of trouble\" is a subjective view and self-evaluation of a person, a subjective view."}, "sentence_sentiment": {"answer": "Negative", "rationale": "Because the article \"I'm not a person of trouble\" is negative, implying that the interviewee may have some trouble or distress, and therefore is negative emotional."}, "language_style": {"answer": "Calm and direct", "rationale": "The language style of the entry is relatively calm and straightforward, and it clearly expresses the subject of the questionnaire, namely, whether the person surveyed is a person who is often troubled. Moreover, the entry does not use excessive rhetorical techniques or emotional rendering, is more balanced and objective, and therefore can be judged as \"cool and direct\"."}, "time_dimension": {"answer": "Present", "rationale": "The entry expresses the subjective assessment of the subject, using the general current \"no\" and the current state of affairs."}, "sentence_type": "Negative declarative sentence", "pos": {"nouns": {"person": 1, "Trouble": 1}, "verbs": {"No, it's not": 1, "Fill": 1}, "adjectives": {"One": 1}, "numerals": {"One": 2}, "pronouns": {"I": 1}}}, {"content": "I really like most of the people I meet.", "word_count": 13, "label": "", "sentence_structure": "Subject鈥搗erb鈥揷omplement", "subjective_objective": {"answer": "Subjective", "rationale": "Like being patient subjective."}, "sentence_sentiment": {"answer": "Positive", "rationale": "Because the entry is a positive emotion and attitude, i.e. a preference for most of the people encountered."}, "language_style": {"answer": "Tactful and warm", "rationale": "The expression in the entry is more moderate, not directly confirming or rejecting one's attitude to the person it meets, but rather expressing its feelings in a way that is more euphemismal and friendly, as it is \"real likes.\" At the same time, the description \"most people I meet\" is not too absolute or biased, and expresses an attitude of tolerance and understanding, which is more in keeping with the \"false\" language style."}, "time_dimension": {"answer": "Present", "rationale": ""}, "sentence_type": "Affirmative declarative sentence", "pos": {"nouns": {"Investigations": 1, "Questionnaire": 1, "Entry": 1}, "verbs": {"Analysis": 1, "Yeah": 1, "I met him": 1}, "adjectives": {"Really": 1, "Most of them": 1}, "numerals": {"2": 1}, "pronouns": {"I": 1}}}, {"content": "I do not like to waste time in daydreaming.", "word_count": 13, "label": "", "sentence_structure": "Subject鈥搗erb鈥搊bject", "subjective_objective": {"answer": "Subjective", "rationale": "Because \"like to do or not\" is a subjective attitude and choice of a human being."}, "sentence_sentiment": {"answer": "Negative", "rationale": "Because the entry \"not to waste time on daydreams\" is negative."}, "language_style": {"answer": "Calm and direct", "rationale": "The language style of the entry is more concise, not using excessive rhetorical techniques or emotional rendering, but rather directly describing the attitude of the other side to waste time and to daydreaming. This language style is more objective and practical."}, "time_dimension": {"answer": "Present", "rationale": "\"Don't like\" and \"Dreaming Day\" are both current state or behavior, so the entry is asking the testee about his current thoughts and behavior and judging as it is."}, "sentence_type": "Negative declarative sentence", "pos": {"nouns": {"Time": 1, "Daydreams": 1}, "verbs": {"Do it": 1, "Yeah": 1, "Wasted": 1}, "adjectives": {"White": 1, "I don't like it": 2}, "numerals": {"3": 1}, "pronouns": {"I": 1}}}, {"content": "I would doubt and sarcasm the attempts of others.", "word_count": 12, "label": "", "sentence_structure": "Subject鈥搗erb鈥搊bject", "subjective_objective": {"answer": "Subjective", "rationale": "Because the attempt to suspect and sarcastic others is a subjective attitude or view, determined by subjective factors such as the individual ' s perception, emotions, values, etc., rather than by objective facts."}, "sentence_sentiment": {"answer": "Negative", "rationale": "Because the \"suspicion and sarcasm\" in the entry is a negative act."}, "language_style": {"answer": "Tactful and warm", "rationale": "The entry is presented in a more euphemistic way, without a direct critical or negative tone, but in a more moderate and respectful way of expressing a view of the behaviour of the other party in a question-and-answer manner."}, "time_dimension": {"answer": "Past", "rationale": "The keyword \"will\" means a customary act or state of conduct which has existed for some time and can therefore be judged as \"the past\"."}, "sentence_type": "Affirmative declarative sentence", "pos": {"nouns": {"Questionnaire": 1, "Entry": 1, "others": 1, "Attempt": 1}, "verbs": {"Yes": 1, "Doubt": 1, "Ironic": 1}, "numerals": {"4": 1}, "pronouns": {"I": 1}}}, {"content": "5. I am efficient and competent at work.", "word_count": 14, "label": "", "sentence_structure": "Subject鈥揷opula鈥損redicative", "subjective_objective": {"answer": "Subjective", "rationale": "Because this evaluation involves patients ' perception of themselves and self-perception, it is an expression of subjective attitudes or beliefs."}, "sentence_sentiment": {"answer": "Positive", "rationale": "Because the expression in the entry is \"efficient and competent\", it is a positive assessment of an individual's ability to work."}, "language_style": {"answer": "Calm and direct", "rationale": "The entry directly expresses a question about whether the other party is able to perform its tasks efficiently and competently in the performance of its work, without using excessive rhetorical or emotional rendering and with a more objective tone."}, "time_dimension": {"answer": "Present", "rationale": "Among them, \"efficient and competent\" describes self-evaluation by the testee, demonstrating his recognition of his efficiency and competence."}, "sentence_type": "Affirmative declarative sentence", "pos": {"nouns": {"Work": 1, "Efficiency": 1, "Competencies": 1}, "verbs": {"be": 1}, "adjectives": {"Efficient": 1}}}, {"content": "6. I have little fear and anxiety.", "word_count": 10, "label": "", "sentence_structure": "Subject鈥揷opula鈥損redicative", "subjective_objective": {"answer": "Subjective", "rationale": "Because \"fear and anxiety\" is a psychological experience and feeling of a person, a subjective attitude of the patient."}, "sentence_sentiment": {"answer": "Neutral", "rationale": "Because the entry describes the state of personal feelings, there is no clear positive or negative sentiment."}, "language_style": {"answer": "Tactful and warm", "rationale": "The entry uses a positive expression ( \"very rarely feeling fear and anxiety\") and expresses a positive and confident emotional attitude, which is more comforting and supportive and makes the interviewee feel comfortable and relaxed."}, "time_dimension": {"answer": "Present", "rationale": "The keyword is \"very little\" , which means \"low frequency,\" and \"fear and anxiety\" which means psychological state. So the sentence is the person who was tested in the emotional state of the moment, and it does not relate to past or future situations."}, "sentence_type": "Negative declarative sentence", "pos": {"verbs": {"I feel": 1, "Fear": 1, "Anxiety": 1}, "adjectives": {"Very few": 1}}}, {"content": "I like talking to others.", "word_count": 9, "label": "", "sentence_structure": "Subject鈥揷opula鈥損redicative", "subjective_objective": {"answer": "Subjective", "rationale": "Because \"like\" expresses the positive attitude and preferences of patients to talk to people, which is the subjective feeling and perception of patients."}, "sentence_sentiment": {"answer": "Positive", "rationale": "Because the expression \"likes\" in the entry is positive."}, "language_style": {"answer": "Tactful and warm", "rationale": ""}, "time_dimension": {"answer": "Present", "rationale": ""}, "sentence_type": "Affirmative declarative sentence", "pos": {"verbs": {"very": 1, "Yeah": 1, "and": 1, "others": 1, "Talk": 1}, "adjectives": {"Yeah": 1}, "adverbs": {"very": 1}, "pronouns": {"I": 1}}}, {"content": "8. The pattern of nature and art makes me feel very good.", "word_count": 19, "label": "", "sentence_structure": "Subject鈥揷opula鈥損redicative", "subjective_objective": {"answer": "Subjective", "rationale": "Because the sentence is a subjective one, which is the person's feelings and experience."}, "sentence_sentiment": {"answer": "Positive", "rationale": "Because the phrase \"natural and artistic patterns make me feel so good\" in the entry expresses the author's love and appreciation of nature and art, and positive feelings."}, "language_style": {"answer": "Tactful and warm", "rationale": ""}, "time_dimension": {"answer": "Present", "rationale": "The sentence expresses the sense of the testee of nature and the pattern of art, which does not relate to the time dimension of the past or the future, and can therefore be judged as now."}, "sentence_type": "Affirmative declarative sentence", "pos": {"nouns": {"Nature": 1, "Arts": 1, "Pattern": 1, "Form": 1, "Omei": 1}, "verbs": {"鈾?And I'm gonna be 鈾?: 1, "I feel": 1, "Very": 1}, "adjectives": {"Omei": 1}, "adverbs": {"Extreme": 1}}}, {"content": "I believe that many people would do so if you allowed others to take advantage of you.", "word_count": 23, "label": "", "sentence_structure": "Subject鈥搗erb鈥搊bject", "subjective_objective": {"answer": "Objective", "rationale": "Because it is an observation and inference of the behaviour of others, it does not involve subjective attitudes or feelings of individuals. \""}, "sentence_sentiment": {"answer": "Negative", "rationale": "Because the idea of \"convincing if anyone is allowed to take advantage of you\" in the entry is negative."}, "language_style": {"answer": "Tactful and warm", "rationale": "The expression is more personal and interactive, in a gentle and inviting tone, rather than in direct statements or orders. Moreover, the tone of the article is one of persuasion rather than direct accusations of the behaviour of the other, which is also consistent with the euphemism of the language style."}, "time_dimension": {"answer": "Present", "rationale": "\"Believe\" and \"Permissive\" are verbs describing current attitudes or beliefs, indicating that the testee is thinking and holding such views. Thus, the entry expresses the current situation."}, "sentence_type": "Affirmative declarative sentence", "pos": {"nouns": {"Yes": 1, "person": 2, "you": 2, "Cheap": 1, "A lot": 1, "Do it": 1}, "verbs": {"Allow": 1, "Jim": 1, "Yes": 2}, "adjectives": {"Here": 1, "Sample": 1}, "adverbs": {"Both": 1, "If": 1}, "numerals": {"Nine": 1, "A lot": 1}, "pronouns": {"you": 2, "they": 1}}}, {"content": "I will keep my things neat and clean.", "word_count": 13, "label": "", "sentence_structure": "Subject鈥搗erb鈥揷omplement", "subjective_objective": {"answer": "Subjective", "rationale": "Because keeping the item in order and cleaning is the subjective attitude and choice of the individual. While this may also be due to acts caused by environmental requirements or other external factors, it is described in this context as an individual ' s decision or conduct and is therefore viewed from a subjective perspective."}, "sentence_sentiment": {"answer": "Positive", "rationale": "Because the behaviour in the entry, \"maintain the things in order and clean\" is positive, and it is a sign of responsibility and good habits."}, "language_style": {"answer": "Tactful and warm", "rationale": "The language style of the entry is more euphemistic and moderate, and it is not directly ordered or critical, but rather directed to the respondent by means of inquiries, which are more acceptable and cooperative."}, "time_dimension": {"answer": "Present", "rationale": "The word \"will\" in the entry indicates a future intention or plan, but it is a personal custom by using the verb of the first person ' s verb. Thus, the temporal dimension is now judged."}, "sentence_type": "Affirmative declarative sentence", "pos": {"nouns": {"Object": 1, "Clean": 1, "Cleaning": 1}, "verbs": {"Yes": 1, "Hold on": 1, "Quite neat": 1, "and": 1}, "adjectives": {"Nice and neat": 1, "Clean": 1}, "numerals": {"10": 1}}}, {"content": "I am often nervous and unstable.", "word_count": 12, "label": "", "sentence_structure": "Subject鈥搗erb鈥揷omplement", "subjective_objective": {"answer": "Subjective", "rationale": "Because it's a subjective experience, \"often nervous and unstable\" is a description of the patient's subjective feeling."}, "sentence_sentiment": {"answer": "Negative", "rationale": "Because \"stretch and frenzy\" is a negative emotional state."}, "language_style": {"answer": "Tactful and warm", "rationale": "The entry is more proficient and interactive by speaking in a gentle and inviting manner rather than by direct statement or order. At the same time, the article is more euphemistic, warm and does not cause undue psychological pressure on the interviewees by using the terms \"stress\" and \"deepness\"."}, "time_dimension": {"answer": "Present", "rationale": "The keyword \"often feels\" indicates that it is an ongoing act or state of affairs that is taking place."}, "sentence_type": "Affirmative declarative sentence", "pos": {"nouns": {"I": 1, "Nervous": 1, "Mind": 1, "Uncertainty": 1}, "verbs": {"Sensor": 1, "and": 1, "Freeze": 1}, "adverbs": {"Regular": 1}, "numerals": {"11": 1}}}, {"content": "I like the many people around me.", "word_count": 10, "label": "", "sentence_structure": "Subject鈥搗erb鈥揷omplement", "subjective_objective": {"answer": "Subjective", "rationale": "Because \"like\" is a subjective feeling and attitude of a person, a personal perception and assessment of the surrounding environment, and therefore a subjective perspective."}, "sentence_sentiment": {"answer": "Neutral", "rationale": "Because the entry is not clearly emotional, it merely describes a fact."}, "language_style": {"answer": "Tactful and warm", "rationale": "The entry is asked in an invitation-style, softer language, less than a too direct or hard language, which is more comfortable and willing to participate. At the same time, the question of the purpose of the article is not stressful or uncomfortable, and therefore can be judged as a soft and warm language."}, "time_dimension": {"answer": "Present", "rationale": "Now the entry describes the current emotional state and preferences of the testees, i.e. whether they like a lot of people around them. Because emotions and preferences usually change with time and circumstances, the time dimension of the entry is now."}, "sentence_type": "Affirmative declarative sentence", "pos": {"nouns": {"person": 1, "Around": 1}, "verbs": {"Yeah": 1, "in": 1}, "numerals": {"A lot": 1}, "classifiers": {"individual": 1}, "pronouns": {"I": 1}}}, {"content": "I have only a little or no feelings about poetry.", "word_count": 16, "label": "", "sentence_structure": "Subject鈥揷opula鈥損redicative", "subjective_objective": {"answer": "Subjective", "rationale": "Because \"feel\" and \"involved\" are the subjective feelings of the patient."}, "sentence_sentiment": {"answer": "Negative", "rationale": "Because the expression \"only a little feeling or even indifference to poetry\" has a negative mood."}, "language_style": {"answer": "Calm and direct", "rationale": "The entry is more calm and objective in its language, with no excessive emotional or rhetorical style. It directly asks the respondents about their feelings about poetry and clearly expresses both \"slightly feeling\" or \"indifferent.\""}, "time_dimension": {"answer": "Present", "rationale": "In the entry, \"sweetly\" and \"indifferently\" indicate the feelings of the subjects to the poem, which are present, present, so the temporal dimension is now."}, "sentence_type": "Negative declarative sentence", "pos": {"nouns": {"Poetry": 1, "Feel": 1, "Move": 1}, "verbs": {"have": 1, "to": 1, "Nothing": 1}, "adjectives": {"A little bit": 1, "Even": 1}, "adverbs": {"I mean": 1}, "numerals": {"13": 1}, "pronouns": {"I": 1}}}, {"content": "14. If needed, I would manipulate others to what I wanted.", "word_count": 19, "label": "", "sentence_structure": "Subject鈥搗erb鈥搊bject", "subjective_objective": {"answer": "Subjective", "rationale": "Because \"whom I want to be able to manipulate\" is the patient's will and attitude, and it's subjective."}, "sentence_sentiment": {"answer": "Negative", "rationale": "Because the \"manipulation\" in the entries is negative."}, "language_style": {"answer": "Calm and direct", "rationale": "The entry is a direct statement asking the other party whether it is willing to manipulate others to achieve its own goals, and the language style is more calm and objective."}, "time_dimension": {"answer": "Present", "rationale": "Now the word \"will\" in the entry indicates possible future behaviour, but in this sentence, the condition \"if necessary\" makes it possible. So, although this action has not yet taken place, it is a statement with future orientation, so the temporal dimension is now judged."}, "sentence_type": "Affirmative declarative sentence", "pos": {"verbs": {"Yes": 4, "Go on": 1, "Manipulation": 1, "others": 1, "Achieved": 1}, "adverbs": {"If": 1}, "numerals": {"14": 1}, "pronouns": {"I": 1}}}, {"content": "I am not a methodical person.", "word_count": 13, "label": "", "sentence_structure": "Subject鈥揷opula鈥損redicative", "subjective_objective": {"answer": "Subjective", "rationale": "Because \"not a methodical person\" is an individual's perception and assessment of his or her own, and it is subjective."}, "sentence_sentiment": {"answer": "Negative", "rationale": "Because the expression \"not being a methodical person\" in the entry is a negative assessment of the individual's abilities and has a negative emotional connotation."}, "language_style": {"answer": "Calm and direct", "rationale": "The entry describes the person surveyed in a direct and clear manner his or her own assessment, without using excessive rhetorical or emotional colours, and with a more objective and calm tone."}, "time_dimension": {"answer": "Present", "rationale": "The entry describes the personality characteristics or customary behaviour of the person being tested, so the temporal dimension is now judged."}, "sentence_type": "Negative declarative sentence", "pos": {"nouns": {"person": 1}, "verbs": {"be": 1, "Do it": 1, "Orderly": 1, "Say it": 1}, "adjectives": {"No, it's not": 1}, "adverbs": {"One": 1, "have": 1, "individual": 1}, "numerals": {"15": 1}}}, {"content": "16. I am often angry at the way I have been treated.", "word_count": 15, "label": "", "sentence_structure": "Subject鈥揷opula鈥損redicative", "subjective_objective": {"answer": "Subjective", "rationale": "Because the emotional reaction to \"others treat me\" and \"I'm angry\" is from the patient's own perspective, and it's the patient's subjective feeling."}, "sentence_sentiment": {"answer": "Negative", "rationale": "Because the \"I'm Anger\" emotion in the entry is negative."}, "language_style": {"answer": "Calm and direct", "rationale": "The point is simple: the question is not one of rhetorical or emotional clarity, but of directness to ask if the interviewee often feels anger about the words and actions of others in interpersonal relationships. This language style is more direct, objective and rational, and is quite different from the euphemism of the warm language."}, "time_dimension": {"answer": "Past", "rationale": "The output result is \"uncertain.\""}, "sentence_type": "Affirmative declarative sentence", "pos": {"nouns": {"others": 1, "I": 1, "Modalities": 1, "Anger": 1}, "verbs": {"Treatment": 1, "鈾?And I'm gonna be 鈾?: 1, "I feel": 1}, "adverbs": {"Regular": 1}}}, {"content": "17. When I read a poem or appreciate an art, I sometimes get excited or surprised.", "word_count": 27, "label": "", "sentence_structure": "Subject鈥揷opula鈥損redicative", "subjective_objective": {"answer": "Subjective", "rationale": "Because \"excited\" and \"surprise\" feel is the patient's subjective experience."}, "sentence_sentiment": {"answer": "Positive", "rationale": "Because \"excited or surprised\" in the entry is a positive emotional reaction."}, "language_style": {"answer": "Tactful and warm", "rationale": ""}, "time_dimension": {"answer": "Present", "rationale": "In the entry, \"When I read a poem or appreciate an art\" means what is being done, while \"sometimes I get excited or surprised\" means it's a regular emotional experience. So the time dimension of this questionnaire is now."}, "sentence_type": "Affirmative declarative sentence", "pos": {"nouns": {"First": 1, "Poetry": 1, "Annex": 1, "Artwork": 1}, "verbs": {"Reading": 1, "Enjoy": 1, "Yes": 2, "I feel": 1, "Excited": 1, "Surprise": 1}, "adjectives": {"Sometimes": 1}, "numerals": {"17": 1, "1": 2}, "pronouns": {"I": 1}}}, {"content": "I have always liked working alone.", "word_count": 9, "label": "", "sentence_structure": "Subject鈥搗erb鈥揷omplement", "subjective_objective": {"answer": "Subjective", "rationale": "Because a person's preference is a subjective feeling."}, "sentence_sentiment": {"answer": "Neutral", "rationale": "Because the entry does not have any emotional color."}, "language_style": {"answer": "Calm and direct", "rationale": "The language style of the entry is \"cool and direct.\" It raises the question in a concise manner, without using excessive rhetorical or emotional rendering."}, "time_dimension": {"answer": "Present", "rationale": "The keyword \"always likes\" indicates that it is a customary or tendentious description, while \"separate work\" refers to the current state of work or behaviour, and therefore judges it as now."}, "sentence_type": "Affirmative declarative sentence", "pos": {"verbs": {"Always": 1, "Working alone": 1}, "adjectives": {"Alone": 1}, "adverbs": {"Always": 1}, "pronouns": {"I": 1}}}, {"content": "19. Some people feel that I am selfish and self-centred.", "word_count": 13, "label": "", "sentence_structure": "Subject鈥揷opula鈥損redicative", "subjective_objective": {"answer": "Subjective", "rationale": "This is a perception of my evaluation or opinion and is a subjective judgement and assessment of others."}, "sentence_sentiment": {"answer": "Negative", "rationale": "Because it expresses the negative behavioural characteristic \"self-serving and self-centered\" and is a negative description."}, "language_style": {"answer": "Tactful and warm", "rationale": "The entry uses a euphemism, avoiding situations that directly accuse or provoke resentment. At the same time, the first and second person are used to make the person questioned more receptive and interactive."}, "time_dimension": {"answer": "Past", "rationale": "The keyword \"some feel\" indicates that it is the view or assessment of the past by some, and therefore the entry is of a temporal dimension of the past."}, "sentence_type": "Negative declarative sentence", "pos": {"nouns": {"person": 1, "Self-centred": 1}, "verbs": {"I think": 1, "Selfish": 1}, "numerals": {"19": 1}, "pronouns": {"I": 1}}}, {"content": "20. I seem to be unable to organize things in a structured manner.", "word_count": 17, "label": "", "sentence_structure": "Subject鈥揷opula鈥損redicative", "subjective_objective": {"answer": "Subjective", "rationale": "Because \"always\" means that it's been the case, in the patient's psychological state and subjective feeling."}, "sentence_sentiment": {"answer": "Negative", "rationale": "Because the \"can't get things organized\" act in the entry is negative."}, "language_style": {"answer": "Calm and direct", "rationale": "The entry presents the problems of the respondents in management matters in an objective and concise manner, with no excessive rhetoric and emotional rendering, and with a more straightforward tone."}, "time_dimension": {"answer": "Present", "rationale": "Now, \"like\" in the entry is a subjective feeling or impression, and \"always\" indicates that the state persists. These words point to the current situation, so it can be judged that the entry is an inquiry into the present behaviour or mentality of the subject."}, "sentence_type": "Negative declarative sentence", "pos": {"nouns": {"Things": 1, "Arrangements": 1, "The well is organized": 1}, "verbs": {"Always": 1, "- Put it on": 1}, "adverbs": {"I think so": 1, "Total": 1}, "pronouns": {"I": 1}}}, {"content": "21. I rarely feel lonely or depressed.", "word_count": 10, "label": "", "sentence_structure": "Subject鈥揷opula鈥損redicative", "subjective_objective": {"answer": "Subjective", "rationale": "Because words like \"fear\" and \"very little\" indicate that it's a patient's subjective feeling and experience."}, "sentence_sentiment": {"answer": "Positive", "rationale": "Because the description \"very rarely feeling lonely or depressed\" in the entry is positive and indicates a better emotional state."}, "language_style": {"answer": "Tactful and warm", "rationale": "The entry is more softly phrased. It uses a softly-inquiriesed tone, which allows the interviewee to think and express his thoughts and feelings in a gentle and inviting manner, rather than direct orders or statements. This expression is more personal and interactive. At the same time, the entry does not use too many rhetorical or emotional expressions, but presents the problem in a more objective and neutral way, which is also a reflection of its softly-speaking style."}, "time_dimension": {"answer": "Present", "rationale": "The keywords \"very few\" and \"sorrowthy or depressed\" indicate that less of these negative emotions are present in the testee's current state."}, "sentence_type": "Negative declarative sentence", "pos": {"verbs": {"I feel": 1, "Loneliness": 1, "Blue": 1}, "adjectives": {"Very few": 1}}}, {"content": "I would rather be a leader than a leader.", "word_count": 17, "label": "", "sentence_structure": "Subject鈥搗erb鈥搊bject", "subjective_objective": {"answer": "Subjective", "rationale": "Because it is the patient's subjective idea and attitude."}, "sentence_sentiment": {"answer": "Negative", "rationale": "Because the behavior and attitude of \"unwilling to be leader\" is negative."}, "language_style": {"answer": "Calm and direct", "rationale": "The language style of the entry is more direct, more concise, and there is no excessive rhetoric or emotional rendering. It opens up to the question whether the person surveyed prefers to act independently rather than lead others, and is clear and free of any subjective emotions or preferences."}, "time_dimension": {"answer": "Present", "rationale": ""}, "sentence_type": "Negative declarative sentence", "pos": {"nouns": {"I": 2, "I'm a good guy": 1, "others": 1, "Leader": 1}, "verbs": {"I'd rather": 1, "Yeah": 1, "I don't want to": 1, "Be": 1, "Okay": 1}, "adverbs": {"of": 2, "not": 1}, "numerals": {"22": 1}}}, {"content": "I have paid little attention to my emotions or feelings in different contexts.", "word_count": 19, "label": "", "sentence_structure": "Subject鈥搗erb鈥揷omplement", "subjective_objective": {"answer": "Subjective", "rationale": "Because \"less attention\" and \"your emotions or feelings\" are subjective perceptions and descriptions of how people feel about themselves."}, "sentence_sentiment": {"answer": "Negative", "rationale": "Because the expression \"less attention\" conveys a pattern of neglect and negligence, this is often considered negative."}, "language_style": {"answer": "Calm and direct", "rationale": "The type of questionnaire is more appropriate for data collection and analysis, and allows for a more accurate understanding of the interviewee鈥檚 psychological situation and needs."}, "time_dimension": {"answer": "Present", "rationale": ""}, "sentence_type": "Negative declarative sentence", "pos": {"nouns": {"Questionnaire": 1, "Entry": 1, "Different": 1, "Environment": 1, "Emotion": 1, "Feel": 1}, "verbs": {"Attention": 1, "I'm under": 1, "self": 1}, "adjectives": {"Very few": 1}, "numerals": {"23": 1}, "pronouns": {"I": 1}}}, {"content": "24. Some people feel that I am cold and docile.", "word_count": 12, "label": "", "sentence_structure": "Subject鈥揷opula鈥損redicative", "subjective_objective": {"answer": "Subjective", "rationale": "Because \"fear,\" \"crazy\" and \"love\" are the evaluations and perceptions of others, and the subjective attitudes and views of patients."}, "sentence_sentiment": {"answer": "Negative", "rationale": "Because the description of \"apathy and a love for thought\" in the entries is negative."}, "language_style": {"answer": "Calm and direct", "rationale": "The entry is based on a direct language style, without a rhetorical or emotional rendering, but rather on a straightforward expression of the perception and assessment of the potential existence of the interviewee, which is more objective and quiet."}, "time_dimension": {"answer": "Present", "rationale": "The entry describes the current behaviour or characteristics of the subject, and the keyword is \"like\", indicating that it is the view or evaluation of another person, and the temporal dimension is now judged."}, "sentence_type": "Negative declarative sentence", "pos": {"nouns": {"person": 1, "I think": 1, "Coldness": 1, "Count it out": 1}, "verbs": {"I think": 1, "Cold and unkind": 1}, "adjectives": {"Coldness": 1, "Count it out": 1}, "adverbs": {"Some": 1}, "pronouns": {"I": 1}}}, {"content": "25. I will do everything in my power to do the job assigned to me.", "word_count": 17, "label": "", "sentence_structure": "Subject鈥搗erb鈥搊bject", "subjective_objective": {"answer": "Subjective", "rationale": "Because \"will do everything you can\" is a subjective expression of the patient's attitude and will."}, "sentence_sentiment": {"answer": "Positive", "rationale": "Because the \"do your best\" behavior and attitude in the entry is positive."}, "language_style": {"answer": "Tactful and warm", "rationale": "The language style of the entry is more euphemismal, because it uses a positive and encouraging expression, and is not too direct or harsh. For example, the words \"will do your best\" and \"all assigned to me\" are used to convey a sense of responsibility for the job, which makes it easier for the person under investigation to feel comfortable and cooperative."}, "time_dimension": {"answer": "Present", "rationale": "The keyword \"will do everything\" and \"complete\" all imply a constant, current state of behaviour."}, "sentence_type": "Affirmative declarative sentence", "pos": {"nouns": {"Work": 1}, "verbs": {"Yes": 1, "Do your best": 1, "Completed": 1, "Assign": 1}, "adverbs": {"Here": 1, "I": 1}, "pronouns": {"Everything": 1}}}, {"content": "I sometimes feel that I am completely worthless.", "word_count": 13, "label": "", "sentence_structure": "Subject鈥揷opula鈥損redicative", "subjective_objective": {"answer": "Subjective", "rationale": "Because \"I feel completely worthless\" is the patient's subjective feeling and experience."}, "sentence_sentiment": {"answer": "Negative", "rationale": "Because the mood of feeling worthless in the entry is negative."}, "language_style": {"answer": "Tactful and warm", "rationale": "The entry asks the interviewees in a more moderate way whether they have experienced a low sense of self-worth, using the vague expression \"sometimes\" and not directly rejecting or blaming each other's feelings, and is more linguistically sensitive and thoughtful."}, "time_dimension": {"answer": "Past", "rationale": "The entry expresses the feelings of the testee at a certain point in time, and the dimension of time is a passing judgement."}, "sentence_type": "Negative declarative sentence", "pos": {"nouns": {"Time": 1, "I": 1, "self": 1, "Value": 1}, "verbs": {"I feel": 1, "be": 1, "It's not worth it": 1}, "adjectives": {"Total": 1, "It's worthless": 1}, "numerals": {"26": 1}}}, {"content": "27. I often feel energetic.", "word_count": 9, "label": "", "sentence_structure": "Subject鈥搗erb鈥揷omplement", "subjective_objective": {"answer": "Subjective", "rationale": "Because \"energy is a feeling and experience, a subjective view of the patient."}, "sentence_sentiment": {"answer": "Positive", "rationale": "Because the expression \"often feeling energetic\" in the entry is positive."}, "language_style": {"answer": "Tactful and warm", "rationale": "The entry uses a relatively euphemistic formulation, by asking the other side whether he or she is often feeling energetically about the other side鈥檚 physical and psychological state, and avoiding direct questioning may be uncomfortable for the person under investigation. At the same time, the sentence is simple and easy to understand, making the questionnaire more friendly."}, "time_dimension": {"answer": "Present", "rationale": ""}, "sentence_type": "Affirmative declarative sentence", "pos": {"verbs": {"Common sense": 1, "I feel": 1, "- The energy": 1, "Wang-seok": 1}, "adjectives": {"Often": 1}}}, {"content": "28. When I find the right way to do things, I will insist on doing so.", "word_count": 24, "label": "", "sentence_structure": "Subject鈥搗erb鈥搊bject", "subjective_objective": {"answer": "Subjective", "rationale": "Because \"will insist\" reflects the patient's personal attitudes and decisions, which are seen from a subjective perspective."}, "sentence_sentiment": {"answer": "Positive", "rationale": "Since the wording of the article was positive, the acceptance of the correct approach and the persistence of the approach were emphasized."}, "language_style": {"answer": "Tactful and warm", "rationale": "The language style of the entry is more euphemistic, because it does not ask the other side's views or attitudes in the form of an order or direct statement, but rather in the form of an invitation and concern. For example, the phrase \"when I find the right way to do things\" is used to guide the respondents to think about their situation without asking them directly or instructing them how to answer it. Moreover, the positive expression \"I will insist on this approach\" gives people a positive, positive feeling, not a command tone."}, "time_dimension": {"answer": "Present", "rationale": "And by the words \"I found the right way to do things,\" it's a way of describing the person's current mentalities and behavior. So the time dimension of this questionnaire entry is now."}, "sentence_type": "Affirmative declarative sentence", "pos": {"nouns": {"Methodology": 1, "Things": 1}, "verbs": {"Found it": 1, "Hold on": 1, "Adopt": 1}, "adjectives": {"Correct": 1}, "numerals": {"28": 1}, "pronouns": {"I": 1}}}, {"content": "29. I usually try to be thoughtful and thoughtful.", "word_count": 13, "label": "", "sentence_structure": "Subject鈥揷opula鈥損redicative", "subjective_objective": {"answer": "Subjective", "rationale": "Because \"considerate and thoughtful\" is the patient's subjective attitude and behaviour."}, "sentence_sentiment": {"answer": "Positive", "rationale": "Because the behavior in the entry is positive, \"as thoughtful as possible\"."}, "language_style": {"answer": "Tactful and warm", "rationale": "The entry uses more moderate language, \"usually try\" and \"considerately considerately\" , which give the impression that the interviewee is positive and willing to show it in his interpersonal contacts. Moreover, the entire sentence is not directed or required, but is expressed in a question-and-answer manner, which is more acceptable and willing to answer questions. The language style of the questionnaire is thus described as euphemism."}, "time_dimension": {"answer": "Present", "rationale": ""}, "sentence_type": "Affirmative declarative sentence", "pos": {"verbs": {"Yes": 1, "Try": 1, "Kind": 1, "and": 1, "Concerns": 1, "That's very thoughtful": 1}, "adjectives": {"Usually": 1}}}, {"content": "I sometimes fail to be credible or credible as I should be.", "word_count": 16, "label": "", "sentence_structure": "Subject鈥搗erb鈥揷omplement", "subjective_objective": {"answer": "Subjective", "rationale": "Because the ability to be reliable/reliability depends on the patient's subjective attitude and behaviour.\""}, "sentence_sentiment": {"answer": "Negative", "rationale": "Because the entry \"can't be reliable or credible\" expresses negative emotional attitudes."}, "language_style": {"answer": "Tactful and warm", "rationale": "The language style of the entry is more euphemistic, by using the expression \"sometimes\" to express the confusion and impatience of the interviewee, while avoiding direct accusations and attacks. At the same time, \"my due credibility or credibility\" leaves enough room for reflection and reflection to give the interviewee the opportunity to proactively seek ways to improve."}, "time_dimension": {"answer": "Present", "rationale": "Now the entry \"sometimes\" means that the act is not a constant, but not a single one in the past. It implies that the testees should take into account their current state and recent behaviour in answering the question. Moreover, the description \"failure to be reliable or credible\" also indicates that it is an expression of behaviour or state, not a specific event or result. Thus, the entry mainly reflects the present situation."}, "sentence_type": "Negative declarative sentence", "pos": {"nouns": {"I": 2, "Time": 1, "Maybe": 1, "Do it": 1, "You should": 1, "Reliable": 1, "Trustable": 1}, "verbs": {"No, I can't": 1, "Do it": 1, "be": 1, "or": 1}, "adjectives": {"Maybe": 1, "You should": 1, "Reliable": 1, "Trustable": 1}, "adverbs": {"Sometimes": 1}}}, {"content": "31. I have rarely been depressed or frustrated.", "word_count": 10, "label": "", "sentence_structure": "Subject鈥揷opula鈥損redicative", "subjective_objective": {"answer": "Subjective", "rationale": "Because \"depressed or frustrated\" is a psychological state of the individual that needs to be examined through the patient's own perception and description."}, "sentence_sentiment": {"answer": "Positive", "rationale": "Because \"very rarely feeling depressed or depressed\" expresses a positive mood."}, "language_style": {"answer": "Calm and direct", "rationale": "The entry is in the form of a direct interview, with a clear language style, which does not have too many emotional or rhetorical features and appears to be relatively calm and objective."}, "time_dimension": {"answer": "Present", "rationale": "\"I'm rarely depressed or frustrated\" is the expression of the current state and feeling, so the temporal dimension is now."}, "sentence_type": "Negative declarative sentence", "pos": {"verbs": {"I feel": 1, "Blue": 1, "Frustration": 1}, "adjectives": {"Less": 1}, "adverbs": {"very": 1}, "numerals": {"31": 1}}}, {"content": "32. My life is moving fast.", "word_count": 8, "label": "", "sentence_structure": "Subject鈥揷opula鈥損redicative", "subjective_objective": {"answer": "Subjective", "rationale": "The basis for the judgement: the pace of life is the patient ' s subjective feelings and experience of his or her own living situation."}, "sentence_sentiment": {"answer": "Neutral", "rationale": "Because the entry does not have any emotional color."}, "language_style": {"answer": "Calm and direct", "rationale": "The entry presents the characteristics of the pace of life of the person under investigation in a concise and clear manner, without excessive qualifier or emotional rendering, and is more objective and direct in tone. \""}, "time_dimension": {"answer": "Present", "rationale": ""}, "sentence_type": "Affirmative declarative sentence", "pos": {"verbs": {"Life": 1, "Spacing": 1}, "adjectives": {"Come on": 1}, "numerals": {"32": 1}}}, {"content": "I often try new and foreign food.", "word_count": 15, "label": "", "sentence_structure": "Subject鈥搗erb鈥搊bject", "subjective_objective": {"answer": "Subjective", "rationale": "Because \"often\" and \"new\" and \"foreign\" food are all the choices and experiences of patients themselves, and are a reflection of their subjective attitudes."}, "sentence_sentiment": {"answer": "Positive", "rationale": "Because the \"to try new and foreign food\" act is positive."}, "language_style": {"answer": "Tactful and warm", "rationale": "This is a more personal and interactive expression that attracts the active participation of the interviewees."}, "time_dimension": {"answer": "Present", "rationale": ""}, "sentence_type": "Affirmative declarative sentence", "pos": {"nouns": {"Questionnaire": 1, "Entry": 1, "Food": 1}, "verbs": {"Yes": 1, "Go on": 1, "Try": 1, "New": 1, "and": 1, "Foreign": 1}, "adverbs": {"Regular": 1}, "numerals": {"33": 1}}}, {"content": "Most of the people who know me like me.", "word_count": 12, "label": "", "sentence_structure": "Subject鈥搗erb鈥揷omplement", "subjective_objective": {"answer": "Subjective", "rationale": "Because \"like\" is a subjective feeling and evaluation."}, "sentence_sentiment": {"answer": "Positive", "rationale": "Because the phrase \"most of those who know me like me\" in the entry is a positive emotional expression."}, "language_style": {"answer": "Tactful and warm", "rationale": "The entry uses a more subjective expression to ask the other side about their personal relationship, while making the respondent more willing to share his/her feelings through questioning. Moreover, the sentence is soft, friendly, comfortable and welcome. The language style of the questionnaire is therefore \"precipitous.\""}, "time_dimension": {"answer": "Present", "rationale": "Now the entry asks the testee what he feels and what he's going through, the key words are \"most of the people who know me\" and \"like me,\" all pointing to the situation."}, "sentence_type": "Affirmative declarative sentence", "pos": {"nouns": {"Most of them": 1, "person": 2, "Yes": 1, "I": 2, "Yeah": 1}, "verbs": {"Yes": 1, "Yeah": 1}, "adverbs": {"Most of them": 1}, "numerals": {"34": 1}, "pronouns": {"they": 1}}}, {"content": "When I made a commitment, I usually had the ability to follow through.", "word_count": 14, "label": "", "sentence_structure": "Subject鈥搗erb鈥搊bject", "subjective_objective": {"answer": "Subjective", "rationale": "For it is a subjective belief or attitude to commit and to be able to implement."}, "sentence_sentiment": {"answer": "Positive", "rationale": "Because the \"implementing\" is positive."}, "language_style": {"answer": "Calm and direct", "rationale": "The entry directly expresses the subject of questioning the other party ' s behaviour in respect of compliance with the commitment and uses a positive statement, which is not overly pedantic or emotional and which is more objective in tone."}, "time_dimension": {"answer": "Present", "rationale": "The key word \"normally\" makes it possible to judge the dimension of time for now. So the answer is \"now\"."}, "sentence_type": "Affirmative declarative sentence", "pos": {"verbs": {"Do it": 1, "Commitments": 1, "Follow-up": 1, "What the hell": 1}, "adjectives": {"Usually": 1}, "numerals": {"35": 1}}}, {"content": "36. Many times, when things are not right, I feel frustrated and want to give up.", "word_count": 21, "label": "", "sentence_structure": "Subject鈥揷opula鈥損redicative", "subjective_objective": {"answer": "Subjective", "rationale": "Because \"fear defeat and want to give up\" is the patient's subjective emotional experience."}, "sentence_sentiment": {"answer": "Negative", "rationale": "Because the \"frustration and desire to give up\" in the entry is negative."}, "language_style": {"answer": "Calm and direct", "rationale": "The language style of the entry is calm and straightforward. It uses a direct inquiry approach, without too many emotional adorations or rhetorical techniques, but rather an objective, concise way of expressing the questions, which allows respondents to understand and answer directly. This language style is common in the questionnaire, because it helps researchers to obtain more accurate and reliable data."}, "time_dimension": {"answer": "Present", "rationale": "\"Feel\" and \"want to give up\" means that it's the person who's been tested now, so the entry belongs to now."}, "sentence_type": "Affirmative declarative sentence", "pos": {"nouns": {"Time": 2, "Things": 1, "Frustration": 1, "Abandon": 1}, "verbs": {"Yes": 2, "I feel": 1, "Abandon": 1}, "adjectives": {"Something's wrong": 1, "Many times": 1}, "numerals": {"36": 1}}}, {"content": "I am a very active person.", "word_count": 10, "label": "", "sentence_structure": "Subject鈥揷opula鈥損redicative", "subjective_objective": {"answer": "Subjective", "rationale": "Because \"very active\" is a description of the patient himself, a subjective attitude of the patient."}, "sentence_sentiment": {"answer": "Positive", "rationale": "Because the description of \"very active\" in the entry is positive, indicating that the person has a lively and open character."}, "language_style": {"answer": "Calm and direct", "rationale": "The entry uses a direct phrase, \"I'm a very active person\", without adding too many embellishments or rhetorical techniques, and with a calmer and more direct tone."}, "time_dimension": {"answer": "Present", "rationale": ""}, "sentence_type": "Affirmative declarative sentence", "pos": {"verbs": {"be": 1, "One": 1}, "adjectives": {"Tens": 1, "Active": 1}}}, {"content": "38. I like to think and play with theory or abstraction.", "word_count": 16, "label": "", "sentence_structure": "Subject鈥搗erb鈥揷omplement", "subjective_objective": {"answer": "Subjective", "rationale": "Because \"like\" is the patient's personal feeling and attitude, and it's subjective."}, "sentence_sentiment": {"answer": "Positive", "rationale": "Because the act of \"like to think and play abstract ideas\" is positive."}, "language_style": {"answer": "Tactful and warm", "rationale": ""}, "time_dimension": {"answer": "Present", "rationale": "The entry is an expression of the testee ' s preference or customary behaviour, which is generally the current state of affairs and can therefore be judged as the present."}, "sentence_type": "Affirmative declarative sentence", "pos": {"nouns": {"Think": 1, "Play": 1, "Theory": 1, "Abstract": 1, "Perceptions": 1}, "verbs": {"Yeah": 1}, "numerals": {"38": 1}}}, {"content": "I prefer to work with people rather than compete.", "word_count": 14, "label": "", "sentence_structure": "Subject鈥搗erb鈥搊bject", "subjective_objective": {"answer": "Subjective", "rationale": "Because \"I prefer to\" is a subjective view of the values and attitudes of the patient."}, "sentence_sentiment": {"answer": "Positive", "rationale": "Because the expression \"better to work with people\" in the entry is positive attitudes and behaviour."}, "language_style": {"answer": "Tactful and warm", "rationale": "The language style of the entry is more euphemistic and warm, and leads the interviewee to express his/her attitude in a way that is not an order but a question."}, "time_dimension": {"answer": "Present", "rationale": "The entry describes a person's current preferences or attitudes, which is, \"I prefer to work with people rather than compete with them.\""}, "sentence_type": "Affirmative declarative sentence", "pos": {"nouns": {"Questionnaire": 1, "Entry": 1, "person": 2, "Cooperation": 1, "Competition": 1}, "verbs": {"I'd rather": 1, "and": 3, "Cooperation": 1, "I don't want to": 1, "Competition": 1}, "adverbs": {"Down there": 1, "Please": 1, "Analysis": 1, "and": 2, "not": 1}, "numerals": {"39": 1}, "pronouns": {"I": 2}}}, {"content": "I have a clear set of objectives and can work systematically towards it.", "word_count": 22, "label": "", "sentence_structure": "Subject鈥搗erb鈥揷omplement", "subjective_objective": {"answer": "Subjective", "rationale": "Because the purpose and effort are the subjective will and behaviour of the individual."}, "sentence_sentiment": {"answer": "Positive", "rationale": "The entry is based on a positive attitude and quality, i.e., having a clear objective and being able to move in an orderly way towards that goal. It can therefore be judged as positive emotional."}, "language_style": {"answer": "Calm and direct", "rationale": "The entry uses a relatively simple language structure and vocabulary, with no excessive emotional or rhetorical style. At the same time, it does not use any doubt or plea, but rather expresses a specific view or fact in the form of a statement, so that the language style is more calm and straightforward."}, "time_dimension": {"answer": "Past", "rationale": "The entry describes only one of the capabilities and state of the person tested, and is not relevant to time."}, "sentence_type": "Affirmative declarative sentence", "pos": {"nouns": {"Objective": 2, "Entry": 1, "Work": 1}, "verbs": {"have": 1, "Dynamite": 1, "Work": 1}, "adjectives": {"Clear": 1, "It's a little organized": 1}, "numerals": {"40": 1}, "pronouns": {"I": 1}}}, {"content": "Sometimes I feel ashamed to hide.", "word_count": 11, "label": "", "sentence_structure": "Subject鈥搗erb鈥揷omplement", "subjective_objective": {"answer": "Subjective", "rationale": "Because \"smuggling\" and \"wanting to hide\" are the feelings and attitudes of the patient, the entry is viewed from the patient's subjective point of view."}, "sentence_sentiment": {"answer": "Negative", "rationale": "Because shame is a negative sentiment."}, "language_style": {"answer": "Tactful and warm", "rationale": "The entry is more personal and interactive when it is spoken in a gentle and inviting manner, rather than in direct statements or orders. At the same time, the issue is set up more closely to the actual emotional experience of the person under investigation, and can easily resonate and share."}, "time_dimension": {"answer": "Past", "rationale": "It is a behaviour or emotion that describes the past experience, based on the phrase \"sometimes\" and \"will\", and therefore the temporal dimension is the past."}, "sentence_type": "Affirmative declarative sentence", "pos": {"nouns": {"Time": 1, "I": 1}, "verbs": {"Yes": 1, "Shame": 1, "Hide": 1, "Get up": 1}, "adverbs": {"Sometimes": 1}, "numerals": {"41": 1}}}, {"content": "42. I like to go through the same situation and be in the same situation.", "word_count": 14, "label": "", "sentence_structure": "Subject鈥搗erb鈥揷omplement", "subjective_objective": {"answer": "Subjective", "rationale": "Because \"like\" is a subjective feeling or attitude."}, "sentence_sentiment": {"answer": "Positive", "rationale": "Because the attitude of \"like to go through the field\" in the entry is positive."}, "language_style": {"answer": "Tactful and warm", "rationale": "The language style of the entry is euphemism, because it asks the interviewees, in a gentle and inviting tone, whether they like to be involved and experience events. This expression gives the respondents a sense of respect and understanding and may encourage them to be more willing to share their experiences and views."}, "time_dimension": {"answer": "Present", "rationale": "The entry is a statement of the testee's personal orientation, and the emotional word \"likes\" indicates that this is a description of the behaviour of the general current, and the temporal dimension of this questionnaire entry is now."}, "sentence_type": "Affirmative declarative sentence", "pos": {"nouns": {"I": 1, "鈾?And the way it goes 鈾?: 1, "Events": 1}, "verbs": {"Yeah": 1, "鈾?And in the midst of 鈾?: 1}, "adverbs": {"鈾?And the 鈾?: 1}, "numerals": {"42": 1}}}, {"content": "I am not interested in thinking about the patterns of the universe or the human condition.", "word_count": 18, "label": "", "sentence_structure": "Subject鈥搗erb鈥搊bject", "subjective_objective": {"answer": "Subjective", "rationale": "Because \"Is there an interest\" or \"thinking\" is the patient's own feelings and thoughts, and the entry is viewed from the patient's subjective point of view."}, "sentence_sentiment": {"answer": "Negative", "rationale": "Because the \"no interest\" in the entry is a negative attitude to thinking about the patterns of the universe or human conditions."}, "language_style": {"answer": "Calm and direct", "rationale": "The language style of the entry is more calm and straightforward, without excessive rhetoric or emotional rendering, simply stating a fact. This expression is more objective and clear, and can convey the meaning of the problem directly."}, "time_dimension": {"answer": "Present", "rationale": "The keyword \"no interest\" indicates current, ongoing movement or state, and \"43.\" It also suggests that this is a matter of current state."}, "sentence_type": "Negative declarative sentence", "pos": {"nouns": {"The universe": 1, "Pattern": 1, "Humans": 1}, "verbs": {"No, I'm not": 1, "Interest": 1, "Thinking": 1}, "adjectives": {"Mine": 1, "of": 2}, "numerals": {"43": 1}, "pronouns": {"I": 1}}}, {"content": "If I don't like a person, I'll let him know.", "word_count": 16, "label": "", "sentence_structure": "Subject鈥搗erb鈥搊bject", "subjective_objective": {"answer": "Subjective", "rationale": "Because it is the patient ' s subjective attitude and decision."}, "sentence_sentiment": {"answer": "Negative", "rationale": "Because the behavior in the entry \"to let the other party know that he does not like\" can cause harm or discomfort to the other party, it is negative emotional."}, "language_style": {"answer": "Calm and direct", "rationale": "The language style of the entry is relatively cold and straightforward, with no excessive rhetorical or emotional rendering, and it is more calm and objective. It expresses a code of conduct in a clear statement that when you don't like someone, you tell each other about it. This language style is more about facts and logic than emotional and emotional."}, "time_dimension": {"answer": "Past", "rationale": "The sentence expresses past actions, \"if I do not like a person\" indicates that this is a hypothetical situation, and \"will let him know\" indicates that action has been taken. The temporal dimension of the article is therefore past."}, "sentence_type": "Affirmative declarative sentence", "pos": {"nouns": {"person": 1, "Questionnaire Entry": 1, "Jean": 1}, "verbs": {"Yes": 3, "Yeah": 1}, "numerals": {"44": 1}, "pronouns": {"I": 1}}}, {"content": "I have worked hard to achieve my objectives.", "word_count": 9, "label": "", "sentence_structure": "Subject鈥搗erb鈥搊bject", "subjective_objective": {"answer": "Subjective", "rationale": "Because \"work hard\" and \"objective\" are patients' personal perceptions and assumptions, subjective perspectives."}, "sentence_sentiment": {"answer": "Positive", "rationale": "Because the entry \"work hard\" expresses positive actions and attitudes."}, "language_style": {"answer": "Calm and direct", "rationale": "The language style of the entry is more straightforward, concise and not overly rhetorical or emotional, with greater emphasis on objective descriptions of the facts and direct presentation of the issues."}, "time_dimension": {"answer": "Past", "rationale": ""}, "sentence_type": "Affirmative declarative sentence", "pos": {"nouns": {"Questionnaire": 1, "Entry": 1, "Objective": 1}, "verbs": {"Work hard to finish": 1, "Give": 1}, "numerals": {"45": 1}}}, {"content": "46. I often feel inferior to others.", "word_count": 11, "label": "", "sentence_structure": "Subject鈥搗erb鈥揷omplement", "subjective_objective": {"answer": "Subjective", "rationale": "Because \"I feel less than anyone else\" is a subjective feeling and evaluation."}, "sentence_sentiment": {"answer": "Negative", "rationale": "Because the description of \"often feeling inferior to others\" in the entry expresses negative assessments and a comparative mentality of the person and is negative."}, "language_style": {"answer": "Tactful and warm", "rationale": "The language style of the entry is more euphemistic and warm, and it does not directly point to problems or deficiencies that the person under investigation may have, but rather asks in a more caring and understanding way to understand the person鈥檚 inner feelings. This expression is more likely to resonate with the person under investigation and help them to respond more readily to the questions."}, "time_dimension": {"answer": "Past", "rationale": "The term \"often feels\" denotes a description of the subjective past, so the temporal dimension of the entry is the past."}, "sentence_type": "Negative declarative sentence", "pos": {"nouns": {"Questionnaire": 1, "Entry": 1, "person": 1}, "verbs": {"Regular": 1, "I feel": 1, "Why don't you": 1}, "adjectives": {"others": 1}, "numerals": {"46": 1}, "pronouns": {"I": 1}}}, {"content": "I am not an optimist.", "word_count": 11, "label": "", "sentence_structure": "Subject鈥揷opula鈥損redicative", "subjective_objective": {"answer": "Subjective", "rationale": "Because optimism is patient subjectivity."}, "sentence_sentiment": {"answer": "Negative", "rationale": "Because the expression \"not optimists\" in the entry is negative emotional."}, "language_style": {"answer": "Calm and direct", "rationale": "The entry is more concise and has no excessive emotional rendering or rhetorical techniques to express the views of the respondents in a direct presentation."}, "time_dimension": {"answer": "Present", "rationale": "The word \"is\" in the entry is a positive judgement, while \"is not\" is a negative reverse judgement, and \"optimist\" is an adjective phrase describing the attributes of the dominant language. The entry is therefore a term that describes the current mindset and view of the testee, and the temporal dimension is now."}, "sentence_type": "Negative declarative sentence", "pos": {"nouns": {"Questionnaire": 1, "Entry": 1, "Optimism": 1, "Who": 1}, "verbs": {"be": 1, "No, it's not": 1, "Yes": 1}, "adjectives": {"Optimistic": 1, "of": 2}, "adverbs": {"and": 1}, "numerals": {"47": 1}, "pronouns": {"I": 1}}}, {"content": "48. I am curious about the thought-provoking.", "word_count": 12, "label": "", "sentence_structure": "Subject鈥揷opula鈥損redicative", "subjective_objective": {"answer": "Subjective", "rationale": "Because \"quiet about the thought-provoking\" expresses the feelings and attitudes of the patient's individual, and is a subjective examination."}, "sentence_sentiment": {"answer": "Positive", "rationale": "Because the entry \"quiet about thought\" expresses positive attitudes and behavior."}, "language_style": {"answer": "Tactful and warm", "rationale": "The language style of the entry is more euphemistic and warm. It uses the expression \"to be curious,\" rather than \"to think,\" which is more personal and interactive. Moreover, \"thinking\" is a more abstract concept, and it is more resonant with the other. So the language style of the entry is euphemism."}, "time_dimension": {"answer": "Future", "rationale": "The keyword \"quiet about the thought-provoking\" indicates that the testees have the will and the intention to explore and understand these things in the future. The temporal dimension of this article is therefore the future."}, "sentence_type": "Affirmative declarative sentence", "pos": {"nouns": {"Thinking": 1, "Things": 1, "Curiosity": 1}, "verbs": {"to": 1, "Fill": 1, "Think": 1}, "numerals": {"48": 1}}}, {"content": "49. I often have disputes with family and colleagues.", "word_count": 12, "label": "", "sentence_structure": "Subject鈥搗erb鈥搊bject", "subjective_objective": {"answer": "Subjective", "rationale": "Because \"controversial with family and colleagues\" is a subjective attitude or an expression of experience."}, "sentence_sentiment": {"answer": "Negative", "rationale": "Because the \"drive\" in the entry is negative."}, "language_style": {"answer": "Calm and direct", "rationale": "The language style of the entry is relatively calm and straightforward, with no excessive emotional accoutrements or rhetorical techniques, but rather directly expresses the subject of questioning whether the other party is often in dispute with family and colleagues, allowing the interviewee to quickly understand and answer the questions."}, "time_dimension": {"answer": "Present", "rationale": "The entry describes the current behaviour or state of the subject, namely, \"always arguing with family and colleagues\" and can therefore be judged as \"now\"."}, "sentence_type": "Affirmative declarative sentence", "pos": {"nouns": {"I": 1, "Family": 1, "Co-workers": 1, "Arguments": 1}, "verbs": {"Often": 1, "and": 1, "Rise": 1}, "adverbs": {"and": 1}, "numerals": {"49": 1}}}, {"content": "I must pursue excellence in everything.", "word_count": 8, "label": "", "sentence_structure": "Subject鈥揷opula鈥損redicative", "subjective_objective": {"answer": "Subjective", "rationale": "For the pursuit of excellence is a subjective attitude or objective and is not an objective fact or act."}, "sentence_sentiment": {"answer": "Positive", "rationale": "Because the \"prosperity pursuit\" in the entry is a positive attitude and behavior."}, "language_style": {"answer": "Calm and direct", "rationale": "The entry is based on a straightforward language style, with no excessive rhetoric or emotional colour, and is more objective and calm. It expresses the subject of questioning each other in a concise and clear manner about their attitude to the pursuit of excellence."}, "time_dimension": {"answer": "Present", "rationale": "There is no clear time indicator or phrase in the entry indicating the time dimension of the past, present or future, but \"I must pursue excellence in every way\" is an expression of attitude and conviction that usually persists and influences behaviour. Therefore, the entry is more biased towards describing the status and values of the subjects and judging them as it is now."}, "sentence_type": "Affirmative declarative sentence", "pos": {"verbs": {"Seek": 1, "Excellent": 1}, "adverbs": {"Yes": 1}, "numerals": {"50": 1}}}, {"content": "51. I often feel helpless and hope that someone will be able to solve my problems.", "word_count": 19, "label": "", "sentence_structure": "Subject鈥搗erb鈥搊bject", "subjective_objective": {"answer": "Subjective", "rationale": "Because \"Feel helpless\" and \"I want someone to solve my problems\" are the personal feelings and aspirations of the patients, reflecting their subjective feelings."}, "sentence_sentiment": {"answer": "Negative", "rationale": "Because the words \"fearless\" and \"seeing someone to solve my problems\" both imply an emotional need for help, which is usually considered negative."}, "language_style": {"answer": "Calm and direct", "rationale": "The entry is more calm and straightforward in language, has no excessive emotional rendering or speech skills, and is more objective in tone. It expresses the subject of asking each other whether they are often helpless in a simple and clear way."}, "time_dimension": {"answer": "Present", "rationale": "I often feel that \"often\" means \"higher-frequency behaviour,\" and \"like\" uses the current state of affairs, which means that it has not only happened in the past, but may still be going on. And \"I hope someone can solve my problems\" also shows the current state of the needs and emotions of the subjects, so the entry reflects the current mood and mentality."}, "sentence_type": "Affirmative declarative sentence", "pos": {"nouns": {"Helpless": 1, "Problem": 1}, "verbs": {"I feel": 1, "Hope": 1, "Resolve": 1}, "adverbs": {"Regular": 1}, "numerals": {"51": 1}, "pronouns": {"I": 1}}}, {"content": "52. I am a happy and happy person.", "word_count": 10, "label": "", "sentence_structure": "Subject鈥揷opula鈥損redicative", "subjective_objective": {"answer": "Subjective", "rationale": "Because the feelings of \"happy\" and \"happy\" are described in terms of patients' subjective feelings, subjective attitudes and emotional experiences."}, "sentence_sentiment": {"answer": "Positive", "rationale": "Because the words \"happy\" and \"happy\" express positive emotional attitudes."}, "language_style": {"answer": "Tactful and warm", "rationale": "The entry uses positive emotional terms for \"happy\" and \"happy\" and is not expressed in too direct or strong a language, but in a gentle and encouraging way. Such language styles are more comfortable and acceptable and help create a good investigation atmosphere."}, "time_dimension": {"answer": "Present", "rationale": ""}, "sentence_type": "Affirmative declarative sentence", "pos": {"nouns": {"person": 1, "Happy": 2}, "verbs": {"be": 1}, "adjectives": {"One": 1, "Happy": 2}, "numerals": {"52": 1}, "pronouns": {"I": 1}}}, {"content": "53. I believe that giving students access to controversial speeches can only confuse and mislead their thinking.", "word_count": 26, "label": "", "sentence_structure": "Subject鈥搗erb鈥搊bject", "subjective_objective": {"answer": "Subjective", "rationale": "Because \"convincing\" is the patient's personal opinion and opinion, and it's subjective."}, "sentence_sentiment": {"answer": "Negative", "rationale": "Because the term \"disorder and misleading\" in the entry is negative and indicates an attitude against students listening to controversial speeches."}, "language_style": {"answer": "Calm and direct", "rationale": "The language style of the entry is more straightforward, with no excessive rhetorical or emotional overtones, but rather the presentation of personal views in objective, calm language. At the same time, the phrase \"only\" is used to express strong judgement and confidence, with a more positive and resolute tone."}, "time_dimension": {"answer": "Present", "rationale": ""}, "sentence_type": "Negative declarative sentence", "pos": {"nouns": {"Students": 1, "Argumentative": 1, "Speeches": 1, "Ideas": 1}, "verbs": {"Yes": 1, "Jean": 1, "Listen": 1, "Confusion": 1, "Misdirection": 1}, "adjectives": {"Only": 1}, "adverbs": {"Only": 1}, "numerals": {"53": 1}, "pronouns": {"they": 1}}}, {"content": "54. I have already made a very high assessment.", "word_count": 10, "label": "", "sentence_structure": "Subject鈥搗erb鈥揷omplement", "subjective_objective": {"answer": "Subjective", "rationale": "Because the sentence relates to the assessment of one ' s own, it is an expression of the individual ' s subjective consciousness."}, "sentence_sentiment": {"answer": "Positive", "rationale": "Because the entry \"A high rating of self\" expresses a self-assertion and self-confidence, and is a positive emotion."}, "language_style": {"answer": "Tactful and warm", "rationale": "The entry is more gentle and friendly in an encouraging tone to ask respondents about self-evaluation. This expression makes it easier for respondents to relax, more willing to answer questions honestly and more interactive."}, "time_dimension": {"answer": "Present", "rationale": "The entry expresses the current behaviour or state, i.e. the testee has a high rating of himself and the temporal dimension is now judged."}, "sentence_type": "Affirmative declarative sentence", "pos": {"verbs": {"to": 1, "have": 1, "Here": 1}, "adjectives": {"very": 1, "High": 1}, "numerals": {"54": 1}, "pronouns": {"I": 1}}}, {"content": "55. I am well placed to move things right in the right direction.", "word_count": 17, "label": "", "sentence_structure": "Subject鈥搗erb鈥揷omplement", "subjective_objective": {"answer": "Subjective", "rationale": "Because \"the pace of self-madeness\" and \"the timely completion of things\" are subjective perceptions and behavioural descriptions of patients."}, "sentence_sentiment": {"answer": "Positive", "rationale": "Because the \"can get things right\" act in the entry is positive and expresses a sense of self-management and responsibility."}, "language_style": {"answer": "Tactful and warm", "rationale": "The entry is more euphemistic and encouraging. It uses the positive word \"sweet,\" implying that the respondent is doing well in this regard. Moreover, it does not ask the respondent too directly about self-evaluation, but expresses the issue in a positive and supportive manner. It is therefore considered \"precipitous\" to be the language style."}, "time_dimension": {"answer": "Present", "rationale": "The entry describes the current behaviour or abilities of the testee, in which terms such as \"capable\", \"fitting your own pace\" and \"doing it on time\" are all words of the current state. Thus, the temporal dimension is now."}, "sentence_type": "Affirmative declarative sentence", "pos": {"nouns": {"Step": 1, "Things": 1}, "verbs": {"Press": 1, "Done": 1}, "adjectives": {"self": 1, "Very good": 1}, "numerals": {"55": 1}, "pronouns": {"I": 1}}}, {"content": "When I'm under great stress, sometimes I feel like I'm having a nervous breakdown.", "word_count": 24, "label": "", "sentence_structure": "Subject鈥揷opula鈥損redicative", "subjective_objective": {"answer": "Subjective", "rationale": "Because experience like a nervous breakdown is a subjective feeling and emotional reaction. While stress is an objective thing, it is perceived and responded to differently, and therefore it is more subjective."}, "sentence_sentiment": {"answer": "Negative", "rationale": "Because the description of \"psychological breakdown\" in the entry is negative."}, "language_style": {"answer": "Tactful and warm", "rationale": "The language style of the entry is more euphemistic, using metaphors to describe the feelings of stress, rather than direct statements or orders, which are more personal and interactive. Moreover, the entry is not overly emotionally qualified, but rather questions are asked in an objective and calm manner, which helps the interviewee to reflect his or her psychological condition more honestly."}, "time_dimension": {"answer": "Present", "rationale": ""}, "sentence_type": "Affirmative declarative sentence", "pos": {"nouns": {"Pressure": 1, "Spirit": 1, "Crash": 1}, "verbs": {"in": 1, "I feel": 1, "Yeah": 1}, "adjectives": {"Great": 1}, "numerals": {"56": 1}, "pronouns": {"I": 1}}}, {"content": "I'm easy to laugh at.", "word_count": 5, "label": "", "sentence_structure": "Subject鈥揷opula鈥損redicative", "subjective_objective": {"answer": "Subjective", "rationale": "Because it's a person's subjective feeling and reaction."}, "sentence_sentiment": {"answer": "Positive", "rationale": "Because \"easy to laugh\" is usually considered a positive quality, which suggests that this man is more optimistic and more open."}, "language_style": {"answer": "Tactful and warm", "rationale": "The entry uses descriptive language, which does not directly evaluate or judge the emotional state of the person interviewed, but is presented in a more objective and moderate way."}, "time_dimension": {"answer": "Present", "rationale": ""}, "sentence_type": "Affirmative declarative sentence", "pos": {"verbs": {"Smile": 1}, "adjectives": {"Easy": 1}, "pronouns": {"I": 1}}}, {"content": "58. I believe that when decisions are taken on ethical issues, we should follow religious authority.", "word_count": 22, "label": "", "sentence_structure": "Subject鈥搗erb鈥搊bject", "subjective_objective": {"answer": "Subjective", "rationale": "Because \"I think\" is a subjective expression of the respondent's own view and conviction, the entry on this questionnaire is a subjective approach to the interviewee."}, "sentence_sentiment": {"answer": "Negative", "rationale": "Because \"religious authority\" in this entry may be seen by some as a restriction of personal freedom, there is a certain negative emotional colour."}, "language_style": {"answer": "Calm and direct", "rationale": "The entry expresses a point of view in a direct presentation, without excessive rhetorical or emotional rendering, and with a more objective tone."}, "time_dimension": {"answer": "Present", "rationale": "I think the word \"concept\" expresses a subjective attitude and expression of opinion, and the word \"should obey religious authority\" indicates that the attitude of the speaker towards religious authority is now, that is, that this view is formed now."}, "sentence_type": "Affirmative declarative sentence", "pos": {"nouns": {"Ethical issues": 1, "Decision": 1, "Religious authority": 1}, "verbs": {"I think": 1, "Do it": 1, "Reactions": 1, "Compliance": 1}, "adverbs": {"in": 1, "Go, go, go": 1, "Time": 1}, "numerals": {"58": 1}, "pronouns": {"we": 1}}}, {"content": "59. Attitude is uncompromising.", "word_count": 12, "label": "", "sentence_structure": "Subject鈥揷opula鈥損redicative", "subjective_objective": {"answer": "Subjective", "rationale": "Because \"recalculous\" is a subjective attitude or character description."}, "sentence_sentiment": {"answer": "Negative", "rationale": "Because \"dilemma\" is negative."}, "language_style": {"answer": "Calm and direct", "rationale": "The entry expressed a point in a direct presentation, with no excessive rhetorical or emotional rendering, and a more objective tone."}, "time_dimension": {"answer": "Present", "rationale": "\"I am stubborn\" means the present attitude and conviction, so the entry is the present."}, "sentence_type": "Affirmative declarative sentence", "pos": {"verbs": {"be": 1, "Recalculable": 1}, "adjectives": {"Obstinate": 1, "Uncompromising": 1}, "numerals": {"59": 1}}}, {"content": "60. It took me a lot of time to get back to work.", "word_count": 15, "label": "", "sentence_structure": "Subject鈥搗erb鈥揷omplement", "subjective_objective": {"answer": "Subjective", "rationale": "Because \"it takes a lot of time to get settled\" is a personal feeling and thought, and it is therefore a subjective examination."}, "sentence_sentiment": {"answer": "Negative", "rationale": "Because the \"slow time to settle\" in the entry is negative."}, "language_style": {"answer": "Calm and direct", "rationale": "The language style of the entry is more calm and straightforward, and there is no excessive emotional rendering or rhetorical technique. It simply and succinctly describes the fact that \"I need to spend a lot of time adapting to my new job,\" which is objective and easy to understand."}, "time_dimension": {"answer": "Present", "rationale": "The entry uses \"want\" and \"thoussance\", which means that it is the person being tested's current behavior or mentality and therefore belongs to the present."}, "sentence_type": "Affirmative declarative sentence", "pos": {"nouns": {"Time": 1, "Work": 1}, "verbs": {"Flowers": 1, "Settle down": 1}, "adverbs": {"very": 1}, "numerals": {"60": 1}, "pronouns": {"I": 1}}}]}

{"questionnaire_title": "Personal trust table", "questionnaire_category": "Mental health", "item_count": 25, "emotion_shift_overall": "From negative to positive", "language_style_overall": "Calm and direct", "items": [{"content": "1. In our society, hypocrisy is on the rise.", "word_count": 18, "label": "", "sentence_structure": "Subject鈥損redicate", "subjective_objective": {"answer": "Objective", "rationale": "Because the title describes social phenomena, which are objective."}, "sentence_sentiment": {"answer": "Negative", "rationale": "Because it describes a negative social phenomenon."}, "language_style": {"answer": "Tactful and warm", "rationale": "The entry does not directly indicate whether the other party agrees with the existence of this phenomenon, but is presented in an objective manner, in a more moderate tone, without excessive emotional or subjective judgement, which makes the respondents more free and comfortable in their responses."}, "time_dimension": {"answer": "Past", "rationale": "The entries \"in our society\" and \"in our society\" point to a past trend or change, and they judge as the past."}, "sentence_type": "Affirmative declarative sentence", "pos": {"nouns": {"phenomena": 2, "Hypocrisy": 1, "Social": 1}, "verbs": {"in": 1, "More and more": 1, "have": 1}, "adjectives": {"Hypocrisy": 1}, "adverbs": {"More and more": 1}, "numerals": {"1": 3, "2": 1}, "classifiers": {"individual": 2}}}, {"content": "2. When dealing with strangers, you should be careful unless they provide a basis on which to prove that they are trustworthy.", "word_count": 31, "label": "", "sentence_structure": "Subject鈥搗erb鈥搊bject", "subjective_objective": {"answer": "Subjective", "rationale": "Objective: Objective basis of judgement: this is the objective fact of the recommendation, and what should be done."}, "sentence_sentiment": {"answer": "Neutral", "rationale": "Because the entry gives only one suggestion and does not express positive or negative emotional expressions."}, "language_style": {"answer": "Calm and direct", "rationale": "The entry is based on a direct expression and is more serious. It clearly conveys the view that care should be taken when dealing with strangers, unless the other person can provide sufficient grounds for trust. This is more straightforward, without excessive rhetoric and emotional rendering, and is consistent with the calm and direct style."}, "time_dimension": {"answer": "Present", "rationale": "The entry describes the attitude or behaviour that should be taken when dealing with strangers, without addressing the specifics of the past or the future. Thus, the temporal dimension is judged as \"now\"."}, "sentence_type": "Affirmative declarative sentence", "pos": {"nouns": {"Stranger": 1, "Dealing": 1, "Basis": 1}, "verbs": {"I'd better": 1, "Take it out": 1, "Prove it": 1, "Trust": 1}, "adverbs": {"Watch out": 1}, "numerals": {"2": 1}, "pronouns": {"they": 1}}}, {"content": "3. Unless we attract more people into the political arena, the future of the country will be bleak.", "word_count": 26, "label": "", "sentence_structure": "Subject鈥搗erb鈥搊bject", "subjective_objective": {"answer": "Subjective", "rationale": "Because this is a person ' s view and prediction, based on their convictions and expectations, and not an objective fact."}, "sentence_sentiment": {"answer": "Negative", "rationale": "Because the expression \"the future will be bleak\" is used in the negative emotional expression of the article and expresses a sense of anxiety and pessimism, it is a negative emotional expression."}, "language_style": {"answer": "Calm and direct", "rationale": "This expression gives readers some sense of pressure and urgency, indicating their concern and dissatisfaction with the current political situation."}, "time_dimension": {"answer": "Past", "rationale": ""}, "sentence_type": "Negative declarative sentence", "pos": {"nouns": {"Questionnaire Entry": 1, "person": 2, "Politics": 1, "Country": 2, "Future": 1}, "verbs": {"More attraction": 1, "Intruder": 1, "Dark": 1}, "adjectives": {"Tens": 1}, "adverbs": {"Unless": 1}, "numerals": {"3": 1}, "pronouns": {"Here": 1}}}, {"content": "4. Fear, social shame or punishment, rather than conscience, prevent the majority from breaking the law.", "word_count": 25, "label": "", "sentence_structure": "Subject鈥揷opula鈥損redicative", "subjective_objective": {"answer": "Subjective", "rationale": "This view is an expression of the subjective view of the researcher of the reasons why the majority is prevented from breaking the law."}, "sentence_sentiment": {"answer": "Negative", "rationale": "Because the words \"fear, social shame or punishment\" in the entry are negative and do not mention positive factors."}, "language_style": {"answer": "Calm and direct", "rationale": "The entry is presented in an objective manner, which describes the reasons for preventing the majority from breaking the law."}, "time_dimension": {"answer": "Present", "rationale": "The sentence is mainly a description of a general phenomenon or view, and does not clearly point to the past or the future, and thus is judged to be the present."}, "sentence_type": "Affirmative declarative sentence", "pos": {"nouns": {"person": 1, "Law": 1, "Fear": 1, "Society is a shame": 1, "Punishment": 1, "Conscience": 1}, "verbs": {"Stop": 1, "Infractions": 1}, "numerals": {"Most": 1, "4": 1}}}, {"content": "5. The failure of teachers to conduct examinations at the time of the examination could lead to more fraud.", "word_count": 21, "label": "", "sentence_structure": "Subject鈥搗erb鈥搊bject", "subjective_objective": {"answer": "Subjective", "rationale": "Because this is based on the subjective assumption that researchers or questionnaire designers do not all cheat without being tested. This view requires empirical research to verify whether it is accurate."}, "sentence_sentiment": {"answer": "Negative", "rationale": "Because the behavior \"false\" in the entry is negative."}, "language_style": {"answer": "Calm and direct", "rationale": ""}, "time_dimension": {"answer": "Present", "rationale": "The entry is a description of a possible phenomenon or situation, \"when tested\" as a time limit, while \"may lead\" implies that this is a possibility, without specifying whether it has occurred. It can therefore be judged that the entry is primarily a discussion of the current situation rather than of past or future."}, "sentence_type": "Affirmative declarative sentence", "pos": {"nouns": {"Examinations": 1, "Teacher": 1, "The exam": 1, "person": 2, "Cheating": 1}, "verbs": {"Here we go": 1, "Examination": 1, "Result": 1, "More": 1, "Yes": 1}, "adverbs": {"Maybe": 1}, "numerals": {"5": 1, "More": 1}}}, {"content": "Parents are usually trustworthy in keeping their promises.", "word_count": 17, "label": "", "sentence_structure": "Subject鈥揷opula鈥損redicative", "subjective_objective": {"answer": "Objective", "rationale": "Because the credibility of the subject is an objective fact of the evaluation (parents), it is not the patient ' s own subjective feeling or attitude."}, "sentence_sentiment": {"answer": "Positive", "rationale": "This item expresses confidence in the parents ' commitment and is a positive attitude and evaluation."}, "language_style": {"answer": "Tactful and warm", "rationale": "The sentence is not a strong expression of opinion, but rather a more peaceful one. At the same time, it seeks to inspire the confidence of the interviewee in his parents' respect for their promises, which is more accompanied and interactive and belongs to a euphemism and warmer language."}, "time_dimension": {"answer": "Present", "rationale": "The entry describes a status quo or a normal situation, namely, that \"parents are usually trustworthy in keeping their promises\", implying that it is a general phenomenon and does not address the temporal dimensions of the past or the future."}, "sentence_type": "Affirmative declarative sentence", "pos": {"nouns": {"parents": 1, "Promises": 1}, "verbs": {"Compliance": 1, "You can trust it": 1}, "adverbs": {"Usually": 1}, "numerals": {"6": 1}}}, {"content": "7. The United Nations will never be an effective force for world peace.", "word_count": 21, "label": "", "sentence_structure": "Subject鈥揷opula鈥損redicative", "subjective_objective": {"answer": "Subjective", "rationale": "Because the sentence expresses the subjective view of the subject (i.e. the interviewee) as to whether the United Nations can be an effective force for world peace."}, "sentence_sentiment": {"answer": "Negative", "rationale": "The entries express negative attitudes and negative predictions about the United Nations."}, "language_style": {"answer": "Calm and direct", "rationale": "The entry presents in a clear and concise manner the view of the United Nations as to whether it can effectively maintain world peace, with a direct linguistic style, without too much emotional overturning or rhetorical. At the same time, the view is subjective, without any justification or justification to support the argument, and therefore has a calm and objective tone.\""}, "time_dimension": {"answer": "Future", "rationale": "The keyword is \"never\" , which indicates that the future prediction and assumption that the United Nations cannot be an effective force for world peace. Moreover, the word \"will\" adopts the general future pattern, emphasizing that this is a possibility that has not yet occurred and that of the future. The entry therefore relates to the future and judges it as the future."}, "sentence_type": "Negative declarative sentence", "pos": {"nouns": {"UNITED NATIONS": 1, "World peace": 1, "Power": 1}, "verbs": {"Be": 1, "Maintenance": 1, "It works": 1}, "adverbs": {"Forever": 1}}}, {"content": "8. The Court is a place where we can all be treated fairly.", "word_count": 16, "label": "", "sentence_structure": "Subject鈥揷opula鈥損redicative", "subjective_objective": {"answer": "Objective", "rationale": "Because it is an opinion or belief in the universality of the Court, it does not involve subjective attitudes or experience of individuals."}, "sentence_sentiment": {"answer": "Positive", "rationale": "Because \"just treatment\" is positive."}, "language_style": {"answer": "Calm and direct", "rationale": "The language style of the entry is more direct and objective, and there is no excessive rhetorical or emotional rendering, and a fact or view is expressed in a concise manner. \""}, "time_dimension": {"answer": "Present", "rationale": "The entry \"we\" and \"we\" all show that this is a statement of the current state or status, that is, that in the present case all people are treated fairly by the courts. So the time dimension is now."}, "sentence_type": "Affirmative declarative sentence", "pos": {"nouns": {"Courts": 1, "we": 1, "Justice": 1, "Treatment": 1, "Places": 1}, "verbs": {"be": 1, "Yes": 1, "Received": 1, "Treatment": 1}, "adjectives": {"Fair": 1}, "numerals": {"8": 1}, "pronouns": {"EMPTY": 1}}}, {"content": "9. Most would be surprised to learn how much news the public heard and saw had been distorted.", "word_count": 30, "label": "", "sentence_structure": "Subject鈥搗erb鈥搊bject", "subjective_objective": {"answer": "Subjective", "rationale": "Because this is the patient ' s perception of what the public may feel, it is a subjective assumption."}, "sentence_sentiment": {"answer": "Negative", "rationale": "Because the entry expresses the majority of people who would be surprised at the distortion of the news, which is a negative emotional attitude."}, "language_style": {"answer": "Calm and direct", "rationale": "The language style of the entry is more direct, is not overly phrasing, is clearly worded and is expressed in a concise manner, and therefore can be judged as being a calm and straightforward language style."}, "time_dimension": {"answer": "Present", "rationale": "In the sentence, the words \"if known\" are about the current or recent situation, namely, \"how much news the public has heard and seen has been distorted\", and the latter description is about the possible impact of this situation, that is, \"the majority would be surprised.\" So the time dimension of this entry is now judged."}, "sentence_type": "Affirmative declarative sentence", "pos": {"nouns": {"Questionnaire Entry": 1, "Public": 1, "Public information": 2, "How much": 1, "Misdirection": 1, "Most people": 1, "I feel": 1, "Surprise": 1}, "verbs": {"I know": 1, "Yes": 3, "Yeah": 1, "Already": 1, "By": 1, "Misdirection": 1, "I feel": 1, "Surprise": 1}, "numerals": {"9": 1, "How much": 1}, "classifiers": {"individual": 1}, "pronouns": {"If": 1, "Public": 1, "How much": 1, "Most people": 1}}}, {"content": "10. Whatever the statements, it is desirable to consider that the majority is primarily concerned with its own well-being.", "word_count": 26, "label": "", "sentence_structure": "Subject鈥損redicate", "subjective_objective": {"answer": "Subjective", "rationale": "Because \"think\" and \"main concern\" are patients' subjective judgments and attitudes."}, "sentence_sentiment": {"answer": "Negative", "rationale": "Because the expression \"preferably or not, most people are primarily concerned with their own happiness\" in the entry is a skeptical and negative attitude, with some negative emotional connotations."}, "language_style": {"answer": "Tactful and warm", "rationale": "The entry uses a recommendatory tone, not direct orders or assertions. It expresses the author鈥檚 view of the majority and invites readers to reflect on and agree with this view, rather than to insist on its acceptance. This expression is more personal and interactive."}, "time_dimension": {"answer": "Present", "rationale": ""}, "sentence_type": "Affirmative declarative sentence", "pos": {"nouns": {"People": 2, "Statement": 1, "Most people": 2, "I'm happy": 1}, "verbs": {"I think": 1, "Care": 1, "be": 1, "I'd better": 1, "Or is it": 1, "Whatever": 1, "What": 1, "Expression": 1}, "adjectives": {"Most": 2}, "adverbs": {"Main": 1}, "numerals": {"10": 1, "2": 2}, "pronouns": {"Other": 1}}}, {"content": "11. Although news is available in newspapers, radio and television, it is difficult to obtain objective coverage of public events.", "word_count": 36, "label": "", "sentence_structure": "Subject鈥搗erb鈥搊bject", "subjective_objective": {"answer": "Objective", "rationale": "This statement is a statement of the fact that it is difficult to obtain objective reporting on public events, which is an objective situation that does not depend on a subjective view of the individual."}, "sentence_sentiment": {"answer": "Negative", "rationale": "Because the phrase \"we have difficulty getting objective reports of public events\" in the entry is negative."}, "language_style": {"answer": "Calm and direct", "rationale": "The article is more straightforward, and there is no euphemism or emotional expression. It simply describes a fact and presents a point that, despite the many channels of access to news, it is difficult to obtain objective coverage of public events. This straightforward language helps readers to understand the author鈥檚 views clearly without interference."}, "time_dimension": {"answer": "Present", "rationale": ""}, "sentence_type": "Negative declarative sentence", "pos": {"nouns": {"Newspapers": 1, "Radio": 1, "Television": 1, "Public information": 2, "Public events": 1, "Objective reporting": 1}, "verbs": {"Yeah": 1, "Got it": 1, "Coverage": 1}, "adjectives": {"Objective": 1}, "adverbs": {"It's hard": 1}, "numerals": {"11": 1, "1": 2}, "pronouns": {"we": 1}}}, {"content": "12. The future seemed promising.", "word_count": 8, "label": "", "sentence_structure": "Subject鈥揷opula鈥損redicative", "subjective_objective": {"answer": "Subjective", "rationale": "Because \"Hope\" is a subjective feeling and expectation, it is a subjective view of the patient's personal vision and expectations for the future."}, "sentence_sentiment": {"answer": "Positive", "rationale": "It is an expression of optimism and positive attitude towards the future."}, "language_style": {"answer": "Tactful and warm", "rationale": "The language style of the entry is more euphemistic, using the word \"seemingly\" to express expectations for the future, and not too directly or with any certainty that the future will be filled with hope. This expression is more subtle and objective."}, "time_dimension": {"answer": "Future", "rationale": "The keyword is \"the future\", which expresses vision and expectations for the future, and the temporal dimension is judged to be the future."}, "sentence_type": "Affirmative declarative sentence", "pos": {"verbs": {"have": 1, "Seems like": 1}, "adjectives": {"very": 1, "There's hope": 1}}}, {"content": "13. If there is real awareness of the political events taking place at the international level, then there are reasons for the public to be more concerned than now.", "word_count": 33, "label": "", "sentence_structure": "Subject鈥搗erb鈥搊bject", "subjective_objective": {"answer": "Subjective", "rationale": "Because \"there are reasons to worry more\" is the perception or perception of the interviewee, not the objective reality. This involves the subjectivity and emotional reaction of the interviewee."}, "sentence_sentiment": {"answer": "Negative", "rationale": "Because the emotional color of \"fear\" in the entry is negative."}, "language_style": {"answer": "Calm and direct", "rationale": "The entry presents the subject in a direct manner, without any rhetorical or emotional presentation and with a more objective tone."}, "time_dimension": {"answer": "Present", "rationale": "The phrase \"if\" indicates the scenario, \"positive\" and \"incurred\" indicates that these events are currently being carried out, so the sentence describes what is happening."}, "sentence_type": "Affirmative declarative sentence", "pos": {"nouns": {"International": 1, "Political events": 1, "Public": 1, "Rationale": 1}, "verbs": {"Roger that": 1, "Come on": 1, "I'm worried": 1, "have": 1}, "adjectives": {"Really": 1}, "adverbs": {"More": 1}, "numerals": {"13": 1, "Present": 1}, "pronouns": {"Well": 1, "- Yeah": 1}}}, {"content": "14. The promises made by most elected officials in the campaign are sincere.", "word_count": 17, "label": "", "sentence_structure": "Subject鈥揷opula鈥損redicative", "subjective_objective": {"answer": "Objective", "rationale": "Because the \"promise of elected officials in the election campaign\" is an objective fact, the sentence is a description or judgement of that objective fact and is therefore an objective one."}, "sentence_sentiment": {"answer": "Positive", "rationale": "Because the entry expresses the honest promise of most elected officials in the campaign, which is a positive description of behaviour, the emotional colour of expression is positive."}, "language_style": {"answer": "Calm and direct", "rationale": "It presents a fact in an objective and clear manner, without too much ado or emotional, with a more neutral tone, and with a direct message that the investigators want to know."}, "time_dimension": {"answer": "Present", "rationale": ""}, "sentence_type": "Affirmative declarative sentence", "pos": {"nouns": {"Questionnaire Entry": 1, "Officials": 1, "Elections": 1, "Promises": 1, "-Significant": 1}, "verbs": {"Selected": 1, "be": 2, "in": 1, "The campaign is underway": 1, "of": 2, "Promises": 1, "Be honest": 1}, "adjectives": {"Most": 1, "-Significant": 1}, "numerals": {"14": 1}}}, {"content": "15. Many major national sporting events are subject to some form of manipulation and exploitation.", "word_count": 25, "label": "", "sentence_structure": "Subject鈥搗erb鈥搊bject", "subjective_objective": {"answer": "Objective", "rationale": "Because it is a description of a phenomenon, not a subjective feeling or attitude of the patient."}, "sentence_sentiment": {"answer": "Negative", "rationale": "Because the \"manipulation and exploitation\" is negative."}, "language_style": {"answer": "Calm and direct", "rationale": "The language style of the entry is calm and objective, and does not use too many emotional language or rhetorical techniques. It expresses concern and concern about the manipulation and use of the phenomenon in national sports games in a direct way."}, "time_dimension": {"answer": "Present", "rationale": "The keyword \"was\" denotes a state or behaviour currently in progress, and the existence of \"manipulation and utilization\" refers to a problem at present. Thus, the entry describes the current situation and judges it as now."}, "sentence_type": "Negative declarative sentence", "pos": {"nouns": {"National": 1, "Sports competitions": 1, "Manipulation": 1, "Utilization": 1}, "verbs": {"Received": 1, "Form": 1}, "adjectives": {"Significant": 1, "Some kind of": 1}, "numerals": {"15": 1}}}, {"content": "16. Most expert statements about the limitations of their knowledge are credible.", "word_count": 19, "label": "", "sentence_structure": "Subject鈥揷opula鈥損redicative", "subjective_objective": {"answer": "Subjective", "rationale": "This is a subjective assessment because it involves patients ' perceptions and perceptions of specialists."}, "sentence_sentiment": {"answer": "Positive", "rationale": "Because the entry expresses a positive view that most experts are self-aware and are willing to acknowledge their knowledge limitations."}, "language_style": {"answer": "Calm and direct", "rationale": "The language style of the entry is more objective and calm, and there is no excessive use of emotional vocabulary or rhetorical techniques. It conveys the views in a direct way, is neutral and makes sense."}, "time_dimension": {"answer": "Present", "rationale": "The entry is for the purpose of asking the testee about the attitude and perception of a certain view or belief, and the temporal dimension is now judged."}, "sentence_type": "Affirmative declarative sentence", "pos": {"nouns": {"Experts": 1, "Limitations": 1, "Knowledge": 1}, "verbs": {"Related": 1, "Statement": 1, "be": 1, "Trustable": 1}, "adverbs": {"Most": 1}, "numerals": {"16": 1}}}, {"content": "17. The threat of punishment by most parents is credible.", "word_count": 17, "label": "", "sentence_structure": "Subject鈥揷opula鈥損redicative", "subjective_objective": {"answer": "Objective", "rationale": "Because the credibility of the threat of punishment is a fact that can be confirmed by investigation and data. This is an objective question, not a subjective attitude or feeling of the patient."}, "sentence_sentiment": {"answer": "Negative", "rationale": "Because the purpose of the \"punishment\" in the entry is negative."}, "language_style": {"answer": "Calm and direct", "rationale": "The entry presents a view in a direct, concise language, with no excessive emotional or rhetorical style, which is straightforward and calm."}, "time_dimension": {"answer": "Present", "rationale": "The key words \"most parents\" and \"threat of punishment\" and the verb \"yes\" indicate that this is a description of the current phenomenon."}, "sentence_type": "Affirmative declarative sentence", "pos": {"nouns": {"Most parents": 1, "Enforcement of penalties": 1, "Threats": 1}, "verbs": {"About": 1, "It's credible": 1}, "adjectives": {"Trustable": 1}, "numerals": {"17": 1}}}, {"content": "18. Most people will come to terms with their intentions.", "word_count": 19, "label": "", "sentence_structure": "Subject鈥搗erb鈥搊bject", "subjective_objective": {"answer": "Subjective", "rationale": "The statement of its intentions will certainly fulfil the subjective view and conviction of one person."}, "sentence_sentiment": {"answer": "Positive", "rationale": "Because the behavior in the entry \"show your intentions and will come true\" is positive."}, "language_style": {"answer": "Calm and direct", "rationale": "The entry presents a point in a direct, concise way, with no excessive emotional rendering or rhetorical style, and is more objective."}, "time_dimension": {"answer": "Present", "rationale": "The keywords \"speak\" and \"defunct\" both indicate that it is a customary act or state and describe the current situation, so the temporal dimension of this entry is now."}, "sentence_type": "Affirmative declarative sentence", "pos": {"nouns": {"person": 1, "I'm going to": 1, "Achieved": 1}, "verbs": {"Say it": 1, "Yes": 1, "Go on": 1}, "adverbs": {"Most": 1}}}, {"content": "In this age of competition, you could be taken advantage of if you don't keep your guard.", "word_count": 26, "label": "", "sentence_structure": "Subject鈥損redicate", "subjective_objective": {"answer": "Subjective", "rationale": "Because this phrase expresses patients ' perception and attitude to being vigilant in a competitive environment, it is a subjective judgement."}, "sentence_sentiment": {"answer": "Negative", "rationale": "Because the phrase \"whose attention is not likely to take advantage of you\" in the entry implies a negative competitive attitude and a sense of mistrust."}, "language_style": {"answer": "Calm and direct", "rationale": "The language style of the entry is more direct and clear, and not too much rhetorical or euphemistic, but rather a message of the need for constant vigilance in a competitive society, in a more direct and objective language."}, "time_dimension": {"answer": "Future", "rationale": "The keyword \"In this age of competition\" suggests that this is a possible future, and the description \"If you are not vigilant, you may be taken advantage of\" suggests that it is a vision and a expectation for the future. The entry is therefore a future issue."}, "sentence_type": "Affirmative declarative sentence", "pos": {"nouns": {"Age": 1, "Watch out": 2, "others": 2, "Cheap": 1}, "verbs": {"Hold on": 1, "Jim": 1}, "adjectives": {"Competing": 1}, "adverbs": {"It's possible": 1}, "numerals": {"19": 1}, "pronouns": {"you": 1}}}, {"content": "20. Most idealists acted in good faith and in accordance with the tenets they themselves preached.", "word_count": 26, "label": "", "sentence_structure": "Subject鈥揷opula鈥損redicative", "subjective_objective": {"answer": "Subjective", "rationale": "Because words like \"in good faith\" and \"acting according to the doctrine that you preach\" describe the character and behaviour of the idealists, all of which are viewed from the subjective point of view of the researchers."}, "sentence_sentiment": {"answer": "Positive", "rationale": "Because the idealists' qualities and behaviour, as expressed in this entry, are positive, as are their own messages."}, "language_style": {"answer": "Calm and direct", "rationale": "The entry is based on a judgement that expresses in an objective, concise manner the view that \"most idealists act in good faith and in accordance with the tenets they themselves preach\" and that there is no excessive rhetorical or emotional rendering, which is more straightforward and direct. Thus, the language style of the article can be described as being calm and straightforward."}, "time_dimension": {"answer": "Past", "rationale": "The entry describes the behaviour characteristics and characteristics of \"most idealists\" that were already present in the past and widely accepted. Thus, in terms of time dimension, the content of the questionnaire is mainly related to the past."}, "sentence_type": "Affirmative declarative sentence", "pos": {"nouns": {"Most": 1, "Idealist": 1, "-Significant": 1, "Letters": 1, "Act": 1}, "verbs": {"be": 1, "According to": 1, "Promotion": 1}, "adjectives": {"They're themselves": 1}, "numerals": {"20": 1}}}, {"content": "Most promoters are honest in describing their products.", "word_count": 19, "label": "", "sentence_structure": "Subject鈥揷opula鈥損redicative", "subjective_objective": {"answer": "Objective", "rationale": "Because honesty is an objective code of conduct or attitude, and the process of describing products is an objective fact. While the promoters may have subjective views or motives, their description is based on objective facts."}, "sentence_sentiment": {"answer": "Neutral", "rationale": "As the entry merely stated a fact, it did not express any feelings or attitudes."}, "language_style": {"answer": "Calm and direct", "rationale": "The language style of the entry is more direct, objective and does not involve too many rhetorical or emotional renderings, which are used primarily to present a view or a fact."}, "time_dimension": {"answer": "Present", "rationale": ""}, "sentence_type": "Affirmative declarative sentence", "pos": {"nouns": {"Questionnaire Entry": 1, "Sales staff": 1, "Products": 1}, "verbs": {"Description": 1, "be": 1, "Honesty": 1}, "numerals": {"21": 1}}}, {"content": "22. Most students do not cheat even when they are certain they will not be discovered.", "word_count": 20, "label": "", "sentence_structure": "Subject鈥損redicate", "subjective_objective": {"answer": "Subjective", "rationale": "Because the descriptions of \"most students\" and \"when sure they won't be found\" are based on the surveyor's perspective, \"no cheating\" is the student's subjective attitude or behaviour. So the entry to the questionnaire is viewed from a subjective perspective."}, "sentence_sentiment": {"answer": "Positive", "rationale": "Because this entry describes the integrity of most students."}, "language_style": {"answer": "Tactful and warm", "rationale": "The entry is more proficient and interactive when it is spoken in a gentle and inviting manner, rather than in direct statements or orders. At the same time, it does not use excessive rhetorical or emotional renderings, which are more objective and sound."}, "time_dimension": {"answer": "Present", "rationale": "The entry contains a current action \"no cheating\" and a normal adverb \"most students\" and a hyphen \"even\" indicating the hypothesis. This information points to the current state or behaviour. It can therefore be judged to be the present situation."}, "sentence_type": "Negative declarative sentence", "pos": {"nouns": {"Students": 1, "Hold on": 1, "Found": 1, "Cheating": 1}, "verbs": {"Yes": 1, "not": 1, "Do it": 1}, "adjectives": {"Most": 1, "have": 1}, "adverbs": {"Even if": 1, "in": 1, "Yeah": 1}, "numerals": {"22": 1}}}, {"content": "Most maintenance staff do not charge more if they consider themselves to be without your expertise.", "word_count": 24, "label": "", "sentence_structure": "Subject鈥搗erb鈥搊bject", "subjective_objective": {"answer": "Objective", "rationale": "Because it is an observation and description of the behaviour of maintenance personnel, not a patient鈥檚 subjective feeling or attitude. It describes how most maintenance personnel act in the circumstances, without involving the patient鈥檚 personal experience and views."}, "sentence_sentiment": {"answer": "Positive", "rationale": "Because the behavior in the entry \"most maintenance staff don't charge more if you don't have their expertise\" is positive."}, "language_style": {"answer": "Tactful and warm", "rationale": "The entry, which is being dialogueled in a more moderate sense, uses the \"even if not\" concessionary tone, expressing a sense of concern and understanding. At the same time, the possibility of direct criticism or criticism is being avoided, making the questionnaire more personal and interactive."}, "time_dimension": {"answer": "Present", "rationale": "The key words \"most maintenance staff\" and \"conceive that you do not have the expertise and are not expensive\" are all possible scenarios under the current circumstances."}, "sentence_type": "Negative declarative sentence", "pos": {"nouns": {"Maintenance staff": 1, "Expertise": 1, "Costs": 1}, "verbs": {"I think": 1, "No, I won't": 2, "Overrear": 1, "Fees": 1}, "adjectives": {"Most": 1, "Other": 1}, "adverbs": {"Even if": 1}, "numerals": {"23": 1}, "pronouns": {"you": 1}}}, {"content": "24. A significant part of the charges against insurance companies are false.", "word_count": 17, "label": "", "sentence_structure": "Subject鈥揷opula鈥損redicative", "subjective_objective": {"answer": "Objective", "rationale": "This is an objective reality for insurance companies. While some of the charges may not be entirely false, at least some of the charges are fraudulent or misleading, so the sentence describes the situation objectively."}, "sentence_sentiment": {"answer": "Negative", "rationale": "Since the statement of purpose of the article concerns false claims by insurance companies, it implies unfaithful behaviour by insurance companies and therefore has a negative emotional connotation."}, "language_style": {"answer": "Calm and direct", "rationale": "The entry is more direct in language style, with no excessive emotional accommodation or euphemism. It presents the view that a significant part of the insurance company鈥檚 complaint is false, is objective and does not contain obvious subjective sentiments, and therefore can be judged as a calm and direct language style."}, "time_dimension": {"answer": "Present", "rationale": "The keyword \"a substantial part\" indicates the current situation, and \"false\" is the general current, so the temporal dimension of the article is now."}, "sentence_type": "Negative declarative sentence", "pos": {"nouns": {"Insurance companies": 1, "The complaint": 1, "Part": 1}, "verbs": {"have": 1, "be": 1}, "adjectives": {"Pretty much": 1, "Fake": 1}, "numerals": {"24": 1}}}, {"content": "25. Most people answered the questions in the polls honestly.", "word_count": 16, "label": "", "sentence_structure": "Subject鈥搗erb鈥搊bject", "subjective_objective": {"answer": "Objective", "rationale": "Because most people honestly answer questions from polls about observed facts, they do not relate to subjective attitudes or perceptions of the interviewees."}, "sentence_sentiment": {"answer": "Positive", "rationale": "Because the \"an honest answer\" in the entry is positive."}, "language_style": {"answer": "Calm and direct", "rationale": "The language style of the entry is more direct, with no excessive adoration or emotional colour, but simply expresses the subject matter of the investigation. This style is usually more appropriate in a formal setting or in situations where a message needs to be delivered quickly.\""}, "time_dimension": {"answer": "Present", "rationale": "This entry asks the respondent ' s opinion as to whether the majority is responding honestly to the questions in the polls, without addressing past or future behaviour or mentality. The time dimension of this article is therefore now judged."}, "sentence_type": "Affirmative declarative sentence", "pos": {"nouns": {"Problem": 1, "A poll": 1, "Most people": 1}, "verbs": {"Honesty": 1, "Answer": 1}, "adjectives": {"Most": 1, "Honest": 1}, "numerals": {"25": 1}}}]}

{"questionnaire_title": "Chinese version of the Family Environment Sheet (FES-CV)", "questionnaire_category": "Emotion and affect", "item_count": 90, "emotion_shift_overall": "From positive to negative", "language_style_overall": "Tactful and warm", "items": [{"content": "We, the family members, always offer each other the greatest help and support.", "word_count": 21, "label": "", "sentence_structure": "Subject鈥搗erb鈥搊bject", "subjective_objective": {"answer": "Subjective", "rationale": "This is a subjective examination of the patient ' s assessment and perception of his or her family situation."}, "sentence_sentiment": {"answer": "Positive", "rationale": "Because the entry \"to give each other the greatest help and support\" expresses a positive family relationship and interaction."}, "language_style": {"answer": "Tactful and warm", "rationale": "This language style is effective in building emotional ties with the interviewee and in enabling them to share more information about family life."}, "time_dimension": {"answer": "Present", "rationale": "And by the word \"always\" and \"one another,\" we can tell that the sentence describes a customary, recurrent act, whereby family members have been giving each other the greatest help and support. So the time dimension can be judged now."}, "sentence_type": "Affirmative declarative sentence", "pos": {"nouns": {"Family members": 2, "Support": 1, "Help": 1}, "verbs": {"Here": 1, "Total": 1, "be": 1, "- Yeah, yeah": 1, "Give it to me": 1, "Max": 1}, "adjectives": {"Largest": 1}, "adverbs": {"Both": 1}, "classifiers": {"individual": 1}, "pronouns": {"we": 1}}}, {"content": "Family members always hide their feelings in their hearts and do not reveal them to other family members.", "word_count": 26, "label": "", "sentence_structure": "Subject鈥搗erb鈥搊bject", "subjective_objective": {"answer": "Objective", "rationale": "The emotional state and manner of expression of family members are objective facts."}, "sentence_sentiment": {"answer": "Negative", "rationale": "Because the entry \"hidden your feelings in your heart without revealing them to other family members\" is negative."}, "language_style": {"answer": "Calm and direct", "rationale": "The entry presents the problem in a straightforward manner, without too many rhetorical or emotional renderings, and is more objective in tone."}, "time_dimension": {"answer": "Past", "rationale": "The behaviour of family members who had hidden their feelings in their hearts for some time in the past has been \"always\" indicative of the persistence of such behaviour."}, "sentence_type": "Affirmative declarative sentence", "pos": {"nouns": {"Family members": 2, "Emotion": 2, "In my heart": 1, "Other": 1, "Family": 1}, "verbs": {"Always": 1, "- Put it on": 1, "Hide": 1, "Disclosure": 1, "In": 1}, "adverbs": {"in": 1, "not": 1}, "numerals": {"2": 1}, "pronouns": {"Other": 1}}}, {"content": "The family often fights.", "word_count": 6, "label": "", "sentence_structure": "Subject鈥搗erb鈥揷omplement", "subjective_objective": {"answer": "Objective", "rationale": "Since the fights were an objective occurrence, not related to the patient ' s preference, they were an objective examination."}, "sentence_sentiment": {"answer": "Negative", "rationale": "The judgment is based on the fact that the \"challenge\" is negative."}, "language_style": {"answer": "Calm and direct", "rationale": "The entry directly asked about the conflict in the family of the interviewee, was not overly emotional or rhetorical, and was relatively calm and objective."}, "time_dimension": {"answer": "Present", "rationale": "According to the sentence, \"three homes often fight,\" we can judge that the entry is about the family status of the person being tested at some point or for a period of time (for example, over the past year). So we can classify the entry as \"the past\"."}, "sentence_type": "Affirmative declarative sentence", "pos": {"nouns": {"Family": 3, "Argument": 3}, "verbs": {"Regular": 1, "have": 1}, "numerals": {"3": 1}}}, {"content": "In our homes, we rarely act alone.", "word_count": 13, "label": "", "sentence_structure": "Subject鈥搗erb鈥揷omplement", "subjective_objective": {"answer": "Subjective", "rationale": "Because the sentence describes the individual feelings and experiences of patients, who rarely engage in activities alone in the home, is a subjective experience and judgement."}, "sentence_sentiment": {"answer": "Negative", "rationale": "Because the \"very few activities on their own\" act in the entry is negative."}, "language_style": {"answer": "Tactful and warm", "rationale": "Because the entry uses the words \"less\" and \"self-sole activities\" to convey more euphemistically the less dynamic interaction of family members. Such expression does not make the respondents feel too direct or offensive, helps to establish good investigative relationships and makes it easier to obtain real and objective information."}, "time_dimension": {"answer": "Present", "rationale": ""}, "sentence_type": "Negative declarative sentence", "pos": {"nouns": {"Home": 1, "Medium": 1, "we": 1, "self": 1, "Alone": 1, "Activities": 1}, "verbs": {"in": 1, "Less": 1, "Do it": 1}, "adverbs": {"Very few": 1}, "numerals": {"4": 1}}}, {"content": "The family members do everything they can.", "word_count": 18, "label": "", "sentence_structure": "Subject鈥搗erb鈥揷omplement", "subjective_objective": {"answer": "Objective", "rationale": "Because \"whatever the family members do is done to their best efforts\" describes the behaviour of the family members, which is an objective fact. While the sentence also contains a subjective view (that is, that the conduct is commendable), it looks at the veracity or objectivity of the statement in this topic, and we therefore classify it as an objective one."}, "sentence_sentiment": {"answer": "Positive", "rationale": "Because the \"best of all\" approach in the entry is positive."}, "language_style": {"answer": "Tactful and warm", "rationale": "The language style of the entry is more euphemistic, as it uses encouraging and supportive language, which expresses recognition and praise for the efforts of the respondents and their families."}, "time_dimension": {"answer": "Present", "rationale": ""}, "sentence_type": "Affirmative declarative sentence", "pos": {"nouns": {"Family members": 1, "Things": 1}, "verbs": {"Do it": 1, "Do what you can": 1}, "numerals": {"5": 1}}}, {"content": "Our family has often spoken about political and social issues.", "word_count": 14, "label": "", "sentence_structure": "Subject鈥搗erb鈥搊bject", "subjective_objective": {"answer": "Subjective", "rationale": "It is considered subjectively because it is a family ' s choice of behaviour that reflects the values and attitudes of family members."}, "sentence_sentiment": {"answer": "Neutral", "rationale": "Because the entry merely describes a fact, it does not show a positive or negative emotional attitude."}, "language_style": {"answer": "Tactful and warm", "rationale": "The entry is more personal and interactive when it is presented in a gentle and inviting tone, rather than in direct statements or orders. At the same time, by asking the families of the other party whether political and social issues are frequently discussed, the interviewee is more willing to cooperate with the questionnaire by feeling the atmosphere of concern and respect."}, "time_dimension": {"answer": "Past", "rationale": "The keyword \"Our family is talking about\" indicates that it is a customary act and that it has existed for some time, and thus is judged to be a past."}, "sentence_type": "Affirmative declarative sentence", "pos": {"nouns": {"Family": 1, "Political": 1, "Social": 1}, "verbs": {"Discussion": 1, "Regular": 1}, "adverbs": {"Regular": 1}, "numerals": {"6": 1}, "pronouns": {"we": 1}}}, {"content": "Most weekends and evenings are spent at home by family members, without going out to social and recreational events.", "word_count": 32, "label": "", "sentence_structure": "Subject鈥搗erb鈥搊bject", "subjective_objective": {"answer": "Objective", "rationale": "Because \"getting home\" and \"get out of social and recreational activities\" are the description of objective facts."}, "sentence_sentiment": {"answer": "Neutral", "rationale": "Because the entry does not express positive or negative feelings, attitudes or behaviours. It describes the fact that most people are at home and at night at weekends, not outside."}, "language_style": {"answer": "Calm and direct", "rationale": "The language style of the entry is a calm and straightforward one. It presents a fact, not using excessive emotional accolades or subjective evaluations. This expression is often more objective and authentic, and is suitable for the survey of such situations as the need for accurate data.\""}, "time_dimension": {"answer": "Past", "rationale": "The time dimension of this questionnaire entry is therefore judged to be past."}, "sentence_type": "Affirmative declarative sentence", "pos": {"nouns": {"Weekend": 1, "Night": 1, "Family members": 1, "Home": 2, "Medium": 1, "Recreational activities": 1}, "verbs": {"Pass": 1, "Participation": 1, "Social": 1}, "adjectives": {"Most of them": 1, "not": 1}, "numerals": {"7": 1}}}, {"content": "We all believe that, no matter how difficult, children should first meet the needs of the elderly.", "word_count": 27, "label": "", "sentence_structure": "Subject鈥搗erb鈥搊bject", "subjective_objective": {"answer": "Subjective", "rationale": "Because the sentence expresses the subjective view and expectation that children should meet the needs of the elderly. This view is based on social and ethical values, not on objective facts or realities."}, "sentence_sentiment": {"answer": "Positive", "rationale": "The view expressed in the entry is an expression of respect and concern for older persons, and emphasizes that children should do their best to meet the needs of older persons."}, "language_style": {"answer": "Tactful and warm", "rationale": "The entry uses the word \"we think\" to introduce the idea and the word \"should\" to express a sense of duty and responsibility. This expression is more friendly and persuasive, and it is easier to make the interviewer accept and resonate, and therefore it can be considered a mild and warm expression."}, "time_dimension": {"answer": "Past", "rationale": "The entry describes the views and attitudes of a certain concept or behaviour and does not address the specific temporal dimensions of the past, present or future, and therefore does not allow for a time judgement."}, "sentence_type": "Affirmative declarative sentence", "pos": {"nouns": {"Difficulties": 1, "Children": 1, "Requirements": 1}, "verbs": {"I think": 1, "Satisfied": 1}, "adjectives": {"Large": 1}, "adverbs": {"Both": 1, "First of all": 1}, "numerals": {"8": 1}, "classifiers": {"Plant": 1}, "pronouns": {"Various": 1}}}, {"content": "The larger activities in the family are carefully arranged.", "word_count": 16, "label": "", "sentence_structure": "Subject鈥揷opula鈥損redicative", "subjective_objective": {"answer": "Subjective", "rationale": "Because it is a subjective statement, it describes the individual attitude or conviction of the patient in planning and organizing family activities."}, "sentence_sentiment": {"answer": "Neutral", "rationale": "Because the entry does not have any emotional color."}, "language_style": {"answer": "Tactful and warm", "rationale": "The language style of the entry appears to be more euphemistic and less lax, by using a comparative expression of \"larger activities in the home\" and emphasizing that these activities are \"fully organized\" rather than directly claiming that all activities are. This way reduces some of the language pressure, makes readers more receptive and willing to learn more about them."}, "time_dimension": {"answer": "Present", "rationale": "In the sentence, \"The Biggest Activity in the Home\" is a past event, but \"the carefully arranged\" indicates that the activity is in the state in which it was being carried out or completed while speaking. Thus, the entry describes the current state."}, "sentence_type": "Affirmative declarative sentence", "pos": {"nouns": {"Home": 1, "Activities": 2, "Arrangements": 1}, "verbs": {"be": 1, "Yes": 1, "Careful": 1, "Bigger": 1}, "adjectives": {"Careful": 1, "Bigger": 1}, "numerals": {"9": 1}, "classifiers": {"Home": 1}}}, {"content": "Family members rarely force other family members to comply with family rules.", "word_count": 17, "label": "", "sentence_structure": "Subject鈥搗erb鈥搊bject", "subjective_objective": {"answer": "Objective", "rationale": "Because \"family compliance\" is an observed fact."}, "sentence_sentiment": {"answer": "Neutral", "rationale": "Since the entry merely describes a fact, it does not involve positive or negative emotional tendencies."}, "language_style": {"answer": "Tactful and warm", "rationale": "The language style of the entry is euphemistic, because it raises questions in a gentle, question-and-answer manner without direct orders or accusations. At the same time, it uses positive words (such as \"very few demands\"), which give people a sense of openness and support. Such questionnaires are designed to be more easily accessible to respondents for real feedback and active participation."}, "time_dimension": {"answer": "Present", "rationale": "\"Low\" means that such behaviour occurs to some extent, and \"very few families force other family members to comply with the family code\" refers to the current family situation and is therefore a description of the current situation."}, "sentence_type": "Affirmative declarative sentence", "pos": {"nouns": {"Family members": 2, "Family rules": 2, "Home": 1}, "verbs": {"Force": 1, "Compliance": 1}, "adjectives": {"Very few": 1}, "numerals": {"10": 1}, "pronouns": {"Other": 1, "they": 1}}}, {"content": "Eleven at home we were bored.", "word_count": 10, "label": "", "sentence_structure": "Subject鈥揷opula鈥損redicative", "subjective_objective": {"answer": "Subjective", "rationale": "This is because of the patient ' s feelings about the family and because it is a manifestation of subjective attitudes."}, "sentence_sentiment": {"answer": "Negative", "rationale": "Because the expression \"distressed\" in the entry has a negative emotional color."}, "language_style": {"answer": "Tactful and warm", "rationale": "The entry is more personal and interactive, using a gentle and inviting tone of speech rather than a direct statement or order. At the same time, the entry is not overly emotional or rhetorical, and is more objective and concise."}, "time_dimension": {"answer": "Past", "rationale": "Past."}, "sentence_type": "Affirmative declarative sentence", "pos": {"nouns": {"Home": 1, "we": 1, "Boring": 1}, "verbs": {"I feel": 1, "Boring": 1}, "adverbs": {"very": 1}, "numerals": {"11": 1}}}, {"content": "Twelve at home we can say whatever we want.", "word_count": 15, "label": "", "sentence_structure": "Subject鈥搗erb鈥搊bject", "subjective_objective": {"answer": "Subjective", "rationale": "Because \"whatever you can say\" expresses what the patient expects and thinks at home, is the patient's subjective attitude."}, "sentence_sentiment": {"answer": "Positive", "rationale": "Because the purpose of the entry is to \"do whatever you want to say.\""}, "language_style": {"answer": "Tactful and warm", "rationale": "The entry is more personal and interactive by speaking in a gentle and inviting manner, rather than by direct statement or order. At the same time, the \"home\" setting adds a comfortable and private atmosphere to the language style of the article."}, "time_dimension": {"answer": "Present", "rationale": "The entry describes the situation in the family today, where the words \"in\" and \"may\" indicate that this is an ongoing state and therefore is the current state of affairs."}, "sentence_type": "Affirmative declarative sentence", "pos": {"nouns": {"Home": 1, "Talk": 1}, "verbs": {"I'd like to say": 1, "Yeah": 1}, "adverbs": {"Just": 1, "What": 2}, "numerals": {"12": 1, "Both": 1}, "pronouns": {"we": 1}}}, {"content": "Family members rarely publicly anger each other.", "word_count": 14, "label": "", "sentence_structure": "Subject鈥搗erb鈥揷omplement", "subjective_objective": {"answer": "Subjective", "rationale": "Because anger is a subjective act and feeling among family members, the title describes their subjective attitude or perception."}, "sentence_sentiment": {"answer": "Neutral", "rationale": ""}, "language_style": {"answer": "Tactful and warm", "rationale": ""}, "time_dimension": {"answer": "Past", "rationale": "The entry \"very rarely publicly angry\" describes past behaviour or state, so it can be judged that this happened in the past. At the same time, \"family members\" is a persistent group relationship, suggesting that such behaviour may be a permanent situation. So it can be considered as a description of family interactions in the past."}, "sentence_type": "Affirmative declarative sentence", "pos": {"nouns": {"Family members": 1, "Anger": 1}, "verbs": {"You too": 1, "- Get angry": 1, "Very few": 1}, "adjectives": {"Public": 1}, "adverbs": {"very": 1}, "numerals": {"13": 1, "1": 2}}}, {"content": "We all encourage the independence of our families.", "word_count": 16, "label": "", "sentence_structure": "Subject鈥搗erb鈥搊bject", "subjective_objective": {"answer": "Subjective", "rationale": "Because \"very encouraged\" expresses the subjective attitude and the inclination of researchers."}, "sentence_sentiment": {"answer": "Positive", "rationale": "Because \"Encouraging a Family to Be Independent\" is a positive attitude and behavior."}, "language_style": {"answer": "Tactful and warm", "rationale": "The entry is more adept and interactive when it is spoken in a gentle and inviting manner, rather than by direct statements or orders."}, "time_dimension": {"answer": "Future", "rationale": "This entry expresses the expectation and hope for the future that the behaviour or attitude of \"very encouraging\" family members with an independent spirit will be promoted and practiced in the future."}, "sentence_type": "Affirmative declarative sentence", "pos": {"nouns": {"Home": 1, "Lee": 1, "person": 1, "Independence": 1}, "verbs": {"Encourage": 1, "Yes": 1}, "adverbs": {"Very": 1}, "numerals": {"14": 1}}}, {"content": "Family members have spent almost all their energy on a promising future.", "word_count": 21, "label": "", "sentence_structure": "Subject鈥搗erb鈥搊bject", "subjective_objective": {"answer": "Subjective", "rationale": "Because \"all the energy\" is a subjective assessment and judgement, not an objective fact. This reflects a subjective view and attitude of the respondents to the efforts of family members for their own future."}, "sentence_sentiment": {"answer": "Positive", "rationale": "Because the entry \"for a good future\" expresses a positive will and attitude."}, "language_style": {"answer": "Tactful and warm", "rationale": "The entry presents, in a caring and understanding manner, the efforts of family members for the future development of their children, as well as the respect and interest of the family of the interviewee, and the euphemism and warmth of the language."}, "time_dimension": {"answer": "Present", "rationale": ""}, "sentence_type": "Affirmative declarative sentence", "pos": {"nouns": {"Future": 1, "Family members": 1, "- The energy": 1}, "verbs": {"Flowers": 1, "have": 1, "Both": 1, "Almost": 1}, "adjectives": {"Okay": 1}, "adverbs": {"All": 1}, "numerals": {"15": 1, "Both": 1}, "classifiers": {"individual": 1}, "pronouns": {"they": 1}}}, {"content": "We rarely go out to listen to lectures, to see movies or to museums and to watch exhibitions.", "word_count": 22, "label": "", "sentence_structure": "Subject鈥搗erb鈥搊bject", "subjective_objective": {"answer": "Objective", "rationale": "Because \"very few outsides to listen to lectures, to see movies or to go to museums and to see exhibitions\" are the objective behaviour and experience of patients."}, "sentence_sentiment": {"answer": "Negative", "rationale": "Because the \"very little out\" act in the entry is negative."}, "language_style": {"answer": "Calm and direct", "rationale": "This expression is more objective and easy to understand and accept."}, "time_dimension": {"answer": "Past", "rationale": "\"Low\" means that the frequency of the past is lower, \"outdoors to listen to lectures, to see movies or to go to museums and to see exhibitions\" is the behaviour of the past."}, "sentence_type": "Negative declarative sentence", "pos": {"nouns": {"Lectures": 1, "Film": 1, "Museums": 1, "Exhibition": 1}, "verbs": {"Out": 1, "Listen": 1, "Look": 2}, "adverbs": {"Very few": 1}, "numerals": {"16": 1}, "classifiers": {"individual": 1}}}, {"content": "Family members often go out to friends' homes to play and eat together.", "word_count": 19, "label": "", "sentence_structure": "Subject鈥搗erb鈥搊bject", "subjective_objective": {"answer": "Subjective", "rationale": "Because the sentence describes the patient ' s domestic behaviour and preferences, it is the patient ' s subjective view."}, "sentence_sentiment": {"answer": "Positive", "rationale": "Because the purpose of the entry is to \"family members often go out to friends' houses and eat together\"."}, "language_style": {"answer": "Tactful and warm", "rationale": "The language style of the questionnaire entry is euphemistic, as it uses an invitation tone to ask if the interviewees are often travelling with their families. This expression is more personal and interactive, which is better resonance with the interviewee and does not make them feel investigated or pressured."}, "time_dimension": {"answer": "Past", "rationale": "The entry \"often\" refers to past customary behaviour, \"out to friends' homes and to eat together\" which happened in the past and is therefore judged as past."}, "sentence_type": "Affirmative declarative sentence", "pos": {"nouns": {"Family members": 2, "Friends": 1, "Rice": 1}, "verbs": {"- I'm out": 1, "Go play": 1, "Let's eat together": 1}, "adverbs": {"and": 1}, "numerals": {"17": 1}}}, {"content": "Family members agreed that they should act in a manner that was socially appropriate.", "word_count": 16, "label": "", "sentence_structure": "Subject鈥搗erb鈥搊bject", "subjective_objective": {"answer": "Subjective", "rationale": "This view is an expression of the patient ' s subjective attitude and conviction."}, "sentence_sentiment": {"answer": "Neutral", "rationale": "Because the entry was not of a clear emotional orientation, it was merely a statement of the facts."}, "language_style": {"answer": "Calm and direct", "rationale": "The language style of the entry is calm and straightforward. It presents a point in a concise manner, without using excessive emotional or rhetorical techniques and with an objective and neutral tone."}, "time_dimension": {"answer": "Present", "rationale": "The entry in the past described an attitude of acceptance of the social culture at a certain point in time (specifically, for how long it was not mentioned) and thus judged as \"the past\"."}, "sentence_type": "Affirmative declarative sentence", "pos": {"nouns": {"Family members": 1, "Yes": 1, "Social climate": 1}, "verbs": {"I think": 1, "Do it": 1, "Reactions": 1, "Complementarity": 1}, "adverbs": {"Both": 1}, "numerals": {"18": 1}, "classifiers": {"individual": 1}}}, {"content": "In general, we are all concerned to keep our homes in order.", "word_count": 20, "label": "", "sentence_structure": "Subject鈥搗erb鈥揷omplement", "subjective_objective": {"answer": "Objective", "rationale": "Because \"all\" and \"all\" are objective factual descriptions."}, "sentence_sentiment": {"answer": "Positive", "rationale": "Because the purpose of the entry is to get home organized."}, "language_style": {"answer": "Tactful and warm", "rationale": ""}, "time_dimension": {"answer": "Present", "rationale": "In the sentence, both \"general\" and \"general\" indicate that this is a common habit, while \"care\" indicates that the act is of active interest. So the temporal dimension of this questionnaire entry is now judged."}, "sentence_type": "Affirmative declarative sentence", "pos": {"nouns": {"Home": 1, "Pack up": 1, "The well is organized": 1}, "verbs": {"Attention": 1, "- Put it on": 1, "Okay": 1}, "adjectives": {"General": 1}, "adverbs": {"Both": 1}, "numerals": {"19": 1}, "pronouns": {"All of us": 1}}}, {"content": "There are few regular living patterns and family rules in 20 households.", "word_count": 15, "label": "", "sentence_structure": "Subject鈥搗erb鈥搊bject", "subjective_objective": {"answer": "Objective", "rationale": "Because the existence of the laws and the rules of life is an objective fact."}, "sentence_sentiment": {"answer": "Neutral", "rationale": "Because the entry does not have any emotional color."}, "language_style": {"answer": "Tactful and warm", "rationale": "This is more polite, euphemistic and more responsive to the person under investigation."}, "time_dimension": {"answer": "Present", "rationale": "The keyword \"almost rarely\" indicates the current state or situation, does not relate to the past time frame, nor does it refer to future time plans or intentions, so the temporal dimension of the article is now."}, "sentence_type": "Negative declarative sentence", "pos": {"nouns": {"Company": 2, "Family": 1}, "verbs": {"Existence": 1, "Provisions": 1}, "adjectives": {"Steady": 1, "Normal": 1}, "numerals": {"Twenty": 1, "Very few": 1}, "classifiers": {"Home": 1, "Entry": 1}}}, {"content": "Family members are willing to devote a lot of effort to their family's work.", "word_count": 17, "label": "", "sentence_structure": "Subject鈥搗erb鈥搊bject", "subjective_objective": {"answer": "Subjective", "rationale": "Because the attitude of \"yes\" is the patient's own subjective feeling and perception."}, "sentence_sentiment": {"answer": "Neutral", "rationale": "Because the entry does not contain any apparent emotional tendency or evaluation. It simply describes the fact that family members are willing to devote considerable effort to domestic matters."}, "language_style": {"answer": "Tactful and warm", "rationale": "The basis for the judgement: 1. Language temperature and: \"a great amount of energy\" are used, which is a more euphemistic expression, avoiding direct criticism or orders and giving people a sense of proximity and understanding. 2. There is less subjective: the entry does not contain too much personal subjective or emotional content, but rather an objective description of a factual situation."}, "time_dimension": {"answer": "Present", "rationale": "The words \"will\" and \"willing\" in this questionnaire entry indicate that it is a description or assessment of the current state, and therefore the judgement is now."}, "sentence_type": "Affirmative declarative sentence", "pos": {"nouns": {"Family members": 1, "- The energy": 1, "Home": 1, "Yes": 1}, "verbs": {"Yes": 1, "Flowers": 1, "Do it": 1}, "adjectives": {"Large": 1}, "numerals": {"21": 1}}}, {"content": "It is easy to irritate family members by complaining at home.", "word_count": 13, "label": "", "sentence_structure": "Subject鈥搗erb鈥搊bject", "subjective_objective": {"answer": "Subjective", "rationale": "Because \"Easier to tire families\" is a subjective judgement, depending on the views and feelings of the observers."}, "sentence_sentiment": {"answer": "Negative", "rationale": "Because the \"float\" in the entry is negative."}, "language_style": {"answer": "Calm and direct", "rationale": "The entry conveys the possible consequences in an objective and direct manner, without excessive emotional or rhetorical tact, and therefore can be judged as \"cool and direct\"."}, "time_dimension": {"answer": "Past", "rationale": "The keywords \"at home\", \"sorture\" and \"obstinate\" indicate that the entry describes a past experience or situation."}, "sentence_type": "Negative declarative sentence", "pos": {"nouns": {"22": 1, "Home": 1, "Family": 1, "Complaints": 1, "Boring": 1}, "verbs": {"鈾?And I'm gonna be 鈾?: 1, "in": 1, "Easy": 1, "Medium": 1}, "adjectives": {"very": 1}, "adverbs": {"Regular": 1}, "numerals": {"22": 1}, "classifiers": {"Home": 1, "person": 1, "Boring": 1}, "pronouns": {"he": 1}}}, {"content": "Sometimes family members fall when they are angry.", "word_count": 12, "label": "", "sentence_structure": "Subject鈥搗erb鈥搊bject", "subjective_objective": {"answer": "Objective", "rationale": "Because the incident of \"thumping something when family members are angry\" has already happened, and it is an objective fact."}, "sentence_sentiment": {"answer": "Negative", "rationale": "Because the act of \"thumping something when angry\" in the entry is negative."}, "language_style": {"answer": "Calm and direct", "rationale": "The entry is presented in a direct manner, explicitly asking the interviewee whether family members would fall in some cases, in a calm and objective language."}, "time_dimension": {"answer": "Present", "rationale": ""}, "sentence_type": "Affirmative declarative sentence", "pos": {"nouns": {"Family members": 1, "Anger": 1, "Something": 1}, "verbs": {"- Get angry": 1, "Fall": 1}, "adverbs": {"Sometimes": 1}, "numerals": {"23": 1}}}, {"content": "Family members are independent in their thinking.", "word_count": 11, "label": "", "sentence_structure": "Subject鈥搗erb鈥搊bject", "subjective_objective": {"answer": "Objective", "rationale": "Because the question of whether a family member is independent is an objective fact and can be ascertained by observation or investigation."}, "sentence_sentiment": {"answer": "Neutral", "rationale": "It is a description of the way family members think."}, "language_style": {"answer": "Tactful and warm", "rationale": "The entry is more euphemistic, not too strong or too direct. By using the word \"both\" it is a phrase that all family members have the ability to think independently, with a temperature and a positive orientation. At the same time, the expression \"independent thinking\" is also more objective, without prejudice or subjective emotional."}, "time_dimension": {"answer": "Present", "rationale": "The entry describes the way family members think, and does not address past or future situations, so the temporal dimension is now judged."}, "sentence_type": "Affirmative declarative sentence", "pos": {"nouns": {"Family members": 1, "Problem": 1}, "verbs": {"Independent thinking": 1}, "numerals": {"24": 1}}}, {"content": "Family members agree that raising the standard of living is more important than anything else.", "word_count": 24, "label": "", "sentence_structure": "Subject鈥搗erb鈥搊bject", "subjective_objective": {"answer": "Objective", "rationale": "Because \"the idea that raising living standards is more important than anything else\" is the objective existence of the views and opinions of family members, and it is their subjective idea, not the patient's subjective attitude."}, "sentence_sentiment": {"answer": "Positive", "rationale": "Because the goal of \"achieving better standards of living\" is positive."}, "language_style": {"answer": "Calm and direct", "rationale": ""}, "time_dimension": {"answer": "Future", "rationale": ""}, "sentence_type": "Affirmative declarative sentence", "pos": {"nouns": {"Family members": 1, "Standard of living": 1, "Things": 1}, "verbs": {"I think": 1, "鈾?And I'm gonna be 鈾?: 1, "Increase": 1, "Important": 1}, "adverbs": {"Both": 2}, "numerals": {"25": 1}, "classifiers": {"individual": 1}}}, {"content": "We all believe that learning new knowledge is more important than anything else.", "word_count": 20, "label": "", "sentence_structure": "Subject鈥搗erb鈥搊bject", "subjective_objective": {"answer": "Subjective", "rationale": "Because \"all of us think\" expresses the subjective attitude and opinion of the interviewees."}, "sentence_sentiment": {"answer": "Positive", "rationale": "Because the entry indicates that learning new knowledge is important and positive emotional."}, "language_style": {"answer": "Tactful and warm", "rationale": "The language style of the entry is relatively warm, and it uses the words \"both\" and \"important\" to express the views, rather than direct command or emphasis. At the same time, the sentence structure is simpler and easier to understand. It is more acceptable and acceptable to express it, and it can inspire a resonance."}, "time_dimension": {"answer": "Present", "rationale": "The word \"all\" in the entry represents a consensus among all, emphasizing the general status quo, and thus the temporal dimension is now judged."}, "sentence_type": "Affirmative declarative sentence", "pos": {"nouns": {"we": 1, "Learning": 1, "Knowledge": 1}, "verbs": {"I think": 1, "Learn": 1}, "adjectives": {"New": 1, "Any": 1}, "adverbs": {"Both": 2}, "numerals": {"26": 1}}}, {"content": "No one in the family has taken part in any sports activities.", "word_count": 12, "label": "", "sentence_structure": "Subject鈥搗erb鈥揷omplement", "subjective_objective": {"answer": "Objective", "rationale": "This is an objective description of whether family members are involved in sports activities, and does not involve subjective attitudes or feelings of patients."}, "sentence_sentiment": {"answer": "Negative", "rationale": "Because the entry describes a negative situation, \"no one in the family takes part in any sporting activity\", which may mean that family members lack exercise habits or are in poor physical health."}, "language_style": {"answer": "Calm and direct", "rationale": "The language style of the entry is more direct, with no excessive adorations and emotional colours, but rather expresses the content and intent of the survey in simple and clear language, which meets the objective and accurate requirements of the questionnaire study."}, "time_dimension": {"answer": "Past", "rationale": ""}, "sentence_type": "Negative declarative sentence", "pos": {"nouns": {"Home": 1, "person": 1, "Sports activities": 1}, "verbs": {"Participation": 1, "Medium": 1, "Nobody": 1}, "numerals": {"27": 1}}}, {"content": "Family members often help the elderly and disabled around them.", "word_count": 22, "label": "", "sentence_structure": "Subject鈥搗erb鈥搊bject", "subjective_objective": {"answer": "Objective", "rationale": "The frequency, manner and the manner in which such acts occur are objective."}, "sentence_sentiment": {"answer": "Positive", "rationale": "Because the behaviour in the entry \"often helps the elderly and the disabled around them\" is positive, it expresses the sense of social responsibility and public good of the family members."}, "language_style": {"answer": "Tactful and warm", "rationale": "The language style of the entry is more euphemistic, expressing concern and respect for the behaviour of the person under investigation by asking questions. At the same time, the description of \"often helping\" conveys a positive, good-faith message with a sense of contagion and peace."}, "time_dimension": {"answer": "Present", "rationale": ""}, "sentence_type": "Affirmative declarative sentence", "pos": {"nouns": {"Family members": 1, "Life": 1, "Around": 1, "Older persons": 1, "Persons with disabilities": 1}, "verbs": {"Help": 1, "Regular": 1, "in": 1}, "adjectives": {"28": 1}}}, {"content": "In our homes, it is often not found when something needs to be used.", "word_count": 20, "label": "", "sentence_structure": "Subject鈥搗erb鈥搊bject", "subjective_objective": {"answer": "Objective", "rationale": "Because \"none of what is needed at home\" is an objective phenomenon that does not require subjective assessment or attitude on the part of the patient."}, "sentence_sentiment": {"answer": "Negative", "rationale": "Because \"often\" is negative."}, "language_style": {"answer": "Tactful and warm", "rationale": "The entry is more intimate and interactive in terms of a more moderate and inviting tone of dialogue, expressing a common life phenomenon, and not a direct tone of accusation or orders."}, "time_dimension": {"answer": "Present", "rationale": ""}, "sentence_type": "Negative declarative sentence", "pos": {"nouns": {"Our home": 1, "Time": 1, "Something": 1}, "verbs": {"Use": 1, "Find": 1, "Often": 1}, "adverbs": {"in": 1, "But": 1}, "numerals": {"29": 1}, "pronouns": {"When": 1}}}, {"content": "The time spent eating and sleeping in our house is constant.", "word_count": 19, "label": "", "sentence_structure": "Subject鈥揷opula鈥損redicative", "subjective_objective": {"answer": "Objective", "rationale": "This is an objective description of family life habits and there is no subjective judgement or emotional colour."}, "sentence_sentiment": {"answer": "Neutral", "rationale": "Since the entry merely stated a fact, it did not contain any emotional colour and was therefore neutral."}, "language_style": {"answer": "Calm and direct", "rationale": "The language style of the entry, which is of a direct type, does not describe the facts using any rhetorical or euphemistic expression, but rather expresses the fact that the regular eating and sleeping time in family life is a more objective, clear and direct feeling.\""}, "time_dimension": {"answer": "Present", "rationale": "According to the phrase \"all are static\", it can be inferred that the act has remained unchanged in the past, in the present and in the future. The temporal dimension of the article is therefore now."}, "sentence_type": "Affirmative declarative sentence", "pos": {"nouns": {"30": 1, "Our home": 1, "Rice": 1, "Sleep": 1, "Time": 1}, "verbs": {"Eat": 1, "Sleep": 1, "Change": 1}, "adjectives": {"It's the same": 1}, "numerals": {"30": 1}, "classifiers": {"individual": 1}}}, {"content": "There is a climate of harmony in our home.", "word_count": 15, "label": "", "sentence_structure": "Subject鈥揷opula鈥損redicative", "subjective_objective": {"answer": "Objective", "rationale": "The family environment is an objective environment, independent of the patient ' s own subjective feelings."}, "sentence_sentiment": {"answer": "Positive", "rationale": "Because the description of \"a climate of harmony\" in the entry is positive."}, "language_style": {"answer": "Tactful and warm", "rationale": "The language style of the entry is more euphemistic, warm, expressed in a descriptive rather than an order or inquiry, and is somewhat subjective and emotional. This expression is more likely to bring the interviewee closer to the researcher, and to make them feel respected and understood."}, "time_dimension": {"answer": "Present", "rationale": "The keyword \"yes\" and \"one\" means that it is a description of the current state, so the temporal dimension is now judged."}, "sentence_type": "Affirmative declarative sentence", "pos": {"nouns": {"The atmosphere": 1, "Harmony": 1}, "verbs": {"in": 1, "have": 1}, "adjectives": {"Our family": 1, "One": 1}, "numerals": {"31": 1}}}, {"content": "Each person in the family can speak of his or her difficulties and problems.", "word_count": 19, "label": "", "sentence_structure": "Subject鈥搗erb鈥搊bject", "subjective_objective": {"answer": "Objective", "rationale": "Because this sentence is a fact or a situation in which everyone in 32 families has the right to tell their families about their difficulties and problems. This does not involve subjective feelings or attitudes of individuals, and is therefore objective."}, "sentence_sentiment": {"answer": "Neutral", "rationale": "Because the entry does not have any emotional color."}, "language_style": {"answer": "Tactful and warm", "rationale": "The language style of the entry is euphemism. It uses the expression \"everyone\" to emphasize that everyone has the right to speak about his or her difficulties and worries, and to speak with a more caring, caring and understanding tone. Moreover, \"all can say\" also conveys an open and supportive message that makes people comfortable and accepted."}, "time_dimension": {"answer": "Present", "rationale": "The entry describes a current state or fact that \"everyone can say\" indicates that it is a universal act, not a past experience or future plan. Thus, the temporal dimension is now judged."}, "sentence_type": "Affirmative declarative sentence", "pos": {"nouns": {"Family": 1, "person": 1, "Difficulties": 1, "Trouble": 1}, "verbs": {"Yeah": 1, "Tell me": 1, "have": 1}, "numerals": {"32 units": 1}, "classifiers": {"Every one of them": 1}, "pronouns": {"self": 1}}}, {"content": "Little anger was reported among 33 family members.", "word_count": 11, "label": "", "sentence_structure": "Subject鈥搗erb鈥揷omplement", "subjective_objective": {"answer": "Objective", "rationale": "Because \"very little anger among family members\" is a fact that can be observed and measured, and does not depend on a subjective feeling or attitude of the individual. This is therefore an objective examination entry."}, "sentence_sentiment": {"answer": "Neutral", "rationale": "Because the entry is not emotional, it merely describes a factual situation."}, "language_style": {"answer": "Tactful and warm", "rationale": ""}, "time_dimension": {"answer": "Present", "rationale": ""}, "sentence_type": "Affirmative declarative sentence", "pos": {"nouns": {"Family members": 1, "- Grumpy": 1}, "verbs": {"Very few": 1, "Let's go": 1}, "adverbs": {"Very few": 1}, "numerals": {"33": 1}}}, {"content": "Everyone in our family is completely free to go.", "word_count": 16, "label": "", "sentence_structure": "Subject鈥搗erb鈥揷omplement", "subjective_objective": {"answer": "Objective", "rationale": "The fact of the family members ' access is objective."}, "sentence_sentiment": {"answer": "Neutral", "rationale": "Because the entry merely describes a fact and does not involve positive or negative emotional aspects."}, "language_style": {"answer": "Tactful and warm", "rationale": "The language style in the entry is euphemism. It uses a gentle and polite language to express the freedom of access of family members, rather than direct statements or orders. This way allows readers to feel a more friendly and comfortable atmosphere, and it also makes it easier to resonate and participate."}, "time_dimension": {"answer": "Present", "rationale": "According to \"34 Our Home\" in the title, it can be seen that this sentence describes the current situation and judges it as the present. Moreover, the difference between \"everyone\" is \"full freedom\" and is not a past or future act, confirming the answer further."}, "sentence_type": "Affirmative declarative sentence", "pos": {"nouns": {"Home": 1, "Everyone": 1, "Access": 1}, "verbs": {"be": 1, "Total": 1, "Freedom": 1}, "adverbs": {"34": 1}, "numerals": {"of": 2, "Every": 1}, "pronouns": {"we": 1}}}, {"content": "We all believe that competition is good in any case.", "word_count": 16, "label": "", "sentence_structure": "Subject鈥搗erb鈥搊bject", "subjective_objective": {"answer": "Subjective", "rationale": "Because \"we believe\" is an expression of subjective attitudes that reflect the perception and belief of the respondents in competition."}, "sentence_sentiment": {"answer": "Positive", "rationale": "Because \"competition\" is described in the entry as \"goodness,\" it conveys a positive emotional attitude."}, "language_style": {"answer": "Tactful and warm", "rationale": "Although the phrase \"we believe\" expresses a competitive view, it makes it more euphemistic, not too much of a point of view, but rather an attempt to make it acceptable and relevant to the interviewees. At the same time, \"in any case\" also shows the inclusiveness and universality of this view, which enhances the relevance of the questionnaire."}, "time_dimension": {"answer": "Present", "rationale": "Now the entry describes the current conviction of the testee that \"we all believe that competition is good in any case\" 鈥?a statement of perceptions or attitudes, and therefore the judgement is now."}, "sentence_type": "Affirmative declarative sentence", "pos": {"nouns": {"we": 1, "Situation": 1, "Competition": 1}, "verbs": {"Yes": 1, "be": 1}, "adjectives": {"Any": 1, "Good news": 1}, "numerals": {"35": 1}}}, {"content": "We are not so interested in cultural activities.", "word_count": 13, "label": "", "sentence_structure": "Subject鈥搗erb鈥揷omplement", "subjective_objective": {"answer": "Subjective", "rationale": "Because \"we are not so interested in cultural activities\" is a subjective assessment or view that reflects the attitude or perception of the patient, not the objective facts or results."}, "sentence_sentiment": {"answer": "Negative", "rationale": "Because \"less interested\" expresses a negative attitude."}, "language_style": {"answer": "Calm and direct", "rationale": "The language style of the entry is a calm and straightforward type, and it expresses the interest of the respondents in cultural activities in a concise and straightforward manner."}, "time_dimension": {"answer": "Past", "rationale": ""}, "sentence_type": "Negative declarative sentence", "pos": {"nouns": {"Cultural activities": 1}, "verbs": {"Not so interested": 1}, "adjectives": {"we": 1, "to": 1, "Well": 1, "Interested": 1}, "numerals": {"36": 1}}}, {"content": "We watch movies or sports games, outings, etc.", "word_count": 16, "label": "", "sentence_structure": "Subject鈥搗erb鈥揷omplement", "subjective_objective": {"answer": "Objective", "rationale": "Because watching a film or sports game, or travelling on a field trip, is an objective experience and behaviour of the patient, it is not a subjective feeling or attitude of the patient."}, "sentence_sentiment": {"answer": "Positive", "rationale": "Because the entries are all about the kind of leisure that people like, and they have positive emotions."}, "language_style": {"answer": "Tactful and warm", "rationale": "The language style of the entry is a euphemism. First, using the expression \"We're often\" makes the issue more open and friendly, and leads the interviewee to share his experience and feelings; second, \"seeing a movie or sports game, out-of-country, etc.\" is a more specific description, allowing the interviewee to better understand and answer the questions. Moreover, the whole sentence is more euphemismal, not overly emotional, and is consistent with the euphemism of the euphemism."}, "time_dimension": {"answer": "Present", "rationale": ""}, "sentence_type": "Affirmative declarative sentence", "pos": {"nouns": {"Film": 1, "Sports competitions": 1, "A field trip": 1}, "verbs": {"Look": 1, "Regular": 1, "Out": 1, "External": 1, "Play": 1}, "adjectives": {"Wait": 1}, "adverbs": {"37": 1}, "numerals": {"37": 1}}}, {"content": "We consider active and passive bribery to be an acceptable phenomenon.", "word_count": 18, "label": "", "sentence_structure": "Subject鈥搗erb鈥搊bject", "subjective_objective": {"answer": "Subjective", "rationale": "Because \"concept\" indicates that it is a subjective view and attitude of the patient, not an objective reality or phenomenon."}, "sentence_sentiment": {"answer": "Negative", "rationale": "Because the notion that active and passive bribery is acceptable is negative and corrupt."}, "language_style": {"answer": "Calm and direct", "rationale": "The entry expresses the view of \"admissibility of active and passive bribery\" in a direct and objective manner, without too many rhetorical or emotional expressions and in a more calm and straightforward manner."}, "time_dimension": {"answer": "Present", "rationale": "The entry will contain visions and expectations for the future, with the keywords \"considering\" , \"acceptable\" and \"resistence\" pointing to situations that have not yet occurred and that may arise in the future. The temporal dimension of the entry is therefore judged to be the future."}, "sentence_type": "Negative declarative sentence", "pos": {"nouns": {"phenomena": 1, "Bribing": 1, "Bribery": 1, "We think": 1}, "verbs": {"be": 1, "Yeah": 1, "Accept": 1}, "adjectives": {"38": 1}}}, {"content": "39 In our home, it is important to be punctual.", "word_count": 12, "label": "", "sentence_structure": "Subject鈥搗erb鈥搊bject", "subjective_objective": {"answer": "Objective", "rationale": "Because \"a lot of attention is paid to punctuality\" is a family value, attitude or code of conduct, and it is an objective fact."}, "sentence_sentiment": {"answer": "Positive", "rationale": "Because \"heavy\" expresses a positive attitude and emotional tendency, and \"be on time\" is a positive behavioral habit or quality."}, "language_style": {"answer": "Tactful and warm", "rationale": "The entry uses descriptive language rather than direct orders or evaluations, making the respondents more comfortable and comfortable in answering questions, thus making it easier to obtain real feedback. At the same time, the words are soft and respectful, giving people a friendly and cordial feeling and increasing the participants ' sense of participation and trust."}, "time_dimension": {"answer": "Present", "rationale": "The entry describes current family values and behaviour, indicating that there is a notion or expectation that \"in our house\" is to do things on time. So the time dimension is now."}, "sentence_type": "Affirmative declarative sentence", "pos": {"nouns": {"39": 1, "Our home": 1, "Yes": 1}, "verbs": {"Value": 1, "Do it": 1, "On time": 1}, "adjectives": {"very": 1}, "numerals": {"39": 1}, "pronouns": {"Here": 1}}}, {"content": "There's a fixed way to do everything in our family.", "word_count": 14, "label": "", "sentence_structure": "Subject鈥搗erb鈥搊bject", "subjective_objective": {"answer": "Objective", "rationale": "This is a description of the facts as they relate to the family and practice."}, "sentence_sentiment": {"answer": "Neutral", "rationale": "Because the entry does not have any emotional color."}, "language_style": {"answer": "Tactful and warm", "rationale": "The entry is more personal and interactive when it is spoken in a gentle and inviting manner, rather than in direct statements or orders. At the same time, the issue is not too direct in its examination of the family, but rather leads to the sharing of one鈥檚 views by describing that family does everything in a regular way, which is more exploratory and open."}, "time_dimension": {"answer": "Present", "rationale": "The entry contains a description of \"our home\" which indicates that it is a description of the current family situation, and \"there is a fixed way\" that indicates that the state is continuous. So, the time dimension of the entry is now."}, "sentence_type": "Affirmative declarative sentence", "pos": {"nouns": {"Modalities": 1, "Things": 1}, "verbs": {"Do it": 1, "have": 1, "Fixed": 1}, "adverbs": {"Any": 1}, "numerals": {"40": 1}, "pronouns": {"Our home": 1}}}, {"content": "Few people volunteer to do so when they have a family problem.", "word_count": 13, "label": "", "sentence_structure": "Subject鈥搗erb鈥揷omplement", "subjective_objective": {"answer": "Subjective", "rationale": "Because \"very few people volunteer to do it\" is the patient's perception and feelings of the family, and it's subjective."}, "sentence_sentiment": {"answer": "Negative", "rationale": "Because the entry describes the fact that few people volunteer to help at home, it implies a negative social climate."}, "language_style": {"answer": "Calm and direct", "rationale": "The entry is based on a simple and clear statement of a phenomenon or fact, not using excessive rhetoric or emotional rendering. Nor is it in any doubt, but in the form of a statement, which is more objective, direct and acquitous."}, "time_dimension": {"answer": "Present", "rationale": "According to the phrase \"very few people\" and \"willing to do it\", it can be judged to be the current situation, that is, what happens in the family. So the time dimension of the questionnaire entry is now."}, "sentence_type": "Negative declarative sentence", "pos": {"nouns": {"Home": 1, "Yes": 1}, "verbs": {"have": 1, "Go on": 1, "Do it": 1, "Yes": 1}, "adjectives": {"Less": 1}, "adverbs": {"very": 1}, "numerals": {"41": 1}, "classifiers": {"person": 1}, "pronouns": {"Here": 1}}}, {"content": "Family members often express their feelings in public.", "word_count": 18, "label": "", "sentence_structure": "Subject鈥搗erb鈥搊bject", "subjective_objective": {"answer": "Subjective", "rationale": "Because \"always expressing each other's feelings in public\" is a subjective feeling and attitude that requires the patient's own answers."}, "sentence_sentiment": {"answer": "Positive", "rationale": "Because the \"public expression of mutual feelings\" in the entry is positive."}, "language_style": {"answer": "Tactful and warm", "rationale": "The entry is more euphemistic and sweet, expressing concern for emotional communication between family members of the interviewee in a gentle and intimate way, and emphasizing the importance of public expression of feelings. This expression is relatively more intimate and warm, and is better able to attract the attention of the interviewee and make the questions more acceptable and more answerable."}, "time_dimension": {"answer": "Present", "rationale": "The word \"often\" in the entry indicates that it is a customary act, and that the incident of \"the public expression of feelings among family members\" also occurs now. So, the answer is now."}, "sentence_type": "Affirmative declarative sentence", "pos": {"nouns": {"Family members": 1, "Emotion": 1}, "verbs": {"Publicly": 1}, "adjectives": {"Regular": 1, "To each other": 1}, "numerals": {"42": 1}}}, {"content": "Family members often blame and criticize each other.", "word_count": 14, "label": "", "sentence_structure": "Subject鈥搗erb鈥搊bject", "subjective_objective": {"answer": "Objective", "rationale": "Because \"accusations and criticisms between family members\" are objective phenomena that can be observed, recorded and validated. While this may cause psychological stress and distress to patients, it is an objective description in itself, not a subjective feeling or idea of the patient."}, "sentence_sentiment": {"answer": "Negative", "rationale": "Because \"competence and criticism\" in the entries is a negative act that can trigger family conflicts."}, "language_style": {"answer": "Calm and direct", "rationale": "The entry describes the problem in an objective and direct manner, without excessive emotional or subjective judgement, and is consistent with the calm and direct language style."}, "time_dimension": {"answer": "Present", "rationale": ""}, "sentence_type": "Affirmative declarative sentence", "pos": {"nouns": {"Family members": 2, "Blame": 1, "Criticism": 1}, "verbs": {"Regular": 1, "- Yeah, yeah": 1, "Blame": 1, "Criticism": 1}, "adverbs": {"Regular": 1, "- Yeah, yeah": 1}, "numerals": {"43": 1}}}, {"content": "44 (Family members rarely take into account the views of the rest of the family in their work.", "word_count": 19, "label": "", "sentence_structure": "Subject鈥搗erb鈥揷omplement", "subjective_objective": {"answer": "Subjective", "rationale": "The subjective perception and perception of the patient is that the family member is acting with the views of others in mind."}, "sentence_sentiment": {"answer": "Negative", "rationale": "Because the behaviour of family members described in the entry is selfish and disrespectful and has a negative emotional connotation."}, "language_style": {"answer": "Calm and direct", "rationale": "This language style is more appropriate for objective and accurate data collection and analysis."}, "time_dimension": {"answer": "Past", "rationale": "The entry describes the way and attitude of family members in doing their work over a certain period of time, and therefore the temporal dimension is judged to be the past."}, "sentence_type": "Negative declarative sentence", "pos": {"nouns": {"Family members": 2, "Observations": 2}, "verbs": {"Work": 1, "Consider": 1, "Present": 1}, "adjectives": {"Other": 1, "of": 4}, "numerals": {"44": 1}}}, {"content": "We have been constantly reflecting on ourselves and forcing ourselves to do what it takes to do better than once.", "word_count": 27, "label": "", "sentence_structure": "Subject鈥搗erb鈥揷omplement", "subjective_objective": {"answer": "Subjective", "rationale": "Because \"always constantly reflect on yourself\" and \"forced to do things better than once\" are the thoughts and behaviours of the patient, and are the expression of subjective attitudes."}, "sentence_sentiment": {"answer": "Positive", "rationale": "Because the \"rethinking yourself\" and \"doing what you can to do better\" behaviours and attitudes in the entries are positive."}, "language_style": {"answer": "Calm and direct", "rationale": "The entry presents the subject in a direct presentation, without excessive rhetorical or emotional rendering, and is more objective in tone."}, "time_dimension": {"answer": "Past", "rationale": ""}, "sentence_type": "Affirmative declarative sentence", "pos": {"nouns": {"we": 1, "Retrospect": 1, "self": 2, "Things": 1, "Once": 1, "- Yeah": 1}, "verbs": {"Always": 1, "Constantly": 1, "Forced": 1, "Try": 1, "Do it": 1}, "adjectives": {"Okay": 1}, "adverbs": {"Coercion": 1, "One at a time": 1}, "numerals": {"45": 1}}}, {"content": "We rarely discuss issues related to scientific and technological knowledge.", "word_count": 17, "label": "", "sentence_structure": "Subject鈥搗erb鈥搊bject", "subjective_objective": {"answer": "Objective", "rationale": "Because \"very little discussion of the issues related to scientific and technological knowledge\" is an act or fact that has already occurred and is an objective examination."}, "sentence_sentiment": {"answer": "Negative", "rationale": "Because the \"very little discussion\" in the entry is negative."}, "language_style": {"answer": "Calm and direct", "rationale": "The entry is open to dialogue in a direct question-and-answer manner, without too much qualifier or emotional rendering and with a more objective and incipient tone."}, "time_dimension": {"answer": "Past", "rationale": "The \"very little discussion\" in the entry indicates that such conduct was more common in the past at some point in time and that the temporal dimension can therefore be judged to be past."}, "sentence_type": "Negative declarative sentence", "pos": {"nouns": {"we": 1, "Problem": 1}, "verbs": {"Discussion": 1, "Related": 1, "In terms of": 1}, "adjectives": {"Very few": 1, "Scientific and technical knowledge": 1}, "numerals": {"46": 1}}}, {"content": "Each of us is particularly interested in one or two recreational activities.", "word_count": 18, "label": "", "sentence_structure": "Subject鈥搗erb鈥搊bject", "subjective_objective": {"answer": "Objective", "rationale": "Because hobby is an objective thing, it doesn't depend on a subjective view.\""}, "sentence_sentiment": {"answer": "Neutral", "rationale": "Because the entry is merely a description of a fact and does not involve any emotional tendencies or attitudes."}, "language_style": {"answer": "Tactful and warm", "rationale": "The language style of the entry is more euphemistic, making the issue more intimate and cozy by using the expression \"everyone in our family.\" At the same time, the expression \"special interest\" is more appropriate, neither too strongly negative or positive, nor too emotionally worded, making the language style of the problem more balanced and objective."}, "time_dimension": {"answer": "Present", "rationale": "The keyword \"everyone in our family\" and \"all\" represent the current state of interest of family members, so the temporal dimension of the article is now judged."}, "sentence_type": "Affirmative declarative sentence", "pos": {"nouns": {"Our home": 1, "Everyone": 1, "Recreational activities": 1, "1锝?": 1, "Item": 1}, "verbs": {"Both": 1, "to": 1, "Very interested": 1}, "adverbs": {"47": 1}, "numerals": {"1锝?": 1, "1": 1, "2": 1}, "classifiers": {"Item": 1}, "pronouns": {"Here": 1}}}, {"content": "We believe that, in any case, the elders should be persuaded by the elders.", "word_count": 21, "label": "", "sentence_structure": "Subject鈥搗erb鈥搊bject", "subjective_objective": {"answer": "Subjective", "rationale": "Because \"think\" expresses a personal view and opinion, it is viewed from the patient's subjective perspective."}, "sentence_sentiment": {"answer": "Positive", "rationale": "Because the attitudes in the entries are positive, it is believed that the elders should be advised by the elders, whatever they may be."}, "language_style": {"answer": "Calm and direct", "rationale": "The entry uses a direct presentation, expressing the opinion of the surveyer that the older generation should be persuaded by the older generation. The language style is simpler, with no excessive emotional colours and rhetorical techniques, and therefore can be judged as a \"cool straight\" language style."}, "time_dimension": {"answer": "Present", "rationale": ""}, "sentence_type": "Affirmative declarative sentence", "pos": {"nouns": {"we": 1, "Sir": 1, "Senior": 1, "Construction": 1}, "verbs": {"I think": 1, "I think so": 1, "Accept": 1, "Give": 1}, "adjectives": {"How's it going": 1}, "numerals": {"48": 1}}}, {"content": "49 (Our family often changes their plans.", "word_count": 14, "label": "", "sentence_structure": "Subject鈥搗erb鈥揷omplement", "subjective_objective": {"answer": "Subjective", "rationale": "Because \"often\" is a subjective term of description that reflects the view of the respondents of the phenomenon of changes in family planning."}, "sentence_sentiment": {"answer": "Negative", "rationale": "Because the \"often changing their plans\" act in the entry is negative."}, "language_style": {"answer": "Tactful and warm", "rationale": "The entry uses a more euphemistic language, using \"our family\" instead of \"the people\" to say \"direct blame or criticism\" and to describe the problem with \"often changing their plans\" instead of \"they are not responsible or self-sufficient.\" This expression is more moderate and inclusive, and it facilitates acceptance and collaboration by the respondents to the questionnaire."}, "time_dimension": {"answer": "Present", "rationale": "The time-form phrase in the sentence, \"often\", can be judged to describe a constant act, \"Our people often change their plans.\" So the time dimension of the questionnaire entry should be now."}, "sentence_type": "Affirmative declarative sentence", "pos": {"nouns": {"person": 2, "Planned": 1}, "verbs": {"Often": 1, "Change": 1, "they": 1, "of": 1}, "adverbs": {"Often": 1}, "numerals": {"49": 1}, "pronouns": {"Our home": 1}}}, {"content": "Our family places great emphasis on adherence to regular living patterns and family rules.", "word_count": 20, "label": "", "sentence_structure": "Subject鈥搗erb鈥搊bject", "subjective_objective": {"answer": "Objective", "rationale": "Because \"compliance with fixed living patterns and family rules\" is a family's norm and requirement, not a patient's subjective attitude or idea. The issue is therefore considered from an objective perspective."}, "sentence_sentiment": {"answer": "Positive", "rationale": "Because it expresses the importance attached to and the preservation of family order, it is a positive attitude."}, "language_style": {"answer": "Calm and direct", "rationale": "The language style of the entry is more objective and straightforward, with no excessive rhetorical or emotional colours, but rather a simple expression of the importance attached to the observance of life norms and family rules."}, "time_dimension": {"answer": "Present", "rationale": "The entry describes the current situation, and the keywords are \"Our family places great emphasis on\" and \"abiding with fixed living patterns and family rules\", which represent the current state and practice.\""}, "sentence_type": "Affirmative declarative sentence", "pos": {"nouns": {"Home": 1, "Lifestyle": 1, "Family rules": 1}, "verbs": {"Emphasizing": 1, "Compliance": 1, "have": 1}, "adjectives": {"Very": 1}, "numerals": {"50": 1}, "pronouns": {"we": 1}}}, {"content": "Family members are always supportive of each other with sincerity.", "word_count": 14, "label": "", "sentence_structure": "Subject鈥搗erb鈥揷omplement", "subjective_objective": {"answer": "Objective", "rationale": "Because \"family members always support each other sincerely\" is a descriptive statement, a fact of observed behaviour and relationships among family members, it is objective."}, "sentence_sentiment": {"answer": "Positive", "rationale": "Because the act of \"support one another with heart\" is positive, indicating that family members are in harmony and mutual love."}, "language_style": {"answer": "Tactful and warm", "rationale": "The entry depicts relationships between family members in positive terms, and uses the words \"thanky\" and \"support each other\" to express the warmth and harmony of the family atmosphere, which gives people a comfort and comfort. Such language is more in keeping with the needs of emotional investigation and better guides respondents to truly express their feelings and experiences."}, "time_dimension": {"answer": "Past", "rationale": "The entry describes the state of relations between family members, which existed in the past and therefore is judged to be the past."}, "sentence_type": "Affirmative declarative sentence", "pos": {"nouns": {"Family members": 1, "Support": 1}, "verbs": {"be": 1, "Always": 1, "And I'm gonna be honest with you": 1, "Support": 2}, "adjectives": {"I'm not sure": 1}, "adverbs": {"Always": 1, "Land": 1}, "numerals": {"51": 1}}}, {"content": "If you say at home that you are dissatisfied with family matters, some feel uncomfortable.", "word_count": 21, "label": "", "sentence_structure": "Subject鈥搗erb鈥搊bject", "subjective_objective": {"answer": "Subjective", "rationale": "Because \"someone will feel uncomfortable\" is a subjective judgement that reflects the attitude and perception of the interviewee on such topics. While this perception is based on objective family circumstances (e.g., relationships between family members), the interviewee鈥檚 feelings and perceptions remain subjective."}, "sentence_sentiment": {"answer": "Negative", "rationale": "It is negative because it is an expression of the fact that it is uncomfortable to say at home that it is not satisfied with family matters."}, "language_style": {"answer": "Tactful and warm", "rationale": "The entry uses a soft, invitation-style tone, which allows the interviewee to wonder whether it would be uncomfortable to say discontent at home, be more objective, and not overemphasis or blame. This euphemism is more resonant and self-reflective."}, "time_dimension": {"answer": "Present", "rationale": "The act of \"if you say you're not happy with family matters\" is a situation under hypothetical and virtual conditions, whereas the sentence uses the usual current state of affairs, which suggests that this can happen even in virtual situations. Thus, the temporal dimension of this questionnaire entry is now judged."}, "sentence_type": "Affirmative declarative sentence", "pos": {"nouns": {"Family matters": 1, "Dissatisfied": 1, "person": 1, "- Yeah": 1}, "verbs": {"Say it": 1, "I think": 1}, "adverbs": {"If": 1, "Yes": 2, "At home": 1}, "numerals": {"52": 1}, "pronouns": {"Someone's here": 1}}}, {"content": "Family members sometimes fight each other.", "word_count": 10, "label": "", "sentence_structure": "Subject鈥搗erb鈥搊bject", "subjective_objective": {"answer": "Objective", "rationale": "Because \"family members sometimes fight each other\" is an objective fact describing the family situation, it is not necessary to judge by the patient's subjective attitude or feelings."}, "sentence_sentiment": {"answer": "Negative", "rationale": "Because the \"fights\" in the entries are negative."}, "language_style": {"answer": "Calm and direct", "rationale": "The entry is clear and succinct, directly expressing the core of the problem, without adding too many rhetorical or emotional techniques to give people an objective and straightforward sense of purpose."}, "time_dimension": {"answer": "Present", "rationale": "The keyword \"sometimes fighting each other\" represents the dynamic relationship between current family members, so the time dimension of the entry is now."}, "sentence_type": "Affirmative declarative sentence", "pos": {"nouns": {"Family members": 2, "Fight": 1}, "verbs": {"Sometimes": 1, "- Yeah, yeah": 1}, "adverbs": {"Sometimes": 1}, "numerals": {"53": 1}}}, {"content": "Family members rely on the help of their families to resolve their difficulties.", "word_count": 22, "label": "", "sentence_structure": "Subject鈥搗erb鈥搊bject", "subjective_objective": {"answer": "Objective", "rationale": "It is an objective fact that family members rely on family help to resolve difficulties."}, "sentence_sentiment": {"answer": "Negative", "rationale": "Because the action of \"depending on family help\" in the entry is negative."}, "language_style": {"answer": "Tactful and warm", "rationale": "This way the person is comfortable and willing to share the true situation."}, "time_dimension": {"answer": "Past", "rationale": "This sentence describes the fact that at some point in the past 54 family members had relied on their families to help solve the problem."}, "sentence_type": "Affirmative declarative sentence", "pos": {"nouns": {"Family members": 1, "Family": 1, "Difficulties": 1}, "verbs": {"Dependency": 1, "Resolve": 1, "I met him": 1}, "numerals": {"54": 1}}}, {"content": "Family members are less concerned about promotion, learning achievement, etc.", "word_count": 19, "label": "", "sentence_structure": "Subject鈥搗erb鈥搊bject", "subjective_objective": {"answer": "Objective", "rationale": "Because \"less concerned\" is an objective description that reflects the attitude and perception of family members on issues such as promotion, academic achievement, and not the subjective or emotional experience of the patient."}, "sentence_sentiment": {"answer": "Negative", "rationale": "Because the \"not very concerned\" attitude in the entries is negative."}, "language_style": {"answer": "Calm and direct", "rationale": "The entry is a concise expression of the low level of interest of the family members of the subjects in issues such as promotion, learning achievement, the absence of excessive qualifier or emotional colours and the relative objectivity and idiosyncratic style of the subjects."}, "time_dimension": {"answer": "Past", "rationale": "The entry describes the situation over time and therefore judges \"the past\"."}, "sentence_type": "Negative declarative sentence", "pos": {"nouns": {"Family members": 1, "Functions": 1, "Achievements": 1}, "verbs": {"Not really": 1}, "adjectives": {"55": 1}}}, {"content": "There are people playing instruments at home.", "word_count": 7, "label": "", "sentence_structure": "Subject鈥搗erb鈥搊bject", "subjective_objective": {"answer": "Objective", "rationale": "Because \"someone plays an instrument\" is an objective fact, independent of the patient's subjective attitude, and therefore an objective examination."}, "sentence_sentiment": {"answer": "Neutral", "rationale": "As the entry merely presents a fact, it does not express any positive or negative feelings, attitudes, behaviour, purpose or quality."}, "language_style": {"answer": "Calm and direct", "rationale": "The language style of the entry is more objective, direct, and there is no excessive emotional colour or qualifier, but simply a simple and clear statement of the fact that in these 56 families there are parts of the family where members play musical instruments."}, "time_dimension": {"answer": "Present", "rationale": "The sentence is a status quo, where 56 families are currently playing with instruments. This is happening now, and does not involve future or past events, so it is possible to judge the time dimension now."}, "sentence_type": "Affirmative declarative sentence", "pos": {"nouns": {"person": 1, "Instrument": 1}, "verbs": {"have": 1, "Play": 1}, "numerals": {"56": 1}, "classifiers": {"Home": 1}}}, {"content": "Family members do not often engage in recreational activities except for work studies.", "word_count": 18, "label": "", "sentence_structure": "Subject鈥搗erb鈥搊bject", "subjective_objective": {"answer": "Objective", "rationale": "This is because it describes the behaviour of family members, is an objective fact and is not influenced by the subjective attitude of the individual."}, "sentence_sentiment": {"answer": "Negative", "rationale": "Because family members described in the entries lack recreational activities, such behaviour may affect their quality of life and social relations and therefore have a negative impact."}, "language_style": {"answer": "Calm and direct", "rationale": "The entry is presented in the form of a factual description, without excessive emotional or subjective evaluation, and is directly and objectively designed to obtain information about the recreational activities of the family members of the interviewee."}, "time_dimension": {"answer": "Present", "rationale": "The keywords \"other than work learning\" and \"unusual\" indicate that the act is a state of custom at this stage, so the temporal dimension is now judged."}, "sentence_type": "Negative declarative sentence", "pos": {"nouns": {"Family members": 1, "Work": 1, "Learning": 1, "Recreational activities": 1}, "verbs": {"Divide": 1, "Regular": 1}, "adverbs": {"not": 1}, "numerals": {"57": 1}}}, {"content": "Family members voluntarily maintain public sanitation.", "word_count": 15, "label": "", "sentence_structure": "Subject鈥搗erb鈥搊bject", "subjective_objective": {"answer": "Subjective", "rationale": "Because \"voluntary maintenance of public sanitation\" is a subjective attitude and behaviour of a human being, which belongs to the inner world and to the mind of the individual."}, "sentence_sentiment": {"answer": "Positive", "rationale": "Because the entry expresses a positive behaviour and attitude 鈥?a practice that family members voluntarily maintain for their sanitation 鈥?is commendable and encouraging."}, "language_style": {"answer": "Tactful and warm", "rationale": "The language style of the entry is more euphemistic, using the words \"voluntary\" and \"maintenance\" and the words \"preserve\" are more kind and gentle, expressing an attitude of encouragement and support and giving people a positive sense of direction."}, "time_dimension": {"answer": "Present", "rationale": "The entry now describes the current situation, namely \"58 family members voluntarily maintain public sanitation\", which indicates that this is the case."}, "sentence_type": "Affirmative declarative sentence", "pos": {"nouns": {"Family members": 1, "Public sanitation": 1}, "verbs": {"Both": 1, "Voluntary": 1, "Maintenance": 1}, "adverbs": {"58": 1}}}, {"content": "The family members carefully kept their rooms clean.", "word_count": 16, "label": "", "sentence_structure": "Subject鈥搗erb鈥揷omplement", "subjective_objective": {"answer": "Objective", "rationale": "Because \"careful maintenance of the cleanness of their room\" is an objective act of family members, not involving their subjective attitude or feelings."}, "sentence_sentiment": {"answer": "Positive", "rationale": "Because the act of \"carefully keeping his room clean\" is positive and shows that this person has a responsibility and a good life habit."}, "language_style": {"answer": "Calm and direct", "rationale": "The language style of the entry is calm, objective, straightforward, and does not involve too many rhetorical or emotional expressions, which directly describe the attitude of family members to room cleaning."}, "time_dimension": {"answer": "Present", "rationale": ""}, "sentence_type": "Affirmative declarative sentence", "pos": {"nouns": {"Family members": 1, "Room": 1, "Clean": 1}, "verbs": {"Hold on": 1, "Seriously": 1, "Land": 1}, "adjectives": {"Seriously": 1}, "adverbs": {"Land": 1}, "numerals": {"59": 1}}}, {"content": "Family members may leave at night without prior consultation with their families.", "word_count": 21, "label": "", "sentence_structure": "Subject鈥搗erb鈥搊bject", "subjective_objective": {"answer": "Objective", "rationale": "This is an objective fact."}, "sentence_sentiment": {"answer": "Negative", "rationale": "Because the \"no prior consultation with family\" in the entry may raise security and communication issues, it is negative."}, "language_style": {"answer": "Calm and direct", "rationale": "The entry expresses the required content in a concise and clear language, without too many rhetorical and emotional renderings, directness and clarity."}, "time_dimension": {"answer": "Present", "rationale": "The entry describes the current rules for family members to leave, and the temporal dimension is now judged."}, "sentence_type": "Negative declarative sentence", "pos": {"nouns": {"Family": 2, "Members": 1, "Night": 1, "External": 1, "Home": 1, "person": 1, "- Let's talk": 1}, "verbs": {"Yeah": 2, "Whatever": 1, "Out": 1, "No, I don't": 1, "Before": 1, "and": 1}, "adverbs": {"60": 1}, "numerals": {"60": 1}, "classifiers": {"individual": 1}, "pronouns": {"they": 2}}}, {"content": "There is little collective spirit in our family.", "word_count": 10, "label": "", "sentence_structure": "Subject鈥搗erb鈥揷omplement", "subjective_objective": {"answer": "Subjective", "rationale": "Since \"collective spirit\" is an abstract concept that cannot be directly measured or observed, it needs to be viewed from the subjective perspective of the patient."}, "sentence_sentiment": {"answer": "Negative", "rationale": "Because \"very few\" in the entries indicate a lack of solidarity and cooperation among family members, which is a negative phenomenon."}, "language_style": {"answer": "Calm and direct", "rationale": "The language style of the entry is calm and straightforward, and it is more objective in its tone, as it directly expresses the collective mental deficiency of the family of the subject, without any use of rhetorical or emotional colours to describe it."}, "time_dimension": {"answer": "Present", "rationale": "The sentence describes the current situation, which is \"our home\" which means that the subject is a member of the family now, and \"very few\" which means that this is not common or sufficient, so the temporal dimension is now."}, "sentence_type": "Negative declarative sentence", "pos": {"nouns": {"Our home": 1, "Collective spirit": 1}, "verbs": {"Less": 1, "have": 1}, "adjectives": {"A lot": 1}, "numerals": {"61": 1}}}, {"content": "Our families can speak openly about their economic problems.", "word_count": 18, "label": "", "sentence_structure": "Subject鈥搗erb鈥揷omplement", "subjective_objective": {"answer": "Objective", "rationale": "It is an objective fact that family economics can be discussed openly as a concrete expression of the family environment and atmosphere."}, "sentence_sentiment": {"answer": "Neutral", "rationale": "Because the entry is a statement of fact and does not contain any emotional colour."}, "language_style": {"answer": "Tactful and warm", "rationale": "The language style of the entry is more euphemistic, using the means of inviting each other to discuss rather than direct orders or statements, which are more personal and interactive. It also implies that the family ' s atmosphere of communication should be open and honest, so that family members can better understand and support each other ' s economic needs and decision-making."}, "time_dimension": {"answer": "Present", "rationale": "The keyword \"can speak openly\" indicates that this is a situation or habit in the current family."}, "sentence_type": "Affirmative declarative sentence", "pos": {"nouns": {"Family": 1, "Economic issues": 1}, "verbs": {"Yeah": 1, "Talk openly": 1, "Discussion": 1}, "adverbs": {"Openly": 1}, "numerals": {"62": 1}, "pronouns": {"we": 1}}}, {"content": "When the views of the 63 family members diverged, we all avoided it in order to maintain peace.", "word_count": 25, "label": "", "sentence_structure": "Subject鈥搗erb鈥搊bject", "subjective_objective": {"answer": "Objective", "rationale": "This is a description of the behaviour of family members when their opinions are divided, an objective fact, and a review of their behaviour rather than subjective ideas."}, "sentence_sentiment": {"answer": "Negative", "rationale": "Because the \"shun it\" act in the entry is negative."}, "language_style": {"answer": "Tactful and warm", "rationale": "The language style of the entry tends to be euphemistic. In describing the differences among family members, the words \"both\" and \"always\" are used to emphasize consistency, while avoiding direct identification of problems. Moreover, the reference to \"peaceful peace\" implies a peaceful solution. These terms and expressions are relatively mild, giving people a sense of affection and warmth."}, "time_dimension": {"answer": "Past", "rationale": "And the word \"both\" means that this is a common situation in the family and can therefore also be considered a past experience or state of affairs. Thus, the entry can be judged as \"old or present\"."}, "sentence_type": "Negative declarative sentence", "pos": {"nouns": {"Family members": 1, "Observations": 1, "Difference": 1, "# And the twilight #": 1}, "verbs": {"Generate": 1, "Avoidance": 1, "Both": 1, "Hold on": 1}, "adverbs": {"Always": 1}, "numerals": {"63": 1}, "classifiers": {"individual": 1}}}, {"content": "Family members want their families to solve the problem independently.", "word_count": 15, "label": "", "sentence_structure": "Subject鈥搗erb鈥搊bject", "subjective_objective": {"answer": "Subjective", "rationale": "The idea of family members is a subjective one."}, "sentence_sentiment": {"answer": "Positive", "rationale": "Because the behaviour and attitude of \"family solves the problem\" in the entry is positive."}, "language_style": {"answer": "Calm and direct", "rationale": "The language style of the entry is relatively calm and straightforward, describing the core of the problem in an objective and concise manner, without too much emotional or rhetorical style. This expression makes it easier for readers to understand and accept information."}, "time_dimension": {"answer": "Present", "rationale": "The time line in the sentence is \"hope,\" which implies that the speaker expects the present or future behaviour, and thus judges the current state of affairs. At the same time, \"family solves the problem\" is a description of the current situation or the future goal, which supports the outcome."}, "sentence_type": "Affirmative declarative sentence", "pos": {"nouns": {"Family members": 1, "Problem": 1}, "verbs": {"Hope": 1, "Resolve": 1, "Independence": 1}, "adjectives": {"64": 1}, "numerals": {"64": 1}, "classifiers": {"individual": 1}}}, {"content": "Our family is not so active in achieving what has been achieved.", "word_count": 16, "label": "", "sentence_structure": "Subject鈥搗erb鈥揷omplement", "subjective_objective": {"answer": "Subjective", "rationale": "This is a subjective feeling and perception of the patient, not an objective fact."}, "sentence_sentiment": {"answer": "Negative", "rationale": "Because the \"less positive\" attitude in the entry is negative."}, "language_style": {"answer": "Tactful and warm", "rationale": "This is a more harmonious and supportive way of expressing the views of the family of the person interviewed about the achievements."}, "time_dimension": {"answer": "Present", "rationale": "The temporal dimension of the article can be judged by the phrase \"family\" and \"not so active\" as it is now, i.e., the current attitude of family members."}, "sentence_type": "Negative declarative sentence", "pos": {"nouns": {"Family": 1, "Achievements": 1}, "verbs": {"to": 1, "Access": 1, "No, it's not": 1, "Well": 1, "Positive": 1}, "adverbs": {"Well": 1}, "numerals": {"65": 1}}}, {"content": "Family members often go to the library.", "word_count": 9, "label": "", "sentence_structure": "Subject鈥搗erb鈥搊bject", "subjective_objective": {"answer": "Objective", "rationale": "Because \"family members often go to libraries\" is an objective fact, independent of individual subjective consciousness."}, "sentence_sentiment": {"answer": "Neutral", "rationale": "Because the entry describes a fact and does not involve positive or negative emotional tendencies."}, "language_style": {"answer": "Tactful and warm", "rationale": "The entry is more adept and interactive when it is spoken in a gentle and inviting manner, rather than by direct statements or orders."}, "time_dimension": {"answer": "Past", "rationale": "The term \"always go\" in this entry indicates a pattern of past behaviour and therefore judgement is a past."}, "sentence_type": "Affirmative declarative sentence", "pos": {"nouns": {"Family members": 1, "Library": 1}, "verbs": {"Always": 1}, "numerals": {"66": 1}}}, {"content": "Family members sometimes take recreational studies according to their preferences or interests.", "word_count": 21, "label": "", "sentence_structure": "Subject鈥搗erb鈥搊bject", "subjective_objective": {"answer": "Subjective", "rationale": "Since the entry relates to the family life and personal preferences of the patient, it is examined from a subjective perspective."}, "sentence_sentiment": {"answer": "Positive", "rationale": "Because the act of \"participating in recreational learning according to personal preferences or interests\" is positive and can have a positive impact."}, "language_style": {"answer": "Tactful and warm", "rationale": "The language style of the entry is relatively euphemistic, friendly, and does not imply direct orders or acrimony, but rather raises questions in a form of invitation and advice."}, "time_dimension": {"answer": "Past", "rationale": "The entry \"sometimes\" indicates that it is a past experience or behaviour and describes the daily activities and decision-making of family members. Thus, the time dimension is the past."}, "sentence_type": "Affirmative declarative sentence", "pos": {"nouns": {"Family members": 1, "Learning": 1}, "verbs": {"Participation": 1, "Recreational": 1}, "adjectives": {"Personal": 1, "Hobbies": 1, "Interest": 1}, "adverbs": {"Sometimes": 1}, "numerals": {"67": 1, "individual": 1}}}, {"content": "Family members agreed to act in a moral capacity.", "word_count": 17, "label": "", "sentence_structure": "Subject鈥搗erb鈥搊bject", "subjective_objective": {"answer": "Objective", "rationale": "Because this entry describes the attitude and behaviour of a family member towards the tenets of morality, it is an objective statement, not a subjective feeling or attitude of the patient."}, "sentence_sentiment": {"answer": "Neutral", "rationale": "Because the entry does not address any positive or negative emotional attitudes, behaviour or purpose. It simply describes the views of family members and does not contain any evaluative language."}, "language_style": {"answer": "Calm and direct", "rationale": "The entry is based on the direct statement of the family member ' s views, the absence of a speech technique or emotional rendering, the more objective tone of the speech, and the fact that the language style is so calm and straightforward."}, "time_dimension": {"answer": "Past", "rationale": "The entry describes a past state of affairs and view that family members have in the past considered it necessary to adhere to the moral precepts of the past. The temporal dimension is therefore a past."}, "sentence_type": "Affirmative declarative sentence", "pos": {"nouns": {"Family members": 1, "Ethical precepts": 1, "Operations": 1}, "verbs": {"I think": 1, "Stay put": 1}, "numerals": {"68": 1}}}, {"content": "The division of labour among everyone in our family is clear.", "word_count": 14, "label": "", "sentence_structure": "Subject鈥搗erb鈥揷omplement", "subjective_objective": {"answer": "Objective", "rationale": "Because the distribution of roles among family members is an objective fact, even if the patient may have different views or feelings about it."}, "sentence_sentiment": {"answer": "Neutral", "rationale": "Since the entry describes the division of labour within the family, it does not contain any obvious emotional colours."}, "language_style": {"answer": "Tactful and warm", "rationale": "The entry is more adept and interactive when it is spoken in a gentle and inviting manner, rather than by direct statements or orders."}, "time_dimension": {"answer": "Present", "rationale": "The entry describes the division of labour between family members today, does not cover past or future information and therefore is judged to be the present."}, "sentence_type": "Affirmative declarative sentence", "pos": {"nouns": {"69": 1, "Our home": 1, "Everyone": 1, "Division of labour": 1}, "verbs": {"be": 1, "Clear": 1}, "numerals": {"69": 1}, "classifiers": {"individual": 1}, "pronouns": {"Our home": 1}}}, {"content": "There are no strict rules in our house to bind us.", "word_count": 16, "label": "", "sentence_structure": "Subject鈥搗erb鈥搊bject", "subjective_objective": {"answer": "Objective", "rationale": "Because the existence of a rule is an objective fact and is not affected by the subjective will of the individual."}, "sentence_sentiment": {"answer": "Neutral", "rationale": "Because the entry describes an objective situation and does not contain any positive or negative emotional colours."}, "language_style": {"answer": "Tactful and warm", "rationale": "The language style of the entry is more euphemistic, and conveys the message of a relatively relaxed and free family environment by using a description such as \"no strict rules\". At the same time, the whole phrase is more moderate, without too many negative emotions or coercive remarks, and is more acceptable and understandable."}, "time_dimension": {"answer": "Present", "rationale": "The terms \"no strict rules\" and \"no binding us\" indicate that the act is in progress and that there is no obvious point of reference for the past or future. The temporal dimension of the article should therefore be judged now."}, "sentence_type": "Negative declarative sentence", "pos": {"nouns": {"Our home": 1, "Rule": 1}, "verbs": {"No, I'm not": 1, "Constraints": 1}, "adjectives": {"Strict": 1}, "adverbs": {"in": 1}, "numerals": {"70": 1}, "pronouns": {"we": 2}}}, {"content": "Family members have always been able to get along with each other.", "word_count": 14, "label": "", "sentence_structure": "Subject鈥搗erb鈥揷omplement", "subjective_objective": {"answer": "Subjective", "rationale": "Because \"compatibility\" is a subjective feeling and an assessment, different standards may differ."}, "sentence_sentiment": {"answer": "Positive", "rationale": "Because the description \"family members always fit in with each other\" in the entry is positive."}, "language_style": {"answer": "Tactful and warm", "rationale": "This entry is used to communicate with people in question, not directly, but by asking questions about their views and feelings. This expression makes them more comfortable and natural and more accessible to people."}, "time_dimension": {"answer": "Future", "rationale": "The term \"always agree\" suggests that relations between family members are durable and stable, and it is therefore possible to judge that the act or state will continue in the future. The temporal dimension of the article is therefore the future."}, "sentence_type": "Affirmative declarative sentence", "pos": {"nouns": {"Family members": 2, "You too": 1}, "verbs": {"Always": 1, "It's a good fit": 1}, "adverbs": {"Both": 1}, "numerals": {"71": 1}}}, {"content": "Family members speak between themselves with great care to avoid hurting each other ' s feelings.", "word_count": 22, "label": "", "sentence_structure": "Subject鈥搗erb鈥揷omplement", "subjective_objective": {"answer": "Objective", "rationale": "The basis for the determination: this questionnaire entry is an examination of the way family members behave, i.e., whether they are very careful to avoid harming the other party鈥檚 feelings when speaking. Such behaviour exists objectively and does not need to be assessed on the patient鈥檚 subjective views or feelings."}, "sentence_sentiment": {"answer": "Positive", "rationale": "This behaviour is positive and positive because the entry describes the good state of family members who are very sensitive to each other ' s feelings in their communication."}, "language_style": {"answer": "Tactful and warm", "rationale": ""}, "time_dimension": {"answer": "Present", "rationale": "The words \"intermediate\" in \"spokes between family members\" indicate interaction between family members, and words such as \"both\" and \"fully careful\" indicate that the behaviour is a normality in the context of the person speaking. At the same time, the description \"avoiding to hurt the feelings of the other person\" implies that the act is a state of current conduct, so it can be judged that the entry is asking about the situation."}, "sentence_type": "Affirmative declarative sentence", "pos": {"nouns": {"Family members": 1, "Emotion": 1}, "verbs": {"Speech": 1, "Attention": 1, "Avoid": 1, "Hurt": 1}, "adverbs": {"Both": 1, "very": 1}, "numerals": {"72": 1}}}, {"content": "Family members often want to outnumber each other.", "word_count": 12, "label": "", "sentence_structure": "Subject鈥搗erb鈥揷omplement", "subjective_objective": {"answer": "Subjective", "rationale": "This is because of the patient ' s observation and subjective evaluation of the family situation."}, "sentence_sentiment": {"answer": "Negative", "rationale": "Because the \"want to beat the other\" attitude in the entry is negative."}, "language_style": {"answer": "Calm and direct", "rationale": "The entry describes a phenomenon in a concise language, with no excessive emotional or rhetorical modification, and a more direct and objective language style."}, "time_dimension": {"answer": "Past", "rationale": "This sentence describes the behaviour of \"73 family members\", in which the word \"normal\" indicates that this custom may have existed, present and future. Thus, the temporal dimension of the article is a matter of the past."}, "sentence_type": "Affirmative declarative sentence", "pos": {"nouns": {"Family members": 1, "The other side": 1}, "verbs": {"More than you can imagine": 1}, "adverbs": {"Regular": 1}, "numerals": {"73": 1}}}, {"content": "If family members often act alone, they can hurt the feelings of others in the family.", "word_count": 22, "label": "", "sentence_structure": "Subject鈥搗erb鈥搊bject", "subjective_objective": {"answer": "Subjective", "rationale": "Because the subject is to examine the patient鈥檚 subjective view and judgment that 鈥渋f family members often act alone, it can hurt the feelings of others in the family.鈥?This is the patient鈥檚 view, attitude or conviction, not an objective fact."}, "sentence_sentiment": {"answer": "Negative", "rationale": "Because the result of the entry \"will hurt the feelings of the rest of the family\" is negative."}, "language_style": {"answer": "Tactful and warm", "rationale": "The entry is more personal and interactive when it is spoken in a gentle and alert tone, rather than in direct statements or orders. It does not use overly strong emotional terms or language, but rather raises a potential problem in an objective, neutral tone. It is therefore a warmer language that can be judged."}, "time_dimension": {"answer": "Present", "rationale": "The term \"if...\" is now used in the entry, which indicates that it is a hypothetical situation, but does not specify whether it is past, present or future. At the same time, \"often acting alone\" and \"harming the feelings of others in the family\" are the language that describes current behaviour and results, so it is possible to judge the time dimension of the entry."}, "sentence_type": "Negative declarative sentence", "pos": {"nouns": {"Family members": 1, "Activities": 1, "Emotion": 1}, "verbs": {"Regular": 1, "Alone": 1, "Hurt": 1}, "adverbs": {"Yes": 1, "I'm sorry": 1}, "numerals": {"74": 1}}}, {"content": "75 is an old habit of our family to work and enjoy.", "word_count": 14, "label": "", "sentence_structure": "Subject鈥搗erb鈥搊bject", "subjective_objective": {"answer": "Objective", "rationale": "This is an objective situation and a habit for the family."}, "sentence_sentiment": {"answer": "Positive", "rationale": "Because the aim of the act \"75 Work and Enjoy\" is positive."}, "language_style": {"answer": "Tactful and warm", "rationale": "The entry presents the traditional concept of a family in a more honest and objective way, without overly emotional expressions or emphasises, which are more acclaimable and acceptable."}, "time_dimension": {"answer": "Past", "rationale": "The temporal dimension of the purpose of the article is therefore judged to be the past."}, "sentence_type": "Affirmative declarative sentence", "pos": {"nouns": {"I'll get to work and enjoy myself": 1, "Our home": 1, "Old habits": 1}, "verbs": {"be": 1, "Work": 1, "Enjoy": 1}, "numerals": {"75": 1}, "pronouns": {"Here": 1}}}, {"content": "We're watching TV in our house more than reading.", "word_count": 13, "label": "", "sentence_structure": "Subject鈥揷opula鈥損redicative", "subjective_objective": {"answer": "Subjective", "rationale": "Because \"watching television is more important than reading\" is a subjective evaluation and judgement, not an objective fact or behaviour. Different people may have different views and assessments, so this is viewed from the patient's subjective point of view."}, "sentence_sentiment": {"answer": "Negative", "rationale": "Because the expression \"watching television is more important than reading\" in the entry is a demeaning expression of the importance of reading, it is a negative emotional attitude."}, "language_style": {"answer": "Calm and direct", "rationale": "The language style of the entry is more straightforward, there is no excessive rhetorical or emotional rendering, and the tone is more calm and objective. At the same time, the purpose statement of the article is more subjective, expressing the views and positions of the speaker."}, "time_dimension": {"answer": "Present", "rationale": "It is a description of current family life habits, and therefore of current behaviour, based on the phrase \"in our home\". At the same time, there is no mention of past or future specific events or situations, so the temporal dimension of the article is now."}, "sentence_type": "Affirmative declarative sentence", "pos": {"nouns": {"Television": 1, "Books": 1, "Importance": 1}, "verbs": {"Look": 1, "Read it": 1, "Comparison": 1}, "adjectives": {"More": 1}, "numerals": {"76": 1}, "pronouns": {"Our home": 1}}}, {"content": "Family members often participate in social events outside the home during their leisure time.", "word_count": 21, "label": "", "sentence_structure": "Subject鈥搗erb鈥搊bject", "subjective_objective": {"answer": "Objective", "rationale": "Since `often', `participating in social activities outside the home' are specific behaviours or events, not subjective ideas or feelings of the patient, this is seen from an objective perspective."}, "sentence_sentiment": {"answer": "Positive", "rationale": "Because the behavior in the entry \"to participate in social events outside the home\" is positive."}, "language_style": {"answer": "Tactful and warm", "rationale": "\"The language style of the survey is euphemism. Because it uses more objective and neutral expressions, it does not put too much emphasis on personal feelings and views. At the same time, the terms \"normal\" and \"extrasocial activities\" are more polite and gentle, making it easier to accept and answer questions."}, "time_dimension": {"answer": "Present", "rationale": "\"Attent\" is a regular act, \"participation\" is a historical pattern, which means that it has occurred in the past, but does not indicate whether it continues to this date. The entry is therefore mainly a description of the current situation."}, "sentence_type": "Affirmative declarative sentence", "pos": {"nouns": {"Family members": 1, "Amateur Time": 1, "Social events": 1}, "verbs": {"Participation": 1}, "adjectives": {"Always": 1, "Outside": 1}, "numerals": {"77": 1}}}, {"content": "We believe that divorce is immoral, whatever it is.", "word_count": 16, "label": "", "sentence_structure": "Subject鈥搗erb鈥搊bject", "subjective_objective": {"answer": "Subjective", "rationale": "This is a subjective examination of the patient ' s personal views and convictions."}, "sentence_sentiment": {"answer": "Negative", "rationale": "Because of the view in the entry that divorce is immoral, the value of which is clearly negative."}, "language_style": {"answer": "Calm and direct", "rationale": "The language style of the entry is more calm and straightforward, and there is no excessive emotional or rhetorical style. It clearly expresses its view of divorce 鈥?immorality 鈥?which is a very decisive and firm attitude."}, "time_dimension": {"answer": "Present", "rationale": "The entry expresses the individual views and opinions of the speaker, uses the current state of affairs and does not address past or future situations."}, "sentence_type": "Negative declarative sentence", "pos": {"nouns": {"we": 1, "How's it going": 1, "Divorce": 1, "Ethics": 1}, "verbs": {"I think": 1, "be": 1, "not": 1, "of": 1}, "adjectives": {"Whatever": 1}, "numerals": {"78": 1}}}, {"content": "79 of us have no plans.", "word_count": 9, "label": "", "sentence_structure": "Subject鈥搗erb鈥揷omplement", "subjective_objective": {"answer": "Subjective", "rationale": "Because the sentence is a subjective judgement that \"our family\" lacks a plan to spend money. It is an assessment based on personal experience and attitudes, and therefore subjective."}, "sentence_sentiment": {"answer": "Negative", "rationale": "Because \"no plan\" is negative."}, "language_style": {"answer": "Calm and direct", "rationale": "The language style of the entry is relatively calm and straightforward, and a simple and clear description of a situation (no money plan at home) is not too much accompanied or emotional."}, "time_dimension": {"answer": "Present", "rationale": "In summary, the time dimension of this set of questionnaire entries can be divided into past, present and future."}, "sentence_type": "Negative declarative sentence", "pos": {"nouns": {"Our home": 1, "Money": 1, "Planned": 1}, "verbs": {"No, I'm not": 1, "Flowers": 1}, "numerals": {"79": 1}}}, {"content": "Our family rules or family rules cannot be changed.", "word_count": 17, "label": "", "sentence_structure": "Subject鈥揷opula鈥損redicative", "subjective_objective": {"answer": "Subjective", "rationale": "Because \"not to change\" is the patient's subjective view and attitude to family life."}, "sentence_sentiment": {"answer": "Neutral", "rationale": "Because the entry describes a fact, it does not show a positive, negative or neutral emotional tendency."}, "language_style": {"answer": "Calm and direct", "rationale": "The purpose of this article is expressed more directly, without excessive rhetoric or emotional rendering, and in a more objective and balanced manner. At the same time, the entry does not raise the question in a euphemism, but rather directly presents a point of view or a rule, which can be judged as a calm and straightforward language style."}, "time_dimension": {"answer": "Present", "rationale": ""}, "sentence_type": "Negative declarative sentence", "pos": {"nouns": {"80": 1, "Lifestyle": 1, "Family rules": 1}, "verbs": {"be": 1, "Yes": 1, "Change": 1}, "numerals": {"80": 1}, "pronouns": {"we": 1}}}, {"content": "Every member of the family has always received adequate attention.", "word_count": 17, "label": "", "sentence_structure": "Subject鈥搗erb鈥揷omplement", "subjective_objective": {"answer": "Objective", "rationale": "Because \"Every member of the family has always been given sufficient attention\" is an objective fact of family life, independent of the subjective perception of the individual."}, "sentence_sentiment": {"answer": "Positive", "rationale": "Because the \"full care\" in the entry expresses positive emotions and attitudes."}, "language_style": {"answer": "Tactful and warm", "rationale": "This language is more friendly and sweet and gives people a positive emotional feeling."}, "time_dimension": {"answer": "Present", "rationale": "The keyword \"always\" and \"both\" indicates that the entry describes the current state or behaviour and is directed at all family members. Thus, the judgement is now."}, "sentence_type": "Affirmative declarative sentence", "pos": {"nouns": {"Family": 1, "Members": 2, "Care": 1}, "verbs": {"Got it": 1, "Full": 1}, "adjectives": {"Every one": 1}, "numerals": {"81": 1}, "pronouns": {"Both": 1}}}, {"content": "Our family often talks spontaneously about sensitive family issues.", "word_count": 18, "label": "", "sentence_structure": "Subject鈥搗erb鈥搊bject", "subjective_objective": {"answer": "Subjective", "rationale": "Because \"snarrating\" and \"sensitive\" are subjectively felt by patients."}, "sentence_sentiment": {"answer": "Neutral", "rationale": "Because the entry describes a family phenomenon, it does not express positive or negative emotional sentiments."}, "language_style": {"answer": "Tactful and warm", "rationale": "The entry, by using the terms \"autonomous\" and \"sensitive questions\", describes the attitude and atmosphere of family members when discussing such topics, preferring to lead respondents to questions in a gentle, understanding and supportive manner, thus making language more euphemismal."}, "time_dimension": {"answer": "Present", "rationale": "The keyword \"often\" denotes the frequency and habit of the action, while the word \"talking\" uses the general current, indicating that the act has not only occurred in the past but is still ongoing. The entry therefore describes the current act or state."}, "sentence_type": "Affirmative declarative sentence", "pos": {"nouns": {"Our home": 1, "Problem": 1, "Family": 1}, "verbs": {"Regular": 1, "Auto": 1, "Talk": 1, "Sensitive": 1}, "adjectives": {"very": 1}, "adverbs": {"Land": 1}, "numerals": {"82": 1}}}, {"content": "The 83 families sometimes argued loudly when they had a conflict.", "word_count": 13, "label": "", "sentence_structure": "Subject鈥搗erb鈥搊bject", "subjective_objective": {"answer": "Objective", "rationale": "The purpose of this questionnaire is to describe the phenomenon of conflict and disputes between family members, which is an objective fact. Even if the patient has acted with a high degree of intensity, such as arguing loudly, it is still an objective description."}, "sentence_sentiment": {"answer": "Negative", "rationale": "Because the \"screaming\" in the entries is negative."}, "language_style": {"answer": "Calm and direct", "rationale": "The entry describes the person under investigation in a direct and objective language style with a loud argument in the family, without too much emotional overtones or personal subjective evaluation."}, "time_dimension": {"answer": "Present", "rationale": "The keyword \"sometimes\" indicates that it is a regular, customary act and takes place in the present or the nearest period, so that the entry is in its current state of affairs."}, "sentence_type": "Affirmative declarative sentence", "pos": {"nouns": {"Family members": 1, "Paradoxious": 1, "Argument": 1}, "verbs": {"have": 1, "Louder": 1, "Argument": 2}, "adverbs": {"Sometimes": 1}, "numerals": {"83": 1}, "classifiers": {"individual": 1}}}, {"content": "In our home, it is true that members are encouraged to move freely.", "word_count": 15, "label": "", "sentence_structure": "Subject鈥搗erb鈥搊bject", "subjective_objective": {"answer": "Objective", "rationale": "It is an objective fact that \"84 encourages members to move freely at home\" is a specific rule or act in the family. It is subjective to look at whether the act of this rule has occurred and at its subjective attitude."}, "sentence_sentiment": {"answer": "Positive", "rationale": "Because the behaviour and attitude of \"encouraging members to move freely\" in the entry is positive."}, "language_style": {"answer": "Tactful and warm", "rationale": "The entry uses a more moderate expression, which emphasizes its authenticity by the word \"sure\" and expresses its attitude to family members' behaviour by \"encouraging\" rather than by ordering. This language style is more friendly and interactive, and is acceptable and resonant."}, "time_dimension": {"answer": "Present", "rationale": "Now, \"84\" in the sentence refers to a particular point or event, while \"do encourage\" and \"all to move freely\" indicate that this is a persistent phenomenon or behaviour. So, the entry to the questionnaire describes the current situation and is a current state of affairs."}, "sentence_type": "Affirmative declarative sentence", "pos": {"nouns": {"we": 1, "Home": 1, "Members": 1, "Activities": 1}, "verbs": {"Encourage": 1, "Both": 1, "Freedom": 1}, "adverbs": {"Indeed": 1}, "numerals": {"84": 1}, "pronouns": {"we": 1}}}, {"content": "Family members often compare to others to see who is doing good work.", "word_count": 19, "label": "", "sentence_structure": "Subject鈥搗erb鈥搊bject", "subjective_objective": {"answer": "Subjective", "rationale": "Because \"often\" is patient subjectivity, and \"often compared to others\" is patient thinking."}, "sentence_sentiment": {"answer": "Negative", "rationale": "Because the \"comparison\" in the entry is negative and may lead to a lower sense of self-worth and excessive competition."}, "language_style": {"answer": "Tactful and warm", "rationale": "The language style of the entry is more euphemistic and moderate. It does not directly criticize or accuse 85 family members of their behaviour, but rather raises an observation and asks questions about whether it exists. It is more acceptable to the other side and more likely to generate dialogue and reflection. At the same time, the use of the term \"often\" also suggests that it is a more general phenomenon, not a few cases, which makes the language style more neutral and peaceful."}, "time_dimension": {"answer": "Present", "rationale": "And in conclusion, the answer is \"now\"."}, "sentence_type": "Affirmative declarative sentence", "pos": {"nouns": {"Family members": 1, "Learning": 1, "Work": 1}, "verbs": {"Often": 1, "and": 1, "Comparison": 1, "Look": 1, "Who": 1, "Okay": 1}, "adverbs": {"85": 1}}}, {"content": "Family members are very fond of music, art and literature.", "word_count": 14, "label": "", "sentence_structure": "Subject鈥搗erb鈥搊bject", "subjective_objective": {"answer": "Objective", "rationale": "Because \"a lot of music, art and literature\" is an objective description, and it does not require subjective evaluation by patients."}, "sentence_sentiment": {"answer": "Positive", "rationale": "Because \"like\" in the entry is a positive emotional attitude."}, "language_style": {"answer": "Tactful and warm", "rationale": "The language style of the entry is more euphemistic, as it uses the invitation tone ( \"Please analyse below\") to guide the reader to the questions, while the interviewee is positively described and praised without direct criticism or denial. Moreover, it does not use too complex or hard language, making it easier for readers to understand and accept."}, "time_dimension": {"answer": "Present", "rationale": "The entry represents an objective fact, i.e. the preferences of family members, which do not relate to past or future behaviour or expectations, and therefore the judgement is now."}, "sentence_type": "Affirmative declarative sentence", "pos": {"nouns": {"Family members": 1, "Music": 1, "Arts": 1, "Literature": 1}, "verbs": {"Yeah": 1}, "adverbs": {"very": 1}, "numerals": {"86": 1}}}, {"content": "Our entertainment activities are conducted by watching television, listening to radio, not by going out.", "word_count": 23, "label": "", "sentence_structure": "Subject鈥搗erb鈥搊bject", "subjective_objective": {"answer": "Objective", "rationale": "Because the title describes a certain form of conduct, it is a statement of objective facts."}, "sentence_sentiment": {"answer": "Negative", "rationale": "Because the entries \"watch TV, listen to radio\" are more favourable to negative indoor modes of activity than \"out-of-the-air activities\"."}, "language_style": {"answer": "Tactful and warm", "rationale": "The language style of the entry is more euphemistic, and dialogue is conducted in an invitation-style, rather than directly speaking or ordering. The \"but\" in the entry is an expression of a contrast and a turn-over, implying that there may be a different way of entertaining the other, which is more moderate, friendly and more acceptable to the respondents."}, "time_dimension": {"answer": "Present", "rationale": "The entry describes the way the testee is entertaining now, using \"Yes\" and \"No\" , which indicates that it is a statement of fact, without reference to past or future circumstances. Thus, the temporal dimension is now judged."}, "sentence_type": "Affirmative declarative sentence", "pos": {"nouns": {"we": 1, "Recreational activities": 1, "Modalities": 1, "Television": 1, "Radio": 1, "Outgoing activities": 1}, "verbs": {"be": 1, "Look": 1, "Listen": 1, "Out": 1}, "adverbs": {"87": 1, "Not": 1}, "numerals": {"87": 1}, "classifiers": {"individual": 1}, "pronouns": {"we": 1}}}, {"content": "We believe that improving the standard of living in the family is more important than upholding ethical standards.", "word_count": 24, "label": "", "sentence_structure": "Subject鈥搗erb鈥揷omplement", "subjective_objective": {"answer": "Subjective", "rationale": "Objective is an objective fact that examines the patient ' s experience and considers it to be subjective."}, "sentence_sentiment": {"answer": "Negative", "rationale": "Because the idea that \"a higher standard of living in the family is more important than strict observance of moral standards\" in the entry is negative."}, "language_style": {"answer": "Calm and direct", "rationale": "This entry directly expresses the researchers鈥?view of the priority between the standard of living and the moral standard of the family, without using excessive rhetoric or emotional rendering. Such language style is more objective and practical.\""}, "time_dimension": {"answer": "Present", "rationale": "\"Between\" and \"Between\" are the general currents, expressing the current views, attitudes or behaviour. The entry therefore describes the views and views of the testees."}, "sentence_type": "Affirmative declarative sentence", "pos": {"nouns": {"Living standards at home": 1, "Strictly ethical": 1}, "verbs": {"Increase": 1, "- Yeah": 1, "And": 1, "It's important": 1}, "adverbs": {"88, we think": 1}, "numerals": {"88": 1}}}, {"content": "We have to have someone to wash the dishes right after dinner.", "word_count": 14, "label": "", "sentence_structure": "Subject鈥搗erb鈥揷omplement", "subjective_objective": {"answer": "Subjective", "rationale": "Because \"there must be someone who washes the dishes\" is the patient's subjective will or rule."}, "sentence_sentiment": {"answer": "Negative", "rationale": "Because the requirement that \"there must be someone who must wash the dishes\" is ordered and mandatory, may cause stress and resentment among family members."}, "language_style": {"answer": "Tactful and warm", "rationale": "The basis for the judgement: although the entry concerns the division of household tasks, it is not directly ordered or required in terms of language, but rather expressed in terms of \"must\" . At the same time, the expression \"one has to wash the dishes\" appears to be more euphemistic and gentle, respects the feelings of each family member and gives some autonomy, so that the overall language style is more euphemismal."}, "time_dimension": {"answer": "Past", "rationale": ""}, "sentence_type": "Imperative sentence", "pos": {"nouns": {"Our home": 1, "After dinner": 1, "person": 1, "Wash dishes": 1}, "verbs": {"I have to": 1, "Go on": 1, "Wash": 1}, "adverbs": {"Immediately": 1}, "numerals": {"89": 1}, "pronouns": {"Here": 1}}}, {"content": "Those who break home rules are severely criticized.", "word_count": 16, "label": "", "sentence_structure": "Subject鈥搗erb鈥搊bject", "subjective_objective": {"answer": "Objective", "rationale": "Since criticism is an objective fact, not controlled by the will of the individual, objective results are being examined."}, "sentence_sentiment": {"answer": "Negative", "rationale": "Because the \"severe criticism\" in the entry is negative."}, "language_style": {"answer": "Calm and direct", "rationale": "The entry clearly expresses the message that when a person violates family rules, he or she will be severely criticized, and that he or she does not use excessive rhetorical techniques or emotional renderings, which are more straightforward and direct."}, "time_dimension": {"answer": "Present", "rationale": "The questionnaire entry describes a general situation in which \"breachers at home\" are subject to criticism. This is irrelevant to the past or the future, and therefore the temporal dimension is now judged. At the same time, this situation is not only one person but one that is a general rule or circumstance, and therefore does not involve a specific personal pronoun or verb."}, "sentence_type": "Affirmative declarative sentence", "pos": {"nouns": {"Home": 1, "A family man": 1, "Criticism": 1}, "verbs": {"Yes": 1, "Received": 1, "Hard": 1}, "numerals": {"90": 1}}}]}

{"questionnaire_title": "- I'm not sure.", "questionnaire_category": "Mental health", "item_count": 15, "emotion_shift_overall": "From positive to negative", "language_style_overall": "Tactful and warm", "items": [{"content": "I am often nervous, even at informal gatherings.", "word_count": 17, "label": "", "sentence_structure": "Subject鈥揷opula鈥損redicative", "subjective_objective": {"answer": "Subjective", "rationale": "Because stress is a subjective emotional experience, determined by the patient鈥檚 own feelings and perceptions. Even if the meeting is an objective occasion, the perception and reaction to it is an individual subjective experience."}, "sentence_sentiment": {"answer": "Negative", "rationale": "Because the act of \"stretching\" in the entry is negative."}, "language_style": {"answer": "Calm and direct", "rationale": "The basis of the judgement is as follows: the entry asks the interviewees in a concise and clear manner whether they are often nervous at informal gatherings. It does not use excessive rhetorical or emotional overtones, nor does it attempt to induce the interviewees to give specific answers by asking questions. This direct inquiry makes the question more objective and practical."}, "time_dimension": {"answer": "Present", "rationale": "According to the phrase \"I often feel\" it can be judged to be an act or feeling describing the current state, and the word \"often\" indicates that this happens regularly, so it can be established that it is an act or attitude about the present."}, "sentence_type": "Negative declarative sentence", "pos": {"nouns": {"Party": 1, "Event": 1}, "verbs": {"I feel": 1, "Nervous": 1}, "adjectives": {"Informal": 1}, "numerals": {"1": 2}, "pronouns": {"I": 1}}}, {"content": "I am usually uncomfortable with a group of people I don't know.", "word_count": 20, "label": "", "sentence_structure": "Subject鈥揷opula鈥損redicative", "subjective_objective": {"answer": "Subjective", "rationale": "Because feeling uncomfortable is a subjective feeling and experience of an individual, not an objective fact."}, "sentence_sentiment": {"answer": "Negative", "rationale": "Because the description \"frightfully\" is negative."}, "language_style": {"answer": "Calm and direct", "rationale": "The entry uses a clear observation or feeling in a simple and clear language, with no excessive emotional adoration or rhetorical techniques. Such language style is common in psychological questionnaires, which are designed to accurately capture the subjective experiences and ideas of the interviewee."}, "time_dimension": {"answer": "Present", "rationale": "The entry now describes the feelings and experiences of the person tested in the current circumstances, with the time dimension being the present."}, "sentence_type": "Affirmative declarative sentence", "pos": {"nouns": {"person": 1, "Group": 1, "No, I don't": 1, "Together": 1, "Time": 1}, "verbs": {"I feel": 1, "Usually": 1, "in": 1, "Time": 1}, "adjectives": {"I'm not comfortable": 1}, "adverbs": {"Together": 1}, "numerals": {"2": 1}, "classifiers": {"Group": 1, "individual": 1}, "pronouns": {"I": 1}}}, {"content": "I usually feel comfortable talking to a heterosexual.", "word_count": 16, "label": "", "sentence_structure": "Subject鈥揷opula鈥損redicative", "subjective_objective": {"answer": "Subjective", "rationale": "Because \"easy\" is an emotional experience, which belongs to the patient's own psychological state and feelings, and is a subjective examination."}, "sentence_sentiment": {"answer": "Positive", "rationale": "Because the \"easiness\" in the entry is positive."}, "language_style": {"answer": "Tactful and warm", "rationale": "The entry is expressed in a relatively accessible way, using a first person's expression to bring the person under investigation closer together, while avoiding the use of too direct or strong language to give a friendly and comfortable atmosphere for dialogue."}, "time_dimension": {"answer": "Present", "rationale": ""}, "sentence_type": "Affirmative declarative sentence", "pos": {"nouns": {"Heterosexual": 1, "Talk": 1}, "verbs": {"I feel": 1, "Usually": 1, "Easy": 1}, "adjectives": {"One": 1, "bit": 1}, "adverbs": {"in": 1, "Time": 1}, "numerals": {"3": 1}, "pronouns": {"I": 1}}}, {"content": "I was nervous when I had to talk to teachers or supervisors.", "word_count": 17, "label": "", "sentence_structure": "Subject鈥搗erb鈥揷omplement", "subjective_objective": {"answer": "Subjective", "rationale": "Because feeling nervous is a subjective feeling for one."}, "sentence_sentiment": {"answer": "Negative", "rationale": "Because the \"sweet\" sentiment in the entry is negative."}, "language_style": {"answer": "Calm and direct", "rationale": "The entry asks the interviewee whether he or she is nervous when talking to a teacher or supervisor in a direct and concise manner, without using any rhetorical or emotional colours, and with a more calm and objective language style."}, "time_dimension": {"answer": "Present", "rationale": "The entry is for the purpose of asking the testee how he feels when he must speak to the teacher or supervisor, which usually occurs and is experienced in the current situation, and thus judged as now."}, "sentence_type": "Affirmative declarative sentence", "pos": {"nouns": {"Teacher": 1, "Sir": 1, "Talk": 2}, "verbs": {"I feel": 1, "Nervous": 1, "I have to": 1, "Same": 1, "When we talk": 1, "Say it": 1}, "adverbs": {"in": 1, "I'm sure": 1, "- It's tense": 1}, "numerals": {"4": 1}}}, {"content": "The parties often make me anxious and uncomfortable.", "word_count": 14, "label": "", "sentence_structure": "Subject鈥搗erb鈥揷omplement", "subjective_objective": {"answer": "Subjective", "rationale": "Because anxiety and discomfort are subjective emotional experiences, it reflects patients ' perceptions and feelings of attending a meeting."}, "sentence_sentiment": {"answer": "Negative", "rationale": "Because \"anxiety\" and \"inhabitation\" in the entries are negative emotional experiences."}, "language_style": {"answer": "Tactful and warm", "rationale": "The language style of the entry is more euphemistic, with little direct criticism or blame, but rather a sense of understanding and concern to guide the respondents to express their feelings. This language style allows respondents to be more open to sharing their thoughts and feelings and helps to create a good atmosphere of communication."}, "time_dimension": {"answer": "Past", "rationale": "In the entry, both \"party\" and \"fearful and uncomfortable\" acts occurred in the past, and thus judged to be the past."}, "sentence_type": "Negative declarative sentence", "pos": {"nouns": {"Party": 1, "Anxiety": 1, "I'm not comfortable": 1}, "verbs": {"鈾?And I'm gonna be 鈾?: 1, "I feel": 1}, "adjectives": {"Often": 1, "and": 1}, "numerals": {"5": 1}}}, {"content": "I may be less shy in social relations than most people.", "word_count": 20, "label": "", "sentence_structure": "Subject鈥揷opula鈥損redicative", "subjective_objective": {"answer": "Subjective", "rationale": "Because \"less shyness\" is a personal feeling or self-evaluation, which is a subjective view of the patient."}, "sentence_sentiment": {"answer": "Positive", "rationale": "Because the expression \"less shyness\" in the entry is positive, it is a high indicator of self-evaluation."}, "language_style": {"answer": "Tactful and warm", "rationale": ""}, "time_dimension": {"answer": "Present", "rationale": ""}, "sentence_type": "Affirmative declarative sentence", "pos": {"nouns": {"person": 1, "Social interaction": 1, "Shame": 1}, "verbs": {"in": 1, "Maybe": 1, "Less": 1}, "adjectives": {"Most of them": 1, "and": 1}, "numerals": {"6": 1}, "classifiers": {"individual": 1}}}, {"content": "I was often nervous when I spoke to same-sex people that I was not familiar with.", "word_count": 20, "label": "", "sentence_structure": "Subject鈥搗erb鈥揷omplement", "subjective_objective": {"answer": "Subjective", "rationale": "Because feeling nervous is a subjective feeling and experience."}, "sentence_sentiment": {"answer": "Negative", "rationale": "Because \"frightful\" in the entries expresses negative emotions and attitudes."}, "language_style": {"answer": "Calm and direct", "rationale": "The entry directly expresses the subject of questioning whether the other party is often nervous when talking to less familiar same-sex persons, and is not using excessive rhetorical or emotional rendering, which is more objective and sound."}, "time_dimension": {"answer": "Present", "rationale": "The word \"I'm often\" denotes a regular, customary act, while \"i'm nervous\" uses the normal current, which means that it has not only happened in the past but may still be ongoing. So, the entry asks the testee about the current behavior or mentality, and the temporal dimension is now."}, "sentence_type": "Affirmative declarative sentence", "pos": {"nouns": {"7": 1, "Same-sex": 1, "Talk": 1, "Nervous": 1}, "verbs": {"in": 1, "I feel": 1, "I don't know": 1, "Regular": 1}, "adverbs": {"Often": 1, "Not really": 1}, "numerals": {"7": 1}, "pronouns": {"I": 1}}}, {"content": "I'm nervous about job interviews.", "word_count": 11, "label": "", "sentence_structure": "Subject鈥揷opula鈥損redicative", "subjective_objective": {"answer": "Subjective", "rationale": "Because \"stretch\" is a subjective emotional reaction."}, "sentence_sentiment": {"answer": "Negative", "rationale": "Because the \"stretch\" in the entry is negative."}, "language_style": {"answer": "Tactful and warm", "rationale": "The entry is more adept and interactive when it is spoken in a gentle and inviting manner, rather than by direct statements or orders."}, "time_dimension": {"answer": "Present", "rationale": "The sentence \"that's nervous\" and \"that's when you're interviewing for work\" suggests that the entry describes the current situation, that is, the sense of tension that will arise during job interviews."}, "sentence_type": "Affirmative declarative sentence", "pos": {"nouns": {"8": 1, "Job interviews": 1}, "verbs": {"Yes": 1, "Nervous": 1, "of": 1}}}, {"content": "I hope I have more confidence in social life.", "word_count": 17, "label": "", "sentence_structure": "Subject鈥搗erb鈥揷omplement", "subjective_objective": {"answer": "Subjective", "rationale": "Hope is a subjective attitude and expectation that reflects patients ' subjective perceptions of their expectations and needs."}, "sentence_sentiment": {"answer": "Positive", "rationale": "Because the entries \"hope\" and \"confidence\" are more positive expressions of attitude and desire."}, "language_style": {"answer": "Tactful and warm", "rationale": "The language style of the entry is more euphemistic, raising questions in the form of hope, and not directly accusing or criticizing the interviewee. This expression is more easily accepted and answered by the interviewee. At the same time, the expression \"confidence is more positive\" encourages the interviewee to improve his social performance."}, "time_dimension": {"answer": "Present", "rationale": "The word \"favorite\" in the entry expresses subjective desires and expectations, while \"confidence\" is a manifestation of state or capacity, meaning that such expectations are at the current point of time and are therefore judged to be the present."}, "sentence_type": "Imperative sentence", "pos": {"nouns": {"Questionnaire Entry": 1, "Hope": 1, "self": 1, "Socially": 1, "Confidence": 1, "Event": 1}, "verbs": {"Hope": 1, "in": 1, "More": 1, "Some": 1}, "adjectives": {"Self-confident": 1}, "numerals": {"9": 1}, "pronouns": {"self": 1}}}, {"content": "I'm not very anxious about it in social settings.", "word_count": 13, "label": "", "sentence_structure": "Subject鈥搗erb鈥揷omplement", "subjective_objective": {"answer": "Subjective", "rationale": "Because \"very little anxiety\" is a personal feeling and experience, a subjective view of the patient."}, "sentence_sentiment": {"answer": "Positive", "rationale": "Because the description \"very rarely anxiety\" in the entry is positive."}, "language_style": {"answer": "Tactful and warm", "rationale": "The entry leads the respondents to the questions in a question and concern manner, using more compassionate expressions that make them more acceptable and willing to share their true feelings. At the same time, the statement does not directly reveal or accuse the respondents of social anxiety, but rather stimulates reflection and communication by asking questions."}, "time_dimension": {"answer": "Present", "rationale": "The entry describes the current state of the testees, in which they are rarely anxious in social settings. The keyword \"now\" indicates that it is a current statement, not a past or future state."}, "sentence_type": "Affirmative declarative sentence", "pos": {"nouns": {"Socially": 1, "Anxiety": 1}, "verbs": {"I feel": 1, "Very few": 1}, "adjectives": {"10": 1, "In social settings": 1}, "numerals": {"10": 1, "In social settings": 1}}}, {"content": "I'm generally a shy person.", "word_count": 12, "label": "", "sentence_structure": "Subject鈥揷opula鈥損redicative", "subjective_objective": {"answer": "Subjective", "rationale": "Because shyness is a subjective attitude or emotional experience."}, "sentence_sentiment": {"answer": "Neutral", "rationale": "Because the entry does not express positive or negative attitudes, behaviour, purpose or quality. It merely describes the fact that the interviewee is usually shy."}, "language_style": {"answer": "Tactful and warm", "rationale": "The entry uses a euphemism, rather than a direct statement or evaluation of the personality of the other, which avoids causing discomfort or resistance on the part of the person under investigation and increases his or her motivation and initiative to participate in the questionnaire. At the same time, the use of the term \"general\" leaves the person under investigation some room for more truthful and accurate answers."}, "time_dimension": {"answer": "Past", "rationale": "The entry contains the term \"weakness\" describing the character of the individual and uses the term \"general\" to denote the description normally, implying that the speaker is describing his or her personality and thus judging the dimension of time as the past."}, "sentence_type": "Affirmative declarative sentence", "pos": {"nouns": {"11": 1, "General": 1, "person": 1, "Shy": 1}, "verbs": {"be": 1}, "adverbs": {"鈾?And the 鈾?: 1, "General": 1}, "numerals": {"11": 1}, "pronouns": {"I": 1}}}, {"content": "I'm always nervous when I talk to a charming heterosexual.", "word_count": 19, "label": "", "sentence_structure": "Subject鈥搗erb鈥搊bject", "subjective_objective": {"answer": "Subjective", "rationale": "Because \"stretch\" is a subjective or emotional experience."}, "sentence_sentiment": {"answer": "Negative", "rationale": "Because \"stretch\" in the entry is negative."}, "language_style": {"answer": "Tactful and warm", "rationale": "The entry is more personal and interactive when it is spoken in a gentle and inviting tone, rather than in direct statements or orders. At the same time, the adjective \"character\" is used to depict the characteristics of the opposite sex and gives people a sense of affection and friendship."}, "time_dimension": {"answer": "Present", "rationale": "The keyword \"in conversation with a charming heterosexual person\" indicates behaviour in a given situation, and \"I'm often nervous\" suggests that this tension is a common experience. Since this information is a description of the current situation, it can be concluded that it is an assessment and description of the current state, that is, \"now.\""}, "sentence_type": "Affirmative declarative sentence", "pos": {"nouns": {"12": 1, "Heterosexual": 1, "Talk": 1}, "verbs": {"in": 1, "I feel": 1, "Nervous": 1}, "adjectives": {"Fascinating": 1}, "numerals": {"One": 1}, "pronouns": {"I": 1}}}, {"content": "I'm usually nervous when I call someone I don't know.", "word_count": 17, "label": "", "sentence_structure": "Subject鈥搗erb鈥揷omplement", "subjective_objective": {"answer": "Subjective", "rationale": "Since stress is a personal feeling and experience, it is a subjective element of the examination."}, "sentence_sentiment": {"answer": "Negative", "rationale": "Because \"stretch\" is negative."}, "language_style": {"answer": "Calm and direct", "rationale": "The entry directly expresses the subject of questioning the other party about his or her stress when calling someone who is not familiar, and does not use excessive rhetoric or emotional rendering, which is more objective and balanced."}, "time_dimension": {"answer": "Present", "rationale": "\"Calling someone less familiar\" is a behavior or state, and \"I usually feel nervous\" is a subjective feeling of that behavior or state. So the temporal dimension of the subject is now."}, "sentence_type": "Affirmative declarative sentence", "pos": {"nouns": {"person": 1, "Telephone": 1, "Nervous": 1}, "verbs": {"Here": 1, "Fight": 1, "I think": 1}, "adjectives": {"Not really": 1}, "adverbs": {"Usually": 1}, "numerals": {"13": 1}}}, {"content": "I was nervous when I spoke to the authorities.", "word_count": 14, "label": "", "sentence_structure": "Subject鈥揷opula鈥損redicative", "subjective_objective": {"answer": "Subjective", "rationale": "Because it is a personal feeling of the patient, it is a subjective experience and perception."}, "sentence_sentiment": {"answer": "Negative", "rationale": "Because the \"frightful\" sentiment in the entry is negative."}, "language_style": {"answer": "Calm and direct", "rationale": "The entry is more calm and objective in its language, and does not have too much embellishment or emotional rendering. It directly raises the question of whether the interviewee is nervous when talking to an authority. This expression is simpler and easier to understand."}, "time_dimension": {"answer": "Present", "rationale": "Now the entry describes the feelings and experiences of the person being tested in the current situation, which is \"I'm nervous\" and can therefore be judged as now. At the same time, \"in talking to authorities\" means that it is a feeling that occurs in a given situation, and can be seen as a concrete expression of the present."}, "sentence_type": "Affirmative declarative sentence", "pos": {"nouns": {"I": 1, "Authority": 1, "Talk": 1, "Nervous": 1}, "verbs": {"in": 1, "I feel": 1, "Talk": 1}, "adverbs": {"14": 1}, "numerals": {"14": 1}}}, {"content": "Even in a group quite different from me, I usually feel relaxed.", "word_count": 25, "label": "", "sentence_structure": "Subject鈥搗erb鈥搊bject", "subjective_objective": {"answer": "Subjective", "rationale": "Because \"fright relax\" is a personal feeling or experience, a patient's subjective attitude."}, "sentence_sentiment": {"answer": "Neutral", "rationale": "Because the entry only describes a situation, it does not express a positive or negative emotional attitude."}, "language_style": {"answer": "Tactful and warm", "rationale": ""}, "time_dimension": {"answer": "Present", "rationale": "\"Even in a group quite different from me,\" which indicates that the testee is describing a situation or state, and \"normally I feel relaxed\" means that it is a constant feeling or experience. So, the entry to the questionnaire is mainly about the current situation of the testee, that is, how often they feel the relaxion occurs, and should be in the current state of affairs."}, "sentence_type": "Affirmative declarative sentence", "pos": {"nouns": {"Population": 1, "Relax": 1}, "verbs": {"in": 1, "I feel": 1}, "adjectives": {"Pretty much": 1, "Different": 1}, "adverbs": {"Still": 1, "Usually": 1, "Centre": 1}, "numerals": {"15": 1}, "pronouns": {"I": 1}}}]}

{"questionnaire_title": "DISS personality test", "questionnaire_category": "Behavioral questionnaire", "item_count": 161, "emotion_shift_overall": "From positive to negative", "language_style_overall": "Neutral", "items": [{"content": "1 For risk-taking: for those who are willing to face new things and to take control of them with determination;", "word_count": 21, "label": "", "sentence_structure": "Subject鈥搗erb鈥搊bject", "subjective_objective": {"answer": "Subjective", "rationale": "Because \"willing to face new things and be determined to master\" is the patient's personal attitude and behavioural orientation, and is subjective."}, "sentence_sentiment": {"answer": "Positive", "rationale": "Because the expression of \"risky\" and \"determinately determined\" attitudes and behaviour is positive, it is an expression of the spirit of adventure and the determination of this person to act decisively."}, "language_style": {"answer": "Tactful and warm", "rationale": "The entry is more euphemistic and friendly, using the words \"will\" and \"bold\" to express appreciation and encouragement for risk-taking rather than direct emphasis or command. At the same time, it is used to describe this quality in more common and understandable language, to bring it closer to the interviewee ' s life experience and feelings and to increase the motivation of his or her response."}, "time_dimension": {"answer": "Future", "rationale": "The words \"really risky\", \"willing to face new things\" and \"willing to take control\" all relate to future visions and expectations, and the entry is judged to be future."}, "sentence_type": "Affirmative declarative sentence", "pos": {"nouns": {"Adventure": 1, "person": 1}, "verbs": {"Yes": 1, "Face it": 1, "How dare you": 1, "Make up your mind": 1, "Got it": 1}, "adjectives": {"Richer than": 1}, "numerals": {"1": 1}}}, {"content": "2. Be resilient: easily adapted to any environment;", "word_count": 14, "label": "", "sentence_structure": "Subject鈥搗erb鈥揷omplement", "subjective_objective": {"answer": "Subjective", "rationale": "For a person ' s resilience is a subjective feeling and assessment, not an objective reality."}, "sentence_sentiment": {"answer": "Positive", "rationale": "Because the words \"resilient\" and \"easy adaptation to any environment\" in the entries are positive."}, "language_style": {"answer": "Tactful and warm", "rationale": "The entry uses descriptive language to describe the characteristics of \"resilient\" rather than direct statements or orders, which are more personal and interactive, making it easier for the respondents to accept and be willing to answer questions. At the same time, positive language and positive tone are used, giving the respondents a positive impression and encouragement to be more willing to participate in the questionnaire study."}, "time_dimension": {"answer": "Present", "rationale": "The entry describes the characteristics that are now present in the testee, namely, that it is adaptable and able to adapt easily to the various environments, so the temporal dimension is now judged."}, "sentence_type": "Affirmative declarative sentence", "pos": {"nouns": {"Adaptive": 1, "Environment": 1}, "verbs": {"Strong": 1, "Adaptation": 1, "鈾?As if I was 鈾?: 1, "Easy": 1}, "adjectives": {"Strong": 1, "Any": 1, "鈾?As if I was 鈾?: 1, "Easy": 1}, "adverbs": {"Any": 1}}}, {"content": "Liver: Vibrant, vivid, gestures;", "word_count": 13, "label": "", "sentence_structure": "Subject鈥搗erb鈥揷omplement", "subjective_objective": {"answer": "Subjective", "rationale": "Because \"vigorating\" is a subjective word of evaluation, describing the attitude and behaviour of patients in communicating with others. Although the extent of the patient鈥檚 vibrancy can be assessed by observing his face and gestures, it is based on the subjective impression of the observer."}, "sentence_sentiment": {"answer": "Positive", "rationale": "According to the description in the entry, vivid expression and multiple gestures are usually considered positive qualities or attitudes."}, "language_style": {"answer": "Tactful and warm", "rationale": "The language style of the entry is euphemistic, contagious and encouraging, without too direct or hard-line tone and language, but rather a moderate way to direct the respondents to share their own situation. Moreover, the terms \"viorous\", \"expressive\" and \"multi-portrait\" also give positive, enthusiastic and more conducive motivation and participation."}, "time_dimension": {"answer": "Present", "rationale": ""}, "sentence_type": "Affirmative declarative sentence", "pos": {"nouns": {"Vivid": 2}, "verbs": {"Fill": 1, "Emoticons": 1, "More": 1, "Gestures": 1}, "numerals": {"3": 1}}}, {"content": "4 Good at analysis: It is a good idea to look at the logic and correct relationship between the various components.", "word_count": 22, "label": "", "sentence_structure": "Subject鈥搗erb鈥揷omplement", "subjective_objective": {"answer": "Subjective", "rationale": "Because \"like\" is a patient's description of an individual's preferences or attitudes that are good at analysis, it's a subjective view."}, "sentence_sentiment": {"answer": "Positive", "rationale": "Because \"analytical\" and \"like to study the logic and correct relationship between the parts\" in the entries are positive qualitative descriptions."}, "language_style": {"answer": "Tactful and warm", "rationale": "This entry is used in a question-and-answer tone, and in a positive tone, it expresses the strengths of the interviewee, not the direct statement or order."}, "time_dimension": {"answer": "Present", "rationale": "The entry describes the testee's current personality or pattern of behaviour, the keywords being \"like\" and \"good\" , which all indicate a state or custom rather than an act that has occurred in the past. At the same time, \"4\" in \"goodly analysed\" is also a clear sign that it is an evaluation or a score of individual characteristics or behaviour. Thus, the entry describes the current situation."}, "sentence_type": "Affirmative declarative sentence", "pos": {"nouns": {"It's a good analysis": 1, "Logical": 1, "Relations": 1}, "verbs": {"Yeah": 1, "Research": 1}, "adjectives": {"Good at it": 1, "Between parts": 1, "Correct": 1}, "numerals": {"4": 1}}}, {"content": "1 Perseverance: the things that are available can be done;", "word_count": 18, "label": "", "sentence_structure": "Subject鈥搗erb鈥搊bject", "subjective_objective": {"answer": "Subjective", "rationale": "Because \"continuing\" is a subjective attitude or belief that indicates the resilience of a person in pursuing a goal. While this may affect a person's behaviour and decision-making, it is a personal view itself and is therefore subjective."}, "sentence_sentiment": {"answer": "Positive", "rationale": "Because the words \"continuing\" and \"doing what is done before doing something new\" are positive, positive attitudes and qualities."}, "language_style": {"answer": "Calm and direct", "rationale": "The entry expresses a concept or principle in a concise and clear statement, without too many rhetorical or emotional renderings, and with a more calm and straightforward language style."}, "time_dimension": {"answer": "Present", "rationale": "The keyword \"to do what is available before doing something new\" represents an act or state that is now in progress and therefore is judged to be now."}, "sentence_type": "Affirmative declarative sentence", "pos": {"nouns": {"Keep it up": 1, "Yes": 2, "Things": 1}, "verbs": {"Yes": 1, "Completed": 1, "Talent": 1, "Do it": 1}, "adverbs": {"Existing": 1}, "numerals": {"One": 1}}}, {"content": "2 Entertainment: joyful and funny;", "word_count": 14, "label": "", "sentence_structure": "Subject鈥損redicate", "subjective_objective": {"answer": "Subjective", "rationale": "Because entertainment is a subjective feeling and attitude, it reflects personal preferences and emotional reactions to recreational activities."}, "sentence_sentiment": {"answer": "Positive", "rationale": "Because the entries \"happy, fun and humor\" are positive emotions and attitudes."}, "language_style": {"answer": "Tactful and warm", "rationale": "The language style of the entry is more euphemismal. It first uses the term \"favourable entertainment\" to describe the subject of the survey, then expresses positive emotions through the words \"joy,\" \"joyful\" and \"joy\" and \"homicity\" and uses semicolons to separate different options to make the questionnaire more intuitive. These language features make the entry more gentle, friendly and attractive, which helps to increase the enthusiasm and interest of respondents to fill out the questionnaire."}, "time_dimension": {"answer": "Present", "rationale": "The entry \"2-Presentation\" shows that it's a personal survey of pleasure, and \"happy and funny\" is the current entertainment and characteristic of the testee, so the temporal dimension is now judged."}, "sentence_type": "Affirmative declarative sentence", "pos": {"nouns": {"I like it": 1, "Recreation": 1, "Happy": 1, "Fun": 1, "Humor": 1}, "adjectives": {"Full": 1}, "adverbs": {"Fill": 1}}}, {"content": "(c) Convince: to serve with logic and facts without authority and authority;", "word_count": 20, "label": "", "sentence_structure": "Subject鈥搗erb鈥搊bject", "subjective_objective": {"answer": "Objective", "rationale": "Because \"good to convince\" is a ability and skill to influence the views or behaviour of others through logic and facts, not through authority or power. It is an objective criterion that can be observed and assessed, independent of the subjective views of individuals."}, "sentence_sentiment": {"answer": "Positive", "rationale": "Because the purpose of the \"goodly persuaded\" act in the entry is positive, it is an indication that a person has good communication skills and ability to solve problems."}, "language_style": {"answer": "Tactful and warm", "rationale": ""}, "time_dimension": {"answer": "Present", "rationale": "The entry describes a capability or skill that is not clearly time-directed and therefore is judged to be the present."}, "sentence_type": "Affirmative declarative sentence", "pos": {"nouns": {"Convince": 1, "Logical": 1, "The facts": 1, "Powerful": 1, "Powers": 1}, "verbs": {"Use": 2, "Conserve": 1}, "adjectives": {"Good at it": 1}, "numerals": {"3": 1}}}, {"content": "4 Peace: Peace and calm in the conflict.", "word_count": 14, "label": "", "sentence_structure": "Subject鈥搗erb鈥揷omplement", "subjective_objective": {"answer": "Objective", "rationale": "Because \"peace\" is a state or characteristic, a manifestation of whether or not the conflict is calm and free from external factors. This state or characteristic is not seen from the patient鈥檚 own point of view."}, "sentence_sentiment": {"answer": "Positive", "rationale": "Basis: Undisturbed and calm in conflict is a positive response."}, "language_style": {"answer": "Calm and direct", "rationale": "The entry is a straightforward expression of the theme 鈥?to remain calm in conflict, to be free of excessive rhetoric or emotional rendering and to be more objective in tone."}, "time_dimension": {"answer": "Present", "rationale": "The entry describes a state of affairs or ability that people should have in the face of conflict, namely, \"in the event of conflict, without interference, and in order to maintain calm\", which is an expression of behaviour or attitude. Since the title does not use the term \"situational or future\" the term \"time dimension of the article is now."}, "sentence_type": "Affirmative declarative sentence", "pos": {"nouns": {"Peace": 1, "Conflict": 1, "Interference": 1, "Calm down": 1}, "verbs": {"Yes": 1, "Hold on": 1}, "adverbs": {"Not": 1}, "numerals": {"4": 1}}}, {"content": "1 Consistency: the perception and preference of others is easy to accept and do not hold back;", "word_count": 18, "label": "", "sentence_structure": "Subject鈥搗erb鈥揷omplement", "subjective_objective": {"answer": "Subjective", "rationale": "Because \"accept other people's views\" and \"not to hold on to one's views\" are based on personal thoughts and attitudes."}, "sentence_sentiment": {"answer": "Negative", "rationale": "Because \"subordination\" in the entries can deprive people of their self-advocacy and self-defeating thinking."}, "language_style": {"answer": "Tactful and warm", "rationale": "Warm and warm."}, "time_dimension": {"answer": "Present", "rationale": ""}, "sentence_type": "Affirmative declarative sentence", "pos": {"nouns": {"Congratulate": 1, "Views": 1, "I like it": 1, "I'm afraid so": 1}, "verbs": {"Accept": 1, "Hold on": 1}, "adverbs": {"Easy": 1}}}, {"content": "Self-sacrifice: willingness to give up an individual opinion in the interest of others;", "word_count": 17, "label": "", "sentence_structure": "Subject鈥搗erb鈥揷omplement", "subjective_objective": {"answer": "Subjective", "rationale": "Because self-sacrifice is a subjective attitude and choice of one."}, "sentence_sentiment": {"answer": "Positive", "rationale": "Because the purpose of the entry is to \"willing to give up a personal opinion in the interest of others\"."}, "language_style": {"answer": "Tactful and warm", "rationale": "The reference is based on the following: - The entry is based on the theme of \"self-sacrifice\" and is not directly stated or ordered. - The question is \"willing to give up a personal opinion\" and this expression is more personal and interactive. - Overall, the entry focuses more on guiding the interviewee to think and reflect than simply seeking answers."}, "time_dimension": {"answer": "Present", "rationale": "The descriptions of \"self-sacrifice\" and \"willing to give up an individual opinion in the interest of others\" in this entry point to the present behaviour or attitude and thus to the present."}, "sentence_type": "Affirmative declarative sentence", "pos": {"nouns": {"Self-sacrifice": 1, "Others": 1, "Interest": 1, "Individual opinions": 1}, "verbs": {"Abandon": 1, "Yes": 1}, "pronouns": {"you": 1}}}, {"content": "3 Socialization: consider it fun to be with people, not a challenge or business opportunity;", "word_count": 24, "label": "", "sentence_structure": "Subject鈥搗erb鈥搊bject", "subjective_objective": {"answer": "Subjective", "rationale": "Because \"getting along with people is fun\" is a subjective assessment and perception of the patient's subjective feelings and perceptions of his/her personal relationships. And \"not a challenge or a business opportunity\" is also judged on the basis of the patient's personal views and attitudes, so the questionnaire is based on the patient's subjective perspective."}, "sentence_sentiment": {"answer": "Positive", "rationale": "Because the words \"socially good\" and \"regarding being fun\" in the entries are all positive emotional."}, "language_style": {"answer": "Tactful and warm", "rationale": "The entry is more personal and interactive when it is spoken in a gentle and inviting tone, rather than in direct statements or orders. At the same time, it uses descriptive terms such as \"sociality\" and \"conceives fun with people\" to depict the character characteristics of the person under investigation, the temperature of the language and the euphemism."}, "time_dimension": {"answer": "Present", "rationale": "The sentence \"conception\" and \"yes\" makes it possible to judge that the item is being asked about the person's current thoughts and perceptions, so the temporal dimension is now. At the same time, \"socialization\" and \"conceivement\" are fun, not challenges or business opportunities\" are also depicting the character and behaviour of the person being tested, further confirming that this is a survey of the state of affairs."}, "sentence_type": "Affirmative declarative sentence", "pos": {"nouns": {"Social": 1, "person": 2, "Get along": 1, "Challenges": 1, "Business opportunities": 1}, "verbs": {"I think": 1, "be": 2, "Play": 1, "and": 1, "Get along": 1, "No, it's not": 1, "Be": 1}, "adjectives": {"Good at it": 1}, "adverbs": {"3": 1, "Fun": 1}, "pronouns": {"Here": 1}}}, {"content": "4 Strong will: determination to do things in their own way.", "word_count": 14, "label": "", "sentence_structure": "Subject鈥搗erb鈥揷omplement", "subjective_objective": {"answer": "Subjective", "rationale": "For the determination to do things in their own way is the patient ' s subjective attitude and conviction, not the objective fact or result."}, "sentence_sentiment": {"answer": "Positive", "rationale": "For the determination is a positive quality, and it is an expression of individual determination and perseverance to achieve its goals."}, "language_style": {"answer": "Calm and direct", "rationale": "The language style of the entry is more direct, more concise, less emotive or euphemistic, but rather conveys the theme \"Determined to do things in your own way\" through a simple sentence. This way conveys the message more accurately, while making it easier for respondents to understand and answer questions."}, "time_dimension": {"answer": "Present", "rationale": "The entry expresses a customary attitude or belief, and the phrases \"resolved\" and \"doing things in your own way\" indicate that this is the present description."}, "sentence_type": "Affirmative declarative sentence", "pos": {"nouns": {"Will": 1, "Firm": 1, "Resolve": 1, "Modalities": 1}, "verbs": {"Do it": 1}, "numerals": {"4": 1}}}, {"content": "1 Identity: recognition by virtue of personality or character;", "word_count": 16, "label": "", "sentence_structure": "Subject鈥搗erb鈥搊bject", "subjective_objective": {"answer": "Subjective", "rationale": "Because it's the patient's own thoughts and feelings that are being examined."}, "sentence_sentiment": {"answer": "Positive", "rationale": "Because the result of \"conception\" is usually seen as a positive effect."}, "language_style": {"answer": "Tactful and warm", "rationale": "The entry uses a more moderate, invited language style, which leads the interviewee to think and express by asking if the other person is recognized because of his or her personality or character. It is also more natural in tone, with some degree of affection and interaction."}, "time_dimension": {"answer": "Present", "rationale": "The entry \"Acknowledging by personality or character\" describes a phenomenon that is often present in the current social environment and does not relate to past or future situations. Thus, the temporal dimension is now judged."}, "sentence_type": "Affirmative declarative sentence", "pos": {"nouns": {"person": 2, "Character": 1, "Charm": 1}, "verbs": {"Yes": 1, "Letting": 1}, "numerals": {"1": 2}}}, {"content": "2. Care of the feelings and needs of others;", "word_count": 12, "label": "", "sentence_structure": "Subject鈥搗erb鈥揷omplement", "subjective_objective": {"answer": "Subjective", "rationale": "Because \"sensitive\" and \"careful about the feelings and needs of others\" are subjective attitudes and ideas of patients, and are viewed from a subjective perspective."}, "sentence_sentiment": {"answer": "Positive", "rationale": "Because \"care for the feelings and needs of others\" is a positive act, a manifestation of respect and love for others."}, "language_style": {"answer": "Tactful and warm", "rationale": "The language style of the entry is more gentle and friendly, using the words \"care\" and \"need\" to express the affection and care of the other. At the same time, the question is not directed or asked to be sensitive to others, but rather to engage in a conversation in a way that is more acceptable and responsive."}, "time_dimension": {"answer": "Present", "rationale": "The entry asks the testee whether he has the character of being \"sensitive\" and the time dimension is now judged."}, "sentence_type": "Affirmative declarative sentence", "pos": {"nouns": {"Kind": 1, "person": 1, "Feel": 1, "Yes": 1}, "verbs": {"Care": 1}}}, {"content": "3 Competitive: the desire to win is always strong, as a competition;", "word_count": 20, "label": "", "sentence_structure": "Subject鈥搗erb鈥揷omplement", "subjective_objective": {"answer": "Subjective", "rationale": "Because \"a serious desire for win\" is the patient's personal emotion and attitude, and it's subjective."}, "sentence_sentiment": {"answer": "Negative", "rationale": "Because the entries \"competitive\" and \"serious desire for winning\" have negative emotional aspects."}, "language_style": {"answer": "Calm and direct", "rationale": "The entry is more straightforward in language style, with no excessive rhetorical or emotional rendering. It clearly describes a character character that is characterized by a strong desire for competition and a desire for victory, and is expressed in terms such as \"competitive\" and \"serious desire for winning\" and appears to be more incipient and objective."}, "time_dimension": {"answer": "Present", "rationale": "The keyword \"always\" means that the act is in its current state of custom and that \"serious desire for victory\" also describes the current attitude and circumstances, and therefore is judged to be \"now\"."}, "sentence_type": "Affirmative declarative sentence", "pos": {"nouns": {"Competitiveness": 1, "Everything": 1, "Competition": 1, "Serious": 1, "Desire": 1}, "verbs": {"Consider": 1, "be": 1, "have": 1}, "adjectives": {"of": 5, "Always": 1}, "adverbs": {"Always": 1, "have": 1}, "classifiers": {"One": 1}, "pronouns": {"Here": 1}}}, {"content": "4 Self-control: control of your emotions, rarely exposed.", "word_count": 14, "label": "", "sentence_structure": "Subject鈥搗erb鈥揷omplement", "subjective_objective": {"answer": "Subjective", "rationale": "Because the degree of self-control is the patient ' s subjective judgement and perception."}, "sentence_sentiment": {"answer": "Negative", "rationale": "Because the purpose of \"controlling your emotions\" may be to discourage negative emotions, and \"very little disclosure\" may indicate that this person is not good at expressing his true feelings. All of this may imply a negative emotional state or attitude."}, "language_style": {"answer": "Tactful and warm", "rationale": "The entry is more euphemistic, by asking the other side about self-control rather than directly pointing to the inadequate emotional expression."}, "time_dimension": {"answer": "Present", "rationale": ""}, "sentence_type": "Affirmative declarative sentence", "pos": {"nouns": {"Self-control": 1, "Emotion": 1, "The exposed": 1}, "verbs": {"Control": 1, "have": 1, "Very few": 1}, "adjectives": {"Your own": 1}, "adverbs": {"Very few": 1}, "numerals": {"4": 1}}}, {"content": "One is a wake-up call: to give him fresh and exciting excitement;", "word_count": 14, "label": "", "sentence_structure": "Subject鈥搗erb鈥揷omplement", "subjective_objective": {"answer": "Subjective", "rationale": "Because the description involves emotional influence, it needs to be judged on the basis of the individual ' s feelings and experiences as being a refreshing and therefore subjective one."}, "sentence_sentiment": {"answer": "Positive", "rationale": "Because \"pull people\" and \"creative new excitement\" in the entries are positive emotional words."}, "language_style": {"answer": "Tactful and warm", "rationale": "The language style of the entry is more euphemistic. It describes the feeling of \"gentle and refreshing\" in descriptive terms, which is more gentle, friendly and more resonant with the reader. At the same time, the entry does not contain command or direct guiding language, but rather gives advice to the other on the basis of the statement of facts, which is more euphemistic and respectful."}, "time_dimension": {"answer": "Present", "rationale": "In this questionnaire entry, \"Creating a fresh and exciting stimulus\" refers to current or recent behaviour or mentality, so the temporal dimension is now judged."}, "sentence_type": "Imperative sentence", "pos": {"nouns": {"person": 1, "Exciting": 1}, "verbs": {"鈾?And I'm gonna be 鈾?: 1, "Come on": 1, "Here": 1, "# Fresh and refreshing #": 1}, "numerals": {"1": 2}}}, {"content": "Respect for others: respect for honesty;", "word_count": 10, "label": "", "sentence_structure": "Subject鈥搗erb鈥搊bject", "subjective_objective": {"answer": "Subjective", "rationale": "Because \"respect for others\" is the patient's perception and attitude about how to get along with people, and it's a subjective view."}, "sentence_sentiment": {"answer": "Positive", "rationale": "Because the entry emphasizes honest respect for others, it is a positive quality and attitude."}, "language_style": {"answer": "Tactful and warm", "rationale": "The entry is more personal and interactive in its expression of conversation in a gentle and inviting tone, rather than a direct statement or order. At the same time, it conveys positive values, which are interesting and supportive, through the use of terms such as \"honesty\" and \"respect\"."}, "time_dimension": {"answer": "Present", "rationale": "The entry describes an act or attitude, with no clear time indication or time-related description, and therefore the judgement is now."}, "sentence_type": "Imperative sentence", "pos": {"nouns": {"Respect": 2, "person": 1, "Honesty": 1}, "verbs": {"Respect": 1, "Yeah": 1, "Honesty": 1}, "numerals": {"2": 1}}}, {"content": "3. Be responsive: effective response to any situation;", "word_count": 18, "label": "", "sentence_structure": "Subject鈥搗erb鈥揷omplement", "subjective_objective": {"answer": "Subjective", "rationale": "Because being well attuned to the patient ' s own coping capacity, attitude and manner is a subjective aspect."}, "sentence_sentiment": {"answer": "Positive", "rationale": "Because the description of \"adopportunity, effective response\" in the entry is positive."}, "language_style": {"answer": "Tactful and warm", "rationale": "The entry is a more gentle and friendly expression of the characteristic of \"comprehensive\" in an encouraging and positive way, by emphasizing an effective response and conveying a positive upward and problem-solving message."}, "time_dimension": {"answer": "Future", "rationale": "The entry uses the word \"capable\" to express the acquisition of a capability or skill, which usually occurs at some point in the future. At the same time, the phrase \"any situation can respond effectively\" also indicates that the testee needs to have this ability to respond to change in the future and can therefore be judged as a future behavior and mindset."}, "sentence_type": "Affirmative declarative sentence", "pos": {"nouns": {"Change": 1, "Response": 1}, "verbs": {"Good at it": 1, "Make it": 2}, "adjectives": {"It works": 1}, "adverbs": {"Any": 1, "Both": 1}}}, {"content": "4 Implicit: self-restrainting and enthusiasm.", "word_count": 11, "label": "", "sentence_structure": "Subject鈥搗erb鈥揷omplement", "subjective_objective": {"answer": "Subjective", "rationale": "Because the words \"insinuated,\" \"self-restraint\", \"emotionality and enthusiasm\" describe the patient's own identity or orientation, require the patient's subjective cognitive and emotional experience to answer, and are therefore subjective in nature."}, "sentence_sentiment": {"answer": "Positive", "rationale": "Because \"self-restrainting emotions and enthusiasm\" are positive qualities and attitudes."}, "language_style": {"answer": "Tactful and warm", "rationale": "The language style of the entry is more euphemistic and moderate, expressing concern about individual emotional management capabilities in a less lax way, and encouraging respondents to show positive emotional attitudes. This expression reduces the pressure on respondents and leads them to share real ideas and feelings."}, "time_dimension": {"answer": "Present", "rationale": "The keywords \"self-restraint\" and \"hotness\" represent an attitude or behaviour of the individual that is now being carried out. It is therefore the present implicit expression of the testee that can be judged."}, "sentence_type": "Affirmative declarative sentence", "pos": {"nouns": {"Intense": 1, "Self-restraint": 1, "Emotion": 1, "Ardent": 1}, "adjectives": {"Intense": 1, "Self-restraint": 1}, "numerals": {"4": 1}}}, {"content": "Live and live: life and excitement;", "word_count": 12, "label": "", "sentence_structure": "Subject鈥搗erb鈥揷omplement", "subjective_objective": {"answer": "Subjective", "rationale": "Because \"vitable\" is a subjective feeling and experience, and it depends on individual perceptions, emotions, and attitudes. Although \"invigorating and excited\" is an objective state of things, the description emphasizes subjective feelings, and the answer is subjective."}, "sentence_sentiment": {"answer": "Positive", "rationale": "Because \"vio-vio\" means life and excitement, and it's a positive adjective."}, "language_style": {"answer": "Tactful and warm", "rationale": "The entry describes the \"vigorating\" character in a positive and encouraging manner, using a graphic metaphor that allows respondents to better understand and feel the meaning of the concept. This expression is more moderate, friendly and powerful, and helps to stimulate the motivation and initiative of the respondents."}, "time_dimension": {"answer": "Present", "rationale": "The entry now describes a state or feature, referring to the current feeling or situation, so the temporal dimension is now judged."}, "sentence_type": "Affirmative declarative sentence", "pos": {"nouns": {"- It's alive": 1, "Life Force": 1, "Excited": 1}, "verbs": {"Fill": 1}}}, {"content": "2. Satisfied: easy to accept any situation and environment;", "word_count": 13, "label": "", "sentence_structure": "Subject鈥搗erb鈥揷omplement", "subjective_objective": {"answer": "Subjective", "rationale": "Because \"Easy to accept any situation and the environment\" is a personal feeling and attitude that belongs to the subjective perspective of the patient."}, "sentence_sentiment": {"answer": "Positive", "rationale": "Because the \"easy to accept anything and the environment\" attitude is positive."}, "language_style": {"answer": "Tactful and warm", "rationale": "The language style of the entry is more euphemistic and warm, and it is more soft and friendly to express, in an encouraging tone, the characteristics that the other party can easily adapt to in the face of different circumstances and circumstances."}, "time_dimension": {"answer": "Present", "rationale": "The terms \"easy to accept\" and \"any circumstances and the environment\" indicate that this is a description of the current state and can therefore be judged as it is."}, "sentence_type": "Affirmative declarative sentence", "pos": {"nouns": {"2 Satisfied": 1, "Situation": 1, "Environment": 1}, "verbs": {"Accept": 1}, "adjectives": {"Easy": 1}, "adverbs": {"Any": 1}}}, {"content": "3 Sensitivity: excessive attention to surrounding personnel;", "word_count": 12, "label": "", "sentence_structure": "Subject鈥搗erb鈥揷omplement", "subjective_objective": {"answer": "Subjective", "rationale": "Because \"excessive care\" is the patient's subjective attitude and feelings, and it is a subjective assessment of the surrounding people."}, "sentence_sentiment": {"answer": "Negative", "rationale": "Because the \"too much care\" act in the entry is negative."}, "language_style": {"answer": "Tactful and warm", "rationale": "The language style of the entry is more euphemistic. It does not directly point to the shortcomings or shortcomings of the person surveyed, but rather uses an implicit expression of what needs to be investigated, making it easier for the person surveyed to accept and answer questions."}, "time_dimension": {"answer": "Present", "rationale": "The entry describes the person's current feelings and behaviour, the keywords being \"sensitive\" and \"excessively caring\" and all point to the current state or situation. Thus, the temporal dimension of the article is now judged."}, "sentence_type": "Affirmative declarative sentence", "pos": {"nouns": {"Sensitive": 1, "Around": 1, "Personnel": 1}, "verbs": {"Care": 1}, "adjectives": {"That's too much": 1}}}, {"content": "Self-reliance: independence is strong and relies on its own abilities, judgement and talents.", "word_count": 19, "label": "", "sentence_structure": "Subject鈥搗erb鈥揷omplement", "subjective_objective": {"answer": "Subjective", "rationale": "Because \"independent, self-reliant, judgement-based and intellectual\" are self-evaluations and attitudes of individual patients and are subject to subjective examination."}, "sentence_sentiment": {"answer": "Positive", "rationale": "Because the entries \"reliance\" and \"reliance on your own ability, judgment, and intelligence\" express a positive quality or attitude."}, "language_style": {"answer": "Tactful and warm", "rationale": "The language style of the entry is relatively gentle and friendly, and there is no too direct or strong language, but is expressed in a way that encourages and suggests, and is more readily acceptable and cooperative to the respondents."}, "time_dimension": {"answer": "Present", "rationale": ""}, "sentence_type": "Affirmative declarative sentence", "pos": {"nouns": {"Self-reliance": 1, "Independence": 1, "Capacity": 1, "Judgement": 1, "Genius": 1}, "verbs": {"Strong": 1, "Dependence": 1, "Only": 1, "be": 1}, "adjectives": {"self": 1}, "numerals": {"4": 1}}}, {"content": "Planner 1: First, to make a detailed plan and to proceed strictly without changing it;", "word_count": 22, "label": "", "sentence_structure": "Subject鈥搗erb鈥搊bject", "subjective_objective": {"answer": "Subjective", "rationale": "Because it is the patient ' s subjective attitude to like to plan and to apply it strictly."}, "sentence_sentiment": {"answer": "Positive", "rationale": "Because \"do a detailed plan\" and \"clausely require a plan\" in the entries are positive."}, "language_style": {"answer": "Calm and direct", "rationale": "The language style of the entry is more direct, not too much adoration or emotional, and more factual and objective. It expresses the characteristics of the group of \"planners\" in a simple language, which is more than detailed planning before action is taken, and is strictly enforced. The language style of the entry can therefore be described as \"cool and straight.\""}, "time_dimension": {"answer": "Past", "rationale": "\"Preliminarily planned, and strictly planned, without changing the words \"first done,\" \"and\" and \"yes\" in the words \"first done\" means that it is a completed or ongoing act or state, so the entry describes past behaviour."}, "sentence_type": "Affirmative declarative sentence", "pos": {"nouns": {"Planner": 1, "Planned": 1}, "verbs": {"Do it": 1, "Conduct": 1, "Yes": 1, "Changes": 1}, "adjectives": {"Detailed": 1, "Strict": 1}}}, {"content": "(b) Patience: without delay, calm and tolerant;", "word_count": 15, "label": "", "sentence_structure": "Subject鈥搗erb鈥揷omplement", "subjective_objective": {"answer": "Subjective", "rationale": "Because it involves the patient ' s inner feelings and attitudes, it is subjective."}, "sentence_sentiment": {"answer": "Positive", "rationale": "Because the \"no delay\" and \"cool and tolerated\" in the entries are positive."}, "language_style": {"answer": "Tactful and warm", "rationale": "Warm and warm."}, "time_dimension": {"answer": "Present", "rationale": "The questionnaire entry asks if the person tested has such qualities, so the time dimension is now judged."}, "sentence_type": "Affirmative declarative sentence", "pos": {"nouns": {"Patience": 1, "Delay": 1}, "verbs": {"Not due": 1, "I'm upset": 1, "It's okay": 1, "Calm down": 1}, "adjectives": {"2": 1, "and": 1, "Calm down": 1, "Tolerance": 1}, "adverbs": {"Not due": 1}, "numerals": {"2": 1}}}, {"content": "3 Active: Believing in their ability to turn their lives into security;", "word_count": 14, "label": "", "sentence_structure": "Subject鈥搗erb鈥揷omplement", "subjective_objective": {"answer": "Subjective", "rationale": "Because this is a patient ' s assessment of his confidence and attitude, it is subjective."}, "sentence_sentiment": {"answer": "Positive", "rationale": "Because \"believing in your ability to turn to safety\" is a positive attitude and belief."}, "language_style": {"answer": "Calm and direct", "rationale": "The entry uses a concise language style, which directly expresses the meaning and requirements of the problem, without excessive ado and emotional colours.\""}, "time_dimension": {"answer": "Present", "rationale": ""}, "sentence_type": "Affirmative declarative sentence", "pos": {"nouns": {"Capacity": 1}, "verbs": {"Yes": 1, "have": 1, "\"and return to safety.\"": 1}, "adjectives": {"Positive": 1}, "numerals": {"3": 1}}}, {"content": "4 Promoters: use character charms or encourage others to participate.", "word_count": 16, "label": "", "sentence_structure": "Subject鈥搗erb鈥搊bject", "subjective_objective": {"answer": "Subjective", "rationale": "Because \"using character charm or encouraging others to participate\" is the attitude and behaviour of the patient himself, the subjective perspective is being examined."}, "sentence_sentiment": {"answer": "Positive", "rationale": "Because \"invited character\" and \"encouraged participation\" are positive behaviours that inspire others to participate actively."}, "language_style": {"answer": "Tactful and warm", "rationale": "The language style of the entry is more euphemistic, and it invites or leads the interviewee to participate actively by using positive words such as \"use\", \"encourage\" and other forms of direct command or threat."}, "time_dimension": {"answer": "Present", "rationale": "The entry describes a way of acting in the current situation (i.e. \"with character charm\" and \"to encourage others to participate\"), and thus judges \"now\"."}, "sentence_type": "Affirmative declarative sentence", "pos": {"nouns": {"Promoter": 1, "Personal charm": 1, "Encourage": 1, "others": 1, "Participation": 1}, "verbs": {"Utilization": 1, "or": 1}, "numerals": {"4": 1}}}, {"content": "1 Positive: confidence, little hesitation or wavering;", "word_count": 12, "label": "", "sentence_structure": "Subject鈥搗erb鈥揷omplement", "subjective_objective": {"answer": "Subjective", "rationale": "Because self-confidence, hesitation or wavering are subjective feelings and attitudes of patients."}, "sentence_sentiment": {"answer": "Positive", "rationale": "Because \"confident\" and \"very little hesitation or wavering\" in the entries are positive qualities."}, "language_style": {"answer": "Tactful and warm", "rationale": "The language style of the entry is more euphemistic. First, it starts with the word \"yes,\" which shows that it is a positive attitude and an assessment that gives a good first impression. Second, it is expressed in a relatively moderate way, without too direct or hard language, and gives people a comfortable feeling. Finally, \"confidence, little hesitation or wavering\" is also described as a sense of encouragement and support, and of understanding and respect."}, "time_dimension": {"answer": "Present", "rationale": "The key words \"Acknowledging: confidence\" and \"very little hesitation or wavering\" can be used to judge that the entry describes the emotional state or character characteristics of the person being tested."}, "sentence_type": "Affirmative declarative sentence", "pos": {"nouns": {"Questionnaire Entry": 1, "Confidence": 1, "Whispering": 1, "Shake": 1}, "verbs": {"Sure": 1}, "adjectives": {"Very few": 1}}}, {"content": "2 Free from restrictions: do not like or are held hostage by a plan;", "word_count": 18, "label": "", "sentence_structure": "Subject鈥搗erb鈥揷omplement", "subjective_objective": {"answer": "Subjective", "rationale": "This is a description of the patient ' s habits and attitudes and a reflection of subjective perceptions and perceptions."}, "sentence_sentiment": {"answer": "Neutral", "rationale": "Because the entry does not have any emotional color."}, "language_style": {"answer": "Tactful and warm", "rationale": ""}, "time_dimension": {"answer": "Present", "rationale": "The entry describes the current state and preferences of the testee, the key words \"free\" , \"not like advance planning\" and \"be held hostage\" point to the present situation."}, "sentence_type": "Affirmative declarative sentence", "pos": {"nouns": {"Questionnaire Entry": 1, "Constraints": 1, "Planned": 2, "Hold": 1}, "verbs": {"Yeah": 1, "Advance": 1, "Planned": 1, "Hold": 1}, "adjectives": {"Free and free": 1}, "numerals": {"2": 1}}}, {"content": "3 Humiliation: quiet, not good at talking;", "word_count": 9, "label": "", "sentence_structure": "Subject鈥搗erb鈥揷omplement", "subjective_objective": {"answer": "Subjective", "rationale": "Because shame is a subjective assessment, it describes the character and behaviour of the patient."}, "sentence_sentiment": {"answer": "Negative", "rationale": "Because the word \"silent\" is often seen as a negative character that means that this person is reluctant to communicate with others."}, "language_style": {"answer": "Tactful and warm", "rationale": "Warm and warm."}, "time_dimension": {"answer": "Past", "rationale": ""}, "sentence_type": "Affirmative declarative sentence", "pos": {"nouns": {"Humiliation": 1, "Quiet": 1, "Talk": 1}, "adjectives": {"Humiliation": 1, "Quiet": 1, "Not good": 1}, "numerals": {"3": 1}}}, {"content": "Timeliness: Life depends on schedule, and does not like plans being interfered with.", "word_count": 22, "label": "", "sentence_structure": "Subject鈥搗erb鈥搊bject", "subjective_objective": {"answer": "Subjective", "rationale": "Because \"time-sensitive\" and \"not like plans being interfered with\" are subjective evaluations and interpretations of patients' behaviour and attitudes."}, "sentence_sentiment": {"answer": "Negative", "rationale": "Because the attitude of \"not like the plan being interfered with\" in the entry is negative."}, "language_style": {"answer": "Calm and direct", "rationale": "The entry is more directly written, without too many qualifiers or rhetorical techniques. It explicitly asks whether the interviewee prefers to schedule his or her life and does not like others disrupting his or her own plans, which is more objective and direct and which is also more calm."}, "time_dimension": {"answer": "Present", "rationale": "The entry describes a current pattern of behaviour or tendency, i.e. \"dependent schedules\" and \"not like plans being interfered with\", and does not address past or future behaviour. The questionnaire entry can therefore be judged as now."}, "sentence_type": "Affirmative declarative sentence", "pos": {"nouns": {"Timescale": 1, "Schedule": 1, "person": 1, "Planned": 1}, "verbs": {"- It's a matter of action": 1, "Dependence": 1, "Yeah": 1, "By": 1, "Interference": 1}, "adverbs": {"have": 1, "not": 1}, "numerals": {"4": 1}, "classifiers": {"individual": 1}, "pronouns": {"person": 1}}}, {"content": "1 Relocation: to change itself to act in coordination with others and at their request in a short time;", "word_count": 23, "label": "", "sentence_structure": "Subject鈥搗erb鈥揷omplement", "subjective_objective": {"answer": "Subjective", "rationale": "Because the terms \"change yourself\" and \"do as others require\" relate to the patient's own decision-making and behavioural choices, this is viewed from the patient's subjective perspective."}, "sentence_sentiment": {"answer": "Positive", "rationale": "Because the \"replacement\" act in the entry, while seemingly making compromises and concessions, is in fact a compromise and concession for the purpose of reaching consensus and building good relations. Such behaviour can be seen as positive in some cases, because it helps to promote harmonious human relations."}, "language_style": {"answer": "Tactful and warm", "rationale": ""}, "time_dimension": {"answer": "Present", "rationale": ""}, "sentence_type": "Affirmative declarative sentence", "pos": {"nouns": {"Relocation": 1, "self": 1, "Others": 2, "Coordination": 1, "Time": 1, "Request": 1}, "verbs": {"Change": 1, "Press": 1, "Act": 1}, "adverbs": {"Short term": 1}, "numerals": {"1": 4}, "pronouns": {"I": 1}}}, {"content": "2 A structured one: a systematic and structured one;", "word_count": 16, "label": "", "sentence_structure": "Subject鈥搗erb鈥搊bject", "subjective_objective": {"answer": "Subjective", "rationale": "Because \"situation\" is an adjective term used to describe human characteristics or behavioural characteristics, which is usually based on subjective observation and evaluation. Thus, \"situation\" is subjective."}, "sentence_sentiment": {"answer": "Neutral", "rationale": "Because the entry does not have any emotional color."}, "language_style": {"answer": "Tactful and warm", "rationale": "The entry is presented in descriptive language, emphasizing the characteristics of a person who has a systematic and structured approach to the matter. At the same time, a more moderate and encouraging tone is used, without direct criticism or direction of how the other party should do it, which is more personal and interactive."}, "time_dimension": {"answer": "Past", "rationale": "Past, present"}, "sentence_type": "Affirmative declarative sentence", "pos": {"nouns": {"The well is organized": 1, "person": 1}, "verbs": {"have": 1, "Arrangements": 1}, "adjectives": {"System": 1, "Structure": 1}, "classifiers": {"individual": 1}}}, {"content": "3 Frank: without reservations, speak frankly;", "word_count": 10, "label": "", "sentence_structure": "Subject鈥搗erb鈥揷omplement", "subjective_objective": {"answer": "Subjective", "rationale": "Because \"clarity\" is a subjective attitude or character characteristic of a person, it reflects whether a person is willing to express his thoughts and feelings in a genuine manner. This is irrelevant to objective facts and is therefore subjective."}, "sentence_sentiment": {"answer": "Positive", "rationale": "For frankness, unreservedness and the quality of statements are positive."}, "language_style": {"answer": "Tactful and warm", "rationale": "The entry is relatively euphemistic and is described in terms such as \"confident\" and \"unreserved\" but is presented in a more moderate and less direct or harsh manner. At the same time, through the introduction of questions, respondents are encouraged to speak actively, and the questionnaire is more interactive and participatory."}, "time_dimension": {"answer": "Present", "rationale": "According to the expression \"scientific\" and \"unreserved, frank statements\", the entry describes an attitude or behaviour that is not directly related to past or future conduct, and therefore the temporal dimension is now judged."}, "sentence_type": "Imperative sentence", "pos": {"nouns": {"- Frank": 1, "Presentations": 1}, "verbs": {"No reservation": 1, "- Frank": 1}}}, {"content": "4 Optimistic: Make others and themselves believe that everything will be better.", "word_count": 18, "label": "", "sentence_structure": "Subject鈥搗erb鈥搊bject", "subjective_objective": {"answer": "Subjective", "rationale": "Because optimism is an individual subjective attitude or belief that does not depend on whether a specific event occurs or not. Therefore, it is the subjective view of the patient that makes people and themselves believe that anything will improve."}, "sentence_sentiment": {"answer": "Positive", "rationale": "Because the word \"optimistic\" in the entry expresses a positive mood and the description of the purpose of the article tends to be positive."}, "language_style": {"answer": "Tactful and warm", "rationale": "The entry uses positive and encouraging language to describe optimism, that is, to give positive results by convincing people and themselves that anything can change. This language style is more moderate, friendly and inspiring, more resonant and willing to cooperate with the questionnaire."}, "time_dimension": {"answer": "Present", "rationale": "The entry asks the testee about his current attitude or conviction, and the keywords \"optimistic\" and \"confident that everything will improve\" point to the current attitude and perception. Thus, the time dimension of the entry is now judged."}, "sentence_type": "Affirmative declarative sentence", "pos": {"nouns": {"Optimistic": 1, "Others": 1, "self": 1, "Things": 1, "Better": 1}, "verbs": {"You're": 1, "Yes": 1}, "adverbs": {"Any": 1}, "numerals": {"4": 1}}}, {"content": "1 Forced: giving orders and forcing others to obey;", "word_count": 13, "label": "", "sentence_structure": "Subject鈥搗erb鈥揷omplement", "subjective_objective": {"answer": "Objective", "rationale": "The basis for the judgement is to examine whether the patient's subjective attitude or objective facts are. For the entry in the questionnaire, \"Forcibleness: Issue orders, compel others to listen to them; \" , the observation is that it describes an act or situation and should therefore be objective. So the answer is \"Objective.\""}, "sentence_sentiment": {"answer": "Negative", "rationale": "Because the words \"order\" and \"coercion\" in the entries are negative."}, "language_style": {"answer": "Calm and direct", "rationale": "The language style of the entry is relatively calm and straightforward, and it clearly identifies a behaviour characteristic - \"ordering\" and \"coercing others to listen to\" - without too many words or qualms, and with objectivity and simplicity. This type of description usually applies to issues that require a clear statement or warning, and can convey information directly, reducing misunderstandings and ambiguity."}, "time_dimension": {"answer": "Past", "rationale": "The entry describes a behavioural characteristic or character that does not involve the temporal dimension of the past, present or future and therefore cannot be judged by past/present/future."}, "sentence_type": "Imperative sentence", "pos": {"nouns": {"Forced": 1, "Order": 1, "Others": 1}, "verbs": {"Forced": 1, "Listen": 1}}}, {"content": "2 Faithful: consistent, reliable, faithful and sometimes unfounded;", "word_count": 19, "label": "", "sentence_structure": "Subject鈥搗erb鈥揷omplement", "subjective_objective": {"answer": "Subjective", "rationale": "Because the description of \"loyalty\" is primarily concerned with the patient's personal qualities and attitudes, which are usually examined from a subjective perspective. While this quality may in some cases affect objective results, in evaluating this entry it is more concerned with whether the patient's attitude and behaviour are consistent with this qualitative definition."}, "sentence_sentiment": {"answer": "Positive", "rationale": "Because \"sustained, faithful\" is a positive quality, and \"sometimes unfounded\" indicates that this person is willing to give for others and is a positive emotional attitude."}, "language_style": {"answer": "Tactful and warm", "rationale": "The entry uses a more euphemistic and implied language style rather than a direct statement or order. For example, the words \"consistently reliable\" and \"faithful\" express a positive and positive attitude and values, but do not overemphasize or criticize them, but rather in a moderate way to guide the interviewee to reflect on whether his behaviour and attitude are consistent with those standards. Moreover, \"sometimes, unfounded dedication\" also uses a more euphemism, expressing understanding of and respect for loyalty, without directly accusing or criticizing the shortcomings of the interviewee, and displaying a higher level of linguistic and artistic skills."}, "time_dimension": {"answer": "Past", "rationale": "The words \"consistent\" and \"sometimes\" in the entry describe past experiences and behaviours, and therefore judge the past."}, "sentence_type": "Affirmative declarative sentence", "pos": {"nouns": {"Faith": 1, "Reliable": 1, "Faithful": 1, "Give": 1}, "verbs": {"Always": 1, "be": 1, "I'm not sure I'm gonna do this": 1, "Give": 1}, "adjectives": {"Reliable": 1, "Faithful": 1}, "adverbs": {"Sometimes": 1}}}, {"content": "3 Interesting: funny, funny, making everything a good story;", "word_count": 20, "label": "", "sentence_structure": "Subject鈥揷opula鈥損redicative", "subjective_objective": {"answer": "Subjective", "rationale": "The blogger says that the government is not a party to the law, because it is a subjective attitude or behaviour of individuals who are funny, funny and telling stories."}, "sentence_sentiment": {"answer": "Positive", "rationale": "Basis: The words \"joy\" and \"joy\" are all positive emotional and attitudinal expressions."}, "language_style": {"answer": "Tactful and warm", "rationale": "The entry is more euphemistic and gentle, asking the interviewees in an invitation tone whether they have such characteristics. At the same time, the entry uses positive terms such as \"interest\", \"interest\", \"joke\", \"joy,\" etc., which help the interviewee to feel comfortable and to answer questions."}, "time_dimension": {"answer": "Present", "rationale": ""}, "sentence_type": "Affirmative declarative sentence", "pos": {"nouns": {"Story": 1}, "verbs": {"鈾?Turn into 鈾?: 1, "Speak": 1}, "adjectives": {"Interesting": 1, "Funny": 1, "Humor": 1}}}, {"content": "4 Friendly: No talking, no argument.", "word_count": 11, "label": "", "sentence_structure": "Subject鈥搗erb鈥揷omplement", "subjective_objective": {"answer": "Objective", "rationale": "Because this is a description of the patient's behaviour, it does not involve subjective attitudes or feelings of the patient. \"No talking, no arguing\" is an expression of objective behaviour."}, "sentence_sentiment": {"answer": "Negative", "rationale": "Because the expression \"no initiative\" and \"no argument\" in this article are both negative."}, "language_style": {"answer": "Calm and direct", "rationale": "This is a relatively calm, objective and direct description that is consistent with the serene style of alienation."}, "time_dimension": {"answer": "Present", "rationale": ""}, "sentence_type": "Affirmative declarative sentence", "pos": {"nouns": {"Friendly": 1, "Talk": 1, "Arguments": 1}, "verbs": {"No initiative": 1, "Love": 1}, "numerals": {"4": 1}}}, {"content": "1 Brave: dare to take risks without fear;", "word_count": 10, "label": "", "sentence_structure": "Subject鈥損redicate", "subjective_objective": {"answer": "Subjective", "rationale": "Because bravery is a subjective attitude and personality of a human being."}, "sentence_sentiment": {"answer": "Positive", "rationale": "Because the word \"bold\" is often seen as a positive quality or attitude, a sign of fear of danger and difficulty and a dare to challenge oneself."}, "language_style": {"answer": "Calm and direct", "rationale": "This definition is expressed in positive language, without the use of negative terms or descriptions, such as fear, cowardice, etc., giving a positive and encouraging feeling. Moreover, the article is formulated in a concise, easy to understand, and without too many complex professional terms and concepts, which helps respondents to accept and answer questions more easily. Thus, the language style of the article can be judged \" brave\" according to the above analysis."}, "time_dimension": {"answer": "Past", "rationale": "The temporal dimension of the purpose of the article cannot therefore be judged as past, present or future, but rather describes a static personality."}, "sentence_type": "Affirmative declarative sentence", "pos": {"nouns": {"Brave": 1, "Adventure": 1, "Fearless": 1}, "adjectives": {"How dare you": 1}}}, {"content": "2. Be considerate: be respectful and patient;", "word_count": 9, "label": "", "sentence_structure": "Subject鈥搗erb鈥揷omplement", "subjective_objective": {"answer": "Subjective", "rationale": "Because \"good\" and \"good\" and \"satisfaction\" are subjective attitudes or behaviour."}, "sentence_sentiment": {"answer": "Positive", "rationale": "Because \"be nice,\" \"be nice,\" \"be patient\" are positive qualities."}, "language_style": {"answer": "Tactful and warm", "rationale": "The language style of the entry is more euphemistic, because it uses the terms \"thanky,\" \"goody\" and \"satisfactionful\" , which give people a warm, warm feeling. At the same time, the entry does not use direct orders or criticism, but requests are made in a way that is encouraging and supportive, and that is more acceptable and followed."}, "time_dimension": {"answer": "Present", "rationale": "The keywords \"to be decent\" and \"to be patient\" describe a behaviour that is carried out in the current life and is therefore judged by the time dimension of the article."}, "sentence_type": "Affirmative declarative sentence", "pos": {"nouns": {"Kind": 1, "person": 1, "Patience": 1}, "verbs": {"- Wait": 1}, "adjectives": {"Nice": 1}}}, {"content": "3 Note the details: observations are minimal and things are done in an orderly manner;", "word_count": 15, "label": "", "sentence_structure": "Subject鈥搗erb鈥揷omplement", "subjective_objective": {"answer": "Subjective", "rationale": "Because \"watch the details\", \"watch the intuitive\" and \"do things in a methodical manner\" are terms that describe subjective feelings or attitudes such as the patient's own mentalities, behavioral habits, etc."}, "sentence_sentiment": {"answer": "Positive", "rationale": "Because \"watch the details\", \"observation in the micro\" and \"doing things in a methodical manner\" are positive terms."}, "language_style": {"answer": "Tactful and warm", "rationale": "The entry is more adept and interactive when it is spoken in a gentle and inviting manner, rather than by direct statements or orders."}, "time_dimension": {"answer": "Present", "rationale": "The entry describes the habits or characteristics of the person being tested and does not address past or future acts or events and is therefore present."}, "sentence_type": "Imperative sentence", "pos": {"nouns": {"Attention": 1, "Details": 1, "Observation": 1, "Things": 1, "Structure": 1}, "verbs": {"Attention": 1, "Do it": 1, "Observation": 1, "It's a little organized": 1}, "adjectives": {"Micro-introducing": 1, "Structure": 1}, "adverbs": {"Attention": 2, "It's a little organized": 1}, "classifiers": {"3": 1, "individual": 1}}}, {"content": "4 Cute: Happy, fun to have with others.", "word_count": 13, "label": "", "sentence_structure": "Subject鈥搗erb鈥揷omplement", "subjective_objective": {"answer": "Subjective", "rationale": "Because \"happy\" and \"happy to be with others\" are subjective feelings and attitudes of patients."}, "sentence_sentiment": {"answer": "Positive", "rationale": "Because the entries \"happy\" and \"joyful\" are positive words."}, "language_style": {"answer": "Tactful and warm", "rationale": "The adjective \"Cute\" is used to describe the feelings of the interviewees, giving them a warm, warm feeling, a more euphemistic style of language. At the same time, it makes the whole issue more positive and warm by expressing the pleasure and pleasure of the interviewees in their interpersonal interactions."}, "time_dimension": {"answer": "Present", "rationale": "The entry asks the testee for the definition and understanding of \"cute\" and the temporal dimension is now judged."}, "sentence_type": "Affirmative declarative sentence", "pos": {"nouns": {"Questionnaire Entry": 1, "Cute": 1, "Happy": 1, "Others": 1, "Get along": 1, "Fun": 1}, "verbs": {"Analysis": 1, "and": 1}, "adjectives": {"4": 1}, "numerals": {"4": 1}}}, {"content": "One is happy: to be dynamic and to pass happiness on to others;", "word_count": 16, "label": "", "sentence_structure": "Subject鈥揷opula鈥損redicative", "subjective_objective": {"answer": "Subjective", "rationale": "Because \"vio\" and \"transmit happiness to others\" are patients' subjective feelings and behaviour of happiness, and that is the subjective point of view."}, "sentence_sentiment": {"answer": "Positive", "rationale": "Because the entry \"Four and pass happiness on to others\" expresses a positive emotional attitude."}, "language_style": {"answer": "Tactful and warm", "rationale": "The entry is more personal and interactive when it is spoken in a gentle and inviting tone, rather than in direct statements or orders. At the same time, positive expressions such as \"viable\" and \"transforming happiness to others\" provide the interviewee with a pleasant and comfortable response atmosphere."}, "time_dimension": {"answer": "Present", "rationale": ""}, "sentence_type": "Affirmative declarative sentence", "pos": {"nouns": {"Questionnaire Entry": 1, "person": 2, "Happy": 4, "Dynamic": 1}, "verbs": {"Fill": 1, "Pass": 1}, "adjectives": {"- I'm sorry": 1, "Happy": 1, "Vibrant": 1}, "numerals": {"1": 3}}}, {"content": "2. Cultural upbringing: special interest in art, such as theatre and symphony;", "word_count": 19, "label": "", "sentence_structure": "Subject鈥搗erb鈥揷omplement", "subjective_objective": {"answer": "Subjective", "rationale": "Because love of art and academia is a subjective attitude or hobby."}, "sentence_sentiment": {"answer": "Positive", "rationale": "Because the words \"special hobby\" and \"art science\" are all positive emotional expressions."}, "language_style": {"answer": "Tactful and warm", "rationale": "The language style of the entry is more euphemistic and, by asking questions, leads the respondents to share their artistic and academic preferences and express them in a more friendly and intimate manner."}, "time_dimension": {"answer": "Present", "rationale": "The entry asks the testee about the current level of cultural upbringing and hobby, so the temporal dimension is now judged."}, "sentence_type": "Affirmative declarative sentence", "pos": {"nouns": {"Cultural rehabilitation": 1, "Arts": 1, "Academic": 1, "Theatre": 1, "Symphony": 1}, "verbs": {"Hobbies": 1}, "numerals": {"2": 1}}}, {"content": "3. Self-confidence: confident of one ' s individual abilities and success;", "word_count": 13, "label": "", "sentence_structure": "Subject鈥搗erb鈥揷omplement", "subjective_objective": {"answer": "Subjective", "rationale": "Because \"confidence\" is an individual belief and attitude, and it is a subjective dimension of thinking and feeling."}, "sentence_sentiment": {"answer": "Positive", "rationale": "Because \"convincing one's own abilities and success\" is a positive, positive emotion and attitude."}, "language_style": {"answer": "Tactful and warm", "rationale": "The language style of the entry is more euphemistic and emotional, and by using positive words such as \"confirming\" and \"success\" it attempts to encourage the interviewee to express his or her self-confidence, without direct orders or criticism. This type of questionnaire is usually easier to obtain the cooperation and trust of the interviewee.\""}, "time_dimension": {"answer": "Present", "rationale": "The keywords \"convinced\" and \"personal ability and success\" emphasize the subjective feelings and attitudes of the testee, which is a description of the degree of confidence that the testee has at present. Moreover, the word \"confident\" implies that this is a question of mental state. In conclusion, the entry is to question the testee in his current state of mind, that is, now."}, "sentence_type": "Affirmative declarative sentence", "pos": {"nouns": {"Confidence": 1, "Success": 1}, "verbs": {"Sure": 1}}}, {"content": "4 Follow-up: steady and consistent.", "word_count": 15, "label": "", "sentence_structure": "Subject鈥搗erb鈥揷omplement", "subjective_objective": {"answer": "Objective", "rationale": "Because \"suspensive\" and \"salary\" are observed behaviors or states, and are objective descriptions."}, "sentence_sentiment": {"answer": "Positive", "rationale": "Because \"smooth\" and \"continue\" are positive behavioural qualities."}, "language_style": {"answer": "Tactful and warm", "rationale": ""}, "time_dimension": {"answer": "Present", "rationale": ""}, "sentence_type": "Affirmative declarative sentence", "pos": {"nouns": {"Follow-up": 1, "Emotion": 1, "Things": 1}, "verbs": {"Steady": 1, "Do it": 1, "Hold on": 1, "Follow-up": 1}, "adverbs": {"Always": 1}}}, {"content": "Ideasism: to imagine new things by its own perfect standards;", "word_count": 20, "label": "", "sentence_structure": "Subject鈥搗erb鈥搊bject", "subjective_objective": {"answer": "Subjective", "rationale": "This is a reflection of patients ' self-evaluation and subjective attitudes."}, "sentence_sentiment": {"answer": "Positive", "rationale": "Because \"idealism\" in the entries is often considered a positive attitude and quality, indicating a desire for new things and a vision. While the \"perfect criteria\" in the entries may be interpreted as negative, throughout the description, this attitude is more seen as a positive force."}, "language_style": {"answer": "Calm and direct", "rationale": "The definition of \"idealism\" is directly illustrated by the contents of the entry, which is not based on any rhetorical or emotional rendering, and is more objective in tone. 2. The entry presents a definition of the concept in a concise and quick expression, rather than in vague or ambiguous language, which is consistent with the calm and straightforward language style."}, "time_dimension": {"answer": "Present", "rationale": "The entry describes a tendency or preference to \"presume the measure of new things by its own perfect criteria.\" This tendency or preference is present, and thus judgment is now."}, "sentence_type": "Affirmative declarative sentence", "pos": {"nouns": {"Idealism": 1, "Standard": 1, "Scenario": 1, "Measurement": 1, "Things": 1}, "verbs": {"Here": 1, "Imagine": 1, "Measurement": 1}, "adjectives": {"Perfect": 1}, "numerals": {"1": 2}}}, {"content": "Independence: self-sufficient, self-sufficient and self-sufficient, without the need for help;", "word_count": 17, "label": "", "sentence_structure": "Subject鈥搗erb鈥揷omplement", "subjective_objective": {"answer": "Subjective", "rationale": "Because independent descriptions are based more on the views and feelings of the patient than on objective facts or behaviour."}, "sentence_sentiment": {"answer": "Positive", "rationale": "Because the words \"independent self-confidence\" and \"without the help of others\" in the entries are all positive emotions and attitudes."}, "language_style": {"answer": "Tactful and warm", "rationale": "The entry is more euphemistic. It does not use a direct negative or prescriptive language, but rather an invitation to encourage respondents to think about their independence and self-confidence, and provides a self-evaluation perspective. Nor does it use too negative or offensive terms to give people a friendly and friendly feeling."}, "time_dimension": {"answer": "Present", "rationale": "The keywords \"independence\" and \"self-sufficiency\" indicate that this is a description of the person being tested in his or her current state or character, and therefore judgement is now."}, "sentence_type": "Affirmative declarative sentence", "pos": {"nouns": {"Independence": 2, "Confidence": 1, "Others": 1}, "verbs": {"Yes": 1, "Help": 1}, "numerals": {"2": 1}}}, {"content": "3 Non-aggressive: not to say or do things that may cause dissatisfaction and opposition;", "word_count": 23, "label": "", "sentence_structure": "Subject鈥搗erb鈥揷omplement", "subjective_objective": {"answer": "Objective", "rationale": "Because offensiveness is a subjective attitude or behaviour, \"to say nothing or do something that may cause dissatisfiedness and opposition\" is an objective fact that describes such attitudes or behaviour."}, "sentence_sentiment": {"answer": "Negative", "rationale": "Because the entry's behaviour \"does not say or do anything that may cause disaffection and opposition\" is negative and expresses an attitude of avoiding conflict and maintaining harmony."}, "language_style": {"answer": "Calm and direct", "rationale": ""}, "time_dimension": {"answer": "Present", "rationale": "According to the description \"no offensive\" of \"speech or do\" it can be seen that the entry is asking about the behaviour of the person tested. At the same time, \"what may cause dissatisfaction and opposition\" also indicates that it is an assessment of the present conduct. Thus, the time dimension of the article is now."}, "sentence_type": "Affirmative declarative sentence", "pos": {"nouns": {"Aggressive": 1, "Things": 1}, "verbs": {"Say it": 1, "Do it": 1, "Cause": 1, "Dissatisfied": 1, "Objection": 1}, "adjectives": {"None": 1}, "numerals": {"3": 1}, "classifiers": {"individual": 1}}}, {"content": "4 is inspiring: encouraging others to participate, join and make everything interesting.", "word_count": 22, "label": "", "sentence_structure": "Subject鈥搗erb鈥揷omplement", "subjective_objective": {"answer": "Subjective", "rationale": "Because descriptions like \"inspiring\" and \" making everything interesting\" are based on patients' personal views and attitudes. These descriptions reflect patients' views on how to encourage others and make work or activity interesting, so they are subjective."}, "sentence_sentiment": {"answer": "Positive", "rationale": "Because the entry \"Encourages others to participate, join and make everything interesting\" shows that the person has a positive influence and social ability to engage others actively and is therefore a positive act or quality."}, "language_style": {"answer": "Tactful and warm", "rationale": "The language style of the entry is euphemism. First, the phrase \"inspiration\" conveys positive emotional and encouraging meanings, giving people a warm feeling. Second, \"incentives to other people to participate, to join\" and \"get everything interesting,\" which are more gentle and contagious, more resonant and supportive, and fit the warmness of language style."}, "time_dimension": {"answer": "Present", "rationale": "The actions of \"encourage\" and \"participate\" and \"getting interesting\" are all happening now, so the time dimension of this article is now."}, "sentence_type": "Imperative sentence", "pos": {"nouns": {"Incentives": 2, "others": 1, "Participation": 1, "Add": 1, "Things": 1, "Interesting": 1}, "verbs": {"Encourage": 1, "鈾?And become 鈾?: 1}, "adjectives": {"Rich": 1}, "numerals": {"4": 1}}}, {"content": "1. Emotional exposure: Never concealing feelings... preferences, and often speaking with people without having to contact them;", "word_count": 24, "label": "", "sentence_structure": "Subject鈥搗erb鈥揷omplement", "subjective_objective": {"answer": "Subjective", "rationale": "Because emotions, preferences and behaviours are subjective experiences and manifestations of patients."}, "sentence_sentiment": {"answer": "Positive", "rationale": "Because the words \"emotional appearance\" and \"never disguise emotion, pleasure\" in the entries are positive and express an open and frank attitude."}, "language_style": {"answer": "Tactful and warm", "rationale": "The language style of the entry is more euphemistic and warm, and it triggers a resonance with the other. In the form of expression, the other person is not asked directly or ordered to answer, but is guided by a gentle and inviting tone to reflect on the behaviour of the other. This makes the person surveyed more comfortable and relaxed, and thus more willing to reflect his or her situation."}, "time_dimension": {"answer": "Present", "rationale": "The entry describes the sexual behaviour of the person being tested, which often touches people in conversations without control and often does not conceal his feelings and preferences. Such behaviour is taking place and therefore falls within the context of the present state of affairs."}, "sentence_type": "Affirmative declarative sentence", "pos": {"nouns": {"Emotion": 2, "Cover it up": 1, "I like it": 1, "Talk": 1, "Others": 1}, "verbs": {"not": 2, "Exposure": 1}, "adjectives": {"Show": 1, "I can't help it": 1}, "numerals": {"Regular": 1}, "classifiers": {"Body": 1}, "pronouns": {"self": 1}}}, {"content": "Deep: deep and often in-house, and aversion to superficial conversations and entertainment;", "word_count": 20, "label": "", "sentence_structure": "Subject鈥搗erb鈥揷omplement", "subjective_objective": {"answer": "Subjective", "rationale": "Deepness is the patient's subjective attitude."}, "sentence_sentiment": {"answer": "Negative", "rationale": "Because the entry \"to hate superficial conversation, to entertain\" is a negative emotional expression."}, "language_style": {"answer": "Tactful and warm", "rationale": "Warm and warm."}, "time_dimension": {"answer": "Past", "rationale": "In conclusion, the output is: \"Now/Before\"."}, "sentence_type": "Affirmative declarative sentence", "pos": {"nouns": {"Deep": 2, "Province": 1, "Talk": 1, "Fun": 1, "Disgusting": 1}, "adjectives": {"Often": 1}, "adverbs": {"Yes": 1}, "numerals": {"2": 1}}}, {"content": "Decisive: the ability to make judgements and conclusions quickly;", "word_count": 15, "label": "", "sentence_structure": "Subject鈥搗erb鈥揷omplement", "subjective_objective": {"answer": "Subjective", "rationale": "Because \"decisive\" is a human character or ability, it needs to be understood by observation or self-reporting, and it is the patient's subjective perspective."}, "sentence_sentiment": {"answer": "Positive", "rationale": "Because \"decisive\" is a positive quality that shows that individuals have the capacity to make rapid decisions and to act."}, "language_style": {"answer": "Calm and direct", "rationale": "The language of the entry is clear and concise, without too many rhetorical or emotional features, but rather directly describes the ability required by the characteristic of \"decisive\" to make a quick judgement and draw conclusions. Such expression is more objective and accurate and meets the professional requirements of the questionnaire survey."}, "time_dimension": {"answer": "Present", "rationale": "The keyword \"with\" and \"capable\" indicates that the entry describes the characteristics or state of the person currently tested, so the temporal dimension is now judged."}, "sentence_type": "Affirmative declarative sentence", "pos": {"nouns": {"Decisive": 1, "Judgement": 1, "Conclusions": 1}, "verbs": {"have": 1, "Do it": 1, "Capacity": 1}, "adverbs": {"very": 1}, "numerals": {"3": 1}}}, {"content": "4 Humor: A nice and calm sense of humor.", "word_count": 13, "label": "", "sentence_structure": "Subject鈥搗erb鈥揷omplement", "subjective_objective": {"answer": "Objective", "rationale": "Because humor is an expression or style, and it is a description based on facts, it is objective."}, "sentence_sentiment": {"answer": "Positive", "rationale": "Because the quality of humor is positive. Although the phrases are described as \"peaceful\" and \"cool\", they are not negative emotional, but rather a compliment for humor. So the emotional color of the article is positive."}, "language_style": {"answer": "Tactful and warm", "rationale": "The language style of the entry is euphemism. It uses the terms \"peaceful\" and \"cool\" to describe humor, to give people a sense of moderation, and to suggest that humor requires calm and rationality, which makes the whole description more friendly and friendly. Moreover, the word \"speech\" shows respect for and attention to the feelings and attitudes of the respondents, which further increases the warmth of the questionnaire."}, "time_dimension": {"answer": "Future", "rationale": "The entry expresses expectations of the testee's future behaviour or attitude, and the keyword \"will\" indicates that the act has not yet taken place but is expected to occur in the future."}, "sentence_type": "Affirmative declarative sentence", "pos": {"nouns": {"Humor": 2, "- You're a good guy": 1, "Calm down": 1}, "verbs": {"have": 1}, "adjectives": {"Peace": 1, "Calm down": 1}, "adverbs": {"And": 1, "Land": 1}, "numerals": {"4": 1}}}, {"content": "1 Mediator: the parties are often in the middle of reconciling different opinions in order to avoid conflict between them;", "word_count": 22, "label": "", "sentence_structure": "Subject鈥搗erb鈥搊bject", "subjective_objective": {"answer": "Subjective", "rationale": "Because the role and conduct of the mediator are determined by his subjective judgement and will."}, "sentence_sentiment": {"answer": "Positive", "rationale": "Because the mediators are active and aim to resolve conflicts and promote harmony."}, "language_style": {"answer": "Tactful and warm", "rationale": "The entry uses descriptive terms such as \"mediators\", \"regulars\", \"moderns\", \"different opinions\", etc., and positive emotional colours (avoiding conflict between the two sides), which are more moderate and friendly in their expression and facilitate closer proximity between the interviewees and the investigators."}, "time_dimension": {"answer": "Present", "rationale": ""}, "sentence_type": "Affirmative declarative sentence", "pos": {"nouns": {"Mediator": 1, "Observations": 1, "Conflict": 1}, "verbs": {"Centred": 1, "Reconciliation": 1, "Avoid": 1}, "adverbs": {"Regular": 1}}}, {"content": "Musical: A hobby of participation and a deeper appreciation, because of the artistic nature of music, not because of the pleasure of performance", "word_count": 33, "label": "", "sentence_structure": "Subject鈥搗erb鈥搊bject", "subjective_objective": {"answer": "Subjective", "rationale": "Because love for music is the patient's subjective attitude."}, "sentence_sentiment": {"answer": "Positive", "rationale": "The entry shows a positive emotional attitude towards the love and deep appreciation of the artistic nature of music."}, "language_style": {"answer": "Tactful and warm", "rationale": "The entry is more personal and interactive when it is spoken in a gentle and inviting manner, rather than in direct statements or orders. It attempts to understand whether the interviewee has a deep interest in and appreciation of music, and emphasizes art rather than the pleasure of performance, which is both polite and acceptable."}, "time_dimension": {"answer": "Present", "rationale": "According to the description of the time words \"now\" and \"with greater appreciation\" in the input sentence, the entry is to ask the testee's current situation or habits and can therefore be judged as \"now\"."}, "sentence_type": "Affirmative declarative sentence", "pos": {"nouns": {"Musical": 1, "Hobbies": 1, "Participation": 1, "Capacity": 1, "Artistic": 1, "Fun": 1}, "verbs": {"I'm sure you'll find it very interesting": 1, "Because": 2, "- A showman": 1}, "adjectives": {"Deeper": 1, "Artistic": 1}, "numerals": {"2": 1}, "pronouns": {"Because": 1}}}, {"content": "3. Sponsor: efficient promoter, leader of others, unable to exercise his or her functions;", "word_count": 20, "label": "", "sentence_structure": "Subject鈥搗erb鈥揷omplement", "subjective_objective": {"answer": "Subjective", "rationale": "However, if the questionnaire is intended to assess the objective characteristics of the patient鈥檚 social skills, leadership, etc., then the entry may require more information to prove whether the sponsor is really the efficient promoter and the leader of others. In this case, the entry may be considered subjective, as it is based on the author鈥檚 perception and description."}, "sentence_sentiment": {"answer": "Positive", "rationale": "This item is a reflection of the high leadership and motivation of the sponsors."}, "language_style": {"answer": "Calm and direct", "rationale": "The entry describes the characteristics and behaviour of the initiator in a concise and clear language, without too many emotional colours or qualifiers."}, "time_dimension": {"answer": "Present", "rationale": "The purpose of the questionnaire is to describe the characteristics and behaviour of the initiators, using the current state of the art as \"means that it is a description of the current state and that the entry should therefore be classified as \"now\"."}, "sentence_type": "Affirmative declarative sentence", "pos": {"nouns": {"Sponsor": 1, "Efficiency": 1, "Promoter": 1, "Others": 1, "Leader": 1, "I can't help it": 1}, "verbs": {"be": 1, "Promotion": 1, "Leadership": 1}, "adjectives": {"Efficient": 1}, "adverbs": {"I can't help it": 1}, "pronouns": {"he": 1}}}, {"content": "4-Gayfriends: Love to be around a party, and make new friends who don't treat anyone as strangers", "word_count": 25, "label": "", "sentence_structure": "Subject鈥搗erb鈥搊bject", "subjective_objective": {"answer": "Subjective", "rationale": "Because it's the patient's subjective attitude to like to go around and make new friends."}, "sentence_sentiment": {"answer": "Positive", "rationale": "Because the article is positive, it expresses attitudes and preferences towards friendship, without negative emotions or negative behaviour."}, "language_style": {"answer": "Tactful and warm", "rationale": "The entry is a soft, inviting conversation, expressing a love for socialization and a good understanding of new people. This expression is more comfortable and acceptable to the interviewees and is willing to share their real thoughts and feelings."}, "time_dimension": {"answer": "Present", "rationale": "The keyword \"likes to be a new friend\" and \"good friends\" happens now or recently, so time dimensions are now judged."}, "sentence_type": "Affirmative declarative sentence", "pos": {"nouns": {"friends": 2, "Party": 1, "Stranger": 1}, "verbs": {"Hand it over": 1, "Yeah": 1, "Good friends": 1, "Round": 1, "Not right": 1}, "adjectives": {"New": 1}, "adverbs": {"No, I'm not": 1}, "numerals": {"4": 1}, "pronouns": {"Anyone": 1}}}, {"content": "1 Considers it thoughtful: understanding people, helping people, remembering special days;", "word_count": 19, "label": "", "sentence_structure": "Subject鈥搗erb鈥揷omplement", "subjective_objective": {"answer": "Subjective", "rationale": "Because \"good people\" and \"help others\" and \"remember special days\" are subjective behaviour and attitudes of patients."}, "sentence_sentiment": {"answer": "Positive", "rationale": "Because the words \"considerate\" and \"cute\" and \"helping others\" and \"remembering special days\" are all positive."}, "language_style": {"answer": "Tactful and warm", "rationale": "The entry uses the active words \"thank you\" and \"help others\" and \"remember special days\" and uses the term \"considered\" as the general theme, which is more sweet, encouraging and interesting."}, "time_dimension": {"answer": "Present", "rationale": "The entry describes a behaviour or attitude characteristic, namely, \"considerate\" and gives specific examples. There are no clear time indicators in these examples, but normally we assume that the act is a continuous state or customary conduct, and therefore can be judged as now.\""}, "sentence_type": "Imperative sentence", "pos": {"nouns": {"That's very thoughtful": 1, "It's a good understanding": 1, "person": 2, "Day": 1}, "verbs": {"Consider": 1, "Help": 1, "Remember": 1, "Special": 1}, "adjectives": {"It's a good understanding": 1, "Special": 1}, "adverbs": {"That's very thoughtful": 1}, "numerals": {"1": 6}, "classifiers": {"individual": 2}, "pronouns": {"others": 1}}}, {"content": "2: Do not fulfil their objectives and do not desist;", "word_count": 10, "label": "", "sentence_structure": "Subject鈥損redicate", "subjective_objective": {"answer": "Subjective", "rationale": "Because it is a subjective attitude and mentality, a strong pursuit of and adherence to goals. While such an attitude may affect objective behaviour and outcomes, it is essentially a subjective psychological state."}, "sentence_sentiment": {"answer": "Positive", "rationale": "Because the behavior in the entry \"failed, never stopped\" is positive."}, "language_style": {"answer": "Calm and direct", "rationale": "The entry describes a personality characteristic in a concise and clear language 鈥?adherence, no rhetorical or emotional rendering, and a more objective tone."}, "time_dimension": {"answer": "Present", "rationale": ""}, "sentence_type": "Affirmative declarative sentence", "pos": {"verbs": {"Hold on": 1, "Da-da": 1, "Stop": 1}, "adjectives": {"not": 2}, "adverbs": {"Decision": 1, "No, I won't": 1}}}, {"content": "(a) More than three words: constant talk, jokes to entertain others, and the embarrassment of silence should be avoided;", "word_count": 30, "label": "", "sentence_structure": "Subject鈥搗erb鈥搊bject", "subjective_objective": {"answer": "Subjective", "rationale": "Because \"the embarrassment of feeling the silence should be avoided\" is the patient's subjective idea."}, "sentence_sentiment": {"answer": "Positive", "rationale": "Because the \"speak and laugh and entertain\" act is positive."}, "language_style": {"answer": "Tactful and warm", "rationale": "\"Slow and warm.\""}, "time_dimension": {"answer": "Past", "rationale": "The entry describes the testee's past or customary behaviour, namely, \"continuous speech\" and \"negatively avoids silence\", and therefore judges the past."}, "sentence_type": "Affirmative declarative sentence", "pos": {"nouns": {"Word": 2, "A joke": 1, "Recreation": 1, "Others": 1, "Silence": 1, "Awkward": 1}, "verbs": {"Keep talking": 1, "Speak": 1, "I think": 1, "Should be avoided": 1, "Bring it here": 1}, "adverbs": {"I'm gonna have to talk to you": 1, "I think so": 1, "Avoid": 1}, "numerals": {"3": 1}}}, {"content": "Tolerance: People ' s ideas and opinions are easy to accept and do not need to object or change.", "word_count": 23, "label": "", "sentence_structure": "Subject鈥搗erb鈥揷omplement", "subjective_objective": {"answer": "Subjective", "rationale": "Because \"acceptable\" and \"no need to object or change\" attitudes are patients' subjective feelings and choices."}, "sentence_sentiment": {"answer": "Positive", "rationale": "For tolerance is a positive quality, which expresses understanding and acceptance of the different views and ideas of others."}, "language_style": {"answer": "Tactful and warm", "rationale": "The entry is more intimate and interactive when it is spoken in a gentle and inviting manner, rather than in direct statements or orders. At the same time, the entry is worded in more polite and polite terms, without using too strong or direct language, and more in line with the investigative context and requirements of psychological questionnaires."}, "time_dimension": {"answer": "Present", "rationale": "The entry describes the current attitude and behaviour of the testee, the keywords being \"acceptable\", \"no objection\" and \"change,\" all implying the current state. So, the time dimension of this questionnaire entry is now."}, "sentence_type": "Affirmative declarative sentence", "pos": {"nouns": {"Tolerance": 1, "Thoughts": 1, "Observations": 1, "Others": 1}, "verbs": {"Admissible": 1, "Yes": 1, "Objection": 1, "Change": 1}, "adjectives": {"4": 1}}}, {"content": "1 Listener: Listeners are willing to listen to others;", "word_count": 10, "label": "", "sentence_structure": "Subject鈥搗erb鈥揷omplement", "subjective_objective": {"answer": "Subjective", "rationale": "Because \"willing to listen to others\" is a subjective attitude and choice of a person, not an objective fact or behaviour."}, "sentence_sentiment": {"answer": "Positive", "rationale": "Because the act of \"willing to hear others\" in the entry is a positive attitude, that is, a caring, supportive attitude."}, "language_style": {"answer": "Tactful and warm", "rationale": "The language style of the entry is euphemistic. First, the term \"listen\" gives a positive impression that the other person is needed to be the object of a talk. Second, the sentence as a whole uses an invitation tone, expressing the desire to listen to the other person's needs and expectations, and is more gentle, friendly and friendly."}, "time_dimension": {"answer": "Present", "rationale": "The words \"will\" and \"hear\" in the entry indicate that this is a current act, i.e. whether the person tested is willing to listen to others. So, the time dimension is now."}, "sentence_type": "Affirmative declarative sentence", "pos": {"nouns": {"Listener": 1, "Speech": 1}, "verbs": {"Yes": 1, "Listen": 1}}}, {"content": "(b) The absolute loyalty of loyalty to one ' s own ideals, friends and work, sometimes without even a reason;", "word_count": 26, "label": "", "sentence_structure": "Subject鈥搗erb鈥揷omplement", "subjective_objective": {"answer": "Subjective", "rationale": "Because \"absolutely faithful, sometimes without a reason\" is the patient's subjective attitude."}, "sentence_sentiment": {"answer": "Positive", "rationale": "Because the behavior in the entry is positive: \"Favoritely faithful to your ideals, friends, work.\""}, "language_style": {"answer": "Tactful and warm", "rationale": "The language style of the entry is more moderate, using words such as \"absolute loyalty\", \"not even justification\" to express loyalty and commitment to the ideal, and more softer. Moreover, the use of the words \"friends, jobs\" makes the entry more humane and universal, and more resonant and identity-based."}, "time_dimension": {"answer": "Present", "rationale": "The entry now describes an act or attitude of the person being tested, namely \"loyalty\". Although there is no clear time indicator, the general current state of affairs is used to express such an attitude. Thus, the temporal dimension of the entry is now judged."}, "sentence_type": "Affirmative declarative sentence", "pos": {"nouns": {"Faithful": 1, "Ideal": 1, "friends": 1, "Work": 1, "Rationale": 1}, "verbs": {"to": 2, "self": 2, "I'm absolutely faithful": 1, "Not even": 1}, "adverbs": {"Sometimes": 1}, "pronouns": {"self": 2}}}, {"content": "3. Leaders: born leadership, who do not believe that others are better than themselves;", "word_count": 21, "label": "", "sentence_structure": "Subject鈥搗erb鈥搊bject", "subjective_objective": {"answer": "Subjective", "rationale": "Because \"breed leadership\" and \"no one's abilities are greater than they are\" are based on the subjective views of the questionnaire designers, and are not objective facts."}, "sentence_sentiment": {"answer": "Negative", "rationale": "Because the leadership style described in the entry is self-centered and distrustful, which is a negative attitude."}, "language_style": {"answer": "Calm and direct", "rationale": "The entry presents a characteristic of the type of leader in a concise language style, with no excessive rhetorical or emotional rendering, and a more objective tone."}, "time_dimension": {"answer": "Present", "rationale": "The keyword \"innate leadership\", \"inbelief of others is more than self\" suggests that it is a description of a view or phenomenon that currently exists, and the temporal dimension of the article is now."}, "sentence_type": "Affirmative declarative sentence", "pos": {"nouns": {"Leader": 2, "Capacity": 1}, "verbs": {"Yes": 1, "- Yeah": 1}, "adjectives": {"It's natural": 1, "Your own": 1}, "numerals": {"3": 1}}}, {"content": "4 Vibrant: dynamic, energetic.", "word_count": 12, "label": "", "sentence_structure": "Subject鈥揷opula鈥損redicative", "subjective_objective": {"answer": "Subjective", "rationale": "Because \"vio\" and \"energy\" are the feelings and experiences of the patient, and they are subjective."}, "sentence_sentiment": {"answer": "Positive", "rationale": "Because \"vio\" and \"energy\" are positive adjectives, which are healthy and energetic."}, "language_style": {"answer": "Tactful and warm", "rationale": "The language style of the entry is more euphemistic and warm. It uses active vocabulary, \"viable\" and \"viable,\" and expresses attitudes towards the interviewee in a way that encourages and supports it. This moderate tone makes the questionnaire more attractive and more acceptable."}, "time_dimension": {"answer": "Present", "rationale": "Now the entry describes the current state of the testee, \"viocity\" and \"energy\" are adjectives used to describe the current state or feeling. So it's now."}, "sentence_type": "Affirmative declarative sentence", "pos": {"nouns": {"Dynamic": 2, "鈾?Full of 鈾?: 2}, "verbs": {"Fill": 1, "- The energy": 1, "鈾?Full of 鈾?: 1}, "adjectives": {"He's alive": 1}}}, {"content": "1. Sufficient: to satisfy what is in his possession, and to envy little;", "word_count": 15, "label": "", "sentence_structure": "Subject鈥搗erb鈥揷omplement", "subjective_objective": {"answer": "Subjective", "rationale": "Because satisfaction is the patient's subjective attitude."}, "sentence_sentiment": {"answer": "Positive", "rationale": "Because the \"satisfaction\" attitude is positive, indicating a sense of satisfaction and gratitude."}, "language_style": {"answer": "Tactful and warm", "rationale": "The entry is more personal and interactive in its expression of conversation in a gentle and encouraging tone, rather than a direct statement or order. At the same time, by using a description like \"very little envy\", it highlights a sense of well-being and avoids too direct an assessment, making respondents feel more comfortable and comfortable."}, "time_dimension": {"answer": "Present", "rationale": "The entry represents a subjective attitude or feeling and does not involve past or future conduct, so the temporal dimension is now judged."}, "sentence_type": "Affirmative declarative sentence", "pos": {"nouns": {"Satisfied": 2, "self": 1, "Possession": 1, "others": 1}, "verbs": {"Envious": 1}, "adverbs": {"Very few": 1}, "numerals": {"1": 2}, "pronouns": {"self": 1}}}, {"content": "2 Chiefs: call for leadership and follow by others;", "word_count": 13, "label": "", "sentence_structure": "Subject鈥搗erb鈥搊bject", "subjective_objective": {"answer": "Subjective", "rationale": "Because \"require leadership and follow\" is a subjective attitude and behaviour of a person, it reflects the subjective ideas and demands of patients for authority, control and interpersonal relations."}, "sentence_sentiment": {"answer": "Negative", "rationale": "Because the \"requirement of leadership\" may involve a tendency to abuse power and control others, which are negative."}, "language_style": {"answer": "Calm and direct", "rationale": "The language style of the entry is calm and straightforward, describing the characteristics of type 2 leaders through simple words such as \"request\", \"leadership\" and \"following.\" There are no excessive rhetorical or emotional colours."}, "time_dimension": {"answer": "Present", "rationale": "According to the keywords \"required\" and \"in the process of being held/owned\", the entry describes the person being tested in the present state or behaviour and thus judged to be now."}, "sentence_type": "Imperative sentence", "pos": {"nouns": {"Chief": 1, "Leadership": 1, "others": 1, "Follow": 1}, "verbs": {"Request": 1, "Follow": 1}, "numerals": {"2": 1}}}, {"content": "3 Mapers: use graphic figures to organize life and solve problems;", "word_count": 17, "label": "", "sentence_structure": "Subject鈥搗erb鈥搊bject", "subjective_objective": {"answer": "Objective", "rationale": "The way and method of behaviour of cartographers are viewed from an objective perspective, by using charts and data to manage and solve problems in life. While this approach may be influenced by individual subjective ideas, it is here that the actual actions and behaviour choices of cartographers are more important than their subjective attitudes or feelings."}, "sentence_sentiment": {"answer": "Positive", "rationale": "Because the action of \"organizing life with graphics, solving problems\" is positive."}, "language_style": {"answer": "Tactful and warm", "rationale": "For the given entry \"3 cartographer: organise life and solve problems with graphic numbers,\" it can be considered as a calm and direct expression. It is more concise, not too much accommodative or emotional, and more focused on communicating information and functional descriptions. At the same time, use symbols; it also shows some calm and reason, not a soft and warm language style. So, in sum, the language style of the purpose is considered to be straightforward."}, "time_dimension": {"answer": "Present", "rationale": "The entry describes the use of graphs and figures by the \"magramographer\" to deal with life problems, without specifying whether the act has occurred or will occur in the past. Thus, the time dimension is now."}, "sentence_type": "Affirmative declarative sentence", "pos": {"nouns": {"Graphicsman": 2, "Chart": 1, "Numbers": 1, "Life": 1, "Problem": 1}, "verbs": {"Use": 1, "Come on": 1, "Organizations": 1, "Resolve": 1}, "numerals": {"3": 1}}}, {"content": "4 Lovers: People's focus, favorite.", "word_count": 15, "label": "", "sentence_structure": "Subject鈥搗erb鈥揷omplement", "subjective_objective": {"answer": "Subjective", "rationale": "The entry describes what people think of them, and what people think and feel is usually subjective. So the assessment of this entry should also be subjective. On the other hand, while \"hot\" can be considered an objective criterion or characteristic (for example, a person may indeed be susceptible to attention because of their appearance, character or other characteristics), it emphasizes this subjective feeling or evaluation (i.e. the level of preference for the patient)."}, "sentence_sentiment": {"answer": "Positive", "rationale": "Because \"fun\" and \"fun\" are positive emotional expressions, indicating that the person being evaluated has a popular and popular character."}, "language_style": {"answer": "Tactful and warm", "rationale": "The language style of the entry is more euphemistic. It uses the positive term \"hot-to-pop\" and emphasizes people's attention and preferences, and the way people express themselves is more natural, and there is no too direct or too cold. This expression helps to create a good atmosphere of communication, making respondents more willing to participate in the questionnaire."}, "time_dimension": {"answer": "Present", "rationale": "The entry describes the person's own state or characteristics, and the keyword is \"like\", indicating that this is a present situation, not a past or a future."}, "sentence_type": "Affirmative declarative sentence", "pos": {"nouns": {"person": 2, "Centre": 1}, "verbs": {"Attention": 1, "Yeah": 1}, "adjectives": {"It's so sweet": 1}, "numerals": {"4": 1}}}, {"content": "1 Perfectist: Order is high for yourself, for others, for everything;", "word_count": 22, "label": "", "sentence_structure": "Subject鈥搗erb鈥揷omplement", "subjective_objective": {"answer": "Subjective", "rationale": "Because of the patient ' s subjective assessment and perception of himself."}, "sentence_sentiment": {"answer": "Positive", "rationale": "Because the \"perfectist\" described in the entry is demanding for himself and others, which is usually considered a positive attitude. And the reference to \"ordered things\" in the entry is a positive feature. The purpose of the article is therefore expressed in a positive emotional way."}, "language_style": {"answer": "Calm and direct", "rationale": ""}, "time_dimension": {"answer": "Past", "rationale": "So the term \"perfectist\" is the subject of the article or the keyword, and \"high standards, orderly things\" is the way it is developed and described. Time dimensions are not judged, because the entry does not clearly indicate whether this feature has been, is being, or will be."}, "sentence_type": "Affirmative declarative sentence", "pos": {"nouns": {"Perfect": 1, "self": 1, "others": 1, "Things": 1, "Order": 1}, "verbs": {"have": 2}, "adjectives": {"High": 1, "Standard": 1, "Order": 1}, "numerals": {"1": 4}, "pronouns": {"they": 1}}}, {"content": "2-Assist: Easy to live, easy to speak, easy to approach;", "word_count": 13, "label": "", "sentence_structure": "Subject鈥搗erb鈥揷omplement", "subjective_objective": {"answer": "Subjective", "rationale": "Because \"soft\" is a character of a human being, a subjective attitude of a patient."}, "sentence_sentiment": {"answer": "Positive", "rationale": "Because the descriptions in the entries are positive terms, this quality is positive."}, "language_style": {"answer": "Tactful and warm", "rationale": "The language style of the entry is mainly descriptive, and the positive and positive messages are conveyed by using terms such as \"coherent\" and \"spoken\" and are not too much direct instruction or negative evaluation, so that the euphemism of language can be judged as warm."}, "time_dimension": {"answer": "Present", "rationale": "The entry describes the personality character \"2 and gas\" of the individual, in which the verbs \"coherent\", \"cobalt\" and \"sophisticate\" are the general current, indicating that it is the individual's current behaviour or characteristic."}, "sentence_type": "Affirmative declarative sentence", "pos": {"nouns": {"2-Assist": 1}, "verbs": {"Easy": 3, "Jean": 1}, "adjectives": {"person": 1, "Close": 1}, "numerals": {"2": 1}}}, {"content": "3 Work: work, work, and no rest;", "word_count": 16, "label": "", "sentence_structure": "Subject鈥搗erb鈥揷omplement", "subjective_objective": {"answer": "Subjective", "rationale": "Because \"working\" is a subjective attitude and behavioral habit of a human being."}, "sentence_sentiment": {"answer": "Negative", "rationale": "Because \"stop-work\" and \"no rest\" have negative emotional aspects."}, "language_style": {"answer": "Calm and direct", "rationale": "The language style of the entry is more straightforward and realistic in describing a behaviour characteristic 鈥?hard work. It does not have too much emotional accoutrements, nor does it use euphemism to guide respondents to answer questions, but is presented in an objective way, so language style is calm and straightforward."}, "time_dimension": {"answer": "Present", "rationale": ""}, "sentence_type": "Affirmative declarative sentence", "pos": {"nouns": {"I'm very hardworking": 1, "Work": 2, "Tasks": 1, "Rest": 1}, "verbs": {"鈾?And it's not stopping 鈾?: 1, "Completed": 1, "Yes": 1, "Rest": 1}, "adjectives": {"3": 1}, "numerals": {"3": 1}}}, {"content": "4 Welcome: the soul figure at the gathering, the welcome guest.", "word_count": 17, "label": "", "sentence_structure": "Subject鈥搗erb鈥揷omplement", "subjective_objective": {"answer": "Subjective", "rationale": "Because the words \"popular,\" \"soul figure,\" and \"guest\" express the subjective attitude of patients who think they are liked and valued by others."}, "sentence_sentiment": {"answer": "Positive", "rationale": "Because the entries describe \"soul characters\" and \"popular guests\", these words are usually considered positive."}, "language_style": {"answer": "Tactful and warm", "rationale": "The language style of the entry is euphemism, because it describes a certain type of person in a friendly and respectful manner, without directly pointing to their shortcomings or errors. This expression is more acceptable and comfortable."}, "time_dimension": {"answer": "Present", "rationale": "The entry describes a current situation or characteristic, namely, \"soul figures at a gathering and are welcome\", and can therefore be judged as now."}, "sentence_type": "Affirmative declarative sentence", "pos": {"nouns": {"Party": 1, "Spiritual characters": 1, "Guests": 2}, "verbs": {"Received": 1, "Welcome": 2}, "adjectives": {"Hot": 1}, "numerals": {"4": 1}}}, {"content": "1 Jump: dynamic and dynamic;", "word_count": 12, "label": "", "sentence_structure": "Subject鈥搗erb鈥揷omplement", "subjective_objective": {"answer": "Subjective", "rationale": "This description evaluates the attitudes and feelings of the patient and is a subjective one."}, "sentence_sentiment": {"answer": "Positive", "rationale": "Because the description of \"vio-vio-vio\" in the entry is positive and expresses positive emotional attitudes."}, "language_style": {"answer": "Tactful and warm", "rationale": ""}, "time_dimension": {"answer": "Present", "rationale": "The entry describes the characteristics or characteristics of the person being tested, and does not indicate a clear time-line, so it can be judged as now."}, "sentence_type": "Affirmative declarative sentence", "pos": {"nouns": {"Jump": 1, "Dynamic": 1, "- Yes, he's very lively": 1}, "adjectives": {"Fill": 1}}}, {"content": "2 Fearless: bold progress without risk;", "word_count": 10, "label": "", "sentence_structure": "Subject鈥搗erb鈥揷omplement", "subjective_objective": {"answer": "Subjective", "rationale": "Because \"Bold move forward, fear of adventure\" is the patient's subjective attitude and thoughts, self-evaluation of the fearlessness."}, "sentence_sentiment": {"answer": "Positive", "rationale": "Great. Because the words \"dauntless,\" \"boldly moving forward\" and \"never risking\" in the entries are positive words, expressing a positive and courageous attitude.\""}, "language_style": {"answer": "Tactful and warm", "rationale": "The language style of the entry is euphemistic, using the encouraging words \"bold move forward\", \"no risk\" and this expression can inspire the active attitude and willingness of the respondents to participate."}, "time_dimension": {"answer": "Present", "rationale": "The keywords \"dauntless,\" \"boldly moving forward\" and \"not afraid of risk\" all indicate a positive attitude or behaviour, all describing the person's current state or ability. So, the entry primarily asks about the person's current character or behavioural orientation, and the temporal dimension is now."}, "sentence_type": "Imperative sentence", "pos": {"nouns": {"Fearless": 1, "Forward": 1, "Adventure": 1}, "verbs": {"Fearless": 1, "Forward": 1, "Yes": 1}}}, {"content": "3. Normative: always upholding a moral code of consent;", "word_count": 21, "label": "", "sentence_structure": "Subject鈥搗erb鈥搊bject", "subjective_objective": {"answer": "Subjective", "rationale": "Because \"identity\" represents the values and beliefs of the individual and is a subjective view of the individual."}, "sentence_sentiment": {"answer": "Positive", "rationale": "Because the entry expresses a positive quality, that is, the individual should observe and insist on ethical behaviour."}, "language_style": {"answer": "Tactful and warm", "rationale": "The entry uses positive language, expressing expectations and encouragement to the interviewees in an invitation manner, rather than direct orders or criticism. At the same time, the term \"time and time\" also demonstrates a long-standing concern and continuity of the behaviour of the interviewees, with a certain degree of moderation and accompaniment."}, "time_dimension": {"answer": "Past", "rationale": "The entry describes the behaviour of the testee, i.e., adherence to accepted ethical norms at all times."}, "sentence_type": "Affirmative declarative sentence", "pos": {"nouns": {"Normative": 1, "Behave": 1, "Ethics": 1}, "verbs": {"Hold on": 1, "Yes": 1}, "adjectives": {"Time": 1, "Your own": 1}, "numerals": {"3": 1}}}, {"content": "Balance: Steady, intermediate.", "word_count": 9, "label": "", "sentence_structure": "Subject鈥搗erb鈥揷omplement", "subjective_objective": {"answer": "Objective", "rationale": "Because \"balance\" is a stable state, neutral, impartial, objective state. And \"stabilize, go the middle way\" is an objective expression of that state. So the point of view of this questionnaire is objective."}, "sentence_sentiment": {"answer": "Neutral", "rationale": "Because the entry does not express positive or negative feelings, attitudes, behaviours, aims or qualities. It merely describes a characteristic or characteristic."}, "language_style": {"answer": "Tactful and warm", "rationale": "The term \"stabilized\" and \"intermediate\" are used to convey the concept of balance, and are not too direct or rigid. This moderate and implicit approach helps to create a harmonious, friendly atmosphere that is more likely to resonate and respond to the respondents."}, "time_dimension": {"answer": "Future", "rationale": "The entry describes the expectations and pursuits of an ideal state of affairs, namely, to maintain stability and to find the best compromise among the various options, which involves future plans and actions. The temporal dimension is therefore judged in terms of the future."}, "sentence_type": "Affirmative declarative sentence", "pos": {"nouns": {"Balance": 1, "Steady": 1, "Intermediate route": 1}, "verbs": {"Let's go": 1}, "adjectives": {"Ping": 1, "Medium": 1, "Steady": 1}, "numerals": {"4": 1}}}, {"content": "1 Boring: dead and indignant;", "word_count": 10, "label": "", "sentence_structure": "Subject鈥揷opula鈥損redicative", "subjective_objective": {"answer": "Subjective", "rationale": "Because boredom is a subjective feeling and evaluation."}, "sentence_sentiment": {"answer": "Negative", "rationale": "Because \"silent\" means that things lack vitality and appeal, it's a negative expression."}, "language_style": {"answer": "Tactful and warm", "rationale": "The entry uses the relatively euphemistic term \"silent\" to describe a situation, without directly using negative or offensive language. Moreover, the expression \"silent, infuriated\" is also a somewhat subjective and emotional expression, with more temperature and humanized features. It is therefore considered to be a euphemism."}, "time_dimension": {"answer": "Present", "rationale": "The entry describes a sense or attitude to something, does not clearly point to time, and therefore the judgement is now."}, "sentence_type": "Negative declarative sentence", "pos": {"nouns": {"Boring": 1, "Dead": 1, "Angry": 1}, "adjectives": {"Boring": 1, "He's dead": 1, "Missing": 1}}}, {"content": "2-: To avoid attention and to avoid naturality;", "word_count": 19, "label": "", "sentence_structure": "Subject鈥搗erb鈥搊bject", "subjective_objective": {"answer": "Objective", "rationale": "Because \"sweet\", \"shunting people's attention\", \"unnaturally\" are the behaviour of patients, not their subjective feelings or attitudes, and this is seen from an objective perspective."}, "sentence_sentiment": {"answer": "Negative", "rationale": "Because the behavior in the entries \"shun people's attention\" and \"unnaturally under the eyes of people\" are all negative."}, "language_style": {"answer": "Calm and direct", "rationale": "The entry describes the definition of \"single\" in a concise language, without using too many speech-writing or emotional renderings, and is more objective in tone. It also does not use euphemism to wrap the question up, but rather to direct the subject on which researchers would like to know, making the questionnaire more intuitive and understandable. The language style of the article is therefore judged to be calm."}, "time_dimension": {"answer": "Past", "rationale": "The actions of \"scramble\" and \"scrambling\" occurred at some point in the past, so the entry describes the behaviour or mentality of the person tested in the past."}, "sentence_type": "Definition", "pos": {"nouns": {"Zenium": 1, "others": 1, "Attention": 2, "The crowd": 1, "person": 1}, "verbs": {"Hide": 1, "I'm under": 1, "It's not natural": 1}, "adjectives": {"Zenium": 1, "of": 1, "Someone else's": 1, "Attention": 2, "People": 1}, "adverbs": {"EMPTY": 2}, "classifiers": {"individual": 1}, "pronouns": {"others": 1}}}, {"content": "3 Proud: good performance, pompousness and loud voice;", "word_count": 12, "label": "", "sentence_structure": "Subject鈥搗erb鈥揷omplement", "subjective_objective": {"answer": "Subjective", "rationale": "Your description of \"exhibited\" is an adjective used by patients in self-evaluations, which expresses their views on their behaviour and characteristics, and therefore this part is subjective."}, "sentence_sentiment": {"answer": "Negative", "rationale": "Because the entries are characterized as \"showing,\" \"good performance\", \"fat\" and \"sounding\" are all negative."}, "language_style": {"answer": "Calm and direct", "rationale": "The language style of the entry is more calm and straightforward, and not too much rhetorical or emotional, but rather a simple and clear term to characterize the person surveyed. This way helps the interviewee to understand the question more accurately and to answer the question more quickly."}, "time_dimension": {"answer": "Present", "rationale": "The entry describes the current state or behaviour of the person being tested, using the words \"good behaviour\", \"fatal\" and \"sounding loud\" and thus judging as now."}, "sentence_type": "Affirmative declarative sentence", "pos": {"nouns": {"Shower": 1, "Performance": 1, "鈾?The twilight 鈾?: 1, "Sound": 1}, "adjectives": {"Okay": 1, "Large": 1}, "numerals": {"3": 1}}}, {"content": "4 Obscene: the euphoria is at hand, sometimes with a slight arrogance.", "word_count": 13, "label": "", "sentence_structure": "Subject鈥搗erb鈥揷omplement", "subjective_objective": {"answer": "Subjective", "rationale": "Because \"arbitrary\" and \"insolent\" are all the people's own evaluations and feelings, and they're subjective."}, "sentence_sentiment": {"answer": "Negative", "rationale": "Because the terms \"adult,\" \"opportunity\" and \"slightly arrogant\" are negative descriptions, suggesting that the attitudes and behaviour of the persons under investigation at the time of the tendency are negative."}, "language_style": {"answer": "Tactful and warm", "rationale": "The entry describes \"arbitrary\" in a more moderate and guiding language, without being overly direct or negative. At the same time, \"holy command\" and \"slightly arrogant\" are also more neutral and objective expressions, reflecting the euphemism and warmness of the language style of the questionnaire."}, "time_dimension": {"answer": "Past", "rationale": "The entry describes a character of the testee (i.e. \"apostate\") and uses a past pattern (i.e. \"honey commands, sometimes somewhat arrogant\". The time dimension of the entry is therefore judged to be the past."}, "sentence_type": "Affirmative declarative sentence", "pos": {"nouns": {"Obsessive": 1, "Command": 1, "Rule": 1, "Arrogance": 1}, "verbs": {"Hi, Hi": 1, "Slightly": 1}, "adjectives": {"Obscene": 1, "Arrogant": 1}, "numerals": {"4": 1}}}, {"content": "1 Dispersion: living in an disorderly manner;", "word_count": 9, "label": "", "sentence_structure": "Subject鈥搗erb鈥揷omplement", "subjective_objective": {"answer": "Subjective", "rationale": "Because scatteredness is an individual attitude or tendency, it is not an objective situation."}, "sentence_sentiment": {"answer": "Negative", "rationale": "Because \"dispersion\" and \"life insolence\" in the entries are negative."}, "language_style": {"answer": "Calm and direct", "rationale": "The entry uses a more direct language style, which describes the meaning of the concept of \"dispersion\" in a concise and clear manner, without using excessive rhetorical or emotional language."}, "time_dimension": {"answer": "Present", "rationale": ""}, "sentence_type": "Negative declarative sentence", "pos": {"nouns": {"Wandering": 1, "Life": 1, "Order": 1}, "verbs": {"I'm not gonna let you go": 1, "None": 1}}}, {"content": "2 No compassion: problems and problems of others are not easily understood;", "word_count": 16, "label": "", "sentence_structure": "Subject鈥搗erb鈥揷omplement", "subjective_objective": {"answer": "Subjective", "rationale": "Because understanding and compassion are patients ' subjective attitudes or emotional reactions."}, "sentence_sentiment": {"answer": "Negative", "rationale": "Because the term \"uncompassionate\" is often seen as a negative quality, which means a lack of understanding and caring for the feelings of others."}, "language_style": {"answer": "Calm and direct", "rationale": "The language style of the entry is more direct and concise, with no excessive rhetorical or emotional vocabulary, and a more calm and objective tone. At the same time, the subject and purpose of the study are defined by listing phenomena (intelligible problems and problems) and results (no compassion)."}, "time_dimension": {"answer": "Present", "rationale": "The entry describes the current behaviour or attitude of the testee, i.e., difficulties in understanding others ' problems and problems, and does not show sufficient compassion. Therefore, the temporal dimension is now judged."}, "sentence_type": "Negative declarative sentence", "pos": {"nouns": {"2": 1, "Compassion": 1}, "verbs": {"None": 1, "Understood": 1, "have": 1}, "adjectives": {"It's hard": 1}, "numerals": {"One": 1}}}, {"content": "Lack of enthusiasm: it is not easy to get excited and often it is difficult to do good;", "word_count": 16, "label": "", "sentence_structure": "Subject鈥揷opula鈥損redicative", "subjective_objective": {"answer": "Subjective", "rationale": ""}, "sentence_sentiment": {"answer": "Negative", "rationale": "Because the words \"lack of enthusiasm\", \"unexplored\" and \"hard to do\" in the entries are negative."}, "language_style": {"answer": "Tactful and warm", "rationale": "The entry uses descriptive language to express the question, not directly pointing to the existence of problems or criticisms of the other, but rather to ask questions in a gentle and caring manner, allowing the respondent to answer the question more easily, so it can be judged that the language style of the questionnaire is euphemism."}, "time_dimension": {"answer": "Present", "rationale": ""}, "sentence_type": "Negative declarative sentence", "pos": {"nouns": {"鈾?Hot": 2, "Excited": 1, "Yes": 1}, "verbs": {"Lack": 1, "I feel": 1, "Good": 1}, "adverbs": {"It's hard": 1, "Regular": 1}, "numerals": {"3": 1, "One": 2}}}, {"content": "No forgiveness: no forgiveness, no forgetting the harm others have done to themselves, and no envy.", "word_count": 21, "label": "", "sentence_structure": "Subject鈥搗erb鈥搊bject", "subjective_objective": {"answer": "Subjective", "rationale": "This is the subjective view and attitude of the patient, since the ease with which others are forgiven and forgotten and the ease with which jealousy is felt are the emotional and psychological state of the individual."}, "sentence_sentiment": {"answer": "Negative", "rationale": "Because \"unforgivable and forgetful\" and \"enviable\" are negative emotions and attitudes in the entries."}, "language_style": {"answer": "Calm and direct", "rationale": "The entry expresses the problem in a more direct and objective way, not using excessive rhetorical or emotional techniques, but simply asking the question. Such expression is more understandable and can be effectively accessed."}, "time_dimension": {"answer": "Past", "rationale": "In the entry, \"unforgivable and forgotten harm to others\" and \"enviable\" are expressions of an individual's past behaviour or mentality, and are therefore judged as past."}, "sentence_type": "Negative declarative sentence", "pos": {"nouns": {"Questionnaire Entry": 1, "Hurt": 1, "Forgiveness": 2, "Forget it": 1, "others": 1, "Jealousy": 1}, "verbs": {"not": 4, "Easy": 2, "Forgiveness and forget": 2, "Here": 1, "to": 1, "Cause": 1}, "adjectives": {"Tolerance": 1}, "numerals": {"4": 1}}}, {"content": "1 Reservations: unwillingness to participate, especially when matters are complex;", "word_count": 16, "label": "", "sentence_structure": "Subject鈥搗erb鈥揷omplement", "subjective_objective": {"answer": "Subjective", "rationale": "Because \"resistance\" is a subjective attitude or emotional reaction, not a description or assessment of objective facts. Although complex things may be an objective fact, the choice of \"resistance\" is based on personal feelings and subjective judgment. So, \"Reservation: unwilling to participate, especially when things are complicated\" is the patient's subjective attitude."}, "sentence_sentiment": {"answer": "Negative", "rationale": "The basis for the judgment: because the expressions \"resistance to participate\" and \"when things are complicated\" all have a negative, evasive attitude."}, "language_style": {"answer": "Calm and direct", "rationale": "The basis of the judgement is as follows: the entry directly indicates that the respondent may in some cases be \"sent to participate\" in response, especially when the matter becomes complex. The expression is not based on rhetorical or emotional representation, but rather on a direct and objective statement of the facts. The language style of the entry can therefore be described as direct and concise."}, "time_dimension": {"answer": "Past", "rationale": "The expression \"resistance to participate\" and \"especially when things are complicated\" in this entry relate to past experiences or attitudes and can therefore be judged as questioning the testee's past behaviour or mentality."}, "sentence_type": "Negative declarative sentence", "pos": {"nouns": {"Reservations": 1, "Participation": 1, "Things": 1}, "verbs": {"Yes": 1, "Special": 1}, "adjectives": {"Complex": 1}}}, {"content": "2 Hate: often put into mind the affronts of others, actual or perceived;", "word_count": 23, "label": "", "sentence_structure": "Subject鈥搗erb鈥揷omplement", "subjective_objective": {"answer": "Subjective", "rationale": "The hate is an offensive subjective assessment and feeling."}, "sentence_sentiment": {"answer": "Negative", "rationale": "Because the descriptions of \"hate\" and \"insulting others often in the heart\" are negative emotional reactions."}, "language_style": {"answer": "Calm and direct", "rationale": "The entry describes the definition and characteristics of \"hate\" in a more objective and direct way, without too much emotional or rhetorical style. Such expression can convey the message accurately and make it easier for the interviewee to understand the meaning of the problem."}, "time_dimension": {"answer": "Past", "rationale": "The entry is about the person who was tested for his or her perception and reaction to the behaviour of others, and relates to the emotional experience of the past and can therefore be judged as \"the past\"."}, "sentence_type": "Affirmative declarative sentence", "pos": {"nouns": {"Hate": 1, "others": 1, "I'm sorry": 1, "鈾?In the heart 鈾?: 1}, "verbs": {"- Put it on": 1, "Fire": 1, "Regular": 1}, "adverbs": {"Actual": 1, "I imagined it": 1}}}, {"content": "3. Retrogression: resistance, or refusal to accept the methods of others, stubbornness;", "word_count": 19, "label": "", "sentence_structure": "Subject鈥搗erb鈥搊bject", "subjective_objective": {"answer": "Subjective", "rationale": "Persistence is the patient ' s subjective attitude and behaviour, because of the resistance or rejection of others."}, "sentence_sentiment": {"answer": "Negative", "rationale": "Because \"resist\" and \"suspensive\" are all negative."}, "language_style": {"answer": "Calm and direct", "rationale": ""}, "time_dimension": {"answer": "Past", "rationale": "The entry describes a past act or attitude of the person tested, namely, \"the resistance, or the refusal to accept another person's methods, to hold a hold.\" While such behaviour may persist in some cases, the time reference point for this entry is the past."}, "sentence_type": "Definition sentence", "pos": {"nouns": {"Reverse": 1, "Resist": 1, "Methodology": 1, "Obstinent": 1}, "verbs": {"Accept": 1, "Resistance": 1}, "numerals": {"3": 1}}}, {"content": "4-D: Repeating the same thing or story, forgetting that it has been repeated many times, and always looking for the subject.", "word_count": 30, "label": "", "sentence_structure": "Subject鈥損redicate", "subjective_objective": {"answer": "Objective", "rationale": "Because the patient is describing his or her behaviour, it is the patient ' s objective attitude and behaviour, not his or her subjective thoughts or feelings."}, "sentence_sentiment": {"answer": "Negative", "rationale": "Because the word \"nosing\" usually has a negative connotation, meaning that it is too loud and repeated, and the description in the entry also emphasizes repeating the same thing or story, forgetting the repeated and constant talk-talking behaviour, which can be judged as negative."}, "language_style": {"answer": "Tactful and warm", "rationale": ""}, "time_dimension": {"answer": "Present", "rationale": "The entry describes the current behaviour or state of the subject, \"repeated to the same thing or story\", \"forget to repeat\" and \"always to keep talking about it,\" which are current behavioral expressions. So, the judgment is \"now\"."}, "sentence_type": "Affirmative declarative sentence", "pos": {"nouns": {"- No, no, no": 1, "Things": 1, "Story": 1, "Topic": 1}, "verbs": {"Speak": 2, "Repeat": 2, "Forget it": 1, "Find": 1, "Say it": 1}, "adverbs": {"Always": 1, "Constantly": 1}, "numerals": {"4": 1}}}, {"content": "Twenty-four,", "word_count": 3, "label": "", "sentence_structure": "Subject鈥損redicate", "subjective_objective": {"answer": "Subjective", "rationale": "Please provide more details so that I can answer your questions."}, "sentence_sentiment": {"answer": "Neutral", "rationale": "Basis of judgement: The entry is not of any specific emotional colour, but provides a number."}, "language_style": {"answer": "Calm and direct", "rationale": "Please provide a text of specific questions for my analysis, if you lack the content of the questionnaire entries that require judgement."}, "time_dimension": {"answer": "Present", "rationale": "If you are missing a sentence, please provide the relevant statement for analysis."}, "sentence_type": "Interrogative sentence", "pos": {"nouns": {"Questionnaire Entry": 1}, "numerals": {"Twenty": 1, "Four": 1}, "classifiers": {"individual": 1}}}, {"content": "1 Chosen: stick to the details of the trifle, always picking out the ones that are not enough;", "word_count": 14, "label": "", "sentence_structure": "Subject鈥搗erb鈥揷omplement", "subjective_objective": {"answer": "Subjective", "rationale": "Because it is the behaviour or attitude of individuals to insist on the details of the minor, the lack of selection, etc., and the feelings and perceptions of the patients themselves."}, "sentence_sentiment": {"answer": "Negative", "rationale": "Because \"choo-hoo-hoo-hoo-hoo\" and \"choo-hoo-hoo-hoo-hoo\" are negative terms, which are a negative attitude or behaviour."}, "language_style": {"answer": "Tactful and warm", "rationale": "The entry is in the form of a euphemism that leads the interviewee to reflect on his behaviour, rather than a direct statement or order, and is relatively gentle and friendly. At the same time, the expression \"always prefers to be insufficiently \" is not too straightforward or negative, but is based on objective facts, leaving the interviewee some room for self-reflection."}, "time_dimension": {"answer": "Present", "rationale": "The entry describes a current behavior or state, where \"choo-hoo-hoo-hoo-hoo\" and \"choo-hoo-hoo-hoo-hoo-hoo\" are all actions of the present. Thus, the temporal dimension of the article should be judged as now."}, "sentence_type": "Negative declarative sentence", "pos": {"nouns": {"Picky": 1, "Details": 1, "Insufficient": 1}, "verbs": {"Hold on": 1, "Yeah": 1}, "adverbs": {"Total": 1}}}, {"content": "2 timid: often feeling serious anxiety and grief;", "word_count": 15, "label": "", "sentence_structure": "Subject鈥搗erb鈥揷omplement", "subjective_objective": {"answer": "Subjective", "rationale": "Because \"coward\" is a subjective feeling and attitude, describing the patient鈥檚 perception and assessment of his emotional state. While this fear and anxiety may have been caused by objective circumstances or events, the description of \"coward\" focuses more on the patient鈥檚 subjective emotional experience."}, "sentence_sentiment": {"answer": "Negative", "rationale": "Because \"timid\" and \"awesome\" are negative emotional attitudes."}, "language_style": {"answer": "Tactful and warm", "rationale": ""}, "time_dimension": {"answer": "Present", "rationale": ""}, "sentence_type": "Affirmative declarative sentence", "pos": {"nouns": {"Coward": 2, "I'm worried": 1, "Anxiety": 1, "Sadness": 1}, "adjectives": {"Serious": 1}, "adverbs": {"Regular": 1}, "numerals": {"2": 1}}}, {"content": "3 Memories: lack of self-regulation, leading to forgetting and a reluctance to remember boring things;", "word_count": 22, "label": "", "sentence_structure": "Subject鈥搗erb鈥揷omplement", "subjective_objective": {"answer": "Subjective", "rationale": "Because \"lack of self-restraint\" and \"resistance of boring things\" are subjective attitudes or feelings of patients."}, "sentence_sentiment": {"answer": "Negative", "rationale": "Because the words \"forgettable\" and \"lack of self-restraint\" in the entries all have a negative meaning, describing a bad behavior or quality."}, "language_style": {"answer": "Tactful and warm", "rationale": "The entry is more euphemistic and attempts to describe the situation in a sense of understanding and support. It does not directly criticize or accuse the other of his behaviour, but rather tries to understand and help them improve the situation. Moreover, the use of positive terms (lack of self-restraint) and query-style words (resistance to remember boring things) can also be considered a warm expression of euphemism."}, "time_dimension": {"answer": "Present", "rationale": "The entry describes a behaviour that is manifesting and the consequences of being forgotten due to lack of self-regulation, and that such behaviour is currently being carried out. Thus, the temporal dimension is now judged."}, "sentence_type": "Affirmative declarative sentence", "pos": {"nouns": {"Forgetful": 2, "Self-restraint": 1, "Memories": 1, "Things": 1}, "verbs": {"Result": 1, "Yes": 1}, "adjectives": {"It's boring": 1}, "numerals": {"3": 1}, "pronouns": {"I": 1}}}, {"content": "4 Probate: speak out and express their views directly.", "word_count": 15, "label": "", "sentence_structure": "Subject鈥搗erb鈥揷omplement", "subjective_objective": {"answer": "Objective", "rationale": "Because straightness refers to a character or behaviour, not to the patient ' s subjective feelings or attitudes."}, "sentence_sentiment": {"answer": "Positive", "rationale": "Because the act and attitude of \"invocacy, expressing one's views directly\" is positive, demonstrating the courage and frankness of a person to express his thoughts."}, "language_style": {"answer": "Calm and direct", "rationale": "The language style of the entry is of a calm and straightforward type, which clearly describes a particular character of the person under investigation, without using excessive rhetoric or emotional rendering, and with a fair and objective tone."}, "time_dimension": {"answer": "Present", "rationale": "The entry is based on the description of \"into the plain\" and \"in the direct expression\" in the title, and it is asked whether the testee is inclined to express himself in the present way of acting. The temporal dimension is therefore now."}, "sentence_type": "Affirmative declarative sentence", "pos": {"nouns": {"Straight": 1, "- I'm not saying anything": 1, "Observations": 1}, "verbs": {"Expression": 1}}}, {"content": "1 Not patient: I cannot bear to wait for others;", "word_count": 11, "label": "", "sentence_structure": "Subject鈥搗erb鈥揷omplement", "subjective_objective": {"answer": "Subjective", "rationale": "Because \"dreaminess\" is a subjective assessment that reflects the attitude and emotional state of the individual, not the objective reality. While waiting may be objective, it is subjective to feel impatient."}, "sentence_sentiment": {"answer": "Negative", "rationale": "Because the character of \"dragging\" in the entry is negative."}, "language_style": {"answer": "Tactful and warm", "rationale": "The entry is more euphemistic and warm, and asks the other side whether it is impatient, rather than directly critical or accusing, by using the more objective term of \"dryness.\" At the same time, the expression \"unbearable waiting\" is relatively implicit and mild, making the question more acceptable and more open to answer."}, "time_dimension": {"answer": "Present", "rationale": "The entry describes the current status or characteristics of the subjects, i.e. whether they are \"unsatisfactory\" at this stage."}, "sentence_type": "Negative declarative sentence", "pos": {"nouns": {"Patience": 1, "Wait": 1, "person": 1}, "verbs": {"have": 1, "Take it": 1, "By": 1}, "adjectives": {"Nope": 1, "Hard": 1}, "numerals": {"1": 2}}}, {"content": "2 Insecurity: fear and self-confidence;", "word_count": 13, "label": "", "sentence_structure": "Subject鈥揷opula鈥損redicative", "subjective_objective": {"answer": "Subjective", "rationale": "Because \"insecurity\", \"fear\" and \"inconfidence\" are personal feelings and perceptions of patients, subjectively."}, "sentence_sentiment": {"answer": "Negative", "rationale": "Because both \"fear\" and \"innocent\" in the entries have negative emotional aspects."}, "language_style": {"answer": "Calm and direct", "rationale": "The language style of the entry is a calm and straightforward one, which directly and clearly describes the subject (insecurity) and does not have too many rhetorical and emotional renderings.\""}, "time_dimension": {"answer": "Present", "rationale": ""}, "sentence_type": "Affirmative declarative sentence", "pos": {"nouns": {"Safe": 1, "I'm worried": 1, "Self-confidence": 1}, "verbs": {"I feel": 1, "have": 1}, "adjectives": {"None": 2}, "numerals": {"2": 1}}}, {"content": "3 Indecisive: difficult to decide;", "word_count": 9, "label": "", "sentence_structure": "Subject鈥搗erb鈥搊bject", "subjective_objective": {"answer": "Subjective", "rationale": "Because \"indecisiveness\" is a personal characteristic or behaviour that reflects the attitude and preferences of patients in making decisions."}, "sentence_sentiment": {"answer": "Negative", "rationale": "Because \"indecisive\" and \"hard to decide\" have negative meanings."}, "language_style": {"answer": "Calm and direct", "rationale": "The entry uses a simple and clear language to describe the subject matter, without too much emotional or rhetorical style, and is more intuitive than straightforward."}, "time_dimension": {"answer": "Present", "rationale": "The entry describes the current behaviour of the testee, and \"hard\" and \"decided\" are all terms relevant to the present, and therefore the judgement is now."}, "sentence_type": "Negative declarative sentence", "pos": {"nouns": {"Entry": 1, "Indecisiveness": 1, "Decision-making": 1}, "verbs": {"Analysis": 1, "Down": 1}, "adjectives": {"It's hard": 1}, "numerals": {"3": 1}}}, {"content": "4 Good to interrupt: a loud speaker is not a good listener, and he does not pay attention to what others say.", "word_count": 26, "label": "", "sentence_structure": "Subject鈥搗erb鈥揷omplement", "subjective_objective": {"answer": "Subjective", "rationale": "Because this phrase describes the behaviour and attitudes of a person, such as \"shut-out\" and \"not paying attention to others\", which describe his behaviour and ideas from his own perspective, the entry is a subjective one to examine the patient."}, "sentence_sentiment": {"answer": "Negative", "rationale": "Because the description of \"not a good listener\" and \"not to notice the words of others\" in the entry is negative."}, "language_style": {"answer": "Calm and direct", "rationale": "The entry is based on a simple and straightforward presentation of issues and views, not using rhetorical techniques or excessive emotional colours, but rather in a direct, objective manner. This language style is more suitable for official settings such as questionnaires, which conveys clearly what researchers want to know and avoid creating misunderstandings or prejudice among the respondents."}, "time_dimension": {"answer": "Past", "rationale": "The answer is as follows: The entry describes behaviour or habits that are common to a person in communication, past/present/in the future, and does not address the temporal dimensions of the past, present or future."}, "sentence_type": "Negative declarative sentence", "pos": {"nouns": {"- I'm not": 1, "Spokesperson": 1, "Audience": 1, "person": 2, "Speech": 1}, "verbs": {"Okay": 1, "I'm gonna be a big fan": 1, "Attention": 1, "Say it": 1}, "adverbs": {"No, it's not": 1, "Okay": 1}, "classifiers": {"One": 2}}}, {"content": "1 Unwelcome: rejection of people for serious requirements of perfection;", "word_count": 17, "label": "", "sentence_structure": "Subject鈥搗erb鈥揷omplement", "subjective_objective": {"answer": "Subjective", "rationale": "Because \"require perfection\" and \"rejection\" are the psychological state that the patient feels and belong to the patient's subjective idea."}, "sentence_sentiment": {"answer": "Negative", "rationale": "Because the \"rejection\" in the entry is negative."}, "language_style": {"answer": "Tactful and warm", "rationale": "For the given input sentence, \"1 is unwelcome: it is rejected for great demands of perfection;\" I think its language style is more \"cool and direct.\" It is a direct statement of a fact 鈥?because it is perfect, and it discourages others from approaching you. Although it uses some aphrodisiac techniques (such as metaphors), it is more direct and objective in general, and there is no excessive emotional overtones or euphemism. So I think the language style of this entry is straightforward."}, "time_dimension": {"answer": "Past", "rationale": ""}, "sentence_type": "Negative declarative sentence", "pos": {"nouns": {"Welcome": 1, "Request": 1, "Perfect": 1, "Lee": 1}, "verbs": {"not": 1, "Reject": 1, "Yes": 1, "A thousand miles": 1}, "adjectives": {"Tight": 1}, "adverbs": {"Yes": 1}}}, {"content": "2 Not to participate: not to join, not to participate, not to be interested in the lives of others;", "word_count": 20, "label": "", "sentence_structure": "Subject鈥搗erb鈥揷omplement", "subjective_objective": {"answer": "Objective", "rationale": "Because \"no\" and \"no\" and \"no interest in other people's lives\" describe the behaviour or attitude of patients in social matters, not their subjective feelings or ideas. So, these entries look at patients from an objective perspective."}, "sentence_sentiment": {"answer": "Negative", "rationale": "The basis for the judgement: the expression \"indulgentness\" in the article, \"involvement\" and \"involvement\" in other people's lives\" have negative emotional connotations."}, "language_style": {"answer": "Tactful and warm", "rationale": "The language style of the entry is more moderate, with relatively polite language, respect for the will and feelings of the other, and no excessive coercive or negative emotional colour, and prefers to treat the choice of the subject in an open and understanding manner. At the same time, expressions such as \"indulgent\" and \"not to participate\" also meet the neutral and objective requirements of the questionnaire survey and do not exert undue psychological pressure on the person under investigation."}, "time_dimension": {"answer": "Present", "rationale": ""}, "sentence_type": "Negative declarative sentence", "pos": {"nouns": {"Questionnaire": 1, "Entry": 1, "Participants": 1, "Interest": 1}, "verbs": {"Participation": 1, "Add": 1, "I'd love to": 1, "Interested": 1}, "adjectives": {"No, I don't": 1, "not": 2, "others": 1}, "numerals": {"2": 1}, "pronouns": {"Here": 1}}}, {"content": "3 Unpredictable: time and excitement, time and again, or always not keeping promises;", "word_count": 19, "label": "", "sentence_structure": "Subject鈥搗erb鈥揷omplement", "subjective_objective": {"answer": "Subjective", "rationale": "Because the description involves emotional fluctuations and commitment, these are the inherent feelings and attitudes of the patient and are therefore subjective in nature."}, "sentence_sentiment": {"answer": "Negative", "rationale": "Because the references to \"hard to predict\", \"time to get excited, time to get low\" and \"always to keep promises\" are negative."}, "language_style": {"answer": "Tactful and warm", "rationale": "The language style of the entry is relatively euphemistic, allowing the interviewee to assess his/her own emotional volatility and commitment. At the same time, the use of the words \"time and always \" is more objective and neutral, without direct criticism or criticism. This expression is more easily understood and accepted by the interviewee."}, "time_dimension": {"answer": "Present", "rationale": "The entry describes the behaviour of the person who was tested over a period of time, and the keywords \"time and always\" indicate that the state of affairs does not persist, but is intermittent and therefore judged to be the present."}, "sentence_type": "Affirmative declarative sentence", "pos": {"nouns": {"Unpredictable": 1, "Excited": 1, "Down": 1, "Promises": 1}, "verbs": {"be": 1, "Cash": 1}, "adjectives": {"Time": 2, "Always": 1}, "numerals": {"3": 1}}}, {"content": "4 Lack of compassion: it is difficult to express publicly feelings for the weak or the victims.", "word_count": 21, "label": "", "sentence_structure": "Subject鈥搗erb鈥揷omplement", "subjective_objective": {"answer": "Subjective", "rationale": "Because \"hard to express in public\" is the patient's own feeling and assessment, and it's a subjective attitude and emotional reaction."}, "sentence_sentiment": {"answer": "Negative", "rationale": "Because the expressions \"lack of compassion\" and \"difficulties to express feelings for the weak or the victims\" are all negative emotional expressions."}, "language_style": {"answer": "Tactful and warm", "rationale": "The entry is more personal and interactive when it is spoken in a gentle and inviting manner, rather than in direct statements or orders. At the same time, it attempts to understand whether the interviewees lack compassion in their dealings with others, and that such compassion is difficult to express in public."}, "time_dimension": {"answer": "Present", "rationale": "First, the word \"very hard to express in public\" indicates the severity and frequency of this situation, a term that describes current or recent behaviour. So we can assume that the entry is asking the testee about the current behaviour or attitude. Second, the \"emotionality to the weak or the victim\" suggests that the entry is asking the testee about compassion, which is a reflection of emotion and attitude, and that compassion usually needs to be expressed through certain actions. So we can further speculate that the entry may be asking the testee whether the testee has in the past tried to express publicly sympathy for the weak or the victims. In the light of the information provided in the above-mentioned section, I think that the time frame for the questionnaire is now, i.e., asking the testee about the behaviour and attitude of the testee."}, "sentence_type": "Negative declarative sentence", "pos": {"nouns": {"Questionnaire": 1, "Entry": 1, "Compassion": 1, "The weak": 1, "Victims": 1, "Emotion": 1}, "verbs": {"Public expression": 1}, "adjectives": {"It's hard": 1}, "numerals": {"4": 1}}}, {"content": "1 Perseverance: to remain in line with their own views and not to listen to their differences;", "word_count": 18, "label": "", "sentence_structure": "Subject鈥搗erb鈥揷omplement", "subjective_objective": {"answer": "Subjective", "rationale": "Because \"obstinate\" is an individual attitude or behavioural orientation, a subjective attitude of the patient's own view and handling of the situation."}, "sentence_sentiment": {"answer": "Negative", "rationale": "Because the term \"obstinacy\" is often seen as a negative feature, indicating reluctance to accept new ideas or changes."}, "language_style": {"answer": "Calm and direct", "rationale": "The language style of the entry is relatively simple and not overly accommodative or emotional, directly expressing the character of the firm execution of the person under investigation and the relatively objective and calm tone of the speech."}, "time_dimension": {"answer": "Present", "rationale": "The entry describes a character of the testee, namely whether they prefer to hold back their views and this tendency may still exist at this time. \""}, "sentence_type": "Affirmative declarative sentence", "pos": {"nouns": {"Obstinacy": 1, "Observations": 2, "self": 1, "Different": 1}, "verbs": {"Hold on": 1, "Photo": 1, "Okay": 1, "Listen": 1}, "adverbs": {"very": 1, "not": 1}, "numerals": {"1": 2}, "pronouns": {"self": 1}}}, {"content": "2. Following: Unusual and randomly doing things;", "word_count": 15, "label": "", "sentence_structure": "Subject鈥搗erb鈥揷omplement", "subjective_objective": {"answer": "Subjective", "rationale": "This is a subjective assessment of the patient ' s description and perception of his or her behaviour."}, "sentence_sentiment": {"answer": "Negative", "rationale": "Because the expressions \"funct\" and \"unusual\" in the entries are negative, indicating a lack of planning and targeting."}, "language_style": {"answer": "Tactful and warm", "rationale": "The language style of the entry is more euphemistic and gentle. Using the word \"fun\" to express the customary characteristics of the other, rather than directly criticizing or accusing, is easier for the interviewees to accept and to be willing to continue their investigation. At the same time, the whole sentence is more relaxed, friendly and friendly."}, "time_dimension": {"answer": "Present", "rationale": "The entry asks the testee about his behaviour habits or preferences, i.e. whether they have a certain regularity and consistency in their work or whether they have randomity and arbitrariness. Therefore, in terms of time dimension, the entry does not refer explicitly to the past, present or future period, but rather to a description of a general state or characteristic."}, "sentence_type": "Imperative sentence", "pos": {"nouns": {"Things": 2, "Random": 1, "Coherence": 1}, "verbs": {"Do it": 2, "have": 1}, "adjectives": {"Whatever": 1}, "numerals": {"2": 1}}}, {"content": "3 Hard to please: it is difficult to please others because of the high demands;", "word_count": 18, "label": "", "sentence_structure": "Subject鈥搗erb鈥揷omplement", "subjective_objective": {"answer": "Objective", "rationale": "Because it was observed by the patient, i.e. whether others find it difficult to please."}, "sentence_sentiment": {"answer": "Negative", "rationale": "Because the description of \"difficult to please\" in the entry is negative."}, "language_style": {"answer": "Calm and direct", "rationale": "The entry is in plain and clear language, and the interviewee may in some cases be difficult to please. It is not too much rhetoric or emotional, but rather is expressed in a more objective and direct way."}, "time_dimension": {"answer": "Present", "rationale": "The term \"because of the high demands\" and \"to make it difficult for others to please\" allows the entry to be judged by the current situation of the person being tested, and therefore the time dimension is now."}, "sentence_type": "Affirmative declarative sentence", "pos": {"nouns": {"3": 1, "It's hard to please": 1, "Because": 1, "Request": 1, "Too high": 1, "others": 1}, "verbs": {"鈾?And I'm gonna be 鈾?: 1}, "adjectives": {"High": 1}, "numerals": {"3": 1, "Too much": 1}, "pronouns": {"Here": 1}}}, {"content": "Slow down: delayed, uneasy or always slow.", "word_count": 22, "label": "", "sentence_structure": "Subject鈥搗erb鈥揷omplement", "subjective_objective": {"answer": "Subjective", "rationale": "Because \"slow action\" is a patient's self-perception and subjective evaluation, it is his own psychological state and attitude that is examined."}, "sentence_sentiment": {"answer": "Negative", "rationale": "Because \"slow action\", \"slow action\" and \"unable participation or slow action\" are negative descriptions in the entries."}, "language_style": {"answer": "Calm and direct", "rationale": "The language style of the entry is relatively calm and straightforward, it clearly describes the problems that the person under investigation may have, does not use any rhetorical or emotional means of rendering, and is more objective. This language style helps to ensure the accuracy and validity of the questionnaire, as it avoids any language interference that could lead to a misresponse."}, "time_dimension": {"answer": "Past", "rationale": "The entry describes the behaviour of the person tested during a certain period of time, i.e., slow movement."}, "sentence_type": "Affirmative declarative sentence", "pos": {"nouns": {"Action": 1, "Slow down": 1, "Participation": 1, "Shoot": 1}, "verbs": {"Action": 1, "Slow down": 1, "Participation": 1, "Always Slow": 1}, "adjectives": {"Slow down": 1, "Easy": 1, "Half": 1}, "adverbs": {"Slow down": 1, "Always": 1}, "numerals": {"4": 1, "Half": 1}}}, {"content": "1 flat: flat, low, middle, no low, little emotional;", "word_count": 21, "label": "", "sentence_structure": "Subject鈥搗erb鈥揷omplement", "subjective_objective": {"answer": "Objective", "rationale": "Because \"silent\" is a descriptive term that is an objective description of a person's emotions and attitudes, not of the patient's own subjective feelings or experiences."}, "sentence_sentiment": {"answer": "Negative", "rationale": "Because the word \"silent\" in the entry is neutral or negative."}, "language_style": {"answer": "Calm and direct", "rationale": "The language style of the entry is calm and straightforward. It clearly describes the \"silent\" character, without any rhetorical or emotional rendering, and is objective. It conveys the message accurately, making it easier for readers to understand and accept."}, "time_dimension": {"answer": "Present", "rationale": ""}, "sentence_type": "Affirmative declarative sentence", "pos": {"nouns": {"Flat": 1}, "verbs": {"Show": 1}, "adjectives": {"Flat": 1, "Apathy": 1, "Centre": 1, "None": 1, "High": 1, "Very few": 1, "Emotion": 1}, "adverbs": {"very": 1}, "numerals": {"1": 2}}}, {"content": "2 Pessimism: while expectations are best, they often begin to see disadvantages;", "word_count": 21, "label": "", "sentence_structure": "Subject鈥搗erb鈥搊bject", "subjective_objective": {"answer": "Subjective", "rationale": "Because \"pessimism\" is a personal view and attitude that reflects the patient's assessment and perception of things, it is viewed from a subjective perspective."}, "sentence_sentiment": {"answer": "Negative", "rationale": "Because \"pessimism\" is negative."}, "language_style": {"answer": "Tactful and warm", "rationale": "The entry is more adept and interactive when it is spoken in a gentle and inviting manner, rather than by direct statements or orders."}, "time_dimension": {"answer": "Present", "rationale": "The entry describes a psychological orientation or behavioural pattern of the person tested, and \"the best expectation, but often the first to see the disadvantage\" is a constant, customary psychological state, and thus the judgment is now."}, "sentence_type": "Affirmative declarative sentence", "pos": {"nouns": {"Pessimism": 1, "Things": 1, "Negative": 1}, "verbs": {"Look forward to it": 1, "Yeah": 1}, "adverbs": {"First of all": 1, "Often": 1}, "classifiers": {"2": 1}}}, {"content": "3 Self-esteem: self-evaluation is high and considers itself the best candidate;", "word_count": 17, "label": "", "sentence_structure": "Subject鈥搗erb鈥搊bject", "subjective_objective": {"answer": "Subjective", "rationale": "Because \"self-absorbed\" is a personal, subjective feeling and perception."}, "sentence_sentiment": {"answer": "Negative", "rationale": "Because the characteristic of \"self-righteousness\" is often considered negative, it can lead to overconfidence among individuals and neglect of others' views and feedback, thereby affecting their own performance and development."}, "language_style": {"answer": "Calm and direct", "rationale": "The language style of the entry is more straightforward, with no excessive rhetorical or emotional tact, and is primarily an expression of concern and discussion about the psychological identity of the interviewee."}, "time_dimension": {"answer": "Present", "rationale": "The keywords are \"self-evaluation\" and \"conception,\" which both imply the testee's assessment and perception of himself in the current thinking and behaviour. And the adverb \"best\" in the sentence indicates the testee's confidence and self-awareness, which is also a current-day behaviour."}, "sentence_type": "Affirmative declarative sentence", "pos": {"nouns": {"- You're so arrogant": 1, "Self-evaluation": 1, "Selection": 1}, "verbs": {"I think": 1, "be": 1}, "adjectives": {"Highest": 1}, "numerals": {"3": 1}, "pronouns": {"self": 2}}}, {"content": "4 Leave: to do what he likes, to please others and to advocate for himself.", "word_count": 27, "label": "", "sentence_structure": "Subject鈥搗erb鈥搊bject", "subjective_objective": {"answer": "Subjective", "rationale": "Because \"leave\" and \"permit\" people to do what they like to be nice to others and to be able to advocate themselves\" are subjective ideas and behaviours of patients."}, "sentence_sentiment": {"answer": "Negative", "rationale": "Because the entries are \"false\" and \"benevolent and preaching\" and all have negative emotional aspects."}, "language_style": {"answer": "Tactful and warm", "rationale": ""}, "time_dimension": {"answer": "Past", "rationale": "This attitude and reaction happened in the past, so it was judged to be the past. So the answer is \"the past.\""}, "sentence_type": "Affirmative declarative sentence", "pos": {"nouns": {"person": 2, "Things": 2, "Beloved": 1, "others": 3, "self": 1, "Questionnaire Entry": 1}, "verbs": {"Do it": 1, "Let's do it": 1, "Hsui": 1, "Yeah": 1, "Advocacy": 1}, "adjectives": {"EMPTY": 2, "he": 1}, "numerals": {"4": 1}, "classifiers": {"individual": 1}, "pronouns": {"self": 1}}}, {"content": "1 Easier: mundane, childish, excite, forgotten immediately after;", "word_count": 18, "label": "", "sentence_structure": "Subject鈥揷opula鈥損redicative", "subjective_objective": {"answer": "Subjective", "rationale": "Because \"freaky,\" \"creature,\" \"child personality\" and \"excited\" are all words that describe patient's subjective attitudes, and \"forget it\" is an objective observation of the results of these emotional reactions. So, \"one anger: mundane, child's personality, emotional, and then forget it immediately;\" The main thing that is examined is the patient's subjective feelings and emotional state, so it's a subjective entry."}, "sentence_sentiment": {"answer": "Negative", "rationale": "Because words like \"repulsive,\" \"corruptible\" and \"excited\" are negative descriptions, which means that the person is more unstable and prone to anger."}, "language_style": {"answer": "Calm and direct", "rationale": "The entry uses simple and quick language to describe the \"frightful\" character, does not use excessive rhetorical or emotional rendering, and is more balanced and objective. At the same time, it does not contain any euphemisms, such as emotional vocabulary or metaphors, and the overall language style tends to be more calm and straightforward."}, "time_dimension": {"answer": "Present", "rationale": "According to the terms \"repulsive,\" \"climate,\" \"excited,\" it can be seen that the entry describes the current behaviour and emotional state of the person being tested. At the same time, \"forgetting it soon\" indicates that these events occur less frequently than for a long time, so it is the behaviour or mentality that is being questioned by this questionnaire entry."}, "sentence_type": "Affirmative declarative sentence", "pos": {"nouns": {"Easier than I thought": 1, "It's a change": 1, "Child personality": 1, "Excited": 1}, "verbs": {"Easy": 1, "Remember": 1, "Forget it": 1}, "adjectives": {"Come on": 1}, "numerals": {"1": 3}}}, {"content": "2 No objectives: not interested in, nor interested in, targeting;", "word_count": 14, "label": "", "sentence_structure": "Subject鈥搗erb鈥搊bject", "subjective_objective": {"answer": "Subjective", "rationale": "Because \"like\" and \"intentionally set a goal\" are personal attitudes or emotional experiences of patients and are subjectively viewed."}, "sentence_sentiment": {"answer": "Negative", "rationale": "Because the phrase \"not interested in, and not interested in, targeting\" indicates a lack of motivation and commitment and is negative."}, "language_style": {"answer": "Calm and direct", "rationale": "The language style of the entry is relatively calm and straightforward, and there is no excessive emotional or rhetorical approach, which simply and clearly expresses the subject of questioning the respondents about whether they have a clear objective."}, "time_dimension": {"answer": "Present", "rationale": ""}, "sentence_type": "Negative declarative sentence", "pos": {"nouns": {"Objective": 2, "I don't like it": 1, "No, I didn't mean to": 1}, "verbs": {"Staple": 1, "Yeah": 1}, "adverbs": {"Yeah": 1}, "numerals": {"2": 1}}}, {"content": "3 Good argument: it is easy to argue with people, and feel right about everything;", "word_count": 21, "label": "", "sentence_structure": "Subject鈥搗erb鈥揷omplement", "subjective_objective": {"answer": "Subjective", "rationale": ""}, "sentence_sentiment": {"answer": "Negative", "rationale": "The \"easy to argue\" and \"right to feel right to anything\" in the entries represent a more adversarial attitude and over-confident behaviour that can be considered negative features or behaviours."}, "language_style": {"answer": "Tactful and warm", "rationale": ""}, "time_dimension": {"answer": "Past", "rationale": "The entry describes the practices and behaviours of the testee in the past, namely, \"Easy to argue\" and \"Easy to argue\" and \"Easy to feel right about anything.\" Both of these features refer to what happened over time. So we can judge the time dimension of the entry is the past."}, "sentence_type": "Affirmative declarative sentence", "pos": {"nouns": {"Disputes": 1, "Debate": 1, "Argument": 1, "Yes": 1}, "verbs": {"Okay": 1, "Arguments": 1, "I think": 1, "be": 1}, "adjectives": {"Easy": 1, "Right": 1}}}, {"content": "4 Unanimous: I feel easily alienated, often insecure or afraid that others do not like to be with myself.", "word_count": 31, "label": "", "sentence_structure": "Subject鈥搗erb鈥搊bject", "subjective_objective": {"answer": "Subjective", "rationale": "Because the descriptions of \"easy to be alienated\", \"often insecure\" and \"so worried about people not having to live with themselves\" are based on the patient's inner feeling, the questionnaire entry is subjective."}, "sentence_sentiment": {"answer": "Negative", "rationale": "Because the entries \"feared out\" and \"insecurity\" and \"fearing that people don't like being with themselves\" all have negative emotional features."}, "language_style": {"answer": "Tactful and warm", "rationale": "The entry is more adept and interactive when it is spoken in a gentle and inviting manner, rather than by direct statements or orders."}, "time_dimension": {"answer": "Present", "rationale": "The entry describes the current state of behavior and mental state of the testee, which is now being experienced by words such as \"easy feeling\" and \"frequent absence\". Thus, the temporal dimension is now judged."}, "sentence_type": "Affirmative declarative sentence", "pos": {"nouns": {"I'm so sorry": 1, "Safe": 1, "Disconnection": 1, "I'm worried": 1, "others": 1, "Get along": 1}, "verbs": {"I feel": 1, "By": 2, "Regular": 1, "No, I'm not": 1, "Yeah": 1, "self": 1}, "adjectives": {"I'm so sorry": 1, "Disconnection": 1, "Unsettled": 1, "I'm worried": 1}, "adverbs": {"Easy": 1, "Regular": 1, "No, I'm not": 1}, "numerals": {"4": 1, "1": 7}, "classifiers": {"individual": 1, "self": 1}, "pronouns": {"self": 1}}}, {"content": "One innocence: childish innocence, and lack of understanding of the meaning of life;", "word_count": 16, "label": "", "sentence_structure": "Subject鈥搗erb鈥揷omplement", "subjective_objective": {"answer": "Subjective", "rationale": "Because the word \"innocent\" describes the patient's subjective feelings and attitudes and is the patient's perception and assessment of his or her behaviour, feelings, etc., or that of others."}, "sentence_sentiment": {"answer": "Neutral", "rationale": "The entry describes naive features and does not involve positive or negative emotional colours."}, "language_style": {"answer": "Tactful and warm", "rationale": "The entry depicts the concept of \"innocent\" in a descriptive sense, without directly pointing to a good or bad quality. At the same time, it uses a myths like \"childlike\" and \"ununderstanding the truth of life\" that make language more vivid and contagious. Thus, its language style is more euphemistic and warm."}, "time_dimension": {"answer": "Past", "rationale": "The entry describes a character or state, namely, \"Innocence.\" \"Childly simple\" and \"ununderstanding the truth of life\" are all descriptions of the past. So, the time dimension of the entry is the past."}, "sentence_type": "Affirmative declarative sentence", "pos": {"nouns": {"Naughty": 1, "Son": 1, "Pure": 1, "Life": 1, "Shinji": 1}, "verbs": {"Understood": 1}, "numerals": {"1": 2}, "pronouns": {"not": 1}}}, {"content": "2 Negative: often seeing the negative side of things with little positive attitude;", "word_count": 23, "label": "", "sentence_structure": "Subject鈥搗erb鈥揷omplement", "subjective_objective": {"answer": "Subjective", "rationale": "Because the entry in the questionnaire examines patients ' perception of and attitude to things and is subjective."}, "sentence_sentiment": {"answer": "Negative", "rationale": "The basis for the judgement: the position described in the entry is that \"the negative side of things is often seen in the dark side of the picture\" and that this attitude is negative. At the same time, no positive act or purpose is mentioned, nor is there a neutral description, so that the answer can be determined to be negative."}, "language_style": {"answer": "Calm and direct", "rationale": "The entry describes in an objective and clear manner the attitude or preference of the person under investigation, does not use excessive qualifiers or emotionally coloured language, and therefore the language style is considered to be straightforward and dispassionate."}, "time_dimension": {"answer": "Present", "rationale": "The keyword \"often\" expresses a constant attitude, \"see\" and \"unique\" both adopt the general current, meaning that the description is a description of the current behaviour or state. The entry is therefore an inquiry as to whether the testee has a negative side in the current attitude."}, "sentence_type": "Affirmative declarative sentence", "pos": {"nouns": {"Negative": 2, "Things": 1, "Noodles": 2, "Attitude": 1}, "verbs": {"Yeah": 1, "Often": 1, "have": 1}, "adjectives": {"Dark": 1, "Positive": 1}, "adverbs": {"And": 1, "Not much": 1}}}, {"content": "3 Be reckless: confident and bold but always inappropriate;", "word_count": 15, "label": "", "sentence_structure": "Subject鈥搗erb鈥揷omplement", "subjective_objective": {"answer": "Subjective", "rationale": "Because recklessness is a character or attitude of a human being and is the perception and assessment of the patient himself, it is subjective."}, "sentence_sentiment": {"answer": "Negative", "rationale": "Because the term \"reckless\" is often seen as negative and indicates a lack of caution and thoughtfulness. Although it may sometimes be characterized as an act of self-confidence and courage, it is described as \"inappropriate\" and implies that it may have negative effects or consequences. The emotional colour of the questionnaire is therefore negative."}, "language_style": {"answer": "Calm and direct", "rationale": "The language style of the entry is more direct, concise and without any reticulation or emotional accent, and is mainly an objective description of the expression of the character character of \"reckless\"."}, "time_dimension": {"answer": "Present", "rationale": ""}, "sentence_type": "Affirmative declarative sentence", "pos": {"nouns": {"You're reckless": 1, "Confidence": 1, "- Brave": 1, "Problem": 1}, "verbs": {"Fill": 1, "have": 1, "Always": 1, "Appropriate": 1}, "adjectives": {"not": 2, "Appropriate": 1}, "adverbs": {"Total": 1}, "pronouns": {"I": 1}}}, {"content": "4 Apathy: Ignorance, pass.", "word_count": 10, "label": "", "sentence_structure": "Subject鈥損redicate", "subjective_objective": {"answer": "Objective", "rationale": "Because apathy is an objective expression of the patient."}, "sentence_sentiment": {"answer": "Negative", "rationale": "Because the expression \"indifferentness\" and \"excessiveness\" are negative emotions, attitudes or behaviours."}, "language_style": {"answer": "Calm and direct", "rationale": "The language style of the entry is more straightforward, with no excessive emotional accompaniment or euphemism, and more focused on communicating information and describing facts. This tone applies to situations that need to be clearly expressed, such as the description of certain character characteristics or behaviour habits in the questionnaire."}, "time_dimension": {"answer": "Present", "rationale": "The entry describes the current attitude or behaviour of the testee, \"indifferentness\" and \"excessiveness\" are all descriptions of the current mood and attitude of the testee. Thus, the temporal dimension is now judged."}, "sentence_type": "Negative declarative sentence", "pos": {"nouns": {"Coldness": 1, "I'm not concerned": 1, "It's a good thing": 1}, "verbs": {"be": 1}, "adjectives": {"4": 1}, "numerals": {"4": 1}}}, {"content": "1 Fear: at times, uncertainty, anxiety, anxiety;", "word_count": 13, "label": "", "sentence_structure": "Subject鈥搗erb鈥搊bject", "subjective_objective": {"answer": "Subjective", "rationale": "Because fear is a description of a subjective emotional state, reflecting the feeling of anxiety and fear within the patient."}, "sentence_sentiment": {"answer": "Negative", "rationale": "Because the entries \"fear,\" \"uncertain,\" \"anxiety\" and \"fear\" have negative emotional aspects."}, "language_style": {"answer": "Calm and direct", "rationale": "The language style of the entry is more direct, not using excessive rhetorical or emotional rendering, but presented in an objective and factual presentation, which is obvious."}, "time_dimension": {"answer": "Present", "rationale": "The entry is for the purpose of asking if the person under examination currently has a \"fear\" emotional state, which is now judged in terms of time dimension."}, "sentence_type": "Affirmative declarative sentence", "pos": {"nouns": {"Fear": 1, "Anxiety": 1, "I'm upset": 1}, "verbs": {"I feel": 1}, "adverbs": {"Time and time": 1}, "classifiers": {"1": 1}}}, {"content": "2 Poorly connected: always picking people up, not being liked;", "word_count": 16, "label": "", "sentence_structure": "Subject鈥搗erb鈥揷omplement", "subjective_objective": {"answer": "Subjective", "rationale": "Because \"showing people\" and \"not liked\" are situations that patients feel or experience themselves, and are subjectively viewed."}, "sentence_sentiment": {"answer": "Negative", "rationale": "Because the \"showing, not liking\" in the entry is negative."}, "language_style": {"answer": "Tactful and warm", "rationale": "The language style of the entry is relatively mild, not directly critical of or accusing the other of his behaviour or personality, but rather of leading the conversation by describing the problems and grievances that the other party may have. At the same time, the language is softer and objective, such as \"always prefers to pick people up\" and \"not like\" , which is more a source of reflection and change."}, "time_dimension": {"answer": "Present", "rationale": "\"As always picking up people's problems\" and \"not liked\" are words that describe the current state or characteristics, so the entry asks about the current social behaviour and relationships of the subjects."}, "sentence_type": "Negative declarative sentence", "pos": {"nouns": {"Communication": 1, "Mistake": 1, "person": 1, "Yeah": 1}, "verbs": {"Not good": 1, "Always": 1, "Pick": 1, "By": 1}, "adjectives": {"Yes": 1, "Communication": 1, "of": 2}, "adverbs": {"Total": 1}, "numerals": {"2": 1}, "pronouns": {"I": 1}}}, {"content": "Worker 3: Working and ashamed to rest for reward or sense of achievement, rather than perfection, and therefore for setting ambitious goals.", "word_count": 36, "label": "", "sentence_structure": "Subject鈥搗erb鈥搊bject", "subjective_objective": {"answer": "Subjective", "rationale": "Because \"work crazy,\" \"great goals\", \"shambling rest\" and so on, it's a subjective view."}, "sentence_sentiment": {"answer": "Negative", "rationale": "Because the \"shambles\" in the entry is negative."}, "language_style": {"answer": "Calm and direct", "rationale": "The language style of the questionnaire entry is calm and straightforward. It clearly identifies \"work-crazy\" and does not have too many emotional or rhetorical tacts to qualify. It also uses a simple and clear language to enable respondents to quickly understand and answer questions."}, "time_dimension": {"answer": "Past", "rationale": "In the entry, \"workaholic\" is a description of behaviour, which is a description of past behaviour or habit. And \"a great goal is working.\" It is a move or state of performance in the past, so it can be judged to be a past entry."}, "sentence_type": "Affirmative declarative sentence", "pos": {"nouns": {"Workaholic": 1, "Rewards": 1, "A sense of achievement": 1, "Perfect": 1, "The great target": 1, "Rest": 1}, "verbs": {"Establishment": 1, "Work": 2, "Shame on": 1}, "adjectives": {"Constantly": 1, "Perfect": 1}, "numerals": {"3": 1}}}, {"content": "4 Yes: It is necessary to recognize the appreciation, like an actor.", "word_count": 15, "label": "", "sentence_structure": "Subject鈥搗erb鈥搊bject", "subjective_objective": {"answer": "Subjective", "rationale": "Because the idea of being recognized and appreciated is a subjective feeling and idea of the patient."}, "sentence_sentiment": {"answer": "Positive", "rationale": "Because \"honey to be\" and \"need to be appreciated\" are positive and eager to be recognized."}, "language_style": {"answer": "Tactful and warm", "rationale": "The language style of this entry is more euphemism and gentle. First, \"honor to be\" shows a desire to be accepted by others, rather than a direct emphasis on self-centre or narcissism. Second, \"need to be appreciated, like an actor\" expresses a need, but instead of being in a strong, or command-style, way of visualizing the emotional state, using metaphorically, making the language softer, warmer and more attractive."}, "time_dimension": {"answer": "Present", "rationale": "The entry describes the current state of the person's mind and needs, and the word \"need\" indicates that this is a present demand. At the same time, the sentence also contains a recollection of the past, which is \"I liked it...\", but that does not affect our classification as a current category."}, "sentence_type": "Affirmative declarative sentence", "pos": {"nouns": {"Happy": 1, "Yes": 2, "The others": 1, "- I'm impressed": 1, "Actor": 1}, "numerals": {"4": 1}}}, {"content": "1 Excessive sensitivity: overreaction to things and being offended when misunderstood;", "word_count": 21, "label": "", "sentence_structure": "Subject鈥搗erb鈥揷omplement", "subjective_objective": {"answer": "Subjective", "rationale": "Because \"excessively sensitive\" and \"inflicted when misunderstood\" are subjective feelings of patients."}, "sentence_sentiment": {"answer": "Negative", "rationale": "Because \"excessively sensitive\" in the entry, \"invited when misunderstood\" is negative emotions and attitudes."}, "language_style": {"answer": "Tactful and warm", "rationale": ""}, "time_dimension": {"answer": "Present", "rationale": "The keywords \"excessive sensitivity\", \"excessive reaction\" and \"invited when misunderstood\" all indicate the behaviour or mentality that may arise at present. The entry therefore asks the testee about the behaviour or mental characteristics of the present and the temporal dimension judge it as now."}, "sentence_type": "Affirmative declarative sentence", "pos": {"nouns": {"That's too much": 2, "Sensitive": 1, "Things": 1, "Response": 1, "person": 1, "Misunderstanding": 1, "I'm sorry": 1}, "verbs": {"to": 1, "I feel": 1, "By": 3, "When misconstrued": 1, "(a) Intruding": 1}, "adjectives": {"It's too much": 1}, "numerals": {"1": 6}, "pronouns": {"you": 1}}}, {"content": "2 Unpretty and sophisticated: often expressing themselves in an offensive or poorly considered manner;", "word_count": 22, "label": "", "sentence_structure": "Subject鈥搗erb鈥揷omplement", "subjective_objective": {"answer": "Subjective", "rationale": "Because \"untouchable and sophisticated\" and \"insulting or misconsidering\" are the descriptions of patients who are subjectively evaluating and taking attitudes towards themselves."}, "sentence_sentiment": {"answer": "Negative", "rationale": "Because the \"expression of oneself in an offensive or misconceived manner\" in the entry is negative."}, "language_style": {"answer": "Calm and direct", "rationale": "The entry is more directly written, has no excessive speech or emotional rendering, and is speaking in a balanced and objective manner. It asks whether the interviewee often expresses his views and feelings in an offensive or unconsidered manner.\""}, "time_dimension": {"answer": "Present", "rationale": "\"Recurring\" means that the act is a customary, repeated act, occurring at the present time and therefore falling within the present context."}, "sentence_type": "Negative declarative sentence", "pos": {"nouns": {"Not smooth and seasoned": 1, "I'm sorry": 1, "Modalities": 1, "self": 1}, "verbs": {"Use": 1, "Expression": 1}, "adjectives": {"Regular": 1, "I'm not thinking": 1}, "numerals": {"2": 1}, "pronouns": {"self": 1}}}, {"content": "3 Coward: having difficulty retreating;", "word_count": 8, "label": "", "sentence_structure": "Subject鈥搗erb鈥揷omplement", "subjective_objective": {"answer": "Objective", "rationale": "Because timidity is a manifestation or characteristic, not the patient ' s own perception or feeling, it is objective."}, "sentence_sentiment": {"answer": "Negative", "rationale": "Because the act of \"difficult retreat\" in the entry is negative."}, "language_style": {"answer": "Calm and direct", "rationale": "The entry is more directly linguistically, with no excessive rhetorical or emotional rendering, but rather directly describes the behaviour characteristics that the person under investigation may have (i.e., retreats in case of difficulties)."}, "time_dimension": {"answer": "Past", "rationale": "The keyword \"difficult to retreat\" in the entry describes a past act or state, and the temporal dimension of the subject can therefore be judged to be the past."}, "sentence_type": "Negative declarative sentence", "pos": {"nouns": {"Difficulties": 1, "Frightened": 1}, "verbs": {"I met him": 1, "Retrenched": 1}, "numerals": {"3": 1}}}, {"content": "4-Talk: Unmanageable, unstoppable, unheard.", "word_count": 18, "label": "", "sentence_structure": "Subject鈥搗erb鈥揷omplement", "subjective_objective": {"answer": "Subjective", "rationale": "\"The difficulty of control, the lack of access to others\" is the subjective behaviour and attitude of the patient, and the entry is therefore viewed from a subjective perspective."}, "sentence_sentiment": {"answer": "Negative", "rationale": "Because \"crowding\" and \"not listening to others\" are negative behavioural features."}, "language_style": {"answer": "Calm and direct", "rationale": "The language style of the entry is calm and straightforward. It describes a behaviour characteristic in a simple and straightforward way -- like to talk, not to listen to others, not to use excessive rhetorical techniques or emotional rendering, and to be more objective in tone."}, "time_dimension": {"answer": "Present", "rationale": "The time dimension of this questionnaire entry is now, because it describes the current behaviour or state of the subjects, i.e. whether they are often unable to control their own words and whether they can effectively listen to others."}, "sentence_type": "Negative declarative sentence", "pos": {"nouns": {"Questionnaire": 1, "Entry": 1, "- You're talking": 1, "I'm gonna be a big fan": 1, "Listen": 1, "others": 1}, "verbs": {"Analysis": 1}, "adverbs": {"Hard": 1, "No, I can't": 1}, "classifiers": {"individual": 1}}}, {"content": "1 : Uncertainty and lack of confidence in what is being done;", "word_count": 17, "label": "", "sentence_structure": "Subject鈥揷opula鈥損redicative", "subjective_objective": {"answer": "Subjective", "rationale": "Because \"sweet\" is what patients say about themselves and feel about themselves, and it's subjective."}, "sentence_sentiment": {"answer": "Negative", "rationale": "Because \"things\" and \"suspects\" and \"lack of confidence\" are all words that describe negative emotions or quality."}, "language_style": {"answer": "Tactful and warm", "rationale": "The term \"sweet\" is depicted in descriptive terms, without any direct definition or evaluation, and is a mild expression that makes it easier for the respondents to accept and understand the questions. At the same time, the article is more acrimony, giving attention to the feelings and feelings of the respondents, making them more comfortable and relaxed in answering the questions."}, "time_dimension": {"answer": "Present", "rationale": "Now the entry describes a character or behaviour of the person being tested, namely, \"snap\" and uses the general current, so it can be judged as now."}, "sentence_type": "Negative declarative sentence", "pos": {"nouns": {"Boom": 1, "- The thing": 1, "Confidence": 1}, "verbs": {"I'm not sure": 1, "Do it": 1}, "numerals": {"1": 2}}}, {"content": "2. Life disorders: lack of capacity to organize life;", "word_count": 13, "label": "", "sentence_structure": "Subject鈥搗erb鈥揷omplement", "subjective_objective": {"answer": "Subjective", "rationale": "Because lack of capacity to organize life is the patient鈥檚 subjective feeling and perception. While this may affect the patient鈥檚 living conditions, it is a subjective experience and assessment itself."}, "sentence_sentiment": {"answer": "Negative", "rationale": "Because the description of \"lack of ability to organize\" in this entry is negative and implies problems in the management of life by individuals."}, "language_style": {"answer": "Tactful and warm", "rationale": "The language style of the entry is euphemism. It uses a mild term \"lack of ability to organize life\" without direct criticism or criticism of the other, making it easier to accept and cooperate with the investigation. Moreover, it is more caring and understanding, and more close to the respondent."}, "time_dimension": {"answer": "Past", "rationale": "The entry describes a situation in the life of the testee, where \"lack of the ability to organize\" is a problem that the testee has experienced in the past and is therefore judged to be \"the past\"."}, "sentence_type": "Negative declarative sentence", "pos": {"nouns": {"Life": 1, "Capacity": 1}, "verbs": {"Lack": 1, "Arrangements": 1}, "adjectives": {"Disorder": 1}, "numerals": {"2": 1}}}, {"content": "3rd Zone: impulsive control of things and others and command of others;", "word_count": 16, "label": "", "sentence_structure": "Subject鈥搗erb鈥搊bject", "subjective_objective": {"answer": "Subjective", "rationale": "Because \"inspiring control of things and others, directing others\" is the subjective expression of the patient's behaviour and is therefore subjective in nature."}, "sentence_sentiment": {"answer": "Negative", "rationale": "Because in the entries, \"destroyed\" and \"involved control\" and \"directed other people\" are all negative emotional."}, "language_style": {"answer": "Calm and direct", "rationale": "The term \"control\" and \"command\" are used in the entry, which directly describes the behaviour of a person, does not use excessive rhetorical or emotional rendering, and is more objective in tone. It is therefore considered that the language style of the article is straightforward and calm."}, "time_dimension": {"answer": "Present", "rationale": "The entry describes the current behaviour or character characteristics of the testee, i.e. whether they often control things and others and direct them. Therefore, the temporal dimension is now. \""}, "sentence_type": "Adjective", "pos": {"nouns": {"Zone": 1, "Expedition": 1, "Things": 1, "others": 1, "Command": 1, "Others": 1}, "verbs": {"Control": 1}, "adverbs": {"3": 1}, "classifiers": {"individual": 1}}}, {"content": "4 Depression: Often low mood.", "word_count": 8, "label": "", "sentence_structure": "Subject鈥揷opula鈥損redicative", "subjective_objective": {"answer": "Subjective", "rationale": "Because often the low mood is a subjective feeling, it is the emotional experience within the patient."}, "sentence_sentiment": {"answer": "Negative", "rationale": "Because the description \"often depressed\" in the entries is negative."}, "language_style": {"answer": "Calm and direct", "rationale": ""}, "time_dimension": {"answer": "Present", "rationale": ""}, "sentence_type": "Affirmative declarative sentence", "pos": {"nouns": {"Depression": 1, "Emotion": 1, "Down": 1}, "verbs": {"Often": 1}, "numerals": {"4": 1}, "classifiers": {"individual": 1}}}, {"content": "(a) Lack of perseverance: volatility, contradictions and irrationality;", "word_count": 21, "label": "", "sentence_structure": "Subject鈥搗erb鈥揷omplement", "subjective_objective": {"answer": "Subjective", "rationale": "Because \"indulgent\" is a term that describes patients' subjective attitudes, expressing their feelings and perceptions. This emotional state is determined by their own mental state and perceptions, and does not require external evidence to confirm or refute it."}, "sentence_sentiment": {"answer": "Negative", "rationale": "Because the words \"transparent,\" \"controversial\", \"unsatisfactory\" and \"irregularity of emotions and actions\" in the entries all have negative emotional connotations."}, "language_style": {"answer": "Calm and direct", "rationale": "The entry is a direct illustration of the specific manifestations of \"indulgentness\" 鈥?\"transformation\", \"controversial\" and \"unlogical emotions and actions\" 鈥?which are not over-modified by rhetoric or emotional colours, and are more objective and concise. Thus, the language style of the article can be considered as a straightforward type of calm."}, "time_dimension": {"answer": "Present", "rationale": "The entry of the questionnaire is a question about the behaviour, mentality and the time dimension of the testee. It is therefore not possible to judge in the past/present/future manner."}, "sentence_type": "Negative declarative sentence", "pos": {"nouns": {"Fortitude": 1, "Instability": 1, "Paradoxious": 1, "Emotion": 1, "Action": 1, "Logical": 1}, "verbs": {"Lack": 1}, "adverbs": {"- Yeah, yeah": 1}, "numerals": {"1": 2}}}, {"content": "Introverted: Living in their own world, thinking and interest are in their hearts;", "word_count": 19, "label": "", "sentence_structure": "Subject鈥搗erb鈥揷omplement", "subjective_objective": {"answer": "Subjective", "rationale": "Because inward orientation is a subjective attitude that reflects the character of the individual."}, "sentence_sentiment": {"answer": "Negative", "rationale": "Because the word \"introvert\" usually has a negative connotation. While people who refer to inward orientation may take their thoughts and interests to heart, this is not necessarily a positive description, because it implies that they may lack social skills or be unwilling to share their ideas with others."}, "language_style": {"answer": "Tactful and warm", "rationale": "The language style of the entry is relatively euphemistic and emotional, using visualized expressions to describe the characteristics of the inner person. At the same time, this moderate language makes it easier for the interviewee to open his heart and to share his thoughts and feelings."}, "time_dimension": {"answer": "Present", "rationale": "In conclusion, for the input sentence, \"2 Introvert: Living in your own world, thinking and interest are in your heart;\" the output should be \"now\"."}, "sentence_type": "Affirmative declarative sentence", "pos": {"nouns": {"Inner": 1, "World": 1, "Ideas": 1, "Interest": 1}, "verbs": {"Live": 1, "Fire": 1}, "adjectives": {"Your own": 1, "In my heart": 1}, "numerals": {"2": 1}}}, {"content": "3 Intolerance: the view, attitude and way of doing things cannot be tolerated;", "word_count": 20, "label": "", "sentence_structure": "Subject鈥搗erb鈥搊bject", "subjective_objective": {"answer": "Subjective", "rationale": "Because \"impermissible\" is a subjective attitude or feeling that describes patients' reactions to the views, attitudes and ways of doing things. Such reactions are based on the patient's personal experience and values, and are not necessarily objective."}, "sentence_sentiment": {"answer": "Negative", "rationale": "Because the expression \"impermissible\" in the entry is negative."}, "language_style": {"answer": "Calm and direct", "rationale": "The language style of the entry is more blunt, with no excessive emotional and rhetorical style. It clearly indicates the unacceptability of some acts, and does not attempt to hide the fact in euphemism or gentle language. It may be more serious and serious."}, "time_dimension": {"answer": "Present", "rationale": "The entry describes the perception of the testee in the current behaviour or mentality of \"impermissible views, attitudes and ways of doing things\" and thus judges as now."}, "sentence_type": "Negative declarative sentence", "pos": {"nouns": {"Tolerance": 1, "Others": 1, "Views": 1, "Attitude": 1, "Modalities": 1}, "verbs": {"No, I can't": 2, "Take it": 1}, "adjectives": {"Intolerance": 1}, "numerals": {"3": 1}}}, {"content": "4 No objection: Ignorance on many matters.", "word_count": 12, "label": "", "sentence_structure": "Subject鈥搗erb鈥揷omplement", "subjective_objective": {"answer": "Subjective", "rationale": "Because \"indifferentness\" is the patient's subjective attitude and feelings."}, "sentence_sentiment": {"answer": "Negative", "rationale": "Because \"indifferentness\" in the entries is a negative emotional attitude, indicating that there is no interest or concern in the matter."}, "language_style": {"answer": "Calm and direct", "rationale": "The entry expresses the subject in a simple and clear phrase - \"indifferent to many things\" - without too much rhetorical or emotional presentation, and is more objective in tone. At the same time, it is more in line with the formal and professional requirements of psychological questionnaire research."}, "time_dimension": {"answer": "Present", "rationale": "\"No objection\" indicates the positive attitude of the testee to this matter, and \"indifferentness to many things\" is a concrete expression of this attitude, which points to the current behaviour or state. Thus, the temporal dimension of the article is now judged."}, "sentence_type": "Affirmative declarative sentence", "pos": {"nouns": {"Things": 2, "I'm not concerned": 1}, "verbs": {"No objection": 1}, "numerals": {"4": 1}}}, {"content": "1 Discretion: living in an irregular environment, often without access to anything;", "word_count": 18, "label": "", "sentence_structure": "Subject鈥搗erb鈥搊bject", "subjective_objective": {"answer": "Objective", "rationale": "Because chaos is an objective description of the patient's living environment.\""}, "sentence_sentiment": {"answer": "Negative", "rationale": "The basis for the judgement: \"discretionary\" living environments usually lead to increased psychological stress, and it is therefore negative."}, "language_style": {"answer": "Calm and direct", "rationale": "This is more easily understood and answered by the interviewee."}, "time_dimension": {"answer": "Present", "rationale": "The keywords \"unordered living environment\" and \"often undiscovered\" describe a status quo or customary behaviour, and therefore judge it as now."}, "sentence_type": "Negative declarative sentence", "pos": {"nouns": {"Life": 1, "Environment": 1, "Order": 1, "Something": 1}, "verbs": {"None": 2, "Find": 1, "Chaos": 1}, "adjectives": {"Chapter": 1, "Regular": 1}, "adverbs": {"None": 1}, "numerals": {"1": 3}, "classifiers": {"individual": 1}}}, {"content": "2 Emotional: emotional insurgencies are not easily high and when they feel unappreciated, they are easily downplayed;", "word_count": 21, "label": "", "sentence_structure": "Subject鈥搗erb鈥揷omplement", "subjective_objective": {"answer": "Subjective", "rationale": "Because emotional insurgencies and depression are not easy, it is the subjective feelings and experiences of patients."}, "sentence_sentiment": {"answer": "Negative", "rationale": "Because \"unpredictable\" and \"unappreciated when you feel unappreciated\" are both negative emotional states."}, "language_style": {"answer": "Tactful and warm", "rationale": ""}, "time_dimension": {"answer": "Present", "rationale": "\"Femaleness\" and \"Easy to be down when you feel unappreciated\" indicate the current state of emotion and feeling, so the entry is asking about the current behavior or mentality."}, "sentence_type": "Affirmative declarative sentence", "pos": {"nouns": {"Emotional": 1, "Emotion": 1, "High": 1, "Enjoy": 1, "Down": 1}, "verbs": {"It's hard": 1, "I feel": 1, "Easy": 1}, "adverbs": {"2": 1, "very": 1}, "numerals": {"1": 3}}}, {"content": "3 Mumbling: whispering, not being inconvenient;", "word_count": 15, "label": "", "sentence_structure": "Subject鈥搗erb鈥揷omplement", "subjective_objective": {"answer": "Subjective", "rationale": "Because mumbling is a monologue of a human being, a subjective expression of thought and emotion. Although it may be triggered by objective factors such as anxiety, stress, etc., this expression is still subjective."}, "sentence_sentiment": {"answer": "Negative", "rationale": "Because the act of \"mumbling\" in the entry is negative, it may indicate personal anxiety, anxiety or frustration."}, "language_style": {"answer": "Calm and direct", "rationale": "The language style of the entry is relatively simple and not overly phrasing or emotional, describing the behaviour of the subject in a direct and objective manner. \""}, "time_dimension": {"answer": "Past", "rationale": "[output sentence] Past/Now"}, "sentence_type": "Imperative sentence", "pos": {"nouns": {"3": 1, "鈾?Mumbling": 1, "- Keep your voice down": 1, "I care": 1, "Clear": 1}, "verbs": {"鈾?Mumbling": 1, "Talk": 1, "I don't care": 1}, "adjectives": {"Quiet": 1, "Clear": 1}, "adverbs": {"Mumbling": 1, "I don't care": 1}, "numerals": {"3": 1}}}, {"content": "4-Governed: Smartly handled, manipulated, and made good for yourself.", "word_count": 17, "label": "", "sentence_structure": "Subject鈥搗erb鈥搊bject", "subjective_objective": {"answer": "Subjective", "rationale": "Because \"making it work for yourself\" is the patient's subjective view and expectation of the purpose and outcome of the act."}, "sentence_sentiment": {"answer": "Positive", "rationale": "Because the entries \"manipulation\", \"smartness\" and \"making it in his own interest\" are both positive and have a certain tendency to pursue his own interests."}, "language_style": {"answer": "Tactful and warm", "rationale": ""}, "time_dimension": {"answer": "Past", "rationale": "\"It is a description of the person's behaviour or attitude, which does not relate to the time dimension of the past, present or future. Thus, it can be judged that the entry is a description of the person's current or current behaviour or attitude, that is, \"now\"."}, "sentence_type": "Affirmative declarative sentence", "pos": {"nouns": {"He likes to manipulate": 1, "Things": 1}, "verbs": {"Manipulation": 2, "- It's a matter of action": 1, "鈾?And I'm gonna be 鈾?: 1, "Good": 1}, "adjectives": {"Smart": 1}, "numerals": {"4": 1}}}, {"content": "1. Slow: slow thinking and excessive trouble;", "word_count": 14, "label": "", "sentence_structure": "Subject鈥搗erb鈥揷omplement", "subjective_objective": {"answer": "Subjective", "rationale": "Because \"slow\" is patient subjectively felt and evaluated."}, "sentence_sentiment": {"answer": "Negative", "rationale": "Because the entry \"the idea of action is slow, it's too cumbersome\" describes a negative behaviour and attitude."}, "language_style": {"answer": "Calm and direct", "rationale": "The entry is a straightforward expression of the characteristics of the person being tested in terms of action, thinking, and does not use excessive rhetoric or emotional rendering. Thus, its language style is a straightforward type of calm."}, "time_dimension": {"answer": "Present", "rationale": "The entry now describes the current state or orientation of the testee, i.e., that both movement and thought are slow and tend to become too cumbersome. This description is usually used to assess the behaviour of individuals in their daily lives, so the temporal dimension is now judged."}, "sentence_type": "Negative declarative sentence", "pos": {"nouns": {"Action": 1, "Ideas": 1}, "verbs": {"Comparison": 1, "Slow": 1, "That's too much": 1, "Trouble": 1}, "adjectives": {"Slow": 1}}}, {"content": "2 Recalculable: determination to act on its own will, and not easily persuaded;", "word_count": 17, "label": "", "sentence_structure": "Subject鈥搗erb鈥揷omplement", "subjective_objective": {"answer": "Subjective", "rationale": "Because \"durance\" and \"sure to act on their own will, and not easily convinced\" are the attitudes and behaviour of the patient, they are subjective."}, "sentence_sentiment": {"answer": "Negative", "rationale": "Because \"dreaming\" is often considered a negative quality, it is difficult to change and unwilling to accept the views of others."}, "language_style": {"answer": "Calm and direct", "rationale": "The entry is in a simple and clear language, which directly expresses the individual's self-preservation and incompetence in decision-making."}, "time_dimension": {"answer": "Present", "rationale": ""}, "sentence_type": "Adjective", "pos": {"nouns": {"Obstinate": 1, "Resolve": 1, "Will": 1}, "verbs": {"Ei": 1, "Act": 1, "It's hard": 1, "By": 1, "Convince": 1}, "adverbs": {"2": 1, "Obstinate": 1}, "numerals": {"2": 1}}}, {"content": "Good performance: To attract people, it needs to become a focus of attention;", "word_count": 20, "label": "", "sentence_structure": "Subject鈥搗erb鈥搊bject", "subjective_objective": {"answer": "Subjective", "rationale": "Because it is the patient's subjective attitude that is at the centre of attention."}, "sentence_sentiment": {"answer": "Positive", "rationale": "Because the entries \"to attract\" and \"to be a center of attention\" are all positive."}, "language_style": {"answer": "Tactful and warm", "rationale": "Warm and warm."}, "time_dimension": {"answer": "Past", "rationale": "The entry is a description of a person ' s performance and self-awareness, and does not contain clear time indicators or time statements, and therefore cannot be judged past, present or future."}, "sentence_type": "Imperative sentence", "pos": {"nouns": {"Performance": 2, "Attractive": 1, "Centre": 1}, "verbs": {"Yes": 1, "Be": 1, "Attention": 1}, "adjectives": {"Okay": 1}, "numerals": {"3": 1}, "pronouns": {"self": 1, "person": 1}}}, {"content": "4. The key to the prevention is that it is not easy to believe that the real motivation behind the language exists as its main title.", "word_count": 22, "label": "", "sentence_structure": "Subject鈥搗erb鈥搊bject", "subjective_objective": {"answer": "Subjective", "rationale": "Because the sentence concerns the belief and attitude of individuals, namely, \"unconvinced\" and \"predominantly available,\" it is judged by factors such as their experience, perceptions and values. The questionnaire is therefore viewed from the subjective perspective of the patient."}, "sentence_sentiment": {"answer": "Negative", "rationale": "Because the entries contain expressions of \"unconvinced\" and \"presence\", implying negative feelings of doubt, mistrust, etc."}, "language_style": {"answer": "Calm and direct", "rationale": "The entry expresses in a direct presentation areas of concern to the interviewees, with no excessive rhetoric or emotional colour, and is more objective and calm."}, "time_dimension": {"answer": "Present", "rationale": ""}, "sentence_type": "Affirmative declarative sentence", "pos": {"nouns": {"Watch your step": 1, "Motive": 1, "Subject鈥損redicate": 1}, "verbs": {"have": 1, "Yes": 1, "Existence": 1}, "adjectives": {"It's hard": 1}, "numerals": {"4": 1}}}, {"content": "1 Insistence: it takes a great deal of time to be alone and avoid crowds;", "word_count": 15, "label": "", "sentence_structure": "Subject鈥搗erb鈥揷omplement", "subjective_objective": {"answer": "Subjective", "rationale": "Because \"insolent\" is a personal feeling and experience, and the time and behaviour of avoiding people is based on such feelings and experiences, the perspective of the questionnaire entry is subjective."}, "sentence_sentiment": {"answer": "Negative", "rationale": "Because the adjective \"silent\" in the entry is a negative emotion or attitude."}, "language_style": {"answer": "Calm and direct", "rationale": "The language style of the entry is a calm and straightforward type. It describes in a concise and clear language a psychological characteristic of the subject - isolation, the absence of excessive emotional modification or rhetorical techniques, and a more objective tone."}, "time_dimension": {"answer": "Present", "rationale": "The entry describes a behavioural characteristic, i.e., that individuals take a great deal of time and isolation to avoid crowds. The characteristic of such behaviour can be considered as the current, present state or orientation, and therefore judged as \"now\"."}, "sentence_type": "Declarative sentence", "pos": {"nouns": {"I'm alone": 1, "Time": 1, "Population": 1}, "verbs": {"Yes": 1, "Alone": 1, "Avoid": 1}, "numerals": {"Mass": 1}, "classifiers": {"individual": 1}}}, {"content": "2 The desire to rule: no hesitation in expressing their right or control;", "word_count": 20, "label": "", "sentence_structure": "Subject鈥搗erb鈥揷omplement", "subjective_objective": {"answer": "Subjective", "rationale": "Because \"dominantness\" is a subjective emotion or tendency, a self-assessment of whether the patient has a certain quality."}, "sentence_sentiment": {"answer": "Positive", "rationale": "For the expression \"without hesitation to express its right or control\" reflects a strong sense of confidence and a dominant will, which is a positive emotion, attitude or behaviour."}, "language_style": {"answer": "Calm and direct", "rationale": "The entry \"Cool and Direct\" directly shows whether the respondents tend to show their authority or desire to control the problem, and the language style is more objective and calm, with no excessive adoration and emotional rendering."}, "time_dimension": {"answer": "Present", "rationale": ""}, "sentence_type": "Affirmative declarative sentence", "pos": {"nouns": {"The desire to rule": 1, "Control capacity": 1}, "verbs": {"Organisation": 1, "Without hesitation": 1}, "adjectives": {"Correct": 1}, "adverbs": {"Land": 2, "Without hesitation": 1}}}, {"content": "3 Lazy: always measure how much effort is needed to do the best;", "word_count": 21, "label": "", "sentence_structure": "Subject鈥搗erb鈥揷omplement", "subjective_objective": {"answer": "Subjective", "rationale": "Because \"always measure how much effort things cost\" and \"doing what is best\" is the patient's perception and attitude to his own behaviour, and it is subjective."}, "sentence_sentiment": {"answer": "Negative", "rationale": "Because what is expressed in the entries as \"always measuring how much effort is needed\" and \"not doing the best\" is a lazy, unpositive attitude, which is a negative emotional color."}, "language_style": {"answer": "Tactful and warm", "rationale": ""}, "time_dimension": {"answer": "Present", "rationale": "The words \"always\" and \"best\" indicate that this is a normal form of customary behaviour. Thus, the time dimension of the article is now."}, "sentence_type": "Negative declarative sentence", "pos": {"nouns": {"Lazy": 1, "Things": 2, "- The energy": 1}, "verbs": {"Always": 1, "Qualification": 1, "Cost": 1, "Do it": 1}, "adverbs": {"First": 1, "I'd better": 1}, "numerals": {"3": 1}}}, {"content": "4 Big Voices: Talking and laughter always cover people.", "word_count": 14, "label": "", "sentence_structure": "Subject鈥搗erb鈥搊bject", "subjective_objective": {"answer": "Objective", "rationale": "Because \"talking and laughter always cover people\" is the behaviour of patients in their daily lives, not their subjective feelings or thoughts, and therefore is an objective examination."}, "sentence_sentiment": {"answer": "Negative", "rationale": "Because \"speaks and laughs always cover up\" in the entry is a negative expression of disrespect."}, "language_style": {"answer": "Calm and direct", "rationale": ""}, "time_dimension": {"answer": "Past", "rationale": ""}, "sentence_type": "Affirmative declarative sentence", "pos": {"nouns": {"- The voice": 2, "Sound": 1, "Laughing": 1, "Others": 1}, "verbs": {"Talk": 1, "Smile": 1, "Yeah": 1}, "adjectives": {"Large": 1}, "numerals": {"4": 1}}}, {"content": "1 Delay: slow start, need for momentum;", "word_count": 12, "label": "", "sentence_structure": "Subject鈥搗erb鈥揷omplement", "subjective_objective": {"answer": "Subjective", "rationale": "Because delay is a subjective choice of conduct and attitude, not an objective fact or result. Everyone can delay, depending on their personal preferences, emotional state and their perception of the mission. Therefore, it is subjective to look at the delay, not just to describe objectively what happened."}, "sentence_sentiment": {"answer": "Negative", "rationale": "Because the delay is negative."}, "language_style": {"answer": "Tactful and warm", "rationale": "The entry uses a more euphemistic formulation, which does not refer to the respondent鈥檚 problems too directly or harshly, but rather leads them to think about their behaviour in a moderate way. At the same time, the term 鈥渆verything starts slowly, needs momentum鈥?is used to make the questionnaire more relevant to the real life scene and helps the interviewee to better understand and answer the questions."}, "time_dimension": {"answer": "Present", "rationale": "The keywords are \"slow start\" and \"need for drive\", which describe the person being tested in his or her current behaviour or state. The entry therefore refers to the current behaviour or state."}, "sentence_type": "Affirmative declarative sentence", "pos": {"nouns": {"Delay": 1, "Yes": 1}, "verbs": {"Start": 1, "Yes": 1, "Driver": 1}, "adverbs": {"鈾?Where 鈾?: 1, "Slow": 1}, "numerals": {"1": 2}}}, {"content": "2- Over-suspect: suspicious and untrustworthy;", "word_count": 11, "label": "", "sentence_structure": "Subject鈥搗erb鈥揷omplement", "subjective_objective": {"answer": "Subjective", "rationale": "Because doubt and mistrust are patients' subjective attitudes."}, "sentence_sentiment": {"answer": "Negative", "rationale": "Because the words \"suspicious\" and \"suspicious\" in the entries are negative."}, "language_style": {"answer": "Calm and direct", "rationale": ""}, "time_dimension": {"answer": "Present", "rationale": ""}, "sentence_type": "Affirmative declarative sentence", "pos": {"nouns": {"Yes": 1, "Doubt": 1, "others": 1}, "verbs": {"be": 1, "not": 1, "Yes": 1}, "adjectives": {"I'm suspicious": 1}, "numerals": {"2": 1}}}, {"content": "3 Easier: irritation and anger when action is not fast or when the assigned task cannot be completed;", "word_count": 23, "label": "", "sentence_structure": "Subject鈥搗erb鈥揷omplement", "subjective_objective": {"answer": "Subjective", "rationale": "Because anger is a emotional reaction, it is the patient鈥檚 subjective feeling and attitude. While this may be caused by objective circumstances, such as a lack of speed or failure to complete the assigned work, patients鈥?response to these situations remains subjective."}, "sentence_sentiment": {"answer": "Negative", "rationale": "Because the entry describes the negative mood of anger and has a negative emotional tone."}, "language_style": {"answer": "Calm and direct", "rationale": "The language style of the entry is relatively calm and straightforward, with no excessive qualifier or emotional colour. It clearly lists the problematic behaviour that may exist in the person under investigation and uses objective and precise language to describe it, which is obvious."}, "time_dimension": {"answer": "Past", "rationale": "The entry describes the reaction of the person tested in a given situation (inappropriate action or inability to complete the assigned task), which has already occurred and is therefore a temporal dimension of the past."}, "sentence_type": "Affirmative declarative sentence", "pos": {"nouns": {"Easier than I thought": 1, "Action": 1, "Work": 1}, "verbs": {"Completed": 1, "Assign": 1, "I'm so upset": 1, "- Get angry": 1}, "numerals": {"3": 1}}}, {"content": "4 Not focused: not able to focus or concentrate.", "word_count": 15, "label": "", "sentence_structure": "Subject鈥搗erb鈥揷omplement", "subjective_objective": {"answer": "Subjective", "rationale": "Because of the subjective attitude or mental state of focus, it is up to the patient to determine whether he or she is able to concentrate or concentrate."}, "sentence_sentiment": {"answer": "Negative", "rationale": "Because the \"can't concentrate\" in the entry is negative."}, "language_style": {"answer": "Tactful and warm", "rationale": "The entry is more moderate in language style, using a descriptive phrase to express the problems that the other side may have, without strong language or critical tone. This is more acceptable and understandable and helps to create a good atmosphere of communication."}, "time_dimension": {"answer": "Past", "rationale": "In the entry, \"not\" expresses a problem that has arisen in the past and is therefore judged to be \"the past\"."}, "sentence_type": "Negative declarative sentence", "pos": {"nouns": {"Focus": 1, "Strength": 1}, "verbs": {"Cannot": 1, "Focus": 2}, "adverbs": {"not": 1, "Or": 1}, "numerals": {"4": 1}}}, {"content": "1. Retributive: To hate and punish those who offend themselves;", "word_count": 14, "label": "", "sentence_structure": "Subject鈥搗erb鈥揷omplement", "subjective_objective": {"answer": "Subjective", "rationale": "Because reprisals are an emotional response, they reflect patients ' subjective feelings and attitudes towards the behaviour of others."}, "sentence_sentiment": {"answer": "Negative", "rationale": "Because the words \"retaliation,\" \"hate\" and \"punishment\" are negative."}, "language_style": {"answer": "Calm and direct", "rationale": "The entry clearly describes the concept of \"retributive\" in simple language, without too many rhetorical or emotional renderings and with a more objective and direct tone."}, "time_dimension": {"answer": "Present", "rationale": "The keyword \"hate and punish\" represents an emotional reaction and behaviour that may be occurring or already have occurred, and therefore the temporal dimension is now judged."}, "sentence_type": "Affirmative declarative sentence", "pos": {"nouns": {"Retribution": 1, "Hate": 1, "Punishment": 1, "I'm sorry": 1, "self": 1, "person": 1}, "verbs": {"have": 1}, "adverbs": {"and": 1}, "numerals": {"One": 2}, "pronouns": {"self": 1}}}, {"content": "2 Disturbing: new and old, not to do the same thing for long periods of time;", "word_count": 18, "label": "", "sentence_structure": "Subject鈥搗erb鈥揷omplement", "subjective_objective": {"answer": "Subjective", "rationale": "Because \"a new age\" and \"a new age\" and \"a new age\" are personal feelings and preferences of the patient and are subjective."}, "sentence_sentiment": {"answer": "Negative", "rationale": "Because the words \"sympathetic\" and \"not like doing the same thing for a long time\" in the entries all convey a negative sentiment."}, "language_style": {"answer": "Tactful and warm", "rationale": "The entry is more personal and interactive when it is spoken in a gentle and inviting manner, rather than in direct statements or orders. It is also not too much rhetorical or emotionally recasting, and is more objective and accessible to the interviewees."}, "time_dimension": {"answer": "Present", "rationale": "\"Infuries\" is a term for the current mood of the testees, and \"invited\" and \"involved in doing the same thing for a long time\" are also a term for the present. So the temporal dimension of this article is now."}, "sentence_type": "Affirmative declarative sentence", "pos": {"nouns": {"I'm so upset": 1, "鈾?New and old 鈾?: 1, "Things": 1}, "verbs": {"Yeah": 1, "Do it": 1, "Tired": 1}, "adverbs": {"Long time": 1}, "numerals": {"2": 1}}}, {"content": "3 Barely: unwilling to participate or to input;", "word_count": 12, "label": "", "sentence_structure": "Subject鈥搗erb鈥揷omplement", "subjective_objective": {"answer": "Objective", "rationale": "This is a description of the patient ' s behaviour in a given situation, not a subjective perception or attitude of the patient."}, "sentence_sentiment": {"answer": "Negative", "rationale": "Because the word \"absorbent\" usually indicates reluctance or reluctance, it is a negative term."}, "language_style": {"answer": "Calm and direct", "rationale": "The entry expresses the requirement of attitude towards the subject in a concise and clear language, is not overly emotional or rhetorical and is more directly acrimony."}, "time_dimension": {"answer": "Past", "rationale": "The purpose of the questionnaire is to describe the attitudes or preferences of the person being tested in a given situation, without a clear temporal dimension, and therefore to determine precisely whether the behaviour or mentality of the past, present or future is present."}, "sentence_type": "Negative declarative sentence", "pos": {"nouns": {"Questionnaire Entry": 1, "I barely": 1, "Input": 1}, "verbs": {"Yes": 1, "Participation": 1, "Say it": 1}, "adjectives": {"not": 1, "of": 2}, "adverbs": {"very": 1}, "numerals": {"3": 1}}}, {"content": "4 Indiscretion: impatience, lack of thought and hasty action.", "word_count": 15, "label": "", "sentence_structure": "Subject鈥搗erb鈥搊bject", "subjective_objective": {"answer": "Objective", "rationale": "Because hastyness is an objective manifestation, not related to the patient ' s subjective attitude."}, "sentence_sentiment": {"answer": "Negative", "rationale": "Because \"negative\" behavior in the entries is negative."}, "language_style": {"answer": "Tactful and warm", "rationale": "The language style of the entry is mild and euphemistic. It uses descriptive terms \"discretionary\" to express criticism or evaluation of certain acts, and to emphasize that the motivation behind such behaviour may be indulging in patience and reflection. Such expressions are more reflective and self-improvement than direct accusations."}, "time_dimension": {"answer": "Present", "rationale": "The entry now describes the current orientation or behaviour of the testee, the keywords being \"discretionary\", \"insatiable\" and \"insatisfactional\", which are describing the current state and characteristics."}, "sentence_type": "Negative declarative sentence", "pos": {"nouns": {"Insensitive": 1, "Patience": 1, "Think": 1, "Action": 1}, "verbs": {"No, I'm not": 1, "The long run": 1, "Scratch": 1, "Analysis": 1}, "adjectives": {"4": 1, "Insensitive": 1, "None": 1, "Patience": 1, "Think": 1, "Scratch": 1}, "numerals": {"4": 1}, "classifiers": {"individual": 1}}}, {"content": "1 Compromise: to avoid contradictions, even if they are right, and to give up their positions;", "word_count": 24, "label": "", "sentence_structure": "Subject鈥搗erb鈥揷omplement", "subjective_objective": {"answer": "Subjective", "rationale": "Because \"compromise\" and \"rejection\" are patients' subjective attitudes and behaviours."}, "sentence_sentiment": {"answer": "Negative", "rationale": "Because \"compromise\" and \"release your position\" in the entries are both negative."}, "language_style": {"answer": "Calm and direct", "rationale": "The entry is a direct indication of the definition of compromise, does not use excessive rhetorical or emotional rendering, is more balanced and objective, and therefore can be judged to be a calm and straightforward language style."}, "time_dimension": {"answer": "Present", "rationale": "The keyword \"to avoid contradictions, even if it is right, and to give up its position\" describes a customary act of the person being tested, which has not only occurred in the past but may still be ongoing. Thus, the temporal dimension of the article is now judged."}, "sentence_type": "Negative declarative sentence", "pos": {"nouns": {"Compromise": 1, "Paradoxious": 1, "Position": 1}, "verbs": {"Avoid": 1, "Abandon": 1}, "adverbs": {"Even if": 1}}}, {"content": "Good criticism: constant measurement and judgement, often considering making objections;", "word_count": 22, "label": "", "sentence_structure": "Subject鈥搗erb鈥揷omplement", "subjective_objective": {"answer": "Objective", "rationale": "Because \"good criticism\" is a personal character or behaviour, an objective fact that the patient has, not a subjective feeling or experience."}, "sentence_sentiment": {"answer": "Negative", "rationale": "Because the act of \"continuous measurement and judgement, often considering making objections\" in the entry is negative."}, "language_style": {"answer": "Calm and direct", "rationale": "The language style of the entry is more direct and objective, without too much emotional or rhetorical, simply describing a characteristic or behaviour, and using a clear adjective to describe it. So I think the language style of the entry is \"cool and straight.\""}, "time_dimension": {"answer": "Present", "rationale": "The words \"continuous measurement and judgement\" and \"regarding objection\" suggest that the entry is asking the testee about his attitude and behaviour towards criticism and can therefore be judged as now."}, "sentence_type": "Affirmative declarative sentence", "pos": {"nouns": {"Criticism": 1, "Constantly": 1, "Measurement": 1, "Judgement": 1, "Regular": 1, "Present": 1, "Objection": 1}, "verbs": {"That's very critical": 1, "Down": 1, "Consider": 1, "Present": 1}, "adverbs": {"Land": 1, "and": 1, "鈾?Keep going 鈾?: 1, "Regular": 1}, "numerals": {"2": 1}}}, {"content": "3 Sly: smart, always able to achieve the goal;", "word_count": 13, "label": "", "sentence_structure": "Subject鈥搗erb鈥揷omplement", "subjective_objective": {"answer": "Subjective", "rationale": "Because slyness is a human character or behaviour that is viewed from a subjective perspective."}, "sentence_sentiment": {"answer": "Negative", "rationale": "The basis for the judgement: the term \"soft\" is usually derogatory, indicating negative behaviour and quality, such as deception, dishonesty, etc. So the entry \"3 Sweat: Smart, There is always a Way to Reach the Goal\" is a negative quality."}, "language_style": {"answer": "Calm and direct", "rationale": "The entry is more straightforward, describing the character of a person, and does not use too much accommodative language and is more concise and quick."}, "time_dimension": {"answer": "Present", "rationale": "The entry describes an individual's identity or character, and the terms \"soft\" , \"smart\" and \"thousands to achieve an end\" indicate that this is a current pattern of behaviour. At the same time, these acts may have existed in the past, but in this case, more emphasis is placed on the current state and performance."}, "sentence_type": "Affirmative declarative sentence", "pos": {"nouns": {"Sly": 1, "Smart": 1, "Approach": 1, "Purpose": 1}, "verbs": {"Achieved": 1}, "adjectives": {"Sly": 2, "Smart": 1, "Always": 1}, "numerals": {"3": 1}}}, {"content": "4 Be a good change: short-term, child-like attention, needing changes, fear of boredom.", "word_count": 20, "label": "", "sentence_structure": "Subject鈥搗erb鈥搊bject", "subjective_objective": {"answer": "Subjective", "rationale": "Because change is a subjective characteristic and feeling of the patient, it is a subjective description."}, "sentence_sentiment": {"answer": "Negative", "rationale": "Because the words \"sweet\" and \"fear of boredom\" in the entries are all derogatory and negative emotional expressions, indicating a bad attitude or quality."}, "language_style": {"answer": "Tactful and warm", "rationale": "The entry uses a more moderate tone, describing it by analogy \"like a child\" to make it more friendly and understandable. At the same time, the purpose of the article is expressed in a more euphemistic manner, without direct criticism or criticism, but rather in an attempt to lead each other to reflect and reflect on their own behaviour in a positive way."}, "time_dimension": {"answer": "Present", "rationale": ""}, "sentence_type": "Affirmative declarative sentence", "pos": {"nouns": {"It's a change": 1, "Son": 1, "Attention": 1, "Change": 1, "Boring": 1}, "verbs": {"Yeah": 1, "Yes": 2}, "adjectives": {"Short": 1}, "numerals": {"4": 1}}}]}

{"questionnaire_title": "Summary of health surveys", "questionnaire_category": "Emotion and affect", "item_count": 11, "emotion_shift_overall": "No obvious trend", "language_style_overall": "Calm and direct", "items": [{"content": "1. In general, your health status is as follows:", "word_count": 11, "label": "", "sentence_structure": "Subject鈥揷opula鈥損redicative", "subjective_objective": {"answer": "Subjective", "rationale": "The general health situation is subjective, as it is a person ' s own feeling and assessment."}, "sentence_sentiment": {"answer": "Neutral", "rationale": "Because the entry does not have any emotional color."}, "language_style": {"answer": "Tactful and warm", "rationale": ""}, "time_dimension": {"answer": "Present", "rationale": "The entry asks the testee about his current state of health and the time dimension is now judged."}, "sentence_type": "Interrogative sentence", "pos": {"nouns": {"Overall": 1, "Health status": 1}, "verbs": {"be": 1}}}, {"content": "2 and 1 year ago, you felt your health condition was as follows:", "word_count": 16, "label": "", "sentence_structure": "Subject鈥搗erb鈥揷omplement", "subjective_objective": {"answer": "Subjective", "rationale": "This is a subjective examination, since the patient has a personal perception and evaluation of his or her health status."}, "sentence_sentiment": {"answer": "Neutral", "rationale": "Because the entry does not express positive or negative feelings, it merely asks about changes in personal feelings."}, "language_style": {"answer": "Tactful and warm", "rationale": ""}, "time_dimension": {"answer": "Past", "rationale": ""}, "sentence_type": "Interrogative sentence", "pos": {"nouns": {"Year": 1, "Before": 1, "self": 1, "Health status": 1}, "verbs": {"I think": 1, "be": 1}, "adjectives": {"1 year ago": 1}, "numerals": {"2": 1}, "pronouns": {"you": 1}}}, {"content": "3. The following questions are all related to daily activities.", "word_count": 43, "label": "", "sentence_structure": "Subject鈥揷opula鈥損redicative", "subjective_objective": {"answer": "Subjective", "rationale": "The problem is the extent to which the patient ' s subjective health affects his or her daily life."}, "sentence_sentiment": {"answer": "Neutral", "rationale": "As this entry merely asks about the restrictions on daily activities imposed by the health status of the person interviewed, it does not express any emotional colour or attitude."}, "language_style": {"answer": "Calm and direct", "rationale": "This is a more calm, professional and targeted way of expressing itself, which is suitable for use in questionnaires, so that accurate data can be collected."}, "time_dimension": {"answer": "Present", "rationale": "The entry asks whether the present physical health of the person tested affects their daily activities and requires the person tested to assess the extent of the impact, which is now judged in terms of time dimension."}, "sentence_type": "Interrogative sentence", "pos": {"nouns": {"Problem": 2, "Day": 1, "Activities": 2, "Health status": 1, "Limits": 1, "Level": 1}, "verbs": {"Think about it": 1, "Is it possible": 1, "Limited": 1, "If": 1, "have": 1, "How far are you": 1}, "adverbs": {"Both": 1, "Day": 1}, "numerals": {"3": 1}, "pronouns": {"Yours": 2}}}, {"content": "Have you, in the last four weeks, had the following questions arisen in your work and daily activities for reasons of health?", "word_count": 36, "label": "", "sentence_structure": "Subject鈥搗erb鈥搊bject", "subjective_objective": {"answer": "Subjective", "rationale": "The question is to examine the patient's subjective feelings, so the answer should be \"subjective\"."}, "sentence_sentiment": {"answer": "Neutral", "rationale": "The entry is merely an inquiry as to whether the person tested had an impact on work and daily life for health reasons, and it does not have any emotional colour."}, "language_style": {"answer": "Tactful and warm", "rationale": "This expression is more personal and humanistic and helps to establish a good investigative relationship."}, "time_dimension": {"answer": "Past", "rationale": "The entry is for the purpose of questioning the behaviour of the subject over the past four weeks, and the time dimension is judged to be the past."}, "sentence_type": "Interrogative sentence", "pos": {"nouns": {"Past": 1, "4 weeks": 1, "Work": 1, "Daily life": 1, "Reason": 1, "Problem": 2}, "verbs": {"Come on": 1, "Because": 1, "Physical health": 1}, "adjectives": {"Below": 1, "These": 1}, "numerals": {"Four": 1}, "pronouns": {"you": 1, "of": 2}}}, {"content": "Have you, in the last four weeks, been able to do your work and your daily activities to the following questions for emotional reasons (such as depression or anxiety)?", "word_count": 40, "label": "", "sentence_structure": "Subject鈥搗erb鈥搊bject", "subjective_objective": {"answer": "Subjective", "rationale": "Because \"emotional causes\" and \"pression or anxiety\" are the subjective feelings and thoughts of patients."}, "sentence_sentiment": {"answer": "Neutral", "rationale": "The entry merely asked whether the past four weeks had affected work and everyday life for emotional reasons, without any emotional colour."}, "language_style": {"answer": "Calm and direct", "rationale": "In a direct query, the entry clearly stated that the survey was about the effect of emotions on work and daily life, and listed specific issues, with a more objective and calm language style."}, "time_dimension": {"answer": "Past", "rationale": "The entry asks about the behaviour of the person tested in the last four weeks, and the keywords are \"for the last four weeks\" and \"causes of emotion (such as depression or anxiety)\" and \"the following problems\". These point to the past."}, "sentence_type": "Interrogative sentence", "pos": {"nouns": {"Past": 1, "4 weeks": 1, "Work": 1, "Daily activities": 1, "Reason": 1, "Repression": 1, "Fear": 1, "Problem": 1}, "verbs": {"Come on": 1}, "adverbs": {"Yes": 1}, "numerals": {"Four": 1}, "classifiers": {"individual": 1}}}, {"content": "6. To what extent has your health or emotional infirmity affected your normal social interaction with your family, friends, neighbours or groups over the past four weeks?", "word_count": 43, "label": "", "sentence_structure": "Subject鈥搗erb鈥搊bject", "subjective_objective": {"answer": "Objective", "rationale": "Since the actual behaviour and situation of the patient over the past four weeks have been examined, it is an objective perspective."}, "sentence_sentiment": {"answer": "Negative", "rationale": "Because \"health or emotional discomfort\" is a negative situation in the entry, it may have a negative impact on social interaction."}, "language_style": {"answer": "Calm and direct", "rationale": "The entry is based on the following: 1. The entry directly asks the respondents how far their health or emotional state has affected their social activities over the past four weeks. 2. Instead of using excessive rhetorical or emotional rendering, the entry presents the problem in a more objective and direct way, allowing the respondents to understand the question and answer it clearly. 3. The entry is also more concise in its language, without lengthy sentences or complex terms, which are easily understood and accepted by the wider population."}, "time_dimension": {"answer": "Past", "rationale": ""}, "sentence_type": "Interrogative sentence", "pos": {"nouns": {"Past": 1, "4 weeks": 1, "Health": 1, "Emotion": 2, "Level": 1, "Family": 1, "friends": 1, "Neighbors": 1, "Collective": 1, "Normal": 1, "Social interaction": 1}, "verbs": {"It's affecting": 1}, "adverbs": {"How much": 1, "Lee": 2}, "numerals": {"Four": 1}, "pronouns": {"you": 1, "they": 1}}}, {"content": "7 - Have you had any physical pain in the last 4 weeks?", "word_count": 14, "label": "", "sentence_structure": "Subject鈥損redicate", "subjective_objective": {"answer": "Objective", "rationale": "Because the physical pain is the physical state of the patient, it is an objective fact that can be directly observed and measured."}, "sentence_sentiment": {"answer": "Neutral", "rationale": "Because the entry does not have any emotional color."}, "language_style": {"answer": "Calm and direct", "rationale": "The language style of the entry is relatively simple, there is no excessive emotional rendering or speech writing, and the tone is more calm and objective. It is a typical way of asking questions directly about the physical pain that the person under investigation has experienced in the past four weeks."}, "time_dimension": {"answer": "Past", "rationale": "The entry is for the testee ' s physical condition over the past four weeks, and the time dimension is judged to be past."}, "sentence_type": "Interrogative sentence", "pos": {"nouns": {"Past": 1, "4": 1, "Week": 1, "Body": 1, "Pain": 1}, "verbs": {"have": 1}, "numerals": {"4": 1}, "classifiers": {"individual": 1}}}, {"content": "Have your physical pains affected your work and your housework in the last four weeks?", "word_count": 24, "label": "", "sentence_structure": "Subject鈥搗erb鈥搊bject", "subjective_objective": {"answer": "Objective", "rationale": "This is an objective fact because it is an inquiry into the impact of the patient ' s past physical condition on work and household work."}, "sentence_sentiment": {"answer": "Negative", "rationale": "The basis for the judgement is that \"physical pain affects work and household chores\" is a negative situation."}, "language_style": {"answer": "Calm and direct", "rationale": "The entry is more straightforward, has no excessive rhetoric or emotional resonance, and is primarily intended to ask the interviewees whether their physical condition has affected their work and household chores over the past four weeks and can therefore be described as \"cooling straight\"."}, "time_dimension": {"answer": "Past", "rationale": "The entry asks whether the testee ' s physical pain has affected his work and housework over the past four weeks, and the temporal dimension is a matter of past."}, "sentence_type": "Interrogative sentence", "pos": {"nouns": {"Past": 1, "Week": 1, "Body": 1, "Pain": 1, "Work": 1, "Domestic work": 1}, "verbs": {"Impact": 1}, "adverbs": {"Four": 1, "Lee": 1}, "numerals": {"8銆?: 1}}}, {"content": "The following questions are about your own feelings over the past month, what is your situation with regard to each question?", "word_count": 40, "label": "", "sentence_structure": "Subject鈥搗erb鈥搊bject", "subjective_objective": {"answer": "Subjective", "rationale": "Because the question is about the patient ' s feelings and circumstances over the past month, it is about the patient ' s personal subjective experience and perception."}, "sentence_sentiment": {"answer": "Neutral", "rationale": "Because the entry merely asked the respondents what they had felt over the past month, it had not been of any emotional colour."}, "language_style": {"answer": "Calm and direct", "rationale": "The language style of the entry is straightforward. It opens the door to research questions, not using too many rhetorical or emotional techniques, but in plain and plain language. Moreover, it is not subject to subjective guidance, but rather to the objective and impartial nature of the interviewee鈥檚 response, based on his own reality."}, "time_dimension": {"answer": "Past", "rationale": "The entry is for the testee ' s experience over the past month and the time dimension is judged to be the past."}, "sentence_type": "Interrogative sentence", "pos": {"nouns": {"Problem": 2, "Past": 1, "In the month": 1, "self": 1, "Feel": 1, "Things": 1, "Situation": 1}, "verbs": {"It's about": 1, "Say it": 1, "EMPTY": 2}, "adjectives": {"Details": 1}, "numerals": {"9": 1, "One": 3, "Month": 1}, "classifiers": {"individual": 1}, "pronouns": {"you": 1}}}, {"content": "10: Inhealth affects your social activities (e.g., visiting friends and family):", "word_count": 17, "label": "", "sentence_structure": "Subject鈥搗erb鈥搊bject", "subjective_objective": {"answer": "Subjective", "rationale": "Because the subject is one of the patients鈥?own feelings and perceptions, i.e. whether ill health affects their own social activities, it is not an objective fact."}, "sentence_sentiment": {"answer": "Negative", "rationale": "Because the expression \"inhealth affects your social activities\" in the entry is negative."}, "language_style": {"answer": "Calm and direct", "rationale": "The language style of the entry is calm and straightforward, and it raises a simple and straightforward question, which does not contain any rhetorical or emotional colours, but rather asks the interviewees in an objective and neutral manner about the actual situation."}, "time_dimension": {"answer": "Present", "rationale": ""}, "sentence_type": "Negative declarative sentence", "pos": {"nouns": {"Ill health": 1, "Impact": 1, "Social activities": 1, "Go visit your friends": 1}, "verbs": {"Impact": 1, "Go visit your friends": 1}, "numerals": {"10": 1}}}, {"content": "11 - Please see each of the following questions, which answer is best suited to your situation?", "word_count": 21, "label": "", "sentence_structure": "Subject鈥搗erb鈥搊bject", "subjective_objective": {"answer": "Subjective", "rationale": "Because the entry is asked about the views and feelings of the interviewees themselves and is a subjective examination."}, "sentence_sentiment": {"answer": "Neutral", "rationale": "As the entry does not express positive or negative feelings, attitudes, behaviour, purpose or quality."}, "language_style": {"answer": "Calm and direct", "rationale": "The language style of the entry is more straightforward, with no excessive adoration or emotional colour, and with calm and objective tone, and is intended to allow the interviewee to express his views and ideas as accurately as possible."}, "time_dimension": {"answer": "Present", "rationale": ""}, "sentence_type": "Interrogative sentence", "pos": {"nouns": {"Questionnaire": 1, "Entry": 1, "Problem": 1, "Answer": 1, "Situation": 1}, "verbs": {"Look": 1, "Match": 1}, "numerals": {"11": 1, "Every one": 2}, "classifiers": {"Plant": 1, "Which one": 2, "Below": 1}, "pronouns": {"you": 1}}}]}

{"questionnaire_title": "Sarason Test Anxious Sheets", "questionnaire_category": "Mental health", "item_count": 37, "emotion_shift_overall": "From positive to negative", "language_style_overall": "Neutral", "items": [{"content": "I've always thought that people are smarter than me when a big test comes.", "word_count": 25, "label": "", "sentence_structure": "Subject鈥揷opula鈥損redicative", "subjective_objective": {"answer": "Subjective", "rationale": "Because \"always thinking\" is a reflection of the patient's personal feelings and attitudes."}, "sentence_sentiment": {"answer": "Negative", "rationale": "Because the phrase \"always thinking of others being much smarter than me\" in the entry is negative, expressing self-confidence and envy for others."}, "language_style": {"answer": "Calm and direct", "rationale": "The entry is in a straightforward language style, is not over-articulated or emotional, and is more objective in tone.\""}, "time_dimension": {"answer": "Past", "rationale": "The entry describes a mentality or feeling that does not involve specific past, present or future behaviour and therefore does not allow for a judgement on its temporal dimension."}, "sentence_type": "Negative declarative sentence", "pos": {"nouns": {"Number of times": 1, "Significant": 1, "Examinations": 1, "Time": 1, "person": 1, "Smart": 2}, "verbs": {"It's a temporary call": 1, "Always thinking": 1}, "adjectives": {"More": 1}, "adverbs": {"- Yeah": 1, "Much more": 1}, "pronouns": {"I": 1}}}, {"content": "If I'm going to have to do a smart test, I'm very anxious before I do it.", "word_count": 22, "label": "", "sentence_structure": "Subject鈥損redicate", "subjective_objective": {"answer": "Subjective", "rationale": "Because anxiety is an emotional experience, it's the patient's subjective feeling."}, "sentence_sentiment": {"answer": "Negative", "rationale": "Because the emotional attitude of \"very anxious\" in the entry is negative."}, "language_style": {"answer": "Calm and direct", "rationale": "The entry is asked whether the person surveyed was anxious before performing the smart test, without using excessive adoration or emotional expressions, and with a more calm and direct language style."}, "time_dimension": {"answer": "Present", "rationale": "The \"will\" in the entry indicates that it is an act of hypotheticality, and the description \"I'll do a smart test\" indicates that it is an imminent situation. So the time dimension of this questionnaire entry is now."}, "sentence_type": "Affirmative declarative sentence", "pos": {"nouns": {"Smart Test": 1, "Before": 1, "Anxiety": 1}, "verbs": {"Yes": 1, "Do it": 1, "Very": 1}, "adjectives": {"Anxiety": 1}, "adverbs": {"Yes, I'm going": 1, "in": 1, "Very": 1}, "pronouns": {"I": 1}}}, {"content": "If I knew there would be a smart test, I'd feel confident and relaxed before that.", "word_count": 27, "label": "", "sentence_structure": "Subject鈥搗erb鈥搊bject", "subjective_objective": {"answer": "Subjective", "rationale": "Because feeling self-confidence and ease is the subjective feeling of the patient when facing the test, not the objective fact."}, "sentence_sentiment": {"answer": "Positive", "rationale": "Because the description \"fears confident, easy\" is positive emotional."}, "language_style": {"answer": "Tactful and warm", "rationale": "The entry is more euphemistic, using the hypothetical phrase \"if I know\" to express a possibility and, in the following part, to express positive feelings, such as \"I'm confident and relaxed.\" This expression neither puts pressure or threat on the person interviewed, nor gives rise to positive emotions and self-assurance."}, "time_dimension": {"answer": "Past", "rationale": "Output: Past"}, "sentence_type": "Affirmative declarative sentence", "pos": {"nouns": {"Questionnaire Entry": 1, "Smart Test": 1, "Before": 1, "Confidence": 1, "Easy": 1}, "verbs": {"Yes": 2, "There will be": 1, "I feel": 1, "very": 2, "be": 1, "in": 1, "have": 1}, "adjectives": {"A lot": 1}, "adverbs": {"Before": 1, "very": 2}, "numerals": {"3": 1, "Once": 1}, "pronouns": {"I": 1}}}, {"content": "I'll sweat a lot when I take the big test.", "word_count": 13, "label": "", "sentence_structure": "Subject鈥搗erb鈥揷omplement", "subjective_objective": {"answer": "Objective", "rationale": "Because sweating is a physical reaction that does not involve subjective feelings or will of the individual."}, "sentence_sentiment": {"answer": "Negative", "rationale": "The basis for the judgement: sweating may be a cause of tension and anxiety during major examinations, and therefore it is a negative act."}, "language_style": {"answer": "Calm and direct", "rationale": "The entry is a direct statement of the fact that the major examinations are sweaty, without any qualifier or emotional colour, and that the tone is more objective. The language style is therefore \"cool and straight\"."}, "time_dimension": {"answer": "Present", "rationale": "\"As part of a major test\" means a certain time scenario, and \"I will sweat a lot\" describes the physical reaction that occurs in this time scenario, so the entry reflects the current behavior or state."}, "sentence_type": "Affirmative declarative sentence", "pos": {"nouns": {"Major examinations": 1, "Khan": 1}, "verbs": {"Yes": 1}, "adverbs": {"A lot": 1}, "pronouns": {"I": 1}}}, {"content": "During the exam, I found myself thinking things that had nothing to do with the examination.", "word_count": 24, "label": "", "sentence_structure": "Subject鈥搗erb鈥搊bject", "subjective_objective": {"answer": "Subjective", "rationale": "Because \"thinking something that has nothing to do with the test\" is the patient's subjective idea."}, "sentence_sentiment": {"answer": "Negative", "rationale": "Because the \"always thinking about something that has nothing to do with the content of the exam\" in the entry is negative and may affect the performance of the exam."}, "language_style": {"answer": "Calm and direct", "rationale": "The language style of the entry is relatively calm and objective, and directly expresses the question of whether the other party is easily distracted during the examination, without excessive rhetorical or emotional rendering."}, "time_dimension": {"answer": "Present", "rationale": "The entry is for the purpose of asking the respondent about the state of attention during the examination, and the time dimension is now judged."}, "sentence_type": "Affirmative declarative sentence", "pos": {"nouns": {"Examination period": 1, "self": 1, "Examination content": 1, "Yes": 1}, "verbs": {"Found": 1, "I'm thinking": 1, "Yes": 1}, "adverbs": {"Always": 1, "Not relevant": 1}, "pronouns": {"I": 1}}}, {"content": "When a sudden attack came, I was scared.", "word_count": 19, "label": "", "sentence_structure": "Subject鈥搗erb鈥揷omplement", "subjective_objective": {"answer": "Subjective", "rationale": "Because \"fear\" is the patient's subjective feeling, and it's psychological experience."}, "sentence_sentiment": {"answer": "Negative", "rationale": "Because the \"fear\" attitude in the entry is negative."}, "language_style": {"answer": "Tactful and warm", "rationale": "The language style of the entry is more moderate, and it is more personal and interactive, by speaking with a voice that cares about the other person's feelings, rather than by direct statements or orders. At the same time, the \"shock-and-shock test\" in the entry gives a sense of urgency, which further increases emotionality."}, "time_dimension": {"answer": "Past", "rationale": "\"Feared\" means that the testee's fear of the test in his past experience is judged by the time dimension of the past."}, "sentence_type": "Negative declarative sentence", "pos": {"nouns": {"Examinations": 1, "Attack": 1}, "verbs": {"I feel": 1, "Come on": 1, "Yes": 1}, "adjectives": {"Suddenly": 1}, "numerals": {"6": 1}, "pronouns": {"I": 1}}}, {"content": "I often thought of failure during the exam.", "word_count": 12, "label": "", "sentence_structure": "Subject鈥搗erb鈥搊bject", "subjective_objective": {"answer": "Subjective", "rationale": "Because \"I often think of failure during exams\" is a person's inner feeling and thinking, subjective."}, "sentence_sentiment": {"answer": "Negative", "rationale": "Because the idea of \"thinking failure\" in the entry is negative and could have a negative impact on the results of the examination."}, "language_style": {"answer": "Calm and direct", "rationale": "The entry is more straightforward, concise, with no excessive accommodation or emotional rendering, and is more neutral and objective. It asks whether the respondents often think of failure during the examination, and does not use such a terminologies as euphemism or metaphors. It is therefore judged to be \"cool and direct.\""}, "time_dimension": {"answer": "Present", "rationale": "Because the title appears in the time-frame of the \"test\" and it represents the behaviour or mentality of the time period and is in the process of being judged to be the present."}, "sentence_type": "Negative declarative sentence", "pos": {"nouns": {"Examination period": 1, "Failed": 1}, "verbs": {"Yes": 1, "Present": 1}, "adverbs": {"Regular": 1}}}, {"content": "I've been nervous since the big exams, and I've had a stomach problem.", "word_count": 18, "label": "", "sentence_structure": "Subject鈥搗erb鈥揷omplement", "subjective_objective": {"answer": "Subjective", "rationale": "Because \"frightful\" and \"tummy indisposed\" are the feelings and experiences of the patient themselves, and are subjectively viewed."}, "sentence_sentiment": {"answer": "Negative", "rationale": "Because \"the stomach is not well\" in the entry is a negative sign."}, "language_style": {"answer": "Tactful and warm", "rationale": "The language style of the entry is a warmer type. It uses the words \"often\" and \"and so on\" and expresses the feeling of stress and stomach discomfort after the examination, which is more intimate and sympathetic."}, "time_dimension": {"answer": "Present", "rationale": "The keyword \"after significant examinations\" indicates that the act occurred after a certain point in time in the past, but it is still possible and therefore judged to be the present."}, "sentence_type": "Affirmative declarative sentence", "pos": {"nouns": {"Examinations": 1, "Stomach": 1, "Nervous": 1, "I'm not well": 1}, "verbs": {"I feel": 2, "And so": 1}, "adjectives": {"Significant": 1}, "adverbs": {"Regular": 1}, "pronouns": {"I": 1}}}, {"content": "I've been getting a little bit confused about things like smart and finals.", "word_count": 20, "label": "", "sentence_structure": "Subject鈥搗erb鈥搊bject", "subjective_objective": {"answer": "Subjective", "rationale": "Because \"fun\" is a subjective feeling or emotional reaction."}, "sentence_sentiment": {"answer": "Negative", "rationale": "Because the emotional attitude in the entry, \"Assisting on things like smart and final exams,\" is negative."}, "language_style": {"answer": "Tactful and warm", "rationale": "The language style of the entry is euphemistic. Using the word \"speech\" expression, the interviewee senses the attention and understanding of researchers about their feelings, while avoiding too direct or negative expressions, making the questionnaire more personal and interactive."}, "time_dimension": {"answer": "Present", "rationale": "\"I'm always feeling a little bit of a bit of a mental and final test,\" describes the current psychological state of the person being tested, so it can be judged that the entry is about the present."}, "sentence_type": "Negative declarative sentence", "pos": {"nouns": {"Smart test": 1, "Final examination": 1, "Yes": 1}, "verbs": {"I feel": 1, "- What": 1}, "adjectives": {"Always": 1}, "numerals": {"9": 1}}}, {"content": "10. A good achievement in one test does not seem to increase my confidence in the second.", "word_count": 29, "label": "", "sentence_structure": "Subject鈥搗erb鈥搊bject", "subjective_objective": {"answer": "Subjective", "rationale": "Because it involves expectations and confidence for the future, it is a subjective view of the individual."}, "sentence_sentiment": {"answer": "Negative", "rationale": "Because the phrase \"can't increase my confidence in the second exam\" in the entry is negative."}, "language_style": {"answer": "Calm and direct", "rationale": "The entry expresses a point of view in a direct, simple language, without too much embellishment or emotional rendering. This is more easily understood and accepted by the interviewees and effectively conveys the subject or issue that the researchers want to explore.\""}, "time_dimension": {"answer": "Past", "rationale": "So we can classify it as \"the past/now\"."}, "sentence_type": "Negative declarative sentence", "pos": {"nouns": {"Examinations": 2, "Achievements": 1, "Confidence": 1}, "verbs": {"Access": 1, "Increase": 1, "Seems like": 1, "Yes": 1}, "adjectives": {"Okay": 1}, "adverbs": {"No, I can't": 1, "In the second exam": 1}, "numerals": {"10": 1}, "pronouns": {"I": 1}}}, {"content": "I sometimes feel like my heart beats fast during a major exam.", "word_count": 16, "label": "", "sentence_structure": "Subject鈥揷opula鈥損redicative", "subjective_objective": {"answer": "Subjective", "rationale": "Because \"heart beats fast\" is a subjective feeling or experience, not an objective fact."}, "sentence_sentiment": {"answer": "Negative", "rationale": "Because \"heart beats fast\" is a physical reaction, usually related to stress or anxiety, which is usually negative."}, "language_style": {"answer": "Tactful and warm", "rationale": "Because the article is more euphemistic in its purpose, and uses vague words like \"sometimes\" that do not express feelings too directly or strongly or ask respondents to reveal their own state of mind. At the same time, language style is more sweet, friendly, and emotional with caring and understanding."}, "time_dimension": {"answer": "Present", "rationale": "The keyword \"sometimes feel\" indicates that it is a regular, customary behaviour and may appear again during future examinations."}, "sentence_type": "Negative declarative sentence", "pos": {"nouns": {"Major examination period": 1, "Heart rate": 1}, "verbs": {"I feel": 1, "Faster": 1}, "adjectives": {"Come on": 1}, "adverbs": {"Sometimes": 1}, "numerals": {"11": 1}}}, {"content": "I always felt better after the exam than I actually did.", "word_count": 20, "label": "", "sentence_structure": "Subject鈥揷opula鈥損redicative", "subjective_objective": {"answer": "Subjective", "rationale": "This is a subjective examination of the patient ' s assessment and perception of his or her own performance."}, "sentence_sentiment": {"answer": "Negative", "rationale": "Because the psychological state of the entry, \"which always feels better than it actually is\", may reflect excessive demands and self-criticalness and have a certain negative emotional tone."}, "language_style": {"answer": "Tactful and warm", "rationale": "The entry allows the interviewee to reflect on himself and express his feelings in a way that is not directly rebukeed or evaluated, and is more moderate in language style. At the same time, the article ' s context is also inspired and understood, and it is more resonant with the interviewee."}, "time_dimension": {"answer": "Present", "rationale": "The entry describes the feelings and thoughts of the testee after the examination, which are present and do not relate to the time dimension of the past or the future. The time dimension is therefore judged as \"now\"."}, "sentence_type": "Affirmative declarative sentence", "pos": {"nouns": {"Examinations": 1, "End": 1, "Always": 1, "I think": 1, "Yeah": 1, "- Yeah": 1, "Actually": 1, "Do it": 1, "Better": 1}, "verbs": {"Conduct": 1, "I think": 1, "- Yeah": 1, "Do it": 1, "Better": 1}, "adverbs": {"Always": 1}, "numerals": {"12": 1}, "pronouns": {"I": 1}}}, {"content": "I'm always depressed after the exams.", "word_count": 13, "label": "", "sentence_structure": "Subject鈥揷opula鈥損redicative", "subjective_objective": {"answer": "Subjective", "rationale": "Because \"frightly depressed\" is a subjective emotional experience."}, "sentence_sentiment": {"answer": "Negative", "rationale": "Because \"distressed\" in the entry is a negative emotional expression."}, "language_style": {"answer": "Calm and direct", "rationale": "The entry directly expresses the subject of asking whether the other party is often depressed after the examination, without using excessive rhetorical or emotional rendering, and is more objective in tone. \""}, "time_dimension": {"answer": "Past", "rationale": ""}, "sentence_type": "Negative declarative sentence", "pos": {"nouns": {"Examinations": 1, "Depression": 1}, "verbs": {"Over": 1, "I feel": 1}, "adjectives": {"very": 1}, "numerals": {"13": 1}, "pronouns": {"I": 1}}}, {"content": "I've always had a feeling of tension before every final exam.", "word_count": 20, "label": "", "sentence_structure": "Subject鈥揷opula鈥損redicative", "subjective_objective": {"answer": "Subjective", "rationale": "Because \"frightful feeling\" is the patient's subjective experience and feeling."}, "sentence_sentiment": {"answer": "Negative", "rationale": "Because \"sweet\" feels a negative emotion."}, "language_style": {"answer": "Tactful and warm", "rationale": "The language style of the entry is more euphemistic. It uses the expression \"there is always a\" to avoid direct accusations or ordering the interviewee's emotional reaction, while at the same time directing the interviewee to think and share his feelings by asking him whether he has a similar experience, making the whole question-and-answer process more intimate and natural."}, "time_dimension": {"answer": "Future", "rationale": "The temporal dimension of the purpose of the article is therefore judged to be the future."}, "sentence_type": "Affirmative declarative sentence", "pos": {"nouns": {"Final examination": 1, "Feel": 1}, "verbs": {"have": 1, "Total": 1, "Here": 1}, "adjectives": {"Nervous": 1, "Unsettled": 1}, "numerals": {"Every time": 1}, "pronouns": {"I": 1}}}, {"content": "My emotional reaction won't interfere with my exams at the 15th.", "word_count": 16, "label": "", "sentence_structure": "Subject鈥揷opula鈥損redicative", "subjective_objective": {"answer": "Subjective", "rationale": "Because \"emotional reaction\" and \"inviolability\" are subjective assessments and feelings of patients, and they are subjective."}, "sentence_sentiment": {"answer": "Positive", "rationale": "Because the description of \"emotional reaction won't interfere with my exam\" in the entry is positive."}, "language_style": {"answer": "Calm and direct", "rationale": "The language style of the entry is calm and straightforward. It clearly raises a question and asks respondents whether emotional reactions during the examination will affect their performance. It does not use any rhetorical or emotional rendering, but rather is simple, direct and clear-cut, so that the respondents to the questionnaire can express their views and feelings accurately and clearly."}, "time_dimension": {"answer": "Present", "rationale": "The entry \"at the time of the examination\" indicates a specific point or period of time, while \"the emotional reaction will not interfere with my examination\" is a description of the status quo or the state, so it can be judged that the entry is asking the person who was tested about his current behaviour or state."}, "sentence_type": "Negative declarative sentence", "pos": {"nouns": {"Examinations": 2, "Emotional response": 1}, "verbs": {"No, I won't": 1, "Interference": 1, "Examinations": 1}, "adverbs": {"EMPTY": 2}, "numerals": {"15": 1, "Yes": 1}, "pronouns": {"I": 1}}}, {"content": "I was always nervous during the exam, so I forgot what I knew.", "word_count": 22, "label": "", "sentence_structure": "Subject鈥搗erb鈥揷omplement", "subjective_objective": {"answer": "Subjective", "rationale": "Because \"heat\" and \"forgot\" are patients' subjective feelings and experiences."}, "sentence_sentiment": {"answer": "Negative", "rationale": "Because in the entry, \"I was always nervous during the exam, so that what I knew was forgotten,\" the behavior and the results were negative."}, "language_style": {"answer": "Calm and direct", "rationale": "The decision-making basis: The article is more straightforward, with no excessive emotional accolades or rhetorical techniques. It asks directly whether the interviewee is prone to stress during the examination, and points out that such tension may lead to the forgotten knowledge that is already known. This simple and clear expression is consistent with the calm and straightforward language style."}, "time_dimension": {"answer": "Present", "rationale": "The entry can be judged by the phrases \"during the examination\" and \"often under stress\". At the same time, it can be established that the item is asking about the current state or experience, as the person is used to describe the behaviour normally present (e.g. \"often under stress\"."}, "sentence_type": "Negative declarative sentence", "pos": {"nouns": {"Examination period": 1, "Nervous": 1, "Yes": 1, "Something": 1}, "verbs": {"Regular": 1, "very": 1, "So": 1, "Forget it": 1}, "adjectives": {"Nervous": 1}, "adverbs": {"very": 1, "Regular": 1}, "numerals": {"16": 1}, "pronouns": {"I": 1}}}, {"content": "17. Retrieve of important examinations seems to be a big challenge to me.", "word_count": 21, "label": "", "sentence_structure": "Subject鈥揷opula鈥損redicative", "subjective_objective": {"answer": "Subjective", "rationale": "Because the word \"challenge\" reflects patients' feelings and perceptions of the exam, and is a subjective attitude."}, "sentence_sentiment": {"answer": "Negative", "rationale": "Because the description of \"the big challenge\" in the entry is negative."}, "language_style": {"answer": "Calm and direct", "rationale": "The language style of the entry is relatively calm and straightforward, with no excessive qualifiers or emotional expressions, but rather with a more objective view of the challenges that the respondents may face in retracing important examinations."}, "time_dimension": {"answer": "Present", "rationale": "The entry describes an experience or experience that is a great challenge when studying important examinations. As this feeling is a current one, it can be judged as now."}, "sentence_type": "Affirmative declarative sentence", "pos": {"nouns": {"Review": 1, "Examinations": 1, "I": 1, "Challenges": 1}, "verbs": {"Seems like": 1, "be": 1, "Come on": 1, "to": 1}, "adjectives": {"Important": 1, "Large": 1}, "numerals": {"17": 1}}}, {"content": "I'm confused by the more I try to review one of the exams.", "word_count": 17, "label": "", "sentence_structure": "Subject鈥搗erb鈥揷omplement", "subjective_objective": {"answer": "Subjective", "rationale": "Because the entry describes the patient's subjective feelings and emotional reactions, namely, \"sweeting\" , which cannot be directly derived from objective observation or measurement. At the same time, \"more effort to review\" is an expression of subjective effort. The point of view of the questionnaire is therefore subjective."}, "sentence_sentiment": {"answer": "Negative", "rationale": "Because the phrase \"the more you try to get confused\" in the entry is negative."}, "language_style": {"answer": "Calm and direct", "rationale": "The entry expresses confusion about the examination review in an objective and direct manner, with no excessive emotional or rhetorical modification and a more straightforward tone."}, "time_dimension": {"answer": "Present", "rationale": "The entry to the questionnaire is to ask the testee what he is, \"A test for a given test\" shows that this is a particular situation, and \"I'm getting confused by the effort to review\" is the experience of the testee, which points to the current dimension of time."}, "sentence_type": "Negative declarative sentence", "pos": {"nouns": {"One": 1, "Door": 1, "Examinations": 1, "Tsk": 1, "Try": 1, "Review": 2, "I feel": 1, "Confused": 1}, "verbs": {"to": 1, "be": 1, "I feel": 1}, "adverbs": {"Tsk": 2}, "numerals": {"18": 1}, "classifiers": {"One": 1}, "pronouns": {"I": 1}}}, {"content": "19. Once the exam was over, I tried to stop worrying, but I couldn't.", "word_count": 20, "label": "", "sentence_structure": "Subject鈥搗erb鈥搊bject", "subjective_objective": {"answer": "Subjective", "rationale": "Because it is the patient鈥檚 subjective feeling and thinking. Although the examination is over, the patient is still unable to stop worrying, which is the subjective emotional experience within them."}, "sentence_sentiment": {"answer": "Negative", "rationale": "Because the \"alleged fear but unable\" in the entry is negative."}, "language_style": {"answer": "Calm and direct", "rationale": "The language style of the entry is calm and straightforward. The entry is based on direct inquiries and is sound and objective. It does not use excessive rhetorical or emotional rendering, but rather presents the subject directly. It is more in line with the questionnaire study, which requires as much information as possible on the person surveyed."}, "time_dimension": {"answer": "Past", "rationale": "The entry describes the behaviour and mentality of the person tested after an examination, and thus the temporal dimension can be judged to be past."}, "sentence_type": "Negative declarative sentence", "pos": {"nouns": {"Door": 1, "Examinations": 2, "One": 1, "End": 1, "Fear": 1}, "verbs": {"Some": 1, "Try": 1, "Stop": 1, "Do it": 1}, "adverbs": {"But": 1}, "numerals": {"9": 1, "1": 4}, "pronouns": {"I": 2}}}, {"content": "I sometimes wonder if I can finish college during the exam.", "word_count": 19, "label": "", "sentence_structure": "Subject鈥搗erb鈥搊bject", "subjective_objective": {"answer": "Subjective", "rationale": "Because \"I can finish college\" is a personal thought and a concern of the patient, a subjective examination."}, "sentence_sentiment": {"answer": "Negative", "rationale": "Because the \"sometimes I wonder if I can finish college\" behavior in the entry is a matter of concern and uncertainty, and it's a negative emotion."}, "language_style": {"answer": "Tactful and warm", "rationale": "The language style of the entry is more euphemistic and carries some self-doubt sentiment, using the expression \"sometimes want\" and is more sensitive and comprehensible than direct statements, making it easier for interviewees to open their hearts and answer questions."}, "time_dimension": {"answer": "Present", "rationale": "In this entry, \"the examination period\" means a specific period of time, while \"sometimes you want\" indicates that it is a customary act, so the entry asks the person who was tested for his or her mental state and thoughts during the current period, and so judges now."}, "sentence_type": "Interrogative sentence", "pos": {"nouns": {"Examination period": 1, "I": 1, "University studies": 1}, "verbs": {"Yes": 2, "Completed": 1}, "adverbs": {"Sometimes": 1}, "numerals": {"20": 1}}}, {"content": "I'd rather write a paper than take an exam as a result of a course.", "word_count": 26, "label": "", "sentence_structure": "Subject鈥搗erb鈥搊bject", "subjective_objective": {"answer": "Subjective", "rationale": "Because \"I'd rather write a paper than take an exam\" is the patient's personal opinion and choice."}, "sentence_sentiment": {"answer": "Positive", "rationale": "Because the entry \"I prefer to write a paper instead of take a test\" is positive and indicates a preference for writing a paper."}, "language_style": {"answer": "Tactful and warm", "rationale": "The language style of the entry is more euphemistic, it expresses its preference in a contrast, without directly criticizing or blaming the other party's examination system. Moreover, the use of the term \"better\" makes the tone more soft and avoids direct rejection or denial."}, "time_dimension": {"answer": "Present", "rationale": "\"Better\" and \"but\" means comparison and choice, implying that this is a situation that happens now. At the same time, \"Accomplishment as a course\" suggests that it is a task or a requirement that is relevant now, so the temporal dimension of the article is now."}, "sentence_type": "Affirmative declarative sentence", "pos": {"nouns": {"Questionnaire Entry": 1, "Title": 1, "Papers": 1, "Number of times": 1, "Examinations": 1, "Courses": 1, "Achievements": 1}, "verbs": {"I'd rather": 1, "Write": 1, "Participation": 1, "As": 1}, "adverbs": {"Some": 1}, "numerals": {"21": 1}, "pronouns": {"I": 1}}}, {"content": "I wish the exam would be less annoying.", "word_count": 12, "label": "", "sentence_structure": "Subject鈥搗erb鈥揷omplement", "subjective_objective": {"answer": "Subjective", "rationale": "Because hope is a subjective emotion and will."}, "sentence_sentiment": {"answer": "Negative", "rationale": "Because \"Stamp less annoying\" in the entry is an expression of an irritation and dissatisfaction with the exam, and a negative sentiment."}, "language_style": {"answer": "Calm and direct", "rationale": "The language style of the article ' s purpose can therefore be judged as being straightforward and straightforward."}, "time_dimension": {"answer": "Present", "rationale": "The entry asks the testee about the current test, and the time dimension is now judged. The keyword \"I want\" indicates a desire or a desire, and it points to the current situation, and therefore is in the current period."}, "sentence_type": "Exclamatory sentence", "pos": {"nouns": {"Examinations": 1, "Trouble": 1}, "verbs": {"Hope": 1, "Don't": 1, "Boring": 1}, "adjectives": {"True": 1}, "numerals": {"22": 1}, "pronouns": {"I": 1}}}, {"content": "I'm sure I'd be better off if I took the exam alone and without a time limit.", "word_count": 28, "label": "", "sentence_structure": "Subject鈥搗erb鈥搊bject", "subjective_objective": {"answer": "Subjective", "rationale": "Because \"rebel\" and \"better\" are patients' subjective ideas."}, "sentence_sentiment": {"answer": "Positive", "rationale": "Because the words \"rebel\" and \"better\" in the entry convey a sense of confidence and optimism, it can be judged positive emotional tendencies."}, "language_style": {"answer": "Calm and direct", "rationale": "The basis for the judgement: the purpose of the article is more straightforward and objective, without any emotional or rhetorical use. It directly illustrates the belief of the interviewees that taking the examination alone without a time limit would achieve better results. This expression is more of a statement of fact than a subjective assessment, and therefore it is clear that its language style is straightforward."}, "time_dimension": {"answer": "Present", "rationale": "The entry describes a hypothetical situation and expresses a belief or attitude that individuals will perform better under certain conditions (i.e., taking an examination alone without a time limit). These conditions and results occur in the present or in the likely future, and therefore are judged to be the present."}, "sentence_type": "Affirmative declarative sentence", "pos": {"nouns": {"Examinations": 1, "Time limits": 1}, "verbs": {"Yes": 1, "Participation": 1, "Better": 1}, "adverbs": {"Alone": 1, "If": 1, "And": 1, "No, I'm not": 1}, "numerals": {"23": 1}, "pronouns": {"I": 1}}}, {"content": "Thinking about how much I can score in my exams, it's affecting my exams and my exams.", "word_count": 22, "label": "", "sentence_structure": "Subject鈥搗erb鈥搊bject", "subjective_objective": {"answer": "Subjective", "rationale": "The idea of a single person's subjective idea of what to score affects the review and examination, which is also based on personal feelings and judgements."}, "sentence_sentiment": {"answer": "Negative", "rationale": "Because the \"I'm Affecting my Review and Examination\" behavior in the entry is negative."}, "language_style": {"answer": "Tactful and warm", "rationale": "The language style of the entry is euphemistic, and it explores whether the idea of the interviewee is affecting his or her review and performance by asking him or her whether he or she is too concerned about his or her possible scores before the examination. At the same time, the question also shows concern and understanding of the interviewee without direct criticism or criticism."}, "time_dimension": {"answer": "Present", "rationale": "\"Thinking\" is an indication of the current move, \"how much can be scored in the exam, which affects my review and the examination.\" The entry describes the current situation and impact. The entry is therefore asking whether the testee is now having an impact on his review and the examination by considering his or her results."}, "sentence_type": "Affirmative declarative sentence", "pos": {"nouns": {"Grandpa": 1, "Examinations": 1, "min": 1, "I": 2, "Review": 1}, "verbs": {"Yes": 1, "Got it": 1, "It's affecting": 1, "Review": 1, "Examinations": 1}, "adverbs": {"More": 1, "How much": 1}, "numerals": {"24": 1, "How much": 1}, "pronouns": {"I": 1}}}, {"content": "If the exam can be abolished, I think I can learn better.", "word_count": 17, "label": "", "sentence_structure": "Subject鈥搗erb鈥搊bject", "subjective_objective": {"answer": "Subjective", "rationale": "Because \"the exam can be abolished\" and \"I can learn better\" are subjective views and expectations of patients."}, "sentence_sentiment": {"answer": "Positive", "rationale": "This entry expresses positive attitudes and expectations about the elimination of examinations, which can help them learn better."}, "language_style": {"answer": "Tactful and warm", "rationale": "The entry is more adept and interactive when it is spoken in a gentle and inviting manner, rather than by direct statements or orders."}, "time_dimension": {"answer": "Future", "rationale": "The entry contains a vision and expectations of the future possibility, namely, \"if the examination can be abolished\", and expresses the desire to achieve better learning in the future. The temporal dimension is therefore judged in the future."}, "sentence_type": "Affirmative declarative sentence", "pos": {"nouns": {"Examinations": 1, "Abolition": 1, "Word": 1, "I": 1, "Better": 1}, "verbs": {"Yes": 3, "Learn": 1, "Abolition": 1}, "adverbs": {"If": 1}, "numerals": {"25": 1}}}, {"content": "I'm not worried about the test: I don't understand it now.", "word_count": 23, "label": "", "sentence_structure": "Subject鈥揷opula鈥損redicative", "subjective_objective": {"answer": "Subjective", "rationale": "This is the subjective idea of the patient鈥檚 attitude to the examination and his/her perception. While this attitude may be influenced by objective factors (such as learning), the patient鈥檚 own assessment is subjective."}, "sentence_sentiment": {"answer": "Neutral", "rationale": "Because the entry merely describes attitudes towards the examination and does not express positive or negative emotional sentiments."}, "language_style": {"answer": "Tactful and warm", "rationale": ""}, "time_dimension": {"answer": "Present", "rationale": ""}, "sentence_type": "Affirmative declarative sentence", "pos": {"nouns": {"Examinations": 1, "Attitude": 1, "Present": 1}, "verbs": {"Hug": 1, "Yes": 1, "I'm worried": 1}, "adjectives": {"Here": 1, "of": 2}, "adverbs": {"But": 1, "No, it's not": 1}, "numerals": {"26": 1}, "pronouns": {"I": 1}}}, {"content": "I don't understand why some people are so nervous about the exam.", "word_count": 18, "label": "", "sentence_structure": "Subject鈥搗erb鈥搊bject", "subjective_objective": {"answer": "Subjective", "rationale": "Because tension is a subjective emotional state, different people may have different views and feelings."}, "sentence_sentiment": {"answer": "Negative", "rationale": "Because the entries contain the derogatory word \"strained\" and express negative attitudes."}, "language_style": {"answer": "Calm and direct", "rationale": "The entry uses direct questioning to ask why the other party is nervous about the examination, without too many emotional modifications or euphemisms, and with a more calm and direct language style."}, "time_dimension": {"answer": "Present", "rationale": "The key words are \"I don't get it\" and \"why are some people so nervous about the exam\" and they all describe the feelings and thoughts that the testees are feeling right now."}, "sentence_type": "Interrogative sentence", "pos": {"nouns": {"Entry": 1, "person": 1, "Examinations": 1}, "verbs": {"Understood": 1, "Nervous": 1, "Yeah, it's... it's nervous": 1}, "adjectives": {"I don't understand": 1}, "numerals": {"27": 1}, "pronouns": {"I": 1}}}, {"content": "My bad thoughts will interfere with my performance in the exam.", "word_count": 18, "label": "", "sentence_structure": "Subject鈥揷opula鈥損redicative", "subjective_objective": {"answer": "Subjective", "rationale": "Because it is a subjective assessment and perception of one ' s abilities, ideas and ideas."}, "sentence_sentiment": {"answer": "Negative", "rationale": "Because the phrase \"I'm bad thinking will interfere with my performance in the exam\" in the entry is negative, expressing doubts and denials about my abilities."}, "language_style": {"answer": "Calm and direct", "rationale": "The entry is based on a specific question expressed in a concise and clear statement, without using excessive rhetorical or emotional descriptions. It is speaking in a balanced and objective manner, and it asks directly whether the interviewee was affected by a bad idea in the examination. The language style of the question is more calm and straightforward."}, "time_dimension": {"answer": "Present", "rationale": "The keywords \"bad thoughts\" and \"disturbing\" indicate that the act is affecting the current behaviour of the testee, and therefore judging as now."}, "sentence_type": "Negative declarative sentence", "pos": {"nouns": {"Thoughts": 1, "Examinations": 1, "Performance": 1}, "verbs": {"very": 1, "Bad": 1, "Yes": 1, "Interference": 1, "in": 1}, "adjectives": {"Bad": 1}, "adverbs": {"very": 1}, "numerals": {"28": 1}, "pronouns": {"I": 1}}}, {"content": "I'm not much more effective at studying finals than I am at the usual exams.", "word_count": 19, "label": "", "sentence_structure": "Subject鈥搗erb鈥揷omplement", "subjective_objective": {"answer": "Subjective", "rationale": "Because \"more hard\" is a subjective assessment and feeling."}, "sentence_sentiment": {"answer": "Negative", "rationale": "Because the behavior in the entry is \"no more powerful than the retinal exams\" is negative."}, "language_style": {"answer": "Calm and direct", "rationale": "This is a more calm and straightforward language, with simple statements expressing the individual view of the respondent鈥檚 attitude to learning, and not too much emotional or rhetorical."}, "time_dimension": {"answer": "Present", "rationale": "The entry describes the present retrospectal attitudes and behaviour of the subject, and the words \"no more than\" suggest a comparison, referring to the current situation."}, "sentence_type": "Negative declarative sentence", "pos": {"nouns": {"Final examination": 1, "-A usual exam": 1}, "verbs": {"Review": 2, "Work hard": 1}, "adverbs": {"Not like that": 1, "More": 1}, "numerals": {"29": 1}, "classifiers": {"individual": 1}, "pronouns": {"I": 1}}}, {"content": "I'm still anxious, though I'm doing a good job of studying some exams.", "word_count": 20, "label": "", "sentence_structure": "Subject鈥揷opula鈥損redicative", "subjective_objective": {"answer": "Subjective", "rationale": "Because \"agitation\" is a subjective emotional experience, reflecting the patient鈥檚 concern and anxiety about his or her performance. Although it is an objective fact to review a particular examination well, it is the individual鈥檚 feeling that determines the good and bad, so the entry looks at the patient鈥檚 subjective attitude and feelings, which are subjective."}, "sentence_sentiment": {"answer": "Negative", "rationale": "Because \"anxiety\" is negative emotional."}, "language_style": {"answer": "Tactful and warm", "rationale": "The expression \"though it is still\" is used, which shows the respondents' efforts to review and express their concerns and concerns about the examination. It is more gentle, polite, avoids too direct or hard language, comforts and understanding. It also contains emotional terms such as \"anxiety,\" increases the protege and interaction of the language, and makes people more willing to answer questions and share their real feelings."}, "time_dimension": {"answer": "Present", "rationale": ""}, "sentence_type": "Negative declarative sentence", "pos": {"nouns": {"Tests": 1, "Examinations": 1, "Anxiety": 1}, "verbs": {"Review": 1, "I feel": 1}, "adjectives": {"Okay": 1, "Some door": 1}, "adverbs": {"Still": 1}, "numerals": {"30": 1}, "pronouns": {"I": 1}}}, {"content": "I'm not good before the big test.", "word_count": 10, "label": "", "sentence_structure": "Subject鈥搗erb鈥揷omplement", "subjective_objective": {"answer": "Subjective", "rationale": "Because \"eating bad\" is a personal feeling and experience, and it's a subjective view of the patient."}, "sentence_sentiment": {"answer": "Negative", "rationale": "Because the \"eating\" act in the entry is negative."}, "language_style": {"answer": "Calm and direct", "rationale": ""}, "time_dimension": {"answer": "Present", "rationale": "The entry is a question about the behaviour of the testee before a major examination, and the time dimension is a past and a future, because \"test\" is a potential future event. Moreover, \"I don't like eating\" is a symptom of the past, suggesting that the problem may have arisen in the past. So the answer is \"The past/the future\"."}, "sentence_type": "Negative declarative sentence", "pos": {"nouns": {"Major examinations": 1, "Hong": 1}, "verbs": {"Eat": 1, "Okay": 1}, "adjectives": {"not": 1}, "numerals": {"31": 1}, "pronouns": {"I": 1}}}, {"content": "I found my arm shaking before the big test.", "word_count": 16, "label": "", "sentence_structure": "Subject鈥搗erb鈥揷omplement", "subjective_objective": {"answer": "Subjective", "rationale": "Because finding one ' s own physical reaction is an individual ' s feeling and experience and is a subjective observation."}, "sentence_sentiment": {"answer": "Negative", "rationale": "Because the physical reaction of \"arms shaking\" in the entry is negative."}, "language_style": {"answer": "Tactful and warm", "rationale": ""}, "time_dimension": {"answer": "Past", "rationale": "The entry asks about the behaviour or symptoms of the person tested prior to the major examination, and the temporal dimension is judged to be past."}, "sentence_type": "Negative declarative sentence", "pos": {"nouns": {"Examinations": 1, "Arms": 1}, "verbs": {"Found": 1, "Wreck": 1}, "adjectives": {"Significant": 1}, "numerals": {"32": 1}, "pronouns": {"I": 1}}}, {"content": "I don't have much to ask for \"temporary paganism\" before I take the exam.", "word_count": 16, "label": "", "sentence_structure": "Subject鈥搗erb鈥揷omplement", "subjective_objective": {"answer": "Subjective", "rationale": "Because \"very few\" is a subjective judgment and feeling."}, "sentence_sentiment": {"answer": "Neutral", "rationale": "Because the entry does not have any emotional color."}, "language_style": {"answer": "Calm and direct", "rationale": "The language style of the entry is a straightforward type of calm, and it presents in an objective and concise manner whether the subject is often in need of temporary dementia before the examination."}, "time_dimension": {"answer": "Past", "rationale": "The term \"very little\" and \"pre-test\" can be judged to have occurred in the past and to have ended, so the temporal dimension of the article is the past."}, "sentence_type": "Negative declarative sentence", "pos": {"nouns": {"Examinations": 1, "Buddha's Foot": 1}, "verbs": {"have": 1, "Hug": 1, "Yes": 1}, "adjectives": {"Temporary": 1, "Less": 1}, "numerals": {"33": 1}}}, {"content": "34. The school should realize that some students are anxious about the exam, which affects their grades.", "word_count": 29, "label": "", "sentence_structure": "Subject鈥搗erb鈥搊bject", "subjective_objective": {"answer": "Subjective", "rationale": "This view reflects the subjective view and recommendation of the surveyer that the school should be aware of the impact of the student anxiety on the results of the examination and take measures to help them."}, "sentence_sentiment": {"answer": "Negative", "rationale": "Because the entry expresses a negative attitude and emotion 鈥?students are anxious and upset. While the entry refers to a possible solution (i.e. schools should recognize and address this), it itself expresses a negative emotion and attitude."}, "language_style": {"answer": "Calm and direct", "rationale": "The entry presents in a direct manner the question that the surveyer wanted to understand - whether students were affected by the stress of the examination, the use of too much acrimonym and expression, and the more calm and straightforward language style."}, "time_dimension": {"answer": "Present", "rationale": "The entry describes the current situation, i.e., that some students are anxious about the examination, which may affect their examination results. \""}, "sentence_type": "Affirmative declarative sentence", "pos": {"nouns": {"School": 1, "Students": 2, "Examinations": 2, "Anxiety": 1, "Achievements": 1}, "verbs": {"I should be aware of that": 1, "Impact": 1}, "adjectives": {"Some": 1}, "numerals": {"34": 1}, "pronouns": {"they": 1}}}, {"content": "I don't think it's that much of a stressful time during the exam.", "word_count": 18, "label": "", "sentence_structure": "Subject鈥搗erb鈥揷omplement", "subjective_objective": {"answer": "Subjective", "rationale": "Because \"thinks\" is an expression of subjective attitude."}, "sentence_sentiment": {"answer": "Negative", "rationale": "Because the entry \"should not get so nervous\" expresses a negative emotional attitude, implying that the examination should be easy to treat, rather than overstretched and anxious."}, "language_style": {"answer": "Tactful and warm", "rationale": "The language style of the entry is euphemistic, because it uses some polite, respectful and caring words to express opinions, such as \"seemingly\" or \"not\" rather than direct orders or criticism. Such expressions are more resonant with the respondents and make them more willing to participate in the questionnaire."}, "time_dimension": {"answer": "Present", "rationale": "\"Assisting\" that this is the subjective view of the testee, \"during the examination\" and \"should\" and \"strained\" are all descriptions of the present situation, so the entry reflects the present situation."}, "sentence_type": "Affirmative declarative sentence", "pos": {"nouns": {"Examination period": 1, "Nervous": 1}, "verbs": {"I think": 1, "Fuck": 1, "Okay": 1, "I think so": 1, "Well": 1}, "adjectives": {"I think so": 1, "Nervous": 1}, "adverbs": {"Seems like": 1, "Period": 1}, "numerals": {"35": 1}, "pronouns": {"I": 1}}}, {"content": "I feel uncomfortable when I get into the test papers.", "word_count": 17, "label": "", "sentence_structure": "Subject鈥揷opula鈥損redicative", "subjective_objective": {"answer": "Subjective", "rationale": "Because \"sweetly uncomfortable\" is an expression of subjective feeling."}, "sentence_sentiment": {"answer": "Negative", "rationale": "The term \"inhabited\" is judged to be negative."}, "language_style": {"answer": "Calm and direct", "rationale": "The entry is described directly, without using euphemism or warm words. It simply and succinctly expresses the feeling of the subject when looking at the paper, which is more objective and calm."}, "time_dimension": {"answer": "Present", "rationale": ""}, "sentence_type": "Negative declarative sentence", "pos": {"nouns": {"Tests": 1, "I": 2}, "verbs": {"Exposure": 1, "Send": 1, "I think": 1, "very": 2, "I'm not comfortable": 1}, "adjectives": {"I'm not comfortable": 1}, "adverbs": {"Just": 1}, "pronouns": {"One": 1}}}, {"content": "I hate teachers who like to take \"sudden attack\" exams.", "word_count": 18, "label": "", "sentence_structure": "Subject鈥搗erb鈥搊bject", "subjective_objective": {"answer": "Subjective", "rationale": "Because the words \"disguised\" and \"like\" are the emotional attitudes of the patient, the subjective feelings."}, "sentence_sentiment": {"answer": "Negative", "rationale": "It's a negative emotional color because it's a reflection of the dislike and aversion to teachers."}, "language_style": {"answer": "Tactful and warm", "rationale": "The language style of the entry is relatively moderate and euphemistic, allowing respondents to express their views and feelings about a situation in a way that is asked. At the same time, the expression \"bout of attack\" is not too strong or aggressive, but rather uses a more neutral and objective term, which is more acceptable to the interviewees and is willing to answer the question."}, "time_dimension": {"answer": "Present", "rationale": "\"I hate teacher's tendency to use the verbs in the \"sudden attack\" test class, which is the general current, which means that the tester's current attitude and feelings. So the temporal dimension is now."}, "sentence_type": "Negative declarative sentence", "pos": {"nouns": {"I": 1, "Teacher": 1, "A surprise attack": 1, "Examinations": 1, "Courses": 1}, "verbs": {"Shit": 1, "Yeah": 1, "Fuck": 1, "Pattern": 1}, "adjectives": {"Attack": 1}, "numerals": {"37": 1}}}]}

{"questionnaire_title": "Eisenke's emotional stability test.", "questionnaire_category": "Emotion and affect", "item_count": 210, "emotion_shift_overall": "From positive to negative", "language_style_overall": "Neutral", "items": [{"content": "001, do you think you can do it like most people do?", "word_count": 15, "label": "", "sentence_structure": "Subject鈥搗erb鈥搊bject", "subjective_objective": {"answer": "Subjective", "rationale": "Because the issue requires a subjective view and evaluation of the patient ' s abilities, it is a subjective one."}, "sentence_sentiment": {"answer": "Neutral", "rationale": "Because the entry asks about the individual ' s perception of his/her abilities, there is no apparent emotional tendency."}, "language_style": {"answer": "Tactful and warm", "rationale": "The language style of the entry is more euphemistic, and the question is directed to the question-and-answerer, and the negative or positive tone is not used strongly or directly."}, "time_dimension": {"answer": "Present", "rationale": "Key words \"consider\" and \"can\" represent an assessment of current self-capacity and performance, implying that it is a subjective view, and not a reference to past experience or future plans. So the time dimension is now."}, "sentence_type": "Interrogative sentence", "pos": {"nouns": {"you": 1, "person": 2, "Behaviour": 1}, "verbs": {"I think": 1, "Yes": 2, "Act": 1}, "numerals": {"001": 1, "More": 1}, "pronouns": {"That way": 1}}}, {"content": "002, you seem to have had a bad day.", "word_count": 9, "label": "", "sentence_structure": "Subject鈥搗erb鈥揷omplement", "subjective_objective": {"answer": "Subjective", "rationale": "Because \"bad luck\" is a subjective feeling or perception, different people may experience and evaluate differently. The questionnaire therefore aims at looking at subjective attitudes or experiences of patients."}, "sentence_sentiment": {"answer": "Negative", "rationale": "Because \"bad things\" in the entries are negative descriptions."}, "language_style": {"answer": "Calm and direct", "rationale": "The language style of the entry is relatively calm and straightforward, and there is no excessive emotional rendering or rhetorical use, and the tone is objective and concise in expressing the subject of whether the subject of the inquiry is often confronted with unfortunate events in life."}, "time_dimension": {"answer": "Present", "rationale": "The keywords are \"like\" and \"bad things,\" which indicate that the testee is describing the current state or feeling, and thus the temporal dimension is now."}, "sentence_type": "Affirmative declarative sentence", "pos": {"nouns": {"Questionnaire": 1, "Entry": 1, "Bad luck": 1}, "verbs": {"I've got it": 1}, "adjectives": {"Seems like": 1, "End of story": 1}, "numerals": {"002": 1}}}, {"content": "003. Are you more blushing than most people?", "word_count": 12, "label": "", "sentence_structure": "Subject鈥搗erb鈥搊bject", "subjective_objective": {"answer": "Subjective", "rationale": "Because \"Easier Face\" is the person's own feeling and experience, and it's a subjective attitude."}, "sentence_sentiment": {"answer": "Neutral", "rationale": "The entry is not a positive or negative emotional expression."}, "language_style": {"answer": "Tactful and warm", "rationale": "The language style of the entry is more euphemistic, as it uses a more gentle tone to ask each other about the situation, rather than a too direct or command-style tone. It also does not reveal any information that might make the other person uncomfortable. This expression is more likely to receive positive responses from the interviewees."}, "time_dimension": {"answer": "Present", "rationale": "The keyword \"Are you more red than most people?\" denotes current or recent behaviour, so the entry asks about the person's current behaviour or state."}, "sentence_type": "Interrogative sentence", "pos": {"nouns": {"Questionnaire": 1, "Entry": 1, "person": 1}, "verbs": {"Easy": 1, "Red": 1, "- Yeah": 1, "Most": 1, "Compare": 1}, "adjectives": {"Large": 1, "More": 1}, "numerals": {"003": 1, "Most": 1}, "pronouns": {"you": 1}}}, {"content": "004. Sometimes a thought is in your head, and you want to get rid of it, but you can't.", "word_count": 25, "label": "", "sentence_structure": "Subject鈥搗erb鈥揷omplement", "subjective_objective": {"answer": "Subjective", "rationale": "Because this is about the patient鈥檚 own thoughts and feelings, including their inability to control themselves. These are examined from the patient鈥檚 subjective point of view."}, "sentence_sentiment": {"answer": "Negative", "rationale": "Because the expression \"can't stop\" is negative."}, "language_style": {"answer": "Calm and direct", "rationale": "The entry is more directly linguistically, with no excessive rhetoric or emotional rendering. It expresses in an objective, concise manner whether the subject has had experience of over-thinking an issue, in line with the characteristics and requirements of the questionnaire."}, "time_dimension": {"answer": "Present", "rationale": ""}, "sentence_type": "Affirmative declarative sentence", "pos": {"nouns": {"Ideas": 1, "In the brain": 1}, "verbs": {"Repeatedly": 1, "Yes": 2, "- Stop it": 1, "Do it": 1}, "adverbs": {"Sometimes": 1}, "numerals": {"004": 1, "One": 1}, "pronouns": {"you": 1}}}, {"content": "005. Do you want to quit or quit your bad hobby?", "word_count": 17, "label": "", "sentence_structure": "Subject鈥搗erb鈥搊bject", "subjective_objective": {"answer": "Subjective", "rationale": "Because \"want to be removed or to be removed\" is the patient's personal will and is subjective."}, "sentence_sentiment": {"answer": "Positive", "rationale": "Because the \"exhibit or stop bad hobby\" act in the entry is positive, it expresses the desire of the interviewees to change their bad habits."}, "language_style": {"answer": "Calm and direct", "rationale": "This type of question and answer help researchers to collect more accurate and specific data."}, "time_dimension": {"answer": "Past", "rationale": "The keyword \"want to stop\" and \"bad hobby\" suggests that it is a question of whether the person tested wanted to change a certain behaviour. The time dimension is that the entry is a question of the will and the ideas of the present or the past, and can therefore be judged as past/now."}, "sentence_type": "Interrogative sentence", "pos": {"nouns": {"Grandpa": 1, "Questionnaire": 1, "Entry": 1, "Bad": 1, "A hobby": 1, "Smoke": 1}, "verbs": {"Yes": 1, "Detachment": 1, "Quit": 1, "have": 1}, "adjectives": {"of": 5}, "numerals": {"005": 1, "One": 2}, "classifiers": {"individual": 1}}}, {"content": "006. Are you always feeling good and energetic?", "word_count": 14, "label": "", "sentence_structure": "Subject鈥搗erb鈥搊bject", "subjective_objective": {"answer": "Subjective", "rationale": "Because \"fears well\" and \"energy is\" is the patient's own feelings and experiences, and it's subjective."}, "sentence_sentiment": {"answer": "Positive", "rationale": "Because \"fears well and energy well\" is a positive attitude and emotional state."}, "language_style": {"answer": "Tactful and warm", "rationale": "It is because it talks in a gentle, inviting tone, not in direct statements or orders. Such expressions are more personal and interactive, allowing the respondents to feel respect and understanding, and to open their hearts and answer questions in a real way."}, "time_dimension": {"answer": "Present", "rationale": "The entry asks about the current state and feelings of the person being tested, and about the present experience and state.\""}, "sentence_type": "Interrogative sentence", "pos": {"nouns": {"Investigations": 1, "Entry": 1, "- The energy": 1, "鈾?Full of 鈾?: 1}, "verbs": {"Feel": 1, "Always": 1}, "adjectives": {"Good": 1}, "numerals": {"006": 1}}}, {"content": "007, do you often get upset about guilt?", "word_count": 11, "label": "", "sentence_structure": "Subject鈥搗erb鈥搊bject", "subjective_objective": {"answer": "Subjective", "rationale": "Because the words \"often\" and \"fear\" relate to the feelings and feelings of the patient, they are subjectively viewed."}, "sentence_sentiment": {"answer": "Negative", "rationale": "Because the \"guilty\" sentiment in the entry is negative."}, "language_style": {"answer": "Calm and direct", "rationale": "This language style is appropriate for a critical situation such as psychological questionnaires, which help the interviewees understand and answer questions more accurately."}, "time_dimension": {"answer": "Present", "rationale": "The keyword \"always\" and the verb \"involved by guilt\" suggest that this is a question of the current state of affairs, so the temporal dimension of the article is now."}, "sentence_type": "Interrogative sentence", "pos": {"nouns": {"007": 1, "Bury": 1, "Trouble": 1}, "verbs": {"Often": 1, "Yes": 1, "Trouble": 1}, "adverbs": {"Regular": 1}, "numerals": {"007": 1}}}, {"content": "008, do you feel a little proud?", "word_count": 10, "label": "", "sentence_structure": "Subject鈥損redicate", "subjective_objective": {"answer": "Subjective", "rationale": "Because pride is a manifestation of subjective emotions or attitudes."}, "sentence_sentiment": {"answer": "Negative", "rationale": "Because pride is usually seen as a negative character."}, "language_style": {"answer": "Tactful and warm", "rationale": "The entry is more personal and interactive in its expression, in a gentle and inviting tone, rather than in direct statements or orders. At the same time, the phrase \"You feel a little proud?\" focuses more on the search for what is inside the interviewer than on giving a direct answer, either positive or negative, which reflects the concern and respect of the questionnaire designer for the mental health of the interviewee."}, "time_dimension": {"answer": "Present", "rationale": "The entry asks the testee about his current emotional feelings, and the time dimension is now judged. The keyword \"slightly proud\" indicates a subjective emotional experience and uses the current state of affairs, so it can be inferred that the item is about the present situation."}, "sentence_type": "Interrogative sentence", "pos": {"nouns": {"Proud": 1}, "verbs": {"I think": 1, "have": 1}, "adjectives": {"A little": 1, "Proud": 1}, "numerals": {"008": 1}, "pronouns": {"you": 1}}}, {"content": "When you wake up in the morning, do you often feel depressed?", "word_count": 16, "label": "", "sentence_structure": "Subject鈥損redicate", "subjective_objective": {"answer": "Subjective", "rationale": "Because \"smuggling\" is a subjective emotional experience that requires the patient to feel and describe itself."}, "sentence_sentiment": {"answer": "Negative", "rationale": "Because \"depressive\" is negative."}, "language_style": {"answer": "Tactful and warm", "rationale": "Warm and warm."}, "time_dimension": {"answer": "Present", "rationale": ""}, "sentence_type": "Interrogative sentence", "pos": {"nouns": {"Morning": 1, "Wake up": 1, "Time": 1, "you": 1, "Is it possible": 1, "Regular": 1, "I feel": 1, "The mood": 1, "Depression": 1}, "verbs": {"EMPTY": 1, "have": 1}, "adjectives": {"EMPTY": 1}, "adverbs": {"EMPTY": 1, "Regular": 1}, "numerals": {"009": 1, "1": 1}, "classifiers": {"individual": 1}, "pronouns": {"you": 1}}}, {"content": "010, even when you're sad, you're rarely insomnia?", "word_count": 15, "label": "", "sentence_structure": "Subject鈥搗erb鈥搊bject", "subjective_objective": {"answer": "Subjective", "rationale": "Because insomnia is a subjective feeling and experience of a human being."}, "sentence_sentiment": {"answer": "Neutral", "rationale": "Because the entry is a question of a situation and there is no apparent emotional tendency."}, "language_style": {"answer": "Tactful and warm", "rationale": "The basis for the judgement: the question is asked in a more moderate way, using an invitation expression that does not directly present or command the other party to answer. This expression is more personal and interactive, and more resonant and participatory with the person under investigation."}, "time_dimension": {"answer": "Present", "rationale": "The keyword \"freak\" and \"very little insomnia\" indicate that it is an inquiry into the current state of the subject, and therefore judgement is now."}, "sentence_type": "Interrogative sentence", "pos": {"nouns": {"Time": 1, "Sad": 1, "Insomnia": 1}, "verbs": {"Even if": 1, "Very few": 1, "Sleep": 1}, "adjectives": {"Extreme": 1}, "adverbs": {"Yeah": 1, "in": 1}, "numerals": {"010": 1}}}, {"content": "011, do you often feel the alarm sounds so gruesome and unbearable?", "word_count": 19, "label": "", "sentence_structure": "Subject鈥搗erb鈥搊bject", "subjective_objective": {"answer": "Subjective", "rationale": "Because \"scrambling\" and \"unbearable\" are patients' subjective feelings about the sound of alarm clocks."}, "sentence_sentiment": {"answer": "Negative", "rationale": "Because the description \"the alarm clock is so loud and unbearable\" in the entry is negative."}, "language_style": {"answer": "Calm and direct", "rationale": "The language style of the entry is calm and straightforward. It uses a simple and straightforward inquiry, without too much rhetorical or emotional overturning, but rather a direct question about whether the subject of alarm alarm sounds are often felt by the testees. This tone is more objective, practical, and clearly conveys the message and intent of the problem, which helps to collect accurate data."}, "time_dimension": {"answer": "Present", "rationale": "The entry is for the current behaviour and feelings of the testee, and the time dimension is now judged."}, "sentence_type": "Interrogative sentence", "pos": {"nouns": {"Alarm clock": 1, "Sound": 1, "- That's a good ear": 1, "Hard": 1, "Take it": 1}, "verbs": {"I feel": 1, "Zenium": 1, "Tens": 1, "- That's a good ear": 1, "Hard": 1, "Take it": 1}, "adjectives": {"- That's a good ear": 2, "Hard": 1}, "adverbs": {"Often": 1, "Tens": 1}, "numerals": {"011": 1}}}, {"content": "012, do you want to learn and enjoy the game that looks like you're good at it?", "word_count": 25, "label": "", "sentence_structure": "Subject鈥搗erb鈥搊bject", "subjective_objective": {"answer": "Subjective", "rationale": "Because \"to learn and enjoy it\" is the patient's subjective attitude and desire."}, "sentence_sentiment": {"answer": "Positive", "rationale": "Because the \"want to learn and enjoy it\" act is positive in the entry."}, "language_style": {"answer": "Tactful and warm", "rationale": "The entry uses a more euphemistic tone and a questionable way of talking, which is to understand whether the other party is interested in learning and enjoying the fun of a game that appears to be less than his/her own."}, "time_dimension": {"answer": "Future", "rationale": ""}, "sentence_type": "Interrogative sentence", "pos": {"nouns": {"Game": 1, "Fun": 1}, "verbs": {"Yes": 2, "Learn": 1, "Enjoy": 1}, "adjectives": {"Looks like it": 1, "very": 2, "Yes, I'm good": 1}, "numerals": {"012": 1}, "pronouns": {"you": 1}}}, {"content": "Are you hungry?", "word_count": 7, "label": "", "sentence_structure": "Subject鈥搗erb鈥搊bject", "subjective_objective": {"answer": "Subjective", "rationale": "According to the description of the subject, \"failure\" is a symptom or a feeling, a patient's subjective experience and observation, so the question is to examine subjective ideas. So the answer is \"subjective.\""}, "sentence_sentiment": {"answer": "Negative", "rationale": "Because \"bad appetite\" is negative."}, "language_style": {"answer": "Tactful and warm", "rationale": "According to the description of the question, \"013, are you hungry?\" By using an open and caring question, the interviewee answers that there is no too much subjective judgement or emotional language, and therefore can be classified as \"sweetly\" language style."}, "time_dimension": {"answer": "Present", "rationale": ""}, "sentence_type": "Interrogative sentence", "pos": {"nouns": {"Questionnaire": 1, "Entry": 1, "A appetite": 1}, "verbs": {"Is it possible": 1, "Bad": 1}, "numerals": {"013": 1}}}, {"content": "014, when you're actually right, do you often look for yourself?", "word_count": 23, "label": "", "sentence_structure": "Subject鈥搗erb鈥搊bject", "subjective_objective": {"answer": "Subjective", "rationale": "Because looking for yourself is not a subjective idea and behaviour of one person."}, "sentence_sentiment": {"answer": "Negative", "rationale": "Because the actions in the entries are self-criticizing and negative, and have a negative emotional connotation."}, "language_style": {"answer": "Tactful and warm", "rationale": "The language style of the entry is more euphemistic, using a more mild form of questioning to guide respondents to answer questions rather than to make direct statements or orders. At the same time, by raising open questions such as \"if often\" the respondents are given the opportunity to reflect and express their thoughts and feelings in greater depth."}, "time_dimension": {"answer": "Past", "rationale": "The temporal dimension of the purpose of the article is therefore judged to be past."}, "sentence_type": "Interrogative sentence", "pos": {"nouns": {"014": 1, "self": 1}, "verbs": {"Find": 1, "be": 1, "No, I'm not": 1}, "adjectives": {"Regular": 1, "Wrong": 1}, "numerals": {"One": 2, "0": 1}, "pronouns": {"you": 1}}}, {"content": "Do you often feel like a loser?", "word_count": 12, "label": "", "sentence_structure": "Subject鈥搗erb鈥搊bject", "subjective_objective": {"answer": "Subjective", "rationale": "I think it's the patient's subjective attitude."}, "sentence_sentiment": {"answer": "Negative", "rationale": "Because the attitude of \"often feeling like a loser\" in the entry is negative."}, "language_style": {"answer": "Calm and direct", "rationale": "The entry directly asked whether the person surveyed had experienced a feeling of being a loser, without too much qualifier or emotional colour, and with a more objective and straightforward language style."}, "time_dimension": {"answer": "Past", "rationale": "So the time dimension is now judged."}, "sentence_type": "Interrogative sentence", "pos": {"nouns": {"self": 1, "Loser": 1}, "verbs": {"I think": 1}, "adjectives": {"Often": 1}, "numerals": {"015": 1}}}, {"content": "016 鈥?In general, are you satisfied with your life?", "word_count": 14, "label": "", "sentence_structure": "Subject鈥搗erb鈥搊bject", "subjective_objective": {"answer": "Subjective", "rationale": "Because satisfaction is a subjective experience and feeling."}, "sentence_sentiment": {"answer": "Positive", "rationale": "Because the \"satisfaction\" attitude in the entries is positive."}, "language_style": {"answer": "Calm and direct", "rationale": "The entry is based on a straightforward language style, with no over-modified or emotional expressions, and it appears objective and neutral to ask the interviewee directly about his satisfaction with life."}, "time_dimension": {"answer": "Present", "rationale": "The entry is asking the testee what he feels and how he is now, and the keyword is \"generally\" and \"satisfaction\" and they point to the situation."}, "sentence_type": "Interrogative sentence", "pos": {"nouns": {"Overall": 1, "you": 2, "Life": 1}, "verbs": {"Satisfied": 1, "be": 1}, "adverbs": {"Overall": 1}, "numerals": {"016": 1}}}, {"content": "017, are you usually calm and not easily disturbed?", "word_count": 13, "label": "", "sentence_structure": "Subject鈥搗erb鈥搊bject", "subjective_objective": {"answer": "Subjective", "rationale": "The subject is examined in terms of the patient ' s emotional state and subjective feelings, which are viewed from the patient ' s subjective perspective."}, "sentence_sentiment": {"answer": "Positive", "rationale": "Because \"quiet, not too easy to be disturbed\" is a positive quality."}, "language_style": {"answer": "Tactful and warm", "rationale": "The entry uses querying and uses descriptive terms (e.g. \"quiet\" and \"uneasy to be disturbed\") to try to understand the personality of the other. This expression is relatively gentle and euphemism, avoids the language of direct orders or criticism, is more acceptable and is willing to answer questions."}, "time_dimension": {"answer": "Past", "rationale": "The entry is a limitation on the extent of time dimension not clearly defined in the context of the question of the emotional state and the manner of coping with stress of the person being tested, so it is not possible to determine whether it is past, present or future."}, "sentence_type": "Interrogative sentence", "pos": {"nouns": {"you": 1, "Usually": 1, "Calm down": 1, "It's not easy": 1, "Disturbed": 1}, "verbs": {"be": 1, "Do it": 0, "Here": 0}, "adjectives": {"Calm down": 1, "Easy": 1}, "adverbs": {"Usually": 1, "not": 1}, "numerals": {"017": 1}, "classifiers": {"individual": 0}, "pronouns": {"you": 1}}}, {"content": "018. When reading, do you find it difficult to ascertain the meaning of the sentence if the marker is wrong?", "word_count": 28, "label": "", "sentence_structure": "Subject鈥搗erb鈥搊bject", "subjective_objective": {"answer": "Objective", "rationale": "Because it is an observation and inquiry into the error of the mark during reading, it is not a subjective feeling or attitude of the patient. It is therefore an objective response and experience to examine the person interviewed."}, "sentence_sentiment": {"answer": "Neutral", "rationale": "As this entry does not express positive or negative emotions."}, "language_style": {"answer": "Calm and direct", "rationale": "The entry is based on direct questions, is not based on excessive rhetoric or emotional rendering and is more objective in tone."}, "time_dimension": {"answer": "Present", "rationale": ""}, "sentence_type": "Interrogative sentence", "pos": {"nouns": {"Time": 1, "Reading": 1, "Punctuation Error": 1, "Sentence": 1, "Meaning": 1}, "verbs": {"Found": 1, "I think": 1, "It's hard": 1, "I know": 1}, "adjectives": {"Error": 1}, "adverbs": {"If": 1}, "numerals": {"018": 1, "One": 2}, "pronouns": {"you": 1}}}, {"content": "Do you systematically control body formation through exercise or restriction of diet?", "word_count": 21, "label": "", "sentence_structure": "Subject鈥搗erb鈥搊bject", "subjective_objective": {"answer": "Subjective", "rationale": "Because \"sporting\", \"cutting\" and \"systemic control\" are the actions and thoughts of the patient, and therefore the subjective angle."}, "sentence_sentiment": {"answer": "Positive", "rationale": "The purpose of the act of \"activation or restriction of diet to control body formation in a systematic manner\" in the entry is positive and does not have any negative emotional connotations."}, "language_style": {"answer": "Tactful and warm", "rationale": ""}, "time_dimension": {"answer": "Present", "rationale": "The entry describes the behaviour or state of the person being tested, \"whether\" means that it is a selective question, while \"systematic control of body form through exercise or restriction of diet\" is a description of the specific behaviour, pointing to the current situation."}, "sentence_type": "Interrogative sentence", "pos": {"nouns": {"Entry": 1, "Questionnaire": 1, "Body": 1}, "verbs": {"Pass": 1, "Exercise": 1, "Limits": 1, "Diet": 1, "Come on": 1, "Systematic control": 1}, "adverbs": {"Is it possible": 1}, "numerals": {"019": 1}, "classifiers": {"One": 1, "individual": 2}}}, {"content": "020, are your skin very sensitive and painful?", "word_count": 12, "label": "", "sentence_structure": "Subject鈥損redicate", "subjective_objective": {"answer": "Subjective", "rationale": "Because skin sensitivity and fear of pain are subjective feelings and experiences."}, "sentence_sentiment": {"answer": "Negative", "rationale": "This is a negative situation because the entries describe skin sensitivity and fear of pain."}, "language_style": {"answer": "Calm and direct", "rationale": "The entry directly asked whether the person surveyed was sensitive to his skin and was not sensitive to pain and did not use euphemism or reproducing language, and therefore could be judged to be calm and straightforward."}, "time_dimension": {"answer": "Present", "rationale": "\"Is your skin very sensitive and painful?\" The phrase \"now\" in \"is it asking the testee about the current state or feelings, the key word being \"your skin is very sensitive and pain-prone\" refers to the current state or feelings."}, "sentence_type": "Interrogative sentence", "pos": {"nouns": {"020": 1, "Skin": 1, "Sensitive": 1, "It hurts": 1}, "adverbs": {"Very": 1}}}, {"content": "021, do you ever feel like your life is disappointing to your parents?", "word_count": 20, "label": "", "sentence_structure": "Subject鈥搗erb鈥搊bject", "subjective_objective": {"answer": "Subjective", "rationale": "Because \"you feel\" is the patient's subjective feeling and understanding, disappointment with parents is not an objective reality."}, "sentence_sentiment": {"answer": "Negative", "rationale": "Because the \"defeating parents\" act in the entry is negative."}, "language_style": {"answer": "Tactful and warm", "rationale": "The entry is more personal and interactive when it is spoken in a gentle and inviting manner, rather than in direct statements or orders. At the same time, the issue involves personal family relations, and the use of a soft and warm language style better protects the privacy and feelings of the person under investigation, avoiding unnecessary stress and harm."}, "time_dimension": {"answer": "Past", "rationale": "In the entry, the words \"sometimes\", \"fear\" and \"disappointing\" indicate that this is a memory of the past or a reflection of it, and therefore judgement is the past."}, "sentence_type": "Interrogative sentence", "pos": {"nouns": {"Entry": 1, "Life": 1, "parents": 1}, "verbs": {"I think": 1, "鈾?And I'm gonna be 鈾?: 1}, "numerals": {"021": 1}, "classifiers": {"individual": 1}, "pronouns": {"you": 2}}}, {"content": "- Are you upset about your low self?", "word_count": 11, "label": "", "sentence_structure": "Subject鈥搗erb鈥搊bject", "subjective_objective": {"answer": "Subjective", "rationale": "Because \"fear\" is the patient's subjective feeling and attitude."}, "sentence_sentiment": {"answer": "Negative", "rationale": "Because the words \"subsistence\" and \"probate\" in the entries are negative emotional terms."}, "language_style": {"answer": "Tactful and warm", "rationale": "The entry is more personal and interactive when it is spoken in a gentle and inviting manner, rather than in direct statements or orders. At the same time, \"probable\" is replaced by \"problem\" to make the respondents feel more comfortable and willing to answer questions more truthfully."}, "time_dimension": {"answer": "Present", "rationale": "Now both \"you\" and \"freak\" are using the current state of affairs to indicate that the act or feeling is currently under way, so the entry is asking the person who was tested for the act or feeling."}, "sentence_type": "Interrogative sentence", "pos": {"nouns": {"I'm so low": 1, "Trouble": 1}, "verbs": {"Yes": 1}, "adverbs": {"- You're not": 1}, "numerals": {"022": 1}}}, {"content": "In life, do you find many pleasures?", "word_count": 16, "label": "", "sentence_structure": "Subject鈥損redicate", "subjective_objective": {"answer": "Subjective", "rationale": "Because finding happy things requires subjective perception and evaluation."}, "sentence_sentiment": {"answer": "Positive", "rationale": "Because the entry describes something that can be found happy in life, it is a positive attitude or quality."}, "language_style": {"answer": "Tactful and warm", "rationale": ""}, "time_dimension": {"answer": "Present", "rationale": "The entry asks if the testee can find many pleasant things in his current life, and the time dimension is now judged."}, "sentence_type": "Interrogative sentence", "pos": {"nouns": {"Life": 1, "Yes": 1, "Have a nice day": 1}, "verbs": {"Yes": 1, "Let's go": 1, "Current": 1, "Do it": 1}, "adjectives": {"Many": 1}, "numerals": {"023": 1}}}, {"content": "Have you ever felt that you have many difficulties that you cannot overcome?", "word_count": 18, "label": "", "sentence_structure": "Subject鈥搗erb鈥搊bject", "subjective_objective": {"answer": "Subjective", "rationale": "This is because of the patient ' s personal feelings and perceptions and is subjective."}, "sentence_sentiment": {"answer": "Negative", "rationale": "Because the expression \"many insurmountable difficulties\" in the entry is negative."}, "language_style": {"answer": "Calm and direct", "rationale": "The entry is more calm and straightforward in language, and it asks whether the interviewee has at some point felt that there are many insurmountable difficulties, that the description is simple and not overly embellished or emotional. It is therefore clear that the language style is calm and straightforward."}, "time_dimension": {"answer": "Present", "rationale": "The entry asks the testee about the current state and state of the person, and the time dimension is now judged. The term \"time\" indicates a regular, customary behaviour, while the word \"yes\" adopts the general current state of affairs, which means that the behaviour has not only occurred in the past but may continue now. Thus, the time dimension of the questionnaire entry is now."}, "sentence_type": "Interrogative sentence", "pos": {"nouns": {"Questionnaire Entry": 1, "Difficulties": 1}, "verbs": {"have": 1, "I think": 1, "Overcoming": 1}, "adjectives": {"Many": 1, "Cannot": 1}, "numerals": {"024": 1}, "pronouns": {"you": 1}}}, {"content": "025, do you ever force your hand wash yourself, even though you know your hands are clean?", "word_count": 24, "label": "", "sentence_structure": "Subject鈥搗erb鈥搊bject", "subjective_objective": {"answer": "Subjective", "rationale": "Since the question concerns the behaviour and ideas of the patients themselves, it is asked whether they have the impulse or behaviour to force them to wash their hands, which is the subjective feeling and attitude of the patients."}, "sentence_sentiment": {"answer": "Negative", "rationale": "Because the \"forced hand washing\" in the entry is negative. It may be a form of coercion, though clean hands are also forced to wash hands."}, "language_style": {"answer": "Tactful and warm", "rationale": "The language style of the entry is relatively warm. Using a soft \"Do you have time\" form rather than a direct statement or order, the respondents are more willing to share their thoughts and feelings. Moreover, the expression \"Although you know your hands are clean\" avoids too strong or critical a tone, with greater emphasis on communication and understanding with the respondents."}, "time_dimension": {"answer": "Present", "rationale": ""}, "sentence_type": "Interrogative sentence", "pos": {"nouns": {"you": 2, "Time": 1, "Forced": 1, "self": 1, "Wash your hands": 1, "鈾?Though 鈾?: 1, "I know": 1, "Hands": 1, "very": 1, "Clean": 1}, "verbs": {"Is it possible": 1, "have": 1, "Wash": 1, "Yes": 1}, "adverbs": {"Sometimes": 1, "Yes, I do": 1, "鈾?Though 鈾?: 1}, "numerals": {"025": 1}}}, {"content": "Are you sure your character has been determined by childhood experience and therefore cannot be changed?", "word_count": 26, "label": "", "sentence_structure": "Subject鈥揷opula鈥損redicative", "subjective_objective": {"answer": "Subjective", "rationale": "Because \"rebel\" is the patient's personal conviction and opinion."}, "sentence_sentiment": {"answer": "Negative", "rationale": "Because the purpose of the article is negative, it expresses a view of character that cannot be changed, and that may be a source of despair and helplessness."}, "language_style": {"answer": "Tactful and warm", "rationale": ""}, "time_dimension": {"answer": "Present", "rationale": "The words \"rebel,\" \"decided\" and \"decided\" are the state or beliefs of the moment, while \"childhood experience\" refers to past events. The questionnaire entry is therefore an inquiry into the testee's opinion as to whether personality can change the issue, and the time dimension is now."}, "sentence_type": "Interrogative sentence", "pos": {"nouns": {"Character": 1, "Childhood": 1, "Experience": 1}, "verbs": {"Yes": 1, "Decision": 1, "Change": 1}, "adjectives": {"Already": 1}, "adverbs": {"Cannot": 1}, "numerals": {"026": 1}}}, {"content": "027, do you often feel dizzy?", "word_count": 11, "label": "", "sentence_structure": "Subject鈥損redicate", "subjective_objective": {"answer": "Objective", "rationale": "Because \"dizziness\" is a physical symptom or experience, it can be observed and measured and is therefore objectively viewed."}, "sentence_sentiment": {"answer": "Negative", "rationale": "Because \"druck\" is a negative physical condition."}, "language_style": {"answer": "Tactful and warm", "rationale": "The language style of the entry is euphemism. The entry takes the form of a question sentence, asking whether the other party is often dizzy, expressing it in a mild and caring manner. At the same time, the term \"temporal\" is used to describe frequency, without directly indicating the seriousness or harm of the problem, and reflects respect and care for the person under investigation. This euphemism and caring language makes it easier for the person under investigation to feel trust and comfort and thus be more willing to share his real feelings."}, "time_dimension": {"answer": "Present", "rationale": "The entry is for the purpose of asking whether the person being tested is currently experiencing a frequent feeling of vertigo and therefore judging as now."}, "sentence_type": "Interrogative sentence", "pos": {"nouns": {"027": 1, "Brain": 1, "- Oh, my God": 1}, "verbs": {"I feel": 1}, "numerals": {"027": 1}}}, {"content": "Do you feel guilty for something you can't forgive?", "word_count": 16, "label": "", "sentence_structure": "Subject鈥搗erb鈥搊bject", "subjective_objective": {"answer": "Subjective", "rationale": "The fact that he thinks he has committed an unforgivable crime is the subjective feeling and perception of the patient."}, "sentence_sentiment": {"answer": "Negative", "rationale": "Because the phrase \"inexcusable sin\" in the entry is negative."}, "language_style": {"answer": "Calm and direct", "rationale": "The entry directly expresses the subject of questioning the other party about whether he or she has been inexcusable, without using excessive rhetoric or emotional rendering, and with a more objective tone."}, "time_dimension": {"answer": "Present", "rationale": "The keyword \"likes to have committed an unforgivable crime\" expresses the current emotional and cognitive state of the person being tested, and thus the judgment is now."}, "sentence_type": "Interrogative sentence", "pos": {"nouns": {"self": 1, "Sin": 1}, "verbs": {"I think": 1, "Yes": 1, "Forgive me": 1}, "adjectives": {"I'm sorry": 1}, "numerals": {"028": 1}}}, {"content": "- Are you confident, in general?", "word_count": 10, "label": "", "sentence_structure": "Subject鈥揷opula鈥損redicative", "subjective_objective": {"answer": "Subjective", "rationale": "Because the entry is an interview with the patient ' s self-evaluation and feelings and is a subjective examination."}, "sentence_sentiment": {"answer": "Positive", "rationale": "Because the expression \"very confident\" has a positive emotional color."}, "language_style": {"answer": "Tactful and warm", "rationale": "The entry uses a more euphemistic approach, avoiding asking directly whether the other party has some kind of specialty or ability. At the same time, the term \"very confident\" also uses a more flexible language, making it more soft. It is therefore judged that the language style of the questionnaire entry is euphemism."}, "time_dimension": {"answer": "Present", "rationale": ""}, "sentence_type": "Interrogative sentence", "pos": {"nouns": {"Overall": 1, "Confidence": 1}, "verbs": {"Is it possible": 1, "very": 1}, "adverbs": {"Both": 1}, "numerals": {"029": 1}}}, {"content": "030. Sometimes you don't care what happens in the future.", "word_count": 10, "label": "", "sentence_structure": "Subject鈥搗erb鈥搊bject", "subjective_objective": {"answer": "Subjective", "rationale": "Because the subject is examined by the patient's subjective attitude and perception, namely, \"Whether or not it cares about the future\"."}, "sentence_sentiment": {"answer": "Negative", "rationale": "Because the entry \"no matter what happens in the future\" expresses a negative attitude."}, "language_style": {"answer": "Calm and direct", "rationale": "The entry is in the form of a question and question-and-answer statement, which is more straightforward in tone, and does not involve excessive rhetorical or emotional rendering, and is of a more calm and direct language style."}, "time_dimension": {"answer": "Future", "rationale": "The future."}, "sentence_type": "Interrogative sentence", "pos": {"nouns": {"030": 1, "Future": 1}, "verbs": {"I care": 1, "What": 1}, "adverbs": {"Sometimes": 1}}}, {"content": "031, do you always feel very nervous about life?", "word_count": 13, "label": "", "sentence_structure": "Subject鈥搗erb鈥搊bject", "subjective_objective": {"answer": "Subjective", "rationale": "Because stress is a subjective feeling and experience, everyone can feel and react differently to stress and stress. The problem is therefore one of subjective feelings and experiences of patients."}, "sentence_sentiment": {"answer": "Negative", "rationale": "Because the attitude of \"frightful life\" is negative."}, "language_style": {"answer": "Calm and direct", "rationale": "The entry is more directly written, with no excessive qualifier or emotional colour, but rather directly asks the interviewee whether he or she feels the stress and stress of life."}, "time_dimension": {"answer": "Present", "rationale": "The phrase \"always feeling\" in the entry indicates the current state and feelings of the person being tested, which is the current state of affairs."}, "sentence_type": "Interrogative sentence", "pos": {"nouns": {"Questionnaire": 1, "Entry": 1, "Life": 1, "Nervous": 1}, "verbs": {"I feel": 1}, "adjectives": {"Very": 1}, "numerals": {"031": 1}}}, {"content": "032, do you sometimes get upset about the little things that are always in your mind?", "word_count": 24, "label": "", "sentence_structure": "Subject鈥搗erb鈥搊bject", "subjective_objective": {"answer": "Subjective", "rationale": "Because \"fear\" is a subjective emotional response."}, "sentence_sentiment": {"answer": "Negative", "rationale": "Because the behavior in the entries \"twirling in mind\" is negative."}, "language_style": {"answer": "Tactful and warm", "rationale": ""}, "time_dimension": {"answer": "Present", "rationale": "The words \"sometimes\" and \"sorts in mind and troubles\" suggest that this is a description of current behaviour or feelings, so the entry is a judgement of the past."}, "sentence_type": "Interrogative sentence", "pos": {"nouns": {"Details": 1, "It's nothing": 1, "Ideas": 1}, "verbs": {"- A entanglement": 1, "Trouble": 1, "Time": 1, "Yes": 1}, "adjectives": {"Quick End": 1}, "adverbs": {"Total": 1, "And": 1}, "numerals": {"032": 1}}}, {"content": "033, no matter what others say, do you always follow your own decisions?", "word_count": 19, "label": "", "sentence_structure": "Subject鈥損redicate", "subjective_objective": {"answer": "Subjective", "rationale": "Because \"always acting on your own decisions\" is a personal attitude or belief that belongs to the patient's subjective perspective."}, "sentence_sentiment": {"answer": "Positive", "rationale": "Because the \"do as you like\" act is positive."}, "language_style": {"answer": "Tactful and warm", "rationale": ""}, "time_dimension": {"answer": "Present", "rationale": "The entry asks the testee about his current behaviour or preference, i.e. whether they will insist on their own decisions when facing a different opinion."}, "sentence_type": "Interrogative sentence", "pos": {"nouns": {"person": 2, "self": 1, "Decision": 1, "Behaviour": 1}, "verbs": {"Say it": 1, "Press": 1, "Okay": 1}, "adverbs": {"Always": 1}, "numerals": {"033": 1}, "pronouns": {"you": 1}}}, {"content": "034, do you have more headaches than most people?", "word_count": 11, "label": "", "sentence_structure": "Subject鈥搗erb鈥搊bject", "subjective_objective": {"answer": "Subjective", "rationale": "Because headaches are a subjective feeling and experience of individuals."}, "sentence_sentiment": {"answer": "Negative", "rationale": "Because the \"headache\" in the entry is negative."}, "language_style": {"answer": "Calm and direct", "rationale": "The language style of the entry is calm and straightforward, and it raises questions in simple and clear language, without too many emotional or rhetorical techniques. This expression allows the respondents to understand the question more accurately and to give a true answer."}, "time_dimension": {"answer": "Present", "rationale": "The entry asks the testee's current state of health, \"034\" is a number or code, \"Are you more likely to have headaches than most people?\" It is the specific question that points to the current situation. So the time dimension is now."}, "sentence_type": "Interrogative sentence", "pos": {"nouns": {"Questionnaire": 1, "Entry": 1, "person": 1, "Headache": 1}, "verbs": {"- Yeah": 1, "Easy": 1}, "numerals": {"034": 1, "Most": 1}}}, {"content": "Do you often have a serious wish to repent for what you did?", "word_count": 21, "label": "", "sentence_structure": "Subject鈥搗erb鈥搊bject", "subjective_objective": {"answer": "Subjective", "rationale": "Because \"serious wishes\" are the feelings and thoughts of the patient himself."}, "sentence_sentiment": {"answer": "Negative", "rationale": "The entry is based on a judgement that indicates whether a person has a psychological state of guilt about what he or she has done and wants to be saved, which is usually a negative emotional or attitude."}, "language_style": {"answer": "Calm and direct", "rationale": "The entry is more directly phrased, is not overly emotional or qualifier, but is simple enough to ask the other side whether it regrets its actions."}, "time_dimension": {"answer": "Past", "rationale": "Repent is a move in the past, and \"serious wish\" is an expectation of the future, so the entry relates to the past and the next two temporal dimensions."}, "sentence_type": "Interrogative sentence", "pos": {"nouns": {"you": 1, "self": 1, "What I did": 1, "Repent": 1, "Serious wishes": 1}, "verbs": {"have": 1, "Regular": 1, "Conduct": 1}, "adverbs": {"Regular": 1, "- You're not": 1}, "numerals": {"035": 1}}}, {"content": "036. Do you often wish you were someone else?", "word_count": 15, "label": "", "sentence_structure": "Subject鈥搗erb鈥搊bject", "subjective_objective": {"answer": "Subjective", "rationale": "Because \"hope is another person\" is a personal thought and desire, a subjective attitude of the patient."}, "sentence_sentiment": {"answer": "Negative", "rationale": "Because the entry expresses a desire to change its mindset, it may be negative feelings such as dissatisfaction with the status quo or lack of confidence."}, "language_style": {"answer": "Tactful and warm", "rationale": "The entry uses a soft question, not directly asking the other side whether it has feelings of self-doubt or dissatisfaction, but rather leads the other side to think about it in a more moderate way. This expression is more respectful of the other side鈥檚 feelings and privacy, and more open to the interviewee."}, "time_dimension": {"answer": "Present", "rationale": ""}, "sentence_type": "Interrogative sentence", "pos": {"nouns": {"self": 1, "person": 2}, "verbs": {"Hope": 1, "be": 1, "Regular": 1}, "adverbs": {"Regular": 1}, "numerals": {"036": 1}, "classifiers": {"individual": 1}}}, {"content": "You feel energetic at 037?", "word_count": 10, "label": "", "sentence_structure": "Subject鈥損redicate", "subjective_objective": {"answer": "Subjective", "rationale": "Because it's a subjective assessment of how a patient feels about herself."}, "sentence_sentiment": {"answer": "Positive", "rationale": "Because the entry is asked about subjective perceptions of individuals, it is a positive attitude and state of affairs."}, "language_style": {"answer": "Tactful and warm", "rationale": "This expression is more likely to enjoy the trust and cooperation of the interviewee, and also to make the questionnaire process more smooth and pleasant."}, "time_dimension": {"answer": "Present", "rationale": "The entry asks the testee's current state, the keywords being \"normal\" and \"absorbed of energy,\" all pointing to the present situation. So the judgement is now."}, "sentence_type": "Interrogative sentence", "pos": {"nouns": {"- The energy": 1, "鈾?Full of 鈾?: 1}, "verbs": {"I feel": 1}, "numerals": {"037": 1}}}, {"content": "038, were you afraid of the dark when you were a kid?", "word_count": 9, "label": "", "sentence_structure": "Subject鈥搗erb鈥搊bject", "subjective_objective": {"answer": "Subjective", "rationale": "Because \"fear of darkness\" is a subjective feeling and emotional reaction, and it is the patient's personal experience and experience. Although darkness is objective, people's perception and reaction to it is subjective. So the problem is the patient's subjective experience and feelings."}, "sentence_sentiment": {"answer": "Negative", "rationale": "Because the act of \"fearing darkness\" in the entry is negative."}, "language_style": {"answer": "Tactful and warm", "rationale": "The language style of the entry is more euphemistic, using the term \"childhood\" to resonate and resonate with each other and to engage in dialogue in a way that is more accessible and answers the questions."}, "time_dimension": {"answer": "Past", "rationale": ""}, "sentence_type": "Interrogative sentence", "pos": {"nouns": {"When I was little": 1, "Darkness": 1}, "verbs": {"Scared": 1}, "numerals": {"038": 1}}}, {"content": "039. Are you interested in some kind of superstition to avoid such unlucky things as the cracking of walking?", "word_count": 33, "label": "", "sentence_structure": "Subject鈥搗erb鈥搊bject", "subjective_objective": {"answer": "Subjective", "rationale": "Because superstition, \"avoid bad luck\" are patients' subjective attitudes and ideas."}, "sentence_sentiment": {"answer": "Negative", "rationale": "Basis for judgement: because the entries refer to \"superstitious rituals\" and \"avoiding unlucky things\", these acts are usually considered to be negative or negative emotional."}, "language_style": {"answer": "Tactful and warm", "rationale": ""}, "time_dimension": {"answer": "Present", "rationale": "The keyword \"are interested in\" suggests that it is a selective issue to know whether the subjects are interested in some superstition and often engage in such activities in order to avoid bad luck."}, "sentence_type": "Interrogative sentence", "pos": {"nouns": {"Superstitious": 1, "Ceremony": 1, "Walk": 1, "Sound": 1, "Scratch": 1, "Good luck": 1}, "verbs": {"I'm so excited": 1, "Avoid": 1, "Send": 1}, "adverbs": {"Something like that": 1}, "classifiers": {"Plant": 1}, "pronouns": {"you": 1}}}, {"content": "040. Do you think it's hard to control weight?", "word_count": 10, "label": "", "sentence_structure": "Subject鈥損redicate", "subjective_objective": {"answer": "Subjective", "rationale": "Because \"the feeling\" expresses the patient's personal feelings and views, it's a subjective examination."}, "sentence_sentiment": {"answer": "Negative", "rationale": "Because it implies that individuals have challenges and problems in weight control."}, "language_style": {"answer": "Calm and direct", "rationale": "The entry is based on a direct, concise presentation, which is not too much rhetorical or emotional, but rather on a direct inquiry as to whether the respondent finds it difficult to control weight. This straightforward language style is usually more common in psychological questionnaires, as it provides a more objective understanding of the feelings and attitudes of the interviewee."}, "time_dimension": {"answer": "Present", "rationale": "The entry asks about the current feelings and attitudes of the person being tested and is therefore current."}, "sentence_type": "Interrogative sentence", "pos": {"nouns": {"Weight": 1, "Difficulties": 1}, "verbs": {"I think": 1, "Control": 1}, "numerals": {"040": 1}}}, {"content": "041. Do you feel a twitch in the face, head, shoulder?", "word_count": 15, "label": "", "sentence_structure": "Subject鈥搗erb鈥搊bject", "subjective_objective": {"answer": "Subjective", "rationale": "Because it's the patient's subjective feelings and experiences."}, "sentence_sentiment": {"answer": "Neutral", "rationale": "Because the entry is asked about physical symptoms and there is no apparent emotional tendency."}, "language_style": {"answer": "Tactful and warm", "rationale": "The entry is more personal and interactive, speaking in a gentle and inviting tone, rather than directly speaking or ordering. At the same time, the article is more balanced, not too emotional, comforting and relaxed. So I think the language style of the questionnaire is euphemism."}, "time_dimension": {"answer": "Present", "rationale": "\"Feeling facial, head, shoulder convulsions\" is a regular, customary and ongoing act in response to this question, so the time dimension of this questionnaire entry is now."}, "sentence_type": "Interrogative sentence", "pos": {"nouns": {"Face": 1, "Head": 1, "Shoulder": 1, "Twitches": 1}, "verbs": {"I feel": 1, "Time": 1}, "numerals": {"041": 1, "Sometimes": 1}}}, {"content": "You think people are always hard on you?", "word_count": 12, "label": "", "sentence_structure": "Subject鈥損redicate", "subjective_objective": {"answer": "Subjective", "rationale": "Because \"I feel like someone's not gonna be hard on themselves\" is a subjective feeling."}, "sentence_sentiment": {"answer": "Negative", "rationale": "Because the expression \"always felt that you were not being challenged\" expressed a negative emotion and attitude that felt that you were often criticized or blamed."}, "language_style": {"answer": "Tactful and warm", "rationale": "So we can judge that the language style is \"false warmness\"."}, "time_dimension": {"answer": "Present", "rationale": "Based on the general current state of \"always\" and \"like\" in \"doesn't you often feel\" it can be inferred that the entry is asking the testee about the current feelings or behaviour. Thus, the temporal dimension of the article is now."}, "sentence_type": "Interrogative sentence", "pos": {"nouns": {"Questionnaire": 1, "Entry": 1, "you": 2, "Regular": 1, "others": 1, "to": 1, "It's not hard": 1}, "verbs": {"I think": 1, "be": 1, "Regular": 1, "It's not hard": 1}, "adverbs": {"Regular": 1}, "numerals": {"042": 1}, "pronouns": {"you": 2}}}, {"content": "Does public discourse make you uncomfortable?", "word_count": 14, "label": "", "sentence_structure": "Subject鈥搗erb鈥搊bject", "subjective_objective": {"answer": "Subjective", "rationale": "It is a personal subjective feeling that feels uncomfortable."}, "sentence_sentiment": {"answer": "Negative", "rationale": "Because the expression \"sweetly uncomfortable\" in the entry is negative."}, "language_style": {"answer": "Calm and direct", "rationale": "The language style of the entry is relatively straightforward, and the absence of too many emotional colours or qualifiers is a more objective way of describing it. It makes the questionnaire more practical by asking if the interviewees are nervous or uncomfortable with the subject of speaking in public, by obtaining answers to simple and clear questions."}, "time_dimension": {"answer": "Past", "rationale": "The word \"is it uncomfortable?\" in the words \"is it uncomfortable?\" indicates that this has happened in the past, so it can be judged that the item is asking the testee about his past experiences and feelings. At the same time, \" very uncomfortable\" is also describing the emotional reaction of the past, further reinforcing the judgement that this is a question of the past."}, "sentence_type": "Interrogative sentence", "pos": {"nouns": {"Public speaking": 1, "I feel": 1, "I'm not comfortable": 1}, "verbs": {"鈾?And I'm gonna be 鈾?: 1}, "numerals": {"043": 1}}}, {"content": "Have you ever felt miserable for no reason?", "word_count": 17, "label": "", "sentence_structure": "Subject鈥搗erb鈥搊bject", "subjective_objective": {"answer": "Subjective", "rationale": "Because \"I feel miserable\" is the patient's subjective feeling and thoughts."}, "sentence_sentiment": {"answer": "Negative", "rationale": "Because the idea of \"felt miserable\" is negative."}, "language_style": {"answer": "Calm and direct", "rationale": "The language style of the entry is more straightforward, not using too many qualifiers or emotional colours, but asking the person surveyed whether he or she has had a tragic experience without a reason."}, "time_dimension": {"answer": "Present", "rationale": "\"At one time\" means a movement or state that happened in the past, and \"and feel miserable for no reason\" describes an emotional state, so the temporal dimension of this sentence is now."}, "sentence_type": "Interrogative sentence", "pos": {"nouns": {"044": 1, "Questionnaire": 1, "Entry": 1, "Tragic": 1}, "verbs": {"I think": 1, "For no reason": 1, "self": 1, "very": 1}, "adjectives": {"For no reason": 1}, "adverbs": {"Once": 1, "very": 1}, "pronouns": {"you": 1, "self": 1}}}, {"content": "045. Do you often get busy and seem to be asking for something, and actually don't know what you're asking for?", "word_count": 21, "label": "", "sentence_structure": "Subject鈥搗erb鈥搊bject", "subjective_objective": {"answer": "Subjective", "rationale": "Because the question refers to subjective expression like \"Are you often\" and asks about personal feelings and experiences. This requires patients to answer questions from their own perspective, and therefore is subjective."}, "sentence_sentiment": {"answer": "Negative", "rationale": "Because it expresses a negative attitude that does not know what it really wants."}, "language_style": {"answer": "Calm and direct", "rationale": ""}, "time_dimension": {"answer": "Past", "rationale": ""}, "sentence_type": "Interrogative sentence", "pos": {"nouns": {"you": 1, "045": 1, "Busy": 1, "I don't know what I'm asking for": 1}, "verbs": {"Regular": 1, "Seems like": 1, "I'm gonna need something": 1, "Actual": 1, "Yes": 1, "Please": 1, "be": 1}, "adjectives": {"Often": 1, "Busy": 1, "I don't know what I'm asking for": 1}, "numerals": {"045": 1, "Regular": 1}, "classifiers": {"individual": 1, "I don't know what I'm asking for": 1}, "pronouns": {"you": 1}}}, {"content": "046. Do you often worry about locking drawers, windows, doors, boxes?", "word_count": 19, "label": "", "sentence_structure": "Subject鈥搗erb鈥搊bject", "subjective_objective": {"answer": "Objective", "rationale": "It is an objective fact that the patient is concerned about the locking of drawers, windows, doors, boxes, etc."}, "sentence_sentiment": {"answer": "Neutral", "rationale": "Because the entry was not clearly emotional, it was merely asking the other party whether it was concerned."}, "language_style": {"answer": "Calm and direct", "rationale": "This is a language style that is more direct, concise, less curated and emotional, and more focused on the description of objective facts and the communication of the purpose of the investigation."}, "time_dimension": {"answer": "Present", "rationale": "The word \"normal\" denotes regular, customary behaviour, while the word \"fear\" and \"lock\" describe the person's current psychological state. The entry therefore determines the current dimension of the person being tested in terms of the current psychological state."}, "sentence_type": "Interrogative sentence", "pos": {"nouns": {"The drawer": 1, "The window": 1, "Door": 1, "Box": 1}, "verbs": {"Lock it up": 1, "I'm worried": 1}, "adverbs": {"Yes": 1}, "numerals": {"046": 1, "1": 1}}}, {"content": "047. Do you believe in God, destiny and supernatural forces that control your life and your death?", "word_count": 24, "label": "", "sentence_structure": "Subject鈥搗erb鈥搊bject", "subjective_objective": {"answer": "Subjective", "rationale": "Because it is subjective to believe that supernatural power is the individual belief and attitude of the patient."}, "sentence_sentiment": {"answer": "Neutral", "rationale": "Because the entry does not have any emotional color."}, "language_style": {"answer": "Calm and direct", "rationale": "The entry asks the respondents, in a direct and clear manner, whether they believe in the supernatural forces of God, destiny, and not in too many rhetorical or emotional language, with a greater focus on the transmission of and access to information."}, "time_dimension": {"answer": "Present", "rationale": "\"Believe\" is the person who was tested, the idea or belief, and therefore the judgment is now. And \"God, fate, supernatural forces that control your life and your illness\" is a specific description of that faith, but they are not themselves expressions of behaviour or attitude, but exist as background information, and therefore do not participate in the determination of the time dimension."}, "sentence_type": "Interrogative sentence", "pos": {"nouns": {"Jesus": 1, "Destiny": 1, "Power": 1, "Old and sick": 1}, "verbs": {"Yes": 1, "Control": 1}, "adverbs": {"Is it possible": 1}, "numerals": {"047": 1}}}, {"content": "048, are you worried about your illness?", "word_count": 9, "label": "", "sentence_structure": "Subject鈥搗erb鈥搊bject", "subjective_objective": {"answer": "Subjective", "rationale": "Because \"scared\" reflects the patient's subjective feelings and fears."}, "sentence_sentiment": {"answer": "Negative", "rationale": "Because the \"scared\" attitude in the entry is negative."}, "language_style": {"answer": "Calm and direct", "rationale": "This is a straightforward way of asking questions, without too much rhetorical or emotional presentation, and is more objective in tone. This way makes it easier for respondents to understand the intentions of the questions and to answer them accurately."}, "time_dimension": {"answer": "Present", "rationale": "The words \"scared\" and \"diseased\" in the sentence make it clear that the entry is asking the testee what he feels and what he feels now, so the dimension of time is now."}, "sentence_type": "Interrogative sentence", "pos": {"nouns": {"Diseases": 1, "self": 1}, "verbs": {"Okay": 1, "I'm worried": 1}, "adverbs": {"very": 1}, "numerals": {"048": 1}, "pronouns": {"you": 1}}}, {"content": "049. Do you believe that the happiness that comes at this moment will eventually have to be repaid?", "word_count": 22, "label": "", "sentence_structure": "Subject鈥搗erb鈥搊bject", "subjective_objective": {"answer": "Subjective", "rationale": "Because \"convincing\" is a subjective attitude or opinion."}, "sentence_sentiment": {"answer": "Negative", "rationale": "Because it expresses a negative sentiment and attitude that happiness must be paid."}, "language_style": {"answer": "Calm and direct", "rationale": "The language style of the entry is \"cool and direct.\" It is a clear and direct question, without any rhetoric or emotional rendering. It simply asks the interviewee whether he believes that the happiness they currently receive will eventually be rewarded or compensated. This straightforward expression helps the interviewee to provide clear and clear answers, without needing too much explanation or interpretation."}, "time_dimension": {"answer": "Present", "rationale": "The entry asks about the current attitude and conviction of the testee that \"the happiness that comes at this moment will eventually have to be repaid.\" The time dimension is now."}, "sentence_type": "Interrogative sentence", "pos": {"nouns": {"Happiness": 1, "Reimbursement": 1}, "verbs": {"Yes": 1, "Got it": 1}, "adverbs": {"At this moment": 1, "Eventually": 1}, "numerals": {"049": 1}}}, {"content": "050 If possible, will you change yourself in many ways?", "word_count": 18, "label": "", "sentence_structure": "Subject鈥搗erb鈥搊bject", "subjective_objective": {"answer": "Subjective", "rationale": "The question is about the patient ' s subjective will and attitude."}, "sentence_sentiment": {"answer": "Neutral", "rationale": "Because the entry does not have any positive or negative emotional connotations."}, "language_style": {"answer": "Tactful and warm", "rationale": "Warm and warm."}, "time_dimension": {"answer": "Present", "rationale": "The entry asks the testee about changing his/her attitude, which does not have a clear time frame or the context of the event, and therefore makes it difficult to classify as past, present or future."}, "sentence_type": "Interrogative sentence", "pos": {"nouns": {"In terms of": 1, "self": 1}, "verbs": {"Maybe": 1, "Change": 2}, "adjectives": {"Many": 1}, "adverbs": {"If": 1}, "numerals": {"050": 1, "Will": 1}, "pronouns": {"you": 1}}}, {"content": "051. Do you think you're looking forward to it?", "word_count": 10, "label": "", "sentence_structure": "Subject鈥搗erb鈥搊bject", "subjective_objective": {"answer": "Subjective", "rationale": "Because \"optimistic\" is a subjective assessment and view, everyone's views are different."}, "sentence_sentiment": {"answer": "Positive", "rationale": "Because the \"optimistic\" approach in the entry is positive."}, "language_style": {"answer": "Calm and direct", "rationale": "The entry is a direct and clear query about the person ' s future, does not involve excessive rhetorical or emotional rendering, and is more calm and objective in language style."}, "time_dimension": {"answer": "Future", "rationale": "The entry asks the testees about their future and their expectations, and the key words are \"their own future\" and \"optimistic\", all pointing to situations that have not yet occurred and that may arise in the future."}, "sentence_type": "Interrogative sentence", "pos": {"nouns": {"Future": 1, "Optimistic": 1}, "verbs": {"self": 1, "I think": 1}, "numerals": {"051": 1}}}, {"content": "052, are you shaking and sweating in the face of a difficult task?", "word_count": 15, "label": "", "sentence_structure": "Subject鈥搗erb鈥搊bject", "subjective_objective": {"answer": "Objective", "rationale": "Because shaking and sweating are physical reactions to a given situation and are objective."}, "sentence_sentiment": {"answer": "Negative", "rationale": "Because the physical reactions of \"shock and sweat\" in the entries are usually considered negative reactions to stress."}, "language_style": {"answer": "Calm and direct", "rationale": "The language style of the entry is more straightforward, with no excessive rhetorical or emotional rendering, and a more calm and objective tone.\""}, "time_dimension": {"answer": "Present", "rationale": "The entry is an expression of behaviour in the current moment, i.e., in the answer to the question, and can therefore be judged as \"now\"."}, "sentence_type": "Interrogative sentence", "pos": {"nouns": {"Tasks": 1, "Hard": 1, "# Wresh #": 1, "Sweat": 1}, "verbs": {"Yes": 3, "# Wresh #": 1, "Sweat": 1}, "adjectives": {"Hard": 2}, "numerals": {"052": 1}}}, {"content": "Before you go to bed, do you always check all the lights, gear and pipes in accordance with the procedure?", "word_count": 28, "label": "", "sentence_structure": "Subject鈥搗erb鈥搊bject", "subjective_objective": {"answer": "Objective", "rationale": "This is an objective fact because of the actual behaviour of the patient before he goes to bed at night."}, "sentence_sentiment": {"answer": "Positive", "rationale": "Because \"the procedure for checking all lights, appliances and pipes is carefully and emotionally positive.\""}, "language_style": {"answer": "Calm and direct", "rationale": "The language style of the entry is relatively calm and straightforward, and there is no excessive rhetorical or emotional overtones, and direct questioning of the behaviour of the person under investigation in the form of a question-and-answer period."}, "time_dimension": {"answer": "Past", "rationale": "\"Before bed\" indicates that this behaviour is usually performed while the person is ready for rest, and that \"the procedure for checking all lights, appliances and pipes for good\" is an inquiry into the behaviour of the person at a certain point in time, so the entry looks at the behaviour of the person who was tested."}, "sentence_type": "Interrogative sentence", "pos": {"nouns": {"Go to bed": 1, "Procedures": 1, "Lights": 1, "Use the gear": 1, "Pipes": 1}, "verbs": {"Press regularly": 1, "Inspection": 1, "Close it": 1, "No, I'm not": 1}, "adverbs": {"Before": 1}, "numerals": {"053": 1, "All": 1}, "classifiers": {"individual": 1}}}, {"content": "If something goes wrong, do you often attribute it to bad luck rather than bad luck?", "word_count": 29, "label": "", "sentence_structure": "Subject鈥搗erb鈥搊bject", "subjective_objective": {"answer": "Subjective", "rationale": "Because the failure of the matter is attributed to bad luck or inappropriate methods of interpretation, it is the patient ' s subjective attitude."}, "sentence_sentiment": {"answer": "Negative", "rationale": "Because the \"unlucky\" behavior in the entry is negative."}, "language_style": {"answer": "Calm and direct", "rationale": "The entry asks whether the respondents often shift responsibility to bad luck rather than to their own methods when they have problems. It is more objective and straightforward. There is no excessive emotional accommodation or euphemism, which is typical of \"cool and straight\" language styles."}, "time_dimension": {"answer": "Past", "rationale": "The keyword \"is it\" means that it is an option that requires judgement from the testee; and \"unlucky\" and \"inappropriate methodology\" point to two possible attributions, respectively. As the issue does not have a clear temporal dimension, it is not possible to determine whether it is past, present or future behaviour or mentality."}, "sentence_type": "Interrogative sentence", "pos": {"nouns": {"Yes": 2, "Error": 1, "Lucky": 1, "Methodology": 1, "Questionnaire Entry": 1}, "verbs": {"Out": 1, "They're often classified as": 1, "be": 1, "Not right": 1}, "adjectives": {"Bad": 1, "Appropriate": 1}, "adverbs": {"If": 1}, "numerals": {"054": 1}, "pronouns": {"you": 1}}}, {"content": "055. Even if you think you're just cold, do you have to go to see the doctor?", "word_count": 22, "label": "", "sentence_structure": "Subject鈥搗erb鈥搊bject", "subjective_objective": {"answer": "Objective", "rationale": "Since access to a doctor is a specific action required of the patient, it is considered from an objective perspective."}, "sentence_sentiment": {"answer": "Positive", "rationale": "Because the proposal for the entry is to go to the hospital, it is a positive attitude towards health."}, "language_style": {"answer": "Tactful and warm", "rationale": "The words are more euphemistic, and the expression \"must also\" is used to guide respondents to think and answer questions, and it is more gentle and convincing. And the expression \"even if it cools\" is more sweet, does not overemphasize the severity of the disease, and it is more friendly and understanding."}, "time_dimension": {"answer": "Present", "rationale": "The entry asks the testee's choice in the current situation, which is, \"Do you have to go to see the patient even if you think you're just cold?\" This is the choice. So the temporal dimension is now."}, "sentence_type": "Interrogative sentence", "pos": {"nouns": {"It's cold": 1, "Diseases": 1}, "verbs": {"I think": 1, "Look": 1, "I'm sure": 1}, "adverbs": {"Just": 1, "Yeah": 1, "Yes": 1}, "numerals": {"055": 1}, "pronouns": {"you": 2}}}, {"content": "056, do you care if you live better than most people around you?", "word_count": 22, "label": "", "sentence_structure": "Subject鈥搗erb鈥搊bject", "subjective_objective": {"answer": "Subjective", "rationale": "This is because of patients ' subjective feelings and self-evaluation."}, "sentence_sentiment": {"answer": "Positive", "rationale": "Because it expresses a positive attitude, i.e. a comparison and concern for one ' s own life."}, "language_style": {"answer": "Tactful and warm", "rationale": ""}, "time_dimension": {"answer": "Present", "rationale": "The keywords \"are concerned\" and \"are they better than most people around us\" indicate that the subject is thinking about it at this moment, so it can be judged that the entry relates to current behaviour or attitudes."}, "sentence_type": "Interrogative sentence", "pos": {"nouns": {"you": 1, "self": 1, "Around": 1, "Most people": 1, "Life": 1, "Better": 1}, "verbs": {"I care": 1, "- Yeah": 1}, "adjectives": {"Most of them": 1}, "adverbs": {"More": 1}, "numerals": {"056": 1}}}, {"content": "057. In general, do you feel like you're well received?", "word_count": 20, "label": "", "sentence_structure": "Subject鈥搗erb鈥搊bject", "subjective_objective": {"answer": "Subjective", "rationale": "Since this is a question of asking patients about their subjective feelings, it is the patient ' s own perception of his or her popularity."}, "sentence_sentiment": {"answer": "Positive", "rationale": "Because it expresses a positive emotional attitude."}, "language_style": {"answer": "Tactful and warm", "rationale": "The terms of the questionnaire are soft, friendly, and in a question-and-answer manner, free to express their views, rather than direct orders or denial. At the same time, the use of the term \"popular\" is a more euphemism of the level of popularity, and does not overemphasize or criticize the level of self-confidence of the interviewee."}, "time_dimension": {"answer": "Present", "rationale": "The entry asks what the testee feels in the current state of affairs, and the keywords are \"general\" and \"you think\" and they all indicate that this is a description of the current situation. So we can judge the time dimension of the article is now."}, "sentence_type": "Interrogative sentence", "pos": {"nouns": {"Situation": 1, "self": 1, "Welcome": 1}, "verbs": {"I think": 1, "Yes": 1, "be": 1}, "adjectives": {"General": 1}, "numerals": {"057": 1, "Pong": 1}, "pronouns": {"Everyone": 1}}}, {"content": "Have you ever thought you'd be worse off dead?", "word_count": 15, "label": "", "sentence_structure": "Subject鈥搗erb鈥搊bject", "subjective_objective": {"answer": "Subjective", "rationale": "Because a description of \"if\" is based on the patient's own feelings and thoughts and is subjective."}, "sentence_sentiment": {"answer": "Negative", "rationale": "Because the idea of \"I'm not better off dead\" in the entry is negative."}, "language_style": {"answer": "Tactful and warm", "rationale": "The language style of the entry is more euphemistic, sensitive and understanding of the other鈥檚 feelings, using a question-and-answer approach rather than a direct statement or order. This way allows respondents to express their true situation without being unduly embarrassed or pressured, and more reflective of the researcher鈥檚 concerns and respect."}, "time_dimension": {"answer": "Past", "rationale": "The \"had\" behaviour occurred in the past and the question was about past experiences and feelings."}, "sentence_type": "Interrogative sentence", "pos": {"nouns": {"Thoughts": 1, "self": 1}, "verbs": {"have": 1}, "numerals": {"058": 1}, "pronouns": {"you": 1}}}, {"content": "059, even if you knew you wouldn't be hurt, would you be afraid of some people or things?", "word_count": 24, "label": "", "sentence_structure": "Subject鈥搗erb鈥搊bject", "subjective_objective": {"answer": "Subjective", "rationale": "Because it is the patient鈥檚 subjective feeling and attitude, that is, his fear and fear of some people or things. While these things may not cause substantial harm to himself, the emotional response is his own perception and experience."}, "sentence_sentiment": {"answer": "Negative", "rationale": "Because the \"fear\" in the entry is negative."}, "language_style": {"answer": "Calm and direct", "rationale": "This is a more direct and objective language style, which does not have too many emotional or rhetorical features, and helps to obtain accurate and true answers."}, "time_dimension": {"answer": "Present", "rationale": ""}, "sentence_type": "Interrogative sentence", "pos": {"nouns": {"Hurt": 1, "person": 1, "Yes": 1}, "verbs": {"Yes": 2, "have": 1, "I'm scared": 1}, "adverbs": {"Even if": 1}, "numerals": {"059": 1}, "pronouns": {"you": 2}}}, {"content": "060, Do you carefully store food or food at home to prevent food shortages?", "word_count": 26, "label": "", "sentence_structure": "Subject鈥搗erb鈥搊bject", "subjective_objective": {"answer": "Objective", "rationale": "The objective reality is that the entry is concerned with an act (storage of food at home) and an outcome (preventing food shortages)."}, "sentence_sentiment": {"answer": "Neutral", "rationale": "Because the entry does not have any emotional color."}, "language_style": {"answer": "Tactful and warm", "rationale": ""}, "time_dimension": {"answer": "Present", "rationale": "The keywords \"are you careful\" , \"stores\" and \"prevent food shortages\" point to the present behaviour and attitude of the testees."}, "sentence_type": "Interrogative sentence", "pos": {"nouns": {"Questionnaire": 1, "Entry": 1, "Home": 1, "Food": 3, "Food shortages": 1}, "verbs": {"Storage": 1, "Protection": 1}, "adjectives": {"Watch your wings": 1}, "adverbs": {"Land": 1}, "numerals": {"060": 1, "Some": 1, "1": 2}, "classifiers": {"Some": 1}, "pronouns": {"you": 1}}}, {"content": "061. Have you ever felt a bad mind to control you?", "word_count": 16, "label": "", "sentence_structure": "Subject鈥搗erb鈥搊bject", "subjective_objective": {"answer": "Subjective", "rationale": "Because it is a subjective experience and feeling of a human being, it is based on the inner world of the individual."}, "sentence_sentiment": {"answer": "Negative", "rationale": "Because \"bad thoughts\" in the entries are negative words, expressing negative feelings."}, "language_style": {"answer": "Tactful and warm", "rationale": "The language style of the entry is a euphemism. It uses the expression \"I felt\" to emphasize neither the existence of the problem nor the possibility of denying it. Moreover, the question \"a bad idea dominates you\" is more moderate, guided, and does not directly accuse or attack the person under investigation. Thus, the language style of the article is more friendly and inclusive and more acceptable to and co-opted by the person under investigation."}, "time_dimension": {"answer": "Past", "rationale": "The keywords are \"had felt\" and \"a bad idea dominated you\", which all indicate an experience or behaviour that has already occurred, and the entry is therefore judged to be the past."}, "sentence_type": "Interrogative sentence", "pos": {"nouns": {"you": 2, "Bad thinking": 1, "Rule": 1}, "verbs": {"I feel": 1, "have": 1}, "numerals": {"061": 1}}}, {"content": "062. Do you often feel exhausted?", "word_count": 10, "label": "", "sentence_structure": "Subject鈥損redicate", "subjective_objective": {"answer": "Subjective", "rationale": "Basis for the determination: The entry asks about the patient ' s subjective perception that he/she is often exhausted and therefore subject to subjective examination."}, "sentence_sentiment": {"answer": "Negative", "rationale": "Because the \"float\" in the entry is negative."}, "language_style": {"answer": "Calm and direct", "rationale": "The entry is based on a simple question of whether the person interviewed is often exhausted, without any rhetorical or emotional presentation, and is more balanced and objective, and therefore can be judged to be calm and straightforward."}, "time_dimension": {"answer": "Present", "rationale": "The keyword \"always\" denotes a regular act, \"facilitated\" is the current feeling or state, so the entry asks about the present experience."}, "sentence_type": "Interrogative sentence", "pos": {"nouns": {"Questionnaire": 1, "Entry": 1, "Exhausted": 1}, "verbs": {"I always feel": 1, "Is it possible": 1, "Tired": 1}, "adverbs": {"Regular": 1}, "numerals": {"062": 1}}}, {"content": "063, have you done something that makes you feel sorry for your life?", "word_count": 15, "label": "", "sentence_structure": "Subject鈥搗erb鈥搊bject", "subjective_objective": {"answer": "Subjective", "rationale": "It is the subjective feeling and experience of the patient to do something that is regrettable."}, "sentence_sentiment": {"answer": "Negative", "rationale": "Because \"what makes you regret forever\" is negative."}, "language_style": {"answer": "Calm and direct", "rationale": "It raises a clear question directly, without too much rhetorical or emotional overtones, and with a more objective tone, in order to know whether the interviewee has ever had something that he deeply regrets."}, "time_dimension": {"answer": "Past", "rationale": "The entry asks if the person tested has done anything in the past that leads to regret and therefore the time dimension is a matter of the past."}, "sentence_type": "Interrogative sentence", "pos": {"nouns": {"Questionnaire Entry": 1, "you": 2, "Yes": 2, "Life": 1, "Sorry": 1}, "verbs": {"Do it": 1, "鈾?And I'm gonna be 鈾?: 1}, "adverbs": {"063": 1, "Some": 1}, "numerals": {"063": 1, "One": 2, "Two": 1}, "pronouns": {"you": 2}}}, {"content": "064, do you always have confidence in your decisions?", "word_count": 15, "label": "", "sentence_structure": "Subject鈥搗erb鈥搊bject", "subjective_objective": {"answer": "Subjective", "rationale": "Because \"confident\" is a psychological state, reflecting the individual feelings and attitudes of the patient, and is subjective."}, "sentence_sentiment": {"answer": "Positive", "rationale": "Because the \"confident\" attitude in the entries is positive."}, "language_style": {"answer": "Calm and direct", "rationale": "It asked the interviewees in a direct and clear manner whether they were always confident in their decisions, without too many rhetorical or emotional expressions and with a more objective tone."}, "time_dimension": {"answer": "Present", "rationale": "The entry asks the testee about his attitude and feelings at this moment, and the keyword is \"always\" and \"confident,\" all pointing to the state of the moment. So the time dimension is now."}, "sentence_type": "Interrogative sentence", "pos": {"nouns": {"Decision": 1, "Confidence": 1}, "verbs": {"be": 1, "Fill": 1}, "adverbs": {"Always": 1}, "numerals": {"064": 1}}}, {"content": "065, do you often feel frustrated?", "word_count": 7, "label": "", "sentence_structure": "Subject鈥損redicate", "subjective_objective": {"answer": "Subjective", "rationale": "Because feeling frustrated is a subjective emotional experience."}, "sentence_sentiment": {"answer": "Negative", "rationale": "Because the \"often frustrated\" act in the entry is negative."}, "language_style": {"answer": "Calm and direct", "rationale": "The entry directly asked the respondents whether they were often frustrated about the subject, did not use excessive rhetorical techniques or emotional rendering, and was more objective in tone."}, "time_dimension": {"answer": "Present", "rationale": "The entry uses the word \"normal\" to denote a regular act and the general current state of affairs, which means that the act has not only occurred in the past, but may still be ongoing. Thus, it can be judged as now."}, "sentence_type": "Interrogative sentence", "pos": {"nouns": {"Frustration": 1}, "verbs": {"I feel": 1}, "numerals": {"065": 1}}}, {"content": "066, are you more anxious than the rest of the world?", "word_count": 12, "label": "", "sentence_structure": "Subject鈥搗erb鈥搊bject", "subjective_objective": {"answer": "Subjective", "rationale": "Because \"Easy anxiety\" and \"Easy anxiety\" are feelings and perceptions of the patient, and are manifestations of subjective attitudes."}, "sentence_sentiment": {"answer": "Neutral", "rationale": "Because the entry asks whether the individual is more anxious than others and has no apparent emotional tendency."}, "language_style": {"answer": "Calm and direct", "rationale": "The entry asks the respondents, in a direct and clear manner, whether they are less likely to be in an anxiety than others, are not overtranslation or emotionally retroverted and are more objective and calm."}, "time_dimension": {"answer": "Past", "rationale": "The entry asks about the testee's past experience or characteristics, and the keyword is \"more difficult to be anxious than others\" and it involves comparison and evaluation of the past."}, "sentence_type": "Interrogative sentence", "pos": {"nouns": {"Questionnaire": 1, "Entry": 1, "person": 1, "Anxiety": 1}, "verbs": {"- Yeah": 1, "More": 1, "Easy": 1, "Anxiety": 1}, "adjectives": {"Other": 1}, "numerals": {"066": 1, "Others": 1}, "pronouns": {"you": 1}}}, {"content": "067. Are you particularly afraid and repulsive of dirty things?", "word_count": 12, "label": "", "sentence_structure": "Subject鈥搗erb鈥搊bject", "subjective_objective": {"answer": "Subjective", "rationale": "Because fear and aversion to dirty things are the feelings and attitudes of the individual patient and are subjective."}, "sentence_sentiment": {"answer": "Negative", "rationale": "Because \"special fear and disgust\" is a negative adjective."}, "language_style": {"answer": "Calm and direct", "rationale": "The entry directly asks whether the other party has a particular fear or aversion to dirty things, which is more directly spoken and is not over-refuted."}, "time_dimension": {"answer": "Present", "rationale": "The keywords \"you\" and \"special fear and disgust\" indicate that this is an inquiry into the current behaviour or feelings of the subject, so it is now."}, "sentence_type": "Interrogative sentence", "pos": {"nouns": {"Questionnaire Entry": 1, "Dirty things": 1}, "verbs": {"Very scared": 1, "Disgusting": 1, "- You're not": 1, "be": 1, "and": 1}, "adjectives": {"Special": 1}, "adverbs": {"- You're not": 1, "and": 1, "Special": 1}, "numerals": {"067": 1}}}, {"content": "068. Do you often feel that you are the victim of some outsider ' s control?", "word_count": 22, "label": "", "sentence_structure": "Subject鈥搗erb鈥搊bject", "subjective_objective": {"answer": "Subjective", "rationale": "As the entry asks the patient ' s own feelings and perceptions, i.e. whether he considers himself to be a victim of some outside control and therefore to be viewed from the patient ' s subjective perspective."}, "sentence_sentiment": {"answer": "Negative", "rationale": "Because the expression \"victims of uncontrolled external force\" in the entry gives people negative feelings and expresses a controlled emotional and passive state."}, "language_style": {"answer": "Calm and direct", "rationale": ""}, "time_dimension": {"answer": "Past", "rationale": "The entry asks the testee about past behaviour or experience and therefore determines past."}, "sentence_type": "Interrogative sentence", "pos": {"nouns": {"Questionnaire": 1, "Entry": 1, "068": 1, "self": 1, "External force": 1, "Victims": 1}, "verbs": {"I feel": 1, "Regular": 1, "be": 1, "Control": 1, "of": 1, "Cannot": 1, "Victims": 1}, "adverbs": {"Regular": 1, "Cannot": 1}, "numerals": {"068": 1}, "pronouns": {"you": 1, "self": 1}}}, {"content": "069. Are you considered to be a sick and infirm?", "word_count": 14, "label": "", "sentence_structure": "Subject鈥搗erb鈥搊bject", "subjective_objective": {"answer": "Subjective", "rationale": "Because the word \"considered\" reflects whether the person interviewed is considered a sick and infirm person from another perspective, and is subjective."}, "sentence_sentiment": {"answer": "Negative", "rationale": "Because the expression \"infirmity\" in the entry is negative."}, "language_style": {"answer": "Calm and direct", "rationale": "This article is more objective, does not have too many rhetorical or emotional accents, and asks whether the person surveyed is considered to be infirm."}, "time_dimension": {"answer": "Past", "rationale": "The entry asks the testee's past experience and assessment, i.e. whether they were ever considered to be \"a sick and infirm person\". The temporal dimension is therefore a past."}, "sentence_type": "Interrogative sentence", "pos": {"nouns": {"069": 1, "person": 2, "I'm sick and sick": 1}, "verbs": {"I think": 1, "be": 1, "have": 1}, "adjectives": {"Infirm and sick": 1}, "numerals": {"069": 1, "you": 1, "One": 2, "Infirm and sick": 1}, "pronouns": {"you": 1}}}, {"content": "Are you often blamed and punished without any reason?", "word_count": 16, "label": "", "sentence_structure": "Subject鈥搗erb鈥搊bject", "subjective_objective": {"answer": "Subjective", "rationale": "Because \"acquirement and punish without cause\" is the patient's subjective feeling of the environment in which he is living."}, "sentence_sentiment": {"answer": "Negative", "rationale": "Because the entry \"often being blamed and punished without any reason\" is negative."}, "language_style": {"answer": "Calm and direct", "rationale": "The language style of the entry is more straightforward, there is no excessive adoration or emotional colour, and a question is raised in a concise manner as to whether the other party is often subject to unwarranted accusations and punishment."}, "time_dimension": {"answer": "Present", "rationale": "The keyword \"often\" denotes a regular, customary act, \"accusal and punishment\" is an event that has already occurred, so the entry is a description of the situation of the person being tested."}, "sentence_type": "Interrogative sentence", "pos": {"nouns": {"Blame": 1, "Punishment": 1}, "verbs": {"Received": 1, "For no reason": 1}, "adjectives": {"Often": 1}, "adverbs": {"Land": 1}, "numerals": {"070": 1}}}, {"content": "071, do you think you're very insightful?", "word_count": 11, "label": "", "sentence_structure": "Subject鈥損redicate", "subjective_objective": {"answer": "Subjective", "rationale": "It is a subjective feeling and a perception that is very insightful."}, "sentence_sentiment": {"answer": "Neutral", "rationale": "Because the entry does not have any emotional color."}, "language_style": {"answer": "Calm and direct", "rationale": "The entry directly asks the interviewees whether they have a perception of themselves, and the language style is more straightforward and concise, without too much ado or emotional colour."}, "time_dimension": {"answer": "Present", "rationale": "The keyword \"I feel\" and \"Yes\" both occurs at the moment, so the entry describes the person's current feelings and views."}, "sentence_type": "Interrogative sentence", "pos": {"nouns": {"071": 1, "See, uh": 1}, "verbs": {"I think": 1, "have": 1}}}, {"content": "072, is there always hope for you?", "word_count": 13, "label": "", "sentence_structure": "Subject鈥搗erb鈥搊bject", "subjective_objective": {"answer": "Subjective", "rationale": "Because \"no hope\" is a subjective assessment."}, "sentence_sentiment": {"answer": "Negative", "rationale": "Because the description \"something never works out\" in the entry is negative."}, "language_style": {"answer": "Calm and direct", "rationale": "The point is based on the following: the purpose of this article is to use the negative expression of doubt, and to ask the interviewee whether he or she considers that things are always hopeless. This expression is more direct and clear, is not overly emotional or rhetorical, and is more objective and calm."}, "time_dimension": {"answer": "Present", "rationale": "The entry asks the testee about the current situation and feelings, and the keywords \"for you\" and \"for you?\" are all questions about the current state."}, "sentence_type": "Interrogative sentence", "pos": {"nouns": {"Things": 1, "Hope": 1}, "verbs": {"be": 1, "have": 1, "to": 1}, "adjectives": {"Nope": 1, "Always": 1}, "adverbs": {"No, I'm not": 1}, "numerals": {"072": 1}, "pronouns": {"you": 1}}}, {"content": "073, do you often worry about something unrealistic without a reason?", "word_count": 19, "label": "", "sentence_structure": "Subject鈥搗erb鈥搊bject", "subjective_objective": {"answer": "Subjective", "rationale": "Because \"unjustified fear for something unrealistic\" is the patient's subjective feeling and thoughts, and it looks at the patient's anxiety level and way of thinking."}, "sentence_sentiment": {"answer": "Negative", "rationale": "Because the entry \"for no reason, for something unrealistic\" is negative."}, "language_style": {"answer": "Calm and direct", "rationale": "The language style of the entry is more straightforward, not in euphemism or poetry, but in a simple question form, asking whether the respondents are often worried about unrealistic things. This is more straightforward, simple, easy to understand and answer."}, "time_dimension": {"answer": "Present", "rationale": "It can be deduced from the word \"normal\" in the sentence and the word \"is\" in the general sense of the present, that the sentence asks the testee about the present customary behaviour, i.e. whether the testee is often unprovokedly worried about something that is unrealistic. The entry is therefore about the present behavior or mentality."}, "sentence_type": "Interrogative sentence", "pos": {"nouns": {"Entry": 1, "Questionnaire": 1, "Something": 1}, "verbs": {"Regular": 1, "For no reason": 1, "I'm worried": 1, "Yes": 1, "be": 1}, "adjectives": {"It's not realistic": 1}, "adverbs": {"Land": 1}, "numerals": {"073": 1}}}, {"content": "074, outside. If there was a fire, did you plan on how to escape?", "word_count": 19, "label": "", "sentence_structure": "Subject鈥搗erb鈥搊bject", "subjective_objective": {"answer": "Subjective", "rationale": "The question is subjective because it is about the patient ' s personal views and plans."}, "sentence_sentiment": {"answer": "Positive", "rationale": "Because the \"how to get out first\" thing in the entry is positive."}, "language_style": {"answer": "Calm and direct", "rationale": "The purpose of this question is to know whether the person under investigation has a pre-existing escape plan in case of a fire, so that he or she can be informed and educated about fire safety."}, "time_dimension": {"answer": "Present", "rationale": "The entry asks how the person tested would act in the current environment (i.e. \"outside\"), in case of fire. The keywords \"if there is a fire\" and \"if there is a plan to escape\" indicate that this is a question of current or future circumstances, and therefore the word \"now\"."}, "sentence_type": "Interrogative sentence", "pos": {"nouns": {"Outside": 1, "Fire": 1, "you": 2, "Planned": 1, "What": 1, "Run away": 1}, "verbs": {"I met him": 1, "Is it possible": 1, "First": 1, "Planned": 1, "What": 1, "Run away": 1}, "adverbs": {"If": 1, "First": 1}, "numerals": {"074": 1}}}, {"content": "Do you always design a clear plan before you do anything, instead of taking chances?", "word_count": 23, "label": "", "sentence_structure": "Subject鈥搗erb鈥搊bject", "subjective_objective": {"answer": "Subjective", "rationale": "Because \"design a clear plan\" and \"take chances\" are subjective attitudes or behaviour of patients, not objective facts or results."}, "sentence_sentiment": {"answer": "Positive", "rationale": "Because the \"design a clear plan\" in the entry is positive, indicating purpose and planning."}, "language_style": {"answer": "Calm and direct", "rationale": "The reason for this is: - The question is whether it is \"if\" in the form of a question, there is no excessive emotional rendering or rhetorical writing, and the tone is more objective and calm. The entry directly asks whether the person surveyed always has a clear plan before doing his or her business, expressing direct concern and exploration of the issue."}, "time_dimension": {"answer": "Present", "rationale": "The key words \"pre-action\" and \"always\" in the entry to the questionnaire indicate that the act is a customary, regular act. Thus, the temporal dimension of the article can be judged to be the present."}, "sentence_type": "Interrogative sentence", "pos": {"nouns": {"Work": 1, "Front": 1, "Planned": 1, "Lucky": 1}, "verbs": {"Design": 1, "Do it": 1, "be": 1, "Always": 1, "Pong": 1}, "adjectives": {"Clear": 1}, "adverbs": {"not": 1, "And": 1, "It's": 1}, "numerals": {"075": 1, "One": 1}}}, {"content": "076. Do you have a small medicine box at your house to keep the rest of your medications?", "word_count": 25, "label": "", "sentence_structure": "Subject鈥損redicate", "subjective_objective": {"answer": "Objective", "rationale": "Because having a small medicine box is the patient's objective state."}, "sentence_sentiment": {"answer": "Neutral", "rationale": "Because the entry does not have any emotional color."}, "language_style": {"answer": "Tactful and warm", "rationale": "The entry is relatively euphemistic in its language, because it uses the tone of the inquiry and leads the interviewee to answer the question in the form of \"Does your family have any...\". Moreover, it does not directly express any evaluation or recommendation, but rather respects the views and choices of the interviewee. Such expression is more likely to create good interaction and enhance the cooperation of the interviewee."}, "time_dimension": {"answer": "Present", "rationale": "The keywords \"yes\" and \"store\" indicate that the act was being or had been performed by the testee, so the temporal dimension is now judged."}, "sentence_type": "Interrogative sentence", "pos": {"nouns": {"076": 1, "Home": 1, "Lee": 1, "A small medicine box": 1, "Various": 1, "Drugs": 1}, "verbs": {"Save": 1, "Look": 1, "Remaining": 1, "have": 1}, "adverbs": {"Before": 1}, "numerals": {"One": 2, "Various": 1}, "classifiers": {"Come on": 1, "of": 3, "Both": 1, "- You're not": 1}, "pronouns": {"you": 1}}}, {"content": "077, if someone is going to reprimand you, will you go to heart?", "word_count": 13, "label": "", "sentence_structure": "Subject鈥損redicate", "subjective_objective": {"answer": "Subjective", "rationale": "Because this is the patient ' s subjective feeling and attitude, whether or not to take someone else ' s reprimand depends on the patient ' s personal emotional response."}, "sentence_sentiment": {"answer": "Negative", "rationale": "Because the \"touch to the heart\" act in the entry is negative."}, "language_style": {"answer": "Calm and direct", "rationale": "The entry is more direct, concise, without too much emotional or accommodative, or any euphemism. It raises a question directly and expects the interviewee to give a clear answer, so it can be judged as \"cool and direct.\""}, "time_dimension": {"answer": "Present", "rationale": "The part \"If someone is scolding you\" contains a hypothetical condition, \"Do you mind?\" is a question of asking the subject how he reacts to and feels about this condition, so the entry is now."}, "sentence_type": "Interrogative sentence", "pos": {"nouns": {"person": 1, "Censorship": 1, "In my heart": 1}, "verbs": {"Go on": 2}, "adverbs": {"If": 1}, "numerals": {"077": 1}, "pronouns": {"you": 1}}}, {"content": "078, do you often feel ashamed of something you've done?", "word_count": 17, "label": "", "sentence_structure": "Subject鈥搗erb鈥搊bject", "subjective_objective": {"answer": "Subjective", "rationale": "This is a self-perception and evaluation of the patient and is a subjective examination."}, "sentence_sentiment": {"answer": "Negative", "rationale": "Because the \"smear\" sentiment in the entry is negative."}, "language_style": {"answer": "Tactful and warm", "rationale": "The entry uses a gentle and caring language style, which is \"often ashamed of something you've done\" and which does not directly point to or dictates the feelings of each other. It is more personal and interactive, and it is easier for the interviewee to open his heart and provide a true answer."}, "time_dimension": {"answer": "Present", "rationale": "The term \"always\" indicates that it is a question of the person's current or recent behaviour or feelings, and it is therefore possible to judge the time dimension of the article as it is now."}, "sentence_type": "Interrogative sentence", "pos": {"nouns": {"Things": 2, "you": 2, "Shame": 1}, "verbs": {"Do it": 1, "I feel": 1}, "numerals": {"078": 1}}}, {"content": "079. Do you laugh like most people?", "word_count": 10, "label": "", "sentence_structure": "Subject鈥搗erb鈥搊bject", "subjective_objective": {"answer": "Subjective", "rationale": "Because this is the patient ' s subjective description of himself, the feeling of being a laughing person is a personal subjective view."}, "sentence_sentiment": {"answer": "Neutral", "rationale": "Because the entry is merely asking if the interviewee likes to laugh, and it does not contain any emotional colours."}, "language_style": {"answer": "Calm and direct", "rationale": "The language style of the entry is straightforward, because it expresses the subject in a direct question, without using a rhetorical or emotional language."}, "time_dimension": {"answer": "Present", "rationale": "The keyword \"like most people\" and \"love\" indicate that the act took place on the subject, using the usual current, and therefore judging it as now."}, "sentence_type": "Interrogative sentence", "pos": {"nouns": {"Questionnaire Entry": 1, "person": 1, "Smile": 1}, "verbs": {"Love": 1, "Sample": 1, "Smile": 1}, "numerals": {"079": 1, "More": 1}, "classifiers": {"individual": 1}, "pronouns": {"you": 1, "and": 1, "Most people are the same": 1}}}, {"content": "080, for most of the time, you've been worried about someone or something?", "word_count": 20, "label": "", "sentence_structure": "Subject鈥搗erb鈥搊bject", "subjective_objective": {"answer": "Subjective", "rationale": "Because \"crazy\" is a subjective feeling and emotion."}, "sentence_sentiment": {"answer": "Negative", "rationale": "Because \"crazy\" in the entries is an expression of a negative emotion and attitude."}, "language_style": {"answer": "Calm and direct", "rationale": "The entry directly expresses the subject of questioning whether the other party is often concerned about someone or something, and does not use excessive rhetoric or emotional rendering, which is more objective and balanced."}, "time_dimension": {"answer": "Past", "rationale": "The entry asks about the psychological state of the person tested at most times, which involves a review and assessment of the psychological condition over time, and therefore is based on the past."}, "sentence_type": "Interrogative sentence", "pos": {"nouns": {"Time": 1, "person": 1, "Yes": 1}, "verbs": {"I feel": 1, "I'm worried": 1}, "adjectives": {"Most": 1, "of": 2}, "adverbs": {"Both": 1}, "numerals": {"080": 1}}}, {"content": "081, are you upset because you put things in the wrong place?", "word_count": 18, "label": "", "sentence_structure": "Subject鈥搗erb鈥搊bject", "subjective_objective": {"answer": "Subjective", "rationale": "It is the patient's subjective feeling to be upset because of the wrong place."}, "sentence_sentiment": {"answer": "Negative", "rationale": "Because \"freaky\" is negative."}, "language_style": {"answer": "Tactful and warm", "rationale": "The language style of the entry is more gentle, and the person surveyed is guided by questioning to share his or her feelings, without a direct blame or criticism."}, "time_dimension": {"answer": "Present", "rationale": "The keyword \"will\" uses the general current, which means that it is a customary act or state, occurring in the current or past period. At the same time, \"something is in the wrong place and is in the wrong place\" describes a feeling or state, which also indicates that it is a description of the present. So, the entry describes whether the person who was tested now has a difficult experience because the thing was in the wrong place."}, "sentence_type": "Interrogative sentence", "pos": {"nouns": {"Local": 1, "I'm so upset": 1, "It's hard": 1}, "verbs": {"Yes": 1, "Wrong": 1, "I'm so upset": 1}, "numerals": {"081": 1}}}, {"content": "Have you ever made decisions by throwing coins or a similar total chance?", "word_count": 24, "label": "", "sentence_structure": "Subject鈥搗erb鈥搊bject", "subjective_objective": {"answer": "Objective", "rationale": "Because the subject of the question is \"you,\" but the question is whether or not you have made decisions by throwing coins or a similar purely on-the-spot method, and it is being examined objectively."}, "sentence_sentiment": {"answer": "Positive", "rationale": "Because the purpose of the article is not expressed in any negative emotional terms, and the act of \"decision-making with a coin or similar purely amenity\" is not clearly a good or a bad thing. The entry can therefore be considered neutral."}, "language_style": {"answer": "Tactful and warm", "rationale": "The language style of the entry is relatively euphemistic, using the term \"had\" and \"like\" to avoid a direct denial or blame. At the same time, the method of \"decision-making with coins or similar purely price-driven methods\" is not overrated, but is objective in questioning."}, "time_dimension": {"answer": "Present", "rationale": "The entry used the term \"had\" to indicate that the act had already occurred and was not directly related to the present. It should therefore be judged as having been done in the past."}, "sentence_type": "Interrogative sentence", "pos": {"nouns": {"Questionnaire Entry": 1, "Coin": 1, "Decision-making": 1}, "verbs": {"Use": 1, "Do it": 1, "Throw it": 1, "Similar": 1, "Yeah": 1, "Rate": 1}, "adverbs": {"Once": 1, "Total": 1}, "numerals": {"082": 1}}}, {"content": "083, are you very worried about your health?", "word_count": 10, "label": "", "sentence_structure": "Subject鈥搗erb鈥搊bject", "subjective_objective": {"answer": "Subjective", "rationale": "Because \"very worried\" is a description of subjective feelings or attitudes, which represent the emotions and perceptions inside the patient."}, "sentence_sentiment": {"answer": "Negative", "rationale": "Because the emotional attitude of \"very worried\" is negative."}, "language_style": {"answer": "Calm and direct", "rationale": "The entry asks whether the other party is very concerned about his health, is clear and clear, and does not use any qualifier or emotional colours, which makes it more calm and straightforward."}, "time_dimension": {"answer": "Present", "rationale": "The entry asks the subject about his current anxiety, the keywords are \"very worried\" and \"your health\", all pointing to the state of the moment."}, "sentence_type": "Interrogative sentence", "pos": {"nouns": {"you": 1, "Health": 1}, "verbs": {"Very worried": 1, "- You're not": 1}, "adverbs": {"Very": 1}, "numerals": {"083": 1}}}, {"content": "084, if you had an accident, would you think it was your punishment?", "word_count": 22, "label": "", "sentence_structure": "Subject鈥搗erb鈥搊bject", "subjective_objective": {"answer": "Subjective", "rationale": "Because \"reward\" is a personal belief or opinion, and it is subjective."}, "sentence_sentiment": {"answer": "Negative", "rationale": "Because the word \"retribution\" in the entry is derogatory and implies a negative mood and attitude."}, "language_style": {"answer": "Calm and direct", "rationale": "The entry is based on the following: it is a direct question about whether the other party considers itself a reward in the event of an accident, is not speaking too much or is emotionally retroverted, and is more objective. It is a direct question that better captures the real thoughts and feelings of the interviewee and avoids the bias that may be associated with the design of an overly guided question."}, "time_dimension": {"answer": "Past", "rationale": "The entry asks the views of the testee on past accidents, and therefore the temporal dimension is a past."}, "sentence_type": "Interrogative sentence", "pos": {"nouns": {"Accidents": 1, "Retribution": 1}, "verbs": {"Come on": 1, "I think": 1}}}, {"content": "When you look at your own photos, do you feel embarrassed and complain that people are never treating you fairly?", "word_count": 31, "label": "", "sentence_structure": "Subject鈥搗erb鈥搊bject", "subjective_objective": {"answer": "Subjective", "rationale": "Because this is a question of asking the patient how and how he feels."}, "sentence_sentiment": {"answer": "Negative", "rationale": "Because the entries \"suffering\" and \"complaining people never treat you fairly\" have negative feelings."}, "language_style": {"answer": "Tactful and warm", "rationale": "The entry is more adept and interactive when it is spoken in a gentle and inviting manner, rather than by direct statements or orders."}, "time_dimension": {"answer": "Present", "rationale": "The entry asks the subject about emotional and mental reactions in a given situation. Key words include \"watching,\" \"photo,\" \"distressed,\" \"complaining,\" all of which point to the current situation, and therefore judge it as now.\""}, "sentence_type": "Interrogative sentence", "pos": {"nouns": {"Photos": 1, "self": 1, "People": 1}, "verbs": {"Watch": 1, "I feel": 2, "- He's complaining": 1, "Fair treatment": 1, "No, I can't": 1}, "adjectives": {"I'm embarrassed": 1}, "adverbs": {"Total": 1}, "numerals": {"085": 1}, "pronouns": {"you": 2, "Here": 1}}}, {"content": "086. Do you often feel inexorable and tired for no reason?", "word_count": 18, "label": "", "sentence_structure": "Subject鈥搗erb鈥搊bject", "subjective_objective": {"answer": "Subjective", "rationale": "Because the descriptions of \"incompetent\" and \"tired\" are subjective."}, "sentence_sentiment": {"answer": "Negative", "rationale": "Because the description of \"incompetent and tired\" in the entries is negative."}, "language_style": {"answer": "Tactful and warm", "rationale": "The entry is more euphemistic, and it does not use strong tone or critical language to ask the respondents about their situation. Rather, it raises questions in a more moderate way, hoping that the respondents will be free to express their true feelings. This expression is more likely to create a good atmosphere of communication and to get real feedback."}, "time_dimension": {"answer": "Present", "rationale": "The entry contains the descriptive language \"often\" and the verb \"means\" which indicates that this is a frequent phenomenon and that it is the present. Thus, the time dimension of the questionnaire entry is now."}, "sentence_type": "Interrogative sentence", "pos": {"nouns": {"Reason": 1, "I'm not good at it": 1, "Tired": 1}, "verbs": {"I feel": 1, "Nothing": 1, "Regular": 1, "have": 1}, "adverbs": {"Land": 1}, "numerals": {"086": 1}}}, {"content": "If you're ugly in a social setting, can you easily forget it?", "word_count": 20, "label": "", "sentence_structure": "Subject鈥搗erb鈥搊bject", "subjective_objective": {"answer": "Subjective", "rationale": "Because it looks at patients' attitudes or perceptions of a situation, they ask them whether they can \"remember easily\" the awkward things that happen in a social setting. Such attitudes or perceptions are subjective, because they reflect individual experience, feelings and views and may vary from person to person."}, "sentence_sentiment": {"answer": "Positive", "rationale": "Because the \"can easily forget it\" attitude in the entry is positive."}, "language_style": {"answer": "Tactful and warm", "rationale": "The language style of the entry is euphemistic, using questionwords to guide respondents to think and express their views. At the same time, the expression \"always to forget\" is relatively mild and not overly direct or negative."}, "time_dimension": {"answer": "Present", "rationale": "The keyword \"can easily\" indicates that the act is a habit or a normal practice, and therefore it can be considered a present manifestation."}, "sentence_type": "Interrogative sentence", "pos": {"nouns": {"Socially": 1, "Ugly": 1}, "verbs": {"Yes": 2, "Forget it": 1, "Out": 1}, "adverbs": {"very": 1, "Easy": 1}, "numerals": {"087": 1}}}, {"content": "088, do you keep a detailed account of all your expenses?", "word_count": 15, "label": "", "sentence_structure": "Subject鈥搗erb鈥搊bject", "subjective_objective": {"answer": "Subjective", "rationale": "Because \"the details of the account\" is a subjective act and attitude, related to the patient's feelings."}, "sentence_sentiment": {"answer": "Positive", "rationale": "Because the actions in the entries are \"detailed accounting\", this is usually seen as an expression of responsibility and financial competence, and therefore has a positive emotional colour."}, "language_style": {"answer": "Calm and direct", "rationale": "The language style of the entry is more straightforward, with no excessive rhetoric or emotional rendering, but simply asking if the other party would record all the costs in detail and be more objective in tone."}, "time_dimension": {"answer": "Present", "rationale": "The word \"all\" in \"do you keep a detailed account\"? The word \"all\" indicates that this has been done, and the word \"bookkeeping\" uses the usual current pattern, which means that it has not only happened in the past, but may still be going on. So the entry asks if the person who was tested is currently accustomed to recording his own expenses in detail, and the time dimension is now judged."}, "sentence_type": "Interrogative sentence", "pos": {"nouns": {"you": 2, "All": 1, "Cost": 1, "Account": 1}, "verbs": {"Remember": 1, "Both": 1, "Detail": 1, "Fine": 1}, "adjectives": {"Details": 1}, "adverbs": {"All": 1}, "numerals": {"088": 1}}}, {"content": "Are your actions often contrary to custom and the wishes of parents?", "word_count": 20, "label": "", "sentence_structure": "Subject鈥搗erb鈥搊bject", "subjective_objective": {"answer": "Subjective", "rationale": "Because it's the patient's own feeling, it's subjective."}, "sentence_sentiment": {"answer": "Negative", "rationale": "Because the \"conformity with custom and parents' wishes\" in the entry is negative."}, "language_style": {"answer": "Calm and direct", "rationale": "The language style of the entry is relatively cold and straightforward, using a direct question-and-answer approach, without too many emotional expressions or qualifiers. At the same time, the stronger term \"contradite\" is used to give people a serious, formal sense of the word."}, "time_dimension": {"answer": "Past", "rationale": "The keyword \"doing what\" and \"always contradicting custom and parents' wishes\" are all descriptions and assessments of the past. The problem is therefore one of past behaviour or mentality."}, "sentence_type": "Interrogative sentence", "pos": {"nouns": {"you": 1, "What I did": 1, "Customs": 1, "parents": 1, "Hope": 1}, "verbs": {"Regular": 1, "Contravention": 1}, "adverbs": {"089": 1}, "numerals": {"089": 1, "One": 1}}}, {"content": "090, severe pain and suffering, making it impossible for you to focus on your work?", "word_count": 26, "label": "", "sentence_structure": "Subject鈥搗erb鈥搊bject", "subjective_objective": {"answer": "Subjective", "rationale": "Because it is the subjective feeling of the patients that they feel that pain prevents them from focusing on their work."}, "sentence_sentiment": {"answer": "Negative", "rationale": "Because the emotional experience of \"serious pain and suffering\" in the entry is negative and leads to a lack of focus on the work."}, "language_style": {"answer": "Calm and direct", "rationale": "The language style of the entry is more calm and straightforward, rather than using excessive rhetorical or emotional rendering, and instead directly asks the other side whether it is unable to concentrate because of severe pain and suffering in the course of its work, in the form of clear and concise questions. \""}, "time_dimension": {"answer": "Present", "rationale": ""}, "sentence_type": "Interrogative sentence", "pos": {"nouns": {"Pain": 2, "Attention": 1, "Work": 1}, "verbs": {"鈾?And I'm gonna be 鈾?: 1, "Focus": 1}, "adjectives": {"Serious": 1}, "adverbs": {"Impossible": 1}, "numerals": {"090": 1}}}, {"content": "091. Do you regret your premature sexual behavior?", "word_count": 14, "label": "", "sentence_structure": "Subject鈥搗erb鈥搊bject", "subjective_objective": {"answer": "Subjective", "rationale": "Because \"regrets\" are a manifestation of subjective emotion, so it's subjective."}, "sentence_sentiment": {"answer": "Negative", "rationale": "Because the \"premature sex\" in the entry is negative."}, "language_style": {"answer": "Calm and direct", "rationale": ""}, "time_dimension": {"answer": "Present", "rationale": "\"Are you sorry for your premature sexual activity?\" The term \"you\" refers to the person who was tested, and whether \"is\" means that it is a question of choice, and that requires an answer, yes or no. So the time dimension of this article is now."}, "sentence_type": "Interrogative sentence", "pos": {"nouns": {"Sexuality": 1, "Regret": 1}, "verbs": {"be": 1, "For you": 1, "And": 1}, "adverbs": {"It's too early": 1}, "numerals": {"091": 1}}}, {"content": "092. Do some members of your family make you feel better than you do?", "word_count": 18, "label": "", "sentence_structure": "Subject鈥搗erb鈥搊bject", "subjective_objective": {"answer": "Subjective", "rationale": "Because of the problem, the patient ' s subjective feelings were based on his own values and self-evaluation, and whether he felt that he was not good enough."}, "sentence_sentiment": {"answer": "Negative", "rationale": "Because the entries contain elements that devalue self, they create negative emotions."}, "language_style": {"answer": "Tactful and warm", "rationale": "The language style of the entry is more euphemistic, dialogues in a question-and-answer manner, and uses vague language such as \"Are there some members\" to avoid direct accusations or harm to one's self-esteem. At the same time, the term \"not good\" also expresses the subject of the question more implicitly than directly."}, "time_dimension": {"answer": "Present", "rationale": "\"The home\" indicates that this is the current situation, \"makes you feel\" means a feeling or an emotion, which points to the situation now or in the recent period, and therefore to the present."}, "sentence_type": "Interrogative sentence", "pos": {"nouns": {"Members": 1, "self": 1, "Not good enough": 1}, "verbs": {"I feel": 1, "have": 1}, "numerals": {"092": 1}, "classifiers": {"Some": 1}, "pronouns": {"you": 1}}}, {"content": "093, do you get disturbed by noise?", "word_count": 10, "label": "", "sentence_structure": "Subject鈥搗erb鈥搊bject", "subjective_objective": {"answer": "Subjective", "rationale": "Because it's the patient's subjective feelings and experiences that are disturbed by noise."}, "sentence_sentiment": {"answer": "Negative", "rationale": "Because \"noise disturbance\" in the entry is a negative effect."}, "language_style": {"answer": "Calm and direct", "rationale": "The entry is directly asked in a way that is not overly phrasing or emotionally strong and that is more objective and neutral."}, "time_dimension": {"answer": "Present", "rationale": "Based on the word \"normal\" in the sentence and the general current, the action of \"acceptance\" is taking place at this stage, so it's now."}, "sentence_type": "Interrogative sentence", "pos": {"nouns": {"Noise": 1, "Excuse me": 1}, "verbs": {"Copy that": 1}, "adjectives": {"Regular": 1}, "numerals": {"093": 1}}}, {"content": "You're easy to relax when you sit or lie down?", "word_count": 13, "label": "", "sentence_structure": "Subject鈥搗erb鈥搊bject", "subjective_objective": {"answer": "Subjective", "rationale": "Because \"relax\" is a subjective feeling."}, "sentence_sentiment": {"answer": "Positive", "rationale": "Because \"easy to relax\" is positive."}, "language_style": {"answer": "Tactful and warm", "rationale": ""}, "time_dimension": {"answer": "Present", "rationale": "The description of \"sitting\" and \"sitting down\" is for current behavior or state, \"Do you relax easily?\" Ask the testee if he can relax in these circumstances, and judge now."}, "sentence_type": "Interrogative sentence", "pos": {"nouns": {"Sit down": 1, "Lie down": 1}, "verbs": {"Relax": 1, "Easy": 1, "Sit down": 1, "Lie down": 1}, "adjectives": {"very": 1}, "adverbs": {"very": 1, "Easy": 1}, "numerals": {"094": 1}}}, {"content": "095, are you worried about the infection of bacteria in public?", "word_count": 18, "label": "", "sentence_structure": "Subject鈥搗erb鈥搊bject", "subjective_objective": {"answer": "Subjective", "rationale": "Because \"fear\" is a subjective feeling and attitude."}, "sentence_sentiment": {"answer": "Negative", "rationale": "Because \"scared\" is negative."}, "language_style": {"answer": "Tactful and warm", "rationale": "The entry is more personal and interactive when it is spoken in a gentle and inviting manner, rather than in direct statements or orders. At the same time, the words are more common and easy to understand, and do not create undue tension or pressure on the respondents to obtain a true answer."}, "time_dimension": {"answer": "Present", "rationale": "The entry asks the subject about his current fears and feelings, and the keywords are \"are he worried\" and \"in public\" and they point to the state of the present."}, "sentence_type": "Interrogative sentence", "pos": {"nouns": {"095": 1, "you": 1, "I'm worried": 1, "Public": 1, "Infection": 1, "Bacteria": 1}, "verbs": {"Is it possible": 1, "very": 1, "Lee": 1, "Go, go, go": 1}, "adverbs": {"very": 1}, "numerals": {"095": 1}, "pronouns": {"you": 1}}}, {"content": "096, when you're alone, are you trying to be nice?", "word_count": 17, "label": "", "sentence_structure": "Subject鈥搗erb鈥搊bject", "subjective_objective": {"answer": "Subjective", "rationale": "The entry of the questionnaire is viewed from the patient's subjective point of view and asks if the patient has any attempt to be friendly with the person when he or she feels alone."}, "sentence_sentiment": {"answer": "Positive", "rationale": "Because the \"work to be nice\" act in the entry is positive."}, "language_style": {"answer": "Tactful and warm", "rationale": "The entry is more euphemistic, using the phrase \"whether or not\" to guide the interviewee to think and answer questions, and to create a friendly and friendly atmosphere by asking the interviewee how he feels. Moreover, the expression \"friendly\" is more positive and positive, and it inspires the interviewee to seek positive solutions rather than being alone."}, "time_dimension": {"answer": "Present", "rationale": ""}, "sentence_type": "Interrogative sentence", "pos": {"nouns": {"Loneliness": 1, "Friendly": 1, "person": 1}, "verbs": {"I feel": 1, "Try": 1, "- Wait": 1}, "adverbs": {"Is it possible": 2}, "numerals": {"096": 1}}}, {"content": "097. Do you often get upset about the unbearable itching?", "word_count": 16, "label": "", "sentence_structure": "Subject鈥搗erb鈥搊bject", "subjective_objective": {"answer": "Subjective", "rationale": "Because \"the unbearable itch\" is a subjective feeling that requires patients to judge their own gravity and impact."}, "sentence_sentiment": {"answer": "Negative", "rationale": "Because the \"unbearably itching\" in the entry is negative, causing pain."}, "language_style": {"answer": "Calm and direct", "rationale": "The language style of the entry is calm, as it clearly asks whether the person surveyed is troubled by itching. It does not use any euphemism or rhetoric to hide the nature of the problem, but rather expresses the intention of the researcher directly and succinctly. This language style helps to ensure that the person surveyed understands the questionnaire accurately and can answer the questions."}, "time_dimension": {"answer": "Present", "rationale": "The term \"often\" is used to indicate the frequency of the act, and the expression \"problems for ...\" also implies that it is a state of continuity, so it can be judged that the temporal dimension of the purpose of the article is now."}, "sentence_type": "Interrogative sentence", "pos": {"nouns": {"Problem": 1, "Entry": 1, "Itches": 1, "Trouble": 1}, "verbs": {"be": 1, "Regular": 1, "Yes": 1, "Take it": 1, "And": 1, "Trouble": 1}, "adverbs": {"Regular": 1, "Hard": 2, "Very": 1}, "numerals": {"097": 1}}}, {"content": "098, do you have some unforgivable bad habits?", "word_count": 14, "label": "", "sentence_structure": "Subject鈥搗erb鈥搊bject", "subjective_objective": {"answer": "Subjective", "rationale": "Because the existence of some \"impermissible bad habits\" is the patient's personal opinion and attitude."}, "sentence_sentiment": {"answer": "Negative", "rationale": "Because the term \"imperceptible bad habits\" is negative."}, "language_style": {"answer": "Tactful and warm", "rationale": "The language style of this entry is more euphemistic and warm, and by using the expression \"absolute bad habits\" it leads the respondents to reflect on their behaviour and provides a more tolerant and understanding attitude that encourages the respondents to answer questions more frankly."}, "time_dimension": {"answer": "Past", "rationale": "\"There are certain unforgivable bad habits\" that indicate that these habits exist and may have existed for some time, and the entry is therefore more inclined to question the testee's past experiences and behaviour."}, "sentence_type": "Interrogative sentence", "pos": {"nouns": {"Custom": 1, "Bad habits": 1}, "verbs": {"have": 1, "Forgiveness": 1}, "adjectives": {"No way": 1}, "numerals": {"098": 1}}}, {"content": "099. Are you very unhappy if someone criticizes you?", "word_count": 16, "label": "", "sentence_structure": "Subject鈥搗erb鈥搊bject", "subjective_objective": {"answer": "Subjective", "rationale": "Because \"very unhappy\" is a subjective emotional reaction that reflects the individual feelings and attitudes of the interviewees towards criticism."}, "sentence_sentiment": {"answer": "Negative", "rationale": "Because the emotional expression \"very unhappy\" in the entry is negative."}, "language_style": {"answer": "Tactful and warm", "rationale": "The entry is more euphemistic, using the \"Whether or not\" expression, which expresses concern about the person being interviewed by asking questions, while avoiding the context of direct orders or evaluations."}, "time_dimension": {"answer": "Present", "rationale": "The entry is for the purpose of asking the respondent about the reaction and feelings of criticism, and the time dimension is now judged."}, "sentence_type": "Interrogative sentence", "pos": {"nouns": {"person": 1, "Criticism": 1, "Very": 1, "Not fast": 1}, "verbs": {"I feel": 1, "be": 1, "have": 1, "Criticism": 1, "Is it possible": 1}, "adjectives": {"Very": 1}, "numerals": {"099": 1}, "pronouns": {"you": 1}}}, {"content": "Do you often feel that you are being treated unfairly?", "word_count": 18, "label": "", "sentence_structure": "Subject鈥搗erb鈥搊bject", "subjective_objective": {"answer": "Subjective", "rationale": "Because \"think\" is the patient's subjective feeling."}, "sentence_sentiment": {"answer": "Negative", "rationale": "Because the description of \"inequity in life\" is negative, implying that the interviewee may have negative emotional experiences."}, "language_style": {"answer": "Calm and direct", "rationale": "The entry is dialogueable in a direct query, without excessive rhetorical or emotional rendering, and is more objective in tone."}, "time_dimension": {"answer": "Present", "rationale": "The keyword \"often feels\" indicates a customary feeling or idea, implying that the act has occurred in the past. Thus, the time dimension of the entry is now judged."}, "sentence_type": "Interrogative sentence", "pos": {"nouns": {"self": 1, "Life": 1, "Treatment": 1}, "verbs": {"I think": 1, "Received": 1, "It's not fair": 1}, "adjectives": {"Fair": 1}, "numerals": {"100": 1}, "pronouns": {"you": 1}}}, {"content": "101, are you easily surprised by the presence of unexpected people?", "word_count": 20, "label": "", "sentence_structure": "Subject鈥損redicate", "subjective_objective": {"answer": "Subjective", "rationale": "Because it is the feelings and attitudes of individuals that are easily surprised, it is the subject matter of subjective examination."}, "sentence_sentiment": {"answer": "Positive", "rationale": "Since the entry indicates unexpected experience, which may be surprising and exciting."}, "language_style": {"answer": "Tactful and warm", "rationale": "Warm and warm."}, "time_dimension": {"answer": "Present", "rationale": ""}, "sentence_type": "Interrogative sentence", "pos": {"nouns": {"101": 1, "person": 2, "Come on": 1}, "verbs": {"Easy": 1, "Yes": 1, "Surprise": 1}, "adjectives": {"I'm surprised": 1}, "adverbs": {"very": 1}, "numerals": {"101": 1}, "classifiers": {"individual": 2}}}, {"content": "102, do you always give it back with care, even if it's too little?", "word_count": 21, "label": "", "sentence_structure": "Subject鈥搗erb鈥搊bject", "subjective_objective": {"answer": "Subjective", "rationale": "Because \"always very carefully returning the debit\" is the patient's attitude and behaviour, which is the patient's subjective idea. Even if it is \"very small\" it is the patient's subjective attitude that is examined."}, "sentence_sentiment": {"answer": "Positive", "rationale": "Because the act of \"returning borroweds carefully\" is positive and expresses a responsible attitude on the part of a person."}, "language_style": {"answer": "Tactful and warm", "rationale": "The entry is more euphemistic and warm, asking the respondents in a gentle and inviting tone. Instead of direct statements or orders, it uses a kind and friendly expression that makes the respondents comfortable and relaxed. Moreover, the issue does not put too much pressure or judgement on the respondents and is more focused on understanding the actual situation and attitudes of the respondents."}, "time_dimension": {"answer": "Present", "rationale": "\"Always\" means that the behaviour is very frequent, and \"even if the money is insignificant\" means that it exists in all circumstances, so the entry is to ask the person who was tested for the current conduct."}, "sentence_type": "Interrogative sentence", "pos": {"nouns": {"102": 1, "you": 1, "Borrows": 1, "Money": 1}, "verbs": {"Always": 1, "Very carefully": 1, "Return": 1, "Borrow": 1, "Less than": 1, "It's insignificant": 1, "- You're not": 1}, "adjectives": {"Careful": 1}, "adverbs": {"Always": 1, "Very carefully": 1}, "numerals": {"102": 1, "1": 4, "Less than": 1, "It's insignificant": 1}, "classifiers": {"Objects": 1}, "pronouns": {"you": 1}}}, {"content": "103. Do you feel like you can't influence what's happening around you?", "word_count": 18, "label": "", "sentence_structure": "Subject鈥搗erb鈥搊bject", "subjective_objective": {"answer": "Subjective", "rationale": "This is a subjective approach, since it is a description of what the patient feels for himself."}, "sentence_sentiment": {"answer": "Negative", "rationale": "Because the expression \"can't be left or left\" in the entry is negative."}, "language_style": {"answer": "Calm and direct", "rationale": "The language style of the entry is calm and straightforward. It clearly asks the interviewee whether he feels he cannot control the things around him, without too much emotional adoration or rhetoric, and in an objective and clear manner."}, "time_dimension": {"answer": "Present", "rationale": "The entry asks the testee's current feelings and experiences, the keywords being \"doesn't feel\" and \"can't be left\", all of which indicate that this is a state of experience. So, the temporal dimension is now."}, "sentence_type": "Interrogative sentence", "pos": {"nouns": {"Things": 2}, "verbs": {"I feel": 1, "Around": 1, "Come on": 1}, "adjectives": {"No, I can't": 1}, "numerals": {"103": 1, "you": 1}, "pronouns": {"you": 2}}}, {"content": "104, are you healthy?", "word_count": 7, "label": "", "sentence_structure": "Subject鈥損redicate", "subjective_objective": {"answer": "Objective", "rationale": "Because physical health is an objective physiological condition, not a subjective feeling or attitude."}, "sentence_sentiment": {"answer": "Neutral", "rationale": "Because the entry is merely a question of health status, there is no apparent emotional tendency."}, "language_style": {"answer": "Tactful and warm", "rationale": "This is a way to make the interviewer more receptive and more willing to respond to questions."}, "time_dimension": {"answer": "Present", "rationale": "The entry asks about the present state of the person being tested, does not relate to past or future information, and therefore is judged to be \"now\"."}, "sentence_type": "Interrogative sentence", "pos": {"nouns": {"Health": 1, "Body": 1}, "verbs": {"be": 1}, "numerals": {"104": 1}}}, {"content": "105. Do you suffer from conscience?", "word_count": 10, "label": "", "sentence_structure": "Subject鈥搗erb鈥搊bject", "subjective_objective": {"answer": "Subjective", "rationale": "Because the conscience of the person is a person ' s own feeling and is subjective."}, "sentence_sentiment": {"answer": "Negative", "rationale": "Because the act of \"conscientious torture\" in the entry is negative."}, "language_style": {"answer": "Calm and direct", "rationale": "The entry asks in a concise manner whether the interviewees are often subjected to conscience distress or suffering, without excessive emotional reticulation or subjective evaluation, and with a more straightforward and calm language style."}, "time_dimension": {"answer": "Present", "rationale": "In this entry, \"You're often\" means a regular act, \"Culture of conscience\" is a state or experience, so it is the current situation of the testee that can be judged. So, time dimension is now."}, "sentence_type": "Interrogative sentence", "pos": {"nouns": {"Conscience": 1, "Torture": 1}, "verbs": {"I'm tired": 1}, "adverbs": {"Regular": 1}, "numerals": {"105": 1}}}, {"content": "Are you used by people?", "word_count": 15, "label": "", "sentence_structure": "Subject鈥搗erb鈥搊bject", "subjective_objective": {"answer": "Subjective", "rationale": "This is usually based on the patient鈥檚 own observations and understanding, and is therefore subjective."}, "sentence_sentiment": {"answer": "Negative", "rationale": "Because it expresses the attitude of others towards you and is negative."}, "language_style": {"answer": "Calm and direct", "rationale": "The entry, in the form of a direct inquiry, clearly sets out a theme, without adding any rhetorical or emotional colour, and is based on a linguistic bias towards objectivity and calmness."}, "time_dimension": {"answer": "Present", "rationale": "\"Whether\" means an inquiry into the current state, \"used\" is an act or state that has occurred or is in progress, so the question is whether the person tested is being used by others."}, "sentence_type": "Interrogative sentence", "pos": {"nouns": {"People": 1, "Object": 2}, "verbs": {"- Put it on": 1, "Do it": 1, "Utilization": 1}, "numerals": {"106": 1, "they": 1}, "pronouns": {"you": 1}}}, {"content": "107. Do you think people actually don't care about you?", "word_count": 15, "label": "", "sentence_structure": "Subject鈥搗erb鈥搊bject", "subjective_objective": {"answer": "Subjective", "rationale": "Because \"think\" is a subjective judgement that reflects the views and attitudes of the person under investigation. The entry looks at the subjective feelings of the person under investigation, not the objective facts."}, "sentence_sentiment": {"answer": "Negative", "rationale": "Because the expression \"people don't really care about you\" in the entry is negative emotional."}, "language_style": {"answer": "Tactful and warm", "rationale": "The entry is more adept and interactive when it is spoken in a gentle and inviting manner, rather than by direct statements or orders."}, "time_dimension": {"answer": "Past", "rationale": ""}, "sentence_type": "Interrogative sentence", "pos": {"nouns": {"Questionnaire": 1, "Problem": 1, "person": 2, "Care": 1}, "verbs": {"I think": 1, "I don't care": 1}, "adjectives": {"Actually": 1}, "numerals": {"107": 1, "2": 1}, "classifiers": {"individual": 1}, "pronouns": {"you": 1}}}, {"content": "108, sit still for a while. Is it hard for you?", "word_count": 16, "label": "", "sentence_structure": "Subject鈥搗erb鈥搊bject", "subjective_objective": {"answer": "Subjective", "rationale": "Because \"sit quietly for a while\" is a personal feeling and experience, a patient's subjective view."}, "sentence_sentiment": {"answer": "Neutral", "rationale": "Because the entry does not have any emotional color."}, "language_style": {"answer": "Calm and direct", "rationale": "The entry is a direct question-and-answer dialogue, without too much qualifier or emotional colour, and is more balanced and objective in tone, and more suitable for use in such contexts as research."}, "time_dimension": {"answer": "Present", "rationale": "\"Sit quietly for a while\" is a current movement or state, so the question is about the person being tested's current feelings and behaviour. So the answer is now."}, "sentence_type": "Interrogative sentence", "pos": {"nouns": {"Quiet": 1, "Sit down": 1, "Just a minute": 1}, "verbs": {"Stay": 1, "have": 1, "I think": 1}, "adverbs": {"Land": 1, "not": 1, "Easy": 1}, "numerals": {"108": 1}, "pronouns": {"you": 1, "Here": 1}}}, {"content": "Do you often bow to others and not believe that others can do it?", "word_count": 21, "label": "", "sentence_structure": "Subject鈥搗erb鈥搊bject", "subjective_objective": {"answer": "Subjective", "rationale": "Because the attitude of the person must be respectful is the subjective attitude of the patient."}, "sentence_sentiment": {"answer": "Negative", "rationale": "Because the attitude of \"no trust\" in the entries is negative."}, "language_style": {"answer": "Tactful and warm", "rationale": "The language style of the entry is more euphemistic, and it does not ask directly whether the person under investigation has excessive interference in the affairs of others, but rather whether they have excessive interference in certain circumstances. This way of asking questions respects the person under investigation more and can trigger more in-depth reflection and answers."}, "time_dimension": {"answer": "Present", "rationale": "The temporal dimension of the purpose of the article is therefore now judged."}, "sentence_type": "Interrogative sentence", "pos": {"nouns": {"Questionnaire": 1, "Entry": 1, "person": 1, "Things": 1}, "verbs": {"Do it": 1, "Yes": 1}, "adjectives": {"Often": 1, "I'll do it": 1, "others": 1}, "numerals": {"109": 1}, "pronouns": {"you": 1}}}, {"content": "110. Are you easy to convince?", "word_count": 10, "label": "", "sentence_structure": "Subject鈥搗erb鈥搊bject", "subjective_objective": {"answer": "Subjective", "rationale": "Because \"easy to be persuaded\" is the patient's personal feelings and attitude, and it's subjective."}, "sentence_sentiment": {"answer": "Negative", "rationale": "Basis of judgement: being easily persuaded may mean a lack of independent thinking and decision-making capacity, and is a negative feature."}, "language_style": {"answer": "Calm and direct", "rationale": "The language style of the entry is relatively calm and straightforward, with no excessive use of rhetorical techniques or emotional rendering, and with a clear and objective tone, the testee is asked whether he or she is easily persuaded by others."}, "time_dimension": {"answer": "Past", "rationale": "The entry asks the testee about past behaviour or habits, and the temporal dimension is judged to be past. The keyword \"always persuaded\" refers to whether the testee has had experience or tendency to be vulnerable to others in the past."}, "sentence_type": "Interrogative sentence", "pos": {"nouns": {"110": 1, "you": 1, "others": 1, "Convince": 1}, "adjectives": {"very": 1}, "adverbs": {"Easy": 1}, "numerals": {"1": 2}}}, {"content": "111, is there any intestine disorder in your family?", "word_count": 15, "label": "", "sentence_structure": "Subject鈥搗erb鈥搊bject", "subjective_objective": {"answer": "Subjective", "rationale": "Because it is the patient's subjective feelings and perceptions that are being questioned."}, "sentence_sentiment": {"answer": "Negative", "rationale": "The entry is concerned with the health of the family, which may cause anxiety and negative feelings."}, "language_style": {"answer": "Tactful and warm", "rationale": ""}, "time_dimension": {"answer": "Past", "rationale": "The entry asks about the health of the family members of the person tested in the past and therefore the temporal dimension is judged to be the past."}, "sentence_type": "Interrogative sentence", "pos": {"nouns": {"you": 1, "Family": 1, "- The stomach": 1, "Mistake": 1}, "verbs": {"Is it possible": 1, "have": 1}, "numerals": {"111": 1}}}, {"content": "Do you think you've lost your youth?", "word_count": 14, "label": "", "sentence_structure": "Subject鈥搗erb鈥搊bject", "subjective_objective": {"answer": "Subjective", "rationale": "Because \"desolation\" is a subjective assessment, depending on the individual's feelings and perceptions."}, "sentence_sentiment": {"answer": "Negative", "rationale": "Because the \"defeating your youth\" act is negative."}, "language_style": {"answer": "Tactful and warm", "rationale": "The entry is more adept and interactive when it is spoken in a gentle and inviting manner, rather than by direct statements or orders."}, "time_dimension": {"answer": "Past", "rationale": "The entry asks about the subjective perception and evaluation of the past by the testee, the keywords being \"desolation\" and \"their youth\", which describe events that have occurred."}, "sentence_type": "Interrogative sentence", "pos": {"nouns": {"you": 1, "Youth": 1}, "verbs": {"Waste": 1, "I think": 1}, "numerals": {"112": 1}, "classifiers": {"individual": 1}}}, {"content": "113, do you like to ask some questions about your own worth as a human being?", "word_count": 24, "label": "", "sentence_structure": "Subject鈥搗erb鈥搊bject", "subjective_objective": {"answer": "Subjective", "rationale": "Because \"like\" is a subjective feeling and attitude of a person."}, "sentence_sentiment": {"answer": "Neutral", "rationale": "Because the entry does not have any emotional color."}, "language_style": {"answer": "Calm and direct", "rationale": "The entry clearly expresses the subject of the investigation in a direct inquiry 鈥?the attitude of the other party to raising the question of its own value. It is not too much rhetorical or emotional, and is more objective in tone, so it can be judged as 鈥渃ool and direct鈥?"}, "time_dimension": {"answer": "Present", "rationale": "The entry asks the testee's current behaviour or state, i.e. whether they like to raise questions about their own value, and therefore judge it now."}, "sentence_type": "Interrogative sentence", "pos": {"nouns": {"Questionnaire Entry": 1, "Problem": 2, "self": 1, "Value": 1}, "verbs": {"Yeah": 1, "T": 1, "As": 1}}}, {"content": "114. Do you often feel lonely?", "word_count": 8, "label": "", "sentence_structure": "Subject鈥搗erb鈥搊bject", "subjective_objective": {"answer": "Subjective", "rationale": "Because loneliness is a subjective emotional experience, it is the psychological state and feelings of the patient."}, "sentence_sentiment": {"answer": "Negative", "rationale": "Because the word \"single\" in the entry has a negative emotional connotation, which means that the person under investigation often feels alone."}, "language_style": {"answer": "Tactful and warm", "rationale": "The language style of the entry is more euphemistic, avoiding direct statements or orders and being more gentle and caring by asking if the other side is often alone. This expression is more likely to trigger the interviewee鈥檚 resonance and share his feelings, and to gain the interviewer鈥檚 trust and cooperation."}, "time_dimension": {"answer": "Present", "rationale": ""}, "sentence_type": "Interrogative sentence", "pos": {"nouns": {"you": 1, "Loneliness": 1, "Questionnaire": 1, "Entry": 1}, "verbs": {"I feel": 1, "Regular": 1}, "adverbs": {"Often": 1}, "numerals": {"114": 1}}}, {"content": "115, are you too worried about money?", "word_count": 11, "label": "", "sentence_structure": "Subject鈥搗erb鈥搊bject", "subjective_objective": {"answer": "Objective", "rationale": "Because the question of whether to worry too much about money is an objective fact that can be observed and measured. Patients' subjective attitude can be understood by their answers, but that does not change the fact that \"too much to worry about money\" is objective in itself."}, "sentence_sentiment": {"answer": "Negative", "rationale": "The basis for the judgment is that the act of \"excessive anxiety\" is negative."}, "language_style": {"answer": "Calm and direct", "rationale": "The entry asks the other side whether it is too economic, is not using too much qualifier or emotional language and is more objective and direct."}, "time_dimension": {"answer": "Present", "rationale": "The keywords are \"excessive\" and \"fear\", which represent the current behaviour or state of the subject, and the entry should therefore be classified as \"now\"."}, "sentence_type": "Interrogative sentence", "pos": {"nouns": {"Problem": 1, "Money": 1}, "verbs": {"I'm worried too much": 1}, "adjectives": {"That's too much": 1, "Land": 1}, "adverbs": {"It's too much": 1}, "numerals": {"115": 1}}}, {"content": "116, would you rather go under the railing than go around the road?", "word_count": 24, "label": "", "sentence_structure": "Subject鈥搗erb鈥搊bject", "subjective_objective": {"answer": "Subjective", "rationale": "This is because of the patient ' s choice and attitude and is viewed from a subjective perspective."}, "sentence_sentiment": {"answer": "Negative", "rationale": "Because the behaviour in the entry \"penetrating under the railing\" is risky and irresponsible, it may raise security concerns."}, "language_style": {"answer": "Calm and direct", "rationale": "This entry is a direct and clear query about the interviewee鈥檚 risk-taking habits, is simple, has no excessive rhetorical or emotional rendering. This expression is more easily understood and answered by the interviewee and is more in line with the questionnaire."}, "time_dimension": {"answer": "Past", "rationale": "Keywords include \"better\" or \"not\" etc. Besides, \"blow under the railway\" and \"turning\" are behavioural descriptions, which take place in the present or in the future, rather than at a specific point in time in the past."}, "sentence_type": "Interrogative sentence", "pos": {"nouns": {"Road": 1, "Bar": 1}, "verbs": {"Ningcheng": 1, "Go through": 1, "No, I don't": 1, "Go around": 1, "Let's go": 1}, "adverbs": {"From": 1, "Down there": 1, "And": 1}, "numerals": {"116": 1}, "classifiers": {"individual": 1}}}, {"content": "117. Do you often feel like life is too much for you?", "word_count": 11, "label": "", "sentence_structure": "Subject鈥損redicate", "subjective_objective": {"answer": "Subjective", "rationale": "This is a question of patients ' subjective feelings and perceptions of life, not a description of objective facts."}, "sentence_sentiment": {"answer": "Negative", "rationale": "Because the \"unmanageable\" in the entry is negative."}, "language_style": {"answer": "Calm and direct", "rationale": "The entry raises the question in a direct and clear manner, without excessive rhetorical or emotional rendering, and with more objectivity and calm tone."}, "time_dimension": {"answer": "Present", "rationale": "The keyword is \"You often feel\" , which suggests that the testee needs to remember his past and answer him. And \"life is hard to cope\" is a descriptive phrase that has no direct connection to the past or the future. So this entry should be classified as \"now.\""}, "sentence_type": "Interrogative sentence", "pos": {"nouns": {"Life": 1, "Payable": 1}, "verbs": {"I feel": 1, "Regular": 1}, "adverbs": {"Hard": 1, "very": 1}, "numerals": {"117": 1, "One": 2}, "pronouns": {"you": 1}}}, {"content": "118. Do others show sympathy when you are not feeling well?", "word_count": 14, "label": "", "sentence_structure": "Subject鈥搗erb鈥搊bject", "subjective_objective": {"answer": "Subjective", "rationale": "Because the question is one of asking patients about their subjective feelings and observations, that is, how others react when they are not feeling well. The question is therefore viewed from a subjective perspective."}, "sentence_sentiment": {"answer": "Positive", "rationale": "Because the expression of sympathy is positive, expressing concern and understanding for others."}, "language_style": {"answer": "Tactful and warm", "rationale": "The entry uses a more mild question-and-question phrase and ends with a question mark, expressing concern and concern about the interviewee, while avoiding the language of direct command or evaluation, and is of good kinship and interaction."}, "time_dimension": {"answer": "Present", "rationale": "In this entry, \"uncomfortable\" refers to the current state of the person being tested, and \"others expressing sympathy\" is the reaction of others to this situation and can therefore be judged as questioning the person being tested."}, "sentence_type": "Interrogative sentence", "pos": {"nouns": {"you": 1, "I'm not feeling well": 1, "others": 1, "Compassion": 1}, "verbs": {"Is it possible": 1, "Organisation": 1, "I'm not feeling well": 1}, "numerals": {"118": 1, "One": 1}, "pronouns": {"they": 1}}}, {"content": "119. Do you think you don't deserve the trust and friendship of others?", "word_count": 19, "label": "", "sentence_structure": "Subject鈥搗erb鈥搊bject", "subjective_objective": {"answer": "Subjective", "rationale": "Because \"think\" means the patient's subjective idea."}, "sentence_sentiment": {"answer": "Negative", "rationale": "Because the attitude of \"not worthy of trust and friendship\" in the entry is negative."}, "language_style": {"answer": "Tactful and warm", "rationale": "The language style of the entry is euphemism. While asking the other side whether it has a self-denial view, it does not use a direct negative or accusatory tone, but rather a respectful and encouraging attitude towards the other, which makes it easier to open the door to the interviewee and helps to obtain more real and effective feedback."}, "time_dimension": {"answer": "Present", "rationale": "The entry contains a description of the current emotional state of the testee, \"Do you think \"does it\" means a double-checked attitude, while \"thinks\" uses the general current, meaning that the idea may be the present psychological feeling of the testee. Thus, the temporal dimension of the article is now."}, "sentence_type": "Interrogative sentence", "pos": {"nouns": {"self": 1, "others": 1, "Trust": 1, "Friendship": 2}, "verbs": {"I think": 1, "Got it": 1, "It's good enough": 1}, "adjectives": {"not": 1}, "numerals": {"119": 1}, "pronouns": {"you": 1}}}, {"content": "120. Do you think people are complimenting you when they say your virtues?", "word_count": 21, "label": "", "sentence_structure": "Subject鈥搗erb鈥搊bject", "subjective_objective": {"answer": "Subjective", "rationale": "Because the problem involves the patient ' s perception of and reaction to others ' own evaluations, it is the patient ' s subjective attitude."}, "sentence_sentiment": {"answer": "Neutral", "rationale": "Since the entry did not express any emotional preference, it merely asked the respondents about the perception of others as to their own merits."}, "language_style": {"answer": "Calm and direct", "rationale": "The language style of the entry is more direct, more concise, and there is no excessive rhetorical or emotional rendering. It asks the other side in a more objective way about the other's assessment of his or her own merits, without too much emotional or subjective assumptions. Thus, the entry can be classified as a \"cool and straight\" language style."}, "time_dimension": {"answer": "Past", "rationale": "\"When people talk about your virtues\" means a past or present experience, \"Do you think they're complimenting you?\" is asking the testee about feelings or attitudes, so the entry is about the present."}, "sentence_type": "Interrogative sentence", "pos": {"nouns": {"People": 1, "Advantages": 1, "Time": 1, "Congratulations": 1}, "verbs": {"Say it": 1, "Rise": 1, "I think": 2, "in": 1, "they": 1, "Is it possible": 1, "you": 2, "be": 1, "Yes": 1}, "numerals": {"120銆?: 1, "One": 1}, "classifiers": {"EMPTY": 1}, "pronouns": {"When": 1, "Here": 1}}}, {"content": "Do you think you have contributed to the world and a meaningful life?", "word_count": 23, "label": "", "sentence_structure": "Subject鈥搗erb鈥搊bject", "subjective_objective": {"answer": "Subjective", "rationale": "Since the problem involves the patient ' s self-awareness and perception, it needs to be viewed from the patient ' s subjective perspective."}, "sentence_sentiment": {"answer": "Positive", "rationale": "Because the entry \"Contribution to the world and lead a meaningful life\" is a positive quality."}, "language_style": {"answer": "Tactful and warm", "rationale": ""}, "time_dimension": {"answer": "Present", "rationale": "Key words \"consider\" and \"are\" mean that this is a matter of the current state of affairs, asking the testees what they feel and what they think. At the same time, the words \"does or doesn't contribute\" and \"meaning life\" also indicate that it is an assessment and reflection of the state of life."}, "sentence_type": "Interrogative sentence", "pos": {"nouns": {"World": 1, "Contribution": 1, "Life": 1}, "verbs": {"I think": 1, "have": 1, "- Yes": 1}, "adjectives": {"Yes": 1, "Meaningful": 1}, "numerals": {"121": 1}}}, {"content": "122. Are you easy to sleep?", "word_count": 8, "label": "", "sentence_structure": "Subject鈥損redicate", "subjective_objective": {"answer": "Subjective", "rationale": "Because sleep-altering is a subjective feeling or experience."}, "sentence_sentiment": {"answer": "Neutral", "rationale": "Because the entry does not have any emotional color."}, "language_style": {"answer": "Tactful and warm", "rationale": "The entry is more adept and interactive when it is spoken in a gentle and inviting manner, rather than by direct statements or orders."}, "time_dimension": {"answer": "Present", "rationale": "The entry asks the testee about his current state of sleep, and the time dimension is now judged."}, "sentence_type": "Interrogative sentence", "pos": {"nouns": {"you": 1, "It's easy": 1, "Sleep": 1}, "verbs": {"Is it possible": 1, "Easy": 1, "Enter": 1}, "adverbs": {"very": 1}, "numerals": {"122": 1}}}, {"content": "123, you're not gonna make a fuss?", "word_count": 6, "label": "", "sentence_structure": "Subject鈥搗erb鈥揷omplement", "subjective_objective": {"answer": "Subjective", "rationale": "Because the \"unlimited section\" is an evaluative term, it describes the character of a person, which is a subjective idea and perception of the individual."}, "sentence_sentiment": {"answer": "Negative", "rationale": "Because the act of \"no-no-no-no-no\" may be considered reckless or irresponsible in some circumstances, it is negative."}, "language_style": {"answer": "Tactful and warm", "rationale": "The language style of the entry is more euphemistic. It makes the respondents feel more comfortable and free to express their views and attitudes in a way that is asked, rather than directly ordering or evaluating. At the same time, the use of words is more neutral, without too strong negative emotions or encouraging rhetoric, and it is more natural to feel more friendly."}, "time_dimension": {"answer": "Present", "rationale": "The entry asks the testee for current behavioural or character characteristics, and the time dimension is now judged."}, "sentence_type": "Interrogative sentence", "pos": {"nouns": {"Subsection": 1, "you": 1}, "verbs": {"I'm free": 1}, "adjectives": {"Small": 1, "Large": 1}, "adverbs": {"Both": 1}, "numerals": {"123": 1, "1": 1, "2": 1, "3": 1}, "pronouns": {"Here": 1}}}, {"content": "124, can most of what you do make people happy?", "word_count": 16, "label": "", "sentence_structure": "Subject鈥搗erb鈥搊bject", "subjective_objective": {"answer": "Subjective", "rationale": "Because this is a highly subjective expression, it involves the individual ' s performance and the feelings of others, requires the patient to evaluate for himself the impact of his or her behaviour on others and is therefore subject to subjective scrutiny."}, "sentence_sentiment": {"answer": "Positive", "rationale": "Because the action in the entry \"makes people happy\" is positive."}, "language_style": {"answer": "Tactful and warm", "rationale": "The language style of the entry is more euphemistic, because it uses a verb of inquiry rather than direct orders or statements. At the same time, it uses terms that are comforting and friendly, such as \"most\" and \"happy\" that help respondents to better accept and answer the question."}, "time_dimension": {"answer": "Present", "rationale": "The keyword \"most of the things done\" means that the testee needs to take into account his general behaviour over time, not a specific act of the past. At the same time, \"are you happy?\" is an assessment of the testee's current behaviour, so the temporal dimension is now."}, "sentence_type": "Interrogative sentence", "pos": {"nouns": {"Yes": 2, "Objects": 1, "person": 3}, "verbs": {"Do it": 1, "Yes": 1, "鈾?And I'm gonna be 鈾?: 1}, "adjectives": {"Large": 1, "Department": 1, "More": 1, "Come on": 1, "Pleasant": 2}, "adverbs": {"Most": 1, "Both": 1}, "numerals": {"124": 1}, "classifiers": {"Number of times": 1}, "pronouns": {"you": 1}}}, {"content": "125. Do you have a long constipation?", "word_count": 6, "label": "", "sentence_structure": "Subject鈥損redicate", "subjective_objective": {"answer": "Objective", "rationale": "The fact that the patient has experienced chronic constipation is an objective fact."}, "sentence_sentiment": {"answer": "Negative", "rationale": "Basis of judgement: constipation is usually considered a negative physical condition."}, "language_style": {"answer": "Calm and direct", "rationale": "The language style of the entry is more straightforward and concise, and it is not too much rhetorical or emotional, but rather questions in an objective and neutral tone. It is therefore judged to be \"cool and straight.\""}, "time_dimension": {"answer": "Past", "rationale": "The entry asks about the health of the person tested in the past, especially with regard to long-term constipation, and therefore the temporal dimension is a matter of the past."}, "sentence_type": "Interrogative sentence", "pos": {"nouns": {"125": 1, "Decency": 1}, "verbs": {"Long term": 1}, "numerals": {"125": 1}}}, {"content": "126. Do you always think about what happened in the past and regret that you have not done better?", "word_count": 25, "label": "", "sentence_structure": "Subject鈥搗erb鈥搊bject", "subjective_objective": {"answer": "Subjective", "rationale": "The question is asked about the patient ' s past thoughts and feelings, which need to be answered on the basis of the patient ' s personal experience, and is therefore subjective."}, "sentence_sentiment": {"answer": "Negative", "rationale": "Because the entry \" always considers what happened in the past and regrets that it did not do better\" is negative and expresses regret and dissatisfaction with its own past."}, "language_style": {"answer": "Tactful and warm", "rationale": "The entry is more personal and interactive when it is spoken in a gentle, inquisitive manner rather than directly speaking or ordering."}, "time_dimension": {"answer": "Present", "rationale": ""}, "sentence_type": "Interrogative sentence", "pos": {"nouns": {"Things": 1, "Past": 1, "It happened in the past": 1, "self": 1, "I'm sorry": 1}, "verbs": {"Do it": 1, "Always": 1, "Consider": 1, "I can't": 1, "Better": 1}, "adverbs": {"Always": 1, "More": 1}, "numerals": {"126": 1}, "classifiers": {"individual": 1}, "pronouns": {"self": 1}}}, {"content": "127. Have you sometimes concealed your views for fear of being mocked or criticized?", "word_count": 22, "label": "", "sentence_structure": "Subject鈥搗erb鈥搊bject", "subjective_objective": {"answer": "Subjective", "rationale": "This is a question of the patient鈥檚 subjective perspective, as it asks whether the patient has a mental state, i.e., concealing his or her opinion in fear of ridicule or criticism."}, "sentence_sentiment": {"answer": "Negative", "rationale": "Because the behavior in the entry \"hidden your opinion\" is negative."}, "language_style": {"answer": "Tactful and warm", "rationale": "The language style of the entry is more euphemistic, using expressions such as \"sometimes\" or \"disguised opinion for fear of ridicule or criticism\", which avoids direct accusations or orders to ask questions in a more understanding and inclusive tone. This tone helps to create a good survey atmosphere and to increase the co-operation and comfort of the interviewee."}, "time_dimension": {"answer": "Present", "rationale": "The time dimension of this questionnaire entry is therefore judged now."}, "sentence_type": "Interrogative sentence", "pos": {"nouns": {"Observations": 1, "others": 1, "Scoff": 1, "Criticism": 1}, "verbs": {"Concealed": 1, "be": 1, "Yes": 1, "Sometimes": 1}, "adverbs": {"or": 1}, "numerals": {"127": 1}, "pronouns": {"self": 1}}}, {"content": "128. Do you think that no one in the world loves you?", "word_count": 14, "label": "", "sentence_structure": "Subject鈥搗erb鈥揷omplement", "subjective_objective": {"answer": "Subjective", "rationale": "Because \"the feeling\" is the subjective view and feeling of the patient."}, "sentence_sentiment": {"answer": "Negative", "rationale": "Because the phrase \"no one loves you\" in the entry is negative."}, "language_style": {"answer": "Tactful and warm", "rationale": "The language style of the entry is more euphemistic, with questions being asked in a question-and-answer manner, which is relatively soft, gives people a sense of affection and attention. At the same time, this way of asking questions encourages respondents to think and express their views, which facilitates more information and feedback."}, "time_dimension": {"answer": "Present", "rationale": "The entry asks about the present subjective feelings and perceptions of the subject and thus the judgement is now."}, "sentence_type": "Rhetorical question", "pos": {"nouns": {"person": 1, "Love": 1, "you": 1, "World": 1}, "verbs": {"I think": 1, "No, I'm not": 1, "Love": 1}, "adverbs": {"Really": 1}, "numerals": {"128": 1, "Alone": 1}, "pronouns": {"you": 1}}}, {"content": "129, in social settings, are you easily embarrassed?", "word_count": 15, "label": "", "sentence_structure": "Subject鈥搗erb鈥搊bject", "subjective_objective": {"answer": "Subjective", "rationale": "Because of the embarrassment is a subjective feeling and experience."}, "sentence_sentiment": {"answer": "Negative", "rationale": "Because the emotional experience of being embarrassed in the entries is usually negative."}, "language_style": {"answer": "Tactful and warm", "rationale": ""}, "time_dimension": {"answer": "Present", "rationale": ""}, "sentence_type": "Interrogative sentence", "pos": {"nouns": {"Questionnaire": 1, "Entry": 1, "Event": 1, "I'm embarrassed": 1}, "verbs": {"I feel": 1, "Easy": 1}, "adverbs": {"very": 1, "Medium": 1, "in": 1}, "numerals": {"129": 1}}}, {"content": "130. Do you keep used items for future use?", "word_count": 19, "label": "", "sentence_structure": "Subject鈥搗erb鈥搊bject", "subjective_objective": {"answer": "Subjective", "rationale": "Because the idea and decision whether to retain used or not is the patient's subjective attitude."}, "sentence_sentiment": {"answer": "Neutral", "rationale": "Because the entry does not have any emotional color."}, "language_style": {"answer": "Tactful and warm", "rationale": "The language style of the entry is more euphemistic and moderate, and the person interviewed is guided by a question-and-answer approach, rather than by direct orders or statements, which is more personal and interactive."}, "time_dimension": {"answer": "Future", "rationale": "The entry is for questioning the behaviour or attitude of the testee in the future, and the keyword \"use of the future\" indicates that this is the vision and expectation for the future."}, "sentence_type": "Interrogative sentence", "pos": {"nouns": {"Waste": 1, "Items": 1, "Future": 1, "Use the field": 1}, "verbs": {"Keep it": 1, "Send it": 1}, "numerals": {"130": 1}, "classifiers": {"individual": 1}}}, {"content": "Do you believe your future is in your hands?", "word_count": 15, "label": "", "sentence_structure": "Subject鈥搗erb鈥搊bject", "subjective_objective": {"answer": "Subjective", "rationale": "Because it is a subjective view or belief that believes that the future is in the hands of itself."}, "sentence_sentiment": {"answer": "Positive", "rationale": "Because the attitude in the entry, \"Believe that your future is in your hands\" is positive."}, "language_style": {"answer": "Tactful and warm", "rationale": "The entry is more personal and interactive in a gentle and encouraging way, asking the counterparty whether they believe they can take control of the future rather than directly making statements or orders."}, "time_dimension": {"answer": "Future", "rationale": "The keywords \"Believe\" and \"Behind your hand\" indicate the vision and expectations for the future, so the entry is judged to be the future."}, "sentence_type": "Interrogative sentence", "pos": {"nouns": {"Questionnaire Entry": 1, "The future": 1, "Hands": 1}, "verbs": {"Got it": 1, "in": 1, "Yes": 1, "have": 1}, "adverbs": {"you": 2, "- You're not": 1}, "numerals": {"131": 1, "One": 1}}}, {"content": "132. Have you ever had a nervous breakdown?", "word_count": 10, "label": "", "sentence_structure": "Subject鈥損redicate", "subjective_objective": {"answer": "Subjective", "rationale": "Because the existence of nervous weakness is a patient ' s own experience and perception, and belongs to the patient ' s subjective feeling."}, "sentence_sentiment": {"answer": "Negative", "rationale": "Since \"neurosis\" is usually considered a negative health condition, the purpose of this article is negative."}, "language_style": {"answer": "Calm and direct", "rationale": "The entry uses a more formal language style, with objective tone, with no excessive emotional rendering or rhetorical techniques, and is a calm and direct language style."}, "time_dimension": {"answer": "Past", "rationale": "The entry asks if the person tested had had experience of \"neurological decay\", which usually occurred in the past and is therefore judged to be past."}, "sentence_type": "Interrogative sentence", "pos": {"nouns": {"Nerve failure": 1}, "verbs": {"have": 1}, "numerals": {"132": 1}}}, {"content": "133. Do you have some kind of guilt hidden inside you and fear that one day someone will know?", "word_count": 28, "label": "", "sentence_structure": "Subject鈥搗erb鈥搊bject", "subjective_objective": {"answer": "Subjective", "rationale": "Because the subject is examined by the patient's subjective feelings and thoughts."}, "sentence_sentiment": {"answer": "Negative", "rationale": "The reference to \"a fear that one day someone will know\" in the entry is negative."}, "language_style": {"answer": "Tactful and warm", "rationale": "The entry uses a soft, invitation-style conversation, which is expressed in a non-direct statement or order, which is more personal and interactive. At the same time, the words \"criticism\" and \"fear\" are used to express concern and concern about what the other person feels inside, and the language style is more euphemistic."}, "time_dimension": {"answer": "Past", "rationale": "So the temporal dimension of this questionnaire entry is judged as past, present, future."}, "sentence_type": "Interrogative sentence", "pos": {"nouns": {"Inside": 1, "Guilt": 1, "I'm worried": 1, "One day": 1, "others": 1, "Yes": 1}, "verbs": {"Hide": 1, "be": 1, "I'm sure I will": 1}, "adverbs": {"Total": 1}, "numerals": {"Thirteen-three": 1}, "pronouns": {"you": 1}}}, {"content": "134. Are you ashamed in social settings and realize that you are so shy?", "word_count": 23, "label": "", "sentence_structure": "Subject鈥搗erb鈥搊bject", "subjective_objective": {"answer": "Subjective", "rationale": "The answer to this questionnaire is subjective: the question is about the subjective feelings and perceptions of individuals in the social sphere, namely, \"Do you feel shy and realize that you are shy?\""}, "sentence_sentiment": {"answer": "Negative", "rationale": "Because the \"smugness\" in the entry is negative."}, "language_style": {"answer": "Tactful and warm", "rationale": "The entry is more euphemistic, using the word \"whether or not\" to guide the question, to express concern and respect for the interviewee. At the same time, it describes a feeling rather than a direct act, avoiding undue pressure or discomfort on the interviewee."}, "time_dimension": {"answer": "Present", "rationale": "Because the phrases that appear are generally the current state of \"reception\" and \"self-aware\" that means that the act occurred at the current point in time, so it is now."}, "sentence_type": "Interrogative sentence", "pos": {"nouns": {"Socially": 1, "Shy": 2, "self": 1}, "verbs": {"I feel": 1, "I'm aware of that": 1}, "numerals": {"134": 1}, "pronouns": {"you": 1}}}, {"content": "135. Do you think bringing a child into the world is a difficult thing?", "word_count": 24, "label": "", "sentence_structure": "Subject鈥搗erb鈥搊bject", "subjective_objective": {"answer": "Subjective", "rationale": "Because the question is about the patient ' s personal views and attitudes to a particular matter, it is a subjective matter."}, "sentence_sentiment": {"answer": "Negative", "rationale": "Because the idea that \"to bring a child into the world is a difficult thing\" is a negative emotional attitude."}, "language_style": {"answer": "Tactful and warm", "rationale": "The basis for the judgement: the entry is a soft, invitation-style conversation, not a direct statement or order. First, \"You think that the use of this doubt phrase makes the question more open and interactive, and leads the interviewees to express their views and opinions rather than being forced to accept a particular view or position. Second, \"Is it difficult to bring a child to the world?\" The question does not give any clear answer or preference, but rather raises the question in an objective and neutral way, in which the interviewee himself thinks and answers, which is more personal and interactive. The language style of the article is thus described as euphemism."}, "time_dimension": {"answer": "Future", "rationale": "The entry asks the testee about his future and his expectations, \"Do you think\" and \"will it be a difficult thing?\" It shows vision and prediction of the future, and therefore judges it as the future."}, "sentence_type": "Interrogative sentence", "pos": {"nouns": {"Things": 1, "Son": 1, "World": 1}, "verbs": {"Bring it": 1, "I think": 1, "It's hard": 1}, "adjectives": {"Hard": 1}, "adverbs": {"very": 1}, "numerals": {"135": 1}}}, {"content": "136. Are you easily overwhelmed if things are not proceeding as planned?", "word_count": 25, "label": "", "sentence_structure": "Subject鈥搗erb鈥搊bject", "subjective_objective": {"answer": "Subjective", "rationale": "Because \"discretion\" is the patient's subjective feeling and reaction to something that is not being done as expected."}, "sentence_sentiment": {"answer": "Negative", "rationale": "The basis for the judgment: because \"easy to feel helpless\" is a negative emotional reaction."}, "language_style": {"answer": "Tactful and warm", "rationale": "The entry, which is \"cool and direct\", directly asks the respondents about their emotional reactions in the event of a violation, and is more objective and direct."}, "time_dimension": {"answer": "Present", "rationale": "The entry uses the time mark \"now\", indicating that the person being questioned is in the current state or habitually."}, "sentence_type": "Interrogative sentence", "pos": {"nouns": {"Things": 1, "Book": 1, "Planned": 1}, "verbs": {"Conduct": 1, "I feel": 1, "Easy": 1, "I'm in a hurry": 1}, "adjectives": {"No. No": 1}, "numerals": {"136銆?: 1}, "classifiers": {"individual": 1}}}, {"content": "When the room is in a mess, are you uncomfortable?", "word_count": 14, "label": "", "sentence_structure": "Subject鈥搗erb鈥搊bject", "subjective_objective": {"answer": "Subjective", "rationale": "Because \"incomfortable\" is the patient's subjective feeling of the room being messy."}, "sentence_sentiment": {"answer": "Negative", "rationale": "Because the description of \"rooms in disarray\" in the entries is usually considered to be a negative environmental factor, it can cause uncomfortable emotional reactions."}, "language_style": {"answer": "Calm and direct", "rationale": "Basis for the determination: The entry is asked in a direct and concise manner whether the person surveyed is uncomfortable when the room is in a state of disarray, is not presented with excessive rhetorical or emotional rendering, and is more objective in tone."}, "time_dimension": {"answer": "Present", "rationale": "The entry \"when the room is in a state of disarray\" describes the current environment, and \"are you not feeling well?\" The question is the subjective perception of the situation by the testee. Thus, the temporal dimension of the article is now judged."}, "sentence_type": "Interrogative sentence", "pos": {"nouns": {"Room": 1, "Inside": 1, "Chaos": 1, "Time": 1, "you": 1, "I feel": 1, "I'm not feeling well": 1}, "verbs": {"be": 1, "Is it possible": 1, "I feel": 1}, "adjectives": {"very": 1, "Chaos": 1}, "adverbs": {"very": 1}, "numerals": {"137": 1}, "pronouns": {"you": 1}}}, {"content": "138. Do you have the same will as anyone else?", "word_count": 11, "label": "", "sentence_structure": "Subject鈥搗erb鈥搊bject", "subjective_objective": {"answer": "Subjective", "rationale": "Because will is an individual's subjective feeling and experience."}, "sentence_sentiment": {"answer": "Positive", "rationale": "Because the act of \"will\" in the entry is positive and indicates the firm determination and perseverance of the individual."}, "language_style": {"answer": "Tactful and warm", "rationale": "Although the subject directly asks the question \"Are you as willing as anyone else?\", it asks questions in a more euphemistic way. By using the word \"are you\" it seems more like a conversation than a one-sided inquiry. And the phrase \"Are you like others\" is more kind and gentle and better able to guide respondents to questions."}, "time_dimension": {"answer": "Present", "rationale": "The word \"now\" in the sentence makes it clear that the entry is asking the testee about the current behaviour or state, and that the time dimension is now."}, "sentence_type": "Interrogative sentence", "pos": {"nouns": {"Questionnaire Entry": 1, "Will": 1}, "verbs": {"Did you": 1, "have": 1}, "numerals": {"138": 1, "Same thing": 1}, "pronouns": {"others": 1}}}, {"content": "139. Do you often feel upset?", "word_count": 7, "label": "", "sentence_structure": "Subject鈥搗erb鈥搊bject", "subjective_objective": {"answer": "Subjective", "rationale": "Since heart palpation is the patient ' s subjective feeling, it is up to the patient to determine whether or not heart palpation is felt."}, "sentence_sentiment": {"answer": "Negative", "rationale": "Because the heart attack is a negative physical discomfort in the entry."}, "language_style": {"answer": "Calm and direct", "rationale": "The entry explicitly asks the respondents whether they are often in a state of disquiet, with a more direct language style, without too much ado or emotional colour."}, "time_dimension": {"answer": "Present", "rationale": "The entry asks whether the testee is currently feeling a sense of disheartening, and therefore judges \"now\"."}, "sentence_type": "Interrogative sentence", "pos": {"nouns": {"Heartbreak": 1}, "verbs": {"I always feel": 1}, "numerals": {"139": 1}}}, {"content": "Do you believe in evil?", "word_count": 8, "label": "", "sentence_structure": "Subject鈥損redicate", "subjective_objective": {"answer": "Subjective", "rationale": "Believe is subjective."}, "sentence_sentiment": {"answer": "Positive", "rationale": "For \"rebel of evil and evil\" is a positive value and attitude that expresses a firm belief in justice and fairness."}, "language_style": {"answer": "Calm and direct", "rationale": "The entry asks in a straightforward way, \"Do you believe in evil and evil?\" It does not add any qualifier or emotional colour, and the language style is relatively calm and straightforward."}, "time_dimension": {"answer": "Present", "rationale": "The entry is an inquiry into the person ' s beliefs or attitudes to moral values, which do not have a clear time-pointing point and therefore does not allow an accurate judgement of the time dimension."}, "sentence_type": "Interrogative sentence", "pos": {"nouns": {"you": 1, "There's a bad deal": 1}, "verbs": {"Yes": 1}, "numerals": {"140": 1}}}, {"content": "141. Do you feel inferior to the person you met, even though objectively you are no worse than him?", "word_count": 26, "label": "", "sentence_structure": "Subject鈥搗erb鈥搊bject", "subjective_objective": {"answer": "Subjective", "rationale": "The basis for the judgement: the question is examined by the person's feelings and attitude, that is, subjective. Although the reference to \"objectively you are no worse than he\" is made in the query, the comparison is based on the person's self-evaluation and therefore remains subjective."}, "sentence_sentiment": {"answer": "Negative", "rationale": "Because the attitude of feeling low in the entry is negative."}, "language_style": {"answer": "Tactful and warm", "rationale": ""}, "time_dimension": {"answer": "Present", "rationale": ""}, "sentence_type": "Interrogative sentence", "pos": {"nouns": {"person": 2, "You're a low man": 1, "Subjective": 1, "Objective": 1, "Entry": 1, "Questionnaire": 1}, "verbs": {"I met him": 1, "I feel": 1, "- Yeah": 1, "Bad": 1, "Compare": 1}, "adjectives": {"Subjectively": 1, "Objectively": 1}, "numerals": {"141": 1}, "pronouns": {"you": 2, "he": 1}}}, {"content": "142. In general, have you succeeded in achieving your goals in life?", "word_count": 19, "label": "", "sentence_structure": "Subject鈥搗erb鈥搊bject", "subjective_objective": {"answer": "Subjective", "rationale": "This is a subjective question of the achievement of the objectives of an individual ' s life and requires an answer from the patient according to his or her circumstances."}, "sentence_sentiment": {"answer": "Positive", "rationale": "Because \"successful achievement of life's goals\" in the entry is a positive expression."}, "language_style": {"answer": "Calm and direct", "rationale": "This is not too much rhetorical or emotional, and is more objective and practical."}, "time_dimension": {"answer": "Present", "rationale": "The entry asks about the current living conditions and experiences of the subjects and judges them now, based on the keywords \"generally\" and \"successfully\"."}, "sentence_type": "Interrogative sentence", "pos": {"nouns": {"Entry": 1, "142": 1, "General": 1, "you": 2, "Life": 1, "Objective": 1}, "verbs": {"Achieved": 1, "It worked": 1}, "adjectives": {"Speak": 1}, "adverbs": {"General": 1, "Success": 1}, "numerals": {"142": 1, "Two": 1}, "pronouns": {"you": 2, "Life": 1, "Objective": 1}}}, {"content": "143. Do you often wake up from nightmares and sweat?", "word_count": 16, "label": "", "sentence_structure": "Subject鈥搗erb鈥搊bject", "subjective_objective": {"answer": "Subjective", "rationale": "The question of whether the nightmares are happening and whether they are frightened is the patient's subjective feeling."}, "sentence_sentiment": {"answer": "Negative", "rationale": "Because the \"wrecked nightmares and sweating\" act in the entry is negative."}, "language_style": {"answer": "Calm and direct", "rationale": "The entry appears in the form of a straightforward question, with no excessive rhetorical or emotional rendering, and is more objective and cold, so it can be judged that its language style is \"cool and straight\"."}, "time_dimension": {"answer": "Past", "rationale": "Thus, the entry contains both a question about the past (i.e. whether the person tested in the past woke up to nightmares and scared up the sweat) and a question about the present (i.e. whether the person tested now often woke up to nightmares and scared up the sweat)."}, "sentence_type": "Interrogative sentence", "pos": {"nouns": {"Nightmares": 1, "Wake up": 1, "He's sweating": 1}, "verbs": {"Always": 1, "It's scary": 1}, "adverbs": {"Regular": 1}, "numerals": {"143": 1}}}, {"content": "144. Do you feel sick if someone else's dog licks your face?", "word_count": 16, "label": "", "sentence_structure": "Subject鈥搗erb鈥搊bject", "subjective_objective": {"answer": "Objective", "rationale": "Because it's the patient's feelings and reactions to the objective fact that the dog licks his face."}, "sentence_sentiment": {"answer": "Negative", "rationale": "Because the behavior in the entry is negative."}, "language_style": {"answer": "Calm and direct", "rationale": ""}, "time_dimension": {"answer": "Present", "rationale": "The word \"if\" indicates a hypothetical situation, \"others' dog licks your face\" is an action that has not yet happened, and \"Are you sick?\" It is a reaction to and feeling of the situation. The entry therefore primarily asks the subject about the current feelings and reactions, and does not address the time dimension of the past or the future."}, "sentence_type": "Interrogative sentence", "pos": {"nouns": {"Dog": 1, "Face": 1}, "verbs": {"Lick it": 1, "Sensor": 1, "Present": 1, "Gross": 1}, "adjectives": {"Someone else's": 1}, "numerals": {"144": 1}}}, {"content": "145. Because something has always been bothering you, you have to change your plan. Do you think making plans is a waste of time?", "word_count": 33, "label": "", "sentence_structure": "Subject鈥搗erb鈥搊bject", "subjective_objective": {"answer": "Subjective", "rationale": "The idea that planning is a waste of time is the patient's subjective idea."}, "sentence_sentiment": {"answer": "Negative", "rationale": "Because the idea that planning is a waste of time in the entry is negative."}, "language_style": {"answer": "Calm and direct", "rationale": "Although it asks each other's views in the way you say \"you feel\", it does not have too much euphemism or supposition, but rather it asks the other side directly whether it is wasting time on the plan. So I think its language style is calm and straightforward."}, "time_dimension": {"answer": "Past", "rationale": "The entry asks about the testee's past feelings and experience, i.e. his subjective view of the plan.\""}, "sentence_type": "Interrogative sentence", "pos": {"nouns": {"Things": 1, "Planned": 2, "Time": 1}, "verbs": {"Interference": 1, "鈾?And I'm gonna be 鈾?: 1, "I think": 1, "Staple": 1, "Wasted": 1}, "adjectives": {"Total": 1, "Some": 1, "I had to": 1}, "numerals": {"145": 1, "2": 1, "1": 3}, "pronouns": {"you": 1}}}, {"content": "146. Do you always worry that your family will get sick?", "word_count": 12, "label": "", "sentence_structure": "Subject鈥搗erb鈥搊bject", "subjective_objective": {"answer": "Subjective", "rationale": "The question is a subjective one, as it raises questions about the patient ' s emotional and ideological orientation."}, "sentence_sentiment": {"answer": "Negative", "rationale": "Because the attitude in the entry, \"A fear of family getting sick,\" is negative."}, "language_style": {"answer": "Tactful and warm", "rationale": "The entry uses the euphemism of asking questions, using the word \"always\" to express concern and understanding of the person being investigated, while also directing the answer to questions in a way that \"are you worried that the family will get sick?\" The language style is more acrimonymous and gentle."}, "time_dimension": {"answer": "Present", "rationale": "The keyword \"always\" indicates that this is a constant concern, and \"family sickness\" is a possible future event, but it is in this sentence the object of concern, emphasizing the emotional response. So, in sum, the time dimension of this questionnaire entry is now."}, "sentence_type": "Interrogative sentence", "pos": {"nouns": {"you": 1, "Family": 1, "Diseases": 1, "Fan": 1}, "verbs": {"Always worried": 1}, "adverbs": {"- You're not": 1}, "numerals": {"146": 1}}}, {"content": "147. If you do something morally condemnable, can you quickly forget it and look to the future?", "word_count": 27, "label": "", "sentence_structure": "Subject鈥搗erb鈥搊bject", "subjective_objective": {"answer": "Subjective", "rationale": "It is a subjective attitude and psychological state to see whether patients can face and correct their own wrongs in a positive way, rather than being insulated from past mistakes."}, "sentence_sentiment": {"answer": "Positive", "rationale": "Because the article is expressed as a question of a positive quality for a person 鈥?a quick excuse for himself and for looking forward."}, "language_style": {"answer": "Tactful and warm", "rationale": "The language style of this entry is euphemism, because it uses the \"can\" and more moderate words to give a sense of affection and concern when asking the respondents whether they can quickly forget the morally reprehensible behaviour and look to the future. Moreover, the question does not directly point to the fact that the acts are wrong or immoral, but rather is expressed in a more subtle way, so that the respondents can feel relatively relaxed and comfortable in their responses."}, "time_dimension": {"answer": "Future", "rationale": "The key word \"can you forget quickly and look to the future\" expresses vision and expectations for the future."}, "sentence_type": "Interrogative sentence", "pos": {"nouns": {"Yes": 2, "The future": 1}, "verbs": {"Do it": 1, "Yes": 1, "Forget it": 1, "Look at it": 1}, "adjectives": {"Moral condemnation": 1}, "adverbs": {"very": 1}, "numerals": {"147": 1}, "pronouns": {"you": 2}}}, {"content": "148. Do you usually think you can achieve what you want?", "word_count": 18, "label": "", "sentence_structure": "Subject鈥搗erb鈥搊bject", "subjective_objective": {"answer": "Subjective", "rationale": "Because the subject was asked about the feelings and attitudes of the interviewees, that is, their belief in their abilities and objectives, and therefore their subjective perspective."}, "sentence_sentiment": {"answer": "Positive", "rationale": "Because the goal of \"to achieve what you want\" in the entry is positive, and it is a manifestation of one's confidence and determination."}, "language_style": {"answer": "Calm and direct", "rationale": "The basis for the judgement: the article is formulated in a concise manner, without the use of too many qualifiers or emotional terms. The question is simply whether the interviewee is able to achieve his or her goals and his or her views, and is speaking in a more objective and calm manner. It can therefore be concluded that the language style of the article is \"cool and direct.\""}, "time_dimension": {"answer": "Present", "rationale": "The keyword \"usually\" indicates a normal view, and \"to achieve what you want\" implies the degree of confidence of the testee and his ability to achieve what he wants. So the entry is primarily about the behaviour of the testee in the current mindset and mentality."}, "sentence_type": "Interrogative sentence", "pos": {"nouns": {"Questionnaire": 1, "Entry": 1, "Objective": 1}, "verbs": {"I think": 1, "Yes": 3, "Achieved": 2}, "adverbs": {"Usually": 1}, "numerals": {"148銆?: 1}, "pronouns": {"you": 1}}}, {"content": "149. Do you always get sentimental?", "word_count": 7, "label": "", "sentence_structure": "Subject鈥損redicate", "subjective_objective": {"answer": "Subjective", "rationale": "Because \"fearful\" is a subjective emotional experience and judgment."}, "sentence_sentiment": {"answer": "Negative", "rationale": "Because the word \"mental\" usually has some negative emotions."}, "language_style": {"answer": "Tactful and warm", "rationale": "The entry is more personal and interactive when it is spoken in a gentle and inviting tone, rather than in direct statements or orders. At the same time, it is not overly emotional, speaking in a more objective manner, and makes the interviewee comfortable and comfortable. So the language style of the questionnaire is warm."}, "time_dimension": {"answer": "Present", "rationale": "\"At times\" is a customary emotional experience, and \"memotional\" is a state of emotion that is now in a state of modernity, so the time dimension of the questionnaire entry is now."}, "sentence_type": "Interrogative sentence", "pos": {"nouns": {"Grandpa": 1, "Questionnaire": 1, "Entry": 1, "I'm sorry": 1}, "verbs": {"Regular": 1, "Easy": 1, "Do it": 1}, "adjectives": {"of": 2}, "numerals": {"149": 1}, "classifiers": {"individual": 1}}}, {"content": "150, will your voice become awe when you talk to someone and want to be impressed?", "word_count": 31, "label": "", "sentence_structure": "Subject鈥搗erb鈥搊bject", "subjective_objective": {"answer": "Subjective", "rationale": "Because \"especially to impress\" is the patient's subjective attitude."}, "sentence_sentiment": {"answer": "Negative", "rationale": "Because \"the sound becomes awe\" is usually caused by negative emotions such as stress or lack of confidence."}, "language_style": {"answer": "Tactful and warm", "rationale": "The entry is more adept and interactive when it is spoken in a gentle and inviting manner, rather than by direct statements or orders."}, "time_dimension": {"answer": "Present", "rationale": "The entry asks if the person tested has performed specifically in communicating with others, and the time dimension is now judged."}, "sentence_type": "Interrogative sentence", "pos": {"nouns": {"person": 2, "Talk": 1, "Sound": 2, "Impression": 1, "Entry": 1}, "verbs": {"Yes": 2, "鈾?And become 鈾?: 1, "Stay": 1, "Talk": 1, "Special": 1}, "adjectives": {"Deep": 1, "A good shake": 1}, "adverbs": {"In particular": 1}, "numerals": {"150": 1, "Two": 1}, "pronouns": {"you": 2, "others": 1, "When": 1}}}, {"content": "Are you the kind of person who doesn't ask for anything?", "word_count": 13, "label": "", "sentence_structure": "Subject鈥搗erb鈥搊bject", "subjective_objective": {"answer": "Subjective", "rationale": "Because \"Is it the kind of person who doesn't ask for anything\" is a question of the patient's personal qualities, of his subjective attitude and values."}, "sentence_sentiment": {"answer": "Neutral", "rationale": "Because it doesn't involve emotional colour."}, "language_style": {"answer": "Calm and direct", "rationale": "The entry is based on a direct question, without any rhetorical or emotional rendering. This expression is simple and easy to understand and answer questions, and therefore is a calm and direct language style."}, "time_dimension": {"answer": "Present", "rationale": "The entry asks the testee about his current behaviour or state, i.e. whether he is the kind of person who doesn't want anything. Therefore, the time dimension is now judged."}, "sentence_type": "Interrogative sentence", "pos": {"nouns": {"Yes": 1, "person": 2}, "verbs": {"Please": 1}, "adjectives": {"Thousand": 1}, "adverbs": {"not": 1}, "numerals": {"151": 1}, "pronouns": {"you": 1}}}, {"content": "152. Do you prefer the kind of work that others decide and tell you what to do?", "word_count": 23, "label": "", "sentence_structure": "Subject鈥搗erb鈥搊bject", "subjective_objective": {"answer": "Subjective", "rationale": "Because it is subjective to like or dislike to be determined by the patient ' s own attitudes and feelings."}, "sentence_sentiment": {"answer": "Negative", "rationale": "Because the act of \"decision-making by others and telling you what to do\" in the entry is negative, it means that the person under investigation does not like to work too passively."}, "language_style": {"answer": "Tactful and warm", "rationale": "The language style of the entry is euphemism, because it uses a question-and-answer tone, without telling the interviewee what job to choose. At the same time, the \"fluent\" tone makes it easier for the interviewee to accept the problem and to share his real thoughts and feelings."}, "time_dimension": {"answer": "Past", "rationale": ""}, "sentence_type": "Interrogative sentence", "pos": {"nouns": {"Work": 1, "Decision-making": 1, "Tell": 1}, "verbs": {"Yeah": 1, "By": 1, "Do it": 1}, "adjectives": {"Others": 1}, "numerals": {"152": 1}, "pronouns": {"you": 1}}}, {"content": "153. Do you always get cold feet even when it's warm?", "word_count": 17, "label": "", "sentence_structure": "Subject鈥搗erb鈥搊bject", "subjective_objective": {"answer": "Subjective", "rationale": "Because the question is whether the patient feels himself cold and is a subjective one."}, "sentence_sentiment": {"answer": "Negative", "rationale": "Because the physical phenomenon of \"cold hands and feet\" is usually considered uncomfortable, the entry expresses a negative emotion or attitude."}, "language_style": {"answer": "Tactful and warm", "rationale": "The language style of the entry is euphemism, as it uses an invitation tone and asks the other side for a specific situation, rather than direct orders or statements. Moreover, it is more moderate in its expression and does not make the person under investigation feel pressured or uncomfortable."}, "time_dimension": {"answer": "Present", "rationale": "\"Do you often get cold?\" In \"At times\" the word \"At times\" is a regular, customary act, and \"Is it?\" The word \"At times\" is in the form of a general question sentence, which means that the act may still be ongoing. So, the entry to the questionnaire asks about the person who was tested."}, "sentence_type": "Interrogative sentence", "pos": {"nouns": {"Weather": 1, "Time": 1, "you": 1, "Hands and feet": 1, "Cold": 1}, "verbs": {"be": 1, "Yeah": 1, "Often": 1, "Warm": 1, "have": 1}, "adjectives": {"Even": 1}, "numerals": {"153": 1}, "pronouns": {"you": 1}}}, {"content": "154. Do you often pray for forgiveness?", "word_count": 14, "label": "", "sentence_structure": "Subject鈥搗erb鈥搊bject", "subjective_objective": {"answer": "Subjective", "rationale": "Because \"pray\" is a personal belief and attitude, and it is the patient's subjective behaviour."}, "sentence_sentiment": {"answer": "Neutral", "rationale": "Because the entry does not have any emotional color."}, "language_style": {"answer": "Tactful and warm", "rationale": "The language style of the entry is more moderate, using a way of asking, not directly, questions, using polite language and expressing respect for the privacy of the interviewee. At the same time, the issue does not overemphasize the religious colour, but rather asks the interviewee鈥檚 views in a more open and inclusive manner."}, "time_dimension": {"answer": "Past", "rationale": "The entry \"Do you often ask forgiveness through prayer?\" indicates that it is a pattern of past behaviour or habit, and therefore the dimension of time is a past."}, "sentence_type": "Interrogative sentence", "pos": {"nouns": {"Questionnaire Entry": 1, "Pray": 1, "Forgiveness": 1}, "verbs": {"Regular": 1, "All": 1, "Pass": 1, "Request": 1, "Got it": 1, "- You're not": 1}, "adverbs": {"Regular": 1}, "numerals": {"154": 1}, "classifiers": {"individual": 1}}}, {"content": "155. Are you satisfied with your looks?", "word_count": 11, "label": "", "sentence_structure": "Subject鈥搗erb鈥搊bject", "subjective_objective": {"answer": "Subjective", "rationale": "Since satisfaction is a subjective assessment, different people have different perceptions and criteria for their own appearance."}, "sentence_sentiment": {"answer": "Neutral", "rationale": "Because the entry was not emotional, it was merely an inquiry into the satisfaction of the person surveyed with his or her own appearance."}, "language_style": {"answer": "Calm and direct", "rationale": "The entry is brief and clear in asking the respondents whether they are satisfied with their appearance and the language style is more calm and objective."}, "time_dimension": {"answer": "Present", "rationale": "The entry asks if the testee is currently satisfied with his or her appearance, and the time dimension is now judged."}, "sentence_type": "Interrogative sentence", "pos": {"nouns": {"Look at her": 1, "Satisfaction": 1}, "verbs": {"I feel": 1, "Satisfied": 1}, "numerals": {"155": 1}}}, {"content": "156. Do you think people always get lucky?", "word_count": 14, "label": "", "sentence_structure": "Subject鈥搗erb鈥搊bject", "subjective_objective": {"answer": "Subjective", "rationale": "Because \"think\" is the patient's own subjective feeling."}, "sentence_sentiment": {"answer": "Negative", "rationale": "Because it expresses a sense of jealousy or envy."}, "language_style": {"answer": "Calm and direct", "rationale": "The language style of the entry is more direct, and there is no excessive rhetorical or emotional rendering, but rather a simple question about whether the interviewee feels that way. This tone is more appropriate for a more formal or scientific research environment.\""}, "time_dimension": {"answer": "Present", "rationale": "The entry asks about the current feelings and perceptions of the subject and does not address past or future acts or events, and therefore the judgement is now."}, "sentence_type": "Interrogative sentence", "pos": {"nouns": {"you": 1, "others": 1, "Lucky": 1}, "verbs": {"I think": 1, "I've got it": 1}, "adverbs": {"Always": 1}, "numerals": {"156": 1}}}, {"content": "157, can you stay calm in an emergency?", "word_count": 13, "label": "", "sentence_structure": "Subject鈥損redicate", "subjective_objective": {"answer": "Subjective", "rationale": "Because it's a question of whether the patient is capable of keeping calm in an emergency."}, "sentence_sentiment": {"answer": "Neutral", "rationale": "Because the entry asks about a capability and does not involve emotional colour."}, "language_style": {"answer": "Calm and direct", "rationale": "The entry uses a straightforward language style, asking the respondent's ability to respond in an emergency, without any rhetorical or emotional modification, and is simple and targeted. Thus, the language style is \"cool and direct\"."}, "time_dimension": {"answer": "Present", "rationale": "The entry asks the testee's capabilities or state under the current circumstances, so the temporal dimension is now judged. At the same time, the word \"capable\" also indicates that it is a potential capability or behaviour that may need to be applied and validated in the future."}, "sentence_type": "Interrogative sentence", "pos": {"nouns": {"Situation": 1, "Emergency": 1, "Calm down": 1}, "verbs": {"Yes": 1, "Hold on": 1, "Stay calm": 1}, "adverbs": {"in": 1, "In case of emergency": 1}, "numerals": {"157": 1}}}, {"content": "158. Do you have all the dates and all the things that have to be done on the same day on the book?", "word_count": 25, "label": "", "sentence_structure": "Subject鈥搗erb鈥搊bject", "subjective_objective": {"answer": "Objective", "rationale": "Because \"Is it all on the book and all what must be done on the same day\" is the actual behaviour of the patient, and the behaviour of the patient is examined, the entry is considered from an objective perspective."}, "sentence_sentiment": {"answer": "Positive", "rationale": "Because the behavior in the entry \"all dates and all that has to be done on the same day\" is an organized and planned act, which is usually considered positive."}, "language_style": {"answer": "Calm and direct", "rationale": "The basis for the determination: The entry is in the form of a direct query, a simple and straightforward query about whether the other party is accustomed to recording all dates and all the things that must be done on the same day. This question is made in a manner that is not based on any rhetorical or emotional overtones, is more objective in tone and is a straightforward language style."}, "time_dimension": {"answer": "Present", "rationale": ""}, "sentence_type": "Interrogative sentence", "pos": {"nouns": {"Grandpa": 1, "Date": 1, "Yes": 2, "The book": 1}, "verbs": {"be": 1, "Remember": 1, "- Put it on": 1, "Both": 1, "Do it": 1}, "adverbs": {"All": 1, "Same day": 1}, "numerals": {"158": 1, "1": 4, "2": 1}, "classifiers": {"Grandpa": 1, "individual": 1}, "pronouns": {"you": 1}}}, {"content": "159. Do you feel that changing the environment in life is also futile?", "word_count": 18, "label": "", "sentence_structure": "Subject鈥搗erb鈥搊bject", "subjective_objective": {"answer": "Subjective", "rationale": "Because it is the patient's own subjective feelings and ideas that feel that life is in vain."}, "sentence_sentiment": {"answer": "Negative", "rationale": "Because it contains a \"fun\" negative sentiment."}, "language_style": {"answer": "Calm and direct", "rationale": "The language style of the entry is straightforward, as it raises a clear question directly, and does not use excessive emotional colours or rhetorical techniques."}, "time_dimension": {"answer": "Past", "rationale": "The entry asks about the testee's past behaviour or experience, i.e. whether there was ever a feeling that \"the change of environment in life is also futile\". The temporal dimension is therefore a past."}, "sentence_type": "Interrogative sentence", "pos": {"nouns": {"Life": 1, "Environment": 1, "In vain": 1}, "verbs": {"Change": 1, "I feel": 1}, "numerals": {"159": 1}}}, {"content": "160. Do you often feel like you're having trouble breathing?", "word_count": 9, "label": "", "sentence_structure": "Subject鈥損redicate", "subjective_objective": {"answer": "Subjective", "rationale": "Since the difficulty of breathing is the patient ' s physical and subjective experience, the perspective of this questionnaire entry is based on the patient ' s subjective perspective."}, "sentence_sentiment": {"answer": "Negative", "rationale": "Since \"respiration difficulties\" are generally considered as a disease or health problem, the expression of the purpose of the article is negative."}, "language_style": {"answer": "Calm and direct", "rationale": "The entry is asked in simple and direct language, without too much ado or emotional, with an objective and calm tone, and easy to understand and answer by the interviewee."}, "time_dimension": {"answer": "Past", "rationale": ""}, "sentence_type": "Interrogative sentence", "pos": {"nouns": {"160": 1, "I can't breathe": 1}, "verbs": {"I feel": 1}, "adverbs": {"Regular": 1}, "numerals": {"160": 1}, "classifiers": {"individual": 1}}}, {"content": "Do you feel embarrassed when you hear dirty stories?", "word_count": 13, "label": "", "sentence_structure": "Subject鈥搗erb鈥搊bject", "subjective_objective": {"answer": "Subjective", "rationale": "Because of the emotional reaction, the patient's subjective feeling."}, "sentence_sentiment": {"answer": "Negative", "rationale": "Because \"distressed\" in the entry is a negative emotional experience."}, "language_style": {"answer": "Tactful and warm", "rationale": "The language style of the entry is euphemism. It uses an indirect question method to understand how people feel when they hear dirty stories about the subject. At the same time, the whole sentence is less polite, less critical or accusing, and gives people a comfortable feeling of affection."}, "time_dimension": {"answer": "Present", "rationale": "\"As you hear a dirty story,\" \"hearing\" uses the usual current state of affairs, which suggests that it is a description of current or customary behaviour. And \"Are you in a state of embarrassment?\" is asking the testee about his current feelings or state, and judging it as now."}, "sentence_type": "Interrogative sentence", "pos": {"nouns": {"Story": 1, "I'm embarrassed": 1}, "verbs": {"Listen": 1, "I feel": 1}, "adjectives": {"Naughty": 1}, "numerals": {"161": 1}}}, {"content": "162. Do you remain silent about people you don't like?", "word_count": 15, "label": "", "sentence_structure": "Subject鈥搗erb鈥搊bject", "subjective_objective": {"answer": "Subjective", "rationale": "Because \"like\" and \"not like\" are subjective attitudes of patients, the view of this questionnaire is subjective."}, "sentence_sentiment": {"answer": "Negative", "rationale": "Because the act of \"silence\" in the entry is negative."}, "language_style": {"answer": "Calm and direct", "rationale": "The basis of the judgement is as follows: The entry directly asks the respondent about whether the person he does not like will remain silent. Although the problem itself is somewhat subjective and emotional, it is expressed in a more balanced and objective manner, without too many adorations or emotional expressions, and therefore its linguistic style can be considered as being straightforward and straightforward."}, "time_dimension": {"answer": "Present", "rationale": "The entry \"Are you\" indicates an inquiry into the state of the moment, while the \"silence\" implies that it is a continuing act or state. So, the time dimension of the entry is now."}, "sentence_type": "Interrogative sentence", "pos": {"nouns": {"person": 1, "Silence": 1}, "verbs": {"Yeah": 1, "Hold on": 1, "Is it possible": 1}, "adjectives": {"not": 1}, "adverbs": {"Yes": 1, "you": 2}, "numerals": {"162": 1}}}, {"content": "163. Do you feel like you can't handle your surroundings for a long time?", "word_count": 20, "label": "", "sentence_structure": "Subject鈥搗erb鈥搊bject", "subjective_objective": {"answer": "Subjective", "rationale": "This perception is the patient ' s subjective experience and perception and is judged by the emotions and attitudes within him."}, "sentence_sentiment": {"answer": "Negative", "rationale": "Because the description \"can't control your surroundings\" in the entry is negative."}, "language_style": {"answer": "Tactful and warm", "rationale": "The language style of the entry is euphemism, because it uses a tone of inquiry and concern, without direct criticism or criticism of the other. It sends a message to the interviewees that they may be experiencing difficulties and trying to encourage them to face and solve them. Moreover, the issue uses the term \"long\" and \"unmanageable\" to describe emotional state, rather than using strong language to express emotions. These features all indicate that the language style of the article is more gentle and close."}, "time_dimension": {"answer": "Past", "rationale": ""}, "sentence_type": "Interrogative sentence", "pos": {"nouns": {"Long time": 1, "you": 2, "Environment": 1}, "verbs": {"I feel": 1, "Handle": 1, "Cannot": 1}, "adverbs": {"very": 1}, "numerals": {"163": 1}}}, {"content": "164. Do you feel nervous and confused when you think about the difficulties you face?", "word_count": 27, "label": "", "sentence_structure": "Subject鈥搗erb鈥搊bject", "subjective_objective": {"answer": "Subjective", "rationale": "Because of stress and confusion, patients feel subjectively when they face difficulties."}, "sentence_sentiment": {"answer": "Negative", "rationale": "Because the description of \"frightful and unattractive\" in the entry is negative."}, "language_style": {"answer": "Tactful and warm", "rationale": "Warm and warm."}, "time_dimension": {"answer": "Present", "rationale": ""}, "sentence_type": "Interrogative sentence", "pos": {"nouns": {"Difficulties": 1, "Time": 1, "you": 2, "self": 1, "Faced": 1, "Nervous": 1, "I don't know what to do": 1}, "verbs": {"Yes": 1, "I feel": 1, "Is it possible": 1, "Sometimes": 1, "I think": 1, "Face it": 1, "be": 1, "I don't know what to do": 1, "Faced": 1}, "adjectives": {"Nervous": 1, "I don't know what to do": 1}, "adverbs": {"Time": 1, "Is it possible": 1, "Sometimes": 1}, "numerals": {"164": 1, "1": 3}, "pronouns": {"you": 2, "self": 1}}}, {"content": "Do you always have to do your hair and clothes before you come in?", "word_count": 22, "label": "", "sentence_structure": "Subject鈥搗erb鈥搊bject", "subjective_objective": {"answer": "Subjective", "rationale": "Because the preparation of hair and clothing is the patient ' s personal habits and attitudes, it is a subjective act."}, "sentence_sentiment": {"answer": "Neutral", "rationale": "Because the entry describes a person ' s behaviour prior to visiting another person, there is no apparent positive or negative emotional colour."}, "language_style": {"answer": "Tactful and warm", "rationale": "The entry is more personal and interactive when it is spoken in a gentle, inquisitive manner, rather than in direct statements or orders. It also does not use any negative terminology or stress that the other party must do so in the prescribed manner, but rather in a more understanding and supportive manner."}, "time_dimension": {"answer": "Present", "rationale": "The keyword \"visiting\" and \"before entering\" and \"whether or not to always\" indicate that this is a question of what is happening so often. It can therefore be concluded that the entry to the questionnaire looks at the present behaviour or habits of the person being tested."}, "sentence_type": "Interrogative sentence", "pos": {"nouns": {"165": 1, "Visiting": 1, "others": 1, "Door": 1, "Hair": 1, "Clothes": 1}, "verbs": {"Collapse": 1, "In": 1}, "adverbs": {"It's gotta be": 1}, "numerals": {"165": 1}, "classifiers": {"Number of times": 1}}}, {"content": "166. Do you often find it difficult to control your life?", "word_count": 17, "label": "", "sentence_structure": "Subject鈥搗erb鈥搊bject", "subjective_objective": {"answer": "Subjective", "rationale": "Because it is subjective for the patient to feel and feel about his or her own living conditions."}, "sentence_sentiment": {"answer": "Negative", "rationale": "Because the attitude of \"hard to control the direction of life\" in the entries is negative."}, "language_style": {"answer": "Calm and direct", "rationale": "The language style of the entry is calmer and more direct, without the use of excessive rhetorical or emotional characterizations. It is more concise and clear in its ability to obtain accurate information in an objective and clear tone that asks whether the interviewees often feel unable to control their own direction of life."}, "time_dimension": {"answer": "Present", "rationale": "\"Do you often find it difficult to control your life?\" The word \"normal\" in \"always\" denotes a regular, customary behaviour, and \"thinks\" adopts the general current, which means that this behaviour has not only happened in the past, but may still be ongoing. So the entry asks the testee about the current behaviour and mindset, and the temporal dimension is now."}, "sentence_type": "Interrogative sentence", "pos": {"nouns": {"Questionnaire Entry": 1, "you": 1, "Life course": 1}, "verbs": {"I think": 1, "Control": 1}, "adjectives": {"Regular": 1, "Hard": 2, "of": 2}, "numerals": {"166": 1}, "classifiers": {"individual": 1}}}, {"content": "167. Do you think it is a waste of time to go to see the disease because of mild discomfort, such as coughing, cold, cold?", "word_count": 27, "label": "", "sentence_structure": "Subject鈥搗erb鈥搊bject", "subjective_objective": {"answer": "Subjective", "rationale": "Because the question relates to patients' subjective perception and attitude about whether they are wasting their time with doctors."}, "sentence_sentiment": {"answer": "Negative", "rationale": "Because the idea in the entry that \"a slight discomfort, such as cough, cold, cold, go to see a sick person, is a waste of time\" is negative."}, "language_style": {"answer": "Calm and direct", "rationale": "The entry is based on a direct question-and-answer dialogue, without too much rhetorical or emotional presentation, and is more objective in tone. At the same time, the question is not one in which any euphemism is used, but rather one in which the respondent is guided by a clear question."}, "time_dimension": {"answer": "Present", "rationale": "The keyword \"is it\" indicates that it is a question of the testee's current perception and attitude, and both \"for a slight discomfort\" and \"for a medical visit is a waste of time\" point to the present situation."}, "sentence_type": "Interrogative sentence", "pos": {"nouns": {"Questionnaire": 1, "Entry": 1, "Time": 1, "I'm not feeling well": 2, "Cough": 1, "It's cold": 1, "Cold": 1, "- I'm in the hospital": 1}, "verbs": {"I think": 1, "be": 1, "Wasted": 1}, "adjectives": {"Light": 1, "I'm not feeling well": 2}, "numerals": {"167": 1, "2": 1}, "classifiers": {"individual": 1}}}, {"content": "168. Do you often feel like you have done something wrong, even though there is no real basis for that feeling?", "word_count": 28, "label": "", "sentence_structure": "Subject鈥搗erb鈥搊bject", "subjective_objective": {"answer": "Subjective", "rationale": "Because it is the subjective feeling of the patient that feels wrong. Although there is no real basis for it, it is still generated and experienced by the patient himself, and therefore is subjective in its perspective."}, "sentence_sentiment": {"answer": "Negative", "rationale": "Because the \"wrong\" feeling in the entry is negative."}, "language_style": {"answer": "Calm and direct", "rationale": "This is often used in such contexts as questionnaires, psychological tests, etc., to maintain objectivity and accuracy."}, "time_dimension": {"answer": "Past", "rationale": "\"At times, it is a feeling\" of past customary behaviour, \"although it is not true?\" It is a feeling that has existed for some time. The entry therefore asks about the behaviour and feelings of the person who was tested in the past."}, "sentence_type": "Interrogative sentence", "pos": {"nouns": {"168": 1, "Things": 2, "Based on": 1}, "verbs": {"I feel": 1, "Wrong": 1, "I think so": 1, "have": 1, "Indeed": 1}, "adverbs": {"Often": 1, "鈾?Though 鈾?: 1}, "numerals": {"168": 1, "2": 1, "1": 3}, "classifiers": {"Annex": 1}, "pronouns": {"you": 1, "I feel like": 1}}}, {"content": "169. Do you find it very difficult to do things to win the attention and praise of others?", "word_count": 24, "label": "", "sentence_structure": "Subject鈥搗erb鈥搊bject", "subjective_objective": {"answer": "Subjective", "rationale": "Because the words \"fright\" and \"very difficult\" are the feelings and perceptions of the patient personally and are subjective."}, "sentence_sentiment": {"answer": "Negative", "rationale": "Because the purpose of \"to gain the attention and praise of others\" in the entry is described as \"very difficult\", which is a negative emotional attitude."}, "language_style": {"answer": "Tactful and warm", "rationale": ""}, "time_dimension": {"answer": "Present", "rationale": "The entry asks about the current feelings and perceptions of the subject, the keyword \"you think\" and \"very difficult\" and all point to what is happening. So the answer is \"now\"."}, "sentence_type": "Interrogative sentence", "pos": {"nouns": {"Questionnaire": 1, "Entry": 1, "others": 1, "Attention": 1, "- Congratulations": 1, "Work": 2}, "verbs": {"I think": 1, "Winning": 1, "Very": 1, "Difficulties": 1}, "adverbs": {"For": 1, "Land": 1}, "numerals": {"169": 1}, "pronouns": {"you": 1}}}, {"content": "170, look back. Do you feel deceived?", "word_count": 13, "label": "", "sentence_structure": "Subject鈥搗erb鈥搊bject", "subjective_objective": {"answer": "Subjective", "rationale": "The subject is the patient鈥檚 feelings and perceptions of memory, and therefore his subjective thoughts. Even if the facts of the deception are objective, the sense and perception of deception are individual subjective experiences, and therefore the subject matter of this article is subjective."}, "sentence_sentiment": {"answer": "Negative", "rationale": "Because the \"smuggling\" experience in the entry is negative."}, "language_style": {"answer": "Calm and direct", "rationale": "The entry answers the question in a direct and clear tone, without using excessive rhetorical or emotional rendering, expressing it in a more objective and idiosyncratic manner."}, "time_dimension": {"answer": "Past", "rationale": "In this entry, \"recalling back\" means looking back and thinking about the past, and \"does it feel deceived?\" is an evaluation of the past's behaviour or events. Thus, the temporal dimension of the article can be judged to be the past."}, "sentence_type": "Interrogative sentence", "pos": {"nouns": {"The old days": 1, "- Liar": 1}, "verbs": {"Look back": 1, "I think": 1}, "numerals": {"170": 1}}}, {"content": "171. Has it been a long time since you were humiliated?", "word_count": 13, "label": "", "sentence_structure": "Subject鈥搗erb鈥搊bject", "subjective_objective": {"answer": "Subjective", "rationale": "Because it's a subjective feeling and emotional experience to feel uncomfortable."}, "sentence_sentiment": {"answer": "Negative", "rationale": "Because the \"shampering you for a long time\" in the entry is negative."}, "language_style": {"answer": "Tactful and warm", "rationale": "The entry is more euphemistic, is more intrusive and interactive, and gives respondents greater willingness to cooperate and to respond to questions. At the same time, it uses more objective language to describe the impact of humiliation on people and avoids unnecessary psychological burdens on the interviewee."}, "time_dimension": {"answer": "Present", "rationale": "The entry \"shambleded\" and \"worried you for a long time\"? describes an act and its effect on the mood of the testee, and the temporal dimension is now."}, "sentence_type": "Interrogative sentence", "pos": {"nouns": {"Humiliation": 1, "Sorry": 1, "It's been a long time": 1}, "verbs": {"鈾?And I'm gonna be 鈾?: 1, "Yes": 1, "It's hard": 1, "Long": 1}, "adverbs": {"very": 2}, "numerals": {"171": 1}}}, {"content": "172, do you always try to correct the grammatical error of others when you talk to others, although courtesy may not allow it?", "word_count": 34, "label": "", "sentence_structure": "Subject鈥搗erb鈥搊bject", "subjective_objective": {"answer": "Subjective", "rationale": "This is a description of the specific behaviour and attitudes of patients in social interaction."}, "sentence_sentiment": {"answer": "Negative", "rationale": "Because the word \"correcting other people's grammatical errors\" in the entry is critical and negative. Although it is mentioned in the context of the words \"biteness may not allow it,\" it is still negative in itself."}, "language_style": {"answer": "Calm and direct", "rationale": "The language style of the entry is a calm and direct type, because it raises a question in a direct way and asks the interviewee to answer. It does not use any rhetorical or emotional colour to attract the reader鈥檚 interest or emotional reaction. On the contrary, it is neutral and objective, and asks the interviewee in a direct way whether they have a specific pattern of behaviour."}, "time_dimension": {"answer": "Present", "rationale": "The entry describes the behaviour of the person who was tested during the communication with others, i.e., attempts to correct the grammatical error of others. Although such behaviour may be considered uncivil, the person who was tested still chooses to do it. So the expressions \"always try\" and \"even though courtesy may not allow it to do so\" indicate that this is what is happening now.\""}, "sentence_type": "Interrogative sentence", "pos": {"nouns": {"person": 2, "others": 1, "Talk": 1, "you": 1, "Always": 1, "Try": 1, "Correct": 1, "Syntax Error": 1, "鈾?Though 鈾?: 1, "Courage": 1, "Maybe": 1, "Allow": 1, "Do it": 1, "- You're not": 1, "EMPTY": 1}, "verbs": {"and": 1, "Say it": 1, "Try": 1, "Correct": 1, "Allow": 1, "Do it": 1}, "adjectives": {"Always": 1, "Courage": 1, "Maybe": 1}, "adverbs": {"Is it possible": 1, "鈾?Though 鈾?: 1}, "numerals": {"172": 1}, "pronouns": {"you": 1}}}, {"content": "173. Do you think things are so unpredictable that they can't be found?", "word_count": 25, "label": "", "sentence_structure": "Subject鈥搗erb鈥搊bject", "subjective_objective": {"answer": "Subjective", "rationale": "Because it is the patient's feeling and understanding of the current situation."}, "sentence_sentiment": {"answer": "Negative", "rationale": "Because the entries \"uncertain\" and \"unable to find the law\" express a negative attitude."}, "language_style": {"answer": "Tactful and warm", "rationale": ""}, "time_dimension": {"answer": "Present", "rationale": "The keywords \"what's happening now\" and \"what's not happening\" indicate that it's asking the testee what it feels and what it feels like, and it's judging now."}, "sentence_type": "Interrogative sentence", "pos": {"nouns": {"Things": 1, "Change": 1, "Pattern": 1}, "verbs": {"I think": 1, "I can't find it": 1}, "adjectives": {"I don't know": 1, "It's just": 1}, "numerals": {"173": 1}, "pronouns": {"you": 1}}}, {"content": "174, if you have a cold, will you go to bed right away?", "word_count": 16, "label": "", "sentence_structure": "Subject鈥損redicate", "subjective_objective": {"answer": "Subjective", "rationale": "Because it is a subjective judgement and behavioural choice of the patient to have a cold and to go to bed immediately."}, "sentence_sentiment": {"answer": "Positive", "rationale": "Because the behavior in the entry \"right to bed\" is positive, indicating a concern for and care for their physical condition."}, "language_style": {"answer": "Calm and direct", "rationale": "The language style of the entry is more calm and straightforward, and there is no excessive emotional rendering or rhetorical technique, but rather questions are asked in a concise manner that allows the person surveyed to understand and respond with clarity."}, "time_dimension": {"answer": "Past", "rationale": "The entry is for the testee ' s behavioural reaction when assuming that he/she has a cold, and the temporal dimension is a past."}, "sentence_type": "Interrogative sentence", "pos": {"nouns": {"Cold": 1, "The bed": 1, "Rest": 1}, "verbs": {"Okay": 1, "Go, go, go": 2, "If": 1, "you": 3, "Now": 1}, "adverbs": {"Is it possible": 1, "Now": 1}, "numerals": {"174": 1}, "pronouns": {"you": 3}}}, {"content": "175. Are you disappointed that your teacher did not have enough to prepare for his classes?", "word_count": 22, "label": "", "sentence_structure": "Subject鈥搗erb鈥搊bject", "subjective_objective": {"answer": "Objective", "rationale": "Because the questionnaire entry asks about the patient's feelings about the teacher's inadequate preparation, which is an objective fact, not about the patient's subjective attitude or feelings. The answer is therefore \"objective\"."}, "sentence_sentiment": {"answer": "Negative", "rationale": "Because the \"disappointed with the teacher\" act in the entry is negative."}, "language_style": {"answer": "Tactful and warm", "rationale": "This is a more intrusive form of expression that avoids direct accusations or criticisms of teachers."}, "time_dimension": {"answer": "Past", "rationale": "\"Was Disappointing during Classes\" is a past event, so the time dimension of this entry is the past."}, "sentence_type": "Interrogative sentence", "pos": {"nouns": {"175": 1, "you": 1, "Teacher": 1, "Disappointment": 1}, "verbs": {"I feel": 1, "Disappointment": 1, "Is it possible": 1, "Because": 1, "Classes": 1, "he": 1, "And": 1, "Disappointed": 2}, "adjectives": {"Full": 1}, "numerals": {"175": 1}, "pronouns": {"you": 1, "Teacher": 1, "he": 1}}}, {"content": "176. Do you often think that you are better than you actually are?", "word_count": 17, "label": "", "sentence_structure": "Subject鈥搗erb鈥搊bject", "subjective_objective": {"answer": "Subjective", "rationale": "Because \"think better than you're actually.\" It's the patient's subjective idea."}, "sentence_sentiment": {"answer": "Negative", "rationale": "Because the \"self-imagining\" in the entry is a fictional and not a practical reality."}, "language_style": {"answer": "Tactful and warm", "rationale": "\"Slow and warm.\""}, "time_dimension": {"answer": "Present", "rationale": ""}, "sentence_type": "Interrogative sentence", "pos": {"nouns": {"Questionnaire": 1, "Entry": 1, "person": 1}, "verbs": {"Scenario": 1, "- Yeah": 1, "Okay": 1}, "adjectives": {"Actual": 1}, "adverbs": {"Often": 1, "More": 1}, "numerals": {"176": 1}, "pronouns": {"self": 1}}}, {"content": "177. Are you happy with the rest of the world?", "word_count": 12, "label": "", "sentence_structure": "Subject鈥搗erb鈥搊bject", "subjective_objective": {"answer": "Subjective", "rationale": "Because the question is whether the patient feels as happy as others in his or her life, it is a subjective feeling."}, "sentence_sentiment": {"answer": "Neutral", "rationale": "Because the entry does not have any emotional color."}, "language_style": {"answer": "Tactful and warm", "rationale": "The language style of the entry is euphemism. It uses a gentle and inviting tone, which is more personal and interactive, by asking the interviewee about his living conditions in an open manner, rather than directly speaking or ordering. It also does not have too much rhetorical or emotional presentation, which is more objective and gives people a sense of sincerity and goodwill."}, "time_dimension": {"answer": "Present", "rationale": "\"Are you happy with living like everyone else?\" The question is mainly about the current living conditions of the testees, so it is possible to judge the entry as it is."}, "sentence_type": "Interrogative sentence", "pos": {"nouns": {"you": 1, "others": 1, "Life": 1, "Happy": 1}, "verbs": {"Live": 1}, "adverbs": {"and": 1, "Same thing": 1}, "numerals": {"177": 1}}}, {"content": "178. Can you know yourself by describing yourself?", "word_count": 15, "label": "", "sentence_structure": "Subject鈥搗erb鈥搊bject", "subjective_objective": {"answer": "Subjective", "rationale": "The subject is examined by the patient ' s own self-awareness and is subjective."}, "sentence_sentiment": {"answer": "Neutral", "rationale": "Because the entry does not have any emotional color."}, "language_style": {"answer": "Tactful and warm", "rationale": "The entry is more adept and interactive when it is spoken in a gentle and inviting manner, rather than by direct statements or orders."}, "time_dimension": {"answer": "Present", "rationale": "The entry asks if the person tested is able to understand his or her situation by describing himself, and thus judges it as \"now\"."}, "sentence_type": "Interrogative sentence", "pos": {"nouns": {"178": 1, "self": 2}, "verbs": {"Yes": 2, "Pass": 1}, "adverbs": {"Come on": 1}, "numerals": {"1": 1}, "pronouns": {"you": 1}}}, {"content": "179. Do you describe yourself as a perfect person?", "word_count": 15, "label": "", "sentence_structure": "Subject鈥搗erb鈥搊bject", "subjective_objective": {"answer": "Subjective", "rationale": "Because those who answer \"perfect\" or \"incompetent\" are describing their views and feelings, which are the expression of their subjective perception."}, "sentence_sentiment": {"answer": "Negative", "rationale": "Because the word \"perfect\" in the entries is often considered too ideal and has a negative emotional connotation."}, "language_style": {"answer": "Calm and direct", "rationale": "The entry is made in a simple and clear language, without too much rhetorical or emotional overturning, and asks whether the interviewee considers himself to be a perfect individual. This straightforward inquiry is more objective and professional, avoiding the interference of subjective factors and facilitating the collection of more accurate data."}, "time_dimension": {"answer": "Present", "rationale": "The keyword \"indicates\" and \"in-person\" both to the current behaviour or state, so the entry asks the person who was tested for the present behaviour or state."}, "sentence_type": "Interrogative sentence", "pos": {"nouns": {"Entry": 1, "person": 1, "Perfect": 1}, "verbs": {"- Put it on": 1, "Description": 1}, "adjectives": {"Perfect": 1}, "numerals": {"179": 1}, "pronouns": {"you": 1}}}, {"content": "180. Do you always have a clear purpose in life?", "word_count": 12, "label": "", "sentence_structure": "Subject鈥搗erb鈥揷omplement", "subjective_objective": {"answer": "Subjective", "rationale": "The approach and belief of individuals in the life goals are viewed from a subjective perspective."}, "sentence_sentiment": {"answer": "Positive", "rationale": "Because the entry \"Always have a clear purpose for life\" expresses positive qualities and attitudes of the individual."}, "language_style": {"answer": "Calm and direct", "rationale": "The entry asks the respondents in a straightforward manner whether they often set and pursue their life goals, and the language style is more calm and objective, without too much emotional overturning or euphemism."}, "time_dimension": {"answer": "Present", "rationale": "The entry is asking the testee about his current state of life and attitude, the keyword being \"always\" and \"have a clear life goal\", which all indicate the current situation."}, "sentence_type": "Interrogative sentence", "pos": {"nouns": {"Life": 1, "Objective": 1}, "verbs": {"Always": 1, "have": 1}, "adverbs": {"Always": 1}, "numerals": {"180, you": 1}}}, {"content": "181, did you see the color of your tongue often in the morning?", "word_count": 14, "label": "", "sentence_structure": "Subject鈥搗erb鈥搊bject", "subjective_objective": {"answer": "Subjective", "rationale": "Because \"watching the tongue colour in the morning\" is the patient's own choice and habit, not the condition of objective existence."}, "sentence_sentiment": {"answer": "Neutral", "rationale": "Because the entry does not have any emotional color."}, "language_style": {"answer": "Calm and direct", "rationale": "The entry raises questions in a straightforward manner, without using euphemism or vague language. This simple and clear expression is more likely to allow respondents to understand and answer questions with precision, and therefore to be in a calm and straightforward language style."}, "time_dimension": {"answer": "Present", "rationale": ""}, "sentence_type": "Interrogative sentence", "pos": {"nouns": {"Morning": 1, "you": 2, "tongue": 1, "Colour": 1}, "verbs": {"I'm always watching": 1}, "adverbs": {"Is it possible": 1, "Regular": 1}, "numerals": {"181銆?: 1, "1": 1}, "pronouns": {"you": 2}}}, {"content": "182. Do you often think that you've been too bad at people before when you remember the past?", "word_count": 23, "label": "", "sentence_structure": "Subject鈥搗erb鈥搊bject", "subjective_objective": {"answer": "Subjective", "rationale": "Because the subject is about the patient's own perception and feelings, which are \"like to feel too bad about others before,\" it's an examination of subjective attitudes or emotional experiences."}, "sentence_sentiment": {"answer": "Negative", "rationale": "Because the entry \"likes to feel bad about others\" expresses a negative and self-responsible attitude towards yourself."}, "language_style": {"answer": "Tactful and warm", "rationale": ""}, "time_dimension": {"answer": "Past", "rationale": "In terms of time dimension, the entry mainly relates to the past period. The output should therefore be \"the past\"."}, "sentence_type": "Interrogative sentence", "pos": {"nouns": {"Memories": 1, "Past": 1, "self": 1, "others": 1}, "verbs": {"I think": 1, "Treatment": 1, "Too much": 1, "Not good": 1}, "adverbs": {"Regular": 1}, "numerals": {"182": 1}, "pronouns": {"you": 1}}}, {"content": "183, do you ever feel like you've never done anything good?", "word_count": 17, "label": "", "sentence_structure": "Subject鈥搗erb鈥搊bject", "subjective_objective": {"answer": "Subjective", "rationale": "Because feeling like you're not doing good is a subjective feeling or an idea."}, "sentence_sentiment": {"answer": "Negative", "rationale": "Because the phrase \"never done anything good\" in the entry is negative, expressing self-denial and negative feelings."}, "language_style": {"answer": "Calm and direct", "rationale": "The language style of the entry is more direct, concise, and not too much qualifier or emotional colour, but rather a simple and straightforward question of whether the other person has such a feeling."}, "time_dimension": {"answer": "Present", "rationale": "The question \"Do you ever feel that the word \"time\" means \"frequency of behavior,\" \"You've never done anything good?\" suggests that the situation is being thought and assessed, so the entry is about the situation now."}, "sentence_type": "Interrogative sentence", "pos": {"nouns": {"183": 1, "you": 2, "Time": 1, "Yes": 1, "Good news": 1}, "verbs": {"I think": 1, "Do it": 1}, "adverbs": {"Sometimes": 1}, "numerals": {"183": 1, "2": 1, "1": 4}, "pronouns": {"you": 2, "Any": 1}}}, {"content": "184. Do you often feel like you're a redundant person in life?", "word_count": 16, "label": "", "sentence_structure": "Subject鈥搗erb鈥搊bject", "subjective_objective": {"answer": "Subjective", "rationale": "Because it is a subjective feeling and a perception that it is superfluous."}, "sentence_sentiment": {"answer": "Negative", "rationale": "Because the idea of \"I feel you're a redundant person in life\" in the entry is negative."}, "language_style": {"answer": "Tactful and warm", "rationale": "The entry uses a more euphemistic approach, not directly asking the other side whether it feels that it is superfluous, but rather, in an understanding and caring attitude, to guide the interviewee to think and express his feelings. Such expression is more acceptable and can reduce the resistance that the interviewee may have caused by being offended."}, "time_dimension": {"answer": "Present", "rationale": ""}, "sentence_type": "Interrogative sentence", "pos": {"nouns": {"184": 1, "you": 2, "person": 1, "Life": 1, "Overload": 1}, "verbs": {"I feel": 1, "be": 2, "Overload": 1}, "adjectives": {"Regular": 1}, "numerals": {"184": 1, "2": 1}, "pronouns": {"you": 2, "Life": 1, "person": 1}}}, {"content": "185. Are you worried about what might happen?", "word_count": 18, "label": "", "sentence_structure": "Subject鈥搗erb鈥搊bject", "subjective_objective": {"answer": "Subjective", "rationale": "Because this issue concerns the patient ' s fears and anxiety for the future, it is his subjective idea."}, "sentence_sentiment": {"answer": "Negative", "rationale": "Because the \"mind the unnecessary\" act in the entry is negative."}, "language_style": {"answer": "Calm and direct", "rationale": "The language style of the entry is of a calm and direct type, and it directly asks the interviewee whether there are situations of fear or anxiety, and there is no excessive emotional overturning or use of euphemism.\""}, "time_dimension": {"answer": "Present", "rationale": "The entry asks whether the testee has a current habit or tendency to be too concerned about what might happen in the future, whether the keyword \"is\" indicates that it is selective, while \"is going to happen\" indicates that it is a future expectation. Thus, the temporal dimension of the article can be judged as it is now."}, "sentence_type": "Interrogative sentence", "pos": {"nouns": {"Yes": 2, "Heart": 2}, "verbs": {"Come on": 1, "Fuck": 1, "Maybe": 1}, "adjectives": {"It's not necessary": 1}, "adverbs": {"Yes": 1}, "numerals": {"185": 1}}}, {"content": "186. When trouble prevents you from sleeping, do you leave the bed as you have done?", "word_count": 24, "label": "", "sentence_structure": "Subject鈥搗erb鈥搊bject", "subjective_objective": {"answer": "Objective", "rationale": "Since the question is about the behaviour of the patient (whether or not there is a habit of leaving the bed), it is about the patient ' s objective behaviour, not his subjective thoughts or feelings. The purpose of this article is therefore to be objective."}, "sentence_sentiment": {"answer": "Negative", "rationale": "Because \"fearful things\" and \"leave the bed\" in the entries imply negative emotions and behavior."}, "language_style": {"answer": "Tactful and warm", "rationale": ""}, "time_dimension": {"answer": "Present", "rationale": "\"When the trouble is making you sleep\" is part of a description of a status quo or customary behaviour, which shows that the person tested has a certain pattern of behaviour when facing sleep problems. So, \"now\" is the temporal dimension of this questionnaire entry."}, "sentence_type": "Interrogative sentence", "pos": {"nouns": {"Things": 1, "Trouble": 1, "Sleeping in the bed": 1}, "verbs": {"鈾?And I'm gonna be 鈾?: 1, "Cannot": 1, "Sleep": 1, "According to": 1, "Leave": 1}, "adjectives": {"Custom": 1}, "numerals": {"186": 1}}}, {"content": "187, do you often feel that people are using you?", "word_count": 13, "label": "", "sentence_structure": "Subject鈥搗erb鈥搊bject", "subjective_objective": {"answer": "Subjective", "rationale": "Because it is a subjective feeling or opinion that is used."}, "sentence_sentiment": {"answer": "Negative", "rationale": "Because the expression \"likes someone else is using you\" in the entry implies negative emotions and attitudes."}, "language_style": {"answer": "Calm and direct", "rationale": "The language style of the entry is more calm and straightforward, and the interviewee is asked whether he or she is often perceived to be used by others."}, "time_dimension": {"answer": "Present", "rationale": ""}, "sentence_type": "Interrogative sentence", "pos": {"nouns": {"you": 1, "person": 1, "Entry": 1, "Utilization": 1}, "verbs": {"Feel": 1, "Often": 1, "be": 1}, "adverbs": {"Often": 1}, "numerals": {"187": 1}}}, {"content": "188. Do you weigh every day?", "word_count": 8, "label": "", "sentence_structure": "Subject鈥搗erb鈥搊bject", "subjective_objective": {"answer": "Objective", "rationale": "Because \"weight every day\" is an objective act or fact, it is not a subjective attitude of the patient. This is therefore considered from an objective perspective."}, "sentence_sentiment": {"answer": "Neutral", "rationale": "Because the entry does not address emotions, attitudes or behaviour, it merely asks about a daily custom."}, "language_style": {"answer": "Calm and direct", "rationale": "The tone of the question is more objective, and it is not too emotional or rhetorical to ask whether the person surveyed is weighing every day. This is a straightforward way to make the question more clear to the interviewer and to answer it more quickly."}, "time_dimension": {"answer": "Past", "rationale": "The entry asks about the testee ' s past habits or behaviour, i.e. whether they weigh weight every day, and therefore the temporal dimension is a past."}, "sentence_type": "Interrogative sentence", "pos": {"nouns": {"Oh, my God": 1, "Weight": 1}, "verbs": {"Both": 1, "Claims": 1, "- You're not": 1}, "adverbs": {"Chile": 1}, "numerals": {"188": 1}}}, {"content": "189. Do you expect God to punish you in the next life?", "word_count": 16, "label": "", "sentence_structure": "Subject鈥搗erb鈥搊bject", "subjective_objective": {"answer": "Subjective", "rationale": "Because \"expectation\" is patient's subjective attitude."}, "sentence_sentiment": {"answer": "Negative", "rationale": "Because the attitude in the entry, \"I hope God will punish you in the next life,\" is negative."}, "language_style": {"answer": "Calm and direct", "rationale": "The language style of the entry is \"cool and direct\" and it asks the interviewee questions about religious beliefs and ethics in a direct and clear manner, without too much emotional or rhetorical."}, "time_dimension": {"answer": "Future", "rationale": ""}, "sentence_type": "Interrogative sentence", "pos": {"nouns": {"Jesus": 1, "In the next life": 1, "Sin": 1}, "verbs": {"Expectations": 1, "Punishment": 1}, "numerals": {"189": 1}}}, {"content": "190. Do you often question your sexual abilities?", "word_count": 12, "label": "", "sentence_structure": "Subject鈥損redicate", "subjective_objective": {"answer": "Subjective", "rationale": "Because suspicion is a subjective emotion or attitude."}, "sentence_sentiment": {"answer": "Negative", "rationale": "Because the attitude of \"suspecting your sexual ability\" is negative."}, "language_style": {"answer": "Calm and direct", "rationale": "The language style of the entry is more direct and explicit in asking the other side whether it has self-doubt about sexual ability, without excessive ado or emotional colours, and with more objective and quiet tone."}, "time_dimension": {"answer": "Present", "rationale": "\"Always\" denotes a regular act, and \"suspicious\" uses the general current, which means that the act has not only occurred in the past but may still be ongoing. The entry is therefore an inquiry into the behaviour and mentality of the person being tested."}, "sentence_type": "Interrogative sentence", "pos": {"nouns": {"Sexuality": 1, "Doubt": 1, "you": 2}, "verbs": {"be": 1, "Often": 1, "have": 1}, "adverbs": {"very": 1, "Often": 1}, "numerals": {"190": 1}, "pronouns": {"Yours": 1}}}, {"content": "191. Is your sleep usually irregular?", "word_count": 12, "label": "", "sentence_structure": "Subject鈥搗erb鈥搊bject", "subjective_objective": {"answer": "Subjective", "rationale": "Because sleep patterns are a personal habit and a sense of feeling, a subjective view of the patient."}, "sentence_sentiment": {"answer": "Negative", "rationale": "Because the word \"irregular\" in the entry expresses a negative emotional tone and implies poor sleep quality."}, "language_style": {"answer": "Tactful and warm", "rationale": "The entry is more proficient and interactive in its expression of a moderate and inviting tone of speech, rather than a direct statement or order. At the same time, terms such as \"usually\" also reflect euphemism, without overly subjective or accusing the other party of his or her actions."}, "time_dimension": {"answer": "Present", "rationale": "The entry is an inquiry into the current behaviour or state of the person being tested, and the keyword \"usually\" indicates that it is a normal practice, not a temporary act, and therefore the judgement is now."}, "sentence_type": "Interrogative sentence", "pos": {"nouns": {"Sleep": 1, "Usually": 1, "No, no, no, no": 1}, "verbs": {"be": 1, "- You're not": 1}, "adjectives": {"- You're not": 1}, "numerals": {"191": 1}, "classifiers": {"Usually": 1, "of": 2, "you": 1}, "pronouns": {"you": 1}}}, {"content": "192. Do you often get excited for no reason?", "word_count": 15, "label": "", "sentence_structure": "Subject鈥搗erb鈥搊bject", "subjective_objective": {"answer": "Subjective", "rationale": "Because the description \"often without cause\" involves the patient's own emotional experience and behavioural reaction, and is the patient's subjective feeling."}, "sentence_sentiment": {"answer": "Negative", "rationale": "Because the \"excited without cause\" act in the entry is negative."}, "language_style": {"answer": "Calm and direct", "rationale": "The entry asks the other in a direct and objective manner whether the other party is in a state of emotional breakdown, without too many rhetorical or emotional colours, and is more calm in tone."}, "time_dimension": {"answer": "Present", "rationale": "The keywords \"often\" and \"beginning\" indicate that it is a regular or customary act without a clear time reference point and should therefore be judged now."}, "sentence_type": "Interrogative sentence", "pos": {"nouns": {"Questionnaire Entry": 1, "you": 1, "Reason": 1, "Excited": 1}, "verbs": {"鈾?And become 鈾?: 1, "have": 1}, "adjectives": {"For no reason": 1}, "numerals": {"192": 1}}}, {"content": "193. Is it essential for you to keep it neat and orderly?", "word_count": 17, "label": "", "sentence_structure": "Subject鈥揷opula鈥損redicative", "subjective_objective": {"answer": "Subjective", "rationale": "Because the question is one of personal perceptions and attitudes, it requires the patient to respond to the situation on his or her own merits and is therefore subjective."}, "sentence_sentiment": {"answer": "Positive", "rationale": "Because keeping it neat and orderly is a positive attitude and behaviour."}, "language_style": {"answer": "Tactful and warm", "rationale": "The entry is more adept and interactive when it is spoken in a gentle and inviting manner, rather than by direct statements or orders."}, "time_dimension": {"answer": "Present", "rationale": "The entry asks the testee about his or her attitude and perception of maintaining a clean and orderly approach, which is the current state and the thinking, and thus the judgement is the present."}, "sentence_type": "Interrogative sentence", "pos": {"nouns": {"Clean": 1, "Order": 1}, "verbs": {"Hold on": 1}, "adjectives": {"Important": 1, "you": 1}, "adverbs": {"Come on": 1, "be": 1, "to": 1}, "numerals": {"193": 1, "To the end": 1}}}, {"content": "194. Have you ever been influenced by advertising to buy something you don't actually want to buy?", "word_count": 26, "label": "", "sentence_structure": "Subject鈥搗erb鈥搊bject", "subjective_objective": {"answer": "Subjective", "rationale": "The question is whether the patient himself is affected by advertising to purchase unwanted goods, and is an examination of the patient ' s subjective ideas and behaviour."}, "sentence_sentiment": {"answer": "Negative", "rationale": "Because the \"getting what you don't need because of advertising\" in the entry is negative."}, "language_style": {"answer": "Calm and direct", "rationale": "The entry is directly asked in a simple and clear language, without too many rhetorical or emotional renderings, and is more calm and direct in language style.\""}, "time_dimension": {"answer": "Present", "rationale": ""}, "sentence_type": "Interrogative sentence", "pos": {"nouns": {"Questionnaire": 1, "Entry": 1, "Impact": 2, "person": 1, "Something": 2}, "verbs": {"Yes": 2, "Buy": 2, "be": 1}, "adjectives": {"Actual": 1, "and": 1, "Yes": 1}, "adverbs": {"Sometimes": 1, "And": 1}, "numerals": {"194": 1, "Some": 1}, "pronouns": {"you": 2}}}, {"content": "195. Are you often upset by noise?", "word_count": 11, "label": "", "sentence_structure": "Subject鈥搗erb鈥搊bject", "subjective_objective": {"answer": "Subjective", "rationale": "Because \"fear\" is the feeling and the thought inside the patient, and it's subjective."}, "sentence_sentiment": {"answer": "Negative", "rationale": "Because the \"noise-freak\" behavior in the entry is negative."}, "language_style": {"answer": "Calm and direct", "rationale": "The entry raises questions in a concise and clear statement, without too many rhetorical or emotional colours, and appears to be calm and objective.\""}, "time_dimension": {"answer": "Present", "rationale": "\"195\" is a number, \"Do you often\" means the person who was tested, his habits or feelings, and \"noise-fearing\" is the dominant word and the word, which are key words for the person being tested's present state and behaviour. The entry therefore asks about the person's current state and behaviour, which is the current state."}, "sentence_type": "Interrogative sentence", "pos": {"nouns": {"Noise": 1, "Trouble": 1}, "verbs": {"Regular": 1, "Yes": 2, "Trouble": 1}, "adverbs": {"Often": 1}, "numerals": {"195": 1, "1": 4}}}, {"content": "196. If you encounter setbacks in interpersonal relations, do you always blame yourself?", "word_count": 20, "label": "", "sentence_structure": "Subject鈥搗erb鈥搊bject", "subjective_objective": {"answer": "Subjective", "rationale": "Because it's the patient's subjective attitude or idea."}, "sentence_sentiment": {"answer": "Negative", "rationale": "Because the \"criticizing\" act in the entry is negative."}, "language_style": {"answer": "Tactful and warm", "rationale": "The entry is presented in a gentle and understanding way, rather than directly critical or accusing, which is more a source of self-reflection and honest answers from the interviewees."}, "time_dimension": {"answer": "Present", "rationale": "\"If you encounter a setback in interpersonal interaction\" is a hypothetical scenario, and \"always blame yourself\" is a constant mentality or behaviour, so the entry asks about the person's current interpersonal psychological state. So it's now."}, "sentence_type": "Interrogative sentence", "pos": {"nouns": {"Human contact": 1, "Frustration": 1, "Blame": 1}, "verbs": {"I met him": 1, "be": 1, "Blame": 1}, "adverbs": {"Always": 1}, "numerals": {"196": 1}, "pronouns": {"you": 1}}}, {"content": "197, do you have a minimum self-esteem?", "word_count": 9, "label": "", "sentence_structure": "Subject鈥損redicate", "subjective_objective": {"answer": "Subjective", "rationale": "Because self-esteem is the inner feeling and attitude of a person, it is subjective."}, "sentence_sentiment": {"answer": "Positive", "rationale": "Because \"self-esteem\" is a positive emotional attitude, expressing respect for and valuing for yourself."}, "language_style": {"answer": "Calm and direct", "rationale": "The language style of the entry is more straightforward, rather than excessive rhetoric or emotional colours, and the expression is more objective and explicit by asking the respondents directly in a concise and clear language about their self-esteem."}, "time_dimension": {"answer": "Present", "rationale": "The keyword \"you\" and \"yes\" context suggests that the entry is to question the present attitude or state of the subject, so it is judged now."}, "sentence_type": "Interrogative sentence", "pos": {"nouns": {"Self-esteem": 1}, "verbs": {"have": 1, "At least": 1}, "adverbs": {"you": 1, "- You're not": 1}, "numerals": {"197": 1}}}, {"content": "198. Do you often feel lonely even when you're with someone else?", "word_count": 20, "label": "", "sentence_structure": "Subject鈥搗erb鈥搊bject", "subjective_objective": {"answer": "Subjective", "rationale": "Because loneliness is a subjective feeling, it depends on the emotional and psychological state of the individual."}, "sentence_sentiment": {"answer": "Negative", "rationale": "Because \"often feeling alone\" in this entry is a negative emotional experience."}, "language_style": {"answer": "Tactful and warm", "rationale": "The language style of the entry is a soft and warm type. It asks in a gentle way, avoiding direct accusations or criticisms. It is more sensitive and interactive, and it is easier to resonate and respond to the interviewee. Nor is the subject too strongly rhetorical or emotionally retroverted, but rather in simpler and plain language, to allow the interviewee to understand and answer the question more intuitively."}, "time_dimension": {"answer": "Present", "rationale": ""}, "sentence_type": "Interrogative sentence", "pos": {"nouns": {"you": 2, "Others": 1, "Time": 1, "Loneliness": 1, "Questionnaire": 1, "Entry": 1}, "verbs": {"Common sense": 1, "Together": 1, "I feel": 1}, "adjectives": {"Other": 1, "Loneliness": 1}, "adverbs": {"Even if": 1, "Yeah": 2}, "numerals": {"198": 1}, "pronouns": {"and": 1}}}, {"content": "199. Did you ever think you needed some sedatives?", "word_count": 15, "label": "", "sentence_structure": "Subject鈥搗erb鈥搊bject", "subjective_objective": {"answer": "Subjective", "rationale": "Because the need for sedatives is a subjective feeling and demand."}, "sentence_sentiment": {"answer": "Negative", "rationale": "Because the act of \"needing some sedatives\" is negative and suggests that there may be mental health problems."}, "language_style": {"answer": "Calm and direct", "rationale": ""}, "time_dimension": {"answer": "Past", "rationale": "The term \" was\" in the entry indicates that it is an inquiry into the testee's past experience and therefore the temporal dimension is a matter of the past."}, "sentence_type": "Interrogative sentence", "pos": {"nouns": {"you": 1, "Once": 1, "Yes": 1, "- Yeah": 1, "Some": 1, "Depressants": 1}, "verbs": {"I think": 1}, "adverbs": {"Once": 1}, "numerals": {"199": 1}, "classifiers": {"Some": 1}, "pronouns": {"you": 1}}}, {"content": "200. Do you feel very unhappy if your life schedule is disrupted by unexpected events?", "word_count": 29, "label": "", "sentence_structure": "Subject鈥搗erb鈥搊bject", "subjective_objective": {"answer": "Subjective", "rationale": "Because \"very bad feeling\" is the patient's subjective emotional reaction."}, "sentence_sentiment": {"answer": "Negative", "rationale": "Because the emotional reaction of \"very unhappy\" in the entry is negative."}, "language_style": {"answer": "Tactful and warm", "rationale": "The language style of the entry is more moderate, and the feelings of the other are not directly questioned or ordered, but rather are expressed in an invitation-style tone, which is more personal and interactive."}, "time_dimension": {"answer": "Present", "rationale": "Now the entry asks the testee's feelings and reactions to unexpected events in the current life, and the time dimension is now judged. The keyword \"if you're being disturbed by unexpected events\" suggests that this is a hypothetical situation, but is it very unpleasant because you used the current state of affairs before?\" So it can be inferred that this is a reality that the testee may encounter."}, "sentence_type": "Interrogative sentence", "pos": {"nouns": {"Life": 1, "Schedule": 1, "Things": 1, "Break it up": 1, "Feel": 1, "Very": 1, "Not fast": 1}, "verbs": {"By": 1, "Break it up": 1, "I feel": 1}, "adjectives": {"Unexpected": 1}, "adverbs": {"200, if you": 1, "- Yeah": 1}, "numerals": {"200": 1}, "pronouns": {"you": 1}}}, {"content": "201, do you predict your future through the vagaries?", "word_count": 17, "label": "", "sentence_structure": "Subject鈥搗erb鈥搊bject", "subjective_objective": {"answer": "Subjective", "rationale": "Because the charade is a subjective act and attitude, it is the patient who chooses it, and it does not affect objective reality."}, "sentence_sentiment": {"answer": "Neutral", "rationale": "Because the entry does not have any emotional color."}, "language_style": {"answer": "Calm and direct", "rationale": "The entry is based on a simple question, which is whether the other party is often engaged in a rhetorical or emotional overtones. This expression is more objective and straightforward, and is consistent with the calm and direct language style."}, "time_dimension": {"answer": "Future", "rationale": "The keyword \"predict your future\" points to a situation that has not yet occurred and that may occur in the future, so the entry is in the future."}, "sentence_type": "Interrogative sentence", "pos": {"nouns": {"Questionnaire": 1, "Entry": 1, "201": 1, "self": 1, "The future": 1}, "verbs": {"Is it possible": 1, "Pass": 1, "- So, guess what": 1, "Projections": 1}, "numerals": {"201": 1}, "pronouns": {"you": 1}}}, {"content": "202. Do you feel something stuck in your throat?", "word_count": 14, "label": "", "sentence_structure": "Subject鈥搗erb鈥搊bject", "subjective_objective": {"answer": "Subjective", "rationale": "Because \"Feel something stuck in your throat\" is the patient's subjective feeling."}, "sentence_sentiment": {"answer": "Neutral", "rationale": "Because it does not involve emotional colour."}, "language_style": {"answer": "Calm and direct", "rationale": "The language style of the entry is a calm and straightforward type, and it asks the questions whether the interviewee feels that there are aliens stuck in his throat, that there are no excessive emotional or speech-writing features, and that the tone is more objective and direct. This type of questionnaire is usually applied to situations where information is quickly collected, and answers are quantified."}, "time_dimension": {"answer": "Present", "rationale": "It is therefore possible to judge that the entry is a description of the current behaviour or mentality."}, "sentence_type": "Interrogative sentence", "pos": {"nouns": {"202": 1, "you": 1, "Something": 1, "Throat": 1}, "verbs": {"I feel": 1, "- Yeah": 1, "in": 1}, "numerals": {"202": 1}, "classifiers": {"Blocks": 1}, "pronouns": {"you": 1}}}, {"content": "203, do you sometimes get sick of your own sexual desires and fantasies?", "word_count": 21, "label": "", "sentence_structure": "Subject鈥搗erb鈥搊bject", "subjective_objective": {"answer": "Subjective", "rationale": "Because feelings of sexual desire and sexual fantasy are the expression of individual subjective experiences."}, "sentence_sentiment": {"answer": "Negative", "rationale": "Because the mood of \"repulsion of your sexual desires and fantasies\" in the entry is negative."}, "language_style": {"answer": "Calm and direct", "rationale": "The entry is in a straightforward manner asking the respondents whether they are averse to their sexual desires and fantasies, without using excessive ados or emotional colours, and with a more calm and straightforward language style."}, "time_dimension": {"answer": "Present", "rationale": "The entry is for the testee ' s current behaviour or mentality, and the time dimension is now judged."}, "sentence_type": "Interrogative sentence", "pos": {"nouns": {"self": 1, "Sex": 1, "Sex fantasy": 1}, "verbs": {"I feel": 1, "Disgusting": 1}, "numerals": {"203": 1}, "pronouns": {"you": 1}}}, {"content": "204. Do you think your personality is attractive to the opposite sex?", "word_count": 15, "label": "", "sentence_structure": "Subject鈥搗erb鈥搊bject", "subjective_objective": {"answer": "Subjective", "rationale": "This is a question of self-awareness of patients and concerns their subjective perception and evaluation of their character."}, "sentence_sentiment": {"answer": "Positive", "rationale": "Because the entry describes the attraction of one ' s personality to the opposite sex, it is usually considered attractive as a positive quality."}, "language_style": {"answer": "Tactful and warm", "rationale": "The entry is based on an invitation-based approach, which is not directly stated or ordered, but rather a moderate approach to dialogue, which is more personal and interactive. At the same time, the expression \"you think\" gives respondents more space for reflection and freedom of expression, and greater respect for the subject's subjective feelings and personal perceptions."}, "time_dimension": {"answer": "Future", "rationale": "The entry contains visions and expectations for the future, and the keywords are \"you think\" and \"will\" and they all point to situations that have not yet occurred and that may occur in the future."}, "sentence_type": "Interrogative sentence", "pos": {"nouns": {"Personality": 1, "Heterosexual": 1}, "verbs": {"I think": 1, "Attractive": 1}, "adjectives": {"It's attractive": 1}, "numerals": {"204": 1}, "pronouns": {"you": 1}}}, {"content": "205, in most times, do you feel calm and content?", "word_count": 17, "label": "", "sentence_structure": "Subject鈥搗erb鈥搊bject", "subjective_objective": {"answer": "Subjective", "rationale": "This is a question of subjective perceptions and evaluation of the patient ' s own feelings."}, "sentence_sentiment": {"answer": "Positive", "rationale": "As the entry asks whether the interviewee has felt calm and satisfied in his or her heart for most of the time, it is a positive description aimed at understanding the mental health status of the interviewee."}, "language_style": {"answer": "Tactful and warm", "rationale": "The entry is more personal and interactive when it is spoken in a gentle and inviting manner, rather than in direct statements or orders. At the same time, it is not too much rhetorical or emotional to make, simply asking the interviewee what he feels inside, and speaking in a more objective and objective manner."}, "time_dimension": {"answer": "Future", "rationale": "The entry asks the testee about the future of his or her inner feelings, and the key words are \"most of the time\" and \"will feel\" and they point to the future."}, "sentence_type": "Interrogative sentence", "pos": {"nouns": {"Time": 1, "Inside": 1, "Peace": 1, "Satisfied": 1}, "verbs": {"I feel": 1, "be": 1}, "adjectives": {"Most": 1}, "numerals": {"205": 1}, "classifiers": {"Lee": 1, "you": 1}}}, {"content": "206. Are you a neurotic man?", "word_count": 10, "label": "", "sentence_structure": "Subject鈥搗erb鈥搊bject", "subjective_objective": {"answer": "Subjective", "rationale": "Because neurons are the subjective feeling and self-awareness of individuals."}, "sentence_sentiment": {"answer": "Negative", "rationale": "Because \"nervous\" is usually considered a negative characteristic."}, "language_style": {"answer": "Calm and direct", "rationale": "The entry asks the respondents directly and explicitly whether they have neurotic characteristics, do not have excessive emotional colours or qualifiers, and the language style is relatively calm and straightforward."}, "time_dimension": {"answer": "Present", "rationale": "The keyword \"you\" suggests that it is an assessment of the subject, and the description of \"a neurotic person\" is based on current observation or self-awareness, so the answer is now."}, "sentence_type": "Interrogative sentence", "pos": {"nouns": {"Nerves": 1, "person": 1}, "verbs": {"be": 1}, "adjectives": {"One": 1}, "numerals": {"206": 1}}}, {"content": "207, do you often spend a lot of time collating your manuscripts so you can know where they are when you need them?", "word_count": 33, "label": "", "sentence_structure": "Subject鈥搗erb鈥搊bject", "subjective_objective": {"answer": "Subjective", "rationale": "Since the problem concerns the patient ' s personal habits and behaviour choices, the frequency and purpose of the patient ' s preparation are examined and are therefore subjective."}, "sentence_sentiment": {"answer": "Positive", "rationale": "Because the entry \"often takes a lot of time to sort out the manuscripts so you can know where they are when you need them\" is a positive act both in purpose and effect."}, "language_style": {"answer": "Tactful and warm", "rationale": "The language style of the entry is more euphemistic, using the words \"often\" and \"many\" to describe the frequency and extent of behaviour, and also in the form of a question-and-answer period, allowing respondents to answer the question from their own perspective. These factors make the language style of the question more gentle, friendly and interactive."}, "time_dimension": {"answer": "Present", "rationale": ""}, "sentence_type": "Interrogative sentence", "pos": {"nouns": {"Time": 1, "Scripts": 1, "Yes": 1}, "verbs": {"Collapse": 1, "Flowers": 1, "Yes": 1, "in": 1}, "adjectives": {"Mass": 1, "Often": 1, "There": 1}, "numerals": {"207": 1}, "pronouns": {"you": 1}}}, {"content": "208. Do you always have to decide what movie or program you're watching?", "word_count": 20, "label": "", "sentence_structure": "Subject鈥搗erb鈥搊bject", "subjective_objective": {"answer": "Subjective", "rationale": "The question is examined from a subjective point of view because it is the patient ' s own thoughts and feelings."}, "sentence_sentiment": {"answer": "Negative", "rationale": "Because the \"other person decides\" act in the entry is passive, implying a lack of self-determination and a certain degree of negative emotion."}, "language_style": {"answer": "Calm and direct", "rationale": "The entry is conducted in a direct question-and-answer manner, with a calm and concise language style, without too many rhetorical and emotional renderings.\""}, "time_dimension": {"answer": "Present", "rationale": "The entry asks about the current status of the subjects, i.e. whether they are often determined by others to watch a film or a programme. The keyword \"always\" and \"others\" and \"what you see\" point to current behaviour and circumstances."}, "sentence_type": "Interrogative sentence", "pos": {"nouns": {"you": 1, "Film": 1, "Programs": 1}, "verbs": {"be": 1, "Look": 1, "Decision": 1}, "adverbs": {"Always": 1}, "numerals": {"208": 1}}}, {"content": "209. Have you ever felt cold and hot?", "word_count": 11, "label": "", "sentence_structure": "Subject鈥損redicate", "subjective_objective": {"answer": "Objective", "rationale": "Because the feeling of cold and heat is the experience and perception of the patient, and it is subjective."}, "sentence_sentiment": {"answer": "Negative", "rationale": "Because \"sweet cold and hot\" is a feeling of instability, which is usually considered negative."}, "language_style": {"answer": "Tactful and warm", "rationale": "The entry uses a more euphemistic expression that leads the interviewee to answer questions by asking him whether he has ever felt a sense of being a member of the other party, without directly expressing his views or attitudes. This expression is usually considered more friendly and friendly, making it easier for the interviewee to feel comfortable and to share his thoughts."}, "time_dimension": {"answer": "Present", "rationale": "The keyword \"Feeling cold and hot\" suggests that the subject asks the testee whether he has experienced it recently, so the temporal dimension is now judged."}, "sentence_type": "Interrogative sentence", "pos": {"nouns": {"Questionnaire": 1, "Entry": 1, "Feel": 1}, "verbs": {"have": 1, "鈾?And the cold and the heat 鈾?: 1}, "numerals": {"209": 1}}}, {"content": "210, can you easily forget what you did?", "word_count": 15, "label": "", "sentence_structure": "Subject鈥搗erb鈥搊bject", "subjective_objective": {"answer": "Subjective", "rationale": "Because it is subjective to think and to think about whether or not people are easy to forget about doing wrong."}, "sentence_sentiment": {"answer": "Negative", "rationale": "Because the act of \"doing wrong\" in the entry is negative, forgetting it may be seen as a sign of evading responsibility or not taking the issue seriously. The purpose of the questionnaire is therefore somewhat negative."}, "language_style": {"answer": "Tactful and warm", "rationale": "The entry is more adept and interactive when it is spoken in a gentle and inviting manner, rather than by direct statements or orders."}, "time_dimension": {"answer": "Past", "rationale": ""}, "sentence_type": "Interrogative sentence", "pos": {"nouns": {"Yes": 1, "Questionnaire": 1, "Entry": 1}, "verbs": {"Yes": 1, "Forget it": 1, "Do it": 1, "Wrong": 1}, "adverbs": {"very": 1, "Easy": 2}, "numerals": {"210": 1}, "pronouns": {"you": 1}}}]}

{"questionnaire_title": "Fear of negative evaluation (FNE)", "questionnaire_category": "Mental health", "item_count": 12, "emotion_shift_overall": "No obvious trend", "language_style_overall": "Neutral", "items": [{"content": "I'm worried about what people think of me, even though I know it doesn't matter.", "word_count": 21, "label": "", "sentence_structure": "Subject鈥搗erb鈥搊bject", "subjective_objective": {"answer": "Subjective", "rationale": "Because \"I'm worried,\" \"I know it doesn't matter\" is the patient's personal feelings and thoughts, so it's subjective."}, "sentence_sentiment": {"answer": "Negative", "rationale": "Because the words \"fear\" and \"no matter\" in the entries convey a negative emotion and attitude."}, "language_style": {"answer": "Calm and direct", "rationale": "The entry expresses, in a calm and objective manner, the attitude of the subject to self-awareness, is more flat in tone and does not involve too many rhetorical or emotional accents, and therefore can be judged as \"cool and direct\"."}, "time_dimension": {"answer": "Present", "rationale": "The entry uses the now completed time \"knows\" to indicate a perception of the past, while also describing a current anxiety and anxiety. The entry thus mainly reflects the current state of the person being tested."}, "sentence_type": "Interrogative sentence", "pos": {"nouns": {"Questionnaire": 1, "Entry": 1, "He's": 1, "Problem": 1, "Observations": 1, "It's important": 1}, "verbs": {"I'm worried": 1, "Look": 1, "Yes": 1, "have": 1}, "adjectives": {"What": 1, "How much": 1}, "adverbs": {"鈾?Though 鈾?: 1}, "numerals": {"1": 2}, "pronouns": {"I": 1}}}, {"content": "I don't care if I know people are creating an impression against me.", "word_count": 24, "label": "", "sentence_structure": "Subject鈥搗erb鈥搊bject", "subjective_objective": {"answer": "Subjective", "rationale": "The attitude and feelings of the patient are examined and therefore subjective."}, "sentence_sentiment": {"answer": "Negative", "rationale": "Because \"not caring\" in the entry is an expression of the author's indifference and indifference to negative evaluations, which is a manifestation of negative emotion."}, "language_style": {"answer": "Calm and direct", "rationale": "The entry is more straightforward, concise, not used too much rhetorical or emotionally retrofitting, and is more objective and calm. It directly asks the interviewee about his attitude to the negative impression, and is not too euphemistic or polite."}, "time_dimension": {"answer": "Past", "rationale": "The use of the keyword \"even if you know\" and \"not care\" suggests that it is a description of past memories or experiences, and therefore the entry belongs to the past."}, "sentence_type": "Interrogative sentence", "pos": {"nouns": {"Questionnaire": 1, "Entry": 1, "person": 1, "Impression": 1}, "verbs": {"Yes": 1, "Form": 1, "I care": 1}, "adjectives": {"Negative": 1}, "adverbs": {"Even if": 1}, "pronouns": {"I": 2}}}, {"content": "I'm always afraid people will notice my weaknesses.", "word_count": 15, "label": "", "sentence_structure": "Subject鈥搗erb鈥揷omplement", "subjective_objective": {"answer": "Subjective", "rationale": "Because the words \"fear\" and \"care\" are the patient's subjective feelings and judgment about his or her own shorts."}, "sentence_sentiment": {"answer": "Negative", "rationale": "Because the attitude in the entry, \"A fear that others will notice my weaknesses,\" is negative."}, "language_style": {"answer": "Tactful and warm", "rationale": "The entry is more euphemistic and gentle, allowing the respondent to express his or her views and feelings in a way that is asked, rather than direct orders or accusations. At the same time, the more implicit term \"short spots\" is used to describe the shortcomings of the individual and to avoid undue pressure and discomfort on the person under investigation."}, "time_dimension": {"answer": "Past", "rationale": ""}, "sentence_type": "Negative declarative sentence", "pos": {"nouns": {"I": 1, "others": 1, "Short": 1}, "verbs": {"Scared": 1, "Attention": 1, "Present": 1, "have": 1}, "adverbs": {"Regular": 1}, "numerals": {"3": 1}}}, {"content": "I'm hardly concerned about the impression I've made.", "word_count": 18, "label": "", "sentence_structure": "Subject鈥搗erb鈥搊bject", "subjective_objective": {"answer": "Subjective", "rationale": "The subjective attitude of the patient, i.e. whether he or she is often worried about their perception, is being examined."}, "sentence_sentiment": {"answer": "Neutral", "rationale": "Because the entry does not have any emotional color."}, "language_style": {"answer": "Tactful and warm", "rationale": "The entry is more gentle and soft in language, with relatively soft words, and no overly direct or hard expression. At the same time, it gives the person surveyed a free answer in a way that gives a sense of affection and respect.\""}, "time_dimension": {"answer": "Present", "rationale": "According to the above-mentioned input and judgment, \"4. I'm hardly concerned about what I'm giving the impression\" is about the state or behaviour of the moment, so the answer is \"now\"."}, "sentence_type": "Negative declarative sentence", "pos": {"nouns": {"I": 2, "others": 1, "Impression": 1}, "verbs": {"Mind your own business": 1, "Stay": 1}, "adjectives": {"Almost": 1}, "numerals": {"4": 1}}}, {"content": "I'm afraid people will disagree with me.", "word_count": 10, "label": "", "sentence_structure": "Subject鈥揷opula鈥損redicative", "subjective_objective": {"answer": "Subjective", "rationale": "Because fear is the patient's subjective mood and attitude."}, "sentence_sentiment": {"answer": "Negative", "rationale": "Because the \"fear that others will disagree with me\" attitude in the entry is negative."}, "language_style": {"answer": "Tactful and warm", "rationale": "This language style reduces the psychological pressure of the interviewee and helps to obtain more realistic and accurate answers."}, "time_dimension": {"answer": "Present", "rationale": "Because \"Fear\" is an emotional state that is now in progress, \"Will anyone agree with me\" is also a concern and a expectation for the current situation. Therefore, the entry should be classified as \"Now\"."}, "sentence_type": "Negative declarative sentence", "pos": {"nouns": {"Questionnaire": 1, "Entry": 1, "person": 1, "Yes": 1}, "verbs": {"Scared": 1, "Yes": 2, "not": 1}, "adverbs": {"others": 1, "Yes, I will": 1, "Don't": 1, "not": 1}, "classifiers": {"individual": 1}, "pronouns": {"I": 1}}}, {"content": "I'm afraid people will find out what's wrong with me.", "word_count": 12, "label": "", "sentence_structure": "Subject鈥搗erb鈥揷omplement", "subjective_objective": {"answer": "Subjective", "rationale": "Because \"fear\" is a subjective emotional reaction."}, "sentence_sentiment": {"answer": "Negative", "rationale": "Because the emotional expression \"fear\" in the entry and the context of \"others will find my fault\" indicate a negative emotional attitude."}, "language_style": {"answer": "Tactful and warm", "rationale": "The entry raises questions in a way that is concerned and understood, rather than directly critical or accusing, and that is more easily expressed in a way that allows the respondents to open their minds and answer questions in a true manner."}, "time_dimension": {"answer": "Past", "rationale": "The actions of \"fear\" and \"discovery\" in this entry have occurred at some point in the past or now, but do not specify a specific time frame. So, it can be judged by context as past or present. Common sense and context dictate that people are usually afraid of past mistakes, and therefore tend to be more inclined to past times."}, "sentence_type": "Negative declarative sentence", "pos": {"nouns": {"Questionnaire": 1, "Entry": 1, "person": 1, "Wrong": 1}, "verbs": {"Scared": 1, "Found": 1}, "adverbs": {"Down there": 1, "Yes": 1}, "numerals": {"6": 1}, "pronouns": {"I": 1}}}, {"content": "I'm not worried about what people think of me.", "word_count": 14, "label": "", "sentence_structure": "Subject鈥揷opula鈥損redicative", "subjective_objective": {"answer": "Subjective", "rationale": "Because worry is the patient's subjective feeling."}, "sentence_sentiment": {"answer": "Neutral", "rationale": "Because the entry does not have any emotional color."}, "language_style": {"answer": "Calm and direct", "rationale": "Although the entry does not use any negative emotional vocabulary, it is more directly worded, emphasizing individual attitudes and positions rather than euphemism or softly guiding each other. This language style is usually more authoritative and persuasive and suitable for use in situations where a clear statement or decision is needed."}, "time_dimension": {"answer": "Present", "rationale": "\"No\" in \"not to be upset about what others think of me\" indicates that the act is not a normal past or future. At the same time, \"disturbing\" uses the current state of affairs, indicating that the act is taking place or has taken place. Taken together, the questionnaire entry asks about the person's current state of mind, so the answer is \"now.\""}, "sentence_type": "Negative declarative sentence", "pos": {"nouns": {"I": 1, "Observations": 1}, "verbs": {"Not for": 1, "I'm worried": 1}, "adverbs": {"and": 1, "Yes": 1, "of": 1}}}, {"content": "When I talk to someone, I'm worried about what they'll think of me.", "word_count": 18, "label": "", "sentence_structure": "Subject鈥揷opula鈥損redicative", "subjective_objective": {"answer": "Subjective", "rationale": "Because it's the patient's subjective feeling and concern."}, "sentence_sentiment": {"answer": "Negative", "rationale": "Because the \"worried what they'd think of me\" in the entry is negative."}, "language_style": {"answer": "Tactful and warm", "rationale": "The entry uses a more moderate and encouraging expression to ask the respondents whether they are disturbed and worried in their social contacts, which makes it easier for the respondents to open their minds and answer honestly."}, "time_dimension": {"answer": "Present", "rationale": "The key word \"now talking to others\" and \"so worried about what they'll look at me\" suggests that this is a description of the current situation."}, "sentence_type": "Negative declarative sentence", "pos": {"nouns": {"Time": 1, "I": 2, "others": 1, "Talk": 1, "they": 1, "Observations": 1}, "verbs": {"I'm worried": 1, "Yes": 1, "Look": 1, "Same": 1}, "adverbs": {"When": 1, "What": 1}, "pronouns": {"Here": 1}}}, {"content": "I'm usually worried about what I've made of people.", "word_count": 21, "label": "", "sentence_structure": "Subject鈥搗erb鈥搊bject", "subjective_objective": {"answer": "Subjective", "rationale": "Because \"fear\" and \"impression\" are patients' subjective feelings and attitudes."}, "sentence_sentiment": {"answer": "Negative", "rationale": "Because the behavior in the entry is negative."}, "language_style": {"answer": "Calm and direct", "rationale": "\"Calm down straight.\""}, "time_dimension": {"answer": "Present", "rationale": "\"I usually\" means a customary, recurring situation, \"always worrying\" always means \"a continuous act,\" so the entry describes the current state and behaviour of the subject."}, "sentence_type": "Interrogative sentence", "pos": {"nouns": {"Questionnaire": 1, "Entry": 1, "person": 1, "Impression": 1}, "verbs": {"Always": 1, "I'm worried": 1, "Stay": 1, "Here": 1}, "adverbs": {"What the hell": 1, "More": 1}, "numerals": {"9": 1}, "classifiers": {"individual": 1}, "pronouns": {"I": 2}}}, {"content": "I don't care if I know someone who's judging me.", "word_count": 19, "label": "", "sentence_structure": "Subject鈥搗erb鈥搊bject", "subjective_objective": {"answer": "Subjective", "rationale": "This is because the patient ' s self-perception and attitude towards others is subjective."}, "sentence_sentiment": {"answer": "Negative", "rationale": "Because \"not caring\" is negative."}, "language_style": {"answer": "Calm and direct", "rationale": "The entry describes a scene in a simple and clear language, where when someone evaluates himself, his attitude is unimportant. The language style is straightforward, and there is no excessive emotional overtones or rhetorical techniques that give people a sense of calm and rationality."}, "time_dimension": {"answer": "Present", "rationale": "The key word \"if I know\" means \"the condition or the scenario, \"not care\" is a personal emotional and attitudinal reaction, which is a description of the current situation. So, the entry describes the emotional state of the person who was tested when he knew his person's assessment of himself, and can be judged as now."}, "sentence_type": "Negative declarative sentence", "pos": {"nouns": {"person": 1, "Evaluation": 1}, "verbs": {"Yes": 1, "I care": 1}, "adjectives": {"A little": 1}, "adverbs": {"not": 1, "in": 1, "Time": 1}, "numerals": {"10": 1}, "pronouns": {"I": 1}}}, {"content": "I think I care too much about what people think of me.", "word_count": 17, "label": "", "sentence_structure": "Subject鈥揷opula鈥損redicative", "subjective_objective": {"answer": "Subjective", "rationale": "Because \"too much care about what people think of me\" is a subjective feeling and attitude."}, "sentence_sentiment": {"answer": "Negative", "rationale": "Because \"too much care\" in the entry expresses a negative emotional attitude to the denial and displeasure of others about themselves."}, "language_style": {"answer": "Tactful and warm", "rationale": "Reason: The expression used in this article uses words such as \"sometimes\" and \"too caring\" to express some concern and self-questioning about one another. This expression is relatively moderate and incriminating, without direct accusations or criticism, and is more acceptable and resonant. Thus, the language style of the entry on the questionnaire can be considered as euphemistic."}, "time_dimension": {"answer": "Present", "rationale": "Sometimes \"sometimes\" means \"sometimes\" that the behaviour is less frequent, occasional, and the word \"want\" that is the general current, means that it not only happened in the past, but may still be going on. And at the same time, \"too much care for what others think about me\" is also a description of what is happening."}, "sentence_type": "Negative declarative sentence", "pos": {"nouns": {"Time": 1, "I": 1, "Thoughts": 1}, "verbs": {"Yes": 1, "I care": 2, "to": 1}, "adjectives": {"Too much": 1}, "adverbs": {"Sometimes": 1, "Yes": 1}, "numerals": {"11": 1}, "pronouns": {"others": 1}}}, {"content": "I've always been afraid I'd say the wrong thing or do the wrong thing.", "word_count": 14, "label": "", "sentence_structure": "Subject鈥損redicate", "subjective_objective": {"answer": "Subjective", "rationale": "Because \"fear\" is an expression of subjective emotion and attitude, a feeling inside the patient."}, "sentence_sentiment": {"answer": "Negative", "rationale": "Because the mood for \"fears to say the wrong thing or do the wrong thing\" in the entry is negative."}, "language_style": {"answer": "Calm and direct", "rationale": "The entry is based on a direct and objective tone of inquiry as to whether the person under investigation is often concerned about his/her own mistakes or wrongs, and is not overly emotional or rhetorical, and can therefore be judged as \"cool and straight\"."}, "time_dimension": {"answer": "Present", "rationale": ""}, "sentence_type": "Negative declarative sentence", "pos": {"nouns": {"Questionnaire Entry": 1, "Word": 1, "Yes": 1}, "verbs": {"I'm worried": 1, "Say it": 1, "Do it": 1}, "adverbs": {"Often": 1}, "numerals": {"12": 1}, "pronouns": {"I": 1}}}]}

{"questionnaire_title": "Questionnaire on Type A Behaviour Types", "questionnaire_category": "Behavioral questionnaire", "item_count": 60, "emotion_shift_overall": "From positive to negative", "language_style_overall": "Calm and direct", "items": [{"content": "I feel like a carefree, free-minded person.", "word_count": 18, "label": "", "sentence_structure": "Subject鈥揷opula鈥損redicative", "subjective_objective": {"answer": "Subjective", "rationale": "Because the entry expresses the patient ' s perception and feelings of his or her own quality, it is a subjective description of the patient from his or her own perspective."}, "sentence_sentiment": {"answer": "Positive", "rationale": "Because the entry is a positive attitude and emotion, and considers itself a carefree, casual person."}, "language_style": {"answer": "Tactful and warm", "rationale": "The entry is more euphemistic, using descriptive terms (such as \"no worries\" and \"no idleness\") to express the person surveyed's sense of his or her personality, without using direct statements or commands. At the same time, this expression is more natural and can resonate with the person under investigation and stimulate their positive response."}, "time_dimension": {"answer": "Present", "rationale": "The entry is for the testee ' s self-characterization, and the time dimension is now judged."}, "sentence_type": "Affirmative declarative sentence", "pos": {"nouns": {"self": 1, "person": 1, "No worries": 1, "I'm just gonna have a little time": 1}, "verbs": {"I think": 1, "be": 1}, "adjectives": {"No worries": 1, "I'm just gonna have a little time": 1}, "pronouns": {"One": 1}}}, {"content": "I walk fast, even if it's nothing important.", "word_count": 15, "label": "", "sentence_structure": "Subject鈥搗erb鈥揷omplement", "subjective_objective": {"answer": "Subjective", "rationale": "Because it is the patient ' s own description of himself, reflecting his own subjective feelings and attitudes."}, "sentence_sentiment": {"answer": "Neutral", "rationale": "Because the entry does not have any emotional color."}, "language_style": {"answer": "Calm and direct", "rationale": "The entry is in plain and clear language and directly asks the interviewees about their habits and behaviour as they walk, without excessive ado or emotional colours, and is expressed more directly."}, "time_dimension": {"answer": "Present", "rationale": ""}, "sentence_type": "Affirmative declarative sentence", "pos": {"verbs": {"Let's go": 1, "have": 1, "Come on": 1}, "adjectives": {"It's important": 1}, "adverbs": {"Yeah": 1}, "pronouns": {"I": 1}}}, {"content": "I often feel that there's too much to do and that there's pressure.", "word_count": 15, "label": "", "sentence_structure": "Subject鈥揷opula鈥損redicative", "subjective_objective": {"answer": "Subjective", "rationale": "Because \"the feeling of being too much, the stress\" is the patient's personal feelings and thoughts, and it's a subjective observation."}, "sentence_sentiment": {"answer": "Negative", "rationale": "Because the mood \"Feeling too much to do, having pressure\" in the entry is negative."}, "language_style": {"answer": "Calm and direct", "rationale": "The entry is presented in a simple narrative, without any rhetorical or emotional rendering, with a more objective tone, and directly conveying the subject of questioning whether the other party is under constant pressure. It also does not use any euphemism or subtle language to guide the respondent, but rather takes a more direct and explicit approach, which is consistent with the straight style of calm."}, "time_dimension": {"answer": "Past", "rationale": "The entry describes the current state of the person being tested, and \"I often feel\" indicates that this feeling is long-term and it exists now. So the time dimension of the entry is a past."}, "sentence_type": "Negative declarative sentence", "pos": {"nouns": {"I": 1, "Yes": 1, "Pressure": 1}, "verbs": {"I feel": 1, "Do it": 1, "I think so": 1}, "adverbs": {"Regular": 1, "Too much": 1}, "numerals": {"3": 1}}}, {"content": "It's hard for me to change my mind about what I decide.", "word_count": 17, "label": "", "sentence_structure": "Subject鈥搗erb鈥揷omplement", "subjective_objective": {"answer": "Subjective", "rationale": "The entry emphasizes the individual鈥檚 subjective consciousness and autonomy, i.e. the degree of individual adherence to his or her own decision."}, "sentence_sentiment": {"answer": "Neutral", "rationale": "According to the scene described in the title, the entry simply describes the fact that individuals are not easily influenced by others in their own decision-making, and does not express positive or negative emotional sentiments. The purpose of the article is therefore emotional polarization."}, "language_style": {"answer": "Tactful and warm", "rationale": "The language style of the entry is euphemistic. It begins with respect and understanding of the other, and attempts to create an atmosphere of dialogue rather than directly expressing one's views or ordering others to do it. Moreover, the entry uses moderate language, \"It's hard to change my mind\" and emphasizes my decision-making, but also shows an open and inclusive mindset, and is open to others. This language style is more resonating with the other, facilitating effective communication and communication."}, "time_dimension": {"answer": "Present", "rationale": "This entry describes the attitude and tendency of the testees in making decisions, that is, they are more willing to make decisions themselves and are not vulnerable to influence. This description happens now, so the answer is \"now.\""}, "sentence_type": "Affirmative declarative sentence", "pos": {"nouns": {"Yes": 1, "Ideas": 1}, "verbs": {"Decision": 1, "Jean": 1, "Change": 1}, "adjectives": {"self": 1, "Hard": 1}, "numerals": {"4": 1}, "classifiers": {"individual": 1}, "pronouns": {"I": 1}}}, {"content": "5. Some people and things are always making me angry.", "word_count": 13, "label": "", "sentence_structure": "Subject鈥搗erb鈥揷omplement", "subjective_objective": {"answer": "Objective", "rationale": "The existence of certain persons or things that upset people is objective, while the feeling of anger is subjective."}, "sentence_sentiment": {"answer": "Negative", "rationale": "Because the entry \"freak\" expresses a negative emotional attitude."}, "language_style": {"answer": "Calm and direct", "rationale": "The entry directly asks the respondents whether they have any disturbing persons or things, does not use excessive rhetorical techniques or emotional rendering, and is more calm and objective."}, "time_dimension": {"answer": "Present", "rationale": "The keyword \"make\" indicates the current state or situation, \"often irritating me\" indicates that it is a constant, present feeling or state, and the entry is now."}, "sentence_type": "Affirmative declarative sentence", "pos": {"nouns": {"person": 1, "Yes": 1}, "verbs": {"鈾?And I'm gonna be 鈾?: 1, "I'm angry": 1}, "adverbs": {"Often": 1}, "numerals": {"5": 1}, "pronouns": {"I": 1}}}, {"content": "When I need to buy something, but I'm in line, I'd rather not buy it.", "word_count": 18, "label": "", "sentence_structure": "Subject鈥搗erb鈥搊bject", "subjective_objective": {"answer": "Subjective", "rationale": "Because \"I'd rather not buy\" reflects patients' subjective choices and attitudes."}, "sentence_sentiment": {"answer": "Negative", "rationale": "Because the \"better than buy\" act in the entry is negative."}, "language_style": {"answer": "Calm and direct", "rationale": "The entry expresses a view or behavioural preference through a simple and clear statement, does not have too much emotional or rhetorical style and is more calm and straightforward."}, "time_dimension": {"answer": "Present", "rationale": "The entry contains a hypothetical scenario, \"I need to buy something but I want to line up\" , which is a scenario that may happen in the future. And \"I prefer not to buy\" is a situation where the tester's attitude and choice towards the situation, which also points to future behavior or mentality. So the temporal dimension of the article is the future."}, "sentence_type": "Negative declarative sentence", "pos": {"nouns": {"Something": 1, "Line up": 1}, "verbs": {"I need it": 1, "Buy": 1, "Line": 1, "I'd rather not": 1, "No, I'm not buying": 1}, "adjectives": {"Long": 1}, "adverbs": {"But": 1, "And": 1, "Urgent": 1, "I'd rather": 1}, "numerals": {"6": 1}, "pronouns": {"I": 2}}}, {"content": "I can't get any work done, I can't get any work done. I'm just gonna have to make time.", "word_count": 21, "label": "", "sentence_structure": "Subject鈥搗erb鈥搊bject", "subjective_objective": {"answer": "Objective", "rationale": "This is a description of the stress and difficulty that patients feel in their work and is their subjective feeling."}, "sentence_sentiment": {"answer": "Negative", "rationale": "Because the phrase \"some jobs I can't arrange\" in the entry is negative."}, "language_style": {"answer": "Calm and direct", "rationale": ""}, "time_dimension": {"answer": "Present", "rationale": "The keywords \"not at all\" and \"only to be done temporarily\" indicate that the act is currently being performed and may continue. The time dimension of the article is therefore now."}, "sentence_type": "Exclamatory sentence", "pos": {"nouns": {"Work": 1, "Time": 1}, "verbs": {"Arrangements": 1, "Do it": 2, "Squeeze": 1}, "adjectives": {"Not at all": 1, "Temporary": 1}, "numerals": {"7": 1}}}, {"content": "I'm never late for work or a date.", "word_count": 13, "label": "", "sentence_structure": "Subject鈥搗erb鈥揷omplement", "subjective_objective": {"answer": "Objective", "rationale": "The delay is an objective examination, as it is a behaviour of the patient that can be observed and recorded."}, "sentence_sentiment": {"answer": "Positive", "rationale": "Because the entry expresses a positive attitude and habit."}, "language_style": {"answer": "Calm and direct", "rationale": "The entry uses a simple and clear language to ask if the other party is never late for the subject at work or on a date, without excessive adoration and emotional colour, and is more direct and objective."}, "time_dimension": {"answer": "Present", "rationale": "The entry \" never\" denotes a situation that never occurred, so it can be judged as describing the person being tested for the current act. At the same time, the expression \"at work or on a date\" also indicates that it is describing a regular, customary act."}, "sentence_type": "Affirmative declarative sentence", "pos": {"verbs": {"Work": 1, "Date": 1, "Never": 1, "Late": 1}, "adverbs": {"not": 2}}}, {"content": "When I'm doing something, I'm always upset who bothers me, whether I'm doing it or not.", "word_count": 25, "label": "", "sentence_structure": "Subject鈥搗erb鈥搊bject", "subjective_objective": {"answer": "Subjective", "rationale": "Because \"freak\" is a subjective feeling or emotional reaction that requires patient reporting or experience to be measured. Although disturbed is an objective event, the response to it is subjective, and therefore it is subjective."}, "sentence_sentiment": {"answer": "Negative", "rationale": "Because the \"freak\" act in the entry is negative."}, "language_style": {"answer": "Calm and direct", "rationale": "The entry is a blunt, blunt language expression of the other鈥檚 feelings of disgust and anger at being disturbed. The question is simple and straightforward, without using euphemism or tact to highlight the core of the problem."}, "time_dimension": {"answer": "Present", "rationale": "The entry describes the feelings and reactions of the subjects in their current state, i.e., when they are doing their work, they are upset if they are disturbed. So, the time dimension is now judged."}, "sentence_type": "Affirmative declarative sentence", "pos": {"nouns": {"Yes": 1, "person": 1}, "verbs": {"Do it": 1, "Quiet": 1, "Sensor": 1}, "adjectives": {"Meaning": 1, "None": 1, "Total": 1, "I'm upset": 1, "Fire": 1}, "adverbs": {"Like": 1, "Just": 1}, "pronouns": {"Who": 1}}}, {"content": "I'm not used to slow-sense, slow-shartered people.", "word_count": 17, "label": "", "sentence_structure": "Subject鈥搗erb鈥搊bject", "subjective_objective": {"answer": "Subjective", "rationale": "Because I'm not comfortable with other people's behavior, it's patient's subjective attitude."}, "sentence_sentiment": {"answer": "Negative", "rationale": "Because the term \"slow, slow and slow\" in the entry is the object of denigration and denial, it has a negative emotional colour."}, "language_style": {"answer": "Calm and direct", "rationale": "The language style of the entry is a cold-blooded type, as it uses direct criticism and negative language to describe the target population. It can be offensive or uncomfortable, but it can also express clearly the attitudes and positions of the speaker."}, "time_dimension": {"answer": "Present", "rationale": "Now the words \"always\" in the entry are a customary act, while the descriptions \"unsatisfied\" and \"slowly rational, unslowly\" indicate that this attitude is now. So, the entry is now."}, "sentence_type": "Affirmative declarative sentence", "pos": {"nouns": {"Entry": 1, "person": 2, "Slow-Sensey": 1, "It's not that slow": 1}, "verbs": {"I don't like it": 1, "Total": 1, "Look": 1}, "adjectives": {"Slow-Sensey": 1, "It's not that slow": 1}, "adverbs": {"Total": 1, "not": 2}, "numerals": {"10": 1}, "pronouns": {"I": 1}}}, {"content": "I'm too busy to breathe because I've done too much.", "word_count": 20, "label": "", "sentence_structure": "Subject鈥揷opula鈥損redicative", "subjective_objective": {"answer": "Subjective", "rationale": "Because \"often too busy to breathe\" is a feeling or experience, a patient's subjective attitude."}, "sentence_sentiment": {"answer": "Negative", "rationale": "Because the words \"too busy\" and \"too much\" in the entries have negative feelings."}, "language_style": {"answer": "Calm and direct", "rationale": "The entry is more calm and straightforward, with no excessive rhetoric or emotional colour, and is more objective. It expresses the subject of the problem in a simple and clear way, i.e. whether the respondents are often under stress and too much is not being done."}, "time_dimension": {"answer": "Present", "rationale": "The word \"I'm often\" means a regular, customary act, and the word \"sustained\" means that it is currently being done. So, the entry to the questionnaire asks about the current state and feelings of the testee, and the temporal dimension is now."}, "sentence_type": "Affirmative declarative sentence", "pos": {"nouns": {"Questionnaire": 1, "Entry": 1, "Time": 1, "Things": 1}, "verbs": {"Often": 1, "Busy": 1, "I can't breathe": 1, "Because": 1, "It's time to do it": 1, "Too much": 1}, "adverbs": {"Often": 1, "The": 1}, "numerals": {"11": 1, "The": 1, "Too much": 1}, "pronouns": {"I": 1}}}, {"content": "I'd like to do something more important alone, even if I work with someone else.", "word_count": 23, "label": "", "sentence_structure": "Subject鈥搗erb鈥搊bject", "subjective_objective": {"answer": "Subjective", "rationale": "Because \"always\" describes the patient's thoughts and attitudes, and it's subjective."}, "sentence_sentiment": {"answer": "Negative", "rationale": "Because the attitude of \"always trying to do something more important alone\" in the entry is negative, emphasizing individualism and unwillingness to share responsibility with others."}, "language_style": {"answer": "Calm and direct", "rationale": "The language style of the entry is a calm, straightforward presentation, with no excessive rhetorical and emotional colours."}, "time_dimension": {"answer": "Present", "rationale": "\"Wanted\" means a constant thought or tendency, \"work with someone\" is an event that has already happened, so the sentence describes the situation."}, "sentence_type": "Affirmative declarative sentence", "pos": {"nouns": {"Questionnaire": 1, "Entry": 1, "person": 1, "Cooperation": 1, "Part": 1}, "verbs": {"Call": 1, "Yes": 2, "Completed": 1, "Alone": 1}, "adjectives": {"Important": 1}, "numerals": {"12": 1}, "classifiers": {"Some": 1}, "pronouns": {"I": 1}}}, {"content": "Sometimes I really want to scold people.", "word_count": 7, "label": "", "sentence_structure": "Subject鈥搗erb鈥揷omplement", "subjective_objective": {"answer": "Subjective", "rationale": "Because \"I really want to curse\" is a person's inner emotion and thoughts, and it's subjective."}, "sentence_sentiment": {"answer": "Negative", "rationale": "Because the \"scolding\" in the entry is negative."}, "language_style": {"answer": "Calm and direct", "rationale": "The language style of the entry is relatively cold and direct, indicating a situation where the person concerned is asked whether he or she is in a state of uncontrollable emotion, wants to vent, and is more direct and not too emotional."}, "time_dimension": {"answer": "Present", "rationale": ""}, "sentence_type": "Imperative sentence", "pos": {"verbs": {"True": 1, "Yes": 1, "Scolded": 1}, "adjectives": {"Sometimes": 1}}}, {"content": "I always like to slow down and think before I do anything.", "word_count": 21, "label": "", "sentence_structure": "Subject鈥搗erb鈥揷omplement", "subjective_objective": {"answer": "Subjective", "rationale": "Because this is the subjective idea and attitude of the patients, they describe the way they deal with the problem."}, "sentence_sentiment": {"answer": "Negative", "rationale": "Because \"slow\" and \"sweet, unsustainable\" in the entries are negative behaviors or attitudes."}, "language_style": {"answer": "Tactful and warm", "rationale": "The language style of the entry is relatively warm, and by describing the way individuals behave and think, rather than directly criticizing or evaluating, the interviewee is more willing to reveal his real thoughts in his response. At the same time, the phrase is somewhat instructive, encouraging the interviewee to reflect and examine his or her own behaviour."}, "time_dimension": {"answer": "Present", "rationale": "The \"always\" and \"uncertainty\" in the past entry indicate that this is a pattern of customary behaviour, which may have lasted for some time and is therefore judged to be the past."}, "sentence_type": "Negative declarative sentence", "pos": {"nouns": {"Things": 2, "Ideas": 1}, "verbs": {"Do it": 1, "Yeah": 1, "Take your time": 1, "Think before you think about it": 1, "I can't hold it": 1}, "adjectives": {"Yeah": 1, "Slow": 1, "Think before you think about it": 1}, "adverbs": {"Always": 1, "And": 1}, "numerals": {"14": 1}, "pronouns": {"I": 1}}}, {"content": "15. If someone were to buy something, I would have to accuse him or interfere.", "word_count": 25, "label": "", "sentence_structure": "Subject鈥搗erb鈥搊bject", "subjective_objective": {"answer": "Subjective", "rationale": "Because the questionnaire item looks at patients' reactions and attitudes in a given situation."}, "sentence_sentiment": {"answer": "Negative", "rationale": "Because \"stigmatization\" and \"interference\" in the entries are both negative."}, "language_style": {"answer": "Calm and direct", "rationale": "Reason: The entry directly describes the situation of \"queuing for something\" and clearly outlines its own reaction when faced with \"some man has to buy\" situations, without excessive rhetoric or emotional rendering, and is more objective. While the final reference to the acts of accusing and interfering is not the subject of the article, it is the author's reaction."}, "time_dimension": {"answer": "Present", "rationale": "The entry \"if\" and \"if\" indicates that this is a hypothetical situation, while \"suspect him or intervene\" describes the reaction of the person tested in the event of such a situation. Since it is a hypothetical issue and describes the current behaviour orientation of the person being tested, it is possible to judge the time dimension of the entry as it is now."}, "sentence_type": "Exclamatory sentence", "pos": {"nouns": {"Line up": 1, "person": 2, "Blame": 1, "Interference": 1}, "verbs": {"Buy": 1, "Gaz茅": 1, "Shino-kun": 1, "Stay": 1, "Blame": 1, "Come out": 1, "Interference": 1}, "adverbs": {"If": 1, "Just": 2, "I can't help it": 1}, "numerals": {"15": 1}, "pronouns": {"he": 1}}}, {"content": "I always try to convince people to agree with me.", "word_count": 15, "label": "", "sentence_structure": "Subject鈥搗erb鈥搊bject", "subjective_objective": {"answer": "Subjective", "rationale": "Because trying to convince others to agree with their own views is a subjective act and idea of one's own."}, "sentence_sentiment": {"answer": "Positive", "rationale": "Because the purpose of the entry is to convince others to agree with me."}, "language_style": {"answer": "Calm and direct", "rationale": "The entry asks in a straightforward manner whether the respondents often try to convince others to accept their views, and the language style is relatively calm and direct."}, "time_dimension": {"answer": "Past", "rationale": "The keyword \"always\" indicates that it is a regular, customary act with no clear future direction, so the time dimension of this entry is past and present."}, "sentence_type": "Affirmative declarative sentence", "pos": {"nouns": {"I": 1, "person": 1, "Convince": 1, "others": 1, "Views": 1}, "verbs": {"Try": 1, "Agreed": 1}, "adverbs": {"Always": 1}, "numerals": {"16锛?: 1}}}, {"content": "I sometimes feel like I'm worried more than I should be.", "word_count": 27, "label": "", "sentence_structure": "Subject鈥搗erb鈥搊bject", "subjective_objective": {"answer": "Subjective", "rationale": "Because the degree of feeling, worrying, etc. are the individual feelings and assessments of the patient, and it's subjective."}, "sentence_sentiment": {"answer": "Negative", "rationale": "The basis of the judgment: the behaviour or quality of \"what I'm concerned about is far more than what I should be concerned about\" is negative."}, "language_style": {"answer": "Tactful and warm", "rationale": "The language style of the entry is more euphemistic and friendly, leads to answers by asking each other what they feel, and uses subjective expressions like \"I think\" that increase interaction with the person under investigation. Moreover, the expression \"what I should worry about is far more than what I should care about\" is more implicit, without direct accusations or criticism, leaving more room for self-reflection."}, "time_dimension": {"answer": "Present", "rationale": "The keyword \"I feel,\" \"I worry about\" suggests that it is a state and a feeling. And \"more than I should care about\" is a description of the status quo. So the entry is about the current behavior or mentality of the testee, and the time dimension is now."}, "sentence_type": "Imperative sentence", "pos": {"nouns": {"Time": 1, "self": 1, "Yes": 2, "Scope": 1}, "verbs": {"I think": 1, "Mind your own business": 2, "More than": 1, "I think so": 1, "Sou-san": 1, "Company": 1}, "adjectives": {"Far": 1, "More": 1}, "adverbs": {"Far": 1, "Company": 1, "Both": 1}, "numerals": {"17": 1}, "pronouns": {"I": 2}}}, {"content": "I don't care what I do, even if I'm worse than others.", "word_count": 17, "label": "", "sentence_structure": "Subject鈥損redicate", "subjective_objective": {"answer": "Subjective", "rationale": "Because \"no matter\" is a subjective feeling and attitude, reflecting the patient鈥檚 perception and appreciation of self-worth. Although the sentence refers to comparisons with others, it is only a situational description, with emphasis on the patient鈥檚 subjective attitude."}, "sentence_sentiment": {"answer": "Negative", "rationale": "Because the attitude of the entry is \"I don't care,\" and this attitude is usually considered negative because it shows a lack of self-proclaimment and motivation."}, "language_style": {"answer": "Tactful and warm", "rationale": "The entry raises questions in a way that encourages and supports, rather than directly criticizing or challenging each other, a language that is comfortable and inspiring. At the same time, it conveys a tolerant and receptive attitude that helps to build good relationships and foster positive dialogue."}, "time_dimension": {"answer": "Present", "rationale": ""}, "sentence_type": "Negative declarative sentence", "pos": {"nouns": {"Yes": 1, "person": 1}, "verbs": {"Do it": 1, "- Yeah": 1, "Bad": 1, "None": 1}, "adjectives": {"Don't": 1, "Okay": 1}, "adverbs": {"Both": 1, "Yeah": 1, "It doesn't matter": 1}, "pronouns": {"I": 1}}}, {"content": "I'm not in a hurry. I'm in no hurry. I'm in no hurry.", "word_count": 23, "label": "", "sentence_structure": "Subject鈥搗erb鈥揷omplement", "subjective_objective": {"answer": "Subjective", "rationale": "Because it's the patient's description and perception of his emotions."}, "sentence_sentiment": {"answer": "Neutral", "rationale": "Because the entry does not have any emotional color."}, "language_style": {"answer": "Calm and direct", "rationale": "The entry describes the attitude and approach of the other in the face of the matter in an objective and calm manner, without using excessive rhetorical or emotional overtook. It is more plain, direct, and it feels like it is telling the truth rather than expressing feelings or opinions. It is therefore considered that the language style of the article is straightforward."}, "time_dimension": {"answer": "Present", "rationale": ""}, "sentence_type": "Negative declarative sentence", "pos": {"nouns": {"Yes": 3, "- It's urgent": 1, "It works": 1, "Wrong": 1}, "verbs": {"Do it": 1, "Urgent": 2, "Use": 1, "Error": 1, "Yeah": 3}, "adjectives": {"not": 4}, "adverbs": {"not": 4, "Urgent": 2}, "numerals": {"19": 1}, "pronouns": {"I": 1}}}, {"content": "I never thought I'd do what I wanted.", "word_count": 15, "label": "", "sentence_structure": "Subject鈥搗erb鈥搊bject", "subjective_objective": {"answer": "Subjective", "rationale": "This is a subjective view and idea of the patient and an assessment of self-activism."}, "sentence_sentiment": {"answer": "Negative", "rationale": "Because the attitude of \"not thinking about doing what you want\" in the entry is negative."}, "language_style": {"answer": "Tactful and warm", "rationale": "The entry is a soft and warm language. It uses a question-and-answer tone, not a direct statement or order. At the same time, the entry does not express criticism or accusations against the person under investigation, but rather engages in a softly invited dialogue. This expression is more intimate and interactive, helping to create a good investigation climate and to better attract respondents to the questionnaire."}, "time_dimension": {"answer": "Past", "rationale": ""}, "sentence_type": "Negative declarative sentence", "pos": {"nouns": {"Questionnaire entries": 1, "Thoughts": 1, "Things": 1}, "verbs": {"Yes": 1, "Do it": 1}, "adverbs": {"Never": 1, "self": 2, "Yes": 1}, "classifiers": {"individual": 1}, "pronouns": {"I": 1}}}, {"content": "21. Every day, everything makes me nervous.", "word_count": 14, "label": "", "sentence_structure": "Subject鈥揷opula鈥損redicative", "subjective_objective": {"answer": "Subjective", "rationale": "Because the sentence describes the patient ' s own feelings and experiences and is a subjective observation."}, "sentence_sentiment": {"answer": "Negative", "rationale": "Because the description \"sweetly nervous\" shows a negative emotional attitude."}, "language_style": {"answer": "Calm and direct", "rationale": "The entry uses direct and concise language, which clearly expresses the subject of asking the other side whether they are feeling very nervous every day, without too many rhetorical or emotional renderings, and is more objective in tone."}, "time_dimension": {"answer": "Present", "rationale": ""}, "sentence_type": "Affirmative declarative sentence", "pos": {"nouns": {"Things": 1, "Spirit": 1, "Tens": 1, "Nervous": 1}, "verbs": {"鈾?And I'm gonna be 鈾?: 1}, "adverbs": {"Chile": 1, "Both": 1, "Tens": 1}, "numerals": {"21": 1}}}, {"content": "22. I'm going to play, like, the park, and I always finish reading it, waiting for the people I'm with.", "word_count": 22, "label": "", "sentence_structure": "Subject鈥搗erb鈥搊bject", "subjective_objective": {"answer": "Subjective", "rationale": "Because \"I\" is the patient's own subjective feeling and behavior."}, "sentence_sentiment": {"answer": "Positive", "rationale": "Because the behavior in the entries is positive, and the purpose is to wait for the same person."}, "language_style": {"answer": "Tactful and warm", "rationale": "Warm and warm."}, "time_dimension": {"answer": "Present", "rationale": ""}, "sentence_type": "Affirmative declarative sentence", "pos": {"nouns": {"Park": 1}, "verbs": {"- Go on": 1, "Look": 1, "Wait": 1, "Come on": 1}, "adverbs": {"Yeah": 1, "Always": 1}, "numerals": {"22": 1}, "classifiers": {"individual": 1}, "pronouns": {"I": 1, "The same people": 1}}}, {"content": "I can't always tolerate people's shortcomings and problems.", "word_count": 15, "label": "", "sentence_structure": "Subject鈥損redicate", "subjective_objective": {"answer": "Subjective", "rationale": "Because intolerance is the patient's subjective attitude."}, "sentence_sentiment": {"answer": "Negative", "rationale": "Because \"intolerant of others' shortcomings and problems\" in the entry is a negative emotion and attitude."}, "language_style": {"answer": "Calm and direct", "rationale": "The entry is presented in direct language, with no excessive emotional accommodations or euphemisms, and therefore the language style is more incriminating and direct."}, "time_dimension": {"answer": "Present", "rationale": "The keyword is \"I can't often\" to indicate that it's the person who's been tested, or that it's not always\" to indicate that it's a regular act, so the temporal dimension is now."}, "sentence_type": "Negative declarative sentence", "pos": {"nouns": {"Questionnaire Entry": 1, "Disadvantages": 1, "Mistake": 1}, "verbs": {"Often": 1, "No, I can't": 1, "Tolerance": 1}, "numerals": {"23": 1}, "pronouns": {"I": 1}}}, {"content": "24. Of all the people I know, I like them all.", "word_count": 13, "label": "", "sentence_structure": "Subject鈥搗erb鈥搊bject", "subjective_objective": {"answer": "Subjective", "rationale": "Because it is the patient's emotional attitude towards others and his assessment, whether or not he likes it, that is, the individual's subjective feeling."}, "sentence_sentiment": {"answer": "Neutral", "rationale": "Because the entry describes the general attitude of individuals towards the people they know, it does not express a positive or negative emotional profile."}, "language_style": {"answer": "Tactful and warm", "rationale": "The language style of the entry is more euphemistic and implicit, the term is soft and polite, and does not directly express a strong emotional tendency. At the same time, the expression \"one by one\" is used to emphasize a comprehensive and inclusive approach. This makes the questionnaire more acceptable to respondents and encourages them to answer questions in good faith."}, "time_dimension": {"answer": "Present", "rationale": "The keyword \"persons I know\" means that it's a description of the current human relationship, and \"I like everyone\" means that it's a general attitude or behavior, so the time dimension of this article is now."}, "sentence_type": "Affirmative declarative sentence", "pos": {"nouns": {"person": 1, "Yeah": 1}, "verbs": {"Yes": 1, "be": 1, "Both": 1}, "adverbs": {"- A few": 1}, "numerals": {"24锛?: 1}, "pronouns": {"I": 1}}}, {"content": "I always wanted to correct him immediately when I heard people say incorrect things.", "word_count": 22, "label": "", "sentence_structure": "Subject鈥搗erb鈥揷omplement", "subjective_objective": {"answer": "Subjective", "rationale": "Because \"go right now and fix him\" is the patient's psychological activity and attitude, and it's subjective."}, "sentence_sentiment": {"answer": "Positive", "rationale": "Because the purpose of the entry is to correct him immediately."}, "language_style": {"answer": "Calm and direct", "rationale": "The entry is directly presented in order to ask the respondents about their attitudes and behaviour in the face of incorrect opinions of others, without excessive rhetoric or emotional rendering, and with a more calm and objective language style."}, "time_dimension": {"answer": "Present", "rationale": "The keyword \"I always want to correct him immediately\" means it's a statement describing the person being tested for his current behavior. Time dimension is now."}, "sentence_type": "Imperative sentence", "pos": {"nouns": {"person": 2, "Observations": 1, "Correct": 1}, "verbs": {"Yes": 1, "Publication": 1, "I want to go": 1, "Correct": 1}, "adverbs": {"Immediately": 1}, "numerals": {"25": 1}, "pronouns": {"I": 1, "he": 1}}}, {"content": "I'm a little faster than anyone else, no matter what.", "word_count": 14, "label": "", "sentence_structure": "Subject鈥揷opula鈥損redicative", "subjective_objective": {"answer": "Subjective", "rationale": "It is the patient's subjective attitude, who thinks he is faster than others."}, "sentence_sentiment": {"answer": "Positive", "rationale": "Because the description \"all sooner\" in the entry is positive."}, "language_style": {"answer": "Calm and direct", "rationale": "The entry describes a subjective feeling in a concise and clear language, without using a rhetorical or emotional projection, which is more calm and objective."}, "time_dimension": {"answer": "Present", "rationale": "\"I am faster than others\" is an assessment of personal abilities, does not relate to past or future situations, and therefore the judgement is now."}, "sentence_type": "Affirmative declarative sentence", "pos": {"nouns": {"Things": 1, "person": 1}, "verbs": {"Do it": 1, "- Yeah": 1, "Come on": 1}, "adjectives": {"Come on": 1}, "adverbs": {"Both": 1}, "numerals": {"26": 1, "One": 1}, "pronouns": {"I": 1, "others": 1}}}, {"content": "I'm considered to be a simple, profitable, efficient person.", "word_count": 17, "label": "", "sentence_structure": "Subject鈥揷opula鈥損redicative", "subjective_objective": {"answer": "Subjective", "rationale": "Because this part of the \"conception\" contains the views and assessments of others and is subjective."}, "sentence_sentiment": {"answer": "Positive", "rationale": "Because the descriptions of \"pull,\" \"profit\" and \"efficient\" in the entries are positive qualities."}, "language_style": {"answer": "Calm and direct", "rationale": "The entry uses objective descriptive terms such as \"pull,\" \"profit\" and \"efficient\", without too much emotional or pedigree language, and is more straightforward and calm."}, "time_dimension": {"answer": "Present", "rationale": "\"The entry describes the perception and evaluation of the person tested, based on the knowledge and understanding of the moment, so the temporal dimension is now judged.\""}, "sentence_type": "Affirmative declarative sentence", "pos": {"nouns": {"person": 1, "Questionnaire Entry": 1, "Well, let's just say": 1, "Leaning": 1, "Efficiency": 1}, "verbs": {"I think": 1, "be": 1, "I": 1, "One": 1, "Be": 1}, "adjectives": {"Well, let's just say": 1, "Leaning": 1, "Efficiency": 1}, "numerals": {"27锛?: 1}, "classifiers": {"individual": 1}, "pronouns": {"People": 1}}}, {"content": "I always felt like I was capable of doing everything right.", "word_count": 15, "label": "", "sentence_structure": "Subject鈥搗erb鈥揷omplement", "subjective_objective": {"answer": "Subjective", "rationale": "Because this is the patient ' s perception and assessment of himself, it is subjective feeling and attitude."}, "sentence_sentiment": {"answer": "Positive", "rationale": "Because it expresses a self-acceptance and self-confidence and positive emotional colours."}, "language_style": {"answer": "Tactful and warm", "rationale": "The entry is asked in a positive and encouraging manner, with a more positive and optimistic language style, without directly expressing criticism or challenge, and gives a sense of affection and encouragement."}, "time_dimension": {"answer": "Present", "rationale": "In the entry, \"always feel\" expresses a constant feeling or belief, and \"with the ability to do everything well\" describes a state of the moment or a capability, and therefore judges it as now."}, "sentence_type": "Affirmative declarative sentence", "pos": {"nouns": {"Capacity": 1, "Things": 1}, "verbs": {"I always thought": 1, "It can": 1, "Do it": 1}, "adjectives": {"Everything": 1}, "numerals": {"28锛?: 1}, "pronouns": {"I": 2}}}, {"content": "I'm always anxious to say what I think and even interrupt people's voices.", "word_count": 24, "label": "", "sentence_structure": "Subject鈥搗erb鈥揷omplement", "subjective_objective": {"answer": "Subjective", "rationale": "Because \"suspecting to say what you think\" and \"staggering\" are subjective behaviors and attitudes of patients."}, "sentence_sentiment": {"answer": "Negative", "rationale": "Because \"suspect to say what you think\" and \"stagger\" are negative."}, "language_style": {"answer": "Tactful and warm", "rationale": "The entry uses the first-person pronoun \"I\" and introduces emotional colours (such as \"always eager\" to \"even interrupt\") in describing behaviour, which makes it easier for readers to resonate and also more intimate and relevant. Moreover, \"talking\" also emphasizes that it is a matter of interpersonal interaction, not of personal habits or character defects, which makes the questionnaire more constructive and positive guidance."}, "time_dimension": {"answer": "Present", "rationale": "The words \"always\" and \"interrupted words\" denote the behaviour of the subjects in the chat, which have the current state of affairs, and the topic should therefore be the present."}, "sentence_type": "Negative declarative sentence", "pos": {"nouns": {"Chat": 1, "Thoughts": 1, "Word": 2}, "verbs": {"Say it": 3, "Interrupt": 1, "Out": 1}, "adverbs": {"Total": 1, "Yeah": 1, "Urgent": 1, "Even": 1}, "numerals": {"29": 1}, "classifiers": {"individual": 1}, "pronouns": {"self": 1, "others": 1}}}, {"content": "I'm considered to be quiet, sunk, patient.", "word_count": 16, "label": "", "sentence_structure": "Subject鈥揷opula鈥損redicative", "subjective_objective": {"answer": "Subjective", "rationale": "Because my assessment is a subjective view."}, "sentence_sentiment": {"answer": "Positive", "rationale": "Because the words \"quiet, sunk, patient\" in the entry are all positive."}, "language_style": {"answer": "Calm and direct", "rationale": "The language style of the entry is a calm and straightforward type, because it directly presents the perception of \"me\" and is not rendered by the use of rhetorical or emotional colours. This expression is usually objective and direct, and can effectively convey the message."}, "time_dimension": {"answer": "Past", "rationale": "So the time dimension of this entry is past."}, "sentence_type": "Affirmative declarative sentence", "pos": {"nouns": {"person": 2, "Patience": 1, "鈾?Sinking 鈾?: 1, "Quiet": 1}, "verbs": {"I think": 1, "be": 1}, "adjectives": {"individual": 1, "have": 1, "Be quiet": 1, "It's heavy": 1, "Patience": 1}, "numerals": {"30": 1}, "pronouns": {"I": 1}}}, {"content": "I think there are few people I know who deserve my trust and admiration.", "word_count": 25, "label": "", "sentence_structure": "Subject鈥揷opula鈥損redicative", "subjective_objective": {"answer": "Subjective", "rationale": "Because \"I feel\" expresses the patient's subjective opinion."}, "sentence_sentiment": {"answer": "Negative", "rationale": "Because the phrase \"very few people I trust and admire\" in the entry is an expression of a negative emotional attitude."}, "language_style": {"answer": "Calm and direct", "rationale": "The language style of the entry is one of a calm and direct type. Instead of using too many rhetorical techniques or emotional renderings, it presents the matter in an objective and direct manner, allowing the person surveyed to understand and answer the question clearly. Moreover, the article is more semantic and has no overly emotional tone, making it easier for the person surveyed to express his or her true feelings and views."}, "time_dimension": {"answer": "Past", "rationale": "The entry expresses the views and opinions of the testees on their past, and the keywords are \"and very little\" among the people I know, all pointing to the past."}, "sentence_type": "Negative declarative sentence", "pos": {"nouns": {"person": 2, "I": 1, "Trust": 1, "I admire that": 1, "Centre": 1}, "verbs": {"I think": 1, "It's worth it": 1, "Yes": 1, "Not much": 1}, "adverbs": {"Really": 1}, "numerals": {"31": 1}}}, {"content": "I have many ideas and plans for the future, and I always want to be able to do it as soon as possible.", "word_count": 21, "label": "", "sentence_structure": "Subject鈥搗erb鈥搊bject", "subjective_objective": {"answer": "Subjective", "rationale": "Because the attitude of \"thinks and intentions about the future\" and \"realization\" are personal thoughts and aspirations of patients and are subjective in nature."}, "sentence_sentiment": {"answer": "Positive", "rationale": "Because the attitude in the entry, \"There are many ideas and intentions for the future and always want to be realized as soon as possible\", is positive and expresses expectations and expectations for the future."}, "language_style": {"answer": "Tactful and warm", "rationale": "This entry is expressed in a message of encouragement and understanding, asking if the other side has many plans and expectations for the future, and wants to be able to do so as soon as possible. This expression is more personal and interactive than direct orders or questioning."}, "time_dimension": {"answer": "Future", "rationale": ""}, "sentence_type": "Affirmative declarative sentence", "pos": {"nouns": {"The future": 1, "Thoughts": 1, "I'm going to": 1}, "verbs": {"have": 1, "Always": 1, "Yes": 1, "As soon as possible": 1, "Achieved": 1}, "adverbs": {"and": 1}, "numerals": {"32": 1}, "pronouns": {"I": 1}}}, {"content": "I can talk about people sometimes.", "word_count": 11, "label": "", "sentence_structure": "Subject鈥搗erb鈥揷omplement", "subjective_objective": {"answer": "Subjective", "rationale": "Because \"talking\" is a subjective act, which reflects the mind and attitude of people."}, "sentence_sentiment": {"answer": "Negative", "rationale": "Because this \"speaks the gossip\" is generally considered a negative act, damaging human relationships."}, "language_style": {"answer": "Calm and direct", "rationale": "The entry uses a simple and clear language style, which does not add too much emotional or rhetorical style, and is more objective. It asks the respondents directly whether they have had any experience with gossip, and is plain and simple to understand. So the language style for this purpose is straightforward and calm."}, "time_dimension": {"answer": "Present", "rationale": "Therefore, the content of this questionnaire entry can be judged as now in terms of time dimension."}, "sentence_type": "Exclamatory sentence", "pos": {"nouns": {"Time": 1, "I": 2, "He's": 1, "Baloney": 1}, "verbs": {"Yes": 1, "Say it": 1}, "adverbs": {"Yeah": 1, "Yes": 1}, "numerals": {"33锛?: 1}}}, {"content": "34. I eat fast, despite the time available.", "word_count": 12, "label": "", "sentence_structure": "Subject鈥搗erb鈥搊bject", "subjective_objective": {"answer": "Subjective", "rationale": "Because the entry is primarily concerned with patients鈥?subjective perception and perception of their own behaviour, i.e., their perception of and attitude to their eating habits. Although this behaviour occurs objectively (i.e., at a fast pace of eating over a wide period of time), the surveyer is concerned with the patient鈥檚 subjective perception and perception, and therefore is subjective in nature."}, "sentence_sentiment": {"answer": "Negative", "rationale": "Because the \"Eat fast\" act in the entry is negative."}, "language_style": {"answer": "Calm and direct", "rationale": "The entry describes the habits of the person under investigation in a straightforward manner, with no excessive rhetoric or emotional rendering, and is more objective in tone."}, "time_dimension": {"answer": "Present", "rationale": ""}, "sentence_type": "Affirmative declarative sentence", "pos": {"nouns": {"Time": 1, "Rice": 1}, "verbs": {"Yu Yu": 1, "Eat": 1, "Come on": 1}, "adjectives": {"very": 1}, "adverbs": {"鈾?Though 鈾?: 1}, "numerals": {"34": 1, "very": 1}}}, {"content": "I'm in a hurry to hear a speech or report, but I'm not sure I can tell you!", "word_count": 26, "label": "", "sentence_structure": "Subject鈥搗erb鈥揷omplement", "subjective_objective": {"answer": "Subjective", "rationale": "Because \"very anxious\" and \"so much to say!\" are subjective reactions and evaluations of people who speak or report."}, "sentence_sentiment": {"answer": "Negative", "rationale": "Because the words \"very anxious\" and \"like to say!\" in the entries reflect dissatisfaction with the expression of self-worth and are negative emotions and attitudes."}, "language_style": {"answer": "Tactful and warm", "rationale": "The entry is more personal and interactive when it is presented in a soft, inviting tone, rather than in direct statements or orders. At the same time, it is used in a personalized language that gives respondents a sense of interest and understanding, increasing the participation and effectiveness of the questionnaire."}, "time_dimension": {"answer": "Present", "rationale": "The entry describes the emotional reactions and thoughts of the subjects when they hear others speak or report bad, which are currently being carried out and are therefore judged in terms of time dimension."}, "sentence_type": "Affirmative declarative sentence", "pos": {"nouns": {"person": 1, "Report": 1, "Speech": 1, "I'm in a hurry": 1}, "verbs": {"Listen": 1, "Speak": 2, "Urgent": 1}, "adverbs": {"Like": 1, "Very": 1}, "numerals": {"35": 1}, "pronouns": {"Just": 1, "Always": 1, "Why don't you": 1}}}, {"content": "Even if someone bullied me, I don't care.", "word_count": 13, "label": "", "sentence_structure": "Subject鈥搗erb鈥搊bject", "subjective_objective": {"answer": "Subjective", "rationale": "Because \"not caring\" is the patient's own thoughts and attitudes, and it's subjective."}, "sentence_sentiment": {"answer": "Negative", "rationale": "Because the attitude of \"not caring\" in the entries is negative."}, "language_style": {"answer": "Calm and direct", "rationale": "The language style of the entry appears to be a little indifferent and unsure, and shows a lack of sensitivity to the bullying, reflecting no emotional fluctuations or warmth, and therefore can be judged as \"crazy\"."}, "time_dimension": {"answer": "Present", "rationale": "The entry is an inquiry into the current behaviour and mentality of the testee, and the description of \"not caring\" and \"not caring\" indicates that this is an attitude of concern about the current situation, and the temporal dimension is now."}, "sentence_type": "Negative declarative sentence", "pos": {"nouns": {"Bullying": 1, "I": 2}, "verbs": {"Bullying": 1, "I care": 1}, "adverbs": {"Even if": 1}}}, {"content": "I sometimes drag things up to tomorrow.", "word_count": 17, "label": "", "sentence_structure": "Subject鈥損redicate", "subjective_objective": {"answer": "Subjective", "rationale": "This is a statement of the patient ' s self-perception, which relates to the perception of personal habits and preferences and is subjective."}, "sentence_sentiment": {"answer": "Negative", "rationale": "Because the entry \"Tow what we should do today to tomorrow\" is negative."}, "language_style": {"answer": "Calm and direct", "rationale": "The use of a simple language directly raises the question, without adding too many rhetorical or emotional techniques and with a more balanced and objective tone."}, "time_dimension": {"answer": "Past", "rationale": "So the entry for the questionnaire is to ask the testee about the behaviour of the present or the past, and the time dimension is judged as present and the past."}, "sentence_type": "Exclamatory sentence", "pos": {"nouns": {"Yes": 1, "Oh, my God": 1}, "verbs": {"Do it": 1, "Drag": 1, "have": 1}, "adverbs": {"Sometimes": 1, "Yes": 1}, "numerals": {"37锛?: 1}, "pronouns": {"I": 1}}}, {"content": "I'm not polite to him when others are rude to me.", "word_count": 15, "label": "", "sentence_structure": "Subject鈥搗erb鈥揷omplement", "subjective_objective": {"answer": "Subjective", "rationale": "Because the judgment and attitude of the patient is subjective in the case of indecent assault."}, "sentence_sentiment": {"answer": "Negative", "rationale": "Because the action in the entry \"is also unkind to him\" is negative and represents a revenge and hostility."}, "language_style": {"answer": "Calm and direct", "rationale": "The article is a strong and direct expression, without excessive emotional or euphemism, which directly indicates a reaction to the indecent behaviour of others. Moreover, it is more objective and neutral, without any emotional or subjective conjecture, and therefore can be considered as a calm and straightforward language style."}, "time_dimension": {"answer": "Past", "rationale": ""}, "sentence_type": "Affirmative declarative sentence", "pos": {"nouns": {"person": 2, "he": 1, "Insolent": 1, "I": 2, "You're welcome": 1}, "verbs": {"When": 1, "to": 2, "be": 1, "Yeah": 1, "Insolent": 1}, "adjectives": {"Don't": 1}, "adverbs": {"Time": 1}, "numerals": {"38锛?: 1}}}, {"content": "39. When someone is asking questions about me or my work, it's easy to discourage me.", "word_count": 24, "label": "", "sentence_structure": "Subject鈥搗erb鈥搊bject", "subjective_objective": {"answer": "Subjective", "rationale": "Because the sentence expresses the feelings and attitudes of the patient when facing criticism from others, it falls within the realm of subjective emotion."}, "sentence_sentiment": {"answer": "Negative", "rationale": "Because the \"Easy to Violate my Motivation\" act in the entry is negative."}, "language_style": {"answer": "Calm and direct", "rationale": ""}, "time_dimension": {"answer": "Past", "rationale": ""}, "sentence_type": "Negative declarative sentence", "pos": {"nouns": {"person": 1, "Active": 1, "Work": 1}, "verbs": {"To me": 1, "- You're a liar": 1, "Frustration": 1, "Easy": 1, "have": 1}, "adverbs": {"very": 1}, "numerals": {"39": 1, "One": 2}, "classifiers": {"individual": 1}, "pronouns": {"I": 1}}}, {"content": "I often feel late, but I'm early to see the watch.", "word_count": 18, "label": "", "sentence_structure": "Subject鈥揷opula鈥損redicative", "subjective_objective": {"answer": "Subjective", "rationale": "Because of the difference in the time and actual time, it is the patient ' s subjective feeling."}, "sentence_sentiment": {"answer": "Neutral", "rationale": "Because the entry describes a situation, it does not express any positive or negative emotional tendencies."}, "language_style": {"answer": "Tactful and warm", "rationale": "The entry is more personal and interactive when it is presented in a gentle and inviting tone, rather than in direct statements or orders. At the same time, it uses a daily language style that is more understandable and acceptable to the interviewees."}, "time_dimension": {"answer": "Present", "rationale": "I often feel that the word \"normal\" means \"regular, customary behaviour,\" that it's taking the current state of affairs, that it's happening now. So the time dimension of this questionnaire entry is now."}, "sentence_type": "Negative declarative sentence", "pos": {"nouns": {"Time": 2, "Table": 1}, "verbs": {"Sensor": 1, "Look": 1, "Late": 1, "Morning": 1}, "adjectives": {"Often": 1, "Already": 1, "And": 1}, "adverbs": {"Yeah": 1}, "numerals": {"40": 1}, "pronouns": {"I": 1}}}, {"content": "I feel like I'm a very sensitive person.", "word_count": 18, "label": "", "sentence_structure": "Subject鈥揷opula鈥損redicative", "subjective_objective": {"answer": "Subjective", "rationale": "Because \"sensitive\" is a subjective feeling and self-awareness."}, "sentence_sentiment": {"answer": "Positive", "rationale": "Because the \"very sensitive\" character of the entry is usually seen as a positive quality."}, "language_style": {"answer": "Tactful and warm", "rationale": "The entry, which is based on the first person's first name, uses the descriptive adjective \"very sensitive\" to express a more subjective feeling. At the same time, the whole sentence is relatively gentle and friendly, with little emphasis or criticism, and is more acceptable to the interviewees."}, "time_dimension": {"answer": "Present", "rationale": ""}, "sentence_type": "Affirmative declarative sentence", "pos": {"nouns": {"I": 1, "person": 2, "Yes": 1}, "verbs": {"I think": 1, "be": 1, "to": 2}, "adjectives": {"Very": 1, "Sensitive": 1}, "numerals": {"41锛?: 1}}}, {"content": "I've always been in a hurry, trying to do as much as I can with as little time as I can.", "word_count": 25, "label": "", "sentence_structure": "Subject鈥搗erb鈥搊bject", "subjective_objective": {"answer": "Subjective", "rationale": "Because \"hurry\" and \"as many things as possible\" are the feelings and thoughts of the patient, so subjective."}, "sentence_sentiment": {"answer": "Negative", "rationale": "Because the \"hurry\" act in the entry is negative."}, "language_style": {"answer": "Calm and direct", "rationale": "The entry uses a simple and clear language, which directly expresses the subject of asking the other party whether it is often in haste to do things and trying to do as many tasks as possible in the shortest time."}, "time_dimension": {"answer": "Present", "rationale": "The entry describes the present behaviour of the testee, and the keywords \"always\" and \"attempt\" indicate that it is a general, customary act and therefore the judgement is now."}, "sentence_type": "Affirmative declarative sentence", "pos": {"nouns": {"Yes": 2, "Time": 1, "Problem": 1}, "verbs": {"Do it": 1, "be": 1, "Hurry up": 1, "OPERATIONS": 1, "Try": 1, "Use": 1, "At least": 1, "As much as you can": 1}, "adjectives": {"It's a rush": 1}, "adverbs": {"Always": 1, "Try": 1}, "numerals": {"42": 1, "More": 1}, "classifiers": {"individual": 1}, "pronouns": {"I": 1}}}, {"content": "If you make a mistake, I'll admit it.", "word_count": 17, "label": "", "sentence_structure": "Subject鈥損redicate", "subjective_objective": {"answer": "Subjective", "rationale": "It is subjective to admit that the decision to admit the error is left to the patient himself."}, "sentence_sentiment": {"answer": "Positive", "rationale": "Because \"prosperately admit\" is positive."}, "language_style": {"answer": "Calm and direct", "rationale": "The language style of the entry is relatively calm and objective, with no excessive rhetoric or emotional rendering, and a point of view is presented directly."}, "time_dimension": {"answer": "Present", "rationale": "The keywords \"performed wrong\", \"ever size\" and \"all-in-out admission\" indicate that it describes the present attitude and behaviour of the testee, so it is judged now."}, "sentence_type": "Negative declarative sentence", "pos": {"nouns": {"Error": 1, "Perpetrators": 1, "Size": 1}, "verbs": {"All": 1, "Both": 1, "Initiatives": 1, "Recognition": 1}, "adverbs": {"Whatever": 1}, "numerals": {"43": 1}, "pronouns": {"I": 1}}}, {"content": "When I was on a bus, I always felt too slow, even though I was driving fast.", "word_count": 23, "label": "", "sentence_structure": "Subject鈥搗erb鈥揷omplement", "subjective_objective": {"answer": "Subjective", "rationale": "Because \"I feel too slow to drive\" is a personal subjective feeling of the patient."}, "sentence_sentiment": {"answer": "Negative", "rationale": "Because the attitude of \"I feel too slow\" in the entry is negative."}, "language_style": {"answer": "Calm and direct", "rationale": "The entry is more straightforward in language style, with no excessive rhetorical or emotional rendering. It opens up to a question and expresses the subject of the investigation in a more objective and calm manner by the guiding phrase \"I often feel too slow to drive very fast.\""}, "time_dimension": {"answer": "Past", "rationale": "\"The car is driving fast\" and \"often feels\" indicates that the act has been repeated in the past and is therefore judged to be past."}, "sentence_type": "Negative declarative sentence", "pos": {"nouns": {"Bus": 1, "Car": 2, "person": 1}, "verbs": {"Sit down": 1, "Open": 3, "Sensor": 1}, "adjectives": {"Come on": 1, "Slow": 1}, "adverbs": {"Too much": 2}, "numerals": {"44": 1, "One": 1, "Two": 1}, "classifiers": {"Vehicles": 1, "individual": 1}, "pronouns": {"I": 1}}}, {"content": "I don't want to do anything for him, even if I'm not good at it.", "word_count": 24, "label": "", "sentence_structure": "Subject鈥損redicate", "subjective_objective": {"answer": "Objective", "rationale": "Because the content of the entry is a description of a person's behaviour or attitude, which does not involve the patient's subjective feelings or ideas, but is based on objective facts. For example, \"Looking at someone else's bad\" and \"doing no want for him\" are both observable acts or facts, and the entry is therefore viewed from an objective perspective."}, "sentence_sentiment": {"answer": "Negative", "rationale": "Because the \"no want to do for him\" act in the entry reflects a negative attitude and emotional colour."}, "language_style": {"answer": "Calm and direct", "rationale": "The language style of the entry is more straightforward and not too much adoration or emotional, but rather a more objective and rational way of expressing, aimed at understanding the attitude of the person under investigation towards the behaviour of others."}, "time_dimension": {"answer": "Past", "rationale": "The keyword \"do whatever\" and \"I don't want to do it for him, even if I look at others, \" is a description of the situation, without any reference to the past, or any vision or expectation of the future. So the entry to the questionnaire is the time dimension of the present."}, "sentence_type": "Negative declarative sentence", "pos": {"nouns": {"Yes": 2, "others": 1, "he": 1}, "verbs": {"Do it": 3, "Watch": 1, "Yes": 1, "Substitute": 1, "Take it": 1}, "adverbs": {"Whatever": 1, "What": 1}, "numerals": {"45": 1}, "pronouns": {"I": 1}}}, {"content": "I'm often worried about the work that's not done.", "word_count": 20, "label": "", "sentence_structure": "Subject鈥搗erb鈥搊bject", "subjective_objective": {"answer": "Subjective", "rationale": "Because \"often\" and \"fear\" are words that describe patients' own subjective feelings and attitudes."}, "sentence_sentiment": {"answer": "Negative", "rationale": "Because the \"fear\" emotion in the entry is negative."}, "language_style": {"answer": "Tactful and warm", "rationale": "The entry is based on a more moderate expression of the subject of queries about whether the other party is often worried about the incompleteness of the work, is more objective in tone, is not directly stated or ordered, is more personal and interactive. Moreover, the article begins with the use of \"You are often\" , which makes it easier to resonate with the respondents, increases the effectiveness and accuracy of the questionnaire."}, "time_dimension": {"answer": "Past", "rationale": "The entry is therefore an inquiry into the present behaviour and mentality of the person being tested, and the time dimension is now judged."}, "sentence_type": "Affirmative declarative sentence", "pos": {"nouns": {"Work": 1, "One day": 1, "Past": 1}, "verbs": {"Do it": 1, "I feel": 1, "Fear": 1}, "adverbs": {"Often": 1, "And": 1}, "numerals": {"46锛?: 1}}}, {"content": "If I'm responsible for a lot of things, it's much better than now.", "word_count": 20, "label": "", "sentence_structure": "Subject鈥揷opula鈥損redicative", "subjective_objective": {"answer": "Subjective", "rationale": "This expression is a reflection of patients ' subjective attitudes and self-evaluation."}, "sentence_sentiment": {"answer": "Positive", "rationale": "Because the phrase \"I'll take care of it, it'll be much better.\" It's positive."}, "language_style": {"answer": "Tactful and warm", "rationale": "The language style of the entry is euphemism. Using hypothetical words like \"if I take responsibility\", it expresses a positive but modest attitude. At the same time, the sentence contains no language of direct accusation or criticism, but presents its views in an objective and moderate way. This language style can better attract the cooperation and participation of the respondents and facilitate access to real and accurate information."}, "time_dimension": {"answer": "Present", "rationale": ""}, "sentence_type": "Affirmative declarative sentence", "pos": {"nouns": {"Things": 1, "I": 1, "Responsible": 1}, "verbs": {"Come on": 1, "- Yeah": 1, "More": 1}, "adjectives": {"A lot": 1, "Situation": 1, "Much better": 1}, "numerals": {"47": 1}}}, {"content": "I sometimes think of bad ideas that I can't say.", "word_count": 16, "label": "", "sentence_structure": "Subject鈥搗erb鈥揷omplement", "subjective_objective": {"answer": "Subjective", "rationale": "Because it's the patient's subjective idea or the feeling inside."}, "sentence_sentiment": {"answer": "Negative", "rationale": "Because the \"bad thinking\" act in the entry is negative."}, "language_style": {"answer": "Calm and direct", "rationale": "The entry is a straightforward query to the interviewees whether they have had some unspoken and unspoken bad thoughts, and the language style is more direct and objective."}, "time_dimension": {"answer": "Present", "rationale": "The keyword \"sometimes\" indicates that it is describing the current behaviour or state of the subject, and that it is a continuing act, \"in the mind of some unspoken bad thoughts.\" The entry therefore determines the present dimension of the testee's mental state."}, "sentence_type": "Affirmative declarative sentence", "pos": {"nouns": {"Time": 1, "Some": 1, "Thinking": 1}, "verbs": {"Yes": 3, "Present": 1, "Say it": 1, "No exit": 1, "Bad": 1}, "adjectives": {"I can't say": 1, "Bad": 1}, "numerals": {"48": 1}, "pronouns": {"I": 1}}}, {"content": "49. I can obey and cooperate even if my leadership is weak and low-level.", "word_count": 25, "label": "", "sentence_structure": "Subject鈥搗erb鈥搊bject", "subjective_objective": {"answer": "Subjective", "rationale": "Because words like \"even\" and \"can\" indicate that it's the attitude and belief of the patient."}, "sentence_sentiment": {"answer": "Negative", "rationale": "The evaluation of \"poor leadership and low level\" in the entry is negative."}, "language_style": {"answer": "Calm and direct", "rationale": "The entry is based on a direct statement of the subject, without any qualifier or emotional rendering. It does not attempt to resonate or react to the person under investigation, but rather asks questions and waits for answers. This language style is more objective and practical than personalized emotional expression."}, "time_dimension": {"answer": "Present", "rationale": "The word \"capable\" in this entry uses the general current, indicating that it is the current attitude and behaviour of the testee. At the same time, the expression \"even if...\" emphasizes the persistence and stability of such attitudes and behaviour, and further supports this judgement."}, "sentence_type": "Negative declarative sentence", "pos": {"nouns": {"person": 2, "Capacity": 1, "Horizontal": 1, "Leadership": 1, "I": 1}, "verbs": {"Bad": 1, "Low": 1, "How's it going": 1, "Obey": 1, "Cooperation": 1}, "adjectives": {"Bad": 1, "Low": 1}, "numerals": {"49": 1}, "classifiers": {"individual": 1}, "pronouns": {"I": 1}}}, {"content": "I've always been impatient and impatient when I had to wait for something.", "word_count": 20, "label": "", "sentence_structure": "Subject鈥搗erb鈥搊bject", "subjective_objective": {"answer": "Subjective", "rationale": "Because \"crazy\" and \"insatiable\" are the subjective feelings and attitudes of the patients."}, "sentence_sentiment": {"answer": "Negative", "rationale": "Because \"crazy\" and \"insatiable\" are negative emotional terms."}, "language_style": {"answer": "Calm and direct", "rationale": "The language style of the entry is more direct, rather than over-modified or emotional, but it is rather an objective description of the person being investigated ' s inner feelings when it has to wait for something, which appears calm and direct."}, "time_dimension": {"answer": "Present", "rationale": "The keyword \"always\" indicates that it is a constant state and feeling, while the verb \"and so on\" implies that the act is taking place or has already taken place. The entry therefore asks about the person's current behaviour or mental state."}, "sentence_type": "Affirmative declarative sentence", "pos": {"nouns": {"Time": 1, "Wait": 1, "It's a rush": 1, "Patience": 1}, "verbs": {"I have to": 1, "Wait": 1, "be": 1, "Always": 1, "Lack": 1}, "adjectives": {"It's a rush": 1}, "adverbs": {"Always": 1}, "numerals": {"50锛?: 1}}}, {"content": "I often feel like I'm not capable of doing anything so I just want to quit when things go wrong.", "word_count": 29, "label": "", "sentence_structure": "Subject鈥搗erb鈥搊bject", "subjective_objective": {"answer": "Subjective", "rationale": "Because the subject's thoughts and feelings are described in terms of \"Esperating that you are not capable of doing well\", \"I want to quit when I have not done well\" and so on, the entry is examined from a subjective perspective."}, "sentence_sentiment": {"answer": "Negative", "rationale": "The basis for the judgement: the entries \"perception of lack of capacity\" and \"in case of a failure to do things, want to quit\" are both negative and express a lack of confidence and determination in their abilities and perseverance."}, "language_style": {"answer": "Tactful and warm", "rationale": "The language style of the entry is more euphemistic, and it guides the interviewee to share his or her feelings in the way he or she is asked, rather than to direct accusations or criticism. Moreover, the use of expressions such as \"often\" and \"inconvenient to abandon\" avoids overly emotional sentiments, making it easier for the interviewee to accept and share their thoughts and feelings."}, "time_dimension": {"answer": "Present", "rationale": ""}, "sentence_type": "Negative declarative sentence", "pos": {"nouns": {"Capacity": 1, "Things": 1, "It's not going well": 1, "Abandon": 1}, "verbs": {"I feel": 1, "Yes": 2, "Abandon": 1, "Do it": 1, "I met him": 1}, "adjectives": {"Often": 1, "Not enough": 1, "It's not good": 1}, "numerals": {"51": 1}, "pronouns": {"I": 1}}}, {"content": "I watch TV every day, and I watch movies, or I feel sick.", "word_count": 21, "label": "", "sentence_structure": "Subject鈥搗erb鈥搊bject", "subjective_objective": {"answer": "Subjective", "rationale": "Since it is the patient ' s choice and decision whether or not to read, read and duration on a daily basis, it is subjective."}, "sentence_sentiment": {"answer": "Positive", "rationale": "Because the entries are used to watch television, movies and are not used to television, films make people uncomfortable, it can be inferred that the respondents have a certain reliance on and enjoy television and films, which is a positive sentiment."}, "language_style": {"answer": "Tactful and warm", "rationale": "The language style of the entry is euphemistic and warm, expressing one鈥檚 needs and expectations by describing one鈥檚 feelings and habits, rather than directly ordering or asking the other to make some change. At the same time, the phrase 鈥渋nconveniently uncomfortable鈥?also reflects some emotional colours, making dialogue more personal."}, "time_dimension": {"answer": "Present", "rationale": "\"Everyday\", \"at the same time\" and \"or \"at least in a state of habitual, regular behavior or mental illness\" indicate that it occurs at the present time. Thus, the entry is now judged."}, "sentence_type": "Affirmative declarative sentence", "pos": {"nouns": {"Television": 1, "Film": 1, "Heart": 1}, "verbs": {"Look": 3, "I'm not feeling well": 1}, "adverbs": {"Chile": 1, "Yeah": 1, "Otherwise": 1}, "numerals": {"52": 1}, "pronouns": {"I": 1}}}, {"content": "I'll never delay anything I've been asked to do.", "word_count": 17, "label": "", "sentence_structure": "Subject鈥損redicate", "subjective_objective": {"answer": "Objective", "rationale": "Since the delay is an objective result, the subjective approach is subjective."}, "sentence_sentiment": {"answer": "Positive", "rationale": "Because the \"never delay\" in the entry is positive."}, "language_style": {"answer": "Calm and direct", "rationale": "The entry describes a person's attitude and habits in a more straightforward and concise manner, without excessive emotional accommodation or rhetoric. Thus, its language style can be described as \"cool straight\"."}, "time_dimension": {"answer": "Past", "rationale": "At the same time, the conditional \"as long as you have promised\" statement indicates that the act is based on a promise or a decision of the past. So the entire sentence is a customary summary of past behaviour, which is consistent with the characteristics of the past."}, "sentence_type": "Affirmative declarative sentence", "pos": {"nouns": {"Yes": 1, "Promise": 1, "Delay": 1}, "verbs": {"Tot": 1, "OPERATIONS": 1, "Promise": 1, "Delay": 1}, "adverbs": {"Never": 1}, "numerals": {"53": 1}, "pronouns": {"I": 1, "others": 1}}}, {"content": "People say I'm patient. I don't have to rush anything.", "word_count": 17, "label": "", "sentence_structure": "Subject鈥揷opula鈥損redicative", "subjective_objective": {"answer": "Subjective", "rationale": "Because \"someone says I'm patient\" is someone else's opinion or perception of himself, subjectively."}, "sentence_sentiment": {"answer": "Positive", "rationale": "Because the entries \"suspensive\" and \"no rush\" are both positive descriptions."}, "language_style": {"answer": "Tactful and warm", "rationale": "This entry expresses each other's characteristics in a complimentary and positive way and adds more persuasive language by \"people say\". This language style is more moderate and friendly, and it can effectively increase the motivation and collaboration of respondents to the questionnaire."}, "time_dimension": {"answer": "Past", "rationale": "So the answer is \"the past\"."}, "sentence_type": "Affirmative declarative sentence", "pos": {"nouns": {"person": 1, "Patience": 1, "Yes": 1}, "verbs": {"Say it": 1, "have": 1, "Fuck": 1, "No hurry": 1}, "adjectives": {"very": 2, "Both": 1, "Urgent": 1}, "adverbs": {"Both": 1}, "numerals": {"54": 1}, "pronouns": {"I": 1}}}, {"content": "I'm rarely late when I'm in a car, boat or time for work. I'm upset if the other party's late.", "word_count": 29, "label": "", "sentence_structure": "Subject鈥損redicate", "subjective_objective": {"answer": "Subjective", "rationale": "Because it's the patient's own subjective experience and emotional response."}, "sentence_sentiment": {"answer": "Negative", "rationale": "Because \"very rarely late\" is neutral, and \"if the other party gets upset\" is a negative emotional attitude."}, "language_style": {"answer": "Calm and direct", "rationale": "The language style of the entry is calm and straightforward, because it asks the respondents whether they will be angry or angry in certain circumstances. It is not too emotionally overt or euphemistic, but rather presents the problem in a straightforward way. The style is objective and clear, enabling the respondents to quickly understand the questions and answer them accurately."}, "time_dimension": {"answer": "Present", "rationale": ""}, "sentence_type": "Affirmative declarative sentence", "pos": {"nouns": {"Out": 1, "- By car": 1, "Boat": 1, "person": 2, "Time": 1, "Operations": 1, "Yes": 1}, "verbs": {"I": 3, "Very few": 1, "Late": 1, "Hold on": 1, "I'm angry": 1, "A promise": 1}, "adjectives": {"Very few": 1}, "adverbs": {"very": 2}, "numerals": {"55": 1, "1": 7}, "classifiers": {"individual": 1}, "pronouns": {"I": 3}}}, {"content": "I say a lie or two sometimes.", "word_count": 11, "label": "", "sentence_structure": "Subject鈥搗erb鈥揷omplement", "subjective_objective": {"answer": "Subjective", "rationale": "Because it is the patient ' s subjective attitude and choice to say a lie or a lie."}, "sentence_sentiment": {"answer": "Negative", "rationale": "Because the \"false\" act in the entry is negative."}, "language_style": {"answer": "Tactful and warm", "rationale": "The language style of the entry is more euphemistic and moderate, rather than asking whether the other party is often lying, but rather directing the respondents to share their true situation by asking questions, which is more personal and interactive."}, "time_dimension": {"answer": "Present", "rationale": "The keyword \"frequently\" and \"also\" indicates that the act is present and may not occur frequently, so the entry describes the person being tested for the act or habit."}, "sentence_type": "Affirmative declarative sentence", "pos": {"nouns": {"That's a lie": 1, "Questionnaire Entry": 1}, "verbs": {"Yes": 1, "Sometimes": 1, "Yeah": 1}, "adverbs": {"Sometimes": 1, "Yes": 1, "Yeah": 1}, "numerals": {"56锛?: 1}, "pronouns": {"I": 1}}}, {"content": "There are many things that we could have shared, but I like to do it alone.", "word_count": 20, "label": "", "sentence_structure": "Subject鈥搗erb鈥搊bject", "subjective_objective": {"answer": "Subjective", "rationale": "Because it is a subjective feeling and attitude of one person. While this behaviour involves the affairs of others, the decision of those who choose to assume it alone is based on their own ideas and values and is therefore still viewed from a subjective point of view."}, "sentence_sentiment": {"answer": "Negative", "rationale": "Because the \"I like to fuck one person\" thing in the entry is negative."}, "language_style": {"answer": "Calm and direct", "rationale": "The entry expresses in a direct manner the preference of the interviewee for a mandate on his own, and the language style is more objective and calm, with no excessive rhetorical or emotional colours."}, "time_dimension": {"answer": "Present", "rationale": "The entry now describes the sexual behaviour or preference of the person being tested, i.e. the preference for a person to perform the task, which is an act now being performed."}, "sentence_type": "Affirmative declarative sentence", "pos": {"nouns": {"Yes": 2, "person": 3, "It's a burden": 1, "Everyone": 1, "Fuck": 1}, "verbs": {"Yeah": 1, "Go on": 1, "Share": 1}, "adverbs": {"Many": 1, "It was": 1}, "numerals": {"57锛?: 1}, "pronouns": {"I": 1}}}, {"content": "I think people understand me too slowly, not even me.", "word_count": 25, "label": "", "sentence_structure": "Subject鈥揷opula鈥損redicative", "subjective_objective": {"answer": "Subjective", "rationale": "Because \"I think\" means that it's the patient's subjective feeling and perception."}, "sentence_sentiment": {"answer": "Negative", "rationale": "Because the description \"others understand my words too slowly, or even as I mean\" in the entry expresses a negative, disgruntled feeling."}, "language_style": {"answer": "Tactful and warm", "rationale": "The entry expresses a subjective feeling by using the subjective expression \"I think\" and the negative description \"too slow\" or \"not even understanding\" but does not directly blame or criticize others, but rather raises the question in a more moderate way. Thus, its language style can be considered as euphemism."}, "time_dimension": {"answer": "Present", "rationale": "The entry is therefore a question of the subjective feelings and views of the testee, and the temporal dimension is now judged."}, "sentence_type": "Negative declarative sentence", "pos": {"nouns": {"others": 1, "Word": 1, "Understood": 2, "Meaning": 1}, "verbs": {"I think": 1, "Too slow": 1, "I don't understand": 1}, "adverbs": {"Too slow": 1, "Like": 1}, "numerals": {"58锛?: 1}, "pronouns": {"I": 2}}}, {"content": "I'm a sexual obscenity.", "word_count": 10, "label": "", "sentence_structure": "Subject鈥揷opula鈥損redicative", "subjective_objective": {"answer": "Subjective", "rationale": "Because \"sex is violent\" is a human character based on a subjective perception and evaluation of the individual. It is not objective, but it is formed and manifested in the individual's cognitive framework. So the phrase \"I am a sexual man\" expresses the individual's subjective perception and assessment of himself and is subjective."}, "sentence_sentiment": {"answer": "Negative", "rationale": "Because the description of \"sexy manic\" in the entry is negative."}, "language_style": {"answer": "Calm and direct", "rationale": "The language style of the entry is relatively simple and does not involve excessive rhetorical or emotional rendering, but rather expresses the subject of questioning the other party in a direct and objective manner whether it has a temperamental character."}, "time_dimension": {"answer": "Present", "rationale": "The main phrase in \"I am a sexual man\" is the first person to call \"I\" \"I\" and the verb \"Yes\" means the subject's perception and appreciation of self, so this is happening at this point in time."}, "sentence_type": "Affirmative declarative sentence", "pos": {"nouns": {"person": 1, "Sex": 1, "- Routine": 1}, "adjectives": {"- Routine": 1}, "numerals": {"59": 1}, "pronouns": {"I": 1}}}, {"content": "I'm always easy to see people's weaknesses and ignore others' strengths.", "word_count": 20, "label": "", "sentence_structure": "Subject鈥搗erb鈥搊bject", "subjective_objective": {"answer": "Subjective", "rationale": "The advantage of neglecting others because they are often easily visible is the patient ' s subjective thoughts and attitudes."}, "sentence_sentiment": {"answer": "Negative", "rationale": "Because the attitude of \"regarding the strengths of others by being easy to see\" in the entries is negative."}, "language_style": {"answer": "Tactful and warm", "rationale": "The language style of the entry is euphemism. It leads the respondents to self-reflection, not direct statements or orders, by means of a quiz. At the same time, the words are softer and not too emotional. This expression makes the respondents more willing to participate in the questionnaire and more open to the views and suggestions of the questioners."}, "time_dimension": {"answer": "Present", "rationale": "So the time dimension of this questionnaire entry is now."}, "sentence_type": "Negative declarative sentence", "pos": {"nouns": {"Questionnaire": 1, "Entry": 1, "Short": 1, "others": 2, "Strengths": 1}, "verbs": {"Yeah": 1, "Neglect": 1, "Regular": 1, "Easy": 1}, "adjectives": {"Often": 1, "Someone else's": 2, "Easy": 1, "Short": 1}, "numerals": {"60": 1}, "classifiers": {"individual": 1}, "pronouns": {"I": 1}}}]}

{"questionnaire_title": "Family-based upbringing assessment questionnaire", "questionnaire_category": "Behavioral questionnaire", "item_count": 13, "emotion_shift_overall": "From positive to negative", "language_style_overall": "Tactful and warm", "items": [{"content": "One, when the child is unhappy, I'll try to adjust the child's mood.", "word_count": 19, "label": "", "sentence_structure": "Subject鈥搗erb鈥搊bject", "subjective_objective": {"answer": "Subjective", "rationale": "Because \"will try to adjust the mood of the child\" is the patient's subjective attitude and treatment."}, "sentence_sentiment": {"answer": "Positive", "rationale": "Because the entry is \"to try to adjust the child's emotions\", it's a positive act, showing that parents care about the child's feelings and take action to help them deal with emotional problems."}, "language_style": {"answer": "Tactful and warm", "rationale": "Because it uses a gentle and inviting tone to ask if the other side agrees with this idea of parenting, rather than direct orders or statements. Moreover, the expression \"want to adjust\" is also full of concern and understanding, giving people a warm sense of affection. Thus, the whole phrase is soft, friendly and is in keeping with the warm and gentle language style."}, "time_dimension": {"answer": "Present", "rationale": ""}, "sentence_type": "Affirmative declarative sentence", "pos": {"nouns": {"Son": 2, "Emotion": 1}, "verbs": {"in": 1, "Yes": 2, "Methodology": 1, "Adjustment": 1}, "adjectives": {"It's not good": 1}, "numerals": {"1": 2}, "pronouns": {"I": 1}}}, {"content": "I'm patiently listening to the child's words.", "word_count": 14, "label": "", "sentence_structure": "Subject鈥搗erb鈥揷omplement", "subjective_objective": {"answer": "Subjective", "rationale": "Because \"the patient hearing the child say what the child needs\" is the researcher asking the testee about attitudes and perceptions, it is subjective."}, "sentence_sentiment": {"answer": "Positive", "rationale": "Because it expresses concern and respect for the child, it has a positive emotional colour."}, "language_style": {"answer": "Tactful and warm", "rationale": "The entry is more euphemistic and warm. It uses more moderate language, such as \"suffering\" and \"the child needs to say\" , emphasizing listening to and understanding the child's feelings and needs, and giving people a sense of care. At the same time, this expression is more personal and interactive, and it can attract resonance and positive responses from the interviewees."}, "time_dimension": {"answer": "Present", "rationale": "Now both \"hear\" and \"say\" in the entry use the general current, which suggests that it is a description of current behaviour, not a vision of past or future. Thus, the judgement is based on the time dimension of the present."}, "sentence_type": "Affirmative declarative sentence", "pos": {"nouns": {"Son": 2, "Word": 2}, "verbs": {"Listen": 1, "Say it": 1, "Yes": 1}, "adjectives": {"Patience": 1}, "adverbs": {"I": 1}, "numerals": {"2": 1}}}, {"content": "I'll check if the kid's done his homework.", "word_count": 14, "label": "", "sentence_structure": "Subject鈥搗erb鈥揷omplement", "subjective_objective": {"answer": "Objective", "rationale": "Since it is an objective fact, not subjective attitude or feeling, that parents need to check whether the child has completed his or her homework."}, "sentence_sentiment": {"answer": "Neutral", "rationale": "Because the entry does not have any emotional color."}, "language_style": {"answer": "Calm and direct", "rationale": "The entry directly asks the other party whether it is concerned with the child's homework, does not have too many rhetorical or emotional renderings, and expresses a more objective and neutral view."}, "time_dimension": {"answer": "Present", "rationale": "The entry now asks about the behaviour currently being performed, namely, \"the child is being checked for completion of homework\". The time dimension therefore is now judged."}, "sentence_type": "Interrogative sentence", "pos": {"nouns": {"Son": 1, "Homework": 1}, "verbs": {"Inspection": 1, "Completed": 1}, "adverbs": {"I": 1}, "numerals": {"3": 1}}}, {"content": "I know where the kids are after class.", "word_count": 12, "label": "", "sentence_structure": "Subject鈥搗erb鈥搊bject", "subjective_objective": {"answer": "Subjective", "rationale": "This is based on subjective judgement and observation. Although this information can be obtained objectively (e.g. by interviewing others or by looking at surveillance), the patient鈥檚 perception and understanding is subjective."}, "sentence_sentiment": {"answer": "Neutral", "rationale": "Since the entry does not contain any emotional colour, it is a factual statement and asks whether the subjects of the investigation know the place of the child ' s post-school activities."}, "language_style": {"answer": "Tactful and warm", "rationale": "The language style of the entry is euphemism, affectionate, and the positive expression \"I know\" is used to express a caring and supportive attitude, rather than direct orders or inquiries. At the same time, the term \"child\" is used to increase the resonance of affection and emotion. Overall, the entry conveys concern and love for the child in a gentle language."}, "time_dimension": {"answer": "Present", "rationale": "The keyword \"know\" means the state of consciousness, \"where the child is after school\" is the current situation, so the entry should be judged now."}, "sentence_type": "Affirmative declarative sentence", "pos": {"nouns": {"Son": 1, "After class": 1, "Local": 1}, "verbs": {"Yes": 1, "in": 1, "Where": 1}, "numerals": {"4": 1}, "pronouns": {"I": 1}}}, {"content": "I like the way kids are like this.", "word_count": 10, "label": "", "sentence_structure": "Subject鈥揷opula鈥損redicative", "subjective_objective": {"answer": "Subjective", "rationale": "Because \"like\" represents personal preferences and attitudes, and it is a subjective element."}, "sentence_sentiment": {"answer": "Positive", "rationale": "Because \"like the child now\" expresses positive emotional attitudes."}, "language_style": {"answer": "Tactful and warm", "rationale": "The entry uses a descriptive language style, which is not too straightforward or personal, but rather a moderate expression that gives the respondents a sense of respect and understanding, which is more likely to generate resonance and positive feedback from the interviewees."}, "time_dimension": {"answer": "Present", "rationale": "The entry \"likes the child now\" represents a preference for and attitude towards the current situation, does not relate to past or future behaviour or mentality, and therefore judgement is now."}, "sentence_type": "Imperative sentence", "pos": {"nouns": {"Questionnaire": 1, "Entry": 1, "Son": 1}, "verbs": {"Yeah": 1}, "adjectives": {"Present": 1, "鈾?Looks like": 1}, "numerals": {"5": 1}}}, {"content": "I asked about the things that children do with friends.", "word_count": 15, "label": "", "sentence_structure": "Subject鈥搗erb鈥搊bject", "subjective_objective": {"answer": "Subjective", "rationale": "As this is the question of the child by the researcher, the content and manner of the interview are the subjective judgement and the conduct of the researcher."}, "sentence_sentiment": {"answer": "Neutral", "rationale": "Because the entry does not have any emotional color."}, "language_style": {"answer": "Calm and direct", "rationale": "This entry directly asks the respondents about their behaviour with their children and friends, the temperature of the language and the fact that no euphemism or emotionalization is used. This makes the answer more straightforward."}, "time_dimension": {"answer": "Present", "rationale": "The entry is about the present behaviour or activity of the subject, the keywords being \"Question now\" and \"Doing what\" and they all point to what is currently happening and are therefore judged to be \"now\"."}, "sentence_type": "Interrogative sentence", "pos": {"nouns": {"Son": 1, "friends": 1, "Things": 1}, "verbs": {"Ask": 1, "Do it": 1}, "adverbs": {"Six": 1}, "numerals": {"6": 1}, "pronouns": {"they": 1}}}, {"content": "I'll be sure when the kids do something right.", "word_count": 17, "label": "", "sentence_structure": "Subject鈥搗erb鈥揷omplement", "subjective_objective": {"answer": "Subjective", "rationale": "Because \"sure\" is the patient's subjective behavior and attitude."}, "sentence_sentiment": {"answer": "Positive", "rationale": "Because the \"sure\" act is positive in the entry."}, "language_style": {"answer": "Tactful and warm", "rationale": "The entry uses the positive and encouraging language of \"yes\" and expresses a moderate tone and attitude that gives the respondents attention and support. It also implies that the questionnaire is about how parents treat their children, with greater emphasis on emotional communication and understanding."}, "time_dimension": {"answer": "Present", "rationale": "The entry describes the current behaviour of the speaker, which is that of affirming and commending a child when doing something. Thus, in terms of time dimension, the behaviour is the present."}, "sentence_type": "Affirmative declarative sentence", "pos": {"nouns": {"Son": 1, "Things": 1, "Sure": 1}, "verbs": {"Do it": 1, "Okay": 1, "Yes": 1, "Here": 1, "Give it to me": 1}, "adverbs": {"Some": 1, "Okay": 1}, "numerals": {"7": 1}, "pronouns": {"When": 1, "Time": 1}}}, {"content": "I'd like to know what the problems the kids have.", "word_count": 12, "label": "", "sentence_structure": "Subject鈥搗erb鈥搊bject", "subjective_objective": {"answer": "Subjective", "rationale": "Hope and understanding are subjective attitudes of patients."}, "sentence_sentiment": {"answer": "Positive", "rationale": "Because the act of \"know what the problems the child is facing\" in the entry expresses a caring and caring attitude and is positive emotional."}, "language_style": {"answer": "Tactful and warm", "rationale": "The language style of the entry is more euphemistic and expresses to each other an attitude of concern and understanding about the child ' s issues, without showing an overly direct or rigid emotional tone."}, "time_dimension": {"answer": "Present", "rationale": "The entry asks what the person currently wishes to know, the keyword \"I want to know\", which indicates that it is an ongoing or planned act or aspiration and therefore falls within the current temporal dimension."}, "sentence_type": "Interrogative sentence", "pos": {"nouns": {"Son": 1, "Problem": 2, "Hope": 1, "Roger that": 1}, "verbs": {"I met him": 1, "have": 1}, "numerals": {"8": 1}, "pronouns": {"I": 1}}}, {"content": "I'm gonna make sure the kids get to bed on time.", "word_count": 12, "label": "", "sentence_structure": "Subject鈥搗erb鈥揷omplement", "subjective_objective": {"answer": "Subjective", "rationale": "Because \"must\" expresses the patient's subjective attitude."}, "sentence_sentiment": {"answer": "Positive", "rationale": "Because the purpose of the act \"must have children in bed on time\" is positive and is a manifestation of the parents' concern and sense of responsibility for their children's health."}, "language_style": {"answer": "Calm and direct", "rationale": "The entry uses a more direct and explicit language style, expressing a strong demand for children to go to bed on time, without too many emotional renderings or rhetorical techniques, and is more objective and balanced."}, "time_dimension": {"answer": "Present", "rationale": ""}, "sentence_type": "Imperative sentence", "pos": {"nouns": {"Son": 1, "The bed": 1, "I'm not sure": 1}, "verbs": {"I'm sure": 1, "Yes": 1, "On time": 1, "Go, go, go": 1, "Sleep": 1}, "adverbs": {"Nine": 1}, "numerals": {"9": 1}}}, {"content": "I'm happy with the kids' words and behaviour.", "word_count": 10, "label": "", "sentence_structure": "Subject鈥搗erb鈥揷omplement", "subjective_objective": {"answer": "Subjective", "rationale": "As satisfaction is a patient ' s perception and assessment of the child, it is a subjective attitude."}, "sentence_sentiment": {"answer": "Positive", "rationale": "Because \"satisfaction with the child's words and behaviour\" expresses a positive attitude, namely, satisfaction with the child's performance."}, "language_style": {"answer": "Tactful and warm", "rationale": "The expression is more personal and interactive, in a gentle and inviting tone of dialogue rather than direct statements or orders. At the same time, the word \"satisfaction\" implies positive recognition and encouragement of the person under investigation, further enhancing the sensitivity of the language."}, "time_dimension": {"answer": "Present", "rationale": "\"10 I'm satisfied with the child's words and behaviour\" and \"speech and behaviour\" indicate that this is a statement that evaluates the child's current behaviour, so time dimension is now judged."}, "sentence_type": "Imperative sentence", "pos": {"nouns": {"Son": 1, "Behave yourself": 1}, "verbs": {"Satisfied": 1, "be": 1}, "numerals": {"10": 1}}}, {"content": "I have to have the kid tell me where he's going.", "word_count": 15, "label": "", "sentence_structure": "Subject鈥搗erb鈥揷omplement", "subjective_objective": {"answer": "Subjective", "rationale": "Because \"must the child tell me where he's going\" is the patient's subjective will and decision, not the objective fact."}, "sentence_sentiment": {"answer": "Positive", "rationale": "Because the words \"must\" and \"Tell me where he's going\" in the entry express a strong sense of responsibility and concern, which is a positive emotional attitude."}, "language_style": {"answer": "Tactful and warm", "rationale": "The language style of the entry is more euphemistic and gentle, expressing concern for and love for the child, and using an invitation to feel respected and understood. This expression is more conducive to establishing good parenting and to making children more open to their parents."}, "time_dimension": {"answer": "Past", "rationale": "The \"said\" and \"go\" actions in the entry occurred at some point in the past and had an impact on the present, so the judgement was the past."}, "sentence_type": "Imperative sentence", "pos": {"nouns": {"Son": 1, "Local": 1}, "verbs": {"Tell": 1, "Go on": 1}, "adverbs": {"I'm sure": 1, "Yes": 1}, "numerals": {"11": 1}, "pronouns": {"he": 1}}}, {"content": "I'm making the time the child must go home.", "word_count": 12, "label": "", "sentence_structure": "Subject鈥搗erb鈥揷omplement", "subjective_objective": {"answer": "Subjective", "rationale": "The fact that the time when the child must return home is the decision and expectation of the parents reflects a subjective attitude."}, "sentence_sentiment": {"answer": "Neutral", "rationale": "Because the entry does not have any emotional color."}, "language_style": {"answer": "Calm and direct", "rationale": "The entry is expressed in clear and direct language, meaning that the child is required to return home at a given time, without excessive rhetoric or emotional colours and in a less expressive manner."}, "time_dimension": {"answer": "Present", "rationale": "The entry asks about the way the testee organizes and manages the child in a day-to-day manner, which involves words such as \"regulation\", \"must\", and emphasizes control, restraint, etc., and therefore can be judged as now."}, "sentence_type": "Imperative sentence", "pos": {"nouns": {"Son": 1, "Time": 1}, "verbs": {"Provisions": 1, "Home": 1}, "adverbs": {"I have to": 1}, "numerals": {"12": 1}, "classifiers": {"individual": 1}}}, {"content": "The rules I set for the kids are to follow.", "word_count": 11, "label": "", "sentence_structure": "Subject鈥搗erb鈥揷omplement", "subjective_objective": {"answer": "Subjective", "rationale": "Because the words \"rules\" and \"must obey\" reflect the subjective attitude and expectations of the questionnaire designers."}, "sentence_sentiment": {"answer": "Negative", "rationale": "Because the requirement of \"must comply\" in the entry is mandatory, it may cause stress and dissatisfaction among children."}, "language_style": {"answer": "Calm and direct", "rationale": "The entry expresses the subject of the requirement that children comply with their own rules in a concise and clear language, without too much qualifier or emotional colour, and is more direct and explicit."}, "time_dimension": {"answer": "Present", "rationale": "The time dimension of this entry can therefore be judged as now."}, "sentence_type": "Imperative sentence", "pos": {"nouns": {"Rules": 1, "Son": 1}, "verbs": {"Freeze": 1, "I have to": 1, "Compliance": 1}, "numerals": {"13": 1}}}]}

{"questionnaire_title": "SAS Axis Self-Measuring Table", "questionnaire_category": "Mental health", "item_count": 20, "emotion_shift_overall": "From negative to positive", "language_style_overall": "Neutral", "items": [{"content": "One, feeling more nervous and anxious than usual.", "word_count": 12, "label": "", "sentence_structure": "Subject鈥搗erb鈥揷omplement", "subjective_objective": {"answer": "Subjective", "rationale": "Because the verb \"think\" suggests that it's a patient's subjective feeling or experience."}, "sentence_sentiment": {"answer": "Negative", "rationale": "Because \"stretch and rush\" in the entry is a negative emotional reaction, expressing a negative emotional attitude."}, "language_style": {"answer": "Calm and direct", "rationale": "The language style of the entry is more straightforward, there is no excessive rhetorical or emotional rendering, and the tone is more objective, and the interviewee is asked whether he or she is more nervous and anxious in his or her daily life."}, "time_dimension": {"answer": "Present", "rationale": "The entry is for the current feelings or state of the subject, and the keyword \"felts more normal\" represents a change from the usual situation and therefore the judgement is now."}, "sentence_type": "Imperative sentence", "pos": {"nouns": {"Questionnaire": 1, "Entry": 1, "Nervous": 1, "- It's urgent": 1}, "verbs": {"I think": 1, "- Yeah": 1, "Easy": 1, "Nervous": 1, "I'm in a hurry": 1}, "adverbs": {"Normal": 1}}}, {"content": "2. Fear without cause", "word_count": 9, "label": "", "sentence_structure": "Subject鈥搗erb鈥揷omplement", "subjective_objective": {"answer": "Subjective", "rationale": "Because the feeling of \"no reason\" is usually a subjective feeling of a person."}, "sentence_sentiment": {"answer": "Negative", "rationale": "Because the \"fear without cause\" sentiment in the entry is negative."}, "language_style": {"answer": "Tactful and warm", "rationale": "The entry is more euphemistic, using the phrase \"for no reason\" to describe the problems that the other side may have, without directly accusing or ordering the other to change. Moreover, the purpose of the article is soft and caring, as it is listening to the other side, and it is therefore judged to be euphemistic and warm."}, "time_dimension": {"answer": "Present", "rationale": "The entry is judged by the keyword \"unprovoked fear\", which asks whether the person tested was unprovoked by fear of behaviour or emotion at the current point of time, and therefore is now."}, "sentence_type": "Imperative sentence", "pos": {"nouns": {"For no reason": 1, "Scared": 1}, "verbs": {"I feel": 1}, "adverbs": {"Land": 1}}}, {"content": "Three, easy to upset or panic.", "word_count": 11, "label": "", "sentence_structure": "Subject鈥搗erb鈥揷omplement", "subjective_objective": {"answer": "Subjective", "rationale": "Because \"easy to be upset or frightened\" is an emotional and subjective experience that reflects the emotional state and the inner experience of the patient. This feeling is the patient's own subjective experience, not the objective fact or the result of his behaviour. So the answer to this questionnaire should be \"subjective.\""}, "sentence_sentiment": {"answer": "Negative", "rationale": "Because the words \"deep in the mind\" and \"frightful\" in the entries are both negative."}, "language_style": {"answer": "Tactful and warm", "rationale": "The entry uses euphemisms, \"easy\" and \"fright\" to avoid too strong a negative mood, and also expresses the interest and understanding of the investigator of the person under investigation. The language style of the entry can therefore be judged as euphemism."}, "time_dimension": {"answer": "Past", "rationale": "The entry is for the purpose of asking the person who was tested whether there was a psychological problem and the temporal dimension is a past."}, "sentence_type": "Interrogative sentence", "pos": {"nouns": {"Psychological": 1, "I'm so upset": 1, "Fear": 1}, "verbs": {"I think": 1, "Easy": 1, "鈾?And I'm gonna be 鈾?: 1}}}, {"content": "Four, I think it might go crazy.", "word_count": 7, "label": "", "sentence_structure": "Subject鈥搗erb鈥揷omplement", "subjective_objective": {"answer": "Subjective", "rationale": "Because it is a subjective feeling and a fear that reflects the emotional and psychological state of the patient, not the objective fact."}, "sentence_sentiment": {"answer": "Negative", "rationale": "Because the term \"crazy\" is negative emotional."}, "language_style": {"answer": "Calm and direct", "rationale": "The entry directly expresses the subject of questioning the other party about the possibility of madness, without excessive rhetoric or emotional rendering, and with a more objective tone."}, "time_dimension": {"answer": "Past", "rationale": "The basis for the judgement: - \"Feel\" means subjective feelings or experiences, which are a continuous process, both in the past and in the present. - \"Present to go mad\" suggests that feeling is a speculation about the possibilities of the future, but also a reflection of the state of the present. Thus, the entry relates to both the past and present feelings of the testee and what may happen in the future. In sum, the entry can be seen as asking whether the testee has experienced similar feelings in the past or whether the testee is feeling such stress or anxiety."}, "sentence_type": "Affirmative declarative sentence", "pos": {"nouns": {"Crazy": 1}, "verbs": {"I think": 1, "Let's go": 1}, "adverbs": {"Maybe": 1}}}, {"content": "Five, I think everything's fine. Nothing bad happens.", "word_count": 16, "label": "", "sentence_structure": "Subject鈥揷opula鈥損redicative", "subjective_objective": {"answer": "Subjective", "rationale": "Because it is not an objective fact that it is a subjective feeling and perception of a person that everything is good and that nothing is going to happen."}, "sentence_sentiment": {"answer": "Neutral", "rationale": "Because the entry is not clearly emotional, it merely describes a vision of the future."}, "language_style": {"answer": "Tactful and warm", "rationale": "The language style of the entry is more euphemistic, active, more positive and positive, and not too direct or command-style, more like an encouragement and affirmation."}, "time_dimension": {"answer": "Present", "rationale": ""}, "sentence_type": "Affirmative declarative sentence", "pos": {"nouns": {"Things": 1, "Everything": 1}, "verbs": {"Come on": 1}, "adjectives": {"Okay": 1, "Unfortunately": 1}, "adverbs": {"Both": 1, "Yeah": 1}, "numerals": {"5": 1}, "pronouns": {"you": 1}}}, {"content": "Six, hands and feet shaking.", "word_count": 6, "label": "", "sentence_structure": "Subject鈥搗erb鈥揷omplement", "subjective_objective": {"answer": "Objective", "rationale": "Because tremors are a description of the patient鈥檚 physical symptoms, which are objective. Although they may be related to the patient鈥檚 psychological state, they are directly observed and reported by the patient when completing the questionnaire, and are therefore considered objective."}, "sentence_sentiment": {"answer": "Negative", "rationale": "Because the act of tremors is usually associated with negative emotions such as stress, fear, etc."}, "language_style": {"answer": "Calm and direct", "rationale": "The entry directly asked the interviewees whether there were cases of \"shocking hands and feet\" and did not use excessive rhetoric or emotional rendering, and was more calm and objective in language style."}, "time_dimension": {"answer": "Present", "rationale": "The keyword \"fisting hands and feet\" indicates current physiological symptoms or reactions, and therefore the judgement is now."}, "sentence_type": "Exclamatory sentence", "pos": {"nouns": {"Hands and feet": 1, "# Wresh #": 1, "# Shatter #": 1}}}, {"content": "7 - A headache, a headache and a back.", "word_count": 13, "label": "", "sentence_structure": "Subject鈥搗erb鈥揷omplement", "subjective_objective": {"answer": "Objective", "rationale": "The objective facts that patients experience because of headaches, headaches and back pains."}, "sentence_sentiment": {"answer": "Negative", "rationale": "Because it describes a feeling of pain."}, "language_style": {"answer": "Calm and direct", "rationale": "The entry raises the question in an objective and direct manner, does not have too much emotional or rhetorical style and is more calm and straightforward."}, "time_dimension": {"answer": "Past", "rationale": "The entry, \"Worried by headaches, neck pains and back pains\", describes an experience that has already occurred and is judged by the time dimension of the past."}, "sentence_type": "Affirmative declarative sentence", "pos": {"nouns": {"Headache": 2, "Backache": 1, "Trouble": 1}, "verbs": {"Because": 1, "And": 1, "Trouble": 1}, "numerals": {"7": 1}}}, {"content": "8. Feeling vulnerable to weakness and fatigue", "word_count": 9, "label": "", "sentence_structure": "Subject鈥搗erb鈥揷omplement", "subjective_objective": {"answer": "Subjective", "rationale": "Because \"feeling vulnerable and tired\" is the patient's own feeling and experience, and it's the result of subjective observations."}, "sentence_sentiment": {"answer": "Negative", "rationale": "Because \"weakness and fatigue\" is a negative emotional and physical state, the statement of the questionnaire is negative."}, "language_style": {"answer": "Tactful and warm", "rationale": ""}, "time_dimension": {"answer": "Present", "rationale": "According to the title, \"Easy and fatigue\" is a current feeling or state, so it can be judged that the entry is an inquiry into the present behaviour or mentality of the person being tested."}, "sentence_type": "Negative declarative sentence", "pos": {"nouns": {"Feel": 1, "Weakness": 1, "Weary": 1}, "verbs": {"Easy": 1, "Here": 1}}}, {"content": "Nine. Feel calm and sit quietly.", "word_count": 15, "label": "", "sentence_structure": "Subject鈥搗erb鈥揷omplement", "subjective_objective": {"answer": "Subjective", "rationale": "Because feeling, sitting quiet is the feeling and subjectivity of the patient."}, "sentence_sentiment": {"answer": "Positive", "rationale": "Because \"salent\" and \"salary\" in the entries are positive emotions or attitudes."}, "language_style": {"answer": "Tactful and warm", "rationale": "The entry uses more gentle and emotional terms, such as \"peaceful\" and \"silently\" to try to guide the respondents to feel a calm and comfortable state of affairs in answering questions. This expression is more intimate and interactive, making it easier for the respondents to feel comfortable and to cooperate with the questionnaire."}, "time_dimension": {"answer": "Present", "rationale": "The questionnaire entry asks about the current feelings and state of the subject, the keyword \"and feel\" and \"sit quietly\" and points to the person's current feelings. So the time dimension of the entry is now."}, "sentence_type": "Affirmative declarative sentence", "pos": {"nouns": {"Calm down": 1, "Quiet": 1}, "verbs": {"I think": 1, "Sit down": 1}, "adverbs": {"Easy": 1, "And": 1}, "numerals": {"9": 1}}}, {"content": "I feel like my heart is beating fast.", "word_count": 7, "label": "", "sentence_structure": "Subject鈥搗erb鈥揷omplement", "subjective_objective": {"answer": "Subjective", "rationale": "Because heart rate is a subjective experience."}, "sentence_sentiment": {"answer": "Negative", "rationale": "Because \"the heart beats fast\" is a sign of physical discomfort, which is usually considered a negative situation."}, "language_style": {"answer": "Calm and direct", "rationale": "The entry is a simple question, in a straightforward manner, about whether the other party often feels an acceleration of the heart rate, does not have too many rhetorical or emotional renderings, and is expressed in a relatively calm and objective manner."}, "time_dimension": {"answer": "Present", "rationale": "In the entry, \"Feel\" and \"heart beats fast\" describe the experience and experience of the testee right now, so the temporal dimension is now."}, "sentence_type": "Interrogative sentence", "pos": {"nouns": {"Heart rate": 1}, "verbs": {"I think": 1, "Come on": 1}, "numerals": {"10": 1}}}, {"content": "Eleven. A moment of dizziness.", "word_count": 10, "label": "", "sentence_structure": "Subject鈥搗erb鈥揷omplement", "subjective_objective": {"answer": "Subjective", "rationale": "The pain of a moment of dizziness is the patient's subjective feelings and experiences."}, "sentence_sentiment": {"answer": "Negative", "rationale": "Because the attitude of \"probable\" in the entry is negative."}, "language_style": {"answer": "Calm and direct", "rationale": "Although the entry asks whether the other party has a problem of dizziness, it is more direct and objective, is not over-modified or inflective and is therefore more appropriate in the language style of \"cool and direct\"."}, "time_dimension": {"answer": "Past", "rationale": "In the entry, \"show of dizziness\" describes the symptoms of the past, indicating that the person tested had experienced dizziness and was troubled. The time dimension of the article is therefore the past."}, "sentence_type": "Affirmative declarative sentence", "pos": {"nouns": {"Dizziness": 1, "Trouble": 1}, "verbs": {"Because": 1, "And": 1, "Trouble": 1}, "numerals": {"One": 2}, "pronouns": {"For a while": 1, "you": 1}}}, {"content": "12, fainting, or feeling fainting.", "word_count": 13, "label": "", "sentence_structure": "Subject鈥搗erb鈥揷omplement", "subjective_objective": {"answer": "Objective", "rationale": "Because it is the feeling and experience of the patient, it is an objective fact, that a fainting or feeling fainting is the same."}, "sentence_sentiment": {"answer": "Negative", "rationale": "Because the \"stuned\" in the entry is negative."}, "language_style": {"answer": "Calm and direct", "rationale": "The language style of the entry is relatively calm and objective, and the interviewee is asked whether he has a fainting seizure and is presented in a simple and clear language."}, "time_dimension": {"answer": "Past", "rationale": "The entry, which is an inquiry into the past experiences and feelings of the subject, contains the keyword \"had\" and \"like feeling faint\", which points to what has happened. The temporal dimension of the article can therefore be judged to be past."}, "sentence_type": "Interrogative sentence", "pos": {"nouns": {"鈾?And the attacks 鈾?: 1, "I'm fainted": 2, "I think": 1}, "verbs": {"have": 1, "Yes": 1, "I'm fainted": 1, "Like": 1}, "adverbs": {"I think": 1}, "numerals": {"12": 1}, "classifiers": {"individual": 1}}}, {"content": "It's easy to breathe.", "word_count": 10, "label": "", "sentence_structure": "Subject鈥揷opula鈥損redicative", "subjective_objective": {"answer": "Subjective", "rationale": "The sentence is supposed to be seen from the point of view of the patient, whether it is subjective or objective. By analysing the phrase \"sipping out\" and \"easy\" in the sentence, it is possible to conclude that the patient feels his/her own health, and is therefore subjective."}, "sentence_sentiment": {"answer": "Neutral", "rationale": "Because the entry describes a biological phenomenon and does not have an emotional character."}, "language_style": {"answer": "Tactful and warm", "rationale": "The entry is more adept and interactive when it is spoken in a gentle and inviting manner, rather than by direct statements or orders."}, "time_dimension": {"answer": "Present", "rationale": "\"Easy to breathe\" describes the person being tested for the moment's feelings or experiences, so the entry is the current time dimension."}, "sentence_type": "Affirmative declarative sentence", "pos": {"nouns": {"Questionnaire Entry": 1, "Breathe in": 1, "Breathe out": 1}, "verbs": {"I feel": 1, "Easy": 1}, "numerals": {"13": 1, "Both": 1}}}, {"content": "14: A numbness and sting.", "word_count": 7, "label": "", "sentence_structure": "Subject鈥搗erb鈥搊bject", "subjective_objective": {"answer": "Objective", "rationale": "Because \"smoke and sting\" is a physical feeling, an objective fact that patients can feel directly."}, "sentence_sentiment": {"answer": "Negative", "rationale": "Because \"smoke and sting\" are symptoms of physical discomfort, they are often considered negative experiences."}, "language_style": {"answer": "Calm and direct", "rationale": "The entry directly asks the other party whether it has symptoms of numbness and stinging, and is not speaking in a balanced and objective manner using excessive qualifier or emotional expressions."}, "time_dimension": {"answer": "Past", "rationale": ""}, "sentence_type": "Interrogative sentence", "pos": {"nouns": {"Hands and feet": 1, "I'm numb": 1, "Stings": 1}}}, {"content": "15: Worried by stomach pain and indigestion", "word_count": 12, "label": "", "sentence_structure": "Subject鈥搗erb鈥揷omplement", "subjective_objective": {"answer": "Subjective", "rationale": "Because stomach pain and indigestion are the personal experiences and feelings of patients."}, "sentence_sentiment": {"answer": "Negative", "rationale": "Because the words \"attendance pain and indigestion\" and \"profun\" in the entries are negative emotional expressions, which are negative."}, "language_style": {"answer": "Calm and direct", "rationale": "The entry directly expresses the question whether the subject is troubled by stomach pain and indigestion, and is not couched in excessive terms or emotionally, and is more objective in tone."}, "time_dimension": {"answer": "Past", "rationale": "The keyword \"because of stomach pain and digestive indigestion\" suggests that it is a question of past experience or state. Moreover, \"and distress\" also indicates that it is an unpleasant experience and a description of the past. So the entry asks if the person tested was troubled by stomach pain and digestion."}, "sentence_type": "Affirmative declarative sentence", "pos": {"nouns": {"Stomach pain": 1, "Indigestion": 1}, "verbs": {"And the pain": 1}, "numerals": {"15": 1}}}, {"content": "16, often pee.", "word_count": 5, "label": "", "sentence_structure": "Subject鈥搗erb鈥揷omplement", "subjective_objective": {"answer": "Objective", "rationale": "Because \"often pee\" is an observed physiological phenomenon, not a patient's subjective feeling or idea."}, "sentence_sentiment": {"answer": "Negative", "rationale": "Because the act of \"often peeing\" can cause discomfort and distress and thus have some negative effect."}, "language_style": {"answer": "Calm and direct", "rationale": "The language style of the entry is more objective and calm, and the interviewee is asked whether he/she is often in need of urination, and there is no excessive emotional or rhetorical style. Such expression makes it easier for the interviewee to understand the meaning of the problem and is more conducive to the collection of reliable survey data."}, "time_dimension": {"answer": "Present", "rationale": ""}, "sentence_type": "Imperative sentence", "pos": {"nouns": {"Pee": 1}, "verbs": {"Always": 1}}}, {"content": "17, hands are often dry and warm.", "word_count": 9, "label": "", "sentence_structure": "Subject鈥揷opula鈥損redicative", "subjective_objective": {"answer": "Objective", "rationale": "The situation of the hand is an objective fact that can be observed directly."}, "sentence_sentiment": {"answer": "Neutral", "rationale": "Because the entry only describes the state of the hand and does not contain any emotional colours."}, "language_style": {"answer": "Tactful and warm", "rationale": "The entry uses descriptive language, which describes the hand state as \"dry warm\", does not directly express any evaluation or recommendation, is softer and neutral, and is more friendly. At the same time, it can generate resonance and recollection among the respondents and makes them more willing to participate in the questionnaire."}, "time_dimension": {"answer": "Present", "rationale": "The entry describes the current state of the testee, so the temporal dimension is judged to be \"now\"."}, "sentence_type": "Affirmative declarative sentence", "pos": {"nouns": {"Hands": 1, "Dry and warm": 1}, "verbs": {"Often": 1}}}, {"content": "It's red.", "word_count": 4, "label": "", "sentence_structure": "Subject鈥搗erb鈥揷omplement", "subjective_objective": {"answer": "Objective", "rationale": "Because of the red fever that patients experience."}, "sentence_sentiment": {"answer": "Neutral", "rationale": "Because the entry does not address specific emotions, attitudes, behaviours, purposes or qualities, it merely describes a biological phenomenon."}, "language_style": {"answer": "Tactful and warm", "rationale": "The entry is more adept and interactive when it is spoken in a gentle and inviting manner, rather than by direct statements or orders."}, "time_dimension": {"answer": "Past", "rationale": "The entry asks if the person tested had had a red fever in his past experience and the temporal dimension is judged to be past."}, "sentence_type": "Imperative sentence", "pos": {"nouns": {"Face": 1, "Hot": 1}, "verbs": {"Red": 1, "Heat": 1}}}, {"content": "It's easy to sleep and it's good.", "word_count": 10, "label": "", "sentence_structure": "Subject鈥揷opula鈥損redicative", "subjective_objective": {"answer": "Subjective", "rationale": "Because it is the patient ' s subjective feeling and assessment that sleeps easily and well, it is not an objective fact."}, "sentence_sentiment": {"answer": "Positive", "rationale": "Because the entry \"sleep-easy and well\" is a positive quality, that is, a good quality of sleep."}, "language_style": {"answer": "Tactful and warm", "rationale": "The language style of the entry is euphemistic, conveys positive meaning by using words like \"easy\" and \"good\" and gives people a sense of affection and comfort. Such expression makes it easier for respondents to relax and share the real situation."}, "time_dimension": {"answer": "Present", "rationale": "The entry describes the current state of the testee, the key words being \"sleep easy\" and \"sleep well,\" which both indicate the present state. And \"and\" also indicates that there are two simultaneous states. So the temporal dimension is now."}, "sentence_type": "Affirmative declarative sentence", "pos": {"nouns": {"Problem": 1, "Item": 1, "Easy": 1, "Enter": 1, "Sleep": 1, "And": 1, "very": 1}, "verbs": {"Sleep": 2, "Okay": 1}, "adverbs": {"And": 1}, "numerals": {"19": 1, "1": 2}, "classifiers": {"individual": 1}}}, {"content": "20, nightmares.", "word_count": 3, "label": "", "sentence_structure": "Subject鈥搗erb鈥揷omplement", "subjective_objective": {"answer": "Subjective", "rationale": "Because nightmares are an individual subjective feeling and experience, they are the subject of subjective observations."}, "sentence_sentiment": {"answer": "Negative", "rationale": "Because nightmares usually lead to negative emotional reactions."}, "language_style": {"answer": "Calm and direct", "rationale": "The entry directly expresses the subject of questioning the other party in a concise and clear language about whether he or she has had a nightmare, without using excessive rhetoric or emotional rendering, and with a more balanced and objective tone."}, "time_dimension": {"answer": "Present", "rationale": "The entry used to ask if the person tested had any past experience - having nightmares. Time dimensions judge the past."}, "sentence_type": "Exclamatory sentence", "pos": {"nouns": {"Nightmare": 1}, "verbs": {"Do it": 1}}}]}

{"questionnaire_title": "Chinese version of the Family Environment Sheet (FES-CV)", "questionnaire_category": "Emotion and affect", "item_count": 90, "emotion_shift_overall": "From positive to negative", "language_style_overall": "Tactful and warm", "items": [{"content": "We, the family members, always offer each other the greatest help and support.", "word_count": 21, "label": "", "sentence_structure": "Subject鈥搗erb鈥搊bject", "subjective_objective": {"answer": "Subjective", "rationale": "This sentence describes the patient ' s subjective perception and feelings about his/her family member relationship."}, "sentence_sentiment": {"answer": "Positive", "rationale": "Because the behaviour and attitude of \"best help and support to each other\" in the entry is positive."}, "language_style": {"answer": "Tactful and warm", "rationale": "The entry expresses a positive attitude and expectations towards family relations by using the words \"always\" and \"best help and support to each other\", which are more moderate and friendly, and which facilitate the acceptance of the respondents and their willingness to answer questions."}, "time_dimension": {"answer": "Present", "rationale": ""}, "sentence_type": "Affirmative declarative sentence", "pos": {"nouns": {"Family members": 1, "Max": 1, "Help": 1, "Support": 1}, "verbs": {"Give it to me": 1}, "adjectives": {"Always": 1, "- Yeah, yeah": 1}, "classifiers": {"Both": 1, "Most": 1}}}, {"content": "Family members always hide their feelings in their hearts and do not reveal them to other family members.", "word_count": 26, "label": "", "sentence_structure": "Subject鈥搗erb鈥搊bject", "subjective_objective": {"answer": "Subjective", "rationale": "Because \" always hide your feelings from the heart, not to the rest of the family\" is a description of subjective behaviour and attitude."}, "sentence_sentiment": {"answer": "Negative", "rationale": "Because the entry \"Having feelings in your heart without revealing them to other family members\" is negative."}, "language_style": {"answer": "Tactful and warm", "rationale": ""}, "time_dimension": {"answer": "Present", "rationale": "The answer to this question by the testee is the behaviour or circumstances of the second family member, and the words \"always\" and \"not to reveal to other family members\" indicate that the conduct has been ongoing and that the temporal dimension is now judged."}, "sentence_type": "Imperative sentence", "pos": {"nouns": {"Family members": 2, "Emotion": 1}, "verbs": {"Always": 1, "- Put it on": 1, "Hide": 1, "Disclosure": 1}, "adverbs": {"In my heart": 1, "Other": 1}, "numerals": {"2": 1}, "pronouns": {"self": 1, "they": 1}}}, {"content": "The family often fights.", "word_count": 6, "label": "", "sentence_structure": "Subject鈥搗erb鈥揷omplement", "subjective_objective": {"answer": "Objective", "rationale": "Because \"often fighting\" is an objective fact of family status, not a subjective feeling or attitude of the patient."}, "sentence_sentiment": {"answer": "Negative", "rationale": "Because the fight is negative."}, "language_style": {"answer": "Calm and direct", "rationale": "The title is simple and clear, and there are no too many reticent words, which are straightforward, clear and clear, and which give a clear picture of what the problem means.\""}, "time_dimension": {"answer": "Past", "rationale": "The keyword \"in\" denotes the state of affairs within the family, while the word \"normal\" indicates that such quarrels are frequent and not accidental, and the entry asks whether the person tested had a regular history of quarrels in family life in the past."}, "sentence_type": "Affirmative declarative sentence", "pos": {"nouns": {"Home": 1, "Fight": 1}, "verbs": {"Regular": 1}, "numerals": {"3": 1}}}, {"content": "In our homes, we rarely act alone.", "word_count": 13, "label": "", "sentence_structure": "Subject鈥搗erb鈥揷omplement", "subjective_objective": {"answer": "Subjective", "rationale": "Because \"very few activities on their own\" are subjective feelings and experiences, not objective facts or situations. This may reflect the patient's family environment, social habits, or other personal factors. The questionnaire is therefore subjective in its perspective."}, "sentence_sentiment": {"answer": "Negative", "rationale": "Because the \"very few activities on their own\" act in the entry is negative."}, "language_style": {"answer": "Calm and direct", "rationale": ""}, "time_dimension": {"answer": "Present", "rationale": "The entry describes the current state of behaviour, and the keywords \"very few\" and \"single activity\" indicate that this occurs at home and is the current state, so it is judged now."}, "sentence_type": "Negative declarative sentence", "pos": {"nouns": {"Home": 1, "Activities": 1}, "verbs": {"Very few": 1, "self": 1, "Alone": 1, "Conduct": 1}, "adverbs": {"Very few": 1, "Alone": 1}, "numerals": {"4": 1}}}, {"content": "The family members do everything they can.", "word_count": 18, "label": "", "sentence_structure": "Subject鈥揷opula鈥損redicative", "subjective_objective": {"answer": "Objective", "rationale": "Because \"family members do everything they can\" is the patient's family situation, which is a fact that can be observed and verified, it is objective."}, "sentence_sentiment": {"answer": "Positive", "rationale": "This item expresses the positive attitudes and behaviour of family members."}, "language_style": {"answer": "Tactful and warm", "rationale": "The entry uses the expression \"best-case\" and does not directly evaluate or criticize the behaviour of family members, which is more moderate, non-subjective and more easily accepted and answered by the interviewee. The language style of the purpose is therefore considered to be euphemism."}, "time_dimension": {"answer": "Present", "rationale": "Keywords include \"family members\", \"whatever\" and \"do what you can\", pointing to the attitudes and behaviours of the testees in their current family life."}, "sentence_type": "Affirmative declarative sentence", "pos": {"nouns": {"Family members": 2, "Things": 1, "Try": 1}, "verbs": {"Do it": 1, "be": 1, "Yes": 1}, "adjectives": {"What": 1, "Both": 1, "of": 3}, "adverbs": {"Whatever": 1, "Both": 1}, "pronouns": {"they": 1}}}, {"content": "Our family has often spoken about political and social issues.", "word_count": 14, "label": "", "sentence_structure": "Subject鈥搗erb鈥搊bject", "subjective_objective": {"answer": "Subjective", "rationale": "Because \"often talking about political and social issues\" is a family's behaviour and habits, and it is a subjective situation."}, "sentence_sentiment": {"answer": "Neutral", "rationale": "Because the entry is merely a description of the behaviour of a family and does not express any emotional tendencies."}, "language_style": {"answer": "Tactful and warm", "rationale": "The language style of the entry is more moderate and euphemistic and the expression of invitation is used to ask whether political and social issues are regularly discussed in the family of the other, which is more acceptable and answered."}, "time_dimension": {"answer": "Present", "rationale": "The words \"often\" and \"talk\" in the entry indicate that this is a customary, ongoing act, so the temporal dimension is now judged."}, "sentence_type": "Affirmative declarative sentence", "pos": {"nouns": {"Our home": 1, "Political": 1, "Social issues": 1}, "verbs": {"Regular": 1, "Discussion": 1}, "adverbs": {"Regular": 1}, "numerals": {"6": 1}, "classifiers": {"individual": 1}}}, {"content": "Most weekends and evenings are spent at home by family members, without going out to social and recreational events.", "word_count": 32, "label": "", "sentence_structure": "Subject鈥搗erb鈥搊bject", "subjective_objective": {"answer": "Objective", "rationale": "This requires an objective result through investigation or observation."}, "sentence_sentiment": {"answer": "Negative", "rationale": "Because the behaviour in the entry, \"invisibility for social and recreational activities\", is negative and may imply a lack of interaction and communication between family members."}, "language_style": {"answer": "Tactful and warm", "rationale": "The words \"most\" and \"at home\" are used in the sentence, without emphasis or criticism, giving people a sense of understanding and support, and a warmer language style."}, "time_dimension": {"answer": "Past", "rationale": "The expressions \"mostly\" , \"coustics and evenings\" and \"involvement in social and recreational activities\" all indicate that this is a past situation, and therefore the temporal dimension of the article is the past."}, "sentence_type": "Affirmative declarative sentence", "pos": {"nouns": {"Weekend": 2, "Night": 1, "Family members": 2, "Home": 1, "Medium": 1, "Out": 1, "Participation": 1, "Social": 1, "Recreation": 1, "Activities": 1}, "verbs": {"be": 2, "Pass": 1, "not": 1, "Outings": 1}, "adverbs": {"Most of them": 1, "Both": 1, "And": 1, "not": 1}, "numerals": {"7": 1}, "classifiers": {"individual": 1}, "pronouns": {"Family members": 2}}}, {"content": "We all believe that, no matter how difficult, children should first meet the needs of the elderly.", "word_count": 27, "label": "", "sentence_structure": "Subject鈥搗erb鈥搊bject", "subjective_objective": {"answer": "Subjective", "rationale": "This is a subjective view of the relationship between children and the elderly."}, "sentence_sentiment": {"answer": "Positive", "rationale": "Because this entry expresses respect and care for the elderly and has a positive emotional tendency."}, "language_style": {"answer": "Tactful and warm", "rationale": "The language style of the entry is more euphemistic. It does not directly dictate or oblige respondents to agree to this view, but rather leads them to think and answer questions in a mild way that is more acceptable and willing to share their views."}, "time_dimension": {"answer": "Present", "rationale": "The sentence is generally the present, and thus is the present, expressing a view or attitude on the current situation."}, "sentence_type": "Affirmative declarative sentence", "pos": {"nouns": {"Difficulties": 1, "Children": 1, "Requirements": 1}, "verbs": {"I think": 1, "I think so": 2, "Satisfied": 1}, "adjectives": {"Large": 1, "Various": 1}, "adverbs": {"First of all": 1}, "numerals": {"8": 1}, "pronouns": {"we": 1, "they": 1}}}, {"content": "The larger activities in the family are carefully arranged.", "word_count": 16, "label": "", "sentence_structure": "Subject鈥搗erb鈥揷omplement", "subjective_objective": {"answer": "Subjective", "rationale": "This is because patients have subjective knowledge and description of their family activities."}, "sentence_sentiment": {"answer": "Neutral", "rationale": "Because the entry does not have any emotional color."}, "language_style": {"answer": "Tactful and warm", "rationale": "The entry uses a more euphemistic language, which indicates that most large events are carefully planned, and does not stress this too directly or forcefully. Such expressions are relatively mild and more acceptable."}, "time_dimension": {"answer": "Past", "rationale": ""}, "sentence_type": "Affirmative declarative sentence", "pos": {"nouns": {"Activities": 2, "Home": 1, "Arrangements": 1}, "verbs": {"be": 1, "Yes": 1, "Careful": 1}, "adjectives": {"Bigger": 1, "Details": 1}, "numerals": {"9": 1}}}, {"content": "Family members rarely force other family members to comply with family rules.", "word_count": 17, "label": "", "sentence_structure": "Subject鈥搗erb鈥揷omplement", "subjective_objective": {"answer": "Objective", "rationale": "The fact that families rarely force other family members to comply with the family code is a description of the objective phenomenon and examines the development and implementation of family rules."}, "sentence_sentiment": {"answer": "Neutral", "rationale": "Because the entry does not have any emotional color."}, "language_style": {"answer": "Tactful and warm", "rationale": "The language style of the entry is more euphemistic, and it is more intimate and friendly by asking the respondents whether they are rarely obliged to comply with family rules, rather than by direct orders or statements. At the same time, the expression \"family\" is used to increase verbality and intimacy."}, "time_dimension": {"answer": "Present", "rationale": "The use of the keywords \"very few\" and \"compliance\" suggests that the entry describes current family conditions or behaviour. At the same time, \"very few\" suggests that this may not always be the case, and that it may have occurred differently in the past."}, "sentence_type": "Affirmative declarative sentence", "pos": {"nouns": {"Family rules": 1, "Family": 1}, "verbs": {"Very few": 1, "Compliance": 1, "Force": 1}, "adverbs": {"A lot": 1}, "numerals": {"10": 1}, "classifiers": {"individual": 1}}}, {"content": "Eleven at home we were bored.", "word_count": 10, "label": "", "sentence_structure": "Subject鈥揷opula鈥損redicative", "subjective_objective": {"answer": "Subjective", "rationale": "Because \"distressed\" is a subjective feeling and experience that is felt by the patient himself, and does not reflect objective facts."}, "sentence_sentiment": {"answer": "Negative", "rationale": "Because it expresses a negative emotional state, i.e. bored at home."}, "language_style": {"answer": "Tactful and warm", "rationale": "The language style of the entry tends to be euphemistic, because it asks questions in a sensitive manner and does not directly criticize or accuse the family environment of boredom."}, "time_dimension": {"answer": "Past", "rationale": "The entry describes the feelings or experiences of the interviewee in a given situation, and the time dimension is not clear about the past, present or future, and therefore no definitive time judgement can be made."}, "sentence_type": "Affirmative declarative sentence", "pos": {"nouns": {"Home": 1, "Boring": 1}, "verbs": {"I feel": 1, "Boring": 1, "Home": 1}, "adverbs": {"very": 1}, "numerals": {"11": 1}}}, {"content": "Twelve at home we can say whatever we want.", "word_count": 15, "label": "", "sentence_structure": "Subject鈥搗erb鈥搊bject", "subjective_objective": {"answer": "Subjective", "rationale": "Because this description involves the patients ' inner feelings and attitudes in the family environment, i.e. they feel that they are free in the home and can express their own thoughts, which is the patient ' s subjective perception and feelings."}, "sentence_sentiment": {"answer": "Neutral", "rationale": "The entry is not an expression of any positive or negative emotional tendencies."}, "language_style": {"answer": "Tactful and warm", "rationale": "The entry is more euphemistic and sweet, asking the interviewee in an invitation speech whether he/she is free to express his/her opinion at home, rather than direct orders or judgment. At the same time, the use of the term \"home\" as a friendly language adds to the feeling of affection and affection."}, "time_dimension": {"answer": "Present", "rationale": "The time indicator \"at home\" in that sentence indicated the current situation, while the verb \"into say yes\" implied the continuity of the situation and the time dimension of the questionnaire entry could therefore be judged to be the present."}, "sentence_type": "Affirmative declarative sentence", "pos": {"nouns": {"Home": 1, "Lee": 1}, "verbs": {"Yes": 1, "Say it": 2, "Just": 1, "Yeah": 1}, "adverbs": {"Both": 1}, "numerals": {"12": 1}, "pronouns": {"we": 1, "What": 1, "Well": 1}}}, {"content": "Family members rarely publicly anger each other.", "word_count": 14, "label": "", "sentence_structure": "Subject鈥搗erb鈥揷omplement", "subjective_objective": {"answer": "Objective", "rationale": "Because \"very few public anger\" is an objective expression of behaviour among family members, there is no need for subjective perceptions or attitudes on the part of the patient."}, "sentence_sentiment": {"answer": "Positive", "rationale": "Because the entry \"very few family members are publicly angry with each other\" indicates a harmonious family relationship, which is a positive description."}, "language_style": {"answer": "Tactful and warm", "rationale": ""}, "time_dimension": {"answer": "Present", "rationale": ""}, "sentence_type": "Affirmative declarative sentence", "pos": {"nouns": {"Family members": 1, "You too": 1, "Anger": 1}, "verbs": {"Let's go": 1, "Less": 1}, "adjectives": {"Public": 1}, "adverbs": {"very": 1}, "numerals": {"13": 1}}}, {"content": "We all encourage the independence of our families.", "word_count": 16, "label": "", "sentence_structure": "Subject鈥搗erb鈥搊bject", "subjective_objective": {"answer": "Subjective", "rationale": "Because \"all encourage\" shows the attitude and the perception of the researchers about the spirit of independence, which is a subjective view."}, "sentence_sentiment": {"answer": "Positive", "rationale": "Because the purpose of the entry is to \"better encourage\" the behavior."}, "language_style": {"answer": "Calm and direct", "rationale": "The entry presents a point in a clear, straightforward tone, with no excessive adoration or emotional colour, and a more calm and straightforward language style."}, "time_dimension": {"answer": "Present", "rationale": "Now the entry uses the word \"extraordinary\" to express strong encouragement and the word \"both\" to indicate that it applies to all, which points to the current situation. At the same time, \"independentness\" is a description of behaviour, which is a current act or attitude. So, I think the time dimension of this questionnaire is now."}, "sentence_type": "Affirmative declarative sentence", "pos": {"nouns": {"we": 1, "Family": 1, "Independence": 1}, "verbs": {"Both": 1, "Encourage": 1, "Yes": 1}, "adverbs": {"Very": 1}, "numerals": {"14": 1}}}, {"content": "Family members have spent almost all their energy on a promising future.", "word_count": 21, "label": "", "sentence_structure": "Subject鈥搗erb鈥搊bject", "subjective_objective": {"answer": "Objective", "rationale": "Because \"good future\" and \"family members spend almost all their energy\" are objective facts. Although these acts and attitudes may be influenced by patients' subjective influence, they are considered objective outcomes or situations in this matter."}, "sentence_sentiment": {"answer": "Positive", "rationale": "Because the words \"for a good future\" and \"for a good future\" are all expressions of positive attitudes and behavior."}, "language_style": {"answer": "Calm and direct", "rationale": "The entry presents the fact in an objective and straightforward way that family members have invested a lot of effort in order to have a better future."}, "time_dimension": {"answer": "Past", "rationale": ""}, "sentence_type": "Affirmative declarative sentence", "pos": {"nouns": {"Future": 1, "Family members": 1, "- The energy": 1}, "verbs": {"Flowers": 1, "have": 1}, "adjectives": {"Okay": 1, "Almost": 1}, "numerals": {"15": 1}, "classifiers": {"Both": 1}}}, {"content": "We rarely go out to listen to lectures, to see movies or to museums and to watch exhibitions.", "word_count": 22, "label": "", "sentence_structure": "Subject鈥搗erb鈥搊bject", "subjective_objective": {"answer": "Objective", "rationale": "Because it is a habit or experience of the patient, it is an objective fact."}, "sentence_sentiment": {"answer": "Neutral", "rationale": "Because the entry does not have any emotional color."}, "language_style": {"answer": "Calm and direct", "rationale": "The entry is more calm and objective, and does not have too many emotional or oral techniques."}, "time_dimension": {"answer": "Present", "rationale": "The entry describes the present behaviour of the testee, in which the words \"very few\" and \"out of the house\" indicate that this is a description of the current situation. So the temporal dimension is now."}, "sentence_type": "Negative declarative sentence", "pos": {"nouns": {"we": 1, "Lectures": 1, "Film": 1, "Museums": 1, "Exhibition": 1}, "verbs": {"Out": 1, "Listen": 1, "Look": 3, "Go on": 2}, "adjectives": {"Very few": 1}, "numerals": {"16": 1}, "classifiers": {"individual": 1}}}, {"content": "Family members often go out to friends' homes to play and eat together.", "word_count": 19, "label": "", "sentence_structure": "Subject鈥搗erb鈥揷omplement", "subjective_objective": {"answer": "Objective", "rationale": "Because \"family members often go out to friends' houses and eat together\" is an objective fact that actually happens, not the thoughts or feelings of patients."}, "sentence_sentiment": {"answer": "Positive", "rationale": "Because the behaviour in the entries (family members often go out to friends ' homes and eat together) is positive, it emphasizes communication and interaction between family members."}, "language_style": {"answer": "Calm and direct", "rationale": "The entry is based on an objective and concise description of the fact that family members of the person interviewed often go out to their friends ' homes and eat together, without adding too much ado or emotional colour, and with a more straightforward tone."}, "time_dimension": {"answer": "Present", "rationale": ""}, "sentence_type": "Affirmative declarative sentence", "pos": {"nouns": {"Family members": 1, "friends": 1, "Rice": 1}, "verbs": {"Regular": 1, "Present": 1, "Go play": 1, "in": 1, "Together": 1, "Eat": 1}, "adverbs": {"and": 1, "Regular": 1}, "numerals": {"17": 1}}}, {"content": "Family members agreed that they should act in a manner that was socially appropriate.", "word_count": 16, "label": "", "sentence_structure": "Subject鈥搗erb鈥搊bject", "subjective_objective": {"answer": "Subjective", "rationale": "Because it relates to the concept and values of the family of the individual, it is subjective."}, "sentence_sentiment": {"answer": "Neutral", "rationale": "Because the entry does not have any emotional color."}, "language_style": {"answer": "Calm and direct", "rationale": "Calm down straight."}, "time_dimension": {"answer": "Past", "rationale": ""}, "sentence_type": "Affirmative declarative sentence", "pos": {"nouns": {"Family members": 2, "Social climate": 1}, "verbs": {"I think": 1, "Reactions": 1, "Do it": 1, "Complementarity": 1}, "adverbs": {"Both": 1}, "numerals": {"18": 1}}}, {"content": "In general, we are all concerned to keep our homes in order.", "word_count": 20, "label": "", "sentence_structure": "Subject鈥搗erb鈥揷omplement", "subjective_objective": {"answer": "Subjective", "rationale": "Because \"all\" and \"care to clean up the house\" are subjective judgments and ideas of the patients."}, "sentence_sentiment": {"answer": "Positive", "rationale": "Because the \"scrutinizing home-cleaning\" act in the entry is positive."}, "language_style": {"answer": "Tactful and warm", "rationale": "The entry uses a more euphemistic and invitationable tone rather than a direct statement or order, which is more gentle and friendly and more resonant with the respondents and their participation."}, "time_dimension": {"answer": "Present", "rationale": "Now the entry describes the general, current behavior or state of affairs, which is, \"We're all concerned about keeping our homes together.\" So it's now."}, "sentence_type": "Affirmative declarative sentence", "pos": {"nouns": {"19": 1, "Everyone": 1, "Home": 1, "Pack up": 1, "The well is organized": 1}, "verbs": {"Attention": 1, "- Put it on": 1}, "adverbs": {"General": 1, "Both": 1}, "numerals": {"19": 1}, "pronouns": {"we": 1}}}, {"content": "There are few regular living patterns and family rules in 20 households.", "word_count": 15, "label": "", "sentence_structure": "Subject鈥搗erb鈥搊bject", "subjective_objective": {"answer": "Objective", "rationale": "Because of the objective facts of a regular living pattern and family rules that patients experience, \"very rarely\" means that patients are seen and described."}, "sentence_sentiment": {"answer": "Negative", "rationale": "Because the behaviour of \"very few fixed living patterns and family rules\" in the entries is detrimental to the harmonious and stable development of families and has a negative emotional connotation."}, "language_style": {"answer": "Tactful and warm", "rationale": "The language style of the entry is more euphemistic, and it uses the means of questioning rather than direct statements or orders."}, "time_dimension": {"answer": "Present", "rationale": "The keyword \"very rarely\" indicates the current state of affairs, and \"fixed living patterns and family rules\" is an aspect of the current state of life, and thus it is judged to be the present."}, "sentence_type": "Negative declarative sentence", "pos": {"nouns": {"Family": 1, "Family rules": 1, "Pattern": 1}, "verbs": {"Very few": 1, "have": 1, "Fixed": 1}, "adjectives": {"Medium": 1}, "numerals": {"20": 1}}}, {"content": "Family members are willing to devote a lot of effort to their family's work.", "word_count": 17, "label": "", "sentence_structure": "Subject鈥搗erb鈥搊bject", "subjective_objective": {"answer": "Subjective", "rationale": "Because \"will\" is a subjective expression of the individual's will and emotional orientation, which is the subject of subjective considerations."}, "sentence_sentiment": {"answer": "Neutral", "rationale": "Because the entry does not have any emotional color."}, "language_style": {"answer": "Tactful and warm", "rationale": "The entry is more personal and interactive when it is spoken in an invitation-style tone, rather than in direct statements or orders. At the same time, the term \"great energy\" is used to make the question more open and inclusive and to make it easier for respondents to reflect and answer in depth about family relations."}, "time_dimension": {"answer": "Future", "rationale": ""}, "sentence_type": "Affirmative declarative sentence", "pos": {"nouns": {"Family members": 1, "- The energy": 1, "Home": 1, "Lee": 1, "Yes": 1}, "verbs": {"Yes": 1, "Flowers": 1, "Do it": 1}, "adjectives": {"very": 1, "Large": 1}, "numerals": {"21": 1}}}, {"content": "It is easy to irritate family members by complaining at home.", "word_count": 13, "label": "", "sentence_structure": "Subject鈥搗erb鈥搊bject", "subjective_objective": {"answer": "Subjective", "rationale": "Because \"facilitating family\" is a subjective judgement and feeling, depending on the family's reaction and attitude, it is not an objective reality."}, "sentence_sentiment": {"answer": "Negative", "rationale": "Because it expresses a negative emotion and attitude, \"family boredom\" is a negative reaction."}, "language_style": {"answer": "Calm and direct", "rationale": "The language style of the entry is more straightforward, with no excessive adoration or rendering, and is more calm and objective, expressing a direct statement of the issue."}, "time_dimension": {"answer": "Present", "rationale": "The key words of this entry are \"sorture at home\" and \"easy to cause\", all of which point to what is happening now. The entry therefore describes the current behaviour or state of the person being tested, which can be judged as present behaviour or mental state."}, "sentence_type": "Negative declarative sentence", "pos": {"nouns": {"22": 1, "Home": 1, "Complaints": 1, "Family": 1, "Boring": 1}, "verbs": {"鈾?And I'm gonna be 鈾?: 1}, "adverbs": {"very": 1}, "numerals": {"22": 1}, "classifiers": {"individual": 1}, "pronouns": {"He/she": 1}}}, {"content": "Sometimes family members fall when they are angry.", "word_count": 12, "label": "", "sentence_structure": "Subject鈥搗erb鈥搊bject", "subjective_objective": {"answer": "Objective", "rationale": "Because the fall of something when angry is an objective fact, is the behaviour of family members."}, "sentence_sentiment": {"answer": "Negative", "rationale": "Because the fall in the entry is negative."}, "language_style": {"answer": "Calm and direct", "rationale": "The language style of the entry is relatively straightforward and concise, and there is no excessive emotional or rhetorical approach, but rather a simple and clear description of the phenomenon, which is more understandable and acceptable."}, "time_dimension": {"answer": "Past", "rationale": "This entry describes the behaviour that often occurs in a family, i.e., that family members fall in certain circumstances."}, "sentence_type": "Affirmative declarative sentence", "pos": {"nouns": {"Family members": 1, "Anger": 1, "Something": 1}, "verbs": {"- Get angry": 1, "Fall": 1}, "adverbs": {"Sometimes": 1}, "numerals": {"23": 1}}}, {"content": "Family members are independent in their thinking.", "word_count": 11, "label": "", "sentence_structure": "Subject鈥搗erb鈥揷omplement", "subjective_objective": {"answer": "Objective", "rationale": "Since the question of whether family members are independent is an objective fact that can be understood by observation or enquiry."}, "sentence_sentiment": {"answer": "Positive", "rationale": "Because \"independent thinking\" is a positive quality."}, "language_style": {"answer": "Tactful and warm", "rationale": "The entry is more personal and interactive by speaking in a gentle and inviting manner, rather than by direct statement or order. At the same time, the word \"both\" is used to reflect the equal treatment of each family member and to give a warmer sense of affection."}, "time_dimension": {"answer": "Present", "rationale": "The entry describes the current situation and situation, where all members of the tested family are able to think independently at this point in time."}, "sentence_type": "Affirmative declarative sentence", "pos": {"nouns": {"Family members": 1, "Problem": 1}, "verbs": {"Both": 1, "Independent thinking": 1}, "numerals": {"24": 1}}}, {"content": "Family members agree that raising the standard of living is more important than anything else.", "word_count": 24, "label": "", "sentence_structure": "Subject鈥搗erb鈥搊bject", "subjective_objective": {"answer": "Subjective", "rationale": "The views of family members are subjective and different people have different values and perceptions."}, "sentence_sentiment": {"answer": "Positive", "rationale": "This attitude is positive because the expression of this article reflects the importance and desire of family members for an improved standard of living."}, "language_style": {"answer": "Calm and direct", "rationale": "The language style of the entry is relatively simple and not too much phrasing or emotional rendering, but rather a direct statement of the fact that 25 family members consider it most important to improve their standard of living."}, "time_dimension": {"answer": "Present", "rationale": ""}, "sentence_type": "Affirmative declarative sentence", "pos": {"nouns": {"Family members": 1, "Standard of living": 1, "Things": 1}, "verbs": {"I think": 1, "Increase": 1}, "adjectives": {"Any": 2, "Important": 1}, "numerals": {"25": 1}, "pronouns": {"they": 1}}}, {"content": "We all believe that learning new knowledge is more important than anything else.", "word_count": 20, "label": "", "sentence_structure": "Subject鈥搗erb鈥搊bject", "subjective_objective": {"answer": "Objective", "rationale": "Because \"we all think it's more important to learn new knowledge than anything else\" is a universal view or belief, and does not involve the patient's own subjective feelings or ideas. So, it's an objective way of looking at it."}, "sentence_sentiment": {"answer": "Positive", "rationale": "Because the expression \"learning new knowledge is more important than anything else\" in the entry is positive."}, "language_style": {"answer": "Tactful and warm", "rationale": "The language style of the entry is relatively euphemistic, implying that it is a generally accepted view, not a unilateral one, by using the phrase \"we all believe\" . Such expression is more acceptable and participatory."}, "time_dimension": {"answer": "Future", "rationale": "The entry expresses the expectation and the notion that in the future more attention will be given to the importance of learning new knowledge. While this perception may exist in the present, it is more expressed here as a trend towards the future. The answer is therefore \"the future\"."}, "sentence_type": "Affirmative declarative sentence", "pos": {"nouns": {"we": 1, "New": 1, "Knowledge": 1, "Yes": 1}, "verbs": {"I think": 1, "Learn": 1, "- Yeah": 1, "Both": 1, "Important": 1}, "adjectives": {"Any": 1}, "numerals": {"26": 1}, "pronouns": {"Other": 1}}}, {"content": "No one in the family has taken part in any sports activities.", "word_count": 12, "label": "", "sentence_structure": "Subject鈥搗erb鈥揷omplement", "subjective_objective": {"answer": "Objective", "rationale": "Because \"no one in the family takes part in sports activities\" is a fact that is a matter of fact and is an objective observation."}, "sentence_sentiment": {"answer": "Negative", "rationale": "Because the \"no one takes part in any sporting activity\" in the entry is negative."}, "language_style": {"answer": "Tactful and warm", "rationale": "The entry uses a soft and inviting tone for dialogue rather than direct statements or orders, which is more personal and interactive. At the same time, it avoids terms and tone that may cause resistance to the respondents, making the questionnaire more acceptable and accessible."}, "time_dimension": {"answer": "Present", "rationale": ""}, "sentence_type": "Negative declarative sentence", "pos": {"nouns": {"Home": 2, "person": 1, "Sports activities": 1}, "verbs": {"Participation": 1}, "adverbs": {"Various": 1}, "numerals": {"27": 1}}}, {"content": "Family members often help the elderly and disabled around them.", "word_count": 22, "label": "", "sentence_structure": "Subject鈥搗erb鈥搊bject", "subjective_objective": {"answer": "Objective", "rationale": "Because \"family members often help the elderly and disabled around them in their lives\" is an objective fact. Although this behaviour may be influenced by subjective factors such as the values, beliefs, etc. of the patient, it is itself objective and can be judged by observation and documentation."}, "sentence_sentiment": {"answer": "Positive", "rationale": "Because the behaviour in the entry is to help the elderly and disabled around them, it is a positive social act."}, "language_style": {"answer": "Tactful and warm", "rationale": "The entry is more euphemistic and sweet. It is more personal and interactive in an invitation to ask the respondents whether they are often helping the elderly and disabled around them in their lives."}, "time_dimension": {"answer": "Present", "rationale": "The entry describes the current behaviour of family members, the key words being \"life\" and \"often helping\" , all of which point to the conduct currently being performed. At the same time, \"now\" also indicates that the behaviour is a state of normality or custom."}, "sentence_type": "Affirmative declarative sentence", "pos": {"nouns": {"Family members": 1, "Life": 1, "Older persons": 1, "Persons with disabilities": 1}, "verbs": {"in": 1, "Help": 1, "Around": 1}, "adverbs": {"Regular": 1}, "numerals": {"28": 1}}}, {"content": "In our homes, it is often not found when something needs to be used.", "word_count": 20, "label": "", "sentence_structure": "Subject鈥搗erb鈥搊bject", "subjective_objective": {"answer": "Objective", "rationale": "Because it describes objective facts in the family, such as the placement of objects, it does not relate to the feelings or thoughts of the patient."}, "sentence_sentiment": {"answer": "Negative", "rationale": "Because \"unreceived\" in the entry is negative."}, "language_style": {"answer": "Tactful and warm", "rationale": "This is a more euphemistic approach, not directly accusing or complaining about the situation in the family, but rather allowing the person under investigation to express his or her situation freely in a way that is more personal and interactive."}, "time_dimension": {"answer": "Present", "rationale": "The entry \"often\" indicates that it is a customary act, that \"need\" and \"not found\" are described as a state of the moment, so it can be judged that it is a question of the current behaviour or state."}, "sentence_type": "Negative declarative sentence", "pos": {"nouns": {"Home": 1, "Something": 1}, "verbs": {"Use": 1, "Find": 1}, "adjectives": {"Often": 1}, "numerals": {"29": 1}}}, {"content": "The time spent eating and sleeping in our house is constant.", "word_count": 19, "label": "", "sentence_structure": "Subject鈥搗erb鈥揷omplement", "subjective_objective": {"answer": "Objective", "rationale": "This is an objective fact because of the patient ' s description of his/her family life habits."}, "sentence_sentiment": {"answer": "Neutral", "rationale": "Because the entry is a mere statement of fact and does not contain any emotional colour."}, "language_style": {"answer": "Calm and direct", "rationale": "The language style of the entry is more straightforward, not using too many rhetorical techniques or emotional renderings, but simply stating a fact. It is therefore a calm and straightforward language style."}, "time_dimension": {"answer": "Past", "rationale": "At the same time, the main phrase of the article, \"our home\", indicates that the problem is a description of an already existing and fixed situation, and therefore can be judged in terms of time dimension."}, "sentence_type": "Affirmative declarative sentence", "pos": {"nouns": {"Time": 1, "Home": 1, "Rice": 1, "I'm not sure": 1}, "verbs": {"Eat": 1, "Sleep": 1, "be": 1, "Change": 1}, "adjectives": {"It's the same": 1}, "numerals": {"30": 1}, "pronouns": {"we": 1}}}, {"content": "There is a climate of harmony in our home.", "word_count": 15, "label": "", "sentence_structure": "Subject鈥搗erb鈥揷omplement", "subjective_objective": {"answer": "Subjective", "rationale": "Because \"a climate of harmony\" is a subjective feeling and experience that requires the patient to feel and describe itself."}, "sentence_sentiment": {"answer": "Positive", "rationale": "Because the description of \"a climate of harmony\" in the entry is positive."}, "language_style": {"answer": "Tactful and warm", "rationale": "The entry conveys a positive emotional colour by describing \"a climate of harmony\" and uses a softer and gentler language, so that its language style can be judged as euphemism."}, "time_dimension": {"answer": "Present", "rationale": ""}, "sentence_type": "Affirmative declarative sentence", "pos": {"nouns": {"31": 1, "The atmosphere": 1, "Family": 1}, "verbs": {"have": 1, "One": 1, "Hold on": 1}, "adjectives": {"Harmony": 1}, "numerals": {"One": 2, "Plant": 1}, "classifiers": {"individual": 1}, "pronouns": {"we": 1}}}, {"content": "Each person in the family can speak of his or her difficulties and problems.", "word_count": 19, "label": "", "sentence_structure": "Subject鈥搗erb鈥搊bject", "subjective_objective": {"answer": "Subjective", "rationale": "Because the sentence describes a possible situation, it does not specify whether everyone has such an opportunity or whether they will. It is therefore a subjective statement."}, "sentence_sentiment": {"answer": "Positive", "rationale": "Because it expresses an attitude of support, understanding and acceptance, and encourages everyone to share their difficulties and worries, a positive emotional colour."}, "language_style": {"answer": "Tactful and warm", "rationale": "The language style of the entry is more euphemistic and warm, encouraging respondents to share their thoughts and feelings by using the expression \"Everyone can speak of his or her difficulties and worries\", which is relatively gentle and friendly and increases the openness and participation of the interviewees."}, "time_dimension": {"answer": "Present", "rationale": ""}, "sentence_type": "Affirmative declarative sentence", "pos": {"nouns": {"Home": 1, "person": 1, "Difficulties": 1, "Trouble": 1}, "verbs": {"Tell me": 1, "Yeah": 1}, "adverbs": {"Every one of them": 1, "Both": 1}, "numerals": {"32": 1}}}, {"content": "Little anger was reported among 33 family members.", "word_count": 11, "label": "", "sentence_structure": "Subject鈥搗erb鈥揷omplement", "subjective_objective": {"answer": "Subjective", "rationale": "Because \"very little anger\" is the patient's subjective assessment and perception of family relationships."}, "sentence_sentiment": {"answer": "Positive", "rationale": "Because \"very few\" in the entry means that family relations are harmonious and a climate is a positive quality."}, "language_style": {"answer": "Tactful and warm", "rationale": "The expression \"very rarely has a temperament\" gives a more positive and harmonious sense. So the language style of this questionnaire entry is euphemistic."}, "time_dimension": {"answer": "Present", "rationale": "The entry describes the current state of coexistence between family members and therefore is judged to be \"now\"."}, "sentence_type": "Affirmative declarative sentence", "pos": {"nouns": {"Family members": 1, "- Grumpy": 1}, "verbs": {"Very few": 1, "Let's go": 1}, "adjectives": {"33": 1}, "numerals": {"33": 1}}}, {"content": "Everyone in our family is completely free to go.", "word_count": 16, "label": "", "sentence_structure": "Subject鈥搗erb鈥揷omplement", "subjective_objective": {"answer": "Subjective", "rationale": "This is the subjective view of the patient of his or her family situation and involves the knowledge, evaluation and understanding of the behaviour and rules of family members."}, "sentence_sentiment": {"answer": "Neutral", "rationale": "Because the entry does not have any emotional color."}, "language_style": {"answer": "Tactful and warm", "rationale": "The language style of the entry is more euphemistic, using the expression \"everyone in our family\" to express intimate relationships between family members and the phrase \"full freedom\" to describe the openness and inclusion of the family environment. Such language styles are more likely to resonate and trust the interviewee, and thus to be more willing to participate in the questionnaire and to give a true and objective answer."}, "time_dimension": {"answer": "Present", "rationale": "The entry describes the current state or situation, i.e., that everyone in the family of the speaker is free to enter and leave without any restrictions or restrictions."}, "sentence_type": "Affirmative declarative sentence", "pos": {"nouns": {"Our home": 1, "Everyone": 1, "Access": 1}, "verbs": {"be": 1, "Total": 1, "Freedom": 1}, "numerals": {"34": 1}}}, {"content": "We all believe that competition is good in any case.", "word_count": 16, "label": "", "sentence_structure": "Subject鈥搗erb鈥搊bject", "subjective_objective": {"answer": "Subjective", "rationale": "Because the word \"rebel\" expresses the views and attitudes of the patient."}, "sentence_sentiment": {"answer": "Positive", "rationale": "Because the words \"both believe\" and \"good\" convey positive emotional attitudes."}, "language_style": {"answer": "Calm and direct", "rationale": ""}, "time_dimension": {"answer": "Present", "rationale": ""}, "sentence_type": "Affirmative declarative sentence", "pos": {"nouns": {"Situation": 1, "Competition": 2, "Good news": 1}, "verbs": {"Yes": 1, "be": 1}, "adjectives": {"Any": 1}, "numerals": {"35": 1}, "pronouns": {"we": 1}}}, {"content": "We are not so interested in cultural activities.", "word_count": 13, "label": "", "sentence_structure": "Subject鈥搗erb鈥搊bject", "subjective_objective": {"answer": "Subjective", "rationale": "Because interest is an individual subjective feeling and attitude."}, "sentence_sentiment": {"answer": "Negative", "rationale": "Because \"less interested\" expresses a negative emotional attitude."}, "language_style": {"answer": "Calm and direct", "rationale": "The language style of the \"cool straight\" article is more straightforward, is less over-modified or emotional, and is more factual, and is easy to understand and respond to."}, "time_dimension": {"answer": "Present", "rationale": "Depending on the context, \"36\" may be a number or serial number, indicating that it is a specific question in the questionnaire. And \"We are not so interested in cultural activities\" is the answer given to this question by the testee, which is a description of the behaviour or attitude of the moment."}, "sentence_type": "Negative declarative sentence", "pos": {"nouns": {"Cultural activities": 1, "Interest": 1}, "verbs": {"to": 1, "Interested": 1}, "adjectives": {"Well": 1}, "numerals": {"36": 1}}}, {"content": "We watch movies or sports games, outings, etc.", "word_count": 16, "label": "", "sentence_structure": "Subject鈥搗erb鈥搊bject", "subjective_objective": {"answer": "Objective", "rationale": "Because watching movies, sports games and field trips are objective facts that patients experience."}, "sentence_sentiment": {"answer": "Positive", "rationale": "Because \"seeing a movie or sports game, going out on a field trip\" is a positive way of entertainment and leisure."}, "language_style": {"answer": "Tactful and warm", "rationale": "The language style of the entry is more moderate, using advice or invitation to the respondent to answer the question rather than direct orders or coercion. At the same time, the sentence also expresses a positive emotional tone and encourages the respondent to engage in a rich variety of recreational and leisure activities."}, "time_dimension": {"answer": "Present", "rationale": "And as can be seen from the entry sentence, \"We watch movies or sports, go out on a field trip, etc.,\" it's a description of current customary behaviour, so the temporal dimension is now."}, "sentence_type": "Affirmative declarative sentence", "pos": {"nouns": {"Film": 1, "Sports competitions": 1, "A field trip": 1}, "verbs": {"Look": 1, "Regular": 1, "Go on": 1}, "adverbs": {"Regular": 1}, "numerals": {"37": 1}}}, {"content": "We consider active and passive bribery to be an acceptable phenomenon.", "word_count": 18, "label": "", "sentence_structure": "Subject鈥搗erb鈥搊bject", "subjective_objective": {"answer": "Subjective", "rationale": "Because \"think\" is a subjective term, which indicates the attitude and opinion of the person interviewed. While active and passive bribery is an objective phenomenon, the extent to which the person interviewed accepts it is subjective and not objective."}, "sentence_sentiment": {"answer": "Negative", "rationale": "Because the act of \"bribery and bribery\" is negative, it is also a negative attitude to consider it acceptable."}, "language_style": {"answer": "Calm and direct", "rationale": "The language style of the entry is calm and straightforward. It directly asks the respondents about the phenomenon of bribery, which is not overly emotional or acrimony."}, "time_dimension": {"answer": "Present", "rationale": "Now the keyword \"concept\" indicates that it is a present idea or view, that \"acceptable\" expresses an attitude or view of active and passive bribery, and does not address past or future situations. The entry therefore describes the current thinking and attitude of the testee."}, "sentence_type": "Negative declarative sentence", "pos": {"nouns": {"phenomena": 1, "Bribery": 1, "Bribing": 1}, "verbs": {"I think": 1, "Accept": 1}, "adverbs": {"Yeah": 1}, "numerals": {"38": 1}}}, {"content": "39 In our home, it is important to be punctual.", "word_count": 12, "label": "", "sentence_structure": "Subject鈥搗erb鈥搊bject", "subjective_objective": {"answer": "Objective", "rationale": "The importance of punctuality is an expression of values or family values and an objective reality."}, "sentence_sentiment": {"answer": "Positive", "rationale": "Because the expression \"are very serious\" expresses a positive attitude, namely, the importance and acceptance of doing things on time."}, "language_style": {"answer": "Tactful and warm", "rationale": "The entry makes the context more natural by using the first person's pronoun, \"we\", while using positive tone to express the importance of \"just on time\" rather than direct command or criticism. The language style of the entry is therefore more euphemismal."}, "time_dimension": {"answer": "Past", "rationale": "The keywords \"are very important\" and \"do things on time\" indicate that they are a description or a view of the past."}, "sentence_type": "Affirmative declarative sentence", "pos": {"nouns": {"39": 1, "Home": 1, "Yes": 2, "Do it": 1, "Time": 1}, "verbs": {"It's important": 1, "Do it": 1, "Be on time": 1}, "adjectives": {"On time": 1}, "numerals": {"39": 1}}}, {"content": "There's a fixed way to do everything in our family.", "word_count": 14, "label": "", "sentence_structure": "Subject鈥搗erb鈥搊bject", "subjective_objective": {"answer": "Subjective", "rationale": "This approach is subjective, as it is developed by the family of the patient."}, "sentence_sentiment": {"answer": "Neutral", "rationale": "Since the entry merely stated a fact, it did not contain any apparent emotional tendencies."}, "language_style": {"answer": "Calm and direct", "rationale": "The entry is based on objective representations, which do not use emotional language, so it can be judged as being calm and straightforward. Such expressions help the interviewee to understand the problem more accurately, without being too euphemistic or vague to be misleading. In short, the language style of the questionnaire entry is calm and straightforward."}, "time_dimension": {"answer": "Present", "rationale": "In the future, the entry describes the vision and expectations for the future, with the keywords \"do anything\" and \"have a fixed way\" pointing to what may happen in the future."}, "sentence_type": "Affirmative declarative sentence", "pos": {"nouns": {"Our home": 1, "Yes": 1, "Modalities": 1}, "verbs": {"Do it": 1, "have": 1, "Fixed": 1}, "numerals": {"40": 1}}}, {"content": "Few people volunteer to do so when they have a family problem.", "word_count": 13, "label": "", "sentence_structure": "Subject鈥搗erb鈥揷omplement", "subjective_objective": {"answer": "Subjective", "rationale": "Because \"very few people volunteer to do it\" reflects the attitude and perception of the patient towards family members and is the subjective feeling and view of the patient."}, "sentence_sentiment": {"answer": "Negative", "rationale": "Because \"very few people volunteer to do it\" in the entry is a negative attitude."}, "language_style": {"answer": "Tactful and warm", "rationale": "The entry uses an invitation to ask if the other party has been offered help in family matters, which is more personal and interactive, making it easier for people to open their hearts and answer questions."}, "time_dimension": {"answer": "Present", "rationale": ""}, "sentence_type": "Negative declarative sentence", "pos": {"nouns": {"Yes": 1, "person": 2}, "verbs": {"have": 1, "Do it": 1}, "adjectives": {"Very few": 1, "Voluntary": 1}, "numerals": {"41": 1}}}, {"content": "Family members often express their feelings in public.", "word_count": 18, "label": "", "sentence_structure": "Subject鈥搗erb鈥揷omplement", "subjective_objective": {"answer": "Subjective", "rationale": "Whether family members often express their feelings in public is a reflection of subjective feelings and attitudes."}, "sentence_sentiment": {"answer": "Positive", "rationale": "Because the act of \"public expression of mutual feelings\" is positive."}, "language_style": {"answer": "Calm and direct", "rationale": "The sentence of this article is clearly structured and is clear, and it does not use too many emotional terms or rhetorical techniques, but rather asks the respondents whether they agree with the view that \"family members often express their feelings in public\" and therefore the language style is straightforward and calm."}, "time_dimension": {"answer": "Present", "rationale": ""}, "sentence_type": "Affirmative declarative sentence", "pos": {"nouns": {"Family members": 1, "Emotion": 1}, "verbs": {"Expression": 1, "Public": 1, "Between": 1}, "adjectives": {"- A little bit": 1}, "numerals": {"42": 1}}}, {"content": "Family members often blame and criticize each other.", "word_count": 14, "label": "", "sentence_structure": "Subject鈥搗erb鈥搊bject", "subjective_objective": {"answer": "Subjective", "rationale": "Because \"often blame and criticize each other\" is the subjective perception and judgement of a patient about his family relationship. While this is an objective reality, this subjective perception and evaluation is the focus of the questionnaire."}, "sentence_sentiment": {"answer": "Negative", "rationale": "Because the \"competence and criticism\" in the entry is negative."}, "language_style": {"answer": "Calm and direct", "rationale": "The entry is presented in an objective and direct manner, not with too much adoration or emotional colour, but with a simple and clear description of a phenomenon. It is therefore more of a calmer language style."}, "time_dimension": {"answer": "Present", "rationale": ""}, "sentence_type": "Affirmative declarative sentence", "pos": {"nouns": {"Family members": 1, "- Yeah, yeah": 1, "Blame": 1, "Criticism": 1}, "verbs": {"Regular": 1, "Mutual blame and criticism": 1}, "adverbs": {"Regular": 1}, "numerals": {"43": 1}}}, {"content": "Family members rarely take into account the views of the rest of the family.", "word_count": 19, "label": "", "sentence_structure": "Subject鈥搗erb鈥揷omplement", "subjective_objective": {"answer": "Objective", "rationale": "The sentence describes the objective behaviour and circumstances of family members when doing their work, without involving subjective attitudes or feelings of the patient."}, "sentence_sentiment": {"answer": "Negative", "rationale": "Because the purpose of the article is to state that \"very little consideration is given to the views of the rest of the family\" is a negative act."}, "language_style": {"answer": "Calm and direct", "rationale": "The entry presents the problem in a factual manner, without using a rhetorical or emotional rendering, and is more objective in tone. At the same time, it directly points to a specific phenomenon that enables the person surveyed to understand the meaning of the problem more accurately."}, "time_dimension": {"answer": "Past", "rationale": "The entry describes a problem that family members have experienced in some past behaviour or attitude, i.e. they rarely take into account the views of the rest of the family when they do their work."}, "sentence_type": "Negative declarative sentence", "pos": {"nouns": {"Family members": 1, "Yes": 2, "person": 1, "Home": 1}, "verbs": {"Do it": 2, "Consider": 1, "Say it": 1}, "adjectives": {"Very few": 1, "Other": 1}, "numerals": {"44": 1}, "pronouns": {"Home": 1}}}, {"content": "We have been constantly reflecting on ourselves and forcing ourselves to do what it takes to do better than once.", "word_count": 27, "label": "", "sentence_structure": "Subject鈥搗erb鈥揷omplement", "subjective_objective": {"answer": "Subjective", "rationale": "This is a reflection of patients ' subjective ideas and attitudes."}, "sentence_sentiment": {"answer": "Positive", "rationale": "Because \"regarding yourself\" and \"doing what you can to do better than you\" are positive attitudes and behaviours."}, "language_style": {"answer": "Calm and direct", "rationale": "The entry describes in a direct and clear language the habit that people often have to reflect and work to improve themselves. This language style does not use excessive rhetoric or emotional colours, but rather objectively presents a fact and can therefore be classified as a \"cool and direct\" language style."}, "time_dimension": {"answer": "Present", "rationale": ""}, "sentence_type": "Affirmative declarative sentence", "pos": {"nouns": {"Retrospect": 1, "Things": 1, "Coercion": 1}, "verbs": {"Always": 1, "Constantly": 1, "Retrospect": 2, "Try": 1, "Do it": 2, "Better once than once": 1}, "adverbs": {"self": 2, "Coercion": 1, "Always": 1, "Constantly": 1, "Better once than once": 1}, "numerals": {"45": 1}, "pronouns": {"we": 1}}}, {"content": "We rarely discuss issues related to scientific and technological knowledge.", "word_count": 17, "label": "", "sentence_structure": "Subject鈥搗erb鈥搊bject", "subjective_objective": {"answer": "Subjective", "rationale": "The sentence is a subjective view of whether they discuss scientific and technological knowledge between themselves and others. The word \"very rarely\" also indicates that it is a subjective assessment of the patient, not an objective fact that can be measured or observed."}, "sentence_sentiment": {"answer": "Neutral", "rationale": "Because the entry is not clearly emotional, it merely describes a fact."}, "language_style": {"answer": "Calm and direct", "rationale": "The language style of the entry is more straightforward, with no excessive emotional or rhetorical tact, but simply a statement of fact or opinion, and can therefore be judged as \"cool and direct\"."}, "time_dimension": {"answer": "Present", "rationale": "The sentence indicates the current situation, where \"very little discussion\" indicates that such behaviour has been and is less frequent."}, "sentence_type": "Negative declarative sentence", "pos": {"nouns": {"we": 1, "Problem": 1}, "verbs": {"Discussion": 1, "Related": 1, "In terms of": 1}, "adjectives": {"Very few": 1, "Scientific and technical knowledge": 1}, "numerals": {"46": 1}}}, {"content": "Each of us is particularly interested in one or two recreational activities.", "word_count": 18, "label": "", "sentence_structure": "Subject鈥搗erb鈥搊bject", "subjective_objective": {"answer": "Subjective", "rationale": "Because \"special interest\" is a subjective assessment or feeling that reflects the attitude and preferences of family members for recreational activities."}, "sentence_sentiment": {"answer": "Positive", "rationale": "Because the entry expresses the fact that family members share a common hobby, which is a positive emotional color."}, "language_style": {"answer": "Tactful and warm", "rationale": "The entry is more euphemistic, is not expressed in a strong tone or in a command style, but is presented in an invitation and discussion format."}, "time_dimension": {"answer": "Present", "rationale": "And by the phrase \"we're all very interested,\" it's a description of the current situation. And \"1-2 entertainment\" also shows that it's a constant hobby, so the entry should be on the current time scale."}, "sentence_type": "Affirmative declarative sentence", "pos": {"nouns": {"Our home": 1, "person": 1, "Recreational activities": 1, "Interest": 1}, "verbs": {"to": 1, "Special": 1, "Interested": 1}, "adverbs": {"47": 1, "One to two": 1}, "numerals": {"47": 1, "1": 1, "2": 1}, "classifiers": {"Item": 1}}}, {"content": "We believe that, in any case, the elders should be persuaded by the elders.", "word_count": 21, "label": "", "sentence_structure": "Subject鈥搗erb鈥搊bject", "subjective_objective": {"answer": "Subjective", "rationale": "The purpose of this questionnaire is to refer to the values and beliefs of individuals and therefore to the patient ' s perspective."}, "sentence_sentiment": {"answer": "Positive", "rationale": "Because it conveys a positive value, namely, respect for and obedience to the opinions of elders."}, "language_style": {"answer": "Tactful and warm", "rationale": "The entry uses the phrase \"we believe\" to indicate that it is the author's view and to \"should\" express the word \"advisory\" rather than an order or a direct demand. This expression is more euphemism, more acceptable and understandable. At the same time, the notion that \"adults should be persuaded by their elders\" is more positive and positive, without too much negative emotion or evaluation, and has some kind of acrimony and interaction."}, "time_dimension": {"answer": "Present", "rationale": "Now the entry represents the individual views and attitudes of the speaker, with no clear temporal dimensions to indicate past, present or future. It expresses current ideas and views."}, "sentence_type": "Affirmative declarative sentence", "pos": {"nouns": {"we": 1, "Sir": 2, "Senior": 1, "Construction": 1}, "verbs": {"I think": 1, "I think so": 1, "Accept": 1, "Here": 1}, "adverbs": {"Whatever": 1}, "numerals": {"48": 1}, "pronouns": {"Here": 1}}}, {"content": "Our family often changes their plans.", "word_count": 14, "label": "", "sentence_structure": "Subject鈥搗erb鈥搊bject", "subjective_objective": {"answer": "Subjective", "rationale": "Basis for the judgement: This phrase describes the phenomenon of a family member who often changes their plans, based on the observations and experiences of the speaker, and therefore is subjective."}, "sentence_sentiment": {"answer": "Negative", "rationale": "Because \"often changing their plans\" is negative."}, "language_style": {"answer": "Tactful and warm", "rationale": "The entry is more euphemistic, using a more moderate tone to ask the respondents whether there are frequent changes in the plan, and there are no overly direct or hard-line expressions that leave a feeling of more affection and comfort."}, "time_dimension": {"answer": "Past", "rationale": "As can be seen from the sentence, \"often changing their plans\" is a description of a customary, past event, so the time dimension of this questionnaire entry should be the past."}, "sentence_type": "Affirmative declarative sentence", "pos": {"nouns": {"person": 1, "Planned": 1}, "verbs": {"Change": 1, "Often": 1}, "adverbs": {"Often": 1}, "numerals": {"49": 1}, "pronouns": {"we": 1}}}, {"content": "Our family places great emphasis on adherence to regular living patterns and family rules.", "word_count": 20, "label": "", "sentence_structure": "Subject鈥搗erb鈥搊bject", "subjective_objective": {"answer": "Objective", "rationale": "Because \"compliance with fixed living rules and family rules\" is a family code or rule of conduct, it is objective. And it is the patient's subjective attitude to stress compliance with these rules."}, "sentence_sentiment": {"answer": "Neutral", "rationale": "Because the entry is not of a clear emotional nature, it merely presents a fact."}, "language_style": {"answer": "Tactful and warm", "rationale": "The entry uses a more euphemistic and moderate expression, using the phrase \"highly emphasis\" to express the values of the family without direct orders or demands from the other. Such language styles are more acceptable and understandable and more friendly and sweet."}, "time_dimension": {"answer": "Present", "rationale": "The \"Our Family is Strongly Focused\" in the entry indicates that it is a behavior that is now being upheld, and that it is also a behaviour that is now being performed by \"to respect the rules of life and family.\" So the time dimension of this questionnaire entry is now judged."}, "sentence_type": "Affirmative declarative sentence", "pos": {"nouns": {"50": 1, "Our home": 1, "Lifestyle": 1, "Family rules": 1}, "verbs": {"Emphasizing": 1, "Compliance": 1}, "adverbs": {"Very": 1}, "numerals": {"50": 1}, "classifiers": {"individual": 1}}}, {"content": "Family members are always supportive of each other with sincerity.", "word_count": 14, "label": "", "sentence_structure": "Subject鈥搗erb鈥揷omplement", "subjective_objective": {"answer": "Subjective", "rationale": "Because \"thank you\" and \"support each other\" are subjective feelings and attitudes of patients."}, "sentence_sentiment": {"answer": "Positive", "rationale": "Because the \"regardless support\" in the entry is positive."}, "language_style": {"answer": "Tactful and warm", "rationale": "The language style of the entry is euphemistic, because it uses words such as \"creative support for each other\" and is a moderate, invited expression of positive assessment and concern about the family relationship of the interviewee. This expression is personal and interactive, and it makes it easier for the interviewee to resonate and to share his feelings."}, "time_dimension": {"answer": "Past", "rationale": "In the entry, \"all\" indicates that this has been and continues at some point in the past, and therefore can be judged to be the past. At the same time, the description also relates to relations and support between family members, which usually refers to social relations and patterns of behaviour that have developed over the long term and over the past and present periods."}, "sentence_type": "Affirmative declarative sentence", "pos": {"nouns": {"51": 1, "Family members": 1, "Support": 1}, "verbs": {"Always": 1, "I'm sorry": 1, "- Yeah, yeah": 1}, "adverbs": {"Both": 1}, "numerals": {"51": 1}}}, {"content": "If you say at home that you are dissatisfied with family matters, some feel uncomfortable.", "word_count": 21, "label": "", "sentence_structure": "Subject鈥搗erb鈥搊bject", "subjective_objective": {"answer": "Subjective", "rationale": "Because \"incomprehensible\" is a subjective feeling and experience, the point of view of this questionnaire is subjective."}, "sentence_sentiment": {"answer": "Negative", "rationale": "Because the emotional tendency of the phrase \"dissatisfaction with family matters makes others uncomfortable\" in the entry is negative."}, "language_style": {"answer": "Calm and direct", "rationale": "The entry expresses the view, in an objective and concise manner, that public expression of dissatisfaction with household chores may be uncomfortable for others."}, "time_dimension": {"answer": "Past", "rationale": "The words \"in the family\", \"said\" and \"discomforts\" indicate that this is a past event or situation. The entry therefore describes a possible past occurrence."}, "sentence_type": "Affirmative declarative sentence", "pos": {"nouns": {"Home": 1, "Dissatisfied": 1, "Family matters": 1}, "verbs": {"Say it": 1, "I think": 1, "Yes": 2}, "adjectives": {"- Yeah": 1}, "numerals": {"52": 1}, "pronouns": {"person": 1}}}, {"content": "Family members sometimes fight each other.", "word_count": 10, "label": "", "sentence_structure": "Subject鈥搗erb鈥搊bject", "subjective_objective": {"answer": "Objective", "rationale": "Because \"family members sometimes fight each other\" is an objective fact, not a subjective feeling or attitude of the patient. Although the patient may be troubled or under stress, this situation is not controlled by the patient, but is determined by objective factors."}, "sentence_sentiment": {"answer": "Negative", "rationale": "Because the \"fights\" in the entries are negative."}, "language_style": {"answer": "Calm and direct", "rationale": "The language style of the entry appears to be cold, with direct reference to fighting between family members, and no excessive emotional accompaniment or euphemism."}, "time_dimension": {"answer": "Present", "rationale": "According to the phrase \"sometimes\", it can be seen that the act is occurring or is recurrent, so the entry describes the current state or behaviour."}, "sentence_type": "Affirmative declarative sentence", "pos": {"nouns": {"Family members": 1, "Fight": 1}, "verbs": {"Sometimes": 1, "- Yeah, yeah": 1}, "adverbs": {"Sometimes": 1}, "numerals": {"53": 1}}}, {"content": "Family members rely on the help of their families to resolve their difficulties.", "word_count": 22, "label": "", "sentence_structure": "Subject鈥搗erb鈥搊bject", "subjective_objective": {"answer": "Objective", "rationale": "It is an objective fact that family members are facing difficulties and seeking help from their families."}, "sentence_sentiment": {"answer": "Negative", "rationale": "Because the \"all rely on family help\" in the entry is negative."}, "language_style": {"answer": "Tactful and warm", "rationale": "The reference to the questionnaire is relatively euphemistic, and it does not directly blame or criticize family members for their behaviour, but rather describes the situation in an objective and understandable manner. Moreover, the entry uses positive terms (e.g. \"dependent\", \"help\" etc.), which help to alleviate possible negative sentiments."}, "time_dimension": {"answer": "Present", "rationale": "The words \"family members\", \"dependentness\" and \"help\" indicate that this is a statement describing the current state of affairs, so that the temporal dimension of the article should be the present."}, "sentence_type": "Affirmative declarative sentence", "pos": {"nouns": {"Family members": 1, "Family": 1, "Help": 1, "Difficulties": 1}, "verbs": {"Dependency": 1, "Resolve": 1}, "numerals": {"54": 1}, "pronouns": {"they": 1}}}, {"content": "Family members are less concerned about promotion, learning achievement, etc.", "word_count": 19, "label": "", "sentence_structure": "Subject鈥搗erb鈥搊bject", "subjective_objective": {"answer": "Objective", "rationale": "Because \"family members are not concerned about promotion, academic achievement, etc.\" is an objective fact describing the attitudes and behaviour of family members."}, "sentence_sentiment": {"answer": "Negative", "rationale": "Because the \"not very concerned\" attitude in the entries is negative."}, "language_style": {"answer": "Calm and direct", "rationale": ""}, "time_dimension": {"answer": "Present", "rationale": "The \"less concerned\" in the entry indicates the current state of affairs, which means the feelings or attitudes of the person who was tested in response to the question, and thus the judgement is now."}, "sentence_type": "Negative declarative sentence", "pos": {"nouns": {"Family members": 1, "Promotion": 1, "Learning achievement": 1}, "verbs": {"Not really": 1}, "adjectives": {"55": 1}}}, {"content": "There are people playing instruments at home.", "word_count": 7, "label": "", "sentence_structure": "Subject鈥搗erb鈥搊bject", "subjective_objective": {"answer": "Objective", "rationale": "Because it is a fact that people play their instruments at home, they can observe and measure the objective. This is not a manifestation of patients鈥?subjective feelings or attitudes, but rather a fact that can be observed directly."}, "sentence_sentiment": {"answer": "Neutral", "rationale": "Because the entry does not have any emotional color."}, "language_style": {"answer": "Tactful and warm", "rationale": "The language style of the entry is more euphemistic, because it asks the other person in an invitation tone whether someone in his home plays an instrument, without direct orders or statements. At the same time, the \"56 family\" also uses a more intimate expression, giving people a sense of accessibility."}, "time_dimension": {"answer": "Past", "rationale": ""}, "sentence_type": "Affirmative declarative sentence", "pos": {"nouns": {"Home": 1, "person": 2, "Instrument": 1}, "verbs": {"Play": 1}, "numerals": {"56": 1}}}, {"content": "Family members do not often engage in recreational activities except for work studies.", "word_count": 18, "label": "", "sentence_structure": "Subject鈥搗erb鈥搊bject", "subjective_objective": {"answer": "Objective", "rationale": "The objective idea is subjective, and therefore not the point of view of the questionnaire."}, "sentence_sentiment": {"answer": "Negative", "rationale": "Because the \"unusual recreational activity\" in the entry is negative."}, "language_style": {"answer": "Calm and direct", "rationale": "The language style of the entry is more direct, with no excessive qualifier or emotional expression, but rather a direct statement of a fact or view. At the same time, the tone of the article is more objective, without a combination of personal emotions or subjective judgements, and more in line with the language requirements and norms of the questionnaire."}, "time_dimension": {"answer": "Past", "rationale": "The phrase \"other than...\" is used in the entry to express the situation before a certain point in time, so that it can be judged as a description of past conduct or circumstances."}, "sentence_type": "Negative declarative sentence", "pos": {"nouns": {"Family members": 1, "Work": 1, "Learning": 1, "Recreational activities": 1}, "verbs": {"Divide": 1, "Conduct": 1}, "adjectives": {"57": 1}, "adverbs": {"Not often": 1}, "classifiers": {"individual": 1}}}, {"content": "Family members voluntarily maintain public sanitation.", "word_count": 15, "label": "", "sentence_structure": "Subject鈥搗erb鈥搊bject", "subjective_objective": {"answer": "Objective", "rationale": "It is an objective fact that the voluntary maintenance of public sanitation by family members can be observed and verified."}, "sentence_sentiment": {"answer": "Positive", "rationale": "Because \"voluntary maintenance of public sanitation\" is a positive behaviour and attitude that demonstrates respect for and love for the community environment."}, "language_style": {"answer": "Tactful and warm", "rationale": "The entry leads the respondents to share their family ' s environmental actions in a positive and encouraging manner, without directly accusing or ordering the other party to do something, and in a more soft and friendly manner."}, "time_dimension": {"answer": "Present", "rationale": "The entry \"all of them voluntarily\" indicates a current movement or state, and \"maintenance of public sanitation\" is the content of that action or state, so the temporal dimension of the article can be judged to be the present."}, "sentence_type": "Affirmative declarative sentence", "pos": {"nouns": {"Family members": 1, "Public sanitation": 1}, "verbs": {"Both": 1, "Voluntary": 1, "Maintenance": 1}, "numerals": {"58": 1}}}, {"content": "The family members carefully kept their rooms clean.", "word_count": 16, "label": "", "sentence_structure": "Subject鈥搗erb鈥搊bject", "subjective_objective": {"answer": "Subjective", "rationale": "Family members ' ideas and behaviour are subjective, and their choice to keep their rooms clean is a manifestation of subjective attitudes."}, "sentence_sentiment": {"answer": "Positive", "rationale": "Because the act of \"carefully keeping your room clean\" is positive and reflects a sense of responsibility and self-discipline towards yourself and your family."}, "language_style": {"answer": "Tactful and warm", "rationale": "The language style of the entry is relatively euphemistic and gentle, using an inquisitorial tone, without direct orders or coercion to perform the task. This is more acceptable and helps to create a good communication climate."}, "time_dimension": {"answer": "Present", "rationale": "\"Scrutinizing\" and \"maintenance\" indicate that the act is in progress, and \"cleaning your room\" is a description of the status or condition of the object being observed. So, the sentence is a state of view that is currently under way."}, "sentence_type": "Affirmative declarative sentence", "pos": {"nouns": {"Family members": 1, "My room": 1, "Clean": 1}, "verbs": {"Keep it real": 1}, "adverbs": {"59": 1, "Land": 1}, "numerals": {"59": 1}}}, {"content": "Family members may leave at night without prior consultation with their families.", "word_count": 21, "label": "", "sentence_structure": "Subject鈥搗erb鈥搊bject", "subjective_objective": {"answer": "Objective", "rationale": "Because \"out\" is an objective fact, the question is examined by the views or attitudes of the interviewees on that objective fact."}, "sentence_sentiment": {"answer": "Neutral", "rationale": "Because the entry is only a description of a phenomenon and does not express any positive or negative emotional colours."}, "language_style": {"answer": "Tactful and warm", "rationale": "The language style of the entry is euphemistic. First, the entry starts with the first person's name \"you\" and uses a language of courtesy and respect, such as \"please analyse below,\" which shows that the surveyer wishes the interviewee to be free to express his or her views and to respect them. Meanwhile, the phrase \"without prior consultation with his or her family\" does not direct or require the other party to do so, but rather makes a suggestion. Moreover, the entry does not use any negative vocabulary or tone in the language, but rather presents the facts in a smooth and neutral way. This euphemism helps the interviewee to feel more comfortable and relaxed, thus making it easier to provide real feedback."}, "time_dimension": {"answer": "Present", "rationale": ""}, "sentence_type": "Negative declarative sentence", "pos": {"nouns": {"Family members": 1, "Night": 1}, "verbs": {"Yeah": 1, "Out": 1, "No, I don't": 1, "- Let's talk": 1}, "adverbs": {"Out of the house at random": 1}, "numerals": {"60": 1}, "classifiers": {"individual": 1}}}, {"content": "There is little collective spirit in our family.", "word_count": 10, "label": "", "sentence_structure": "Subject鈥搗erb鈥揷omplement", "subjective_objective": {"answer": "Subjective", "rationale": "Because \"the collective spirit of our family is very little\" is a subjective perception and assessment of the situation of our families."}, "sentence_sentiment": {"answer": "Negative", "rationale": "Because the word \"very rarely\" in the entry conveys negative emotional colours."}, "language_style": {"answer": "Calm and direct", "rationale": "This is a simple and clear language, which is used to ask the respondents whether they are in a family that is collective, not too much adour or emotional."}, "time_dimension": {"answer": "Present", "rationale": "The entry describes the current situation, which means that \"the collective spirit of our family\" is rarely present, so the temporal dimension is now judged."}, "sentence_type": "Negative declarative sentence", "pos": {"nouns": {"Home": 1, "Collective spirit": 1}, "verbs": {"Less": 1}, "adjectives": {"61": 1}}}, {"content": "Our families can speak openly about their economic problems.", "word_count": 18, "label": "", "sentence_structure": "Subject鈥搗erb鈥搊bject", "subjective_objective": {"answer": "Subjective", "rationale": "Because it is subjective to discuss openly whether the economics of the family is a matter of the views and feelings of the individual."}, "sentence_sentiment": {"answer": "Positive", "rationale": "Since the entry expresses an open and frank approach and behaviour of communication among family members, this means that it helps to build a relationship of mutual trust and support and is therefore positive."}, "language_style": {"answer": "Tactful and warm", "rationale": "The language style of the entry is more euphemistic, using the expression \"can speak openly\" rather than direct orders or demands, giving people a more intimate and respectful sense of respect. At the same time, the words \"our home\" make the respondents more comfortable and comfortable, and more open their minds to real answers."}, "time_dimension": {"answer": "Present", "rationale": "The keyword \"can speak openly\" denotes an act or state that is now in progress, so the time dimension of the entry is judged to be now."}, "sentence_type": "Affirmative declarative sentence", "pos": {"nouns": {"Family": 1, "Economic issues": 1}, "verbs": {"Yeah": 1, "Talk": 1}, "adverbs": {"Openly": 1}, "numerals": {"62": 1}}}, {"content": "When the views of the 63 family members diverged, we all avoided it in order to maintain peace.", "word_count": 25, "label": "", "sentence_structure": "Subject鈥搗erb鈥搊bject", "subjective_objective": {"answer": "Objective", "rationale": "Because it is a description of an objective situation 鈥?one where differences of opinion exist among family members and where they have taken some form of dealing with the situation in order to maintain harmonious relations."}, "sentence_sentiment": {"answer": "Negative", "rationale": "Because the \"divide\" act in the entry is negative."}, "language_style": {"answer": "Tactful and warm", "rationale": "The words \"all the time\" and \"to keep the peace\" are used in this entry, in a more moderate and slow way, without directly pointing to the seriousness of the problem or accusing anyone, but rather emphasizing that everyone has been trying to maintain harmonious relations. This expression is more euphemism, more thoughtful, and helps to reduce tensions and improve understanding and communication among family members."}, "time_dimension": {"answer": "Past", "rationale": "The keyword \"continues to evade\" indicates that such acts have continued over a period of time, so we can judge that the entry relates to past conduct or state."}, "sentence_type": "Affirmative declarative sentence", "pos": {"nouns": {"Family members": 1, "Observations": 1, "Difference": 1, "# And the twilight #": 1}, "verbs": {"Generate": 1, "Avoidance": 1, "Hold on": 1}, "adverbs": {"Always": 1}, "classifiers": {"63": 1}}}, {"content": "Family members want their families to solve the problem independently.", "word_count": 15, "label": "", "sentence_structure": "Subject鈥搗erb鈥搊bject", "subjective_objective": {"answer": "Subjective", "rationale": "The thoughts and expectations of family members are subjective."}, "sentence_sentiment": {"answer": "Positive", "rationale": "Because the entry \"I want my family to solve this problem\" expresses a positive expectation and attitude."}, "language_style": {"answer": "Calm and direct", "rationale": "The language style of the entry is relatively calm and straightforward, and it is not too emotional or rhetorical, but rather a clear statement of the fact that most family members expect their families to be able to solve the problem independently."}, "time_dimension": {"answer": "Future", "rationale": "The keyword is \"favorite,\" which means that the testee has a vision and expectations for the future. \"family solves the problem independently\" is also a future act or state, so the temporal dimension of the article is the future."}, "sentence_type": "Affirmative declarative sentence", "pos": {"nouns": {"Family": 1, "Members": 1, "Problem": 1}, "verbs": {"Hope": 1, "Resolve": 1}, "adjectives": {"Independence": 1}, "numerals": {"64": 1}, "pronouns": {"Family": 1}}}, {"content": "Our family is not so active in achieving what has been achieved.", "word_count": 16, "label": "", "sentence_structure": "Subject鈥搗erb鈥揷omplement", "subjective_objective": {"answer": "Subjective", "rationale": "This is a subjective description of the patient ' s attitude and perception of his family."}, "sentence_sentiment": {"answer": "Negative", "rationale": "Because the \"not so positive about achievement\" attitude in the entry is negative."}, "language_style": {"answer": "Calm and direct", "rationale": "The entry expresses the attitude of family members to achievement in a concise and clear language, and does not have too much emotional rendering or rhetorical techniques, so that the language style is more calm and straightforward."}, "time_dimension": {"answer": "Present", "rationale": "The keyword \"Our family is less active in achieving achievements\" expresses current family attitudes and perceptions."}, "sentence_type": "Negative declarative sentence", "pos": {"nouns": {"Family": 1, "Achievements": 1}, "verbs": {"Access": 1, "Positive": 1}, "adverbs": {"Not so much": 1}, "numerals": {"65": 1}}}, {"content": "Family members often go to the library.", "word_count": 9, "label": "", "sentence_structure": "Subject鈥搗erb鈥搊bject", "subjective_objective": {"answer": "Objective", "rationale": "It is not the patient ' s subjective attitude or feelings that are the objective fact that he or she is often in the library."}, "sentence_sentiment": {"answer": "Neutral", "rationale": "Because the entry does not have any emotional color."}, "language_style": {"answer": "Tactful and warm", "rationale": "The language style of the entry is more euphemistic, and the interviewee is asked in an invitation tone about whether their family members often go to the library."}, "time_dimension": {"answer": "Present", "rationale": "\"66 Family members often go to the library\" to indicate regular, customary behaviour, and the word \"go\" to the general current means that this behaviour has not only occurred in the past, but may still be ongoing. Thus, the entry is for questioning the current behaviour of the person being tested, and the temporal dimension is now judged."}, "sentence_type": "Affirmative declarative sentence", "pos": {"nouns": {"Family members": 1, "Library": 1}, "verbs": {"Always": 1}, "numerals": {"66": 1}}}, {"content": "Family members sometimes take recreational studies according to their preferences or interests.", "word_count": 21, "label": "", "sentence_structure": "Subject鈥搗erb鈥搊bject", "subjective_objective": {"answer": "Subjective", "rationale": "This is a description of the patient ' s subjective observation and evaluation of family members."}, "sentence_sentiment": {"answer": "Positive", "rationale": "Because the purpose of the entry is to \"take recreational studies according to the preferences or interests of the individual\"."}, "language_style": {"answer": "Tactful and warm", "rationale": "The language style of the entry is a warmer type, and it is more accommodative and invited to ask the family members of the interviewee if they are sometimes involved in recreational studies in accordance with their personal preferences."}, "time_dimension": {"answer": "Present", "rationale": "The entry asks if the person tested is involved in recreational studies based on his/her interests and preferences."}, "sentence_type": "Affirmative declarative sentence", "pos": {"nouns": {"Family members": 1, "Entertainment studies": 1}, "verbs": {"Participation": 1, "Press": 1, "Personal hobby": 1, "Interest": 1}, "adverbs": {"Sometimes": 1}, "numerals": {"67": 1}}}, {"content": "Family members agreed to act in a moral capacity.", "word_count": 17, "label": "", "sentence_structure": "Subject鈥搗erb鈥搊bject", "subjective_objective": {"answer": "Subjective", "rationale": "Because it is the patient's subjective feeling and attitude."}, "sentence_sentiment": {"answer": "Negative", "rationale": "Because the purpose of the act of \"damn morality\" in the entry is negative."}, "language_style": {"answer": "Tactful and warm", "rationale": "The language style of the entry is more euphemistic, using the term \"conceptive\" to express attitudes and views of the interviewee, without too much subjective or command-style speech."}, "time_dimension": {"answer": "Present", "rationale": "The entry in the past described a past fact, namely that 68 family members had in the past considered that they were to follow the code of ethics."}, "sentence_type": "Affirmative declarative sentence", "pos": {"nouns": {"Family members": 1, "Ethical precepts": 1, "Operations": 1}, "verbs": {"I think": 1, "Stay put": 1}, "adverbs": {"Both": 1}, "numerals": {"68": 1}, "pronouns": {"they": 1}}}, {"content": "The division of labour among everyone in our family is clear.", "word_count": 14, "label": "", "sentence_structure": "Subject鈥搗erb鈥揷omplement", "subjective_objective": {"answer": "Objective", "rationale": "Because the division of labour among individuals at home is an objective distribution, determined by family members ' consultations or traditional customs, and not by the patient ' s personal subjective attitude."}, "sentence_sentiment": {"answer": "Neutral", "rationale": "Because the entry does not have any emotional color."}, "language_style": {"answer": "Tactful and warm", "rationale": "The term \"clear\" is a clear and clear message, but it is not too much retrospect or evaluation, and it is more objective and neutral."}, "time_dimension": {"answer": "Past", "rationale": ""}, "sentence_type": "Affirmative declarative sentence", "pos": {"nouns": {"Our home": 1, "Everyone": 1, "Division of labour": 1, "Clear": 1}, "verbs": {"be": 1, "min": 1, "Ming": 1}, "numerals": {"69": 1}, "pronouns": {"Well": 1}}}, {"content": "There are no strict rules in our house to bind us.", "word_count": 16, "label": "", "sentence_structure": "Subject鈥搗erb鈥搊bject", "subjective_objective": {"answer": "Subjective", "rationale": "Because \"there are no strict rules to bind us\" is a subjective view and feeling of one person and an individual's subjective attitude."}, "sentence_sentiment": {"answer": "Neutral", "rationale": "As the entry is a mere statement of fact, it does not express a positive or negative emotional attitude."}, "language_style": {"answer": "Calm and direct", "rationale": "The language style of the entry is relatively simple and straightforward, presenting a fact directly, without too much embellishment or emotional addition, and therefore can be judged as a calm and direct language style."}, "time_dimension": {"answer": "Present", "rationale": ""}, "sentence_type": "Negative declarative sentence", "pos": {"nouns": {"Family": 1, "Rule": 1}, "verbs": {"have": 1, "Constraints": 1}, "adjectives": {"Strict": 1, "No, I'm not": 1}, "adverbs": {"in": 1, "Our home": 1}, "numerals": {"70": 1}, "classifiers": {"individual": 1}, "pronouns": {"we": 2}}}, {"content": "Family members have always been able to get along with each other.", "word_count": 14, "label": "", "sentence_structure": "Subject鈥搗erb鈥揷omplement", "subjective_objective": {"answer": "Subjective", "rationale": "It is the patient ' s subjective feeling that family members fit in."}, "sentence_sentiment": {"answer": "Positive", "rationale": "Because the relationships between family members expressed in the entry are positive."}, "language_style": {"answer": "Tactful and warm", "rationale": "The entry uses a more moderate and euphemistic description, which does not express feelings or assessments too directly or strongly, but rather presents the facts in an objective and neutral way. This way better guides respondents to relax their mentalities and to answer questions more honestly and naturally."}, "time_dimension": {"answer": "Past", "rationale": "The entry describes the past, and \"still fit\" indicates that this state of affairs has existed for some time and is still ongoing and can therefore be judged to be the past."}, "sentence_type": "Affirmative declarative sentence", "pos": {"nouns": {"Family members": 1}, "verbs": {"Always": 1, "It's a good fit": 1}, "adverbs": {"Both": 1}, "numerals": {"71": 1}}}, {"content": "Family members speak between themselves with great care to avoid hurting each other ' s feelings.", "word_count": 22, "label": "", "sentence_structure": "Subject鈥搗erb鈥搊bject", "subjective_objective": {"answer": "Subjective", "rationale": "The way in which family members speak is a subjective attitude and behaviour and is therefore subjective in nature."}, "sentence_sentiment": {"answer": "Positive", "rationale": "Because the purpose of the entry is to \"do not hurt the other person's feelings.\""}, "language_style": {"answer": "Tactful and warm", "rationale": "The language style of the entry is more euphemistic, because it expresses a theme 鈥?the way family members interact 鈥?in a way of attention, respect and understanding. It does not point directly to the problem or accuse someone of his or her behaviour, but rather conveys the message through positive descriptions, which can reduce the defensive spirit of the other party and facilitate a smooth dialogue."}, "time_dimension": {"answer": "Present", "rationale": "\"When speaking, care is taken to avoid hurting the other party's feelings\" describes the state and behaviour of the present, so the temporal dimension is now."}, "sentence_type": "Affirmative declarative sentence", "pos": {"nouns": {"Family members": 1, "Speech": 1, "Emotion": 1}, "verbs": {"Attention": 1, "Avoid": 1, "Hurt": 1, "Speak": 1}, "adjectives": {"very": 1, "Both": 1}, "numerals": {"72": 1}}}, {"content": "Family members often want to outnumber each other.", "word_count": 12, "label": "", "sentence_structure": "Subject鈥搗erb鈥揷omplement", "subjective_objective": {"answer": "Subjective", "rationale": "The idea that family members often want to out-think each other is viewed from the patient ' s subjective perspective."}, "sentence_sentiment": {"answer": "Negative", "rationale": "Because the \"often wanting to outplay each other\" act in the entry is negative."}, "language_style": {"answer": "Calm and direct", "rationale": "The entry describes a phenomenon in a concise and clear language, does not add too much emotional colour or acrimonym, and is more calm and objective."}, "time_dimension": {"answer": "Present", "rationale": "In that sentence, \"often\" means regular, customary behaviour, and \"thinking one another better than the other\" is also present. So, the sentence describes the state of the present, judging by \"now\"."}, "sentence_type": "Affirmative declarative sentence", "pos": {"nouns": {"Family members": 1, "The other side": 1}, "verbs": {"Regular": 1, "Yes": 1, "More than anything": 1}, "adverbs": {"You too": 1}, "numerals": {"73": 1}}}, {"content": "If family members often act alone, they can hurt the feelings of others in the family.", "word_count": 22, "label": "", "sentence_structure": "Subject鈥搗erb鈥搊bject", "subjective_objective": {"answer": "Objective", "rationale": "Because it is an objective fact that family members often work alone, it is the result of the behaviour that hurts the feelings of others in the family."}, "sentence_sentiment": {"answer": "Negative", "rationale": "Because \"harming the feelings of the rest of the family\" is negative."}, "language_style": {"answer": "Tactful and warm", "rationale": "This is a more euphemistic way to alert respondents to the problems of interaction and interaction between family members, rather than directly accusing or ordering."}, "time_dimension": {"answer": "Future", "rationale": "In conclusion, the output is \"the future\"."}, "sentence_type": "Affirmative declarative sentence", "pos": {"nouns": {"Family members": 1, "Activities": 1, "Emotion": 1}, "verbs": {"Regular": 1, "Alone": 1, "Hurt": 1, "Yes": 1}, "adverbs": {"Other": 1}, "numerals": {"74": 1}, "pronouns": {"Home": 1}}}, {"content": "75 is an old habit of our family to work and enjoy.", "word_count": 14, "label": "", "sentence_structure": "Subject鈥搗erb鈥搊bject", "subjective_objective": {"answer": "Subjective", "rationale": "Because \"old habits\" are the views and attitudes of the patient personally and are subjective."}, "sentence_sentiment": {"answer": "Neutral", "rationale": "The article is not an emotional expression."}, "language_style": {"answer": "Tactful and warm", "rationale": "The entry is more personal and interactive in its expression of a gentle and inviting tone, rather than a direct statement or order. At the same time, it gives a sense of affection and affection to the person under investigation, using the \"old habits of our family\"."}, "time_dimension": {"answer": "Past", "rationale": "\"75 Work before Enjoyment\" is a description of past behaviour or custom, and the entry is therefore about past behaviour or mentality."}, "sentence_type": "Affirmative declarative sentence", "pos": {"nouns": {"75": 1, "Work": 1, "Enjoy": 1, "Our home": 1, "Old habits": 1}, "verbs": {"be": 1, "I'll get to work and enjoy myself": 1}, "adjectives": {"of": 2}, "numerals": {"75": 1}, "pronouns": {"Our home": 1}}}, {"content": "We're watching TV in our house more than reading.", "word_count": 13, "label": "", "sentence_structure": "Subject鈥搗erb鈥揷omplement", "subjective_objective": {"answer": "Subjective", "rationale": "Because \"watching television is more important than reading\" is a personal value and preference, and it is an individual's subjective attitude."}, "sentence_sentiment": {"answer": "Negative", "rationale": "Because it expresses the contempt for reading and the importance attached to television, it has a negative emotional tendency."}, "language_style": {"answer": "Tactful and warm", "rationale": "The language style of this questionnaire entry is euphemism. It does not directly criticize or accuse the interviewee's family of neglect of reading, but rather expresses a problem through a gentle, invitation-style tone, which reduces the personality of the problem and makes it easier to engage in dialogue. Such expression is more personal and interactive."}, "time_dimension": {"answer": "Present", "rationale": "In this questionnaire entry, \"watching television is more important than reading\" is a description of the current situation or attitude, without information about the past or the future, and therefore the temporal dimension is judged as \"now\"."}, "sentence_type": "Affirmative declarative sentence", "pos": {"nouns": {"Television": 1, "Read": 1, "Home": 1, "we": 1}, "verbs": {"Look": 1, "- Yeah": 1, "Important": 1}, "adverbs": {"76": 1}, "numerals": {"76": 1}, "pronouns": {"I": 1}}}, {"content": "Family members often participate in social events outside the home during their leisure time.", "word_count": 21, "label": "", "sentence_structure": "Subject鈥搗erb鈥搊bject", "subjective_objective": {"answer": "Objective", "rationale": "Because \"family members often participate in social activities outside the home during their leisure time\" is a description of the situation by the patient's family and is an objective fact."}, "sentence_sentiment": {"answer": "Positive", "rationale": "Because the act of \"participating in social activities outside the family\" can enhance social ties and mutual feelings among family members, it is positive."}, "language_style": {"answer": "Tactful and warm", "rationale": "The language style of the entry is more euphemistic and gentle, and the person is asked about his family life, rather than directly ordering or making statements. This expression is more intimate, natural, and more likely to trigger the resonance and collaboration of the person interviewed."}, "time_dimension": {"answer": "Present", "rationale": "In the phrase \"77 family members often participate in social activities outside the home during their leisure time\", the word \"often\" denotes a regular, customary behaviour, while \"participation\" adopts the general current pattern, meaning that the behaviour has occurred in the past and may continue to exist. Thus, the entry describes the current behaviour or state."}, "sentence_type": "Affirmative declarative sentence", "pos": {"nouns": {"Family members": 1, "Amateur Time": 1, "Social events": 1}, "verbs": {"Participation": 1, "Always": 1}, "adverbs": {"Outside": 1}, "numerals": {"77": 1}}}, {"content": "We believe that divorce is immoral, whatever it is.", "word_count": 16, "label": "", "sentence_structure": "Subject鈥搗erb鈥搊bject", "subjective_objective": {"answer": "Subjective", "rationale": "Because \"thinks\" represents the patient's subjective attitude."}, "sentence_sentiment": {"answer": "Negative", "rationale": "Because the notion of \"divorce is immoral\" expresses a negative assessment of divorce."}, "language_style": {"answer": "Calm and direct", "rationale": "The language style of the entry is more calm and objective, and it does not use excessive emotional aesthetic or euphemism. It expresses the author鈥檚 view of divorce as immoral."}, "time_dimension": {"answer": "Past", "rationale": "The entry contains views and assessments on divorce, but does not address past, present or future behaviour or mentality, and therefore does not belong to any situation past, present or future."}, "sentence_type": "Negative declarative sentence", "pos": {"nouns": {"we": 1, "Divorce": 1, "Ethics": 1}, "verbs": {"I think": 1, "be": 1, "not": 1, "of": 2}, "adjectives": {"How's it going": 1}, "adverbs": {"Whatever": 1}, "numerals": {"78": 1}}}, {"content": "79 of us have no plans.", "word_count": 9, "label": "", "sentence_structure": "Subject鈥搗erb鈥揷omplement", "subjective_objective": {"answer": "Objective", "rationale": "Because \"there's no plan for spending money at home\" is an objective situation that does not need to be described by the patient's subjective judgement or perception."}, "sentence_sentiment": {"answer": "Negative", "rationale": "Because \"no plan\" is negative."}, "language_style": {"answer": "Calm and direct", "rationale": "The language style of the entry is a calm and direct type. It directly raises the question of whether the family has a reasonable consumption plan, is clear and stated, and does not have any rhetorical or emotional rendering."}, "time_dimension": {"answer": "Present", "rationale": "\"Our family pays no money\" describes the current situation or state of affairs, where households lack planning and budgetary arrangements for consumption. Thus, the entry is in the present category."}, "sentence_type": "Negative declarative sentence", "pos": {"nouns": {"Our home": 1, "Money": 1, "Planned": 1}, "verbs": {"have": 1, "No, I'm not": 1}, "numerals": {"79": 1}}}, {"content": "Our family rules or family rules cannot be changed.", "word_count": 17, "label": "", "sentence_structure": "Subject鈥揷opula鈥損redicative", "subjective_objective": {"answer": "Objective", "rationale": "Because \"the family's life patterns or family rules cannot be changed\" is a factual statement describing the family rules, and is objective, not subjective, or attitude of the patient."}, "sentence_sentiment": {"answer": "Neutral", "rationale": "Because the entry merely presents a fact, it does not show a positive or negative emotional tendency."}, "language_style": {"answer": "Tactful and warm", "rationale": ""}, "time_dimension": {"answer": "Present", "rationale": "The entry indicates the attitude and perception of the speaker to their family life patterns or rules, that is, they believe that these rules are unchanged. The description is made at the current point of time and does not relate to the future or to any period in the past. The time dimension of the entry is therefore \"now\"."}, "sentence_type": "Negative declarative sentence", "pos": {"nouns": {"Lifestyle": 1, "Family rules": 1}, "verbs": {"be": 1, "Change": 1}, "adjectives": {"No, I can't": 1, "80": 1}, "numerals": {"80": 1}, "pronouns": {"we": 1}}}, {"content": "Every member of the family has always received adequate attention.", "word_count": 17, "label": "", "sentence_structure": "Subject鈥搗erb鈥揷omplement", "subjective_objective": {"answer": "Objective", "rationale": "It is an objective fact that a family member is adequately cared for and can be proved or perjured."}, "sentence_sentiment": {"answer": "Positive", "rationale": "The expression of the purpose of the article, which emphasizes the full interest of every family member, implies a positive family environment and atmosphere."}, "language_style": {"answer": "Tactful and warm", "rationale": "The words \"every member\" and \"full care\" are used to convey positive messages. Such expressions increase the acceptance of the person surveyed and facilitate real and effective feedback."}, "time_dimension": {"answer": "Present", "rationale": "The sentence expresses a constant, customary behaviour whereby every person in the family is given sufficient attention and thus the temporal dimension is judged to be the present."}, "sentence_type": "Affirmative declarative sentence", "pos": {"nouns": {"Family": 1, "Members": 1, "Care": 1}, "verbs": {"Got it": 1, "Always": 1, "Full": 1}, "adjectives": {"81": 1}, "numerals": {"Every one": 1}}}, {"content": "Our family often talks spontaneously about sensitive family issues.", "word_count": 18, "label": "", "sentence_structure": "Subject鈥搗erb鈥搊bject", "subjective_objective": {"answer": "Subjective", "rationale": "Because the words \"often\" and \"autonomously\" indicate that it is subjectively felt or acted upon by the patient or interviewee."}, "sentence_sentiment": {"answer": "Positive", "rationale": "Because \"prosperity\" and \"family sensitivity\" are positive words and behaviours in the entries. This shows that family members are very close and that they are open to controversial topics."}, "language_style": {"answer": "Tactful and warm", "rationale": "The language style of the entry is more euphemistic and warm, and by using the phrase \"speak spontaneously\" it highlights the intimate relationship between family members and the openness with which they face sensitive issues. Such expressions make it easier for respondents to feel respected and understood and to open their minds to questions."}, "time_dimension": {"answer": "Past", "rationale": ""}, "sentence_type": "Affirmative declarative sentence", "pos": {"nouns": {"Our home": 1, "Family": 1, "Problem": 1}, "verbs": {"Regular": 1, "It's spontaneous": 1}, "adjectives": {"Sensitive": 1}, "numerals": {"82": 1}}}, {"content": "The 83 families sometimes argued loudly when they had a conflict.", "word_count": 13, "label": "", "sentence_structure": "Subject鈥搗erb鈥揷omplement", "subjective_objective": {"answer": "Subjective", "rationale": "The basis for the judgement: the title \"Sometimes loudly quarrels\" is a description of the patient's personal experience and feelings and is therefore viewed from a subjective perspective."}, "sentence_sentiment": {"answer": "Negative", "rationale": "Because the \"showing\" act is negative, indicating tension and conflict in family relations."}, "language_style": {"answer": "Calm and direct", "rationale": "The entry describes a family conflict in a concise manner, without adding too many qualifiers or emotional colours and with a more direct and objective tone."}, "time_dimension": {"answer": "Past", "rationale": ""}, "sentence_type": "Affirmative declarative sentence", "pos": {"nouns": {"Family": 1, "Paradoxious": 1, "Argument": 1}, "verbs": {"Sometimes": 1, "Louder": 1, "Argument": 1}, "adverbs": {"Louder": 1, "- I do": 1}, "numerals": {"83": 1}}}, {"content": "In our home, it is true that members are encouraged to move freely.", "word_count": 15, "label": "", "sentence_structure": "Subject鈥搗erb鈥搊bject", "subjective_objective": {"answer": "Objective", "rationale": "Because \"84 does encourage members to move freely in our home\" is an objective statement that describes the rules and culture of a family. Such rules and cultures exist and can be observed and recorded. While family members may have subjective views or feelings about these rules, the sentence itself does not directly examine their subjective ideas, but rather provides an objective statement of fact."}, "sentence_sentiment": {"answer": "Positive", "rationale": "Because the entry expresses a positive and positive attitude, i.e., encouraging free movement."}, "language_style": {"answer": "Tactful and warm", "rationale": "The entry is more adept and interactive by opening up a dialogue in a gentle and inviting tone, emphasizing the openness and freedom of the family atmosphere, without direct orders or criticism."}, "time_dimension": {"answer": "Present", "rationale": "The entry describes the current situation and state of affairs, namely, \"in our house\" the conduct or situation that is being performed is \"to encourage members to move freely\"."}, "sentence_type": "Affirmative declarative sentence", "pos": {"nouns": {"84": 1, "Our home": 1, "Members": 1, "Free movement": 1}, "verbs": {"Encourage": 1, "Both": 1, "Activities": 1}, "adverbs": {"Indeed": 1}, "numerals": {"84": 1}, "pronouns": {"Our home": 1, "Members": 1}}}, {"content": "Family members often compare to others to see who is doing good work.", "word_count": 19, "label": "", "sentence_structure": "Subject鈥搗erb鈥搊bject", "subjective_objective": {"answer": "Objective", "rationale": "Because \"family members are often compared to others\" is an objective thing, and it is an objective fact."}, "sentence_sentiment": {"answer": "Negative", "rationale": "Because the behaviour of \"often comparing\" in the entries can lead to negative emotions and competitive attitudes, not positive behaviour."}, "language_style": {"answer": "Tactful and warm", "rationale": "The language style of the entry is euphemism. It is not directly critical or critical, but expressed in a way that is asked and understood, making it easier for the respondents to accept and to answer questions. Moreover, using the term \"family member\" gives a warmer sense of affection and is more conducive to a positive investigation."}, "time_dimension": {"answer": "Present", "rationale": "\"Now.\""}, "sentence_type": "Affirmative declarative sentence", "pos": {"nouns": {"Family members": 1, "others": 1, "Learning": 2}, "verbs": {"Regular": 1, "and": 1, "- Yeah": 1, "Look": 1, "Okay": 1}, "adverbs": {"Often": 1}, "numerals": {"85": 1}}}, {"content": "Family members are very fond of music, art and literature.", "word_count": 14, "label": "", "sentence_structure": "Subject鈥搗erb鈥搊bject", "subjective_objective": {"answer": "Objective", "rationale": "Because \"family members are very fond of music, art and literature\" is an objective fact and does not involve subjective attitudes or experiences of patients."}, "sentence_sentiment": {"answer": "Positive", "rationale": "Because the words \"like music, art and literature\" in the entries express positive emotional attitudes."}, "language_style": {"answer": "Tactful and warm", "rationale": "The language style of the entry is more euphemistic, using positive descriptions and positive language, such as \"like\" and \"many\", expressing love and respect for the family of the subject. Moreover, the description of \"86 family members\" is also more objective and neutral."}, "time_dimension": {"answer": "Present", "rationale": "The entry describes a state of conduct or behaviour in progress, or \"likes it.\" At the same time, \"86 family members\" also indicate that it is a state or behaviour that is currently being performed. Thus, the temporal dimension of the purpose of the article can be judged to be the present."}, "sentence_type": "Affirmative declarative sentence", "pos": {"nouns": {"Family members": 1, "Music": 1, "Arts": 1, "Literature": 1}, "verbs": {"Yeah": 1}, "adjectives": {"very": 1}, "numerals": {"86": 1}}}, {"content": "Our entertainment activities are conducted by watching television, listening to radio, not by going out.", "word_count": 23, "label": "", "sentence_structure": "Subject鈥搗erb鈥搊bject", "subjective_objective": {"answer": "Objective", "rationale": "Because the title describes the patient ' s way of recreational activity, it is an objective description, not the patient ' s subjective attitude or ideas."}, "sentence_sentiment": {"answer": "Negative", "rationale": "Because the entries \"watch TV, listen to radio\" are not as active as \"out of the house.\""}, "language_style": {"answer": "Calm and direct", "rationale": "The language style of the entry is more straightforward, not using too much qualifier or emotional expression, but rather a simple statement of the fact that most people prefer to find entertainment in their home TV and radio rather than choosing to go out."}, "time_dimension": {"answer": "Present", "rationale": ""}, "sentence_type": "Affirmative declarative sentence", "pos": {"nouns": {"Recreational activities": 1, "Modalities": 1, "Television": 1, "Radio": 1, "Out": 1, "Activities": 1}, "verbs": {"be": 1, "Look": 1, "Listen": 1, "Not": 1, "Out": 1}, "numerals": {"87": 1}, "classifiers": {"individual": 1}}}, {"content": "We believe that improving the standard of living in the family is more important than upholding ethical standards.", "word_count": 24, "label": "", "sentence_structure": "Subject鈥搗erb鈥搊bject", "subjective_objective": {"answer": "Subjective", "rationale": "Because this view involves the evaluation and trade-off of \"family life\" and \"ethics\" and belongs to individual subjective perceptions and values."}, "sentence_sentiment": {"answer": "Negative", "rationale": "Because the idea in the entry that \"a higher standard of living in the family is more important than strict observance of ethical standards\" may lead to neglect of moral and ethical norms, with negative social consequences."}, "language_style": {"answer": "Tactful and warm", "rationale": "The language style of the entry is more euphemistic and moderate, not directly emphasizing or denigrating the values of either party, but rather presenting a point of view and inviting each other to think and discuss. This expression makes it easier for respondents to accept and be willing to engage in dialogue."}, "time_dimension": {"answer": "Present", "rationale": "The entry describes a view or belief of the respondent, which reflects current attitudes and perceptions and does not address past or future acts or events. Thus, the temporal dimension is now judged."}, "sentence_type": "Affirmative declarative sentence", "pos": {"nouns": {"Family": 1, "Life": 2, "Horizontal": 1, "Standard": 1}, "verbs": {"Increase": 1, "Hold tight": 1}, "adjectives": {"Important": 2}, "numerals": {"88": 1}, "classifiers": {"individual": 1}}}, {"content": "We have to have someone to wash the dishes right after dinner.", "word_count": 14, "label": "", "sentence_structure": "Subject鈥搗erb鈥揷omplement", "subjective_objective": {"answer": "Objective", "rationale": "Since it is an objective fact whether or not a dishwashing is necessary, it is not a diversion of the will of the human person."}, "sentence_sentiment": {"answer": "Negative", "rationale": "Because the expression \"must be immediately\" in the entry gives people a sense of coercion, some sense of depression and some negative emotions."}, "language_style": {"answer": "Calm and direct", "rationale": "The language style of this entry is very direct and clear, with no euphemism or qualifier, and is strong and prescriptive. This cold language style may give people a harsh or unpopular feeling, but it also conveys clearly the desired results."}, "time_dimension": {"answer": "Present", "rationale": "The word \"we must\" means something that has to be done, and the word \"we must\" means that the general current is used, which means that this behaviour has not only happened in the past, but may still be ongoing. The entry therefore determines the time dimension of the testee ' s current family habits and lifestyle."}, "sentence_type": "Imperative sentence", "pos": {"nouns": {"Home": 1, "Rice": 1, "person": 1, "The bowl": 1}, "verbs": {"Go on": 1, "Wash": 1}, "adverbs": {"I have to": 1, "Immediately": 1}, "numerals": {"89": 1}, "pronouns": {"we": 1}}}, {"content": "Those who break home rules are severely criticized.", "word_count": 16, "label": "", "sentence_structure": "Subject鈥搗erb鈥搊bject", "subjective_objective": {"answer": "Objective", "rationale": "Because it is an objective fact to violate the rules of the family and to be criticized, it is not related to the patient ' s own subjective attitude."}, "sentence_sentiment": {"answer": "Negative", "rationale": "Because the \"severe criticism\" in the entry is an expression of a negative emotional attitude."}, "language_style": {"answer": "Calm and direct", "rationale": "The entry is more directly linguistically, with no excessive adoration and emotional rendering, directly presenting a fact or rule, and more serious and decisive."}, "time_dimension": {"answer": "Past", "rationale": "The word \"will\" is used to indicate future actions, but the main phrase \"90 in violation of family rules\" is a description of a past fact or state. Thus, the temporal dimension of this questionnaire entry is a past."}, "sentence_type": "Affirmative declarative sentence", "pos": {"nouns": {"Home": 1, "Family rules": 1, "Criticism": 1}, "verbs": {"Violations": 1, "Yes": 1, "Accept": 1}, "adjectives": {"Hard": 1}, "numerals": {"90": 1}}}]}

{"questionnaire_title": "Sarason Test Anxious Sheets", "questionnaire_category": "Mental health", "item_count": 37, "emotion_shift_overall": "From positive to negative", "language_style_overall": "Calm and direct", "items": [{"content": "- When a big test comes, I think people are smarter than me.", "word_count": 25, "label": "", "sentence_structure": "Subject鈥揷opula鈥損redicative", "subjective_objective": {"answer": "Subjective", "rationale": "Because the idea of being smarter than others is a subjective assessment and comparison, reflecting individual feelings and perceptions."}, "sentence_sentiment": {"answer": "Negative", "rationale": "Because the expression uses a language that demeans itself, \"brainer\" is a negative emotional color."}, "language_style": {"answer": "Calm and direct", "rationale": "The entry is based on a direct statement asking the other person whether he or she is in a psychological condition when facing a major test, i.e., that he or she is too worried about his or her abilities being inadequate compared to others."}, "time_dimension": {"answer": "Present", "rationale": ""}, "sentence_type": "Imperative sentence", "pos": {"nouns": {"Number of times": 1, "Significant": 1, "Examinations": 1, "Time": 1, "person": 1, "More": 1}, "verbs": {"Yes": 1, "Come on": 1, "Always thinking": 1, "- Yeah": 1, "Smart": 1}, "adjectives": {"Large": 1, "Heavy": 1}, "adverbs": {"When": 1, "Total": 1, "Both": 1, "Okay": 1, "More": 1}, "numerals": {"One": 2}, "pronouns": {"I": 1}}}, {"content": "If I'm going to do a smart test, I'll be very anxious before I do it.", "word_count": 22, "label": "", "sentence_structure": "Subject鈥搗erb鈥搊bject", "subjective_objective": {"answer": "Subjective", "rationale": "Because anxiety is a subjective emotional reaction, it is caused by the emotional state of the individual."}, "sentence_sentiment": {"answer": "Negative", "rationale": "Because the \"very anxious\" attitude in the entry is negative."}, "language_style": {"answer": "Calm and direct", "rationale": "The language style of the entry is more direct, without any emotional or accompanimental language, but simply a description of a fact or situation. Such language style is usually used to describe an objective phenomenon, convey information or conduct a question-finding. \""}, "time_dimension": {"answer": "Present", "rationale": "If the word \"if\" denotes a hypothetical situation, the word \"will\" adopts the general future pattern, meaning that the act will occur in the future. The temporal dimension of the article is therefore present and future."}, "sentence_type": "Affirmative declarative sentence", "pos": {"nouns": {"Test": 1, "Before": 1, "Anxiety": 1}, "verbs": {"Do it": 2, "Yes": 1}, "adjectives": {"Smart": 1, "Very": 1}, "numerals": {"2": 1}, "pronouns": {"I": 1}}}, {"content": "If I knew there was gonna be a smart test, I felt confident and relaxed before that.", "word_count": 27, "label": "", "sentence_structure": "Subject鈥搗erb鈥揷omplement", "subjective_objective": {"answer": "Subjective", "rationale": "Because \"I feel confident and relaxed\" is the subjective feeling and attitude of patients to the intelligent tests they're about to take."}, "sentence_sentiment": {"answer": "Positive", "rationale": "Because the description \"sweetly confident and easy\" in the entry is positive and indicates a positive attitude on the part of individuals."}, "language_style": {"answer": "Tactful and warm", "rationale": "The entry leads the interviewee to share his feelings in a way that is more acceptable and willing to answer than direct orders or statements. At the same time, the words \"very confident\" and \"very easy\" are used to convey positive and optimistic emotional sentiments, making the language style more euphemismal."}, "time_dimension": {"answer": "Present", "rationale": "\"If\" and \"before\" indicate that this is a hypothetical situation, but it may have happened in the past and may have affected the testees now. And \"I'll feel confident and easy\" suggests that this is the current emotional state of the testees, and therefore the judgment is \"now.\""}, "sentence_type": "Exclamatory sentence", "pos": {"nouns": {"Smart Test": 1, "Confidence": 1, "Relax": 1}, "verbs": {"Yes": 2, "Will": 1, "have": 1, "I feel": 1, "very": 2, "in": 1, "Before": 1, "I": 1}, "adjectives": {"Confidence": 1, "Easy": 1}, "adverbs": {"very": 2}}}, {"content": "I'll sweat a lot when I take the big test.", "word_count": 13, "label": "", "sentence_structure": "Subject鈥搗erb鈥揷omplement", "subjective_objective": {"answer": "Subjective", "rationale": "Because sweating feelings and reactions are patients' subjective experiences."}, "sentence_sentiment": {"answer": "Negative", "rationale": "Because \"will sweat a lot\" is negative."}, "language_style": {"answer": "Calm and direct", "rationale": "The entry directly expresses the subject of questioning whether the other party will sweat when taking a major examination, without using excessive rhetorical or emotional rendering, and is more objective in tone."}, "time_dimension": {"answer": "Present", "rationale": "The entry is therefore a question of the behaviour or mentality of the person tested at the major examination, the temporal dimension of which is now judged."}, "sentence_type": "Affirmative declarative sentence", "pos": {"nouns": {"Major examinations": 1, "Khan": 1}, "verbs": {"Yes": 1}, "adjectives": {"A lot": 1}, "numerals": {"4": 1}}}, {"content": "During the five exams, I found myself thinking things that had nothing to do with the exam.", "word_count": 24, "label": "", "sentence_structure": "Subject鈥搗erb鈥搊bject", "subjective_objective": {"answer": "Subjective", "rationale": "Because \"thinking something that has nothing to do with the test\" is the patient's subjective feeling and experience."}, "sentence_sentiment": {"answer": "Negative", "rationale": "Because the entry \"always thinking about something that has nothing to do with the test\" is negative."}, "language_style": {"answer": "Calm and direct", "rationale": "The entry directly expresses the fact that the subjects are more distracted during the examination in a concise and straightforward manner, without too much emotional or rhetorical use, and with a more objective and straightforward tone."}, "time_dimension": {"answer": "Present", "rationale": "The words \"during the examination\" and \"discovery\" all indicate that this is a description of the current state, not of the past or of the future. Thus, the temporal dimension of the article is now."}, "sentence_type": "Affirmative declarative sentence", "pos": {"nouns": {"Examinations": 2, "self": 1, "Yes": 1}, "verbs": {"Found": 1, "I'm thinking": 1}, "adjectives": {"Not relevant": 1}, "adverbs": {"Always": 1}, "numerals": {"5": 1}, "pronouns": {"I": 1}}}, {"content": "When a sudden attack test came, I was scared.", "word_count": 19, "label": "", "sentence_structure": "Subject鈥搗erb鈥揷omplement", "subjective_objective": {"answer": "Subjective", "rationale": "Because fear is the patient's subjective feeling."}, "sentence_sentiment": {"answer": "Negative", "rationale": "Because the \"fear\" in the entry is negative."}, "language_style": {"answer": "Calm and direct", "rationale": "The entry directly expresses the subject of questioning the other party ' s feelings when it comes to the test, without excessive rhetorical or emotional rendering, and is more objective in tone."}, "time_dimension": {"answer": "Present", "rationale": ""}, "sentence_type": "Negative declarative sentence", "pos": {"nouns": {"Number of times": 1, "Attack": 1, "Examinations": 1, "I": 1}, "verbs": {"Come on": 1, "I feel": 1, "Yes": 1}, "adjectives": {"Suddenly": 1}, "numerals": {"6": 1}}}, {"content": "I always thought I'd fail during the seven.", "word_count": 12, "label": "", "sentence_structure": "Subject鈥搗erb鈥揷omplement", "subjective_objective": {"answer": "Subjective", "rationale": "Because \"failure\" is a person's inner thought and concern, and it's part of the subjective sense."}, "sentence_sentiment": {"answer": "Negative", "rationale": "Because the act of \"I often thought I'd fail during the exam\" expresses concern and anxiety about the exam, and it is a negative sentiment."}, "language_style": {"answer": "Calm and direct", "rationale": "The language style of the entry is \"cool and direct\". It expresses the subject in a clear statement, without using a rhetorical or emotional modification."}, "time_dimension": {"answer": "Present", "rationale": "The keyword \"frequent thought\" indicates that it is a customary, recurring exercise of thought, and the entry is therefore judged by the person being questioned about the behaviour or mentality of the person being tested."}, "sentence_type": "Negative declarative sentence", "pos": {"nouns": {"Examinations": 2, "Period": 1, "Failed": 1}, "verbs": {"Regular": 1, "Yes": 2}, "numerals": {"7": 1}, "pronouns": {"I": 1}}}, {"content": "I've been nervous since the 8th major exam, and I've had a stomach problem.", "word_count": 18, "label": "", "sentence_structure": "Subject鈥揷opula鈥損redicative", "subjective_objective": {"answer": "Subjective", "rationale": "As the stress and stomach disorders are felt by the patient himself, they are subjective."}, "sentence_sentiment": {"answer": "Negative", "rationale": "Because \"heat\" and \"the stomach discomfort\" are negative emotional experiences in the entries."}, "language_style": {"answer": "Calm and direct", "rationale": "The entry directly asked the respondents about their physical reactions after the important examinations, the language was clear and not too much emotional rendering or speech-writing, and therefore the language style was considered to be calm and straightforward."}, "time_dimension": {"answer": "Past", "rationale": "The keyword \"after the 8th Major Examination\" indicates that the events that have passed, \"often nervous, and so bad in the stomach\" describes the effects of the events on the subjects, so the entry asks about the behaviour or mental state of the past."}, "sentence_type": "Affirmative declarative sentence", "pos": {"nouns": {"Examinations": 2, "Stomach": 1}, "verbs": {"I feel": 1, "Nervous": 1, "I'm not well": 1, "- Yeah": 1}, "adverbs": {"Regular": 1}, "numerals": {"8": 1}, "pronouns": {"I": 2}}}, {"content": "I'm always getting a little bit confused about smart and finals.", "word_count": 20, "label": "", "sentence_structure": "Subject鈥搗erb鈥揷omplement", "subjective_objective": {"answer": "Subjective", "rationale": "Because fear is the patient's subjective feeling."}, "sentence_sentiment": {"answer": "Negative", "rationale": "Because the word \"fun\" in the entry conveys a negative emotional attitude, indicating that the interviewee is afraid of the smart and final examinations."}, "language_style": {"answer": "Calm and direct", "rationale": "The entry is a straightforward expression of the question about fear of the subject in the smart and final examinations, and is not based on excessive rhetorical or emotional rendering, and is more objective in tone."}, "time_dimension": {"answer": "Present", "rationale": "The \"always feeling\" in the entry indicates a constant, customary feeling, and \"fE\" uses the general current, meaning that this feeling has not only happened in the past, but may still be ongoing. So, the time dimension of the questionnaire entry is now."}, "sentence_type": "Negative declarative sentence", "pos": {"nouns": {"Smart test": 1, "Final examination": 1, "Yes": 2, "- What": 1}, "verbs": {"to": 1, "Sensor": 1, "I feel": 1, "Total": 1, "- What": 1}, "adjectives": {"I'm not sure": 1, "Scared": 1}, "adverbs": {"Total": 1}, "numerals": {"9": 1}, "pronouns": {"I": 1}}}, {"content": "10 good results in one test doesn't seem to increase my confidence in the second.", "word_count": 29, "label": "", "sentence_structure": "Subject鈥搗erb鈥揷omplement", "subjective_objective": {"answer": "Subjective", "rationale": "Because the feeling of \"good achievement increases confidence\" is the patient's subjective judgement and emotional experience, not the objective fact."}, "sentence_sentiment": {"answer": "Negative", "rationale": "Because the entry indicates that the impact of the examination results on confidence is negative."}, "language_style": {"answer": "Calm and direct", "rationale": "The entry is a simple and straightforward expression of the lack of correlation between good achievement and self-confidence, and does not involve too much adoration or emotional rendering.\""}, "time_dimension": {"answer": "Present", "rationale": "\""}, "sentence_type": "Negative declarative sentence", "pos": {"nouns": {"Once": 2, "Examinations": 2, "Good grades": 1, "Second": 1, "Confidence": 1}, "verbs": {"Access": 1, "Yes": 1, "Increase": 1, "in": 1, "Medium": 3}, "adjectives": {"Seems like": 1}, "adverbs": {"No, it's not": 1, "I'm sure": 1}, "numerals": {"10": 1}, "pronouns": {"I": 1}}}, {"content": "I sometimes feel like my heart beats fast during the big exams.", "word_count": 16, "label": "", "sentence_structure": "Subject鈥搗erb鈥揷omplement", "subjective_objective": {"answer": "Subjective", "rationale": "Because \"fearing a heartbeat\" is a subjective experience and feeling. Although it may be associated with some physical reaction (e.g. stress), it is subjective in itself and may vary from person to person."}, "sentence_sentiment": {"answer": "Negative", "rationale": "Because \"heart beats fast\" is a negative physical reaction."}, "language_style": {"answer": "Calm and direct", "rationale": "The entry is based on a direct and concise approach, without too much rhetorical or emotional overtook, and is more objective in tone. Nor is it intended to direct respondents to respond in a specific direction, but rather to invite them to share their experiences and feelings on the basis of the facts."}, "time_dimension": {"answer": "Present", "rationale": ""}, "sentence_type": "Negative declarative sentence", "pos": {"nouns": {"Examination period": 1, "Heart rate": 1}, "verbs": {"I feel": 1, "have": 1}, "adjectives": {"Significant": 1, "Sometimes": 1}, "numerals": {"11": 1}}}, {"content": "I always thought I'd do better after the 12th exam than I actually did.", "word_count": 20, "label": "", "sentence_structure": "Subject鈥揷opula鈥損redicative", "subjective_objective": {"answer": "Subjective", "rationale": "Because \"it's always felt better than it actually is the patient's subjective idea and feeling. Although it is based on the actual performance of the examination, the patient believes that he or she is capable of doing better, which is a personal expectation and self-evaluation, and is therefore a subjective examination."}, "sentence_sentiment": {"answer": "Negative", "rationale": "Because the attitude in the entry that \"it's always better to do than it actually does\" is negative."}, "language_style": {"answer": "Tactful and warm", "rationale": "The entry asks the interviewees in a spirit of encouragement and support, using the words \"always\" and \"may\" to express a positive upward and self-affirming attitude. This language style usually makes the interviewees feel understood and encouraged, and it is easier to open their minds and share their thoughts and feelings."}, "time_dimension": {"answer": "Past", "rationale": "The entry indicates a feeling or mentality of the person who was tested after the examination, and the keywords \"after the examination\" and \"at times feel better than actually\" point to what has happened in the past and judge it as the past."}, "sentence_type": "Affirmative declarative sentence", "pos": {"nouns": {"Examinations": 1, "End": 1, "I think": 1}, "verbs": {"be": 1, "Yeah": 2, "Do it": 1, "Okay": 1, "Better": 1}, "adverbs": {"Always": 1}, "numerals": {"12": 1}, "pronouns": {"I": 1}}}, {"content": "I'm always depressed after the 13th.", "word_count": 13, "label": "", "sentence_structure": "Subject鈥揷opula鈥損redicative", "subjective_objective": {"answer": "Subjective", "rationale": "Because \"frightful\" is the experience of the patient's own feelings and emotions, and it's a subjective examination."}, "sentence_sentiment": {"answer": "Negative", "rationale": "Because depression is a negative emotional experience, expressing negative attitudes."}, "language_style": {"answer": "Calm and direct", "rationale": "The language style is more common in psychological questionnaires, which help researchers to understand the psychological state and feelings of the subjects more accurately."}, "time_dimension": {"answer": "Past", "rationale": "And \"after the exam\" means that the event happened in the past, and \"I'm always depressed\" means that this emotional reaction continues to this day, so the time dimension of this entry is both past and present."}, "sentence_type": "Negative declarative sentence", "pos": {"nouns": {"Examinations": 1, "Depression": 1}, "verbs": {"Over": 1, "I feel": 1, "be": 1}, "adjectives": {"Always": 1}, "numerals": {"13": 1}, "pronouns": {"I": 1}}}, {"content": "I always had a feeling of tension before the 14 finals.", "word_count": 20, "label": "", "sentence_structure": "Subject鈥揷opula鈥損redicative", "subjective_objective": {"answer": "Subjective", "rationale": "Because \"feel\" is a subjective experience and a sense."}, "sentence_sentiment": {"answer": "Negative", "rationale": "Because \"frightful\" is negative."}, "language_style": {"answer": "Tactful and warm", "rationale": "The language style of the entry is more euphemistic and warm, and it asks the interviewees in a gentle and caring manner whether they are often nervous before the final exam, which is more acclaimable and acceptable and helps to create a good atmosphere of communication."}, "time_dimension": {"answer": "Present", "rationale": ""}, "sentence_type": "Affirmative declarative sentence", "pos": {"nouns": {"Every time": 1, "Final examination": 1, "Feel": 1}, "verbs": {"There's always one": 1, "have": 1, "Examination": 1}, "adjectives": {"Nervous": 1, "Unsettled": 1}, "numerals": {"14": 1}}}, {"content": "I'm not gonna get my emotional reaction when I'm 15 years old.", "word_count": 16, "label": "", "sentence_structure": "Subject鈥搗erb鈥揷omplement", "subjective_objective": {"answer": "Subjective", "rationale": "Because emotional reaction is a subjective feeling and experience of one person, different people may react differently to the same situation. So \"emotional reaction does not interfere with my test\" is the subjective perception and attitude of the patient."}, "sentence_sentiment": {"answer": "Positive", "rationale": "Because the entry \"Fellow Reaction will not interfere with my exam\" means a positive attitude and behavior."}, "language_style": {"answer": "Calm and direct", "rationale": "The entry is presented directly, without too many qualifiers or emotional colours, with a more flat and objective tone, and therefore its linguistic style can be classified as \"cool and direct\"."}, "time_dimension": {"answer": "Past", "rationale": "The keyword \"15 Tests\" shows that this entry asks about past experience, i.e. whether the emotional reaction of the testee has influenced their examination at some point in the past. So the answer is \"the past.\""}, "sentence_type": "Negative declarative sentence", "pos": {"nouns": {"Examinations": 2, "Emotional response": 1}, "verbs": {"Yes": 1, "Interference": 1, "Examinations": 1}, "adverbs": {"not": 1}, "numerals": {"15": 1}, "pronouns": {"I": 1}}}, {"content": "I was always nervous during the 16th exam, so I forgot what I knew.", "word_count": 22, "label": "", "sentence_structure": "Subject鈥搗erb鈥搊bject", "subjective_objective": {"answer": "Subjective", "rationale": "Because \"Esperate\" and \"what you know is forgotten\" are subjective descriptions of the patient's emotional and cognitive state."}, "sentence_sentiment": {"answer": "Negative", "rationale": "Because \"sweet\" and \"whatever you know is forgotten\" are negative expressions in the entries."}, "language_style": {"answer": "Calm and direct", "rationale": "The entry is a direct expression of the tension that is often experienced during the examination, and that has affected memory ability. It is more balanced and objective, and does not use excessive rhetorical or emotional rendering. It is therefore considered to be a calm and straightforward language style."}, "time_dimension": {"answer": "Present", "rationale": "The keyword \"during the exam\" indicates that this was an act that occurred over time, and \"often nervous\" and \"or forget what was known\" is a description of the act and the result. So, the entry describes the behaviour and the mentality of the testee at some stage of the past, so the temporal dimension is now judged."}, "sentence_type": "Affirmative declarative sentence", "pos": {"nouns": {"Examination period": 1, "I": 2, "Something": 1}, "verbs": {"Regular": 1, "very": 1, "Yes": 1, "Forget it": 1}, "adjectives": {"Nervous": 1}, "adverbs": {"And so": 1}, "numerals": {"16": 1}}}, {"content": "17, I've had a big challenge in studying important exams.", "word_count": 21, "label": "", "sentence_structure": "Subject鈥揷opula鈥損redicative", "subjective_objective": {"answer": "Subjective", "rationale": "Because \"the feeling is a big challenge\" reflects the patient's subjective feelings and attitudes."}, "sentence_sentiment": {"answer": "Negative", "rationale": "Because the expression \"big challenge\" in the entry has negative emotions."}, "language_style": {"answer": "Calm and direct", "rationale": "The language style of the entry is relatively calm and straightforward, with no excessive emotional or rhetorical tact, and is a straightforward expression of the challenges faced by respondents in their review of important examinations."}, "time_dimension": {"answer": "Present", "rationale": "The entry \"now\" indicates the current situation or state, that is, the person being tested is experiencing this situation and feels that this is a challenge."}, "sentence_type": "Affirmative declarative sentence", "pos": {"nouns": {"Review": 1, "Important": 1, "Examinations": 1, "I": 1, "Challenges": 1}, "verbs": {"Seems like": 1, "be": 1, "One": 1, "have": 1}, "adjectives": {"Big": 1}, "numerals": {"17": 1}}}, {"content": "I'm confused about the more I try to review one of the exams.", "word_count": 17, "label": "", "sentence_structure": "Subject鈥搗erb鈥揷omplement", "subjective_objective": {"answer": "Subjective", "rationale": "Because \"maginalization\" is a subjective feeling that cannot be proved or refuted, but can only be described by the patient himself. While trying to review is an objective act, it is the person ' s own feelings and experiences that are confused, so the entry is viewed from the patient ' s subjective perspective."}, "sentence_sentiment": {"answer": "Negative", "rationale": "Because the expression \"the more you try to read it, the more you get confused\" has negative emotional, attitude, behaviour, purpose or quality."}, "language_style": {"answer": "Calm and direct", "rationale": "The entry \"Cool and Direct\" directly expresses the confusion that the subject is being felt as the other party is studying a particular examination, without excessive rhetorical or emotional rendering, and with a more balanced and objective tone."}, "time_dimension": {"answer": "Present", "rationale": ""}, "sentence_type": "Affirmative declarative sentence", "pos": {"nouns": {"18": 1, "One test": 1, "I": 1, "Confused": 1}, "verbs": {"Review": 1, "I feel": 1}, "adjectives": {"Tsk": 2, "Try": 1}, "adverbs": {"Tsk": 2}, "numerals": {"18": 1}, "classifiers": {"One": 1}, "pronouns": {"I": 1}}}, {"content": "I tried to stop worrying about it, but I couldn't.", "word_count": 20, "label": "", "sentence_structure": "Subject鈥搗erb鈥揷omplement", "subjective_objective": {"answer": "Subjective", "rationale": "Because \"failure\" is the patient's subjective feeling and experience, and it's the patient's inner reaction."}, "sentence_sentiment": {"answer": "Negative", "rationale": "Because the \"can't\" is both negative and emotional."}, "language_style": {"answer": "Calm and direct", "rationale": "The language style of the entry is calm and straightforward, without too much emotional rendering or rhetorical, and is objective and expresses the psychological state and feelings of the other party directly after an incident."}, "time_dimension": {"answer": "Present", "rationale": ""}, "sentence_type": "Negative declarative sentence", "pos": {"nouns": {"The door exam": 1, "Fear": 2}, "verbs": {"End": 1, "Try": 1, "Stop": 1, "Do it": 1}, "adverbs": {"Some": 1, "One": 1, "But": 1}, "numerals": {"19": 1}, "pronouns": {"I": 1}}}, {"content": "I sometimes wonder if I can finish college during the 20th.", "word_count": 19, "label": "", "sentence_structure": "Subject鈥搗erb鈥搊bject", "subjective_objective": {"answer": "Subjective", "rationale": "Because sometimes I wonder if I can finish college is the patient's subjective idea during the 20th exam."}, "sentence_sentiment": {"answer": "Negative", "rationale": "Because the purpose of the entry is to \"sometimes wonder if I can finish college.\""}, "language_style": {"answer": "Tactful and warm", "rationale": "The language style of the entry is more euphemistic. It does not directly express the other ' s anxiety or anxiety, but rather leads the other to think and share his thoughts and feelings by asking questions, which are more gentle, friendly and acceptable."}, "time_dimension": {"answer": "Present", "rationale": "The entry contains a specific point of time for \"the examination\" and uses the first person's expression \"I\" to indicate that it is a description of the current state.\""}, "sentence_type": "Interrogative sentence", "pos": {"nouns": {"Examinations": 1, "Period": 1, "I": 2, "Time": 1, "University": 1, "Studies": 1}, "verbs": {"Yes": 3, "Completed": 1}, "adverbs": {"Sometimes": 1}, "numerals": {"20": 1}}}, {"content": "I'd rather write a paper than take an exam as a result of a course.", "word_count": 26, "label": "", "sentence_structure": "Subject鈥搗erb鈥搊bject", "subjective_objective": {"answer": "Subjective", "rationale": "This is because patients prefer to write thesis or to take the examination as individuals ' choice and attitude."}, "sentence_sentiment": {"answer": "Positive", "rationale": "Because the behavior in the entry \"will rather write a paper\" and \"the purpose\" as a achievement in a given course is positive."}, "language_style": {"answer": "Calm and direct", "rationale": "The language style of the entry is straightforward and clearly expresses the attitude of the respondents to writing the papers and taking the examination, without excessive emotional or rhetorical use."}, "time_dimension": {"answer": "Present", "rationale": "The entry describes the current mindset and preferences of the testee and therefore judges \"now\"."}, "sentence_type": "Affirmative declarative sentence", "pos": {"nouns": {"Papers": 1, "Examinations": 1, "Door": 1, "Courses": 1, "Achievements": 1}, "verbs": {"I'd rather": 1, "Write": 1, "Participation": 1}, "adverbs": {"21": 1, "Not": 1}, "classifiers": {"One": 3}, "pronouns": {"I": 1}}}, {"content": "I wish I could stop being so annoying about the exam.", "word_count": 12, "label": "", "sentence_structure": "Subject鈥搗erb鈥揷omplement", "subjective_objective": {"answer": "Subjective", "rationale": "Because \"I hope the exam is not so annoying\" is about personal expectations and emotions, and it's subjective."}, "sentence_sentiment": {"answer": "Negative", "rationale": "Because the entry \"Stamp less annoying\" expresses a negative mood and attitude."}, "language_style": {"answer": "Tactful and warm", "rationale": "Warm and warm."}, "time_dimension": {"answer": "Future", "rationale": "The keywords \"hope\" and \"take not so annoying\" point to situations that have not yet occurred and that may arise in the future, and the entry is therefore a future act or mentality."}, "sentence_type": "Interrogative sentence", "pos": {"nouns": {"22": 1, "Examinations": 1, "Trouble": 1}, "verbs": {"Hope": 1, "Yes": 1, "Don't": 1}, "adjectives": {"It's annoying": 1}, "numerals": {"22": 1}, "pronouns": {"I": 1}}}, {"content": "23 I'm sure I'd be better off if I took the exam alone and without a time limit.", "word_count": 28, "label": "", "sentence_structure": "Subject鈥搗erb鈥搊bject", "subjective_objective": {"answer": "Subjective", "rationale": "Because this phrase expresses the convictions and expectations of the patient, and is based on individual subjective experience. Even if it is true, it is still a subjective view."}, "sentence_sentiment": {"answer": "Negative", "rationale": "Because the assumption in the entry that the act \"if there is no time limit\" is negative, it suggests that the speaker may be afraid of time pressure."}, "language_style": {"answer": "Calm and direct", "rationale": "The article is more objective and direct, without any rhetorical or emotional replicating. It clearly expresses a person's belief that he or she will behave better without time and pressure, without using euphemism or vague language to express that view. So I think the language style of the purpose is a straightforward and calm type."}, "time_dimension": {"answer": "Future", "rationale": "The entry expresses the expectations and visions of the testee for the future, and the keywords are \"will be better\" and \"if I sit alone and there is no time limit\" all point to situations that have not yet occurred and that may arise in the future."}, "sentence_type": "Affirmative declarative sentence", "pos": {"nouns": {"23": 1, "Examinations": 1, "Time": 1, "Limits": 1, "I": 1}, "verbs": {"Yes": 1, "Participation": 1, "- You're a good student": 1}, "adjectives": {"Alone": 1, "Better": 1}, "numerals": {"23": 1}, "classifiers": {"Examinations": 1, "Limits": 1}, "pronouns": {"If": 1, "I": 2}}}, {"content": "I'm thinking about how much I can score in my exams, which affects my studies and my exams.", "word_count": 22, "label": "", "sentence_structure": "Subject鈥搗erb鈥搊bject", "subjective_objective": {"answer": "Subjective", "rationale": "Because the subject is to examine the patient's own thoughts and emotions, which are subjective, namely, \"Thinking about how much I can score in the exam.\" Although this idea may affect the patient's review and examination, it is only the result, still subjectively."}, "sentence_sentiment": {"answer": "Negative", "rationale": "Because the \"impacting the review and examination\" act in the entry is negative."}, "language_style": {"answer": "Calm and direct", "rationale": "The entry is more directly expressed, without too much rhetoric or emotional overspewing, and is also very objective. It asks whether respondents often over-think the score before the examination and whether this concern affects their learning and performance."}, "time_dimension": {"answer": "Present", "rationale": "\"Thinking about how much I can score in the exam\" describes the person's current state of mind, \"I've influenced my studies and exams\" and describes the consequences or results of this state of mind, so the entry asks the person's current behavior or mental state."}, "sentence_type": "Exclamatory sentence", "pos": {"nouns": {"24": 1, "Examinations": 2, "min": 1, "I": 1, "Review": 1}, "verbs": {"Think about it": 1, "Okay": 1, "Impact": 1, "of": 3}, "numerals": {"How much": 1}}}, {"content": "If I can get the exam canceled, I think I can do better.", "word_count": 17, "label": "", "sentence_structure": "Subject鈥搗erb鈥搊bject", "subjective_objective": {"answer": "Subjective", "rationale": "Because \"I want\" expresses the patient's thoughts and wishes, and it's subjective."}, "sentence_sentiment": {"answer": "Negative", "rationale": "Because the precondition of \"if the exam can be abolished\" implies dissatisfaction with the examination system and negative attitudes. And the latter phrase, \"I think I can learn better,\" is based on this negative premise, so the emotional tendency of the whole sentence is negative."}, "language_style": {"answer": "Tactful and warm", "rationale": "The entry is a hypothetical and self-reflective dialogue, expressing dissatisfaction with the current examination system and the desire for a more liberal way of learning, with a relatively euphemism and a certain subjective emotional tone."}, "time_dimension": {"answer": "Present", "rationale": "The entry expresses the subjective views and expectations of the interviewees regarding the current status quo, without reference to past or future situations."}, "sentence_type": "Affirmative declarative sentence", "pos": {"nouns": {"Examinations": 1, "Word": 1}, "verbs": {"Yes": 2, "Abolition": 1, "Learn": 1, "Okay": 1, "Better": 1}, "adverbs": {"If": 1, "If you": 1, "More": 1}, "numerals": {"25": 1}, "pronouns": {"I": 1}}}, {"content": "I'm not worried about the test. I don't understand it right now.", "word_count": 23, "label": "", "sentence_structure": "Subject鈥搗erb鈥搊bject", "subjective_objective": {"answer": "Subjective", "rationale": "Because \"I'm not worried\" reflects the patient's personal perception and emotional reaction. Although the patient is confused about the test now, they are positive and think they have the ability to overcome the difficulties."}, "sentence_sentiment": {"answer": "Positive", "rationale": "Because the attitudes in the entries are positive, they demonstrate confidence in learning and progress."}, "language_style": {"answer": "Calm and direct", "rationale": "The language style of the entry is relatively calm and straightforward, and it shows in a narrative way the attitude towards the examination, with no excessive rhetorical or emotional rendering."}, "time_dimension": {"answer": "Present", "rationale": "Answer: Now."}, "sentence_type": "Affirmative declarative sentence", "pos": {"nouns": {"Examinations": 1, "Attitude": 1}, "verbs": {"Hug": 1, "Yes": 1, "I'm not worried": 1}, "adjectives": {"26": 1}, "adverbs": {"to": 1, "Present": 1, "But": 1, "No, it's not": 1, "Both": 1}, "numerals": {"26": 1}, "pronouns": {"I": 1}}}, {"content": "I don't understand why some people are so nervous about the exam.", "word_count": 18, "label": "", "sentence_structure": "Subject鈥搗erb鈥揷omplement", "subjective_objective": {"answer": "Subjective", "rationale": "Because words like \"not understand\" and \"why\" indicate that it's the thoughts and feelings inside the patient, and it's a subjective examination."}, "sentence_sentiment": {"answer": "Negative", "rationale": "Because the expression \"so nervous about the exam\" in the entry is negative."}, "language_style": {"answer": "Calm and direct", "rationale": "The entry presents the observed phenomenon in a direct and objective manner, with no excessive emotional or rhetorical colours and a more calm and straightforward language style."}, "time_dimension": {"answer": "Present", "rationale": ""}, "sentence_type": "Interrogative sentence", "pos": {"nouns": {"person": 1, "Examinations": 1}, "verbs": {"Understood": 1, "Why": 1, "to": 1, "Nervous": 1}, "adjectives": {"True": 1}, "adverbs": {"Well": 1}, "numerals": {"27": 1}, "pronouns": {"I": 1}}}, {"content": "My bad ideas interfere with my performance in the exam.", "word_count": 18, "label": "", "sentence_structure": "Subject鈥揷opula鈥損redicative", "subjective_objective": {"answer": "Subjective", "rationale": "This is a self-evaluation and idea of the patient and a subjective examination."}, "sentence_sentiment": {"answer": "Negative", "rationale": "Because the expression \"bad ideas can interfere with my performance in the exam\" is negative, and it is a negative expression of a negative attitude to my abilities, which may affect their normal level of performance in the exam."}, "language_style": {"answer": "Tactful and warm", "rationale": "The entry is more euphemistic, expressing doubts and concerns about its abilities by using the description of \"bad ideas\" and not directly denying its value or ability. At the same time, the use of the word \"disturbing\" is neutral, avoiding an overly negative emotional tone. Such a presentation can make people more comfortable and receptive and facilitate the effective conduct of questionnaires."}, "time_dimension": {"answer": "Present", "rationale": "The key words \"bad thoughts\", \"disturbing\" and \"in the exam\" point to the situation, so they say \"now\"."}, "sentence_type": "Negative declarative sentence", "pos": {"nouns": {"Thoughts": 1, "Examinations": 1}, "verbs": {"very": 1, "Bad": 1, "Interference": 1, "in": 1, "Medium": 1, "Yes": 1}, "adjectives": {"Bad": 1}, "adverbs": {"very": 1}, "numerals": {"28": 1}}}, {"content": "I'm not doing much better than I'm doing the final exam.", "word_count": 19, "label": "", "sentence_structure": "Subject鈥搗erb鈥揷omplement", "subjective_objective": {"answer": "Subjective", "rationale": "Because \"more labour\" is a description of subjective attitudes and feelings, of the assessment and perception of self-behaviour by the candidates."}, "sentence_sentiment": {"answer": "Negative", "rationale": "Because the \"no more than a review of the usual test\" in the entry is negative."}, "language_style": {"answer": "Calm and direct", "rationale": "The entry directly expresses the desire of the researchers to know whether the respondents are trying to review the final exam as hard as they were during the refresher exams, in a more straightforward and concise language style."}, "time_dimension": {"answer": "Present", "rationale": "\"I'm studying\" in \"29\" means that what's happening is not more powerful than the \"Tramp Time\" test, which means that it's a comparative level, which means that the act is going. So this entry describes the behavior or state that is now under way, that is, \"now\"."}, "sentence_type": "Exclamatory sentence", "pos": {"nouns": {"Final examination": 1, "-A usual exam": 1, "Review": 2}, "verbs": {"No, it's not like that": 1, "Review": 1, "Work hard": 1}, "adjectives": {"End of period": 1, "Normal": 1}, "adverbs": {"and": 1}, "numerals": {"I'm 29": 1}}}, {"content": "I'm still worried about some exams, though I'm good at some exams.", "word_count": 20, "label": "", "sentence_structure": "Subject鈥搗erb鈥揷omplement", "subjective_objective": {"answer": "Subjective", "rationale": "Despite my thorough review, the anxiety is the patient's own feelings and attitudes."}, "sentence_sentiment": {"answer": "Negative", "rationale": "Because \"anxiety\" in this entry is a negative emotional attitude. Although the first part expresses positive behaviour (good reading of a test), the negative emotional attitude of anxiety negates the first part of the motivation."}, "language_style": {"answer": "Tactful and warm", "rationale": "The entry, by describing the individual ' s feelings, leads the interviewee to share his thoughts and experiences, without asking questions or ordering the other party to answer, and therefore the language style is more euphemismal and warmer."}, "time_dimension": {"answer": "Present", "rationale": "\"Now.\""}, "sentence_type": "Affirmative declarative sentence", "pos": {"nouns": {"30": 1, "Door": 1, "Examinations": 1, "Review": 1, "Anxiety": 1}, "verbs": {"to": 1, "I feel": 1, "be": 1, "Review": 1}, "adjectives": {"Okay": 1}, "adverbs": {"鈾?Though 鈾?: 1, "Still": 1}, "numerals": {"30": 1}, "pronouns": {"I": 1}}}, {"content": "I'm not good before the big test.", "word_count": 10, "label": "", "sentence_structure": "Subject鈥搗erb鈥揷omplement", "subjective_objective": {"answer": "Subjective", "rationale": "Because \"eating bad\" is a subjective feeling and experience, related to the emotional state of the individual, psychological stress, etc. It is the patient's own feeling and experience, and it is a subjective view."}, "sentence_sentiment": {"answer": "Negative", "rationale": "Because the term \"favourful\" is usually a negative experience, implying anxiety or stress."}, "language_style": {"answer": "Calm and direct", "rationale": "The entry asks the other person directly whether he or she feels abated before the important examination, without too many rhetorical or emotional renderings, and with a more calm and straightforward language style."}, "time_dimension": {"answer": "Past", "rationale": "The keyword \"before the major exam\" means that it happened at some point in the past, and the description \"sniffed\" suggests that it is an end in itself, so the entry asks the testee about his past behavior."}, "sentence_type": "Negative declarative sentence", "pos": {"nouns": {"Examinations": 1, "Significant": 1}, "verbs": {"Eat": 1, "Hong": 1}, "adverbs": {"in": 1, "Front": 1}, "numerals": {"31": 1}}}, {"content": "I found my arm shaking before the big test.", "word_count": 16, "label": "", "sentence_structure": "Subject鈥搗erb鈥搊bject", "subjective_objective": {"answer": "Objective", "rationale": "Because arm shaking is an objective fact that patients experience, it is not influenced by patients鈥?subjective consciousness, but by the natural reaction of organisms to outside stimulation. While patients鈥?subjective feelings may affect their behaviour and emotions, arm shaking is not subjective in itself, but an objective phenomenon."}, "sentence_sentiment": {"answer": "Negative", "rationale": "Because the \"arms will shake\" act is negative."}, "language_style": {"answer": "Calm and direct", "rationale": ""}, "time_dimension": {"answer": "Past", "rationale": "The entry describes a specific situation (i.e. \"before major examinations\") that has occurred in the past and is therefore judged to be past."}, "sentence_type": "Affirmative declarative sentence", "pos": {"nouns": {"Major examinations": 1, "Arms": 1}, "verbs": {"Found": 1, "Wreck": 1}, "adjectives": {"Front": 1, "32": 1}, "numerals": {"32": 1}}}, {"content": "I don't have much to ask for \"temporary possession\" before I take the exam.", "word_count": 16, "label": "", "sentence_structure": "Subject鈥搗erb鈥揷omplement", "subjective_objective": {"answer": "Subjective", "rationale": "Because \"very few need to be improvised\" is a subjective assessment of the patient's situation."}, "sentence_sentiment": {"answer": "Positive", "rationale": "Because the \"very little need for a temporary Buddha's Foot\" act in the entry is positive."}, "language_style": {"answer": "Calm and direct", "rationale": "The entry describes the customary behaviour of the person under investigation in an objective and concise manner, without excessive emotional colours or rhetorical techniques, and with a calm and straightforward linguistic style."}, "time_dimension": {"answer": "Present", "rationale": ""}, "sentence_type": "Negative declarative sentence", "pos": {"nouns": {"33": 1, "Examinations": 1, "Buddha's Foot": 1}, "verbs": {"have": 1, "Hug": 1, "Yes": 1}, "adjectives": {"Very few": 1, "Yeah": 1}, "numerals": {"33": 1}, "pronouns": {"I": 1}}}, {"content": "34 schools should realize that some students are anxious about the exam, which affects their grades.", "word_count": 29, "label": "", "sentence_structure": "Subject鈥搗erb鈥搊bject", "subjective_objective": {"answer": "Subjective", "rationale": "Because \"some students are more anxious about the exam\" and \"which affects their test results\" are the views or assumptions of researchers, not the objective facts. These statements reflect the researchers' views and explanations of the problem, and are therefore subjective."}, "sentence_sentiment": {"answer": "Negative", "rationale": "Because \"a little anxious about the exam\" and \"which will affect their test results\" are negative descriptions."}, "language_style": {"answer": "Tactful and warm", "rationale": "The language style of the entry is more moderate, respectful, and does not imply direct accusations or orders. It is an invitation and advice form, which is intended to make schools aware of the emotional problems of students and to take appropriate measures to help them to ease anxiety."}, "time_dimension": {"answer": "Present", "rationale": "The entry now describes the current situation, in which schools should be aware that some students may be affected by their examination performance due to their anxiety. In it, \"know\" means an attitude or action that should be taken to show that the problem exists and needs attention and resolution."}, "sentence_type": "Affirmative declarative sentence", "pos": {"nouns": {"School": 2, "Students": 2, "Examinations": 2, "Anxiety": 1, "Achievements": 1}, "verbs": {"Recognizing": 1, "Impact": 1, "to": 1}, "adjectives": {"Compare": 1}, "adverbs": {"Reactions": 1}, "numerals": {"34": 1}, "pronouns": {"Here": 1, "they": 1}}}, {"content": "I don't think it's that much of a stress disorder during the exam.", "word_count": 18, "label": "", "sentence_structure": "Subject鈥搗erb鈥揷omplement", "subjective_objective": {"answer": "Subjective", "rationale": "Because \"think\" and \"should\" are both thinking and judging from the perspective of the patient."}, "sentence_sentiment": {"answer": "Negative", "rationale": "Because the entry \"should not make it so tense\" expresses a negative attitude and emotion."}, "language_style": {"answer": "Tactful and warm", "rationale": "The entry presents its views in a more moderate sense, rather than directly making accusations or orders, which is more personal and interactive. At the same time, the language style of the article is relatively easy, natural, and not overly emotional, and is more in line with the context of the questionnaire."}, "time_dimension": {"answer": "Present", "rationale": ""}, "sentence_type": "Affirmative declarative sentence", "pos": {"nouns": {"I": 1, "Examination period": 1, "Nervous": 1}, "verbs": {"I think": 1, "I think so": 1, "Fuck": 1, "Okay": 1}, "adjectives": {"Well": 1}, "adverbs": {"Seems like": 1}, "numerals": {"35": 1}}}, {"content": "I felt uncomfortable when I came into contact with the papers that were sent.", "word_count": 17, "label": "", "sentence_structure": "Subject鈥搗erb鈥揷omplement", "subjective_objective": {"answer": "Subjective", "rationale": "It's a sense of self-restraint that is the subjective feeling and emotional reaction of the individual."}, "sentence_sentiment": {"answer": "Negative", "rationale": "Because the \"frightful\" attitude in the entry is negative."}, "language_style": {"answer": "Calm and direct", "rationale": "The language style of the entry is calm and straightforward, and it does not use too many emotional qualifiers or expressions to describe how the person is feeling when he sees the paper. This simple description is easier to understand and can accurately convey the feelings of the person interviewed.\""}, "time_dimension": {"answer": "Present", "rationale": "The entry now describes the feelings and reactions of the testee when exposed to the test papers, and is a current situation, and thus the time dimension is now judged."}, "sentence_type": "Affirmative declarative sentence", "pos": {"nouns": {"Tests": 1, "I feel": 1}, "verbs": {"Exposure": 1, "Issue": 1, "I think": 1, "I'm not comfortable": 1}, "adverbs": {"Just": 1}, "numerals": {"36": 1}, "pronouns": {"I": 1}}}, {"content": "I hate teachers who like to take \"Sudden Strike\" exams.", "word_count": 18, "label": "", "sentence_structure": "Subject鈥搗erb鈥搊bject", "subjective_objective": {"answer": "Subjective", "rationale": "Because the title says, \"I hate it,\" it's the patient's own subjective idea."}, "sentence_sentiment": {"answer": "Negative", "rationale": "Because the entries \"disguised\" and \"shocked attack\" are negative terms, expressing a negative emotional attitude."}, "language_style": {"answer": "Calm and direct", "rationale": "The entry describes directly the grievances of the respondents in respect of certain courses, with no excessive adorations and emotional colours, and with a more objective language style.\""}, "time_dimension": {"answer": "Present", "rationale": "Based on the description of \"disguised\" and \"liked\" in the sentence, and the verb \"fuck\" (means the conduct of the act), it is a sentence describing the current feeling and attitude. So, the time dimension of this entry is now."}, "sentence_type": "Negative declarative sentence", "pos": {"nouns": {"Teacher": 1, "Attack": 1, "Examinations": 1, "Courses": 1}, "verbs": {"Shit": 1, "Yeah": 1, "Fuck": 1, "Attack": 1, "Conduct": 1}, "adjectives": {"Suddenly": 1}, "numerals": {"37": 1}, "pronouns": {"I": 1}}}]}

{"questionnaire_title": "Concise psychiatric scale", "questionnaire_category": "Mental health", "item_count": 20, "emotion_shift_overall": "From positive to negative", "language_style_overall": "Calm and direct", "items": [{"content": "1. Care for physical health", "word_count": 6, "label": "", "sentence_structure": "Subject鈥搗erb鈥揷omplement", "subjective_objective": {"answer": "Subjective", "rationale": "Because \"care for health\" is a subjective attitude or perception, it reflects the degree of importance that patients attach to their health and is part of their subjective perception and attitude."}, "sentence_sentiment": {"answer": "Positive", "rationale": "Because \"care for health\" is a positive attitude, a concern for health and protection."}, "language_style": {"answer": "Tactful and warm", "rationale": "The term \"care\" is used to guide the respondents to reflect on their level of concern for their health, rather than direct orders or criticism. At the same time, \"health\" is a relatively broad and abstract concept, which avoids creating resentment and resistance among the respondents."}, "time_dimension": {"answer": "Present", "rationale": "The keywords are \"care\" and \"health,\" which are all about the current state and attitude, so the temporal dimension of the article is now."}, "sentence_type": "Imperative sentence", "pos": {"nouns": {"Physical health": 1}, "verbs": {"Care": 1}}}, {"content": "2. Anxiety", "word_count": 2, "label": "", "sentence_structure": "Subject鈥損redicate", "subjective_objective": {"answer": "Subjective", "rationale": "Because anxiety is what people feel and experience inside."}, "sentence_sentiment": {"answer": "Negative", "rationale": "Because the word \"anxiety\" is usually seen as a negative emotion or experience."}, "language_style": {"answer": "Calm and direct", "rationale": "The entry directly asks the interviewees whether they are often anxious, have no excessive rhetoric or emotional rendering, speak with a more objective tone and are in a calm and direct language style."}, "time_dimension": {"answer": "Present", "rationale": "The entry does not have a clear time indicator or time statement, and therefore it is tacitly asked about the current state of the person being tested. The keyword \"anxiety\" indicates an emotional reaction, implying that the person being tested may experience some kind of stress or distress, and needs to assess his or her current psychological condition."}, "sentence_type": "Interrogative sentence", "pos": {"nouns": {"Anxiety": 1}}}, {"content": "3. Emotional barriers", "word_count": 6, "label": "", "sentence_structure": "Subject鈥搗erb鈥揷omplement", "subjective_objective": {"answer": "Subjective", "rationale": "The emotional communication barrier is a subjective psychological experience, which refers to the difficulties that patients experience in establishing and maintaining relationships in social relationships."}, "sentence_sentiment": {"answer": "Negative", "rationale": "Because the issue of \"emotional communication disorder\" is indicative of the psychological problems that may exist in an individual and has some negative emotions."}, "language_style": {"answer": "Calm and direct", "rationale": "The entry directly asked the respondents whether they had emotional problems, did not use excessive rhetoric or emotional rendering and was more objective and calm."}, "time_dimension": {"answer": "Present", "rationale": ""}, "sentence_type": "Imperative sentence", "pos": {"nouns": {"Emotional communication disorder": 1}}}, {"content": "4. Confusion of concepts", "word_count": 4, "label": "", "sentence_structure": "Subject鈥搗erb鈥揷omplement", "subjective_objective": {"answer": "Subjective", "rationale": "Because \"mutual confusion\" is a subjective judgement that reflects the patient's perception and feelings about his cognitive state."}, "sentence_sentiment": {"answer": "Negative", "rationale": "Because the description of \"mutual confusion\" is negative, it may indicate that the testee has difficulties or ambiguities in understanding a problem or knowledge point."}, "language_style": {"answer": "Calm and direct", "rationale": "The entry is presented in simple terms, with no excessive emotional or rhetorical style, and is more in line with the calm and straightforward language style."}, "time_dimension": {"answer": "Present", "rationale": "The entry describes the current situation or state of the subjects, i.e. whether they have a problem of confusion. Therefore, the temporal dimension is now judged."}, "sentence_type": "Interrogative sentence", "pos": {"nouns": {"Concept": 1, "Chaos": 1}, "numerals": {"4": 1}}}, {"content": "5. The concept of evil", "word_count": 4, "label": "", "sentence_structure": "Subject鈥損redicate", "subjective_objective": {"answer": "Subjective", "rationale": "Because the idea of evil is the patient's personal opinion and conviction."}, "sentence_sentiment": {"answer": "Negative", "rationale": "Because \"criminal\" is a negative attitude or quality."}, "language_style": {"answer": "Tactful and warm", "rationale": ""}, "time_dimension": {"answer": "Past", "rationale": "The keyword is \"criminal notions\", which describe ideas or ideas that were or still exist in the past, and the temporal dimension of the article is therefore the past."}, "sentence_type": "Interrogative sentence", "pos": {"nouns": {"Sin": 1, "Perceptions": 1}}}, {"content": "6-Escalation", "word_count": 2, "label": "", "sentence_structure": "Subject鈥損redicate", "subjective_objective": {"answer": "Subjective", "rationale": "Because stress is a subjective feeling and emotional experience."}, "sentence_sentiment": {"answer": "Negative", "rationale": "Basis of judgement: \"stretch\" in the entry is usually seen as a negative sentiment, indicating anxiety or anxiety in certain cases."}, "language_style": {"answer": "Calm and direct", "rationale": "The language style of the entry is more straightforward, there is no excessive phrasing or emotional expression, and it appears calm and straightforward. It asks the respondents directly whether they are nervous, express themselves clearly, have a high amount of information and practicality. The language style of the entry is therefore \"cool and direct\"."}, "time_dimension": {"answer": "Present", "rationale": "The keyword \"stretch\" indicates the current state of the subject's mental state, so the temporal dimension is now judged."}, "sentence_type": "Imperative sentence", "pos": {"adjectives": {"Nervous": 1}}}, {"content": "7. Fake and act", "word_count": 5, "label": "", "sentence_structure": "Subject鈥損redicate", "subjective_objective": {"answer": "Subjective", "rationale": "Because \"dressing and acting\" is a person's behaviour and is the expression of a subjective individual's consciousness."}, "sentence_sentiment": {"answer": "Negative", "rationale": "Because \"dressing and acting\" is often considered hypocritical, unfaithful behaviour and therefore negative emotional attitudes."}, "language_style": {"answer": "Tactful and warm", "rationale": "The entry is based on a more moderate term, \"dress\" and \"act\", rather than a direct accusation or attack, and is not expressed in a way that is too intense or emotional. It is more personal and more responsive to the other party."}, "time_dimension": {"answer": "Past", "rationale": "So the answer is \"no apparent state of affairs\"."}, "sentence_type": "Affirmative declarative sentence", "pos": {"nouns": {"Marriage": 1, "State": 1}, "verbs": {"Load": 1, "Do it": 1}}}, {"content": "8. Exaggeration", "word_count": 2, "label": "", "sentence_structure": "Subject鈥損redicate", "subjective_objective": {"answer": "Subjective", "rationale": "Because \"exaggeration\" is an individual's subjective feeling and attitude."}, "sentence_sentiment": {"answer": "Negative", "rationale": "According to the information provided, the act of \"exaggeration\" is usually considered negative, as it involves an inaccurate description of the facts or an exaggeration. The emotional colour of this purpose is therefore negative."}, "language_style": {"answer": "Calm and direct", "rationale": "The entry uses a simple and clear language to describe the survey, does not contain any accommodative language or emotional vocabulary, and therefore it can be judged as \"cool and direct\"."}, "time_dimension": {"answer": "Present", "rationale": "The keyword \"exaggered\" denotes the person's current behaviour or attitude, so the entry describes the current situation."}, "sentence_type": "Interrogative sentence", "pos": {"verbs": {"Quail": 1, "Large": 1}}}, {"content": "9. Heart depression", "word_count": 4, "label": "", "sentence_structure": "Subject鈥搗erb鈥揷omplement", "subjective_objective": {"answer": "Subjective", "rationale": "Because depression is a subjective emotional experience."}, "sentence_sentiment": {"answer": "Negative", "rationale": "Because \"deepness\" is usually considered a negative emotional and psychological state, contrary to positive emotions."}, "language_style": {"answer": "Calm and direct", "rationale": "The entry directly expresses the subject of questioning the other party for the existence of depression, without excessive rhetorical or emotional rendering, and is more objective in tone."}, "time_dimension": {"answer": "Present", "rationale": "The entry is for the purpose of asking the person tested for a certain psychological state, which is now judged by the temporal dimension."}, "sentence_type": "Affirmative declarative sentence", "pos": {"nouns": {"Heartland": 1, "Depression": 1}}}, {"content": "10. Hostile", "word_count": 3, "label": "", "sentence_structure": "Subject鈥損redicate", "subjective_objective": {"answer": "Objective", "rationale": "Since hostility is the expression of behaviour of others, it is an objective fact."}, "sentence_sentiment": {"answer": "Negative", "rationale": "Because the entry \"enemy\" expresses a negative attitude."}, "language_style": {"answer": "Tactful and warm", "rationale": ""}, "time_dimension": {"answer": "Present", "rationale": "Depending on the format of the subject, the entry needs to be judged to ask the respondent whether it has been or is, or will be, an act or mentality. However, the term \"hostility\" does not have a clear temporal dimension. It is a term describing the individual character that may exist, present and future. It is therefore not possible to determine precisely the temporal dimension of the item. In the light of the foregoing, it is not certain whether \"hostility\" is an act or mentality of the past, present or future."}, "sentence_type": "Interrogative sentence", "pos": {"nouns": {"Hostile": 1}}}, {"content": "11. Doubts", "word_count": 2, "label": "", "sentence_structure": "Subject鈥損redicate", "subjective_objective": {"answer": "Subjective", "rationale": "Because \"suspection\" is a psychological or emotional experience, it usually requires self-observation and assessment by the patient."}, "sentence_sentiment": {"answer": "Negative", "rationale": "Because \"suspection\" is often seen as a negative emotion or attitude, it can lead to unnecessary tension and anxiety."}, "language_style": {"answer": "Tactful and warm", "rationale": "It uses an open-ended question-and-answer approach, avoiding direct criticism or accusations against the person under investigation. At the same time, more common psychological terminology is used, making it easier for the person under investigation to understand the meaning of the problem and to share his/her thoughts and feelings."}, "time_dimension": {"answer": "Future", "rationale": "There is no clear time indicator for the entry, but the emotional state of \"suspection\" is usually related to the future, because people usually have doubts and concerns about what is not happening. Thus, we can judge the temporal dimension of the article as being the future."}, "sentence_type": "Interrogative sentence", "pos": {"nouns": {"Doubt": 1}}}, {"content": "12, hallucinating.", "word_count": 2, "label": "", "sentence_structure": "Subject鈥搗erb鈥揷omplement", "subjective_objective": {"answer": "Subjective", "rationale": "The illusion is usually considered to be a sensory experience resulting from the abnormal handling of information in the brain, which may involve a misreading of sound, light, colour, shape, touch, etc. So, \"the illusion\" is usually viewed from the patient's subjective angle. For example, patients may describe the hallucinations they see or hear."}, "sentence_sentiment": {"answer": "Negative", "rationale": "Because the symptoms of \"sight\" in the entry are usually considered to be abnormal or pathological, it expresses a negative emotion."}, "language_style": {"answer": "Calm and direct", "rationale": "The entry directly asks the other side whether there is a illusion, which is relatively straightforward and objective, and which does not have too much adoration or emotional colour, and which is consistent with the calm and direct language style."}, "time_dimension": {"answer": "Past", "rationale": "The entry asks if the person tested has a hallucination and the temporal dimension is judged to be past or present."}, "sentence_type": "Interrogative sentence", "pos": {"nouns": {"Imagination": 1}}}, {"content": "13, slow motion.", "word_count": 4, "label": "", "sentence_structure": "Subject鈥搗erb鈥揷omplement", "subjective_objective": {"answer": "Objective", "rationale": "Because slow movement is a symptom of the patient and an objective fact."}, "sentence_sentiment": {"answer": "Negative", "rationale": "Because the \"slow\" act is negative."}, "language_style": {"answer": "Calm and direct", "rationale": "The entry directly expresses in a concise and clear language the question of whether the person under investigation is slow, is not over-modified or emotional, and is of a calm and straightforward style."}, "time_dimension": {"answer": "Past", "rationale": "The entry describes the symptoms or state of the past, i.e. \"slowness\"."}, "sentence_type": "Interrogative sentence", "pos": {"nouns": {"Actions": 1, "Slow down": 1}, "numerals": {"13": 1}}}, {"content": "14. Non-cooperation", "word_count": 3, "label": "", "sentence_structure": "Subject鈥損redicate", "subjective_objective": {"answer": "Subjective", "rationale": "Because \"non-cooperation\" is a subjective attitude or behaviour that reflects the patient's reluctance to cooperate with others."}, "sentence_sentiment": {"answer": "Negative", "rationale": "Because \"non-cooperation\" in the entry is a negative behavior or attitude."}, "language_style": {"answer": "Calm and direct", "rationale": "The entry directly expresses a question about the tendency of the other party to refrain from cooperating, without excessive rhetorical or emotional rendering, and with a more objective tone."}, "time_dimension": {"answer": "Future", "rationale": "The keyword \"non-cooperation\" indicates that the entry is asked about the person's possible future behaviour or attitude and is therefore judged to be future."}, "sentence_type": "Negative declarative sentence", "pos": {"nouns": {"Cooperation": 1}, "verbs": {"not": 1, "Do it": 1}}}, {"content": "15. The content of abnormal thinking", "word_count": 6, "label": "", "sentence_structure": "Subject鈥損redicate", "subjective_objective": {"answer": "Objective", "rationale": "Because the content of abnormal thinking is the actual state of mind of the patient and is a description of objective facts."}, "sentence_sentiment": {"answer": "Neutral", "rationale": "Because the entry describes the content of abnormal thinking, it does not in itself give a single word of praise."}, "language_style": {"answer": "Tactful and warm", "rationale": "The entry is more personal and interactive in its expression, in a gentle and inviting tone, rather than in direct statements or orders. Moreover, the subject of \"outright thinking\" is itself more sensitive and personal privacy, and the euphemism is more likely to protect the dignity and interests of the person under investigation."}, "time_dimension": {"answer": "Future", "rationale": "The entry contains ideas and expectations of possible abnormal thinking, the keywords being \"abnormal thinking\" and \"emergence\" and all pointing to situations that have not yet occurred and that may occur in the future."}, "sentence_type": "Imperative sentence", "pos": {"nouns": {"Unusual": 1, "Thinking": 1, "Contents": 1}, "numerals": {"15": 1}}}, {"content": "16: Emotional peace", "word_count": 4, "label": "", "sentence_structure": "Subject鈥搗erb鈥揷omplement", "subjective_objective": {"answer": "Subjective", "rationale": "Because emotions are the experience and feelings inside the patient, they are subjective."}, "sentence_sentiment": {"answer": "Neutral", "rationale": "Because the entry does not express any positive or negative emotional tendencies, it is merely a description of the emotional state of the person under investigation."}, "language_style": {"answer": "Calm and direct", "rationale": "The language style of the entry is a calm and straightforward type. The reasons are as follows: - The purpose of the article is simple and clear, not to overdo the adoration or emotional rendering, but to question directly the emotional state of the person interviewed, which is more objective and direct. - Moreover, the purpose of the questionnaire as a whole is probably to understand the psychological condition of the person interviewed, whereas the description of emotional calm is to question the question of \"emotional\" and therefore requires a calm and objective attitude in order to produce more accurate results."}, "time_dimension": {"answer": "Future", "rationale": "The entry asks the testee about the future emotional state or planning, and therefore judges the future. While the term \"emotional peace\" describes the current or past state, in this context it is used more as a projection of the future or as a expectation."}, "sentence_type": "Negative declarative sentence", "pos": {"nouns": {"Emotion": 1, "Flat": 1}}}, {"content": "17, excavated.", "word_count": 2, "label": "", "sentence_structure": "Subject鈥搗erb鈥揷omplement", "subjective_objective": {"answer": "Subjective", "rationale": "Because \"excited\" is the emotional state that the patient feels, and it's the subjective angle of the patient."}, "sentence_sentiment": {"answer": "Positive", "rationale": "Because \"excited\" is positive emotional expression."}, "language_style": {"answer": "Calm and direct", "rationale": "The entry is more directly written, with no excessive rhetorical or emotional rendering, but rather asks the interviewee whether he or she is excited in a given situation in a calm and objective manner. This expression is usually more common in psychological questionnaires, which help researchers to obtain more accurate information about the interviewee鈥檚 psychological condition and feelings."}, "time_dimension": {"answer": "Present", "rationale": "The entry is for the interview of the testee's feelings and experience of the word \"excited,\" which is now judged in time dimension. The key word is \"excited,\" which represents an emotional state or experience that has nothing to do with past and future behavior or mentalities."}, "sentence_type": "Exclamatory sentence", "pos": {"verbs": {"Excited": 1}}}, {"content": "18. Targeted barriers", "word_count": 4, "label": "", "sentence_structure": "Subject鈥搗erb鈥揷omplement", "subjective_objective": {"answer": "Objective", "rationale": "Because \"directive disorder\" is a medical term, it refers to the fact that the patient's cognitive function is problematic and that it is impossible to determine the direction or spatial location correctly, which is an objective symptom."}, "sentence_sentiment": {"answer": "Negative", "rationale": "Because \"directive disorder\" usually means the inability to judge direction, it may be a manifestation of mental illness or brain damage and can therefore be considered a negative quality or behaviour."}, "language_style": {"answer": "Calm and direct", "rationale": "The language style of the entry is calm and straightforward. It clearly points to the subject of the study 鈥?orientation barriers 鈥?which are not based on too many rhetorical or emotional expressions, but rather on plain and plain language, which enables readers to quickly understand the meaning and importance of the subject. Such language styles help to improve the effectiveness and credibility of the questionnaire, while also making the respondents more willing to participate in the survey."}, "time_dimension": {"answer": "Present", "rationale": "The entry describes whether the person tested has a psychological problem - a directional disorder - which is a symptom or state that may arise at the moment and therefore is judged to be the present."}, "sentence_type": "Interrogative sentence", "pos": {"nouns": {"Targeted barriers": 1}}}, {"content": "Self-awareness barriers", "word_count": 5, "label": "", "sentence_structure": "Subject鈥搗erb鈥揷omplement", "subjective_objective": {"answer": "Subjective", "rationale": "Self-awareness means the patient鈥檚 ability to understand and understand his or her mental state."}, "sentence_sentiment": {"answer": "Negative", "rationale": "Because self-absorbed disorders are a mental illness, they are often manifested in negative emotional attitudes that do not allow for objective understanding and assessment of their behaviour and thinking."}, "language_style": {"answer": "Calm and direct", "rationale": "The language style of the entry is straightforward. It uses simple words to describe a subject, namely, whether an individual has a self-knowledge barrier, and not too much of a rhetorical or emotional style, but rather an objective neutral description.\""}, "time_dimension": {"answer": "Past", "rationale": "The entry is for the purpose of asking the testee whether there is a self-awareness barrier, and there is no clear temporal dimension, and therefore cannot be classified as any time frame past, present or future."}, "sentence_type": "Exclamatory sentence", "pos": {"nouns": {"Self-awareness barrier": 1}}}, {"content": "20 - Can't work", "word_count": 4, "label": "", "sentence_structure": "Subject鈥搗erb鈥揷omplement", "subjective_objective": {"answer": "Subjective", "rationale": "Since the ability to work is determined by the patient ' s own abilities and wishes, it is a subjective judgement."}, "sentence_sentiment": {"answer": "Negative", "rationale": "Because the \"not working\" act is negative."}, "language_style": {"answer": "Tactful and warm", "rationale": "The entry raises questions in question and uses a positive-negative structure to guide each other ' s thinking, which is relatively mild and acceptable, reflecting respect and interest for the person under investigation."}, "time_dimension": {"answer": "Present", "rationale": "According to the phrase \"not working\", the entry asks if the person tested is currently unable to work, and therefore judges \"now\"."}, "sentence_type": "Negative declarative sentence", "pos": {"nouns": {"Work": 1}, "verbs": {"No, I can't": 1}, "numerals": {"20": 1}}}]}

{"questionnaire_title": "Household Environment Volume Table (FES)", "questionnaire_category": "Mental health", "item_count": 90, "emotion_shift_overall": "From positive to negative", "language_style_overall": "Tactful and warm", "items": [{"content": "1. We family members always give each other the greatest help and support.", "word_count": 21, "label": "", "sentence_structure": "Subject鈥搗erb鈥揷omplement", "subjective_objective": {"answer": "Subjective", "rationale": "Because the words \"always\" and \"maximum\" indicate the subjective attitude and evaluation of the patient, this is viewed from a subjective perspective."}, "sentence_sentiment": {"answer": "Positive", "rationale": "Because the \"best help and support to each other\" act in the entry is positive."}, "language_style": {"answer": "Tactful and warm", "rationale": "The language style of the entry is more euphemistic. It uses the words \"to each other\" and \"to help and support the most\" to give people a sense of comfort and solidarity. At the same time, the tone is less nuanced, less emphasis or criticism."}, "time_dimension": {"answer": "Present", "rationale": ""}, "sentence_type": "Affirmative declarative sentence", "pos": {"nouns": {"we": 1, "Family members": 1, "Help": 2, "Support": 1}, "verbs": {"Total": 1, "be": 1, "Here": 1, "Give it to me": 1, "- Yeah, yeah": 1}, "adjectives": {"Max": 1}, "adverbs": {"Always": 1}, "classifiers": {"Both": 1, "- Yeah, yeah": 1}, "pronouns": {"we": 1}}}, {"content": "2. Family members always hide their feelings from other family members", "word_count": 26, "label": "", "sentence_structure": "Subject鈥搗erb鈥搊bject", "subjective_objective": {"answer": "Subjective", "rationale": "Whether the feelings of family members are hidden is the subjective feeling and perception of the patient."}, "sentence_sentiment": {"answer": "Negative", "rationale": "Because the entry \"hidden feelings in your heart without telling the rest of your family\" is negative."}, "language_style": {"answer": "Tactful and warm", "rationale": "The entry uses a soft, invitation-style tone for dialogue, rather than direct statements or orders, which is more personal and interactive. At the same time, the issue encourages respondents to share their views and experiences, making them feel more comfortable and free."}, "time_dimension": {"answer": "Past", "rationale": "The basis for the judgment: \"Always\" in \"always\" means \"always\" in \"hidden\" to other family members, and \"no\" is a negative, so it can be considered a mixture of past and present."}, "sentence_type": "Negative declarative sentence", "pos": {"nouns": {"Family members": 2, "Emotion": 1, "In my heart": 1, "Other family members": 1}, "verbs": {"Always": 1, "- Put it on": 1, "Hide": 1, "Disclosure": 1}, "adverbs": {"in": 1, "To Other": 1}, "numerals": {"2": 1}, "pronouns": {"Your own": 1}}}, {"content": "3. Frequent fighting at home", "word_count": 6, "label": "", "sentence_structure": "Subject鈥揷opula鈥損redicative", "subjective_objective": {"answer": "Objective", "rationale": "Because \"recurring quarrels at home\" are an objective situation, it is not influenced by the patient's subjective feelings and only by observation can it be concluded."}, "sentence_sentiment": {"answer": "Negative", "rationale": "Because the \"often fighting\" in the entry is negative."}, "language_style": {"answer": "Calm and direct", "rationale": "It asks whether the interviewee is often in a family in which he or she has a quarrel, in order to understand the family situation and possible problems. Such expressions are more calm and objective and help to gather more accurate information."}, "time_dimension": {"answer": "Present", "rationale": "The entry describes the current family situation, with the keywords \"home\" and \"often fighting\", which indicate that this situation has existed for some time and has had an impact on the life of the person being tested, so it can be judged that this is what is happening."}, "sentence_type": "Negative declarative sentence", "pos": {"nouns": {"Home": 1, "Fight": 1}, "verbs": {"Regular": 1, "have": 1}, "adverbs": {"Regular": 1}}}, {"content": "We rarely act alone in our homes.", "word_count": 13, "label": "", "sentence_structure": "Subject鈥搗erb鈥搊bject", "subjective_objective": {"answer": "Subjective", "rationale": "Because \"very few people are acting alone\" is the patient's subjective feeling and behavioral habits."}, "sentence_sentiment": {"answer": "Negative", "rationale": "Because the \"very few activities on their own\" act in the entry is negative."}, "language_style": {"answer": "Tactful and warm", "rationale": "The entry is more adept and interactive when it is spoken in a gentle and inviting manner, rather than by direct statements or orders."}, "time_dimension": {"answer": "Present", "rationale": "The time-style phrase \"at home\" in the sentence and the verb \"very rarely act alone\" suggest that the entry is to ask the subject about the present behaviour and state of the person being tested, and therefore to be exported as \"now\"."}, "sentence_type": "Negative declarative sentence", "pos": {"nouns": {"Home": 1, "Activities": 1}, "verbs": {"Very few": 1, "self": 1, "Alone": 1, "Conduct": 1}, "adverbs": {"Very few": 1, "self": 1, "Alone": 1}, "numerals": {"4": 1}, "pronouns": {"we": 1}}}, {"content": "5. Family members do everything they can.", "word_count": 17, "label": "", "sentence_structure": "Subject鈥揷opula鈥損redicative", "subjective_objective": {"answer": "Objective", "rationale": "For `whatever it is, it is done' is a narrative fact that does not require the patient's subjective attitude or opinion and is therefore objective."}, "sentence_sentiment": {"answer": "Positive", "rationale": "Because the entry \"do your best\" expresses positive attitudes and behaviour, meaning that family members do everything they can to do."}, "language_style": {"answer": "Tactful and warm", "rationale": "The entry asks the respondents, in an encouraging and supportive manner, about the behaviour of their family members, using positive and positive language, expressing concern and understanding about the respondents. At the same time, the temperature of the words and respect for each other ' s feelings are more likely to resonate and inspire the confidence of the respondents."}, "time_dimension": {"answer": "Present", "rationale": "The entry describes the attitude and perception of the testee to the work of his family members, and the word \"whatever\" in it indicates that this is a general situation and is a current, ongoing state of affairs. Thus, the temporal dimension of the entry is now judged."}, "sentence_type": "Affirmative declarative sentence", "pos": {"nouns": {"Family members": 1, "What's up": 1}, "verbs": {"Do it": 1, "be": 1, "Do what you can": 1}, "classifiers": {"Both": 1}}}, {"content": "6. Political and social issues are often discussed in our home.", "word_count": 14, "label": "", "sentence_structure": "Subject鈥搗erb鈥搊bject", "subjective_objective": {"answer": "Subjective", "rationale": "Because talking is a subjective act, expressing the will and attitude of the patient."}, "sentence_sentiment": {"answer": "Neutral", "rationale": "Because the entry does not have any emotional color."}, "language_style": {"answer": "Tactful and warm", "rationale": "The entry is more moderate in language style, not in a strong or direct way, but in a more friendly and interactive way. It is easier to express the words in a more comfortable and relaxed way, and more willing to share their views and experiences."}, "time_dimension": {"answer": "Past", "rationale": "According to the phrase \"speaks often\" it can be judged as a description of the past, so the output is \"the past\"."}, "sentence_type": "Affirmative declarative sentence", "pos": {"nouns": {"Home": 1, "Political": 1, "Social": 1, "Problem": 1}, "verbs": {"Talk": 1, "be": 1}, "adverbs": {"Regular": 1}, "numerals": {"6": 1}}}, {"content": "7. Most family members spend their weekends and nights at home without going out to social or recreational events", "word_count": 32, "label": "", "sentence_structure": "Subject鈥搗erb鈥搊bject", "subjective_objective": {"answer": "Objective", "rationale": "This is an objective description of the patient鈥檚 current state of life."}, "sentence_sentiment": {"answer": "Neutral", "rationale": "Because the entry merely describes a fact and does not express any emotional colour."}, "language_style": {"answer": "Tactful and warm", "rationale": "The language style of the entry is euphemistic, and the fact that the interviewer is asked to share his/her thoughts by asking questions, without direct orders or mandatory requests for answers, is more likely to resonate and cooperate with the interviewee."}, "time_dimension": {"answer": "Present", "rationale": "\"The entry is to question the current behaviour of the subject, and the time dimension is now judged.\""}, "sentence_type": "Affirmative declarative sentence", "pos": {"nouns": {"Weekend": 1, "Night": 1, "Family members": 1, "Social": 1, "Recreational activities": 1}, "verbs": {"Pass": 1, "Participation": 1}, "adverbs": {"Most of them": 1, "Both": 1, "And": 1, "not": 1}, "numerals": {"7": 1}, "classifiers": {"individual": 1}}}, {"content": "8. We all believe that, no matter how difficult, children should be the first to meet the needs of the elderly.", "word_count": 28, "label": "", "sentence_structure": "Subject鈥搗erb鈥搊bject", "subjective_objective": {"answer": "Subjective", "rationale": "Because \"we think\" expresses a subjective view or view."}, "sentence_sentiment": {"answer": "Neutral", "rationale": "The purpose of the article is expressed without any positive or negative emotional, attitudinal, behavioural, purpose or quality, and it is therefore neutral."}, "language_style": {"answer": "Tactful and warm", "rationale": "The entry uses the expression \"we\" and \"first\" to emphasize a common view and priority rather than a sense of coercion or order; 2. The conditional phrase \"no matter how difficult\" also shows respect and understanding, without demeaning the needs of the elderly; 3. The expression \"to meet the needs of the elderly\" does not specify what needs are, but rather emphasizes comprehensive and inclusive consideration. These are all expressions of euphemism."}, "time_dimension": {"answer": "Present", "rationale": "The entry contains a description and endorsement of the current view, \"We all believe\" and, at the same time, \"the needs of older persons should be met first\" expresses the requirements or expectations of behaviour or attitude and points to the current social perception. Thus, the judgement is based on the present."}, "sentence_type": "Affirmative declarative sentence", "pos": {"nouns": {"Difficulties": 1, "Children": 1, "Old man": 1, "Various": 1, "Requirements": 1}, "verbs": {"I think": 1, "I think so": 1, "Satisfied": 1, "have": 1}, "adverbs": {"How old": 1, "First of all": 1}, "pronouns": {"we": 1}}}, {"content": "The larger activities at home are carefully arranged.", "word_count": 16, "label": "", "sentence_structure": "Subject鈥揷opula鈥損redicative", "subjective_objective": {"answer": "Subjective", "rationale": "Because \"after careful arrangement\" is a subjective judgement that reflects the views and attitudes of the respondents, not the objective facts."}, "sentence_sentiment": {"answer": "Neutral", "rationale": "The entry describes a form of behaviour in the context of family activities and does not contain any emotional or attitudinal colour."}, "language_style": {"answer": "Calm and direct", "rationale": ""}, "time_dimension": {"answer": "Present", "rationale": "The entry indicates that in the current family life, more important family activities require detailed planning and preparation, and therefore it is now judged."}, "sentence_type": "Affirmative declarative sentence", "pos": {"nouns": {"Home": 1, "Medium": 1, "Bigger": 1, "Activities": 1, "Both": 1, "After that": 1, "- Be careful": 1, "Arrangements": 1}, "verbs": {"be": 1, "Yes": 2}, "adjectives": {"Bigger": 1, "Careful": 1}, "numerals": {"9": 1}, "classifiers": {"individual": 1}}}, {"content": "Family members rarely force other family members to comply with family rules", "word_count": 17, "label": "", "sentence_structure": "Subject鈥搗erb鈥搊bject", "subjective_objective": {"answer": "Subjective", "rationale": "Because \"compliance with the rules of the family\" is a thing that family members should do and that they need their will and action to achieve. The degree of compliance with this behaviour therefore depends on the subjective attitude and choice of the individual."}, "sentence_sentiment": {"answer": "Neutral", "rationale": "Because the entry does not have any emotional color."}, "language_style": {"answer": "Tactful and warm", "rationale": "The entry is presented in question form and the word \"very rarely\" is used to express a relatively relaxed tone, giving a comfortable and friendly feeling of the person and a warm and gentle language style."}, "time_dimension": {"answer": "Present", "rationale": "The keywords \"family rarely demands\" and \"compliance with family rules\" suggest that the act is taking place at the moment, so it can be judged that it is an inquiry into the conduct of the present."}, "sentence_type": "Negative declarative sentence", "pos": {"nouns": {"Family": 1, "Family rules": 1}, "verbs": {"Force": 1, "Compliance": 1}, "adverbs": {"Very few": 1}, "numerals": {"10": 1}, "classifiers": {"Family members": 1}}}, {"content": "We're bored at home.", "word_count": 10, "label": "", "sentence_structure": "Subject鈥揷opula鈥損redicative", "subjective_objective": {"answer": "Subjective", "rationale": "Because boredom is a subjective experience and feeling of a human being."}, "sentence_sentiment": {"answer": "Negative", "rationale": "Because \"distressed\" expresses a negative sentiment."}, "language_style": {"answer": "Tactful and warm", "rationale": "The entry uses more euphemistic language, which attempts to make the person surveyed more free and comfortable to express his/her feelings, rather than to force him/her to give some kind of answer. At the same time, \"at home\" implies that this is a family-based problem, and that the language is filled with gentleness and understanding, which is more acceptable and willing to share his/her own thoughts."}, "time_dimension": {"answer": "Present", "rationale": "The keyword \"at home\" means that this is a specific situation, and \"uninterested\" is the subjective feeling of the testee, which all indicate that it is a description and an assessment of the current situation."}, "sentence_type": "Negative declarative sentence", "pos": {"nouns": {"Home": 1, "we": 1}, "verbs": {"I feel": 1, "Boring": 1}, "adjectives": {"very": 1}, "numerals": {"11": 1}}}, {"content": "12. At home, we can say whatever we want.", "word_count": 15, "label": "", "sentence_structure": "Subject鈥損redicate", "subjective_objective": {"answer": "Subjective", "rationale": "Because \"What we can say at home\" is the patient's subjective thoughts and feelings, reflecting their way of communicating and the family atmosphere."}, "sentence_sentiment": {"answer": "Neutral", "rationale": "Because the entry does not express positive or negative feelings, it merely presents a fact."}, "language_style": {"answer": "Tactful and warm", "rationale": "The language style of the entry is more euphemistic, not too much emphasis or command, but rather a moderate and inviting tone of dialogue, which is more acceptable and participatory."}, "time_dimension": {"answer": "Present", "rationale": "The keywords \"at home\" and \"may\" indicate that this is a description of the situation, and \"if you want to say anything\" expresses the free expression of the person in the family, so the entry reflects the behaviour or mentality of the moment."}, "sentence_type": "Affirmative declarative sentence", "pos": {"nouns": {"Home": 1, "we": 1, "Talk": 1}, "verbs": {"Yes": 1, "Say it": 2}, "adverbs": {"Just": 1}, "numerals": {"12": 1}, "classifiers": {"individual": 1}, "pronouns": {"What": 1}}}, {"content": "13. Little public anger among family members", "word_count": 14, "label": "", "sentence_structure": "Subject鈥揷opula鈥損redicative", "subjective_objective": {"answer": "Subjective", "rationale": "Whether or not family members are publicly angry with each other is a subjective criterion for evaluation, and the respondents are required to respond to it in the light of their own observations and feelings."}, "sentence_sentiment": {"answer": "Neutral", "rationale": "Because the entry is not clearly emotional, it merely describes a fact."}, "language_style": {"answer": "Tactful and warm", "rationale": "The entry is more personal and interactive when it is spoken in a gentle and inviting manner, rather than in direct statements or orders. At the same time, the language of the question is neutral and objective, and does not contain any obvious value judgement or emotional colour, allowing the interviewee to answer the questions more freely and comfortably."}, "time_dimension": {"answer": "Past", "rationale": "The key words in the sentence are \"very few\" and \"public anger.\" These two words express an attitude or behaviour of family members in their interaction, that is, avoiding public debate or excavating emotions. So, this entry describes a current situation. In the light of the above, the time dimension of the questionnaire is now."}, "sentence_type": "Negative declarative sentence", "pos": {"nouns": {"Family members": 2, "You too": 1, "Public anger": 1}, "verbs": {"Very few": 1}, "adverbs": {"very": 1}, "numerals": {"13": 1}}}, {"content": "We all encourage the family to be independent.", "word_count": 16, "label": "", "sentence_structure": "Subject鈥搗erb鈥搊bject", "subjective_objective": {"answer": "Subjective", "rationale": "Because \"very encouraged\" is an attitude or view and reflects the subjective view of researchers."}, "sentence_sentiment": {"answer": "Positive", "rationale": "Because the \"very encouraged\" in the entry is a positive attitude towards the spirit of independence."}, "language_style": {"answer": "Tactful and warm", "rationale": "The language style of the entry is euphemistic, because it uses the phrase \"we are all very encouraged\" to express a positive attitude and expectations towards family members. It also does not direct orders or instructions, but rather leads the interviewee to think and answer questions in a moderate way. This makes the questionnaire more personal and interactive, and helps the interviewee to better engage and express his views."}, "time_dimension": {"answer": "Present", "rationale": "The entry asks the testee's attitude to \"whether the family should have an independent spirit\", does not address the time dimension of the past, present or future, and is therefore judged neutral."}, "sentence_type": "Affirmative declarative sentence", "pos": {"nouns": {"we": 1, "Home": 1, "Lee": 1, "person": 1, "Spirit": 1}, "verbs": {"Encourage": 1, "Yes": 1}, "adjectives": {"Both": 1, "Very": 1}, "numerals": {"14": 1}, "pronouns": {"Well": 1}}}, {"content": "15. Family members have spent almost all their energy on a future.", "word_count": 21, "label": "", "sentence_structure": "Subject鈥搗erb鈥搊bject", "subjective_objective": {"answer": "Subjective", "rationale": "The reason for the energy spent by family members is the patient ' s own subjective judgement and expectations."}, "sentence_sentiment": {"answer": "Negative", "rationale": "Because the description of \"almost energy\" shows that family members have sacrificed a lot to have a good future, which may have some negative effects and consequences."}, "language_style": {"answer": "Tactful and warm", "rationale": "The language style of the entry is relatively warm. Although strong language is used in describing the great efforts of family members to pursue a better future, it is not direct command or blame, but rather by asking questions to stimulate resonance and reflection."}, "time_dimension": {"answer": "Present", "rationale": "The reference in the past to \"all of the energy spent\" indicates that this is an event of the past, describing the energy that family members have devoted to a better future. The time dimension of this entry is therefore the past."}, "sentence_type": "Affirmative declarative sentence", "pos": {"nouns": {"Future": 1, "Family members": 2, "- The energy": 1}, "verbs": {"They're all spent": 1, "have": 1, "Almost": 1, "All": 1}, "adjectives": {"Okay": 1}, "adverbs": {"Both": 1, "Almost": 1}, "numerals": {"15": 1, "All of it": 1}, "pronouns": {"Family members": 2, "they": 1}}}, {"content": "We rarely go out to talk, watch plays or go to museums and watch exhibitions.", "word_count": 21, "label": "", "sentence_structure": "Subject鈥搗erb鈥搊bject", "subjective_objective": {"answer": "Objective", "rationale": "Because \"very few outsides listen to lectures, see theatres or go to museums and see exhibitions\" are objective acts that have occurred in patients, the subjective idea of the patient is subjective."}, "sentence_sentiment": {"answer": "Negative", "rationale": "Because the \"very little out\" act in the entry is negative."}, "language_style": {"answer": "Calm and direct", "rationale": "The entry is based on a direct statement asking the respondent whether he or she is often involved in cultural activities."}, "time_dimension": {"answer": "Past", "rationale": "\"Low time is spent outside the lecture, the play or the museum and the exhibition\" practice has been and may have stopped. The temporal dimension is therefore a matter of the past."}, "sentence_type": "Negative declarative sentence", "pos": {"nouns": {"Lectures": 1, "The play": 1, "Museums": 1, "Exhibition": 1}, "verbs": {"Out": 1, "Listen": 1, "Look": 2, "Go on": 1}, "adjectives": {"Very few": 1}, "numerals": {"16": 1}}}, {"content": "17. Family members often go out to friends ' homes and have dinner together.", "word_count": 19, "label": "", "sentence_structure": "Subject鈥搗erb鈥揷omplement", "subjective_objective": {"answer": "Objective", "rationale": "Because it is an objective behaviour of family members to go out to friends and eat together."}, "sentence_sentiment": {"answer": "Positive", "rationale": "Because the entry is a close family relationship, the act of having a party and having dinner together is usually considered an active and pleasant social event."}, "language_style": {"answer": "Tactful and warm", "rationale": "The language style of the questionnaire entry is euphemistic, because it asks the family members of the person under investigation whether they often go out to their friends鈥?homes and eat together. This expression brings them closer to the person under investigation and makes them feel more comfortable and willing to share their true situation."}, "time_dimension": {"answer": "Present", "rationale": ""}, "sentence_type": "Affirmative declarative sentence", "pos": {"nouns": {"Family members": 1, "Friends": 1, "Rice": 1}, "verbs": {"Regular": 1, "Go play": 1, "Together": 1, "Eat": 1}, "adverbs": {"and": 1, "Together": 1}, "numerals": {"17": 1}, "classifiers": {"individual": 1}}}, {"content": "18. Family members agree that they should be responsive to the social climate.", "word_count": 16, "label": "", "sentence_structure": "Subject鈥搗erb鈥搊bject", "subjective_objective": {"answer": "Subjective", "rationale": "Because \"thinks\" is the patient's view and opinion of the situation and is subjective."}, "sentence_sentiment": {"answer": "Neutral", "rationale": "Because the entry describes a certain perception or attitude of family members, it does not express a positive or negative emotional tendency."}, "language_style": {"answer": "Calm and direct", "rationale": "So I think the language style of this questionnaire entry is \"cool straight.\""}, "time_dimension": {"answer": "Present", "rationale": ""}, "sentence_type": "Affirmative declarative sentence", "pos": {"nouns": {"Family members": 1, "Work": 1, "Social climate": 1}, "verbs": {"I think": 1, "Reactions": 1, "Complementarity": 1}, "numerals": {"18": 1}}}, {"content": "In general, we're all concerned about keeping our homes together.", "word_count": 20, "label": "", "sentence_structure": "Subject鈥搗erb鈥揷omplement", "subjective_objective": {"answer": "Subjective", "rationale": "The care to keep the house in order is the patient ' s subjective attitude and behaviour."}, "sentence_sentiment": {"answer": "Neutral", "rationale": "Because the entry does not have any emotional color."}, "language_style": {"answer": "Calm and direct", "rationale": "It is presented in a short question, asking the respondents whether they are often concerned with household hygiene and cleaning, without excessive accoutrements or emotional colours. At the same time, this clear and concise expression is more in keeping with the linguistic characteristics of the questionnaire and facilitates access to objective and accurate data."}, "time_dimension": {"answer": "Present", "rationale": "The term \"general\" in this entry indicates a regular, customary behaviour, and \"care\" adopts the general current, meaning that the act has not only occurred in the past, but may still be ongoing. \"Setting the house in order\" describes a current state of behaviour, and can therefore be judged as the present."}, "sentence_type": "Affirmative declarative sentence", "pos": {"nouns": {"General": 1, "Everyone": 1, "Home": 1, "Attention": 1, "Pack up": 1, "The well is organized": 1}, "verbs": {"Both": 1}, "adverbs": {"Generally": 1}, "pronouns": {"we": 1}}}, {"content": "20. There are few regular living patterns and family rules in the family.", "word_count": 15, "label": "", "sentence_structure": "Subject鈥揷opula鈥損redicative", "subjective_objective": {"answer": "Subjective", "rationale": "This is because it is subjective for the patient to have his own opinion of whether there is a regular living order and family discipline in the family."}, "sentence_sentiment": {"answer": "Negative", "rationale": "Because the behaviour of \"very few fixed living patterns and family rules\" in the entries is negative and may lead to disorder in the family order and lack of discipline."}, "language_style": {"answer": "Tactful and warm", "rationale": ""}, "time_dimension": {"answer": "Present", "rationale": ""}, "sentence_type": "Negative declarative sentence", "pos": {"nouns": {"Home": 1, "Lifestyle": 1, "Family rules": 1}, "verbs": {"have": 1, "Less": 1, "Fixed": 1, "Development": 1}, "adjectives": {"Very few": 1, "Fixed": 1}, "numerals": {"20": 1, "Very few": 1}}}, {"content": "21. Family members are willing to devote a lot of effort to their family.", "word_count": 17, "label": "", "sentence_structure": "Subject鈥搗erb鈥揷omplement", "subjective_objective": {"answer": "Subjective", "rationale": "The willingness of family members to devote considerable effort to domestic work is a subjective issue, as it is based on the attitudes and perceptions of the respondents towards family members."}, "sentence_sentiment": {"answer": "Positive", "rationale": "For the behaviour of \"family members are willing to devote a great deal of their energy to the family\" in the entry is a sign of active participation and responsibility of family members, a positive attitude or quality."}, "language_style": {"answer": "Tactful and warm", "rationale": "The language style of the entry is more euphemistic, warm, and dialogue is conducted in a question-and-answer tone, rather than direct command or statement. At the same time, the description of \"absorbed of great energy\" is positive, and gives people a more comfortable and inspiring feeling. So I think the entry uses a soft language style."}, "time_dimension": {"answer": "Present", "rationale": "The time dimension of the article could therefore be judged to be the present."}, "sentence_type": "Affirmative declarative sentence", "pos": {"nouns": {"Family members": 1, "- The energy": 1, "Things": 1}, "verbs": {"Yes": 1, "Flowers": 1, "Do it": 1}, "adjectives": {"It's big": 1}, "numerals": {"21": 1}, "classifiers": {"very": 1}}}, {"content": "22. Prosecuting at home can be a pain in the family.", "word_count": 13, "label": "", "sentence_structure": "Subject鈥揷opula鈥損redicative", "subjective_objective": {"answer": "Objective", "rationale": "Because it is an objective result that the attitudes and reactions of families depend on whether they are bored with their families, and the feelings or ideas of the patients are subjective."}, "sentence_sentiment": {"answer": "Negative", "rationale": "The basis for the judgement: \"the fact that the complaint is easily boring the family\" is negative."}, "language_style": {"answer": "Calm and direct", "rationale": "The language style of the entry is \"cool and straight.\" Instead of using too many qualifiers or emotional colours, it presents a fact in a simple and clear way and raises a question that needs to be answered."}, "time_dimension": {"answer": "Present", "rationale": "The entry describes a recurring family situation and therefore allows for a judgement of the time dimension."}, "sentence_type": "Negative declarative sentence", "pos": {"nouns": {"Home": 1, "Complaints": 1, "Family": 1, "Boring": 1}, "verbs": {"鈾?And I'm gonna be 鈾?: 1}, "adverbs": {"very": 1}, "numerals": {"22": 1}}}, {"content": "23. Sometimes family members fall when they're angry.", "word_count": 12, "label": "", "sentence_structure": "Subject鈥搗erb鈥揷omplement", "subjective_objective": {"answer": "Objective", "rationale": "Because \"thumping something in anger\" is a specific act of behaviour that can be observed and documented objectively."}, "sentence_sentiment": {"answer": "Negative", "rationale": "Because the act of \"thumping something when angry\" in the entry is negative."}, "language_style": {"answer": "Tactful and warm", "rationale": "The entry is more gentle and friendly in its language, using the means of questioning, which makes the respondent more willing to express his or her true thoughts and feelings. Nor does it use too direct or strong language to guide or control the respondent's answers, making the questionnaire more objective and impartial."}, "time_dimension": {"answer": "Present", "rationale": "The entry is a time dimension that determines the past when the person tested has witnessed or experienced the anger and fall of a family member in the family."}, "sentence_type": "Affirmative declarative sentence", "pos": {"nouns": {"Family members": 1, "Anger": 1, "Something": 1}, "verbs": {"- Get angry": 1, "Fall": 1}, "adverbs": {"Sometimes": 1}, "numerals": {"23": 1}}}, {"content": "24. Family members think independently.", "word_count": 11, "label": "", "sentence_structure": "Subject鈥揷opula鈥損redicative", "subjective_objective": {"answer": "Objective", "rationale": "Because the question of whether family members are thinking independently is an objective fact and is not subject to personal subjective influence."}, "sentence_sentiment": {"answer": "Positive", "rationale": "Because the \"independent thinking\" in the entry is positive."}, "language_style": {"answer": "Tactful and warm", "rationale": "The entry uses an invitation-style tone, whereby respondents self-assess their family members ' self-assessment of whether they are independent and more responsive. At the same time, the language style of the article is more moderate, without the use of overly direct or strong emotional terms, giving people a comfortable feeling and easier to receive positive responses from the interviewees."}, "time_dimension": {"answer": "Past", "rationale": "The entry describes the family situation over time (uncertainly specific length of time) and therefore determines \"the past\"."}, "sentence_type": "Affirmative declarative sentence", "pos": {"nouns": {"Family members": 1, "Independence": 1, "Think": 1, "Problem": 1}, "verbs": {"Both": 1}, "numerals": {"24": 1}}}, {"content": "25. Family members believe that it is more important to raise living standards than anything else.", "word_count": 24, "label": "", "sentence_structure": "Subject鈥搗erb鈥搊bject", "subjective_objective": {"answer": "Subjective", "rationale": "The views of family members are subjective."}, "sentence_sentiment": {"answer": "Positive", "rationale": "The entry is not negative or neutral because it is expressed in terms of the importance attached by family members to improving their standard of living."}, "language_style": {"answer": "Calm and direct", "rationale": "The entry is based on a direct and clear expression of the family member鈥檚 view of the improvement of living standards, without the use of excessive rhetorical or emotional rendering, and in a more objective manner. This expression enables the person surveyed to understand the question with precision and without creating any ambiguity."}, "time_dimension": {"answer": "Present", "rationale": "The keyword \"family members agree\" indicates that it is a current attitude or view, whereas the entire sentence describes a general phenomenon or view and therefore the judgement is now."}, "sentence_type": "Affirmative declarative sentence", "pos": {"nouns": {"Family members": 1, "Standard of living": 1, "Things": 1}, "verbs": {"I think": 1, "鈾?And I'm gonna be 鈾?: 1, "Increase": 1}, "adverbs": {"Both": 1}, "numerals": {"25": 1}, "pronouns": {"Any other": 1}}}, {"content": "We all believe that learning new knowledge is more important than anything else.", "word_count": 20, "label": "", "sentence_structure": "Subject鈥揷opula鈥損redicative", "subjective_objective": {"answer": "Subjective", "rationale": "Because \"think\" is the patient's subjective attitude."}, "sentence_sentiment": {"answer": "Positive", "rationale": "Because the idea of learning new knowledge is more important than anything else in the entry is positive."}, "language_style": {"answer": "Calm and direct", "rationale": "The entry expresses the subject in a direct and clear way 鈥?the importance of learning new knowledge, without using rhetorical techniques or excessive emotional rendering. This is more objective and clear."}, "time_dimension": {"answer": "Present", "rationale": "In this sentence, \"We all think\" is the expression of the current attitude and perception, and \"learning new knowledge is more important than anything else\" emphasizes the importance of learning new knowledge at the moment. So, it can be judged that the entry is about the present."}, "sentence_type": "Affirmative declarative sentence", "pos": {"nouns": {"we": 1, "Knowledge": 1}, "verbs": {"I think": 1, "Learn": 1, "- Yeah": 1, "Both": 2}, "adjectives": {"New": 1, "Anything else": 1}, "adverbs": {"Important": 1}, "numerals": {"26": 1, "Anything else": 1}}}, {"content": "27. No one in the family participates in sports activities", "word_count": 12, "label": "", "sentence_structure": "Subject鈥揷opula鈥損redicative", "subjective_objective": {"answer": "Objective", "rationale": "Because \"no one takes part in any sporting activity at home\" is an objective fact for the family of the patient and does not involve the patient's subjective attitude."}, "sentence_sentiment": {"answer": "Negative", "rationale": "Because the act of \"no one in the family takes part in sports activities\" is negative and may reflect a lack of sports habits or a lack of attention to sports by family members."}, "language_style": {"answer": "Tactful and warm", "rationale": "This entry uses a soft, invitation-style tone to engage in dialogue, asking if family members of the other side are involved in various sporting activities, and not in direct statements or orders."}, "time_dimension": {"answer": "Present", "rationale": "The keyword \"no one at home\" denotes the current state or situation, so the time dimension of the article is now."}, "sentence_type": "Negative declarative sentence", "pos": {"nouns": {"person": 1, "Sports activities": 1}, "verbs": {"Participation": 1}, "numerals": {"27": 1}, "classifiers": {"Plant": 1}, "pronouns": {"Home": 1, "Nope": 1}}}, {"content": "28. Family members often help the elderly and disabled around them in their lives", "word_count": 22, "label": "", "sentence_structure": "Subject鈥搗erb鈥搊bject", "subjective_objective": {"answer": "Objective", "rationale": "Since \"family members often help the elderly and disabled around them in their lives\" are descriptions of objective behaviour, it is the patient's behaviour that is being examined in accordance with this criterion, not their subjective thoughts or feelings."}, "sentence_sentiment": {"answer": "Positive", "rationale": "Because the behaviour in the entry \"family members often help the elderly and disabled around them in their lives\" is positive and represents a caring and helpful quality."}, "language_style": {"answer": "Tactful and warm", "rationale": ""}, "time_dimension": {"answer": "Present", "rationale": "Now, because \"often helping\" indicates that it is a customary act, and it is now happening. The sentence does not address past or future events or situations."}, "sentence_type": "Affirmative declarative sentence", "pos": {"nouns": {"Family members": 1, "Life": 1, "Help": 1, "Around": 1, "Older persons": 1, "Persons with disabilities": 1}, "verbs": {"in": 1, "Regular": 1, "Help": 2}, "adverbs": {"Regular": 1}, "numerals": {"28": 1}, "classifiers": {"individual": 1}}}, {"content": "29. In our house, when you need something to use, you don't always find it.", "word_count": 19, "label": "", "sentence_structure": "Subject鈥搗erb鈥搊bject", "subjective_objective": {"answer": "Objective", "rationale": "Because \"at home\" and \"when needing something\" and \"when unable to find\" are objective facts or conditions, it is objective circumstances."}, "sentence_sentiment": {"answer": "Negative", "rationale": "Because \"unreceived\" in the entries is negative."}, "language_style": {"answer": "Tactful and warm", "rationale": "The entry is more adept and interactive when it is spoken in a gentle and inviting manner, rather than by direct statements or orders."}, "time_dimension": {"answer": "Present", "rationale": "The term \"often\" denotes customary behaviour, which is a constant problem in describing family life, and the temporal dimension of the article is therefore the present."}, "sentence_type": "Negative declarative sentence", "pos": {"nouns": {"Our home": 1, "Something": 1, "Time": 1}, "verbs": {"Use": 1, "Find": 1}, "adverbs": {"Often": 1}, "numerals": {"29": 1}}}, {"content": "30. The time of eating and sleeping in our house is the same.", "word_count": 19, "label": "", "sentence_structure": "Subject鈥揷opula鈥損redicative", "subjective_objective": {"answer": "Objective", "rationale": "This is an objective fact, as it describes the patient ' s family life patterns."}, "sentence_sentiment": {"answer": "Neutral", "rationale": "Because the entry does not have any emotional color."}, "language_style": {"answer": "Calm and direct", "rationale": "The language style of the entry is calm and straightforward. It expresses the question of the consistency of the diet and sleep time in the interviewee鈥檚 family in a concise and clear manner, without using euphemism or rhetoric to decorate the sentence."}, "time_dimension": {"answer": "Present", "rationale": "This sentence refers to the situation of our family, which is that \"the time of eating and sleeping is the same.\" So the answer is now."}, "sentence_type": "Affirmative declarative sentence", "pos": {"nouns": {"Time": 1, "Home": 1, "Rice": 1, "I'm not sure": 1}, "verbs": {"Eat": 1, "Sleep": 1, "Change": 1}, "adjectives": {"- Ten percent": 1}, "numerals": {"30": 1}, "classifiers": {"Both": 1}, "pronouns": {"we": 1}}}, {"content": "31. There is a harmony in our home.", "word_count": 14, "label": "", "sentence_structure": "Subject鈥揷opula鈥損redicative", "subjective_objective": {"answer": "Subjective", "rationale": "Because \"a climate of harmony\" is a family atmosphere that patients feel, and it is their subjective perception and feelings."}, "sentence_sentiment": {"answer": "Positive", "rationale": "Because the \"climate of harmony\" in the entry is a positive emotional expression."}, "language_style": {"answer": "Tactful and warm", "rationale": "The entry uses descriptive language to convey the subject of the survey - the family environment - without asking or ordering the interviewee to answer the questions directly. Moreover, the term \"harmonization\" is used to make people feel comfortable, comfortable and in a soft and warm language style."}, "time_dimension": {"answer": "Present", "rationale": "The entry describes the current family climate, the situation in which \"now\" is. The keywords are \"a harmonious atmosphere\" and \"in our house,\" which all indicate that the item is asking about the present situation."}, "sentence_type": "Affirmative declarative sentence", "pos": {"nouns": {"The atmosphere": 1, "Home": 1, "Unanimously": 1}, "verbs": {"have": 1, "Form": 1}, "adjectives": {"Harmony": 1}, "numerals": {"31": 1}, "pronouns": {"we": 1}}}, {"content": "32. Everyone in the family can tell us about their difficulties and their problems.", "word_count": 18, "label": "", "sentence_structure": "Subject鈥搗erb鈥搊bject", "subjective_objective": {"answer": "Subjective", "rationale": "The thoughts and feelings of each individual in the family are subjective and can be said to be their subjective attitude."}, "sentence_sentiment": {"answer": "Positive", "rationale": "Because the entry \"can speak of your difficulties and troubles\" is positive and encourages open communication."}, "language_style": {"answer": "Tactful and warm", "rationale": "The language style of the entry is euphemism. It raises questions in a friendly and caring manner, encouraging the interviewee to speak openly about the problems and problems he/she encounters in his/her family. This gentle and respectful language creates a safe and supportive environment that makes the interviewee feel more willing to share his/her feelings and experiences."}, "time_dimension": {"answer": "Present", "rationale": "The entry asks about the current state of behaviour of family members, i.e. whether everyone can express their difficulties and distress at home."}, "sentence_type": "Affirmative declarative sentence", "pos": {"nouns": {"Home": 1, "Everyone": 1, "Difficulties": 1, "Trouble": 1}, "verbs": {"Yeah": 2, "Tell me": 1}, "adverbs": {"Both": 1}, "numerals": {"32": 1}}}, {"content": "33. Little anger among family members", "word_count": 11, "label": "", "sentence_structure": "Subject鈥搗erb鈥揷omplement", "subjective_objective": {"answer": "Objective", "rationale": "Because \"very little anger among family members\" is an observed phenomenon or fact, it is objective because it is available through, for example, surveys or records."}, "sentence_sentiment": {"answer": "Neutral", "rationale": "Because the entry is merely a description of a fact and does not express positive or negative emotional sentiments."}, "language_style": {"answer": "Calm and direct", "rationale": "The entry asks the respondent, in a more direct and objective manner, whether he has been in a state of frequent agitation in family relations, without excessive ado or emotional colour, and in a more honest manner."}, "time_dimension": {"answer": "Present", "rationale": ""}, "sentence_type": "Negative declarative sentence", "pos": {"nouns": {"Family members": 1, "- Grumpy": 1}, "verbs": {"Let's go": 1, "Very few": 1}, "adjectives": {"Very few": 1}, "numerals": {"33": 1}}}, {"content": "34. Everyone in our family is completely free to go in and out.", "word_count": 15, "label": "", "sentence_structure": "Subject鈥搗erb鈥揷omplement", "subjective_objective": {"answer": "Subjective", "rationale": "Because \"everyone in our family is completely free\" is a subjective judgement or opinion that reflects the respondent's perception and assessment of the degree of freedom of access for his or her family members."}, "sentence_sentiment": {"answer": "Neutral", "rationale": "Since the entry only objectively describes the free movement of family members, it does not involve any emotional colour or value judgement."}, "language_style": {"answer": "Calm and direct", "rationale": "The language style of the entry is more straightforward, it is not based on excessive rhetorical or emotional rendering, and it is more objective in tone. It simply expresses the fact that family members are free to travel to and from their homes."}, "time_dimension": {"answer": "Present", "rationale": "The entry describes \"everyone in our family is completely free to enter or leave\", and does not address past or future behaviors or mentalities, so it is judged as \"now\"."}, "sentence_type": "Affirmative declarative sentence", "pos": {"nouns": {"person": 1, "Access": 1, "Freedom": 1}, "verbs": {"be": 1, "Total": 2}, "numerals": {"34": 1}, "pronouns": {"Everyone in our family": 1}}}, {"content": "We all believe that competition is good in any case.", "word_count": 16, "label": "", "sentence_structure": "Subject鈥搗erb鈥搊bject", "subjective_objective": {"answer": "Subjective", "rationale": "Because \"all believe\" means that people are subjective and believe."}, "sentence_sentiment": {"answer": "Positive", "rationale": "Because it expresses a positive attitude and belief in \"competition\"."}, "language_style": {"answer": "Calm and direct", "rationale": "This language style is more consistent with the rigour and objectivity of the questionnaire."}, "time_dimension": {"answer": "Present", "rationale": "Now the entry asks the testee about his present perception and beliefs, both \"both\" and \"convincing\" suggest that this is a subjective attitude or belief, and therefore a temporal dimension of the present. At the same time, the expression \"competitive is good in any case\" also shows that this is a view of a universal phenomenon, not limited to past or future situations."}, "sentence_type": "Affirmative declarative sentence", "pos": {"nouns": {"Situation": 1, "Competition": 2, "Yes": 1}, "verbs": {"Letter": 1, "be": 1, "Okay": 1, "in": 1, "Both": 1, "Any": 1, "Yes": 1}, "adverbs": {"Any": 1}, "numerals": {"35": 1}, "pronouns": {"we": 1, "Here": 1}}}, {"content": "We're not so interested in cultural activities.", "word_count": 13, "label": "", "sentence_structure": "Subject鈥搗erb鈥揷omplement", "subjective_objective": {"answer": "Subjective", "rationale": "This is a subjective examination because of the patient ' s personal feelings and perceptions."}, "sentence_sentiment": {"answer": "Negative", "rationale": "Because the \"less interested\" attitude in the entry is negative."}, "language_style": {"answer": "Tactful and warm", "rationale": "The entry is more personal and interactive when it is presented in a gentle and inviting tone, rather than in direct statements or orders. At the same time, the expression \"less interested in cultural activities\" is relatively neutral and objective, avoiding undue pressure or discomfort on the person under investigation."}, "time_dimension": {"answer": "Present", "rationale": "The keyword is \"we are not so interested in cultural activities\" and it is a current situation or attitude."}, "sentence_type": "Negative declarative sentence", "pos": {"nouns": {"Cultural activities": 1}, "verbs": {"Not so interested": 1}, "adverbs": {"Well": 1, "not": 1}, "numerals": {"36": 1}}}, {"content": "We watch movies or sports, go out on a field trip, etc.", "word_count": 16, "label": "", "sentence_structure": "Subject鈥搗erb鈥搊bject", "subjective_objective": {"answer": "Objective", "rationale": "Because it is an objective fact describing the behaviour, not the subjective attitude or feeling of the patient."}, "sentence_sentiment": {"answer": "Positive", "rationale": "Because the entries are positive, such as watching movies, sports games, outings, etc."}, "language_style": {"answer": "Tactful and warm", "rationale": "The language style of the entry is more euphemistic and warm. It asks each other in a convenient way whether they are often involved in social events, such as watching movies, sports games, etc., and expresses an invitation and encouragement that makes people feel friendly and comfortable. Moreover, it reduces the resistance of respondents and makes them more accessible to their cooperation and support."}, "time_dimension": {"answer": "Present", "rationale": "The word \"normally\" in \"we watch movies or sports games, go out on the field, etc.\" means that there is a regular, customary behaviour, and the phrase as a whole uses the general current, which means that this behaviour has not only occurred in the past, but may still be ongoing. So the time dimension of this questionnaire entry is now."}, "sentence_type": "Affirmative declarative sentence", "pos": {"nouns": {"Film": 1, "Sports competitions": 1, "A field trip": 1}, "verbs": {"Look": 1, "Regular": 1, "Out": 1}, "adverbs": {"Wait": 1}, "numerals": {"37": 1}}}, {"content": "38. We consider bribery to be an acceptable phenomenon.", "word_count": 16, "label": "", "sentence_structure": "Subject鈥損redicate", "subjective_objective": {"answer": "Subjective", "rationale": "Because \"think\" is an individual's view and attitude, it's a subjective description."}, "sentence_sentiment": {"answer": "Negative", "rationale": "Because \"bribery\" is against the laws and regulations and morality, it is unacceptable."}, "language_style": {"answer": "Calm and direct", "rationale": "The entry expresses a view of the phenomenon of bribery in a direct and non-emotional language, without using euphemism or subtlety, and is more objective and calm."}, "time_dimension": {"answer": "Past", "rationale": "Based on the time words \"considered\" and \"acceptable phenomenon\" in the input sentence, it can be inferred that the entry relates to the views of the respondent on the phenomenon of bribery in the past and is therefore judged to be the past."}, "sentence_type": "Negative declarative sentence", "pos": {"nouns": {"Bribing": 1, "phenomena": 1}, "verbs": {"I think": 1, "Accept": 1}, "numerals": {"38": 1}}}, {"content": "39. We're very concerned about punctuality.", "word_count": 12, "label": "", "sentence_structure": "Subject鈥揷opula鈥損redicative", "subjective_objective": {"answer": "Subjective", "rationale": "This is because patients have subjective perceptions of their family."}, "sentence_sentiment": {"answer": "Neutral", "rationale": ""}, "language_style": {"answer": "Tactful and warm", "rationale": "This is a more euphemistic expression that guides respondents to think about whether they have a similar view by describing family values, rather than directly asking or ordering each other to complete their mission on time."}, "time_dimension": {"answer": "Present", "rationale": "The keywords are \"are very sensitive\" and \"do things on time\", which indicate attitudes and requirements of conduct, and these may be ongoing or customary. Therefore, the entry should be judged as \"now\"."}, "sentence_type": "Affirmative declarative sentence", "pos": {"nouns": {"Our home": 1, "Yes": 1}, "verbs": {"Do it": 1, "Yes": 1, "Value": 1, "On time": 1}, "numerals": {"39": 1}}}, {"content": "There's a certain way we do anything in our family.", "word_count": 14, "label": "", "sentence_structure": "Subject鈥搗erb鈥揷omplement", "subjective_objective": {"answer": "Objective", "rationale": "The objective fact is not about the patient鈥檚 subjective attitude or thoughts. The entry is therefore objective."}, "sentence_sentiment": {"answer": "Neutral", "rationale": "The entry describes a fact that describes the habits and ways in which the family does things, without expressing a positive or negative emotional tendency."}, "language_style": {"answer": "Calm and direct", "rationale": "The language style of this entry is not too much speech-tale or emotional, and is more objective. It is a direct expression of the subject of asking the other family whether there are some regular ways of acting, and it is not expressed in euphemism, in subtle or vague language. In the light of the above, the language style of this questionnaire entry can be judged as \"cool and direct.\""}, "time_dimension": {"answer": "Present", "rationale": ""}, "sentence_type": "Affirmative declarative sentence", "pos": {"nouns": {"Modalities": 1, "Yes": 1, "Home": 1, "Fixed": 1}, "verbs": {"have": 1, "Do it": 1}, "adverbs": {"Fixed": 1}, "numerals": {"40": 1}, "pronouns": {"we": 1}}}, {"content": "When there's a family problem, few people volunteer.", "word_count": 13, "label": "", "sentence_structure": "Subject鈥搗erb鈥搊bject", "subjective_objective": {"answer": "Objective", "rationale": "Because \"when there is a family problem, few people volunteer to do it\" is an observable phenomenon or fact, and it is objective. It is subjective to examine patients' subjective thoughts, such as their feelings and perceptions."}, "sentence_sentiment": {"answer": "Negative", "rationale": "Because \"very few people volunteer\" in the entry is negative."}, "language_style": {"answer": "Calm and direct", "rationale": "The entry expresses the theme \"very few people come home to help themselves\" in a direct manner, without too much reticent language and with a more objective and direct tone."}, "time_dimension": {"answer": "Present", "rationale": "The entry asks if the person tested is often confronted with a lack of active help in family matters, so the time is now judged. The basis of the judgement is: \"very few people volunteer to do it\" describes a situation of status or custom, not past experience or future results."}, "sentence_type": "Negative declarative sentence", "pos": {"nouns": {"Things": 1, "person": 2}, "verbs": {"Voluntary": 1, "Do it": 1}, "adjectives": {"Very few": 1}, "numerals": {"41": 1}, "pronouns": {"Home": 1}}}, {"content": "42. Family members often express their feelings in public", "word_count": 18, "label": "", "sentence_structure": "Subject鈥搗erb鈥揷omplement", "subjective_objective": {"answer": "Subjective", "rationale": "The emotional expression of family members is the patient ' s subjective feeling and understanding."}, "sentence_sentiment": {"answer": "Positive", "rationale": "Because it expresses the ability to express feelings among family members in public, it is a positive act."}, "language_style": {"answer": "Tactful and warm", "rationale": "The language style of the entry is more euphemistic and sweet, and by using the term \"often expressing feelings in public\" it makes questions more gentle and sensitive to emotional communication. It makes people comfortable and encourages respondents to share their thoughts and feelings more openly."}, "time_dimension": {"answer": "Present", "rationale": "\"Real\" and \"public expression\" in \"family members often express their feelings in public\" indicate that this is a customary act and that it is happening, so we can judge that it is a problem now."}, "sentence_type": "Affirmative declarative sentence", "pos": {"nouns": {"Family members": 1, "Emotion": 1}, "verbs": {"Regular": 1, "Openly": 1, "Expression": 1, "Between them": 1}, "adjectives": {"Public": 1}, "adverbs": {"Regular": 1}, "numerals": {"42": 1}}}, {"content": "43. Frequent mutual blame and criticism among family members", "word_count": 14, "label": "", "sentence_structure": "Subject鈥搗erb鈥揷omplement", "subjective_objective": {"answer": "Objective", "rationale": "Because blame and criticism are objective events, even if the patient considers this to be a negative family environment."}, "sentence_sentiment": {"answer": "Negative", "rationale": "Because the \"mutual blame and criticism\" in the entry is negative."}, "language_style": {"answer": "Tactful and warm", "rationale": "The entry seeks to create a safe and comfortable atmosphere, through the use of moderate language and expression, in which respondents are encouraged to share their thoughts and feelings. At the same time, the language of direct accusations or criticism is avoided, making the questionnaire more acceptable and accessible."}, "time_dimension": {"answer": "Present", "rationale": "The word \"normal\" in the entry denotes a regular, customary act, and the \"mutual blame and criticism\" act not only has occurred in the past, but may still be ongoing. Thus, the temporal dimension is now judged."}, "sentence_type": "Negative declarative sentence", "pos": {"nouns": {"Family members": 1, "Blame": 1, "Criticism": 1}, "verbs": {"Regular": 1, "- Yeah, yeah": 1, "Blame": 1, "Criticism": 1}, "adverbs": {"Regular": 1, "- Yeah, yeah": 1}, "numerals": {"43": 1}}}, {"content": "44. Family members rarely take into account the views of the rest of the family", "word_count": 19, "label": "", "sentence_structure": "Subject鈥搗erb鈥揷omplement", "subjective_objective": {"answer": "Subjective", "rationale": "Because \"very little consideration for the views of the rest of the family\" is subjective observation and evaluation of the patient."}, "sentence_sentiment": {"answer": "Negative", "rationale": "Because the \"very little consideration for the views of the rest of the family\" in the entry is negative."}, "language_style": {"answer": "Calm and direct", "rationale": "The entry is presented in a direct manner, is not subject to too many rhetorical or emotional renderings, and is more concise. At the same time, the article is more objective, does not evaluate or criticize the interviewee, making the question more neutral and professional. It is therefore judged that the language style of the entry is \"cool and direct\"."}, "time_dimension": {"answer": "Present", "rationale": "The entry describes the current family situation of the person being tested, and the keyword \"very few\" and \"consider\" indicates that the act is occurring now. So the time dimension of the entry is now."}, "sentence_type": "Negative declarative sentence", "pos": {"nouns": {"Family members": 1, "Things": 1, "Observations": 1}, "verbs": {"Do it": 1, "Consider": 1}, "adjectives": {"Less": 1, "Other": 1}, "adverbs": {"Very few": 1}, "numerals": {"44": 1}}}, {"content": "We're always thinking about ourselves and forcing ourselves to do what we can to do better than once.", "word_count": 27, "label": "", "sentence_structure": "Subject鈥損redicate", "subjective_objective": {"answer": "Subjective", "rationale": "Because \"thinking about yourself\" and \"forced yourself to do better than once\" are the thoughts and attitudes of the patients."}, "sentence_sentiment": {"answer": "Positive", "rationale": "The article ' s purpose is to express positive attitudes and behaviour that are self-promoting and endeavouring to advance."}, "language_style": {"answer": "Calm and direct", "rationale": "The language is more consistent with the rigour and scientific nature of the questionnaire, and more able to guide the interviewee to reflect and give real and accurate answers."}, "time_dimension": {"answer": "Present", "rationale": "\"We always\" means regular, customary behaviour, \"research ourselves\" and \"forced ourselves to do what is best for one time\" are acts that are currently being done. So, the questionnaire entry is to ask the testee about the behaviour and mindset of the moment, and the time dimension is now."}, "sentence_type": "Affirmative declarative sentence", "pos": {"nouns": {"Retrospect": 1, "self": 2, "Things": 1}, "verbs": {"Do it": 2, "鈾?And I'm gonna be 鈾?: 1, "Okay": 2, "Coercion": 1, "Try": 1, "- Put it on": 2, "One at a time": 1}, "adverbs": {"Always": 1, "Constantly": 1, "One at a time": 1}, "numerals": {"45": 1}, "classifiers": {"individual": 1}, "pronouns": {"we": 1}}}, {"content": "46. We rarely discuss issues related to scientific and technological knowledge.", "word_count": 17, "label": "", "sentence_structure": "Subject鈥搗erb鈥搊bject", "subjective_objective": {"answer": "Subjective", "rationale": "According to the given definition, the question is subjective if it concerns the patient's subjective feelings or attitudes; if it is a description of objective facts, it is objective. Thus, \"We rarely discuss issues related to scientific and technological knowledge\" is a subjective issue, as it concerns the patient's attitudes and perceptions."}, "sentence_sentiment": {"answer": "Neutral", "rationale": "Because the entry does not have any emotional color."}, "language_style": {"answer": "Calm and direct", "rationale": "The language style of the entry is more straightforward, with no excessive rhetoric or emotional colour, and an objective statement of fact, which is more calm."}, "time_dimension": {"answer": "Past", "rationale": "Time dimension: The key words of the past: little discussion, little explanation of the problems associated with scientific and technological knowledge: The entry asks about the behaviour of the subjects in the past, i.e. whether they often discuss issues related to scientific and technological knowledge. Because the word \"very rarely\" is used to indicate that such behaviour was not common in the past. The answer is therefore the past."}, "sentence_type": "Negative declarative sentence", "pos": {"nouns": {"we": 1, "Scientific and technical knowledge": 1, "Problem": 1}, "verbs": {"Discussion": 1, "Related": 1, "Very few": 1}, "adjectives": {"Technology": 1}, "adverbs": {"In terms of": 1}, "numerals": {"46": 1}, "pronouns": {"Here": 1}}}, {"content": "47. Everyone in our family is particularly interested in one or two entertainment events.", "word_count": 18, "label": "", "sentence_structure": "Subject鈥搗erb鈥搊bject", "subjective_objective": {"answer": "Objective", "rationale": "The interest of family members is objective, and their subjective attitude is not to be taken into account."}, "sentence_sentiment": {"answer": "Positive", "rationale": "This item is a manifestation of the family ' s solidarity and harmony, as it expresses the interests shared by the family."}, "language_style": {"answer": "Tactful and warm", "rationale": "The entry is more gentle and considerate in its tone of being asked by a friendly and caring interview of the interests of the family members of the other party, and is not expressed in direct orders or criticism."}, "time_dimension": {"answer": "Present", "rationale": "The keyword \"we\" and \"special interest\" all indicate that this is a description of the current family situation, and therefore the judgement is now."}, "sentence_type": "Affirmative declarative sentence", "pos": {"nouns": {"person": 1, "Recreational activities": 1}, "verbs": {"Special": 1, "Interested": 1}, "adjectives": {"Every one": 1, "Both": 1}, "numerals": {"47": 1, "1锝?": 1}, "classifiers": {"Item": 2}, "pronouns": {"Our home": 1}}}, {"content": "48. We believe that, whatever the case, the elders should be advised by the elders.", "word_count": 21, "label": "", "sentence_structure": "Subject鈥搗erb鈥搊bject", "subjective_objective": {"answer": "Subjective", "rationale": "Because \"think\" expresses the subjective attitude and the perception of researchers."}, "sentence_sentiment": {"answer": "Negative", "rationale": "Because the entry expresses a mandatory attitude that, whatever the circumstances, the ascendants must accept the advice of the elders, an attitude that may create pressure and feelings of discomfort for the ascendants."}, "language_style": {"answer": "Calm and direct", "rationale": "The entry is more straightforward, not using too many rhetorical or euphemisms, but rather presenting the point in a clear and concise way. This is more in keeping with the characteristics of the questionnaire, which can convey the nature and intent of the problem directly."}, "time_dimension": {"answer": "Present", "rationale": "The entry describes a view or belief, does not address a specific time frame and therefore the temporal dimension cannot be judged as past, present or future."}, "sentence_type": "Affirmative declarative sentence", "pos": {"nouns": {"we": 1, "Sir": 1, "Senior": 1, "Construction": 1}, "verbs": {"I think": 1, "I think so": 2, "Accept": 1, "Give": 1}, "adjectives": {"How's it going": 1}, "adverbs": {"Both": 1}, "numerals": {"48": 1}, "pronouns": {"Here": 1}}}, {"content": "Our family often changes their plans.", "word_count": 14, "label": "", "sentence_structure": "Subject鈥搗erb鈥搊bject", "subjective_objective": {"answer": "Subjective", "rationale": "Because \"often changing their plans\" is the patient's observation and understanding of his family members, and it's a subjective idea and perception."}, "sentence_sentiment": {"answer": "Negative", "rationale": "Because the \"often changing their plans\" act in the entry is negative."}, "language_style": {"answer": "Tactful and warm", "rationale": "It uses a more gentle and inviting tone, rather than a direct statement or order, which is more personal and interactive. And it is more natural to say \"our family.\""}, "time_dimension": {"answer": "Present", "rationale": ""}, "sentence_type": "Affirmative declarative sentence", "pos": {"nouns": {"Our family": 1, "they": 1, "Planned": 1}, "verbs": {"Often": 1, "Change": 1}, "adverbs": {"Often": 1}, "numerals": {"49": 1}, "pronouns": {"Our home": 1}}}, {"content": "Our family has a strong emphasis on living by regular rules and family rules.", "word_count": 20, "label": "", "sentence_structure": "Subject鈥搗erb鈥搊bject", "subjective_objective": {"answer": "Objective", "rationale": "Because \"emphasis on the observance of fixed living patterns and family rules\" is a rule or requirement of the family and is determined by the family members together, it is considered from an objective perspective."}, "sentence_sentiment": {"answer": "Positive", "rationale": "Because the purpose of the action \"to emphasize adherence to fixed living patterns and family rules\" in the entry is positive, it can foster a good habits and a sense of discipline for children."}, "language_style": {"answer": "Tactful and warm", "rationale": "The entry uses a more euphemistic language style, such as \"Our Home\" instead of \"Your Home\" in general, and emphasizes the \"very stressful\" tone. This expression is more friendly and easier for respondents to accept and cooperate with the investigation."}, "time_dimension": {"answer": "Past", "rationale": "The keyword \"Our Family is Strongly Focused\" indicates that the entry describes the past of the family, and the temporal dimension is the past."}, "sentence_type": "Affirmative declarative sentence", "pos": {"nouns": {"Family": 1, "Lifestyle": 1, "Family rules": 1}, "verbs": {"Emphasizing": 1, "Yes": 1, "Compliance": 1, "have": 1}, "adjectives": {"Very": 1}, "numerals": {"50": 1}, "pronouns": {"we": 1}}}, {"content": "Family members always support each other with heart.", "word_count": 14, "label": "", "sentence_structure": "Subject鈥搗erb鈥揷omplement", "subjective_objective": {"answer": "Subjective", "rationale": "Because \" wholehearted support\" is an emotional attitude that is subjectively felt and evaluated by patients. While this support may be objectively real, the subject is one of patient attitudes and perceptions about family relations, and therefore subject to subjective scrutiny."}, "sentence_sentiment": {"answer": "Positive", "rationale": "Because the act of \"regardless solidarity\" reflects a positive family atmosphere and emotional ties between members, and is positive."}, "language_style": {"answer": "Tactful and warm", "rationale": "This entry, in a moderate and positive language, expresses praise and encouragement for the practice of mutual support among family members, is more positive and is not too direct or hard. Such expression makes it easier for the interviewee to feel comfortable and to answer questions."}, "time_dimension": {"answer": "Present", "rationale": ""}, "sentence_type": "Affirmative declarative sentence", "pos": {"nouns": {"Family members": 2, "Support": 1}, "verbs": {"Both": 1, "Always": 1, "I'm not sure": 1, "- Yeah, yeah": 1, "Support": 1}, "adjectives": {"I'm not sure": 1}, "adverbs": {"Always": 1}, "numerals": {"51": 1}}}, {"content": "If you say you're unhappy about your family, someone will feel uncomfortable.", "word_count": 21, "label": "", "sentence_structure": "Subject鈥揷opula鈥損redicative", "subjective_objective": {"answer": "Subjective", "rationale": "Because \"can feel uncomfortable\" is a subjective feeling."}, "sentence_sentiment": {"answer": "Negative", "rationale": "Because the emotional reaction in the entry is negative."}, "language_style": {"answer": "Tactful and warm", "rationale": "The entry is more softly phrased, using a way of asking questions rather than directly speaking or ordering. It raises questions in a way that is concerned and understanding, and that allows the interviewee to share his thoughts and feelings, which is more likely to trigger resonance and active participation by the interviewee. At the same time, this euphemism avoids the possibility that language that is too direct or conflicting may lead to a misgiving or aversion."}, "time_dimension": {"answer": "Present", "rationale": "The entry uses the word \"will\" to express a hypothetical situation in which, in the current family environment, it may be uncomfortable to raise grievances. The questionnaire entry is therefore asking about the current behaviour or mentality of the person being tested."}, "sentence_type": "Negative declarative sentence", "pos": {"nouns": {"Home": 1, "Dissatisfied": 1, "person": 1, "Family matters": 1}, "verbs": {"Say it": 1, "I think": 1, "I'm not feeling well": 1}, "numerals": {"52": 1}}}, {"content": "53. Family members sometimes fight each other.", "word_count": 10, "label": "", "sentence_structure": "Subject鈥搗erb鈥揷omplement", "subjective_objective": {"answer": "Objective", "rationale": "Since the existence of conflict and fighting between family members is an objective fact, even if the patient has subjective feelings and perceptions of it, the reality is objective."}, "sentence_sentiment": {"answer": "Negative", "rationale": "Because the \"fights\" in the entries are negative."}, "language_style": {"answer": "Tactful and warm", "rationale": "The entry uses a more moderate formulation, avoiding direct accusations or conflicting attacks on family members, and instead leads the respondents to the question in a way that is both exploratory and understandable, making it easier to accept and answer. At the same time, the term \"sometimes\" also indicates that this phenomenon is not common, further reducing the possibility of negative emotions."}, "time_dimension": {"answer": "Present", "rationale": "The keyword \"sometimes\" indicates that the act is not a regular one, but it is not an occasional one, but a situation that sometimes occurs, so the temporal dimension is now judged."}, "sentence_type": "Negative declarative sentence", "pos": {"nouns": {"Family members": 1, "Fight": 1}, "verbs": {"Sometimes": 1, "- Yeah, yeah": 1, "Fight": 1}, "adverbs": {"Sometimes": 1}, "numerals": {"53": 1}}}, {"content": "54. Family members rely on family help to resolve their difficulties.", "word_count": 22, "label": "", "sentence_structure": "Subject鈥搗erb鈥揷omplement", "subjective_objective": {"answer": "Subjective", "rationale": "Because \"dependance on family help\" is the subjective attitude and perception of the patient towards family members."}, "sentence_sentiment": {"answer": "Negative", "rationale": "Because the action of \"dependent family help\" in the entry is negative."}, "language_style": {"answer": "Tactful and warm", "rationale": "The entry is not based on a strong emotional or command tone, but rather on a moderate and understandable expression of concern and concern for the family situation of the interviewee. This expression is more likely to trigger resonance and positive responses from the interviewee."}, "time_dimension": {"answer": "Present", "rationale": ""}, "sentence_type": "Affirmative declarative sentence", "pos": {"nouns": {"Family members": 1, "Family": 1, "Help": 1, "Difficulties": 1}, "verbs": {"Dependency": 1, "Resolve": 1}, "numerals": {"54": 1}}}, {"content": "55. Family members are less concerned about promotion, academic achievement, etc.", "word_count": 19, "label": "", "sentence_structure": "Subject鈥搗erb鈥搊bject", "subjective_objective": {"answer": "Objective", "rationale": "Because \"family members are not concerned about promotion, learning achievement\" is what the surveyer observes, not about the patient's subjective feelings or attitudes."}, "sentence_sentiment": {"answer": "Negative", "rationale": "Because the \"less concerned\" in the entry expresses negative emotions and attitudes."}, "language_style": {"answer": "Calm and direct", "rationale": "The entry presents a point in the form of a direct statement that family members are less concerned about the promotion and academic achievement of individuals."}, "time_dimension": {"answer": "Past", "rationale": "At the same time, the keyword \"family member\" and \"less care\" point to what happened in the past."}, "sentence_type": "Negative declarative sentence", "pos": {"nouns": {"Family members": 1, "Promotion": 1, "Learning achievement": 1}, "verbs": {"Not really": 1}, "adverbs": {"very": 1}, "numerals": {"55": 1}}}, {"content": "56. There are people playing instruments in their homes.", "word_count": 7, "label": "", "sentence_structure": "Subject鈥搗erb鈥揷omplement", "subjective_objective": {"answer": "Objective", "rationale": "Because \"someone plays an instrument\" is an objective fact for family members."}, "sentence_sentiment": {"answer": "Neutral", "rationale": "Because the entry does not have any emotional color."}, "language_style": {"answer": "Tactful and warm", "rationale": "The entry uses a more euphemistic approach to the question, \"Is anyone playing with an instrument at home?\", which is relatively polite and gentle, and is more acceptable and answered. At the same time, the language style of the article does not have too much emotional or direct command expression, giving people a sense of affection and friendship."}, "time_dimension": {"answer": "Present", "rationale": "In the entry, \"Someone at home plays an instrument\" describes an action or state that is currently in progress and therefore judged to be now."}, "sentence_type": "Affirmative declarative sentence", "pos": {"nouns": {"Home": 1, "person": 1, "Instrument": 1}, "verbs": {"Play": 1}, "numerals": {"56": 1}}}, {"content": "57. Family members do not often engage in recreational activities except for work studies", "word_count": 18, "label": "", "sentence_structure": "Subject鈥搗erb鈥揷omplement", "subjective_objective": {"answer": "Objective", "rationale": "Since the subject is described as the recreational activities of family members, it does not involve the patient ' s own subjective feelings or attitudes and is therefore an objective examination."}, "sentence_sentiment": {"answer": "Negative", "rationale": "Because the entry 鈥渦nfrequent recreational activities鈥?expresses a negative attitude or behaviour implying that family members lack time for recreation and relaxation, which may affect their physical and mental health and family relations."}, "language_style": {"answer": "Calm and direct", "rationale": "The language style of the entry is relatively simple, the issue of research is directly expressed, there is no excessive rhetorical and emotional rendering, and the tone is more calm and objective."}, "time_dimension": {"answer": "Present", "rationale": ""}, "sentence_type": "Negative declarative sentence", "pos": {"nouns": {"Family members": 1, "Work": 1, "Learning": 1, "Recreational activities": 1}, "verbs": {"Divide": 1, "Conduct": 1}, "adverbs": {"Not often": 1}, "numerals": {"57": 1}}}, {"content": "58. Family members voluntarily go to public sanitation", "word_count": 15, "label": "", "sentence_structure": "Subject鈥搗erb鈥搊bject", "subjective_objective": {"answer": "Objective", "rationale": "Since it is an objective fact that family members are voluntarily involved in public sanitation, there is no need for subjective judgement or emotional colour of the patient."}, "sentence_sentiment": {"answer": "Positive", "rationale": "Because the entry \"family members voluntarily go to public sanitation\" is positive."}, "language_style": {"answer": "Tactful and warm", "rationale": "The entry is more adept and interactive when it is spoken in a gentle and inviting manner, rather than by direct statements or orders."}, "time_dimension": {"answer": "Present", "rationale": "According to the description of \"all of them voluntarily\" and \"public sanitation\", this is a description of the current behaviour or state, so it can be judged that the entry is an inquiry into the present behaviour or condition of the person being tested."}, "sentence_type": "Affirmative declarative sentence", "pos": {"nouns": {"Family members": 1, "Public sanitation": 1}, "verbs": {"Both": 1, "Go on": 1, "Do it": 1}, "adverbs": {"Voluntary": 1}, "numerals": {"58": 1}}}, {"content": "59. Family members carefully keep their rooms clean.", "word_count": 16, "label": "", "sentence_structure": "Subject鈥搗erb鈥揷omplement", "subjective_objective": {"answer": "Objective", "rationale": "Because it is an objective fact to keep their rooms clean, the seriousness of family members is merely the patient ' s subjective attitude."}, "sentence_sentiment": {"answer": "Positive", "rationale": "Because the entry \"family members carefully keep their rooms clean\" is positive."}, "language_style": {"answer": "Tactful and warm", "rationale": "The language style of the entry is euphemistic. It guides the interviewer to reflect on his or her behaviour in a gentle and inviting tone, rather than directly ordering or criticizing his or her behaviour. Moreover, the expression \"resisting seriously\" has an encouraging and positive meaning, aimed at making the interviewee feel the importance of being in harmony with the family and of personal responsibility."}, "time_dimension": {"answer": "Past", "rationale": "The keywords \"serious\" and \"maintenance\" are all describing the current state of the subject, without any obvious past or future hints, and can therefore be defined as current behaviour or attitude."}, "sentence_type": "Affirmative declarative sentence", "pos": {"nouns": {"Family members": 1, "self": 1, "Room": 1, "Clean": 1}, "verbs": {"Keep it real": 1}, "adverbs": {"Land": 1}, "numerals": {"59": 1}}}, {"content": "60. Family members are free to leave at night without prior consultation with their families.", "word_count": 21, "label": "", "sentence_structure": "Subject鈥搗erb鈥揷omplement", "subjective_objective": {"answer": "Objective", "rationale": "This is an objective description of the rules or regulations governing the family and does not involve subjective attitudes or feelings of the patient."}, "sentence_sentiment": {"answer": "Negative", "rationale": "The act of \"without prior consultation with family\" in the entry may result in tension in family relations and jeopardize the harmonious stability of the family."}, "language_style": {"answer": "Calm and direct", "rationale": "The language style of the entry is a calm and straightforward type. It uses objective, concise language, which directly describes the content of the survey, without excessive emotional or rhetorical tact, and gives people a sense of reason and seriousness. At the same time, it is sound, without any subjective bias, and meets the basic requirements of the questionnaire study."}, "time_dimension": {"answer": "Future", "rationale": ""}, "sentence_type": "Negative declarative sentence", "pos": {"nouns": {"Family members": 2, "Night": 1, "- Let's talk": 1}, "verbs": {"Yeah": 1, "Out of the house at random": 1, "Before": 1, "With family": 1}, "adverbs": {"No, I don't": 1}, "numerals": {"60": 1}, "pronouns": {"they": 1}}}, {"content": "61. The collective spirit of our family is very small.", "word_count": 10, "label": "", "sentence_structure": "Subject鈥搗erb鈥揷omplement", "subjective_objective": {"answer": "Subjective", "rationale": "Because \"the collective spirit of our family is rarely\" is a subjective assessment or opinion."}, "sentence_sentiment": {"answer": "Negative", "rationale": "Because the entry is a negative expression of \"a little collective spirit\"."}, "language_style": {"answer": "Tactful and warm", "rationale": "The entry is more personal and interactive in its expression, in a gentle and inviting tone, rather than in direct statements or orders. At the same time, the first person 's proxies 'we' make the problem more personal and domestic, and more resonant and thought-provoking for the respondents."}, "time_dimension": {"answer": "Present", "rationale": "According to the analysis of the context, the entry of the questionnaire asked the testee about his or her collective spirit, which is judged by the temporal dimension as the present state and description."}, "sentence_type": "Negative declarative sentence", "pos": {"nouns": {"Our home": 1, "Collective spirit": 1}, "verbs": {"Very few": 1}, "numerals": {"61": 1}}}, {"content": "Our family can talk openly about the economy of our family.", "word_count": 17, "label": "", "sentence_structure": "Subject鈥搗erb鈥揷omplement", "subjective_objective": {"answer": "Subjective", "rationale": "It is a subjective examination, as it is the perception and attitude of individuals that speak of openness."}, "sentence_sentiment": {"answer": "Positive", "rationale": "Because the \"open talk about the economy of the family\" is positive."}, "language_style": {"answer": "Tactful and warm", "rationale": "The entry is based on the following: - The phrase is used in an invitation tone, with a softer word, and in a way that makes it easier for readers to resonate and participate. - The phrase \"publicly talk\" also demonstrates the author's attitude in trying to create an open and transparent communication environment, not direct orders or demands. This expression is more euphemismal and warmer in language style."}, "time_dimension": {"answer": "Present", "rationale": ""}, "sentence_type": "Affirmative declarative sentence", "pos": {"nouns": {"Family": 1, "Economy": 1, "Problem": 1}, "verbs": {"Talk openly": 1}, "adverbs": {"Yeah": 1}, "numerals": {"62": 1}, "pronouns": {"Our home": 1}}}, {"content": "63. When the views of family members diverged, we avoided it to keep it peaceful.", "word_count": 25, "label": "", "sentence_structure": "Subject鈥搗erb鈥搊bject", "subjective_objective": {"answer": "Subjective", "rationale": "This is the patient ' s perception and attitude to the way his or her family is treated when they are facing a disagreement."}, "sentence_sentiment": {"answer": "Negative", "rationale": "Because the purpose of the act of \"avoiding disagreement\" in the entry is to avoid conflict, it is somewhat negative."}, "language_style": {"answer": "Tactful and warm", "rationale": "The entry is more euphemistic and gentle. It uses an invitation tone to guide the interviewee to reflect on his behaviour and does not directly state or order the interviewee to do so. This expression is more personal and interactive, and it is better able to resonate and think."}, "time_dimension": {"answer": "Past", "rationale": "So the answer is \"the past\"."}, "sentence_type": "Negative declarative sentence", "pos": {"nouns": {"Family members": 1, "Observations": 1, "Difference": 1, "# And the twilight #": 1}, "verbs": {"Generate": 1, "Avoidance": 1, "Hold on": 1}, "adverbs": {"Always": 1, "Both": 1}, "numerals": {"63": 1}, "pronouns": {"it": 1}}}, {"content": "64. Family members want their families to solve their problems independently", "word_count": 15, "label": "", "sentence_structure": "Subject鈥搗erb鈥搊bject", "subjective_objective": {"answer": "Objective", "rationale": "Because the hope is the subjective attitude of family members, the subject is objective in examining whether such attitudes affect the patient ' s thinking."}, "sentence_sentiment": {"answer": "Neutral", "rationale": "Because the entry describes a situation and does not express positive or negative feelings."}, "language_style": {"answer": "Tactful and warm", "rationale": "The language style of this entry is more euphemistic and respectful, with more polite and gentle language, and with an attitude of understanding and support, without direct orders or criticism, which is more conducive to the positive responses of respondents."}, "time_dimension": {"answer": "Present", "rationale": "The \"hope\" in this entry represents a current state or expectation, does not relate to past or future time dimensions and therefore is judged to be the present."}, "sentence_type": "Affirmative declarative sentence", "pos": {"nouns": {"Family members": 1, "Problem": 1, "Home": 1, "person": 2}, "verbs": {"Hope": 1, "Resolve": 1, "Independence": 1}, "numerals": {"64": 1}, "pronouns": {"Lee": 1, "Home": 1}}}, {"content": "Our family is not very active in achieving what they have done.", "word_count": 16, "label": "", "sentence_structure": "Subject鈥搗erb鈥揷omplement", "subjective_objective": {"answer": "Subjective", "rationale": "Because \"positive\" is a subjective attitude and view, the questionnaire is intended to describe the attitude of the family members of the patient towards achievement and is therefore a subjective examination."}, "sentence_sentiment": {"answer": "Negative", "rationale": "Because the expression \"not so active\" is negative."}, "language_style": {"answer": "Calm and direct", "rationale": "The entry presents in an objective and direct manner the attitude of the members of the family of the person under investigation towards achievement, without excessive rhetoric and emotional rendering."}, "time_dimension": {"answer": "Present", "rationale": "The keywords \"our family\" and \"achievement\" and \"not so active\" point to the current family situation."}, "sentence_type": "Negative declarative sentence", "pos": {"nouns": {"Our home": 1, "person": 1, "Achievements": 1}, "verbs": {"to": 1, "Access": 1, "Not so much": 1, "Positive": 1}, "adverbs": {"Well": 1}, "numerals": {"65": 1}}}, {"content": "66. Family members often go to libraries", "word_count": 9, "label": "", "sentence_structure": "Subject鈥搗erb鈥搊bject", "subjective_objective": {"answer": "Objective", "rationale": "The blogger says that the government is not going to be able to get a doctor to take care of the patients because of the objective facts that patients experience in the library."}, "sentence_sentiment": {"answer": "Positive", "rationale": "Because the \"Relationship to Library\" is positive."}, "language_style": {"answer": "Tactful and warm", "rationale": "Because the entry leads the respondents to share information related to family members in an interview-based manner, the language is more accommodative and moderate and does not imply direct criticism or evaluation, and it is easier to obtain real feedback."}, "time_dimension": {"answer": "Present", "rationale": "The \"Retired\" move occurs now, and \"family members\" is the dominant expression, which means that the regular behaviour of family members is now taking place. So, the entry describes the current situation."}, "sentence_type": "Affirmative declarative sentence", "pos": {"nouns": {"Family members": 1, "Library": 1}, "verbs": {"Always": 1}, "adverbs": {"Regular": 1}, "numerals": {"66": 1}}}, {"content": "67. Family members sometimes take recreational studies according to their preferences or interests.", "word_count": 21, "label": "", "sentence_structure": "Subject鈥搗erb鈥搊bject", "subjective_objective": {"answer": "Objective", "rationale": "Because it is the objective behaviour of family members to participate in recreational studies according to their preferences or interests."}, "sentence_sentiment": {"answer": "Positive", "rationale": "Because the purpose of the entry is to \"take recreational studies according to the preferences or interests of the individual\"."}, "language_style": {"answer": "Tactful and warm", "rationale": "The entry is more personal and interactive when it is spoken in a gentle and inviting tone, rather than in direct statements or orders. At the same time, the word \"sometimes\" also reflects the inclusiveness and understanding of the questionnaire designers, which makes the interviewee comfortable and respectful."}, "time_dimension": {"answer": "Present", "rationale": "According to the keyword \"sometimes\" and \"personal hobbies or interests to participate in recreational studies\", the entry asks whether it is possible for family members of the person being tested to participate in recreational learning activities in accordance with their interests and preferences at this time, and therefore the temporal dimension of the purpose of the article can be judged to be the present."}, "sentence_type": "Affirmative declarative sentence", "pos": {"nouns": {"Family members": 1, "Personal hobby": 1, "Interest": 2, "Entertainment studies": 1}, "verbs": {"Participation": 1, "Press": 1}, "adverbs": {"Sometimes": 1}, "numerals": {"67": 1}}}, {"content": "Family members think they're gonna do what they're supposed to do.", "word_count": 17, "label": "", "sentence_structure": "Subject鈥搗erb鈥搊bject", "subjective_objective": {"answer": "Subjective", "rationale": "The question of whether the need to act in a moral capacity is considered personal and opinional by the interviewees."}, "sentence_sentiment": {"answer": "Positive", "rationale": "Because the purpose and value orientation of the act of \"damn morality\" in the entry is positive."}, "language_style": {"answer": "Tactful and warm", "rationale": "The language style of the entry is more euphemistic, expressing the views of family members by the word \"thinking\" without direct orders or requiring that the other party must comply with the moral precepts. At the same time, the term \"dependance\" is more moderate, expressing a insistence than a tough demand. The language style of the entry is therefore more euphemismal."}, "time_dimension": {"answer": "Past", "rationale": "The entry describes past perceptions or behaviour of family members, and the keyword \"considers\" and \"resistence\" indicates that this is a description of the past."}, "sentence_type": "Affirmative declarative sentence", "pos": {"nouns": {"Family members": 1, "Ethical precepts": 1, "Yes": 1}, "verbs": {"I think": 1, "Stay put": 1, "Go on": 1}, "adverbs": {"Both": 1}, "numerals": {"68": 1}}}, {"content": "69. In our home, the division of labour is clear.", "word_count": 14, "label": "", "sentence_structure": "Subject鈥揷opula鈥損redicative", "subjective_objective": {"answer": "Objective", "rationale": "Because \"a clear division of labour for everyone\" is the objective state and rule of family life."}, "sentence_sentiment": {"answer": "Neutral", "rationale": "Because the entry does not have any emotional color."}, "language_style": {"answer": "Tactful and warm", "rationale": "It does not directly ask about the feelings or circumstances of individuals, but rather describes a phenomenon or situation in a more objective and neutral way. It does not use a too strong or command-style tone, but rather adds more elements of care and understanding to its expression. This way makes it easier for the interviewee to feel comfortable and relaxed, and thus to complete the questionnaire."}, "time_dimension": {"answer": "Present", "rationale": "The word \"in our house\" indicates the current situation, and \"clear\" describes the current state of the division of family roles, which is the time dimension of the topic."}, "sentence_type": "Affirmative declarative sentence", "pos": {"nouns": {"Family": 1, "Everyone": 1, "Division of labour": 1}, "verbs": {"be": 1, "Clear": 1, "min": 1, "Workers": 1}, "adverbs": {"in": 1, "Our home": 1, "Every": 1, "individual": 1}, "numerals": {"69": 1}}}, {"content": "In our house, there are no strict rules to bind us.", "word_count": 16, "label": "", "sentence_structure": "Subject鈥搗erb鈥揷omplement", "subjective_objective": {"answer": "Subjective", "rationale": "Because strict rules are objective facts of a family, patients believe that they have no such rules in their family."}, "sentence_sentiment": {"answer": "Neutral", "rationale": "Because the entry describes a family situation, there is no apparent positive or negative emotional tendency."}, "language_style": {"answer": "Tactful and warm", "rationale": "The language style of the entry is more euphemistic, using terms such as \"we\" and \"no strict rules to bind us\", expressing a more relaxed, free atmosphere and family culture, and being friendly and sweet."}, "time_dimension": {"answer": "Present", "rationale": "The entry describes the current state of the family rules, using the expressions \"in our house\" and \"no strict rules to bind us\", which means that this is the current situation. So the time dimension of the entry is now."}, "sentence_type": "Negative declarative sentence", "pos": {"nouns": {"Our home": 1, "Rule": 1, "Constraints": 1}, "verbs": {"have": 1, "Strict": 1, "Come on": 1, "Constraints": 2}, "adjectives": {"Strict": 1}, "numerals": {"70": 1}, "pronouns": {"we": 1}}}, {"content": "71. Family members always get along with each other.", "word_count": 14, "label": "", "sentence_structure": "Subject鈥搗erb鈥揷omplement", "subjective_objective": {"answer": "Subjective", "rationale": "Because \"all the time you get along with each other\" is a subjective feeling and an evaluation that reflects the patient's perception of family relationships."}, "sentence_sentiment": {"answer": "Positive", "rationale": "Because the expression \"family members always fit in with each other\" is positive and indicates harmony and harmony in family relations."}, "language_style": {"answer": "Calm and direct", "rationale": "The entry directly asks if the relationship between the interviewee and family members is harmonious, is not overly adorational or emotional, and is more objective and calm."}, "time_dimension": {"answer": "Present", "rationale": ""}, "sentence_type": "Affirmative declarative sentence", "pos": {"nouns": {"Family members": 1, "You too": 1, "Always": 1}, "verbs": {"It's a good fit": 1}, "adverbs": {"Both": 1}, "numerals": {"71": 1}}}, {"content": "72. Family members speak to each other with great care to avoid hurting each other ' s feelings.", "word_count": 22, "label": "", "sentence_structure": "Subject鈥搗erb鈥揷omplement", "subjective_objective": {"answer": "Objective", "rationale": "Because the words and actions of family members are objective facts, not the patient ' s own subjective views."}, "sentence_sentiment": {"answer": "Positive", "rationale": "Because the action of \"Beware of hurting the other\" in the entry is positive."}, "language_style": {"answer": "Tactful and warm", "rationale": "This is a way to get the interviewee to resonate and cooperate."}, "time_dimension": {"answer": "Present", "rationale": "The entry asks how the family members of the testee live together, and the time dimension is now judged."}, "sentence_type": "Affirmative declarative sentence", "pos": {"nouns": {"Family members": 1, "Speech": 1, "Emotion": 1}, "verbs": {"Attention": 1, "Avoid": 1, "Hurt": 1}, "adverbs": {"Both": 1}, "numerals": {"72": 1}, "pronouns": {"The other side": 1}}}, {"content": "Family members often want to out-think each other.", "word_count": 12, "label": "", "sentence_structure": "Subject鈥搗erb鈥揷omplement", "subjective_objective": {"answer": "Subjective", "rationale": "Because \"one wants the other better\" is a subjective feeling and attitude of one person, an assessment of family relations, not an objective fact."}, "sentence_sentiment": {"answer": "Negative", "rationale": "Because the \"often wanting to outplay each other\" act in the entry is negative."}, "language_style": {"answer": "Calm and direct", "rationale": "The language style of the entry is calm and straightforward. It asks the interviewees in a straightforward way about their family relationship, without using too many rhetorical or emotional expressions, and in a more objective way."}, "time_dimension": {"answer": "Present", "rationale": "The time dimension of the topic is therefore now."}, "sentence_type": "Negative declarative sentence", "pos": {"nouns": {"Family members": 1, "You too": 1}, "verbs": {"Yes": 1, "More than anything": 1}, "adjectives": {"Regular": 1}, "numerals": {"73": 1}}}, {"content": "74. If family members often act alone, it hurts the feelings of others in the family.", "word_count": 22, "label": "", "sentence_structure": "Subject鈥搗erb鈥搊bject", "subjective_objective": {"answer": "Subjective", "rationale": "Because \"harming the feelings of the rest of the family\" is a patient's subjective judgment and concern."}, "sentence_sentiment": {"answer": "Negative", "rationale": "Because the \"harming the feelings of the rest of the family\" in the entry is negative."}, "language_style": {"answer": "Tactful and warm", "rationale": "The entry uses a more euphemistic language style to express the possible negative consequences and to try to draw the attention and reflection of the interviewees, rather than direct orders or accusations of the behaviour of the interviewees. This mild tone is more likely to make the interviewees more receptive and willing to change their behaviour."}, "time_dimension": {"answer": "Present", "rationale": "The keywords \"often acting alone\" and \"injuring the feelings of other family members\" indicate that this is a description of the current behaviour or state. Thus, the entry \"if family members often act alone, it hurts the feelings of other family members\" is about the present, that is, it refers to the current situation."}, "sentence_type": "Negative declarative sentence", "pos": {"nouns": {"Family members": 1, "Activities": 1, "Emotion": 1}, "verbs": {"Regular": 1, "Alone": 1, "Wounded": 1, "Home": 1, "Other": 1}, "adverbs": {"If": 1, "Yes": 1}, "numerals": {"74": 1}}}, {"content": "75. It's our old habit to work and enjoy.", "word_count": 14, "label": "", "sentence_structure": "Subject鈥揷opula鈥損redicative", "subjective_objective": {"answer": "Objective", "rationale": "Because \"the old habits of working and enjoying our family\" are a descriptive fact that does not require the patient's personal judgement or attitude and is therefore an objective examination."}, "sentence_sentiment": {"answer": "Neutral", "rationale": "Because the entry does not have any emotional color."}, "language_style": {"answer": "Calm and direct", "rationale": "The entry is more directly written, without any syllable or emotional accent, but simply describes the fact that \"the first work and the first enjoy is their old habits\". This is usually more objective, calm and direct."}, "time_dimension": {"answer": "Present", "rationale": "The keyword \"work before enjoying\" and \"old habits of our family\" suggest that this behaviour is the norm in family life today, so the entry describes the state of affairs."}, "sentence_type": "Affirmative declarative sentence", "pos": {"nouns": {"Work": 1, "Enjoy": 1, "we": 1, "Home": 1, "Custom": 1}, "verbs": {"be": 1, "Work first": 1, "Enjoy the rest": 1, "Old": 1, "have": 1}, "adverbs": {"Old": 1}, "numerals": {"75": 1}}}, {"content": "76. Television is more important in our home than reading.", "word_count": 13, "label": "", "sentence_structure": "Subject鈥揷opula鈥損redicative", "subjective_objective": {"answer": "Subjective", "rationale": "Because this entry relates to the situation of my home and the preference of the individual for values, it is a subjective examination."}, "sentence_sentiment": {"answer": "Negative", "rationale": "Because the entry expresses the importance attached to reading in contempt and television, which is a negative emotional tendency."}, "language_style": {"answer": "Calm and direct", "rationale": "The entry is simple, is not based on too many rhetorical or emotional expressions, is more objective and expresses the subject matter in a straightforward manner."}, "time_dimension": {"answer": "Present", "rationale": "Now the entry is describing current behavior or views, the keywords being \"in our house\" and \"watching television is more important than reading\" and all point to activities or perceptions that are now under way."}, "sentence_type": "Negative declarative sentence", "pos": {"nouns": {"Television": 1, "Read it": 1, "Books": 1}, "verbs": {"Look": 1, "- Yeah": 1, "More": 1, "Heavy": 1}, "adverbs": {"in": 1}, "numerals": {"76": 1}, "pronouns": {"we": 1}}}, {"content": "77. Family members often participate in social events outside the home during their leisure time", "word_count": 21, "label": "", "sentence_structure": "Subject鈥搗erb鈥搊bject", "subjective_objective": {"answer": "Objective", "rationale": "Because it is the behaviour of family members that is being examined, not their subjective idea."}, "sentence_sentiment": {"answer": "Positive", "rationale": "Because the behaviour of \"family members often participate in social activities outside the home during their leisure time\" is positive. This shows that family members have social needs and abilities and are willing to engage in social activities proactively, contributing to family cohesion and the development of relationships are positive effects."}, "language_style": {"answer": "Calm and direct", "rationale": "The entry is more directly linguistically, with no excessive emotional or rhetorical style, and is designed to explicitly ask whether family members of the person under investigation are often involved in social activities outside the home."}, "time_dimension": {"answer": "Present", "rationale": "The word \"common\" in \"family members often participate in social events outside the home during their leisure time\" denotes regular, customary behaviour, while the current pattern of \"participating\" and \"part-time\" is the general one, meaning that this behaviour has occurred in the past and may continue to occur."}, "sentence_type": "Affirmative declarative sentence", "pos": {"nouns": {"Family members": 1, "Amateur Time": 1, "Social events": 1}, "verbs": {"Participation": 1, "Always": 1}, "adverbs": {"Outside": 1, "of": 2, "Regular": 1}, "numerals": {"77": 1}}}, {"content": "78. We think that divorce is immoral, whatever it is.", "word_count": 16, "label": "", "sentence_structure": "Subject鈥搗erb鈥揷omplement", "subjective_objective": {"answer": "Subjective", "rationale": "This is a subjective opinion, because it is a personal value and ethical standard of the interviewees."}, "sentence_sentiment": {"answer": "Negative", "rationale": "This entry is negative and emotional because it expresses a negative and derogatory attitude towards divorce."}, "language_style": {"answer": "Calm and direct", "rationale": "The entry directly expresses the subjective position that divorce is immoral, that there is no excessive emotional or euphemism and that the language style is more direct."}, "time_dimension": {"answer": "Past", "rationale": "It merely expresses a view or attitude and does not relate to past, present or future acts or events."}, "sentence_type": "Negative declarative sentence", "pos": {"nouns": {"Divorce": 1, "Dow": 1, "Deok-hoon": 1}, "verbs": {"Recognition": 1, "Yes": 2, "Off": 1}, "adjectives": {"not": 1, "Dow": 1, "Deok-hoon": 1}, "adverbs": {"None": 1, "Comment": 1, "What": 1, "Sample": 1}, "numerals": {"78": 1}}}, {"content": "We don't have a plan for the money.", "word_count": 9, "label": "", "sentence_structure": "Subject鈥搗erb鈥揷omplement", "subjective_objective": {"answer": "Subjective", "rationale": "The idea of whether or not to spend money is a patient's subjective idea."}, "sentence_sentiment": {"answer": "Negative", "rationale": "Because the \"no plan\" in the entry is negative."}, "language_style": {"answer": "Calm and direct", "rationale": "The entry is a direct expression of the need to ask the other family whether it has been unplanned, not to use excessive rhetorical techniques or emotional rendering, and to be more objective in terms of tone."}, "time_dimension": {"answer": "Present", "rationale": "So, the time dimension should be now. So the output is: \"Now.\""}, "sentence_type": "Negative declarative sentence", "pos": {"nouns": {"Money": 1, "Planned": 1}, "verbs": {"Flowers": 1, "have": 1}, "adverbs": {"No, I'm not": 1}, "numerals": {"79": 1}, "pronouns": {"Our home": 1}}}, {"content": "80. The family rules and family rules are not changed.", "word_count": 17, "label": "", "sentence_structure": "Subject鈥揷opula鈥損redicative", "subjective_objective": {"answer": "Objective", "rationale": "Because the existence of family rules and their ability to change are objective facts, not subjective feelings or attitudes of patients."}, "sentence_sentiment": {"answer": "Negative", "rationale": "Because the expression \"can't change\" is negative."}, "language_style": {"answer": "Calm and direct", "rationale": "The language style of the entry is calm, straightforward, and there is no excessive rhetorical or emotional rendering. It describes a rule in an objective and clear manner, and does not attempt to persuade or induce the interviewee to accept it.\""}, "time_dimension": {"answer": "Present", "rationale": "\"Our laws or family rules\" is a terminological phrase describing the current state of the family, and \"not changing\" indicates that it is a well-established fact and can therefore be judged as the present."}, "sentence_type": "Negative declarative sentence", "pos": {"nouns": {"Our home": 1, "Lifestyle": 1, "Family rules": 1}, "verbs": {"be": 2, "Change": 1}, "adverbs": {"not": 1}, "numerals": {"80": 1}, "pronouns": {"I": 1}}}, {"content": "81. Every member of the family has always been given full attention.", "word_count": 17, "label": "", "sentence_structure": "Subject鈥搗erb鈥揷omplement", "subjective_objective": {"answer": "Objective", "rationale": "Because the title is to describe the fact that every member of the family is sufficiently concerned. It does not involve the individual ' s feelings or perceptions, and is therefore objective."}, "sentence_sentiment": {"answer": "Positive", "rationale": "Because the description \"Every member has always been given due attention\" in the entry is positive."}, "language_style": {"answer": "Tactful and warm", "rationale": "The entry uses a positive, individualized language style, emphasizing that every member of the family should be given adequate attention, with a high temperature and emotional tone of encouragement and support."}, "time_dimension": {"answer": "Present", "rationale": "The entry in the future expresses expectations and visions for the future, and the key words are \"every member has always been\" and \"fully concerned.\" They point to situations that have not yet occurred and that may arise in the future."}, "sentence_type": "Affirmative declarative sentence", "pos": {"nouns": {"Family": 1, "Every one": 1, "Members": 1, "Care": 1}, "verbs": {"Got it": 1, "Always": 1, "Care": 1}, "adjectives": {"Full": 1}, "numerals": {"81": 1}}}, {"content": "Our family often discusses family issues on their own.", "word_count": 18, "label": "", "sentence_structure": "Subject鈥搗erb鈥搊bject", "subjective_objective": {"answer": "Subjective", "rationale": "Because \"sponsible discussion\" and \"sensitive questions\" reflect patients' subjective attitudes and feelings."}, "sentence_sentiment": {"answer": "Positive", "rationale": "Because the purpose of the entry is to \"discuss family sensitivity\" spontaneously, which is conducive to communication and understanding among family members."}, "language_style": {"answer": "Tactful and warm", "rationale": "The entry uses a milder tone, does not directly evaluate or blame the problem, but rather is a proactive approach to channel the interviewee's experience and views. At the same time, by using the term \"our home\" it gives a warmer sense of affection and ease of engagement."}, "time_dimension": {"answer": "Present", "rationale": "The \"often\" in \"our house\" means \"high frequency,\" and the \"self-discussion\" and \"sensitive\" describe the behaviour or state that is occurring, so it can be judged that the entry is about the current behavior or mentality."}, "sentence_type": "Affirmative declarative sentence", "pos": {"nouns": {"Home": 1, "Problem": 1}, "verbs": {"Discussion": 1, "Auto-symmetrical": 1, "very": 1}, "adjectives": {"Sensitive": 1}, "adverbs": {"Regular": 1, "Land": 1}, "numerals": {"82": 1}}}, {"content": "83. Family quarrels sometimes out loud.", "word_count": 13, "label": "", "sentence_structure": "Subject鈥搗erb鈥搊bject", "subjective_objective": {"answer": "Objective", "rationale": "Since the entry in the questionnaire describes the behaviour that a patient may have in the face of a family conflict, it is an objective fact."}, "sentence_sentiment": {"answer": "Negative", "rationale": "Because the \"screaming\" in the entries is negative."}, "language_style": {"answer": "Tactful and warm", "rationale": ""}, "time_dimension": {"answer": "Past", "rationale": "The entry is for questioning the behaviour of the person who was tested in the event of a conflict between his family and the key word is \"sometimes\" and \"showing\" indicating that the act occurred in the past and may still be ongoing, and therefore can be judged as \"now\"."}, "sentence_type": "Affirmative declarative sentence", "pos": {"nouns": {"Family": 1, "Paradoxious": 1, "Argument": 1}, "verbs": {"Sometimes": 1, "Yes": 1, "Louder": 1}, "adverbs": {"Sometimes": 1, "Large": 1}, "numerals": {"83": 1}}}, {"content": "84. It is true that in our home members are encouraged to move freely.", "word_count": 15, "label": "", "sentence_structure": "Subject鈥搗erb鈥搊bject", "subjective_objective": {"answer": "Subjective", "rationale": "Because \"encouraging members to move freely\" is a view or attitude, it is a subjective perspective."}, "sentence_sentiment": {"answer": "Positive", "rationale": "Because the entry \"Encourage Free Activity\" expresses a positive attitude."}, "language_style": {"answer": "Tactful and warm", "rationale": "The language style of the entry is euphemistic, warmer, and the expression of respect for and support for the free activities of members in the family is more gentle and friendly by using the terms \"recognizing\" and \"freedom of movement\". At the same time, \"members\" emphasize that such encouragement is directed at all, and that it enhances the tenderness and inclusiveness of expression."}, "time_dimension": {"answer": "Present", "rationale": "The entry now uses the current completion pattern ( \"do encourage\") and refers to the current state and behaviour of the family, and thus judges it as now."}, "sentence_type": "Affirmative declarative sentence", "pos": {"nouns": {"Members": 1, "Free movement": 1}, "verbs": {"Encourage": 1}, "adverbs": {"Indeed": 1}, "numerals": {"84": 1}, "pronouns": {"we": 1}}}, {"content": "85. Family members often compare to others, depending on who works well.", "word_count": 19, "label": "", "sentence_structure": "Subject鈥搗erb鈥搊bject", "subjective_objective": {"answer": "Subjective", "rationale": "Family members are a subjective attitude and behaviour compared to others."}, "sentence_sentiment": {"answer": "Negative", "rationale": "Because the \"often comparing\" behavior in the entry is negative."}, "language_style": {"answer": "Calm and direct", "rationale": "The language style of the entry is a straightforward expression of the subject of questioning whether family members are overcomparisonous, with no excessive rhetorical or emotional rendering, and with a more objective tone."}, "time_dimension": {"answer": "Present", "rationale": "The entry \"often\" denotes regular, customary behaviour, \"comparison\", \"who learns well at work\" is an ongoing act or state, and therefore judgement is now a behavior."}, "sentence_type": "Affirmative declarative sentence", "pos": {"nouns": {"Family members": 1, "others": 1, "Work": 1, "Learning": 1}, "verbs": {"Comparison": 1, "Look": 1, "Do it": 1}, "adverbs": {"Often": 1}, "numerals": {"85": 1}, "pronouns": {"Who": 1}}}, {"content": "86. Family members love music, art and literature.", "word_count": 14, "label": "", "sentence_structure": "Subject鈥搗erb鈥揷omplement", "subjective_objective": {"answer": "Subjective", "rationale": "The attitude of family members, preferences, etc. are subjective descriptions."}, "sentence_sentiment": {"answer": "Neutral", "rationale": "Because the entry merely stated a fact and did not contain any emotional colour."}, "language_style": {"answer": "Tactful and warm", "rationale": "This expression is more comfortable for the person surveyed and is willing to participate in the questionnaire."}, "time_dimension": {"answer": "Present", "rationale": "The entry indicates a description of the current family member ' s hobby, so the temporal dimension is now judged."}, "sentence_type": "Affirmative declarative sentence", "pos": {"nouns": {"Family members": 1, "Music": 1, "Arts": 1, "Literature": 1}, "adjectives": {"very": 2}, "numerals": {"86": 1}}}, {"content": "Our main way of entertaining is watching TV, listening to radio, not going out.", "word_count": 25, "label": "", "sentence_structure": "Subject鈥搗erb鈥搊bject", "subjective_objective": {"answer": "Objective", "rationale": "This is a description of the patient ' s current lifestyle and does not involve subjective attitudes or feelings of the patient."}, "sentence_sentiment": {"answer": "Negative", "rationale": "Because the tendency in the entry to \"predominantly watch television, listen to radio, not go out of the house\" is negative and lacks outdoor exercise and social activities."}, "language_style": {"answer": "Calm and direct", "rationale": "The entry uses a direct presentation, which clearly indicates that the entertainment activities of the subjects of the survey are mainly indoor, do not involve excessive rhetorical and emotional colours and appear to be relatively calm and objective."}, "time_dimension": {"answer": "Present", "rationale": "At the same time, the expression \"main\" and \"not\" implies that it is a statement or an assessment of the current situation."}, "sentence_type": "Affirmative declarative sentence", "pos": {"nouns": {"we": 2, "Recreational activities": 1, "Main modalities": 1, "Television": 1, "Radio": 1, "Outgoing activities": 1}, "verbs": {"be": 2, "Look": 1, "Listen": 1, "Not": 1, "Out": 1}, "adjectives": {"87": 1}, "numerals": {"87": 1}, "classifiers": {"Plant": 1}, "pronouns": {"we": 2}}}, {"content": "88. We believe that raising the standard of living in the family is more important than upholding ethical standards.", "word_count": 24, "label": "", "sentence_structure": "Subject鈥搗erb鈥搊bject", "subjective_objective": {"answer": "Subjective", "rationale": "Because the words \"consider\" and \"important\" express the subjective attitude and values of the person under investigation, which are viewed from the perspective of the patient."}, "sentence_sentiment": {"answer": "Negative", "rationale": "Because the idea in the entry that \"a higher standard of living in the family is more important than strict observance of moral standards\" is demeaning and has a negative emotional connotation."}, "language_style": {"answer": "Calm and direct", "rationale": "The entry is based on the following criteria: it is a direct expression of the subject matter, is not based on excessive rhetoric or emotional rendering, and is more objective in tone. It is also not expressed in any euphemism or invitation, but rather in a direct statement."}, "time_dimension": {"answer": "Past", "rationale": "The \"concept\" in the entry to the questionnaire represents a view or attitude of the past, while the \"important\" in the latter also indicates that it is an already developed perception or perception and can therefore be judged as the past."}, "sentence_type": "Affirmative declarative sentence", "pos": {"nouns": {"we": 1, "Home": 1, "Standard of living": 1, "Ethical standards": 1}, "verbs": {"I think": 1, "Increase": 1, "Yes": 2, "Hold tight": 1}, "adjectives": {"Important": 1}, "adverbs": {"And": 1}, "numerals": {"88": 1}}}, {"content": "We need someone to wash the dishes right after dinner.", "word_count": 14, "label": "", "sentence_structure": "Subject鈥搗erb鈥揷omplement", "subjective_objective": {"answer": "Subjective", "rationale": "Because whether \"one must wash the dishes immediately after eating\" is the patient's personal opinion and opinion and is subjective."}, "sentence_sentiment": {"answer": "Negative", "rationale": "Because the requirement \"must be immediately\" may create some negative effects on the respondents by creating pressure and dissatisfaction."}, "language_style": {"answer": "Calm and direct", "rationale": "The language style of the entry is more straightforward, there is no excessive speech or emotional rendering, and the tone is more objective. It clearly sets out a specific requirement in family life that people must wash dishes immediately after eating, an expression that is highly indicative and executive."}, "time_dimension": {"answer": "Present", "rationale": "The entry indicates a rule or customary sexual act, i.e., a person who needs to wash dishes immediately after the meal, which occurs and continues."}, "sentence_type": "Imperative sentence", "pos": {"nouns": {"Our home": 1, "After dinner": 1, "person": 1, "The bowl": 1}, "verbs": {"I have to": 1, "Go on": 1, "Wash": 1}, "adverbs": {"Immediately": 1}, "numerals": {"89": 1}}}, {"content": "90. Those who break home rules are severely criticized.", "word_count": 16, "label": "", "sentence_structure": "Subject鈥搗erb鈥搊bject", "subjective_objective": {"answer": "Objective", "rationale": "This is a description of the family rules and the manner in which the punishment is imposed, and does not involve the patient鈥檚 own subjective attitude or feelings."}, "sentence_sentiment": {"answer": "Negative", "rationale": "Because the \"severe criticism\" in the entry is negative."}, "language_style": {"answer": "Calm and direct", "rationale": "The language style of the entry is calm and straightforward, and it clearly indicates that there is no excessive use of rhetoric or emotional rendering for persons who violate family rules."}, "time_dimension": {"answer": "Present", "rationale": "The entry describes the current family rules and the treatment of offenders and thus the judgement is now."}, "sentence_type": "Affirmative declarative sentence", "pos": {"nouns": {"Home": 1, "A family man": 1, "Criticism": 1}, "verbs": {"Violations": 1, "Received": 1, "Hard": 1}, "adjectives": {"Hard": 1}, "numerals": {"90": 1}}}]}

{"questionnaire_title": "The CCSAS adaptation schedule for Chinese university students", "questionnaire_category": "Behavioral questionnaire", "item_count": 59, "emotion_shift_overall": "From positive to negative", "language_style_overall": "Tactful and warm", "items": [{"content": "There are always things in life that are of interest to me.", "word_count": 16, "label": "", "sentence_structure": "Subject鈥揷opula鈥損redicative", "subjective_objective": {"answer": "Subjective", "rationale": "Because \"interested\" is the patient's subjective feeling and attitude, not the objective fact or the result."}, "sentence_sentiment": {"answer": "Positive", "rationale": "Because the phrase \"there's always something I'm interested in\" in the entry shows positive attitudes and emotions."}, "language_style": {"answer": "Tactful and warm", "rationale": "The basis for the judgement: the entry is a positive and invited tone of dialogue, which expresses an attitude of encouragement and certainty, and is not expressed in a strong manner, such as direct command or negative evaluation. Thus, the language style of the entry is more in keeping with the \"false and warm\" character."}, "time_dimension": {"answer": "Present", "rationale": "The entry describes the current living conditions of the person tested and thus judges it as now."}, "sentence_type": "Imperative sentence", "pos": {"nouns": {"Life": 1, "Things": 1}, "verbs": {"have": 1, "Always": 1, "Interested": 1}, "adjectives": {"Every day": 1, "I'm interested": 1}, "numerals": {"1锛?: 1}, "pronouns": {"I": 1}}}, {"content": "If I were to choose again, I would still live as I am now.", "word_count": 20, "label": "", "sentence_structure": "Subject鈥損redicate", "subjective_objective": {"answer": "Subjective", "rationale": "This is an expression of patients' subjective views and attitudes towards their lives."}, "sentence_sentiment": {"answer": "Neutral", "rationale": "Because the entry describes only one ' s personal thoughts and attitudes, it does not display positive or negative emotional aspects."}, "language_style": {"answer": "Tactful and warm", "rationale": "The language style of the entry is euphemismal. It gives the interviewer a space for reflection and expression by presenting assumptions (if I choose again) that lead him to think about his life and satisfaction, and that he does not directly criticize or assess his lifestyle, but rather engages in a moderate and open-minded dialogue."}, "time_dimension": {"answer": "Present", "rationale": "The \"if I choose again\" in this entry is a hypothetical conditional statement, in which \"now\" refers to the current state of life. Therefore, depending on the time dimension, the content of this entry should be classified as \"now\"."}, "sentence_type": "Affirmative declarative sentence", "pos": {"nouns": {"Number of times": 1, "Selection": 1, "Life": 1}, "verbs": {"Jean": 1, "You choose": 1, "Yeah": 1, "Live": 1}, "adverbs": {"Again": 1, "And": 1, "Present": 1}, "numerals": {"2": 1}, "pronouns": {"I": 1}}}, {"content": "I am always happy.", "word_count": 9, "label": "", "sentence_structure": "Subject鈥揷opula鈥損redicative", "subjective_objective": {"answer": "Subjective", "rationale": "Because \"always happy\" is a personal feeling or attitude, a subjective view."}, "sentence_sentiment": {"answer": "Positive", "rationale": "Because the expression \"happiness\" is positive emotional."}, "language_style": {"answer": "Tactful and warm", "rationale": "The language style of the entry is euphemism. It is a gentle and friendly way of asking questions, which makes it easier for respondents to feel focused and understood, and to answer questions. At the same time, it does not directly ask the interviewee about his or her state of feeling or emotion, but rather, it is a way of responding by describing his or her mood. This avoids stress or discomfort and makes him or her more willing to share their real thoughts and experiences."}, "time_dimension": {"answer": "Present", "rationale": ""}, "sentence_type": "Affirmative declarative sentence", "pos": {"verbs": {"I feel": 1, "Always": 1, "Have a nice day": 1}, "adjectives": {"Have a nice day": 1}}}, {"content": "4. I usually read professional-related books.", "word_count": 12, "label": "", "sentence_structure": "Subject鈥搗erb鈥搊bject", "subjective_objective": {"answer": "Subjective", "rationale": "Because \"often prefers to talk to people to ease trouble\" is a description of personal behaviour and feelings, and therefore a subjective one. And \"Please analyse the following questionnaire entry: \"4 I read professionally about it\" is an objective fact-finding exercise to ask the respondents about their behaviour."}, "sentence_sentiment": {"answer": "Positive", "rationale": "Because the \"commonly professional books\" act positively in the entries."}, "language_style": {"answer": "Calm and direct", "rationale": "The language style of the entry is relatively simple, there is no excessive emotional colour or rhetorical technique, and the respondents are asked whether they regularly read professional books and have a high degree of objectivity and practicality."}, "time_dimension": {"answer": "Present", "rationale": ""}, "sentence_type": "Affirmative declarative sentence", "pos": {"nouns": {"I": 1, "Normal": 1, "Professional": 1, "Books": 1}, "verbs": {"Regular": 1, "Look": 1, "Related": 1}, "numerals": {"4锛?: 1}}}, {"content": "5. I have rarely understood the needs of society for talent.", "word_count": 14, "label": "", "sentence_structure": "Subject鈥搗erb鈥搊bject", "subjective_objective": {"answer": "Objective", "rationale": "Because \"know the needs of society for talent\" is an objective act or behaviour that can be measured and observed by facts and data. Even if a patient is unwilling to engage in such behaviour or activity, it remains an objective phenomenon."}, "sentence_sentiment": {"answer": "Negative", "rationale": "Because the \"very little to know\" act in the entry is negative."}, "language_style": {"answer": "Tactful and warm", "rationale": "The language style of the entry is more euphemistic, using the relatively mild term \"very few\" to avoid outright denial or blame. At the same time, the word \"understanding\" also expresses a proactive attitude that encourages the other to explore and learn, rather than to receive information passively."}, "time_dimension": {"answer": "Past", "rationale": "The entry \"very little\" means that it is not very frequent, and understanding the needs of society for talent requires some time and effort, so it can be inferred that this has occurred over time, but may not be frequent. At the same time, \"understanding\" is an exercise that has been completed in the past, and can be further confirmed."}, "sentence_type": "Negative declarative sentence", "pos": {"nouns": {"Questionnaire Entry": 1, "Requirements": 1}, "verbs": {"Roger that": 1, "Go on": 1}, "adjectives": {"Less": 1}, "adverbs": {"very": 1, "Very few": 1}, "classifiers": {"individual": 1}}}, {"content": "I have often been overwhelmed by frustration.", "word_count": 14, "label": "", "sentence_structure": "Subject鈥搗erb鈥揷omplement", "subjective_objective": {"answer": "Subjective", "rationale": "Because \"always one can't\" describes patients' subjective feelings and thoughts."}, "sentence_sentiment": {"answer": "Negative", "rationale": "Because the behavior in the entry is negative."}, "language_style": {"answer": "Calm and direct", "rationale": "The entry is expressed in more direct, concise language, without too much emotional or rhetorical style. It raises an inquiry question in an objective, neutral way, that is, how the interviewee reacts to a difficult situation. Such language styles are usually considered calm and direct."}, "time_dimension": {"answer": "Present", "rationale": ""}, "sentence_type": "Negative declarative sentence", "pos": {"nouns": {"Things": 1, "Desperate": 1, "I can't get a chance": 1}, "verbs": {"I met him": 1, "Regular": 1, "be": 1}, "adjectives": {"Desperate": 1}, "adverbs": {"Often": 1}, "classifiers": {"individual": 1}}}, {"content": "7. I am pleased with the current university life.", "word_count": 12, "label": "", "sentence_structure": "Subject鈥揷opula鈥損redicative", "subjective_objective": {"answer": "Subjective", "rationale": "Because satisfaction is a subjective feeling and evaluation."}, "sentence_sentiment": {"answer": "Positive", "rationale": "Because the attitude expressed in the entry is positive."}, "language_style": {"answer": "Tactful and warm", "rationale": "The entry describes the interviewee鈥檚 feelings of current university life in a positive and positive manner, rather than directly expressing satisfaction or dissatisfaction, which is more moderate and friendly. At the same time, the article鈥檚 purpose is easier and more natural, is not overly emotional, and is more resonant and active."}, "time_dimension": {"answer": "Present", "rationale": "The entry is for the purpose of asking the respondent about the satisfaction of the current ( \"now\") university life, the time dimension of which is now judged."}, "sentence_type": "Affirmative declarative sentence", "pos": {"nouns": {"University life": 1}, "verbs": {"Satisfied": 1}, "adverbs": {"very": 1}}}, {"content": "8. I am well aware of whether it is time to continue to study or work after graduation.", "word_count": 18, "label": "", "sentence_structure": "Subject鈥搗erb鈥搊bject", "subjective_objective": {"answer": "Objective", "rationale": "It is an objective fact to know whether to continue to study or work after graduation, and it is not necessary to judge the patient ' s subjective attitude or feelings."}, "sentence_sentiment": {"answer": "Neutral", "rationale": "Because the entry does not have any emotional color."}, "language_style": {"answer": "Calm and direct", "rationale": "This questionnaire entry asks the respondents in a clear and direct way about their intentions and plans for the future employment direction, and there is no excessive emotional overturn or euphemism."}, "time_dimension": {"answer": "Present", "rationale": "The entry describes the current cognitive state and attitude of the testees, i.e. whether they have made it clear whether they should continue their studies or choose to work after graduation. Thus, in terms of time dimension, they are judged as \"now\"."}, "sentence_type": "Affirmative declarative sentence", "pos": {"nouns": {"After graduation": 1, "Further education": 1, "Work": 1}, "verbs": {"I know": 1, "It's time to continue": 1, "Or is it": 1, "Work": 1}, "adverbs": {"Clear": 1}, "numerals": {"8锛?: 1}}}, {"content": "9. I am very enthusiastic about the current learning.", "word_count": 13, "label": "", "sentence_structure": "Subject鈥搗erb鈥揷omplement", "subjective_objective": {"answer": "Subjective", "rationale": "Because \"high passion\" is the patient's subjective attitude or feeling."}, "sentence_sentiment": {"answer": "Positive", "rationale": "Because it expresses positive attitudes and feelings."}, "language_style": {"answer": "Tactful and warm", "rationale": "The entry is more personal and interactive when it is spoken in a positive, encouraging tone rather than by direct questioning or command. At the same time, the entry does not use any negative terminology or tone, but gives the interviewee comfort and welcome by means of positive and commendable statements."}, "time_dimension": {"answer": "Present", "rationale": "The entry asks the subject about his current emotional attitude, and the keywords \"learning now\" and \"high enthusiasm\" indicate that the dimension of time is now."}, "sentence_type": "Affirmative declarative sentence", "pos": {"nouns": {"I": 1, "Present": 1, "Learning": 1}, "verbs": {"have": 1, "High": 1, "鈾?Hot": 1}, "adjectives": {"very": 1}, "numerals": {"9": 1}}}, {"content": "10. I believe that my strengths outweigh those of others.", "word_count": 12, "label": "", "sentence_structure": "Subject鈥搗erb鈥搊bject", "subjective_objective": {"answer": "Subjective", "rationale": "Because \"thinks\" is a subjective term, which means that it is the patient's personal view and opinion."}, "sentence_sentiment": {"answer": "Positive", "rationale": "Because the expression \"more than \" in the entry is positive."}, "language_style": {"answer": "Tactful and warm", "rationale": "The language style of the entry is more euphemistic and moderate, and it is not overly direct or emotional. Using this subjective evaluation, I think, gives the interviewee the opportunity to express his/her own identity and avoids judgement or evaluation of the interviewee. This approach is more likely to trigger resonance among the interviewee and make him/her more willing to participate in the questionnaire."}, "time_dimension": {"answer": "Present", "rationale": "The entry is the testee ' s self-perception assessment and refers to current and present feelings and ideas."}, "sentence_type": "Affirmative declarative sentence", "pos": {"nouns": {"Advantages": 1, "Disadvantages": 1}, "verbs": {"I think": 1, "More than": 1}, "adjectives": {"self": 1}, "numerals": {"10锛?: 1}, "pronouns": {"I": 1}}}, {"content": "11. Many people have asked me to play with them.", "word_count": 12, "label": "", "sentence_structure": "Subject鈥搗erb鈥搊bject", "subjective_objective": {"answer": "Subjective", "rationale": "Because \"play with them\" is the patient's subjective feeling and attitude, his self-awareness of his social situation."}, "sentence_sentiment": {"answer": "Positive", "rationale": "Because the \"play with them\" act in the entry expresses a positive attitude towards social relations with others."}, "language_style": {"answer": "Tactful and warm", "rationale": "The language style in the entry is more euphemistic, using the first person's pronoun, \"I\", and using an invitation tone, which gives readers a sense of interest and popularity. At the same time, the purpose of the article is expressed more implicitly, without directly expressing an assessment or criticism of the behaviour of the other party and with a greater emphasis on good interaction with the other."}, "time_dimension": {"answer": "Present", "rationale": "The entry describes a state of current behaviour, and the words \"both\" and \"see me\" indicate that the act is ongoing and therefore judged to be the present."}, "sentence_type": "Affirmative declarative sentence", "pos": {"nouns": {"person": 2, "Questionnaire": 1, "Entry": 1, "Together": 1, "Play": 1}, "verbs": {"Both": 1, "Find": 1, "and": 1, "they": 1, "Together": 1, "Play": 1}, "adverbs": {"very": 1}, "numerals": {"11": 1}, "classifiers": {"individual": 1}, "pronouns": {"I": 1}}}, {"content": "12. I have never learned about the characteristics and requirements of different occupations by reading books about career choices.", "word_count": 29, "label": "", "sentence_structure": "Subject鈥搗erb鈥搊bject", "subjective_objective": {"answer": "Subjective", "rationale": "Because \"never\" is patient's subjective attitude."}, "sentence_sentiment": {"answer": "Neutral", "rationale": "The basis for the determination: the entry is simply a description of the individual ' s behaviour and does not express any emotional colour and is therefore neutral."}, "language_style": {"answer": "Calm and direct", "rationale": ""}, "time_dimension": {"answer": "Present", "rationale": "The sentence says \"never\" and \"read\" to judge that the item is asked about the current behaviour of the person being tested, so the temporal dimension is now. At the same time, \"books about choice of profession to understand the characteristics and requirements of different occupations\" are also a specific description of the act or activity, which further supports this determination."}, "sentence_type": "Negative declarative sentence", "pos": {"nouns": {"Entry": 1, "Reading": 1, "Books": 1, "Different": 1, "Occupation": 1, "Characteristics": 1, "Request": 1}, "verbs": {"Never": 1, "Pass": 1, "Roger that": 1, "Employment": 1, "Come on": 1}, "adjectives": {"Various": 1, "Related": 1}, "adverbs": {"Never": 1}, "numerals": {"12": 1, "1": 2}, "classifiers": {"Plant": 1}, "pronouns": {"I": 1}}}, {"content": "13. When I do not want to do things alone, I can always find someone to accompany me.", "word_count": 17, "label": "", "sentence_structure": "Subject鈥搗erb鈥搊bject", "subjective_objective": {"answer": "Subjective", "rationale": "Because \"Don't want to do things alone\" and \"Don't always find someone with me\" are subjective feelings and behaviour. These are the aspects that are examined from a subjective perspective."}, "sentence_sentiment": {"answer": "Positive", "rationale": "Because the \" always find someone with me\" act is positive, which means that this person has good social skills and personal relationships."}, "language_style": {"answer": "Tactful and warm", "rationale": ""}, "time_dimension": {"answer": "Present", "rationale": ""}, "sentence_type": "Affirmative declarative sentence", "pos": {"nouns": {"person": 2, "Yes": 1}, "verbs": {"Do it": 1, "Find": 1, "Stay": 1}, "adjectives": {"not": 1, "Alone": 1}, "numerals": {"13": 1}, "pronouns": {"I": 1}}}, {"content": "I know what I am fit to do.", "word_count": 12, "label": "", "sentence_structure": "Subject鈥搗erb鈥搊bject", "subjective_objective": {"answer": "Subjective", "rationale": "Because knowing what is appropriate for oneself requires judgement based on the subjective knowledge and experience of the individual."}, "sentence_sentiment": {"answer": "Positive", "rationale": "Because \"know\" and \"fit\" in the entries are all positive words."}, "language_style": {"answer": "Calm and direct", "rationale": ""}, "time_dimension": {"answer": "Present", "rationale": "The key words are \"know\" and \"suitable\" , which point to situations that have not yet occurred and that may arise in the future. At the same time, \"what work\" also implies planning and expectations for future career development, and further confirms that this is a judgement about the future."}, "sentence_type": "Affirmative declarative sentence", "pos": {"nouns": {"Work": 1, "self": 1}, "verbs": {"Yes": 1, "Do it": 1}, "numerals": {"14": 1}, "classifiers": {"individual": 1}, "pronouns": {"I": 1}}}, {"content": "I never felt alone.", "word_count": 7, "label": "", "sentence_structure": "Subject鈥揷opula鈥損redicative", "subjective_objective": {"answer": "Subjective", "rationale": "Because feeling alone is a subjective feeling."}, "sentence_sentiment": {"answer": "Neutral", "rationale": "As the entry does not express positive or negative feelings, attitudes, behaviour, purpose or quality."}, "language_style": {"answer": "Calm and direct", "rationale": "The language style of the entry is more direct, clear, with no excessive rhetorical or emotional rendering, and is more objective in tone, with the aim of understanding whether the person under investigation often feels alone."}, "time_dimension": {"answer": "Present", "rationale": ""}, "sentence_type": "Affirmative declarative sentence", "pos": {"verbs": {"I feel": 1, "Loneliness": 1}, "adjectives": {"Never": 1}}}, {"content": "16. I always find my strengths and encourage myself in them.", "word_count": 19, "label": "", "sentence_structure": "Subject鈥搗erb鈥揷omplement", "subjective_objective": {"answer": "Subjective", "rationale": "This is because of patients ' subjective attitude and behaviour towards self-evaluation."}, "sentence_sentiment": {"answer": "Positive", "rationale": "Because it expresses a positive attitude and behaviour, i.e., to exploit its merits and thereby encourage itself."}, "language_style": {"answer": "Tactful and warm", "rationale": "The language style of the entry is more euphemism and encouragement, not too direct or hard-line, but rather to direct the interviewee to his own strengths in a positive and upward-minded manner and encourage them to use it to inspire themselves. This expression is more personal and interactive."}, "time_dimension": {"answer": "Present", "rationale": ""}, "sentence_type": "Affirmative declarative sentence", "pos": {"nouns": {"Advantages": 1, "self": 2, "Encourage": 1}, "verbs": {"Found": 1, "Always": 1, "Go on": 1, "Here": 1, "Come on": 1, "and": 1}, "adverbs": {"Total": 1}}}, {"content": "17. My amateur life is rich and no change is required.", "word_count": 17, "label": "", "sentence_structure": "Subject鈥揷opula鈥損redicative", "subjective_objective": {"answer": "Subjective", "rationale": "Because the descriptions of \"rich\" and \"no change\" are the subjective feelings and attitudes of the patient."}, "sentence_sentiment": {"answer": "Neutral", "rationale": "Because the entry does not express positive or negative emotional expressions, it simply presents a fact."}, "language_style": {"answer": "Tactful and warm", "rationale": "The entry is more euphemistic and gentle, not directly expressing accusations or suggestions about the person under investigation, but rather asking the person in question whether he or she is satisfied with his or her amateur life in an invitation manner. This language is more respectful of the person鈥檚 subjective feelings and wishes and is more likely to receive a positive response from the person under investigation."}, "time_dimension": {"answer": "Present", "rationale": ""}, "sentence_type": "Affirmative declarative sentence", "pos": {"nouns": {"Amateur life": 1, "Change": 1}, "verbs": {"Yes": 1, "Do it": 1, "Rich": 1}, "adjectives": {"very": 1}, "numerals": {"17锛?: 1}}}, {"content": "I do not know how to compliment others.", "word_count": 10, "label": "", "sentence_structure": "Subject鈥搗erb鈥搊bject", "subjective_objective": {"answer": "Subjective", "rationale": "Because \"I don't know how to compliment others\" is a personal experience and experience of the patient, and it's a subjective examination."}, "sentence_sentiment": {"answer": "Negative", "rationale": "Because the description of \"I don't know how to compliment\" in the entry is negative."}, "language_style": {"answer": "Tactful and warm", "rationale": "The language style of the entry is euphemism and warmness. It uses a verb of inquiry, expressing concern and understanding about the difficulties that the other party may encounter in praising others, and encouraging them to try to share their thoughts. Such expression does not put pressure or criticism on the interviewees, nor does it make them feel respected and understood."}, "time_dimension": {"answer": "Present", "rationale": "The entry asks about the current situation of the testee, that is, he/she does not know how to compliment others on the issue. Although the problem may have occurred at some point in the past, it is mainly concerned with the current state of the testee. The time dimension of the entry is therefore \"now\"."}, "sentence_type": "Negative declarative sentence", "pos": {"nouns": {"Grandpa": 1, "Questionnaire": 1, "Entry": 1, "person": 1, "others": 1}, "verbs": {"Yes": 1, "What": 1, "I'm flattered": 1}, "adverbs": {"I": 1, "What": 1}, "numerals": {"18锛?: 1}}}, {"content": "I will not plan for the achievement of my own career goals.", "word_count": 18, "label": "", "sentence_structure": "Subject鈥搗erb鈥搊bject", "subjective_objective": {"answer": "Subjective", "rationale": "This is an expression of the patient ' s personal convictions and attitudes and is considered subjective."}, "sentence_sentiment": {"answer": "Negative", "rationale": "Because the behaviour \"not planned\" in the entries is negative and may affect the career development and achievement of the individual's goals."}, "language_style": {"answer": "Calm and direct", "rationale": "The language style of the entry is straightforward, not using excessive emotional accompaniment or euphemism, but rather presenting facts and asking questions."}, "time_dimension": {"answer": "Present", "rationale": "The keyword \"does not\" indicate that this is a description of behaviour in the general modern sense, that \"realizing your own career\" and \"programming\" are in the present state of behaviour or state."}, "sentence_type": "Negative declarative sentence", "pos": {"nouns": {"self": 1, "Occupation": 1, "Objective": 1, "Planned": 1}, "verbs": {"Development": 1, "Achieved": 1, "Yes": 1}, "adverbs": {"No, I won't": 1}, "numerals": {"19": 1}, "pronouns": {"I": 1}}}, {"content": "20. I do not know what to do when there is a conflict with others.", "word_count": 16, "label": "", "sentence_structure": "Subject鈥搗erb鈥搊bject", "subjective_objective": {"answer": "Subjective", "rationale": "The point is based on the subjective feelings and thoughts of patients, who are overwhelmed when they have conflicts with others. It is a subjective issue, because it involves the emotions and attitudes of individuals, not an objective fact that can be observed or measured."}, "sentence_sentiment": {"answer": "Negative", "rationale": "Because the \"I don't know what to do\" in the entry is a helpless and confused sentiment, and it's negative."}, "language_style": {"answer": "Tactful and warm", "rationale": "The language style of the entry is euphemismal. It asks the interviewees in a gentle and caring manner about the problems they experience in the face of conflict, tries to create an atmosphere of support and understanding, and encourages the interviewees to share their thoughts and feelings, rather than directly ordering or criticizing them for not knowing how to deal with the conflict."}, "time_dimension": {"answer": "Present", "rationale": ""}, "sentence_type": "Interrogative sentence", "pos": {"nouns": {"Conflict": 1, "others": 1}, "verbs": {"Come on": 1, "Yes": 1, "OPERATIONS": 1}, "adverbs": {"not": 1, "What": 1, "of": 2}, "pronouns": {"I": 1}}}, {"content": "21. I have rarely been nervous or anxious.", "word_count": 10, "label": "", "sentence_structure": "Subject鈥揷opula鈥損redicative", "subjective_objective": {"answer": "Subjective", "rationale": "Because \"very rarely\" is a subjective experience and feeling, not an objective act or event."}, "sentence_sentiment": {"answer": "Negative", "rationale": "Because \"very little tension or anxiety\" is a negative description, which suggests that this person is often nervous or anxious."}, "language_style": {"answer": "Calm and direct", "rationale": "The entry is a simple and clear expression of the subject of questioning whether the other party is often nervous or anxious, and is more objective in tone."}, "time_dimension": {"answer": "Present", "rationale": ""}, "sentence_type": "Negative declarative sentence", "pos": {"nouns": {"Nervous": 1, "Anxiety": 1}, "verbs": {"I feel": 1, "Very few": 1}, "adverbs": {"very": 1}, "numerals": {"21": 1}}}, {"content": "22. I will develop some hobbyes in the light of my own situation.", "word_count": 19, "label": "", "sentence_structure": "Subject鈥搗erb鈥揷omplement", "subjective_objective": {"answer": "Subjective", "rationale": "Because the words \"will\" and \"according to your own reality\" indicate that this is a patient's subjective attitude and behavioral plan."}, "sentence_sentiment": {"answer": "Positive", "rationale": "Because the entries \"will be based on your own reality\" and \"bringing out amateurs\" are positive expressions."}, "language_style": {"answer": "Tactful and warm", "rationale": "The entry is more euphemistic, raising questions in a gentle and caring manner, so that the interviewee is comfortable and respectful in his response. At the same time, the article ' s words are simple and clear, and there is no excessive rhetorical approach that makes it easy to understand the intent.\""}, "time_dimension": {"answer": "Present", "rationale": "The term \"will\" in this entry represents a future will, but at this point there is no real action, and it is now judged. At the same time, the act \"bringing some hobbyes in your own hands\" may require action in the future, and thus will have some future. Taken together, the time dimension of the entry can be judged to be present and future."}, "sentence_type": "Interrogative sentence", "pos": {"nouns": {"Situation": 1, "Amateurity": 1}, "verbs": {"Yes": 1, "Based on": 1, "Cultivation": 1}, "adverbs": {"self": 1, "Actual": 1}, "numerals": {"22": 1}, "pronouns": {"I": 1}}}, {"content": "I always compare my own strengths with others.", "word_count": 16, "label": "", "sentence_structure": "Subject鈥搗erb鈥搊bject", "subjective_objective": {"answer": "Subjective", "rationale": "This is because the patient describes his or her psychological state, and it involves personal feelings and perceptions, and it is subjective."}, "sentence_sentiment": {"answer": "Negative", "rationale": "Because the \"comparison of your own shorts\" in the entry is negative."}, "language_style": {"answer": "Calm and direct", "rationale": "The language style of the entry is the \"cool and direct\" type. The basis of the judgement is: the words of the entry are simple, not too much adoration and emotional, and are straightforward in expressing the subject of the habit of asking if the other side has a better or less advantage than the other. So it is judged to be \"cool and straight.\""}, "time_dimension": {"answer": "Past", "rationale": ""}, "sentence_type": "Negative declarative sentence", "pos": {"nouns": {"Entry": 1, "self": 1, "Short": 1, "others": 1, "Strengths": 1}, "verbs": {"Take it": 1, "- Yeah": 1}, "numerals": {"23": 1}, "pronouns": {"I": 1}}}, {"content": "24. I have often reflected on learning.", "word_count": 10, "label": "", "sentence_structure": "Subject鈥損redicate", "subjective_objective": {"answer": "Subjective", "rationale": "This is because patients have a subjective view and perception of themselves as they are constantly reflecting."}, "sentence_sentiment": {"answer": "Positive", "rationale": "Because the purpose of the \"Rethinking Learning\" act in the entry is positive."}, "language_style": {"answer": "Tactful and warm", "rationale": ""}, "time_dimension": {"answer": "Present", "rationale": "The keyword \"rethinking learning\" suggests that it is a statement describing the current behaviour of the testee, and therefore it is now judged."}, "sentence_type": "Affirmative declarative sentence", "pos": {"nouns": {"Learning": 1, "Reflections": 1}, "verbs": {"Conduct": 1}, "adverbs": {"Regular": 1}, "numerals": {"24": 1}}}, {"content": "When I was hit, I thought of my own good side.", "word_count": 17, "label": "", "sentence_structure": "Subject鈥搗erb鈥揷omplement", "subjective_objective": {"answer": "Subjective", "rationale": "Because thinking about your own good is a subjective way of thinking and reaction."}, "sentence_sentiment": {"answer": "Positive", "rationale": "Because the \"will think of yourself as a good side\" in the entry is positive."}, "language_style": {"answer": "Tactful and warm", "rationale": "The language style of the entry is more euphemistic, not using too direct or strong emotional terms, but rather, in a message of encouragement and comfort, to guide the interviewee to reflect on his strengths and positive aspects, which are more comfortable and acceptable."}, "time_dimension": {"answer": "Present", "rationale": ""}, "sentence_type": "Affirmative declarative sentence", "pos": {"nouns": {"Strike": 1, "One side": 1}, "verbs": {"Yes": 4, "Present": 1, "- It's a hit": 1, "Received": 1}, "adjectives": {"Okay": 1}, "numerals": {"25": 1}, "pronouns": {"I": 1}}}, {"content": "26. Although my amateur life is poor, I do not know how to change this situation.", "word_count": 24, "label": "", "sentence_structure": "Subject鈥搗erb鈥搊bject", "subjective_objective": {"answer": "Subjective", "rationale": "Because \"I don't know how to change\" is the patient's subjective idea and attitude."}, "sentence_sentiment": {"answer": "Negative", "rationale": "Because the entry \"Don't know how to change this\" expresses a feeling of helplessness and confusion, with a certain negative emotional color."}, "language_style": {"answer": "Tactful and warm", "rationale": "The entry expresses the difficulty of the other in an understanding and encouragement, and raises the question of how to guide the interviewer to think and explore how to change the situation. This tone is more euphemismal, warmer, and more stimulating for the interviewee to actively participate and change himself."}, "time_dimension": {"answer": "Past", "rationale": "The \"not knowing\" in the entry shows the testee's hesitation and confusion about changing the status quo, which is a description of the past. And the structure of \"though...\" also suggests that the testee may have tried some of the changes, but the effect is not obvious. So the time dimension of this questionnaire entry can be judged as the past."}, "sentence_type": "Negative declarative sentence", "pos": {"nouns": {"Amateur life": 1, "Status": 1}, "verbs": {"Yes": 1, "Change": 1}, "adjectives": {"Poverty": 1}, "adverbs": {"very": 1, "But": 1}, "classifiers": {"26": 1}, "pronouns": {"I": 1}}}, {"content": "I am always energetic and full of energy.", "word_count": 11, "label": "", "sentence_structure": "Subject鈥揷opula鈥損redicative", "subjective_objective": {"answer": "Subjective", "rationale": "Because the words \"absorbed\" and \"satisfaction\" describe the feelings and perceptions of the patient himself, they are subjective."}, "sentence_sentiment": {"answer": "Positive", "rationale": "Because the expression \"always energetic, full of spirit\" conveys a positive sentiment, indicating that the individual is in a better state of mind."}, "language_style": {"answer": "Tactful and warm", "rationale": "The entry describes the characteristics of the person under investigation in a positive way, rather than directly questioning or criticizing, which is more acceptable and willing to share their ideas. Moreover, the use of the term \"always\" adds positive tone and makes people feel more positive."}, "time_dimension": {"answer": "Present", "rationale": ""}, "sentence_type": "Affirmative declarative sentence", "pos": {"nouns": {"- I'm very energetic": 1, "Full of spirit": 1}, "verbs": {"Always": 1}}}, {"content": "I do not know how to allocate time for learning.", "word_count": 12, "label": "", "sentence_structure": "Subject鈥搗erb鈥搊bject", "subjective_objective": {"answer": "Subjective", "rationale": "Because I don't know how to allocate time for study, which reflects the attitude and feelings of patients about how they manage their time, is a subjective exercise."}, "sentence_sentiment": {"answer": "Neutral", "rationale": "Because the entry does not express any positive or negative feelings, attitudes, behaviour, purpose or quality. It simply describes a fact."}, "language_style": {"answer": "Tactful and warm", "rationale": "The entry is more adept and interactive when it is spoken in a gentle and inviting manner, rather than by direct statements or orders."}, "time_dimension": {"answer": "Past", "rationale": "The entry is for questioning the testee about confusion and problems in the current learning state, and does not address future behaviour or expectations, so it is judged as \"the past\"."}, "sentence_type": "Interrogative sentence", "pos": {"nouns": {"Learning time": 1}, "verbs": {"Yes": 1, "Allocation": 1}, "adverbs": {"How's that": 1}, "numerals": {"28": 1}, "pronouns": {"I": 1}}}, {"content": "I feel better than others.", "word_count": 12, "label": "", "sentence_structure": "Subject鈥揷opula鈥損redicative", "subjective_objective": {"answer": "Subjective", "rationale": "Because it's the patient's subjective attitude."}, "sentence_sentiment": {"answer": "Positive", "rationale": "Because it expresses a positive attitude of sentiment and confidence in itself."}, "language_style": {"answer": "Tactful and warm", "rationale": "The entry uses a more euphemistic and inviting language style, which does not directly express an assessment or comparison of the abilities of others, but rather is more proximate and interactive in a more objective and neutral way of asking the respondents about their abilities."}, "time_dimension": {"answer": "Present", "rationale": "The entry represents the subjective feeling or conviction of the person being tested and the temporal dimension is now judged."}, "sentence_type": "Affirmative declarative sentence", "pos": {"nouns": {"self": 1, "Capacity": 1, "others": 1}, "verbs": {"I think": 1, "- Yeah": 1, "Strong": 1}, "numerals": {"29": 1}, "pronouns": {"self": 1}}}, {"content": "30. I am very tired of learning now.", "word_count": 10, "label": "", "sentence_structure": "Subject鈥揷opula鈥損redicative", "subjective_objective": {"answer": "Subjective", "rationale": "Because \"very bored\" is a personal feeling or emotion that is seen and described from the patient's subjective point of view."}, "sentence_sentiment": {"answer": "Negative", "rationale": "Because the \"very tired\" attitude in the entries is negative."}, "language_style": {"answer": "Calm and direct", "rationale": "The entry is based on a direct language style, a concise expression of the main elements of the problem, and there is no excessive rhetorical or emotional rendering. This expression is more objective and accurate and helps to obtain real feedback from the interviewees."}, "time_dimension": {"answer": "Present", "rationale": "Now the entry describes the subjective perception of the subject of the current learning, and \"I'm very tired\" of expressing the negative emotions and attitudes of the testee, so it can be judged as it is."}, "sentence_type": "Interrogative sentence", "pos": {"nouns": {"Learning": 1}, "verbs": {"Boring": 1}, "adverbs": {"Very": 1}}}, {"content": "I am well-informed when compared to my peers.", "word_count": 12, "label": "", "sentence_structure": "Subject鈥揷opula鈥損redicative", "subjective_objective": {"answer": "Subjective", "rationale": "Because \"favouring\" is the patient's personal feeling and attitude, and it's subjective."}, "sentence_sentiment": {"answer": "Positive", "rationale": "Because the entry \"favoritely\" expresses a positive emotional attitude."}, "language_style": {"answer": "Tactful and warm", "rationale": "The entry uses a comparative and emotional vocabulary of \"satisfaction\" and a first person's perspective, which is a more euphemistic language style. At the same time, it gives people a sense of proximity and resonance."}, "time_dimension": {"answer": "Present", "rationale": "The keyword \"as compared to peers\" shows that the testee has reached the conclusion after comparing with his peers, and \"felt well\" expresses the subjective feeling of the testee. The entry therefore primarily looks at the mood and values of the testee in his current state."}, "sentence_type": "Affirmative declarative sentence", "pos": {"nouns": {"Questionnaire Entry": 1, "Peer": 1, "Satisfied": 1}, "verbs": {"I feel": 1, "- Yeah": 1, "Compare": 1}, "adjectives": {"very": 1}, "numerals": {"31": 1}, "pronouns": {"I": 1}}}, {"content": "32. I am not used to school-set hours.", "word_count": 13, "label": "", "sentence_structure": "Subject鈥揷opula鈥損redicative", "subjective_objective": {"answer": "Subjective", "rationale": "Because \"not used\" is the patient's subjective feeling and attitude."}, "sentence_sentiment": {"answer": "Negative", "rationale": "Because the attitude of \"not used\" in the entries is negative."}, "language_style": {"answer": "Tactful and warm", "rationale": ""}, "time_dimension": {"answer": "Present", "rationale": ""}, "sentence_type": "Exclamatory sentence", "pos": {"nouns": {"I": 1, "School regulations": 1, "Time of rest": 1}, "verbs": {"I'm not used to it": 1}, "numerals": {"32锛?: 1}}}, {"content": "I am not troubled by my appearance.", "word_count": 11, "label": "", "sentence_structure": "Subject鈥揷opula鈥損redicative", "subjective_objective": {"answer": "Subjective", "rationale": "This is because of patients' subjective views and attitudes towards their own appearance."}, "sentence_sentiment": {"answer": "Positive", "rationale": "Because \"not bothered by your appearance\" is a positive attitude and quality."}, "language_style": {"answer": "Calm and direct", "rationale": "The language style of the entry is more straightforward, without excessive rhetorical or emotional rendering, simply stating a point of view and being more objective in tone."}, "time_dimension": {"answer": "Past", "rationale": "The phrase \"I\" indicates that it is a description of the individual, while \"not bothered by his appearance\" indicates that this emotion or attitude existed in the past."}, "sentence_type": "Negative declarative sentence", "pos": {"nouns": {"I": 1, "Looks": 1, "Trouble": 1}, "verbs": {"No, I'm not": 1, "Trouble": 1}, "adverbs": {"And": 1}, "numerals": {"33": 1}}}, {"content": "34. I have always set different learning goals at different stages of learning.", "word_count": 20, "label": "", "sentence_structure": "Subject鈥搗erb鈥搊bject", "subjective_objective": {"answer": "Subjective", "rationale": "This is a target set by the patient himself and is subjective and thought-provoking."}, "sentence_sentiment": {"answer": "Positive", "rationale": "Because the \"always set different learning goals\" behavior is positive."}, "language_style": {"answer": "Calm and direct", "rationale": "The entry expresses the content of the question in direct, concise language, without using rhetorical or emotionally strong descriptions and in a calm and objective manner."}, "time_dimension": {"answer": "Future", "rationale": "The keyword \"always\" denotes customary behaviour, \"at different stages of learning\" and \"different learning objectives\" indicate that this is a future planning or decision. The entry therefore describes future behaviour or mindset."}, "sentence_type": "Affirmative declarative sentence", "pos": {"nouns": {"Study stage": 1, "Learning objectives": 1}, "verbs": {"Development": 1, "Always": 1}, "adverbs": {"Different": 2}, "numerals": {"34": 1}}}, {"content": "35. When I had difficulties, there were many people who were willing to help me.", "word_count": 16, "label": "", "sentence_structure": "Subject鈥搗erb鈥揷omplement", "subjective_objective": {"answer": "Subjective", "rationale": "This is a subjective attitude and perception of the patient, and therefore is subjective."}, "sentence_sentiment": {"answer": "Positive", "rationale": "Because the phrase \"many people will help me\" in the entry is positive."}, "language_style": {"answer": "Tactful and warm", "rationale": "The language style of the entry is euphemism, because it uses the expression \"willing to help\" to convey a positive, positive sentiment and to express a sense of trust and gratitude to others. At the same time, the sentence is not in a strong or direct language, but in a gentle way, giving people a warm feeling."}, "time_dimension": {"answer": "Present", "rationale": "The keyword \"when difficult\" indicates that this is a context describing the current situation, and the entry therefore indicates the present behaviour or state of affairs."}, "sentence_type": "Affirmative declarative sentence", "pos": {"nouns": {"Difficulties": 1, "person": 2}, "verbs": {"have": 1, "Yes": 1, "Help": 1}, "adjectives": {"A lot": 1}, "numerals": {"35锛?: 1}, "pronouns": {"I": 1}}}, {"content": "36. I have clear career objectives.", "word_count": 9, "label": "", "sentence_structure": "Subject鈥搗erb鈥搊bject", "subjective_objective": {"answer": "Subjective", "rationale": "Because the explicit professional objective is the patient ' s personal attitude and convictions, it is a subjective issue."}, "sentence_sentiment": {"answer": "Positive", "rationale": "Because the behaviour and attitude of the entry, \"with a clear career objective\", is positive."}, "language_style": {"answer": "Tactful and warm", "rationale": "The language style of the entry is more euphemistic, leads the respondents to questions in a question-and-answer manner, and gives positive and positive expression (e.g. \"yes\"), which is easier to open the interviewer's heart and answer in good faith."}, "time_dimension": {"answer": "Present", "rationale": "\"Specific career goals\" is a statement describing the current state of the testee, so the temporal dimension is now judged."}, "sentence_type": "Affirmative declarative sentence", "pos": {"nouns": {"Occupation": 1, "Objective": 1}, "verbs": {"have": 1, "Clear": 1}, "numerals": {"36": 1}}}, {"content": "I know how to care for others.", "word_count": 9, "label": "", "sentence_structure": "Subject鈥搗erb鈥搊bject", "subjective_objective": {"answer": "Subjective", "rationale": "Because this is the patient ' s assessment of his or her abilities, it is subjective thinking and attitude."}, "sentence_sentiment": {"answer": "Positive", "rationale": "Because the \"know how to care for others\" act is positive."}, "language_style": {"answer": "Tactful and warm", "rationale": "The entry uses an invitation-style tone, asking if the other party knows how to care for others, and expresses in a more moderate, friendly and interactive way. At the same time, it avoids the use of too direct or command-like language, which makes people feel more comfortable."}, "time_dimension": {"answer": "Present", "rationale": ""}, "sentence_type": "Affirmative declarative sentence", "pos": {"nouns": {"Questionnaire": 1, "Entry": 1}, "verbs": {"Yes": 1, "Care": 1}, "adverbs": {"How's that": 1}, "numerals": {"37锛?: 1}}}, {"content": "My amateur life is boring.", "word_count": 10, "label": "", "sentence_structure": "Subject鈥揷opula鈥損redicative", "subjective_objective": {"answer": "Subjective", "rationale": "Because \"amateur life is boring\" is the patient's subjective perception and assessment of his current situation."}, "sentence_sentiment": {"answer": "Negative", "rationale": "Because the description \"single-tip\" in the entry is negative."}, "language_style": {"answer": "Tactful and warm", "rationale": ""}, "time_dimension": {"answer": "Present", "rationale": "The entry describes the current living conditions of the testees, without any mention of past or future. The keywords \"my amateur life\" and \"single bored\" are descriptions of current life, indicating that this is the current situation of the testees, and therefore judging as the present."}, "sentence_type": "Negative declarative sentence", "pos": {"nouns": {"Amateur life": 1, "It's boring": 1}, "adjectives": {"Mine": 1, "Boring": 1, "Undo": 1}, "adverbs": {"38锛?: 1}, "numerals": {"38锛?: 1}}}, {"content": "I have often adjusted my emotional state by diverting my attention.", "word_count": 19, "label": "", "sentence_structure": "Subject鈥搗erb鈥揷omplement", "subjective_objective": {"answer": "Subjective", "rationale": "Because diversion is a subjective form of behaviour."}, "sentence_sentiment": {"answer": "Positive", "rationale": "Because the purpose of the behavior \"resort to the emotional state\" in the entry is positive."}, "language_style": {"answer": "Calm and direct", "rationale": "The language style of the entry is relatively direct, and there is no excessive use of emotional qualifiers or rhetorical techniques, with the preponderance of factual statements, and the tone of the speech is calm.\""}, "time_dimension": {"answer": "Present", "rationale": ""}, "sentence_type": "Affirmative declarative sentence", "pos": {"nouns": {"Questionnaire Entry": 1, "Emotional state": 1}, "verbs": {"Transfer": 1, "Adjustment": 1}, "adverbs": {"Often": 1}, "numerals": {"39": 1}}}, {"content": "40. I rarely summarised the previous stage of learning.", "word_count": 15, "label": "", "sentence_structure": "Subject鈥搗erb鈥揷omplement", "subjective_objective": {"answer": "Subjective", "rationale": "Because \"summary\" is a subjective act that requires the patient to act on his own initiative, and the frequency of the act is also a subjective judgement of the patient."}, "sentence_sentiment": {"answer": "Negative", "rationale": "Because the \"very little summarizing of the previous period's learning\" in the entry is negative."}, "language_style": {"answer": "Tactful and warm", "rationale": "This is a more personal and interactive way of expressing the questions."}, "time_dimension": {"answer": "Present", "rationale": "The keyword \"very rarely\" denotes a less frequent act, \"summation\" is an act of action, using the general current, so this sentence describes the situation of the person being tested."}, "sentence_type": "Negative declarative sentence", "pos": {"nouns": {"Phase": 1, "Learning": 1}, "verbs": {"Conduct": 1, "Summary": 1}, "adjectives": {"Very few": 1}, "numerals": {"40": 1}}}, {"content": "41. I will combine the various factors to determine my career choice.", "word_count": 18, "label": "", "sentence_structure": "Subject鈥搗erb鈥搊bject", "subjective_objective": {"answer": "Subjective", "rationale": "Because this reflects the individual values of the patient and the way in which he or she makes decisions, it is based on his or her subjective views and judgements."}, "sentence_sentiment": {"answer": "Positive", "rationale": "Because the act of \"compatibility of factors\" is rational and comprehensive and facilitates informed choices."}, "language_style": {"answer": "Calm and direct", "rationale": "The entry, in a clear and concise language style, directly describes the diversity of factors that the person under investigation would consider in determining his or her career objectives, without excessive emotional or rhetorical overtones, giving a calm and objective sense of humor."}, "time_dimension": {"answer": "Future", "rationale": "The keyword \"will\" indicates that this is a future plan and plan, and the entry is therefore an inquiry into the future career planning and targeting of the person being tested."}, "sentence_type": "Affirmative declarative sentence", "pos": {"nouns": {"Factors": 1, "Employment": 1, "Objective": 1}, "verbs": {"Yes": 1, "Synthesis": 1, "Sure": 1}, "numerals": {"41锛?: 1}}}, {"content": "42. I do not know how to accept myself.", "word_count": 16, "label": "", "sentence_structure": "Subject鈥損redicate", "subjective_objective": {"answer": "Subjective", "rationale": "Because \"accept yourself\" is a subjective attitude and emotional experience."}, "sentence_sentiment": {"answer": "Negative", "rationale": "Because \"acceptance\" in the entry is a positive attitude and behaviour, but the use of the expression \"no idea what to do\" implies that the interviewees encounter difficulties in achieving such attitudes and behaviour, thereby giving a negative feeling."}, "language_style": {"answer": "Tactful and warm", "rationale": "The language style of the entry is more moderate and considerate, and the \"I\" is the main language that guides the other to self-thinking and exploring, encouraging them to find a way to accept themselves, rather than giving instructions or evaluations directly. At the same time, the use of question sentences can also generate a sense of participation and resonance, increasing the effectiveness and interaction of questionnaires."}, "time_dimension": {"answer": "Present", "rationale": "The entry \"not knowing\" is an expression of uncertainty and confusion about the current situation, and \"accepting yourself\" is an expression of self-awareness and attitude. So the basis of the judgement is now."}, "sentence_type": "Negative declarative sentence", "pos": {"nouns": {"Questionnaire Entry": 1, "Approach": 1, "self": 2}, "verbs": {"Yes": 1, "鈾?And I'm gonna be 鈾?: 1, "Admission": 1}, "adverbs": {"What": 1}, "numerals": {"42锛?: 1}, "pronouns": {"I": 1}}}, {"content": "43. Many people are willing to go out with me.", "word_count": 10, "label": "", "sentence_structure": "Subject鈥搗erb鈥搊bject", "subjective_objective": {"answer": "Subjective", "rationale": "Because \"willing to be with me\" is a subjective feeling or attitude, not an objective fact or result of behaviour."}, "sentence_sentiment": {"answer": "Positive", "rationale": "Because the purpose of the act of \"willing to be with me\" in the entry is positive."}, "language_style": {"answer": "Tactful and warm", "rationale": "The entry uses a more euphemistic and polite language, which does not directly emphasize its merits or abilities, but rather uses an implicit way of asking the other side about himself."}, "time_dimension": {"answer": "Present", "rationale": "Based on the phrase \"all willing to \"to \" engage, it can be inferred that this is a description of the social situation of the person being tested, so the temporal dimension is now."}, "sentence_type": "Affirmative declarative sentence", "pos": {"nouns": {"person": 1, "- To be with someone": 1}, "verbs": {"Both": 1, "Yes": 1, "and": 1}, "adjectives": {"A lot": 1}, "numerals": {"43锛?: 1}, "pronouns": {"I": 1}}}, {"content": "I love my profession very much.", "word_count": 10, "label": "", "sentence_structure": "Subject鈥揷opula鈥損redicative", "subjective_objective": {"answer": "Subjective", "rationale": "Because \"like\" is a subjective emotional attitude."}, "sentence_sentiment": {"answer": "Positive", "rationale": "Because the word \"very much\" expresses a positive emotional attitude."}, "language_style": {"answer": "Tactful and warm", "rationale": "The entry expresses, in a positive and positive manner, the other party ' s love for his or her profession, is more acrimony and encouragemental and contagious."}, "time_dimension": {"answer": "Present", "rationale": "The keyword \"very\" and \"like\" means the current state of attitude and emotion, so the entry asks about the person's current behaviour or mentality."}, "sentence_type": "Interrogative sentence", "pos": {"verbs": {"Yeah": 1, "Professional": 1}, "adjectives": {"Very": 1}, "numerals": {"44": 1}}}, {"content": "I do not know what will cheer me up.", "word_count": 17, "label": "", "sentence_structure": "Subject鈥搗erb鈥揷omplement", "subjective_objective": {"answer": "Subjective", "rationale": "Because this expression expresses the patient ' s perception and perception of his or her own internal state, it examines the patient ' s subjective attitudes and emotional experience."}, "sentence_sentiment": {"answer": "Negative", "rationale": "Because \"I don't know what to do to make myself happy\" expresses a negative mood and attitude."}, "language_style": {"answer": "Tactful and warm", "rationale": "The entry is more adept and interactive when it is spoken in a gentle and inviting manner, rather than by direct statements or orders."}, "time_dimension": {"answer": "Past", "rationale": ""}, "sentence_type": "Interrogative sentence", "pos": {"nouns": {"Things": 2, "self": 1, "Happy": 1, "Get up": 1}, "verbs": {"Do it": 1, "鈾?And I'm gonna be 鈾?: 1}, "adverbs": {"not": 1, "What": 1}, "numerals": {"45": 1}, "pronouns": {"Where": 1}}}, {"content": "46. I like places of recreation, leisure or exercise in schools.", "word_count": 15, "label": "", "sentence_structure": "Subject鈥搗erb鈥搊bject", "subjective_objective": {"answer": "Subjective", "rationale": "Because it's the patient's subjective attitude."}, "sentence_sentiment": {"answer": "Positive", "rationale": "Because \"like\" in the entries expresses positive emotional attitudes."}, "language_style": {"answer": "Tactful and warm", "rationale": "The expression \"Sweet Warm\" is more personal and interactive when it is spoken in a gentle and inviting manner, rather than directly speaking or ordering."}, "time_dimension": {"answer": "Present", "rationale": "The entry describes the degree of preferences of the person tested for places of recreation, leisure or exercise in schools, which is a current state and emotional attitude."}, "sentence_type": "Affirmative declarative sentence", "pos": {"nouns": {"School": 1, "Recreation": 1, "Leisure": 1, "Places": 1}, "verbs": {"Yeah": 1}, "adverbs": {"of": 2, "or": 1}, "numerals": {"46锛?: 1}}}, {"content": "47. I have always been learning from the failures of the examinations.", "word_count": 14, "label": "", "sentence_structure": "Subject鈥搗erb鈥揷omplement", "subjective_objective": {"answer": "Subjective", "rationale": "Because lessons learned from examination failures are subjective behaviour and attitudes of a person, and are based on individual perceptions, experience and thinking. While these lessons may relate to objective facts and circumstances, the process of summarizing and learning is itself subjective."}, "sentence_sentiment": {"answer": "Positive", "rationale": "Because the action of \"Backing the Lessons of Examination Failure\" in the entry is positive, it shows the respondents' determination to reflect and progress themselves."}, "language_style": {"answer": "Calm and direct", "rationale": "The entry is in a concise manner asking respondents whether they often draw lessons from the failed examinations, without too many rhetorical or emotional colours and with more objective and direct tone."}, "time_dimension": {"answer": "Present", "rationale": "The keyword \"always\" and the verb \"resumed lessons from failed examinations\" means that it is a customary, frequent movement or state, and therefore is judged to be \"now\"."}, "sentence_type": "Affirmative declarative sentence", "pos": {"nouns": {"Lessons learned": 1, "Examinations": 1}, "verbs": {"Summary": 1, "Failed": 1}, "adverbs": {"Always": 1}, "numerals": {"47": 1}, "pronouns": {"I": 1}}}, {"content": "48. I believe that there are many unsatisfactory places in university life.", "word_count": 18, "label": "", "sentence_structure": "Subject鈥損redicate", "subjective_objective": {"answer": "Subjective", "rationale": "Because \"think\" is the patient's personal opinion and feelings."}, "sentence_sentiment": {"answer": "Negative", "rationale": "Because the description \"unsatisfactory\" is negative."}, "language_style": {"answer": "Calm and direct", "rationale": "The language style of the entry is calm and straightforward. It clearly expresses the researchers鈥?queries about the views of the subjects surveyed, without using excessive emotional colours or rhetorical techniques, and is more objective in tone."}, "time_dimension": {"answer": "Present", "rationale": "The entry is an inquiry into the subjective perception of the respondent ' s dissatisfaction with university life, and the temporal dimension is now judged."}, "sentence_type": "Affirmative declarative sentence", "pos": {"nouns": {"University life": 1, "Local": 1}, "verbs": {"I think": 1, "have": 1, "Not exactly": 1, "Do it": 1}, "adjectives": {"A lot": 1, "Not exactly": 1}, "numerals": {"48": 1}, "pronouns": {"I": 1}}}, {"content": "49. I have always sought to improve learning skills in memory, attention and so forth.", "word_count": 20, "label": "", "sentence_structure": "Subject鈥搗erb鈥揷omplement", "subjective_objective": {"answer": "Subjective", "rationale": "Because the ways and means of \"enhancement of memory, attention, etc.\" are chosen and implemented by the patient himself and are subjective behaviour of the patient."}, "sentence_sentiment": {"answer": "Positive", "rationale": "Because the purpose and attitude of the action \"to improve the ability to learn from memory, attention, etc.\" in the entry is positive."}, "language_style": {"answer": "Tactful and warm", "rationale": "The language style of the entry is more friendly and encouraging, and the other side is asked whether the other side has ideas and behaviour that improves its learning ability through various methods, rather than direct command or criticism."}, "time_dimension": {"answer": "Present", "rationale": ""}, "sentence_type": "Affirmative declarative sentence", "pos": {"nouns": {"Approach": 1, "Memory": 1, "Attention": 1, "Learning ability": 1}, "verbs": {"Increase": 1, "Come on": 1, "be": 1, "Yes": 1}, "adverbs": {"Always": 1, "Wait": 1}, "numerals": {"49": 1}, "pronouns": {"I": 1}}}, {"content": "50. I am good at communicating with others in words.", "word_count": 13, "label": "", "sentence_structure": "Subject鈥搗erb鈥揷omplement", "subjective_objective": {"answer": "Subjective", "rationale": "Because it is the subjective attitude or ability of a person to communicate with others, it is a personal quality test."}, "sentence_sentiment": {"answer": "Positive", "rationale": "Because \"good at communicating with others in words\" is positive."}, "language_style": {"answer": "Tactful and warm", "rationale": "The entry is more euphemistic and gentle, using the positive word \"good\" to describe the capabilities of the person under investigation and not directly indicating whether the other person actually possesses such capabilities. Nor is there any criticism or negative evaluation used to make the tone more peaceful."}, "time_dimension": {"answer": "Present", "rationale": "The entry describes the current capabilities of the testees, and \"I'm good\" is a general current phrase that indicates that this is their current state of capability."}, "sentence_type": "Affirmative declarative sentence", "pos": {"nouns": {"I": 1, "Words": 1, "Communications": 1}, "verbs": {"Good at it": 1, "Conduct": 1}, "adverbs": {"very": 1}, "numerals": {"50": 1}}}, {"content": "51. I have often been conscious in preparing for future work through participation in social practice activities.", "word_count": 26, "label": "", "sentence_structure": "Subject鈥搗erb鈥搊bject", "subjective_objective": {"answer": "Subjective", "rationale": "Because it is the subjective attitude of the patient that is the conscious act."}, "sentence_sentiment": {"answer": "Positive", "rationale": "The basis for the judgement: the behaviour in the entry \"consciously preparing for future work through participation in social practice activities\" is positive."}, "language_style": {"answer": "Tactful and warm", "rationale": "The language style of the entry is more moderate and euphemistic, for example, by using the phrase \"consciously\" to express the purpose of participation in social practice, and by using the phrase \"preparation for future work\" to give a positive and upward feeling. This language style can be perceived as respectful and understandable, and helps to increase the completion rate of questionnaires and the quality of data."}, "time_dimension": {"answer": "Future", "rationale": "The entry contains visions and expectations for the future, and the keyword \"work for the future\" refers to situations that have not yet occurred and that may arise in the future."}, "sentence_type": "Affirmative declarative sentence", "pos": {"nouns": {"Questionnaire": 1, "Entry": 1, "Awareness": 1, "Pass": 1, "Participation": 1, "Social practice activities": 1, "Work": 1, "Future": 1, "Ready": 1}, "verbs": {"have": 1, "Consciously": 1, "Do it": 1, "Ready": 1}, "adverbs": {"Regular": 1, "Yes": 1}, "numerals": {"51锛?: 1}, "classifiers": {"individual": 1}}}, {"content": "52. I have been very well adapted to my university life.", "word_count": 11, "label": "", "sentence_structure": "Subject鈥揷opula鈥損redicative", "subjective_objective": {"answer": "Subjective", "rationale": "Because \"adaptation\" is an expression of subjective feelings and attitudes."}, "sentence_sentiment": {"answer": "Positive", "rationale": "Because it expresses a positive feeling and satisfaction with university life."}, "language_style": {"answer": "Tactful and warm", "rationale": "The language style of the entry is more euphemistic, using the expression \"very well adapted\" to give people a sense of positive, confident, not direct statement or command. Moreover, it is more balanced, objective, less emotional, and more natural. So, the language style of the questionnaire is warm."}, "time_dimension": {"answer": "Present", "rationale": ""}, "sentence_type": "Affirmative declarative sentence", "pos": {"verbs": {"Adaptation": 1, "Life": 1}, "adjectives": {"Very": 1}}}, {"content": "53. When I am unhappy, I will only complain.", "word_count": 9, "label": "", "sentence_structure": "Subject鈥搗erb鈥搊bject", "subjective_objective": {"answer": "Subjective", "rationale": "Because \"sort\" is an expression of patients' subjective attitude and treatment when they are unhappy."}, "sentence_sentiment": {"answer": "Negative", "rationale": "Because the \"only complain\" in the entry is negative."}, "language_style": {"answer": "Calm and direct", "rationale": ""}, "time_dimension": {"answer": "Present", "rationale": "\"Unhappiness\" suggests that this is an event in a given situation, and \"only complain\" emphasizes this as a customary reaction, so the time dimension of this questionnaire entry is now."}, "sentence_type": "Affirmative declarative sentence", "pos": {"nouns": {"Time": 1, "- He's complaining": 1}, "verbs": {"I'm not happy": 1, "Yes": 1, "Just": 1, "- He's complaining": 1}, "adverbs": {"Only": 1, "Yes": 1}, "numerals": {"53": 1}}}, {"content": "54. I find the dormitories comfortable.", "word_count": 11, "label": "", "sentence_structure": "Subject鈥揷opula鈥損redicative", "subjective_objective": {"answer": "Subjective", "rationale": "Because \"like\" means it's a subjective feeling for the patient."}, "sentence_sentiment": {"answer": "Positive", "rationale": "Because the description \"I feel comfortable with the dormitories\" in the entry is positive, expressing the author's satisfaction with the dormitory environment."}, "language_style": {"answer": "Tactful and warm", "rationale": "This is a more acceptable expression by asking each other about the evaluation of the current dormitories, and by engaging in a more polite and moderate dialogue without directly criticizing or commending them."}, "time_dimension": {"answer": "Present", "rationale": "The entry represents the subjective perception and evaluation of the current dormitories by the testee, and the temporal dimension is now judged."}, "sentence_type": "Affirmative declarative sentence", "pos": {"nouns": {"Accommodation": 1, "Comfortable": 1}, "verbs": {"I think": 1}, "adjectives": {"Present": 1}, "numerals": {"54": 1}, "pronouns": {"I": 1}}}, {"content": "55. When I met strangers, I did not know how to talk to them.", "word_count": 17, "label": "", "sentence_structure": "Subject鈥搗erb鈥搊bject", "subjective_objective": {"answer": "Subjective", "rationale": "The basis for the decision: This question concerns the patient鈥檚 personal feelings and experience, that is, the patient does not know how to talk to strangers when he or she is faced with them. This is a subjective statement because it reflects the patient鈥檚 own inner and emotional state."}, "sentence_sentiment": {"answer": "Negative", "rationale": "Because the \"I don't know how to talk to them\" act is negative."}, "language_style": {"answer": "Calm and direct", "rationale": "The entry expresses the core of the problem directly in a concise and clear language, is not overly accommodative or emotional and is more objective and incriminating."}, "time_dimension": {"answer": "Present", "rationale": ""}, "sentence_type": "Negative declarative sentence", "pos": {"nouns": {"Stranger": 1, "Talk": 1}, "verbs": {"Yes": 1, "How's that": 1, "and": 1, "they": 1, "Talk": 1}, "adverbs": {"I met him": 1, "Time": 1}, "numerals": {"55锛?: 1}, "pronouns": {"I": 1}}}, {"content": "When I want to talk, I can always find someone to talk to.", "word_count": 16, "label": "", "sentence_structure": "Subject鈥搗erb鈥搊bject", "subjective_objective": {"answer": "Subjective", "rationale": "Because finding someone to talk is the patient's subjective wish and action."}, "sentence_sentiment": {"answer": "Positive", "rationale": "Because the entry \"can talk with people\" expresses a positive attitude and behavior."}, "language_style": {"answer": "Tactful and warm", "rationale": "The entry is more personal and interactive when it is spoken in a gentle and inviting manner, rather than in direct statements or orders. At the same time, the expression \"you always find people\" conveys a positive atmosphere of optimism and good human relations."}, "time_dimension": {"answer": "Present", "rationale": "The keywords \"want to talk,\" \" always able,\" \"get someone\" and \"talk together\" indicate that the act is in progress, so it's now."}, "sentence_type": "Affirmative declarative sentence", "pos": {"nouns": {"I": 1, "person": 2, "Chat": 2}, "verbs": {"Yes": 1, "Find": 1, "Together": 1, "Talk": 1}, "adverbs": {"Total": 1}, "numerals": {"56锛?: 1}}}, {"content": "57. I feel increasingly confused about what I will do in the future.", "word_count": 20, "label": "", "sentence_structure": "Subject鈥揷opula鈥損redicative", "subjective_objective": {"answer": "Subjective", "rationale": "Because \"I feel increasingly confused\" is a subjective feeling and thought."}, "sentence_sentiment": {"answer": "Negative", "rationale": "Because the entry is a lost and confused sentiment and is negative."}, "language_style": {"answer": "Calm and direct", "rationale": ""}, "time_dimension": {"answer": "Past", "rationale": "\"Feel\" means that it is a description and perception of the past, and \"getting confused\" means that it has been worsening or exacerbating for some time. The entry is therefore asking the testee about past behavior or mental changes and judging by past."}, "sentence_type": "Affirmative declarative sentence", "pos": {"nouns": {"self": 1, "The future": 1, "Work": 1}, "verbs": {"I think": 1, "Active": 1, "Frustration": 1}, "adverbs": {"Tsk": 1}, "numerals": {"57锛?: 1}}}, {"content": "58. When I am in a bad mood, I go out and get my head blown.", "word_count": 13, "label": "", "sentence_structure": "Subject鈥搗erb鈥揷omplement", "subjective_objective": {"answer": "Subjective", "rationale": "This is because the patient chooses the behaviour and expresses their subjective attitude towards how to deal with the bad mood."}, "sentence_sentiment": {"answer": "Positive", "rationale": "Because the \"out\" act in the entry is positive."}, "language_style": {"answer": "Tactful and warm", "rationale": "The entry is more personal and interactive in its expression of conversation in a gentle and inviting manner, rather than in direct statements or orders. At the same time, it implies an attitude of support and understanding that allows respondents to feel the attention and respect."}, "time_dimension": {"answer": "Present", "rationale": ""}, "sentence_type": "Affirmative declarative sentence", "pos": {"nouns": {"The mood": 1, "Time": 1, "Heart": 1}, "verbs": {"Yes": 2, "Get out": 1, "Spread out": 1}, "adjectives": {"Not good": 1}, "adverbs": {"Time": 1}, "numerals": {"58": 1}, "classifiers": {"individual": 1}, "pronouns": {"I": 1}}}, {"content": "59. I feel that I have become more and more adapted to university studies.", "word_count": 16, "label": "", "sentence_structure": "Subject鈥揷opula鈥損redicative", "subjective_objective": {"answer": "Subjective", "rationale": "Because \"accommodation\" is a personal feeling and a cognitive expression of the patient's subjective attitude and mental state."}, "sentence_sentiment": {"answer": "Positive", "rationale": "Because the \"increasing adaptation to university studies\" attitude in the entries is positive."}, "language_style": {"answer": "Tactful and warm", "rationale": "The entry uses a self-evaluation approach, which expresses its progress in a more \"increasing\" level of comparison and is more moderate and positive. Such language styles can easily resonate and identify with each other and help to create a good communication climate."}, "time_dimension": {"answer": "Present", "rationale": ""}, "sentence_type": "Affirmative declarative sentence", "pos": {"nouns": {"self": 1, "University": 1, "Learning": 1}, "verbs": {"I think": 1, "Adaptation": 1, "More and more": 1}, "adjectives": {"More and more": 1}, "adverbs": {"Tsk": 1}, "numerals": {"59": 1}}}]}

{"questionnaire_title": "Alcohol Dependency Questionnaire (MAST)", "questionnaire_category": "Behavioral questionnaire", "item_count": 24, "emotion_shift_overall": "No obvious trend", "language_style_overall": "Calm and direct", "items": [{"content": "I. Do you think you're normal in drinking?", "word_count": 12, "label": "", "sentence_structure": "Subject鈥損redicate", "subjective_objective": {"answer": "Subjective", "rationale": "Because \"you think\" is about the patient's personal views and attitudes, and it's a subjective examination."}, "sentence_sentiment": {"answer": "Neutral", "rationale": "Because the entry does not express any positive or negative emotional attitude, the interviewee was asked whether his or her drinking habits were normal."}, "language_style": {"answer": "Calm and direct", "rationale": "The entry is based on a simple question, which is direct, and asks the interviewee about his drinking habits."}, "time_dimension": {"answer": "Present", "rationale": "The entry asks the testee about his current drinking habits and the time dimension is now judged."}, "sentence_type": "Interrogative sentence", "pos": {"nouns": {"Drinking habits": 1, "Normal": 1}, "verbs": {"I think": 1, "be": 1}}}, {"content": "Have you ever had a drink the first night, and you wake up the next day and you can't remember what happened the first night?", "word_count": 28, "label": "", "sentence_structure": "Subject鈥搗erb鈥搊bject", "subjective_objective": {"answer": "Objective", "rationale": "Since it is an objective fact that patients experience first-hand whether to drink and forget part of their experience, it is subjective to examine subjective ideas."}, "sentence_sentiment": {"answer": "Negative", "rationale": "Because the entry \"Drunk-to-Drunk Can't Think About Part of the Life\" is negative."}, "language_style": {"answer": "Calm and direct", "rationale": ""}, "time_dimension": {"answer": "Present", "rationale": "The word \"had\" in it indicates the act that occurred in the past, and the description \"can't remember\" points to the memory of the past. The temporal dimension of this article is therefore a matter of the past."}, "sentence_type": "Interrogative sentence", "pos": {"nouns": {"Oh, my God": 2, "Drink": 1, "Next": 1, "Part of it": 1, "Things": 1}, "verbs": {"Yes": 1, "I don't remember": 1, "Wake up": 1}, "adverbs": {"First night": 1, "Tsang": 1, "Number of times": 1}, "numerals": {"2锛?: 1, "One": 2}}}, {"content": "Have your spouses, parents or other close relatives ever feared or complained about your drinking?", "word_count": 22, "label": "", "sentence_structure": "Subject鈥搗erb鈥搊bject", "subjective_objective": {"answer": "Subjective", "rationale": "Because fear or complaint of drinking is a subjective feeling and assessment of a person, it does not necessarily reflect objective facts."}, "sentence_sentiment": {"answer": "Negative", "rationale": "Because \"fear\" and \"complaint\" in the entries are negative emotional terms, which indicate that family members have a negative attitude towards your drinking behaviour."}, "language_style": {"answer": "Calm and direct", "rationale": "The article is based on the following criteria: the question is clear, and there is no excessive rhetorical or emotional overture. At the same time, an objective and neutral language is used, which is not preferred to either side. Such language is more in line with the professional requirements of the questionnaire and can produce more accurate and realistic results."}, "time_dimension": {"answer": "Present", "rationale": ""}, "sentence_type": "Interrogative sentence", "pos": {"nouns": {"Spouse": 1, "parents": 1, "Other": 1, "Close relatives": 1, "Drink": 1, "I'm worried": 1, "- He's complaining": 1}, "verbs": {"Yes, I did": 1}, "numerals": {"3锛?: 1}, "pronouns": {"you": 1}}}, {"content": "4. When you drink one or two glasses, can you stop drinking without trying?", "word_count": 22, "label": "", "sentence_structure": "Subject鈥搗erb鈥搊bject", "subjective_objective": {"answer": "Subjective", "rationale": "It is the patient ' s subjective attitude and ability to examine whether he or she can control his or her alcohol behaviour."}, "sentence_sentiment": {"answer": "Neutral", "rationale": "Because the entry does not express positive or negative feelings, attitudes, behaviour, purpose or quality, it merely raises a question."}, "language_style": {"answer": "Calm and direct", "rationale": "The language style of the entry is straightforward. It asks the interviewee about his or her ability to control himself after drinking, without using excessive emotional or rhetorical techniques, and with a sound and objective tone, making the problem more relevant and effective."}, "time_dimension": {"answer": "Present", "rationale": "The entry is for the testee ' s ability to act with control over alcohol, and the time dimension is now judged."}, "sentence_type": "Interrogative sentence", "pos": {"nouns": {"you": 1, "1": 1, "2": 1, "Cups": 2, "Wine": 1, "Restraint": 1, "self": 1, "Stop": 1}, "verbs": {"Drink": 1, "Yes": 1, "It's not hard": 1, "Just": 1, "Stop": 1}, "adjectives": {"None": 1}, "adverbs": {"Just": 1}, "numerals": {"1": 1, "2": 1}, "classifiers": {"Cups": 2}, "pronouns": {"self": 1}}}, {"content": "Have you ever felt guilty about drinking?", "word_count": 10, "label": "", "sentence_structure": "Subject鈥搗erb鈥搊bject", "subjective_objective": {"answer": "Subjective", "rationale": "Because guilt is a subjective feeling and emotional experience."}, "sentence_sentiment": {"answer": "Negative", "rationale": "Because the \"guilty\" sentiment in the entry is negative."}, "language_style": {"answer": "Tactful and warm", "rationale": "The language style of the entry is more euphemistic, and the question is expressed by using the word \"had\" as a reminder of and reflection on the past, rather than simply asking whether the other side still feels guilty. At the same time, the question is not too direct or prescriptive, but rather in a more friendly and understanding way."}, "time_dimension": {"answer": "Present", "rationale": "In the entry, the word \"had\" denotes past behaviour, but the question itself is whether the current situation is still guilty of drinking. The temporal dimension of the article should therefore be judged now."}, "sentence_type": "Interrogative sentence", "pos": {"nouns": {"Drink": 1, "Guilt": 1}, "verbs": {"Tsang": 1, "I feel": 1}, "adverbs": {"- You're not": 1}, "numerals": {"5": 1}}}, {"content": "Do your relatives and friends think you're normal in drinking?", "word_count": 15, "label": "", "sentence_structure": "Subject鈥搗erb鈥搊bject", "subjective_objective": {"answer": "Objective", "rationale": "This is an objective question, since it is an objective one to evaluate the normality of drinking habits from the perspective of others."}, "sentence_sentiment": {"answer": "Neutral", "rationale": "Because the entry does not express any positive or negative emotional attitude, it merely asks the other party for its views."}, "language_style": {"answer": "Calm and direct", "rationale": "The entry asks the other person, in a direct question, whether he or she considers his or her drinking habits normal, simpler and less vocal, and not too much rhetorical or emotional rendering."}, "time_dimension": {"answer": "Past", "rationale": "The entry asks about the views of the person ' s relatives and friends about his past drinking habits and thus the temporal dimension can be judged as past."}, "sentence_type": "Interrogative sentence", "pos": {"nouns": {"Friends": 1, "Custom": 1, "Drink": 1}, "verbs": {"I think": 1, "Normal": 1, "Drinking": 1}, "numerals": {"6锛?: 1}, "pronouns": {"you": 2}}}, {"content": "When you're not going to drink, can you do it?", "word_count": 16, "label": "", "sentence_structure": "Subject鈥損redicate", "subjective_objective": {"answer": "Subjective", "rationale": "The entry of the questionnaire examines the patient ' s subjective attitude and behavioural control and is therefore subjective in its perspective."}, "sentence_sentiment": {"answer": "Negative", "rationale": "Because the behaviour in the entry \"no alcohol\" is positive, but the words \"can we do it?\" indicate that it is challenging, needs to be overcome to achieve it, and therefore has a negative emotional tone."}, "language_style": {"answer": "Tactful and warm", "rationale": "The more euphemistic and warm language of the entry, which uses the means of questioning, and the more gentle way in which the respondents are asked, make it easier for them to respond with more willingness to share their real thoughts and feelings and to create a good atmosphere of communication."}, "time_dimension": {"answer": "Present", "rationale": "So, in terms of time dimension, this entry is now."}, "sentence_type": "Interrogative sentence", "pos": {"nouns": {"Time": 2, "you": 1, "Wine": 1}, "verbs": {"I'm going to": 1, "Do it": 1, "Yeah": 1}, "adverbs": {"not": 1}, "numerals": {"7锛?: 1}, "pronouns": {"When": 1}}}, {"content": "Have you ever been to an AA?", "word_count": 10, "label": "", "sentence_structure": "Subject鈥搗erb鈥搊bject", "subjective_objective": {"answer": "Objective", "rationale": "The basis for the judgement: participation in an event to stop drinking is a concrete act, an objective fact that the patient has experienced, not the patient鈥檚 thoughts or attitudes. This is therefore viewed from an objective perspective."}, "sentence_sentiment": {"answer": "Neutral", "rationale": "The entry is merely an inquiry about whether or not to have participated in alcohol abdication activities, and does not express any positive or negative emotional tendencies."}, "language_style": {"answer": "Tactful and warm", "rationale": "The entry is more euphemistic, in which the interviewee is asked to think and answer questions, without direct orders or judgment of the other party鈥檚 behaviour, and more gentle. This makes the interviewee more willing to share his/her true situation and enhances the effectiveness and credibility of the questionnaire."}, "time_dimension": {"answer": "Present", "rationale": ""}, "sentence_type": "Interrogative sentence", "pos": {"nouns": {"A clean drink": 1, "Activities": 1}, "verbs": {"Participation": 1}}}, {"content": "Did you ever fight after drinking?", "word_count": 10, "label": "", "sentence_structure": "Subject鈥損redicate", "subjective_objective": {"answer": "Objective", "rationale": "This is objective because it is an inquiry into past events and does not involve subjective feelings or attitudes of the patient."}, "sentence_sentiment": {"answer": "Negative", "rationale": "Because the \"battering\" in the entry is negative."}, "language_style": {"answer": "Calm and direct", "rationale": "The entry directly and explicitly asks the person who was tested whether he or she had had a conflict with another person after drinking, without using a terminological or euphemism, with a temperature and objectiveness, and meets the basic requirements of the questionnaire survey."}, "time_dimension": {"answer": "Present", "rationale": "The entry is an inquiry into whether the person tested had ever been involved in a fight after drinking, and the temporal dimension is a priori. The use of the word \"had\" in the title suggests that the issue was investigated and evaluated in relation to past events."}, "sentence_type": "Interrogative sentence", "pos": {"nouns": {"Drink": 1, "Fighting": 1, "person": 1}, "verbs": {"Tsang": 1, "and": 1, "Fighting": 1}, "adverbs": {"Back": 1}}}, {"content": "Have you ever had a conflict with your spouse, parents or other close relatives over alcohol consumption?", "word_count": 25, "label": "", "sentence_structure": "Subject鈥搗erb鈥搊bject", "subjective_objective": {"answer": "Subjective", "rationale": "This is because the patient is asked whether he has had such a subjective experience and is examined for his subjective feelings and behaviour."}, "sentence_sentiment": {"answer": "Negative", "rationale": "Because the entry \"on the basis of a conflict over drinking\" is negative."}, "language_style": {"answer": "Calm and direct", "rationale": "The entry asks the respondents in a direct and clear manner whether they have had any experience of having problems with their families because of alcohol problems. The temperature of the words and the lack of excessive emotional rendering or speech skills.\""}, "time_dimension": {"answer": "Past", "rationale": "The entry asks about past behaviour and state of affairs, and therefore is judged on the basis of the \"old\"."}, "sentence_type": "Interrogative sentence", "pos": {"nouns": {"Problem": 1, "Paradoxious": 1, "Close relatives": 1, "Spouse": 1, "parents": 1, "Drink": 1}, "verbs": {"Zeng Zhen": 1, "Generate": 1}, "adverbs": {"you": 1, "And": 1}, "numerals": {"10锛?: 1}}}, {"content": "Has your spouse (or other family members) ever sought help from others for your drinking?", "word_count": 25, "label": "", "sentence_structure": "Subject鈥搗erb鈥搊bject", "subjective_objective": {"answer": "Subjective", "rationale": "The questionnaire is intended to cover the patient鈥檚 personal behaviour and family relations and is therefore a subjective examination."}, "sentence_sentiment": {"answer": "Negative", "rationale": "Because the behaviour of the entries is \"drinking\" and the need to turn to others is all negative."}, "language_style": {"answer": "Calm and direct", "rationale": "The entry uses a direct question, and clearly asks whether the person surveyed and his/her spouse (or other family members) have sought help from others because of alcohol problems."}, "time_dimension": {"answer": "Past", "rationale": ""}, "sentence_type": "Interrogative sentence", "pos": {"nouns": {"Spouse": 1, "Family members": 1, "Things": 1, "Others": 1}, "verbs": {"Tsang": 1, "Yes": 1, "Help": 1}, "numerals": {"11": 1}, "pronouns": {"you": 1}}}, {"content": "Have you ever broken up with a friend because you drank?", "word_count": 14, "label": "", "sentence_structure": "Subject鈥搗erb鈥搊bject", "subjective_objective": {"answer": "Subjective", "rationale": "It is the patient's subjective experience and feelings to break up with his best friend because of the alcohol."}, "sentence_sentiment": {"answer": "Negative", "rationale": "Because the \"cause breakup with a friend\" act is negative."}, "language_style": {"answer": "Calm and direct", "rationale": "The entry is in a concise manner asking the interviewees whether they broke up with friends because of alcohol consumption, in plain language, without excessive ado or emotional rendering. \""}, "time_dimension": {"answer": "Past", "rationale": "The entry is for the purpose of judging the past by asking whether the person who was tested had broken up with a friend because of drinking."}, "sentence_type": "Interrogative sentence", "pos": {"nouns": {"Questionnaire": 1, "Entry": 1, "Alcohol": 1, "Friends": 1, "Break up": 1}, "verbs": {"Tsang": 1, "Result": 1}, "adverbs": {"Because": 1, "- You're not": 1}, "numerals": {"12": 1}}}, {"content": "Have you had problems at work and in your studies because you drank?", "word_count": 16, "label": "", "sentence_structure": "Subject鈥搗erb鈥搊bject", "subjective_objective": {"answer": "Objective", "rationale": "Because questions about work and learning are objective facts, they are not related to the patient ' s subjective attitude."}, "sentence_sentiment": {"answer": "Negative", "rationale": "Because \"problems at work, learning\" is a negative behavior."}, "language_style": {"answer": "Calm and direct", "rationale": "The entry directly asks whether the other party has had problems with alcohol consumption at work or in study, which is more direct and objective, and does not use over-recorative language."}, "time_dimension": {"answer": "Present", "rationale": "The entry now asks whether the person tested at some point in the past (i.e. after the last drink) has caused problems at work or learning because of alcohol consumption, and thus it is now judged."}, "sentence_type": "Interrogative sentence", "pos": {"nouns": {"Problem": 1, "Work": 1, "Learning": 1, "Drink": 1}, "verbs": {"Out": 1}, "numerals": {"Tsang": 1, "13": 1}}}, {"content": "Have you been subjected to excessive chastisement, warning or dismissal for drinking?", "word_count": 18, "label": "", "sentence_structure": "Subject鈥搗erb鈥搊bject", "subjective_objective": {"answer": "Objective", "rationale": "This is to be seen from an objective perspective, since the imposition of excessive sanctions, warnings or dismissals is based on objective facts and is not influenced by the patient鈥檚 subjective attitude."}, "sentence_sentiment": {"answer": "Negative", "rationale": "Because the act of \"excessive punishment, warning or dismissal\" in the entry is negative."}, "language_style": {"answer": "Calm and direct", "rationale": "The entry is based on a direct question about whether the person interviewed has been disciplined, warned or expelled for drinking, in a clear and euphemistic manner."}, "time_dimension": {"answer": "Past", "rationale": "The entry \"had\" denoted a past experience, while \"had\" indicated that it was a completed move and thus judged to be a past."}, "sentence_type": "Interrogative sentence", "pos": {"nouns": {"Drink": 1, "Disposition": 1, "Warning": 1, "Dismissed": 1}, "verbs": {"Received": 1, "That's too much": 1, "Zeng Zhen": 1}, "adverbs": {"very": 1}, "numerals": {"14": 1}}}, {"content": "Have you been drinking for more than two days and without taking care of your family?", "word_count": 23, "label": "", "sentence_structure": "Subject鈥搗erb鈥搊bject", "subjective_objective": {"answer": "Subjective", "rationale": "It is subjective in view of the fact that it involves the thoughts and behaviour of the patient."}, "sentence_sentiment": {"answer": "Negative", "rationale": "Because the entry \"for drinking, for more than two days, delayed work or did not take care of the family\" is negative."}, "language_style": {"answer": "Calm and direct", "rationale": "The entry is based on a direct question as to whether the person surveyed was delayed in his or her work or did not take care of the family for more than two days. The sentence is straightforward and does not contain any qualifier or emotional colour."}, "time_dimension": {"answer": "Present", "rationale": "The entry is for questioning the testee's past behaviour, the temporal dimension of which is judged to be the past. \"At one point, \"was an event in the past, and \"drinking causes more than two days of work or neglecting the family\" is a specific act of the past. Thus, the purpose of the article is to judge the past."}, "sentence_type": "Interrogative sentence", "pos": {"nouns": {"you": 1, "Drink": 1, "Ongoing": 1, "Two days": 1, "Above": 1, "Hold on": 1, "Work": 1, "or": 1, "No care": 1, "Family": 1}, "verbs": {"Zeng Zhen": 1, "Drinking wine": 1, "Ongoing": 1, "Hold on": 1, "Care": 1}, "adverbs": {"Two days or more": 1}, "numerals": {"15": 1, "1": 1, "2": 1}, "classifiers": {"individual": 1}}}, {"content": "Do you drink often in the morning?", "word_count": 9, "label": "", "sentence_structure": "Subject鈥損redicate", "subjective_objective": {"answer": "Subjective", "rationale": "Because the drink is the patient's subjective choice."}, "sentence_sentiment": {"answer": "Negative", "rationale": "Because \"drinking\" is a negative act."}, "language_style": {"answer": "Calm and direct", "rationale": "The entry asks the respondents in a direct and clear manner whether they are in a habit of drinking, in a clear language, with no excessive emotional or orality, and is typical of a calm and direct expression."}, "time_dimension": {"answer": "Present", "rationale": "The emergence of the keywords \"frequent\" and \"morning\" indicates that the act is occurring frequently within the current time frame and that the specific time at which the act occurs is morning, so the entry describes the present act or habit."}, "sentence_type": "Interrogative sentence", "pos": {"nouns": {"a.m": 1, "Drink up": 1}, "verbs": {"Regular": 1, "in": 1, "Drink": 1, "Spray": 1}, "adverbs": {"you": 1}, "numerals": {"16": 1}}}, {"content": "Did the doctor say you had liver problems or cirrhosis?", "word_count": 16, "label": "", "sentence_structure": "Subject鈥搗erb鈥搊bject", "subjective_objective": {"answer": "Objective", "rationale": "This is a question of whether a patient is diagnosed with liver disease by a doctor, based on the professional judgement and medical records of the doctor, rather than on the patient ' s subjective feelings."}, "sentence_sentiment": {"answer": "Negative", "rationale": "Because \"hepatic problems or cirrhosis\" in the entry is negative."}, "language_style": {"answer": "Calm and direct", "rationale": "The entry directly asks whether the person interviewed has been informed by a doctor of liver problems or of cirrhosis, is more objective and direct in language style, and does not have too much emotional or oral skills."}, "time_dimension": {"answer": "Past", "rationale": "The entry asks if the person tested had past medical diagnosis of liver problems or experience of cirrhosis, and therefore judged to be past."}, "sentence_type": "Interrogative sentence", "pos": {"nouns": {"Doctor": 1, "Problem": 1, "Hepatic cirrhosis": 1}, "verbs": {"I said": 1, "have": 2}, "adverbs": {"Tsang": 1, "or": 1}, "numerals": {"17": 1}, "pronouns": {"you": 1}}}, {"content": "Have you ever had a twilight after drinking in large quantities? 18' Have you ever had a serious tremor or a vision after drinking in large quantities?", "word_count": 35, "label": "", "sentence_structure": "Subject鈥搗erb鈥搊bject", "subjective_objective": {"answer": "Objective", "rationale": "Because these symptoms are objective phenomena that patients may have after drinking in large quantities, they are not subjective."}, "sentence_sentiment": {"answer": "Neutral", "rationale": "Basis of judgement: The entry describes only behaviours and phenomena that may occur after extensive drinking, and does not have any emotional colour."}, "language_style": {"answer": "Calm and direct", "rationale": "Both questions are straightforward and clear, and are not expressed in too much qualifier or emotional terms. They ask the respondents directly whether they have experienced certain specific symptoms after drinking a lot, and are more objective and calm.\""}, "time_dimension": {"answer": "Past", "rationale": "The entry contains two parts, respectively, about the testee's past and present history of alcohol abuse. The first part, \"Have you ever had a haze after a lot of alcohol? \", is about past alcohol abuse and the symptoms that this has caused; the second part, \"Have you ever had a severe tremor or hearing illusion after a lot of alcohol?\" is about the persistence of past alcohol abuse. Thus, the time dimension of the article is both past and present."}, "sentence_type": "Interrogative sentence", "pos": {"nouns": {"Drink": 2, "You're a good man": 1, "Serious": 1, "鈾?The tremors 鈾?: 1, "I'm hearing things": 1, "Vision": 1}, "verbs": {"- Yes": 3}, "numerals": {"Mass": 2}}}, {"content": "Have you ever sought help from anyone for problems caused by drinking?", "word_count": 17, "label": "", "sentence_structure": "Subject鈥搗erb鈥搊bject", "subjective_objective": {"answer": "Subjective", "rationale": "The question concerns the patient鈥檚 subjective attitude and behaviour choices, i.e. whether he has sought help from others. While the question concerns the objective fact of drinking, it is primarily about the patient鈥檚 subjective reaction to the problem of drinking."}, "sentence_sentiment": {"answer": "Negative", "rationale": "Because the incident \"problems arising from drinking\" was itself negative, and because the question whether the interviewees sought help from others was based on that fact, the emotional colour of the article was negative."}, "language_style": {"answer": "Tactful and warm", "rationale": "The entry is more euphemistic and sensitive to the individual's feelings, using the guiding term \"you were\" instead of direct orders or inquiries, making it easier for the respondents to accept and answer questions. At the same time, the description \"Questions arising from drinking\" is more objective and neutral, and does not make the respondents feel stigmatized or ashamed."}, "time_dimension": {"answer": "Past", "rationale": "According to the keyword \"had\" and \"help with others\", it can be seen that the entry asks about past behaviour or experience, and therefore is judged to be \"the past\"."}, "sentence_type": "Interrogative sentence", "pos": {"nouns": {"Problem": 1, "Others": 1}, "verbs": {"Zeng Zhen": 1, "Cause": 1, "Help": 1, "Go on": 1}, "adverbs": {"Tsang": 1}, "numerals": {"19": 1}, "pronouns": {"you": 1}}}, {"content": "Have you ever been hospitalized for problems caused by drinking?", "word_count": 16, "label": "", "sentence_structure": "Subject鈥搗erb鈥搊bject", "subjective_objective": {"answer": "Objective", "rationale": "It is a fact that can be proved whether or not to be hospitalized because of problems caused by alcohol consumption."}, "sentence_sentiment": {"answer": "Negative", "rationale": "Because the result of \"hospital\" is negative."}, "language_style": {"answer": "Calm and direct", "rationale": "This language style is more in line with the questionnaire and helps researchers to obtain more accurate answers from the respondents."}, "time_dimension": {"answer": "Past", "rationale": "The entry asked whether the person tested had been hospitalized in the past for drinking and the time dimension was judged to be past."}, "sentence_type": "Interrogative sentence", "pos": {"nouns": {"Problem": 1, "Hospital": 1, "Drink": 1}, "verbs": {"I've lived here": 1, "Cause": 1}, "numerals": {"20": 1}}}, {"content": "Have you ever been hospitalized in psychiatric departments in psychiatric hospitals or general hospitals because of problems caused by drinking?", "word_count": 28, "label": "", "sentence_structure": "Subject鈥搗erb鈥搊bject", "subjective_objective": {"answer": "Objective", "rationale": "This is an objective fact of the patient ' s experience."}, "sentence_sentiment": {"answer": "Negative", "rationale": "Because of the negative consequences of the act and the result of \"hospitalization in psychiatric wards or general hospitals\"."}, "language_style": {"answer": "Calm and direct", "rationale": "The test is based on: - The question-and-answer approach used for this article is a direct question-and-answer language rather than a euphemism. - The entries are also sensitive and personal, and therefore the use of direct questions is more effective in collecting relevant information."}, "time_dimension": {"answer": "Past", "rationale": "The entry \"had\" indicated that it was a past act, and therefore the temporal dimension was a past."}, "sentence_type": "Interrogative sentence", "pos": {"nouns": {"Problem": 1, "Alcohol": 1, "The court": 2, "Hospitalization": 1, "Psychiatric facility": 1, "General hospital": 1, "Psychiatric": 1}, "verbs": {"Cause": 1, "in": 1, "I've lived here": 1, "Tsang": 1}, "adverbs": {"Because": 1, "Tsang": 1}, "numerals": {"21": 1, "1": 6}, "classifiers": {"individual": 1}, "pronouns": {"you": 1}}}, {"content": "You have sought psychiatric, other doctors, social workers, psychological counselling for emotional problems caused by alcohol consumption.", "word_count": 32, "label": "", "sentence_structure": "Subject鈥搗erb鈥搊bject", "subjective_objective": {"answer": "Subjective", "rationale": "This is the situation in which the patient has experienced seeking help and is the patient ' s subjective feeling and behaviour."}, "sentence_sentiment": {"answer": "Negative", "rationale": "Because the entry describes the negative situation of the need for help, which is caused by emotional problems caused by alcohol consumption."}, "language_style": {"answer": "Calm and direct", "rationale": "The entry uses a simple and clear language and asks the interviewee whether he or she has sought professional help because of the emotional problems caused by alcohol consumption."}, "time_dimension": {"answer": "Past", "rationale": ""}, "sentence_type": "Interrogative sentence", "pos": {"nouns": {"Alcohol dependency": 1, "Emotional problems": 1, "Psychiatric": 1, "Doctor": 1, "Social workers": 1, "Consultants": 1, "Help": 1}, "verbs": {"Result": 1, "Help": 2}, "numerals": {"22": 1}}}, {"content": "Have you ever been detained for drinking or driving? (If so, how many times?)", "word_count": 23, "label": "", "sentence_structure": "Subject鈥搗erb鈥搊bject", "subjective_objective": {"answer": "Objective", "rationale": "The question is whether the patient has ever acted in such a way, not their subjective thoughts or feelings."}, "sentence_sentiment": {"answer": "Negative", "rationale": "The entry \"Detention for drinking or driving drunk\" is negative."}, "language_style": {"answer": "Calm and direct", "rationale": "The entry uses direct and clear language to ask if the person interviewed has been detained for driving alcohol and further requests."}, "time_dimension": {"answer": "Past", "rationale": "In the entry, the word \"had\" indicates that there was a past movement, and that there was a past act of \"detention.\" And, \"had\" and \"how many times had it been?\" was a question of quantitative information about the past, which further confirmed that the act had occurred in the past. Thus, the temporal dimension of the article covers both the past and the present."}, "sentence_type": "Interrogative sentence", "pos": {"nouns": {"you": 1, "Drink": 1, "Drunk": 1, "Drive": 1, "Detention": 1, "How many times": 1}, "verbs": {"Zeng Zhen": 1, "Yes": 1, "Detention": 1}, "adverbs": {"or": 1, "Back": 1, "Drunk": 1, "Total": 1}, "numerals": {"23": 1, "How many times": 1}}}, {"content": "Have you been detained for other drinking for hours? (If so, how many times?)", "word_count": 25, "label": "", "sentence_structure": "Subject鈥搗erb鈥搊bject", "subjective_objective": {"answer": "Objective", "rationale": "This is an objective fact-finding exercise of the patient ' s past experience."}, "sentence_sentiment": {"answer": "Negative", "rationale": "Because the \"detention\" in the entry is negative."}, "language_style": {"answer": "Calm and direct", "rationale": "The entry is based on a simple and straightforward inquiry into whether the person interviewed has been detained for drinking and the number of times, with no excessive adoration or emotional colours, and a more objective tone."}, "time_dimension": {"answer": "Present", "rationale": "The entry is an inquiry into whether the person was detained for drinking in the past, but it is not clear whether the time frame is past, present or future. Therefore, the answer to the question needs to be judged by the actual situation of the person. If the person who was tested is responding to the previous situation, the time dimension of the entry can be judged to be past; if the person is responding to the present situation, the time dimension of the entry can be judged to be present."}, "sentence_type": "Interrogative sentence", "pos": {"nouns": {"Alcohol behaviour": 1, "Detention": 1, "Hours": 1}, "verbs": {"By": 1, "Yes": 1, "Total": 1, "How many times": 1}, "adverbs": {"Zeng Zhen": 1}, "numerals": {"How many": 1, "Once": 1}, "classifiers": {"individual": 1}, "pronouns": {"you": 1}}}]}

{"questionnaire_title": "Professional character test scale", "questionnaire_category": "Mental health", "item_count": 186, "emotion_shift_overall": "From negative to positive", "language_style_overall": "Neutral", "items": [{"content": "I am aware of the instructions of this test:", "word_count": 10, "label": "", "sentence_structure": "Subject鈥搗erb鈥搊bject", "subjective_objective": {"answer": "Subjective", "rationale": "Because the \"very clear\" evaluation is usually based on individual understanding and feelings, it is viewed from the patient's subjective perspective."}, "sentence_sentiment": {"answer": "Neutral", "rationale": "Because the entry does not have any emotional color."}, "language_style": {"answer": "Calm and direct", "rationale": "\"Calm down straight.\""}, "time_dimension": {"answer": "Present", "rationale": ""}, "sentence_type": "Interrogative sentence", "pos": {"nouns": {"Test": 1, "Annotations": 1}, "verbs": {"very": 1, "I see": 1}}}, {"content": "2. I can answer every question of this test honestly: if I had the opportunity, I would:", "word_count": 31, "label": "", "sentence_structure": "Subject鈥搗erb鈥搊bject", "subjective_objective": {"answer": "Subjective", "rationale": "The entry is based on the patient ' s attitude and behaviour choices when filling out the questionnaire and is the patient ' s subjective thinking and behaviour."}, "sentence_sentiment": {"answer": "Positive", "rationale": "Because the approach in the entry is positive, the intention is to answer the questions honestly and actively participate in the testing."}, "language_style": {"answer": "Tactful and warm", "rationale": ""}, "time_dimension": {"answer": "Present", "rationale": ""}, "sentence_type": "Interrogative sentence", "pos": {"nouns": {"Test": 1, "Problem": 1, "Opportunities": 1}, "verbs": {"Do it": 1, "Answer": 2, "Yes": 1}, "adjectives": {"Honestly": 1, "Every one of them": 1}, "adverbs": {"If": 1, "Both": 1, "If you": 1}, "numerals": {"2": 1}, "pronouns": {"I": 3}}}, {"content": "3. I am capable of coping with difficulties:", "word_count": 10, "label": "", "sentence_structure": "Subject鈥損redicate", "subjective_objective": {"answer": "Subjective", "rationale": "This is because patients evaluate and perceive their abilities and fall within the realm of subjective attitudes and beliefs."}, "sentence_sentiment": {"answer": "Neutral", "rationale": "The basis for the determination: the entry is a description of competence, does not involve emotional or attitudinal colours and is therefore neutral."}, "language_style": {"answer": "Tactful and warm", "rationale": "The entry is more adept and interactive when it is spoken in a gentle and inviting manner, rather than by direct statements or orders."}, "time_dimension": {"answer": "Present", "rationale": "The keyword \"I'm now\" is a state or condition of the moment, so the entry describes the current level of capability of the person being tested."}, "sentence_type": "Affirmative declarative sentence", "pos": {"nouns": {"Capacity": 1, "Difficulties": 1}, "verbs": {"have": 1, "Payable": 1}, "numerals": {"3": 1}}}, {"content": "4. Even the beasts in the cages are a source of concern:", "word_count": 22, "label": "", "sentence_structure": "Subject鈥損redicate", "subjective_objective": {"answer": "Subjective", "rationale": "Because \"fearing\" is the patient's subjective feeling and emotional reaction."}, "sentence_sentiment": {"answer": "Negative", "rationale": "Because the behavior in the entry is \"fearful\" is negative."}, "language_style": {"answer": "Calm and direct", "rationale": "The entry is presented in a direct presentation, without any aesthetic or emotional rendering. This language style is calm and objective, and it accurately conveys the information that researchers want to understand."}, "time_dimension": {"answer": "Present", "rationale": "The keyword \"even\" means \"even\" is a hypothetical situation, and \"see\" means \"is an act that is being committed.\" So the entry describes the person's current state of feeling and emotion."}, "sentence_type": "Affirmative declarative sentence", "pos": {"nouns": {"Iron cage": 1, "Beast": 1}, "verbs": {"Off": 1, "See": 1, "Yes": 1, "I feel": 1, "I'm not sure": 1}, "adjectives": {"Iron": 1, "- Yeah": 1, "Unsettled": 1}, "numerals": {"4": 1}}}, {"content": "I was always afraid to criticize others:", "word_count": 14, "label": "", "sentence_structure": "Subject鈥搗erb鈥揷omplement", "subjective_objective": {"answer": "Subjective", "rationale": "Because the idea of being afraid and critical of others is the patient's subjective attitude."}, "sentence_sentiment": {"answer": "Negative", "rationale": "Because the entry \"failed to criticize others' words and deeds\" expresses a negative emotional attitude, namely, fear or lack of courage to express their views and opinions."}, "language_style": {"answer": "Calm and direct", "rationale": "The entry asks in a direct and clear manner whether the other party often feels afraid to criticize others for their words and deeds, without excessive ado or emotional rendering.\""}, "time_dimension": {"answer": "Present", "rationale": "The entry describes a customary act, \"always dared not to criticize others' behaviour\", which has not only happened in the past, but may still be ongoing. Thus, the temporal dimension is now judged."}, "sentence_type": "Negative declarative sentence", "pos": {"nouns": {"Criticism": 1, "others": 1, "Words and lines": 1}, "verbs": {"Always": 1, "I dare you": 1, "How dare you": 1, "Criticism": 1}, "adverbs": {"Always": 1, "I dare you": 1, "How dare you": 1}, "numerals": {"5": 1}, "pronouns": {"I": 1}}}, {"content": "My thoughts seem to be that:", "word_count": 6, "label": "", "sentence_structure": "Subject鈥揷opula鈥損redicative", "subjective_objective": {"answer": "Subjective", "rationale": "Because the idea of \"me\" is patient subjective experience and understanding."}, "sentence_sentiment": {"answer": "Neutral", "rationale": "The entry is not marked by any emotional, attitudinal or behavioural tendencies."}, "language_style": {"answer": "Calm and direct", "rationale": "The questionnaire entries are more calm and straightforward in language, and are not pediculated with excessive rhetorical or emotional colours. It asks respondents in simple and straightforward questions about their state of mind, which is more objective and direct, and is unlikely to lead them."}, "time_dimension": {"answer": "Present", "rationale": "The entry is for the purpose of questioning the subjective feelings and way of thinking of the subject, and the keywords \"think\" and \"seemingly\" point to a description of the individual's mental state, and therefore the judgement is now."}, "sentence_type": "Interrogative sentence", "pos": {"verbs": {"Seems like": 1}, "adjectives": {"Mine": 1, "Ideas": 1}}}, {"content": "I'm not good at jokes, but I'm good at funny things:", "word_count": 12, "label": "", "sentence_structure": "Subject鈥搗erb鈥搊bject", "subjective_objective": {"answer": "Subjective", "rationale": "Because \"not good\" and \"jokes\" and \"jokes\" and \"fun\" are all about the ability and interest of individuals in terms of their subjective views, and about the assessment and feelings of patients."}, "sentence_sentiment": {"answer": "Negative", "rationale": "Because the expression \"not good at jokes, telling funny things\" in the entry is negative."}, "language_style": {"answer": "Calm and direct", "rationale": "The language style of the entry is relatively simple and does not involve excessive rhetorical or emotional rendering, presenting the survey in an objective and direct manner."}, "time_dimension": {"answer": "Past", "rationale": "The keyword \"not good\" is about past experience and the current state of affairs."}, "sentence_type": "Negative declarative sentence", "pos": {"nouns": {"A joke": 1, "Yes": 1}, "verbs": {"Good at it": 1, "Speak": 1}, "adjectives": {"Interesting": 1}, "numerals": {"7": 1}}}, {"content": "When I saw a neighbor or a new friend arguing, I always:", "word_count": 15, "label": "", "sentence_structure": "Subject鈥搗erb鈥搊bject", "subjective_objective": {"answer": "Subjective", "rationale": "This is the patient鈥檚 subjective attitude and way of responding, and the feeling and behavioral orientation of the person in the context of the argument."}, "sentence_sentiment": {"answer": "Neutral", "rationale": "As the entry does not describe any positive or negative behaviour, attitude or emotion, it raises a question."}, "language_style": {"answer": "Calm and direct", "rationale": "The entry raises questions directly and clearly gives the option of choice to the interviewee, which is more direct and objective."}, "time_dimension": {"answer": "Present", "rationale": "\"I always\" means customary behaviour, and \"when I see a fight\" indicates a constant, continuous pattern. So the entry describes the present behavioural orientation of the testee."}, "sentence_type": "Interrogative sentence", "pos": {"nouns": {"Neighbors": 1, "Argument": 1, "New Friends": 1}, "verbs": {"Yes": 1, "Always": 1}}}, {"content": "10. During the mass rally, I:", "word_count": 7, "label": "", "sentence_structure": "Subject鈥損redicate", "subjective_objective": {"answer": "Subjective", "rationale": "Because this is a question of how the patient feels and what he thinks."}, "sentence_sentiment": {"answer": "Neutral", "rationale": "Because the entry describes only the behaviour of a person and does not contain any emotional colour or evaluation."}, "language_style": {"answer": "Calm and direct", "rationale": "The entry uses direct and concise language, does not have too many rhetorical or emotional colours and asks the interviewees in a clear manner about their behaviour during the mass rally. \""}, "time_dimension": {"answer": "Present", "rationale": "The entry is for the present behaviour or state of the subject, and the keywords \"gang\" and \"me\" indicate that this is an issue of current status and can be judged as now."}, "sentence_type": "Imperative sentence", "pos": {"nouns": {"Mass rallies": 1, "I": 1}, "verbs": {"in": 1}}}, {"content": "I would like to make one:", "word_count": 6, "label": "", "sentence_structure": "Subject鈥搗erb鈥搊bject", "subjective_objective": {"answer": "Subjective", "rationale": "Because \"will\" is a word for the patient's will, and it's in the realm of subjective attitudes."}, "sentence_sentiment": {"answer": "Neutral", "rationale": "Since the entry does not describe specific behaviour, attitudes or feelings, it merely raises a question."}, "language_style": {"answer": "Tactful and warm", "rationale": "The language style of the article could therefore be considered \"precipitous\"."}, "time_dimension": {"answer": "Present", "rationale": "According to the key word in the sentence, \"I do\", it can be seen that the entry asks about the attitude and will of the person being tested in one respect, so it can be judged that the time dimension is now."}, "sentence_type": "Interrogative sentence", "pos": {"nouns": {"Questionnaire": 1, "Entry": 1}, "verbs": {"Yes": 1, "Do it": 1, "Here": 1}, "numerals": {"11": 1}, "pronouns": {"One": 1}}}, {"content": "12. When reading, I like to choose:", "word_count": 8, "label": "", "sentence_structure": "Subject鈥搗erb鈥搊bject", "subjective_objective": {"answer": "Subjective", "rationale": "Because it is the personal interest and preference of the patient who is interested in reading, this is a manifestation of his subjective attitude."}, "sentence_sentiment": {"answer": "Neutral", "rationale": "Because the entry is merely a question of the reading habits of the other party, and it does not have any emotional colour."}, "language_style": {"answer": "Calm and direct", "rationale": "The entry is a clear and direct question, asking which way the interviewee prefers to read."}, "time_dimension": {"answer": "Present", "rationale": "The entry asks the testee for his current behaviour or preferences, so the time dimension is now judged."}, "sentence_type": "Interrogative sentence", "pos": {"nouns": {"Reading": 1, "Yeah": 1, "Select to Read": 1}, "verbs": {"Select to Read": 1}, "numerals": {"12": 1}}}, {"content": "I think that many people are a little bit out of their mind, but they are reluctant to admit that:", "word_count": 22, "label": "", "sentence_structure": "Subject鈥搗erb鈥搊bject", "subjective_objective": {"answer": "Subjective", "rationale": "Because \"conception\" and \"resistance\" are subjective attitudes or perceptions of patients."}, "sentence_sentiment": {"answer": "Negative", "rationale": "Because the phrase \"a lot of people are out of their minds\" in the entry is negative."}, "language_style": {"answer": "Calm and direct", "rationale": "The language style of the entry is relatively direct and frank, with no excessive rhetoric or emotional qualification, and gives a brief and straightforward expression of concern and perception of the mental health of the population."}, "time_dimension": {"answer": "Present", "rationale": "The keywords \"I think\" and \"both\" are expressed in some ways, indicating that it is the present view or opinion of the speaker and is subjective. At the same time, \"only they are unwilling to admit\" also indicates a phenomenon or state, not past experience or future expectations, so it can be judged that the entry describes the present situation."}, "sentence_type": "Interrogative sentence", "pos": {"nouns": {"person": 2, "In my heart": 1, "A lot": 1, "It's not normal": 1}, "verbs": {"I think": 1, "Some": 1, "have": 1, "I'm willing to admit it": 1}, "adjectives": {"Normal": 1, "Both": 1}, "adverbs": {"Just": 1}, "numerals": {"13": 1}, "classifiers": {"Some": 1}, "pronouns": {"they": 1}}}, {"content": "14. I hope that my lover is good at communication and does not need to be able to:", "word_count": 19, "label": "", "sentence_structure": "Subject鈥搗erb鈥揷omplement", "subjective_objective": {"answer": "Subjective", "rationale": "Because hope is patient's subjective attitude."}, "sentence_sentiment": {"answer": "Positive", "rationale": "Because the attitude of \"I hope my lover is good at making love\" is positive."}, "language_style": {"answer": "Tactful and warm", "rationale": "The entry uses euphemism, attempts to convey the expectations and preferences of the investigator and avoid direct orders or criticism."}, "time_dimension": {"answer": "Present", "rationale": "The entry asks the testee about his/her lover ' s expectations, and the time dimension is now judged."}, "sentence_type": "Interrogative sentence", "pos": {"nouns": {"Lover": 1, "Communication": 1, "Arts talent": 1}, "verbs": {"Hope": 1, "Yes": 1}, "numerals": {"14": 1}, "pronouns": {"I": 1}}}, {"content": "15. I can still treat with courtesy those who are impulsive and angry:", "word_count": 19, "label": "", "sentence_structure": "Subject鈥搗erb鈥搊bject", "subjective_objective": {"answer": "Subjective", "rationale": "Because being polite is patient's subjective attitude."}, "sentence_sentiment": {"answer": "Neutral", "rationale": "Because the entry does not express a positive or negative emotional profile. While it refers to the description of \"sexual impatience\" and \"freakyness,\" this does not mean that the attitude towards such persons is negative or positive. The purpose of the article is to show that even when faced with such a person, it can be treated with courtesy, which is more a description of behaviour or quality than emotional."}, "language_style": {"answer": "Tactful and warm", "rationale": "This is a more friendly way of speaking, which is more gentle, using polite, respectful and understanding language to express attitudes towards people who are impatient and angry."}, "time_dimension": {"answer": "Past", "rationale": "The entry describes the behaviour or attitude of the testee in the past, which is \"I can still treat you with courtesy\"."}, "sentence_type": "Affirmative declarative sentence", "pos": {"nouns": {"- A personality": 1, "Routine": 1, "person": 2, "I": 1}, "verbs": {"Let's go": 1, "Yes": 1, "Here": 1, "Stay with each other": 1}, "adjectives": {"Routine": 1}, "adverbs": {"Still": 1, "Yes": 1}, "numerals": {"15": 1}, "classifiers": {"individual": 2}, "pronouns": {"I": 1}}}, {"content": "I am often disturbed when I am served:", "word_count": 12, "label": "", "sentence_structure": "Subject鈥搗erb鈥揷omplement", "subjective_objective": {"answer": "Subjective", "rationale": "Because \"susperate\" is a subjective feeling and a psychological state of mind."}, "sentence_sentiment": {"answer": "Negative", "rationale": "Because the entry \"suspicion\" expresses a negative attitude."}, "language_style": {"answer": "Calm and direct", "rationale": "The language style of the entry is calmer and more direct, with no excessive rhetorical or emotional rendering, but rather a direct presentation of the subject of the inquiry, i.e. whether it is disturbing to be served. This tone is more objective and clear, helping the interviewee to understand the question accurately and to give the correct answer."}, "time_dimension": {"answer": "Present", "rationale": "Now the word \"time\" in \"time of service\" means behaviour at a certain moment, while \"often\" means frequent, customary behaviour. Thus, \"when I am often disturbed when served, \" describes a normal state of mind or feeling, which refers to situations in which the person interviewed is often nervous and uncomfortable when served by others, a situation that can be and continues to be."}, "sentence_type": "Imperative sentence", "pos": {"nouns": {"person": 1, "It's a little disturbing": 1}, "verbs": {"Yes": 1, "- The service": 1, "Often": 1}, "adjectives": {"Ann": 1}, "adverbs": {"Stimulation": 1}, "classifiers": {"individual": 1}}}, {"content": "17. After performing physical or intellectual work, I always need more rest time from others to maintain the efficiency of my work:", "word_count": 35, "label": "", "sentence_structure": "Subject鈥搗erb鈥搊bject", "subjective_objective": {"answer": "Subjective", "rationale": "Because it's the patient's subjective perception of his or her situation."}, "sentence_sentiment": {"answer": "Negative", "rationale": "Because the entry is a situation where more rest time is required, this may affect the efficiency of the work and therefore have a negative emotional connotation."}, "language_style": {"answer": "Calm and direct", "rationale": "The entry is more direct and objective in its language style, with no excessive rhetorical or emotional rendering. It asks whether the interviewee needs more time to recover energy after manual or intellectual work in order to maintain efficiency, speech temperature and straightforwardness, without too much subjective emotion or personal colour."}, "time_dimension": {"answer": "Present", "rationale": "The entry describes the behaviour or state of the person being tested, which is now often present, i.e., the need for more time to restore and maintain work efficiency after manual or intellectual work. The answer is therefore \"now\"."}, "sentence_type": "Affirmative declarative sentence", "pos": {"nouns": {"Physical strength": 1, "Brain": 1, "Labour": 1, "person": 1, "others": 1, "Time": 1, "Efficiency": 1}, "verbs": {"have": 1, "Yes": 2, "Hold on": 1}, "adjectives": {"More": 1, "of": 3, "Yes": 1}, "adverbs": {"Always": 1}, "numerals": {"17": 1}, "pronouns": {"I": 1}}}, {"content": "I woke up in the middle of the night, often in a state of anxiety, unable to sleep:", "word_count": 17, "label": "", "sentence_structure": "Subject鈥損redicate", "subjective_objective": {"answer": "Subjective", "rationale": "Because \"often\" means that it's a patient's own experience and feelings, and it's a subjective experience."}, "sentence_sentiment": {"answer": "Negative", "rationale": "Because the \"sleepless for all kinds of unease\" act in the entry is negative."}, "language_style": {"answer": "Tactful and warm", "rationale": ""}, "time_dimension": {"answer": "Present", "rationale": "The entry uses descriptions of \"often\" and \"not sleeping\" and the keyword refers to the situation that the person being tested is experiencing on a regular basis. The time dimension of the article is therefore judged to be now."}, "sentence_type": "Affirmative declarative sentence", "pos": {"nouns": {"At midnight": 1, "Wake up": 1, "All of them": 1, "Unsettled": 1, "Sleep": 1}, "verbs": {"Regular": 1, "Yes": 1}, "adverbs": {"Often": 1, "Both": 1}, "numerals": {"18": 1, "One": 1}, "classifiers": {"individual": 1}, "pronouns": {"I": 2}}}, {"content": "19. When things went wrong, I often had to cry and talk:", "word_count": 18, "label": "", "sentence_structure": "Subject鈥損redicate", "subjective_objective": {"answer": "Subjective", "rationale": "Because \"sweeting\" is the subjective reaction and emotional expression of patients when they encounter problems."}, "sentence_sentiment": {"answer": "Negative", "rationale": "Because the \"salary communication\" in the entry is a negative emotional reaction."}, "language_style": {"answer": "Calm and direct", "rationale": "The entry describes the individual's behaviour and reaction when things go wrong. There is no qualifier or emotional rendering, and the language is very straightforward and objective.\""}, "time_dimension": {"answer": "Present", "rationale": "\"When things go wrong\" indicates that this act happened in the past and now, and that the word \"often\" in \"I'm often rushing to communicate with aldicarb\" indicates regular, customary behaviour, while the word \"hurt to communicate with the aldicarb\" uses the current state of affairs, which means that this act may have occurred many times in the past or to date. So, this entry describes the current behaviour."}, "sentence_type": "Affirmative declarative sentence", "pos": {"nouns": {"Yes": 1, "Love": 1, "Conduct": 1, "Time": 2, "Regular": 1, "Tears": 1, "Communications": 1}, "verbs": {"not": 1, "Good": 1, "I'm in a hurry": 1, "Aldicarb-tear communication": 1}, "adverbs": {"Often": 1}, "numerals": {"19": 1}, "classifiers": {"individual": 1}}}, {"content": "20. I thought that divorce was not bound by traditional beliefs, provided that both parties agreed:", "word_count": 23, "label": "", "sentence_structure": "Subject鈥搗erb鈥搊bject", "subjective_objective": {"answer": "Subjective", "rationale": "Because \"I thought\" expressed the patient's thoughts and opinions, it was subjective."}, "sentence_sentiment": {"answer": "Neutral", "rationale": "Since this entry expresses the views and perceptions of individuals on divorce, it does not involve positive or negative emotional aspects."}, "language_style": {"answer": "Tactful and warm", "rationale": "The entry expresses an understanding and respect by asking the respondents about divorce and highlighting the importance of consent. At the same time, it expresses its views in terms of \"free from traditional attitudes\", without directly criticizing or rejecting traditional views, and is more moderate and euphemismal."}, "time_dimension": {"answer": "Present", "rationale": "The keywords \"conception\" and \"consent to divorce\" and \"free from traditional attitudes\" all indicate that this is the perception and attitude of the testees towards the idea of divorce, and it describes the situation as it is."}, "sentence_type": "Affirmative declarative sentence", "pos": {"nouns": {"On one side": 2, "Divorce": 1, "Tie": 1, "Perceptions": 1}, "verbs": {"I thought": 1, "Agreed": 1, "Yes": 1, "Yeah": 1}, "adjectives": {"Traditional": 1}, "numerals": {"20": 1}, "pronouns": {"Both sides": 1}}}, {"content": "21. My interest in people or things can easily change:", "word_count": 14, "label": "", "sentence_structure": "Subject鈥揷opula鈥損redicative", "subjective_objective": {"answer": "Subjective", "rationale": "Because interest is easily changed by the subjective feeling and experience of a person."}, "sentence_sentiment": {"answer": "Neutral", "rationale": "As this entry does not express a clear positive or negative emotional expression, it describes the characteristics of personal interest and is an objective statement."}, "language_style": {"answer": "Tactful and warm", "rationale": "The language style of the entry is more euphemistic, rather than asking whether the person surveyed is susceptible to change, but rather using a mild, question-and-answer approach that leads the person to think and express himself."}, "time_dimension": {"answer": "Present", "rationale": "The entry describes a customary behaviour whereby the testee's interest in a person or object is susceptible to change and the change of interest may continue until now.\""}, "sentence_type": "Affirmative declarative sentence", "pos": {"nouns": {"Interest": 1, "person": 1, "Objects": 1}, "verbs": {"Change": 1}, "adjectives": {"Easy": 1}}}, {"content": "22. In the course of my work, I would like to:", "word_count": 6, "label": "", "sentence_structure": "Subject鈥搗erb鈥搊bject", "subjective_objective": {"answer": "Subjective", "rationale": "This is a test of patients ' subjective perception and attitude to their will."}, "sentence_sentiment": {"answer": "Neutral", "rationale": "Because the entry is merely a question of working attitudes, it does not involve any positive or negative emotional aspects."}, "language_style": {"answer": "Tactful and warm", "rationale": "The entry is more personal and interactive, using a question-and-answer approach, which is conducted in a moderate and encouraging tone rather than a direct statement or order. At the same time, the issue reflects respect and concern for the attitudes of the interviewees, who feel valued and understood."}, "time_dimension": {"answer": "Present", "rationale": "The entry asks the attitude and will of the person being tested in the current work and thus judges it as now."}, "sentence_type": "Interrogative sentence", "pos": {"verbs": {"Yes": 1}, "adjectives": {"Work": 1}, "numerals": {"22": 1, "I": 1}}}, {"content": "23. I often speak to myself without cause:", "word_count": 12, "label": "", "sentence_structure": "Subject鈥搗erb鈥揷omplement", "subjective_objective": {"answer": "Subjective", "rationale": "Because \"always self-speaking without reason\" is a personal experience and feeling of the patient, and it is a subjective act. Although this may be related to the patient's psychological state, it is itself based on the subjective experience of the individual."}, "sentence_sentiment": {"answer": "Neutral", "rationale": "Because the entry simply describes an act, there is no apparent emotional colour or value judgement."}, "language_style": {"answer": "Calm and direct", "rationale": ""}, "time_dimension": {"answer": "Past", "rationale": ""}, "sentence_type": "Affirmative declarative sentence", "pos": {"nouns": {"I": 1, "Reason": 1, "You're talking to yourself": 1}, "verbs": {"Regular": 1, "For no reason": 1, "Speak to yourself": 1}, "adjectives": {"For no reason": 1}, "adverbs": {"Often": 1}, "numerals": {"23": 1}}}, {"content": "24. Whether it be work, diet or outing, I always:", "word_count": 15, "label": "", "sentence_structure": "Subject鈥搗erb鈥揷omplement", "subjective_objective": {"answer": "Subjective", "rationale": "Because this sentence describes the feelings and preferences of the patient himself, and is a subjective expression."}, "sentence_sentiment": {"answer": "Positive", "rationale": "Because this entry expresses a positive attitude and behaviour in all respects."}, "language_style": {"answer": "Tactful and warm", "rationale": "The language style of the entry is euphemism. First, it uses polite language, \"please\" to introduce the problem and to question the other party in an invitation and respectful manner. Second, it does not directly express an assessment or criticism of the other party ' s behaviour, but rather adopts an objective and neutral attitude that allows the interviewees to express their views and ideas freely, thus enhancing the credibility and reliability of the questionnaire."}, "time_dimension": {"answer": "Present", "rationale": "\"Whether or not\" always indicates that it is a continuous custom or behaviour, and the entry is therefore an inquiry into the present habits or behaviour of the person being tested."}, "sentence_type": "Interrogative sentence", "pos": {"nouns": {"Work": 1, "Diet": 1, "Traveling": 1}, "verbs": {"be": 1}, "adverbs": {"Always": 1}}}, {"content": "25. I also wonder if there is a real interest in my words and deeds:", "word_count": 19, "label": "", "sentence_structure": "Subject鈥搗erb鈥搊bject", "subjective_objective": {"answer": "Subjective", "rationale": "Because the perception of doubt and real interest in my words and deeds is the patient's subjective feeling."}, "sentence_sentiment": {"answer": "Negative", "rationale": "Because it expresses a negative sentiment, which is \"suspicion,\" and which is a reaction to others. It can therefore be judged to be negative emotional."}, "language_style": {"answer": "Calm and direct", "rationale": "The language style of the entry is more calm and objective, and there is no excessive emotional rendering or rhetorical technique, but rather a more direct way of asking the person surveyed whether he or she has ever had doubts about the reaction of others to his or her own words and actions."}, "time_dimension": {"answer": "Past", "rationale": "The entry describes a feeling or thought of the person tested, and \"suspect\" indicates that such a feeling or idea has existed or continued in the past, and thus is judged to be \"the past\"."}, "sentence_type": "Interrogative sentence", "pos": {"nouns": {"I": 1, "others": 1, "Interest": 1}, "verbs": {"鈾?And I'm gonna be 鈾?: 1, "Doubt": 1, "have": 1}, "adverbs": {"Really": 1, "Is it possible": 2}, "numerals": {"25": 1}, "pronouns": {"to": 1}}}, {"content": "If I work in a factory, I would do the following:", "word_count": 12, "label": "", "sentence_structure": "Subject鈥搗erb鈥搊bject", "subjective_objective": {"answer": "Subjective", "rationale": "Because the attitude of \"yes\" belongs to the patient's own subjective feelings and wishes, the questionnaire entry is viewed from the patient's subjective perspective."}, "sentence_sentiment": {"answer": "Neutral", "rationale": "Because the entry does not have any emotional color."}, "language_style": {"answer": "Tactful and warm", "rationale": "The language style of the questionnaire entry is relatively warm, it is used in a more moderate way to ask questions and in an invitation form to make the interviewee feel more friendly and comfortable. At the same time, it is more conducive to obtaining a true answer from the interviewee."}, "time_dimension": {"answer": "Past", "rationale": "The entry uses the conditional phrase \"if I work in the factory\", which indicates that it is hypothetical, not relevant for the present and for the future. At the same time, the verb \"will do\" indicates that it is a description of past preferences or attitudes. So the temporal dimension of the entry is past."}, "sentence_type": "Imperative sentence", "pos": {"nouns": {"Plant": 1, "Work": 1, "Yes": 1}, "verbs": {"Do it": 1}}}, {"content": "27. In reading, I would like to read:", "word_count": 8, "label": "", "sentence_structure": "Subject鈥搗erb鈥搊bject", "subjective_objective": {"answer": "Subjective", "rationale": "Because it is the patient ' s subjective will and preference."}, "sentence_sentiment": {"answer": "Neutral", "rationale": "Because the entry does not express any emotional colour, it merely asks the interviewees about their reading preferences."}, "language_style": {"answer": "Tactful and warm", "rationale": "The language style of the entry is more euphemistic and gentle, and it guides the interviewee to express his preferences in an interview, while being friendly and close, and not too hard or straightforward. The entry can therefore be considered as euphemism and warm."}, "time_dimension": {"answer": "Present", "rationale": ""}, "sentence_type": "Interrogative sentence", "pos": {"nouns": {"Reading": 1}, "verbs": {"May I": 1, "Reading": 1}, "numerals": {"27": 1}}}, {"content": "28. Three words are listed after this topic, the one that is different from the other two.", "word_count": 22, "label": "", "sentence_structure": "Subject鈥搗erb鈥搊bject", "subjective_objective": {"answer": "Subjective", "rationale": "Because of the need for comparison and judgement, it is a subjective approach."}, "sentence_sentiment": {"answer": "Neutral", "rationale": ""}, "language_style": {"answer": "Calm and direct", "rationale": "The entry is designed to be simple and clear, and meets the basic requirements of the psychological questionnaire study."}, "time_dimension": {"answer": "Future", "rationale": "The keywords are \"afterward listing\" and \"different categories\", all of which point to situations that have not yet occurred and that may arise in the future."}, "sentence_type": "Interrogative sentence", "pos": {"nouns": {"This is the subject": 1, "Words": 3, "Other": 1}, "verbs": {"List": 1, "Different": 1, "Category": 1}, "numerals": {"28": 1, "Three": 1}}}, {"content": "If I can get to a new environment, I want to:", "word_count": 13, "label": "", "sentence_structure": "Subject鈥損redicate", "subjective_objective": {"answer": "Subjective", "rationale": "Because this is the imagination and expectation of the patient for the new environment, it is subjective."}, "sentence_sentiment": {"answer": "Neutral", "rationale": "Because the entry does not have any emotional color."}, "language_style": {"answer": "Calm and direct", "rationale": "The language style of the article ' s purpose may therefore be neutral or objective, and there is no excessive emotional or tendentious expression."}, "time_dimension": {"answer": "Present", "rationale": "The key words in the entry are \"if I can\" and \"if I want,\" which implies that it is a vision and a expectation of the state of the moment, not a past experience or a future outcome. And \"new environment\" also shows that the problem is related to the living environment. So the time dimension of this questionnaire should be now."}, "sentence_type": "Interrogative sentence", "pos": {"nouns": {"Environment": 1, "I": 1}, "verbs": {"Yes": 1, "Present": 1}, "adverbs": {"Yes": 1, "If": 1}, "numerals": {"29": 1}}}, {"content": "30. Throughout my life, I always felt that I could achieve what I had hoped for:", "word_count": 19, "label": "", "sentence_structure": "Subject鈥搗erb鈥搊bject", "subjective_objective": {"answer": "Subjective", "rationale": "This entry examines patients鈥?subjective feelings and beliefs about whether they believe they can achieve the desired goals. It is therefore viewed from a subjective perspective."}, "sentence_sentiment": {"answer": "Positive", "rationale": "Because the \"I always feel I can achieve what I want\" attitude is positive."}, "language_style": {"answer": "Tactful and warm", "rationale": "The entry is more personal and interactive when it is presented in a gentle and inviting tone, rather than in direct statements or orders. At the same time, the language style of the article is more positive, expressing optimism about the future of the interviewee."}, "time_dimension": {"answer": "Present", "rationale": ""}, "sentence_type": "Interrogative sentence", "pos": {"nouns": {"Life": 1, "I": 2, "Objective": 1}, "verbs": {"Achieved": 1, "I think": 1}, "adjectives": {"Projected": 1}, "numerals": {"30": 1}}}, {"content": "31. When I lie, I feel ashamed not to look at each other:", "word_count": 18, "label": "", "sentence_structure": "Subject鈥揷opula鈥損redicative", "subjective_objective": {"answer": "Subjective", "rationale": "Because \"rest of shame\" and \"failure to look at each other\" are subjective feelings and behaviours of patients, looking at their emotional experiences and their internal state."}, "sentence_sentiment": {"answer": "Negative", "rationale": "Because the \"deep ashamed\" act in the entry is negative."}, "language_style": {"answer": "Calm and direct", "rationale": "The entry presents a straightforward question, which is more objective and cold, is less emotional and rhetorical and is more in line with the language style of the psychological questionnaire."}, "time_dimension": {"answer": "Present", "rationale": ""}, "sentence_type": "Interrogative sentence", "pos": {"nouns": {"Lies": 1, "Inside": 1, "Shame": 1, "The other side": 1}, "verbs": {"Say it": 1, "I think": 1, "Total": 1, "Look": 1}, "adjectives": {"Shame": 1}, "adverbs": {"I'm afraid": 1}, "numerals": {"31": 1}, "pronouns": {"I": 1}}}, {"content": "32. If I had a pistol in my hand with a bullet, I would have to take it out to reassure myself:", "word_count": 29, "label": "", "sentence_structure": "Subject鈥搗erb鈥搊bject", "subjective_objective": {"answer": "Subjective", "rationale": "Because it reflects a mental state or feeling of anxiety and fear in the face of a pistol."}, "sentence_sentiment": {"answer": "Negative", "rationale": "Because it expresses a fear and a sense of disquiet about its own security, it has a negative emotional connotation."}, "language_style": {"answer": "Calm and direct", "rationale": "The entry raises questions in a straightforward manner, without too many adorations and emotional expressions, and with a more calm and objective tone."}, "time_dimension": {"answer": "Present", "rationale": "The words \"presumably\" and \"must\" suggest that this is a hypothetical situation, implying that the interviewee is imagining a possible reaction in an extreme situation. The entry is therefore an inquiry into the potential risk level or coping strategy of the interviewee. The time dimension is now judged."}, "sentence_type": "Affirmative declarative sentence", "pos": {"nouns": {"Pistols": 1, "Bullets": 2}, "verbs": {"Take it": 1, "Fire": 1}, "numerals": {"One": 1, "Two": 1}, "pronouns": {"I": 1}}}, {"content": "33. Most people think I'm a funny person:", "word_count": 15, "label": "", "sentence_structure": "Subject鈥搗erb鈥搊bject", "subjective_objective": {"answer": "Subjective", "rationale": "Because \"I'm a funny person\" is what others think of me, what others think/evaluate, and what they look at from a subjective perspective."}, "sentence_sentiment": {"answer": "Positive", "rationale": "Because most people rate the person evaluated positively, indicating that he or she has a funny character."}, "language_style": {"answer": "Tactful and warm", "rationale": "The language style of the entry is more euphemistic. It asks the interviewee in a positive and friendly way, comforting and accommodating the interviewee and encouraging him to share his/her views."}, "time_dimension": {"answer": "Present", "rationale": "The keyword \"and\" is \"mostly\" to indicate that the question is asking the interviewee about his current state or state, so the answer is \"now\"."}, "sentence_type": "Affirmative declarative sentence", "pos": {"nouns": {"person": 2, "Talk": 1, "Funny": 1}, "verbs": {"I think": 1, "be": 1, "One": 1, "of": 3}, "adjectives": {"Most people": 1, "Talk": 1, "Funny": 1}, "adverbs": {"Both": 1}, "numerals": {"33": 1}, "classifiers": {"individual": 1}, "pronouns": {"I": 1}}}, {"content": "34. If people knew what I was thinking, they would be surprised:", "word_count": 19, "label": "", "sentence_structure": "Subject鈥搗erb鈥搊bject", "subjective_objective": {"answer": "Subjective", "rationale": "Because the sentence describes the patient ' s own feelings and thoughts, not the objective facts."}, "sentence_sentiment": {"answer": "Negative", "rationale": "Because it implies the existence of stereotypes and prejudices against people, it is a negative emotional colour."}, "language_style": {"answer": "Calm and direct", "rationale": "The entry uses a direct, concise language style, without any rhetorical or emotional rendering, which describes the subject of the survey in a factual and straightforward way. This language style is usually used in questionnaires as a way of avoiding the need to direct respondents to the responses to ensure objectivity and accuracy of the results."}, "time_dimension": {"answer": "Present", "rationale": "The entry describes a hypothesis or fear of the testee that he or she would be surprised when someone learned what was inside them. This psychological state is current, immediate, and therefore judgment is now."}, "sentence_type": "Affirmative declarative sentence", "pos": {"nouns": {"person": 2, "Inside": 1, "Stereotypes": 1, "Surprise": 1}, "verbs": {"Yes": 3, "Eat": 1, "I'm surprised": 1}, "adverbs": {"If": 1, "very": 1}, "numerals": {"34": 1, "One": 2}}}, {"content": "In public, if I suddenly become the center of attention, I feel agitated.", "word_count": 27, "label": "", "sentence_structure": "Subject鈥揷opula鈥損redicative", "subjective_objective": {"answer": "Subjective", "rationale": "Because this feeling is the patient ' s inner experience and thoughts, and it is a subjective perspective."}, "sentence_sentiment": {"answer": "Negative", "rationale": "Because the \"frightful\" act in the entry is negative."}, "language_style": {"answer": "Tactful and warm", "rationale": "The entry is more euphemistic and not used too direct or strong language. It expresses the subject in a question-and-answer manner, giving the interviewee greater autonomy, peace and sense of peace, and more acrimony and abating tone."}, "time_dimension": {"answer": "Present", "rationale": ""}, "sentence_type": "Affirmative declarative sentence", "pos": {"nouns": {"Public": 1, "Centre": 1, "It's a little disturbing": 1}, "verbs": {"Yes": 1, "Be": 1, "I feel": 1, "Attention": 1}, "adjectives": {"Suddenly": 1, "Attention, everyone": 1}, "numerals": {"35": 1}, "pronouns": {"I": 1}}}, {"content": "I always like to attend big parties or gatherings:", "word_count": 16, "label": "", "sentence_structure": "Subject鈥搗erb鈥搊bject", "subjective_objective": {"answer": "Subjective", "rationale": "Because \"a man likes\" is a person's attitude and tendency, a patient's subjective feeling."}, "sentence_sentiment": {"answer": "Positive", "rationale": "Because the \"like to attend\" in the entry shows that the author's attitude towards participation in a large party or assembly is positive."}, "language_style": {"answer": "Calm and direct", "rationale": "The entry is based on a more concise and clear presentation of the theme, \"I always like to attend a large party or gathering\", without too much rhetorical or emotional retrofitting. At the same time, it is more objective and does not express any particular emotional colour. It is therefore considered to be a calm and straightforward language."}, "time_dimension": {"answer": "Present", "rationale": ""}, "sentence_type": "Affirmative declarative sentence", "pos": {"nouns": {"Questionnaire Entry": 1, "Party": 1, "Gathering": 1}, "verbs": {"Yeah": 1, "Participation": 1}, "adjectives": {"Big": 1}, "adverbs": {"Total": 1}, "numerals": {"36": 1}, "pronouns": {"I": 1}}}, {"content": "37. In the discipline, I like:", "word_count": 7, "label": "", "sentence_structure": "Subject鈥搗erb鈥搊bject", "subjective_objective": {"answer": "Subjective", "rationale": "Because it is a subjective attitude and emotional reaction of a person, not an objective reality or fact."}, "sentence_sentiment": {"answer": "Neutral", "rationale": "Because the entry merely asks the respondents about their preferences for the subject, it does not involve any emotion or attitude."}, "language_style": {"answer": "Tactful and warm", "rationale": "The entry leads the interviewee to share his/her preferences for the discipline rather than direct orders or requests for answers. This expression is more accommodative and friendly, and it can easily trigger a positive response from the interviewee. At the same time, the question is not in too strong language or evaluable vocabulary, but rather uses a neutral and objective query that allows the interviewee to express his/her views and feelings freely, without feeling constrained or constrained."}, "time_dimension": {"answer": "Present", "rationale": "The entry is now asked about the current preferences or preferences of the person being tested, does not address the time dimension of the past or the future and is therefore in the present context."}, "sentence_type": "Affirmative declarative sentence", "pos": {"nouns": {"Subjects": 1, "Yeah": 1}, "verbs": {"be": 1}, "numerals": {"37": 1}}}, {"content": "I often doubt the honesty of those who, unexpectedly, are too kind to me.", "word_count": 28, "label": "", "sentence_structure": "Subject鈥搗erb鈥搊bject", "subjective_objective": {"answer": "Subjective", "rationale": "Because doubting such attitudes and feelings is the patient ' s own subjective view."}, "sentence_sentiment": {"answer": "Negative", "rationale": "Because the act of \"suspecting the honesty of the motives of others\" in the entry is negative and expresses mistrust and suspicion."}, "language_style": {"answer": "Calm and direct", "rationale": ""}, "time_dimension": {"answer": "Present", "rationale": "The keyword \"often\" indicates that the act is recurrent, that the description of \"unsurprisingly too kind to me\" indicates that the act has already occurred in the past, and that \"in honesty\" points to the circumstances that may arise in the future. Thus, the temporal dimension of the article is now judged."}, "sentence_type": "Interrogative sentence", "pos": {"nouns": {"Questionnaire": 1, "Entry": 1, "person": 1, "Motive": 1, "Honesty": 1}, "verbs": {"Often": 1, "Doubt": 1, "From": 1, "Too much": 1, "Friendly": 1, "Is it possible": 1, "be": 1, "Honest": 1}, "adjectives": {"That's too much": 1, "Friendly": 1, "Honesty": 1}, "adverbs": {"Often": 1, "Too much": 1}, "numerals": {"38": 1}, "pronouns": {"I": 2}}}, {"content": "I'd like to make my life a living:", "word_count": 14, "label": "", "sentence_structure": "Subject鈥搗erb鈥揷omplement", "subjective_objective": {"answer": "Subjective", "rationale": "Because \"I'm willing\" shows that it's a patient's personal will or attitude, and it's a subjective examination."}, "sentence_sentiment": {"answer": "Neutral", "rationale": "Because the entry does not have any emotional color."}, "language_style": {"answer": "Tactful and warm", "rationale": "The entry is more euphemistic and gentle, using metaphors to describe the lifestyle of the person surveyed, and does not directly express any clear requirements or instructions."}, "time_dimension": {"answer": "Present", "rationale": "The entry asks the current living conditions and habits of the person tested, and does not refer to past or future situations, and therefore judges it as now."}, "sentence_type": "Interrogative sentence", "pos": {"nouns": {"Life": 1, "Arrangements": 1}, "verbs": {"Yes": 1, "- Put it on": 1, "Yeah": 1}, "adverbs": {"Okay": 1, "Yeah": 1}, "numerals": {"39": 1}, "pronouns": {"I": 1}}}, {"content": "40. What I think is needed at this time is:", "word_count": 10, "label": "", "sentence_structure": "Subject鈥搗erb鈥搊bject", "subjective_objective": {"answer": "Subjective", "rationale": "Because \"I think\" indicates that this is the patient's personal opinion and opinion, and it's a subjective attitude."}, "sentence_sentiment": {"answer": "Neutral", "rationale": "Because the entry does not have any emotional color."}, "language_style": {"answer": "Calm and direct", "rationale": "The language style of the entry is more calm and straightforward, and the interviewer鈥檚 views or needs about the current needs are asked in a concise and clear manner, without using excessive rhetorical or emotional colours."}, "time_dimension": {"answer": "Present", "rationale": "The entry contains the keyword \"current\", which refers to the current point of time of the speaker, and thus the judgement is now."}, "sentence_type": "Affirmative declarative sentence", "pos": {"nouns": {"I think": 1, "Yes": 1, "Something": 1}, "verbs": {"be": 1}, "adverbs": {"Current": 1}, "numerals": {"40": 1, "One": 1}, "pronouns": {"I": 1}}}, {"content": "I sometimes feel like I need a lot of physical work:", "word_count": 16, "label": "", "sentence_structure": "Subject鈥損redicate", "subjective_objective": {"answer": "Subjective", "rationale": "Because \"I need to work hard\" is a personal feeling and a will, a patient's subjective attitude."}, "sentence_sentiment": {"answer": "Negative", "rationale": "Because the act \"I need intense manual labour\" in the entry may have been caused by anxiety, stress, etc., and therefore has some negative emotional connotations."}, "language_style": {"answer": "Calm and direct", "rationale": "The entry directly asks the interviewees whether they are sometimes in need of intense physical labour, without excessive emotional or rhetorical styles, and with a more calm and objective language style."}, "time_dimension": {"answer": "Present", "rationale": "This sentence indicates that the testees sometimes feel a strong need for manual labour, a feeling that is either current or experienced, and therefore the time dimension is now judged."}, "sentence_type": "Interrogative sentence", "pos": {"nouns": {"Time": 1, "I think": 1, "Manual labour": 1}, "verbs": {"Yes": 1, "I think": 1}, "adjectives": {"Extreme": 1}, "numerals": {"41": 1}}}, {"content": "I'm willing to go out with educated people rather than rude people:", "word_count": 22, "label": "", "sentence_structure": "Subject鈥搗erb鈥搊bject", "subjective_objective": {"answer": "Subjective", "rationale": "Because \"resistance to engage with rude people\" is a subjective attitude of the patient, the point of view of this questionnaire is subjective."}, "sentence_sentiment": {"answer": "Positive", "rationale": "Because the entry \"willing to go with a cultured person\" expresses a positive attitude."}, "language_style": {"answer": "Tactful and warm", "rationale": "The words \"will\" and \"no\" are used to express themselves, to make the respondents feel comfortable and natural in answering questions, rather than to use strong negative or positive tone. At the same time, the descriptions of \"educated\" and \"brubber\" are also more objective and neutral, and do not favour one side or use negative evaluation terms, so the language style of the article as a whole is a warmer type."}, "time_dimension": {"answer": "Present", "rationale": "Now the entry asks the testee about his preferences for social objects, the keywords being \"will\" and \"no\" , which indicate the current attitude or behavioural orientation. At the same time, the concepts of \"bred person\" and \"black person\" are described in the current social context. Thus, the time dimension of the entry can be judged as it is."}, "sentence_type": "Affirmative declarative sentence", "pos": {"nouns": {"person": 2, "- To be with someone": 1}, "verbs": {"Yes": 2, "Travel": 1, "No, I don't": 1, "Same": 1}, "adjectives": {"- Yes, sir": 1, "Naughty": 1}, "numerals": {"42": 1}, "pronouns": {"I": 1}}}, {"content": "43. In matters that must be handled with wisdom:", "word_count": 15, "label": "", "sentence_structure": "Subject鈥搗erb鈥搊bject", "subjective_objective": {"answer": "Subjective", "rationale": "This is because it examines the subjective views and attitudes of the patients."}, "sentence_sentiment": {"answer": "Neutral", "rationale": "Because the entry describes a situation without any apparent emotional or attitudinal bias."}, "language_style": {"answer": "Calm and direct", "rationale": "The entry expresses the questions that need answers in a short and clear manner, without too much embellishment or emotional addition, and with a more direct and objective language style."}, "time_dimension": {"answer": "Present", "rationale": "The entry does not specify the past or future state of affairs, but rather describes a current behaviour or state of conduct, i.e. whether the person tested is capable of dealing with certain matters that require wisdom. Thus, the basis of the judgement is \"now\"."}, "sentence_type": "Affirmative declarative sentence", "pos": {"nouns": {"Questionnaire": 1, "Entry": 1, "Service": 1}, "verbs": {"Processing": 1, "By virtue": 1, "Wisdom": 1}, "adverbs": {"in": 1, "Medium": 1}, "numerals": {"43": 1}}}, {"content": "When the leader called me, I:", "word_count": 8, "label": "", "sentence_structure": "Subject鈥搗erb鈥揷omplement", "subjective_objective": {"answer": "Subjective", "rationale": "Because \"me\" reactions and feelings are subjective experiences and attitudes of patients."}, "sentence_sentiment": {"answer": "Negative", "rationale": "Because the situation of being summoned by the leadership is usually stressful or stressful, it carries some negative sentiment."}, "language_style": {"answer": "Calm and direct", "rationale": "The entry is presented in an objective question form, which does not contain too many emotional or rhetorical techniques, is more semantic and direct and is more in line with the linguistic style of the psychological questionnaire."}, "time_dimension": {"answer": "Present", "rationale": "The words \"when\" in the entry indicate the current or recent period, while the verbs \"call\" and \"I\" indicate that this is a description of the current experience or feelings of the person being tested. The temporal dimension of the entry is therefore judged as now."}, "sentence_type": "Declarative sentence", "pos": {"nouns": {"Leadership": 1, "I": 2}, "verbs": {"Call": 1}, "numerals": {"44": 1}}}, {"content": "45. If I have a generous offer, I am willing to do the following work in the care of a mentally ill person:", "word_count": 19, "label": "", "sentence_structure": "Subject鈥損redicate", "subjective_objective": {"answer": "Subjective", "rationale": "Because \"will\" is a subjective attitude or will, and it is the individual feeling and choice of the patient, the answer to this questionnaire is subjective."}, "sentence_sentiment": {"answer": "Neutral", "rationale": "Because the entry does not have any emotional color."}, "language_style": {"answer": "Calm and direct", "rationale": "The language style of the entry is more straightforward, with no excessive rhetoric or emotional rendering, but rather with a simple statement expressing a view or a will. Nor is it expressed in any euphemism or courtesy, so that it can be judged as a \"cool straight\" language style."}, "time_dimension": {"answer": "Present", "rationale": "The entry indicates whether the person tested is willing to work with a mentally ill person when he or she is in a situation of \"good treatment\"."}, "sentence_type": "Affirmative declarative sentence", "pos": {"nouns": {"Treatment": 1, "Work": 1, "Nursing": 1, "Patients": 1, "Mental patients": 1}, "verbs": {"- It's thick": 1, "Yes": 1, "Do it": 1}, "numerals": {"45": 1}, "pronouns": {"I": 1}}}, {"content": "46. I like reading in newspapers:", "word_count": 7, "label": "", "sentence_structure": "Subject鈥搗erb鈥搊bject", "subjective_objective": {"answer": "Subjective", "rationale": "Like being patient subjective."}, "sentence_sentiment": {"answer": "Neutral", "rationale": "Because the entry does not have any emotional color."}, "language_style": {"answer": "Tactful and warm", "rationale": "The entry is more personal and interactive, by speaking in a gentle, inviting manner, asking the other person about his preferences in reading the newspaper. At the same time, it can be more respected and valued by the interviewee by using the question phrase."}, "time_dimension": {"answer": "Past", "rationale": "The entry asks the type of content that the person is interested in reading in the newspaper, which is not clearly time-directed, and therefore does not address past, present or future behaviour or mindset."}, "sentence_type": "Interrogative sentence", "pos": {"nouns": {"Report": 1}, "verbs": {"Yeah": 1, "Read it": 2}, "numerals": {"46": 1}}}, {"content": "47. In accepting difficult tasks, I have always:", "word_count": 11, "label": "", "sentence_structure": "Subject鈥搗erb鈥揷omplement", "subjective_objective": {"answer": "Subjective", "rationale": "Because the situation and feelings of \"in the face of difficult tasks\" are usually assessed on the basis of the attitudes and emotions of individuals, this is a subjective issue."}, "sentence_sentiment": {"answer": "Negative", "rationale": "Because the act of \"acceptance of difficult tasks\" is positive in the entry, \"always\" means a constant act, implying that the person may have negative emotions or attitudes when facing difficult tasks."}, "language_style": {"answer": "Calm and direct", "rationale": "The entry uses objective, concise language to describe the attitude of the person tested in the face of difficult tasks, without adding too much adour or emotional. The language style is therefore judged to be calm and straightforward."}, "time_dimension": {"answer": "Present", "rationale": "The entry asks the testee about the current behaviour or attitude, and therefore the temporal dimension is now judged."}, "sentence_type": "Interrogative sentence", "pos": {"nouns": {"Accept": 1, "Hard tasks": 1}, "verbs": {"be": 1, "Total": 1}, "adverbs": {"Always": 1}, "numerals": {"47": 1}}}, {"content": "48. On tour, I would rather watch a painter ' s writer than listen to the debate:", "word_count": 25, "label": "", "sentence_structure": "Subject鈥搗erb鈥揷omplement", "subjective_objective": {"answer": "Subjective", "rationale": "Because the patient is expressing his/her preferences and preferences, it is subjective."}, "sentence_sentiment": {"answer": "Negative", "rationale": "Because the attitude expressed in the entries is \"I'd rather watch than listen,\" there's a certain negative tendency."}, "language_style": {"answer": "Calm and direct", "rationale": "The entry is a direct indication of the author ' s tendency to travel, not using excessive rhetorical techniques or emotional rendering, and is more objective."}, "time_dimension": {"answer": "Past", "rationale": "In the entry, the structure \"better than\" expresses the testee's preference and choice for a given past scene, and therefore judges the past."}, "sentence_type": "Affirmative declarative sentence", "pos": {"nouns": {"Tour": 1, "Painter": 1, "Writer": 1, "Everyone": 1, "Debate": 1}, "verbs": {"in": 1, "Watch": 1, "I'd rather": 1, "Listen": 1}}}, {"content": "I'm a little bit nervous, and I'm a little bit stunned:", "word_count": 15, "label": "", "sentence_structure": "Subject鈥搗erb鈥揷omplement", "subjective_objective": {"answer": "Subjective", "rationale": "Because the words \"shock\" imply the patient's own subjective feelings and experiences."}, "sentence_sentiment": {"answer": "Negative", "rationale": "Because the terms \"nervous\" and \"shock\" have negative emotional connotations."}, "language_style": {"answer": "Calm and direct", "rationale": "The language style of the entry is of a calm and direct type, with no excessive qualifier or emotional colour, but rather a simple and straightforward presentation of a fact."}, "time_dimension": {"answer": "Past", "rationale": "The keyword \"my nervous weakness\", \"a little thrill, a bit of a chill\" is a description of my own experience, which usually happens in the past. So, the entry is a past."}, "sentence_type": "Interrogative sentence", "pos": {"nouns": {"Nerves": 1, "Exciting": 1}, "verbs": {"Weakness": 1, "Warm": 1}, "adjectives": {"A little": 1, "Points": 1}, "adverbs": {"Just": 1}, "numerals": {"49": 1}}}, {"content": "50. Up in the morning, there was often fatigue:", "word_count": 12, "label": "", "sentence_structure": "Subject鈥揷opula鈥損redicative", "subjective_objective": {"answer": "Subjective", "rationale": "Because \"often weary\" is the patient's subjective feeling, it's the patient's subjective experience."}, "sentence_sentiment": {"answer": "Negative", "rationale": "Because the expression \"often tired\" in the entry is negative."}, "language_style": {"answer": "Tactful and warm", "rationale": "The entry is more personal and interactive in its expression of a soft, invitation-style conversation, rather than a direct statement or order. It also increases the willingness of respondents to share their feelings and ideas, through the use of question-words, thereby increasing the effectiveness and reliability of the questionnaire."}, "time_dimension": {"answer": "Present", "rationale": "The keywords are \"getting up in the morning\" and \"often feeling\" and they all point to a state or situation in progress, so the entry is in its current state."}, "sentence_type": "Affirmative declarative sentence", "pos": {"nouns": {"Morning": 1, "Weary": 1, "Shit": 1}, "verbs": {"Get up": 1, "I feel": 1}, "adverbs": {"Often": 1}, "numerals": {"50": 1}}}, {"content": "51. If the treatment is the same, I would choose to:", "word_count": 10, "label": "", "sentence_structure": "Subject鈥搗erb鈥搊bject", "subjective_objective": {"answer": "Subjective", "rationale": "Because the will to choose is the patient's subjective attitude."}, "sentence_sentiment": {"answer": "Neutral", "rationale": "Because the entry does not relate to specific persons or matters, nor does it express any emotional colour."}, "language_style": {"answer": "Calm and direct", "rationale": "The language style of the entry is relatively straightforward, not using too much qualifier or emotionally strong expression, but simply raising a question and giving a choice. This language style is more consistent with the objective and neutral character of the questionnaire."}, "time_dimension": {"answer": "Past", "rationale": "The entry asks what jobs the testee would choose to do under the same conditions, which requires consideration of the historical experience and preferences of the testee and therefore belongs to a review of the past."}, "sentence_type": "Interrogative sentence", "pos": {"nouns": {"Treatment": 1, "Choose": 1, "Do it": 1}, "verbs": {"May I": 1}, "numerals": {"51": 1}}}, {"content": "52. Every time a holiday is celebrated or a marriage is celebrated, I:", "word_count": 13, "label": "", "sentence_structure": "Subject鈥搗erb鈥揷omplement", "subjective_objective": {"answer": "Subjective", "rationale": "Because the word \"I\" indicates that it's personal feelings and attitudes of the patient."}, "sentence_sentiment": {"answer": "Positive", "rationale": "Because the behavior in the entry \"attends every year of the holidays or at marriage of a relative\" is an active social act."}, "language_style": {"answer": "Tactful and warm", "rationale": "The language style of the entry is more euphemistic, beginning with a \"everytime\" expression of a situation common to everyday life, and then asking questions, which leads the respondents to think and answer, rather than to direct statements or orders."}, "time_dimension": {"answer": "Present", "rationale": "The keywords are \"everytime\" and \"time\", which indicate that it is a regular, customary act, judging in time as now."}, "sentence_type": "Declarative sentence", "pos": {"nouns": {"New Year's Eve": 1, "Marriage": 1}, "verbs": {"Every time": 1, "I": 1, "Both": 1, "Yes": 1}, "adverbs": {"Both": 1}, "numerals": {"52": 1}, "pronouns": {"I": 1}}}, {"content": "53. Three figures are included after this topic, which is different from the other two figures:", "word_count": 23, "label": "", "sentence_structure": "Subject鈥搗erb鈥搊bject", "subjective_objective": {"answer": "Objective", "rationale": "Because the subject requires comparison between the categories of numbers, not the subjective feelings or attitudes of the patients."}, "sentence_sentiment": {"answer": "Neutral", "rationale": "Because the entry does not have any emotional color."}, "language_style": {"answer": "Calm and direct", "rationale": "The language style of the entry is calm, as it is clear and succinct, and does not use any rhetorical or emotional colour to influence the reader's answers. In addition, the structure of the question is clear, easy to understand and answer."}, "time_dimension": {"answer": "Present", "rationale": "The topic was to enable the testee to identify and indicate a number that was different from the other two and that was currently in the process of conduct or state."}, "sentence_type": "Interrogative sentence", "pos": {"nouns": {"This is the subject": 1, "Three": 1, "Numbers": 2, "One": 1, "The other two": 1, "Not the same": 1}, "verbs": {"Included": 1, "That": 1}, "adverbs": {"Back": 1}, "numerals": {"Five": 1, "Three": 1, "One": 1, "Two": 1}, "pronouns": {"Where": 1, "individual": 2, "Two": 1}}}, {"content": "Cats and fish are like cattle and:", "word_count": 7, "label": "", "sentence_structure": "Subject鈥搗erb鈥搊bject", "subjective_objective": {"answer": "Subjective", "rationale": "Because the question gave respondents a metaphor, expressed their personal views and ways of thinking, it was examined from a subjective point of view."}, "sentence_sentiment": {"answer": "Neutral", "rationale": "Because the entry is not emotional, it raises only a question of fact."}, "language_style": {"answer": "Tactful and warm", "rationale": "The entry is more personal and interactive when it is spoken in a gentle and inviting manner, rather than in direct statements or orders. At the same time, the issue is used in more humorous, relaxed, and without too serious or tense tone."}, "time_dimension": {"answer": "Present", "rationale": "The entry is now to ask the subject of the testee ' s subjective view or opinion on an issue, and the temporal dimension is now judged."}, "sentence_type": "Interrogative sentence", "pos": {"nouns": {"Cat": 1, "Fish": 1, "Cow": 1, "Yeah": 1}, "numerals": {"54": 1}}}, {"content": "55. The teacher I admired in primary school still deserves my admiration:", "word_count": 20, "label": "", "sentence_structure": "Subject鈥搗erb鈥搊bject", "subjective_objective": {"answer": "Subjective", "rationale": "This is a reflection of subjective evaluation, as patients view and approach the people who have experienced the past."}, "sentence_sentiment": {"answer": "Positive", "rationale": "Because the description \"the admired teacher, still worthy of my admiration\" is positive."}, "language_style": {"answer": "Tactful and warm", "rationale": "The language style of this questionnaire is more euphemism, more subtle, and more subtle, using the expression \"to this day I am admired\" to express respect for teachers than simply saying that teachers are great or the best. This language style gives people a warmer and more respectful feeling of respect, and makes it easier for respondents to share their thoughts and experiences."}, "time_dimension": {"answer": "Past", "rationale": ""}, "sentence_type": "Affirmative declarative sentence", "pos": {"nouns": {"Primary": 1, "Respect": 2, "Teacher": 1, "Present": 1}, "verbs": {"in": 1, "It's worth it": 1}, "adjectives": {"Still": 1}, "numerals": {"55": 1}, "pronouns": {"I": 1}}}, {"content": "I think I do have some qualities that are not beyond the reach of others:", "word_count": 19, "label": "", "sentence_structure": "Subject鈥揷opula鈥損redicative", "subjective_objective": {"answer": "Subjective", "rationale": "This is a self-evaluation and self-perception of patients and a subjective attitude and perception."}, "sentence_sentiment": {"answer": "Positive", "rationale": "Because the expression \"unvisible quality\" is positive."}, "language_style": {"answer": "Tactful and warm", "rationale": "Warm and warm."}, "time_dimension": {"answer": "Present", "rationale": ""}, "sentence_type": "Interrogative sentence", "pos": {"nouns": {"I": 2, "Some": 1, "others": 1, "Quality": 1}, "verbs": {"I think": 1, "have": 1, "and": 1}, "adjectives": {"Indeed": 1}, "adverbs": {"Not as much as I can": 1}, "numerals": {"56": 1}, "classifiers": {"individual": 1}}}, {"content": "57. I can be relieved that, according to my abilities, even if I do something ordinary:", "word_count": 24, "label": "", "sentence_structure": "Subject鈥搗erb鈥搊bject", "subjective_objective": {"answer": "Subjective", "rationale": "Because it is a subjective idea that it is the patient ' s own perception and attitude to his or her abilities that needs to be examined."}, "sentence_sentiment": {"answer": "Positive", "rationale": "Because the expression \"even if I do something ordinary, I'll be reassured\" in the entry is positive and expresses confidence in and acceptance of my abilities."}, "language_style": {"answer": "Tactful and warm", "rationale": "The entry uses a more euphemistic and encouraging language style, without directly presenting or ordering the other party to perform a particular task. At the same time, the effectiveness and appeal of the questionnaire is enhanced by giving the interviewee a sense of interest and respect by describing the individual ' s abilities and attitude to work."}, "time_dimension": {"answer": "Future", "rationale": "The keywords \"in my power\", \"even if I do something ordinary\" and \"I'll be safe\" indicate the expectations and confidence of the testees in their future career development and are therefore part of the future behavior or mentality."}, "sentence_type": "Affirmative declarative sentence", "pos": {"nouns": {"Capacity": 1, "Work": 2, "Things": 1}, "verbs": {"Jean": 1, "Do it": 1, "Yes": 2, "An Xin": 1}, "adjectives": {"Normal": 1}, "adverbs": {"Even if": 1}, "numerals": {"57": 1, "Some": 1}, "pronouns": {"I": 2}}}, {"content": "58. I like to watch movies or other entertainment events:", "word_count": 18, "label": "", "sentence_structure": "Subject鈥搗erb鈥搊bject", "subjective_objective": {"answer": "Subjective", "rationale": "This is a subjective examination, since the number of times a person prefers to watch a film or other recreational activity is determined by the patient ' s subjective will and preferences and belongs to the patient ' s own attitudes and feelings."}, "sentence_sentiment": {"answer": "Neutral", "rationale": "Because the entry merely asks about the frequency of a person ' s interest in watching a film or participating in other entertainment activities, it is not emotional."}, "language_style": {"answer": "Calm and direct", "rationale": "The language style of the entry is relatively simple, and the interviewee is asked directly about the frequency of the film or other entertainment, without excessive ado and emotional colour."}, "time_dimension": {"answer": "Past", "rationale": "It therefore does not belong to any period of time."}, "sentence_type": "Interrogative sentence", "pos": {"nouns": {"Film": 1, "Recreational activities": 1, "Number": 1}, "verbs": {"Yeah": 1, "Look": 1, "Participation": 1, "Number": 1}, "numerals": {"58": 1}, "classifiers": {"or": 1, "EMPTY": 2}}}, {"content": "59. I like to do things that require sophisticated technology:", "word_count": 14, "label": "", "sentence_structure": "Subject鈥搗erb鈥搊bject", "subjective_objective": {"answer": "Subjective", "rationale": "Because I like to do jobs that require sophisticated technology is my subjective attitude."}, "sentence_sentiment": {"answer": "Neutral", "rationale": "Since the entry does not address any positive or negative behaviour, attitude, emotion, etc., it merely presents the fact that it prefers to perform work that requires sophisticated technology."}, "language_style": {"answer": "Calm and direct", "rationale": "The entry directly asks whether the other party prefers to perform work that requires sophisticated technology, without adding excessive adoration or emotional colour, and with a more calm and straightforward language style."}, "time_dimension": {"answer": "Present", "rationale": ""}, "sentence_type": "Affirmative declarative sentence", "pos": {"nouns": {"Work": 1, "Technology": 1}, "verbs": {"Yeah": 1, "Active": 1}, "adjectives": {"Precision": 1}, "numerals": {"59": 1}}}, {"content": "60. I am always more cautious in the face of persons of prestige and stature:", "word_count": 20, "label": "", "sentence_structure": "Subject鈥搗erb鈥揷omplement", "subjective_objective": {"answer": "Subjective", "rationale": "This is a description of the patient ' s reaction and attitude in a given situation, and relates to the patient ' s self-perception and mental state, which is subjective."}, "sentence_sentiment": {"answer": "Negative", "rationale": "Because the \"promoting caution\" in the entry is negative."}, "language_style": {"answer": "Calm and direct", "rationale": "The entry is more objective and neutral in terms of direct questioning of the respondent ' s behaviour in a particular situation, without using excessive qualifiers or rhetorical techniques."}, "time_dimension": {"answer": "Present", "rationale": ""}, "sentence_type": "Interrogative sentence", "pos": {"nouns": {"Honor": 1, "Status": 1, "person": 2, "Promotive": 1, "Careful": 1}, "verbs": {"be": 1, "in": 1, "have": 2, "More": 1, "Promotive": 1, "Careful": 1}, "adverbs": {"Always": 1}, "numerals": {"60": 1}, "pronouns": {"I": 1}}}, {"content": "61. For me, seeing or performing in front of the public is a difficult task:", "word_count": 20, "label": "", "sentence_structure": "Subject鈥搗erb鈥搊bject", "subjective_objective": {"answer": "Subjective", "rationale": "Because it is the patient ' s own feeling and perception, it is subjective."}, "sentence_sentiment": {"answer": "Negative", "rationale": "Because the expression \"a difficult thing\" in the entry is negative."}, "language_style": {"answer": "Calm and direct", "rationale": ""}, "time_dimension": {"answer": "Present", "rationale": ""}, "sentence_type": "Affirmative declarative sentence", "pos": {"nouns": {"The public": 1, "Noodles": 2, "See": 1, "Performing": 1}, "verbs": {"be": 1, "Do it": 1}, "adjectives": {"One": 1, "Hard": 1}, "adverbs": {"Yes": 1, "I'll tell you what": 2}, "numerals": {"61": 1}, "classifiers": {"individual": 1}}}, {"content": "62. I would like to:", "word_count": 3, "label": "", "sentence_structure": "Subject鈥損redicate", "subjective_objective": {"answer": "Subjective", "rationale": "Because \"will\" is a word for the patient's subjective will."}, "sentence_sentiment": {"answer": "Neutral", "rationale": "Because the entry does not express positive or negative feelings, attitudes, behaviour, purpose or quality."}, "language_style": {"answer": "Tactful and warm", "rationale": "The language style of the entry is more euphemistic and polite and gives people a sense of respect and attention, while leaving room for imagination and self-expression."}, "time_dimension": {"answer": "Present", "rationale": "The entry indicates the attitude or will of the testee to a particular matter, does not address the time dimension of the past or the future, and therefore the judgement is now."}, "sentence_type": "Imperative sentence", "pos": {"nouns": {"I": 1, "Yes": 1}}}, {"content": "63. Even if I did something that made people laugh, I would be able to do it:", "word_count": 21, "label": "", "sentence_structure": "Subject鈥搗erb鈥搊bject", "subjective_objective": {"answer": "Subjective", "rationale": "This is a subjective attitude and perception of the patient, a perception and an assessment of his or her behaviour."}, "sentence_sentiment": {"answer": "Positive", "rationale": "Because the entry \"can be honest\" expresses a positive attitude."}, "language_style": {"answer": "Calm and direct", "rationale": "The article is more straightforward, not using too many rhetorical techniques or emotional renderings, and is more objective and calm. At the same time, the issue is not one of euphemism, invitation, but is presented in a direct interview, which places greater emphasis on the seriousness and importance of the issue."}, "time_dimension": {"answer": "Present", "rationale": "The keywords \"even\" and \"even\" and \"can\" mean that it is a customary act and attitude, and the temporal dimension is now judged."}, "sentence_type": "Affirmative declarative sentence", "pos": {"nouns": {"Yes": 1, "person": 1}, "verbs": {"Do it": 1, "Jean": 1, "Smile": 1, "Be nice": 1}, "adverbs": {"Even if": 1}, "numerals": {"63": 1}, "pronouns": {"I": 1}}}, {"content": "I think that no one would be happy to have the trouble:", "word_count": 19, "label": "", "sentence_structure": "Subject鈥搗erb鈥搊bject", "subjective_objective": {"answer": "Subjective", "rationale": "Since the entry relates to the patient ' s judgement and understanding of the mental state of another person, this perception is the patient ' s subjective feelings and attitudes."}, "sentence_sentiment": {"answer": "Negative", "rationale": "Because \"happiness\" is a negative sentiment and it is immoral to wish others to experience difficulties, the emotional colour of the article is negative."}, "language_style": {"answer": "Calm and direct", "rationale": "The language style of the entry is more calm and straightforward, not using too many words or emotional renderings, but rather expressing the views and attitudes of the interviewee through a simple presentation."}, "time_dimension": {"answer": "Present", "rationale": "\"I think\" indicates that this is the view and attitude of the interviewee, and \"no one would be happy to see me in trouble\" also expresses the view of the interviewer about the reality, which points to the situation. The time dimension of this article is therefore now."}, "sentence_type": "Imperative sentence", "pos": {"nouns": {"person": 2, "Hope": 1, "I": 2, "I met him": 1, "Difficulties": 1}, "verbs": {"I think": 1, "To be happy": 1, "Yes": 1, "Hope": 1}, "adjectives": {"Nobody": 1}, "numerals": {"64": 1}}}, {"content": "65. A person should consider the real meaning of life:", "word_count": 14, "label": "", "sentence_structure": "Subject鈥搗erb鈥搊bject", "subjective_objective": {"answer": "Subjective", "rationale": "Because \"thinking what life really means\" is a personal idea or opinion that belongs to the patient's subjective perspective."}, "sentence_sentiment": {"answer": "Neutral", "rationale": "Because the entry does not have any emotional color."}, "language_style": {"answer": "Tactful and warm", "rationale": "The entry uses a question-oriented format rather than a direct statement or order, which is more likely to trigger reflection and self-reflection by the other party and gives a sense of care and understanding, so that the language style is more euphemistic."}, "time_dimension": {"answer": "Present", "rationale": "The entry asks whether the testee should consider the real meaning of life at the current point of time, without addressing the past or future time dimension, and therefore judge it as now."}, "sentence_type": "Imperative sentence", "pos": {"nouns": {"person": 1, "Life": 1, "Real meaning": 1}, "verbs": {"Consider": 1, "be": 1}, "numerals": {"65": 1}}}, {"content": "When I am very happy, there is always a feeling of 鈥渟lowness鈥?", "word_count": 18, "label": "", "sentence_structure": "Subject鈥搗erb鈥揷omplement", "subjective_objective": {"answer": "Subjective", "rationale": "Because it's the patient's own subjective thoughts and feelings."}, "sentence_sentiment": {"answer": "Negative", "rationale": "Because the feeling of \"goodness is not long\" in the entries is negative."}, "language_style": {"answer": "Tactful and warm", "rationale": "The entry is more personal and interactive when it is spoken in a gentle and inviting manner, rather than in direct statements or orders. Moreover, the expression \"goodness is not long\" is also a euphemism, implying that the interviewee may have some degree of anxiety or anxiety."}, "time_dimension": {"answer": "Present", "rationale": ""}, "sentence_type": "Affirmative declarative sentence", "pos": {"nouns": {"Happy": 1, "Feel": 1}, "verbs": {"have": 1, "Total": 1, "be": 1, "I think": 1}, "adjectives": {"Very": 1, "It's not long": 1}, "adverbs": {"Time": 1}, "numerals": {"67": 1}}}, {"content": "68. In general, I am optimistic in difficult circumstances:", "word_count": 15, "label": "", "sentence_structure": "Subject鈥搗erb鈥揷omplement", "subjective_objective": {"answer": "Subjective", "rationale": "Because \"always keep optimism\" is a personal attitude or perception, a patient's subjective feeling."}, "sentence_sentiment": {"answer": "Positive", "rationale": "Because \"still optimistic\" is a positive attitude."}, "language_style": {"answer": "Calm and direct", "rationale": "The entry uses simple and straightforward language to describe the subject matter, without adding too many words or emotional renderings, and is more objective. This expression allows the respondents to understand the problem more clearly and facilitates subsequent data analysis and interpretation."}, "time_dimension": {"answer": "Present", "rationale": ""}, "sentence_type": "Affirmative declarative sentence", "pos": {"nouns": {"Situation": 1, "Difficulties": 1, "Optimistic": 1}, "verbs": {"Yes": 1, "Hold on": 1, "Total": 1}, "adjectives": {"General": 1}, "adverbs": {"Medium": 1}, "numerals": {"68": 1}}}, {"content": "69. Relocation is a very unpleasant thing:", "word_count": 11, "label": "", "sentence_structure": "Subject鈥揷opula鈥損redicative", "subjective_objective": {"answer": "Subjective", "rationale": "Because \"extremely unhappy\" is the patient's subjective feeling of the objective event of moving."}, "sentence_sentiment": {"answer": "Negative", "rationale": "Because the phrase \"relocation is a very unpleasant thing\" in the entry is negative."}, "language_style": {"answer": "Calm and direct", "rationale": "The entry directly expresses the subject of questioning the other party ' s views on relocation, does not use excessive rhetoric or emotional rendering, and is more objective in tone."}, "time_dimension": {"answer": "Present", "rationale": "The keywords are \"relocation\" and \"extremely unpleasant\" and they are all expressions of the present, and therefore the present."}, "sentence_type": "Negative declarative sentence", "pos": {"nouns": {"Relocation": 1, "Yes": 1}, "verbs": {"be": 1, "It's a bad time": 1}, "adjectives": {"It's a very unpleasant thing": 1}, "adverbs": {"Extreme": 1}, "numerals": {"69": 1}}}, {"content": "70. When I was young, when my parents and I had different opinions:", "word_count": 17, "label": "", "sentence_structure": "Subject鈥損redicate", "subjective_objective": {"answer": "Subjective", "rationale": "The entry is a question of the patient's subjective experience and feelings when he is young."}, "sentence_sentiment": {"answer": "Neutral", "rationale": "This entry describes the situation of individuals who, at a young age, disagree with their parents and does not involve positive or negative emotional aspects."}, "language_style": {"answer": "Calm and direct", "rationale": "This topic is more direct, objective, with no excessive emotional rendering or rhetorical techniques, and questions about the attitude and handling of the interviewee in the face of a disagreement with his or her parents."}, "time_dimension": {"answer": "Present", "rationale": "Now, the term \"youth\" is used to say the time frame, but the term \"when my parents and I disagree\" is about the present or the past, so the best term is \"now\"."}, "sentence_type": "Interrogative sentence", "pos": {"nouns": {"Young": 1, "Time": 1, "parents": 1, "Observations": 2, "Different": 1}, "verbs": {"and": 1, "have": 1, "Time": 1}, "adverbs": {"When": 1}, "numerals": {"70": 1}, "pronouns": {"I": 1}}}, {"content": "71. I wish to bring my family:", "word_count": 8, "label": "", "sentence_structure": "Subject鈥搗erb鈥揷omplement", "subjective_objective": {"answer": "Subjective", "rationale": "Because hope is a subjective emotion and attitude."}, "sentence_sentiment": {"answer": "Neutral", "rationale": "The entry is not of a clear emotional orientation."}, "language_style": {"answer": "Tactful and warm", "rationale": "The language style of the entry appears to be more moderate, friendly and guiding, and it is easier to understand the views of the family by asking the other side about their thoughts and wishes, which makes the interviewee comfortable and willing to answer questions. At the same time, the word \"hope\" expresses positive expectations and encouragement, adding to the friendly nature of the questionnaire."}, "time_dimension": {"answer": "Present", "rationale": "The entry is an inquiry into the testee's expectations or aspirations for the future, and therefore the future is judged. Key words \"hope\" and \"take my family\" point to the future that has not yet happened."}, "sentence_type": "Imperative sentence", "pos": {"nouns": {"Family": 1}, "verbs": {"Hope": 1, "- Put it on": 1}}}, {"content": "72. In my efforts to solve the problem, I have relied on:", "word_count": 10, "label": "", "sentence_structure": "Subject鈥搗erb鈥搊bject", "subjective_objective": {"answer": "Subjective", "rationale": "This issue relates to patients ' preferences and habits in solving problems and is therefore a subjective one."}, "sentence_sentiment": {"answer": "Neutral", "rationale": ""}, "language_style": {"answer": "Calm and direct", "rationale": "The language style of the entry is more direct, clear, with no excessive adoration or emotional colour, and more focused on the presentation of the issue itself and the way in which the question is asked."}, "time_dimension": {"answer": "Present", "rationale": "The entry describes the manner in which the person tested acted in resolving a specific problem, and the temporal dimension is now judged."}, "sentence_type": "Interrogative sentence", "pos": {"nouns": {"Problem": 1, "Resolve": 1, "鈾?With the help of": 1}, "verbs": {"Time": 1, "More": 1}, "adverbs": {"More": 1}, "numerals": {"72": 1}, "classifiers": {"individual": 1}}}, {"content": "73. When needed to be quick to decide, I always:", "word_count": 11, "label": "", "sentence_structure": "Subject鈥搗erb鈥搊bject", "subjective_objective": {"answer": "Subjective", "rationale": "The subject is a subjective one, since it is a question of the patient ' s own behaviour and attitude."}, "sentence_sentiment": {"answer": "Neutral", "rationale": "Because the entry is just a question, and it is not emotional."}, "language_style": {"answer": "Calm and direct", "rationale": ""}, "time_dimension": {"answer": "Present", "rationale": ""}, "sentence_type": "Imperative sentence", "pos": {"nouns": {"Questionnaire": 1, "Entry": 1, "Now, let's get this straight": 1}, "verbs": {"be": 1, "Total": 1}, "adverbs": {"in": 1, "Always": 1}, "numerals": {"73": 1}}}, {"content": "74. Recently, on one or two matters, I feel I have been innocent:", "word_count": 19, "label": "", "sentence_structure": "Subject鈥揷opula鈥損redicative", "subjective_objective": {"answer": "Subjective", "rationale": "Because feeling innocent is the patient's subjective feeling and thinking."}, "sentence_sentiment": {"answer": "Negative", "rationale": "Because the expression \"innocently tired\" in this entry has a negative emotional connotation."}, "language_style": {"answer": "Tactful and warm", "rationale": "The language style of the entry is euphemistic, using the term \"innocently tired\" in a relatively mild tone, without direct accusations or offensive words, and helps the interviewee to open his heart to questions. At the same time, the sentence ends with a false mark, indicating that he will then ask specific questions or explanations, increasing the interviewee's expectations and participation."}, "time_dimension": {"answer": "Present", "rationale": "\"Recently\" means the recent, current, \"I\" means the person's own behaviour or state, and the entry describes the person's feelings and experience at the time."}, "sentence_type": "Interrogative sentence", "pos": {"nouns": {"Things": 2, "person": 1}, "verbs": {"I think": 1, "be": 1, "I'm tired": 1}, "adjectives": {"Innocent": 1, "One or two": 1}, "adverbs": {"Most recently": 1}, "numerals": {"74": 1, "2": 1}}}, {"content": "I am good at controlling my expression:", "word_count": 9, "label": "", "sentence_structure": "Subject鈥搗erb鈥揷omplement", "subjective_objective": {"answer": "Subjective", "rationale": "Because \"controllable\" is the patient's subjective feeling and perception, not the objective fact."}, "sentence_sentiment": {"answer": "Neutral", "rationale": "Because the entry does not have any emotional color."}, "language_style": {"answer": "Calm and direct", "rationale": "The entry is in a concise manner, asking the interviewee whether he is good at controlling his face, and the language style is more calm and objective, without using excessive emotional colours or rhetorical techniques."}, "time_dimension": {"answer": "Present", "rationale": "The words \"good\" , \"control\" and \"expression\" indicate that the person being tested is describing a customary act or attitude, which is now, in time. Thus, the purpose of this article is now time-dimensional."}, "sentence_type": "Interrogative sentence", "pos": {"nouns": {"Questionnaire": 1, "Entry": 1, "Emoticons": 1}, "verbs": {"Good at it": 1, "Control": 1}, "numerals": {"75": 1}, "pronouns": {"I": 1}}}, {"content": "76. If the treatment is the same, I would like to make one:", "word_count": 11, "label": "", "sentence_structure": "Subject鈥搗erb鈥搊bject", "subjective_objective": {"answer": "Subjective", "rationale": "The question is one of personal preferences and wishes of the patient and is one of subjective attitudes."}, "sentence_sentiment": {"answer": "Neutral", "rationale": "Because the entry is merely an inquiry into the wishes of the interviewees and does not involve any positive or negative emotional aspects."}, "language_style": {"answer": "Calm and direct", "rationale": "Please provide more information for judgement purposes."}, "time_dimension": {"answer": "Past", "rationale": "The entry uses the virtual expression \"I'd like to do a...\" and suggests that the interviewee is assuming a situation when he answers the question, which does not mean that they are going through it now. So, judging by the dimensions of time, the content of the entry is in the past."}, "sentence_type": "Interrogative sentence", "pos": {"nouns": {"Treatment": 1, "I": 1}, "verbs": {"Do it": 1}, "numerals": {"76": 1}}}, {"content": "To take the example of 鈥渟urprising鈥?and 鈥渘ew鈥? the following is considered to be a combination of 鈥渇ear鈥?and () fear:", "word_count": 17, "label": "", "sentence_structure": "Subject鈥搗erb鈥搊bject", "subjective_objective": {"answer": "Subjective", "rationale": "Because the subject is a personal combination of views and is subjective."}, "sentence_sentiment": {"answer": "Negative", "rationale": "Because it's about fear, it's a negative mood and attitude."}, "language_style": {"answer": "Calm and direct", "rationale": "The question is presented in a more objective and concise manner, without excessive emotional or rhetorical styles, and in a more flat tone, by asking the other side what it considers to be a combination of a term."}, "time_dimension": {"answer": "Future", "rationale": "The entry asks the respondent about the views and ideas of an issue, not about the specific circumstances of the past or the present, but about the possibilities and expectations of the future."}, "sentence_type": "Interrogative sentence", "pos": {"nouns": {"Surprise": 1, "- New": 1, "Match": 2, "Fear": 1}, "verbs": {"Recognition": 1, "Yes": 1}, "adverbs": {"and": 2}}}, {"content": "78. Three fractions are given after this topic, which is different from the other two fractions:", "word_count": 24, "label": "", "sentence_structure": "Subject鈥搗erb鈥搊bject", "subjective_objective": {"answer": "Objective", "rationale": "Because this question is an examination of the respondents ' cognitive ability to classify numbers, it does not need to involve subjective attitudes or experiences of individuals."}, "sentence_sentiment": {"answer": "Neutral", "rationale": "Because the entry does not have any emotional color."}, "language_style": {"answer": "Calm and direct", "rationale": "The language style of the entry is calm and straightforward. It does not use any rhetorical or emotional colour to attract the attention of the respondents, but rather asks the respondents in a concise and precise manner, enabling them to answer quickly and accurately. At the same time, this cold and objective language style makes the questionnaire more neutral and impartial, avoiding prejudice or misleading the respondents."}, "time_dimension": {"answer": "Present", "rationale": "The entry was used to ask the respondent about the perception or reaction of a situation that had already occurred and was therefore a matter of past practice."}, "sentence_type": "Interrogative sentence", "pos": {"nouns": {"Problem": 1, "min": 2, "Number": 2, "Answer": 1, "Category": 1}, "verbs": {"List": 1, "Which one": 1, "Same": 1, "Different": 1}, "numerals": {"III": 1}, "classifiers": {"individual": 2}, "pronouns": {"Here": 1}}}, {"content": "79. Some people, somehow, have been avoiding or indifferent to me:", "word_count": 16, "label": "", "sentence_structure": "Subject鈥搗erb鈥搊bject", "subjective_objective": {"answer": "Subjective", "rationale": "Because \"for some reason\" suggests that it is a patient's subjective feeling and perception, not an objective fact."}, "sentence_sentiment": {"answer": "Negative", "rationale": "Because the \"scrambling or cold\" act is negative."}, "language_style": {"answer": "Calm and direct", "rationale": ""}, "time_dimension": {"answer": "Past", "rationale": "And it can be inferred from the phrase \"always avoiding or imitating me\" that this is a description of what happened over time, and therefore a judgement of the past."}, "sentence_type": "Interrogative sentence", "pos": {"nouns": {"person": 1, "Avoidance": 1, "Cold": 1}, "verbs": {"Yes": 1, "Total": 1, "be": 1, "Avoid": 1, "Cold": 1, "Light": 1}, "adjectives": {"Some": 1, "None": 1}, "adverbs": {"Why": 1, "What": 1, "Total": 1}, "numerals": {"79": 1}, "pronouns": {"I": 1}}}, {"content": "80. I am very kind, but I often do not get a good reward:", "word_count": 15, "label": "", "sentence_structure": "Subject鈥搗erb鈥搊bject", "subjective_objective": {"answer": "Subjective", "rationale": "The basis for the judgement: \"I am kind\" is patient self-evaluation and subjective thinking; and \"but often I am not well rewarded\" reflects patient expectations of the outcome of his behaviour, and is also patient's subjective feeling. So, this questionnaire entry is more viewed from the patient's subjective perspective."}, "sentence_sentiment": {"answer": "Negative", "rationale": "Because the \"often bad\" result is negative."}, "language_style": {"answer": "Tactful and warm", "rationale": "The language style of the entry is more euphemismal. It uses first-person expressions that express their feelings and experiences in a sincere and self-serving manner, and that do not over-opt or directly blame others, but rather think more from their own perspective. Such expressions are more likely to resonate and understand the other side, and can create a good atmosphere of communication."}, "time_dimension": {"answer": "Past", "rationale": "The entry describes the experience and feelings of the testee in the past, the key words being \"good-will\" and \"often bad-for-good\" 鈥?all of which have happened in the past. The time dimension of the questionnaire is therefore past."}, "sentence_type": "Interrogative sentence", "pos": {"nouns": {"Grandpa": 1, "Questionnaire": 1}, "verbs": {"Please": 1, "Analysis": 1, "Got it": 1}, "adjectives": {"Down there": 1, "Good idea": 1}, "adverbs": {"Although": 1, "But": 1, "Often": 1}, "numerals": {"80": 1}, "pronouns": {"I": 2, "person": 1}}}, {"content": "I don't like the best:", "word_count": 10, "label": "", "sentence_structure": "Subject鈥搗erb鈥揷omplement", "subjective_objective": {"answer": "Subjective", "rationale": "Because \"not like\" is patient's subjective attitude."}, "sentence_sentiment": {"answer": "Negative", "rationale": "Because the description \"not like the winner\" has negative feelings."}, "language_style": {"answer": "Calm and direct", "rationale": "This is a language style that is more objective, rational and suitable for describing or communicating factual information."}, "time_dimension": {"answer": "Present", "rationale": "The word \"no\" in the entry indicates a negative act or attitude, and the \"not like\" indicates a subjective feeling of the act, all pointing to the current situation."}, "sentence_type": "Interrogative sentence", "pos": {"nouns": {"person": 1, "You're a winner": 1}, "verbs": {"Yeah": 1}, "adjectives": {"I don't like it": 1}}}, {"content": "82. My friends are indeed too few compared to the average:", "word_count": 14, "label": "", "sentence_structure": "Subject鈥揷opula鈥損redicative", "subjective_objective": {"answer": "Subjective", "rationale": "Because the entry compares the number of friends of patients with the general population, it relates to the patient ' s subjective perception of himself."}, "sentence_sentiment": {"answer": "Negative", "rationale": "Because the phrase \"friends are really too few\" in the entry is negative."}, "language_style": {"answer": "Calm and direct", "rationale": "The language style of the entry is more straightforward, with no over-doping or emotional rendering, more about presenting a fact or asking a question. This language style is usually more acceptable and understandable."}, "time_dimension": {"answer": "Present", "rationale": "The entry is describing the state or feeling of the moment, and the keyword \"as opposed to the average person\" and \"as opposed to the general person\" all indicate that this is a subjective assessment, which occurs at the present time. So, the temporal dimension is now."}, "sentence_type": "Negative declarative sentence", "pos": {"nouns": {"General": 1, "person": 1, "friends": 1}, "verbs": {"- Yeah": 1, "Too much": 1}, "adjectives": {"More": 1, "Less": 1}, "numerals": {"82": 1}, "pronouns": {"I": 1}}}, {"content": "83. I have always avoided engaging in remunerated activities, without the necessity of doing so:", "word_count": 23, "label": "", "sentence_structure": "Subject鈥搗erb鈥搊bject", "subjective_objective": {"answer": "Subjective", "rationale": "Because words like \"must\" and \"always\" show that this is the patient's subjective attitude."}, "sentence_sentiment": {"answer": "Negative", "rationale": "Because the act of avoiding gainful activity in the entry is negative."}, "language_style": {"answer": "Calm and direct", "rationale": "The entry is more straightforward, concise, and has no excessive accrimation or emotional colour. It asks whether the person surveyed, in circumstances where it is unnecessary, avoids engaging in remunerated activities, and expresses it in a more objective and neutral manner."}, "time_dimension": {"answer": "Past", "rationale": "The term \"always\" indicates the person who was tested in the past, and \"not as a last resort\" indicates that such conduct is not a temporary decision or occasional circumstance, but is recurrent or customary. The entry therefore primarily asks about the person who was tested in the past."}, "sentence_type": "Negative declarative sentence", "pos": {"nouns": {"Situation": 1, "Payability": 1, "Activities": 1}, "verbs": {"Avoidance": 1, "Participation": 1}, "adjectives": {"I'll have to": 1}, "adverbs": {"Always": 1}, "numerals": {"83": 1}}}, {"content": "84. I believe that leadership is more important than performance:", "word_count": 18, "label": "", "sentence_structure": "Subject鈥搗erb鈥搊bject", "subjective_objective": {"answer": "Subjective", "rationale": "Because \"think\" is the patient's own view and attitude, it's a subjective examination."}, "sentence_sentiment": {"answer": "Negative", "rationale": "Because the \"good for leadership\" act is negative."}, "language_style": {"answer": "Calm and direct", "rationale": "The language style of the entry is relatively simple, there is no excessive rhetorical or emotional rendering, and it is more objective and direct in its tone to ask the question whether the other party considers that the orientation of the leader is more important than the performance of the job."}, "time_dimension": {"answer": "Present", "rationale": "The entry uses the current state of the art \"considers\" and the descriptive adjective \"important\" to indicate the current attitude and perception of the subject."}, "sentence_type": "Affirmative declarative sentence", "pos": {"nouns": {"Questionnaire": 1, "Entry": 1, "Leadership": 1, "Work": 1, "Performance": 1}, "verbs": {"I think": 1, "- I'm glad you're here": 1, "That's right": 1, "- Yeah": 1, "More important": 1}, "adverbs": {"More": 1}, "numerals": {"84": 1}, "pronouns": {"I": 1}}}, {"content": "85. In participating in the competition, I have focused on the activities of the competition, regardless of its success or failure:", "word_count": 23, "label": "", "sentence_structure": "Subject鈥搗erb鈥搊bject", "subjective_objective": {"answer": "Subjective", "rationale": "Because the focus on activity rather than success is the patient's own psychological state and attitude."}, "sentence_sentiment": {"answer": "Positive", "rationale": "Because the behavior and attitude in the entry, \"focusing on the competition\" is positive."}, "language_style": {"answer": "Calm and direct", "rationale": "The language style of the entry is calm and straightforward. It expresses the attitude and behaviour of the competition in a concise and clear manner, does not contain too much emotional or rhetorical style, and is more objective and rational."}, "time_dimension": {"answer": "Present", "rationale": ""}, "sentence_type": "Affirmative declarative sentence", "pos": {"nouns": {"Competition": 2, "Activities": 1, "Success or failure": 1}, "verbs": {"Participation": 1, "Focus": 1, "Count": 1}, "adverbs": {"Always": 1, "not": 1}, "numerals": {"85": 1}}}, {"content": "86. As I personally wish, the work I would like to do is:", "word_count": 16, "label": "", "sentence_structure": "Subject鈥搗erb鈥搊bject", "subjective_objective": {"answer": "Subjective", "rationale": "Because \"it is my will\" to show that it is the patient's personal will and expectations, and it is subjective."}, "sentence_sentiment": {"answer": "Neutral", "rationale": "Because the entry does not express any emotional colour or attitude, it merely presents an individual wish."}, "language_style": {"answer": "Tactful and warm", "rationale": "This entry is more euphemistic, does not ask or order the other person to answer the question, but rather engages in a dialogue in a way that is personal and desired."}, "time_dimension": {"answer": "Past", "rationale": ""}, "sentence_type": "Imperative sentence", "pos": {"nouns": {"Personal": 1, "Will": 1, "Work": 1}, "verbs": {"Hope": 1, "Do it": 1}, "adverbs": {"According to": 1, "of": 2, "be": 1}, "numerals": {"86": 1}, "pronouns": {"I": 1}}}, {"content": "The performance of the staff is adjusted at any time", "word_count": 10, "label": "", "sentence_structure": "Subject鈥搗erb鈥揷omplement", "subjective_objective": {"answer": "Objective", "rationale": "Because work performance is a fact that can be observed and measured, not the patient ' s own subjective feelings or attitudes."}, "sentence_sentiment": {"answer": "Negative", "rationale": "Because the article's formulation contains the phrase 鈥渁t any time adjusted鈥? it implies that poor performance may result in negative consequences such as layoffs or salary reductions."}, "language_style": {"answer": "Tactful and warm", "rationale": "This entry is a soft, inviting expression of dialogue, expression of concern and respect for each other's performance and provides a positive, problem-solving attitude. Such expression is more personal and interactive and better promotes communication and cooperation."}, "time_dimension": {"answer": "Present", "rationale": "The entry \"Performance at any time\" refers to the behaviour or state of the person being tested, and thus the judgement is now."}, "sentence_type": "Interrogative sentence", "pos": {"nouns": {"Performance": 1, "Adjustment": 1}, "verbs": {"And at any time": 1}, "adverbs": {"Anytime": 1}}}, {"content": "87. I would like to read:", "word_count": 5, "label": "", "sentence_structure": "Subject鈥搗erb鈥搊bject", "subjective_objective": {"answer": "Subjective", "rationale": "Because \"will\" is a word of will, attitude, and it is subjective."}, "sentence_sentiment": {"answer": "Positive", "rationale": "Because \"willing to read\" expresses a positive attitude."}, "language_style": {"answer": "Tactful and warm", "rationale": "The entry is a positive expression of \"willing\" to guide respondents to answer questions and to use \"read\" in the context of the language, creating a comfortable and relaxed atmosphere, so that the language style is more euphemismal and warm."}, "time_dimension": {"answer": "Present", "rationale": "The entry is for the purpose of questioning the person being tested for his current behaviour, and the keyword \"willing to read\" indicates that this is a description of the present will and therefore judgement is now."}, "sentence_type": "Imperative sentence", "pos": {"nouns": {"Questionnaire": 1, "Entry": 1, "Reading": 1}, "verbs": {"Yes": 1}, "adverbs": {"Down there": 1, "Please": 1}, "numerals": {"87": 1}}}, {"content": "88. I believe that the fear of punishment is the main reason why many people are afraid to commit crimes:", "word_count": 24, "label": "", "sentence_structure": "Subject鈥搗erb鈥搊bject", "subjective_objective": {"answer": "Subjective", "rationale": "Because \"think\" means the patient's own subjective views and opinions."}, "sentence_sentiment": {"answer": "Negative", "rationale": "Because the entries \"daunted crime\" and \"feared punishment\" both indicate a negative emotional attitude."}, "language_style": {"answer": "Calm and direct", "rationale": "The language style of the entry is calm, as it expresses the subject directly, \"The main reason why many do not dare to commit crimes\", without any rhetorical or emotional modification. At the same time, it uses a simple and clear language that enables readers to understand its meaning quickly."}, "time_dimension": {"answer": "Present", "rationale": ""}, "sentence_type": "Interrogative sentence", "pos": {"nouns": {"88": 1, "person": 1, "Crime": 1, "Reason": 1, "Main": 1, "Punished": 1}, "verbs": {"I think": 1, "have": 1, "I'm afraid": 1, "be": 1, "Yes": 1}, "adjectives": {"Many": 1, "Main": 1}, "adverbs": {"鈾?And the reason 鈾?: 1}, "numerals": {"88": 1}, "classifiers": {"individual": 1}, "pronouns": {"Other": 1}}}, {"content": "89. My parents never strictly asked me to comply:", "word_count": 16, "label": "", "sentence_structure": "Subject鈥搗erb鈥搊bject", "subjective_objective": {"answer": "Objective", "rationale": "This is an objective statement of the fact that the patient is examining his or her own family environment."}, "sentence_sentiment": {"answer": "Negative", "rationale": "Because the description of the act \"Parents never strictly asked me to obey\" in the entry is negative."}, "language_style": {"answer": "Calm and direct", "rationale": "The basis for the determination: the entry directly expresses the subject of questioning the parents of the other parent about their behaviour, without excessive rhetorical or emotional rendering, and is more objective in tone."}, "time_dimension": {"answer": "Past", "rationale": "The entry describes the situation of the tested person with his parents, and the temporal dimension is a past."}, "sentence_type": "Imperative sentence", "pos": {"nouns": {"parents": 2, "- The thing": 1, "Submissive": 1}, "verbs": {"Request": 1, "Strict": 1, "Never": 1, "not": 2}, "adjectives": {"Never": 1, "Strict": 1}, "adverbs": {"not": 2, "EMPTY": 1}, "numerals": {"89": 1}, "pronouns": {"I": 1}}}, {"content": "The spirit of 鈥渞esisting on and on鈥?is often overlooked:", "word_count": 19, "label": "", "sentence_structure": "Subject鈥搗erb鈥揷omplement", "subjective_objective": {"answer": "Subjective", "rationale": "This subjective judgement is based on the author's personal experience and observations, and does not objectively describe or evaluate others' experiences and observations. Thus, the questionnaire entry is a subjective one."}, "sentence_sentiment": {"answer": "Positive", "rationale": "Because \"remaining, building on\" is a positive state of mind and attitude."}, "language_style": {"answer": "Tactful and warm", "rationale": "The language style of the entry is a euphemism. First, the word \"often\" implies the importance of the spirit, but does not make a direct determination; second, \"neglected\" shows the existence of the problem, but does not blame or criticize anyone; and, finally, \"dependant, building on\" is described in a more optimistic way, without too negative or negative language. These factors make the language style of the article more profane and encouraging."}, "time_dimension": {"answer": "Past", "rationale": "This sentence is more of a point or observation, and is not clearly time-bound."}, "sentence_type": "Negative declarative sentence", "pos": {"nouns": {"Spirit": 1, "Guys": 1}, "verbs": {"Often": 1, "By": 1, "Ignore": 1, "Keep going": 1, "It's all over": 1}, "adverbs": {"Regular": 1, "Again": 2}, "numerals": {"90": 1}}}, {"content": "When I get angry, I always:", "word_count": 11, "label": "", "sentence_structure": "Subject鈥搗erb鈥搊bject", "subjective_objective": {"answer": "Subjective", "rationale": "Because \"always\" shows that it is the patient's subjective attitude and response, not the objective facts or results."}, "sentence_sentiment": {"answer": "Negative", "rationale": "Because the behavior in the entry \"I'm always angry\" can be offensive or scary."}, "language_style": {"answer": "Calm and direct", "rationale": "The language style of the entry is more calm. It presents a specific situation (when someone is angry with me) and asks the interviewee what action is usually taken in this case. This question-and-answer approach is relatively direct and clear, and does not involve too much adoration or emotional expression. So, \"cool directly\" is more of a linguistic description of the entry."}, "time_dimension": {"answer": "Present", "rationale": ""}, "sentence_type": "Interrogative sentence", "pos": {"nouns": {"person": 1, "Fire": 1}, "verbs": {"Let's go": 1, "Always": 1, "to": 1}, "adverbs": {"When": 1}, "numerals": {"91": 1}, "pronouns": {"I": 1}}}, {"content": "92. I want people to be friendly:", "word_count": 11, "label": "", "sentence_structure": "Subject鈥損redicate", "subjective_objective": {"answer": "Subjective", "rationale": "Hope is a subjective attitude and expectation."}, "sentence_sentiment": {"answer": "Positive", "rationale": "Because the entry \"I want people to be friendly\" expresses positive emotions and attitudes."}, "language_style": {"answer": "Tactful and warm", "rationale": "The entry conveys a desire and expectation for goodwill by using positive language ( \"friendly living\") and \"favourable\" expressions. At the same time, it does not directly order or compel the interviewee to do so, but is expressed in an invitation, gentleness, and makes people more receptive to and think about the idea in their answers, reflecting a warmer language style."}, "time_dimension": {"answer": "Present", "rationale": "The words \"hope\", \"want\" and \"friendly living\" in this entry indicate that it is an expectation or expectation, referring to the present situation and the future direction. We can therefore judge it as an act or mentality of the present."}, "sentence_type": "Imperative sentence", "pos": {"nouns": {"Hope": 1, "People": 1, "Friendly": 1, "Get along": 1}, "verbs": {"Yes": 1, "Both": 1}, "adjectives": {"Friendly": 1}, "numerals": {"92": 1}}}, {"content": "93. I rarely feel afraid, whether on very high roofs or in deep tunnels:", "word_count": 28, "label": "", "sentence_structure": "Subject鈥搗erb鈥搊bject", "subjective_objective": {"answer": "Subjective", "rationale": "Because feeling afraid is a subjective feeling and emotional reaction."}, "sentence_sentiment": {"answer": "Positive", "rationale": "Because the description in the entry shows that individuals have the qualities and attitudes to face the challenge with courage, it is a positive emotional experience."}, "language_style": {"answer": "Calm and direct", "rationale": "This language style is more calm and objective, and allows for accurate information."}, "time_dimension": {"answer": "Past", "rationale": "Past."}, "sentence_type": "Interrogative sentence", "pos": {"nouns": {"Rooftops": 1, "Tunnel": 1}, "verbs": {"I feel": 2, "Frightened": 1, "Unsettled": 1}, "adjectives": {"Very high": 1, "Very few": 1, "Extremely": 1}, "adverbs": {"Very few": 1, "Whether it's in there": 1}, "numerals": {"93": 1}}}, {"content": "94. As long as there is no fault in it, I can always be comfortable with what others say:", "word_count": 20, "label": "", "sentence_structure": "Subject鈥搗erb鈥揷omplement", "subjective_objective": {"answer": "Subjective", "rationale": "Because \"well-meaning\" is a subjective feeling and attitude."}, "sentence_sentiment": {"answer": "Positive", "rationale": "Because the attitude in the entries is self-confidence, firmness and positiveness, it can maintain self-confidence even in the face of criticism or questioning by others and feel that it has not done anything wrong."}, "language_style": {"answer": "Tactful and warm", "rationale": "The language style of the entry is more euphemistic, as it uses words that express feelings and attitudes, such as \"good-hearted\" and \"good-for-nothing\". In addition, the entry uses a more positive and positive tone, expressing trust and respect for the interviewee's self-evaluation."}, "time_dimension": {"answer": "Present", "rationale": "The entry is about the person's inner state and attitude, the key words being \"as long as there is no fault\" and \"as always\" and they all point to the person's current feelings and thoughts. So the time dimension of the entry is now."}, "sentence_type": "Imperative sentence", "pos": {"nouns": {"Wrong": 1, "person": 1}, "verbs": {"Always": 1, "You're all right": 1}, "adverbs": {"Whatever": 1}, "numerals": {"94": 1}, "pronouns": {"I": 1}}}, {"content": "95. I believe that sometimes the power to deal with issues that cannot be solved with reason has to be relied upon:", "word_count": 27, "label": "", "sentence_structure": "Subject鈥損redicate", "subjective_objective": {"answer": "Subjective", "rationale": "Because \"think\" is the patient's subjective attitude."}, "sentence_sentiment": {"answer": "Negative", "rationale": "Because the \"power to deal with\" in the entry is negative."}, "language_style": {"answer": "Calm and direct", "rationale": "The language style of the entry is so blunt that it uses tough words such as \"must\" and \"power\" and is not overly pedantic or euphemistic, showing a more resolute attitude."}, "time_dimension": {"answer": "Future", "rationale": "The keyword \"sometimes it has to be managed by power\" expresses vision and expectations for the future, and therefore the temporal dimension is judged to be the future."}, "sentence_type": "Affirmative declarative sentence", "pos": {"nouns": {"Problem": 1, "Reason": 1, "Power": 1}, "verbs": {"I think": 1, "Resolve": 1, "Processing": 1}, "adverbs": {"Cannot": 1, "Sometimes": 1, "I had to": 1}, "numerals": {"95": 1}, "pronouns": {"I": 1}}}, {"content": "96. When I was young, I dated a friend of the opposite sex:", "word_count": 14, "label": "", "sentence_structure": "Subject鈥搗erb鈥搊bject", "subjective_objective": {"answer": "Subjective", "rationale": "This is because patients describe and evaluate their past behaviour and because it is an individual subjective experience."}, "sentence_sentiment": {"answer": "Neutral", "rationale": "It simply asks whether the interviewee had been with a heterosexual friend when he was young."}, "language_style": {"answer": "Tactful and warm", "rationale": "Because the language style of the entry is more accommodative, using the expression \"youth\" and using the theme \"to have a heterosexual friend\" as the subject of the survey, it makes it easier for the respondents to accept and to answer questions."}, "time_dimension": {"answer": "Past", "rationale": ""}, "sentence_type": "Affirmative declarative sentence", "pos": {"nouns": {"Young": 1, "Time": 1, "Fae friend": 1, "- To be with someone": 1}, "verbs": {"in": 1, "have": 1}, "numerals": {"96": 1}}}, {"content": "97. I am an activist in community activities:", "word_count": 14, "label": "", "sentence_structure": "Subject鈥損redicate", "subjective_objective": {"answer": "Subjective", "rationale": "Because \"activists\" are patients' own self-evaluations or subjective perceptions of them, they are subjective."}, "sentence_sentiment": {"answer": "Positive", "rationale": "Because the expression \"activists\" is usually seen as a positive quality or behaviour, it is characterized by active participation in community activities, active speaking, etc."}, "language_style": {"answer": "Tactful and warm", "rationale": "The entry is more euphemistic and moderate, asking about the role of the other party in the activities of the association and not directly presenting or ordering, and is more expressive and interactive."}, "time_dimension": {"answer": "Past", "rationale": ""}, "sentence_type": "Affirmative declarative sentence", "pos": {"nouns": {"Association activities": 1, "Activists": 1}, "verbs": {"be": 1}, "adjectives": {"One": 1}, "numerals": {"97": 1}, "pronouns": {"I": 1}}}, {"content": "98. In the midst of the loud voices, I can still concentrate on my work without interference:", "word_count": 17, "label": "", "sentence_structure": "Subject鈥搗erb鈥揷omplement", "subjective_objective": {"answer": "Objective", "rationale": "Since this statement relates to a person ' s ability to work with a focus on noise, it is a fact or result that can be quantified and is therefore objective."}, "sentence_sentiment": {"answer": "Positive", "rationale": "Because the behavior in the entry \"without interference, focus on work\" is positive."}, "language_style": {"answer": "Calm and direct", "rationale": "The entry is more straightforward in language style, with no excessive emotional colour and rhetorical skills. It simply asks the interviewee whether he or she can concentrate on working in a noisy environment, and is more objective and calm."}, "time_dimension": {"answer": "Present", "rationale": "The entry is for the purpose of asking the testee about the focus in a given environment, and the time dimension is now judged."}, "sentence_type": "Affirmative declarative sentence", "pos": {"nouns": {"Voice": 1, "Loud": 1, "Interference": 1, "Focus": 1, "Work": 1}, "verbs": {"Yes": 1, "Not": 1, "Impact": 1, "Focus": 1, "Work": 1}, "adjectives": {"Loud": 1, "Interference": 1}, "numerals": {"98": 1}}}, {"content": "99. In some contexts, I have often put my work on hold because of confusion:", "word_count": 25, "label": "", "sentence_structure": "Subject鈥搗erb鈥揷omplement", "subjective_objective": {"answer": "Subjective", "rationale": "Because confusion and delusion are the patient鈥檚 subjective feelings and way of thinking, and putting work on hold is the patient鈥檚 reaction. These reflect the patient鈥檚 inner state and behavioral choices, and are therefore subjective in nature."}, "sentence_sentiment": {"answer": "Negative", "rationale": "The \"suspecting of the job and putting it on hold\" in the entry is negative."}, "language_style": {"answer": "Calm and direct", "rationale": "The language style of the entry is calm and straightforward. It expresses a theme in a concise and clear language, namely, that in a given context, the interviewee may be confused and put on hold. It is not too much rhetorical or emotional to describe, and it is more objective in tone."}, "time_dimension": {"answer": "Present", "rationale": "Now, the keywords \"often\" and \"inflicted and put the work on hold\" indicate that it is a narrative of the present, not of past or future behaviour."}, "sentence_type": "Affirmative declarative sentence", "pos": {"nouns": {"Heartland": 1, "Confused": 1, "I'm not sure": 1, "Work": 1, "Hold it": 1}, "verbs": {"Often": 1, "Into the": 1, "Because": 1, "Will": 1}, "adjectives": {"Some": 1, "Down": 1}, "numerals": {"99": 1}, "pronouns": {"I": 1}}}, {"content": "100. I rarely use embarrassing language to stabbing people ' s feelings:", "word_count": 17, "label": "", "sentence_structure": "Subject鈥損redicate", "subjective_objective": {"answer": "Subjective", "rationale": "Because \"very few words are used to stab people's feelings\" are subjective attitudes and behaviours of patients."}, "sentence_sentiment": {"answer": "Neutral", "rationale": "Because the entry does not express a clear emotional colour, it simply describes the behaviour of a person."}, "language_style": {"answer": "Tactful and warm", "rationale": "This is more polite and friendly and more easily to gain the trust and cooperation of the person under investigation."}, "time_dimension": {"answer": "Present", "rationale": "The entry describes a customary act of the testee, which is \"very rarely stabbing someone's feelings in embarrassing language.\" Using the verb \"less\" in the general current state of affairs, it indicates that the act is frequent and may still exist. So the time dimension of the entry is now."}, "sentence_type": "Affirmative declarative sentence", "pos": {"nouns": {"Questionnaire": 1, "Entry": 1, "Languages": 1, "Stab": 1, "others": 1, "Emotion": 1}, "verbs": {"Use": 1, "I'm embarrassed": 1, "Go on": 1, "Stab": 1}, "adjectives": {"Very few": 1, "I'm embarrassed": 1}, "adverbs": {"very": 1}, "numerals": {"100": 1}, "pronouns": {"I": 1, "others": 1}}}, {"content": "If I choose, I prefer to choose:", "word_count": 11, "label": "", "sentence_structure": "Subject鈥搗erb鈥搊bject", "subjective_objective": {"answer": "Subjective", "rationale": "Because it is the patient ' s subjective will and preference."}, "sentence_sentiment": {"answer": "Neutral", "rationale": "Since the entry does not express a clear positive or negative emotional tendency, it merely provides a question for the respondents to answer."}, "language_style": {"answer": "Calm and direct", "rationale": "The entry is based on direct questioning of the respondent ' s preferences, the absence of euphemism or emotional acrimony, and the more objective and cold tone of the speech."}, "time_dimension": {"answer": "Present", "rationale": "Now the entry uses the conditional phrase \"If I choose, I prefer to do\" to express a hypothetical situation, where the testee has a tendency or choice to face a situation that does not clearly point to the past or the future, and therefore the judgement is now."}, "sentence_type": "Imperative sentence", "pos": {"nouns": {"Questionnaire": 1, "Entry": 1}, "verbs": {"Jean": 1, "Choose": 2, "Do it": 1}, "adverbs": {"I'd rather not": 1}, "numerals": {"101": 1}, "classifiers": {"individual": 1}, "pronouns": {"I": 1}}}, {"content": "鈥淚ncomprehensible鈥?means:", "word_count": 8, "label": "", "sentence_structure": "Subject鈥搗erb鈥搊bject", "subjective_objective": {"answer": "Subjective", "rationale": "Because \"inexactly\" is a subjective view or opinion, and it is not an objective fact or a verifiable result."}, "sentence_sentiment": {"answer": "Neutral", "rationale": "The reference is based on the fact that the term \"inexactly\" is defined and does not express any emotional colour."}, "language_style": {"answer": "Calm and direct", "rationale": "The entry gives a direct meaning to the term, does not have too many rhetorical or emotional colours and is more direct and concise in language style."}, "time_dimension": {"answer": "Past", "rationale": "The question is to ask the subject about the understanding or memory of a term, without any reference to future behaviour or expectations. At the same time, the issue is based on traditional expressions of Chinese culture, not language expressions that are now widely used. Thus, in terms of time dimension, the entry can be judged as past."}, "sentence_type": "Declarative sentence", "pos": {"nouns": {"Meaning": 1, "Ry": 1, "Win": 1, "Word": 1}, "verbs": {"be": 1}, "adverbs": {"not": 1}, "numerals": {"102": 1}}}, {"content": "To take the example of the combination of 鈥渢ort鈥?and 鈥渄rilling鈥? I think the match between 鈥渃leaver鈥?and () knife:", "word_count": 18, "label": "", "sentence_structure": "Subject鈥搗erb鈥搊bject", "subjective_objective": {"answer": "Subjective", "rationale": "This is the subjective association and matching of particular terms of the respondents."}, "sentence_sentiment": {"answer": "Neutral", "rationale": "Because the entry is merely a question of matching, and it does not contain any emotional colour."}, "language_style": {"answer": "Calm and direct", "rationale": "Based on this logic, the language style of the questionnaire entries can be considered euphemism, because it does not directly tell respondents what to fill in, but rather leads them to think through one example and encourages them to use their creativity and imagination to fill out the blanks. Moreover, it is more interactive and participatory, because the respondents need to think and choose, rather than simply accept or reject a question. This increases the appeal and effectiveness of the questionnaire, and it also makes the respondents feel more respected and valued."}, "time_dimension": {"answer": "Present", "rationale": "The \"I think\" in the entry indicates that this is an expression of subjective opinion, and that the match between the knife and the other word is an act that has not yet happened and that requires imagination and reflection by the testee, so the temporal dimension is now judged."}, "sentence_type": "Interrogative sentence", "pos": {"nouns": {"Iron Curtain": 1, "Digging": 1, "Knife": 1, "Match": 2}, "numerals": {"103": 1}, "pronouns": {"I": 1}}}, {"content": "I've been on the street, avoiding people I don't want to say hello to:", "word_count": 19, "label": "", "sentence_structure": "Subject鈥損redicate", "subjective_objective": {"answer": "Subjective", "rationale": "Because \"the person I hate to say hello\" is the patient's subjective perception and attitude."}, "sentence_sentiment": {"answer": "Negative", "rationale": "Because the \"shield\" act in the entry is negative."}, "language_style": {"answer": "Calm and direct", "rationale": "The language style of the entry is more straightforward and has no excessive rhetoric or emotional colour, the main purpose being to obtain the behaviour of the person under investigation in a given situation."}, "time_dimension": {"answer": "Present", "rationale": "The keyword \"in the street\" and \"often avoid\" means that the act is frequently committed in the current time frame. So the time dimension of this questionnaire entry is now."}, "sentence_type": "Affirmative declarative sentence", "pos": {"nouns": {"On the street": 1, "I": 2, "person": 2}, "verbs": {"in": 1, "Avoid": 1, "No, I don't": 1, "Say hello": 1}, "adjectives": {"Often": 1, "I don't want to": 1}, "numerals": {"104": 1, "1": 3}, "pronouns": {"I don't want to": 1}}}, {"content": "When I listen to music, I think that if someone is around to talk,", "word_count": 22, "label": "", "sentence_structure": "Subject鈥搗erb鈥揷omplement", "subjective_objective": {"answer": "Subjective", "rationale": "This is the subjective experience and feeling of patients in emotional reactions and diversions in specific situations."}, "sentence_sentiment": {"answer": "Negative", "rationale": "Because the \"someone talks loudly\" in the entry is a negative emotional effect by interrupting the listener's attention to music."}, "language_style": {"answer": "Calm and direct", "rationale": "The basis for the determination: There is no rhetorical or emotional overture in the entry, but rather a situation is presented directly and questions are posed to the interviewee. This language style is more objective, is not reflected in too much emotional or subjective attitudes, is more factual and logical, and is consistent with the linguistic characteristics of the questionnaire."}, "time_dimension": {"answer": "Present", "rationale": "The entry describes a scene where people suddenly talk about music when the subjects are listening to music with their attention. This is what is happening, so time horizons are now judged."}, "sentence_type": "Interrogative sentence", "pos": {"nouns": {"Music": 1, "person": 1}, "verbs": {"Get some sleep": 1, "Listen": 1, "If you're not gonna do it, you're gonna do it": 1, "It's right next to me": 1, "I'm talking about it": 1}, "adverbs": {"When": 1, "Time": 1}, "numerals": {"105": 1}, "pronouns": {"I": 1}}}, {"content": "106. In the classroom, if my opinion is different from that of the teacher, I often:", "word_count": 18, "label": "", "sentence_structure": "Subject鈥搗erb鈥搊bject", "subjective_objective": {"answer": "Subjective", "rationale": "This is a subjective experience of the patient, since it describes the patient ' s personal attitude or reaction in a given situation."}, "sentence_sentiment": {"answer": "Negative", "rationale": "Because the premise that \"if my opinion is different from that of a teacher\" implies a potential contradiction and conflict, which may lead to negative emotional reactions. And \"often\" emphasizes the frequency of this situation, further enhancing its potential for negative effects."}, "language_style": {"answer": "Calm and direct", "rationale": "The entry is in a clear language, asking the interviewee about his/her behaviour in the classroom, and does not use euphemisms such as euphemism or hints."}, "time_dimension": {"answer": "Past", "rationale": "\"In class\" indicates that this is a description of the past, \"if my opinion is different from that of the teacher\" indicates that it is a possible event, and \"often\" indicates that this behaviour is frequent. So the entry to the questionnaire asks about the frequency of the testee's past behaviour, that is, \"in class, if my opinion is different from that of the teacher\"."}, "sentence_type": "Interrogative sentence", "pos": {"nouns": {"Classroom": 1, "Observations": 1, "Teacher": 1}, "verbs": {"If": 1, "Different": 1, "Often": 1}, "adverbs": {"Often": 1}, "numerals": {"106": 1}}}, {"content": "107. When I speak to the opposite sex alone, it is not natural:", "word_count": 15, "label": "", "sentence_structure": "Subject鈥搗erb鈥搊bject", "subjective_objective": {"answer": "Subjective", "rationale": "Because \"unnatural\" is a feeling or experience, a subjective view of the patient himself."}, "sentence_sentiment": {"answer": "Negative", "rationale": "Because it expresses the feeling of discomfort and negative emotion when talking to opposite sex."}, "language_style": {"answer": "Calm and direct", "rationale": "Calm down straight."}, "time_dimension": {"answer": "Present", "rationale": "The keyword \"always\" denotes a regular act, while the sentence is generally in the current state, so the entry describes the current state of conduct of the testee and should be judged as the present."}, "sentence_type": "Interrogative sentence", "pos": {"nouns": {"Heterosexual": 1, "Talk": 1}, "verbs": {"I": 2, "It seems": 1, "It's not natural": 1}, "adjectives": {"Alone": 1}, "numerals": {"107": 1}}}, {"content": "108. I have certainly not been very successful in the area of handling:", "word_count": 14, "label": "", "sentence_structure": "Subject鈥搗erb鈥搊bject", "subjective_objective": {"answer": "Subjective", "rationale": "Because \"less successful\" is a subjective evaluation that reflects a patient's self-assessment of his/her ability to be treated."}, "sentence_sentiment": {"answer": "Negative", "rationale": "Because the expression \"not very successful\" is negative."}, "language_style": {"answer": "Tactful and warm", "rationale": "The entry is expressed in a query, without directly accusing or evaluating the individual ' s performance, and is more euphemistic and moderate. At the same time, the language is more neutral, avoiding the use of too harsh a term, which makes people comfortable and comfortable."}, "time_dimension": {"answer": "Present", "rationale": "So, the time dimension is now. So the output is: Now."}, "sentence_type": "Interrogative sentence", "pos": {"nouns": {"In terms of": 1, "- I'm in the middle of something": 1, "I": 2}, "verbs": {"in": 1, "- Wait": 1, "Take it": 1, "Success": 1}, "adjectives": {"Not really": 1}, "numerals": {"108": 1}}}, {"content": "When I work as a difficult person, I always do:", "word_count": 13, "label": "", "sentence_structure": "Subject鈥搗erb鈥搊bject", "subjective_objective": {"answer": "Subjective", "rationale": "This sentence is an expression of patients ' subjective attitudes and behavioural choices when they face difficult work."}, "sentence_sentiment": {"answer": "Negative", "rationale": "Because \"hard work\" is usually a stressful and challenging expression, it tends to be negative."}, "language_style": {"answer": "Calm and direct", "rationale": "The entry directly asks the respondents about their behaviour in the face of difficult work, without excessive rhetorical or emotional rendering, and is more objective in tone."}, "time_dimension": {"answer": "Present", "rationale": "The entry uses the word \"always\" to indicate the testee's customary behaviour in the face of difficult work, and thus to judge it as now."}, "sentence_type": "Imperative sentence", "pos": {"nouns": {"Annex": 1, "Work": 1}, "verbs": {"Do it": 1, "Always": 1}, "adverbs": {"EMPTY": 1}, "numerals": {"109": 1}}}, {"content": "I've made friends, men and women are half:", "word_count": 13, "label": "", "sentence_structure": "Subject鈥搗erb鈥揷omplement", "subjective_objective": {"answer": "Objective", "rationale": "This is a description of the sex ratio, not the individual feelings or attitudes of the patient."}, "sentence_sentiment": {"answer": "Neutral", "rationale": "Because the entry does not have any emotional color."}, "language_style": {"answer": "Tactful and warm", "rationale": "The entry is more euphemistic, polite and gentle in its language. It does not indicate directly the sex ratio of the person surveyed, but it is based on the assumption that the person surveyed has more respect for and inclusion of the privacy and personal choices of the person surveyed by asking about the sex ratio of the person himself in the relationship. \""}, "time_dimension": {"answer": "Present", "rationale": "The keywords \"in my friendship\" and \"in half of each sex\" indicate that the testee is describing his current situation, so the time dimension of the entry is now."}, "sentence_type": "Affirmative declarative sentence", "pos": {"nouns": {"110": 1, "I": 1, "friends": 1, "Men and women": 2}, "verbs": {"Deal": 1, "Jim": 1}, "adjectives": {"Half": 1}, "numerals": {"Each": 1, "One": 2}}}, {"content": "111. In terms of making friends:", "word_count": 8, "label": "", "sentence_structure": "Subject鈥搗erb鈥揷omplement", "subjective_objective": {"answer": "Subjective", "rationale": "Because making friends is a subjective attitude and behaviour of one."}, "sentence_sentiment": {"answer": "Neutral", "rationale": "Because the entry merely asks about your situation in terms of making friends, it does not have some assessment or emotional orientation."}, "language_style": {"answer": "Calm and direct", "rationale": "This entry uses a direct, specific approach, explicitly asking the respondents about their specific circumstances and experiences in making friends, and does not have too many rhetorical or emotional expressions."}, "time_dimension": {"answer": "Present", "rationale": "The entry asks the person who was tested for his current social status or ability, and the keyword \"I am making friends\" points to the current situation."}, "sentence_type": "Interrogative sentence", "pos": {"nouns": {"friends": 1}, "verbs": {"Deal": 1}, "numerals": {"111": 1, "I": 1}}}, {"content": "112. I am willing to be a social scientist rather than a mechanical engineer:", "word_count": 22, "label": "", "sentence_structure": "Subject鈥搗erb鈥搊bject", "subjective_objective": {"answer": "Subjective", "rationale": "Because \"I'm willing to be a social scientist, not a mechanical engineer\" is the patient's personal opinion and choice."}, "sentence_sentiment": {"answer": "Neutral", "rationale": "As this entry merely describes the individual ' s professional orientation, it does not show positive or negative emotional connotations."}, "language_style": {"answer": "Calm and direct", "rationale": "The entry directly enquired about the respondent ' s orientation towards career choices, which was not accompanied by excessive rhetorical or emotional rendering, and was more objective in tone."}, "time_dimension": {"answer": "Present", "rationale": "The entry is now judged on the time dimension by asking the testee ' s current will and attitude."}, "sentence_type": "Affirmative declarative sentence", "pos": {"nouns": {"Social scientists": 1, "Mechanical engineers": 1}, "verbs": {"Yes": 1, "I don't want to": 1}, "adverbs": {"One": 2}, "numerals": {"112": 1}, "pronouns": {"I": 1}}}, {"content": "113. If I find the shortcomings of others, I often make accusations in a desperate manner:", "word_count": 22, "label": "", "sentence_structure": "Subject鈥搗erb鈥搊bject", "subjective_objective": {"answer": "Subjective", "rationale": "Because \"always, you can't blame\" is the patient's subjective attitude and behaviour."}, "sentence_sentiment": {"answer": "Negative", "rationale": "Because the \"neglectful accusations\" in the entry are negative."}, "language_style": {"answer": "Calm and direct", "rationale": "The entry uses direct, concise language to express the subject, does not have too many rhetorical or emotional renderings, and is more objective and cold.\""}, "time_dimension": {"answer": "Past", "rationale": "The entry \"often\" indicates a pattern of behaviour that is constant, and that both \"discovering other people's shortcomings\" and \"discretionary accusations\" have occurred in the past, so it can be judged that this is an assessment of the testee's past behaviour."}, "sentence_type": "Interrogative sentence", "pos": {"nouns": {"Disadvantages": 1, "Note": 1, "Blame": 1}, "verbs": {"Found": 1, "I'll give up": 1, "Present": 1}, "adverbs": {"Often": 1, "Everything": 1}, "numerals": {"113": 1}, "pronouns": {"I": 1, "others": 1}}}, {"content": "114. I like to try to influence the announcement of working with me, which is the purpose of my plan:", "word_count": 29, "label": "", "sentence_structure": "Subject鈥搗erb鈥搊bject", "subjective_objective": {"answer": "Subjective", "rationale": "Because it's the patient's subjective attitude."}, "sentence_sentiment": {"answer": "Positive", "rationale": "Because \"I like to try to influence the notice of working with me, it's what they can help me with.\" The purpose of this act is positive."}, "language_style": {"answer": "Calm and direct", "rationale": "The entry is based on a simple and clear description of the respondent鈥檚 attitude towards those who work with him, i.e., preferring to try to influence them to help achieve their goals. This expression is direct and concise, without excessive rhetoric or emotional rendering, and is consistent with the straight style of calm."}, "time_dimension": {"answer": "Present", "rationale": "This questionnaire entry is about the current behaviour or preferences of the person being tested, and \"likes to try to influence the notice to work with me\" and \"helping me to plan the purpose\" are all expressions of the current state of affairs."}, "sentence_type": "Interrogative sentence", "pos": {"nouns": {"114": 1, "I": 2, "Work": 1, "Announcements": 1, "Assistance": 1, "Purpose": 1}, "verbs": {"Yeah": 1, "Try": 1, "Impact": 1, "Together": 1, "Work": 1, "Yes": 1, "Assistance": 1, "Planned": 1}, "adverbs": {"and": 1, "Sou-san": 1}, "numerals": {"114": 1, "I": 2}, "pronouns": {"they": 1}}}, {"content": "I like to do music, dance, or press interviews, etc.:", "word_count": 17, "label": "", "sentence_structure": "Subject鈥搗erb鈥搊bject", "subjective_objective": {"answer": "Subjective", "rationale": "Because \"like\" is an individual's subjective feeling and attitude."}, "sentence_sentiment": {"answer": "Neutral", "rationale": "Because the entry is only a description of a person ' s preferences and interests, it does not involve any emotional colour, nor does it involve positive or negative behaviour, attitudes or objectives. The entry is therefore neutral."}, "language_style": {"answer": "Tactful and warm", "rationale": "The entry is more personal and interactive in a gentle and inviting tone of conversation, rather than in direct statements or orders. At the same time, it offers a wide range of possible options that allow respondents to choose their own answers that are appropriate to their circumstances, reflecting the respect and understanding of the questionnaire designers."}, "time_dimension": {"answer": "Present", "rationale": "So the time dimension of this entry is judged now."}, "sentence_type": "Affirmative declarative sentence", "pos": {"nouns": {"Work": 3, "Music": 1, "Dance": 1, "Press interviews": 1}, "verbs": {"Yeah": 1, "Do it": 2, "or": 2, "Wait": 1}, "adverbs": {"Very": 1}, "numerals": {"115": 1}}}, {"content": "When people praise me, I am ashamed:", "word_count": 17, "label": "", "sentence_structure": "Subject鈥搗erb鈥揷omplement", "subjective_objective": {"answer": "Subjective", "rationale": "Because shame is a subjective feeling and attitude."}, "sentence_sentiment": {"answer": "Negative", "rationale": "Because the emotional attitude of \"always ashamed\" in the entry is negative."}, "language_style": {"answer": "Tactful and warm", "rationale": ""}, "time_dimension": {"answer": "Present", "rationale": ""}, "sentence_type": "Negative declarative sentence", "pos": {"nouns": {"Time": 1, "People": 1, "Praise": 1}, "verbs": {"I think": 1, "Shame and shame": 2}, "adverbs": {"When": 1}, "numerals": {"116": 1}, "pronouns": {"I": 1}}}, {"content": "117. I believe that the issues most needed to be addressed by a country are:", "word_count": 16, "label": "", "sentence_structure": "Subject鈥搗erb鈥搊bject", "subjective_objective": {"answer": "Subjective", "rationale": "Because the answer to the question depends on the patient ' s thoughts and views, it is examined from a subjective point of view."}, "sentence_sentiment": {"answer": "Neutral", "rationale": "Because the entry is personal, it does not express a positive or negative attitude."}, "language_style": {"answer": "Calm and direct", "rationale": ""}, "time_dimension": {"answer": "Present", "rationale": ""}, "sentence_type": "Declarative sentence", "pos": {"nouns": {"Problem": 2, "Country": 1}, "verbs": {"I think": 1, "It needs to be addressed": 1}, "numerals": {"117": 1, "Most": 1}, "pronouns": {"I": 1}}}, {"content": "118. Sometimes I have the fear of a great catastrophe without cause:", "word_count": 18, "label": "", "sentence_structure": "Subject鈥搗erb鈥揷omplement", "subjective_objective": {"answer": "Subjective", "rationale": "Because \"fear generation\" is a subjective feeling and experience."}, "sentence_sentiment": {"answer": "Negative", "rationale": "Because the \"fear of great evil\" in the entry is a negative emotional attitude."}, "language_style": {"answer": "Calm and direct", "rationale": "The entry directly expresses the subject of questioning the other party for undue fear of great harm, without excessive rhetoric or emotional rendering and with more objective tone."}, "time_dimension": {"answer": "Past", "rationale": "The entry is for the purpose of asking whether the person tested has ever had or is not present \"sometimes, without cause, a fear of great harm\", the temporal dimension of which is judged by past or present."}, "sentence_type": "Affirmative declarative sentence", "pos": {"nouns": {"Questionnaire": 1, "Entry": 1, "Time": 1, "I": 1, "Fear": 1}, "verbs": {"Yes": 1, "Production": 1, "Fan": 1, "Faced": 1}, "adjectives": {"It's a disaster": 1, "For no reason": 1}, "adverbs": {"Sometimes": 1}}}, {"content": "119. In my childhood, I was afraid of the darkness:", "word_count": 12, "label": "", "sentence_structure": "Subject鈥搗erb鈥揷omplement", "subjective_objective": {"answer": "Objective", "rationale": "Because \"the fear of darkness\" is a quantifiable fact, it does not take into account the subjective attitude or feelings of the patient."}, "sentence_sentiment": {"answer": "Neutral", "rationale": "Because the entry merely asked the extent of the person ' s fear of darkness in childhood, it did not contain any emotional colour."}, "language_style": {"answer": "Calm and direct", "rationale": "The entry was asked by direct questioners in a concise and straightforward manner about their fear of darkness during childhood, and was more calm and direct in language style."}, "time_dimension": {"answer": "Past", "rationale": ""}, "sentence_type": "Declarative sentence", "pos": {"nouns": {"Childhood": 1, "Darkness": 1, "Number": 1}, "verbs": {"Scared": 1}, "numerals": {"119": 1}}}, {"content": "120. In leisure, I like:", "word_count": 9, "label": "", "sentence_structure": "Subject鈥搗erb鈥搊bject", "subjective_objective": {"answer": "Subjective", "rationale": "Likeness is a subjective attitude, and the subject is therefore viewed from the patient ' s subjective perspective."}, "sentence_sentiment": {"answer": "Neutral", "rationale": "According to the sentence, the entry is simply a question of the preferences of an individual in his or her leisure time and does not clearly express positive or negative feelings, attitudes, behaviour, purpose or quality. It can therefore be judged neutral."}, "language_style": {"answer": "Tactful and warm", "rationale": "The language style of the entry is more euphemistic and gentle, giving people a sense of affection. It uses an invitation-style question-and-answer approach rather than direct orders or statements, which makes the respondents more willing to share their thoughts and feelings and more easily participate in the questionnaire-filling process. At the same time, this expression can ease the tension and anxiety of the respondents, making them more comfortable and comfortable."}, "time_dimension": {"answer": "Present", "rationale": "The entry asks about the present preferences and habits of the testee, and the time dimension is now judged. The keyword \"in leisure\" indicates that this is the routine behaviour of the testee, while \"like\" is a description or evaluation of the behaviour. Thus, the time frame of the entry is mainly now."}, "sentence_type": "Declarative sentence", "pos": {"nouns": {"Time": 1, "Yeah": 1}, "verbs": {"in": 1, "- Leisure": 1}, "numerals": {"120": 1}}}, {"content": "121. When people criticize me for being weird, I:", "word_count": 13, "label": "", "sentence_structure": "Subject鈥搗erb鈥搊bject", "subjective_objective": {"answer": "Subjective", "rationale": "Because this is a subjective approach to the patient ' s own feelings and reactions."}, "sentence_sentiment": {"answer": "Negative", "rationale": "Because the \"others criticize me for being abnormal\" act is negative."}, "language_style": {"answer": "Tactful and warm", "rationale": "The entry is more proficient and interactive when it is spoken in a gentle and inviting tone, rather than in direct statements or orders. At the same time, the issue is couched in more neutral terms, with no overly emotional or subjective judgement, and in a soft and warm language style."}, "time_dimension": {"answer": "Present", "rationale": "The keyword \"when\" means a point or a particular event, and \"I\" means the main word, that is, the testee is doing it himself. The entire sentence describes the reaction of the testee when faced with criticism from others, and therefore the judgment is now."}, "sentence_type": "Interrogative sentence", "pos": {"nouns": {"People": 1, "Criticism": 1, "I": 2, "Weird": 1, "It's not normal": 1}, "verbs": {"When": 1, "be": 1}, "adjectives": {"Weird": 1, "It's not normal": 1}, "numerals": {"121": 1}, "pronouns": {"I": 2}}}, {"content": "When I come to a new city to find an address, I often:", "word_count": 16, "label": "", "sentence_structure": "Subject鈥損redicate", "subjective_objective": {"answer": "Subjective", "rationale": "Customary practice was a subjective attitude, and it was the free will of the individual to choose how to act."}, "sentence_sentiment": {"answer": "Neutral", "rationale": "Because the entry does not have any emotional color."}, "language_style": {"answer": "Tactful and warm", "rationale": ""}, "time_dimension": {"answer": "Past", "rationale": "And \"when you come to a new city to find an address\" describes the circumstances and conditions under which the act occurs, which is the expression of the current state of affairs, emphasizing the current impact of past behaviour. The time dimension of this questionnaire entry should therefore be judged as now."}, "sentence_type": "Interrogative sentence", "pos": {"nouns": {"Questionnaire": 1, "Entry": 1, "New": 1, "Urban": 1, "Address": 1}, "verbs": {"Come on": 1, "Find": 1, "Time": 1, "Often": 1}, "adverbs": {"Often": 1}, "numerals": {"122": 1}}}, {"content": "123. When friends say she wants to rest at home, I always try to encourage her to go out with me:", "word_count": 29, "label": "", "sentence_structure": "Subject鈥搗erb鈥搊bject", "subjective_objective": {"answer": "Subjective", "rationale": "Because the entry is an inquiry into patients鈥?subjective behaviour and thoughts about whether they will try to convince friends to change their mind when they do not want to go out. This is seen from the patient鈥檚 perspective."}, "sentence_sentiment": {"answer": "Positive", "rationale": "Because the behavior in the entry \"tried to encourage friends to go out\" is positive."}, "language_style": {"answer": "Tactful and warm", "rationale": "The entry is more euphemistic and respectful of the other. Although it asks whether the interviewee will try to persuade his friend to leave, it does not use command or coercive language. Rather, it raises questions in a mild manner and gives the interviewee sufficient space to express his thoughts and feelings."}, "time_dimension": {"answer": "Present", "rationale": ""}, "sentence_type": "Interrogative sentence", "pos": {"nouns": {"friends": 1, "she": 1, "Home": 1, "Rest": 1, "Time": 1, "Try": 1, "Incentives": 1, "Same": 1, "I": 2, "Together": 1, "Outside": 1, "Play": 1}, "verbs": {"Statement": 1, "Yes": 1, "in": 1, "Rest": 1, "Always": 1, "Try": 1, "Incentives": 1, "Same": 1, "I": 2, "Go on": 1, "Play": 1}, "adverbs": {"When": 1, "Always": 1}, "numerals": {"123": 1, "1": 2, "2": 1, "3": 1}, "classifiers": {"individual": 1}, "pronouns": {"I": 2}}}, {"content": "124. In bedtime, I often:", "word_count": 7, "label": "", "sentence_structure": "Subject鈥搗erb鈥搊bject", "subjective_objective": {"answer": "Subjective", "rationale": "Because the word \"often\" shows that it's a subjective attitude, a description of the patient's experience."}, "sentence_sentiment": {"answer": "Neutral", "rationale": "Because the entry does not have any emotional color."}, "language_style": {"answer": "Calm and direct", "rationale": "The language style of the entry is a straightforward type of calm, as it is a simple question, and it is a question of asking the person who is being questioned what happens when he or she sleeps."}, "time_dimension": {"answer": "Present", "rationale": "The entry is a question of the current habits or circumstances of the person being tested, \"in bed\" indicating that it is a specific point or period of time, and \"often\" indicates that it is a regular or customary act. Thus, the temporal dimension of the entry is now judged."}, "sentence_type": "Interrogative sentence", "pos": {"nouns": {"In bed": 1, "I": 1}, "verbs": {"Often": 1}, "numerals": {"124": 1}}}, {"content": "125. When I was disturbed, I:", "word_count": 7, "label": "", "sentence_structure": "Subject鈥搗erb鈥揷omplement", "subjective_objective": {"answer": "Subjective", "rationale": "Because when someone bothers me, the situation is judged by the patient's subjective feelings."}, "sentence_sentiment": {"answer": "Negative", "rationale": "Since the term \"disturbing\" in the entry is usually understood to have a negative connotation, the option may express a negative attitude."}, "language_style": {"answer": "Tactful and warm", "rationale": ""}, "time_dimension": {"answer": "Present", "rationale": "The entry is for the purpose of asking the testee about the behaviour or attitude of the person in the event of trouble, and the temporal dimension is now judged."}, "sentence_type": "Imperative sentence", "pos": {"verbs": {"Disturbing": 1, "have": 1}, "numerals": {"125": 1}}}, {"content": "126. If the treatment is the same, I would like to make one:", "word_count": 11, "label": "", "sentence_structure": "Subject鈥搗erb鈥搊bject", "subjective_objective": {"answer": "Subjective", "rationale": "Because \"will\" is a subjective will and attitude of a person."}, "sentence_sentiment": {"answer": "Neutral", "rationale": "Because the entry merely asks about the will of the individual, it does not involve any emotional or behavioural tendencies."}, "language_style": {"answer": "Calm and direct", "rationale": "It is not expressed in too many terms or emotional colours, but rather it is asked directly what the respondents would choose to do in the same conditions. So my judgment is \"cool straight.\""}, "time_dimension": {"answer": "Present", "rationale": "The entry asks the testees, in the same case, what they prefer to do, and therefore its time dimension is now."}, "sentence_type": "Interrogative sentence", "pos": {"nouns": {"Treatment": 1, "person": 1}, "verbs": {"Do it": 2}, "numerals": {"126": 1, "Same": 1}, "pronouns": {"I": 1}}}, {"content": "鈥淭ime becomes eternal鈥?is a metaphor:", "word_count": 11, "label": "", "sentence_structure": "Subject鈥搗erb鈥搊bject", "subjective_objective": {"answer": "Subjective", "rationale": "Because it is a metaphor, it is necessary to judge the meaning of the patient by understanding his or her thoughts and feelings, rather than observing the actual events or facts."}, "sentence_sentiment": {"answer": "Neutral", "rationale": "Because the entry does not have any emotional color."}, "language_style": {"answer": "Tactful and warm", "rationale": "The entry is more proficient and interactive in terms of a gentle and inviting tone of speech, rather than a direct statement or order. At the same time, this metaphor is used to guide respondents to thinking, making language style more attractive and inspiring."}, "time_dimension": {"answer": "Present", "rationale": "The entry is an inquiry into the victim's understanding and perception of the metaphor \"time becomes eternal\" and does not address past or future behaviour or mentality. The entry therefore describes the current state or view."}, "sentence_type": "Interrogative sentence", "pos": {"nouns": {"Time": 1, "Forever": 1, "- I'm sorry": 1}, "verbs": {"鈾?Turn into 鈾?: 1}, "numerals": {"127": 1}}}, {"content": "Which one of the items should be taken up after the phrase 鈥渋n the year 2000鈥?, "word_count": 13, "label": "", "sentence_structure": "Subject鈥搗erb鈥搊bject", "subjective_objective": {"answer": "Subjective", "rationale": "This is because the patient ' s choice of options is subjective and subjective."}, "sentence_sentiment": {"answer": "Neutral", "rationale": "Because the entry is an objective issue and does not express any emotional colour."}, "language_style": {"answer": "Calm and direct", "rationale": "The language style of the entry is more straightforward, and there is no excessive rhetorical or emotional rendering, but rather a clear and concise language that allows readers to quickly understand its meaning and requirements. At the same time, the tone is more objective and neutral, and there is no excessive emotional colour. Thus, it is judged that the language style of the entry is straightforward and calm."}, "time_dimension": {"answer": "Future", "rationale": "The content of the questionnaire entry is about the steps of an operation or activity, which relates to the expression \"which should be followed by this topic\", implying that this step needs to be implemented at some point in the future. The temporal dimension is therefore judged in terms of the future."}, "sentence_type": "Interrogative sentence", "pos": {"nouns": {"Problem": 1, "Item": 1, "Back": 1}, "verbs": {"Analysis": 1, "Answer": 1}, "numerals": {"128": 1}, "classifiers": {"Which one": 1}, "pronouns": {"This is the subject": 1}}}, {"content": "129. Wherever I go, I can clearly identify the direction:", "word_count": 17, "label": "", "sentence_structure": "Subject鈥搗erb鈥揷omplement", "subjective_objective": {"answer": "Objective", "rationale": "Because wherever it is, it is clear that the objective facts that are experienced by the patient in the direction of the patient can be identified."}, "sentence_sentiment": {"answer": "Positive", "rationale": "Because the entry expresses personal abilities, there is no negative or neutral emotional colour."}, "language_style": {"answer": "Calm and direct", "rationale": "The entry is more objective, direct, and does not have too much emotional or rhetorical style. It simply raises a question and asks the person ' s attitude or opinion in a direct way to enable him to understand the question and answer it more clearly."}, "time_dimension": {"answer": "Present", "rationale": "The entry describes a capability or habit that, regardless of location, clearly identifies direction, which occurs now."}, "sentence_type": "Affirmative declarative sentence", "pos": {"nouns": {"Local": 1, "Direction": 1}, "verbs": {"Present": 1, "Yes": 1, "Identification": 1}, "adverbs": {"Whatever": 1, "Both": 1, "Clear": 1}, "numerals": {"129": 1}, "pronouns": {"I": 1}}}, {"content": "130. I love the professions and the jobs I have learned:", "word_count": 16, "label": "", "sentence_structure": "Subject鈥搗erb鈥搊bject", "subjective_objective": {"answer": "Subjective", "rationale": "Because love is the subjective feeling and attitude of a person, it reflects the preferences, interest and commitment to his or her profession and work."}, "sentence_sentiment": {"answer": "Positive", "rationale": "Because the word \"love\" conveys positive, positive emotions and expresses love and support for the profession and work that is studied."}, "language_style": {"answer": "Tactful and warm", "rationale": "The language style of the entry is more euphemistic, encouraging and supportive, expressing a positive attitude and conviction, and trying to inspire the interviewees to feel enthusiastic and proud of their profession and work. This expression makes them more comfortable and willing to share their real thoughts and feelings."}, "time_dimension": {"answer": "Present", "rationale": ""}, "sentence_type": "Interrogative sentence", "pos": {"nouns": {"I": 2, "Professional": 1, "Work": 1}, "verbs": {"Love": 1}, "adjectives": {"Learn": 1, "Committed": 1}, "numerals": {"130": 1}}}, {"content": "131. I think it is okay if I am anxious to borrow from a friend and when a friend is not at home:", "word_count": 32, "label": "", "sentence_structure": "Subject鈥損redicate", "subjective_objective": {"answer": "Subjective", "rationale": "Because it is perceived as subjective."}, "sentence_sentiment": {"answer": "Negative", "rationale": "Because the attitude in the entry \"it's okay not to say anything\" is negative."}, "language_style": {"answer": "Calm and direct", "rationale": "The language style of the entry is calm and straightforward, because it is clear, and it is not too much emotionally refined or rhetorical. It asks the respondents in a direct way about the \"no-said\" behaviour and makes it clear that the situation is in the absence of friends. This type of questionnaire entry is usually used to collect objective and neutral data for further research and analysis."}, "time_dimension": {"answer": "Present", "rationale": "The entry uses the \"already\" and the \"generally\" \"concept\" of the current state of affairs, which indicates that it is a constant thought or attitude, which is the present behavior or mentality."}, "sentence_type": "Affirmative declarative sentence", "pos": {"nouns": {"friends": 2, "Something": 1}, "verbs": {"Borrow": 1, "in": 1, "I think": 1, "Call": 1, "Remove": 1, "have": 1}, "adjectives": {"Urgent": 1}, "adverbs": {"not": 2}, "numerals": {"131": 1, "One": 1}, "pronouns": {"I": 1}}}, {"content": "132. I like to tell my friends some interesting personal stories:", "word_count": 18, "label": "", "sentence_structure": "Subject鈥搗erb鈥搊bject", "subjective_objective": {"answer": "Subjective", "rationale": "Because \"like\" and \"explaining personal interesting experiences\" are subjective attitudes and behaviours of patients."}, "sentence_sentiment": {"answer": "Positive", "rationale": "Because the behavior in the entry \"likes to tell friends some interesting personal experiences\" expresses a positive emotional attitude and quality."}, "language_style": {"answer": "Tactful and warm", "rationale": "The language style of the entry tends to be euphemistic, because it allows respondents to share their stories by means of questioning, rather than by direct orders or requests. This way more respects and encourages respondents to participate in questionnaires."}, "time_dimension": {"answer": "Present", "rationale": "The words \"like\" and \"team\" in the entry describe the subjective feelings and behaviour of the day, so it's now."}, "sentence_type": "Affirmative declarative sentence", "pos": {"nouns": {"Questionnaire Entry": 1, "friends": 1, "Experience": 1}, "verbs": {"Yeah": 1, "Tell me": 1, "Here": 1}, "adverbs": {"Some": 1, "Personal": 1, "Interesting": 1}, "numerals": {"132": 1}, "pronouns": {"I": 1}}}, {"content": "I'd rather do one:", "word_count": 6, "label": "", "sentence_structure": "Subject鈥搗erb鈥搊bject", "subjective_objective": {"answer": "Subjective", "rationale": "Since this is a matter of choice of individual preferences and preferences, it is the subjective attitudes and values of the patient that are examined."}, "sentence_sentiment": {"answer": "Positive", "rationale": "Because \"better\" is a motivation for subjective choice."}, "language_style": {"answer": "Tactful and warm", "rationale": "The entry is more personal and interactive when it is spoken in a gentle and inviting manner, rather than in direct statements or orders. Moreover, the language style of the article is relatively simple and not too much rhetorical to be understood and answered by the interviewees."}, "time_dimension": {"answer": "Present", "rationale": "The entry for the questionnaire is an inquiry into the personal preferences or values of the person being tested, and the temporal dimension is now judged."}, "sentence_type": "Interrogative sentence", "pos": {"verbs": {"Do it": 1, "I'd rather not": 1}, "adjectives": {"individual": 1}, "numerals": {"133": 1}, "pronouns": {"I": 1}}}, {"content": "134. I always arrange for leisure time to be spent:", "word_count": 17, "label": "", "sentence_structure": "Subject鈥搗erb鈥搊bject", "subjective_objective": {"answer": "Subjective", "rationale": "This is a subjective perspective, as it is a patient ' s self-evaluation and attitude."}, "sentence_sentiment": {"answer": "Positive", "rationale": "Because the behaviour in the entry \"to arrange for time not to be wasted\" is positive, indicating that the respondents are serious and positive about time."}, "language_style": {"answer": "Calm and direct", "rationale": "According to this purpose, it asked the respondents about the management of their time off, which is more straightforward, and does not use excessive rhetorical or emotional rendering. At the same time, the entry does not use euphemism or a doubt sentence, but is presented in a statement with a more objective tone. It therefore makes it possible to judge the language style of the purpose as \"cooling straight.\""}, "time_dimension": {"answer": "Present", "rationale": ""}, "sentence_type": "Affirmative declarative sentence", "pos": {"nouns": {"Amateur Time": 1, "Arrangements": 1, "Time": 2, "Wasted": 1}, "verbs": {"Do it": 1, "鈾?And I'm gonna be 鈾?: 1, "Okay": 1}, "adverbs": {"Always": 1, "not": 1}, "numerals": {"134": 1}, "classifiers": {"One": 2}}}, {"content": "135. In my dealings with others, I often have a sense of inferiority, without cause:", "word_count": 23, "label": "", "sentence_structure": "Subject鈥搗erb鈥搊bject", "subjective_objective": {"answer": "Subjective", "rationale": "Because it's the patient's subjective emotional experience."}, "sentence_sentiment": {"answer": "Negative", "rationale": "Because the emotional color of the description \"often produces a sense of inferiority without reason\" in the entry is negative."}, "language_style": {"answer": "Tactful and warm", "rationale": ""}, "time_dimension": {"answer": "Present", "rationale": "\"Always\" indicates that it is a customary act, \"a sense of inferiority\" also implies a continuing state of emotion, and therefore is judged to be the present behaviour or mentality."}, "sentence_type": "Interrogative sentence", "pos": {"nouns": {"- To be with someone": 1, "person": 1, "I'm so low": 1}, "verbs": {"Generate": 1, "Yes": 1, "Sensor": 1}, "adjectives": {"For no reason": 1}, "numerals": {"135": 1}, "pronouns": {"I": 1}}}, {"content": "136. Talk to someone who is not familiar with me:", "word_count": 12, "label": "", "sentence_structure": "Subject鈥搗erb鈥揷omplement", "subjective_objective": {"answer": "Subjective", "rationale": "Because \"for me\" means that it's a patient's own feeling or perception, subjective."}, "sentence_sentiment": {"answer": "Negative", "rationale": "Because \"unknown people talk\" can make people uncomfortable or nervous, and it's a negative emotion."}, "language_style": {"answer": "Tactful and warm", "rationale": "This expression is more friendly and helps to improve the comfort and participation of the interviewee."}, "time_dimension": {"answer": "Present", "rationale": "The entry describes the experience or perception of the testee of the current state, i.e., the situation in which he or she is talking to someone he or she is not familiar, so the temporal dimension is now judged."}, "sentence_type": "Declarative sentence", "pos": {"nouns": {"person": 1, "Talk": 1}, "verbs": {"Tell me": 1, "be": 1}, "adjectives": {"I don't know him": 1}, "adverbs": {"and": 1, "Both": 2}, "numerals": {"136": 1, "Here": 1, "individual": 1}, "pronouns": {"I": 1}}}, {"content": "137. My favorite music is:", "word_count": 8, "label": "", "sentence_structure": "Subject鈥揷opula鈥損redicative", "subjective_objective": {"answer": "Subjective", "rationale": "Because \"the music that you like\" is a personal preference, a patient's subjective attitude."}, "sentence_sentiment": {"answer": "Neutral", "rationale": "Because the entry does not have any emotional color."}, "language_style": {"answer": "Calm and direct", "rationale": "The entry uses a simple and clear language, and asks the other person directly what his favorite type of music is, without too many emotional renderings or rhetorical techniques, and the language style is straightforward and calm."}, "time_dimension": {"answer": "Past", "rationale": ""}, "sentence_type": "Affirmative declarative sentence", "pos": {"nouns": {"I": 1, "Music": 2}, "verbs": {"Yeah": 1}, "numerals": {"137": 1}}}, {"content": "138. I like to think about Africa:", "word_count": 6, "label": "", "sentence_structure": "Subject鈥搗erb鈥搊bject", "subjective_objective": {"answer": "Subjective", "rationale": "Because \"inflicted\" is a psychological activity or emotional experience, it is a feeling and thought inside the patient and is therefore a subjective examination."}, "sentence_sentiment": {"answer": "Positive", "rationale": "Because the description of \"love is not true\" is positive and expresses imaginative, creative attitudes and qualities."}, "language_style": {"answer": "Tactful and warm", "rationale": "The language style of the entry is more euphemistic, and investigations are conducted in the poetic expression \"I love to think about it,\" while avoiding the use of too direct or command-style language. This expression makes it easier for the respondents to feel comfortable and comfortable and to be more willing to participate in the questionnaire."}, "time_dimension": {"answer": "Present", "rationale": "The keyword \"I love to think wrong\" expresses a customary or psychological preference of the subjects, which suggests that they often have such an idea at this stage. So the time dimension of this entry is now judged."}, "sentence_type": "Imperative sentence", "pos": {"nouns": {"138": 1, "I": 1, "I'm not gonna get into this": 1}}}, {"content": "139. I believe that the world situation will improve over the next two decades:", "word_count": 17, "label": "", "sentence_structure": "Subject鈥搗erb鈥搊bject", "subjective_objective": {"answer": "Subjective", "rationale": "Because \"think\" expresses patients' subjective views or predictions about the future."}, "sentence_sentiment": {"answer": "Positive", "rationale": "Because the attitude in the entries is that the world situation will improve over the next two decades, it is a positive view."}, "language_style": {"answer": "Calm and direct", "rationale": "The entry raises questions and presents them in a direct manner, does not use excessive rhetorical or emotional rendering, and is more calm and objective in its language style."}, "time_dimension": {"answer": "Future", "rationale": "The keywords \"The world situation for the next 20 years\" and \"the world will definitely be better\" indicate that the entry is discussing what has not happened and what is likely to happen in the future."}, "sentence_type": "Interrogative sentence", "pos": {"nouns": {"The world situation": 1, "The next 20 years": 1}, "verbs": {"I think": 1, "It's gonna be better": 1}, "adverbs": {"I'm sure": 1}, "numerals": {"Twenty-two": 1}}}, {"content": "140. In childhood, I love reading:", "word_count": 9, "label": "", "sentence_structure": "Subject鈥搗erb鈥搊bject", "subjective_objective": {"answer": "Subjective", "rationale": "Because \"like reading\" is a subjective feeling and choice of a person."}, "sentence_sentiment": {"answer": "Neutral", "rationale": "Because the entry does not express positive or negative attitudes, behaviours, aims or qualities. It simply describes the fact that reading is a thing that is preferred in childhood."}, "language_style": {"answer": "Tactful and warm", "rationale": "The entry is more proficient and interactive by engaging in dialogue in a gentle and inviting manner, rather than by direct statements or orders. It also avoids the resistance or discomfort that may arise from the use of overly direct language."}, "time_dimension": {"answer": "Past", "rationale": ""}, "sentence_type": "Exclamatory sentence", "pos": {"nouns": {"Childhood": 1, "Yeah": 1, "Reading": 1}, "verbs": {"be": 1, "I": 1}, "numerals": {"140": 1}}}, {"content": "I've always been interested in machines, cars, etc.", "word_count": 13, "label": "", "sentence_structure": "Subject鈥搗erb鈥揷omplement", "subjective_objective": {"answer": "Subjective", "rationale": "Because \"I'm interested in machines, cars, etc.\" It's a personal perception of the patient and a preference for it, it's a subjective observation."}, "sentence_sentiment": {"answer": "Neutral", "rationale": "Because the entry does not have any emotional color."}, "language_style": {"answer": "Tactful and warm", "rationale": "The entry is more adept and interactive when it is spoken in a gentle and inviting manner, rather than by direct statements or orders."}, "time_dimension": {"answer": "Present", "rationale": ""}, "sentence_type": "Affirmative declarative sentence", "pos": {"nouns": {"Mechanical": 1, "Car": 1, "Interest": 1}, "verbs": {"Come on": 1, "Always": 1}, "adverbs": {"Wait": 1}, "numerals": {"141": 1}, "pronouns": {"I": 1}}}, {"content": "142. Even if I were to be the manager of a suspended offender, I would have done a great job:", "word_count": 28, "label": "", "sentence_structure": "Subject鈥搗erb鈥搊bject", "subjective_objective": {"answer": "Subjective", "rationale": "Because this is a subjective assessment of the patient ' s abilities."}, "sentence_sentiment": {"answer": "Neutral", "rationale": "This entry does not address information such as emotions, attitudes or behaviours, but rather questions the respondent ' s willingness to work in a particular position."}, "language_style": {"answer": "Tactful and warm", "rationale": "The entry uses an invitation expression rather than a direct statement or order. By using the phrase \"even if\" to make the respondents more willing to answer questions and share their ideas, the questionnaire is more valid and reliable. At the same time, the phrase \"good work\" is more positive and positive, and does not create negative emotions or stress. The language style of the article is therefore euphemism."}, "time_dimension": {"answer": "Present", "rationale": ""}, "sentence_type": "Affirmative declarative sentence", "pos": {"nouns": {"Probation": 1, "Release": 1, "Perpetrators": 1, "Manager": 1, "Work": 1}, "verbs": {"Do it": 2, "Yes": 1, "Fuck": 1}, "adverbs": {"Even if": 1, "Yeah": 1}, "numerals": {"142": 1}}}, {"content": "I'm just a man who can work hard and make a little difference.", "word_count": 22, "label": "", "sentence_structure": "Subject鈥搗erb鈥揷omplement", "subjective_objective": {"answer": "Subjective", "rationale": "Because the words \"regarded\" and \"only\" in the sentence indicate the views and feelings of the interviewees, which are viewed from a subjective perspective."}, "sentence_sentiment": {"answer": "Negative", "rationale": "Because the expression \"only those who are perceived to be \" and \"were only a few of the hard work\" has a negative and modest emotional colour, it can be considered negative."}, "language_style": {"answer": "Calm and direct", "rationale": "This language style gives people a cold, direct feeling. So it can be described as \"cool and straight.\""}, "time_dimension": {"answer": "Past", "rationale": "\"143\" means that it is a serial number, and judging from the whole point of view, the main phrase of this sentence is me, the term being considered, and the ben is just a person who can work hard and achieve a little. Among them, \"I am just thought\" is an expression of a past experience or state, which has not changed. So the time dimension of this entry is the past."}, "sentence_type": "Negative declarative sentence", "pos": {"nouns": {"Questionnaire": 1, "Entry": 1, "person": 1, "Achievements": 1}, "verbs": {"I think": 1, "be": 1, "Yes": 1, "- Oh, my God": 1, "A little": 1}, "adjectives": {"Just": 1, "One": 2, "And": 1}, "adverbs": {"It's just": 1}, "numerals": {"143": 1}, "classifiers": {"individual": 1}, "pronouns": {"I": 1}}}, {"content": "144. I am still in a state of unhappiness:", "word_count": 19, "label": "", "sentence_structure": "Subject鈥搗erb鈥揷omplement", "subjective_objective": {"answer": "Subjective", "rationale": "Because \"stagger\" is a subjective expression of attitude."}, "sentence_sentiment": {"answer": "Positive", "rationale": "Because the \"I can still keep up the spirit in the bad circumstances\" act in the entry is positive."}, "language_style": {"answer": "Tactful and warm", "rationale": "The entry is more proficient and interactive when it is spoken in a gentle and inviting manner, rather than in direct statements or orders. At the same time, it does not use excessive rhetorical or emotional rendering, which is more objective and consistent with the linguistic style of the questionnaire survey."}, "time_dimension": {"answer": "Present", "rationale": "The entry indicates that the testee is able to maintain a spirit of excitement even in the face of a difficult situation. The tester is therefore judged to be a current behavioural or mental condition."}, "sentence_type": "Affirmative declarative sentence", "pos": {"nouns": {"Situation": 1, "Down": 1, "Spirit": 1, "Exhilaration": 1}, "verbs": {"Hold on": 1, "Still can": 1, "be": 1}, "adjectives": {"It's not going well": 1}, "numerals": {"144": 1}}}, {"content": "145. I consider birth control to be an important condition for solving economic and peace problems:", "word_count": 22, "label": "", "sentence_structure": "Subject鈥搗erb鈥搊bject", "subjective_objective": {"answer": "Subjective", "rationale": "This is a subjective attitude to the question of whether \"restrictive parenthood is an essential condition for solving economic and peace problems\"."}, "sentence_sentiment": {"answer": "Positive", "rationale": "Because the idea that birth control is an important condition for solving economic and peace problems is positive in the entry."}, "language_style": {"answer": "Calm and direct", "rationale": "The language style of the entry is more straightforward, and is not retroverted with excessive rhetorical or emotional colours, but rather with an objective and rational approach."}, "time_dimension": {"answer": "Present", "rationale": "The entry shows the current personal views and convictions of the testee, and therefore the temporal dimension is now judged."}, "sentence_type": "Affirmative declarative sentence", "pos": {"nouns": {"Problem": 2, "Fertility": 1, "Conditions": 1, "Resolve": 1, "Economy": 1, "Peace": 1, "Moderate": 1}, "verbs": {"I think": 1, "be": 1, "Important": 1, "Resolve": 1}, "adjectives": {"Economy": 1, "Peace": 1}, "numerals": {"145": 1}, "pronouns": {"I": 1}}}, {"content": "At work, I like to plan on my own, and I don't want to be interfered with:", "word_count": 18, "label": "", "sentence_structure": "Subject鈥搗erb鈥搊bject", "subjective_objective": {"answer": "Subjective", "rationale": "Because \"like\" and \"resisting from others\" are the personal attitudes and feelings of the patients."}, "sentence_sentiment": {"answer": "Negative", "rationale": "Because the \"resistance to be interfered\" attitude in the entries is negative."}, "language_style": {"answer": "Calm and direct", "rationale": "The language style of the entry is relatively objective, concise, and there is no excessive rhetorical or emotional overture. The interviewee is asked whether he/she prefers to plan his/her work alone and is not willing to be interfered with."}, "time_dimension": {"answer": "Present", "rationale": "The descriptions of \"like\" and \"resisting from interference\" in the entry are directed at current behaviour or attitudes and therefore judge as now."}, "sentence_type": "Affirmative declarative sentence", "pos": {"nouns": {"Work": 1, "I": 1}, "verbs": {"Yeah": 1, "Planning": 1, "Yes": 1, "Interference": 1}, "adverbs": {"Alone": 1, "I don't want to": 1}, "numerals": {"146": 1}}}, {"content": "147. Despite some of the gays and my disagreement, she was able to be united:", "word_count": 22, "label": "", "sentence_structure": "Subject鈥搗erb鈥揷omplement", "subjective_objective": {"answer": "Subjective", "rationale": "Despite the patient ' s subjective attitude."}, "sentence_sentiment": {"answer": "Neutral", "rationale": "Because the entry describes an objective fact, it does not carry any positive or negative emotional colour."}, "language_style": {"answer": "Calm and direct", "rationale": "The entry is more straightforward, more concise, less speech-making or emotional, more focused on the expression of facts and views. At the same time, the entry does not use too many complex or remote terms that are easily understood. Thus, its language style can be classified as \"cool and straight.\""}, "time_dimension": {"answer": "Present", "rationale": "The entry is about the current situation, where gay people can remain united, even though they disagree with \"me.\" The keyword \"though,\" but \"are\" means that it is an ongoing move or state, so the entry should be classified as now."}, "sentence_type": "Affirmative declarative sentence", "pos": {"nouns": {"Comrade": 2, "Observations": 1, "Solidarity": 1}, "verbs": {"Call": 1, "Fuck": 1, "Yes": 1}, "adjectives": {"Yes": 1, "It's not": 1}, "adverbs": {"Still": 1}, "numerals": {"147": 1}, "pronouns": {"she": 1, "I": 1}}}, {"content": "148. I have always kept myself from carelessness and from neglecting details in my work and learning:", "word_count": 23, "label": "", "sentence_structure": "Subject鈥搗erb鈥揷omplement", "subjective_objective": {"answer": "Objective", "rationale": "Because \"always making yourself careless, not neglecting details\" is a behaviour that is a reality for patients in their work and studies and is therefore an objective examination."}, "sentence_sentiment": {"answer": "Positive", "rationale": "Because the entry \"does not careless, don't ignore details\" is positive."}, "language_style": {"answer": "Tactful and warm", "rationale": "Because the entry does not directly express criticism or demands, it is a message of encouragement and support to allow the respondents to express their attitudes and behaviour. At the same time, more moderate and friendly language is used, which makes it easier to resonate and respond positively to the respondents."}, "time_dimension": {"answer": "Present", "rationale": "The entry describes the testee ' s customary behaviour in work and learning, using the general current, and thus judging the present behaviour or mentality."}, "sentence_type": "Affirmative declarative sentence", "pos": {"nouns": {"Work": 1, "Learning": 1, "self": 2, "I'm not sure I'm gonna be able to help you": 1, "Ignore": 1, "Details": 1}, "verbs": {"鈾?And I'm gonna be 鈾?: 1}, "adverbs": {"Always": 1}, "numerals": {"148": 1}}}, {"content": "149. After arguing or being in danger, I often display tremors and fatigues that I cannot work:", "word_count": 29, "label": "", "sentence_structure": "Subject鈥搗erb鈥揷omplement", "subjective_objective": {"answer": "Subjective", "rationale": "Because \"showing a tremor, exhausted, and not working\" is the subjective experience and feeling of the patient."}, "sentence_sentiment": {"answer": "Negative", "rationale": "Because the reactions described in the entry, such as tremors, exhaustion and lack of peace of mind, are all negative emotions and experiences, indicating that the person suffered a negative psychological impact after arguing or having suffered an accident."}, "language_style": {"answer": "Calm and direct", "rationale": "The language style of the entry is more calm and straightforward, with no excessive rhetorical or emotional rendering, and directs the person to ask about the person鈥檚 performance and feelings in a given situation."}, "time_dimension": {"answer": "Present", "rationale": ""}, "sentence_type": "Negative declarative sentence", "pos": {"nouns": {"Questionnaire": 1, "Entry": 1, "Argument": 1, "Accidents": 1, "鈾?The tremors 鈾?: 1, "I'm exhausted": 1, "An Xin": 1, "Work": 1}, "verbs": {"Show it": 1, "have": 1, "It's a bad idea": 1}, "adjectives": {"Often": 1, "No, I can't": 1}, "numerals": {"149": 1}}}, {"content": "150. I never take drugs without a prescription from a doctor:", "word_count": 14, "label": "", "sentence_structure": "Subject鈥損redicate", "subjective_objective": {"answer": "Objective", "rationale": "Because the question is whether the patient is using drugs as recommended by the doctor, it is an objective fact and is not related to the patient ' s subjective attitude."}, "sentence_sentiment": {"answer": "Positive", "rationale": "Because it expresses a positive attitude, i.e., never taking drugs."}, "language_style": {"answer": "Calm and direct", "rationale": "The entry is in a concise manner, asking the interviewee whether he or she is taking drugs without a medical prescription, and the language style is more direct and objective."}, "time_dimension": {"answer": "Present", "rationale": ""}, "sentence_type": "Affirmative declarative sentence", "pos": {"nouns": {"Doctor": 1, "Prescription": 1, "Medicine": 1}, "verbs": {"be": 2, "Eat up": 1, "Food": 1}, "adverbs": {"Never": 1}, "numerals": {"150": 1}, "pronouns": {"I": 1}}}, {"content": "151. Based on my personal interest, I would like to participate in:", "word_count": 13, "label": "", "sentence_structure": "Subject鈥搗erb鈥搊bject", "subjective_objective": {"answer": "Subjective", "rationale": "Because \"personal interest\" and \"willing to participate\" are patients' own subjective attitudes and wishes."}, "sentence_sentiment": {"answer": "Neutral", "rationale": "Because the entry is merely an inquiry into the interests of the other party, it does not involve any positive or negative emotional attitude."}, "language_style": {"answer": "Tactful and warm", "rationale": "The language style of the entry is more euphemistic, it asks each other about their interests in a more gentle manner and expresses the hope that they will be actively involved. At the same time, the entry does not use too many direct instructions or strong tone to make the dialogue more intimate and friendly."}, "time_dimension": {"answer": "Present", "rationale": "The keywords \"personal interest\" and \"willing to participate\" indicate that this is a survey of the current interest and willingness of the subjects, so the temporal dimension is now judged."}, "sentence_type": "Imperative sentence", "pos": {"nouns": {"Personal": 1, "Interest": 1}, "verbs": {"Yes": 1, "Participation": 1}, "numerals": {"151": 1}}}, {"content": "152. I think the combination of fire and stun is the following:", "word_count": 18, "label": "", "sentence_structure": "Subject鈥搗erb鈥搊bject", "subjective_objective": {"answer": "Subjective", "rationale": "Since the title requires the following words, \"I think\", to be answered in the light of the individual's understanding and opinion, it is the subjective perspective of the patient that is examined."}, "sentence_sentiment": {"answer": "Neutral", "rationale": "According to the sentence given, \"I think appeasement and () pair\" does not express any emotional or attitude, and the purpose of the article is therefore neutral."}, "language_style": {"answer": "Calm and direct", "rationale": "This is because the purpose of the article is very straightforward, clearly raising the issue of the need to fill the vacancy, and not using excessive emotional or rhetorical techniques, but maintaining an objective and calm tone."}, "time_dimension": {"answer": "Present", "rationale": "The main word for the entry in the questionnaire is \"I,\" which expresses the individual views and views of the speaker. And the verb \"considers\" indicates that this is a current view and view, and therefore the judgement is now."}, "sentence_type": "Interrogative sentence", "pos": {"nouns": {"Starfire and Shaowon": 1, "鈾?To forgive and to forgive 鈾?: 1}, "verbs": {"I think": 1, "Match": 2}}}, {"content": "The relationship between the 鈥渃lock clock鈥?and 鈥渢ime鈥?is similar to that between 鈥渄resser鈥?and ():", "word_count": 16, "label": "", "sentence_structure": "Subject鈥搗erb鈥搊bject", "subjective_objective": {"answer": "Objective", "rationale": "The issue is an objective one, since it is an examination of the understanding of the relationship between the subjects and does not involve subjective attitudes or experience of the persons interviewed."}, "sentence_sentiment": {"answer": "Neutral", "rationale": "Because the entry does not have any emotional color."}, "language_style": {"answer": "Tactful and warm", "rationale": "The language style of the entry is more euphemistic, it raises problems by metaphor, it is more gentle and it is more friendly. It is also guiding, making it easier for respondents to understand and think about the problem."}, "time_dimension": {"answer": "Past", "rationale": ""}, "sentence_type": "Interrogative sentence", "pos": {"nouns": {"Watch": 1, "Time": 1, "Relations": 2, "A tailor": 1, "Sewn": 1}, "verbs": {"and": 3}, "numerals": {"153": 1}}}, {"content": "154. Living dreams often interfere with my sleep:", "word_count": 13, "label": "", "sentence_structure": "Subject鈥搗erb鈥揷omplement", "subjective_objective": {"answer": "Subjective", "rationale": "Because a living dream is a subjective experience of the patient."}, "sentence_sentiment": {"answer": "Negative", "rationale": "Because the description \"often interferes with my sleep\" in the entry is negative."}, "language_style": {"answer": "Tactful and warm", "rationale": ""}, "time_dimension": {"answer": "Present", "rationale": "In this entry, both \"virtual dreams\" and \"often interfere with my sleep\" refer to the state or situation of the present, so the temporal dimension of the article can be judged to be the present."}, "sentence_type": "Interrogative sentence", "pos": {"nouns": {"Dreams": 1, "Sleep": 1}, "verbs": {"Vivid": 1, "Interference": 1, "Often": 1, "Mine": 1}, "adjectives": {"Often": 1, "It's vivid": 1}, "numerals": {"154": 1}, "classifiers": {"individual": 1}, "pronouns": {"I": 1}}}, {"content": "I love to be upset:", "word_count": 6, "label": "", "sentence_structure": "Subject鈥搗erb鈥搊bject", "subjective_objective": {"answer": "Subjective", "rationale": "Because I love to be upset is a subjective feeling and attitude, looking at the personal values and emotional tendencies of the patient."}, "sentence_sentiment": {"answer": "Positive", "rationale": "Because the \"fault\" in the entry reflects a sense of justice and the act of aiding human pleasure, with positive emotional colours."}, "language_style": {"answer": "Calm and direct", "rationale": "The language style of the entry is relatively simple, and the interviewee is asked whether he likes to be indifferent. There is no excessive rhetorical or emotional colour that gives a calm and objective impression.\""}, "time_dimension": {"answer": "Past", "rationale": "The entry \"I love to be upset\" denotes the past experience or emotional state, and therefore judges the past."}, "sentence_type": "Interrogative sentence", "pos": {"verbs": {"Fight": 1, "I'm not happy": 1}, "numerals": {"155": 1}, "pronouns": {"I": 1}}}, {"content": "156. If I go to a new city, I will:", "word_count": 13, "label": "", "sentence_structure": "Subject鈥損redicate", "subjective_objective": {"answer": "Subjective", "rationale": "This is because of patients ' subjective view of the individual ' s will and intentions to go to the new city."}, "sentence_sentiment": {"answer": "Neutral", "rationale": "Because the entry does not have any emotional color."}, "language_style": {"answer": "Calm and direct", "rationale": "The entry uses a direct question form and does not contain any emotional accoutrements or guiding language, so that its language style can be considered as calm and straightforward."}, "time_dimension": {"answer": "Future", "rationale": ""}, "sentence_type": "Imperative sentence", "pos": {"verbs": {"Go on": 1, "Yes": 2}, "adjectives": {"New": 1}, "numerals": {"156": 1}, "pronouns": {"I": 1}}}, {"content": "157. I like to wear plain clothes, not fancy clothes:", "word_count": 16, "label": "", "sentence_structure": "Subject鈥搗erb鈥揷omplement", "subjective_objective": {"answer": "Subjective", "rationale": "Because \"love to wear\" and \"not to wear\" are patients' subjective attitudes."}, "sentence_sentiment": {"answer": "Negative", "rationale": "Because the entry expresses rejection and dislike of gorgeous clothing, it is a negative emotional attitude."}, "language_style": {"answer": "Calm and direct", "rationale": "The entry expresses the preferences of the respondent in the form of a direct statement, does not use excessive rhetorical or emotional rendering, and is more objective in tone."}, "time_dimension": {"answer": "Present", "rationale": "\"I like to wear plain clothes, I don't want to wear fancy clothes\" describes the taste and attitude of the testees today and therefore belongs to the time dimension of the present."}, "sentence_type": "Exclamatory sentence", "pos": {"nouns": {"Clothes": 2, "- It's gorgeous": 1, "Clothing": 1}, "verbs": {"Yes": 1, "May I": 1}, "adjectives": {"PARK Soo-soo": 1}, "numerals": {"157": 1}, "pronouns": {"I": 1}}}, {"content": "158. I think that quiet entertainment is far better than a party:", "word_count": 17, "label": "", "sentence_structure": "Subject鈥搗erb鈥搊bject", "subjective_objective": {"answer": "Subjective", "rationale": "Because \"think\" means that it's a patient's personal opinion and attitude, it's a subjective entry."}, "sentence_sentiment": {"answer": "Neutral", "rationale": "Since the entry merely expresses a point of view, it does not express a positive or negative emotional tendency."}, "language_style": {"answer": "Calm and direct", "rationale": "The language style of the entry is a calm and straightforward type. It expresses the view of the respondents on the form of entertainment in a concise and clear language, with no excessive emotional rendering or rhetorical techniques, language temperature and objectivity, and is intended to capture the real thoughts of the person under investigation."}, "time_dimension": {"answer": "Present", "rationale": "The entry is for the views and ideas of the testee, and the keyword \"considers\" indicates that it is a subjective judgement, without reference to specific past or future acts or events. Thus, the temporal dimension is now judged."}, "sentence_type": "Interrogative sentence", "pos": {"nouns": {"Recreation": 1, "The party": 1}, "verbs": {"I think": 1, "More than anything": 1}, "adjectives": {"Quiet": 1, "It's hilarious": 1}, "numerals": {"158": 1}, "pronouns": {"I": 1}}}, {"content": "159. I am not willing to accept criticisms of others, knowing that I have shortcomings:", "word_count": 18, "label": "", "sentence_structure": "Subject鈥搗erb鈥搊bject", "subjective_objective": {"answer": "Subjective", "rationale": "Because the purpose of the questionnaire is to examine patients鈥?subjective attitudes and perceptions, i.e. whether they are willing to accept criticism from others. While this situation is objective (i.e., the patient has shortcomings and others may criticize him), in this case, the subjective feelings and attitudes of the patient are more of a concern and therefore fall within the scope of subjective examination."}, "sentence_sentiment": {"answer": "Negative", "rationale": "Because the \"unwillingness to accept criticism\" in the entry is negative."}, "language_style": {"answer": "Tactful and warm", "rationale": ""}, "time_dimension": {"answer": "Present", "rationale": "In time, this is a current-day statement describing the current state and behaviour of the talking person. So, the entry is now."}, "sentence_type": "Negative declarative sentence", "pos": {"nouns": {"Disadvantages": 1, "Criticism": 1}, "verbs": {"Yes": 1, "Accept": 1, "others": 1}, "adjectives": {"Ming": 1}, "adverbs": {"But": 1}, "numerals": {"159": 1}, "pronouns": {"self": 1}}}, {"content": "I always use the word 鈥測es, no, no, no, no, no, no, no, no, no, no, no, no, no, no, no, no, no, no, no, no, no, no, no, no, no, no.", "word_count": 17, "label": "", "sentence_structure": "Subject鈥搗erb鈥搊bject", "subjective_objective": {"answer": "Subjective", "rationale": "This is a self-evaluation and attitude of patients and is a subjective expression."}, "sentence_sentiment": {"answer": "Positive", "rationale": "Because the treatment principle in the entry is considered positive."}, "language_style": {"answer": "Calm and direct", "rationale": "The entry directly asks whether the person surveyed is following the principle of \"Yes, yes, yes, yes, yes, yes, yes, yes, yes, yes, yes, yes, yes, yes, yes, yes, yes, yes\" in dealing with the matter."}, "time_dimension": {"answer": "Present", "rationale": "The keyword \"always\" indicates that it is the tester's current customary behaviour, so it should be now."}, "sentence_type": "Affirmative declarative sentence", "pos": {"nouns": {"Principles": 1}, "verbs": {"Always": 1, "- Put it on": 1, "Consider": 1, "Processing": 1}, "adverbs": {"Always": 1, "Yes, no, no, no, no": 1}, "numerals": {"160": 1}, "pronouns": {"I": 1}}}, {"content": "161. When I work, I don't like people visiting me:", "word_count": 19, "label": "", "sentence_structure": "Subject鈥搗erb鈥搊bject", "subjective_objective": {"answer": "Subjective", "rationale": "Because it's the attitude and feelings that patients don't like when people are visiting."}, "sentence_sentiment": {"answer": "Negative", "rationale": "Because the \"not like people visiting around\" act in the entry expresses negative attitudes."}, "language_style": {"answer": "Calm and direct", "rationale": "The language style of the entry is simpler, not using too many rhetorical techniques or emotional renderings. It is a direct description of a behaviour or feeling, and it is more direct, with little euphemism or hint. It is more suitable for a questionnaire-based interview, because it helps the interviewees to better understand the problem and reduces the possibility of ambiguity and misunderstanding."}, "time_dimension": {"answer": "Present", "rationale": "The entry is therefore an inquiry into the current behaviour and feelings of the person being tested, based on the present."}, "sentence_type": "Negative declarative sentence", "pos": {"nouns": {"Work": 1, "person": 2, "Next to it": 1, "Visitors": 1}, "verbs": {"Yeah": 1, "have": 1, "in": 1, "Visiting side by side": 1}, "adjectives": {"Many": 1}, "adverbs": {"not": 1}, "numerals": {"161": 1}, "classifiers": {"individual": 1}}}, {"content": "162. I believe that it is not appropriate to insult those who are educated, even if they are wrong, such as doctors, teachers, etc.:", "word_count": 32, "label": "", "sentence_structure": "Subject鈥搗erb鈥搊bject", "subjective_objective": {"answer": "Subjective", "rationale": "Because the entry is a patient's perception and assessment of his or her subjective attitude, it is \"subjective\"."}, "sentence_sentiment": {"answer": "Neutral", "rationale": "Because the entry expresses a view or belief and does not contain any emotional colour."}, "language_style": {"answer": "Tactful and warm", "rationale": "This entry expresses opinions in question, and uses polite and respectful language to avoid direct orders or accusations."}, "time_dimension": {"answer": "Past", "rationale": ""}, "sentence_type": "Affirmative declarative sentence", "pos": {"nouns": {"person": 3, "Error": 1, "Cultural upbringing": 1, "Doctor": 1, "Teachers": 1}, "verbs": {"Insulting": 2, "I think": 1, "have": 1, "Like": 1, "Yeah": 1, "Not supposed to": 1}, "adverbs": {"Even if": 1}, "numerals": {"162": 1, "1": 4}, "pronouns": {"Those": 1, "Like": 1, "I": 1}}}, {"content": "163. In the various courses, I like:", "word_count": 9, "label": "", "sentence_structure": "Subject鈥搗erb鈥搊bject", "subjective_objective": {"answer": "Subjective", "rationale": "Because \"like\" is a subjective emotional attitude."}, "sentence_sentiment": {"answer": "Neutral", "rationale": "Because the entry does not have any emotional color."}, "language_style": {"answer": "Tactful and warm", "rationale": "The entry is more euphemistic in its language style, as it opens in a question-and-answer manner and encourages respondents to share their personal preferences and interests."}, "time_dimension": {"answer": "Present", "rationale": "The keyword is \"like\", which is the testee's attitude or preference to the current situation, so the subject is asking about the current behaviour or preference."}, "sentence_type": "Declarative sentence", "pos": {"nouns": {"Courses": 1, "Yeah": 1}, "verbs": {"in": 1}, "adverbs": {"Various": 1}, "numerals": {"163": 1}}}, {"content": "164. The self-righteous, profane people have made me angry:", "word_count": 16, "label": "", "sentence_structure": "Subject鈥搗erb鈥搊bject", "subjective_objective": {"answer": "Subjective", "rationale": "Because \"self-righteous, beautiful\" and \"freaking\" are emotional reactions and perceptions of patients."}, "sentence_sentiment": {"answer": "Negative", "rationale": "Because the emotional reaction to \"freaking\" in the entry is negative."}, "language_style": {"answer": "Calm and direct", "rationale": ""}, "time_dimension": {"answer": "Present", "rationale": "\"164. Those who think that they are so, so good, so angry at me:\" This questionnaire entry asks whether the person who was tested was ever angry at having met someone who was so proud and so good. From a temporal point of view, it may be relevant to the past (e.g. the person who was tested in the past was so angry at the behaviour of some) or to the present or future (e.g. the person who was tested is so angry at some people, or is expected to meet such people and be so angry). So it can be answered simply: it contains content about feelings and emotions, and does not have a clear time-line to point at them."}, "sentence_type": "Affirmative declarative sentence", "pos": {"nouns": {"person": 2, "It's a beautiful place": 1, "You're so full of yourself": 1}, "verbs": {"鈾?And I'm gonna be 鈾?: 1, "Angry": 1}, "adjectives": {"Those": 1}, "numerals": {"164": 1}}}, {"content": "165. Talk to the right people:", "word_count": 9, "label": "", "sentence_structure": "Subject鈥搗erb鈥搊bject", "subjective_objective": {"answer": "Subjective", "rationale": "Because \"go to the right\" is a subjective criterion, and the subject is what is required to talk to, the subject is the patient's subjective attitude."}, "sentence_sentiment": {"answer": "Neutral", "rationale": "As the entry does not address any positive or negative emotional, attitudinal, behavioural, purpose or quality, it merely describes a factual situation."}, "language_style": {"answer": "Calm and direct", "rationale": "The language style of the entry is calm and straightforward. It is a simple question, which directly asks the interviewee about the feelings or experiences of communicating with a regular person, without using too many rhetorical or emotional terms to guide the answer, with a more straightforward tone, with a more precise tone."}, "time_dimension": {"answer": "Present", "rationale": "The entry describes the current behaviour or state of the subject, \"and\" as an ongoing move, and \"correspondent person talking\" is the specific element of the act, so it can be judged that the dimension of time is \"now\"."}, "sentence_type": "Interrogative sentence", "pos": {"nouns": {"Questionnaire": 1, "Entry": 1, "You're on the right track": 1, "person": 2, "Talk": 1}, "verbs": {"and": 1, "Talk": 1}, "adjectives": {"You're on the right track": 1}, "numerals": {"165": 1}}}, {"content": "I like:", "word_count": 3, "label": "", "sentence_structure": "Subject鈥搗erb鈥搊bject", "subjective_objective": {"answer": "Subjective", "rationale": "Because I like being patient subjective."}, "sentence_sentiment": {"answer": "Neutral", "rationale": "Because the entry is merely an inquiry into the preferences of the interviewees for something, it does not involve emotional attitudes or behavioural tendencies."}, "language_style": {"answer": "Tactful and warm", "rationale": "The language style of the entry is euphemistic, as it is presented in a way that gives people a warm sense of affection and does not use direct or command language. This way encourages respondents to express their thoughts and feelings actively and sincerely, thus obtaining more accurate research data."}, "time_dimension": {"answer": "Present", "rationale": "The entry asks about the testee ' s preference or preference for something, which is now being experienced or thought about, and therefore the temporal dimension is now judged."}, "sentence_type": "Affirmative declarative sentence", "pos": {"adjectives": {"Yeah": 1}, "numerals": {"166": 1}}}, {"content": "167. If I am consulted, I agree:", "word_count": 11, "label": "", "sentence_structure": "Subject鈥搗erb鈥搊bject", "subjective_objective": {"answer": "Subjective", "rationale": "Because the entry is concerned with the patient ' s own views and opinions and is a subjective one of the patients."}, "sentence_sentiment": {"answer": "Positive", "rationale": "The expression of the purpose of the article, which indicates the willingness of the interviewees to provide their own observations and recommendations, is a positive attitude."}, "language_style": {"answer": "Tactful and warm", "rationale": "The language style of the entry is relatively euphemistic, because it uses an invitation-style tone to ask each other about their views, and it uses a positive expression, \"I agree.\" This expression is relatively soft, friendly and easier to make interviewees comfortable and willing to share their views."}, "time_dimension": {"answer": "Present", "rationale": "The entry asks the testee about his or her attitude and position in the face of a request for advice or advice, i.e. whether he or she prefers to agree. In terms of time dimension, the question is about future behaviour or attitude, so the answer is \"the future\"."}, "sentence_type": "Imperative sentence", "pos": {"nouns": {"Observations": 1, "I": 2}, "verbs": {"Request": 1, "Yes": 1}, "adverbs": {"If": 1, "of": 1}, "numerals": {"167": 1}}}, {"content": "168. Sometimes I get depressed for no reason and cry:", "word_count": 15, "label": "", "sentence_structure": "Subject鈥搗erb鈥搊bject", "subjective_objective": {"answer": "Subjective", "rationale": "Because \"cry\" and \"disordered without cause\" are the feelings and experiences of the patient, which are subjective."}, "sentence_sentiment": {"answer": "Negative", "rationale": "Because the act of \"deep and weeping without cause\" in the entry is negative."}, "language_style": {"answer": "Tactful and warm", "rationale": "The language style of the entry is more euphemistic, using words like \"sometimes\" and \"for no reason\" to express emotions, rather than simply saying \"I'm always feeling frustration and pain.\" This expression is mild, and it reduces each other's resistance."}, "time_dimension": {"answer": "Present", "rationale": "The entry contains words such as \"sometimes\", \"will\" and other words indicating frequency of time and customary behaviour, which indicate that this is a description of the person's current or recent feelings or state. Thus, the temporal dimension of the article can be judged to be now."}, "sentence_type": "Exclamatory sentence", "pos": {"nouns": {"Time": 1, "I": 1, "For no reason": 1, "Frustration": 1, "Crying": 1}, "verbs": {"Yes": 1, "I feel": 1, "Crying": 1}, "adjectives": {"For no reason": 1, "Frustration": 1}, "adverbs": {"Sometimes": 1}}}, {"content": "169. When arguing against the opposite position, I argue that:", "word_count": 14, "label": "", "sentence_structure": "Subject鈥搗erb鈥搊bject", "subjective_objective": {"answer": "Subjective", "rationale": "Because \"promise\" is a person's attitude or opinion, subjective."}, "sentence_sentiment": {"answer": "Positive", "rationale": "Because the word \"advocacy\" in the entry expresses a positive attitude of holding one's own and insisting on expressing the argument of those who are opposed to their position."}, "language_style": {"answer": "Calm and direct", "rationale": "The language style of the entry is more straightforward, and there is no rhetorical or emotional rendering. It opens up to a specific point and asks the person under investigation to choose an attitude or behaviour. This expression is very clear, concise, without too much ambiguity or ambiguity, and therefore belongs to the \"cool straight\" language style."}, "time_dimension": {"answer": "Future", "rationale": "The entry is an inquiry into the position and attitude of the testee when he or she disputes with those who have different positions in the future, and the keyword \"when he or she is arguing with those who have the opposite position\" and \"I advocate\" point to situations that have not yet occurred and that may arise in the future. The judgement is therefore based on the future."}, "sentence_type": "Interrogative sentence", "pos": {"nouns": {"person": 1, "Argument": 1, "Position": 1}, "verbs": {"When": 1, "Claim": 1}, "adjectives": {"On the contrary": 1}, "numerals": {"169": 1}, "pronouns": {"I": 1}}}, {"content": "170. I have always been emotional rather than rational, and my views have often been uncertain:", "word_count": 23, "label": "", "sentence_structure": "Subject鈥搗erb鈥搊bject", "subjective_objective": {"answer": "Subjective", "rationale": "Because \"relationship without reason\" and \"swaying views\" are patients' subjective attitudes and ideas."}, "sentence_sentiment": {"answer": "Negative", "rationale": "Because the expression \"persistently uncertain\" is negative and represents an unstable and unreliable attitude."}, "language_style": {"answer": "Tactful and warm", "rationale": "The entry is more personal and interactive when it is spoken in a gentle and inviting manner, rather than in direct statements or orders. At the same time, it is not too strong in tone or emotional, but rather a more objective description of the emotional tendencies and ways of thinking of the person under investigation."}, "time_dimension": {"answer": "Present", "rationale": "\"I have always\" expressed a long-term customary behaviour, \"heavy without reason\" and \"swaying views\" describe the current situation. The entry therefore asks about the current state or orientation of the person being tested, judging by the current time dimension."}, "sentence_type": "Affirmative declarative sentence", "pos": {"nouns": {"Emotion": 1, "Reason": 1, "Views": 1, "鈾?Unstable 鈾?: 1}, "verbs": {"Heavy": 2, "Not Heavy": 1, "And so": 1, "Regular": 1}, "adjectives": {"It's about emotion, not reason": 1}, "numerals": {"170": 1}, "pronouns": {"I": 1}}}, {"content": "171. My studies are based on:", "word_count": 7, "label": "", "sentence_structure": "Subject鈥搗erb鈥搊bject", "subjective_objective": {"answer": "Subjective", "rationale": "Because \"my studies\" involve the individual attitudes and behaviour of patients, it is a subjective perspective."}, "sentence_sentiment": {"answer": "Neutral", "rationale": "Because the entry does not have any emotional color."}, "language_style": {"answer": "Calm and direct", "rationale": "The language style of the entry is a calm and direct type. The basis of the judgement is that the purpose of the article is very straightforward, without too much rhetorical or emotional publicity. It asks the interviewee what is relied upon to study in a way that is essentially objective, and therefore the language style is more calm and straightforward."}, "time_dimension": {"answer": "Present", "rationale": "Now the entry is asking the testee about the current learning style, and the keywords \"my learning\" and \"so much\" point to what is happening now. So the answer is \"now.\""}, "sentence_type": "Imperative sentence", "pos": {"nouns": {"Learning": 1, "鈾?L-l-l-l-l-l-l-l-l-l-l-l-l-l-l-I-I-I-I-I-I-I-I-I-I-I-I-I-I-I-I-I-I-I-I-I-I-I-": 1}, "verbs": {"be": 1}}}, {"content": "172. I prefer to choose a job with higher wages, not with security, rather than a job with low wages.", "word_count": 33, "label": "", "sentence_structure": "Subject鈥搗erb鈥搊bject", "subjective_objective": {"answer": "Subjective", "rationale": "Because \"better choice\" and \"responsible\" are subjective attitudes and ideas of the patient."}, "sentence_sentiment": {"answer": "Positive", "rationale": "Because the behaviour in the entry \"choice a higher-paid job\" expresses a positive attitude and willingness to pursue higher incomes."}, "language_style": {"answer": "Calm and direct", "rationale": "The language style of the entry is more straightforward, not excessive rhetoric or emotional rendering, but rather an objective, straightforward expression of the information the investigator would like to know."}, "time_dimension": {"answer": "Present", "rationale": "The entry is therefore concerned primarily with the present attitude or behaviour of the person being tested."}, "sentence_type": "Affirmative declarative sentence", "pos": {"nouns": {"Work": 2, "Wages": 2, "Safeguards": 1}, "verbs": {"Selection": 1, "I care": 1, "Do it": 1, "I'd rather": 1}, "adjectives": {"High": 1, "Low Fixed": 1}, "adverbs": {"I'd rather not": 1, "I don't want to": 1}, "numerals": {"172": 1}, "pronouns": {"I": 1}}}, {"content": "173. I have always been able to grasp my position in the discussions:", "word_count": 17, "label": "", "sentence_structure": "Subject鈥搗erb鈥搊bject", "subjective_objective": {"answer": "Subjective", "rationale": "Because the title says \"I can always take my position\", the word \"I\" here refers to the patient's own thoughts and behaviour, and it is subjective."}, "sentence_sentiment": {"answer": "Neutral", "rationale": "Because the entry describes only the behaviour of a person in the context of a discussion, it does not express a positive or negative emotional profile."}, "language_style": {"answer": "Calm and direct", "rationale": "Because the purpose of this article is simple and clear, rather than using too many rhetorical techniques or emotional rendering, it directly raises a clear question and asks the respondents to answer whether they can take their position when they participate in the discussion. Such language styles are more objective and direct, and are suitable for scientific research or data collection."}, "time_dimension": {"answer": "Present", "rationale": "The keyword \"always\" denotes customary, regular behaviour, \"take\" and \"self\" denotes behaviour that is taking place, so the temporal dimension is now judged."}, "sentence_type": "Interrogative sentence", "pos": {"nouns": {"Discussion": 1, "Position": 1}, "verbs": {"Participation": 1, "Yes": 1, "Hold on": 1, "be": 1}, "adverbs": {"Always": 1}, "numerals": {"173": 1, "Your own": 1}, "pronouns": {"I": 1}}}, {"content": "174. I am often disturbed by little things that matter:", "word_count": 15, "label": "", "sentence_structure": "Subject鈥搗erb鈥揷omplement", "subjective_objective": {"answer": "Subjective", "rationale": "Because \"often disturbed by little things that matter\" is the personal feeling and experience of the patient, it is subjectively viewed."}, "sentence_sentiment": {"answer": "Negative", "rationale": "Because the entry \"smuggling by little things\" expresses a negative emotional attitude."}, "language_style": {"answer": "Tactful and warm", "rationale": ""}, "time_dimension": {"answer": "Present", "rationale": ""}, "sentence_type": "Negative declarative sentence", "pos": {"nouns": {"Small": 1, "Yes": 1}, "verbs": {"By": 1, "Disturbing": 1}, "adjectives": {"It doesn't matter": 1}, "adverbs": {"Often": 1}, "classifiers": {"Some": 1}}}, {"content": "175. I prefer to live in a noisy urban area rather than in a quiet area:", "word_count": 21, "label": "", "sentence_structure": "Subject鈥搗erb鈥搊bject", "subjective_objective": {"answer": "Subjective", "rationale": "Because \"better\" is patient's subjective attitude."}, "sentence_sentiment": {"answer": "Negative", "rationale": "Because \"the noisy urban areas\" are usually associated with negative factors such as noise, pollution, the emotional color of the article is negative."}, "language_style": {"answer": "Tactful and warm", "rationale": "The language style of the question is more euphemistic, because it uses a soft approach to questioning, rather than direct orders or statements. Moreover, it attempts to direct respondents to share their preferences and feelings in order to better understand their needs and expectations. It may make the interviewees more comfortable and relaxed, and thus more willing to participate in the investigation and provide a true answer."}, "time_dimension": {"answer": "Present", "rationale": "\"I prefer\" to indicate a subjective will, while \"residence\" to the general current, to indicate that this behaviour has not only happened in the past, but may still be ongoing. So, \"175. I prefer to live in a crowded city rather than in a quiet area:\" describes the current preferences and attitudes."}, "sentence_type": "Affirmative declarative sentence", "pos": {"nouns": {"Urban": 1, "Region": 1}, "verbs": {"Stay": 2, "I'd rather": 1, "I don't want to": 1}, "adjectives": {"Loud": 1, "Invisibility": 1}, "adverbs": {"I'd rather not": 1, "And": 1}, "numerals": {"175": 1}, "pronouns": {"I": 1}}}, {"content": "176. I would like to do the following if I were to be left to choose:", "word_count": 15, "label": "", "sentence_structure": "Subject鈥搗erb鈥搊bject", "subjective_objective": {"answer": "Subjective", "rationale": "This is because of patients ' subjective attitude towards different jobs and choices."}, "sentence_sentiment": {"answer": "Neutral", "rationale": "Because the entry does not have any emotional color."}, "language_style": {"answer": "Tactful and warm", "rationale": "The entry is more personal and interactive by speaking in a gentle, inviting tone, rather than by direct statement or order. At the same time, this euphemism increases the willingness of respondents to share their ideas and opinions."}, "time_dimension": {"answer": "Future", "rationale": "The entry asks the testee about the will and expectations of the future, the keywords \"if I choose\" and \"if I wish to do\" point to the possibility of the future that has not yet occurred. The entry is therefore a time dimension of the future."}, "sentence_type": "Imperative sentence", "pos": {"nouns": {"Work": 2, "Selection": 1}, "verbs": {"Do it": 1}, "numerals": {"176": 1}, "pronouns": {"I": 1}}}, {"content": "177. One person () is a person who is tired of:", "word_count": 7, "label": "", "sentence_structure": "Subject鈥搗erb鈥搊bject", "subjective_objective": {"answer": "Objective", "rationale": "For \"one person, everyone is tired\" is a description of the facts and is not influenced by the subjective attitude of the individual."}, "sentence_sentiment": {"answer": "Negative", "rationale": "Because the result of the \"sick\" in the entry is negative."}, "language_style": {"answer": "Tactful and warm", "rationale": "The language style of the entry is euphemism and warmth, and it is one person, and everyone is tired of it, and not too hard or direct. It is more acceptable and willing to participate in the survey, reflecting the interest and respect of the questionnaire designers."}, "time_dimension": {"answer": "Past", "rationale": "The entry does not have a clear pointer to the temporal dimension and therefore cannot judge whether it is past, present or future."}, "sentence_type": "Imperative sentence", "pos": {"nouns": {"person": 2, "Yes": 1}, "verbs": {"I'm tired": 1}, "adverbs": {"One": 1}, "numerals": {"177": 1, "One person": 1}, "classifiers": {"individual": 1}}}, {"content": "178. The parents of the Goncho Jackie are often (or) encouraged by:", "word_count": 12, "label": "", "sentence_structure": "Subject鈥搗erb鈥揷omplement", "subjective_objective": {"answer": "Subjective", "rationale": "The \"prospect\" and \"often\" and \"promoting\" are subjective descriptions and evaluations, looking at parents' attitudes and perspectives and therefore at the subjective angle."}, "sentence_sentiment": {"answer": "Negative", "rationale": "The basis for the judgement: The parents of the son Jackie Hope often interfere too much in the growth of their children, using high pressure to force them to develop in accordance with their expectations. This \"smoking\" behaviour is detrimental to the healthy growth of the child and is therefore a negative attitude and behaviour."}, "language_style": {"answer": "Calm and direct", "rationale": "The entry raises the question in a direct manner, without using rhetorical or emotional language, which is more objective and calm.\""}, "time_dimension": {"answer": "Present", "rationale": "The temporal dimension of this purpose is therefore now, as it describes a phenomenon or conduct that currently exists."}, "sentence_type": "Interrogative sentence", "pos": {"nouns": {"Jackie": 1, "Parents": 1, "Mio-Pong": 1}, "adjectives": {"Often": 1}, "numerals": {"178": 1}}}, {"content": "179. Climate change does not affect my emotions:", "word_count": 13, "label": "", "sentence_structure": "Subject鈥搗erb鈥揷omplement", "subjective_objective": {"answer": "Objective", "rationale": "Because climate change is an objective fact, it is not influenced by individual subjective sentiments."}, "sentence_sentiment": {"answer": "Negative", "rationale": "Because the expression \"not affected\" in this entry means that the interviewees lack attention and attention to climate change and may have negative impacts on environmental protection."}, "language_style": {"answer": "Calm and direct", "rationale": "The language style of the entry is calm and straightforward, and it does not use too many rhetorical techniques or emotional terms, but rather expresses a view in an objective and concise manner. This style is common in psychological questionnaires, which reduces subjective bias and emotional interference by the testees, making the results more accurate and reliable."}, "time_dimension": {"answer": "Past", "rationale": "So it can be classified as \"the past and the present\"."}, "sentence_type": "Affirmative declarative sentence", "pos": {"nouns": {"Climate": 1, "Change": 1, "Emotion": 1}, "verbs": {"Impact": 1}}}, {"content": "180. Because I have some ideas on all issues, I am considered by all to be a wise person:", "word_count": 30, "label": "", "sentence_structure": "Subject鈥揷opula鈥損redicative", "subjective_objective": {"answer": "Subjective", "rationale": "Because \"I'm considered to be a wise person\" came to conclusions based on the feelings and thoughts of the interviewees, it's a subjective perspective."}, "sentence_sentiment": {"answer": "Positive", "rationale": "Because the \"considered wise\" is positive."}, "language_style": {"answer": "Tactful and warm", "rationale": "Although the entry describes a fact (that is, others think that the person who speaks is wise), its tone and wording are more euphemistic. For example, the expression \"because of ...\" is used to guide the other person to thinking, without directly speaking verbally or critically. Moreover, the terms used are more moderate, such as \"people\" and \"suspicious people\" , and are more objective and neutral. So, the language style of the article is more like euphemism."}, "time_dimension": {"answer": "Present", "rationale": "The entry is for self-evaluation or other opinion of the person being tested, the temporal dimension of which cannot be judged and therefore the present."}, "sentence_type": "Interrogative sentence", "pos": {"nouns": {"Problem": 1, "Observations": 1, "person": 1, "Brain": 1}, "verbs": {"Both": 1, "Recognition": 1, "be": 2, "have": 1, "I think": 1}, "adjectives": {"Some": 1, "Smart": 1}, "adverbs": {"So": 1, "Because": 1}, "numerals": {"180": 1}, "pronouns": {"I": 1, "Everyone": 1}}}, {"content": "181. The voice of my speech:", "word_count": 6, "label": "", "sentence_structure": "Subject鈥搗erb鈥揷omplement", "subjective_objective": {"answer": "Subjective", "rationale": "Because \"sound\" is usually felt subjectively by individuals, different people may have different views and feelings, and therefore it is subjective in its perspective."}, "sentence_sentiment": {"answer": "Neutral", "rationale": "As the entry does not address any emotion, attitude, behaviour, purpose or quality, it merely describes the voice of a person."}, "language_style": {"answer": "Tactful and warm", "rationale": "The language style of the entry is euphemistic, because it uses an invitation-style question-and-answer approach to guide respondents to think about their voice characteristics rather than to speak or order directly. This expression is more personal and interactive. At the same time, the question is not too professional or technical to be understood and answered by the respondents."}, "time_dimension": {"answer": "Past", "rationale": "The entry asks if the testee's past behavior or state, i.e., the voice of my speech, has ever been expressed in a particular way. The temporal dimension is therefore a matter of the past."}, "sentence_type": "Declarative sentence", "pos": {"nouns": {"Sound": 1, "Speech": 1}, "verbs": {"have": 1}}}, {"content": "182. I was considered by the class to be a passionate person:", "word_count": 16, "label": "", "sentence_structure": "Subject鈥搗erb鈥搊bject", "subjective_objective": {"answer": "Subjective", "rationale": "Because the entry is an inquiry into the patient ' s own perception of himself, it is a subjective examination."}, "sentence_sentiment": {"answer": "Positive", "rationale": "Because the expression \"active enthusiasm\" in the entry is a positive assessment that the person is considered by the class to be a dynamic and friendly person."}, "language_style": {"answer": "Tactful and warm", "rationale": "This language style helps create a more harmonious and positive atmosphere of communication."}, "time_dimension": {"answer": "Present", "rationale": "The entry describes the current perception and perception of people, and therefore the temporal dimension is now judged."}, "sentence_type": "Affirmative declarative sentence", "pos": {"nouns": {"A squad": 1, "I": 1}, "verbs": {"I think": 1, "be": 1, "One": 2, "A passionate man": 1}, "adjectives": {"Active": 1, "鈾?Hot 鈾?: 1}, "numerals": {"Class one": 1, "Both": 1}, "classifiers": {"individual": 1, "person": 1}}}, {"content": "I like to do the worst jobs:", "word_count": 13, "label": "", "sentence_structure": "Subject鈥搗erb鈥搊bject", "subjective_objective": {"answer": "Subjective", "rationale": "Because like a subjective attitude of a person."}, "sentence_sentiment": {"answer": "Positive", "rationale": "Because the entries express a preference for jobs with more opportunities for travel, they are positive emotional attitudes."}, "language_style": {"answer": "Calm and direct", "rationale": "This is a more straightforward language style, with no over-modified or emotional expression, and direct queries about the respondent鈥檚 attitude to the opportunity to travel to work."}, "time_dimension": {"answer": "Present", "rationale": "In the entry, \"I like\" is a current attitude and tendency, and \"job with more opportunities for travel\" is a descriptive term that shows the characteristics of work. So the time dimension of the entry is now."}, "sentence_type": "Affirmative declarative sentence", "pos": {"nouns": {"Work": 1, "Travel opportunities": 1}, "verbs": {"Yeah": 1, "Do it": 1, "Out": 1}, "adverbs": {"Compare": 1}, "numerals": {"183": 1}, "classifiers": {"individual": 1}}}, {"content": "184. I have done very strictly to try to do everything right:", "word_count": 16, "label": "", "sentence_structure": "Subject鈥搗erb鈥揷omplement", "subjective_objective": {"answer": "Subjective", "rationale": "Because \"doing things strictly and trying to be good\" is a personal attitude and behavioural tendency of the patient and is subjective."}, "sentence_sentiment": {"answer": "Positive", "rationale": "Because the words \"strictly\" and \"excellently perfect\" in the entries are positive adjectives, which indicate a serious and responsible attitude towards excellence."}, "language_style": {"answer": "Calm and direct", "rationale": "The language style of the entry is more objective and straightforward, with no excessive rhetorical or emotional colours, and the attitude and pursuit of the other in doing business is expressed in plain and clear language."}, "time_dimension": {"answer": "Present", "rationale": "The entry asks the person who was tested for his current behaviour or mentality, and the word \"do\" adopts the general current, meaning that the act has not only occurred in the past but may still continue."}, "sentence_type": "Affirmative declarative sentence", "pos": {"nouns": {"I": 1, "Yes": 1, "Strict": 1, "Seek": 1, "Put things in the way": 1, "OPERATIONS": 1, "Perfect": 1}, "verbs": {"Do it": 1, "Seek": 1, "Put things in the way": 1, "OPERATIONS": 1}, "adjectives": {"Strict": 1, "Perfect": 1}, "adverbs": {"EMPTY": 1}, "pronouns": {"I": 1}}}, {"content": "185. When recovering or returning borrowed items, I always carefully checked to see whether they remained the same:", "word_count": 26, "label": "", "sentence_structure": "Subject鈥搗erb鈥搊bject", "subjective_objective": {"answer": "Objective", "rationale": "Because this is a behaviour that the patient is describing, which has already occurred and can be observed and verified, it is therefore objective."}, "sentence_sentiment": {"answer": "Positive", "rationale": "Because \"check carefully\" and \"maintain\" both express positive attitudes and qualities."}, "language_style": {"answer": "Calm and direct", "rationale": "The entry is in a concise language, asking the respondents whether they carefully examine the contents of the borrowed items and ensure that they remain as they are."}, "time_dimension": {"answer": "Present", "rationale": ""}, "sentence_type": "Affirmative declarative sentence", "pos": {"nouns": {"Something": 1, "Inspection": 1}, "verbs": {"Remove": 1, "Return": 1, "Hold on": 1}, "adjectives": {"Careful": 1, "As is": 1}, "numerals": {"185": 1}, "classifiers": {"individual": 1}}}, {"content": "I'm usually a very energetic, busy person:", "word_count": 12, "label": "", "sentence_structure": "Subject鈥搗erb鈥揷omplement", "subjective_objective": {"answer": "Subjective", "rationale": "Because words like \"normally\" and \"absorbed of energy\" and \"bridled of things\" express patients' subjective feelings and attitudes."}, "sentence_sentiment": {"answer": "Positive", "rationale": "Because the description \"absorbed, busy\" is positive, positive attitude and behaviour."}, "language_style": {"answer": "Tactful and warm", "rationale": "The entry is more adept and interactive when it is spoken in a gentle and inviting manner, rather than by direct statements or orders."}, "time_dimension": {"answer": "Present", "rationale": "According to the description of \"normally\" and \"scramblingly\" it's a description of the current state of the interviewee, so the answer is \"now\"."}, "sentence_type": "Affirmative declarative sentence", "pos": {"nouns": {"Questionnaire": 1, "Entry": 1, "- The energy": 1, "鈾?Full of 鈾?: 1, "Busy": 1, "It's a lot of things": 1}, "verbs": {"Usually": 1}, "adjectives": {"Busy": 1, "More": 1, "鈾?Full of 鈾?: 1}, "adverbs": {"Usually": 1}, "numerals": {"186": 1}, "classifiers": {"individual": 1}}}, {"content": "187. I am sure that I did not omit or hesitate to answer any of the questions above:", "word_count": 22, "label": "", "sentence_structure": "Subject鈥搗erb鈥搊bject", "subjective_objective": {"answer": "Subjective", "rationale": "The reason for this is that it is the patient ' s subjective attitude to believe that he did not omit or respond carelessly."}, "sentence_sentiment": {"answer": "Neutral", "rationale": ""}, "language_style": {"answer": "Calm and direct", "rationale": "The language style of the entry is relatively calm and straightforward, and there is no excessive emotional or rhetorical approach, which directly expresses expectations and demands for answers to questions from the respondents."}, "time_dimension": {"answer": "Past", "rationale": "The entry \"any question above\" refers to the question before, and the question is whether the person tested has omitted or inadvertently answered the question in the past."}, "sentence_type": "Affirmative declarative sentence", "pos": {"nouns": {"Problem": 2}, "verbs": {"Sure": 1, "No, I'm not": 1, "Missing": 1, "I'm not sure I can": 1, "Answer": 1, "Up there": 1}, "adjectives": {"Any": 1}, "adverbs": {"Indeed": 1, "You're a good man": 1}, "numerals": {"187": 1, "2": 1}, "pronouns": {"I": 1}}}]}

{"questionnaire_title": "Diagram of defects", "questionnaire_category": "Self-cognition", "item_count": 36, "emotion_shift_overall": "From positive to negative", "language_style_overall": "Tactful and warm", "items": [{"content": "Do you often feel that you are not as good as most people you know?", "word_count": 21, "label": "", "sentence_structure": "Subject鈥搗erb鈥搊bject", "subjective_objective": {"answer": "Subjective", "rationale": "This is a subjective feeling and comparison of the patient and does not represent the real situation of others."}, "sentence_sentiment": {"answer": "Negative", "rationale": "Because it expresses a self-denial, comparative and inferior feeling."}, "language_style": {"answer": "Calm and direct", "rationale": "The entry is a direct question-and-answer dialogue, with a more calm and objective language style, with no excessive rhetorical or emotional overtones. It asks whether the interviewee has a psychological state of self-evaluation that is less straightforward and more straightforward in order to obtain accurate answers."}, "time_dimension": {"answer": "Present", "rationale": "The keyword \"often\" indicates that this feeling is frequent, not occasional or one-off."}, "sentence_type": "Interrogative sentence", "pos": {"nouns": {"Questionnaire": 1, "Entry": 1, "person": 1}, "verbs": {"I think": 1, "Not as good as it is": 2}, "adjectives": {"Most": 1}, "adverbs": {"Regular": 1, "I know him": 1}, "classifiers": {"individual": 1, "of": 2}, "pronouns": {"you": 1}}}, {"content": "Have you ever considered yourself a worthless person?", "word_count": 17, "label": "", "sentence_structure": "Subject鈥搗erb鈥搊bject", "subjective_objective": {"answer": "Subjective", "rationale": "Because \"thinking that you are a worthless person\" is a personal feeling and perception, a subjective view of the patient."}, "sentence_sentiment": {"answer": "Negative", "rationale": "Because the idea of \"thinking that you're a worthless person\" in the entry is negative."}, "language_style": {"answer": "Tactful and warm", "rationale": "This language style helps to reduce the tension and resistance of the interviewees and is more willing to answer questions honestly."}, "time_dimension": {"answer": "Past", "rationale": "The entry asks about the experience and feelings of the subject, and the key words are \"was it ever\" and \"the worthless person\", all of which point to what happened in the past."}, "sentence_type": "Interrogative sentence", "pos": {"nouns": {"Questionnaire": 1, "Entry": 1, "person": 1, "It's worthless": 1}, "verbs": {"I think": 1, "be": 1}, "adjectives": {"Once": 1}, "adverbs": {"2": 1}}}, {"content": "How confident will you be of the respect you have for someone you know?", "word_count": 25, "label": "", "sentence_structure": "Subject鈥搗erb鈥搊bject", "subjective_objective": {"answer": "Subjective", "rationale": "The subject is a subjective attitude of the patient, as it is asked about his expectations and confidence in the future."}, "sentence_sentiment": {"answer": "Positive", "rationale": "Because \"confidence\" in the entry is a positive emotional attitude, expressing expectations and trust in the future."}, "language_style": {"answer": "Tactful and warm", "rationale": "The entry is more friendly and interactive when it is spoken in a gentle and inviting manner, rather than in direct statements or orders. At the same time, the context of the issue is more natural and easily acceptable to the interviewees."}, "time_dimension": {"answer": "Present", "rationale": "The expression \"you have confidence in\" suggests that the question is to ask the testee about his current attitude or opinion, so the temporal dimension is now."}, "sentence_type": "Interrogative sentence", "pos": {"nouns": {"person": 2, "Confidence": 1}, "verbs": {"Yes": 1, "Look": 1, "Respect": 1, "have": 1, "Okay": 1, "Large": 1, "Faith": 1}, "adverbs": {"One day": 1}, "numerals": {"3": 1, "One": 2}, "pronouns": {"you": 2}}}, {"content": "Have you been so disheartened that you are beginning to wonder if you are a worthy person?", "word_count": 29, "label": "", "sentence_structure": "Subject鈥搗erb鈥搊bject", "subjective_objective": {"answer": "Subjective", "rationale": "Because the perception of self-evaluation and self-worth is a subjective feeling within the individual."}, "sentence_sentiment": {"answer": "Negative", "rationale": "Because the expressions \"depressed\" and \"suspecting yourself for value\" in the entries are negative, implying negative tendencies in individual emotional state and self-awareness."}, "language_style": {"answer": "Tactful and warm", "rationale": "The entry uses the words \"depressed\" and \"suspecting yourself as a valuable person\" to describe possible negative emotional experiences, but instead of directly blaming or criticizing each other, it leads the respondents to share their feelings in an understanding and acceptance. And the \"have you ever\" expression is more friendly and respectful of the interviewee, which makes it more comfortable and reassuring."}, "time_dimension": {"answer": "Past", "rationale": "The entry is for the purpose of asking the person who was tested for a certain period of time (i.e., in the past) whether he or she had been frustrated, suspected of his or her own experience and therefore belonged to the past."}, "sentence_type": "Interrogative sentence", "pos": {"nouns": {"Questionnaire Entry": 1, "person": 2, "Value": 1}, "verbs": {"Is it possible": 3, "Tsang": 1, "I'm so depressed": 1, "Doubt": 1, "be": 1, "individual": 2}, "adjectives": {"It's worth it": 1}, "adverbs": {"So much so that": 1}, "pronouns": {"you": 1, "self": 3}}}, {"content": "Do you often feel averse to yourself?", "word_count": 11, "label": "", "sentence_structure": "Subject鈥搗erb鈥搊bject", "subjective_objective": {"answer": "Subjective", "rationale": "Because \"always feeling sick\" is an individual's subjective feeling and emotional experience, not an objective reality or behaviour. Different people may have different answers, so the question is to look at the patient's subjective attitude and emotional state."}, "sentence_sentiment": {"answer": "Negative", "rationale": "Because \"hatting yourself\" is a negative emotional experience, the expression is negative."}, "language_style": {"answer": "Calm and direct", "rationale": "The entry directly asks the other side whether it is self-loathing, is more linguistically direct and does not use excessive rhetorical techniques or emotional rendering."}, "time_dimension": {"answer": "Present", "rationale": "The entry now asks the testee about his current and ongoing feelings and thus judges it now."}, "sentence_type": "Interrogative sentence", "pos": {"nouns": {"Questionnaire": 1, "Entry": 1, "self": 1}, "verbs": {"I feel": 1, "Shit": 1}, "adverbs": {"Regular": 1}, "numerals": {"5": 1}, "pronouns": {"you": 1}}}, {"content": "6. In general, how confident are you in your abilities?", "word_count": 16, "label": "", "sentence_structure": "Subject鈥搗erb鈥搊bject", "subjective_objective": {"answer": "Subjective", "rationale": "Because self-confidence is a subjective feeling and attitude of the individual."}, "sentence_sentiment": {"answer": "Positive", "rationale": "Because the entry asks about the level of confidence in its abilities, it is a self-affirmative attitude and is therefore positive."}, "language_style": {"answer": "Tactful and warm", "rationale": "The entry is more euphemistic. It is more compassionate and interactive, using a question-and-answer tone rather than direct orders or statements. At the same time, the question does not overemphasize the importance of individual confidence, but rather raises a question in a more objective way that allows respondents to respond to their own reality, which also makes it easier to resonate and think."}, "time_dimension": {"answer": "Present", "rationale": "The entry asks about the current feelings and attitudes of the testee and does not address past or future acts or events, and therefore the judgement is now."}, "sentence_type": "Interrogative sentence", "pos": {"nouns": {"Questionnaire Entry": 1, "Confidence": 1}, "verbs": {"General": 1, "have": 1}, "adjectives": {"Large": 1, "Your own": 1}, "adverbs": {"More": 1}, "numerals": {"6": 1}}}, {"content": "Do you often feel that you can't do anything well?", "word_count": 16, "label": "", "sentence_structure": "Subject鈥搗erb鈥搊bject", "subjective_objective": {"answer": "Subjective", "rationale": "The problem is examined by the patient ' s own feelings and perspectives and by the subjective perspective."}, "sentence_sentiment": {"answer": "Negative", "rationale": "Because the expression \"likes that you can't do anything well\" is filled with negative sentiments, expressing doubts about and denial of self-capacity, is a negative attitude or emotion."}, "language_style": {"answer": "Calm and direct", "rationale": "The entry asks in a direct, clear language style whether the other party has a regular experience of self-denial, without too many emotional rendering or rhetorical techniques, and is more calm and objective."}, "time_dimension": {"answer": "Present", "rationale": "The entry asks the subject about the current mood and experience, the keyword being \"frequent\" and \"fE\" and all point to the state of the process now under way."}, "sentence_type": "Interrogative sentence", "pos": {"nouns": {"you": 1, "Yes": 2}, "verbs": {"I always feel": 1, "I can't do it": 1}, "adverbs": {"The long run": 1}, "numerals": {"7": 1}}}, {"content": "How worried are you about getting along with others?", "word_count": 16, "label": "", "sentence_structure": "Subject鈥搗erb鈥搊bject", "subjective_objective": {"answer": "Subjective", "rationale": "Because the level of concern is a subjective feeling and assessment of one."}, "sentence_sentiment": {"answer": "Neutral", "rationale": "Because the entry does not have any emotional color."}, "language_style": {"answer": "Tactful and warm", "rationale": "The language style of the entry is euphemistic, and it enhances interaction by using the term \"concern\" to express concern about the other side, while avoiding direct inquiries about issues that may cause discomfort."}, "time_dimension": {"answer": "Present", "rationale": "The entry asks about the present mood and feelings of the subject, so it can be judged that the time dimension of the article is \"now\"."}, "sentence_type": "Interrogative sentence", "pos": {"nouns": {"Questionnaire": 1, "Entry": 1, "Others": 1}, "verbs": {"to": 1, "Get along": 1, "I'm worried": 1}, "adjectives": {"Large": 1}, "adverbs": {"How's that": 1}, "numerals": {"8": 1}, "pronouns": {"you": 1}}}, {"content": "9. Do you often fear criticism from teachers or employers for what you have done?", "word_count": 24, "label": "", "sentence_structure": "Subject鈥搗erb鈥搊bject", "subjective_objective": {"answer": "Subjective", "rationale": "Because \"fear\" is the patient's subjective sentiment."}, "sentence_sentiment": {"answer": "Negative", "rationale": "Because the mood for \"fear of criticism\" in the entry is negative."}, "language_style": {"answer": "Tactful and warm", "rationale": "The entry is more personal and interactive when it is spoken in a gentle and inviting manner, rather than in direct statements or orders. At the same time, the expression \"invited criticism from teachers or employers\" is more euphemismal and avoids pressure or negative feelings towards the interviewees."}, "time_dimension": {"answer": "Present", "rationale": "The temporal dimension of the purpose of the article should therefore be judged as present and future."}, "sentence_type": "Interrogative sentence", "pos": {"nouns": {"you": 1, "Regular": 1, "I'm worried": 1, "By": 1, "What I did": 1, "Teacher": 1, "Employers": 1, "Criticism": 1}, "verbs": {"Is it possible": 1, "Present": 1}, "adverbs": {"Regular": 1}, "numerals": {"9": 1}, "pronouns": {"you": 1}}}, {"content": "10. When you walked into one room, where you had gathered others and were talking, did you ever feel fear and anxiety?", "word_count": 33, "label": "", "sentence_structure": "Subject鈥搗erb鈥搊bject", "subjective_objective": {"answer": "Subjective", "rationale": "Because \"fear and anxiety\" is a subjective emotional reaction."}, "sentence_sentiment": {"answer": "Negative", "rationale": "Because the \"fear and anxiety\" described in the entry is a negative emotional experience."}, "language_style": {"answer": "Calm and direct", "rationale": "The entry is in the form of a direct question-and-answer period, with no excessive emotional rendering or accoutrements. At the same time, negative emotional terms such as \"fear and anxiety\" clearly indicate the subject matter. Therefore, the language style can be considered as calm and straightforward."}, "time_dimension": {"answer": "Past", "rationale": "The entry asks if the person tested has experienced some emotion or feeling in a particular situation (i.e., entering a room where others are talking)."}, "sentence_type": "Interrogative sentence", "pos": {"nouns": {"Room": 1, "person": 2, "Talk": 1, "Scared": 1, "Anxiety": 1}, "verbs": {"Walk in": 1, "Gather": 1, "I feel": 1, "Talk": 1}, "adjectives": {"Other": 1, "Already": 1}, "numerals": {"10": 1, "2": 1}, "classifiers": {"individual": 1}, "pronouns": {"you": 2}}}, {"content": "Do you often feel disturbed?", "word_count": 11, "label": "", "sentence_structure": "Subject鈥搗erb鈥搊bject", "subjective_objective": {"answer": "Subjective", "rationale": "Because \"frightful\" is the patient's subjective feeling and experience."}, "sentence_sentiment": {"answer": "Negative", "rationale": "Because the feeling of \"frightfulness\" in the entries is negative."}, "language_style": {"answer": "Tactful and warm", "rationale": "Warm and warm."}, "time_dimension": {"answer": "Present", "rationale": "The entry \"Does it feel often\" represents an inquiry and an evaluation of the current state, and therefore a judgement is now made."}, "sentence_type": "Interrogative sentence", "pos": {"nouns": {"you": 1, "Regular": 1, "I feel": 1, "It's a little disturbing": 1}, "adverbs": {"Is it possible": 1, "Regular": 1}, "numerals": {"11": 1}}}, {"content": "12. How much fear do you have in your work or your studies of others seeing you as a success or a loser?", "word_count": 32, "label": "", "sentence_structure": "Subject鈥搗erb鈥搊bject", "subjective_objective": {"answer": "Subjective", "rationale": "The problem is subjective because it relates to the level of the patient ' s inner feelings and fears."}, "sentence_sentiment": {"answer": "Negative", "rationale": "Because the word \"fear\" in the entry is negative."}, "language_style": {"answer": "Tactful and warm", "rationale": ""}, "time_dimension": {"answer": "Present", "rationale": "The entry asks the extent to which the person who is tested is concerned about the perception of others in the current work or study, without reference to past or future behaviour or mindset."}, "sentence_type": "Interrogative sentence", "pos": {"nouns": {"Work": 1, "Learning": 1, "person": 1, "Consider": 1, "Successor": 1, "Loser": 1, "I'm worried": 1}, "verbs": {"have": 1, "be": 1}, "adjectives": {"Other": 1}, "adverbs": {"How old": 1}, "numerals": {"12": 1}, "pronouns": {"you": 1}}}, {"content": "13. Is it difficult for you to find a decent conversation among the crowd?", "word_count": 22, "label": "", "sentence_structure": "Subject鈥搗erb鈥搊bject", "subjective_objective": {"answer": "Subjective", "rationale": "Because this is a question of asking patients whether they find it difficult to find the right topic in the population. This requires their personal experience, emotional and intellectual ability to answer, and therefore is a subjective approach."}, "sentence_sentiment": {"answer": "Negative", "rationale": "Because the expression \"to find a decent conversation is difficult for you\" has a negative emotional effect."}, "language_style": {"answer": "Tactful and warm", "rationale": "The language style of the entry is euphemistic. It uses more euphemistic and friendly language to ask whether it is difficult for the respondents to find suitable topics for conversation in the population, avoiding direct accusations or criticism."}, "time_dimension": {"answer": "Present", "rationale": "The entry is for the testee to ask about the current behaviour and mindset, and the time dimension is now judged."}, "sentence_type": "Interrogative sentence", "pos": {"nouns": {"Population": 1, "Talk": 1}, "verbs": {"Found it": 1, "It's hard": 1}, "adjectives": {"That's right": 1}, "numerals": {"13": 1}}}, {"content": "How long will it take to forget something that makes you look stupid when you make an embarrassing mistake or do something that makes you look stupid?", "word_count": 43, "label": "", "sentence_structure": "Subject鈥搗erb鈥搊bject", "subjective_objective": {"answer": "Subjective", "rationale": "Because this question is about how long it takes patients to forget their own mistakes or stupid behaviour, it is about personal feelings and experiences and is therefore subjective."}, "sentence_sentiment": {"answer": "Negative", "rationale": "Because the entries \"make an embarrassing mistake\" and \"look stupid\" have negative emotional connotations."}, "language_style": {"answer": "Tactful and warm", "rationale": ""}, "time_dimension": {"answer": "Past", "rationale": "The dimension of time is the past and the future. Because this can happen at any time, and since everyone has different views on how to forget it, there is no clear point of reference for time."}, "sentence_type": "Interrogative sentence", "pos": {"nouns": {"Problem": 1, "Error": 2, "Time": 1, "Things": 1, "It's so sad": 1, "Stupid": 1, "you": 2, "Cost": 1}, "verbs": {"Yes": 2, "Do it": 1, "鈾?And I'm gonna be 鈾?: 1, "Looks like": 1, "Flowers": 1, "I'm not sure": 1, "Okay": 1}, "adjectives": {"I'm embarrassed": 1, "Stupid": 1}, "numerals": {"14": 1, "2": 1, "1": 5}, "classifiers": {"individual": 1}, "pronouns": {"it": 1}}}, {"content": "Do you often feel uncomfortable when you meet strangers?", "word_count": 14, "label": "", "sentence_structure": "Subject鈥搗erb鈥搊bject", "subjective_objective": {"answer": "Subjective", "rationale": "Because \"often feeling uncomfortable\" is a subjective experience and feeling."}, "sentence_sentiment": {"answer": "Negative", "rationale": "Because the entry \"frightfully\" expresses a negative emotional attitude."}, "language_style": {"answer": "Tactful and warm", "rationale": "The language style of the entry is more euphemistic and warm, making the question more gentle and easy to answer by asking the person who is not feeling comfortable with the person in question when he meets him, rather than directly accusing or criticizing him. This expression helps to increase the interaction and trust of the person under investigation."}, "time_dimension": {"answer": "Present", "rationale": "The keyword \"when meeting a stranger\", \"frightfully\" means the person who was tested in his current state or feeling, and the entry describes the person's current behaviour and mentality."}, "sentence_type": "Interrogative sentence", "pos": {"nouns": {"Stranger": 1, "Entry": 1}, "verbs": {"I met him": 1, "Common sense": 1, "I'm not comfortable": 1, "- You're not": 1}, "adjectives": {"Regular": 1, "I'm not comfortable": 1}, "adverbs": {"- You're not": 1}, "numerals": {"15": 1}}}, {"content": "16. Do you often worry that others will not be willing to be with you?", "word_count": 19, "label": "", "sentence_structure": "Subject鈥搗erb鈥搊bject", "subjective_objective": {"answer": "Subjective", "rationale": "Because \"care\" is a person's inner feeling and thoughts, and it's subjective."}, "sentence_sentiment": {"answer": "Negative", "rationale": "Because the emotional expression \"regarding\" in the entry is negative."}, "language_style": {"answer": "Tactful and warm", "rationale": "Warm and warm."}, "time_dimension": {"answer": "Present", "rationale": "The entry uses the general current and asks the testee about his current behaviour or state, and therefore is judged to be \"now\"."}, "sentence_type": "Interrogative sentence", "pos": {"nouns": {"Questionnaire": 1, "Entry": 1, "person": 2, "Together": 1}, "verbs": {"be": 1, "Yes": 1, "I'm worried": 1, "Same": 1, "Together": 1}, "adverbs": {"Regular": 1}, "numerals": {"16": 1}, "pronouns": {"you": 1}}}, {"content": "Do you often get bothered by shame?", "word_count": 11, "label": "", "sentence_structure": "Subject鈥搗erb鈥搊bject", "subjective_objective": {"answer": "Subjective", "rationale": "Because shyness is the emotion that patients feel and is a subjective observation."}, "sentence_sentiment": {"answer": "Negative", "rationale": "Because \"weakness\" is usually seen as a negative emotion or quality."}, "language_style": {"answer": "Calm and direct", "rationale": "The language style of the entry is more direct, there is no excessive rhetoric or emotional rendering, and it is objective and clear."}, "time_dimension": {"answer": "Present", "rationale": ""}, "sentence_type": "Interrogative sentence", "pos": {"nouns": {"Shame": 1, "Disturbing": 1}, "verbs": {"Is it possible": 1, "Regular": 1, "Yes": 1}, "adverbs": {"Sou-san": 1, "of": 2}, "numerals": {"17": 1}, "pronouns": {"you": 1}}}, {"content": "18. How much concern or credit do you have when you think that some of the people you meet have a bad opinion of you?", "word_count": 28, "label": "", "sentence_structure": "Subject鈥搗erb鈥搊bject", "subjective_objective": {"answer": "Subjective", "rationale": "Because \"fear\" is the patient's subjective feeling and attitude."}, "sentence_sentiment": {"answer": "Negative", "rationale": "Because the entries \"bad perception of you\" and \"assurance\" have negative emotional aspects."}, "language_style": {"answer": "Tactful and warm", "rationale": "The entry is more proficient and interactive by speaking in a gentle, inquisitive manner, rather than by direct statement or order. At the same time, the term \"magnitude\" also reflects the care that the questionnaire designer seeks to understand what the interviewer feels inside."}, "time_dimension": {"answer": "Past", "rationale": "The keyword \"facing,\" \"depressive\" or \"too much concern\" points to past experience or what it feels now. The entry therefore contains both past information and present circumstances, and can be judged as past/now."}, "sentence_type": "Interrogative sentence", "pos": {"nouns": {"Some": 2, "person": 2, "Observations": 1, "Bad": 1}, "verbs": {"I met him": 1, "Attention": 1, "You're a good man": 1}, "adverbs": {"More": 1}, "numerals": {"18": 1}, "classifiers": {"individual": 2}, "pronouns": {"you": 1}}}, {"content": "Have you often been anxious or disturbed by what others think of you?", "word_count": 22, "label": "", "sentence_structure": "Subject鈥搗erb鈥搊bject", "subjective_objective": {"answer": "Subjective", "rationale": "Because it is the patient ' s feelings and attitudes towards the views of others that are examined and the subjective perspective."}, "sentence_sentiment": {"answer": "Negative", "rationale": "Because the entry \"anxiety or anxiety\" expresses a negative mood and attitude."}, "language_style": {"answer": "Tactful and warm", "rationale": "The entry is more intimate and interactive by means of a dialogue by means of inquiry rather than direct statements or orders, and by way of a more moderate and inviting expression."}, "time_dimension": {"answer": "Present", "rationale": "The keyword \"often\" means that it is a customary, repetitive act, while \"fearing or uneasy\" describes the person's immediate emotional state and psychological experience. So, the entry asks about the person's current feelings and behaviour, judging by the present."}, "sentence_type": "Interrogative sentence", "pos": {"nouns": {"Questionnaire Entry": 1, "Observations": 1, "Anxiety": 1, "Unsettled": 1}, "verbs": {"Is it possible": 2, "Regular": 1, "I feel": 1, "Because": 1, "to": 1, "Anxiety or anxiety": 1}, "adjectives": {"Other": 1, "Yours": 2}, "numerals": {"19": 1}, "pronouns": {"you": 2}}}, {"content": "How worried or anxious are you if you have to read an article in class and understand its meaning?", "word_count": 31, "label": "", "sentence_structure": "Subject鈥搗erb鈥搊bject", "subjective_objective": {"answer": "Subjective", "rationale": "Because this is a matter of emotional reaction and attitude."}, "sentence_sentiment": {"answer": "Negative", "rationale": "Because the mood of \"fear or anxiety\" in the entry is negative."}, "language_style": {"answer": "Tactful and warm", "rationale": ""}, "time_dimension": {"answer": "Present", "rationale": "The time frame of the entry is assessed in the light of the current situation, i.e. the degree of anxiety and feelings that students experience when reading and understanding articles. The entry should therefore be classified as \"now\"."}, "sentence_type": "Interrogative sentence", "pos": {"nouns": {"Classroom": 1, "Articles": 1, "Meaning": 1, "I'm worried": 1, "Anxiety": 1}, "verbs": {"Reading": 1, "I have to": 1, "Get it": 1}, "adjectives": {"Here": 1, "This": 1}, "numerals": {"20": 1}, "pronouns": {"you": 1}}}, {"content": "21. How worried or anxious are you when you have to write an opinion to convince teachers who may have different views from you?", "word_count": 34, "label": "", "sentence_structure": "Subject鈥搗erb鈥搊bject", "subjective_objective": {"answer": "Subjective", "rationale": "Because anxiety or anxiety is a subjective feeling and expectation of a possible future event."}, "sentence_sentiment": {"answer": "Negative", "rationale": "Because \"fear or anxiety\" is negative."}, "language_style": {"answer": "Tactful and warm", "rationale": "The language style of the entry is euphemistic. The basis of the judgement is that it first highlights the difference between \"your\" views and \"teachers who may have different views\" in order to stimulate the thoughts of the interviewees. Then questions are asked whether the interviewees will be worried or anxious about such situations, without expressing their own attitude or giving answers. Moreover, the purpose of the article is more peaceful, friendly, without harsh or accusatory language, and more emphasizes objectivity and neutrality of the investigation. Such language makes the interviewees more willing to share their true ideas and to gain their trust and cooperation."}, "time_dimension": {"answer": "Present", "rationale": "\"21. How worried or anxious are you when you have to write an opinion to convince teachers who may have different views from you?\" This entry is to question the current emotional state of the testee, and therefore the time dimension is now."}, "sentence_type": "Interrogative sentence", "pos": {"nouns": {"Observations": 2, "Maybe": 1, "Teacher": 1}, "verbs": {"Write": 1, "I have to": 1, "Convince": 1, "have": 1, "I'm worried": 1, "Anxiety": 1}, "adverbs": {"When": 1, "This": 1}, "numerals": {"21": 1}, "classifiers": {"Grandpa": 1}}}, {"content": "22. Do you often find it difficult to complete such exercises as the expression of your views in writing?", "word_count": 28, "label": "", "sentence_structure": "Subject鈥搗erb鈥搊bject", "subjective_objective": {"answer": "Subjective", "rationale": "This is a subjective question."}, "sentence_sentiment": {"answer": "Negative", "rationale": "Because the description of \"difficult\" in the entry is negative."}, "language_style": {"answer": "Tactful and warm", "rationale": "So the entry can be judged as \"sweetly\" language style."}, "time_dimension": {"answer": "Present", "rationale": ""}, "sentence_type": "Interrogative sentence", "pos": {"nouns": {"Questionnaire": 1, "Entry": 1, "Views": 1, "Operations": 1, "Difficulties": 1}, "verbs": {"Completed": 1, "Expression": 1, "I feel": 1, "Regular": 1}, "adjectives": {"Written form": 1, "Your own": 1}, "numerals": {"22": 1}}}, {"content": "23. How often do difficulties arise in classroom reading and understanding exercises?", "word_count": 22, "label": "", "sentence_structure": "Subject鈥搗erb鈥搊bject", "subjective_objective": {"answer": "Objective", "rationale": "Because \"how often difficult it is\" is a quantifiable question that can be answered by real data. At the same time, it does not look at the patient's thoughts, feelings or subjective attitudes, but rather directly asks about the facts that are objectively present."}, "sentence_sentiment": {"answer": "Neutral", "rationale": "Because the entry merely describes an phenomenon objectively, it does not involve positive or negative emotional colours."}, "language_style": {"answer": "Calm and direct", "rationale": "The entry asked the interviewees in a direct and clear manner about the actual situation, without excessive emotional or rhetorical tacts and with a more objective and calm tone."}, "time_dimension": {"answer": "Present", "rationale": "\"Now\" is an entry that asks the subject about the experience and behaviour that occur in the current situation, that is, the frequency of difficulties encountered in the classroom reading and understanding exercise. The temporal dimension is therefore judged as \"now\"."}, "sentence_type": "Interrogative sentence", "pos": {"nouns": {"Classroom": 1, "Read understanding": 1, "Practice": 1, "Situation": 1, "Difficulties": 1}, "verbs": {"have": 1, "Pong": 1, "Present": 1}, "adjectives": {"Frequent": 1}, "numerals": {"23": 1}, "classifiers": {"individual": 1}}}, {"content": "Do you often imagine that you are less capable of learning than your classmates?", "word_count": 18, "label": "", "sentence_structure": "Subject鈥搗erb鈥搊bject", "subjective_objective": {"answer": "Subjective", "rationale": "Because \"imagina\" is patient's subjective attitude."}, "sentence_sentiment": {"answer": "Negative", "rationale": "Because the phrase \"less than the classmates\" in the entry is negative."}, "language_style": {"answer": "Calm and direct", "rationale": "This language style is more concise and understandable and suitable for use in such situations as questionnaires, where a great deal of information is required."}, "time_dimension": {"answer": "Present", "rationale": ""}, "sentence_type": "Interrogative sentence", "pos": {"nouns": {"self": 1, "Learning ability": 1, "Students": 1}, "verbs": {"Is it possible": 1, "Imagine": 1, "Bad": 1}, "adverbs": {"Regular": 1}, "numerals": {"24": 1}}}, {"content": "25. When you presented a paper on a major job, did you think you did a lot of good?", "word_count": 29, "label": "", "sentence_structure": "Subject鈥搗erb鈥搊bject", "subjective_objective": {"answer": "Subjective", "rationale": "Because \"think\" is the patient's subjective attitude."}, "sentence_sentiment": {"answer": "Positive", "rationale": "Because the \"excellent\" in the entry is a positive assessment of your behavior."}, "language_style": {"answer": "Tactful and warm", "rationale": "The language style of this entry is more euphemistic, using positive words like \"excellent\" and \"multiple\" and leading the respondents to think and answer by asking questions. This expression is more personal and inclusive than direct orders or criticism."}, "time_dimension": {"answer": "Present", "rationale": "The entry asks the testee for an evaluation of the current status and therefore belongs to the present."}, "sentence_type": "Interrogative sentence", "pos": {"nouns": {"Grandpa": 1, "Important jobs": 1, "Thesis": 1, "Number": 1}, "verbs": {"I think": 1, "You did a great job": 1}, "adverbs": {"More": 1}, "numerals": {"25": 1}, "pronouns": {"self": 1}}}, {"content": "26. Do you feel that you must learn more to achieve the same results as your fellow students?", "word_count": 36, "label": "", "sentence_structure": "Subject鈥搗erb鈥搊bject", "subjective_objective": {"answer": "Subjective", "rationale": "This is a subjective assessment of the patient鈥檚 level of effort in learning, which cannot be measured by objective data."}, "sentence_sentiment": {"answer": "Negative", "rationale": "Because the \"must learn to work harder\" is negative emotional."}, "language_style": {"answer": "Tactful and warm", "rationale": "The entry is more personal and interactive when it is presented in a gentle and inviting tone, rather than in direct statements or orders. It also does not use too strong or negative terms, making the problem more amenable and making it easier for respondents to share their own situations."}, "time_dimension": {"answer": "Present", "rationale": "The entry is therefore an inquiry into the present behaviour or mentality of the person being tested."}, "sentence_type": "Interrogative sentence", "pos": {"nouns": {"Situation": 1, "Students": 1, "Achievements": 1}, "verbs": {"I feel": 1, "I have to": 1, "Learning": 2, "Access": 1, "Compare": 1, "Try harder": 1, "Talent": 1, "Same thing": 1, "More": 1}, "adverbs": {"Same as them": 1, "More": 1}, "numerals": {"26": 1}, "pronouns": {"you": 1, "self": 1, "they": 1}}}, {"content": "Have you ever been ashamed of your physical or image?", "word_count": 16, "label": "", "sentence_structure": "Subject鈥搗erb鈥搊bject", "subjective_objective": {"answer": "Subjective", "rationale": "Because shame is a manifestation of subjective emotions and feelings, it is the subjective assessment of the patient ' s body image."}, "sentence_sentiment": {"answer": "Negative", "rationale": "Because the expression of feeling \"smear\" in the entry is negative."}, "language_style": {"answer": "Tactful and warm", "rationale": "Warm and warm."}, "time_dimension": {"answer": "Past", "rationale": ""}, "sentence_type": "Interrogative sentence", "pos": {"nouns": {"Body": 1, "Image": 1, "self": 1}, "verbs": {"Tsang": 1, "Yes": 1, "I feel": 1, "Shame": 1}, "adverbs": {"- You're not": 1}, "numerals": {"27": 1}, "pronouns": {"you": 1}}}, {"content": "Do you often feel that most of your friends or companions are physically more attractive than you?", "word_count": 26, "label": "", "sentence_structure": "Subject鈥搗erb鈥搊bject", "subjective_objective": {"answer": "Subjective", "rationale": "Because this is what patients are subjective about themselves."}, "sentence_sentiment": {"answer": "Negative", "rationale": "Because the phrase \"often feels more attractive to your friends or companions than to yourself\" in the entry is negative."}, "language_style": {"answer": "Tactful and warm", "rationale": "This language style is more friendly and gentle, helping to create a good atmosphere of communication and easier access to the trust and cooperation of the interviewee."}, "time_dimension": {"answer": "Past", "rationale": "The entry is for the testee's past feelings and experiences, and the keyword \"often feels\" indicates that it is a description of past behaviour. The temporal dimension is therefore a matter of past."}, "sentence_type": "Interrogative sentence", "pos": {"nouns": {"Questionnaire": 1, "Entry": 1, "friends": 1, "Companion": 1, "Body": 1, "Charm": 1}, "verbs": {"I feel": 1, "- Yeah": 1, "More": 1}, "adjectives": {"Often": 1}, "numerals": {"28": 1}}}, {"content": "Do you often wish or fantasize to be more beautiful?", "word_count": 17, "label": "", "sentence_structure": "Subject鈥搗erb鈥搊bject", "subjective_objective": {"answer": "Subjective", "rationale": "Because \"hope or fantasies\" are patients' subjective attitudes and ideas."}, "sentence_sentiment": {"answer": "Positive", "rationale": "Because the \"be prettier\" expectation in the entry is positive."}, "language_style": {"answer": "Tactful and warm", "rationale": "This language style makes it easier for the interviewee to feel comfortable and to share his real thoughts."}, "time_dimension": {"answer": "Present", "rationale": "The word \"normal\" in \"you often\" indicates regular, customary behaviour, while \"hope\" and \"fanta\" adopt the general current, meaning that this behaviour has not only happened in the past, but may still be ongoing. So the temporal dimension of this questionnaire entry is now."}, "sentence_type": "Interrogative sentence", "pos": {"nouns": {"Questionnaire Entry": 1, "Nice": 1}, "verbs": {"Hope": 1, "Fantasy": 1, "Change": 1}, "adverbs": {"Often": 1}, "numerals": {"29": 1}}}, {"content": "Have you ever been worried or anxious about your ability to attract heterosexuals?", "word_count": 22, "label": "", "sentence_structure": "Subject鈥搗erb鈥搊bject", "subjective_objective": {"answer": "Subjective", "rationale": "This is because patients feel and assess their abilities."}, "sentence_sentiment": {"answer": "Negative", "rationale": "Because the \"fear or anxiety\" in the entry is negative."}, "language_style": {"answer": "Tactful and warm", "rationale": "The entry leads the interviewer to think about his feelings by asking questions, which is relatively mild, and does not directly express his assessment or judgment. At the same time, the more intimate term \"the opposite sex\" is used to describe the target population, which is more resonant and open to the interviewee. So I think the language style of the purpose is euphemism."}, "time_dimension": {"answer": "Past", "rationale": ""}, "sentence_type": "Interrogative sentence", "pos": {"nouns": {"Heterosexual": 1, "self": 2, "Capacity": 1, "I'm worried": 1, "Anxiety": 1}, "verbs": {"Attractive": 1, "I feel": 1, "I was worried": 1, "I'm worried": 1}, "adverbs": {"Tsang": 1}, "numerals": {"30": 1}, "pronouns": {"you": 1}}}, {"content": "What is your confidence in what others think you are attractive?", "word_count": 23, "label": "", "sentence_structure": "Subject鈥搗erb鈥搊bject", "subjective_objective": {"answer": "Subjective", "rationale": "Because the problem is examined by the patient ' s subjective attitude and confidence."}, "sentence_sentiment": {"answer": "Positive", "rationale": "Since the entry asks about the level of confidence, which is usually considered a positive emotion and attitude."}, "language_style": {"answer": "Calm and direct", "rationale": "The entry asks the interviewee in a direct and clear way about his degree of external appeal and confidence, without excessive embellishment or emotional rendering."}, "time_dimension": {"answer": "Present", "rationale": "The entry asks the testee's current state or feeling, which is, \"How confident do you feel about your appearance?\" and therefore belongs to the time dimension of the present."}, "sentence_type": "Interrogative sentence", "pos": {"nouns": {"Questionnaire": 1, "Entry": 1, "person": 2, "Appearance": 1, "Attractive": 1, "Confidence": 1, "Level": 1}, "verbs": {"have": 1, "I think": 1, "be": 1, "How much": 1, "Large": 1, "It's attractive": 1, "I think you're attractive": 1}, "adjectives": {"Other": 1}, "adverbs": {"More": 1}, "numerals": {"31": 1}, "pronouns": {"you": 1}}}, {"content": "Have you ever thought about your physical incoherence?", "word_count": 14, "label": "", "sentence_structure": "Subject鈥搗erb鈥搊bject", "subjective_objective": {"answer": "Subjective", "rationale": "Because \"want\" is a subjective verb, which means the patient's inner feeling or thinking."}, "sentence_sentiment": {"answer": "Negative", "rationale": "Because the expression \"physical disharmony\" in the entry is negative, implying dissatisfaction with or inferiority to self-image."}, "language_style": {"answer": "Tactful and warm", "rationale": "The entry is more adept and interactive when it is spoken in a gentle and inviting manner, rather than by direct statements or orders."}, "time_dimension": {"answer": "Present", "rationale": "In the past, the entry asked the person tested whether there had been any misalignment or concern for his body, and the temporal dimension was judged to be past."}, "sentence_type": "Interrogative sentence", "pos": {"nouns": {"self": 1, "Body": 1, "Coordination": 1}, "verbs": {"Yes": 1, "Pass": 1}, "numerals": {"32": 1}, "pronouns": {"you": 1}}}, {"content": "33. Do you feel less capable of sports than most others?", "word_count": 23, "label": "", "sentence_structure": "Subject鈥搗erb鈥搊bject", "subjective_objective": {"answer": "Subjective", "rationale": "Because \"fear\" is the patient's personal feeling and perception, it's a subjective question."}, "sentence_sentiment": {"answer": "Negative", "rationale": "Because the expression \"unlike most other people\" in the entry contains negative feelings, implying a sense of weakness and self-demeaning that is tried in sports."}, "language_style": {"answer": "Calm and direct", "rationale": "The entry expresses a direct language of comparison of the sports abilities of the respondents, with no excessive adoration or emotional colour, and with a more objective tone."}, "time_dimension": {"answer": "Present", "rationale": "The entry asks the testee's current feelings and attitudes, does not relate to the past or future time dimension, and therefore the judgement is now."}, "sentence_type": "Interrogative sentence", "pos": {"nouns": {"you": 1, "Sports": 1, "Capacity": 1, "Others": 1}, "verbs": {"I feel": 1, "Why don't you": 1, "Other": 1}, "adjectives": {"Most of them": 1}, "numerals": {"33": 1}}}, {"content": "34. When you take part in sports events that require physical coordination, you're often worried about yourself being bad?", "word_count": 30, "label": "", "sentence_structure": "Subject鈥搗erb鈥搊bject", "subjective_objective": {"answer": "Subjective", "rationale": "Because \"fear\" is the patient's personal emotion and subjective feeling."}, "sentence_sentiment": {"answer": "Negative", "rationale": "Because the attitude of \"a fear of being bad\" in the entry is negative."}, "language_style": {"answer": "Tactful and warm", "rationale": "The entry is more softly phrased. It uses a question-and-answer approach to ask if the interviewees are worried about participating in sports, which is more resonant and participatory than direct statements. At the same time, the question-and-answer type of \"You're often afraid you're not doing well?\" gives the interviewee the space to express his thoughts without creating a sense of pressure or blame."}, "time_dimension": {"answer": "Present", "rationale": "Both the verbs \"participating\" and \"respect\" follow the current state of affairs, and the word \"normal\" denotes a regular, customary behaviour, all of which point to the current situation. The entry therefore asks the testee about his current behaviour or mentality and judges it as the present."}, "sentence_type": "Interrogative sentence", "pos": {"nouns": {"Sports activities": 1, "Physical coordination": 1}, "verbs": {"Participation": 1, "I'm worried": 1, "I can't do it": 1}, "adverbs": {"Regular": 1}, "numerals": {"34": 1}, "pronouns": {"you": 2}}}, {"content": "Have you ever thought about your lack of dancing skills or your lack of long search for physical coordination?", "word_count": 33, "label": "", "sentence_structure": "Subject鈥搗erb鈥搊bject", "subjective_objective": {"answer": "Subjective", "rationale": "Because lack of dance skills and physical coherency of physical exercise is the patient ' s own feeling and perception, it is a subjective perspective."}, "sentence_sentiment": {"answer": "Negative", "rationale": "Because the words \"lack of dancing skills\" and \"not good at physical harmony\" are all negative emotional expressions in the entries."}, "language_style": {"answer": "Tactful and warm", "rationale": "Warm and warm."}, "time_dimension": {"answer": "Past", "rationale": "The entry asks whether the person tested had had or was not confident about his or her ability to dance or his or her ability to coordinate his or her physical exercise. The keyword \"have you ever thought of\" and \"have you ever had the ability to dance, or are not good at sports involving physical coordination?\" all points to past or present experiences and feelings."}, "sentence_type": "Interrogative sentence", "pos": {"nouns": {"you": 2, "That's right": 1, "Dance": 2, "Sports": 1, "Coordination": 1}, "verbs": {"Yes": 2, "Missing": 1, "to": 1, "Chief": 1, "Involving": 1}, "adjectives": {"self": 1, "Body": 1}, "numerals": {"35": 1, "One": 2}}}, {"content": "36. Do you feel uneasy or frightened when you try to be good at a sports event and you know that others are watching?", "word_count": 40, "label": "", "sentence_structure": "Subject鈥搗erb鈥搊bject", "subjective_objective": {"answer": "Subjective", "rationale": "This is a subjective feeling and emotional reaction of the patient, not an objective fact."}, "sentence_sentiment": {"answer": "Negative", "rationale": "Because the act of \"disturbing or frightened\" in the entry is negative."}, "language_style": {"answer": "Calm and direct", "rationale": "The entry is more calm and straightforward in language, it clearly presents a specific scene (i.e., in competitions) and asks if the interviewee feels uneasy or frightened in such a situation."}, "time_dimension": {"answer": "Present", "rationale": "The key words \"will\" and \"watching\" are both descriptions of the situation."}, "sentence_type": "Interrogative sentence", "pos": {"nouns": {"Sports activities": 1, "Others": 1}, "verbs": {"Try": 1, "Yes": 2, "- Show some respect": 1, "Watch": 1, "It seems": 1, "Unsettled": 1, "Fear": 1}, "adverbs": {"Yes": 2, "In the process of": 1}, "numerals": {"36": 1}, "pronouns": {"you": 1}}}]}

{"questionnaire_title": "A table of subjective well-being for adolescents", "questionnaire_category": "Emotion and affect", "item_count": 36, "emotion_shift_overall": "From negative to positive", "language_style_overall": "Tactful and warm", "items": [{"content": "One, my friends respect me.", "word_count": 9, "label": "", "sentence_structure": "Subject鈥搗erb鈥搊bject", "subjective_objective": {"answer": "Subjective", "rationale": "Because \"fully respectful of me\" is a patient's self-evaluation and perception, a subjective description."}, "sentence_sentiment": {"answer": "Positive", "rationale": "Basis: The expression \"very respectful of me\" is included in the statement of the purpose of this article, which expresses a positive feeling of respect by others."}, "language_style": {"answer": "Tactful and warm", "rationale": "The entry is more personal and interactive by means of a gentle and inviting tone of dialogue rather than by direct statements or orders. At the same time, the article ' s tone is more positive, free of negative emotional influences, and more likely to arouse the resonance and willingness of the person under investigation to respond."}, "time_dimension": {"answer": "Present", "rationale": "The keyword \"my friends respect me\" means that we're in the process of doing something or in the state."}, "sentence_type": "Interrogative sentence", "pos": {"nouns": {"friends": 1, "Respect": 1}, "verbs": {"very": 1, "Both": 1}, "adjectives": {"Mine": 1, "very": 1}, "classifiers": {"One": 1}}}, {"content": "I like being with my parents.", "word_count": 11, "label": "", "sentence_structure": "Subject鈥搗erb鈥搊bject", "subjective_objective": {"answer": "Subjective", "rationale": "Because it is the emotional attitude of the individual, whether or not you like it, it is subjective."}, "sentence_sentiment": {"answer": "Positive", "rationale": "Because the \"likes to be with my parents\" behavior in the entry is positive."}, "language_style": {"answer": "Tactful and warm", "rationale": "The entry is more personal and interactive in a more euphemistic way in which the other party is asked whether he or she prefers to be with his or her parents rather than to make a direct statement or order."}, "time_dimension": {"answer": "Present", "rationale": "The entry \"likes to be with my parents\" expresses a current behavior or state, so it's judged now."}, "sentence_type": "Interrogative sentence", "pos": {"nouns": {"Questionnaire": 1, "Entry": 1, "I like it": 1, "parents": 1}, "verbs": {"Yeah": 1, "in": 1, "Together": 1}, "classifiers": {"2銆?: 1}}}, {"content": "I'm not feeling well at school.", "word_count": 10, "label": "", "sentence_structure": "Subject鈥揷opula鈥損redicative", "subjective_objective": {"answer": "Subjective", "rationale": "The subject is a matter of the patient ' s feelings in school and is a subjective one."}, "sentence_sentiment": {"answer": "Negative", "rationale": "Because the \"uncomfortable\" mood in the entry is negative."}, "language_style": {"answer": "Tactful and warm", "rationale": "The entry, in a caring and caring way, asks about the other person鈥檚 feelings in school, without direct accusations or criticism, reflects respect for and understanding of the person鈥檚 emotions. At the same time, this euphemism increases the willingness of the person to share his or her true thoughts and feelings."}, "time_dimension": {"answer": "Past", "rationale": "In the entry, \"disturbed at school\" describes events that have occurred in the past and therefore judge them as \"the past\"."}, "sentence_type": "Affirmative declarative sentence", "pos": {"nouns": {"School": 1, "I'm not feeling well": 1}, "verbs": {"in": 1, "I feel": 1}}}, {"content": "I want to live elsewhere, not now.", "word_count": 19, "label": "", "sentence_structure": "Subject鈥搗erb鈥揷omplement", "subjective_objective": {"answer": "Subjective", "rationale": "Because \"hope\" represents patient's subjective attitude."}, "sentence_sentiment": {"answer": "Negative", "rationale": "Because the attitude of \"I want to live elsewhere\" expresses an unsatisfied or unsatisfactory feeling."}, "language_style": {"answer": "Calm and direct", "rationale": "The entry directly expresses the other party ' s dissatisfaction with the current place of residence and desire for change, with a more straightforward language style, without any aesthetic or emotional reverence."}, "time_dimension": {"answer": "Future", "rationale": "The entry expresses the testee's expectations and vision for the future, and the keywords are \"hope\", \"residence\" and \"not where it is\" , all pointing to situations that have not yet occurred and that may arise in the future. Thus, the judgement is based on the future."}, "sentence_type": "Affirmative declarative sentence", "pos": {"nouns": {"Local": 2, "self": 1}, "verbs": {"Hope": 1, "Stay": 1, "in": 1, "be": 1, "And": 1}, "adjectives": {"Don't": 1, "Current": 1}, "adverbs": {"More": 1, "of": 2, "not": 1}, "numerals": {"4": 1}, "pronouns": {"self": 1, "Here": 1}}}, {"content": "5. I was basically not forced to do what I didn't like.", "word_count": 18, "label": "", "sentence_structure": "Subject鈥搗erb鈥搊bject", "subjective_objective": {"answer": "Objective", "rationale": "Because \"no one basically forces me to do something I don't like\" is an objective description, involving behaviour and facts that can be tested by observation or data. While this may reflect the patient鈥檚 subjective feelings or attitudes, in this particular situation the entry is intended to collect objective information, not to assess the patient鈥檚 subjective attitudes or emotional state."}, "sentence_sentiment": {"answer": "Neutral", "rationale": "Because the entry does not clearly express a positive or negative emotional tendency, it is merely a description of an objective reality."}, "language_style": {"answer": "Calm and direct", "rationale": "The language style of the entry is calm and straightforward. It clearly presents a fact or view, is not expressed in a euphemism or indirect manner and is more objective and neutral."}, "time_dimension": {"answer": "Present", "rationale": "The entry \"basically not\" indicates that the past has been going on until now, and \"no one has forced me to do what I don't like\" indicates a normal state of affairs, and can therefore be judged as now."}, "sentence_type": "Affirmative declarative sentence", "pos": {"nouns": {"person": 1, "Yes": 2}, "verbs": {"Forced": 1, "Do it": 2, "Yeah": 1}, "adjectives": {"Basic": 1, "Nope": 1}, "adverbs": {"From": 1, "Both": 1}, "numerals": {"5": 1}, "pronouns": {"I": 1}}}, {"content": "6. I have achieved the desired academic achievement.", "word_count": 13, "label": "", "sentence_structure": "Subject鈥搗erb鈥揷omplement", "subjective_objective": {"answer": "Subjective", "rationale": "Since this entry relates to the individual ' s feelings and perceptions that he or she has achieved the desired academic achievement, it is examined from the patient ' s subjective perspective."}, "sentence_sentiment": {"answer": "Positive", "rationale": "Because the expression \"the best achievement in school\" is positive."}, "language_style": {"answer": "Tactful and warm", "rationale": "This entry presents the academic achievements of the respondents in a positive and encouraging manner, is more acclaimable, friendly, and is not too direct or rigid. Such expression can more easily resonate with the respondents and is more likely to receive real feedback."}, "time_dimension": {"answer": "Present", "rationale": ""}, "sentence_type": "Imperative sentence", "pos": {"nouns": {"Studies": 1, "Achievements": 1, "Ideal": 1}, "verbs": {"Access": 1}, "numerals": {"6": 1}}}, {"content": "Seven, I have many friends.", "word_count": 6, "label": "", "sentence_structure": "Subject鈥搗erb鈥搊bject", "subjective_objective": {"answer": "Subjective", "rationale": "Because \"many friends\" are subjective self-evaluations and perceptions, involving patients' satisfaction and satisfaction with their social circle. Although the existence of friends is an objective fact, the evaluation and perception of these facts is based on patients' subjective considerations."}, "sentence_sentiment": {"answer": "Positive", "rationale": "Because the entry \"a lot of friends\" expresses a positive emotion and attitude."}, "language_style": {"answer": "Tactful and warm", "rationale": "The entry is more personal and interactive by asking the other side whether there are many friends, by using a more moderate approach to the topic, without direct statements or orders."}, "time_dimension": {"answer": "Present", "rationale": "Now the entry is to ask the person who was tested about the current social situation, the keyword \"is\" indicates the persistence of the state, while the sentence is now in the normal state, which may be continuing. So the temporal dimension is now."}, "sentence_type": "Affirmative declarative sentence", "pos": {"nouns": {"friends": 1}, "verbs": {"have": 1}, "numerals": {"A lot": 1}}}, {"content": "8. My family is a happy family.", "word_count": 12, "label": "", "sentence_structure": "Subject鈥揷opula鈥損redicative", "subjective_objective": {"answer": "Subjective", "rationale": "Because happiness is a subjective feeling and an assessment, not an objective fact that can be measured."}, "sentence_sentiment": {"answer": "Positive", "rationale": "Because the expression \"happy family\" in the entry is positive."}, "language_style": {"answer": "Tactful and warm", "rationale": "The entry uses the word \"happy\" to describe the family, expresses the blessing and respect of the family of the interviewee and gives a warm and loving feeling."}, "time_dimension": {"answer": "Present", "rationale": "The entry describes the subjective feelings of the testee to his family, and the temporal dimension is now judged."}, "sentence_type": "Affirmative declarative sentence", "pos": {"nouns": {"Family": 1, "Happiness": 1}, "verbs": {"be": 1}}}, {"content": "I don't like many things at school.", "word_count": 12, "label": "", "sentence_structure": "Subject鈥損redicate", "subjective_objective": {"answer": "Subjective", "rationale": "School things are objective, and \"not like\" is the patient's subjective feeling about them."}, "sentence_sentiment": {"answer": "Negative", "rationale": "Because the \"not like\" attitude in the entry is negative."}, "language_style": {"answer": "Calm and direct", "rationale": "The entry expresses a displeasure with many things in schools in a directly explicit language, without using euphemism or implicit expressions, and therefore the language style is more calm and straightforward."}, "time_dimension": {"answer": "Present", "rationale": "The keyword \"not likes\" indicates negative attitudes of the subjects and may reflect their dissatisfaction or distress with school life."}, "sentence_type": "Negative declarative sentence", "pos": {"nouns": {"School": 1, "A lot": 1, "Things": 1, "I like it": 1}, "verbs": {"Both": 1, "I don't like it": 1}, "numerals": {"9": 1}, "classifiers": {"individual": 1}, "pronouns": {"I": 1}}}, {"content": "There are many things that are not good around my environment.", "word_count": 17, "label": "", "sentence_structure": "Subject鈥搗erb鈥揷omplement", "subjective_objective": {"answer": "Subjective", "rationale": "Because \"unsatisfactory\" is the subjective perception and assessment of the person's environment."}, "sentence_sentiment": {"answer": "Negative", "rationale": "Because the entry \"many things are not good\" expresses negative emotions."}, "language_style": {"answer": "Tactful and warm", "rationale": "By describing a situation, the entry does not directly ask the other side about his/her feelings or attitudes, but rather leaves the interviewee to think and judge himself. At the same time, the term is more moderate and neutral, without too strong an emotional colour or value judgement, and is more acceptable and more responsive."}, "time_dimension": {"answer": "Present", "rationale": ""}, "sentence_type": "Affirmative declarative sentence", "pos": {"nouns": {"Life": 1, "Environment": 1, "Things": 2}, "verbs": {"have": 1, "Not good enough": 1}, "adjectives": {"Many": 1}, "numerals": {"10": 1}}}, {"content": "11, I can basically do what I want.", "word_count": 15, "label": "", "sentence_structure": "Subject鈥搗erb鈥搊bject", "subjective_objective": {"answer": "Subjective", "rationale": "Because \"acting according to your wishes\" is a manifestation of personal will and attitude, and it is the patient's subjective feeling."}, "sentence_sentiment": {"answer": "Positive", "rationale": "Because the act of \"can act according to your wishes\" in the entry is positive, expressing a sense of autonomy and satisfaction."}, "language_style": {"answer": "Tactful and warm", "rationale": "The entry is more personal and interactive when it is spoken in a question-and-answer manner rather than directly speaking or ordering. At the same time, it leaves the respondent a space for an autonomous choice of answer, increasing the appeal and participation of the questionnaire."}, "time_dimension": {"answer": "Present", "rationale": "The keyword \"can\" means a capability or skill to act as you wish. At the same time, \"basically\" also indicates that such behaviour is frequent and continues to this day. Thus, the temporal dimension of the article is now judged."}, "sentence_type": "Affirmative declarative sentence", "pos": {"verbs": {"Yes": 1, "According to": 1, "self": 2, "Will": 1, "Act": 1}, "adjectives": {"Basic": 1}, "numerals": {"11": 1}}}, {"content": "I'm more than satisfied with my academic status.", "word_count": 10, "label": "", "sentence_structure": "Subject鈥揷opula鈥損redicative", "subjective_objective": {"answer": "Subjective", "rationale": "Because \"satisfaction\" is a subjective attitude, which expresses the patient's assessment and feelings about his or her academic status."}, "sentence_sentiment": {"answer": "Positive", "rationale": "Because the \"satisfaction\" attitude in the entry is positive."}, "language_style": {"answer": "Calm and direct", "rationale": "The entry is straightforward, does not have too many rhetorical or emotional renderings, and asks the subject of satisfaction with the academic status of the other party, which is more linguistically sound and objective."}, "time_dimension": {"answer": "Present", "rationale": "The entry is for the testee ' s satisfaction with the current academic status, and the time dimension is now judged."}, "sentence_type": "Affirmative declarative sentence", "pos": {"verbs": {"I": 1, "Satisfied": 1}, "adjectives": {"More": 1}, "numerals": {"12": 1}}}, {"content": "13, my friends will help me if I need them.", "word_count": 15, "label": "", "sentence_structure": "Subject鈥搗erb鈥搊bject", "subjective_objective": {"answer": "Subjective", "rationale": "Because this view is a subjective judgement and expectation of the interviewee about his personal relationship. While \"friends help\" is an objective possibility, whether or not they really help depends on the circumstances and the actual circumstances of the relationship, the answer to this question focuses more on the subjective attitude and expectations of the interviewee."}, "sentence_sentiment": {"answer": "Positive", "rationale": "Because it expresses a positive quality, that is, friends are willing to help themselves."}, "language_style": {"answer": "Tactful and warm", "rationale": "This is a more euphemistic and warm expression that can be more resonant with the interviewee. At the same time, it is more respectful and sensitive."}, "time_dimension": {"answer": "Present", "rationale": "The entry expresses the current feeling or perception of the testees that their friends will help themselves if they need it. So the time dimension is now."}, "sentence_type": "Affirmative declarative sentence", "pos": {"nouns": {"Help": 1, "friends": 1}, "verbs": {"Yes": 2, "Provision": 1}, "adverbs": {"If": 1}, "numerals": {"13": 1}, "pronouns": {"I": 1}}}, {"content": "I like the way parents are educated most of the time.", "word_count": 15, "label": "", "sentence_structure": "Subject鈥搗erb鈥搊bject", "subjective_objective": {"answer": "Subjective", "rationale": "Because likeness is an individual attitude and perception, it is subjective."}, "sentence_sentiment": {"answer": "Positive", "rationale": "Because the \"like\" attitude in the entry is positive."}, "language_style": {"answer": "Tactful and warm", "rationale": "The language style of the entry is more euphemistic, warm, using a positive term like \"like\" and not directly expressing praise or criticism of the way parents are educated, but rather describing their feelings in a more objective manner. It is more proficient and inclusive, making the respondents more comfortable and natural in their answers."}, "time_dimension": {"answer": "Present", "rationale": "The entry now asks the testee's current attitude or perception, \"most of the time\", and emphasizes the current state and orientation."}, "sentence_type": "Affirmative declarative sentence", "pos": {"nouns": {"Time": 1, "Yeah": 1, "Parents": 1, "Educational approach": 1}, "verbs": {"Mostly": 1, "I like it": 2}, "adverbs": {"Most": 1, "Okay": 1}, "numerals": {"14": 1, "1": 3}}}, {"content": "I like going to school.", "word_count": 6, "label": "", "sentence_structure": "Subject鈥搗erb鈥搊bject", "subjective_objective": {"answer": "Subjective", "rationale": "Because \"like\" is a subjective attitude or emotional experience, which belongs to the patient's inner feeling and perception, and is therefore subjective."}, "sentence_sentiment": {"answer": "Positive", "rationale": "Because \"like\" expresses a positive emotional attitude."}, "language_style": {"answer": "Tactful and warm", "rationale": "The language style of the entry is more moderate, it is expressed in a positive way and does not contain any negative emotions or evaluation, giving a positive upward feeling."}, "time_dimension": {"answer": "Present", "rationale": ""}, "sentence_type": "Affirmative declarative sentence", "pos": {"nouns": {"School": 1}, "verbs": {"Yeah": 1, "Go on": 1}, "numerals": {"15": 1}}}, {"content": "16 I live in a place where social security is good.", "word_count": 11, "label": "", "sentence_structure": "Subject鈥揷opula鈥損redicative", "subjective_objective": {"answer": "Objective", "rationale": "The social security situation is not the objective fact of life for the patient, but rather the subjective attitude or feelings of the patient."}, "sentence_sentiment": {"answer": "Positive", "rationale": "Because the description of \"good social security\" is positive."}, "language_style": {"answer": "Tactful and warm", "rationale": "The entry is more personal and interactive when it is spoken in a gentle and inviting manner, rather than in direct statements or orders. While the theme is security and social security, the language style retains its euphemism and warmth."}, "time_dimension": {"answer": "Present", "rationale": "The entry indicates that the testee's observation and evaluation of his current living environment, \"where I live\" and \"good social security\" are all descriptions of the state of affairs. So the temporal dimension is now."}, "sentence_type": "Affirmative declarative sentence", "pos": {"nouns": {"Local": 1, "Social security": 1}, "verbs": {"Life": 1}, "adjectives": {"Okay": 1}, "numerals": {"16": 1, "I": 1}}}, {"content": "17, basically I have the freedom to choose.", "word_count": 12, "label": "", "sentence_structure": "Subject鈥揷opula鈥損redicative", "subjective_objective": {"answer": "Subjective", "rationale": "Because it is a subjective assessment and feeling of the patient ' s state of life."}, "sentence_sentiment": {"answer": "Positive", "rationale": "Because the expression \"freedom of choice\" in the entry is positive, it is an expression of the individual's right and freedom to decide."}, "language_style": {"answer": "Tactful and warm", "rationale": "The entry uses a euphemistic and inquisitive language style rather than a direct statement or order, which is more personal and interactive. At the same time, the use of the word \"basic\" makes the question more open and non-judgmental, and gives the respondents a sense of self-centered answers."}, "time_dimension": {"answer": "Present", "rationale": "The keyword \"basically\" indicates that it is a general description, while the verb \"yes\" adopts the general current, meaning that this situation still exists. So the entry \"essentially I have the freedom to choose\" is a question of the current behaviour or state."}, "sentence_type": "Affirmative declarative sentence", "pos": {"verbs": {"have": 1, "Choose": 1}, "adjectives": {"Basic": 1, "From": 2}, "adverbs": {"Go, go, go": 1, "Both": 1, "Freedom": 1}, "numerals": {"17": 1}, "pronouns": {"I": 1}}}, {"content": "I have developed more comprehensively in school than most of my classmates.", "word_count": 17, "label": "", "sentence_structure": "Subject鈥揷opula鈥損redicative", "subjective_objective": {"answer": "Subjective", "rationale": "Because the entry is about the patient's assessment of himself, \"I'm developing more comprehensively in school\", which is a subjective judgement."}, "sentence_sentiment": {"answer": "Positive", "rationale": "The phrase \"more comprehensive development in school\" in the entry is positive, meaning that individuals perform better in school than most people."}, "language_style": {"answer": "Tactful and warm", "rationale": "The language style of the entry is euphemism. It does not directly commend or criticize the interviewee, but rather describes the comprehensiveness of the interviewee鈥檚 development in the school in a more objective manner and uses more gentle and polite terms that give people a sense of affection and respect."}, "time_dimension": {"answer": "Present", "rationale": ""}, "sentence_type": "Affirmative declarative sentence", "pos": {"nouns": {"Development": 1, "Students": 1, "School": 1}, "verbs": {"and": 1, "- Yeah": 1, "Compare": 1, "in": 1}, "adjectives": {"Most": 1, "Comprehensive": 1}, "adverbs": {"Compare": 1}, "numerals": {"18": 1}}}, {"content": "19 - My friends are very kind to me.", "word_count": 9, "label": "", "sentence_structure": "Subject鈥搗erb鈥揷omplement", "subjective_objective": {"answer": "Subjective", "rationale": "Because it is the patient's own assessment of friends, it is subjective feeling and view."}, "sentence_sentiment": {"answer": "Positive", "rationale": "Because the expression \"friends are good to me\" is positive."}, "language_style": {"answer": "Tactful and warm", "rationale": "The entry is presented in a positive way, praises the good of friends and does not directly ask each other about their feelings or assessments, and is more gentle and friendly in language style."}, "time_dimension": {"answer": "Present", "rationale": ""}, "sentence_type": "Affirmative declarative sentence", "pos": {"nouns": {"Friends": 1, "I": 2}, "verbs": {"to": 1, "Okay": 1}, "numerals": {"19": 1}}}, {"content": "My family is very nice together.", "word_count": 12, "label": "", "sentence_structure": "Subject鈥搗erb鈥揷omplement", "subjective_objective": {"answer": "Subjective", "rationale": "Because \"conciliation\" is the patient's feelings and assessment of family relations, and it's subjective."}, "sentence_sentiment": {"answer": "Positive", "rationale": "Because the \"family harmony\" is positive in the entry."}, "language_style": {"answer": "Tactful and warm", "rationale": "The entry describes, in a positive and positive tone, the harmony of relationships with family, without too much negative emotion or criticism, but rather with an affirmation and praise. This language style is more encouraging and positive, and it gives people comfort and comfort."}, "time_dimension": {"answer": "Present", "rationale": "The keywords \"my family,\" \"get together\" and \"very nice\" point to the current situation."}, "sentence_type": "Affirmative declarative sentence", "pos": {"nouns": {"Family": 1, "Get along": 1, "Harmony": 1}, "verbs": {"in": 1, "Together": 1, "very": 1}, "adverbs": {"very": 1}, "numerals": {"20": 1}}}, {"content": "I like school life.", "word_count": 7, "label": "", "sentence_structure": "Subject鈥搗erb鈥搊bject", "subjective_objective": {"answer": "Subjective", "rationale": "The view of the subjectivity of a person is that the purpose of the questionnaire is to consider the subjective approach of the patient."}, "sentence_sentiment": {"answer": "Positive", "rationale": "Because the attitude of \"like school life\" in the entries is positive."}, "language_style": {"answer": "Tactful and warm", "rationale": "The entry, which guides the interviewee in an encouraging and positive tone to share their feelings about school life, does not directly ask or order, which makes the interviewee more comfortable and relaxed and more willing to participate in the investigation."}, "time_dimension": {"answer": "Present", "rationale": "The entry expresses the degree of interest of the testee in the current (i.e. \"now\" ) and the keyword is \"like\", which means that the testee has a positive feeling at the moment."}, "sentence_type": "Affirmative declarative sentence", "pos": {"nouns": {"School life": 1}, "verbs": {"Yeah": 1}}}, {"content": "I live in a good social climate.", "word_count": 11, "label": "", "sentence_structure": "Subject鈥搗erb鈥揷omplement", "subjective_objective": {"answer": "Subjective", "rationale": "This is a subjective judgement and perception of the patient, and it is a reflection of his or her perception and assessment of the social climate in which he or she lives."}, "sentence_sentiment": {"answer": "Neutral", "rationale": "Because the entry describes objective facts, there is no apparent emotional tendency."}, "language_style": {"answer": "Tactful and warm", "rationale": "The language style of the entry is euphemistic. It uses positive and complimentary words, rather than direct criticism or denial, so that respondents feel respected and understood, are more willing to participate in the investigation and share real ideas and feelings. At the same time, using this language style makes the questionnaire more friendly and friendly, and makes the survey more attractive and credible."}, "time_dimension": {"answer": "Past", "rationale": "The entry describes the life of the past, and the keyword \"where I live\" indicates that it is a memory and an assessment of the past, and therefore the dimension of time is a judgement of the past."}, "sentence_type": "Affirmative declarative sentence", "pos": {"nouns": {"Local": 1, "Social climate": 1}, "verbs": {"I": 1, "Life": 1, "I think": 1}, "adjectives": {"Okay": 1}, "numerals": {"22": 1, "One": 1}}}, {"content": "23, I can do what I like when I'm out of school.", "word_count": 16, "label": "", "sentence_structure": "Subject鈥搗erb鈥搊bject", "subjective_objective": {"answer": "Subjective", "rationale": "Because \"like\" is the patient's subjective feeling, and \"can do what he likes\" is the patient's own decision and choice."}, "sentence_sentiment": {"answer": "Positive", "rationale": "Because \"do what you like\" is positive."}, "language_style": {"answer": "Tactful and warm", "rationale": "The language style of the entry is more euphemistic and warm, using more neutral terms such as \"excurricular time\" and \"the thing you like\" and asking each other in a way that is caring and understanding whether there is enough time and space to do what you like. Such expression makes it easier for the interviewee to feel comfortable and to share his thoughts and feelings."}, "time_dimension": {"answer": "Present", "rationale": "In the entry, \"after school time\" means an act that is currently being performed or being considered, and therefore judgement is now. At the same time, \"can do what you like\" implies that it is an act of initiative, of choice, not of coercion."}, "sentence_type": "Affirmative declarative sentence", "pos": {"nouns": {"After-school hours": 1, "Things": 1}, "verbs": {"Yes": 1, "Yeah": 1}, "adjectives": {"Your own": 1}, "adverbs": {"in": 1}, "numerals": {"23": 1}, "pronouns": {"I": 1}}}, {"content": "I've received more honours in school than my classmates.", "word_count": 19, "label": "", "sentence_structure": "Subject鈥揷opula鈥損redicative", "subjective_objective": {"answer": "Subjective", "rationale": "Because the entry relates to the patient ' s perception and comparison of himself, it is the patient ' s subjective judgement."}, "sentence_sentiment": {"answer": "Positive", "rationale": "Because the phrase \"rewarded more honor\" in the entry is positive."}, "language_style": {"answer": "Tactful and warm", "rationale": "By comparing the amount of honour that you have received in school, the entry is not a direct expression of pride or complacency, but is presented in a relatively peaceful way, which is more acceptable. This language style gives people a more cordial and moderate sense of affection and more likely to resonate and cooperate with the person under investigation."}, "time_dimension": {"answer": "Present", "rationale": "鈥渋n respect of鈥?the article is usually used to compare the differences or advantages of the two, so the temporal dimension of the article is now."}, "sentence_type": "Affirmative declarative sentence", "pos": {"nouns": {"Students": 1, "Honours": 1, "School": 1}, "verbs": {"Comparison": 1, "Okay": 1}, "adverbs": {"More": 1}, "numerals": {"24": 1}, "pronouns": {"I": 1}}}, {"content": "I'm a very good fellow among myself.", "word_count": 12, "label": "", "sentence_structure": "Subject鈥揷opula鈥損redicative", "subjective_objective": {"answer": "Subjective", "rationale": "Because \"trust\" is a subjective feeling and perception, which reflects the status, influence and respect of the person interviewed in his or her own companion. This perception and perception may be based on the behaviour, characteristics, speech, etc. of the person interviewed, or may be influenced by other factors, such as sociocultural background, interpersonal relationships, etc. So, \"I am very well trusted among my peers\" is a subjective description."}, "sentence_sentiment": {"answer": "Positive", "rationale": "Because the \"very powerful\" in the entry indicates that it has some influence and status among its peers, and is a positive emotional attitude."}, "language_style": {"answer": "Tactful and warm", "rationale": "The entry is used to ask the other side about their status as peers, to learn about their social abilities and self-confidence in a more euphemistic way, and to give them recognition and encouragement."}, "time_dimension": {"answer": "Present", "rationale": "The keyword \"I\" indicates that it is a description of itself, and \"acceptance\" is an assessment or perception of itself. These keywords point to the person who is being tested, and so the entry reflects the person who is being tested, and the person who is being tested, so that is the person who is. And, in conclusion, the answer is \"now.\""}, "sentence_type": "Affirmative declarative sentence", "pos": {"nouns": {"25": 1, "Companion": 1, "Power": 1}, "verbs": {"have": 1}, "numerals": {"25": 1}}}, {"content": "26, my parents treat me equally.", "word_count": 11, "label": "", "sentence_structure": "Subject鈥搗erb鈥搊bject", "subjective_objective": {"answer": "Subjective", "rationale": "Because \"Equality me\" is an evaluative statement that involves patients' subjective feelings and views."}, "sentence_sentiment": {"answer": "Positive", "rationale": "Because \"equal treatment\" expresses positive attitudes and qualities."}, "language_style": {"answer": "Tactful and warm", "rationale": "This is a more acceptable language and a more accessible one."}, "time_dimension": {"answer": "Present", "rationale": "The entry describes the current situation of the subjects, i.e. whether they believe that their parents can treat themselves equally and therefore judge them as now."}, "sentence_type": "Affirmative declarative sentence", "pos": {"nouns": {"parents": 1, "I": 1}, "verbs": {"Yes": 1, "Equality": 1, "- Wait": 1}, "adjectives": {"to": 1}, "numerals": {"26": 1}}}, {"content": "I love school life.", "word_count": 8, "label": "", "sentence_structure": "Subject鈥搗erb鈥搊bject", "subjective_objective": {"answer": "Subjective", "rationale": "I like the patient's subjective attitude."}, "sentence_sentiment": {"answer": "Positive", "rationale": "Because the entry \"likes school life\" expresses a positive emotional attitude and behavioral intent."}, "language_style": {"answer": "Tactful and warm", "rationale": "The entry expresses, in a positive and positive way, the degree of interest in school life and the more verbically sweet and inspiring style of the person surveyed. At the same time, this expression can better bring the person surveyed closer to the point of the investigation and improve its effectiveness."}, "time_dimension": {"answer": "Present", "rationale": "\"like\" action takes place now, so time dimension is judged now."}, "sentence_type": "Affirmative declarative sentence", "pos": {"nouns": {"Life": 1, "School": 1}, "verbs": {"Yeah": 1}, "numerals": {"27": 1}}}, {"content": "28 Our world is peaceful and peaceful.", "word_count": 13, "label": "", "sentence_structure": "Subject鈥揷opula鈥損redicative", "subjective_objective": {"answer": "Objective", "rationale": "Because whether the world is peaceful is determined by reality is an objective fact. Although individuals may have different views or feelings, this does not change the world鈥檚 reality."}, "sentence_sentiment": {"answer": "Neutral", "rationale": "The entry describes an objective state of existence and does not express positive or negative emotional expressions."}, "language_style": {"answer": "Calm and direct", "rationale": "The language style of the entry is relatively calm and objective, and there is no excessive emotional rendering or rhetorical technique, and a point is made directly."}, "time_dimension": {"answer": "Past", "rationale": "The entry is an old fact that \"the world we live in was once \"peaceful.\""}, "sentence_type": "Affirmative declarative sentence", "pos": {"nouns": {"World": 1, "we": 1}, "verbs": {"Survive": 1, "be": 1, "Peace and peace": 1, "of": 1}, "adjectives": {"Peace": 1}, "numerals": {"28": 1}}}, {"content": "29, basically, nobody interferes with my life.", "word_count": 12, "label": "", "sentence_structure": "Subject鈥揷opula鈥損redicative", "subjective_objective": {"answer": "Objective", "rationale": "Because it is an objective fact to have anyone interfered."}, "sentence_sentiment": {"answer": "Neutral", "rationale": "Because the entry does not have any emotional color."}, "language_style": {"answer": "Calm and direct", "rationale": "The entry directly expresses the subject of a question about whether the other party is regularly subjected to interference in life, without excessive rhetoric or emotional rendering and with a more objective tone."}, "time_dimension": {"answer": "Present", "rationale": "The keyword \"no one's basically interfering in my life\" means the current state and feelings of the testees, so the temporal dimension is now."}, "sentence_type": "Affirmative declarative sentence", "pos": {"nouns": {"person": 1, "Interference": 1, "Life": 1}, "verbs": {"No, I'm not": 1, "Interference": 1, "Pass": 1}, "adjectives": {"Basic": 1}, "adverbs": {"Go, go, go": 1, "Both": 1}, "numerals": {"29": 1}, "pronouns": {"I": 1}}}, {"content": "I feel like I'm a good fellow.", "word_count": 13, "label": "", "sentence_structure": "Subject鈥揷opula鈥損redicative", "subjective_objective": {"answer": "Subjective", "rationale": "Because \"sweet\" is a subjective expression, it looks at the patient's subjective attitude and emotional experience."}, "sentence_sentiment": {"answer": "Positive", "rationale": "Because \"Hand of Honor\" in the entry expresses positive emotional attitudes."}, "language_style": {"answer": "Tactful and warm", "rationale": "The language style of the entry is more euphemistic and uses a more subjective expression that guides the answers to be tried in a more moderate and personal way."}, "time_dimension": {"answer": "Present", "rationale": ""}, "sentence_type": "Affirmative declarative sentence", "pos": {"nouns": {"self": 1, "Companion": 1, "Face": 1}, "verbs": {"I think": 1, "have": 1}, "numerals": {"30": 1}}}, {"content": "I like to make friends with different people.", "word_count": 13, "label": "", "sentence_structure": "Subject鈥搗erb鈥搊bject", "subjective_objective": {"answer": "Subjective", "rationale": "Because it is a subjective attitude and a preference to like new friends."}, "sentence_sentiment": {"answer": "Positive", "rationale": "Because the desire to have new friends is often seen as a positive emotion and attitude."}, "language_style": {"answer": "Tactful and warm", "rationale": "The language style of the entry is more moderate, with no direct orders or criticism, but rather a dialogue in a way that involves inquiries and suggestions, which makes it easier to generate positive answers and interaction. Moreover, using a phrase such as \"unlike today\" gives people a sense of openness, inclusion and better relationships."}, "time_dimension": {"answer": "Present", "rationale": "So this entry can be judged now."}, "sentence_type": "Affirmative declarative sentence", "pos": {"nouns": {"31": 1, "friends": 1}, "verbs": {"Yeah": 1, "Deal": 1, "and": 1, "Present": 1, "Different": 1}, "adjectives": {"Different": 1}, "numerals": {"31": 1}}}, {"content": "32. My family members spoke to each other very nicely.", "word_count": 15, "label": "", "sentence_structure": "Subject鈥搗erb鈥揷omplement", "subjective_objective": {"answer": "Subjective", "rationale": "Because \"good speech\" is the patient's subjective feeling and evaluation."}, "sentence_sentiment": {"answer": "Positive", "rationale": "Because the \"speaks friendly\" act in the entry is positive."}, "language_style": {"answer": "Tactful and warm", "rationale": "The language style of the entry is more euphemistic and warm, using the term \"inter se\" to give a sense of comfort and harmony, without directly expressing the rating or a clear affirmation or denial, and therefore the tone is more moderate."}, "time_dimension": {"answer": "Present", "rationale": "The entry describes the interaction between the family members of the testee, and the key words are \"me,\" \"family members\" and \"friendly to each other,\" which all indicate that it is a description of the state of the moment, and therefore the judgment is \"now\"."}, "sentence_type": "Affirmative declarative sentence", "pos": {"nouns": {"Family members": 1, "Speech": 1, "Friendly": 1}, "verbs": {"To each other": 1, "Speak": 1}, "adjectives": {"very": 1}, "numerals": {"32": 1}}}, {"content": "I'm having fun in school.", "word_count": 10, "label": "", "sentence_structure": "Subject鈥揷opula鈥損redicative", "subjective_objective": {"answer": "Subjective", "rationale": "Because \"interest\" is a subjective feeling and evaluation that reflects the personal experience and perceptions of school life."}, "sentence_sentiment": {"answer": "Positive", "rationale": "Because the entry expresses a positive emotional attitude."}, "language_style": {"answer": "Tactful and warm", "rationale": "Warm and warm."}, "time_dimension": {"answer": "Present", "rationale": "The keyword \"life in school is fun\" expresses the description and the feeling of the state of affairs, and therefore the judgment is the present."}, "sentence_type": "Affirmative declarative sentence", "pos": {"nouns": {"School": 1, "Life": 1, "Fun": 1}, "verbs": {"in": 1, "Pass": 1}, "adjectives": {"very": 1, "Interesting": 1}, "numerals": {"33": 1}, "pronouns": {"I": 1}}}, {"content": "I have a great sense of achievement in school.", "word_count": 10, "label": "", "sentence_structure": "Subject鈥揷opula鈥損redicative", "subjective_objective": {"answer": "Subjective", "rationale": "Because \"the sense of achievement is\" is the patient's subjective experience, so it's \"subjective.\""}, "sentence_sentiment": {"answer": "Positive", "rationale": "Because the \"sense of achievement\" in the entry is a positive emotional attitude."}, "language_style": {"answer": "Tactful and warm", "rationale": "The entry describes, in a positive tone, the individual鈥檚 sense of academic achievement, without over-direct or over-emphasis on it, making the interviewee comfortable and natural in his response. At the same time, the use of the term 鈥渧ery鈥?also shows that this feeling is strong and persistent, making the problem more deep and open."}, "time_dimension": {"answer": "Present", "rationale": "The entry describes the current situation, \"at school\" indicating that the field of study is, and \"very sense of achievement\" is an assessment of the current state, so the answer is \"now\"."}, "sentence_type": "Affirmative declarative sentence", "pos": {"nouns": {"I": 1, "Studies": 1}, "verbs": {"in": 1, "have": 1, "Achievements": 1}, "adjectives": {"very": 1, "Done": 1, "Fountain": 1}, "numerals": {"34": 1}}}, {"content": "I have a lot of fun with my friends.", "word_count": 14, "label": "", "sentence_structure": "Subject鈥搗erb鈥搊bject", "subjective_objective": {"answer": "Subjective", "rationale": "Because \"a lot of fun\" is a subjective feeling or evaluation that reflects the patient's perception of his/her relationship with friends."}, "sentence_sentiment": {"answer": "Positive", "rationale": "Because the description \"a lot of fun\" is positive."}, "language_style": {"answer": "Tactful and warm", "rationale": "The entry is more personal and interactive when it is spoken in a gentle and inviting tone, rather than in direct statements or orders. Moreover, the first person 's pronoun 'I' is used to give people a sense of being closer and to show that it is a sharing of personal experience."}, "time_dimension": {"answer": "Present", "rationale": ""}, "sentence_type": "Affirmative declarative sentence", "pos": {"nouns": {"friends": 1, "Interesting": 1}, "verbs": {"in": 1, "have": 1}, "adverbs": {"A lot": 1}, "numerals": {"35": 1}, "pronouns": {"I": 1}}}, {"content": "I'm talking to my parents with pleasure.", "word_count": 15, "label": "", "sentence_structure": "Subject鈥搗erb鈥搊bject", "subjective_objective": {"answer": "Subjective", "rationale": "Because \"happy talk\" is a subjective feeling and an evaluation that requires the patient to feel and describe himself."}, "sentence_sentiment": {"answer": "Positive", "rationale": "Because the behaviour and attitude described in the entry is positive, namely, \"happy talk\" and indicates good relations with parents."}, "language_style": {"answer": "Tactful and warm", "rationale": "The entry uses a more subjective expression, which describes the way in which the person is communicating with his or her parents, using a first person ' s perspective, which makes it easier for the person surveyed to resonate and to be more sensitive and humane. Moreover, the tone of the entry is relatively mild and less intrusive, and there is no too direct or hard-line questioning of the other person, and this euphemism is more acceptable."}, "time_dimension": {"answer": "Present", "rationale": ""}, "sentence_type": "Affirmative declarative sentence", "pos": {"nouns": {"I": 1, "parents": 1}, "verbs": {"and": 1, "in": 1, "Yes": 1, "Talk": 1}, "adjectives": {"Happy": 1}, "numerals": {"36": 1}, "classifiers": {"individual": 1}}}]}

{"questionnaire_title": "Mental health control source table", "questionnaire_category": "Self-cognition", "item_count": 28, "emotion_shift_overall": "From negative to positive", "language_style_overall": "Calm and direct", "items": [{"content": "1. A person with a high intellectual capacity of 160 is abnormal in terms of the normal distribution of statistics.", "word_count": 26, "label": "", "sentence_structure": "Subject鈥揷opula鈥損redicative", "subjective_objective": {"answer": "Objective", "rationale": "Because it's objective from a statistical point of view."}, "sentence_sentiment": {"answer": "Negative", "rationale": "Because the description of \"unusual\" in the entry is a negative and derogatory term, it means that the individual is rated negatively."}, "language_style": {"answer": "Calm and direct", "rationale": "The entry presents a fact in a scientificly rigorous tone, does not add too much personal emotion or rhetorical techniques and can therefore be judged as a calm and direct language style."}, "time_dimension": {"answer": "Present", "rationale": "\"Statistically normal distribution is abnormal\" and describes the whole group, involving the concepts of probability and statistics, so that it can be judged to be a description of present or future behaviour or mentality."}, "sentence_type": "Negative declarative sentence", "pos": {"nouns": {"person": 2, "Statistics": 1, "Normal distribution": 1, "Angle": 1, "In terms of": 1}, "verbs": {"in": 1, "Look": 1, "be": 1, "Yeah": 1}, "adjectives": {"Ji-ho": 1, "Normal": 1}, "numerals": {"160": 1}, "classifiers": {"individual": 1}}}, {"content": "2. Psychotherapy is for people who can't solve their problems themselves and who need to find someone stronger than themselves to support them.", "word_count": 34, "label": "", "sentence_structure": "Subject鈥搗erb鈥搊bject", "subjective_objective": {"answer": "Subjective", "rationale": "This statement is an expression of the author ' s subjective view and definition of the subject of psychological treatment."}, "sentence_sentiment": {"answer": "Negative", "rationale": "Because the entry describes those who need help to solve the problem, it has the effect of derogatory and exclusive."}, "language_style": {"answer": "Calm and direct", "rationale": "The language style of calming down the article is one of the cool and straightforward types. It clearly identifies the target group for psychological treatment, namely, those who cannot solve the problem independently, need external support and help."}, "time_dimension": {"answer": "Present", "rationale": ""}, "sentence_type": "Affirmative declarative sentence", "pos": {"nouns": {"Psychotherapy": 1, "Object": 1, "Problem": 1, "Dependence": 1}, "verbs": {"be": 1, "Find": 1}, "adjectives": {"No, I can't": 1, "self": 2, "Strong": 1}, "numerals": {"Those": 1, "Some": 1}, "pronouns": {"Those": 1, "Here": 1}}}, {"content": "3. To recover from a serious psychological problem, you must first be willing to temporarily hand over yourself to an experienced professional, who will be fully responsible.", "word_count": 43, "label": "", "sentence_structure": "Subject鈥搗erb鈥搊bject", "subjective_objective": {"answer": "Subjective", "rationale": "This is the patient鈥檚 choice and perception of the treatment, and is subject to subjective scrutiny."}, "sentence_sentiment": {"answer": "Positive", "rationale": "Because the entry emphasizes the importance of seeking professional help and expresses a positive attitude. While it concerns \"handing yourself over to someone else\", this is considered a necessary and positive step."}, "language_style": {"answer": "Calm and direct", "rationale": "The language style of the entry is more direct and clear, with no rhetorical or emotional recitation, and more factually and logically based statements, which are more persuasive and professional."}, "time_dimension": {"answer": "Past", "rationale": "The keyword \"must\" and \"first\" and the verb \"show yourself temporarily to an experienced professional with full responsibility\", indicating that the proposal is based on the current situation, not on past or future scenarios. So the temporal dimension is now."}, "sentence_type": "Affirmative declarative sentence", "pos": {"nouns": {"Serious": 1, "Psychological problems": 1, "Recovery": 1, "self": 1, "Experience": 1, "Professional": 1, "Comprehensive": 1, "Responsible": 1}, "verbs": {"Yes": 1, "Here": 1, "Responsible": 1}, "adjectives": {"I'm happy": 1, "For now": 1, "Experienced": 1}, "adverbs": {"First of all": 1}}}, {"content": "4. According to psychoanalytical theory, the stage of development in the emergence of hysteria is the period of punishment we know well ... (penalstage)", "word_count": 29, "label": "", "sentence_structure": "Subject鈥搗erb鈥搊bject", "subjective_objective": {"answer": "Objective", "rationale": "Because this view is based on psychoanalytical theory, it is an objective fact."}, "sentence_sentiment": {"answer": "Neutral", "rationale": "Because the entry does not have any emotional color."}, "language_style": {"answer": "Calm and direct", "rationale": "The entry presents a psychological theory in an objective manner, with no excessive emotional or oral verb, which is more calm and direct."}, "time_dimension": {"answer": "Future", "rationale": "The entry contains a description of the time when parental conflict occurs in psychoanalytical theory, noting that it is a possible phenomenon at a certain stage of development (punitive period). Thus, it is primarily about future or future possibilities."}, "sentence_type": "Affirmative declarative sentence", "pos": {"nouns": {"Questionnaire Entry": 1, "Psychoanalytical theory": 1, "The conflict between pedophiles": 1, "Developmental": 1, "Period of punishment": 1, "penalstage": 1}, "verbs": {"Come on": 1, "I know you well": 1}, "numerals": {"4": 1}}}, {"content": "5. Persons with mental problems should play a major role in developing their own treatment programmes.", "word_count": 23, "label": "", "sentence_structure": "Subject鈥搗erb鈥搊bject", "subjective_objective": {"answer": "Subjective", "rationale": "Because the subject \"Persons with mental problems\" and \"therapeutic program\" are subjective acts and ideas of patients."}, "sentence_sentiment": {"answer": "Positive", "rationale": "Because the expression \"the person with a mental problem has a major role to play in the development of his or her own treatment programme\", reflects respect for and trust in patients, their participation in treatment decision-making and their adherence to the idea of central treatment in modern psychology."}, "language_style": {"answer": "Tactful and warm", "rationale": "The words of the article also reflect respect and support for people with mental problems, encouraging them to participate proactively in their own treatment programming, and to be more humane and caring in their communication style."}, "time_dimension": {"answer": "Present", "rationale": ""}, "sentence_type": "Affirmative declarative sentence", "pos": {"nouns": {"Psychological problems": 1, "person": 2, "Treatment programme": 1}, "verbs": {"Reactions": 1, "Rise": 1, "Production": 1, "Freeze": 1}, "adverbs": {"self": 1}, "numerals": {"5": 1}}}, {"content": "6. Those who receive psychiatric treatment should not take any significant decisions before they are heard.", "word_count": 29, "label": "", "sentence_structure": "Subject鈥搗erb鈥搊bject", "subjective_objective": {"answer": "Subjective", "rationale": "Because \"the person receiving the psychiatric shivering\" is the patient, and \"no major decision should be taken before hearing the opinion\" is a recommendation to the patient, it is examined from the patient's subjective point of view."}, "sentence_sentiment": {"answer": "Neutral", "rationale": "Because the entry does not have any emotional color."}, "language_style": {"answer": "Calm and direct", "rationale": "The entry presents a straightforward view that no major decision should be taken before the person receiving treatment for mental illness is heard."}, "time_dimension": {"answer": "Present", "rationale": "The description \"before the person receiving the treatment for mental shaking has heard\" implies a status quo or a rule that would require advice or guidance during treatment and should therefore be judged as now."}, "sentence_type": "Negative declarative sentence", "pos": {"nouns": {"person": 2, "Observations": 1, "Decision": 2}, "verbs": {"Accept": 1, "Give": 1, "Make": 1}, "adjectives": {"Significant": 1}, "adverbs": {"I think so": 1}, "numerals": {"6": 1}, "pronouns": {"they": 1}}}, {"content": "7. Reactive schizophrenia is slow, hidden.", "word_count": 17, "label": "", "sentence_structure": "Subject鈥揷opula鈥損redicative", "subjective_objective": {"answer": "Objective", "rationale": "Because the process of onset is characterized by an objective fact."}, "sentence_sentiment": {"answer": "Negative", "rationale": "Because the words \"incident\" and \"since\" in the entries are all words that describe disease, and disease is usually considered to be negative. So the emotional color of the article is negative."}, "language_style": {"answer": "Calm and direct", "rationale": "The entry describes the symptoms of reactive schizophrenia in a concise and clear language, without excessive emotional or rhetorical use. The language style of the article can therefore be described as being calm and straightforward."}, "time_dimension": {"answer": "Past", "rationale": "The entry describes the symptoms of reactive schizophrenia, and the \"slow, hidden\" description suggests that the occurrence of the disease is a gradual process, not a sudden occurrence. The temporal dimension of the entry is therefore the past."}, "sentence_type": "Affirmative declarative sentence", "pos": {"nouns": {"Reactive schizophrenia": 1, "I'm sick": 1, "Class Slow": 1, "Infiltration": 1}, "verbs": {"be": 1}, "numerals": {"7": 1}}}, {"content": "8. If a mental patient intends to take new action, the professional should decide what he/she should do first.", "word_count": 33, "label": "", "sentence_structure": "Subject鈥搗erb鈥搊bject", "subjective_objective": {"answer": "Objective", "rationale": "Since the actions in this topic are judged and decided by professional medical personnel, they are objective in their perspective."}, "sentence_sentiment": {"answer": "Neutral", "rationale": "Because the entry does not have any obvious emotional colour."}, "language_style": {"answer": "Calm and direct", "rationale": "The entry raises the question in a straightforward and straightforward way, without too much emotional or rhetorical, more logical and practical. Thus, its language style is a straightforward, calm form of speech."}, "time_dimension": {"answer": "Present", "rationale": "The \"if\" in the entry indicates the hypothetical condition, \"shall decide\" action occurs now or in the future. So the temporal dimension is now."}, "sentence_type": "Imperative sentence", "pos": {"nouns": {"Mental patients": 1, "Action": 1, "Professional": 1, "First of all": 1}, "verbs": {"I'm going to": 1, "Adoption": 1, "Decision": 1, "Do it": 1}, "adjectives": {"New": 1}, "numerals": {"8": 1}, "pronouns": {"he": 1, "she": 1}}}, {"content": "9. The decision as to when to terminate psychiatric treatment should depend on the patient, not the person who treated it.", "word_count": 22, "label": "", "sentence_structure": "Subject鈥搗erb鈥搊bject", "subjective_objective": {"answer": "Objective", "rationale": "This is an objective fact, independent of the subjective influence of the individual."}, "sentence_sentiment": {"answer": "Positive", "rationale": "Because the emphasis on respect for the patient ' s choices and interests is a positive attitude."}, "language_style": {"answer": "Tactful and warm", "rationale": "The entry is more euphemistic. It does not direct orders or criticize the therapist, but rather proposes that when decisions about when to terminate psychotherapy should take into account the patient鈥檚 feelings and needs, which is more respectful of the patient and enables good patient relationships to be established."}, "time_dimension": {"answer": "Present", "rationale": "The keywords \"determinating when to terminate psychiatric treatment\" and \"dependantly, not the person who treats it\" describe the current situation or behaviour and therefore judge the present."}, "sentence_type": "Affirmative declarative sentence", "pos": {"nouns": {"Psychotherapy": 2, "Patients": 1, "Therapist": 1}, "verbs": {"Decision": 1, "Termination": 1, "Reactions": 1, "It depends": 1, "be": 1}, "adverbs": {"When": 1, "Not": 1}, "numerals": {"9": 1, "Two": 1}, "pronouns": {"This": 1}}}, {"content": "The lives of people with mental problems are so complex that they have little idea what they should do to improve their situation.", "word_count": 37, "label": "", "sentence_structure": "Subject鈥揷opula鈥損redicative", "subjective_objective": {"answer": "Subjective", "rationale": "Because \"what you should do to improve your situation\" is a patient's judgment and expectation of his or her own situation, it is subjective."}, "sentence_sentiment": {"answer": "Negative", "rationale": "Because the descriptions in the entries contain negative terms of \"life is too complex\" and \"near possible to think\" suggesting that people with mental problems are unable to deal with their problems."}, "language_style": {"answer": "Tactful and warm", "rationale": ""}, "time_dimension": {"answer": "Present", "rationale": ""}, "sentence_type": "Negative declarative sentence", "pos": {"nouns": {"Psychological problems": 1, "person": 2, "Life": 1, "Complex": 1, "Situation": 1}, "verbs": {"have": 1, "Too much": 1, "I've got it": 1, "Do it": 1, "Improvement": 1}, "adjectives": {"Complex": 1}, "adverbs": {"Almost": 1}, "numerals": {"10": 1}, "pronouns": {"they": 1}}}, {"content": "11. If psychological treatment is like building a house, a good healer should not only provide you with tools, but also design the house for you.", "word_count": 41, "label": "", "sentence_structure": "Subject鈥搗erb鈥搊bject", "subjective_objective": {"answer": "Subjective", "rationale": "Since the entry emphasizes the role and responsibility of the treatmenter, the treatment plan needs to be designed in accordance with the patient ' s specific circumstances, as a result of the patient ' s subjective judgement and decision-making."}, "sentence_sentiment": {"answer": "Neutral", "rationale": "The entry does not display a positive or negative emotional profile, but simply presents the view that good healers should provide both tools and design houses for their clients."}, "language_style": {"answer": "Tactful and warm", "rationale": "The language style of the entry is more euphemistic. By comparing psychological therapy to house-building, it is emphasized that a good therapist is important. This euphemism is more acceptable to the interviewee and is more conducive to a positive communication atmosphere."}, "time_dimension": {"answer": "Past", "rationale": "However, we can classify it as \"now\" or \"for the future\" based on the content of the sentence. As can be seen from the sentence, \"good healers should not only provide you with tools, but also design this house for you.\" It is the expectation and demand of the current therapist that the present therapist should have these skills. So the entry can be \"now.\""}, "sentence_type": "Affirmative declarative sentence", "pos": {"nouns": {"Psychotherapy": 1, "Tools": 1, "House": 2, "Therapist": 1}, "verbs": {"Should be provided": 1, "Should be designed": 1}, "adverbs": {"Not only": 1}, "numerals": {"11": 1}, "pronouns": {"he": 1}}}, {"content": "12. The homeSzas considered mental illness a mystery.", "word_count": 10, "label": "", "sentence_structure": "Subject鈥搗erb鈥搊bject", "subjective_objective": {"answer": "Subjective", "rationale": "This is the view and attitude of Thomesazas and is a subjective examination."}, "sentence_sentiment": {"answer": "Negative", "rationale": "Since the subject of \"mental illness\" is usually considered a negative situation or state, the expression of the purpose of the article may be considered negative."}, "language_style": {"answer": "Calm and direct", "rationale": "The entry is more directly linguistically, without excessive rhetorical or emotional rendering, simply presenting the point of view of the homeSzas. This expression is more objective, neutral and factual."}, "time_dimension": {"answer": "Present", "rationale": "The entry is a question of the views and attitudes of the person being tested and does not contain a clear reference to the temporal dimension, so it is judged to be neutral."}, "sentence_type": "Negative declarative sentence", "pos": {"nouns": {"ThomeSzas": 1, "Mental illness": 1, "The riddle": 1}, "verbs": {"I think": 1, "be": 1}, "numerals": {"12": 1}}}, {"content": "13. Psychopaths should tell their patients how to adopt a healthy lifestyle, and not whether they can find it themselves.", "word_count": 38, "label": "", "sentence_structure": "Subject鈥搗erb鈥搊bject", "subjective_objective": {"answer": "Subjective", "rationale": "This is a subjective assessment of the behaviour that should be taken by the psychotherapists."}, "sentence_sentiment": {"answer": "Positive", "rationale": "Because the suggestion that the patient should be informed by the person who is the therapist in the entry is positive and useful."}, "language_style": {"answer": "Calm and direct", "rationale": "The entry is strong and clear, expressing the duties and responsibilities of the psychologist, without any euphemism or warmer a very direct feeling."}, "time_dimension": {"answer": "Present", "rationale": "Keywords include \"should\" and \"should not\" , which indicate that it is a guiding recommendation for current behaviour. Moreover, the \"said\" in \"tell them\" also uses the general current, implying that this is an ongoing task."}, "sentence_type": "Affirmative declarative sentence", "pos": {"nouns": {"Psychopaths": 1, "Patients": 2, "Lifestyle": 1, "Health": 1, "Sit down": 1, "self": 1}, "verbs": {"Should": 1, "Tell": 1, "Adoption": 1, "Can you": 1, "Found it": 1}, "adjectives": {"Health": 1}, "adverbs": {"Should": 1}, "numerals": {"13": 1}, "pronouns": {"they": 1, "Theirs": 1}}}, {"content": "14. Patients should try to get the views of the treatmenter on the merits.", "word_count": 19, "label": "", "sentence_structure": "Subject鈥搗erb鈥搊bject", "subjective_objective": {"answer": "Subjective", "rationale": "Because \"the view of the person receiving treatment about what is right or wrong\" is an attitude or behaviour that the patient should adopt, it is the patient's subjective idea that is being examined."}, "sentence_sentiment": {"answer": "Negative", "rationale": "Because the \"best efforts\" in the entry are positive, but the addition of \"the views of the therapist about what is right or wrong\" may make patients feel compelled to accept some of the views they do not share, and thus have some negative side effects."}, "language_style": {"answer": "Tactful and warm", "rationale": "The entry is more euphemistic and warm, and it does not have direct orders or a mandatory tone, but rather provides a way of encouraging and advising respondents to answer questions. At the same time, the entry conveys a positive message that patients should be treated on the basis of the views of the person, which is also a relatively mild statement, avoiding expressions that may lead to negative emotions or conflict."}, "time_dimension": {"answer": "Present", "rationale": "\"This sentence describes the situation, which relates to patient behaviour and attitudes towards health workers. So the time dimension of the questionnaire entry is \"now\"."}, "sentence_type": "Affirmative declarative sentence", "pos": {"nouns": {"Patients": 1, "Therapist": 1, "Views": 1}, "verbs": {"Reactions": 1, "Accept": 1, "Try": 1, "About": 1, "What's the difference": 1}, "adverbs": {"Try": 1}, "numerals": {"14": 1}}}, {"content": "15. If a person has recourse to a treatment person, he or she should be largely responsible for improving the condition.", "word_count": 25, "label": "", "sentence_structure": "Subject鈥搗erb鈥搊bject", "subjective_objective": {"answer": "Subjective", "rationale": "Because the entry looks at the views and attitudes of the therapists, i.e. whether they believe that the patient is primarily responsible for his or her own improvement, which is the subjective view of the therapist."}, "sentence_sentiment": {"answer": "Negative", "rationale": "Because the phrase \"should be largely responsible for improving the condition\" in the entry is mandatory and recalcitrant and negative."}, "language_style": {"answer": "Calm and direct", "rationale": "The entry directly describes the attribution of responsibility, does not overdo the adoration or emotional rendering, and is more calm and direct in language style.\""}, "time_dimension": {"answer": "Present", "rationale": "The section \"If someone has recourse to a treatment person\" describes a hypothetical scenario, which refers to current or future events. And the \"most responsibility for improving the condition\" expresses the behaviour or attitude that should be taken in this case, which is also a current concept. Thus, the entire entry describes the current situation and the norms that should be followed, and the temporal dimension is now judged."}, "sentence_type": "Imperative sentence", "pos": {"nouns": {"person": 2, "Therapist": 1, "Medical conditions": 1, "You're a good man": 1}, "verbs": {"In": 1, "Help": 1, "Should": 1, "Improvement": 1, "Negative": 1, "Most of them": 1}, "adverbs": {"If": 1}, "numerals": {"15": 1, "Large": 1}, "classifiers": {"individual": 1}, "pronouns": {"Here": 1}}}, {"content": "16. Rationality is the avoidance of thinking that leads to anxiety.", "word_count": 17, "label": "", "sentence_structure": "Subject鈥搗erb鈥揷omplement", "subjective_objective": {"answer": "Subjective", "rationale": "This is because the attitude and interpretation of the patient to anxiety is subjective."}, "sentence_sentiment": {"answer": "Negative", "rationale": "Because the \"anxiety\" and \"aversion is rationalization\" in the entries are negative."}, "language_style": {"answer": "Tactful and warm", "rationale": "The entry is more euphemistic, is expressed in a more soft language, is not too much in direct, command language, but is more gentle in inviting the other party to answer questions with some degree of affection."}, "time_dimension": {"answer": "Present", "rationale": "The entry in the future contains a description of the coping strategy or attitude that may occur in the future, namely \"avoiding,\" which is an expectation and an idea for the future. The keywords are \"thinking of anxiety\" and \"scrutinizing consciously\" , which point to the possibility of the future. At the same time, \"rationalizing\" is also an idea of the way forward."}, "sentence_type": "Affirmative declarative sentence", "pos": {"nouns": {"Anxiety": 1, "Thinking": 1, "There's a renegade": 1, "Rationalization": 1}, "verbs": {"Result": 1, "Avoidance": 1}}}, {"content": "17. In psychotherapy, the treatmenter does not think what the patient thinks.", "word_count": 21, "label": "", "sentence_structure": "Subject鈥揷opula鈥損redicative", "subjective_objective": {"answer": "Subjective", "rationale": "Because the entry \"the healer's mind\" and \"the patient's mind\" are both subjectively viewed as the process of psychotherapy. The subjective idea is subjective, so the entry is subjective."}, "sentence_sentiment": {"answer": "Positive", "rationale": "Because the entry \"the treatmenter does not think as important as the patient thinks\" indicates that the treatment takes the patient's thoughts and feelings seriously and is a positive attitude."}, "language_style": {"answer": "Tactful and warm", "rationale": "The entry uses a more euphemistic language style to express the intention of the treatmenter to respect the patient鈥檚 inner feelings and thoughts, rather than to directly emphasize his or her importance. This is a more moderate and acceptable language, which allows for better interaction with the interviewee."}, "time_dimension": {"answer": "Present", "rationale": "The keyword \"in the process of treatment\" means that this is happening now, so it should be judged now."}, "sentence_type": "Negative declarative sentence", "pos": {"nouns": {"Psychotherapy": 1, "Therapist": 1, "Patient": 2}, "verbs": {"I'm thinking": 2, "Important": 1}, "numerals": {"17": 1}, "pronouns": {"Other": 1}}}, {"content": "18. Most of the mental patients discharged should be under close observation for a period of time.", "word_count": 23, "label": "", "sentence_structure": "Subject鈥搗erb鈥搊bject", "subjective_objective": {"answer": "Objective", "rationale": "Because \"de-institutionalization\" and \"psychiatrically\" and \"observation\" are objective facts or situations."}, "sentence_sentiment": {"answer": "Positive", "rationale": "Because the behavior in the entry is \"observation\" to ensure the patient's recovery and health, and it's a positive behavior."}, "language_style": {"answer": "Tactful and warm", "rationale": "The language style of the entry is relatively euphemistic and cautious, with no too direct or hard-line tone, but rather with a moderate and suggested approach, with some degree of protege and protege."}, "time_dimension": {"answer": "Present", "rationale": "\"should\" indicates that this is a recommendatory or guiding phrase that applies to the current or future situation. The condition of \"mental patients discharged from hospital\" is presented in the context of the current circumstances, and therefore the temporal dimension is now judged."}, "sentence_type": "Affirmative declarative sentence", "pos": {"nouns": {"Discharged": 1, "Mental patients": 1, "Period": 1, "Keep your eyes open": 1}, "verbs": {"Accept": 1, "Should": 1, "Give": 1}, "adverbs": {"Most of them": 1}, "numerals": {"One": 1}, "classifiers": {"Period": 1}}}, {"content": "19. The objectives of psychological care should be set by the patient, not the therapist.", "word_count": 20, "label": "", "sentence_structure": "Subject鈥搗erb鈥搊bject", "subjective_objective": {"answer": "Subjective", "rationale": "Because the setting of the goals requires the patient鈥檚 own personal experience and needs, which is the patient鈥檚 subjective feeling and decision. The role of the therapist is to help and support the patient in achieving their goals, and it is also to be seen from the patient鈥檚 subjective perspective."}, "sentence_sentiment": {"answer": "Positive", "rationale": "This item emphasizes the substance and participation of patients, reflects respect for and interest in their rights and interests and is a positive attitude."}, "language_style": {"answer": "Calm and direct", "rationale": "This is a more objective and direct expression, which is not too emotional or rhetorical, but rather directly suggests the idea that the objective of psychotherapy should be set by the patient."}, "time_dimension": {"answer": "Present", "rationale": ""}, "sentence_type": "Affirmative declarative sentence", "pos": {"nouns": {"Psychotherapy": 1, "Objective": 1, "Patient": 1, "Therapist": 1}, "verbs": {"Should": 1, "Development": 1}, "adverbs": {"By": 1}, "numerals": {"19": 1}, "pronouns": {"Here": 1}}}, {"content": "20. The individuals who benefit most from group therapy are almost always those who pay the most attention to group leaders.", "word_count": 31, "label": "", "sentence_structure": "Subject鈥搗erb鈥搊bject", "subjective_objective": {"answer": "Subjective", "rationale": "Because \"the best\" and \"the best care\" are patients' subjective feelings and attitudes."}, "sentence_sentiment": {"answer": "Neutral", "rationale": ""}, "language_style": {"answer": "Calm and direct", "rationale": "The entry is presented with objective data, without too much subjective evaluation or emotional colour. At the same time, the purpose of the article is expressed in a more direct, explicit way, without the use of language that is too complex or convoluted, and therefore more language styles are more incriminating and direct."}, "time_dimension": {"answer": "Present", "rationale": "The entry describes a current behaviour or state where those who benefit most from group therapy tend to be more sensitive to group leaders."}, "sentence_type": "Affirmative declarative sentence", "pos": {"nouns": {"Group therapy": 1, "Personal": 1, "Group leaders": 1}, "verbs": {"Benefit": 1, "Watch out": 1}, "adjectives": {"Most": 2, "Large": 1, "to": 1}, "numerals": {"20": 1, "Most": 2}, "pronouns": {"Those": 1, "Here": 1}}}, {"content": "21. Mental patients should not be encouraged to be left to take care of their daily needs.", "word_count": 20, "label": "", "sentence_structure": "Subject鈥搗erb鈥搊bject", "subjective_objective": {"answer": "Subjective", "rationale": "Because \"not\" and \"encourage\" are subjective terms of evaluation, expressing opposition to or support for a certain behaviour."}, "sentence_sentiment": {"answer": "Negative", "rationale": "Because the expression \"not to encourage\" in this entry is negative in its emotional connotation, implying that the mentally ill should not be left to take care of their daily lives. This is contrary to the basic principle of caring for and respecting the mentally ill, and is therefore negative."}, "language_style": {"answer": "Calm and direct", "rationale": "The language style of the entry is cold, without any euphemism or warm adoration, and presents the point directly, with some stigma and negative colour. This language style may be offensive or uncomfortable."}, "time_dimension": {"answer": "Present", "rationale": "The entry is a statement of a principle or guideline that should be followed and does not address past or future conduct or circumstances, and therefore the judgement is now."}, "sentence_type": "Negative declarative sentence", "pos": {"nouns": {"Mental patients": 1, "others": 1, "Care": 1, "Daily requirements": 1}, "verbs": {"Encourage": 1, "Jean": 1, "Come on": 1}, "adverbs": {"No, I shouldn't": 1}, "numerals": {"21": 1}}}, {"content": "22. If a mental patient is convinced that he/she is cured and no longer need medication, he/she can do so.", "word_count": 30, "label": "", "sentence_structure": "Subject鈥搗erb鈥搊bject", "subjective_objective": {"answer": "Subjective", "rationale": "Because \"sure\" is the patient's own subjective judgment and idea."}, "sentence_sentiment": {"answer": "Negative", "rationale": "Because the entries relate to misunderstandings and disrespect of the mentally ill, they are considered to be free to decide whether or not to continue taking medication, which is in fact a very dangerous and irresponsible act."}, "language_style": {"answer": "Calm and direct", "rationale": "The entry is a simple and clear statement of a fact, which is not overly adouristic or emotional. It is intended to convey a message or alert the interviewee to a situation, rather than to attempt to influence the interviewee鈥檚 attitude or emotions. It is therefore more calm and straightforward in its language."}, "time_dimension": {"answer": "Present", "rationale": "In the entry, the \"may do so\" expresses a presumption and a choice, which suggests that the testee needs to make a decision or act now, and therefore judge now.\""}, "sentence_type": "Affirmative declarative sentence", "pos": {"nouns": {"Mental patients": 1, "Diseases": 1, "Sure": 1, "Healing": 1, "Medicine": 1}, "verbs": {"Suppose": 1, "Yeah": 1, "Do it": 1}, "adjectives": {"Spirit": 1, "Healing": 1}, "numerals": {"22": 1}, "pronouns": {"he": 2, "she": 1}}}, {"content": "23. Persons seeking psychological care seek advice from specialists and do so accordingly.", "word_count": 26, "label": "", "sentence_structure": "Subject鈥搗erb鈥搊bject", "subjective_objective": {"answer": "Objective", "rationale": "Because the question of the inquiry is the purpose of the person seeking psychological treatment, it is an objective fact and not the patient ' s subjective attitude."}, "sentence_sentiment": {"answer": "Negative", "rationale": "Because the purpose of the act expressed in this entry is somewhat restrictive, there is a lack of openness and a positive element of self-exploration."}, "language_style": {"answer": "Calm and direct", "rationale": "The entry states in direct and concise language that the purpose of recourse to psychological treatment is to obtain expert advice and to do so, without any use of rhetorical or emotional techniques, speech temperature and objectivity."}, "time_dimension": {"answer": "Present", "rationale": ""}, "sentence_type": "Affirmative declarative sentence", "pos": {"nouns": {"Purpose": 1, "person": 1, "Appeal": 1, "Psychotherapy": 1, "Observations": 1, "Experts": 1, "Do as you're told": 1}, "verbs": {"Yeah": 1, "Request": 1, "and": 1, "Do it": 1}, "numerals": {"23": 1}}}, {"content": "24. As a general principle, a psychiatrist should be clear in his/her decisions on behalf of the patient.", "word_count": 24, "label": "", "sentence_structure": "Subject鈥搗erb鈥搊bject", "subjective_objective": {"answer": "Subjective", "rationale": "Because \"into the heart\" is a personal feeling or attitude, a subjective view of the patient."}, "sentence_sentiment": {"answer": "Neutral", "rationale": "Because the entry is a statement of fact or of a code of conduct, it is not emotional."}, "language_style": {"answer": "Calm and direct", "rationale": "The language style of the questionnaire entry is more calm and straightforward, and it offers a clear view that it does not use too many rhetorical techniques or emotional rendering. Moreover, the tone of the sentence is more objective and not subjective. So we can describe the language style of the questionnaire entry as \"cool and straight.\""}, "time_dimension": {"answer": "Present", "rationale": "The entry describes a behaviour or attitude, i.e. the mental health doctor鈥檚 mental state of mind that should be in order to make a decision. This mentality is the current code of conduct or moral code, and the basis for the judgement is now."}, "sentence_type": "Interrogative sentence", "pos": {"nouns": {"General principles": 1, "- A psychiatrist": 1, "Decision": 1, "Patients": 1}, "verbs": {"Representative": 1, "Do it": 2, "Reactions": 1, "Tanya": 1}, "adjectives": {"In my heart": 1}, "numerals": {"24": 1}}}, {"content": "25. Disputes about normal and unusual behaviour acquired in a similar manner are at the heart of learning theories that address poor adaptation.", "word_count": 37, "label": "", "sentence_structure": "Subject鈥搗erb鈥搊bject", "subjective_objective": {"answer": "Objective", "rationale": "Because the entry describes the basic concepts and theories of a discipline, not subjective attitudes or experiences involving patients. This descriptive phrase is usually objective."}, "sentence_sentiment": {"answer": "Neutral", "rationale": ""}, "language_style": {"answer": "Calm and direct", "rationale": "The entry describes the central position of normal and abnormal behaviour acquisition in adaptive learning theory in a more formal and professional language style. This expression is relatively objective and calm, and does not have too many emotional or rhetorical features."}, "time_dimension": {"answer": "Past", "rationale": ""}, "sentence_type": "Affirmative declarative sentence", "pos": {"nouns": {"Normal": 1, "Behaviour": 2, "Disputes": 1, "Core": 1, "Part": 1, "Learning Theory": 1, "Poor adaptation": 1}, "verbs": {"Related": 1, "be": 2, "Involving": 1, "Access": 1}, "adjectives": {"Similar": 1, "Unusual": 1}, "numerals": {"25": 1}}}, {"content": "26. A good therapist would encourage patients to make their own decisions about what they should do.", "word_count": 25, "label": "", "sentence_structure": "Subject鈥搗erb鈥搊bject", "subjective_objective": {"answer": "Subjective", "rationale": "It is the subjective attitude and behaviour of the psychotherapist that encourages the patient to make his or her own decisions."}, "sentence_sentiment": {"answer": "Positive", "rationale": "Because the behaviour of encouraging patients to make their own decisions is positive and helps them to build their capacity to make decisions and solve problems."}, "language_style": {"answer": "Calm and direct", "rationale": "The entry is based on a judgement that is clear and concise, that the competence and duties of the psychologist are presented directly, that is, not in the form of excessive rhetorical or emotional expressions, but in the form of objective and clear expressions. The language style of the entry can therefore be described as \"cool and direct\"."}, "time_dimension": {"answer": "Past", "rationale": "The entry describes the ideal characteristics and behaviour of a therapist and does not specifically address the present or future performance of the person tested. The temporal dimension is therefore a past."}, "sentence_type": "Affirmative declarative sentence", "pos": {"nouns": {"Psychopaths": 1, "Patients": 1, "Ideas": 1}, "verbs": {"Yes": 1, "Encourage": 1, "Do it": 1, "Take it": 1}, "adverbs": {"Okay": 1}, "numerals": {"26": 1}, "pronouns": {"self": 1}}}, {"content": "It is much better to ask professionals questions about education than to talk to friends, since professional opinions are more valuable.", "word_count": 34, "label": "", "sentence_structure": "Subject鈥搗erb鈥搊bject", "subjective_objective": {"answer": "Subjective", "rationale": "The sentence expresses the view that professional opinions are more valuable than those of friends. This is a subjective judgement, as it reflects the views and convictions of the authors."}, "sentence_sentiment": {"answer": "Positive", "rationale": "Because the expression \"professional opinion is more valuable\" in the entry is positive emotional."}, "language_style": {"answer": "Calm and direct", "rationale": "The entry expresses, in a direct and clear manner, the affirmation and importance attached to the search for professional opinion, without excessive ado or emotional, and appears to be more calm and straightforward in tone.\""}, "time_dimension": {"answer": "Past", "rationale": "The content of \"to the professionals\" and \"to the friends\" and \"to the professionals' opinions\" are all past experiences and views, so the temporal dimension of this questionnaire entry is past."}, "sentence_type": "Affirmative declarative sentence", "pos": {"nouns": {"Professionals": 1, "Problem": 1, "friends": 1, "Observations": 1}, "verbs": {"To consult": 1, "Talk to": 2, "Much better": 1, "It's more valuable": 1}, "adjectives": {"Professional": 1}, "adverbs": {"More": 2}, "numerals": {"27": 1}}}, {"content": "When a psychological problem is encountered, one cannot find a solution for itself.", "word_count": 22, "label": "", "sentence_structure": "Subject鈥搗erb鈥搊bject", "subjective_objective": {"answer": "Subjective", "rationale": "Because it is a subjective view or belief of a person that they believe cannot solve their psychological problems alone."}, "sentence_sentiment": {"answer": "Negative", "rationale": "Because the idea that a person cannot find a solution by himself is negative in the entry."}, "language_style": {"answer": "Tactful and warm", "rationale": "The entry uses a more euphemistic language style, avoiding directly denying the possibility that individuals can solve their psychological problems independently. It leads respondents to think and accept a general view, which most people need to help with when they face psychological problems."}, "time_dimension": {"answer": "Present", "rationale": "The entry indicates that the testee believes that they need external help to solve the problem in the face of psychological problems. It is a description of the state of the present, not a vision of the future or the past. So the time dimension of the entry is now."}, "sentence_type": "Negative declarative sentence", "pos": {"nouns": {"Psychological problems": 1, "Alone": 1, "Solutions": 1}, "verbs": {"I've got it": 1, "Maybe": 1, "Found it": 1}, "adjectives": {"self": 1}, "adverbs": {"When": 1, "Impossible": 1}, "numerals": {"28": 1}}}]}

{"questionnaire_title": "Internal - external psycho-control source table", "questionnaire_category": "Self-cognition", "item_count": 25, "emotion_shift_overall": "From negative to positive", "language_style_overall": "Calm and direct", "items": [{"content": "1. The problem with most children today is that parents are too indulgent in them.", "word_count": 24, "label": "", "sentence_structure": "Subject鈥搗erb鈥搊bject", "subjective_objective": {"answer": "Subjective", "rationale": "Because this entry presents the subjective view and opinion of researchers on the phenomenon of \"parents giving up their children\"."}, "sentence_sentiment": {"answer": "Negative", "rationale": "Because the word \"leave\" in the entry is negative and implies that parents are not doing their share of responsibility."}, "language_style": {"answer": "Calm and direct", "rationale": "The entry is presented in a straightforward manner, with no excessive qualifier or emotional expression and a calm and objective tone."}, "time_dimension": {"answer": "Present", "rationale": "The entry is a description of the current situation, in which the words \"now\" and \"most\" point to the present. At the same time, the problem arises as it indicates that the problem is happening and is the reflection of the current state of affairs."}, "sentence_type": "Affirmative declarative sentence", "pos": {"nouns": {"Problem": 2, "Son": 1, "Parents": 2, "Let's do it": 1}, "verbs": {"Come on": 1, "be": 1, "It's": 1, "Let's do it": 1}, "adjectives": {"Most of them": 1, "Now": 1}, "numerals": {"One": 1, "Two": 1}, "pronouns": {"they": 1}}}, {"content": "2. The human tragedy is the result of the mistakes they have made.", "word_count": 15, "label": "", "sentence_structure": "Subject鈥揷opula鈥損redicative", "subjective_objective": {"answer": "Subjective", "rationale": "Because \"the mistake people make\" is a subjective judgment, different people may have different views and understandings."}, "sentence_sentiment": {"answer": "Neutral", "rationale": "Since the entry did not express feelings or attitudes, it merely stated one view."}, "language_style": {"answer": "Calm and direct", "rationale": "The entry uses a very direct and clear language style, without any accommodative vocabulary or emotional colour, giving a calm and rational sense of mind and therefore can be judged as a cold and straightforward type."}, "time_dimension": {"answer": "Present", "rationale": "The entry expresses current social perceptions or prevailing views, with no clear time-pointing. It describes the view that the source of misfortune is usually perceived as being due to its own mistakes, and therefore the temporal dimension is now judged."}, "sentence_type": "Affirmative declarative sentence", "pos": {"nouns": {"People": 1, "Unfortunately": 1, "Causes": 1, "Error": 1}, "verbs": {"Yes": 1}}}, {"content": "3. No matter how hard people try to stop it, war will always happen.", "word_count": 17, "label": "", "sentence_structure": "Subject鈥搗erb鈥搊bject", "subjective_objective": {"answer": "Objective", "rationale": "Because the occurrence of war does not depend on the degree of effort of the people."}, "sentence_sentiment": {"answer": "Negative", "rationale": "Because the expression \"war always happens\" in the entry conveys a pessimism and a negative sentiment."}, "language_style": {"answer": "Calm and direct", "rationale": "The entry is presented in a more objective and factual way, without too much emotional rendering or rhetorical, and with a relatively calm and straightforward language style."}, "time_dimension": {"answer": "Present", "rationale": "The entry describes a general phenomenon in which war is repeated, i.e., it is not helpful to try to prevent it. This is not clear when it is directed, and it can be judged as \"now\"."}, "sentence_type": "Affirmative declarative sentence", "pos": {"nouns": {"War": 1, "People": 1, "Stop": 1}, "verbs": {"Try": 1, "Yes": 1, "Come on": 1}, "adverbs": {"Whatever": 1, "What": 1, "Always": 1}, "numerals": {"3": 1}}}, {"content": "4. Unfortunately, no matter how hard a person tries, his value will be largely unrecognized.", "word_count": 25, "label": "", "sentence_structure": "Subject鈥搗erb鈥搊bject", "subjective_objective": {"answer": "Subjective", "rationale": "Because \"driving\" , \"value\" and \"recognized\" evaluations are based on individual subjective perceptions and views. While such evaluations may be influenced by objective circumstances, in this sentence these factors are considered subjective."}, "sentence_sentiment": {"answer": "Negative", "rationale": "Because the result of \"unrecognized\" in the entry is negative."}, "language_style": {"answer": "Tactful and warm", "rationale": "The entry uses a more euphemistic expression, which \"unfortunately\" guides the interviewee into the situation and describes the problem in a more objective and neutral language, which \"no matter how hard a person tries, his value will not be recognized.\" This language helps the interviewer to answer the questions more openly in a psychological questionnaire, while avoiding a disproportionate emotional reaction."}, "time_dimension": {"answer": "Past", "rationale": "The expressions \"unfortunately\" in the entry, \"no matter how hard a person tries\" and \"no matter how much of his value is not recognized\" suggest that this has happened or is happening. It therefore falls within the context of describing the past."}, "sentence_type": "Negative declarative sentence", "pos": {"nouns": {"person": 2, "Try": 1, "Value": 1, "Recognition": 1}, "verbs": {"be": 1, "I'll get it": 1, "Not available": 1}, "adjectives": {"Unfortunately": 1}, "adverbs": {"How's that": 1}, "numerals": {"One": 2}, "pronouns": {"he": 1}}}, {"content": "5. Most students are unaware that their scores are to some extent influenced by the element of accident.", "word_count": 29, "label": "", "sentence_structure": "Subject鈥搗erb鈥搊bject", "subjective_objective": {"answer": "Subjective", "rationale": "Because this phrase describes the students ' perceptions and views, it is subjective."}, "sentence_sentiment": {"answer": "Negative", "rationale": "Because the \"not recognizing\" in the entry is negative."}, "language_style": {"answer": "Tactful and warm", "rationale": "The entry uses a more moderate and objective language style, and presents it to the interviewees in a way that is caring, understanding and supportive. It does not directly criticize or accuse students of misconceptions, but rather presents its views in an open and inclusive manner and attempts to guide the interviewees to think. This expression is more personal and interactive, helping to create a good communication climate and to increase the rate and effectiveness of questionnaires."}, "time_dimension": {"answer": "Present", "rationale": ""}, "sentence_type": "Negative declarative sentence", "pos": {"nouns": {"Students": 2, "Score": 2, "Level": 1, "Accident factor": 1, "Impact": 1}, "verbs": {"Both": 1, "I didn't realize": 1, "Yes": 1}, "adjectives": {"Most of them": 1}, "numerals": {"5": 1, "In a way": 1}, "pronouns": {"they": 1}}}, {"content": "Those who are capable are not able to be leaders because they are unable to take advantage of opportunities.", "word_count": 24, "label": "", "sentence_structure": "Subject鈥搗erb鈥搊bject", "subjective_objective": {"answer": "Subjective", "rationale": "Because the descriptions of \"capable people\" and \"failure to be leaders\" are based on the views and assessments of the respondents, and the reasons for \"failure to take advantage of the opportunities\" are subjective assumptions of the respondents. The perspective of this questionnaire is therefore subjective."}, "sentence_sentiment": {"answer": "Positive", "rationale": "Because the words \"capable people\" and \"failure to be leaders\" in the entries are positive expressions, and \"failure to take advantage of opportunities\" are negative expressions."}, "language_style": {"answer": "Tactful and warm", "rationale": "The entry is more personal, interactive and suitable for psychological questionnaires. At the same time, it is more gentle and euphemistic."}, "time_dimension": {"answer": "Present", "rationale": "So, in terms of time dimension, this entry is more about the current situation, that is, why those who are capable are not leaders. So the answer is:"}, "sentence_type": "Negative declarative sentence", "pos": {"nouns": {"Capacity": 1, "person": 2, "Leader": 1, "Opportunities": 1}, "verbs": {"have": 1, "Yes": 2, "Not": 2, "Be": 1, "Utilization": 1}, "adjectives": {"of": 4, "People with abilities": 1}, "numerals": {"6": 1}, "pronouns": {"they": 1}}}, {"content": "8. How to be alone with someone who can't be attracted to himself.", "word_count": 24, "label": "", "sentence_structure": "Subject鈥搗erb鈥搊bject", "subjective_objective": {"answer": "Subjective", "rationale": "Because it is the views and attitudes of patients in situations where they cannot be favoured."}, "sentence_sentiment": {"answer": "Negative", "rationale": "Because the entries \"can't let others have feelings for themselves\" and \"not to be with others\" have negative emotional aspects."}, "language_style": {"answer": "Calm and direct", "rationale": "The entry is presented directly to the interviewees in order to ask them about the way in which people do not get to be in a good mood for others, and the language style is more calm and objective and does not have too many rhetorical and emotional overtones."}, "time_dimension": {"answer": "Present", "rationale": "The entry describes the current behaviour and attitude of the testees, i.e. they cannot give them a favourable feeling and therefore encounter difficulties in interpersonal relations."}, "sentence_type": "Interrogative sentence", "pos": {"nouns": {"Questionnaire Entry": 1, "I'm so glad": 1, "person": 2, "Get along": 1}, "verbs": {"Jean": 1, "to": 1, "have": 2, "No, I can't": 1, "and": 2, "bit": 1}, "adjectives": {"Other": 1}, "numerals": {"8": 1}, "pronouns": {"Those": 1, "self": 1, "others": 1}}}, {"content": "9. The life experience of a man determines what he is.", "word_count": 19, "label": "", "sentence_structure": "Subject鈥揷opula鈥損redicative", "subjective_objective": {"answer": "Subjective", "rationale": "Because \"a person's life experience\" is a subjective experience and understanding of a person, not an objective fact or something that happens. And \"a person's life\" is based on a person's subjective perception and judgment. So this questionnaire entry is viewed from the patient's subjective perspective."}, "sentence_sentiment": {"answer": "Neutral", "rationale": "Because the entry describes an objective fact, i.e. the impact of an individual ' s life experience on his or her personality, does not express a positive or negative emotional profile."}, "language_style": {"answer": "Tactful and warm", "rationale": "The language style of the entry is euphemistic and warm, and is mainly reflected in a gentle, invitation-style conversation, rather than in direct statements or orders. Moreover, terms such as \"life experience\" and \"decision\" are not expressed in too strong terms, but rather in a more personal way that leads respondents to reflect and share their own views, which is more personal and interactive."}, "time_dimension": {"answer": "Past", "rationale": "The entry describes the impact of a person ' s life experience on an individual, highlighting the relationship between past experience and the characteristics that it embodies."}, "sentence_type": "Affirmative declarative sentence", "pos": {"nouns": {"Life experience": 1, "person": 2, "Decision": 1}, "verbs": {"be": 1, "What": 1, "What's up": 1, "of": 1}, "numerals": {"One": 2}, "classifiers": {"individual": 1}, "pronouns": {"he": 1}}}, {"content": "11. The subject of the test has often been irrelevant to the content of the lecture, and the study has been useless.", "word_count": 28, "label": "", "sentence_structure": "Subject鈥搗erb鈥搊bject", "subjective_objective": {"answer": "Subjective", "rationale": "Because the sense of \"testing is always irrelevant\" and \"retrospect is useless\" is the patient's subjective view."}, "sentence_sentiment": {"answer": "Negative", "rationale": "Because the phrase \"tests are always irrelevant to the content of the lectures, the re-study of the subject is useless\" and it expresses negative attitudes and emotions."}, "language_style": {"answer": "Calm and direct", "rationale": "The entry describes in a concise and clear language the views of the interviewees on the relevance of the subjects and the content of the classroom and the effects of the refresher study, which are not overly rhetorical or emotional, and are more objective and balanced. Its language style is therefore straightforward."}, "time_dimension": {"answer": "Present", "rationale": "Now the entry describes the problems that the subject faces in the current learning process, and the keywords \"many\" and \"always\" indicate that the problem is long-standing, while \"no study\" reveals the specific manifestations of the problem. Thus, the time dimension of the entry can be judged as now."}, "sentence_type": "Negative declarative sentence", "pos": {"nouns": {"Time": 1, "Test question": 1, "Lectures": 1, "Re-learning": 1}, "verbs": {"be": 1, "Same": 1, "It's not relevant": 1, "have": 1, "Use": 1, "No, I'm not": 1}, "adjectives": {"A lot": 1, "A little": 1}, "adverbs": {"Always": 1}, "classifiers": {"individual": 1}, "pronouns": {"Here": 1}}}, {"content": "12. The world was dominated by a few people in power, and little could be done by the small.", "word_count": 26, "label": "", "sentence_structure": "Subject鈥揷opula鈥損redicative", "subjective_objective": {"answer": "Subjective", "rationale": "Because it expresses the patient ' s vision and views of the world, it is a manifestation of subjective attitudes."}, "sentence_sentiment": {"answer": "Negative", "rationale": "Because the words \"a few manipulations of power\" and \"no small person can do anything about it\" in the entries are negative emotional."}, "language_style": {"answer": "Calm and direct", "rationale": ""}, "time_dimension": {"answer": "Present", "rationale": "The entry did not have clear time indicators or verbs, but it could be understood as a description or view of the past. It expressed an opinion of the current state of the world, that it is controlled by a few people and that ordinary people cannot change that. So we can judge it as a past."}, "sentence_type": "Negative declarative sentence", "pos": {"nouns": {"World": 1, "person": 1, "Manipulation": 1, "Little man": 1}, "verbs": {"Main": 1, "By": 1, "Do it": 1}, "adjectives": {"Less": 1, "How many": 1, "The power": 1}, "adverbs": {"For that": 1, "No, I'm fine": 1}, "numerals": {"12": 1, "How many": 1}, "pronouns": {"Here": 1}}}, {"content": "13. Pre-planneding is not always the best way to do it, because many things end up as a very lucky product.", "word_count": 31, "label": "", "sentence_structure": "Subject鈥揷opula鈥損redicative", "subjective_objective": {"answer": "Subjective", "rationale": "Because the words \"just\" and \"good luck\" indicate that it's the patient's thoughts and feelings, not the objective fact."}, "sentence_sentiment": {"answer": "Negative", "rationale": "Because the phrase \"just a lucky product\" in the entry is negative and negative emotional."}, "language_style": {"answer": "Calm and direct", "rationale": "The entry describes in an objective and direct manner the view that advance planning is not always the best strategy, and that there is no excessive emotional or rhetorical style and a calmer tone."}, "time_dimension": {"answer": "Past", "rationale": "Thus, in terms of time dimension, it can be considered either as a past experience or status (i.e., some may consider that planning is not always effective) or as a possible outcome in the future (i.e., in some cases success may be due to luck rather than planning). In conclusion, the time frame for the purpose of this article is, and will be, possible."}, "sentence_type": "Negative declarative sentence", "pos": {"nouns": {"Things": 1, "Good luck": 1, "Production": 1}, "verbs": {"Order": 1, "Planned": 1, "be": 2, "Best practice": 1, "Always": 1, "In the end": 1, "Just": 1, "Lucky": 1, "Generate": 1}, "adverbs": {"Not really": 1, "Always": 1}, "numerals": {"13": 1}, "pronouns": {"Here": 1}}}, {"content": "14. Everyone has a good side:", "word_count": 10, "label": "", "sentence_structure": "Subject鈥搗erb鈥揷omplement", "subjective_objective": {"answer": "Subjective", "rationale": "Because it is the patient ' s perception of others that is the expression of individual subjective judgment."}, "sentence_sentiment": {"answer": "Neutral", "rationale": "Because the entry merely stated a point of view, it did not address emotional or behavioural tendencies."}, "language_style": {"answer": "Tactful and warm", "rationale": "The entry expresses a positive view of everyone in a spirit of encouragement and support, rather than directly criticizing or rejecting. This expression is more likely to create a positive and harmonious atmosphere that will facilitate the collection of more real and positive answers."}, "time_dimension": {"answer": "Present", "rationale": "The entry describes the prevailing phenomena or facts, has no specific time frame or future expectations and therefore is now judged."}, "sentence_type": "Affirmative declarative sentence", "pos": {"nouns": {"Everyone": 1, "Okay": 1, "One side": 1}, "verbs": {"have": 1, "be": 1}, "numerals": {"14": 1}}}, {"content": "15. Many times we prefer to make decisions by throwing coins.", "word_count": 16, "label": "", "sentence_structure": "Subject鈥搗erb鈥搊bject", "subjective_objective": {"answer": "Subjective", "rationale": "Because it is a subjective choice and attitude of one to decide whether to flip a coin."}, "sentence_sentiment": {"answer": "Neutral", "rationale": ""}, "language_style": {"answer": "Calm and direct", "rationale": "The language style of the entry is more calm and straightforward, with the presentation of the facts being the main one, and there is no excessive emotional rendering or euphemism. This style clearly conveys what the investigator wants to know.\""}, "time_dimension": {"answer": "Present", "rationale": "The keyword \"many times\" indicates that it is a regular, customary act, and \"better\" it means that the tester prefers to make decisions in this way, which points to the conduct that is now being carried out. Thus, the entry describes the situation."}, "sentence_type": "Affirmative declarative sentence", "pos": {"nouns": {"Time": 1, "we": 1, "Coin": 1, "Decision": 1}, "verbs": {"I'd rather": 1, "Throw": 1, "Do it": 1}, "adverbs": {"A lot": 1, "Many times": 1}, "numerals": {"15": 1}, "pronouns": {"you": 1}}}, {"content": "16. It depends on people ' s abilities to do the right job, and luck has nothing to do with it.", "word_count": 27, "label": "", "sentence_structure": "Subject鈥搗erb鈥搊bject", "subjective_objective": {"answer": "Objective", "rationale": "Since capacity is an objective element, and luck is an uncontrollable factor, whether it allows people to do the right job does not depend on luck."}, "sentence_sentiment": {"answer": "Positive", "rationale": "Because the approach in the entries is positive, it is believed that the achievements of people should be judged by their abilities and not by luck."}, "language_style": {"answer": "Calm and direct", "rationale": "The entry is more direct and rational, has no excessive emotional or oral style, and is more calm and objective. It expresses the view that \"to do the right job for yourself depends on your abilities\", and that luck is not decisive here."}, "time_dimension": {"answer": "Past", "rationale": "It is therefore not possible to determine whether it is past, present or future behaviour or mentality."}, "sentence_type": "Affirmative declarative sentence", "pos": {"nouns": {"People": 2, "Work": 1, "Capacity": 1, "Lucky": 1}, "verbs": {"Jean": 1, "Do it": 1, "It depends": 1}, "adjectives": {"Suits": 1, "Relations": 1}, "adverbs": {"This": 1, "Nothing": 1}, "numerals": {"16": 1}, "pronouns": {"they": 1}}}, {"content": "17. People could control the world ' s affairs by participating actively in political and social affairs.", "word_count": 25, "label": "", "sentence_structure": "Subject鈥搗erb鈥搊bject", "subjective_objective": {"answer": "Subjective", "rationale": "Because \"manage the world\" is the subjective perception and expectation of the participants, not the objective fact."}, "sentence_sentiment": {"answer": "Positive", "rationale": "Because the entry emphasizes the positive role of active participation in political and social affairs in the mastery of the world, it conveys a positive attitude and emotional colour."}, "language_style": {"answer": "Calm and direct", "rationale": "The entry presents a point directly, without using any stylistic or emotional presentation, and is more objective in tone. It does not ask the respondents' views, nor does it express any euphemism or encouragement, and therefore belongs to the \"cool and direct\" language style."}, "time_dimension": {"answer": "Present", "rationale": "In the future, the entry \"Can control the world\" expresses a vision of the future, while \"active participation in political and social affairs\" is the action required to achieve that goal. The time dimension of the sentence is therefore judged to be the future."}, "sentence_type": "Affirmative declarative sentence", "pos": {"nouns": {"person": 2, "Political": 1, "Social": 1, "Service": 1, "World": 1, "Yes": 1}, "verbs": {"Yes": 1, "Participation": 1, "Control": 1}, "adjectives": {"Just": 1, "Positive": 1}, "numerals": {"17": 1}, "pronouns": {"People": 1}}}, {"content": "There is no such thing as 鈥渓uck鈥?", "word_count": 10, "label": "", "sentence_structure": "Subject鈥揷opula鈥損redicative", "subjective_objective": {"answer": "Subjective", "rationale": "Because luck is a subjective view and conviction, different people may have different views. So, \"18. There's no such thing as \"luck.\" \"It's the patient's subjective attitude and conviction that is being examined."}, "sentence_sentiment": {"answer": "Neutral", "rationale": "Since the purpose of the article is expressed as objective facts and does not involve emotional tendencies or value judgements, it is neutral."}, "language_style": {"answer": "Tactful and warm", "rationale": "The language style of the entry is more euphemistic. It does not directly negate the existence of \"luck\" but expresses its views in a more subtle way, which is more likely to trigger the reflection and discussion of the respondents and increase the participation of the questionnaire."}, "time_dimension": {"answer": "Future", "rationale": "The entry is a subjective judgement about whether the person tested has a certain view or belief in the future, and the temporal dimension is the future."}, "sentence_type": "Negative declarative sentence", "pos": {"nouns": {"Lucky": 1}, "verbs": {"No, I'm not": 1, "be": 1, "have": 1}, "adjectives": {"Not at all": 1}, "adverbs": {"There's no such thing as a": 1}}}, {"content": "19. The best way to disguise mistakes is usually to cover them.", "word_count": 11, "label": "", "sentence_structure": "Subject鈥揷opula鈥損redicative", "subjective_objective": {"answer": "Objective", "rationale": "Because \"smearing the wrong\" is a specific act or fact, not the patient's mind or feeling, it is an objective view."}, "sentence_sentiment": {"answer": "Negative", "rationale": "Because the \"disguised error\" is negative."}, "language_style": {"answer": "Calm and direct", "rationale": "The language style of the entry is calm and straightforward. It clearly states the view that \"the disguise of error is usually the best way\", and it is not expressed in any rhetorical or emotional terms. It is more objective, direct and clear, and less subjective."}, "time_dimension": {"answer": "Past", "rationale": "The entry describes a view or belief ( \"the best way to disguise a mistake\"), but it does not clearly indicate whether it is past, present or future behaviour or mentality, and therefore cannot determine its temporal dimension."}, "sentence_type": "Negative declarative sentence", "pos": {"verbs": {"Cover it up": 1, "Error": 1, "Usually": 1}, "adjectives": {"Best": 1}, "adverbs": {"be": 1}}}, {"content": "20. How many friends you have depends on how much you're dealing with.", "word_count": 16, "label": "", "sentence_structure": "Subject鈥搗erb鈥搊bject", "subjective_objective": {"answer": "Subjective", "rationale": "The number of friends is determined by the characteristics and character of the individual, which is the patient ' s personal view and feelings, and is therefore subjectively viewed."}, "sentence_sentiment": {"answer": "Neutral", "rationale": "Because the entry does not have any emotional color."}, "language_style": {"answer": "Tactful and warm", "rationale": "The language style of the entry is euphemism. It talks in a quiz and uses a more subjective term \"depending on what you are.\" It is more personal and interactive. At the same time, it does not overemphasize or dictate what the other side should do, but rather allows the interviewee to respond to the questionnaire according to his own circumstances, making the questionnaire more human."}, "time_dimension": {"answer": "Present", "rationale": ""}, "sentence_type": "Affirmative declarative sentence", "pos": {"nouns": {"friends": 1, "person": 2}, "verbs": {"have": 1, "Remove": 1}, "adjectives": {"Here": 1, "Sample": 1}, "adverbs": {"More": 1}, "numerals": {"One": 1, "Some": 1}, "pronouns": {"you": 2}}}, {"content": "21. Most of the misfortunes were caused by lack of talent, ignorance and inertia.", "word_count": 20, "label": "", "sentence_structure": "Subject鈥揷opula鈥損redicative", "subjective_objective": {"answer": "Subjective", "rationale": "Because it involves the patient's subjective view of the definition and attribution of \"unfortunateness\"."}, "sentence_sentiment": {"answer": "Negative", "rationale": "Because the phrase \"unfortunately caused by lack of talent, ignorance and laziness\" in the entry is negative and demeaning."}, "language_style": {"answer": "Calm and direct", "rationale": "The entry presents the point in an objective and clear manner, without excessive rhetorical or emotional colouring, and with a more calm and straightforward tone."}, "time_dimension": {"answer": "Past", "rationale": "The sentence describes a general view or belief, with no clear time-pointing, and therefore the temporal dimension is judged to be the past."}, "sentence_type": "Affirmative declarative sentence", "pos": {"nouns": {"Unfortunately": 1, "That's right": 1, "Yes": 1, "Lazy": 1}, "verbs": {"be": 1, "Missing": 1, "Zhu": 1}, "adjectives": {"Large": 1, "More": 1, "not": 1, "Less": 1, "Lucky": 1}, "numerals": {"21": 1}}}, {"content": "It was too difficult to control the work of politicians in the office.", "word_count": 23, "label": "", "sentence_structure": "Subject鈥搗erb鈥搊bject", "subjective_objective": {"answer": "Subjective", "rationale": "Because the entry is a reflection of the views and feelings of people, which are that it is difficult to control the behaviour of politicians. This is a subjective observation and reflection."}, "sentence_sentiment": {"answer": "Negative", "rationale": "Because the expression \"control of politicians in the office\" in the entry is too difficult to do, it has a negative emotional tone, which shows that people feel helpless and powerless."}, "language_style": {"answer": "Calm and direct", "rationale": "The language style of the entry is so calm that it is a direct expression of the view of the difficulty of controlling the behaviour of politicians, without too much ado or emotional."}, "time_dimension": {"answer": "Past", "rationale": ""}, "sentence_type": "Interrogative sentence", "pos": {"nouns": {"People": 1, "Politicians": 1, "Office": 1, "- A trick": 1}, "verbs": {"Yes": 1, "Control": 1, "Fuck": 1}, "adjectives": {"It's too hard": 1}, "numerals": {"22": 1}}}, {"content": "23. I have learned that there is a direct link between merit and merit.", "word_count": 17, "label": "", "sentence_structure": "Subject鈥搗erb鈥搊bject", "subjective_objective": {"answer": "Subjective", "rationale": "Because the sentence expresses a view or view that there is a direct link between \"learning for good\" and \"good and bad.\" This is a subjective judgement and perception of the individual. While this relationship is based to some extent on objective facts (e.g. learning efforts can improve performance), the perception and conviction of such a connection remain subjective."}, "sentence_sentiment": {"answer": "Positive", "rationale": "Because it expresses a positive belief that learning can bring good results."}, "language_style": {"answer": "Calm and direct", "rationale": "The language style of the entry is more direct and concise, with no excessive adoration or emotional rendering, with facts and logic as the main expression, and more scientific and objective. At the same time, the sentence clearly expresses the relationship between the level of effort and the achievement of learning, and is clear and understandable."}, "time_dimension": {"answer": "Present", "rationale": "The entry expresses the views and beliefs of the speaker on the relationship between learning and achievement, does not address past or future behaviour or state and therefore is judged to be \"now\"."}, "sentence_type": "Interrogative sentence", "pos": {"nouns": {"Learning": 1, "Fountain": 1, "Achievements": 1, "It's bad": 1, "Contact": 1}, "verbs": {"be": 1, "have": 1, "Direct": 1, "and": 1, "Learn": 1}, "adverbs": {"Yes": 1}, "numerals": {"23": 1}, "pronouns": {"I": 1}}}, {"content": "A good leader would give a clear division of labour to everyone.", "word_count": 19, "label": "", "sentence_structure": "Subject鈥搗erb鈥搊bject", "subjective_objective": {"answer": "Objective", "rationale": "Because \"specify division of labour for everyone\" is a responsibility and behaviour that should be assigned to the leader, and it is objective and not the patient's own subjective idea."}, "sentence_sentiment": {"answer": "Positive", "rationale": "Because the \"specified division of labour\" in the entry is positive, it shows that leaders have good organizational and managerial capacity."}, "language_style": {"answer": "Calm and direct", "rationale": "The language style of the entry is calm and straightforward, and it presents a view or fact in a concise and clear manner, with no excessive emotional or rhetorical style. This language style is common in questionnaires, as it helps respondents to better understand and answer questions without misunderstanding or confusion over language complexity."}, "time_dimension": {"answer": "Present", "rationale": "The keyword \"will\" uses the general current, indicating that such behaviour occurs now or frequently. The entry therefore asks the testee about the views of current or past leaders and does not address future situations."}, "sentence_type": "Affirmative declarative sentence", "pos": {"nouns": {"Leader": 1, "person": 1, "Division of labour": 1}, "verbs": {"Here": 1, "Do it": 1}, "adjectives": {"Okay": 1, "Clear": 1}, "numerals": {"24": 1}, "pronouns": {"Every": 1}}}, {"content": "25. I would never believe that opportunity or luck could play an important role in my life.", "word_count": 24, "label": "", "sentence_structure": "Subject鈥搗erb鈥搊bject", "subjective_objective": {"answer": "Subjective", "rationale": "Because \"never believe\" is a subjective attitude or belief of a human being."}, "sentence_sentiment": {"answer": "Negative", "rationale": "Because the entry expresses mistrust and negative attitudes towards opportunity and luck."}, "language_style": {"answer": "Calm and direct", "rationale": "The language style of the entry is more calm, and it does not use too many rhetorical techniques or emotional renderings, but rather expresses the other side鈥檚 views of opportunity or luck in a straightforward way. This expression is more objective and rational."}, "time_dimension": {"answer": "Past", "rationale": "\"No belief\" in \"fundamental\" expresses a strong negative attitude and view of the past, while \"convincing\" adopts the general current, indicating that this attitude is still present. The entry therefore asks about the behaviour and mentality of the subjects in the past."}, "sentence_type": "Interrogative sentence", "pos": {"nouns": {"Opportunities": 1, "Lucky": 1, "Life": 1}, "verbs": {"Yes": 3, "in": 1, "Rise": 1, "Role": 1}, "adjectives": {"Not at all": 1, "very": 1}, "adverbs": {"No, I won't": 1, "or": 1}, "numerals": {"25": 1}, "classifiers": {"individual": 1}, "pronouns": {"I": 1}}}, {"content": "It's no use trying to please others. People who like you will like you.", "word_count": 23, "label": "", "sentence_structure": "Subject鈥搗erb鈥搊bject", "subjective_objective": {"answer": "Subjective", "rationale": "Because the sentence emphasizes \"who likes you, will like you.\" It's the patient's personal opinion and feelings, so it's subjective."}, "sentence_sentiment": {"answer": "Positive", "rationale": "Because it expresses a positive attitude that a person is not a person's preference but a natural emotion."}, "language_style": {"answer": "Tactful and warm", "rationale": "The entry offers proposals in a euphemismal and encouraging way to express the idea that \"do not take too seriously others' views, be true and be appreciated.\" Such language styles are more acceptable and understandable, helping respondents to relax and answer questions in a frank manner."}, "time_dimension": {"answer": "Present", "rationale": ""}, "sentence_type": "Affirmative declarative sentence", "pos": {"nouns": {"others": 1, "person": 1, "Beloved": 1, "Useful": 1, "Yeah": 2, "you": 2}, "verbs": {"Try": 1, "Nothing": 1, "Yes": 1}, "adverbs": {"It's natural": 1}, "numerals": {"26": 1}}}, {"content": "27. Sport is an excellent way of shaping character.", "word_count": 19, "label": "", "sentence_structure": "Subject鈥揷opula鈥損redicative", "subjective_objective": {"answer": "Subjective", "rationale": "Because it's a question of whether sport can shape character. It's subjective."}, "sentence_sentiment": {"answer": "Positive", "rationale": "Because the phrase \"sport is the perfect way\" in the entry is positive."}, "language_style": {"answer": "Tactful and warm", "rationale": "The entry is a soft, invited expression of the positive impact of sport on character formation, without direct command or evaluation of the other party鈥檚 consent. Such expression is more personal and interactive, and more easily engages and shares the views of the interviewees."}, "time_dimension": {"answer": "Present", "rationale": "The entry expresses a view or view of the current situation, which does not relate to past or future acts or events. The keyword \"is\" and \"a\" both indicates that this is the current statement and therefore the judgement is the present."}, "sentence_type": "Affirmative declarative sentence", "pos": {"nouns": {"Sports": 1, "Character": 1, "Modalities": 1}, "verbs": {"be": 1, "Shape": 1}, "adjectives": {"Excellent": 1}, "numerals": {"27": 1}}}]}

{"questionnaire_title": "Core self-evaluation tables", "questionnaire_category": "Self-cognition", "item_count": 10, "emotion_shift_overall": "From positive to negative", "language_style_overall": "Tactful and warm", "items": [{"content": "I believe I can succeed in life.", "word_count": 14, "label": "", "sentence_structure": "Subject鈥搗erb鈥搊bject", "subjective_objective": {"answer": "Subjective", "rationale": "Because \"convincing\" is the patient's subjective attitude, not the objective fact."}, "sentence_sentiment": {"answer": "Positive", "rationale": "Because \"believing in your life to succeed\" is a positive mentality and conviction."}, "language_style": {"answer": "Tactful and warm", "rationale": "The language style of the entry is euphemism. The judgment is based on the idea that the article begins with a positive and positive expression, \"I believe\" rather than directly asking or ordering the interviewee to believe. Moreover, \"life is a positive set of goals, not a negative pressure. Overall, the questionnaire entry is more moderate, friendly and encouraging in its expression and more in keeping with the euphemism of the language style."}, "time_dimension": {"answer": "Present", "rationale": "The entry expresses the belief or attitude of the testee to his or her own, which is not relevant to the past, present and future time dimensions, and therefore cannot be judged by \"the past\", \"the present\" or \"the future\"."}, "sentence_type": "Affirmative declarative sentence", "pos": {"nouns": {"Life": 1, "Success": 1}, "verbs": {"Yes": 2, "Access": 1}, "pronouns": {"self": 1}}}, {"content": "I'm always feeling depressed.", "word_count": 9, "label": "", "sentence_structure": "Subject鈥搗erb鈥揷omplement", "subjective_objective": {"answer": "Subjective", "rationale": "Because \"often feeling depressed\" is a description of an emotional state, of the patient's subjective experience and feelings."}, "sentence_sentiment": {"answer": "Negative", "rationale": "Because the entry \"frightfully low\" is an expression of a negative emotional state."}, "language_style": {"answer": "Calm and direct", "rationale": "The entry directly expresses the subject of questioning whether the other party often feels depressed, does not use excessive rhetoric or emotional rendering, and is more objective in tone."}, "time_dimension": {"answer": "Present", "rationale": "\"I often feel\" means \"often\" a high-frequency behavior, and \"like\" uses the normal current, which means that this behavior has not only occurred in the past, but may still be ongoing. So the entry describes the current situation, where the testees are often feeling depressed at the present time."}, "sentence_type": "Affirmative declarative sentence", "pos": {"nouns": {"Emotion": 1, "Down": 1}, "verbs": {"I feel": 1, "Regular": 1}, "numerals": {"2": 1}, "pronouns": {"I": 1}}}, {"content": "When I failed, I felt useless.", "word_count": 11, "label": "", "sentence_structure": "Subject鈥搗erb鈥揷omplement", "subjective_objective": {"answer": "Subjective", "rationale": "Because it feels like a subjective emotion and attitude."}, "sentence_sentiment": {"answer": "Negative", "rationale": "Because the entry \"feels useless\" expresses a negative and depressed mood."}, "language_style": {"answer": "Tactful and warm", "rationale": "The entry is asked in a caring and supportive manner, trying to understand the emotional experience of the subject in the face of failure, and is expressed in a more moderate and encouraging manner."}, "time_dimension": {"answer": "Present", "rationale": "Key words \"feel\" and \"self is useless\" are descriptions of the state of the moment, which means the emotional experience of the person who was tested when he or she was unsuccessful."}, "sentence_type": "Negative declarative sentence", "pos": {"nouns": {"Failed": 1, "Feel": 1, "self": 1, "It's useless": 1}, "verbs": {"Yes": 1, "I think": 1, "Yeah": 1}, "numerals": {"3": 1}, "pronouns": {"I": 1}}}, {"content": "I can do all the work.", "word_count": 11, "label": "", "sentence_structure": "Subject鈥搗erb鈥揷omplement", "subjective_objective": {"answer": "Subjective", "rationale": "Because \"success\" is a subjective judgement, it's a patient's self-evaluation of his/her abilities."}, "sentence_sentiment": {"answer": "Positive", "rationale": "Because it expresses a sense of confidence and competence."}, "language_style": {"answer": "Tactful and warm", "rationale": "The entry uses positive language to ask the other side whether it can successfully accomplish its tasks, with a sense of encouragement and support, which is more euphemism and moderation and can be respected and valued."}, "time_dimension": {"answer": "Present", "rationale": "The keyword \"successful completion of tasks\" indicates that the person tested is either on the way or capable of fulfilling his or her mission, which is a matter of current status. The temporal dimension therefore is now judged."}, "sentence_type": "Affirmative declarative sentence", "pos": {"nouns": {"I": 1, "Success": 1, "Mandates": 1}, "verbs": {"Yes": 1, "Completed": 1}, "adverbs": {"Okay": 1}, "numerals": {"4": 1}}}, {"content": "I feel like I'm not sure about work.", "word_count": 14, "label": "", "sentence_structure": "Subject鈥揷opula鈥損redicative", "subjective_objective": {"answer": "Subjective", "rationale": "I think that personal knowledge and feelings about work (learning) are subjective."}, "sentence_sentiment": {"answer": "Negative", "rationale": "Because \"uncertainty\" in the entries expresses a negative emotional attitude, namely, lack of self-confidence in their work or learning."}, "language_style": {"answer": "Tactful and warm", "rationale": "The article is relatively soft in tone, not in a strong negative or critical tone, but in a mild way that expresses the doubts and concerns of the other party, which is more likely to resonate and respond positively."}, "time_dimension": {"answer": "Present", "rationale": ""}, "sentence_type": "Negative declarative sentence", "pos": {"nouns": {"self": 1, "Work": 1, "Learning": 1}, "verbs": {"I think": 1, "have": 1, "Hold on": 1}, "adverbs": {"5": 1}, "numerals": {"I": 1}}}, {"content": "I'm satisfied with myself, in general.", "word_count": 10, "label": "", "sentence_structure": "Subject鈥揷opula鈥損redicative", "subjective_objective": {"answer": "Subjective", "rationale": "As satisfaction is a subjective feeling of one person and an assessment of one ' s own. While this assessment may be influenced by external factors, it is still the result of individual subjectivity."}, "sentence_sentiment": {"answer": "Positive", "rationale": "Because the term \"satisfaction\" is positive and the evaluation is self-evaluation and has a positive emotional tone."}, "language_style": {"answer": "Tactful and warm", "rationale": "The entry is used in a more moderate way to ask the respondents how they feel about their overall satisfaction, rather than directly demanding or ordering, which makes them more comfortable and acceptable. At the same time, the term \"general\" is used to describe the issues, making them more open and inclusive and to gain the real feelings of the interviewees."}, "time_dimension": {"answer": "Present", "rationale": "The word \"6\" indicates that the entry is the sixth question, and the word \"general\" in \"generally\" represents the overall, comprehensive picture, while the word \"come\" adopts the general current, meaning that this evaluation has not only occurred in the past, but may still be ongoing. Thus, the time dimension of the entry is judged to be the present."}, "sentence_type": "Affirmative declarative sentence", "pos": {"nouns": {"Overall": 1, "self": 1}, "verbs": {"Satisfied": 1}, "adverbs": {"6": 1, "Overall": 1}}}, {"content": "I doubt my ability.", "word_count": 8, "label": "", "sentence_structure": "Subject鈥搗erb鈥揷omplement", "subjective_objective": {"answer": "Subjective", "rationale": "Because \"suspecting one's own abilities\" is a personal, subjective idea and feeling. Although this idea may be based on some objective facts or experience, it is still subjective in itself."}, "sentence_sentiment": {"answer": "Negative", "rationale": "Because the \"suspect of your abilities\" emotion in the entry is negative."}, "language_style": {"answer": "Calm and direct", "rationale": "Because the entry raises the question in a more objective manner, does not have too many rhetorical or emotional expressions, is more plain and therefore belongs to the \"cool and straight\" language style."}, "time_dimension": {"answer": "Past", "rationale": "Past."}, "sentence_type": "Interrogative sentence", "pos": {"nouns": {"self": 1, "Capacity": 1}, "verbs": {"Doubt": 1}, "adverbs": {"Down": 1, "Most": 1}, "numerals": {"7": 1}}}, {"content": "I feel like I'm not sure I'm gonna succeed.", "word_count": 16, "label": "", "sentence_structure": "Subject鈥揷opula鈥損redicative", "subjective_objective": {"answer": "Subjective", "rationale": "Because \"I feel uncertain about my career success\" is a subjective feeling and view of a person, not an objective reality."}, "sentence_sentiment": {"answer": "Negative", "rationale": "Because the \"unsure\" attitude in the entry is negative."}, "language_style": {"answer": "Calm and direct", "rationale": "The language style of the entry is calm and straightforward, and it uses the subjective term \"thinking himself\" and the specific description \"unsure of career success\" to express the attitudes and feelings of the person under investigation. This expression is more objective and direct, less emotional or rhetorical, and makes it easier for the person under investigation to give a true answer."}, "time_dimension": {"answer": "Past", "rationale": "The entry describes the current mindset and feelings of the testee, relates to the current state and the ideas and is therefore in the context of the past."}, "sentence_type": "Negative declarative sentence", "pos": {"nouns": {"Career": 1, "Success": 1}, "verbs": {"I think": 1, "have": 1}, "adjectives": {"self": 1}, "adverbs": {"Nope": 1, "No, I'm not": 1}, "numerals": {"8": 1}}}, {"content": "I have the ability to handle most of my own problems.", "word_count": 14, "label": "", "sentence_structure": "Subject鈥搗erb鈥搊bject", "subjective_objective": {"answer": "Subjective", "rationale": "Because \"capability\" is a self-assessment of an individual and is a subjective judgement."}, "sentence_sentiment": {"answer": "Positive", "rationale": "Because the entry expresses a positive attitude that it is capable of solving the problem independently."}, "language_style": {"answer": "Tactful and warm", "rationale": "The language style of this entry is more euphemistic and positive and encouraging, is plain and sweet, and is not expressed in too direct or strong a tone. It also includes trust and certainty in the interviewee鈥檚 self-capacity, making it more acceptable and willing to answer questions."}, "time_dimension": {"answer": "Present", "rationale": ""}, "sentence_type": "Interrogative sentence", "pos": {"nouns": {"Capacity": 1, "Problem": 2}, "verbs": {"have": 1, "Processing": 1, "self": 2, "of": 3, "Most of them": 1}, "numerals": {"9": 1}, "classifiers": {"individual": 1}, "pronouns": {"I": 1}}}, {"content": "I feel terrible about 10 things. I don't have hope.", "word_count": 15, "label": "", "sentence_structure": "Subject鈥搗erb鈥揷omplement", "subjective_objective": {"answer": "Subjective", "rationale": "Because \"think\" is the patient's subjective feeling and attitude."}, "sentence_sentiment": {"answer": "Negative", "rationale": "Because the words \"bad, hopeless\" in the entry are negative."}, "language_style": {"answer": "Calm and direct", "rationale": "The entry is in plain and simple language and directly expresses the question whether the other party often feels that much of life is bad, that there is no hope for the subject, that there is no excessive rhetoric or emotional rendering, and that the tone is more objective."}, "time_dimension": {"answer": "Present", "rationale": "The keyword \"thinks\" means a subjective feeling, and \"many\" and \"all\" implies the prevalence and continuity of such emotions, so the temporal dimension is now judged."}, "sentence_type": "Negative declarative sentence", "pos": {"nouns": {"Things": 2, "Hope": 1}, "verbs": {"I think": 1, "very": 2, "No, I'm not": 1, "Shit": 1}, "adverbs": {"A lot": 1, "Both": 1}, "numerals": {"10": 1}, "classifiers": {"individual": 1}, "pronouns": {"I": 1}}}]}

{"questionnaire_title": "Self-measurement table for depression (SDS)", "questionnaire_category": "Mental health", "item_count": 20, "emotion_shift_overall": "From negative to positive", "language_style_overall": "Tactful and warm", "items": [{"content": "I'm bored and depressed.", "word_count": 11, "label": "", "sentence_structure": "Subject鈥揷opula鈥損redicative", "subjective_objective": {"answer": "Subjective", "rationale": ""}, "sentence_sentiment": {"answer": "Negative", "rationale": "Because \"silent\" and \"unsatisfied\" in the entries are terms that describe negative emotions."}, "language_style": {"answer": "Tactful and warm", "rationale": "The entry uses a more euphemistic and gentle tone, without directly accusing or ordering the other party about what to do, but rather asking questions in a caring and understanding manner. Moreover, the words \"fell\" and \"silent\" convey a more intimate and caring emotional colour, making the questionnaire more personal and interactive."}, "time_dimension": {"answer": "Present", "rationale": "Now the entry expresses the current feelings and state of the subject, and the key words \"frightful\" and \"silent\" point to the moment."}, "sentence_type": "Negative declarative sentence", "pos": {"nouns": {"I think": 1, "It's boring": 1, "I'm depressed": 1}, "adjectives": {"It's boring": 1, "Low": 1}, "adverbs": {"I think": 1}}}, {"content": "I think it's best in the morning.", "word_count": 11, "label": "", "sentence_structure": "Subject鈥揷opula鈥損redicative", "subjective_objective": {"answer": "Subjective", "rationale": "Because \"think\" represents the patient's personal feelings and perspectives, and it's seen from a subjective perspective."}, "sentence_sentiment": {"answer": "Positive", "rationale": "Because the attitude in the entries is positive, and it's the best time in the morning."}, "language_style": {"answer": "Tactful and warm", "rationale": "The language style of the entry is more euphemistic and emotional, making the respondents more natural and real when expressing their views by using subjectively defined terms such as \"I feel\" and also making the interviewee feel more friendly and comfortable."}, "time_dimension": {"answer": "Present", "rationale": "The entry indicates the view or feeling of the testee at a certain point in time, i.e. \"I feel\" and is expressed at the moment, and therefore the judgement is now."}, "sentence_type": "Affirmative declarative sentence", "pos": {"nouns": {"Oh, my God": 1, "Morning": 1}, "verbs": {"I think": 1, "Okay": 1}, "adjectives": {"Morning": 1}, "numerals": {"2": 1}, "pronouns": {"I": 1}}}, {"content": "I cried in the field for a while or I tried to cry.", "word_count": 12, "label": "", "sentence_structure": "Subject鈥搗erb鈥揷omplement", "subjective_objective": {"answer": "Subjective", "rationale": "Because \"a moment of position cries out or wants to cry\" describes the patient's own emotional reactions and experiences, which are subjective."}, "sentence_sentiment": {"answer": "Negative", "rationale": "Because the act of crying out of the entry is negative."}, "language_style": {"answer": "Tactful and warm", "rationale": "The entry expresses concern about the emotional state of the other party in a more euphemistic way than by direct questioning or command, which is more accommodative and caring expression."}, "time_dimension": {"answer": "Past", "rationale": "The keywords are \"cry out of a position\" and \"want to cry\", which describe the behaviour or thoughts of the subjects over time and therefore judge as the past."}, "sentence_type": "Affirmative declarative sentence", "pos": {"nouns": {"Questionnaire": 1, "Entry": 1, "Position": 1}, "verbs": {"Cry": 2, "I'm crying": 1}, "adverbs": {"A little bit of position": 1}, "numerals": {"3": 1}}}, {"content": "I'm not sleeping well at night.", "word_count": 7, "label": "", "sentence_structure": "Subject鈥揷opula鈥損redicative", "subjective_objective": {"answer": "Subjective", "rationale": "Because good night sleep is the patient's subjective feeling and experience."}, "sentence_sentiment": {"answer": "Negative", "rationale": "Because the \"night sleep\" behavior in the entry is negative."}, "language_style": {"answer": "Calm and direct", "rationale": "The entry is more straightforward in its language style, does not have too many speech techniques or emotional renderings, is more calm and objective in tone and expresses the factual theme of asking the other party whether there is a sleep problem."}, "time_dimension": {"answer": "Past", "rationale": ""}, "sentence_type": "Exclamatory sentence", "pos": {"nouns": {"Night": 1, "Sleep": 1}, "verbs": {"not": 1, "Okay": 1}, "adverbs": {"4": 1}}}, {"content": "I eat as much as I eat.", "word_count": 9, "label": "", "sentence_structure": "Subject鈥搗erb鈥揷omplement", "subjective_objective": {"answer": "Objective", "rationale": "Because \"eating\" is an objective act or state, \"almost as in peace\" is a description of an objective fact. This is not an examination of patients' subjective attitudes or feelings, but simply a report of an objective phenomenon. So the entry to the questionnaire is considered from an objective perspective."}, "sentence_sentiment": {"answer": "Neutral", "rationale": "Because the entry only describes the food intake of the person surveyed, it is not clearly emotional."}, "language_style": {"answer": "Calm and direct", "rationale": "It does not have too much emotional colour or rhetorical style, but rather expresses in a concise and clear way the degree to which the respondents are similar to normal eating habits. This language style reduces the influence of subjective factors and makes the results more credible."}, "time_dimension": {"answer": "Present", "rationale": ""}, "sentence_type": "Interrogative sentence", "pos": {"nouns": {"5": 1, "I": 1, "Eat": 1, "Normal": 1, "More": 1}, "adverbs": {"Same thing": 1}, "numerals": {"One": 2}, "classifiers": {"individual": 1}}}, {"content": "I was as happy with the opposite sex as I ever was.", "word_count": 16, "label": "", "sentence_structure": "Subject鈥搗erb鈥揷omplement", "subjective_objective": {"answer": "Subjective", "rationale": "Because happiness is a subjective feeling, it is determined by the patient ' s own mental state and is not influenced by objective external factors."}, "sentence_sentiment": {"answer": "Positive", "rationale": "Because \"happy\" is a positive emotional experience."}, "language_style": {"answer": "Tactful and warm", "rationale": "The entry is more personal and interactive in its expression of conversation in a gentle and inviting tone, rather than direct statements or orders. At the same time, the term \"happy\" also expresses a positive emotional attitude, making the context more friendly."}, "time_dimension": {"answer": "Present", "rationale": "At the same time, the immediate temporal dimension can be considered, as this experience and experience are now being observed and assessed. In conclusion, the temporal dimension of the article should be present and immediate."}, "sentence_type": "Affirmative declarative sentence", "pos": {"nouns": {"Heterosexual": 1, "Exposure": 1, "Have a nice day": 1, "Previous": 1}, "verbs": {"and": 1, "I feel": 1}, "adverbs": {"Same thing": 1}, "numerals": {"6": 1}, "pronouns": {"I": 1}}}, {"content": "I noticed my weight was falling.", "word_count": 10, "label": "", "sentence_structure": "Subject鈥搗erb鈥揷omplement", "subjective_objective": {"answer": "Subjective", "rationale": "Because weight change is a subjective perception and perception of a person, not an objective fact. While weight change may be true, it does not mean that the person is losing weight or is having problems with his health, and it is subjective."}, "sentence_sentiment": {"answer": "Negative", "rationale": "This situation is described as negative because of the health problems that may result from weight loss."}, "language_style": {"answer": "Tactful and warm", "rationale": "The entry makes the question more friendly and inspiring by using the first-person pronoun \"I\" to establish personal contact with the interviewees, while the active word \"reduce\" to express the subject. Such language style is more accessible to the interviewees than direct questioning."}, "time_dimension": {"answer": "Past", "rationale": "The entry is a description of the testee ' s finding of a loss of weight, and the time dimension is a past."}, "sentence_type": "Affirmative declarative sentence", "pos": {"nouns": {"I": 1, "Weight": 1}, "verbs": {"I realized": 1, "Down": 1}, "numerals": {"7": 1}}}, {"content": "I have a problem with constipation.", "word_count": 7, "label": "", "sentence_structure": "Subject鈥搗erb鈥揷omplement", "subjective_objective": {"answer": "Subjective", "rationale": "Because constipation is a feeling of physical discomfort, it is the patient ' s own subjective feelings and experiences, not an objective state of existence."}, "sentence_sentiment": {"answer": "Negative", "rationale": "Because the description of \"simplified pain\" in the entry is negative."}, "language_style": {"answer": "Tactful and warm", "rationale": "The language style of the entry is more euphemistic, avoiding the use of language that is too straightforward or offensive, giving a gentle, friendly feeling and is more suitable for use in psychological questionnaire studies."}, "time_dimension": {"answer": "Past", "rationale": "So the answer is \"the past\"."}, "sentence_type": "Affirmative declarative sentence", "pos": {"nouns": {"Decency": 1, "Trouble": 1}, "verbs": {"have": 1}, "adverbs": {"8": 1}}}, {"content": "Nine of my heart beats faster than usual.", "word_count": 7, "label": "", "sentence_structure": "Subject鈥揷opula鈥損redicative", "subjective_objective": {"answer": "Subjective", "rationale": "Because the patient ' s own perception is required to conclude that it is the patient ' s subjective feeling and falls within the scope of the subjective examination."}, "sentence_sentiment": {"answer": "Negative", "rationale": "Because \"heart beats faster than usual\" in the entries are usually considered to be a physical discomfort and are therefore negative."}, "language_style": {"answer": "Calm and direct", "rationale": "The language style of the entry is more straightforward, with no excessive rhetoric or emotional rendering, but rather a concise, direct language presentation, which is more objective and practical."}, "time_dimension": {"answer": "Present", "rationale": "According to the information given, \"heart beat faster than usual\" is a description of the current state of feeling and state, so the temporal dimension of the article should be judged as \"now\"."}, "sentence_type": "Affirmative declarative sentence", "pos": {"nouns": {"Heart rate": 1, "Normal": 1}, "verbs": {"- Yeah": 1, "Come on": 1}, "adverbs": {"More than usual": 1}, "numerals": {"9": 1}}}, {"content": "I'm tired for no reason.", "word_count": 9, "label": "", "sentence_structure": "Subject鈥搗erb鈥揷omplement", "subjective_objective": {"answer": "Subjective", "rationale": "Because fatigue is a subjective feeling."}, "sentence_sentiment": {"answer": "Negative", "rationale": "Because the \"difficult for no reason\" sentiment in the entry is negative."}, "language_style": {"answer": "Tactful and warm", "rationale": ""}, "time_dimension": {"answer": "Present", "rationale": "The entry is for the current emotional state of the testee, the temporal dimension of which is now judged. The keyword \"unprovoked fatigue\" expresses the present physical and emotional experience of the testee and is therefore in the present context."}, "sentence_type": "Negative declarative sentence", "pos": {"nouns": {"10": 1, "Weary": 1}, "verbs": {"I feel": 1, "For no reason": 1}, "adverbs": {"For no reason": 1}, "numerals": {"10": 1}}}, {"content": "My mind is as clear as ever.", "word_count": 11, "label": "", "sentence_structure": "Subject鈥揷opula鈥損redicative", "subjective_objective": {"answer": "Objective", "rationale": "Because the sobering mind is an observable fact, the patient鈥檚 subjective judgment is not required. The question is therefore examined from an objective perspective."}, "sentence_sentiment": {"answer": "Neutral", "rationale": "Because the entry does not have any emotional color."}, "language_style": {"answer": "Tactful and warm", "rationale": "This entry is used in a gentle and inviting manner to talk about the other鈥檚 mental state at a given point in time, without direct statements or orders."}, "time_dimension": {"answer": "Present", "rationale": "The entry is for the current state of the testee ' s mind, and the time dimension is now judged."}, "sentence_type": "Affirmative declarative sentence", "pos": {"nouns": {"Brain": 1, "Normal": 1}, "verbs": {"Clear": 1}, "adverbs": {"Same thing": 1}, "numerals": {"11": 1}, "pronouns": {"I": 1}}}, {"content": "I don't think it's hard to do things that often.", "word_count": 14, "label": "", "sentence_structure": "Subject鈥揷opula鈥損redicative", "subjective_objective": {"answer": "Subjective", "rationale": "Because \"the feeling\" expresses a subjective sense and view of individuals, it is their subjective view that respondents believe that there are no difficulties in doing what they often do."}, "sentence_sentiment": {"answer": "Positive", "rationale": "Because it expresses a positive attitude that it is capable of meeting the challenges of life."}, "language_style": {"answer": "Tactful and warm", "rationale": "The entry uses a moderate and encouraging expression, rather than a direct denial or criticism, which makes it easier for the interviewee to accept and share his thoughts and feelings. At the same time, the purpose of the article is designed more liberally, giving the interviewee more freedom to answer."}, "time_dimension": {"answer": "Present", "rationale": ""}, "sentence_type": "Affirmative declarative sentence", "pos": {"nouns": {"Things": 1, "Difficulties": 1}, "verbs": {"I think": 1, "Do it": 1, "have": 1}, "adjectives": {"Regular": 1, "The long run": 1}, "adverbs": {"and": 1, "No, I'm not": 1}, "numerals": {"12": 1}, "pronouns": {"I": 1}}}, {"content": "I feel restless and uneasy.", "word_count": 11, "label": "", "sentence_structure": "Subject鈥搗erb鈥揷omplement", "subjective_objective": {"answer": "Subjective", "rationale": "Because it is the patient's subjective feeling that is uneasy and unsettled."}, "sentence_sentiment": {"answer": "Negative", "rationale": "Because \"uneasy\" is a negative emotion."}, "language_style": {"answer": "Tactful and warm", "rationale": "The language style of the entry is more euphemistic and gentle, rather than directly expressing emotions or attitudes, but rather, in a more soft way, leads the interviewee to think about his/her feelings."}, "time_dimension": {"answer": "Present", "rationale": "The entry \"Fright and still\" expresses the current psychological state and feelings of the person being tested, so the temporal dimension is now judged."}, "sentence_type": "Affirmative declarative sentence", "pos": {"nouns": {"13": 1, "Unsettled": 1, "Calm down": 2, "Get down": 1}, "verbs": {"I think": 1}, "adjectives": {"Ann": 1, "Quiet": 1}, "numerals": {"13": 1}, "pronouns": {"I": 1}}}, {"content": "I have hope for the future.", "word_count": 8, "label": "", "sentence_structure": "Subject鈥搗erb鈥揷omplement", "subjective_objective": {"answer": "Subjective", "rationale": "Because \"give hope\" is a subjective attitude."}, "sentence_sentiment": {"answer": "Positive", "rationale": "It expresses a positive attitude and expectations for the future."}, "language_style": {"answer": "Tactful and warm", "rationale": ""}, "time_dimension": {"answer": "Future", "rationale": "The entry contains visions and expectations for the future, and the keywords are \"for the future\" and \"for the future\", which point to situations that have not yet occurred and that may arise in the future."}, "sentence_type": "Affirmative declarative sentence", "pos": {"nouns": {"Future": 1, "Hope": 1}, "verbs": {"Have": 1}, "adverbs": {"to": 1}, "numerals": {"14": 1}}}, {"content": "I'm more excited than usual.", "word_count": 8, "label": "", "sentence_structure": "Subject鈥揷opula鈥損redicative", "subjective_objective": {"answer": "Subjective", "rationale": "Because \"funner than usual\" is a patient's subjective feeling and experience."}, "sentence_sentiment": {"answer": "Negative", "rationale": "Because \"Easy Excited\" is negative."}, "language_style": {"answer": "Tactful and warm", "rationale": "The entry is more personal and interactive in its expression of a gentle and inviting tone, rather than a direct statement or order. And the description \"I am more excited than usual\" is also more objective and neutral, and there is no excessive emotional rendering or rhetorical technique, which makes people feel more friendly and comfortable."}, "time_dimension": {"answer": "Present", "rationale": "The entry uses a comparative term such as \"more than usual\" to indicate that the respondent has to assess in response to their normal situation, and therefore the temporal dimension is now judged."}, "sentence_type": "Affirmative declarative sentence", "pos": {"nouns": {"15": 1, "I": 1}, "verbs": {"- Yeah": 1, "Easy": 1, "Excited": 1}, "adjectives": {"Normal": 1}, "numerals": {"15": 1}, "pronouns": {"I": 1}}}, {"content": "16, I think it's easy to make a decision.", "word_count": 11, "label": "", "sentence_structure": "Subject鈥揷opula鈥損redicative", "subjective_objective": {"answer": "Subjective", "rationale": "Because it's the patient's subjective attitude."}, "sentence_sentiment": {"answer": "Positive", "rationale": "Because the \"Easy decision-making\" attitude in the entry is positive."}, "language_style": {"answer": "Calm and direct", "rationale": "The entry is in a concise language style asking each other about the ease of decision-making, without adding too much qualifier or emotional colour and with a more objective and cold language."}, "time_dimension": {"answer": "Present", "rationale": "\"I think \"the feeling\" indicates that it is a subjective feeling or emotional experience, and \"decisions are easy\" is a descriptive statement that the speaker considers it easy to make a decision at some point in time. So this entry reflects the views and feelings of the subject."}, "sentence_type": "Affirmative declarative sentence", "pos": {"nouns": {"Decision": 1}, "verbs": {"Do it": 1, "I think": 1, "Easy": 1}, "adjectives": {"Easy": 1}, "numerals": {"16": 1}, "pronouns": {"I": 1}}}, {"content": "I feel like I'm a useful person. Someone needs me.", "word_count": 16, "label": "", "sentence_structure": "Subject鈥揷opula鈥損redicative", "subjective_objective": {"answer": "Subjective", "rationale": "Because \"think\" is patient subjective and self-evaluation."}, "sentence_sentiment": {"answer": "Positive", "rationale": "Because the entry describes people who think they are useful, the self-evaluation that someone needs is a positive emotion and attitude."}, "language_style": {"answer": "Tactful and warm", "rationale": "The language style of the entry is euphemism, because it uses a first person鈥檚 perspective and encourages the interviewee to think about his or her strengths and contributions by expressing a sense of self-worth and demand. Moreover, it does not use a strong negative or command tone, but rather leads the interviewee to the questionnaire in a moderate way."}, "time_dimension": {"answer": "Present", "rationale": "The keyword \"think\" and \"yes\" both uses the general current, indicating the current self-awareness and state. And, \" somebody needs me\" also refers to the current situation or feeling. So, the entry to the questionnaire primarily looks at the current thoughts and feelings of the testee."}, "sentence_type": "Affirmative declarative sentence", "pos": {"nouns": {"17": 1, "person": 2}, "verbs": {"I think": 1, "be": 1, "Yes": 1}, "adjectives": {"It works": 1}, "numerals": {"individual": 1}, "pronouns": {"self": 1, "I": 1}}}, {"content": "I've had a really interesting life.", "word_count": 10, "label": "", "sentence_structure": "Subject鈥揷opula鈥損redicative", "subjective_objective": {"answer": "Subjective", "rationale": "Because \"interesting\" is a subjective feeling."}, "sentence_sentiment": {"answer": "Positive", "rationale": "Because \"life is fun\" expresses positive attitudes and feelings."}, "language_style": {"answer": "Tactful and warm", "rationale": "The entry uses a more subjective language style and is more positive, giving people a sense of encouragement and affection and a more euphemism and affectionate expression."}, "time_dimension": {"answer": "Present", "rationale": ""}, "sentence_type": "Affirmative declarative sentence", "pos": {"nouns": {"Life": 1, "Meaning": 1}, "verbs": {"Pass": 1, "have": 1}, "adjectives": {"very": 1, "More": 1}, "adverbs": {"of": 2}, "pronouns": {"I": 1}}}, {"content": "I think it would be better if I died.", "word_count": 17, "label": "", "sentence_structure": "Subject鈥搗erb鈥揷omplement", "subjective_objective": {"answer": "Subjective", "rationale": "Because it's the patient's subjective attitude and thoughts that think that if I die, someone else will live better."}, "sentence_sentiment": {"answer": "Negative", "rationale": "Basis for judgement: The formulation in the entry is negative, as it expresses a feeling that people will live better after their death and that they will deny themselves."}, "language_style": {"answer": "Calm and direct", "rationale": "The entry asks each other ' s views in a direct and clear manner, does not have too many rhetorical or emotional renderings and is more objective in tone."}, "time_dimension": {"answer": "Present", "rationale": "The entry is to ask the views and attitudes of the person being tested, and the time dimension is now judged."}, "sentence_type": "Affirmative declarative sentence", "pos": {"nouns": {"19": 1, "I think": 1, "If": 1, "I": 2, "Dead": 1, "others": 1, "Life": 1, "More": 1, "Better": 1}, "verbs": {"Yes": 1}, "adverbs": {"More": 1}, "numerals": {"19": 1}, "classifiers": {"If": 1}, "pronouns": {"If": 1, "I": 2, "Dead": 1, "others": 1}}}, {"content": "I'm still interested in 20 things that I'm usually interested in.", "word_count": 15, "label": "", "sentence_structure": "Subject鈥搗erb鈥揷omplement", "subjective_objective": {"answer": "Subjective", "rationale": "Because \"interest\" is the individual feeling and attitude of the patient, the entry is viewed from the patient's subjective perspective."}, "sentence_sentiment": {"answer": "Positive", "rationale": "Because the \"I'm still interested in things\" act positive in the entry."}, "language_style": {"answer": "Tactful and warm", "rationale": "The language style of the entry is more euphemistic, using the words \"normal\" and \"still the same\" to express the other's continuing interest in certain things. At the same time, this expression is relatively positive and gives a positive emotional look."}, "time_dimension": {"answer": "Present", "rationale": ""}, "sentence_type": "Exclamatory sentence", "pos": {"nouns": {"Yes": 1, "Interest": 1}, "verbs": {"Interested": 1, "Sample": 1}, "adjectives": {"Normal": 1}, "adverbs": {"Still": 1}, "numerals": {"20": 1}, "pronouns": {"I": 1}}}]}

{"questionnaire_title": "Defense Mode Questionnaire (DSQ)", "questionnaire_category": "Behavioral questionnaire", "item_count": 88, "emotion_shift_overall": "From positive to negative", "language_style_overall": "Neutral", "items": [{"content": "One, I get satisfaction from helping others, and if I don't, I get depressed.", "word_count": 26, "label": "", "sentence_structure": "Subject鈥搗erb鈥搊bject", "subjective_objective": {"answer": "Subjective", "rationale": "Because \"I get satisfaction from helping others\" and \"I get depressed if I don't do this\" are subjective thoughts and feelings of patients."}, "sentence_sentiment": {"answer": "Positive", "rationale": "Because \"helping others to be content\" is positive, showing a positive attitude and behavior that is helpful. And \"if I don't do this, I'm going to be depressed\" is positive, demonstrating the importance of helping others to their mental health."}, "language_style": {"answer": "Tactful and warm", "rationale": "The entry expresses the positive meaning of helping others in a moderate way, and suggests that failure to do so could have negative emotional effects. This expression not only attracts attention and reflection, but also creates a friendly and caring atmosphere."}, "time_dimension": {"answer": "Present", "rationale": ""}, "sentence_type": "Affirmative declarative sentence", "pos": {"nouns": {"I": 2, "Others": 1, "Satisfied": 1, "Depression": 1}, "verbs": {"From": 1, "Help": 1, "Access": 1, "It's gonna be": 1, "If": 1, "Just": 1}, "adjectives": {"Emotion": 1}, "numerals": {"1": 6}}}, {"content": "Two, I'm often called a temperamental man.", "word_count": 13, "label": "", "sentence_structure": "Subject鈥揷opula鈥損redicative", "subjective_objective": {"answer": "Subjective", "rationale": "This is because it is a subjective assessment of the patient."}, "sentence_sentiment": {"answer": "Negative", "rationale": "Because the description of \"grumpy\" in the entry is negative."}, "language_style": {"answer": "Calm and direct", "rationale": "The entry presents the character characteristics of the person being evaluated in a direct descriptive statement, without excessive emotional accoutrements or explanations, and is more oriented towards an objective statement."}, "time_dimension": {"answer": "Present", "rationale": "The entry describes the image of the person being tested in the eyes of others and uses the term \"people say it\" to indicate that this is a recurring view, so the temporal dimension is now judged."}, "sentence_type": "Affirmative declarative sentence", "pos": {"nouns": {"person": 1, "- Grumpy": 1, "- Routine": 1, "Problem": 1, "Investigations": 1}, "verbs": {"Say it": 1, "be": 1, "Regular": 1, "Do it": 1}, "adjectives": {"- He's a bitch": 1}, "adverbs": {"Regular": 1}, "numerals": {"2": 1, "individual": 1}, "pronouns": {"I": 1}}}, {"content": "Three, I can put it aside when I don't have time to deal with a difficult thing.", "word_count": 25, "label": "", "sentence_structure": "Subject鈥搗erb鈥搊bject", "subjective_objective": {"answer": "Objective", "rationale": "The content of this questionnaire is about the response strategy of patients to difficult problems, and is about the individual鈥檚 perception and behaviour. This is the subjective idea and attitude of patients, and therefore is the subject of subjective scrutiny. And the results of the implementation of this strategy (e.g. whether or not to solve the problem successfully, or whether or not it affects follow-up, etc.) are objective facts, so the entry 鈥淚 can set aside鈥?in this questionnaire when I have no time to deal with a difficult matter."}, "sentence_sentiment": {"answer": "Neutral", "rationale": "Because the entry does not have any emotional color."}, "language_style": {"answer": "Calm and direct", "rationale": "The entry is presented in an objective narrative that is more concise, free of emotional or rhetorical expressions, and more calm and straightforward."}, "time_dimension": {"answer": "Past", "rationale": "\"I\" represents the testee himself, and \"there's no time to deal with a difficult matter\" is a past experience or situation, and \"can set it aside\" is a past act. So this entry describes the way the testee has dealt with similar issues in the past."}, "sentence_type": "Affirmative declarative sentence", "pos": {"nouns": {"I": 1, "Time": 1, "Processing": 1, "Things": 1, "- It's a problem": 1}, "verbs": {"Yeah": 2, "Hold it": 1, "On the side": 1}, "adverbs": {"No, I'm not": 1, "Some": 1}, "numerals": {"3": 1}, "classifiers": {"individual": 1}}}, {"content": "Four. People treat me unfairly.", "word_count": 11, "label": "", "sentence_structure": "Subject鈥搗erb鈥揷omplement", "subjective_objective": {"answer": "Subjective", "rationale": "Because \"people are treated with me\" is a subjective feeling or view, a perception and experience within the patient, not an objective fact. So \"4\" is a subjective entry."}, "sentence_sentiment": {"answer": "Negative", "rationale": "Because the \"people always treat me unfairly\" in the entry is negative."}, "language_style": {"answer": "Calm and direct", "rationale": "The sentence is based on the following: the entry presents a point in the form of a direct statement, that \"people are always unfair to me.\" This approach is not based on too many rhetorical or emotional expressions, but on an objective and straightforward way of expressing it. Moreover, the entry does not use any question or question to direct the respondents to answer the question, but rather a statement."}, "time_dimension": {"answer": "Present", "rationale": "Now the \"always\" in the entry indicates that it is a recurrent phenomenon, while \"unfairly treated me\" is a subjective feeling and an assessment, all of which implies that it is a description of the current situation and therefore a judgement of the present."}, "sentence_type": "Negative declarative sentence", "pos": {"nouns": {"person": 1, "Problem": 1, "Questionnaire": 1, "Entry": 1}, "verbs": {"Total": 1, "be": 1, "Treatment": 1}, "adjectives": {"It's not fair": 1, "Land": 1}, "adverbs": {"Always": 1}, "numerals": {"4": 1}, "pronouns": {"I": 1}}}, {"content": "Five, I'm free of my anxiety by doing positive or predictable things, drawing, doing carpentry work, etc.", "word_count": 34, "label": "", "sentence_structure": "Subject鈥搗erb鈥搊bject", "subjective_objective": {"answer": "Subjective", "rationale": "Because the title \"I\" shows that it's the patient's subjective view and behavior, that is, they choose to respond to anxiety through active activity."}, "sentence_sentiment": {"answer": "Positive", "rationale": "Because the acts described in the entries are positive and they help to ease anxiety."}, "language_style": {"answer": "Tactful and warm", "rationale": "This is a more euphemistic language style, which does not direct guidance or orders to the interviewee. At the same time, positive and predictable terminology is used to motivate the interviewee to take action to ease anxiety and anxiety."}, "time_dimension": {"answer": "Present", "rationale": "In the entry, words like \"I'm now\" and \"I'm usually\" indicate that this is a description of the person's current state and behaviour. Besides, \"e.g. painting, carpentry work, etc.\" also highlights that these positive acts are now being carried out, so the entry should be judged as \"now\"."}, "sentence_type": "Affirmative declarative sentence", "pos": {"nouns": {"Questionnaire Entry": 1, "Anxiety": 1, "Things": 2, "Positive": 1, "Predictability": 1, "Painting": 1, "Carpentry": 1}, "verbs": {"Get rid of it": 1, "Do it": 3}, "adverbs": {"Some": 1, "Like": 1}, "numerals": {"5": 1}, "pronouns": {"I": 1}}}, {"content": "I've been deferring some of the things I should do today to tomorrow.", "word_count": 19, "label": "", "sentence_structure": "Subject鈥搗erb鈥搊bject", "subjective_objective": {"answer": "Subjective", "rationale": "Because \"deferring something to tomorrow\" is a patient's behavior and is subjective."}, "sentence_sentiment": {"answer": "Negative", "rationale": "Because delaying work is a negative act."}, "language_style": {"answer": "Tactful and warm", "rationale": "The term \"frequently\" is used to suggest that it is not normal, that it is spoken with a temperature and without a sense of blame, that it makes it easier to accept and answer questions. At the same time, the description \"delaying something to do today\" is also more euphemismal and does not make people feel criticized or blamed."}, "time_dimension": {"answer": "Past", "rationale": "According to the phrase \"accidentally\" and \"delaying today's work to tomorrow\", it can be judged to be a description of the testee's past or present behaviour, so the answer is \"the past/now\"."}, "sentence_type": "Affirmative declarative sentence", "pos": {"nouns": {"Things": 1, "Today": 1, "Tomorrow": 1}, "verbs": {"- Put it on": 1, "Postponement": 1, "Do it": 1}, "adjectives": {"Some": 1, "The": 1, "of": 2}, "adverbs": {"Sometimes": 1}, "pronouns": {"I": 1}}}, {"content": "Seven, I don't know why I'm always in the same situation.", "word_count": 18, "label": "", "sentence_structure": "Subject鈥搗erb鈥搊bject", "subjective_objective": {"answer": "Subjective", "rationale": "Because \"the same frustrations\" are the patients' own feelings and experiences, and therefore the subjective angle."}, "sentence_sentiment": {"answer": "Negative", "rationale": "Because the entry \"Don't know why it's always the same frustration\" expresses a feeling of helplessness and confusion, and it's negative."}, "language_style": {"answer": "Calm and direct", "rationale": "This is a simple and straightforward way of describing the situation in which the person under investigation is often subjected to the same frustration in his or her life, without using excessive rhetorical or emotional overtook, and with more objective and direct tone."}, "time_dimension": {"answer": "Present", "rationale": "Now, based on the words \"always\" and \"attractively\" in the sentence, it can be judged to be a description of the current state or experience, and therefore of the current behavior or mentality. At the same time, \"no idea\" also indicates that it is a question or a problem, further illustrating the current state of mind."}, "sentence_type": "Interrogative sentence", "pos": {"nouns": {"7": 1, "I don't know": 1, "Why": 1, "Same": 1, "Frustration": 1, "Situation": 1}, "verbs": {"I met him": 1, "Total": 1}, "adverbs": {"Always": 1}, "numerals": {"7": 1}, "pronouns": {"I": 1}}}, {"content": "I can laugh at myself quite easily.", "word_count": 13, "label": "", "sentence_structure": "Subject鈥搗erb鈥揷omplement", "subjective_objective": {"answer": "Subjective", "rationale": "Because I can laugh at myself quite easily as a subjective judgement that reflects the attitude and perception of the patient to self-awareness."}, "sentence_sentiment": {"answer": "Positive", "rationale": "Because the description of \"smoke myself\" in the entry is positive, showing confidence and self-scoffing."}, "language_style": {"answer": "Tactful and warm", "rationale": ""}, "time_dimension": {"answer": "Present", "rationale": "The entry \"I can laugh at myself quite easily\" shows that it is a customary act or attitude, usually occurring now or in the past, and probably going on in the future. So the time dimension is now."}, "sentence_type": "Affirmative declarative sentence", "pos": {"verbs": {"Yes": 1, "Scoff": 1, "I": 2}, "adjectives": {"Pretty much": 1, "Easy": 1}}}, {"content": "Nine. When I get frustrated, I act like a child.", "word_count": 13, "label": "", "sentence_structure": "Subject鈥搗erb鈥揷omplement", "subjective_objective": {"answer": "Subjective", "rationale": "Because frustration is manifested in patients ' subjective reactions and emotional experiences."}, "sentence_sentiment": {"answer": "Negative", "rationale": "Because the description of \"expression as a child\" in the entry may indicate that an individual is emotional, indefatigable and derogatory in the face of frustration."}, "language_style": {"answer": "Tactful and warm", "rationale": "The language style of the entry is more euphemistic, and it is more personal and interactive, by speaking with an invitation rather than directly speaking or ordering. At the same time, the phrase \"like a child\" has a somewhat metaphorical character, which increases the performance and vitality of the sentence."}, "time_dimension": {"answer": "Past", "rationale": "The word \"I\" in \"I'm frustrated\" means that the individual's own behavior or emotional experience occurred in the past, and the description \"expression is like a child\" is also a reflection of the past. The entry is therefore an inquiry into the testee's performance in the past, and the temporal dimension is a judgement of the past."}, "sentence_type": "Affirmative declarative sentence", "pos": {"nouns": {"Frustration": 1, "Son": 1}, "verbs": {"Yes": 1, "Performance": 1}, "adverbs": {"Like": 1}, "numerals": {"9": 1}}}, {"content": "I'm ashamed to be a pious man in my interests.", "word_count": 16, "label": "", "sentence_structure": "Subject鈥搗erb鈥搊bject", "subjective_objective": {"answer": "Subjective", "rationale": "Because shame is a subjective emotional experience."}, "sentence_sentiment": {"answer": "Negative", "rationale": "Because the act of \"smuggling\" in the entries is negative, it expresses a reluctance to fight for its own rights."}, "language_style": {"answer": "Tactful and warm", "rationale": "The language style of the entry is more euphemistic, soft in language and relatively soft in expression, and does not express the views that are intended to be emphasized too directly or strongly, and is more personal and interactive."}, "time_dimension": {"answer": "Present", "rationale": "In \"in the defence of my interests,\" the structure of \"in the context of\" shows that it is an ongoing act or state, while \"I'm ashamed\" indicates the feelings of the speaker for this act, so the entire entry describes a current situation."}, "sentence_type": "Negative declarative sentence", "pos": {"nouns": {"Interest": 1, "Count": 1}, "verbs": {"Maintenance": 1, "Shame": 1, "With people": 1}, "adjectives": {"Mine": 1}, "numerals": {"10": 1}}}, {"content": "Eleven. I'm better than most of the people I know.", "word_count": 13, "label": "", "sentence_structure": "Subject鈥揷opula鈥損redicative", "subjective_objective": {"answer": "Subjective", "rationale": "Because the entry relates to evaluation and comparison, it is the patient ' s subjective perception and attitude."}, "sentence_sentiment": {"answer": "Positive", "rationale": "Because the expression in the entry is self-affirming, positive emotional."}, "language_style": {"answer": "Tactful and warm", "rationale": "The entry is presented in a comparative manner, rather than directly presenting its merits or strengths, which is usually more euphemistic and respectful of the attitudes and feelings of the interviewees in the psychological questionnaire."}, "time_dimension": {"answer": "Present", "rationale": "The entry is a description of the present self-perception and comparative object of the testee, and the time dimension is now judged."}, "sentence_type": "Affirmative declarative sentence", "pos": {"nouns": {"Questionnaire": 1, "Entry": 1, "person": 1}, "verbs": {"Yes": 1, "- Yeah": 2, "Strong": 1}, "adjectives": {"Most of them": 1}, "numerals": {"11": 1}, "pronouns": {"I": 1}}}, {"content": "People abuse me.", "word_count": 7, "label": "", "sentence_structure": "Subject鈥搗erb鈥揷omplement", "subjective_objective": {"answer": "Subjective", "rationale": "Because it's the patient's subjective feeling and perception."}, "sentence_sentiment": {"answer": "Negative", "rationale": "Because \"abuse\" in the entry is a term with a negative meaning."}, "language_style": {"answer": "Tactful and warm", "rationale": "The entry uses the first person's single-digit verb \"I\" to resonate with the person under investigation, while the descriptive verb \"I'm mistreated\" to convey a negative mood and situation. This expression is more moderate and thoughtful, helping to create a good atmosphere of communication and to make the person under investigation more willing to share his or her feelings."}, "time_dimension": {"answer": "Past", "rationale": "The keyword \"often\" indicates the events that have occurred in the past, and \"abuse\" is a review of the person's description and experience. The entry therefore primarily asks whether the person who was tested has been abused in the past."}, "sentence_type": "Negative declarative sentence", "pos": {"nouns": {"People": 1, "I": 1}, "verbs": {"Abuse": 1}, "numerals": {"12": 1}}}, {"content": "13, if someone lied to me or stole my money, I'd rather he get help than punishment.", "word_count": 27, "label": "", "sentence_structure": "Subject鈥損redicate", "subjective_objective": {"answer": "Subjective", "rationale": "Because it reflects the subjective attitude and values of patients, which tend to excuse others ' mistakes rather than merely punish them."}, "sentence_sentiment": {"answer": "Positive", "rationale": "Because the attitude in the entries is positive, it is hoped that the understanding and sympathy of the fraudster will be addressed by helping them."}, "language_style": {"answer": "Tactful and warm", "rationale": "The language style of the entry is more moderate and emotional, and it does not directly express a harsh accusation of fraud and theft, but rather leads the other to think about the relationship between honesty and forgiveness by asking a hypothetical question. This expression is more reflective of the humanistic care of investigators and respect for the attitudes of the interviewees."}, "time_dimension": {"answer": "Present", "rationale": "The keyword \"If someone cheats me or steals my money\" and \"I prefer that he be helped rather than punished\" are all describing the current thinking and attitude, so this entry is about the present."}, "sentence_type": "Affirmative declarative sentence", "pos": {"nouns": {"person": 2, "Money": 1, "Help": 1, "Punishment": 1}, "verbs": {"Liar": 1, "Steal": 1, "I'd rather": 1, "Got it": 1, "Yes": 1}, "numerals": {"13": 1, "One": 1}, "classifiers": {"individual": 1}, "pronouns": {"Some": 1, "he": 2, "I": 1}}}, {"content": "I think sometimes something so bad can't be said.", "word_count": 16, "label": "", "sentence_structure": "Subject鈥搗erb鈥揷omplement", "subjective_objective": {"answer": "Subjective", "rationale": "Because \"want\" is a psychological activity or experience, and it's usually a subjective angle."}, "sentence_sentiment": {"answer": "Negative", "rationale": "Because the entry \"bad things that cannot be said\" expresses negative feelings."}, "language_style": {"answer": "Calm and direct", "rationale": "The entry is more straightforward in language style, with no excessive rhetoric or emotional rendering, and asks if the other side is ever thinking of something very bad and speaking in a more objective manner."}, "time_dimension": {"answer": "Present", "rationale": "The keyword \"frequently\" and \"in the hope\" both indicate that it is a question of describing the person's current behaviour or feelings, so the entry is asking what the person's current thoughts or feelings are."}, "sentence_type": "Affirmative declarative sentence", "pos": {"nouns": {"Things": 1}, "verbs": {"Yes": 1}, "adjectives": {"Bad": 1, "I can't say it": 1}, "adverbs": {"Sometimes": 1}}}, {"content": "I laugh at some dirty jokes.", "word_count": 14, "label": "", "sentence_structure": "Subject鈥搗erb鈥揷omplement", "subjective_objective": {"answer": "Subjective", "rationale": "Because the laugh is because of the patient's subjective feelings."}, "sentence_sentiment": {"answer": "Positive", "rationale": "And it's neutral because it describes the individual laughter response, and it doesn't involve any negative emotions or behavior."}, "language_style": {"answer": "Tactful and warm", "rationale": "The language style of the entry is a mild and warm type. First, the question leads the respondents to answer in a mild way, using the word \"frequently\" without being too strong or directly required. Second, by describing the way the respondents react (screeched by some dirty jokes), the question appears to be more cordial and sweet, and does not involve too much judgement or criticism of the behaviour."}, "time_dimension": {"answer": "Past", "rationale": ""}, "sentence_type": "Affirmative declarative sentence", "pos": {"nouns": {"A joke": 1, "Smile": 1}, "verbs": {"be": 1, "have": 1, "Sometimes": 1, "Because": 1, "And": 1}, "adjectives": {"Nasty": 1}, "adverbs": {"Sometimes": 1}, "numerals": {"15": 1}}}, {"content": "16 I was said to be like a camel bird, burying my head in the sand, in other words, I was often deliberately neglecting some unpleasant things.", "word_count": 37, "label": "", "sentence_structure": "Subject鈥搗erb鈥搊bject", "subjective_objective": {"answer": "Subjective", "rationale": "Because \"into the sand\" and \"intentionally ignoring some unpleasant things\" are subjective ideas and behaviours of patients."}, "sentence_sentiment": {"answer": "Negative", "rationale": "Because the act of \"neglecting something unpleasant\" is negative."}, "language_style": {"answer": "Tactful and warm", "rationale": "The entry uses metaphoric terms and a softer description of the behaviour of the person surveyed, which is more euphemistic and gentle. Such language can reduce resistance and increase motivation and accuracy in the questionnaire."}, "time_dimension": {"answer": "Present", "rationale": "\"I'm like a camel bird, burying my head in the sand\" with the word \"speak\" and \"burial\" in the general current, which means that this act has not only happened in the past, but may still be going on. So the time dimension of this questionnaire entry is now."}, "sentence_type": "Affirmative declarative sentence", "pos": {"nouns": {"People": 1, "A camel bird": 1, "Head": 1, "Sand": 1, "Things": 1}, "verbs": {"Say it": 1, "Elephant": 1, "Bury it": 1, "Neglect": 2, "Intentionally": 1}, "adjectives": {"It's not pleasant": 1}, "numerals": {"16": 1}, "classifiers": {"Only": 1}, "pronouns": {"I": 1}}}, {"content": "I can't compete with people at all costs.", "word_count": 14, "label": "", "sentence_structure": "Subject鈥搗erb鈥揷omplement", "subjective_objective": {"answer": "Subjective", "rationale": "Because \"can't do everything\" is the patient's subjective feeling and attitude."}, "sentence_sentiment": {"answer": "Negative", "rationale": "Because the \"can't compete with people\" act in the entry is negative."}, "language_style": {"answer": "Calm and direct", "rationale": "The language style of the entry is relatively calm and straightforward, and it explicitly asks whether the interviewee lacks a dedicated attitude to competition and does not use excessive rhetorical or emotional rendering. This expression helps the interviewee to understand the problem more clearly and to provide an accurate answer."}, "time_dimension": {"answer": "Present", "rationale": ""}, "sentence_type": "Negative declarative sentence", "pos": {"nouns": {"I": 1, "Strength": 1, "person": 1, "Competition": 1}, "verbs": {"Do your best": 1, "and": 1, "Competition": 1}, "adverbs": {"Often": 1}}}, {"content": "I've always felt better than the people I'm with.", "word_count": 13, "label": "", "sentence_structure": "Subject鈥揷opula鈥損redicative", "subjective_objective": {"answer": "Subjective", "rationale": "Because feeling better than anyone else is a personal subjective feeling and evaluation."}, "sentence_sentiment": {"answer": "Negative", "rationale": "Because the expression \"more than the person I'm with\" in the entry implies a sense of self- superiority, with a certain negative emotional colour."}, "language_style": {"answer": "Calm and direct", "rationale": "The entry is a direct statement asking whether the interviewees often consider themselves stronger in their dealings with others, with a more objective and quiet language style, and with no excessive rhetorical or emotional colours.\""}, "time_dimension": {"answer": "Past", "rationale": "The keyword \"I often feel\" suggests that it is a constant subjective feeling, without reference to past or future time references, and it can be judged as a current description and evaluation."}, "sentence_type": "Affirmative declarative sentence", "pos": {"nouns": {"Questionnaire": 1, "Entry": 1, "person": 1}, "verbs": {"I always feel": 1, "- Yeah": 1, "in": 1, "Strong": 1}, "adjectives": {"Together": 1}, "numerals": {"18": 1}, "pronouns": {"I": 2}}}, {"content": "I'm trying to take everything I've got.", "word_count": 14, "label": "", "sentence_structure": "Subject鈥搗erb鈥揷omplement", "subjective_objective": {"answer": "Subjective", "rationale": "Because \"think\" is a subjective verb, describing the imagination and the thoughts inside a person, not an objective fact."}, "sentence_sentiment": {"answer": "Negative", "rationale": "Because the actions in the entries are deprivation, with negative emotional colours."}, "language_style": {"answer": "Calm and direct", "rationale": "The entry is strongly worded, clearly expressing the core of the problem, and does not ease the tone by adoration or euphemism. It therefore prefers to be more calm and straightforward."}, "time_dimension": {"answer": "Present", "rationale": ""}, "sentence_type": "Negative declarative sentence", "pos": {"nouns": {"Someone": 1, "Everything": 1}, "verbs": {"Deprivation": 1, "Got it": 1}, "numerals": {"19": 1}, "pronouns": {"I": 1}}}, {"content": "I get angry sometimes.", "word_count": 5, "label": "", "sentence_structure": "Subject鈥搗erb鈥揷omplement", "subjective_objective": {"answer": "Subjective", "rationale": "The blogger says that the government is not a party to the law, because anger is the patient's subjective emotional experience and expression."}, "sentence_sentiment": {"answer": "Negative", "rationale": "Because the act of \"greed\" in the entry is negative."}, "language_style": {"answer": "Tactful and warm", "rationale": ""}, "time_dimension": {"answer": "Present", "rationale": "The entry is asking the testee about his current behaviour or state, because it uses the terms \"sometimes\" and \"excited.\" So the time dimension is now."}, "sentence_type": "Affirmative declarative sentence", "pos": {"verbs": {"- Get angry": 1}, "adverbs": {"Time": 1, "Sometimes": 1}}}, {"content": "21 - I've often acted on my own initiative, driven by some kind of inner force.", "word_count": 24, "label": "", "sentence_structure": "Subject鈥搗erb鈥揷omplement", "subjective_objective": {"answer": "Subjective", "rationale": "Because \"inherent force\" and \"involved behaviour\" are subjective feelings and experiences of patients."}, "sentence_sentiment": {"answer": "Neutral", "rationale": "As the entry does not express any positive or negative emotional attitude, behaviour, purpose or quality, it merely describes a possible phenomenon."}, "language_style": {"answer": "Tactful and warm", "rationale": ""}, "time_dimension": {"answer": "Present", "rationale": "\"I often\" indicates that this is a customary act, and \"uninvolved\" means that it is spontaneous and not controlled by subjective will. So \"I often do something by some internal force, and I do something by myself\" describes the person being tested in his present state or pattern of behaviour."}, "sentence_type": "Affirmative declarative sentence", "pos": {"nouns": {"Plant": 1, "Inside power": 1, "Driver": 1, "Behaviour": 1}, "verbs": {"Often": 1, "Make": 2}, "adverbs": {"I can't help it": 1}, "numerals": {"21": 1}, "pronouns": {"I": 1}}}, {"content": "I'd rather starve to death than be forced to eat.", "word_count": 12, "label": "", "sentence_structure": "Subject鈥搗erb鈥揷omplement", "subjective_objective": {"answer": "Subjective", "rationale": "Because \"I'd rather starve\" and \"I don't want to be forced to eat\" are patients' personal thoughts and attitudes, subjectively."}, "sentence_sentiment": {"answer": "Negative", "rationale": "The article is intended to express aversion and resistance to being forced to eat."}, "language_style": {"answer": "Calm and direct", "rationale": "The language style of the entry is more straightforward, concise, and does not involve too many rhetorical or emotional renderings, but simply expresses the attitude of the other party to eating. Thus, the language style of the article can be judged as \"cool and direct\"."}, "time_dimension": {"answer": "Present", "rationale": "The temporal dimension of the purpose of the article is therefore now judged."}, "sentence_type": "Interrogative sentence", "pos": {"nouns": {"Starving": 1, "Rice": 1}, "verbs": {"I'd rather": 1, "Starving": 1, "I don't want to": 1, "Forced": 1, "Eat": 1}, "adverbs": {"I'd rather not": 1, "not": 2}, "numerals": {"22": 1}, "pronouns": {"I": 1}}}, {"content": "23, I've often been deliberately ignoring some of the dangers.", "word_count": 18, "label": "", "sentence_structure": "Subject鈥搗erb鈥揷omplement", "subjective_objective": {"answer": "Subjective", "rationale": "Because the title refers to \"I have often deliberately ignored some dangers\", which is the patient's own psychological activity and attitude, and is subjective."}, "sentence_sentiment": {"answer": "Negative", "rationale": "Because the act in the entry \"intentionally neglecting some of the dangers\" is a potentially dangerous act, it is somewhat negative in nature."}, "language_style": {"answer": "Calm and direct", "rationale": "The entry is more direct, has no emotional or accommodative tone, giving people a calm and rational sense. It asks whether the interviewees often ignore danger and suggests that they may have a \"superhuman\" psychological state. Such an approach may be painful or uncomfortable, but it is straightforward and clear."}, "time_dimension": {"answer": "Present", "rationale": "The word \"I'm often\" means \"recurring, customary\" and \"neglecting some danger\" is a current act, so the time dimension of the article is now."}, "sentence_type": "Affirmative declarative sentence", "pos": {"nouns": {"Danger": 1, "Superman": 1}, "verbs": {"Neglect": 1, "be": 1}, "adjectives": {"Often": 1}, "adverbs": {"On purpose": 1}, "numerals": {"23": 1}, "pronouns": {"I": 1}}}, {"content": "I'm proud of my ability to denigrate others.", "word_count": 15, "label": "", "sentence_structure": "Subject鈥搗erb鈥搊bject", "subjective_objective": {"answer": "Subjective", "rationale": "Because pride and the ability to debase others are the feelings and the assessments of the patients themselves, it is subjective."}, "sentence_sentiment": {"answer": "Negative", "rationale": "Because the expression \"the ability to debase others\" is offensive and negative."}, "language_style": {"answer": "Calm and direct", "rationale": "The entry is based on a straightforward approach to the subject of questioning the other party for derogatory behaviour, without using excessive rhetorical or emotional rendering, and is more objective in tone. At the same time, it does not attempt to arouse the resonance of the person under investigation or to induce him to agree with a certain view, but rather to ask the other person ' s views and behaviour directly, so that his or her language style can be judged as calm."}, "time_dimension": {"answer": "Future", "rationale": "The entry contains visions and expectations for the future, and the key words are \"I'm proud\" and \"I'm proud\" and they all point to situations that have not yet occurred and that may occur in the future."}, "sentence_type": "Affirmative declarative sentence", "pos": {"nouns": {"I": 1, "Demeaning": 1, "Capacity": 1, "Honor": 1}, "verbs": {"Here": 1, "Proud": 1}, "numerals": {"24": 1}}}, {"content": "25, people tell me I always feel killed.", "word_count": 13, "label": "", "sentence_structure": "Subject鈥搗erb鈥搊bject", "subjective_objective": {"answer": "Subjective", "rationale": "Because this is the way others evaluate and observe themselves, it is a subjective perspective."}, "sentence_sentiment": {"answer": "Negative", "rationale": "Because the expression \"always feeling killed\" implies fear and anxiety for your own safety, and it has a negative emotional tone."}, "language_style": {"answer": "Tactful and warm", "rationale": "The entry uses a second person's word \"you\" and verb \"Tell\" that the person under investigation feels part of the conversation, not the subject of direct questioning or accusations. At the same time, the subject \"always feeling killed\" is expressed in a relatively euphemism, without being too direct or too emotional. It is therefore said that the language style of the article is euphemism."}, "time_dimension": {"answer": "Present", "rationale": ""}, "sentence_type": "Affirmative declarative sentence", "pos": {"nouns": {"People": 1, "Feel": 1, "Shit": 1}, "verbs": {"Tell": 1, "have": 1}, "numerals": {"25": 1}, "pronouns": {"I": 1}}}, {"content": "26, sometimes I get angry when I feel bad.", "word_count": 12, "label": "", "sentence_structure": "Subject鈥損redicate", "subjective_objective": {"answer": "Subjective", "rationale": "Because \"fear bad\" and \"freak bad\" are both the feelings and emotional reactions of patients, and are the expression of subjective attitudes."}, "sentence_sentiment": {"answer": "Negative", "rationale": "Because it's negative in the entry."}, "language_style": {"answer": "Tactful and warm", "rationale": "The entry uses the words \"sometimes,\" \"me,\" and the more moderate expression \"when feeling bad,\" rather than directly describing emotions or blaming each other. These features make the language style more euphemismal and friendly."}, "time_dimension": {"answer": "Present", "rationale": "The entry describes the current behavior or feelings of the testee, \"sometimes it feels bad\" and \"I'll be angry\" indicating that this is his current customary behaviour, and therefore judging now."}, "sentence_type": "Affirmative declarative sentence", "pos": {"nouns": {"Time": 1, "- Grumpy": 1}, "verbs": {"Feel": 1, "Let's go": 1}, "adjectives": {"Okay": 1}, "adverbs": {"Just": 1, "Time": 2, "Not good": 1}, "numerals": {"26": 1}, "pronouns": {"I": 1}}}, {"content": "I often do things on my own when something bothers me.", "word_count": 23, "label": "", "sentence_structure": "Subject鈥損redicate", "subjective_objective": {"answer": "Subjective", "rationale": "Because it is the patient who describes his/her subjective feelings and behavioural reactions."}, "sentence_sentiment": {"answer": "Negative", "rationale": "Because the description of \"some act of an autonomous nature\" in the entry implies that such conduct is negative and may be a way of inadvertently avoiding problems or pressures."}, "language_style": {"answer": "Calm and direct", "rationale": "The entry is presented in direct, concise language, without excessive emotional modifications or euphemism. This makes it easier for the respondents to understand and answer questions and to reflect more accurately the real situation of the respondents."}, "time_dimension": {"answer": "Past", "rationale": "\"Recurring behaviour\" in \"often\" is often expressed by \"involved acts\" and \"in making\" is a general use of the present state of affairs, which means that the act has not only occurred in the past but may still be ongoing. Thus, the entry is to question the testee about past behavioral habits and temporal dimensions."}, "sentence_type": "Affirmative declarative sentence", "pos": {"nouns": {"Yes": 1, "Objects": 1, "鈾?And I'm gonna be 鈾?: 1, "I": 1, "Regular": 1, "Love": 1, "Do it": 1, "Some": 1, "Okay": 1}, "verbs": {"Trouble": 1, "not": 1, "By": 1, "Lord": 1, "From": 1, "Okay": 1, "Out": 1, "Do it": 2}, "adverbs": {"Regular": 1, "Some": 1}, "numerals": {"27": 1}, "pronouns": {"I": 2, "Here": 1}}}, {"content": "28, when things go wrong, I get sick.", "word_count": 12, "label": "", "sentence_structure": "Subject鈥揷opula鈥損redicative", "subjective_objective": {"answer": "Subjective", "rationale": "Because \"pregnant\" is a patient's subjective feeling and explanation, not an objective fact."}, "sentence_sentiment": {"answer": "Negative", "rationale": "Because the \"sick\" act in the entry is negative."}, "language_style": {"answer": "Calm and direct", "rationale": "The language style of the entry is more direct and concise, without excessive embellishment or emotional rendering, and instead describes the relationship between behaviour and outcome in an objective and calm manner."}, "time_dimension": {"answer": "Present", "rationale": ""}, "sentence_type": "Negative declarative sentence", "pos": {"nouns": {"Yes": 1, "It's not easy": 1, "Diseases": 1}, "verbs": {"Yes": 1, "Fan": 1}, "adjectives": {"Yes": 1}, "numerals": {"28": 1}, "pronouns": {"I": 1}}}, {"content": "I'm a very self-sufficient man.", "word_count": 11, "label": "", "sentence_structure": "Subject鈥揷opula鈥損redicative", "subjective_objective": {"answer": "Subjective", "rationale": "Because \"have control\" is a personal character or attitude that requires patient assessment and awareness."}, "sentence_sentiment": {"answer": "Positive", "rationale": "Because the entry describes a person with good self-control, which is a positive quality."}, "language_style": {"answer": "Calm and direct", "rationale": ""}, "time_dimension": {"answer": "Present", "rationale": "\"I am a very self-sufficient person\" shows that the person who speaks has a self-perception of his personality and that this character exists at the current point of time, so it is possible to judge the time dimension of the article as it is."}, "sentence_type": "Affirmative declarative sentence", "pos": {"nouns": {"person": 1, "Self-restraint": 1}, "verbs": {"be": 1, "have": 1}, "adjectives": {"very": 1}, "numerals": {"29": 1}, "pronouns": {"I": 1}}}, {"content": "I'm like an undesired artist.", "word_count": 16, "label": "", "sentence_structure": "Subject鈥揷opula鈥損redicative", "subjective_objective": {"answer": "Subjective", "rationale": "Because \"undesired artist\" is a subjective assessment and feeling that reflects the patient's own psychological state and emotional experience."}, "sentence_sentiment": {"answer": "Negative", "rationale": "Because the metaphor \"unsatisfactory artist\" is derogatory and expresses negative emotional expressions."}, "language_style": {"answer": "Tactful and warm", "rationale": "The language style of the entry is more euphemistic and warm, using metaphoric techniques (similar to a \"unsatisfactory artist\") and highlighting personal feelings and emotions. This expression is more friendly and acceptable."}, "time_dimension": {"answer": "Past", "rationale": "The entry uses the metaphor \"like...\" to compare itself with \"undesperable artist\" and to describe the past and the feelings."}, "sentence_type": "Affirmative declarative sentence", "pos": {"nouns": {"Artist": 1}, "verbs": {"Yeah": 1, "You're right": 1, "Same thing": 1}, "adjectives": {"It's just": 1, "I'm not": 1}, "numerals": {"30": 1}, "pronouns": {"I": 1}}}, {"content": "I don't always tell the truth.", "word_count": 7, "label": "", "sentence_structure": "Subject鈥損redicate", "subjective_objective": {"answer": "Subjective", "rationale": "Because it is a subjective attitude and behavioural choice of a person to tell the truth."}, "sentence_sentiment": {"answer": "Negative", "rationale": "Because the entry is \"not always telling the truth\", it is implied that it may be false, negative attitudinal or qualitative."}, "language_style": {"answer": "Tactful and warm", "rationale": "The language style of the entry is euphemistic. By using negative forms, \"not always,\" the speaker does not make it clear that he always speaks the truth, but leaves room for room and possibility. At the same time, this expression is relatively mild and avoids direct accusations or attacks on others, making it more acceptable and willing to engage in further communication."}, "time_dimension": {"answer": "Present", "rationale": "I hear \"always\" often in the words \"recurringly,\" and \"hearing\" in the general current, which means that this is not only happening in the past, but may still be going on. Again, \"not always telling the truth\" also indicates that it is a present act or tendency."}, "sentence_type": "Negative declarative sentence", "pos": {"verbs": {"Always": 1, "Say it": 1, "The truth": 1}, "adjectives": {"not": 1}}}, {"content": "I'll avoid it when I feel my pride is hurt.", "word_count": 16, "label": "", "sentence_structure": "Subject鈥搗erb鈥搊bject", "subjective_objective": {"answer": "Subjective", "rationale": "Because the response \"I'll avoid\" is the patient's subjective attitude and behavior. Although self-esteem is an objective event, it is the patient's own decision how to react, so it is subjective."}, "sentence_sentiment": {"answer": "Negative", "rationale": "Because the \"divide\" act in the entry is negative."}, "language_style": {"answer": "Calm and direct", "rationale": ""}, "time_dimension": {"answer": "Present", "rationale": ""}, "sentence_type": "Exclamatory sentence", "pos": {"nouns": {"Self-esteem": 1, "Hurt": 1, "Avoidance": 1}, "verbs": {"I feel": 1, "Yes": 1, "Avoidance": 1}, "adverbs": {"Just": 1}, "numerals": {"32": 1}, "pronouns": {"I": 1}}}, {"content": "I've often been forced to do things so much too much that others had to restrain me.", "word_count": 31, "label": "", "sentence_structure": "Subject鈥搗erb鈥搊bject", "subjective_objective": {"answer": "Subjective", "rationale": "Because the words \"compulsive,\" \"necessaryly limited\" are the subjective feelings and judgments of the patient."}, "sentence_sentiment": {"answer": "Negative", "rationale": "Because the entries \"pushing yourself to do something too much\" and \"the others have to limit me\" are negative emotional acts."}, "language_style": {"answer": "Calm and direct", "rationale": "The entry is more straightforward, is not too much rhetorical or emotional, but rather describes the problems that the person under investigation may have. Nor does it use any guiding language to make the person under investigation more free and honest in answering. So I think the language style for this purpose is straightforward and calm."}, "time_dimension": {"answer": "Present", "rationale": "\"I often do not have the autonomy to say that this is a regular, customary act, and the words \"forced myself to do something too excessive\" and \"must limit me\" indicate that this is still happening. So, the time dimension of this purpose is now."}, "sentence_type": "Negative declarative sentence", "pos": {"nouns": {"Entry": 1, "Questionnaire": 1, "person": 2, "Things": 1, "Limits": 1}, "verbs": {"Analysis": 1, "Forced": 1, "Fuck": 1, "I had to": 1, "Limits": 1}, "adverbs": {"Often": 1, "I can't help it": 1, "Overwhelming": 1}, "numerals": {"33": 1, "Others": 1}, "pronouns": {"I": 1}}}, {"content": "34 My friends think I'm a country boy.", "word_count": 12, "label": "", "sentence_structure": "Subject鈥搗erb鈥搊bject", "subjective_objective": {"answer": "Subjective", "rationale": "Because \"how people see themselves\" is the patient's subjective feeling, which is seen from the patient's perspective."}, "sentence_sentiment": {"answer": "Negative", "rationale": "Because the description of \"becoming a country boy\" in the entry is negative."}, "language_style": {"answer": "Tactful and warm", "rationale": "The entry uses a more euphemistic formulation that asks if the other person has been seen by friends as a \"squad.\" This expression avoids direct denial or criticism, is more respectful and understanding and facilitates good interaction."}, "time_dimension": {"answer": "Present", "rationale": "According to the sentence given, \"I\" is considered by friends as \"the countryman.\" The phrase is a description of the current situation, and therefore the temporal dimension is now. At the same time, the passive tone of \"in the opinion of friends\" implies that it is a question of describing what others think of me or of evaluating me, not of \"I\" to evaluate its own behaviour or character, so the subjectivity of the purpose of the article can be classified as a subjective attitude."}, "sentence_type": "Negative declarative sentence", "pos": {"nouns": {"friends": 1, "Country boy": 1}, "verbs": {"- Put it on": 1, "Look at it": 1}, "numerals": {"34": 1}, "pronouns": {"I": 1}}}, {"content": "I've been avoiding it a lot when I'm angry.", "word_count": 12, "label": "", "sentence_structure": "Subject鈥搗erb鈥揷omplement", "subjective_objective": {"answer": "Subjective", "rationale": "Because I often avoid it as a personal subjective feeling and reaction. Although it may be influenced by external circumstances (e.g. in cases of anger), it still looks at the problem from the perspective of the patient."}, "sentence_sentiment": {"answer": "Negative", "rationale": "Because the act of \"scramble\" is usually considered to be a way to avoid problems or to counter negative responses, it is emotionally negative."}, "language_style": {"answer": "Calm and direct", "rationale": "The language style of the entry is more straightforward, with no excessive rhetorical or emotional rendering, but rather a simple description of a pattern of behaviour. This style is easier for respondents to understand and answer."}, "time_dimension": {"answer": "Present", "rationale": "Now that I often avoid the word \"normal\" in \"recurring, customary behaviour\" and \"neglect\" in the general current, it means that this behaviour has not only occurred in the past, but may still be ongoing. So, the questionnaire entry is asking the testee about the behaviour and mentality of the subject at present."}, "sentence_type": "Negative declarative sentence", "pos": {"nouns": {"Time": 1, "Anger": 1}, "verbs": {"Avoidance": 1}, "adverbs": {"Often": 1}, "numerals": {"35": 1}}}, {"content": "I'm always more vigilant than I should be with those who are really nice to me.", "word_count": 30, "label": "", "sentence_structure": "Subject鈥搗erb鈥搊bject", "subjective_objective": {"answer": "Subjective", "rationale": "This reflects patients ' subjective attitudes and observations."}, "sentence_sentiment": {"answer": "Negative", "rationale": "Because the \"severe alert\" act in the entry is negative."}, "language_style": {"answer": "Tactful and warm", "rationale": "The language style of the entry is more euphemism and moderation, and the author does not express his emotions or position directly. He/she tries to guide the respondents in a calm and objective way to reflect on their attitudes to interpersonal relations and to raise a question that needs to be reflected. This tone helps to create an open environment for discussion and makes the respondents more willing to share their thoughts and experiences."}, "time_dimension": {"answer": "Present", "rationale": "The keyword \"I\" often means \"a regular act,\" and \"a higher level of vigilance\" is the current attitude or habit of the testee. So the entry is mainly about the current behaviour and attitude of the testee."}, "sentence_type": "Affirmative declarative sentence", "pos": {"nouns": {"Questionnaire item": 1, "Problem": 2, "person": 4, "Friendly": 1, "Vigilance": 1}, "verbs": {"Often": 1, "to": 1, "Hold on": 1, "Doubt": 1}, "adjectives": {"High": 1, "I should": 1}, "adverbs": {"More": 1, "Indeed": 1}, "numerals": {"36": 1, "4": 1}, "classifiers": {"individual": 2}, "pronouns": {"I": 1}}}, {"content": "I've learned a lot of talent to live my life without any problems.", "word_count": 22, "label": "", "sentence_structure": "Subject鈥搗erb鈥搊bject", "subjective_objective": {"answer": "Subjective", "rationale": "Because \"special talent learned\" and \"life-free\" are subjective assessments of the patient's abilities and future."}, "sentence_sentiment": {"answer": "Positive", "rationale": "Because the words \"special talent\" and \"life without problems\" in the entries are all positive emotional."}, "language_style": {"answer": "Tactful and warm", "rationale": "The language style of the entry is euphemismal, because it attempts to share with the interviewees, in a way that they ask and encourage, the special talents they already possess, and implies that they can help them learn more in their future lives. This tone is so friendly and non-threatening that it is easier to open their minds and engage in dialogue."}, "time_dimension": {"answer": "Past", "rationale": "The word \"a\" in the entry indicates that a past move or state has been completed and has had an impact on the present. At the same time, the word \"a learned\" is one of the hallmarks of the past, indicating that this \"special talent\" was acquired at some point in the past. It is therefore possible to judge that the entry is an inquiry into whether the person tested had acquired a particular ability in the past and that this ability has already had an impact on his life."}, "sentence_type": "Affirmative declarative sentence", "pos": {"nouns": {"Talent": 1, "Problem": 1, "Fan": 1}, "verbs": {"鈾?And I'm gonna be 鈾?: 1, "Pass": 1, "I can learn": 1}, "adjectives": {"Special": 1, "Enough": 1}, "adverbs": {"None": 1}, "numerals": {"37": 1}, "pronouns": {"I": 1}}}, {"content": "I sometimes choose people I hardly know when I'm voting.", "word_count": 22, "label": "", "sentence_structure": "Subject鈥搗erb鈥搊bject", "subjective_objective": {"answer": "Subjective", "rationale": "Since voting at the time of the election is a subjective choice of the patient, although it may be influenced by external factors."}, "sentence_sentiment": {"answer": "Negative", "rationale": "Because the behavior in the entry \"selection of those I hardly know\" can lead to irrational decision-making and bad results, with negative aspects."}, "language_style": {"answer": "Calm and direct", "rationale": "The entry raises the question in an objective and direct manner, without any rhetorical or emotional rendering, and focuses more on the accuracy of the transmission of information."}, "time_dimension": {"answer": "Past", "rationale": "The words \"sometimes\" and \"in the election\" and \"often\" indicate that this is a description of the past, so the temporal dimension of this entry is the past."}, "sentence_type": "Negative declarative sentence", "pos": {"nouns": {"Time": 1, "Elections": 1, "person": 1}, "verbs": {"Choose": 1, "Roger that": 1, "Often": 1}, "adjectives": {"Some": 1, "Almost": 1}, "adverbs": {"in": 1, "Both": 1}, "numerals": {"38": 1}, "pronouns": {"I": 2}}}, {"content": "I've often been late for appointments.", "word_count": 9, "label": "", "sentence_structure": "Subject鈥搗erb鈥揷omplement", "subjective_objective": {"answer": "Subjective", "rationale": "Because \"failure to make time\" is a subjective feeling and experience."}, "sentence_sentiment": {"answer": "Negative", "rationale": "Because the \"failure to make the appointment\" in the entry is negative."}, "language_style": {"answer": "Calm and direct", "rationale": "The entry is in a straightforward and clear manner asking the other party whether it is often not on time, without using excessive rhetorical techniques or emotional rendering, and with a more balanced and objective tone."}, "time_dimension": {"answer": "Present", "rationale": "The keyword \"often late\" denotes a customary act in which \"often\" indicates that it is frequent, and \"failure to do so\" is a specific move that indicates that the act has not only occurred in the past but may still be ongoing. Thus, the entry is now judged."}, "sentence_type": "Negative declarative sentence", "pos": {"nouns": {"Questionnaire": 1, "Entry": 1}, "verbs": {"Analysis": 1, "Regular": 1, "Yes": 1, "Date": 1, "On time": 1}, "adverbs": {"Often": 1}, "numerals": {"39": 1}}}, {"content": "I've got a lot of fantasies, but I can't do much in real life.", "word_count": 15, "label": "", "sentence_structure": "Subject鈥搗erb鈥揷omplement", "subjective_objective": {"answer": "Subjective", "rationale": "Because \"fanta\" and \"do\" are based on the patient's subjective vision or practice, not on objectively observed facts. Thus, this is viewed from the patient's subjective perspective."}, "sentence_sentiment": {"answer": "Negative", "rationale": "Because the \"fantasy, can't do much\" in the entry is negative."}, "language_style": {"answer": "Tactful and warm", "rationale": ""}, "time_dimension": {"answer": "Present", "rationale": "The entry asks the testee about the relationship between fantasy and actual action. Although it contains the past ( \"more fantasy\"), its main meaning is to describe it now. So we can judge it as now."}, "sentence_type": "Affirmative declarative sentence", "pos": {"nouns": {"Fantasy": 1, "Real life": 1}, "verbs": {"Fantasy": 1, "Do it": 1, "In... in": 1}, "adjectives": {"More": 1, "Less": 1}, "numerals": {"40": 1}}}, {"content": "I'm ashamed to deal with people.", "word_count": 8, "label": "", "sentence_structure": "Subject鈥搗erb鈥揷omplement", "subjective_objective": {"answer": "Subjective", "rationale": "Because \"shaming\" is a emotional attitude of a human being, which is subjective."}, "sentence_sentiment": {"answer": "Negative", "rationale": "Because \"shaming\" is a negative emotion."}, "language_style": {"answer": "Calm and direct", "rationale": "The entry directly expresses the subject of asking the other party whether it is ashamed to deal with people, without excessive rhetoric or emotional rendering, and with a more objective tone."}, "time_dimension": {"answer": "Past", "rationale": "The keyword is \"shammy\", which is an emotional attitude, usually referring to reactions to past experiences or events. The temporal dimension of the article is therefore judged to be the past."}, "sentence_type": "Negative declarative sentence", "pos": {"nouns": {"I": 1, "person": 1, "Dealing": 1}, "verbs": {"Shame": 1, "Hand it over": 1}, "adverbs": {"Shame": 1}, "numerals": {"41": 1}}}, {"content": "42, I'm not afraid of anything.", "word_count": 6, "label": "", "sentence_structure": "Subject鈥搗erb鈥揷omplement", "subjective_objective": {"answer": "Subjective", "rationale": "Because \"no fear\" is a subjective feeling and attitude of the individual, it reflects the existence or intensity of the fear of something."}, "sentence_sentiment": {"answer": "Neutral", "rationale": "Since the entry does not have a clear emotional tendency or value judgement, it is merely a narrative statement."}, "language_style": {"answer": "Calm and direct", "rationale": "The entry asks questions in a direct and clear way, \"I am afraid of nothing,\" which is very direct and categorical, without any rhetorical or emotional replication. This expression allows respondents to understand the question more clearly and does not create confusion or misunderstanding in their answers. Moreover, the language style of the purpose is somewhat challenging. It challenges the respondents to their beliefs or attitudes by asking questions. This way gives the interviewees the opportunity to re-examine their thoughts and behaviour, and may trigger their deep reflections and reflections."}, "time_dimension": {"answer": "Past", "rationale": "The entry is not clear on the point of time and can be understood to cover both past situations and present and future situations, and therefore it is not possible to determine the specific time dimension."}, "sentence_type": "Negative declarative sentence", "pos": {"verbs": {"Not afraid": 1}, "adverbs": {"Both": 1, "not": 1}, "numerals": {"42": 1}, "pronouns": {"I": 1}}}, {"content": "43, sometimes I think I'm an angel, sometimes I think I'm a demon.", "word_count": 20, "label": "", "sentence_structure": "Subject鈥損redicate", "subjective_objective": {"answer": "Subjective", "rationale": "Because \"think I'm an angel\" and \"think I'm a demon\" are the patients' own thoughts, and they're subjective."}, "sentence_sentiment": {"answer": "Neutral", "rationale": "Because the entry is a description of a person's self-perception and does not contain any emotional colour."}, "language_style": {"answer": "Tactful and warm", "rationale": "The entry uses the word \"think\" to express the self-perception of the speaker and to use the opposing image of \"angels\" and \"demons\" as metaphors. This expression neither directly negates or affirms one's behaviour or quality, but gives people the opportunity to think and understand their inner world in greater depth, and therefore has a certain euphemism and warmness."}, "time_dimension": {"answer": "Past", "rationale": "Past and future"}, "sentence_type": "Affirmative declarative sentence", "pos": {"nouns": {"I": 2, "Angel": 1, "Demon": 1}, "verbs": {"I think": 2, "be": 2}, "adjectives": {"Sometimes": 2}, "numerals": {"43": 1}}}, {"content": "I won't lose, I won't lose.", "word_count": 12, "label": "", "sentence_structure": "Subject鈥搗erb鈥揷omplement", "subjective_objective": {"answer": "Subjective", "rationale": "Because \"I can only win but not lose\" is a psychological feeling or attitude, a patient's subjective view of the success."}, "sentence_sentiment": {"answer": "Negative", "rationale": "Because the attitude in the entries is \"winner than lose\", it is too absolute and utilitarian and can easily lead to negative sentiments such as excessive competition and anxiety."}, "language_style": {"answer": "Calm and direct", "rationale": "The entry uses strong emotional language, which expresses the extreme desire for victory and the unacceptability of failure, and therefore it can be judged as \"cool and direct\"."}, "time_dimension": {"answer": "Present", "rationale": "At the same time, \"I can only win, I cannot lose\" expresses an overly strong expectation of results, which may require further exploration of the psychological state and motivation of the person being tested."}, "sentence_type": "Negative declarative sentence", "pos": {"nouns": {"The game": 1, "I": 1}, "verbs": {"Win": 1, "Lose": 1, "in": 1}, "adjectives": {"Only": 1}, "numerals": {"44": 1}}}, {"content": "45, when I'm angry, I'm ready to be a sarcasm.", "word_count": 15, "label": "", "sentence_structure": "Subject鈥搗erb鈥揷omplement", "subjective_objective": {"answer": "Subjective", "rationale": "Because \"willing to stutter\" is an emotional state and attitude, a subjective feeling for the patient."}, "sentence_sentiment": {"answer": "Negative", "rationale": "Basis: The words \"irror\" and \"scrawling\" in the entry are negative emotions and behaviours and can therefore be judged negative."}, "language_style": {"answer": "Calm and direct", "rationale": "The entry directly expresses the subject of questioning whether the other party will be bitter in the event of anger, without excessive rhetoric or emotional rendering, and is more objective in tone."}, "time_dimension": {"answer": "Present", "rationale": "Now, \"I'm ready to be bitter\" \"to be\" means that it's a change of state, and that change happens when I'm angry, so it's a present, immediate act. And \"I'm angry\" means that it's a state of emotion, and it's happening now, so the whole entry describes the situation."}, "sentence_type": "Exclamatory sentence", "pos": {"nouns": {"I": 1, "Time": 1, "person": 1}, "verbs": {"鈾?And become 鈾?: 1, "Scratch": 1}, "adjectives": {"Anger": 1, "very": 1}, "numerals": {"45": 1}}}, {"content": "I'm a public counterattack when my self-esteem is hurt.", "word_count": 15, "label": "", "sentence_structure": "Subject鈥搗erb鈥搊bject", "subjective_objective": {"answer": "Subjective", "rationale": "Because \"public counterattack\" is the patient's response and response, and it is subjective and behavioural."}, "sentence_sentiment": {"answer": "Negative", "rationale": "Because the \"public counterattack\" in the entry is potentially conflict- and contradictory and has a negative emotional tone."}, "language_style": {"answer": "Calm and direct", "rationale": "The entry uses a direct language style, without too many acrimony or rhetoric, expressing attitudes and positions that would openly counter the act in the event of an injury to self-esteem.鈥?}, "time_dimension": {"answer": "Present", "rationale": "The entry is a description of the person's current behaviour or attitude, as judged by the keyword \"publicly counter\" ."}, "sentence_type": "Affirmative declarative sentence", "pos": {"nouns": {"I": 1, "Self-esteem": 1}, "verbs": {"Just": 1, "Public": 1, "Fight back": 1}, "adverbs": {"in": 1, "When injured": 1}, "numerals": {"46": 1}}}, {"content": "47, I think I should be turned over when I get hurt.", "word_count": 15, "label": "", "sentence_structure": "Subject鈥搗erb鈥搊bject", "subjective_objective": {"answer": "Subjective", "rationale": "Because the word \"thinks\" indicates that it is the patient's personal opinion and attitude."}, "sentence_sentiment": {"answer": "Negative", "rationale": "Because \"injured\" and \"retarded\" are negative behaviors or emotions."}, "language_style": {"answer": "Calm and direct", "rationale": "The language style of the entry is more straightforward, and not too euphemistic or vague. It expresses the attitude and behaviour that should be followed in the event of injury in a straightforward manner, without excessive emotional or rhetorical style, and is more objective. Therefore, the language style of the entry is straightforward and calm."}, "time_dimension": {"answer": "Present", "rationale": "The term \"think\" and \"just\" makes it possible to judge that the entry is the present mindset or belief of the testee, and thus the temporal dimension is now."}, "sentence_type": "Affirmative declarative sentence", "pos": {"nouns": {"Questionnaire": 1, "Entry": 1, "person": 2, "Hurt": 1, "Face": 1}, "verbs": {"I think": 1, "Yes": 1, "I think so": 1, "Flip": 1}, "adverbs": {"When": 1}, "numerals": {"47": 1}, "pronouns": {"I": 2}}}, {"content": "48, I read every day. Not every page.", "word_count": 14, "label": "", "sentence_structure": "Subject鈥搗erb鈥搊bject", "subjective_objective": {"answer": "Subjective", "rationale": "This is because the patient's description of his or her reading habits is a manifestation of subjective attitudes."}, "sentence_sentiment": {"answer": "Negative", "rationale": "Because the entry expresses a attitude of abandonment or incomplete reading."}, "language_style": {"answer": "Tactful and warm", "rationale": "The entry is more adept and interactive when it is spoken in a gentle and inviting manner, rather than by direct statements or orders."}, "time_dimension": {"answer": "Present", "rationale": "The keyword \"day\" and \"time of reading\" means it's a sentence describing the person's current customary sexual behaviour, so the time dimension is now."}, "sentence_type": "Imperative sentence", "pos": {"verbs": {"Read it": 2, "Read the newspaper": 1}, "adverbs": {"Chile": 1, "No, it's not": 1, "Both": 1}, "numerals": {"48": 1}, "pronouns": {"I": 1}}}, {"content": "49, when I'm depressed, I avoid.", "word_count": 8, "label": "", "sentence_structure": "Subject鈥搗erb鈥揷omplement", "subjective_objective": {"answer": "Subjective", "rationale": "Because \"shield\" is a patient's subjective reaction and behavioral choice, reflecting the patient's response to frustration."}, "sentence_sentiment": {"answer": "Negative", "rationale": "Because \"shield\" in the entry means avoiding negative emotions, and it's a negative act."}, "language_style": {"answer": "Calm and direct", "rationale": "The entry directly asked the respondents how they responded to the mood when they were depressed, in a clear and simple language, without too much embellishment or emotional rendering."}, "time_dimension": {"answer": "Present", "rationale": "In the future, the entry contains visions and expectations for the future, with the key words \"if you are frustrated\" and \"if you are avoided\", all pointing to situations that have not yet occurred and that may arise in the future."}, "sentence_type": "Negative declarative sentence", "pos": {"nouns": {"Frustration": 1, "Avoid": 1}, "verbs": {"Yes": 2}, "adverbs": {"Time": 1, "Just": 1}, "numerals": {"49": 1}}}, {"content": "I'm shy about sex.", "word_count": 9, "label": "", "sentence_structure": "Subject鈥揷opula鈥損redicative", "subjective_objective": {"answer": "Subjective", "rationale": "Because being shy is a subjective feeling and attitude."}, "sentence_sentiment": {"answer": "Negative", "rationale": "Because the entry \"scrambling\" expresses a negative emotional attitude."}, "language_style": {"answer": "Tactful and warm", "rationale": ""}, "time_dimension": {"answer": "Present", "rationale": ""}, "sentence_type": "Negative declarative sentence", "pos": {"nouns": {"I": 1, "Sexual issues": 1}, "verbs": {"I feel": 1, "Shy": 1}, "numerals": {"50": 1}}}, {"content": "I always felt like someone I knew was a protector.", "word_count": 18, "label": "", "sentence_structure": "Subject鈥搗erb鈥揷omplement", "subjective_objective": {"answer": "Subjective", "rationale": "Because \"fear\" is a subjective feeling, which means the patient's personal opinion and feelings."}, "sentence_sentiment": {"answer": "Positive", "rationale": "Because the image of \"protector\" in the entries is usually positive, giving people a sense of security and dependence."}, "language_style": {"answer": "Tactful and warm", "rationale": "The entry is more personal and interactive when it is spoken in a gentle and inviting manner, rather than in direct statements or orders. Moreover, the language style of the article is more subtle, not too emotional, but rather a calm way of directing the interviewee to share his feelings and experiences."}, "time_dimension": {"answer": "Present", "rationale": "The keyword \"always\" means that it is a constant act or condition, and the status of the dominant word \"I\" is open-ended, so it is possible to judge the temporal dimension of the article as it is."}, "sentence_type": "Affirmative declarative sentence", "pos": {"nouns": {"person": 1, "Protect the gods": 1}, "verbs": {"I feel": 1, "Yes": 1, "Elephant": 1}, "adverbs": {"Always": 1}, "numerals": {"51": 1}, "pronouns": {"I": 1, "Someone": 1}}}, {"content": "52 My philosophies are: 鈥淒o not believe, do not do, do not look.鈥?, "word_count": 19, "label": "", "sentence_structure": "Subject鈥搗erb鈥搊bject", "subjective_objective": {"answer": "Subjective", "rationale": "Because \"my philosophies are...\" means that it's a personal view and attitude, and it's subjective."}, "sentence_sentiment": {"answer": "Negative", "rationale": "Because the expression \"no reason, no reason, no reason\" contains a negative and exclusive attitude and is a negative emotional expression."}, "language_style": {"answer": "Calm and direct", "rationale": "The entry expresses directly the subject of a philosophic inquiry in a concise language, without using too many qualifiers or emotional expressions, and is more balanced and objective."}, "time_dimension": {"answer": "Present", "rationale": ""}, "sentence_type": "Affirmative declarative sentence", "pos": {"nouns": {"Philosophy of the world": 1}, "verbs": {"be": 1, "I'm not gonna believe this": 1, "I don't want to do it": 1, "I don't want to see": 1}, "numerals": {"52銆?: 1}}}, {"content": "53 I think that people have a good or a bad side.", "word_count": 9, "label": "", "sentence_structure": "Subject鈥搗erb鈥搊bject", "subjective_objective": {"answer": "Subjective", "rationale": "This is because of the patient ' s personal opinion or conviction and is the subject of subjective attitudes."}, "sentence_sentiment": {"answer": "Neutral", "rationale": "Because the entry expresses a person ' s view, there is no apparent emotional tendency."}, "language_style": {"answer": "Calm and direct", "rationale": "The entry is presented in a straightforward manner, without too many rhetorical or emotional renderings, and in a more calm and objective language. It directly asks the respondents about the classification of persons, making the issue clearer and more specific."}, "time_dimension": {"answer": "Present", "rationale": "The entry asks the testee about the perception and perception of \"people have good or bad\" and does not have clear time limits or references to past experiences, and therefore it is now judged."}, "sentence_type": "Affirmative declarative sentence", "pos": {"nouns": {"person": 1, "Okay": 1, "Bad": 1}, "verbs": {"I think": 1, "min": 1}, "numerals": {"53": 1}}}, {"content": "I'd be in trouble at work or a seamstress if my boss got me mad.", "word_count": 29, "label": "", "sentence_structure": "Subject鈥搗erb鈥搊bject", "subjective_objective": {"answer": "Subjective", "rationale": "Because the questionnaire's purpose is to address the individual feelings and reactions of the interviewees, including dissatisfaction with the superiors and possible ways of acting. These are subjective thoughts and attitudes of the interviewees, and the questionnaire is therefore based on the subjective perspective of the respondents."}, "sentence_sentiment": {"answer": "Negative", "rationale": "Because the entry \"problem or grinder\" is negative, it displays dissatisfaction and revenge on the superiors and is detrimental to the harmonious development of the working atmosphere and human relations."}, "language_style": {"answer": "Calm and direct", "rationale": "The basis for the determination: This entry expresses the possible consequences and behaviour in a direct way, i.e., when the respondent is disgruntled, they may respond to the superiors by negative behaviour. This expression is not too much accommodative or obscure, but rather a clear message. The language style of the entry can therefore be described as being straightforward."}, "time_dimension": {"answer": "Past", "rationale": "The words \"may\" and \"may\" are a possible and hypothetical description, which is usually a function of speculation or retroactivity with respect to past acts or situations, depending on the context and manner in which the article is expressed, and therefore the entry may be judged to relate to past acts or mentalities."}, "sentence_type": "Negative declarative sentence", "pos": {"nouns": {"Sir": 1, "I": 2, "Work": 1, "Trouble": 1, "Revenge": 1}, "verbs": {"Fuck": 1, "Angry": 1, "Find": 1, "A seaman": 1}, "adverbs": {"Maybe": 1}, "numerals": {"54": 1}}}, {"content": "55, everybody's against me.", "word_count": 9, "label": "", "sentence_structure": "Subject鈥搗erb鈥揷omplement", "subjective_objective": {"answer": "Subjective", "rationale": "Because this is a subjective examination of the patients ' own feelings and perceptions."}, "sentence_sentiment": {"answer": "Negative", "rationale": "Because it expresses a negative emotion and attitude that others are always against themselves."}, "language_style": {"answer": "Calm and direct", "rationale": "The language style of the entry is more straightforward, not using excessive rhetorical techniques or emotional rendering, but rather a direct expression of whether the investigator wants to know whether there is a confrontation between all of us."}, "time_dimension": {"answer": "Present", "rationale": ""}, "sentence_type": "Negative declarative sentence", "pos": {"nouns": {"person": 1, "I": 1}, "verbs": {"to": 1, "Here": 1, "Fuck": 1}, "adjectives": {"Both": 1}, "adverbs": {"Every": 1, "individual": 1}, "numerals": {"55": 1}}}, {"content": "I'm always friendly with people I hate.", "word_count": 15, "label": "", "sentence_structure": "Subject鈥搗erb鈥揷omplement", "subjective_objective": {"answer": "Subjective", "rationale": "Because \"obsessed\" and \"friendly\" are patients' subjective feelings and attitudes."}, "sentence_sentiment": {"answer": "Negative", "rationale": "Because the description of \"the hateful person\" in the entry is negative, the latter act, in contrast to the \"good\" act, highlights the unnaturality and tact of the act, and thus has a negative emotional connotation."}, "language_style": {"answer": "Tactful and warm", "rationale": "The language style of the entry is more euphemistic than that of a directly cold or offensive language. The use of the expression \"often\" also shows that this is a relatively common but not absolute situation, making the issue more objective and open. At the same time, the expression \"I hate people who express friendship\" does not overemphasize dissatisfaction with these people, but rather focuses more on the description of individual behaviour, which also reflects its euphemism."}, "time_dimension": {"answer": "Present", "rationale": ""}, "sentence_type": "Negative declarative sentence", "pos": {"nouns": {"person": 1, "Friendly": 1}, "verbs": {"Often": 1, "Organisation": 1, "Shit": 1}, "adverbs": {"to": 1, "Those": 1}, "numerals": {"56": 1}}}, {"content": "57, if one of the engines on my plane fails, I'm very nervous.", "word_count": 23, "label": "", "sentence_structure": "Subject鈥搗erb鈥搊bject", "subjective_objective": {"answer": "Subjective", "rationale": "Because \"very nervous\" is the patient's subjective feeling and reaction to this situation."}, "sentence_sentiment": {"answer": "Negative", "rationale": "Because the \"very nervous\" emotion in the entry is negative."}, "language_style": {"answer": "Calm and direct", "rationale": "The entry uses a clear and straightforward language to express emotional reactions to the potential of engine failure, without excessive embellishment or euphemism, and therefore language style is straightforward and dispassionate."}, "time_dimension": {"answer": "Present", "rationale": ""}, "sentence_type": "Exclamatory sentence", "pos": {"nouns": {"Aircraft": 1, "Engines": 1, "Nervous": 1}, "verbs": {"Take it": 1, "It's not working": 1, "Yes": 1, "Very": 1, "Nervous": 1}, "adverbs": {"If": 1, "Just": 1, "Very": 1}, "numerals": {"57": 1}, "pronouns": {"I": 2}}}, {"content": "58, I know a man who can do anything and does what he does right.", "word_count": 22, "label": "", "sentence_structure": "Subject鈥搗erb鈥搊bject", "subjective_objective": {"answer": "Subjective", "rationale": "Because it is subjective for the patient to know and evaluate a person."}, "sentence_sentiment": {"answer": "Positive", "rationale": "Because the entry describes a person who can be reasonably honest, it is a positive quality."}, "language_style": {"answer": "Tactful and warm", "rationale": "The entry is more personal and interactive when it is spoken in a gentle and inviting manner, rather than in direct statements or orders. It also implies whether the person interviewed has ever known such a person and emphasizes the competence and integrity of the person, with the aim of leading the interviewee to remember his or her experiences and reflect on the quality of his or her interactions with such persons."}, "time_dimension": {"answer": "Past", "rationale": "In conclusion, the questionnaire contains a description of the testee ' s past."}, "sentence_type": "Affirmative declarative sentence", "pos": {"nouns": {"person": 1, "Yes": 1, "Reasonable": 1, "Integrity": 1}, "verbs": {"Yes": 1, "Do it": 2}, "adjectives": {"Here": 1, "Both": 1, "Yes": 1, "Reasonable": 1, "Integrity": 1}, "numerals": {"58": 1}, "classifiers": {"One": 2}, "pronouns": {"he": 1}}}, {"content": "59, if my emotional loss is preventing me from doing what I'm doing, then I can control it.", "word_count": 27, "label": "", "sentence_structure": "Subject鈥搗erb鈥搊bject", "subjective_objective": {"answer": "Subjective", "rationale": "The entry examines the attitude and beliefs of the patient in relation to his or her emotional management capacity, which are subjective. For example, it is the patient鈥檚 subjective perception and attitude that he or she believes that he or she is capable of controlling his or her emotions and inhibiting them because of fear that their emotional exposure will hinder his or her career."}, "sentence_sentiment": {"answer": "Positive", "rationale": "Because the \"control\" behavior in the entries is positive."}, "language_style": {"answer": "Calm and direct", "rationale": ""}, "time_dimension": {"answer": "Present", "rationale": ""}, "sentence_type": "Affirmative declarative sentence", "pos": {"nouns": {"Emotion": 1, "- Out": 1, "Career": 1}, "verbs": {"Yes": 1, "- Intrusive": 1, "Control": 1}, "adverbs": {"If": 1, "Well": 1, "Just": 1}, "numerals": {"59": 1}, "classifiers": {"individual": 1}, "pronouns": {"I": 2}}}, {"content": "60, some people are planning to kill me.", "word_count": 10, "label": "", "sentence_structure": "Subject鈥搗erb鈥搊bject", "subjective_objective": {"answer": "Subjective", "rationale": "This is a subjective fear and imagination of the patient, not an objective reality."}, "sentence_sentiment": {"answer": "Negative", "rationale": "Because the \"mixed intent\" in the entry is negative."}, "language_style": {"answer": "Calm and direct", "rationale": "The entry asks the person under investigation, in a direct and clear manner, whether he has encountered a conspiracy to harm himself, and is harsher and more direct.\""}, "time_dimension": {"answer": "Past", "rationale": "In the entry, \"some people are\" means that the act is taking place or has just taken place, so the temporal dimension is a past."}, "sentence_type": "Affirmative declarative sentence", "pos": {"nouns": {"person": 2, "Shit": 1, "I": 1}, "verbs": {"In the process of": 1, "Conspiracy": 1, "Shit": 1}, "numerals": {"60": 1}}}, {"content": "61, I usually see the good side of the bad.", "word_count": 15, "label": "", "sentence_structure": "Subject鈥搗erb鈥搊bject", "subjective_objective": {"answer": "Subjective", "rationale": "Because seeing the good side of the bad is a subjective perception and perception of one person, is based on his own experience and emotional experience."}, "sentence_sentiment": {"answer": "Positive", "rationale": "Because the \"seeing the good side of the bad\" in the entry is positive and expresses optimism and positive views about life."}, "language_style": {"answer": "Tactful and warm", "rationale": "The entry is more personal and interactive when it is spoken in a gentle and inviting tone, rather than in direct statements or orders. At the same time, the subject of \"seeing the good side of the bad\" is itself more subjective and emotional, so using a soft language style is more reflective of the respect and understanding of the questionnaire designers."}, "time_dimension": {"answer": "Present", "rationale": ""}, "sentence_type": "Affirmative declarative sentence", "pos": {"nouns": {"Bad times": 1, "Noodles": 1}, "verbs": {"Yeah": 1, "have": 1}, "adverbs": {"Usually": 1, "Medium": 1, "Okay": 1}, "numerals": {"61": 1}, "pronouns": {"I": 1}}}, {"content": "I had to do something I didn't want to do, and I had a headache.", "word_count": 20, "label": "", "sentence_structure": "Subject鈥揷opula鈥損redicative", "subjective_objective": {"answer": "Subjective", "rationale": "Because \"headache\" is a subjective feeling, it looks at the psychological response of the patient when faced with something that he does not want to do."}, "sentence_sentiment": {"answer": "Negative", "rationale": "Because the \"headache\" act in the entry is negative."}, "language_style": {"answer": "Calm and direct", "rationale": "The entry expresses the subject in a simple and clear language, without too much emotional or rhetorical presentation, and the language style is straightforward and calm."}, "time_dimension": {"answer": "Past", "rationale": "\"When I have to do something I do not want to do,\" indicates that the entry relates to what happened in the past, and \"headache\" is the result or effect. Thus, the temporal dimension of the article is the past."}, "sentence_type": "Negative declarative sentence", "pos": {"nouns": {"I": 1, "Yes": 1, "Headache": 1}, "verbs": {"Do it": 1, "Go on": 1, "Okay": 1, "not": 1, "May I": 1, "Ouch": 1}, "adjectives": {"I had to": 1}, "adverbs": {"Just": 1}, "numerals": {"62": 1}}}, {"content": "I often find myself very friendly with those who deserve to hate.", "word_count": 20, "label": "", "sentence_structure": "Subject鈥搗erb鈥搊bject", "subjective_objective": {"answer": "Subjective", "rationale": "Because feeling friendly is an emotional reaction, a subjective experience."}, "sentence_sentiment": {"answer": "Negative", "rationale": "Because the purpose of the act \"to express a good will towards those who deserve to be hated\" in the entry is unclear, it may lead to negative consequences and is therefore considered negative."}, "language_style": {"answer": "Tactful and warm", "rationale": "The entry is based on the following: 1. The entry conveys the reflection and description of the other party's behaviour through the words \"often\" and \"very friendly\", without directly accusing or criticizing others; 2. It uses more moderate and polite language, such as \"persons who deserve to be hated\", rather than offensive terms, such as \"enemy\" directly; 3. It also displays emotional colours, indicating that the author feels about his or her own behaviour and wants to be improved."}, "time_dimension": {"answer": "Present", "rationale": "So, the output is: \"Now.\""}, "sentence_type": "Affirmative declarative sentence", "pos": {"nouns": {"Entry": 1, "Questionnaire": 1, "person": 1}, "verbs": {"Found": 1, "to": 2, "Organisation": 1, "Hate": 1, "Friendly": 1}, "adverbs": {"Often": 1}, "numerals": {"63": 1}, "pronouns": {"I": 1}}}, {"content": "I think \"everyone has good will\" doesn't exist. If you're not, you're not.", "word_count": 27, "label": "", "sentence_structure": "Subject鈥搗erb鈥搊bject", "subjective_objective": {"answer": "Subjective", "rationale": "Because it expresses the views and convictions of the patient, it is subjective."}, "sentence_sentiment": {"answer": "Negative", "rationale": "Because the attitudes and beliefs in the entries (which think that \"good will for all\" does not exist) are negative."}, "language_style": {"answer": "Tactful and warm", "rationale": "The language style of this entry is more direct and somewhat pessimistic, and it expresses a questioning and negative view of humanity. It uses the term \"thinking\" in the description, and emphasizes the views of individuals without too much discussion or exploration of other possibilities. It is relatively calm, but it may also be depressing or uncomfortable for the respondents."}, "time_dimension": {"answer": "Present", "rationale": ""}, "sentence_type": "Negative declarative sentence", "pos": {"nouns": {"person": 2, "Good will": 1}, "verbs": {"I think": 1, "Existence": 1, "Not good": 3, "Everything": 1}, "adjectives": {"Good will": 1}, "adverbs": {"Both": 1, "not": 2}, "numerals": {"64": 1, "Two": 1}, "pronouns": {"you": 2, "I": 1}}}, {"content": "I'm not gonna be angry at those people I hate.", "word_count": 16, "label": "", "sentence_structure": "Subject鈥搗erb鈥搊bject", "subjective_objective": {"answer": "Subjective", "rationale": "Because \"never\" or \"not\" are personal attitudes and emotional expressions of patients, and are viewed from a subjective perspective."}, "sentence_sentiment": {"answer": "Negative", "rationale": "Because the behavior in the entry, \"I will never be angry at those I hate,\" is negative."}, "language_style": {"answer": "Calm and direct", "rationale": "The language style of the entry is calm and straightforward. It clearly expresses the subject of asking the other side whether to express anger in the face of a person who hates it, without using euphemism or rhetoric. This expression is clear, concise and easy to understand."}, "time_dimension": {"answer": "Present", "rationale": "\"I will never\" in \"never\" will express a strong determination and will and will, which is usually used to describe the current state or behaviour, so the temporal dimension of the purpose of the article is now judged."}, "sentence_type": "Negative declarative sentence", "pos": {"nouns": {"I": 2, "person": 1}, "verbs": {"Never": 1, "Organisation": 1, "to": 1, "Shit": 1, "Anger": 1}, "adjectives": {"Those": 1}, "adverbs": {"Never": 1}, "numerals": {"65": 1}}}, {"content": "66, I'm sure life is unjust to me.", "word_count": 12, "label": "", "sentence_structure": "Subject鈥揷opula鈥損redicative", "subjective_objective": {"answer": "Subjective", "rationale": "Because \"conviction\" is a subjective attitude or belief."}, "sentence_sentiment": {"answer": "Negative", "rationale": "Because the \"convinced life is unjust to me\" attitude in the entry is negative."}, "language_style": {"answer": "Calm and direct", "rationale": "The entry is relatively straightforward in language style, and does not use too many qualifiers or emotional terms. It expresses a belief or view in a direct and clear way, namely, that life is unjust to me. This style is more objective and calm."}, "time_dimension": {"answer": "Present", "rationale": "\"Sure\" means the current belief in life and the \"unfair\" expresses a negative sentiment or perception, so the temporal dimension of this questionnaire entry is now judged."}, "sentence_type": "Negative declarative sentence", "pos": {"nouns": {"Life": 1, "I": 2, "Injustice": 1}, "verbs": {"Sure": 1, "to": 1, "be": 1}, "adverbs": {"not": 1}, "numerals": {"66": 1}}}, {"content": "67, with a severe blow, I'll fall.", "word_count": 13, "label": "", "sentence_structure": "Subject鈥揷opula鈥損redicative", "subjective_objective": {"answer": "Subjective", "rationale": "Because this statement reflects the patient ' s self-perception and self-evaluation, it is a subjective attitude or emotional reaction."}, "sentence_sentiment": {"answer": "Negative", "rationale": "Because the \"falling\" act in the entry is negative."}, "language_style": {"answer": "Calm and direct", "rationale": "The entry uses a concise language describing the possible reactions of individuals in the event of severe blows, without excessive embellishment or emotional rendering, and with a more direct and calm language style."}, "time_dimension": {"answer": "Present", "rationale": "According to the description of \"under severe blow\", it can be assumed that this is a state of affairs or experience; the word \"as it is\" indicates that there will be a collapse in the event of a severe blow. Thus, \"now\" is the time frame for the purpose of the article."}, "sentence_type": "Negative declarative sentence", "pos": {"nouns": {"Strike": 1}, "verbs": {"Yes": 1, "Falling down": 1}, "adjectives": {"Serious": 1}, "numerals": {"67": 1}, "pronouns": {"I": 1}}}, {"content": "68, when I realized I had to face a difficult situation, like exams, trade unions. I tried to imagine what it would do and planned to deal with it.", "word_count": 46, "label": "", "sentence_structure": "Subject鈥搗erb鈥搊bject", "subjective_objective": {"answer": "Subjective", "rationale": "This is a self-presentation of patients, an expression of their imagination and coping strategies to the plight, subjective perceptions and attitudes based on personal experience and feelings."}, "sentence_sentiment": {"answer": "Positive", "rationale": "Because the expression of the purpose of the article is positive, it is a positive emotional expression, i.e., a proactive strategy to anticipate and plan solutions in the face of difficulties."}, "language_style": {"answer": "Calm and direct", "rationale": "The language style of the entry is calmer and straightforward. It describes in an objective way a response to a dilemma, without too much emotional or rhetorical. This expression is clearer, easy to understand and accept."}, "time_dimension": {"answer": "Present", "rationale": "The entry is for the present behaviour and mindset of the testee, and the time dimension is now judged."}, "sentence_type": "Affirmative declarative sentence", "pos": {"nouns": {"The dilemma": 1, "Examinations": 1, "Recruitment": 1, "Meetings": 1, "Imagine": 1, "Methodology": 1}, "verbs": {"Faced": 1, "I'm aware of that": 1, "Try": 1, "Planned": 1, "Payable": 1}, "adverbs": {"Just": 1, "Like": 1, "Yes": 1, "I had to": 1}, "numerals": {"68": 1}, "pronouns": {"I": 1}}}, {"content": "69, the doctors will never really figure out what I'm suffering from.", "word_count": 17, "label": "", "sentence_structure": "Subject鈥搗erb鈥揷omplement", "subjective_objective": {"answer": "Subjective", "rationale": "Because the \"realization\" of a disease depends on the professional knowledge and skills of the doctor, the complexity of the patient's condition, which involves the judgement and perception of many subjects and is therefore subjective."}, "sentence_sentiment": {"answer": "Negative", "rationale": "Basis of judgement: The statement of purpose is characterized by a negative and pessimistic sentiment, implying that doctors are unable to accurately diagnose the disease and may give rise to negative feelings of lack of confidence in the health system."}, "language_style": {"answer": "Calm and direct", "rationale": "The language style of the entry is calm and direct. It asks the other side whether, in a direct way, the doctor believes that he or she is not fully certain of their condition, that he or she is not using excessive speech techniques or emotional rendering, and that it is more objective. This style is usually used in investigations or research to collect as accurate and useful data as possible."}, "time_dimension": {"answer": "Past", "rationale": "The entry \"never\" expresses a strong negative attitude and past experience, so it can be judged to be a description of the past."}, "sentence_type": "Negative declarative sentence", "pos": {"nouns": {"Doctors": 1, "Diseases": 1}, "verbs": {"Never": 1, "I know": 1, "Patient": 1}, "adverbs": {"Really": 1}, "numerals": {"69": 1}, "pronouns": {"I": 1}}}, {"content": "I'm not sad when someone close to me dies.", "word_count": 18, "label": "", "sentence_structure": "Subject鈥搗erb鈥揷omplement", "subjective_objective": {"answer": "Subjective", "rationale": "Because emotional expression is subjective."}, "sentence_sentiment": {"answer": "Negative", "rationale": "Because the \"I'm not sad\" act in the entry is negative."}, "language_style": {"answer": "Calm and direct", "rationale": "The entry is a direct question-and-answer dialogue, with no excessive rhetorical or emotional accents, and is more objective in tone."}, "time_dimension": {"answer": "Present", "rationale": "The word \"when\" denotes an act at a moment or at a time, and \"not sad\" indicates that the act is in its present state, so it can be judged that the entry describes the present feeling or state."}, "sentence_type": "Negative declarative sentence", "pos": {"nouns": {"person": 1, "Sad": 1}, "verbs": {"Die": 1, "not": 1, "Sad": 1}, "adjectives": {"very": 1, "Close": 1}, "adverbs": {"When": 1}, "numerals": {"70": 1}, "pronouns": {"I": 1}}}, {"content": "71, I apologize for my rudeness after I fought for my own good.", "word_count": 26, "label": "", "sentence_structure": "Subject鈥搗erb鈥搊bject", "subjective_objective": {"answer": "Subjective", "rationale": "Because \"brubber\" and \"poaching\" are subjective feelings and behaviours of patients."}, "sentence_sentiment": {"answer": "Negative", "rationale": "The article ' s formulation includes negative acts such as fighting and rudeness."}, "language_style": {"answer": "Tactful and warm", "rationale": "The language style of this entry is more euphemistic and implicit, rather than directly expressing the negative consequences of the struggle and the wrong behaviour of one's own, it uses more moderate terminology and tone to reduce offensive and conflicting attitudes towards the other. This expression is more friendly, friendly and acceptable and helps to build better interpersonal relationships."}, "time_dimension": {"answer": "Past", "rationale": "The entries \"after I fight for my interests\" and \"I apologize to people for my rudeness\" describe past behavior or state and therefore are based on past."}, "sentence_type": "Affirmative declarative sentence", "pos": {"nouns": {"Interest": 1, "person": 2, "Fighting": 1, "Apologize": 1}, "verbs": {"in": 1, "For": 1, "Fighting": 1, "Apologize": 1}, "adjectives": {"Naughty": 1}, "numerals": {"71": 1}, "pronouns": {"I": 2}}}, {"content": "72, most of what happened to me wasn't my responsibility.", "word_count": 19, "label": "", "sentence_structure": "Subject鈥揷opula鈥損redicative", "subjective_objective": {"answer": "Subjective", "rationale": "Because \"accident,\" \"mostly\" and \"not my responsibility\" are the subjective views of patients."}, "sentence_sentiment": {"answer": "Negative", "rationale": "Because it expresses an attitude of evading responsibility and has a negative emotional connotation."}, "language_style": {"answer": "Calm and direct", "rationale": "The entry expresses the subject in a straightforward manner, without excessive rhetorical or emotional rendering, and is more balanced and objective."}, "time_dimension": {"answer": "Present", "rationale": "The keywords \"occupying\" and \"most things are not my responsibility\" indicate that the subject holds this view in his current state of mind."}, "sentence_type": "Negative declarative sentence", "pos": {"nouns": {"Things": 1, "Responsibility": 1}, "verbs": {"Let's go": 1, "be": 1, "have": 1}, "adjectives": {"Most of them": 1, "It's about me": 1}, "adverbs": {"and": 1, "Not": 1, "Marriage": 1}, "numerals": {"72": 1}, "pronouns": {"I": 1}}}, {"content": "73, when I feel depressed or anxious, eat something that makes me feel better.", "word_count": 26, "label": "", "sentence_structure": "Subject鈥搗erb鈥搊bject", "subjective_objective": {"answer": "Subjective", "rationale": "Since the subject is the thoughts and behaviour of patients in the event of emotional depression or anxiety, such ideas and behaviour are determined by the patient himself and are therefore subjective in nature."}, "sentence_sentiment": {"answer": "Negative", "rationale": "Because the act of eating can cause physical health problems and does not really solve emotional problems."}, "language_style": {"answer": "Tactful and warm", "rationale": "This is a more gentle and caring expression, asking if the person interviewed has a habit of eating and eating to relieve stress."}, "time_dimension": {"answer": "Past", "rationale": "The keywords \"Feeling emotional depression or anxiety\" and \"I feel better\" refer to the past behavior or state. Thus, it can be judged that the entry is asking whether the person who was tested had in the past reduced the feeling of emotional depression or anxiety by eating something."}, "sentence_type": "Affirmative declarative sentence", "pos": {"nouns": {"Emotion": 1, "Repression": 1, "Anxiety": 1, "Unsettled": 1, "Food": 1, "Something": 1, "Feel": 2, "Better": 1}, "verbs": {"Eat": 1, "鈾?And I'm gonna be 鈾?: 1, "Feel": 1}, "adjectives": {"Repression": 1, "Anxiety": 1, "Unsettled": 1}, "adverbs": {"When": 1, "Yeah": 1, "Some": 1}, "numerals": {"73": 1}, "classifiers": {"Points": 1}, "pronouns": {"I": 1}}}, {"content": "74, hard work makes me feel better.", "word_count": 10, "label": "", "sentence_structure": "Subject鈥揷opula鈥損redicative", "subjective_objective": {"answer": "Subjective", "rationale": "Because \"fear better\" is the patient's subjective feeling and judgment."}, "sentence_sentiment": {"answer": "Positive", "rationale": "The basis for the judgement: \"I feel better working hard\" in the entry expresses a positive attitude and feeling that through hard work I can achieve and satisfy."}, "language_style": {"answer": "Tactful and warm", "rationale": "The entry expresses, in an encouraging and supportive tone, the recognition and appreciation of the positive working attitude of the interviewees, whose soft and warm use of words does not put pressure or discomfort on the interviewees. At the same time, this language style makes it easier for the interviewees to open their minds and answer questions."}, "time_dimension": {"answer": "Present", "rationale": "The word \"makes me feel better\" indicates the effect of past behavior at a certain point in time, while \"favorite\" is a description of the current state. So the sentence asks the testee in its current state, and the temporal dimension is now."}, "sentence_type": "Affirmative declarative sentence", "pos": {"nouns": {"Work": 1, "Feel": 1}, "verbs": {"鈾?And I'm gonna be 鈾?: 1, "I'm working hard": 1, "Better": 1}, "adverbs": {"More": 1}, "numerals": {"74": 1}}}, {"content": "75, the doctor can't really help me with this.", "word_count": 12, "label": "", "sentence_structure": "Subject鈥搗erb鈥搊bject", "subjective_objective": {"answer": "Subjective", "rationale": "Because \"can\" or \"can\" is the patient's personal view and judgment, it is a patient's perspective, and the answer is subjective."}, "sentence_sentiment": {"answer": "Negative", "rationale": "Because the phrase \"can't really help me solve the problem\" in the entry is negative."}, "language_style": {"answer": "Calm and direct", "rationale": "The language style of the entry is relatively simple, with no excessive rhetorical or emotional rendering, and more focus on the transmission of information and the presentation of problems. This expression is more in line with the needs of actual research and research."}, "time_dimension": {"answer": "Past", "rationale": "The entry is an inquiry into the views of the testees on the experiences and feelings of the past, and the keyword \"can't really help me solve the problem\" means that this is an event that has already occurred, and it is therefore judged to be the past."}, "sentence_type": "Negative declarative sentence", "pos": {"nouns": {"Doctor": 1, "Problem": 1}, "verbs": {"Yes": 1, "Help": 1, "Resolve": 1}, "adjectives": {"True": 1}, "numerals": {"75": 1}}}, {"content": "I've heard a lot about my feelings.", "word_count": 15, "label": "", "sentence_structure": "Subject鈥搗erb鈥搊bject", "subjective_objective": {"answer": "Subjective", "rationale": "Because \"all the time people say\" belongs to others' evaluations and perceptions, and it's the patient's subjective feeling."}, "sentence_sentiment": {"answer": "Negative", "rationale": "Because the \"not to expose your feelings\" act in the entry is negative."}, "language_style": {"answer": "Calm and direct", "rationale": "The entry is more direct and concise in language style, and does not have too many rhetorical or emotional renderings. It clearly expresses the question whether the other party is often asked about the subject of the comment that he is not good at expressing feelings, and is more objective and neutral."}, "time_dimension": {"answer": "Present", "rationale": "The entry asks if the testees often hear people say they are not good at expressing their feelings. Key words include \"often\" and \"hear people say\" , which indicate that the act is customary and frequent, but also relates to the situation today. So, the output is: \"Now\"."}, "sentence_type": "Negative declarative sentence", "pos": {"nouns": {"Questionnaire": 1, "Entry": 1, "person": 2, "Emotion": 1}, "verbs": {"Listen": 1, "Say it": 1, "Not exposed": 1, "Regular": 1}, "adverbs": {"Regular": 1}, "numerals": {"76": 1}, "pronouns": {"I": 2, "People": 1}}}, {"content": "77 I think people are watching movies, plays or books that mean more to the senses than they want to be in these works.", "word_count": 35, "label": "", "sentence_structure": "Subject鈥搗erb鈥搊bject", "subjective_objective": {"answer": "Subjective", "rationale": "Because \"concept\" is the patient's subjective attitude, \"the people are watching movies, theatres, or books that mean more to the senses than these works are meant to express.\" And the patient's view is based on his own experience, thinking, and feeling. So the questionnaire is viewed from the patient's subjective perspective."}, "sentence_sentiment": {"answer": "Positive", "rationale": "It is a positive attitude because the entry considers that people have a better understanding of films, theatres or books than the work itself is intended to convey."}, "language_style": {"answer": "Tactful and warm", "rationale": "The language style of the entry is euphemism and moderation, using a number of expressions to guide respondents to think and share their views, such as \"I think\" and \"people can learn more in...\" . Such expressions are easier to resonate and interact with respondents."}, "time_dimension": {"answer": "Present", "rationale": ""}, "sentence_type": "Affirmative declarative sentence", "pos": {"nouns": {"People": 1, "Film": 1, "Theatre": 1, "Books": 1, "Meaning": 2, "I've learned": 1, "Works": 1}, "verbs": {"I think": 1, "Look": 1, "Learn": 1, "Expression": 1}, "adjectives": {"More": 1}, "numerals": {"77": 1}, "pronouns": {"These": 1}}}, {"content": "78, I feel like I have some custom or ritual behavior I have to do, and it's causing me a lot of trouble.", "word_count": 32, "label": "", "sentence_structure": "Subject鈥搗erb鈥搊bject", "subjective_objective": {"answer": "Subjective", "rationale": "Because the entry asks about the feelings and experiences of the patient and about the patient ' s subjective feelings."}, "sentence_sentiment": {"answer": "Negative", "rationale": "Because the entries \"some custom or ritual act not by choice\" and \"problems of trouble\" are negative descriptions."}, "language_style": {"answer": "Tactful and warm", "rationale": "This entry is expressed in a mild and understandable way, asking the interviewee whether he or she has certain habits or behaviours that have caused himself or herself inconvenience."}, "time_dimension": {"answer": "Present", "rationale": "The keywords \"fear,\" \"some,\" \"involved\" indicate that this feeling is the experience of the moment, while \"problems a lot\" indicate that the act has an impact on the person being tested. So, the entry is to ask the person who is being tested for his current behaviour and feelings."}, "sentence_type": "Affirmative declarative sentence", "pos": {"nouns": {"I": 2, "Some": 1, "It's not up to you": 1, "Custom": 1, "Ceremonial acts": 1, "A lot": 1, "Trouble": 1}, "verbs": {"Feel it": 1, "have": 1, "Do it": 1, "Bring it": 1}, "adjectives": {"It's not up to you": 1, "of": 3}, "numerals": {"78": 1, "One": 2}}}, {"content": "79, when I'm nervous, I drink or take pills.", "word_count": 11, "label": "", "sentence_structure": "Subject鈥損redicate", "subjective_objective": {"answer": "Subjective", "rationale": "Because this is a patient鈥檚 own description, and is an expression of subjective feelings and behaviour. While drinking and taking medication are objective facts, patients choose to respond in this way when they are nervous, based on their own subjective judgement and decision-making."}, "sentence_sentiment": {"answer": "Negative", "rationale": "Because the behavior in the entry \"drinking or taking medication\" is negative."}, "language_style": {"answer": "Calm and direct", "rationale": "The entry describes a behaviour pattern in a concise manner, without too much emotional rendering or rhetorical. The tone is more objective and direct, and is intended to reveal how the interviewee reacts to stress, rather than attempting to induce or influence the response of the interviewee."}, "time_dimension": {"answer": "Past", "rationale": ""}, "sentence_type": "Affirmative declarative sentence", "pos": {"nouns": {"Nervous": 1, "Wine": 1, "Medicine": 1}, "verbs": {"Drink": 1, "Eat": 1}, "numerals": {"79": 1}}}, {"content": "80, I want to stay with someone when I'm in a bad mood.", "word_count": 17, "label": "", "sentence_structure": "Subject鈥搗erb鈥揷omplement", "subjective_objective": {"answer": "Subjective", "rationale": "Because \"want to stay with someone\" is the patient's inner will, the patient's subjective feeling."}, "sentence_sentiment": {"answer": "Positive", "rationale": "Because the purpose of the \"staying with someone\" act in the entry is to alleviate the negative mood of yourself, and it is positive."}, "language_style": {"answer": "Tactful and warm", "rationale": "It uses a gentle tone and expresses a desire to communicate with others, rather than directly expressing its emotions or needing help. This expression is more accompanimental and can easily trigger resonance and reaction from the respondents."}, "time_dimension": {"answer": "Present", "rationale": ""}, "sentence_type": "Affirmative declarative sentence", "pos": {"nouns": {"The mood": 1, "Have a nice day": 1, "others": 1}, "verbs": {"Yes": 1, "Stay together": 1}, "numerals": {"80": 1}, "pronouns": {"I": 1}}}, {"content": "81, if I could feel a sense of frustration, I could handle it better.", "word_count": 23, "label": "", "sentence_structure": "Subject鈥搗erb鈥搊bject", "subjective_objective": {"answer": "Subjective", "rationale": "Because the sentence describes the patient ' s own subjective feelings and coping methods, it is a subjective examination."}, "sentence_sentiment": {"answer": "Positive", "rationale": "Because the purpose of the entry is to \"presume and deal with frustration\"."}, "language_style": {"answer": "Tactful and warm", "rationale": ""}, "time_dimension": {"answer": "Future", "rationale": "The keywords \"if\" and \"can\" indicate that this is an act under hypothetical conditions, that is, only if such conditions are met. And \"if you are frustrated\" refers to what may happen in the future, so the entry is judged to be the future."}, "sentence_type": "Imperative sentence", "pos": {"nouns": {"Word": 1, "Frustration": 1}, "verbs": {"Yes": 4, "Foreground": 1, "Payable": 1}, "adverbs": {"If": 1, "More": 1}, "numerals": {"81": 1}, "pronouns": {"I": 1}}}, {"content": "82, no matter how much I complain, I never get a satisfactory result.", "word_count": 18, "label": "", "sentence_structure": "Subject鈥損redicate", "subjective_objective": {"answer": "Subjective", "rationale": "Because \"satisfaction\" is a subjective feeling and attitude, and it is based on individual experience and expectations. The purpose of this questionnaire entry is therefore to see whether patients feel that their complaints are satisfactorily resolved, which is a subjective view."}, "sentence_sentiment": {"answer": "Negative", "rationale": "Because the result of the entry \"never had a satisfactory result\" was negative."}, "language_style": {"answer": "Calm and direct", "rationale": "The language style of the entry is more calm and direct, and there is no excessive emotional rendering or rhetorical technique. It is more practical and practical to ask the person under investigation in an objective, concise manner."}, "time_dimension": {"answer": "Past", "rationale": "\"How to complain\" and \"how to get a satisfactory result\" all indicate that this is an experience that has already happened. The time dimension of this questionnaire entry should therefore be the past."}, "sentence_type": "Negative declarative sentence", "pos": {"nouns": {"82": 1, "Scoff": 1, "Result": 1}, "verbs": {"Got it": 1, "Let's go": 1, "Not": 1, "Satisfied": 1}, "adverbs": {"What": 1, "Never": 1}, "numerals": {"82": 1}, "pronouns": {"I": 1}}}, {"content": "83, I often find myself insensitive when the environment is about to cause a serious emotional reaction.", "word_count": 27, "label": "", "sentence_structure": "Subject鈥搗erb鈥搊bject", "subjective_objective": {"answer": "Subjective", "rationale": "Because \"insensitive\" is an emotional state, a patient's subjective experience and feelings."}, "sentence_sentiment": {"answer": "Negative", "rationale": "Because the \"insensitive\" act in the entry is negative."}, "language_style": {"answer": "Tactful and warm", "rationale": "The language style of the entry is more euphemistic, by using the expression \"I often find\" to make the question more objective and neutral, and to make it easier for the interviewee to accept and answer. Moreover, the description \"insensitive\" does not directly accuse or attack the interviewee's emotional state, but rather leads the interviewee to reflect on his emotional management capacity in a more subtle way."}, "time_dimension": {"answer": "Past", "rationale": "So the time dimension of this entry is judged now."}, "sentence_type": "Negative declarative sentence", "pos": {"nouns": {"Environment": 1, "Emotional response": 1, "Insensitive": 1}, "verbs": {"Found": 1, "Cause": 1, "Yes": 1}, "adjectives": {"Serious": 1}, "adverbs": {"Often": 1}, "numerals": {"83": 1}, "pronouns": {"I": 1}}}, {"content": "84, work without me, free me from emotional depression and anxiety.", "word_count": 19, "label": "", "sentence_structure": "Subject鈥搗erb鈥搊bject", "subjective_objective": {"answer": "Subjective", "rationale": "Because \"forget work\" is a subjective attitude or behavioural choice, which does not reflect objective facts, but rather the subjective intent of the patient to change his emotional state in this way."}, "sentence_sentiment": {"answer": "Positive", "rationale": "Because \"forgetting to work\" can make people forget some unpleasant emotions and have a relaxing effect, the emotional color of the article is positive."}, "language_style": {"answer": "Calm and direct", "rationale": "The entry is a direct expression of the view that forgetting my work can help to alleviate emotional distress, without too many rhetorical or emotional expressions, and with a more objective tone."}, "time_dimension": {"answer": "Present", "rationale": "In the past, the entry \"forgetting to work\" represents the past experience or behavior, and \"to free me from emotional depression and anxiety\" describes the outcome of this behaviour, and it also happens in the past. Thus, the entry is a description of the dimensions of the past time."}, "sentence_type": "Affirmative declarative sentence", "pos": {"nouns": {"Work": 1, "Emotion": 1, "Blue": 1, "Anxiety": 1}, "verbs": {"Forget about me": 1, "鈾?And I'm gonna be 鈾?: 2, "Get rid of it": 1}, "adjectives": {"It's so sad": 1, "Anxious": 1}, "adverbs": {"Forget about me": 1}, "numerals": {"84": 1}}}, {"content": "I smoke when I'm nervous.", "word_count": 9, "label": "", "sentence_structure": "Subject鈥損redicate", "subjective_objective": {"answer": "Subjective", "rationale": "As stress is an emotional experience, smoking is a personal habit or coping strategy that is the subject of subjective attitudes and behaviour choices."}, "sentence_sentiment": {"answer": "Negative", "rationale": "Because the \"smoking when you're nervous\" act in the entry is negative."}, "language_style": {"answer": "Calm and direct", "rationale": "The language style of the entry is more straightforward, concise, and there is no excessive qualifier or emotional colour, more so as to convey a fact or behaviour, which appears to be calmer and more direct."}, "time_dimension": {"answer": "Past", "rationale": "The sentence, judged by the keyword \"smoking\" , describes the conduct of the person being tested at some point or time in the past and is therefore a historical one."}, "sentence_type": "Affirmative declarative sentence", "pos": {"nouns": {"Time": 1, "I": 1}, "verbs": {"Just smoke": 1}, "adjectives": {"Nervous": 1}, "numerals": {"85": 1}}}, {"content": "86, if I get into some kind of crisis, I'll look for someone else who has the same fate as me.", "word_count": 28, "label": "", "sentence_structure": "Subject鈥搗erb鈥搊bject", "subjective_objective": {"answer": "Subjective", "rationale": "Because this is the idea and belief of the patients themselves, it reflects their views on how to deal with the crisis. It is not an objective fact, but rather influenced by the personal experience and values of the patients."}, "sentence_sentiment": {"answer": "Negative", "rationale": "Because the entry \"when in some kind of crisis\" and \"researching for another person of my own destiny\" has a negative emotional connotation."}, "language_style": {"answer": "Tactful and warm", "rationale": "The language style of the entry is more euphemistic, as it uses an invitation to question the other side's ideas and avoids direct orders or statements. Moreover, the entry uses emotional language such as \"crisis\" and \"fate\" to resonate and focus the other side. These elements make the article more gentle, friendly and responsive."}, "time_dimension": {"answer": "Future", "rationale": "So the answer is \"the future\"."}, "sentence_type": "Imperative sentence", "pos": {"nouns": {"Crisis": 1, "person": 2, "Destiny": 1}, "verbs": {"Into the": 1, "Search": 1, "Yes": 1}, "adverbs": {"Place": 1, "Same thing": 1}, "numerals": {"86": 1}, "pronouns": {"I": 2}}}, {"content": "87, if I did something wrong, I can't be blamed.", "word_count": 13, "label": "", "sentence_structure": "Subject鈥揷opula鈥損redicative", "subjective_objective": {"answer": "Subjective", "rationale": "Because \"not to be blamed\" is a subjective feeling and attitude, not an objective fact or behaviour."}, "sentence_sentiment": {"answer": "Negative", "rationale": "Because the \"not to be blamed\" attitude in the entry is negative."}, "language_style": {"answer": "Tactful and warm", "rationale": "The language style of the entry is more euphemistic and uses an invitation expression to guide respondents to answer questions. At the same time, the expression \"no blame\" has some comfort and understanding, which is more in keeping with the euphemism of the language."}, "time_dimension": {"answer": "Present", "rationale": "The entry describes a personal feeling or belief, which does not relate to past or future situations, but to the current state of mind. The keyword \"doing wrong\" and \"not being blamed\" all indicate the psychological response and the idea."}, "sentence_type": "Imperative sentence", "pos": {"nouns": {"Things": 1, "Blame": 1}, "verbs": {"Wrong": 1, "Yes": 1}, "adverbs": {"If": 1, "No, I can't": 1}, "numerals": {"87": 1}, "pronouns": {"I": 1}}}, {"content": "88, if I had an attack on someone, I felt like I needed something to move that idea.", "word_count": 31, "label": "", "sentence_structure": "Subject鈥搗erb鈥搊bject", "subjective_objective": {"answer": "Subjective", "rationale": "Since the purpose of the questionnaire is to describe the person ' s feelings and thoughts, it is to examine the subjective aspect."}, "sentence_sentiment": {"answer": "Negative", "rationale": "Because the idea and behavior of \"attacking someone\" in the entry is negative."}, "language_style": {"answer": "Tactful and warm", "rationale": "The language style of the entry is more euphemistic, and the interviewer is guided by the means of questioning to express his feelings and thoughts, without directly accusing or ordering the other."}, "time_dimension": {"answer": "Present", "rationale": "Now the entry describes the feelings and thoughts of the testee at the moment, \"if I have an attack on the idea\" is a hypothetical sentence, which suggests that it is a possible situation, and \"I feel the need to do something to divert the idea\" is the result or behaviour of this scenario. So, the entry describes the feelings and ideas that are now, and is related to the current behaviour."}, "sentence_type": "Affirmative declarative sentence", "pos": {"nouns": {"Thoughts": 2, "Others": 1, "Transfer": 1}, "verbs": {"have": 1, "Feel": 1, "Do it": 1, "Yes": 1, "Attack": 1, "Transfer": 1}, "adverbs": {"If": 1, "Just": 2}, "numerals": {"88": 1}, "pronouns": {"I": 1}}}]}

{"questionnaire_title": "Unanimous Self-Assessment Table (UCLA)", "questionnaire_category": "Mental health", "item_count": 20, "emotion_shift_overall": "From positive to negative", "language_style_overall": "Tactful and warm", "items": [{"content": "Do you often feel in harmony with the people around you?", "word_count": 14, "label": "", "sentence_structure": "Subject鈥搗erb鈥搊bject", "subjective_objective": {"answer": "Subjective", "rationale": "Because the question is about the patient's own feelings and is a subjective experience."}, "sentence_sentiment": {"answer": "Positive", "rationale": "The article is positive and expresses the desire of the interviewee to maintain good personal relations."}, "language_style": {"answer": "Tactful and warm", "rationale": "The language style of the entry is euphemismal. By using a question-and-answer format, dialogue is conducted in a gentle and inviting tone, which makes the interviewee comfortable and respectful. Moreover, the issue is not directly critical or critical, but rather positively leads the interviewee to reflect on his personal relationship."}, "time_dimension": {"answer": "Present", "rationale": "The keyword \"often feels\" indicates that it is a question of what it is now, so the time dimension of the article is now.\""}, "sentence_type": "Interrogative sentence", "pos": {"nouns": {"you": 1, "person": 1, "Relations": 1, "Harmony": 1}, "verbs": {"I always feel": 1}, "adverbs": {"Regular": 1}}}, {"content": "Do you often feel the lack of partners?", "word_count": 9, "label": "", "sentence_structure": "Subject鈥損redicate", "subjective_objective": {"answer": "Subjective", "rationale": "Because it is subjective to feel that there is no partner."}, "sentence_sentiment": {"answer": "Negative", "rationale": "Because the \"lack of partners\" in the entry is an expression of a negative emotion."}, "language_style": {"answer": "Tactful and warm", "rationale": "The entry is more adept and interactive when it is spoken in a gentle and inviting manner, rather than by direct statements or orders."}, "time_dimension": {"answer": "Present", "rationale": "This entry uses the word \"normal\" to indicate a constant pattern of situations, indicating that the issue is being asked, and that the person tested has any such feelings. Thus, it is a state of affairs."}, "sentence_type": "Interrogative sentence", "pos": {"nouns": {"Partners": 1}, "verbs": {"I always feel": 1, "Missing": 1}, "adverbs": {"Regular": 1}}}, {"content": "Do you often feel that no one can trust?", "word_count": 11, "label": "", "sentence_structure": "Subject鈥搗erb鈥搊bject", "subjective_objective": {"answer": "Subjective", "rationale": "Because no one can trust the subjective feeling and perception of a person."}, "sentence_sentiment": {"answer": "Negative", "rationale": "Because the expression \"no one can trust\" in the entry is negative and expresses an emotion of lack of trust."}, "language_style": {"answer": "Tactful and warm", "rationale": "The entry is more adept and interactive when it is spoken in a gentle and inviting manner, rather than by direct statements or orders."}, "time_dimension": {"answer": "Past", "rationale": "The entry asks whether the testee often has a feeling of no one to trust, a feeling that may have occurred in the past or may still exist. The temporal dimension is therefore judged by the past."}, "sentence_type": "Interrogative sentence", "pos": {"nouns": {"person": 1, "Trust": 1}, "verbs": {"I feel": 1, "Yeah": 1, "Trust": 1}, "adverbs": {"Regular": 1}, "numerals": {"3": 1}, "pronouns": {"you": 1}}}, {"content": "Do you often feel lonely?", "word_count": 7, "label": "", "sentence_structure": "Subject鈥損redicate", "subjective_objective": {"answer": "Subjective", "rationale": "Because \"slept\" is an emotional experience, a patient's subjective feelings and perceptions."}, "sentence_sentiment": {"answer": "Negative", "rationale": "Because \"slept\" is a negative emotional state."}, "language_style": {"answer": "Tactful and warm", "rationale": "The language style of the entry is more euphemistic, soft, and does not directly ask if the other side has ever felt lonely, but rather leads to reflection and answers in a way that is more sensitive and sensitive. This expression is more likely to enjoy the trust and cooperation of the interviewee and contributes to the effectiveness and accuracy of the investigation."}, "time_dimension": {"answer": "Past", "rationale": "\"Do you often feel lonely?\" The word \"always\" indicates a regular, customary behaviour, and \"fEssure\" uses the usual current state of affairs, which means that this behaviour has not only occurred in the past, but may still be ongoing. The entry therefore asks whether the tested person has often felt lonely in the past and has time dimensions to judge the past."}, "sentence_type": "Interrogative sentence", "pos": {"nouns": {"Questionnaire": 1, "Entry": 1, "Loneliness": 1}, "verbs": {"Common sense": 1, "Present": 1}, "numerals": {"4": 1}}}, {"content": "Do you often feel like one of your friends?", "word_count": 14, "label": "", "sentence_structure": "Subject鈥搗erb鈥搊bject", "subjective_objective": {"answer": "Subjective", "rationale": "Because it is a subjective experience and feeling that one of our friends feels like he is."}, "sentence_sentiment": {"answer": "Positive", "rationale": "Because the phrase \"often feels like a friend\" in the entry expresses a positive attitude."}, "language_style": {"answer": "Tactful and warm", "rationale": "The entry is more adept and interactive when it is spoken in a gentle and inviting manner, rather than by direct statements or orders."}, "time_dimension": {"answer": "Present", "rationale": "The keyword \"often feels\" and \"belongs to one of our friends\" means that the act is present, continuous, and therefore the temporal dimension is now judged."}, "sentence_type": "Interrogative sentence", "pos": {"nouns": {"you": 1, "Guys": 1, "friends": 1, "One": 1}, "verbs": {"Common sense": 1, "belong": 1, "on": 1, "I feel": 1}, "adverbs": {"Regular": 1}, "numerals": {"5": 1}, "classifiers": {"individual": 1}}}, {"content": "Do you often feel that you have much in common with the people around you?", "word_count": 16, "label": "", "sentence_structure": "Subject鈥搗erb鈥搊bject", "subjective_objective": {"answer": "Subjective", "rationale": "Because it is a personal feeling and perception that there is often a sense of common ground with the people around us, it is a subjective examination."}, "sentence_sentiment": {"answer": "Positive", "rationale": "Because the description \"often feels much in common with the people around\" in the entry is positive and expresses a positive and optimistic attitude."}, "language_style": {"answer": "Tactful and warm", "rationale": "The entry is more moderate in language, and it is a question-word that leads respondents to wonder if they often feel like they have something in common with the people around them, and the temperature of the language is easy to accept. At the same time, the issue is not expressed in too direct or sharp language, which is a soft and warm language."}, "time_dimension": {"answer": "Present", "rationale": "So the time dimension is now judged."}, "sentence_type": "Interrogative sentence", "pos": {"nouns": {"Questionnaire Entry": 1, "you": 2, "Around": 1, "person": 1, "Common ground": 1}, "verbs": {"I always feel": 1, "have": 1}, "adverbs": {"Many": 1}, "numerals": {"6": 1}}}, {"content": "Do you often feel intimacy to anyone?", "word_count": 14, "label": "", "sentence_structure": "Subject鈥損redicate", "subjective_objective": {"answer": "Subjective", "rationale": "Because it is a subjective emotion and experience, it requires patients to report themselves in order to obtain answers."}, "sentence_sentiment": {"answer": "Negative", "rationale": "Because the description \"fearing in intimacy\" in the entry is negative."}, "language_style": {"answer": "Tactful and warm", "rationale": "The entry is more euphemistic in language style, because it uses the means of questioning and \"Does it often feel\" to guide respondents to answer questions. Moreover, it uses emotional terms (such as \"nearness\") to convey a feeling of concern and understanding rather than directly expressing the meaning of blame or criticism. Thus, the language style of the article can be considered more euphemism."}, "time_dimension": {"answer": "Present", "rationale": "The entry asks the current feelings and state of the subject, and the keyword \"often feels\" indicates that this is a constant feeling and therefore the judgement is now."}, "sentence_type": "Interrogative sentence", "pos": {"nouns": {"Questionnaire": 1, "Entry": 1, "person": 1, "Intimacy": 1}, "verbs": {"I feel": 1, "Regular": 1, "and": 1, "Both": 1, "not": 1, "- You're not": 1}, "adjectives": {"Any": 1}, "numerals": {"7": 1}, "pronouns": {"you": 1}}}, {"content": "Do you often feel that your interest and thoughts are different from those of the people around you?", "word_count": 20, "label": "", "sentence_structure": "Subject鈥搗erb鈥搊bject", "subjective_objective": {"answer": "Subjective", "rationale": "Because the words \"often\" and \"interest and thought\" relate to the patient's psychological feelings and perceptions, and are subjective experiences and perspectives of the patient."}, "sentence_sentiment": {"answer": "Neutral", "rationale": "The entry simply asks whether the interviewees often have different interests and ideas than others and does not express any positive or negative emotional sentiments."}, "language_style": {"answer": "Tactful and warm", "rationale": "The language style of the entry is euphemism, because it is a soft, invitation-style conversation, not a direct statement or order. The interviewer uses the expression \"Please analyse\" at the beginning of the sentence, showing respect and concern for the interviewee. Then, the phrases \"commonly felt\" and \"unlikely than anyone around you\" are used in the narrative, which are mild, have no strong negative emotions or critical language, thus reducing the resistance of the interviewee and increasing the effectiveness of the questionnaire."}, "time_dimension": {"answer": "Past", "rationale": "You often feel that the word \"always\" means a regular, customary act, and the word \"fear\" uses the past, which means that it has happened in the past. And the description \"unlike people around you\" also points to the past experience and feelings, so the entry contains both a review of the past and a current feeling."}, "sentence_type": "Interrogative sentence", "pos": {"nouns": {"Interest": 1, "Thoughts": 1, "person": 1}, "verbs": {"I feel": 1, "Same thing": 1}, "adverbs": {"Regular": 1, "and": 2}, "pronouns": {"you": 1}}}, {"content": "Do you often feel like having friends and friends?", "word_count": 15, "label": "", "sentence_structure": "Subject鈥搗erb鈥搊bject", "subjective_objective": {"answer": "Subjective", "rationale": "Because \"want\" is the patient's subjective will and feelings."}, "sentence_sentiment": {"answer": "Positive", "rationale": "Because the attitude of \"wanting to have friends and friends\" in the entries is positive."}, "language_style": {"answer": "Tactful and warm", "rationale": "The entry is more personal and interactive in terms of the expression."}, "time_dimension": {"answer": "Present", "rationale": "The keyword \"often feels\" means \"recurring, customary behaviour, \"want\" and \"to be with and have friends\" expresses expectations or goals for the future. So the time dimension of this entry is now."}, "sentence_type": "Interrogative sentence", "pos": {"nouns": {"Questionnaire": 1, "Entry": 1, "person": 2, "friends": 1}, "verbs": {"Common sense": 1, "Yes": 2, "Travel": 1, "Deal": 1}, "adverbs": {"Regular": 1}, "numerals": {"9": 1}, "pronouns": {"you": 1}}}, {"content": "Do you often feel close to others?", "word_count": 9, "label": "", "sentence_structure": "Subject鈥搗erb鈥揷omplement", "subjective_objective": {"answer": "Subjective", "rationale": "Because \"often\" and \"intimate\" are the subjective experiences and feelings of the patient."}, "sentence_sentiment": {"answer": "Neutral", "rationale": "Because the entry does not have any emotional color."}, "language_style": {"answer": "Tactful and warm", "rationale": "The entry is more personal and interactive when it is spoken in a gentle and inviting manner, rather than in direct statements or orders. At the same time, the use of terms such as \"common sense\" is more euphemism, avoiding the question of whether the other side always feels close to the person."}, "time_dimension": {"answer": "Present", "rationale": "The word \"always\" in \"doesn't feel close to someone\" denotes a regular, customary behaviour, while \"sense\" adopts the general current, which means that the behaviour has not only occurred in the past but may still persist. The questionnaire entry is therefore asking about the current behaviour or feelings of the person being tested, and is based on the present."}, "sentence_type": "Interrogative sentence", "pos": {"nouns": {"Questionnaire": 1, "Entry": 1, "Close": 1}, "verbs": {"I feel": 1, "Regular": 1, "person": 1}, "adverbs": {"and": 1}, "numerals": {"10": 1}, "pronouns": {"you": 1}}}, {"content": "Do you often feel left alone?", "word_count": 9, "label": "", "sentence_structure": "Subject鈥搗erb鈥搊bject", "subjective_objective": {"answer": "Subjective", "rationale": "Because being left alone is a subjective feeling and experience."}, "sentence_sentiment": {"answer": "Negative", "rationale": "Because the \"screw\" emotion in the entry is negative."}, "language_style": {"answer": "Tactful and warm", "rationale": "The entry is in the form of a question sentence and in the form of a \"you\" as the main word, expressing concern and concern for the insider, and speaking in a relatively gentle and friendly manner. At the same time, the question does not directly indicate whether the other side has been left out, but rather, it is a way of giving the respondent a sense of euphemism and a warmer way of answering the questions."}, "time_dimension": {"answer": "Present", "rationale": "The term \"normal\" in the entry denotes a regular act, while \"the feeling\" adopts the general current, so that the temporal dimension of the purpose of the article can be judged to be the present."}, "sentence_type": "Interrogative sentence", "pos": {"nouns": {"person": 1, "Cold": 1}, "verbs": {"I feel": 1}, "adverbs": {"Regular": 1, "- You're not": 1}, "numerals": {"11": 1, "you": 1}}}, {"content": "Do you often feel that you have no sense in being with someone else?", "word_count": 15, "label": "", "sentence_structure": "Subject鈥搗erb鈥搊bject", "subjective_objective": {"answer": "Subjective", "rationale": "Because the subject is asked about the patient ' s views and feelings regarding his/her dealings with others, it is a subjective one."}, "sentence_sentiment": {"answer": "Negative", "rationale": "Because the \"often feeling meaningless\" sentiment in the entry is negative."}, "language_style": {"answer": "Calm and direct", "rationale": "The language style of the entry is direct and clear, and it does not use euphemism or implied expression, but rather is a straightforward question about whether the interviewee often feels that he or she has no sense of dealing with others."}, "time_dimension": {"answer": "Present", "rationale": "\"You often feel that the word \"normal\" means \"recurrent, customary\" behaviour, and \"fEalse\" uses the general current, which means that this behaviour has not only occurred in the past, but may still be ongoing. Thus, the entry asks the testee about the current behaviour and feelings, and the temporal dimension is now."}, "sentence_type": "Interrogative sentence", "pos": {"nouns": {"Questionnaire": 1, "Entry": 1, "person": 1, "Transactions": 1, "Meaning": 1}, "verbs": {"I feel": 1, "Regular": 1, "Nothing": 1}, "adverbs": {"Regular": 1, "Nothing": 1}, "numerals": {"12": 1}}}, {"content": "Do you often feel that no one knows you well?", "word_count": 12, "label": "", "sentence_structure": "Subject鈥搗erb鈥搊bject", "subjective_objective": {"answer": "Subjective", "rationale": "The problem is a subjective one, because it concerns the feelings and perceptions of the patient himself."}, "sentence_sentiment": {"answer": "Negative", "rationale": "Because \"Nobody knows me well\" in the entry expresses a negative emotional attitude."}, "language_style": {"answer": "Tactful and warm", "rationale": "The language style of the entry is more euphemistic, using the means of questioning rather than direct statements or orders, which makes it more friendly and acceptable. In addition, the entry involves the individual ' s inner feeling, which is more easily expressed in a way that allows the interviewee to open his heart and answer questions."}, "time_dimension": {"answer": "Past", "rationale": "The entry asked the testee whether he had felt that no one was well aware of his situation and that the time dimension was a past."}, "sentence_type": "Interrogative sentence", "pos": {"nouns": {"Questionnaire Entry": 1, "person": 1, "Understood": 1}, "verbs": {"I feel": 1, "Regular": 1, "Nobody": 1, "very": 1, "Roger that": 1}, "adverbs": {"Regular": 1, "very": 1}, "numerals": {"13": 1}, "classifiers": {"individual": 1}, "pronouns": {"you": 1}}}, {"content": "Do you often feel isolated from others?", "word_count": 11, "label": "", "sentence_structure": "Subject鈥損redicate", "subjective_objective": {"answer": "Subjective", "rationale": "Because this is a patient's description of what he feels inside, it's a subjective examination."}, "sentence_sentiment": {"answer": "Negative", "rationale": "Because the behavior and attitude of \"separating\" in the entries is negative."}, "language_style": {"answer": "Tactful and warm", "rationale": "The entry uses a soft, invitation-style tone for dialogue rather than direct statements or orders, which is more personal and interactive. At the same time, it is not overly emotional, but rather an objective and neutral way of asking about the person under investigation."}, "time_dimension": {"answer": "Present", "rationale": ""}, "sentence_type": "Interrogative sentence", "pos": {"nouns": {"Questionnaire": 1, "Entry": 1, "person": 1, "Separate": 1}, "verbs": {"I always feel": 1}, "adverbs": {"Regular": 1, "Yes": 1}, "numerals": {"14": 1}, "pronouns": {"you": 1}}}, {"content": "Do you often feel like you can find your partner when you want?", "word_count": 16, "label": "", "sentence_structure": "Subject鈥搗erb鈥搊bject", "subjective_objective": {"answer": "Subjective", "rationale": "Because \"if you can find a partner\" is the patient's subjective feeling and judgment."}, "sentence_sentiment": {"answer": "Positive", "rationale": "Because the entry \"Find a partner\" expresses the expectations and positive attitude of people about social relationships."}, "language_style": {"answer": "Tactful and warm", "rationale": "This is a more euphemistic way of giving people a sense of resonance and trust, and helps to create a good atmosphere of interview."}, "time_dimension": {"answer": "Present", "rationale": "The keyword \"often feels\" indicates that it is a customary state description, and \"getting a partner\" is a present act or experience. So the entry describes the situation of the person being tested, not the past or the future."}, "sentence_type": "Interrogative sentence", "pos": {"nouns": {"Questionnaire Entry": 1, "Partners": 1, "Time": 1}, "verbs": {"I feel": 1, "I can find it": 1, "Yes": 1, "Common sense": 1}, "adverbs": {"Just": 1, "Yes": 1, "Time": 1}, "numerals": {"15": 1, "1": 2, "2": 1}, "classifiers": {"individual": 1}, "pronouns": {"you": 1, "When": 1}}}, {"content": "Do you often feel that someone really knows you?", "word_count": 12, "label": "", "sentence_structure": "Subject鈥損redicate", "subjective_objective": {"answer": "Subjective", "rationale": "Because \"fear\" is a subjective feeling and perception, the question is to examine the subjective feeling of the patient."}, "sentence_sentiment": {"answer": "Neutral", "rationale": "Because the entry does not have any emotional color."}, "language_style": {"answer": "Tactful and warm", "rationale": "The entry is more personal and interactive when it is spoken in a gentle and inviting manner, rather than in direct statements or orders. At the same time, it is used to guide the other side to answer, and to make the answer more comfortable and natural in the way it ends with questions."}, "time_dimension": {"answer": "Present", "rationale": ""}, "sentence_type": "Interrogative sentence", "pos": {"nouns": {"person": 1, "Roger that": 1}, "verbs": {"I always feel": 1}, "adverbs": {"Really": 1}, "numerals": {"16": 1}, "pronouns": {"you": 1}}}, {"content": "Do you often feel shy?", "word_count": 7, "label": "", "sentence_structure": "Subject鈥損redicate", "subjective_objective": {"answer": "Subjective", "rationale": "Because being shy is a subjective feeling and emotional experience."}, "sentence_sentiment": {"answer": "Negative", "rationale": "Because the \"weak\" mood in the entry is negative."}, "language_style": {"answer": "Tactful and warm", "rationale": "The entry is more adept and interactive when it is spoken in a gentle and inviting manner, rather than by direct statements or orders."}, "time_dimension": {"answer": "Present", "rationale": "Now, \"Do you often feel shy?\", the word \"always\" means \"recurring, habitually\" and \"fEaling\" means \"the present\" means that it not only happened in the past, but may still be going on. So, this entry asks the testee about the behaviour and feelings of the person."}, "sentence_type": "Interrogative sentence", "pos": {"nouns": {"Shame": 1}, "verbs": {"I always feel": 1, "- You're not": 1}, "numerals": {"17": 1}}}, {"content": "Do you often feel surrounded but not concerned about you?", "word_count": 16, "label": "", "sentence_structure": "Subject鈥搗erb鈥搊bject", "subjective_objective": {"answer": "Subjective", "rationale": "It is a subjective examination as it is felt that someone is surrounding himself but not concerned about the feelings and experiences of the individual who is the patient."}, "sentence_sentiment": {"answer": "Negative", "rationale": "Because the \"someone around you but doesn't care about you\" is negative."}, "language_style": {"answer": "Tactful and warm", "rationale": "The entry is more adept and interactive when it is spoken in a gentle and inviting manner, rather than by direct statements or orders."}, "time_dimension": {"answer": "Past", "rationale": "Past."}, "sentence_type": "Interrogative sentence", "pos": {"nouns": {"person": 2, "Care": 1, "you": 3}, "verbs": {"Round and round": 1, "I feel": 1, "Care": 1}, "adjectives": {"Regular": 1, "not": 1, "But": 1}, "adverbs": {"very": 1}, "numerals": {"18": 1, "2": 1, "1": 4}, "pronouns": {"you": 3}}}, {"content": "Do you often feel like someone wants to talk to you?", "word_count": 13, "label": "", "sentence_structure": "Subject鈥搗erb鈥搊bject", "subjective_objective": {"answer": "Subjective", "rationale": "Because \"willing to talk to you\" is a patient's subjective feeling."}, "sentence_sentiment": {"answer": "Neutral", "rationale": "Because the entry does not involve any emotional or value judgement, it merely asks whether the interviewees often feel that they are being talked to around them."}, "language_style": {"answer": "Tactful and warm", "rationale": "The language style of the entry is euphemistic, because the question is asked in an invitation and in a friendly manner, rather than in direct command or inquiry. This expression makes the interviewee more comfortable and relaxed and more likely to answer the question in a genuine manner."}, "time_dimension": {"answer": "Present", "rationale": "The entry asks whether the testees often feel that they are being talked to, and the time dimension is now. The keyword \"often feels\" indicates that it is a regular feeling, while the verb \"talking\" uses the usual current pattern, meaning that the act may still be occurring."}, "sentence_type": "Interrogative sentence", "pos": {"nouns": {"person": 1, "Talk": 1}, "verbs": {"Sensor": 1, "May I": 1, "Hand it over": 1}, "adverbs": {"Regular": 1}, "numerals": {"9": 1}, "classifiers": {"individual": 1}, "pronouns": {"you": 1}}}, {"content": "Do you often feel that someone deserves your trust?", "word_count": 12, "label": "", "sentence_structure": "Subject鈥搗erb鈥搊bject", "subjective_objective": {"answer": "Subjective", "rationale": "Because the problem is one of the feelings and perceptions of the patient himself, it is a subjective one."}, "sentence_sentiment": {"answer": "Positive", "rationale": "Because the quality of \"trustworthy\" in the entry is positive."}, "language_style": {"answer": "Tactful and warm", "rationale": "The language style of the entry is more euphemistic, and the way in which the person is asked is to lead the person to think about his or her emotional state, rather than to express or order directly, is more personal and interactive."}, "time_dimension": {"answer": "Present", "rationale": "Because the \"common sense\" in the sentence indicates that it is a regular, customary act, while the \"fEs\" is a general current, indicating that the state of affairs persists, so the entry asks about the current feeling."}, "sentence_type": "Interrogative sentence", "pos": {"nouns": {"you": 1, "person": 1, "Trust": 1}, "verbs": {"Common sense": 1, "Value": 1, "Okay": 1}, "adverbs": {"Regular": 1}, "numerals": {"20": 1}, "classifiers": {"individual": 1}}}]}

{"questionnaire_title": "Emotional Balance Sheet", "questionnaire_category": "Mental health", "item_count": 10, "emotion_shift_overall": "From positive to negative", "language_style_overall": "Tactful and warm", "items": [{"content": "Is there something particularly keen or interested?", "word_count": 13, "label": "", "sentence_structure": "Subject鈥揷opula鈥損redicative", "subjective_objective": {"answer": "Subjective", "rationale": "Because \"hot\" and \"interest\" are subjective attitudes or feelings of patients."}, "sentence_sentiment": {"answer": "Positive", "rationale": "Because \"hot\" and \"interest\" in entries are positive emotional terms."}, "language_style": {"answer": "Tactful and warm", "rationale": "This is more personal and interactive, and better able to guide the interviewee to self-reflection and answer questions."}, "time_dimension": {"answer": "Present", "rationale": "The entry is an inquiry into the current situation and feelings of the person being tested, and \"sweet or particularly interested in something\" is a possible state of behaviour or emotion that exists at the present time, and therefore the temporal dimension is now judged."}, "sentence_type": "Interrogative sentence", "pos": {"nouns": {"Yes": 1, "A passion": 1, "Interest": 1}, "verbs": {"to": 1, "Special": 2, "Interested": 1}, "adverbs": {"Special": 1}, "numerals": {"One": 2}}}, {"content": "- You're not comfortable?", "word_count": 6, "label": "", "sentence_structure": "Subject鈥揷opula鈥損redicative", "subjective_objective": {"answer": "Subjective", "rationale": "/Objectively, refer to the above example, because it is a subjective feeling to feel uneasy, so the output is \"subjective\"."}, "sentence_sentiment": {"answer": "Negative", "rationale": "Because the \"sitting\" act in the entry is negative."}, "language_style": {"answer": "Calm and direct", "rationale": "According to the information provided, the expression \"B. is restless\" is relatively simple and clear, has no excessive emotional or rhetorical style and can therefore be judged as being calm and straightforward."}, "time_dimension": {"answer": "Past", "rationale": "Past, present"}, "sentence_type": "Interrogative sentence", "pos": {"verbs": {"I feel": 1, "Sit tight": 1}}}, {"content": "C. Proud of what others have done to you?", "word_count": 16, "label": "", "sentence_structure": "Subject鈥搗erb鈥搊bject", "subjective_objective": {"answer": "Subjective", "rationale": "Because \"proud\" is the subjective feeling and attitude of patients to the praise of others."}, "sentence_sentiment": {"answer": "Positive", "rationale": "Because the behavior in the entry is \"proud\" positive."}, "language_style": {"answer": "Tactful and warm", "rationale": "The entry is more personal and interactive by speaking in a gentle and inviting manner, rather than by direct statement or order. At the same time, this euphemism reduces the resistance of the respondents and makes it easier to obtain real and accurate data feedback."}, "time_dimension": {"answer": "Present", "rationale": "This entry asks whether the testees are proud of their work at the present time and therefore the time dimension is now judged."}, "sentence_type": "Interrogative sentence", "pos": {"nouns": {"others": 1, "Work": 1, "- Congratulations": 1, "Proud": 1}, "verbs": {"to": 1, "I feel": 1}, "adverbs": {"Because": 1, "And": 1}, "pronouns": {"you": 1}}}, {"content": "D. Very lonely or far away?", "word_count": 9, "label": "", "sentence_structure": "Subject鈥損redicate", "subjective_objective": {"answer": "Subjective", "rationale": "Because \"lsolation\" and \"detached from others\" are subjective experiences of patients that cannot be objectively measured or observed."}, "sentence_sentiment": {"answer": "Negative", "rationale": "Because the act of \"very alone or far away from others\" in the entry is negative, it suggests that the interviewee may have social problems or problems of personal tension."}, "language_style": {"answer": "Tactful and warm", "rationale": "The language style of the entry is euphemistic. By using words like \"very\" and \"single\" to express concern and understanding of the person being investigated, the description \"touch away\" also suggests that communication with others may be problematic, and that expression is more caring and personal."}, "time_dimension": {"answer": "Past", "rationale": "The keyword \"very lonely or far away from others\" indicates that the person tested has often felt alone or away from others at some point in the past or at present, and the purpose of this article is therefore to question the person tested about his or her past or present feelings."}, "sentence_type": "Interrogative sentence", "pos": {"nouns": {"Loneliness": 1, "Others": 1}, "verbs": {"Stay away": 1, "Loneliness": 1}, "numerals": {"Ten": 1, "min": 1}, "classifiers": {"individual": 1}}}, {"content": "E. Happy because of the work done?", "word_count": 14, "label": "", "sentence_structure": "Subject鈥揷opula鈥損redicative", "subjective_objective": {"answer": "Subjective", "rationale": "The fact that it is a personal feeling or emotional experience to be happy to do the job is a subjective examination."}, "sentence_sentiment": {"answer": "Positive", "rationale": "Because the \"happy\" mood in the entry is positive."}, "language_style": {"answer": "Tactful and warm", "rationale": "The article is based on a soft, inviting expression that engages in dialogue in a kind and interactive way, rather than directly speaking or ordering. At the same time, it does not use excessive rhetorical or emotional overtones, which are more objective. Such language makes it easier for the interviewee to feel comfortable and to share his thoughts and feelings."}, "time_dimension": {"answer": "Present", "rationale": "The keywords are \"happy\" and \"completed\", which point to acts or situations that are ongoing or new, so it can be concluded that this is an investigation of the current mood."}, "sentence_type": "Interrogative sentence", "pos": {"nouns": {"Work": 1, "Have a nice day": 1}, "verbs": {"Completed": 1, "I feel": 1}}}, {"content": "F. Disturbing?", "word_count": 2, "label": "", "sentence_structure": "Subject鈥搗erb鈥揷omplement", "subjective_objective": {"answer": "Subjective", "rationale": "Because heartache is a subjective emotional experience, reflecting the feelings of the patient."}, "sentence_sentiment": {"answer": "Negative", "rationale": "Because \"freak\" is a negative emotional state."}, "language_style": {"answer": "Calm and direct", "rationale": "The entry uses simple, plain language to describe the problem, and there is no excessive rhetorical or emotional colour, so the language style is calm and straightforward."}, "time_dimension": {"answer": "Present", "rationale": "The entry is for the testee's current state or feeling, and \"fear\" is an adjective, which is the state of emotion at the moment, so it's judged to be \"now\"."}, "sentence_type": "Interrogative sentence", "pos": {"verbs": {"I'm upset": 1}, "adjectives": {"Boring": 1}}}, {"content": "G. As if he was at the top of the world?", "word_count": 16, "label": "", "sentence_structure": "Subject鈥搗erb鈥揷omplement", "subjective_objective": {"answer": "Subjective", "rationale": "This is a personal psychological experience of the patient and is a subjective examination."}, "sentence_sentiment": {"answer": "Positive", "rationale": "Because the description \"floating feeling\" has a positive emotional color."}, "language_style": {"answer": "Tactful and warm", "rationale": "The words of the entry are warm and warm, using metaphoric rhetoric, linking the \"tops of the world\" to \"floating feeling\" and creating a positive, confident atmosphere. At the same time, the use of direct orders or strongly emotional language is avoided, making the questions more acceptable and answerable."}, "time_dimension": {"answer": "Present", "rationale": "According to the words \"like\" and \"in\" and \"in\" in the sentence, the entry is to ask the person who was tested for his or her current feelings or state, so the time dimension is now judged."}, "sentence_type": "Interrogative sentence", "pos": {"nouns": {"World": 1, "Top": 1, "Feel": 1}, "verbs": {"Location": 1, "in": 1, "鈾?Fly": 1}, "adjectives": {"It's like": 1}, "adverbs": {"have": 1}, "pronouns": {"Here": 1}}}, {"content": "H. Optimal or very unhappy?", "word_count": 8, "label": "", "sentence_structure": "Subject鈥搗erb鈥揷omplement", "subjective_objective": {"answer": "Subjective", "rationale": "Because \"blow\" and \"very unhappy\" are the feelings and experiences of the patient himself, they are subjective."}, "sentence_sentiment": {"answer": "Negative", "rationale": "Because \"black\" and \"very unhappy\" are negative words, which are expressions of negative emotion."}, "language_style": {"answer": "Tactful and warm", "rationale": "The entry uses a more euphemistic approach to ask the respondents whether they have experienced periods of depression or extreme discomfort, and to avoid direct questioning of the embarrassment and resistance that personal experiences may bring."}, "time_dimension": {"answer": "Future", "rationale": "The entry asks if the person tested will feel \"better or very unhappy\" in the future and therefore judges it as the future."}, "sentence_type": "Interrogative sentence", "pos": {"nouns": {"Blue": 1, "Very": 1, "Happiness": 1}, "verbs": {"Luxury": 1, "Very": 1}}}, {"content": "I. Things are going according to your wishes?", "word_count": 10, "label": "", "sentence_structure": "Subject鈥揷opula鈥損redicative", "subjective_objective": {"answer": "Subjective", "rationale": "Because the questionnaire entry asks the respondents' feelings and thoughts, namely, \"Is the matter going according to your wishes\", which requires the interviewee to judge and answer for himself, and is therefore subjective."}, "sentence_sentiment": {"answer": "Positive", "rationale": ""}, "language_style": {"answer": "Calm and direct", "rationale": "The language of the entry is relatively simple and not too much rhetorical or emotional, but rather direct the respondents to the question and to the point of being more calm and objective."}, "time_dimension": {"answer": "Present", "rationale": "The entry uses \"in the process\" to indicate an ongoing move, \"in your will\" indicating that the current situation is proceeding according to individual expectations, so the time dimension of the article is now."}, "sentence_type": "Interrogative sentence", "pos": {"nouns": {"Things": 1, "Will": 1}, "verbs": {"Press": 1, "Development": 1}, "adverbs": {"you": 1, "of": 2}, "pronouns": {"Here": 1}}}, {"content": "J. Disturbed by someone's criticism?", "word_count": 12, "label": "", "sentence_structure": "Subject鈥揷opula鈥損redicative", "subjective_objective": {"answer": "Subjective", "rationale": "Because \"uneasy\" is the patient's subjective feeling."}, "sentence_sentiment": {"answer": "Negative", "rationale": "Because \"uneasy\" in the entries is an expression of negative emotions and attitudes."}, "language_style": {"answer": "Tactful and warm", "rationale": ""}, "time_dimension": {"answer": "Past", "rationale": "The entry asks whether the testee was ever disturbed by criticism, and the temporal dimension is the past. The keyword \"disturbed by someone's criticism\" suggests that this is an issue of past experience, which is not directly related to the present or the future."}, "sentence_type": "Interrogative sentence", "pos": {"nouns": {"Someone": 1, "Criticism": 1, "Unsettled": 1}, "verbs": {"I feel": 1, "be": 1}, "adjectives": {"of": 2}, "pronouns": {"And": 1}}}]}

{"questionnaire_title": "Family function assessment", "questionnaire_category": "Mental health", "item_count": 60, "emotion_shift_overall": "From negative to positive", "language_style_overall": "Neutral", "items": [{"content": "1. The misunderstandings that we have with each other have made it difficult to organize some family activities.", "word_count": 18, "label": "", "sentence_structure": "Subject鈥搗erb鈥搊bject", "subjective_objective": {"answer": "Subjective", "rationale": "This is the subjective perception and perception of the patients of the misunderstandings that occur in the family activity."}, "sentence_sentiment": {"answer": "Negative", "rationale": "Because the entries \"misunderstanding\" and \"difficult to organize family activities\" have a negative emotional effect."}, "language_style": {"answer": "Calm and direct", "rationale": "This is a more straightforward and objective expression, which is not overly accommodative or emotional, and which directly addresses the problem of family activities that are difficult to organize because of misunderstanding."}, "time_dimension": {"answer": "Present", "rationale": "The words \"arranged\" in the entry, which for reasons of \"expressed\", denoted behaviour, indicated that the issue described the current situation or state and was therefore about the present."}, "sentence_type": "Interrogative sentence", "pos": {"nouns": {"we": 2, "You too": 1, "Misunderstanding": 1, "Some": 1, "Family activities": 1}, "verbs": {"Because": 1, "Arrangements": 1, "Understood": 1}, "numerals": {"One": 4, "Some": 1}, "classifiers": {"individual": 1}, "pronouns": {"we": 2, "You too": 1}}}, {"content": "2. We resolve most of our daily problems near our accommodation", "word_count": 16, "label": "", "sentence_structure": "Subject鈥搗erb鈥搊bject", "subjective_objective": {"answer": "Objective", "rationale": "Since \"resolving most everyday problems near the place of residence\" is an objective fact experienced by the patient, it is the objective situation that is being examined."}, "sentence_sentiment": {"answer": "Neutral", "rationale": "Because the entry does not express positive or negative feelings, attitudes, behaviour, purpose or quality, it merely states a fact."}, "language_style": {"answer": "Tactful and warm", "rationale": "The entry is more personal and interactive when it is spoken in an invitation-style tone, rather than directly speaking or ordering. At the same time, the expression \"we solve most everyday problems near our place of residence\" is not overemphasis or criticism, but rather provides an objective factual description that gives people a sense of neutrality."}, "time_dimension": {"answer": "Present", "rationale": "\"We\" solves most of the day-to-day problems near the house, and the words \"near the house\" and \"most\" indicate that this is an ongoing act, so the entry describes the current situation."}, "sentence_type": "Interrogative sentence", "pos": {"nouns": {"Accommodation": 1, "Day-to-day issues": 1}, "verbs": {"Resolve": 1, "in": 1}, "numerals": {"Mostly": 1, "Day": 1}, "classifiers": {"individual": 1}, "pronouns": {"we": 1}}}, {"content": "When there are people in the family who are upset, others know why he is upset.", "word_count": 19, "label": "", "sentence_structure": "Subject鈥搗erb鈥搊bject", "subjective_objective": {"answer": "Objective", "rationale": "The basis for the determination is the objective fact that is examined, namely, whether there are other persons who know of the anguish of someone in the family."}, "sentence_sentiment": {"answer": "Positive", "rationale": "The purpose of this article is to encourage attention and support for the mental health of others and to have a positive emotional dimension."}, "language_style": {"answer": "Tactful and warm", "rationale": "The entry is more personal and interactive when it is spoken in a gentle and inviting manner, rather than in direct statements or orders. It attempts to understand whether the interviewee is well-off in the family to attend and support the emotional needs of his family, and the answers to such questions can also reflect the level of care that the interviewee has for his family."}, "time_dimension": {"answer": "Present", "rationale": "The entry now describes the situation in the immediate family, i.e. whether there is a member of the family who can understand and know the reasons for the troubles of others."}, "sentence_type": "Affirmative declarative sentence", "pos": {"nouns": {"Home": 1, "person": 2, "Trouble": 2}, "verbs": {"Yes": 1, "have": 1, "Why": 1, "Trouble": 1}, "numerals": {"3": 1}, "pronouns": {"Other": 1, "he": 1}}}, {"content": "When you ask someone to do something, you must check whether they did it.", "word_count": 22, "label": "", "sentence_structure": "Subject鈥搗erb鈥搊bject", "subjective_objective": {"answer": "Objective", "rationale": "Because it is not the patient ' s subjective idea that someone is required to do something and to check whether they have acted objectively."}, "sentence_sentiment": {"answer": "Neutral", "rationale": "Because the entry does not have any emotional color."}, "language_style": {"answer": "Tactful and warm", "rationale": ""}, "time_dimension": {"answer": "Past", "rationale": ""}, "sentence_type": "Imperative sentence", "pos": {"nouns": {"person": 1, "Something": 1, "Things": 1}, "verbs": {"Request": 1, "Do it": 2, "Inspection": 1, "Did you do it or not": 1}, "adverbs": {"EMPTY": 1}, "numerals": {"Some": 1}, "classifiers": {"individual": 1}, "pronouns": {"you": 1, "they": 1}}}, {"content": "5. If someone is in trouble, others are too concerned.", "word_count": 17, "label": "", "sentence_structure": "Subject鈥損redicate", "subjective_objective": {"answer": "Subjective", "rationale": "Because this is the subjective view and perception of the behaviour of others."}, "sentence_sentiment": {"answer": "Negative", "rationale": "Because the \"excessive concern\" in the entry is negative."}, "language_style": {"answer": "Tactful and warm", "rationale": "The language style of the entry is more moderate, not directly expressing blame or criticism of \"others\", but rather describing a possible phenomenon in a more objective and understandable way. It avoids creating feelings of resentment or resistance on the part of the other side and helps to create a good atmosphere of communication."}, "time_dimension": {"answer": "Present", "rationale": "The keywords \"in trouble\" and \"over-care\" describe a state of affairs or phenomenon, so the entry is to ask the views or experiences of the person being tested in the present circumstances."}, "sentence_type": "Affirmative declarative sentence", "pos": {"nouns": {"person": 2, "Trouble": 1}, "verbs": {"I met him": 1, "Excessive attention": 1}, "adjectives": {"Other": 1}, "adverbs": {"If": 1}, "numerals": {"5": 1}}}, {"content": "6. In times of crisis, we can support each other.", "word_count": 12, "label": "", "sentence_structure": "Subject鈥搗erb鈥搊bject", "subjective_objective": {"answer": "Subjective", "rationale": "The sentence describes subjective attitudes and mutually supportive behaviour in the face of crisis. While such behaviour may occur in an objective reality (such as a natural disaster or a health crisis), it focuses more on the subjective thinking and emotional response of people. Thus, the entry to the questionnaire looks at subjective views and attitudes."}, "sentence_sentiment": {"answer": "Positive", "rationale": "Because the \"support each other\" act is positive."}, "language_style": {"answer": "Tactful and warm", "rationale": "The entry uses a positive term \"support each other\" and starts with \"we can\" and expresses a cooperative and cooperative approach. This language style is more encouraging, stimulating the motivation and participation of the respondents, and making them feel more comfortable and respectful."}, "time_dimension": {"answer": "Present", "rationale": ""}, "sentence_type": "Imperative sentence", "pos": {"nouns": {"Crisis": 1, "Support": 1}, "verbs": {"Come on": 1, "Yes": 1, "To each other": 1}}}, {"content": "7. When unexpected surprises occur, we are overwhelmed.", "word_count": 18, "label": "", "sentence_structure": "Subject鈥損redicate", "subjective_objective": {"answer": "Subjective", "rationale": "Because \"we're not able to help ourselves\" is the patient's subjective feeling and reaction."}, "sentence_sentiment": {"answer": "Negative", "rationale": "Because the act of \"handlessness\" is a negative emotion and a sense of helplessness."}, "language_style": {"answer": "Calm and direct", "rationale": "The entry is a simple and straightforward expression of the subject that people may feel overwhelmed with incipient and simple language in the face of an unexpected situation. There is no excessive emotional rendering or rhetorical technique, so it can be judged as \"cooling straight.\""}, "time_dimension": {"answer": "Future", "rationale": "\"Unforeseen accidents\" are a possible future scenario, and \"unpreparedness\" is a reaction and expectation to this situation, so the entry describes future behaviour or mentality."}, "sentence_type": "Affirmative declarative sentence", "pos": {"nouns": {"Accident": 1, "Brothers": 1, "Yeah": 1}, "verbs": {"Come on": 1, "It's unexpected": 1, "we": 1, "I'm in a hurry": 1}, "adjectives": {"It's unexpected": 1}, "numerals": {"7": 1}, "pronouns": {"we": 1}}}, {"content": "8. Our home often uses everything we need.", "word_count": 17, "label": "", "sentence_structure": "Subject鈥搗erb鈥揷omplement", "subjective_objective": {"answer": "Objective", "rationale": "Because \"soft\" is an objective fact, it does not require subjective attitudes or opinions of the patient."}, "sentence_sentiment": {"answer": "Negative", "rationale": "Because the \"what is needed is out\" behavior is negative."}, "language_style": {"answer": "Calm and direct", "rationale": "This item presents a situation or question in a more objective and straightforward way, without excessive rhetorical or emotional rendering."}, "time_dimension": {"answer": "Present", "rationale": "The keywords are \"frequent\" and \"necessary\" and they both represent a customary act or state of affairs, so the temporal dimension of the article is now."}, "sentence_type": "Affirmative declarative sentence", "pos": {"nouns": {"Home": 1, "Something": 2}, "verbs": {"- Put it on": 1, "Yes": 1, "Use the light": 1}, "adverbs": {"Often": 1}, "numerals": {"8": 1}, "classifiers": {"individual": 1}, "pronouns": {"we": 2}}}, {"content": "We are not willing to reveal our feelings to each other.", "word_count": 15, "label": "", "sentence_structure": "Subject鈥搗erb鈥揷omplement", "subjective_objective": {"answer": "Subjective", "rationale": "Because \"will\" or \"will\" to reveal feelings is the patient's subjective will and attitude, and it is a subjective view."}, "sentence_sentiment": {"answer": "Negative", "rationale": "Because the entry expresses an attitude of reluctance to express feelings, with some negative tendencies."}, "language_style": {"answer": "Calm and direct", "rationale": "The entry expresses the subject directly in a concise and clear language, with no excessive rhetoric or emotional rendering, and is more objective in tone."}, "time_dimension": {"answer": "Present", "rationale": "The entry describes the current situation and feelings, uses the general current and emphasizes the relationship between the two sides of the dominant word \"we\", so it can be judged that the dimension of time is now.\""}, "sentence_type": "Negative declarative sentence", "pos": {"nouns": {"we": 2, "Emotion": 1}, "verbs": {"I don't want to": 2, "The exposed": 1, "Show": 1}, "adjectives": {"- A little bit": 1}, "numerals": {"9": 1}, "pronouns": {"self": 1}}}, {"content": "We affirm that family members have done their part in the family.", "word_count": 19, "label": "", "sentence_structure": "Subject鈥搗erb鈥搊bject", "subjective_objective": {"answer": "Subjective", "rationale": "Because the word \"yes\" indicates that it is the patient's subjective view and attitude to whether family members are doing their part."}, "sentence_sentiment": {"answer": "Positive", "rationale": "Because the expression \"recognizing that family members have done their own family duties\" in the entry is positive and expresses recognition and praise of family members."}, "language_style": {"answer": "Tactful and warm", "rationale": "The entry is expressed in a more euphemistic and moderate manner, without direct criticism or criticism, and therefore in a more euphemistic manner."}, "time_dimension": {"answer": "Present", "rationale": ""}, "sentence_type": "Affirmative declarative sentence", "pos": {"nouns": {"Questionnaire Entry": 1, "Family members": 1, "Each one": 1, "Family duties": 1}, "verbs": {"Sure": 1, "- All of it": 2}, "adverbs": {"Both": 1}, "numerals": {"10": 1, "Each one": 1}, "classifiers": {"Grandpa": 1}}}, {"content": "11. We cannot talk about our sorrows.", "word_count": 13, "label": "", "sentence_structure": "Subject鈥搗erb鈥搊bject", "subjective_objective": {"answer": "Objective", "rationale": "Because \"we can't talk to each other\" is a statement of fact that describes people's behaviour or their limitations. It's seen from an objective perspective, not as subjective thoughts or feelings of patients."}, "sentence_sentiment": {"answer": "Negative", "rationale": "Because the entry expresses a depressed, negative exchange attitude and is negative."}, "language_style": {"answer": "Calm and direct", "rationale": "The language style of the entry is of a more direct and objective type, with no excessive emotional rendering or rhetorical techniques, but rather a clear expression of the researcher鈥檚 desire that he or she should not talk to the testee."}, "time_dimension": {"answer": "Past", "rationale": "The entry uses the words \"not\" and \"talk\" to convey a negative description of the past and can therefore be judged to be the past."}, "sentence_type": "Negative declarative sentence", "pos": {"nouns": {"we": 2, "Talk": 1, "Sad": 1}, "verbs": {"No, I can't": 1, "To each other": 1, "Talk": 1}, "adverbs": {"No, I can't": 1, "To each other": 1}, "numerals": {"11": 1, "we": 2, "Sad": 1}, "pronouns": {"Ours": 1}}}, {"content": "We often act on our decisions on the issue.", "word_count": 16, "label": "", "sentence_structure": "Subject鈥搗erb鈥搊bject", "subjective_objective": {"answer": "Subjective", "rationale": "Because \"We often act on our decisions about the problem\" describes the behaviour and attitude of individuals and is subjective."}, "sentence_sentiment": {"answer": "Positive", "rationale": "Because the \"deact by decision\" in the entry is a positive attitude, indicating purposeful and proactive behaviour."}, "language_style": {"answer": "Tactful and warm", "rationale": "The language style of the entry is a euphemism. It uses a kind of interview-style tone, which is more gentle and friendly, without direct orders or criticism. This way makes the interviewee comfortable and respectful, more open to investigation and provides real answers."}, "time_dimension": {"answer": "Present", "rationale": ""}, "sentence_type": "Affirmative declarative sentence", "pos": {"nouns": {"Problem": 1, "Decision": 1, "Action": 1}, "verbs": {"Based on": 1, "Go on": 1, "Regular": 1, "Do it": 1}, "adverbs": {"very": 1}, "numerals": {"12": 1}, "pronouns": {"we": 1}}}, {"content": "13. Your affairs are of interest to others only if they are important to them.", "word_count": 19, "label": "", "sentence_structure": "Subject鈥搗erb鈥揷omplement", "subjective_objective": {"answer": "Subjective", "rationale": "This is because patients ' perception and assessment of others ' attitudes towards their own affairs are part of the subjective sense of the individual."}, "sentence_sentiment": {"answer": "Negative", "rationale": "Because the entry \"they will be interested only if they are important to others\", the behaviour and attitude are negative and express the concern and support of others as important to individuals."}, "language_style": {"answer": "Calm and direct", "rationale": "The language style of the entry is straightforward, and it is not expressed by the presentation of a point of view using excessive rhetorical or emotional colours. This tone is usually objective and rational and applies to situations where logical reasoning and analysis are required."}, "time_dimension": {"answer": "Present", "rationale": "The entry asks the testees about their current situation, i.e. whether they find their own things in their experience only interesting when they are important to others. Therefore, the time dimension is now judged."}, "sentence_type": "Affirmative declarative sentence", "pos": {"nouns": {"Yes": 2, "person": 2, "others": 1, "Interest": 1}, "verbs": {"Only": 1, "to": 1, "That's right": 2, "Yes": 1, "Interested": 1}, "adverbs": {"Only": 1, "Both": 1}, "numerals": {"13": 1, "2": 2, "1": 4}, "pronouns": {"they": 1}}}, {"content": "14. From what those people are talking about, you don't understand what one of them is thinking.", "word_count": 24, "label": "", "sentence_structure": "Subject鈥搗erb鈥搊bject", "subjective_objective": {"answer": "Subjective", "rationale": "Because one's idea is to be understood through observation and understanding, it is to be seen from the subjective perspective of the patient."}, "sentence_sentiment": {"answer": "Neutral", "rationale": "Because the entry does not have any emotional color."}, "language_style": {"answer": "Tactful and warm", "rationale": "The language style of this entry is more euphemistic, and it is investigated by asking if the other person has ever encountered a lack of understanding of the other person's thoughts in a conversation. At the same time, the phrase \"please analyse\" reflects a polite and polite tone that makes the questionnaire more friendly and acceptable."}, "time_dimension": {"answer": "Past", "rationale": ""}, "sentence_type": "Negative declarative sentence", "pos": {"nouns": {"person": 2, "Word": 1, "Yes": 1}, "verbs": {"From": 1, "I'm talking": 1, "I don't understand": 1}, "numerals": {"14": 1, "Alone": 1}, "pronouns": {"you": 1}}}, {"content": "15. Domestic matters are not adequately shared by family members.", "word_count": 14, "label": "", "sentence_structure": "Subject鈥搗erb鈥揷omplement", "subjective_objective": {"answer": "Subjective", "rationale": "Because \"full sharing\" is a relative subjective assessment, depending on the views and criteria of different people."}, "sentence_sentiment": {"answer": "Negative", "rationale": "Because the entry \"Domestic affairs are not adequately shared by family members\" is negative."}, "language_style": {"answer": "Tactful and warm", "rationale": "The language style of the entry is euphemistic. It uses a relatively neutral and objective form of question, and does not directly criticize or accuse a family member, but rather raises a question that needs to be discussed and improved, which makes the respondents more willing to share their views and experiences."}, "time_dimension": {"answer": "Past", "rationale": ""}, "sentence_type": "Negative declarative sentence", "pos": {"nouns": {"Domestic matters": 1, "Family members": 2, "Share": 1}, "verbs": {"have": 1, "Full": 1}, "numerals": {"15": 1}}}, {"content": "16. Everyone was what they were and could be recognized.", "word_count": 15, "label": "", "sentence_structure": "Subject鈥搗erb鈥搊bject", "subjective_objective": {"answer": "Subjective", "rationale": "Because it is about people's perception and acceptance of their own, it is a subjective feeling or belief."}, "sentence_sentiment": {"answer": "Positive", "rationale": "Because it expresses the idea of universal human values and respect for individual differences."}, "language_style": {"answer": "Tactful and warm", "rationale": "The language style of the entry is euphemism. It uses positive words, \"to be accepted by others\" and emphasizes each individual's individuality, \"anything.\" This expression encourages respondents to actively present their personality characteristics when completing questionnaires, and to think that each person is unique and valuable. It is softer and more friendly, helping to create a good atmosphere of interaction."}, "time_dimension": {"answer": "Present", "rationale": "The entry describes a prevailing belief or view that people have their own characteristics and characteristics that are acceptable and acceptable to others. In terms of time dimension, the entry does not relate to past or future situations and therefore is now judged."}, "sentence_type": "Affirmative declarative sentence", "pos": {"nouns": {"person": 2, "others": 1, "Approval": 1}, "verbs": {"be": 1, "Yes": 1, "By": 1, "Approval": 1}, "adjectives": {"Sample": 1}, "numerals": {"Every": 1, "individual": 1, "Both": 1}, "pronouns": {"self": 1}}}, {"content": "17. You do not follow the rules, but you are easy to escape.", "word_count": 14, "label": "", "sentence_structure": "Subject鈥搗erb鈥搊bject", "subjective_objective": {"answer": "Subjective", "rationale": "Because \"unusual\" and \"exemptive\" are subjective perceptions or views of patients."}, "sentence_sentiment": {"answer": "Negative", "rationale": "It is negative because it expresses a non-compliance with the rules, irresponsible behaviour and implies that such behaviour can lead to good results."}, "language_style": {"answer": "Calm and direct", "rationale": "The entry, which uses a straightforward language style, expresses inquiries about the ability of the interviewee to evade punishment without using excessive qualifying or emotional expressions, and is more balanced and objective."}, "time_dimension": {"answer": "Present", "rationale": "The entry describes the routine behaviour and attitude of the testee and uses the current state of affairs ( \"Unusual\" and \"evading punishment\". It therefore determines its temporal dimension as it is now."}, "sentence_type": "Negative declarative sentence", "pos": {"nouns": {"Rules": 1, "Disposition": 1}, "verbs": {"No, I don't": 1, "Operations": 1, "Escapeable": 1}, "adjectives": {"Easy": 1}, "numerals": {"17": 1}}}, {"content": "18. Everyone put things on the table, not by implication.", "word_count": 20, "label": "", "sentence_structure": "Subject鈥搗erb鈥揷omplement", "subjective_objective": {"answer": "Objective", "rationale": "Because \"everyone puts things on the table, without implying\" is the way they behave or communicate, an objective fact that can be observed or documented, and is not influenced by individual subjective attitudes."}, "sentence_sentiment": {"answer": "Neutral", "rationale": "Because the entry does not have any emotional color."}, "language_style": {"answer": "Tactful and warm", "rationale": "The language style of this entry is euphemism. It uses a more gentle, inviting tone to ask if the interviewee agrees that people can be honest with each other, rather than being implied. This expression is more friendly and interactive, and it makes it easier for the interviewee to answer questions and feel comfortable."}, "time_dimension": {"answer": "Present", "rationale": "The keyword \"all\" indicates that this is the current situation, and the sentence as a whole describes an act or state, so it is judged as now."}, "sentence_type": "Affirmative declarative sentence", "pos": {"nouns": {"Things": 1, "Desktop": 1, "Methodology": 1}, "verbs": {"Huh": 1, "Say it": 1, "Use": 1, "Insinuations": 1}, "adverbs": {"Both": 1}, "numerals": {"18": 1}, "classifiers": {"individual": 1}, "pronouns": {"Everyone": 1}}}, {"content": "19. Some of us lacked feelings.", "word_count": 10, "label": "", "sentence_structure": "Subject鈥揷opula鈥損redicative", "subjective_objective": {"answer": "Subjective", "rationale": "According to three examples given, \"subjective\" refers to the patient's subjective attitude or feelings, and \"objective\" to the factual results or external conditions experienced by the patient. The sentence in the title \"19. Some of us lack feelings\" can be understood as a subjective assessment or opinion, because lack of feelings is a subjective feeling and perception, not an objective fact. The answer to this questionnaire should therefore be \"subjective.\""}, "sentence_sentiment": {"answer": "Negative", "rationale": "Because the description \"lack of emotion\" is negative."}, "language_style": {"answer": "Calm and direct", "rationale": "This entry is a direct observation that some people lack feelings. This expression is more objective, direct, less over-modified and emotional, and more in line with the linguistic style of the study."}, "time_dimension": {"answer": "Present", "rationale": "The keyword \"some of us\" says that the description applies to the present situation, that \"lack of emotion\" is a state description, and it happens now, so the entry is about the present."}, "sentence_type": "Negative declarative sentence", "pos": {"nouns": {"person": 2, "Emotion": 1}, "verbs": {"Lack": 1}, "numerals": {"Some": 1, "19": 1}, "classifiers": {"Medium": 1}, "pronouns": {"we": 1}}}, {"content": "20. When there are surprises, we know how to deal with them.", "word_count": 16, "label": "", "sentence_structure": "Subject鈥搗erb鈥搊bject", "subjective_objective": {"answer": "Objective", "rationale": "This is the objective fact of the description, i.e. knowing how to respond to an emergency, rather than the patient ' s subjective attitude or feelings."}, "sentence_sentiment": {"answer": "Neutral", "rationale": "Because the entry does not have any emotional color."}, "language_style": {"answer": "Calm and direct", "rationale": "The language style of the entry is a calm and straightforward one. It provides a simple and clear question about the ability of respondents to respond to sudden-onset situations, with no excessive emotional rendering and rhetorical techniques to make it easy for readers to understand and answer questions."}, "time_dimension": {"answer": "Present", "rationale": "The entry \"know how to handle\" refers to the current state or capability, and does not address the past point of time or future direction, so it is judged to be now."}, "sentence_type": "Affirmative declarative sentence", "pos": {"nouns": {"Events": 1, "Processing": 1}, "verbs": {"Yes": 1, "I met him": 1, "What": 1, "Processing": 1}, "adverbs": {"Suddenly": 1}}}, {"content": "We avoid talking about what we fear and concern.", "word_count": 15, "label": "", "sentence_structure": "Subject鈥搗erb鈥搊bject", "subjective_objective": {"answer": "Subjective", "rationale": "Because \"fear\" and \"care\" are the feelings inside the patient, and they're subjective."}, "sentence_sentiment": {"answer": "Negative", "rationale": "Because \"do not talk\" in the entry is negative."}, "language_style": {"answer": "Tactful and warm", "rationale": "The language style of the entry is more euphemistic, using the words \"avoid\" and \"talk\" and is relatively mild, without directly expressing a strong emotional or command tone. This euphemism is more readily acceptable to the respondents and is more in keeping with the nature of the psychological questionnaire."}, "time_dimension": {"answer": "Past", "rationale": "Such acts may have occurred, present and future, without a clear time limit. They can therefore be classified as \"now\"."}, "sentence_type": "Negative declarative sentence", "pos": {"nouns": {"Yes": 1, "we": 1}, "verbs": {"Avoid": 1, "I'm talking": 1, "Scared": 1, "Attention": 1}, "adverbs": {"and": 1, "Both": 1}, "numerals": {"21": 1}, "pronouns": {"we": 1}}}, {"content": "22. It is rare for us to speak of warm feelings.", "word_count": 13, "label": "", "sentence_structure": "Subject鈥搗erb鈥揷omplement", "subjective_objective": {"answer": "Subjective", "rationale": "Because \"speak each other's warm feelings\" is a subjective expression and emotional experience that belongs to the feelings and attitudes inside the patient."}, "sentence_sentiment": {"answer": "Positive", "rationale": "Because the article is positive, it emphasizes the warm and emotional exchanges among people."}, "language_style": {"answer": "Tactful and warm", "rationale": "The language style of the entry is euphemism. It is a gentle and inviting tone of conversation, with the emphasis on \"unusual\" and the expression that both sides should communicate and express their gratitude. This expression is more personal and interactive."}, "time_dimension": {"answer": "Present", "rationale": "The term \"said\" in the entry uses the general current, which means that it is a statement describing the state of the moment, and \"unusual to say\" indicates that the act is occurring less frequently in the present. So the temporal dimension is now."}, "sentence_type": "Affirmative declarative sentence", "pos": {"nouns": {"we": 1, "Feel": 1}, "verbs": {"Say it": 1, "To each other": 1, "Okay": 1, "Hard": 1}, "adjectives": {"It's rare": 1, "Temperature": 1}, "numerals": {"22": 1}}}, {"content": "We are experiencing economic difficulties.", "word_count": 8, "label": "", "sentence_structure": "Subject鈥損redicate", "subjective_objective": {"answer": "Objective", "rationale": "Because economic difficulties are an objective situation, whether the patient is aware of it or not."}, "sentence_sentiment": {"answer": "Negative", "rationale": "Because \"economically difficult\" is a negative situation."}, "language_style": {"answer": "Tactful and warm", "rationale": "The entry uses a gentle and caring tone, conveying to the respondents an understanding of and concern for the economic difficulties they experience, rather than directly presenting the facts or ordering the other party to do so. This expression is more likely to resonate with the respondents and helps to create a good investigation climate."}, "time_dimension": {"answer": "Past", "rationale": "The entry describes an event or situation that has occurred, i.e., \"distressed by economic difficulties\", and therefore the temporal dimension is a matter of the past."}, "sentence_type": "Negative declarative sentence", "pos": {"nouns": {"Economic difficulties": 1}, "verbs": {"I met him": 1}}}, {"content": "24. After our family tried to solve a problem, we usually discussed whether it had been resolved.", "word_count": 30, "label": "", "sentence_structure": "Subject鈥搗erb鈥搊bject", "subjective_objective": {"answer": "Subjective", "rationale": "This is a mixture of patient subjective attitudes and observations. The question is whether the problem has been solved, and after trying to solve a problem, we usually discuss it. So the questionnaire's purpose is to look at both patient subjective attitudes and objective observations."}, "sentence_sentiment": {"answer": "Neutral", "rationale": "Because the entry does not have any emotional color."}, "language_style": {"answer": "Tactful and warm", "rationale": "The entry uses more euphemistic terms such as \"tried\" and \"normal\" and includes human emotions in the description (discussed whether or not the problem has been solved), which increases the motivation of the respondents to participate and the willingness of the respondents to fill out the questionnaire."}, "time_dimension": {"answer": "Present", "rationale": "\"Try to solve\" and \"discussed whether\" are all descriptions of actions or states that are in progress or that have occurred, so the time dimension of this entry is now."}, "sentence_type": "Interrogative sentence", "pos": {"nouns": {"Problem": 2, "we": 2, "Discussion": 1, "Resolve": 2}, "verbs": {"Try": 1, "Resolve": 2, "Usually": 1, "Yes": 1, "Discussion": 1, "Is it possible": 1, "Already": 1}, "adverbs": {"After": 1, "Usually": 1, "Is it possible": 1}, "numerals": {"24": 1}, "pronouns": {"Here": 1}}}, {"content": "25. We're so self-centred.", "word_count": 10, "label": "", "sentence_structure": "Subject鈥揷opula鈥損redicative", "subjective_objective": {"answer": "Subjective", "rationale": "Because \"self-centred\" is a manifestation of subjective attitudes or perceptions."}, "sentence_sentiment": {"answer": "Negative", "rationale": "Because the expression \"too self-centred\" in the entry is negative."}, "language_style": {"answer": "Tactful and warm", "rationale": "The entry uses a euphemism to ask questions, not directly accusing or criticizing, but expressing concern and understanding of the person under investigation in a moderate and inclusive manner. Such expression is more likely to resonate with the person under investigation and to increase their participation and motivation."}, "time_dimension": {"answer": "Present", "rationale": "\"We are too self-centred\" describes a constant, customary phenomenon that has not only happened in the past, but may still be ongoing.\""}, "sentence_type": "Negative declarative sentence", "pos": {"nouns": {"we": 1, "Centre": 1}, "verbs": {"Too much": 1, "Here": 1, "For the center": 1}, "adverbs": {"Yes": 1}, "numerals": {"25": 1}}}, {"content": "26. We can express our feelings to each other.", "word_count": 13, "label": "", "sentence_structure": "Subject鈥搗erb鈥搊bject", "subjective_objective": {"answer": "Subjective", "rationale": "The basis for the judgement: because \"expression of one's feelings\" is an emotional communication that requires the patient's own will and attitude to achieve it, it is subjective."}, "sentence_sentiment": {"answer": "Neutral", "rationale": "Because the entry does not have any emotional color."}, "language_style": {"answer": "Tactful and warm", "rationale": "The entry is more proficient and interactive by engaging in dialogue in a moderate and inviting manner, rather than by direct statements or orders. At the same time, the article is neutral in its purpose, does not have too much emotional or positional orientation and is more focused on objective descriptions."}, "time_dimension": {"answer": "Present", "rationale": "In the future, the word \"capable\" in the entry indicates a possibility or expectation, and the keywords are \"expression\" and \"favouring\" and all point to the situation that may arise in the future. Thus, it can be judged that the entry is about the future."}, "sentence_type": "Affirmative declarative sentence", "pos": {"nouns": {"Questionnaire Entry": 1, "Feel": 1}, "verbs": {"Expression": 1, "To each other": 1, "Yes": 1}, "adverbs": {"self": 1}, "numerals": {"26": 1}}}, {"content": "27. We have no clear requirements for dressmaking habits.", "word_count": 14, "label": "", "sentence_structure": "Subject鈥損redicate", "subjective_objective": {"answer": "Objective", "rationale": "Since it is an objective fact that dressmaking habits are required to be independent of the patient ' s individual subjective attitude."}, "sentence_sentiment": {"answer": "Neutral", "rationale": "Because the entry does not address any clearly emotional content such as emotions, attitudes or behaviours, it merely describes the requirements of dressmaking habits."}, "language_style": {"answer": "Calm and direct", "rationale": "The language style of the entry is more straightforward, and it is not modified by any means of rhetoric or emotional colour, expressing the fact that there is no specific requirement for dressmaking habits."}, "time_dimension": {"answer": "Present", "rationale": "The entry describes the current behaviour or state, and \"no express requirement for dress dressing habits\" indicates that there is no explicit provision for dress dressing habits."}, "sentence_type": "Exclamatory sentence", "pos": {"nouns": {"we": 1, "It's a dress-up habit": 1}, "verbs": {"have": 1, "Request": 1}, "adjectives": {"Clear": 1}, "numerals": {"27": 1}}}, {"content": "We don't love each other.", "word_count": 10, "label": "", "sentence_structure": "Subject鈥搗erb鈥揷omplement", "subjective_objective": {"answer": "Subjective", "rationale": "Because \"we don't love each other\" is a subjective feeling and attitude, a subjective view of the patient's relationship with others."}, "sentence_sentiment": {"answer": "Negative", "rationale": "Because the \"no love\" in the entry expresses a negative emotion and attitude, implying a lack of emotional connection and interaction."}, "language_style": {"answer": "Calm and direct", "rationale": "The language style of the entry is more straightforward, with no excessive rhetorical or emotional rendering, and a greater focus on the expression of facts and information."}, "time_dimension": {"answer": "Present", "rationale": "\"We don't love each other\" describes a current state of behaviour or habit, and therefore judges it as now."}, "sentence_type": "Negative declarative sentence", "pos": {"nouns": {"Questionnaire Entry": 1, "Love": 1}, "verbs": {"Organisation": 1, "And then, you know, between you and me": 1}, "numerals": {"28": 1}}}, {"content": "We speak straight, not round the corner.", "word_count": 15, "label": "", "sentence_structure": "Subject鈥損redicate", "subjective_objective": {"answer": "Subjective", "rationale": "Because \"we speak straight, not round the corner\" is the patient's subjective attitude or belief, which reflects the patient's perception of how he communicates with others. This view is not necessarily objective, and it is therefore subjective."}, "sentence_sentiment": {"answer": "Positive", "rationale": "Because the \"just say\" in the entry is positive."}, "language_style": {"answer": "Calm and direct", "rationale": "The entry conveys the attitude and manner in which the other person communicates with the other person in a concise and clear statement, without any means of rhetoric or emotional rendering, and expresses it more directly and objectively.\""}, "time_dimension": {"answer": "Past", "rationale": "The words \"we\" and \"all\" indicate that this is a description of an event or phenomenon that is widespread in the past, and that \"talking without turning the corner\" also reflects the pattern of past behaviour."}, "sentence_type": "Affirmative declarative sentence", "pos": {"nouns": {"Questionnaire": 1, "Entry": 1, "person": 1, "Talk": 1, "Bend": 1, "Zipt": 1}, "verbs": {"to": 1, "Say it": 2, "Just say it": 1, "Turn around and rub the horns": 1}, "adjectives": {"Both": 1, "Straight": 1}, "numerals": {"29": 1}}}, {"content": "30. Each of us has a specific mandate and responsibility.", "word_count": 15, "label": "", "sentence_structure": "Subject鈥搗erb鈥搊bject", "subjective_objective": {"answer": "Subjective", "rationale": "Since the mandate and the responsibility are duties and responsibilities that the individual considers to be his or her own, the task and responsibility are subjective."}, "sentence_sentiment": {"answer": "Neutral", "rationale": "Because the entry does not have any emotional color."}, "language_style": {"answer": "Calm and direct", "rationale": "The language style of the entry is straightforward and calm, and it clearly expresses the fixed duties and tasks of each individual, without excessive emotional colours or qualifiers."}, "time_dimension": {"answer": "Present", "rationale": "The entry describes a prevailing fact or situation, with no clear time limits or time indicators, and therefore is judged to be the present."}, "sentence_type": "Affirmative declarative sentence", "pos": {"nouns": {"Tasks": 1, "Duties": 1, "person": 1}, "verbs": {"have": 1, "Freeze": 1, "Special": 1}, "adjectives": {"Every": 1}, "numerals": {"30": 1}, "pronouns": {"we": 1}}}, {"content": "31. The emotional atmosphere of the family is very poor.", "word_count": 10, "label": "", "sentence_structure": "Subject鈥揷opula鈥損redicative", "subjective_objective": {"answer": "Subjective", "rationale": "Because \"fun\" or \"fun\" are personal feelings and experiences, subjective."}, "sentence_sentiment": {"answer": "Negative", "rationale": "Because the entry \"unsure mood\" is an expression of a negative emotional tendency."}, "language_style": {"answer": "Tactful and warm", "rationale": "The language style of the entry is more euphemistic and moderate, starting with the positive description of \"fun\" and then using \"fail\" to express negative situations. This way leads respondents to think and answer questions more objectively, while reducing the discomfort or defensive psychologicalities that may arise."}, "time_dimension": {"answer": "Past", "rationale": "In the entry, \"unsure mood\" describes the past experience and feelings. The keyword \"family mood\" shows the past of the family as a whole."}, "sentence_type": "Affirmative declarative sentence", "pos": {"nouns": {"Emotional atmosphere": 1, "Family": 1}, "verbs": {"It's not good": 1}, "adverbs": {"very": 1}}}, {"content": "32. We have the principle of punishing human beings.", "word_count": 9, "label": "", "sentence_structure": "Subject鈥搗erb鈥搊bject", "subjective_objective": {"answer": "Objective", "rationale": "The principle of whether or not there is a penalty is a rule established by an organization or society and is not a fact that an individual can change subjectively."}, "sentence_sentiment": {"answer": "Positive", "rationale": "For the purpose of the act of \"the principle of punishment\" in the entry is to preserve social order and justice."}, "language_style": {"answer": "Calm and direct", "rationale": "The language style of the entry is \"cool and direct\". It expresses a view or principle in the form of a simple and clear statement, without too much emotional rendering or embellishment. This style is usually used in a formal and professional environment, which conveys messages clearly and avoids unnecessary confusion or misunderstanding."}, "time_dimension": {"answer": "Present", "rationale": "The keyword \"we have\" means the state of things, and the \"Punishment Principles\" is a principle that has been set and accepted, so the entry describes the current situation."}, "sentence_type": "Affirmative declarative sentence", "pos": {"nouns": {"person": 1, "Principles": 1}, "verbs": {"have": 1, "Punishment": 1}, "numerals": {"32": 1}}}, {"content": "33. We participate only when something interests us all.", "word_count": 20, "label": "", "sentence_structure": "Subject鈥搗erb鈥搊bject", "subjective_objective": {"answer": "Subjective", "rationale": "It is the patient鈥檚 subjective attitude or perception, as it is based on his preference for and interest in certain things. While this may be an observed phenomenon, it reflects more of the patient鈥檚 feelings and attitudes."}, "sentence_sentiment": {"answer": "Neutral", "rationale": "Because the entry describes only one condition, it does not display a positive or negative emotional attitude."}, "language_style": {"answer": "Tactful and warm", "rationale": "The language style of the entry is more euphemistic and gentle, not to express its own ideas or demands directly, but to direct the respondents to share their views or experiences in a more open and polite manner. Moreover, the entry uses some polite words, such as \"talents\", to enhance its prot茅g茅 and interaction."}, "time_dimension": {"answer": "Present", "rationale": "The entry is for the purpose of asking the person ' s attitude or opinion, and the time dimension is now judged."}, "sentence_type": "Affirmative declarative sentence", "pos": {"nouns": {"Things": 1, "Interest": 1, "Participation": 1}, "verbs": {"鈾?And I'm gonna be 鈾?: 1, "Jean": 0, "Interested": 1, "Join us": 1}, "adjectives": {"Both": 1, "we": 2, "of": 3, "Interested": 1}, "adverbs": {"Only": 1}, "numerals": {"33": 1}, "pronouns": {"Something": 1, "we": 2}}}, {"content": "34. There was no time to do what was of interest to him.", "word_count": 13, "label": "", "sentence_structure": "Subject鈥搗erb鈥揷omplement", "subjective_objective": {"answer": "Subjective", "rationale": "Because \"interest\" is the patient's personal preference and preference, and belongs to subjective feelings and attitudes. And \"no time\" is an objective fact or situation. The purpose of the questionnaire is therefore subjective."}, "sentence_sentiment": {"answer": "Negative", "rationale": "Because the description \"no time\" in the entry is negative."}, "language_style": {"answer": "Tactful and warm", "rationale": "The language style of the entry is more euphemistic and moderate, using the means of inquiry rather than direct orders or accusations, which are more readily acceptable to the interviewees and are willing to share their real ideas. In addition, the entry contains positive elements, such as \"do what you want\" , which is also an encouragement and support to make the questionnaire more friendly and friendly."}, "time_dimension": {"answer": "Present", "rationale": "The entry \"does not have time to do\" indicates the current reality and therefore the time dimension can be judged as it is now."}, "sentence_type": "Negative declarative sentence", "pos": {"nouns": {"Time": 1, "Yes": 1}, "verbs": {"Do it": 1}, "adjectives": {"No, I'm not": 1, "Interested": 1}, "numerals": {"34": 1}}}, {"content": "35. We often do not speak out about our own ideas.", "word_count": 13, "label": "", "sentence_structure": "Subject鈥損redicate", "subjective_objective": {"answer": "Subjective", "rationale": "This is because the patient observes and describes his or her behaviour and belongs to the subjective experience or feelings of the individual."}, "sentence_sentiment": {"answer": "Negative", "rationale": "Because the \"don't say what you think\" in the entry is negative."}, "language_style": {"answer": "Tactful and warm", "rationale": "The entry is more euphemistic, because it is asked in an invitation-like manner, without direct orders or pressure. Nor is it too emotional or rhetorical to influence the other. This expression is more gentle and friendly, and it is more resonant and cooperative."}, "time_dimension": {"answer": "Present", "rationale": "Now the word \"normal\" in the entry denotes a regular, customary act, while the word \"speak\" adopts the normal current state of affairs, which means that the act has not only occurred in the past, but may still be ongoing. So, the temporal dimension is now."}, "sentence_type": "Negative declarative sentence", "pos": {"nouns": {"Thoughts": 1, "self": 1}, "verbs": {"Say it": 1, "Come out": 1, "Regular": 1}, "adverbs": {"not": 1}, "numerals": {"35": 1}}}, {"content": "We feel that we can be tolerated.", "word_count": 12, "label": "", "sentence_structure": "Subject鈥揷opula鈥損redicative", "subjective_objective": {"answer": "Subjective", "rationale": "Because \"fear\" is a subjective experience, and \"cable\" is based on personal feelings and perceptions."}, "sentence_sentiment": {"answer": "Positive", "rationale": "Because the attitude of being tolerated in the entries is positive."}, "language_style": {"answer": "Tactful and warm", "rationale": "The language style of the entry is more euphemistic, allowing the interviewee to express his thoughts and feelings more freely by using the expression \"we feel\" as a subjective expression. At the same time, the subject \"can be tolerated\" is also raised in a more objective, moderate way, avoiding too direct or mandatory language."}, "time_dimension": {"answer": "Present", "rationale": "The keyword \"fear\" and \"can\" are all ongoing moves, so the entry is asking the person who's been tested for what he feels."}, "sentence_type": "Affirmative declarative sentence", "pos": {"nouns": {"we": 1, "person": 1, "Tolerance": 1}, "verbs": {"I feel": 1, "Yes": 1, "By": 1, "Tolerance": 1}, "numerals": {"36": 1}, "pronouns": {"we": 1}}}, {"content": "37. We are interested in each other only when something is in the individual interest.", "word_count": 20, "label": "", "sentence_structure": "Subject鈥揷opula鈥損redicative", "subjective_objective": {"answer": "Subjective", "rationale": "Because it expresses the view that the relationship between human beings depends on whether there is a profit, which is the patient ' s subjective attitude."}, "sentence_sentiment": {"answer": "Negative", "rationale": "Because the entry expresses a utilitarian attitude that people are concerned only with what is in their own interest, which may trigger indifference and selfishness towards others."}, "language_style": {"answer": "Calm and direct", "rationale": "The entry describes a view or belief in a straightforward language, without over-modified language or emotional expression, so that the language style can be described as \"cooling straight\"."}, "time_dimension": {"answer": "Present", "rationale": "The description \"we are interested in each other only when something is in the interest of the individual\" is an illustration of a general phenomenon or view, without clear time-lined sexual orientation, and can therefore be judged as the present behaviour or mentality."}, "sentence_type": "Affirmative declarative sentence", "pos": {"nouns": {"Yes": 1, "Personal": 1, "Lee": 1}, "verbs": {"have": 1, "That's right": 1, "Interested": 1}, "adjectives": {"- Yeah, yeah": 1}, "adverbs": {"When": 1, "Only": 2}, "numerals": {"37": 1}}}, {"content": "38. We can address most of the emotional concerns.", "word_count": 14, "label": "", "sentence_structure": "Subject鈥搗erb鈥搊bject", "subjective_objective": {"answer": "Subjective", "rationale": "Because \"resolving most emotional distress\" is a subjective view or belief, not an objective fact. Different individuals may have different understandings and experiences."}, "sentence_sentiment": {"answer": "Positive", "rationale": "Because the expression \"can solve most emotional troubles\" in the entry is positive."}, "language_style": {"answer": "Tactful and warm", "rationale": "The language style of the entry is euphemism, because it conveys the message by using some polite and euphemistic expression, and is not too direct or too tough. For example, the phrase \"we can resolve most emotional distress\" is more euphemism than direct orders or promises like \"we will help you solve problems.\" This type of language style is often more acceptable and understood, and can create a more harmonious and harmonious atmosphere of dialogue."}, "time_dimension": {"answer": "Present", "rationale": "The words \"can\" and \"cancellation\" in the entry indicate that it is a sentence describing the current state or capability, and therefore the judgement is now."}, "sentence_type": "Affirmative declarative sentence", "pos": {"nouns": {"Emotion": 1, "Trouble": 1}, "verbs": {"Resolve": 1}, "adjectives": {"Most of them": 1}, "adverbs": {"Go, go, go": 1}, "numerals": {"38": 1}, "classifiers": {"individual": 1}}}, {"content": "39. In our home, intimacy and warmness are secondary.", "word_count": 14, "label": "", "sentence_structure": "Subject鈥搗erb鈥搊bject", "subjective_objective": {"answer": "Subjective", "rationale": "Because \"intimate and warmness is the patient's subjective view of his family's status.\""}, "sentence_sentiment": {"answer": "Negative", "rationale": "Because the expression \"intimate and warmness is secondary\" in the entry implies that family relationships are not close enough and cozy enough to be a negative emotional color."}, "language_style": {"answer": "Calm and direct", "rationale": "The entry describes the relative position of \"intimacy and warmness\" in the family in a more objective and direct manner, with no excessive aesthetic or emotional colours and a more honest tone."}, "time_dimension": {"answer": "Present", "rationale": ""}, "sentence_type": "Affirmative declarative sentence", "pos": {"nouns": {"Intimacy": 1, "Temperature": 1, "Status": 1}, "verbs": {"Housing": 1}, "adverbs": {"Main": 1}, "numerals": {"39": 1}, "pronouns": {"we": 1}}}, {"content": "We discuss who does the housework.", "word_count": 8, "label": "", "sentence_structure": "Subject鈥搗erb鈥搊bject", "subjective_objective": {"answer": "Subjective", "rationale": "The question of who does the housework is subjective in view of the subjective opinions and choices of the patient and others."}, "sentence_sentiment": {"answer": "Neutral", "rationale": "Because the entry merely raised a topic for discussion, it did not involve any positive or negative emotional aspects."}, "language_style": {"answer": "Calm and direct", "rationale": "The language style of the entry is a straightforward and calm type. It presents a specific problem in a concise manner, with little too much embellishment or emotional expression, and is more objective and neutral."}, "time_dimension": {"answer": "Present", "rationale": "The entry is for the current discussion of the respondent, and the time dimension is now judged."}, "sentence_type": "Interrogative sentence", "pos": {"nouns": {"Domestic work": 1, "we": 1}, "verbs": {"Discussion": 1, "Do it": 1}, "numerals": {"40": 1}}}, {"content": "41. It is difficult to make decisions in our home.", "word_count": 15, "label": "", "sentence_structure": "Subject鈥揷opula鈥損redicative", "subjective_objective": {"answer": "Objective", "rationale": "Because it's an objective fact that examines the difficulties of family decision-making."}, "sentence_sentiment": {"answer": "Negative", "rationale": "Because the expression \"difficult\" in the entry is negative."}, "language_style": {"answer": "Tactful and warm", "rationale": "The language style of the entry is more euphemistic, using the implicit and more euphemistic expression \"it is difficult to make a decision about things,\" avoiding the tone of direct criticism or accusations. At the same time, the first person's proxy, \"we,\" gives people a sense of affection and interaction."}, "time_dimension": {"answer": "Present", "rationale": "The keywords \"in our house\" and \"is difficult\" point to the current family situation, indicating that the respondents consider it difficult to make decisions in the family at the present time. So the time dimension of this article is now."}, "sentence_type": "Negative declarative sentence", "pos": {"nouns": {"Yes": 1, "Love": 1, "Decision": 1}, "verbs": {"Do it": 1, "Out": 1, "Difficulties": 1}, "adjectives": {"Home": 1, "we": 1}, "numerals": {"41": 1}, "pronouns": {"be": 1}}}, {"content": "42. Our family cares for each other only when it is in their own interest.", "word_count": 19, "label": "", "sentence_structure": "Subject鈥搗erb鈥搊bject", "subjective_objective": {"answer": "Subjective", "rationale": "This is because the patient ' s perception and evaluation of the relationship between family members is subjective."}, "sentence_sentiment": {"answer": "Negative", "rationale": "Because the behavior in the entries \"only care for one another in his own favor\" expresses the emotional colour of selfishness and indifference to others."}, "language_style": {"answer": "Calm and direct", "rationale": "The language style of the entry is so cold that it presents the subject in a direct, concise manner that does not use emotional accoutrements or rhetorical techniques to emphasize the tone. This expression is usually considered to be an objective, formal language style that may be more acceptable and understandable in certain contexts."}, "time_dimension": {"answer": "Present", "rationale": ""}, "sentence_type": "Interrogative sentence", "pos": {"nouns": {"Our home": 1, "person": 2, "self": 1}, "verbs": {"have": 1, "That's right": 1, "Take care": 1}, "adjectives": {"Good": 1}, "numerals": {"42": 1}, "pronouns": {"Here": 1, "Time": 1}}}, {"content": "43. We are all frank with each other.", "word_count": 9, "label": "", "sentence_structure": "Subject鈥搗erb鈥揷omplement", "subjective_objective": {"answer": "Subjective", "rationale": "Because frankness is the patient's subjective attitude."}, "sentence_sentiment": {"answer": "Positive", "rationale": "Because \"bright one\" in the entries is a positive human relationship and communication attitude."}, "language_style": {"answer": "Tactful and warm", "rationale": "The language style of the entry is more euphemistic, using the expression \"we're one another\" to give people a sense of equality, harmony, and not too direct or hard-line. At the same time, the word \"confident\" implies an open and sincere attitude that makes it easier to feel comfortable and willing to participate in the investigation."}, "time_dimension": {"answer": "Present", "rationale": "The entry is an inquiry into the attitudes or views of the person being tested, and the time dimension does not allow for a judgement of past, present or future, and the output is therefore \"no time reference\". At the same time, the entry does not have a clear time frame or indication to indicate a particular point of time, and therefore no specific time reference."}, "sentence_type": "Affirmative declarative sentence", "pos": {"verbs": {"To each other": 1, "STAGE": 1, "Both": 1, "very": 1, "- Frank": 1}, "numerals": {"43": 1}}}, {"content": "44. We do not comply with any rules and standards.", "word_count": 12, "label": "", "sentence_structure": "Subject鈥搗erb鈥搊bject", "subjective_objective": {"answer": "Subjective", "rationale": "Because this expression expresses the patient ' s attitude towards rules and standards, it is a subjective idea or view."}, "sentence_sentiment": {"answer": "Negative", "rationale": "Because the purpose of the article was expressed in a negative manner, it was a sign of a lack of compliance with rules and standards."}, "language_style": {"answer": "Calm and direct", "rationale": "The entry directly expresses the subject of questions about compliance with rules and standards, does not use excessive rhetorical or emotional rendering, and is more objective in tone."}, "time_dimension": {"answer": "Past", "rationale": "The entry does not have a clear temporal dimension, but in terms of context, \"non-compliance with any rules or standards\" describes an attitude or a state of behaviour that may be past or present. But, given that the issue is investigating the current way of thinking and belief systems, we can consider it as a present expression."}, "sentence_type": "Negative declarative sentence", "pos": {"verbs": {"Compliance": 1, "Give": 1}, "adjectives": {"Any": 1, "Rule": 1, "Standard": 1}, "numerals": {"44": 1}}}, {"content": "45. If people are to do something, they often need to be reminded.", "word_count": 17, "label": "", "sentence_structure": "Subject鈥損redicate", "subjective_objective": {"answer": "Subjective", "rationale": "Because it is the patient's subjective feeling and judgment to remind someone to do something."}, "sentence_sentiment": {"answer": "Neutral", "rationale": "Because the entry describes only a phenomenon, it does not express positive or negative emotional tendencies."}, "language_style": {"answer": "Tactful and warm", "rationale": ""}, "time_dimension": {"answer": "Present", "rationale": "The keywords \"to\" and \"to be\" mean that it is a customary act and takes place in the moment where the person speaking is present."}, "sentence_type": "Affirmative declarative sentence", "pos": {"nouns": {"Yes": 1, "person": 2, "Synchronising folder": 1}, "verbs": {"Do it": 1, "Yes": 1, "Synchronising folder": 1}, "adverbs": {"Always": 1}, "numerals": {"45": 1}, "pronouns": {"they": 1}}}, {"content": "46. We can decide how to resolve the problem.", "word_count": 15, "label": "", "sentence_structure": "Subject鈥搗erb鈥搊bject", "subjective_objective": {"answer": "Subjective", "rationale": "Since \"how to solve problems\" is a process that requires reflection and judgement, involving subjective factors such as the perception of the individual, values and so forth, it is viewed from the perspective of the patient."}, "sentence_sentiment": {"answer": "Positive", "rationale": "Because the \"problem\" in the entry is positive."}, "language_style": {"answer": "Calm and direct", "rationale": "The entry is a direct expression of the question of whether the other party can decide how to solve the problem, without using too many rhetorical or emotional means of rendering it to be more objective in tone. At the same time, it is worded in a simple and clear manner, conveys the meaning of the problem, and is not too retouched to fit the language style of calmness and directness."}, "time_dimension": {"answer": "Present", "rationale": "The keyword \"can make a decision on how to solve the problem\" represents current or frequent behaviour, and therefore the temporal dimension is now judged."}, "sentence_type": "Affirmative declarative sentence", "pos": {"nouns": {"Problem": 1, "Decision": 1}, "verbs": {"Yes": 1, "Resolve": 1, "Make it": 1}, "adverbs": {"How's that": 1}, "numerals": {"46": 1}, "pronouns": {"we": 1}}}, {"content": "47. If the principle is broken, we don't know what's going to happen.", "word_count": 19, "label": "", "sentence_structure": "Subject鈥搗erb鈥搊bject", "subjective_objective": {"answer": "Subjective", "rationale": "This is the patient ' s subjective view and concern about what might happen after the principle has been broken."}, "sentence_sentiment": {"answer": "Negative", "rationale": "Because the \"principles are broken\" in the entry is negative."}, "language_style": {"answer": "Calm and direct", "rationale": "The language style of the entry is more calm and direct, with no excessive emotional or oral verb, and is more objective and clear. It succinctly conveys the greater warning that principles can be broken to the extent that they cause uncertainty and risk."}, "time_dimension": {"answer": "Future", "rationale": "The entry contains a statement of \"if\" and \"will happen\", which indicates that it is a projection or expectation of the future. The entry therefore relates to future behaviour or attitudes, based on the future of the temporal dimension."}, "sentence_type": "Negative declarative sentence", "pos": {"nouns": {"Principles": 1, "Yes": 1}, "verbs": {"Break": 1, "Yes": 1, "Come on": 1}, "adverbs": {"If": 1, "Yes, I will": 1, "What": 1}, "numerals": {"47": 1}, "pronouns": {"we": 1}}}, {"content": "48. Anything can work in our home.", "word_count": 11, "label": "", "sentence_structure": "Subject鈥揷opula鈥損redicative", "subjective_objective": {"answer": "Objective", "rationale": "This is a description of the family by the patient, a statement of the facts, not a subjective feeling or attitude of the patient."}, "sentence_sentiment": {"answer": "Positive", "rationale": "Because the expression \"anything works\" in this entry conveys a positive and optimistic attitude."}, "language_style": {"answer": "Tactful and warm", "rationale": "The language style of the entry is more euphemistic and sweet, using subjective expressions, and gives people a comfortable and comfortable feeling. It helps to create a good atmosphere of communication, making respondents more willing to share their thoughts and feelings."}, "time_dimension": {"answer": "Present", "rationale": "The entry describes an attitude or belief of the interviewees that \"anything works in our house\", which exists now and is not related to past and future events. Thus, the judgement is based on the present."}, "sentence_type": "Affirmative declarative sentence", "pos": {"verbs": {"It's gonna work": 1}, "adjectives": {"Any": 1, "we": 1, "Home": 1}, "adverbs": {"Both": 1}, "numerals": {"48": 1}}}, {"content": "49. We'll show it in warm air.", "word_count": 9, "label": "", "sentence_structure": "Subject鈥搗erb鈥揷omplement", "subjective_objective": {"answer": "Subjective", "rationale": "Because \"expressing warmness\" is a subjective attitude or choice, not just a description of objective facts."}, "sentence_sentiment": {"answer": "Positive", "rationale": "Because \"the warm expression\" in the entry is positive, and it is a warm, caring emotion."}, "language_style": {"answer": "Tactful and warm", "rationale": "The entry expresses positive emotional sentiments in the term \"temperature\" and uses the term \"expression\" to guide respondents to share their feelings and ideas, a more intimate, friendly language with an incentive and support."}, "time_dimension": {"answer": "Present", "rationale": "The entry \"will\" indicates what will happen in the future, and \"temperate expression\" is an ongoing move, so the sentence describes the behaviour of the subject."}, "sentence_type": "Affirmative declarative sentence", "pos": {"nouns": {"Temperature": 1}, "verbs": {"Expression": 1, "Come out": 1}, "adverbs": {"Will": 1}, "numerals": {"49": 1}}}, {"content": "We calm down on the ground to emotional problems.", "word_count": 14, "label": "", "sentence_structure": "Subject鈥搗erb鈥揷omplement", "subjective_objective": {"answer": "Subjective", "rationale": "Because \"we calm down to emotional problems\" is the patient's personal attitude and psychological state, which is subjective."}, "sentence_sentiment": {"answer": "Neutral", "rationale": "The entry is not of a clear emotional orientation."}, "language_style": {"answer": "Tactful and warm", "rationale": "The entry uses a mild and invitational tone, without directly stating or ordering the other party to do so, but rather in a way that allows the other party to think and answer questions in a way that is either suggested or directed. Moreover, the term \"silent\" gives a calm and calm sense of calm, emphasizing the need to maintain a rational and objective attitude on matters involving feelings."}, "time_dimension": {"answer": "Present", "rationale": "In the future, the entry uses the future state of \"we're going\", which presupposes that the act or state will occur or exist in the future, and thus will be judged to be future."}, "sentence_type": "Affirmative declarative sentence", "pos": {"nouns": {"Problem": 1}, "verbs": {"Calm down, ground": 1, "Involving": 1}, "adjectives": {"Calm down": 1}, "adverbs": {"Land": 2}, "numerals": {"50": 1}, "pronouns": {"we": 1}}}, {"content": "51. We cannot live together in harmony.", "word_count": 8, "label": "", "sentence_structure": "Subject鈥搗erb鈥揷omplement", "subjective_objective": {"answer": "Subjective", "rationale": "Because \"failure to live together\" is a subjective feeling and perception that reflects the patient's assessment and perception of his/her relationship with others."}, "sentence_sentiment": {"answer": "Negative", "rationale": "Because the entry \"not living together\" expresses negative emotions and attitudes."}, "language_style": {"answer": "Calm and direct", "rationale": "The entry uses direct, concise language to express the subject matter, without excessive ado or emotional colour, so that the language style is \"cool and straight\"."}, "time_dimension": {"answer": "Present", "rationale": "The entry describes the current situation, and \"failure to live together\" is an ongoing act and thus the judgement is now."}, "sentence_type": "Imperative sentence", "pos": {"nouns": {"we": 1, "Harmony": 1}, "verbs": {"No, I can't": 1}, "numerals": {"51": 1}}}, {"content": "52. We have lived a lifetime without talking to each other.", "word_count": 11, "label": "", "sentence_structure": "Subject鈥搗erb鈥揷omplement", "subjective_objective": {"answer": "Objective", "rationale": "This is because patients describe their behaviour and experiences and is a reflection of objective facts."}, "sentence_sentiment": {"answer": "Negative", "rationale": "Because \"lifeful, quiet\" is a negative act in the entry."}, "language_style": {"answer": "Calm and direct", "rationale": "The entry uses a simple and quick language style to present a code of conduct or opinion, without excessive qualifiers or emotional colours, and is more direct and objective in tone."}, "time_dimension": {"answer": "Past", "rationale": "The entry describes an event or state that has been experienced, and the words \"lifeful life\" and \"non-spoken\" indicate that this is a memory of the past."}, "sentence_type": "Affirmative declarative sentence", "pos": {"nouns": {"Life": 1, "Gas": 1, "- Yeah, yeah": 1, "Speech": 1}, "verbs": {"Yes": 1, "Fan": 1, "Speak": 1}, "adverbs": {"Just": 1}, "numerals": {"52": 1}}}, {"content": "53. Generally, we are not satisfied with the domestic work assigned to ourselves.", "word_count": 22, "label": "", "sentence_structure": "Subject鈥搗erb鈥搊bject", "subjective_objective": {"answer": "Subjective", "rationale": "Because satisfaction is a subjective feeling and assessment within a person."}, "sentence_sentiment": {"answer": "Negative", "rationale": "Because the attitude of \"unsatisfactory\" in the entries is negative."}, "language_style": {"answer": "Calm and direct", "rationale": "The entry expresses the subject of the survey in a concise language, without excessive rhetoric or emotional rendering, and is more objective and direct."}, "time_dimension": {"answer": "Present", "rationale": "\"General\" means the usual, normal situation, and \"unsatisfied\" describes the current feelings and attitudes of the person being tested, so the entry asks the person who is being questioned about the current feelings."}, "sentence_type": "Negative declarative sentence", "pos": {"nouns": {"General": 1, "Domestic work": 1}, "verbs": {"Assign": 1, "I feel": 1, "Satisfied": 1}, "adjectives": {"self": 1}, "adverbs": {"Both": 1, "not": 1}, "numerals": {"53": 1}}}, {"content": "54. Despite our good intentions, we have intervened too much in each other ' s lives.", "word_count": 22, "label": "", "sentence_structure": "Subject鈥搗erb鈥搊bject", "subjective_objective": {"answer": "Subjective", "rationale": "Because \"too much interference in each other's lives\" is a subjective assessment that reflects the patient's perception of his or her behaviour and that of others."}, "sentence_sentiment": {"answer": "Negative", "rationale": "Because \"too much interference in each other's lives\" is negative."}, "language_style": {"answer": "Tactful and warm", "rationale": "The entry is based on a more euphemistic approach: it offers the view that, despite their good intentions, they interfere too much in each other鈥檚 lives. It is not too direct or hard to express themselves, but rather more gentle and polite to convey the message by implication and reminder. At the same time, the article is simple and easy to understand, which is also in keeping with the euphemism of the language style."}, "time_dimension": {"answer": "Present", "rationale": ""}, "sentence_type": "Negative declarative sentence", "pos": {"nouns": {"we": 2, "Meaning": 1, "Life": 1}, "verbs": {"Intention": 1, "Intervention": 1, "Life": 1}, "adjectives": {"Good": 1, "More": 1}, "adverbs": {"鈾?Though 鈾?: 1}, "numerals": {"54": 1}, "pronouns": {"You too": 1}}}, {"content": "55. We have the principle of responding to situations of risk.", "word_count": 12, "label": "", "sentence_structure": "Subject鈥搗erb鈥搊bject", "subjective_objective": {"answer": "Objective", "rationale": "Because the Principles are objective, our standards or guidelines for dealing with dangerous situations, and are not influenced by individual subjective factors."}, "sentence_sentiment": {"answer": "Neutral", "rationale": "As this entry does not address any positive or negative behaviour, attitude or emotional colour."}, "language_style": {"answer": "Tactful and warm", "rationale": "The language style of the entry is a warmer one. Although the entry asks the other about the principle of responding to a risk situation, it is expressed in an invitation rather than in a direct order or statement. This expression is more personal and interactive, and makes people feel more willing to participate and share their views and experiences."}, "time_dimension": {"answer": "Present", "rationale": "The entry uses the current state of the art \"Yes\", describing the current situation or fact, i.e. whether the investigator has a principle of response to the risk situation. The judgement is therefore now."}, "sentence_type": "Affirmative declarative sentence", "pos": {"nouns": {"Principles": 1, "Situation": 1, "Danger": 1}, "verbs": {"Payable": 1}, "adjectives": {"have": 1, "we": 1}, "numerals": {"55": 1}}}, {"content": "We trust each other.", "word_count": 6, "label": "", "sentence_structure": "Subject鈥搗erb鈥搊bject", "subjective_objective": {"answer": "Subjective", "rationale": "Because mutual trust is a subjective feeling and an evaluation, it needs to be answered by the patient's own feelings, not by objective facts."}, "sentence_sentiment": {"answer": "Neutral", "rationale": "Because the entry does not express positive or negative emotional expressions, it is describing a state of interpersonal relations."}, "language_style": {"answer": "Tactful and warm", "rationale": "The language style of the entry is more euphemistic, not directly speaking or ordering, but by asking questions to guide the interviewee to think about whether he can trust one another in human relationships. This gentle and inviting tone is more friendly and interactive, and contributes to a good investigation atmosphere."}, "time_dimension": {"answer": "Present", "rationale": "The entry expresses the state of relations between the speaker and the other, that is, the sense of trust that exists between them and that is the state that is now in existence."}, "sentence_type": "Affirmative declarative sentence", "pos": {"nouns": {"Trust": 1}, "verbs": {"To each other": 1, "Trust": 1}, "numerals": {"56": 1}}}, {"content": "We cried in public.", "word_count": 8, "label": "", "sentence_structure": "Subject鈥搗erb鈥揷omplement", "subjective_objective": {"answer": "Subjective", "rationale": "Because \"cry out\" is a subjective expression of a human emotion and behaviour, not an objective thing or phenomenon."}, "sentence_sentiment": {"answer": "Negative", "rationale": "Because the act of \"public crying\" is often considered negative and may affect the image and self-esteem of others."}, "language_style": {"answer": "Tactful and warm", "rationale": "The article is more euphemistic, moderate, and does not have too much emotional and command-style expression. It guides the interviewee to share his experiences and feelings in an invitation manner, rather than directly asking if he has cried in a public place. It is more acceptable and stimulates the interviewee鈥檚 motivation and participation."}, "time_dimension": {"answer": "Present", "rationale": "The keyword is \"we cry in public,\" which describes a past or present behavior or state, and the temporal dimension is now."}, "sentence_type": "Affirmative declarative sentence", "pos": {"nouns": {"we": 1, "Public": 1, "Sound": 1}, "verbs": {"Cry": 1, "Out": 1}, "adverbs": {"Come on": 1}, "numerals": {"57": 1}}}, {"content": "58. We do not have the right means of transportation.", "word_count": 11, "label": "", "sentence_structure": "Subject鈥損redicate", "subjective_objective": {"answer": "Objective", "rationale": "Since the availability of suitable means of transport is an objective fact, it is irrelevant to the patient ' s subjective attitude."}, "sentence_sentiment": {"answer": "Negative", "rationale": "Since the purpose of the article is to say \"no suitable means of transport\", it is a negative description of the situation."}, "language_style": {"answer": "Tactful and warm", "rationale": "The language style of the entry is more euphemistic, using the expression \"We don't have\" to give people an objective, neutral sense of being not directly critical or accusing anyone, but also expressing their needs and expectations. Such euphemism makes each other more receptive and willing to explore solutions."}, "time_dimension": {"answer": "Present", "rationale": "The entry describes a state of current status or condition, i.e., the testees do not think they have the right means of transport. Thus, the judgement is based on \"we do not have\" and \"transport\", which point to the state or condition."}, "sentence_type": "Negative declarative sentence", "pos": {"nouns": {"Transport": 1}, "verbs": {"have": 1, "Suits": 1}, "numerals": {"58": 1}}}, {"content": "59. When we do not like what others do, we point them out.", "word_count": 24, "label": "", "sentence_structure": "Subject鈥搗erb鈥搊bject", "subjective_objective": {"answer": "Subjective", "rationale": "Because the words \"like\" and \"hate\" reflect the subjective attitude of the individual, the entry is viewed from the subjective point of view of the patient."}, "sentence_sentiment": {"answer": "Negative", "rationale": "Because the behavior in the entry \"shows others what they don't like\" can lead to conflict and conflict, with negative emotional aspects."}, "language_style": {"answer": "Calm and direct", "rationale": "The entry directly describes the fact that people do not like the behaviour of others, and does not use excessive rhetorical or emotional rendering, which is more objective and balanced. The language style of the purpose is therefore straightforward and calm."}, "time_dimension": {"answer": "Present", "rationale": "The entry is for the purpose of asking the testee how to observe and react to the behaviour of others, and the time dimension is now judged."}, "sentence_type": "Affirmative declarative sentence", "pos": {"nouns": {"we": 2, "person": 1, "What you did": 1, "Entry": 1}, "verbs": {"Yeah": 1, "Here": 1, "Note": 1}, "adverbs": {"Just": 1, "Yes": 2}, "numerals": {"59": 1}, "pronouns": {"he": 1}}}, {"content": "60. We have tried to solve the problem in every way possible.", "word_count": 13, "label": "", "sentence_structure": "Subject鈥搗erb鈥搊bject", "subjective_objective": {"answer": "Subjective", "rationale": "Because \"doing all you can\" is a subjective act and attitude that shows that patients are trying to solve problems."}, "sentence_sentiment": {"answer": "Positive", "rationale": "The positive formulation of the article is indicative of their proactive attitude and action in the face of problems."}, "language_style": {"answer": "Tactful and warm", "rationale": "The language style of the entry is euphemistic and sweet, and by using the expression \"We do everything we can\" it makes the question more abated, friendly, and not subject to stress or discomfort. At the same time, the theme of \"problem\" is also positive, helping to mobilize the interest and participation of the respondents."}, "time_dimension": {"answer": "Present", "rationale": "The entry \"we want\" means the current action or state, and the \"how to solve the problem\" is also an ongoing move, so it is possible to judge the time dimension of the entry as it is."}, "sentence_type": "Affirmative declarative sentence", "pos": {"nouns": {"Approach": 1, "Problem": 1}, "verbs": {"Yes": 1, "End of story": 1, "Come on": 1, "Resolve": 1}, "adverbs": {"Various": 1}, "numerals": {"60": 1}}}]}

{"questionnaire_title": "Trust Scale", "questionnaire_category": "Self-cognition", "item_count": 18, "emotion_shift_overall": "From positive to negative", "language_style_overall": "Tactful and warm", "items": [{"content": "I know what his/her companions will do, and he/she will never behave as I expected.", "word_count": 22, "label": "", "sentence_structure": "Subject鈥搗erb鈥搊bject", "subjective_objective": {"answer": "Subjective", "rationale": "Because the sentence contains \"I know,\" \"I know,\" \"I know,\" and it's all subjective and judgmental."}, "sentence_sentiment": {"answer": "Positive", "rationale": "Because it expresses knowledge and trust in peers, it is a positive attitude."}, "language_style": {"answer": "Calm and direct", "rationale": "The entry is based on a straightforward approach to the interviewee ' s prognosis of his companion ' s behaviour, without any use of rhetorical or emotional colours, and is more calm and objective in language style."}, "time_dimension": {"answer": "Present", "rationale": "The entry expresses a perception and understanding of the current situation, namely, \"know what your peers will do,\" based on current information and experience, not on future or past circumstances. Moreover, \"he/she does what I think is necessary\" also indicates that this is a current act or state, not a past experience or future scenario. So, the time dimension of this questionnaire is now."}, "sentence_type": "Affirmative declarative sentence", "pos": {"nouns": {"Companion": 1, "he": 1, "Work": 1, "I thought so": 1}, "verbs": {"Yes": 1, "Do it": 1, "Out": 1}, "adjectives": {"Always": 1, "not": 1, "I thought so": 1}, "adverbs": {"What will happen": 1, "What": 1}, "pronouns": {"I": 2}}}, {"content": "2. I found my companion to be a fully dependent person, especially in the event of a major incident.", "word_count": 25, "label": "", "sentence_structure": "Subject鈥搗erb鈥搊bject", "subjective_objective": {"answer": "Subjective", "rationale": "Because \"totally reliable\" and \"especially in case of a major incident\" are patients' subjective assessments and perceptions of peers."}, "sentence_sentiment": {"answer": "Positive", "rationale": "Because the description of \"a fellow person is a person who can rely on it\" in the entry is positive."}, "language_style": {"answer": "Tactful and warm", "rationale": "The entry is more adept and interactive when it is spoken in a gentle and inviting manner, rather than by direct statements or orders."}, "time_dimension": {"answer": "Present", "rationale": "The use of the words \"I find\" and \"fully reliable\" and \"when a major event occurs\" in the questionnaire entries suggests that this is a description or a feeling of the current situation, and therefore the judgement is now."}, "sentence_type": "Affirmative declarative sentence", "pos": {"nouns": {"Companion": 1, "person": 1, "Major incidents": 1}, "verbs": {"Found": 1, "be": 1, "Yeah": 1, "Dependence": 1}, "adjectives": {"Total": 1}, "numerals": {"2": 1}, "pronouns": {"I": 1}}}, {"content": "3. My companions ' behaviour has changed in a certain way, and I cannot always predict what he/she would do next.", "word_count": 34, "label": "", "sentence_structure": "Subject鈥搗erb鈥搊bject", "subjective_objective": {"answer": "Subjective", "rationale": "Because I am a character that describes the behaviour of one's peers through experience and perception, which involves subjective judgment and understanding of the behaviour of others."}, "sentence_sentiment": {"answer": "Negative", "rationale": "Because the entries describe a negative situation, namely, \"uncertain behaviour\" and \"unforeseen\", the words are all in the mood of denial and anxiety."}, "language_style": {"answer": "Calm and direct", "rationale": "The reason is that the entry is presented in a more straightforward and objective way, without excessive rhetoric or emotional colour, and rather directly describes the problems encountered by the subjects of the survey in their interpersonal contacts. Moreover, the tone of the article is relatively calm and calm, and there is no strong emotional tendency."}, "time_dimension": {"answer": "Present", "rationale": "The entry describes the sense and understanding of peer behaviour of the testee, in which \"always\" and \"also\" means that it is a recurring phenomenon, and the temporal dimension is now judged."}, "sentence_type": "Affirmative declarative sentence", "pos": {"nouns": {"Companion": 1, "Behaviour": 1, "Change": 1, "I don't know": 1, "Next time": 1, "Yes": 1}, "verbs": {"be": 1, "Do it": 1, "Out": 1, "You're": 1, "Surprise": 1}, "adjectives": {"None": 1, "I'm not sure": 1, "Next time": 1}, "adverbs": {"Always": 1, "And": 1}, "numerals": {"One": 1, "Number of times": 1}, "pronouns": {"he": 1, "she": 1}}}, {"content": "4. Although time is running out and no one can be sure of the future, I am confident that my colleagues will give me strength whatever happens.", "word_count": 39, "label": "", "sentence_structure": "Subject鈥搗erb鈥搊bject", "subjective_objective": {"answer": "Subjective", "rationale": "This is an expression of the patient ' s conviction and expectations, which are subjective."}, "sentence_sentiment": {"answer": "Positive", "rationale": "Because the entry \"Believes that no matter what happens, my fellow man will give me strength\" expresses a positive belief and attitude."}, "language_style": {"answer": "Tactful and warm", "rationale": "The entry uses expressions like \"trust\" and \"power\" and is filled with positive attitudes and trust and expectations of others, with a more euphemism and warmer language. At the same time, the use of \"but\" turns to reflect an optimistic attitude to face difficulties and a sense of kindness."}, "time_dimension": {"answer": "Present", "rationale": "The phrase \"I am sure that my fellow man will give me strength whatever happens\" expresses a belief and confidence that his fellow man will support and help in times of difficulty. So the key word and the time dimension of this entry is now."}, "sentence_type": "Affirmative declarative sentence", "pos": {"nouns": {"Time": 1, "鈾?And it's gone 鈾?: 1, "The future": 1, "Companion": 1, "Power": 1}, "verbs": {"have": 1, "Come on": 1, "Here": 1, "Yes": 1}, "adjectives": {"Sure": 1}, "adverbs": {"鈾?And keep on 鈾?: 1, "Both": 1}, "numerals": {"4": 1}, "pronouns": {"I": 1}}}, {"content": "5. Based on past experience, I cannot fully trust the commitment of my peers.", "word_count": 21, "label": "", "sentence_structure": "Subject鈥搗erb鈥搊bject", "subjective_objective": {"answer": "Subjective", "rationale": "This is because patients ' subjective assessment of past experience and attitude to it is not entirely dependent on the commitment of their peers to him."}, "sentence_sentiment": {"answer": "Negative", "rationale": "Because the idea of \"not fully trusting my fellow people in my commitment\" in the entry expresses negative emotions and attitudes."}, "language_style": {"answer": "Calm and direct", "rationale": "The entry is asked in an objective and direct manner, without any use of rhetorical or emotional acrimony, and is clear and clear. At the same time, it reflects respect for the privacy and feelings of the interviewee. \""}, "time_dimension": {"answer": "Past", "rationale": "The entry contains the phrase \"senior experience\" which indicates that it is asking the testee about the past experience and feelings. The temporal dimension of the article is therefore \"the past\"."}, "sentence_type": "Negative declarative sentence", "pos": {"nouns": {"Experience": 1, "Companion": 1, "Commitments": 1}, "verbs": {"Based on": 1, "Trust": 1, "Here": 1}, "adjectives": {"Past": 1, "Total": 1}, "numerals": {"5": 1}, "pronouns": {"I": 1}}}, {"content": "6. Sometimes it is difficult for me to be absolutely certain that my peers will always look after me, that the future is too uncertain and that our relationship will change considerably as time passes.", "word_count": 44, "label": "", "sentence_structure": "Subject鈥搗erb鈥搊bject", "subjective_objective": {"answer": "Subjective", "rationale": "This is a subjective assumption and concern of patients about future changes in relationships, and is based on their personal experience and emotional state, and is not an objective fact."}, "sentence_sentiment": {"answer": "Negative", "rationale": "Because the words \"it's hard to be absolutely certain that my partner will always take care of me\" and \"the future is too uncertain that our relationship will change considerably as time passes\" are both negative emotional."}, "language_style": {"answer": "Tactful and warm", "rationale": "The language style of the entry is euphemism. By using words such as \"sometimes,\" \"difficult to be absolutely certain\" , a strong and direct expression of questioning or accusations was avoided; at the same time, the expressions \"too uncertain for the future\" and \"too much will change in our relationship as time passes\" also reflect the author's understanding and respect for the interviewee, without overemphasis on his position or opinion."}, "time_dimension": {"answer": "Future", "rationale": "The term \"future\" denotes a situation that has not yet occurred and that is about to happen. The entry describes the uncertainty and concern of the testee about future relations, so it can be judged to be a question about the future."}, "sentence_type": "Negative declarative sentence", "pos": {"nouns": {"Sometimes": 1, "Companion": 1, "Care": 1, "The future": 2, "Time": 1, "鈾?And it's gone 鈾?: 1, "Relations": 1}, "verbs": {"It's hard": 1, "Absolutely": 1, "It's gonna happen": 1}, "adjectives": {"I'm so confused": 1}, "adverbs": {"Always": 1}, "numerals": {"6": 1, "It's big": 1}, "pronouns": {"I": 1}}}, {"content": "7. My companion is a very honest man, and even if he says something that is unbelievably believed, others will believe that he is telling the truth.", "word_count": 37, "label": "", "sentence_structure": "Subject鈥揷opula鈥損redicative", "subjective_objective": {"answer": "Subjective", "rationale": "Because this is a subjective assessment of the patient ' s honesty to others."}, "sentence_sentiment": {"answer": "Neutral", "rationale": "Because the entry describes the character characteristics of the peers and does not express positive or negative emotional aspects."}, "language_style": {"answer": "Tactful and warm", "rationale": "The language of the entry is more euphemistic and warm, and the expression \"very honest person\" and \"unbelievable person will believe\" are used to describe the character of the person, which is more acceptable and can inspire resonance with the other."}, "time_dimension": {"answer": "Past", "rationale": "The entry describes the peer evaluation and perception of others by the testee, all of which refer to past experience and perceptions. While reference is made to \"now\", the term \"now\" here refers to the current context, not the present. The temporal dimension of the article is therefore the past."}, "sentence_type": "Affirmative declarative sentence", "pos": {"nouns": {"Companion": 1, "person": 1, "Word": 2, "The facts": 1}, "verbs": {"be": 2, "Say it": 4, "Yes": 4, "others": 1}, "adjectives": {"Tens": 1, "Honesty": 1, "Unbelievable": 1}, "numerals": {"VII": 1}, "pronouns": {"I": 1}}}, {"content": "My companions are difficult to grasp, and people sometimes have no idea how he's going to do it.", "word_count": 24, "label": "", "sentence_structure": "Subject鈥搗erb鈥搊bject", "subjective_objective": {"answer": "Subjective", "rationale": "Because words like \"unpredictable\" and \"uncertainty\" describe patients' subjective feelings and judgment about their peers."}, "sentence_sentiment": {"answer": "Negative", "rationale": "Because words such as \"unpredictable\" and \"uncertainty\" in the entries imply that peer behaviour is difficult to predict and control, they can lead to negative emotions or outcomes."}, "language_style": {"answer": "Tactful and warm", "rationale": "The entry expresses a subjective feeling in descriptive language, without directly criticizing or accusing others. At the same time, the words \"untouchable\" and \"uncertainty\" are used to describe this feeling, which is more mild."}, "time_dimension": {"answer": "Present", "rationale": "The entry for the questionnaire is to ask the testee about his peers and to evaluate them, and to describe them on the basis of the current situation. The keyword \"unpredictable\" and \"uncertainly how he will behave\" are expressions of current behaviour or characteristics, and therefore the judgement is now."}, "sentence_type": "Affirmative declarative sentence", "pos": {"nouns": {"Companion": 1, "person": 1, "Behaviour": 1}, "verbs": {"Jean": 1, "- A touch": 1, "Sure": 1, "Act": 1}, "adjectives": {"It's hard": 1}, "adverbs": {"Sometimes": 1}, "pronouns": {"he": 1}}}, {"content": "9. My companion has proved to be a trustworthy person, and whoever he/she marries will never do anything unfaithful (even if he/she is not caught with absolute certainty).", "word_count": 47, "label": "", "sentence_structure": "Subject鈥搗erb鈥搊bject", "subjective_objective": {"answer": "Subjective", "rationale": "Because the words \"credible\" and \"unfaithful\" all reflect the subjective judgement and assessment of the interviewees, they are not objective facts."}, "sentence_sentiment": {"answer": "Neutral", "rationale": "Because the entry is a statement of fact and does not express any emotional colour."}, "language_style": {"answer": "Tactful and warm", "rationale": "The language style of the entry is more euphemistic, using positive and positive terms such as \"proven\" and \"trustworthy\" to describe peers. At the same time, the expression of this part of the article \"never he/she marries anyone who would have done anything unfaithful (even if he/she had absolute certainty that he/she would not be caught\") is more subtle, avoiding direct accusations or attacks against the other, and enhancing the friendly and acceptance of the questionnaire."}, "time_dimension": {"answer": "Future", "rationale": "The entry contains visions and expectations for the future, the key words being \"My companion has proved to be a trustworthy person\" and \"never do anything unfaithful,\" all pointing to situations that have not yet occurred and that may arise in the future. And the word \"marriage\" implies that it is a planning or vision for the future."}, "sentence_type": "Affirmative declarative sentence", "pos": {"nouns": {"Companion": 1, "person": 2, "Things": 2, "Yes": 1, "Sure": 1, "Marriage": 1, "Absolutely": 1, "Hold on": 1, "Catch": 1}, "verbs": {"Prove it": 1, "Yes": 2, "Do it": 1, "Make": 1, "Never": 1, "have": 1, "By": 3, "Hold on": 1}, "adjectives": {"Trusted": 1, "Absolutely": 1}, "adverbs": {"Already": 1, "Both": 1, "Unfaithful": 1, "Even if": 1}, "numerals": {"9": 1, "One": 2, "Two": 1}, "pronouns": {"he": 1, "she": 1, "I": 1}}}, {"content": "I never thought that the inexorable conflict and the serious tensions would undermine our relationship, which is capable of being tested by any fundamentals.", "word_count": 47, "label": "", "sentence_structure": "Subject鈥搗erb鈥搊bject", "subjective_objective": {"answer": "Subjective", "rationale": "This is the subjective attitude and conviction of patients who believe that their relationship can withstand the challenges and difficulties. This perception does not reflect objective facts, but rather reflects patients鈥?perceptions and beliefs about their relationship."}, "sentence_sentiment": {"answer": "Positive", "rationale": "Because it expresses trust and tenacity in relationships, it is a positive emotional colour."}, "language_style": {"answer": "Calm and direct", "rationale": "The entry is strong, clearly stated, and does not use rhetorical or euphemistic expressions. It directly expresses the surveyer鈥檚 view of the resilience of human relationships, arguing that they are not affected by the relationship even in the face of conflict and tension. This language style usually appears on serious issues or discussions, in order to maintain professionalism and objectivity."}, "time_dimension": {"answer": "Present", "rationale": ""}, "sentence_type": "Affirmative declarative sentence", "pos": {"nouns": {"Conflict": 1, "Nervous": 1, "Relations": 2, "Test": 1}, "verbs": {"I think": 1, "It's gonna hurt": 1, "It's gonna be okay": 1, "Preside": 1, "Come on": 1}, "adjectives": {"Serious": 1, "Unpredictable": 1, "Any one of them": 1}, "adverbs": {"Never": 1, "we": 2}, "numerals": {"10": 1}}}, {"content": "I am familiar with the behaviour of my peers, who always have certain rules to follow.", "word_count": 28, "label": "", "sentence_structure": "Subject鈥搗erb鈥揷omplement", "subjective_objective": {"answer": "Subjective", "rationale": "Basis of judgement: The entry on the questionnaire relates to the perception and feeling of \"me\" and is therefore subjective."}, "sentence_sentiment": {"answer": "Positive", "rationale": "Because the entry expresses knowledge and trust in peer behaviour and is a positive attitude."}, "language_style": {"answer": "Tactful and warm", "rationale": "The entry is worded in more moderate terms, without directly stating whether the other party's behaviour is correct or appropriate. Instead, the level of understanding and familiarity with the other party's behaviour is expressed in a question-and-answer format, which is more likely to trigger positive responses and in-depth communication from the other party and helps to build good interaction."}, "time_dimension": {"answer": "Present", "rationale": "The entry is for the purpose of asking the testee about the degree of observation and understanding of peer behaviour, which is now judged in terms of time dimension."}, "sentence_type": "Affirmative declarative sentence", "pos": {"nouns": {"Questionnaire Entry": 1, "Companion": 1, "Behaviour": 1, "Things": 1, "Rules": 1}, "verbs": {"I know you": 1, "Do it": 2, "have": 1, "Go ahead": 1}, "adjectives": {"Tens": 1}, "numerals": {"11": 1, "One": 3, "I'm sure": 1}, "classifiers": {"individual": 1}, "pronouns": {"he": 1, "or": 1, "she": 1, "Here": 1}}}, {"content": "12. If I have never faced a particular problem with my fellow man before, I may be concerned that he or she may not take my feelings into account.", "word_count": 38, "label": "", "sentence_structure": "Subject鈥損redicate", "subjective_objective": {"answer": "Subjective", "rationale": "Because this is about the patient ' s own thoughts and feelings, it is based on the patient ' s subjective experience and personal perceptions."}, "sentence_sentiment": {"answer": "Negative", "rationale": "Because the idea that he or she might not take my feelings into account in the entry is negative."}, "language_style": {"answer": "Tactful and warm", "rationale": "The language style of the entry is euphemism. It uses a tone of inquiry and invitation, not an order or a direct statement. This expression is more intimate and interactive, and it is easier to direct respondents to answer questions. Moreover, the entry does not use strong emotional terms or negative evaluations, but rather questions in a balanced and rational way."}, "time_dimension": {"answer": "Present", "rationale": "Now that the entry \"if I have never faced a particular problem with my peers before\" and \"I may be concerned that he or she may not take my feelings into account\" suggests that this is a hypothetical scenario for what might have happened in the past, and that the behaviour is not clearly defined now, and therefore the temporal dimension is now judged."}, "sentence_type": "Affirmative declarative sentence", "pos": {"nouns": {"Companion": 1, "Problem": 1, "Emotion": 1}, "verbs": {"Faced": 1, "I'm worried": 1, "Account": 1}, "adverbs": {"Maybe": 1}, "numerals": {"12": 1}, "pronouns": {"I": 2, "He or she": 1}}}, {"content": "13. Even in familiar situations, I am not fully sure that my peers will repeat the last pattern of behaviour.", "word_count": 29, "label": "", "sentence_structure": "Subject鈥搗erb鈥搊bject", "subjective_objective": {"answer": "Subjective", "rationale": "Because \"not sure how one's partner repeats the last behavior\" is the patient's subjective expectation and perception of the behaviour of a fellow in a familiar environment. This reflects the patient's uncertainty and fear of others' reactions in interpersonal relationships, and is a subjective emotional experience and cognitive assessment."}, "sentence_sentiment": {"answer": "Negative", "rationale": "Because the expression \"were not absolutely certain that the other person will repeat the last act\" in the entry has negative emotional aspects such as uncertainty, mistrust, etc."}, "language_style": {"answer": "Tactful and warm", "rationale": "The language style of the entry is more euphemistic, and dialogue is conducted in a gentle and inviting tone, rather than in direct statements or commands. This expression is more personal and interactive, and better guides respondents to questions and to a good atmosphere of communication."}, "time_dimension": {"answer": "Present", "rationale": "\"Even if\" in \"even\" a familiar occasion indicates a condition, \"the familiar occasion\" is a context that describes the current situation, so the temporal dimension of the article is now."}, "sentence_type": "Negative declarative sentence", "pos": {"nouns": {"Event": 1, "Companion": 1, "Behaviour": 1}, "verbs": {"Yes": 1, "Sure": 1, "Repeat": 1}, "adjectives": {"I know it": 1, "Totally": 2}, "adverbs": {"Yeah": 1}, "numerals": {"13": 1}, "pronouns": {"I": 1}}}, {"content": "14. I feel very secure in the face of the new environment that is unknown, because I know that my peers will never let me lose.", "word_count": 32, "label": "", "sentence_structure": "Subject鈥揷opula鈥損redicative", "subjective_objective": {"answer": "Subjective", "rationale": "Because knowing that my peers are never going to lose is a subjective idea and belief of one person. While this may be based on past experience and interaction, it is still a personal perception and perception of the situation. So this entry is viewed from a subjective perspective."}, "sentence_sentiment": {"answer": "Positive", "rationale": "Because the entry expresses the sense of security and trust in peers in the face of new and unknown circumstances, these feelings and attitudes are positive."}, "language_style": {"answer": "Tactful and warm", "rationale": "It uses a gentle and inviting tone of speech to engage in dialogue, rather than direct statements or orders, which is more personal and interactive. At the same time, it conveys a message of mutual cooperation, support and care, which is comfortable and reassuring, by emphasizing that \"the companion will never let me lose.\""}, "time_dimension": {"answer": "Future", "rationale": ""}, "sentence_type": "Affirmative declarative sentence", "pos": {"nouns": {"Environment": 1, "Companion": 1, "Clear": 1}, "verbs": {"Faced": 1, "I feel": 1, "Yes": 1, "Jean": 1, "I'm sorry": 1}, "adjectives": {"Unknown": 1, "Tens": 1}, "adverbs": {"Never": 1, "Yes": 1, "Because": 1}, "numerals": {"14": 1}, "classifiers": {"individual": 1}, "pronouns": {"I": 1, "be": 1}}}, {"content": "15. My companions are not necessarily trustworthy, and I can recall several instances of untrustworthy behaviour.", "word_count": 33, "label": "", "sentence_structure": "Subject鈥搗erb鈥揷omplement", "subjective_objective": {"answer": "Objective", "rationale": "This is a subjective assessment of the patient鈥檚 perception of his/her partner鈥檚 trustworthiness. However, the patient鈥檚 response is based on the specific behaviour and experience they observe, which is an objective fact."}, "sentence_sentiment": {"answer": "Negative", "rationale": "Because the expression \"he has several untrustworthy acts\" in the entry is negative emotional."}, "language_style": {"answer": "Calm and direct", "rationale": "The entry is more calm and straightforward in its language style, which is not based on excessive rhetorical or emotional colours, but on an objective and clear expression of a view. Moreover, the entry is not in question form, but rather a statement of a fact. It can therefore be considered as being calm and straightforward."}, "time_dimension": {"answer": "Present", "rationale": "The entry describes the person ' s observation and evaluation of others, in which the key word \"remember\" indicates that it is a memory and description of past experiences, but there is no clear time limit and therefore can be judged as now."}, "sentence_type": "Negative declarative sentence", "pos": {"nouns": {"Companion": 1, "person": 1, "Behaviour": 1}, "verbs": {"I'm sure": 1, "Yeah": 1, "Let's get": 1, "Yes": 1, "I remember": 1, "have": 2, "Untrustworthy": 1}, "adjectives": {"Trustful": 1}, "adverbs": {"not": 1}, "numerals": {"15": 1, "he": 1, "A few times": 1}, "classifiers": {"One": 1, "Number of times": 1}, "pronouns": {"I": 1}}}, {"content": "16. I'm not comfortable sometimes thinking about my relationship, because I can't feel any better about the future.", "word_count": 35, "label": "", "sentence_structure": "Subject鈥揷opula鈥損redicative", "subjective_objective": {"answer": "Subjective", "rationale": "Since the questionnaire is intended to understand the patient ' s subjective feelings and perceptions, the entry describes the patient ' s subjective perception of investing in emotional relationships and is therefore subjective."}, "sentence_sentiment": {"answer": "Negative", "rationale": "Because the entries \"uncomfortable\" and \"unable to rest in the future\" are all negative emotional expressions of concern and discomfort."}, "language_style": {"answer": "Tactful and warm", "rationale": "The entry uses expressions such as \"think\" and \"accomplishment\" with a more moderate, caring tone, rather than directly accusing or ordering each other. At the same time, by describing their feelings, the other side is able to understand and respond more easily to the problem, reflecting equality and respect between the two sides in the dialogue."}, "time_dimension": {"answer": "Present", "rationale": "The entry describes the emotional experience and feelings of the subject, in which \"I am occasionally uncomfortable\" and \"because I have difficulty in feeling fully comfortable with the future\" indicate that this emotion or feeling is happening. So the temporal dimension is now."}, "sentence_type": "Affirmative declarative sentence", "pos": {"nouns": {"Questionnaire Entry": 1, "Relationship investment": 1, "Two people": 1, "The future": 1}, "verbs": {"I thought": 1, "I feel": 1, "It's hard": 1, "Don't worry": 1}, "adjectives": {"I'm not feeling well": 1, "Total": 1}, "adverbs": {"Sometimes": 1}, "pronouns": {"I": 2}}}, {"content": "17. My colleagues have not proved to be always trustworthy in the past, and I have hesitated on several occasions to engage in activities that have easily brought down my weaknesses.", "word_count": 42, "label": "", "sentence_structure": "Subject鈥搗erb鈥搊bject", "subjective_objective": {"answer": "Subjective", "rationale": "This entry examines patients鈥?subjective thoughts and attitudes, namely, the level of trust in their peers and their willingness to participate in certain activities. While these situations are objective, such perceptions and decisions are subjective judgements of patients based on their own experience and observations."}, "sentence_sentiment": {"answer": "Negative", "rationale": "Because the entries \"My companions were not always trusted\" and \"I was hesitant to let them go into activities that would easily set me in a weak spot\" are all negative emotional."}, "language_style": {"answer": "Calm and direct", "rationale": "The entry uses direct language to describe the problem and to make clear the impact of the other party鈥檚 behaviour on the subject."}, "time_dimension": {"answer": "Past", "rationale": "Moreover, \"proved\" shows that these things have happened and have been proven or confirmed, further reinforcing the past."}, "sentence_type": "Affirmative declarative sentence", "pos": {"nouns": {"The facts": 1, "Companion": 1, "Number": 1, "Activities": 1, "Weaknesses": 1}, "verbs": {"Prove it": 1, "Always": 1, "It's worth it": 1, "Trust": 1, "Whispering": 1, "Let's get him in": 2, "The curtain": 1}, "adverbs": {"Past": 1, "Not really": 1, "Always": 1, "Easy": 1}, "numerals": {"17": 1, "1": 3}, "classifiers": {"Number of times": 1}, "pronouns": {"Mine": 1}}}, {"content": "18. My fellow men act in a coherent manner.", "word_count": 11, "label": "", "sentence_structure": "Subject鈥揷opula鈥損redicative", "subjective_objective": {"answer": "Objective", "rationale": "Because \"consistent\" describes the behaviour of the patient's companion, it's an objective fact."}, "sentence_sentiment": {"answer": "Neutral", "rationale": "Because the entry does not have any emotional color."}, "language_style": {"answer": "Tactful and warm", "rationale": "The entry is dialogueable in a sense of inquiry and inquiry and avoids the use of strong negative or positive terms by using expressions such as \"with\" , a soft and invitationary tone that is more natural and easy to relax and actively engage the respondents in the questionnaire."}, "time_dimension": {"answer": "Past", "rationale": "The keyword \"consistent\" denotes an evaluation of the past behaviour of peers and therefore the entry is a past."}, "sentence_type": "Affirmative declarative sentence", "pos": {"nouns": {"Behaviour": 1, "Coherence": 1}, "verbs": {"Yes": 1, "Companion": 1, "Behaviour": 1}, "numerals": {"18": 1}, "pronouns": {"I": 1}}}]}

{"questionnaire_title": "- A depression questionnaire.", "questionnaire_category": "Mental health", "item_count": 66, "emotion_shift_overall": "From positive to negative", "language_style_overall": "Tactful and warm", "items": [{"content": "I set my goals as commercially as possible.", "word_count": 13, "label": "", "sentence_structure": "Subject鈥搗erb鈥揷omplement", "subjective_objective": {"answer": "Subjective", "rationale": "Because \"as far as possible\" and \"as long as you can\" are subjective attitudes and behavioral behaviour of patients."}, "sentence_sentiment": {"answer": "Positive", "rationale": "Because the purpose of the action \"targeting\" in the entry is positive."}, "language_style": {"answer": "Tactful and warm", "rationale": "The language style of the entry is more euphemistic and warm, and it is a soft, invitation-style conversation, rather than a direct statement or order. For example, the words \"as far as possible\" and \"as much as possible to set a goal for yourself\" are used to make the tone more sensitive and interactive, and to stimulate the thoughts and answers of the interviewees."}, "time_dimension": {"answer": "Past", "rationale": "In it, \"as far as possible\" means that it is a proactive mentality and contains visions for the future; \"targeting\" involves planning and action for the future, and therefore is a future act. So, in combination, this questionnaire entry covers the time dimension of the past, the present and the future."}, "sentence_type": "Affirmative declarative sentence", "pos": {"nouns": {"Objective": 1, "self": 1}, "verbs": {"Set": 1, "Agreed": 1}, "adjectives": {"Maybe": 1, "Land": 2}, "numerals": {"1": 3}, "pronouns": {"I": 1}}}, {"content": "Without the support of those around me, I would feel isolated.", "word_count": 17, "label": "", "sentence_structure": "Subject鈥搗erb鈥揷omplement", "subjective_objective": {"answer": "Subjective", "rationale": "Because it is the patient ' s own subjective feeling that determines whether or not he or she feels alone, he or she is not influenced by others."}, "sentence_sentiment": {"answer": "Negative", "rationale": "Because the feeling of being alone in the entry is negative."}, "language_style": {"answer": "Tactful and warm", "rationale": "The language style of the entry is euphemism. It does not directly express the feelings that the other person may feel alone and helpless, but rather asks questions in a more moderate way, leaving the interviewee with room for self-reflection and choice, and an atmosphere of understanding and care."}, "time_dimension": {"answer": "Present", "rationale": "The keyword \"no\" suggests that it describes the current situation, and \"will feel\" suggests that it is an expected feeling, so the entry is to ask what the testee feels when there is a lack of support from the surroundings, and the time dimension is now."}, "sentence_type": "Negative declarative sentence", "pos": {"nouns": {"People around": 1, "Support": 1, "I'm alone": 1}, "verbs": {"No, I'm not": 1, "I feel": 1}, "adjectives": {"I'm alone": 1}, "numerals": {"2": 1}, "pronouns": {"I": 1}}}, {"content": "3. I am easily satisfied with the current plans and objectives and never pursue higher goals.", "word_count": 24, "label": "", "sentence_structure": "Subject鈥揷opula鈥損redicative", "subjective_objective": {"answer": "Subjective", "rationale": "Because the words \"easy to satisfy\" and \"no higher goals\" express a subjective attitude and idea of the patient."}, "sentence_sentiment": {"answer": "Negative", "rationale": "Because the \"no higher goal\" act is negative."}, "language_style": {"answer": "Tactful and warm", "rationale": ""}, "time_dimension": {"answer": "Present", "rationale": ""}, "sentence_type": "Negative declarative sentence", "pos": {"nouns": {"Planned": 1, "Objective": 2, "Seek": 1, "Higher": 1}, "verbs": {"I": 1, "Easy": 1, "Satisfied with": 1, "Never": 1, "Go on": 1}, "adverbs": {"Current": 1}, "numerals": {"3": 1}}}, {"content": "I sometimes feel tall and sometimes I feel small.", "word_count": 21, "label": "", "sentence_structure": "Subject鈥搗erb鈥揷omplement", "subjective_objective": {"answer": "Subjective", "rationale": "Because \"I feel tall\" and \"I feel small\" are the subjective feelings inside the patient."}, "sentence_sentiment": {"answer": "Neutral", "rationale": "Because the entry does not have a clear emotional orientation or evaluative language, it only describes the self-perception of the interviewees in different contexts."}, "language_style": {"answer": "Tactful and warm", "rationale": "The language style of the entry is more euphemistic and uses the expression \"sometimes feel\" to make the issue more subtle and gentle, to stimulate resonance and reflection among the respondents and to give them respect and understanding."}, "time_dimension": {"answer": "Present", "rationale": "The keyword \"sometimes feel very big\" and \"sometimes feel very small\" means that the testee is answering this question by describing the current feeling or state. So, the time dimension of this entry is now."}, "sentence_type": "Affirmative declarative sentence", "pos": {"nouns": {"I": 2, "self": 3, "Tall": 1, "Small": 1}, "verbs": {"I think": 2}, "adjectives": {"Sometimes": 2}}}, {"content": "5. When I have a close relationship with others, I have never felt afraid of losing.", "word_count": 25, "label": "", "sentence_structure": "Subject鈥揷opula鈥損redicative", "subjective_objective": {"answer": "Subjective", "rationale": "Because \"I've never felt like losing\" is the patient's subjective attitude and emotional experience."}, "sentence_sentiment": {"answer": "Positive", "rationale": "Because the entry expresses the psychological burden of no fear of loss after establishing a close relationship, it is a positive attitude and emotion."}, "language_style": {"answer": "Tactful and warm", "rationale": "The language style of the entry is euphemism. It uses the expression \"never feeling of loss\" , which is mild, not direct, and gives the impression of comfort and affection. In addition, positive words, such as \"close relationships\" are used in the entry, which makes the entire entry more inspiring."}, "time_dimension": {"answer": "Present", "rationale": "The entry is therefore about current behaviour and mindset."}, "sentence_type": "Affirmative declarative sentence", "pos": {"nouns": {"Relations": 1, "Feel": 1}, "verbs": {"Form": 1, "have": 1, "Lose": 1}, "adjectives": {"Close": 1, "Never": 1, "Fearless": 1}, "adverbs": {"Never": 2}, "pronouns": {"I": 1}}}, {"content": "I urgently need something that can be provided only by others.", "word_count": 16, "label": "", "sentence_structure": "Subject鈥搗erb鈥揷omplement", "subjective_objective": {"answer": "Subjective", "rationale": "Because \"urgently needed,\" \"what only others can offer\" is the patient's own feelings and expectations, a subjective description."}, "sentence_sentiment": {"answer": "Negative", "rationale": "Because the attitude of \"necessary need for something that only others can provide\" is negative."}, "language_style": {"answer": "Calm and direct", "rationale": "The language style of the entry is more straightforward, not using too many qualifiers or euphemisms, but rather presenting a fact directly. This expression is usually used in psychological questionnaires to obtain more realistic and accurate answers."}, "time_dimension": {"answer": "Present", "rationale": "In the entry, \"emergency\" is a strong demand or desire for something, a state of affairs, and therefore judgement is now. And \"what can be provided by someone else\" is meant to be directed to outsiders or things, which further underscores the current situation."}, "sentence_type": "Affirmative declarative sentence", "pos": {"nouns": {"person": 1, "Something": 1}, "verbs": {"Provision": 1, "Yes": 1}, "adjectives": {"Urgent": 1}, "pronouns": {"I": 1}}}, {"content": "I often find myself unable to act on my own standards or ideals.", "word_count": 19, "label": "", "sentence_structure": "Subject鈥搗erb鈥搊bject", "subjective_objective": {"answer": "Subjective", "rationale": "Because \"can't act on your own standards or ideals\" is a subjective feeling and experience."}, "sentence_sentiment": {"answer": "Negative", "rationale": "Because the act of \"can't act according to your own standards or ideals\" is negative."}, "language_style": {"answer": "Tactful and warm", "rationale": "The entry is more adept and interactive when it is spoken in a gentle and inviting manner, rather than by direct statements or orders."}, "time_dimension": {"answer": "Present", "rationale": "The keywords \"often\" and \"self\" do not indicate that it is a regular, customary act, which does not specify a specific point or time and can therefore be judged as now."}, "sentence_type": "Negative declarative sentence", "pos": {"nouns": {"Standard": 1, "Ideal": 1, "self": 2}, "verbs": {"I realized": 1, "Press": 1, "Act": 1}, "adverbs": {"Regular": 1}, "pronouns": {"I": 1}}}, {"content": "I feel that I can always reach my full potential.", "word_count": 15, "label": "", "sentence_structure": "Subject鈥揷opula鈥損redicative", "subjective_objective": {"answer": "Subjective", "rationale": "Because \"realizing your potential\" is self-evaluation and the feeling of the patient, it is a subjective approach."}, "sentence_sentiment": {"answer": "Positive", "rationale": "Because the entry \"Full your potential\" expresses personal confidence and motivation, and is a positive emotional attitude."}, "language_style": {"answer": "Tactful and warm", "rationale": ""}, "time_dimension": {"answer": "Present", "rationale": ""}, "sentence_type": "Affirmative declarative sentence", "pos": {"nouns": {"Questionnaire Entry": 1, "Potential": 1}, "verbs": {"I feel": 1, "Play": 1, "Yes": 2}, "adverbs": {"Total": 1}, "pronouns": {"I": 2}}}, {"content": "9. The lack of long-standing relationships does not give me any good reason to think.", "word_count": 15, "label": "", "sentence_structure": "Subject鈥揷opula鈥損redicative", "subjective_objective": {"answer": "Subjective", "rationale": "Because \"not to worry\" is the patient's subjective feeling and attitude."}, "sentence_sentiment": {"answer": "Neutral", "rationale": "Because the entry does not have any emotional color."}, "language_style": {"answer": "Tactful and warm", "rationale": "The language style of the entry is relatively euphemistic and warm. By using the expression \"do not worry me\" the possibility of outright denying or attacking others is avoided. At the same time, it is more friendly and acceptable."}, "time_dimension": {"answer": "Future", "rationale": "The keyword \"lack of a long-term relationship\" and \"without my care\" point to what may happen in the future. So, the entry asks if the testee is worried about the lack of long-term human relations in the future."}, "sentence_type": "Negative declarative sentence", "pos": {"nouns": {"Relations": 1, "Fear": 1}, "verbs": {"Missing": 1, "Jean": 1}, "adjectives": {"Long": 1, "Precautionary": 1}, "pronouns": {"I": 1}}}, {"content": "I'll feel worthless if I don't meet my expectations.", "word_count": 19, "label": "", "sentence_structure": "Subject鈥揷opula鈥損redicative", "subjective_objective": {"answer": "Subjective", "rationale": "Because it involves the patient ' s assessment of his or her self-worth, it is the psychological state and feelings of the individual and is therefore subjective."}, "sentence_sentiment": {"answer": "Negative", "rationale": "Because the expression \"will feel worthless\" in the entry is negative."}, "language_style": {"answer": "Calm and direct", "rationale": "The language style of the entry is relatively calm and straightforward, and the attitude or emotion of the interviewee when he or she encounters a failure to meet his or her expectations is expressed in a concise and clear statement. At the same time, the question of the entry is abstract, avoiding the use of too many specific situations or emotional colours, making it more universal and objective."}, "time_dimension": {"answer": "Present", "rationale": ""}, "sentence_type": "Negative declarative sentence", "pos": {"nouns": {"Value": 1, "Expectations": 1}, "verbs": {"Yes": 2, "I think": 1, "Achieved": 1, "have": 1}, "adjectives": {"Your own": 1}, "adverbs": {"If": 1, "No, I'm not": 1}, "numerals": {"10": 1}, "pronouns": {"I": 1}}}, {"content": "11. I feel helpless on many occasions.", "word_count": 11, "label": "", "sentence_structure": "Subject鈥揷opula鈥損redicative", "subjective_objective": {"answer": "Subjective", "rationale": "Because it's a subjective experience."}, "sentence_sentiment": {"answer": "Negative", "rationale": "Because the emotional experience of being alone in the entries is negative."}, "language_style": {"answer": "Tactful and warm", "rationale": "The language style of the entry is euphemism, because it uses a gentle and inviting tone to engage in dialogue, rather than a direct statement or order. At the same time, the expression \"single-free\" conveys a sense of concern and understanding, which is more in line with the attitude that psychological questionnaire researchers should have in conducting their investigations."}, "time_dimension": {"answer": "Present", "rationale": ""}, "sentence_type": "Negative declarative sentence", "pos": {"nouns": {"Time": 1, "I'm alone": 1}, "verbs": {"I think": 1}, "adjectives": {"Many": 1}, "numerals": {"11": 1}}}, {"content": "12. I have little fear that my words and actions will be taken against me.", "word_count": 15, "label": "", "sentence_structure": "Subject鈥搗erb鈥揷omplement", "subjective_objective": {"answer": "Objective", "rationale": "The content of the entry is an objective fact or description of the situation and does not involve subjective attitudes or feelings of the patient."}, "sentence_sentiment": {"answer": "Neutral", "rationale": "Because the entry is not clearly emotional."}, "language_style": {"answer": "Calm and direct", "rationale": "The language style of the entry is more calm and straightforward, with no excessive qualifier or rhetorical techniques, but rather a simple and clear expression of a fact or view. At the same time, this expression is more objective and neutral, and does not provoke feelings of resentment or resistance on the part of the person under investigation."}, "time_dimension": {"answer": "Present", "rationale": "The keyword \"very rarely\" suggests that it is a constant state of affairs or customary behaviour, and that \"fearing that one's words or actions will be wrong\" is a current-day act. The entry is therefore focused on the person's current state and feelings."}, "sentence_type": "Negative declarative sentence", "pos": {"verbs": {"I'm worried": 1, "Suffered": 1}, "adjectives": {"Your own": 1, "Less": 1}, "adverbs": {"very": 1}, "numerals": {"12": 1}, "classifiers": {"One": 1}, "pronouns": {"I": 1}}}, {"content": "There is a considerable distance between my current situation and my hopes.", "word_count": 21, "label": "", "sentence_structure": "Subject鈥揷opula鈥損redicative", "subjective_objective": {"answer": "Subjective", "rationale": "Because this phrase expresses the feelings and perceptions of patients between their current situation and expectations, it is a subjective examination."}, "sentence_sentiment": {"answer": "Negative", "rationale": "Because the description \"there's a considerable distance\" in the entry is negative."}, "language_style": {"answer": "Tactful and warm", "rationale": "The entry uses a more moderate language style, which is not directly critical or complaining, but rather expresses the respondent鈥檚 dissatisfaction and confusion in a euphemism. At the same time, the article is more balanced, with no excessive emotional overtones or strong subjective. Such expression is more likely to give the interviewee the sense of respect and understanding, and thus more likely to cooperate with the completion of the questionnaire."}, "time_dimension": {"answer": "Present", "rationale": ""}, "sentence_type": "Negative declarative sentence", "pos": {"nouns": {"Status": 1, "Hope": 1, "Distance": 1}, "verbs": {"have": 1, "in": 1, "be": 1}, "adjectives": {"It's quite a lot": 1}, "numerals": {"13": 1}, "pronouns": {"I": 1}}}, {"content": "14. I was happy in the fierce competition.", "word_count": 12, "label": "", "sentence_structure": "Subject鈥揷opula鈥損redicative", "subjective_objective": {"answer": "Subjective", "rationale": "Because feeling happy is a subjective experience and feeling."}, "sentence_sentiment": {"answer": "Positive", "rationale": "Because the attitude of \"happy in a fierce competition\" in the entry is positive."}, "language_style": {"answer": "Calm and direct", "rationale": "The entry presents a point in a concise and quick way, that is, happiness in a fierce competition. There is no excessive rhetorical or emotional rendering, with a more objective tone, with a direct expression, and without any emotional colour. It can therefore be considered as a calm and direct language style."}, "time_dimension": {"answer": "Present", "rationale": "Keywords include \"me,\" \"fEE\" and \"joy,\" which suggest that the person tested is in a state of mind at this point."}, "sentence_type": "Affirmative declarative sentence", "pos": {"nouns": {"Competition": 1, "Happy": 1}, "verbs": {"I feel": 1}, "adjectives": {"Strong": 1}, "numerals": {"14": 1}}}, {"content": "I feel that there are many responsibilities that must be assumed.", "word_count": 12, "label": "", "sentence_structure": "Subject鈥揷opula鈥損redicative", "subjective_objective": {"answer": "Subjective", "rationale": "Because the word \"thinks\" shows that it is a personal view and feeling of the patient, not an objective reality."}, "sentence_sentiment": {"answer": "Positive", "rationale": "Because the \"I feel obliged to take responsibility\" attitude in the entry is positive."}, "language_style": {"answer": "Tactful and warm", "rationale": "This language style makes it easier to establish good communication with the person under investigation."}, "time_dimension": {"answer": "Present", "rationale": "The keyword is \"the feeling\", which is the subjective feeling and the idea of the subject; \"many responsibilities\" suggests that these responsibilities may be present, so the entry is now."}, "sentence_type": "Affirmative declarative sentence", "pos": {"nouns": {"Responsibility": 1}, "verbs": {"I think": 1, "I have to": 2}, "adverbs": {"Many": 1}, "numerals": {"15": 1}}}, {"content": "Sometimes I feel 鈥渆mpty鈥?inside.", "word_count": 9, "label": "", "sentence_structure": "Subject鈥揷opula鈥損redicative", "subjective_objective": {"answer": "Subjective", "rationale": "Because feeling empty is a subjective emotional experience."}, "sentence_sentiment": {"answer": "Negative", "rationale": "Because the word \"empty\" in the entry is an expression of a negative emotion or feeling."}, "language_style": {"answer": "Tactful and warm", "rationale": "The entry is more personal and interactive when it is spoken in a moderate and concerned manner, rather than in direct statements or orders. At the same time, the word \"empty\" is surrounded by the use of quotation marks, emphasizing that it is a subjective feeling, not an objective fact, that makes the interviewee feel more comfortable and comfortable."}, "time_dimension": {"answer": "Present", "rationale": "The \"sometimes feel\" in the entry indicates that the testee is describing his current feelings, and thus judging the time dimension as now."}, "sentence_type": "Negative declarative sentence", "pos": {"nouns": {"Inside": 1, "Empty": 1}, "verbs": {"I feel": 1}, "adjectives": {"Sometimes": 1}}}, {"content": "17. I am not easily satisfied with the status quo.", "word_count": 8, "label": "", "sentence_structure": "Subject鈥搗erb鈥揷omplement", "subjective_objective": {"answer": "Subjective", "rationale": "Because unsatisfied is a subjective feeling and attitude."}, "sentence_sentiment": {"answer": "Negative", "rationale": "Because the attitude of \"not easy to satisfy the status quo\" in the entries is negative."}, "language_style": {"answer": "Tactful and warm", "rationale": "The language style of the article is between euphemism and calm. Although the issue expresses a negative view (i.e. the interviewee is not easily satisfied with the status quo), it does not use strong negative language or emotional expressions. Rather, it raises questions in a more neutral and peaceful way and invites the interviewee to share his views."}, "time_dimension": {"answer": "Present", "rationale": "The entry \"unsatisfactory to the status quo\" indicates that the testee is not satisfied with the current situation and indicates that the situation may continue and therefore is judged to be the present."}, "sentence_type": "Negative declarative sentence", "pos": {"verbs": {"Satisfied": 1, "Status": 1}, "adjectives": {"It's hard": 1}}}, {"content": "I do not care whether the demands of others are met.", "word_count": 14, "label": "", "sentence_structure": "Subject鈥搗erb鈥揷omplement", "subjective_objective": {"answer": "Subjective", "rationale": "Because \"not care whether or not other people's demands are met\" is a personal attitude or feeling, and therefore subjective."}, "sentence_sentiment": {"answer": "Negative", "rationale": "Because the entry \"not care whether or not other people's demands are met\" is negative."}, "language_style": {"answer": "Tactful and warm", "rationale": "The language style in the entry is more euphemistic, follows the expression \"I don't care\" and does not directly deny or attack others, but articulates its views in a more self-righteous and independent manner. Such language styles are more acceptable to respondents in the questionnaire, which helps to increase the rate and effectiveness of the questionnaire."}, "time_dimension": {"answer": "Present", "rationale": "The entry indicates the attitude of the interviewees to meeting the requirements of others, which is a current and present situation and should therefore be classified as \"now\"."}, "sentence_type": "Negative declarative sentence", "pos": {"verbs": {"Achieved": 1, "Request": 1}, "adjectives": {"I don't care": 1}, "pronouns": {"others": 1}}}, {"content": "I'm scared when I feel the silence.", "word_count": 12, "label": "", "sentence_structure": "Subject鈥揷opula鈥損redicative", "subjective_objective": {"answer": "Subjective", "rationale": "Because feeling lonely is a subjective feeling, panic is also the patient ' s own emotional reaction and is therefore subjective."}, "sentence_sentiment": {"answer": "Negative", "rationale": "Because the act of \"frightful when you feel lonely\" in the entry is negative."}, "language_style": {"answer": "Calm and direct", "rationale": "The entry directly describes the reactions that respondents may have when they feel lonely 鈥?panic, language styles that are relatively direct and objective."}, "time_dimension": {"answer": "Present", "rationale": "\"will\" means that the future is complete, indicating that it is a customary act, often occurring now. The words \"silent\" and \"crazed\" suggest that this is an emotional reaction, so the entry is mainly an inquiry into the current behaviour and emotional state of the person being tested."}, "sentence_type": "Affirmative declarative sentence", "pos": {"nouns": {"Silent": 1, "Panic": 1}, "verbs": {"Yes": 1, "Change": 1}, "adjectives": {"Panic": 1}, "adverbs": {"very": 1}, "pronouns": {"I": 1}}}, {"content": "20. If I lose a close friend, I feel like I have lost some important part of myself.", "word_count": 32, "label": "", "sentence_structure": "Subject鈥揷opula鈥損redicative", "subjective_objective": {"answer": "Subjective", "rationale": "Because losing a close friend is a subjective experience and feeling. While it is based on objective facts (i.e. the existence of friends), its impact and significance are generated in the individual ' s subjective sense."}, "sentence_sentiment": {"answer": "Negative", "rationale": "Because the expression in the entry \"lost a close friend, I feel like I've lost some important part of myself\" conveys a negative emotional attitude."}, "language_style": {"answer": "Tactful and warm", "rationale": "The language style of the entry is a warmer type, because it is not directly expressed in a strong emotional or harsh manner, but in a gentle and caring way."}, "time_dimension": {"answer": "Past", "rationale": "\"If you lose\" means it's hypothetical, and \"lost\" means it's something that's already happened. So, \"20. If you lose a close friend, I feel like I've lost some important part of myself.\" It describes the behavior or mentality of the past."}, "sentence_type": "Affirmative declarative sentence", "pos": {"nouns": {"friends": 1, "Self": 1}, "verbs": {"Lose": 2, "I think": 1, "Elephant": 1}, "adjectives": {"Intimate": 1, "Important": 1}, "adverbs": {"very": 1}, "pronouns": {"I": 1}}}, {"content": "21. No matter how many mistakes I have made, I will not be turned away.", "word_count": 20, "label": "", "sentence_structure": "Subject鈥搗erb鈥搊bject", "subjective_objective": {"answer": "Subjective", "rationale": "Because \"people won't shut me out\" is a subjective expectation or belief, not an objective fact."}, "sentence_sentiment": {"answer": "Positive", "rationale": "Because the phrase \"no one will shut me out\" in the entry is positive and expresses the attitude towards my acceptance and inclusion."}, "language_style": {"answer": "Tactful and warm", "rationale": "The language style of the entry is more euphemistic, as it uses an invitation-style tone, and does not directly reject or order the interviewee."}, "time_dimension": {"answer": "Present", "rationale": "And in the future, the entry expresses the testee's expectations and vision of what might happen in the future, in which \"no matter how many mistakes I made\" are past experiences and reflections, \"no one will turn me away\" is an imagination and a vision of the future. So, the dimension of time is the future."}, "sentence_type": "Negative declarative sentence", "pos": {"nouns": {"Error": 1, "person": 1, "Door": 1}, "verbs": {"Yes": 2, "Will": 1, "- I'm not going anywhere": 1}, "adverbs": {"Whatever": 1, "How much": 1}, "numerals": {"21": 1}, "pronouns": {"I": 1}}}, {"content": "22. It is difficult for me to break up the relationship that is displeased.", "word_count": 13, "label": "", "sentence_structure": "Subject鈥搗erb鈥揷omplement", "subjective_objective": {"answer": "Subjective", "rationale": "Because \"hard to break\" is a description of subjective feelings and attitudes, the result of the interviewee's self-perception."}, "sentence_sentiment": {"answer": "Negative", "rationale": "Because the entry describes \"the difficulty of breaking up\" and this is negative."}, "language_style": {"answer": "Calm and direct", "rationale": "The language style of the entry is straightforward, as it is presented in a clear question form, without any rhetorical or emotional resonance to the interviewee."}, "time_dimension": {"answer": "Present", "rationale": "The keyword \"hard to break\" suggests that it is a question of the present or recent behaviour or state of the person being tested, and that \"unhappy relationships\" are also a past or present situation. Thus, the time dimension of the article is now."}, "sentence_type": "Negative declarative sentence", "pos": {"nouns": {"Relations": 1, "It's not nice": 1, "Interrupt": 1}, "verbs": {"Hard": 1, "鈾?And I'm gonna be 鈾?: 1}, "adjectives": {"It's not pleasant": 1}, "adverbs": {"Hard": 1}, "numerals": {"22": 1}}}, {"content": "I'm often afraid of losing my close friends.", "word_count": 16, "label": "", "sentence_structure": "Subject鈥揷opula鈥損redicative", "subjective_objective": {"answer": "Subjective", "rationale": "Because \"care\" is a subjective sentiment."}, "sentence_sentiment": {"answer": "Negative", "rationale": "Because the emotional expression \"fearing of losing a close friend\" in the entry is negative."}, "language_style": {"answer": "Tactful and warm", "rationale": "The entry is expressed in a more moderate and euphemistic language, which does not directly address or command the other party to answer questions, but rather leads the other side to think and respond in an invitation and concern manner. Such language styles are more resonant with the other side and contribute to a good atmosphere of interaction."}, "time_dimension": {"answer": "Present", "rationale": ""}, "sentence_type": "Affirmative declarative sentence", "pos": {"nouns": {"Questionnaire Entry": 1, "23": 1, "Danger": 1, "Close friends": 1}, "verbs": {"I'm worried": 1, "have": 1, "Lose": 1}, "adjectives": {"Often": 1, "of": 2}, "numerals": {"23": 1}, "pronouns": {"I": 1}}}, {"content": "24. I was being asked too much.", "word_count": 8, "label": "", "sentence_structure": "Subject鈥揷opula鈥損redicative", "subjective_objective": {"answer": "Subjective", "rationale": "Because the idea that \"others are asking me too much\" is the person who was interviewed's own feelings and perceptions, and it's a subjective view."}, "sentence_sentiment": {"answer": "Negative", "rationale": "Because the phrase \"others are asking too much of me\" in the entry is negative emotional."}, "language_style": {"answer": "Tactful and warm", "rationale": "The language style of the entry is euphemistic. It uses more moderate language to express the interest and understanding of the subject, without direct blame or criticism. It is more likely to create a good communication climate and make the subject more willing to share his thoughts and feelings."}, "time_dimension": {"answer": "Present", "rationale": ""}, "sentence_type": "Negative declarative sentence", "pos": {"nouns": {"others": 1, "I": 1, "Request": 1, "Standard": 1}, "verbs": {"to": 1, "Too high": 1}, "adjectives": {"High": 1}, "numerals": {"24": 1}}}, {"content": "With others, I can easily underestimate or 鈥渟old鈥?myself.", "word_count": 16, "label": "", "sentence_structure": "Subject鈥搗erb鈥揷omplement", "subjective_objective": {"answer": "Subjective", "rationale": "Because \"basket\" or \"underestimation\" are the feelings and attitudes of the patients themselves, a subjective description."}, "sentence_sentiment": {"answer": "Negative", "rationale": "Because the attitude of \"underestimating or \"selling yourself\" in the entry is negative."}, "language_style": {"answer": "Tactful and warm", "rationale": ""}, "time_dimension": {"answer": "Present", "rationale": "The term \"easy to underestimate or \"sell yourself cheap\" in this entry describes the current state and behaviour of the testee, and therefore the temporal dimension is now judged."}, "sentence_type": "Negative declarative sentence", "pos": {"nouns": {"others": 1, "Together": 1, "self": 1}, "verbs": {"Easy": 1, "Underestimation": 1, "Cheap": 1}, "adverbs": {"Call": 1}, "numerals": {"25": 1}}}, {"content": "I do not care much how people repay.", "word_count": 11, "label": "", "sentence_structure": "Subject鈥搗erb鈥揷omplement", "subjective_objective": {"answer": "Subjective", "rationale": "Because the words \"not care\" and \"how to repay\" reflect the patient's subjective attitude."}, "sentence_sentiment": {"answer": "Neutral", "rationale": "The entry describes the attitude of a person who is less concerned with the way others are repaying them, and who is not clearly motivated by positive or negative emotional tendencies."}, "language_style": {"answer": "Calm and direct", "rationale": "The entry expresses the subject of questioning the other party ' s attitude to return in a direct presentation, with no excessive rhetorical or emotional rendering, and is more objective in tone."}, "time_dimension": {"answer": "Present", "rationale": "\"Not much care\" indicates the attitude and behaviour of the testee, which is ongoing when speaking, and therefore the temporal dimension is now judged."}, "sentence_type": "Negative declarative sentence", "pos": {"verbs": {"I care": 1, "Refund": 1}, "adjectives": {"Not much": 1}, "pronouns": {"I": 1}}}, {"content": "27. There will be friction and conflict between the two people, however close they may be.", "word_count": 20, "label": "", "sentence_structure": "Subject鈥揷opula鈥損redicative", "subjective_objective": {"answer": "Objective", "rationale": "Because \"relationship\" and \"relationship and conflict\" are objective facts that are not influenced by patients' subjective attitudes."}, "sentence_sentiment": {"answer": "Negative", "rationale": "Because the act of \"there will be friction and conflict\" in the entry is negative."}, "language_style": {"answer": "Tactful and warm", "rationale": "This entry is a simple and neutral entry, which does not express criticism or praise directly, but is an objective statement of a widespread phenomenon."}, "time_dimension": {"answer": "Present", "rationale": "The word \"yes\" indicates that the current state of affairs is complete, that the act has occurred in the past and has an impact on the present. Thus, the temporal dimension of the sentence is now judged."}, "sentence_type": "Affirmative declarative sentence", "pos": {"nouns": {"Two": 1, "Relations": 1, "Grid": 1, "Conflict": 1}, "verbs": {"have": 1, "Whatever": 1}, "adverbs": {"How": 1}, "numerals": {"27": 1}}}, {"content": "I am very sensitive to the suggestion that someone else has rejected.", "word_count": 14, "label": "", "sentence_structure": "Subject鈥揷opula鈥損redicative", "subjective_objective": {"answer": "Subjective", "rationale": "Because sensitivity is the patient ' s subjective feeling and attitude."}, "sentence_sentiment": {"answer": "Negative", "rationale": "Because the expression \"very sensitive to the suggestion that someone else has rejected\" has negative emotions and attitudes."}, "language_style": {"answer": "Tactful and warm", "rationale": "The entry is more adept and interactive when it is spoken in a gentle and inviting manner, rather than by direct statements or orders."}, "time_dimension": {"answer": "Present", "rationale": "The entry describes the current behaviour or feelings of the individual, i.e., \"I\" is very sensitive to the suggestion that someone else has rejected, so the temporal dimension is now."}, "sentence_type": "Affirmative declarative sentence", "pos": {"nouns": {"I": 1, "person": 1, "- And then they turned it down": 1, "Insinuations": 1}, "verbs": {"to": 1, "Sensitive": 1, "Reject": 1}, "adjectives": {"Very": 1, "Sensitive": 1}, "numerals": {"28": 1}}}, {"content": "29. My success is important for the family.", "word_count": 10, "label": "", "sentence_structure": "Subject鈥揷opula鈥損redicative", "subjective_objective": {"answer": "Subjective", "rationale": "Because success, family is what patients think is important."}, "sentence_sentiment": {"answer": "Positive", "rationale": "Because \"success\" and \"important\" are positive emotional terms in the entries and emphasize the close connection between individual success and family."}, "language_style": {"answer": "Tactful and warm", "rationale": ""}, "time_dimension": {"answer": "Present", "rationale": "The entry expresses the idea that the dominant language considers its success to be very important, that this attitude is subjective, that it is present and that it relates to individual values and objectives, and that it can therefore be judged as \"now\"."}, "sentence_type": "Affirmative declarative sentence", "pos": {"nouns": {"Success": 1, "Family": 1}, "verbs": {"It's... it's important": 1}, "numerals": {"29": 1}}}, {"content": "I often feel disappointed.", "word_count": 11, "label": "", "sentence_structure": "Subject鈥揷opula鈥損redicative", "subjective_objective": {"answer": "Subjective", "rationale": "Because \"I think\" is a subjective feeling and view, which reflects the self-evaluation and emotional state of the patient's heart."}, "sentence_sentiment": {"answer": "Negative", "rationale": "Because the \"frightful\" attitude in the entry is negative."}, "language_style": {"answer": "Tactful and warm", "rationale": "The entry uses a more euphemistic and moderate tone to ask if the other side often feels disappointed. It does not directly point to a question or order a certain behaviour on the part of the respondent, but rather engages in a more encouraging and interactive tone of conversation. This expression makes it easier for the respondent to relax and to share his real feelings."}, "time_dimension": {"answer": "Present", "rationale": "The entry \"I often feel\" expresses a constant feeling and mentality that is occurring at the moment and may have existed for some time. So, the entry relates to the current behavior or mentality.\""}, "sentence_type": "Negative declarative sentence", "pos": {"nouns": {"self": 1, "Disappointment": 1}, "verbs": {"I think": 1, "You're": 1, "Disappointment": 1}, "adverbs": {"Often": 1}, "numerals": {"30": 1}, "pronouns": {"I": 1}}}, {"content": "31. When someone else pisses me off, I let him know how I feel.", "word_count": 19, "label": "", "sentence_structure": "Subject鈥搗erb鈥搊bject", "subjective_objective": {"answer": "Subjective", "rationale": "Because \"to let others know what they feel\" is a subjective attitude or reaction that patients may take in the face of conflict. This depends on the patient鈥檚 personality, how they manage their emotions, and how they perceive the situation and what they expect."}, "sentence_sentiment": {"answer": "Positive", "rationale": "Because the behavior in the entry \"Let someone know how they feel\" expresses positive attitudes and behavior."}, "language_style": {"answer": "Calm and direct", "rationale": "The entry is based on a simple and direct expression of the question whether the other party will be willing to inform the other party of the subject of his or her feelings at this point in the encounter with the other person鈥檚 anger. The tone is not based on too many rhetorical or emotional overtones, and is expressed in a more objective and straightforward way, which is a straightforward way of calming."}, "time_dimension": {"answer": "Future", "rationale": "The word \"will\" denotes a future action or state, so the temporal dimension of the article is the future."}, "sentence_type": "Affirmative declarative sentence", "pos": {"nouns": {"others": 1, "Fire": 1, "he": 1, "she": 1, "Feel": 1}, "verbs": {"Fuck": 1, "Yes": 3, "Jean": 1}, "adverbs": {"When": 1, "Time": 1, "very": 1}, "numerals": {"31": 1}, "pronouns": {"I": 1}}}, {"content": "32. I have been constant and have spared no effort to please or help those around me.", "word_count": 19, "label": "", "sentence_structure": "Subject鈥搗erb鈥揷omplement", "subjective_objective": {"answer": "Subjective", "rationale": "Because \"please or help the people around\" are subjective behaviour and attitudes of the patient, the character and motivation of the patient are examined."}, "sentence_sentiment": {"answer": "Positive", "rationale": "Because the quality of the act \"continuously, unstintingly, unstintingly, to please or help the people around\" in the entry is positive."}, "language_style": {"answer": "Tactful and warm", "rationale": "The entry is more personal and interactive in its expression, in a gentle and inviting tone, rather than in direct statements or orders. Moreover, it uses positive terms (such as \"continuous\", \"exhaustible\" and \"pleasure\" and \"help\"), which demonstrate the author's desire for positive and optimistic answers from the interviewees."}, "time_dimension": {"answer": "Past", "rationale": "\"I am persistent\" and \"resistent\" indicate that this act has been and continues for some time, and is therefore judged to be past and present."}, "sentence_type": "Affirmative declarative sentence", "pos": {"nouns": {"Questionnaire": 1, "Entry": 1, "person": 1}, "verbs": {"Always": 1, "To please": 1, "Help": 1, "Make your best effort": 1}, "adjectives": {"Around": 1}, "pronouns": {"I": 1}}}, {"content": "I am energetic (capable, powerful).", "word_count": 9, "label": "", "sentence_structure": "Subject鈥揷opula鈥損redicative", "subjective_objective": {"answer": "Subjective", "rationale": "Because of the energy that people have, they are subjectively aware of and evaluate their own situation."}, "sentence_sentiment": {"answer": "Positive", "rationale": "Because \"energy\" in the entry is a positive character or state of the individual."}, "language_style": {"answer": "Tactful and warm", "rationale": "The entry is more euphemistic in its language style, because it uses an invitation tone to make interviewees feel respected and cared for. At the same time, it uses positive terms, such as \"energy\" to create a positive atmosphere and encourage respondents to share their feelings and ideas."}, "time_dimension": {"answer": "Past", "rationale": "The entry asks the testee about his current state of feeling and time dimension, which is now judged. Although the word \"pleasant\" has some persistence, it still describes the current state, not past experience or future expectations."}, "sentence_type": "Affirmative declarative sentence", "pos": {"nouns": {"Questionnaire": 1, "Entry": 1, "- The energy": 1, "Capacity": 1, "Power": 1}, "verbs": {"鈾?Full of 鈾?: 1}, "numerals": {"33": 1}}}, {"content": "34: I find it difficult to say no to friends' requests.", "word_count": 13, "label": "", "sentence_structure": "Subject鈥搗erb鈥搊bject", "subjective_objective": {"answer": "Subjective", "rationale": "As the sentence relates to personal feelings and attitudes, it is viewed from a subjective perspective."}, "sentence_sentiment": {"answer": "Negative", "rationale": "Because the \"no\" act in the entry is negative to a friend's request."}, "language_style": {"answer": "Tactful and warm", "rationale": "The language style of the entry is more euphemistic and thoughtful, and the difficulty of asking the other side whether it is difficult to refuse a friend ' s request in a more moderate manner is a more acceptable expression and is willing to answer the question."}, "time_dimension": {"answer": "Present", "rationale": ""}, "sentence_type": "Negative declarative sentence", "pos": {"nouns": {"friends": 1, "Request": 1}, "verbs": {"I realized": 1, "Yes": 1, "Reject": 1}, "adjectives": {"Hard": 1}, "adverbs": {"very": 1}, "numerals": {"34": 1}}}, {"content": "35. I will never feel truly secure in a close relationship.", "word_count": 19, "label": "", "sentence_structure": "Subject鈥揷opula鈥損redicative", "subjective_objective": {"answer": "Subjective", "rationale": "Because the subject is asked about the patient ' s feelings and attitudes, it is examined from a subjective point of view."}, "sentence_sentiment": {"answer": "Negative", "rationale": "Because the phrase \"never really feel safe\" in the entry is negative."}, "language_style": {"answer": "Tactful and warm", "rationale": "The language style of the entry is relatively euphemistic, soft, and it avoids too direct or too strong a expression, giving comfort and peace of approach. At the same time, it uses an open-ended question-and-answer format that gives more space for speech and thought."}, "time_dimension": {"answer": "Present", "rationale": "The keyword \"I will never\" and \"I really feel safe\" indicates that it is a emotional experience and feeling, so the entry is a current state of affairs."}, "sentence_type": "Negative declarative sentence", "pos": {"nouns": {"Relations": 1, "Clear": 1}, "verbs": {"Yes": 1, "I feel": 1, "Never": 1}, "adjectives": {"Close": 1}, "adverbs": {"Really": 1}, "numerals": {"35": 1}}}, {"content": "36. I often change my own perception of myself, sometimes feel perfect, sometimes see my own shortcomings and feel my own incompetence.", "word_count": 39, "label": "", "sentence_structure": "Subject鈥揷opula鈥損redicative", "subjective_objective": {"answer": "Subjective", "rationale": "Because \"the perception of self changes\" and \"the feeling of perfection or nothing\" are personal feelings and thoughts of the patient and are subjective."}, "sentence_sentiment": {"answer": "Neutral", "rationale": "Because the entry does not express a clear positive or negative emotional expression, it describes a change in a person ' s perception of himself."}, "language_style": {"answer": "Tactful and warm", "rationale": "The language style of the entry is euphemism. The words \"often change\" and \"sometimes feel\" are used to express views and feelings, rather than simply \"always\" or \"totally wrong\" , which is more moderate, inclusive and respectful and which better guides respondents to share their true thoughts."}, "time_dimension": {"answer": "Present", "rationale": "The perspective and attitude of \"I\" are a description of the current situation, and the keywords are \"views of ...\" and \"often changes\" and all point to the current state."}, "sentence_type": "Affirmative declarative sentence", "pos": {"nouns": {"Observations": 1, "self": 2, "Perfect": 1, "Insufficient": 1, "Nothing": 1}, "verbs": {"Change": 1, "I feel": 2, "Yeah": 1, "I think": 3}, "adjectives": {"Often": 1, "Perfect": 1, "Insufficient": 1, "Nothing": 1}, "numerals": {"36": 1}, "pronouns": {"I": 2}}}, {"content": "37. I am often afraid of changing circumstances.", "word_count": 10, "label": "", "sentence_structure": "Subject鈥搗erb鈥揷omplement", "subjective_objective": {"answer": "Objective", "rationale": "Because change of circumstances is an objective fact, fear is the subjective feeling of the patient of that objective fact."}, "sentence_sentiment": {"answer": "Negative", "rationale": "Because the mood of fear in the entries is negative."}, "language_style": {"answer": "Tactful and warm", "rationale": "The entry uses the descriptive language \"I am often afraid of changing circumstances\" rather than directly blaming or evaluating the other. At the same time, the \"sweet\" language style emphasizes communication and understanding with the respondents, which is more conducive to good interaction and to a higher rate of recovery of questionnaires."}, "time_dimension": {"answer": "Present", "rationale": "Now the keyword \"always\" denotes a regular, customary behaviour, and \"fearful of changing circumstances\" describes an emotional state, which suggests that the testees, in answering this question, describe their current or recent feelings. The entry is therefore to ask the testee about his current behaviour and mental state."}, "sentence_type": "Negative declarative sentence", "pos": {"nouns": {"Situation": 1, "Change": 1, "Fear": 1}, "verbs": {"Common cause": 1, "Fear": 1}, "adverbs": {"Common cause": 2}, "numerals": {"37": 1}}}, {"content": "38. Even if the closest are about to leave, I will be able to live on my own.", "word_count": 22, "label": "", "sentence_structure": "Subject鈥搗erb鈥搊bject", "subjective_objective": {"answer": "Subjective", "rationale": "Because it is a subjective assessment of the patient ' s abilities and attitude to his or her abilities."}, "sentence_sentiment": {"answer": "Negative", "rationale": "Because the situation in the entry, \"even if the closest person is leaving,\" is negative, and the subsequent act, \"I can live on my own,\" expresses an attitude of independent living, which may be positive, but it may also be a negative response that is forced to be taken because of lack of emotional support."}, "language_style": {"answer": "Tactful and warm", "rationale": "The entry expresses understanding and support for the interviewee in a more moderate and encouraging way, rather than directly questioning or criticizing. Such language styles can reduce the tension and anxiety of the interviewee, increase his/her participation and trust."}, "time_dimension": {"answer": "Past", "rationale": "The entry describes a situation or attitude and uses past tenses to express it, and therefore to judge it as past."}, "sentence_type": "Affirmative declarative sentence", "pos": {"nouns": {"person": 1, "Let's go": 1, "Life": 2}, "verbs": {"Yes": 1, "self": 1, "Get down": 1}, "adjectives": {"The closest": 1}, "adverbs": {"Same thing": 1}, "numerals": {"38": 1}, "pronouns": {"I": 1}}}, {"content": "39. People must persevere in their pursuit of the love of others: that is, love must be fought for.", "word_count": 24, "label": "", "sentence_structure": "Subject鈥損redicate", "subjective_objective": {"answer": "Subjective", "rationale": "Because the title refers to \"must\", which is an expression of subjective attitudes and beliefs."}, "sentence_sentiment": {"answer": "Positive", "rationale": "Because it expresses positive attitudes and behaviour, namely, \"suspending to love\" and \"to love\"."}, "language_style": {"answer": "Tactful and warm", "rationale": "The language style of the entry is more euphemistic, not using direct orders or threats to express its views, but rather using a more moderate and inviting tone, trying to stimulate the thoughts and resonance of the respondents and encouraging them to actively pursue the love of others. Moreover, the words \"continuous\" and \"promoting\" have a positive motivation and inspirational character, making the language style of the article more positive."}, "time_dimension": {"answer": "Present", "rationale": "The entry now describes a general perception in modern society that people need to work constantly to get love and love from others. This emphasis is on proactive attitudes and actions to get a sense of satisfaction and happiness. Thus, the temporal dimension is \"now\"."}, "sentence_type": "Affirmative declarative sentence", "pos": {"nouns": {"person": 1, "Love": 2, "Hold on": 1, "Seek": 1, "Say it": 1, "Fight": 1}, "verbs": {"I have to": 1, "I'm not going to let you go": 1, "Yeah": 1, "Fight": 1}, "adverbs": {"I have to": 1, "And you keep on": 1}, "numerals": {"39": 1}}}, {"content": "40. I am particularly sensitive to the feelings of others about their words and deeds.", "word_count": 16, "label": "", "sentence_structure": "Subject鈥揷opula鈥損redicative", "subjective_objective": {"answer": "Subjective", "rationale": "Because this is a description of what the patient feels for himself, it is a subjective view and emotional experience."}, "sentence_sentiment": {"answer": "Negative", "rationale": "Because the entry \"specially sensitive to others' feelings of their own words and deeds\" is negative."}, "language_style": {"answer": "Tactful and warm", "rationale": "The language style of the entry is more euphemistic and implicit, using the expression of inquiry rather than direct statements, which is more resonant with the interviewee and increases his participation. At the same time, the description \"specially sensitive to the feelings of others about their words and deeds\" is presented in a more objective and neutral way, avoiding the use of overly subjective or biased language."}, "time_dimension": {"answer": "Present", "rationale": "The keywords \"I,\" \"I'm very sensitive to others\" and \"I'm particularly sensitive to my own words and actions\" indicate that the entry is describing the current attitudes and behaviour of the subjects."}, "sentence_type": "Affirmative declarative sentence", "pos": {"nouns": {"Others": 1, "self": 1, "Words and lines": 1, "Feel": 1, "Sensitive": 1}, "verbs": {"to": 1, "Special": 1, "Sensitive": 1}, "adverbs": {"Special": 1}, "numerals": {"40": 1}, "classifiers": {"individual": 1}}}, {"content": "41. I often feel guilty for what I have said and done.", "word_count": 11, "label": "", "sentence_structure": "Subject鈥揷opula鈥損redicative", "subjective_objective": {"answer": "Subjective", "rationale": "Because guilt is the patient ' s subjective assessment and perception of himself, not the objective facts observed."}, "sentence_sentiment": {"answer": "Negative", "rationale": "Because guilt is a negative sentiment."}, "language_style": {"answer": "Tactful and warm", "rationale": "The entry is more personal and interactive in its expression of a moderate, caring and supportive expression of concern for the person under investigation, rather than directly criticizing or accusing him."}, "time_dimension": {"answer": "Past", "rationale": ""}, "sentence_type": "Affirmative declarative sentence", "pos": {"nouns": {"Behaviour": 1, "self": 1, "Guilt": 1}, "verbs": {"Because": 1, "Regular": 1, "And": 1, "I feel": 1}, "adverbs": {"Regular": 1, "And": 1}, "numerals": {"41": 1}, "pronouns": {"I": 1}}}, {"content": "42. I am a very independent person.", "word_count": 10, "label": "", "sentence_structure": "Subject鈥揷opula鈥損redicative", "subjective_objective": {"answer": "Subjective", "rationale": "Because independence is the subjective characteristic and self-evaluation of an individual, one person has a perception and perception of one ' s own."}, "sentence_sentiment": {"answer": "Positive", "rationale": "Because the description of \"independent\" in the entry is positive."}, "language_style": {"answer": "Calm and direct", "rationale": "The language style of the entry is calm and straightforward. It directly illustrates the core of the problem 鈥?whether the subject is independent and does not use too much embellishment or emotional expression. This is simple and easy to understand."}, "time_dimension": {"answer": "Present", "rationale": "The keyword \"Yes\" indicates that the person being tested is describing his or her character characteristics and is a statement of the current situation."}, "sentence_type": "Affirmative declarative sentence", "pos": {"nouns": {"person": 1}, "verbs": {"be": 1, "have": 1}, "adjectives": {"Independence": 1, "Strong": 1}, "numerals": {"42": 1}, "pronouns": {"I": 1}}}, {"content": "43. I often feel guilty.", "word_count": 6, "label": "", "sentence_structure": "Subject鈥搗erb鈥揷omplement", "subjective_objective": {"answer": "Subjective", "rationale": "This statement relates to self-evaluation and feelings of patients and is a subjective examination."}, "sentence_sentiment": {"answer": "Negative", "rationale": "Because the feeling of guilt is negative."}, "language_style": {"answer": "Calm and direct", "rationale": "The language style of the entry is calm and straightforward. It is simple to ask the interviewees whether they have often felt guilty, without using too many qualifiers or emotional colours, and in a more objective and direct way."}, "time_dimension": {"answer": "Past", "rationale": "The \"common sense\" in this entry indicates a constant, customary psychological state, while \"guilty\" is a subjective feeling, describing the feelings that the person tested has had for some time. Thus, the temporal dimension of the article is judged by the past."}, "sentence_type": "Negative declarative sentence", "pos": {"verbs": {"I": 1, "Common sense": 1, "Guilty": 1}, "adverbs": {"Regular": 1}, "numerals": {"43": 1}}}, {"content": "I think I'm a very complex person, a person with a 鈥渕ulti-faceted鈥?dimension.", "word_count": 21, "label": "", "sentence_structure": "Subject鈥揷opula鈥損redicative", "subjective_objective": {"answer": "Subjective", "rationale": "Because the phrase \"I feel\" is about the patient's subjective feelings and self-awareness."}, "sentence_sentiment": {"answer": "Neutral", "rationale": "The entry does not express any positive or negative emotional connotations, but rather describes the individual ' s perception and understanding of himself, without any apparent emotional tendencies."}, "language_style": {"answer": "Tactful and warm", "rationale": "This is a more moderate, encouraging and interactive way of developing a good communication environment."}, "time_dimension": {"answer": "Present", "rationale": "The keywords \"want,\" \"complex\" and \"multi-faceted\" all indicate that this is the current feeling and understanding of the testees."}, "sentence_type": "Affirmative declarative sentence", "pos": {"nouns": {"Questionnaire Entry": 1, "person": 2, "Side": 1}, "verbs": {"Yes": 2, "be": 1}, "adjectives": {"Complex": 1, "Multiple": 1}, "numerals": {"44": 1}, "pronouns": {"I": 1}}}, {"content": "I am very worried that I will offend or harm those close to me.", "word_count": 17, "label": "", "sentence_structure": "Subject鈥搗erb鈥揷omplement", "subjective_objective": {"answer": "Subjective", "rationale": "Fear is a subjective feeling, and it is closely related to the emotions and attitudes inside the individual. Even if some of the fears may be based on objective facts, the fear itself remains a subjective experience."}, "sentence_sentiment": {"answer": "Negative", "rationale": "Because the attitude in the entry, \"A fear of offending or hurting\", is negative."}, "language_style": {"answer": "Tactful and warm", "rationale": "The entry raises questions in an understanding and concern for the feelings of the other, expressing concern and understanding of the problems that the interviewees may cause to close people, and the language style is more euphemism and warmer."}, "time_dimension": {"answer": "Present", "rationale": "\"I'm very worried\" indicates that the testee is experiencing this emotional state of concern, and this is an assessment of the current situation. The questionnaire entry is therefore asking the testee about his current behaviour and mental state."}, "sentence_type": "Negative declarative sentence", "pos": {"nouns": {"Questionnaire": 1, "Entry": 1, "I'm sorry": 1, "Hurt": 1, "person": 2}, "verbs": {"I'm worried": 1, "Yes": 1, "I'm sorry": 1, "Hurt": 1}, "adjectives": {"Tens": 1, "Close": 1}, "numerals": {"45": 1}, "pronouns": {"I": 2}}}, {"content": "46. I would be frightened by anger.", "word_count": 9, "label": "", "sentence_structure": "Subject鈥揷opula鈥損redicative", "subjective_objective": {"answer": "Subjective", "rationale": "Because \"I'm scared\" is a patient's sense of feeling and subjective emotional reaction. It reflects the patient's inner experience and emotional state, which is a subjective approach."}, "sentence_sentiment": {"answer": "Negative", "rationale": "Because the \"fright will panic me\" act in the entry is negative."}, "language_style": {"answer": "Calm and direct", "rationale": "The entry describes the consequences of anger in simple and clear language, with no excessive rhetorical or emotional rendering, and a more objective tone. The language style is therefore a straightforward, calm expression."}, "time_dimension": {"answer": "Present", "rationale": ""}, "sentence_type": "Affirmative declarative sentence", "pos": {"nouns": {"Anger": 1, "Frightened": 1, "I'm lost": 1}, "verbs": {"Let's go": 1, "鈾?And I'm gonna be 鈾?: 1}, "numerals": {"46": 1}}}, {"content": "47. What matters is not your identity, but your achievements.", "word_count": 18, "label": "", "sentence_structure": "Subject鈥揷opula鈥損redicative", "subjective_objective": {"answer": "Objective", "rationale": "Because it's subjective to think that \"it's not your identity that matters, but your achievements,\" but it's objective to say that \"your identity\" and \"your achievements\" exist."}, "sentence_sentiment": {"answer": "Positive", "rationale": "The emphasis in the entries on individual achievements can inspire motivation and motivation."}, "language_style": {"answer": "Calm and direct", "rationale": "The entry expresses the purpose of the survey in a concise and clear manner, without too much qualifier or emotional colour, and with a direct and clear tone."}, "time_dimension": {"answer": "Present", "rationale": "The sentence is a statement of a view that does not address past or future acts or events. The keyword \"and\" is not a \"show that the speaker has emphasized \"achieving\" now, not \"identity.\" The entry is therefore about the present situation."}, "sentence_type": "Affirmative declarative sentence", "pos": {"nouns": {"Identity": 1, "Achievements": 1}, "verbs": {"Important": 1, "be": 2, "Access": 1}, "adverbs": {"not": 1, "of": 3}, "numerals": {"47": 1, "you": 2}, "pronouns": {"you": 2}}}, {"content": "48. I feel good whether I have succeeded or failed.", "word_count": 14, "label": "", "sentence_structure": "Subject鈥搗erb鈥揷omplement", "subjective_objective": {"answer": "Subjective", "rationale": "Because \"fears well\" is a subjective feeling and attitude, not an objective fact or result."}, "sentence_sentiment": {"answer": "Positive", "rationale": "Because it expresses a positive attitude that can be maintained both in terms of success and failure."}, "language_style": {"answer": "Tactful and warm", "rationale": "The entry is more euphemistic, using the term \"frightful\" to express positive emotional attitudes. Nor is it too direct or intense to give people a sense of comfort. So I think it is a euphemism."}, "time_dimension": {"answer": "Present", "rationale": ""}, "sentence_type": "Affirmative declarative sentence", "pos": {"verbs": {"Feel": 2, "Success": 1, "Failed": 1}, "adjectives": {"Good": 1}, "numerals": {"48": 1}}}, {"content": "49. I can easily put aside my feelings and problems and give full attention to others.", "word_count": 31, "label": "", "sentence_structure": "Subject鈥搗erb鈥搊bject", "subjective_objective": {"answer": "Subjective", "rationale": "Because the words \"easy\" and \"cared with all intent\" in the title indicate the patient鈥檚 own attitude and emotional orientation. The subjective attitude of the patient is subjective."}, "sentence_sentiment": {"answer": "Positive", "rationale": "Because the behaviour in the entries is positive, it expresses concern and concern for others."}, "language_style": {"answer": "Tactful and warm", "rationale": "The entry uses euphemism to ask the other person whether he or she is easily concerned about the feelings and problems of the other person, rather than asking the other person directly whether there are problems or if he or she needs help. This way makes people feel more friendly and respectful and more likely to get real answers."}, "time_dimension": {"answer": "Present", "rationale": "The word \"I'm Easy\" means \"a high frequency, constant behavior, \"a concern about the feelings and problems of others\" indicates that this behavior is not only happening in the past, but may still be going on. So, the entry describes the current behavior and attitude."}, "sentence_type": "Affirmative declarative sentence", "pos": {"nouns": {"Questionnaire": 1, "Entry": 1, "Feel": 2, "Problem": 2, "self": 1, "others": 1}, "verbs": {"Fire": 1, "Care": 1}, "adjectives": {"All in mind": 1}, "adverbs": {"very": 1, "Land": 2}, "numerals": {"49": 1}, "pronouns": {"I": 1}}}, {"content": "50. If a man of my concern is angry at me, I fear he will leave me.", "word_count": 24, "label": "", "sentence_structure": "Subject鈥揷opula鈥損redicative", "subjective_objective": {"answer": "Subjective", "rationale": "This is because the patient ' s fears and thoughts about the possible reactions and behaviour of others are a manifestation of subjective attitudes."}, "sentence_sentiment": {"answer": "Negative", "rationale": "Because the expression \"so worried he'd leave me\" in the entry is a negative emotional attitude."}, "language_style": {"answer": "Tactful and warm", "rationale": "This entry is more euphemistic and gentle in language style by describing a possible situation and expressing concerns about the outcome. This expression is more resonant and understanding and facilitates the effectiveness of the questionnaire."}, "time_dimension": {"answer": "Present", "rationale": "The entry describes the fear and fear of a person who is \"my concern\" when he is angry with his voice. It is a description of the current or recent situation, so the time dimension is now."}, "sentence_type": "Affirmative declarative sentence", "pos": {"nouns": {"person": 1, "I": 2, "he": 2, "Fire": 1, "Off": 1, "Go on": 1}, "verbs": {"Yes": 1, "Charge": 1, "Let's go": 2, "I'm worried": 1}, "adverbs": {"Suppose": 1, "Of concern": 1}, "numerals": {"50": 1}}}, {"content": "51. I feel uncomfortable when I have to take on important responsibilities.", "word_count": 16, "label": "", "sentence_structure": "Subject鈥揷opula鈥損redicative", "subjective_objective": {"answer": "Subjective", "rationale": "Because feeling uncomfortable is a subjective feeling or emotional reaction."}, "sentence_sentiment": {"answer": "Negative", "rationale": "Because the emotional reaction of \"frightful\" in the entries is negative."}, "language_style": {"answer": "Tactful and warm", "rationale": "The entry is more adept and interactive when it is spoken in a gentle and inviting manner, rather than by direct statements or orders."}, "time_dimension": {"answer": "Present", "rationale": "The entry describes the emotional reaction that the testee may feel in the current circumstances, namely, \"when it comes to taking on important responsibilities\", and therefore the judgement is \"now\"."}, "sentence_type": "Negative declarative sentence", "pos": {"nouns": {"Responsibility": 1, "Pressure": 1}, "verbs": {"Take it": 1, "Yes": 1, "I feel": 1, "I'm not comfortable": 1}, "adjectives": {"Important": 1}, "adverbs": {"- Yes, sir": 1}, "numerals": {"51": 1}}}, {"content": "52. After fighting with friends, I must admit my mistakes as soon as possible.", "word_count": 15, "label": "", "sentence_structure": "Subject鈥搗erb鈥搊bject", "subjective_objective": {"answer": "Objective", "rationale": "The question is whether the purpose of the questionnaire is to determine whether the patient鈥檚 subjective attitude or objective facts are the subject of the questionnaire."}, "sentence_sentiment": {"answer": "Positive", "rationale": "Because the \"acknowledgement of error\" act is positive and expresses a responsible attitude towards itself and the other side."}, "language_style": {"answer": "Calm and direct", "rationale": "The entry uses direct and clear language to describe the response of the interviewee after the argument, is not overly emotional or rhetorical and is more objective in tone."}, "time_dimension": {"answer": "Present", "rationale": "Now the entry \"must admit mistakes as soon as possible\" suggests that this is a current and obligatory code of conduct, and therefore the temporal dimension is now judged. At the same time, the \"after fighting with friends\" suggests that the act occurred in a particular situation, i.e., that an apology is required at some point after the argument has occurred."}, "sentence_type": "Affirmative declarative sentence", "pos": {"nouns": {"friends": 1, "Argument": 1, "Error": 1}, "verbs": {"Recognition": 1, "Fight": 1, "I have to": 1, "As soon as possible": 1}, "adverbs": {"Back": 1}, "numerals": {"52": 1}, "pronouns": {"I": 1}}}, {"content": "I am reluctant to admit my weaknesses.", "word_count": 10, "label": "", "sentence_structure": "Subject鈥損redicate", "subjective_objective": {"answer": "Subjective", "rationale": "The lack of willingness to acknowledge its own weaknesses is a subjective attitude of the patient."}, "sentence_sentiment": {"answer": "Negative", "rationale": "Because the \"unrecognizing\" attitude in the entry is negative."}, "language_style": {"answer": "Calm and direct", "rationale": "The entry conveys the subject in a straightforward and clear presentation, without too much rhetorical or emotional projection, and with a more calm and direct language style."}, "time_dimension": {"answer": "Present", "rationale": ""}, "sentence_type": "Negative declarative sentence", "pos": {"nouns": {"Self": 1, "Weaknesses": 1}, "verbs": {"I don't want to": 1, "Recognition": 1}, "numerals": {"53": 1}}}, {"content": "54. It was important that I liked my work, not whether it was being commended.", "word_count": 25, "label": "", "sentence_structure": "Subject鈥揷opula鈥損redicative", "subjective_objective": {"answer": "Subjective", "rationale": "Because \"like your job\" is a subjective feeling or attitude."}, "sentence_sentiment": {"answer": "Positive", "rationale": "This is positive because the entries express attitudes and beliefs that favour their own work."}, "language_style": {"answer": "Tactful and warm", "rationale": "The language style of the entry is more euphemismal. It does not directly express a negative attitude to being praised, but rather, by emphasizing that it likes its work, it expresses a sense of self-confidence and self-acceptance. This way is more likely to trigger the resonance of the person under investigation, making it easier for him to answer questions with open mind."}, "time_dimension": {"answer": "Present", "rationale": "The words \"like\" and \"are commended\" in this entry describe the current or past situation and do not address the future time dimension. Thus, the judgement is now."}, "sentence_type": "Imperative sentence", "pos": {"nouns": {"Work": 2, "- Congratulations": 1}, "verbs": {"Yeah": 1, "Got it": 1}, "adjectives": {"Important": 1, "self": 1}, "adverbs": {"No, it's not": 1, "Is it possible": 1}, "numerals": {"54": 1}, "pronouns": {"I": 1}}}, {"content": "I feel very lonely after a fight.", "word_count": 13, "label": "", "sentence_structure": "Subject鈥揷opula鈥損redicative", "subjective_objective": {"answer": "Subjective", "rationale": "Because \"single feeling\" is a subjective feeling, reflecting the psychological response of the patient after the argument."}, "sentence_sentiment": {"answer": "Negative", "rationale": "Because the result of the entry \"Feeling very lonely\" is negative."}, "language_style": {"answer": "Tactful and warm", "rationale": "The entry uses a soft, invitation-style tone for dialogue rather than direct statements or orders, which is more personal and interactive. At the same time, the description of \"very lonely\" implies that the other party may feel depressed after a quarrel, which is more likely to trigger resonance and participation by the person under investigation."}, "time_dimension": {"answer": "Past", "rationale": "The entry describes the feelings of the person who was tested after an argument, and the temporal dimension is the past."}, "sentence_type": "Negative declarative sentence", "pos": {"nouns": {"Argument": 1, "Loneliness": 1}, "verbs": {"Yes": 1, "I feel": 1}, "adjectives": {"Very": 1}, "numerals": {"55": 1}}}, {"content": "56. In my dealings with others, I am mindful of what others can give me.", "word_count": 19, "label": "", "sentence_structure": "Subject鈥搗erb鈥揷omplement", "subjective_objective": {"answer": "Subjective", "rationale": "This is a subjective attitude of patients and reflects their perception and expectations of interpersonal relations."}, "sentence_sentiment": {"answer": "Negative", "rationale": "Because the entry \"are careful what others can give me\" expresses a demand attitude and does not reflect positive mutual assistance or behaviour."}, "language_style": {"answer": "Tactful and warm", "rationale": ""}, "time_dimension": {"answer": "Present", "rationale": "The entry asks the testee about the current way of thinking or values, and the time dimension is judged to be the present. The keyword \"care\" indicates that it is a constant, continuous act, while \"what can I be given to someone else\" reflects the testee's self-centre and exchange of ideas, which implies that it is a present act or attitude."}, "sentence_type": "Negative declarative sentence", "pos": {"nouns": {"- To be with someone": 1, "person": 1, "others": 2, "I": 1, "What": 1}, "verbs": {"Attention": 1, "Here": 1}, "adverbs": {"very": 1}, "numerals": {"56": 1}, "pronouns": {"Here": 1}}}, {"content": "I rarely think about my family.", "word_count": 9, "label": "", "sentence_structure": "Subject鈥揷opula鈥損redicative", "subjective_objective": {"answer": "Subjective", "rationale": "Because \"I rarely think about my family\" is a subjective feeling and attitude that reflects the level of thought and concern of patients about their families."}, "sentence_sentiment": {"answer": "Negative", "rationale": "Because the tendency to act like \" hardly think about my family\" in the entry is negative."}, "language_style": {"answer": "Calm and direct", "rationale": "The language style of the entry is straightforward, as it simply asks the respondents whether they think very little about their families and do not use any rhetorical or emotional colour to qualify the problem. Moreover, this type of survey is usually designed to obtain objective information, and therefore requires neutral and direct language styles."}, "time_dimension": {"answer": "Past", "rationale": "The entry describes the conduct of the person who was tested in the past, using the term \"very rarely\" to indicate that such conduct was rare or unusual in the past."}, "sentence_type": "Negative declarative sentence", "pos": {"nouns": {"Family": 1}, "verbs": {"Yes": 1}, "adjectives": {"Less": 1}, "adverbs": {"Very few": 1}, "numerals": {"57": 1}}}, {"content": "58. My feelings for relatives and friends have changed from time to time: sometimes I feel angry, sometimes tender and in love.", "word_count": 31, "label": "", "sentence_structure": "Subject鈥搗erb鈥揷omplement", "subjective_objective": {"answer": "Subjective", "rationale": "/Objective: Subjective, because it's a description of what patients feel, reflects their emotional changes, is a manifestation of subjective."}, "sentence_sentiment": {"answer": "Neutral", "rationale": "According to the article, \"I often change my feelings of my friends and my loved ones\" , a statement that neither has a clear emotional color (e.g. love, disgust, etc.) nor specific behaviour or quality. So it can be judged neutral. And \"sometimes I feel angry, sometimes tender, but soft\" but not clearly emotional, so I can also be considered neutral."}, "language_style": {"answer": "Tactful and warm", "rationale": "The language style of the entry is more euphemistic and sensitive, it expresses its emotional state by describing changes in the feelings of relatives and friends, and it uses metaphoric techniques (such as \"floating\" and \"sweet like water\"), making the words more poetic and warm. This expression is more likely to generate resonance and a sense of identity among the respondents."}, "time_dimension": {"answer": "Past", "rationale": "The words \"sometimes\" and \"sometimes\" indicate that this is a sentence describing past and present behaviour, so the entry covers both present and past."}, "sentence_type": "Affirmative declarative sentence", "pos": {"nouns": {"Friends": 2, "Feel": 1, "鈾?And the fury 鈾?: 1, "鈾?Like water 鈾?: 1, "Love": 1, "Sponge": 1}, "verbs": {"It changes every now and then": 1}, "adjectives": {"鈾?And the fury 鈾?: 1, "鈾?Like water 鈾?: 2, "Love is so sweet": 1}, "adverbs": {"Sometimes": 2, "And then again": 1}, "pronouns": {"I": 1}}}, {"content": "59. My words and actions have a great impact on the people around me.", "word_count": 13, "label": "", "sentence_structure": "Subject鈥揷opula鈥損redicative", "subjective_objective": {"answer": "Subjective", "rationale": "Because it is not an objective fact to influence the subjective feelings and judgements of one person."}, "sentence_sentiment": {"answer": "Positive", "rationale": "Because the behaviour and attitude of the entries are positive, \"the impact on the people around them is significant\" indicating that individuals have influence in social interaction."}, "language_style": {"answer": "Calm and direct", "rationale": "\"Calm down straight.\""}, "time_dimension": {"answer": "Past", "rationale": "It is therefore impossible to judge with precision past, present or future behaviour or mentality, and should be labelled as \"other\"."}, "sentence_type": "Affirmative declarative sentence", "pos": {"nouns": {"Behaviour": 1, "person": 1, "Impact": 1}, "verbs": {"to": 1, "It's big": 1, "have": 1}, "adverbs": {"very": 2}, "numerals": {"59": 1, "Large": 1}, "classifiers": {"individual": 1}}}, {"content": "I sometimes feel like I'm \"special.\"", "word_count": 10, "label": "", "sentence_structure": "Subject鈥搗erb鈥揷omplement", "subjective_objective": {"answer": "Subjective", "rationale": "Because feeling \"extraordinary\" is the patient's subjective feeling and experience, and it is a description and evaluation of his emotional state."}, "sentence_sentiment": {"answer": "Negative", "rationale": "Because the expression \"special gas\" in the entry is negative."}, "language_style": {"answer": "Tactful and warm", "rationale": "The entry is more personal and interactive by engaging in dialogue in a gentle and inviting manner, rather than by direct statements or orders. At the same time, the more verbal expression \"special \" is used to make the questionnaire more pro-people and more acceptable."}, "time_dimension": {"answer": "Present", "rationale": "\"I sometimes feel \"sometimes\" means \"sometimes\" and \"sometimes\" means \"sometimes\" means that the general state of affairs is used, which means that this behaviour has not only happened in the past, but may still be ongoing. So, the entry to the questionnaire is to ask the person who was tested about his or her current feelings and experiences, and to judge now."}, "sentence_type": "Exclamatory sentence", "pos": {"nouns": {"self": 1, "Gas": 1}, "verbs": {"I feel": 1, "be": 1}, "adjectives": {"Special": 1}, "numerals": {"60": 1}, "pronouns": {"I": 1}}}, {"content": "I grew up in an extremely closed family.", "word_count": 14, "label": "", "sentence_structure": "Subject鈥揷opula鈥損redicative", "subjective_objective": {"answer": "Subjective", "rationale": "Because I think I grew up in a closed family, which is the patient's personal perception and subjective evaluation."}, "sentence_sentiment": {"answer": "Negative", "rationale": "Because the description of \"extremely closed\" in the entry is negative."}, "language_style": {"answer": "Calm and direct", "rationale": "The entry presents the characteristics of the family environment in the course of the person ' s upbringing in a direct and explicit manner, without the use of overly reverbative language or emotional language to make the answer more objective and accurate."}, "time_dimension": {"answer": "Past", "rationale": "The keyword \"Breath\" shows that this is a description of an individual's past experience, while \"extremely closed families\" provides a specific context, further indicating that this is a description of the family environment in the past. The questionnaire is therefore an inquiry into the past of the person tested, and the temporal dimension is a past."}, "sentence_type": "Affirmative declarative sentence", "pos": {"nouns": {"Family": 1, "Grow": 1, "Extreme": 1, "Closed": 1}, "verbs": {"in": 1, "be": 1}, "adjectives": {"Closed": 1, "Extreme": 1}, "numerals": {"61": 1}, "pronouns": {"I": 1}}}, {"content": "62. I am very satisfied with my own achievements and those of my own.", "word_count": 14, "label": "", "sentence_structure": "Subject鈥揷opula鈥損redicative", "subjective_objective": {"answer": "Subjective", "rationale": "The entry is about patients' subjective perception of self-awareness."}, "sentence_sentiment": {"answer": "Positive", "rationale": "Because the \"satisfaction with your achievements\" approach in the entry is positive."}, "language_style": {"answer": "Calm and direct", "rationale": "The entry uses simple and clear language to express the subject matter, without too many rhetorical or emotional colours, and is more straightforward. It directly asks the interviewee about his satisfaction with his or her own achievements, which is an objective, direct expression."}, "time_dimension": {"answer": "Present", "rationale": "The entry shows the attitude of the respondent in responding to the questionnaire, indicating that he is now very satisfied with himself and his achievements."}, "sentence_type": "Affirmative declarative sentence", "pos": {"verbs": {"to": 1, "self": 1, "of": 2, "Achievements": 1, "Tens": 1, "Satisfied": 1}, "adjectives": {"Tens": 1}, "adverbs": {"Tens": 1}, "numerals": {"62": 1}, "pronouns": {"I": 1}}}, {"content": "63. I hope to get a lot from my relatives and friends.", "word_count": 15, "label": "", "sentence_structure": "Subject鈥搗erb鈥搊bject", "subjective_objective": {"answer": "Subjective", "rationale": "Because \"favorite\" is a subjective desire or expectation of a person, not an objective fact or result."}, "sentence_sentiment": {"answer": "Positive", "rationale": "This is a positive attitude, since it expresses the desire to establish close relationships with people and thereby to gain support and help."}, "language_style": {"answer": "Tactful and warm", "rationale": "The entry is presented in the form of \"hope\" and expresses the interviewee's expectations and wishes for support from relatives and friends, in a more gentle and friendly manner, and is not too direct or commandive, and is more friendly."}, "time_dimension": {"answer": "Present", "rationale": "The keywords \"hope\" and \"can\" indicate that this is the current mindset and expectations of the testees, and therefore the judgement is now."}, "sentence_type": "Imperative sentence", "pos": {"nouns": {"Questionnaire": 1, "Entry": 1, "Friends": 1, "Something": 1}, "verbs": {"Hope": 1, "Okay": 1}, "adjectives": {"Many": 1}, "numerals": {"63": 1}, "pronouns": {"I": 1}}}, {"content": "I tend to be too harsh on myself.鈥?, "word_count": 11, "label": "", "sentence_structure": "Subject鈥揷opula鈥損redicative", "subjective_objective": {"answer": "Subjective", "rationale": "Because \"preferance\" is the patient's own subjective perception and feeling."}, "sentence_sentiment": {"answer": "Negative", "rationale": "Because the \"too harsh\" act is negative."}, "language_style": {"answer": "Tactful and warm", "rationale": "The language style of the entry is euphemism, and it is not directly critical or commanding. This expression is more friendly and respectful, and encourages respondents to share real ideas and feelings."}, "time_dimension": {"answer": "Present", "rationale": ""}, "sentence_type": "Affirmative declarative sentence", "pos": {"verbs": {"Preferative": 1, "That's too much": 1, "Hard": 1}, "adjectives": {"self": 1}, "adverbs": {"That's too much": 1}, "numerals": {"64": 1}, "pronouns": {"I": 1}}}, {"content": "It does not bother me at all to be alone.", "word_count": 12, "label": "", "sentence_structure": "Subject鈥搗erb鈥揷omplement", "subjective_objective": {"answer": "Subjective", "rationale": "Because being alone is not a disconcerting feeling or attitude."}, "sentence_sentiment": {"answer": "Neutral", "rationale": "Because the entry does not have any emotional color."}, "language_style": {"answer": "Calm and direct", "rationale": "The entry is in a concise language, which directly expresses the respondents鈥?feelings of being alone, and does not have too many rhetorical or emotional renderings."}, "time_dimension": {"answer": "Present", "rationale": "\"Sitting alone\" means a state of custom or a regular occurrence, \"not bothering me at all\" means a description of the feeling or emotional experience of the state, both of which imply that it is the feeling or behaviour of the moment. So, the entry is now."}, "sentence_type": "Affirmative declarative sentence", "pos": {"verbs": {"You're": 1, "I'm upset": 1}, "adjectives": {"Not at all": 1}, "adverbs": {"Alone": 1}}}, {"content": "I always use guidelines or targets against myself.", "word_count": 14, "label": "", "sentence_structure": "Subject鈥搗erb鈥搊bject", "subjective_objective": {"answer": "Subjective", "rationale": "This is a subjective attitude to patient self-evaluation."}, "sentence_sentiment": {"answer": "Positive", "rationale": "Because the behaviour in the entries \"often with guidelines or targets against themselves\" is positive, with self-proclaimed and progressive motivation."}, "language_style": {"answer": "Calm and direct", "rationale": "\"Calm down straight.\""}, "time_dimension": {"answer": "Present", "rationale": "\"Recurringly\" indicates that the act is customary and that it has not only occurred in the past, but is still ongoing. \"Count yourself with norms or targets\" is also a description of the current state and behaviour. The entry to the questionnaire therefore asks about the current behaviour and state of the person being tested, which is the current state."}, "sentence_type": "Affirmative declarative sentence", "pos": {"nouns": {"Guidelines": 1, "Objective": 1, "Contrast": 1, "self": 1}, "verbs": {"Use": 1, "Regular": 1}, "adverbs": {"Come on": 1}, "numerals": {"66": 1}, "pronouns": {"I": 2}}}]}

{"questionnaire_title": "Defense Mode Questionnaire (DSQ)", "questionnaire_category": "Behavioral questionnaire", "item_count": 88, "emotion_shift_overall": "From positive to negative", "language_style_overall": "Neutral", "items": [{"content": "One, I get satisfaction from helping others, and if I don't, I get depressed.", "word_count": 26, "label": "", "sentence_structure": "Subject鈥搗erb鈥揷omplement", "subjective_objective": {"answer": "Subjective", "rationale": "Because the sentence expresses the patient ' s views and feelings in helping others and the emotional reactions that may arise if the act is not realized, it is the patient ' s subjective attitude."}, "sentence_sentiment": {"answer": "Positive", "rationale": "Because the behavior \"helping others\" and the purpose \"getting satisfied\" in the entry are positive. And the condition \"if you would\" also shows that it is important for the emotional health of the individual, and further emphasizes its positive significance."}, "language_style": {"answer": "Tactful and warm", "rationale": "The language style of the entry is more euphemistic. First, it uses a mild tone of expression to express the subject, and the positive statement that I am satisfied by helping others, rather than simply saying that it is not. Second, it highlights the consequences of not doing it, i.e. depression, which makes it more concerned and considerate. Overall, the entry gives people a feeling of warmth and caring, which is more acceptable and willing to answer questions."}, "time_dimension": {"answer": "Present", "rationale": "The keyword \"I get satisfaction from helping others\" and \"if...\" means that it is a constant act or tendency and is related to the current emotional state. So, this entry describes the current behavior and mentality."}, "sentence_type": "Affirmative declarative sentence", "pos": {"nouns": {"I": 2, "Others": 1, "Satisfied": 1, "If": 1, "Here": 1, "Just": 1, "鈾?And become 鈾?: 1, "Emotional depression": 1}, "verbs": {"From": 1, "Help": 1, "Access": 1, "Yes": 2, "鈾?And become 鈾?: 1, "Here": 1}, "adjectives": {"Satisfied": 1, "Emotional depression": 1}, "numerals": {"1": 7}, "pronouns": {"I": 2}}}, {"content": "Two, I'm often called a temperamental man.", "word_count": 13, "label": "", "sentence_structure": "Subject鈥揷opula鈥損redicative", "subjective_objective": {"answer": "Subjective", "rationale": "Because the description of \"grumpy\" is based on the observation and evaluation of others, not on the subjective feeling of the patient."}, "sentence_sentiment": {"answer": "Negative", "rationale": "Because the term \"grumpy\" is usually considered negative."}, "language_style": {"answer": "Calm and direct", "rationale": "The entry uses a specific fact as a reference and then asks the other side directly about it, in a fair and objective manner, without excessive emotional rendering or rhetorical techniques."}, "time_dimension": {"answer": "Past", "rationale": "And, \"I'm a grumpy person,\" shows that the testee is describing his character, a personal statement, with no specific point in the time dimension. Taken together, the time dimension of the questionnaire is mainly the past."}, "sentence_type": "Affirmative declarative sentence", "pos": {"nouns": {"People": 1, "- Grumpy": 1, "- Routine": 1, "person": 1}, "verbs": {"I always say": 1, "be": 1}, "adjectives": {"- Routine": 1}, "pronouns": {"I": 1}}}, {"content": "Three, I can put it aside when I don't have time to deal with a difficult thing.", "word_count": 25, "label": "", "sentence_structure": "Subject鈥搗erb鈥搊bject", "subjective_objective": {"answer": "Subjective", "rationale": "Because it is the patient's subjective attitude and choice. Although \"no time\" is an objective fact, it is the patient's subjective decision whether or not to put things aside."}, "sentence_sentiment": {"answer": "Neutral", "rationale": "Because the entry does not have any emotional color."}, "language_style": {"answer": "Tactful and warm", "rationale": ""}, "time_dimension": {"answer": "Past", "rationale": "Thus, the entry describes both the past (i.e. \"when there is no time to deal with a difficult matter\") and the possible future conduct (i.e. \"it can be set aside\". In combination, the temporal dimension of the article should be present and future."}, "sentence_type": "Affirmative declarative sentence", "pos": {"nouns": {"Time": 1, "Things": 1}, "verbs": {"Processing": 1, "Hold it": 1, "On the side": 1}, "adjectives": {"It's a problem": 1}, "adverbs": {"No, I'm not": 1, "Yeah": 1}, "numerals": {"3": 1}, "pronouns": {"I": 1}}}, {"content": "Four. People treat me unfairly.", "word_count": 11, "label": "", "sentence_structure": "Subject鈥搗erb鈥揷omplement", "subjective_objective": {"answer": "Subjective", "rationale": "Because \"always treating me unfairly\" is a subjective feeling and perception of a person, based on his own experience and perception. While this perception may be influenced by objective factors, it is still a subjective judgement in general."}, "sentence_sentiment": {"answer": "Negative", "rationale": "Because the \"unfairly\" act in the entry is negative."}, "language_style": {"answer": "Calm and direct", "rationale": ""}, "time_dimension": {"answer": "Present", "rationale": "The entry does not have a clear time dimension reference point, so it can be judged to be the current state of affairs. The keyword \"people\" indicates that this situation is repeated and may persist. At the same time, the terms \"unfairness\" and \"unfair treatment\" imply that the person being tried feels that some form of injustice is being done, and this information indicates that the entry describes the present situation."}, "sentence_type": "Negative declarative sentence", "pos": {"nouns": {"person": 1, "Fair": 1, "Treatment": 1}, "verbs": {"Always": 1, "not": 1, "Treatment": 1}, "adjectives": {"It's not fair": 1}, "adverbs": {"Land": 1, "Always": 1}, "classifiers": {"4": 1}, "pronouns": {"I": 1}}}, {"content": "Five, I'm free of my anxiety by doing positive or predictable things, drawing, doing carpentry work, etc.", "word_count": 34, "label": "", "sentence_structure": "Subject鈥搗erb鈥搊bject", "subjective_objective": {"answer": "Subjective", "rationale": "It is the patient ' s subjective behaviour and thoughts to do something positive or predictable to get away from anxiety."}, "sentence_sentiment": {"answer": "Positive", "rationale": "Because the description of the article refers to \"doing something positive or predictable\" and \"leave your own anxiety\", these acts are positive."}, "language_style": {"answer": "Tactful and warm", "rationale": "The entry is more euphemistic and friendly, uses a recommendatory tone, and is softly used, which makes people feel respected and understood. In addition, it provides concrete examples of how respondents can understand and answer questions, which is also a positive way of interacting."}, "time_dimension": {"answer": "Present", "rationale": "The entry is for the purpose of asking the testee about his current behaviour and mental state and can therefore be judged as now."}, "sentence_type": "Affirmative declarative sentence", "pos": {"nouns": {"Things": 2, "Positive": 1, "Predictability": 1, "Anxiety": 1, "Painting": 1, "Carpentry": 1}, "verbs": {"Get rid of it": 1, "Pass": 1, "Do some": 1}, "adjectives": {"Your own": 1}, "numerals": {"5": 1}, "classifiers": {"Wait": 1}}}, {"content": "I've been deferring some of the things I should do today to tomorrow.", "word_count": 19, "label": "", "sentence_structure": "Subject鈥搗erb鈥搊bject", "subjective_objective": {"answer": "Objective", "rationale": "It is an act and an objective fact to postpone today's events to tomorrow."}, "sentence_sentiment": {"answer": "Negative", "rationale": "Because the \"delaying things\" act in the entry is negative."}, "language_style": {"answer": "Calm and direct", "rationale": "The question was whether respondents often deferred what they should have done today to tomorrow in a direct manner, without too many rhetorical or emotional accents, and with a more calm and direct language style."}, "time_dimension": {"answer": "Present", "rationale": "Now, the entry \"deferring to tomorrow some of what is to be done today\" means that the testee is describing his current behaviour or habits and thus judging now."}, "sentence_type": "Affirmative declarative sentence", "pos": {"nouns": {"Questionnaire Entry": 1, "Things": 2}, "verbs": {"- Put it on": 1, "Postponement": 1, "Do it": 1}, "adjectives": {"Sometimes": 1}, "adverbs": {"Today": 1, "Tomorrow": 1}, "numerals": {"6": 1, "Some": 1}, "classifiers": {"Entry": 1}, "pronouns": {"I": 1}}}, {"content": "Seven, I don't know why I'm always in the same situation.", "word_count": 18, "label": "", "sentence_structure": "Subject鈥搗erb鈥搊bject", "subjective_objective": {"answer": "Subjective", "rationale": "Because \"Don't know why\" is part of the patient's subjective feeling and perception."}, "sentence_sentiment": {"answer": "Neutral", "rationale": "Because the entry is not clearly emotional."}, "language_style": {"answer": "Tactful and warm", "rationale": "This entry, which is asked in a caring and supportive manner, highlights the frustrations that the interviewees may encounter and encourages them to share more information."}, "time_dimension": {"answer": "Present", "rationale": "The words \"always\" and \"attached\" in the entry indicate the act or situation at this stage, while the words \"unknown\" indicate confusion and confusion about the current state. Thus, the time dimension of the entry is now."}, "sentence_type": "Interrogative sentence", "pos": {"nouns": {"Frustration": 1, "Situation": 1}, "verbs": {"I met him": 1, "Yes": 1, "be": 1}, "adverbs": {"Always": 1, "Same": 1}, "numerals": {"7": 1}, "pronouns": {"I": 1, "Why": 1}}}, {"content": "I can laugh at myself quite easily.", "word_count": 13, "label": "", "sentence_structure": "Subject鈥搗erb鈥揷omplement", "subjective_objective": {"answer": "Subjective", "rationale": "Because \"can laugh at myself quite easily\" is a subjective self-awareness and attitude."}, "sentence_sentiment": {"answer": "Positive", "rationale": "Because the \"scoffing\" in the entry is positive, and it's self-admission and sense of humor."}, "language_style": {"answer": "Tactful and warm", "rationale": "The language style of the entry is euphemism. It uses a gentle and inviting tone to make the respondents feel respected and understood. And the use of the term \"scoffing\" makes the questionnaire more comfortable and less stressful or uncomfortable."}, "time_dimension": {"answer": "Present", "rationale": "The keyword \"can laugh at myself quite easily\" means the present attitude and abilities of the subject, and the entry is therefore an inquiry into the present situation."}, "sentence_type": "Affirmative declarative sentence", "pos": {"nouns": {"self": 1}, "verbs": {"Yes": 1, "Easy": 1, "Scoff": 1}, "adverbs": {"Pretty much": 1, "Land": 2}, "pronouns": {"I": 1}}}, {"content": "Nine. When I get frustrated, I act like a child.", "word_count": 13, "label": "", "sentence_structure": "Subject鈥揷opula鈥損redicative", "subjective_objective": {"answer": "Subjective", "rationale": "Because of the frustration and the appearance that the child is the patient ' s own observation and description, it is a subjective view."}, "sentence_sentiment": {"answer": "Negative", "rationale": "Because \"when you get frustrated, act like a child\" is negative."}, "language_style": {"answer": "Tactful and warm", "rationale": "By analogy, the entry euphemisms the psychological state of an individual in the face of frustration, avoiding too direct or strong a language expression, and being more intimate and interactive."}, "time_dimension": {"answer": "Present", "rationale": "The keywords \"when frustrated\" and \"expression like a child\" indicate that this is a personal mode of reaction, and does not involve the time dimension of the past or the future. So the time of this entry is now."}, "sentence_type": "Interrogative sentence", "pos": {"nouns": {"Frustration": 1, "Performance": 1, "Son": 1}, "verbs": {"Yes": 1, "Elephant": 1}, "adverbs": {"Just": 1}, "pronouns": {"I": 1}}}, {"content": "I'm ashamed to be a pious man in my interests.", "word_count": 16, "label": "", "sentence_structure": "Subject鈥搗erb鈥搊bject", "subjective_objective": {"answer": "Subjective", "rationale": "Because the words \"shamble\" and \"scheme\" are a description of the patient's behaviour and attitude, reflecting his subjective feelings."}, "sentence_sentiment": {"answer": "Negative", "rationale": "The basis for the judgment is that the act of \"shammy against others\" is negative."}, "language_style": {"answer": "Calm and direct", "rationale": "The entry allows the respondents to express their views in a direct question, without too many rhetorical or emotional expressions, and with a more objective tone. At the same time, the question \"if\" phrase is more straightforward and clear."}, "time_dimension": {"answer": "Present", "rationale": "The keyword \"scoffed\" means that it is a state of behaviour, and \"in the defence of my interests\" points to the circumstances and purpose in which it occurs. \"Are I now ashamed of being compared to others, in the defence of my interests?\""}, "sentence_type": "Negative declarative sentence", "pos": {"nouns": {"Interest": 1, "In terms of": 1}, "verbs": {"Maintenance": 1, "Count": 1}, "adjectives": {"Shame": 1}, "adverbs": {"With people": 1}, "numerals": {"10": 1}}}, {"content": "Eleven. I'm better than most of the people I know.", "word_count": 13, "label": "", "sentence_structure": "Subject鈥揷opula鈥損redicative", "subjective_objective": {"answer": "Subjective", "rationale": "Because this involves patient self-evaluation and comparison, it is subjective thinking and judgement."}, "sentence_sentiment": {"answer": "Neutral", "rationale": "Because the entry does not have any emotional color."}, "language_style": {"answer": "Calm and direct", "rationale": "The language style of the entry is more calm and straightforward, with no excessive rhetorical or emotional rendering, but rather a direct expression of the subject of asking the other side whether they think they are stronger than most people around them. This expression is more objective and accurate and more effective in obtaining the real thoughts of the person being tested."}, "time_dimension": {"answer": "Present", "rationale": "The entry describes the self-evaluation of the person tested, who considers himself better than most of the people they know. This perception is taking place now, and does not address the time dimension of the past or the future."}, "sentence_type": "Affirmative declarative sentence", "pos": {"nouns": {"Questionnaire Entry": 1, "person": 2, "Most of them": 1}, "verbs": {"Yes": 1, "- Yeah": 1, "Strong": 1}, "adjectives": {"Strong": 1}, "numerals": {"11": 1, "I": 1}, "classifiers": {"Both": 1}, "pronouns": {"I": 1, "of": 1}}}, {"content": "People abuse me.", "word_count": 7, "label": "", "sentence_structure": "Subject鈥搗erb鈥搊bject", "subjective_objective": {"answer": "Subjective", "rationale": "Because \"abuse\" is a subjective description that reflects the patient's perception and feelings of being treated by others."}, "sentence_sentiment": {"answer": "Negative", "rationale": "Because the word \"abuse\" in the entry is clearly negative emotional."}, "language_style": {"answer": "Tactful and warm", "rationale": "Warm and warm."}, "time_dimension": {"answer": "Present", "rationale": "The keyword \"people\" often means that such behaviour occurs frequently or customarily, and \"ill-treating me\" means that it is a situation that is happening, so the time dimension of the purpose is now."}, "sentence_type": "Negative declarative sentence", "pos": {"nouns": {"People": 1, "Abuse": 1}, "verbs": {"Often": 1}, "numerals": {"12": 1}}}, {"content": "13, if someone lied to me or stole my money, I'd rather he get help than punishment.", "word_count": 27, "label": "", "sentence_structure": "Subject鈥搗erb鈥搊bject", "subjective_objective": {"answer": "Subjective", "rationale": "Because the phrase \"better\" expresses the values and attitudes of the individual, which are viewed from the patient's subjective point of view. Although the phrase refers to objective events (swindled or money stolen), the attitude towards them is subjective. Thus, the questionnaire's purpose is subjective."}, "sentence_sentiment": {"answer": "Positive", "rationale": "Because the purpose of the action in the entry is positive, i.e., to expect the deceptor to be helped rather than punished."}, "language_style": {"answer": "Tactful and warm", "rationale": "This language style is more likely to lead respondents to think about issues from a human, emotional, rather than direct statement or order."}, "time_dimension": {"answer": "Present", "rationale": "\"If someone lied to me or stole my money,\" is hypothetical, but the main phrase \"I\" indicates that the speaker is thinking and judging at this moment. So, this entry is asking the testee's current attitude and opinion."}, "sentence_type": "Negative declarative sentence", "pos": {"nouns": {"Someone": 1, "Liar": 1, "Money": 1, "Help": 1, "Punishment": 1}, "verbs": {"I'd rather": 1, "Got it": 1, "Steal": 1}, "adverbs": {"If": 1, "Some": 1, "or": 1, "Both": 1}, "numerals": {"13": 1}, "pronouns": {"I": 1}}}, {"content": "I think sometimes something so bad can't be said.", "word_count": 16, "label": "", "sentence_structure": "Subject鈥搗erb鈥揷omplement", "subjective_objective": {"answer": "Subjective", "rationale": "Because \"Thinking bad is not the way to say it\" is a human mind and mind, and it's a subjective view."}, "sentence_sentiment": {"answer": "Negative", "rationale": "Because the \"thinking something so bad\" in the entry is negative."}, "language_style": {"answer": "Calm and direct", "rationale": "The entry directly asked the interviewees whether they had any negative ideas, were not over-prescriptive or emotional, and were speaking in a balanced and objective manner."}, "time_dimension": {"answer": "Present", "rationale": "The keyword \"wanted\" denotes the conduct currently being carried out, and \"bad enough\" describes what the person under examination is thinking at this point in time, and can therefore be judged as now."}, "sentence_type": "Affirmative declarative sentence", "pos": {"nouns": {"Things": 2, "Bad": 1, "mouth": 1}, "verbs": {"Yes": 1, "Say it": 1}, "adjectives": {"No, I can't": 1, "Sometimes": 1}, "adverbs": {"Okay": 1}, "numerals": {"14": 1, "Some": 1}}}, {"content": "I laugh at some dirty jokes.", "word_count": 14, "label": "", "sentence_structure": "Subject鈥搗erb鈥揷omplement", "subjective_objective": {"answer": "Subjective", "rationale": "Because \"smoke for dirty jokes\" is a patient's subjective feeling and behaviour, and it's a subjective examination of the mental state."}, "sentence_sentiment": {"answer": "Negative", "rationale": "Because the \"smuggle of the dirty\" is negative."}, "language_style": {"answer": "Tactful and warm", "rationale": "The entry is more gentle, elegant, and not too direct or rude. Moreover, it does not use a strong tone to guide respondents to answer questions, but rather in a more relaxed and natural way. This makes the questionnaire more friendly and friendly and more acceptable to respondents."}, "time_dimension": {"answer": "Present", "rationale": "At the same time, the \"smoke\" suggests that it is a specific act, and therefore it can also be considered as a current state of affairs. In conclusion, the time dimension of this questionnaire entry should be the present."}, "sentence_type": "Affirmative declarative sentence", "pos": {"nouns": {"Questionnaire Entry": 1, "A joke": 1}, "verbs": {"Because": 1, "Smile": 1}, "adjectives": {"Naughty": 1, "Sometimes": 1}, "adverbs": {"And": 1}, "numerals": {"15": 1}}}, {"content": "16 I was said to be like a camel bird, burying my head in the sand, in other words, I was often deliberately neglecting some unpleasant things.", "word_count": 37, "label": "", "sentence_structure": "Subject鈥搗erb鈥搊bject", "subjective_objective": {"answer": "Subjective", "rationale": "Because the title refers to \"people say\" and examines the views and assessments of others, it is the patient's subjective attitude."}, "sentence_sentiment": {"answer": "Negative", "rationale": "Because the entries \"smear your head in the sand\" and \"intentionally ignore\" are negative."}, "language_style": {"answer": "Tactful and warm", "rationale": "Warm and warm."}, "time_dimension": {"answer": "Present", "rationale": "The term \"speaks\" in \"I'm like a camelbird\" uses the usual past, indicating that the act occurred in the past. And the description \"I tend to ignore unpleasant things\" suggests that it is a customary and ongoing act, and therefore the temporal dimension is now."}, "sentence_type": "Affirmative declarative sentence", "pos": {"nouns": {"People": 1, "A camel bird": 1, "self": 1, "Head": 1, "Sand": 1, "Things": 1}, "verbs": {"Say it": 1, "Elephant": 1, "Bury it": 1, "Neglect": 1, "Intentionally": 1, "Ignore": 1}, "adjectives": {"It's not good": 1}, "adverbs": {"Total": 1, "In other words": 1}, "numerals": {"16": 1}, "classifiers": {"Some": 1}, "pronouns": {"I": 2}}}, {"content": "I can't compete with people at all costs.", "word_count": 14, "label": "", "sentence_structure": "Subject鈥搗erb鈥揷omplement", "subjective_objective": {"answer": "Subjective", "rationale": "Because \"can't do everything\" is a subjective feeling and an evaluation."}, "sentence_sentiment": {"answer": "Negative", "rationale": "Because the \"cannot compete with people\" act in the entry is negative, implying that individuals are not competitive and motivated."}, "language_style": {"answer": "Calm and direct", "rationale": "The entry directly expresses a question about whether the other party is often unable to focus on the subject at all when competing with others, and is not using excessive rhetorical or emotional rendering, which is more objective and balanced."}, "time_dimension": {"answer": "Present", "rationale": "The keywords \"often\" and \"not\" both mean that it is a state or behaviour of the moment, so it is judged as now."}, "sentence_type": "Negative declarative sentence", "pos": {"nouns": {"Questionnaire Entry": 1, "17": 1, "I": 2, "person": 1, "Competition": 1}, "verbs": {"No, I can't": 1, "Do your best": 1, "and": 1, "Competition": 1}, "adverbs": {"Often": 1}, "numerals": {"17": 1}}}, {"content": "I've always felt better than the people I'm with.", "word_count": 13, "label": "", "sentence_structure": "Subject鈥揷opula鈥損redicative", "subjective_objective": {"answer": "Subjective", "rationale": "Because \"reception of being better than others\" is patient's subjective attitude or self-evaluation, and it is subjective."}, "sentence_sentiment": {"answer": "Positive", "rationale": "Because it is positive because it expresses a sense of confidence and self-assertion."}, "language_style": {"answer": "Calm and direct", "rationale": "The language style of the entry is more direct and objective, and there is no excessive emotional rendering or rhetorical technique, but rather direct queries about whether the person interviewed feels this feeling, which is more calm and direct."}, "time_dimension": {"answer": "Present", "rationale": "The word \"often\" in the entry denotes a regular, customary behaviour, and the word \"sension\" adopts the general current, meaning that the act has not only occurred in the past, but may still be ongoing.\""}, "sentence_type": "Affirmative declarative sentence", "pos": {"nouns": {"person": 1, "Questionnaire": 1, "Entry": 1, "18": 1}, "verbs": {"Sensor": 1, "- Yeah": 1, "in": 1, "Together": 1, "Regular": 1}, "adjectives": {"Strong": 1}, "numerals": {"18": 1}, "pronouns": {"I": 2, "and": 1}}}, {"content": "I'm trying to take everything I've got.", "word_count": 14, "label": "", "sentence_structure": "Subject鈥搗erb鈥揷omplement", "subjective_objective": {"answer": "Subjective", "rationale": "Because \"someone is trying to take away everything I've got\" is the patient's imagination and fear, not the objective fact."}, "sentence_sentiment": {"answer": "Negative", "rationale": "Because the entry \"deprive me everything\" expresses negative emotions and attitudes."}, "language_style": {"answer": "Calm and direct", "rationale": "The entry is simple, is not too much rhetorical or emotional, and asks the interviewee whether he has ever been tried to deprive himself of his own. This calm, direct language style facilitates more realistic and objective information."}, "time_dimension": {"answer": "Present", "rationale": "The time keyword \"is\" in the sentence is judged to be the current feeling or state of the testee, so the answer is \"now\"."}, "sentence_type": "Negative declarative sentence", "pos": {"nouns": {"person": 1, "I": 2, "Everything": 1}, "verbs": {"I'm thinking": 1, "Deprivation": 1, "Got it": 1}, "numerals": {"Some": 1, "19": 1}}}, {"content": "I get angry sometimes.", "word_count": 5, "label": "", "sentence_structure": "Subject鈥搗erb鈥揷omplement", "subjective_objective": {"answer": "Subjective", "rationale": "Because \"freak\" is an emotional reaction, it is a subjective feeling and experience of the patient, and it is therefore subjective."}, "sentence_sentiment": {"answer": "Negative", "rationale": "Because \"freak\" is a negative emotion."}, "language_style": {"answer": "Tactful and warm", "rationale": "The entry is more personal and interactive when it is spoken in a gentle and inviting tone, rather than in direct statements or orders. At the same time, the expression \"I am sometimes angry\" is not too harsh or negative, making it easier for interviewees to respond and share real ideas."}, "time_dimension": {"answer": "Present", "rationale": "In the entry, \"sometimes\" means occasional, occasional behaviour, while \"irror\" is a description of past behaviour, so it can be judged that it is a sentence that is now complete in its current state, describing the testee's experience of one or more of his angers over a certain period of time."}, "sentence_type": "Affirmative declarative sentence", "pos": {"verbs": {"- Get angry": 1}, "adverbs": {"Sometimes": 1}}}, {"content": "21 - I've often acted on my own initiative, driven by some kind of inner force.", "word_count": 24, "label": "", "sentence_structure": "Subject鈥揷opula鈥損redicative", "subjective_objective": {"answer": "Subjective", "rationale": "This is a subjective feeling and experience of the patient and an inherently driven behaviour."}, "sentence_sentiment": {"answer": "Neutral", "rationale": "Because the entry only describes a phenomenon, it does not express a positive or negative emotional profile."}, "language_style": {"answer": "Tactful and warm", "rationale": ""}, "time_dimension": {"answer": "Present", "rationale": "Now the \"frequent\" in the entry indicates that the act is recurrent and in its present state, so the temporal dimension is now judged."}, "sentence_type": "Affirmative declarative sentence", "pos": {"nouns": {"Inside power": 1, "Driver": 1, "Behaviour": 1}, "verbs": {"Often": 1, "Make": 1, "I can't help it": 1}, "adverbs": {"in": 1, "Plant": 1}, "numerals": {"21": 1}, "classifiers": {"Some": 1}}}, {"content": "I'd rather starve to death than be forced to eat.", "word_count": 12, "label": "", "sentence_structure": "Subject鈥搗erb鈥揷omplement", "subjective_objective": {"answer": "Subjective", "rationale": "Because \"I'd rather starve than be forced to eat\" is the patient's subjective attitude and will."}, "sentence_sentiment": {"answer": "Negative", "rationale": "Because the attitude in the entry \"I'd rather starve than be forced to eat\" is negative."}, "language_style": {"answer": "Calm and direct", "rationale": "The entry expresses the respondent ' s feelings of resentment about being forced to eat in a direct and clear manner, without using euphemism or implicit language, and is more firmly worded."}, "time_dimension": {"answer": "Past", "rationale": "The entry describes a tendency or attitude of the person being tested, which is that he or she would rather starve to death than surrender in the face of being forced to eat. The tendency or attitude is based on personal values and experiences and is not limited by time."}, "sentence_type": "Negative declarative sentence", "pos": {"nouns": {"I": 1, "Starving": 1, "Forced": 1, "Rice": 1}, "verbs": {"I'd rather": 1, "Starving": 1, "I don't want to": 1, "Forced": 1, "Eat": 1}, "adverbs": {"I'd rather not": 1, "not": 1}, "numerals": {"22": 1}}}, {"content": "23, I've often been deliberately ignoring some of the dangers.", "word_count": 18, "label": "", "sentence_structure": "Subject鈥搗erb鈥搊bject", "subjective_objective": {"answer": "Subjective", "rationale": "Because the title refers to \"intentional neglect of some dangers\", it is the patient's subjective attitude and behaviour."}, "sentence_sentiment": {"answer": "Negative", "rationale": "Because the act in the entry \"intentionally neglecting some danger\" is negative."}, "language_style": {"answer": "Calm and direct", "rationale": "The language style of the entry is relatively calm, is not overly acrimonym or emotional, and is simple and straightforward, expressing the question whether the subject is often deliberately ignored."}, "time_dimension": {"answer": "Present", "rationale": ""}, "sentence_type": "Affirmative declarative sentence", "pos": {"nouns": {"Danger": 1, "Superman": 1}, "verbs": {"Regular": 1, "Ignoring": 1}, "adverbs": {"Seems like": 1}, "numerals": {"23": 1}}}, {"content": "I'm proud of my ability to denigrate others.", "word_count": 15, "label": "", "sentence_structure": "Subject鈥揷opula鈥損redicative", "subjective_objective": {"answer": "Subjective", "rationale": "For pride is a subjective feeling and attitude."}, "sentence_sentiment": {"answer": "Negative", "rationale": "The basis for the judgement: the \"capability to debase others\" in the entry is a negative act and the emotional attitude expressed is therefore negative."}, "language_style": {"answer": "Calm and direct", "rationale": "The entry directly expresses the subject of asking the other party whether it is proud of its ability to demean others, and it appears that the tone is more objective than the excessive use of rhetorical techniques or emotional rendering, so that the language style is straightforward and calm."}, "time_dimension": {"answer": "Present", "rationale": "The term \"now\" in the sentence makes it possible to judge that the entry is concerned with the current attitude and behaviour of the person being tested, and that the temporal dimension is now."}, "sentence_type": "Affirmative declarative sentence", "pos": {"nouns": {"Capacity": 1, "others": 1, "Honor": 1}, "verbs": {"have": 1, "Demeaning": 1, "Proud": 1}, "numerals": {"24": 1}, "pronouns": {"I": 1}}}, {"content": "25, people tell me I always feel killed.", "word_count": 13, "label": "", "sentence_structure": "Subject鈥搗erb鈥搊bject", "subjective_objective": {"answer": "Subjective", "rationale": "This is because the patient is assessed by others, and the patient ' s subjective feelings are viewed from the perspective of others and are therefore subject matter."}, "sentence_sentiment": {"answer": "Negative", "rationale": "Because the \"always feeling killed\" in the entry is an expression of a negative sentiment."}, "language_style": {"answer": "Tactful and warm", "rationale": "The entry, in terms of first person's description, uses the phrase \"people tell me\" to guide each other to share their feelings, which is more respectful. Moreover, the expression \"I always feel killed\" is more euphemismal, avoiding direct accusations or criticism of each other's psychology."}, "time_dimension": {"answer": "Present", "rationale": "\"Tell me the word \"in\" used the usual past, which means that the message has been passed on to the testee for some time, and \"I always feel killed\" used the usual current, which shows that it continues. So, the entry mainly concerns the experience and feeling of the present."}, "sentence_type": "Affirmative declarative sentence", "pos": {"nouns": {"People": 1, "Feel": 1}, "verbs": {"Tell": 1, "have": 1, "Total": 1}, "adjectives": {"Shit": 1}, "numerals": {"25": 1}, "pronouns": {"I": 1}}}, {"content": "26, sometimes I get angry when I feel bad.", "word_count": 12, "label": "", "sentence_structure": "Subject鈥損redicate", "subjective_objective": {"answer": "Subjective", "rationale": "Because this is the patient ' s inner feeling and attitude, it is subjective."}, "sentence_sentiment": {"answer": "Negative", "rationale": "Because it's negative in the entry."}, "language_style": {"answer": "Calm and direct", "rationale": "The entry is directly asked whether the person interviewed is prone to a temperamental disorder in a bad mood, is not expressing it more directly and objectively using excessive rhetorical or emotional rendering."}, "time_dimension": {"answer": "Present", "rationale": ""}, "sentence_type": "Affirmative declarative sentence", "pos": {"nouns": {"Time": 1, "Not good": 1, "- Grumpy": 1}, "verbs": {"Feel": 1, "Just": 1, "Let's go": 1}, "adverbs": {"Sometimes": 1}, "numerals": {"26": 1}}}, {"content": "I often do things on my own when something bothers me.", "word_count": 23, "label": "", "sentence_structure": "Subject鈥搗erb鈥揷omplement", "subjective_objective": {"answer": "Subjective", "rationale": "Because \"something is done by the person\" is the patient's subjective feeling and experience."}, "sentence_sentiment": {"answer": "Negative", "rationale": "Because the expression \"somethings are done by yourself\" in the entry gives a negative feeling."}, "language_style": {"answer": "Calm and direct", "rationale": "The language style of the entry is more calm and straightforward, and it presents an objective way of expressing the problem that people may encounter, i.e., they do something unwittingly when they are troubled."}, "time_dimension": {"answer": "Present", "rationale": "Therefore, the time dimension of the entry is judged now."}, "sentence_type": "Affirmative declarative sentence", "pos": {"nouns": {"Yes": 1, "Objects": 1, "Behaviour": 1}, "verbs": {"鈾?And I'm gonna be 鈾?: 1, "Do it": 1, "Often": 1, "It's not up to you": 1}, "adjectives": {"Trouble": 1}, "adverbs": {"Some": 1, "Land": 2}, "numerals": {"27": 1}, "pronouns": {"I": 1}}}, {"content": "28, when things go wrong, I get sick.", "word_count": 12, "label": "", "sentence_structure": "Subject鈥揷opula鈥損redicative", "subjective_objective": {"answer": "Subjective", "rationale": "This is the patient鈥檚 subjective attitude, since it is the patient鈥檚 own subjective feeling that determines whether or not he or she is ill."}, "sentence_sentiment": {"answer": "Negative", "rationale": "Because the result of \"sick\" in the entry is negative."}, "language_style": {"answer": "Tactful and warm", "rationale": "The entry uses a first-person expression and expresses an emotional feeling, which is relatively soft and friendly and reflects a warm and euphemism of language."}, "time_dimension": {"answer": "Past", "rationale": "The 28th question of the questionnaire, \"When things go wrong\" and \"I get sick,\" are all about the past. So the time dimension of this questionnaire entry is the past."}, "sentence_type": "Imperative sentence", "pos": {"nouns": {"Yes": 1, "Diseases": 1}, "verbs": {"Yes": 1, "On your own": 1, "Just": 1, "Fan": 1}, "adjectives": {"not": 1, "Yes": 1}, "numerals": {"28": 1}, "pronouns": {"I": 1}}}, {"content": "I'm a very self-sufficient man.", "word_count": 11, "label": "", "sentence_structure": "Subject鈥揷opula鈥損redicative", "subjective_objective": {"answer": "Subjective", "rationale": "Because \"I'm a very self-sufficient person\" is a patient's self-evaluation, reflecting patients' subjective attitudes and perceptions."}, "sentence_sentiment": {"answer": "Positive", "rationale": "Because the quality of the expression in the entry is positive."}, "language_style": {"answer": "Tactful and warm", "rationale": "The entry describes the capacity of the interviewee in positive language, does not use any strong negative or critical approach, and is expressed in a more moderate and encouraging manner. At the same time, the article is more easy and friendly and more easily elicits the positive response and participation of the interviewee."}, "time_dimension": {"answer": "Present", "rationale": "According to the sentence, \"I am one,\" it is a statement describing a personal quality, \"self-restraint\" is the specific content of that character, and \"very\" is the extent of that quality. Because it is a description of the current state, it is possible to judge that the item is being asked about the current behaviour or characteristics of the subject, that is, now."}, "sentence_type": "Affirmative declarative sentence", "pos": {"nouns": {"person": 1, "Self-restraint": 1}, "verbs": {"be": 1, "Do it": 1}, "adjectives": {"very": 1, "have": 1}, "numerals": {"29": 1}, "pronouns": {"I": 1}}}, {"content": "I'm like an undesired artist.", "word_count": 16, "label": "", "sentence_structure": "Subject鈥揷opula鈥損redicative", "subjective_objective": {"answer": "Subjective", "rationale": "Because \"unsatisfactory\" and \"same as an artist\" are personal feelings and assessments of patients, subjective views."}, "sentence_sentiment": {"answer": "Negative", "rationale": "Because the description of \"undesirable\" in the entry is negative."}, "language_style": {"answer": "Calm and direct", "rationale": "The language style of the entry is more direct, it is not over-modified or emotional, but rather a simple and straightforward expression of the person surveyed as if he were an undesired artist.\""}, "time_dimension": {"answer": "Present", "rationale": "According to the two keywords in the input sentence, \"it's like \"and\" it can be seen that the entry describes the current situation or state of the subject, so it's now."}, "sentence_type": "Affirmative declarative sentence", "pos": {"nouns": {"Artist": 1}, "verbs": {"Yeah": 1, "You're right": 1}, "adjectives": {"It's just": 1, "I'm not": 1}, "numerals": {"30": 1}, "pronouns": {"I": 1}}}, {"content": "I don't always tell the truth.", "word_count": 7, "label": "", "sentence_structure": "Subject鈥搗erb鈥揷omplement", "subjective_objective": {"answer": "Subjective", "rationale": "Because the silence is the patient ' s own psychological activity and attitude, it is subjective."}, "sentence_sentiment": {"answer": "Negative", "rationale": "Because the \"not always telling the truth\" act in the entry is negative, indicating that the person interviewed is lying."}, "language_style": {"answer": "Tactful and warm", "rationale": "The entry is more adept and interactive when it is spoken in a gentle and inviting manner, rather than by direct statements or orders."}, "time_dimension": {"answer": "Present", "rationale": "The keyword \"I hear \" often \" denotes a regular, customary act, while the word \"hear\" uses the normal current, which means that it has not only happened in the past, but may still be ongoing. Likewise, \"not always\" in \"not always telling the truth\" indicates that this is also the case at the present time. So, the entry is asking the subject about the current behaviour or habits, so the answer is \"now\"."}, "sentence_type": "Negative declarative sentence", "pos": {"nouns": {"I": 1, "The truth": 1}, "verbs": {"Say it": 1, "Total": 1, "be": 1, "not": 1}, "adverbs": {"Always": 1, "not": 1}, "numerals": {"31": 1}}}, {"content": "I'll avoid it when I feel my pride is hurt.", "word_count": 16, "label": "", "sentence_structure": "Subject鈥搗erb鈥搊bject", "subjective_objective": {"answer": "Subjective", "rationale": "Because \"avoidance\" is a choice of behaviour that needs to be determined by the subjective will of the individual. Even when he or she is injured by self-esteem, some may choose to face it rather than to avoid it. So \"shiding\" is the patient's subjective reaction and behavioural choice."}, "sentence_sentiment": {"answer": "Negative", "rationale": "Because the \"divide\" act in the entry is negative."}, "language_style": {"answer": "Calm and direct", "rationale": "The entry directly expresses the subject of questioning the other party ' s reaction to a self-esteem injury, without excessive rhetoric or emotional rendering, and with a more objective tone."}, "time_dimension": {"answer": "Past", "rationale": "The entry therefore falls into the category of present or past."}, "sentence_type": "Exclamatory sentence", "pos": {"nouns": {"Self-esteem": 1, "Hurt": 1}, "verbs": {"Yes": 1, "Avoidance": 1}, "adverbs": {"Just": 1}, "numerals": {"32": 1, "I": 1}, "pronouns": {"When": 1}}}, {"content": "I've often been forced to do things so much too much that others had to restrain me.", "word_count": 31, "label": "", "sentence_structure": "Subject鈥搗erb鈥搊bject", "subjective_objective": {"answer": "Subjective", "rationale": "Because the words \"absolutely compelled\" and \"others have to limit\" describe patients' subjective feelings and behaviour, the entry is viewed from a subjective perspective."}, "sentence_sentiment": {"answer": "Negative", "rationale": "Because both the entries \"pushing yourself to do something too much\" and \"others have to limit me\" have negative emotional aspects."}, "language_style": {"answer": "Tactful and warm", "rationale": ""}, "time_dimension": {"answer": "Present", "rationale": ""}, "sentence_type": "Negative declarative sentence", "pos": {"nouns": {"Things": 1, "person": 1, "Limits": 1}, "verbs": {"I'm not gonna let you do it": 1, "Fuck": 1, "We have to limit it": 1}, "adjectives": {"Overwhelming": 1}, "adverbs": {"Often": 1, "Automatically": 1}, "numerals": {"33": 1}, "pronouns": {"I": 1}}}, {"content": "34 My friends think I'm a country boy.", "word_count": 12, "label": "", "sentence_structure": "Subject鈥搗erb鈥搊bject", "subjective_objective": {"answer": "Subjective", "rationale": "Because this entry relates to the assessment and perception of patients by friends, it is viewed from the patient ' s subjective perspective."}, "sentence_sentiment": {"answer": "Negative", "rationale": "Because the expression \"regarded as a hillbilly\" in the entry is negative and carries a certain sense of derogatory and discriminatory feelings."}, "language_style": {"answer": "Tactful and warm", "rationale": "The entry uses a euphemism to express the image of the person in question among friends, without directly denying or attacking, but rather to direct the person in question to answer questions with questioning and concern. Such expression is more moderate and respectful of the emotional experience of the person interviewed, and is more conducive to obtaining accurate and true feedback."}, "time_dimension": {"answer": "Past", "rationale": ""}, "sentence_type": "Negative declarative sentence", "pos": {"nouns": {"Friends": 1, "Country boy": 1}, "verbs": {"Look at it": 1}, "numerals": {"34": 1}, "pronouns": {"I": 1}}}, {"content": "I've been avoiding it a lot when I'm angry.", "word_count": 12, "label": "", "sentence_structure": "Subject鈥搗erb鈥揷omplement", "subjective_objective": {"answer": "Subjective", "rationale": "Because \"scramble\" is a subjective response, which reflects how patients deal with their emotions and behaviour when they are angry."}, "sentence_sentiment": {"answer": "Negative", "rationale": "Because the \"divide\" act in the entry is negative."}, "language_style": {"answer": "Calm and direct", "rationale": "The language style of the entry is relatively calm and straightforward, with no excessive rhetorical or emotional rendering, and directly expresses the subject of questioning the behaviour of the other party in the event of anger."}, "time_dimension": {"answer": "Present", "rationale": "Now, \"at the time of...\" means time limits, and \"I often\" means that this is a constant habit. So, the sentence is a description of the person being tested for current behaviour and habits."}, "sentence_type": "Negative declarative sentence", "pos": {"nouns": {"I": 1, "Anger": 1}, "verbs": {"Often": 1, "Avoidance": 1}, "adverbs": {"in": 1}, "numerals": {"35": 1}}}, {"content": "I'm always more vigilant than I should be with those who are really nice to me.", "word_count": 30, "label": "", "sentence_structure": "Subject鈥搗erb鈥搊bject", "subjective_objective": {"answer": "Subjective", "rationale": "Because this entry describes patients ' subjective views and attitudes towards others."}, "sentence_sentiment": {"answer": "Negative", "rationale": "Because the behavior in the entry \"to be more vigilant towards my friendly people\" may lead to misunderstanding and unnecessary vigilance, and is therefore negative."}, "language_style": {"answer": "Calm and direct", "rationale": "The entry is presented in direct statements, with no excessive rhetorical or emotional rendering, and is more objective in tone. It asks whether the person surveyed is more vigilant about his friends, which is a calm and rational way of asking questions."}, "time_dimension": {"answer": "Present", "rationale": ""}, "sentence_type": "Affirmative declarative sentence", "pos": {"nouns": {"Vigilance": 1, "person": 2, "Friendly": 1, "Doubt": 1}, "verbs": {"Hold on": 1, "Watch out": 1, "to": 2, "Friendly": 1, "I think so": 1, "Doubtful": 1}, "adjectives": {"Indeed": 1}, "adverbs": {"Often": 1}, "numerals": {"36": 1, "Higher": 1}, "pronouns": {"I": 2}}}, {"content": "I've learned a lot of talent to live my life without any problems.", "word_count": 22, "label": "", "sentence_structure": "Subject鈥損redicate", "subjective_objective": {"answer": "Subjective", "rationale": "Because \"I have learned a special talent that will enable me to live my life without problems\" is the subjective idea and belief of the patient. It reflects the patient ' s assessment and expectations, and does not reflect the effects of the objective facts or the external environment. So the questionnaire entry is subjective."}, "sentence_sentiment": {"answer": "Positive", "rationale": "Because the expression \"special talent has been learned\" in the entry is positive."}, "language_style": {"answer": "Calm and direct", "rationale": "The entry is based on a more direct and explicit expression of the subject matter and the content of the problem, without using excessive rhetoric or emotional colours."}, "time_dimension": {"answer": "Past", "rationale": ""}, "sentence_type": "Affirmative declarative sentence", "pos": {"nouns": {"Talent": 1, "Problem": 1, "Fan": 1}, "verbs": {"I can learn": 1, "鈾?And I'm gonna be 鈾?: 2, "Pass": 1}, "adjectives": {"Special": 1, "Nothing": 1}, "numerals": {"37": 1}, "pronouns": {"I": 1}}}, {"content": "I sometimes choose people I hardly know when I'm voting.", "word_count": 22, "label": "", "sentence_structure": "Subject鈥搗erb鈥搊bject", "subjective_objective": {"answer": "Subjective", "rationale": "Because \"selection of those I hardly know\" is the patient's choice and opinion, and it's subjective."}, "sentence_sentiment": {"answer": "Negative", "rationale": "Because the action of choosing those I hardly know is somewhat negative."}, "language_style": {"answer": "Calm and direct", "rationale": "The entry directly expresses the subject of an inquiry as to whether the other party will choose a candidate who is not well known at the time of the election, in a balanced manner and without too much rhetorical or emotional overtones."}, "time_dimension": {"answer": "Present", "rationale": "In the future, the entry describes the behaviour or attitude of the testee in the future, i.e., he may choose someone he does not know at the election. Although the sentence refers to \"I am often\" , the word \"always\" here does not refer to past customary behaviour, but to future behavioural trends. Thus, the judgement is the future."}, "sentence_type": "Negative declarative sentence", "pos": {"nouns": {"Time": 1, "Elections": 1, "person": 2, "I don't know": 1}, "verbs": {"Choose": 1, "Roger that": 1}, "adverbs": {"Sometimes": 1, "Often": 1}, "numerals": {"38": 1}, "pronouns": {"I": 2}}}, {"content": "I've often been late for appointments.", "word_count": 9, "label": "", "sentence_structure": "Subject鈥搗erb鈥揷omplement", "subjective_objective": {"answer": "Subjective", "rationale": "Since the patient ' s ability to travel on time is about the patient ' s personal behaviour, it is his own subjective attitude and behaviour."}, "sentence_sentiment": {"answer": "Negative", "rationale": "Because the \"failure to make the appointment\" in the entry is negative."}, "language_style": {"answer": "Calm and direct", "rationale": "The entry is more directly linguistically, with no excessive emotional or rhetorical style, and directly addresses the time management problems of the subject. This expression is more concise and clear, and helps to obtain accurate data information."}, "time_dimension": {"answer": "Past", "rationale": "Since the key word in the title \"often cannot\" denotes a past customary act, the temporal dimension of the article is the past."}, "sentence_type": "Negative declarative sentence", "pos": {"nouns": {"Questionnaire Entry": 1, "About": 1}, "verbs": {"Often": 1, "Yes": 1, "Date": 1}, "adverbs": {"not": 1}, "numerals": {"39": 1}}}, {"content": "I've got a lot of fantasies, but I can't do much in real life.", "word_count": 15, "label": "", "sentence_structure": "Subject鈥搗erb鈥揷omplement", "subjective_objective": {"answer": "Objective", "rationale": "Because \"fanta\" and \"acts in real life\" are objective facts, and the title is just a description of the phenomenon, and it does not examine the subjective ideas of the patient."}, "sentence_sentiment": {"answer": "Negative", "rationale": "Because the \"fantasy, the less to be done in real life\" behavior and attitude in the entries are negative."}, "language_style": {"answer": "Tactful and warm", "rationale": "This is a relatively friendly expression that encourages the interviewee to respond in a real way."}, "time_dimension": {"answer": "Present", "rationale": "The \"fanta\" and \"real life\" in the entry are descriptions of the current situation, so the temporal dimension is now."}, "sentence_type": "Negative declarative sentence", "pos": {"nouns": {"I": 2, "Fantasy": 1, "Life": 1, "Reality": 1}, "verbs": {"Fantasy": 1, "Do it": 1, "be": 1}, "adverbs": {"Yeah": 1}, "numerals": {"40": 1}}}, {"content": "I'm ashamed to deal with people.", "word_count": 8, "label": "", "sentence_structure": "Subject鈥搗erb鈥揷omplement", "subjective_objective": {"answer": "Subjective", "rationale": "Because \"shammy\" is the expression of the patient's inner feelings and attitudes, and it's subjective."}, "sentence_sentiment": {"answer": "Negative", "rationale": "Because the act of \"smiling with people\" is negative and represents a lack of social skills and confidence."}, "language_style": {"answer": "Tactful and warm", "rationale": ""}, "time_dimension": {"answer": "Present", "rationale": "By the way, \"smuggling\" and \"dealing with people\" are the description of the person's current state and behaviour, so the entry indicates the current situation."}, "sentence_type": "Negative declarative sentence", "pos": {"nouns": {"I": 1, "person": 1, "Dealing": 1}, "verbs": {"Shame": 1, "Hand it over": 1}, "adverbs": {"Shame": 1}, "numerals": {"41": 1}}}, {"content": "42, I'm not afraid of anything.", "word_count": 6, "label": "", "sentence_structure": "Subject鈥搗erb鈥揷omplement", "subjective_objective": {"answer": "Subjective", "rationale": "Because fear is a manifestation of subjective emotion."}, "sentence_sentiment": {"answer": "Neutral", "rationale": "Because the entry is merely a description of the attitude of the interviewee and is not emotional."}, "language_style": {"answer": "Calm and direct", "rationale": "The language style of the entry is more straightforward, and there is no excessive adoration or emotional rendering, and the subject of asking the other side whether it is \"no fear of anything\" is expressed in a simple language. This tone makes one feel more objective, clear and understandable."}, "time_dimension": {"answer": "Present", "rationale": ""}, "sentence_type": "Negative declarative sentence", "pos": {"verbs": {"Not afraid": 1}, "adjectives": {"Not even": 1, "What": 1}, "numerals": {"42": 1}}}, {"content": "43, sometimes I think I'm an angel, sometimes I think I'm a demon.", "word_count": 20, "label": "", "sentence_structure": "Subject鈥搗erb鈥揷omplement", "subjective_objective": {"answer": "Subjective", "rationale": "Because \"think\" shows that patients are expressing their subjective views and feelings."}, "sentence_sentiment": {"answer": "Negative", "rationale": "Because the expression \"thinks you're the devil\" in the entry is negative."}, "language_style": {"answer": "Tactful and warm", "rationale": "The language style of this entry is more euphemistic, because it uses metaphoric rhetoric to express feelings and feelings. It is usually more moderate than direct statements, and it triggers resonance and reflection among the respondents. At the same time, the images of \"angels\" and \"demons\" are more abstract and subjective, and thus provide a better understanding of the emotional state and the inner world of the respondents."}, "time_dimension": {"answer": "Present", "rationale": "Sometimes it is considered that \"sometimes\" means \"recurrent, customary behaviour\" and \"consider\" takes the normal current state of affairs, which means that the act has not only occurred in the past but may still be ongoing. So, the entry to the questionnaire asks what the person is thinking and feeling."}, "sentence_type": "Affirmative declarative sentence", "pos": {"nouns": {"Time": 2, "I think": 2, "I": 2, "individual": 2, "Angel": 1, "Demon": 1}, "verbs": {"be": 2}, "adverbs": {"Sometimes": 2}, "numerals": {"43": 1}}}, {"content": "I won't lose, I won't lose.", "word_count": 12, "label": "", "sentence_structure": "Subject鈥搗erb鈥揷omplement", "subjective_objective": {"answer": "Subjective", "rationale": "Because \"win, not lose\" is a personal idea or attitude that is a manifestation of the patient's own mental state."}, "sentence_sentiment": {"answer": "Negative", "rationale": "Because the \"win-win\" attitude in the entries is competitive and dissuasive, it may lead to an excessive pursuit of victory and neglect of the process and enjoyment of the competition, and is therefore negative."}, "language_style": {"answer": "Calm and direct", "rationale": "The language style of the entry is more straightforward, not using rhetorical techniques or highly emotional terms, but rather a direct expression of the subject of questioning whether the other party is seeking victory and not accepting failure. Such language style gives people an objective, calm feeling that it is better to conduct a questionnaire to investigate situations that require neutrality and objectivity."}, "time_dimension": {"answer": "Future", "rationale": "The entry describes an expectation or belief in what may happen in the future, the key words being \"when the game is played\" and \"when the game is won, not lost.\" Although these things may not have happened, the testees express their expectation that they will always win in the future. So, judging by the dimensions of time, the future is the future."}, "sentence_type": "Negative declarative sentence", "pos": {"nouns": {"The game": 1, "I": 2, "Win": 1, "Lose": 1}, "verbs": {"in": 1, "Yes": 2, "Win": 1, "Lose": 1}, "adverbs": {"Only": 1}, "numerals": {"44": 1}}}, {"content": "45, when I'm angry, I'm ready to be a sarcasm.", "word_count": 15, "label": "", "sentence_structure": "Subject鈥搗erb鈥揷omplement", "subjective_objective": {"answer": "Subjective", "rationale": "Because \"getting\" means that it's a patient's subjective feeling and reaction in a given situation."}, "sentence_sentiment": {"answer": "Negative", "rationale": "Because the \"getting ready to squawk\" act in the entry is negative."}, "language_style": {"answer": "Calm and direct", "rationale": "The entry uses a direct language style, explicitly asking the respondents whether they are easily gruesome in anger, without adding too much emotional colour or rhetoric, and in a more objective and calm manner.\""}, "time_dimension": {"answer": "Present", "rationale": "\"When I'm angry\" shows that this is an emotional state, which may have happened in the current or the recent past, so the temporal dimension is now."}, "sentence_type": "Exclamatory sentence", "pos": {"nouns": {"Time": 1, "I": 2, "Anger": 1, "person": 1}, "verbs": {"鈾?And become 鈾?: 1, "Yes": 1, "Scratch": 1}, "adverbs": {"very": 1}, "numerals": {"45": 1}}}, {"content": "I'm a public counterattack when my self-esteem is hurt.", "word_count": 15, "label": "", "sentence_structure": "Subject鈥搗erb鈥搊bject", "subjective_objective": {"answer": "Subjective", "rationale": "Because \"public counteraction\" is a behaviour that is the patient's reaction to an injury to his or her self-esteem, his or her subjective attitude and choice."}, "sentence_sentiment": {"answer": "Negative", "rationale": "Because the \"public counterattack\" in the entry is negative."}, "language_style": {"answer": "Calm and direct", "rationale": "The article is more straightforward, is not couched in too many terms or emotional terms, and is more objective. It directly asks what the person will do when his or her self-esteem is injured, without attempting to channel the answer in a euphemism, so the language style is more calm and direct."}, "time_dimension": {"answer": "Present", "rationale": "The keyword \"publicly counter\" suggests that the tester will respond publicly when describing the current customary behaviour, i.e., the immediate self-esteem injury. Thus, the time dimension of the entry is now judged."}, "sentence_type": "Affirmative declarative sentence", "pos": {"nouns": {"Self-esteem": 1, "Hurt": 1, "Fight back": 1}, "verbs": {"Just": 1, "Public": 1, "Yes": 1}, "adverbs": {"In me": 1}, "numerals": {"46": 1}}}, {"content": "47, I think I should be turned over when I get hurt.", "word_count": 15, "label": "", "sentence_structure": "Subject鈥搗erb鈥搊bject", "subjective_objective": {"answer": "Subjective", "rationale": "Because \"think\" and \"think\" are words of personal attitude or opinion, it is viewed from the patient's subjective perspective."}, "sentence_sentiment": {"answer": "Negative", "rationale": "Because the act of \"turning face when injured\" in the entry is negative, it expresses a negative reaction to the injury of others."}, "language_style": {"answer": "Calm and direct", "rationale": "The entry presents its views in a direct presentation, without using any rhetorical or emotional rendering to express calm and clear language."}, "time_dimension": {"answer": "Present", "rationale": "The keyword \"considers\" and \"should\" indicates that it is a view or attitude without explicit reference; at the same time, \"at the time of injury\" means that it is an act in a particular situation, not a long-term habit. Thus, the entry describes the current view and attitude of the subject, which is judged in time dimension."}, "sentence_type": "Affirmative declarative sentence", "pos": {"nouns": {"Hurt": 1, "You're a fucking liar": 1}, "verbs": {"I think": 1, "Just": 1, "I think so": 1, "Yes": 1}, "numerals": {"47": 1}, "pronouns": {"I": 1}}}, {"content": "48, I read every day. Not every page.", "word_count": 14, "label": "", "sentence_structure": "Subject鈥搗erb鈥揷omplement", "subjective_objective": {"answer": "Subjective", "rationale": "Because reading and not reading are patients' subjective choices and behaviours."}, "sentence_sentiment": {"answer": "Positive", "rationale": "Because the entry expresses a reading habit and does not bring any emotional colour."}, "language_style": {"answer": "Calm and direct", "rationale": "This entry is based on a direct statement asking the interviewee whether he or she does not read all the pages of the newspaper."}, "time_dimension": {"answer": "Present", "rationale": ""}, "sentence_type": "Negative declarative sentence", "pos": {"nouns": {"I": 1, "Report": 1, "Board": 1}, "verbs": {"Read it": 2}, "adverbs": {"Chile": 1, "Both": 1}, "numerals": {"48": 1}}}, {"content": "49, when I'm depressed, I avoid.", "word_count": 8, "label": "", "sentence_structure": "Subject鈥搗erb鈥揷omplement", "subjective_objective": {"answer": "Objective", "rationale": "Because \"depression\" and \"exclude\" are objective facts that patients experience, and they do not involve subjective attitudes or feelings."}, "sentence_sentiment": {"answer": "Negative", "rationale": "Because the behavior \"shield\" in the entry is negative."}, "language_style": {"answer": "Calm and direct", "rationale": "The entry is designed to guide the respondents to answer questions by a specific description of \"I'm frustrated,\" without too much rhetorical or emotional rendering, and is more objective. So, its language style is straightforward and calm."}, "time_dimension": {"answer": "Present", "rationale": ""}, "sentence_type": "Negative declarative sentence", "pos": {"nouns": {"Frustration": 1, "Avoidance": 1}, "verbs": {"Yes": 1, "Avoid": 1}, "adverbs": {"Just": 1}, "numerals": {"49": 1}}}, {"content": "I'm shy about sex.", "word_count": 9, "label": "", "sentence_structure": "Subject鈥揷opula鈥損redicative", "subjective_objective": {"answer": "Subjective", "rationale": "Because shyness is a subjective emotional experience."}, "sentence_sentiment": {"answer": "Negative", "rationale": "Because the word \"screech\" expresses negative emotional attitudes."}, "language_style": {"answer": "Tactful and warm", "rationale": "The entry is more adept and interactive when it is spoken in a gentle and inviting manner, rather than by direct statements or orders."}, "time_dimension": {"answer": "Present", "rationale": "The main word for the entry is \"I\" and the word \"weak of sex\" is a statement of the current feeling or state, so the temporal dimension is now."}, "sentence_type": "Negative declarative sentence", "pos": {"nouns": {"Sexual issues": 1, "Shy": 1}, "verbs": {"Sensor": 1, "Present": 1}, "numerals": {"50": 1}}}, {"content": "I always felt like someone I knew was a protector.", "word_count": 18, "label": "", "sentence_structure": "Subject鈥搗erb鈥搊bject", "subjective_objective": {"answer": "Subjective", "rationale": "Because \"fear\" means that it's the patient's own feeling and perception, and it's subjective."}, "sentence_sentiment": {"answer": "Positive", "rationale": "Because \"protector\" is usually seen as a positive image and symbol of the person's role in helping and supporting the interviewee."}, "language_style": {"answer": "Tactful and warm", "rationale": "Warm and warm."}, "time_dimension": {"answer": "Present", "rationale": "\"I always feel \"always\" means \"recurring, customary\" behaviour, and \"fEalse\" means that it not only happened in the past, but may still be going on. So this entry describes what the tester feels now."}, "sentence_type": "Affirmative declarative sentence", "pos": {"nouns": {"Someone": 1, "Protect the gods": 1}, "verbs": {"I feel": 1, "Elephant": 1, "be": 1}, "adjectives": {"Total": 1}, "numerals": {"51": 1}, "pronouns": {"I": 2, "I know him": 1}}}, {"content": "52 My philosophies are: 鈥淒o not believe, do not do, do not look.鈥?, "word_count": 19, "label": "", "sentence_structure": "Subject鈥搗erb鈥揷omplement", "subjective_objective": {"answer": "Subjective", "rationale": "Because the philosophy of the world is the idea and the idea that is deep inside the patient, it is subjective."}, "sentence_sentiment": {"answer": "Negative", "rationale": "Because the \"unjustified, unjustified\" attitudes and behaviour in the entries are negative."}, "language_style": {"answer": "Calm and direct", "rationale": "The language style of the entry is more straightforward, and it is not based on too many rhetorical techniques or emotional renderings. It sets out in a logical and clear way the core philosophy of a human being鈥檚 life philosophy, namely, to believe in and practice what is reasonable and to avoid unreasonable audio-visual behavior. Such expression is more calm and objective, and helps readers to understand the meaning of the questionnaire entries."}, "time_dimension": {"answer": "Present", "rationale": "The entry asks about the current philosophies of the testee and thus judges it now."}, "sentence_type": "Affirmative declarative sentence", "pos": {"nouns": {"Philosophy of the world": 1, "I'm not": 3, "Letter": 1, "Do it": 1, "View": 1}, "verbs": {"EMPTY": 2}, "adverbs": {"EMPTY": 4}, "numerals": {"52": 1}}}, {"content": "53 I think that people have a good or a bad side.", "word_count": 9, "label": "", "sentence_structure": "Subject鈥搗erb鈥搊bject", "subjective_objective": {"answer": "Subjective", "rationale": "Because \"think\" means that it's a patient's personal opinion and conviction, and it's a subjective view."}, "sentence_sentiment": {"answer": "Negative", "rationale": "Because the entries are divided into \"good\" and \"bad\", this dichotomy of classification can lead to discrimination and prejudice and is negative."}, "language_style": {"answer": "Calm and direct", "rationale": "The language style of the entry is the \"cool and direct\" type. It is a narrative view, with no excessive adoration or emotional colour, and is more objective and straightforward. It is suitable for use in, for example, questionnaires, so that the interviewee can understand the questions with precision and give a true answer."}, "time_dimension": {"answer": "Present", "rationale": ""}, "sentence_type": "Affirmative declarative sentence", "pos": {"nouns": {"person": 1, "It's bad": 1}, "verbs": {"I think": 1}, "numerals": {"53": 1}}}, {"content": "I'd be in trouble at work or a seamstress if my boss got me mad.", "word_count": 29, "label": "", "sentence_structure": "Subject鈥搗erb鈥搊bject", "subjective_objective": {"answer": "Subjective", "rationale": "Because the expression \"I may\" involves the patient's personal thoughts and feelings, and it's subjective."}, "sentence_sentiment": {"answer": "Negative", "rationale": "Because the entries \"problem\" and \"marber\" are negative."}, "language_style": {"answer": "Calm and direct", "rationale": "The entry uses direct language to express dissatisfaction and possible behaviour of the superior, does not use a rhetorical or other euphemism, and therefore appears to be more calm and straightforward in language style.\""}, "time_dimension": {"answer": "Future", "rationale": "The key word \"if\" and \"may\" is to judge that the act has not yet taken place, so it is for the future."}, "sentence_type": "Negative declarative sentence", "pos": {"nouns": {"Problem": 1, "Sir": 1, "I": 2, "Work": 1, "Trouble": 1, "A seaman": 1, "Revenge": 1}, "verbs": {"Maybe": 1, "Find": 1, "Fuck": 1, "Angry": 1, "in": 1, "Do it": 1, "or": 1}, "adverbs": {"If": 1, "Just": 1, "Maybe": 2}, "numerals": {"54": 1}}}, {"content": "55, everybody's against me.", "word_count": 9, "label": "", "sentence_structure": "Subject鈥搗erb鈥揷omplement", "subjective_objective": {"answer": "Subjective", "rationale": "This is a subjective examination, as it is the patient ' s own perception and understanding of whether he is doing something to himself."}, "sentence_sentiment": {"answer": "Negative", "rationale": "Because the expression \"everyone and I are doing it\" in the entry has a certain negative sentiment, which means that people are acting in a way that makes them uncomfortable or troubled."}, "language_style": {"answer": "Tactful and warm", "rationale": "The language style of the entry is more euphemistic. It raises questions in a question-and-answer manner, with no direct blame or assault, and reflects respect and care for the person interviewed. At the same time, the expression \"everyone\" is used to make the issue more inclusive and universal, and to stimulate reflection and resonance among the person interviewed."}, "time_dimension": {"answer": "Present", "rationale": "The entry can be judged to describe the current state by reference to the word \"all\" in the sentence and to the absence of a clear time word (such as the verb of the past or the future state of the situation), and thus the temporal dimension is now."}, "sentence_type": "Negative declarative sentence", "pos": {"nouns": {"person": 1, "I": 1}, "verbs": {"- To the front": 1}, "numerals": {"55": 1}}}, {"content": "I'm always friendly with people I hate.", "word_count": 15, "label": "", "sentence_structure": "Subject鈥搗erb鈥揷omplement", "subjective_objective": {"answer": "Subjective", "rationale": "Because \"hate\" and \"friendly\" are patients' emotional attitudes and patients' subjective feelings."}, "sentence_sentiment": {"answer": "Negative", "rationale": "Because the entry \"I hate people\" is negative, and the \"expression\" is contradictory, so the emotional color is negative."}, "language_style": {"answer": "Calm and direct", "rationale": "The entry \"Calm down straight\" is a direct and clear query about the behaviour of the person under investigation, with no excessive rhetorical or emotional rendering, and is more objective in tone."}, "time_dimension": {"answer": "Past", "rationale": "So the output should read: \"The Past\"."}, "sentence_type": "Negative declarative sentence", "pos": {"nouns": {"Questionnaire": 1, "Entry": 1}, "verbs": {"Organisation": 1, "Friendly": 1, "Shit": 1}, "adjectives": {"Friendly": 1, "Shit": 1}, "adverbs": {"Often": 1}, "numerals": {"56": 1}}}, {"content": "57, if one of the engines on my plane fails, I'm very nervous.", "word_count": 23, "label": "", "sentence_structure": "Subject鈥損redicate", "subjective_objective": {"answer": "Subjective", "rationale": "Because feeling nervous is an emotional reaction, it is the patient's subjective feeling."}, "sentence_sentiment": {"answer": "Negative", "rationale": "Because \"very nervous\" in the entry is a negative emotional attitude."}, "language_style": {"answer": "Calm and direct", "rationale": "The entry presents a hypothetical situation in a straightforward manner and asks the other side for emotional reactions in the face of such a situation, without excessive rhetorical or emotional rendering, and with a more balanced and objective tone. \""}, "time_dimension": {"answer": "Present", "rationale": "Now, the entry \"I'll be very nervous\" means a hypothetical emotional reaction, not a description of the past or the future. So the temporal dimension is now."}, "sentence_type": "Exclamatory sentence", "pos": {"nouns": {"Aircraft": 1, "Engines": 1, "Nervous": 1}, "verbs": {"Take it": 1, "It's not working": 1, "Yes": 1, "Very": 1}, "adverbs": {"If": 1, "Just": 1, "Very": 1}, "numerals": {"57": 1}, "classifiers": {"One": 2}, "pronouns": {"I": 1}}}, {"content": "58, I know a man who can do anything and does what he does right.", "word_count": 22, "label": "", "sentence_structure": "Subject鈥搗erb鈥搊bject", "subjective_objective": {"answer": "Subjective", "rationale": "Because \"he can do anything and do justly\" is the patient's subjective view and evaluation."}, "sentence_sentiment": {"answer": "Positive", "rationale": "Because \"can do\" and \"reasonable integrity\" are all positive descriptions."}, "language_style": {"answer": "Tactful and warm", "rationale": "The entry uses a euphemism to stimulate each other's thinking about their own situation by describing the experience of \"I know such a person.\" At the same time, the assessment of \"doing and doing what is right\" is also positive and positive emotional, increasing the tenderness and appeal of language."}, "time_dimension": {"answer": "Present", "rationale": "The entry describes the expectations and assumptions of what might happen in the future, and \"I know such a person\" suggests that this person has not yet appeared or exists in the future. So the judgement is based on the words of the key to the future in terms of time dimension, \"know such a person\" and \"does everything he can and does it with integrity\" that relate to things that have not happened."}, "sentence_type": "Affirmative declarative sentence", "pos": {"nouns": {"person": 1, "Yes": 1, "Reasonable": 1, "Integrity": 1}, "verbs": {"Yes": 1, "Do it": 2}, "adjectives": {"Both": 1, "Reasonable": 1, "Integrity": 1}, "numerals": {"58": 1, "One": 2}, "pronouns": {"he": 1}}}, {"content": "59, if my emotional loss is preventing me from doing what I'm doing, then I can control it.", "word_count": 27, "label": "", "sentence_structure": "Subject鈥損redicate", "subjective_objective": {"answer": "Subjective", "rationale": "Because the problem involves patient emotional control and behavioral decision-making, it depends on patient鈥檚 subjective judgement and ability to regulate. While the venting of emotions may affect the career being performed, how to deal with this impact depends on the patient鈥檚 own decision and ability."}, "sentence_sentiment": {"answer": "Positive", "rationale": "Because the behavior and attitude of \"manage\" in the entries is positive."}, "language_style": {"answer": "Calm and direct", "rationale": "The language style of the entry is more calm and straightforward, and there is no excessive emotional rendering or rhetorical technique. It presents a simple question and gives an answer direction. It is appropriate for official situations such as questionnaires."}, "time_dimension": {"answer": "Present", "rationale": ""}, "sentence_type": "Negative declarative sentence", "pos": {"nouns": {"Emotion": 2, "- Out": 1, "- Intrusive": 1, "Career": 1, "Control": 1}, "verbs": {"Yes": 2, "鈾?And I'm gonna be 鈾?: 1, "Control": 1, "- Intrusive": 1}, "adverbs": {"Just": 1, "Well": 1, "of": 3}, "numerals": {"59": 1}, "pronouns": {"I": 1}}}, {"content": "60, some people are planning to kill me.", "word_count": 10, "label": "", "sentence_structure": "Subject鈥搗erb鈥搊bject", "subjective_objective": {"answer": "Subjective", "rationale": "Because \"some people are planning to kill me\" is the patient's subjective idea, not the objective fact."}, "sentence_sentiment": {"answer": "Negative", "rationale": "Because the conspiracy to kill me is negative, it means that there is a risk of harm or threat, and it has a certain negative emotional connotation."}, "language_style": {"answer": "Calm and direct", "rationale": "The entry raises the question in a direct and clear manner, without excessive rhetorical or emotional rendering, and is more balanced and objective, so that the language style is \"cool and straight\"."}, "time_dimension": {"answer": "Present", "rationale": "The keyword \"is\" means that the act is in progress, while the term \"scheming to harm me\" concerns the situation and feelings."}, "sentence_type": "Affirmative declarative sentence", "pos": {"nouns": {"person": 1, "Shit": 1, "I": 1}, "verbs": {"Conspiracy": 1}, "numerals": {"60": 1, "Some": 1}}}, {"content": "61, I usually see the good side of the bad.", "word_count": 15, "label": "", "sentence_structure": "Subject鈥揷opula鈥損redicative", "subjective_objective": {"answer": "Subjective", "rationale": "Because the feelings and thoughts of seeing are the subjective perceptions of the patient."}, "sentence_sentiment": {"answer": "Positive", "rationale": "Because the purpose of the entry is to see the good side of the bad."}, "language_style": {"answer": "Tactful and warm", "rationale": "The entry is more adept and interactive when it is spoken in a gentle and inviting manner, rather than by direct statements or orders."}, "time_dimension": {"answer": "Present", "rationale": "The keyword \"usually\" indicates that it is a regular, customary act or attitude, while \"seeing the good side of the bad\" indicates a subjective view or reaction to the current state of affairs, so the entry describes the present behaviour or attitude of the testee."}, "sentence_type": "Affirmative declarative sentence", "pos": {"nouns": {"Bad times": 1, "Noodles": 1}, "verbs": {"Yeah": 1, "When": 1, "Do it": 1}, "adjectives": {"Okay": 1}, "numerals": {"61": 1, "I": 1, "Usually": 1, "It's good in the bad": 2, "Medium": 1, "One side": 1}}}, {"content": "I had to do something I didn't want to do, and I had a headache.", "word_count": 20, "label": "", "sentence_structure": "Subject鈥搗erb鈥揷omplement", "subjective_objective": {"answer": "Subjective", "rationale": "Because headaches are a subjective feeling and experience of the patient, depending on the patient鈥檚 attitude and emotional reaction to what is done. While this feeling may have been caused by some physical reason, in this case it appears as a subjective evaluation."}, "sentence_sentiment": {"answer": "Negative", "rationale": "Because the expression \"I have to do something I don't want to do\" has a passive emotional color and the result of \"headache\" is also negative."}, "language_style": {"answer": "Calm and direct", "rationale": "The entry describes in a more direct language style the link between the headache of the person under investigation and the unwillingness to do something, with no excessive emotional rendering or euphemism, and with greater objectivity and relevance."}, "time_dimension": {"answer": "Past", "rationale": "The entry therefore contains a description of the past and a possible projection of the future, with the temporal dimension judged as the past and the future."}, "sentence_type": "Negative declarative sentence", "pos": {"nouns": {"I": 1, "Things": 1, "Headache": 1}, "verbs": {"Do it": 1, "Go on": 1, "Okay": 1, "not": 1, "May I": 1, "Ouch": 1, "have": 1}, "adjectives": {"Yes": 2, "None": 1, "May I": 1, "of": 2, "Headache": 1}, "adverbs": {"Just": 1}, "numerals": {"62": 1}}}, {"content": "I often find myself very friendly with those who deserve to hate.", "word_count": 20, "label": "", "sentence_structure": "Subject鈥搗erb鈥搊bject", "subjective_objective": {"answer": "Subjective", "rationale": "The basis for the judgement: this is the attitude and behaviour of the patient himself, which is described by the patient, and which is his subjective thoughts and feelings."}, "sentence_sentiment": {"answer": "Negative", "rationale": "Because the \"expression of friendship\" in the entry is against common sense and therefore has a negative emotional connotation."}, "language_style": {"answer": "Tactful and warm", "rationale": "This entry uses euphemism to express the behaviour of the person who is being investigated who may be friendly to the person whom he should hate. This language style is not too direct or radical to be too resonant and to be more open to the person being investigated."}, "time_dimension": {"answer": "Past", "rationale": "\"Always\" and \"for those who deserve to be hated\" suggest that this is a regular practice which has occurred many times in the past and can therefore be judged to be the past."}, "sentence_type": "Affirmative declarative sentence", "pos": {"nouns": {"I": 1, "person": 2, "Questionnaire": 1, "Entry": 1}, "verbs": {"Often": 1, "Found": 1, "to": 2, "Organisation": 1, "Hate": 1, "Friendly": 1}, "adjectives": {"I suppose so": 1, "very": 1}, "numerals": {"63": 1}}}, {"content": "I think \"everyone has good will\" doesn't exist. If you're not, you're not.", "word_count": 27, "label": "", "sentence_structure": "Subject鈥搗erb鈥搊bject", "subjective_objective": {"answer": "Subjective", "rationale": "Because the title \"I think\" shows that it's a personal opinion and conviction of the patient, and it's a subjective view."}, "sentence_sentiment": {"answer": "Negative", "rationale": "Because the idea in the entry that everyone has good will is not there to be."}, "language_style": {"answer": "Calm and direct", "rationale": "The entry directly expresses the author's scepticism about the idea that everyone has good will, and that if a person is not good, then everything is bad. Such language is calm, objective, and not too emotional or rhetorical."}, "time_dimension": {"answer": "Past", "rationale": ""}, "sentence_type": "Negative declarative sentence", "pos": {"nouns": {"64": 1, "I think": 1, "Good will": 2, "person": 1, "Existence": 1, "If": 1, "Not good": 2, "Everything": 1}, "verbs": {"be": 1, "have": 1, "I think": 1, "Existence": 1, "If you": 1, "not": 1, "Okay": 2, "Well": 1, "Both": 1, "It doesn't exist": 1}, "adjectives": {"Good will": 2, "Not good": 2}, "numerals": {"64": 1, "Two": 1}, "classifiers": {"individual": 1}, "pronouns": {"you": 2, "Everything": 1}}}, {"content": "I'm not gonna be angry at those people I hate.", "word_count": 16, "label": "", "sentence_structure": "Subject鈥搗erb鈥搊bject", "subjective_objective": {"answer": "Subjective", "rationale": "Because \"like\" and \"hate\" are patients' subjective attitudes."}, "sentence_sentiment": {"answer": "Negative", "rationale": "Because the entry \"never angry with those I hate\" suggests that you hide your emotions from those who hate, which can lead to an accumulation of depression and discontent, which is detrimental to the mental health of the individual."}, "language_style": {"answer": "Calm and direct", "rationale": "The language style of the entry is relatively direct, not using too many acrimonyms or euphemisms, but rather expressing a direct attitude towards objects that do not want to express anger."}, "time_dimension": {"answer": "Past", "rationale": "\"never\" expresses a firm decision or attitude, and \"Expressing anger at those I hate\" is an act, so the entry is to question the testee's past behavior."}, "sentence_type": "Negative declarative sentence", "pos": {"nouns": {"person": 1, "Anger": 1}, "verbs": {"Never": 1, "to": 1, "Organisation": 1, "Ew": 1}, "adjectives": {"Boring": 1, "Hate": 1}, "adverbs": {"Never": 1}, "numerals": {"65": 1}, "pronouns": {"I": 1}}}, {"content": "66, I'm sure life is unjust to me.", "word_count": 12, "label": "", "sentence_structure": "Subject鈥揷opula鈥損redicative", "subjective_objective": {"answer": "Subjective", "rationale": "Because \"I'm sure\" represents patient's subjective attitude and conviction."}, "sentence_sentiment": {"answer": "Negative", "rationale": "Because the expression \"life is unjust to me\" in the entry is a negative emotion and attitude."}, "language_style": {"answer": "Calm and direct", "rationale": "The language style of the entry is more calm and objective, and there is no excessive emotional rendering or rhetorical technique, but rather a direct expression of the attitude to the subject of asking about the fairness of life. And the expression \"I am sure\" is also more firm and direct."}, "time_dimension": {"answer": "Present", "rationale": "The entry expresses the views and beliefs of the testee, without reference to past or future behaviour or mentality. The keyword \"I am sure\" indicates that the testee is convinced of his own views, and \"life is unjust to me\" is the subjective feeling and conviction of the testee. So, the time dimension of the questionnaire entry is now."}, "sentence_type": "Negative declarative sentence", "pos": {"nouns": {"I": 2, "Life": 1, "Justice": 1}, "verbs": {"Sure": 1, "be": 1, "to": 1}, "adjectives": {"not": 1}, "numerals": {"66": 1}}}, {"content": "67, with a severe blow, I'll fall.", "word_count": 13, "label": "", "sentence_structure": "Subject鈥揷opula鈥損redicative", "subjective_objective": {"answer": "Subjective", "rationale": "Because \"falling down\" is a description of a person's sense and subjective attitude, and it's the patient's own subjective experience."}, "sentence_sentiment": {"answer": "Negative", "rationale": "Because the \"falling\" act in the entry is negative."}, "language_style": {"answer": "Calm and direct", "rationale": "The language style of the entry is more calm and straightforward, and more objective terms are used to express the theme \"I will fall under a severe blow.\" This language style is usually used in surveys or research questionnaires to avoid directing respondents to specific answers."}, "time_dimension": {"answer": "Present", "rationale": "\"will\" is a verb of a state, which indicates a preference or possibility that such an act may occur in the future and therefore is judged to be the present."}, "sentence_type": "Negative declarative sentence", "pos": {"nouns": {"Serious": 1, "Strike": 1, "I": 1, "Falling": 1}, "verbs": {"Yes": 1, "Get down": 1}, "adjectives": {"Down there": 1}, "numerals": {"67": 1}}}, {"content": "68, when I realized I had to face a difficult situation, like exams, trade unions. I tried to imagine what it would do and planned to deal with it.", "word_count": 46, "label": "", "sentence_structure": "Subject鈥搗erb鈥搊bject", "subjective_objective": {"answer": "Objective", "rationale": "Because the sentence describes an act and a response strategy in a given situation, it is an objective result that examines the patient ' s behavioural reaction rather than subjective thinking."}, "sentence_sentiment": {"answer": "Positive", "rationale": "The purpose of the article is to express positive attitudes and strategic behaviour in dealing with dilemmas."}, "language_style": {"answer": "Calm and direct", "rationale": "The entry describes in a straightforward and clear way the coping strategies of people in situations of distress, with no excessive emotional or rhetorical tact, and is more objective and incriminating."}, "time_dimension": {"answer": "Present", "rationale": "In the entry, \"I realize\" and \"I'm trying to imagine what it's going to be and to plan some way to deal with it\" means that the action or state that is currently under way, so I'm judging now."}, "sentence_type": "Affirmative declarative sentence", "pos": {"nouns": {"The dilemma": 1, "Examinations": 1, "Recruitment fair": 1, "Time": 2, "Methodology": 1}, "verbs": {"I'm aware of that": 1, "Faced": 1, "Imagine": 1, "Plan": 1, "Payable": 1}, "adverbs": {"Just": 1, "Yes": 1}, "numerals": {"68": 1}, "pronouns": {"I": 1}}}, {"content": "69, the doctors will never really figure out what I'm suffering from.", "word_count": 17, "label": "", "sentence_structure": "Subject鈥搗erb鈥揷omplement", "subjective_objective": {"answer": "Subjective", "rationale": "Because \"never\" is a subjective judgement, not an objective statement based on facts."}, "sentence_sentiment": {"answer": "Negative", "rationale": "The basis for the judgment: \"never really ascertain\" in the entry expresses a question about the ability of a doctor and a lack of trust, and it is a negative emotion."}, "language_style": {"answer": "Tactful and warm", "rationale": "The language style of the entry is more euphemistic, and it asks the respondents, in a sense of care and understanding, whether they can really understand their condition."}, "time_dimension": {"answer": "Present", "rationale": "The words \"know\" and \"disease\" suggest that this attitude is in the current or past, so the temporal dimension is now."}, "sentence_type": "Negative declarative sentence", "pos": {"nouns": {"Doctors": 1, "Diseases": 1}, "verbs": {"Never": 1, "I know": 1, "Patient": 1}, "adverbs": {"Really": 1}, "numerals": {"69": 1}, "pronouns": {"I": 1}}}, {"content": "I'm not sad when someone close to me dies.", "word_count": 18, "label": "", "sentence_structure": "Subject鈥揷opula鈥損redicative", "subjective_objective": {"answer": "Subjective", "rationale": "Because it is the patient's subjective feeling and attitude."}, "sentence_sentiment": {"answer": "Negative", "rationale": "Because the attitude of \"not sad\" in the entry is negative."}, "language_style": {"answer": "Calm and direct", "rationale": ""}, "time_dimension": {"answer": "Present", "rationale": ""}, "sentence_type": "Negative declarative sentence", "pos": {"nouns": {"person": 1, "Sad": 1}, "verbs": {"Die": 1, "not": 1, "Sad": 1}, "adjectives": {"Close": 1}, "adverbs": {"When": 1, "No, it's not": 1}, "numerals": {"70銆?: 1}, "pronouns": {"I": 1}}}, {"content": "71, I apologize for my rudeness after I fought for my own good.", "word_count": 26, "label": "", "sentence_structure": "Subject鈥搗erb鈥搊bject", "subjective_objective": {"answer": "Subjective", "rationale": "This is the patient鈥檚 subjective attitude or post-acting self-perception. Although the fight is an objective fact, the apology is subjective, as is the cause (brubbery)."}, "sentence_sentiment": {"answer": "Negative", "rationale": "Because the actions in the entries are negative."}, "language_style": {"answer": "Tactful and warm", "rationale": "The language style of the entry is euphemism. It expresses the common nature of behaviour, while avoiding direct accusations or criticism, by using words such as \"often\". Moreover, the expression \"poll\" is also accompanied by a self-reflectional and respectful attitude."}, "time_dimension": {"answer": "Past", "rationale": "The phrase \"after\" is included in the sentence, which indicates what happened in the past; at the same time, it is also suggested that it is a regular act. Thus, the entry describes the conduct or habits of the person who was tested in the past."}, "sentence_type": "Affirmative declarative sentence", "pos": {"nouns": {"Interest": 1, "person": 2, "Fighting": 1, "Apologize": 1}, "verbs": {"in": 1, "For": 1, "Often": 1, "Because": 1, "Naughty": 1, "In": 1, "Apologize": 1}, "adjectives": {"Naughty": 1}, "adverbs": {"After": 1, "Often": 1}, "numerals": {"71": 1}, "pronouns": {"I": 2}}}, {"content": "72, most of what happened to me wasn't my responsibility.", "word_count": 19, "label": "", "sentence_structure": "Subject鈥揷opula鈥損redicative", "subjective_objective": {"answer": "Subjective", "rationale": "Because this phrase expresses the patient ' s perception and attitude as to whether he is responsible for certain things, it is subjective."}, "sentence_sentiment": {"answer": "Negative", "rationale": "Because the entry expresses the negative attitude of the parties to their environment, and considers that most matters are not their own responsibility, tend to blame others and to avoid responsibility."}, "language_style": {"answer": "Tactful and warm", "rationale": "The language style of the entry is more moderate, friendly, and does not directly blame or criticize the other side for its lack of responsibility, but rather leads to the other side in a more inclusive and understanding way. This expression is more personal and interactive."}, "time_dimension": {"answer": "Present", "rationale": "The entry describes the current feelings and thoughts of the testee, and the words \"occupying\" and \"most\" indicate that this is a fact or situation that is happening, so the temporal dimension is now judged."}, "sentence_type": "Negative declarative sentence", "pos": {"nouns": {"Situation": 1, "Responsibility": 2, "Things": 2}, "verbs": {"Come on": 1, "be": 1, "Related": 1}, "adverbs": {"Most of them": 1, "No, it's not": 1}, "numerals": {"72": 1}, "pronouns": {"I": 1}}}, {"content": "73, when I feel depressed or anxious, eat something that makes me feel better.", "word_count": 26, "label": "", "sentence_structure": "Subject鈥搗erb鈥搊bject", "subjective_objective": {"answer": "Subjective", "rationale": "Because the idea and feeling of eating is the patient's subjective feeling and thought."}, "sentence_sentiment": {"answer": "Positive", "rationale": "Because the \"Eat something\" in the entry may be a useful way to ease emotions in some cases. While it does not solve the underlying problems, it can help people forget their worries and ease their negative emotions for a short time. So I think it is a positive expression."}, "language_style": {"answer": "Tactful and warm", "rationale": "The entry is more personal and interactive in its expression of a gentle and inviting tone, rather than a direct statement or order. At the same time, the expression \"make me feel better\" is more euphemistic, rather than too tough to point out the need for a certain act, but rather gives people a sense of self-selection."}, "time_dimension": {"answer": "Present", "rationale": "The words \"fear,\" \"eat,\" \"may\" all indicate that this is a context of the situation at hand, so the entry is now."}, "sentence_type": "Affirmative declarative sentence", "pos": {"nouns": {"Emotion": 1, "Repression": 1, "Anxiety": 1, "Unsettled": 1, "Something": 1}, "verbs": {"Feel": 2, "鈾?And I'm gonna be 鈾?: 1, "Eat": 1}, "adjectives": {"Better": 1}, "numerals": {"73": 1}, "classifiers": {"Points": 1}, "pronouns": {"I": 1}}}, {"content": "74, hard work makes me feel better.", "word_count": 10, "label": "", "sentence_structure": "Subject鈥搗erb鈥揷omplement", "subjective_objective": {"answer": "Subjective", "rationale": "Because \"fear better\" is the patient's feelings and experience of working hard, and it's a subjective assessment."}, "sentence_sentiment": {"answer": "Positive", "rationale": "Because \"working hard\" and \"favouring better\" both express positive emotional attitudes."}, "language_style": {"answer": "Tactful and warm", "rationale": "The entry uses a mild expression like \"favorite\" without directly describing strong emotional terms such as \"happy\" and \"fulfilling\" and expressing a more incisive and implicit attitude. Moreover, the act of \"working hard\" is recognized and praised, and gives a sense of encouragement and support in general, and the language style is more euphemistic and warm."}, "time_dimension": {"answer": "Present", "rationale": "In conclusion, according to the entry entered, \"74, hard work makes me feel better\" can be judged as being current behavior or mentality."}, "sentence_type": "Affirmative declarative sentence", "pos": {"nouns": {"Work hard": 1, "Feel": 1}, "verbs": {"鈾?And I'm gonna be 鈾?: 1, "Work": 1, "Feel": 1, "Okay": 1}, "numerals": {"74": 1}}}, {"content": "75, the doctor can't really help me with this.", "word_count": 12, "label": "", "sentence_structure": "Subject鈥揷opula鈥損redicative", "subjective_objective": {"answer": "Subjective", "rationale": "Because \"can really help me solve problems\" is a subjective assessment that reflects patients' perceptions and expectations of doctors' abilities."}, "sentence_sentiment": {"answer": "Negative", "rationale": "Because the phrase \"can't really help me solve the problem\" in the entry is negative and shows that there's little trust in doctors."}, "language_style": {"answer": "Calm and direct", "rationale": "The entry conveys information in a direct presentation, without excessive rhetorical or emotional rendering, and is more calm and objective in its linguistic style."}, "time_dimension": {"answer": "Present", "rationale": ""}, "sentence_type": "Negative declarative sentence", "pos": {"nouns": {"Problem": 1, "Doctor": 1}, "verbs": {"Help": 1, "Resolve": 1}, "adverbs": {"True": 1}, "numerals": {"75": 1}}}, {"content": "I've heard a lot about my feelings.", "word_count": 15, "label": "", "sentence_structure": "Subject鈥搗erb鈥搊bject", "subjective_objective": {"answer": "Subjective", "rationale": "Because \"not to expose your feelings\" is an emotional expression that requires a judgement of the patient's personal feelings and experience, it is subjective."}, "sentence_sentiment": {"answer": "Negative", "rationale": "Because the expression of the entry is negative, i.e. it is often said that it does not reveal its feelings."}, "language_style": {"answer": "Tactful and warm", "rationale": "The entry is more adept and interactive when it is spoken in a gentle and inviting manner, rather than by direct statements or orders."}, "time_dimension": {"answer": "Present", "rationale": "The word \"always\" in \"I hear\" denotes a regular, customary act, while the word \"hear\" uses the usual current pattern, meaning that this act has not only happened in the past, but may still be ongoing."}, "sentence_type": "Negative declarative sentence", "pos": {"nouns": {"76": 1, "Questionnaire": 1, "Entry": 1, "person": 1, "Emotion": 1}, "verbs": {"Listen": 1, "Say it": 2, "Exposure": 1}, "adverbs": {"Regular": 1}, "numerals": {"76": 1}, "classifiers": {"individual": 1}, "pronouns": {"I": 1}}}, {"content": "77 I think people are watching movies, plays or books that mean more to the senses than they want to be in these works.", "word_count": 35, "label": "", "sentence_structure": "Subject鈥搗erb鈥搊bject", "subjective_objective": {"answer": "Subjective", "rationale": "Because the title \"I think\" shows that it's a patient's subjective view."}, "sentence_sentiment": {"answer": "Positive", "rationale": "Because the entry expresses the positive view that artistic works may be understood more than the author intended, which is positive."}, "language_style": {"answer": "Tactful and warm", "rationale": "The entry presents a point in an invitation and uses a comparison to express the strength of the view, rather than direct command or assertion. This language style is more acclaimable, gentler, and easily resonates and reflects the reader."}, "time_dimension": {"answer": "Present", "rationale": "The entry is for the purpose of asking the respondent about the understanding and understanding of the viewing of films, theatre or books, and the temporal dimension is now judged."}, "sentence_type": "Affirmative declarative sentence", "pos": {"nouns": {"People": 1, "Film": 1, "Theatre": 1, "Books": 1, "Meaning": 3, "Works": 1}, "verbs": {"I think": 1, "Look": 1, "Learn": 1, "To express": 1}, "numerals": {"77": 1}, "pronouns": {"Here": 1}}}, {"content": "78, I feel like I have some custom or ritual behavior I have to do, and it's causing me a lot of trouble.", "word_count": 32, "label": "", "sentence_structure": "Subject鈥搗erb鈥搊bject", "subjective_objective": {"answer": "Subjective", "rationale": "Because \"no one can be free\" and \"customary\" and \"problem\" are the feelings and experiences of the patient, so it's subjective."}, "sentence_sentiment": {"answer": "Negative", "rationale": "Because the \"problems\" act in the entry is negative."}, "language_style": {"answer": "Tactful and warm", "rationale": ""}, "time_dimension": {"answer": "Present", "rationale": "The entry describes the current feelings and experiences of the testee and includes the ongoing exercise of \"some custom or ritual behaviour that is not self-determined\" and therefore the temporal dimension is now judged."}, "sentence_type": "Affirmative declarative sentence", "pos": {"nouns": {"I": 2, "Some": 1, "It's not up to you": 1, "Custom": 1, "or": 1, "Ceremonial acts": 1, "and": 1, "Here": 1, "Bring it": 1, "A lot": 1, "Trouble": 1}, "verbs": {"Feel it": 1, "have": 1, "Go on": 2, "Do it": 2, "Give it to me": 1}, "adverbs": {"I can't help it": 1, "very": 1}, "numerals": {"78": 1, "Some": 1, "A lot": 1}, "classifiers": {"individual": 1}, "pronouns": {"I": 2}}}, {"content": "79, when I'm nervous, I drink or take pills.", "word_count": 11, "label": "", "sentence_structure": "Subject鈥搗erb鈥揷omplement", "subjective_objective": {"answer": "Subjective", "rationale": "Because \"I\" shows that it's the patient's own behavior and attitude."}, "sentence_sentiment": {"answer": "Negative", "rationale": "Because \"when you're nervous\" and \"drinking or taking medication\" are negative behavior."}, "language_style": {"answer": "Calm and direct", "rationale": "The language style of the entry is more calm and objective, and it does not use excessive rhetorical techniques or emotional rendering. It directly expresses the way in which individuals can react when they are nervous, which is more direct and concise in the questionnaire."}, "time_dimension": {"answer": "Present", "rationale": "According to the title \"Drinking or taking medication\", \"when\"... it can be seen as describing the current tension and coping, and thus judging as now."}, "sentence_type": "Affirmative declarative sentence", "pos": {"nouns": {"I": 1, "Wine": 1, "Medicine": 1}, "verbs": {"Drink": 1, "Eat": 1, "Nervous": 1}, "numerals": {"79": 1}}}, {"content": "80, I want to stay with someone when I'm in a bad mood.", "word_count": 17, "label": "", "sentence_structure": "Subject鈥搗erb鈥揷omplement", "subjective_objective": {"answer": "Subjective", "rationale": "Because \"want\" means a state of mind, a subjective feeling of the patient."}, "sentence_sentiment": {"answer": "Positive", "rationale": "Because the \"want to stay with someone\" act is positive."}, "language_style": {"answer": "Tactful and warm", "rationale": "The entry uses moderate language to ask the interviewees whether they have a need to be with others in the event of a poor mood, without direct orders or criticism, showing respect and understanding of the interviewees, and a more euphemismal style."}, "time_dimension": {"answer": "Present", "rationale": "The word \"when\" in \"when I'm not happy\" indicates the existence of conditions or conditions, and \"discomfort\" is a phrase describing the state of affairs; \"in the case of wanting to stay with someone else\" indicates the tendency or habit of the person being tested in the case. The entry is therefore an inquiry into the person being tested for behaviour or reaction in the present circumstances."}, "sentence_type": "Affirmative declarative sentence", "pos": {"nouns": {"I": 1, "Time": 1}, "verbs": {"Yes": 1, "and": 1, "Stay together": 1}, "adjectives": {"It's not good": 1}, "numerals": {"80": 1}}}, {"content": "81, if I could feel a sense of frustration, I could handle it better.", "word_count": 23, "label": "", "sentence_structure": "Subject鈥搗erb鈥搊bject", "subjective_objective": {"answer": "Subjective", "rationale": "Because it is the patient's subjective perception and judgment to be frustrated in advance."}, "sentence_sentiment": {"answer": "Positive", "rationale": "Because the \"better\" behavior in the entry is positive."}, "language_style": {"answer": "Tactful and warm", "rationale": "The language style of the entry is relatively warm. It is more friendly and interactive to speak in a gentle and inviting tone, rather than to speak or order directly. By using the words \"if\" and \"predict\" and so forth, it gives the interviewees more space to think and encourages them to share their ideas. It helps create a positive and comfortable atmosphere for questionnaire-filling, and makes the respondents more willing to participate and answer questions honestly."}, "time_dimension": {"answer": "Future", "rationale": "The keywords are \"if I can feel\" and \"just\" , which point to a hypothesis or scenario, implying that this has not happened, but may be achieved in the future. The entry therefore asks the testee about the expectations and plans for the future's ability to cope with frustration, the time dimension being judged in the future."}, "sentence_type": "Imperative sentence", "pos": {"nouns": {"I": 2, "Frustration": 1, "Word": 1, "Entry": 1}, "verbs": {"Yes": 1, "Foreground": 1, "Payable": 1}, "adjectives": {"Better": 1}, "adverbs": {"If": 1}, "numerals": {"81": 1}}}, {"content": "82, no matter how much I complain, I never get a satisfactory result.", "word_count": 18, "label": "", "sentence_structure": "Subject鈥損redicate", "subjective_objective": {"answer": "Subjective", "rationale": "Since the expression and behaviour of a person is a matter of protest, the satisfaction of the outcome is also based on his or her own feelings and is therefore examined from a subjective perspective."}, "sentence_sentiment": {"answer": "Negative", "rationale": "Because \"shut\" and \"unsatisfied\" in this entry are both negative behaviours and results."}, "language_style": {"answer": "Calm and direct", "rationale": "The entry uses a more direct negative expression, emphasizing that in some cases, the result of the act is not ideal. The expression is simple and clear, and is not too emotional and is of a blunt and straightforward language style."}, "time_dimension": {"answer": "Past", "rationale": "The entry describes events that the subject has experienced, and the result \"never had a satisfactory result\" occurred in a certain period of time in the past. Thus, the basis of the judgement is \"in the past\"."}, "sentence_type": "Negative declarative sentence", "pos": {"nouns": {"Result": 1, "Scoff": 1}, "verbs": {"Got it": 1, "Let's go": 1, "Here": 1}, "adjectives": {"Satisfied": 1}, "adverbs": {"Whatever": 1, "What": 1}, "numerals": {"82": 1}, "pronouns": {"I": 1}}}, {"content": "83, I often find myself insensitive when the environment is about to cause a serious emotional reaction.", "word_count": 27, "label": "", "sentence_structure": "Subject鈥揷opula鈥損redicative", "subjective_objective": {"answer": "Subjective", "rationale": "Because \"I often find\" and \"insensitive\" are described from the patient's subjective point of view."}, "sentence_sentiment": {"answer": "Negative", "rationale": "Because the \"insensitive\" act in the entry is negative."}, "language_style": {"answer": "Tactful and warm", "rationale": ""}, "time_dimension": {"answer": "Present", "rationale": "\"I'm often\" means regular, customary behaviour, while \"insensitive\" implies that this state of affairs occurs under certain circumstances and may still exist. The entry therefore describes a state of the subject's current status or experience."}, "sentence_type": "Negative declarative sentence", "pos": {"nouns": {"Emotional response": 1, "Environment": 1, "Serious": 1, "I": 2}, "verbs": {"Yes": 1, "Found": 1, "Insensitive": 1}, "adjectives": {"Often": 1}, "numerals": {"83": 1}}}, {"content": "84, work without me, free me from emotional depression and anxiety.", "word_count": 19, "label": "", "sentence_structure": "Subject鈥揷opula鈥損redicative", "subjective_objective": {"answer": "Subjective", "rationale": "The purpose of this article is to describe the subjective way in which patients feel that working can help them to escape from emotional problems."}, "sentence_sentiment": {"answer": "Positive", "rationale": "Because \"forgetting to work\" in the entry can bring about emotional improvement, and it is positive."}, "language_style": {"answer": "Tactful and warm", "rationale": "The entry is more euphemistic and soft, does not speak directly about the subject of work that can be free from emotional problems, but rather leads the respondents to share their feelings in an invitation. At the same time, the entry does not use too much emotional or rhetorical style, which is more verbistic."}, "time_dimension": {"answer": "Present", "rationale": "According to the description of \"Unforgetting to work\" in the title, it is a state of behavior or custom, and \"to free me from emotional depression and anxiety\" is the result or effect of this behaviour. The entry therefore primarily asks whether the person tested is now engaged in such behaviour and whether it helps to alleviate negative emotions."}, "sentence_type": "Affirmative declarative sentence", "pos": {"nouns": {"Work": 1, "Emotion": 1, "Blue": 1, "Anxiety": 1}, "verbs": {"Forget me": 1, "鈾?And I'm gonna be 鈾?: 2, "Get rid of it": 1}, "adjectives": {"of": 4}, "numerals": {"84": 1}}}, {"content": "I smoke when I'm nervous.", "word_count": 9, "label": "", "sentence_structure": "Subject鈥損redicate", "subjective_objective": {"answer": "Subjective", "rationale": "Because I smoke is the patient's subjective choice and behavior, reflecting their inner feelings and coping. It's about their emotional state, and it's therefore subjective."}, "sentence_sentiment": {"answer": "Negative", "rationale": "Because the \"smoking when you're nervous\" act in the entry is negative."}, "language_style": {"answer": "Calm and direct", "rationale": "The entry directly expresses the subject of asking the other party whether to use smoking to ease emotions in times of stress, without using excessive rhetoric or emotional rendering, and is more objective in tone."}, "time_dimension": {"answer": "Present", "rationale": ""}, "sentence_type": "Affirmative declarative sentence", "pos": {"nouns": {"Time": 1, "Nervous": 1, "Smoke": 1}, "verbs": {"Just suck it": 1}, "adverbs": {"Just": 1}, "numerals": {"85": 1}}}, {"content": "86, if I get into some kind of crisis, I'll look for someone else who has the same fate as me.", "word_count": 28, "label": "", "sentence_structure": "Subject鈥搗erb鈥搊bject", "subjective_objective": {"answer": "Subjective", "rationale": "This is the patient ' s subjective idea and expectation that problems can be solved by finding people who are in the same situation."}, "sentence_sentiment": {"answer": "Negative", "rationale": "Because the words \"when in some crisis\" and \"seeing another person of my own destiny\" in the entries are negative emotional."}, "language_style": {"answer": "Tactful and warm", "rationale": "The language style of the entry is more euphemistic, asking questions in a more friendly and understanding manner, without direct criticism or criticism."}, "time_dimension": {"answer": "Present", "rationale": "The keyword \"local search\" suggests that this is a description of the current situation, while \"in a crisis\" indicates the preconditions for the act to occur. The entry therefore asks if the person being tested will act in the present, and the time dimension is now."}, "sentence_type": "Imperative sentence", "pos": {"nouns": {"Crisis": 1, "person": 2, "Destiny": 1}, "verbs": {"Into the": 1, "Search": 1, "Yes": 1}, "adverbs": {"Place": 1}, "numerals": {"86": 1}, "pronouns": {"I": 2}}}, {"content": "87, if I did something wrong, I can't be blamed.", "word_count": 13, "label": "", "sentence_structure": "Subject鈥揷opula鈥損redicative", "subjective_objective": {"answer": "Subjective", "rationale": "Because \"not to be blamed\" is an expression of personal feelings and attitudes, which are subjective thoughts of patients."}, "sentence_sentiment": {"answer": "Negative", "rationale": "Because the requirement of \"no blame\" is negative and reflects a evading of responsibility and reflection."}, "language_style": {"answer": "Calm and direct", "rationale": "The entry uses a direct and explicit expression, asking if the interviewee believes that he should not be blamed for his mistakes. This language style is more objective, has less emotional or accompaniment, and is more focused on transmitting information and obtaining answers."}, "time_dimension": {"answer": "Present", "rationale": "In the future, the entry asks the testee about the future vision and expectations of whether criticism or responsibility can be accepted in the face of a mistake. While the sentence contains a former assumption ( \"if I do something wrong,\" it is actually exploring the possible future, and thus judging it as the future."}, "sentence_type": "Imperative sentence", "pos": {"nouns": {"Things": 1, "Blame": 1}, "verbs": {"Wrong": 1, "Yes": 1}, "numerals": {"87": 1}, "pronouns": {"I": 1}}}, {"content": "88, if I had an attack on someone, I felt like I needed something to move that idea.", "word_count": 31, "label": "", "sentence_structure": "Subject鈥搗erb鈥搊bject", "subjective_objective": {"answer": "Objective", "rationale": "This is an examination of the patient ' s own feelings and reactions, a subjective attitude or subjective experience of the patient and therefore subjective."}, "sentence_sentiment": {"answer": "Negative", "rationale": "Because \"There's an idea of attacking people\" and \"There's something to do to divert it\" in the entries is negative."}, "language_style": {"answer": "Calm and direct", "rationale": "The language of the article is clear, and there is no excessive emotional or rhetoricality. It raises a question directly and gives a concrete observation, without trying to guide the respondents in a euphemism. This language style is more in line with the objective and accurate requirements of the psychological questionnaire."}, "time_dimension": {"answer": "Present", "rationale": "The keyword \"if\" and \"feel\" both means the situation, and \"need to do something\" also indicates that it is a current experience. So the entry describes the behavioral reaction that the person being tested may have in the current psychological state."}, "sentence_type": "Affirmative declarative sentence", "pos": {"nouns": {"88": 1, "Attacking someone": 2, "Thoughts": 2, "Transfer": 1, "Yes": 1, "Things": 1}, "verbs": {"have": 1, "Feel": 1, "Do it": 1}, "adverbs": {"If": 1, "Just": 2}, "numerals": {"88": 1, "Two": 1, "One": 3}, "classifiers": {"Plant": 1, "individual": 1}, "pronouns": {"I": 1}}}]}

{"questionnaire_title": "Internal control, powerful others and opportunity tables", "questionnaire_category": "Behavioral questionnaire", "item_count": 24, "emotion_shift_overall": "From negative to positive", "language_style_overall": "Calm and direct", "items": [{"content": "1. My ability to lead in this position depends primarily on my ability.", "word_count": 14, "label": "", "sentence_structure": "Subject鈥搗erb鈥搊bject", "subjective_objective": {"answer": "Subjective", "rationale": "Because \"is it a subjective idea or goal\" and \"mostly my ability\" is a self-perception and assessment of the individual, this is viewed from a subjective perspective."}, "sentence_sentiment": {"answer": "Neutral", "rationale": "Because the entry merely stated a point of view and did not contain any emotional tendencies or evaluation, it was neutral."}, "language_style": {"answer": "Calm and direct", "rationale": "The language style of the entry is calm and straightforward. It expresses the theme in a concise and clear way, \"Can I lead primarily by my ability\" and does not use too many rhetorical or emotional techniques. This expression is clear, accurate, and applies to such scenarios as research."}, "time_dimension": {"answer": "Present", "rationale": "The entry is for the purpose of asking the testee ' s opinion as to whether he or she is qualified for a leadership position, and the time dimension is now judged."}, "sentence_type": "Interrogative sentence", "pos": {"nouns": {"Capacity": 1, "Leadership": 1}, "verbs": {"On top of it": 1, "Shit": 1}, "pronouns": {"I": 1}}}, {"content": "2. My life is largely influenced by accidents.", "word_count": 17, "label": "", "sentence_structure": "Subject鈥搗erb鈥揷omplement", "subjective_objective": {"answer": "Subjective", "rationale": "Because \"a lot of the surprises\" are subjective feelings and evaluations."}, "sentence_sentiment": {"answer": "Negative", "rationale": "Because the expression \"by accident\" in the entry indicates that life is in a precarious state, with some negative tendencies."}, "language_style": {"answer": "Calm and direct", "rationale": "The language style of the entry is calm and straightforward. It raises questions directly to the respondents through a simple and concise presentation, without using excessive rhetorical or emotional rendering, and with a more objective tone."}, "time_dimension": {"answer": "Past", "rationale": "The entry describes the current living conditions of the subject, the keywords \"to a large extent\" and \"to be influenced by\" and suggests that this is a description of the past."}, "sentence_type": "Affirmative declarative sentence", "pos": {"nouns": {"Life": 1, "Accident": 1, "Impact": 1}, "verbs": {"Yes": 1, "Around": 1}, "adjectives": {"A lot": 1}}}, {"content": "I feel that what happens in my life is in the hands of powerful people.", "word_count": 22, "label": "", "sentence_structure": "Subject鈥揷opula鈥損redicative", "subjective_objective": {"answer": "Subjective", "rationale": "Because \"fear\" means that it's a patient's personal subjective feeling and perception."}, "sentence_sentiment": {"answer": "Negative", "rationale": "Because the expression \"life is controlled by powerful people\" in the entry is negative."}, "language_style": {"answer": "Calm and direct", "rationale": "The entry is more straightforward in its language, is not subject to much rhetorical or emotional overture, and is more neutral and objective in tone."}, "time_dimension": {"answer": "Present", "rationale": "The word \"I feel\" means the current psychological feeling, while the word \"to\" means that it has existed in the past, but may still be in existence. So, the entry describes the current psychological state and feeling."}, "sentence_type": "Interrogative sentence", "pos": {"nouns": {"Yes": 1, "Life": 1, "person": 2, "Power": 1}, "verbs": {"I feel": 1, "Let's go": 1, "Fan": 1, "Got it": 1}, "adjectives": {"It's powerful": 1}, "numerals": {"3": 1}, "pronouns": {"I": 2}}}, {"content": "4. Whether or not I was in a car accident depends primarily on my driving skills.", "word_count": 17, "label": "", "sentence_structure": "Subject鈥搗erb鈥搊bject", "subjective_objective": {"answer": "Subjective", "rationale": "Because \"or not to have an accident\" is an individual subjective feeling and judgment, not an objective fact."}, "sentence_sentiment": {"answer": "Neutral", "rationale": "Because the entry is a mere statement of fact and does not contain any emotional colour."}, "language_style": {"answer": "Calm and direct", "rationale": "The language style of the entry is more direct and clear, not expressed in excessive rhetorical or emotional terms, but in fact-based, objective and neutral terms."}, "time_dimension": {"answer": "Present", "rationale": "The entry uses the current state of \"whether or not\" as a general act or state, in which the testee considers that his or her probability of having a car accident depends primarily on his or her current driving technology. The temporal dimension therefore determines the present."}, "sentence_type": "Interrogative sentence", "pos": {"nouns": {"Car accident": 1, "Driving techniques": 1}, "verbs": {"Is it possible": 1, "Out": 1, "It depends": 1, "Driving": 1}, "numerals": {"4": 1}, "pronouns": {"I": 1}}}, {"content": "I was almost certain I could do it when I made the plan.", "word_count": 15, "label": "", "sentence_structure": "Subject鈥搗erb鈥揷omplement", "subjective_objective": {"answer": "Subjective", "rationale": "Because \"enforceable\" is a subjective judgement and confidence of the patient, it is viewed from a subjective perspective."}, "sentence_sentiment": {"answer": "Positive", "rationale": "Because the \"I can plan\" act is positive."}, "language_style": {"answer": "Tactful and warm", "rationale": "The language style of the entry is more euphemistic, using the means of questioning and using the words \"almost certain\" to express confidence, be gentler and be kind. Such expressions reduce the resistance of the person surveyed and make him more willing to answer the questions."}, "time_dimension": {"answer": "Present", "rationale": ""}, "sentence_type": "Affirmative declarative sentence", "pos": {"nouns": {"Planned": 1}, "verbs": {"Staple": 1, "Implementation": 1}, "adjectives": {"I'm sure it is": 1}, "adverbs": {"Almost": 1, "Yes": 1}}}, {"content": "6. When bad luck comes, I often do not have the opportunity to protect my interests.", "word_count": 20, "label": "", "sentence_structure": "Subject鈥搗erb鈥搊bject", "subjective_objective": {"answer": "Subjective", "rationale": "Because \"in the event of a bad thing\" and \"in the event of a lack of opportunity to protect their interests\" are described from the subjective point of view of the patient, the entry on the questionnaire is subjective."}, "sentence_sentiment": {"answer": "Negative", "rationale": "Because the description of the act \"often without the opportunity to protect his interests\" in the entry is negative."}, "language_style": {"answer": "Calm and direct", "rationale": "The language style of the entry is calm and straightforward, without any qualifier or emotional expression, but simply asks the interviewee whether he or she is capable of protecting his or her interests in the event of an unfortunate incident."}, "time_dimension": {"answer": "Present", "rationale": ""}, "sentence_type": "Interrogative sentence", "pos": {"nouns": {"Bad luck": 1, "Opportunities": 1, "self": 1, "Interest": 1}, "verbs": {"- I'll meet you": 1, "Regular": 1, "No, I'm not": 1, "Protection": 1}, "adjectives": {"Shit": 1}, "adverbs": {"Often": 1, "No, I'm not": 1}, "numerals": {"6": 1}, "pronouns": {"I": 1}}}, {"content": "I got what I was after, usually because I was lucky.", "word_count": 19, "label": "", "sentence_structure": "Subject鈥搗erb鈥搊bject", "subjective_objective": {"answer": "Subjective", "rationale": "Because \"I got what I was after\" is a patient's subjective feeling and explanation. Though luck may have worked, the patient still thinks it's the result of his efforts. So, \"usually because I'm lucky\" is a reflection of the patient's subjective view."}, "sentence_sentiment": {"answer": "Negative", "rationale": "Because the entry expresses a negative attitude that its success is based primarily on luck rather than effort."}, "language_style": {"answer": "Calm and direct", "rationale": "The language style of the entry is a calm, straightforward, non-pleasant, rhetorical and emotional presentation. It develops the subject by stating directly the fact that \"I get what I seek, usually because I get lucky\" rather than by drawing emotional or attitudeal guidance to elicit the responses of the respondents."}, "time_dimension": {"answer": "Present", "rationale": "The keyword \"getting\" and \"what I'm after\" and \"because\" usually means what happens now or in the past, so the entry indicates that the testees think that they're successful on the basis of luck. The temporal dimension judges now."}, "sentence_type": "Affirmative declarative sentence", "pos": {"nouns": {"Questionnaire Entry": 1, "Something": 1, "Good luck": 1}, "verbs": {"Access": 1, "Seek": 1, "Usually": 1, "Lucky": 1}, "adverbs": {"I": 2}, "numerals": {"7": 1}}}, {"content": "8. Although I am well-equipped, I cannot be entrusted with a heavy burden if I do not bring those in high places together.", "word_count": 31, "label": "", "sentence_structure": "Subject鈥搗erb鈥搊bject", "subjective_objective": {"answer": "Subjective", "rationale": "This is a subjective judgement and expectation of the patient and involves an evaluation of his or her abilities and the behaviour of others."}, "sentence_sentiment": {"answer": "Negative", "rationale": "Because the expression \"if I do not bring those in high places together, I cannot be held responsible\" in the entry is clearly negative and emotional, indicating the notion of the acquisition of power and status through improper means."}, "language_style": {"answer": "Calm and direct", "rationale": "The language style of the entry is calm and straightforward. It clearly expresses the view that you cannot get a job or a post if you do not bring people in high places together, and that you do not use too much emotional or rhetorical to qualify sentences. This is usually more persuasive and practical."}, "time_dimension": {"answer": "Future", "rationale": "It is therefore possible to judge that this is the future."}, "sentence_type": "Negative declarative sentence", "pos": {"nouns": {"Capacity": 1, "person": 2, "Heavy task": 1}, "verbs": {"Zoom in": 1, "\"Attack.\"": 1, "If": 1, "Maybe": 1, "Good": 1}, "adjectives": {"High": 1}, "adverbs": {"Just": 1, "Impossible": 1}, "numerals": {"8": 1}, "pronouns": {"I": 1}}}, {"content": "9. The amount of friends I have depends on how I am.", "word_count": 18, "label": "", "sentence_structure": "Subject鈥揷opula鈥損redicative", "subjective_objective": {"answer": "Subjective", "rationale": "Because \"how I am\" is a patient's personal feeling and self-evaluation, and it's therefore subjectively viewed."}, "sentence_sentiment": {"answer": "Neutral", "rationale": "Because the entry does not have any emotional color."}, "language_style": {"answer": "Tactful and warm", "rationale": ""}, "time_dimension": {"answer": "Present", "rationale": "The keywords are \"possession\" and \"dependency\" , which point to the current state or behaviour. At the same time, the phrase \"how I am\" implies that this is a subjective, self-evaluation issue, so it can be judged that the entry is asking the testee what he thinks and feels."}, "sentence_type": "Affirmative declarative sentence", "pos": {"nouns": {"Questionnaire Entry": 1, "friends": 1, "person": 2}, "verbs": {"Hug": 1, "It depends": 1, "How's it going": 1}, "adverbs": {"How much": 1}, "numerals": {"9": 1}, "pronouns": {"I": 2}}}, {"content": "I often find that what is about to happen is destined to happen.", "word_count": 17, "label": "", "sentence_structure": "Subject鈥搗erb鈥搊bject", "subjective_objective": {"answer": "Subjective", "rationale": "Because \"receiving\" is a subjective feeling or attitude, and \"that's what's about to happen\" is a belief or an opinion, which is the subjective idea of the patient."}, "sentence_sentiment": {"answer": "Negative", "rationale": "Because the idea of \"desired to happen\" in the entry is negative."}, "language_style": {"answer": "Calm and direct", "rationale": "The entry directly expresses the subject of questioning the other party about his or her beliefs in fate, without excessive rhetoric or emotional rendering, and is more objective in tone."}, "time_dimension": {"answer": "Future", "rationale": "The entry \"that will happen\" and \"that will happen\" point to what is not yet happening and what may happen in the future. The temporal dimension therefore is the future."}, "sentence_type": "Affirmative declarative sentence", "pos": {"nouns": {"Questionnaire": 1, "Entry": 1, "Come on": 2}, "verbs": {"Found": 1, "Yes, I'm going": 1, "Yes": 1, "Destiny": 1}, "adverbs": {"Often": 1}, "numerals": {"10": 1, "2": 1}, "pronouns": {"Those": 1}}}, {"content": "My life is dominated by those who are powerful.", "word_count": 15, "label": "", "sentence_structure": "Subject鈥搗erb鈥搊bject", "subjective_objective": {"answer": "Subjective", "rationale": "Because \"the control of those who have power\" is a subjective perception and feeling, a subjective assessment of life. While such control may exist objectively, individual feelings are subjective."}, "sentence_sentiment": {"answer": "Negative", "rationale": "Because the \"man-man-man-man-man-man\" act in the entry is negative."}, "language_style": {"answer": "Calm and direct", "rationale": "Because the wording of the entry is clear, concise and does not use euphemism or rhetoric, the interviewees were directly asked whether they were influenced by the powerful in their own lives."}, "time_dimension": {"answer": "Present", "rationale": "The entry describes the current situation of the testees, where they feel that their lives are controlled by powerful people. Thus, the time dimension is now judged."}, "sentence_type": "Negative declarative sentence", "pos": {"nouns": {"Life": 1, "Power": 2, "person": 1}, "verbs": {"Control": 1}, "numerals": {"11": 1}, "pronouns": {"I": 1}}}, {"content": "12. The question of whether I had a car accident was mostly one of luck.", "word_count": 15, "label": "", "sentence_structure": "Subject鈥揷opula鈥損redicative", "subjective_objective": {"answer": "Subjective", "rationale": "Because \"the accident\" is a subjective experience, and \"the question of whether it's mainly a matter of luck\" is also a question of the patient's perception or interpretation of the issue, so it is a subjective question."}, "sentence_sentiment": {"answer": "Neutral", "rationale": "Because the entry does not express a positive, negative or neutral emotional expression. It is merely asking a fact, and is not motivated by any emotional orientation."}, "language_style": {"answer": "Tactful and warm", "rationale": "The language style of the entry is more euphemistic and warm, expressing the question of whether it is \"in\" in question, and using moderate language to guide respondents to answer questions. At the same time, this euphemism makes the questionnaire more personal and interactive."}, "time_dimension": {"answer": "Present", "rationale": "\"Whether or not to hit a car accident\" means asking about and recalling past events, but \"mostly the problem of luck\" shows the current attitude, perception or conviction of the testee and therefore the judgment is now."}, "sentence_type": "Interrogative sentence", "pos": {"nouns": {"I": 1, "Car accident": 1, "Problem": 1}, "verbs": {"Is it possible": 1, "- I'll meet you": 1, "Mostly": 1}, "adjectives": {"Lucky": 1}, "numerals": {"12": 1}}}, {"content": "13. When there is conflict with powerful groups, people like me rarely have the opportunity to protect their personal interests.", "word_count": 36, "label": "", "sentence_structure": "Subject鈥搗erb鈥搊bject", "subjective_objective": {"answer": "Subjective", "rationale": "Because the sentence relates to the individual ' s perception and perception of the individual ' s ability to protect his or her interests, it is a subjective attitude and emotional expression."}, "sentence_sentiment": {"answer": "Negative", "rationale": "Because the expression \"very few opportunities to protect their personal interests\" in the entry is negative."}, "language_style": {"answer": "Calm and direct", "rationale": "The entry describes in an objective and clear language the problems and difficulties that individuals may encounter in facing powerful groups, without excessive emotional accolades or subjective evaluations. Its linguistic style can therefore be classified as calm and straightforward."}, "time_dimension": {"answer": "Past", "rationale": "Therefore, judging from the temporal dimension, the entry relates to past and future behaviour or mindsets, while reflecting the social problems and challenges of reality."}, "sentence_type": "Negative declarative sentence", "pos": {"nouns": {"Same": 1, "Power": 1, "Gang": 1, "Conflict": 1, "person": 2, "Opportunities": 1, "Protection": 1, "self": 1, "Personal": 1, "Interest": 1}, "verbs": {"Come on": 1, "Yes": 1, "Protection": 1}, "adjectives": {"Very few": 1}, "adverbs": {"When": 1, "Yeah": 1}, "numerals": {"13": 1}, "pronouns": {"I": 1}}}, {"content": "14. In my opinion, it is not always wise to plan early, because many things have proved to be a matter of luck or bad luck.", "word_count": 39, "label": "", "sentence_structure": "Subject鈥搗erb鈥搊bject", "subjective_objective": {"answer": "Subjective", "rationale": "According to the information provided, \"as I see it\" indicates that this is the patient's personal view and opinion. The view of the questionnaire is therefore subjective."}, "sentence_sentiment": {"answer": "Negative", "rationale": "Since the entry states that \"early planning is not always wise\", this view is somewhat negative."}, "language_style": {"answer": "Calm and direct", "rationale": "The entry is expressed in a direct manner, without excessive rhetorical or emotional rendering. It is sound and objective and calm."}, "time_dimension": {"answer": "Past", "rationale": ""}, "sentence_type": "Interrogative sentence", "pos": {"nouns": {"Planned": 1, "Problem": 1}, "verbs": {"Prove it": 1, "be": 2, "Morning": 1, "Order": 1, "Lucky": 1, "Bad luck": 1, "By": 1, "Staple": 1, "Looks like it": 1}, "adjectives": {"Smart": 1}, "adverbs": {"Always": 1}, "numerals": {"14": 1}, "classifiers": {"One": 1}, "pronouns": {"I": 1}}}, {"content": "15. To get what I want, I need to be nicer to those who are stronger than me.", "word_count": 21, "label": "", "sentence_structure": "Subject鈥搗erb鈥搊bject", "subjective_objective": {"answer": "Subjective", "rationale": "Because \"good\" and \"powerful people\" are patients' subjective views and attitudes to the issue."}, "sentence_sentiment": {"answer": "Negative", "rationale": "Because the act of \"need to please someone more powerful than me\" in the entry is negative, with a clear attitude of accommodation and compromise."}, "language_style": {"answer": "Calm and direct", "rationale": "The language style of the entry is more direct, there is no excessive qualifier or emotional colour, and the tone is more objective and neutral and more suitable for investigation and study."}, "time_dimension": {"answer": "Past", "rationale": "The entries included \"to get what you want\" and \"to be more powerful than I\" are experiences or ideas of the past, and they are therefore judged as past."}, "sentence_type": "Affirmative declarative sentence", "pos": {"nouns": {"Something": 1, "Power": 1, "person": 1}, "verbs": {"Yes": 3, "Got it": 1, "Beloved": 1}, "numerals": {"15": 1, "- Yeah": 1}, "pronouns": {"I": 1}}}, {"content": "16. My ability to be a judge depends on my being lucky enough to be in the right place at the right time.", "word_count": 28, "label": "", "sentence_structure": "Subject鈥揷opula鈥損redicative", "subjective_objective": {"answer": "Subjective", "rationale": "This is a patient鈥檚 subjective attitude, because the possibility of being a judge is determined by many factors, including the ability, effort, and opportunity of the individual. While luck may have played a role in certain cases, this does not mean that a person鈥檚 ability to be a judge is entirely dependent on his or her own luck."}, "sentence_sentiment": {"answer": "Neutral", "rationale": "Because the entry does not express a positive or negative emotional tendency. It simply states the view that the individual ' s ability to be a judge may be linked to luck, without evaluating or responding to that view."}, "language_style": {"answer": "Calm and direct", "rationale": "The language style of the entry is more direct and objective, and does not involve too many emotional or euphemistic expressions. It clearly presents the view that there is a correlation between \"competence\" and \"good luck and right time and place\" and appears to be calmer and direct."}, "time_dimension": {"answer": "Present", "rationale": "The entry is an inquiry into the current thinking or belief of the subject, without reference to past or future circumstances. In it, the words \"is it the official\" , \"is it the judge\" and \"is in the right place at the right time\" indicate that this is the current conviction or view."}, "sentence_type": "Interrogative sentence", "pos": {"nouns": {"Officer": 1, "Time": 1, "Seats": 1}, "verbs": {"When": 1, "It depends": 1, "Lucky": 1, "in": 1, "In": 1}, "adjectives": {"Appropriate": 2}, "adverbs": {"Enough": 1}, "numerals": {"16": 1}, "pronouns": {"I": 2}}}, {"content": "17. If important people don ' t like me, I may not be able to make too many friends.", "word_count": 22, "label": "", "sentence_structure": "Subject鈥搗erb鈥搊bject", "subjective_objective": {"answer": "Subjective", "rationale": "Because it involves the patient's own subjective feelings and perceptions. The \"vital person doesn't like me\" and \"probably not having many friends\" are all subjective aspects of the patient's perception of his social abilities and of the attitude of others towards himself."}, "sentence_sentiment": {"answer": "Negative", "rationale": "Because the entry \"If important people don't like me, I'm probably not gonna make too many friends\" expresses a sense of anxiety and negative sentiment."}, "language_style": {"answer": "Tactful and warm", "rationale": ""}, "time_dimension": {"answer": "Present", "rationale": "\"if\" leads to a hypothesis that means that this is a possible situation; and the \"possibly\" behind it is a prediction and extrapolation of the current outcome. So the entire sentence describes what the speaker thinks and feels now, and the temporal dimension is now."}, "sentence_type": "Affirmative declarative sentence", "pos": {"nouns": {"Important people": 1, "person": 1, "friends": 1}, "verbs": {"Yeah": 1, "Turn it in": 1}, "adjectives": {"Too much": 1, "More": 1}, "adverbs": {"If": 1, "Maybe": 1}, "numerals": {"17": 1, "Too much": 1}, "pronouns": {"I": 1}}}, {"content": "I am perfectly capable of controlling everything in my life.", "word_count": 14, "label": "", "sentence_structure": "Subject鈥搗erb鈥揷omplement", "subjective_objective": {"answer": "Subjective", "rationale": "Because \"all that can govern my life\" is a subjective attitude and conviction of a man, and it doesn't mean that life is actually completely controlled."}, "sentence_sentiment": {"answer": "Positive", "rationale": "Because the expression \"dominant in my life\" in the entry is positive."}, "language_style": {"answer": "Calm and direct", "rationale": "The entry is a straightforward and clear question to the interviewees about whether they think they can control everything in life, and the language is simple and not too much rhetorical and emotional."}, "time_dimension": {"answer": "Present", "rationale": ""}, "sentence_type": "Interrogative sentence", "pos": {"verbs": {"Lord": 1, "Life": 1, "Everything": 1}, "adjectives": {"Total": 1}, "pronouns": {"I": 1}}}, {"content": "19. I am generally able to protect my interests.", "word_count": 12, "label": "", "sentence_structure": "Subject鈥揷opula鈥損redicative", "subjective_objective": {"answer": "Subjective", "rationale": "This statement reflects the respondents ' self-evaluation and views on their own and is a subjective expression."}, "sentence_sentiment": {"answer": "Neutral", "rationale": "Because the entry does not express positive or negative emotional sentiments, it merely describes a de facto act."}, "language_style": {"answer": "Calm and direct", "rationale": "The entry is presented in a direct, objective and non-emotional manner and is therefore a direct and calm expression. \""}, "time_dimension": {"answer": "Present", "rationale": "The entry describes the current state and capabilities of the testees, indicating that they can normally protect their interests, and therefore the temporal dimension is now judged."}, "sentence_type": "Affirmative declarative sentence", "pos": {"nouns": {"Interest": 1}, "verbs": {"Protection": 1, "Yes": 1}, "adverbs": {"General": 1}, "pronouns": {"I": 1}}}, {"content": "20. Whether I will have a car accident depends primarily on the other drivers.", "word_count": 17, "label": "", "sentence_structure": "Subject鈥搗erb鈥搊bject", "subjective_objective": {"answer": "Objective", "rationale": "It is an objective fact that the act of the other driver determines whether a car accident will occur."}, "sentence_sentiment": {"answer": "Negative", "rationale": "Because the act of \"car crash\" in the entry is negative."}, "language_style": {"answer": "Tactful and warm", "rationale": "The entry uses a more euphemistic tone, which attempts to lead the interviewee to reflect on the relationship between his behaviour and attitude and traffic accidents by asking questions. This is more polite than speaking directly and respecting the views of the interviewee, and is more likely to generate reflection and resonance."}, "time_dimension": {"answer": "Present", "rationale": "This expression does not address past or future events, and therefore the temporal dimension is now judged."}, "sentence_type": "Interrogative sentence", "pos": {"nouns": {"Car accident": 1, "Driver": 1}, "verbs": {"Yes": 1, "It depends": 1}, "adverbs": {"Main": 1}, "numerals": {"20": 1}, "pronouns": {"Other": 1, "I": 1}}}, {"content": "I got what I wanted, usually because I worked hard for it.", "word_count": 22, "label": "", "sentence_structure": "Subject鈥搗erb鈥搊bject", "subjective_objective": {"answer": "Subjective", "rationale": "This is the subjective idea of the patient, involving self-evaluation and attribution."}, "sentence_sentiment": {"answer": "Positive", "rationale": "Because the words \"get what you want\" and \"work hard\" are positive emotions and attitudes."}, "language_style": {"answer": "Calm and direct", "rationale": "The basis for the judgement: The entry expresses the view that people usually get what they want because of their hard work. This is more direct, objective, with no excessive emotional rendering or rhetorical style, consistent with the \"cool and straight\" language style."}, "time_dimension": {"answer": "Present", "rationale": ""}, "sentence_type": "Affirmative declarative sentence", "pos": {"nouns": {"Questionnaire": 1, "Entry": 1, "I": 3, "Got it": 1, "Yes": 1, "Usually": 1, "Because": 1, "鈾?And the 鈾?: 1, "Hard work": 1}, "verbs": {"be": 1, "Yes": 1, "Got it": 1, "Because": 1, "For": 1, "Hard work": 1, "Pass": 1}, "adverbs": {"Yes": 2, "Usually": 1}, "classifiers": {"individual": 1}, "pronouns": {"I": 3}}}, {"content": "22. In order to implement my plans, I want to be sure that they are in line with the taste of those who are more powerful than me.", "word_count": 30, "label": "", "sentence_structure": "Subject鈥搗erb鈥搊bject", "subjective_objective": {"answer": "Subjective", "rationale": "Because the existence of \"the person who is more powerful than me\" is a subjective assumption of the patient."}, "sentence_sentiment": {"answer": "Negative", "rationale": "Because the \"fits the taste of those who are more powerful than me\" in the entry has a demeaning effect and is negative."}, "language_style": {"answer": "Calm and direct", "rationale": "The entry directly expresses the subject of a question about whether the other party is often concerned about past events, and does not use excessive rhetoric or emotional rendering, which is more objective and balanced."}, "time_dimension": {"answer": "Present", "rationale": "Now the entry describes the current state of thinking and behaviour of the testee, the word \"to\" indicates an intention or a decision, which means that it is a matter of consideration or decision. Moreover, the expression \"persons with greater power than me\" shows that it is a reality, not a future or a past vision. So, the time dimension of the entry is now."}, "sentence_type": "Interrogative sentence", "pos": {"nouns": {"Planned": 2, "Power": 2, "person": 1, "Taste": 1}, "verbs": {"Implementation": 1, "Sure": 1, "Match": 1}, "adverbs": {"- Yeah": 1, "I": 1}, "numerals": {"22": 1}, "pronouns": {"Those": 1}}}, {"content": "23. My life is determined by my actions.", "word_count": 12, "label": "", "sentence_structure": "Subject鈥搗erb鈥搊bject", "subjective_objective": {"answer": "Subjective", "rationale": "This is a subjective examination of the patient ' s perception and understanding of his or her own life."}, "sentence_sentiment": {"answer": "Neutral", "rationale": "Because the entry does not have any emotional color."}, "language_style": {"answer": "Calm and direct", "rationale": "The language style of the entry is more calm and straightforward, rather than using excessive rhetorical techniques or emotional rendering, it expresses its views in a concise and clear manner. It emphasizes the influence of the individual's behaviour on his or her life, not on external factors, the temperature of the language and the firmness of the language."}, "time_dimension": {"answer": "Present", "rationale": "The entry is an expression of the belief or view that the person tested believes that his or her life is determined by his or her current behaviour. The temporal dimension of the past or the future is not addressed."}, "sentence_type": "Affirmative declarative sentence", "pos": {"nouns": {"Life": 1, "Behaviour": 1}, "verbs": {"Decision": 1}, "numerals": {"23": 1}, "pronouns": {"I": 1}}}, {"content": "24. The problem of having more or less friends is primarily one of fate.", "word_count": 16, "label": "", "sentence_structure": "Subject鈥揷opula鈥損redicative", "subjective_objective": {"answer": "Subjective", "rationale": "Because \"how much of a friend\" is a subjective feeling and perception of a person, which is considered to be a subjective view and belief of the individual, determined by fate."}, "sentence_sentiment": {"answer": "Neutral", "rationale": "Since the entry does not express any apparent emotional or attitudinal preference, it merely presents a point of view."}, "language_style": {"answer": "Tactful and warm", "rationale": "The entry is more euphemistic and somewhat subjective. By expressing the view that \"the main issue is fate\" it implies some sort of impropriety or passivity in social relations among the subjects of the inquiry, without being too direct in rejecting or accusing, and generally being mild."}, "time_dimension": {"answer": "Past", "rationale": ""}, "sentence_type": "Affirmative declarative sentence", "pos": {"nouns": {"24": 1, "I": 1, "Problem": 1}, "verbs": {"Possession": 1, "Main": 1, "be": 1, "of": 2}, "adjectives": {"More": 1, "Less": 1, "friends": 1, "Destiny": 1}, "adverbs": {"or": 1, "Yes": 1}, "numerals": {"24": 1}, "pronouns": {"Here": 1}}}]}

{"questionnaire_title": "Social evasive and distressing tables", "questionnaire_category": "Behavioral questionnaire", "item_count": 28, "emotion_shift_overall": "From negative to positive", "language_style_overall": "Tactful and warm", "items": [{"content": "1. I feel relaxed even in unfamiliar social settings.", "word_count": 21, "label": "", "sentence_structure": "Subject鈥揷opula鈥損redicative", "subjective_objective": {"answer": "Subjective", "rationale": "Because feeling relaxed is a subjective feeling."}, "sentence_sentiment": {"answer": "Positive", "rationale": "According to the title, \"I still feel relaxed in unfamiliar social settings\" is a positive attitude or quality, because it shows that the interviewee has the ability to adapt to the new environment and remain calm. The emotional color of the article is therefore positive."}, "language_style": {"answer": "Tactful and warm", "rationale": "The entry is more euphemistic, friendly, and attempts to make the interviewee more natural in answering questions, by using the words \"even\" and \"still\" and giving people a friendly and understanding atmosphere. Moreover, \"whether\" is also a more gentle way to ask questions, rather than a direct statement or order, further reinforces this feeling of comfort."}, "time_dimension": {"answer": "Present", "rationale": "The entry asks if the testee feels relaxed in the current social setting, so the time dimension is now judged. \""}, "sentence_type": "Interrogative sentence", "pos": {"nouns": {"Socially": 1, "I": 1}, "verbs": {"I feel": 1, "Relax": 1, "be": 1}, "adjectives": {"I don't know": 1}, "adverbs": {"Still": 1}}}, {"content": "2. I shall endeavour not to force me to participate in the work due.", "word_count": 19, "label": "", "sentence_structure": "Subject鈥搗erb鈥搊bject", "subjective_objective": {"answer": "Subjective", "rationale": "Because the words \"avoid\" and \"participate in the relationship\" indicate the patient's subjective attitude and behaviour, and are the expression of the patient's subjective perception and action."}, "sentence_sentiment": {"answer": "Negative", "rationale": "Because the \"avoid\" act in the entry is negative."}, "language_style": {"answer": "Calm and direct", "rationale": "\""}, "time_dimension": {"answer": "Present", "rationale": "The entry \"I try to avoid\" indicates the behaviour or habit currently being performed and contains the keyword \"necessarily avoid\", which indicates that the testee is trying to control his behaviour, and that this effort may be continuing in the near future. So the time dimension of the entry is now."}, "sentence_type": "Negative declarative sentence", "pos": {"nouns": {"Professional relations": 1}, "verbs": {"Participation": 1, "Avoid": 1, "鈾?And I'm gonna be 鈾?: 1}, "adjectives": {"Try": 1}, "adverbs": {"Situation": 1}, "pronouns": {"I": 1}}}, {"content": "I'm easy to relax when I'm with strangers.", "word_count": 16, "label": "", "sentence_structure": "Subject鈥揷opula鈥損redicative", "subjective_objective": {"answer": "Subjective", "rationale": "Because \"Easy to relax\" is a subjective feeling and experience of a person, involving subjective factors such as an individual's emotional, psychological state, not just an objective situation. So the entry \"Easy to relax when I'm with strangers\" looks at the subjective feelings and experiences of the person interviewed, which are subjective."}, "sentence_sentiment": {"answer": "Neutral", "rationale": "Because the entry does not have any emotional color."}, "language_style": {"answer": "Tactful and warm", "rationale": "The language style of the entry is more euphemistic, using the expression \"easy\" to describe a person's state in a strange environment, without too much emphasis or judgement. And the question \"if\" is also more gentle and gives a sense of affection."}, "time_dimension": {"answer": "Present", "rationale": "The entry describes the feelings and experiences of the subject in its current state, the keywords being \"I'm with strangers\" and \"Easy to relax.\" Both of these keywords point to the current situation. So the time dimension of this questionnaire entry should be \"Now\"."}, "sentence_type": "Interrogative sentence", "pos": {"nouns": {"Stranger": 1, "Together": 1}, "verbs": {"Easy": 1, "Relax": 1, "in": 1, "Same": 1}, "adjectives": {"Strange": 1, "very": 1}, "adverbs": {"Time": 1}, "pronouns": {"I": 1}}}, {"content": "I don't really want to avoid people.", "word_count": 13, "label": "", "sentence_structure": "Subject鈥損redicate", "subjective_objective": {"answer": "Subjective", "rationale": "Because the purpose of the questionnaire is to describe the feelings and perceptions of the patient, such as \"not particularly wanting to avoid people\", which are personal thoughts and attitudes of the patient and are subjective."}, "sentence_sentiment": {"answer": "Neutral", "rationale": "Because the entry does not contain a clear description of emotional orientation or behaviour, it merely presents a fact."}, "language_style": {"answer": "Tactful and warm", "rationale": "The language style of the entry is more euphemistic, because it uses the expression \"not particularly want to avoid people,\" rather than simply \"I hate people.\" It is more soft, friendly and acceptable."}, "time_dimension": {"answer": "Past", "rationale": "The keyword \"is not particularly designed to avoid\" means a description of the past, and \"is it\" is a question of the past, so the temporal dimension of the article is a past."}, "sentence_type": "Interrogative sentence", "pos": {"nouns": {"Questionnaire Entry": 1, "person": 2}, "verbs": {"To avoid": 1}, "adverbs": {"Special": 1, "not": 1}, "numerals": {"4": 1}, "pronouns": {"I": 1}}}, {"content": "5. I usually find social settings disturbing.", "word_count": 17, "label": "", "sentence_structure": "Subject鈥損redicate", "subjective_objective": {"answer": "Subjective", "rationale": "Because it is a subjective experience of patients ' feelings and perceptions of social settings."}, "sentence_sentiment": {"answer": "Negative", "rationale": "Because the expression \"disturbing\" in the entry has negative emotions."}, "language_style": {"answer": "Calm and direct", "rationale": "The language style of the entry is more concise, not using too many qualifiers or emotional expressions, but rather directly asking the respondents whether they are distraught in social settings, which is more direct and objective."}, "time_dimension": {"answer": "Present", "rationale": "The keyword \"I usually find\" means current, habitual behaviour, so the purpose of the article is now time-dimensional. At the same time, the purpose of the article involves social settings and falls within the realm of personal emotional responses."}, "sentence_type": "Interrogative sentence", "pos": {"nouns": {"Questionnaire": 1, "Entry": 1, "Socially": 1, "I'm upset": 1}, "verbs": {"Found": 1, "You're": 1, "be": 1}, "adjectives": {"Usually": 1}, "adverbs": {"It's disturbing": 1}, "pronouns": {"I": 1}}}, {"content": "6. I usually feel calm and comfortable in social settings.", "word_count": 17, "label": "", "sentence_structure": "Subject鈥揷opula鈥損redicative", "subjective_objective": {"answer": "Subjective", "rationale": "The sense of calm and comfort is the patient's subjective feeling."}, "sentence_sentiment": {"answer": "Positive", "rationale": "Because the entries express calm and comfort in social settings, this emotion and feeling is usually positive."}, "language_style": {"answer": "Calm and direct", "rationale": "The entry is a direct query about the person ' s feelings in the social setting, is not overly emotional or isomanic and is more calm and objective."}, "time_dimension": {"answer": "Present", "rationale": "The entry asks if the person tested feels calm and comfortable in the current social setting, does not relate to past or future behaviour or mentality, and therefore judges it as now."}, "sentence_type": "Interrogative sentence", "pos": {"nouns": {"Questionnaire Entry": 1, "Socially": 1, "I": 2, "Calm down": 1, "Comfortable": 1}, "verbs": {"Feel": 1, "Usually": 1, "be": 1, "and": 1}, "adjectives": {"Calm down": 1, "Comfortable": 1}, "adverbs": {"Usually": 1, "Is it possible": 1}, "pronouns": {"I": 2}}}, {"content": "7. When talking to people of the opposite sex, I usually feel relaxed.", "word_count": 16, "label": "", "sentence_structure": "Subject鈥揷opula鈥損redicative", "subjective_objective": {"answer": "Subjective", "rationale": "Because \"feel relaxed\" is a patient's experience, a subjective approach."}, "sentence_sentiment": {"answer": "Positive", "rationale": "Because the \"feel relaxing\" in the entry is an emotional positive attitude."}, "language_style": {"answer": "Tactful and warm", "rationale": "The entry uses query language, which allows dialogue to take place in a soft and inviting manner, rather than direct statements or orders, which are more friendly and interactive, reflecting a soft and warm language style. At the same time, the term \"relax\" conveys a positive emotional colour, further increasing the appeal and participation of the questionnaire."}, "time_dimension": {"answer": "Present", "rationale": "\"In the conversation of the opposite sex\" means the person's current situation or feelings, and \"I usually feel relaxed\" means the subjective experience and attitude of the person. Thus, the temporal dimension of the article is now judged."}, "sentence_type": "Interrogative sentence", "pos": {"nouns": {"Questionnaire Entry": 1, "Same-sex": 1, "Talk": 2, "Feel": 2, "Relax": 1, "Is it possible": 1}, "verbs": {"in": 1, "Usually": 1, "Feel": 1, "Relax": 1, "be": 1}, "adjectives": {"Same-sex": 1, "Heterosexual": 1, "Relax": 1}, "adverbs": {"Usually": 1}, "numerals": {"7": 1}, "classifiers": {"individual": 1}, "pronouns": {"I": 1}}}, {"content": "I try to avoid speaking to others unless I am particularly familiar.", "word_count": 18, "label": "", "sentence_structure": "Subject鈥搗erb鈥搊bject", "subjective_objective": {"answer": "Subjective", "rationale": "Because expressions such as \"necessarily avoid\" and \"unless specially known\" are subjective attitudes and feelings of patients, they are subject to subjective scrutiny."}, "sentence_sentiment": {"answer": "Negative", "rationale": "Because the behavior in the entry \"as little as possible to avoid talking to people\" is negative."}, "language_style": {"answer": "Calm and direct", "rationale": "The entry is a direct, concise interview with the interviewees about their habits or preferences in interpersonal contacts, with no excessive emotional accoutrements or rhetorical techniques, and with a relatively calm and straightforward language style."}, "time_dimension": {"answer": "Present", "rationale": "The keywords are \"I try to avoid\" and \"unless I am particularly familiar\" , which point to an ongoing, present state or conduct. \"Whether\" indicates a selective confirmation or denial of the act, further emphasizing that this is asking about the current act or situation."}, "sentence_type": "Interrogative sentence", "pos": {"nouns": {"Questionnaire": 1, "Entry": 1, "Speech": 1}, "verbs": {"Try to avoid it": 1, "With others": 1, "Is it possible": 1}, "adjectives": {"Special": 1}, "adverbs": {"Unless": 1}, "pronouns": {"I": 2}}}, {"content": "9. If there were opportunities to meet with new people, I would seize them.", "word_count": 18, "label": "", "sentence_structure": "Subject鈥損redicate", "subjective_objective": {"answer": "Subjective", "rationale": "This is because of the patient ' s personal views and attitudes and is a subjective examination."}, "sentence_sentiment": {"answer": "Positive", "rationale": "It is an expression of a positive attitude and behavioural tendency to seize the opportunity to meet a new person."}, "language_style": {"answer": "Tactful and warm", "rationale": "The entry uses a conditional phrase like \"if...\" to express a proposal or request, while being gentler in tone, giving people a sense of affection, understanding and support, and therefore a warm and euphemism of language."}, "time_dimension": {"answer": "Future", "rationale": "The entry contains visions and expectations for the future, and the keywords are \"if there is a chance to meet a new person\" and \"if I will catch it\" and they all point to situations that have not yet occurred and that may occur in the future. So the judgement is the future."}, "sentence_type": "Interrogative sentence", "pos": {"nouns": {"New guy": 1, "Opportunities": 1, "Grab him": 1}, "verbs": {"have": 1, "Meeting": 1, "I'll catch you": 2}, "adverbs": {"If": 1, "of": 3}, "numerals": {"9": 1}, "pronouns": {"I": 1}}}, {"content": "10. I usually feel strung and nervous about the presence of different sexes at informal gatherings.", "word_count": 26, "label": "", "sentence_structure": "Subject鈥損redicate", "subjective_objective": {"answer": "Subjective", "rationale": "Because \"frightful and nervous\" is the patient's feeling or attitude to a particular situation, and it is the expression of subjective experience."}, "sentence_sentiment": {"answer": "Negative", "rationale": "Because \"flash\" and \"stretch\" are negative emotional experiences."}, "language_style": {"answer": "Tactful and warm", "rationale": "The term \"are there\" is more euphemistic and not overly direct or strongly expressed. For example, the term \"are there\" is more euphemistic and does not cause undue pressure or discomfort on the person interviewed. At the same time, the article is more acrimonym and careful, and seeks to understand the feelings and experiences of the person interviewed, thereby improving the follow-up research and analysis."}, "time_dimension": {"answer": "Present", "rationale": "Now the entry describes the behaviour or feelings of the person being tested in a given situation, i.e., the feeling of having a different sex at an informal meeting, without a clear time pointing to the past or the future. Thus, the temporal dimension is judged to be \"now\"."}, "sentence_type": "Interrogative sentence", "pos": {"nouns": {"Party": 1, "Heterosexual": 1, "Fingerprint": 1, "Nervous": 1}, "verbs": {"have": 1, "Participation": 1, "I think": 1}, "adjectives": {"Informal": 1}, "adverbs": {"Usually": 1, "If": 1, "and": 1}, "numerals": {"10": 1}, "pronouns": {"I": 1}}}, {"content": "I am usually anxious to be with people unless they are particularly familiar.", "word_count": 24, "label": "", "sentence_structure": "Subject鈥揷opula鈥損redicative", "subjective_objective": {"answer": "Subjective", "rationale": "Because it describes the feelings and reactions of patients in social settings, it is subjective and therefore subjective, as it is the patient ' s subjective thoughts and experiences."}, "sentence_sentiment": {"answer": "Negative", "rationale": "Because the \"anxiety with people\" in the entry is negative."}, "language_style": {"answer": "Tactful and warm", "rationale": "The entry is a soft, invited conversation, using the \"whether or not\" formula, and not directly expressing the negative emotional or psychological state of the other, but using a more euphemism and accommodative language style to guide respondents to answer. Moreover, the expression \"unless they are particularly familiar\" implies that social anxiety may be caused by unfamiliar or uncomfortable interpersonal relationships, which is more understandable and acceptable."}, "time_dimension": {"answer": "Present", "rationale": ""}, "sentence_type": "Interrogative sentence", "pos": {"nouns": {"People": 2, "Anxiety": 1, "Together": 1, "Time": 1, "Unless": 1, "Yes": 1, "Is it possible": 1}, "verbs": {"I feel": 1, "in": 1, "and": 3, "Special": 1, "Yes": 1, "be": 1}, "adjectives": {"Usually": 1, "Anxiety": 1}, "adverbs": {"Unless": 1, "Special": 1}, "numerals": {"11": 1, "2": 1}, "classifiers": {"individual": 1}, "pronouns": {"I": 1}}}, {"content": "I usually feel relaxed when I'm with a group.", "word_count": 17, "label": "", "sentence_structure": "Subject鈥揷opula鈥損redicative", "subjective_objective": {"answer": "Subjective", "rationale": "The question is asked about the patient ' s feelings and experience and about the patient ' s subjective view."}, "sentence_sentiment": {"answer": "Positive", "rationale": "Because the behavior in the entry \"Easy to Feel\" is positive."}, "language_style": {"answer": "Tactful and warm", "rationale": ""}, "time_dimension": {"answer": "Present", "rationale": "The keyword \"often feels relaxed\" means that it is a customary feeling, and the word \"whether\" indicates that the question is a question of the person's current feelings. Thus, the time dimension of the article is now."}, "sentence_type": "Interrogative sentence", "pos": {"nouns": {"Questionnaire": 1, "person": 1, "Group": 1, "Together": 1, "Time": 1, "Relax": 1}, "verbs": {"I feel": 1, "in": 1, "Together": 1, "Usually": 1, "Is it possible": 1}, "adjectives": {"Relax": 1}, "adverbs": {"Together": 1}, "numerals": {"12": 1}, "pronouns": {"I": 1}}}, {"content": "I often want to leave the crowd.", "word_count": 10, "label": "", "sentence_structure": "Subject鈥損redicate", "subjective_objective": {"answer": "Subjective", "rationale": "Because this is a subjective approach to the patient ' s own inner feelings and thoughts."}, "sentence_sentiment": {"answer": "Negative", "rationale": "Because the \"want to leave the crowd\" act in the entry is negative."}, "language_style": {"answer": "Tactful and warm", "rationale": "The entry is more proficient and interactive by using a soft, invitation-style tone of dialogue rather than direct statements or orders. At the same time, the language style of the article is more euphemistic, with no overly emotional tone or direct questioning of the attitudes of the other party."}, "time_dimension": {"answer": "Present", "rationale": "\"Relationship with the crowd\" indicates the current behaviour or feelings of the testee, and \"whether\" indicates that this is a selective behaviour that requires the testee to answer itself whether it is in accordance with his or her actual situation, and therefore the temporal dimension is now judged."}, "sentence_type": "Interrogative sentence", "pos": {"nouns": {"Population": 1, "Questionnaire": 1, "Entry": 1}, "verbs": {"Yes": 1, "Leave": 1, "Is it possible": 1}, "adverbs": {"Regular": 1}, "numerals": {"13": 1}, "pronouns": {"I": 1}}}, {"content": "I am usually uncomfortable with people I don't know.", "word_count": 22, "label": "", "sentence_structure": "Subject鈥揷opula鈥損redicative", "subjective_objective": {"answer": "Subjective", "rationale": "Because it's a patient's subjective experience."}, "sentence_sentiment": {"answer": "Negative", "rationale": "Because the entry describes \"unsatisfied\" negative emotions, which indicate a sense of unease and tension among strangers, and a negative emotional tone."}, "language_style": {"answer": "Tactful and warm", "rationale": "The language style of the entry is euphemism, because it uses an invitation tone and attempts to stimulate each other鈥檚 thinking about their own situation. At the same time, it does not use any language or vocabulary that could lead to negative emotions or stress. Rather, it uses a gentle and caring attitude to guide interlocutors to share their feelings."}, "time_dimension": {"answer": "Present", "rationale": "Whether the entry \"is to ask the testee what is happening now, and the time dimension is judged now."}, "sentence_type": "Interrogative sentence", "pos": {"nouns": {"Population": 1, "I": 2}, "verbs": {"Stand up": 1, "I feel": 1, "I'm not comfortable": 1, "Yes": 1}, "adjectives": {"Usually": 1}, "adverbs": {"Time": 1, "Is it possible": 1}, "numerals": {"14": 1}}}, {"content": "15. When I first met someone, I usually relaxed.", "word_count": 18, "label": "", "sentence_structure": "Subject鈥揷opula鈥損redicative", "subjective_objective": {"answer": "Subjective", "rationale": "Because \"relax\" is a subjective feeling, different people may feel different about the same situation and react differently."}, "sentence_sentiment": {"answer": "Neutral", "rationale": "As the entry only describes a situation, it does not express a positive or negative emotional tendency."}, "language_style": {"answer": "Calm and direct", "rationale": "The entry is based on a direct and clear inquiry into the feelings of the person when he first met, without excessive rhetorical or emotional rendering, and is more balanced and objective, and therefore can be judged as being calm and straightforward."}, "time_dimension": {"answer": "Present", "rationale": "The keyword \"usually\" and \"relax\" describes the person's current state, so it can be judged that the entry is a question of the behaviour or mentality of the present."}, "sentence_type": "Interrogative sentence", "pos": {"nouns": {"Initial": 1, "I met him": 1, "Some people": 1, "Relax": 1}, "verbs": {"be": 1, "Usually": 1}, "adverbs": {"in": 1}, "numerals": {"15": 1}, "pronouns": {"I": 1}}}, {"content": "16. Whether being introduced has made me nervous and anxious.", "word_count": 18, "label": "", "sentence_structure": "Subject鈥揷opula鈥損redicative", "subjective_objective": {"answer": "Subjective", "rationale": "Because stress and anxiety are subjective emotional experiences of patients."}, "sentence_sentiment": {"answer": "Negative", "rationale": "Because \"heavy and anxiety\" are both negative emotional experiences."}, "language_style": {"answer": "Calm and direct", "rationale": "The entry is based on the following: The person interviewed is asked directly whether he or she is nervous and anxious when he or she is introduced to others, and is not speaking with too many emotional renderings or speech techniques, which are more objective and direct."}, "time_dimension": {"answer": "Present", "rationale": "The keyword \"presented\" indicates a current state of behaviour, \"stress and anxiety\" is the emotional or emotional reaction that the act brings, so the temporal dimension of the entry should be the present."}, "sentence_type": "Interrogative sentence", "pos": {"nouns": {"Introduction": 1, "person": 1, "Feel": 1, "Nervous": 1, "Anxiety": 2}, "verbs": {"鈾?Make": 1, "I feel": 1}, "numerals": {"16": 1}}}, {"content": "17. Although the rooms are full of human beings, I may still go in.", "word_count": 20, "label": "", "sentence_structure": "Subject鈥損redicate", "subjective_objective": {"answer": "Subjective", "rationale": "The question is asked about the patient ' s behavioural orientation and willingness in a given situation, i.e. his attitude."}, "sentence_sentiment": {"answer": "Positive", "rationale": "Because the \"I may have gone in despite being a living person\" act in the entry shows a brave and risky attitude, a positive emotional attitude."}, "language_style": {"answer": "Tactful and warm", "rationale": "The phrase \"I may go in despite being alive\" is more personal and interactive. The phrase \"even though the room is full of human beings,\" is also relatively euphemism, with no too strong a negative or positive tone, and makes the respondents more relaxed when they answer."}, "time_dimension": {"answer": "Present", "rationale": "And in conclusion, the answer is \"now\"."}, "sentence_type": "Interrogative sentence", "pos": {"nouns": {"Room": 1, "Livers": 1}, "verbs": {"Yes": 1, "In": 1}, "adverbs": {"鈾?Though 鈾?: 1}, "pronouns": {"I": 1}}}, {"content": "18. I would avoid going forward to add people to a large group of people.", "word_count": 19, "label": "", "sentence_structure": "Subject鈥損redicate", "subjective_objective": {"answer": "Subjective", "rationale": "Because it is the patient's subjective feeling and attitude."}, "sentence_sentiment": {"answer": "Negative", "rationale": "Because the entry \"to avoid going to the top of the top to the top of the top\" is negative."}, "language_style": {"answer": "Tactful and warm", "rationale": "The entry is more euphemistic and gentle, and the interviewer is guided by an invitation to answer, without being too direct or hard-lined. At the same time, the description of behaviour \"avoiding to go forward and join a large group\" is relatively objective and neutral, and does not have too much emotional colour. Thus, the language style of the article can be judged as \"pretty warm.\""}, "time_dimension": {"answer": "Future", "rationale": "The entry \"will\" indicate a will or a preference, and \"avoid to go to the top of the crowd\" is an action that has not yet happened and will be taken. The time dimension of the entry is therefore the future."}, "sentence_type": "Interrogative sentence", "pos": {"nouns": {"I": 1, "A whole bunch of people": 1}, "verbs": {"Yes": 1, "Avoid": 1, "Step forward": 1, "Add": 1}, "adverbs": {"Is it possible": 1}}}, {"content": "19. When my supervisor wanted to talk to me, I was pleased to talk to him.", "word_count": 19, "label": "", "sentence_structure": "Subject鈥搗erb鈥揷omplement", "subjective_objective": {"answer": "Subjective", "rationale": "Because \"happily talking to him\" is the patient's personal feeling and attitude, and it's subjective."}, "sentence_sentiment": {"answer": "Positive", "rationale": "Basis: The act in the entry \"happily talking to him\" expresses a positive attitude."}, "language_style": {"answer": "Tactful and warm", "rationale": ""}, "time_dimension": {"answer": "Present", "rationale": "The keywords \"when my superior wants to talk to me\" and \"I'm happy to talk to him\" indicate that the act is a current situation or attitude, so the time dimension of the article is now."}, "sentence_type": "Interrogative sentence", "pos": {"nouns": {"Sir": 1, "Talk": 2}, "verbs": {"Yes": 1, "Same": 1, "I'm glad": 1, "and": 1, "Talk": 1}, "adjectives": {"Happy": 1}, "adverbs": {"very": 1}, "pronouns": {"I": 1}}}, {"content": "20. When I am with a group, I usually feel uncomfortable.", "word_count": 20, "label": "", "sentence_structure": "Subject鈥揷opula鈥損redicative", "subjective_objective": {"answer": "Subjective", "rationale": "As this entry relates to the feelings and experiences of patients in the population, it is examined from the patient ' s subjective perspective."}, "sentence_sentiment": {"answer": "Negative", "rationale": "Because the \"frightful\" emotion in the entry is negative."}, "language_style": {"answer": "Tactful and warm", "rationale": "The language style of the entry is euphemism. It is more personal and interactive in its expression, in a gentle and inviting tone, rather than in its direct statement or order. At the same time, the term \"sweet\" is used to express emotions, and to reflect the subtlety and tenderness of language."}, "time_dimension": {"answer": "Present", "rationale": "The term \"usually\" in this entry indicates a constant situation, \"frightful\" and \"whether\" are descriptions of the current state of mind, so it can be judged that the time dimension of the article is now."}, "sentence_type": "Interrogative sentence", "pos": {"nouns": {"Group": 1, "I'm not sure": 1}, "verbs": {"Feel": 1}, "adverbs": {"Usually": 1, "Together": 1, "Is it possible": 1}, "classifiers": {"individual": 1}, "pronouns": {"I": 1}}}, {"content": "I like to avoid crowds.", "word_count": 9, "label": "", "sentence_structure": "Subject鈥搗erb鈥揷omplement", "subjective_objective": {"answer": "Subjective", "rationale": "Because it is the subjective attitude and feelings of the patient that is attracted or not, it is a subjective view."}, "sentence_sentiment": {"answer": "Negative", "rationale": "Because the \"scramble\" in the entry is negative."}, "language_style": {"answer": "Tactful and warm", "rationale": "The language style of the entry is more euphemistic, using an invitation to ask questions rather than a direct statement or order."}, "time_dimension": {"answer": "Present", "rationale": "The keywords \"like to avoid crowds\" and \"whether\" point to the person being tested in his current state or behaviour, so it can be judged that the temporal dimension of the article is now."}, "sentence_type": "Interrogative sentence", "pos": {"nouns": {"Population": 1, "Questionnaire": 1, "Entry": 1, "I": 1}, "verbs": {"Yeah": 1, "Get out of the way": 1, "be": 1, "Yes": 1}, "numerals": {"21": 1}}}, {"content": "22. Whether talking to people at night or at social gatherings is not a problem for me.", "word_count": 22, "label": "", "sentence_structure": "Subject鈥搗erb鈥搊bject", "subjective_objective": {"answer": "Subjective", "rationale": "Because it is the patient's subjective feeling and attitude."}, "sentence_sentiment": {"answer": "Positive", "rationale": "Because the behavior in the entry \"talking to people\" is positive and without any negative emotional aspects, it can be judged positive."}, "language_style": {"answer": "Tactful and warm", "rationale": "The language style of the entry is euphemistic, and the person surveyed is guided by the means of questioning to express his or her views, without directly evaluating or judging them."}, "time_dimension": {"answer": "Present", "rationale": "The entry asks about the current behaviour or state of the subject, the keyword is \"at night or at social parties\" and \"talking to me is not a problem.\" So the time dimension of this questionnaire entry is now judged."}, "sentence_type": "Interrogative sentence", "pos": {"nouns": {"Night": 1, "Social parties": 1, "People": 1, "Talk": 1}, "verbs": {"be": 1, "It's not a problem": 2}, "adverbs": {"not": 1}, "numerals": {"22": 1}, "pronouns": {"I": 1}}}, {"content": "23. I rarely feel comfortable among a large group.", "word_count": 17, "label": "", "sentence_structure": "Subject鈥搗erb鈥搊bject", "subjective_objective": {"answer": "Objective", "rationale": "Because the sentence describes a person鈥檚 feelings in a group of people, it is an objective fact. Although this feeling may be influenced by individual subjective perceptions, the sentence itself does not clearly express the subjective attitude or ideas of the patient and is therefore an objective examination."}, "sentence_sentiment": {"answer": "Negative", "rationale": "Because the phrase \"very rarely feel comfortable\" in the entry is negative."}, "language_style": {"answer": "Tactful and warm", "rationale": "The language style of the entry is more euphemistic, using a softer expression like \"You rarely feel comfortable\" and not directly questioning or accusing each other, but directing each other to think and answer questions in a gentle and caring way. This expression makes it easier to make the interviewee comfortable and receptive, and thus more willing to answer questions honestly."}, "time_dimension": {"answer": "Past", "rationale": "In terms of time dimension, this entry does not clearly point to past, present or future, and can therefore be judged to be of a general nature."}, "sentence_type": "Interrogative sentence", "pos": {"nouns": {"Population": 1, "Centre": 1, "I'm comfortable": 1}, "verbs": {"Yes": 1, "I feel": 1, "be": 1, "Very few": 1, "I can feel it": 1}, "adverbs": {"in": 1, "A lot of them": 1, "Centre": 1, "Extreme": 1, "Less": 1}, "numerals": {"23": 1}, "pronouns": {"I": 1}}}, {"content": "24. I have often come up with excuses to avoid social activities.", "word_count": 18, "label": "", "sentence_structure": "Subject鈥損redicate", "subjective_objective": {"answer": "Subjective", "rationale": "Because \"preventing some excuses\" is patient's subjective thinking and decision-making, and it's subjective."}, "sentence_sentiment": {"answer": "Negative", "rationale": "Because the \"scrambling social activities\" act is negative."}, "language_style": {"answer": "Tactful and warm", "rationale": "The entry uses the \"whether\" as open, gives the respondent a space for free expression and the term is more moderate, friendly, without direct orders or acrimonyms, and is more readily acceptable to the person under investigation and willing to share his/her thoughts."}, "time_dimension": {"answer": "Present", "rationale": ""}, "sentence_type": "Interrogative sentence", "pos": {"nouns": {"- An excuse": 1, "Social events": 1}, "verbs": {"I've got it": 1, "Avoidance": 1}, "adverbs": {"Regular": 1, "Some": 1}, "pronouns": {"I": 1}}}, {"content": "25. I sometimes act as a person ' s introduction to one another.", "word_count": 17, "label": "", "sentence_structure": "Subject鈥損redicate", "subjective_objective": {"answer": "Subjective", "rationale": "Because the description of \"acting as\" and \"role\" involves subjective perceptions and behaviour of patients, it is a subjective approach."}, "sentence_sentiment": {"answer": "Positive", "rationale": "Because the \"acting as a person's introductory role\" in the entry is positive, it is a positive quality to indicate that the person is willing to help others to establish contact."}, "language_style": {"answer": "Tactful and warm", "rationale": "The entry is more personal and interactive in terms of a soft tone, which is used in a query, without direct statements or orders."}, "time_dimension": {"answer": "Present", "rationale": "In the future, the entry is to ask the testee about the behaviour or attitude that may occur in the future, and the temporal dimension of the keyword \"sometimes\" and \"acting\" points to the situation that has not occurred and is therefore judged to be the future."}, "sentence_type": "Interrogative sentence", "pos": {"nouns": {"Role": 1, "To each other": 1}, "verbs": {"Act as": 1, "Introduction": 1}, "adverbs": {"Sometimes": 1, "be": 1}, "numerals": {"25": 1}, "pronouns": {"I": 1}}}, {"content": "26. I try to avoid formal social occasions.", "word_count": 14, "label": "", "sentence_structure": "Subject鈥揷opula鈥損redicative", "subjective_objective": {"answer": "Subjective", "rationale": "Because \"I try\" means the patient's subjective efforts and will."}, "sentence_sentiment": {"answer": "Negative", "rationale": "Because the \"negative as far as possible\" behavior in the entries is negative, it is a sign of unwillingness to participate in social settings."}, "language_style": {"answer": "Calm and direct", "rationale": "The entry is presented in a clear question form, with no excessive emotional rendering or rhetorical techniques, and is more objective in tone, so that the language style is of a straightforward type of calm."}, "time_dimension": {"answer": "Present", "rationale": "Because the words \"as far as possible\" and \"as far as possible\" in the entry indicate an act that has occurred or is occurring, and \"if\" is an inquiry or confirmation of a past act, the temporal dimension of the entry should be the past."}, "sentence_type": "Interrogative sentence", "pos": {"nouns": {"Questionnaire Entry": 1, "Event": 1}, "verbs": {"Avoid": 1, "Social": 1}, "adjectives": {"Formal": 1}, "adverbs": {"Try": 1, "be": 1}, "pronouns": {"I": 1}}}, {"content": "27. I usually participate in the various social contacts in which I can participate. I usually go wherever I want to.", "word_count": 36, "label": "", "sentence_structure": "Subject鈥搗erb鈥搊bject", "subjective_objective": {"answer": "Subjective", "rationale": "Because the subject is \"I\" what it usually does, it's a subjective idea and behavioral habit of the patient, and it's therefore a subjective perspective."}, "sentence_sentiment": {"answer": "Positive", "rationale": "Because the entry expresses an attitude and behaviour that actively engages in social interaction, it does not display negative emotions or tendencies."}, "language_style": {"answer": "Tactful and warm", "rationale": "The language style of the entry is more euphemistic and friendly, using expressions such as \"normal\" or \"whether\" or \"whether\" and being more moderate and inclusive, making people more comfortable and willing to cooperate in completing the questionnaire."}, "time_dimension": {"answer": "Present", "rationale": "The keywords \"normally\", \"generally\" and \"go\" all indicate that this is a regular, customary act, which is still ongoing, and it is therefore possible to judge that the entry describes the present behaviour or mentality."}, "sentence_type": "Interrogative sentence", "pos": {"nouns": {"27": 1, "I": 2, "Various": 1, "Social interaction": 1, "Social events": 1, "General": 1, "Is it possible": 1}, "verbs": {"Participation": 1, "Yes, I can": 1}, "adverbs": {"Usually": 2, "Whatever": 1, "Both": 1}, "numerals": {"27": 1}, "classifiers": {"Various": 1, "Social events": 1}, "pronouns": {"I": 2}}}, {"content": "I found it easy to relax when I was with someone else.", "word_count": 15, "label": "", "sentence_structure": "Subject鈥搗erb鈥搊bject", "subjective_objective": {"answer": "Subjective", "rationale": "Because the words \"discovery\" and \"easy\" are all described in the patient's subjective sense."}, "sentence_sentiment": {"answer": "Positive", "rationale": "Because finding \"relaxion when with others is easy\" is a positive emotional experience and attitude."}, "language_style": {"answer": "Tactful and warm", "rationale": "The entry is more personal and interactive when it is spoken in a gentle and inviting tone, rather than in direct statements or orders. At the same time, the words \"discovery\" and \"easy\" convey a positive and relaxed atmosphere."}, "time_dimension": {"answer": "Present", "rationale": "The keyword \"discovered\" and \"when with other people\" are described as being present or present, so the entry indicates the situation of the person being tested."}, "sentence_type": "Interrogative sentence", "pos": {"nouns": {"Problem": 1, "Entry": 1, "person": 1, "Relax": 1}, "verbs": {"Found": 1, "Easy": 1, "In": 1}, "adjectives": {"Others": 1, "Together": 1, "This": 1}, "adverbs": {"very": 1}, "numerals": {"28": 1, "One": 2}, "classifiers": {"individual": 1}, "pronouns": {"I": 1}}}]}

{"questionnaire_title": "UCLA Lonely Measure", "questionnaire_category": "Mental health", "item_count": 20, "emotion_shift_overall": "From negative to positive", "language_style_overall": "Tactful and warm", "items": [{"content": "Do you often feel in harmony with the people around you?", "word_count": 14, "label": "", "sentence_structure": "Subject鈥搗erb鈥搊bject", "subjective_objective": {"answer": "Subjective", "rationale": "Because it involves the patient ' s feelings about his/her relationship with others, it is a manifestation of subjective attitudes."}, "sentence_sentiment": {"answer": "Positive", "rationale": "Because \"harmonious\" relationships are positive emotions, and they express positive attitudes."}, "language_style": {"answer": "Tactful and warm", "rationale": "The language style of the entry is more euphemismal, because it asks the interviewees in a gentle and friendly manner, trying to create a comfortable and pleasant atmosphere. At the same time, it does not ask the interviewees too directly about their personal feelings, but rather uses more neutral and objective language to guide the discussion."}, "time_dimension": {"answer": "Present", "rationale": ""}, "sentence_type": "Interrogative sentence", "pos": {"nouns": {"you": 1, "People around": 1, "Relations": 1, "Harmony": 1}, "verbs": {"Common sense": 1, "I feel": 1, "Harmony of relations": 1}, "adjectives": {"Harmony": 1}, "adverbs": {"Regular": 1}, "pronouns": {"you": 1}}}, {"content": "Do you often feel the lack of partners?", "word_count": 9, "label": "", "sentence_structure": "Subject鈥損redicate", "subjective_objective": {"answer": "Subjective", "rationale": "Since the problem involved patients ' feelings and subjective experiences, they were asked whether they often felt that they lacked partners and therefore were subjective in nature."}, "sentence_sentiment": {"answer": "Negative", "rationale": "Because the \"lack of partners\" attitude in the entries is negative."}, "language_style": {"answer": "Tactful and warm", "rationale": "The entry is more personal and interactive when it is presented in a way that is in a question and concern rather than in a direct statement or order. At the same time, the term is more euphemistic and avoids the use of strong words that may be offensive to the other party."}, "time_dimension": {"answer": "Present", "rationale": "The entry asks if the testees often feel the lack of partners in their current lives, emphasizing the current state of their feelings and status, so the time dimension is now judged."}, "sentence_type": "Interrogative sentence", "pos": {"nouns": {"Partners": 1, "Questionnaire": 1}, "verbs": {"I feel": 1, "Missing": 1}, "classifiers": {"individual": 1}}}, {"content": "Do you often feel that no one can trust?", "word_count": 11, "label": "", "sentence_structure": "Subject鈥搗erb鈥搊bject", "subjective_objective": {"answer": "Subjective", "rationale": "Because it is a subjective feeling and feeling that no one can trust."}, "sentence_sentiment": {"answer": "Negative", "rationale": "Because the emotional description of \"no one can trust\" in the entry is negative."}, "language_style": {"answer": "Calm and direct", "rationale": "The entry is a direct question-and-answer dialogue, is not overly rhetorical or emotional, is more objective in tone and asks whether the other side often feels that no one can be trusted."}, "time_dimension": {"answer": "Past", "rationale": "\"You often feel that the word \"normal\" indicates a regular, customary behaviour, while the word \"sension\" uses the general current, which means that the act has not only occurred in the past, but may still be ongoing. The entry therefore asks whether the person tested has often experienced a history of people who feel that they are not able to trust."}, "sentence_type": "Interrogative sentence", "pos": {"nouns": {"person": 1, "Trust": 1}, "verbs": {"I feel": 1, "Yeah": 1}, "adverbs": {"Regular": 1}, "numerals": {"3": 1}}}, {"content": "Do you often feel the silence?", "word_count": 7, "label": "", "sentence_structure": "Subject鈥搗erb鈥揷omplement", "subjective_objective": {"answer": "Subjective", "rationale": "Because feeling lonely is a subjective feeling, not a fact that can be directly observed."}, "sentence_sentiment": {"answer": "Negative", "rationale": "Because \"slonelitude\" in the entry is a negative emotion, expressing a negative attitude."}, "language_style": {"answer": "Tactful and warm", "rationale": "The language style of the entry is more euphemistic and friendly, and the fact that the other party is not feeling lonely, and is not using direct or hard language, is more likely to resonate and share their feelings."}, "time_dimension": {"answer": "Present", "rationale": "The word \"normal\" in \"common sense\" denotes a regular, customary behaviour, while the word \"sension\" adopts the general current, meaning that this behaviour has not only occurred in the past, but may still be ongoing. The entry is therefore an inquiry into the present feelings of the person being tested, and the temporal dimension is now judged."}, "sentence_type": "Interrogative sentence", "pos": {"nouns": {"Silent": 1}, "verbs": {"Sensor": 1, "Regular": 1}, "adjectives": {"Silent": 1}, "adverbs": {"Regular": 1}, "numerals": {"4": 1}}}, {"content": "Do you often feel like one of your friends?", "word_count": 14, "label": "", "sentence_structure": "Subject鈥搗erb鈥搊bject", "subjective_objective": {"answer": "Subjective", "rationale": "Because the subject \"often feels\" emphasizes the subjective feelings and experiences of patients, i.e. whether they feel that they are part of a group of friends. This subjective feelings and experiences cannot be concluded by objective observation or measurement, and therefore this is a subjective entry."}, "sentence_sentiment": {"answer": "Positive", "rationale": "Because the \"Feeling a friend\" attitude in the entry is positive."}, "language_style": {"answer": "Tactful and warm", "rationale": "The entry uses a question-and-answer tone rather than a direct statement or order, which is more adept and interactive. At the same time, the question provides a more open answer space ( \"yes\" or \"no\"), encourages respondents to express their true feelings, and does not use excessive rhetorical or emotional rendering, which is more objective. So I think the language style of this questionnaire is euphemism."}, "time_dimension": {"answer": "Past", "rationale": "Past, present"}, "sentence_type": "Interrogative sentence", "pos": {"nouns": {"Questionnaire": 1, "Entry": 1, "Friends": 1}, "verbs": {"I feel": 1, "belong to": 1}, "adverbs": {"Regular": 1, "One of them": 1}, "numerals": {"5": 1}}}, {"content": "Do you often feel that you have much in common with the people around you?", "word_count": 16, "label": "", "sentence_structure": "Subject鈥搗erb鈥搊bject", "subjective_objective": {"answer": "Subjective", "rationale": "Because feeling in common with others is a subjective idea or feeling, not an objective fact."}, "sentence_sentiment": {"answer": "Neutral", "rationale": "Because the entry does not have any emotional color."}, "language_style": {"answer": "Tactful and warm", "rationale": "The language style of the entry is more euphemistic, friendly, and leads the other to think and express his/her feelings by asking questions, rather than directly giving judgment or demands. Such language styles can be perceived as respectful and understandable, helping to create a good atmosphere of investigation and interaction."}, "time_dimension": {"answer": "Present", "rationale": "The keyword \"often feels\" indicates that it is a question of the current feeling, and \"many things in common\" indicates the social context, so the temporal dimension of the article is now."}, "sentence_type": "Interrogative sentence", "pos": {"nouns": {"person": 1, "Common ground": 1}, "verbs": {"I feel": 1, "have": 1}, "adjectives": {"Many": 1}, "adverbs": {"Regular": 1}, "numerals": {"6": 1}, "classifiers": {"individual": 1}, "pronouns": {"you": 1}}}, {"content": "Do you often feel intimacy to anyone?", "word_count": 14, "label": "", "sentence_structure": "Subject鈥損redicate", "subjective_objective": {"answer": "Subjective", "rationale": "Because \"feel\" is the patient's personal subjective feeling, and \"intimateness with anyone\" is also the conclusion based on the patient's own observations and experiences."}, "sentence_sentiment": {"answer": "Negative", "rationale": "Because the description of intimacy in the entries is negative, indicating emotional isolation and alienation."}, "language_style": {"answer": "Tactful and warm", "rationale": "The entry is more personal and interactive when it is spoken in a gentle and inviting manner, rather than in direct statements or orders. At the same time, it does not use too strong negative terms, but rather uses a more neutral formulation to ask the interviewee whether he feels alienated and therefore judge the euphemism of the language."}, "time_dimension": {"answer": "Present", "rationale": "\"You often feel that the word \"always\" indicates a regular, customary act, while the word \"sension\" uses the usual current pattern, which means that the act not only has occurred in the past, but may still be ongoing. So this entry asks the testee about the situation."}, "sentence_type": "Interrogative sentence", "pos": {"nouns": {"person": 2, "Intimacy": 1, "Questionnaire": 1}, "verbs": {"I feel": 1, "Both": 1, "not": 1, "- You're not": 1}, "adverbs": {"Regular": 1}, "pronouns": {"you": 1}}}, {"content": "Do you often feel that your interest and thoughts are different from those of the people around you?", "word_count": 20, "label": "", "sentence_structure": "Subject鈥搗erb鈥搊bject", "subjective_objective": {"answer": "Subjective", "rationale": "The issue is subjective because it relates to the feelings and perceptions of the patient himself."}, "sentence_sentiment": {"answer": "Neutral", "rationale": "Because the entry is about personal feelings and does not have a clear emotional orientation."}, "language_style": {"answer": "Tactful and warm", "rationale": "The entry uses a more euphemistic expression, which leads the interviewee to share his feelings rather than to make a direct statement or order. At the same time, the entry does not have a strong emotional tone, but rather a calm and understanding approach to the question, so the language style tends to be more euphemistic."}, "time_dimension": {"answer": "Present", "rationale": "The keyword \"You often feel\" means \"a regular, customary act,\" which means \"a different from the person around you\" means that it has not only happened in the past, but may still be ongoing. So, the entry to the questionnaire is to ask the subject about his current behaviour and state, and the time dimension is now."}, "sentence_type": "Interrogative sentence", "pos": {"nouns": {"Questionnaire": 1, "Entry": 1, "Interest": 1, "Thoughts": 1, "Around": 1, "person": 1}, "verbs": {"I feel": 1, "Regular": 1, "It's different": 1}, "adjectives": {"Different": 1}, "adverbs": {"Regular": 1}, "numerals": {"8": 1}}}, {"content": "Do you often feel like having friends and friends?", "word_count": 15, "label": "", "sentence_structure": "Subject鈥搗erb鈥搊bject", "subjective_objective": {"answer": "Subjective", "rationale": "Because the entry of the questionnaire asks patients whether they often have the intention to be with people and to have friends, it is the patient ' s subjective attitude."}, "sentence_sentiment": {"answer": "Positive", "rationale": "Because the \"want to be friends and have friends\" attitude in this entry is positive."}, "language_style": {"answer": "Tactful and warm", "rationale": ""}, "time_dimension": {"answer": "Present", "rationale": ""}, "sentence_type": "Interrogative sentence", "pos": {"nouns": {"Questionnaire Entry": 1, "person": 2, "Travel": 1, "Deal": 1, "friends": 1}, "verbs": {"I always feel": 1, "Yes": 1, "and": 1}, "adverbs": {"Regular": 1, "you": 1}, "numerals": {"9": 1}}}, {"content": "Do you often feel close to others?", "word_count": 9, "label": "", "sentence_structure": "Subject鈥損redicate", "subjective_objective": {"answer": "Subjective", "rationale": "Because \"fear\" is a subjective emotional experience that reflects the patient's perception and feelings about his relationship with others."}, "sentence_sentiment": {"answer": "Positive", "rationale": "Because the \"near\" act in the entry is positive."}, "language_style": {"answer": "Tactful and warm", "rationale": "The entry is more adept and interactive when it is spoken in a gentle and inviting manner, rather than by direct statements or orders."}, "time_dimension": {"answer": "Present", "rationale": "The word \"often\" denotes a regular, customary behaviour, while the word \"sense\" adopts the general current, which means that the behaviour has not only occurred in the past, but may still be ongoing. The entry therefore asks about the current emotional state of the person being tested, which is judged by the time dimension."}, "sentence_type": "Interrogative sentence", "pos": {"nouns": {"person": 1, "Close": 1}, "verbs": {"Sensor": 1, "Present": 1}, "adverbs": {"Regular": 1}, "numerals": {"10": 1}}}, {"content": "Do you often feel left alone?", "word_count": 9, "label": "", "sentence_structure": "Subject鈥搗erb鈥搊bject", "subjective_objective": {"answer": "Subjective", "rationale": "Because \"fearing to be left alone\" is the patient's own subjective feeling and experience, and it's a subjective angle."}, "sentence_sentiment": {"answer": "Negative", "rationale": "Because the feeling of being left behind in the entries is negative."}, "language_style": {"answer": "Tactful and warm", "rationale": "The entry is more personal and interactive in terms of dialogue in a moderate and caring tone of the other鈥檚 feelings, rather than direct statements or orders. At the same time, questions are asked in a more friendly and caring manner, which makes it easier for respondents to feel the goodwill and respect of the surveyer and contributes to the effectiveness and response rate of the questionnaire."}, "time_dimension": {"answer": "Present", "rationale": ""}, "sentence_type": "Interrogative sentence", "pos": {"nouns": {"Questionnaire": 1, "Entry": 1, "person": 1, "Cold": 1}, "verbs": {"I always feel": 1}, "adverbs": {"Regular": 1, "- You're not": 1}, "numerals": {"11": 1, "you": 1}}}, {"content": "Do you often feel that you have no sense in being with someone else?", "word_count": 15, "label": "", "sentence_structure": "Subject鈥損redicate", "subjective_objective": {"answer": "Subjective", "rationale": "Because the problem is one of patient subjective feelings and ideas."}, "sentence_sentiment": {"answer": "Negative", "rationale": "Because the description of \"no sense\" in the entry is negative."}, "language_style": {"answer": "Calm and direct", "rationale": "The language style of the entry is more straightforward, and there is no excessive rhetoric or emotional colour to direct the interviewee to express his thoughts and feelings by asking questions."}, "time_dimension": {"answer": "Past", "rationale": "The word \"normal\" denotes a regular, customary act, while \"perception\" adopts the general current, which means that the act has not only occurred in the past but may still be ongoing. Thus, the entry contains an inquiry into what the person was and is feeling."}, "sentence_type": "Interrogative sentence", "pos": {"nouns": {"you": 1, "Regular": 1, "I feel": 1, "others": 1, "Travel": 1, "It's pointless": 1}, "verbs": {"I always feel": 1}, "adverbs": {"Regular": 1}, "numerals": {"12": 1}, "pronouns": {"you": 1}}}, {"content": "Do you often feel that no one knows you well?", "word_count": 12, "label": "", "sentence_structure": "Subject鈥損redicate", "subjective_objective": {"answer": "Subjective", "rationale": "Because \"fear\" and \"understanding\" are subjective feelings or experiences of patients."}, "sentence_sentiment": {"answer": "Negative", "rationale": "Because \"no one knows\" in the entry is an isolated, neglected negative feeling."}, "language_style": {"answer": "Tactful and warm", "rationale": "The language style of the entry is more euphemistic, allowing the interviewee to reflect and share his/her feelings, without directly expressing any assessment or opinion. This expression allows the interviewee to be more open to questions and to feel more comfortable and respectful in his/her conversation."}, "time_dimension": {"answer": "Present", "rationale": "\"You often feel that the word \"normal\" means a regular, customary act, while the word \"sension\" uses the normal current state of affairs, which means that it not only happened in the past, but may still be ongoing. So, the entry is primarily about the emotional experience and feelings of the person being tested."}, "sentence_type": "Interrogative sentence", "pos": {"nouns": {"person": 1, "you": 1, "Roger that": 1}, "verbs": {"I feel": 1, "have": 1}, "adverbs": {"Regular": 1}, "numerals": {"13": 1}, "pronouns": {"very": 1}}}, {"content": "Do you often feel isolated from others?", "word_count": 11, "label": "", "sentence_structure": "Subject鈥損redicate", "subjective_objective": {"answer": "Subjective", "rationale": "Because \"fEat\" is a subjective feeling."}, "sentence_sentiment": {"answer": "Negative", "rationale": "Because \"separating from others\" is a negative emotional experience."}, "language_style": {"answer": "Tactful and warm", "rationale": "So I'm judging the language style of this questionnaire entry as \"precipitous and warm.\""}, "time_dimension": {"answer": "Present", "rationale": "The keyword \"always feel\" and \"segregated\" means a constant state of mind or feeling, so it can be judged that the entry describes the situation of the person being tested."}, "sentence_type": "Interrogative sentence", "pos": {"nouns": {"14": 1, "you": 1, "Don't": 1, "person": 1, "Interval": 1}, "verbs": {"Regular": 1, "I feel": 1, "Separate": 1}, "adverbs": {"- You're not": 1}, "numerals": {"14": 1}, "pronouns": {"you": 1}}}, {"content": "Do you often feel like you can find your partner when you want?", "word_count": 16, "label": "", "sentence_structure": "Subject鈥搗erb鈥搊bject", "subjective_objective": {"answer": "Subjective", "rationale": "Because it is a personal feeling and attitude to find a partner, it is the subjective view of the patient."}, "sentence_sentiment": {"answer": "Positive", "rationale": "Because the purpose of the action of finding a partner in the entry is positive."}, "language_style": {"answer": "Tactful and warm", "rationale": ""}, "time_dimension": {"answer": "Present", "rationale": ""}, "sentence_type": "Interrogative sentence", "pos": {"nouns": {"you": 1, "Partners": 1}, "verbs": {"I always feel": 1, "Yes": 1, "Find": 1}, "adjectives": {"Yes": 1}, "adverbs": {"When": 1, "Time": 1}, "numerals": {"15": 1}}}, {"content": "Do you often feel that someone really knows you?", "word_count": 12, "label": "", "sentence_structure": "Subject鈥搗erb鈥搊bject", "subjective_objective": {"answer": "Subjective", "rationale": "The answer is subjective, since the question concerns whether the patient feels understood, which is a subjective feeling and experience."}, "sentence_sentiment": {"answer": "Neutral", "rationale": "Since the entry was not of any emotional colour, it was merely asking the person under investigation about a fact."}, "language_style": {"answer": "Tactful and warm", "rationale": "The language style of the entry is euphemistic, warm, and it uses the expression \"often felt\" to give people a warm, warm feeling, while at the same time showing concern and understanding. It is more resonant and responsive, and more suitable for the use of questionnaires, etc."}, "time_dimension": {"answer": "Present", "rationale": "The entry asks the testee how he feels now, and the keywords are \"often feeling\" and \"does anyone really know you?\" both indicate that it is a general, customary act or state."}, "sentence_type": "Interrogative sentence", "pos": {"nouns": {"person": 1, "Roger that": 1}, "verbs": {"I feel": 1, "Really": 1, "Roger that": 1}, "adjectives": {"Regular": 1, "Really": 1}, "numerals": {"16": 1}, "pronouns": {"you": 1}}}, {"content": "Do you often feel shy?", "word_count": 7, "label": "", "sentence_structure": "Subject鈥損redicate", "subjective_objective": {"answer": "Subjective", "rationale": "Because being shy is a subjective emotional experience."}, "sentence_sentiment": {"answer": "Negative", "rationale": "Because \"weak\" is a negative emotional attitude."}, "language_style": {"answer": "Tactful and warm", "rationale": "The language style of the entry is relatively gentle and friendly, and the choice of words is not too direct or offensive, giving people a friendly and warm feeling. At the same time, the issue is presented in a more humanistic way, aimed at knowing whether the interviewees are often shy in their lives, rather than merely concerned about whether they are in such a situation."}, "time_dimension": {"answer": "Present", "rationale": ""}, "sentence_type": "Interrogative sentence", "pos": {"nouns": {"Shame": 1, "Questionnaire": 1, "Entry": 1}, "verbs": {"I feel": 1, "Regular": 1, "Shame": 1, "you": 1, "Questionnaire": 1, "Entry": 1}, "adjectives": {"Regular": 1}, "adverbs": {"- You're not": 1}, "numerals": {"17": 1}, "pronouns": {"you": 1}}}, {"content": "Do you often feel like people are around you, but they don't care about you?", "word_count": 16, "label": "", "sentence_structure": "Subject鈥搗erb鈥搊bject", "subjective_objective": {"answer": "Subjective", "rationale": "Because this is the patient ' s subjective experience and judgment."}, "sentence_sentiment": {"answer": "Negative", "rationale": "Because the description \"people around you but don't care about you\" in the entry is negative."}, "language_style": {"answer": "Tactful and warm", "rationale": "The entry uses a more euphemistic approach to questions, uses the term \"often feeling\" to express the emotional state of the interviewee and adds a counter-questioning form, allowing the interviewee to think and answer questions on his own. This way is more gentle and respectful of the interviewer鈥檚 feelings than the direct interview."}, "time_dimension": {"answer": "Present", "rationale": "\"You often feel\" means \"recurring, \"customary\" behaviour, and \"fEalse\" uses the usual current pattern, which means that this behaviour has not only happened in the past, but may still be ongoing."}, "sentence_type": "Interrogative sentence", "pos": {"nouns": {"person": 1, "Problem": 1, "Care": 1}, "verbs": {"Common sense": 1, "Round and round": 1, "- No, it's not": 1}, "adverbs": {"Regular": 1, "But": 1}, "numerals": {"18": 1}, "pronouns": {"you": 2}}}, {"content": "Do you often feel like someone wants to talk to you?", "word_count": 13, "label": "", "sentence_structure": "Subject鈥搗erb鈥搊bject", "subjective_objective": {"answer": "Subjective", "rationale": "Because \"fEs\" is the patient's subjective feeling."}, "sentence_sentiment": {"answer": "Positive", "rationale": "Because the \"someone wants to talk to you\" act is positive."}, "language_style": {"answer": "Tactful and warm", "rationale": "So the language style of the entry is \"slow and warm\"."}, "time_dimension": {"answer": "Present", "rationale": "The keyword \"always\" denotes regular, customary behaviour, \"you often feel\" using the usual current state of affairs, which means that this feeling may have occurred in the past, but it still continues. So the temporal dimension of this article is now."}, "sentence_type": "Interrogative sentence", "pos": {"nouns": {"person": 1, "Talk": 1}, "verbs": {"Yes": 1, "Common sense": 1, "and": 1, "Talk": 1}, "adverbs": {"Regular": 1, "you": 1}, "pronouns": {"you": 1}}}, {"content": "Do you often feel that someone deserves your trust?", "word_count": 12, "label": "", "sentence_structure": "Subject鈥搗erb鈥搊bject", "subjective_objective": {"answer": "Subjective", "rationale": "Because the entry examines whether the patient feels that someone deserves to be trusted, it is the patient ' s subjective feeling and attitude."}, "sentence_sentiment": {"answer": "Positive", "rationale": "Because the \"someone deserves your trust\" in the entry is positive and expresses the need for a relationship of trust."}, "language_style": {"answer": "Tactful and warm", "rationale": "The entry is more intimate and interactive when it is spoken in a gentle and inviting manner, rather than in direct statements or orders. At the same time, the article is more objective and has no excessive rhetorical or emotional accents, which are more friendly and comfortable."}, "time_dimension": {"answer": "Present", "rationale": "The content of this questionnaire entry is therefore mainly to ask whether the person being tested currently has a reliable presence."}, "sentence_type": "Interrogative sentence", "pos": {"nouns": {"Questionnaire": 1, "Entry": 1, "person": 1, "Trust": 1}, "verbs": {"I always feel": 1, "It's worth it": 1, "Trust": 1}, "adverbs": {"Regular": 1}, "numerals": {"20": 1}, "classifiers": {"individual": 1}, "pronouns": {"you": 1}}}]}

{"questionnaire_title": "General Hospital Anxiety/depression (HAD) Emotional Test Form", "questionnaire_category": "Mental health", "item_count": 7, "emotion_shift_overall": "From negative to positive", "language_style_overall": "Tactful and warm", "items": [{"content": "I feel nervous or painful.", "word_count": 8, "label": "", "sentence_structure": "Subject鈥揷opula鈥損redicative", "subjective_objective": {"answer": "Subjective", "rationale": "Because the word \"fEs\" shows that it is a subjective feeling and experience of the patient, not an objective reality."}, "sentence_sentiment": {"answer": "Negative", "rationale": "Because \"frightful or painful\" in the entry is a negative emotional experience."}, "language_style": {"answer": "Tactful and warm", "rationale": ""}, "time_dimension": {"answer": "Past", "rationale": "Because the word \"fE felt\" in the sentence is used to indicate the effect or result of past actions on the present, the entry to the questionnaire is in the current state of the subject and is of the current temporal dimension."}, "sentence_type": "Interrogative sentence", "pos": {"nouns": {"I": 1, "Nervous": 1, "Pain": 1}, "verbs": {"Sensor": 1, "Present": 1, "be": 1}, "adverbs": {"or": 1}}}, {"content": "I'm a little scared, like I'm expecting something terrible to happen.", "word_count": 23, "label": "", "sentence_structure": "Subject鈥搗erb鈥揷omplement", "subjective_objective": {"answer": "Subjective", "rationale": "Because \"fear\" and \"preception\" are all subjective feelings and thoughts of patients."}, "sentence_sentiment": {"answer": "Negative", "rationale": "Because \"fear\" and \"negative things are going to happen\" are negative emotional expressions."}, "language_style": {"answer": "Tactful and warm", "rationale": "The entry is more personal and interactive when it is presented in a gentle and inviting tone, rather than in direct statements or orders. At the same time, it is presented in a first person's name, making it easier for the interviewees to resonate and to feel a sense of identity."}, "time_dimension": {"answer": "Present", "rationale": "\"As if\" and \"awesome\" indicate that it is a current, instant emotional experience. \"As if\" in \"aforesees, \"as if\" expresses a feeling of uncertainty or speculation, \"a feeling of \"presumptance\" is a vision of the future, which shows that the testees do not now experience anything terrible directly, but rather that they are concerned or afraid of the future based on some clues. So the entry asks what the tester feels and how he feels."}, "sentence_type": "Interrogative sentence", "pos": {"nouns": {"I": 1, "Things": 1}, "verbs": {"I feel": 1, "Foreground": 1, "Come on": 1}, "adjectives": {"Scared": 1}, "adverbs": {"Some": 1}}}, {"content": "Three, I'm in trouble.", "word_count": 7, "label": "", "sentence_structure": "Subject鈥揷opula鈥損redicative", "subjective_objective": {"answer": "Subjective", "rationale": "Because \"inner\" is a personal feeling and experience, and it is a subjective assessment."}, "sentence_sentiment": {"answer": "Negative", "rationale": "Because the feeling of \"involved\" is negative."}, "language_style": {"answer": "Calm and direct", "rationale": "The entry directly expresses the subject of questioning the other party for any distress, does not use excessive rhetorical or emotional rendering, and is more objective in tone."}, "time_dimension": {"answer": "Present", "rationale": "The keyword \"I'm troubled\" suggests that it is a description of the state of the mood, and therefore the judgment is now."}, "sentence_type": "Affirmative declarative sentence", "pos": {"nouns": {"鈾?In the heart 鈾?: 1, "Trouble": 1}, "verbs": {"Fill": 1}, "adverbs": {"3": 1}}}, {"content": "I can sit down in a free and easy manner.", "word_count": 11, "label": "", "sentence_structure": "Subject鈥搗erb鈥揷omplement", "subjective_objective": {"answer": "Subjective", "rationale": "Because \"can sit free and easy\" is a subjective feeling for a person."}, "sentence_sentiment": {"answer": "Positive", "rationale": "Because \"free and easy\" means a positive, relaxed mentality and state of affairs."}, "language_style": {"answer": "Tactful and warm", "rationale": "The entry is more personal and interactive when it is spoken in a gentle and inviting tone, rather than in direct statements or orders. At the same time, the easier words of \"relax\" and \"easy\" are used, and give people a warm, comfortable feeling."}, "time_dimension": {"answer": "Present", "rationale": "Now the entry is asking about the current capabilities or state of the person being tested, and \"I can sit free and easy\" describes a state of readiness, with no clear time limits or the impact of past experience. So the time dimension of the entry is now judged."}, "sentence_type": "Affirmative declarative sentence", "pos": {"nouns": {"I": 1, "- No, no, no": 1, "Easy": 1, "Seats": 1}, "verbs": {"Yes": 1, "Sit down": 1}, "adjectives": {"Have a nice time": 1, "Easy": 1}, "adverbs": {"And": 1, "Land": 1}, "classifiers": {"individual": 1}}}, {"content": "I feel a terrible fear.", "word_count": 12, "label": "", "sentence_structure": "Subject鈥揷opula鈥損redicative", "subjective_objective": {"answer": "Subjective", "rationale": "Because \"feel\" is a subjective feeling, which describes the patient's own inner experience and emotional response, it's a subjective perspective."}, "sentence_sentiment": {"answer": "Negative", "rationale": "Because the \"shocking fear\" in the entry is an expression of a negative emotion."}, "language_style": {"answer": "Tactful and warm", "rationale": "The entry is based on the following: it expresses a strong, unusual negative sentiment that the person under investigation experienced at a given point in time, using a graphic language that describes \"shocking fear.\" This expression is more direct and specific, but it does not have too much emotional overturning or rhetorical techniques, so it can be judged as a calm, direct language style."}, "time_dimension": {"answer": "Present", "rationale": "\"I feel a shaking fear\" in \"fear\" suggests that it is a instant experience of feeling or emotional experience, so time dimension is now judged."}, "sentence_type": "Exclamatory sentence", "pos": {"nouns": {"Fear": 1}, "verbs": {"I feel": 1, "# Wresh #": 1}, "adjectives": {"One": 1, "It's a shivering thing": 1}, "numerals": {"5": 1}, "pronouns": {"I": 1}}}, {"content": "I'm a little restless. I feel like I have to move.", "word_count": 17, "label": "", "sentence_structure": "Subject鈥搗erb鈥揷omplement", "subjective_objective": {"answer": "Subjective", "rationale": "Because \"fright to move\" is the patient's subjective feeling and idea."}, "sentence_sentiment": {"answer": "Negative", "rationale": "Because the descriptions of \"silent\" and \"must be active\" are negative, indicating that people feel uncomfortable or anxious."}, "language_style": {"answer": "Tactful and warm", "rationale": "The language style of the entry is more moderate and uses an inquisitorial tone to engage in dialogue, giving people a sense of proximity and friendship."}, "time_dimension": {"answer": "Present", "rationale": "The keyword \"a little restless\" and \"as if it was necessary to act\" all indicate that this is an act or mentality that the testee is in progress or has been in place for some time to come, and therefore belongs to the present."}, "sentence_type": "Affirmative declarative sentence", "pos": {"nouns": {"Sit tight": 1, "Activities": 1}, "verbs": {"have": 1, "I feel": 1, "Not": 1, "Yes": 1, "No way": 1}, "adjectives": {"A little": 1}, "numerals": {"6": 1}}}, {"content": "I suddenly felt fear.", "word_count": 7, "label": "", "sentence_structure": "Subject鈥搗erb鈥揷omplement", "subjective_objective": {"answer": "Subjective", "rationale": "Because fear is an individual's subjective feeling."}, "sentence_sentiment": {"answer": "Negative", "rationale": "Because the feeling of fear in the entry is negative."}, "language_style": {"answer": "Calm and direct", "rationale": "The entry is based on the fact that the article is simple and clear, and does not use too many emotional terms or rhetorical techniques to describe the emotional experience of the person under investigation. At the same time, the entry does not attempt to direct the person under investigation to answer, but rather directly asks them whether they have a sudden sense of fear, which is more objective and direct."}, "time_dimension": {"answer": "Present", "rationale": ""}, "sentence_type": "Exclamatory sentence", "pos": {"nouns": {"Fear": 1}, "verbs": {"Suddenly": 1, "have": 1}, "numerals": {"7": 1}}}]}

{"questionnaire_title": "General Happiness Sheet (GWB)", "questionnaire_category": "Emotion and affect", "item_count": 25, "emotion_shift_overall": "From negative to positive", "language_style_overall": "Neutral", "items": [{"content": "How do you feel in general (in the past month)?", "word_count": 16, "label": "", "sentence_structure": "Subject鈥搗erb鈥搊bject", "subjective_objective": {"answer": "Subjective", "rationale": "Because the general feeling is one person's subjective feeling and evaluation."}, "sentence_sentiment": {"answer": "Neutral", "rationale": "As the entry merely asked about the overall perception of the past month, it did not address any positive or negative behaviour, attitude or sentiment."}, "language_style": {"answer": "Tactful and warm", "rationale": "The entry is more adept and interactive when it is spoken in a gentle and inviting manner, rather than by direct statements or orders."}, "time_dimension": {"answer": "Past", "rationale": ""}, "sentence_type": "Interrogative sentence", "pos": {"nouns": {"Overall feeling": 1, "Past": 1, "A month": 1}, "verbs": {"What": 1}, "adverbs": {"in": 1}, "numerals": {"One": 2}, "classifiers": {"individual": 1}, "pronouns": {"you": 1}}}, {"content": "Are you upset about your own neurotic or 鈥減sychiatric鈥?(in the past month)?", "word_count": 26, "label": "", "sentence_structure": "Subject鈥搗erb鈥搊bject", "subjective_objective": {"answer": "Subjective", "rationale": "Because \"challenge\" is a subjective feeling and attitude. Although this may be related to objective circumstances, such as whether or not it is misunderstood or encountered by others, it is in itself a subjective experience of the patient."}, "sentence_sentiment": {"answer": "Negative", "rationale": "Because the expression \"nervous or \"neurological\" in the entry is negative."}, "language_style": {"answer": "Tactful and warm", "rationale": "Warm and warm."}, "time_dimension": {"answer": "Past", "rationale": "The time unit mentioned in the entry was \"the past month\" and therefore judged to be the past."}, "sentence_type": "Exclamatory sentence", "pos": {"nouns": {"Questionnaire Entry": 1, "self": 1, "Nerves": 1, "Crazy": 1, "Past": 1, "A month": 1}, "verbs": {"I feel": 1, "Trouble": 1}, "adjectives": {"of": 2, "or": 1, "Past": 1}, "adverbs": {"Is it possible": 1, "Both": 1}, "numerals": {"1": 4}, "classifiers": {"individual": 1}, "pronouns": {"you": 1}}}, {"content": "Have you been in firm control of your behaviour, thinking, emotions or feelings (for the past month)?", "word_count": 31, "label": "", "sentence_structure": "Subject鈥搗erb鈥搊bject", "subjective_objective": {"answer": "Objective", "rationale": "Since control is the patient ' s observation and assessment of himself, it is subjective; whereas the behaviour, thinking, emotion or feeling of the past month is an objective fact that can be quantified."}, "sentence_sentiment": {"answer": "Negative", "rationale": "Because the description of \"concealed control\" in the entries gives the impression that it is too strict and depressing and can lead to negative emotions and attitudes."}, "language_style": {"answer": "Calm and direct", "rationale": "The entry uses a direct question-and-answer approach to ask the respondents whether they have been able to control their behaviour, thinking, emotions or feelings for the past month, and the language style is more concise, with no excessive adoration or emotional colours, and is a calm and direct expression."}, "time_dimension": {"answer": "Past", "rationale": "The sentence \"in the past month\" makes it possible to judge the entry by asking the person who was tested for his or her behaviour and state in the last month, and therefore the temporal dimension is a past."}, "sentence_type": "Interrogative sentence", "pos": {"nouns": {"Behaviour": 1, "Thinking": 1, "Emotion": 1, "Feel": 1, "Past": 1, "A month": 1}, "verbs": {"Controlled": 1}, "adverbs": {"Always": 1, "In the hole": 1}, "numerals": {"3": 1}}}, {"content": "4. Are you skeptical of anything worth doing (in the past month) because of grief, loss of confidence, disappointment or many problems?", "word_count": 39, "label": "", "sentence_structure": "Subject鈥搗erb鈥搊bject", "subjective_objective": {"answer": "Subjective", "rationale": "Basis of judgement: to examine the patient ' s subjective feelings and thoughts."}, "sentence_sentiment": {"answer": "Negative", "rationale": "Because the moods of \"pity, loss of confidence, disappointment\" in the entries are all negative, and because they may lead to questions about the value and meaning of life, the expression of the article is negative."}, "language_style": {"answer": "Calm and direct", "rationale": "The entry uses a direct question-and-answer approach, asking if the interviewees have wondered whether they have been in a state of grief, loss of confidence, disappointment or a lot of trouble for the past month, whether there is anything worth doing. This approach is simple and clear, without too much emotional overturning and rhetorical use, and is more calm and straightforward."}, "time_dimension": {"answer": "Past", "rationale": ""}, "sentence_type": "Interrogative sentence", "pos": {"nouns": {"Sad": 1, "Confidence": 1, "Disappointment": 1, "A lot of trouble": 1, "Things": 1, "January": 1}, "verbs": {"Doubt": 1, "It's worth it": 1, "Do it": 1}, "adjectives": {"Lose": 1, "Any": 1}, "classifiers": {"individual": 1}, "pronouns": {"you": 1}}}, {"content": "Are you being or ever being subjected to any constraint, stimulus or pressure (in the past month)?", "word_count": 29, "label": "", "sentence_structure": "Subject鈥搗erb鈥搊bject", "subjective_objective": {"answer": "Subjective", "rationale": "Because \"restricted, irritated or pressured\" is a situation that is felt by a patient, it is the subjective experience and feelings of the patient that are examined."}, "sentence_sentiment": {"answer": "Negative", "rationale": "Because the act of \"restricted, irritated or pressured\" in the entry is negative."}, "language_style": {"answer": "Calm and direct", "rationale": "The entry is based on a simple and clear language: it is asked whether the interviewee has been restrained, stimulated or pressured in the last month. There is no rhetorical or emotional rendering, it is more objective, it is not too much acrimony and emotional, and it is more focused on the effective transmission of information. So, I think, its language style is straightforward."}, "time_dimension": {"answer": "Past", "rationale": "The entry asks if the person tested has been subjected to constraints, irritation or pressure during the past month and therefore is of a temporal dimension of the past."}, "sentence_type": "Interrogative sentence", "pos": {"nouns": {"Questionnaire Entry": 1, "Constraints": 1, "Exciting": 1, "Pressure": 1, "Past": 1, "A month": 1}, "verbs": {"Received": 2, "I've been receiving it": 1, "- We're on it": 1}, "adjectives": {"Any": 1}, "numerals": {"5": 1}}}, {"content": "6. Is your life happy, satisfied or happy (for the past month)?", "word_count": 21, "label": "", "sentence_structure": "Subject鈥搗erb鈥搊bject", "subjective_objective": {"answer": "Subjective", "rationale": "Because happiness, satisfaction or pleasure is the patient's subjective feeling and assessment of life over the past month."}, "sentence_sentiment": {"answer": "Positive", "rationale": "Because the entry asks about the individual ' s happiness and satisfaction, it is a positive emotional attitude."}, "language_style": {"answer": "Tactful and warm", "rationale": "The entry uses a euphemism, by asking the person under investigation about his or her life experience over the past month, allowing the other person to respond more freely by expressing his or her own inner thoughts and by speaking in a gentle and inviting manner, rather than directly speaking or ordering. This expression is more personal and interactive."}, "time_dimension": {"answer": "Past", "rationale": "The entry is for questioning the behaviour and mentality of the person tested over the past month, and the time dimension is judged to be past."}, "sentence_type": "Interrogative sentence", "pos": {"nouns": {"you": 2, "Life": 1, "Happiness": 1, "Satisfied": 1, "Have a nice day": 1, "A month": 1}, "verbs": {"be": 2, "Is it possible": 1, "Satisfactory or": 1}, "adjectives": {"Happiness": 1, "Satisfied": 1, "Have a nice day": 1}, "adverbs": {"in": 1, "In the past month": 1}, "numerals": {"Six": 1}, "classifiers": {"individual": 1}}}, {"content": "7. Do you have reason to suspect that you have lost your mind or control over behaviour, conversation, mind or memory (in the past month)?", "word_count": 39, "label": "", "sentence_structure": "Subject鈥搗erb鈥搊bject", "subjective_objective": {"answer": "Subjective", "rationale": "This is because the patient has a subjective judgement and feeling that he or she has lost his or her sense of reason or has no control over his or her behaviour, conversation, thinking or memory. This view is based on the patient鈥檚 personal experience and perception, which is not an objective fact."}, "sentence_sentiment": {"answer": "Negative", "rationale": "Because \"unsane\" and \"unscrutinized behavior, conversation, mind or memory\" in the entries are negative behaviors and emotions."}, "language_style": {"answer": "Calm and direct", "rationale": "The language style of the entry is relatively calm and straightforward, and access to relevant information is obtained by explicitly asking the other party whether there has been a decline in self-control over a period of time. This expression is simple and clear, and there is no excessive emotional rendering or adoration, which is conducive to the interviewee ' s understanding and answers accurately and quickly."}, "time_dimension": {"answer": "Past", "rationale": "The entry is about the experience and feelings of the testees over the past month, and the temporal dimension is about the past. At the same time, the key words \"unthinking\" and \"lost control over behavior, conversation, thinking or memory\" also imply that this experience has happened in the past."}, "sentence_type": "Interrogative sentence", "pos": {"nouns": {"Rationale": 1, "Reason": 1, "Behaviour": 1, "Talk": 1, "Thinking": 1, "Memory": 1, "Control": 1, "Past": 2, "A month": 1}, "verbs": {"Doubt": 1, "Lose": 2, "have": 1, "Reason": 1, "Behaviour": 1, "Talk": 1, "Thinking": 1, "Memory": 1, "Control": 1}, "numerals": {"A month": 1, "7": 1}, "classifiers": {"individual": 1}, "pronouns": {"self": 1}}}, {"content": "8. Are you worried, worried or uneasy (for the past month)?", "word_count": 20, "label": "", "sentence_structure": "Subject鈥搗erb鈥搊bject", "subjective_objective": {"answer": "Subjective", "rationale": "The patient feels the same way because of anxiety, anxiety or anxiety."}, "sentence_sentiment": {"answer": "Negative", "rationale": "Because the words \"anxiety, fear or unease\" in the entry express a negative sentiment."}, "language_style": {"answer": "Calm and direct", "rationale": "The entry uses a direct language style to ask the interviewees whether they have been anxious, worried or disturbed in the last month, without too many rhetorical or emotional accents and with a more objective tone."}, "time_dimension": {"answer": "Past", "rationale": "The entry asks about the feelings and experiences of the person tested over the past month, and the time dimension is judged to be past."}, "sentence_type": "Interrogative sentence", "pos": {"nouns": {"Anxiety": 1, "I'm worried": 1, "Unsettled": 1, "A month": 1}, "verbs": {"I feel": 1}, "numerals": {"One": 1}, "classifiers": {"individual": 1}}}, {"content": "9. Do you feel clear and energetic after you wake up (in the past month)?", "word_count": 26, "label": "", "sentence_structure": "Subject鈥搗erb鈥搊bject", "subjective_objective": {"answer": "Objective", "rationale": "The subject is a question of the patient ' s situation during the past month, not of the patient ' s subjective feelings."}, "sentence_sentiment": {"answer": "Positive", "rationale": "Because the entries are a positive feeling, that is, a clear and energetic awakening."}, "language_style": {"answer": "Tactful and warm", "rationale": ""}, "time_dimension": {"answer": "Past", "rationale": ""}, "sentence_type": "Interrogative sentence", "pos": {"nouns": {"you": 1, "Brain": 1, "- The energy": 1, "Past": 1, "A month": 1}, "verbs": {"Wake up": 1, "I feel": 1, "Clear": 1, "鈾?Full of 鈾?: 1}, "adjectives": {"Wake up": 1, "鈾?Full of 鈾?: 1}, "numerals": {"Nine": 1, "One": 2}}}, {"content": "10. Have you been troubled by illness, physical discomfort, pain or fear of disease (in the past month)?", "word_count": 32, "label": "", "sentence_structure": "Subject鈥搗erb鈥搊bject", "subjective_objective": {"answer": "Objective", "rationale": "This is an objective examination of whether the patient has been troubled by objective conditions such as illness and physical discomfort during the past month."}, "sentence_sentiment": {"answer": "Negative", "rationale": "Because the negative factors in the entry, \"disease, discomfort, pain or fear of disease\", can cause people to be troubled and negative."}, "language_style": {"answer": "Calm and direct", "rationale": "This entry is in the form of a clear question, asking whether the person surveyed has been troubled by illness, infirmity, pain or fear of illness during the past month."}, "time_dimension": {"answer": "Past", "rationale": "The entry asks about the behaviour of the subject over the past month, so it can be judged as the past. At the same time, the description of \"probable by the troubles\" also points to the past situation and feelings."}, "sentence_type": "Interrogative sentence", "pos": {"nouns": {"Diseases": 1, "Body": 1, "I'm not well": 1, "Pain": 1, "Fear of disease": 1, "Trouble": 1}, "verbs": {"Because": 1, "And": 1, "Trouble": 1}, "adverbs": {"In the past month": 1}}}, {"content": "Is your daily life filled with things that interest you?", "word_count": 28, "label": "", "sentence_structure": "Subject鈥搗erb鈥搊bject", "subjective_objective": {"answer": "Subjective", "rationale": "Because it's the patient's subjective feeling and perception."}, "sentence_sentiment": {"answer": "Positive", "rationale": "Because the entry \"shows you interest\" expresses positive attitudes and behavior."}, "language_style": {"answer": "Tactful and warm", "rationale": "The entry is more personal and interactive by speaking in a gentle and inviting manner, rather than by direct statement or order. At the same time, by using open questions such as \"are you\" to guide respondents to think and answer, the questionnaire is made more flexible and free and has easier access to real and accurate information."}, "time_dimension": {"answer": "Past", "rationale": ""}, "sentence_type": "Interrogative sentence", "pos": {"nouns": {"Life": 1, "Things": 2, "Past": 1, "A month": 1}, "verbs": {"Fill": 1, "Jean": 1, "Interested": 1}, "adverbs": {"Chile": 1}, "numerals": {"11": 1, "1": 3}, "pronouns": {"you": 1}}}, {"content": "12. Have you been depressed and depressed (in the past month)?", "word_count": 18, "label": "", "sentence_structure": "Subject鈥搗erb鈥搊bject", "subjective_objective": {"answer": "Subjective", "rationale": "Because it's the emotional state that the patient feels, and it's subjective."}, "sentence_sentiment": {"answer": "Negative", "rationale": "Because the description \"frustration and depression\" in the entry is negative."}, "language_style": {"answer": "Calm and direct", "rationale": "The language style of the entry is calm and straightforward. It asks the respondents in a concise and straightforward manner whether they have been frustrated and depressed in the past month, without using excessive rhetorical techniques or emotional rendering.\""}, "time_dimension": {"answer": "Past", "rationale": ""}, "sentence_type": "Interrogative sentence", "pos": {"nouns": {"Questionnaire Entry": 1, "Frustration": 1, "Blue": 1, "Past": 1, "A month": 1}, "verbs": {"I feel": 1, "be": 1}, "adverbs": {"Is it possible": 1}, "numerals": {"12": 1}}}, {"content": "Are you stable and capable of grasping yourself (for the past month)?", "word_count": 22, "label": "", "sentence_structure": "Subject鈥搗erb鈥搊bject", "subjective_objective": {"answer": "Subjective", "rationale": "Because the subject is asking the patient about his or her own feelings and experiences, it is the patient ' s subjective perspective."}, "sentence_sentiment": {"answer": "Positive", "rationale": "Because the entry \"emotional stability and control\" means positive emotions and attitudes."}, "language_style": {"answer": "Tactful and warm", "rationale": "The entry is more euphemistic and gentle, asking each other about emotional stability over the past month in a less questionable manner, and not using too direct or mandatory language. At the same time, the expression \"hold your hand\" is more implicit in expressing concern and concern about emotional management capacity, making the questionnaire more personal and human."}, "time_dimension": {"answer": "Past", "rationale": ""}, "sentence_type": "Interrogative sentence", "pos": {"nouns": {"Emotion": 1, "person": 1, "A month": 1}, "verbs": {"Is it possible": 1, "Steady": 1, "Hold on": 1}, "adjectives": {"self": 1}, "numerals": {"13": 1, "One": 2, "individual": 1}, "pronouns": {"you": 1}}}, {"content": "14. Are you tired, overtired, powerless or exhausted (for the past month)?", "word_count": 24, "label": "", "sentence_structure": "Subject鈥搗erb鈥搊bject", "subjective_objective": {"answer": "Subjective", "rationale": "The patient feels tired, tired, weak or exhausted."}, "sentence_sentiment": {"answer": "Negative", "rationale": "Because the symptoms described in the entries are negative and represent physical or psychological discomfort."}, "language_style": {"answer": "Calm and direct", "rationale": "This is a more accurate way for respondents to express their feelings and for researchers to obtain real data."}, "time_dimension": {"answer": "Past", "rationale": "\"The past.\""}, "sentence_type": "Interrogative sentence", "pos": {"nouns": {"Tired": 1, "I'm tired": 1, "- I'm weak": 1, "I'm exhausted": 1, "Past": 1, "A month": 1}, "verbs": {"I feel": 1, "or": 1}, "adverbs": {"Yes": 1}, "numerals": {"14": 1}}}, {"content": "15. How much care or credit have you given to your own health (in the past month)?", "word_count": 24, "label": "", "sentence_structure": "Subject鈥搗erb鈥搊bject", "subjective_objective": {"answer": "Subjective", "rationale": "The subject is a subjective perception and evaluation of the patient, as it asks him about the extent of his health concerns."}, "sentence_sentiment": {"answer": "Positive", "rationale": "This is a positive attitude, as the entry expresses health concerns and concerns."}, "language_style": {"answer": "Tactful and warm", "rationale": "The language style of the entry tends to be euphemistic, as it uses an inquisitorial tone to guide respondents to share their views and feelings. At the same time, by using the terms \"care\" and \"concern\" to express their concern and respect for the interviewees, the issue is closer to the lives and feelings of the interviewees, thus making it easier to obtain real and effective feedback."}, "time_dimension": {"answer": "Past", "rationale": "The keyword in the entry is \"for the past month\" and indicates that the entry is asked about the behaviour or state of the person who was tested during the past month, and therefore the temporal dimension is judged to be past."}, "sentence_type": "Interrogative sentence", "pos": {"nouns": {"self": 1, "Health": 1, "Level": 1, "Past": 1, "One": 1, "Month": 1}, "verbs": {"Care": 1, "You're a good man": 1}, "adjectives": {"How's that": 1}, "adverbs": {"Past": 1, "of": 2, "in": 1, "Lee": 1}, "numerals": {"15": 1, "A month": 1}, "classifiers": {"individual": 1}, "pronouns": {"you": 1}}}, {"content": "How relaxed or nervous do you feel?", "word_count": 21, "label": "", "sentence_structure": "Subject鈥揷opula鈥損redicative", "subjective_objective": {"answer": "Subjective", "rationale": "Because \"relax or nervous\" is a patient's subjective feeling."}, "sentence_sentiment": {"answer": "Neutral", "rationale": "Because the entry merely asks about a person ' s feelings over the past month, it does not express positive or negative emotional tendencies."}, "language_style": {"answer": "Tactful and warm", "rationale": ""}, "time_dimension": {"answer": "Past", "rationale": ""}, "sentence_type": "Interrogative sentence", "pos": {"nouns": {"Questionnaire": 1, "Level": 2, "Month": 1}, "verbs": {"I feel": 1, "Relax": 1, "Nervous": 1, "How's that": 1}, "adverbs": {"Past": 1, "One": 1}, "numerals": {"16": 1}, "classifiers": {"Lee": 1}}}, {"content": "How do you feel about your energy, spirit and energy?", "word_count": 23, "label": "", "sentence_structure": "Subject鈥搗erb鈥揷omplement", "subjective_objective": {"answer": "Subjective", "rationale": "Because \"fear,\" \"how energy, spirit and energy\" are personal feelings and subjective assessments of patients."}, "sentence_sentiment": {"answer": "Positive", "rationale": "Because the words \"energy, spirit and vitality\" in the entry are positive, indicating that people are in good health and psychological well-being."}, "language_style": {"answer": "Tactful and warm", "rationale": ""}, "time_dimension": {"answer": "Past", "rationale": "The entry contains the time frame \"the past month\", and therefore judged to be the past."}, "sentence_type": "Interrogative sentence", "pos": {"nouns": {"self": 1, "- The energy": 1, "Spirit": 1, "Dynamic": 1, "Past": 1, "One": 1, "In the month": 1}, "verbs": {"Feel": 1}, "numerals": {"17": 1}}}, {"content": "How depressed or happy are you (in the past month)?", "word_count": 19, "label": "", "sentence_structure": "Subject鈥搗erb鈥搊bject", "subjective_objective": {"answer": "Subjective", "rationale": "The question item, \"How depressed or happy are you?\" is a subjective view of the patient's emotional state and is therefore subjective."}, "sentence_sentiment": {"answer": "Positive", "rationale": "Because the \"happy\" attitude is positive."}, "language_style": {"answer": "Tactful and warm", "rationale": "The entry is more adept and interactive when it is spoken in a gentle and inviting manner, rather than by direct statements or orders."}, "time_dimension": {"answer": "Past", "rationale": "The entry is for the psychological state of the person tested over the past month, and the temporal dimension is judged to be the past."}, "sentence_type": "Interrogative sentence", "pos": {"nouns": {"Level": 1, "Blue": 1, "Happy": 1, "One": 1, "Month": 1}, "verbs": {"be": 1, "Like": 1, "What": 1, "Past": 1}, "adverbs": {"in": 1, "Lee": 1}, "numerals": {"18": 1}}}, {"content": "19. Do you feel that you need help because of serious personality, emotional, behavioural or mental problems (in the past year)?", "word_count": 33, "label": "", "sentence_structure": "Subject鈥搗erb鈥搊bject", "subjective_objective": {"answer": "Subjective", "rationale": "The problem is seen from a subjective perspective because it relates to the person ' s own feelings and perceptions."}, "sentence_sentiment": {"answer": "Negative", "rationale": "Because the description of \"serious emotional, behavioural or mental problems\" in the entry is negative."}, "language_style": {"answer": "Tactful and warm", "rationale": "The entry is more euphemistic, using a more neutral expression such as \"needs for help\" to avoid undue pressure and discomfort on the respondents. At the same time, the interest and concern of the respondents is demonstrated by asking about the situation over the past year."}, "time_dimension": {"answer": "Past", "rationale": ""}, "sentence_type": "Interrogative sentence", "pos": {"nouns": {"Character": 1, "Emotion": 1, "Behaviour": 1, "Spirit": 1, "Problem": 1, "Help": 1, "One year": 1}, "verbs": {"I feel": 1, "Yes": 1}, "adjectives": {"Serious": 1}, "numerals": {"One": 2}, "classifiers": {"individual": 1}}}, {"content": "Have you ever felt like you were going to be or near being?", "word_count": 20, "label": "", "sentence_structure": "Subject鈥搗erb鈥搊bject", "subjective_objective": {"answer": "Subjective", "rationale": "Because feeling a mental breakdown or being close to a mental breakdown is a subjective feeling of the patient."}, "sentence_sentiment": {"answer": "Negative", "rationale": "Because the feeling in the entry that \"the feeling is going to collapse or is approaching a breakdown\" is negative."}, "language_style": {"answer": "Calm and direct", "rationale": "The entry directly asks if the interviewee has experienced a mental breakdown, without using euphemism or accommodation. This language style is more direct and objective.\""}, "time_dimension": {"answer": "Past", "rationale": "The entry is for questioning the testee ' s past behaviour or mental state, and the time dimension is judged to be past."}, "sentence_type": "Interrogative sentence", "pos": {"nouns": {"Mental breakdown": 1, "Close": 1}, "verbs": {"I feel": 1, "Mental breakdown": 1, "Close": 1, "Crash": 1, "Tsang": 1}, "adverbs": {"Yes, I'm going": 1, "Close to": 1}, "numerals": {"20": 1}}}, {"content": "Have you ever had a nervous breakdown?", "word_count": 10, "label": "", "sentence_structure": "Subject鈥搗erb鈥搊bject", "subjective_objective": {"answer": "Subjective", "rationale": "Because a mental breakdown is a subjective feeling and experience of a person, different people may react differently to the same situation. The problem is therefore a subjective one for the patient."}, "sentence_sentiment": {"answer": "Negative", "rationale": "Because the \"psychological breakdown\" in the entry is negative."}, "language_style": {"answer": "Calm and direct", "rationale": "The entry directly asked the interviewees whether they had experienced a psychological breakdown, not much rhetorical or emotional rendering, and a relatively calm and objective language style."}, "time_dimension": {"answer": "Past", "rationale": "The word \"had\" in the entry indicates that it was an experience or event, so the temporal dimension is a past."}, "sentence_type": "Interrogative sentence", "pos": {"nouns": {"Mental breakdown": 1}, "verbs": {"have": 1, "Pass": 1}}}, {"content": "Have you ever been treated in a psychiatric hospital, a psychiatric ward of a general hospital or a mental health clinic because of character, character, behaviour or mental problems?", "word_count": 41, "label": "", "sentence_structure": "Subject鈥搗erb鈥搊bject", "subjective_objective": {"answer": "Objective", "rationale": "The question is whether the patient has ever received the relevant medical treatment, and it does not involve subjective feelings or ideas."}, "sentence_sentiment": {"answer": "Negative", "rationale": "Since the entry relates to mental health problems and describes previous treatment experiences, it may give rise to some negative emotions and memories."}, "language_style": {"answer": "Calm and direct", "rationale": "The entry directly asks if the person interviewed has had any experience of treatment in a medical institution such as a psychiatric hospital, which is more concise, has no excessive emotional colour or accommodative language and is therefore a calm and direct language style."}, "time_dimension": {"answer": "Past", "rationale": ""}, "sentence_type": "Interrogative sentence", "pos": {"nouns": {"Character": 1, "Love": 1, "Behaviour": 1, "Mental problems": 1, "Mental hospital": 1, "General hospital": 1, "Psychiatric ward": 1, "Mental health clinics": 1, "Treatment": 1}, "verbs": {"Tsang": 1, "in": 1, "Because": 1, "Character, character, behaviour or mental problems": 1, "Treatment": 1, "In psychiatric hospitals, psychiatric wards of general hospitals or mental health clinics": 1}, "adverbs": {"Tsang": 1}, "numerals": {"22": 1}, "classifiers": {"individual": 1}}}, {"content": "Have you ever sought a psychiatrist, psychologist or psychologist for character, emotional, behavioural or mental problems?", "word_count": 29, "label": "", "sentence_structure": "Subject鈥搗erb鈥搊bject", "subjective_objective": {"answer": "Subjective", "rationale": "This is a subjective experience and attitude of the patient, who is required to recall past behaviour and describe his or her emotional and psychological state."}, "sentence_sentiment": {"answer": "Neutral", "rationale": "The entry does not show positive or negative emotional characteristics, but merely asks the interviewee whether he has had a specific behavioural experience."}, "language_style": {"answer": "Calm and direct", "rationale": "The language style of the entry is one of calm and straightforward, and it directly asks whether the person under investigation has had any experience of seeking the help of a psychiatrist, psychologist, or has not used excessive emotional qualifiers or rhetoric to guide the person under investigation."}, "time_dimension": {"answer": "Present", "rationale": "The entry asks whether the person tested had had previous experience of seeking help from a psychiatrist or psychologist for reasons of character, emotional, behavioural or mental problems, and therefore the temporal dimension is now judged."}, "sentence_type": "Interrogative sentence", "pos": {"nouns": {"Character": 1, "Emotion": 1, "Behaviour": 1, "Spirit": 1, "Problem": 1, "Psychiatrist": 1, "Psychologists": 1}, "verbs": {"Tsang": 1, "Appeal": 1}, "adverbs": {"Because": 1}, "numerals": {"23": 1, "1": 6}}}, {"content": "24. Have you sought assistance from the following persons for reasons of character, emotional, behavioural or mental problems?", "word_count": 23, "label": "", "sentence_structure": "Subject鈥搗erb鈥搊bject", "subjective_objective": {"answer": "Subjective", "rationale": "The questionnaire entry is a subjective one. It asks about the patients鈥?self-awareness and subjective feelings, whether they seek help because of their personality, emotional, behavioural or mental problems."}, "sentence_sentiment": {"answer": "Neutral", "rationale": "Because the entry is merely an inquiry about the existence of recourse to a particular person and does not involve any emotional colour."}, "language_style": {"answer": "Calm and direct", "rationale": "The language style of the entry is a calm and straightforward one. It clearly raises the question of whether the interviewee has had experience seeking help from others because of his character, emotional, behavioural or mental problems, and does not have too many rhetorical techniques or emotional renderings. This tone is more objective and more accurate in obtaining the answers of the interviewee."}, "time_dimension": {"answer": "Past", "rationale": "The entry is for questioning the testee ' s past behaviour and the temporal dimension is a past."}, "sentence_type": "Interrogative sentence", "pos": {"nouns": {"Character": 1, "Emotion": 1, "Behaviour": 1, "Mental problems": 1, "Help": 1, "Personnel": 1}, "verbs": {"Is it possible": 1, "Because": 1, "on": 1, "Below": 1}, "numerals": {"24": 1}}}, {"content": "Have you ever talked to family members or friends about your problems?", "word_count": 19, "label": "", "sentence_structure": "Subject鈥搗erb鈥搊bject", "subjective_objective": {"answer": "Subjective", "rationale": "The question is whether the patient has had such experience, which relates to his or her thoughts and feelings and is therefore subjective."}, "sentence_sentiment": {"answer": "Positive", "rationale": "Because the \"talking about your own problems\" act is positive."}, "language_style": {"answer": "Tactful and warm", "rationale": "Warm and warm."}, "time_dimension": {"answer": "Present", "rationale": "\"As a person has expressed his past behaviour, \"family members or friends\" refers to social objects common to people in their daily lives, so the entry is asking if the person tested has had communication with others in the past. At the same time, \"talking about his own\" implies that this communication involves emotional experience and thinking within the individual, which is consistent with the current mental health concerns, so it can be judged that the time dimension of the article is now."}, "sentence_type": "Interrogative sentence", "pos": {"nouns": {"Problem": 1, "Family members": 1, "friends": 1}, "verbs": {"Talk": 1, "be": 1}, "adverbs": {"Tsang": 1, "Your own": 1}, "numerals": {"25": 1, "One": 1}}}]}

{"questionnaire_title": "Table for evaluation of parenting", "questionnaire_category": "Behavioral questionnaire", "item_count": 66, "emotion_shift_overall": "From negative to positive", "language_style_overall": "Tactful and warm", "items": [{"content": "I feel like my parents interfered with everything I did.", "word_count": 15, "label": "", "sentence_structure": "Subject鈥揷opula鈥損redicative", "subjective_objective": {"answer": "Subjective", "rationale": "It is a reflection of a subjective attitude to the examination of the patient ' s own subjective feelings."}, "sentence_sentiment": {"answer": "Negative", "rationale": "Because the entry \"Parents Intervest in Everything I Do\" expresses a feeling of control, restraint, negative emotion."}, "language_style": {"answer": "Calm and direct", "rationale": "The language style of the entry is calm and straightforward. It asks the interviewee whether he or she feels that their parents are interfering with everything they do, without too much emotional adornment or rhetorical techniques, and with a more balanced and objective tone."}, "time_dimension": {"answer": "Present", "rationale": "The entry expresses the experience and experience of the testee at the moment, that is, the \"think\" of parents interfering with everything they do, and therefore judging as now."}, "sentence_type": "Interrogative sentence", "pos": {"nouns": {"I": 1, "parents": 1, "Something": 1}, "verbs": {"I think": 1, "Interference": 1, "Do it": 1}, "adverbs": {"Every": 1}, "classifiers": {"One": 2}}}, {"content": "I can feel him/her very much in his/her words and in his/her face.", "word_count": 19, "label": "", "sentence_structure": "Subject鈥搗erb鈥揷omplement", "subjective_objective": {"answer": "Subjective", "rationale": "Because \"favours\" are subjective feelings and understandings, not objective facts. Although parents' behaviour may be objective, it is an individual's subjective experience and perception."}, "sentence_sentiment": {"answer": "Positive", "rationale": "Because it's a positive emotional attitude, and \"love\" is a positive emotional color."}, "language_style": {"answer": "Tactful and warm", "rationale": "The purpose of this article is to be more balanced, without direct orders or emphasis, and to invite the interviewee to reflect on his or her feelings in a more moderate way. The entry uses specific terms such as \"parents' words, expressions\" to make it more visible to the interviewer, and to interact and communicate emotionally in the family environment. The purpose of the article is to encourage the interviewee to reflect on himself, to focus more on the feelings and experiences of his or her heart than on the mere fact that he or she is concerned with."}, "time_dimension": {"answer": "Present", "rationale": ""}, "sentence_type": "Interrogative sentence", "pos": {"nouns": {"parents": 1, "Talk": 1, "Emoticons": 1, "he": 1, "she": 1}, "verbs": {"Yes": 1, "Feel": 1, "Yeah": 1}, "adverbs": {"very": 2}, "numerals": {"2": 1}, "pronouns": {"I": 1}}}, {"content": "Parents love me more than my brothers and sisters.", "word_count": 15, "label": "", "sentence_structure": "Subject鈥搗erb鈥揷omplement", "subjective_objective": {"answer": "Subjective", "rationale": "Because the entry examines the patient ' s perception and feelings of his or her own experience, i.e. whether the parents prefer themselves, it is a subjective judgement."}, "sentence_sentiment": {"answer": "Negative", "rationale": "Because the entry shows that there is unfair treatment in family relations, that is, parents prefer \"me\", which may cause jealousy and dissatisfaction among siblings."}, "language_style": {"answer": "Calm and direct", "rationale": "The entry presents a point of view and is expressed in a comparative manner, with no excessive emotional or pedigree language, and therefore can be judged as a calm and direct language style."}, "time_dimension": {"answer": "Past", "rationale": "The entries \"more than my brothers and sisters\" and \"parents love me\" describe past experiences or feelings, and therefore judge as past."}, "sentence_type": "Affirmative declarative sentence", "pos": {"nouns": {"Questionnaire Entry": 1, "Brothers and sisters": 1, "parents": 1, "Pity": 1}, "verbs": {"Comparison": 1, "Pity": 1}, "numerals": {"3": 1}, "pronouns": {"I": 1}}}, {"content": "I can feel the love of my parents.", "word_count": 11, "label": "", "sentence_structure": "Subject鈥揷opula鈥損redicative", "subjective_objective": {"answer": "Subjective", "rationale": "Because feeling the love of parents is the patient's subjective feeling."}, "sentence_sentiment": {"answer": "Positive", "rationale": "Because the attitude of feeling the parents love me in the entry is positive."}, "language_style": {"answer": "Tactful and warm", "rationale": ""}, "time_dimension": {"answer": "Present", "rationale": ""}, "sentence_type": "Affirmative declarative sentence", "pos": {"nouns": {"parents": 1, "Love it": 1}, "verbs": {"I can feel it": 1, "to": 1}, "adverbs": {"I": 1}, "pronouns": {"they": 1}}}, {"content": "5. Even with small negligence, my parents punished me.", "word_count": 14, "label": "", "sentence_structure": "Subject鈥搗erb鈥搊bject", "subjective_objective": {"answer": "Subjective", "rationale": "Because \"punishment\" is based on parents' subjective views and attitudes towards patients."}, "sentence_sentiment": {"answer": "Negative", "rationale": "Because the \"punishment\" in the entry is negative."}, "language_style": {"answer": "Calm and direct", "rationale": "The entry describes in a direct manner the manner in which the person under investigation was punished in his or her upbringing, in a clear and concise language, without excessive emotional or rhetorical colours, and can therefore be judged as \"cool and direct\"."}, "time_dimension": {"answer": "Past", "rationale": "The entry describes how and how often the person who was tested grew up to be punished for the error, which has already occurred and has had an impact on the test. The time dimension of the entry is therefore judged to be past."}, "sentence_type": "Negative declarative sentence", "pos": {"nouns": {"Error": 1, "parents": 2}, "verbs": {"Punishment": 1, "be": 1, "have": 1}, "adjectives": {"Small": 1}, "numerals": {"5": 1}, "pronouns": {"I": 1}}}, {"content": "Parents always try to influence me in a silent way, making me a remarkable person.", "word_count": 23, "label": "", "sentence_structure": "Subject鈥搗erb鈥揷omplement", "subjective_objective": {"answer": "Objective", "rationale": "This sentence describes what patients feel, that is, the expectations and influence of parents over the patient, rather than the patient ' s subjective attitude or feelings."}, "sentence_sentiment": {"answer": "Positive", "rationale": "Because the purpose of the entry, \"Parents always try to influence me in a sublime way,\" is positive, that is, parents want their children to be better."}, "language_style": {"answer": "Tactful and warm", "rationale": "The entry uses expressions such as \"always trying\" and \"silently influencing\" and is soft and emotional, rather than a direct statement or order, which makes it easier for the interviewee to accept and answer questions."}, "time_dimension": {"answer": "Present", "rationale": "And it's a key word in the sentence, \"to make me.\" It's a description of the state or behaviour."}, "sentence_type": "Affirmative declarative sentence", "pos": {"nouns": {"parents": 1, "Smuggle": 1, "Impact": 1, "person": 1}, "verbs": {"Try": 1, "鈾?And I'm gonna be 鈾?: 1, "Be": 1}, "adjectives": {"It's a good one": 1}, "adverbs": {"Total": 1, "Land": 1}, "classifiers": {"individual": 1}}}, {"content": "7. I feel that my parents have given me some uniqueness in some respects.", "word_count": 18, "label": "", "sentence_structure": "Subject鈥搗erb鈥揷omplement", "subjective_objective": {"answer": "Subjective", "rationale": "Because \"think\" is the patient's subjective feeling and perception, it's a subjective view."}, "sentence_sentiment": {"answer": "Positive", "rationale": "Because it expresses a positive emotional attitude - the sense that parents have understanding and respect for themselves."}, "language_style": {"answer": "Tactful and warm", "rationale": "The entry is more euphemistic and gentle. It uses the first person's pronoun, \"I,\" and expresses in a question form the view of the interviewee as to whether he or she is recognized by his or her parents for his or her uniqueness in some respects. This expression is more friendly, makes the interviewee more comfortable and more accessible."}, "time_dimension": {"answer": "Present", "rationale": "The entry represents the subjective perception and perception of the situation of the person being tested, and thus the temporal dimension is now judged."}, "sentence_type": "Affirmative declarative sentence", "pos": {"nouns": {"parents": 1, "I": 2, "In terms of": 1, "Where I come from": 1}, "verbs": {"I think": 1, "Allow": 1, "have": 1}, "adverbs": {"Some": 1}, "numerals": {"7": 1}}}, {"content": "8. Parents can give me what other siblings are not.", "word_count": 19, "label": "", "sentence_structure": "Subject鈥搗erb鈥搊bject", "subjective_objective": {"answer": "Subjective", "rationale": "Because \"getting what other siblings don't get\" is a personal feeling and an assessment, it's a subjective perspective."}, "sentence_sentiment": {"answer": "Positive", "rationale": "Because the act of \"Parents can give me what other siblings can't get\" in the entry is an expression of a positive emotion."}, "language_style": {"answer": "Tactful and warm", "rationale": "The entry uses an invitation tone to engage in dialogue, not directly speaking or ordering, which is more personal and interactive. At the same time, the entry is not overly emotional, but rather questions in a gentle and objective manner, reflecting the scientific and professional nature of the questionnaire design."}, "time_dimension": {"answer": "Present", "rationale": "The entry asks about the family situation of the person currently tested and therefore the judgement is now."}, "sentence_type": "Affirmative declarative sentence", "pos": {"nouns": {"parents": 1, "Brothers and sisters": 2, "Something": 1}, "verbs": {"Jean": 1, "Got it": 1}, "numerals": {"8": 1}}}, {"content": "9. The punishment of my parents is fair and proportionate.", "word_count": 14, "label": "", "sentence_structure": "Subject鈥揷opula鈥損redicative", "subjective_objective": {"answer": "Subjective", "rationale": "This sentence describes the patient ' s perception and feelings about the punishment imposed by his parents, which is his subjective knowledge."}, "sentence_sentiment": {"answer": "Positive", "rationale": "Because the words in the entry are that the punishment of the child by the parents is fair and appropriate, and that is positive emotion and attitude."}, "language_style": {"answer": "Calm and direct", "rationale": "At the same time, it does not involve too much emotional or subjective evaluation, but rather questions based on facts, and can therefore be considered as a calm and straightforward language style."}, "time_dimension": {"answer": "Past", "rationale": "The entry relates to the person who was tested for the evaluation and judgement of his past experience and can therefore be judged as the past."}, "sentence_type": "Affirmative declarative sentence", "pos": {"nouns": {"parents": 1, "Punishment": 1, "Impartiality": 1, "Reasonability": 1}, "verbs": {"be": 1, "Fair": 1, "Appropriate": 1}, "adverbs": {"Appropriate": 1}, "pronouns": {"I": 1}}}, {"content": "I feel that my parents are tough on me.", "word_count": 10, "label": "", "sentence_structure": "Subject鈥揷opula鈥損redicative", "subjective_objective": {"answer": "Subjective", "rationale": "Because \"think\" is a term for subjective feelings, which conveys the patient's perception and feelings about the attitude of parents."}, "sentence_sentiment": {"answer": "Negative", "rationale": "Because the description \"very hard\" in the entry is negative."}, "language_style": {"answer": "Calm and direct", "rationale": ""}, "time_dimension": {"answer": "Past", "rationale": "The keyword \"thinks\" and \"severe\" is a term for describing the current state, without reference to past or future circumstances. The temporal dimension of the article is therefore now."}, "sentence_type": "Affirmative declarative sentence", "pos": {"nouns": {"Questionnaire": 1, "Entry": 1, "parents": 1, "I": 2}, "verbs": {"I think": 1, "to": 1, "very": 1, "Hard": 1}, "numerals": {"10": 1}}}, {"content": "Parents always influence what I should wear or what I should dress up.", "word_count": 22, "label": "", "sentence_structure": "Subject鈥搗erb鈥搊bject", "subjective_objective": {"answer": "Subjective", "rationale": "Because \"always right and left\" is the patient's subjective feeling and perception."}, "sentence_sentiment": {"answer": "Negative", "rationale": "Because the \"parents always decide what I should wear or what I should dress\" in the entry is a negative expression of restraint and control."}, "language_style": {"answer": "Tactful and warm", "rationale": "The entry is more personal and interactive when it is spoken in a gentle and inviting manner, rather than in direct statements or orders. It does not blame or criticize the person under investigation, but rather expresses the desire of the researcher to know what the person is feeling and experience in family relations."}, "time_dimension": {"answer": "Present", "rationale": "The phrase \"always\" and \"that\" makes it possible to judge that the act is recurrent and relates to the current situation. So, the time dimension of this entry is now."}, "sentence_type": "Affirmative declarative sentence", "pos": {"nouns": {"parents": 1, "Clothes": 1, "Get dressed": 1}, "verbs": {"Total": 1, "Around": 1, "Put it on": 1, "It's time to dress": 1}, "adverbs": {"Always": 1}, "numerals": {"11": 1}, "pronouns": {"I": 1}}}, {"content": "12. Parents do not allow me to do something other children can do because they fear that something will happen to me.", "word_count": 29, "label": "", "sentence_structure": "Subject鈥搗erb鈥搊bject", "subjective_objective": {"answer": "Subjective", "rationale": "Because the words \"permissive\" and \"fear\" are patients' subjective feelings and perceptions of their parents' behaviour and attitudes."}, "sentence_sentiment": {"answer": "Negative", "rationale": "Because the description \"not allowing me to do something other than what other children can do\" and \"fear of something\" in the entries are negative and express the emotional attitude of parents to overprotect and worry about their children."}, "language_style": {"answer": "Tactful and warm", "rationale": "This language style is often more easily trusted and coordinated by the interviewee."}, "time_dimension": {"answer": "Past", "rationale": "The reference to \"not allowed\" and \"fear\" in the entry suggests that this is a past event or situation. And \"because\" leads to a cause-form and implies that it is a past event or act. So the temporal dimension of this questionnaire entry is the past."}, "sentence_type": "Negative declarative sentence", "pos": {"nouns": {"parents": 1, "Son": 1, "Things": 1, "they": 1, "I": 1}, "verbs": {"Allow": 1, "Do it": 1, "Something's wrong": 1}, "adjectives": {"Other": 1, "Scared": 1}, "adverbs": {"Yeah": 1, "Yes": 2}, "numerals": {"12": 1, "Some": 1}, "pronouns": {"Here": 1, "they": 1}}}, {"content": "13. When I was a child, my parents beat me or reprimanded me in front of others.", "word_count": 19, "label": "", "sentence_structure": "Subject鈥搗erb鈥搊bject", "subjective_objective": {"answer": "Subjective", "rationale": "Because \"striking me in front of others\" is a subjective experience of memory."}, "sentence_sentiment": {"answer": "Negative", "rationale": "Because the words \"beat\" and \"criticism\" in the entries are negative."}, "language_style": {"answer": "Calm and direct", "rationale": "The entry is more objective and direct in its language, does not have too many rhetorical or emotional expressions, and directly asks the interviewee whether he or she had been beaten or reprimanded in front of others by his or her parents when he or she was a child."}, "time_dimension": {"answer": "Past", "rationale": ""}, "sentence_type": "Affirmative declarative sentence", "pos": {"nouns": {"Time": 1, "parents": 2, "person": 1, "Noodles": 1, "Censorship": 1}, "verbs": {"Fight": 1, "In front of you": 1, "others": 1, "Tsang": 1}, "adverbs": {"very": 1, "In me": 1}, "numerals": {"13": 1}, "pronouns": {"I": 1}}}, {"content": "Parents are always concerned about what I do at night.", "word_count": 13, "label": "", "sentence_structure": "Subject鈥搗erb鈥搊bject", "subjective_objective": {"answer": "Objective", "rationale": "This is a statement of fact, not a subjective feeling or attitude of the patient."}, "sentence_sentiment": {"answer": "Negative", "rationale": "The entry is an expression of parents ' excessive concern for the privacy of the child and has a negative emotional tendency."}, "language_style": {"answer": "Calm and direct", "rationale": "The language style of the entry is calm and straightforward. It expresses the core of the problem in a simple and clear language, without too many rhetorical or emotional resonance.\""}, "time_dimension": {"answer": "Present", "rationale": "The keyword \"always concerned\" indicates that it is a continuous, customary act, with no clear reference point for the past or future time, and therefore it is now judged."}, "sentence_type": "Affirmative declarative sentence", "pos": {"nouns": {"parents": 2, "Night": 1, "What": 1}, "verbs": {"Always": 1, "Attention": 1, "Fuck": 1}, "adverbs": {"very": 1}, "numerals": {"14": 1, "I": 1}, "classifiers": {"individual": 1}}}, {"content": "When things go wrong, I feel that my parents are encouraging me and giving me some comfort.", "word_count": 29, "label": "", "sentence_structure": "Subject鈥揷opula鈥損redicative", "subjective_objective": {"answer": "Subjective", "rationale": "Because \"I feel that my parents are trying to encourage me, to give me some comfort\" is the patient's subjective feeling and understanding."}, "sentence_sentiment": {"answer": "Positive", "rationale": "Because the phrase \"can feel my parents trying to encourage me, to get some comfort\" in the entry expresses positive emotions and attitudes."}, "language_style": {"answer": "Tactful and warm", "rationale": "The language style of the entry is more euphemism and warm. It gives people a warm, comfortable feeling by using expressions like \"I can feel my parents encouraging me\" and \"I can get some comfort.\" It also helps to create a good atmosphere for questionnaire filling and makes the respondents more willing to share their true situation."}, "time_dimension": {"answer": "Present", "rationale": ""}, "sentence_type": "Affirmative declarative sentence", "pos": {"nouns": {"Yes": 1, "person": 2, "parents": 1, "Encourage": 1, "Consolation": 1}, "verbs": {"I met him": 1, "I feel": 1, "End of story": 1, "鈾?And I'm gonna be 鈾?: 1}, "adjectives": {"It's not easy": 1, "Children": 1}, "numerals": {"15": 1, "Some": 1}, "pronouns": {"I": 1}}}, {"content": "Parents are always too worried about my health.", "word_count": 12, "label": "", "sentence_structure": "Subject鈥揷opula鈥損redicative", "subjective_objective": {"answer": "Subjective", "rationale": "Because \"excessive fear\" is a subjective feeling or evaluation, not an objective description. Such a perception and assessment may be patients' perception of and reaction to their parents' behaviour and therefore be subjective in nature."}, "sentence_sentiment": {"answer": "Negative", "rationale": "Because the expression \"too much to worry\" in the entry is negative."}, "language_style": {"answer": "Tactful and warm", "rationale": "The entry is more personal and interactive in its expression of a gentle and inviting tone, rather than a direct statement or order. At the same time, it expresses concern and understanding about the interviewee ' s experience, and gives the interviewee the sense of respect and acceptance."}, "time_dimension": {"answer": "Past", "rationale": "The term \"always\" in this entry indicates a situation that has existed in the past, and \"excessive fear\" shows that this behaviour has been significant over time. Thus, the temporal dimension of this entry is judged to be past."}, "sentence_type": "Affirmative declarative sentence", "pos": {"nouns": {"parents": 1, "Health": 1}, "verbs": {"Total": 1, "I'm worried too much": 1, "be": 1}, "adverbs": {"Always": 1}, "pronouns": {"I": 1}}}, {"content": "17. Parents often punish me more than I deserve.", "word_count": 17, "label": "", "sentence_structure": "Subject鈥搗erb鈥揷omplement", "subjective_objective": {"answer": "Subjective", "rationale": "Because \"often beyond what I deserve\" is a subjective description of the person interviewed, which is subjective."}, "sentence_sentiment": {"answer": "Negative", "rationale": "The fact that the article states that \"parents often punish me more than I should\" indicates that the parents' punishment is excessive and is a negative act."}, "language_style": {"answer": "Tactful and warm", "rationale": ""}, "time_dimension": {"answer": "Present", "rationale": "The entry described the experience of the person tested, namely, \"Pension of parents to me\", and used the expression \"often more than I deserve\", indicating that this was an event that had occurred in the past and had occurred. The time frame of the article was therefore the past."}, "sentence_type": "Affirmative declarative sentence", "pos": {"nouns": {"parents": 1, "I": 2, "Level": 1}, "verbs": {"to": 1, "Punishment": 1, "Super": 1, "Pass": 1, "Yes": 1}, "adjectives": {"Often": 1}, "numerals": {"17": 1}}}, {"content": "18. If I do not do what I say at home, parents get angry.", "word_count": 16, "label": "", "sentence_structure": "Subject鈥損redicate", "subjective_objective": {"answer": "Objective", "rationale": "The basis for the decision: The questionnaire entry describes an objective fact that may happen, i.e. that when a patient does not follow his or her parents鈥?orders in his or her home, they may be upset. This is not a subjective attitude or feeling of the patient, but rather an observable phenomenon."}, "sentence_sentiment": {"answer": "Negative", "rationale": "Because \"freak\" is negative."}, "language_style": {"answer": "Calm and direct", "rationale": "The language style of the entry is a calm and straightforward one, which leads the respondents to answer questions by presenting facts directly, without excessive emotional or rhetorical means."}, "time_dimension": {"answer": "Present", "rationale": "\"If I do not do what I say at home\" is a hypothetical situation, which means that the act may or has occurred in the future. And \"parents will be angry\" is the result of this act, and it happens now or in the past. So the entire sentence describes a state and situation."}, "sentence_type": "Affirmative declarative sentence", "pos": {"nouns": {"Home": 1, "Lee": 1, "parents": 1, "Fire": 1}, "verbs": {"Listen": 1, "Yes": 1, "Let's go": 1}, "adjectives": {"not": 2}, "pronouns": {"I": 1}}}, {"content": "19. If I do something wrong, my parents always give me a sense of guilt or guilt in a sad way.", "word_count": 32, "label": "", "sentence_structure": "Subject鈥搗erb鈥搊bject", "subjective_objective": {"answer": "Subjective", "rationale": "Because the title \"Making a sense of guilt or guilt\" describes the feelings and thoughts of the patient, it is subjective."}, "sentence_sentiment": {"answer": "Negative", "rationale": "Because the \"crime or guilt\" emotion in the entry is negative."}, "language_style": {"answer": "Tactful and warm", "rationale": "\"Slow and warm.\""}, "time_dimension": {"answer": "Present", "rationale": "Past"}, "sentence_type": "Affirmative declarative sentence", "pos": {"nouns": {"Yes": 1, "parents": 2, "One": 1, "Look": 1, "Feel": 1, "- A sense of guilt": 1, "Guilt": 1}, "verbs": {"Wrong": 1, "鈾?And I'm gonna be 鈾?: 1, "have": 1}, "adjectives": {"Sad": 1, "of": 3}, "numerals": {"One": 2, "Two": 1}, "pronouns": {"I": 1}}}, {"content": "20. I find it difficult for parents to get close.", "word_count": 9, "label": "", "sentence_structure": "Subject鈥揷opula鈥損redicative", "subjective_objective": {"answer": "Subjective", "rationale": "Because \"think\" is a subjective expression of feeling, which is the subjective feeling and perception of parents."}, "sentence_sentiment": {"answer": "Negative", "rationale": "Because the expression \"hard to get close\" in the entry is negative."}, "language_style": {"answer": "Calm and direct", "rationale": "\"Calm down straight.\""}, "time_dimension": {"answer": "Past", "rationale": "The keyword \"I find it difficult for parents to get close\" indicates that the person tested had felt or experienced it at some point in the past, so the entry could be classified as past."}, "sentence_type": "Negative declarative sentence", "pos": {"nouns": {"parents": 1, "Close": 1}, "verbs": {"I think": 1, "Hard": 1, "Answer": 1}, "adverbs": {"20": 1, "Both": 1}, "numerals": {"20": 1}, "pronouns": {"I": 1}}}, {"content": "21. I am embarrassed by the fact that parents have been in front of others talking about something I have said or done.", "word_count": 30, "label": "", "sentence_structure": "Subject鈥搗erb鈥揷omplement", "subjective_objective": {"answer": "Subjective", "rationale": "Because being embarrassed is a subjective emotional reaction."}, "sentence_sentiment": {"answer": "Negative", "rationale": "Because the entry describes an embarrassing experience and is a negative emotion and attitude."}, "language_style": {"answer": "Tactful and warm", "rationale": "The entry is more euphemistic, sensitive, and concerned about the feelings and inner experience of the person under investigation. It uses the first person's proxy, \"I,\" to give people a warm, real feeling; and it describes their emotional reactions with \"unsure\" rather than directly blaming or criticizing their parents, in a more moderate way."}, "time_dimension": {"answer": "Present", "rationale": "Now the entry describes the experience of the testee, the key word being \"I feel embarrassed\" , which means that it is affecting the emotional and psychological state of the testee. At the same time, the act of \"some words or things I have said\" has happened in front of others, but its impact on the testee is still ongoing, so the time dimension of the article is now."}, "sentence_type": "Affirmative declarative sentence", "pos": {"nouns": {"parents": 1, "person": 1, "Word": 2, "Yes": 1, "I": 1, "In terms of": 1, "Here": 1, "I feel": 1, "I'm embarrassed": 1}, "verbs": {"Yes": 1, "- No, no, no": 1, "Say it": 1, "Do it": 1, "鈾?And I'm gonna be 鈾?: 1}, "adverbs": {"very": 1}, "numerals": {"21": 1}}}, {"content": "22. I think that my parents prefer me to my brothers and sisters.", "word_count": 18, "label": "", "sentence_structure": "Subject鈥揷opula鈥損redicative", "subjective_objective": {"answer": "Subjective", "rationale": "Because \"think\" shows that it's a subjective feeling or perception of the patient."}, "sentence_sentiment": {"answer": "Positive", "rationale": "Because the expression \"I prefer\" in the entry is a positive emotional expression."}, "language_style": {"answer": "Tactful and warm", "rationale": "This is a soft phrase and an interview, which does not directly accuse or express one鈥檚 feelings, but rather attempts to understand the feelings and thoughts of the person under investigation. Such a style of language is better able to guide the person under investigation to think and answer, while avoiding stimulating language that may cause the other to feel or conflict."}, "time_dimension": {"answer": "Present", "rationale": "The keyword \"like\" and \"like\" point to the current subjective feelings and judgment, so the entry describes the current feelings and perceptions of the testee."}, "sentence_type": "Affirmative declarative sentence", "pos": {"nouns": {"parents": 1, "I": 1, "Brothers and sisters": 1}, "verbs": {"More like it": 1, "be": 1, "I think": 1}, "adverbs": {"More": 1}, "numerals": {"22": 1}}}, {"content": "23. Parents are small in terms of what I need.", "word_count": 16, "label": "", "sentence_structure": "Subject鈥搗erb鈥揷omplement", "subjective_objective": {"answer": "Subjective", "rationale": "Because \"smugness\" is the patient's subjective feeling and evaluation."}, "sentence_sentiment": {"answer": "Negative", "rationale": "Because the description of \"smugness\" in the entry is negative emotional."}, "language_style": {"answer": "Calm and direct", "rationale": "The decision is based on the following: The purpose is more objective, direct, and does not involve too much rhetorical or emotional overtones. It directly expresses the opinion of the surveyed parents about their needs, without any euphemism or ambiguity. It can therefore be concluded that the entry has a calm and direct language style."}, "time_dimension": {"answer": "Present", "rationale": "The entry describes the feelings and views of the testee, the word \"yes\" being used to describe the general current, as an expression of the current situation. Although this view may have been developed in the past, in this questionnaire it is more reflective of the present feelings and perceptions of the testee."}, "sentence_type": "Negative declarative sentence", "pos": {"nouns": {"Something": 1, "parents": 2}, "verbs": {"Satisfied": 1, "be": 1, "Here": 1}, "adjectives": {"- You're a little bit of a jerk": 1}, "numerals": {"23": 1}, "pronouns": {"I": 1}}}, {"content": "24. Parents often care about my score.", "word_count": 12, "label": "", "sentence_structure": "Subject鈥揷opula鈥損redicative", "subjective_objective": {"answer": "Objective", "rationale": "Because \"care\" is a subjective emotion, and the subject is to ask the objective fact that parents often care about points. So the sentence is to look at the objective fact."}, "sentence_sentiment": {"answer": "Positive", "rationale": "Because in the entry, \"Parents often care about my score\" is a positive act."}, "language_style": {"answer": "Tactful and warm", "rationale": "The entry is more modest and encouraging by describing phenomena rather than directly evaluating them."}, "time_dimension": {"answer": "Present", "rationale": "The entry is for the purpose of asking the testee about the attitude of his parents towards academic achievement, and the temporal dimension is now judged."}, "sentence_type": "Affirmative declarative sentence", "pos": {"nouns": {"parents": 1, "Score": 1}, "verbs": {"Often": 1, "I care": 1, "Access": 1}, "adverbs": {"very": 1}, "numerals": {"24": 1}}}, {"content": "25. I can feel the support of parents if I face a difficult task.", "word_count": 22, "label": "", "sentence_structure": "Subject鈥揷opula鈥損redicative", "subjective_objective": {"answer": "Subjective", "rationale": "Because it is a subjective feeling and perception that individuals experience and judge themselves, it is an objective fact whether the task is difficult and whether parents really support it. The main objective of this questionnaire is to look at the subjective attitudes and feelings of the patient."}, "sentence_sentiment": {"answer": "Positive", "rationale": "Because the expression \"can feel support from parents\" in the entry is positive."}, "language_style": {"answer": "Tactful and warm", "rationale": "The language style of the entry is more euphemistic, it uses the word \"empirical\" and emphasizes the support of parents rather than simply how difficult the task is. This expression gives respondents attention and support, and is more acceptable and answers to questions."}, "time_dimension": {"answer": "Present", "rationale": ""}, "sentence_type": "Affirmative declarative sentence", "pos": {"nouns": {"Tasks": 1, "Difficulties": 1, "Support": 1, "parents": 1}, "verbs": {"Faced": 1, "Yes": 1, "I feel": 1}, "adverbs": {"If": 1}, "numerals": {"25": 1}, "pronouns": {"I": 1}}}, {"content": "I am often used at home as a 鈥渟acrifice鈥?or 鈥渁 herd of evil鈥?", "word_count": 17, "label": "", "sentence_structure": "Subject鈥揷opula鈥損redicative", "subjective_objective": {"answer": "Subjective", "rationale": "Because being \"responsible\" or \"bad-damn horse\" is a subjective feeling and perception."}, "sentence_sentiment": {"answer": "Negative", "rationale": "Because the description of \"bet\" or \"dangerous horse\" in the entry is negative."}, "language_style": {"answer": "Tactful and warm", "rationale": ""}, "time_dimension": {"answer": "Present", "rationale": ""}, "sentence_type": "Negative declarative sentence", "pos": {"nouns": {"Home": 1, "Lee": 1, "A scapegoat": 1, "The bad guys": 1}, "verbs": {"When": 1, "Do it": 1, "By": 2}, "adverbs": {"Often": 1}, "numerals": {"26": 1}, "pronouns": {"I": 1}}}, {"content": "Parents always pick on friends I like.", "word_count": 13, "label": "", "sentence_structure": "Subject鈥搗erb鈥搊bject", "subjective_objective": {"answer": "Subjective", "rationale": "Because \"Parents always pick on my favorite friends\" is the patient's subjective feelings and perceptions of his own experience."}, "sentence_sentiment": {"answer": "Negative", "rationale": "Because the \"parents always pick out friends I like\" act in the entry is a denial of the right of the child to choose friends, and it's a negative emotion."}, "language_style": {"answer": "Tactful and warm", "rationale": "Warm and warm."}, "time_dimension": {"answer": "Past", "rationale": ""}, "sentence_type": "Negative declarative sentence", "pos": {"nouns": {"parents": 1, "friends": 1}, "verbs": {"Always": 1, "Picky": 1, "Yeah": 1}, "adverbs": {"Sou-san": 1, "I": 1}, "numerals": {"27": 1}}}, {"content": "Parents always think that I caused their discomfort.", "word_count": 16, "label": "", "sentence_structure": "Subject鈥搗erb鈥揷omplement", "subjective_objective": {"answer": "Subjective", "rationale": "Because the word \"always thinks\" indicates that it is the patient's subjective feeling and perception."}, "sentence_sentiment": {"answer": "Negative", "rationale": "Because the expression \"parents always think their discomfort is caused by me\" in the entry is a negative emotional tendency."}, "language_style": {"answer": "Tactful and warm", "rationale": "The entry is more personal and interactive when it is spoken in a gentle and inviting manner, rather than in direct statements or orders. At the same time, the article is more neutral and has no excessive emotional colours, which makes the interviewee more comfortable and comfortable."}, "time_dimension": {"answer": "Present", "rationale": "The entry describes the current situation, that is, the thoughts and perceptions of the parents of the interviewee. The word \"general\" indicates that the phenomenon persists, while the word \"caused by me\" explains the idea, that is, it is related to the interviewee. So, the time dimension of the entry is now judged."}, "sentence_type": "Negative declarative sentence", "pos": {"nouns": {"parents": 1, "they": 1, "Not fast": 1, "I": 1}, "verbs": {"Total": 1, "I thought": 1, "Cause": 1}, "adverbs": {"By": 1}, "numerals": {"28": 1}}}, {"content": "29. Parents always try to encourage me to become the best.", "word_count": 15, "label": "", "sentence_structure": "Subject鈥搗erb鈥揷omplement", "subjective_objective": {"answer": "Subjective", "rationale": "Because \"encourage\" and \"make me the best\" are the subjective feelings of the patient."}, "sentence_sentiment": {"answer": "Positive", "rationale": "Because the purpose of \"encourage\" and \"make the best of them\" is positive."}, "language_style": {"answer": "Tactful and warm", "rationale": "The entry describes, in a moderate manner, the expectations and support of parents for their children, and is more cordial and sweet, without direct use of orders or mandatory language."}, "time_dimension": {"answer": "Past", "rationale": "The entry is a question about the perception of the person who was tested about the attitudes and behaviour of parents as they grow up, and the temporal dimension is a past."}, "sentence_type": "Affirmative declarative sentence", "pos": {"nouns": {"parents": 1, "Good man": 1}, "verbs": {"Total": 1, "Encourage": 1, "鈾?And I'm gonna be 鈾?: 1, "Be": 1}, "adverbs": {"Always trying": 1}, "numerals": {"29": 1}, "pronouns": {"I": 1}}}, {"content": "30. Parents always told me they loved me.", "word_count": 13, "label": "", "sentence_structure": "Subject鈥揷opula鈥損redicative", "subjective_objective": {"answer": "Subjective", "rationale": "Because \"parents always say they love me\" is the patient's subjective perception and interpretation of parental behaviour, so the answer is \"subjective\"."}, "sentence_sentiment": {"answer": "Positive", "rationale": "Because the expression \"love me\" in the entry is positive."}, "language_style": {"answer": "Tactful and warm", "rationale": "Warm and warm."}, "time_dimension": {"answer": "Present", "rationale": ""}, "sentence_type": "Affirmative declarative sentence", "pos": {"nouns": {"parents": 2, "I": 1, "Love": 1}, "verbs": {"Total": 1, "Organisation": 1, "be": 1}, "adverbs": {"Always": 1}, "numerals": {"30": 1}}}, {"content": "31. Parents have confidence in me and allow me to do something alone.", "word_count": 18, "label": "", "sentence_structure": "Subject鈥揷opula鈥損redicative", "subjective_objective": {"answer": "Subjective", "rationale": "Because \"very trusting and allowing\" and \"doing something alone\" are the attitudes and behaviours of parents described from the perspective of patients, the entry is viewed from a subjective perspective."}, "sentence_sentiment": {"answer": "Positive", "rationale": "Because \"trust\" and \"permissive to do things alone\" are positive emotions and attitudes."}, "language_style": {"answer": "Tactful and warm", "rationale": "The language style of the entry is euphemistic and sweet, using the words \"very trusting\" and \"permissively allowing me to do something alone\" to give people a comfortable, respectful feeling, not direct orders or accusations. This is more likely to trigger resonance and active participation by the respondents."}, "time_dimension": {"answer": "Present", "rationale": "The entry describes the relationship between the testee and his parents and the status of the testee's autonomy, where the words \"very trusting\" and \"permissively allowing me to do something alone\" indicate that these things are happening or already exist. The temporal dimension of the article should therefore be judged now."}, "sentence_type": "Affirmative declarative sentence", "pos": {"nouns": {"parents": 1, "I": 1, "Something": 1}, "verbs": {"to": 1, "very": 1, "Trust": 1, "and": 1, "Allow": 1, "Completed": 1, "Alone": 1}, "adjectives": {"Trusted and allowed": 1}, "adverbs": {"very": 1, "Alone": 1}, "numerals": {"31": 1}, "pronouns": {"they": 1}}}, {"content": "32. I think parents are very thin in my view.", "word_count": 12, "label": "", "sentence_structure": "Subject鈥搗erb鈥揷omplement", "subjective_objective": {"answer": "Subjective", "rationale": "Because words like \"think\" and \"think\" usually express a subjective view or feeling of an individual."}, "sentence_sentiment": {"answer": "Negative", "rationale": "Because the entry \"parents are thin\" expresses a negative feeling and attitude, implying subjective feelings of contempt or disrespect."}, "language_style": {"answer": "Calm and direct", "rationale": "The wording of this article is simple and clear, without excessive rhetorical or emotional overtones, and gives a direct view of the value the person is giving to his parents, and is more objective and direct."}, "time_dimension": {"answer": "Past", "rationale": "The entry uses the old-temporal \"I feel\" to indicate that it is a description of the past's feelings or experiences."}, "sentence_type": "Negative declarative sentence", "pos": {"nouns": {"I": 1, "parents": 1, "Views": 1}, "verbs": {"I think": 1, "Heavy": 1}, "adjectives": {"Thin": 1}, "numerals": {"32": 1}}}, {"content": "33. I feel that parents are very happy with me.", "word_count": 13, "label": "", "sentence_structure": "Subject鈥揷opula鈥損redicative", "subjective_objective": {"answer": "Subjective", "rationale": "Because the verb \"thinks\" indicates that it is the patient's subjective perception and feelings about his relationship with his parents."}, "sentence_sentiment": {"answer": "Positive", "rationale": "Because the words in the entry, \"Parents are willing to be with me,\" express positive emotions and attitudes."}, "language_style": {"answer": "Tactful and warm", "rationale": "The entry uses the first person's pronoun, \"I\" and expresses in a positive tone their views of parents, so that the interviewee feels respected and understood, and is not too direct or hard. This euphemism and warmer language is more appropriate for psychological questionnaire research."}, "time_dimension": {"answer": "Present", "rationale": ""}, "sentence_type": "Affirmative declarative sentence", "pos": {"nouns": {"parents": 1, "Together": 1}, "verbs": {"I think": 1, "May I": 1, "in": 1}, "adjectives": {"very": 1}, "adverbs": {"Call": 1, "I": 1}, "numerals": {"33": 1}}}, {"content": "34. I feel that my parents are so mean and mean to me.", "word_count": 13, "label": "", "sentence_structure": "Subject鈥揷opula鈥損redicative", "subjective_objective": {"answer": "Subjective", "rationale": "The blogger says that the government is not a party to the law, because it feels like it is a description of the patient's subjective feelings."}, "sentence_sentiment": {"answer": "Negative", "rationale": "Because the word \"simply, stingy\" in the entry is a derogatory word, expressing negative emotions."}, "language_style": {"answer": "Tactful and warm", "rationale": "The entry uses a more objective formulation, which does not directly blame or criticize the behaviour of the other party, but rather expresses its own feelings. This language style makes it easier to accept and understand, while avoiding unnecessary conflicts or disputes."}, "time_dimension": {"answer": "Past", "rationale": ""}, "sentence_type": "Negative declarative sentence", "pos": {"nouns": {"parents": 2, "I": 1}, "verbs": {"I think": 1, "- You're a little bit of a jerk": 1, "Stingy": 1, "to": 1}, "adjectives": {"very": 2, "Stingy": 1}, "numerals": {"34": 1}}}, {"content": "Parents always say to me things like that: 鈥淚f you do this, I will be very sad鈥?", "word_count": 24, "label": "", "sentence_structure": "Subject鈥搗erb鈥搊bject", "subjective_objective": {"answer": "Subjective", "rationale": "Because \"Parents always say something like that to me\" is the patient's subjective experience and feelings."}, "sentence_sentiment": {"answer": "Negative", "rationale": "Because the action in the entry, \"If I do this, you'll be very sad,\" could cause a negative emotional reaction to the child."}, "language_style": {"answer": "Calm and direct", "rationale": "The entry presents the subject matter in a direct presentation, does not have too many rhetorical or emotional renderings, is more objective and is more suitable for data analysis and assessment."}, "time_dimension": {"answer": "Present", "rationale": "The entry is an inquiry into the feelings and reactions of the person who is being tested to similar words often spoken by parents, the temporal dimension of which is now judged. Although the words of the past are \"always\" in their language, in practice the term is more a reference to a customary or conventional act than to a specific period of time in the past. Therefore, the entry should be designated as now, as required by the title."}, "sentence_type": "Affirmative declarative sentence", "pos": {"nouns": {"parents": 2, "Word": 1, "Do it": 1, "Yes": 1, "If": 1, "Sad": 1}, "verbs": {"Total": 1, "In": 1, "Say it": 1, "It's gonna be": 1, "Sad": 1}, "adverbs": {"Always": 1, "very": 1}, "numerals": {"35": 1, "If": 1}, "pronouns": {"you": 1, "I": 1}}}, {"content": "36. Parents have to tell me what I am doing when I ask to return to my family.", "word_count": 23, "label": "", "sentence_structure": "Subject鈥搗erb鈥搊bject", "subjective_objective": {"answer": "Subjective", "rationale": "The examination of this issue is based on the patient ' s subjective view of the parents ' claims."}, "sentence_sentiment": {"answer": "Neutral", "rationale": "Because the entry does not express any positive or negative emotional colours, it is merely a description of a fact."}, "language_style": {"answer": "Tactful and warm", "rationale": "The language style of the entry is euphemism. It raises questions in the form of inquiries and uses the polite expression \"please\" to express respect for the interviewee, a gentle way that is more acceptable and answered. Moreover, the entries do not directly order or accuse the interviewee of reporting to his parents, but rather offer a suggestion or expectation that the expression is more harmonious and friendly."}, "time_dimension": {"answer": "Past", "rationale": "According to the title description, \"Dequire me\" and \"have to explain to them what I am doing\" point to a certain point or period of time in the past, so the entry is to question the testee's past behaviour."}, "sentence_type": "Affirmative declarative sentence", "pos": {"nouns": {"parents": 1, "Home": 1, "Things": 1}, "verbs": {"Request": 1, "Back": 1, "Okay": 1, "Say it": 1, "Do it": 1}, "adverbs": {"I have to": 1}, "numerals": {"36": 1}, "pronouns": {"I": 1}}}, {"content": "37. I think that my parents are doing their best to make my youth more meaningful and diverse (e.g. by buying me a lot of books, arranging me to go to summer camps or to join clubs).", "word_count": 43, "label": "", "sentence_structure": "Subject鈥搗erb鈥搊bject", "subjective_objective": {"answer": "Subjective", "rationale": "Because it's the subjective perception and feelings of the patient that are being examined."}, "sentence_sentiment": {"answer": "Neutral", "rationale": "Because the entry describes the acts and actions of parents without reference to their effects or subjective feelings. It is therefore not emotional and is neutral."}, "language_style": {"answer": "Tactful and warm", "rationale": ""}, "time_dimension": {"answer": "Past", "rationale": "The entry describes the senses and memories of the past, specifically about the efforts of parents to make them meaningful and diverse adolescences as children. Therefore, in terms of time dimension, the entry should be judged as \"the past\"."}, "sentence_type": "Affirmative declarative sentence", "pos": {"nouns": {"parents": 2, "Youth": 1, "Books": 1, "Summer camp": 1, "Club": 1}, "verbs": {"I think": 1, "Do your best": 1, "Buy": 1, "Arrangements": 1, "Participation": 1}, "adjectives": {"Meaningful": 1, "It's a lot of color": 1}, "numerals": {"37": 1, "Lots": 1}, "pronouns": {"I": 1}}}, {"content": "Parents often say to me the same thing: 鈥淚s this the reward we get for your daily work?鈥?, "word_count": 32, "label": "", "sentence_structure": "Subject鈥搗erb鈥搊bject", "subjective_objective": {"answer": "Objective", "rationale": "Because the patient鈥檚 parents say this, their views and behaviour, not their own subjective feelings or ideas. The patient鈥檚 feelings about such acts or statements are subjective."}, "sentence_sentiment": {"answer": "Negative", "rationale": "Because the entries are about parents' dissatisfaction and blame, they have a negative emotional connotation."}, "language_style": {"answer": "Calm and direct", "rationale": "This is a way to ask questions that are more specific and accurate."}, "time_dimension": {"answer": "Present", "rationale": ""}, "sentence_type": "Interrogative sentence", "pos": {"nouns": {"parents": 1, "Words": 1, "Refund": 1, "you": 1, "Day": 1, "- Oh, fuck": 1}, "verbs": {"Presentation": 1, "All day long": 1, "Got it": 1, "be": 1, "In": 1}, "adjectives": {"Similar": 1}, "adverbs": {"Regular": 1, "Here": 1}, "numerals": {"38": 1, "One": 1}, "pronouns": {"we": 1}}}, {"content": "Parents often use the excuse of not being comfortable with me as an excuse for not meeting my demands.", "word_count": 19, "label": "", "sentence_structure": "Subject鈥搗erb鈥搊bject", "subjective_objective": {"answer": "Subjective", "rationale": "Because the sentence expresses the patient ' s perception and feelings about his or her parent ' s behaviour, it is a subjective examination."}, "sentence_sentiment": {"answer": "Negative", "rationale": "Because the entry \"I can't be used to justify not meeting my demands\" has a negative emotional connotation."}, "language_style": {"answer": "Tactful and warm", "rationale": "This is a more personal and interactive way of expressing itself, and is easier for the interviewees to accept and answer questions."}, "time_dimension": {"answer": "Past", "rationale": "The term \"normal\" in this entry indicates the pattern of past behaviour, \"not being used as an excuse to fail to meet my demands\" describes what happened in the past. Thus, the temporal dimension of this questionnaire entry is a matter of the past."}, "sentence_type": "Negative declarative sentence", "pos": {"nouns": {"parents": 2, "I": 2, "- An excuse": 1, "Request": 1}, "verbs": {"Always": 1, "No, I can't": 1, "Cozy": 1, "Yes": 1, "Satisfied": 1}, "adverbs": {"Regular": 1, "not": 1}, "numerals": {"39": 1}}}, {"content": "40. I am concerned that my parents have not done what they expected.", "word_count": 25, "label": "", "sentence_structure": "Subject鈥搗erb鈥搊bject", "subjective_objective": {"answer": "Subjective", "rationale": "The basis for the judgement: This is a self-evaluation and feeling of the patient, expressing his/her belief that he/she needs to act in accordance with the parents鈥?demands to ease the pressure and anxiety."}, "sentence_sentiment": {"answer": "Negative", "rationale": "Because the result of \"clemental anxiety\" is negative and expresses an emotional sense of anxiety and guilt."}, "language_style": {"answer": "Tactful and warm", "rationale": "The language style of the questionnaire entry is euphemism. It uses a gentle and inviting tone, emphasizing that acting in the way parents want, can bring about a sense of inner peace, not direct orders or threats. Moreover, using the expression \"clemental unease\" also demonstrates the concern and concern that the person under investigation feels psychologically."}, "time_dimension": {"answer": "Past", "rationale": "In this entry, \"if you do not do what your parents want\" means \"the past's experience or circumstances,\" makes me feel very uncomfortable about my conscience. It also means the past's feelings and experiences. So the time dimension of this questionnaire is the past."}, "sentence_type": "Interrogative sentence", "pos": {"nouns": {"Questionnaire Entry": 1, "parents": 1, "Expectations": 1, "Conscience": 1, "Unsettled": 1}, "verbs": {"Do it": 1, "Yes": 2, "鈾?And I'm gonna be 鈾?: 1, "I feel": 1}, "adjectives": {"Press": 1, "What I expected": 1}, "adverbs": {"very": 1}, "numerals": {"40": 1}, "classifiers": {"individual": 1}, "pronouns": {"I": 1}}}, {"content": "41. I feel that parents have a high demand for my academic achievement, physical activity or similar things.", "word_count": 28, "label": "", "sentence_structure": "Subject鈥搗erb鈥揷omplement", "subjective_objective": {"answer": "Subjective", "rationale": "Because \"think\" shows that it's a patient's perception or feeling."}, "sentence_sentiment": {"answer": "Neutral", "rationale": "The entry describes only one view of the interviewees and does not express positive or negative emotional tendencies."}, "language_style": {"answer": "Calm and direct", "rationale": "The entry uses a concise and clear statement, and does not contain any emotional elements, and expresses the problem in an objective and calm manner. At the same time, the language of the article is consistent with the characteristics of the psychological questionnaire, namely, to be neutral and objective in order to obtain better the real thoughts and feelings of the person under investigation."}, "time_dimension": {"answer": "Present", "rationale": "The entry uses the current state of affairs (the perception), expresses the current feelings and perceptions of the interviewees, and thus the temporal dimension is now judged."}, "sentence_type": "Affirmative declarative sentence", "pos": {"nouns": {"parents": 2, "I": 1, "Learning": 1, "Achievements": 1, "Sports activities": 1, "Things": 1, "Request": 1}, "verbs": {"I think": 1, "have": 1, "Higher": 1, "Request": 1}, "adjectives": {"of": 2, "Achievements": 1, "Sports activities": 1, "Similar": 1}, "adverbs": {"very": 1}, "numerals": {"41": 1}}}, {"content": "42. When I feel sad, I can get comfort from my parents.", "word_count": 19, "label": "", "sentence_structure": "Subject鈥搗erb鈥揷omplement", "subjective_objective": {"answer": "Subjective", "rationale": "The entry is viewed from the patient鈥檚 subjective point of view. It is the emotional experience and need of the patient to feel sad and need comfort, and the ability to get comfort from parents is the result of the patient鈥檚 observation of his or her attitude and behaviour, and is therefore subjective."}, "sentence_sentiment": {"answer": "Positive", "rationale": "Because the purpose of the action in the entry is to get comfort from parents."}, "language_style": {"answer": "Tactful and warm", "rationale": "The entry uses gentle and emotional language, such as \"can be comforted,\" which is more acclaimed, warm and more easily resonated and shared by the person under investigation. At the same time, the title does not directly ask about personal feelings or experiences, but rather reflects a euphemism and individualized language style by providing a context for the person under investigation to remember and think."}, "time_dimension": {"answer": "Present", "rationale": "The entry describes the behaviour of the person who was tested when he or she was sad and uses the general current pattern to indicate that it is a customary act. The entry is therefore the current pattern."}, "sentence_type": "Affirmative declarative sentence", "pos": {"nouns": {"Time": 1, "Sad": 1, "parents": 1, "Consolation": 1}, "verbs": {"I feel": 1, "Got it": 1}, "adverbs": {"Well": 1}, "numerals": {"42": 1}, "pronouns": {"I": 1}}}, {"content": "43. Parents have punished me for no reason.", "word_count": 11, "label": "", "sentence_structure": "Subject鈥搗erb鈥搊bject", "subjective_objective": {"answer": "Subjective", "rationale": "Because \"had punished me for no reason\" is the experience and feeling that the patient remembers, and it is seen from the patient's subjective perspective."}, "sentence_sentiment": {"answer": "Negative", "rationale": "Because \"punishment without cause\" is negative."}, "language_style": {"answer": "Calm and direct", "rationale": "The entry describes a possible experience in direct language, is not expressed in excessive rhetorical or emotional colours and is more objective and calm."}, "time_dimension": {"answer": "Past", "rationale": ""}, "sentence_type": "Negative declarative sentence", "pos": {"nouns": {"parents": 2, "For no reason": 1, "Punishment": 1, "I": 1}, "verbs": {"Tsang": 1, "For no reason": 1, "Punishment": 1, "have": 1, "Say it": 1}, "adjectives": {"For no reason": 1}, "adverbs": {"Land": 1}, "numerals": {"43": 1}}}, {"content": "Parents promised me to do something my friends did.", "word_count": 17, "label": "", "sentence_structure": "Subject鈥搗erb鈥搊bject", "subjective_objective": {"answer": "Subjective", "rationale": "Because it's the patient's subjective feeling and opinion."}, "sentence_sentiment": {"answer": "Positive", "rationale": "Because the \"Parents Promised Me to Do Some Things My Friends\" act positive."}, "language_style": {"answer": "Tactful and warm", "rationale": "The entry is more euphemistic, allowing respondents to express their views in a way that is asked and understood, without too direct or powerful language. At the same time, the expression \"what my friends do\" is more neutral and peaceful, avoiding words or phrases that can provoke negative emotions or counter-intuitive."}, "time_dimension": {"answer": "Present", "rationale": "The entry asks the testee about his current state or behaviour, and the key word is \"Parents promise me to do something my friends do\", which means that the testee is doing something like this or has done something similar."}, "sentence_type": "Affirmative declarative sentence", "pos": {"nouns": {"parents": 1, "I": 1, "Friends": 1, "Things": 1}, "verbs": {"The promise": 1, "Do it": 1}, "adverbs": {"Some": 1}, "numerals": {"44": 1}}}, {"content": "Parents often tell me they don't like my performance at home.", "word_count": 18, "label": "", "sentence_structure": "Subject鈥搗erb鈥搊bject", "subjective_objective": {"answer": "Objective", "rationale": "This is a descriptive statement of the parents鈥?observation and evaluation of their behaviour, rather than of the patients鈥?own feelings or perceptions."}, "sentence_sentiment": {"answer": "Negative", "rationale": "Because the description of \"parents don't like my performance at home\" in the entry is negative."}, "language_style": {"answer": "Tactful and warm", "rationale": "The entry is more euphemistic, soft, polite, and not directly accusing or criticizing each other. At the same time, the issue is presented in a more open and neutral manner, avoiding the possibility of creating negative emotions."}, "time_dimension": {"answer": "Past", "rationale": "The term \"often\" in the entry indicates that it is a customary act and occurs at some point or time in the past. At the same time, the description \"they do not like my behaviour at home\" is based on evaluation and feedback of past behaviour. Thus, the temporal dimension of the article can be judged to be the past."}, "sentence_type": "Negative declarative sentence", "pos": {"nouns": {"parents": 1, "I": 1, "Home": 1, "Performance": 1}, "verbs": {"Say it": 2, "Yeah": 1, "in": 1, "to": 1, "Regular": 1, "I don't like it": 1}, "adjectives": {"Regular": 1}, "adverbs": {"not": 1}, "numerals": {"45": 1}, "pronouns": {"they": 1}}}, {"content": "46. Every time I eat, my parents persuade me or force me to eat more.", "word_count": 20, "label": "", "sentence_structure": "Subject鈥搗erb鈥搊bject", "subjective_objective": {"answer": "Subjective", "rationale": "Because \"prosthetic\" and \"compulsive\" are subjective acts that patients feel."}, "sentence_sentiment": {"answer": "Negative", "rationale": "Because the \"prob me or force me to eat more\" in the entry is negative."}, "language_style": {"answer": "Tactful and warm", "rationale": "The language style of the entry is euphemism, because it expresses a common family phenomenon in a moderate way, without direct orders or accusations."}, "time_dimension": {"answer": "Past", "rationale": ""}, "sentence_type": "Affirmative declarative sentence", "pos": {"nouns": {"Rice": 1, "parents": 1}, "verbs": {"Eat": 1, "- I'm gonna get you out of here": 1, "More": 2, "Forced": 1}, "adverbs": {"Every": 1, "Time": 1}, "numerals": {"46": 1}, "classifiers": {"Some": 1}, "pronouns": {"I": 2}}}, {"content": "47. Parents often criticize me in front of others for being both inert and useless.", "word_count": 19, "label": "", "sentence_structure": "Subject鈥搗erb鈥搊bject", "subjective_objective": {"answer": "Objective", "rationale": "This is a patient ' s assessment and not a patient ' s self-evaluation."}, "sentence_sentiment": {"answer": "Negative", "rationale": "Because the term \"criticism\" in this entry is usually seen as a negative act or attitude that may have a negative impact on the person being evaluated. At the same time, the expression \"unsimplistic and useless\" has a derogatory and negative meaning and a negative emotional connotation."}, "language_style": {"answer": "Calm and direct", "rationale": "The questionnaire entry is based on direct questions, which are given to respondents to answer the questions about the behaviour or situation of the person questioned, without any qualifier or emotional language, and is more objective and calm."}, "time_dimension": {"answer": "Past", "rationale": "The entry describes a past act or state of conduct, in which the parents of the person tested criticized him/her in front of others."}, "sentence_type": "Negative declarative sentence", "pos": {"nouns": {"parents": 2, "others": 1, "Noodles": 1, "Criticism": 1, "I": 3, "Both": 1, "Micro": 1, "Inert": 1, "And": 1, "It's useless": 1}, "verbs": {"In front of you": 1, "Criticism": 1}, "adjectives": {"Badge": 1, "Inert": 1, "It's useless": 1}, "numerals": {"47": 1}}}, {"content": "Parents often focus on what kind of friends I have.", "word_count": 15, "label": "", "sentence_structure": "Subject鈥搗erb鈥搊bject", "subjective_objective": {"answer": "Subjective", "rationale": "Because the concern about what kind of friends are is the subjective attitude or perception of parents, not the objective fact."}, "sentence_sentiment": {"answer": "Positive", "rationale": "Because the \"often focused\" in the entry indicates that parents' interest in and support for their children is a positive emotional attitude."}, "language_style": {"answer": "Tactful and warm", "rationale": "The language style of the entry is euphemism, because it does not use too direct or command-style tone, but rather engages in a conversation with a care and inquiry. It also does not mean blame or criticism, but rather seeks the views and feelings of the other. Such expression is more acceptable and willing to share their own ideas."}, "time_dimension": {"answer": "Past", "rationale": "The \"often\" customary behaviour in the entry implies that it has been occurring for some time in the past. At the same time, the \"care\" action has occurred in the past, so the temporal dimension of the article is the past."}, "sentence_type": "Affirmative declarative sentence", "pos": {"nouns": {"parents": 2, "- To be with someone": 1, "friends": 1}, "verbs": {"Attention": 1}, "adverbs": {"Regular": 1, "Sample": 1}, "numerals": {"48": 1}, "pronouns": {"I": 1}}}, {"content": "49. If anything happens, I am often the only one of my brothers and sisters to be blamed.", "word_count": 25, "label": "", "sentence_structure": "Subject鈥搗erb鈥搊bject", "subjective_objective": {"answer": "Subjective", "rationale": "Since the question is about the individual feelings and experiences of the patient, i.e. whether the patient often feels accused or criticized in the family, it is a subjective one."}, "sentence_sentiment": {"answer": "Negative", "rationale": "For the expression \"often the only one of brothers and sisters to blame\" shows that the respondents are often unfairly accused and criticized, and have negative emotional connotations."}, "language_style": {"answer": "Tactful and warm", "rationale": ""}, "time_dimension": {"answer": "Past", "rationale": ""}, "sentence_type": "Negative declarative sentence", "pos": {"nouns": {"Things": 1, "Brothers and sisters": 1, "Blame": 1}, "verbs": {"Come on": 1, "be": 1, "Yes": 1, "Blame": 1}, "adverbs": {"Often": 1}, "numerals": {"49": 1}, "classifiers": {"One": 1}, "pronouns": {"I": 1}}}, {"content": "50. Parents can let me go along with my natural development.", "word_count": 12, "label": "", "sentence_structure": "Subject鈥搗erb鈥揷omplement", "subjective_objective": {"answer": "Subjective", "rationale": "Because this expression expresses the patient ' s perception and feelings of parental expectations, it is a more subjective expression."}, "sentence_sentiment": {"answer": "Neutral", "rationale": "Because the entry does not have any emotional color."}, "language_style": {"answer": "Tactful and warm", "rationale": "Warm and warm."}, "time_dimension": {"answer": "Present", "rationale": ""}, "sentence_type": "Affirmative declarative sentence", "pos": {"nouns": {"parents": 1, "Development": 1}, "verbs": {"Yes": 1, "Jean": 1, "Let it be": 1}, "adverbs": {"Land": 1}, "numerals": {"50": 1}}}, {"content": "51. Parents often behaved rudely towards me.", "word_count": 10, "label": "", "sentence_structure": "Subject鈥搗erb鈥揷omplement", "subjective_objective": {"answer": "Subjective", "rationale": "Because it's the patient's subjective feeling and perception."}, "sentence_sentiment": {"answer": "Negative", "rationale": "Because the \"parents are often rude to me\" in the entry is negative."}, "language_style": {"answer": "Calm and direct", "rationale": "The entry presents the core of the problem in a straightforward manner, without adding too many rhetorical or emotional features, and with a more calm and objective language style."}, "time_dimension": {"answer": "Present", "rationale": "In this entry, \"often\" indicates that this is a description of the current situation and that \"parents often have rudeness towards me\" is the subjective feeling of the testee of the parent's current behaviour. Thus, the temporal dimension of this article is now judged."}, "sentence_type": "Negative declarative sentence", "pos": {"nouns": {"parents": 1, "I": 1, "Nasty": 1, "Insolent": 1}, "verbs": {"Regular": 1, "to": 1, "Naughty": 1}, "adjectives": {"Nasty": 1, "Insolent": 1}, "numerals": {"51": 1}, "pronouns": {"I": 1}}}, {"content": "52. Parents sometimes punish me harshly for a little bit of a little bit of a thing.", "word_count": 25, "label": "", "sentence_structure": "Subject鈥搗erb鈥搊bject", "subjective_objective": {"answer": "Objective", "rationale": "Because \"punishment\" is a concrete result of behaviour, which is seen from an objective perspective. Even if parents take a harsh attitude and behaviour towards minor things, it is their objective approach, so this entry is viewed from an objective perspective."}, "sentence_sentiment": {"answer": "Negative", "rationale": "Because the \"dramatic punishment\" in the entry is negative."}, "language_style": {"answer": "Tactful and warm", "rationale": "This language style is more acceptable to the interviewees and facilitates access to more realistic and comprehensive information."}, "time_dimension": {"answer": "Past", "rationale": "* Past: it means that parents used to punish me for small things. * Now: \"even\" and \"will\" means that even now, the punishment will still happen. * Future: the entry does not mention the future."}, "sentence_type": "Negative declarative sentence", "pos": {"nouns": {"It's nothing": 1, "Porky, porcelain": 1, "parents": 1, "Punishment": 1}, "verbs": {"Yes": 1, "Hard": 1, "Sometimes": 1}, "adjectives": {"A little": 1}, "adverbs": {"Even": 1}, "numerals": {"52": 1}}}, {"content": "Parents beat me up for no reason.", "word_count": 11, "label": "", "sentence_structure": "Subject鈥搗erb鈥揷omplement", "subjective_objective": {"answer": "Subjective", "rationale": "Because being beaten is a patient's personal experience and a patient's subjective feeling."}, "sentence_sentiment": {"answer": "Negative", "rationale": "Because the \"hit\" incident in the entry was negative and did not mention any positive factors to balance that negative sentiment."}, "language_style": {"answer": "Calm and direct", "rationale": "The entry is of a calm and direct type. The entry specifically asks whether the person surveyed has experienced abusive behaviour by his parents without any reason, does not use excessive qualifier or rhetorical techniques, is honest and objective and aims to obtain information on the actual experience of the person under investigation."}, "time_dimension": {"answer": "Past", "rationale": "In this entry, the word \"had\" denotes a past event and can therefore be judged as a past experience or behaviour."}, "sentence_type": "Affirmative declarative sentence", "pos": {"nouns": {"parents": 1, "I": 1}, "verbs": {"Tsang": 1, "Fight": 1, "For no reason": 1}, "adjectives": {"For no reason": 1}, "adverbs": {"Land": 1}, "numerals": {"53": 1}}}, {"content": "54. Parents usually take part in my amateur activities.", "word_count": 15, "label": "", "sentence_structure": "Subject鈥搗erb鈥搊bject", "subjective_objective": {"answer": "Objective", "rationale": "The basis for the determination: this entry describes whether parents are regularly involved in the patient ' s hobby, which is an objective fact that occurs in the patient ' s life and does not involve the patient ' s subjective feelings or attitudes."}, "sentence_sentiment": {"answer": "Positive", "rationale": "Because \"participation\" in the entry is an expression of parents' support and encouragement for their children and is positive behaviour."}, "language_style": {"answer": "Tactful and warm", "rationale": "The language style of the entry is more euphemistic and emotional. First, it uses a verb rather than a direct statement, which is more interactive. Second, it expresses concern and concern about the family background of the other, making the respondents feel respected and understood when they fill out. So the language style of the entry can be described as \"fluent.\""}, "time_dimension": {"answer": "Present", "rationale": "The entry used to describe the current or previous situation of the person being tested, the keywords being \"normal\" and \"council\" , which all represent customary past behaviour. Besides, \"parents usually take part in my hobby\" has also happened in the past, and therefore the temporal dimension has been a past."}, "sentence_type": "Affirmative declarative sentence", "pos": {"nouns": {"parents": 1, "Amateur activities": 1}, "verbs": {"Participation": 1, "have": 1}, "adverbs": {"Usually": 1}, "numerals": {"54": 1}, "pronouns": {"I": 1}}}, {"content": "55. I was often beaten by my parents.", "word_count": 8, "label": "", "sentence_structure": "Subject鈥搗erb鈥揷omplement", "subjective_objective": {"answer": "Subjective", "rationale": "Because it is a subjective experience and feeling that is beaten by parents."}, "sentence_sentiment": {"answer": "Negative", "rationale": "Because beating is a negative act."}, "language_style": {"answer": "Calm and direct", "rationale": ""}, "time_dimension": {"answer": "Past", "rationale": "The entry \"often\" indicates that this is the past, and \"beating by parents\" is a specific description of the act, and therefore it is judged to be the past."}, "sentence_type": "Negative declarative sentence", "pos": {"nouns": {"I": 1, "parents": 1, "Fight": 1}, "verbs": {"Scrap": 1}, "adverbs": {"Regular": 1}, "numerals": {"55": 1}}}, {"content": "Parents often allow me to go where I like to go, and they do not worry too much.", "word_count": 25, "label": "", "sentence_structure": "Subject鈥搗erb鈥搊bject", "subjective_objective": {"answer": "Subjective", "rationale": "Because it is the patient鈥檚 subjective feeling and perception that parents often allow me to go where I like and do not worry too much. It is an assessment and interpretation of the past, and therefore subjective."}, "sentence_sentiment": {"answer": "Positive", "rationale": "Because the \"Parents often allow me to go where I like\" in the entry is positive."}, "language_style": {"answer": "Tactful and warm", "rationale": "The language style of the entry is more euphemistic and soft, replacing the direct statement \"Parents often allow me\" with \"Parents always leave me\" and adding \"without excessive fear\" after it, both expressing the trust and support of parents and avoiding overemphasis or order. Such expressions are more personal and interactive, making people feel more comfortable and natural."}, "time_dimension": {"answer": "Present", "rationale": "The entry describes the current status and situation of the testees, i.e., parents allow them to go where they like, and there is no undue concern, so the temporal dimension is now judged."}, "sentence_type": "Affirmative declarative sentence", "pos": {"nouns": {"parents": 2, "I": 2, "Local": 1, "I'm worried": 1}, "verbs": {"Allow": 1, "Go on": 1, "Yes": 1, "That's too much": 1}, "adjectives": {"Yeah": 1}, "adverbs": {"Often": 1, "And": 1}, "numerals": {"56": 1, "2": 2, "1": 4}, "pronouns": {"they": 1}}}, {"content": "57. Parents have strict restrictions and no concessions on what I should and should not do.", "word_count": 26, "label": "", "sentence_structure": "Subject鈥搗erb鈥搊bject", "subjective_objective": {"answer": "Subjective", "rationale": "Because \"restrictive\" and \"no concessions\" are patients' feelings and understanding of their parents' behaviour, and they are subjective."}, "sentence_sentiment": {"answer": "Negative", "rationale": "Because the entry \"restricted and uncompromising\" indicates that the parents' discipline is too strict and strong and may have a negative impact on the individual."}, "language_style": {"answer": "Calm and direct", "rationale": ""}, "time_dimension": {"answer": "Past", "rationale": ""}, "sentence_type": "Affirmative declarative sentence", "pos": {"nouns": {"parents": 2, "Yes": 2, "Do it": 2, "I shouldn't": 1, "Both": 1, "have": 1, "Strict": 1, "Limits": 1, "And": 1, "Never": 1, "Compliment": 1}, "verbs": {"The": 1, "Do it": 2, "Jean": 1}, "adjectives": {"Strict": 1, "Absolutely": 1}, "adverbs": {"Both": 1, "Never": 1}, "numerals": {"57": 1, "2": 2}, "pronouns": {"I": 1}}}, {"content": "Parents often treat me in a way that makes me uncomfortable.", "word_count": 17, "label": "", "sentence_structure": "Subject鈥搗erb鈥揷omplement", "subjective_objective": {"answer": "Subjective", "rationale": "This is a form of treatment that is felt by the patient and therefore subjective in nature."}, "sentence_sentiment": {"answer": "Negative", "rationale": "Because \"makes me awkward the way I do\" is negative."}, "language_style": {"answer": "Calm and direct", "rationale": "The language style of the entry is more straightforward and concise, and does not have too many rhetorical or emotional features, but simply asks the respondents directly about the way their parents treat them."}, "time_dimension": {"answer": "Present", "rationale": ""}, "sentence_type": "Negative declarative sentence", "pos": {"nouns": {"parents": 1, "Modalities": 1}, "verbs": {"Treatment": 1, "鈾?And I'm gonna be 鈾?: 1, "very": 1, "I'm embarrassed": 1}, "adjectives": {"Regular": 1}, "adverbs": {"Here": 1}, "numerals": {"One": 2}, "pronouns": {"I": 1}}}, {"content": "59. I find the fear of parents that something may happen to me exaggerated and excessive.", "word_count": 21, "label": "", "sentence_structure": "Subject鈥揷opula鈥損redicative", "subjective_objective": {"answer": "Subjective", "rationale": "Because this is the patient ' s personal perception and feelings, it is subjective."}, "sentence_sentiment": {"answer": "Negative", "rationale": "Because the emotional description of \"parents' fear of possible harm\" in the entry is negative."}, "language_style": {"answer": "Tactful and warm", "rationale": "The language style of the entry is more euphemistic and warm, using the expression \"I think\" and more compassionate and respectful of the views of the interviewees, and is not overly direct or emotional."}, "time_dimension": {"answer": "Present", "rationale": ""}, "sentence_type": "Affirmative declarative sentence", "pos": {"nouns": {"parents": 1, "I": 2, "Problem": 1, "I'm worried": 2, "Fear is exaggerated, it's excessive": 1}, "verbs": {"I think": 1, "Something could happen": 1}, "adjectives": {"Exaggerating": 1, "It's too much": 1}, "numerals": {"59": 1}}}, {"content": "60. I feel a warm, sweet and intimate feeling of love with parents.", "word_count": 21, "label": "", "sentence_structure": "Subject鈥揷opula鈥損redicative", "subjective_objective": {"answer": "Subjective", "rationale": "I think it's subjective to look at the subjective feelings of the patient."}, "sentence_sentiment": {"answer": "Positive", "rationale": "Because the words \"hom, sweet and intimate\" in the entries express positive emotional attitudes."}, "language_style": {"answer": "Tactful and warm", "rationale": "The entry uses a more moderate language describing the relationship between the person under investigation and his parents, and uses positive terms such as \"homic\", \"thank you\" and \"homics\" in an attempt to create a positive and warm emotional atmosphere. Moreover, the expression \"I think\" also shows that it is a subjective assessment, rather than an objective statement, which further strengthens his euphemism."}, "time_dimension": {"answer": "Present", "rationale": "The entry describes the person who is being tested for the present feeling of parental relations, and therefore the judgement is \"now\"."}, "sentence_type": "Affirmative declarative sentence", "pos": {"nouns": {"Questionnaire Entry": 1, "Feel": 1, "parents": 1}, "verbs": {"I think": 1, "Existence": 1, "Warm": 1, "Kind": 1, "Intimacy": 1}, "numerals": {"60": 1}}}, {"content": "61. Parents can tolerate my different views.", "word_count": 15, "label": "", "sentence_structure": "Subject鈥搗erb鈥揷omplement", "subjective_objective": {"answer": "Subjective", "rationale": "Because the phenomenon of \"parents tolerate that I have different views with them\" is a subjective, rather than an objective, perception of the patient. This is mainly about his self-evaluation and understanding of family relationships."}, "sentence_sentiment": {"answer": "Neutral", "rationale": "Because the entry does not have any emotional color."}, "language_style": {"answer": "Tactful and warm", "rationale": "The language style of the entry is more euphemistic, and dialogue is conducted in a question-and-answer tone, rather than in direct statements or orders. By using the terms \"forlerable\" and \"different views\" it creates a positive, understanding and supportive atmosphere. This expression is more personal and interactive, helping respondents to be more willing to participate in the questionnaire and provide real answers."}, "time_dimension": {"answer": "Present", "rationale": ""}, "sentence_type": "Affirmative declarative sentence", "pos": {"nouns": {"parents": 1, "I can live with it": 1, "Different views": 1}, "verbs": {"It's okay": 1}, "numerals": {"61": 1}}}, {"content": "Parents often get mad at me without knowing why.", "word_count": 21, "label": "", "sentence_structure": "Subject鈥搗erb鈥搊bject", "subjective_objective": {"answer": "Objective", "rationale": "Because the words \"often\" and \"unknown reasons\" indicate that this is an objective situation, looking at the behaviour and attitude of parents rather than at the subjective feelings or thoughts of patients."}, "sentence_sentiment": {"answer": "Negative", "rationale": "Because the \"dry\" act in the entry is negative and a negative way of educating the child."}, "language_style": {"answer": "Calm and direct", "rationale": "The entry directly describes the situation of the person under investigation, in which parents often have a temper with him without knowing why. The tone is more objective than the tone of the speech, without using excessive speech techniques or emotional rendering."}, "time_dimension": {"answer": "Past", "rationale": "It can be inferred from the keywords \"often\" and \"without knowing the reason\" that the act has occurred repeatedly and may be repeated in the past."}, "sentence_type": "Affirmative declarative sentence", "pos": {"nouns": {"parents": 1, "Situation": 1, "- Grumpy": 1}, "verbs": {"Often": 1, "Let's go": 1, "to": 1, "Yes": 1}, "adjectives": {"I don't know why": 1}, "adverbs": {"in": 1, "I": 1, "not": 1}, "numerals": {"62": 1}, "pronouns": {"they": 1}}}, {"content": "63. When I did what I felt successful, I felt that my parents were very proud of me.", "word_count": 21, "label": "", "sentence_structure": "Subject鈥搗erb鈥搊bject", "subjective_objective": {"answer": "Subjective", "rationale": "Because \"the feeling\" expresses patients' subjective views of their parents' attitudes when they succeed."}, "sentence_sentiment": {"answer": "Positive", "rationale": "Because the expression \"I feel proud of my parents\" in the entry reflects positive emotions and attitudes."}, "language_style": {"answer": "Tactful and warm", "rationale": ""}, "time_dimension": {"answer": "Present", "rationale": ""}, "sentence_type": "Affirmative declarative sentence", "pos": {"nouns": {"I": 1, "Yes": 1, "Success": 1, "parents": 2}, "verbs": {"I think": 1, "Do it": 1, "Access": 1, "Proud": 1}, "adjectives": {"very": 1}, "numerals": {"63": 1}}}, {"content": "Parents often love me more than my brothers and sisters.", "word_count": 16, "label": "", "sentence_structure": "Subject鈥搗erb鈥搊bject", "subjective_objective": {"answer": "Subjective", "rationale": "Because \"often loving me\" is a patient's self-evaluation of parental attitudes, subjectively and cognitively."}, "sentence_sentiment": {"answer": "Negative", "rationale": "As the entry relates to situations of preference or unequal treatment, it may trigger negative emotional reactions."}, "language_style": {"answer": "Calm and direct", "rationale": "The entry expresses the core of the problem in an objective and direct tone, asking if the interviewees feel that their parents are partial to themselves and their siblings. There is no excessive emotional or rhetorical approach, and more emphasis is placed on obtaining accurate information.\""}, "time_dimension": {"answer": "Present", "rationale": ""}, "sentence_type": "Affirmative declarative sentence", "pos": {"nouns": {"Questionnaire Entry": 1, "Brothers and sisters": 1, "parents": 1}, "verbs": {"Comparison": 1, "Prejudice": 1}, "numerals": {"64": 1}, "pronouns": {"I": 1}}}, {"content": "65. Sometimes even if I was wrong, parents blame their siblings for it.", "word_count": 21, "label": "", "sentence_structure": "Subject鈥搗erb鈥搊bject", "subjective_objective": {"answer": "Subjective", "rationale": "Because the description of \"fault in me\" and \"parents blameing siblings\" is based on the patient's subjective perspective."}, "sentence_sentiment": {"answer": "Negative", "rationale": "Because this entry describes a negative family environment and parent-child relationship."}, "language_style": {"answer": "Calm and direct", "rationale": "The entry is calm and straightforward, asking the respondents whether they have experienced such a mistake (i.e. when they are wrong about themselves, they are blamed by their parents for their siblings) and not using too many euphemisms or gentle language. At the same time, the article is more objective and neutral, and does not display any obvious emotional colours."}, "time_dimension": {"answer": "Past", "rationale": "The sentence describes the behaviour of parents who, in certain circumstances, have devolved responsibility to siblings and may have done so repeatedly, and can therefore be judged to be past."}, "sentence_type": "Affirmative declarative sentence", "pos": {"nouns": {"Time": 1, "Wrong": 1, "Error": 1, "I": 1, "Accountability": 1, "Ren": 1, "Blame": 1, "Home": 1, "Brother": 2, "Sister": 2, "parents": 2}, "verbs": {"in": 1, "- Put it on": 1}, "adverbs": {"Even if": 1}, "numerals": {"65": 1}, "classifiers": {"individual": 1}, "pronouns": {"Well": 1}}}, {"content": "66. Parents often hug me.", "word_count": 7, "label": "", "sentence_structure": "Subject鈥搗erb鈥揷omplement", "subjective_objective": {"answer": "Subjective", "rationale": "Because \"keep\" is the subjective feeling and experience that patients have in their answers."}, "sentence_sentiment": {"answer": "Positive", "rationale": "Because the entry \"Parents often embrace me\" expresses a positive, warm attitude and behaviour of family relationships."}, "language_style": {"answer": "Tactful and warm", "rationale": "Warm and warm."}, "time_dimension": {"answer": "Past", "rationale": ""}, "sentence_type": "Affirmative declarative sentence", "pos": {"nouns": {"parents": 1, "Hug": 1}, "verbs": {"Regular": 1, "be": 1}, "adverbs": {"very": 1}, "pronouns": {"I": 1}}}]}

{"questionnaire_title": "BECK Self-Assessment Questionnaire BDI", "questionnaire_category": "Mental health", "item_count": 52, "emotion_shift_overall": "From positive to negative", "language_style_overall": "Neutral", "items": [{"content": "I'm not depressed.", "word_count": 6, "label": "", "sentence_structure": "Subject鈥揷opula鈥損redicative", "subjective_objective": {"answer": "Subjective", "rationale": "Because \"no depression\" is the patient's subjective feeling and perception, not the objective reality."}, "sentence_sentiment": {"answer": "Neutral", "rationale": "Because the entry simply stated a fact and did not express any emotional tendencies."}, "language_style": {"answer": "Tactful and warm", "rationale": "The language style of the entry is euphemism. It does not make it clear whether the interviewee is really not depressed, but rather uses a more soft and euphemistic way of asking each other about their feelings, which makes it easier to generate resonance and facilitate communication among the interviewee."}, "time_dimension": {"answer": "Present", "rationale": ""}, "sentence_type": "Negative declarative sentence", "pos": {"verbs": {"I feel": 1, "Blue": 1}}}, {"content": "I am depressed or frustrated.", "word_count": 8, "label": "", "sentence_structure": "Subject鈥揷opula鈥損redicative", "subjective_objective": {"answer": "Subjective", "rationale": "Because \"fear\" represents the emotional experience and feelings of the patient, and it is viewed from the patient's subjective perspective."}, "sentence_sentiment": {"answer": "Negative", "rationale": "Because the entries are both \"depressed\" and \"depressed\" with negative emotional expressions."}, "language_style": {"answer": "Tactful and warm", "rationale": "The language style of the entry tends to be euphemistic, because it expresses concern and understanding of each other鈥檚 emotions in a softer tone, and does not use too much strong vocabulary or negative emotions to describe the situation. At the same time, the sentence gives the respondents sufficient space to think and answer questions, rather than forcing them to acknowledge or deny their emotional state."}, "time_dimension": {"answer": "Present", "rationale": "The keyword \"fear\" uses the current state of affairs, \"depression or depression\" describes the current state and emotional experience of the testee, and therefore judges it as now."}, "sentence_type": "Affirmative declarative sentence", "pos": {"nouns": {"Blue": 1, "Frustration": 1}, "verbs": {"I feel": 1}}}, {"content": "Three, I'm depressed all day, I can't get out of it.", "word_count": 9, "label": "", "sentence_structure": "Subject鈥搗erb鈥揷omplement", "subjective_objective": {"answer": "Subjective", "rationale": "Because \"all day\" and \"unresolved\" are patients' subjective feelings and attitudes."}, "sentence_sentiment": {"answer": "Negative", "rationale": "Because the mood of \"depression\" in the entry is negative."}, "language_style": {"answer": "Calm and direct", "rationale": "This entry describes the status of the person under investigation in a straightforward way, with a more calm and objective language style, with no excessive adoration and emotional rendering."}, "time_dimension": {"answer": "Present", "rationale": "The sentence expresses the current situation of the testee, which is \"deep all day, incomprehensible\" and therefore judges as now."}, "sentence_type": "Negative declarative sentence", "pos": {"nouns": {"Questionnaire": 1, "Entry": 1, "Blue": 1, "Get rid of it": 1}, "verbs": {"Analysis": 1, "All day long": 1, "Cannot": 1}, "adjectives": {"All day long": 1, "Blue": 1, "Cannot": 1, "Get rid of it": 1}, "classifiers": {"individual": 1}, "pronouns": {"I": 1}}}, {"content": "I'm depressed and I can't stand it.", "word_count": 11, "label": "", "sentence_structure": "Subject鈥搗erb鈥揷omplement", "subjective_objective": {"answer": "Subjective", "rationale": "Because depression is an emotional state, it describes the patient ' s subjective feelings."}, "sentence_sentiment": {"answer": "Negative", "rationale": "Because the expression \"smallly depressed, I can't stand it\" in the entry is negative."}, "language_style": {"answer": "Calm and direct", "rationale": "The language style of the entry is calm and straightforward. It raises the question in an objective and direct way, without using any rhetorical or emotional rendering, and is more flat and cold. This expression is more likely to give the respondents a clear understanding of the problem and an accurate answer."}, "time_dimension": {"answer": "Past", "rationale": ""}, "sentence_type": "Affirmative declarative sentence", "pos": {"verbs": {"I": 1, "Take it": 1, "I'm not": 1}, "adjectives": {"Tens": 1, "Blue": 1}}}, {"content": "I am not pessimistic about the future.", "word_count": 10, "label": "", "sentence_structure": "Subject鈥搗erb鈥搊bject", "subjective_objective": {"answer": "Subjective", "rationale": "Because a pessimistic attitude of disappointment about the future is a subjective feeling and perception."}, "sentence_sentiment": {"answer": "Positive", "rationale": "Because the \"no pessimism and disappointment\" approach in the entry is positive."}, "language_style": {"answer": "Tactful and warm", "rationale": ""}, "time_dimension": {"answer": "Future", "rationale": "The keyword is \"the future\" , which represents the future of the time dimension. At the same time, the sentence expresses an attitude or emotion that is not pessimistic about the future, which can also be seen as an expectation or a plan, and thus the entry can be judged as a future act or mentality."}, "sentence_type": "Affirmative declarative sentence", "pos": {"verbs": {"to": 1, "No pessimism, no disappointment": 1}, "adjectives": {"Pessimism and disappointment": 1}, "adverbs": {"and": 1}}}, {"content": "I feel less optimistic about the future.", "word_count": 9, "label": "", "sentence_structure": "Subject鈥揷opula鈥損redicative", "subjective_objective": {"answer": "Subjective", "rationale": "Because the word \"fEE\" means that it's a patient's inner feeling, and it's a subjective view."}, "sentence_sentiment": {"answer": "Negative", "rationale": "Because the \"less optimistic future\" in the entry is an expression of a negative mood and attitude."}, "language_style": {"answer": "Tactful and warm", "rationale": "Warm and warm."}, "time_dimension": {"answer": "Future", "rationale": "The entry contains expectations and views for the future, with the keyword \"unsure of the future\" pointing to situations that have not yet occurred and that may arise in the future."}, "sentence_type": "Negative declarative sentence", "pos": {"nouns": {"Future": 1, "Optimistic": 1}, "verbs": {"Sensor": 1, "not": 1, "I feel": 1, "Optimistic": 1}, "adjectives": {"Not really": 1}}}, {"content": "I feel very unsure of the future.", "word_count": 10, "label": "", "sentence_structure": "Subject鈥搗erb鈥揷omplement", "subjective_objective": {"answer": "Subjective", "rationale": "This perception is a personal experience and perception of the patient and is a subjective examination."}, "sentence_sentiment": {"answer": "Negative", "rationale": "Because the emotional expression \"unswept hope for the future\" in the entry is negative."}, "language_style": {"answer": "Calm and direct", "rationale": "The entry expresses the attitude of the other party to the future in a more direct and concise manner, without too much embellishment or rendering, and is more objective and practical."}, "time_dimension": {"answer": "Present", "rationale": ""}, "sentence_type": "Negative declarative sentence", "pos": {"nouns": {"Future": 1}, "verbs": {"I feel": 1, "No hope": 2}}}, {"content": "I feel hopeless and cannot improve.", "word_count": 16, "label": "", "sentence_structure": "Subject鈥揷opula鈥損redicative", "subjective_objective": {"answer": "Subjective", "rationale": "Because it is the subjective emotions and attitudes of the patient."}, "sentence_sentiment": {"answer": "Negative", "rationale": "Because the words \"no hope\" and \"no improvement\" in the entries are negative."}, "language_style": {"answer": "Calm and direct", "rationale": "The entry expresses in a direct manner the questions about the future pessimism of the interviewees, without adding too much adoration or emotional colour, and is relatively straightforward in language style."}, "time_dimension": {"answer": "Future", "rationale": ""}, "sentence_type": "Negative declarative sentence", "pos": {"nouns": {"Hope": 1, "Better": 1}, "verbs": {"I feel": 1, "Maybe": 1, "have": 1, "Come on": 1}, "adjectives": {"Nothing": 1, "Future": 1}, "numerals": {"4": 1}}}, {"content": "I don't feel like losing.", "word_count": 8, "label": "", "sentence_structure": "Subject鈥揷opula鈥損redicative", "subjective_objective": {"answer": "Subjective", "rationale": "Because \"feel\" is usually a subjective experience and attitude of the individual, not an objective fact or situation."}, "sentence_sentiment": {"answer": "Neutral", "rationale": "Because the entry does not have any emotional color."}, "language_style": {"answer": "Tactful and warm", "rationale": "The language style of the entry is euphemistic, avoiding a direct expression of whether or not you have ever been a failure by using the negative word \"not\" and expressing a mild concern about your emotional state. This expression is more personal and interactive, and it is more likely to elicit positive responses and sharing from the interviewees."}, "time_dimension": {"answer": "Present", "rationale": "In this entry, the \"no sense of failure\" status description refers to the current state of feeling or emotion, and therefore the judgement is now."}, "sentence_type": "Negative declarative sentence", "pos": {"verbs": {"None": 1, "Feel": 1}, "adjectives": {"and": 1, "Loser": 1}, "adverbs": {"None": 1}, "pronouns": {"I": 1}}}, {"content": "I feel like I'm failing in comparison to most people.", "word_count": 15, "label": "", "sentence_structure": "Subject鈥揷opula鈥損redicative", "subjective_objective": {"answer": "Subjective", "rationale": "The first of these is the fact that the first time the first time the first time the first time the first time the first time the first time the first time the first time the first time the first time the first time the first time the first time the first time the first time the first time the first time the first time the first time, the second time the first time the first time the first time the first time, the second time the first time the first time the first time, the next time the first time the first time the first time, the next time the first time the first time, the next time the first time the first time, the next time the first time, the next day, the next day, the next day, the next day, the next day, the next day, the next day, the next day, the next day, the next day, the next day, the first, the next, the next."}, "sentence_sentiment": {"answer": "Negative", "rationale": "Because the expression \"thinks that you're failing\" in the entry is negative."}, "language_style": {"answer": "Calm and direct", "rationale": "The language style of the entry is more straightforward, with no use of overly reverbative terms or rhetorical techniques, and a more objective and calm tone, expressing a view of self-evaluation."}, "time_dimension": {"answer": "Present", "rationale": "The \"Feel\" in the entry indicates that it's the current feeling or perception of the testee, and that I'm a failure compared to most people. It's also an perception of the state. So the time dimension of the entry should be judged as now."}, "sentence_type": "Negative declarative sentence", "pos": {"nouns": {"person": 1, "Most people": 1, "Failed": 1}, "verbs": {"I think": 1, "be": 1, "- Yeah": 1, "Compare": 1}, "adjectives": {"Most of them": 1}, "numerals": {"2": 1}, "pronouns": {"I": 1}}}, {"content": "I think it was a series of failures.", "word_count": 17, "label": "", "sentence_structure": "Subject鈥揷opula鈥損redicative", "subjective_objective": {"answer": "Subjective", "rationale": "This is because of the patient ' s personal feelings and views and is subjective."}, "sentence_sentiment": {"answer": "Negative", "rationale": "Because the description of \"a series of failures\" is negative emotional attitudes, it is negative."}, "language_style": {"answer": "Calm and direct", "rationale": "The language style of the entry is more calm and straightforward, with no excessive adoration or emotional colour, and more focused on the objective description of the facts, which gives a sense of the interest and respect of the person being questioned.\""}, "time_dimension": {"answer": "Past", "rationale": "The words \"recall\" and \"life\" and \"a chain of failures\" in the entry all indicate thinking and memories of the past, and therefore judge it as the past."}, "sentence_type": "Affirmative declarative sentence", "pos": {"nouns": {"Fan": 1, "Failed": 2}, "verbs": {"I think": 1, "be": 1, "A series of them": 1, "Recalling": 1}, "adjectives": {"Mine": 1, "Life": 1, "of": 3}, "adverbs": {"Well": 1, "In the middle of the review": 1}, "numerals": {"3": 1}, "pronouns": {"I": 1}}}, {"content": "I feel like a loser.", "word_count": 11, "label": "", "sentence_structure": "Subject鈥揷opula鈥損redicative", "subjective_objective": {"answer": "Subjective", "rationale": "Because \"think\" is a subjective term that describes patient's subjective assessment and perception of themselves."}, "sentence_sentiment": {"answer": "Negative", "rationale": "Because the expression \"failed person\" in the entry is negative, implying low self-evaluation and lack of self-confidence."}, "language_style": {"answer": "Tactful and warm", "rationale": "The entry is more euphemistic and sensitive. It does not directly negate or attack the views of the interviewee, but rather asks him in a gentle and understandable way about his feelings and tries to guide him to think positively. This expression is more likely to make him feel respected and understood, and thus more open to the questionnaire."}, "time_dimension": {"answer": "Present", "rationale": "The entry indicates the current feelings and self-perception of the testee, which is, \"I think I'm a loser.\" So the judgment is based on now."}, "sentence_type": "Negative declarative sentence", "pos": {"nouns": {"I": 1, "person": 1, "Failed": 1}, "verbs": {"I think": 1, "be": 1}, "adjectives": {"One": 2}, "numerals": {"4": 1}}}, {"content": "I don't feel any dissatisfaction.", "word_count": 11, "label": "", "sentence_structure": "Subject鈥損redicate", "subjective_objective": {"answer": "Subjective", "rationale": "Because it's the patient's subjective feeling and perception."}, "sentence_sentiment": {"answer": "Neutral", "rationale": "Because the entry does not have any emotional color."}, "language_style": {"answer": "Calm and direct", "rationale": "The entry expresses the interviewee's satisfaction with his or her life in a straightforward and clear manner. The negative word \"does not feel\" emphasizes the degree of dissatisfaction, and does not use euphemism or vague expression, which is more objective and neutral."}, "time_dimension": {"answer": "Present", "rationale": "The entry is for the current feelings and views of the testee, and the time dimension is now judged."}, "sentence_type": "Negative declarative sentence", "pos": {"verbs": {"I think": 1, "Satisfied": 1}, "adjectives": {"not": 1}, "adverbs": {"and": 1}, "pronouns": {"I": 1}}}, {"content": "2. I feel like I can't enjoy life as usual.", "word_count": 15, "label": "", "sentence_structure": "Subject鈥搗erb鈥揷omplement", "subjective_objective": {"answer": "Subjective", "rationale": "Because it is the patient ' s subjective feeling and assessment that does not enjoy life as usual."}, "sentence_sentiment": {"answer": "Negative", "rationale": "Because the expression \"not to enjoy life as usual\" in the entry has a negative mood."}, "language_style": {"answer": "Tactful and warm", "rationale": "The entry describes the interviewee ' s own feelings in terms of first person ' s perspective, and uses moderate and cogent language, without too much criticism or criticism, and can therefore be considered as euphemism."}, "time_dimension": {"answer": "Present", "rationale": ""}, "sentence_type": "Negative declarative sentence", "pos": {"nouns": {"I": 1, "Life": 1}, "verbs": {"I think": 1, "No, I can't": 1, "Yeah": 1, "Enjoy": 1}, "adjectives": {"Normal": 1}, "adverbs": {"That way": 1}}}, {"content": "3. Nothing can satisfy me.", "word_count": 15, "label": "", "sentence_structure": "Subject鈥搗erb鈥揷omplement", "subjective_objective": {"answer": "Subjective", "rationale": "Because \"not to be satisfied\" is a description of a subjective feeling that reflects patients' perceptions and feelings about something, not objective facts."}, "sentence_sentiment": {"answer": "Negative", "rationale": "Because the expression \"can't make me happy\" is negative."}, "language_style": {"answer": "Calm and direct", "rationale": "The language style of the entry is calm and straightforward. It clearly expresses the question whether the other party is not satisfied with the subject under any circumstances, without too much emotional rendering or rhetorical, and with a more objective and straightforward tone."}, "time_dimension": {"answer": "Future", "rationale": "The keyword \"I cannot satisfy\" expresses expectations and aspirations for the future, and the entry therefore points to future circumstances that have not yet occurred."}, "sentence_type": "Negative declarative sentence", "pos": {"verbs": {"鈾?And I'm gonna be 鈾?: 1, "I feel": 1, "Satisfied": 1, "Some": 1}, "adjectives": {"Any": 1}}}, {"content": "I'm not satisfied with everything.", "word_count": 11, "label": "", "sentence_structure": "Subject鈥揷opula鈥損redicative", "subjective_objective": {"answer": "Subjective", "rationale": "Because it is the patient ' s own subjective feelings and attitudes that are satisfied with things, it is a subjective emotional reaction."}, "sentence_sentiment": {"answer": "Negative", "rationale": "For the purpose of the article, a negative attitude was expressed."}, "language_style": {"answer": "Calm and direct", "rationale": "The language style of the entry is calm and straightforward, because it clearly expresses the dissatisfied attitude of the speaker to everything, without using euphemism or emotional accoutrements. This expression is straightforward and obvious."}, "time_dimension": {"answer": "Present", "rationale": "The keyword \"I am not satisfied with everything\" expresses the individual's current subjective feelings and attitudes, so the temporal dimension of the article is now judged."}, "sentence_type": "Negative declarative sentence", "pos": {"verbs": {"to": 1, "Unsatisfactory": 1}, "adjectives": {"All": 1, "Things": 1}, "adverbs": {"Both": 1}}}, {"content": "I don't feel guilty.", "word_count": 9, "label": "", "sentence_structure": "Subject鈥損redicate", "subjective_objective": {"answer": "Subjective", "rationale": "Because guilt is a subjective emotional experience, the answer is a description of the patient ' s subjective feelings."}, "sentence_sentiment": {"answer": "Neutral", "rationale": "Because the entry does not have any emotional color."}, "language_style": {"answer": "Tactful and warm", "rationale": "Warm and warm."}, "time_dimension": {"answer": "Past", "rationale": "The entry describes the inner state of the person being tested, in which \"without a special sense of guilt\" is a reference to past behaviour or mentalities and is therefore judged as past."}, "sentence_type": "Negative declarative sentence", "pos": {"verbs": {"have": 1, "Special": 1, "Guilt": 1}, "adjectives": {"No, I'm not": 1}}}, {"content": "2. I sometimes feel guilty or feel worthless.", "word_count": 16, "label": "", "sentence_structure": "Subject鈥揷opula鈥損redicative", "subjective_objective": {"answer": "Subjective", "rationale": "Because guilt and self-evaluation are the feelings and thoughts of the patients, and are subjective in nature."}, "sentence_sentiment": {"answer": "Negative", "rationale": "Because the attitude of \"guilty or worthless\" in the entry is negative."}, "language_style": {"answer": "Tactful and warm", "rationale": ""}, "time_dimension": {"answer": "Present", "rationale": ""}, "sentence_type": "Affirmative declarative sentence", "pos": {"nouns": {"Guilt": 1, "Value": 1}, "verbs": {"I feel": 1, "have": 1}, "adverbs": {"Sometimes": 1}, "pronouns": {"I": 1}}}, {"content": "Three, I feel very guilty.", "word_count": 7, "label": "", "sentence_structure": "Subject鈥揷opula鈥損redicative", "subjective_objective": {"answer": "Subjective", "rationale": "Because \"fear\" is a subjective word, which is a personal feeling and emotion, and \"guilty\" is a subjective feeling. So, \"I feel very guilty\" is a patient's subjective idea and emotional experience."}, "sentence_sentiment": {"answer": "Negative", "rationale": "Because guilt is a negative emotion."}, "language_style": {"answer": "Calm and direct", "rationale": "The entry directly expresses the subject of asking the other party whether it feels very guilty, in a concise manner, without using excessive rhetorical or emotional rendering, and with a more balanced and objective tone. At the same time, \"3\" and \"I feel very guilty.\" The expression is very direct and clear, and the interviewee is not being directed to answer questions in a euphemism or indirect way, so I think the language style of the questionnaire is straightforward and straightforward."}, "time_dimension": {"answer": "Present", "rationale": "Based on the word \"fEE\" in the sentence, it can be inferred that the entry is describing the person's current feelings and mental state. So, the temporal dimension is now."}, "sentence_type": "Affirmative declarative sentence", "pos": {"verbs": {"I feel": 1, "Guilt": 1}, "adjectives": {"Very": 1}}}, {"content": "I feel very bad. I'm worthless.", "word_count": 12, "label": "", "sentence_structure": "Subject鈥搗erb鈥揷omplement", "subjective_objective": {"answer": "Subjective", "rationale": "I think it's very bad, and it's not worth a penny that's subjective."}, "sentence_sentiment": {"answer": "Negative", "rationale": "Because the expression \"self is bad, nothing\" in the entry is a negative sentiment and attitude that negates self and demeans itself."}, "language_style": {"answer": "Tactful and warm", "rationale": "The entry allows respondents to share their feelings through self-evaluation and uses relatively decomposition terms such as \"thinking themselves very bad\" and \"no money\" that indicate that the language style of the article is euphemism and warmness. At the same time, this euphemism also helps encourage respondents to share their real thoughts and feelings more openly."}, "time_dimension": {"answer": "Present", "rationale": "The entry expresses the current self-evaluation and mindset of the testees, the keywords being \"felt\" and \"very\" and they all indicate the feelings and attitudes at this moment."}, "sentence_type": "Negative declarative sentence", "pos": {"nouns": {"Questionnaire Entry": 1, "self": 1}, "verbs": {"I think": 1, "Very": 1, "Bad": 1, "It's not worth a penny": 1}, "adjectives": {"Bad": 1, "Very": 1}, "adverbs": {"not": 1}, "numerals": {"4": 1}, "pronouns": {"self": 1}}}, {"content": "I am not disappointed in myself.", "word_count": 10, "label": "", "sentence_structure": "Subject鈥搗erb鈥揷omplement", "subjective_objective": {"answer": "Subjective", "rationale": "Because it is a subjective feeling to be disappointed with one ' s own, it is the patient ' s own judgement."}, "sentence_sentiment": {"answer": "Positive", "rationale": "Because \"no disappointment\" in the entry shows an individual's positive attitude and good self-awareness."}, "language_style": {"answer": "Tactful and warm", "rationale": "The expression of the title in a gentle and encouraging manner, which expresses the recognition and support of the interviewees, is more positive and welcoming than the use of critical or negative language."}, "time_dimension": {"answer": "Past", "rationale": ""}, "sentence_type": "Negative declarative sentence", "pos": {"verbs": {"I feel": 1, "Disappointment": 1}, "adjectives": {"self": 1}, "pronouns": {"I": 1}}}, {"content": "2. I am disappointed in myself.", "word_count": 8, "label": "", "sentence_structure": "Subject鈥搗erb鈥揷omplement", "subjective_objective": {"answer": "Subjective", "rationale": "My disappointment is a subjective feeling."}, "sentence_sentiment": {"answer": "Negative", "rationale": "Because the entry \"disappointment\" expresses a negative emotional attitude."}, "language_style": {"answer": "Tactful and warm", "rationale": "The entry uses a more euphemistic language style, which guides the respondents to answer questions by asking questions, without direct statements or orders, and is more accommodative and acceptable. At the same time, the subject of \"I am disappointed with myself\" is itself relatively sensitive and personal, and the use of euphemisms is a better way to protect the privacy and dignity of the respondents."}, "time_dimension": {"answer": "Present", "rationale": "The entry expresses the feelings and mentality of the testee at the moment, which is \"disappointed with himself\", and the time dimension is now judged."}, "sentence_type": "Negative declarative sentence", "pos": {"verbs": {"I feel": 1, "Disappointment": 1}, "adjectives": {"self": 1}}}, {"content": "I hate myself.", "word_count": 5, "label": "", "sentence_structure": "Subject鈥搗erb鈥揷omplement", "subjective_objective": {"answer": "Subjective", "rationale": "Because \"hatting yourself\" is a subjective feeling or emotional expression."}, "sentence_sentiment": {"answer": "Negative", "rationale": "Because the entry \"Damn myself\" expresses a negative emotional attitude."}, "language_style": {"answer": "Tactful and warm", "rationale": "Instead of using a negative term directly, the entry is a more euphemistic way of asking the other side whether it is dissatisfied or repulsed with certain aspects of its own, which is relatively more moderate and acceptable."}, "time_dimension": {"answer": "Present", "rationale": "The description of \"disguised oneself\" is a personal feeling and mentality, does not relate to a particular situation or behaviour, past or future, and therefore falls within the current temporal dimension."}, "sentence_type": "Negative declarative sentence", "pos": {"nouns": {"Questionnaire": 1, "Entry": 1, "self": 1}, "verbs": {"Shit": 1, "Give": 1}, "numerals": {"3": 1}, "pronouns": {"I": 1}}}, {"content": "Four, I hate myself.", "word_count": 5, "label": "", "sentence_structure": "Subject鈥搗erb鈥揷omplement", "subjective_objective": {"answer": "Subjective", "rationale": "Because \"hate\" is a negative emotional experience, a subjective assessment and attitude of patients towards themselves."}, "sentence_sentiment": {"answer": "Negative", "rationale": "Because \"hate yourself\" in the entry is negative emotion and attitude."}, "language_style": {"answer": "Calm and direct", "rationale": "The language style of the entry is calm, because it clearly expresses the subject of asking the other party whether it is repulsive or disaffected, without excessive rhetorical or emotional recitation. Moreover, the purpose of the article is more objective and not overly emotional, and therefore it can be considered as a direct type of calm."}, "time_dimension": {"answer": "Present", "rationale": "The entry asks the testee about his or her feelings about himself, and the time dimension is now judged. The keyword \"I hate\" expresses a strong negative emotional and self-denial attitude, which may indicate that the testee has some psychological problems or problems and requires further attention and evaluation."}, "sentence_type": "Negative declarative sentence", "pos": {"verbs": {"Hate": 1, "self": 1}}}, {"content": "I didn't mean to hurt myself.", "word_count": 11, "label": "", "sentence_structure": "Subject鈥搗erb鈥搊bject", "subjective_objective": {"answer": "Subjective", "rationale": "Because \"no attempt to hurt yourself\" is a subjective feeling and an idea inside."}, "sentence_sentiment": {"answer": "Neutral", "rationale": "Because the entry does not express any emotional colour, it merely presents a fact."}, "language_style": {"answer": "Calm and direct", "rationale": "The entry is simple and not too simplistic or emotional, and directs the respondents to the questions, which is a calm and direct language style."}, "time_dimension": {"answer": "Present", "rationale": "The keyword \"no\" represents a denial of the current state, while the idea of \"intending to harm yourself\" is an act or state that occurs at the present time, so the entry asks about the current state of the person being tested when completing the questionnaire."}, "sentence_type": "Negative declarative sentence", "pos": {"nouns": {"Thoughts": 1, "self": 1}, "verbs": {"No, I'm not": 1, "To hurt": 1}, "pronouns": {"I": 1}}}, {"content": "I feel better dead.", "word_count": 9, "label": "", "sentence_structure": "Subject鈥揷opula鈥損redicative", "subjective_objective": {"answer": "Subjective", "rationale": "Because it is an expression of subjective feelings and ideas. Although this may not be practical, it reflects the emotional and psychological state of the patient."}, "sentence_sentiment": {"answer": "Negative", "rationale": "Because the entry \"Death\" expresses a negative attitude and behavior."}, "language_style": {"answer": "Calm and direct", "rationale": "The entry directly expresses the subjective feelings of the subject of the inquiry about death in a concise language, without adding too much adoration or emotional colour, and is expressed in a more calm and objective manner.\""}, "time_dimension": {"answer": "Present", "rationale": "In the future, the entry indicates that the testee has a suicidal mind and idea, which is a description of the possible behaviour in the future and therefore is judged to be the future."}, "sentence_type": "Negative declarative sentence", "pos": {"verbs": {"I feel": 1, "Die": 1, "Okay": 1}, "adverbs": {"Or is it": 1}}}, {"content": "I've thought about suicide.", "word_count": 6, "label": "", "sentence_structure": "Subject鈥搗erb鈥搊bject", "subjective_objective": {"answer": "Subjective", "rationale": "Because \"suicides\" are a process of inner activity and thinking, a subjective attitude and emotional experience of the patient."}, "sentence_sentiment": {"answer": "Negative", "rationale": "Because the act of \"consider suicide\" in the entry is negative."}, "language_style": {"answer": "Calm and direct", "rationale": "The entry directs the respondents to the questions, without too much ado and emotional rendering, and is more objective and calm. Such expression avoids unnecessary pressure or misleading the respondents, while helping researchers to understand their psychological state more accurately."}, "time_dimension": {"answer": "Past", "rationale": "In the entry, the \"considered\" pattern is that it was completed in the past, indicating that it had been thought of at some point or time in the past, but not necessarily continued at the present time. The temporal dimension of the article is therefore the past."}, "sentence_type": "Affirmative declarative sentence", "pos": {"nouns": {"Suicide": 1}, "verbs": {"Consider": 1, "Pass": 1}, "numerals": {"III": 1}}}, {"content": "4 I'd kill myself if I had the chance.", "word_count": 12, "label": "", "sentence_structure": "Subject鈥損redicate", "subjective_objective": {"answer": "Subjective", "rationale": "This is because of the patient ' s subjective will and attitude, which expresses the patient ' s concern and impulse to commit suicide."}, "sentence_sentiment": {"answer": "Negative", "rationale": "Because killing yourself is negative."}, "language_style": {"answer": "Tactful and warm", "rationale": "The entry is based on a more direct and strong expression of the idea of whether the other party will commit suicide, which is more direct, and which does not use too much acrimony or consolation, and can therefore be classified as a \"cool straight\" language style."}, "time_dimension": {"answer": "Future", "rationale": "The time dimension is therefore judged to be the future. At the same time, the sentence expresses strong negative sentiments and attitudes and recommends that the questionnaire be designed with caution and that necessary psychological support and assistance be provided."}, "sentence_type": "Affirmative declarative sentence", "pos": {"verbs": {"have": 1, "Kill": 2}, "numerals": {"4": 1}, "pronouns": {"self": 1}}}, {"content": "I'm not losing my interest in seeing others.", "word_count": 12, "label": "", "sentence_structure": "Subject鈥損redicate", "subjective_objective": {"answer": "Subjective", "rationale": "Because loss of interest is a subjective feeling and attitude, not an objective fact."}, "sentence_sentiment": {"answer": "Positive", "rationale": "Because \"not losing interest in engaging with others\" expresses a desire to remain positive in social life."}, "language_style": {"answer": "Tactful and warm", "rationale": "The language style of the entry is more euphemistic and warm. By using the negative language, I express that I have not lost interest in people, and that expression is more friendly and friendly, and more easily gives the respondents the interest and understanding of the survey researchers. Moreover, the whole sentence is more ablaze and positive, giving them a good impression."}, "time_dimension": {"answer": "Present", "rationale": "The entry describes the current behaviour or state of the respondent and can therefore be judged as \"now\"."}, "sentence_type": "Affirmative declarative sentence", "pos": {"nouns": {"I": 1, "Interest": 1, "- To be with someone": 1, "Others": 1}, "verbs": {"I haven't lost it": 1, "Lose": 1, "- To be with someone": 1}, "adverbs": {"Nope": 1, "and": 1}}}, {"content": "2. I have a diminished interest in dealing with others in peace.", "word_count": 18, "label": "", "sentence_structure": "Subject鈥搗erb鈥揷omplement", "subjective_objective": {"answer": "Subjective", "rationale": "Because the decline in interest is a personal feeling and a subjective attitude of the patient."}, "sentence_sentiment": {"answer": "Negative", "rationale": "Because the description \"the interest in dating has diminished\" in the entry is negative."}, "language_style": {"answer": "Tactful and warm", "rationale": "The entry uses a mild, invitation-style expression to ask if the respondents' interest in dealing with people has diminished, and this expression is more personal and interactive. Moreover, the description of \"peacetime is more euphemism than\" avoids directly pointing out that the other side has problems and is more conducive to the trust and cooperation of the interviewees."}, "time_dimension": {"answer": "Present", "rationale": ""}, "sentence_type": "Affirmative declarative sentence", "pos": {"nouns": {"Peacetime comparison": 1, "I": 1, "Others": 1, "- To be with someone": 1, "Interest": 1}, "verbs": {"Yes": 1, "Declining": 1}, "adverbs": {"Normal": 1, "Yes": 1}}}, {"content": "I have lost most of my interest in people, and I have no feelings for them.", "word_count": 22, "label": "", "sentence_structure": "Subject鈥搗erb鈥搊bject", "subjective_objective": {"answer": "Subjective", "rationale": "Because interest in people ' s interaction and feelings belong to the patient ' s personal feelings and attitudes, is subjective."}, "sentence_sentiment": {"answer": "Negative", "rationale": "Because the entries \"lost most of the interest in people\" and \"no feelings for people\" have negative emotional aspects."}, "language_style": {"answer": "Calm and direct", "rationale": "It clearly expresses the fact that the interviewees have lost interest in dealing with people and have no emotional interest in the subject."}, "time_dimension": {"answer": "Past", "rationale": "The entry contains \"lost\" and \"no\", which indicates that this is a description of the emotional state over time and is a temporal dimension of the past."}, "sentence_type": "Negative declarative sentence", "pos": {"nouns": {"person": 2, "Interest": 1, "- To be with someone": 1, "Emotion": 1}, "verbs": {"Lose": 1, "have": 1}, "adjectives": {"Most of them": 1}, "adverbs": {"Already": 1, "No, I'm not": 1}, "numerals": {"3": 1}, "pronouns": {"I": 1}}}, {"content": "I'm not interested in anyone else, and I'm not paying attention to anyone.", "word_count": 16, "label": "", "sentence_structure": "Subject鈥損redicate", "subjective_objective": {"answer": "Subjective", "rationale": "Because the idea that I'm not interested in anyone else, and I'm not paying attention to anyone else, is the patient's own subjective feeling."}, "sentence_sentiment": {"answer": "Negative", "rationale": "Because the entry \"no interest in or disregard for others\" is negative."}, "language_style": {"answer": "Calm and direct", "rationale": "The entry is presented in a straightforward manner, without any rhetorical or emotional colours, and in a calm and objective manner."}, "time_dimension": {"answer": "Present", "rationale": "The words \"I have no interest in other people\" and \"not to pay attention to others\" describe current behaviour and attitudes and therefore judge them as now."}, "sentence_type": "Negative declarative sentence", "pos": {"verbs": {"to": 1, "have": 1, "Nothing": 1, "Interest": 1, "I don't care": 2}, "adjectives": {"- He's a guy": 1, "Totally": 1}, "pronouns": {"I": 1, "others": 1}}}, {"content": "I can make decisions as usual.", "word_count": 11, "label": "", "sentence_structure": "Subject鈥搗erb鈥搊bject", "subjective_objective": {"answer": "Subjective", "rationale": "Because the entry asks about the patient ' s opinion as to whether he or she can make a decision, it is the patient ' s subjective feeling and evaluation."}, "sentence_sentiment": {"answer": "Neutral", "rationale": "The entry is not a positive or negative emotional expression."}, "language_style": {"answer": "Tactful and warm", "rationale": "This article is balanced, natural, without obvious negative emotions or a strong sense of subjectivity. It uses relatively objective and neutral language to describe individual capacity to act, without criticizing or praising anyone or anything. This expression gives people a sense of affection, friendship and trust, which is appropriate for use in, for example, questionnaires."}, "time_dimension": {"answer": "Present", "rationale": "The entry indicates that the testee is describing his current situation, and the word \"capable\" follows the general current, meaning that this is an assessment of current capacity."}, "sentence_type": "Affirmative declarative sentence", "pos": {"nouns": {"Decision": 1, "Normal": 1}, "verbs": {"Make": 1}, "adverbs": {"Same thing": 1}, "pronouns": {"I": 1}}}, {"content": "I tried to avoid making decisions.", "word_count": 8, "label": "", "sentence_structure": "Subject鈥損redicate", "subjective_objective": {"answer": "Subjective", "rationale": "Because trying to avoid making decisions is a subjective attitude or behaviour."}, "sentence_sentiment": {"answer": "Negative", "rationale": "Because the purpose of the \"tried to avoid making a decision\" in the entry is negative."}, "language_style": {"answer": "Calm and direct", "rationale": "The language style of the entry is relatively calm and objective, and there is no excessive rhetorical or emotional rendering, directly expressing the subject of questioning the other party about the behaviour of avoiding a decision."}, "time_dimension": {"answer": "Present", "rationale": "\"I try\" means \"a try\" means \"a move in progress, \"avoid decision\" means the state or result of the act. So, the temporal dimension of this entry is now judged."}, "sentence_type": "Negative declarative sentence", "pos": {"verbs": {"Try": 1, "Do it": 1, "Decision": 1}, "adverbs": {"Try": 1}}}, {"content": "3. For me, it is very difficult to make a decision.", "word_count": 12, "label": "", "sentence_structure": "Subject鈥搗erb鈥揷omplement", "subjective_objective": {"answer": "Subjective", "rationale": "This is a personal feeling and perception of the patient and is a subjective examination."}, "sentence_sentiment": {"answer": "Negative", "rationale": "Because the entry expresses problems and dilemmas of the individual in the decision-making process, it is a negative sentiment."}, "language_style": {"answer": "Calm and direct", "rationale": "The entry is a simple and clear expression of the subject - \"The decision is difficult\" - without too much emotional acquis or euphemism. It is more objective and direct, it is more guiding and it is hoped that the person under investigation will be able to answer in good faith."}, "time_dimension": {"answer": "Present", "rationale": "Now the entry expresses the feelings and thoughts of the testees that it is difficult for him to make a decision in the current state. So the time dimension is now."}, "sentence_type": "Negative declarative sentence", "pos": {"nouns": {"Decision": 1}, "verbs": {"Do it": 1, "be": 1, "Tens": 1, "Difficulties": 1}, "adverbs": {"to": 1, "I'll tell you what": 1}, "pronouns": {"I": 1}}}, {"content": "I can't make any decisions.", "word_count": 9, "label": "", "sentence_structure": "Subject鈥搗erb鈥揷omplement", "subjective_objective": {"answer": "Subjective", "rationale": "For whether or not a decision can be made is a subjective judgement and a manifestation of competence of a person, not just an objective fact."}, "sentence_sentiment": {"answer": "Negative", "rationale": "Because \"no decision\" is a negative emotion and attitude."}, "language_style": {"answer": "Tactful and warm", "rationale": "The language style of the entry tends to be euphemistic. It uses the term \"unable\" to express the degree of difficulty of decision-making, rather than directly stating that it cannot make decisions, thus avoiding pressure and negative emotions, but also conveying its own confusion and need for help. Moreover, \"any decision\" implies the seriousness and urgency of the problem, but does not overemphasize or threaten the other."}, "time_dimension": {"answer": "Present", "rationale": "The entry asks the testee whether, in the current situation, he is able to make a decision without clear time reference and can therefore be considered as the present issue."}, "sentence_type": "Negative declarative sentence", "pos": {"verbs": {"Do it": 1, "Decision": 1}, "adverbs": {"Cannot": 1}, "pronouns": {"I": 1}}}, {"content": "I think I'm no worse than I was before.", "word_count": 14, "label": "", "sentence_structure": "Subject鈥揷opula鈥損redicative", "subjective_objective": {"answer": "Subjective", "rationale": "Because \"like\" is the patient's personal opinion and subjective assessment."}, "sentence_sentiment": {"answer": "Neutral", "rationale": "Because the entry simply stated a fact and did not express any emotional tendencies."}, "language_style": {"answer": "Tactful and warm", "rationale": "The entry is self-evaluation in a self-expressional manner, without direct comparisons or comments from others, and is more accommodative. It is also expressed in a more active and confident manner, with some positive energy. The language style of the questionnaire can therefore be considered as euphemism."}, "time_dimension": {"answer": "Present", "rationale": "It is possible to determine from the sentence that the entry describes the current perception of the testee of his or her self-image. So, the temporal dimension is now."}, "sentence_type": "Affirmative declarative sentence", "pos": {"verbs": {"I think": 1, "Shit": 1}, "adjectives": {"Not at all": 1}, "pronouns": {"I": 1, "Past": 1}}}, {"content": "I'm afraid I look old and not attractive.", "word_count": 14, "label": "", "sentence_structure": "Subject鈥搗erb鈥搊bject", "subjective_objective": {"answer": "Subjective", "rationale": "Because fear is the patient ' s subjective emotional experience, it is the patient ' s assessment of his or her self-image and appearance."}, "sentence_sentiment": {"answer": "Negative", "rationale": "Because the \"I'm afraid I'm getting old and not attracted\" attitude in the entry is negative."}, "language_style": {"answer": "Tactful and warm", "rationale": "According to the context, \"I'm afraid I look old and not attracted to\" this question does not directly use strong or negative terms to express fears and self-demeaning feelings. Instead, it is through a gentle, inviting tone that talks, which makes it easier for the respondents to share their real thoughts and feelings."}, "time_dimension": {"answer": "Present", "rationale": "The phrase \"I\" is the main word, which indicates the person who speaks, the person who speaks, the person who fears, the person who is worried, the person who is not, \"I look old, I don't attract\" is the specific thing that expresses the fear. So, the entry asks about the person who is being tested for behavior and mentalities."}, "sentence_type": "Negative declarative sentence", "pos": {"nouns": {"I": 2, "I'm worried": 1, "Looks like": 1, "Old": 1, "Attractive": 1}, "verbs": {"be": 1, "Attractive": 1, "I'm worried": 1, "Looks like": 1}, "adjectives": {"Old": 1, "Attractive": 1}, "adverbs": {"not": 1, "Yes": 2}, "numerals": {"2": 1}, "pronouns": {"I": 2}}}, {"content": "I think I must have changed my appearance, not to be attractive.", "word_count": 18, "label": "", "sentence_structure": "Subject鈥揷opula鈥損redicative", "subjective_objective": {"answer": "Subjective", "rationale": "Because it's my subjective feeling."}, "sentence_sentiment": {"answer": "Negative", "rationale": "Because the expression in the entry \"the appearance must have changed, become unattractive\" has a negative emotional color."}, "language_style": {"answer": "Tactful and warm", "rationale": "The entry leads to self-reflection by describing itself and expresses a more negative emotional tone in terms such as \"spect\" and \"unattractive.\" This language style is more euphemism and a certain comfort, helping to reduce anxiety and anxiety."}, "time_dimension": {"answer": "Present", "rationale": "The entry describes the current perception and feelings of the subject, and the keyword is \"I think,\" \"I'm sure it's changed,\" which points to the current state."}, "sentence_type": "Negative declarative sentence", "pos": {"nouns": {"Appearance": 1, "Change": 2, "Attractive": 1}, "verbs": {"I think": 1, "Not": 1}, "adjectives": {"Sure": 1, "It's attractive": 1}, "numerals": {"3": 1}, "pronouns": {"I": 1}}}, {"content": "4 I feel ugly and disgusting.", "word_count": 13, "label": "", "sentence_structure": "Subject鈥揷opula鈥損redicative", "subjective_objective": {"answer": "Subjective", "rationale": "Because \"Ugly\" and \"obstinate\" are subjective views of patients about their own image."}, "sentence_sentiment": {"answer": "Negative", "rationale": "Because the description in the entry is negative, the negative sentiment of self-evaluation is pointed out, namely, the perception that the appearance of the person is not good-looking and disgusting."}, "language_style": {"answer": "Calm and direct", "rationale": "The language style of the entry is more direct, rather than using too much accommodative adjectives or adverbs to express emotional colours, it is a concise and clear statement of the subjective perception of the person under investigation of his or her own image."}, "time_dimension": {"answer": "Past", "rationale": "The keyword \"Feel\" indicates that it is a sense of the past and a subjective assessment of the past, and that \"I am ugly and disgusting\" also describes the past self-perception. So, the entry is an inquiry into the feelings and perceptions of the subject in the past."}, "sentence_type": "Negative declarative sentence", "pos": {"nouns": {"Image": 1, "Ugly": 1, "Disgusting": 1}, "verbs": {"I feel": 1}, "adjectives": {"Ugly": 1, "Disgusting": 1}, "numerals": {"4": 1}, "pronouns": {"I": 1}}}, {"content": "I can work like usual.", "word_count": 9, "label": "", "sentence_structure": "Subject鈥損redicate", "subjective_objective": {"answer": "Objective", "rationale": "Because it is an objective situation to work as usual as a patient ' s state of life."}, "sentence_sentiment": {"answer": "Neutral", "rationale": "Because the entry does not have any emotional color."}, "language_style": {"answer": "Tactful and warm", "rationale": "The entry is more accessible and interactive by engaging in dialogue in a question and concern rather than by direct statement or order. At the same time, the wording of the entry is relatively mild and does not use strong or negative terminology, which is more comfortable and reassuring."}, "time_dimension": {"answer": "Present", "rationale": "According to the description of \"can\" and \"can work like usual,\" \"I can work like usual\" is a description of the current situation, and therefore a judgement of the past."}, "sentence_type": "Affirmative declarative sentence", "pos": {"verbs": {"Yes": 1, "Yeah": 1, "That way": 1, "Work": 1}, "pronouns": {"I": 1}}}, {"content": "I'm doing something. It takes extra effort to get started.", "word_count": 15, "label": "", "sentence_structure": "Subject鈥搗erb鈥揷omplement", "subjective_objective": {"answer": "Subjective", "rationale": "Because \"it takes extra effort to start\" is a subjective feeling and experience of a human being."}, "sentence_sentiment": {"answer": "Negative", "rationale": "Because the expression \"to take extra effort to start\" in the entry is negative and indicates negative attitudes and behaviour that may exist among actors when faced with tasks or work, such as delays and idleness."}, "language_style": {"answer": "Calm and direct", "rationale": "The entry, which is directly asked to reflect and answer the respondents, is not overly acrimony or emotional, and is more calm and objective."}, "time_dimension": {"answer": "Present", "rationale": "The two key words \"when doing things\" and \"how much more to start\" all indicate that this is the current behaviour or attitude of the testee, so the answer is now."}, "sentence_type": "Affirmative declarative sentence", "pos": {"nouns": {"Yes": 1, "Try": 1, "Start": 1}, "verbs": {"Do it": 1, "Flowers": 1}, "adverbs": {"Yes": 1, "Time": 1, "That's right": 1, "Extra": 1}, "numerals": {"2": 1}}}, {"content": "I must try to force myself to do it.", "word_count": 13, "label": "", "sentence_structure": "Subject鈥搗erb鈥揷omplement", "subjective_objective": {"answer": "Subjective", "rationale": "Since \"forced self\" is a subjective feeling and behaviour, it is a question of whether the patient believes that he or she needs to be forced to act."}, "sentence_sentiment": {"answer": "Negative", "rationale": "Because the \"hard to force\" act in the entry is negative."}, "language_style": {"answer": "Calm and direct", "rationale": "The entry is more directly linguistically, with little use of rhetorical or emotional qualifiers. It expresses in a concise manner the subject of asking the other side whether it needs to be forced to perform its tasks, and is more objective and rational."}, "time_dimension": {"answer": "Past", "rationale": "The entry expresses the past feelings and experiences of the testee, i.e. the need to \"intense\" himself to perform his tasks or activities, which have occurred in the past."}, "sentence_type": "Affirmative declarative sentence", "pos": {"nouns": {"Questionnaire Entry": 1, "Try": 1, "self": 1}, "verbs": {"I have to": 1, "Forced": 1, "Officers": 1}, "adverbs": {"Zhang": 1}, "numerals": {"3": 1}, "pronouns": {"I": 1}}}, {"content": "Four, I can't do anything at all.", "word_count": 8, "label": "", "sentence_structure": "Subject鈥搗erb鈥揷omplement", "subjective_objective": {"answer": "Subjective", "rationale": "Because \"I can't do anything\" is the patient's subjective feeling and perception, not the objective fact."}, "sentence_sentiment": {"answer": "Negative", "rationale": "Because the \"never do anything\" act in the entry is negative."}, "language_style": {"answer": "Calm and direct", "rationale": "\"Calm down straight.\""}, "time_dimension": {"answer": "Present", "rationale": "The keyword \"total impossibility\" indicates that the person tested is unable to perform any tasks and therefore it can be inferred that the person tested may have problems with his or her physical or psychological state."}, "sentence_type": "Negative declarative sentence", "pos": {"verbs": {"Do it": 1, "Yes": 1}, "adjectives": {"Total": 1}}}, {"content": "I am not tired as ever.", "word_count": 12, "label": "", "sentence_structure": "Subject鈥揷opula鈥損redicative", "subjective_objective": {"answer": "Subjective", "rationale": "Because \"not easily fatigue\" is a subjective feeling and self-assessment, it requires self-evaluation by the patient."}, "sentence_sentiment": {"answer": "Positive", "rationale": "Because the phrase \"not easily tired\" in the entry is positive."}, "language_style": {"answer": "Calm and direct", "rationale": "The entry is an objective and honest expression of the person ' s feelings, without excessive ado or emotional. The purpose of the article is more straightforward, and the euphemism, hint and other techniques are not used to guide the answer."}, "time_dimension": {"answer": "Present", "rationale": "The \"as compared to the past\" in this entry indicates that it compares the current state with the past, that \"not tired\" is a description of the present feelings or experiences of the testee, so it is possible to judge the time dimension of this questionnaire entry as it is now."}, "sentence_type": "Negative declarative sentence", "pos": {"verbs": {"Tired": 1}, "adjectives": {"not": 1, "Previous": 1}, "adverbs": {"and": 1, "Easy": 2}, "pronouns": {"I": 1}}}, {"content": "I'm more tired than I used to be.", "word_count": 10, "label": "", "sentence_structure": "Subject鈥揷opula鈥損redicative", "subjective_objective": {"answer": "Subjective", "rationale": "The entry of the questionnaire examines the patient's subjective feelings, because \"tired\" is a symptom of the patient's self-perception, and past comparisons are based on the patient's personal experience and observations. The answer to this article should therefore be subjective."}, "sentence_sentiment": {"answer": "Negative", "rationale": "Because the \"always tired\" in the entry is a description of the negative state."}, "language_style": {"answer": "Calm and direct", "rationale": "The language style of the entry is more straightforward, not using too many qualifiers or emotional expressions, but using simple and straightforward language to describe the problems that the person surveyed may encounter, a design that is more conducive to obtaining objective and accurate data."}, "time_dimension": {"answer": "Past", "rationale": "\"The past.\""}, "sentence_type": "Affirmative declarative sentence", "pos": {"verbs": {"I think": 1, "Weary": 1}, "adjectives": {"Easy": 1, "Past": 1}}}, {"content": "I'm tired of doing anything.", "word_count": 10, "label": "", "sentence_structure": "Subject鈥揷opula鈥損redicative", "subjective_objective": {"answer": "Subjective", "rationale": "Because \"fear fatigue\" is a subjective feeling and experience of individuals, the subjective feeling of different people may be different."}, "sentence_sentiment": {"answer": "Negative", "rationale": "Because the feeling of \"weary\" in the entries is negative."}, "language_style": {"answer": "Calm and direct", "rationale": "The language style of the entry is more straightforward, with no excessive rhetorical or emotional rendering, and a direct statement of the fact that the other party is tired of doing anything. This expression is more objective and concise, and helps the questionnaire researchers to obtain accurate information."}, "time_dimension": {"answer": "Present", "rationale": "The keyword \"I'm tired of doing anything\" is an expression of a continuous experience and feeling."}, "sentence_type": "Affirmative declarative sentence", "pos": {"verbs": {"Do it": 1, "I feel": 1, "Weary": 1}, "adjectives": {"Any": 1, "Yes": 1}, "adverbs": {"Both": 1}}}, {"content": "I'm too tired to do anything.", "word_count": 12, "label": "", "sentence_structure": "Subject鈥揷opula鈥損redicative", "subjective_objective": {"answer": "Objective", "rationale": "Because fatigue is a physical feeling, an objective fact that can be observed and measured. While patients may think that they are too vulnerable to fatigue, it is still a fact that can be determined by objective measurements (such as blood tests or sleep monitoring). \""}, "sentence_sentiment": {"answer": "Negative", "rationale": "Because the description of the act \"too tired to do anything\" in the entry is negative."}, "language_style": {"answer": "Tactful and warm", "rationale": "The entry uses more euphemistic terms such as \"too tired\" and \"doing nothing\" , which are relatively mild, do not directly deny or criticize the other, but rather lead the other to express his feelings and needs in an understanding and support. At the same time, the sentence is simple and easy to understand. The language style of the article can therefore be described as euphemism."}, "time_dimension": {"answer": "Past", "rationale": "The keywords \"too tired\" and \"can't do anything\" point to the experience and behaviour of the person who was tested over time and thus to the past."}, "sentence_type": "Negative declarative sentence", "pos": {"nouns": {"Weary": 1, "Yes": 1}, "verbs": {"Fuck": 1}, "adjectives": {"Easy": 1, "Tired": 1}, "adverbs": {"Too much": 1}, "numerals": {"4": 1}, "pronouns": {"I": 1}}}, {"content": "I'm not hungry.", "word_count": 9, "label": "", "sentence_structure": "Subject鈥揷opula鈥損redicative", "subjective_objective": {"answer": "Subjective", "rationale": "Because good appetite and bad appetite are a subjective feeling and experience of one person."}, "sentence_sentiment": {"answer": "Neutral", "rationale": "As this entry does not express any positive or negative emotional attitudes or behaviour."}, "language_style": {"answer": "Tactful and warm", "rationale": "The entry is more gentle and friendly, leads the other to answer questions in a question-and-answer manner, and is more easily phrased. At the same time, the use of a phrase such as \"no worse than in the past\" does not put too directly or emphasize individual differences, which are more acceptable and more responsive."}, "time_dimension": {"answer": "Past", "rationale": "The entry is therefore a question of the testee ' s past behaviour or state changes, and concerns a time comparison."}, "sentence_type": "Affirmative declarative sentence", "pos": {"nouns": {"-Adulge": 1, "Past": 1}, "verbs": {"No, it's not like that": 1, "Bad": 1}}}, {"content": "I'm not as good as I used to be.", "word_count": 11, "label": "", "sentence_structure": "Subject鈥搗erb鈥揷omplement", "subjective_objective": {"answer": "Subjective", "rationale": "Because the good appetite is the feelings and experiences of the patient himself, it is a subjective examination."}, "sentence_sentiment": {"answer": "Negative", "rationale": "Because the description \"a better appetite than in the past\" in the entry is negative."}, "language_style": {"answer": "Tactful and warm", "rationale": "The entry uses a more subtle way of expressing the possible appetite for loss, without directly pointing out the problem of the other ' s diet, but rather a relatively mild statement, which makes the language style more euphemism and warmer."}, "time_dimension": {"answer": "Past", "rationale": "The entry is an inquiry into the current state and feelings of the testee, and \"not as good as in the past\" means that the situation is a bit lower than before, so the temporal dimension is now judged."}, "sentence_type": "Negative declarative sentence", "pos": {"nouns": {"-Adulge": 1, "Past": 1}, "verbs": {"have": 1, "Sample": 1}, "adjectives": {"Okay": 1}, "adverbs": {"No, I'm not": 1, "That way": 1}, "pronouns": {"I": 1}}}, {"content": "I'm a lot worse at this time than I used to be.", "word_count": 12, "label": "", "sentence_structure": "Subject鈥搗erb鈥揷omplement", "subjective_objective": {"answer": "Subjective", "rationale": "Because \"attendance\" is a person's own experience, the entry is viewed from the patient's subjective perspective."}, "sentence_sentiment": {"answer": "Negative", "rationale": "Because the description \"a much less appetite\" in the entry is negative."}, "language_style": {"answer": "Tactful and warm", "rationale": "The entry uses a more moderate presentation, which allows respondents to answer questions more naturally by describing \"now\" versus \"the past.\" Moreover, the term \"attendance\" is also relatively objective and neutral, and does not cause discomfort or ostracism among the respondents. Thus, the language of the entry is more euphemismal."}, "time_dimension": {"answer": "Present", "rationale": "The entry uses the word \"now\" to describe the current situation of the subject, and thus to judge the current behaviour or mentality. And, \"I'm much worse at appetite\" is also a description of the current state, which is further confirmation that it is an inquiry into the present situation."}, "sentence_type": "Negative declarative sentence", "pos": {"nouns": {"-Adulge": 1, "Past": 1}, "verbs": {"Bad": 1, "- Yeah": 1, "More": 1}, "adjectives": {"Present": 1}, "numerals": {"3": 1}}}, {"content": "I'm not hungry.", "word_count": 8, "label": "", "sentence_structure": "Subject鈥搗erb鈥揷omplement", "subjective_objective": {"answer": "Subjective", "rationale": "Because appetite is a subjective feeling of one person, the degree of preference for the same food may vary among different people, and is viewed from a subjective perspective."}, "sentence_sentiment": {"answer": "Negative", "rationale": "Because the description \"no appetite\" in the entry is negative."}, "language_style": {"answer": "Calm and direct", "rationale": "The language style of the entry is more direct and concise, with no excessive qualifier or rhetorical use, with a greater focus on factual rather than emotional rendering, and is consistent with the linguistic characteristics of the questionnaire."}, "time_dimension": {"answer": "Present", "rationale": ""}, "sentence_type": "Negative declarative sentence", "pos": {"verbs": {"have": 1}, "adjectives": {"Both": 1, "No, I'm not": 1}, "adverbs": {"A little": 1}}}]}

{"questionnaire_title": "Life satisfaction table", "questionnaire_category": "Mental health", "item_count": 29, "emotion_shift_overall": "From positive to negative", "language_style_overall": "Tactful and warm", "items": [{"content": "When I was old, it seemed better to discover things than I had originally imagined.", "word_count": 20, "label": "", "sentence_structure": "Subject鈥揷opula鈥損redicative", "subjective_objective": {"answer": "Subjective", "rationale": "Because it's the patient's subjective idea."}, "sentence_sentiment": {"answer": "Positive", "rationale": "Because the entry expresses an optimistic and positive attitude that the future will be better than expected."}, "language_style": {"answer": "Tactful and warm", "rationale": "The words of the entry are more euphemistic, using the expression \"when I'm old\" to give people a sense of affection and comfort, while leaving more room for imagination. And, \"It seems better than originally thought,\" the expression is more mild, with no too much emotional or direct command."}, "time_dimension": {"answer": "Future", "rationale": "The entry contains visions and expectations for the future, and the keyword \"when I am old\" points to situations that have not yet occurred and that may arise in the future."}, "sentence_type": "Affirmative declarative sentence", "pos": {"nouns": {"I": 1, "Time": 1, "Things": 2, "Old": 1, "Okay": 1}, "verbs": {"Found": 1, "- Yeah": 1, "Yes": 1, "Look": 1}, "adjectives": {"Original": 1}, "adverbs": {"I think so": 1}}}, {"content": "2. I am better at taking advantage of the opportunities of life than most people I know.", "word_count": 24, "label": "", "sentence_structure": "Subject鈥揷opula鈥損redicative", "subjective_objective": {"answer": "Subjective", "rationale": "Because \"better than most people\" is patient self-evaluation, subjective."}, "sentence_sentiment": {"answer": "Positive", "rationale": "Because the entry describes one ' s abilities and performance in life and is a positive assessment of one ' s own, it can be judged as positive emotional."}, "language_style": {"answer": "Tactful and warm", "rationale": "The language style of the entry is more euphemistic. It expresses the subject in a comparative way, asking the interviewee in a relatively gentle way what he/she is saying about taking advantage of the opportunities of life, and this is more acclaimable and more acceptable."}, "time_dimension": {"answer": "Present", "rationale": "The entry asks the testee about the current time period (i.e., when answering the questionnaire) by comparing his performance with that of most people who know him in terms of taking advantage of the opportunities of life. Thus, the judgement is based on the time period in which \"I am\" is."}, "sentence_type": "Affirmative declarative sentence", "pos": {"nouns": {"Questionnaire Entry": 1, "Opportunities": 1}, "verbs": {"Comparison": 1, "Hold on": 1, "Yes": 1, "Organisation": 1}, "adjectives": {"Most of them": 1, "Better": 2}, "numerals": {"2": 1}, "classifiers": {"individual": 1}, "pronouns": {"I": 1}}}, {"content": "3. This is the most depressing period of my life.", "word_count": 13, "label": "", "sentence_structure": "Subject鈥揷opula鈥損redicative", "subjective_objective": {"answer": "Subjective", "rationale": "Because \"the most dull period\" is a subjective feeling that reflects the emotional state and perception of the patient."}, "sentence_sentiment": {"answer": "Negative", "rationale": "Because it expresses the negative emotions and experiences of life today, the \"most dull period\", and the negative emotional attitude."}, "language_style": {"answer": "Tactful and warm", "rationale": "The language style of the entry is more euphemistic, as it is in the form of a question-and-answer period, without directly saying that the other is in a bad or unhappy state. This expression makes the interviewee more comfortable and receptive and easier to answer questions."}, "time_dimension": {"answer": "Present", "rationale": "The entry is an inquiry into the current feelings of the subject, and the keyword \"now\" indicates that this is a description of the current state."}, "sentence_type": "Negative declarative sentence", "pos": {"nouns": {"Period": 1, "Life": 1, "Boring": 1}, "verbs": {"be": 1, "Most": 1, "Boring": 1}, "adjectives": {"Boring": 1, "Most": 1}, "adverbs": {"Present": 1, "I": 1}, "pronouns": {"Here": 1}}}, {"content": "I am as happy as I was when I was young.", "word_count": 11, "label": "", "sentence_structure": "Subject鈥揷opula鈥損redicative", "subjective_objective": {"answer": "Subjective", "rationale": "Because happiness is a patient's subjective feeling, not an objective fact."}, "sentence_sentiment": {"answer": "Positive", "rationale": "Because the expression \"happiness as young as it is\" is positive and expresses that personal happiness has not changed."}, "language_style": {"answer": "Tactful and warm", "rationale": "The entry is asked in a more subjective way and uses a comparison of \"now\" and \"young\" to stimulate reflection and memory, which is more moderate and friendly and more resonant with the interviewees."}, "time_dimension": {"answer": "Present", "rationale": "The key word is \"I'm happy now\", a statement describing the state of affairs, so the temporal dimension is now."}, "sentence_type": "Affirmative declarative sentence", "pos": {"nouns": {"I'm here": 1, "When I was young": 1, "Happiness": 1}, "verbs": {"Present": 1, "Same thing": 1, "Happiness": 1}, "adverbs": {"and": 1, "Same thing": 1}, "numerals": {"4": 1}, "pronouns": {"I": 1}}}, {"content": "5. My life should have been better.", "word_count": 14, "label": "", "sentence_structure": "Subject鈥揷opula鈥損redicative", "subjective_objective": {"answer": "Subjective", "rationale": "Because \"better\" is a subjective assessment that means that patients think they could have lived better."}, "sentence_sentiment": {"answer": "Positive", "rationale": "Because the expression \"should be better\" expresses an emotion of expectation and hope, and it is a positive attitude."}, "language_style": {"answer": "Tactful and warm", "rationale": "The language style of the entry is more moderate and positive, it is more euphemistic and it encourages each other to think about the possibilities that their lives can be made better. This expression makes it easier for the interviewees to feel comfortable and to share their thoughts."}, "time_dimension": {"answer": "Future", "rationale": "The key word is \"should\" , which is an indication of the vision and expectations for the future, that is, that it should be better to live in the future."}, "sentence_type": "Affirmative declarative sentence", "pos": {"nouns": {"Life": 1, "Original": 1, "Time": 1}, "verbs": {"be": 1, "I think so": 1, "Better": 1}, "adverbs": {"More": 2}, "pronouns": {"I": 1}}}, {"content": "6. This is the best time of my life.", "word_count": 13, "label": "", "sentence_structure": "Subject鈥揷opula鈥損redicative", "subjective_objective": {"answer": "Subjective", "rationale": "Because good is a subjective feeling, it is a subjective subject."}, "sentence_sentiment": {"answer": "Positive", "rationale": "Because the description of \"best times\" conveys positive emotions and attitudes."}, "language_style": {"answer": "Tactful and warm", "rationale": "The language style of the entry is more euphemistic and warm. It uses metaphoric terms to describe the current state as \"the best time of my life\" , which is more subtle and emotional, and more resonant and interactive. At the same time, the issue in this context is more guiding, encouraging respondents to share their experiences and feelings, and facilitating the collection of more rich and real data."}, "time_dimension": {"answer": "Present", "rationale": "Now the entry \"now\" represents the current point of time and asks the testee about his or her current state of view and evaluation, and therefore is a description of the current state."}, "sentence_type": "Affirmative declarative sentence", "pos": {"nouns": {"Time": 1, "One": 1, "Fan": 1, "Good": 1}, "verbs": {"be": 1}, "adjectives": {"Most": 1, "Okay": 1}, "adverbs": {"Present": 1}, "numerals": {"6": 1}, "classifiers": {"individual": 1}}}, {"content": "7. Most of what I have done is boring and boring.", "word_count": 18, "label": "", "sentence_structure": "Subject鈥揷opula鈥損redicative", "subjective_objective": {"answer": "Subjective", "rationale": "Because this is the patient ' s perception and assessment of what he does, it is subjective."}, "sentence_sentiment": {"answer": "Negative", "rationale": "Because the emotional color of the description \"sort and monotonous\" in the entry is negative."}, "language_style": {"answer": "Calm and direct", "rationale": "It expresses in the form of statements that most of the things that the respondents are doing are an annoying and monotonous subject, and that they are not using excessive rhetorical or emotional accents, which are more objective and sound. The advantages of this language style are that it is simple and clear, and that it is easy for the respondents to understand and answer questions; the disadvantages are that it may appear to be a little apathy or lack of interaction, and that it does not well inspire the participants鈥?motivation and interest."}, "time_dimension": {"answer": "Present", "rationale": ""}, "sentence_type": "Negative declarative sentence", "pos": {"nouns": {"Yes": 2, "person": 1}, "verbs": {"Do it": 1, "be": 1, "Boring": 1, "It's boring": 1}, "adjectives": {"It's boring": 2}, "numerals": {"7": 1}, "pronouns": {"I": 1}}}, {"content": "I expect to have had some interesting and pleasant events in the recent past.", "word_count": 19, "label": "", "sentence_structure": "Subject鈥搗erb鈥揷omplement", "subjective_objective": {"answer": "Subjective", "rationale": "Because the expressions \"I think\" and \"estimate\" reflect the patient's personal subjective judgment and prediction."}, "sentence_sentiment": {"answer": "Positive", "rationale": "Because the \"Funny and Happy Things\" in the entry is positive."}, "language_style": {"answer": "Tactful and warm", "rationale": "The entry uses the words \"estimate,\" \"interest,\" and \"happy things\" in a more optimistic tone, and is not too direct or too cold. This language style is more able to attract respondents to the questionnaire and create a friendly, relaxed atmosphere."}, "time_dimension": {"answer": "Present", "rationale": ""}, "sentence_type": "Affirmative declarative sentence", "pos": {"nouns": {"Yes": 1, "Questionnaire": 1, "person": 2}, "verbs": {"estimate": 1, "I met him": 1, "Yes": 1}, "adjectives": {"Interesting": 1, "It's a pleasure": 1}, "adverbs": {"Most recently": 1}, "pronouns": {"I": 1}}}, {"content": "9. What I do now is as interesting as what I have done before.", "word_count": 16, "label": "", "sentence_structure": "Subject鈥揷opula鈥損redicative", "subjective_objective": {"answer": "Subjective", "rationale": "According to the title, \"I'm doing what I've done before\" is the patient's own experience or behaviour, so it can be concluded that the questionnaire entry is a subjective approach to the patient. The answer is therefore \"subjective.\""}, "sentence_sentiment": {"answer": "Neutral", "rationale": "As this entry does not express positive or negative feelings or attitudes."}, "language_style": {"answer": "Tactful and warm", "rationale": "The entry is based on a soft, invitation-style conversation, rather than a direct statement or order. This expression is more personal and interactive, and it is more likely to trigger resonance and sharing among the respondents. At the same time, the entry is not overly rhetorical or emotional, and is more objective in tone."}, "time_dimension": {"answer": "Present", "rationale": "The entry is for the present living condition of the testee and the time dimension is now judged."}, "sentence_type": "Affirmative declarative sentence", "pos": {"nouns": {"Yes": 2, "Before": 1, "Interest": 1}, "verbs": {"Do it": 3, "be": 1, "Present": 1}, "adjectives": {"Interesting": 1}, "adverbs": {"Same thing": 1}, "numerals": {"9": 1}, "pronouns": {"I": 1}}}, {"content": "I feel old and tired. I feel that I am old, but I am not troubled by it.", "word_count": 29, "label": "", "sentence_structure": "Subject鈥揷opula鈥損redicative", "subjective_objective": {"answer": "Subjective", "rationale": "Because feeling old, tired and feeling old is the patient's self-perception and subjective view, but not the pain, it is the patient's attitude and emotional reaction that is subjective.\""}, "sentence_sentiment": {"answer": "Neutral", "rationale": "As the expression in the entry is not emotional, it is an objective description of the individual ' s state."}, "language_style": {"answer": "Tactful and warm", "rationale": "The entry is conducted in a question-and-answer manner, without directly expressing criticism or accusations against the other, but rather by focusing on the other ' s feelings and attitudes, which are more gentle and thoughtful and more resonant with the interviewee."}, "time_dimension": {"answer": "Past", "rationale": "11. The entry now asks the subject what he thinks and what he feels now, the key words being \"I feel I am really old\" and \"but I am not troubled by it\" and all point to the situation as it is."}, "sentence_type": "Affirmative declarative sentence", "pos": {"nouns": {"Questionnaire Entry": 1, "Year": 1, "Age": 1}, "verbs": {"Sensor": 2, "Go, go, go": 1, "Yes": 1, "Trouble": 1}, "adjectives": {"Old": 1, "Tired": 1, "Indeed": 1}, "adverbs": {"self": 1, "No, it's not": 1}, "numerals": {"10": 1, "11": 1}, "classifiers": {"individual": 1}, "pronouns": {"I": 2}}}, {"content": "12. I am quite satisfied to look back.", "word_count": 9, "label": "", "sentence_structure": "Subject鈥揷opula鈥損redicative", "subjective_objective": {"answer": "Subjective", "rationale": "This is a self-evaluation and perception of patients and is a subjective examination."}, "sentence_sentiment": {"answer": "Positive", "rationale": "Because the description \"remembering the past, I'm quite satisfied\" in the entry is positive."}, "language_style": {"answer": "Tactful and warm", "rationale": "The language style of the entry is more euphemistic and optimistic, emphasizing the degree of satisfaction with past experiences, and this expression can be a source of enthusiasm and resonance among the respondents."}, "time_dimension": {"answer": "Past", "rationale": ""}, "sentence_type": "Affirmative declarative sentence", "pos": {"verbs": {"Look back": 1, "Satisfied": 1}, "adjectives": {"The old days": 1, "Pretty much": 1}, "adverbs": {"very": 1}}}, {"content": "13. Even if I could change my past, I would not want to change.", "word_count": 18, "label": "", "sentence_structure": "Subject鈥損redicate", "subjective_objective": {"answer": "Subjective", "rationale": "Because it is patients鈥?subjective attitude and perception of the past. While objectively the past cannot be changed, whether patients are willing to change or depend on their subjective feelings and ideas."}, "sentence_sentiment": {"answer": "Negative", "rationale": "Because the entry expresses an attitude of reluctance to change, which is usually negative."}, "language_style": {"answer": "Calm and direct", "rationale": ""}, "time_dimension": {"answer": "Present", "rationale": "\"even if it can change its past\" means that it is the past's assumptions and imagination, and that \"I will not change\" is the reaction or attitude to that assumption. Thus, the temporal dimension of the article encompasses both the past and the present, and can be judged as the past and the present."}, "sentence_type": "Negative declarative sentence", "pos": {"nouns": {"Past": 1}, "verbs": {"It changes": 1, "have": 1, "I don't want to": 1, "Changed": 1}, "adverbs": {"Even if": 1}, "numerals": {"13": 1}, "pronouns": {"I": 1}}}, {"content": "I have made more stupid decisions than other peers.", "word_count": 21, "label": "", "sentence_structure": "Subject鈥揷opula鈥損redicative", "subjective_objective": {"answer": "Subjective", "rationale": "Because the entry of the questionnaire asks about patients ' subjective perceptions and evaluations of themselves."}, "sentence_sentiment": {"answer": "Negative", "rationale": "Because the \"dick decision\" in the entry is negative."}, "language_style": {"answer": "Tactful and warm", "rationale": "The entry uses a comparative language structure, asking if the respondents have made more mistakes in decision-making than their peers. This expression is more moderate and euphemistic, avoiding language styles that directly blame or negatively evaluate."}, "time_dimension": {"answer": "Past", "rationale": "The entry refers to past experience, \"had\" being an expression of past behaviour and thus a judgement of past."}, "sentence_type": "Affirmative declarative sentence", "pos": {"nouns": {"Peer": 1, "Decision": 2}, "verbs": {"Do it": 1, "Out": 1, "Comparison": 1}, "adjectives": {"Other": 1, "More": 1, "Stupid": 1}, "numerals": {"14": 1, "2": 1}, "classifiers": {"individual": 1}, "pronouns": {"I": 1}}}, {"content": "15. I look younger than other peers.", "word_count": 15, "label": "", "sentence_structure": "Subject鈥揷opula鈥損redicative", "subjective_objective": {"answer": "Subjective", "rationale": "Because \"the appearance is younger\" requires comparison and evaluation by the patient, which is the patient's subjective feeling."}, "sentence_sentiment": {"answer": "Neutral", "rationale": "Because the entry does not have any emotional color."}, "language_style": {"answer": "Tactful and warm", "rationale": "The entry allows respondents to assess their appearance of youth in a comparative way, while avoiding over-direct evaluation by the term \"younger\". This expression is both respectful of the interviewee鈥檚 self-esteem and serves the purpose of the questionnaire designer."}, "time_dimension": {"answer": "Present", "rationale": "The keywords \"as compared to other peers\" and \"younger\" point to a comparison and description of the current situation, so the entry is asking about the current physical features of the person being tested."}, "sentence_type": "Affirmative declarative sentence", "pos": {"nouns": {"Questionnaire": 1, "Peer": 1, "Appearance": 1, "Young": 1}, "verbs": {"Comparison": 1}, "adjectives": {"Other": 1}, "numerals": {"15": 1}, "pronouns": {"I": 1}}}, {"content": "16. I have planned what to do in one month or even one year.", "word_count": 21, "label": "", "sentence_structure": "Subject鈥搗erb鈥搊bject", "subjective_objective": {"answer": "Subjective", "rationale": "Because \"the plan has been drawn up for...\" is a subjective act and decision. This requires conscious thinking and planning of the future, not objective facts or results."}, "sentence_sentiment": {"answer": "Positive", "rationale": "Because the action of \"planing for what to do\" in the entry is positive."}, "language_style": {"answer": "Calm and direct", "rationale": "The language style of the entry is calm, as the question is directly whether the interviewee has planned for the future and has not used too many emotions or rhetoric techniques to guide the interviewee to answer."}, "time_dimension": {"answer": "Future", "rationale": "\"Have\" means that the action occurred in the past, \"have prepared a plan for ...\" denotes future conduct or intention, and the title therefore describes future conduct or intention."}, "sentence_type": "Affirmative declarative sentence", "pos": {"nouns": {"Month": 1, "Year": 1, "Yes": 1, "Planned": 1}, "verbs": {"Development": 1}, "adverbs": {"Already": 1, "Even": 1}, "numerals": {"A month": 1, "One year later": 1}, "pronouns": {"I": 1}}}, {"content": "17. Looking back, I have many things I want to get that I have not.", "word_count": 18, "label": "", "sentence_structure": "Subject鈥搗erb鈥搊bject", "subjective_objective": {"answer": "Subjective", "rationale": "Because what you want, what you don't have are subjective feelings and evaluations of patients."}, "sentence_sentiment": {"answer": "Negative", "rationale": "Because it expresses feelings of regret and loss, it is negative."}, "language_style": {"answer": "Tactful and warm", "rationale": "The language style of the entry is more euphemistic, with a recollection as a reference, and the interviewee is guided to express his regrets and expectations, and is more accommodative and acceptable."}, "time_dimension": {"answer": "Past", "rationale": ""}, "sentence_type": "Negative declarative sentence", "pos": {"nouns": {"The old days": 1, "Something": 2}, "verbs": {"Look back": 1, "Okay": 2}, "adjectives": {"Many": 1, "Not": 1}, "numerals": {"17": 1}, "pronouns": {"I": 1}}}, {"content": "I have suffered too many failures, compared to others.", "word_count": 17, "label": "", "sentence_structure": "Subject鈥搗erb鈥揷omplement", "subjective_objective": {"answer": "Subjective", "rationale": "Because the subject is comparing the number of failures of \"me\" and others, it needs to be considered from the point of view of the patient."}, "sentence_sentiment": {"answer": "Negative", "rationale": "Because the expression \"too many failures\" in the entries has a clear negative emotional connotation."}, "language_style": {"answer": "Calm and direct", "rationale": "The entry uses both comparative and absolute terms, expressing the feelings of the interviewees that they feel very frustrated and frustrated about their failures. This language style is more direct, clear, less over-modified or decomposition, and more in keeping with the immediate and calm features."}, "time_dimension": {"answer": "Past", "rationale": "The keyword \"18 versus the others\" suggests that it is a comparison and reflection of the past, and that \"too many failures\" also describe the past. Thus, the entry is about the past's behavior or mentality."}, "sentence_type": "Negative declarative sentence", "pos": {"nouns": {"18": 1, "I": 1, "Other": 1, "Failed": 1, "Number": 1}, "verbs": {"- Yeah": 1, "Too much": 1}, "adjectives": {"More": 1}, "adverbs": {"- Oh, shit": 1}}}, {"content": "19. I have gained a lot in life that I expected.", "word_count": 18, "label": "", "sentence_structure": "Subject鈥搗erb鈥揷omplement", "subjective_objective": {"answer": "Subjective", "rationale": "Because \"getting quite what I want\" is patient's subjective feeling and judgment."}, "sentence_sentiment": {"answer": "Positive", "rationale": "Because the result of the entry \"getting quite a lot of what I want\" is positive."}, "language_style": {"answer": "Tactful and warm", "rationale": "The language style of the entry is more euphemistic, as it uses positive expressions to ask the other side whether it has been expected in life, rather than simply denying or questioning. Moreover, the sentence contains a qualifier such as \"squirty\" that makes it more soft and respectful."}, "time_dimension": {"answer": "Present", "rationale": "\"getting\" and \"expectation\" are also the verbs of the current state of affairs, which indicate that this phrase describes what is happening now."}, "sentence_type": "Affirmative declarative sentence", "pos": {"nouns": {"Life": 1, "Something": 1}, "verbs": {"Got it": 1, "Expectations": 1}, "adjectives": {"A lot": 1}, "numerals": {"19": 1}, "pronouns": {"I": 1}}}, {"content": "20. Whatever one may say, many ordinary people are getting worse, not better.", "word_count": 25, "label": "", "sentence_structure": "Subject鈥損redicate", "subjective_objective": {"answer": "Subjective", "rationale": "Because this is a subjective view and assessment of authors/researchers, they believe that many people are living worse and worse. This is the conclusion based on their observations, experiences and values, and is therefore a subjective view."}, "sentence_sentiment": {"answer": "Negative", "rationale": "Because the description \"over and over\" in the entry is negative."}, "language_style": {"answer": "Calm and direct", "rationale": "The entry is more straightforward, calm, and does not have too many oral or emotional expressions. It is simple and concise."}, "time_dimension": {"answer": "Past", "rationale": ""}, "sentence_type": "Affirmative declarative sentence", "pos": {"nouns": {"person": 2, "Normal": 1, "Investigations": 1}, "verbs": {"Over": 3, "Say it": 1, "Okay": 1, "It's getting worse": 1, "be": 1}, "adjectives": {"Many": 1, "Shit": 1, "Okay": 1}, "adverbs": {"Whatever": 1, "What": 1}, "numerals": {"20": 1, "Tsk": 2}, "pronouns": {"they": 1}}}, {"content": "What is the most important thing in your life now?", "word_count": 15, "label": "", "sentence_structure": "Subject鈥搗erb鈥搊bject", "subjective_objective": {"answer": "Subjective", "rationale": "The patient ' s current state of life and feelings require a subjective examination."}, "sentence_sentiment": {"answer": "Neutral", "rationale": "Because the entry does not have any emotional color."}, "language_style": {"answer": "Tactful and warm", "rationale": "The language style of the entry is euphemistic, because it asks each other in a gentle and caring way what is most important in their current lives, without direct orders or criticism. This expression is more comfortable and respectful."}, "time_dimension": {"answer": "Present", "rationale": "The entry asks the main concerns or priorities of the person being tested in his current life, and the time dimension is now judged."}, "sentence_type": "Interrogative sentence", "pos": {"nouns": {"Life": 1, "Things": 1}, "verbs": {"be": 1}, "numerals": {"Present": 1, "Most": 1, "of": 2}, "classifiers": {"One": 1}, "pronouns": {"you": 1}}}, {"content": "Are you the happiest person in the world compared to your early life?", "word_count": 15, "label": "", "sentence_structure": "Subject鈥搗erb鈥搊bject", "subjective_objective": {"answer": "Subjective", "rationale": "Because happiness is a subjective feeling, different people may have different definitions and feelings of happiness."}, "sentence_sentiment": {"answer": "Negative", "rationale": "Because the question of \"happiness\" in this entry expresses a subjective perception and evaluation of life, with negative emotional aspects."}, "language_style": {"answer": "Calm and direct", "rationale": "The entry directly asks whether the respondents feel happier now than they did in their past lives, with more direct and concise language styles, and with no excessive rhetorical or emotional rendering."}, "time_dimension": {"answer": "Present", "rationale": "The entry is for the testee's current situation and the time dimension is now judged."}, "sentence_type": "Interrogative sentence", "pos": {"nouns": {"Life": 2, "Present": 1, "Happiness": 1}, "verbs": {"Compare": 1, "- Yeah": 1, "Yes": 1}, "adjectives": {"Early": 1}, "numerals": {"4": 1}, "pronouns": {"you": 1}}}, {"content": "Have you ever feared that you are incapable of what people expect you to do - you are unable to meet what people ask of you?", "word_count": 32, "label": "", "sentence_structure": "Subject鈥搗erb鈥搊bject", "subjective_objective": {"answer": "Subjective", "rationale": "Because \"fear\" and \"failure\" are patients' subjective feelings and thoughts."}, "sentence_sentiment": {"answer": "Negative", "rationale": "Because the mood of \"fearing to fail to meet people's demands\" in the entry is negative."}, "language_style": {"answer": "Tactful and warm", "rationale": "This entry is in the form of a question and question statement, which is more balanced and friendly, and leads the person surveyed to think and answer in a question-and-answer manner, rather than direct orders or statements."}, "time_dimension": {"answer": "Present", "rationale": "The keyword \"had\" and \"have you been unable to do it\" suggests that this is an inquiry about past experience, but the entire sentence uses the usual current state of affairs, indicating that this concern may continue to the present. So this entry is about the current emotional state and experience of the testee, and the time dimension is now."}, "sentence_type": "Interrogative sentence", "pos": {"nouns": {"Questionnaire Entry": 1, "person": 2, "Yes": 2, "Request": 1}, "verbs": {"I'm worried": 1, "Do it": 1, "Satisfied": 1, "Look forward to it": 1}, "adverbs": {"Tsang": 1, "But": 1}, "numerals": {"5": 1, "you": 2}, "pronouns": {"you": 2}}}, {"content": "6. If you want to do what you want, where do you like life best?", "word_count": 24, "label": "", "sentence_structure": "Subject鈥搗erb鈥搊bject", "subjective_objective": {"answer": "Subjective", "rationale": "The problem is that it involves asking the patient about his or her personal preferences and ideals, personal emotional attitudes and imagination, and is viewed from a subjective perspective."}, "sentence_sentiment": {"answer": "Neutral", "rationale": "As the entry does not address any elements of emotion, attitude, behaviour or purpose, it is only a personal preference to ask the interviewee."}, "language_style": {"answer": "Tactful and warm", "rationale": "The entry uses a language that is of a recommendatory and query nature, rather than a direct order or statement, which makes it easier to stimulate reflection and active participation. At the same time, the expression \"whatever you want\" has some positive implication, giving people a sense of encouragement and support."}, "time_dimension": {"answer": "Present", "rationale": "It can therefore be concluded that this entry is for the purpose of asking the present preferences and emotions of the subjects and judging the dimensions of the present."}, "sentence_type": "Interrogative sentence", "pos": {"nouns": {"Questionnaire Entry": 1, "Country name": 1}, "verbs": {"Yes": 4, "Yeah": 1, "Life": 1, "Where": 1}, "numerals": {"6": 1, "Whatever you can do": 1}, "pronouns": {"you": 3}}}, {"content": "How long have you been feeling alone?", "word_count": 11, "label": "", "sentence_structure": "Subject鈥搗erb鈥搊bject", "subjective_objective": {"answer": "Subjective", "rationale": "The point of view of the questionnaire is subjective, because it requires patients to describe and assess their feelings, which is \"how long do you feel alone?\" This is their own perception and experience. While isolation may have some objective elements (such as social circumstances), in this case, the patient鈥檚 answer is more based on their subjective feelings."}, "sentence_sentiment": {"answer": "Neutral", "rationale": "Since the entry is an objective fact, it does not address emotional attitudes or behavioural tendencies."}, "language_style": {"answer": "Calm and direct", "rationale": "The entry uses direct, concise language to ask the person who was interviewed about the time he or she felt alone, without adding too many adorations or emotional colours, and the language style is more calm and straightforward.\""}, "time_dimension": {"answer": "Past", "rationale": "The entry is for the purpose of asking the testee about the past and relates to a description of the past in terms of the time dimension."}, "sentence_type": "Interrogative sentence", "pos": {"nouns": {"Time": 1, "Loneliness": 1}, "verbs": {"I feel": 1, "How much": 1}, "numerals": {"7": 1}}}, {"content": "How long do you feel you're living for no purpose?", "word_count": 14, "label": "", "sentence_structure": "Subject鈥搗erb鈥搊bject", "subjective_objective": {"answer": "Subjective", "rationale": "Because \"fear\" and \"unpurposed\" time are subjective feelings and subjective assessments of patients, it is subjective."}, "sentence_sentiment": {"answer": "Negative", "rationale": "Because \"life without purpose\" is usually seen as a negative emotion or state."}, "language_style": {"answer": "Calm and direct", "rationale": "The entry is based on a simple and clear statement of how much time the interviewee feels in daily life is useless, is simple in language, is not too much rhetorical or emotional in nature, and is more objective in tone. It is therefore judged to be the language style of the article that is straightforward."}, "time_dimension": {"answer": "Present", "rationale": "The key words are \"you now\" and \"you feel\" and they all point to the current situation."}, "sentence_type": "Interrogative sentence", "pos": {"nouns": {"Time": 1, "Life": 1, "Purpose": 1}, "verbs": {"I feel": 1, "have": 1}, "adverbs": {"How much": 1}, "numerals": {"8": 1}}}, {"content": "Do you want to spend more time with your best friends in the future or do you want to spend more time alone?", "word_count": 32, "label": "", "sentence_structure": "Subject鈥搗erb鈥搊bject", "subjective_objective": {"answer": "Subjective", "rationale": "Because the word \"hope\" expresses the patient's subjective attitude."}, "sentence_sentiment": {"answer": "Positive", "rationale": "Because the expectation of \"I hope to spend more time with my best friends\" is positive."}, "language_style": {"answer": "Tactful and warm", "rationale": "The reason is as follows: - The question is guided by \"you want\" to give people a sense of active participation, a more gentle and intimate one; - The question itself is not a neutral answer, either in terms of preference or implication, but rather a neutral one, which makes people comfortable and free to answer."}, "time_dimension": {"answer": "Future", "rationale": "The entry contains visions and expectations for the future, with the key words \"to be with a good friend\" and \"to be alone\" pointing to situations that have not yet occurred and that may arise in the future."}, "sentence_type": "Interrogative sentence", "pos": {"nouns": {"Questionnaire": 1, "Entry": 1, "Future": 1, "Time": 2, "friends": 1, "Alone": 1}, "verbs": {"Hope": 1, "Together": 1, "More": 2}, "adverbs": {"Some": 3}, "numerals": {"9": 1}, "pronouns": {"you": 1}}}, {"content": "How many unfortunate things have you found in your current life?", "word_count": 17, "label": "", "sentence_structure": "Subject鈥搗erb鈥搊bject", "subjective_objective": {"answer": "Subjective", "rationale": "Because \"unlucky things\" are patients' subjective feelings and perceptions."}, "sentence_sentiment": {"answer": "Negative", "rationale": "Because the description of \"unlucky things\" in the entry is negative."}, "language_style": {"answer": "Calm and direct", "rationale": "The entry asks the respondents in a direct question about the number of unfortunate events they encounter in their current lives, and does not have too many emotional renderings or rhetorical techniques."}, "time_dimension": {"answer": "Present", "rationale": "The entry asks the person who was tested for the current life, and the time dimension is now judged."}, "sentence_type": "Interrogative sentence", "pos": {"nouns": {"Life": 1, "Unfortunately": 1, "Things": 1}, "verbs": {"Let's go": 1, "Current": 1}, "numerals": {"More": 1, "10": 1}}}, {"content": "When you're old, is it better or worse than you thought?", "word_count": 19, "label": "", "sentence_structure": "Subject鈥搗erb鈥搊bject", "subjective_objective": {"answer": "Subjective", "rationale": "This is because the patient's subjective view of his future self-status."}, "sentence_sentiment": {"answer": "Neutral", "rationale": "Because the entry does not involve any emotional expression, it is only a statement of the question."}, "language_style": {"answer": "Calm and direct", "rationale": "The entry is presented in a direct question-and-answer format, with no excessive emotional colour or accommodation, and directly asks whether the person surveyed is living better than expected after he/she has reached the age of his/her life."}, "time_dimension": {"answer": "Future", "rationale": "The keyword \"after age\" means that the point of time that has not yet arrived is a future one; and \"Is it better or worse than originally thought\" also relates to future circumstances and expectations. Thus, the entry can be judged as the future."}, "sentence_type": "Interrogative sentence", "pos": {"nouns": {"you": 1, "Old": 1, "Things": 2, "Original": 1, "Imagine": 1, "Okay": 1, "Bad": 1}, "verbs": {"be": 1, "- Yeah": 1}, "adjectives": {"Old": 1}, "adverbs": {"After": 1}, "numerals": {"11": 1}}}, {"content": "12. How happy are you with your life?", "word_count": 13, "label": "", "sentence_structure": "Subject鈥搗erb鈥揷omplement", "subjective_objective": {"answer": "Subjective", "rationale": "Since satisfaction is a subjective self-evaluation, different people have different views and feelings."}, "sentence_sentiment": {"answer": "Neutral", "rationale": "Because the entry does not express a clear positive, negative or emotional attitude, it is asking the respondents about their satisfaction with their lives."}, "language_style": {"answer": "Calm and direct", "rationale": "The entry directly asks the respondents about their satisfaction with their lives, does not have too many emotional colours or qualifiers and is more straightforward."}, "time_dimension": {"answer": "Present", "rationale": "The entry asks about the current life satisfaction of the testee, and the time dimension is judged to be the present. The key word \"how satisfied with...\" indicates that the issue is concerned with the current feelings and attitudes, not with past or future situations."}, "sentence_type": "Interrogative sentence", "pos": {"nouns": {"self": 1, "Life": 1, "Level": 1, "How's that": 1}, "verbs": {"Satisfied": 1}, "adverbs": {"What": 1}, "numerals": {"12": 1}, "pronouns": {"you": 1}}}]}

{"questionnaire_title": "Hamilton depression table.", "questionnaire_category": "Mental health", "item_count": 24, "emotion_shift_overall": "From positive to negative", "language_style_overall": "Neutral", "items": [{"content": "1. Depression", "word_count": 4, "label": "", "sentence_structure": "Subject鈥搗erb鈥揷omplement", "subjective_objective": {"answer": "Subjective", "rationale": "Because \"depressive\" is an emotional state, it is the feeling and experience inside the patient and is therefore subjective."}, "sentence_sentiment": {"answer": "Negative", "rationale": "Because \"depressive\" is a negative emotional state."}, "language_style": {"answer": "Calm and direct", "rationale": "The entry is presented in a concise and clear manner, asking the interviewees whether they are depressed, without too many emotional renderings or rhetorical techniques, and in a calm and straightforward manner."}, "time_dimension": {"answer": "Present", "rationale": "The entry asks about the current emotional state of the subject, the keyword being \"depressive\" and it points to the emotional state that is now going through."}, "sentence_type": "Interrogative sentence", "pos": {"nouns": {"Blue": 1, "Emotion": 1}}}, {"content": "2. Frustration", "word_count": 3, "label": "", "sentence_structure": "Subject鈥搗erb鈥揷omplement", "subjective_objective": {"answer": "Subjective", "rationale": "Because guilt is a feeling and a perception inside a person, a subjective view."}, "sentence_sentiment": {"answer": "Negative", "rationale": "Because guilt is usually a negative emotion."}, "language_style": {"answer": "Calm and direct", "rationale": "The language style of this questionnaire entry is relatively calm and straightforward, and there is no excessive emotional rendering or rhetorical technique. It clearly asks whether the person under investigation has a \"guilty\" of the phenomenon, and it is clear and understandable in a clear and understandable manner."}, "time_dimension": {"answer": "Present", "rationale": "The keyword is \"criticism\" and refers to the current subjective or emotional state of the person, so that it can be judged that the entry is about the act, attitude or mental state of the person."}, "sentence_type": "Interrogative sentence", "pos": {"nouns": {"Perpetrators": 1, "Feel": 1}}}, {"content": "Suicide", "word_count": 2, "label": "", "sentence_structure": "Subject鈥損redicate", "subjective_objective": {"answer": "Subjective", "rationale": "Because \"suicide\" is an idea or a concept, it is an idea inside the patient and is therefore a subjective examination."}, "sentence_sentiment": {"answer": "Negative", "rationale": "Because suicide is a very negative behavior and attitude."}, "language_style": {"answer": "Calm and direct", "rationale": "The entry, which is in direct, concise language, asks the interviewees whether they have committed suicide or any act, is open to the question and helps to obtain more realistic and effective information. \""}, "time_dimension": {"answer": "Past", "rationale": "The entry asks if the person tested has had any experience of suicide and therefore falls under the category of questioning of past behaviour or experience, which is judged by the time dimension."}, "sentence_type": "Imperative sentence", "pos": {"nouns": {"Suicide": 1, "Questionnaire": 1, "Entry": 1}, "verbs": {"Analysis": 1}}}, {"content": "4. Sleeping difficulties", "word_count": 4, "label": "", "sentence_structure": "Subject鈥揷opula鈥損redicative", "subjective_objective": {"answer": "Subjective", "rationale": "Because sleep problems are the subjective feelings and experiences of the patient."}, "sentence_sentiment": {"answer": "Negative", "rationale": "Because the act of sleeping difficulties in the entries is negative."}, "language_style": {"answer": "Tactful and warm", "rationale": "The entry begins with a question-and-answer form, using the objective description of \"hard\" without direct blame or criticism of the other. It is more moderate and acceptable. At the same time, the term \"sleep\" gives people a comfortable and sweet feeling, making the entire language style of the questionnaire more euphemistic."}, "time_dimension": {"answer": "Past", "rationale": "The entry asks if the person tested has problems with sleeping difficulties and the time dimension is a past."}, "sentence_type": "Negative declarative sentence", "pos": {"nouns": {"Problem": 1, "Sleeping difficulties": 1}, "verbs": {"Analysis": 1}, "numerals": {"4": 1}}}, {"content": "5. Sleep deprivation", "word_count": 4, "label": "", "sentence_structure": "Subject鈥揷opula鈥損redicative", "subjective_objective": {"answer": "Subjective", "rationale": "Because sleep is not deep enough for a patient to feel or experience subjectively."}, "sentence_sentiment": {"answer": "Negative", "rationale": "Because the \"sleepless\" behavior is negative and could affect health."}, "language_style": {"answer": "Calm and direct", "rationale": "The entry is more straightforward in language style, does not have too many rhetorical or emotional renderings, is more objective and calm, and asks directly whether the person under investigation has a problem of sleeping."}, "time_dimension": {"answer": "Present", "rationale": "The entry asks about the current state of the person being tested, i.e. whether there is a problem of \"sleep in the present\" and therefore is the present."}, "sentence_type": "Negative declarative sentence", "pos": {"nouns": {"Sleep": 1, "Depth": 1}, "verbs": {"not": 1, "Deep": 1}}}, {"content": "Early awakening", "word_count": 2, "label": "", "sentence_structure": "Subject鈥搗erb鈥揷omplement", "subjective_objective": {"answer": "Subjective", "rationale": "Because early awakening is a subjective feeling and experience of a human being. Although it may be influenced by external factors (such as noise, light, etc.), it is ultimately determined by the subjective state of the individual."}, "sentence_sentiment": {"answer": "Neutral", "rationale": "Because the entry does not have any emotional color."}, "language_style": {"answer": "Calm and direct", "rationale": "The language style of the entry is a calm and straightforward type, with simple questions to obtain information from the respondents, and with no excessive emotional rendering and adoration, which makes it feel more objective and rational."}, "time_dimension": {"answer": "Past", "rationale": "The entry is for the purpose of asking the testee whether he or she is awakening early and the time dimension is judged to be past."}, "sentence_type": "Imperative sentence", "pos": {"nouns": {"Wake up": 1}}}, {"content": "7. Work and interest", "word_count": 5, "label": "", "sentence_structure": "Subject鈥損redicate", "subjective_objective": {"answer": "Subjective", "rationale": "Because the entry asks about the views and feelings of individuals about \"work and interest\" and is a subjective examination."}, "sentence_sentiment": {"answer": "Neutral", "rationale": "The entry does not express any apparent emotional, attitude or behavioural orientation and is therefore neutral."}, "language_style": {"answer": "Tactful and warm", "rationale": "The language style of the entry is euphemism, because it asks the interviewee, in an invitation and caring tone, whether he has a unique view or feeling of work and interest. This expression is more friendly than a direct question, and better guides the interviewee to share his thoughts and experiences freely and openly."}, "time_dimension": {"answer": "Present", "rationale": "The entry does not contain a clear time limit, but describes the current situation of the interviewees and therefore the present."}, "sentence_type": "Interrogative sentence", "pos": {"nouns": {"Work": 1, "Interest": 1}}}, {"content": "8. Blockage", "word_count": 2, "label": "", "sentence_structure": "Subject鈥損redicate", "subjective_objective": {"answer": "Subjective", "rationale": "Because \"retardation\" is a psychological experience or experience that requires self-reporting by the patient, it is subjective."}, "sentence_sentiment": {"answer": "Negative", "rationale": "Because the word \"stopping\" is usually negative emotional."}, "language_style": {"answer": "Calm and direct", "rationale": "In terms of language style, the purpose of the article is relatively straightforward, with no excessive emotional or rhetorical tact, and therefore can be judged as straightforward. At the same time, \"retardation\" as a professional term is more formal in its use and reflects the specialization of questionnaire research."}, "time_dimension": {"answer": "Past", "rationale": "The concept of \"detachment\" involves barriers or problems in psychological behaviour, and in this context, \"detachment\" should be understood as whether the person tested has experienced such a situation in the past and now. Because of the state of the situation, the problem may be both one of the questions about past experience (e.g., whether there has been a situation of overt obstruction) and one of the current state (e.g., whether there is a problem of detachment at this time)."}, "sentence_type": "Imperative sentence", "pos": {"nouns": {"Block": 1}}}, {"content": "9. Extremism", "word_count": 2, "label": "", "sentence_structure": "Subject鈥搗erb鈥揷omplement", "subjective_objective": {"answer": "Subjective", "rationale": "Because \"several\" is a state of emotion, reflecting the feelings and experiences inside the patient, and is a subjective approach."}, "sentence_sentiment": {"answer": "Positive", "rationale": "\"Escalation\" is an adjective, which means emotional excitement, exultation, and is usually used to describe positive emotional state. Thus, for the purposes of the questionnaire, \"extinction\" can be considered a positive term."}, "language_style": {"answer": "Tactful and warm", "rationale": "The term \"excuse\" itself is a more emotional expression of a positive and dynamic state of mind, so I think the language style for this purpose is \"pretentious.\""}, "time_dimension": {"answer": "Past", "rationale": "In conclusion, the temporal dimension of the article would need to be judged on the basis of the responses of the person tested."}, "sentence_type": "Interrogative sentence", "pos": {"adjectives": {"Extremist": 1}}}, {"content": "10. Mental anxiety", "word_count": 5, "label": "", "sentence_structure": "Subject鈥搗erb鈥揷omplement", "subjective_objective": {"answer": "Subjective", "rationale": "The questionnaire is intended to understand whether the patient is anxious and therefore subjective."}, "sentence_sentiment": {"answer": "Negative", "rationale": "Because the word \"anxiety\" is usually seen as a negative emotion or state."}, "language_style": {"answer": "Tactful and warm", "rationale": ""}, "time_dimension": {"answer": "Present", "rationale": "The entry asks the testee whether he has a mental anxiety, and thus can be judged as \"now.\" The keyword \"mental anxiety\" indicates that it is a subjective feeling or emotional state, not a past act or result. Moreover, \"10\" refers to the tenth question that is currently being completed, which further confirms that it is a present, ongoing act."}, "sentence_type": "Negative declarative sentence", "pos": {"nouns": {"Mental anxiety": 1, "Anxiety": 1}, "adjectives": {"Mental": 1}, "numerals": {"10锛?: 1}}}, {"content": "11. Body anxiety", "word_count": 5, "label": "", "sentence_structure": "Subject鈥揷opula鈥損redicative", "subjective_objective": {"answer": "Subjective", "rationale": "Because \"physical anxiety\" is the anxiety that patients experience subjectively."}, "sentence_sentiment": {"answer": "Negative", "rationale": "Because \"anxiety\" is usually considered a negative emotion or experience."}, "language_style": {"answer": "Calm and direct", "rationale": "The language style of the entry is a relatively calm and straightforward one. It uses a simple and clear language, asking directly whether the person interviewed has physical anxiety, without too many rhetorical or emotional renderings.\""}, "time_dimension": {"answer": "Present", "rationale": "The entry asks if the person tested currently has a body anxiety, and the time dimension is now judged."}, "sentence_type": "Exclamatory sentence", "pos": {"nouns": {"Body nature": 1, "Anxiety": 1}, "numerals": {"11": 1}}}, {"content": "12. Gastrointestinal symptoms", "word_count": 5, "label": "", "sentence_structure": "Subject鈥搗erb鈥揷omplement", "subjective_objective": {"answer": "Subjective", "rationale": "The testimonial testimonial is the physical reaction and experience of the patient and is in the realm of objective observation."}, "sentence_sentiment": {"answer": "Negative", "rationale": "Because \"gastrointestinal symptoms\" are usually considered to be uncomfortable, they have negative emotional aspects."}, "language_style": {"answer": "Calm and direct", "rationale": "The entry is in plain and simple language, and the person surveyed is asked whether he or she has symptoms of gastrointestinal disorders, is not overdoing or emotional rendering, and is speaking in a more objective and calm manner."}, "time_dimension": {"answer": "Present", "rationale": "The entry asks if the person tested has a gastrointestinal-related symptoms, and the temporal dimension is now judged."}, "sentence_type": "Interrogative sentence", "pos": {"nouns": {"Stomach": 1, "Symptoms": 1}}}, {"content": "13. All-body symptoms", "word_count": 4, "label": "", "sentence_structure": "Subject鈥搗erb鈥揷omplement", "subjective_objective": {"answer": "Subjective", "rationale": "It's a patient's self-reporting problem, which needs to be viewed from the patient's subjective point of view. So, it's \"subjective.\""}, "sentence_sentiment": {"answer": "Neutral", "rationale": "The entry for this questionnaire is not marked by emotional tendencies, but merely asks if the person surveyed has some physical discomfort."}, "language_style": {"answer": "Tactful and warm", "rationale": "The language style of the entry is euphemistic, leading the interviewee to reflect on his/her physical condition rather than directly informing the questions that need to be answered. The term \"whole body\" is used to give the interviewee a sense of the breadth and importance of the problem, and to increase his/her motivation and initiative in participating in the investigation."}, "time_dimension": {"answer": "Present", "rationale": ""}, "sentence_type": "Affirmative declarative sentence", "pos": {"nouns": {"All-body symptoms": 1}}}, {"content": "14. Sexual symptoms", "word_count": 3, "label": "", "sentence_structure": "Subject鈥搗erb鈥揷omplement", "subjective_objective": {"answer": "Subjective", "rationale": "Because sexual symptoms are usually subjective feelings and experiences of patients."}, "sentence_sentiment": {"answer": "Negative", "rationale": "Because \"sexual symptoms\" are generally associated with physical discomfort or disease, they have some negative emotional effects."}, "language_style": {"answer": "Tactful and warm", "rationale": "The entry uses the term \"sexual symptoms\" and does not address overly sensitive or private topics, but maintains objectivity, warmth and respect by asking the other party whether they exist."}, "time_dimension": {"answer": "Present", "rationale": "In the entry, \"sexual symptoms\" is a term describing the current state of affairs, which means that the testee is now sexually troubled or troubled and can therefore be judged as \"now\"."}, "sentence_type": "Interrogative sentence", "pos": {"nouns": {"Sexual symptoms": 1}}}, {"content": "15. Suspected diseases", "word_count": 2, "label": "", "sentence_structure": "Subject鈥損redicate", "subjective_objective": {"answer": "Subjective", "rationale": "Because \"suspicious\" is usually a patient's excessive fear and speculation of his or her physical condition, and is the patient's subjective attitude and thoughts."}, "sentence_sentiment": {"answer": "Negative", "rationale": "Because \"suspicious\" is often interpreted as an excessive fear and fear of physical health, which is a negative emotional attitude."}, "language_style": {"answer": "Calm and direct", "rationale": "The entry explicitly asks if the person tested is suffering from a suspicious illness, does not use excessive rhetorical or emotional rendering, and is more objective in tone. This direct and concise expression is more conducive to obtaining accurate answers and valid data."}, "time_dimension": {"answer": "Past", "rationale": "The entry asks the testee whether he has symptoms of a suspicious disease and the temporal dimension is a matter of past."}, "sentence_type": "Interrogative sentence", "pos": {"nouns": {"Suspected": 1}}}, {"content": "16. Weight reduction", "word_count": 4, "label": "", "sentence_structure": "Subject鈥搗erb鈥揷omplement", "subjective_objective": {"answer": "Objective", "rationale": "As weight loss is an objective result, it is not possible to judge whether the patient is conscious of weight loss."}, "sentence_sentiment": {"answer": "Negative", "rationale": "Because weight loss may be a manifestation of a health problem, it has a negative emotional effect."}, "language_style": {"answer": "Calm and direct", "rationale": "The entry asks the other in a direct and clear manner whether he has experienced weight loss, does not use excessive rhetorical techniques or emotional rendering, and is more objective and cold."}, "time_dimension": {"answer": "Future", "rationale": "The key word \"the future\" appears in the entry, referring to the possible future scenario, where the respondent may experience a loss of weight in the future."}, "sentence_type": "Affirmative declarative sentence", "pos": {"nouns": {"Weight": 1, "Mitigation": 1}, "verbs": {"Mitigation": 1}}}, {"content": "17. Self-awareness", "word_count": 3, "label": "", "sentence_structure": "Subject鈥搗erb鈥揷omplement", "subjective_objective": {"answer": "Subjective", "rationale": "Because \"self-awareness\" means a person's ability to know and understand his or her own psychological condition, and a person's subjective feelings and perceptions."}, "sentence_sentiment": {"answer": "Neutral", "rationale": "Because the entry does not have any emotional color."}, "language_style": {"answer": "Tactful and warm", "rationale": "The language style of the entry is more euphemistic and gentle, using a more accessible presentation to guide respondents to questions, such as \"self-awareness\" rather than \"know your own situation?\" This expression is more friendly and comfortable and more likely to get real answers."}, "time_dimension": {"answer": "Present", "rationale": "According to the description of the title, \"self-awareness\" is an assessment of the ability or characteristics that requires the testee to evaluate or report in the current state and therefore to judge now."}, "sentence_type": "Interrogative sentence", "pos": {"nouns": {"Self-aware": 1}}}, {"content": "18. Day and night change", "word_count": 4, "label": "", "sentence_structure": "Subject鈥搗erb鈥揷omplement", "subjective_objective": {"answer": "Subjective", "rationale": "Because \"changes of day and night\" describe the patient's own feelings and experiences, and are subjective. While day and night are indeed objective facts, in this question, \"changes of day and night\" emphasize subjective perceptions and experiences."}, "sentence_sentiment": {"answer": "Neutral", "rationale": "Because the entry does not have any emotional color."}, "language_style": {"answer": "Tactful and warm", "rationale": "The entry is more adept and interactive when it is spoken in a gentle and inviting manner, rather than by direct statements or orders."}, "time_dimension": {"answer": "Present", "rationale": "The entry does not clearly indicate the time dimension of past, present or future, and therefore it is difficult to make a specific judgement."}, "sentence_type": "Affirmative declarative sentence", "pos": {"nouns": {"Day": 1, "Night": 1, "Change": 1}, "verbs": {"have": 1}, "numerals": {"18": 1}}}, {"content": "19. Dissolution of personality or reality", "word_count": 7, "label": "", "sentence_structure": "Subject鈥搗erb鈥搊bject", "subjective_objective": {"answer": "Objective", "rationale": "Because the physical or personal disintegration of a person is an objective fact of the patient ' s experience."}, "sentence_sentiment": {"answer": "Negative", "rationale": "Because the notion of \"personal or physical disintegration\" in the entry is often considered a negative expression of mental health problems."}, "language_style": {"answer": "Calm and direct", "rationale": "The entry directly asks whether the person under investigation has a personal or physical breakdown, is not over-repeated or is spoken in a more objective and direct manner. It therefore determines that his language style is \"cool and direct\"."}, "time_dimension": {"answer": "Past", "rationale": "The entry relates to a psychological state or experience that requires the testee to recall the past and therefore judge the past."}, "sentence_type": "Interrogative sentence", "pos": {"nouns": {"Personality": 1, "Reality": 1, "Dissolution": 1}, "verbs": {"have": 1}}}, {"content": "Symptoms of paranoia", "word_count": 4, "label": "", "sentence_structure": "Subject鈥搗erb鈥揷omplement", "subjective_objective": {"answer": "Subjective", "rationale": "Because \"paranoid\" is a subjective psychological experience and cognitive distortion, patients' paranoia is a symptom of their subjective feelings and thinking."}, "sentence_sentiment": {"answer": "Negative", "rationale": "Because the word \"paranoid\" is usually associated with negative emotions and attitudes."}, "language_style": {"answer": "Calm and direct", "rationale": ""}, "time_dimension": {"answer": "Present", "rationale": ""}, "sentence_type": "Interrogative sentence", "pos": {"nouns": {"Paranoia symptoms": 1}}}, {"content": "21. Symptoms of coercion", "word_count": 4, "label": "", "sentence_structure": "Subject鈥搗erb鈥揷omplement", "subjective_objective": {"answer": "Subjective", "rationale": "Because \"compulsory symptoms\" are often mental, emotional or behavioural that the patient feels beyond his control, it is examined from the patient's subjective perspective."}, "sentence_sentiment": {"answer": "Negative", "rationale": "Because \"compulsive symptoms\" are usually a psychological distress, with negative emotional aspects."}, "language_style": {"answer": "Tactful and warm", "rationale": "The language style of the questionnaire entry is euphemism. It uses relatively mild language to guide respondents to reflect on their own situation, rather than asking them directly whether they have problems with the symptoms of coercion. Moreover, it attempts to build a relationship of trust and respect in order to make the respondents more willing to share their feelings."}, "time_dimension": {"answer": "Present", "rationale": "The entry now asks the testee whether the subject is currently suffering from coercion, and therefore the temporal dimension is now judged."}, "sentence_type": "Affirmative declarative sentence", "pos": {"nouns": {"Symptoms of coercion": 1}}}, {"content": "22. A sense of capacity erosion", "word_count": 5, "label": "", "sentence_structure": "Subject鈥揷opula鈥損redicative", "subjective_objective": {"answer": "Subjective", "rationale": "Since the sense of competence is an assessment and perception of one ' s abilities, it is the patient ' s subjective experience and judgement."}, "sentence_sentiment": {"answer": "Negative", "rationale": "Since \"capability loss\" is usually associated with negative sentiments such as reduced self-evaluation and frustration with self-confidence, it can be considered negative."}, "language_style": {"answer": "Calm and direct", "rationale": "The language style of the entry is relatively calm and straightforward, and the interviewer is guided by simple and clear lines of inquiry to reflect and answer questions, without excessive emotional or rhetorical styles, which meet the professional requirements of the psychological questionnaire."}, "time_dimension": {"answer": "Present", "rationale": ""}, "sentence_type": "Negative declarative sentence", "pos": {"nouns": {"Capacity": 1, "I'm feeling less": 1}}}, {"content": "23. Despair", "word_count": 3, "label": "", "sentence_structure": "Subject鈥搗erb鈥揷omplement", "subjective_objective": {"answer": "Subjective", "rationale": "Because despair is an individual feeling and experience, it is a subjective attitude and emotional reaction of the patient."}, "sentence_sentiment": {"answer": "Negative", "rationale": "Because \"depressive\" is a negative emotional state."}, "language_style": {"answer": "Calm and direct", "rationale": ""}, "time_dimension": {"answer": "Present", "rationale": "The entry in the questionnaire asked if the person tested was currently desperate, and the keyword \"difference\" referred to the state of emotion and therefore judged it to be now."}, "sentence_type": "Negative declarative sentence", "pos": {"nouns": {"Despair": 1}}}, {"content": "24. Subjugation", "word_count": 3, "label": "", "sentence_structure": "Subject鈥損redicate", "subjective_objective": {"answer": "Subjective", "rationale": "Because \"self-depression\" is an emotional and psychological state that belongs to the patient's own subjective feelings and experiences."}, "sentence_sentiment": {"answer": "Negative", "rationale": "Because \"the feeling of inferiority\" is a negative emotion or attitude."}, "language_style": {"answer": "Tactful and warm", "rationale": "The question is asked in a more euphemistic and gentle manner, with an encouragement and understanding, without a direct judgement or order, which facilitates the interviewee ' s relief and more truthful answer."}, "time_dimension": {"answer": "Present", "rationale": "The entry is not clear about the time when it is used, nor is it used in past or future times, so it can be judged to describe the current emotional state and feelings of the person being tested as the current temporal dimension."}, "sentence_type": "Negative declarative sentence", "pos": {"nouns": {"I'm so low": 1}}}]}

{"questionnaire_title": "- I'm not sure.", "questionnaire_category": "Mental health", "item_count": 15, "emotion_shift_overall": "From positive to negative", "language_style_overall": "Tactful and warm", "items": [{"content": "I am often nervous, even at informal gatherings.", "word_count": 17, "label": "", "sentence_structure": "Subject鈥揷opula鈥損redicative", "subjective_objective": {"answer": "Subjective", "rationale": "Because \"stretch\" is a subjective feeling or experience, which reflects the patient's psychological state and emotional reaction. Although the meeting is objective, the feelings and reactions to it are determined by the patient himself, and therefore it is a subjective examination."}, "sentence_sentiment": {"answer": "Negative", "rationale": "Because the act of \"stretching\" in the entry is negative."}, "language_style": {"answer": "Calm and direct", "rationale": "The purpose of this article is to be seen as more objective and direct, without too much emotional overture or rhetorical. It clearly asks whether the interviewees are often nervous at informal gatherings, calm and direct, and without too much subjective or recommendatory guidance. This expression is more in line with the form and purpose of the questionnaire and is more conducive to obtaining real feedback from the interviewees."}, "time_dimension": {"answer": "Present", "rationale": "\"and often\" shows that this feeling is recurrent, and \"I usually feel\" is my main word, and it's therefore a description of my state and my emotions."}, "sentence_type": "Negative declarative sentence", "pos": {"nouns": {"Party": 1, "Nervous": 1}, "verbs": {"I feel": 1, "be": 1}, "adjectives": {"Informal": 1}, "adverbs": {"Yeah": 1, "Regular": 1}, "numerals": {"One": 2}, "pronouns": {"I": 1}}}, {"content": "I am usually uncomfortable with a group of people I don't know.", "word_count": 20, "label": "", "sentence_structure": "Subject鈥搗erb鈥揷omplement", "subjective_objective": {"answer": "Subjective", "rationale": "Because feeling uncomfortable is a subjective feeling that reflects the patient's personal experience and attitude to dealing with strangers.\""}, "sentence_sentiment": {"answer": "Negative", "rationale": "Because \"unsatisfied\" is a negative sentiment, which means that the author is uncomfortable with strangers."}, "language_style": {"answer": "Tactful and warm", "rationale": "The language style of the entry is more moderate and attractive, and it does not directly criticize or accuse, but rather leads the respondents to express their feelings and views in a question-and-answer manner. At the same time, the term \"normally\" is used to make sentences more euphemistic and objective, avoiding unnecessary pressure and discomfort on the respondents."}, "time_dimension": {"answer": "Present", "rationale": "The entry in the questionnaire describes a feeling or state of affairs in the current situation, and the keyword \"I usually feel\" indicates that this is a regular situation, so the temporal dimension is now judged."}, "sentence_type": "Affirmative declarative sentence", "pos": {"nouns": {"Population": 1, "person": 2, "Group": 1, "self": 1}, "verbs": {"I feel": 1, "in": 1, "Together": 1, "Yes": 1, "Usually": 1, "I'm not comfortable": 1}, "adjectives": {"I don't know him": 1}, "adverbs": {"Usually": 1}, "numerals": {"2": 1}, "pronouns": {"I": 1}}}, {"content": "I usually feel comfortable talking to a heterosexual.", "word_count": 16, "label": "", "sentence_structure": "Subject鈥搗erb鈥揷omplement", "subjective_objective": {"answer": "Subjective", "rationale": "Because \"easy\" is a subjective feeling and experience, it is a description based on the patient's personal views and feelings and is therefore subjective."}, "sentence_sentiment": {"answer": "Positive", "rationale": "Because the word \"easy\" is a positive emotional attitude."}, "language_style": {"answer": "Tactful and warm", "rationale": "The entry is more personal and interactive when it is spoken in a gentle and inviting manner, rather than in direct statements or orders. At the same time, the way in which it is asked is more intimate and respectful of each other鈥檚 feelings."}, "time_dimension": {"answer": "Present", "rationale": ""}, "sentence_type": "Affirmative declarative sentence", "pos": {"nouns": {"Heterosexual": 1, "Talk": 1, "Time": 1}, "verbs": {"I": 1, "Usually": 1, "I feel": 1, "Easy": 1}, "adjectives": {"Easy": 1}, "adverbs": {"Usually": 1}, "numerals": {"3": 1}, "pronouns": {"One": 1}}}, {"content": "I was nervous when I had to talk to teachers or supervisors.", "word_count": 17, "label": "", "sentence_structure": "Subject鈥搗erb鈥揷omplement", "subjective_objective": {"answer": "Subjective", "rationale": "Because stress is a subjective emotional experience, driven by the patient ' s own inner feelings, not by objective circumstances."}, "sentence_sentiment": {"answer": "Negative", "rationale": "Because the \"strained\" sentiment in the entry is negative."}, "language_style": {"answer": "Calm and direct", "rationale": "The entry is of a direct, clear language style, is not based on excessive rhetorical or emotional rendering, and is more objective in tone."}, "time_dimension": {"answer": "Present", "rationale": "Now the entry indicates the current feelings and state of the testee, judging by the time dimension. The keyword \"when speaking to a teacher or supervisor\" indicates that this is a certain situation, and \"I feel nervous\" is the subjective experience of the testee, pointing to what is happening."}, "sentence_type": "Affirmative declarative sentence", "pos": {"nouns": {"Teacher": 1, "Sir": 1, "Talk": 2}, "verbs": {"I feel": 1, "Nervous": 1}, "adjectives": {"I have to": 1}, "numerals": {"4": 1}, "pronouns": {"I": 1}}}, {"content": "The parties often make me anxious and uncomfortable.", "word_count": 14, "label": "", "sentence_structure": "Subject鈥揷opula鈥損redicative", "subjective_objective": {"answer": "Subjective", "rationale": "Because anxiety and discomfort are the patient ' s own subjective and emotional experience, rather than the objective facts."}, "sentence_sentiment": {"answer": "Negative", "rationale": "Because \"anxiety\" and \"incomprehensible\" are negative emotions."}, "language_style": {"answer": "Tactful and warm", "rationale": ""}, "time_dimension": {"answer": "Present", "rationale": "Now the sentence indicates that the testees are describing their current state and feelings, and the keywords \"meetings regularly\" and \"fearing and uncomfortable\" are all the current situations. So the basis of the judgement is now."}, "sentence_type": "Negative declarative sentence", "pos": {"nouns": {"Party": 1, "Anxiety": 1, "I'm not comfortable": 1}, "verbs": {"鈾?And I'm gonna be 鈾?: 1, "I feel": 1}}}, {"content": "I may be less shy in social relations than most people.", "word_count": 20, "label": "", "sentence_structure": "Subject鈥揷opula鈥損redicative", "subjective_objective": {"answer": "Subjective", "rationale": "Because \"less shyness\" is the patient's subjective feeling and self-evaluation."}, "sentence_sentiment": {"answer": "Positive", "rationale": "Because the description \"less shyness in social interaction\" in the entry is positive."}, "language_style": {"answer": "Tactful and warm", "rationale": ""}, "time_dimension": {"answer": "Present", "rationale": ""}, "sentence_type": "Affirmative declarative sentence", "pos": {"nouns": {"6": 1, "person": 1, "Social interaction": 1, "Shame": 1}, "verbs": {"Maybe": 1, "Less": 1, "In... in": 1}, "adjectives": {"Most people": 1}, "adverbs": {"Compare": 1}, "numerals": {"6": 1, "One": 1}, "classifiers": {"individual": 1}, "pronouns": {"I": 1}}}, {"content": "I was often nervous when I spoke to same-sex people that I was not familiar with.", "word_count": 20, "label": "", "sentence_structure": "Subject鈥搗erb鈥揷omplement", "subjective_objective": {"answer": "Subjective", "rationale": "Because feeling nervous is the patient's subjective emotional reaction."}, "sentence_sentiment": {"answer": "Negative", "rationale": "Because \"strained\" is negative."}, "language_style": {"answer": "Calm and direct", "rationale": "\"Calm down straight.\""}, "time_dimension": {"answer": "Present", "rationale": ""}, "sentence_type": "Affirmative declarative sentence", "pos": {"nouns": {"7": 1, "Same-sex": 1, "Talk": 1}, "verbs": {"in": 1, "I feel": 1, "Nervous": 1}, "adjectives": {"Not really": 1, "I": 1}, "adverbs": {"Often": 1}}}, {"content": "I'm nervous about job interviews.", "word_count": 11, "label": "", "sentence_structure": "Subject鈥搗erb鈥揷omplement", "subjective_objective": {"answer": "Subjective", "rationale": "Because stress is the patient's subjective experience and emotional reaction."}, "sentence_sentiment": {"answer": "Negative", "rationale": "The basis for the judgment: the act of \"climate\" in the entry is negative."}, "language_style": {"answer": "Tactful and warm", "rationale": ""}, "time_dimension": {"answer": "Present", "rationale": "The entry describes the current state of the testees, i.e. whether they are nervous during job interviews, and can therefore be judged as now. The keyword \"will\" represents a customary, recurring situation, which further indicates that this is happening now or recently."}, "sentence_type": "Affirmative declarative sentence", "pos": {"nouns": {"Job interviews": 1, "Nervous": 1}, "verbs": {"Yes": 1, "in": 1, "Interview": 1, "Job seeking": 1}, "numerals": {"8": 1}}}, {"content": "I hope I have more confidence in social life.", "word_count": 17, "label": "", "sentence_structure": "Subject鈥搗erb鈥揷omplement", "subjective_objective": {"answer": "Subjective", "rationale": "Because \"hope\" is a subjective wish or expectation."}, "sentence_sentiment": {"answer": "Positive", "rationale": "Because both hope and confidence in the entries express positive attitudes and emotions."}, "language_style": {"answer": "Tactful and warm", "rationale": "The language style of the entry is more euphemistic, and it does not directly criticize or dictate to the other, but rather expresses hope and advice in a way that encourages and supports it. At the same time, the phrase \"hope\" also has a positive emotional tone, giving people a sense of confidence and strength that is more acceptable and willing to change."}, "time_dimension": {"answer": "Future", "rationale": "The entry contains expectations and visions for the future, and the words \"hope\" and \"confidence\" point to what may have not yet happened and become possible in the future. At the same time, the phrase \"self in the social setting\" implies that this is one of the goals of the future of the individual. Thus, the temporal dimension of the article is to be judged in the future."}, "sentence_type": "Imperative sentence", "pos": {"nouns": {"self": 1, "Socially": 1, "Confidence": 1}, "verbs": {"Hope": 1, "In... in": 1}, "adjectives": {"More": 1, "Foot": 1}, "numerals": {"9": 1}, "pronouns": {"self": 1}}}, {"content": "I'm not very anxious about it in social settings.", "word_count": 13, "label": "", "sentence_structure": "Subject鈥揷opula鈥損redicative", "subjective_objective": {"answer": "Objective", "rationale": "Because in this sentence, \"in social settings\" describes an objective scene or situation, \"very little anxiety\" is a statement of the patient's subjective feelings in a given situation. The entry is therefore objective."}, "sentence_sentiment": {"answer": "Positive", "rationale": "Because it expresses a positive feeling and attitude."}, "language_style": {"answer": "Tactful and warm", "rationale": "The entry is more adept and interactive when it is spoken in a gentle and inviting manner, rather than by direct statements or orders."}, "time_dimension": {"answer": "Present", "rationale": ""}, "sentence_type": "Affirmative declarative sentence", "pos": {"nouns": {"Socially": 1, "Anxiety": 1}, "verbs": {"I feel": 1, "Very few": 1}, "adjectives": {"10": 1}}}, {"content": "I'm generally a shy person.", "word_count": 12, "label": "", "sentence_structure": "Subject鈥揷opula鈥損redicative", "subjective_objective": {"answer": "Subjective", "rationale": "Because \"scrambling\" is a subjective feeling and self-evaluation, the entry on the questionnaire is subjective."}, "sentence_sentiment": {"answer": "Neutral", "rationale": "Because the entry does not have any emotional color."}, "language_style": {"answer": "Tactful and warm", "rationale": "The entry uses a more euphemistic and implicit language style, which does not directly emphasize or negate the character of the person, but is presented by way of self-characterization."}, "time_dimension": {"answer": "Present", "rationale": ""}, "sentence_type": "Imperative sentence", "pos": {"nouns": {"In general": 1, "person": 1}, "verbs": {"be": 1, "Shy": 1}, "adjectives": {"One": 1}, "adverbs": {"In general": 1}, "numerals": {"11": 1}, "pronouns": {"I": 1}}}, {"content": "I'm always nervous when I talk to a charming heterosexual.", "word_count": 19, "label": "", "sentence_structure": "Subject鈥揷opula鈥損redicative", "subjective_objective": {"answer": "Subjective", "rationale": "Because \"stretching\" is a subjective experience and feeling, a patient's emotional state."}, "sentence_sentiment": {"answer": "Negative", "rationale": "Because the \"frightful\" sentiment in the entry is negative."}, "language_style": {"answer": "Calm and direct", "rationale": "The entry is more directly linguistically, without excessive rhetorical or emotional rendering, but only objectively asking the interviewee about the feelings and reactions in a given situation."}, "time_dimension": {"answer": "Present", "rationale": ""}, "sentence_type": "Affirmative declarative sentence", "pos": {"nouns": {"12": 1, "Heterosexual": 1, "Talk": 1, "Nervous": 1}, "verbs": {"in": 1, "I feel": 1}, "adjectives": {"Fascinating": 1}, "numerals": {"One": 1}}}, {"content": "I'm usually nervous when I call someone I don't know.", "word_count": 17, "label": "", "sentence_structure": "Subject鈥搗erb鈥揷omplement", "subjective_objective": {"answer": "Subjective", "rationale": "Because \"stretch\" is a subjective experience, it's a patient's inner feeling, so this questionnaire is viewed from the patient's subjective perspective."}, "sentence_sentiment": {"answer": "Negative", "rationale": "Because the act of \"stretching\" in the entry is negative."}, "language_style": {"answer": "Tactful and warm", "rationale": "The entry is more adept and interactive when it is spoken in a gentle and inviting manner, rather than by direct statements or orders."}, "time_dimension": {"answer": "Present", "rationale": "The keyword \"calling a person who is not familiar\" and \"usually feeling nervous\" point to the current behaviour or state, so the entry describes the person being tested for the present behaviour or feeling."}, "sentence_type": "Negative declarative sentence", "pos": {"nouns": {"Telephone": 1, "person": 1}, "verbs": {"I think": 1, "Fight": 1, "Nervous": 1}, "adjectives": {"Not really": 1, "It's familiar": 1}, "adverbs": {"Usually": 1}, "numerals": {"13": 1}, "pronouns": {"I": 1}}}, {"content": "I was nervous when I spoke to the authorities.", "word_count": 14, "label": "", "sentence_structure": "Subject鈥搗erb鈥揷omplement", "subjective_objective": {"answer": "Subjective", "rationale": "This perception is usually due to the psychological state and experience of the individual, rather than to an assessment based on objective facts."}, "sentence_sentiment": {"answer": "Negative", "rationale": "Because the \"frightful\" sentiment in the entry is negative."}, "language_style": {"answer": "Calm and direct", "rationale": "The entry is in a concise and straightforward manner, asking the interviewees whether they are nervous when talking to authorities, and whether they are more direct and objective in their language style."}, "time_dimension": {"answer": "Present", "rationale": "In the entry, \"I'm nervous\" is a description of the person's current feelings or state, and therefore is the time dimension of the present."}, "sentence_type": "Affirmative declarative sentence", "pos": {"nouns": {"14": 1, "person": 1, "Talk": 1, "Authority": 1}, "verbs": {"in": 1, "I feel": 1, "Nervous": 1}, "numerals": {"14": 1}, "classifiers": {"individual": 1}, "pronouns": {"I": 1}}}, {"content": "I'm usually relaxed even in a group that's quite different from me.", "word_count": 25, "label": "", "sentence_structure": "Subject鈥搗erb鈥揷omplement", "subjective_objective": {"answer": "Subjective", "rationale": "Because the title refers to the feeling of \"me\" and emphasizes the subjective experience of the individual, it is a subjective perspective to look at."}, "sentence_sentiment": {"answer": "Neutral", "rationale": "Because the entry describes a state of affairs, which is not clearly emotional and is neutral."}, "language_style": {"answer": "Calm and direct", "rationale": "The entry is based on the following: - The entry is a point that is open: \"I usually feel relaxed, even in a group that is quite different from me,\" without any rhetorical or emotional colour. - The phrase \"15\" may be a question number or an option number, which is more objective and direct."}, "time_dimension": {"answer": "Present", "rationale": "The keyword \"even\" and \"usually\" indicates that the act is a current norm and that there is a possibility that it will continue to occur in the future."}, "sentence_type": "Affirmative declarative sentence", "pos": {"nouns": {"15": 1, "Population": 2, "I": 2}, "verbs": {"in": 1, "I feel": 1, "Relax": 1}, "adjectives": {"Pretty much": 1, "Different": 1}, "adverbs": {"Usually": 1}, "numerals": {"15": 1, "2": 2}, "classifiers": {"individual": 1}, "pronouns": {"I": 2}}}]}

{"questionnaire_title": "Carroll depression self-assessment", "questionnaire_category": "Mental health", "item_count": 52, "emotion_shift_overall": "From positive to negative", "language_style_overall": "Neutral", "items": [{"content": "I feel good about my spirit.", "word_count": 6, "label": "", "sentence_structure": "Subject鈥揷opula鈥損redicative", "subjective_objective": {"answer": "Subjective", "rationale": "Because \"fEss\" represents the patient's subjective feeling, which is a subjective description."}, "sentence_sentiment": {"answer": "Positive", "rationale": "Because the description of \"frightful\" is a positive emotional state."}, "language_style": {"answer": "Calm and direct", "rationale": ""}, "time_dimension": {"answer": "Present", "rationale": ""}, "sentence_type": "Affirmative declarative sentence", "pos": {"verbs": {"I feel": 1, "Okay": 1}, "adjectives": {"Fine": 1, "God": 1}}}, {"content": "I feel, unfortunately or often, like to cry.", "word_count": 11, "label": "", "sentence_structure": "Subject鈥揷opula鈥損redicative", "subjective_objective": {"answer": "Subjective", "rationale": "Because \"unlucky\" and \"often feeling like crying\" are emotional experiences that patients feel, and are seen from a subjective perspective."}, "sentence_sentiment": {"answer": "Negative", "rationale": "Because \"unfortunate\" and \"often feeling like crying\" in the entries are expressions of negative emotional state."}, "language_style": {"answer": "Tactful and warm", "rationale": "The entry uses a more moderate expression to ask the interviewees whether they are often unhappy or sad, and does not directly use strong negative emotional vocabulary, so the language style is euphemismal and warm. Such a design would make them more willing to share their true feelings, help to better collect data and understand the situation of the interviewees."}, "time_dimension": {"answer": "Past", "rationale": "\"I'm sorry\" means that there's been an unfortunate experience in the past, and \"often feeling like I'm crying\" may be describing the current situation. So the time dimension of this questionnaire entry is related to the past and the present."}, "sentence_type": "Negative declarative sentence", "pos": {"verbs": {"very": 1, "Unfortunately": 1, "Often": 1, "I think": 1, "Cry": 1}, "adjectives": {"Unfortunately": 1}, "adverbs": {"very": 1, "Often": 1}}}, {"content": "I think that I have lost hope in this situation.", "word_count": 12, "label": "", "sentence_structure": "Subject鈥揷opula鈥損redicative", "subjective_objective": {"answer": "Subjective", "rationale": "Because \"I think\" is a subjective expression."}, "sentence_sentiment": {"answer": "Negative", "rationale": "Because the expression \"no more hope\" in the entry has a negative emotional connotation."}, "language_style": {"answer": "Calm and direct", "rationale": "The language style of the entry is relatively straightforward and concise, and it is less speech-based or emotional, and it is more calm and straightforward. At the same time, the context of the article indicates that the person asked wants to know whether the person surveyed is desperate or hopeless about the current situation, which is relatively objective and neutral."}, "time_dimension": {"answer": "Past", "rationale": "The sentence expresses the views and assessments of the testees about their current situation, and the word \"want\" indicates that this is subjective thinking and judgement, while \"the situation is hopeless\" expresses a sense of despair about the current situation, which is a time dimension of the past."}, "sentence_type": "Negative declarative sentence", "pos": {"nouns": {"Situation": 1, "Hope": 1}, "verbs": {"Yes": 2, "have": 1}, "adjectives": {"This one": 1, "Already": 1}, "pronouns": {"I": 1}}}, {"content": "My future can only be unfortunate.", "word_count": 10, "label": "", "sentence_structure": "Subject鈥揷opula鈥損redicative", "subjective_objective": {"answer": "Subjective", "rationale": "Because \"the future can only be unfortunate\" is a personal subjective feeling or belief, not an objective fact."}, "sentence_sentiment": {"answer": "Negative", "rationale": "Because the expression \"can only be unfortunate\" in the entry is filled with negative pessimism."}, "language_style": {"answer": "Calm and direct", "rationale": "The entry is a direct expression of the subject of a question about whether the other party has a pessimistic view of the future, and is not based on too many rhetorical or emotional expressions, which are more objective in tone."}, "time_dimension": {"answer": "Future", "rationale": "The entry expresses expectations and perceptions of the future, and the keywords are \"the future\" and \"only\" , all of which point to situations that have not yet occurred and that may arise in the future. The entry is therefore a time dimension of future behaviour or mentality."}, "sentence_type": "Negative declarative sentence", "pos": {"verbs": {"It will be": 1}, "adjectives": {"Unfortunately": 1}}}, {"content": "I feel like I'm a good person like everyone else.", "word_count": 13, "label": "", "sentence_structure": "Subject鈥揷opula鈥損redicative", "subjective_objective": {"answer": "Subjective", "rationale": "Because \"I feel\" expresses the patient's subjective feelings and evaluation."}, "sentence_sentiment": {"answer": "Neutral", "rationale": "The entry is not positive or negative."}, "language_style": {"answer": "Tactful and warm", "rationale": "The entry uses the words \"like\" and \"like\" to appear subjective and soft, to be more euphemistic and express more natural than direct statements. At the same time, the context of the entry also shows that it is intended to explore the feelings and views of the interviewee ' s self-evaluation, rather than to judge or order the interviewee."}, "time_dimension": {"answer": "Present", "rationale": "The entry now represents the subjective and self-evaluation of the respondent in answering the questions, and thus the temporal dimension is now judged."}, "sentence_type": "Affirmative declarative sentence", "pos": {"nouns": {"I": 1, "others": 1, "Good man": 1}, "verbs": {"I think": 1, "be": 1, "Same": 1}, "adjectives": {"individual": 1}, "numerals": {"5": 1}}}, {"content": "6. I feel worthless and ashamed of myself.", "word_count": 16, "label": "", "sentence_structure": "Subject鈥揷opula鈥損redicative", "subjective_objective": {"answer": "Subjective", "rationale": "Because feeling worthless and ashamed is the patient ' s inner feeling and self-evaluation, it is a subjective examination."}, "sentence_sentiment": {"answer": "Negative", "rationale": "Because the \"Establishing for Self-Defeating and Shame for For Me\" in the entry is negative."}, "language_style": {"answer": "Tactful and warm", "rationale": "The language style of this entry is euphemism. It uses emotional words (such as \"fear\" and \"sham\") and avoids direct accusations or command language. This expression is more likely to create an atmosphere of dialogue and understanding that makes the interviewee more receptive and willing to answer questions."}, "time_dimension": {"answer": "Present", "rationale": "The words \"I feel\" are a description of the emotional state, \"I'm worthless\" and \"I'm ashamed of myself\" are all depictions of the subjective feeling of the subject."}, "sentence_type": "Negative declarative sentence", "pos": {"nouns": {"Value": 1, "self": 2, "Shame": 1}, "verbs": {"Sensor": 2, "have": 1, "Yes": 1}, "numerals": {"6": 1}, "pronouns": {"I": 1}}}, {"content": "Some things in life have upset me and upset me.", "word_count": 16, "label": "", "sentence_structure": "Subject鈥搗erb鈥揷omplement", "subjective_objective": {"answer": "Subjective", "rationale": "Because it is a description of what patients feel, it involves subjective emotional reactions and reflection."}, "sentence_sentiment": {"answer": "Negative", "rationale": "Because \"repentant\" and \"disturbed\" are some negative emotional terms."}, "language_style": {"answer": "Calm and direct", "rationale": "The entry directly expresses the subject of asking the other party whether there are any regrets and concerns in life, and is not based on excessive rhetorical or emotional rendering, which is more objective and balanced."}, "time_dimension": {"answer": "Present", "rationale": "The phrase \"repentant\" and \"involved\" in the sentence is used to describe the person's current feelings or experiences."}, "sentence_type": "Affirmative declarative sentence", "pos": {"nouns": {"Life": 1, "Something": 1, "Regret": 1, "I'm upset": 1}, "verbs": {"鈾?And I'm gonna be 鈾?: 2}, "adjectives": {"Regretful": 1, "I'm upset": 1}, "numerals": {"7": 1}}}, {"content": "I am being punished for the bad deeds of my past life.", "word_count": 18, "label": "", "sentence_structure": "Subject鈥搗erb鈥搊bject", "subjective_objective": {"answer": "Subjective", "rationale": "Because \"punishment\" is a subjective feeling and judgment. Although it may be linked to the objective facts of the past, it still reflects the current mood and conviction of the patient. So, it is a subjective issue."}, "sentence_sentiment": {"answer": "Negative", "rationale": "The basis for the judgement: \"Punishment\" is a negative term, understood by the context, and expresses a negative emotion."}, "language_style": {"answer": "Calm and direct", "rationale": "The entry describes the subject in a straightforward language, without any accrimation or emotional colour, and the language style is a calm one.\""}, "time_dimension": {"answer": "Past", "rationale": "The entry describes the current state and feelings of the testee and refers to \"bad things done in the past\" and therefore its temporal dimension is a matter of the past."}, "sentence_type": "Negative declarative sentence", "pos": {"nouns": {"Life": 1, "Bad things": 1, "Punishment": 1}, "verbs": {"Yes": 1, "Fuck": 1, "In the process of": 1}, "adverbs": {"Past": 1}, "classifiers": {"Sou-san": 1}, "pronouns": {"I": 1}}}, {"content": "I feel that life still makes sense.", "word_count": 9, "label": "", "sentence_structure": "Subject鈥揷opula鈥損redicative", "subjective_objective": {"answer": "Subjective", "rationale": "Because it is a subjective expression of consciousness, it is necessary to judge whether life is meaningful in the light of the individual ' s own views and feelings."}, "sentence_sentiment": {"answer": "Positive", "rationale": "Because the attitude of \"life still makes sense\" is positive."}, "language_style": {"answer": "Tactful and warm", "rationale": "The language style of the entry is euphemistic, soft and not directly positive or negative, but rather an invitation to ask each other about their feelings, which is more acceptable and willing to share their thoughts."}, "time_dimension": {"answer": "Present", "rationale": ""}, "sentence_type": "Affirmative declarative sentence", "pos": {"nouns": {"Life": 1, "Meaning": 1}, "verbs": {"I feel": 1}, "pronouns": {"I": 1}}}, {"content": "I often wish I had died.", "word_count": 11, "label": "", "sentence_structure": "Subject鈥搗erb鈥揷omplement", "subjective_objective": {"answer": "Subjective", "rationale": "Because hope is a subjective expression of emotion and desire, and it looks at the patient鈥檚 inner feelings and thoughts, it is subjective. Although this phrase contains an objective fact 鈥?that the patient is alive 鈥?it is not sufficient to change the subjectivity of this purpose."}, "sentence_sentiment": {"answer": "Negative", "rationale": "Because the idea of \"hope to be dead\" in the entry is negative."}, "language_style": {"answer": "Calm and direct", "rationale": "This entry raises questions in a direct and clear manner, without any euphemism or accommodation, and is more calm and objective."}, "time_dimension": {"answer": "Past", "rationale": ""}, "sentence_type": "Negative declarative sentence", "pos": {"nouns": {"self": 1, "Die": 1}, "verbs": {"Hope": 1, "Yes": 1}, "adverbs": {"Regular": 1}, "numerals": {"10": 1}, "pronouns": {"I": 1}}}, {"content": "I was also thinking about suicide.", "word_count": 9, "label": "", "sentence_structure": "Subject鈥搗erb鈥搊bject", "subjective_objective": {"answer": "Subjective", "rationale": "Because \"thinking how to commit suicide\" reflects the thoughts and intentions of the patient, and is a subjective expression. Although it is an unhealthy idea, it is still the expression of the emotional and psychological state of the individual. It is therefore appropriate to look at the questionnaire from the patient's subjective point of view."}, "sentence_sentiment": {"answer": "Negative", "rationale": "Because suicide is negative."}, "language_style": {"answer": "Calm and direct", "rationale": "The language style of the topic is so cold and the use of a very direct term, \"suicide,\" that it may be a way of expressing the feelings of discomfort and even panic. It is suggested that psychological questionnaires should be designed in such a way as to avoid the use of language that is too direct or that may cause unwelcome."}, "time_dimension": {"answer": "Present", "rationale": "In future, the entry, although it uses the past \"still\" but in terms of context and meaning, expresses the intended and planned future of the testee, i.e., the way they are considering suicide. The time dimension of the questionnaire should therefore be judged as the future. This is also a very serious issue that requires attention and timely measures."}, "sentence_type": "Negative declarative sentence", "pos": {"nouns": {"Think": 1, "Suicide": 1}, "verbs": {"in": 1, "Think": 1, "What": 1, "Suicide": 1}, "adverbs": {"And": 1, "What": 1}, "numerals": {"11": 1}, "pronouns": {"I": 1}}}, {"content": "12. Death is the best way to escape me.", "word_count": 11, "label": "", "sentence_structure": "Subject鈥揷opula鈥損redicative", "subjective_objective": {"answer": "Subjective", "rationale": "Because this is a patient ' s perception of his or her death and a subjective assessment."}, "sentence_sentiment": {"answer": "Negative", "rationale": "Because the article's purpose is to say \"death is the best way to get away with it\", it is a very negative and unhealthy attitude."}, "language_style": {"answer": "Calm and direct", "rationale": "The entry raises the question in a straightforward manner, without any modification or rendering, and with an objective calm tone."}, "time_dimension": {"answer": "Present", "rationale": "The entry expresses the subjective view and conviction of the person being tested and does not mention any information about past or future time. It is therefore a description of the current state of affairs."}, "sentence_type": "Affirmative declarative sentence", "pos": {"nouns": {"Die": 1, "I'd better": 1, "Release": 1}, "verbs": {"be": 1, "Here": 1}, "adjectives": {"I": 1}}}, {"content": "13. At night, I need longer than usual to sleep.", "word_count": 18, "label": "", "sentence_structure": "Subject鈥揷opula鈥損redicative", "subjective_objective": {"answer": "Subjective", "rationale": "Because it's the patient's subjective feeling and experience that requires more time to sleep, it's not necessarily directly related to the objective environment.\""}, "sentence_sentiment": {"answer": "Negative", "rationale": "Because the \"sleeping\" behavior in the entry \"takes longer than usual\" is negative."}, "language_style": {"answer": "Calm and direct", "rationale": "The language style of the entry is relatively simple and does not have too much adoration or emotional colour, and gives an objective and professional impression by asking the other person about his sleep at night."}, "time_dimension": {"answer": "Present", "rationale": "The entry describes the current behaviour and state of the subject, \"at night\" and \"which takes longer than normal to sleep\" and indicates that this is the current situation. The time dimension of the article is therefore the present."}, "sentence_type": "Affirmative declarative sentence", "pos": {"nouns": {"Time": 1, "Sleep": 1}, "verbs": {"Yes": 1, "People sleep": 1}, "adjectives": {"Long": 1, "Normal": 1}, "adverbs": {"More": 1, "That's right": 1}, "numerals": {"13": 1}, "pronouns": {"I": 1}}}, {"content": "14. It takes me more than half an hour to sleep.", "word_count": 15, "label": "", "sentence_structure": "Subject鈥搗erb鈥揷omplement", "subjective_objective": {"answer": "Subjective", "rationale": "Because \"I need more than half an hour to sleep\" is a subjective feeling and experience that reflects the individual's sleep habits and needs."}, "sentence_sentiment": {"answer": "Negative", "rationale": "Because the entry \"it takes more than half an hour to sleep\" is negative."}, "language_style": {"answer": "Tactful and warm", "rationale": "The language style of the entry is relatively mild, using the subjective expression \"I need\" rather than directly accusing or ordering the other to change the sleep habits, making it easier to accept and fill in. At the same time, the tone of the query is used to make people feel respectable and concerned."}, "time_dimension": {"answer": "Present", "rationale": "The keywords are \"needs\" and \"more than half an hour\" and they all point to the current state or behaviour. The answer to this article should therefore be \"now\"."}, "sentence_type": "Affirmative declarative sentence", "pos": {"nouns": {"Time": 1, "Sleep": 1}, "verbs": {"Yes": 1, "Enter": 1, "Sleep": 1}, "adjectives": {"Long": 1}, "adverbs": {"Half": 1, "Go, go, go": 1, "Here": 1, "That's right": 1}, "numerals": {"Half an hour": 1, "14": 1}, "pronouns": {"I": 1}}}, {"content": "I slept in vain and in vain.", "word_count": 11, "label": "", "sentence_structure": "Subject鈥搗erb鈥揷omplement", "subjective_objective": {"answer": "Subjective", "rationale": "Because the feeling of \"insatiable\" and \"inaccurate\" is the patient's subjective experience. Although sleep conditions may be objective facts (e.g., sleep time or quality), it is subjective and may vary from person to person."}, "sentence_sentiment": {"answer": "Negative", "rationale": "Because sleepless, false is a negative physical description."}, "language_style": {"answer": "Tactful and warm", "rationale": "The entry is more personal and interactive when it is spoken in a gentle and inviting manner, rather than in direct statements or orders. At the same time, the first person ' s pronoun 'I' is used, making the interviewee more comfortable with the experience and willing to share his feelings and circumstances."}, "time_dimension": {"answer": "Present", "rationale": ""}, "sentence_type": "Negative declarative sentence", "pos": {"nouns": {"I": 2, "Sleep": 3, "Solve the poverty": 1, "Sleep well": 1, "Minori": 1}, "verbs": {"Sleep": 1, "I'm not tired": 1}, "adjectives": {"not": 1, "Minori": 1}, "adverbs": {"Yeah": 1}, "numerals": {"15": 1, "2": 1, "3": 1}}}, {"content": "I often wake up at night.", "word_count": 7, "label": "", "sentence_structure": "Subject鈥搗erb鈥揷omplement", "subjective_objective": {"answer": "Subjective", "rationale": "Because \"always wake up\" is the patient's subjective feeling and experience."}, "sentence_sentiment": {"answer": "Negative", "rationale": "Because the \"often wake up\" act is negative."}, "language_style": {"answer": "Tactful and warm", "rationale": "The entry asks the other side for sleep problems in a concerned and understanding manner, without direct accusations or judgement, and this expression is more likely to elicit positive responses and sharing from the interviewees."}, "time_dimension": {"answer": "Present", "rationale": ""}, "sentence_type": "Affirmative declarative sentence", "pos": {"verbs": {"Often": 1, "Wake up": 1}, "adverbs": {"Night": 1}}}, {"content": "I woke up earlier than usual in the morning.", "word_count": 9, "label": "", "sentence_structure": "Subject鈥揷opula鈥損redicative", "subjective_objective": {"answer": "Objective", "rationale": "Because \"awakening in the morning before normal\" is the patient's objective experience and observed fact."}, "sentence_sentiment": {"answer": "Neutral", "rationale": "Because the entry does not express positive or negative feelings, attitudes, behaviours, aims or qualities."}, "language_style": {"answer": "Tactful and warm", "rationale": "The entry is presented in the first person's name and the adjective \"early\" is used to describe its behaviour, euphemism and subjectivity. Such expression is more likely to resonate and engage the interviewees and to gain better access to their real feelings and ideas."}, "time_dimension": {"answer": "Past", "rationale": "Past."}, "sentence_type": "Affirmative declarative sentence", "pos": {"verbs": {"I'm awake": 2, "Morning": 1}, "adjectives": {"Normal": 1}, "pronouns": {"I": 1}}}, {"content": "18. I woke up too early in the morning, affecting daily life.", "word_count": 16, "label": "", "sentence_structure": "Subject鈥搗erb鈥揷omplement", "subjective_objective": {"answer": "Subjective", "rationale": "Because \"the morning wakes too early to affect everyday life\" is the patient's subjective feelings and experiences, not the objective facts or behaviour."}, "sentence_sentiment": {"answer": "Negative", "rationale": "Because the act of \"awaking too early in the morning to affect daily life\" in the entry is negative and indicates that life has been negatively affected."}, "language_style": {"answer": "Tactful and warm", "rationale": "The entry is more personal and interactive when it is spoken in a gentle and inviting manner, rather than directly speaking or ordering. At the same time, the expression \"so as to affect everyday life\" is more subtle, does not accuse the other side of his behaviour too directly and makes it easier for the other side to accept and cooperate with the investigation."}, "time_dimension": {"answer": "Past", "rationale": ""}, "sentence_type": "Negative declarative sentence", "pos": {"nouns": {"Morning": 1, "Life": 1}, "verbs": {"Wake up": 1, "Impact": 1}, "adjectives": {"Too much": 1, "Morning": 1}, "numerals": {"18": 1}, "pronouns": {"I": 1}}}, {"content": "I can get happiness and satisfaction from what I do.", "word_count": 17, "label": "", "sentence_structure": "Subject鈥揷opula鈥損redicative", "subjective_objective": {"answer": "Subjective", "rationale": "Because \"to get happiness and satisfaction from what you do\" is the patient's subjective feeling and attitude."}, "sentence_sentiment": {"answer": "Positive", "rationale": "Because the entries express positive attitudes and feelings of happiness and satisfaction with what they do."}, "language_style": {"answer": "Tactful and warm", "rationale": "The entry is more euphemistic and warm, not directly expressing the theme \"Can you get happiness from your work\" but rather directing the respondents to think about it in a gentle, question-like way and give them their personal views. This expression is more friendly and acceptable, and it encourages the respondents to share their views and feelings."}, "time_dimension": {"answer": "Present", "rationale": "The word \"capable\" takes the form of a verb of a state of power or state, and the word \"doing\" is a term of the past that describes past actions. So the entry expresses the feeling that now, it can now be happy and satisfied with what it does."}, "sentence_type": "Affirmative declarative sentence", "pos": {"nouns": {"Yes": 1, "Happy": 1, "Satisfied": 1}, "verbs": {"Yes": 1, "Okay": 1, "Do it": 1}, "adjectives": {"self": 1}, "numerals": {"19": 1}, "classifiers": {"individual": 1}}}, {"content": "I still like to go out and meet people.", "word_count": 10, "label": "", "sentence_structure": "Subject鈥搗erb鈥揷omplement", "subjective_objective": {"answer": "Subjective", "rationale": "Because \"still like\" is the patient's subjective attitude or feeling, not the objective fact or the behaviour that happens."}, "sentence_sentiment": {"answer": "Positive", "rationale": "Because the entry \"likes to meet with people outside\" expresses a positive emotional attitude."}, "language_style": {"answer": "Tactful and warm", "rationale": "The entry is more euphemistic, soft in its language, and does not directly confirm or deny the request for an answer, but rather allows the interviewee to express his thoughts and feelings freely."}, "time_dimension": {"answer": "Present", "rationale": "This sentence indicates the current state or feeling, and the word \"still\" indicates that such a preference has existed for some time and is likely to continue. Thus, the temporal dimension of the article is judged as \"now\"."}, "sentence_type": "Affirmative declarative sentence", "pos": {"nouns": {"Out": 1, "person": 1, "Meeting": 1}, "verbs": {"Yeah": 1, "Out": 1, "See": 1}, "adverbs": {"Still": 1}, "classifiers": {"20": 1}}}, {"content": "21. I have lost many interests.", "word_count": 10, "label": "", "sentence_structure": "Subject鈥搗erb鈥揷omplement", "subjective_objective": {"answer": "Subjective", "rationale": "Because loss of hobby is a subjective feeling and judgement, different people may have different views and feelings."}, "sentence_sentiment": {"answer": "Negative", "rationale": "Because the \"lost many interests\" act in the entry is negative."}, "language_style": {"answer": "Tactful and warm", "rationale": "The entry uses a more euphemistic wording, and \"too many missing\" does not directly raise problems, but rather implicitly expresses the confusion or distress that may exist in the other side. Such expressions are more comfortable and more acceptable and responsive."}, "time_dimension": {"answer": "Past", "rationale": "The entry describes the experience and feelings of individuals prior to a certain point in time and can therefore be judged as past."}, "sentence_type": "Negative declarative sentence", "pos": {"nouns": {"A hobby": 1, "Loss": 1}, "verbs": {"Many": 1, "have": 1, "Lost": 1}, "numerals": {"21": 1}, "pronouns": {"I": 1}}}, {"content": "22. I can still do what I want to do.", "word_count": 13, "label": "", "sentence_structure": "Subject鈥搗erb鈥搊bject", "subjective_objective": {"answer": "Subjective", "rationale": "Because \"hope\" and \"maintenance\" are patients' subjective attitudes or wishes."}, "sentence_sentiment": {"answer": "Positive", "rationale": "Because \"suspensing on doing what you want\" in the entry is a positive attitude and behavior."}, "language_style": {"answer": "Calm and direct", "rationale": "The language style of this entry is calm and straightforward. It is simple to ask whether the person under investigation is still able to do what he wants, without any unnecessary aesthetic or emotional colour. This expression is more objective and neutral and does not guide the person under investigation in the direction of the answer."}, "time_dimension": {"answer": "Present", "rationale": "The entry \"can still\" represents the current situation or state, while \"I can keep doing what I want to do\" emphasizes expectations and plans for the future. So the entry is now about the time dimension."}, "sentence_type": "Affirmative declarative sentence", "pos": {"verbs": {"I can": 1, "Fuck": 1}, "adjectives": {"Hope": 1}, "adverbs": {"Still": 1}, "numerals": {"22": 1}, "pronouns": {"I": 1}}}, {"content": "My thoughts have been clear and fast.", "word_count": 10, "label": "", "sentence_structure": "Subject鈥揷opula鈥損redicative", "subjective_objective": {"answer": "Subjective", "rationale": "Because this phrase describes the subjective perception and feelings of the patient about his or her ability to think, it is viewed from a subjective perspective."}, "sentence_sentiment": {"answer": "Positive", "rationale": "Because the words \"the thought is always clear and fast\" are a description of a person's mental ability or state, a positive attitude or quality."}, "language_style": {"answer": "Calm and direct", "rationale": "The entry expresses the subject of questioning the other party ' s mental ability in a relatively concise manner, with no excessive emotional or rhetorical modus operandi and a more objective tone."}, "time_dimension": {"answer": "Past", "rationale": "The keyword \"have been\" shows that it is a customary description, that the person who was tested has been so, not just a one-time experience, for some time in the past. So, the entry describes the person who was tested in the way he was thinking."}, "sentence_type": "Affirmative declarative sentence", "pos": {"verbs": {"Always": 1, "Clear": 1, "Shortcut": 1}, "adjectives": {"Mine": 1, "Ideas": 1}, "numerals": {"23": 1}}}, {"content": "I was slow to speak and I was not angry.", "word_count": 9, "label": "", "sentence_structure": "Subject鈥搗erb鈥揷omplement", "subjective_objective": {"answer": "Objective", "rationale": "Because slow talking and lack of anger are objective manifestations of the patient, not subjective feelings or attitudes. Even if the patient believes that he is slow to speak and is not angry because of psychological factors, it is still, in the opinion of the observer, objective manifestations.\""}, "sentence_sentiment": {"answer": "Negative", "rationale": "Because \"silent\" and \"no anger\" in the entries have negative meanings."}, "language_style": {"answer": "Tactful and warm", "rationale": "The entry is more adept and interactive when it is spoken in a gentle and inviting manner, rather than by direct statements or orders."}, "time_dimension": {"answer": "Present", "rationale": "The keywords \"silent talking\" and \"not angry\" indicate that this is the description of the testee's current situation."}, "sentence_type": "Negative declarative sentence", "pos": {"nouns": {"Talk": 1, "Angry": 1}, "verbs": {"Say it": 1, "have": 1}, "adjectives": {"Slow": 1, "No, I'm not": 1}, "pronouns": {"I": 1}}}, {"content": "25. I have been unable to do almost anything recently.", "word_count": 12, "label": "", "sentence_structure": "Subject鈥揷opula鈥損redicative", "subjective_objective": {"answer": "Subjective", "rationale": "This is because patients feel and assess their current state of affairs."}, "sentence_sentiment": {"answer": "Negative", "rationale": "Because \"Nothing can be done\" in the entry is an expression of a negative emotional state."}, "language_style": {"answer": "Tactful and warm", "rationale": "The entry uses a moderate tone and a self-presentation approach to ask the interviewee whether he or she is capable of carrying out some tasks. This approach is more likely to trigger the interviewee鈥檚 resonance and reflection than a direct query, and does not create undue pressure or criticism."}, "time_dimension": {"answer": "Present", "rationale": "The entry \"recently\" indicates a recent state of affairs in time, \"almost nothing can be done\" describes the current behaviour of the person being tested and is therefore in the current state of affairs."}, "sentence_type": "Negative declarative sentence", "pos": {"nouns": {"Yes": 1}, "verbs": {"Fuck": 1, "Yes": 1}, "adjectives": {"Almost": 1, "What": 1, "Both": 1, "not": 1}, "numerals": {"25": 1}, "pronouns": {"I": 1}}}, {"content": "I am very slow, and I need help to wash and dress.", "word_count": 16, "label": "", "sentence_structure": "Subject鈥揷opula鈥損redicative", "subjective_objective": {"answer": "Subjective", "rationale": "Because \"very slow\" is patient's subjective feeling and self-evaluation."}, "sentence_sentiment": {"answer": "Negative", "rationale": "Because the \"needs help\" act is negative in the entry."}, "language_style": {"answer": "Tactful and warm", "rationale": "The entry is more adept and interactive when it is spoken in a gentle and inviting manner, rather than by direct statements or orders."}, "time_dimension": {"answer": "Past", "rationale": ""}, "sentence_type": "Negative declarative sentence", "pos": {"nouns": {"Entry": 1, "Questionnaire": 1, "person": 1, "Wash": 1, "Get dressed": 1, "Help": 1}, "verbs": {"Analysis": 1, "Yes": 1, "Help": 1}, "adjectives": {"Very": 1, "Slow": 1}, "numerals": {"26": 1}, "pronouns": {"I": 1}}}, {"content": "I think I look quiet.", "word_count": 10, "label": "", "sentence_structure": "Subject鈥揷opula鈥損redicative", "subjective_objective": {"answer": "Subjective", "rationale": "Because \"I want\" means it's personal feelings or perceptions."}, "sentence_sentiment": {"answer": "Neutral", "rationale": "Because the entry does not express a positive or negative emotional tendency, but simply states the fact that \"I think I look quiet.\""}, "language_style": {"answer": "Tactful and warm", "rationale": "The language style of the entry is relatively euphemistic, describing the description of the person in question in a first person's name, and not clearly expressing satisfaction or dissatisfaction. Such expression is more friendly and natural, and it is easier to generate resonance and participation among the person under investigation."}, "time_dimension": {"answer": "Past", "rationale": "The entry uses the current-day \"idemic\" of the first person called the singular, which suggests that the testee is considering the impression that his appearance is giving to people in answer to questions. Moreover, \"showing\" is a descriptive term, implying that the idea is not a continuing state, but a temporary view or feeling. So we can judge that the time dimension of the article is the past."}, "sentence_type": "Affirmative declarative sentence", "pos": {"nouns": {"Appearance": 1, "Quiet": 1}, "verbs": {"Yes": 1}, "adjectives": {"Mine": 1}, "pronouns": {"I": 1}}}, {"content": "I am restless and uneasy.", "word_count": 9, "label": "", "sentence_structure": "Subject鈥搗erb鈥揷omplement", "subjective_objective": {"answer": "Subjective", "rationale": "Because \"silent, unsettled\" describes the patient's own feelings and experiences, which are subjective in the patient's emotional state."}, "sentence_sentiment": {"answer": "Negative", "rationale": "Because the description of \"sitting\" and \"deep\" in the entries is negative."}, "language_style": {"answer": "Tactful and warm", "rationale": ""}, "time_dimension": {"answer": "Present", "rationale": ""}, "sentence_type": "Exclamatory sentence", "pos": {"nouns": {"Questionnaire": 1, "Entry": 1, "28": 1}, "verbs": {"Sit tight": 1, "I'm not feeling well": 1}, "numerals": {"28": 1}}}, {"content": "29. I am very upset and prone to anger.", "word_count": 11, "label": "", "sentence_structure": "Subject鈥揷opula鈥損redicative", "subjective_objective": {"answer": "Subjective", "rationale": "Because emotional reactions like \"freaky\" and \"irrespondent\" are subjective experiences and feelings of patients."}, "sentence_sentiment": {"answer": "Negative", "rationale": "Because the moods of \"freaky\" and \"freaky\" are negative."}, "language_style": {"answer": "Calm and direct", "rationale": "The entry uses simple and clear language to express directly whether the person under investigation often displays a temperamental, irritating mood, is more objective and neutral, has no excessive rhetorical or emotional overtones and is of a calm and direct linguistic style."}, "time_dimension": {"answer": "Present", "rationale": ""}, "sentence_type": "Affirmative declarative sentence", "pos": {"nouns": {"I'm so upset": 1, "Unsettled": 1, "- Get mad": 1}, "verbs": {"Show": 1, "Okay": 1, "Let's go": 1}, "adjectives": {"Boring": 1, "Ann": 1, "Easy": 1}, "adverbs": {"not": 1, "Regular": 1}, "numerals": {"29": 1}}}, {"content": "30. For most of the time, I had to go around.", "word_count": 14, "label": "", "sentence_structure": "Subject鈥搗erb鈥揷omplement", "subjective_objective": {"answer": "Objective", "rationale": "Because the patient is describing their daily behaviour or habits, not their feelings, attitudes or emotions, this is an objective entry."}, "sentence_sentiment": {"answer": "Negative", "rationale": "Because \"I had to go in and out\" is negative."}, "language_style": {"answer": "Calm and direct", "rationale": "The language style of the entry is more straightforward, with no excessive rhetorical or emotional rendering, and more objective, and is used to ask the other side whether there is a constant pattern of movement."}, "time_dimension": {"answer": "Present", "rationale": "The expression \"most of the time\" and \"must\" indicates that it is a question of describing the current state of the subject, so that time can be judged as now."}, "sentence_type": "Affirmative declarative sentence", "pos": {"verbs": {"I'm gonna go around": 1, "I had to": 1}, "adjectives": {"Most of them": 1}}}, {"content": "31. When reading, my attention is easily focused.", "word_count": 13, "label": "", "sentence_structure": "Subject鈥揷opula鈥損redicative", "subjective_objective": {"answer": "Subjective", "rationale": "Because \"focus\" is an individual's feeling and experience, it is an individual's subjective perception, and it cannot be concluded through objective measurement or observation. The answer to the questionnaire should therefore be subjective."}, "sentence_sentiment": {"answer": "Positive", "rationale": "Because it expresses the advantages of easy focus when reading, it is positive emotional."}, "language_style": {"answer": "Tactful and warm", "rationale": "The language style of the entry is more euphemistic and guides the interviewee to answer questions in an invitation-style manner, rather than direct orders or queries. Moreover, the phrase uses positive expressions, making the interviewee more willing to share his reading habits and experiences."}, "time_dimension": {"answer": "Present", "rationale": ""}, "sentence_type": "Affirmative declarative sentence", "pos": {"nouns": {"Reading": 1, "Attention": 1}, "verbs": {"Focus": 1}, "adjectives": {"Easy": 1}, "adverbs": {"very": 1}}}, {"content": "I feel distracted or disturbed.", "word_count": 12, "label": "", "sentence_structure": "Subject鈥揷opula鈥損redicative", "subjective_objective": {"answer": "Subjective", "rationale": "Because \"the feeling\" shows that it's the patient's subjective feeling."}, "sentence_sentiment": {"answer": "Negative", "rationale": "Because \"deep or uneasy\" is a negative emotional state."}, "language_style": {"answer": "Tactful and warm", "rationale": "The basis of the judgement is as follows: 1. The use of the first person's term \"I\" for questioning makes it easier for the interviewee to resonate and participate; 2. The adjective \"deepness or discomfort\" is used, describing a more general psychological state, avoiding the use of too professional or too remote a vocabulary; 3. The language is relatively flat and natural, and there is no over-modified or emotional term."}, "time_dimension": {"answer": "Present", "rationale": "The keyword \"thinks\" indicates a state of immediate feeling or state, so the time dimension of the article is now."}, "sentence_type": "Affirmative declarative sentence", "pos": {"nouns": {"Mind": 1, "Unsettled": 1}, "verbs": {"I think": 1, "Freeze": 1, "Yeah": 1}, "adjectives": {"Uncertainty": 1}}}, {"content": "33. In many cases, I have been inexplicably afraid.", "word_count": 15, "label": "", "sentence_structure": "Subject鈥搗erb鈥揷omplement", "subjective_objective": {"answer": "Subjective", "rationale": "Because \"fear\" is a subjective feeling."}, "sentence_sentiment": {"answer": "Negative", "rationale": "Because the mood of fear in the entry is negative."}, "language_style": {"answer": "Calm and direct", "rationale": "The entry, which is in direct, concise language, does not have too many emotional renderings or rhetorical techniques, and expresses an objective tone of questioning whether the person under investigation has any hidden fears."}, "time_dimension": {"answer": "Present", "rationale": ""}, "sentence_type": "Affirmative declarative sentence", "pos": {"nouns": {"Situation": 1, "Fear": 1}, "verbs": {"I feel": 1, "I don't know": 2}, "adjectives": {"I don't know": 2}, "numerals": {"33": 1}, "pronouns": {"I": 1}}}, {"content": "I'm in a state of panic, and I'm going crazy.", "word_count": 10, "label": "", "sentence_structure": "Subject鈥揷opula鈥損redicative", "subjective_objective": {"answer": "Subjective", "rationale": "Because the sense of extreme panic and madness is a subjective experience and feeling of a person in a given situation."}, "sentence_sentiment": {"answer": "Negative", "rationale": "Because the description \"extremely panic, seems to be going crazy\" in the entry is full of negative emotions and feelings."}, "language_style": {"answer": "Calm and direct", "rationale": "The entry is based on the following criteria: it is used in direct, concise language to express the emotional state of the subject, without excessive embellishment or emotional rendering. At the same time, the expressions \"extreme panic\" and \"like to go crazy\" are relatively objective, without too much subjective evaluation or speculation, and therefore can be considered as being straightforward in terms of language style."}, "time_dimension": {"answer": "Future", "rationale": "The keyword \"extreme panic\" and \"like to go crazy\" are all descriptions of the psychological conditions that may arise, so the entry is for questioning the future behaviour or mental state of the person being tested."}, "sentence_type": "Affirmative declarative sentence", "pos": {"nouns": {"I": 1, "Panic": 1}, "verbs": {"It looks like": 1, "Yes": 1, "Crazy": 1}, "adjectives": {"Extreme": 1}, "numerals": {"34": 1}}}, {"content": "My digestive condition.", "word_count": 6, "label": "", "sentence_structure": "Subject鈥揷opula鈥損redicative", "subjective_objective": {"answer": "Subjective", "rationale": "Because indigestion is a symptom, the patient ' s feelings or observations are subjective."}, "sentence_sentiment": {"answer": "Negative", "rationale": "Because indigestion is a symptom of physical discomfort, it usually leads to negative perceptions."}, "language_style": {"answer": "Calm and direct", "rationale": "The entry is simple and clear in asking the other side whether it has been indigestion, without any use of rhetorical or emotional rendering, and is more objective in tone. It therefore prefers to be calm and straightforward in its language style."}, "time_dimension": {"answer": "Past", "rationale": "The entry is for the purpose of asking the person who was tested for any indigestion experience, and the time dimension is judged to be past."}, "sentence_type": "Negative declarative sentence", "pos": {"nouns": {"Indigestion": 1}, "numerals": {"35": 1}}}, {"content": "My heart beats faster than usual.", "word_count": 10, "label": "", "sentence_structure": "Subject鈥揷opula鈥損redicative", "subjective_objective": {"answer": "Subjective", "rationale": "Because heart rate is a biological indicator that can be measured, the subject is described as subjective. Although the acceleration of heart rate may be for objective reasons, in this case, we are primarily looking at the feelings and experiences of the patient, so the entry is more subjective."}, "sentence_sentiment": {"answer": "Neutral", "rationale": "Because the entry only describes the acceleration of the heart rate, it does not show positive and negative emotional tendencies."}, "language_style": {"answer": "Tactful and warm", "rationale": "The entry is more personal and interactive when it is spoken in a gentle and inviting manner, rather than in direct statements or orders. Moreover, the issue is designed more broadly, without being too specific or subjectively directed, to give the interviewees more freedom to express their feelings."}, "time_dimension": {"answer": "Present", "rationale": "The entry describes the current heartbeat of the testee, which is judged by the time dimension."}, "sentence_type": "Exclamatory sentence", "pos": {"nouns": {"Heart rate": 1, "Normal": 1}, "verbs": {"Sometimes": 1, "- Yeah": 1, "Come on": 1}, "adverbs": {"Sometimes": 1}, "numerals": {"36": 1}, "pronouns": {"I": 1}}}, {"content": "37. I have a heavier headache and a sense of conceit.", "word_count": 13, "label": "", "sentence_structure": "Subject鈥揷opula鈥損redicative", "subjective_objective": {"answer": "Objective", "rationale": "Because brain palsy and fainting are symptoms or experiences of patients, they are objective facts."}, "sentence_sentiment": {"answer": "Negative", "rationale": "Because \"dumber and faint\" are negative physical symptoms."}, "language_style": {"answer": "Calm and direct", "rationale": "Basis of judgement: The phrase in the article is a simple, objective and direct expression of a phenomenon or symptoms, the description of \"heavier brain constriction and fainting\" being more professional and without any symmetrical or emotional tact, and it is therefore clear that the language style is straightforward and straightforward."}, "time_dimension": {"answer": "Present", "rationale": "Now the entry describes the current state of the person being tested, uses the normal current state of affairs and judges it now. The keyword \"heavy head and fainting\" also suggests that it is the present symptom."}, "sentence_type": "Negative declarative sentence", "pos": {"nouns": {"I'm just a little dizzy": 1, "Fizziness": 1}, "verbs": {"have": 1}, "adjectives": {"Heavy": 1}, "numerals": {"37": 1}}}, {"content": "38. My hands are shaking so much that others can easily detect them.", "word_count": 12, "label": "", "sentence_structure": "Subject鈥搗erb鈥揷omplement", "subjective_objective": {"answer": "Subjective", "rationale": "Because \"freakable\" is a personal perception and experience, a subjective description."}, "sentence_sentiment": {"answer": "Negative", "rationale": "Because the \"hands shaking so hard\" in the entries is negative."}, "language_style": {"answer": "Tactful and warm", "rationale": "The entry uses a more moderate language to describe the situation of the person under investigation, rather than a direct statement or order, so that the pressure or discomfort of the person under investigation is avoided. At the same time, the issue is also dialogueed in an invitation-style manner, more personal and interactive."}, "time_dimension": {"answer": "Present", "rationale": "Now the entry describes the current behaviour or state of the testee, and \"I'm shaking my hand\" means that the act is happening, and \"someone can easily detect\" means that the act is being displayed, so it can be judged to be a description of the testee now."}, "sentence_type": "Negative declarative sentence", "pos": {"nouns": {"Shake your hands": 1, "others": 1, "I realized": 1}, "verbs": {"Okay": 1, "Easy": 1}, "adjectives": {"That's great": 1}, "numerals": {"38": 1}, "pronouns": {"I": 1}}}, {"content": "39. My appetite is as good as usual.", "word_count": 11, "label": "", "sentence_structure": "Subject鈥揷opula鈥損redicative", "subjective_objective": {"answer": "Objective", "rationale": "Because good appetite is the physical state of the patient, not subjective feeling or attitude."}, "sentence_sentiment": {"answer": "Neutral", "rationale": "As the entry merely presents an objective fact, it does not show positive or negative emotional tendencies."}, "language_style": {"answer": "Tactful and warm", "rationale": ""}, "time_dimension": {"answer": "Present", "rationale": ""}, "sentence_type": "Affirmative declarative sentence", "pos": {"nouns": {"-Adulge": 1, "Normal": 1}, "verbs": {"And": 1, "Call": 1, "Same thing": 1, "Okay": 1}, "adverbs": {"not": 1}}}, {"content": "I had to force myself to eat, even a little bit.", "word_count": 18, "label": "", "sentence_structure": "Subject鈥損redicate", "subjective_objective": {"answer": "Subjective", "rationale": "Because \"forcing yourself to eat\" and \"a little bit\" are the patients' own feelings and behaviour, they are viewed from the patient's subjective perspective."}, "sentence_sentiment": {"answer": "Negative", "rationale": "Because the \"forced eating\" act in the entry is negative."}, "language_style": {"answer": "Calm and direct", "rationale": "This is more straightforward and helps to better understand the actual situation of the person under investigation."}, "time_dimension": {"answer": "Present", "rationale": "The entry asks if the person tested is required to force himself to eat, and the time dimension is now judged."}, "sentence_type": "Affirmative declarative sentence", "pos": {"nouns": {"self": 1, "Rice": 1, "A little bit": 1}, "verbs": {"Forced": 1, "Eat": 1}, "adverbs": {"I had to": 1, "Forced": 1}, "numerals": {"40": 1}, "pronouns": {"I": 1}}}, {"content": "41. I am as energetic as I am normally.", "word_count": 12, "label": "", "sentence_structure": "Subject鈥揷opula鈥損redicative", "subjective_objective": {"answer": "Objective", "rationale": "Because it is the patient ' s personal subjective feeling, not the objective fact, that feels energetic."}, "sentence_sentiment": {"answer": "Neutral", "rationale": "The basis of the judgement is that the entry is merely a statement of fact and does not express any positive or negative feelings, attitudes, behaviour, purpose or quality."}, "language_style": {"answer": "Calm and direct", "rationale": ""}, "time_dimension": {"answer": "Present", "rationale": "The keyword \"as usual\" indicates that it is a comparative description, indicating that the current state of energy is generally the same."}, "sentence_type": "Affirmative declarative sentence", "pos": {"nouns": {"- The energy": 1, "鈾?Full of 鈾?: 1}, "verbs": {"I feel": 1, "Same thing": 1, "Same": 1, "be": 1}, "adjectives": {"Normal": 1, "鈾?Full of 鈾?: 1}, "numerals": {"41": 1}, "pronouns": {"I": 1}}}, {"content": "42. For most of the time, I was tired.", "word_count": 12, "label": "", "sentence_structure": "Subject鈥揷opula鈥損redicative", "subjective_objective": {"answer": "Subjective", "rationale": "Because \"weakness\" is a subjective feeling and experience."}, "sentence_sentiment": {"answer": "Negative", "rationale": "Because \"weak\" is a negative state."}, "language_style": {"answer": "Calm and direct", "rationale": "The language style of the entry is more direct, with no excessive use of rhetoric or qualifiers, and more objective and incipient, aimed at obtaining the actual situation of the person under investigation."}, "time_dimension": {"answer": "Present", "rationale": "\"Most of the time\" suggests that this is a description of the current situation, while \"I'm tired\" means that the person tested is in the current state. So the time and content of this questionnaire entry point to the present."}, "sentence_type": "Affirmative declarative sentence", "pos": {"nouns": {"Time": 1, "Weary": 1}, "verbs": {"I feel": 1, "be": 1, "Weary": 1}, "adverbs": {"Large": 1, "Time": 1}, "numerals": {"42": 1}}}, {"content": "43. My sexual desire is the same as before the disease.", "word_count": 9, "label": "", "sentence_structure": "Subject鈥揷opula鈥損redicative", "subjective_objective": {"answer": "Subjective", "rationale": "Because \"sexual desire\" is an inherent feeling and desire of a human being, a subjective experience of a human being."}, "sentence_sentiment": {"answer": "Neutral", "rationale": "Because the entry describes an objective fact, with no obvious emotional colour."}, "language_style": {"answer": "Calm and direct", "rationale": "The entry describes the sexual desire of the person surveyed after the illness in simple and clear language, without excessive rhetorical or emotional rendering, and with a more objective tone."}, "time_dimension": {"answer": "Present", "rationale": "The entry now asks if the testee's sexual desire has changed before or after the onset of the illness, using the expression \"like...\" to indicate that the testee is comparing the current sexual state. The entry therefore describes the current behaviour or state."}, "sentence_type": "Affirmative declarative sentence", "pos": {"nouns": {"Sexuality": 1, "Before you get sick": 1}, "verbs": {"Call": 1, "Same thing": 1}, "adverbs": {"Call": 1, "Same thing": 1}, "numerals": {"43": 1}, "pronouns": {"I": 1}}}, {"content": "44. I lost my sexual appetite after the disease.", "word_count": 12, "label": "", "sentence_structure": "Subject鈥揷opula鈥損redicative", "subjective_objective": {"answer": "Objective", "rationale": "It is an objective fact that the physical reaction of the patient is to be found in the event of loss of sexual desire."}, "sentence_sentiment": {"answer": "Negative", "rationale": "Because the description \"total loss of sexuality\" in the entry is negative."}, "language_style": {"answer": "Calm and direct", "rationale": "The language style of the entry is more direct and objective, and it does not use excessive rhetorical techniques or emotional rendering. It clearly raises a question and wants an exact answer from the other side."}, "time_dimension": {"answer": "Present", "rationale": "The past entry describes the past experience and state of the disease, so the temporal dimension is judged to be the past."}, "sentence_type": "Negative declarative sentence", "pos": {"nouns": {"Sick": 1, "Sexuality": 1}, "verbs": {"Loss": 1, "After the illness": 1}, "numerals": {"44": 1}}}, {"content": "I am too worried about the symptoms of my body.", "word_count": 9, "label": "", "sentence_structure": "Subject鈥揷opula鈥損redicative", "subjective_objective": {"answer": "Subjective", "rationale": "Because \"excessive fear\" is the patient's subjective feeling and attitude."}, "sentence_sentiment": {"answer": "Negative", "rationale": "Because the attitude of \"too much fear\" in the entry is negative."}, "language_style": {"answer": "Tactful and warm", "rationale": ""}, "time_dimension": {"answer": "Present", "rationale": "The entry now indicates that the person being tested is currently acting or feeling \"too worried about the symptoms of the body\", so the temporal dimension is now judged."}, "sentence_type": "Negative declarative sentence", "pos": {"nouns": {"Symptoms": 1, "Body": 1}, "verbs": {"I'm worried too much": 1, "I'm worried": 1}, "adverbs": {"That's too much": 1}, "numerals": {"45": 1}}}, {"content": "46. I am particularly concerned about the functioning of the body.", "word_count": 10, "label": "", "sentence_structure": "Subject鈥搗erb鈥揷omplement", "subjective_objective": {"answer": "Subjective", "rationale": "Because \"special care\" is a subjective attitude or psychological state, which means that patients have a more sensitive mind about their physical health."}, "sentence_sentiment": {"answer": "Positive", "rationale": "Because attention to the functioning of the body is a positive attitude and behaviour."}, "language_style": {"answer": "Tactful and warm", "rationale": "The language style of the entry is more euphemistic and warm. Instead of using a strong tone or command expression directly, it asks if the other side is paying particular attention to the body鈥檚 functions in a more moderate, friendly and understanding way. This expression is more reflective of the researcher鈥檚 concerns and respect and facilitates good interaction."}, "time_dimension": {"answer": "Present", "rationale": "The entry describes the current attitude and behaviour of the testee, namely, \"with particular attention to the functioning of the body\", and is therefore current."}, "sentence_type": "Affirmative declarative sentence", "pos": {"nouns": {"Questionnaire": 1, "Entry": 1, "Body": 1, "Functions": 1}, "verbs": {"Attention": 1}, "adjectives": {"Special": 1}, "numerals": {"46": 1}}}, {"content": "My problems are caused by some serious body disease.", "word_count": 18, "label": "", "sentence_structure": "Subject鈥揷opula鈥損redicative", "subjective_objective": {"answer": "Objective", "rationale": "Because the existence of the problem is an objective fact, the reason is subjective because of the patient ' s understanding and interpretation of the objective reality."}, "sentence_sentiment": {"answer": "Negative", "rationale": "Because the problems mentioned in this entry are \"caused by a serious body disease\", this implies the existence of a physical health condition that may have a negative impact on life."}, "language_style": {"answer": "Calm and direct", "rationale": "The language style of the entry is more direct and objective, and there is no excessive emotional or rhetorical approach, mainly describing the causes of a problem, and it is calm and convincing."}, "time_dimension": {"answer": "Past", "rationale": "The entry \"caused by a serious body disease\" indicates the cause of the problem in the past and is therefore judged to be the past."}, "sentence_type": "Affirmative declarative sentence", "pos": {"nouns": {"Problem": 1, "Serious": 1, "Body disease": 1}, "verbs": {"Cause": 1}, "adjectives": {"Some": 1, "of": 2}, "numerals": {"47": 1}, "pronouns": {"I": 1}}}, {"content": "My body is broken, and it's rotting.", "word_count": 12, "label": "", "sentence_structure": "Subject鈥搗erb鈥揷omplement", "subjective_objective": {"answer": "Subjective", "rationale": "Because \"body is broken, it rots\" is a subjective description and feeling, a subjective perception and a judgement of the patient's condition. This is not an objective fact, but a sense and understanding of the patient's own heart."}, "sentence_sentiment": {"answer": "Negative", "rationale": "Because the narrative in the entries uses negative adjectives, \"fall,\" \"decomposed,\" and expresses negative emotions and attitudes."}, "language_style": {"answer": "Calm and direct", "rationale": "\"Calm down straight.\""}, "time_dimension": {"answer": "Past", "rationale": "The sentence describes an event or state that has already occurred and that the temporal dimension is a word for the past. In it, both \"falling\" and \"decomposed\" are words for the past to complete the time, which means that this state of health has occurred at some point in the past and is now continuing or is over."}, "sentence_type": "Negative declarative sentence", "pos": {"nouns": {"Body": 1, "Rotten": 1}, "verbs": {"It's gone": 1, "Inside": 1}, "numerals": {"48": 1}}}, {"content": "49. I can recover once I have a good rest.", "word_count": 15, "label": "", "sentence_structure": "Subject鈥搗erb鈥揷omplement", "subjective_objective": {"answer": "Subjective", "rationale": "This is a form of expectation or desire of the patient and is subjective."}, "sentence_sentiment": {"answer": "Neutral", "rationale": "Because the entry does not have any emotional color."}, "language_style": {"answer": "Tactful and warm", "rationale": "The language style of the entry is more euphemistic, using positive and optimistic words like \"well rest\" and \"rehabilitate.\" It gives a sense of comfort and encouragement."}, "time_dimension": {"answer": "Future", "rationale": "The entry \"as long as ...\" is a conditional statement indicating some expectation or plan for the future. And \"a good rest\" and \"a complete recovery\" are all future behaviours or states, so the entry asks about the testee's behaviour or attitude to the future. We can therefore judge it as future behavior or mentality."}, "sentence_type": "Affirmative declarative sentence", "pos": {"nouns": {"Rehabilitation": 1, "Line": 1}, "verbs": {"Get some rest": 1, "Yes": 1, "Full recovery": 1}, "adverbs": {"Just": 1}, "numerals": {"49": 1}, "pronouns": {"I": 1}}}, {"content": "50. My illness is caused by the recent bad weather.", "word_count": 14, "label": "", "sentence_structure": "Subject鈥搗erb鈥揷omplement", "subjective_objective": {"answer": "Objective", "rationale": "This sentence examines the patient鈥檚 subjective perception and interpretation, and does not directly examine his attitude."}, "sentence_sentiment": {"answer": "Negative", "rationale": "Since the entry attributes its own illness to external causes, it may have a negative impact on treatment and rehabilitation."}, "language_style": {"answer": "Calm and direct", "rationale": "The entry is directly speech-oriented, does not have too many rhetorical or emotional expressions, and expresses a clear view and reasons, with a more calm and direct language style."}, "time_dimension": {"answer": "Past", "rationale": "The entry's \"recent bad weather\" indicates that this is a description of past events and therefore the temporal dimension is a past."}, "sentence_type": "Affirmative declarative sentence", "pos": {"nouns": {"Diseases": 1, "Weather": 1}, "verbs": {"Okay": 1, "Cause": 1}, "adjectives": {"Most recently": 1, "Bad": 1}, "numerals": {"50": 1}, "pronouns": {"I": 1}}}, {"content": "51. My weight is decreasing.", "word_count": 8, "label": "", "sentence_structure": "Subject鈥搗erb鈥揷omplement", "subjective_objective": {"answer": "Subjective", "rationale": "Because weight loss is the result of subjective observations by patients."}, "sentence_sentiment": {"answer": "Positive", "rationale": "Because the result of \"weight is diminishing\" is positive."}, "language_style": {"answer": "Tactful and warm", "rationale": ""}, "time_dimension": {"answer": "Present", "rationale": "The entry uses the current state of the moment \"is decreasing\", indicating that the act is in progress and judging it as now."}, "sentence_type": "Affirmative declarative sentence", "pos": {"nouns": {"Weight": 1}, "verbs": {"It's slowing down": 1}}}, {"content": "52. My weight can be said to have fallen considerably.", "word_count": 12, "label": "", "sentence_structure": "Subject鈥揷opula鈥損redicative", "subjective_objective": {"answer": "Subjective", "rationale": "Because \"a lot of weight has fallen\" is the patient's subjective perception and judgment. Although this is a measurable fact, the patient's observations and explanations are still subjective."}, "sentence_sentiment": {"answer": "Negative", "rationale": "Because the description of \"a lot of weight loss\" in the entry may suggest that individuals are taking unhealthy measures such as diet."}, "language_style": {"answer": "Tactful and warm", "rationale": "The entry begins in a question-and-answer manner, using the subjective perception of the other side as a starting point to encourage the other to share their own situation. At the same time, the expression \"may be said\" is used to avoid referring directly to sensitive topics (e.g. weight) and to show respect and understanding for the person under investigation. This language style gives people a sense of affection and moderation."}, "time_dimension": {"answer": "Past", "rationale": ""}, "sentence_type": "Affirmative declarative sentence", "pos": {"nouns": {"Weight": 1, "Down": 1}, "verbs": {"Say it": 1, "Yeah": 1, "Many": 1}, "adverbs": {"Many": 1}, "numerals": {"52": 1}}}]}

{"questionnaire_title": "Defense Mode Questionnaire (DSQ)", "questionnaire_category": "Behavioral questionnaire", "item_count": 88, "emotion_shift_overall": "From positive to negative", "language_style_overall": "Neutral", "items": [{"content": "One, I get satisfaction from helping others. If I don't, I get depressed.", "word_count": 26, "label": "", "sentence_structure": "Subject鈥搗erb鈥搊bject", "subjective_objective": {"answer": "Subjective", "rationale": "Because it is a manifestation of subjective feelings and attitudes."}, "sentence_sentiment": {"answer": "Positive", "rationale": "Because the entries \"getting satisfaction from helping others\" and \"doing nothing makes you emotionally depressed\" are both positive and express the positive impact of helping others."}, "language_style": {"answer": "Tactful and warm", "rationale": "The entry is more euphemistic, using the expression \"I would be depressed if I did not do so\" to guide the interviewee to think and share his feelings. At the same time, the entry does not use too direct or command-style tone, but rather asks in a gentle and caring way, which is more likely to arouse resonance and trust among the interviewee."}, "time_dimension": {"answer": "Present", "rationale": "The expression \"I get satisfaction from helping others\" and \"if you do...\" suggests that this is a current or recurrent situation, so this entry is about the current behaviour and mentality of the person being tested."}, "sentence_type": "Affirmative declarative sentence", "pos": {"nouns": {"Satisfied": 1, "Help": 1, "Others": 1, "Emotion": 1, "Depression": 1}, "verbs": {"Access": 1, "鈾?And become 鈾?: 1, "If": 1, "Do it": 1}, "adverbs": {"From": 1, "Suppose": 1}, "numerals": {"One": 2}, "pronouns": {"I": 1}}}, {"content": "Two, I'm often called a temperamental man.", "word_count": 13, "label": "", "sentence_structure": "Subject鈥揷opula鈥損redicative", "subjective_objective": {"answer": "Subjective", "rationale": "Because this is what people say about me, and what they describe from the perspective of others, and what others perceive and perceive my behaviour, and therefore from the subjective perspective."}, "sentence_sentiment": {"answer": "Negative", "rationale": "Because it describes a negative character, \"breeding.\""}, "language_style": {"answer": "Tactful and warm", "rationale": "This expression is more personal and interactive, avoiding the language style of direct accusations or commanding the other, and making it easier to obtain the cooperation and cooperation of the person under investigation. At the same time, \"grumpy\" is a more objective adjective, and there is no excessive emotional vocabulary or rhetorical technique, making the language style of the article ' s purpose more neutral and rational."}, "time_dimension": {"answer": "Present", "rationale": ""}, "sentence_type": "Affirmative declarative sentence", "pos": {"nouns": {"person": 1, "- Grumpy": 1, "- Routine": 1}, "verbs": {"Say it": 1}, "adjectives": {"Regular": 1, "individual": 1, "- He's a bitch": 1}, "pronouns": {"I": 1}}}, {"content": "Three, I can put it aside when I don't have time to deal with a difficult situation.", "word_count": 25, "label": "", "sentence_structure": "Subject鈥搗erb鈥搊bject", "subjective_objective": {"answer": "Subjective", "rationale": "Because the idea of \"no time\" and \"can put it aside\" is patient subjective."}, "sentence_sentiment": {"answer": "Neutral", "rationale": "Because the entry is not clearly emotional."}, "language_style": {"answer": "Calm and direct", "rationale": "The entry is relatively straightforward in language style, and it asks whether the respondent has the capacity to hold it in abeyance in the face of a difficult situation. This description is simple and clear, avoiding excessive rhetoric or emotional overtones and more factual and logical expressions."}, "time_dimension": {"answer": "Past", "rationale": "However, since the sentence uses the general current ( \"able\"), it may mean that the act is recurrent, or even normal, and therefore it can be considered to reflect the current state of affairs. So the final judgement may be present or past."}, "sentence_type": "Affirmative declarative sentence", "pos": {"nouns": {"Situation": 1}, "verbs": {"Processing": 1, "Hold it": 1}, "adjectives": {"It's a problem": 1, "Some": 1}, "numerals": {"3": 1}, "pronouns": {"I": 1}}}, {"content": "Four. People treat me unfairly.", "word_count": 11, "label": "", "sentence_structure": "Subject鈥搗erb鈥揷omplement", "subjective_objective": {"answer": "Subjective", "rationale": "Because \"unfairly treating me\" is the patient's subjective feeling and perception."}, "sentence_sentiment": {"answer": "Negative", "rationale": "Because the \"unfairly\" act in the entry is negative."}, "language_style": {"answer": "Tactful and warm", "rationale": "The entry is more personal and interactive when it is presented in a gentle and inviting tone, rather than in direct statements or orders. At the same time, the word \"always\" is used to channel the feelings of the interviewees and make it easier to resonate with the questionnaire."}, "time_dimension": {"answer": "Present", "rationale": "According to the keyword \"always\" and \"unfair\", it can be seen that the entry is describing a pattern of unfair treatment, so the temporal dimension is now judged."}, "sentence_type": "Negative declarative sentence", "pos": {"nouns": {"person": 1, "Questionnaire": 1, "Entry": 1, "Fair": 1, "Treatment": 1}, "verbs": {"Always": 1, "Unfair": 1, "Treatment": 1}, "adjectives": {"Unfair": 1, "Ping": 1}, "adverbs": {"Land": 1}, "numerals": {"4": 1}, "pronouns": {"I": 1}}}, {"content": "I'm free of my anxiety by doing some positive or foreseeable things, such as painting, carpentry, etc.", "word_count": 34, "label": "", "sentence_structure": "Subject鈥搗erb鈥搊bject", "subjective_objective": {"answer": "Subjective", "rationale": "This is a subjective choice and behaviour. While these activities may help to alleviate the anxiety of the patient, they are still subjective attitudes and behaviour."}, "sentence_sentiment": {"answer": "Positive", "rationale": "Because the behaviour expressed in this entry is positive, i.e., taking action to alleviate anxiety and being positive emotional."}, "language_style": {"answer": "Tactful and warm", "rationale": "The language style of the entry is euphemism, because it uses an invitation tone and asks whether the other side has taken some positive approach, such as painting and carpentry, to ease his anxiety. This expression is more personal and interactive, and it is more resonant and participatory."}, "time_dimension": {"answer": "Past", "rationale": ""}, "sentence_type": "Affirmative declarative sentence", "pos": {"nouns": {"Situation": 2, "Anxiety": 1, "Unsettled": 1, "Painting": 1, "Carpentry": 1}, "verbs": {"Do it": 3, "Get rid of it": 1}, "adjectives": {"Positive": 1, "Predictability": 1}, "numerals": {"5": 1}, "classifiers": {"Wait": 1}, "pronouns": {"I": 1}}}, {"content": "I'll probably postpone some of the things I have to do today until tomorrow.", "word_count": 19, "label": "", "sentence_structure": "Subject鈥搗erb鈥揷omplement", "subjective_objective": {"answer": "Subjective", "rationale": "Because \"deferring to tomorrow some of what is to be done today\" is a patient's subjective behaviour and decision."}, "sentence_sentiment": {"answer": "Negative", "rationale": "Because the \"delay to tomorrow\" act in the entry is negative."}, "language_style": {"answer": "Calm and direct", "rationale": "The entry is more calm and straightforward in language, with no excessive rhetorical or emotional rendering. It asks whether the person surveyed has any information about the delay of today's events until tomorrow, and is clear and simple, easy to understand and answer."}, "time_dimension": {"answer": "Past", "rationale": "The keywords \"what we should do today\" and \"deferring to tomorrow\" indicate that the moment when the act occurred is the past."}, "sentence_type": "Affirmative declarative sentence", "pos": {"verbs": {"- Put it on": 1, "Do it": 2, "Postponement": 1}, "adjectives": {"Some": 1, "Today": 1, "The": 1}, "adverbs": {"Between": 1, "Present": 1, "Tomorrow": 1}, "pronouns": {"I": 1}}}, {"content": "Seven, I don't understand why it's always the same situation.", "word_count": 18, "label": "", "sentence_structure": "Subject鈥搗erb鈥搊bject", "subjective_objective": {"answer": "Subjective", "rationale": "Because I'm confused and puzzled about the reasons why the same frustration is always happening, which is the patient's subjective attitude."}, "sentence_sentiment": {"answer": "Negative", "rationale": "Because \"always encounter the same frustration\" expresses a negative emotion and attitude."}, "language_style": {"answer": "Calm and direct", "rationale": "The entry is more calm and straightforward in language, with no excessive emotional accoutrements or rhetorical techniques. It expresses a problem or confusion in an objective way, and is more plain and understandable."}, "time_dimension": {"answer": "Present", "rationale": ""}, "sentence_type": "Interrogative sentence", "pos": {"nouns": {"Questionnaire": 1, "Entry": 1, "Same": 1, "- Inconvenient": 1}, "verbs": {"Understood": 1, "I met him": 1, "be": 1}, "adjectives": {"Always": 1}, "numerals": {"7": 1}, "pronouns": {"I": 1}}}, {"content": "I can laugh at myself quite easily.", "word_count": 13, "label": "", "sentence_structure": "Subject鈥搗erb鈥揷omplement", "subjective_objective": {"answer": "Subjective", "rationale": "The reason for this is that it is easy to laugh at the subjective attitude and self-awareness of the patient."}, "sentence_sentiment": {"answer": "Positive", "rationale": "Because the \"mock myself\" act in the entry is positive and shows the ability to mock."}, "language_style": {"answer": "Tactful and warm", "rationale": "The entry is more adept and interactive when it is spoken in a gentle and inviting manner, rather than by direct statements or orders."}, "time_dimension": {"answer": "Present", "rationale": "The verb \"can\" in the entry indicates a certain ability, while the verb \"smoke\" and \"self\" indicates an act or state of conduct currently under way, and therefore the judgement is now."}, "sentence_type": "Affirmative declarative sentence", "pos": {"nouns": {"I": 2, "self": 1}, "verbs": {"Yes": 1, "Easy": 1, "Scoff": 1}, "adjectives": {"Pretty much": 1}, "adverbs": {"Land": 1}, "numerals": {"8": 1}}}, {"content": "When I was frustrated, I acted like a child.", "word_count": 13, "label": "", "sentence_structure": "Subject鈥揷opula鈥損redicative", "subjective_objective": {"answer": "Subjective", "rationale": "Because the state and the expression of \"I am frustrated\" is a patient's self-presentation and feelings, it's seen from a subjective perspective."}, "sentence_sentiment": {"answer": "Negative", "rationale": "Because the \"act like a child\" act in the entry is negative."}, "language_style": {"answer": "Tactful and warm", "rationale": "The entry uses a metaphor for the performance of people in the event of frustration, with some degree of tenderness and compassion, and is more gentle and friendly. The language style of the entry can therefore be judged as \"precipitous\"."}, "time_dimension": {"answer": "Past", "rationale": "The entry describes the performance of the testee in the event of a setback, and the keyword \"when I am frustrated\" points to a specific event or situation, and this expression appears to have developed a pattern of customary or fixed behaviour. Thus, the temporal dimension of the entry is a judgement of the past."}, "sentence_type": "Affirmative declarative sentence", "pos": {"nouns": {"Frustration": 1, "Kids": 1}, "verbs": {"Performance": 1}, "pronouns": {"I": 1}}}, {"content": "I'm ashamed to be a pious man in my interests.", "word_count": 16, "label": "", "sentence_structure": "Subject鈥搗erb鈥搊bject", "subjective_objective": {"answer": "Subjective", "rationale": "Because shame is a subjective feeling and attitude that shows that patients may have reservations or hesitations in defending their interests. This is not an objective fact, but a patient鈥檚 inner experience."}, "sentence_sentiment": {"answer": "Negative", "rationale": "Because the attitude of \"smuggling against others\" is negative."}, "language_style": {"answer": "Tactful and warm", "rationale": "The entry is worded in gentle terms and uses an invitation tone, which attempts to direct respondents to share their views and experiences on the issue, rather than direct orders or statements."}, "time_dimension": {"answer": "Present", "rationale": "\"I'm ashamed of being a human being\" indicates current behavior or thinking, so it's about the present."}, "sentence_type": "Negative declarative sentence", "pos": {"nouns": {"Interest": 1, "In terms of": 1, "Count": 1}, "verbs": {"Maintenance": 1, "Shame": 1}, "adverbs": {"With people": 1}, "numerals": {"10": 1}}}, {"content": "Eleven. I'm better than most of the people I know.", "word_count": 13, "label": "", "sentence_structure": "Subject鈥揷opula鈥損redicative", "subjective_objective": {"answer": "Subjective", "rationale": "Because \"I'm better than most people I know\" is a patient's subjective self-evaluation."}, "sentence_sentiment": {"answer": "Negative", "rationale": "Because the entry expresses a self-righteous attitude, which is usually considered negative."}, "language_style": {"answer": "Calm and direct", "rationale": "Because the entry describes a subjective feeling or comparison in a directly explicit language, it does not use too many rhetorical or euphemistic expressions and is more objective and calm."}, "time_dimension": {"answer": "Present", "rationale": "The keyword \"I am better than most of those I know\" means an assessment of my abilities, an act that has occurred in the past but is still ongoing. So the temporal dimension of this article is now judged."}, "sentence_type": "Affirmative declarative sentence", "pos": {"nouns": {"Questionnaire Entry": 1, "person": 1}, "verbs": {"I": 2, "- Yeah": 1, "Yes": 1, "鈾?Strong": 1}, "adjectives": {"Mostly": 1, "Strong": 1}, "numerals": {"11": 1, "Most of them": 1}, "classifiers": {"individual": 1}}}, {"content": "People abuse me.", "word_count": 7, "label": "", "sentence_structure": "Subject鈥搗erb鈥揷omplement", "subjective_objective": {"answer": "Subjective", "rationale": "Because it is the subjective feeling and understanding of the patient of his or her own experience."}, "sentence_sentiment": {"answer": "Negative", "rationale": "Because \"abuse\" is negative."}, "language_style": {"answer": "Tactful and warm", "rationale": "Warm and warm."}, "time_dimension": {"answer": "Present", "rationale": "The keyword \"often\" indicates that it is a regular or customary act, and the act of \"abuse\" also indicates that the act is being or has been committed, so the temporal dimension of the article is now."}, "sentence_type": "Negative declarative sentence", "pos": {"nouns": {"person": 1, "Abuse": 1, "I": 1}, "verbs": {"Often": 1, "Abuse": 1}, "numerals": {"12": 1}}}, {"content": "13, if someone lied to me or stole my money, I'd rather he get help than punishment.", "word_count": 27, "label": "", "sentence_structure": "Subject鈥搗erb鈥搊bject", "subjective_objective": {"answer": "Subjective", "rationale": "Because \"I'd rather get help than punishment\" is the patient's subjective idea."}, "sentence_sentiment": {"answer": "Positive", "rationale": "Because the behavior in the entry is that the other side is expected to be helped, with a positive emotional tendency."}, "language_style": {"answer": "Tactful and warm", "rationale": "The entry uses a more euphemistic and compassionate language style that does not directly express strong dissatisfaction or anger about being deceived or stolen, but rather expresses a more positive and inclusive view. This language style may make the respondents more comfortable and relaxed, and more acceptable and answerable."}, "time_dimension": {"answer": "Present", "rationale": "Now the entry asks what the testee will choose in certain circumstances, and the keywords \"if someone has lied to me or stolen my money\" and \"I prefer that he be helped, not punished\" suggest that the act is something that is going to happen now or in the future. So the temporal dimension is now."}, "sentence_type": "Affirmative declarative sentence", "pos": {"nouns": {"Someone": 1, "I": 1, "Money": 1}, "verbs": {"Liar": 1, "Steal": 1, "Got it": 1, "Yes": 1, "Punishment": 1}, "adverbs": {"I'd rather": 1}, "numerals": {"13": 1}, "pronouns": {"he": 2}}}, {"content": "I think there's something bad that I can't say.", "word_count": 16, "label": "", "sentence_structure": "Subject鈥搗erb鈥揷omplement", "subjective_objective": {"answer": "Subjective", "rationale": "Because \"thinking something so bad that you can't say it\" is the idea and feeling inside the patient, it is examined from a subjective point of view."}, "sentence_sentiment": {"answer": "Negative", "rationale": "Because the idea of \"some bad things that cannot be said\" in the entry is negative."}, "language_style": {"answer": "Calm and direct", "rationale": "The language style of the entry is calm and straightforward, and it does not use euphemism or rhetoric, but rather asks the interviewee whether he or she has considered some cases of bad or inappropriate speech."}, "time_dimension": {"answer": "Present", "rationale": "The temporal dimension of \"intermediate or\" is an occasional, non-frequent movement, which is referred to here as a reflection of these circumstances within a certain time period, and the temporal dimension of the subject can therefore be judged as being present."}, "sentence_type": "Affirmative declarative sentence", "pos": {"nouns": {"Situation": 1}, "verbs": {"Yes": 1, "have": 1}, "adjectives": {"Bad": 1}, "adverbs": {"Between": 1, "No, I can't": 1}, "numerals": {"14": 1}, "pronouns": {"I": 1}}}, {"content": "I laughed at some dirty jokes.", "word_count": 14, "label": "", "sentence_structure": "Subject鈥搗erb鈥揷omplement", "subjective_objective": {"answer": "Subjective", "rationale": "Because laughing is a subjective reaction to dirty jokes."}, "sentence_sentiment": {"answer": "Negative", "rationale": "Because \"dirty jokes\" are negative, and \"glaugh\" may look positive, but in the context, it may not be healthy or appropriate."}, "language_style": {"answer": "Calm and direct", "rationale": "The article is based on a straightforward question about whether the interviewee laughs occasionally because of a dirty joke, without using euphemism or emotional publicity. It is more honest and objective, and it is consistent with the calm and direct language style."}, "time_dimension": {"answer": "Present", "rationale": "\"Sometimes,\" I laugh at, and sometimes, \"I laugh because of some nasty jokes,\" I describe what happened in the past. So, the entry is mainly about whether the testees sometimes laugh at some of these in their current lives."}, "sentence_type": "Affirmative declarative sentence", "pos": {"nouns": {"15": 1, "Between": 1, "A joke": 1}, "verbs": {"Because": 1, "And": 1, "Smile": 1}, "adjectives": {"Nasty": 1}, "adverbs": {"Between": 1}, "numerals": {"15": 1}}}, {"content": "16: I was told to bury my head in the sand like a camel bird, in other words, I was too often deliberately unaware of unpleasant circumstances.", "word_count": 37, "label": "", "sentence_structure": "Subject鈥搗erb鈥搊bject", "subjective_objective": {"answer": "Subjective", "rationale": "Because \"I tend to deliberately ignore some unpleasant situations\" is the patient's subjective attitude."}, "sentence_sentiment": {"answer": "Negative", "rationale": "Because the act of \"neglecting some unpleasantness\" in the entry is negative."}, "language_style": {"answer": "Tactful and warm", "rationale": "The entry uses a metaphorical expression that expresses the opinion of the subject. By comparing the subject to a camel bird, it implies that the other party consciously avoids or ignores unpleasant situations. This expression is neither direct blame nor too strong, and it is more moderate and understanding."}, "time_dimension": {"answer": "Present", "rationale": "The term \"normal\" and \"hearing\" can be judged to describe the frequent behaviour of the person tested. The description of \"into the sand\" and \"intentionally ignoring some unpleasant circumstances\" suggests that this is an assessment of the current behaviour. So, the time dimension of this questionnaire entry is now."}, "sentence_type": "Affirmative declarative sentence", "pos": {"nouns": {"A camel bird": 1, "Sand": 1, "Situation": 1}, "verbs": {"Say it": 1, "Elephant": 1, "- Put it on": 1, "Bury it": 1, "In other words": 1, "Often": 1, "I'm not interested in it": 1}, "adverbs": {"Some": 1, "It's not good": 1}, "numerals": {"6": 1}, "pronouns": {"I": 1}}}, {"content": "I can't compete with people at all costs.", "word_count": 14, "label": "", "sentence_structure": "Subject鈥搗erb鈥揷omplement", "subjective_objective": {"answer": "Subjective", "rationale": "Because \"can't do everything\" is a personal feeling and attitude, subjective."}, "sentence_sentiment": {"answer": "Negative", "rationale": "Because the \"can't compete with people\" act in the entry is negative."}, "language_style": {"answer": "Tactful and warm", "rationale": "The entry is more euphemistic and caring, does not directly accuse or criticize the other party's behaviour, but rather asks in an understanding and support manner. At the same time, the phrase \"do your utmost\" is used to convey a positive attitude and encourage the respondents to continue their efforts to promote themselves in competition."}, "time_dimension": {"answer": "Present", "rationale": "Now the entry uses the term \"always\" and the general current term \"no\", indicating that it is a current, habitual act, and thus the temporal dimension is now judged."}, "sentence_type": "Negative declarative sentence", "pos": {"nouns": {"Entry": 1, "Competition": 1}, "verbs": {"Analysis": 1, "No, I can't": 1, "Do your best": 1, "With people": 1}, "adverbs": {"Often": 1}, "numerals": {"17": 1}}}, {"content": "I've always felt better than the people I'm with.", "word_count": 13, "label": "", "sentence_structure": "Subject鈥揷opula鈥損redicative", "subjective_objective": {"answer": "Subjective", "rationale": "Because \"I feel better than the people I'm with\" is a self-awareness and assessment, a subjective feeling of the patient."}, "sentence_sentiment": {"answer": "Positive", "rationale": "Because the expression \"more than the one I'm with\" in the entry expresses a positive emotional attitude to self-confidence and pride."}, "language_style": {"answer": "Calm and direct", "rationale": "The language style of the entry is calm and straightforward. It does not use euphemism or rhetoric, but rather it directly expresses the theme of whether the person surveyed often feels better than the person with whom he or she is with. This straightforward approach is more in line with the questionnaire and allows for more accurate collection of the views and opinions of the person surveyed."}, "time_dimension": {"answer": "Present", "rationale": ""}, "sentence_type": "Negative declarative sentence", "pos": {"nouns": {"person": 1}, "verbs": {"Common sense": 1, "in": 1}, "adjectives": {"Strong": 1}, "adverbs": {"More than me": 1, "Together": 1}, "numerals": {"18": 1, "and": 1}, "pronouns": {"I": 2}}}, {"content": "I'm trying to take everything I've got.", "word_count": 14, "label": "", "sentence_structure": "Subject鈥搗erb鈥搊bject", "subjective_objective": {"answer": "Subjective", "rationale": "Because it's the heart and feeling of the patient, it's the subjective view."}, "sentence_sentiment": {"answer": "Negative", "rationale": "Because the \"deprive me everything\" in the entry has a negative impact."}, "language_style": {"answer": "Calm and direct", "rationale": "The entry expresses the subject in a straightforward and clear language, \"someone tries to deprive me of everything,\" without using too many euphemisms or emotional terms, which are more objective and calm."}, "time_dimension": {"answer": "Present", "rationale": ""}, "sentence_type": "Negative declarative sentence", "pos": {"nouns": {"person": 1, "Everything": 1}, "verbs": {"Yes": 1, "Deprivation": 1, "Okay": 1}, "pronouns": {"Some": 1, "I": 1}}}, {"content": "I get angry sometimes.", "word_count": 5, "label": "", "sentence_structure": "Subject鈥搗erb鈥揷omplement", "subjective_objective": {"answer": "Subjective", "rationale": "Because anger is an emotional reaction, it belongs to the patient ' s subjective experience and attitude."}, "sentence_sentiment": {"answer": "Negative", "rationale": "Because the \"greed\" act in the entry is negative."}, "language_style": {"answer": "Calm and direct", "rationale": "The entry is more calm and straightforward, and in a concise and straightforward manner, it expresses the question of whether the other party has been angry, is speaking in a balanced and objective manner and does not have too much emotional rendering or rhetorical techniques."}, "time_dimension": {"answer": "Present", "rationale": "The entry now indicates that the person who was tested has had \"sometimes anger\" experiences or behaviour in the present or the recent period, and is therefore judged to be in the present state of affairs."}, "sentence_type": "Affirmative declarative sentence", "pos": {"verbs": {"- Get angry": 1}, "adjectives": {"Time": 1, "Sometimes": 1}}}, {"content": "21 - I've often acted on my own initiative, driven by some kind of inner force.", "word_count": 24, "label": "", "sentence_structure": "Subject鈥搗erb鈥揷omplement", "subjective_objective": {"answer": "Subjective", "rationale": "Because \"inherent strength\" and \"involved behaviour\" are subjective feelings and experiences of patients."}, "sentence_sentiment": {"answer": "Negative", "rationale": "The basis for the judgment: the \"somethings\" in the entry imply a feeling of being unable to control itself, possibly accompanied by some negative emotions or pressures. Thus, this expression can be considered a negative emotional colour."}, "language_style": {"answer": "Tactful and warm", "rationale": "The entry uses a more euphemistic language style that does not directly use strong affirmation or negative tone to express views, but rather describes a phenomenon and directs the interviewee to self-reflection."}, "time_dimension": {"answer": "Present", "rationale": ""}, "sentence_type": "Affirmative declarative sentence", "pos": {"nouns": {"Inside power": 1, "Driver": 1, "Behaviour": 1}, "verbs": {"Often": 1, "Do it": 1, "Out": 1}, "adverbs": {"I can't help it": 1}, "numerals": {"21": 1}, "pronouns": {"I": 1}}}, {"content": "I'd rather starve to death than be forced to eat.", "word_count": 12, "label": "", "sentence_structure": "Subject鈥搗erb鈥揷omplement", "subjective_objective": {"answer": "Subjective", "rationale": "Because the descriptions of \"better\" and \"forced\" are the subjective feelings and attitudes of the patient, and whether or not food is eaten is an objective fact."}, "sentence_sentiment": {"answer": "Negative", "rationale": "The basis for the judgement: because the act of \"forced eating\" is negative, it shows that the person interviewed may be dissatisfied with or afraid of a situation."}, "language_style": {"answer": "Calm and direct", "rationale": "The language style of the entry is more straightforward, with no excessive rhetoric or emotional rendering, but rather the use of concise language to express the subject matter, which allows readers to understand more clearly the meaning of the problem and the purpose of the survey."}, "time_dimension": {"answer": "Present", "rationale": ""}, "sentence_type": "Negative declarative sentence", "pos": {"nouns": {"Starving": 1, "Rice": 1}, "verbs": {"I'd rather": 1, "Starving": 1, "I don't want to": 1, "Forced": 1, "Eat": 1}, "adverbs": {"I'd rather not": 1, "not": 2}, "numerals": {"22": 1}, "pronouns": {"I": 1}}}, {"content": "23, I've been deliberately ignoring some dangers, like I'm a Superman.", "word_count": 18, "label": "", "sentence_structure": "Subject鈥搗erb鈥搊bject", "subjective_objective": {"answer": "Subjective", "rationale": "Because the words \"intentional\" and \"like\" express the patient's own thoughts and attitudes."}, "sentence_sentiment": {"answer": "Negative", "rationale": "Because the act of \"intentional neglect of some danger\" in the entry is negative."}, "language_style": {"answer": "Calm and direct", "rationale": "The article is expressed in a very straightforward manner, without any symmetry or emotional rendering. It clearly asks whether the person under investigation often ignores danger, which is a calm and objective tone."}, "time_dimension": {"answer": "Past", "rationale": "\"Always\" and \"intentionally\" are words that describe customary sexual behaviour that has occurred in the past, and \"neglecting some dangers\" is an expression of past behaviour, so the temporal dimension of this entry is the past."}, "sentence_type": "Affirmative declarative sentence", "pos": {"nouns": {"Danger": 1}, "verbs": {"Ignore": 1, "I think so": 1, "be": 1}, "adjectives": {"Often": 1, "On purpose": 1, "individual": 1, "Superman": 1}, "numerals": {"23": 1}, "pronouns": {"I": 1}}}, {"content": "I'm proud of my ability to denigrate others.", "word_count": 15, "label": "", "sentence_structure": "Subject鈥揷opula鈥損redicative", "subjective_objective": {"answer": "Subjective", "rationale": "Because pride is a subjective assessment and feeling."}, "sentence_sentiment": {"answer": "Negative", "rationale": "Because the \"capable of denigrating others\" in the entry is negative."}, "language_style": {"answer": "Tactful and warm", "rationale": "The language style of the entry appears to be somewhat arrogant, fails to note the importance of respect for others and can easily cause discomfort among the respondents. Thus, the language style of the article can be considered to fall within the category of \"precipitous warmth\" and is more focused on creating a positive and harmonious interviewing atmosphere and enhancing interaction and trust with the respondents."}, "time_dimension": {"answer": "Present", "rationale": "The entry asks the testee about his attitude and his perception of his abilities, which \"I'm proud\" suggests that it is a current emotional experience or self-evaluation. So, in terms of time dimension, it is now."}, "sentence_type": "Affirmative declarative sentence", "pos": {"nouns": {"Capacity": 1, "Demeaning": 1, "others": 1, "Honor": 1}, "verbs": {"Proud": 1}, "adverbs": {"There is": 1}}}, {"content": "25, people tell me I'm always feeling killed.", "word_count": 13, "label": "", "sentence_structure": "Subject鈥搗erb鈥搊bject", "subjective_objective": {"answer": "Subjective", "rationale": "This is because people are assessed as patients and are seen from a subjective perspective."}, "sentence_sentiment": {"answer": "Negative", "rationale": "Because the description of \"the feeling of being killed\" in the entry is negative."}, "language_style": {"answer": "Tactful and warm", "rationale": "The entry is more personal and interactive when it is spoken in a gentle and inviting manner, rather than in direct statements or orders. At the same time, the interrogators do not directly point out whether there is a problem with the other party, but rather give them an opportunity to express their feelings, which is more respectful and understanding."}, "time_dimension": {"answer": "Present", "rationale": ""}, "sentence_type": "Affirmative declarative sentence", "pos": {"nouns": {"person": 1, "Feel": 1}, "verbs": {"Tell": 1, "have": 1}, "adverbs": {"Total": 1}, "numerals": {"25": 1}, "pronouns": {"I": 1}}}, {"content": "I get angry sometimes when I feel bad.", "word_count": 12, "label": "", "sentence_structure": "Subject鈥搗erb鈥揷omplement", "subjective_objective": {"answer": "Subjective", "rationale": "Because feeling is an individual subjective emotional experience, and anger is an individual subjective behaviour."}, "sentence_sentiment": {"answer": "Negative", "rationale": "Because it's negative in the entry."}, "language_style": {"answer": "Calm and direct", "rationale": "The language style of the entry is more straightforward, with no excessive use of rhetorical or emotional refinements, and more objective and cold language, and a tendency to collect the true situation of the other party."}, "time_dimension": {"answer": "Present", "rationale": "Now the entry \"just\" means an act that happens immediately in a given situation, and \"crazy\" is a behavioral move, so the entry describes the situation now."}, "sentence_type": "Affirmative declarative sentence", "pos": {"nouns": {"Feel": 1, "- Grumpy": 1}, "verbs": {"Let's go": 1, "Okay": 1}, "adverbs": {"Sometimes": 1}, "numerals": {"26": 1}}}, {"content": "I often do things on my own when things bother me.", "word_count": 23, "label": "", "sentence_structure": "Subject鈥搗erb鈥揷omplement", "subjective_objective": {"answer": "Subjective", "rationale": "Because \"no one can help themselves\" means that it's a patient's subjective feeling and action."}, "sentence_sentiment": {"answer": "Negative", "rationale": "Because the description of \"I often do something of my own free will\" in the entry may reflect a negative pattern of behaviour. While it does not necessarily mean that this person is always feeling negative or frustrated, it suggests that they may lack effective coping strategies in the face of negative emotions."}, "language_style": {"answer": "Calm and direct", "rationale": "The basis for the judgement: instead of using too many rhetorical techniques or emotional renderings, the entry expresses the phenomenon \"when certain circumstances bother me\" and then describes its possible behaviour in a concise language. This expression is more straightforward and understandable."}, "time_dimension": {"answer": "Present", "rationale": "The word \"make\" indicates the reason, the word \"normal\" indicates a regular, customary act, and the word \"something is done by itself\" occurs now, so the entry describes the act or state of the present."}, "sentence_type": "Affirmative declarative sentence", "pos": {"nouns": {"Situation": 1, "I": 1, "Behaviour": 1}, "verbs": {"鈾?And I'm gonna be 鈾?: 1, "Often": 1, "Do it": 1, "I can't help it": 1}, "adjectives": {"Trouble": 1}, "adverbs": {"Often": 1, "I can't help it": 1}, "numerals": {"27": 1}, "pronouns": {"I": 1}}}, {"content": "28, when things go wrong, I get sick.", "word_count": 12, "label": "", "sentence_structure": "Subject鈥揷opula鈥損redicative", "subjective_objective": {"answer": "Subjective", "rationale": "Because \"I'm gonna get sick\" is a personal subjective feeling and judgment, not an objective fact. It can be influenced by emotions, beliefs and other personal differences. So, this is seen from the patient's subjective perspective."}, "sentence_sentiment": {"answer": "Negative", "rationale": "Because the expression \"I'll get sick when things go wrong\" in the entry is negative."}, "language_style": {"answer": "Tactful and warm", "rationale": "The language style of the entry is more euphemistic, and it is easier to make the interviewer feel comfortable and willing to share his experience and feelings than to ask him directly whether he has experienced similar experiences."}, "time_dimension": {"answer": "Past", "rationale": "The keyword \"incidental\" indicates that it is an outcome or effect, not an act or mentality, and the entry is therefore an inquiry into the person's past behaviour or experience."}, "sentence_type": "Negative declarative sentence", "pos": {"nouns": {"Yes": 1, "Diseases": 1}, "verbs": {"Yes": 2, "On your own": 1, "Fan": 1}, "numerals": {"28": 1}, "pronouns": {"I": 1}}}, {"content": "I'm a very self-sufficient man.", "word_count": 12, "label": "", "sentence_structure": "Subject鈥揷opula鈥損redicative", "subjective_objective": {"answer": "Subjective", "rationale": "Because \"I am a particularly self-sufficient person\" is a person's opinion or perception, a subjective feeling and perception of the individual."}, "sentence_sentiment": {"answer": "Positive", "rationale": "Because the description of \"special self-control\" in the entry is positive and indicates that the individual has positive qualities."}, "language_style": {"answer": "Tactful and warm", "rationale": "The entry uses positive statements to describe the person under investigation, expressing a positive view of the person under investigation, and a more gentle and friendly language. Nor does it use negative or critical language, which does not put pressure or discomfort on the person under investigation, and therefore can be judged as a soft and warm language style."}, "time_dimension": {"answer": "Present", "rationale": "So the time dimension is now judged."}, "sentence_type": "Affirmative declarative sentence", "pos": {"nouns": {"I": 1, "person": 1}, "verbs": {"be": 1, "have": 1}, "adjectives": {"Special": 1, "Self-restraint": 1}, "numerals": {"29": 1}}}, {"content": "I'm like an undesired artist.", "word_count": 16, "label": "", "sentence_structure": "Subject鈥搗erb鈥揷omplement", "subjective_objective": {"answer": "Subjective", "rationale": "Because \"unsatisfactory\" and \"artist\" are patients' subjective feelings and self-evaluations."}, "sentence_sentiment": {"answer": "Negative", "rationale": "Because the word \"unwillful\" in the entry is derogatory and expresses a negative sentiment and attitude."}, "language_style": {"answer": "Tactful and warm", "rationale": "The entry uses a metaphor that compares itself to a \"unsatisfactory artist\" that is both self-scribbling and euphemistic and implicit, and that does not directly make people uncomfortable or offensive. At the same time, the word \"almost\" adds to the sensitivity and interaction of the language, making the questionnaire more friendly and powerful."}, "time_dimension": {"answer": "Present", "rationale": "At the same time, the term \"undesperable artist\" is also an adjective that further highlights the feelings of the testees and thus can be judged to be a description of the situation."}, "sentence_type": "Affirmative declarative sentence", "pos": {"nouns": {"Artist": 1, "I": 1}, "verbs": {"Yeah": 1, "You're right": 1, "Same thing": 1}, "adjectives": {"It's just": 1, "I'm not": 1}, "numerals": {"30": 1}}}, {"content": "I don't always tell the truth.", "word_count": 7, "label": "", "sentence_structure": "Subject鈥損redicate", "subjective_objective": {"answer": "Subjective", "rationale": "Because it is the subjective attitude and choice of one person to tell the truth, not the other. It is the patient鈥檚 perception and assessment of himself, and it is subjective."}, "sentence_sentiment": {"answer": "Negative", "rationale": "Because the \"not always telling the truth\" in the entry is an expression of a negative behaviour."}, "language_style": {"answer": "Tactful and warm", "rationale": "The language style of the entry is more euphemistic and implicit, not expressed in too direct or harsh a language, but in a soft and cursing way, which makes people feel more friendly and friendly. Moreover, the choice of words in the entry is more neutral and less peaceful, without using strong emotional verbs, so it can be judged that the language style is euphemism."}, "time_dimension": {"answer": "Present", "rationale": "The entry is therefore a description of the current state and attitude of the person being tested, and the temporal dimension is now judged."}, "sentence_type": "Negative declarative sentence", "pos": {"nouns": {"Entry": 1, "The truth": 1}, "verbs": {"- No, no, no": 1, "Say it": 1}, "adverbs": {"Always": 1}, "numerals": {"31": 1}, "pronouns": {"I": 1}}}, {"content": "I'll avoid it when I feel my pride is hurt.", "word_count": 16, "label": "", "sentence_structure": "Subject鈥搗erb鈥搊bject", "subjective_objective": {"answer": "Subjective", "rationale": "Because \"shield\" is a subjective act that reflects the way and attitude of patients when they are injured by their self-esteem."}, "sentence_sentiment": {"answer": "Negative", "rationale": "Because the \"divide\" act in the entry is negative."}, "language_style": {"answer": "Tactful and warm", "rationale": "The entry is more adept and interactive when it is spoken in a gentle and inviting manner, rather than by direct statements or orders."}, "time_dimension": {"answer": "Past", "rationale": "The keyword \"I will avoid\" means that it is a response and a mode of behaviour of the past. The temporal dimension of the article is therefore the past."}, "sentence_type": "Exclamatory sentence", "pos": {"nouns": {"Self-esteem": 1, "Hurt": 1}, "verbs": {"Yes": 1, "Avoidance": 1}, "adverbs": {"Just": 1}, "numerals": {"32": 1}}}, {"content": "I often have to do things too much to be self-sufficient, so that others have to limit me.", "word_count": 31, "label": "", "sentence_structure": "Subject鈥搗erb鈥搊bject", "subjective_objective": {"answer": "Subjective", "rationale": "Because the words \"compelling\" and \"overstepping\" are the subjective feelings and behaviours of the patient himself, and they belong to the patient's inner experience and thoughts. So the subject is viewed from the patient's subjective perspective."}, "sentence_sentiment": {"answer": "Negative", "rationale": "Because the entry \"pushing yourself to do something too much, so that others have to limit me\" is negative and negative."}, "language_style": {"answer": "Tactful and warm", "rationale": "The entry, in a more moderate way, asks the respondents whether they are forced to act excessively, using the words \"involvedly\" to express a concern and understanding. Such language is easier to open the interviewee's heart to answer questions."}, "time_dimension": {"answer": "Past", "rationale": "There is no obvious past, present or future time hint on the time dimension of this entry. Therefore, the time dimension should be judged neutral."}, "sentence_type": "Affirmative declarative sentence", "pos": {"nouns": {"Questionnaire": 1, "Entry": 1, "Situation": 1, "Others": 1, "Limits": 1}, "verbs": {"Forced": 2, "I can't do it on my own": 2, "We have to limit it": 1}, "adverbs": {"Often": 1}, "numerals": {"33": 1}, "pronouns": {"self": 1}}}, {"content": "34 My friends think I'm a country boy.", "word_count": 12, "label": "", "sentence_structure": "Subject鈥搗erb鈥揷omplement", "subjective_objective": {"answer": "Subjective", "rationale": "Because \"friends see\" is the patient's subjective feeling."}, "sentence_sentiment": {"answer": "Negative", "rationale": "Because the description of \"countryboy\" in the entry is negative."}, "language_style": {"answer": "Tactful and warm", "rationale": "The entry is more personal and interactive when it is spoken in a gentle and inviting manner, rather than in direct statements or orders. At the same time, the language style of the article is more euphemistic, not too negative, but rather in an objective and balanced manner."}, "time_dimension": {"answer": "Present", "rationale": ""}, "sentence_type": "Negative declarative sentence", "pos": {"nouns": {"Friends": 1, "I": 1, "Country boy": 1}, "verbs": {"- Put it on": 1, "Look": 1, "I'll take it as a treat": 1}, "numerals": {"34": 1}}}, {"content": "I've been avoiding it a lot when I'm angry.", "word_count": 12, "label": "", "sentence_structure": "Subject鈥搗erb鈥揷omplement", "subjective_objective": {"answer": "Subjective", "rationale": "Because \"often evasive\" is a subjective attitude and choice of a person."}, "sentence_sentiment": {"answer": "Negative", "rationale": "Because the act of \"avoiding\" is usually negative, it is a sign of unwillingness to face problems or difficulties."}, "language_style": {"answer": "Calm and direct", "rationale": "The entry is more straightforward in language style, without excessive rhetoric or emotional rendering, simply asking whether the person surveyed would choose to avoid the problem when he/she is angry. This expression is more objective and direct and facilitates access to accurate data."}, "time_dimension": {"answer": "Past", "rationale": ""}, "sentence_type": "Negative declarative sentence", "pos": {"nouns": {"Time": 1, "Anger": 1, "I": 1}, "verbs": {"Avoidance": 1, "Regular": 1}, "adverbs": {"in": 1}, "numerals": {"35": 1}}}, {"content": "I'm always more vigilant than I should be with those who are really nice to me.", "word_count": 30, "label": "", "sentence_structure": "Subject鈥搗erb鈥搊bject", "subjective_objective": {"answer": "Subjective", "rationale": "Because expressions such as \"often\" and \"higher vigilance\" are based on the patient's personal views and experience, and look at patient's subjective attitudes and ideas."}, "sentence_sentiment": {"answer": "Negative", "rationale": "Because it expresses mistrust and has some negative feelings."}, "language_style": {"answer": "Calm and direct", "rationale": "The entry is more straightforward in language style, with no excessive rhetorical or emotional colour. It raises questions in an objective, factual way, so that the interviewee can understand and answer them directly. At the same time, the entry does not use too many complex terms or long sentences, making the questionnaire easier to understand and fill in."}, "time_dimension": {"answer": "Present", "rationale": "The phrase \"often\" and \"maintain\" in the sentence suggests that it is a statement describing habits or tendencies, and therefore is a state of affairs. Moreover, the expression \"the person I should suspect\" implies that it is a state of present or an act. So the answer is \"now\"."}, "sentence_type": "Affirmative declarative sentence", "pos": {"nouns": {"Questionnaire": 1, "Entry": 1, "person": 2, "Vigilance": 1}, "verbs": {"to": 1, "Friendly": 1, "Hold on": 1, "Doubtful": 1}, "adjectives": {"Indeed": 1, "Higher": 1}, "adverbs": {"Often": 1}, "numerals": {"36": 1}, "pronouns": {"I": 2, "Those": 1}}}, {"content": "I've learned a lot of talent to live my life without any problems.", "word_count": 22, "label": "", "sentence_structure": "Subject鈥搗erb鈥搊bject", "subjective_objective": {"answer": "Subjective", "rationale": "This is because it is the subjective view and expectation of the patient of his or her abilities."}, "sentence_sentiment": {"answer": "Positive", "rationale": "Because both the descriptions of \"special talent\" and \"life without problems\" have positive emotional aspects, indicating that this person is confident and hopeful about his future."}, "language_style": {"answer": "Tactful and warm", "rationale": "The entry uses positive expressions, emphasizing that individuals already have special talents that are sufficient to help them cope with challenges and difficulties in their lives. This positive expression is to some extent encouraging and stimulating, and it can easily be accepted and supported by the interviewees."}, "time_dimension": {"answer": "Present", "rationale": "The entry describes a particular talent that the testees have learned and believes can help them to cope with the challenges of their lives. As this is a description of the current situation, the time dimension is now judged."}, "sentence_type": "Affirmative declarative sentence", "pos": {"nouns": {"Talent": 1, "Fan": 1}, "verbs": {"I can learn": 1, "鈾?And I'm gonna be 鈾?: 2, "Pass": 1}, "adjectives": {"Special": 1, "It's fine": 1}, "numerals": {"37": 1, "Life": 1}, "pronouns": {"I": 1}}}, {"content": "I sometimes choose people I hardly know when I'm voting.", "word_count": 22, "label": "", "sentence_structure": "Subject鈥搗erb鈥搊bject", "subjective_objective": {"answer": "Subjective", "rationale": "Because words like \"know\" and \"understand\" are subjective perceptions and feelings of patients."}, "sentence_sentiment": {"answer": "Negative", "rationale": "Because the action of selecting those I hardly know is negative."}, "language_style": {"answer": "Tactful and warm", "rationale": "The language style of the entry is more euphemistic, using the words \"often\" and \"near understood person\" to express their behaviour and ideas, without much direct evaluation or criticism. At the same time, the sentence is also accompanied by a tone of discussion and understanding, which is more suitable for use in questionnaires."}, "time_dimension": {"answer": "Present", "rationale": "The word \"time\" in \"time of election\" means the current point or time, and the word \"select\" means that the act not only has occurred in the past, but may still be ongoing. The entry therefore describes a current act or state that should be judged as \"now\"."}, "sentence_type": "Affirmative declarative sentence", "pos": {"nouns": {"Time": 2, "person": 2, "Elections": 1, "Roger that": 1}, "verbs": {"Choose": 1, "have": 1, "Often": 1, "be": 1}, "adverbs": {"Almost": 1}, "numerals": {"38": 1, "2": 2, "1": 5}, "pronouns": {"I": 2}}}, {"content": "I can't always make time appointments.", "word_count": 9, "label": "", "sentence_structure": "Subject鈥搗erb鈥揷omplement", "subjective_objective": {"answer": "Subjective", "rationale": "Because failure to meet on time is the patient ' s own feeling and experience and is subjective."}, "sentence_sentiment": {"answer": "Negative", "rationale": "Because the \"no time-bound appointment\" in the entry is negative."}, "language_style": {"answer": "Calm and direct", "rationale": "The entry expresses the subject of a question about whether the other party is often unable to attend on time in a straightforward language, without excessive rhetorical or emotional rendering and with a more objective tone."}, "time_dimension": {"answer": "Past", "rationale": "The entry is therefore about asking the testee what the testee is now. Time dimension is now judged."}, "sentence_type": "Negative declarative sentence", "pos": {"nouns": {"I": 1, "A promise": 1, "Date": 1}, "verbs": {"Regular": 1, "Yes": 1, "Freeze": 1, "Go ahead": 1}, "adverbs": {"Often": 1}, "numerals": {"39": 1}}}, {"content": "I've got a lot of fantasies, but I can't do much in real life.", "word_count": 15, "label": "", "sentence_structure": "Subject鈥搗erb鈥揷omplement", "subjective_objective": {"answer": "Subjective", "rationale": "Because \"fanta\" and \"realism\" are both subjectively seen by the patient. Although this is the idea and expectation of the patient himself, it is not necessarily objective and can be considered subjectively."}, "sentence_sentiment": {"answer": "Negative", "rationale": "Because the entry \"supplendently, less can be done in real life\" is a sign of negative attitudes and tendencies."}, "language_style": {"answer": "Tactful and warm", "rationale": "The entry is more friendly and interactive when it is spoken in a gentle and inviting manner, rather than in direct statements or orders. At the same time, questions are designed in a more friendly manner, allowing respondents to answer questions in a relaxed atmosphere."}, "time_dimension": {"answer": "Present", "rationale": "The keyword \"fantasy\" and \"less in reality\" point to behavioural differences in the testee's current state, i.e., that he thinks more, but actually acts less. Thus, the temporal dimension of the article is now judged."}, "sentence_type": "Negative declarative sentence", "pos": {"nouns": {"Fantasy": 1, "Life": 1}, "verbs": {"Fantasy": 1, "Do it": 1}, "adverbs": {"in": 1, "More": 1, "Yeah": 1, "Less": 1}, "numerals": {"40": 1}}}, {"content": "I'm ashamed to deal with people.", "word_count": 8, "label": "", "sentence_structure": "Subject鈥搗erb鈥揷omplement", "subjective_objective": {"answer": "Subjective", "rationale": "Because shame represents the feelings and attitudes of the patient, and is a subjective observation."}, "sentence_sentiment": {"answer": "Negative", "rationale": "Because the entry \"sham\" expresses a negative emotional attitude."}, "language_style": {"answer": "Calm and direct", "rationale": "The language style of the entry is calm and straightforward, and it expresses in plain and clear language the question of whether the other party is ashamed of dealing with people, without too many rhetorical or emotional renderings. This straightforward expression makes the issue clearer and easier to understand."}, "time_dimension": {"answer": "Past", "rationale": "The entry \"shaming\" is a reference to past experience or mentality, and \"dealing with people\" also points to past behaviour or experience, and therefore judgement is a past."}, "sentence_type": "Negative declarative sentence", "pos": {"nouns": {"I": 1, "person": 1, "Dealing": 1}, "verbs": {"Shame": 1, "Hand it over": 1}, "adverbs": {"very": 1}, "numerals": {"41": 1}}}, {"content": "42, I'm not afraid of anything.", "word_count": 6, "label": "", "sentence_structure": "Subject鈥搗erb鈥揷omplement", "subjective_objective": {"answer": "Subjective", "rationale": "Because fear is a subjective feeling."}, "sentence_sentiment": {"answer": "Neutral", "rationale": "Because the entry does not have any emotional color."}, "language_style": {"answer": "Calm and direct", "rationale": "The language style of this entry is calm and straightforward. It asks whether the interviewee is not afraid of anything, is not described in too much qualifier or emotional language, and is expressed in a concise manner."}, "time_dimension": {"answer": "Present", "rationale": "\"I am not afraid of anything\" is a current idea or attitude that shows that the person being tested is in a state of mind that is not afraid, and therefore judges it now."}, "sentence_type": "Negative declarative sentence", "pos": {"verbs": {"Not afraid": 1}, "adjectives": {"Both": 1, "What": 1}, "numerals": {"42": 1}}}, {"content": "43, sometimes I think I'm an angel, sometimes I think I'm a demon.", "word_count": 20, "label": "", "sentence_structure": "Subject鈥損redicate", "subjective_objective": {"answer": "Subjective", "rationale": "Because \"thinking you're an angel\" or \"thinking you're a demon\" are subjective views and self-evaluations of the patient, which are subjective in their view."}, "sentence_sentiment": {"answer": "Neutral", "rationale": "The entry is not a clear expression of emotional preference."}, "language_style": {"answer": "Tactful and warm", "rationale": "The entry is more euphemistic in its language style, using metaphoric techniques to describe how the person was feeling at different times, and a symbolic image of himself as \"angels\" and \"demons.\" This way allows the person to be more willing to share his feelings, or to feel the respect and understanding of the questionnaire designer."}, "time_dimension": {"answer": "Present", "rationale": "The entry describes the person who was tested for his/her perception and feelings at different points in time, and does not have a clear point of time, and thus the judgement is now."}, "sentence_type": "Affirmative declarative sentence", "pos": {"nouns": {"Time": 2, "I": 2, "I think": 2, "individual": 2, "Angel": 1, "Demon": 1}, "verbs": {"be": 4, "have": 1, "I think": 2}, "adjectives": {"Sometimes": 2}, "numerals": {"43": 1}, "classifiers": {"individual": 2}, "pronouns": {"I": 2}}}, {"content": "I won't lose, I won't lose.", "word_count": 12, "label": "", "sentence_structure": "Subject鈥搗erb鈥揷omplement", "subjective_objective": {"answer": "Subjective", "rationale": "Because the idea of \"win\" and \"no lose\" is the patient's subjective attitude."}, "sentence_sentiment": {"answer": "Negative", "rationale": "Because the \"win-no-lose\" attitude in the entries is negative, and shows a mindset that is too ambitious to seek victory and avoid failure."}, "language_style": {"answer": "Calm and direct", "rationale": "The language style of the entry is more direct, not using too many rhetorical or emotional accents, but rather presenting the attitude of the person under investigation towards winning and losing in an objective and clear manner. This language style is usually used in more serious or formal contexts.\""}, "time_dimension": {"answer": "Present", "rationale": "\"In the game\" means that it means the current situation, \"I can only win, not lose\" and it describes the behavior or mentality of the present. So, the time dimension of this entry is now."}, "sentence_type": "Affirmative declarative sentence", "pos": {"nouns": {"The game": 1, "I": 1}, "verbs": {"Win": 1, "Lose": 1}, "numerals": {"44": 1}}}, {"content": "45, when I'm angry, I'm particularly willing to be bitter.", "word_count": 16, "label": "", "sentence_structure": "Subject鈥搗erb鈥揷omplement", "subjective_objective": {"answer": "Subjective", "rationale": "The sentence refers to the patient鈥檚 behaviour and ideas in a given emotional state, which is subjective. While such behaviour may have occurred in an angry situation, it is the patient鈥檚 thoughts and feelings that are examined and therefore subjective."}, "sentence_sentiment": {"answer": "Negative", "rationale": "Because the \"to become especially bitter\" act in the entry is negative."}, "language_style": {"answer": "Calm and direct", "rationale": "The entry directly asked whether the respondents were inclined to sarcasticly or emotionally in anger, without using excessive rhetorical techniques or emotional rendering, and was more objective in tone."}, "time_dimension": {"answer": "Past", "rationale": "The entry describes the emotional state and behaviour of \"when I'm angry\" 鈥?an experience that happened in the past and therefore judged to be the past."}, "sentence_type": "Exclamatory sentence", "pos": {"nouns": {"Time": 1, "person": 1}, "verbs": {"鈾?And become 鈾?: 1, "May I": 1, "Scratch": 1}, "adjectives": {"Special": 1}, "adverbs": {"Special": 1}, "numerals": {"45": 1}}}, {"content": "I'm a public counterattack when my self-esteem is hurt.", "word_count": 15, "label": "", "sentence_structure": "Subject鈥搗erb鈥搊bject", "subjective_objective": {"answer": "Subjective", "rationale": "Because \"public counteraction\" is an act of initiative and is based on the patient's own feelings and attitudes, it is an issue that is examined from the patient's subjective perspective."}, "sentence_sentiment": {"answer": "Negative", "rationale": "Because the \"public counterattack\" in the entry is negative."}, "language_style": {"answer": "Calm and direct", "rationale": "This language style is more objective and direct, and is suitable for the survey of such scenes as the need for clear and accurate information."}, "time_dimension": {"answer": "Present", "rationale": "The keyword \"just\" means that in certain circumstances immediate action is taken, and \"when my self-esteem is injured\" describes a particular situation in which the act is immediately occurring. So the time dimension of the entry is now judged."}, "sentence_type": "Affirmative declarative sentence", "pos": {"nouns": {"Self-esteem": 1, "Hurt": 1}, "verbs": {"In me": 1, "Just": 2, "Public": 1, "Fight back": 1}, "adverbs": {"In me": 1}, "numerals": {"46": 1}, "pronouns": {"I": 1}}}, {"content": "47, I think I should be turned over when I get hurt.", "word_count": 15, "label": "", "sentence_structure": "Subject鈥搗erb鈥搊bject", "subjective_objective": {"answer": "Subjective", "rationale": "Because \"consider\" means that it is a subjective view or attitude of the patient, the entry is a subjective examination."}, "sentence_sentiment": {"answer": "Negative", "rationale": "Because the \"trash\" in the entry is negative."}, "language_style": {"answer": "Calm and direct", "rationale": "The entry is directly explicit, without any rhetorical or emotional rendering, and gives a brief and direct expression to personal views, language temperature and objectivity."}, "time_dimension": {"answer": "Present", "rationale": "The words \"think,\" \"just\" and \"should\" in the entry indicate that this is an opinion or attitude to a situation, which is current. So the temporal dimension of this entry is now."}, "sentence_type": "Affirmative declarative sentence", "pos": {"nouns": {"Questionnaire": 1, "Entry": 1, "I": 1, "Hurt": 1}, "verbs": {"I think": 1, "Just": 1, "I think so": 1, "You're a fucking liar": 1}, "adjectives": {"When": 1}, "numerals": {"47": 1}}}, {"content": "48, I read every day. Not every page.", "word_count": 14, "label": "", "sentence_structure": "Subject鈥搗erb鈥搊bject", "subjective_objective": {"answer": "Subjective", "rationale": "Because \"reading the newspaper every day\" and \"not every page\" are subjective behaviors and attitudes of patients."}, "sentence_sentiment": {"answer": "Negative", "rationale": "Because \"not every page read\" means that readers don't read the paper in its entirety, they probably ignore important information."}, "language_style": {"answer": "Tactful and warm", "rationale": ""}, "time_dimension": {"answer": "Present", "rationale": "Every word in \"I\" means daily, customary behavior, and reading means that it is not only a practice in the past, but may still be a practice. So this entry asks about the testee's current reading habits."}, "sentence_type": "Imperative sentence", "pos": {"nouns": {"Board": 1, "Oh, my God": 1, "Report": 1}, "verbs": {"Read it": 2, "be": 1, "Both": 1}, "adverbs": {"Every one": 1, "not": 1, "Time": 1}, "numerals": {"48": 1, "Every": 1, "individual": 1}, "pronouns": {"I": 1}}}, {"content": "49, when I'm depressed, I avoid.", "word_count": 8, "label": "", "sentence_structure": "Subject鈥搗erb鈥揷omplement", "subjective_objective": {"answer": "Subjective", "rationale": "Because \"shield\" is the patient's own psychological response and response, it is the patient's subjective attitude and experience."}, "sentence_sentiment": {"answer": "Negative", "rationale": "Because the \"shield\" in the entry is negative."}, "language_style": {"answer": "Calm and direct", "rationale": "The entry is a short positive sentence that expresses attitude towards emotional problems, is not overly emotional or rhetorical, and is more objective. It is therefore clear that the language style is straightforward."}, "time_dimension": {"answer": "Present", "rationale": "Now, based on the word \"will\" and the time-forming in the sentence, it can be judged that the entry describes a regular, customary act, which is to avoid frustration. Thus, the temporal dimension is now."}, "sentence_type": "Negative declarative sentence", "pos": {"nouns": {"Frustration": 1, "Time": 1, "Avoid": 1}, "verbs": {"Yes": 1, "Avoid": 1}, "adverbs": {"Just": 1}, "numerals": {"49": 1}}}, {"content": "I'm shy about sex.", "word_count": 9, "label": "", "sentence_structure": "Subject鈥揷opula鈥損redicative", "subjective_objective": {"answer": "Subjective", "rationale": "Because \"smearing\" is a personal subjective and emotional experience that reflects the psychological state and attitude of individuals to sexual problems."}, "sentence_sentiment": {"answer": "Negative", "rationale": "Because the emotional attitude of being shy is negative."}, "language_style": {"answer": "Tactful and warm", "rationale": "The entry is more adept and interactive when it is spoken in a gentle and inviting manner, rather than by direct statements or orders."}, "time_dimension": {"answer": "Present", "rationale": "The keyword \"I'm shy\" means the current emotional state, so the time dimension of the article is now."}, "sentence_type": "Negative declarative sentence", "pos": {"nouns": {"50": 1, "Sexual issues": 1}, "verbs": {"I feel": 1, "Shy": 1}, "numerals": {"50": 1}}}, {"content": "I always felt like someone I knew was a protector.", "word_count": 18, "label": "", "sentence_structure": "Subject鈥搗erb鈥搊bject", "subjective_objective": {"answer": "Subjective", "rationale": "Because \"like a protector\" is a subjective assessment and feeling, and it's a subjective attitude of the patient."}, "sentence_sentiment": {"answer": "Positive", "rationale": "Because the entry \"like a protector\" expresses a positive emotional attitude, which is that this person brings to people a sense of security and help."}, "language_style": {"answer": "Tactful and warm", "rationale": "The language style of the entry is more euphemistic and intimate, and it is not spoken in a strong or direct language. This way of asking questions makes it easier to share ideas and feelings and to build good interaction. At the same time, the metaphor \"like a protector\" is more graphic, more resonant and more interested and willing to answer questions."}, "time_dimension": {"answer": "Past", "rationale": "\"Always\" means \"high frequency,\" and \"like a protective god\" describes a feeling or a perception that has occurred in the past and still has an impact on it. So this entry describes the experience and feelings of the past."}, "sentence_type": "Affirmative declarative sentence", "pos": {"nouns": {"51": 1, "I": 2, "Someone I know": 1, "Protect the gods": 1}, "verbs": {"I feel": 1, "Elephant": 1}, "adverbs": {"Always": 1}, "numerals": {"51": 1}, "classifiers": {"individual": 1}, "pronouns": {"I": 2}}}, {"content": "52 My philosophies are: 鈥淒o not believe, do not do, do not look.鈥?, "word_count": 19, "label": "", "sentence_structure": "Subject鈥搗erb鈥揷omplement", "subjective_objective": {"answer": "Subjective", "rationale": "Because philosophies are a personal conviction and attitude, they are a subjective view of the patient."}, "sentence_sentiment": {"answer": "Negative", "rationale": "Because it expresses a negative attitude towards interpersonal relations, i.e., belief in and adherence to one ' s own logic, rather than trusting others."}, "language_style": {"answer": "Calm and direct", "rationale": "The entry presents a human philosophies in a straightforward manner, with no excessive adoration or emotional rendering, and a more concise language style."}, "time_dimension": {"answer": "Present", "rationale": "The questionnaire entry is about the individual philosophies of the testee, i.e., the belief that he does not trust, does not do or does not look at unreasonable things. The idea may be that the testee has a vision now, or has developed and continues to this day. So, in terms of time dimensions, it is now."}, "sentence_type": "Affirmative declarative sentence", "pos": {"nouns": {"Philosophy of the world": 1, "I'm not gonna believe this": 1, "I don't want to do it": 1, "I don't want to see": 1}, "numerals": {"52": 1}}}, {"content": "53 I think that people have a good or a bad side.", "word_count": 9, "label": "", "sentence_structure": "Subject鈥搗erb鈥搊bject", "subjective_objective": {"answer": "Subjective", "rationale": "Because \"conception\" expresses the patient's subjective view of the issue, and \"people have good or bad\" is an opinion or belief in itself, not an objective fact."}, "sentence_sentiment": {"answer": "Negative", "rationale": "Because the expression \"people have good or bad\" in the entry is discriminatory and biased, and there is a negative perception of human beings."}, "language_style": {"answer": "Calm and direct", "rationale": "The entry presents its views in a straightforward tone, is not over-modified or emotionally rendered and is more calm and objective."}, "time_dimension": {"answer": "Present", "rationale": "According to the input sentence, \"I think...\" \"thinks\" means that it's the tester's present view or attitude, so the judgment is now."}, "sentence_type": "Affirmative declarative sentence", "pos": {"nouns": {"person": 1, "Good and bad": 1}, "verbs": {"I think": 1}, "numerals": {"53": 1}}}, {"content": "I'm afraid my boss would get in trouble at work or a seaman to get back at him.", "word_count": 29, "label": "", "sentence_structure": "Subject鈥搗erb鈥搊bject", "subjective_objective": {"answer": "Subjective", "rationale": "This is the patient ' s subjective idea because it is likely to react to his superiors ' anger."}, "sentence_sentiment": {"answer": "Negative", "rationale": "Because the entry \"Getting In trouble or Grimming to Revenge him\" is negative."}, "language_style": {"answer": "Calm and direct", "rationale": "The entry is straightforward, without any rhetorical or emotional recitation, and expresses a reaction to superior behaviour by presenting the facts."}, "time_dimension": {"answer": "Future", "rationale": "\""}, "sentence_type": "Negative declarative sentence", "pos": {"nouns": {"Sir": 1, "Angry": 1, "Work": 1, "Trouble": 1, "Revenge": 1}, "verbs": {"I think so": 1, "Find": 1, "A seaman": 1}, "adverbs": {"Suppose": 1}, "numerals": {"54": 1}, "classifiers": {"individual": 1}, "pronouns": {"I": 1}}}, {"content": "55, everybody's against me.", "word_count": 9, "label": "", "sentence_structure": "Subject鈥搗erb鈥搊bject", "subjective_objective": {"answer": "Subjective", "rationale": "Because this is the patient ' s subjective idea, it is not what actually happened."}, "sentence_sentiment": {"answer": "Negative", "rationale": "Because the phrase \"all of us and me against the dry\" in the entry is negative and negative emotional and indicates that others are not friendly with me."}, "language_style": {"answer": "Calm and direct", "rationale": "The entry is simple, and the other person is asked whether he or she often feels that the people around him are against himself. There is no excessive rhetorical or emotional rendering, and the tone is more objective.\""}, "time_dimension": {"answer": "Future", "rationale": "The keywords are \"both\" and \"back to back\" , which express a state of anticipation or anticipation of the future, and the entry is therefore a future."}, "sentence_type": "Negative declarative sentence", "pos": {"nouns": {"person": 2, "I": 2, "- To the front": 1}, "verbs": {"and": 1}, "numerals": {"55": 1, "Every": 1}}}, {"content": "I'm always friendly with people I hate.", "word_count": 15, "label": "", "sentence_structure": "Subject鈥搗erb鈥揷omplement", "subjective_objective": {"answer": "Subjective", "rationale": "This is a description of the patient ' s subjective attitude, which often shows behaviour contrary to what he feels inside."}, "sentence_sentiment": {"answer": "Negative", "rationale": "Because the word \"obsessed\" in the entry is an expression of negative emotional attitudes."}, "language_style": {"answer": "Calm and direct", "rationale": "The entry is more direct and concise in language style, is not overly accommodative or emotional, and focuses more on factual descriptions and problem statements, and can therefore be judged as a \"cool straight\" type."}, "time_dimension": {"answer": "Past", "rationale": "In conclusion, for the given psychological questionnaire entry, \"56, I'm always friendly to those I hate,\" the output should be \"now\"."}, "sentence_type": "Negative declarative sentence", "pos": {"nouns": {"Questionnaire": 1, "Entry": 1, "person": 2, "Shit": 1, "Friendly": 1}, "verbs": {"Often": 1, "Organisation": 1}, "adjectives": {"Those": 1}, "numerals": {"56": 1}, "pronouns": {"I": 1}}}, {"content": "57, if one of the engines of the plane I was travelling with failed, I would be particularly nervous.", "word_count": 23, "label": "", "sentence_structure": "Subject鈥搗erb鈥搊bject", "subjective_objective": {"answer": "Subjective", "rationale": "Because it is the patient ' s subjective reaction to and perception of a possible situation, it is an expression of his or her inner experience."}, "sentence_sentiment": {"answer": "Negative", "rationale": "The reference in the entry \"can be particularly tense\" is an expression of a negative emotional attitude."}, "language_style": {"answer": "Calm and direct", "rationale": "The language style of the entry is calm and straightforward. It clearly presents a scenario and asks the interviewee how he or she reacts in such a situation, without using too many euphemisms or emotional expressions."}, "time_dimension": {"answer": "Present", "rationale": "The entry now describes the emotional reaction that the person tested may have in a given situation (a motor of the aircraft failed), namely, \"special stress.\" The entry is therefore asking about the person's current behavior or emotional state."}, "sentence_type": "Exclamatory sentence", "pos": {"nouns": {"Aircraft": 1, "Engines": 1, "Nervous": 1}, "verbs": {"Take it": 1, "It's not working": 1}, "adjectives": {"One": 1, "Special": 1}, "adverbs": {"Suppose": 1, "Just": 1}, "numerals": {"57": 1, "One": 2}, "pronouns": {"I": 1}}}, {"content": "58, I know a man who can do anything and does what he does right.", "word_count": 22, "label": "", "sentence_structure": "Subject鈥搗erb鈥搊bject", "subjective_objective": {"answer": "Subjective", "rationale": "The idea of knowing someone and of seeing him is a human one."}, "sentence_sentiment": {"answer": "Positive", "rationale": "Because the quality of \"reasonable integrity\" in the entry is positive."}, "language_style": {"answer": "Tactful and warm", "rationale": "The entry is more euphemistic, using descriptive terms such as \"same person\" and \"reasonable integrity\" to express praise and approval of a person, without direct command or evaluation of the other. This expression is more personal and interactive."}, "time_dimension": {"answer": "Present", "rationale": "The word \"I know\" in \"I know\" uses the normal current, which means that this behaviour has not only happened in the past, but may still be going on. And, \"He can do anything and do it with integrity\" describes a state or character that exists and therefore judges it now."}, "sentence_type": "Affirmative declarative sentence", "pos": {"nouns": {"Personal": 1, "Yes": 1, "person": 1, "Work": 1, "Justity": 1}, "verbs": {"Yes": 1, "Do it": 2}, "adjectives": {"I see": 1, "Reasonable": 1, "Integrity": 1}, "numerals": {"58": 1}, "classifiers": {"individual": 1}, "pronouns": {"I": 1}}}, {"content": "59, if my feelings are going to get in the way of what I'm doing, then I can control it.", "word_count": 27, "label": "", "sentence_structure": "Subject鈥搗erb鈥搊bject", "subjective_objective": {"answer": "Subjective", "rationale": "This involves self-awareness and management of the patient鈥檚 behaviour and feelings, and is therefore part of the subjective examination."}, "sentence_sentiment": {"answer": "Positive", "rationale": "Because the \"capable\" behavior in the entry is positive."}, "language_style": {"answer": "Calm and direct", "rationale": "The entry expresses the view that self-control is required if the loss of feelings has a negative impact on work. This expression is not too emotional or rhetorical, and is more direct and objective, and corresponds to the cold and direct linguistic style."}, "time_dimension": {"answer": "Present", "rationale": "\"Cutting control\" is the current state of affairs, which suggests that it is a statement of the individual's current abilities. Moreover, the structure \"if it is...\" also shows that it is a hypothetical situation and implies that the testee is trying to control his feelings.\""}, "sentence_type": "Interrogative sentence", "pos": {"nouns": {"Emotion": 1, "- Out": 1, "Career": 1, "Control": 1}, "verbs": {"Suppose": 1, "Obstruction": 1, "Yes": 1}, "numerals": {"59": 1}, "pronouns": {"I": 2}}}, {"content": "60, some people are planning to kill me.", "word_count": 10, "label": "", "sentence_structure": "Subject鈥搗erb鈥搊bject", "subjective_objective": {"answer": "Subjective", "rationale": "Because it's the patient's subjective feeling or concern."}, "sentence_sentiment": {"answer": "Negative", "rationale": "Because the actions in the entries are negative."}, "language_style": {"answer": "Calm and direct", "rationale": "The entry directly expresses a question about whether the other party has ever been framed by a conspiracy, is more objective in tone and does not use excessive rhetorical or emotional rendering."}, "time_dimension": {"answer": "Present", "rationale": "The entry describes a possible event or situation, which is \"scheming to harm me\" which means that some people are planning an act that may occur in the future. So the temporal dimension of the entry is the future."}, "sentence_type": "Affirmative declarative sentence", "pos": {"nouns": {"person": 1, "Shit": 1}, "verbs": {"Conspiracy": 1, "In the process of": 1, "Yes": 1}, "adverbs": {"Some": 1}, "numerals": {"60": 1}}}, {"content": "61, I usually see the good side of the bad.", "word_count": 15, "label": "", "sentence_structure": "Subject鈥搗erb鈥搊bject", "subjective_objective": {"answer": "Subjective", "rationale": "Because the expression \"I can normally see\" is used in the title, it involves the feelings and attitudes of the patient himself, and it is a subjective examination."}, "sentence_sentiment": {"answer": "Positive", "rationale": "Because the entry \"seeing the good side of the bad\" is a positive attitude and perspective."}, "language_style": {"answer": "Tactful and warm", "rationale": "The entry uses the question-and-answer approach to guide respondents to answer questions, is soft in terms, is not directly spoken or ordered, and is more acceptable and resonant. At the same time, the view of \"seeing the good side of the bad\" is more positive, gives people a sense of hope and corresponds to the \"sweetly\" language style."}, "time_dimension": {"answer": "Future", "rationale": "The entry contains visions and expectations for the future, and the term \"normally\" indicates a long-term behavioural tendency or habit, but this behaviour has not yet been fully realized. At the same time, the contrast between \"bad\" and \"good\" implies that the testee wishes to be able to change the current situation and see more positive aspects. The article therefore has a time-bound approach for the future."}, "sentence_type": "Affirmative declarative sentence", "pos": {"nouns": {"Noodles": 1, "Bad times": 1, "Okay": 1}, "verbs": {"Yeah": 1, "Yes": 1, "Do it": 1}, "adverbs": {"Usually": 1, "When": 1, "Medium": 1}, "numerals": {"61": 1}, "pronouns": {"I": 1}}}, {"content": "I had to do something I didn't want to do.", "word_count": 20, "label": "", "sentence_structure": "Subject鈥搗erb鈥揷omplement", "subjective_objective": {"answer": "Subjective", "rationale": "Because the feeling of \"no\" and the experience of headaches are subjective feelings and experiences of patients."}, "sentence_sentiment": {"answer": "Negative", "rationale": "Because the expressions \"incidents\" and \"headaches\" in the entries all have a certain negative emotional connotation that the act is negative."}, "language_style": {"answer": "Calm and direct", "rationale": "The entry is based on a straightforward language, which is open to questions about the headaches of the interviewee when he has to do something he does not want to do. It is not used too much in rhetorical or emotional rendering, and it is more objective and cold, and it is in a calm and direct language style."}, "time_dimension": {"answer": "Present", "rationale": "The entry now describes a situation that the testee is experiencing, i.e., a headache when he has to do something that he does not want to do. So the time dimension is now."}, "sentence_type": "Negative declarative sentence", "pos": {"nouns": {"Situation": 1, "Headache": 1}, "verbs": {"Do it": 2, "Go on": 1, "Okay": 1, "not": 2, "Yes": 1, "Do something": 1, "I": 1}, "adjectives": {"I don't want to": 1}, "adverbs": {"Just": 1}, "numerals": {"62": 1}}}, {"content": "I often find myself very friendly to those who deserve to hate.", "word_count": 21, "label": "", "sentence_structure": "Subject鈥搗erb鈥搊bject", "subjective_objective": {"answer": "Subjective", "rationale": "Because \"expression of special friendship\" is patient's subjective attitude and behaviour."}, "sentence_sentiment": {"answer": "Negative", "rationale": "Because the contrast between \"hate\" and \"specially friendly\" in the entries constitutes a contrast and expresses a negative emotional attitude."}, "language_style": {"answer": "Tactful and warm", "rationale": "The entry is more adept and interactive when it is spoken in a gentle and inviting manner, rather than by direct statements or orders."}, "time_dimension": {"answer": "Past", "rationale": "So this entry is asking the testee about past and present behavior or mentalities, which are the dual dimensions of past and present."}, "sentence_type": "Affirmative declarative sentence", "pos": {"nouns": {"person": 2, "Questionnaire Entry": 1}, "verbs": {"Found": 1, "Organisation": 1, "Hate": 1, "Friendly": 1}, "adjectives": {"Often": 1, "Special": 1}, "numerals": {"63": 1}, "pronouns": {"I": 1}}}, {"content": "64 I think that \"everyone has good will\" does not exist, and if you don't, you can't do anything.", "word_count": 27, "label": "", "sentence_structure": "Subject鈥搗erb鈥搊bject", "subjective_objective": {"answer": "Subjective", "rationale": "Because the title contains \"I think\" which shows that it is the patient's idea and belief, and it is subjective."}, "sentence_sentiment": {"answer": "Negative", "rationale": "Because the \"conception that everyone has good will\" attitude in the entry is negative."}, "language_style": {"answer": "Calm and direct", "rationale": "The entry expresses the author's view in an acclaimed sense that the notion of 鈥渆veryone is good鈥?does not exist and points out that if a person does not have good will himself, they will have problems in other respects. This expression is straightforward and not overly embellished or emotional."}, "time_dimension": {"answer": "Present", "rationale": "The word \"think\" in the entry indicates that the testee is now expressing his views and attitudes, and the latter phrase \"I think\" is a rejection of a general notion that reflects, to some extent, the belief or value of the testee. So the temporal dimension of the entry is now."}, "sentence_type": "Negative declarative sentence", "pos": {"nouns": {"person": 2, "Good will": 1}, "verbs": {"I think": 1, "Existence": 1, "Okay": 2, "Everything": 1, "No way": 2}, "adjectives": {"Both": 1, "Yes": 1}, "numerals": {"64": 1}, "pronouns": {"you": 3, "Well": 1}}}, {"content": "I can't be angry with those I hate.", "word_count": 17, "label": "", "sentence_structure": "Subject鈥搗erb鈥揷omplement", "subjective_objective": {"answer": "Subjective", "rationale": "Because \"no\" and \"no\" are subjective attitudes and judgments of patients."}, "sentence_sentiment": {"answer": "Negative", "rationale": "Because the \"Expressing Anger to Those I hate\" act in the entry is negative."}, "language_style": {"answer": "Calm and direct", "rationale": "The entry uses a more direct and clear expression of the attitudes and principles of the other party in dealing with interpersonal relations, without excessive ado or emotional colour, and therefore can be judged as a calm and direct language style."}, "time_dimension": {"answer": "Past", "rationale": "Moreover, \"the anger expressed by those I hate\" also involves a specific act or emotional reaction, so the entry is asking the testee whether it will or will not take such act or emotional reaction. Because there is no clear time line (such as \"the past\", \"now\", \"the future\" ), it is necessary to judge the time dimension in context. If the context provides a specific time trail, then the time dimension of the article can be determined; if it is not provided, it cannot be determined."}, "sentence_type": "Negative declarative sentence", "pos": {"nouns": {"person": 2, "Anger": 1}, "verbs": {"It's not a sign": 1}, "adjectives": {"Those": 1, "It's disgusting": 1}, "numerals": {"65": 1, "I": 1}}}, {"content": "66, I'm sure life is unjust to me.", "word_count": 12, "label": "", "sentence_structure": "Subject鈥揷opula鈥損redicative", "subjective_objective": {"answer": "Subjective", "rationale": "Because \"convincing\" is patient's subjective attitude and conviction."}, "sentence_sentiment": {"answer": "Negative", "rationale": "Because the expression \"life is unjust to me\" in the entry is full of negative emotions."}, "language_style": {"answer": "Calm and direct", "rationale": "The entry directly expresses the subject of questioning whether life is unfair to them, is more objective and calm, and does not use excessive rhetoric or emotional rendering."}, "time_dimension": {"answer": "Past", "rationale": "The entry is a description of the views and feelings of the testees about the past, the key words being \"conviction\" and \"life is unjust to me\", all pointing to the past experiences and perceptions."}, "sentence_type": "Negative declarative sentence", "pos": {"nouns": {"I": 2, "Life": 1, "Justice": 1}, "verbs": {"be": 1, "Sure": 1, "to": 1}, "adjectives": {"not": 1}, "adverbs": {"very": 1}, "numerals": {"66銆?: 1}}}, {"content": "67, with a severe blow, I'll fall.", "word_count": 13, "label": "", "sentence_structure": "Subject鈥搗erb鈥揷omplement", "subjective_objective": {"answer": "Subjective", "rationale": "Because \"falling\" is a subjective feeling and reaction."}, "sentence_sentiment": {"answer": "Negative", "rationale": "Because the \"falling\" act in the entry is negative."}, "language_style": {"answer": "Calm and direct", "rationale": "The language style of the entry is calm and straightforward, and it is straightforward in asking the individual鈥檚 psychological response to a severe blow, without using too many rhetorical or emotional terms. This simple language style helps the questionnaire researchers to understand the psychological state of the test and how to respond more accurately."}, "time_dimension": {"answer": "Present", "rationale": "In the future, the entry describes a reaction to behaviour that may occur in the face of a severe blow, and although the word \"will\" indicates what may occur, it still points to possible future experience as a whole."}, "sentence_type": "Negative declarative sentence", "pos": {"nouns": {"Serious": 1, "Strike": 1}, "verbs": {"Just": 1, "Falling": 1, "Get down": 1, "Yes": 1}, "adverbs": {"in": 1, "Down": 1}, "numerals": {"67": 1}}}, {"content": "68, when I realized I had to face a difficult situation, like exams, trade unions. I tried to imagine what it would do and planned to deal with it.", "word_count": 46, "label": "", "sentence_structure": "Subject鈥搗erb鈥搊bject", "subjective_objective": {"answer": "Subjective", "rationale": "Because the phrase \"I'm trying to imagine what it's going to be and to plan ways to deal with it\" is a personal thought and plan of action of the patient, a subjective examination."}, "sentence_sentiment": {"answer": "Positive", "rationale": "Because the purpose of the entry is to \"tried to imagine what it will do and plan ways to deal with it.\""}, "language_style": {"answer": "Tactful and warm", "rationale": "The entry is more euphemistic, not speaking too directly or strongly, but rather by describing a scene and asking how the other side responds to a dilemma, thereby triggering the other side鈥檚 reflection and response. This expression is more enlightening and interactive, helping the investigator to understand the inside world and the response."}, "time_dimension": {"answer": "Present", "rationale": "The keyword \"alt\" and \"planned out\" indicates that the act is taking place or has been completed and is therefore the present act or state."}, "sentence_type": "Affirmative declarative sentence", "pos": {"nouns": {"The dilemma": 1, "Examinations": 1, "Recruitment fair": 1, "Entry": 1}, "verbs": {"I'm aware of that": 1, "Faced": 1, "Imagine": 1, "Plan": 1, "Payable": 1}, "adverbs": {"Just": 2}, "numerals": {"68": 1, "it": 1}, "pronouns": {"I": 1}}}, {"content": "69, the doctors can't really figure out what I'm suffering from.", "word_count": 18, "label": "", "sentence_structure": "Subject鈥搗erb鈥揷omplement", "subjective_objective": {"answer": "Subjective", "rationale": "Because \"what's wrong with me\" is a subjective perception of the patient's own physical condition, doctors may not be able to confirm it completely."}, "sentence_sentiment": {"answer": "Negative", "rationale": "Because the expression \"can't really ascertain\" in the entry has a negative emotional colour of suspicion and uncertainty."}, "language_style": {"answer": "Calm and direct", "rationale": "The entry is written in more plain language, with no excessive rhetorical or emotional rendering, and is more objective. It asks whether the other party has a clear problem-oriented and information-transmission attitude to the doctor鈥檚 ability to determine his or her own illness, and therefore prefers to be more calm and straightforward."}, "time_dimension": {"answer": "Past", "rationale": "The use of the keyword \"never\" and \"no doubt\" suggests that this is a description of the past, so the entry relates to the temporal dimensions of the past."}, "sentence_type": "Negative declarative sentence", "pos": {"nouns": {"Doctors": 1, "Diseases": 1}, "verbs": {"Never": 1, "I know": 1, "Patient": 1}, "adjectives": {"Indeed": 1}, "numerals": {"69": 1}, "pronouns": {"I": 1}}}, {"content": "I wasn't sad when someone close to me died.", "word_count": 19, "label": "", "sentence_structure": "Subject鈥揷opula鈥損redicative", "subjective_objective": {"answer": "Subjective", "rationale": "The sentence that was entered, \"I'm not sad when someone who was close to me dies,\" and we can judge it to be subjective in the sense and attitude of the patient, and therefore subjective in the sense that it is."}, "sentence_sentiment": {"answer": "Negative", "rationale": "Because the expression \"I am not sad when someone who is very close to me dies\" in the entry indicates a lack of grief for the loved ones who have died, and may reflect negative emotional attitudes."}, "language_style": {"answer": "Calm and direct", "rationale": "The entry is more calm and straightforward in language, with no amount of emotional rendering or speech. It asks the interviewee in an objective manner about the emotional reactions of his family when they die, and is clear in language."}, "time_dimension": {"answer": "Present", "rationale": "The word \"when\" means a point or a period of time, and \"death\" is what has happened, so it's done in the past. But the whole sentence is a state of present or feeling, that is, now I'm not sad. So it's now."}, "sentence_type": "Negative declarative sentence", "pos": {"nouns": {"person": 1, "Death": 1}, "verbs": {"Die": 1, "It's not sad": 1, "Close": 1}, "adverbs": {"Special": 1}, "numerals": {"70": 1}, "pronouns": {"I": 1}}}, {"content": "71, I apologize for my rudeness after I fought for my own good.", "word_count": 26, "label": "", "sentence_structure": "Subject鈥搗erb鈥搊bject", "subjective_objective": {"answer": "Subjective", "rationale": "Because I am the dominant word, describing the behaviour and ideas of me, and looking at the subjective attitude of the individual."}, "sentence_sentiment": {"answer": "Negative", "rationale": "Because \"fight\" and \"bold\" are both negative."}, "language_style": {"answer": "Tactful and warm", "rationale": ""}, "time_dimension": {"answer": "Past", "rationale": ""}, "sentence_type": "Affirmative declarative sentence", "pos": {"nouns": {"Interest": 1, "person": 2, "Fighting": 1, "Apologize": 1}, "verbs": {"in": 1, "For": 1, "Often": 1, "Because": 1, "Naughty": 1, "In": 1}, "adjectives": {"Mine": 2, "Rude": 1}, "numerals": {"71": 1}, "pronouns": {"I": 1}}}, {"content": "72, most of what happened to me is not my responsibility.", "word_count": 19, "label": "", "sentence_structure": "Subject鈥揷opula鈥損redicative", "subjective_objective": {"answer": "Subjective", "rationale": "It is the patient鈥檚 subjective attitude to consider these circumstances as his own responsibility, since the patient鈥檚 perception and feelings about the events he has experienced."}, "sentence_sentiment": {"answer": "Negative", "rationale": "Because the attitude in the entry that \"most of the cases are not my responsibility\" is negative."}, "language_style": {"answer": "Tactful and warm", "rationale": "The entry is more softly phrased, and it uses a non-direct approach to ask the other side whether or not to agree to a certain view. At the same time, it is more balanced, and does not use too many strong emotional or command language. These are all examples of the questionnaire designers trying to establish good interaction and understand each other鈥檚 intentions."}, "time_dimension": {"answer": "Present", "rationale": "The keywords \"occurring\" and \"mostly not my responsibility\" indicate that it is a perception or belief of the testee in the current state of affairs, and therefore the temporal dimension is now judged."}, "sentence_type": "Negative declarative sentence", "pos": {"nouns": {"Situation": 1, "Responsibility": 1}, "verbs": {"Come on": 1, "be": 1}, "adjectives": {"Most of them": 1, "Related": 1}, "adverbs": {"No, it's not": 1}, "numerals": {"72": 1}, "pronouns": {"I": 1}}}, {"content": "73, when I feel emotional depression or anxiety, eating something makes me feel better.", "word_count": 26, "label": "", "sentence_structure": "Subject鈥搗erb鈥揷omplement", "subjective_objective": {"answer": "Subjective", "rationale": "Because \"I feel better\" is the patient's subjective feeling and experience."}, "sentence_sentiment": {"answer": "Positive", "rationale": "Because the \"Eat something to make me feel better\" act is positive. Although it may have potential risks and problems in itself, in this particular context it is described as a positive response that can help people to ease negative emotions."}, "language_style": {"answer": "Tactful and warm", "rationale": "Warm and warm."}, "time_dimension": {"answer": "Present", "rationale": "The keyword \"Estimating emotional depression or anxiety\" and \"Eating something that makes me feel better\" means the act or the situation, so the time dimension of the article is now."}, "sentence_type": "Affirmative declarative sentence", "pos": {"nouns": {"Emotion": 2, "Repression": 1, "Anxiety": 1, "Unsettled": 1, "Something": 1, "Feel": 1}, "verbs": {"Feel": 2, "Eat": 1, "鈾?And I'm gonna be 鈾?: 1}, "adjectives": {"Better": 1}, "numerals": {"73": 1}, "pronouns": {"I": 1}}}, {"content": "74, hard work makes me feel better.", "word_count": 10, "label": "", "sentence_structure": "Subject鈥揷opula鈥損redicative", "subjective_objective": {"answer": "Subjective", "rationale": "Because \"feel\" is the patient's subjective feeling."}, "sentence_sentiment": {"answer": "Positive", "rationale": "Because the \"work hard to make me feel better\" behavior in the entry is positive."}, "language_style": {"answer": "Tactful and warm", "rationale": "The entry is more adept and interactive when it is spoken in a gentle and inviting manner, rather than by direct statements or orders."}, "time_dimension": {"answer": "Present", "rationale": "The entry describes what the testee feels now, and the word \"make\" indicates that it is the current state or situation, does not relate to the time dimension of the past, and does not predict the future. It therefore falls within the context of the present."}, "sentence_type": "Affirmative declarative sentence", "pos": {"nouns": {"I'm working hard": 1, "Work": 1}, "verbs": {"鈾?And I'm gonna be 鈾?: 1, "Feel": 1, "Better": 1}, "numerals": {"74": 1}}}, {"content": "75, the doctor can't really help me.", "word_count": 12, "label": "", "sentence_structure": "Subject鈥搗erb鈥揷omplement", "subjective_objective": {"answer": "Subjective", "rationale": "Because \"doing the problem\" is a subjective judgement, depending on what the patient wants and experience, not on the actual abilities and performance of the doctor."}, "sentence_sentiment": {"answer": "Negative", "rationale": "Because the phrase \"doctors can't really help me solve problems\" in the entry is negative and expresses denial of the doctor's ability and dissatisfaction."}, "language_style": {"answer": "Tactful and warm", "rationale": "The language style of the entry is euphemism. It expresses the expectations and needs of the interviewee in a gentle and understanding manner, rather than directly criticizing or accusing him, which is more likely to enjoy the trust and cooperation of the interviewee."}, "time_dimension": {"answer": "Past", "rationale": "The \"can't really help me solve the problem\" in the entry is a description of what happened in the past, and therefore judgmental."}, "sentence_type": "Negative declarative sentence", "pos": {"nouns": {"Doctor": 1, "Problem": 1}, "verbs": {"No, I can't": 1, "Indeed": 1, "Help": 1, "Resolve": 1}, "adverbs": {"Indeed": 1}, "numerals": {"75": 1}, "pronouns": {"I": 1}}}, {"content": "I've heard a lot about my feelings.", "word_count": 15, "label": "", "sentence_structure": "Subject鈥搗erb鈥搊bject", "subjective_objective": {"answer": "Subjective", "rationale": "Because \"often listening\" is the patient's subjective feeling, and whether or not to reveal his feelings is the patient's subjective attitude."}, "sentence_sentiment": {"answer": "Negative", "rationale": "Because the \"not to expose your feelings\" act in the entry is negative."}, "language_style": {"answer": "Calm and direct", "rationale": "The language style of the entry is more objective, direct and there is no excessive rhetorical or emotional rendering, and the other party is asked whether it is often perceived and spoken in a more honest manner."}, "time_dimension": {"answer": "Present", "rationale": ""}, "sentence_type": "Negative declarative sentence", "pos": {"nouns": {"Questionnaire": 1, "Entry": 1, "person": 2, "Emotion": 1}, "verbs": {"Listen": 1, "Say it": 1, "not": 1, "Exposure": 1}, "adverbs": {"Regular": 1}, "numerals": {"76": 1}, "pronouns": {"I": 2}}}, {"content": "77 I think people are watching movies, plays or books that mean more to the senses than they want to be in these works.", "word_count": 35, "label": "", "sentence_structure": "Subject鈥搗erb鈥搊bject", "subjective_objective": {"answer": "Subjective", "rationale": "Because \"think\" is the patient's own view and opinion, and it's subjective."}, "sentence_sentiment": {"answer": "Neutral", "rationale": "As the entry merely stated a point of view, there was no apparent emotional tendency or value judgement and was therefore considered neutral."}, "language_style": {"answer": "Tactful and warm", "rationale": "The language style of the entry is euphemistic, because it uses the means of inquiry and dialogue in a gentle and inviting manner. It also emphasizes that people can gain more insight into the work of art, which is more focused on individual feelings and subjective experiences, and gives people a sense of affection, friendship and understanding."}, "time_dimension": {"answer": "Present", "rationale": "The phrase \"considers\" indicates the present perception and understanding of the subject, while \"the significance of watching a film, theatre or book is greater than the meaning of these works\" is a statement and a description of this view, and it is therefore possible to judge that the entry is an inquiry into the present view of the subject."}, "sentence_type": "Affirmative declarative sentence", "pos": {"nouns": {"Film": 1, "Theatre": 1, "Books": 1, "Meaning": 2, "People": 1, "Works": 1}, "verbs": {"I think": 1, "Look": 1, "Learn": 1, "To express": 1}, "adverbs": {"- Yeah": 1}, "numerals": {"77": 1, "More": 1}, "pronouns": {"These": 1}}}, {"content": "78, I feel like I have some custom or ritual behavior I have to do, and it's causing me a lot of trouble.", "word_count": 33, "label": "", "sentence_structure": "Subject鈥揷opula鈥損redicative", "subjective_objective": {"answer": "Subjective", "rationale": "Because \"involved in custom or rituals\" and \"inordinately troubled\" are subjective feelings and experiences of patients, this is viewed from the patient's subjective perspective."}, "sentence_sentiment": {"answer": "Negative", "rationale": "Because the entry \"some custom or ritual act which is not self-contained and causes me extra trouble\" is negative."}, "language_style": {"answer": "Calm and direct", "rationale": "The language style of the entry is \"cool and direct.\" It asks the interviewee whether he feels he has some self-contained habits or rituals that cause himself many problems, which are not accompanied by excessive rhetoric or emotional rendering, and are more balanced and objective."}, "time_dimension": {"answer": "Present", "rationale": ""}, "sentence_type": "Affirmative declarative sentence", "pos": {"nouns": {"Custom": 1, "Ceremony": 1, "Behaviour": 1, "Special": 1, "Trouble": 1}, "verbs": {"I feel": 1, "have": 1, "Do it": 1, "Bring it": 1}, "adverbs": {"It's not up to you": 1}, "numerals": {"78": 1}, "pronouns": {"I": 1}}}, {"content": "79, when I'm nervous, I drink or take pills.", "word_count": 11, "label": "", "sentence_structure": "Subject鈥損redicate", "subjective_objective": {"answer": "Objective", "rationale": "Because \"drinking or taking medication when you're nervous\" is the result of the patient's objective behaviour and is not an individual subjective feeling or attitude."}, "sentence_sentiment": {"answer": "Negative", "rationale": "Because the \"drinking or taking drugs\" act in the entry is negative."}, "language_style": {"answer": "Calm and direct", "rationale": "The entry is presented directly to the interviewee, asking him whether he is used to drinking or taking medication when he is nervous, in a clear and simple language, and without excessive ado and emotional expression."}, "time_dimension": {"answer": "Present", "rationale": "The entry describes current behaviour and habits, i.e., the use of alcohol or medication to relieve emotions when nervous, and thus the judgement is now."}, "sentence_type": "Affirmative declarative sentence", "pos": {"nouns": {"Nervous": 1, "Wine": 1, "Medicine": 1}, "verbs": {"Drink": 1, "Eat": 1}, "adverbs": {"When": 1}, "numerals": {"79": 1}}}, {"content": "80, I want to stay with someone when I'm in a bad mood.", "word_count": 17, "label": "", "sentence_structure": "Subject鈥搗erb鈥搊bject", "subjective_objective": {"answer": "Subjective", "rationale": "Because \"want to be with someone else\" reflects the emotions and needs of patients, and it's a subjective experience. Although it's probably because people feel that they can alleviate their negative emotions by being in contact with others, it's still subjective."}, "sentence_sentiment": {"answer": "Positive", "rationale": "Because the purpose of the entry is to stay with someone else."}, "language_style": {"answer": "Tactful and warm", "rationale": "The entry is more personal and interactive when it is spoken in a gentle and inviting manner, rather than in direct statements or orders. At the same time, the language style of the article is more euphemistic, not expressed in too direct or hard language, and is more suitable for psychological questionnaire surveys, etc."}, "time_dimension": {"answer": "Present", "rationale": "The keyword \"unsatisfied\" and \"wanted to be with someone else\" indicates that the testee is describing a current emotional state and behavioral reaction, so it can be judged that the entry is asking the testee about the current feelings and behaviour."}, "sentence_type": "Affirmative declarative sentence", "pos": {"nouns": {"The mood": 1, "It's not good": 1, "others": 1}, "verbs": {"Yes": 1, "Stay": 1, "in": 2}, "adjectives": {"Have a nice day": 1, "Together": 1}, "numerals": {"80": 1}}}, {"content": "81, if I could feel that I'd be depressed, I could handle it better.", "word_count": 23, "label": "", "sentence_structure": "Subject鈥搗erb鈥搊bject", "subjective_objective": {"answer": "Subjective", "rationale": "Because the expression \"I can handle it better\" appears in the title, which shows that it is the patient's subjective attitude and thoughts."}, "sentence_sentiment": {"answer": "Positive", "rationale": "Because the act of \"presupposing and responding to frustration\" in the entry is positive and expresses the ability to self-regulate and manage emotions."}, "language_style": {"answer": "Tactful and warm", "rationale": ""}, "time_dimension": {"answer": "Future", "rationale": "The entry describes a scenario in which a certain ability (predict frustration) is able to better cope with frustration. As this is a vision and a possibility for the future, the temporal dimension is judged to be the future."}, "sentence_type": "Imperative sentence", "pos": {"nouns": {"I": 2, "Frustration": 1, "Word": 1, "Response": 1}, "verbs": {"Foreground": 1, "Yes": 4, "Okay": 1, "Payable": 1}, "adverbs": {"Suppose": 1, "Land": 1}, "numerals": {"81": 1, "More": 1}}}, {"content": "82, no matter how much I complain, I never get a satisfactory result.", "word_count": 18, "label": "", "sentence_structure": "Subject鈥搗erb鈥揷omplement", "subjective_objective": {"answer": "Subjective", "rationale": "The reaction to the complaints is the patient's subjective feelings and attitudes."}, "sentence_sentiment": {"answer": "Negative", "rationale": "Because the result of the entry, \"never satisfactory\" was negative and expressed a sense of dissatisfaction and disappointment."}, "language_style": {"answer": "Calm and direct", "rationale": "The entry is more straightforward, simpler language style, with no excessive rhetorical or emotional rendering, but rather a message that appears calm and objective by direct asking."}, "time_dimension": {"answer": "Past", "rationale": "The entry describes a past experience, \"however I complain,\" \"however I get satisfied\" is a description of what happened in the past, and therefore a judgement of the past."}, "sentence_type": "Negative declarative sentence", "pos": {"nouns": {"Scoff": 1, "Result": 1}, "verbs": {"Got it": 1, "Let's go": 1, "Never": 1, "I've got it": 1, "Satisfied": 1}, "adverbs": {"Whatever": 1, "How's that": 1}, "numerals": {"82": 1}}}, {"content": "83, I often find myself insensitive when the environment is about to cause a serious emotional reaction.", "word_count": 27, "label": "", "sentence_structure": "Subject鈥揷opula鈥損redicative", "subjective_objective": {"answer": "Subjective", "rationale": "Because \"insensitive\" is the patient's own feeling and experience, it's subjective."}, "sentence_sentiment": {"answer": "Negative", "rationale": "Because \"insensitive\" in the entry is a negative emotional reaction."}, "language_style": {"answer": "Calm and direct", "rationale": "The entry is presented in a concise language, with no excessive rhetoric or emotional colour, and presents the subject in a direct presentation, making it easy for readers to understand and accept."}, "time_dimension": {"answer": "Present", "rationale": "\"I'm often\" indicates that this is a regular act, and \"insensitive\" is a description of the emotional state and behaviour of the person who was tested, or was in the recent period. The entry therefore reflects the current or recent behaviour and mental state of the person being tested."}, "sentence_type": "Negative declarative sentence", "pos": {"nouns": {"Environment": 1, "Emotional response": 1, "Insensitive": 1}, "verbs": {"Found": 1, "Cause": 1, "Yes": 1}, "adverbs": {"Often": 1, "Serious": 1}, "numerals": {"83": 1}, "pronouns": {"I": 2}}}, {"content": "84, work without me, free me from emotional depression and anxiety.", "word_count": 19, "label": "", "sentence_structure": "Subject鈥搗erb鈥揷omplement", "subjective_objective": {"answer": "Objective", "rationale": "Forget I'm the way the patient works, and the emotional reaction that can be objective.\""}, "sentence_sentiment": {"answer": "Positive", "rationale": "Because \"forgetting to work\" in the entry can have the effect of \" getting rid of emotional depression and anxiety,\" and it's positive."}, "language_style": {"answer": "Tactful and warm", "rationale": "The language style of the entry is more euphemistic and encouraging, and it helps people to overcome negative emotions by expressing their own feelings. This sweet and intimate tone makes it easier to get people to feel the same way."}, "time_dimension": {"answer": "Past", "rationale": "The entry \"Working for Me\" is about the past, while \"making me free from emotional depression and anxiety\" is about the effect of past behaviour on the current state of emotion, so it can be judged as a whole."}, "sentence_type": "Affirmative declarative sentence", "pos": {"nouns": {"Forget about me": 1, "Work": 1}, "verbs": {"鈾?And I'm gonna be 鈾?: 1, "Get rid of it": 1, "Blue": 1, "Anxiety": 1}, "adverbs": {"Land": 2}, "numerals": {"84": 1}}}, {"content": "I smoke when I'm nervous.", "word_count": 9, "label": "", "sentence_structure": "Subject鈥搗erb鈥揷omplement", "subjective_objective": {"answer": "Subjective", "rationale": "Because I'm here to represent patients' subjective feelings and behavior."}, "sentence_sentiment": {"answer": "Negative", "rationale": "Because the \"smoking\" act in the entry is negative."}, "language_style": {"answer": "Calm and direct", "rationale": "The entry raises the question in a straightforward manner, without excessive rhetoric or emotional rendering, and with a calm and objective language style."}, "time_dimension": {"answer": "Present", "rationale": ""}, "sentence_type": "Affirmative declarative sentence", "pos": {"nouns": {"Time": 1, "Smoking": 1}, "verbs": {"Just": 1, "Nervous": 1, "Smoking": 1}, "adverbs": {"Just": 1}, "numerals": {"85": 1}}}, {"content": "86, if I get into some kind of crisis, I'm looking for someone else who has the same fate as me.", "word_count": 28, "label": "", "sentence_structure": "Subject鈥搗erb鈥搊bject", "subjective_objective": {"answer": "Subjective", "rationale": "This is a subjective idea and reaction of patients in the face of crisis."}, "sentence_sentiment": {"answer": "Negative", "rationale": "Because the words \"in some crisis\" and \"in search of another person of my own destiny\" in the entries are negative emotional expressions suggesting that the master may not be able to solve the problem independently or escape responsibility."}, "language_style": {"answer": "Tactful and warm", "rationale": "The language style of the entry is a euphemism, which does not directly express expectations or demands for the person under investigation, but rather a soft, invitation-style conversation, which is more friendly and interactive. Moreover, the issue does not use any command or threatening language, which makes people feel more comfortable and relaxed."}, "time_dimension": {"answer": "Past", "rationale": "The entry describes a scenario and uses the past-modern \"fall\" and \"research\" and so on, so the temporal dimension is the past."}, "sentence_type": "Affirmative declarative sentence", "pos": {"nouns": {"Crisis": 1, "person": 2}, "verbs": {"Into the": 1, "Search": 1, "Yes": 1, "Problems": 1}, "adjectives": {"Other": 1, "Similar": 1, "Same thing": 1}, "numerals": {"86": 1}, "pronouns": {"I": 2}}}, {"content": "87, if I'm wrong, I can't be blamed.", "word_count": 13, "label": "", "sentence_structure": "Subject鈥搗erb鈥搊bject", "subjective_objective": {"answer": "Subjective", "rationale": "Because \"not to be blamed\" is a subjective idea or expectation of a person, which is subjective in its perspective."}, "sentence_sentiment": {"answer": "Negative", "rationale": "Basis of judgement: because the act of \"can't be blamed\" in the entry is negative, it represents an evading and irresponsible attitude towards its own mistakes."}, "language_style": {"answer": "Calm and direct", "rationale": "The language style of the entry is calm and straightforward, and there is no excessive emotional rendering or rhetorical technique, but rather a simple and clear statement of the purpose of the inquiry."}, "time_dimension": {"answer": "Present", "rationale": "The entry is about the testee鈥檚 self-awareness and attitude when facing the error. The time dimension, which is not linked to the past or the future, is considered to be a question about the present situation. The basis of the judgement is that the phrase 鈥渋f I have done something wrong鈥?implies a hypothetical situation, while the subsequent phrase 鈥渘ot to be blamed鈥?indicates the testee鈥檚 attitude. This attitude is not related to past or future events, but to current perceptions, so the time horizon is now."}, "sentence_type": "Imperative sentence", "pos": {"nouns": {"Situation": 1, "Blame": 1}, "verbs": {"Wrong": 1, "Yes": 1}, "adverbs": {"Suppose": 1, "not": 1}, "numerals": {"87": 1}, "pronouns": {"I": 1}}}, {"content": "88, if I had an attack on someone, I would feel the need to do something to divert it.", "word_count": 31, "label": "", "sentence_structure": "Subject鈥搗erb鈥搊bject", "subjective_objective": {"answer": "Subjective", "rationale": "Because this phrase describes the feelings and thoughts of the patient himself, it is the patient ' s subjective attitude."}, "sentence_sentiment": {"answer": "Negative", "rationale": "Because the \"think of attacking someone\" in the entry is negative."}, "language_style": {"answer": "Tactful and warm", "rationale": "The language style of the entry is more euphemistic, because it uses the interview-style tone and attempts to guide them to think by understanding each other's feelings. It does not direct command or criticism, but rather provides a more moderate way of exploring the sources and ways of dealing with offensive ideas. This expression helps to build trust relations and encourages respondents to share their thoughts and feelings."}, "time_dimension": {"answer": "Present", "rationale": "The entry describes the feelings and needs of the person under the test in the current state, with the words \"there is\" and \"just feel\" indicating that this is a description of the current psychological state. And, \"there is an idea of attacking others\" is also an ongoing action or state, so the entry corresponds to the time dimension of \"now\"."}, "sentence_type": "Imperative sentence", "pos": {"nouns": {"Attack": 1, "Others": 1, "Thoughts": 2, "Yes": 1, "Situation": 1, "Transfer": 1}, "verbs": {"have": 1, "Feel": 1, "Do something": 1}, "adverbs": {"Suppose": 1}, "numerals": {"88": 1}, "pronouns": {"I": 1, "Here": 2}}}]}

{"questionnaire_title": "Hospital anxiety/depressive (HAD) mood determination", "questionnaire_category": "Mental health", "item_count": 7, "emotion_shift_overall": "From positive to negative", "language_style_overall": "Tactful and warm", "items": [{"content": "I'm still interested in what I've been interested in.", "word_count": 15, "label": "", "sentence_structure": "Subject鈥搗erb鈥揷omplement", "subjective_objective": {"answer": "Subjective", "rationale": "Because \"into the past, or in the past, interest\" is a personal feeling or perception, and is a subjective assessment."}, "sentence_sentiment": {"answer": "Positive", "rationale": "Because the entry is a reflection of a continuing interest in matters of past interest, it is a positive attitude and emotion."}, "language_style": {"answer": "Tactful and warm", "rationale": "The entry is more adept and interactive when it is spoken in a gentle and inviting manner, rather than by direct statements or orders."}, "time_dimension": {"answer": "Present", "rationale": "The entries \"me\" and \"intribution to past interest\" describe the current state, so it can be judged as asking the testee about the current behavior or mentality. The temporal dimension has no clear meaning of past or future."}, "sentence_type": "Interrogative sentence", "pos": {"nouns": {"Things": 2, "Interest": 2}, "verbs": {"to": 1, "Or is it": 1, "have": 1}, "adjectives": {"Previous": 1}, "adverbs": {"very": 1}, "classifiers": {"individual": 1}, "pronouns": {"I": 1}}}, {"content": "I can laugh and see the funny side of things.", "word_count": 17, "label": "", "sentence_structure": "Subject鈥搗erb鈥揷omplement", "subjective_objective": {"answer": "Subjective", "rationale": "\"The funny side of seeing things\" is the patient's subjective feeling and attitude."}, "sentence_sentiment": {"answer": "Positive", "rationale": "Because the entries \"can laugh\" and \"see the interesting side of things\" express a positive, optimistic emotional attitude."}, "language_style": {"answer": "Tactful and warm", "rationale": "The entry is more personal and interactive when it is spoken in a gentle and inviting manner, rather than in direct statements or orders. At the same time, the language style of the entry is more positive and optimistic, and can trigger the echoes of the respondents and make them comfortable and comfortable."}, "time_dimension": {"answer": "Present", "rationale": "The word \"capable\" in the entry indicates a capability or skill that implies that the act exists at the moment, so the temporal dimension is now. And the description \"seeing the interesting side of things\" also shows that it is a description of the current situation, not a future or a past vision. So the answer is \"now.\""}, "sentence_type": "Affirmative declarative sentence", "pos": {"nouns": {"I": 1, "Smile": 1, "Things": 1, "One side": 1}, "verbs": {"Yes": 1, "Yeah": 1, "Smile": 1}, "adjectives": {"Ha ha ha ha ha ha ha ha ha ha ha ha ha ha ha ha ha ha ha ha ha ha ha ha ha ha ha ha ha ha ha ha ha ha ha ha ha ha ha ha ha ha ha ha ha ha ha ha ha ha ha ha ha ha ha ha ha ha ha ha ha ha ha ha ha ha ha ha ha ha ha ha ha ha ha ha ha ha ha ha ha ha ha ha ha ha ha ha ha ha ha ha ha ha ha ha ha ha ha ha ha ha ha ha ha ha ha ha ha ha ha ha ha ha ha ha ha ha ha ha ha ha ha ha ha ha ha ha ha ha ha ha ha ha ha ha ha ha ha ha ha ha ha ha ha ha ha ha ha ha ha ha ha ha ha ha ha ha ha ha ha ha ha ha ha ha ha ha ha ha ha ha ha ha ha ha ha ha ha ha ha ha ha ha ha ha ha ha ha ha ha ha ha ha ha ha ha ha ha ha ha ha ha ha ha ha ha ha ha ha ha ha ha ha ha ha ha ha ha ha ha ha ha ha ha ha ha ha ha ha ha ha ha ha ha ha ha ha ha ha ha ha ha ha ha ha ha ha ha ha ha ha ha ha ha ha": 1, "Interesting": 1}, "numerals": {"2": 1}}}, {"content": "3 had a good time.", "word_count": 4, "label": "", "sentence_structure": "Subject鈥揷opula鈥損redicative", "subjective_objective": {"answer": "Subjective", "rationale": "Because \"favouring\" is a subjective emotional experience."}, "sentence_sentiment": {"answer": "Positive", "rationale": "Because the entry \"happy\" is a positive emotional expression."}, "language_style": {"answer": "Calm and direct", "rationale": "The purpose of the entry is to understand the emotional state of the interviewee and to enable the interviewer to understand and answer questions more quickly in a concise language."}, "time_dimension": {"answer": "Present", "rationale": "The keyword \"happy\" describes the current state of emotion, so the entry asks about the experience and feelings of the subjects."}, "sentence_type": "Affirmative declarative sentence", "pos": {"verbs": {"I feel": 1}, "adjectives": {"Have a nice day": 1}}}, {"content": "I feel like I'm getting retarded.", "word_count": 10, "label": "", "sentence_structure": "Subject鈥揷opula鈥損redicative", "subjective_objective": {"answer": "Subjective", "rationale": "Because it's the patient's subjective experience."}, "sentence_sentiment": {"answer": "Negative", "rationale": "Because the description of \"persons slow\" in the entry is negative."}, "language_style": {"answer": "Tactful and warm", "rationale": "The entry describes the potential existence of the other side in a moderate and caring manner, without direct accusations or criticism, which is more compassionate and understanding."}, "time_dimension": {"answer": "Past", "rationale": "\"I feel like I'm retarded\" and \"like\" means that it's subjective and has changed, so it's in the past."}, "sentence_type": "Affirmative declarative sentence", "pos": {"nouns": {"I": 1, "person": 1}, "verbs": {"I feel": 1, "Slower": 1}, "adverbs": {"I think so": 1}, "numerals": {"4": 1}}}, {"content": "I'm losing interest in my appearance.", "word_count": 15, "label": "", "sentence_structure": "Subject鈥搗erb鈥揷omplement", "subjective_objective": {"answer": "Subjective", "rationale": "Because \"lost interest\" is the patient's subjective attitude."}, "sentence_sentiment": {"answer": "Negative", "rationale": "Because the expression \"lost interest\" in the entry is negative."}, "language_style": {"answer": "Calm and direct", "rationale": "The language of the article \"Quakers and Warmness\" is more euphemism, more euphemism, more compassionate, and less direct negative emotions or critical statements. It leads the respondents to think about their external attitudes and try to understand their underlying emotional state and needs. It reflects both the respect and the interest of the researchers and the increased willingness of the respondents to share their true feelings."}, "time_dimension": {"answer": "Present", "rationale": "The entry \"lost interest\" indicates that the testee's current state is lost interest in his appearance, so the temporal dimension is now judged."}, "sentence_type": "Exclamatory sentence", "pos": {"nouns": {"Appearance": 1, "Get dressed": 1, "self": 1, "Interest": 1}, "verbs": {"to": 1, "Lose": 1}, "numerals": {"5": 1}, "pronouns": {"I": 1}}}, {"content": "I'm looking forward to the future with a pleasant feeling.", "word_count": 12, "label": "", "sentence_structure": "Subject鈥搗erb鈥揷omplement", "subjective_objective": {"answer": "Subjective", "rationale": "Because \"happy mood\" and \"pregnant future\" are patients' subjective feelings and attitudes."}, "sentence_sentiment": {"answer": "Positive", "rationale": "Because \"seeing the future with a pleasant mood\" expresses a positive, optimistic mindset and attitude, without any negative or neutral emotional colours."}, "language_style": {"answer": "Tactful and warm", "rationale": "The language style of the entry is more euphemistic, expressing expectations and hopes for the future by using words such as \"happy mood\" and \"presenting the future,\" which gives people a positive, positive sense of energy. At the same time, the sentence does not directly ask questions or command each other, but rather leads each other to think in an invitation-based way, with more personality and interaction."}, "time_dimension": {"answer": "Future", "rationale": "The entry describes the expectations and expectations of the testees for the future, and the temporal dimensions are judged to be future."}, "sentence_type": "Interrogative sentence", "pos": {"nouns": {"The mood": 1, "Hope": 1, "The future": 1}, "verbs": {"I'm pregnant": 1, "Hope": 1}, "adjectives": {"Happy": 1}, "pronouns": {"I": 1}}}, {"content": "I can appreciate a good book or a good radio or a good TV show.", "word_count": 20, "label": "", "sentence_structure": "Subject鈥搗erb鈥搊bject", "subjective_objective": {"answer": "Subjective", "rationale": "Because \"appear\" is the patient's subjective feeling and attitude."}, "sentence_sentiment": {"answer": "Positive", "rationale": "Because the behavior \"appear\" in the entries is positive, indicating a preference for and acceptance of things."}, "language_style": {"answer": "Tactful and warm", "rationale": "The entry is more adept and interactive when it is spoken in a gentle and inviting manner, rather than by direct statements or orders."}, "time_dimension": {"answer": "Present", "rationale": "Key words \"can\" and \"applied\" indicate that this is a description of current capabilities, and does not involve past or future behaviour or mindsets. Therefore, the temporal dimension is now judged."}, "sentence_type": "Affirmative declarative sentence", "pos": {"nouns": {"I": 1, "Books": 1, "Radio": 1, "Television programmes": 1}, "verbs": {"Yes": 1, "Enjoy": 1}, "adjectives": {"Okay": 3}, "numerals": {"7": 1}}}]}

{"questionnaire_title": "Social support scale (PSSS)", "questionnaire_category": "Emotion and affect", "item_count": 12, "emotion_shift_overall": "No obvious trend", "language_style_overall": "Tactful and warm", "items": [{"content": "I've had problems with people who are around me.", "word_count": 24, "label": "", "sentence_structure": "Subject鈥搗erb鈥搊bject", "subjective_objective": {"answer": "Subjective", "rationale": "The results of this questionnaire are subjective. Because the question of whether a person will be confronted with a problem is based on the subjective perception and perception of the person interviewed and is influenced by many factors, such as the interpersonal relationship of the person interviewed, the level of trust."}, "sentence_sentiment": {"answer": "Positive", "rationale": "Because the purpose of the entry is to be positive \"some people (leaders, relatives, colleagues) who are in my presence when I'm in trouble.\""}, "language_style": {"answer": "Tactful and warm", "rationale": "The entry is more personal and interactive in its expression, by a gentle and inviting tone, rather than by direct statement or order. \""}, "time_dimension": {"answer": "Present", "rationale": "\"I\" and \"I\" in \"when I'm in trouble\" indicate that this is a description of the current situation, so the time dimension of the article is now."}, "sentence_type": "Affirmative declarative sentence", "pos": {"nouns": {"Problem": 1, "person": 3, "Leadership": 1, "Relatives": 1, "Co-workers": 1, "鈾?With me 鈾?: 1}, "verbs": {"Come on": 2, "I met him": 1}, "numerals": {"Some": 1}, "pronouns": {"I": 1}}}, {"content": "I can share pleasure and harm with some people (leaders, relatives, colleagues).", "word_count": 20, "label": "", "sentence_structure": "Subject鈥搗erb鈥搊bject", "subjective_objective": {"answer": "Subjective", "rationale": "This is because of patients' subjective feelings and attitudes about their ability to share happiness and good wounds."}, "sentence_sentiment": {"answer": "Positive", "rationale": "Because the entry describes sharing joy and sorrow with others and is a positive social act."}, "language_style": {"answer": "Tactful and warm", "rationale": "It is because it uses the term \"shared pleasure and sorrow\" to express a positive, optimistic emotional attitude. And it also conveys a sense of proximity and friendship, not a cold or distant language. It is therefore fair to judge that the language style of the article is euphemism."}, "time_dimension": {"answer": "Present", "rationale": ""}, "sentence_type": "Affirmative declarative sentence", "pos": {"nouns": {"Happy": 1, "Yu's injuries": 1}, "verbs": {"Yes": 1, "Share": 1}, "adjectives": {"Some": 1}, "numerals": {"2": 1}, "classifiers": {"person": 1}, "pronouns": {"I": 1}}}, {"content": "My family can help me with practical and concrete work.", "word_count": 15, "label": "", "sentence_structure": "Subject鈥搗erb鈥揷omplement", "subjective_objective": {"answer": "Subjective", "rationale": "It is a subjective attitude of the patient to the question of his or her views and feelings as to whether his or her family can provide practical help to him or her."}, "sentence_sentiment": {"answer": "Positive", "rationale": "Because the expression \"my family can help me with real concretely\" in the entry is positive."}, "language_style": {"answer": "Calm and direct", "rationale": "The language style of the entry is more straightforward, with no excessive embellishment or emotional rendering, and directly expresses the subject of asking the family of the other to be of practical assistance."}, "time_dimension": {"answer": "Present", "rationale": "The entry asks what the testee feels or experiences now, namely, \"Is my family actually able to help me in a concrete way?\" So the time dimension is now."}, "sentence_type": "Affirmative declarative sentence", "pos": {"nouns": {"Family": 1, "Help": 1}, "verbs": {"Here": 1, "Yes": 1, "Specific": 1, "Minori": 1, "Cut": 1, "Land": 1}, "adjectives": {"Practical": 1}, "numerals": {"3": 1}}}, {"content": "4. I have the ability to receive emotional help and support from my family when needed", "word_count": 21, "label": "", "sentence_structure": "Subject鈥搗erb鈥搊bject", "subjective_objective": {"answer": "Subjective", "rationale": "Family help and support are often subjectively felt and evaluated by patients."}, "sentence_sentiment": {"answer": "Positive", "rationale": "This item describes the support that individuals can obtain from the family, which is a positive emotion and behaviour."}, "language_style": {"answer": "Tactful and warm", "rationale": "The language style of the entry is euphemistic and friendly, using questioning rather than direct presentation, emphasizing the support and assistance that families can provide when needed, and using the term \"access\" rather than \"get\" to express a more active and proactive attitude. Such expression is closer to the form of dialogue in everyday life, and makes people feel more comfortable and close."}, "time_dimension": {"answer": "Present", "rationale": "The keyword \"if necessary\", \"capable\" of indicating current or future behaviour, \"getting\" and \"support\" indicate the result of that behaviour, so the entry asks about the current or future status of the person being tested."}, "sentence_type": "Affirmative declarative sentence", "pos": {"nouns": {"Problem": 1, "Answer": 1, "Investigations": 1, "Entry": 1, "Family": 1, "Emotional support": 1}, "verbs": {"Access": 1, "Yes": 2, "Provision": 1, "From": 1, "Got it": 1}, "adjectives": {"Emotionally": 1}, "numerals": {"4": 1, "One": 2}, "classifiers": {"individual": 1}, "pronouns": {"I": 1, "they": 1}}}, {"content": "5. When I have difficulties, some people are the real source of comfort.", "word_count": 24, "label": "", "sentence_structure": "Subject鈥揷opula鈥損redicative", "subjective_objective": {"answer": "Objective", "rationale": "Because some people comfort me with the objective facts I've been through.\""}, "sentence_sentiment": {"answer": "Positive", "rationale": "Because the act of comforting me in the entry is a positive expression of concern and help."}, "language_style": {"answer": "Tactful and warm", "rationale": "The entry is expressed in a more moderate way, using the vague term \"some people\" and the expression \"the real source of comfort\" that makes the sentence more euphemistic and friendly."}, "time_dimension": {"answer": "Present", "rationale": "\"I have a difficult time\" suggests a certain moment or situation, and \"the true source of comfort\" describes the behaviour of others at this time. So the temporal dimension of this sentence is now."}, "sentence_type": "Affirmative declarative sentence", "pos": {"nouns": {"Difficulties": 1, "person": 2, "Leadership": 1, "Relatives": 1, "Co-workers": 1, "Consolation": 1, "Genqua": 1}, "verbs": {"have": 1, "be": 1, "Soothing": 1, "Source": 1}, "numerals": {"5": 1}, "pronouns": {"I": 1}}}, {"content": "My friends can really help me.", "word_count": 12, "label": "", "sentence_structure": "Subject鈥搗erb鈥搊bject", "subjective_objective": {"answer": "Subjective", "rationale": "Because \"My friends can really help me\" is a patient who thinks his friends can help their subjective judgment or feelings."}, "sentence_sentiment": {"answer": "Positive", "rationale": "Because the entry expresses the ability of friends to help, this behaviour is positive."}, "language_style": {"answer": "Tactful and warm", "rationale": "The entry is more adept and interactive when it is spoken in a gentle and inviting manner, rather than by direct statements or orders."}, "time_dimension": {"answer": "Present", "rationale": "The entry uses the current state of affairs or the situation, and \"my friends\" and \"can really help me\" are all describing the situation of the testees."}, "sentence_type": "Affirmative declarative sentence", "pos": {"nouns": {"Friends": 1, "Help": 1}, "verbs": {"Yes": 1, "Really": 1, "of": 2, "Help": 1}, "adjectives": {"Really": 1}, "pronouns": {"I": 1}}}, {"content": "I can count on my friends in times of hardship.", "word_count": 16, "label": "", "sentence_structure": "Subject鈥搗erb鈥搊bject", "subjective_objective": {"answer": "Subjective", "rationale": "It is a subjective view, as it is the subjective choice and behaviour of friends in the face of a dilemma."}, "sentence_sentiment": {"answer": "Positive", "rationale": "Because the \"dependency\" in the entry is positive interpersonal and social skills."}, "language_style": {"answer": "Tactful and warm", "rationale": "The entry is more personal and interactive when it is spoken in a question-and-answer manner, rather than directly speaking or ordering. At the same time, the language style of the article is more euphemistic, without too much negative emotion or pressure, leaving the interviewee with a comfortable feeling."}, "time_dimension": {"answer": "Present", "rationale": "The entry \"in times of difficulty\" indicates a situation or state, while \"may rely on my friends\" is a response strategy or behaviour to this situation or state, so the temporal dimension of the purpose of the article is now judged."}, "sentence_type": "Affirmative declarative sentence", "pos": {"nouns": {"Difficulties": 1, "friends": 2}, "verbs": {"Come on": 1, "Yeah": 2, "Dependence": 1}, "numerals": {"7": 1}, "pronouns": {"I": 1}}}, {"content": "I can talk to my family about my problems.", "word_count": 14, "label": "", "sentence_structure": "Subject鈥搗erb鈥搊bject", "subjective_objective": {"answer": "Subjective", "rationale": "The examination of the patient ' s self-awareness and attitude, i.e., whether he or she believes that he or she can discuss the matter with his or her family, is based on the patient ' s subjective perspective."}, "sentence_sentiment": {"answer": "Positive", "rationale": "Because the purpose of the entry is to talk to my family about my problems."}, "language_style": {"answer": "Tactful and warm", "rationale": "This entry uses language that resonates with the interviewee, beginning with \"I,\" which gives the interviewer a sense of affection and respect, while expressing concern and support for the interviewee."}, "time_dimension": {"answer": "Past", "rationale": "In the entry, \"I\" means the first person's singular subject, indicating that it is a description of the individual's past behavior or experience. The key word \"can talk to his family\" is now the normal time, but its behaviour has occurred in the past, so it can be inferred that the question is whether the person tested was able to discuss their problems with the family in the past."}, "sentence_type": "Affirmative declarative sentence", "pos": {"nouns": {"Family": 1, "Difficulty": 1}, "verbs": {"Yes": 1, "Talk": 1}, "adverbs": {"Your own": 1}, "pronouns": {"I": 1}}}, {"content": "My friends can share their happiness and their injuries.", "word_count": 15, "label": "", "sentence_structure": "Subject鈥搗erb鈥搊bject", "subjective_objective": {"answer": "Subjective", "rationale": "Because sharing happiness and sorrow is the subjective view and expectation of a friend."}, "sentence_sentiment": {"answer": "Neutral", "rationale": "Because the entry describes a fact and does not express positive or negative feelings."}, "language_style": {"answer": "Tactful and warm", "rationale": "The entry is more personal and interactive in its expression of a gentle and inviting tone, in which the person is asked about the feelings and experiences of the other person in a friend relationship."}, "time_dimension": {"answer": "Present", "rationale": "The key word is \"to share pleasure and grief with me,\" which means that the friends of the testees regularly and customarily share happiness and sorrow with them, so the subject is looking at the current situation."}, "sentence_type": "Affirmative declarative sentence", "pos": {"nouns": {"Friends": 1, "Happy": 1, "Yu's injuries": 1}, "verbs": {"Yes": 1, "Share": 1}, "adverbs": {"9": 1}}}, {"content": "There are people in my life who care about my feelings.", "word_count": 23, "label": "", "sentence_structure": "Subject鈥搗erb鈥搊bject", "subjective_objective": {"answer": "Subjective", "rationale": "Because the title refers to \"Care for My Feelings\", which is a subjective feeling and experience, and it looks at the subjective thoughts of the patient."}, "sentence_sentiment": {"answer": "Positive", "rationale": "Because the \"care\" act in the entry is positive, it shows that people are watching and caring for the lives of the interviewees."}, "language_style": {"answer": "Tactful and warm", "rationale": "The language style of the entry is more euphemistic, and it raises questions, which are viewed with concern and respect in a question-and-answer manner. At the same time, the term \"care\" is used to express emotional communication in human relationships, giving people a warm and intimate feeling."}, "time_dimension": {"answer": "Past", "rationale": "The keyword \"10 in my life\" shows that it's a retrospective description, and the act of \"care for my feelings\" has happened in the past and is therefore judged to be the past."}, "sentence_type": "Affirmative declarative sentence", "pos": {"nouns": {"Life": 1, "person": 2, "Leadership": 1, "Relatives": 1, "Co-workers": 1, "Emotion": 1}, "verbs": {"have": 1, "Care": 1}, "numerals": {"10": 1}, "pronouns": {"I": 1}}}, {"content": "My family is willing to help me make decisions.", "word_count": 18, "label": "", "sentence_structure": "Subject鈥搗erb鈥揷omplement", "subjective_objective": {"answer": "Subjective", "rationale": "Because it is the patient's subjective wish to help me make a decision."}, "sentence_sentiment": {"answer": "Positive", "rationale": "Since the family attitude expressed in the entry is positive, it is willing to assist in making decisions."}, "language_style": {"answer": "Tactful and warm", "rationale": "At the same time, the desire to have active and voluntary support from family members rather than coercive or command-type arrangements was emphasized by using the expression \"willing\" and thus more personal and interactive."}, "time_dimension": {"answer": "Present", "rationale": "The serial number \"11\" indicates that this is a survey project, and that \"my family\" refers to the current family situation of the person being tested, so it can be judged that the entry is about the person's current behaviour or state."}, "sentence_type": "Affirmative declarative sentence", "pos": {"nouns": {"Family": 1, "Decision": 2}, "verbs": {"I'd love to": 1, "Assistance": 1, "Make": 1}, "adjectives": {"Various": 1}, "numerals": {"11": 1}, "pronouns": {"I": 1}}}, {"content": "Twelve. I can discuss my problems with my friends.", "word_count": 13, "label": "", "sentence_structure": "Subject鈥搗erb鈥搊bject", "subjective_objective": {"answer": "Subjective", "rationale": "Because it is a subjective hope and expectation to be able to discuss their own dilemmas with friends, it is not an objective situation."}, "sentence_sentiment": {"answer": "Positive", "rationale": "Because the purpose of the entry, \"To discuss your problems with your friends\", is positive, expressing the expectation of support and assistance to friends."}, "language_style": {"answer": "Tactful and warm", "rationale": "The language style of the entry is more euphemistic, it raises questions in a question-and-answer manner and uses the affirmative word \"can\" to express an attitude that wants the other party to answer the question. This expression is more personal and interactive, and it makes the interviewee more willing to share his thoughts and feelings."}, "time_dimension": {"answer": "Present", "rationale": "The word \"capable\" in this entry indicates that the person tested is capable, skilled, and both \"discussion\" and \"problems\" follow the current state of affairs, indicating that such capacity and behaviour are currently being performed."}, "sentence_type": "Affirmative declarative sentence", "pos": {"nouns": {"Problem": 1, "friends": 1, "self": 1}, "verbs": {"Yes": 1, "Discussion": 1}, "adverbs": {"12": 1}, "classifiers": {"individual": 1}, "pronouns": {"I": 1}}}]}

{"questionnaire_title": "Adults' Aexist Scale - Intimacy Experience Table ECR", "questionnaire_category": "Emotion and affect", "item_count": 36, "emotion_shift_overall": "From negative to positive", "language_style_overall": "Tactful and warm", "items": [{"content": "In general, I do not like to let lovers know what they feel inside;", "word_count": 22, "label": "", "sentence_structure": "Subject鈥搗erb鈥搊bject", "subjective_objective": {"answer": "Subjective", "rationale": "Because \"I don't like to let lovers know what they feel inside\" is a subjective attitude and feeling."}, "sentence_sentiment": {"answer": "Negative", "rationale": "Because the attitude of \"not likes\" in the entries is negative, it means having a negative view of the fact that a lover knows what it feels like inside."}, "language_style": {"answer": "Calm and direct", "rationale": "The entry is presented in a direct way, which is a simple and clear expression of the attitude of the subject to the question whether the lover should know what he feels inside. Without too many emotional adorations and rhetorical techniques, the language style is more calm and objective."}, "time_dimension": {"answer": "Present", "rationale": "\"In general\" suggests that this is a description of the general situation, that \"not like single\" is an emotional attitude, and \"to let lovers know\" is a current or recent act, and therefore the judgment is now."}, "sentence_type": "Negative declarative sentence", "pos": {"nouns": {"Overall": 1, "Lovers": 1, "self": 1, "Deep down": 1, "Feel": 1}, "verbs": {"Yeah": 1, "Jean": 1, "Yes": 1}, "adjectives": {"Overall": 1}, "numerals": {"01": 1}, "pronouns": {"I": 1}}}, {"content": "I fear I will be abandoned;", "word_count": 8, "label": "", "sentence_structure": "Subject鈥搗erb鈥揷omplement", "subjective_objective": {"answer": "Subjective", "rationale": "Because \"fear\" is a subjective emotion and feeling, it's the patient's inner experience and thoughts."}, "sentence_sentiment": {"answer": "Negative", "rationale": "Because the emotional expression \"I fear I'll be abandoned\" in the entry is negative."}, "language_style": {"answer": "Tactful and warm", "rationale": "The entry expresses concern about the possible psychological distress of the interviewee in a way that is sensitive and understandable, using softer and more compassionate language, and attempts to create a safe and comfortable atmosphere of communication."}, "time_dimension": {"answer": "Present", "rationale": "The keywords are \"regarded\" and \"will,\" which indicate that it is an expectation or a fear for the future. At the same time, the act \"will be abandoned\" does not occur, but is a possibility that has not yet occurred, and therefore falls within the realm of the past. Taken together, the temporal dimension of this entry is now."}, "sentence_type": "Negative declarative sentence", "pos": {"nouns": {"Questionnaire": 1, "Entry": 1, "Abandon": 1}, "verbs": {"I'm worried": 1, "Yes": 1}, "numerals": {"02": 1}, "pronouns": {"I": 1}}}, {"content": "I think it's a nice thing to be close to a lover.", "word_count": 16, "label": "", "sentence_structure": "Subject鈥揷opula鈥損redicative", "subjective_objective": {"answer": "Subjective", "rationale": "Because \"like\" means it's a subjective feeling for the patient."}, "sentence_sentiment": {"answer": "Positive", "rationale": "Because the entry \"Feeling close to a lover is a pleasant thing\" expresses a positive, positive emotional attitude."}, "language_style": {"answer": "Tactful and warm", "rationale": "The language style of the entry is more euphemistic and sweet, and it raises questions with the interviewees in an invitation-style manner and leads them to express their views and feelings by using the subjective expression \"I think\" as a way to make them feel respected and understood, which is conducive to a good communication climate."}, "time_dimension": {"answer": "Present", "rationale": "The keyword \"Feel\" and \"a pleasant thing\" indicate that it's the feeling and attitude of the interviewee."}, "sentence_type": "Affirmative declarative sentence", "pos": {"nouns": {"Lovers": 1, "Things": 1}, "verbs": {"I think": 1, "Close": 1}, "adjectives": {"Nice": 1}}}, {"content": "I'm worried about my relationship;", "word_count": 10, "label": "", "sentence_structure": "Subject鈥搗erb鈥揷omplement", "subjective_objective": {"answer": "Subjective", "rationale": "Because \"very worried\" is a word for what the patient feels inside, and it's a subjective test."}, "sentence_sentiment": {"answer": "Negative", "rationale": "Because the entry \"I'm worried about my relationship\" expresses a negative mood and attitude."}, "language_style": {"answer": "Tactful and warm", "rationale": "\"Slow and warm.\""}, "time_dimension": {"answer": "Present", "rationale": "Now the entry asks the testee about the current emotional state and fears, and the temporal dimension is now. The keywords \"I\" and \"I'm worried\" and \"I'm in love\" indicate that this is a description of the current emotional situation, not of the past or the future."}, "sentence_type": "Affirmative declarative sentence", "pos": {"nouns": {"Relationships": 1}, "verbs": {"I'm worried": 1}, "adverbs": {"very": 1}}}, {"content": "When my lovers started to be close to me, I found myself retreating;", "word_count": 20, "label": "", "sentence_structure": "Subject鈥搗erb鈥搊bject", "subjective_objective": {"answer": "Subjective", "rationale": "Because \"resort\" is a subjective feeling and reaction of a human being."}, "sentence_sentiment": {"answer": "Negative", "rationale": "Because the act of \"resort\" in the entry is negative."}, "language_style": {"answer": "Tactful and warm", "rationale": ""}, "time_dimension": {"answer": "Present", "rationale": "The keywords \"start\" and \"in retreat\" indicate that this is an ongoing, current emotional reaction, so the entry describes the situation of the person being tested."}, "sentence_type": "Affirmative declarative sentence", "pos": {"nouns": {"Lovers": 1, "self": 1}, "verbs": {"Start": 1, "Close": 1, "Found": 1, "Retrenched": 1}, "adverbs": {"When": 1}, "numerals": {"05": 1}, "pronouns": {"I": 1}}}, {"content": "I fear that lovers will not care for me as much as I do for him (her);", "word_count": 19, "label": "", "sentence_structure": "Subject鈥搗erb鈥搊bject", "subjective_objective": {"answer": "Subjective", "rationale": "Because the questionnaire item describes the patient ' s feelings and fears, it is a manifestation of subjective attitudes."}, "sentence_sentiment": {"answer": "Negative", "rationale": "Because the word \"fear\" in the entry is an expression of a negative emotional attitude."}, "language_style": {"answer": "Tactful and warm", "rationale": ""}, "time_dimension": {"answer": "Present", "rationale": ""}, "sentence_type": "Interrogative sentence", "pos": {"nouns": {"Lovers": 1, "I": 2, "he": 1, "she": 1}, "verbs": {"Yes": 1, "Elephant": 1, "Care": 3}, "adjectives": {"No, I won't": 1, "I": 1}, "adverbs": {"Land": 1}, "pronouns": {"Here": 1}}}, {"content": "When lovers want to be very close to me, I feel uncomfortable;", "word_count": 19, "label": "", "sentence_structure": "Subject鈥搗erb鈥揷omplement", "subjective_objective": {"answer": "Subjective", "rationale": "This is the subjective feeling and attitude of the patient, describing his or her response in a given situation."}, "sentence_sentiment": {"answer": "Negative", "rationale": "Because in this entry, the act of \"frightful\" is negative."}, "language_style": {"answer": "Tactful and warm", "rationale": "The language style of the entry is more euphemistic and gentle, expressing each other's feelings by using the words \"hope\" and \"very\" rather than direct command or criticism. Moreover, using \"me\" as the main word makes it easier for respondents to resonate and share their views. In general, the entry asks respondents' views in a more moderate way, which makes them comfortable and respectful."}, "time_dimension": {"answer": "Present", "rationale": ""}, "sentence_type": "Negative declarative sentence", "pos": {"nouns": {"Lovers": 1, "Very": 1, "Close": 1, "I'm not comfortable": 1}, "verbs": {"Yes": 2, "I think": 1}, "adverbs": {"Very": 1}, "numerals": {"07": 1}, "pronouns": {"I": 1}}}, {"content": "I'm a little worried about losing my lover.", "word_count": 10, "label": "", "sentence_structure": "Subject鈥揷opula鈥損redicative", "subjective_objective": {"answer": "Subjective", "rationale": "Because \"fear\" is the patient's subjective emotions and attitude."}, "sentence_sentiment": {"answer": "Negative", "rationale": "Because the entry is a fear of losing a lover and a negative emotional attitude."}, "language_style": {"answer": "Tactful and warm", "rationale": "The entry uses a relatively euphemism and a kind of tone to ask if the interviewees are worried about losing their lovers, and avoids being too direct or harshly expressed. At the same time, the term \"sweet\" also shows that the fear is not very strong, with some degree of decomposition and inclusiveness, and that it is easier for the interviewees to share their true thoughts."}, "time_dimension": {"answer": "Present", "rationale": "The keyword \"I'm a little worried about losing my lover\" indicates the current fears and mindset of the testee, and therefore the judgment is now."}, "sentence_type": "Interrogative sentence", "pos": {"nouns": {"Lovers": 1}, "verbs": {"Lose": 1, "I'm worried": 1}, "adjectives": {"A little": 1}, "adverbs": {"Yes": 1}, "pronouns": {"I": 1}}}, {"content": "I don't think it's a comfortable thing to be honest with a lover.", "word_count": 20, "label": "", "sentence_structure": "Subject鈥損redicate", "subjective_objective": {"answer": "Subjective", "rationale": "Because \"think\" is a subjective verb, which expresses the feelings and views of the patient. The entry looks at the subjective attitudes and feelings of the patient, not at objective facts or behaviour."}, "sentence_sentiment": {"answer": "Negative", "rationale": "Because the description of \"uncomfortable\" in the entry is negative."}, "language_style": {"answer": "Calm and direct", "rationale": "The language style of the entry is relatively calm and straightforward, and there is no excessive rhetoric or emotional rendering, but rather a simple and clear expression of the opinion of the subject of the inquiry as to openness and impartiality with the lover, which is more objective and neutral."}, "time_dimension": {"answer": "Past", "rationale": "The entry therefore describes the feelings and attitudes of the testees towards their relationship in the past."}, "sentence_type": "Negative declarative sentence", "pos": {"nouns": {"Things": 1, "Lovers": 1, "Open": 1}, "verbs": {"I think": 1, "It's comfy": 1}, "adjectives": {"One": 1}, "numerals": {"09": 1}, "pronouns": {"to": 1}}}, {"content": "I often wish that lovers' feelings for me were as serious as my feelings for lovers;", "word_count": 24, "label": "", "sentence_structure": "Subject鈥搗erb鈥揷omplement", "subjective_objective": {"answer": "Subjective", "rationale": "Because \"hope\" is a subjective term that expresses the thoughts and expectations of patients."}, "sentence_sentiment": {"answer": "Positive", "rationale": "Because \"I hope that lovers take their feelings as seriously as they do their feelings for lovers\" is a positive emotional expression."}, "language_style": {"answer": "Tactful and warm", "rationale": "The entry is more personal and interactive when it is spoken in a gentle and inviting tone, rather than in direct statements or orders. At the same time, by using the word \"favorite\" it expresses a desire for love, and the language style is more euphemism and warmer."}, "time_dimension": {"answer": "Present", "rationale": ""}, "sentence_type": "Affirmative declarative sentence", "pos": {"nouns": {"Questionnaire": 1, "Entry": 1, "Lovers": 2, "Emotion": 2, "I": 3}, "verbs": {"Hope": 1, "to": 2, "Same thing": 1, "Serious": 1}, "adjectives": {"Often": 1}, "numerals": {"10": 1, "Two": 1}, "classifiers": {"individual": 1}}}, {"content": "I want to be close to my lover, but I'm always back on my feet;", "word_count": 17, "label": "", "sentence_structure": "Subject鈥搗erb鈥揷omplement", "subjective_objective": {"answer": "Subjective", "rationale": "Because the words \"think\" and \"rest\" reflect the patient's inner feelings and attitudes and are viewed from a subjective point of view."}, "sentence_sentiment": {"answer": "Negative", "rationale": "Because the act of \"deeping back\" in the entry is negative."}, "language_style": {"answer": "Tactful and warm", "rationale": "The entry is more personal and interactive when it is spoken in a gentle and inviting tone, rather than in direct statements or orders. At the same time, using the word \"but\" turns, it expresses the contradictions and confusion that exist within the respondents, which are more subtle and personal."}, "time_dimension": {"answer": "Present", "rationale": "The use of the keywords \"I want\" and \"Always will\" suggests that it is a description of the person's current feelings and behaviour. In it, \"want\" to express a desire or desire in the heart, and \"always will\" is used to mean a constant, recurring situation, where there is always fear or aversion in the proximity of a lover. So the time dimension of this article is now."}, "sentence_type": "Affirmative declarative sentence", "pos": {"nouns": {"Lovers": 1, "Close": 1, "Retrenched": 1, "Front": 1}, "verbs": {"Yes": 3, "Retrenched": 1, "I'm not going": 1}, "adverbs": {"Always": 1}, "numerals": {"11": 1, "One": 1}, "pronouns": {"I": 1}}}, {"content": "I often want to be in love, but sometimes I scare away.", "word_count": 22, "label": "", "sentence_structure": "Subject鈥搗erb鈥搊bject", "subjective_objective": {"answer": "Subjective", "rationale": "Because \"often thinking\" and \"will scare off lovers\" are patients' subjective thoughts."}, "sentence_sentiment": {"answer": "Neutral", "rationale": "\"To stay with lovers\" is positive, but sometimes it scares them off is negative, so this entry has both positive and negative emotional aspects."}, "language_style": {"answer": "Tactful and warm", "rationale": "The basis for the judgment: the entry starts with \"I often want\" and expresses an idea or emotion that is not directly directed or blamed. And, \"But sometimes it scares off lovers\" in part, which shows the sensitivity and respect for lovers, so the language of the questionnaire is generally more warm."}, "time_dimension": {"answer": "Present", "rationale": "The entry uses the general current pattern of \"often trying\" and \"fright\" behaviour, which means that it has not only happened in the past, but may still be ongoing. At the same time, the word \"but\" suggests that this may be disturbing or uncomfortable for lovers. So the temporal dimension of the entry is now judged."}, "sentence_type": "Affirmative declarative sentence", "pos": {"nouns": {"Questionnaire": 1, "Entry": 1, "Lovers": 2}, "verbs": {"Yes": 1, "鈾?And the shadows are always on the line 鈾?: 1, "- Get out of here": 1}, "adjectives": {"Often": 1}, "adverbs": {"But sometimes": 1}, "numerals": {"12": 1}, "pronouns": {"I": 1}}}, {"content": "When lovers are too close to me, I feel nervous;", "word_count": 20, "label": "", "sentence_structure": "Subject鈥搗erb鈥揷omplement", "subjective_objective": {"answer": "Subjective", "rationale": "Because feeling nervous is a subjective emotional experience, linked to the patient's inner feeling."}, "sentence_sentiment": {"answer": "Negative", "rationale": "Because the \"frightful\" act in the entry is negative."}, "language_style": {"answer": "Calm and direct", "rationale": "Because it describes the emotional response of individuals in a given situation in a direct manner, it does not use excessive rhetorical techniques or emotional rendering, and it is more objective in its tone."}, "time_dimension": {"answer": "Present", "rationale": "The entry describes the immediate feelings and reactions of the subject in his relationship with his lover, and therefore judges \"now\"."}, "sentence_type": "Affirmative declarative sentence", "pos": {"nouns": {"Lovers": 1, "Time": 1, "Inside": 1, "Nervous": 1}, "verbs": {"Yes": 2, "I feel": 1, "Overintimate": 1}, "adjectives": {"When": 1, "鈾?When 鈾?: 1}, "numerals": {"13": 1, "1": 4}, "pronouns": {"I": 1}}}, {"content": "I am afraid of being alone;", "word_count": 8, "label": "", "sentence_structure": "Subject鈥搗erb鈥揷omplement", "subjective_objective": {"answer": "Subjective", "rationale": "Because it's the patient's own feelings and fears."}, "sentence_sentiment": {"answer": "Negative", "rationale": "Because the \"fear of being alone\" in the entry is an expression of a negative sentiment."}, "language_style": {"answer": "Tactful and warm", "rationale": "The language style of the entry is more euphemistic. It asks the person surveyed, in a gentle tone, whether he fears being alone, rather than directly speaking or ordering. This expression is more personal and interactive, making the person surveyed more comfortable and comfortable and easier to open the box."}, "time_dimension": {"answer": "Present", "rationale": "The keyword \"I'm afraid\" means the person's emotions or feelings at this point in time, and \"one person alone\" points to the current situation or state, so the entry asks about the person's current behaviour or mental state."}, "sentence_type": "Affirmative declarative sentence", "pos": {"nouns": {"person": 1, "Alone": 1}, "verbs": {"I": 1, "I'm worried": 1}, "numerals": {"14": 1}}}, {"content": "I'd like to tell my lovers what I think and feel inside, and I think it's a comfortable thing;", "word_count": 29, "label": "", "sentence_structure": "Subject鈥搗erb鈥搊bject", "subjective_objective": {"answer": "Subjective", "rationale": "Because \"think\" is the patient's subjective feeling and attitude."}, "sentence_sentiment": {"answer": "Positive", "rationale": "Because the entries \"willing to tell my thoughts and feelings\" and \"thinking it's a comfortable thing\" express positive emotional attitudes and behavior."}, "language_style": {"answer": "Tactful and warm", "rationale": ""}, "time_dimension": {"answer": "Present", "rationale": ""}, "sentence_type": "Affirmative declarative sentence", "pos": {"nouns": {"Questionnaire": 1, "Entry": 1, "Thoughts": 1, "Feel": 1, "Lovers": 1, "Things": 1}, "verbs": {"Yes": 1, "Tell": 1, "I think": 1, "be": 1, "I'm comfortable": 1}, "adjectives": {"Inside": 1}, "adverbs": {"Here": 2, "One": 1}, "numerals": {"15": 1}, "pronouns": {"I": 1}}}, {"content": "I want to be very close to my lover, sometimes scaring her away;", "word_count": 20, "label": "", "sentence_structure": "Subject鈥搗erb鈥揷omplement", "subjective_objective": {"answer": "Subjective", "rationale": "Because the entry describes the subjective thoughts and feelings of patients (i.e., \"the desire to be very close to a lover\") and the possible consequences of such a desire (i.e., \"sometimes scare a lover away\". This is therefore a highly subjective questionnaire entry."}, "sentence_sentiment": {"answer": "Positive", "rationale": "According to the profile, \"I\" wants to be close to a lover, which is in itself a positive attitude and behaviour. But showing this desire too strongly can scare a lover, and thus has a certain negative side. Overall, the subject of this questionnaire is positive, but needs to be expressed."}, "language_style": {"answer": "Tactful and warm", "rationale": "The entry leads the interviewer to think by describing his feelings, rather than directly asking or accusing the other. This expression is more moderate and inclusive, and it is more resonant and participatory. At the same time, the use of the term \"sometimes\" suggests that it is an occasional situation, and does not cause too much pressure and distress to the interviewee."}, "time_dimension": {"answer": "Present", "rationale": "The keywords \"want\" and \"sometimes\" indicate that this is a current situation or tendency, and the entry should therefore be classified as now."}, "sentence_type": "Interrogative sentence", "pos": {"nouns": {"Wish": 1, "Lovers": 2}, "verbs": {"Yes": 3, "- Get out of here": 1}, "adjectives": {"Very": 1}, "adverbs": {"Sometimes": 1}, "numerals": {"16": 1}, "classifiers": {"individual": 1}, "pronouns": {"I": 1}}}, {"content": "I tried to avoid being too close to my lover;", "word_count": 13, "label": "", "sentence_structure": "Subject鈥搗erb鈥揷omplement", "subjective_objective": {"answer": "Subjective", "rationale": "Because trying to avoid being too close to a lover is the patient's own subjective idea and behaviour."}, "sentence_sentiment": {"answer": "Negative", "rationale": "Because the act of \"avoiding to be too close to lovers\" in the entry is negative."}, "language_style": {"answer": "Tactful and warm", "rationale": "The language style of the entry is more euphemistic, because the questioner does not ask directly whether the other side avoids being close to a lover, but rather leads the interviewee to think and express his/her thoughts by asking. This way gives the interviewee more comfort and freedom to answer the questions more honestly."}, "time_dimension": {"answer": "Present", "rationale": "In the future, the entry contains visions and expectations for the future, with the keywords \"tried to avoid\" and \"too close,\" which point to situations that have not yet occurred and that may arise in the future. At the same time, the use of \"tried\" also indicates that the testee is considering the issue, which has not yet been implemented, and therefore the temporal dimension is judged to be the future."}, "sentence_type": "Negative declarative sentence", "pos": {"nouns": {"Questionnaire Entry": 1, "Lovers": 1}, "verbs": {"Try": 1, "Avoid": 1, "鈾?And become 鈾?: 1, "Close": 1}, "numerals": {"17": 1}, "pronouns": {"I": 1}}}, {"content": "I need my lover to make sure that he/she loves me repeatedly;", "word_count": 18, "label": "", "sentence_structure": "Subject鈥搗erb鈥揷omplement", "subjective_objective": {"answer": "Subjective", "rationale": "Because it's the patient's subjective perception and perception of the needs of lovers."}, "sentence_sentiment": {"answer": "Negative", "rationale": "Because the \"needs to guarantee\" in the entry expresses a feeling of dependency and unease, which is a negative feeling."}, "language_style": {"answer": "Tactful and warm", "rationale": ""}, "time_dimension": {"answer": "Present", "rationale": "The entry describes the expectations and needs of the person being tested for a lover, an emotional experience that is immediate, current state and therefore the time dimension is now judged."}, "sentence_type": "Affirmative declarative sentence", "pos": {"nouns": {"Lovers": 1, "Promise": 1, "He/she": 2, "Love": 1}, "verbs": {"Yes": 1, "be": 1}, "adverbs": {"And again and again": 1}, "numerals": {"18": 1}, "pronouns": {"I": 1}}}, {"content": "I feel that I'm easier to get close to my lovers;", "word_count": 13, "label": "", "sentence_structure": "Subject鈥揷opula鈥損redicative", "subjective_objective": {"answer": "Subjective", "rationale": "Because the word \"think\" indicates that it is a subjective feeling or perception of the patient, and that being close to a lover is the patient's personal experience and perception. The questionnaire item examines the patient's attitude and feelings from a subjective perspective."}, "sentence_sentiment": {"answer": "Positive", "rationale": "Because the attitude in the entry, \"I feel more close to my lover,\" is positive."}, "language_style": {"answer": "Tactful and warm", "rationale": "The entry is more personal and interactive when it is spoken in a gentle and inviting tone, rather than in direct statements or orders. At the same time, the term \"easy\" also expresses the author's positive attitude towards self-awareness, avoiding overly subjective and negative sentiments and being more euphemistic."}, "time_dimension": {"answer": "Present", "rationale": "The keyword \"I feel\" and \"I feel\" is easier, indicating that it is a statement of personal feelings, and that the temporal dimension is now judged."}, "sentence_type": "Affirmative declarative sentence", "pos": {"nouns": {"Questionnaire": 1, "Entry": 1, "Lovers": 1}, "verbs": {"I think": 1, "Easy": 1, "Close": 1}, "numerals": {"19": 1}, "pronouns": {"I": 1}}}, {"content": "I feel like I'm asking my lover to show more feelings and the level of commitment to relationships;", "word_count": 32, "label": "", "sentence_structure": "Subject鈥搗erb鈥搊bject", "subjective_objective": {"answer": "Subjective", "rationale": ""}, "sentence_sentiment": {"answer": "Positive", "rationale": "Because the entry \"I\" thinks that it's making constructive and positive demands, not negative complaints or unreasonable."}, "language_style": {"answer": "Tactful and warm", "rationale": ""}, "time_dimension": {"answer": "Present", "rationale": "The entry shows the current attitude and feeling of the testee, who thinks he is asking for more feelings and input from a lover. The keyword \"felt\" and \"suspect\" point to the current situation, and therefore judge it as now."}, "sentence_type": "Affirmative declarative sentence", "pos": {"nouns": {"I": 1, "Lovers": 1, "Feel": 1, "Relationships": 1, "Level of input": 1}, "verbs": {"I think": 1, "Request": 1, "- Put it on": 1, "Show me": 1}, "adverbs": {"self": 1, "More": 1}, "numerals": {"20": 1}}}, {"content": "I find it difficult to rely on lovers;", "word_count": 17, "label": "", "sentence_structure": "Subject鈥搗erb鈥搊bject", "subjective_objective": {"answer": "Subjective", "rationale": "Because finding me dependent on lovers is a difficult thing, and it's the patient's subjective feeling and judgment."}, "sentence_sentiment": {"answer": "Negative", "rationale": "Because the action of relying on lovers in the entries is negative."}, "language_style": {"answer": "Tactful and warm", "rationale": "The language style of the entry is more euphemistic, expressing reflection and confusion about the issue of dependence on lovers by using terms such as \"discovery\" and \"difficult\" and not using too many negative or direct criticisms. This expression is more conducive to creating a climate of easy and friendly dialogue and to increasing the rate of questionnaire recovery."}, "time_dimension": {"answer": "Present", "rationale": ""}, "sentence_type": "Negative declarative sentence", "pos": {"nouns": {"Found": 1, "Lovers": 1, "Things": 1}, "verbs": {"Jean": 1, "Dependency": 1}, "adjectives": {"Difficulties": 1}, "numerals": {"21": 1}, "pronouns": {"I": 1}}}, {"content": "22. I am not often afraid of being abandoned by lovers;", "word_count": 13, "label": "", "sentence_structure": "Subject鈥揷opula鈥損redicative", "subjective_objective": {"answer": "Subjective", "rationale": "Because \"or the fear of being abandoned by a lover\" is the patient's subjective feeling and thought."}, "sentence_sentiment": {"answer": "Negative", "rationale": "Because \"often worried\" is negative."}, "language_style": {"answer": "Calm and direct", "rationale": "The entry asks the other party in a straightforward manner whether they are often afraid of being abandoned by their lovers, which is more objective in tone and does not have too many rhetorical or emotional renderings."}, "time_dimension": {"answer": "Present", "rationale": ""}, "sentence_type": "Negative declarative sentence", "pos": {"nouns": {"Questionnaire": 1, "Entry": 1, "Lovers": 1, "Abandon": 1}, "verbs": {"be": 1, "I'm worried": 1, "Often": 1, "Abandon": 1}, "adjectives": {"and": 1, "No, it's not": 1, "Often": 1}, "adverbs": {"very": 1}, "numerals": {"22": 1}, "pronouns": {"I": 1}}}, {"content": "I tend not to be too close to lovers;", "word_count": 12, "label": "", "sentence_structure": "Subject鈥搗erb鈥搊bject", "subjective_objective": {"answer": "Subjective", "rationale": "Because \"preferance\" reflects the subjective attitude and preferences of the patient."}, "sentence_sentiment": {"answer": "Negative", "rationale": "Because the \"not being too close to lovers\" act in the entry is negative."}, "language_style": {"answer": "Calm and direct", "rationale": "The entry directly expresses the attitude of asking the other in a relationship, which prefers to remain more alienated, without excessive rhetorical or emotional rendering, and is more objective in tone."}, "time_dimension": {"answer": "Present", "rationale": "The keyword \"prefers not to be too close to lovers\" suggests that it is the current attitude or habitual behaviour of the testee."}, "sentence_type": "Negative declarative sentence", "pos": {"nouns": {"Lovers": 1, "Intimacy": 1}, "verbs": {"Prefer": 1, "not": 1, "Call": 1, "That's too much": 1}, "adjectives": {"That's too much": 1}, "adverbs": {"Orientation": 1}, "numerals": {"23": 1, "1": 4}, "pronouns": {"I": 1}}}, {"content": "24. If I cannot get the attention and attention of my lovers, I will be upset or angry;", "word_count": 25, "label": "", "sentence_structure": "Subject鈥損redicate", "subjective_objective": {"answer": "Subjective", "rationale": "Because \"failure to get the attention and care of a lover\" is the subjective feeling of the patient, and \"disturbing or angry\" is the subjective reaction of the patient to it."}, "sentence_sentiment": {"answer": "Negative", "rationale": "Because \"disturbed\" and \"irregular\" in the entries are negative emotional reactions, expressing emotional dissatisfaction or suffering."}, "language_style": {"answer": "Calm and direct", "rationale": ""}, "time_dimension": {"answer": "Past", "rationale": "Thus, the entry is concerned primarily with present and future behaviour or mentality, rather than with past behaviour or mentality."}, "sentence_type": "Affirmative declarative sentence", "pos": {"nouns": {"Lovers": 1, "Attention": 1, "Care": 1}, "verbs": {"Yes": 1, "Got it": 1, "I'm upset": 1, "Angry": 1}, "adjectives": {"Cannot": 1, "Or": 1}, "adverbs": {"If": 1, "Yes": 1}, "pronouns": {"I": 1}}}, {"content": "I tell my lover everything.", "word_count": 10, "label": "", "sentence_structure": "Subject鈥搗erb鈥揷omplement", "subjective_objective": {"answer": "Subjective", "rationale": "Because it is the patient's subjective feeling and attitude that he thinks he and his lover speak of everything."}, "sentence_sentiment": {"answer": "Positive", "rationale": "Because it expresses a close relationship with lovers to share everything, it is a positive emotion and attitude."}, "language_style": {"answer": "Tactful and warm", "rationale": "The entry uses \"everything says\" to describe the level of communication with a lover, a more euphemistic expression, which does not directly accuse or evaluate a lover, but rather expresses its views in a more peaceful and understanding way. At the same time, the whole sentence is more gentle and gives people a sense of affection."}, "time_dimension": {"answer": "Present", "rationale": ""}, "sentence_type": "Affirmative declarative sentence", "pos": {"nouns": {"Things": 1, "Lovers": 1}, "verbs": {"Speak": 1}, "adverbs": {"Both": 1}, "numerals": {"25": 1}, "pronouns": {"I": 1}}}, {"content": "I find that lovers are not willing to be as close to me as I thought;", "word_count": 20, "label": "", "sentence_structure": "Subject鈥搗erb鈥搊bject", "subjective_objective": {"answer": "Subjective", "rationale": "Because \"I find my lovers not willing to be as close to me as I think\" is the patient's observation and reflection, reflecting their subjective feelings and perceptions."}, "sentence_sentiment": {"answer": "Negative", "rationale": "Because the phrase \"discovering a lover doesn't want to be as close to me as I thought\" in the entry shows a negative emotion and attitude."}, "language_style": {"answer": "Calm and direct", "rationale": "The entry is more directly linguistically, with no excessive rhetorical or emotional accent. It directly expresses the problems and feelings of the subject in a relationship and seeks responses and suggestions from the other party."}, "time_dimension": {"answer": "Past", "rationale": "The entry describes the feelings and behaviour of the person being tested with regard to the lover, and the temporal dimension is a past."}, "sentence_type": "Negative declarative sentence", "pos": {"nouns": {"Lovers": 1, "I": 2, "Close": 1}, "verbs": {"Found": 1, "Yes": 1, "Elephant": 1, "Call": 2}, "adverbs": {"That way": 1}, "numerals": {"26": 1}}}, {"content": "I often discuss with lovers the problems I encounter and the things I care about;", "word_count": 23, "label": "", "sentence_structure": "Subject鈥搗erb鈥搊bject", "subjective_objective": {"answer": "Subjective", "rationale": "Because the content of \"discussion\" and \"care\" is decided by the patient himself, it is seen from the patient's subjective perspective."}, "sentence_sentiment": {"answer": "Positive", "rationale": "Because the entry \"discussing the problems I encounter and what I care about\" is positive."}, "language_style": {"answer": "Tactful and warm", "rationale": ""}, "time_dimension": {"answer": "Present", "rationale": ""}, "sentence_type": "Affirmative declarative sentence", "pos": {"nouns": {"Questionnaire": 1, "Entry": 1, "Lovers": 1, "Problem": 1, "Things": 1}, "verbs": {"Discussion": 1, "I met him": 1, "Care": 1}, "adverbs": {"Regular": 1}, "numerals": {"27": 1}}}, {"content": "I'd be a little anxious and upset if I didn't have a lover.", "word_count": 21, "label": "", "sentence_structure": "Subject鈥搗erb鈥搊bject", "subjective_objective": {"answer": "Subjective", "rationale": "Because \"a little anxiety and anxiety\" is the feeling and attitude inside the patient, and it's subjective."}, "sentence_sentiment": {"answer": "Negative", "rationale": "Because it contains negative emotional terms like \"anxiety and anxiety\"."}, "language_style": {"answer": "Tactful and warm", "rationale": "The basis for the judgment: the hypothetical phrase \"if I have no lover\" in the entry gives people a friendly and sweet feeling. At the same time, the sentence is not expressed in a direct attack or denial of the absence of lovers, but in an understanding and support. This euphemism, warm language, is more acceptable and willing to answer questions."}, "time_dimension": {"answer": "Present", "rationale": "\"If I haven't had a lover,\" says \"if\" that it is a hypothetical condition, that is, anxiety and anxiety in a situation where there is no lover. And the phrase is \"I,\" which means that it is a self-perception. So the time dimension of this purpose should be now."}, "sentence_type": "Affirmative declarative sentence", "pos": {"nouns": {"Lovers": 1, "Anxiety": 1, "Unsettled": 1}, "verbs": {"Yes": 2, "I feel": 1}, "adjectives": {"A little": 1}, "numerals": {"28": 1}, "pronouns": {"I": 1}}}, {"content": "I feel comfortable with relying on lovers;", "word_count": 14, "label": "", "sentence_structure": "Subject鈥揷opula鈥損redicative", "subjective_objective": {"answer": "Subjective", "rationale": "Because it's a very comfortable thing to \"think\" to be attached to a lover, a feeling or a perception within a person, a subjective one."}, "sentence_sentiment": {"answer": "Positive", "rationale": "Because the expression \"felts comfortable to rely on a lover\" expresses a positive emotional attitude, which is to think that it is pleasant and comfortable to rely on a lover."}, "language_style": {"answer": "Tactful and warm", "rationale": "The language style of the questionnaire entry is more euphemistic and warm, and the view of the other party on the degree of dependency in a relationship is expressed in a question-and-answer manner, without being too direct or hard-line. At the same time, the article is more friendly and friendly, and it makes people feel comfortable and natural."}, "time_dimension": {"answer": "Present", "rationale": "Now, the \"I think\" in the questionnaire item shows the testee's current view or feeling, and \"Leave to love is a very comfortable thing\" is a descriptive statement that does not address past or future acts or events. So, the entry is the current temporal dimension."}, "sentence_type": "Affirmative declarative sentence", "pos": {"nouns": {"Lovers": 1, "Things": 1}, "verbs": {"I think": 1, "Dependency": 1, "be": 1}, "adjectives": {"I'm comfortable": 1}, "numerals": {"29": 1}, "pronouns": {"I": 1}}}, {"content": "30. If lovers cannot be with me as I wish, I feel frustrated;", "word_count": 27, "label": "", "sentence_structure": "Subject鈥搗erb鈥搊bject", "subjective_objective": {"answer": "Subjective", "rationale": "Because it's the patient's subjective feeling and the idea."}, "sentence_sentiment": {"answer": "Negative", "rationale": "Because the emotional reaction of \"deep in the heart\" in the entry is negative."}, "language_style": {"answer": "Tactful and warm", "rationale": "The entry is more personal and interactive when it is spoken in a gentle, inquisitive way, rather than directly speaking or ordering. At the same time, it expresses a sense of disappointment and discomfort that a lover cannot be with him, but it is not too negative or intensely emotional, and it is more euphemistic and warm."}, "time_dimension": {"answer": "Present", "rationale": "\"Frustment\" is a subjective feeling, and the entry describes the possible psychological reaction or emotional state of the person being tested and therefore belongs to the present."}, "sentence_type": "Affirmative declarative sentence", "pos": {"nouns": {"Lovers": 1, "I": 2, "Around": 1, "Time": 1, "I'm so depressed": 1}, "verbs": {"No, I can't": 1, "Elephant": 1, "Hope": 1, "in": 1, "I feel": 1}, "adjectives": {"That way": 1}, "adverbs": {"That way": 1}, "numerals": {"30": 1}, "pronouns": {"I": 2}}}, {"content": "I do not care to find comfort from lovers, to listen to counsel and to be helped;", "word_count": 22, "label": "", "sentence_structure": "Subject鈥搗erb鈥揷omplement", "subjective_objective": {"answer": "Subjective", "rationale": "Because \"care\" is a subjective feeling and attitude. The subject is to see if the patient is dependent on the other person for solving the problem or seeking support in a relationship."}, "sentence_sentiment": {"answer": "Negative", "rationale": "Because the \"not mind\" attitude in the entry is negative."}, "language_style": {"answer": "Tactful and warm", "rationale": "The language style of the entry is euphemism. It does not express the cold meaning of \"I don't need the comfort, counsel and help of a lover,\" but rather conveys a more gentle attitude by using the negative word \"not care.\" And it is also \"seeking comfort from a lover, listening to counsel, getting help\" that is more a kind and caring way of speaking, more in a warmer way."}, "time_dimension": {"answer": "Present", "rationale": "The verbs \"not concerned\", \"seeing\", \"hearing\" and \"getting\" in the entry are words that express the current behaviour or state, so it can be judged that the entry is asking the testee about the current attitude or orientation."}, "sentence_type": "Negative declarative sentence", "pos": {"nouns": {"Consolation": 1, "- A word of advice": 1, "Help": 1}, "verbs": {"Search": 1, "Listen": 1, "Got it": 1}, "adverbs": {"No, it's not": 1}, "numerals": {"31": 1}, "pronouns": {"I": 1, "Lovers": 1}}}, {"content": "32. I would be frustrated if, when I need it, my lover was not with me;", "word_count": 23, "label": "", "sentence_structure": "Subject鈥損redicate", "subjective_objective": {"answer": "Subjective", "rationale": "The purpose of this article is therefore to look at the subject in a subjective manner."}, "sentence_sentiment": {"answer": "Negative", "rationale": "Because the entry describes feelings that are frustrating when lovers are not around when they are needed, it is a negative emotional expression."}, "language_style": {"answer": "Tactful and warm", "rationale": "The language style of this entry is euphemism, because it uses words that describe emotions (such as \"difference\") and the expression of inquiry, giving people a sense of affection and understanding. At the same time, it does not directly accuse or dictate that the other side must be with you, but rather expresses your feelings and hopes for understanding and support. Such a tone is more conducive to creating a good atmosphere of communication that makes each other more receptive and willing to change their behaviour."}, "time_dimension": {"answer": "Present", "rationale": ""}, "sentence_type": "Affirmative declarative sentence", "pos": {"nouns": {"Time": 1, "Lovers": 1, "I": 1, "Frustration": 1}, "verbs": {"Yes": 3, "I feel": 1, "in": 1, "Not by me": 1}, "adjectives": {"Frustration": 1}, "numerals": {"32": 1}, "pronouns": {"I": 1}}}, {"content": "33. It is useful for me to ask my lovers when needed;", "word_count": 17, "label": "", "sentence_structure": "Subject鈥搗erb鈥揷omplement", "subjective_objective": {"answer": "Subjective", "rationale": "This is a subjective examination of the patient ' s perception and feelings of seeking help in a relationship."}, "sentence_sentiment": {"answer": "Positive", "rationale": "Because the \"help\" act in the entry is a positive act, which shows the trust and dependence of the person who seeks help."}, "language_style": {"answer": "Tactful and warm", "rationale": ""}, "time_dimension": {"answer": "Present", "rationale": ""}, "sentence_type": "Affirmative declarative sentence", "pos": {"nouns": {"Time": 1, "Lovers": 1, "Help": 1, "It works": 1}, "verbs": {"In": 1, "be": 1, "Help": 1}, "adjectives": {"Yes": 1, "Very": 1}, "numerals": {"33": 1}}}, {"content": "When lovers disagree with me, I feel bad about me;", "word_count": 17, "label": "", "sentence_structure": "Subject鈥揷opula鈥損redicative", "subjective_objective": {"answer": "Subjective", "rationale": "This is the patient ' s subjective idea and attitude."}, "sentence_sentiment": {"answer": "Negative", "rationale": "Because the phrase \"I feel bad about myself\" in the entry is a sign of self-demeaning and negative feelings."}, "language_style": {"answer": "Calm and direct", "rationale": "This entry is a direct statement that indicates the emotional reaction of a person who, in the face of a lover鈥檚 objection, considers himself to be in a problem."}, "time_dimension": {"answer": "Present", "rationale": ""}, "sentence_type": "Negative declarative sentence", "pos": {"nouns": {"Lovers": 1, "Time": 1, "I think": 1, "Indeed": 1, "Okay": 1}, "verbs": {"When": 1, "Yes": 1, "I think": 1, "be": 1, "Not good": 1}, "adverbs": {"Indeed": 1}, "numerals": {"34": 1}, "pronouns": {"I": 1}}}, {"content": "I will seek help from lovers on many matters, including seeking comfort and receiving commitment;", "word_count": 24, "label": "", "sentence_structure": "Subject鈥搗erb鈥搊bject", "subjective_objective": {"answer": "Subjective", "rationale": "This is because of the patient ' s subjective attitude and reflects their behavioural tendency to seek help in a relationship."}, "sentence_sentiment": {"answer": "Positive", "rationale": "Because the actions of \"seeking love\" and \"seeing comfort and being committed\" in the entries indicate dependency and trust in lovers, these can be considered positive emotional expressions."}, "language_style": {"answer": "Tactful and warm", "rationale": "The entry expresses an inclusive and understanding by using terms such as \"many\", \"including\" and attempts to establish a relationship of mutual assistance and support with the interviewees. Moreover, the words \"will\" , \"help\" , \"comfort\" , \"commitment\" and \"commitment\" are soft, have no too strong or direct attitudes, and are also a sign of a warmer language style."}, "time_dimension": {"answer": "Present", "rationale": ""}, "sentence_type": "Affirmative declarative sentence", "pos": {"nouns": {"Things": 1, "Lovers": 1, "Consolation": 1, "Commitments": 1}, "verbs": {"Yes": 2, "In": 1, "Help": 1, "Seek": 1, "Got it": 1}, "numerals": {"35": 1}, "pronouns": {"I": 1}}}, {"content": "36. I feel resentment when lovers don ' t spend time with me;", "word_count": 19, "label": "", "sentence_structure": "Subject鈥揷opula鈥損redicative", "subjective_objective": {"answer": "Subjective", "rationale": "Because \"will feel resentment\" is the subjective feeling and reaction of the patient, and it is a subjective view."}, "sentence_sentiment": {"answer": "Negative", "rationale": "Because \"hate\" in the entry is a negative emotion, a expression of dissatisfaction and hostility towards lovers."}, "language_style": {"answer": "Calm and direct", "rationale": "Calm down straight."}, "time_dimension": {"answer": "Present", "rationale": "The entry describes an emotional reaction or feeling that may occur in the moment, that is, \"when my lovers don't spend time with me,\" there's a \"hate\" mood. So, the dimension of time is now."}, "sentence_type": "Negative declarative sentence", "pos": {"nouns": {"Lovers": 1, "Time": 1, "I": 2, "Together": 1}, "verbs": {"Flowers": 1, "and": 1, "in": 1, "Yes": 1, "I feel": 1, "Hate": 1}, "adverbs": {"When": 1, "not": 1, "Time": 1}, "numerals": {"36": 1}}}]}

{"questionnaire_title": "Questionnaire on personality diagnosis", "questionnaire_category": "Mental health", "item_count": 9, "emotion_shift_overall": "From negative to positive", "language_style_overall": "Calm and direct", "items": [{"content": "I avoid working with people who might criticize myself.", "word_count": 16, "label": "", "sentence_structure": "Subject鈥搗erb鈥搊bject", "subjective_objective": {"answer": "Subjective", "rationale": "Because it is a subjective attitude or experience that the patient feels."}, "sentence_sentiment": {"answer": "Negative", "rationale": "Because the words \"avoid\" and \"criticize yourself\" in the entries imply a negative attitude."}, "language_style": {"answer": "Calm and direct", "rationale": "The entry asks the interviewees directly whether they are avoiding the cooperation of those who might criticize themselves and are not using euphemism or emotional rhetoric, and the language style is relatively simple."}, "time_dimension": {"answer": "Present", "rationale": "\"Now.\""}, "sentence_type": "Negative declarative sentence", "pos": {"nouns": {"Questionnaire Entry": 1, "person": 1}, "verbs": {"Avoid": 1, "Work together": 1}, "pronouns": {"self": 1}}}, {"content": "2. I cannot take a decision without advice or repeated assurances from others.", "word_count": 21, "label": "", "sentence_structure": "Subject鈥揷opula鈥損redicative", "subjective_objective": {"answer": "Subjective", "rationale": "Because \"advisory or repeated assurance\" is a subjective need or expectation."}, "sentence_sentiment": {"answer": "Negative", "rationale": "Because the act of \"I cannot take a decision without the advice or repeated assurances of others\" in the entry indicates a tendency to rely on others, which is negative."}, "language_style": {"answer": "Calm and direct", "rationale": "The term \"just\" is used in this entry to express the relationship of condition, which is simple and not slow. 2. The context of the entry is to describe the need for a person to rely on the advice or repeated assurances of others in making a decision, and to be more objective and calm."}, "time_dimension": {"answer": "Present", "rationale": "The term \"just\" in the entry, which indicates a conditional relationship, and \"can\" which adopts the general current, means that the act has not only occurred in the past but may still be ongoing. Moreover, the expression \"no one else's advice or assurances\" also indicates a normal, recurring situation, so that the temporal dimension of the article is now judged."}, "sentence_type": "Negative declarative sentence", "pos": {"nouns": {"others": 1, "- I'm not sure": 1, "Promise": 1, "Decision": 1}, "verbs": {"No, I'm not": 1, "Do it": 1, "Yes": 1, "Give": 1}, "adverbs": {"If": 1, "or": 1, "Repeatedly": 1}, "numerals": {"2": 1}, "pronouns": {"Just": 1}}}, {"content": "I've often focused too much on detail and neglected the larger aspects.", "word_count": 16, "label": "", "sentence_structure": "Subject鈥揷opula鈥損redicative", "subjective_objective": {"answer": "Subjective", "rationale": "Because \"too much detail, and too much attention to the big\" is the subjective view and attitude of the patient."}, "sentence_sentiment": {"answer": "Negative", "rationale": "Because \"too much detail\" can lead to too much attention to triviality and neglect important and global issues. It is a possible problem or bad habit, and it is therefore negative."}, "language_style": {"answer": "Calm and direct", "rationale": "The entry directly expresses the question whether the other party is often too concerned with detail to ignore the subject of the larger picture, and is not using too many rhetorical techniques or emotional renderings, which are more objective and sound."}, "time_dimension": {"answer": "Present", "rationale": "The word \"always\" in \"I've often focused too much on detail\" denotes regular, customary behaviour, while the words \"too much on detail\" and \"too much on the ground\" describe current or recent behaviour, so the entry asks about the person being tested for his current behaviour or mental state."}, "sentence_type": "Negative declarative sentence", "pos": {"nouns": {"Questionnaire Entry": 1, "Details": 1, "In terms of": 1}, "verbs": {"Analysis": 1, "Attention": 1, "Neglect": 1}, "adjectives": {"That's too much": 1, "Large": 1}, "numerals": {"3": 1}, "pronouns": {"I": 1}}}, {"content": "I need to be at the centre of attention.", "word_count": 27, "label": "", "sentence_structure": "Subject鈥搗erb鈥搊bject", "subjective_objective": {"answer": "Subjective", "rationale": "Because they all reflect patients ' assessment and expectations, they are subjective feelings and attitudes."}, "sentence_sentiment": {"answer": "Positive", "rationale": "2. The phrase \"I need to be at the centre of attention\" is subjective and emotional, expressing personal concern for self-worth and self-esteem, and can be interpreted as positive. 3. The phrase \"I have achieved much more than others have praised me\" also expresses a sense of personal confidence and achievement, and can be interpreted as positive. Combined, the emotional orientation of the questionnaire is positive."}, "language_style": {"answer": "Calm and direct", "rationale": "The entry uses more direct language, explicitly asking the other side whether there is a strong self-centre and considering that the achievements are underestimated. This language style is relatively calm and straightforward, is not overly pedantic or obscure, and is more in line with the objectivity and scientific nature of the psychological questionnaire."}, "time_dimension": {"answer": "Present", "rationale": "The two sentences, which contain \"need\" and \"access\" respectively, describe the current state of mind or behaviour. Therefore, the basis of the judgement is now."}, "sentence_type": "[Affirmative declarative sentence]", "pos": {"nouns": {"Centre": 1, "Achievements": 2, "others": 1, "- I'm impressed": 1}, "verbs": {"Yes": 1, "Be": 1, "Access": 1, "More than": 1, "Sou-san": 1}, "numerals": {"4": 1}, "pronouns": {"I": 2}}}, {"content": "I will use a serious means to stop the people I love from leaving me.", "word_count": 18, "label": "", "sentence_structure": "Subject鈥搗erb鈥搊bject", "subjective_objective": {"answer": "Subjective", "rationale": "Because \"I\" shows that it's the patient's personal opinion and attitude."}, "sentence_sentiment": {"answer": "Negative", "rationale": "Because the \"severe means to stop\" in the entry is negative."}, "language_style": {"answer": "Calm and direct", "rationale": "The entry uses more direct and explicit language to express the problem, without too much emotional rendering or euphemism, and is more serious and rational in tone.\""}, "time_dimension": {"answer": "Present", "rationale": "The entry describes the present mindset and behavioral orientation of the testee, and the keyword \"will\" is a general future pattern, indicating that this may be future behaviour. Moreover, \"the person I love\" indicates that the testee's emotional relationship is also in its present state. So, in the overall judgment, the time dimension of the article is now."}, "sentence_type": "Affirmative declarative sentence", "pos": {"nouns": {"Means": 1, "person": 1}, "verbs": {"Yes": 1, "Stop": 1, "Leave": 1}, "adjectives": {"Serious": 1}, "numerals": {"5": 1}, "pronouns": {"I": 1, "Love": 1}}}, {"content": "I've had legal problems on several occasions.", "word_count": 25, "label": "", "sentence_structure": "Subject鈥搗erb鈥搊bject", "subjective_objective": {"answer": "Objective", "rationale": "Because \"in legal trouble\" and \"in arrest\" are objective facts, and \"sometimes\" are subjective estimates of the patient."}, "sentence_sentiment": {"answer": "Negative", "rationale": "Because \"arrested\" and \"legal troubles\" in the entries are negative terms, which represent a negative situation."}, "language_style": {"answer": "Calm and direct", "rationale": "The entry is directly presented to ask the respondents how often they encounter legal problems, which are more calm and objective in language style, and do not involve excessive emotional rendering and rhetorical techniques."}, "time_dimension": {"answer": "Past", "rationale": ""}, "sentence_type": "Affirmative declarative sentence", "pos": {"nouns": {"I": 2, "Legally": 1, "Trouble": 1, "Catch": 1}, "verbs": {"I met him": 1, "Yes": 1, "Category": 1, "Arrest": 1}, "adverbs": {"How many": 1}, "numerals": {"Number of times": 1, "Category": 1}, "pronouns": {"Here": 1}}}, {"content": "I'm not interested in spending time with family and friends.", "word_count": 17, "label": "", "sentence_structure": "Subject鈥揷opula鈥損redicative", "subjective_objective": {"answer": "Subjective", "rationale": "Because the feeling of spending time with family and friends is a subjective attitude of the patient, it is also a subjective judgement of the individual to be interested."}, "sentence_sentiment": {"answer": "Negative", "rationale": "Because \"I'm not interested in spending time with family and friends\" is negative."}, "language_style": {"answer": "Calm and direct", "rationale": "The entry expresses, in a straightforward manner, a lack of emotional interest in the time spent by family and friends, and is more straightforward and explicit than the use of too many acrimony or euphemisms."}, "time_dimension": {"answer": "Present", "rationale": "I'm not very interested in spending time with family and friends, and I'm not very interested in saying it's subjective, and I'm very uninterested in it. So the entry is now judging by the emotional state of the testee, the time dimension."}, "sentence_type": "Negative declarative sentence", "pos": {"nouns": {"Family": 1, "friends": 1, "Time": 1}, "verbs": {"to": 1, "Common": 1, "Not interested": 1}, "adverbs": {"very": 1}}}, {"content": "I can get special information from what's happening around here.", "word_count": 18, "label": "", "sentence_structure": "Subject鈥搗erb鈥揷omplement", "subjective_objective": {"answer": "Subjective", "rationale": "Because this is the patient ' s personal perception and perception, it involves the ability to feel and understand oneself, and it is a subjective approach."}, "sentence_sentiment": {"answer": "Positive", "rationale": "Because the ability to \"get special information from what happens around\" in the entry is useful and therefore positive."}, "language_style": {"answer": "Tactful and warm", "rationale": ""}, "time_dimension": {"answer": "Present", "rationale": "The keyword \"can get\" from what's happening around you means \"now, now\" is the word \"now\"."}, "sentence_type": "Interrogative sentence", "pos": {"nouns": {"Things": 1, "Information": 1}, "verbs": {"Yes": 1, "Got it": 1, "Come on": 1}, "adjectives": {"Special": 1}, "pronouns": {"I": 1, "From": 1}}}, {"content": "I know that if I do not, people will take advantage of me or try to cheat me.", "word_count": 24, "label": "", "sentence_structure": "Subject鈥搗erb鈥搊bject", "subjective_objective": {"answer": "Subjective", "rationale": "Because the words \"know,\" \"intentional\", \"take advantage\" and \"try to cheat\" are all described from the patient's subjective point of view."}, "sentence_sentiment": {"answer": "Negative", "rationale": "Because \"take advantage\" and \"try to cheat\" in the entries are negative acts that show mistrust and fear."}, "language_style": {"answer": "Tactful and warm", "rationale": "This entry conveys in a more moderate way the information about the potential injustice suffered by the subject, without using direct and harsh language, which is more friendly and understanding."}, "time_dimension": {"answer": "Past", "rationale": "So the answer is \"now\"."}, "sentence_type": "Negative declarative sentence", "pos": {"nouns": {"Questionnaire Entry": 1, "person": 2, "self": 1, "Guys": 1, "Cheap": 1, "- Liar": 1}, "verbs": {"Yes": 2, "Jim": 1, "Try": 1}, "adverbs": {"I didn't mean to": 1}, "numerals": {"9": 1}, "pronouns": {"I": 1}}}]}

{"questionnaire_title": "Self-Description Questionnaire (SDQ)", "questionnaire_category": "Behavioral questionnaire", "item_count": 102, "emotion_shift_overall": "From positive to negative", "language_style_overall": "Neutral", "items": [{"content": "1. Mathematics is one of the best disciplines I have learned.", "word_count": 13, "label": "", "sentence_structure": "Subject鈥揷opula鈥損redicative", "subjective_objective": {"answer": "Subjective", "rationale": "Because the best is based on patients' subjective views and feelings."}, "sentence_sentiment": {"answer": "Positive", "rationale": "Because the expression \"mathematics is one of the best subjects I've learned\" in the entry expresses positive opinions and attitudes."}, "language_style": {"answer": "Tactful and warm", "rationale": "The entry uses self-evaluation to express its mathematical advantages and confidence, without being too direct or positive, to be more moderate and friendly. At the same time, this expression is more acceptable and understandable to the other party."}, "time_dimension": {"answer": "Present", "rationale": ""}, "sentence_type": "Affirmative declarative sentence", "pos": {"nouns": {"Math": 1, "Subjects": 1}, "verbs": {"be": 1, "I can learn": 1}, "adjectives": {"I'd better": 1}, "numerals": {"One": 1}, "classifiers": {"individual": 1}, "pronouns": {"I": 1}}}, {"content": "2. No one thinks I look good.", "word_count": 10, "label": "", "sentence_structure": "Subject鈥搗erb鈥揷omplement", "subjective_objective": {"answer": "Subjective", "rationale": "Because \"no one thinks\" is a subjective judgement, dependent on the views and assessments of others and cannot be proved or denied."}, "sentence_sentiment": {"answer": "Negative", "rationale": "Because the phrase \"no one thinks I look good\" in the entry is negative."}, "language_style": {"answer": "Tactful and warm", "rationale": "The entry uses a Query Language style and uses the negative word \"no\", which is a euphemism for the other party's low self-evaluation of appearances. This expression is more moderate and respectful of each other's feelings, avoiding direct attacks or criticism. At the same time, the issue encourages each other to share their thoughts and feelings and to increase the interaction and participation of questionnaires."}, "time_dimension": {"answer": "Present", "rationale": "The entry indicates that the testee's current state of feeling or \"no one thinks I look good.\""}, "sentence_type": "Negative declarative sentence", "pos": {"nouns": {"person": 1, "Nice": 1}, "verbs": {"I think": 1, "Long": 1}, "pronouns": {"I": 1}}}, {"content": "3. Overall, I have much to be proud of.", "word_count": 15, "label": "", "sentence_structure": "Subject鈥搗erb鈥揷omplement", "subjective_objective": {"answer": "Subjective", "rationale": "This is a subjective assessment that reflects the respondents ' perception and attitude to their strengths."}, "sentence_sentiment": {"answer": "Positive", "rationale": "Because it is an expression of a person ' s affirmation and confidence in himself and of a positive emotional colour."}, "language_style": {"answer": "Tactful and warm", "rationale": "The language style of the entry is euphemistic, because it uses positive forms of statement and positive emotional terms like \"proud\" to try to make respondents feel accepted and encouraged. Moreover, the question is not directly asked about the merits or merits of the individual, but rather, it is a way of generating reflection and resonance among respondents by expressing recognition of each other's achievements, which is more acceptable and willing to share."}, "time_dimension": {"answer": "Present", "rationale": "The use of the keyword \"with\" and \"with a lot of\" indicates that the person being tested is describing their current state or feelings, so the entry reflects the person being tested's current behaviour or mentality."}, "sentence_type": "Affirmative declarative sentence", "pos": {"nouns": {"Overall": 1, "Local": 1}, "verbs": {"have": 1, "It's worth it": 1, "Proud": 1}, "adverbs": {"A lot": 1}, "classifiers": {"individual": 1}}}, {"content": "I sometimes take something from others.", "word_count": 9, "label": "", "sentence_structure": "Subject鈥搗erb鈥搊bject", "subjective_objective": {"answer": "Subjective", "rationale": "Because \"I sometimes take something from someone else\" is the patient's subjective feeling and behavior, and the patient's description of his attitude and behaviour."}, "sentence_sentiment": {"answer": "Negative", "rationale": "Because the entry \"Take something from someone else\" is negative."}, "language_style": {"answer": "Tactful and warm", "rationale": "The entry uses a gentle and inviting tone to ask the other side whether there has been unauthorized use of the other ' s objects, and the language style is euphemism and protege."}, "time_dimension": {"answer": "Past", "rationale": "In terms of the modernity, the entry does not clearly state whether the act occurred, is present or is future, and therefore cannot be accurately classified as one of the past, present or future. But, since the word \"sometimes\" implies that the act is not recurrent, we can consider it a relatively rare act of the present."}, "sentence_type": "Affirmative declarative sentence", "pos": {"nouns": {"Questionnaire": 1, "Entry": 1, "Something": 1}, "verbs": {"Take it": 1}, "adverbs": {"Time": 1, "Sometimes": 1}, "pronouns": {"I": 1}}}, {"content": "I like sports, gymnastics and dances.", "word_count": 15, "label": "", "sentence_structure": "Subject鈥搗erb鈥搊bject", "subjective_objective": {"answer": "Subjective", "rationale": "Because it's the patient's subjective attitude."}, "sentence_sentiment": {"answer": "Positive", "rationale": "The term \"likes\" in the entry expresses a positive attitude, while a variety of sports, gymnastics and dance activities are listed, indicating a positive interest in these activities by the respondents."}, "language_style": {"answer": "Tactful and warm", "rationale": "The language style of the entry is a warmer type. The reason is that it does not use too direct or strong expressions, but rather more moderate and friendly language to guide the interviewee to answer questions. For example, words like \"you like\" give people a warm, comfortable atmosphere, and make them more willing to participate in the investigation and to provide real ideas and feelings. Moreover, the entry avoids the use of sensitive terms that can cause discomfort or controversy, such as \"physics\" and \"dance\", and instead replaces it with \"sports\" to better protect the privacy and dignity of the interviewee."}, "time_dimension": {"answer": "Past", "rationale": "The word \"likes\" in the entry indicates that it is a description of past preferences and behaviour and can therefore be judged as past."}, "sentence_type": "Affirmative declarative sentence", "pos": {"nouns": {"Sports": 1, "Gymnastics": 1, "Dance": 1, "Activities": 1}, "verbs": {"Yeah": 1}, "numerals": {"5": 1}}}, {"content": "6. My language is not well learned.", "word_count": 9, "label": "", "sentence_structure": "Subject鈥揷opula鈥損redicative", "subjective_objective": {"answer": "Subjective", "rationale": "Because \"I\" in this sentence is the subjective view and feeling of the speaker, who believes that his language can no longer be further enhanced. This is not a description of objective facts or a summary of observations, but rather a perception and assessment of self-capacity, and is therefore a subjective examination."}, "sentence_sentiment": {"answer": "Negative", "rationale": "Because the entry \"language is not good\" expresses a negative attitude and a lack of self-confidence."}, "language_style": {"answer": "Calm and direct", "rationale": "The entry is more calm and straightforward in language style, with no excessive speech or emotional rendering, and is more objective in tone. It directly expresses the subject of asking if the other side considers it difficult to learn its own language."}, "time_dimension": {"answer": "Past", "rationale": "The sentence is an assessment of one's past abilities, and the tone is negative. The keyword \"bad learning\" suggests that this is an inalienable fact, namely, the poor learning of languages in the past. It can therefore be judged as the past."}, "sentence_type": "Negative declarative sentence", "pos": {"nouns": {"Languages": 1, "Learn": 1, "Okay": 1, "Yes": 1}, "verbs": {"be": 1, "not": 1}, "adverbs": {"I": 1}}}, {"content": "I have often been more relaxed.", "word_count": 9, "label": "", "sentence_structure": "Subject鈥揷opula鈥損redicative", "subjective_objective": {"answer": "Subjective", "rationale": "Because \"relax\" is the patient's self-perception and subjective attitude, which is viewed from a personal subjective perspective."}, "sentence_sentiment": {"answer": "Neutral", "rationale": "Because the entry does not have any emotional color."}, "language_style": {"answer": "Tactful and warm", "rationale": "The entry expresses a subjective feeling in a light, non-pressure-free way that the interviewee usually relaxes. This expression often appears in psychological questionnaires to encourage the interviewee to respond in a real and natural way. At the same time, using the expression \"always\" makes the answer more flexible and open, reducing the limits on the behaviour patterns of the interviewee."}, "time_dimension": {"answer": "Present", "rationale": "The keyword \"often\" means that it is a regular, customary act, and \"a more relaxed\" describes the attitude or state of the person being tested. The entry is therefore an inquiry into the person's current state, which is judged by the time dimension."}, "sentence_type": "Affirmative declarative sentence", "pos": {"verbs": {"be": 1, "Often": 1, "Comparison": 1, "Relax": 1}, "adverbs": {"very": 1, "of": 2}}}, {"content": "8. My parents are unhappy and disappointed by what I have done.", "word_count": 17, "label": "", "sentence_structure": "Subject鈥搗erb鈥搊bject", "subjective_objective": {"answer": "Objective", "rationale": "Because \"depressing parents and disillusioning\" is a fact, it is the result of the patient's behaviour, and it is therefore considered objectively."}, "sentence_sentiment": {"answer": "Negative", "rationale": "Because the behavior in the entry \"depressing and disillusioning parents\" is negative."}, "language_style": {"answer": "Tactful and warm", "rationale": "This entry uses more euphemistic and moderate language to avoid directly expressing dissatisfaction with parents, but instead describes the consequences of their actions in a more objective and neutral way."}, "time_dimension": {"answer": "Past", "rationale": ""}, "sentence_type": "Negative declarative sentence", "pos": {"nouns": {"parents": 2, "I": 1, "Behaviour": 1, "What you did": 1}, "verbs": {"鈾?And I'm gonna be 鈾?: 1, "Happy": 1, "Disappointment": 1}, "adjectives": {"Regular": 1}, "numerals": {"8": 1}}}, {"content": "In most courses, students come to me for help.", "word_count": 20, "label": "", "sentence_structure": "Subject鈥搗erb鈥搊bject", "subjective_objective": {"answer": "Subjective", "rationale": "Because \"you'll all come to me for help\" is a subjective assessment, which reflects the frequency and extent of other students who are seen to be seeking his help."}, "sentence_sentiment": {"answer": "Positive", "rationale": "Because the behavior in the entry, \"Call me for help,\" is a sign of approval and trust, a positive emotional color."}, "language_style": {"answer": "Tactful and warm", "rationale": "The language style of the entry is more euphemistic, using the words \"most\" and \"to help me\" without directly expressing praise or criticism of the individual. At the same time, the term \"column\" makes it more friendly and friendly and more acceptable to each other's views or suggestions."}, "time_dimension": {"answer": "Present", "rationale": "The entry describes what may happen in the future, i.e., in most future courses, students may come to me for help. So the time dimension is the future."}, "sentence_type": "Affirmative declarative sentence", "pos": {"nouns": {"Questionnaire Entry": 1, "Courses": 1, "Students": 2, "Help": 1}, "verbs": {"Learning": 1, "Find": 1, "Help": 1}, "adverbs": {"Most of them": 1, "Both": 1}, "numerals": {"9": 1}, "pronouns": {"I": 1}}}, {"content": "It's hard for me to be friends with people of the same sex.", "word_count": 14, "label": "", "sentence_structure": "Subject鈥揷opula鈥損redicative", "subjective_objective": {"answer": "Subjective", "rationale": "Because the difficulty of making friends depends on the patient's subjective feelings and experience."}, "sentence_sentiment": {"answer": "Negative", "rationale": "Because the description of \"hard\" is usually considered negative."}, "language_style": {"answer": "Tactful and warm", "rationale": "The entry starts with \"Is it?\" It shows that it is asking the interviewee about feelings or experiences. Moreover, the expression \"I have difficulty making friends with people of the same sex\" is more subtle and euphemismal, not directly expressing negative feelings or accusations, but in a gentle way, directing the interviewee to share his thoughts. These are in keeping with the euphemism of the language style."}, "time_dimension": {"answer": "Present", "rationale": "The keywords \"I'm friends with people of the same sex\" and \"is difficult\" describe the current feelings and experiences of the subjects, so the temporal dimension is now."}, "sentence_type": "Negative declarative sentence", "pos": {"nouns": {"Same sex": 1, "person": 1, "friends": 1}, "verbs": {"and": 1, "Hand it over": 1}, "adjectives": {"Difficulties": 1}, "numerals": {"10": 1}, "pronouns": {"I": 1}}}, {"content": "11. The opposite sex I like, but not me.", "word_count": 13, "label": "", "sentence_structure": "Subject鈥搗erb鈥搊bject", "subjective_objective": {"answer": "Subjective", "rationale": "Because \"like\" is a personal emotion and attitude, it's seen from the patient's subjective perspective."}, "sentence_sentiment": {"answer": "Negative", "rationale": "Because the emotional state of \"like the opposite sex and not like me\" in the entry is negative."}, "language_style": {"answer": "Calm and direct", "rationale": "The language style of the entry is more direct, with no excessive rhetoric or emotional rendering, and the testee is asked directly, in a concise and straightforward manner, whether he or she has ever met a person he or she likes, but not the other person."}, "time_dimension": {"answer": "Present", "rationale": "The sentence \"likes\" and \"buts\" can be judged to describe the current situation and feelings of the testee, and therefore the temporal dimension is now."}, "sentence_type": "Negative declarative sentence", "pos": {"nouns": {"Questionnaire": 1, "Entry": 1, "Heterosexual": 1}, "verbs": {"Yeah": 1, "But": 1}, "adverbs": {"Those": 1}, "numerals": {"11": 1}, "pronouns": {"I": 1}}}, {"content": "12. I often need help in learning mathematics.", "word_count": 13, "label": "", "sentence_structure": "Subject鈥搗erb鈥搊bject", "subjective_objective": {"answer": "Subjective", "rationale": "The basis for the judgement: this statement is an expression of the feelings and experiences of the patient in learning mathematics and is therefore viewed from the patient ' s subjective perspective."}, "sentence_sentiment": {"answer": "Negative", "rationale": "Because the \"needs help\" act is negative in the entry."}, "language_style": {"answer": "Calm and direct", "rationale": "The entry directly expresses the subject of difficulties that the subject of the study may encounter in learning mathematics in a concise language, without using excessive phrasing or emotional colours and with more objective and straightforward tone."}, "time_dimension": {"answer": "Present", "rationale": "\"In mathematics\" means a specific situation or state, and \"I often need help\" means that it's a customary act, so the time dimension of the article is now."}, "sentence_type": "Affirmative declarative sentence", "pos": {"nouns": {"Learning": 1, "Math": 1, "Help": 1}, "verbs": {"I": 1, "Yes": 1, "Provision": 1}, "adverbs": {"Regular": 1}, "numerals": {"12": 1}}}, {"content": "I look good.", "word_count": 8, "label": "", "sentence_structure": "Subject鈥揷opula鈥損redicative", "subjective_objective": {"answer": "Subjective", "rationale": "Because \"good-looking\" is a subjective assessment."}, "sentence_sentiment": {"answer": "Positive", "rationale": "Because the expression \"my face looks good\" in the entry is positive and positive about my appearance."}, "language_style": {"answer": "Tactful and warm", "rationale": "The entry describes in a positive and commendable manner the merits of the profile of the person under investigation, which is softly worded and encouraging, and makes it easier for the person under investigation to feel comfortable and enjoy it."}, "time_dimension": {"answer": "Present", "rationale": "The entry is for the testee鈥檚 view of his or her own appearance, and does not address past or future circumstances."}, "sentence_type": "Affirmative declarative sentence", "pos": {"nouns": {"Face": 1}, "verbs": {"- He's a big boy": 1, "Nice": 1}, "adjectives": {"Good": 1}, "numerals": {"13": 1}}}, {"content": "14. Overall, I feel like I'm a bad person.", "word_count": 12, "label": "", "sentence_structure": "Subject鈥揷opula鈥損redicative", "subjective_objective": {"answer": "Subjective", "rationale": "Because assessing one ' s own capacity is a subjective feeling and perception of the individual."}, "sentence_sentiment": {"answer": "Negative", "rationale": "Because the \"I feel bad\" rating in the entry is negative."}, "language_style": {"answer": "Calm and direct", "rationale": "The entry expresses self-evaluation of the subject in a direct and concise manner, with no excessive qualifier or emotional colour, and is more objective and inclement."}, "time_dimension": {"answer": "Present", "rationale": ""}, "sentence_type": "Negative declarative sentence", "pos": {"verbs": {"I think": 1, "Bad": 1}, "adjectives": {"very": 1}, "numerals": {"14": 1}, "pronouns": {"self": 1}}}, {"content": "I am honest.", "word_count": 5, "label": "", "sentence_structure": "Subject鈥揷opula鈥損redicative", "subjective_objective": {"answer": "Subjective", "rationale": "Because honesty is a subjective attitude or quality."}, "sentence_sentiment": {"answer": "Positive", "rationale": "Because honesty is a positive quality in the entries."}, "language_style": {"answer": "Calm and direct", "rationale": "The entry is more directly written, with no excessive emotional or rhetorical style. It directly asks whether the person surveyed thinks he or she is honest, expressing the researcher鈥檚 trust and respect for the person under investigation. Thus, its language style can be described as 鈥渃ooling straight鈥?"}, "time_dimension": {"answer": "Present", "rationale": ""}, "sentence_type": "Affirmative declarative sentence", "pos": {"nouns": {"I": 1, "Honesty": 1}, "verbs": {"be": 1}}}, {"content": "16. I am required to participate in hard physical exercise.", "word_count": 12, "label": "", "sentence_structure": "Subject鈥搗erb鈥揷omplement", "subjective_objective": {"answer": "Subjective", "rationale": "Because words like \"remember\" or \"remember\" are usually personal subjective feelings and memories."}, "sentence_sentiment": {"answer": "Negative", "rationale": "Because the behavior in the entry \"not to take part in laborious physical exercise\" is negative."}, "language_style": {"answer": "Tactful and warm", "rationale": "The language style of the entry is euphemism. It expresses a gentle and inviting tone of attitude and expectation towards the interviewee ' s involvement in physical exercise, avoiding orders or outright denials, and makes people feel more comfortable and willing to accept it."}, "time_dimension": {"answer": "Present", "rationale": "The entry asks if the person tested is willing to participate in a labour-intensive exercise, which is now judged in terms of time dimension."}, "sentence_type": "Negative declarative sentence", "pos": {"nouns": {"I": 1, "Physical exercise": 1}, "verbs": {"Okay": 1, "Participation": 1, "- It's hard": 1}, "adverbs": {"Badge": 1}, "classifiers": {"of": 2}}}, {"content": "17. I would like to take language classes.", "word_count": 7, "label": "", "sentence_structure": "Subject鈥揷opula鈥損redicative", "subjective_objective": {"answer": "Subjective", "rationale": "Because \"I'd love\" is the patient's personal feelings and attitude."}, "sentence_sentiment": {"answer": "Positive", "rationale": "Because the attitude of \"I'd like to take language classes\" in the entry is positive."}, "language_style": {"answer": "Tactful and warm", "rationale": ""}, "time_dimension": {"answer": "Present", "rationale": "The entry \"intended\" expresses the current emotional state and orientation of the subject, while \"language classes\" refers to the conduct or activity that is being carried out. Thus, the entry relates to the current situation."}, "sentence_type": "Affirmative declarative sentence", "pos": {"nouns": {"Language classes": 1}, "verbs": {"Yes": 1, "Go, go, go": 1}, "adverbs": {"very": 1}}}, {"content": "I'm too careful.", "word_count": 5, "label": "", "sentence_structure": "Subject鈥搗erb鈥揷omplement", "subjective_objective": {"answer": "Subjective", "rationale": "Because worry is a psychological state of a person, it is a subjective feeling."}, "sentence_sentiment": {"answer": "Negative", "rationale": "Because \"too much care\" in the entry indicates a state of excessive concern and is negative."}, "language_style": {"answer": "Calm and direct", "rationale": "The entry directly expresses the question whether the other party is often over-concerned, is not based on excessive rhetorical or emotional rendering, and is more objective in tone."}, "time_dimension": {"answer": "Present", "rationale": "The entry describes the testee's behaviour or mentality as being overly worrying in his current state, and thus judges it as \"now\"."}, "sentence_type": "Negative declarative sentence", "pos": {"nouns": {"Questionnaire": 1, "Entry": 1}, "verbs": {"Analysis": 1, "Too much": 1, "Fear": 1}, "numerals": {"18": 1}}}, {"content": "I have a good relationship with my parents.", "word_count": 9, "label": "", "sentence_structure": "Subject鈥搗erb鈥揷omplement", "subjective_objective": {"answer": "Subjective", "rationale": "The examination is a subjective one, since it is the patient ' s perception and feelings of his/her relationship with his/her parents."}, "sentence_sentiment": {"answer": "Positive", "rationale": "Because the expression \"good with\" is positive."}, "language_style": {"answer": "Calm and direct", "rationale": "The entry uses simple and straightforward language to express the subject of questioning whether the other party is in harmony with his or her parents, and does not use excessive rhetorical or emotional rendering, which is more objective and balanced."}, "time_dimension": {"answer": "Present", "rationale": ""}, "sentence_type": "Affirmative declarative sentence", "pos": {"nouns": {"19": 1, "I": 1, "parents": 1}, "verbs": {"Get along": 1, "Okay": 1}}}, {"content": "I'm stupid, so I can't go to college.", "word_count": 10, "label": "", "sentence_structure": "Subject鈥揷opula鈥損redicative", "subjective_objective": {"answer": "Subjective", "rationale": "Because \"I'm stupid\" is a patient's subjective judgment, not an objective fact."}, "sentence_sentiment": {"answer": "Negative", "rationale": "Because the expression \"very stupid\" in the entry is negative and linked to university enrolment, a negative attitude is expressed in general."}, "language_style": {"answer": "Tactful and warm", "rationale": "The language style of the entry is more euphemistic and personal. It uses self-demeaning expressions that give people a sense of proximity and understanding, and that do not directly deny or accuse each other of their abilities or efforts, but rather try to create a positive atmosphere of support and encouragement. Such expressions are more appropriate for questionnaires or other contexts where the interviewee needs to be respected and understood."}, "time_dimension": {"answer": "Present", "rationale": "The entry is an indication of the present thoughts and beliefs of the testees, the keywords being \"I'm stupid\" and \"I can't go to college\", all pointing to the current situation."}, "sentence_type": "Negative declarative sentence", "pos": {"nouns": {"University": 1, "Stupid": 1}, "verbs": {"I can't get in": 1, "very": 1, "be": 1}, "adjectives": {"Stupid": 1}, "adverbs": {"very": 1}, "numerals": {"20": 1}, "pronouns": {"I": 1}}}, {"content": "21. It is easy for me to make friends with boys.", "word_count": 11, "label": "", "sentence_structure": "Subject鈥揷opula鈥損redicative", "subjective_objective": {"answer": "Subjective", "rationale": "Because it is the patient ' s subjective feeling or attitude to make friends, dealing with boys is also a patient ' s choice and is therefore subjectively viewed."}, "sentence_sentiment": {"answer": "Positive", "rationale": "Because the \"easy to make friends with boys\" act is positive."}, "language_style": {"answer": "Tactful and warm", "rationale": "The language style of the entry is euphemistic. It asks the respondents, in an invitation-style tone, whether it is easy to establish friendships with men, rather than direct orders or statements. Moreover, the use of the term \"friend\" avoids gender stereotypes. This expression respects the individual feelings of the respondents, but also makes them comfortable and willing to share their ideas."}, "time_dimension": {"answer": "Present", "rationale": "\"I'm easy to make friends with boys\" describes the situation, which does not relate to the time dimension of the past or the future. In it, \"I'm easy to express subjective feelings or behavioural tendencies,\" and \"friending boys\" is an objective expression of behaviour."}, "sentence_type": "Affirmative declarative sentence", "pos": {"nouns": {"Questionnaire Entry": 1, "I": 1, "Boys": 1, "friends": 1}, "verbs": {"Easy": 1, "Hand it over": 1}, "adjectives": {"very": 1}}}, {"content": "22. It is easy for me to make friends with girls.", "word_count": 11, "label": "", "sentence_structure": "Subject鈥揷opula鈥損redicative", "subjective_objective": {"answer": "Subjective", "rationale": "Because \"easy\" is the patient's subjective feeling or opinion."}, "sentence_sentiment": {"answer": "Positive", "rationale": "Because the behavior in the entry \"Easy to be friends with girls\" is positive."}, "language_style": {"answer": "Tactful and warm", "rationale": "The words of the entry are more euphemistic, use the word \"easy\" to express its social abilities, and do not directly express self-confidence or superiority. At the same time, the more specific expression of the term \"girl\" rather than \"girl\" is more friendly and friendly."}, "time_dimension": {"answer": "Present", "rationale": "In the future, the entry \"slowly\" expresses hope and confidence in the future, and the key word is \"easy to make friends with girls\", all pointing to situations that have not yet occurred and that may arise in the future."}, "sentence_type": "Exclamatory sentence", "pos": {"nouns": {"Girls": 1, "friends": 1}, "verbs": {"Easy": 1, "Hand it over": 1}, "adjectives": {"very": 1}, "numerals": {"22": 1}, "pronouns": {"I": 1}}}, {"content": "I'd like to take math classes.", "word_count": 7, "label": "", "sentence_structure": "Subject鈥揷opula鈥損redicative", "subjective_objective": {"answer": "Subjective", "rationale": "Because \"I want\" is the patient's subjective feeling and attitude, reflecting the patient's positive feelings and willingness to take math classes."}, "sentence_sentiment": {"answer": "Positive", "rationale": "Because the \"deep\" in the entry is a positive, positive emotional attitude."}, "language_style": {"answer": "Tactful and warm", "rationale": "The language style of the entry is euphemistic, because it uses the expression \"intense\" to convey the attitudes and aspirations of the other party to mathematics classes, without being too direct or strongly required. At the same time, the term \"class\" does not use negative words or negative sentences, making the whole expression more positive and friendly."}, "time_dimension": {"answer": "Present", "rationale": "Now the entry is an expression of the current thoughts and aspirations of the testee, and \"deep\" is a term for a strong emotional expression, which emphasizes the testee's strong desire to attend math classes. So the time dimension of the entry is now judged."}, "sentence_type": "Affirmative declarative sentence", "pos": {"verbs": {"Yes": 1, "Go, go, go": 1}, "adjectives": {"very": 1}, "numerals": {"23": 1}}}, {"content": "Most of my friends look better than me.", "word_count": 14, "label": "", "sentence_structure": "Subject鈥搗erb鈥揷omplement", "subjective_objective": {"answer": "Subjective", "rationale": "Because \"looking better\" is a relative description, depending on the subjective view and criteria of the observer. For different people, the assessment of a person's appearance may differ. So the answer to this questionnaire needs to be based on the subjectivity and opinion of the interviewee himself, and is therefore subjective."}, "sentence_sentiment": {"answer": "Neutral", "rationale": "As the entry does not express a positive, negative or neutral emotional tendency, it merely presents a fact."}, "language_style": {"answer": "Tactful and warm", "rationale": "The language style of the entry is euphemism. It is not \"your friends are better looking than you\" but it is expressed in a more euphemism, so that the person under investigation is not uncomfortable or embarrassed. At the same time, it is more friendly and close and acceptable."}, "time_dimension": {"answer": "Present", "rationale": "The entry describes the current situation and state of the testee, and \"better looking\" is a fact that happened in the past, but evaluates and senses the current state. So the temporal dimension is now."}, "sentence_type": "Affirmative declarative sentence", "pos": {"nouns": {"friends": 1, "person": 1}, "verbs": {"Long": 1, "- Yeah": 1, "Nice": 1}, "adjectives": {"More": 1, "Most of them": 1}, "adverbs": {"More": 1}, "numerals": {"24": 1}, "pronouns": {"I": 1}}}, {"content": "Most of what I did was good.", "word_count": 13, "label": "", "sentence_structure": "Subject鈥搗erb鈥揷omplement", "subjective_objective": {"answer": "Subjective", "rationale": "Because \"good work\" is an evaluation, a subjective view of the patient."}, "sentence_sentiment": {"answer": "Positive", "rationale": "Because the words in the entry, \"I've done most of the right\" are positive emotions and attitudes."}, "language_style": {"answer": "Tactful and warm", "rationale": "The language style of the entry is more euphemistic and soft, using positive tone and expression to guide respondents to answer questions, without too many negatives or criticisms. This expression helps create a positive, friendly and comfortable atmosphere, which makes it easier for respondents to share real ideas and feelings."}, "time_dimension": {"answer": "Present", "rationale": ""}, "sentence_type": "Affirmative declarative sentence", "pos": {"verbs": {"Do it": 2, "Okay": 2}, "adjectives": {"Large": 1, "More": 1}, "adverbs": {"Both": 1}, "numerals": {"25": 1}, "pronouns": {"I": 1}}}, {"content": "26. I lie sometimes to avoid trouble.", "word_count": 10, "label": "", "sentence_structure": "Subject鈥搗erb鈥搊bject", "subjective_objective": {"answer": "Subjective", "rationale": "Subjectivity, because \"false\" is a subjective act, which depends on the individual's intentions and attitudes. While lying may affect objective facts, the question is whether patients prefer to use this method to avoid trouble, which is the result of their subjective ideas and behaviour choices."}, "sentence_sentiment": {"answer": "Negative", "rationale": "Because the entry \"Leave to avoid trouble\" is negative."}, "language_style": {"answer": "Tactful and warm", "rationale": "The language style of the entry is euphemism, because it does not directly accuse or criticize the interviewee for lying about it, but rather expresses the issue in a more moderate and understanding way. Moreover, it uses the term \"avoid trouble,\" which further suggests that people may choose to lie out of fear of conflict or other problems. Overall, the language style of the entry gives people a more cordial and inclusive sense of being afloat."}, "time_dimension": {"answer": "Present", "rationale": "The keywords \"avoid trouble\" and \"sometimes lie\" suggest that it is customary and frequent and should therefore be judged as the present state of affairs."}, "sentence_type": "Negative declarative sentence", "pos": {"nouns": {"Trouble": 1, "Lies": 1}, "verbs": {"Avoid": 1, "Say it": 1}, "adverbs": {"Sometimes": 1}, "numerals": {"26": 1}}}, {"content": "I've been looking for sports, gymnastics and dances.", "word_count": 14, "label": "", "sentence_structure": "Subject鈥搗erb鈥搊bject", "subjective_objective": {"answer": "Subjective", "rationale": "Because I am good at sports, gymnastics and dances, which express the feelings of the patient himself, and from a subjective perspective."}, "sentence_sentiment": {"answer": "Positive", "rationale": "Because the entry \"very good at sports, gymnastics and dance\" is a positive expression and description."}, "language_style": {"answer": "Tactful and warm", "rationale": "The language style of the entry is more euphemistic and personal, using the expression \"very good\" to express the respondent's confidence in and modesty in his or her own evaluation. At the same time, the \"sport, gymnastics and dances\" do not specify which activity, and the open description is more gentle and friendly, consistent with the investigative nature of the subject."}, "time_dimension": {"answer": "Present", "rationale": "The keyword \"very good\" indicates that it describes the current level of skills and abilities, and \"sports, gymnastics, dances, etc.\" is the specific type of activity. So the time dimension of the article is now."}, "sentence_type": "Affirmative declarative sentence", "pos": {"nouns": {"Sports": 1, "Gymnastics": 1, "Dance": 1, "Activities": 1}, "verbs": {"I": 1, "Very good": 1, "Search": 1, "Wait": 1}, "adverbs": {"Long": 1, "Search": 1}, "numerals": {"27": 1}, "classifiers": {"individual": 1}}}, {"content": "I have always been a bad candidate in tests that require reading skills.", "word_count": 17, "label": "", "sentence_structure": "Subject鈥揷opula鈥損redicative", "subjective_objective": {"answer": "Subjective", "rationale": "Because \"always bad\" is the patient's subjective feeling and evaluation."}, "sentence_sentiment": {"answer": "Negative", "rationale": "Because the expression \"always bad in the test of reading skills\" in the entry expresses negative emotions and attitudes."}, "language_style": {"answer": "Calm and direct", "rationale": "The language style of the entry is relatively cold and straightforward, with a preponderant presentation of facts, and there is no excessive emotional or speech-reading, medium-temperature and moderateness, which are suitable for use in official questionnaires."}, "time_dimension": {"answer": "Present", "rationale": "\"Always\" indicates that this is a constant situation, and \"tests of reading skills\" indicates that this is a current situation. The entry is therefore asking the testee about the current reading ability performance, which is the current time dimension."}, "sentence_type": "Negative declarative sentence", "pos": {"nouns": {"Questionnaire": 1, "Entry": 1, "Test": 1, "Capacity": 1, "Always": 1}, "verbs": {"Reading": 1, "Yes": 1, "Examination": 1, "Okay": 1, "be": 1}, "adverbs": {"in": 1, "Medium": 1, "I": 1, "Always": 1, "of": 2, "Not good": 1}, "numerals": {"28": 1}}}, {"content": "29. I am not easily mourning.", "word_count": 6, "label": "", "sentence_structure": "Subject鈥揷opula鈥損redicative", "subjective_objective": {"answer": "Subjective", "rationale": "Because mourning is an emotional response, it is the patient ' s subjective feeling and attitude."}, "sentence_sentiment": {"answer": "Positive", "rationale": "Because the attitude of \"unremarkable mourning\" in the entry is positive."}, "language_style": {"answer": "Calm and direct", "rationale": "The entry uses direct and concise language to ask the interviewees whether they often feel mourning and do not have too many emotional or rhetorical techniques, so that the language style is more calm and straightforward."}, "time_dimension": {"answer": "Future", "rationale": "The keyword \"uneasy\" indicates an attitude or preference that may continue in the future. The entry is therefore to ask if the testee will remain unsatisfied in the future. The temporal dimension is judged in terms of the future."}, "sentence_type": "Negative declarative sentence", "pos": {"verbs": {"Grief": 1}, "adjectives": {"Easy": 1}}}, {"content": "30. I have had difficulty talking to my parents.", "word_count": 9, "label": "", "sentence_structure": "Subject鈥揷opula鈥損redicative", "subjective_objective": {"answer": "Subjective", "rationale": "This is because patients feel and evaluate subjectively speaking to their parents."}, "sentence_sentiment": {"answer": "Negative", "rationale": "Because the phrase \"discussion is difficult\" in the entry is negative."}, "language_style": {"answer": "Tactful and warm", "rationale": "The entry is based on a more moderate expression of the theme, \"I speak with my parents difficult\" , which is not a direct reference to the problem or to the blame, but rather a more neutral and peaceful expression, which is more friendly and friendly, and more easily accessible to the understanding and support of the other. At the same time, the language of the article is more concise, with little redundant information, which enables readers to understand the subject quickly."}, "time_dimension": {"answer": "Past", "rationale": "The verb contained in the entry is \"talk\" , which indicates that the act or state of the past has occurred, and that the adjective \"very difficult\" is used to describe the state of the act, further proving that it is a description of the past. Thus, the temporal dimension of the purpose is a judgement of the past."}, "sentence_type": "Negative declarative sentence", "pos": {"nouns": {"30": 1, "I": 1, "parents": 1, "Talk": 1, "Difficulties": 1}, "adjectives": {"very": 1}}}, {"content": "31. If I really tried to learn, I would be one of the best students in the same grade.", "word_count": 25, "label": "", "sentence_structure": "Subject鈥揷opula鈥損redicative", "subjective_objective": {"answer": "Subjective", "rationale": "Because this expression expresses the individual beliefs and expectations of the patient, it falls within the realm of subjective attitudes."}, "sentence_sentiment": {"answer": "Positive", "rationale": "Because the words \"working hard to learn\" and \"one of the best students\" in the entries are positive emotional."}, "language_style": {"answer": "Tactful and warm", "rationale": "The language style of the entry is euphemism, because it uses the words of advice and encouragement. It does not direct orders or blame each other, but rather offers a possibility and suggests that if the other side works hard to learn, it can achieve that goal. This is more moderate and inspiring."}, "time_dimension": {"answer": "Present", "rationale": "Now, the entry describes the current situation and attitude, the keywords being \"real effort\" and \"will become,\" which all indicate that it is a hypothetical, unprevented act or result. And the time word \"if\" in the sentence indicates that it is a conditional statement, describing the current idea and plan. So the time dimension of this questionnaire entry is now."}, "sentence_type": "Affirmative declarative sentence", "pos": {"nouns": {"Work hard to learn": 1, "Students": 1, "Same grade": 1}, "verbs": {"鈾?Will be 鈾?: 1, "Learning": 1}, "adjectives": {"Best": 2}, "adverbs": {"Really": 1, "One of them": 1}, "numerals": {"31": 1}, "classifiers": {"One": 1, "of": 2}, "pronouns": {"I": 1}}}, {"content": "32. Not many of those of the same sex like me.", "word_count": 13, "label": "", "sentence_structure": "Subject鈥搗erb鈥揷omplement", "subjective_objective": {"answer": "Subjective", "rationale": "Because \"like\" is a subjective emotion, and \"not many of the same sex like me\" is the patient's subjective perception and feelings."}, "sentence_sentiment": {"answer": "Negative", "rationale": "Because the phrase \"not much like me\" in the entry is negative, expressing a negative opinion of yourself."}, "language_style": {"answer": "Calm and direct", "rationale": "The language style of the entry is relatively calm and straightforward, and it clearly expresses the subject of questioning whether the other party has been left out in the same sex population, without excessive rhetoric or emotional rendering."}, "time_dimension": {"answer": "Present", "rationale": "According to the keyword \"like\", it can be seen that the entry is now judged in terms of time dimension by asking the testee's perception and feelings about his popularity among the same sex population."}, "sentence_type": "Negative declarative sentence", "pos": {"nouns": {"Questionnaire": 1, "person": 2, "Gender": 1, "Yeah": 1}, "verbs": {"Medium": 1, "Yeah": 1}, "adjectives": {"Not much": 1}, "adverbs": {"Same": 1}, "numerals": {"32": 1}, "classifiers": {"individual": 1}, "pronouns": {"I": 1}}}, {"content": "33. Not much of a heterosexual person like me.", "word_count": 12, "label": "", "sentence_structure": "Subject鈥搗erb鈥揷omplement", "subjective_objective": {"answer": "Subjective", "rationale": "The problem is viewed from the patient ' s subjective perspective because of the personal feelings and attitudes that a person feels or does not like being a patient."}, "sentence_sentiment": {"answer": "Negative", "rationale": "Because the description of \"likes not much of the opposite sex\" in the entry is negative, suggesting that the interviewee may feel alone or not be admitted."}, "language_style": {"answer": "Calm and direct", "rationale": "The entry asks the other side in a more direct and clear manner whether it feels that it is less different and that it is more objective and neutral in its linguistic style."}, "time_dimension": {"answer": "Present", "rationale": ""}, "sentence_type": "Affirmative declarative sentence", "pos": {"nouns": {"Heterosexual": 1, "- To be with someone": 1}, "verbs": {"Yeah": 1, "Not much": 1}, "adjectives": {"A lot": 1}, "adverbs": {"very": 1}, "numerals": {"33": 1}, "pronouns": {"I": 1}}}, {"content": "34. I have difficulty understanding any question related to mathematics.", "word_count": 16, "label": "", "sentence_structure": "Subject鈥揷opula鈥損redicative", "subjective_objective": {"answer": "Subjective", "rationale": "Because the question of whether to understand mathematics is the subjective and cognitive experience of the patient."}, "sentence_sentiment": {"answer": "Negative", "rationale": "Because the expression \"ununderstandable\" in the entry is negative."}, "language_style": {"answer": "Calm and direct", "rationale": "The entry is more straightforward, less simplistic or emotional, and more objective. It directly expresses the subject of the other side鈥檚 difficulties in mathematics, without euphemism or implicit expression."}, "time_dimension": {"answer": "Present", "rationale": "The entry describes the current status or situation of the person being tested, with no clear time indication, and therefore is judged to be \"now\"."}, "sentence_type": "Negative declarative sentence", "pos": {"nouns": {"Math": 1, "Problem": 2}, "verbs": {"Both": 1, "It's hard to understand": 1}, "adjectives": {"Any": 1, "Relevant": 1}, "adverbs": {"Hard": 1}, "numerals": {"34": 1}}}, {"content": "I look good.", "word_count": 5, "label": "", "sentence_structure": "Subject鈥揷opula鈥損redicative", "subjective_objective": {"answer": "Subjective", "rationale": "Because \"good looking\" is a subjective self-evaluation and perception, different views of the same appearance may differ."}, "sentence_sentiment": {"answer": "Neutral", "rationale": "Because the entry does not express a positive or negative emotional attitude, it merely describes an objective fact."}, "language_style": {"answer": "Calm and direct", "rationale": "The entry directly expresses the subject of questioning whether the other party considers himself to be a good-looking person, without using excessive rhetorical or emotional rendering, and with a more objective tone.\""}, "time_dimension": {"answer": "Past", "rationale": "At the same time, the entry does not contain elements such as behaviour or mentalities that are clearly identified and are therefore not suitable for use in psychological questionnaire studies."}, "sentence_type": "Affirmative declarative sentence", "pos": {"verbs": {"- He's a big boy": 1, "Okay": 1}, "adjectives": {"Nice": 1}}}, {"content": "36. None of what I did seems to be right.", "word_count": 14, "label": "", "sentence_structure": "Subject鈥揷opula鈥損redicative", "subjective_objective": {"answer": "Subjective", "rationale": "This is because it expresses patients ' subjective feelings and perceptions of their abilities or decision-making."}, "sentence_sentiment": {"answer": "Negative", "rationale": "Because the expression \"Nothing is right\" in the entry is negative and implies lower self-evaluation."}, "language_style": {"answer": "Tactful and warm", "rationale": "The language style of the entry is more euphemistic and implicit, giving a sense of affection, understanding and support by asking if the other side has ever thought of it, rather than directly judging or evaluating whether the other side's behaviour is correct. This expression also avoids the possibility of the other side being criticized or blamed, and helps to create a good atmosphere of communication."}, "time_dimension": {"answer": "Present", "rationale": ""}, "sentence_type": "Negative declarative sentence", "pos": {"nouns": {"Yes": 1, "Annex": 1, "Correct": 1}, "verbs": {"Do it": 1, "It looks like": 1, "No, I'm not": 1, "be": 2}, "adjectives": {"Every piece": 1}, "adverbs": {"Both": 1, "It looks like": 1}, "numerals": {"36": 1}, "pronouns": {"I": 1}}}, {"content": "37. I always tell the truth.", "word_count": 6, "label": "", "sentence_structure": "Subject鈥揷opula鈥損redicative", "subjective_objective": {"answer": "Subjective", "rationale": "Because \"always telling the truth\" is a patient's self-evaluation and attitude, and it is a subjective examination."}, "sentence_sentiment": {"answer": "Neutral", "rationale": "Because the entry merely expresses a fact and does not express any feelings or attitudes."}, "language_style": {"answer": "Calm and direct", "rationale": "The language style of the entry is more direct and concise, with no excessive rhetoric or emotional colour, but rather a direct expression of the subject of questioning the other party about whether to speak the truth more often, and is more objective in tone. The language style of the entry is therefore a straightforward type of calm."}, "time_dimension": {"answer": "Present", "rationale": "The keyword is \"always speaking,\" which means it's a customary act of the subject, which happens now."}, "sentence_type": "Affirmative declarative sentence", "pos": {"verbs": {"Speak": 1, "The truth": 1}, "adjectives": {"Total": 1}, "numerals": {"37": 1}}}, {"content": "38. I am clumsy in activities such as sports, gymnastics and dance.", "word_count": 16, "label": "", "sentence_structure": "Subject鈥揷opula鈥損redicative", "subjective_objective": {"answer": "Subjective", "rationale": "Because \"pussy\" is a personal feeling or an assessment, not an objective fact. While some might think that feeling is accurate, it is still a subjective experience."}, "sentence_sentiment": {"answer": "Negative", "rationale": "Because the entries describe their own shortcomings, they are a negative self-evaluation."}, "language_style": {"answer": "Tactful and warm", "rationale": ""}, "time_dimension": {"answer": "Past", "rationale": ""}, "sentence_type": "Negative declarative sentence", "pos": {"nouns": {"Sports": 1, "Gymnastics": 1, "Dance": 1}, "verbs": {"very": 1, "Clumsy": 1}, "numerals": {"38": 1}}}, {"content": "39. It is easy for me to learn languages.", "word_count": 10, "label": "", "sentence_structure": "Subject鈥揷opula鈥損redicative", "subjective_objective": {"answer": "Subjective", "rationale": "Because \"easy\" is a subjective evaluation, which shows how people feel and feel about learning the language."}, "sentence_sentiment": {"answer": "Positive", "rationale": "Because the expression \"it's easy for me to learn languages\" has a positive emotional effect."}, "language_style": {"answer": "Tactful and warm", "rationale": "The entry uses a positive and self-affirming tone of self-confidence and optimism in learning the language, which is often inspiring and inspiring. At the same time, the first person is called \"I\" and increases the interactive nature of the dialogue and gives people a sense of affection."}, "time_dimension": {"answer": "Past", "rationale": ""}, "sentence_type": "Affirmative declarative sentence", "pos": {"nouns": {"I": 1, "Languages": 1}, "verbs": {"Learn": 1, "Easy": 1}, "adverbs": {"very": 1}, "numerals": {"39": 1}}}, {"content": "I am often depressed and worried.", "word_count": 11, "label": "", "sentence_structure": "Subject鈥搗erb鈥揷omplement", "subjective_objective": {"answer": "Subjective", "rationale": "Because emotional and psychological state like depression, anxiety, and so on, is the patient's subjective feeling."}, "sentence_sentiment": {"answer": "Negative", "rationale": "Because the description of \"depressed\" and \"crazed\" in the entries both expressed negative emotional attitudes."}, "language_style": {"answer": "Calm and direct", "rationale": "The language style of the entry is a more straightforward type of calm, as it directly asks whether the person surveyed is often depressed and depressed, without using excessive rhetorical techniques or emotional rendering."}, "time_dimension": {"answer": "Present", "rationale": "In the entry, \"often\" indicates that it is a customary act and that it is happening. \"Deepness, anxiety\" describes the current state of mind and points to the present. So, the time dimension of this questionnaire is now."}, "sentence_type": "Affirmative declarative sentence", "pos": {"nouns": {"I": 1, "Depressive": 1, "I'm worried": 1}, "verbs": {"Regular": 1, "be": 1}, "adjectives": {"Declining": 1, "I'm worried": 1}, "adverbs": {"Regular": 1}, "numerals": {"40": 1}}}, {"content": "41. My parents treated me fairly.", "word_count": 9, "label": "", "sentence_structure": "Subject鈥搗erb鈥揷omplement", "subjective_objective": {"answer": "Objective", "rationale": "Since \"justice\" is a description of the facts, it does not involve personal feelings or attitudes, and is therefore considered from an objective perspective."}, "sentence_sentiment": {"answer": "Positive", "rationale": "The basis for the judgment: \"Parents treat me fairly\" in the entry expresses a positive emotional attitude, which shows that the interviewee believes that his parents are doing justice to themselves. Thus, the emotional color of the purpose is positive."}, "language_style": {"answer": "Tactful and warm", "rationale": "The entry uses a soft tone and positive emotional term \"justice\" designed to make the interviewee feel respected and understood. This euphemism and warm language helps to create a good investigative atmosphere and makes the interviewee more willing to share their views and experiences."}, "time_dimension": {"answer": "Present", "rationale": "The entry expresses the subjective perception of the present state of affairs of the person being tested, describes the present situation and views and therefore judges the present."}, "sentence_type": "Affirmative declarative sentence", "pos": {"nouns": {"parents": 2, "I": 1}, "verbs": {"- Wait": 1, "very": 1, "Justice": 1}, "adjectives": {"Justice": 1}, "adverbs": {"very": 1}, "numerals": {"41": 1}}}, {"content": "42. Most of my courses have had poor results.", "word_count": 10, "label": "", "sentence_structure": "Subject鈥揷opula鈥損redicative", "subjective_objective": {"answer": "Objective", "rationale": "Because it is an objective fact, not a subjective attitude or feeling, that patients are describing their academic performance."}, "sentence_sentiment": {"answer": "Negative", "rationale": "Because the expression \"bad grades\" in the entries is negative."}, "language_style": {"answer": "Calm and direct", "rationale": "The language style of the entry is calm and straightforward, and it directly depicts the performance of the person under investigation, without any rhetorical or emotional rendering."}, "time_dimension": {"answer": "Present", "rationale": "The entry asks about the current learning status of the testee, and the time dimension is now judged. Key words \"I\" and \"most of the poor\" indicate that the testee describes his current academic status, is an answer to the current state, and therefore the judgment is \"now\"."}, "sentence_type": "Negative declarative sentence", "pos": {"nouns": {"Courses": 1, "Achievements": 2, "Bad": 1}, "verbs": {"Access": 1}, "adjectives": {"Most of them": 1}, "numerals": {"42": 1}, "pronouns": {"I": 1}}}, {"content": "43. Boys like me.", "word_count": 8, "label": "", "sentence_structure": "Subject鈥搗erb鈥搊bject", "subjective_objective": {"answer": "Subjective", "rationale": "Because \"like me\" is a subjective feeling and an assessment. Although this sentence refers to the male group, it is based on personal feelings rather than objective facts."}, "sentence_sentiment": {"answer": "Positive", "rationale": "Because the \"like me\" in the entry indicates that the boy's attitude towards me is positive."}, "language_style": {"answer": "Tactful and warm", "rationale": "The entry describes the phenomenon of \"boys like me\" rather than asking the other side whether it has such characteristics or behaviours, expressing concern and curiosity about the subject in a more moderate and implicit way. At the same time, this expression avoids the pressures or discomfort that may arise from the other side, and makes it easier for the other side to share its thoughts and feelings."}, "time_dimension": {"answer": "Present", "rationale": "The sentence expresses the current state or fact and does not contain information about the past or the future, and therefore is judged to be \"now\"."}, "sentence_type": "Affirmative declarative sentence", "pos": {"nouns": {"Boys": 1, "I": 1}, "verbs": {"Yeah": 1}, "numerals": {"43": 1}}}, {"content": "44. The girls like me.", "word_count": 8, "label": "", "sentence_structure": "Subject鈥搗erb鈥揷omplement", "subjective_objective": {"answer": "Subjective", "rationale": "Because it is the subjective feeling and perception of an individual that is attracted or favoured, it is not an objective fact."}, "sentence_sentiment": {"answer": "Positive", "rationale": "Because the expression \"girls like me\" conveys a feeling of love, popularity, positive emotional expression."}, "language_style": {"answer": "Tactful and warm", "rationale": "The entry uses the first person's name, \"I\" to draw the attention of the respondents and to create interaction, while a more subtle and euphemistic expression of the subject that the respondents may like is a female. This expression makes it easier to resonate and engage the respondents, thereby increasing the rate of recovery of questionnaires and effective access to information."}, "time_dimension": {"answer": "Present", "rationale": "The sentence expresses a subjective feeling or view of the person speaking, and the temporal dimension is now judged."}, "sentence_type": "Affirmative declarative sentence", "pos": {"nouns": {"Girls": 1, "I": 1}, "verbs": {"Yeah": 1}, "adverbs": {"Both": 1}, "numerals": {"44": 1}}}, {"content": "I like math.", "word_count": 5, "label": "", "sentence_structure": "Subject鈥損redicate", "subjective_objective": {"answer": "Subjective", "rationale": "Like being patient subjective."}, "sentence_sentiment": {"answer": "Positive", "rationale": "Because \"like\" in the entry is a positive emotional attitude."}, "language_style": {"answer": "Tactful and warm", "rationale": ""}, "time_dimension": {"answer": "Present", "rationale": "\"Assisting on mathematics\" is a current hobby or hobby, so the entry is a description of the person being tested's current behavior or mentality."}, "sentence_type": "Affirmative declarative sentence", "pos": {"nouns": {"Math": 1}, "verbs": {"Yeah": 1}}}, {"content": "I look ugly.", "word_count": 5, "label": "", "sentence_structure": "Subject鈥揷opula鈥損redicative", "subjective_objective": {"answer": "Subjective", "rationale": "Because \"unseemous\" is self-evaluation, subjective."}, "sentence_sentiment": {"answer": "Negative", "rationale": "Because \"unseemous\" in the entries is a negative assessment of their appearance and has a negative emotional connotation."}, "language_style": {"answer": "Calm and direct", "rationale": "The entry directly expresses the question whether the other party feels ugly, does not use excessive rhetoric or emotional rendering, and is more objective in tone."}, "time_dimension": {"answer": "Present", "rationale": "\"A bad look\" is an assessment of the person's appearance, a verb of behaviour that is now a state of the time, and the entry therefore describes the person's current perception and feelings of his or her own appearance."}, "sentence_type": "Negative declarative sentence", "pos": {"nouns": {"Questionnaire Entry": 1, "I": 1, "It's ugly": 1}, "verbs": {"Okay": 1}, "adjectives": {"He looks ugly": 1}}}, {"content": "47. In general, I'm doing the most of the right things.", "word_count": 17, "label": "", "sentence_structure": "Subject鈥揷opula鈥損redicative", "subjective_objective": {"answer": "Subjective", "rationale": "Because this is a subjective attitude to patients ' own assessment and perception."}, "sentence_sentiment": {"answer": "Positive", "rationale": "Because the entry expresses a certain degree of certainty and confidence in their conduct."}, "language_style": {"answer": "Tactful and warm", "rationale": "The entry is presented in a self-proclaimed manner, expressing the positive opinion of the interviewees about themselves and avoiding too much absolute language, making the issue more objective and neutral. Moreover, the use of the question phrase has increased interaction with the respondents and made the questionnaire more friendly."}, "time_dimension": {"answer": "Present", "rationale": ""}, "sentence_type": "Affirmative declarative sentence", "pos": {"nouns": {"Things": 1, "Most of them": 1}, "verbs": {"Do it": 1, "be": 1, "to": 1, "Both": 1}, "adjectives": {"Overall": 1, "Most of them": 1}, "adverbs": {"Extreme": 1, "Both": 1, "of": 3}, "numerals": {"47": 1}, "pronouns": {"I": 1}}}, {"content": "48. Cheating in the examination is possible as long as it is not caught.", "word_count": 16, "label": "", "sentence_structure": "Subject鈥損redicate", "subjective_objective": {"answer": "Subjective", "rationale": "Because the entry examines the attitude and perception of the patient regarding \"test fraud\" and is the patient's subjective view."}, "sentence_sentiment": {"answer": "Negative", "rationale": "Because the \"false in the examination\" in the entry is against ethical and legal norms and has a negative emotional connotation."}, "language_style": {"answer": "Calm and direct", "rationale": "The entry presents the acceptable situation of fraud in a direct and clear language, without any euphemism or ambiguity."}, "time_dimension": {"answer": "Past", "rationale": "48. Cheating in the examination is possible as long as it is not caught."}, "sentence_type": "Affirmative declarative sentence", "pos": {"nouns": {"Tests": 1, "Cheating": 1}, "verbs": {"Yeah": 1, "Arrested": 1, "be": 1, "in": 1}, "adjectives": {"Not": 1}, "adverbs": {"Just": 1}, "numerals": {"48": 1}}}, {"content": "49. I am better than most of my friends in sports, gymnastics and dance.", "word_count": 23, "label": "", "sentence_structure": "Subject鈥揷opula鈥損redicative", "subjective_objective": {"answer": "Subjective", "rationale": "This is a subjective self-evaluation, involving individual feelings and experiences."}, "sentence_sentiment": {"answer": "Positive", "rationale": "The statement in the entry is a positive expression of self-evaluation, i.e., greater than most friends in sports, gymnastics and dance, which expresses self-confidence and positive attitudes towards their abilities."}, "language_style": {"answer": "Tactful and warm", "rationale": "The language style in this entry is relatively gentle and friendly, using a comparative expression that is not directly compared with other friends."}, "time_dimension": {"answer": "Present", "rationale": "The entry \"now\" indicates that the act occurred at the moment, not in relation to past and future behaviour. While it implies that the person tested has strong skills or talents in sports, gymnastics and dance, it focuses on the present situation and does not involve previous experience or future expectations."}, "sentence_type": "Affirmative declarative sentence", "pos": {"nouns": {"Sports": 1, "Gymnastics": 1, "Dance": 1, "Activities": 1, "friends": 1}, "verbs": {"- Yeah": 1, "鈾?Strong": 1}, "adverbs": {"Most of them": 1}, "numerals": {"49": 1}, "pronouns": {"I": 1}}}, {"content": "50. I am not very good at reading.", "word_count": 9, "label": "", "sentence_structure": "Subject鈥揷opula鈥損redicative", "subjective_objective": {"answer": "Subjective", "rationale": "Because \"not good\" is the patient's subjective feeling and evaluation."}, "sentence_sentiment": {"answer": "Negative", "rationale": "Because the expression \"not good\" is negative."}, "language_style": {"answer": "Calm and direct", "rationale": "The language style of the entry is more calm, as it is a direct expression of the subject of asking each other how they read, and there is no excessive emotional rendering or rhetorical technique."}, "time_dimension": {"answer": "Present", "rationale": "This result may have an impact on the daily lives and learning of the person being tested, such as the ability to read when there is a great deal of effort in the examination or work. To improve this situation, it may be necessary to seek the help of teachers or professionals to improve their reading skills."}, "sentence_type": "Negative declarative sentence", "pos": {"nouns": {"I": 1, "Reading": 1}, "verbs": {"in": 1, "Not good": 1}, "adverbs": {"50": 1}}}, {"content": "51. In some cases, others are more vulnerable to mourning than I am.", "word_count": 15, "label": "", "sentence_structure": "Subject鈥揷opula鈥損redicative", "subjective_objective": {"answer": "Subjective", "rationale": "Subjectivity is the subjective view and attitude of patients towards others."}, "sentence_sentiment": {"answer": "Negative", "rationale": "Because the \"silent\" act in the entry is negative."}, "language_style": {"answer": "Calm and direct", "rationale": "The entry is more calm and direct in its language style, does not use excessive speech techniques or emotional rendering, and is also objective in tone. It asks whether the interviewee considers himself to be more frustrated in some ways than others, and is designed to understand the psychological state and character characteristics of the interviewee."}, "time_dimension": {"answer": "Present", "rationale": "The keywords \"something\" and \"others\" and \"silent\" are all about the person being tested, or what he feels. So the time dimension of the article is now."}, "sentence_type": "Affirmative declarative sentence", "pos": {"nouns": {"Things": 1, "Grief": 1}, "verbs": {"- Yeah": 1, "Easy": 1, "Grief": 1}, "adjectives": {"Compare": 1, "Easy": 1}, "adverbs": {"More": 1}, "numerals": {"51": 1, "Some": 1}, "classifiers": {"Some": 1}, "pronouns": {"I": 1}}}, {"content": "52. I often have disputes with my parents.", "word_count": 8, "label": "", "sentence_structure": "Subject鈥搗erb鈥搊bject", "subjective_objective": {"answer": "Subjective", "rationale": "Because the descriptions of \"often\" and \"disputing\" are described from the patient's subjective perspective and do not address specific objective facts or behavioural outcomes."}, "sentence_sentiment": {"answer": "Negative", "rationale": "Because the \"often arguing with parents\" act is negative."}, "language_style": {"answer": "Calm and direct", "rationale": "The entry is more directly written, with no excessive emotional accoutrements or rhetorical techniques, and is asked whether the interviewee is often in dispute with his parents and speaking in a balanced and objective manner."}, "time_dimension": {"answer": "Present", "rationale": "Now the word \"normal\" in the entry denotes a regular, customary act, and the word \"controversial\" is a description and a description of the act, so the temporal dimension is now judged."}, "sentence_type": "Affirmative declarative sentence", "pos": {"nouns": {"Arguments": 1, "parents": 1}, "verbs": {"have": 1, "Constant with": 1}, "adverbs": {"Regular": 1}, "numerals": {"52": 1}}}, {"content": "53. I learned very quickly for most courses.", "word_count": 13, "label": "", "sentence_structure": "Subject鈥搗erb鈥搊bject", "subjective_objective": {"answer": "Subjective", "rationale": "It is subjective to learn quickly because it is the patient's self-perception and perception."}, "sentence_sentiment": {"answer": "Positive", "rationale": "The basis for the judgement is that the expression \"I'm learning very quickly for most of the courses\" in the entry is positive and indicates a strong learning capacity."}, "language_style": {"answer": "Tactful and warm", "rationale": "The language style of the entry is more euphemistic and moderate, using the expression \"for the vast majority of courses\" to avoid directly evaluating or criticizing the learning abilities of the other. At the same time, the sentence expresses the fact that learning is fast, in a positive tone, and in a relatively encouraging tone."}, "time_dimension": {"answer": "Present", "rationale": "The word \"all\" in the entry is a constant act, while the verb \"recognizes\" the general current, meaning that the act not only has occurred in the past, but may still be ongoing. The entry therefore expresses a state and capacity of the present, and the temporal dimension is now judged."}, "sentence_type": "Affirmative declarative sentence", "pos": {"nouns": {"Courses": 1, "Most of them": 1}, "verbs": {"Learn": 1, "Okay": 1, "Come on": 1}, "adjectives": {"Most of them": 1}, "numerals": {"53": 1}, "pronouns": {"I": 1}}}, {"content": "54. I cannot live well with boys.", "word_count": 12, "label": "", "sentence_structure": "Subject鈥搗erb鈥揷omplement", "subjective_objective": {"answer": "Subjective", "rationale": "This is a self-evaluation and subjective view of the patient as to whether he or she can be well with boys."}, "sentence_sentiment": {"answer": "Negative", "rationale": "Because the \"not well with boys\" behavior in the entry is negative."}, "language_style": {"answer": "Calm and direct", "rationale": "The language of the article is a direct expression of the difficulties experienced by the person under investigation in dealing with men, without a euphemism or excessive emotional rendering. At the same time, the language is simple and straightforward, and is directly objective, consistent with the calm and direct linguistic characteristics."}, "time_dimension": {"answer": "Present", "rationale": "The term \"inappropriate\" in the entry indicates the current capacity and state, so the temporal dimension is now judged."}, "sentence_type": "Negative declarative sentence", "pos": {"nouns": {"Questionnaire": 1, "Entry": 1, "Boys": 1, "Relations": 1}, "verbs": {"Yes": 2, "Get along": 1}, "adjectives": {"Okay": 1, "not": 1}, "adverbs": {"very": 1, "Land": 1}, "numerals": {"54": 1}, "pronouns": {"I": 1, "they": 1}}}, {"content": "55. I cannot live well with girls.", "word_count": 12, "label": "", "sentence_structure": "Subject鈥搗erb鈥揷omplement", "subjective_objective": {"answer": "Subjective", "rationale": "This is a personal feeling and subjective perception of the patient, involving self-evaluation and emotional experience."}, "sentence_sentiment": {"answer": "Negative", "rationale": "Because the expression \"failure to get along with girls\" conveys a negative attitude and behaviour that is indicative of a lack of and a problem with their social skills."}, "language_style": {"answer": "Calm and direct", "rationale": "The entry is a direct question-and-answer dialogue, is not overly rhetorical or emotional, is more balanced and objective, and therefore can be judged as being calm and straightforward."}, "time_dimension": {"answer": "Present", "rationale": ""}, "sentence_type": "Negative declarative sentence", "pos": {"nouns": {"Girls": 1, "Get along": 1}, "verbs": {"Not very well": 1}, "adverbs": {"very": 1}, "numerals": {"55": 1}, "pronouns": {"I": 1}}}, {"content": "56. My math test is always bad.", "word_count": 12, "label": "", "sentence_structure": "Subject鈥揷opula鈥損redicative", "subjective_objective": {"answer": "Objective", "rationale": "Because the results of the math tests are objective facts, they do not change with the subjective perception of the human being."}, "sentence_sentiment": {"answer": "Negative", "rationale": "Because \"failure\" in the entries is negative."}, "language_style": {"answer": "Calm and direct", "rationale": "This is a direct expression that helps maintain the objectivity and accuracy of the questionnaire."}, "time_dimension": {"answer": "Present", "rationale": "The entry is for questioning the testee's past behaviour or experience, and the keyword is \"Mathematics test is always bad\", which indicates that it is a continuous act or result that occurred in the past. So the temporal dimension is the past. Answer: The past"}, "sentence_type": "Negative declarative sentence", "pos": {"nouns": {"Mathematics test scores": 1, "Test": 1, "Achievements": 1}, "verbs": {"be": 1, "Okay": 1}, "adverbs": {"Total": 1}, "numerals": {"56": 1}, "pronouns": {"I": 1}}}, {"content": "57. I was thought to be a good-looking person.", "word_count": 9, "label": "", "sentence_structure": "Subject鈥搗erb鈥揷omplement", "subjective_objective": {"answer": "Subjective", "rationale": "Because this part of the questionnaire is concerned with the views and opinions of others, which are not directly accessible from the patient鈥檚 subjective point of view. The purpose of the questionnaire is therefore essentially based on the patient鈥檚 self-perception and subjective feelings, and is therefore subjective."}, "sentence_sentiment": {"answer": "Positive", "rationale": "Because the phrase \"I look good\" in the entry is positive."}, "language_style": {"answer": "Tactful and warm", "rationale": "This expression is more sensitive and sensitive to each other鈥檚 feelings, and helps to create a good atmosphere of communication."}, "time_dimension": {"answer": "Present", "rationale": "In the future, the entry asks the testee about the future expectations or scenarios, \"I look good\" is an evaluation or feedback that may be received in the future, and therefore the temporal dimension is judged in the future."}, "sentence_type": "Affirmative declarative sentence", "pos": {"nouns": {"others": 1, "I": 1, "- He's a big boy": 1, "Nice": 1}, "verbs": {"I think": 1}, "adverbs": {"Really": 1}, "numerals": {"57": 1}}}, {"content": "I have little to be proud of.", "word_count": 12, "label": "", "sentence_structure": "Subject鈥搗erb鈥揷omplement", "subjective_objective": {"answer": "Subjective", "rationale": "Because \"no proud places\" are self-evaluation and subjective feelings of patients."}, "sentence_sentiment": {"answer": "Negative", "rationale": "Because the expression \"no proud place\" in the entry conveys a negative and self-responsible sentiment, and it is negative."}, "language_style": {"answer": "Calm and direct", "rationale": ""}, "time_dimension": {"answer": "Present", "rationale": "The entry is for self-evaluation of the testee and does not contain clear information on the time dimension, so it is not possible to judge its temporal properties."}, "sentence_type": "Negative declarative sentence", "pos": {"nouns": {"Local": 1, "Proud": 1}, "verbs": {"No, I'm not": 1, "It's worth it": 1, "Proud": 1}, "adverbs": {"How much": 1}, "numerals": {"58": 1}, "pronouns": {"I": 1}}}, {"content": "59. Integrity is important to me.", "word_count": 9, "label": "", "sentence_structure": "Subject鈥揷opula鈥損redicative", "subjective_objective": {"answer": "Subjective", "rationale": "Because honesty is a human value and attitude, it belongs to the subjective views and beliefs of the individual."}, "sentence_sentiment": {"answer": "Positive", "rationale": "Because the word \"honest\" is usually seen as a positive quality."}, "language_style": {"answer": "Tactful and warm", "rationale": "The entry is more adept and interactive when it is spoken in a gentle and inviting manner, rather than by direct statements or orders."}, "time_dimension": {"answer": "Present", "rationale": ""}, "sentence_type": "Affirmative declarative sentence", "pos": {"nouns": {"Honesty": 1, "I": 1, "Important": 1}, "verbs": {"Tell me": 1, "be": 1, "Here": 1}, "numerals": {"59": 1}, "pronouns": {"very": 1}}}, {"content": "60. I'm trying to avoid sports and physical education if I can.", "word_count": 19, "label": "", "sentence_structure": "Subject鈥損redicate", "subjective_objective": {"answer": "Subjective", "rationale": "Because \"evading as much as possible\" is the patient's subjective attitude, it describes the aversion of the patient to sports."}, "sentence_sentiment": {"answer": "Negative", "rationale": "Because the \"evading physical education and physical education\" in the entry is negative."}, "language_style": {"answer": "Calm and direct", "rationale": "The entry directly describes the attitudes and behaviour of the person under investigation in a concise and clear language, without any reticulation or emotional rendering. Thus, its language style can be described as \"cool and straight\"."}, "time_dimension": {"answer": "Present", "rationale": "\"I can\" and \"as far as I can\" indicate that it is a normal behavior or attitude, so the entry should be the time dimension of the present."}, "sentence_type": "Affirmative declarative sentence", "pos": {"nouns": {"Sports": 1, "Physical education": 1}, "verbs": {"Run away": 2, "Yes": 1, "Try": 1}, "adjectives": {"Just": 1}, "adverbs": {"Both": 1, "and": 1, "Try": 1}, "numerals": {"60": 1, "I": 1}, "pronouns": {"I": 1}}}, {"content": "61. Languages are one of the best courses I have learned.", "word_count": 13, "label": "", "sentence_structure": "Subject鈥揷opula鈥損redicative", "subjective_objective": {"answer": "Subjective", "rationale": "Because \"best\" is a subjective evaluation that shows that the patient considers language to be one of his/her more effective courses."}, "sentence_sentiment": {"answer": "Positive", "rationale": "For the expression \"languages are one of the best courses I have learned\" expresses the author's self-confidence and positive attitude towards his language."}, "language_style": {"answer": "Tactful and warm", "rationale": ""}, "time_dimension": {"answer": "Past", "rationale": ""}, "sentence_type": "Affirmative declarative sentence", "pos": {"nouns": {"Languages": 1, "Courses": 1}, "verbs": {"be": 1, "I can learn": 1}, "adjectives": {"I'd better": 1, "鈾?And the 鈾?: 1}, "numerals": {"61": 1}}}, {"content": "62. I'm easily nervous.", "word_count": 6, "label": "", "sentence_structure": "Subject鈥揷opula鈥損redicative", "subjective_objective": {"answer": "Subjective", "rationale": "Because \"frightful\" is a patient's self-perception and experience, a subjective description."}, "sentence_sentiment": {"answer": "Negative", "rationale": "Because \"easy stress\" is a negative mood and state."}, "language_style": {"answer": "Calm and direct", "rationale": "The entry is simple and clear, and it is more in the language of the questionnaire to ask directly whether the person surveyed is prone to stress, is not overdoing or rendering too much emotion."}, "time_dimension": {"answer": "Present", "rationale": "The entry describes the current feelings and state of the subject, does not mention past or future events and therefore judges \"now\"."}, "sentence_type": "Affirmative declarative sentence", "pos": {"verbs": {"Easy": 1, "Nervous": 1}, "adjectives": {"very": 1}, "numerals": {"62": 1}}}, {"content": "63. My parents understand me.", "word_count": 7, "label": "", "sentence_structure": "Subject鈥揷opula鈥損redicative", "subjective_objective": {"answer": "Subjective", "rationale": "Because it is a subjective assessment of whether the patient understands his or her parents ' views and feelings."}, "sentence_sentiment": {"answer": "Neutral", "rationale": "Since the entry does not have a clear positive or negative emotional tendency, it merely presents a fact."}, "language_style": {"answer": "Tactful and warm", "rationale": "The entry is more personal and interactive when it is spoken in a gentle and inviting manner, rather than in direct statements or orders. Moreover, the term \"understanding\" is also emotional, implying that the questionnaire designers would like to know about the relationship between the person under investigation and the parents."}, "time_dimension": {"answer": "Past", "rationale": "The keyword is \"My parents understand me\" and it is a way of showing what we've been through."}, "sentence_type": "Affirmative declarative sentence", "pos": {"nouns": {"parents": 2, "I": 1}, "verbs": {"Understood": 1}, "numerals": {"63": 1}}}, {"content": "I'm stupid in most of the courses.", "word_count": 17, "label": "", "sentence_structure": "Subject鈥揷opula鈥損redicative", "subjective_objective": {"answer": "Subjective", "rationale": "Because \"dumb\" is a subjective assessment, not an objective fact."}, "sentence_sentiment": {"answer": "Negative", "rationale": "Because the word \"mute\" expresses negative emotions and attitudes."}, "language_style": {"answer": "Calm and direct", "rationale": "The entry clearly expresses the person under investigation鈥檚 performance in most courses in a direct manner."}, "time_dimension": {"answer": "Present", "rationale": "The keyword \"in most of the course of study\" means that it is a description of the learning situation, and that it's \"unbelievably stupid\" is also about the present. It is therefore a function of the current behavior or mentality that can be judged."}, "sentence_type": "Negative declarative sentence", "pos": {"nouns": {"Learning": 2, "Courses": 1}, "verbs": {"It seems": 1, "Learn": 1}, "adjectives": {"Mostly": 1, "Stupid": 1}, "adverbs": {"Both": 1}, "numerals": {"64": 1}, "pronouns": {"I": 1}}}, {"content": "65. I have some good friends of the same sex.", "word_count": 11, "label": "", "sentence_structure": "Subject鈥搗erb鈥搊bject", "subjective_objective": {"answer": "Objective", "rationale": "Because having a good friend is an objective fact, not a patient's subjective attitude or imagination."}, "sentence_sentiment": {"answer": "Neutral", "rationale": "Because the entry does not have any emotional color."}, "language_style": {"answer": "Tactful and warm", "rationale": "The entry is based on a more acclaimable and moderate expression, which presents information in the form of \"some\" and is not too direct or too strong. It is more likely to generate resonance and trust among the respondents and to enhance the effectiveness of the questionnaire."}, "time_dimension": {"answer": "Present", "rationale": "The entry describes the current social situation of the person tested, i.e. having a friend of the same sex, and does not address the time dimension of the past or the future."}, "sentence_type": "Affirmative declarative sentence", "pos": {"nouns": {"Some": 1, "friends": 1}, "verbs": {"have": 1}, "adjectives": {"Same-sex": 1}, "numerals": {"65": 1}, "pronouns": {"I": 1}}}, {"content": "66. I have many heterosexual friends.", "word_count": 8, "label": "", "sentence_structure": "Subject鈥搗erb鈥揷omplement", "subjective_objective": {"answer": "Subjective", "rationale": "Because \"many heterosexual friends\" are subjective perceptions and choices of individuals, involving aspects of interpersonal relations and personal experience, they are considered subjectively."}, "sentence_sentiment": {"answer": "Neutral", "rationale": "Since the entry does not describe the nature of the relationships between these heterosexual friends, their friendship, and whether they have further development, it cannot be judged by their emotional colours."}, "language_style": {"answer": "Tactful and warm", "rationale": "This is a more moderate way of asking questions, avoiding too direct or hard language. It does not express itself explicitly against or evaluate the person being investigated, but rather leads the person to answer in an inquisitorial and understanding manner. Such language is more acceptable and willing to cooperate in answering questions."}, "time_dimension": {"answer": "Present", "rationale": "The entry is for the testee's current situation, and the time dimension is now. \"Many heterosexual friends\" indicates that the testee now has many friends who are having heterosexual relations. So the output should be \"Now\"."}, "sentence_type": "Affirmative declarative sentence", "pos": {"nouns": {"Fae friend": 1}, "verbs": {"have": 1, "Many": 1}, "adverbs": {"Many": 1}, "numerals": {"66": 1}}}, {"content": "67. My maths are good.", "word_count": 8, "label": "", "sentence_structure": "Subject鈥揷opula鈥損redicative", "subjective_objective": {"answer": "Subjective", "rationale": "Because \"good grades\" are self-evaluation and subjective feelings of patients."}, "sentence_sentiment": {"answer": "Neutral", "rationale": "Because the entry simply objectively presents the mathematical performance of a person and does not involve any emotion, attitude or behaviour."}, "language_style": {"answer": "Calm and direct", "rationale": "The entry is more direct, concise and has no excessive rhetorical or emotional colours. It presents the interviewee鈥檚 mathematical performance directly, with an objective and neutral tone and calmer tone."}, "time_dimension": {"answer": "Present", "rationale": "The entry describes the current state or ability of the testee, and \"my maths are good\" is an expression of the current state of affairs, so it can be judged as now."}, "sentence_type": "Affirmative declarative sentence", "pos": {"nouns": {"Mathematics": 1}, "verbs": {"Good": 1}, "numerals": {"67": 1}}}, {"content": "I'm ugly.", "word_count": 3, "label": "", "sentence_structure": "Subject鈥揷opula鈥損redicative", "subjective_objective": {"answer": "Subjective", "rationale": "Because I'm ugly is a subjective self-evaluation of a person, reflecting the patient's perception and feelings about his or her appearance."}, "sentence_sentiment": {"answer": "Negative", "rationale": "Because the expression \"very ugly\" is negative."}, "language_style": {"answer": "Calm and direct", "rationale": "The language style of the entry is more direct and objective, and there is no excessive rhetorical and emotional overtones, which express the opinion of the person under investigation about his or her appearance and are more balanced in tone."}, "time_dimension": {"answer": "Present", "rationale": "Now the entry indicates that the testee is currently having a negative view of his or her appearance, that is, he or she is ugly. So the judgement is based on the time dimension of \"now\"."}, "sentence_type": "Negative declarative sentence", "pos": {"adjectives": {"Ugly": 1}}}, {"content": "69. I can do as well as most people.", "word_count": 12, "label": "", "sentence_structure": "Subject鈥揷opula鈥損redicative", "subjective_objective": {"answer": "Subjective", "rationale": "As this sentence relates to patient self-evaluation and comparison, it is viewed from the patient ' s subjective perspective."}, "sentence_sentiment": {"answer": "Neutral", "rationale": "Because the entry does not express positive or negative emotional tendencies, it is merely describing a capacity and the relative level of the majority."}, "language_style": {"answer": "Tactful and warm", "rationale": "The language style of the entry is more euphemistic and encouraging, with softer terms, without directly evaluating the other ' s abilities or performance, but rather with positive recognition and support, which is more conducive to the self-esteem and confidence of the testee."}, "time_dimension": {"answer": "Present", "rationale": "The entry describes the attitude of the testee to his/her abilities, without clear time indicators or markers, and therefore can be judged to describe the current state and the ideas."}, "sentence_type": "Affirmative declarative sentence", "pos": {"nouns": {"I": 1, "Most of them": 1, "person": 1}, "verbs": {"Yes": 1, "Do it": 1, "Okay": 2, "and": 1, "Same thing": 1}, "numerals": {"69": 1}}}, {"content": "I lie sometimes.", "word_count": 5, "label": "", "sentence_structure": "Subject鈥搗erb鈥揷omplement", "subjective_objective": {"answer": "Subjective", "rationale": "Because \"fake\" is a subjective act and attitude."}, "sentence_sentiment": {"answer": "Negative", "rationale": "Because \"fake\" is a negative act."}, "language_style": {"answer": "Calm and direct", "rationale": "The entry directly expresses the subject of questioning whether the other party is sometimes lying, does not use excessive rhetorical or emotional rendering, and is more objective in tone."}, "time_dimension": {"answer": "Past", "rationale": ""}, "sentence_type": "Exclamatory sentence", "pos": {"verbs": {"Liar": 1, "person": 1}, "adverbs": {"Sometimes": 1}}}, {"content": "I can run long and long in succession.", "word_count": 11, "label": "", "sentence_structure": "Subject鈥搗erb鈥揷omplement", "subjective_objective": {"answer": "Subjective", "rationale": "Because \"can run far and far\" is an individual's subjective judgement and feeling that cannot be proved or denied by objective facts."}, "sentence_sentiment": {"answer": "Positive", "rationale": "Because the behavior in the entry \"can run long and long\" is positive."}, "language_style": {"answer": "Calm and direct", "rationale": "The entry asks each other about their motor abilities in a direct and concise manner, is not overly pedantic or emotional, and is more calm and objective in language style."}, "time_dimension": {"answer": "Present", "rationale": ""}, "sentence_type": "Affirmative declarative sentence", "pos": {"verbs": {"Yeah": 1, "Run": 1}, "adjectives": {"It's far": 1}, "adverbs": {"Company": 1, "Land": 1, "鈾?And keep on 鈾?: 1}, "numerals": {"71": 1}}}, {"content": "I hate reading.", "word_count": 5, "label": "", "sentence_structure": "Subject鈥搗erb鈥揷omplement", "subjective_objective": {"answer": "Subjective", "rationale": "Because \"obsessed\" is a subjective feeling and attitude, and reading is an objective fact and behaviour."}, "sentence_sentiment": {"answer": "Negative", "rationale": "Because the word \"obsessed\" in the entry is a negative attitude."}, "language_style": {"answer": "Calm and direct", "rationale": "The language style of the entry is more direct and clear, and not too much rhetorical or emotional, but rather a direct expression of the attitude of the subject to reading in a simple and clear language."}, "time_dimension": {"answer": "Past", "rationale": "The entry is about personal feelings and experiences of the past, using the first person's singular form of \"me\" and the expression of the past's temporal expression \"obsessed\" and therefore judging as the past."}, "sentence_type": "Negative declarative sentence", "pos": {"verbs": {"Shit": 1, "Reading": 1}, "adjectives": {"72": 1}}}, {"content": "73. I often feel confused.", "word_count": 9, "label": "", "sentence_structure": "Subject鈥揷opula鈥損redicative", "subjective_objective": {"answer": "Subjective", "rationale": "Because it's the patient's subjective feeling."}, "sentence_sentiment": {"answer": "Negative", "rationale": "Because \"drained\" is a negative sentiment."}, "language_style": {"answer": "Calm and direct", "rationale": "The language style of the entry is straightforward. It directly expresses the subject of asking the other side whether it is often confused, without using too many rhetorical techniques or emotional rendering, and is more objective in tone."}, "time_dimension": {"answer": "Present", "rationale": ""}, "sentence_type": "Affirmative declarative sentence", "pos": {"verbs": {"Common sense": 1, "Chaos": 1}, "adjectives": {"Often": 1}, "numerals": {"73": 1}}}, {"content": "I don't like my parents very much.", "word_count": 10, "label": "", "sentence_structure": "Subject鈥揷opula鈥損redicative", "subjective_objective": {"answer": "Subjective", "rationale": "Because it's personal to like a person."}, "sentence_sentiment": {"answer": "Negative", "rationale": "Because the entry expresses a lack of attachment to parents and is a negative emotional attitude."}, "language_style": {"answer": "Tactful and warm", "rationale": "The language style of the entry is more euphemistic, and leads the interviewee to share their feelings in an interview, rather than directly accusing or expressing subjective opinions. At the same time, the expression \"not likes\" is relatively mild, avoiding the use of too harsh negative terms."}, "time_dimension": {"answer": "Past", "rationale": "The entry indicates a negative sentiment and attitude of the testee towards his parents, which may be the result of past experience or circumstances and can therefore be judged as past."}, "sentence_type": "Negative declarative sentence", "pos": {"nouns": {"I": 1, "parents": 1}, "verbs": {"very": 1, "Yeah": 1}, "adjectives": {"not": 1, "very": 2}, "numerals": {"74": 1}}}, {"content": "75. The vast majority of my courses have performed well.", "word_count": 13, "label": "", "sentence_structure": "Subject鈥揷opula鈥損redicative", "subjective_objective": {"answer": "Subjective", "rationale": "Because \"good grades\" are the result of patients' subjective assessment of their learning performance, so they're subjective."}, "sentence_sentiment": {"answer": "Positive", "rationale": "Because the expression \"good grades\" is positive, indicating good academic results."}, "language_style": {"answer": "Calm and direct", "rationale": "The language style of the entry is more straightforward, there is no excessive rhetorical or emotional rendering, and it is more objective and neutral in its tone, and it is used to express a description of facts or views in a certain respect."}, "time_dimension": {"answer": "Present", "rationale": ""}, "sentence_type": "Affirmative declarative sentence", "pos": {"nouns": {"Achievements": 2, "Courses": 1, "Large": 1}, "verbs": {"Both": 1, "Yeah": 1}, "adjectives": {"Okay": 1, "Extreme": 1}, "adverbs": {"More": 1, "Most of them": 1}, "numerals": {"75": 1}, "pronouns": {"I": 1}}}, {"content": "76. The vast majority of boys have been avoiding me.", "word_count": 11, "label": "", "sentence_structure": "Subject鈥搗erb鈥搊bject", "subjective_objective": {"answer": "Subjective", "rationale": "Because it is a patient ' s self-perception and observation, it expresses their subjective perception and judgement that they are being avoided by others."}, "sentence_sentiment": {"answer": "Negative", "rationale": "Because the \"dive\" act in the entry is negative, implying that boys don't like to go out with her."}, "language_style": {"answer": "Tactful and warm", "rationale": "The entry is more adept and interactive when it is spoken in a gentle and inviting manner, rather than by direct statements or orders."}, "time_dimension": {"answer": "Present", "rationale": "The \"most boys are avoiding me\" in this entry describes a state or state that is in the present, not past or future. So, time dimensions are now judged."}, "sentence_type": "Affirmative declarative sentence", "pos": {"nouns": {"Boys": 1, "I": 1}, "verbs": {"Get out of the way": 1, "Both": 1}, "adjectives": {"Most of them": 1}, "numerals": {"76": 1}}}, {"content": "77. The vast majority of girls are avoiding me.", "word_count": 11, "label": "", "sentence_structure": "Subject鈥搗erb鈥搊bject", "subjective_objective": {"answer": "Subjective", "rationale": "Because \"the vast majority of girls are avoiding me\" is a subjective statement that reflects the views and feelings of the speakers about their social situation, not the objective facts."}, "sentence_sentiment": {"answer": "Negative", "rationale": "Because the word \"scramble\" in the entry conveys a negative emotion and attitude."}, "language_style": {"answer": "Tactful and warm", "rationale": "The sentence is more euphemistic, not directly expressing the negative feelings of the other party to \"shun themselves\" but rather using a relatively less lax formulation; - the phrase \"most girls\" used in the sentence gives people an objective, neutral feeling rather than a subjective emotional description; and the entire sentence gives people a sense of humility and respect for others, which is more acceptable and responsive."}, "time_dimension": {"answer": "Past", "rationale": "Because the sentence contains the time point of the past, \"Most girls avoid me,\" it can be judged that the entry is about asking the subject about his past behavior or experience."}, "sentence_type": "Affirmative declarative sentence", "pos": {"nouns": {"Girls": 1, "I": 1}, "verbs": {"Get out of the way": 1, "Both": 1}, "adjectives": {"Most of them": 1}, "adverbs": {"- Yeah": 1, "Extreme": 1}, "numerals": {"77": 1}}}, {"content": "I never want to go to math again.", "word_count": 10, "label": "", "sentence_structure": "Subject鈥搗erb鈥揷omplement", "subjective_objective": {"answer": "Subjective", "rationale": "Because \"want\" is the patient's subjective idea and attitude."}, "sentence_sentiment": {"answer": "Negative", "rationale": "Because the attitude in the entry \"never want to take math classes again\" is negative."}, "language_style": {"answer": "Calm and direct", "rationale": "The entry expresses a direct, concise and clear language style of dislike or aversion to mathematics, without adding too many rhetorical or emotional expressions and with relatively objective and calm tone."}, "time_dimension": {"answer": "Past", "rationale": "And by the phrase \"I never want to go to math again,\" we can tell that it's about the testee's past learning experience or feelings. So the time dimension of this article is the past."}, "sentence_type": "Negative declarative sentence", "pos": {"nouns": {"Math class": 1}, "verbs": {"Yes": 1, "Go, go, go": 1, "Again": 1}, "adjectives": {"Forever": 1, "not": 1}, "adverbs": {"Again": 1}, "numerals": {"78": 1}, "pronouns": {"I": 1}}}, {"content": "I'm a good-looking guy.", "word_count": 7, "label": "", "sentence_structure": "Subject鈥揷opula鈥損redicative", "subjective_objective": {"answer": "Subjective", "rationale": "Because \"good\" is a personal aesthetic and self-evaluation of the patient, and it is a manifestation of subjective attitudes."}, "sentence_sentiment": {"answer": "Positive", "rationale": "Because \"good-looking\" is a positive description."}, "language_style": {"answer": "Tactful and warm", "rationale": "The language style of the entry is euphemistic. It uses the word \"good\" and puts it behind the main word \"me,\" which can make the interviewee feel accepted and accepted, and does not make them feel pressured or uneasy. Moreover, the whole sentence is more comfortable and is in keeping with the soft and warm language style."}, "time_dimension": {"answer": "Present", "rationale": "The keyword is \"I'm a good-looking person,\" which describes the current state of the person being tested, and therefore judges it now."}, "sentence_type": "Affirmative declarative sentence", "pos": {"verbs": {"Nice": 1}, "adjectives": {"Mine": 1, "very": 1}, "numerals": {"79": 1}}}, {"content": "I feel like my life is worthless.", "word_count": 11, "label": "", "sentence_structure": "Subject鈥揷opula鈥損redicative", "subjective_objective": {"answer": "Subjective", "rationale": "Because \"fear\" is the patient's subjective feeling and perception, this entry is viewed from the patient's subjective perspective."}, "sentence_sentiment": {"answer": "Negative", "rationale": "Because \"no value\" in the entry is an expression of negative emotions and attitudes."}, "language_style": {"answer": "Tactful and warm", "rationale": "The language style of the entry is relatively moderate and friendly, and by using the first person's name \"I\" to guide the interviewee to express his/her own ideas, the sense of interaction and communication with the interviewee is enhanced. At the same time, the phrase \"no value\" is not a direct denial or criticism, but rather a more euphemism, which makes it easier for the interviewee to accept and share his/her real feelings."}, "time_dimension": {"answer": "Present", "rationale": "The entry expresses the current feelings and state of the subject, and the word \"I feel\" indicates that it is a subjective feeling, and the \"I feel\" is the current state of affairs, which means that it is the present. So the temporal dimension of this questionnaire item is now judged."}, "sentence_type": "Negative declarative sentence", "pos": {"nouns": {"Life": 1, "Value": 1}, "verbs": {"I feel": 1, "have": 1}, "adverbs": {"No, I'm not": 1}, "numerals": {"80": 1}, "pronouns": {"I": 1}}}, {"content": "81. I always try to do what I promise.", "word_count": 11, "label": "", "sentence_structure": "Subject鈥搗erb鈥揷omplement", "subjective_objective": {"answer": "Objective", "rationale": "Because consent is itself a behavioural act, an objective fact that can be observed and does not involve the patient ' s actual efforts."}, "sentence_sentiment": {"answer": "Positive", "rationale": "Because the \"I always try to do what I promise\" in the entry is positive."}, "language_style": {"answer": "Calm and direct", "rationale": "The basis for the judgement is as follows: 1. The expression of the article is more straightforward and direct, without too much rhetoric or emotional colour, and it is more objective and idiosyncratic. 2. The entry is clear: a person is asked whether he or she will try to do what he or she promised, to express what is simple and clear, without any qualifier, to enable readers to understand and answer quickly. 3. Emotional orientation is lower: the purpose of the article is calm, without a clear emotional or strong sense of subjectivity."}, "time_dimension": {"answer": "Present", "rationale": "\"Try to do everything possible\" means that the act is in its present state, not in its past or in its future plans, and is therefore judged as \"now\"."}, "sentence_type": "Affirmative declarative sentence", "pos": {"verbs": {"Promise": 1, "Do it": 1, "Try": 1}, "adverbs": {"Always": 1}, "numerals": {"81": 1}, "pronouns": {"I": 1}}}, {"content": "82. I hate sports, gymnastics and dances.", "word_count": 15, "label": "", "sentence_structure": "Subject鈥搗erb鈥搊bject", "subjective_objective": {"answer": "Subjective", "rationale": "Because \"obsessed\" is the individual emotional attitude of patients to sports, gymnastics and dances, and is subjective."}, "sentence_sentiment": {"answer": "Negative", "rationale": "Because \"hate\" is a negative emotional tendency, meaning that it does not like or dislike, the emotional colour of the article is negative."}, "language_style": {"answer": "Calm and direct", "rationale": "The entry answers a question with a clear negative tone, and the language style is more straightforward, with no excessive emotional accommodations or euphemism. It is therefore possible to judge the language style as being straightforward."}, "time_dimension": {"answer": "Present", "rationale": "It can be inferred from the phrases \"I hate\" and \"sport,\" \"mhythmics\" and \"dance\" that the entry describes the current attitude or behaviour of the person being tested and therefore determines \"now\"."}, "sentence_type": "Exclamatory sentence", "pos": {"nouns": {"Sports": 1, "Gymnastics": 1, "Dance": 1, "Activities": 1}, "verbs": {"Shit": 1, "Do it": 1}, "adverbs": {"Category": 1, "of": 2}, "numerals": {"82": 1, "I": 1}}}, {"content": "83. My language has performed well.", "word_count": 8, "label": "", "sentence_structure": "Subject鈥揷opula鈥損redicative", "subjective_objective": {"answer": "Subjective", "rationale": "Because \"my language is very good\" is a self-evaluation, a subjective attitude of the patient."}, "sentence_sentiment": {"answer": "Neutral", "rationale": "Because the entry is not clearly emotional, it is merely a statement of the facts."}, "language_style": {"answer": "Calm and direct", "rationale": "The rationale for the judgement: The purpose of this article is very straightforward and concise, without any word of accrimation or emotional colour. It is simply a simple statement that clearly expresses the good results achieved by the surveyer in the language. It is usually used in situations where reports, data analysis, etc. require a clear message, rather than emotional exchange or storytelling. So, I think the language style of the questionnaire entry is straightforward."}, "time_dimension": {"answer": "Past", "rationale": ""}, "sentence_type": "Affirmative declarative sentence", "pos": {"nouns": {"Achievements": 1, "Languages": 1}, "verbs": {"Okay": 1}, "adjectives": {"very": 1}, "numerals": {"83": 1}}}, {"content": "84. I am easily mourning.", "word_count": 5, "label": "", "sentence_structure": "Subject鈥揷opula鈥損redicative", "subjective_objective": {"answer": "Subjective", "rationale": "Because mourning is an emotional response, it is the patient ' s subjective feeling and attitude."}, "sentence_sentiment": {"answer": "Negative", "rationale": "Because \"silence\" is a negative emotional vocabulary."}, "language_style": {"answer": "Calm and direct", "rationale": "The language style of the entry is relatively simple, there is no excessive rhetorical or emotional rendering, and it is more objective in tone, and it is asked directly whether the other person has a loss-prone situation. So I think the language style of the entry is \"cool and straight.\""}, "time_dimension": {"answer": "Past", "rationale": "The entry is for personal characteristics or customary emotional reactions of the person being tested, which are judged by the time dimension as the past. The keyword \"I am easy\" indicates a normal psychological state, not a temporary emotional response, so it can be judged to describe the past experience and the present state of affairs."}, "sentence_type": "Negative declarative sentence", "pos": {"nouns": {"Questionnaire": 1, "Entry": 1}, "verbs": {"Easy": 1, "Grief": 1}, "numerals": {"84": 1}}}, {"content": "85. My parents do love me.", "word_count": 9, "label": "", "sentence_structure": "Subject鈥揷opula鈥損redicative", "subjective_objective": {"answer": "Objective", "rationale": "Because love is the expression of emotion, it is based on the evaluation of facts and behaviour and is not the subjective feeling of the patient."}, "sentence_sentiment": {"answer": "Positive", "rationale": "Because the expression \"love me very much\" is positive."}, "language_style": {"answer": "Tactful and warm", "rationale": "The entry describes the relationship between the interviewee and the parents in a positive and commendable manner, without directly indicating whether the other party did well or whether there was a lack of sufficient capacity, which reduces the resistance and increases the degree of cooperation of the other party."}, "time_dimension": {"answer": "Past", "rationale": "The keyword \"I do love me\" indicates that it is a description and evaluation of past events, and therefore the entry belongs to the past."}, "sentence_type": "Affirmative declarative sentence", "pos": {"nouns": {"parents": 1, "I": 1}, "verbs": {"Love": 1}, "adjectives": {"Indeed": 1}, "numerals": {"85": 1}}}, {"content": "86. I have difficulty in learning most of the courses.", "word_count": 12, "label": "", "sentence_structure": "Subject鈥搗erb鈥揷omplement", "subjective_objective": {"answer": "Objective", "rationale": "The difficulty of learning is not related to the patient ' s subjective attitude, since it is an objective fact."}, "sentence_sentiment": {"answer": "Negative", "rationale": "The judgement is that the expression \"difficult\" has a clear negative mood."}, "language_style": {"answer": "Tactful and warm", "rationale": "The entry uses a first-person expression, and expresses the problems of the interviewee in learning through a more implicit expression, \"hard.\" This expression is relatively mild, avoiding direct accusations or denial of each other's abilities and efforts, and is more conducive to creating a good atmosphere of communication."}, "time_dimension": {"answer": "Present", "rationale": "The entry asks if the person tested has encountered difficulties in the current learning process, and the time dimension is now judged."}, "sentence_type": "Negative declarative sentence", "pos": {"nouns": {"I": 1, "Courses": 2, "Difficulties": 1}, "verbs": {"Learning": 1, "have": 1}, "adverbs": {"Most": 1, "Large": 1, "More": 1}, "numerals": {"86": 1}}}, {"content": "87. I am easily friends with people of the same sex.", "word_count": 12, "label": "", "sentence_structure": "Subject鈥搗erb鈥揷omplement", "subjective_objective": {"answer": "Subjective", "rationale": "Because it is personal subjective feeling and experience to be easily friends with people of the same sex."}, "sentence_sentiment": {"answer": "Positive", "rationale": "Because this entry indicates the ability of individuals to build friendships with people of the same sex, it is a recognition of social ability."}, "language_style": {"answer": "Tactful and warm", "rationale": "The entry is a soft, invitation-style conversation, which expresses the theme \"Are you easy to be friends with a person of the same sex\" and uses a relatively neutral language style, with no excessive emotional rendering or direct denial/confirmation. This expression is more personal and interactive."}, "time_dimension": {"answer": "Present", "rationale": "The word \"I'm easy\" in the entry indicates that it is the testee's description of his own behaviour, and the word \"easy\" indicates that the act is recurrent or customary, so it can be judged that the temporal dimension of the entry is now."}, "sentence_type": "Affirmative declarative sentence", "pos": {"nouns": {"Questionnaire": 1, "Entry": 1, "person": 1, "Same sex": 1, "friends": 1}, "verbs": {"I": 2, "Easy": 1, "Hand it over": 1}, "adjectives": {"Same-sex": 1}, "numerals": {"87": 1}}}, {"content": "88. I am very much a heterosexual.", "word_count": 8, "label": "", "sentence_structure": "Subject鈥揷opula鈥損redicative", "subjective_objective": {"answer": "Subjective", "rationale": "Because \"very attention to the opposite sex\" is a personal feeling or self-evaluation, a subjective description."}, "sentence_sentiment": {"answer": "Neutral", "rationale": "Because the entry describes the facts and does not involve any emotional colour."}, "language_style": {"answer": "Tactful and warm", "rationale": ""}, "time_dimension": {"answer": "Present", "rationale": ""}, "sentence_type": "Affirmative declarative sentence", "pos": {"verbs": {"It's painful": 1, "Attention": 1}, "adjectives": {"Heterosexual": 1}, "adverbs": {"very": 1}}}, {"content": "My math is always good.", "word_count": 8, "label": "", "sentence_structure": "Subject鈥揷opula鈥損redicative", "subjective_objective": {"answer": "Subjective", "rationale": "Because \"mathematics are always good\" is patient self-evaluation and subjective."}, "sentence_sentiment": {"answer": "Neutral", "rationale": "Because the entry merely objectively describes the mathematical performance of the person under investigation, it does not have any emotional colour."}, "language_style": {"answer": "Tactful and warm", "rationale": "The entry uses the phrase \"always good\" in the subjective description of the individual, does not clearly indicate the specific level of achievement in mathematics or comparison with others, and the language style is more euphemism and humility. At the same time, the first person's proxies \"I\" are used to increase affection and interaction and to better serve the purpose of communicating with the respondents in the questionnaire."}, "time_dimension": {"answer": "Present", "rationale": "The entry now indicates the current state and fact that the testee considers his or her mathematical performance to be good and does not mention past or future information. Therefore, the temporal dimension is now judged."}, "sentence_type": "Affirmative declarative sentence", "pos": {"nouns": {"Math": 1}, "verbs": {"Total": 1, "be": 1, "very": 1, "Okay": 1}, "adverbs": {"Always": 1}, "numerals": {"89": 1}}}, {"content": "90. As long as I do, I can almost do what I want.", "word_count": 18, "label": "", "sentence_structure": "Subject鈥損redicate", "subjective_objective": {"answer": "Subjective", "rationale": "Because it expresses the belief and the perception of the patient about his or her abilities and actions, it is a subjective attitude or view."}, "sentence_sentiment": {"answer": "Positive", "rationale": "Because the entry \"almost can do what you want\" expresses a positive attitude and conviction."}, "language_style": {"answer": "Tactful and warm", "rationale": "The language style of the entry is more euphemistic, positive and encouraging, and the recognition and encouragement of individual abilities is conveyed through the use of terms such as \"real effort\", \"almost can be done\", making it easier for respondents to respond positively."}, "time_dimension": {"answer": "Present", "rationale": ""}, "sentence_type": "Affirmative declarative sentence", "pos": {"nouns": {"Yes": 2, "Strength": 1, "person": 1}, "verbs": {"Yes": 3, "Do it": 1}, "adjectives": {"True": 1, "Try": 1, "Almost": 1}, "numerals": {"90": 1}, "classifiers": {"individual": 1}, "pronouns": {"I": 1}}}, {"content": "91. I lie often.", "word_count": 5, "label": "", "sentence_structure": "Subject鈥損redicate", "subjective_objective": {"answer": "Subjective", "rationale": "Because \"false\" is a behavior and attitude that requires patient self-evaluation and perception, it is subjective."}, "sentence_sentiment": {"answer": "Negative", "rationale": "Because the \"fake\" act is negative."}, "language_style": {"answer": "Calm and direct", "rationale": "The language style of the entry is relatively simple, there is no excessive rhetoric or emotional rendering, and the tone is to ask if the other person is often lying, which makes one feel more objective and realistic."}, "time_dimension": {"answer": "Present", "rationale": "The entry uses the form of \"false already\" at the time of completion, which means that the act began at some point in the past and has an impact on the present. So the time dimension of the entry is now."}, "sentence_type": "Affirmative declarative sentence", "pos": {"verbs": {"Liar": 1}, "adverbs": {"Regular": 1}, "numerals": {"91": 1}}}, {"content": "92. I have always been misinforming in writing.", "word_count": 11, "label": "", "sentence_structure": "Subject鈥搗erb鈥揷omplement", "subjective_objective": {"answer": "Subjective", "rationale": "Because \"I'm always bad at writing\" is patient's subjective feeling and self-evaluation."}, "sentence_sentiment": {"answer": "Negative", "rationale": "Because \"bad\" is negative in the entries, it is a sign of the author's dissatisfaction and frustration with his ability to write."}, "language_style": {"answer": "Tactful and warm", "rationale": "The language style of the entry is more euphemistic and implicit, expressing personal distress and suspicion by the word \"always\" and not directly accusing or criticizing others, but rather focusing more on guiding the other to express their feelings and thoughts, which is more easily acceptable to the respondents and willing to share the true situation."}, "time_dimension": {"answer": "Present", "rationale": ""}, "sentence_type": "Negative declarative sentence", "pos": {"nouns": {"Writing": 1, "Expression": 1}, "verbs": {"Always": 1}, "adjectives": {"not": 1, "Okay": 1}, "numerals": {"92": 1}}}, {"content": "93. I'm a calm person.", "word_count": 8, "label": "", "sentence_structure": "Subject鈥揷opula鈥損redicative", "subjective_objective": {"answer": "Subjective", "rationale": "Because \"cool\" is a self-awareness and evaluation of the patient, a subjective description."}, "sentence_sentiment": {"answer": "Neutral", "rationale": "Because the entry does not have any emotional color."}, "language_style": {"answer": "Tactful and warm", "rationale": "The language style of the entry is euphemism. Although it describes the character characteristics of the person under investigation, it does not use strong vocabulary or direct assertions to make a statement, but rather raises questions in a balanced and respectful manner. This expression is more acceptable and responsive and creates a better atmosphere of communication and interaction."}, "time_dimension": {"answer": "Present", "rationale": ""}, "sentence_type": "Affirmative declarative sentence", "pos": {"nouns": {"person": 1}, "verbs": {"be": 1, "Calm down": 1}, "numerals": {"93": 1}, "pronouns": {"I": 1}}}, {"content": "94. I have learned well in most courses.", "word_count": 12, "label": "", "sentence_structure": "Subject鈥搗erb鈥揷omplement", "subjective_objective": {"answer": "Subjective", "rationale": "Because \"good learning\" is a patient's subjective assessment."}, "sentence_sentiment": {"answer": "Positive", "rationale": "Because the expression \"most of the courses are learned well\" is positive."}, "language_style": {"answer": "Calm and direct", "rationale": "The language style of the entry is more direct, there is no excessive emotional or speech modification, and the tone is objective and neutral, consistent with the formal and serious characteristics of the questionnaire."}, "time_dimension": {"answer": "Present", "rationale": "\"Most\" means the majority of long-term behaviour, which is a time dimension of past or present; \"both\" means a state of existence or continuity, and a time dimension of past or present. Taken together, \"I have learned well in most of my courses.\" In this entry, it is not clear whether what happened in the past or what is currently being done, and therefore the time dimension of this article cannot be determined."}, "sentence_type": "Affirmative declarative sentence", "pos": {"verbs": {"Learn": 1, "Okay": 1}, "adjectives": {"Large": 1, "More": 1}, "adverbs": {"Extreme": 1, "Both": 1, "very": 1}, "numerals": {"94": 1}, "pronouns": {"I": 1}}}, {"content": "95. I have few friends of the same sex.", "word_count": 11, "label": "", "sentence_structure": "Subject鈥損redicate", "subjective_objective": {"answer": "Subjective", "rationale": "Because \"some friends\" are patients' subjective feelings and perceptions."}, "sentence_sentiment": {"answer": "Negative", "rationale": "Because the expression \"no few friends of the same sex\" expresses a lonely, socially inadequate emotional attitude, it is negative."}, "language_style": {"answer": "Calm and direct", "rationale": "The entry expresses the subject in a more direct and objective way 鈥?whether the other side has a small number of friends of the same sex in his social circle."}, "time_dimension": {"answer": "Present", "rationale": ""}, "sentence_type": "Negative declarative sentence", "pos": {"nouns": {"friends": 2, "Gender": 1}, "verbs": {"No, I'm not": 1, "have": 1}, "adjectives": {"Same-sex": 1}, "adverbs": {"How many": 1}, "numerals": {"95": 1}, "classifiers": {"individual": 1}, "pronouns": {"I": 1}}}, {"content": "96. I hate math.", "word_count": 5, "label": "", "sentence_structure": "Subject鈥損redicate", "subjective_objective": {"answer": "Subjective", "rationale": "Because it's a subjective emotional attitude."}, "sentence_sentiment": {"answer": "Negative", "rationale": "Because \"don't like math\" is negative."}, "language_style": {"answer": "Calm and direct", "rationale": "The language style of the entry is calm and straightforward, and it clearly expresses the subject鈥檚 aversion to mathematics, without any euphemism or hint. This expression is clear, concise and helps to obtain accurate information."}, "time_dimension": {"answer": "Past", "rationale": "The entry describes the current subjective feelings and attitudes of the testee, does not relate to future or future plans or expectations, and therefore the temporal dimension is a matter of the past."}, "sentence_type": "Negative declarative sentence", "pos": {"nouns": {"Questionnaire": 1, "Entry": 1, "Math": 1}, "verbs": {"Shit": 1, "Give": 0}, "numerals": {"96": 1}, "pronouns": {"I": 1}}}, {"content": "97. Overall, I am a loser.", "word_count": 11, "label": "", "sentence_structure": "Subject鈥揷opula鈥損redicative", "subjective_objective": {"answer": "Subjective", "rationale": "Because it is an assessment and perception of one ' s own, it is a subjective feeling of the individual."}, "sentence_sentiment": {"answer": "Negative", "rationale": "Because the name \"lost\" is negative."}, "language_style": {"answer": "Tactful and warm", "rationale": "The entry begins with a personal subjective assessment, expressing a general assessment of itself, without directly using a strong term of negative sentiment. The phrase 鈥渋n general鈥?implies a self-reflectional and summationist attitude, rather than a simple emotional release or offensive expression."}, "time_dimension": {"answer": "Present", "rationale": "So the answer is \"now\"."}, "sentence_type": "Negative declarative sentence", "pos": {"nouns": {"person": 1, "Questionnaire": 1, "Loser": 1}, "verbs": {"be": 1, "I think": 1}, "adjectives": {"Overall": 1, "One": 1, "Failed": 1}, "adverbs": {"Land": 1}, "numerals": {"97": 1}, "pronouns": {"I": 1}}}, {"content": "98. One can indeed believe that I can do things right.", "word_count": 15, "label": "", "sentence_structure": "Subject鈥搗erb鈥搊bject", "subjective_objective": {"answer": "Subjective", "rationale": "Because the word \"convinced\" is a patient's own subjective attitude or belief."}, "sentence_sentiment": {"answer": "Positive", "rationale": "It expresses a sense of confidence and optimism that it is capable of fulfilling its mandate."}, "language_style": {"answer": "Tactful and warm", "rationale": "This entry is based on a more euphemistic and encouraging language style, which expresses trust and certainty in the person under investigation, rather than direct orders or criticism."}, "time_dimension": {"answer": "Present", "rationale": "The keyword \"may\" indicates that the purpose of the article is to describe the present capabilities or circumstances, not the past or future."}, "sentence_type": "Affirmative declarative sentence", "pos": {"nouns": {"person": 1, "Things": 1}, "verbs": {"Yes": 1, "Do it": 1}, "adjectives": {"Sure": 1}, "adverbs": {"Indeed": 1, "Yeah": 1, "- Put it on": 1, "Okay": 1}, "numerals": {"98": 1}}}, {"content": "99. I have been learning very quickly in language classes.", "word_count": 10, "label": "", "sentence_structure": "Subject鈥搗erb鈥揷omplement", "subjective_objective": {"answer": "Subjective", "rationale": "Because learning is a subjective feeling and evaluation, it reflects the subjective perception of the patient of his or her performance in language classes."}, "sentence_sentiment": {"answer": "Positive", "rationale": "Because the expression \"teach quickly\" is positive."}, "language_style": {"answer": "Tactful and warm", "rationale": "The expression is more stimulating and positive, given a positive and positive expression of learning, a soft and inspiring expression that does not directly point to strengths or weaknesses."}, "time_dimension": {"answer": "Present", "rationale": ""}, "sentence_type": "Affirmative declarative sentence", "pos": {"nouns": {"I": 1, "Language classes": 1}, "verbs": {"I can learn": 1}, "adjectives": {"Come on": 1}, "adverbs": {"very": 1}, "numerals": {"99": 1}}}, {"content": "100. I am troubled by many things.", "word_count": 10, "label": "", "sentence_structure": "Subject鈥揷opula鈥損redicative", "subjective_objective": {"answer": "Subjective", "rationale": "Because \"strange\" is a subjective feeling and experience."}, "sentence_sentiment": {"answer": "Negative", "rationale": "Because the emotional expression of \"suspicion\" in the entries is negative."}, "language_style": {"answer": "Calm and direct", "rationale": "The language style of the entry is calm, objective, and there is no excessive rhetorical or emotional overtook, and it is a direct expression of the question whether the other side is often worried about the subject."}, "time_dimension": {"answer": "Present", "rationale": ""}, "sentence_type": "Affirmative declarative sentence", "pos": {"nouns": {"I": 1, "Things": 1}, "verbs": {"I feel": 1, "Quiet": 1}, "adjectives": {"Many": 1}, "numerals": {"100": 1}}}, {"content": "101. Most courses are too difficult for me.", "word_count": 11, "label": "", "sentence_structure": "Subject鈥揷opula鈥損redicative", "subjective_objective": {"answer": "Subjective", "rationale": "Because \"most\" and \"too hard\" are patients' own subjective views."}, "sentence_sentiment": {"answer": "Negative", "rationale": "Because the expression \"most of the courses are too hard for me\" is negative."}, "language_style": {"answer": "Tactful and warm", "rationale": "The problem is not one of direct attacks or criticism of the teacher or course content, but of a relatively moderate way of expressing his or her own problems. At the same time, this euphemism helps to build good communication and understanding with the other."}, "time_dimension": {"answer": "Present", "rationale": "The entry describes the current feelings and experiences of the testee, and therefore judges \"now\"."}, "sentence_type": "Negative declarative sentence", "pos": {"nouns": {"Courses": 1, "I": 1}, "verbs": {"Come on": 1, "It's too hard": 1}, "adjectives": {"Most of them": 1}, "adverbs": {"to": 1}, "numerals": {"101": 1}}}, {"content": "102. I am happy to be with friends of the same sex.", "word_count": 14, "label": "", "sentence_structure": "Subject鈥搗erb鈥揷omplement", "subjective_objective": {"answer": "Subjective", "rationale": "Because \"divine\" expresses patients' subjective attitude."}, "sentence_sentiment": {"answer": "Positive", "rationale": "Because the entry expresses a positive attitude and behaviour, i.e. a willingness to interact with friends of the same sex."}, "language_style": {"answer": "Tactful and warm", "rationale": "The entry is more adept and interactive when it is spoken in a gentle and inviting manner, asking if the other side prefers to be with friends of the same sex."}, "time_dimension": {"answer": "Present", "rationale": ""}, "sentence_type": "Affirmative declarative sentence", "pos": {"nouns": {"friends": 1, "Gender": 1}, "verbs": {"I'd love to": 1, "in": 1, "Together": 1}, "adjectives": {"Same-sex": 1}, "numerals": {"102": 1}}}]}

{"questionnaire_title": "Self-exposure and self-hidden", "questionnaire_category": "Mental health", "item_count": 12, "emotion_shift_overall": "From negative to positive", "language_style_overall": "Neutral", "items": [{"content": "I usually talk to friends when I'm sad.", "word_count": 16, "label": "", "sentence_structure": "Subject鈥搗erb鈥搊bject", "subjective_objective": {"answer": "Subjective", "rationale": "Because feeling and talking is a patient's subjective experience."}, "sentence_sentiment": {"answer": "Positive", "rationale": "Because the purpose of the \"confident to friends\" act in the entry is positive and can help to reduce negative emotions."}, "language_style": {"answer": "Tactful and warm", "rationale": "This is a more personal and interactive expression that is better able to attract the attention of the interviewees and increase the rate of recovery of questionnaires."}, "time_dimension": {"answer": "Present", "rationale": ""}, "sentence_type": "Affirmative declarative sentence", "pos": {"nouns": {"Time": 1, "friends": 1, "Speech": 1}, "verbs": {"Use it on": 1, "Sorry": 1, "In": 1, "Speech": 1}, "adverbs": {"Usually": 1}, "classifiers": {"individual": 1}, "pronouns": {"I": 1}}}, {"content": "I do not want to talk about my own problems.", "word_count": 10, "label": "", "sentence_structure": "Subject鈥損redicate", "subjective_objective": {"answer": "Subjective", "rationale": "Because of the reluctance to talk about their own problems, patients' subjective attitude."}, "sentence_sentiment": {"answer": "Negative", "rationale": "Because the \"resisting of talking about your own problems\" act in the entry is negative."}, "language_style": {"answer": "Calm and direct", "rationale": ""}, "time_dimension": {"answer": "Present", "rationale": "The keyword is \"not to talk\" and it means the current state of behaviour. \"Your problem\" is a long-standing problem, so the entry can be classified as the present."}, "sentence_type": "Negative declarative sentence", "pos": {"verbs": {"Yes": 1, "Talk": 1, "self": 1, "Problem": 1}, "adverbs": {"not": 1}, "pronouns": {"I": 1}}}, {"content": "3. When things were not pleasant to me, I used to talk about them.", "word_count": 25, "label": "", "sentence_structure": "Subject鈥搗erb鈥搊bject", "subjective_objective": {"answer": "Subjective", "rationale": "Because the descriptions of \"often\" and \"talking about these things\" are based on patients' own experience and personal feelings and are subjective."}, "sentence_sentiment": {"answer": "Positive", "rationale": "Because the behaviour in the entry \"talking\" is often seen as a positive response that helps to reduce negative emotions and gain support and understanding."}, "language_style": {"answer": "Tactful and warm", "rationale": ""}, "time_dimension": {"answer": "Present", "rationale": "I often find people, which is a customary act, and the movement occurs now or in the past. And, \"When things happen wrong with me,\" also points to current or past events, so the entry describes the current behaviour and mental state of the subject."}, "sentence_type": "Affirmative declarative sentence", "pos": {"nouns": {"Things": 2, "person": 1}, "verbs": {"Come on": 1, "Find": 1, "Talk": 1}, "adjectives": {"It's not good": 1}, "adverbs": {"Regular": 1}, "numerals": {"3": 1}}}, {"content": "I don't usually talk to people about things that upset me.", "word_count": 17, "label": "", "sentence_structure": "Subject鈥搗erb鈥搊bject", "subjective_objective": {"answer": "Subjective", "rationale": "Because it is the patient's subjective attitude to be willing to share his or her worries with others."}, "sentence_sentiment": {"answer": "Negative", "rationale": "Because the \"discussion\" in the entry shows a negative attitude to these things."}, "language_style": {"answer": "Calm and direct", "rationale": "The language style of the entry is relatively calm and straightforward, and it does not use excessive emotional qualifiers or rhetoric techniques, but rather expresses a fact or view in a simple and straightforward way."}, "time_dimension": {"answer": "Present", "rationale": "The entry \"generally not\" means that nothing is normally done, and \"irritating\" means that the current or recent situation. So it concerns the current emotional and behavioural state of the testee.\""}, "sentence_type": "Negative declarative sentence", "pos": {"nouns": {"Things": 2, "person": 1}, "verbs": {"General": 1, "Discussion": 1, "鈾?And I'm gonna be 鈾?: 1, "Sorry": 1}, "adjectives": {"not": 1}, "adverbs": {"Those": 1, "and": 1}, "numerals": {"4": 1}, "pronouns": {"I": 1}}}, {"content": "Five, when I feel depressed or sad, I always take it alone.", "word_count": 19, "label": "", "sentence_structure": "Subject鈥搗erb鈥揷omplement", "subjective_objective": {"answer": "Subjective", "rationale": "Because \"always taking it alone\" is the patient's subjective attitude and behaviour, and it's the patient's choice to deal with negative emotions. Although these emotions may be objective facts, it is the individual's subjective decision to deal with them."}, "sentence_sentiment": {"answer": "Negative", "rationale": "Because the act of \"single\" in the entry is negative, it expresses the lack of an attitude of seeking help and support in the face of difficulties and setbacks."}, "language_style": {"answer": "Calm and direct", "rationale": ""}, "time_dimension": {"answer": "Present", "rationale": "The keywords \"when frustrated or sad\" and \" always taking it alone\" point to the person's current behaviour and mentality, so the entry is asking about the person's current state."}, "sentence_type": "Affirmative declarative sentence", "pos": {"nouns": {"Frustration": 1, "Sorry": 1, "Time": 1, "Undertaking": 1}, "verbs": {"I feel": 1, "be": 1, "Always": 1, "Alone": 1, "Undertaking": 1}, "adverbs": {"When": 1, "or": 1, "Always": 1, "Alone": 1}, "classifiers": {"individual": 1}}}, {"content": "I'll talk to someone about my own problems.", "word_count": 11, "label": "", "sentence_structure": "Subject鈥損redicate", "subjective_objective": {"answer": "Subjective", "rationale": "Because \"talking\" is a subjective will and action of a person, reflecting the individual鈥檚 strategic choice to solve the problem. While this may be influenced by objective factors (such as whether or not there is a suitable person to talk), the final decision-making power is in the individual鈥檚 hands."}, "sentence_sentiment": {"answer": "Positive", "rationale": "Because the purpose of the entry is to get someone to talk about their own problems."}, "language_style": {"answer": "Tactful and warm", "rationale": "The entry is more euphemistic and warm, using an invitation tone to encourage the respondent to seek help and advice from others when he encounters problems, rather than ordering or compelling. At the same time, the entry does not directly ask the person ' s feelings or emotional state, but rather directs the person ' s thoughts and answers by describing the behaviour, which is more moderate and friendly."}, "time_dimension": {"answer": "Present", "rationale": "The word \"will\" denotes the current state or capacity, i.e. the ability of the speaker to fill out the questionnaire, and thus the judgement is now."}, "sentence_type": "Affirmative declarative sentence", "pos": {"nouns": {"person": 1, "Problem": 1}, "verbs": {"I'll find it": 1, "Talk": 1}, "adverbs": {"self": 1}}}, {"content": "Seven, when I'm in a bad mood, I'll talk to my friends.", "word_count": 16, "label": "", "sentence_structure": "Subject鈥搗erb鈥搊bject", "subjective_objective": {"answer": "Subjective", "rationale": "Because \"unwell\" and \"talking to friends\" are the feelings and behaviours of the patient, and they are viewed from a subjective perspective."}, "sentence_sentiment": {"answer": "Positive", "rationale": "Because the purpose of the \"Crying for a Friend\" act in the entry is positive."}, "language_style": {"answer": "Tactful and warm", "rationale": "The language style of the entry is more euphemistic and sweet, as it does not directly order or criticize the behaviour of the other party, but rather asks in an understanding and caring tone. At the same time, by using the word \"me\" the question is more personal and friendly, and it is easier to resonate and share with the respondents."}, "time_dimension": {"answer": "Past", "rationale": "\"I'm in a bad mood\" suggests that this is a description of past or present behavior, and \"to talk to friends\" means that it's a conventional or conventional response to the test. So, the time dimension of this questionnaire entry is past or present."}, "sentence_type": "Affirmative declarative sentence", "pos": {"nouns": {"Time": 1, "friends": 1, "Chat": 1}, "verbs": {"Yes": 2, "Find": 1}, "adjectives": {"Not good": 1}, "numerals": {"7": 1}, "pronouns": {"I": 1}}}, {"content": "If I'm sad, I'm the last person I want to talk to.", "word_count": 15, "label": "", "sentence_structure": "Subject鈥搗erb鈥搊bject", "subjective_objective": {"answer": "Subjective", "rationale": "Because \"no\" is an expression of subjective emotions and attitudes, and \"no one else\" is an expression of behaviour, it is a subjective question."}, "sentence_sentiment": {"answer": "Negative", "rationale": "Because the \"most reluctant to talk to anyone\" act in the entry is negative."}, "language_style": {"answer": "Calm and direct", "rationale": "The language style of the entry is more straightforward, there is no excessive rhetorical or emotional overtook, and the subject of \"resistance to talk\" is expressed in an objective statement, which is more incriminating."}, "time_dimension": {"answer": "Present", "rationale": "The keyword \"I'm sorry\" and \"I'm the last to talk to\" means the person who talks to you is the emotional and behavioural attitude of the person who talks, so the entry is in the current category."}, "sentence_type": "Negative declarative sentence", "pos": {"nouns": {"Sorry": 1, "others": 1, "Speech": 1}, "verbs": {"Yes": 1, "Find": 1, "Speech": 1}, "adverbs": {"I hate it the most": 1}, "numerals": {"8": 1}}}, {"content": "When I encounter difficulties, I rarely talk to anyone about them.", "word_count": 21, "label": "", "sentence_structure": "Subject鈥搗erb鈥搊bject", "subjective_objective": {"answer": "Subjective", "rationale": "Because it is a subjective decision and behaviour of one to talk about difficulties."}, "sentence_sentiment": {"answer": "Negative", "rationale": "Because the \"very few people talk about these difficulties\" act in the entry is negative."}, "language_style": {"answer": "Calm and direct", "rationale": "The language style of the entry is a calm and direct type. It directly describes the fact that the person surveyed has less communication with others in the face of difficulties, and does not use excessive rhetorical or emotional colours to describe the act, but rather to express it in objective and concise language."}, "time_dimension": {"answer": "Past", "rationale": "The keywords \"when in distress\" and \"very rarely talk to others\" point to the conduct and circumstances of the past. The temporal dimension of this article is therefore a matter of the past."}, "sentence_type": "Negative declarative sentence", "pos": {"nouns": {"Hard": 1, "Time": 1, "others": 1, "Talk": 1, "Difficulties": 2}, "verbs": {"Yes": 1, "Find": 1, "Very few": 1, "Talk": 1}, "adjectives": {"Hard": 1, "Location": 1}, "adverbs": {"Less": 1, "When": 1}, "numerals": {"9": 1, "2": 1}, "pronouns": {"I": 1}}}, {"content": "I'm not gonna tell anyone when I'm in pain.", "word_count": 15, "label": "", "sentence_structure": "Subject鈥搗erb鈥揷omplement", "subjective_objective": {"answer": "Subjective", "rationale": "Because \"Tell anyone\" is a choice and a decision of a person, and it is therefore subjective."}, "sentence_sentiment": {"answer": "Negative", "rationale": "Because the \"not telling anyone\" in the entry is negative."}, "language_style": {"answer": "Calm and direct", "rationale": "The language style of the entry is more direct and concise, with no excessive accoutrements or emotional rendering, and conveys the core meaning of the issue mainly in a straightforward manner."}, "time_dimension": {"answer": "Present", "rationale": "The keyword \"never tell anyone\" indicates that it is a customary or recurrent act, occurring when the person tested feels pain, and therefore the temporal dimension is now judged."}, "sentence_type": "Negative declarative sentence", "pos": {"nouns": {"Time": 1, "Pain": 1}, "verbs": {"Yes": 1, "Tell": 1, "Any": 1, "person": 1}, "adjectives": {"of": 2}, "numerals": {"10": 1}, "pronouns": {"I": 1}}}, {"content": "I usually talk to someone when I'm in a bad mood.", "word_count": 16, "label": "", "sentence_structure": "Subject鈥搗erb鈥搊bject", "subjective_objective": {"answer": "Subjective", "rationale": "Like being patient subjective."}, "sentence_sentiment": {"answer": "Positive", "rationale": "Because the purpose of the \"Call for Talk\" in the entry is positive."}, "language_style": {"answer": "Tactful and warm", "rationale": "The language style of the entry is a euphemism. Using the term \"general\" shows that it is a common behaviour, not an absolute situation, while avoiding pressure or discomfort on the person under investigation. Moreover, the description of \"challenge\" is more friendly and inclusive, encouraging the interviewee to share his feelings and experiences, and increasing the relevance and credibility of the questionnaire."}, "time_dimension": {"answer": "Present", "rationale": "The entry indicates that the testee is describing a current behaviour, and that \"general\" means that it is a normal practice, so it can be judged that this is the present situation."}, "sentence_type": "Affirmative declarative sentence", "pos": {"nouns": {"Time": 1, "person": 1, "Chat": 1}, "verbs": {"The mood": 1, "Okay": 1, "Find": 1, "Talk": 1}, "adjectives": {"It's not good": 1}, "numerals": {"11": 1}, "pronouns": {"I": 1}}}, {"content": "I'd like to tell someone I'm not happy.", "word_count": 14, "label": "", "sentence_structure": "Subject鈥搗erb鈥搊bject", "subjective_objective": {"answer": "Subjective", "rationale": "Because \"yes\" is the patient's own mental state and will, and it's a subjective examination."}, "sentence_sentiment": {"answer": "Positive", "rationale": "Since the entry expresses an open and frank attitude and a willingness to share its own negative sentiments, this behaviour is often considered positive."}, "language_style": {"answer": "Tactful and warm", "rationale": "The entry is more euphemistic and gentle, using the means of questioning rather than direct orders or statements, making the respondents more comfortable and willing to share their thoughts and feelings. At the same time, using negative expressions such as \"no\" to question the will of the other party, it is more likely that the respondents will be free to express their true thoughts and feelings."}, "time_dimension": {"answer": "Present", "rationale": "The entry is a survey of the person's current behaviour or attitude, so it can be judged as \"now\"."}, "sentence_type": "Affirmative declarative sentence", "pos": {"nouns": {"Questionnaire": 1, "Entry": 1, "Yes": 1}, "verbs": {"Yes": 1, "Tell": 1}, "adjectives": {"I'm not happy": 1}, "numerals": {"12": 1}, "pronouns": {"I": 1}}}]}

{"questionnaire_title": "Texas Social Behaviour Questionnaire", "questionnaire_category": "Behavioral questionnaire", "item_count": 32, "emotion_shift_overall": "From negative to positive", "language_style_overall": "Tactful and warm", "items": [{"content": "I'm not usually going to talk unless I'm first spoken to.", "word_count": 16, "label": "", "sentence_structure": "Subject鈥搗erb鈥搊bject", "subjective_objective": {"answer": "Subjective", "rationale": "This is because the patient knows and expresses himself in his social habits."}, "sentence_sentiment": {"answer": "Negative", "rationale": "Because the \"no talk\" act in the entry is negative."}, "language_style": {"answer": "Calm and direct", "rationale": "The entry presents the behaviour of the person under investigation in the social setting in a direct and clear language style, without the use of excessive rhetorical or emotional rendering, and with a more objective tone."}, "time_dimension": {"answer": "Past", "rationale": "Past."}, "sentence_type": "Negative declarative sentence", "pos": {"nouns": {"Questionnaire": 1, "Entry": 1, "others": 1, "Speech": 1, "General": 1, "Open up": 1}, "verbs": {"Analysis": 1, "Yes": 1}, "adjectives": {"First": 1, "not": 1}, "adverbs": {"Unless": 1, "General": 1}, "pronouns": {"I": 1}}}, {"content": "2 I think I'm confident.", "word_count": 7, "label": "", "sentence_structure": "Subject鈥揷opula鈥損redicative", "subjective_objective": {"answer": "Subjective", "rationale": "Because \"think\" and \"be sure\" are subjective views of the patient himself."}, "sentence_sentiment": {"answer": "Positive", "rationale": "Because the entry \"I think I'm confident\" expresses a positive attitude and self-awareness."}, "language_style": {"answer": "Calm and direct", "rationale": "The entry was not based on any rhetorical or emotional projection, but rather directly presented the attitudes and views of the person under investigation and was more objective in terms of tone."}, "time_dimension": {"answer": "Present", "rationale": "The words \"conception\" and \"very confident\" in this questionnaire are both descriptions of the state of affairs, \"I think\" means subjective feeling, and \"very confident\" means a level of confidence in their abilities or qualities. So the entry asks the testee's self-evaluation now, that is, a perception of self-confidence."}, "sentence_type": "Affirmative declarative sentence", "pos": {"nouns": {"2": 1, "I think": 1, "very": 1, "Confidence": 1}, "verbs": {"I think": 1, "Confidence": 1}, "adverbs": {"very": 1}, "numerals": {"2": 1}, "pronouns": {"I": 1}}}, {"content": "I'm confident in my appearance.", "word_count": 10, "label": "", "sentence_structure": "Subject鈥搗erb鈥揷omplement", "subjective_objective": {"answer": "Subjective", "rationale": "Because self-confidence is a subjective feeling and attitude, an assessment of the appearance of one ' s own, and an expression of an inner state of mind."}, "sentence_sentiment": {"answer": "Positive", "rationale": "Because it expresses a positive emotional attitude, namely self-identification and self-confidence."}, "language_style": {"answer": "Tactful and warm", "rationale": "The entry is expressed in a spirit of encouragement and support to ask the other side whether they are confident in their own appearance rather than directly questioning or criticizing, and in a way that is more conducive to a positive and positive psychological atmosphere."}, "time_dimension": {"answer": "Present", "rationale": ""}, "sentence_type": "Affirmative declarative sentence", "pos": {"verbs": {"to": 1, "self": 1, "Appearance": 1, "very": 1, "Confidence": 1}, "adjectives": {"of": 1}, "adverbs": {"very": 1}, "pronouns": {"I": 1}}}, {"content": "I'm a good person.", "word_count": 9, "label": "", "sentence_structure": "Subject鈥揷opula鈥損redicative", "subjective_objective": {"answer": "Subjective", "rationale": "Because being a good person is a subjective judgment of personal character."}, "sentence_sentiment": {"answer": "Neutral", "rationale": "The entry describes the characteristics of the personality and does not have a clear emotional tendency."}, "language_style": {"answer": "Tactful and warm", "rationale": "The language style of the entry is euphemistic. It describes itself in self-proclaimed language, and does not use negative or critical language. This expression is usually more comfortable and acceptable."}, "time_dimension": {"answer": "Past", "rationale": "The entry is a statement describing the characteristics or characteristics of the testee, which is judged by the temporal dimension as the past."}, "sentence_type": "Affirmative declarative sentence", "pos": {"verbs": {"be": 1, "Yes": 1, "on": 1, "Hand it over": 1, "International": 1}, "adjectives": {"individual": 1}, "numerals": {"4": 1}, "pronouns": {"I": 1}}}, {"content": "In the crowd, I can hardly think of a proper subject.", "word_count": 14, "label": "", "sentence_structure": "Subject鈥揷opula鈥損redicative", "subjective_objective": {"answer": "Subjective", "rationale": "Because \"the right subject\" is a person's subjective perception and ability, and it's the patient's own feelings and experiences."}, "sentence_sentiment": {"answer": "Negative", "rationale": "Because the \"unable to come up with a decent subject\" in the entry is negative."}, "language_style": {"answer": "Tactful and warm", "rationale": "The language style of the entry is more euphemistic. It guides the interviewee to share his thoughts, not direct orders or criticisms. Moreover, using the term \"unfortunate\" avoids undermining the possibility of the interviewee, but rather expresses understanding and support for them. This expression helps to create a good atmosphere of communication and encourages the interviewee to be more willing to share his feelings and experiences."}, "time_dimension": {"answer": "Present", "rationale": "The keywords \"group\" and \"hard to come to terms\" indicate that the situation is a description of current or recent experience, so the entry is now judged."}, "sentence_type": "Negative declarative sentence", "pos": {"nouns": {"Population": 1, "Topic": 1}, "verbs": {"I've got it": 1, "Nice": 1}, "adjectives": {"Hard": 1}, "adverbs": {"very": 1}, "numerals": {"5": 1}}}, {"content": "In the crowd, I usually do what others need to do, not make recommendations.", "word_count": 23, "label": "", "sentence_structure": "Subject鈥搗erb鈥搊bject", "subjective_objective": {"answer": "Subjective", "rationale": "Because it involves the patient ' s personal orientation and attitude, it is subjective."}, "sentence_sentiment": {"answer": "Negative", "rationale": "Basis of judgement: because the behaviour in the entry \"does not recommend\" may result in the views and ideas of individuals being ignored or rejected, thereby negatively affecting the self-expression and growth of individuals. Thus, the emotional colour of this purpose is negative."}, "language_style": {"answer": "Tactful and warm", "rationale": "The entry is more gentle and friendly by asking about the behaviour of the other party to understand their character and does not directly express an evaluation or recommendation."}, "time_dimension": {"answer": "Present", "rationale": "The keyword \"usually\" means that it is a regular act, and \"in the crowd\" and \"to do what is in the best interests of others\" all indicate that it is a social act. So, this entry asks about the current social behavior habits of the person being tested."}, "sentence_type": "Affirmative declarative sentence", "pos": {"nouns": {"Population": 1, "Yes": 1, "Recommendations": 1}, "verbs": {"Do it": 1, "T": 1, "Go on": 1, "Match": 1}, "adverbs": {"Usually": 1}, "numerals": {"6": 1}, "pronouns": {"I": 1}}}, {"content": "My views are often dominant, in the absence of others.", "word_count": 19, "label": "", "sentence_structure": "Subject鈥搗erb鈥搊bject", "subjective_objective": {"answer": "Subjective", "rationale": "Because it involves the personal views and attitudes of patients, it is a review of their self-perception and beliefs."}, "sentence_sentiment": {"answer": "Positive", "rationale": "Because the article's expression is 鈥渢he preference of the view鈥? which implies that the respondent often wins in the debate, it is a positive expression."}, "language_style": {"answer": "Calm and direct", "rationale": "The entry describes in a straightforward manner the preferences and strong attitudes of a person in the face of different views, the lack of excessive rhetoric or emotional rendering, and the calmer and directness of the language style."}, "time_dimension": {"answer": "Present", "rationale": "\"I am not the same as others\" suggests that this is a particular situation or event, and \"my view is often the dominant\" suggests that it is a constant situation or behaviour, so the entry describes the current or present situation."}, "sentence_type": "Affirmative declarative sentence", "pos": {"nouns": {"7": 1, "person": 1, "Observations": 1, "Advantages": 1}, "verbs": {"in": 1, "Jim": 1, "Time": 1}, "adjectives": {"Other": 1}, "numerals": {"Regular": 1}, "pronouns": {"I": 1}}}, {"content": "I think I am a man who is interested in shaping the situation.", "word_count": 17, "label": "", "sentence_structure": "Subject鈥搗erb鈥搊bject", "subjective_objective": {"answer": "Subjective", "rationale": "Because \"think\" is a personal perception and attitude, and it reflects its own subjective view."}, "sentence_sentiment": {"answer": "Positive", "rationale": "Because the entry \"intentional to influence the situation\" expresses a positive attitude and self-awareness."}, "language_style": {"answer": "Tactful and warm", "rationale": "The entry uses a more euphemistic language style, which does not directly identify the personality characteristics or characteristics of the other, but rather raises questions in a question-and-answer manner, using positive terms such as \"intentional\" and \"right\" to guide the respondents to express their views and attitudes."}, "time_dimension": {"answer": "Present", "rationale": "The sentence describes a current self-perception, which means that the testee is assessing his character and behaviour patterns. So the temporal dimension is now."}, "sentence_type": "Affirmative declarative sentence", "pos": {"nouns": {"self": 1, "person": 1, "Situation": 1}, "verbs": {"I think": 1, "be": 1, "Intent to": 1, "Around": 1}, "adjectives": {"Intent to": 1}, "numerals": {"8": 1}, "pronouns": {"I": 1}}}, {"content": "Nine others saw me.", "word_count": 7, "label": "", "sentence_structure": "Subject鈥搗erb鈥揷omplement", "subjective_objective": {"answer": "Objective", "rationale": "Because it is an objective fact to be seen by others, although it may be influenced by the patient ' s self-perception and subjective feelings."}, "sentence_sentiment": {"answer": "Neutral", "rationale": "Because the entry is not clearly emotional, it is merely a statement of facts."}, "language_style": {"answer": "Tactful and warm", "rationale": ""}, "time_dimension": {"answer": "Present", "rationale": "The entry asks about the current feelings and perceptions of the testee, and the time dimension is now judged."}, "sentence_type": "Affirmative declarative sentence", "pos": {"nouns": {"person": 1, "Look": 1, "Rise": 1}, "verbs": {"Okay": 1, "Rise": 1}, "adjectives": {"Other": 1}, "pronouns": {"I": 1}}}, {"content": "I love social parties, just want to be with people.", "word_count": 16, "label": "", "sentence_structure": "Subject鈥搗erb鈥搊bject", "subjective_objective": {"answer": "Subjective", "rationale": "Because \"like\" represents the feelings and attitudes of the patient, it's a subjective feeling."}, "sentence_sentiment": {"answer": "Neutral", "rationale": "Because the entry does not have any emotional color."}, "language_style": {"answer": "Tactful and warm", "rationale": "Warm and warm."}, "time_dimension": {"answer": "Present", "rationale": "The entry shows that the testee likes social gatherings and is now experiencing them. Although there may be similar experiences or expectations in the past and the future, by the way, \"I love social gatherings, I just want to be with people\" is a current feeling and situation."}, "sentence_type": "Affirmative declarative sentence", "pos": {"nouns": {"Questionnaire": 1, "Entry": 1, "People": 1, "Party": 1}, "verbs": {"Love it": 1, "in": 2, "Together": 1}, "adverbs": {"Just": 1}, "numerals": {"10": 1}, "pronouns": {"I": 1}}}, {"content": "I'm watching someone else's face.", "word_count": 9, "label": "", "sentence_structure": "Subject鈥搗erb鈥揷omplement", "subjective_objective": {"answer": "Subjective", "rationale": "Because it's the patient's subjective attitude to look at other people's faces."}, "sentence_sentiment": {"answer": "Negative", "rationale": "Because the entry describes the tendency of individuals to be sensitive to emotional reactions of others, which may lead to excessive anxiety or over-sensitivity to reactions of others, it is somewhat negative."}, "language_style": {"answer": "Tactful and warm", "rationale": "The entry uses a euphemism, which gives a sense of kindness and moderation by using the descriptive term \"careful\" rather than direct presentation. At the same time, it implies concern and sensitivity for the emotional changes of others, which may make the other feel understood and supported, thus enhancing the effectiveness of the questionnaire and the participation of the respondents."}, "time_dimension": {"answer": "Present", "rationale": "I'm aware that the move is now, and \"other person's face\" is a situational setting, which means that the person who talks is watching someone's face at the moment, so it's now."}, "sentence_type": "Affirmative declarative sentence", "pos": {"verbs": {"Watch out": 1}, "adjectives": {"very": 1, "Someone else's": 1}, "adverbs": {"very": 1}, "numerals": {"11": 1}, "pronouns": {"I": 1}}}, {"content": "I don't seem to be able to get anyone else to notice me.", "word_count": 12, "label": "", "sentence_structure": "Subject鈥揷opula鈥損redicative", "subjective_objective": {"answer": "Subjective", "rationale": "Because \"not getting anyone's attention\" is a personal feeling or observation, it is subjective."}, "sentence_sentiment": {"answer": "Negative", "rationale": "Because the expression \"not able to get anyone to notice me\" in the entry is negative."}, "language_style": {"answer": "Tactful and warm", "rationale": "The entry is more adept and interactive when it is spoken in a gentle and inviting manner, rather than by direct statements or orders."}, "time_dimension": {"answer": "Past", "rationale": ""}, "sentence_type": "Negative declarative sentence", "pos": {"nouns": {"I": 1, "Other": 1, "person": 1, "Attention": 1}, "verbs": {"Seems like": 1, "Jean": 1, "Attention": 1}, "adverbs": {"Cannot": 1, "Seems like": 1}, "numerals": {"12": 1}}}, {"content": "I do not want to be too responsible for others.", "word_count": 12, "label": "", "sentence_structure": "Subject鈥搗erb鈥搊bject", "subjective_objective": {"answer": "Subjective", "rationale": "Because it expresses the views or attitudes of individuals who are unwilling to assume excessive responsibilities, it is their own feeling and thinking."}, "sentence_sentiment": {"answer": "Negative", "rationale": "Because the attitude of \"not taking too much responsibility for others\" in the entry is negative."}, "language_style": {"answer": "Calm and direct", "rationale": "The language style of the entry is more straightforward, and the tone is relatively calm and objective without too many qualifiers or emotional expressions. It asks the interviewee whether he or she is unwilling to be responsible for others' responsibilities, which is more clear about what the problem means and helps researchers to get accurate data feedback."}, "time_dimension": {"answer": "Past", "rationale": "\"No\" and \"N\" are both past movements or states, which means that the entry is to question the person who was tested for past psychological feelings or behaviour."}, "sentence_type": "Negative declarative sentence", "pos": {"nouns": {"Others": 1, "Responsibility": 1}, "verbs": {"I don't want to": 1, "Negative": 1}, "adjectives": {"Too much": 1}, "adverbs": {"not": 1}, "numerals": {"13": 1}, "pronouns": {"I": 1}}}, {"content": "I was comfortable when a powerful man approached me.", "word_count": 17, "label": "", "sentence_structure": "Subject鈥揷opula鈥損redicative", "subjective_objective": {"answer": "Subjective", "rationale": "Because \"favouring\" is a subjective feeling, different people may feel different about the same thing. The answer to this questionnaire is therefore subjective."}, "sentence_sentiment": {"answer": "Negative", "rationale": "Because the description of \"sweet\" in the entry implies a form of worship and submissiveness to power, which is negative."}, "language_style": {"answer": "Calm and direct", "rationale": "The entry asked the interviewees in a direct and objective manner about their feelings when facing powerful people, without excessive rhetoric or emotional rendering, and with no clarity about the temperature of the speech."}, "time_dimension": {"answer": "Past", "rationale": "The temporal dimension of the keyword \"the past\" suggests that the entry is to ask the testee about his past experiences and feelings."}, "sentence_type": "Affirmative declarative sentence", "pos": {"nouns": {"person": 1, "Power": 1}, "verbs": {"Close": 1, "I feel": 1}, "adjectives": {"- Yeah": 1}, "numerals": {"14": 1}, "pronouns": {"I": 1}}}, {"content": "I think I'm indecisive.", "word_count": 9, "label": "", "sentence_structure": "Subject鈥搗erb鈥揷omplement", "subjective_objective": {"answer": "Subjective", "rationale": "Because \"think\" means that it's subjective to the patient."}, "sentence_sentiment": {"answer": "Negative", "rationale": "Because \"indecisive\" is a negative quality in the entry."}, "language_style": {"answer": "Tactful and warm", "rationale": "The entry uses the first-person pronoun, \"I,\" which makes the issue more intimate and interactive. At the same time, \"indecisiveness\" is a negative character description, but it is not directly blamed or criticized in a context, but rather led the testee to reflect on his character in a more euphemistic way. Thus, the language style of the purpose can be described as \"fluent warmness.\""}, "time_dimension": {"answer": "Present", "rationale": ""}, "sentence_type": "Affirmative declarative sentence", "pos": {"nouns": {"I": 1, "Indecisiveness": 1}, "verbs": {"I think": 1}, "adverbs": {"15": 1}, "numerals": {"15": 1}}}, {"content": "I have no doubt about my social skills.", "word_count": 13, "label": "", "sentence_structure": "Subject鈥搗erb鈥揷omplement", "subjective_objective": {"answer": "Subjective", "rationale": "Because it's the patient's assessment and perception of self-socialization.\""}, "sentence_sentiment": {"answer": "Positive", "rationale": "Because the expression \"you can't be sure about your social skills\" has a positive emotional connotation."}, "language_style": {"answer": "Tactful and warm", "rationale": "The language style of the entry is euphemism. It expresses confidence in its social skills in a positive and positive tone, and is not expressed too directly or with too much rigour, but in a moderate way, which is more acceptable and understandable."}, "time_dimension": {"answer": "Present", "rationale": "The keyword \"I am\" is a perception or attitude of the dominant language towards myself, and \"without doubt\" is a sign of the firmness and self-confidence of that attitude. The number \"16\" in \"I\" is understood as a serial word, indicating that the entry is part of the questionnaire. In conclusion, the temporal dimension of the article is now judged."}, "sentence_type": "Affirmative declarative sentence", "pos": {"nouns": {"I": 1, "Social skills": 1}, "verbs": {"to": 1, "No doubt": 2}, "adjectives": {"Your own": 1, "Social": 1}, "adverbs": {"of": 1}}}, {"content": "I think I'm socially clumsy.", "word_count": 12, "label": "", "sentence_structure": "Subject鈥搗erb鈥揷omplement", "subjective_objective": {"answer": "Subjective", "rationale": "Because \"conception\" expresses the patient's personal views and evaluation, it is viewed from the patient's subjective perspective."}, "sentence_sentiment": {"answer": "Negative", "rationale": "Because the expression \"self-mute\" in the entry is derogatory, and it expresses a self-denial sentiment, and it is negative."}, "language_style": {"answer": "Calm and direct", "rationale": "The entry asks the interviewee in an objective and direct manner about his/her social skills, without too many rhetorical or emotional qualifications, and is more calm in his/her language style."}, "time_dimension": {"answer": "Past", "rationale": "The \"conception\" and \"puzzling\" have both adopted the past pattern, which means that it is a self-perception and evaluation of the past, and the temporal dimension of the article is therefore the past."}, "sentence_type": "Negative declarative sentence", "pos": {"nouns": {"self": 1, "Social": 1}, "verbs": {"I think": 1, "Clumsy": 1}, "pronouns": {"I": 1}}}, {"content": "I often find it difficult to defend my own views if they conflict with others.", "word_count": 26, "label": "", "sentence_structure": "Subject鈥搗erb鈥搊bject", "subjective_objective": {"answer": "Subjective", "rationale": "Because the idea that I find myself having difficulty holding my opinion when it's not in line with others is based on personal subjective experience."}, "sentence_sentiment": {"answer": "Negative", "rationale": "Because the behavior in the entry \"hard to defend itself\" is negative."}, "language_style": {"answer": "Tactful and warm", "rationale": ""}, "time_dimension": {"answer": "Past", "rationale": "In conclusion, the temporal dimension of the article is \"now\"."}, "sentence_type": "Affirmative declarative sentence", "pos": {"nouns": {"Conflict": 1, "person": 1, "Views": 1}, "verbs": {"Found": 1, "Maintenance": 1, "Conflict": 1}, "adverbs": {"Regular": 1}, "numerals": {"2": 1}, "pronouns": {"I": 2}}}, {"content": "I'd love to think of myself as a very strong person.", "word_count": 18, "label": "", "sentence_structure": "Subject鈥揷opula鈥損redicative", "subjective_objective": {"answer": "Subjective", "rationale": "Because \"yes\" is about the patient's subjective attitude."}, "sentence_sentiment": {"answer": "Positive", "rationale": "Because the entry \"are willing\" expresses a positive attitude and self-confidence."}, "language_style": {"answer": "Tactful and warm", "rationale": "The language style of the questionnaire entry is euphemistic, as it uses the query expression and the positive word \"willing\" while avoiding too direct or negative language. This expression is more likely to trigger resonance and active participation by the respondents, thus obtaining more accurate results."}, "time_dimension": {"answer": "Present", "rationale": "\"I'd love to see myself as\" means that the testee's current attitude and thoughts, and \"the person with the strong personality\" is the testee's self-awareness and evaluation, so the time dimension of the article is now."}, "sentence_type": "Affirmative declarative sentence", "pos": {"nouns": {"self": 1, "person": 1}, "verbs": {"Look at it": 1, "be": 1}, "adjectives": {"very": 2, "He's a very strong person": 1}, "pronouns": {"One": 1}}}, {"content": "I'd be happy to be in charge if I served on a committee.", "word_count": 18, "label": "", "sentence_structure": "Subject鈥搗erb鈥搊bject", "subjective_objective": {"answer": "Subjective", "rationale": "Because \"I'd love to run\" is the attitude and the will of the patient himself, and it's a subjective examination."}, "sentence_sentiment": {"answer": "Positive", "rationale": "Because the \"divide to be in charge\" act is positive."}, "language_style": {"answer": "Tactful and warm", "rationale": "The language style of the entry is more euphemistic, as it uses polite expressions to raise questions, without direct orders or instructions. At the same time, the word \"deep\" conveys positive attitudes and intentions, making dialogue more friendly and harmonious."}, "time_dimension": {"answer": "Future", "rationale": "\"Staffing in a committee\" and \"I'd love to be in charge\" are both visions and expectations of the future, and therefore judgement is future."}, "sentence_type": "Affirmative declarative sentence", "pos": {"nouns": {"Committees": 1, "Work": 1, "Gladly": 1, "Yes": 1}, "verbs": {"in": 1, "Pipe": 1}, "adverbs": {"If": 1, "In some place": 1, "Medium": 1, "very": 1}, "pronouns": {"I": 1}}}, {"content": "I have always expected success in what I have done.", "word_count": 15, "label": "", "sentence_structure": "Subject鈥搗erb鈥搊bject", "subjective_objective": {"answer": "Subjective", "rationale": "Because \"expectation\" is the individual psychological feeling and expectation of the patient, and it is subjective."}, "sentence_sentiment": {"answer": "Positive", "rationale": "Because the attitude of \"impressing success in what you do\" is positive."}, "language_style": {"answer": "Calm and direct", "rationale": "The entry directly asks the interviewee whether he or she has any expectations of success in what he or she does, the language style is more neutral and objective, and there is no excessive qualifier or emotional colour. It is therefore judged as \"cool and direct\" language style."}, "time_dimension": {"answer": "Past", "rationale": ""}, "sentence_type": "Affirmative declarative sentence", "pos": {"nouns": {"I": 1, "Yes": 1, "Success": 1}, "verbs": {"Expectations": 1, "Access": 1}, "adjectives": {"Regular": 1}, "numerals": {"5": 1}}}, {"content": "I'm comfortable with people who are better off than me.", "word_count": 17, "label": "", "sentence_structure": "Subject鈥搗erb鈥揷omplement", "subjective_objective": {"answer": "Subjective", "rationale": "Because feeling comfortable is a subjective experience and feeling."}, "sentence_sentiment": {"answer": "Positive", "rationale": "Because \"sweet\" is a positive emotional experience."}, "language_style": {"answer": "Tactful and warm", "rationale": "The language style of this entry is more euphemistic. It asks each other in an invitation-style way about how they feel when they interact with someone who is more than their own. It also uses the positive word \"very comfortable\" to show concern and concern for each other. Overall, this expression is more personal and interactive."}, "time_dimension": {"answer": "Present", "rationale": "The entry \"to deal with someone higher than me\" means a regular act, and \"I feel comfortable\" is the psychological state and feeling that it brings. So, the entry describes the situation."}, "sentence_type": "Affirmative declarative sentence", "pos": {"nouns": {"person": 1, "- To be with someone": 1, "Status": 1, "- Yeah": 1}, "verbs": {"I feel": 1, "Dealing": 1, "- Yeah": 1}, "adjectives": {"High": 1, "very": 1}, "numerals": {"6": 1}, "pronouns": {"I": 1}}}, {"content": "I like to be around people and I'm keen on finding social contacts.", "word_count": 19, "label": "", "sentence_structure": "Subject鈥搗erb鈥搊bject", "subjective_objective": {"answer": "Subjective", "rationale": "Because the desire to seek social contact opportunities is the patient ' s personal emotion and attitude and is subject matter."}, "sentence_sentiment": {"answer": "Positive", "rationale": "Because the entries \"like to be in contact\" and \"really looking for opportunities for social contact\" express a positive emotional attitude, namely, the character of a person who likes to be in contact and is socially good."}, "language_style": {"answer": "Tactful and warm", "rationale": "The language style of the entry is more euphemistic, presenting the attitude and preferences of the respondents by using positive words such as \"like\" and \"interest in\" and attractive expressions such as \"seeking social contact\". This approach is more likely to resonate with the respondents and increase their motivation to answer the questionnaire."}, "time_dimension": {"answer": "Present", "rationale": ""}, "sentence_type": "Affirmative declarative sentence", "pos": {"nouns": {"Questionnaire": 1, "Entry": 1, "person": 1, "Exposure": 1, "Opportunities": 1}, "verbs": {"Yeah": 1, "Round": 1, "Search": 1, "A passion": 1, "Social": 1}, "adjectives": {"Down there": 1, "I": 1}, "adverbs": {"and": 1, "on": 1, "With people": 1, "Round": 1, "I'm so excited": 1, "Looking for social contact": 1}, "pronouns": {"Here": 1}}}, {"content": "I'm confident in my own social behavior.", "word_count": 12, "label": "", "sentence_structure": "Subject鈥揷opula鈥損redicative", "subjective_objective": {"answer": "Subjective", "rationale": "Because self-confidence is a subjective attitude or emotional experience, a recognition of one鈥檚 abilities, values, etc. The subject is to see if the patient is confident in his social behaviour, and therefore subjective."}, "sentence_sentiment": {"answer": "Positive", "rationale": "The article is a sign of self-confidence in the social behaviour of the people."}, "language_style": {"answer": "Calm and direct", "rationale": "The entry is a simple and clear expression of the self-confidence of the subject in his or her social behaviour, and does not have too many emotional renderings or rhetorical techniques, so that the language style is straightforward and calm."}, "time_dimension": {"answer": "Present", "rationale": ""}, "sentence_type": "Affirmative declarative sentence", "pos": {"nouns": {"self": 1, "Socialism": 1}, "verbs": {"to": 1, "Confidence": 1}, "adjectives": {"very": 1}, "numerals": {"8": 1}}}, {"content": "I feel I'm sure I'm close to and dealing with anyone I've met.", "word_count": 23, "label": "", "sentence_structure": "Subject鈥揷opula鈥損redicative", "subjective_objective": {"answer": "Subjective", "rationale": "Because the phrase \"I feel\" indicates that it's a patient's subjective feeling."}, "sentence_sentiment": {"answer": "Positive", "rationale": "Because the attitude expressed in the entry is \"sure\" and positive."}, "language_style": {"answer": "Tactful and warm", "rationale": "The entry is more personal and interactive when it is spoken in a gentle and inviting tone, rather than in direct statements or orders. At the same time, it uses positive emotional terms (such as \"sure\" and \"close and deal\"), which makes language style more active, encouraging and supportive."}, "time_dimension": {"answer": "Past", "rationale": "The word \"fE\" follows the current state of completion, indicating that this feeling began to appear at some point in the past and continues to the present, so the entry describes the current feelings and mindset of the testee."}, "sentence_type": "Affirmative declarative sentence", "pos": {"nouns": {"I": 2, "person": 1, "Hold on": 1, "- To be with someone": 1}, "verbs": {"I feel": 1, "have": 1, "Close": 1, "Dealing": 1}, "adjectives": {"Any": 1}, "adverbs": {"Both": 1, "and": 1}, "numerals": {"9": 1}}}, {"content": "I think I'm happy.", "word_count": 8, "label": "", "sentence_structure": "Subject鈥揷opula鈥損redicative", "subjective_objective": {"answer": "Subjective", "rationale": "Because \"think\" expresses a subjective view or feeling, reflecting the patient's attitude and assessment of his or her happiness."}, "sentence_sentiment": {"answer": "Positive", "rationale": "Because the expression \"thinks you're happy\" is positive emotional."}, "language_style": {"answer": "Calm and direct", "rationale": "The language style of the entry is calm and straightforward, as it explicitly asks the respondents about their own happiness, without using any rhetorical or euphemism.\""}, "time_dimension": {"answer": "Present", "rationale": ""}, "sentence_type": "Affirmative declarative sentence", "pos": {"verbs": {"I think": 1, "self": 1, "Happy": 1}, "adjectives": {"very": 1}, "adverbs": {"Come on": 1}, "numerals": {"10": 1}, "pronouns": {"I": 1}}}, {"content": "I like to face a lot of people.", "word_count": 10, "label": "", "sentence_structure": "Subject鈥搗erb鈥搊bject", "subjective_objective": {"answer": "Subjective", "rationale": "Because \"like\" is about personal subjective feelings and tendencies."}, "sentence_sentiment": {"answer": "Positive", "rationale": "Because \"like\" this attitude is positive."}, "language_style": {"answer": "Tactful and warm", "rationale": "The entry is more adept and interactive when it is spoken in a gentle and inviting manner, rather than by direct statements or orders."}, "time_dimension": {"answer": "Present", "rationale": "This entry describes the behaviour or state that the person is currently attracted to, i.e., to a large audience, without reference to the time dimension of the past or the future. Thus, the entry can be judged as \"now\"."}, "sentence_type": "Affirmative declarative sentence", "pos": {"nouns": {"Group listeners": 1, "I": 1}, "verbs": {"Yeah": 1, "Noodles": 1}, "adjectives": {"Large": 1}, "numerals": {"11": 1}}}, {"content": "When I see strangers, I often think he's better than me.", "word_count": 18, "label": "", "sentence_structure": "Subject鈥搗erb鈥揷omplement", "subjective_objective": {"answer": "Subjective", "rationale": "Because \"thinking\" is the patient's subjective feeling and thoughts."}, "sentence_sentiment": {"answer": "Negative", "rationale": "Because the \"better\" attitude in the entry is negative."}, "language_style": {"answer": "Calm and direct", "rationale": "The entry expresses an observation or idea in a more objective and direct way, without too much emotional rendering or rhetorical, with a linguistic temperature and without any ambiguity. Thus, its language style can be described as \"cooling straight.\""}, "time_dimension": {"answer": "Past", "rationale": ""}, "sentence_type": "Affirmative declarative sentence", "pos": {"nouns": {"Stranger": 1, "he": 1}, "verbs": {"Yes": 2}, "adjectives": {"Strong": 1}, "adverbs": {"Often": 1}, "numerals": {"12": 1}, "pronouns": {"I": 1}}}, {"content": "For me, it is difficult to talk to strangers.", "word_count": 15, "label": "", "sentence_structure": "Subject鈥揷opula鈥損redicative", "subjective_objective": {"answer": "Subjective", "rationale": "The phrase \"I\" in the sentence indicates that it is a patient's perspective, and therefore subjective."}, "sentence_sentiment": {"answer": "Negative", "rationale": "Because the entry \"It's hard to talk to strangers\" is negative."}, "language_style": {"answer": "Calm and direct", "rationale": "The language style of the entry is relatively calm and straightforward, and it expresses the problems of the respondents in their interpersonal contacts by means of clear statements, which are not overly emotional or verbal."}, "time_dimension": {"answer": "Present", "rationale": "The entry describes the subjective feelings and experiences of the testee, and \"13 to me\" shows that it is a personal experience and view, while \"hard\" indicates the intensity and persistence of the feeling, and therefore the present behavior or mentality."}, "sentence_type": "Affirmative declarative sentence", "pos": {"nouns": {"13": 1, "I": 1, "Strange": 1, "person": 1}, "verbs": {"Open up": 1, "Talk": 1, "Difficulties": 1}, "adjectives": {"very": 1}, "numerals": {"1": 1}}}, {"content": "When decisions must be made, people will naturally turn to me.", "word_count": 18, "label": "", "sentence_structure": "Subject鈥搗erb鈥搊bject", "subjective_objective": {"answer": "Subjective", "rationale": "This is a description of the patient ' s own abilities, characteristics and subjective perception of the patient."}, "sentence_sentiment": {"answer": "Positive", "rationale": "Because the behavior in the entry \"surely turns to me\" expresses a belief and dependence, and is positive."}, "language_style": {"answer": "Tactful and warm", "rationale": "The entry expresses a theme in a question and invitation way 鈥?others may be inclined to ask for help when faced with decision-making. This language style is more personal and interactive, allowing respondents to share their views and ideas more readily. At the same time, it is more objective in tone and is not overly emotional."}, "time_dimension": {"answer": "Present", "rationale": "\"When a decision must be made\" means that this is often happening in certain circumstances, and therefore it is expressed in the present time; and \"it will naturally turn to me\" describes a customary act or state of conduct of the person being tested, which is in line with the current time frame."}, "sentence_type": "Affirmative declarative sentence", "pos": {"nouns": {"Decision": 1, "person": 1, "Problem": 1}, "verbs": {"Turn": 1, "Make": 1, "Give": 1}, "adjectives": {"Naturally": 1}, "numerals": {"14": 1}, "pronouns": {"they": 1, "I": 1}}}, {"content": "I feel real in the social environment.", "word_count": 12, "label": "", "sentence_structure": "Subject鈥揷opula鈥損redicative", "subjective_objective": {"answer": "Objective", "rationale": "It is a subjective view to see whether or not a person feels that he or she is in a social setting. And whether or not such a feeling or experience actually occurs is an objective fact."}, "sentence_sentiment": {"answer": "Positive", "rationale": "Because \"Feeling strong\" in this entry is a positive emotional attitude."}, "language_style": {"answer": "Tactful and warm", "rationale": "The entry is more adept and interactive when it is spoken in a gentle and inviting manner, rather than by direct statements or orders."}, "time_dimension": {"answer": "Present", "rationale": "The entry can be judged by the sentence's \"fear\" and \"social environment\" as it is asking the testee's current feelings, and the time dimension is now."}, "sentence_type": "Affirmative declarative sentence", "pos": {"nouns": {"Environment": 1, "Social": 1}, "verbs": {"I feel": 1, "Step on it": 1}, "adverbs": {"very": 1}, "numerals": {"15": 1}}}, {"content": "I like to influence others.", "word_count": 13, "label": "", "sentence_structure": "Subject鈥搗erb鈥搊bject", "subjective_objective": {"answer": "Subjective", "rationale": "Because it is the subjective will and attitude of one to prefer to influence others."}, "sentence_sentiment": {"answer": "Negative", "rationale": "Because \"influence\" is usually seen as a manipulation with a certain negative emotion."}, "language_style": {"answer": "Calm and direct", "rationale": "The entry is in a straightforward language style to ask the other party whether it has any behaviour or tendency to influence others, without excessive rhetorical or emotional rendering, and is more objective in tone."}, "time_dimension": {"answer": "Present", "rationale": ""}, "sentence_type": "Affirmative declarative sentence", "pos": {"nouns": {"Yeah": 1, "Others": 1, "I": 1, "Impact": 1}, "verbs": {"Imposition": 1}}}]}

{"questionnaire_title": "Torrance's creative personality self-examining.", "questionnaire_category": "Self-cognition", "item_count": 20, "emotion_shift_overall": "From negative to positive", "language_style_overall": "Tactful and warm", "items": [{"content": "(1) Be able to concentrate when doing things, observing things or listening to people.", "word_count": 18, "label": "", "sentence_structure": "Subject鈥搗erb鈥揷omplement", "subjective_objective": {"answer": "Subjective", "rationale": "Because the entry describes the focus of the individual, needs to be answered in the light of his experience and experience, and is a subject of greater subjectivity."}, "sentence_sentiment": {"answer": "Positive", "rationale": "Because the \"regular\" act in the entry is positive."}, "language_style": {"answer": "Tactful and warm", "rationale": "The language style of the entry is euphemistic, as it uses an invitation tone and a positive term \"to focus\" which makes it easier for respondents to be willing to answer questions and to encourage them to demonstrate better attitudes and behaviour."}, "time_dimension": {"answer": "Present", "rationale": ""}, "sentence_type": "Affirmative declarative sentence", "pos": {"nouns": {"Things": 2, "person": 1}, "verbs": {"OPERATIONS": 1, "Observation": 1, "Listen": 1, "Say it": 1}, "adjectives": {"Focus": 1}}}, {"content": "(2) The method of speaking and writing is often by analogy.", "word_count": 13, "label": "", "sentence_structure": "Subject鈥搗erb鈥搊bject", "subjective_objective": {"answer": "Objective", "rationale": "Because \"the way you speak, write and often say things by analogy\" is the way the patient speaks, and that is an objective fact that can be observed and recorded."}, "sentence_sentiment": {"answer": "Neutral", "rationale": "Because the entry does not have any emotional color."}, "language_style": {"answer": "Tactful and warm", "rationale": "The entry is then used to raise the subject in a question mark and ends with a more moderate and respectful expression, and without too many critical or guiding language. The language style of the entry is therefore judged as \"precipitous\"."}, "time_dimension": {"answer": "Present", "rationale": "\"Now.\""}, "sentence_type": "Affirmative declarative sentence", "pos": {"nouns": {"Talk": 1, "Writing": 1, "Methodology": 1}, "verbs": {"Use": 1, "Similarity": 1}, "numerals": {"2": 1}}}, {"content": "(3) Be able to read, write and paint with full attention.", "word_count": 13, "label": "", "sentence_structure": "Subject鈥搗erb鈥揷omplement", "subjective_objective": {"answer": "Objective", "rationale": "Because it is possible to read, write and paint with a full focus, it is objective that the behaviour can be observed."}, "sentence_sentiment": {"answer": "Positive", "rationale": "Because the entry represents a positive act or quality, i.e. capable of being dedicated to reading, writing and painting."}, "language_style": {"answer": "Tactful and warm", "rationale": "The entry uses positive language, emphasizing the focus of reading, writing and painting, which is more encouraging and stimulating, and more prot茅g茅 and virulent."}, "time_dimension": {"answer": "Present", "rationale": "The entry asks the testee whether he or she can concentrate on some activities, and the time dimension is now judged."}, "sentence_type": "Affirmative declarative sentence", "pos": {"nouns": {"Books": 1, "Writing": 1, "Painting": 1}, "verbs": {"I can read it with all my attention": 1, "Read": 1, "鈾?Write it all with all your attention 鈾?: 1, "Writing": 2, "I'm gonna paint it with all my attention": 1, "Painting": 1}, "adverbs": {"鈾?With all your attention 鈾?: 3}, "classifiers": {"individual": 1}}}, {"content": "(4) There is always a sense of excitement after the completion of the work assigned by the teacher.", "word_count": 17, "label": "", "sentence_structure": "Subject鈥搗erb鈥揷omplement", "subjective_objective": {"answer": "Subjective", "rationale": "Because it is a subjective feeling, different people may feel differently after they have completed their work. While this feeling may have been caused by objective events (e.g., completion of the work), it is itself an inner experience of a human being and is subjective."}, "sentence_sentiment": {"answer": "Positive", "rationale": "Because the \"excited\" mood in the entry is positive."}, "language_style": {"answer": "Tactful and warm", "rationale": "The language style of the entry is euphemistic, because it uses a positive and encouraging tone to ask the respondents how they feel after completing the job that the teacher has set up. This expression not only makes the respondents more comfortable and relaxed, but also stimulates them to respond positively."}, "time_dimension": {"answer": "Present", "rationale": "\"Full\" and \"after\" in \"after completion of the teacher's assignment\" indicate that the act has taken place, and that sense of excitement is the feeling after completion of the job, so the entry describes the situation now."}, "sentence_type": "Affirmative declarative sentence", "pos": {"nouns": {"Teacher": 1, "Operations": 1, "Excited": 1}, "verbs": {"Set": 1, "Completed": 1, "have": 1}, "adjectives": {"Always": 1}, "adverbs": {"Back": 1, "Total": 1}, "numerals": {"4": 1}}}, {"content": "(5) Dare to challenge authority.", "word_count": 7, "label": "", "sentence_structure": "Subject鈥搗erb鈥揷omplement", "subjective_objective": {"answer": "Subjective", "rationale": "Because the challenge to authority depends on the patient ' s subjective attitude and willingness."}, "sentence_sentiment": {"answer": "Positive", "rationale": "Because the entry \"bold to challenge the authority\" shows a positive attitude and courage."}, "language_style": {"answer": "Tactful and warm", "rationale": "This is more personal and interactive because the entry is presented in a moderate and inviting manner, rather than directly speaking or ordering."}, "time_dimension": {"answer": "Present", "rationale": "The entry describes the person's current behaviour or attitude, the keyword \"bold\" to indicate a proactive state of behaviour, and \"challenge\" to indicate that such behaviour involves a certain degree of risk and uncertainty. Thus, the temporal dimension is now."}, "sentence_type": "Imperative sentence", "pos": {"verbs": {"How dare you": 1, "Challenges": 1, "Authority": 1}, "adverbs": {"How dare you": 1}, "numerals": {"5": 1}}}, {"content": "(6) The reasons for being used to looking for things.", "word_count": 12, "label": "", "sentence_structure": "Subject鈥搗erb鈥搊bject", "subjective_objective": {"answer": "Subjective", "rationale": "Because looking for reasons is a subjective way of thinking and behavior. This reflects the cognitive style and mindset of patients, not the objective fact or result."}, "sentence_sentiment": {"answer": "Neutral", "rationale": "Because the entry describes only a habit or behaviour, it does not express a positive or negative emotional tendency."}, "language_style": {"answer": "Calm and direct", "rationale": "The entry directly asks whether the interviewees are used to finding things for a variety of reasons, and does not use excessive rhetoric or emotional colours to describe the problem."}, "time_dimension": {"answer": "Present", "rationale": "The words \"customary\" and \"search\" in the entry indicate that this is an act or state that is now in progress. It is therefore a contemporary expression that can be judged."}, "sentence_type": "Affirmative declarative sentence", "pos": {"nouns": {"Custom": 1, "Various reasons": 1}, "verbs": {"Search": 1}, "adverbs": {"on": 1}, "numerals": {"6": 1}}}, {"content": "(7) can observe things carefully.", "word_count": 8, "label": "", "sentence_structure": "Subject鈥搗erb鈥揷omplement", "subjective_objective": {"answer": "Objective", "rationale": "Because a careful look at things is an expression of behaviour or ability, an objective reality, not a subjective attitude or feeling of the patient."}, "sentence_sentiment": {"answer": "Neutral", "rationale": "Because the entry is not clearly emotional, it merely describes an act or ability."}, "language_style": {"answer": "Tactful and warm", "rationale": "The entry is more euphemistic and moderate, without a direct evaluation or command tone, but rather a question-and-answer approach to the interviewee鈥檚 self-assessment."}, "time_dimension": {"answer": "Present", "rationale": "The entry used to ask the person who was tested about a particular characteristic or habit of the past, which \"can observe things carefully\" means a capacity or habit and therefore is a temporal dimension of the past."}, "sentence_type": "Imperative sentence", "pos": {"verbs": {"Yes": 1, "Look carefully": 1}, "adjectives": {"Seriously": 1}, "adverbs": {"Careful": 1}}}, {"content": "(8) The problems can be identified from other people ' s conversations.", "word_count": 11, "label": "", "sentence_structure": "Subject鈥搗erb鈥揷omplement", "subjective_objective": {"answer": "Subjective", "rationale": "The question of whether or not to find another person is an expression of subjective ability."}, "sentence_sentiment": {"answer": "Positive", "rationale": "The ability to \"discover problems from conversations\" is very helpful in solving problems and promoting self-esteem, since the article contains positive attitudes and behaviour."}, "language_style": {"answer": "Tactful and warm", "rationale": "The entry uses positive statements that can elicit positive responses from the other side, and the narrative is structured in a question-and-answer format, which is more friendly and friendly. At the same time, by using the word \"can\" it is easier to obtain cooperation and cooperation from the other side by expressing recognition and trust in the other side's capabilities."}, "time_dimension": {"answer": "Present", "rationale": "The entry asks if the person tested has the ability to identify the problem in his/her interpersonal interaction. Although the temporal dimension is not explicitly mentioned, it is \"possible\" to indicate that it is a skill or capability, and that such capacity can be used at any time. The temporal dimension of the article is therefore present and future."}, "sentence_type": "Affirmative declarative sentence", "pos": {"verbs": {"Yes": 1, "Found": 1}, "adverbs": {"From": 1}}}, {"content": "(9) Time is often forgotten when creative thinking is being carried out.", "word_count": 17, "label": "", "sentence_structure": "Subject鈥搗erb鈥搊bject", "subjective_objective": {"answer": "Subjective", "rationale": "Because \"Relationship\" is a subjective feeling and experience of a human being. While creative thinking may require a great deal of time and effort, whether \"relationship\" depends on the individual ' s concentration of attention, focus on the task, and individual memory. The answer to this questionnaire is therefore subjective."}, "sentence_sentiment": {"answer": "Negative", "rationale": "Because the \"forget time\" act in the entry is negative."}, "language_style": {"answer": "Tactful and warm", "rationale": "The entry is more adept and interactive when it is spoken in a gentle and inviting manner, rather than by direct statements or orders."}, "time_dimension": {"answer": "Present", "rationale": "The keyword \"conducted\", \"often forgets the time\", indicates that it is a sentence describing the current state or behaviour of the person being tested and therefore belongs to the present."}, "sentence_type": "Imperative sentence", "pos": {"nouns": {"Creative thinking activities": 1, "Time": 1}, "verbs": {"Conduct": 1, "Forget it": 1}, "adverbs": {"Regular": 1}, "numerals": {"9": 1}}}, {"content": "(10) The ability to identify problems on a proactive basis and to identify relationships related to them.", "word_count": 20, "label": "", "sentence_structure": "Subject鈥搗erb鈥揷omplement", "subjective_objective": {"answer": "Objective", "rationale": "Because \"can detect problems and find relationships associated with them\" is a description of individual capabilities and is an objective fact, not a subjective attitude or feeling."}, "sentence_sentiment": {"answer": "Positive", "rationale": "Because \"can find problems\" and \"can find relationships with them\" are positive behaviors and attitudes."}, "language_style": {"answer": "Tactful and warm", "rationale": "The expression is more likely to mobilize the motivation and participation of the respondents than to directly evaluate or criticize the more euphemismal and moderate language of the entry, using more encouraging and glorified terms such as \"in a way that can proactively identify the problem\" and \"can identify the relationships associated with it\"."}, "time_dimension": {"answer": "Present", "rationale": "The keyword \"can identify problems voluntarily\" and \"discover relationships associated with them\" suggests that this is an assessment of the present behaviour or ability of the person being tested."}, "sentence_type": "Affirmative declarative sentence", "pos": {"nouns": {"Problem": 1, "Relationships": 1}, "verbs": {"Found": 1, "Find out": 1}, "adverbs": {"Initiatives": 1}, "numerals": {"10": 1}}}, {"content": "(11) Except for everyday life, most of the time is spent in school.", "word_count": 19, "label": "", "sentence_structure": "Subject鈥搗erb鈥搊bject", "subjective_objective": {"answer": "Subjective", "rationale": "This is because it is the patient ' s subjective attitude to the circumstances under which he or she reports that he or she is able to study."}, "sentence_sentiment": {"answer": "Positive", "rationale": "Because the behavior of \"most of the time you read and learn\" in the entries is positive."}, "language_style": {"answer": "Calm and direct", "rationale": "The sentence is simple and clear, and is not too much rhetorical or emotional, but rather direct."}, "time_dimension": {"answer": "Present", "rationale": "In conclusion, the corresponding answer to this entry is \"now\"."}, "sentence_type": "Affirmative declarative sentence", "pos": {"nouns": {"Daily life": 1, "Time": 1, "Books": 1, "Learning": 1}, "verbs": {"Divide": 1, "in": 2, "Read it": 1, "Learn": 1}, "adverbs": {"Normal": 1, "Most of them": 1}, "numerals": {"11": 1}, "classifiers": {"Number of times": 1}}}, {"content": "(12) Always curious about what's around.", "word_count": 11, "label": "", "sentence_structure": "Subject鈥搗erb鈥揷omplement", "subjective_objective": {"answer": "Subjective", "rationale": "Because it is the patient's subjective attitude to be curious."}, "sentence_sentiment": {"answer": "Positive", "rationale": "Because the \"acceptance\" attitude in the entry is positive."}, "language_style": {"answer": "Tactful and warm", "rationale": "The term \"always\" is used to express the fact that the other party is often curious and to share his or her situation in a way that is more resonant and active in the person under investigation."}, "time_dimension": {"answer": "Future", "rationale": "Although the sentence is based on the phrase \"you\" (indicated as the testee), the entire sentence describes a potential possibility or future behavioural orientation. The keyword \"always\" means a state of continuity or a tendency, while \"quiet\" is an attitude or a value that also points to possible change and development. Thus, depending on the time dimension and the nature of the content, the entry falls into the category of future."}, "sentence_type": "Affirmative declarative sentence", "pos": {"nouns": {"Curiosity": 1, "Heart": 1}, "verbs": {"Hold": 1, "Total": 1}, "adjectives": {"Around": 1, "Yes": 1}, "adverbs": {"Always": 1}, "numerals": {"12": 1}}}, {"content": "(13) When a problem is discovered, there is always a great deal of excitement.", "word_count": 20, "label": "", "sentence_structure": "Subject鈥搗erb鈥揷omplement", "subjective_objective": {"answer": "Subjective", "rationale": "Because it's the patient's subjective feeling that feels excited."}, "sentence_sentiment": {"answer": "Positive", "rationale": "Because the behavior and feeling of \"abnormal excitement\" in the entries is positive."}, "language_style": {"answer": "Tactful and warm", "rationale": "The entry uses a gentle and inviting tone of conversation, is expressed in a more friendly manner and uses the adjective \"abnormally excited\" to give a positive and positive feeling, with a soft and warm style."}, "time_dimension": {"answer": "Present", "rationale": ""}, "sentence_type": "Affirmative declarative sentence", "pos": {"nouns": {"Problem": 1, "Found": 1, "Spirit": 1, "Unusual": 1, "Excited": 1}, "verbs": {"to": 1, "have": 1, "I feel": 1, "New": 1}, "adjectives": {"Some": 1, "New": 1, "Unusual": 1}, "adverbs": {"Total": 1}, "numerals": {"13": 1}}}, {"content": "(14) It is usually possible to predict the outcome of the event and to correctly validate it.", "word_count": 20, "label": "", "sentence_structure": "Subject鈥揷opula鈥損redicative", "subjective_objective": {"answer": "Objective", "rationale": "Because the entry describes competence or characteristics, is based on a statement of fact and does not require a personal feeling or subjective evaluation by the patient."}, "sentence_sentiment": {"answer": "Positive", "rationale": "Because \"can correctly verify this result\" means that it is a positive act that can solve the problem and make the right judgement."}, "language_style": {"answer": "Calm and direct", "rationale": "The entry is based on simple and clear language, is not overly pedigreed or emotional, is more objective and neutral and is more in line with a calm and direct description."}, "time_dimension": {"answer": "Past", "rationale": "The entry asks the testee whether it is capable of predicting and validating the results of the thing, which is a behavior or ability, and therefore determines its temporal dimension."}, "sentence_type": "Affirmative declarative sentence", "pos": {"nouns": {"Questionnaire Entry": 1, "Things": 1, "Result": 2}, "verbs": {"Yes": 2, "Projections": 1, "Authentication": 1}, "adverbs": {"Usually": 1}, "numerals": {"14": 1, "One": 2}, "classifiers": {"individual": 1}}}, {"content": "(15) Even if there are difficulties and setbacks, there is no discouragement.", "word_count": 13, "label": "", "sentence_structure": "Subject鈥損redicate", "subjective_objective": {"answer": "Subjective", "rationale": "Because \"indeed\" is a subjective attitude or emotional reaction."}, "sentence_sentiment": {"answer": "Positive", "rationale": "Because the \"indulgent\" act in the entry is positive."}, "language_style": {"answer": "Calm and direct", "rationale": "The entry directly asks the respondents in a concise and clear language about their attitudes and behaviour in the face of difficulties, without excessive ado or emotional colours, and the language style is a straightforward and calm one."}, "time_dimension": {"answer": "Present", "rationale": "The keywords \"difficult and frustrated\" and \"indeed\" describe the reaction and attitude of the testees in the face of the challenge, which is a customary act, so the temporal dimension is now judged."}, "sentence_type": "Affirmative declarative sentence", "pos": {"nouns": {"Difficulties": 1, "Frustration": 1}, "verbs": {"I met him": 1, "I'm not discouraged": 2}, "adjectives": {"Even if": 1}, "numerals": {"15": 1}}}, {"content": "(16) Thinking about new answers and new results.", "word_count": 14, "label": "", "sentence_structure": "Subject鈥搗erb鈥搊bject", "subjective_objective": {"answer": "Subjective", "rationale": "For thinking about new answers and new results is a subjective way of thinking and attitude."}, "sentence_sentiment": {"answer": "Positive", "rationale": "Because \"Rethinking\" behavior in the entries is positive."}, "language_style": {"answer": "Tactful and warm", "rationale": "The entry uses an invitation tone, which leads the interviewee to reflect on his/her behaviour, language style, relative moderation and affection. At the same time, the words \"often\" and \"new\" emphasize a proactive attitude and further create an atmosphere that encourages reflection and exploration."}, "time_dimension": {"answer": "Present", "rationale": "In the entry, \"often\" means a customary, continuous act, and \"thinking\" also implies that it is an ongoing act. So, the time dimension of this entry is now judged."}, "sentence_type": "Affirmative declarative sentence", "pos": {"nouns": {"Think": 1, "Things": 1, "Answer": 1, "Result": 1}, "verbs": {"Regular": 1, "Think": 1}, "adverbs": {"Regular": 1}, "numerals": {"16": 1}}}, {"content": "(17) A keen observational and problem-solving capability.", "word_count": 17, "label": "", "sentence_structure": "Subject鈥搗erb鈥揷omplement", "subjective_objective": {"answer": "Subjective", "rationale": "Because of the sensitivity of observation and the ability to ask questions, one is subjective in nature and ability."}, "sentence_sentiment": {"answer": "Positive", "rationale": "Because a keen observation and the ability to ask questions are positive qualities."}, "language_style": {"answer": "Tactful and warm", "rationale": "The entry uses more active and inspiring language to describe the competencies required of the person under investigation, such as \"sensitivity\" and \"questioning ability\", and is relatively soft, encouraging and positive, giving a warm and cordial feeling of affection. At the same time, the wording of the entry is somewhat instructive, making it easier for the person under investigation to accept and be willing to participate in the questionnaire."}, "time_dimension": {"answer": "Present", "rationale": "The words \"with\" and \"with a keen observation and the ability to ask questions\" are an indication of the current capabilities or characteristics of the testee, and the temporal dimension of the article can therefore be judged to be the present."}, "sentence_type": "Affirmative declarative sentence", "pos": {"nouns": {"Sensitivity": 1, "Observation": 1, "Capacity to raise questions": 1}, "verbs": {"Yes": 1}, "numerals": {"17": 1}}}, {"content": "(18) Learning with a unique subject of study of its choice and with a unique method of discovery and research.", "word_count": 34, "label": "", "sentence_structure": "Subject鈥搗erb鈥揷omplement", "subjective_objective": {"answer": "Subjective", "rationale": "Because the entry asks about the patient ' s unique research topics and the unique research methods, which are patients ' subjective attitudes and behaviour."}, "sentence_sentiment": {"answer": "Positive", "rationale": "Because the behaviour referred to in the entry \"have its own chosen unique research subject and can adopt its own unique methods of discovery and research\" is positive and indicates that individuals have independent thinking and innovation."}, "language_style": {"answer": "Tactful and warm", "rationale": ""}, "time_dimension": {"answer": "Past", "rationale": "The entry describes the behaviour or mentality of the person who was tested in the past learning experience, referring to \"selected unique research subjects\" and \"the adoption of his own unique methods of discovery and research\", which are descriptions of the past."}, "sentence_type": "Imperative sentence", "pos": {"nouns": {"Learning": 1, "Unique": 1, "Research topics": 1, "Find Methods": 1, "Research methodology": 1}, "verbs": {"have": 1, "Select": 1, "Adoption": 1}, "adjectives": {"self": 2, "Unique": 1}, "numerals": {"18": 1}}}, {"content": "(19) When confronted with problems, possibilities are often explored in many ways, rather than being fixed in one line of thought or limited in one aspect.", "word_count": 34, "label": "", "sentence_structure": "Subject鈥搗erb鈥揷omplement", "subjective_objective": {"answer": "Subjective", "rationale": "Because \"exploring possibilities in many ways\" and \"standing one line or limiting one aspect\" are subjective attitudes and behaviour of patients."}, "sentence_sentiment": {"answer": "Positive", "rationale": "Because the \"exploring possibilities from multiple sources\" in the entry is positive."}, "language_style": {"answer": "Tactful and warm", "rationale": "The entry is asked in a question-and-answer manner, is soft and encouraging, and does not give its own views or orders directly. At the same time, the positive and open attitude of the respondents can be transmitted by using positive terms such as \"diverse possibilities\" to make them more willing to cooperate with the investigation and to produce positive emotional reactions."}, "time_dimension": {"answer": "Present", "rationale": ""}, "sentence_type": "Affirmative declarative sentence", "pos": {"nouns": {"Problem": 1, "In terms of": 2, "Possibilities": 1, "Thinking": 1, "Limits": 1}, "verbs": {"I met him": 1, "Yes": 1, "Explore": 1, "Fixed": 1, "in": 3, "In a certain way": 1}, "adverbs": {"Regular": 1, "Various aspects": 1}, "numerals": {"19": 1}, "classifiers": {"individual": 1}}}, {"content": "(20) There are always new ideas that emerge in the brain and that can emerge even when playing.", "word_count": 24, "label": "", "sentence_structure": "Subject鈥搗erb鈥搊bject", "subjective_objective": {"answer": "Subjective", "rationale": "The questionnaire is intended to describe the subjective feelings and experiences of the patients."}, "sentence_sentiment": {"answer": "Positive", "rationale": "Because the \"new ideas always emerge in the head\" behavior in the entry is positive."}, "language_style": {"answer": "Tactful and warm", "rationale": ""}, "time_dimension": {"answer": "Future", "rationale": ""}, "sentence_type": "Affirmative declarative sentence", "pos": {"nouns": {"Brain": 1, "New vision": 2, "Play": 1}, "verbs": {"鈾?Emergence 鈾?: 1, "Generate": 1, "be": 1}, "adverbs": {"There's always": 1, "Even if": 1}, "numerals": {"20": 1}}}]}

{"questionnaire_title": "The CCSAS adaptation schedule for Chinese university students", "questionnaire_category": "Behavioral questionnaire", "item_count": 60, "emotion_shift_overall": "From negative to positive", "language_style_overall": "Tactful and warm", "items": [{"content": "There are always things in life that are of interest to me.", "word_count": 16, "label": "", "sentence_structure": "Subject鈥揷opula鈥損redicative", "subjective_objective": {"answer": "Subjective", "rationale": "Because interest is the patient's personal feeling and subjective attitude."}, "sentence_sentiment": {"answer": "Positive", "rationale": "Because the phrase \"something that I'm interested in every day\" in the entry conveys a positive emotional attitude."}, "language_style": {"answer": "Tactful and warm", "rationale": "The entry is more personal and interactive when it is spoken in a question-and-answer manner, rather than in direct statements or orders. Moreover, the issue is spoken in positive terms, expressing concern about the positive factors in the lives of the interviewees and has an encouraging effect."}, "time_dimension": {"answer": "Present", "rationale": ""}, "sentence_type": "Affirmative declarative sentence", "pos": {"nouns": {"Life": 1, "Things": 1}, "verbs": {"have": 1, "Total": 1, "be": 1, "Sensor": 1, "Present": 1, "Interest": 1}, "adjectives": {"Every day": 1}, "numerals": {"One": 2}, "pronouns": {"I": 1}}}, {"content": "If I were to choose again, I would still live as I am now.", "word_count": 20, "label": "", "sentence_structure": "Subject鈥損redicate", "subjective_objective": {"answer": "Subjective", "rationale": "This is because patients have subjective attitudes and evaluations of their own lives."}, "sentence_sentiment": {"answer": "Neutral", "rationale": "Since the entry does not express positive or negative emotional expressions, it merely presents a hypothetical situation."}, "language_style": {"answer": "Tactful and warm", "rationale": "The language style of the entry is more euphemistic, not directly expressing feelings or attitudes, but rather asking questions in a relatively neutral and objective manner, which makes it easier for the interviewees to feel comfortable and acceptable and to share their real thoughts."}, "time_dimension": {"answer": "Present", "rationale": "The entry \"Or\" and \"as is\" shows that the testee considers his life to be satisfactory at present and that he will remain so even if he has a chance to re-choice. So the time dimension of the entry is now."}, "sentence_type": "Affirmative declarative sentence", "pos": {"verbs": {"Jean": 1, "Selection": 1, "Life": 1}, "adjectives": {"Yes": 1, "Now, this": 1}, "pronouns": {"I": 2}}}, {"content": "I am always happy.", "word_count": 9, "label": "", "sentence_structure": "Subject鈥揷opula鈥損redicative", "subjective_objective": {"answer": "Subjective", "rationale": "Because \"happiness\" is a subjective feeling and experience."}, "sentence_sentiment": {"answer": "Positive", "rationale": "Because the description of \"always happy\" in the entry expresses a positive emotional attitude."}, "language_style": {"answer": "Tactful and warm", "rationale": "Warm and warm."}, "time_dimension": {"answer": "Present", "rationale": "In this entry, the expression \"always feel\" implies that it is a regular, customary act, and therefore the temporal dimension is now judged."}, "sentence_type": "Affirmative declarative sentence", "pos": {"verbs": {"I feel": 1, "Have a nice day": 1}, "adjectives": {"Total": 1}}}, {"content": "4. I usually read professional-related books.", "word_count": 12, "label": "", "sentence_structure": "Subject鈥搗erb鈥搊bject", "subjective_objective": {"answer": "Objective", "rationale": "Because \"reading a professional book\" is an act that can be observed and recorded, and it is an objective fact."}, "sentence_sentiment": {"answer": "Neutral", "rationale": "Because the entry merely presents a fact, it does not express positive or negative emotional tendencies."}, "language_style": {"answer": "Tactful and warm", "rationale": "The entry is more euphemistic. It does not direct the person to answer the question, but rather directs the person to ask questions about his or her behaviour and to give his or her own answer."}, "time_dimension": {"answer": "Present", "rationale": "The phrase, judged by the terms \"normal\" and \"normal\" in the title, is a reference to current customary behaviour, so the time dimension should be now."}, "sentence_type": "Affirmative declarative sentence", "pos": {"nouns": {"Time": 1, "Entry": 1, "Books": 1}, "verbs": {"I'm always watching": 1}, "adjectives": {"Related": 1}, "adverbs": {"Normal": 1}, "numerals": {"4": 1}}}, {"content": "5. I have rarely understood the needs of society for talent.", "word_count": 14, "label": "", "sentence_structure": "Subject鈥搗erb鈥揷omplement", "subjective_objective": {"answer": "Subjective", "rationale": "Because \"very little to know\" is the subjective attitude and behaviour of the patient, this entry is viewed from a subjective perspective."}, "sentence_sentiment": {"answer": "Negative", "rationale": "Because the behavior in the entries \"very little to understand the needs of society for talent\" is negative."}, "language_style": {"answer": "Tactful and warm", "rationale": "The entry is based on an invitation-style, a soft and open-minded way of asking if the other party has little knowledge of the needs of the human being, and this expression is more personal and interactive. At the same time, the entry is not directly stated or ordered, but rather questions that lead the other to think, which is more in keeping with the euphemism of the language style."}, "time_dimension": {"answer": "Present", "rationale": ""}, "sentence_type": "Negative declarative sentence", "pos": {"nouns": {"Talent": 1, "Requirements": 1}, "verbs": {"To understand": 1, "be": 1}, "adverbs": {"Very few": 1}, "numerals": {"5": 1}, "classifiers": {"individual": 1}, "pronouns": {"I": 1}}}, {"content": "I have often been overwhelmed by frustration.", "word_count": 14, "label": "", "sentence_structure": "Subject鈥搗erb鈥揷omplement", "subjective_objective": {"answer": "Subjective", "rationale": "Because the term \"often\" refers to the individual circumstances and feelings of the patient and is subjective."}, "sentence_sentiment": {"answer": "Negative", "rationale": "Because the \"unsightly\" act in the entry is negative."}, "language_style": {"answer": "Calm and direct", "rationale": "The language style of the entry is calm and straightforward. It asks the interviewee whether he or she feels unprepared when he or she encounters a heart attack, without too much rhetoric and emotional color, and with a temperature and objectivity."}, "time_dimension": {"answer": "Present", "rationale": ""}, "sentence_type": "Negative declarative sentence", "pos": {"nouns": {"Things": 1, "Desperate": 1, "I can't get a chance": 1}, "verbs": {"I met him": 1, "Often": 1}, "adjectives": {"Desperate": 1}, "adverbs": {"Often": 1}, "classifiers": {"individual": 1}, "pronouns": {"I": 1}}}, {"content": "7. I am pleased with the current university life.", "word_count": 12, "label": "", "sentence_structure": "Subject鈥揷opula鈥損redicative", "subjective_objective": {"answer": "Subjective", "rationale": "Because satisfaction is a subjective emotional experience, not an objective fact or behaviour."}, "sentence_sentiment": {"answer": "Positive", "rationale": "Because the \"I'm satisfied\" attitude in the entry is positive."}, "language_style": {"answer": "Tactful and warm", "rationale": "The language style of the entry is euphemism. It asks each other in an encouraging tone about whether they are satisfied with the current university life, without directly emphasizing satisfaction or dissatisfaction, but rather giving each other the opportunity to express their views."}, "time_dimension": {"answer": "Present", "rationale": ""}, "sentence_type": "Affirmative declarative sentence", "pos": {"nouns": {"University": 1, "Life": 1}, "verbs": {"Satisfied": 1}, "adjectives": {"Now": 1}}}, {"content": "8. I am well aware of whether it is time to continue to study or work after graduation.", "word_count": 18, "label": "", "sentence_structure": "Subject鈥搗erb鈥搊bject", "subjective_objective": {"answer": "Subjective", "rationale": "Because it is the patient鈥檚 subjective idea and decision. Although 鈥渢o continue to study or to work after graduation鈥?is a factual question to be considered, the decision taken on this issue is the result of subjective thinking by the patient."}, "sentence_sentiment": {"answer": "Neutral", "rationale": "The entry is merely an inquiry into the clarity of the post-graduation plan and does not express a positive or negative attitude or emotional colour."}, "language_style": {"answer": "Tactful and warm", "rationale": "The entry is a more euphemistic way of giving the interviewee a sense of future planning, and it is not directly ordered or advised to give him more autonomy and space to think. At the same time, the positive phrase, \"I know it well,\" is used to increase the motivation and motivation of the tone and to make people feel respected and understood."}, "time_dimension": {"answer": "Future", "rationale": "The keyword \"after graduation\" refers to the point of the future, and \"to continue to study or work\" is a vision and expectation of the future of the testee. Thus, the entry contains visions and expectations for the future, which are in the context of future behaviour or mindset."}, "sentence_type": "Affirmative declarative sentence", "pos": {"nouns": {"Questionnaire": 1, "Entry": 1, "After graduation": 1, "Further education": 1, "Work": 1}, "verbs": {"Yes": 1, "The": 2, "Go on": 1, "Or is it": 1, "Work": 1}, "adverbs": {"Clear": 1}, "numerals": {"8锛?: 1}, "pronouns": {"I": 1}}}, {"content": "9. I am very enthusiastic about the current learning.", "word_count": 13, "label": "", "sentence_structure": "Subject鈥搗erb鈥揷omplement", "subjective_objective": {"answer": "Subjective", "rationale": "Because \"a great passion for learning now\" is a subjective feeling and attitude of a human being."}, "sentence_sentiment": {"answer": "Positive", "rationale": "Because the entry \"with a high level of enthusiasm\" expresses a positive emotional attitude."}, "language_style": {"answer": "Tactful and warm", "rationale": ""}, "time_dimension": {"answer": "Present", "rationale": ""}, "sentence_type": "Affirmative declarative sentence", "pos": {"nouns": {"鈾?Hot": 1, "Learning": 1}, "verbs": {"have": 1, "High": 1, "very": 1}, "adjectives": {"Hot": 1}, "adverbs": {"to": 1, "Present": 1}}}, {"content": "10. I believe that my strengths outweigh those of others.", "word_count": 12, "label": "", "sentence_structure": "Subject鈥揷opula鈥損redicative", "subjective_objective": {"answer": "Subjective", "rationale": "Because \"think\" represents the patient's subjective judgment and opinion."}, "sentence_sentiment": {"answer": "Positive", "rationale": "Because the attitude in the entry that \"thinks that they have more merit than fault\" is positive and expresses a sense of confidence and optimism."}, "language_style": {"answer": "Tactful and warm", "rationale": "The entry is a positive and positive expression of the person ' s opinion, which is more moderate and more friendly and resonant."}, "time_dimension": {"answer": "Present", "rationale": "The entry expresses a subjective assessment of the testee that his or her strengths outweighs those, which is the current attitude and perception."}, "sentence_type": "Affirmative declarative sentence", "pos": {"nouns": {"Advantages": 1, "Disadvantages": 1, "self": 1}, "verbs": {"I think": 1, "have": 1}, "numerals": {"10": 1}, "pronouns": {"I": 1}}}, {"content": "11. Many people have asked me to play with them.", "word_count": 12, "label": "", "sentence_structure": "Subject鈥搗erb鈥搊bject", "subjective_objective": {"answer": "Subjective", "rationale": "Because it's the patient's subjective attitude to like playing with others."}, "sentence_sentiment": {"answer": "Positive", "rationale": "Because the \"many people come to me with them\" act is positive, indicating that people like to be with me and that they like me."}, "language_style": {"answer": "Tactful and warm", "rationale": "The entry uses a more euphemistic approach to the question of whether the other person wanted to play with him rather than directly asking or ordering, which is more personal and interactive."}, "time_dimension": {"answer": "Present", "rationale": ""}, "sentence_type": "Affirmative declarative sentence", "pos": {"nouns": {"person": 1, "Together": 1}, "verbs": {"Find": 1, "and": 1, "Play": 1}, "numerals": {"Both": 1, "A lot": 1, "11": 1}, "pronouns": {"I": 1}}}, {"content": "12. I have never learned about the characteristics and requirements of different occupations by reading books about career choices.", "word_count": 29, "label": "", "sentence_structure": "Subject鈥搗erb鈥搊bject", "subjective_objective": {"answer": "Objective", "rationale": "This sentence describes the objective fact that the patient has read the relevant books without any subjective attitude or feelings."}, "sentence_sentiment": {"answer": "Neutral", "rationale": "Because the entry does not have any emotional color."}, "language_style": {"answer": "Calm and direct", "rationale": "The entry is presented in a direct manner, without using a rhetorical or emotional style, but rather in a simple and straightforward way."}, "time_dimension": {"answer": "Present", "rationale": "In the entry, \"never\" indicates that it is a customary act, and \"read\" is an ongoing act, so it's judged to be the present. And, \"know\" is the form of a word of the past, but it does not emphasize the past in this sentence and cannot be considered as a past act."}, "sentence_type": "Negative declarative sentence", "pos": {"nouns": {"I": 1, "Book": 1, "Occupation": 2, "Characteristics": 1, "Request": 1}, "verbs": {"Read it": 1, "Yes": 1, "Roger that": 1, "Selection": 1}, "adjectives": {"Related": 1, "Different": 1}, "adverbs": {"Never": 1}, "numerals": {"12": 1}, "classifiers": {"Various": 1}}}, {"content": "13. When I do not want to do things alone, I can always find someone to accompany me.", "word_count": 17, "label": "", "sentence_structure": "Subject鈥搗erb鈥搊bject", "subjective_objective": {"answer": "Subjective", "rationale": "Because \"Don't want to do it alone\" and \"Don't always find someone with me\" are subjective thoughts and feelings of patients, and subjective questions about their social needs and personal preferences."}, "sentence_sentiment": {"answer": "Positive", "rationale": "Because the purpose of the action of finding someone with me in the entry is positive."}, "language_style": {"answer": "Tactful and warm", "rationale": "The article ' s purpose is more intimate and interactive, and expresses the social needs of individuals in a positive manner rather than directly speaking or ordering."}, "time_dimension": {"answer": "Present", "rationale": ""}, "sentence_type": "Affirmative declarative sentence", "pos": {"nouns": {"Yes": 1, "person": 2}, "verbs": {"Yes": 1, "Find": 1, "Stay": 1}, "adverbs": {"When": 1, "Always": 1}, "numerals": {"13": 1}, "pronouns": {"I": 1}}}, {"content": "I know what I am fit to do.", "word_count": 12, "label": "", "sentence_structure": "Subject鈥搗erb鈥搊bject", "subjective_objective": {"answer": "Subjective", "rationale": "Because knowing what is right for you is a subjective judgement and perception. This involves the patient's knowledge, self-evaluation and expectations, which are part of his or her subjective experience and feelings."}, "sentence_sentiment": {"answer": "Neutral", "rationale": "Because the entry expresses an objective perception and does not have a clear emotional colour."}, "language_style": {"answer": "Tactful and warm", "rationale": "The language style of the entry is more euphemistic, and it is more accommodative and encouraging to guide the interviewee to reflect on his or her career orientation, rather than using a cold language such as direct orders or criticism. At the same time, the question is made more relevant by using the first person's term, \"I know,\" which emphasizes the subject's subjective perception and self-understanding."}, "time_dimension": {"answer": "Present", "rationale": "The keyword \"know\" indicates a state of awareness based on current self-awareness and understanding, and therefore judgement is now."}, "sentence_type": "Affirmative declarative sentence", "pos": {"nouns": {"Work": 1}, "verbs": {"Yes": 1, "Do it": 1}, "adverbs": {"self": 1}, "numerals": {"14": 1}, "pronouns": {"What": 1}}}, {"content": "I never felt alone.", "word_count": 7, "label": "", "sentence_structure": "Subject鈥揷opula鈥損redicative", "subjective_objective": {"answer": "Subjective", "rationale": "Because feeling alone is a subjective feeling."}, "sentence_sentiment": {"answer": "Neutral", "rationale": "Because the entry merely stated a fact, and did not have any emotional colour."}, "language_style": {"answer": "Calm and direct", "rationale": "The entry uses a positive tone and is less semantic and does not have too many emotional qualifiers or rhetorical techniques and is a more direct question."}, "time_dimension": {"answer": "Present", "rationale": "This sentence is about the current state of feeling and feeling, and it's about \"never feeling alone\" that means never feeling alone."}, "sentence_type": "Affirmative declarative sentence", "pos": {"verbs": {"Sensor": 1, "Present": 1}, "adjectives": {"Loneliness": 1}, "numerals": {"15": 1}}}, {"content": "16. I always find my strengths and encourage myself in them.", "word_count": 19, "label": "", "sentence_structure": "Subject鈥搗erb鈥揷omplement", "subjective_objective": {"answer": "Subjective", "rationale": "Because \"discovering your own merits\" and \"incentive yourself\" are the personal behaviours and attitudes of the patient and are subjectively viewed."}, "sentence_sentiment": {"answer": "Positive", "rationale": "Because the entry \"discovers its merits and encourages it\" is positive."}, "language_style": {"answer": "Tactful and warm", "rationale": "This is a more positive expression of concern and support for the interviewee鈥檚 ability to assert his/her self-confidence, without direct criticism or criticism, but rather by encouraging and directing the interviewee鈥檚 motivation and self-confidence."}, "time_dimension": {"answer": "Present", "rationale": "The words \"always\" and \"and thus\" in the sentence are the words that describe the current behaviour or state, so it can be judged that this is the current attitude or behaviour of the person being questioned."}, "sentence_type": "Affirmative declarative sentence", "pos": {"nouns": {"I": 1, "Advantages": 1, "self": 2}, "verbs": {"Always to find out": 1, "And encourage it with this": 1}, "adverbs": {"Always": 1}, "numerals": {"16锛?: 1, "This": 1}}}, {"content": "17. My amateur life is rich and no change is required.", "word_count": 17, "label": "", "sentence_structure": "Subject鈥揷opula鈥損redicative", "subjective_objective": {"answer": "Subjective", "rationale": "Because the richness of the amateur life is the patient ' s personal feeling and subjective attitude, it is the patient ' s will that needs to be changed."}, "sentence_sentiment": {"answer": "Neutral", "rationale": "Because the entry does not have any emotional color."}, "language_style": {"answer": "Tactful and warm", "rationale": "The entry is more euphemistic, using the term \"very rich\" to describe the other's amateur life, without directly rejecting or making recommendations, and communicating with the other in a positive encouragement and support. At the same time, the phrase \"no change is required\" is also expressed in a relatively soft way, avoiding feelings that may cause stress or displeasure to the other."}, "time_dimension": {"answer": "Present", "rationale": "The entry describes the current state and feelings of the testee, uses the general current \"very rich\" and \"unnecessary\" and indicates that this is a continuing fact and therefore judges the present."}, "sentence_type": "Affirmative declarative sentence", "pos": {"nouns": {"Amateur life": 1, "Change": 1}, "verbs": {"Yes": 1, "Do it": 1}, "adjectives": {"very": 1, "Rich": 1}, "numerals": {"17": 1}, "pronouns": {"I": 1}}}, {"content": "I do not know how to compliment others.", "word_count": 10, "label": "", "sentence_structure": "Subject鈥損redicate", "subjective_objective": {"answer": "Subjective", "rationale": "Because \"I don't know how to compliment\" is a personal idea or feeling, a subjective examination."}, "sentence_sentiment": {"answer": "Neutral", "rationale": "Because the entry does not have any emotional color."}, "language_style": {"answer": "Tactful and warm", "rationale": "The language style of the entry is more euphemistic, moderate, dialogueable in a way that is asked, and uses the term \"unknowable\" to express its confusion and desire for help. Such a presentation is easier to guide each other to provide effective feedback and support, and to enhance the relevance and interaction of communication."}, "time_dimension": {"answer": "Present", "rationale": "The entry contains the testee's self-characterization \"I don't know how to compliment others\" which indicates that it's the testee's current feeling or situation, and therefore the judgment is \"now\"."}, "sentence_type": "Negative declarative sentence", "pos": {"nouns": {"person": 1, "Entry": 1, "Questionnaire": 1}, "verbs": {"Yes": 1, "I'm flattered": 1, "others": 1}, "numerals": {"18锛?: 1}}}, {"content": "I will not plan for the achievement of my own career goals.", "word_count": 18, "label": "", "sentence_structure": "Subject鈥搗erb鈥揷omplement", "subjective_objective": {"answer": "Objective", "rationale": "Since the formulation of a plan is a practical step, it is an objective fact that it is possible to establish a plan."}, "sentence_sentiment": {"answer": "Negative", "rationale": "Because the \"no plans to achieve their professional goals\" act in the entry is negative."}, "language_style": {"answer": "Calm and direct", "rationale": "The entry is more straightforward in language style, not expressed in too many qualifiers or emotionally coloured words. It expresses, in simple language, the desire to understand whether the subject of the investigation is planning for its own professional goals, with a high level of information and understanding."}, "time_dimension": {"answer": "Past", "rationale": "\"I will not plan for achieving my professional goals\" does not indicate a past behavior or attitude. So the time dimension of this entry is the past."}, "sentence_type": "Negative declarative sentence", "pos": {"nouns": {"Entry": 1, "Questionnaire": 1, "Objective": 1, "Planned": 1}, "verbs": {"Development": 1, "Achieved": 1}, "adverbs": {"No, I won't": 1}, "pronouns": {"self": 1}}}, {"content": "20. I do not know what to do when there is a conflict with others.", "word_count": 16, "label": "", "sentence_structure": "Subject鈥搗erb鈥搊bject", "subjective_objective": {"answer": "Subjective", "rationale": "Subjectivity because \"not knowing what to do\" is the patient's subjective feeling and way of thinking. Although this is a sentence describing situations that conflict with others, it's often a subjective reaction within the individual."}, "sentence_sentiment": {"answer": "Negative", "rationale": "Because the attitude of \"I don't know what to do\" in the entry is negative."}, "language_style": {"answer": "Tactful and warm", "rationale": "The language style of the entry is euphemism, because it asks the person under investigation in a kind and caring manner, rather than in the words of an order or a direct challenge. Moreover, it implies the possibility of seeking help, making the person under investigation more comfortable and willing to share his/her true thoughts."}, "time_dimension": {"answer": "Present", "rationale": "The keyword \"when in conflict with others\" means that this is usually happening right now, and \"I don't know what to do\" indicates the psychological state of the person being tested in this situation, i.e., uncertainty about how to deal with it."}, "sentence_type": "Interrogative sentence", "pos": {"nouns": {"Conflict": 1, "others": 1}, "verbs": {"Come on": 1, "Yes": 1, "OPERATIONS": 1, "FL": 1}, "adverbs": {"not": 1, "The": 1}, "numerals": {"20": 1}, "pronouns": {"I": 1}}}, {"content": "21. I have rarely been nervous or anxious.", "word_count": 10, "label": "", "sentence_structure": "Subject鈥揷opula鈥損redicative", "subjective_objective": {"answer": "Subjective", "rationale": "Because \"very little tension or anxiety\" is a feeling or emotional experience, which is the subjective perspective of the patient."}, "sentence_sentiment": {"answer": "Positive", "rationale": "Because the expression \"very rarely feeling nervous or anxious\" is positive, it is a sign of emotional stability and a sense of calm."}, "language_style": {"answer": "Tactful and warm", "rationale": "The language style of the entry, which is more euphemistic and moderate, and which asks each other about each other in a caring and understanding manner, rather than asking or criticizing directly, is more likely to make the interviewee feel respected and understood and thus more willing to cooperate with the completion of the questionnaire."}, "time_dimension": {"answer": "Present", "rationale": "So the time dimension is now judged."}, "sentence_type": "Negative declarative sentence", "pos": {"verbs": {"I feel": 1, "Nervous": 1, "Anxiety": 1}, "adjectives": {"Very few": 1}}}, {"content": "22. I will develop some hobbyes in the light of my own situation.", "word_count": 19, "label": "", "sentence_structure": "Subject鈥搗erb鈥揷omplement", "subjective_objective": {"answer": "Subjective", "rationale": "Because \"it's your own reality\" that shows that it's the result of patient self-decision and action, with a strong personal and subjective character."}, "sentence_sentiment": {"answer": "Positive", "rationale": "Because the purpose of the act is to \"bring some hobby to your own reality.\""}, "language_style": {"answer": "Tactful and warm", "rationale": "The language style of the entry is euphemistic. It uses the expression \"according to your own circumstances\" to give a gentle advice, and to emphasize the importance of self-determination and individualization. Moreover, \"bringing some hobby\" is an encouraging expression, giving positive positive feelings. In general, the entry leads the interviewee to think and develop his hobby in a moderate and supportive way."}, "time_dimension": {"answer": "Present", "rationale": "The keyword \"will\" indicates that it is a subjective will or intention, and that it is in progress. The entry is therefore an inquiry into the present behaviour or attitude of the person being tested."}, "sentence_type": "Interrogative sentence", "pos": {"nouns": {"self": 1, "Actual": 1, "Amateurity": 1}, "verbs": {"Yes": 1, "Cultivation": 1, "Based on": 1}, "adverbs": {"Actual": 1}, "numerals": {"22": 1}, "classifiers": {"Some": 1}, "pronouns": {"I": 1}}}, {"content": "I always compare my own strengths with others.", "word_count": 16, "label": "", "sentence_structure": "Subject鈥搗erb鈥搊bject", "subjective_objective": {"answer": "Subjective", "rationale": "Because \"to compare your own shorts with others' strengths\" is the patient's subjective feeling and idea."}, "sentence_sentiment": {"answer": "Negative", "rationale": "Because the \"relationship of your own shorts with others\" in the entry is negative and can easily lead to a lower sense of self-worth and low self-esteem."}, "language_style": {"answer": "Calm and direct", "rationale": "Because the language style of the entry is simpler, there is no excessive rhetorical or emotional rendering, and the tone is more objective."}, "time_dimension": {"answer": "Past", "rationale": "The entry \"always\" means repeated actions that occur in a certain period of time in the past and therefore are judged to be past."}, "sentence_type": "Negative declarative sentence", "pos": {"nouns": {"self": 1, "Short": 1, "others": 1, "Strengths": 1}, "verbs": {"Take it": 1, "- Yeah": 1, "Compare": 1}, "adverbs": {"Total": 1}, "numerals": {"23": 1}, "pronouns": {"Here": 1}}}, {"content": "24. I have often reflected on learning.", "word_count": 10, "label": "", "sentence_structure": "Subject鈥損redicate", "subjective_objective": {"answer": "Subjective", "rationale": "Because \"rethinking\" is a subjective idea or behaviour that reflects the patient's self-observation and reflection on his or her learning."}, "sentence_sentiment": {"answer": "Positive", "rationale": "Because the purpose of the act of \"rethinking frequently\" is to improve its learning effectiveness and is a positive learning attitude."}, "language_style": {"answer": "Tactful and warm", "rationale": "The entry is more adept and interactive when it is spoken in a gentle and inviting manner, rather than by direct statements or orders."}, "time_dimension": {"answer": "Present", "rationale": ""}, "sentence_type": "Affirmative declarative sentence", "pos": {"nouns": {"Learning": 1, "Reflections": 1}, "verbs": {"Regular": 1, "Conduct": 1}, "adverbs": {"to": 1}}}, {"content": "When I was hit, I thought of my own good side.", "word_count": 17, "label": "", "sentence_structure": "Subject鈥搗erb鈥揷omplement", "subjective_objective": {"answer": "Subjective", "rationale": "This is a reflection of the patient ' s self-observation and evaluation and of his or her subjective attitude."}, "sentence_sentiment": {"answer": "Positive", "rationale": "Because the behavior in the entries, \"thinking of a good side\" is positive and helps to reduce negative emotions."}, "language_style": {"answer": "Tactful and warm", "rationale": "The entry asks respondents in a way that encourages and supports them, helps them to focus on their strengths and strengths and as a way to address the pressures and challenges."}, "time_dimension": {"answer": "Present", "rationale": ""}, "sentence_type": "Affirmative declarative sentence", "pos": {"nouns": {"Strike": 1, "Noodles": 1}, "verbs": {"Yes": 2}, "adjectives": {"Okay": 1}, "adverbs": {"From": 1}, "classifiers": {"self": 1}}}, {"content": "26. Although my amateur life is poor, I do not know how to change this situation.", "word_count": 24, "label": "", "sentence_structure": "Subject鈥搗erb鈥搊bject", "subjective_objective": {"answer": "Subjective", "rationale": "The reason for this is that it is not clear how to change the subjective perception and feelings that this is for the patient."}, "sentence_sentiment": {"answer": "Negative", "rationale": "Because \"poor\" and \"not knowing how to change\" in the entries are expressions of negative emotions."}, "language_style": {"answer": "Tactful and warm", "rationale": "The entry uses the expression \"no idea how to change\" , which expresses a feeling of help and confusion, is more euphemism and is more friendly. At the same time, it does not directly order or criticize each other, and is relatively gentle. It is therefore a warm word that can be judged."}, "time_dimension": {"answer": "Present", "rationale": "The entry is about the current state and confusion, which is, \"I don't know how to change this, though I have a poor amateur life.\" So the time dimension is now."}, "sentence_type": "Negative declarative sentence", "pos": {"nouns": {"Amateur life": 1, "Status": 1}, "verbs": {"Yes": 1, "Change": 1, "What": 1}, "adjectives": {"very": 1, "Poverty": 1}, "adverbs": {"But": 1, "I don't know": 1}, "numerals": {"26锛?: 1}, "pronouns": {"I": 1}}}, {"content": "I am always energetic and full of energy.", "word_count": 11, "label": "", "sentence_structure": "Subject鈥揷opula鈥損redicative", "subjective_objective": {"answer": "Subjective", "rationale": "Because \"always\" and \"absorbed\" and \"satisfaction\" describe the patient's own subjective feelings and attitudes."}, "sentence_sentiment": {"answer": "Positive", "rationale": "Basis: The behaviour described in the entries is \"always energetic and spiritually full\" positive."}, "language_style": {"answer": "Tactful and warm", "rationale": ""}, "time_dimension": {"answer": "Present", "rationale": ""}, "sentence_type": "Affirmative declarative sentence", "pos": {"nouns": {"I": 1, "- The energy": 1, "鈾?Full of 鈾?: 1, "Spirit": 1, "Full": 1}, "verbs": {"Always": 1, "be": 1}, "adjectives": {"鈾?Full of 鈾?: 1, "Spirit": 1, "Full": 1}, "adverbs": {"Always": 1}, "pronouns": {"I": 1}}}, {"content": "I do not know how to allocate time for learning.", "word_count": 12, "label": "", "sentence_structure": "Subject鈥搗erb鈥搊bject", "subjective_objective": {"answer": "Subjective", "rationale": "Because \"how to allocate time for study\" is a matter to be determined by personal circumstances, it is the patient's subjective attitude."}, "sentence_sentiment": {"answer": "Negative", "rationale": "Because the \"unsure how to allocate time for learning\" behavior in the entries is negative."}, "language_style": {"answer": "Tactful and warm", "rationale": "The entry is more euphemistic and sweet. It uses the means of questioning, not directly accusing or criticizing the interviewees, but expressing concerns about their confusion and distress, which is more readily accepted by the interviewees and is willing to share their true ideas with researchers."}, "time_dimension": {"answer": "Present", "rationale": "According to the entry sentence, \"Don't know how\" and \"Now\", the entry can be judged as asking the testee about his current learning, and therefore as now."}, "sentence_type": "Interrogative sentence", "pos": {"nouns": {"Learning time": 1}, "verbs": {"Allocation": 1, "Yes": 1}, "adverbs": {"How's that": 1}, "numerals": {"28": 1}}}, {"content": "I feel better than others.", "word_count": 12, "label": "", "sentence_structure": "Subject鈥揷opula鈥損redicative", "subjective_objective": {"answer": "Subjective", "rationale": "The perception of being the patient ' s personal perception and feelings is subjective."}, "sentence_sentiment": {"answer": "Positive", "rationale": "Because the entry is about \"self better than others\" evaluation, it is about positive self-awareness and affirmativeness."}, "language_style": {"answer": "Tactful and warm", "rationale": "The language style of the entry is more euphemistic. It uses subjective expression ( \"I feel\") and avoids direct comparison or criticism of others, which reduces the resistance of the respondents and makes them more willing to respond honestly. At the same time, the expression \"more powerful\" is more implicit than that, and does not make the respondents feel too arrogant or arrogant, which would enhance the effectiveness and credibility of the questionnaire."}, "time_dimension": {"answer": "Present", "rationale": "The entry uses the \"Perception\" and \"Perception\" to imply that the testee's view of the current comparison of his or her abilities with others does not relate to past or future behaviour or mentalities. Thus, the time dimension of the entry is now."}, "sentence_type": "Affirmative declarative sentence", "pos": {"nouns": {"Capacity": 1, "self": 1, "others": 1}, "verbs": {"I think": 1, "Strong": 1}, "numerals": {"29": 1}, "pronouns": {"I": 1}}}, {"content": "30. I am very tired of learning now.", "word_count": 10, "label": "", "sentence_structure": "Subject鈥搗erb鈥揷omplement", "subjective_objective": {"answer": "Subjective", "rationale": "Because \"incontinent\" is a subjective emotional experience."}, "sentence_sentiment": {"answer": "Negative", "rationale": "Because the \"very tired\" attitude in the entry is negative."}, "language_style": {"answer": "Calm and direct", "rationale": "The entry directly expresses the subject of questioning the other party ' s attitude to the current state of learning, without excessive rhetorical or emotional rendering and with a more objective tone."}, "time_dimension": {"answer": "Present", "rationale": "The entry indicates that the testee is very tired of learning at this time and does not address the time dimension of the past or the future."}, "sentence_type": "Interrogative sentence", "pos": {"nouns": {"Questionnaire Entry": 1, "Present": 1, "Learning": 1}, "verbs": {"Very": 1, "Boring": 1}, "adverbs": {"Very": 1}, "classifiers": {"individual": 1}, "pronouns": {"I": 1}}}, {"content": "I am well-informed when compared to my peers.", "word_count": 12, "label": "", "sentence_structure": "Subject鈥揷opula鈥損redicative", "subjective_objective": {"answer": "Subjective", "rationale": "Because it's the patient's subjective feeling and comparison."}, "sentence_sentiment": {"answer": "Positive", "rationale": "Because the article is \"satisfied\", it shows that the interviewee has a sense of satisfaction and happiness in his or her own life, and that it is a positive emotional attitude."}, "language_style": {"answer": "Tactful and warm", "rationale": "The language style of the entry is more moderate and friendly, and does not use strong subjective feelings or evaluative terms to describe the subject matter, but is presented in a more objective and neutral manner, which is more acceptable and understandable."}, "time_dimension": {"answer": "Present", "rationale": "The keyword \"as compared to peers\" shows that the problem involves a comparison and comparison, and therefore places greater emphasis on the current feeling. At the same time, \"sure of being well-informed\" is also a highly subjective assessment, so the problem is mainly about the present subjective emotional state, that is, the satisfaction of the testee with his or her own life, achievements, etc."}, "sentence_type": "Affirmative declarative sentence", "pos": {"nouns": {"Peer": 1, "Satisfied": 1}, "verbs": {"Sensor": 1, "- Yeah": 1}, "adverbs": {"very": 1}, "numerals": {"31": 1}}}, {"content": "32. I am not used to school-set hours.", "word_count": 13, "label": "", "sentence_structure": "Subject鈥揷opula鈥損redicative", "subjective_objective": {"answer": "Subjective", "rationale": "Because \"not used\" is a personal feeling and attitude, and it is subjective."}, "sentence_sentiment": {"answer": "Negative", "rationale": "Because the attitude of \"not used\" in the entries is negative."}, "language_style": {"answer": "Tactful and warm", "rationale": "The language style of the entry is more euphemistic, without directly accusing or complaining that the school is not working at a reasonable time, but rather expressing its personal feelings in a relatively peaceful manner, and avoiding possible conflicts and contradictions in the form of a \"not used\" expression. Such language style is more easily a guide to acceptance and understanding of one another's views."}, "time_dimension": {"answer": "Past", "rationale": "The entry describes a custom or attitude of the interviewee, i.e., that the school does not like the time set for the break. Without reference to a specific time frame (e.g. \"the latest month\" or \"for the next year\" etc.), it is not possible to determine whether it is a past experience or a future expectation. However, given that the expression \"not used\" is usually used to describe a custom or preference that has been developed, we can classify it as a past act or attitude."}, "sentence_type": "Exclamatory sentence", "pos": {"nouns": {"Students": 1, "School": 1, "Time of rest": 1}, "verbs": {"Provisions": 1, "Custom": 1}, "numerals": {"32": 1}, "pronouns": {"I": 1}}}, {"content": "I am not troubled by my appearance.", "word_count": 11, "label": "", "sentence_structure": "Subject鈥揷opula鈥損redicative", "subjective_objective": {"answer": "Subjective", "rationale": "For a worry is an emotional reaction, which is generated within the individual and is therefore subjective."}, "sentence_sentiment": {"answer": "Positive", "rationale": "Because the attitude in the entry \"no trouble for its appearance\" is positive."}, "language_style": {"answer": "Tactful and warm", "rationale": "The entry is more adept and interactive when it is spoken in a gentle and inviting manner, rather than by direct statements or orders."}, "time_dimension": {"answer": "Present", "rationale": "The entry indicates the current state or attitude of the person being tested, and \"I\" indicates that the dominant language is the first person and does not refer to past or future time information."}, "sentence_type": "Negative declarative sentence", "pos": {"nouns": {"Looks": 1, "Trouble": 1}, "verbs": {"No, I'm not": 1, "Trouble": 1}, "adverbs": {"not": 1}, "numerals": {"33": 1}}}, {"content": "34. I have always set different learning goals at different stages of learning.", "word_count": 20, "label": "", "sentence_structure": "Subject鈥搗erb鈥搊bject", "subjective_objective": {"answer": "Subjective", "rationale": "Because \"always\" and \"different learning goals\" are patients' personal choices and plans, reflecting their subjective attitudes."}, "sentence_sentiment": {"answer": "Positive", "rationale": "Because the behaviour of \"setting different learning goals\" in the entries is positive, it is an expression that individuals have clear learning plans and goals."}, "language_style": {"answer": "Tactful and warm", "rationale": "The entry expresses concern and understanding about the learning target set by the respondent by means of inquiries rather than direct statements or orders. At the same time, the term \"always\" is used to convey a positive and proactive attitude, making the questionnaire more friendly and interactive, and to reflect a soft and warm language style."}, "time_dimension": {"answer": "Present", "rationale": "The entry indicates that the behaviour or state of the interviewees is present, i.e. they take into account the current learning stage when setting learning goals and set different objectives accordingly. Thus, the key words \"at different learning stages\", \"always\" and \"formulation\" all refer to the present situation."}, "sentence_type": "Affirmative declarative sentence", "pos": {"nouns": {"Different": 2, "Study stage": 1, "Learning objectives": 1}, "verbs": {"Development": 1, "Always": 1}, "adverbs": {"in": 1, "I": 1, "of": 4, "Always": 1, "Different": 2}, "numerals": {"34锛?: 1}, "pronouns": {"I": 1}}}, {"content": "35. When I had difficulties, there were many people who were willing to help me.", "word_count": 16, "label": "", "sentence_structure": "Subject鈥搗erb鈥揷omplement", "subjective_objective": {"answer": "Subjective", "rationale": "Because \"many people are willing to help me\" is a subjective perception of a patient who feels supported and loved. Although it is an objective scenario that can happen (i.e. someone who wants to help), in this sentence it is used to express the feelings and experiences of the patient and is therefore subjective."}, "sentence_sentiment": {"answer": "Positive", "rationale": "Because the \"many people willing to help me\" act is positive."}, "language_style": {"answer": "Tactful and warm", "rationale": "The entry is more adept and interactive when it is spoken in a gentle and inviting manner, rather than by direct statements or orders."}, "time_dimension": {"answer": "Present", "rationale": "The keyword \"difficult\" and \"willing to help\" means that there is a situation, so the answer is \"now\"."}, "sentence_type": "Affirmative declarative sentence", "pos": {"nouns": {"Difficulties": 1, "person": 2, "Help": 1}, "verbs": {"have": 1, "Yes": 1, "Help": 1}, "adverbs": {"A lot": 1}, "numerals": {"35锛?: 1}}}, {"content": "36. I have clear career objectives.", "word_count": 9, "label": "", "sentence_structure": "Subject鈥搗erb鈥揷omplement", "subjective_objective": {"answer": "Subjective", "rationale": "Since the existence of a clear professional objective is the individual ' s perception and attitude, it is subjective."}, "sentence_sentiment": {"answer": "Positive", "rationale": "Because the \"defined career objectives\" in the entry indicate that individuals have a clear desire and planning, it is a positive attitude."}, "language_style": {"answer": "Tactful and warm", "rationale": "The language style of the entry is more euphemistic, using positive positive statements, \"I have a clear career goal,\" and is more encouraging and supportive. Such expressions increase the motivation and participation of the respondents and are more likely to get real answers."}, "time_dimension": {"answer": "Present", "rationale": "The word \"yes\" in it means that there is a state or feature, and \"clear professional objectives\" is a description of the behaviour of the present, so the entry is the current situation of the person being tested."}, "sentence_type": "Affirmative declarative sentence", "pos": {"nouns": {"Career objectives": 1}, "verbs": {"have": 1}}}, {"content": "I know how to care for others.", "word_count": 9, "label": "", "sentence_structure": "Subject鈥搗erb鈥搊bject", "subjective_objective": {"answer": "Subjective", "rationale": "Because knowing how to care for others is a subjective attitude or cognitive ability."}, "sentence_sentiment": {"answer": "Positive", "rationale": "Because \"care\" in the entry is a positive behavior and attitude."}, "language_style": {"answer": "Tactful and warm", "rationale": ""}, "time_dimension": {"answer": "Present", "rationale": "The entry indicates that the testee knows how to care for others and that this ability is currently available and therefore the temporal dimension is now judged."}, "sentence_type": "Affirmative declarative sentence", "pos": {"nouns": {"Questionnaire": 1, "Entry": 1, "Care": 1, "others": 1}, "verbs": {"Yes": 1, "How's that": 1}, "numerals": {"37": 1}}}, {"content": "My amateur life is boring.", "word_count": 10, "label": "", "sentence_structure": "Subject鈥揷opula鈥損redicative", "subjective_objective": {"answer": "Subjective", "rationale": "Because the monotonousness of amateur life is a description of the subjective feeling associated with the patient ' s inner experience."}, "sentence_sentiment": {"answer": "Negative", "rationale": "The basis of the judgment. Because \"single\" in the entries is negative emotions and attitudes."}, "language_style": {"answer": "Tactful and warm", "rationale": "The entry is more adept and interactive when it is spoken in a gentle and inviting manner, rather than by direct statements or orders."}, "time_dimension": {"answer": "Present", "rationale": ""}, "sentence_type": "Negative declarative sentence", "pos": {"nouns": {"Amateur life": 1}, "verbs": {"It's boring": 1}, "adjectives": {"Boring": 1, "Undo": 1}, "numerals": {"38": 1}}}, {"content": "I have often adjusted my emotional state by diverting my attention.", "word_count": 19, "label": "", "sentence_structure": "Subject鈥搗erb鈥揷omplement", "subjective_objective": {"answer": "Subjective", "rationale": "Because \"diversion of your attention\" is a subjective act and decision."}, "sentence_sentiment": {"answer": "Positive", "rationale": "Because the behavior in the entry \"regulating emotional state by diverting your attention\" is positive."}, "language_style": {"answer": "Calm and direct", "rationale": "This language makes the questionnaire more professional and rigorous and avoids possible subjective interference with the outcome."}, "time_dimension": {"answer": "Present", "rationale": "The word \"I'm often\" means regular, customary behaviour, and the \"by ...\" action takes place now, indicating that the person being tested is now adjusting his emotional state in this way. So the time dimension of the article is now."}, "sentence_type": "Affirmative declarative sentence", "pos": {"nouns": {"I": 1, "Attention": 1, "Emotional state": 1}, "verbs": {"Often": 1, "Transfer": 1, "Adjustment": 1}, "adverbs": {"Often": 1}, "numerals": {"39": 1}}}, {"content": "40. I rarely summarised the previous stage of learning.", "word_count": 15, "label": "", "sentence_structure": "Subject鈥損redicate", "subjective_objective": {"answer": "Subjective", "rationale": "This is a self-evaluation of the patient ' s learning habits and a subjective attitude and perception."}, "sentence_sentiment": {"answer": "Negative", "rationale": "Because the \"very little summarizing of the previous period's learning\" in the entry is negative."}, "language_style": {"answer": "Calm and direct", "rationale": "The language style of the entry is more direct, there is no excessive rhetorical or emotional rendering, it is more objective in tone and it is expressed in a simpler and more straightforward manner.\""}, "time_dimension": {"answer": "Past", "rationale": ""}, "sentence_type": "Negative declarative sentence", "pos": {"nouns": {"Phase": 1, "Learning": 1, "Summary": 1}, "verbs": {"Very few": 1, "Conduct": 1}, "adverbs": {"very": 1}, "numerals": {"40锛?: 1}, "pronouns": {"I": 1}}}, {"content": "41. I will combine the various factors to determine my career choice.", "word_count": 18, "label": "", "sentence_structure": "Subject鈥搗erb鈥搊bject", "subjective_objective": {"answer": "Subjective", "rationale": "Because \"the combination of factors to determine\" is the patient's subjective attitude and choice."}, "sentence_sentiment": {"answer": "Positive", "rationale": "Because the action and purpose of the entry is to \"compatible factors to determine their own career choices\"."}, "language_style": {"answer": "Calm and direct", "rationale": "The entry asks the interviewees in a straightforward manner whether they will take into account a variety of factors in determining their career choices, the language style is relatively calm and objective, and there is no excessive emotional rendering or rhetorical technique."}, "time_dimension": {"answer": "Present", "rationale": "The entry indicates the behaviour or attitude of the person being tested in the context of the current consideration and action, and therefore the judgement is now."}, "sentence_type": "Affirmative declarative sentence", "pos": {"nouns": {"Factors": 1, "Targeted employment": 1}, "verbs": {"Yes": 1, "Synthesis": 1, "Sure": 1}, "adverbs": {"Various": 1}, "numerals": {"41锛?: 1}, "pronouns": {"self": 1}}}, {"content": "42. I do not know how to accept myself.", "word_count": 16, "label": "", "sentence_structure": "Subject鈥搗erb鈥搊bject", "subjective_objective": {"answer": "Subjective", "rationale": "Because \"accepting yourself\" is a subjective issue of self-awareness and attitude."}, "sentence_sentiment": {"answer": "Negative", "rationale": "Because the words \"no way to know\" and \"intoto taking oneself\" both imply a confused, uneasy or self-denial mood, the emotional color of the article is negative."}, "language_style": {"answer": "Tactful and warm", "rationale": "This entry uses a more moderate, understanding and supportive tone, expressing to the respondents the concern and respect for their distress and encouraging them to share their feelings and ideas. This expression is easier to create a comfortable and safe questionnaire environment, promoting the participation and honesty of the respondents."}, "time_dimension": {"answer": "Present", "rationale": "The term \"not knowing\" and \"self-admission\" in the sentence makes it possible to judge the current status and feelings of the testee in the questionnaire entry and is therefore part of the current temporal dimension."}, "sentence_type": "Negative declarative sentence", "pos": {"nouns": {"Approach": 1, "Admission": 1, "self": 1}, "verbs": {"Jean": 1, "Yes": 1}, "adverbs": {"What": 1, "Use": 1}, "numerals": {"42": 1}}}, {"content": "43. Many people are willing to go out with me.", "word_count": 10, "label": "", "sentence_structure": "Subject鈥搗erb鈥搊bject", "subjective_objective": {"answer": "Subjective", "rationale": "Because it's the other's view that anyone wants to be with you, not your behavior or your state."}, "sentence_sentiment": {"answer": "Neutral", "rationale": "The entry is not a positive or negative emotional expression."}, "language_style": {"answer": "Tactful and warm", "rationale": "The entry uses a more euphemistic formulation, which asks the respondents about their views in interpersonal relationships, while avoiding the possibility of direct evaluation or accusations. This expression makes people feel more friendly and comfortable and facilitates more genuine feedback."}, "time_dimension": {"answer": "Present", "rationale": "The entry describes a subjective feeling or evaluation of others, does not address the time dimension of past or future, and therefore the judgement is now."}, "sentence_type": "Affirmative declarative sentence", "pos": {"nouns": {"person": 1, "- To be with someone": 1}, "verbs": {"Yes": 1, "and": 1}, "adjectives": {"More": 1}, "adverbs": {"Both": 1}, "numerals": {"43": 1}, "pronouns": {"I": 1}}}, {"content": "I love my profession very much.", "word_count": 10, "label": "", "sentence_structure": "Subject鈥揷opula鈥損redicative", "subjective_objective": {"answer": "Subjective", "rationale": "Because \"like\" is the patient's subjective attitude to his profession."}, "sentence_sentiment": {"answer": "Positive", "rationale": "Because the \"very much\" attitude in the entry is positive."}, "language_style": {"answer": "Tactful and warm", "rationale": "The entry uses positive tone and positive expressions of personal love and approval, which are more personal and inspiring. Nor is it directly accusing or ordering the other, maintaining a moderate and respectful attitude."}, "time_dimension": {"answer": "Present", "rationale": "The entry asks about the testee ' s attitude and emotional state of his or her profession, which does not relate to past or future circumstances, and therefore determines the present."}, "sentence_type": "Affirmative declarative sentence", "pos": {"nouns": {"Professional": 1}, "verbs": {"Yeah": 1}, "adjectives": {"Very": 1, "self": 1}, "numerals": {"44": 1}, "pronouns": {"I": 1}}}, {"content": "I do not know what will cheer me up.", "word_count": 17, "label": "", "sentence_structure": "Subject鈥搗erb鈥搊bject", "subjective_objective": {"answer": "Subjective", "rationale": "Because making yourself happy is the patient's subjective goal and idea."}, "sentence_sentiment": {"answer": "Negative", "rationale": "Because it expresses a negative emotion and attitude, i.e., does not know how to make himself happy."}, "language_style": {"answer": "Tactful and warm", "rationale": "The language style of the entry is more euphemistic and sensitive to each other鈥檚 feelings, and does not directly point to the other鈥檚 shortcomings or problems, but rather leads to sharing emotions and ideas in a way that is asked and cared for. This expression makes each other more receptive and willing to engage in dialogue."}, "time_dimension": {"answer": "Present", "rationale": "The keyword is \"not knowing what to do,\" which means that the person currently tested is in a state of some sort of distress or problem. And \"getting himself happy\" is also a description of the current state, indicating that the person tested wants to change his emotional state. So, the entry to the questionnaire asks about the person currently acting and mentalities."}, "sentence_type": "Interrogative sentence", "pos": {"nouns": {"Things": 1, "self": 1}, "verbs": {"鈾?And I'm gonna be 鈾?: 1, "Happy": 1, "Do it": 1, "Yes": 1}, "adverbs": {"What": 1, "not": 1}, "numerals": {"45锛?: 1}, "pronouns": {"I": 1}}}, {"content": "46. I like places of recreation, leisure or exercise in schools.", "word_count": 15, "label": "", "sentence_structure": "Subject鈥搗erb鈥搊bject", "subjective_objective": {"answer": "Subjective", "rationale": "Because it's the patient's subjective attitude."}, "sentence_sentiment": {"answer": "Positive", "rationale": "Because \"like\" in the entries shows positive attitudes and emotions."}, "language_style": {"answer": "Tactful and warm", "rationale": "This is a softer way of stimulating the audience and enhancing the effectiveness of the questionnaire."}, "time_dimension": {"answer": "Present", "rationale": "The entry contains \"like\" and \"school,\" where \"like\" shows the person's attitude to the situation, while \"school\" points out the specific place where the situation is happening. So the sentence is the person's emotional attitude."}, "sentence_type": "Affirmative declarative sentence", "pos": {"nouns": {"School": 1, "Recreation": 1, "Leisure": 1, "Exercise": 1, "Places": 1}, "verbs": {"Yeah": 1}, "numerals": {"46锛?: 1}}}, {"content": "47. I have always been learning from the failures of the examinations.", "word_count": 14, "label": "", "sentence_structure": "Subject鈥搗erb鈥搊bject", "subjective_objective": {"answer": "Subjective", "rationale": "Because \"thousand lessons from examination failures\" reflect patients' assessment and attitude, and are patients' subjective perceptions."}, "sentence_sentiment": {"answer": "Positive", "rationale": "Because the aim of the entry is to \"take out the lessons from the failed examinations\"."}, "language_style": {"answer": "Tactful and warm", "rationale": "The entry is more euphemistic, expressing each other's habit of reflection and summation by using the words \"always\" and \"lessons learned\" without directly accusing or evaluating. Such expressions are more moderate and contribute to a good atmosphere of communication."}, "time_dimension": {"answer": "Present", "rationale": "So the output should be \"now\"."}, "sentence_type": "Interrogative sentence", "pos": {"nouns": {"Lessons learned": 1, "Examinations": 1}, "verbs": {"Always": 1, "Summary": 1, "Failed": 1, "Lessons learned": 1}, "adverbs": {"Always": 1}, "numerals": {"47": 1}, "pronouns": {"I": 1}}}, {"content": "48. I believe that there are many unsatisfactory places in university life.", "word_count": 18, "label": "", "sentence_structure": "Subject鈥搗erb鈥搊bject", "subjective_objective": {"answer": "Subjective", "rationale": "Because \"think\" is the patient's subjective view."}, "sentence_sentiment": {"answer": "Negative", "rationale": "Because the term \"unsatisfactory\" is negative, expressing dissatisfaction and complaints about university life."}, "language_style": {"answer": "Calm and direct", "rationale": "The entry is presented in a direct way, which clearly expresses the view of the subject of the survey about the disaffection with university life. The expression is simple, straightforward, not overly pedantic or emotional, and is more in line with the objective and neutral principles of the questionnaire."}, "time_dimension": {"answer": "Present", "rationale": "The \"concept\" and \"many\" in the entry indicate that this is a current attitude or view, so the judgment is \"now\"."}, "sentence_type": "Negative declarative sentence", "pos": {"nouns": {"University life": 1, "I'm glad": 1}, "verbs": {"I think": 1, "have": 1, "Local": 1}, "adjectives": {"A lot": 1}, "adverbs": {"very": 1, "Not at all": 1}, "pronouns": {"I": 1}}}, {"content": "49. I have always sought to improve learning skills in memory, attention and so forth.", "word_count": 20, "label": "", "sentence_structure": "Subject鈥搗erb鈥搊bject", "subjective_objective": {"answer": "Subjective", "rationale": "Because \"enhancement of learning skills, memory, attention, etc.\" is a subjective goal of behaviour that requires conscious thinking and practice, rather than objective facts or results."}, "sentence_sentiment": {"answer": "Positive", "rationale": "Because the purpose of the entry is to \"get a way to improve memory, attention, etc. learning skills.\""}, "language_style": {"answer": "Tactful and warm", "rationale": "This is a soft and encouraging language style that makes it easier for respondents to react positively and to remain willing to participate in questionnaires."}, "time_dimension": {"answer": "Present", "rationale": "The key words \"always\" and \"and to increase\" suggest that the testee is doing the act and is likely to continue, so the temporal dimension of the purpose of the article is now judged."}, "sentence_type": "Affirmative declarative sentence", "pos": {"nouns": {"Memory": 1, "Attention": 1, "Learning ability": 1}, "verbs": {"Increase": 1, "Always trying to do it": 1}, "adverbs": {"Always": 1}, "numerals": {"49": 1}}}, {"content": "50. I am good at communicating with others in words.", "word_count": 13, "label": "", "sentence_structure": "Subject鈥搗erb鈥揷omplement", "subjective_objective": {"answer": "Subjective", "rationale": "Because the words \"good\" and \"communication\" describe their abilities and behaviour from the perspective of the patient, they are subjective."}, "sentence_sentiment": {"answer": "Positive", "rationale": "Because the \"good to communicate with others\" act in the entry is positive."}, "language_style": {"answer": "Tactful and warm", "rationale": "The entry uses a more positive expression of \"goodness\" and \"communications with others\" in conjunction with the verb \"and connections\" verb, which expresses a positive attitude. This tone is more cordial and gentle, and it is more resonant and trusting for the respondents and facilitates better completion of the psychological questionnaire study."}, "time_dimension": {"answer": "Present", "rationale": ""}, "sentence_type": "Affirmative declarative sentence", "pos": {"nouns": {"I": 1, "Words": 1, "others": 1, "Communications": 1}, "verbs": {"Good at it": 1, "Conduct": 1}, "adverbs": {"50锛?: 1}, "numerals": {"50": 1}}}, {"content": "51. I have often been conscious in preparing for future work through participation in social practice activities.", "word_count": 26, "label": "", "sentence_structure": "Subject鈥搗erb鈥搊bject", "subjective_objective": {"answer": "Subjective", "rationale": "Because \"I\" is expressing my subjective thoughts and attitudes."}, "sentence_sentiment": {"answer": "Positive", "rationale": "Because the entry \"consciously preparing for future work through participation in social practice activities\" is positive."}, "language_style": {"answer": "Tactful and warm", "rationale": "The questionnaire entries are more euphemistic and warm, use relatively neutral and polite terms, and do not have too many direct or intense emotional features. It asks the respondents in a convenient way whether they are often involved in social practice activities to prepare for future work, and to resonate and cooperate more easily."}, "time_dimension": {"answer": "Future", "rationale": "The entry describes the future behaviour or mentality of the testee, the keywords being \"preparation for future work\" and \"often conscious\", which point to situations that have not yet occurred and that may occur in the future."}, "sentence_type": "Affirmative declarative sentence", "pos": {"nouns": {"Activities": 1, "Work": 2, "Ready": 1}, "verbs": {"# Consciously #": 1, "Participation": 1, "Do it": 1, "Ready": 1}, "adjectives": {"Regular": 1, "Future": 1}, "numerals": {"51": 1}, "pronouns": {"I": 1}}}, {"content": "52. I have been very well adapted to my university life.", "word_count": 11, "label": "", "sentence_structure": "Subject鈥揷opula鈥損redicative", "subjective_objective": {"answer": "Subjective", "rationale": "Because \"adaptation\" is a subjective feeling, a self-evaluation and a perception of one person."}, "sentence_sentiment": {"answer": "Neutral", "rationale": "Because the entry describes the fact that the interviewee is very well adapted to university life and does not display a positive or negative emotional profile."}, "language_style": {"answer": "Tactful and warm", "rationale": ""}, "time_dimension": {"answer": "Present", "rationale": "The keyword \"very well fit\" denotes the current feeling and state, so the entry is to ask the testee what the situation is."}, "sentence_type": "Affirmative declarative sentence", "pos": {"verbs": {"Adaptation": 1, "Life": 1}, "adjectives": {"Very": 1}}}, {"content": "53. When I am unhappy, I will only complain.", "word_count": 9, "label": "", "sentence_structure": "Subject鈥損redicate", "subjective_objective": {"answer": "Subjective", "rationale": "Because \"complaint\" is a subjective emotional reaction that reflects the attitude and behaviour of patients when they are unhappy."}, "sentence_sentiment": {"answer": "Negative", "rationale": "Because \"complaining\" is usually considered a negative emotional expression."}, "language_style": {"answer": "Calm and direct", "rationale": "The entry is presented directly, is not overdoing or emotionally rendering, and is more calm and objective, expressing a more specific behavioural characteristic."}, "time_dimension": {"answer": "Present", "rationale": ""}, "sentence_type": "Affirmative declarative sentence", "pos": {"verbs": {"Yes": 1, "- He's complaining": 1}, "adjectives": {"I'm not happy": 1}, "numerals": {"53": 1}, "pronouns": {"I": 1}}}, {"content": "54. I find the dormitories comfortable.", "word_count": 11, "label": "", "sentence_structure": "Subject鈥揷opula鈥損redicative", "subjective_objective": {"answer": "Subjective", "rationale": "Because it feels like the patient's subjective attitude."}, "sentence_sentiment": {"answer": "Positive", "rationale": "Because \"the dormitory is comfortable now\" is a positive assessment and attitude."}, "language_style": {"answer": "Tactful and warm", "rationale": "The language style of the entry is euphemistic, expressing the interviewee ' s feelings about the present dormitories in a positive and positive tone, without using negative or critical terminology, and more sweet and friendly."}, "time_dimension": {"answer": "Present", "rationale": "The words \"felt\" and \"sweet\" indicate that the person tested felt and evaluated the current accommodation situation, so the entry indicates the current situation."}, "sentence_type": "Affirmative declarative sentence", "pos": {"nouns": {"Accommodation": 1, "Comfortable": 1}, "verbs": {"I think": 1}, "adjectives": {"Present": 1}, "numerals": {"54": 1}, "pronouns": {"I": 1}}}, {"content": "55. When I met strangers, I did not know how to talk to them.", "word_count": 17, "label": "", "sentence_structure": "Subject鈥搗erb鈥搊bject", "subjective_objective": {"answer": "Subjective", "rationale": "Because \"I don't know how to talk to them\" is the patient's subjective feeling and attitude."}, "sentence_sentiment": {"answer": "Negative", "rationale": "Because the \"I don't know how to talk to them\" act is negative."}, "language_style": {"answer": "Tactful and warm", "rationale": "The language style of the entry is more euphemistic, as it raises questions in a question-and-answer manner, without directly accusing or judging the other. It also provides an open question, allowing the interviewee to express his views and feelings freely. Such expression makes it easier for the interviewee to feel comfortable and comfortable, and is more likely to receive real and effective feedback."}, "time_dimension": {"answer": "Past", "rationale": "The entry describes the social problems that the subjects are facing today, and \"not knowing how to talk to them\" is a past or what is happening now, so it can be judged as \"the past\"."}, "sentence_type": "Negative declarative sentence", "pos": {"nouns": {"Stranger": 1, "Talk": 1}, "verbs": {"I met him": 1, "Yes": 1, "How's that": 1, "and": 1, "they": 1, "Talk": 1}, "adverbs": {"How's that": 1}, "numerals": {"55": 1}, "pronouns": {"I": 1}}}, {"content": "When I want to talk, I can always find someone to talk to.", "word_count": 16, "label": "", "sentence_structure": "Subject鈥搗erb鈥搊bject", "subjective_objective": {"answer": "Subjective", "rationale": "Because \"want to talk\" and \"get someone to talk\" are patients' subjective attitudes and feelings."}, "sentence_sentiment": {"answer": "Positive", "rationale": "Because the purpose of the entry is to \"get someone to talk to me.\""}, "language_style": {"answer": "Tactful and warm", "rationale": "The language style of the entry is more euphemistic, warm, and by using the word \"always\" it gives the respondents attention and attention. This expression is more friendly, and it helps to increase the participation of the respondents. At the same time, the expression \"get someone to talk with me\" is relatively positive and optimistic, and it is easily resonant with the respondents."}, "time_dimension": {"answer": "Present", "rationale": ""}, "sentence_type": "Affirmative declarative sentence", "pos": {"nouns": {"I": 1, "person": 1, "Chat": 2}, "verbs": {"Yes": 1, "Find": 1, "Talk": 1}, "adverbs": {"Total": 1}, "numerals": {"56": 1, "One": 1, "Two": 1}, "classifiers": {"individual": 1}}}, {"content": "57. I feel increasingly confused about what I will do in the future.", "word_count": 20, "label": "", "sentence_structure": "Subject鈥揷opula鈥損redicative", "subjective_objective": {"answer": "Subjective", "rationale": "Because \"I think\" is about the patient's own feelings and thoughts, which are subjectively described."}, "sentence_sentiment": {"answer": "Negative", "rationale": "Because the emotional expression \"What's going to happen in the future\" in the entry is negative."}, "language_style": {"answer": "Tactful and warm", "rationale": "The entry is more euphemistic, soft in its language, and not too direct or violent in its expression. It leads the interviewee to share his thoughts and feelings, while showing concern and understanding for the interviewee. So I think the language style of the questionnaire is \"fluent.\""}, "time_dimension": {"answer": "Present", "rationale": "The entry describes the current feelings and mindset of the testee, and therefore judges \"now\"."}, "sentence_type": "Affirmative declarative sentence", "pos": {"nouns": {"The future": 1, "Work": 2, "Frustration": 1}, "verbs": {"I think": 1, "Active": 1, "More and more": 1}, "adjectives": {"self": 1}, "adverbs": {"More and more": 1}, "numerals": {"57": 1}}}, {"content": "58. When I am in a bad mood, I go out and get my head blown.", "word_count": 13, "label": "", "sentence_structure": "Subject鈥搗erb鈥搊bject", "subjective_objective": {"answer": "Subjective", "rationale": "Since emotions are subjective, walking outside is also an act of personal choice, and the subjective perspective is examined in this context."}, "sentence_sentiment": {"answer": "Positive", "rationale": "Because the purpose of the \"out\" act in the entry is positive."}, "language_style": {"answer": "Tactful and warm", "rationale": "The language style of the entry is more euphemistic, leading the respondents to share their emotions and coping strategies in a question-and-answer manner, without directly stating or ordering what the counterparty should do, and this expression is more acceptable and understandable."}, "time_dimension": {"answer": "Past", "rationale": "Because \"I'm not in a bad mood\" is a past situation, and \"I\" is a current customary practice, so this entry covers both the past and the present."}, "sentence_type": "Affirmative declarative sentence", "pos": {"nouns": {"The mood": 1, "I'm going to take a break": 1}, "verbs": {"Yes": 1, "Get out": 1, "Spread out": 1}, "adjectives": {"It's not good": 1}, "adverbs": {"When": 1}}}, {"content": "59. I feel that I have become more and more adapted to university studies.", "word_count": 16, "label": "", "sentence_structure": "Subject鈥揷opula鈥損redicative", "subjective_objective": {"answer": "Subjective", "rationale": "I think it's the patient's subjective attitude."}, "sentence_sentiment": {"answer": "Positive", "rationale": "Because \"the more and more fit for college learning\" expresses a positive attitude."}, "language_style": {"answer": "Tactful and warm", "rationale": "The language style of the entry is more euphemistic, as it uses an encouraging expression that expresses recognition and encouragement for the interviewee to adapt to university studies."}, "time_dimension": {"answer": "Past", "rationale": "In the entry, \"I\" describes the change in the individual's state of life in university studies, from incompetence to gradual adaptation. This change of state has taken place over time, and it is therefore judged to be \"the past.\""}, "sentence_type": "Affirmative declarative sentence", "pos": {"nouns": {"self": 1, "Learning": 1, "University": 1}, "verbs": {"I think": 1, "More and more": 1, "Adaptation": 1}, "adverbs": {"Tsk": 1}, "numerals": {"59": 1}, "pronouns": {"Yes": 1}}}, {"content": "60. I will endeavour to participate in various activities to enrich my amateur life.", "word_count": 19, "label": "", "sentence_structure": "Subject鈥搗erb鈥搊bject", "subjective_objective": {"answer": "Subjective", "rationale": "Because the words \"will try\" and \"participate in a variety of activities\" are subjective attitudes and behaviours of patients."}, "sentence_sentiment": {"answer": "Positive", "rationale": "For the purpose of this article expresses positive behaviour and attitude, namely \"to strive to participate in various activities to enrich my amateur life\"."}, "language_style": {"answer": "Tactful and warm", "rationale": "The language style of the entry is more moderate, leading each other to participate actively in events in a manner that encourages and supports them, and to feel respected and understood. At the same time, the expression \"I will work\" is used in a more positive way and in a more positive way."}, "time_dimension": {"answer": "Present", "rationale": "The entry describes the current behaviour and attitude of the testee, the keywords being \"will\" and \"work\" which indicate that the act is in progress or is planned for the future. At the same time, \"participation in activities\" also indicates that this is what the testee is doing. So, the time dimension of the entry should be now."}, "sentence_type": "Affirmative declarative sentence", "pos": {"nouns": {"Activities": 1, "Amateur life": 1}, "verbs": {"Yes": 1, "Try": 1, "Participation": 1, "Come on": 1, "Rich": 1}, "adverbs": {"Various": 1, "Mine": 1}, "numerals": {"60": 1}}}]}

{"questionnaire_title": "Comprehensive diagnostic table on interpersonal relations", "questionnaire_category": "Behavioral questionnaire", "item_count": 28, "emotion_shift_overall": "From negative to positive", "language_style_overall": "Neutral", "items": [{"content": "1. I can't say what's wrong with myself.", "word_count": 11, "label": "", "sentence_structure": "Subject鈥搗erb鈥揷omplement", "subjective_objective": {"answer": "Subjective", "rationale": "This is because of the patient ' s self-perception and subjective attitude, and because it is his/her description of his/her situation, rather than a reflection of objective facts from outside."}, "sentence_sentiment": {"answer": "Negative", "rationale": "Because the \"speech\" in the entry is a negative emotion."}, "language_style": {"answer": "Tactful and warm", "rationale": "The language style of the entry is euphemism. It asks the interviewee in a kind, understanding and invitation-style way, making him more willing to express his feelings and distress, and not to feel blamed or judged. This moderate tone helps to create a good atmosphere of communication and enhances the trust and comfort of the interviewee."}, "time_dimension": {"answer": "Present", "rationale": ""}, "sentence_type": "Negative declarative sentence", "pos": {"nouns": {"self": 1, "Trouble": 1, "mouth": 1, "That's a good idea": 1}, "verbs": {"have": 1, "Statements": 1}}}, {"content": "2. Unnaturalness in meeting with a living person", "word_count": 11, "label": "", "sentence_structure": "Subject鈥揷opula鈥損redicative", "subjective_objective": {"answer": "Subjective", "rationale": "Because it's not natural to feel a subjective feeling and experience of a human being."}, "sentence_sentiment": {"answer": "Negative", "rationale": "Because \"frightful\" is often interpreted as a sense of stress, anxiety or discomfort, which is usually negative."}, "language_style": {"answer": "Tactful and warm", "rationale": "The entry is more euphemistic, using the word \"feel\" rather than \"unnatural\" directly, which is more likely to trigger resonance and reflection among the respondents. At the same time, the expression \"when meeting with a living person\" is more accommodative and acceptable, giving a warmer sense."}, "time_dimension": {"answer": "Present", "rationale": "The keywords are \"frightful\" and \"when meeting with a living person,\" which all indicate the situation, and therefore judge it as now."}, "sentence_type": "Negative declarative sentence", "pos": {"nouns": {"and": 1, "Livers": 1, "Meet me": 1, "Feel": 1, "It's not natural": 1}, "verbs": {"and": 1, "Meet me": 1, "Feel": 1}, "adjectives": {"It's not natural": 1}}}, {"content": "3. Exult envy and envy.", "word_count": 10, "label": "", "sentence_structure": "Subject鈥損redicate", "subjective_objective": {"answer": "Subjective", "rationale": "Because envy and envy are a subjective emotional experience of patients."}, "sentence_sentiment": {"answer": "Negative", "rationale": "Because \"envious and jealous\" in the entry is a negative emotional attitude."}, "language_style": {"answer": "Tactful and warm", "rationale": "According to the information provided, the language style of the questionnaire entry \"excessive envy and envy\" could be \"precipitous\" or \"coolly straight.\" If the entry uses a more moderate and encouraging expression, such as asking if the other party has ever had feelings of envy or jealousy, and trying to guide them to think about how to deal with them, it is more likely to be \"quietly warm.\" On the contrary, if the entry is more direct and sharp, it simply asks the other person whether he or she is overly envious or jealous of the other's behaviour, without any explanation or advice, then its language style is more likely to be \"cool straight\"."}, "time_dimension": {"answer": "Present", "rationale": "In the entry, \"excessive envy and jealousy\" is a state of present behaviour or mentality that does not address the time dimension of the past or the future, and therefore is judged as \"now\"."}, "sentence_type": "Negative declarative sentence", "pos": {"nouns": {"That's too much": 1, "Envious": 1, "Jealousy": 1, "others": 1}, "adjectives": {"of": 2}, "adverbs": {"Land": 1}}}, {"content": "4. Too little heterosexuality", "word_count": 7, "label": "", "sentence_structure": "Subject鈥搗erb鈥揷omplement", "subjective_objective": {"answer": "Subjective", "rationale": "Because the person who has been involved with the opposite sex is more or less a person ' s personal experience and feelings, it is subjective."}, "sentence_sentiment": {"answer": "Negative", "rationale": "Because the \"too little heterosexuality\" in the entries is negative."}, "language_style": {"answer": "Tactful and warm", "rationale": "The language style of the entry is more euphemistic and moderate, using the expression \"too little interaction\", avoiding direct accusations or criticism of the other, and with a certain degree of orientation and advice, making it more acceptable and reflective of one's own situation. At the same time, it expresses a sense of concern and concern that is valued and respected."}, "time_dimension": {"answer": "Present", "rationale": ""}, "sentence_type": "Negative declarative sentence", "pos": {"nouns": {"Heterosexual": 1, "- To be with someone": 1, "Too little": 1}, "verbs": {"and": 1, "Too much": 1, "Less": 1}, "adjectives": {"Heterosexual": 1, "- To be with someone": 1, "Too little": 1}, "numerals": {"4": 1, "Too much": 1}, "classifiers": {"individual": 1}}}, {"content": "5. Difficulties in the continuation of talks", "word_count": 12, "label": "", "sentence_structure": "Subject鈥搗erb鈥揷omplement", "subjective_objective": {"answer": "Subjective", "rationale": "Because \"difficult\" is a subjective feeling and experience, not an objective fact or situation."}, "sentence_sentiment": {"answer": "Negative", "rationale": "Because the \"difficult\" act in the entry is negative."}, "language_style": {"answer": "Calm and direct", "rationale": "The entry presents a problem or need in a straightforward and concise manner, without too much embellishment or emotional rendering, and appears to be calm and straightforward."}, "time_dimension": {"answer": "Present", "rationale": "The entry asks whether the person being tested is currently experiencing \"difficult meetings\" and can therefore be judged as \"now\"."}, "sentence_type": "Negative declarative sentence", "pos": {"nouns": {"Meetings": 1, "Difficulties": 1}, "verbs": {"I feel": 1, "Continuous": 1}, "adverbs": {"Constantly": 1}, "numerals": {"5": 1}}}, {"content": "6. Tensions in social settings", "word_count": 9, "label": "", "sentence_structure": "Subject鈥揷opula鈥損redicative", "subjective_objective": {"answer": "Subjective", "rationale": "Because \"frightful\" is the patient's own psychological experience and feelings, and it's a subjective examination."}, "sentence_sentiment": {"answer": "Negative", "rationale": "Because the \"frightful\" in the entry is an expression of a negative sentiment."}, "language_style": {"answer": "Calm and direct", "rationale": "The language style of the entry is relatively simple, with no too much emotional or euphemism, but rather a question is raised directly and the respondents are expected to answer. This cold language style helps the respondents to understand the meaning of the question with precision and to quickly give their own answers."}, "time_dimension": {"answer": "Present", "rationale": "The entry is for the purpose of asking if the person tested is nervous in the current social setting and the time dimension is now judged."}, "sentence_type": "Affirmative declarative sentence", "pos": {"nouns": {"Questionnaire": 1, "Entry": 1, "Socially": 1, "Nervous": 1}, "verbs": {"I feel": 1}}}, {"content": "7. Harms are often done.", "word_count": 6, "label": "", "sentence_structure": "Subject鈥搗erb鈥揷omplement", "subjective_objective": {"answer": "Subjective", "rationale": "Because \"too often hurting\" is a person's behaviour, a patient's subjective action."}, "sentence_sentiment": {"answer": "Negative", "rationale": "Because it's negative to hurt others."}, "language_style": {"answer": "Calm and direct", "rationale": "The entry directly asks whether the person surveyed is often harmful to others, does not use a tautological language or emotional colour, and is speaking objectively and clearly."}, "time_dimension": {"answer": "Present", "rationale": "The keyword \"always hurt others\" describes the state of the present or customary behaviour, and therefore the judgment is now."}, "sentence_type": "Negative declarative sentence", "pos": {"nouns": {"Hurt": 1, "others": 1}, "verbs": {"Often": 1}}}, {"content": "8. Unnaturally interacting with heterosexuals", "word_count": 10, "label": "", "sentence_structure": "Subject鈥揷opula鈥損redicative", "subjective_objective": {"answer": "Subjective", "rationale": "Because it is a subjective feeling of an individual, not an objective fact."}, "sentence_sentiment": {"answer": "Negative", "rationale": "Because the expression \"frightful\" in the entry is negative and indicates possible social or psychological problems."}, "language_style": {"answer": "Calm and direct", "rationale": "The language style of the entry is more direct and concise, with no excessive emotional accoutrements or rhetorical techniques, and a greater focus on the explicit presentation and communication of the issue. \""}, "time_dimension": {"answer": "Present", "rationale": ""}, "sentence_type": "Negative declarative sentence", "pos": {"nouns": {"Heterosexual": 1, "Travel": 1, "Feel": 1, "Naturally": 1}, "verbs": {"and": 1, "Travel": 1, "Feel": 1, "Naturally": 1}, "adjectives": {"It's not natural": 1}}}, {"content": "With a large group of friends, often feeling lonely or lost.", "word_count": 17, "label": "", "sentence_structure": "Subject鈥揷opula鈥損redicative", "subjective_objective": {"answer": "Subjective", "rationale": "Because \"sorture or loss\" is a subjective experience of feelings and emotions. Although there may be factors in the external environment (such as a large group of friends), this feeling is mainly due to the patient's own state of mind and emotional reaction. So this entry is viewed from the patient's subjective perspective."}, "sentence_sentiment": {"answer": "Negative", "rationale": "Because the \"sorture or loss\" in the entry is negative."}, "language_style": {"answer": "Tactful and warm", "rationale": "The language style of the entry is more moderate, friendly and guiding, and is intended to make the person surveyed more willing to share his or her true feelings than simply order or criticize. At the same time, the use of \"a large group of friends\" instead of \"a large number of people\" also shows respect for and understanding of individual feelings."}, "time_dimension": {"answer": "Present", "rationale": "The word \"often\" in \"often\" denotes a regular, customary act, while the word \"sense\" adopts the current state of affairs, which means that the act has not only occurred in the past but may still be ongoing. Thus, the entry describes the current state and feelings of the person being tested, and the temporal dimension is now."}, "sentence_type": "Imperative sentence", "pos": {"nouns": {"One": 1, "Big group": 1, "friends": 1, "Loneliness": 1, "Lost": 1}, "verbs": {"Together": 1, "I always feel": 1}, "adverbs": {"or": 1}, "numerals": {"9": 1}}}, {"content": "10. Extreme vulnerability", "word_count": 4, "label": "", "sentence_structure": "Subject鈥揷opula鈥損redicative", "subjective_objective": {"answer": "Subjective", "rationale": "Because \"extreme vulnerability\" is a feeling or attitude that belongs to the patient's subjective perspective."}, "sentence_sentiment": {"answer": "Negative", "rationale": "Because \"vulnerable\" in the entry is a negative act or quality."}, "language_style": {"answer": "Tactful and warm", "rationale": "The entry uses the more euphemistic term \"very easy\" to describe the vulnerability to embarrassment, rather than simply to say that someone is vulnerable to embarrassment, which is more respectful and understanding of the feelings of the interviewee. At the same time, the description of \"sustained\" is more neutral and objective, without too much emotional and subjective judgement, and more in line with the scientific and objective requirements of psychological questionnaire research."}, "time_dimension": {"answer": "Present", "rationale": "The entry is for questioning of the person's current behaviour and mental state, etc., and the keyword \"very vulnerable\" represents a subjective feeling or psychological state, so that the temporal dimension can be judged to be present."}, "sentence_type": "Negative declarative sentence", "pos": {"nouns": {"I'm embarrassed": 1}, "verbs": {"Yes": 1, "Easy": 1}, "adjectives": {"Extreme": 1, "Easy": 1}, "numerals": {"10": 1}}}, {"content": "11. Inability to live in harmony with others", "word_count": 9, "label": "", "sentence_structure": "Subject鈥搗erb鈥揷omplement", "subjective_objective": {"answer": "Subjective", "rationale": "This is a patient ' s subjective perception and self-evaluation, and it examines the patient ' s mental state and interpersonal capacity."}, "sentence_sentiment": {"answer": "Negative", "rationale": "Because the attitude of \"not living together\" in the entry is negative."}, "language_style": {"answer": "Calm and direct", "rationale": "The entry is in a concise language and asks the respondents whether there are situations in which people are not able to live in harmony with others, and is not based on excessive rhetorical or emotional rendering, and is more objective in tone."}, "time_dimension": {"answer": "Present", "rationale": "The entry is a description of the individual character and does not contain a clear temporal dimension judgement."}, "sentence_type": "Negative declarative sentence", "pos": {"nouns": {"person": 2, "Harmony": 1}, "verbs": {"not": 1, "Yes": 1, "Marriage": 1, "Location": 1}, "numerals": {"11": 1, "2": 1}}}, {"content": "12. I wonder how it's possible to stop living with opposite sex.", "word_count": 14, "label": "", "sentence_structure": "Subject鈥搗erb鈥揷omplement", "subjective_objective": {"answer": "Subjective", "rationale": "Because \"just as it is\" is a subjective criterion, different people may have different ways of understanding and dealing with it. The subjective idea and view of the patient is the subjective one."}, "sentence_sentiment": {"answer": "Negative", "rationale": "Because the \"I don't know how to stop\" in the entry is negative."}, "language_style": {"answer": "Tactful and warm", "rationale": "The language style of the entry is euphemism, which leads the interviewee to share his thoughts by using questions, rather than telling the interviewee directly what to do. At the same time, the expression \"just stop\" is more implicit, leaving the interviewee to think and understand himself, without specifying specific behaviours or attitudes."}, "time_dimension": {"answer": "Present", "rationale": "The entry now asks about the situation of the testee in the immediate moment with opposite sex, the key words being \"not knowing\" and \"how to stop.\" Both terms emphasize the current situation and feelings, so we can judge it as a question of the present behavior or mentality."}, "sentence_type": "Exclamatory sentence", "pos": {"nouns": {"Questionnaire": 1, "Entry": 1, "Heterosexual": 1, "Get along": 1, "Stop it": 1}, "verbs": {"Yes": 1, "Analysis": 1}, "adverbs": {"How's that": 1, "I don't know": 1}, "numerals": {"12": 1}}}, {"content": "13. When someone who is not familiar tells himself about his life and feels sorry for himself, he is often uncomfortable.", "word_count": 30, "label": "", "sentence_structure": "Subject鈥搗erb鈥搊bject", "subjective_objective": {"answer": "Subjective", "rationale": "The first is the feeling and reaction of the individual."}, "sentence_sentiment": {"answer": "Negative", "rationale": "Because the \"often uncomfortable\" feeling in the entries is negative."}, "language_style": {"answer": "Tactful and warm", "rationale": ""}, "time_dimension": {"answer": "Present", "rationale": ""}, "sentence_type": "Negative declarative sentence", "pos": {"nouns": {"person": 1, "Life": 1, "Problems": 1, "Compassion": 1, "self": 1}, "verbs": {"to": 2, "Speech": 1, "Sensor": 1, "I'm not comfortable": 1}, "adjectives": {"I know you": 1, "not": 1}, "adverbs": {"Regular": 1}, "numerals": {"13": 1}, "classifiers": {"individual": 1}, "pronouns": {"he": 1}}}, {"content": "14. Fear of what people might think of themselves.", "word_count": 13, "label": "", "sentence_structure": "Subject鈥搗erb鈥揷omplement", "subjective_objective": {"answer": "Subjective", "rationale": "Because fear is a subjective emotional response, it is the patient's fear and imagination of what may happen in the future."}, "sentence_sentiment": {"answer": "Negative", "rationale": "Because the \"fear\" emotion in the entry is negative."}, "language_style": {"answer": "Calm and direct", "rationale": "The entry asks the interviewees whether they are concerned about their external image, with a clear and simple language style, without muddy water, and without any rhetorical or emotional colours."}, "time_dimension": {"answer": "Present", "rationale": "The keyword is \"awesome,\" which means that the testee is experiencing current emotions and feelings, not past or future behaviors or mentalities. Moreover, the word \"yes\" indicates that this is a current state or situation, and further supports this as an entry on the present."}, "sentence_type": "Interrogative sentence", "pos": {"nouns": {"I'm worried": 1, "person": 1, "Bad impression": 1}, "verbs": {"have": 1, "to": 1}, "numerals": {"14": 1}, "pronouns": {"self": 1}}}, {"content": "15. Always try to impress yourself.", "word_count": 11, "label": "", "sentence_structure": "Subject鈥搗erb鈥揷omplement", "subjective_objective": {"answer": "Subjective", "rationale": "Because the words \"best of the mind\" and \"behave yourself\" are the psychological experiences and attitudes of the patient, reflecting the self-perception and social needs of the patient and being subjective."}, "sentence_sentiment": {"answer": "Positive", "rationale": "Because the purpose of the \"best efforts to make others appreciate\" in the entry is positive."}, "language_style": {"answer": "Tactful and warm", "rationale": ""}, "time_dimension": {"answer": "Present", "rationale": ""}, "sentence_type": "Imperative sentence", "pos": {"nouns": {"self": 1, "Try": 1, "Admirable": 1}, "verbs": {"鈾?And I'm gonna be 鈾?: 1, "Always": 1}, "adverbs": {"Try": 1}, "numerals": {"15": 1}}}, {"content": "16. Concealed the opposite sex", "word_count": 6, "label": "", "sentence_structure": "Subject鈥搗erb鈥揷omplement", "subjective_objective": {"answer": "Subjective", "rationale": "Because \"smuggling for opposite sex\" is the heart of the patient, the thought of the patient, the subjective attitude."}, "sentence_sentiment": {"answer": "Negative", "rationale": "Because it's negative to think about the opposite sex."}, "language_style": {"answer": "Calm and direct", "rationale": "\"Calm down straight.\""}, "time_dimension": {"answer": "Future", "rationale": "The entry expresses the idea and expectations that the testees may have of a different sex in the future and can therefore be judged to be the future."}, "sentence_type": "Interrogative sentence", "pos": {"nouns": {"I'm thinking about myself": 1, "Heterosexual": 1}}}, {"content": "17. Often avoid expressing their feelings.", "word_count": 11, "label": "", "sentence_structure": "Subject鈥搗erb鈥揷omplement", "subjective_objective": {"answer": "Subjective", "rationale": "Because \"often avoiding expressing one's feelings\" reflects the patient's self-perception and treatment, and is a subjective view or attitude."}, "sentence_sentiment": {"answer": "Negative", "rationale": "Because the \"show your feelings often\" act is negative."}, "language_style": {"answer": "Tactful and warm", "rationale": "The entry is more adept and interactive when it is spoken in a gentle and inviting manner, rather than by direct statements or orders."}, "time_dimension": {"answer": "Present", "rationale": "\"At times\" is a high frequency and customary behaviour, and the verbs \"avoid\" and \"expression\" are both current and suggest that the behaviour has not only occurred in the past, but may still be ongoing. So the entry to the questionnaire is to ask the testee about the current behaviour, and the temporal dimension is now."}, "sentence_type": "Imperative sentence", "pos": {"nouns": {"Questionnaire": 1, "Entry": 1, "Feel": 1}, "verbs": {"Expression": 1, "Avoid": 1, "Regular": 1}, "adverbs": {"Time": 1}, "numerals": {"17": 1}}}, {"content": "18. Lack of confidence in their own (physical) symptoms", "word_count": 12, "label": "", "sentence_structure": "Subject鈥搗erb鈥揷omplement", "subjective_objective": {"answer": "Subjective", "rationale": "Since the subject is to question patients ' subjective feelings and thoughts, it is to examine self-evaluation of the patient ' s appearance."}, "sentence_sentiment": {"answer": "Negative", "rationale": "Because the \"lack of confidence\" attitude in the entries is negative."}, "language_style": {"answer": "Tactful and warm", "rationale": "Warm and warm."}, "time_dimension": {"answer": "Present", "rationale": "The entry indicates the mental state or attitude that currently exists in the testee, and the temporal dimension is now judged. \"Leave confidence\" describes the subjective feelings and attitudes of the testee, and thus it can be concluded that this is a description of the current psychological condition."}, "sentence_type": "Negative declarative sentence", "pos": {"nouns": {"self": 1, "Instrument": 1, "Looks like a": 1, "Confidence": 1}, "verbs": {"to": 1, "Lack": 1, "have": 1}, "numerals": {"18": 1}, "classifiers": {"individual": 1}, "pronouns": {"Here": 1}}}, {"content": "I hate someone or someone.", "word_count": 11, "label": "", "sentence_structure": "Subject鈥搗erb鈥揷omplement", "subjective_objective": {"answer": "Subjective", "rationale": "It is a subjective sentiment and attitude of the patient because of the person that hates someone or is hated by someone."}, "sentence_sentiment": {"answer": "Negative", "rationale": "Because the \"hot someone\" act in the entry is negative."}, "language_style": {"answer": "Tactful and warm", "rationale": "The entry is relatively gentle, and there is no overly direct or harsh expression. It leads the interviewees to wonder, in a more euphemistic and intrusive way, whether they have ever hated others or been hated, which is more personal and interactive."}, "time_dimension": {"answer": "Past", "rationale": "So the output is \"the past.\""}, "sentence_type": "Negative declarative sentence", "pos": {"nouns": {"Someone": 2, "Shit": 1}, "verbs": {"By": 1, "Shit": 1}, "numerals": {"19": 1}}}, {"content": "20. Disrespect for opposite sex", "word_count": 5, "label": "", "sentence_structure": "Subject鈥搗erb鈥揷omplement", "subjective_objective": {"answer": "Subjective", "rationale": "It is an emotional experience and a subjective feeling of the patient."}, "sentence_sentiment": {"answer": "Negative", "rationale": "Because \"defeating opposite sex\" is a negative attitude."}, "language_style": {"answer": "Calm and direct", "rationale": "The entry is based on a more direct language style, with no excessive rhetorical or emotional rendering, but rather on a clear and direct tone of the person being investigated inquiring about the opposite sex."}, "time_dimension": {"answer": "Present", "rationale": "The entry now asks the testee whether he or she is currently in a position of contempt for opposite sex or tendencies, with no clear time limits, and therefore the temporal dimension is now judged."}, "sentence_type": "Affirmative declarative sentence", "pos": {"nouns": {"Heterosexual": 1, "I'm not looking down": 1}, "verbs": {"I'm not looking down": 1}}}, {"content": "21. Lack of focus", "word_count": 7, "label": "", "sentence_structure": "Subject鈥搗erb鈥揷omplement", "subjective_objective": {"answer": "Subjective", "rationale": "Because \"not listening\" is a subjective experience and perception, reflecting the individual's feelings and judgement about the extent to which he or she is focused."}, "sentence_sentiment": {"answer": "Negative", "rationale": "Because \"not listening\" is negative."}, "language_style": {"answer": "Calm and direct", "rationale": "The language style of the entry is relatively simple and not too much rhetorical or emotional, but rather a direct and objective inquiry into whether the interviewee is unable to listen with attention and is more calm and direct."}, "time_dimension": {"answer": "Present", "rationale": "\"Unable to listen with attention\" is a description of the current state of the person being tested, indicating the experience or feelings that are now occurring, so the temporal dimension of the article is now."}, "sentence_type": "Negative declarative sentence", "pos": {"nouns": {"Focus": 1, "Listen": 1}, "verbs": {"No, I can't": 1}}}, {"content": "22. No one can complain about their own problems.", "word_count": 10, "label": "", "sentence_structure": "Subject鈥搗erb鈥揷omplement", "subjective_objective": {"answer": "Subjective", "rationale": "Because \"no one can complain\" is the patient's subjective feeling and experience."}, "sentence_sentiment": {"answer": "Negative", "rationale": "Because the description \"no one can complain\" itself has a certain negative emotional tone, expressing an inexorable sentiment."}, "language_style": {"answer": "Calm and direct", "rationale": "The entry asked the interviewees in a direct manner whether they felt that they were not entitled to complain when facing their own problems, without using any euphemism, rendering or other means of speech, and with a calmer and more direct language style."}, "time_dimension": {"answer": "Present", "rationale": "The words \"self\", \"fear\" and \"no one can complain\" in this questionnaire entry all indicate that this is a description of the current state of the person being tested, so the temporal dimension is now."}, "sentence_type": "Negative declarative sentence", "pos": {"nouns": {"Trouble": 1, "person": 1, "The complaint": 1}, "verbs": {"have": 1, "self": 1}, "numerals": {"22": 1}}}, {"content": "23. Exclusion and indifference", "word_count": 8, "label": "", "sentence_structure": "Subject鈥搗erb鈥揷omplement", "subjective_objective": {"answer": "Subjective", "rationale": "Because being excluded and apathy is the emotion and experience that patients feel, and it is a subjective view."}, "sentence_sentiment": {"answer": "Negative", "rationale": "Because the experience of being rejected and being aloof usually leads to negative emotional reactions, such as depression, loneliness, etc."}, "language_style": {"answer": "Tactful and warm", "rationale": "The entry is more personal and interactive in its expression of a gentle and inviting tone, rather than a direct statement or order. At the same time, the theme of \"exclusion and indifference\" is sensitive and personalized, and a euphemism can better protect the privacy and dignity of the person under investigation."}, "time_dimension": {"answer": "Present", "rationale": ""}, "sentence_type": "Affirmative declarative sentence", "pos": {"nouns": {"others": 1, "Exclusion": 1, "Coldness": 1}, "verbs": {"Yes": 1}, "numerals": {"23": 1}}}, {"content": "24. Disrespected by opposite sex", "word_count": 6, "label": "", "sentence_structure": "Subject鈥搗erb鈥揷omplement", "subjective_objective": {"answer": "Subjective", "rationale": "Because the words \"deserve\" and \"deserve\" are viewed in the patient's subjective sense and are the patient's assessment of his or her heterosexual identity, the question should be subjective in the sense of the entry."}, "sentence_sentiment": {"answer": "Negative", "rationale": "Because the \"sightened\" act in the entry is negative."}, "language_style": {"answer": "Calm and direct", "rationale": "The article is relatively simple and formulated in a way that is relatively simple, without too much rhetorical or emotional overtones. It asks whether the person interviewed has ever been subjected to contempt or disrespect from a different sex, which is more objective, neutral, does not cause discomfort or resistance to the person interviewed, and more accurately captures their real thoughts and experiences."}, "time_dimension": {"answer": "Present", "rationale": "The entry is for the purpose of asking whether the respondents are currently being treated as inferior to the opposite sex, and the time dimension is now judged."}, "sentence_type": "Negative declarative sentence", "pos": {"nouns": {"Heterosexual": 1, "I'm not looking down": 1}, "adverbs": {"By": 1}}}, {"content": "25. Lack of wide access to opinions and opinions", "word_count": 13, "label": "", "sentence_structure": "Subject鈥搗erb鈥揷omplement", "subjective_objective": {"answer": "Subjective", "rationale": "The lack of a broad range of opinions and opinions is the subjective feeling and attitude of the patient."}, "sentence_sentiment": {"answer": "Negative", "rationale": "Because the act of \"not hearing a wide range of opinions and opinions\" in the entry is negative."}, "language_style": {"answer": "Calm and direct", "rationale": "The entry is more calm and straightforward, has no excessive speech or emotional rendering, and is more objective. It is a simple and clear expression of the subject of asking each other whether they are capable of hearing different views and ideas."}, "time_dimension": {"answer": "Present", "rationale": ""}, "sentence_type": "Negative declarative sentence", "pos": {"nouns": {"Observations": 2}, "verbs": {"Listen": 1, "Broad": 1, "No, I can't": 1}, "adverbs": {"Various": 1}, "numerals": {"25": 1}}}, {"content": "26. He often felt hurt and felt sad.", "word_count": 12, "label": "", "sentence_structure": "Subject鈥搗erb鈥揷omplement", "subjective_objective": {"answer": "Subjective", "rationale": "Because it is a subjective aspect of the patient ' s own feelings and experiences."}, "sentence_sentiment": {"answer": "Negative", "rationale": "Because the \"injured and heartbroken\" act in the entry is negative."}, "language_style": {"answer": "Tactful and warm", "rationale": "The language style of the entry is relatively warm, and it avoids the possibility of direct criticism or accusations by using the word \"self\" rather than directly calling each other. At the same time, the term is soft, like \"smuggling\" and gives people a sense of concern and understanding. This soft language style helps to create a good atmosphere of investigation and makes respondents more willing to share their true feelings."}, "time_dimension": {"answer": "Past", "rationale": ""}, "sentence_type": "Interrogative sentence", "pos": {"nouns": {"self": 1, "Hurt": 1, "Sad": 1}, "verbs": {"Common cause": 1, "Yes": 1, "From the shadows": 1}, "adverbs": {"very": 1}, "numerals": {"26": 1}}}, {"content": "27. People talk about it, they fool me.", "word_count": 8, "label": "", "sentence_structure": "Subject鈥搗erb鈥揷omplement", "subjective_objective": {"answer": "Subjective", "rationale": "Because \"showed, fooled\" is a subjective assessment and feeling that reflects patients鈥?observation and perception of themselves. This experience can affect the emotional state and self-esteem of patients, and is therefore the subject of subjective examination."}, "sentence_sentiment": {"answer": "Negative", "rationale": "Because the \"screw talk, fool\" in the entry is negative."}, "language_style": {"answer": "Tactful and warm", "rationale": "The entry starts with an inquiry and uses the words \"talking\" and \"smuggling\" to enable the interviewee to understand and answer the question. This expression is more euphemism and warmer, and does not cause undue pressure or discomfort on the interviewee."}, "time_dimension": {"answer": "Present", "rationale": "The keyword \"always\" indicates the frequency of the act, while the verb \"talking\" and \"smuggling\" both use the general current, suggesting that the act continues. So, the time attribute of this entry is now."}, "sentence_type": "Negative declarative sentence", "pos": {"nouns": {"person": 1, "Discussion": 1, "Smuggle": 1}, "verbs": {"By": 1}, "adverbs": {"Regular": 1}, "numerals": {"27": 1}}}, {"content": "28. Ignorance of how to get along better with heterosexuals", "word_count": 14, "label": "", "sentence_structure": "Subject鈥搗erb鈥揷omplement", "subjective_objective": {"answer": "Subjective", "rationale": "Because \"I don't know how to get along better\" is the patient's subjective feeling and idea."}, "sentence_sentiment": {"answer": "Negative", "rationale": "Because the attitude of \"I don't know how to get along better\" in the entry is negative."}, "language_style": {"answer": "Tactful and warm", "rationale": ""}, "time_dimension": {"answer": "Present", "rationale": "The entry asks the testee about the current trouble of heterosexual intercourse, and therefore judges it as \"now\"."}, "sentence_type": "Interrogative sentence", "pos": {"nouns": {"Questionnaire": 1, "Entry": 1, "Heterosexual": 1, "- To be with someone": 1, "Get along": 1}, "verbs": {"28": 1, "I don't know": 1, "How's that": 1, "Better": 1, "and": 1, "- To be with someone": 1, "Get along": 1}, "numerals": {"28": 1}, "pronouns": {"and": 1}}}]}

{"questionnaire_title": "Beck's depression questionnaire.", "questionnaire_category": "Behavioral questionnaire", "item_count": 84, "emotion_shift_overall": "From positive to negative", "language_style_overall": "Calm and direct", "items": [{"content": "I'm not sad.", "word_count": 6, "label": "", "sentence_structure": "Subject鈥揷opula鈥損redicative", "subjective_objective": {"answer": "Subjective", "rationale": "Because \"no worry\" is a description of the subjective feeling and emotional state, which is the patient's subjective attitude or emotional experience."}, "sentence_sentiment": {"answer": "Negative", "rationale": "Because the negative form of the expression \"no worries\" expresses a negative emotional attitude."}, "language_style": {"answer": "Calm and direct", "rationale": "The entry is more objective and straightforward in terms of asking the other side whether it is often sad, not using excessive rhetorical techniques or emotional rendering."}, "time_dimension": {"answer": "Present", "rationale": ""}, "sentence_type": "Negative declarative sentence", "pos": {"verbs": {"I don't feel it": 1, "Sad": 1}, "adjectives": {"Sad": 1}}}, {"content": "I'm sad.", "word_count": 5, "label": "", "sentence_structure": "Subject鈥揷opula鈥損redicative", "subjective_objective": {"answer": "Subjective", "rationale": "Because \"sorture\" is the experience and feeling of the patient, and it is a subjective state of personal emotion."}, "sentence_sentiment": {"answer": "Negative", "rationale": "Because \"sorture\" is a negative emotion."}, "language_style": {"answer": "Calm and direct", "rationale": "The entry expresses the subject of inquiries about whether the other party is often troubled in a simple and direct manner, does not use excessive rhetorical techniques or emotional rendering, and is more calm and objective."}, "time_dimension": {"answer": "Past", "rationale": ""}, "sentence_type": "Interrogative sentence", "pos": {"nouns": {"Sad": 1}, "verbs": {"Sensor": 1, "Present": 1}, "pronouns": {"I": 1}}}, {"content": "I've been feeling sad all day and I can't change that mood.", "word_count": 17, "label": "", "sentence_structure": "Subject鈥搗erb鈥揷omplement", "subjective_objective": {"answer": "Subjective", "rationale": "Because \"sorture\" and \"can't change that\" are the feelings and experiences of patients, and so it's subjectively viewed."}, "sentence_sentiment": {"answer": "Negative", "rationale": "Because the emotional attitude of \"all day long\" in the entry is negative."}, "language_style": {"answer": "Calm and direct", "rationale": ""}, "time_dimension": {"answer": "Present", "rationale": "The words \"day and day\" and \"can't change\" suggest that the act or mentality is currently being conducted, so the judgement is now."}, "sentence_type": "Negative declarative sentence", "pos": {"nouns": {"Questionnaire": 1, "Entry": 1, "I": 2, "Tight": 1, "Oh, my God": 1, "Both": 1, "Sensor": 1, "Present": 1, "Sad": 1, "and": 1, "No, I can't": 1, "Change": 1, "This one": 1, "Emotion": 1}, "verbs": {"I feel": 1, "Change": 1}, "adjectives": {"All day long": 1, "Sad": 1}, "adverbs": {"Both": 1, "and": 1}, "numerals": {"2": 1}, "pronouns": {"I": 2}}}, {"content": "I'm so sad and unhappy that I can't stand it.", "word_count": 16, "label": "", "sentence_structure": "Subject鈥揷opula鈥損redicative", "subjective_objective": {"answer": "Subjective", "rationale": "Because grief or discomfort is the subjective feeling inside the patient."}, "sentence_sentiment": {"answer": "Negative", "rationale": "Because the \"unable\" act in the entry is negative."}, "language_style": {"answer": "Calm and direct", "rationale": "The entry directly asked about the emotional state of the interviewee, which was more concise and less curvy and emotional."}, "time_dimension": {"answer": "Present", "rationale": "\"I am very sad or unhappy, so I cannot bear the \"very\" and \"unhappiness\" and \"unsufferable\" all to say that this is the state of the current, ongoing emotional state. So this entry should be classified as a description of the current behavior or mentality."}, "sentence_type": "Negative declarative sentence", "pos": {"nouns": {"Questionnaire Entry": 1, "I": 1, "Sadness": 1, "It's not good": 1}, "verbs": {"Very": 1, "Take it": 1}, "adjectives": {"Sadness": 1, "It's not good": 1}, "adverbs": {"Very": 1}, "classifiers": {"Entry": 1}, "pronouns": {"I": 1}}}, {"content": "I'm not pessimistic about the future.", "word_count": 10, "label": "", "sentence_structure": "Subject鈥揷opula鈥損redicative", "subjective_objective": {"answer": "Subjective", "rationale": "Because \"no pessimism\" is an expression of personal emotions and attitudes, which are the subjective feelings of the patient."}, "sentence_sentiment": {"answer": "Positive", "rationale": "Because the \"no pessimism\" attitude in the entries is positive."}, "language_style": {"answer": "Tactful and warm", "rationale": "Warm and warm."}, "time_dimension": {"answer": "Future", "rationale": "The key word \"the future\" points to this as a vision and a vision for the future, while the negative word \"no\" in the sentence indicates that the testees are optimistic about the future."}, "sentence_type": "Negative declarative sentence", "pos": {"nouns": {"Future": 1, "Pessimism": 1}, "verbs": {"I don't feel it": 1}}}, {"content": "I am pessimistic about the future.", "word_count": 8, "label": "", "sentence_structure": "Subject鈥揷opula鈥損redicative", "subjective_objective": {"answer": "Subjective", "rationale": "Because \"fear pessimism\" is an expression of subjective sentiment."}, "sentence_sentiment": {"answer": "Negative", "rationale": "Because \"pessimism\" is a negative emotional attitude."}, "language_style": {"answer": "Calm and direct", "rationale": "The language style of the entry is \"cool and direct.\" It asks the interviewee directly about the future, without too much emotional overturning or rhetorical. This language style makes the issue more relevant and objective."}, "time_dimension": {"answer": "Present", "rationale": "In the future, the entry contains visions and feelings for the future, and the key words are \"the future\" and \"the pessimism\", all pointing to situations that have not yet occurred and that may arise in the future."}, "sentence_type": "Negative declarative sentence", "pos": {"nouns": {"Future": 1, "Pessimism": 1}, "verbs": {"I feel": 1}}}, {"content": "I feel like I have nothing to hope for.", "word_count": 11, "label": "", "sentence_structure": "Subject鈥揷opula鈥損redicative", "subjective_objective": {"answer": "Subjective", "rationale": "Because \"Esperate that there is nothing to hope\" is the patient's inner feeling and perception, and it's subjective and emotional."}, "sentence_sentiment": {"answer": "Negative", "rationale": "Because \"Nothing to hope\" in the entry expresses a pessimism, frustration, and it's a negative emotional color."}, "language_style": {"answer": "Calm and direct", "rationale": "The entry is more appropriate for psychological questionnaire studies."}, "time_dimension": {"answer": "Present", "rationale": ""}, "sentence_type": "Negative declarative sentence", "pos": {"nouns": {"I": 1, "I hope": 1}, "verbs": {"I feel": 1, "No, I'm not": 1, "Yeah": 1, "Okay": 1}, "adverbs": {"What": 1}}}, {"content": "I feel hopeless and everything is going to be fine.", "word_count": 14, "label": "", "sentence_structure": "Subject鈥揷opula鈥損redicative", "subjective_objective": {"answer": "Subjective", "rationale": "Because \"fear\" is a subjective expression of feeling, reflecting the patient's fears and pessimism about the future."}, "sentence_sentiment": {"answer": "Negative", "rationale": "Because \"Feeling hopeless\" and \"Nothing good\" in the entries is a negative emotional expression."}, "language_style": {"answer": "Tactful and warm", "rationale": "The language style of this entry is more euphemistic and warm, and the language is more moderate, without expressing a strong negative sentiment or accusations directly. At the same time, the expression \"no hope for the future\" and \"nothing can change\" is used implicitly, making it more comfortable and comfortable for respondents to answer questions."}, "time_dimension": {"answer": "Future", "rationale": "Among them, \"Feeling the Unseen\" shows the testees' pessimism towards the future, which they believe will not be better; and \"Nothing changes\" highlights the breadth and persistence of this pessimism."}, "sentence_type": "Negative declarative sentence", "pos": {"nouns": {"I": 1, "Future": 1, "- The thing": 1}, "verbs": {"I feel": 1, "Get better": 1}, "adjectives": {"Hopeless": 1}, "numerals": {"3": 1}}}, {"content": "I don't look like a loser.", "word_count": 8, "label": "", "sentence_structure": "Subject鈥揷opula鈥損redicative", "subjective_objective": {"answer": "Subjective", "rationale": "Because it expresses the patient ' s perception and appreciation of self-worth, it is the patient ' s subjective feeling and attitude."}, "sentence_sentiment": {"answer": "Negative", "rationale": "Because the expression \"not like a loser\" in the entry is negative, and it expresses a self-denial attitude."}, "language_style": {"answer": "Tactful and warm", "rationale": "The entry expresses its recognition of its value in a self-proclaimed manner, rather than directly denying or demeaning itself. It also uses a more euphemistic way of expressing it, avoiding language styles that may cause resentment or negative emotions, and more accompanimental and emotional resonance."}, "time_dimension": {"answer": "Past", "rationale": "According to the sentence entered, \"0. I'm not like a loser\" , which contains both past behaviour (i.e., past performance) and current perceptions and self-evaluations, so the entry contains information on both past and present dimensions."}, "sentence_type": "Negative declarative sentence", "pos": {"nouns": {"Loser": 1}, "verbs": {"No, it doesn't": 1}, "adverbs": {"One": 1}, "pronouns": {"I": 1}}}, {"content": "I think I've failed more than the average person.", "word_count": 15, "label": "", "sentence_structure": "Subject鈥揷opula鈥損redicative", "subjective_objective": {"answer": "Subjective", "rationale": "The first is the fact that the patient has a subjective feeling and is evaluated."}, "sentence_sentiment": {"answer": "Negative", "rationale": "Because \"more failures\" in the entries are negative."}, "language_style": {"answer": "Calm and direct", "rationale": "The language style of the entry is calm and straightforward. It clearly expresses the feeling that the subject is personal failure, not using too many rhetorical techniques or emotional renderings, but in a direct presentation, which is more objective and objective."}, "time_dimension": {"answer": "Present", "rationale": "The entry represents the view of the testee on his or her current situation and does not address past or future behaviour or mentality."}, "sentence_type": "Negative declarative sentence", "pos": {"nouns": {"I": 1, "Number": 1, "General": 1, "person": 1}, "verbs": {"I think": 1, "- Yeah": 1, "Failed": 2}, "adjectives": {"More": 1}, "numerals": {"General": 1, "Some": 1}}}, {"content": "When I look back, I see a lot of failures.", "word_count": 15, "label": "", "sentence_structure": "Subject鈥搗erb鈥揷omplement", "subjective_objective": {"answer": "Subjective", "rationale": "Because \"returning the past\" and \"seeing many failures\" are subjective feelings and perceptions of patients, and therefore subjective perspectives."}, "sentence_sentiment": {"answer": "Negative", "rationale": "Because the term \"failure\" is negative."}, "language_style": {"answer": "Calm and direct", "rationale": "The entry is a straightforward and straightforward way of asking whether the other party has seen many failures in reviewing past experiences, without using excessive rhetorical techniques or emotional rendering."}, "time_dimension": {"answer": "Past", "rationale": "The description of \"When I look back\" shows that this is a context of memories, and the words \"recall\" and \"old\" point to the past, so the entry asks the subject about past behaviour or experience."}, "sentence_type": "Affirmative declarative sentence", "pos": {"nouns": {"Past": 1, "I": 1, "Failed": 1}, "verbs": {"Look back": 1, "Yeah": 1}, "adverbs": {"Many": 1}}}, {"content": "I feel like I'm a total loser.", "word_count": 14, "label": "", "sentence_structure": "Subject鈥揷opula鈥損redicative", "subjective_objective": {"answer": "Subjective", "rationale": "Because \"who has failed completely\" is a subjective assessment that reflects the patient's perception and emotional state of his or her own."}, "sentence_sentiment": {"answer": "Negative", "rationale": "Because the expression \"total failure\" is negative and expresses a rejection of self-worth and frustration."}, "language_style": {"answer": "Tactful and warm", "rationale": "The entry is more euphemistic, using more implicit expressions (such as \"I feel\"), rather than direct statements or orders. Moreover, the description \"who has failed completely\" does not use overly violent or negative emotional terms, but rather conveys emotions in a relatively mild way."}, "time_dimension": {"answer": "Past", "rationale": "The entry is for the purpose of asking the testee about his or her current state and does not address the time dimension of past or future, and therefore the judgement is \"now\"."}, "sentence_type": "Negative declarative sentence", "pos": {"nouns": {"person": 1, "Questionnaire": 1, "Entry": 1, "Failed": 1}, "verbs": {"I feel": 1, "be": 1, "Full": 1, "Yes": 2}, "adjectives": {"Full": 1}, "numerals": {"3": 1, "One": 1, "individual": 2}, "classifiers": {"Grandpa": 1}, "pronouns": {"I": 1}}}, {"content": "I'm as happy as I always am.", "word_count": 11, "label": "", "sentence_structure": "Subject鈥揷opula鈥損redicative", "subjective_objective": {"answer": "Subjective", "rationale": "For satisfaction is a subjective feeling and attitude."}, "sentence_sentiment": {"answer": "Neutral", "rationale": "It is not an emotional entry, but rather a statement that the satisfaction with the matter has not changed."}, "language_style": {"answer": "Calm and direct", "rationale": "The entry is more insensitive in its language style, without any emotional vocabulary or expression. It simply and succinctly asks the respondents about their satisfaction with the matter, lack of emotional and interpersonal tone, and may not be easily attracted to the interest and participation of the respondents."}, "time_dimension": {"answer": "Past", "rationale": "The entry uses terms such as \"hidden\" and \"same\" to indicate the past's feelings and state."}, "sentence_type": "Affirmative declarative sentence", "pos": {"nouns": {"Things": 1, "As usual": 1, "Satisfied": 1}, "verbs": {"to": 1}, "adverbs": {"As usual": 1}}}, {"content": "I'm not as happy about things as I always am.", "word_count": 12, "label": "", "sentence_structure": "Subject鈥揷opula鈥損redicative", "subjective_objective": {"answer": "Subjective", "rationale": "Because this is a patient's description of his or her own feelings and is a subjective examination."}, "sentence_sentiment": {"answer": "Negative", "rationale": "Because the term \"unsatisfactory\" in the entry expresses negative feelings of dissatisfaction, disappointment, etc."}, "language_style": {"answer": "Calm and direct", "rationale": "The entry presents the core of the problem in a concise and clear manner 鈥?whether the respondents are not satisfied with the matter, are not using rhetorical or emotional language, and are relatively objective and calm."}, "time_dimension": {"answer": "Past", "rationale": "The keyword \"not as satisfactory as usual\" indicates that the entry asks the testee about the current mentality or feelings, rather than past or future behaviour or circumstances."}, "sentence_type": "Negative declarative sentence", "pos": {"nouns": {"Things": 1}, "verbs": {"Satisfied": 1, "Elephant": 1}, "adjectives": {"not": 1, "As usual": 1, "of": 1}, "adverbs": {"Same thing": 1}}}, {"content": "I'm not really satisfied with anything anymore.", "word_count": 15, "label": "", "sentence_structure": "Subject鈥揷opula鈥損redicative", "subjective_objective": {"answer": "Subjective", "rationale": "Because this is a patient's description of his or her own feelings, it is a subjective attitude and emotional experience."}, "sentence_sentiment": {"answer": "Negative", "rationale": "Because the expression \"no longer truly satisfied\" in the entry is negative."}, "language_style": {"answer": "Tactful and warm", "rationale": "The entry uses a mild tone of questioning, without directly accusing or criticizing the other party for being dissatisfied with life, but rather in a more friendly and understanding way to guide the interviewee to express his feelings, a language style that is more likely to allow the interviewee to share real ideas."}, "time_dimension": {"answer": "Past", "rationale": "The keyword \"no longer\" indicates that the person tested has abandoned certain feelings or states of the past, and \"anything\" expresses the comprehensiveness and thoroughness of such a waiver, so the temporal dimension of the article is the past."}, "sentence_type": "Negative declarative sentence", "pos": {"nouns": {"Things": 1, "Satisfied": 1}, "verbs": {"Not anymore": 1, "I feel": 1}, "adverbs": {"Any": 1, "Really": 1}, "pronouns": {"I": 1}}}, {"content": "I'm not happy or sick of everything.", "word_count": 12, "label": "", "sentence_structure": "Subject鈥揷opula鈥損redicative", "subjective_objective": {"answer": "Subjective", "rationale": "Because it is a description of the patient ' s subjective attitude and feelings."}, "sentence_sentiment": {"answer": "Negative", "rationale": "Because it expresses dissatisfaction or aversion to everything, it is a negative emotional tendency."}, "language_style": {"answer": "Calm and direct", "rationale": "The entry is a very clear expression of the degree of dissatisfaction with the other party ' s life, without any qualifying words or emotional colour, and is straightforward and concise."}, "time_dimension": {"answer": "Present", "rationale": "The entry now represents the current feelings and attitudes of the testee, using the general current, and thus the judgement is now."}, "sentence_type": "Negative declarative sentence", "pos": {"verbs": {"to": 1, "Unsatisfactory": 1, "Shit": 1}, "adjectives": {"Everything": 1}}}, {"content": "I'm not feeling guilty.", "word_count": 9, "label": "", "sentence_structure": "Subject鈥揷opula鈥損redicative", "subjective_objective": {"answer": "Subjective", "rationale": "Because guilt is a subjective feeling, the question is to see if the patient has had a subjective experience of guilt."}, "sentence_sentiment": {"answer": "Neutral", "rationale": "Because the entry does not express a positive or negative emotional expression, it merely presents a fact."}, "language_style": {"answer": "Calm and direct", "rationale": "The language style of the entry is relatively simple, with no excessive rhetoric or emotional rendering, and is more direct and objective, and is a calm and direct language style."}, "time_dimension": {"answer": "Present", "rationale": "Because the entries use the words \"no,\" \"special\" and \"fear\" to describe a state of feeling."}, "sentence_type": "Negative declarative sentence", "pos": {"verbs": {"No, I'm not": 1, "I feel": 1, "Guilt": 1}}}, {"content": "I felt guilty for a good part of the time.", "word_count": 14, "label": "", "sentence_structure": "Subject鈥搗erb鈥揷omplement", "subjective_objective": {"answer": "Subjective", "rationale": "Because guilt is the patient's subjective and emotional experience."}, "sentence_sentiment": {"answer": "Negative", "rationale": "Because guilt is a negative emotional experience."}, "language_style": {"answer": "Calm and direct", "rationale": "The article is formulated in a straightforward manner, without any supposition or emotional rendering, and in a more objective and objective manner. It does not use any euphemism, encouragement or question-formulation, but rather presents the subject matter and the content of the question. The language style of the questionnaire entry can therefore be classified as being calm and straightforward."}, "time_dimension": {"answer": "Present", "rationale": ""}, "sentence_type": "Negative declarative sentence", "pos": {"nouns": {"Time": 1, "Part": 1, "Guilt": 1}, "verbs": {"I feel": 1, "be": 1}, "adjectives": {"Pretty much": 1}, "numerals": {"One": 2}, "pronouns": {"I": 1}}}, {"content": "I feel guilty for a part of my time.", "word_count": 11, "label": "", "sentence_structure": "Subject鈥揷opula鈥損redicative", "subjective_objective": {"answer": "Subjective", "rationale": "Because guilt is the patient's subjective emotional experience."}, "sentence_sentiment": {"answer": "Negative", "rationale": "Because the \"guilty\" sentiment in the entry is negative."}, "language_style": {"answer": "Tactful and warm", "rationale": "The entry is more adept and interactive when it is spoken in a gentle and inviting manner, rather than by direct statements or orders."}, "time_dimension": {"answer": "Present", "rationale": "\"For a part of time\" refers to a feeling or situation within a certain period of time, and the sentence does not specify whether it is the past, present or future, and therefore can be judged as the present.\""}, "sentence_type": "Negative declarative sentence", "pos": {"nouns": {"Time": 1, "Guilt": 1}, "verbs": {"I feel": 1, "be": 1}, "adverbs": {"In part": 1}, "classifiers": {"Lee": 1}, "pronouns": {"I": 1}}}, {"content": "I feel guilty all the time.", "word_count": 7, "label": "", "sentence_structure": "Subject鈥搗erb鈥揷omplement", "subjective_objective": {"answer": "Subjective", "rationale": "Because guilt is a subjective emotional experience, it means that patients self-evaluation feel guilty for doing something wrong."}, "sentence_sentiment": {"answer": "Negative", "rationale": "Because guilt is a negative emotion, expressing a sense of self-pity and unease."}, "language_style": {"answer": "Tactful and warm", "rationale": ""}, "time_dimension": {"answer": "Present", "rationale": "\"I feel guilty at all times\" means \"stretched at all times\" means \"state and emotion\" and therefore judge as \"now.\""}, "sentence_type": "Negative declarative sentence", "pos": {"verbs": {"I feel": 1, "Guilt": 1}, "adverbs": {"Time": 1}}}, {"content": "I don't feel punished.", "word_count": 10, "label": "", "sentence_structure": "Subject鈥揷opula鈥損redicative", "subjective_objective": {"answer": "Subjective", "rationale": "Because \"no feeling punished\" is a patient's subjective feeling or perception."}, "sentence_sentiment": {"answer": "Neutral", "rationale": "Because the entry does not express positive or negative emotional sentiments, it merely describes a person ' s feelings."}, "language_style": {"answer": "Tactful and warm", "rationale": "The entry uses a relatively soft tone to guide respondents to answer, avoids too direct or hard language, and makes it easier for interviewees to relax and share their feelings."}, "time_dimension": {"answer": "Past", "rationale": "According to the keyword \"no feeling\", it can be seen that the respondent answered the question on the basis of his past experience and feelings and thus judged to be past."}, "sentence_type": "Negative declarative sentence", "pos": {"nouns": {"Punishment": 1}, "verbs": {"I feel": 1, "Yes": 1}, "adverbs": {"Positive": 1}, "numerals": {"0锛?: 1}}}, {"content": "I feel like I may be punished.", "word_count": 9, "label": "", "sentence_structure": "Subject鈥揷opula鈥損redicative", "subjective_objective": {"answer": "Subjective", "rationale": "Because feeling that they may be punished is a subjective emotional and cognitive experience."}, "sentence_sentiment": {"answer": "Negative", "rationale": "Because the entry is a negative sentiment and concern."}, "language_style": {"answer": "Calm and direct", "rationale": "The entry directly expresses fear or fear of punishment, does not use excessive rhetoric or emotional rendering, and is more objective in tone."}, "time_dimension": {"answer": "Present", "rationale": ""}, "sentence_type": "Negative declarative sentence", "pos": {"nouns": {"I": 1, "Punishment": 1}, "verbs": {"I feel": 1, "Yes": 1}}}, {"content": "I'm feeling punished.", "word_count": 7, "label": "", "sentence_structure": "Subject鈥搗erb鈥揷omplement", "subjective_objective": {"answer": "Subjective", "rationale": "Because \"preception\" is a subjective feeling and prediction, it is not a fact that has already occurred, and it is a subjective view."}, "sentence_sentiment": {"answer": "Negative", "rationale": "Because the idea of \"preception is punishable\" in the entry is negative."}, "language_style": {"answer": "Tactful and warm", "rationale": "The entry uses an implicit, implicit expression rather than a directly explicit language, which is more euphemistic and gentle. Moreover, the tone of the article is one of concern and concern, not indifference or severity, which is also a manifestation of its \"fructity\"."}, "time_dimension": {"answer": "Past", "rationale": ""}, "sentence_type": "Affirmative declarative sentence", "pos": {"nouns": {"I": 1, "Punishment": 1}, "verbs": {"Foreground": 1, "Yes": 2}, "numerals": {"2锛?: 1}}}, {"content": "I feel I'm being punished.", "word_count": 9, "label": "", "sentence_structure": "Subject鈥揷opula鈥損redicative", "subjective_objective": {"answer": "Subjective", "rationale": "Because the word \"fEE\" indicates that it is a subjective view of the patient's own feelings and thoughts."}, "sentence_sentiment": {"answer": "Negative", "rationale": "Because \"punishable\" in the entry is an expression of a negative mood and attitude."}, "language_style": {"answer": "Tactful and warm", "rationale": "The entry is more personal and interactive when it is spoken in a gentle and inviting manner, rather than in direct statements or orders. While the issue may involve more sensitive subjects, using a soft and warm language style can avoid uncomfortable or offensive interviewees and encourage them to be more willing to share their feelings and ideas."}, "time_dimension": {"answer": "Present", "rationale": "According to the keyword \"felt\" and \"are\" , the entry is to ask the subject about the current emotional state or feeling, so the time dimension is now."}, "sentence_type": "Affirmative declarative sentence", "pos": {"nouns": {"Punishment": 1}, "verbs": {"I feel": 1, "Yes": 1}, "pronouns": {"I": 1}}}, {"content": "I feel like I'm not a disappointment.", "word_count": 10, "label": "", "sentence_structure": "Subject鈥揷opula鈥損redicative", "subjective_objective": {"answer": "Subjective", "rationale": "Because \"not to disappoint\" is the individual feeling and assessment of the patient, and is subjective."}, "sentence_sentiment": {"answer": "Positive", "rationale": "Because the phrase \"do not disappoint\" in the entry is positive."}, "language_style": {"answer": "Calm and direct", "rationale": "The entry directly asks whether the interviewee believes he will not be disappointed, that the language style is simpler and less verbs or emotional and that therefore the language style can be judged as \"cool and direct\"."}, "time_dimension": {"answer": "Past", "rationale": ""}, "sentence_type": "Negative declarative sentence", "pos": {"nouns": {"I": 1, "person": 1, "Disappointment": 1}, "verbs": {"I feel": 1, "鈾?And I'm gonna be 鈾?: 1}, "adverbs": {"and": 1}}}, {"content": "I'm disappointed in myself.", "word_count": 6, "label": "", "sentence_structure": "Subject鈥揷opula鈥損redicative", "subjective_objective": {"answer": "Subjective", "rationale": "Because disappointment is an expression of subjective emotions and attitudes."}, "sentence_sentiment": {"answer": "Negative", "rationale": "Because the emotional attitude of \"disappointing yourself\" in the entries is negative."}, "language_style": {"answer": "Tactful and warm", "rationale": "The entry uses a more moderate way of asking the other side whether it is often disappointed, rather than directly speaking or ordering, which is more personal and interactive."}, "time_dimension": {"answer": "Present", "rationale": "The entry asks about the current feelings and mindset of the subject, the keywords being \"self\" and \"disappointment\", both of which point to the current state and emotional experience and therefore to the present."}, "sentence_type": "Negative declarative sentence", "pos": {"nouns": {"self": 1}, "verbs": {"Disappointment": 1}}}, {"content": "I hate myself.", "word_count": 5, "label": "", "sentence_structure": "Subject鈥搗erb鈥揷omplement", "subjective_objective": {"answer": "Subjective", "rationale": "Because \"obsessed\" is a subjective feeling or attitude."}, "sentence_sentiment": {"answer": "Negative", "rationale": "Because the \"disgusting yourself\" attitude in the entry is negative."}, "language_style": {"answer": "Calm and direct", "rationale": "The entry directly expresses the question of whether the other party is sick of the subject, does not use excessive rhetorical or emotional rendering, and is more objective in tone."}, "time_dimension": {"answer": "Present", "rationale": ""}, "sentence_type": "Negative declarative sentence", "pos": {"nouns": {"self": 1}, "verbs": {"Shit": 1}}}, {"content": "I hate myself.", "word_count": 5, "label": "", "sentence_structure": "Subject鈥搗erb鈥揷omplement", "subjective_objective": {"answer": "Subjective", "rationale": "Because \"hate\" is a personal feeling or attitude, it is a subjective approach."}, "sentence_sentiment": {"answer": "Negative", "rationale": "Because \"hate yourself\" is a negative emotional attitude, expressing dissatisfaction with yourself and self-denial."}, "language_style": {"answer": "Calm and direct", "rationale": "Because the entry directly expresses the subject of questioning whether the other party is dissatisfied with certain aspects of his/her situation, it does not use excessive rhetoric or emotional rendering, and it is more objective in tone. At the same time, it does not use euphemism, but rather raises questions directly."}, "time_dimension": {"answer": "Present", "rationale": "The entry describes the emotional state of the subject at the moment, and \"I hate\" expresses a strong emotional experience, so it can be judged to be the present."}, "sentence_type": "Negative declarative sentence", "pos": {"nouns": {"Hate": 1, "self": 1}, "verbs": {"Hate": 1, "be": 1, "have": 1}, "numerals": {"III": 1}, "pronouns": {"I": 1}}}, {"content": "I feel like I'm no worse than everyone else.", "word_count": 10, "label": "", "sentence_structure": "Subject鈥揷opula鈥損redicative", "subjective_objective": {"answer": "Subjective", "rationale": "Because the entry looks at self-evaluation and attitude of the patient, it is part of the subjective sense."}, "sentence_sentiment": {"answer": "Neutral", "rationale": "Because the entry is just a description of a feeling, without a clear emotional tendency."}, "language_style": {"answer": "Tactful and warm", "rationale": "The language style of the entry is more euphemistic. First, it guides the interviewee in a positive and positive way, encouraging him to recognize his value and potential, rather than demeaning or denying himself. Second, using the word \"fEAL\" rather than absolute affirmation or denial makes the expression more open and open to different views and feelings. Moreover, the whole sentence is also gentle and friendly, with no too much emotional or stressful accommodation, and is more easily accepted and answered by the interviewee."}, "time_dimension": {"answer": "Present", "rationale": ""}, "sentence_type": "Negative declarative sentence", "pos": {"nouns": {"I": 2, "others": 1}, "verbs": {"Feel": 1, "- Yeah": 1, "Bad": 1}, "adverbs": {"and": 1}, "numerals": {"0": 1}, "classifiers": {"individual": 1}}}, {"content": "I've often been thinking about my shortcomings and mistakes.", "word_count": 15, "label": "", "sentence_structure": "Subject鈥搗erb鈥揷omplement", "subjective_objective": {"answer": "Subjective", "rationale": "Because self-reflection is the patient's subjective attitude."}, "sentence_sentiment": {"answer": "Positive", "rationale": "Because the act of self-reflection in the entry is positive and indicates the right perception and attitude towards yourself."}, "language_style": {"answer": "Calm and direct", "rationale": "The basis for the judgement is that the entry is expressed in a more objective and direct way, is not over-modified or emotionally rendered, and is more calm and rational. This language style allows respondents to understand the question more clearly and reduces the subjectivity of the answers."}, "time_dimension": {"answer": "Past", "rationale": "\"I\" is used to indicate that it is a customary act, both in the past and in the present, so the temporal dimension can be judged as now."}, "sentence_type": "Affirmative declarative sentence", "pos": {"nouns": {"Myself": 1, "Disadvantages": 1, "Error": 1, "Retrospect": 1}, "verbs": {"to": 1, "Regular": 1, "Self": 2}, "adverbs": {"Often": 1}}}, {"content": "I often blame myself for my faults.", "word_count": 10, "label": "", "sentence_structure": "Subject鈥搗erb鈥揷omplement", "subjective_objective": {"answer": "Subjective", "rationale": "Because blame is a subjective attitude and reaction."}, "sentence_sentiment": {"answer": "Negative", "rationale": "Because the \"criticizing\" act in the entry is negative."}, "language_style": {"answer": "Calm and direct", "rationale": "\"Calm down straight.\""}, "time_dimension": {"answer": "Present", "rationale": "The keyword is \"I often blame myself for my fault,\" and \"often\" here means that it is a customary act, and \"accusation\" and \"my fault\" indicate that it is being done. So, this entry describes the person who is being tested for his or her current behavior or mental state."}, "sentence_type": "Negative declarative sentence", "pos": {"nouns": {"Error": 1}, "verbs": {"Blame": 1, "Regular": 1}, "adverbs": {"self": 1}}}, {"content": "I blame myself for every bad thing that happens.", "word_count": 14, "label": "", "sentence_structure": "Subject鈥搗erb鈥搊bject", "subjective_objective": {"answer": "Subjective", "rationale": "The reason is that they are the subjective attitudes and way of thinking of the patients."}, "sentence_sentiment": {"answer": "Negative", "rationale": "The basis for the judgment: the act of \"pride yourself\" in the entry is negative."}, "language_style": {"answer": "Calm and direct", "rationale": "The entry uses direct language to ask whether the respondent is always blaming himself when facing a bad thing. The language style is simpler, not too much rhetorical or emotional, and more in line with the language style of the questionnaire."}, "time_dimension": {"answer": "Present", "rationale": ""}, "sentence_type": "Negative declarative sentence", "pos": {"nouns": {"Yes": 1, "Bad things": 1, "self": 1}, "verbs": {"Both": 1, "Come on": 1, "Blame": 1}, "adjectives": {"Shit": 1}, "adverbs": {"Every time": 1, "Both": 1}, "numerals": {"3锛?: 1}, "pronouns": {"I": 1}}}, {"content": "I don't have any idea of suicide.", "word_count": 10, "label": "", "sentence_structure": "Subject鈥搗erb鈥揷omplement", "subjective_objective": {"answer": "Subjective", "rationale": "The questionnaire's purpose, \"I have no idea of suicide,\" is partly subjective, because it is a question of asking patients about their views and feelings about suicide, not of describing or recording objective facts. Thus, \"no idea of suicide\" is the patient's personal subjective experience and perception, not the objective reality."}, "sentence_sentiment": {"answer": "Neutral", "rationale": "Because the entry does not have any emotional color."}, "language_style": {"answer": "Tactful and warm", "rationale": "Warm and warm."}, "time_dimension": {"answer": "Past", "rationale": ""}, "sentence_type": "Negative declarative sentence", "pos": {"nouns": {"Thoughts": 1, "Suicide": 1}, "verbs": {"have": 1, "Yes": 1}, "adverbs": {"Any": 1}, "classifiers": {"individual": 1}, "pronouns": {"I": 1}}}, {"content": "I have a suicide wish, but I'm not going to do it.", "word_count": 15, "label": "", "sentence_structure": "Subject鈥搗erb鈥揷omplement", "subjective_objective": {"answer": "Subjective", "rationale": "Because the idea of suicide is the patient's subjective idea."}, "sentence_sentiment": {"answer": "Negative", "rationale": "Because, although the person did not actually commit suicide, the idea of suicide itself was a negative sentiment."}, "language_style": {"answer": "Tactful and warm", "rationale": "The entry is more personal and interactive when it is spoken in a gentle and inviting manner, rather than in direct statements or orders. At the same time, the language style of the article is more euphemistic, avoiding the use of too direct a question-and-answer approach that may provoke negative feelings or resistance on the part of the person under investigation."}, "time_dimension": {"answer": "Present", "rationale": "The \"suicide-related thoughts\" and \"no suicides\" both indicate the current state of mind and the thinking, so the temporal dimension is now."}, "sentence_type": "Negative declarative sentence", "pos": {"nouns": {"Thinking": 2, "Suicide": 2}, "verbs": {"have": 1, "Yes": 1, "Go on": 1, "True": 1}, "adjectives": {"of": 3}, "numerals": {"1锛?: 1, "2锛?: 1, "But": 1}, "pronouns": {"I": 1}}}, {"content": "I'd like to kill myself.", "word_count": 5, "label": "", "sentence_structure": "Subject鈥揷opula鈥損redicative", "subjective_objective": {"answer": "Subjective", "rationale": "Because \"I'd like to kill myself\" is the mind and feeling of the patient, and it's a subjective examination."}, "sentence_sentiment": {"answer": "Negative", "rationale": "Because the idea of \"I'd like to kill myself\" in the entry is negative."}, "language_style": {"answer": "Calm and direct", "rationale": "This is a straightforward expression of whether the subject is suicidal, and the language is not euphemistic or emotional, and it is more direct and objective."}, "time_dimension": {"answer": "Present", "rationale": "The keyword \"too much to commit suicide\" expresses a strong state of desire and emotion that may be affecting the current behaviour and mental state of the person being tested."}, "sentence_type": "Exclamatory sentence", "pos": {"nouns": {"Suicide": 1}, "verbs": {"very": 1, "Yes": 1}}}, {"content": "I'd kill myself if I had the chance.", "word_count": 11, "label": "", "sentence_structure": "Subject鈥損redicate", "subjective_objective": {"answer": "Subjective", "rationale": "The problem is primarily one of subjective thinking and attitude of the patient, not of the specific facts or behaviour observed."}, "sentence_sentiment": {"answer": "Negative", "rationale": "Because the act of suicide in the entry is negative."}, "language_style": {"answer": "Calm and direct", "rationale": "The entry directly asks the other party whether it is possible to choose suicide, which is more incorruptible and objective, and does not use excessive emotional colours or qualifiers, making the matter more clear."}, "time_dimension": {"answer": "Present", "rationale": "According to the sentence, \"I would kill myself if I had the chance,\" the word \"into\" is a hypothetical necessity, so the entry is to ask the testee what he thinks or intends in the current circumstances, so the temporal dimension is now."}, "sentence_type": "Negative declarative sentence", "pos": {"nouns": {"Opportunities": 1, "Suicide": 1}, "verbs": {"have": 1, "Yes": 1, "Suicide": 1}, "adverbs": {"Just": 1, "If": 1}, "numerals": {"3": 1}, "pronouns": {"I": 1}}}, {"content": "I'm not crying more than I used to be.", "word_count": 8, "label": "", "sentence_structure": "Subject鈥揷opula鈥損redicative", "subjective_objective": {"answer": "Subjective", "rationale": "Because it's an emotional experience to look at the patient's subjective feelings."}, "sentence_sentiment": {"answer": "Negative", "rationale": "Because the expression \"no more than before\" in the entry is negative, which means that it may be easier to cry now."}, "language_style": {"answer": "Calm and direct", "rationale": "The entry expresses in a direct and clear manner the subject of asking the other party whether it is easier to cry than before, without excessive ado or emotional colour, and with a more calm and straightforward language style."}, "time_dimension": {"answer": "Past", "rationale": "\"No more than before\" indicates that the act has existed at some point in the past and is still present or continues, so that the entry describes the act or state of the past."}, "sentence_type": "Exclamatory sentence", "pos": {"nouns": {"Questionnaire": 1, "Entry": 1, "I": 1}, "verbs": {"- Yeah": 1, "Love": 1, "Cry": 1}, "adverbs": {"Previous": 1}, "numerals": {"0锛?: 1}}}, {"content": "I'm crying more than I used to be.", "word_count": 8, "label": "", "sentence_structure": "Subject鈥揷opula鈥損redicative", "subjective_objective": {"answer": "Subjective", "rationale": "Because \"cry\" is a subjective emotion and experience of the patient, and it's a subjective view."}, "sentence_sentiment": {"answer": "Negative", "rationale": "Because the act of \"love to cry\" in the entry is negative."}, "language_style": {"answer": "Calm and direct", "rationale": "The object of asking the other side whether it is now easier to cry than before is directly expressed in calmness and directly in the entry, without excessive rhetorical or emotional rendering, and in a more objective tone."}, "time_dimension": {"answer": "Past", "rationale": "\"I'm more than I used to\" means comparing with the past, and \"now\" means that the act is a state or act, so the entry is about the judgment of the dimension of time and can be judged as \"now\"."}, "sentence_type": "Interrogative sentence", "pos": {"nouns": {"I": 1, "Present": 1, "Before": 1, "Cry": 1}, "verbs": {"Love": 1, "Cry": 1}, "adverbs": {"- Yeah": 1}, "pronouns": {"Here": 1}}}, {"content": "Now I cry a lot.", "word_count": 6, "label": "", "sentence_structure": "Subject鈥搗erb鈥揷omplement", "subjective_objective": {"answer": "Subjective", "rationale": "Because whether or not \"often crying\" is the patient's own feeling and perception is subjective."}, "sentence_sentiment": {"answer": "Negative", "rationale": "Because the \"often crying\" act in the entry is negative."}, "language_style": {"answer": "Calm and direct", "rationale": "The entry is a simple and straightforward language that directly expresses the subject of asking the other party whether or not to cry frequently, and is not overly emotional or rhetorical, and is more objective and direct."}, "time_dimension": {"answer": "Present", "rationale": ""}, "sentence_type": "Affirmative declarative sentence", "pos": {"nouns": {"Present": 1, "I": 1, "Cry": 1}, "verbs": {"Regular": 1, "Cry": 1}}}, {"content": "I used to cry, but now I can't cry even if I want to.", "word_count": 18, "label": "", "sentence_structure": "Subject鈥搗erb鈥揷omplement", "subjective_objective": {"answer": "Subjective", "rationale": "Because \"want to cry\" and \"can't cry\" are the feelings and experiences of the patient, the view of this questionnaire is subjective."}, "sentence_sentiment": {"answer": "Negative", "rationale": "Because the \"cry can't\" act in the entry is negative."}, "language_style": {"answer": "Calm and direct", "rationale": "The language style of the entry is relatively simple, there is no excessive rhetorical or emotional overture, and it is easy to understand in an objective way. Moreover, the question is in the form of a direct question, not a soft invitation. So I think the language style of the entry is straightforward."}, "time_dimension": {"answer": "Past", "rationale": "The keywords \"old\" and \"but now\" indicate that the period of comparison is past and present. The temporal dimension of the article therefore determines past and present."}, "sentence_type": "Negative declarative sentence", "pos": {"nouns": {"Questionnaire": 1, "Entry": 1, "I": 2, "Present": 1, "Before": 1, "Cry": 2, "Come out": 1}, "verbs": {"Yes": 2, "Cry": 2}, "adjectives": {"Previous": 1, "But": 1, "Yeah": 1, "Present": 1}, "adverbs": {"Even if": 1, "not": 1}, "numerals": {"3锛?: 1}, "pronouns": {"I": 2}}}, {"content": "I'm not as easily pissed off as I ever was.", "word_count": 10, "label": "", "sentence_structure": "Subject鈥揷opula鈥損redicative", "subjective_objective": {"answer": "Subjective", "rationale": "Because \"facilitating\" is a personal experience and experience of the patient, and is a subjective examination."}, "sentence_sentiment": {"answer": "Negative", "rationale": "Because \"fun\" in the entries is a negative word, which means that individual emotions are unstable and angry."}, "language_style": {"answer": "Calm and direct", "rationale": "This is because the purpose of the article is more honest and objective, without too much emotional acquital or euphemism. It directly describes the person being interviewed about his or her emotional changes, asking whether the other person is more susceptible to stimulation or anger than in the past. This expression is more direct and explicit, and is consistent with a straight and calm style of description."}, "time_dimension": {"answer": "Present", "rationale": ""}, "sentence_type": "Negative declarative sentence", "pos": {"nouns": {"I": 1, "Previous": 1}, "verbs": {"- Yeah": 1, "Easy": 1, "- You're a bad guy": 1}, "adjectives": {"and": 1, "not": 1}, "adverbs": {"0锛?: 1}}}, {"content": "I'm more aggressive or angry than ever.", "word_count": 13, "label": "", "sentence_structure": "Subject鈥揷opula鈥損redicative", "subjective_objective": {"answer": "Subjective", "rationale": "Because \"more than ever\" involves comparisons, rather subjective, it is viewed from the subjective perspective of the patient."}, "sentence_sentiment": {"answer": "Negative", "rationale": "Because \"fun\" or \"fun\" in the entry is a negative emotional expression."}, "language_style": {"answer": "Calm and direct", "rationale": "The language style of the entry is \"cool and direct.\" It describes an act or situation in a concise manner, without too many emotional qualifiers or rhetorical techniques. This expression is more objective, accurate and allows the respondents to understand the implications of the problem more clearly."}, "time_dimension": {"answer": "Present", "rationale": "The entry asks the testee about his current state of behaviour, using the comparative form of \"more easily than ever\" to describe recent emotional changes and therefore to judge now.\""}, "sentence_type": "Negative declarative sentence", "pos": {"nouns": {"I": 1, "- You're a bad guy": 1, "Angry": 1}, "verbs": {"- Yeah": 1, "Easy": 2}, "adjectives": {"Previous": 1}}}, {"content": "I'm always angry.", "word_count": 9, "label": "", "sentence_structure": "Subject鈥揷opula鈥損redicative", "subjective_objective": {"answer": "Subjective", "rationale": "Because words like \"often\" and \"freaky\" are the patient's own subjective feelings and attitudes."}, "sentence_sentiment": {"answer": "Negative", "rationale": "Because \"freak\" is a negative emotional attitude."}, "language_style": {"answer": "Tactful and warm", "rationale": ""}, "time_dimension": {"answer": "Present", "rationale": "So the temporal dimension of the article is \"now\"."}, "sentence_type": "Negative declarative sentence", "pos": {"nouns": {"Present": 1, "Fire": 1}, "verbs": {"Regular": 1, "Let's go": 1}, "adverbs": {"Easy": 1}, "pronouns": {"I": 1}}}, {"content": "3. Things that used to piss me off are not gonna piss me off.", "word_count": 22, "label": "", "sentence_structure": "Subject鈥搗erb鈥揷omplement", "subjective_objective": {"answer": "Subjective", "rationale": "Because \"the things that provoke me are not gonna provoke me\" are the subjective feelings and attitudes of the patient, not the objective facts. This change may be caused by changes in the patient's mental state, emotional or cognitive state. So, this is a subjective questionnaire entry."}, "sentence_sentiment": {"answer": "Negative", "rationale": "Because the word \"promoting\" in the entries has a negative emotional connotation."}, "language_style": {"answer": "Calm and direct", "rationale": "The language style of the entry is a calm and straightforward one, which presents the fact in a concise and clear way that what was once able to provoke its emotional reaction cannot now produce the same effect. It is not too much emotional or rhetorical, speaking with a sound and objective tone, suitable for use in, for example, questionnaires."}, "time_dimension": {"answer": "Past", "rationale": ""}, "sentence_type": "Negative declarative sentence", "pos": {"nouns": {"Things": 1, "- You're a bad guy": 2}, "verbs": {"Yes": 1, "Present": 1, ", and completely": 1, "No, I can't": 1}, "adjectives": {"Total": 1}, "adverbs": {"Yes": 1}, "numerals": {"3锛?: 1, "Some": 1}, "classifiers": {"Those": 1}, "pronouns": {"I": 1}}}, {"content": "I'm not losing interest in others.", "word_count": 11, "label": "", "sentence_structure": "Subject鈥搗erb鈥揷omplement", "subjective_objective": {"answer": "Subjective", "rationale": "Because \"interest in others is not diminished\" is the patient's subjective attitude."}, "sentence_sentiment": {"answer": "Neutral", "rationale": "Since the entry simply describes a fact, it does not involve any positive or negative emotional colour."}, "language_style": {"answer": "Tactful and warm", "rationale": "The entry is a soft and warm language by asking the interviewees whether they have changed their interest in others, engaging in a gentle and caring dialogue, and being more friendly and sensitive to each other ' s feelings."}, "time_dimension": {"answer": "Present", "rationale": ""}, "sentence_type": "Negative declarative sentence", "pos": {"nouns": {"Others": 1, "Interest": 1}, "verbs": {"No, I'm not": 1, "Reduction": 1}, "numerals": {"0": 1}, "pronouns": {"I": 1}}}, {"content": "I'm less interested in others than I have ever been.", "word_count": 13, "label": "", "sentence_structure": "Subject鈥揷opula鈥損redicative", "subjective_objective": {"answer": "Subjective", "rationale": "Because \"a lower interest in others than ever before\" is a subjective feeling or attitude that reflects a change in individual subjective thoughts and emotions."}, "sentence_sentiment": {"answer": "Negative", "rationale": "Because the \"reduced interest\" in the entry is negative."}, "language_style": {"answer": "Calm and direct", "rationale": "The entry is not based on rhetorical or emotional rendering, and is more objective. The sentence is simple and clear, and the meaning is clear and not implicit. The article is more formal and professional, and does not have much personal emotional colour. In conclusion, \"I have less interest in others than I have previously\" is a calm and straightforward language."}, "time_dimension": {"answer": "Past", "rationale": ""}, "sentence_type": "Negative declarative sentence", "pos": {"nouns": {"Others": 1, "Interest": 1, "Reduction": 1}, "verbs": {"to": 1, "- He's a guy": 1, "Interested": 1, "Reduction": 1}, "adjectives": {"Previous": 1}, "numerals": {"1锛?: 1}, "pronouns": {"I": 1}}}, {"content": "I lost most of my interest in others.", "word_count": 12, "label": "", "sentence_structure": "Subject鈥揷opula鈥損redicative", "subjective_objective": {"answer": "Subjective", "rationale": "Because \"lost most of the interest\" is a personal feeling and perception, a subjective attitude of the patient."}, "sentence_sentiment": {"answer": "Negative", "rationale": "Because the \"lost majority\" act is negative, indicating a lack of interest or indifference to others."}, "language_style": {"answer": "Tactful and warm", "rationale": "The entry is more personal and interactive by engaging in a soft, inviting tone of dialogue rather than by direct statements or orders. At the same time, the use of the vague term \"most\" avoids asking the respondents too directly and offensive, thereby enhancing the acceptability and reliability of the questionnaire."}, "time_dimension": {"answer": "Present", "rationale": "In the entry, \"I lost someone\" indicates that this loss of interest in others is a current, ongoing situation and can therefore be judged to describe the current behaviour or mentality."}, "sentence_type": "Negative declarative sentence", "pos": {"nouns": {"I": 1, "Others": 1, "Interest": 1}, "verbs": {"to": 1, "Loss": 1, "Lose": 1}, "adjectives": {"Large": 1, "of": 2, "Most of them": 1}, "numerals": {"2": 1}}}, {"content": "I'm not interested in others right now.", "word_count": 10, "label": "", "sentence_structure": "Subject鈥搗erb鈥揷omplement", "subjective_objective": {"answer": "Subjective", "rationale": "Because interest is an inherent thought and feeling of one person, and it is subjective."}, "sentence_sentiment": {"answer": "Negative", "rationale": "Because the description \"no interest in others now\" in the entry shows a negative attitude."}, "language_style": {"answer": "Calm and direct", "rationale": "The entry expresses in a direct, concise language the question of whether the other party is interested in another person at the present time, without using overly reticent language or emotional colours and with a more objective and incriminating tone."}, "time_dimension": {"answer": "Present", "rationale": "The entry \"now\" refers to the current period and describes the attitudes and behaviour of the person being tested at the present time (i.e., at the time of the investigation). The keyword is \"now without interest\" , which shows that the person being tested is not interested or interested in others at the present time."}, "sentence_type": "Negative declarative sentence", "pos": {"nouns": {"I": 1, "Others": 1}, "verbs": {"to": 1, "None": 1, "Interested": 1}, "adverbs": {"Present": 1}, "numerals": {"3": 1}}}, {"content": "I can make a decision as usual.", "word_count": 10, "label": "", "sentence_structure": "Subject鈥揷opula鈥損redicative", "subjective_objective": {"answer": "Subjective", "rationale": "This is a description of the patient ' s subjective perception of whether he or she can make a decision, not an objective fact or result."}, "sentence_sentiment": {"answer": "Neutral", "rationale": "Because the entry does not express a positive or negative emotional attitude, it merely describes an objective fact."}, "language_style": {"answer": "Tactful and warm", "rationale": "The entry is more adept and interactive when it is spoken in a gentle and inviting manner, rather than by direct statements or orders."}, "time_dimension": {"answer": "Present", "rationale": "\"Can make a decision\" is a description of the act or state, and the word \"may\" adopts the general current, meaning that the act has not only occurred in the past, but may still be ongoing. \"Same as before\" means that the act has not changed, and remains normal. So this entry is judging now in terms of the time dimension of asking the testee's current situation."}, "sentence_type": "Affirmative declarative sentence", "pos": {"nouns": {"I": 1, "Decision": 1}, "verbs": {"Do it": 1, "Yes": 1}, "adjectives": {"As always": 1}, "numerals": {"0锛?: 1}}}, {"content": "1. I am taking decisions without previous determinations.", "word_count": 12, "label": "", "sentence_structure": "Subject鈥搗erb鈥揷omplement", "subjective_objective": {"answer": "Subjective", "rationale": "Because the sentence expresses a subjective feeling that the patient feels that his decision-making has decreased. This may be due to a number of factors, such as stress, anxiety, etc., which are the patient鈥檚 personal subjective experience and perception."}, "sentence_sentiment": {"answer": "Negative", "rationale": "Because the act of \"no previous decisive decision\" in the entry is negative."}, "language_style": {"answer": "Calm and direct", "rationale": "The reason is as follows: the entry explores the decision-making capacity of the other party in a more hesitant way than it has in the past. The entire sentence is simple, with no excessive qualifier or accommodative, and is more objective in tone, and therefore can be judged as \"cooling directly\"."}, "time_dimension": {"answer": "Present", "rationale": ""}, "sentence_type": "Negative declarative sentence", "pos": {"nouns": {"Decision": 1, "Present": 1, "Before": 1}, "verbs": {"Do it": 1, "No, I'm not": 1, "Decisive": 1}, "adverbs": {"Comparison": 1}, "pronouns": {"I": 1}}}, {"content": "I'm making decisions much harder than I have ever been.", "word_count": 13, "label": "", "sentence_structure": "Subject鈥揷opula鈥損redicative", "subjective_objective": {"answer": "Subjective", "rationale": "Because \"I'm making decisions now more difficult than ever\" describes how patients feel and what they have."}, "sentence_sentiment": {"answer": "Negative", "rationale": "Because \"it's much harder to make a decision than before\" is negative."}, "language_style": {"answer": "Tactful and warm", "rationale": "The entry is more personal and interactive by using a gentle, invitee tone of speech rather than by direct statement or order. It also implies that the person under investigation may have some problems and challenges in the face of decision-making."}, "time_dimension": {"answer": "Present", "rationale": "\"I\" means that the subject is a person, \"now\" means that the time points to the current state and feeling, and \"decisions are much harder than before\" means that the current state and change of the subject is described. The entry is therefore mainly concerned with the current situation of the subject, i.e. the extent of the difficulty of the subject in making decisions."}, "sentence_type": "Negative declarative sentence", "pos": {"nouns": {"I": 1, "Present": 1, "Decision": 1, "Before": 1}, "verbs": {"Do it": 1, "- Yeah": 1, "Difficulties": 1, "Much more": 1}, "adverbs": {"More": 1}, "numerals": {"2锛?: 1, "More": 1}}}, {"content": "I can't make a decision right now.", "word_count": 10, "label": "", "sentence_structure": "Subject鈥揷opula鈥損redicative", "subjective_objective": {"answer": "Subjective", "rationale": "Because it is the individual feelings and experiences of the patient that make decisions."}, "sentence_sentiment": {"answer": "Negative", "rationale": "Because the \"no decision\" act in the entry is negative."}, "language_style": {"answer": "Calm and direct", "rationale": "The entry uses a concise language that expresses questions about whether the other party is having difficulties in making decisions at the present time, is not over-modified or emotional and is more objective and direct."}, "time_dimension": {"answer": "Present", "rationale": "\"Now.\""}, "sentence_type": "Negative declarative sentence", "pos": {"nouns": {"Decision": 1}, "verbs": {"Do it": 1, "Present": 1, "Total": 1}, "adverbs": {"No, I can't": 1}}}, {"content": "I feel like I'm just like before.", "word_count": 14, "label": "", "sentence_structure": "Subject鈥揷opula鈥損redicative", "subjective_objective": {"answer": "Subjective", "rationale": "Because \"I feel like I'm almost like you\" is a subjective view of a person, a personal perception and experience."}, "sentence_sentiment": {"answer": "Neutral", "rationale": "Because the entry does not have any emotional color."}, "language_style": {"answer": "Tactful and warm", "rationale": "The entry uses a more moderate and encouraging tone to ask questions, rather than a direct statement or order, making it easier for respondents to accept and answer questions. At the same time, the word \"I\" is used to make questions more personal, to give respondents a greater sense of participation and to facilitate more realistic and accurate answers."}, "time_dimension": {"answer": "Present", "rationale": "The entry describes the current feelings of the testees, and the keywords \"like,\" \"looks\" and \"like before\" point to the current state or feeling."}, "sentence_type": "Affirmative declarative sentence", "pos": {"nouns": {"self": 1, "Looks like it": 1, "Before": 1}, "verbs": {"I think": 1, "Almost": 1}, "numerals": {"0": 1}}}, {"content": "I'm afraid I look old or I don't look good.", "word_count": 17, "label": "", "sentence_structure": "Subject鈥揷opula鈥損redicative", "subjective_objective": {"answer": "Subjective", "rationale": "Because \"fear\" is a manifestation of subjective sentiment."}, "sentence_sentiment": {"answer": "Negative", "rationale": "Because the words \"cared\" and \"old\" have negative emotions."}, "language_style": {"answer": "Tactful and warm", "rationale": "The entry uses a tone of concern, asking whether the person surveyed has concerns about his or her appearance, and does not directly criticize or demean the other, which is more moderate and respectful."}, "time_dimension": {"answer": "Present", "rationale": "The entry \"I'm now\" indicates the current state or emotion, that is, the situation in which the person tested is answering the question. So the time dimension is now."}, "sentence_type": "Negative declarative sentence", "pos": {"nouns": {"Questionnaire": 1, "Entry": 1, "I": 2, "I'm worried": 1, "Looks like it": 1, "Old": 1, "Before": 1, "Nice": 1}, "verbs": {"I'm worried": 1, "Looks like it": 1, "Old": 1, "have": 1, "That's good": 1}, "adjectives": {"Old": 1, "That's good": 1}, "numerals": {"1锛?: 1}, "pronouns": {"I": 2}}}, {"content": "I think my appearance has become ugly and permanent.", "word_count": 22, "label": "", "sentence_structure": "Subject鈥揷opula鈥損redicative", "subjective_objective": {"answer": "Subjective", "rationale": "I think it's the patient's subjective view."}, "sentence_sentiment": {"answer": "Negative", "rationale": "Because \"the appearance gets ugly\" and \"permanent change\" are negative descriptions."}, "language_style": {"answer": "Calm and direct", "rationale": "\"Calm down straight.\""}, "time_dimension": {"answer": "Present", "rationale": "The keyword \"I feel\" and \"i have become\" means that the entry is describing the feelings and perceptions of the subject at present, and can therefore be judged as now. And the \"and permanent change\" also implies that this change has taken place or is continuing, which further confirms that it is about the present."}, "sentence_type": "Negative declarative sentence", "pos": {"nouns": {"I": 1, "Looks": 1, "Change": 2}, "verbs": {"I think": 1, "鈾?And become 鈾?: 1, "be": 1}, "adjectives": {"It's not good": 1, "Permanent": 1}, "numerals": {"2锛?: 1}}}, {"content": "I think I look ugly.", "word_count": 10, "label": "", "sentence_structure": "Subject鈥搗erb鈥揷omplement", "subjective_objective": {"answer": "Subjective", "rationale": "This is a subjective examination of the patient ' s personal views and opinions."}, "sentence_sentiment": {"answer": "Negative", "rationale": "Because the idea of \"thinks you're ugly\" is negative."}, "language_style": {"answer": "Calm and direct", "rationale": "The entry is a direct expression of the subject of questioning whether the other party is often concerned about past events, and is not based on excessive rhetoric or emotional rendering, and is more objective in tone."}, "time_dimension": {"answer": "Present", "rationale": ""}, "sentence_type": "Negative declarative sentence", "pos": {"nouns": {"I": 1, "Looks like it": 1, "Ugly": 1}, "verbs": {"I think": 1, "Looks like it": 1, "Ugly": 1}, "adverbs": {"very": 1}}}, {"content": "I can work as usual.", "word_count": 9, "label": "", "sentence_structure": "Subject鈥揷opula鈥損redicative", "subjective_objective": {"answer": "Subjective", "rationale": "This is a self-evaluation of the patient ' s abilities and is a subjective examination."}, "sentence_sentiment": {"answer": "Positive", "rationale": "Because \"can work as usual\" expresses positive attitudes and behavior."}, "language_style": {"answer": "Tactful and warm", "rationale": "The entry uses a more moderate guide, \"Please analyse the following questionnaire entry to start the dialogue, instead of directly presenting or ordering, which is more personal and interactive. Moreover, the article is intended to be presented in a question-and-answer manner without direct conclusions or evaluation, which also reflects its euphemismal language style."}, "time_dimension": {"answer": "Present", "rationale": ""}, "sentence_type": "Affirmative declarative sentence", "pos": {"nouns": {"Questionnaire": 1, "Entry": 1, "Work": 1}, "verbs": {"Yes": 1, "Elephant": 1, "Previous": 1, "Same thing": 1}, "adverbs": {"very": 1, "Sample": 1}, "numerals": {"0锛?: 1}, "pronouns": {"I": 1}}}, {"content": "I'm going to have to work very hard to get to work.", "word_count": 15, "label": "", "sentence_structure": "Subject鈥搗erb鈥揷omplement", "subjective_objective": {"answer": "Subjective", "rationale": "Because this sentence expresses the patient ' s own belief that additional efforts are needed to start working, it is his subjective idea."}, "sentence_sentiment": {"answer": "Negative", "rationale": "Because the entry \"tweetly and particularly hard\" expresses a negative sentiment that requires more effort."}, "language_style": {"answer": "Calm and direct", "rationale": ""}, "time_dimension": {"answer": "Present", "rationale": "The entry is for the present behaviour or mental state of the subject, and the keywords \"want\" and \"now\" indicate the point of time of the problem and therefore the judgement is now."}, "sentence_type": "Imperative sentence", "pos": {"nouns": {"Special efforts": 1, "Yes": 1}, "verbs": {"Yes": 1, "The long run": 1, "Start": 1}, "adjectives": {"A little bit": 1}, "numerals": {"One": 2}, "pronouns": {"I": 1}}}, {"content": "I have to do everything to force myself to do it.", "word_count": 20, "label": "", "sentence_structure": "Subject鈥揷opula鈥損redicative", "subjective_objective": {"answer": "Subjective", "rationale": "Because the words \"must\" and \"forced to do\" express the subjective sense of the patient's behaviour."}, "sentence_sentiment": {"answer": "Negative", "rationale": "Because the act of forcing yourself to do it is a negative attitude and behaviour."}, "language_style": {"answer": "Tactful and warm", "rationale": "The language style of the entry is mild and encouraging, asking if the interviewee has such experience, without direct criticism or criticism."}, "time_dimension": {"answer": "Present", "rationale": "The entry describes the current mental state and attitude of the testee, the key words being \"must do a great deal\" and \"forced to do it\" which indicate that the testee has to do a great deal of effort and feel compelled to do it in carrying out various activities. So the time dimension is now."}, "sentence_type": "Negative declarative sentence", "pos": {"nouns": {"Yes": 1, "Try": 1, "Forced": 1, "self": 1}, "verbs": {"Do it": 2, "I have to": 1, "Go on": 1}, "adjectives": {"Large": 1}, "adverbs": {"very": 1}, "classifiers": {"individual": 1}}}, {"content": "I can't work at all.", "word_count": 7, "label": "", "sentence_structure": "Subject鈥搗erb鈥揷omplement", "subjective_objective": {"answer": "Subjective", "rationale": "Since the entry asks about the patient ' s personal feelings and perceptions, i.e. whether the work can be done, it is examined from the patient ' s subjective perspective."}, "sentence_sentiment": {"answer": "Negative", "rationale": "Because the \"totally incapable of working\" act in the entry is negative."}, "language_style": {"answer": "Tactful and warm", "rationale": "The entry is more euphemistic, warmer, less than a language that is too direct and harsh, but rather uses a more friendly and friendly tone to communicate with the interviewee. This expression is better able to create a good communication climate, make the interviewee more willing to participate in the investigation and have easier access to real and effective feedback."}, "time_dimension": {"answer": "Past", "rationale": "The entry is based on the key words \"totally inoperable\" and \"old\" in the sentence entered. The entry is based on an inquiry into the behaviour or state of the person being tested during a certain period of time. The time dimension is therefore a past judgement."}, "sentence_type": "Imperative sentence", "pos": {"nouns": {"Work": 1}, "verbs": {"Yes": 1, "Work": 1}, "adjectives": {"Total": 1}, "numerals": {"III": 1}}}, {"content": "I'm as good as I used to be.", "word_count": 9, "label": "", "sentence_structure": "Subject鈥揷opula鈥損redicative", "subjective_objective": {"answer": "Objective", "rationale": "Because sleep conditions are an objective state of affairs, not related to the subjective perception of the individual. Although individuals may feel the quality of their sleep, this does not affect their objectivity."}, "sentence_sentiment": {"answer": "Neutral", "rationale": "Because the entry is not emotional, it is a description of a fact."}, "language_style": {"answer": "Tactful and warm", "rationale": "The language style of the entry is euphemistic, because it is expressed in the words \"as before\", which is a mild and objective way of not directly evaluating or criticizing the quality of sleep of the person under investigation. Moreover, the tone of the article is more cordial and friendly, giving a comfortable feeling of comfort, and helps to increase the participation of the person under investigation and the reliability of the questionnaire."}, "time_dimension": {"answer": "Present", "rationale": "In this entry, \"as always\" indicates that the person tested has a comparative reference to the current state of sleep, which is relative to a period of time in the past. It can therefore be judged to be a description of the present statement."}, "sentence_type": "Affirmative declarative sentence", "pos": {"nouns": {"I": 1, "Sleep": 1, "Previous": 1, "Okay": 1}, "verbs": {"Elephant": 1, "Same thing": 1, "have": 1}, "adverbs": {"Usually": 1}}}, {"content": "I'm not sleeping as well as I used to be.", "word_count": 10, "label": "", "sentence_structure": "Subject鈥揷opula鈥損redicative", "subjective_objective": {"answer": "Subjective", "rationale": "Because the improvement in the quality of sleep is the patient ' s own feeling and judgment, it is subjective."}, "sentence_sentiment": {"answer": "Negative", "rationale": "Because it expresses a negative emotional attitude, i.e., sleep is not as good as it used to be."}, "language_style": {"answer": "Calm and direct", "rationale": "The entry is more direct, concise, not overly stale or emotional, and is a calm, direct language style. It describes the sleep of the person under investigation in an objective and neutral manner, with the aim of understanding and collecting relevant information."}, "time_dimension": {"answer": "Past", "rationale": "The keyword \"not as good as ever\" in the entry indicates a decline in the quality of sleep in the past compared to the present and can therefore be judged to have occurred in the past in behaviour or state."}, "sentence_type": "Negative declarative sentence", "pos": {"nouns": {"Sleep": 1, "Previous": 1}, "verbs": {"No, I'm not": 1, "Okay": 1}, "adjectives": {"That way": 1}, "numerals": {"One": 1}, "pronouns": {"I": 1}}}, {"content": "I wake up 1-2 hours earlier than usual and have trouble sleeping again.", "word_count": 14, "label": "", "sentence_structure": "Subject鈥揷opula鈥損redicative", "subjective_objective": {"answer": "Subjective", "rationale": "Because the entry describes the patient ' s own feelings and experiences, it relates to the individual ' s sleep habits and state and is therefore subjective."}, "sentence_sentiment": {"answer": "Negative", "rationale": "Because early awakening and sleep difficulties are negative behaviour."}, "language_style": {"answer": "Calm and direct", "rationale": "The language style of the entry is straightforward and calm, since it describes in objective and concise terms a sleep problem that the person under investigation may encounter without using excessive emotional colours or rhetorical techniques."}, "time_dimension": {"answer": "Present", "rationale": "According to the information provided in the title, \"I wake up one or two hours earlier and have difficulty sleeping again\" is the current state of affairs, so the temporal dimension of this article is \"now\"."}, "sentence_type": "Negative declarative sentence", "pos": {"nouns": {"Problem": 1, "Entry": 1, "As usual": 1, "Hours": 1, "Difficulties": 1}, "verbs": {"Wake up": 1, "Re-in": 1, "have": 2}, "adverbs": {"- Yeah": 1, "Morning": 1}, "numerals": {"l锝?": 1}, "pronouns": {"I": 1}}}, {"content": "I wake up a few hours before I'm ever gonna sleep.", "word_count": 16, "label": "", "sentence_structure": "Subject鈥搗erb鈥揷omplement", "subjective_objective": {"answer": "Subjective", "rationale": "Because early awakening and sleeplessness are the experiences and feelings of the patient himself, they are subjective in nature."}, "sentence_sentiment": {"answer": "Negative", "rationale": "Because the \"early awakening and sleepless\" in the entry is negative."}, "language_style": {"answer": "Calm and direct", "rationale": "The entry raises the sleep problems of the person under investigation in a direct manner, without excessive trimulation or emotional rendering, and with a more objective and clear tone."}, "time_dimension": {"answer": "Present", "rationale": "Present"}, "sentence_type": "Negative declarative sentence", "pos": {"nouns": {"Hours": 1, "Grandpa": 1}, "verbs": {"Wake up": 1, "Re-in": 2}, "adjectives": {"It's earlier than usual": 1, "No, I can't": 1}, "numerals": {"3": 1, "How many": 1, "One": 1}, "pronouns": {"I": 1}}}, {"content": "I'm not more tired than I used to be.", "word_count": 14, "label": "", "sentence_structure": "Subject鈥揷opula鈥損redicative", "subjective_objective": {"answer": "Subjective", "rationale": "Because the description of what I'm feeling now involves personal subjective perceptions and experiences."}, "sentence_sentiment": {"answer": "Negative", "rationale": "Because \"fears to be tired\" is a negative emotional and physical reaction."}, "language_style": {"answer": "Calm and direct", "rationale": "The language style of the entry is relatively simple and clear, with no excessive emotional qualification, and the problem is expressed in an objective and direct manner, allowing the respondents to understand and answer the question clearly and clearly."}, "time_dimension": {"answer": "Present", "rationale": "According to the word \"now\" in the sentence, the entry is to ask the current situation of the person being tested, and the temporal dimension is now."}, "sentence_type": "Negative declarative sentence", "pos": {"nouns": {"I": 1, "Present": 1, "Previous": 1, "Tired": 1}, "verbs": {"Sensor": 1, "- Yeah": 1, "I feel": 1, "Easy": 1, "Tired": 1}, "adverbs": {"No, it's not": 1}, "numerals": {"0": 1}}}, {"content": "I'm a little more tired now than ever.", "word_count": 10, "label": "", "sentence_structure": "Subject鈥揷opula鈥損redicative", "subjective_objective": {"answer": "Subjective", "rationale": "Because this is a self-perception and cognitive description of the patient, it is a subjective description."}, "sentence_sentiment": {"answer": "Negative", "rationale": "Because the expression \"easiness of fatigue\" is negative."}, "language_style": {"answer": "Tactful and warm", "rationale": ""}, "time_dimension": {"answer": "Present", "rationale": "The time reference point in the entry is \"now\", indicating that the person being tested is experiencing \"more fatigue than ever\" and that this is a description of current behaviour or physical condition."}, "sentence_type": "Negative declarative sentence", "pos": {"nouns": {"I": 1, "Tired": 1, "Present": 1}, "verbs": {"- Yeah": 1, "Easy": 1, "Tired": 1}, "adjectives": {"Previous": 1}, "adverbs": {"More": 1}, "classifiers": {"individual": 1}}}, {"content": "I'm tired of doing anything.", "word_count": 10, "label": "", "sentence_structure": "Subject鈥揷opula鈥損redicative", "subjective_objective": {"answer": "Subjective", "rationale": "Because \"I'm tired of doing anything\" is a personal feeling and experience, a patient's subjective view."}, "sentence_sentiment": {"answer": "Negative", "rationale": "The basis for the judgement: the \"retireness\" in the entry is a negative physiological condition, and the emotional expression is therefore negative."}, "language_style": {"answer": "Calm and direct", "rationale": "The entry is relatively calm and straightforward, does not have too many emotional colours or qualifiers, and asks directly whether the interviewee is often tired, more objective and practical."}, "time_dimension": {"answer": "Present", "rationale": "The entry describes the current state of the person under examination, which is \"retired\" as a specific feeling and experience, consistent with the current time dimension."}, "sentence_type": "Affirmative declarative sentence", "pos": {"nouns": {"Yes": 1, "Tired": 1}, "verbs": {"Do it": 1, "Easy": 1}, "adverbs": {"Both": 1}, "pronouns": {"I": 1}}}, {"content": "I'm so tired I can't do anything.", "word_count": 15, "label": "", "sentence_structure": "Subject鈥搗erb鈥揷omplement", "subjective_objective": {"answer": "Subjective", "rationale": "Because I'm too tired to do anything, I'm describing patients' subjective feelings and experiences. It's the result of patients' self-observation and perception, and therefore subjectively."}, "sentence_sentiment": {"answer": "Negative", "rationale": "Because the entry \"I'm too tired to do anything\" expresses a negative mood and state of affairs, indicating that the interviewees are very tired to do anything."}, "language_style": {"answer": "Tactful and warm", "rationale": "The entry uses moderate language and tone, expressing the other鈥檚 feelings in the current state, and not directly accusing or ordering, making the interviewee more receptive and willing to answer questions. At the same time, this language style also allows for the researchers鈥?interest and respect for the interviewee."}, "time_dimension": {"answer": "Past", "rationale": "The entry \"too tired\" and \"not to do anything\" indicate that it is a symptom or state that has occurred, and therefore the temporal dimension is a past."}, "sentence_type": "Negative declarative sentence", "pos": {"nouns": {"Tired": 1, "Things": 1}, "verbs": {"Too much": 1, "Yes": 1}, "adverbs": {"So": 1, "No, I can't": 1}, "numerals": {"I'm so tired I can't do anything": 1}, "pronouns": {"I": 1}}}, {"content": "I'm as hungry as I used to be.", "word_count": 10, "label": "", "sentence_structure": "Subject鈥揷opula鈥損redicative", "subjective_objective": {"answer": "Objective", "rationale": "Because \"eating\" is an objective fact, it's as good as ever an objective description."}, "sentence_sentiment": {"answer": "Neutral", "rationale": "Because the entry does not have any emotional color."}, "language_style": {"answer": "Calm and direct", "rationale": "According to the statement, the phrase \"my appetite is as good as before\" indicates that the respondents did not feel their appetite changed and their tone was relatively flat, so the language style of the entry could be judged as being calm and straightforward."}, "time_dimension": {"answer": "Present", "rationale": "The entry is an inquiry into the current appetite of the person tested, and does not refer to past or future behavioural or mental changes, and therefore the judgement is now."}, "sentence_type": "Affirmative declarative sentence", "pos": {"nouns": {"A appetite": 1, "Before": 1}, "verbs": {"It's just as good": 1}, "adverbs": {"and": 1}, "numerals": {"0锛?: 1}}}, {"content": "I'm not as hungry as I always am.", "word_count": 12, "label": "", "sentence_structure": "Subject鈥揷opula鈥損redicative", "subjective_objective": {"answer": "Subjective", "rationale": "Because the good appetite is the patient's subjective feelings and experiences."}, "sentence_sentiment": {"answer": "Negative", "rationale": "Because the \"fatal is not as good as usual\" behavior in the entries is negative."}, "language_style": {"answer": "Tactful and warm", "rationale": "The entry is more liberal in its language style. It uses the first person's adjective \"I\" to make the interviewee feel friendly and comfortable, while also expressing a self-expressional description that makes the question more open and easy to answer. Moreover, the expression \"not as good as usual\" is not too direct or too negative, but rather reflects the interviewee's feelings more objectively."}, "time_dimension": {"answer": "Present", "rationale": "The main phrase \"I\" is the current state of affairs, \"not as good as usual\" expresses a different state or situation from the past, so the entry describes the experience and feelings of the testees."}, "sentence_type": "Negative declarative sentence", "pos": {"nouns": {"A appetite": 1, "As usual": 1}, "verbs": {"No, I'm not": 1, "Okay": 1}, "adverbs": {"Present": 1, "That way": 1}, "numerals": {"1锛?: 1}}}, {"content": "I'm a little bit less hungry now.", "word_count": 9, "label": "", "sentence_structure": "Subject鈥搗erb鈥揷omplement", "subjective_objective": {"answer": "Subjective", "rationale": "Because the change of appetite is a patient's subjective feeling and experience."}, "sentence_sentiment": {"answer": "Negative", "rationale": "Because the description of \"difficult\" is negative, suggesting that there may be physical discomfort or emotional depression in an individual."}, "language_style": {"answer": "Calm and direct", "rationale": "The entry follows a simple and straightforward language style, directly asking the other party about the current appetite, without excessive adoration or emotional colour, and with a more calm and objective tone."}, "time_dimension": {"answer": "Present", "rationale": "The entry \"now\" indicates that the questionnaire is about the current state of the person being tested, that is, the state of appetite, and therefore the present."}, "sentence_type": "Negative declarative sentence", "pos": {"nouns": {"A appetite": 1}, "verbs": {"Bad": 1, "More": 1}, "adjectives": {"Present": 1, "Yes": 1}, "classifiers": {"individual": 1}, "pronouns": {"I": 1}}}, {"content": "I'm completely out of appetite.", "word_count": 8, "label": "", "sentence_structure": "Subject鈥搗erb鈥揷omplement", "subjective_objective": {"answer": "Subjective", "rationale": "For total lack of appetite is a subjective feeling and experience, not an objective fact."}, "sentence_sentiment": {"answer": "Negative", "rationale": "Because \"totally no appetite\" is a physical discomfort, and is often considered a negative emotional experience."}, "language_style": {"answer": "Calm and direct", "rationale": "The entry is simple and clear, and the person interviewed is asked whether he or she has an appetite, is not over-represented or emotional, and expresses a more objective and calm expression."}, "time_dimension": {"answer": "Past", "rationale": ""}, "sentence_type": "Negative declarative sentence", "pos": {"nouns": {"A appetite": 1, "Not at all": 1}, "verbs": {"have": 2, "Total": 1}, "adjectives": {"None": 1}, "numerals": {"3": 1}}}, {"content": "I haven't lost any weight recently.", "word_count": 12, "label": "", "sentence_structure": "Subject鈥搗erb鈥揷omplement", "subjective_objective": {"answer": "Objective", "rationale": "The fact that the body condition of the patient is due to the weight loss is an objective fact that can be observed."}, "sentence_sentiment": {"answer": "Neutral", "rationale": "Basis of judgement: The entry simply describes recent changes in weight, without expressing positive or negative feelings, attitudes, behaviours, aims or qualities. It can therefore be judged neutral."}, "language_style": {"answer": "Tactful and warm", "rationale": "The language style of the entry is relatively euphemistic and moderate, using easier terminology and expressions such as \"no significant weight loss\" to avoid direct emphasis or criticism. Such expressions are more acceptable to respondents and contribute to the effective implementation of the questionnaire."}, "time_dimension": {"answer": "Present", "rationale": "The keyword \"not visible recently\" indicates that the question is about the recent state of the subject, and that the act of \"weight loss\" did not occur, so the question is a description of the current state or situation."}, "sentence_type": "Negative declarative sentence", "pos": {"nouns": {"Weight loss": 1}, "verbs": {"No, I'm not": 1, "Obviously": 1}, "adjectives": {"Most recently": 1}, "numerals": {"0": 1}, "pronouns": {"I": 1}}}, {"content": "I lost more than five pounds.", "word_count": 8, "label": "", "sentence_structure": "Subject鈥搗erb鈥揷omplement", "subjective_objective": {"answer": "Objective", "rationale": "Because it is an objective fact that patients can quantify whether weight loss exceeds 5 pounds."}, "sentence_sentiment": {"answer": "Negative", "rationale": "Because \"weight loss of more than 5 pounds\" is usually a negative consequence of physical problems such as malnutrition, disease, etc."}, "language_style": {"answer": "Calm and direct", "rationale": "The entry is based on a specific \"five-pound\" criterion, which is a direct question to the respondent whether the weight has dropped above this threshold, without using a rhetorical or emotional rendering, and is more objective in tone and consistent with the calm and direct linguistic style."}, "time_dimension": {"answer": "Past", "rationale": "The entry is for the purpose of determining the past by asking whether the person tested had lost more than 5 pounds of body weight at a given point in the past."}, "sentence_type": "Affirmative declarative sentence", "pos": {"nouns": {"Weight": 1, "- What": 1}, "verbs": {"Down": 1, "More than": 1, "Yes": 1}, "numerals": {"5": 1}}}, {"content": "I lost more than 10 pounds of weight.", "word_count": 8, "label": "", "sentence_structure": "Subject鈥搗erb鈥揷omplement", "subjective_objective": {"answer": "Objective", "rationale": "Because a weight loss of more than 10 pounds is an objective fact, it is not influenced by the patient ' s subjective attitude."}, "sentence_sentiment": {"answer": "Negative", "rationale": "The loss of weight of more than 10 pounds may cause anxiety or concern for your health."}, "language_style": {"answer": "Calm and direct", "rationale": "This entry describes the physical condition of the person under investigation in an objective and direct manner, which is not accompanied by excessive emotional or rhetorical techniques, and is more authentic."}, "time_dimension": {"answer": "Present", "rationale": "The entry uses the past completion pattern ( \"Down by more than 10 pounds\"), describes an action or state that has been completed and should therefore be judged now."}, "sentence_type": "Affirmative declarative sentence", "pos": {"nouns": {"Weight": 1, "Down": 1, "Ten pounds": 1}, "verbs": {"I": 1, "Super": 1, "Pass": 1}}}, {"content": "I lost over 15 pounds of weight. I'm eating diets to reduce weight.", "word_count": 19, "label": "", "sentence_structure": "Subject鈥搗erb鈥搊bject", "subjective_objective": {"answer": "Subjective", "rationale": "The patient鈥檚 subjective behaviour and decision is that of weight loss of over 15 pounds and diet control. Although this may be caused by some objective factors, such as illness, patients鈥?perception of and treatment of these situations is subjective."}, "sentence_sentiment": {"answer": "Positive", "rationale": "Because the behaviour described in the entry as \"Weight loss\" and \"Weight loss through diet control\" is positive, it is indicated that the interviewees are taking positive action to improve their health."}, "language_style": {"answer": "Calm and direct", "rationale": "So I think the language style of this questionnaire entry is straightforward."}, "time_dimension": {"answer": "Present", "rationale": "The entry describes the current situation and behaviour of the person tested, i.e., control of diet to reduce weight and the result of a weight loss of more than 15 pounds, thus judging the time dimension as now."}, "sentence_type": "Affirmative declarative sentence", "pos": {"nouns": {"Weight": 1, "Diet": 1}, "verbs": {"Down": 1, "Control": 1, "Mitigation": 1}, "adjectives": {"More than": 1}, "numerals": {"3": 1, "15": 1}, "classifiers": {"- What": 2}, "pronouns": {"I": 1}}}, {"content": "Not too much fear of health than I used to.", "word_count": 15, "label": "", "sentence_structure": "Subject鈥揷opula鈥損redicative", "subjective_objective": {"answer": "Subjective", "rationale": "This is because patients are subjectively evaluated and compared with their health status."}, "sentence_sentiment": {"answer": "Neutral", "rationale": "Because the entry is a statement of fact and does not contain any emotional colour."}, "language_style": {"answer": "Tactful and warm", "rationale": "The entry is relatively euphemistic and friendly in language, is in question form, is not expressed too directly or strongly, and is more personal and interactive. At the same time, the entry views the health of the individual in a more objective manner, giving a positive and peaceful sense of peace."}, "time_dimension": {"answer": "Past", "rationale": "The key word \"as compared to the past\" in the entry indicates that the question is to ask the testee about the past and therefore to judge it as the past."}, "sentence_type": "Negative declarative sentence", "pos": {"nouns": {"Health": 1, "Body": 1}, "verbs": {"I'm worried": 1, "- Yeah": 1, "It's not too much": 1}, "adjectives": {"Previous": 1}, "adverbs": {"It's too much": 1}, "pronouns": {"I": 1}}}, {"content": "I'm afraid I have a condition like pain, absinthe and constipation.", "word_count": 17, "label": "", "sentence_structure": "Subject鈥搗erb鈥搊bject", "subjective_objective": {"answer": "Subjective", "rationale": "Because \"care\" is patient subjectivity and attitude."}, "sentence_sentiment": {"answer": "Negative", "rationale": "Because the \"fear\" and the list of physical problems in the entry are negative emotions and conditions, and display negative attitudes."}, "language_style": {"answer": "Calm and direct", "rationale": "The language style of the entry is more objective and straightforward, and there is no excessive emotional or rhetorical approach, but rather a factual presentation of the question to the person under investigation."}, "time_dimension": {"answer": "Present", "rationale": "The basis of the judgement is therefore now."}, "sentence_type": "Affirmative declarative sentence", "pos": {"nouns": {"I": 1, "Body": 1, "Mistake": 1, "Pain": 1, "- Nasty": 1, "Decency": 1}, "verbs": {"I'm worried": 1}, "numerals": {"1": 1}}}, {"content": "I'm worried about my physical problems and I'm in the process of thinking about other issues.", "word_count": 19, "label": "", "sentence_structure": "Subject鈥揷opula鈥損redicative", "subjective_objective": {"answer": "Subjective", "rationale": "Because \"I'm anxious\" is the patient's subjective feeling or attitude."}, "sentence_sentiment": {"answer": "Negative", "rationale": "Because \"sweet\" and \"obstructing me from thinking about other things\" in the entries are negative emotional attitudes."}, "language_style": {"answer": "Calm and direct", "rationale": "The article is more direct, with no excessive rhetorical or emotional rendering. It expresses the subject of questioning whether the other party is able to think because of his health problems."}, "time_dimension": {"answer": "Present", "rationale": "The keyword \"I'm in a hurry\" means the current feeling, \"the physical condition prevents me from thinking about other issues\" means the problems that are facing us, so the entry describes the current behavior or state."}, "sentence_type": "Negative declarative sentence", "pos": {"nouns": {"I": 1, "Body": 1, "Mistake": 1, "Think": 1, "Problem": 1}, "verbs": {"very": 1, "Urgent": 1, "Obstruction": 1, "And": 1, "Think": 1, "have": 1}, "adjectives": {"I'm in a hurry": 1}}}, {"content": "I was so desperate for a physical condition that I couldn't think of anything else.", "word_count": 21, "label": "", "sentence_structure": "Subject鈥搗erb鈥揷omplement", "subjective_objective": {"answer": "Subjective", "rationale": "Because \"very anxious\" and \"not thinking about anything else\" are patients' subjective attitudes and feelings."}, "sentence_sentiment": {"answer": "Negative", "rationale": "Because the description \"very anxious for a body disease, so that nothing else can be thought of\" in the entry expresses a negative emotion and attitude."}, "language_style": {"answer": "Calm and direct", "rationale": "The entry is based on a simple and straightforward language that expresses the subject of asking the other about the urgency of the disease, and there is no excessive emotional rendering or speech writing."}, "time_dimension": {"answer": "Past", "rationale": "The entry describes the feelings and experiences of the testees with the past, and the keyword is \"very anxious\" to \"so much that nothing else\" 鈥?all pointing to what has happened, and thus judging as the past."}, "sentence_type": "Interrogative sentence", "pos": {"nouns": {"Physical diseases": 1, "Things": 1}, "verbs": {"I'm in a hurry": 1, "Think": 1}, "adjectives": {"Very": 1}, "adverbs": {"So": 1}, "numerals": {"3": 1}, "pronouns": {"I": 1}}}, {"content": "I've had no change in my sexuality lately.", "word_count": 12, "label": "", "sentence_structure": "Subject鈥搗erb鈥揷omplement", "subjective_objective": {"answer": "Objective", "rationale": "Because \"no changes in sexuality\" are the current state of the patient's physical condition and are objective facts."}, "sentence_sentiment": {"answer": "Neutral", "rationale": "Because the entry only describes a phenomenon and does not contain any emotional colours."}, "language_style": {"answer": "Calm and direct", "rationale": "The language style of the entry is relatively simple and direct, asking the respondents whether their sexual desires have changed in recent times, without excessive emotional or rhetorical styles, which are typical of a calm and direct style."}, "time_dimension": {"answer": "Present", "rationale": ""}, "sentence_type": "Negative declarative sentence", "pos": {"nouns": {"Sexuality": 1, "Change": 1}, "verbs": {"No, I'm not": 1, "have": 1}, "adverbs": {"Most recently": 1}}}, {"content": "I'm less sexually active than ever.", "word_count": 9, "label": "", "sentence_structure": "Subject鈥揷opula鈥損redicative", "subjective_objective": {"answer": "Subjective", "rationale": "Because sexual desire is the subjective feeling and experience of the patient, it is related to the patient ' s own mental state, physical health, etc."}, "sentence_sentiment": {"answer": "Negative", "rationale": "Because \"sexuality is less than ever\" in the entry expresses a negative emotion and attitude, indicating that there are problems with sexual function."}, "language_style": {"answer": "Tactful and warm", "rationale": "The entry raises questions in a more subtle way, avoiding direct questioning of each other's sexual life, expressing them in a more moderate manner and respecting personal privacy. At the same time, \"less than ever\" implies that this is a question of change or comparison, rather than a simple yes or no answer, which would help to obtain more real and specific information."}, "time_dimension": {"answer": "Present", "rationale": "The entry asks the testee about sexual desires at a certain point in time and can therefore be judged as \"now\"."}, "sentence_type": "Negative declarative sentence", "pos": {"nouns": {"Sexuality": 1, "Previous": 1}, "verbs": {"Almost": 1}}}, {"content": "Now my sexual appetite is much lower than ever.", "word_count": 14, "label": "", "sentence_structure": "Subject鈥搗erb鈥揷omplement", "subjective_objective": {"answer": "Subjective", "rationale": "Because sexual desire is a subjective feeling, it is viewed from the patient ' s subjective perspective."}, "sentence_sentiment": {"answer": "Negative", "rationale": "Because the \"sexual deviance\" in the entry is negative."}, "language_style": {"answer": "Tactful and warm", "rationale": "The entry is more personal and interactive when it is spoken in a gentle and inviting manner, rather than in direct statements or orders. At the same time, the issue avoids words that may cause discomfort or embarrassment, and instead presents the content of the investigation in a more euphemism, making it easier for the respondents to be relaxed and open to reply."}, "time_dimension": {"answer": "Present", "rationale": "The entry is about the current state of the testee, and the time dimension is now judged. The keyword \"now\" indicates the current state or behaviour. \"My sexual desire is much lower than ever.\" It shows that it is a description of the current situation, not a speculation of the future or the past.\""}, "sentence_type": "Negative declarative sentence", "pos": {"nouns": {"Sexuality": 1, "Previous": 1}, "verbs": {"Declining": 1}, "adjectives": {"Many": 1}}}, {"content": "I've lost my sexual appetite.", "word_count": 8, "label": "", "sentence_structure": "Subject鈥揷opula鈥損redicative", "subjective_objective": {"answer": "Subjective", "rationale": "Because sexual desire is a subjective feeling and desire of a person, an inherent experience and a psychological state of the individual, the answer to this questionnaire should be subjective."}, "sentence_sentiment": {"answer": "Negative", "rationale": "Because \"total loss of sexuality\" is negative."}, "language_style": {"answer": "Calm and direct", "rationale": "The entry directly asked whether the person surveyed had any lack of sexual desire, without using a rhetorical or emotional correction, and with a more objective and cold tone."}, "time_dimension": {"answer": "Past", "rationale": ""}, "sentence_type": "Negative declarative sentence", "pos": {"nouns": {"Sexuality": 1}, "verbs": {"Loss": 1, "Total": 1}, "numerals": {"3": 1}}}]}

{"questionnaire_title": "Minnesota's Face Personal Test - MMPI", "questionnaire_category": "Mental health", "item_count": 567, "emotion_shift_overall": "From positive to negative", "language_style_overall": "Neutral", "items": [{"content": "I like to read magazines in mechanics.", "word_count": 11, "label": "", "sentence_structure": "Subject鈥搗erb鈥搊bject", "subjective_objective": {"answer": "Subjective", "rationale": "Because \"likes to read mechanical magazines\" is patient's subjective attitude and preferences."}, "sentence_sentiment": {"answer": "Neutral", "rationale": "Because the entry is not of a clear emotional nature, it merely presents a fact."}, "language_style": {"answer": "Tactful and warm", "rationale": "This is a more euphemistic expression, which does not ask the other side鈥檚 interests too directly, but rather leads the interviewee to think and answer in a question-and-answer manner."}, "time_dimension": {"answer": "Present", "rationale": ""}, "sentence_type": "Affirmative declarative sentence", "pos": {"nouns": {"Magazines": 1, "Mechanical": 1}, "verbs": {"Yeah": 1, "Look": 1}}}, {"content": "I'm very hungry.", "word_count": 6, "label": "", "sentence_structure": "Subject鈥揷opula鈥損redicative", "subjective_objective": {"answer": "Subjective", "rationale": "Because appetite is a subjective feeling or evaluation."}, "sentence_sentiment": {"answer": "Positive", "rationale": "Because \"good appetite\" is a positive description."}, "language_style": {"answer": "Calm and direct", "rationale": "The questionnaire entry is very simple and straightforward, and is simply asking the respondents whether they are hungry and do not use any accoutrements or emotional colours, so that the language style is a straightforward type of calm."}, "time_dimension": {"answer": "Present", "rationale": "The entry describes the person's current state of health, which is \"good appetite\", and therefore the judgment is now."}, "sentence_type": "Affirmative declarative sentence", "pos": {"verbs": {"have": 1, "Talk": 1}, "adjectives": {"Okay": 1}}}, {"content": "3. When I woke up in the morning, I felt asleep and awake.", "word_count": 20, "label": "", "sentence_structure": "Subject鈥揷opula鈥損redicative", "subjective_objective": {"answer": "Subjective", "rationale": "Because \"I feel sleep is good, awake\" is a personal feeling and subjective experience. Although it may be due to objective factors, such as good sleep quality, it is subjective in itself."}, "sentence_sentiment": {"answer": "Positive", "rationale": "Because the description \"sleep, awake\" in the entry is positive."}, "language_style": {"answer": "Tactful and warm", "rationale": "Warm and warm."}, "time_dimension": {"answer": "Present", "rationale": "\"I rise in the morning\" means that the testee is in the present situation, and \"mostly sleeps well, awake\" is also a description of the state of affairs. So, the time dimension of the article is now."}, "sentence_type": "Affirmative declarative sentence", "pos": {"nouns": {"Morning": 1, "Time": 1, "Sleep": 1, "Brain": 1, "Wake up": 1}, "verbs": {"Get up": 1, "I think": 1, "Enough": 1, "You're clear": 1}, "adverbs": {"Mostly": 1}}}, {"content": "I think I would like the work of the librarian.", "word_count": 14, "label": "", "sentence_structure": "Subject鈥搗erb鈥搊bject", "subjective_objective": {"answer": "Subjective", "rationale": "Because \"like\" represents the patient's personal feelings and attitudes and is a subjective approach."}, "sentence_sentiment": {"answer": "Positive", "rationale": "Because the attitude in the entry is \"want to like\" and it expresses positive feelings."}, "language_style": {"answer": "Tactful and warm", "rationale": "The entry expresses the potential interest or expectation of the interviewees for the work of the librarian in a more subtle and implicit way, without any direct or negative affirmation. Such expression is more comfortable and acceptable to the interviewees and more able to channel their real thoughts and feelings."}, "time_dimension": {"answer": "Future", "rationale": "The entry expresses the expectations and visions of the subject for a future situation, the key words being \"I want\" and \"I want\" and all point to situations that have not yet occurred and that may occur in the future."}, "sentence_type": "Affirmative declarative sentence", "pos": {"nouns": {"Work": 1, "Librarian": 1}, "verbs": {"Yes": 2, "Yeah": 1}, "adverbs": {"Yes": 1}, "numerals": {"4锛?: 1}, "pronouns": {"I": 1}}}, {"content": "5. I was easily awakened.", "word_count": 7, "label": "", "sentence_structure": "Subject鈥揷opula鈥損redicative", "subjective_objective": {"answer": "Subjective", "rationale": "Because the susceptibility to awakening is a subjective feeling and experience of the patient, it is a subjective examination."}, "sentence_sentiment": {"answer": "Negative", "rationale": "Because the description of \"always woken up\" in the entry is negative."}, "language_style": {"answer": "Tactful and warm", "rationale": "The entry is more personal and interactive when it is spoken in a gentle and inviting manner, rather than in direct statements or orders. At the same time, \"frightful to awakening\" is also a more neutral expression, without overemphasis on personal problems or negative emotions. The language style of the article should therefore be a warmer type."}, "time_dimension": {"answer": "Present", "rationale": ""}, "sentence_type": "Affirmative declarative sentence", "pos": {"verbs": {"Wake up": 1}, "adjectives": {"Easy": 1}}}, {"content": "I like to read the news of crime in the newspapers.", "word_count": 12, "label": "", "sentence_structure": "Subject鈥搗erb鈥搊bject", "subjective_objective": {"answer": "Subjective", "rationale": "Because I like being subjective."}, "sentence_sentiment": {"answer": "Negative", "rationale": "Because the \"like crime news\" act in the entry can trigger negative emotions and attitudes."}, "language_style": {"answer": "Tactful and warm", "rationale": ""}, "time_dimension": {"answer": "Present", "rationale": "The entry is for the present taste or preference of the testee, and the time dimension is now judged."}, "sentence_type": "Affirmative declarative sentence", "pos": {"nouns": {"Newspapers": 1, "Public information": 1, "Crime": 1}, "verbs": {"Yeah": 1, "Look": 1}, "numerals": {"6": 1}}}, {"content": "My hands and feet are often warm.", "word_count": 10, "label": "", "sentence_structure": "Subject鈥揷opula鈥損redicative", "subjective_objective": {"answer": "Objective", "rationale": "Because the warmness of the hands and feet is an objective state, it is not necessary to determine the patient ' s subjective judgment."}, "sentence_sentiment": {"answer": "Positive", "rationale": "Because \"heat hands and feet\" is a feeling of physical comfort, which is usually considered positive emotional experience."}, "language_style": {"answer": "Calm and direct", "rationale": "The entry directly asks the interviewees whether their hands and feet are often warm, with no excessive adoration or emotional colours, and the language style is more objective and calm."}, "time_dimension": {"answer": "Present", "rationale": "The keyword \"often warm\" indicates that it is a statement describing the current state and therefore falls within the context of the present state of affairs."}, "sentence_type": "Affirmative declarative sentence", "pos": {"nouns": {"Hands and feet": 2, "Warm": 1}, "verbs": {"be": 1}, "adjectives": {"Regular": 1}, "numerals": {"7": 1}, "pronouns": {"I": 1}}}, {"content": "8. My daily life is filled with things that interest me.", "word_count": 18, "label": "", "sentence_structure": "Subject鈥揷opula鈥損redicative", "subjective_objective": {"answer": "Subjective", "rationale": "Because the entry asks about the patient ' s personal feelings and views, i.e. whether the patient believes that they are filled with many interesting things in their daily lives, this is a subjective assessment."}, "sentence_sentiment": {"answer": "Positive", "rationale": "Because the expression \"filled with things that interest me\" is positive."}, "language_style": {"answer": "Tactful and warm", "rationale": "This is a more euphemistic and encouraging way of speaking, asking the respondents whether they are well-interested in their daily lives, rather than ordering or evaluating them directly."}, "time_dimension": {"answer": "Present", "rationale": "The phrase \"my daily life\" and \"pleasing\" can be judged to be asking the testee's current state or feelings, so the time dimension is now."}, "sentence_type": "Affirmative declarative sentence", "pos": {"nouns": {"Daily life": 1, "Life": 1, "Things": 1}, "verbs": {"Fill": 1, "鈾?And I'm gonna be 鈾?: 1}, "adjectives": {"It's what interests me": 1}, "adverbs": {"I": 1, "Day": 1}, "pronouns": {"Here": 1}}}, {"content": "9. I have the same ability to work (learning) as before.", "word_count": 17, "label": "", "sentence_structure": "Subject鈥搗erb鈥揷omplement", "subjective_objective": {"answer": "Objective", "rationale": "Because `the ability to work (learning) now' is `a self-evaluation or perception of patients', it is a subjective approach. At the same time, it can be assessed by actual performance, can be observed and judged by others, and therefore has some objectivity."}, "sentence_sentiment": {"answer": "Neutral", "rationale": "Because the entry describes an objective fact and does not involve emotional colour."}, "language_style": {"answer": "Tactful and warm", "rationale": "The entry uses a more euphemism, which is \"almost\" to express the current ability to work and has not changed much compared to before. This expression avoids too direct or aggressive acclaim, which is more conducive to creating a good atmosphere of communication and to the perception of respect and understanding among the interviewees."}, "time_dimension": {"answer": "Present", "rationale": "The entry \"now\" means in time that the current situation or state is in question."}, "sentence_type": "Affirmative declarative sentence", "pos": {"nouns": {"Work": 1, "Learning": 1, "Capacity": 2, "Once upon a time": 1}, "verbs": {"be": 1, "Almost": 1}, "adverbs": {"Present": 1, "Once upon a time": 1}, "numerals": {"9锛?: 1}, "pronouns": {"I": 1}}}, {"content": "10. My throat is always like a block.", "word_count": 17, "label": "", "sentence_structure": "Subject鈥揷opula鈥損redicative", "subjective_objective": {"answer": "Subjective", "rationale": "Because it's the patient's subjective feeling and description."}, "sentence_sentiment": {"answer": "Negative", "rationale": "Because the feeling in the entry, \"There's something stuck in the throat,\" is usually a symptom of physical discomfort or disease, and it's emotionally negative."}, "language_style": {"answer": "Tactful and warm", "rationale": "The entry uses a more euphemistic and moderate formulation, avoiding direct negative evaluation or accusations. At the same time, using vague terms such as \"like\" gives the respondents some space and opportunity to explain themselves, reflecting respect for and concern for the respondents."}, "time_dimension": {"answer": "Present", "rationale": "The keyword is \"my throat,\" which indicates that it is the person who was tested in the moment's feelings and state; and the words \"like\" and \"yes\" also indicate that it is a subjective, sensory description. So this entry should be judged as \"now\"."}, "sentence_type": "Affirmative declarative sentence", "pos": {"nouns": {"Throat": 1, "Something": 1}, "verbs": {"have": 1, "Blocked": 1, "Like": 1}, "numerals": {"Ten": 1, "One": 1}}}, {"content": "11. A person should know his or her dreams and receive guidance and warning.", "word_count": 22, "label": "", "sentence_structure": "Subject鈥搗erb鈥搊bject", "subjective_objective": {"answer": "Subjective", "rationale": "Because \"should\" and \"should be guided and warned\" are patients' subjective attitudes and expectations, knowing their dreams is an act or experience of a person and is therefore objective."}, "sentence_sentiment": {"answer": "Positive", "rationale": "Because the entry emphasizes the importance of self-exploration, it encourages individuals to be inspired and warned through their dreams, and has a positive emotional tone."}, "language_style": {"answer": "Tactful and warm", "rationale": "The language style of the questionnaire entry is relatively euphemistic, as it uses the invitation tone to guide respondents to think about their dreams and to extract inspiration from them, rather than direct orders or statements. Moreover, the sentence emphasizes the importance of self-awareness and self-discovery, which is also reflected in a positive and encouraging attitude."}, "time_dimension": {"answer": "Future", "rationale": "The entry \"A person should know his dream\" offers an expectation or suggestion that people may seek to understand and learn from the dream in the future. Moreover, \"and get guidance and warning from\" suggests that future behaviour or decision-making may be affected by the dream. Thus, the entry is a time dimension for the future."}, "sentence_type": "Affirmative declarative sentence", "pos": {"nouns": {"person": 1, "Dreams": 1, "Guidance": 1, "Warning": 1}, "verbs": {"Reactions": 1, "Go on": 1, "Roger that": 1, "Okay": 1, "Got it": 1}, "adverbs": {"I think so": 1, "From": 1}, "numerals": {"One": 2, "Ten": 1}, "classifiers": {"individual": 1}, "pronouns": {"self": 1}}}, {"content": "12. I like detective novels or mythical novels.", "word_count": 12, "label": "", "sentence_structure": "Subject鈥搗erb鈥搊bject", "subjective_objective": {"answer": "Subjective", "rationale": "Because \"like\" is a subjective attitude or personal preference, the view of this questionnaire entry is subjective."}, "sentence_sentiment": {"answer": "Neutral", "rationale": "Because the entry merely expresses a personal preference and does not involve any emotional colour."}, "language_style": {"answer": "Tactful and warm", "rationale": "The entry is more personal and interactive when it is spoken in a gentle and inviting manner, rather than in direct statements or orders. At the same time, the question is not one of pressure or judgment on the respondent, but of simply asking him about their reading preferences, which is also a more euphemism."}, "time_dimension": {"answer": "Present", "rationale": "The entry expresses the current interest of the testee, and the keyword is \"like\", which indicates that the act has occurred and still exists, so it is now judged."}, "sentence_type": "Affirmative declarative sentence", "pos": {"nouns": {"Detective novels": 1, "Mystery novels": 1}, "verbs": {"Yeah": 1}, "numerals": {"12": 1}}}, {"content": "I have always worked under intense circumstances.", "word_count": 13, "label": "", "sentence_structure": "Subject鈥揷opula鈥損redicative", "subjective_objective": {"answer": "Subjective", "rationale": "Because \"stretch\" is a term for the patient's mental state, reflecting the patient's subjective feelings about work."}, "sentence_sentiment": {"answer": "Negative", "rationale": "Because the \"stretch\" in the entry is negative."}, "language_style": {"answer": "Calm and direct", "rationale": "The entry is a simple and clear statement that directly asks the interviewees whether they are often nervous at work, and that they do not use over-modified language or emotional expressions, so that they can be judged as \"cool and direct \" language styles."}, "time_dimension": {"answer": "Present", "rationale": "The entry asks about the current working state and emotional feelings of the testee, and therefore the time dimension is now judged."}, "sentence_type": "Affirmative declarative sentence", "pos": {"nouns": {"Situation": 1, "Work": 1}, "verbs": {"Always": 1, "Nervous": 1, "Down": 1, "Work": 2}, "adjectives": {"Nervous": 1}, "adverbs": {"very": 1}}}, {"content": "14. I have at least one or two diarrhea per month.", "word_count": 13, "label": "", "sentence_structure": "Subject鈥搗erb鈥揷omplement", "subjective_objective": {"answer": "Objective", "rationale": "The basis for the determination: the entry is to ask whether the patient has periodic symptoms of diarrhoea, which are an objective fact of the patient ' s experience, and the entry is therefore examined from an objective perspective."}, "sentence_sentiment": {"answer": "Negative", "rationale": "Because \"tummy diarrhea\" is a condition of physical discomfort, it is often seen as a negative emotion or experience."}, "language_style": {"answer": "Calm and direct", "rationale": "The entry describes a specific physical condition of the person interviewed in a concise and clear language, which is not overly adour or emotional."}, "time_dimension": {"answer": "Present", "rationale": "The entry describes the testee鈥檚 current behaviour, i.e., they experience diarrhea once or more a month. This behaviour is ongoing and does not involve past or future situations. The temporal dimension of the article should therefore be judged as 鈥渘ow鈥?"}, "sentence_type": "Affirmative declarative sentence", "pos": {"nouns": {"Month": 1, "Number of times": 2, "I'm having a stomach attack": 1}, "verbs": {"have": 1}, "adjectives": {"At least": 1}, "adverbs": {"One, two": 1}, "numerals": {"One": 1, "Two": 1}}}, {"content": "15. Occasionally, I think of something that is so bad that I cannot say.", "word_count": 16, "label": "", "sentence_structure": "Subject鈥搗erb鈥揷omplement", "subjective_objective": {"answer": "Subjective", "rationale": "Because \"thinking\" is a personal process of inner activity and thinking, and it's a patient's subjective feeling and experience."}, "sentence_sentiment": {"answer": "Negative", "rationale": "Because the \"bad things\" in the entry are negative."}, "language_style": {"answer": "Tactful and warm", "rationale": "The entry is more proficient and interactive when it is spoken in a gentle and inviting manner, rather than in direct statements or orders. At the same time, the tone of the entry is neutral, not too much negative emotional, and is better suited for psychological questionnaires."}, "time_dimension": {"answer": "Present", "rationale": "The questionnaire entry asks whether the person tested had a certain psychological state or behaviour at some point, i.e. \"sometimes something that is so bad that it is not clear that the dimension of time points to the past, present or future and therefore it is judged to be the present."}, "sentence_type": "Affirmative declarative sentence", "pos": {"nouns": {"Yes": 1, "Questionnaire": 1, "Entry": 1}, "verbs": {"Yes": 1, "Say it": 1}, "adjectives": {"Bad": 1, "Out": 1, "mouth": 1, "Sometimes": 1}, "numerals": {"15": 1}}}, {"content": "16. I am convinced that life is cruel to me.", "word_count": 11, "label": "", "sentence_structure": "Subject鈥揷opula鈥損redicative", "subjective_objective": {"answer": "Subjective", "rationale": "This is because of the patient ' s personal convictions and views and is subjective."}, "sentence_sentiment": {"answer": "Negative", "rationale": "Because the word \"cruel\" is usually considered a negative emotional word."}, "language_style": {"answer": "Calm and direct", "rationale": "The language style of the entry is a calm and straightforward one. It clearly expresses the view of the subject of the survey about life, without using euphemism or an implicit expression. This straightforward expression helps the researcher to better understand the views and emotional state of the person under investigation."}, "time_dimension": {"answer": "Present", "rationale": "The keyword \"I am convinced\" indicates that it is a belief or attitude of the testee at present, and that the temporal dimension is now judged."}, "sentence_type": "Negative declarative sentence", "pos": {"nouns": {"Life": 1, "I": 2, "Cruel": 1}, "verbs": {"be": 1, "Yes": 1}, "adjectives": {"Deep": 1}, "adverbs": {"I'm sure of that": 1}}}, {"content": "My father was a good man.", "word_count": 9, "label": "", "sentence_structure": "Subject鈥揷opula鈥損redicative", "subjective_objective": {"answer": "Subjective", "rationale": "Because \"my father is a good man\" is a personal opinion and assessment of the father by the interviewees, and is subjective."}, "sentence_sentiment": {"answer": "Positive", "rationale": "Because the expression \"my father is a good man\" is positive."}, "language_style": {"answer": "Tactful and warm", "rationale": "The entry responds positively to the assessment of the father as a good person, which is softer and more positive, making dialogue more friendly and comfortable. At the same time, the expression \"one good person\" is somewhat open-minded, allowing the person questioned more space to speak and think."}, "time_dimension": {"answer": "Past", "rationale": "So I cannot classify it as past, present or future."}, "sentence_type": "Affirmative declarative sentence", "pos": {"nouns": {"I": 1, "Father": 1, "person": 1, "Good man": 1}, "verbs": {"be": 1}, "adjectives": {"One": 1}, "numerals": {"17锛?: 1}}}, {"content": "18. I have few problems with toilet failure.", "word_count": 11, "label": "", "sentence_structure": "Subject鈥搗erb鈥揷omplement", "subjective_objective": {"answer": "Objective", "rationale": "The problem is that the problem of frequent inaccessibility is the patient ' s objective experience."}, "sentence_sentiment": {"answer": "Neutral", "rationale": "Because the entry describes a physical condition and does not contain any apparent emotional tendencies."}, "language_style": {"answer": "Calm and direct", "rationale": "This entry uses a direct and concise language style, explicitly asking the respondents whether they have constipated information, and there is no excessive emotional or rhetorical approach."}, "time_dimension": {"answer": "Present", "rationale": "The phrase \"very little\" denotes low frequency behaviour, while \"incomprehensible problems\" describes a past symptom or state, but does not mean that it is continuing. The entry is therefore a question of the current situation of the person being tested, the temporal dimension being judged as now."}, "sentence_type": "Negative declarative sentence", "pos": {"nouns": {"Shit": 2, "It's not working": 1, "Mistake": 1}, "verbs": {"I": 1, "have": 1, "Very few": 1, "All": 1}, "adverbs": {"very": 1, "Less": 1}, "numerals": {"18": 1, "2": 1}, "classifiers": {"individual": 1}}}, {"content": "When I do a new job, I like to be told who I should be close to.", "word_count": 24, "label": "", "sentence_structure": "Subject鈥搗erb鈥搊bject", "subjective_objective": {"answer": "Subjective", "rationale": "Because it's the patient's subjective attitude and thoughts."}, "sentence_sentiment": {"answer": "Neutral", "rationale": "The entry is not an expression of a clear emotional tendency or attitude."}, "language_style": {"answer": "Tactful and warm", "rationale": "The entry is more gentle in its language style, using a question-and-answer expression and using words such as \"always\" and \"like\" to convey positive emotions. At the same time, the issue is not directed or evaluated by the interviewee, but rather questions in a friendly and open manner, making the interviewee more willing to share his thoughts and feelings."}, "time_dimension": {"answer": "Present", "rationale": "The entry describes a customary act of the testee, in which the person who started a new job seeks guidance and advice from others."}, "sentence_type": "Affirmative declarative sentence", "pos": {"nouns": {"Work": 1, "person": 1, "Things": 1}, "verbs": {"Fuck": 1, "Tell": 1, "Answer": 1, "Near": 1}, "adverbs": {"Total": 1}, "numerals": {"One": 1}, "pronouns": {"I": 1}}}, {"content": "My sex life is satisfactory.", "word_count": 9, "label": "", "sentence_structure": "Subject鈥揷opula鈥損redicative", "subjective_objective": {"answer": "Subjective", "rationale": "Because sex satisfaction is a very subjective feeling, different people have different standards and expectations of sex satisfaction. So, \"my sex satisfaction\" is a patient's subjective approach."}, "sentence_sentiment": {"answer": "Positive", "rationale": "Because the entry expresses a sense of satisfaction."}, "language_style": {"answer": "Tactful and warm", "rationale": "The entry is a more euphemistic way of asking respondents about their satisfaction with their sexual life, without too direct or offensive language, and is more acceptable and responsive."}, "time_dimension": {"answer": "Present", "rationale": "There is no clear past or future time mark in the sentence, but it describes the current situation and can therefore be judged as the present."}, "sentence_type": "Affirmative declarative sentence", "pos": {"nouns": {"Sex": 1, "Satisfied": 1}, "verbs": {"be": 1}}}, {"content": "21. Sometimes I want to leave home.", "word_count": 9, "label": "", "sentence_structure": "Subject鈥損redicate", "subjective_objective": {"answer": "Subjective", "rationale": "Because \"very much wants to leave home\" is the patient's subjective feeling and thoughts."}, "sentence_sentiment": {"answer": "Negative", "rationale": "Because the \"very much wanted to leave home\" act in the entry is negative."}, "language_style": {"answer": "Calm and direct", "rationale": "The language style of the entry is \"cool and direct.\" It asks the interviewee whether he or she has tried to leave his or her home, not using excessive rhetoric or emotional accoutrements, but in an objective and direct manner. This style usually applies to more serious or clearly answered questions."}, "time_dimension": {"answer": "Present", "rationale": "Now the entry describes a current idea or feeling that \"sometimes I want to leave home\" and can therefore be judged by its time dimension."}, "sentence_type": "Affirmative declarative sentence", "pos": {"nouns": {"Time": 1, "I": 1, "Home": 1}, "verbs": {"Very": 1, "Yes": 1, "Leave": 1}, "adverbs": {"Sometimes": 1}}}, {"content": "Sometimes I cry and laugh, and I can't even control myself.", "word_count": 18, "label": "", "sentence_structure": "Subject鈥搗erb鈥揷omplement", "subjective_objective": {"answer": "Subjective", "rationale": "Because the description of \"incontrol\" reflects the emotional experience and subjective feelings of the patient."}, "sentence_sentiment": {"answer": "Negative", "rationale": "Because the \"cry and laugh\" act in the entry is unstable and negative."}, "language_style": {"answer": "Tactful and warm", "rationale": "The entry uses a soft, invitation-style tone for dialogue rather than direct statements or orders, which is more personal and interactive. At the same time, the sentence is simpler and less complex, and easier to understand and answer by the interviewees."}, "time_dimension": {"answer": "Present", "rationale": "Now in the entry, the descriptions of \"sometimes\" and \"uncontrollable\" all refer to the current, present situation, and therefore to the present."}, "sentence_type": "Affirmative declarative sentence", "pos": {"nouns": {"Line": 2, "Tears": 1, "Smile": 1}, "verbs": {"Yes": 1, "Cry": 1, "Smile": 1, "Company": 1, "Control": 1}, "adjectives": {"No, I can't": 1}, "adverbs": {"Sometimes": 1}, "pronouns": {"I": 1}}}, {"content": "I am troubled by the disgusting and vomiting problems.", "word_count": 12, "label": "", "sentence_structure": "Subject鈥搗erb鈥搊bject", "subjective_objective": {"answer": "Subjective", "rationale": ""}, "sentence_sentiment": {"answer": "Negative", "rationale": "Because the description \"the sickness of nausea and vomit makes me bitter\" shows a negative emotional attitude."}, "language_style": {"answer": "Calm and direct", "rationale": "The language style of the entry is more direct, not using too many euphemisms or rhetorical techniques, but rather a direct presentation of the subject, asking if the other side is troubled by the problems of nausea and vomiting. This language style is clearer and more conducive to obtaining accurate answers."}, "time_dimension": {"answer": "Present", "rationale": "The entry is for the purpose of asking the testee whether he is currently sick and vomiting, and the time dimension is now judged."}, "sentence_type": "Affirmative declarative sentence", "pos": {"nouns": {"Disgusting and vomiting": 1, "Mistake": 1, "Trouble": 1}, "verbs": {"鈾?And I'm gonna be 鈾?: 1}, "numerals": {"23": 1}}}, {"content": "24. No one seems to know me.", "word_count": 10, "label": "", "sentence_structure": "Subject鈥揷opula鈥損redicative", "subjective_objective": {"answer": "Subjective", "rationale": "Because \"no one seems to know me\" is the patient's subjective feeling and judgment, reflecting the person's sense of loneliness and the need to be understood."}, "sentence_sentiment": {"answer": "Negative", "rationale": "Because the expression \"no one knows me\" in the entry expresses a lonely, neglected feeling, which is a negative emotion."}, "language_style": {"answer": "Tactful and warm", "rationale": "The entry leads the respondents to express their feelings through a euphemism and compassion, avoiding the resistance that too direct or sharp language can trigger, and the feeling of greater kinship and peace and accessibility."}, "time_dimension": {"answer": "Present", "rationale": "The entry is about the current feelings and experiences of the testee, and the description \"near anyone knows me\" refers to the current situation, so the temporal dimension is now."}, "sentence_type": "Negative declarative sentence", "pos": {"nouns": {"person": 1, "Understood": 1}, "verbs": {"Nope": 1, "have": 1, "Roger that": 1}, "adjectives": {"Not one": 1}, "adverbs": {"Seems like": 1}, "pronouns": {"I": 1}}}, {"content": "I want to be a singer.", "word_count": 8, "label": "", "sentence_structure": "Subject鈥搗erb鈥搊bject", "subjective_objective": {"answer": "Subjective", "rationale": "Because \"want to be a singer\" is the patient's personal will and expectations, and it's subjective."}, "sentence_sentiment": {"answer": "Neutral", "rationale": "Because the entry does not have any emotional color."}, "language_style": {"answer": "Tactful and warm", "rationale": "The language style of the entry is more euphemistic, using the word \"want\" to express a personal desire rather than a goal to be achieved, and softer, while leaving room for reflection and choice."}, "time_dimension": {"answer": "Future", "rationale": "The entry describes a future goal or aspiration, and \"want to be\" indicates that it is a state of mental expectation and aspiration, and therefore the temporal dimension is the future."}, "sentence_type": "Affirmative declarative sentence", "pos": {"nouns": {"Singers": 1}, "verbs": {"I want to be": 1}}}, {"content": "26. When I was in a difficult situation, I felt it would be best to keep my mouth shut.", "word_count": 18, "label": "", "sentence_structure": "Subject鈥搗erb鈥搊bject", "subjective_objective": {"answer": "Subjective", "rationale": "It is better not to speak than to be subjective."}, "sentence_sentiment": {"answer": "Negative", "rationale": "Basis of judgement: The entry \"preferably not to speak\" expresses a negative response and attitude."}, "language_style": {"answer": "Calm and direct", "rationale": "The basis for the judgement: The language of the article is relatively simple and not too much rhetorical or emotional. It is more objective in its tone to ask whether the other party is inclined to remain silent in the face of a difficult situation."}, "time_dimension": {"answer": "Present", "rationale": "The word \"wish best\" is \"takes the usual current state of affairs, which means that it is a state of custom or thought, and \"shut-up\" describes what is most common in this case, so the entry asks about the attitudes and behaviour of the subject."}, "sentence_type": "Negative declarative sentence", "pos": {"nouns": {"Time": 1, "The dilemma": 1}, "verbs": {"I think": 1, "Open up": 1, "I'd better": 1, "be": 1}, "adjectives": {"Difficulties": 1, "of": 2}, "adverbs": {"not": 1, "When": 1}, "numerals": {"26": 1}, "pronouns": {"I": 1}}}, {"content": "Sometimes I feel like I'm possessed by ghosts.", "word_count": 12, "label": "", "sentence_structure": "Subject鈥搗erb鈥揷omplement", "subjective_objective": {"answer": "Subjective", "rationale": "Because the experience of feeling haunted is a subjective feeling and perception of a human being, not an objective reality."}, "sentence_sentiment": {"answer": "Negative", "rationale": "Because the expression \"ghosty\" in the entry is emotional in the sense of fear and anxiety."}, "language_style": {"answer": "Tactful and warm", "rationale": "The entry is more personal and interactive when it is spoken in a gentle and inviting manner, rather than in direct statements or orders. Moreover, it uses a more open and inclusive language, which allows respondents to feel that their feelings are accepted and respected, not challenged or denied."}, "time_dimension": {"answer": "Present", "rationale": "The entry is for the current feelings or experiences of the testee, and the time dimension is now judged."}, "sentence_type": "Affirmative declarative sentence", "pos": {"nouns": {"Ghost": 1, "Body": 1}, "verbs": {"I think": 1, "Attach": 1}, "adverbs": {"Sometimes": 1}, "numerals": {"27": 1}}}, {"content": "When someone else had me, I felt that there was only opportunity to take revenge, and that was for sure.", "word_count": 25, "label": "", "sentence_structure": "Subject鈥搗erb鈥搊bject", "subjective_objective": {"answer": "Subjective", "rationale": "Because \"the feeling of revenge only comes with the chance\" is the patient's psychological thought and opinion. Although this idea may affect the patient's decision-making and behaviour, it is still subjective."}, "sentence_sentiment": {"answer": "Negative", "rationale": "Because the attitude and behaviour in the entry, \"I feel like I should get revenge only at the opportunity,\" is negative."}, "language_style": {"answer": "Calm and direct", "rationale": "The language style of the entry is a calm and direct type. It clearly expresses perceptions and attitudes about each other's behaviour, is not overly emotional or acrimony, and is more objective in tone."}, "time_dimension": {"answer": "Present", "rationale": ""}, "sentence_type": "Negative declarative sentence", "pos": {"nouns": {"person": 1, "Revenge": 1, "Feel": 1, "Opportunities": 1, "Of course": 1}, "verbs": {"If": 1, "I think": 1, "Reactions": 1, "be": 1}, "adverbs": {"When": 1, "Just": 1}, "numerals": {"28": 1}, "pronouns": {"I": 1}}}, {"content": "29. I have stomach problems, which cause me distressingly, many times a week.", "word_count": 21, "label": "", "sentence_structure": "Subject鈥搗erb鈥揷omplement", "subjective_objective": {"answer": "Subjective", "rationale": "Because this is a symptom and a problem that the patient feels for himself, it is a subjective experience."}, "sentence_sentiment": {"answer": "Negative", "rationale": "Because the entry describes \"a problem of excessive stomach acid, which makes me suffer a few times a week\", which shows that the act is negative."}, "language_style": {"answer": "Tactful and warm", "rationale": "The entry uses a more euphemistic expression, avoiding too direct or negative language. And it resonates with the emotional expression \"making me upset\" to make people feel like the same mind and concern as the designer. Such language makes the respondents more willing to respond and to reflect their situation more honestly."}, "time_dimension": {"answer": "Past", "rationale": "\"A problem with too much stomach acid\" and \"a few times a week\" describe the symptoms and experiences of the testee's past. The entry is therefore a past."}, "sentence_type": "Affirmative declarative sentence", "pos": {"nouns": {"Too much stomach acid": 1, "Mistake": 1, "A week": 1, "Yes": 1, "Number": 1, "鈾?Make me 鈾?: 1, "Trouble": 1}, "verbs": {"have": 1, "- Yes, sir": 1}, "adverbs": {"A few times": 1}, "numerals": {"29": 1, "A week": 1, "A few times": 1}, "classifiers": {"Number of times": 1, "individual": 1}, "pronouns": {"I": 1}}}, {"content": "I've been trying to scold people sometimes.", "word_count": 7, "label": "", "sentence_structure": "Subject鈥搗erb鈥揷omplement", "subjective_objective": {"answer": "Subjective", "rationale": "Because \"invite\" is a subjective idea and emotion."}, "sentence_sentiment": {"answer": "Negative", "rationale": "Because the idea of \"I really want to curse\" in the entry is negative."}, "language_style": {"answer": "Calm and direct", "rationale": "The language style of the entry is relatively straightforward, with no excessive rhetoric or emotional rendering, and the mood of the person surveyed is expressed in a concise language."}, "time_dimension": {"answer": "Present", "rationale": ""}, "sentence_type": "Imperative sentence", "pos": {"verbs": {"I'd love to": 1, "Scolded": 1}, "adjectives": {"Sometimes": 1}, "adverbs": {"True": 1}}}, {"content": "31. Every few nights I have nightmares.", "word_count": 12, "label": "", "sentence_structure": "Subject鈥搗erb鈥搊bject", "subjective_objective": {"answer": "Subjective", "rationale": "Because dreams are usually subjective experiences and imaginations, they do not necessarily reflect objective facts. Thus, the intended description of the questionnaire is subjective."}, "sentence_sentiment": {"answer": "Negative", "rationale": "Because \"dreaming\" is a negative act."}, "language_style": {"answer": "Calm and direct", "rationale": "The entry describes the situation that the person under investigation may encounter in a more objective and direct language, and does not use excessive emotional colours or rhetorical techniques."}, "time_dimension": {"answer": "Present", "rationale": "Every few nights, it means customary, frequent behaviour, and I have nightmares, it's a description of the current situation, so the entry reflects the current habits and state of the person being tested."}, "sentence_type": "Affirmative declarative sentence", "pos": {"nouns": {"Night": 1, "Nightmares": 1}, "verbs": {"Yes": 1, "Do it": 1}, "adverbs": {"Separatism": 1, "How many": 1}}}, {"content": "32. I find it difficult to focus on one job.", "word_count": 18, "label": "", "sentence_structure": "Subject鈥搗erb鈥搊bject", "subjective_objective": {"answer": "Subjective", "rationale": "Because \"hard\" and \"focus\" are subjective feelings and evaluations of their abilities."}, "sentence_sentiment": {"answer": "Negative", "rationale": "Because it describes a negative phenomenon, \"difficult to concentrate\", and it expresses a negative emotional state."}, "language_style": {"answer": "Tactful and warm", "rationale": "The language style of the entry is more euphemistic. Using the expression \"I found\" to make the respondents feel more friendly and comfortable, and not too straightforward or too cold. And the expression \"it is difficult to focus on a job\" is also more objective and neutral, and not too emotional."}, "time_dimension": {"answer": "Present", "rationale": ""}, "sentence_type": "Exclamatory sentence", "pos": {"nouns": {"I": 1, "Attention": 1, "Work": 1}, "verbs": {"Found": 1, "- Put it on": 1, "Focus": 1}, "adjectives": {"Hard": 1}, "numerals": {"32": 1}}}, {"content": "I've had a very special and strange experience.", "word_count": 14, "label": "", "sentence_structure": "Subject鈥搗erb鈥揷omplement", "subjective_objective": {"answer": "Subjective", "rationale": "Because the \"very special\" and \"unusual\" description is the patient's subjective feelings and perceptions."}, "sentence_sentiment": {"answer": "Neutral", "rationale": "Because the entry does not have any emotional color."}, "language_style": {"answer": "Tactful and warm", "rationale": "The entry is more personal and interactive in its expression of conversation in a gentle and inviting tone, rather than direct statements or orders. At the same time, the description of \"very special\" and \"unusual\" also more implicitly guides respondents to recall their unique experiences, reflecting the care and care of the questionnaire designers."}, "time_dimension": {"answer": "Past", "rationale": "The entry contains a \"had\" in the past, so it's a \"old.\""}, "sentence_type": "Affirmative declarative sentence", "pos": {"nouns": {"Questionnaire": 1, "Entry": 1, "Experience": 1}, "verbs": {"Yes": 1, "Special": 1, "Strange": 1}, "adjectives": {"very": 2, "Special": 1, "Strange": 1}, "numerals": {"33": 1}, "pronouns": {"I": 1}}}, {"content": "I coughed a lot.", "word_count": 5, "label": "", "sentence_structure": "Subject鈥搗erb鈥揷omplement", "subjective_objective": {"answer": "Subjective", "rationale": "Since cough is a symptom and is caused by a physical reaction, it is examined from the patient ' s subjective perspective."}, "sentence_sentiment": {"answer": "Negative", "rationale": "Because the entry \"cough\" is a pathological phenomenon, which has a negative meaning."}, "language_style": {"answer": "Calm and direct", "rationale": "The entry describes the health of the person under investigation in a direct statement, does not use emotional language or a speech technique, and is air-cold and direct."}, "time_dimension": {"answer": "Past", "rationale": "The term \"frequent cough\" in this entry indicates that it is a symptom or condition of the past and has existed for some time. Although there is no clear time frame (for example, one week, one month, etc.), the term \"frequent\" usually means that the situation has lasted for some time and can therefore be judged to be the past."}, "sentence_type": "Affirmative declarative sentence", "pos": {"verbs": {"Cough": 1}, "adverbs": {"Often": 1}}}, {"content": "I would have achieved much more had it not been for the confrontations with me.", "word_count": 20, "label": "", "sentence_structure": "Subject鈥搗erb鈥揷omplement", "subjective_objective": {"answer": "Subjective", "rationale": "Because \"a greater achievement\" is a expectation or hope, and it's a subjective assessment of the patient himself."}, "sentence_sentiment": {"answer": "Negative", "rationale": "Because the premise in the entry that \"if someone wasn't against me\" is negative."}, "language_style": {"answer": "Calm and direct", "rationale": "The entry describes a point of view in hypothetical form, in which it expresses self-confidence that others are frustrated with their own abilities and that they are hindered."}, "time_dimension": {"answer": "Present", "rationale": "The entry describes the belief or perception of the testees in their present state that they will achieve more if they do not have someone against themselves. This belief reflects a subjective idea or view and does not address the time dimension of the past or the future. So the answer is \"now\"."}, "sentence_type": "Negative declarative sentence", "pos": {"nouns": {"Achievements": 1, "person": 1}, "verbs": {"have": 2, "Yes": 1, "Do it": 1, "to": 1}, "adjectives": {"Large": 1, "More": 1}, "adverbs": {"If not": 1}, "numerals": {"35": 1}, "pronouns": {"I": 2}}}, {"content": "I have little fear for my health.", "word_count": 10, "label": "", "sentence_structure": "Subject鈥揷opula鈥損redicative", "subjective_objective": {"answer": "Subjective", "rationale": "The fear is the patient's subjective feeling or attitude."}, "sentence_sentiment": {"answer": "Positive", "rationale": "Because \"very few fear for their health\" expresses a positive attitude."}, "language_style": {"answer": "Calm and direct", "rationale": "The entry is a question-and-answer session about the level of interest in one鈥檚 health."}, "time_dimension": {"answer": "Present", "rationale": "The keyword \"very few\" indicates that this concern is not frequent or strong and that there may be some optimism among the testers."}, "sentence_type": "Negative declarative sentence", "pos": {"nouns": {"Health": 1}, "verbs": {"I'm worried": 1, "Very few": 1}, "adverbs": {"self": 1}, "numerals": {"36": 1}}}, {"content": "I have never had any trouble for my sexual conduct.", "word_count": 18, "label": "", "sentence_structure": "Subject鈥搗erb鈥搊bject", "subjective_objective": {"answer": "Subjective", "rationale": "Because whether or not this is illegal or risky is a personal judgment and attitude."}, "sentence_sentiment": {"answer": "Neutral", "rationale": "The entry describes a statement of fact and does not express any positive or negative emotional tendencies. It is therefore a neutral entry."}, "language_style": {"answer": "Tactful and warm", "rationale": "The entry is more euphemistic and implicit, not directly addressing sensitive topics of sex, but rather using a more indirect approach to induce respondents to answer questions. At the same time, this expression is more respectful of the privacy and dignity of the person under investigation, and does not impose excessive psychological burdens or embarrassment on the other party."}, "time_dimension": {"answer": "Present", "rationale": "The keyword \"never\" indicates that this is a past-completed sentence, which represents a negative description of the past. But the event \"for my sexual acts\" may continue to exist or be in the future, and therefore not be considered as a complete past, but rather as a present. So this entry describes the present behaviour and attitude of the testee."}, "sentence_type": "Affirmative declarative sentence", "pos": {"nouns": {"Sexual": 1, "Behaviour": 1, "Yes": 1}, "verbs": {"Never": 1, "No, I'm not": 1, "Yes": 1, "Out": 1}, "adverbs": {"Never": 1}, "numerals": {"37锛?: 1}}}, {"content": "When I was a kid, I had a little bit of a thing to do with petty theft.", "word_count": 19, "label": "", "sentence_structure": "Subject鈥損redicate", "subjective_objective": {"answer": "Subjective", "rationale": "Because it involves the patient ' s own past experience and attitudes, it is the patient ' s subjective perspective."}, "sentence_sentiment": {"answer": "Negative", "rationale": "Because the \"sniffing\" act in the entry is negative."}, "language_style": {"answer": "Tactful and warm", "rationale": "The language style of the entry is euphemism, which is not directly accusing or evaluating the behaviour of the other, but rather, in a peaceful way, leads the other to review the past and to express an understanding and acceptance of that part of the experience. This language style helps to create a comfortable atmosphere and enhances the prot茅g茅 and interaction of dialogue."}, "time_dimension": {"answer": "Past", "rationale": "The entry \"I was a little young\" means the past, and \"I've done the petty theft\" means the past, and it's therefore judged to be the past."}, "sentence_type": "Affirmative declarative sentence", "pos": {"nouns": {"Time": 2, "One": 1, "I": 2, "A thief's little hand": 1, "Yes": 1}, "verbs": {"have": 1, "Yes": 1}, "adverbs": {"Small": 2}, "numerals": {"38": 1}, "classifiers": {"One": 2}}}, {"content": "I've been trying to fall sometimes.", "word_count": 8, "label": "", "sentence_structure": "Subject鈥搗erb鈥揷omplement", "subjective_objective": {"answer": "Subjective", "rationale": "Because I want to fall in something that means the patient's own thoughts and emotional state, which is a subjective description."}, "sentence_sentiment": {"answer": "Negative", "rationale": "Because the act of falling something is usually a negative emotional expression, and it is therefore a negative emotional expression."}, "language_style": {"answer": "Calm and direct", "rationale": "The entry directly expresses the subject of an emotional experience of asking the other party whether it had been motivated to fall, without excessive rhetoric or emotional rendering, and with a more objective tone."}, "time_dimension": {"answer": "Present", "rationale": "The entry in the future expresses a desire or idea of the subject, does not have a clear time limit or point to past behaviour, and therefore is judged to be future. At the same time, \"sometimes\" suggests an occasional emotional reaction that may appear in the future."}, "sentence_type": "Exclamatory sentence", "pos": {"nouns": {"Time": 1, "Something": 1}, "verbs": {"Yes": 1, "Fall": 1}, "adjectives": {"Sometimes": 1}, "numerals": {"39": 1}}}, {"content": "There are many times when you prefer to sit in the open and not do anything.", "word_count": 19, "label": "", "sentence_structure": "Subject鈥搗erb鈥搊bject", "subjective_objective": {"answer": "Subjective", "rationale": "Because \"I'd rather sit in the air than do anything\" is the way people feel and think, and it's a subjective angle."}, "sentence_sentiment": {"answer": "Negative", "rationale": "Because the \"I'd rather sit in the air\" act in the entry is negative."}, "language_style": {"answer": "Calm and direct", "rationale": "The language style of the entry is straightforward and it raises a question in a concise manner, without any rhetoric or emotional rendering, and is more objective in tone."}, "time_dimension": {"answer": "Present", "rationale": "The entry describes a customary behaviour or attitude of the person tested, i.e., choosing to choose between empty thoughts and doing things in many cases. Thus, the temporal dimension is now judged."}, "sentence_type": "Negative declarative sentence", "pos": {"nouns": {"Time": 1, "A lot": 1, "Things": 1}, "verbs": {"I'd rather": 1, "Sit down": 1, "I'm not sure": 1, "I don't want to": 1, "Do it": 2}, "adjectives": {"Any": 1}, "adverbs": {"very": 1, "And": 1}, "numerals": {"40": 1}}}, {"content": "41. I have been trying to do nothing for days, weeks and months, because I have been in a state of indecency.", "word_count": 30, "label": "", "sentence_structure": "Subject鈥搗erb鈥搊bject", "subjective_objective": {"answer": "Subjective", "rationale": "That's because \"I can't always lift my mind\" is a subjective feeling and attitude, not an objective description or fact. This reflects the patient's psychological state and emotional experience over time."}, "sentence_sentiment": {"answer": "Negative", "rationale": "Because the behavior and emotional state of the entry, \"No one wants to do anything for days, weeks, months,\" is negative."}, "language_style": {"answer": "Calm and direct", "rationale": "The entry describes an emotional state in a concise language, without excessive rhetorical or emotional rendering, and is more objective in tone. At the same time, the purpose of the article is designed directly and clearly to capture the real feelings and experiences of the person under investigation, rather than to induce or direct his or her answers."}, "time_dimension": {"answer": "Present", "rationale": "The \"some days,\" \"weeks\" and \"months\" that were in the past were all described, and thus judged to be the past. And the \"because\" reasoning also indicates that the act has occurred and ended."}, "sentence_type": "Exclamatory sentence", "pos": {"nouns": {"Questionnaire Entry": 1, "Oh, my God": 2, "Week": 1, "Month": 1, "Spirit": 1}, "verbs": {"Once": 1, "One-man": 2, "Fuck": 1, "T": 1}, "adjectives": {"A few days": 1, "How many": 2, "A few months": 1, "Nothing": 1, "Always": 1}, "numerals": {"41锛?: 1, "2": 1, "3": 1, "4": 1}}}, {"content": "42. My family is not satisfied with the job I have chosen (or the occupation I will choose).", "word_count": 23, "label": "", "sentence_structure": "Subject鈥搗erb鈥搊bject", "subjective_objective": {"answer": "Subjective", "rationale": "Because \"family dissatisfaction with the job I have chosen (or the occupation I will choose)\" is a subjective feeling and idea of the patient. Although this feeling may be influenced by objective reality, here the emphasis is on the patient鈥檚 perception and emotional reaction to the situation, and is therefore classified as a subjective examination."}, "sentence_sentiment": {"answer": "Negative", "rationale": "Because the emotional expression \"family dissatisfied\" in the entry is negative."}, "language_style": {"answer": "Tactful and warm", "rationale": "The language style of the entry is more euphemistic, using the relative intimacy of \"family\" and expressing their dissatisfaction with their career choices rather than directly accusing or ordering. This expression is easier to open the minds of the interviewees and to share their real thoughts and feelings."}, "time_dimension": {"answer": "Present", "rationale": "The phrase uses \"already\" and \"will\" to indicate that it is a description of the current situation or the future, and therefore the judgement is now."}, "sentence_type": "Negative declarative sentence", "pos": {"nouns": {"Family": 1, "Work": 1, "Selection": 2, "Occupation": 1}, "verbs": {"to": 1, "Dissatisfied": 1, "To choose": 1}, "numerals": {"42": 1}, "classifiers": {"individual": 1}}}, {"content": "I was sleeping in a state of discomfort and easily awakened.", "word_count": 10, "label": "", "sentence_structure": "Subject鈥搗erb鈥揷omplement", "subjective_objective": {"answer": "Subjective", "rationale": "Because \"uneasy\" and \"easy to wake up\" are subjective feelings and experiences."}, "sentence_sentiment": {"answer": "Negative", "rationale": "Because \"sleeping\" and \"suspense to wake up\" are negative behaviours or experiences."}, "language_style": {"answer": "Calm and direct", "rationale": "The language style of the entry is straightforward and it directly asks the interviewee, through a simple and clear language, whether there is a state of anxiety and arrogability during sleep, without adding too much emotional colour or acrimony."}, "time_dimension": {"answer": "Past", "rationale": "The entry describes the current behaviour or symptoms of the person being tested, i.e. sleep stress and accommodation, and can therefore be judged as past."}, "sentence_type": "Negative declarative sentence", "pos": {"nouns": {"I": 1, "Sleep": 1, "Wake up": 1}, "verbs": {"Sleep": 1, "Easy": 1, "By": 1, "Surprise": 1}, "adjectives": {"Unsettled": 1}, "numerals": {"43": 1}}}, {"content": "I feel my head is hurting everywhere.", "word_count": 10, "label": "", "sentence_structure": "Subject鈥揷opula鈥損redicative", "subjective_objective": {"answer": "Subjective", "rationale": "Because pain is a subjective feeling and experience of a person, it is impossible to observe or measure directly. The subject is to examine the subjective feelings of the patient, not the objective facts."}, "sentence_sentiment": {"answer": "Negative", "rationale": "Because \"headache\" is usually seen as a negative physical discomfort, expressing a negative emotion or state."}, "language_style": {"answer": "Tactful and warm", "rationale": "The entry uses a more moderate, caring tone of perception, rather than outright denial or stigmatization, which makes respondents more likely to share their experiences and feelings. At the same time, this language style conveys a sense of care and support, which helps to build good interaction."}, "time_dimension": {"answer": "Past", "rationale": ""}, "sentence_type": "Affirmative declarative sentence", "pos": {"nouns": {"Head": 1}, "verbs": {"I think": 1, "Ouch": 1}, "adverbs": {"Both": 1}, "numerals": {"44": 1}}}, {"content": "45. Sometimes I also make false statements.", "word_count": 7, "label": "", "sentence_structure": "Subject鈥損redicate", "subjective_objective": {"answer": "Subjective", "rationale": "Because \"false\" is the patient's subjective behaviour and attitude."}, "sentence_sentiment": {"answer": "Negative", "rationale": "Because \"false\" is negative."}, "language_style": {"answer": "Tactful and warm", "rationale": "The entry uses a first-person expression that gives people a sense of being closer and more real. At the same time, the term \"sometimes\" also indicates that it is reflecting on its own behaviour, without strong accusations or criticisms, and thus presents a more euphemism and gentle language style in general."}, "time_dimension": {"answer": "Present", "rationale": ""}, "sentence_type": "Affirmative declarative sentence", "pos": {"verbs": {"Speak": 1, "Say it": 1}, "adjectives": {"False": 1}, "adverbs": {"Yeah": 1, "Time": 1}, "numerals": {"45": 1}, "pronouns": {"I": 1}}}, {"content": "46. My judgment is better than ever.", "word_count": 14, "label": "", "sentence_structure": "Subject鈥揷opula鈥損redicative", "subjective_objective": {"answer": "Subjective", "rationale": "Because it involves the assessment and perception of the patient ' s judgement, it is a subjective perspective."}, "sentence_sentiment": {"answer": "Positive", "rationale": "This item expresses a positive emotional attitude towards its ability to judge."}, "language_style": {"answer": "Tactful and warm", "rationale": "Warm and warm."}, "time_dimension": {"answer": "Present", "rationale": "The keyword \"anytime now\" indicates that the act is related to the current state of affairs and may describe an ongoing situation or feeling."}, "sentence_type": "Affirmative declarative sentence", "pos": {"nouns": {"- The judgment": 1, "Time": 1}, "verbs": {"- Yeah": 1, "Okay": 1}, "adjectives": {"Any": 1}, "adverbs": {"Previous": 1, "Both": 1}, "numerals": {"46锛?: 1}}}, {"content": "47. Not once or twice a week, I suddenly feel the heat all over me for no reason.", "word_count": 22, "label": "", "sentence_structure": "Subject鈥搗erb鈥搊bject", "subjective_objective": {"answer": "Objective", "rationale": "Because \"suddenly feeling unprovoked fever\" is an objective fact, and an objective fact. Although this feeling may be related to the patient鈥檚 subjective emotions, in this case, the symptoms themselves are the result of objective observations."}, "sentence_sentiment": {"answer": "Neutral", "rationale": "Because the entry describes a physical feeling and does not involve any emotional or attitudinal tendencies."}, "language_style": {"answer": "Calm and direct", "rationale": "The basis for the determination is that the entry is in an objective and neutral tone, asking the interviewees whether they are often \"heated\" symptoms, not using excessive emotional or rhetorical techniques, but rather presenting questions and requesting answers, and therefore being in a calm and direct language style."}, "time_dimension": {"answer": "Past", "rationale": "Therefore, the entry is an inquiry into whether the person tested had had an unprovoked whole-body fever during the past week, which is judged by the time dimension."}, "sentence_type": "Affirmative declarative sentence", "pos": {"nouns": {"Week": 1, "Number of times": 2, "Heat": 1}, "verbs": {"I think": 1, "It's all hot": 1}, "adverbs": {"At least": 1, "For no reason": 1}, "numerals": {"47": 1, "One": 1, "Two": 1}}}, {"content": "48. I am upset to hear people talk about strange things when I'm with people.", "word_count": 25, "label": "", "sentence_structure": "Subject鈥揷opula鈥損redicative", "subjective_objective": {"answer": "Subjective", "rationale": "According to the sentence, \"I'm upset when I hear people talk about strange things,\" we can see that it's a description of subjective feelings and reactions. So, the point of view of this questionnaire is subjective. The basis of the judgment is: \"I\" indicates that it's personal experience or feeling."}, "sentence_sentiment": {"answer": "Negative", "rationale": "Because \"freak\" in the entry is a negative sentiment."}, "language_style": {"answer": "Calm and direct", "rationale": "The entry expresses the problems and distress encountered by the other party in interpersonal relations in a concise manner, without using excessive rhetorical or emotional rendering, and with a more objective tone. Nor does it leave too much room for speculation and direct inquiries as to whether the other party will have similar distress."}, "time_dimension": {"answer": "Present", "rationale": "The word \"just\" in \"just\" can be inferred from the fact that the act is a present, ongoing state of affairs and therefore judged to be now."}, "sentence_type": "Affirmative declarative sentence", "pos": {"nouns": {"I": 1, "person": 2, "Time": 1, "Get along": 1, "others": 1, "Talk": 1, "Yes": 1, "Just": 1, "I'm upset": 1}, "verbs": {"Yes": 1, "Talk": 1, "I'm upset": 1}, "adjectives": {"Weird": 1}, "numerals": {"48": 1}, "classifiers": {"individual": 1}}}, {"content": "49. It would be preferable to take all laws away from them.", "word_count": 13, "label": "", "sentence_structure": "Subject鈥損redicate", "subjective_objective": {"answer": "Subjective", "rationale": "This is because patients have subjective attitudes or perceptions about whether there should be a law."}, "sentence_sentiment": {"answer": "Negative", "rationale": "Because the \"No Law At All\" in the entry is negative."}, "language_style": {"answer": "Calm and direct", "rationale": "The basis for the determination: the entry expresses in a straightforward tone the view that all laws should be repealed in their entirety, without any euphemism, exaggeration or emotional expression to qualify its views. This plain, simple language style is consistent with the immediate condition of calm."}, "time_dimension": {"answer": "Present", "rationale": "\"Better not all laws than \"yes\" and \"no\" are the current state of affairs, indicating that this is a current idea or attitude."}, "sentence_type": "Negative declarative sentence", "pos": {"nouns": {"Law": 2, "All": 2}, "verbs": {"be": 1, "- Put it on": 1, "Yes": 1}, "adjectives": {"All": 1, "I'd better": 1, "Not even a little bit": 1}, "adverbs": {"All": 1}, "numerals": {"49": 1}, "classifiers": {"individual": 2, "Guys": 1}, "pronouns": {"be": 1}}}, {"content": "Sometimes I feel like my soul has left me.", "word_count": 16, "label": "", "sentence_structure": "Subject鈥揷opula鈥損redicative", "subjective_objective": {"answer": "Subjective", "rationale": "Because \"think\" is a subjective feeling and experience, the content of this questionnaire is viewed from the patient's subjective perspective."}, "sentence_sentiment": {"answer": "Negative", "rationale": "Because the feeling of \"I feel my soul leaving my body\" in the entry is negative."}, "language_style": {"answer": "Tactful and warm", "rationale": "The entry is more personal and interactive when it is spoken in a gentle and inviting manner, rather than in direct statements or orders. At the same time, it is used in visualized language to describe personal feelings and to make them more easily understood and answered by the interviewees."}, "time_dimension": {"answer": "Present", "rationale": "The entry indicates that the testee feels this way at the moment he answers the question, so the time dimension is now judged."}, "sentence_type": "Affirmative declarative sentence", "pos": {"nouns": {"Soul": 1, "Body": 1}, "verbs": {"I think": 1, "Leave": 1}, "adverbs": {"Sometimes": 1}, "numerals": {"50锛?: 1}}}, {"content": "51. My health is as healthy as most of my friends.", "word_count": 16, "label": "", "sentence_structure": "Subject鈥揷opula鈥損redicative", "subjective_objective": {"answer": "Subjective", "rationale": "Because \"my body is as healthy as most of my friends\" is patient judgment and feeling."}, "sentence_sentiment": {"answer": "Neutral", "rationale": "Because the entry describes an objective fact, there is no apparent emotional tendency."}, "language_style": {"answer": "Tactful and warm", "rationale": "The language style of the entry is relatively euphemistic and gentle, and it is not too direct or too cold, but rather describes the health of the person surveyed in a more objective and neutral manner, which is more personal and interactive."}, "time_dimension": {"answer": "Present", "rationale": "The entry indicates the testee ' s perception of his/her health and is now described in the state."}, "sentence_type": "Affirmative declarative sentence", "pos": {"nouns": {"Body": 1, "friends": 1, "Health": 1}, "verbs": {"be": 1, "Same thing": 1, "Health": 1}, "adjectives": {"Mostly": 1}, "numerals": {"51": 1}, "pronouns": {"I": 1}}}, {"content": "52. I met my classmates and friends who were not common, unless they first said hello, and I pretended not to see them.", "word_count": 28, "label": "", "sentence_structure": "Subject鈥搗erb鈥搊bject", "subjective_objective": {"answer": "Subjective", "rationale": "The basis for the judgement: the description in the title is the patient's subjective thoughts and behaviour, which involves psychological factors such as the individual's emotions, attitudes and behavioral decisions. For example, the descriptions \"I don't care about him\" and \"I pretend not to see him\" are subjective responses and responses to a particular situation, not an objective fact or an external context."}, "sentence_sentiment": {"answer": "Negative", "rationale": "The basis of the judgment: \"Unless they say hello first, I pretend I didn't see it,\" in the entry, it's negative."}, "language_style": {"answer": "Calm and direct", "rationale": "The entry is relatively straightforward and concise, has no excessive speech skills or emotional rendering, and is more objective in tone. It clearly expresses the desire of researchers to understand how the person under investigation behaves when he meets a fellow student or a friend who is not common."}, "time_dimension": {"answer": "Present", "rationale": "The entry describes the reaction of the person tested in a given situation, i.e. \"when meeting a fellow student or an unusual friend\", which is the current practice and may continue. Thus, the entry belongs to the present."}, "sentence_type": "Affirmative declarative sentence", "pos": {"nouns": {"Students": 1, "friends": 1, "they": 1}, "verbs": {"I met him": 1, "Pretend": 1, "Yeah": 1}, "adjectives": {"Common": 1}, "adverbs": {"Otherwise": 1, "Just": 2}, "pronouns": {"I": 1}}}, {"content": "53. A priest (monasters, monks, priests, imams, etc.) can heal the patient with prayers and hands.", "word_count": 31, "label": "", "sentence_structure": "Subject鈥搗erb鈥搊bject", "subjective_objective": {"answer": "Objective", "rationale": "Because \"a priest (monk, monk, priest, imam, etc.) can heal a patient with prayer and hands on his head\" is a descriptive statement, which expresses a fact or situation that does not require subjective feelings or attitudes of the patient. Thus, it is an objective entry."}, "sentence_sentiment": {"answer": "Neutral", "rationale": "Because the entry does not have any emotional colour. It merely describes a possible treatment and does not express an assessment or attitude to that treatment."}, "language_style": {"answer": "Calm and direct", "rationale": "The entry describes a particular scene in an objective manner, with no excessive rhetorical or emotional rendering, and is more straightforward in tone."}, "time_dimension": {"answer": "Present", "rationale": ""}, "sentence_type": "Affirmative declarative sentence", "pos": {"nouns": {"Reverend": 1, "Monk": 2, "Father": 1, "Grandma": 1, "Cleric": 1, "Diseases": 3, "Pray": 1, "Hands": 1, "Head": 1}, "verbs": {"Yes": 1, "Use": 1, "Fire": 1}, "adverbs": {"in": 1}, "numerals": {"53": 1}, "classifiers": {"bit": 1, "individual": 2}, "pronouns": {"he": 1}}}, {"content": "54. Almost everyone who knew me liked me.", "word_count": 12, "label": "", "sentence_structure": "Subject鈥揷opula鈥損redicative", "subjective_objective": {"answer": "Subjective", "rationale": "Because love is a subjective attitude and whether it is popular and personal, the question is to examine the subjective perspective of the patient."}, "sentence_sentiment": {"answer": "Positive", "rationale": "Because the phrase \"who knows me is almost like me\" in the entry is positive."}, "language_style": {"answer": "Tactful and warm", "rationale": "The term \"almost\" is used in this entry, implying that there may be some uncertainty about the other party's self-perception, while expressing a more modest, unaggressive attitude. Moreover, the expression \"like me\" is softer and does not directly require others to praise or endorse themselves, and that is more acrimonym and inclusive."}, "time_dimension": {"answer": "Present", "rationale": ""}, "sentence_type": "Imperative sentence", "pos": {"nouns": {"I": 2, "person": 1, "Yes": 1}, "verbs": {"Yeah": 1}, "adjectives": {"Almost": 1}, "numerals": {"54": 1}}}, {"content": "I have never been troubled by chest and heartaches.", "word_count": 17, "label": "", "sentence_structure": "Subject鈥揷opula鈥損redicative", "subjective_objective": {"answer": "Objective", "rationale": "This is an objective fact of the patient ' s experience."}, "sentence_sentiment": {"answer": "Negative", "rationale": "The phrase \"never suffered from chest and heart pain\" in the entry suggests that individuals may lack sufficient attention and care in the face of the inaccuracy of such bodies."}, "language_style": {"answer": "Tactful and warm", "rationale": "The language style of the entry is euphemism, because it asks each other about their feelings in an invitation-style tone, and uses a softer expression (such as \"never\") that is not too direct or too tough. It is more closely related to the mood and the psychological state of the interviewee, and increases the motivation and authenticity of the answer."}, "time_dimension": {"answer": "Past", "rationale": "The entry is for the purpose of asking if the person tested had experienced pain or suffering from chest and heart pain, and the temporal dimension was judged to be the past."}, "sentence_type": "Negative declarative sentence", "pos": {"nouns": {"- Boobs": 1, "Heartache": 1, "Trouble": 1}, "verbs": {"Never": 1, "I feel": 1}, "adverbs": {"Never": 1}, "numerals": {"55": 1}, "pronouns": {"I": 1}}}, {"content": "56. When I was a child, I was disciplined by the school for having been a fool.", "word_count": 18, "label": "", "sentence_structure": "Subject鈥搗erb鈥搊bject", "subjective_objective": {"answer": "Subjective", "rationale": "Because \"fun\" and \"resentence\" are based on the interviewee's memory and subjective feelings. These events are past and are self-excluded by the interviewee and are therefore subjectively examined."}, "sentence_sentiment": {"answer": "Negative", "rationale": "Because the \"school discipline\" in the entry is negative."}, "language_style": {"answer": "Calm and direct", "rationale": "The language style of the entry is a calm and straightforward one. It is a simple and straightforward statement of a fact, with no excessive emotional or rhetorical style, and is objective and direct."}, "time_dimension": {"answer": "Past", "rationale": "The reference to \"baby\" and \"had been disciplined by the school for having been a part of the game\" in that sentence were recollections and descriptions of the past, and therefore judged to be the past."}, "sentence_type": "Affirmative declarative sentence", "pos": {"nouns": {"Time": 1, "I": 2, "Disposition": 1}, "verbs": {"Once": 1, "Yes": 1, "- Bullshit": 1}, "adverbs": {"Because": 1, "And": 1}, "numerals": {"56": 1}}}, {"content": "57. I've had a long time with others.", "word_count": 14, "label": "", "sentence_structure": "Subject鈥搗erb鈥揷omplement", "subjective_objective": {"answer": "Objective", "rationale": "Because \"I've been in a long line of meetings with other people\" is a descriptive phrase that reflects the outside expression and attitude of patients to other people and is an objective fact."}, "sentence_sentiment": {"answer": "Neutral", "rationale": "Because the entry does not have any emotional color."}, "language_style": {"answer": "Calm and direct", "rationale": "The language style of the entry is relatively straightforward, with no excessive rhetorical or emotional rendering, and is more calm and objective. It is asking whether the interviewee is easy to establish intimate relationships with others or socialize. It is more appropriate for a psychological questionnaire, because it is able to express clearly the questions that researchers want to understand, while avoiding overly subjective emotional disturbances."}, "time_dimension": {"answer": "Present", "rationale": "The entry is a character characteristic or customary behaviour of the testee, and therefore the temporal dimension is now judged."}, "sentence_type": "Affirmative declarative sentence", "pos": {"nouns": {"others": 1, "See": 1, "Noodles": 1, "Pressure": 1}, "verbs": {"See you": 1, "Press": 1, "Yes": 1, "(Change familiar)": 1}, "adverbs": {"Yes": 1, "and": 1}, "numerals": {"57锛?: 1, "I": 1}}}, {"content": "58. Everything was arranged by God.", "word_count": 13, "label": "", "sentence_structure": "Subject鈥損redicate", "subjective_objective": {"answer": "Subjective", "rationale": "Because it's personal conviction and attitude to think that everything is arranged by God, so it's subjective."}, "sentence_sentiment": {"answer": "Neutral", "rationale": "Because the entry does not express any apparent emotional, attitudinal or behavioural tendencies. It merely presents a point of view, and does not evaluate its positive or negative."}, "language_style": {"answer": "Tactful and warm", "rationale": "The entry uses a more implicit language style, which expresses acceptance of and submission to destiny by quoting the traditional religious belief of God. This language is relatively mild, avoiding overly direct or categorical expressions and making it easier to resonate and identify."}, "time_dimension": {"answer": "Future", "rationale": "The sentence contains visions and expectations for the future, and the key word is \"as arranged by God\" and all point to situations that have not yet occurred and that may arise in the future."}, "sentence_type": "Affirmative declarative sentence", "pos": {"nouns": {"Things": 1, "Master Tin": 1, "Jesus Christ": 1}, "verbs": {"Arrangements": 1}, "adjectives": {"Everything": 1}, "numerals": {"58锛?: 1}}}, {"content": "59. I am often subject to the command of some, who are not as wise as I am.", "word_count": 22, "label": "", "sentence_structure": "Subject鈥搗erb鈥搊bject", "subjective_objective": {"answer": "Subjective", "rationale": "Because \"not as smart as me\" is patient's subjective opinion."}, "sentence_sentiment": {"answer": "Negative", "rationale": "Because it expresses dissatisfaction with and debasement from the command of others and has a negative feeling."}, "language_style": {"answer": "Calm and direct", "rationale": "The entry raises the question in a direct manner, without excessive rhetoric or emotional colours and with a more objective tone."}, "time_dimension": {"answer": "Past", "rationale": "The \"frequent\" in the entry indicates that it is a description of the past, and that \"under the command of certain people\" and \"they are not as smart as I am\" are also events or events of the past. So the time dimension of this questionnaire entry is the past."}, "sentence_type": "Negative declarative sentence", "pos": {"nouns": {"person": 2, "Command": 1, "Why don't you": 1}, "verbs": {"Okay": 1, "Listen": 1, "be": 1}, "adjectives": {"Often": 1, "Some": 1, "Smart": 1}, "numerals": {"59": 1}, "pronouns": {"I": 1}}}, {"content": "60. I do not read every editorial in the newspaper every day.", "word_count": 16, "label": "", "sentence_structure": "Subject鈥搗erb鈥搊bject", "subjective_objective": {"answer": "Subjective", "rationale": "Because \"not every day,\" \"all\" and \"every single\" are patients' own subjective ideas and behaviours."}, "sentence_sentiment": {"answer": "Neutral", "rationale": "Because the entry does not express any positive or negative feelings, attitudes, behaviour, purpose or quality."}, "language_style": {"answer": "Calm and direct", "rationale": ""}, "time_dimension": {"answer": "Present", "rationale": "Now."}, "sentence_type": "Negative declarative sentence", "pos": {"nouns": {"Newspapers": 1, "editorial": 1}, "verbs": {"Look": 1, "Both": 1, "be": 1}, "adjectives": {"Chile": 1}, "adverbs": {"No, it's not": 1, "Go, go, go": 1, "Every single one": 1}, "numerals": {"60锛?: 1}, "pronouns": {"I": 1}}}, {"content": "61. I have never had a normal life.", "word_count": 10, "label": "", "sentence_structure": "Subject鈥損redicate", "subjective_objective": {"answer": "Subjective", "rationale": "Because \"a normal life has never been achieved\" is a subjective feeling and an assessment of the individual's state of life. It involves a number of subjective factors, such as individual emotions, values, etc., which cannot be simply attributed to objective facts or behavioural outcomes."}, "sentence_sentiment": {"answer": "Negative", "rationale": "Because the expression \" never had a normal life\" was full of negative emotions."}, "language_style": {"answer": "Tactful and warm", "rationale": ""}, "time_dimension": {"answer": "Past", "rationale": ""}, "sentence_type": "Negative declarative sentence", "pos": {"nouns": {"Life": 1, "Normal": 1}, "verbs": {"Yes": 1}, "adverbs": {"From the End": 1}, "numerals": {"61": 1}}}, {"content": "62. Certain parts of my body often feel like fire, sting, worm crawling and numbness.", "word_count": 21, "label": "", "sentence_structure": "Subject鈥揷opula鈥損redicative", "subjective_objective": {"answer": "Subjective", "rationale": "The basis for the determination is that the problem concerns the feelings and experiences of the patient and is therefore viewed from the patient ' s subjective perspective."}, "sentence_sentiment": {"answer": "Negative", "rationale": "Because the physical feelings described in this entry are usually negative. These can be pain, discomfort or abnormal, which can have a negative impact on people ' s quality of life."}, "language_style": {"answer": "Tactful and warm", "rationale": ""}, "time_dimension": {"answer": "Past", "rationale": "The entry describes some of the current feelings or experiences of the testee and therefore judges the past."}, "sentence_type": "Affirmative declarative sentence", "pos": {"nouns": {"Part": 1, "Fire": 1, "Stings": 1, "Bug crawling": 1, "I'm numb": 1, "Feel": 1}, "verbs": {"- I'm sure": 1, "Elephant": 1}, "adjectives": {"Fire": 1, "Burn": 1, "Stab": 1, "Bugs": 1, "Ma": 1}, "numerals": {"62锛?: 1}}}, {"content": "My shit is normal and not difficult to control.", "word_count": 10, "label": "", "sentence_structure": "Subject鈥搗erb鈥揷omplement", "subjective_objective": {"answer": "Objective", "rationale": "This is a description of the state of health and is an objective fact of the situation in which the patient is currently living."}, "sentence_sentiment": {"answer": "Neutral", "rationale": "As the entry describes a biological phenomenon, it does not involve an individual ' s emotions, attitudes or behaviour and is therefore neutral."}, "language_style": {"answer": "Tactful and warm", "rationale": "The entry uses a more euphemistic and relaxed tone to describe the excreta of the person surveyed, avoiding direct questioning of issues that may be embarrassing or uncomfortable. At the same time, \"no difficulty\" is also a relatively mild term, avoiding an overly direct assessment of the capacity or state of the person surveyed. The language style of the questionnaire can therefore be considered as euphemistic."}, "time_dimension": {"answer": "Past", "rationale": ""}, "sentence_type": "Affirmative declarative sentence", "pos": {"nouns": {"Shit": 1}, "verbs": {"Normal": 1, "It's not hard": 1, "Control": 1}, "adverbs": {"Normal": 1, "It's not hard": 1}, "numerals": {"63锛?: 1}}}, {"content": "64. Sometimes I do something until others are impatient.", "word_count": 20, "label": "", "sentence_structure": "Subject鈥搗erb鈥搊bject", "subjective_objective": {"answer": "Subjective", "rationale": "Because the \"still work\" and \"others are impatient\" are all based on patients' subjective feelings and attitudes."}, "sentence_sentiment": {"answer": "Negative", "rationale": "Because these acts, \"doing one thing without stopping\" in the entries, \"until someone's impatient,\" show a tendency to be coercive and to interfere with others, and they are negative."}, "language_style": {"answer": "Tactful and warm", "rationale": "Although the entry asks whether there is a pattern of behaviour, it does not directly convey criticism or accusations, but rather uses a more euphemism to initiate dialogue by asking questions. Such expression is more personal and interactive, and it increases the willingness of respondents to share their ideas and experiences."}, "time_dimension": {"answer": "Present", "rationale": ""}, "sentence_type": "Affirmative declarative sentence", "pos": {"nouns": {"Yes": 1, "person": 1}, "verbs": {"Do it": 1, "Yes": 1, "Stop": 1, "to": 1, "Until then": 1, "I'm impatient": 1}, "adjectives": {"鈾?And it's not stopping 鈾?: 1, "of": 2}, "adverbs": {"Sometimes": 1}, "numerals": {"64": 1, "One": 2}, "classifiers": {"Annex": 1}, "pronouns": {"Don't": 1}}}, {"content": "I love my father.", "word_count": 6, "label": "", "sentence_structure": "Subject鈥搗erb鈥搊bject", "subjective_objective": {"answer": "Subjective", "rationale": "Because love is a subjective feeling and attitude."}, "sentence_sentiment": {"answer": "Positive", "rationale": "Because \"love\" is a positive emotional word for love and admiration for the father."}, "language_style": {"answer": "Tactful and warm", "rationale": "The entry expresses love for the father in a gentle and positive way, without too much language or emphasis, but rather a more intuitive but real emotion. At the same time, this euphemism is more acceptable and understandable."}, "time_dimension": {"answer": "Present", "rationale": ""}, "sentence_type": "Affirmative declarative sentence", "pos": {"nouns": {"I": 1, "Father": 1}, "verbs": {"Love": 1}}}, {"content": "66. I can see around me what others do not see, animals and people.", "word_count": 22, "label": "", "sentence_structure": "Subject鈥搗erb鈥揷omplement", "subjective_objective": {"answer": "Subjective", "rationale": "Because this is the patient ' s personal feeling and perception, it is a subjective perception."}, "sentence_sentiment": {"answer": "Positive", "rationale": "Because the description in the entry \"seeing what others can't see, animals and humans\" shows a supernatural observation and sense of motivation."}, "language_style": {"answer": "Tactful and warm", "rationale": "The survey question is asked in a more moderate way whether the person surveyed has special abilities or sensory powers, rather than direct questioning or judgement. At the same time, the descriptive language \"seeing what others cannot see, animals and people\" is used to make it easier for the person surveyed to understand the meaning of the question and to stimulate interest and motivation in answering it."}, "time_dimension": {"answer": "Present", "rationale": "The keyword \"can\" uses the usual current state of affairs, which means that the act has not only happened in the past, but may still be ongoing. Moreover, the word \"seeing\" around me also indicates that it is a description of the current situation. So, the entry is an inquiry into the present perception of the subject."}, "sentence_type": "Affirmative declarative sentence", "pos": {"nouns": {"Questionnaire": 1, "Entry": 1, "Others": 1, "Something": 1, "Animals": 1, "person": 2}, "verbs": {"I can see it": 1, "Yeah": 1}, "adverbs": {"Yes": 1}, "numerals": {"66": 1}, "pronouns": {"I": 1}}}, {"content": "You want me to be as happy as anyone else.", "word_count": 12, "label": "", "sentence_structure": "Subject鈥搗erb鈥揷omplement", "subjective_objective": {"answer": "Subjective", "rationale": "Because hope is a subjective emotion and expectation."}, "sentence_sentiment": {"answer": "Neutral", "rationale": "The entry is not a positive or negative emotional expression."}, "language_style": {"answer": "Tactful and warm", "rationale": "The language style of the entry is more euphemistic, and it allows each other to feel the attention and understanding, while expressing their hopes and expectations."}, "time_dimension": {"answer": "Present", "rationale": "The word \"hope\" in the sentence expresses current wishes or expectations, while the word \"may\" adopts the general current, indicating that such expectations are now being considered and realized. The entry is therefore in the present context."}, "sentence_type": "Affirmative declarative sentence", "pos": {"nouns": {"person": 1, "Happy": 1}, "verbs": {"Hope": 1, "Yes": 1, "Yeah": 1, "That way": 1}, "adverbs": {"very": 1}, "numerals": {"67": 1}, "pronouns": {"I": 1}}}, {"content": "68. I never felt pain behind my neck (circle).", "word_count": 11, "label": "", "sentence_structure": "Subject鈥搗erb鈥揷omplement", "subjective_objective": {"answer": "Objective", "rationale": "Because \"fear\" and \"in pain\" are subjective experiences of patients, and \"never\" are objective descriptions of such experiences. So this is a questionnaire that is examined from an objective perspective."}, "sentence_sentiment": {"answer": "Neutral", "rationale": "It is merely asking the person surveyed whether he or she has had some experience, without evaluating his or her value judgement."}, "language_style": {"answer": "Tactful and warm", "rationale": "The language style of the entry is more euphemistic and sweet, and the expression of a question about whether the other person has experienced a neck pain is expressed by using the negative word \"never\" and is more balanced and friendly. At the same time, this expression leaves the interviewee with a space for self-reflection that allows him to more accurately reflect his situation in answering the question."}, "time_dimension": {"answer": "Present", "rationale": "The entry describes the current feelings and state of the testee, \"never felt\" indicating that it was a continuous, recurrent feeling, and thus the temporal dimension is now judged."}, "sentence_type": "Negative declarative sentence", "pos": {"nouns": {"Neck": 1, "The neck": 1, "Ouch": 2}, "verbs": {"Sensor": 1, "Present": 1, "From": 1, "Not": 1, "I feel": 1, "Ouch": 2}, "adjectives": {"Never": 1}, "numerals": {"68": 1}, "pronouns": {"I": 1}}}, {"content": "69. People of the same sex are a serious attraction to me.", "word_count": 17, "label": "", "sentence_structure": "Subject鈥揷opula鈥損redicative", "subjective_objective": {"answer": "Subjective", "rationale": "Because gender preferences are subjective attitudes of one person."}, "sentence_sentiment": {"answer": "Negative", "rationale": "Because the emotional color of \"severe attraction\" in the entry is negative."}, "language_style": {"answer": "Tactful and warm", "rationale": "The entry uses a euphemismal and leading language style, which allows dialogue by asking the other side's views, without direct statements or orders. This way makes you feel more friendly and comfortable."}, "time_dimension": {"answer": "Present", "rationale": "The sentence is judged by the word \"now\" and the entry is asked about the current thoughts or feelings of the person being tested."}, "sentence_type": "Interrogative sentence", "pos": {"nouns": {"person": 2, "Attractive": 1}, "verbs": {"have": 1, "to": 1, "Attractive": 1}, "adjectives": {"Serious": 1, "Same": 1}, "numerals": {"69": 1}, "classifiers": {"individual": 1}, "pronouns": {"I": 2}}}, {"content": "I used to play the \"paw\" game.", "word_count": 14, "label": "", "sentence_structure": "Subject鈥搗erb鈥搊bject", "subjective_objective": {"answer": "Subjective", "rationale": "Because \"like to play\" is an expression of subjective attitude."}, "sentence_sentiment": {"answer": "Positive", "rationale": "Because the \"like to throw handkerchiefs\" act is positive."}, "language_style": {"answer": "Tactful and warm", "rationale": "The language style of the entry is more euphemistic, it describes past preferences in a recollection, and does not have too many direct statements or orders. This expression is more intimate and interactive, and it feels more sweet and friendly."}, "time_dimension": {"answer": "Past", "rationale": "Based on the keywords \"the past\" and \"the regularity\", we can judge that the subject is a question about the behaviour and habits of the subjects, and therefore the temporal dimension is a matter of the past."}, "sentence_type": "Affirmative declarative sentence", "pos": {"nouns": {"Past": 1, "Drop your handkerchief": 1, "Game": 1}, "verbs": {"Regular": 1, "Yeah": 1, "Play": 1}, "adverbs": {"very": 1, "Regular": 1}, "numerals": {"70": 1}, "pronouns": {"I": 1}}}, {"content": "71. I think many people prefer to exaggerate their misfortunes in order to get sympathy and help from others.", "word_count": 27, "label": "", "sentence_structure": "Subject鈥搗erb鈥搊bject", "subjective_objective": {"answer": "Subjective", "rationale": "\"and felt a manifestation of subjective attitudes.\""}, "sentence_sentiment": {"answer": "Negative", "rationale": "Because the entry expresses a negative assessment of the behaviour of some persons, it is assumed that they exaggerate their misfortunes to achieve a certain purpose and therefore have a certain negative emotional connotation."}, "language_style": {"answer": "Calm and direct", "rationale": "The language style of the entry is a calm and straightforward one. It conveys the author鈥檚 views and attitudes directly through a concise and clear formulation, and is more objective and rational without too much rhetorical or emotional. It also uses the expression of cross-examination, which strengthens the discourse."}, "time_dimension": {"answer": "Present", "rationale": "The entry expresses the views and views of the testee, without clear time-pointing. At the same time, the terms \"felt\" , \"exaggerate\" and \"get\" take the general current state of affairs, indicating that this view is the result of the testee's current thinking. Thus, the temporal dimension of the article is now judged."}, "sentence_type": "Affirmative declarative sentence", "pos": {"nouns": {"Questionnaire": 1, "person": 2, "self": 1, "others": 1, "Compassion": 1, "Help": 1}, "verbs": {"I think": 1, "Yeah": 1, "Exaggeration": 1, "Got it": 1}, "adjectives": {"Unfortunately": 1}, "numerals": {"71": 1}, "pronouns": {"I": 1}}}, {"content": "72. I am troubled by the fact that I feel uncomfortable (attendant) every few days or on a regular basis.", "word_count": 20, "label": "", "sentence_structure": "Subject鈥揷opula鈥損redicative", "subjective_objective": {"answer": "Subjective", "rationale": "Because \"challeng\" is a subjective emotional reaction, reflecting the patient's feelings and attitudes to the stomach/attendance."}, "sentence_sentiment": {"answer": "Negative", "rationale": "Because the entry \"indiscomfort\" is a physical discomfort, usually associated with negative emotions."}, "language_style": {"answer": "Tactful and warm", "rationale": "The entry is more euphemistic, and uses the word \"treasure\" to express concern and understanding of the interviewee. At the same time, it is not a direct statement or order that leads the interviewer to answer questions by asking him whether he is often unwell (attendant)."}, "time_dimension": {"answer": "Present", "rationale": "According to the phrase \"every few days\" and \"often\", it can be inferred that this is a description of the situation of the person being tested, and therefore is judged as \"now.\" And, at the same time, \"the feeling of discomfort and distress\" is also a description of the state of mind."}, "sentence_type": "Affirmative declarative sentence", "pos": {"nouns": {"Questionnaire": 1, "Entry": 1, "Oh, my God": 1, "Stomach": 1, "I'm not feeling well": 1, "Trouble": 1}, "verbs": {"Sensor": 1, "Present": 2, "be": 1, "Yes": 1, "Every": 1, "And": 1, "Answer": 1}, "adverbs": {"Regular": 1, "A few days": 1}, "numerals": {"72锛?: 1, "How many": 1}, "pronouns": {"I": 1}}}, {"content": "I'm a big man.", "word_count": 7, "label": "", "sentence_structure": "Subject鈥揷opula鈥損redicative", "subjective_objective": {"answer": "Subjective", "rationale": "Because it is a subjective assessment and a perception that you are a very important person."}, "sentence_sentiment": {"answer": "Positive", "rationale": "Because the term \"vital person\" is usually seen as a positive self-evaluation."}, "language_style": {"answer": "Tactful and warm", "rationale": ""}, "time_dimension": {"answer": "Present", "rationale": ""}, "sentence_type": "Affirmative declarative sentence", "pos": {"verbs": {"be": 1}, "adjectives": {"Important": 1}, "numerals": {"73": 1}, "pronouns": {"I": 1}}}, {"content": "74. Men: I always wish I was a woman.", "word_count": 11, "label": "", "sentence_structure": "Subject鈥揷opula鈥損redicative", "subjective_objective": {"answer": "Subjective", "rationale": "Because \"favorite\" is a subjective attitude and will of a man."}, "sentence_sentiment": {"answer": "Negative", "rationale": "Because the \"I wish I was a woman\" attitude in the entry is negative."}, "language_style": {"answer": "Tactful and warm", "rationale": "The language style of the entry is more euphemistic, using the expression \"I hope\" to avoid the possibility of directly denying or blaming each other. At the same time, the expression \"I am a woman\" is used to express dissatisfaction with my sex and reflects the respect and concern of speakers for gender equality and diversity."}, "time_dimension": {"answer": "Present", "rationale": "The entry asks about the psychological orientation and feelings of the person being tested, and the words \"all hope\" and \"yes\" indicate that this is a present subjective expression of will or emotion."}, "sentence_type": "Exclamatory sentence", "pos": {"nouns": {"Men": 1, "Women": 1}, "verbs": {"Hope": 1, "be": 1}, "adverbs": {"Total": 1, "Both": 1}, "numerals": {"74锛?: 1}, "pronouns": {"I": 1}}}, {"content": "Woman: I never regret that I am a woman.", "word_count": 14, "label": "", "sentence_structure": "Subject鈥搗erb鈥搊bject", "subjective_objective": {"answer": "Subjective", "rationale": "This is because patients have a subjective attitude towards their sex."}, "sentence_sentiment": {"answer": "Positive", "rationale": "Because the expression \"never regret that I'm a woman\" is positive."}, "language_style": {"answer": "Tactful and warm", "rationale": "The entry asks whether women regret being women in a gentle and respectful manner, is more sensitive and sensitive to each other ' s feelings and does not use direct or mandatory language styles."}, "time_dimension": {"answer": "Present", "rationale": "The key word for the entry in the future is \"never\", which implies the future attitude and behaviour of the testee, i.e., she will not regret being a woman at some point in the future. The temporal dimension therefore judges the future."}, "sentence_type": "Affirmative declarative sentence", "pos": {"nouns": {"Female": 1, "Sorry": 1}, "verbs": {"be": 1, "Because": 2, "And": 1, "Sorry": 1}, "adjectives": {"A woman": 1}, "pronouns": {"I": 1}}}, {"content": "75. I sometimes get angry.", "word_count": 5, "label": "", "sentence_structure": "Subject鈥搗erb鈥揷omplement", "subjective_objective": {"answer": "Subjective", "rationale": "This sentence looks at the patient from the subjective point of view. Because \"excited\" is a subjective emotional reaction that reflects the patient's own emotional state and experience."}, "sentence_sentiment": {"answer": "Negative", "rationale": "Because \"freak\" is a negative emotion."}, "language_style": {"answer": "Tactful and warm", "rationale": "Warm and warm."}, "time_dimension": {"answer": "Present", "rationale": "The entry \"sometimes\" indicates frequency and time uncertainty, but it implies that the person tested has experienced or may be experiencing this situation within a certain period of time. Thus, \"sometimes\" puts the act in any time period past, present or future. However, we cannot be sure whether it is past, present or future, because there is no clear indication of the time."}, "sentence_type": "Affirmative declarative sentence", "pos": {"verbs": {"I": 1, "- Get angry": 1}, "adjectives": {"Sometimes": 1}}}, {"content": "76. I am often pessimistic and disappointed.", "word_count": 9, "label": "", "sentence_structure": "Subject鈥揷opula鈥損redicative", "subjective_objective": {"answer": "Subjective", "rationale": "Because feeling pessimistic and disappointed is a subjective emotional experience."}, "sentence_sentiment": {"answer": "Negative", "rationale": "Because the mood for \"pessimism and disappointment\" is negative."}, "language_style": {"answer": "Tactful and warm", "rationale": ""}, "time_dimension": {"answer": "Present", "rationale": ""}, "sentence_type": "Negative declarative sentence", "pos": {"verbs": {"I feel": 1, "Despair": 1}, "adjectives": {"Pessimism": 1, "Despair": 1}, "adverbs": {"Often": 1}}}, {"content": "I love love novels.", "word_count": 8, "label": "", "sentence_structure": "Subject鈥搗erb鈥搊bject", "subjective_objective": {"answer": "Subjective", "rationale": "Like being patient subjective.\""}, "sentence_sentiment": {"answer": "Positive", "rationale": "Because \"like\" in the entry is a positive emotional attitude."}, "language_style": {"answer": "Tactful and warm", "rationale": "The entry begins with the first person's name, \"I\", which expresses personal preferences, is not too direct or emotional, and is more euphemistic. At the same time, the way in which the questions are asked is friendly and easy, and is consistent with the characteristics of the questionnaire."}, "time_dimension": {"answer": "Present", "rationale": ""}, "sentence_type": "Affirmative declarative sentence", "pos": {"nouns": {"77": 1, "Novel": 1}, "verbs": {"Yeah": 1, "Look": 1}, "adjectives": {"Love": 1}}}, {"content": "I love poetry.", "word_count": 4, "label": "", "sentence_structure": "Subject鈥損redicate", "subjective_objective": {"answer": "Subjective", "rationale": "Because like is a subjective feeling or attitude."}, "sentence_sentiment": {"answer": "Positive", "rationale": "Because \"like\" is a positive attitude."}, "language_style": {"answer": "Tactful and warm", "rationale": "The entry is more personal and interactive when it is presented in a gentle and inviting tone, asking whether the other party likes poetry rather than directly speaking or ordering."}, "time_dimension": {"answer": "Present", "rationale": "The keyword is \"like,\" which means the person's current feelings and attitudes. Moreover, the word \"I\" uses the general current, which means that the act has not only happened in the past but may still be ongoing. Thus, the entry describes the person's current state or orientation."}, "sentence_type": "Affirmative declarative sentence", "pos": {"nouns": {"Questionnaire": 1, "Entry": 1, "Poetry": 1}, "verbs": {"Yeah": 1}, "numerals": {"78": 1}}}, {"content": "79. My feelings cannot be vulnerable.", "word_count": 10, "label": "", "sentence_structure": "Subject鈥揷opula鈥損redicative", "subjective_objective": {"answer": "Subjective", "rationale": "Because the vulnerability of a person is a matter of his or her own feelings and experiences, it is a subjective examination."}, "sentence_sentiment": {"answer": "Neutral", "rationale": "Because the entry describes only a person ' s character and does not involve any emotional colour."}, "language_style": {"answer": "Calm and direct", "rationale": "The language style of the entry is calm and straightforward, as it uses simple, clear language to express the subject, without too many embellishment or emotional descriptions. It asks whether the interviewee finds it difficult to hurt his feelings, and this problem does not need to be asked in a euphemism or warmth, and therefore chooses a calm and direct language style."}, "time_dimension": {"answer": "Future", "rationale": "The entry represents the vision and expectations of the testee for what may happen in the future, and the keyword \"inviolability\" refers to situations that have not yet occurred and that may arise in the future."}, "sentence_type": "Affirmative declarative sentence", "pos": {"nouns": {"Emotion": 1, "Hurt": 1}, "verbs": {"Easy": 1, "Yes": 1}, "adjectives": {"It's not easy": 1}, "numerals": {"79": 1}}}, {"content": "I sometimes play animals.", "word_count": 7, "label": "", "sentence_structure": "Subject鈥搗erb鈥搊bject", "subjective_objective": {"answer": "Subjective", "rationale": "Because \"fun\" is the patient's subjective behavior and attitude."}, "sentence_sentiment": {"answer": "Neutral", "rationale": "The entry describes an act that does not involve feelings, attitudes or values and is therefore neutral."}, "language_style": {"answer": "Tactful and warm", "rationale": ""}, "time_dimension": {"answer": "Past", "rationale": "In the entry, \"sometimes\" denotes behaviour that occurred in the past at a certain point in time, and therefore judgement is past."}, "sentence_type": "Affirmative declarative sentence", "pos": {"nouns": {"Animals": 1}, "verbs": {"- You're kidding me": 1, "have": 1}, "adverbs": {"Sometimes": 1}, "numerals": {"80": 1}, "pronouns": {"I": 1}}}, {"content": "I think I'd like to do a kind of job as a forest manager.", "word_count": 18, "label": "", "sentence_structure": "Subject鈥搗erb鈥搊bject", "subjective_objective": {"answer": "Subjective", "rationale": "Because \"like\" is the patient's subjective attitude and expectations."}, "sentence_sentiment": {"answer": "Positive", "rationale": "Because it expresses personal preferences and work intentions, it has a positive emotional colour."}, "language_style": {"answer": "Tactful and warm", "rationale": "The entry expresses its thoughts and expectations in the first person's name, using the expression \"I want\" to express a more subjective desire and expectation. At the same time, the expression \"I like to be dry\" gives people a sense of kindness, moderation and a more natural flow of communication with the person under investigation."}, "time_dimension": {"answer": "Future", "rationale": "According to \"I want\" and \"The Council\", the entry expresses vision and expectations for the future, and therefore judges the future."}, "sentence_type": "Affirmative declarative sentence", "pos": {"nouns": {"I think": 1, "Yes": 1, "Yeah": 1, "Fuck": 1, "Forest Administrator": 1, "Category I": 1, "Work": 1}, "verbs": {"Yes": 2, "Yeah": 1, "Fuck": 1}, "adverbs": {"81锛?: 1, "What kind of guy": 1}, "numerals": {"81": 1}}}, {"content": "I can't argue with others when they are.", "word_count": 15, "label": "", "sentence_structure": "Subject鈥搗erb鈥搊bject", "subjective_objective": {"answer": "Subjective", "rationale": "This is a subjective examination of the patients ' own feelings and perceptions."}, "sentence_sentiment": {"answer": "Negative", "rationale": "Because the \"no one can compete\" act in the entry is negative."}, "language_style": {"answer": "Calm and direct", "rationale": "The language style of the entry is straightforward and calm, because it raises the question in a concise and clear manner, without using excessive rhetoric or emotional colours.\""}, "time_dimension": {"answer": "Present", "rationale": "In the entry, the word \"normal\" denotes a regular, customary act, while the \"debate\" action occurred at some point in the past and may still be ongoing. Thus, the time dimension of the article is now."}, "sentence_type": "Negative declarative sentence", "pos": {"nouns": {"Time": 1, "others": 2, "Argument": 1}, "verbs": {"Yes": 1}, "adverbs": {"Regular": 1}, "numerals": {"82": 1}, "pronouns": {"I": 1}}}, {"content": "83. Anyone who had the capacity and willingness to work hard could succeed.", "word_count": 22, "label": "", "sentence_structure": "Subject鈥損redicate", "subjective_objective": {"answer": "Objective", "rationale": "Because \"just ...\" expresses an objective relationship of conditionality that does not involve subjective attitudes or feelings of the patient. At the same time, the descriptions of \"capable\" and \"willing to work hard\" are based on objective criteria and assumptions, so the entire sentence is viewed from an objective perspective."}, "sentence_sentiment": {"answer": "Positive", "rationale": "Because the idea of \"whoever is capable and willing to work hard\" in the entry is positive."}, "language_style": {"answer": "Tactful and warm", "rationale": "The entry uses a soft, invitation-style tone for dialogue, emphasizes the importance of individual abilities and will for success and encourages respondents to actively face challenges and pursue goals. This expression is more personal and interactive."}, "time_dimension": {"answer": "Future", "rationale": "The verbs \"may\" and \"will\" indicate that this is about the possibility and the will of the future, and therefore the temporal dimension is to be judged in the future."}, "sentence_type": "Affirmative declarative sentence", "pos": {"nouns": {"Capacity": 1, "person": 2, "Work": 1, "Success": 1}, "verbs": {"Just": 1, "he": 1, "Yes": 4, "Try": 1, "be": 1}, "adverbs": {"Any": 1}, "numerals": {"83": 1}}}, {"content": "84. Recently, I felt it was easy to give up hope for something.", "word_count": 18, "label": "", "sentence_structure": "Subject鈥揷opula鈥損redicative", "subjective_objective": {"answer": "Subjective", "rationale": "Because \"I think\" expresses the feelings and thoughts of the patient himself, and it's a subjective perspective."}, "sentence_sentiment": {"answer": "Negative", "rationale": "Because the \"easy to give up\" act in the entry is negative."}, "language_style": {"answer": "Tactful and warm", "rationale": "The entry raises questions in a caring and understanding manner, using more moderate terms, without too strong emotional or blame, and is more likely to trigger resonance and reflection."}, "time_dimension": {"answer": "Present", "rationale": "The entry \"recently\" indicates the latest period, i.e. the time dimension, is now judged."}, "sentence_type": "Negative declarative sentence", "pos": {"nouns": {"Recently": 1, "I": 1, "Things": 1, "Hope": 1}, "verbs": {"I think": 1, "Easy": 1, "Abandon": 1}, "adverbs": {"very": 1, "Some": 1}, "numerals": {"84": 1}}}, {"content": "85. Sometimes I am attracted to something that is useless to me, such as shoes, gloves, etc., but I always try to touch it or steal it.", "word_count": 44, "label": "", "sentence_structure": "Subject鈥搗erb鈥搊bject", "subjective_objective": {"answer": "Subjective", "rationale": "Because \"to be attracted to something other than what you have\" and \"to try to touch it or steal it\" are subjective thoughts and feelings of patients."}, "sentence_sentiment": {"answer": "Negative", "rationale": "Because the entry \"although these things are useless to me, I always want to touch them or steal them\" is negative."}, "language_style": {"answer": "Calm and direct", "rationale": "The entry is written in plain and simple language, is not used in excessive speech or emotional rendering and is more objective."}, "time_dimension": {"answer": "Past", "rationale": "Because \"sometimes\" indicates that it is a past habit or act, and \"no use\" and \"always\" are also the manifestations of events that have occurred in the past."}, "sentence_type": "Affirmative declarative sentence", "pos": {"nouns": {"person": 1, "Something": 2, "Shoes": 1, "Gloves": 1, "It's useless": 1, "Use": 1}, "verbs": {"By": 1, "Attractive": 1, "have": 1, "Touch": 1, "Steal": 1, "Come on": 1}, "adjectives": {"Serious": 1, "Nothing": 1}, "adverbs": {"Sometimes": 1, "Always": 1}, "numerals": {"85": 1}, "classifiers": {"individual": 1}, "pronouns": {"I": 1}}}, {"content": "86. I do lack self-confidence.", "word_count": 8, "label": "", "sentence_structure": "Subject鈥搗erb鈥揷omplement", "subjective_objective": {"answer": "Subjective", "rationale": "Because lack of self-confidence is a subjective feeling or emotional state."}, "sentence_sentiment": {"answer": "Negative", "rationale": "Because the description of \"lack of self-confidence\" in the entry is negative."}, "language_style": {"answer": "Tactful and warm", "rationale": "The language style of the entry is euphemism. It does not use direct or negative language to describe the lack of confidence of the person under investigation, but rather asks the other person whether he or she really feels this way in a moderate, understanding and supportive manner. This expression is more respectful of and attentive to the psychological state of the person under investigation, and helps to create a better atmosphere of communication and trust."}, "time_dimension": {"answer": "Past", "rationale": "\"A real lack of self-confidence\" indicates that the testee has a lack of confidence and suspicion of his or her abilities at the current point in time, so the entry describes the past."}, "sentence_type": "Negative declarative sentence", "pos": {"nouns": {"Grandpa": 1, "Questionnaire": 1, "Entry": 1, "Self-confidence": 1}, "verbs": {"Analysis": 1, "Missing": 1}, "adverbs": {"Indeed": 1}, "numerals": {"86锛?: 1}}}, {"content": "I'd like to be a florist.", "word_count": 8, "label": "", "sentence_structure": "Subject鈥損redicate", "subjective_objective": {"answer": "Subjective", "rationale": "This is because of the patient ' s personal will and wishes and is subjective."}, "sentence_sentiment": {"answer": "Positive", "rationale": "Because the act of being a florist expresses a positive and positive attitude."}, "language_style": {"answer": "Tactful and warm", "rationale": "The entry is more euphemistic, is not based on direct orders or strong tone. It raises questions in an open and invited manner, giving the interviewee the opportunity to express his or her thoughts and wishes. Moreover, the entry does not assume or pre-set any answers to the interviewee, making it more inclusive and flexible."}, "time_dimension": {"answer": "Future", "rationale": "The entry indicates that the person tested has the will to become a florist in the future and therefore falls within the scope of the future."}, "sentence_type": "Affirmative declarative sentence", "pos": {"nouns": {"Flowers": 1}, "verbs": {"Yes": 1, "Do it": 1}, "numerals": {"87": 1}}}, {"content": "88. I always felt that life was worth it.", "word_count": 11, "label": "", "sentence_structure": "Subject鈥揷opula鈥損redicative", "subjective_objective": {"answer": "Subjective", "rationale": "Because this phrase expresses patients' subjective views and attitudes to life."}, "sentence_sentiment": {"answer": "Positive", "rationale": "Because the attitude of \"life is worth it\" in the entry is positive, indicating optimism and positive perception of life."}, "language_style": {"answer": "Tactful and warm", "rationale": "The language style of the entry is euphemism. It does not directly ask questions or order respondents to answer, but rather expresses their views in an encouraging and open manner and invites respondents to share their views."}, "time_dimension": {"answer": "Present", "rationale": "The keyword \"generally\" denotes a regular, customary act, \"likely\" adopts the general current, which means that the act has not only occurred in the past but may still be ongoing. Thus, the temporal dimension of the purpose of the article is now judged."}, "sentence_type": "Affirmative declarative sentence", "pos": {"verbs": {"I think": 1, "have": 1}, "adjectives": {"It's valuable": 1}, "adverbs": {"Total": 1}, "pronouns": {"I": 1}}}, {"content": "89. There is a debate about the truth to convince the majority of the people.", "word_count": 22, "label": "", "sentence_structure": "Subject鈥搗erb鈥搊bject", "subjective_objective": {"answer": "Subjective", "rationale": "Because \"most people's belief\" is a subjective judgement, and \"to have a debate\" can be considered a strategy or a method. Thus, \"to have most people believe the truth is to have a debate\" is more a subjective expression of the idea that it is based on the views of individuals or some."}, "sentence_sentiment": {"answer": "Neutral", "rationale": "The entry is not clearly emotional or value-based."}, "language_style": {"answer": "Tactful and warm", "rationale": "The language style of the entry is euphemism, which does not speak or order directly, but rather engages in dialogue in a gentle and inviting tone. This expression is more personal and interactive, and it is more likely to attract interest and reflection from the other side. At the same time, the entry refers to the importance of debate, but does not impose or threaten that the other side must participate in the debate, which is more respectful of the other side鈥檚 will and ideas."}, "time_dimension": {"answer": "Present", "rationale": "The entry now describes a widespread phenomenon or view that in modern societies, consensus is often required through debate. This view does not relate to past or future behaviour, and therefore the temporal dimension is now judged."}, "sentence_type": "Affirmative declarative sentence", "pos": {"nouns": {"person": 2, "The facts": 1, "The truth": 1, "Debate": 1}, "verbs": {"鈾?And I'm gonna be 鈾?: 1, "Letter": 1, "Yes": 4}, "adjectives": {"Large": 1}, "numerals": {"89": 1}, "pronouns": {"Yes": 1}}}, {"content": "Sometimes I drag what is supposed to be done today to tomorrow.", "word_count": 17, "label": "", "sentence_structure": "Subject鈥搗erb鈥搊bject", "subjective_objective": {"answer": "Subjective", "rationale": "The entry for this questionnaire is to examine the subjective attitudes and behaviour of the patient and is therefore subjective."}, "sentence_sentiment": {"answer": "Negative", "rationale": "Because the entry \"Tow what should be done today to tomorrow\" is a delayed and negative emotional act."}, "language_style": {"answer": "Calm and direct", "rationale": "The entry uses direct and clear language to express the subject of a question about delays in the conduct of the matter by the interviewees, without too many rhetorical or emotional expressions and with a more objective tone."}, "time_dimension": {"answer": "Present", "rationale": ""}, "sentence_type": "Affirmative declarative sentence", "pos": {"nouns": {"Today": 1, "Yes": 1, "Tomorrow": 1}, "verbs": {"Will": 1, "Do it": 1}, "adverbs": {"Sometimes": 1}, "numerals": {"90锛?: 1}}}, {"content": "I don't care about people. I'm kidding.", "word_count": 11, "label": "", "sentence_structure": "Subject鈥搗erb鈥搊bject", "subjective_objective": {"answer": "Subjective", "rationale": "Because \"no care\" and \"joke\" are personal feelings and attitudes of patients, and they are subjective."}, "sentence_sentiment": {"answer": "Negative", "rationale": "Because the \"no one cares about me\" attitude in the entry is negative."}, "language_style": {"answer": "Calm and direct", "rationale": "The language style of the entry is a calm and straightforward type. The judgement is based on the fact that the entry expresses a view or attitude in the form of a direct statement, namely, that \"I don't care about others' jokes\", that it does not use too much qualifier or rhetorical techniques, and that it is more calm and objective. It also does not contain too much emotional or subjective evaluation, and focuses more on factual description and transmission of information."}, "time_dimension": {"answer": "Present", "rationale": "According to the sentence entered, \"not to care about people making fun of me\" is describing a current attitude or mentality, so the time dimension of the questionnaire entry is now."}, "sentence_type": "Negative declarative sentence", "pos": {"nouns": {"I": 1, "others": 1}, "verbs": {"I don't care": 1, "Open": 1, "A joke": 1}, "adverbs": {"not": 1, "Well": 1, "I care": 1}, "numerals": {"91": 1, "Don't": 1, "individual": 1}}}, {"content": "I want to be a nurse.", "word_count": 6, "label": "", "sentence_structure": "Subject鈥損redicate", "subjective_objective": {"answer": "Subjective", "rationale": "Because \"want to be a nurse\" is a subjective will and desire of a person, a subjective feeling and an idea within the individual."}, "sentence_sentiment": {"answer": "Neutral", "rationale": "Because the entry is not clearly emotional, it is just an idea."}, "language_style": {"answer": "Tactful and warm", "rationale": "The entry is more euphemistic. It expresses the interest or desire of the other side in a way that is asked, without direct orders or requests, making the conversation more interactive and friendly. At the same time, the name of the profession, Nurse, gives a positive feeling of kindness and friendship to the questionnaire."}, "time_dimension": {"answer": "Present", "rationale": "In this entry, the word \"I want\" is a general expression of the present state of affairs, which is the present or the recent desire and thought. Therefore, the time dimension of the article should be the present."}, "sentence_type": "Imperative sentence", "pos": {"nouns": {"Nurse": 1, "I": 1}, "verbs": {"Yes": 1, "When": 1}, "numerals": {"92": 1}}}, {"content": "93. I think most people lie about climbing up.", "word_count": 19, "label": "", "sentence_structure": "Subject鈥搗erb鈥搊bject", "subjective_objective": {"answer": "Subjective", "rationale": "This is a manifestation of subjective attitudes, as patients perceive and evaluate the majority of people."}, "sentence_sentiment": {"answer": "Negative", "rationale": "Because the \"false\" act in the entry is negative."}, "language_style": {"answer": "Calm and direct", "rationale": "The entry presents its views in a direct manner, without using a rhetorical or emotional rendering, which is more calm and objective, allowing the interviewee to understand the problem more directly. \""}, "time_dimension": {"answer": "Present", "rationale": ""}, "sentence_type": "Affirmative declarative sentence", "pos": {"nouns": {"person": 2, "Lies": 1, "Climb": 1, "Questionnaire": 1}, "verbs": {"I think": 1, "For": 1, "Say it": 1, "Whatever": 1}, "adjectives": {"Up": 1, "Most of them": 1}, "numerals": {"93": 1}, "pronouns": {"I": 1}}}, {"content": "94. Many things I regret when I do.", "word_count": 13, "label": "", "sentence_structure": "Subject鈥搗erb鈥揷omplement", "subjective_objective": {"answer": "Subjective", "rationale": "Because \"repension\" is a personal emotional experience and subjective feeling, the question is the subjective perspective of the patient."}, "sentence_sentiment": {"answer": "Negative", "rationale": "Because the \"register after doing\" in the entry is negative."}, "language_style": {"answer": "Tactful and warm", "rationale": "This expression is more likely to bring the questionnaire closer to the respondents and to increase the voluntary and active nature of the questionnaire."}, "time_dimension": {"answer": "Present", "rationale": "The entry asks the testee about the feelings and reflections of what has been done in the past, so the temporal dimension is now judged. At the same time, \"many things\" also indicate that this feeling is directed at a series of specific behaviours rather than a particular event."}, "sentence_type": "Affirmative declarative sentence", "pos": {"nouns": {"Things": 2, "Regret": 1}, "verbs": {"Do it": 1, "Pass": 1}, "adjectives": {"Many": 1}, "adverbs": {"Many": 1}, "numerals": {"94": 1}, "classifiers": {"individual": 1}, "pronouns": {"I": 1}}}, {"content": "95. I go to church (or to temples) almost every week.", "word_count": 15, "label": "", "sentence_structure": "Subject鈥損redicate", "subjective_objective": {"answer": "Subjective", "rationale": "The subject is one of the patient ' s own behaviour and frequency, personal choice and habits, and subjective attitudes."}, "sentence_sentiment": {"answer": "Neutral", "rationale": "Because the entry describes a frequency of behaviour and does not have a clear emotional colour."}, "language_style": {"answer": "Tactful and warm", "rationale": "This entry uses euphemism to ask whether the person interviewed regularly participates in religious activities without direct orders or assertions."}, "time_dimension": {"answer": "Present", "rationale": "The phrase \"almost weekly\" in that sentence denotes a more frequent act, while the word \"go\" uses the usual current state of affairs, meaning that the act has not only occurred in the past but may still continue to be so. Thus, the entry is a description of the act or state of present."}, "sentence_type": "Affirmative declarative sentence", "pos": {"nouns": {"Church": 1, "Temple": 1}, "verbs": {"Go on": 2, "Always": 1}, "adverbs": {"Almost": 1, "Every week": 1}, "numerals": {"95": 1}, "pronouns": {"I": 1}}}, {"content": "96. I hardly ever had any argument with my family.", "word_count": 11, "label": "", "sentence_structure": "Subject鈥搗erb鈥揷omplement", "subjective_objective": {"answer": "Subjective", "rationale": "Because \"almost never loud\" is a subjective assessment that reflects the patient's own perception of relationships with family members."}, "sentence_sentiment": {"answer": "Positive", "rationale": "Because the \"near a lot of family quarrel\" in the entry is positive."}, "language_style": {"answer": "Tactful and warm", "rationale": ""}, "time_dimension": {"answer": "Present", "rationale": "\"I hardly ever had a fight with my family\" means \"almost no\" in frequency, \"almost\" means \"a fight\" means a past act, so it can be judged that the entry is a description of the present situation."}, "sentence_type": "Negative declarative sentence", "pos": {"nouns": {"Home": 1, "person": 2, "mouth": 1}, "verbs": {"Louder": 1}, "adjectives": {"Less": 1, "Almost": 1}, "adverbs": {"Almost": 1}, "numerals": {"96": 1}, "pronouns": {"I": 1}}}, {"content": "97. Sometimes I have a serious impulse to do something amazing or harmful.", "word_count": 22, "label": "", "sentence_structure": "Subject鈥搗erb鈥揷omplement", "subjective_objective": {"answer": "Subjective", "rationale": "Because \"a serious impulse\" and \"doing something amazing or harmful\" are subjective feelings and thoughts of patients."}, "sentence_sentiment": {"answer": "Negative", "rationale": "Because the entry expresses an unhealthy internal impulse and possible harmful behaviour, which has a negative emotional connotation."}, "language_style": {"answer": "Tactful and warm", "rationale": "The entry is more euphemistic, is not directly ordered or questioned about the other party's impulsive behaviour, but invites the other party to answer the question in an understanding and caring manner. At the same time, the expression \"serious\" and \"shocking or harmful\" is used to give greater expression to the concerns and understanding of the person under investigation."}, "time_dimension": {"answer": "Present", "rationale": "Sometimes it's a constant situation, and \"severe impulses\" it means that the act is taking place, so time dimension is now judged."}, "sentence_type": "Affirmative declarative sentence", "pos": {"nouns": {"Plant": 1, "Expedition": 1, "Yes": 2, "Amazing": 1, "Harmful": 1}, "verbs": {"have": 1, "Do it": 2, "Go on": 1}, "adjectives": {"Serious": 1}, "adverbs": {"Sometimes": 1}, "numerals": {"97": 1}}}, {"content": "I believe in good and bad and evil.", "word_count": 11, "label": "", "sentence_structure": "Subject鈥損redicate", "subjective_objective": {"answer": "Subjective", "rationale": "Because the word \"rebel\" represents the patient's subjective attitude or belief."}, "sentence_sentiment": {"answer": "Positive", "rationale": "Because the notion of \"belief for good, for good, for evil\" has positive values."}, "language_style": {"answer": "Tactful and warm", "rationale": "The language style of the entry is more moderate, not too harsh or emotional, but rather a peaceful and inviting tone of the question, more personal and interactive."}, "time_dimension": {"answer": "Present", "rationale": "The entry expresses a conviction or view and does not address past or future acts or events, and therefore the judgement is \"now\"."}, "sentence_type": "Affirmative declarative sentence", "pos": {"nouns": {"Yes": 1, "Good and good": 1, "There's a bad deal": 1}, "verbs": {"I": 1}}}, {"content": "I like to go to a party.", "word_count": 10, "label": "", "sentence_structure": "Subject鈥搗erb鈥揷omplement", "subjective_objective": {"answer": "Subjective", "rationale": "Because \"like\" is patient's subjective attitude."}, "sentence_sentiment": {"answer": "Positive", "rationale": "Because the \"like to party\" act is positive."}, "language_style": {"answer": "Tactful and warm", "rationale": "The entry is more personal and interactive when it is spoken in a gentle and inviting manner, rather than in direct statements or orders. At the same time, the phrase \"I like to participate in a party\" expresses positive attitudes and emotional tendencies, making it more likely that the respondents to the questionnaire are willing to share their real thoughts and feelings."}, "time_dimension": {"answer": "Present", "rationale": "The keyword is \"like to participate\" , which is a sign of current tendencies and behavior. Moreover, \"hot parties\" also suggest that such behaviour may occur in the immediate or near future, so the entry reflects the situation of the subject."}, "sentence_type": "Affirmative declarative sentence", "pos": {"nouns": {"Yeah": 1, "Party": 1}, "verbs": {"Participation": 1, "Yeah": 1}, "adjectives": {"It's hilarious": 1}, "numerals": {"99锛?: 1}}}, {"content": "100. I was hesitant to face a number of questions that were all too far-reaching.", "word_count": 19, "label": "", "sentence_structure": "Subject鈥搗erb鈥揷omplement", "subjective_objective": {"answer": "Subjective", "rationale": "Because hesitation is the subjective feeling and attitude of the patient."}, "sentence_sentiment": {"answer": "Negative", "rationale": "Because the act of \"suspensive\" is negative and expresses a confused and uncertain emotional attitude."}, "language_style": {"answer": "Tactful and warm", "rationale": "The language style of the entry is more euphemistic, using the words \"touching\" and \"suspensive\" to express problems and feelings, rather than using a strong negative or positive word directly, which is more friendly and acceptable. Moreover, the sentence is simpler and less complex, and there are no complex sentences or rhetorical techniques that make it easier for the respondents to understand and answer questions."}, "time_dimension": {"answer": "Present", "rationale": ""}, "sentence_type": "Negative declarative sentence", "pos": {"nouns": {"Problem": 2, "Whispering": 1, "Decision": 1}, "verbs": {"I've got it": 1, "鈾?And I'm gonna be 鈾?: 1, "I feel": 1, "Decision": 1}, "adverbs": {"I've got a lot of things to do": 1, "not": 1}, "numerals": {"100锛?: 1}}}, {"content": "101. I believe that women should have the same freedom in their sexual life as men.", "word_count": 23, "label": "", "sentence_structure": "Subject鈥搗erb鈥搊bject", "subjective_objective": {"answer": "Subjective", "rationale": "Because \"think\" is the patient's subjective attitude."}, "sentence_sentiment": {"answer": "Positive", "rationale": "Because the position expressed in the entries is to support the right of women to freedom and equality in their sexual life, which is positive emotional."}, "language_style": {"answer": "Calm and direct", "rationale": "The entry directly expresses the subject of questioning the other party ' s opinion of the freedom of women in sexual life, without excessive rhetorical or emotional rendering, and is more objective in tone."}, "time_dimension": {"answer": "Present", "rationale": "The entry expresses the current view and attitude of the interviewees, and the words \"considers\" and \"should\" indicate that this is their view, not past or future events."}, "sentence_type": "Affirmative declarative sentence", "pos": {"nouns": {"A woman": 1, "Sex": 1, "In terms of": 1, "You're a good guy": 1, "Freedom": 1}, "verbs": {"I think": 1, "I think so": 1, "have": 1}, "numerals": {"101": 1}, "pronouns": {"I": 1}}}, {"content": "102. I think the hardest to control is myself.", "word_count": 12, "label": "", "sentence_structure": "Subject鈥搗erb鈥搊bject", "subjective_objective": {"answer": "Subjective", "rationale": "Because \"the hardest to control is me\" is the subjective expression of self-awareness by patients."}, "sentence_sentiment": {"answer": "Negative", "rationale": "Because the expression \"most difficult to control\" in the entry is that I express a negative opinion of myself."}, "language_style": {"answer": "Tactful and warm", "rationale": "The language style of the entry is more euphemistic, expressing the individual's views in a way that I think is \"in order\" rather than direct orders or assertions. Moreover, the choice of words in the phrase is more peaceful and not over-repeated by negative emotions. All of this makes the questionnaire's subject more gentle and friendly."}, "time_dimension": {"answer": "Present", "rationale": "In the entry, \"I think\" indicates that this is a statement of a personal subjective feeling, and \"the hardest part is for me\" to describe the current state and the view, and therefore it belongs to the present."}, "sentence_type": "Affirmative declarative sentence", "pos": {"nouns": {"Difficulties": 1, "Control": 1, "self": 1}, "verbs": {"I think": 1, "be": 1}, "adjectives": {"Hard": 1}, "adverbs": {"Most": 1}, "numerals": {"102": 1}, "pronouns": {"I": 1}}}, {"content": "103. I have few muscle cramps or tremors.", "word_count": 14, "label": "", "sentence_structure": "Subject鈥揷opula鈥損redicative", "subjective_objective": {"answer": "Objective", "rationale": "Because \"muscle cramps or tremors\" are a physical phenomenon that can be observed and measured, not the psychological feelings or thoughts of the patient. The problem is therefore the objective physical condition of the patient."}, "sentence_sentiment": {"answer": "Neutral", "rationale": "As the entry describes only a physical condition and does not involve any positive or negative emotions, attitudes or behaviour."}, "language_style": {"answer": "Tactful and warm", "rationale": "The entry is more personal and interactive in its presentation, in a way that is conducted in a way that asks about the other party's state of health, without direct statements or orders. At the same time, the use of the term \"very little\" avoids too positive or negative a phrase that reflects the neutrality and objectivity of the questionnaire survey."}, "time_dimension": {"answer": "Present", "rationale": "The entry indicates the status and behaviour of the respondent in replying to the question, using the general current \"Yes\", indicating the current situation and thus judging the present."}, "sentence_type": "Negative declarative sentence", "pos": {"nouns": {"Muscles": 1, "Twitches": 1, "Wreck": 1, "Mistake": 1}, "verbs": {"I don't have much": 1}, "adverbs": {"Very few": 1, "have": 1}, "numerals": {"103": 1}, "pronouns": {"I": 1}}}, {"content": "104. I seem to care about nothing.", "word_count": 12, "label": "", "sentence_structure": "Subject鈥揷opula鈥損redicative", "subjective_objective": {"answer": "Subjective", "rationale": "Because \"not caring\" is a subjective attitude and feeling, which reflects the patient's psychological state and emotional experience. Although this attitude may be caused by objective factors (e.g. life stress, emotional problems, etc.), in this questionnaire context the description \"not caring\" is a subjective view of the patient, aimed at understanding his emotional reactions and mental health."}, "sentence_sentiment": {"answer": "Negative", "rationale": "Because the attitude of \"not caring\" in the entries is negative."}, "language_style": {"answer": "Calm and direct", "rationale": "The entry is conducted in a direct question-and-answer manner, without the use of too much reverb or emotional language, and is expressed in a more objective and concise manner."}, "time_dimension": {"answer": "Present", "rationale": "The entry describes the current feelings or attitudes of the testee, \"as if\" indicating that the feeling is uncertain or has not yet developed a fixed view, \"does not care about anything\" being the behavior or emotional state of the dominant language, so the temporal dimension of the entry is now judged."}, "sentence_type": "Negative declarative sentence", "pos": {"verbs": {"I care": 1}, "adjectives": {"Seems like": 1, "Both": 1}, "numerals": {"104": 1}}}, {"content": "105. When I'm not feeling well, I sometimes lose my temper.", "word_count": 15, "label": "", "sentence_structure": "Subject鈥揷opula鈥損redicative", "subjective_objective": {"answer": "Subjective", "rationale": "Because \"smuggling\" is the patient's subjective reaction and emotional experience. Although physical incomprehension may be objective, the reaction to it is subjective."}, "sentence_sentiment": {"answer": "Negative", "rationale": "Because the \"fun\" act in the entry is negative."}, "language_style": {"answer": "Tactful and warm", "rationale": "The language style of the entry is more euphemistic and caring. It uses a means of asking questions that allow the person under investigation to express his or her feelings and thoughts freely, rather than directly ordering or accusing them."}, "time_dimension": {"answer": "Present", "rationale": "Now the entry indicates that the testees are describing the current situation, that is, sometimes they are tempered when they are not feeling well. Therefore, the time dimension is now judged."}, "sentence_type": "Affirmative declarative sentence", "pos": {"nouns": {"Time": 1, "Body": 1, "- Grumpy": 1}, "verbs": {"I": 2, "have": 1, "Let's go": 1}, "adjectives": {"not": 1, "- Yeah": 1}, "adverbs": {"Sometimes": 1}, "numerals": {"105锛?: 1}}}, {"content": "I always feel like I've done something wrong or committed something.", "word_count": 21, "label": "", "sentence_structure": "Subject鈥揷opula鈥損redicative", "subjective_objective": {"answer": "Subjective", "rationale": "Because the words \"like\" and \"like\" indicate that it's a patient's own subjective feeling and idea."}, "sentence_sentiment": {"answer": "Negative", "rationale": "Because the idea of \"doing something wrong or committing something wrong\" in the entry is negative."}, "language_style": {"answer": "Tactful and warm", "rationale": "The entry is more adept and interactive when it is spoken in a gentle and inviting manner, rather than by direct statements or orders."}, "time_dimension": {"answer": "Past", "rationale": "\"As always\" means a constant state of emotion, implying that it has existed for some time. Moreover, the expression \"like to have done something wrong or committed something wrong\" also indicates that the sentiment is about past experience or behaviour, so it can be judged that the entry describes the behaviour or mentality of the person tested in the past."}, "sentence_type": "Affirmative declarative sentence", "pos": {"nouns": {"self": 2, "Yes": 1, "Sin": 1}, "verbs": {"The general feeling": 1, "Wrong": 1, "Yes": 1}, "adverbs": {"It looks like": 1}, "numerals": {"106": 1}}}, {"content": "107. I am often happy.", "word_count": 7, "label": "", "sentence_structure": "Subject鈥揷opula鈥損redicative", "subjective_objective": {"answer": "Subjective", "rationale": "Because \"often happy\" is a subjective feeling or attitude, not an objective fact or result of behaviour."}, "sentence_sentiment": {"answer": "Positive", "rationale": "Because the entry \"often happy\" expresses a positive emotional attitude."}, "language_style": {"answer": "Tactful and warm", "rationale": "The language style of the entry is relatively euphemistic, warm, gives a positive emotional and friendly tone by using the term \"often\"."}, "time_dimension": {"answer": "Present", "rationale": ""}, "sentence_type": "Affirmative declarative sentence", "pos": {"verbs": {"Regular": 1, "be": 1, "Happy": 1}, "adjectives": {"Happy": 1}, "adverbs": {"Regular": 1}, "numerals": {"107": 1}}}, {"content": "108. I often feel like my head is swollen.", "word_count": 11, "label": "", "sentence_structure": "Subject鈥揷opula鈥損redicative", "subjective_objective": {"answer": "Subjective", "rationale": "Because this is a self-perception and experience of the patient, it is subjective in its assessment."}, "sentence_sentiment": {"answer": "Negative", "rationale": "Because the \"head-breathing\" symptoms of physical discomfort in the entries are negative."}, "language_style": {"answer": "Tactful and warm", "rationale": "The entry uses descriptive language rather than directly asking the other side whether there is a certain symptom, which is more moderate and respectful of the interviewee ' s privacy, and which also allows him to share his feelings."}, "time_dimension": {"answer": "Present", "rationale": "The keywords are \"and \"heads are sorely swollen\" that they describe the symptoms or experiences of the present, and they judge them as now."}, "sentence_type": "Affirmative declarative sentence", "pos": {"nouns": {"I": 1, "Head": 1, "Boo": 1, "Nose plug": 1}, "verbs": {"I think": 1, "Like": 1}, "adjectives": {"Head swelling, nose plugs": 1}}}, {"content": "109. Some people are too arrogant to fight with them even if I know they are right.", "word_count": 24, "label": "", "sentence_structure": "Subject鈥搗erb鈥搊bject", "subjective_objective": {"answer": "Subjective", "rationale": "Because even if I knew they were right, it was a subjective attitude of the patient."}, "sentence_sentiment": {"answer": "Negative", "rationale": "Because the \"to the hegemonic\" act in the entry is negative and expresses opposition to others."}, "language_style": {"answer": "Calm and direct", "rationale": "The entry is based on a clear and straightforward statement of whether the other party has acted in opposition to others. At the same time, the entry does not use excessive rhetorical or emotional overtook, and is more balanced and objective, so that its language style can be considered as being straightforward and calm."}, "time_dimension": {"answer": "Past", "rationale": ""}, "sentence_type": "Negative declarative sentence", "pos": {"nouns": {"person": 1, "The king": 1, "to": 1, "Fuck": 1}, "verbs": {"Yes": 2, "Right": 1}, "adjectives": {"Too much": 1}, "adverbs": {"Ming": 1}, "numerals": {"109": 1, "One": 2, "Both": 1}, "pronouns": {"I": 1, "they": 2}}}, {"content": "I'm being hurt.", "word_count": 5, "label": "", "sentence_structure": "Subject鈥搗erb鈥揷omplement", "subjective_objective": {"answer": "Subjective", "rationale": "Because \"someone wants to hurt me\" is the patient's subjective idea and feeling."}, "sentence_sentiment": {"answer": "Negative", "rationale": "Because the expression \"someone wants to hurt me\" in the entry is negative and emotional, indicating potential threats and dangers."}, "language_style": {"answer": "Calm and direct", "rationale": "The entry directly expresses the subject of an inquiry into whether the other party has been the victim of malicious attacks or conspiracy, without excessive rhetoric or emotional rendering, and with a more objective tone."}, "time_dimension": {"answer": "Present", "rationale": "The phrase \"someone wants to hurt me\" in that sentence indicates a concern or feeling that exists, and that time is now."}, "sentence_type": "Affirmative declarative sentence", "pos": {"nouns": {"person": 1, "Shit": 1}, "verbs": {"Yes": 1, "Shit": 1}, "numerals": {"110": 1}}}, {"content": "I have never done anything dangerous in the search for stimulus.", "word_count": 17, "label": "", "sentence_structure": "Subject鈥搗erb鈥揷omplement", "subjective_objective": {"answer": "Objective", "rationale": "Because the term \"never\" is the patient's subjective attitude and the objective behaviour of seeking to stimulate and do dangerous things."}, "sentence_sentiment": {"answer": "Neutral", "rationale": "As this entry does not express positive or negative attitudes, behaviour, purpose or quality."}, "language_style": {"answer": "Calm and direct", "rationale": "The language style of the entry is more direct and concise, and there is no excessive rhetorical or emotional rendering, mainly based on objective representations, which are consistent with the linguistic characteristics of the questionnaire."}, "time_dimension": {"answer": "Present", "rationale": "\"Never\" indicates that this is a past-time-completed sentence, that \"doing dangerous things for the purpose of seeking an incentive\" is a description of past behaviour, and it is therefore a question of whether the person tested had done so in the past."}, "sentence_type": "Negative declarative sentence", "pos": {"nouns": {"Investigations": 1, "Exciting": 1, "Danger": 2}, "verbs": {"Do it": 1}, "adverbs": {"Never": 1}, "numerals": {"111": 1}}}, {"content": "112. I often believe that it is necessary to insist on what I believe is right.", "word_count": 18, "label": "", "sentence_structure": "Subject鈥搗erb鈥搊bject", "subjective_objective": {"answer": "Subjective", "rationale": "Because \"I think\" means that it's personal and subjective."}, "sentence_sentiment": {"answer": "Positive", "rationale": "Because the purpose of the act of \"maintenance of what you think is right\" is to be positive and to show positive attitude and quality."}, "language_style": {"answer": "Calm and direct", "rationale": "The entry is dialogueable in a direct interview, with a more calm and objective language style, without excessive rhetorical or emotional rendering.\""}, "time_dimension": {"answer": "Present", "rationale": "\"I'm always\" means \"recurring, \"concepting\" that the general current means that this act has not only happened in the past, but may still be ongoing. \"Consistence\" is also a state of conduct that is currently under way."}, "sentence_type": "Affirmative declarative sentence", "pos": {"nouns": {"Questionnaire Entry": 1, "Yes": 1, "Hold on": 1, "Correct": 1}, "verbs": {"I think": 2, "I have to": 1, "Hold on": 1, "Do it": 1}, "adjectives": {"Often": 1}, "numerals": {"112锛?: 1, "I": 1}, "pronouns": {"Those": 1, "I think": 1}}}, {"content": "113. I believe in the rule of law.", "word_count": 5, "label": "", "sentence_structure": "Subject鈥搗erb鈥搊bject", "subjective_objective": {"answer": "Subjective", "rationale": "Because belief in the rule of law is a subjective attitude or belief."}, "sentence_sentiment": {"answer": "Positive", "rationale": "Because the attitude of \"rebel of the rule of law\" in the entry is positive."}, "language_style": {"answer": "Calm and direct", "rationale": "The entry speaks directly to the subject -- \"I\" believes in the rule of law, does not have too much emotional or rhetorical style, and is more calm and straightforward."}, "time_dimension": {"answer": "Future", "rationale": "The keyword is \"convincing\", indicating vision and expectations for the future; while \"rule of law\" is a future theme or context, the temporal dimension of the article is thus judged to be future."}, "sentence_type": "Affirmative declarative sentence", "pos": {"verbs": {"Yes": 1, "Rule of law": 1}, "numerals": {"113锛?: 1}}}, {"content": "114. I often feel like there is a tight tape on my head.", "word_count": 18, "label": "", "sentence_structure": "Subject鈥揷opula鈥損redicative", "subjective_objective": {"answer": "Subjective", "rationale": "Because it's the patient's subjective feeling that feels so tight."}, "sentence_sentiment": {"answer": "Negative", "rationale": "Because the description in the entry \"like a tight tape on his head\" gives people a feeling of discomfort and negative emotional color."}, "language_style": {"answer": "Tactful and warm", "rationale": ""}, "time_dimension": {"answer": "Present", "rationale": "The sentence describes the current feelings and state of the testee, \"often feeling\" indicating that the feeling is continuous and that it is a current act. So the temporal dimension is now."}, "sentence_type": "Affirmative declarative sentence", "pos": {"nouns": {"Head": 1, "The tape": 1}, "verbs": {"Regular": 1, "I think": 1, "have": 1}, "adjectives": {"Tight": 1}, "numerals": {"Ten": 1}, "pronouns": {"I": 1}}}, {"content": "I believe that there will be 鈥渁gain鈥?after death.", "word_count": 11, "label": "", "sentence_structure": "Subject鈥損redicate", "subjective_objective": {"answer": "Subjective", "rationale": "Because belief is a belief and attitude of the individual, it is subjective."}, "sentence_sentiment": {"answer": "Neutral", "rationale": "Because the entry does not have any emotional color."}, "language_style": {"answer": "Tactful and warm", "rationale": "The language style of the entry is more euphemistic, and it does not directly order or question whether the interviewee believes in a return to life, but rather uses a soft question-and-answer approach. This approach is more likely to induce the interviewee to express his or her views freely, and to enhance the interviewee鈥檚 sense of participation and identity."}, "time_dimension": {"answer": "Future", "rationale": "The entry asks the testee ' s belief in whether there will be a future after death, and its temporal dimension is judged to be the future, as it is a vision and a vision for the future."}, "sentence_type": "Affirmative declarative sentence", "pos": {"nouns": {"person": 2, "In the next life": 1}, "verbs": {"Yes": 1, "have": 1}, "adverbs": {"Yes": 1}, "numerals": {"115锛?: 1}, "pronouns": {"I": 1}}}, {"content": "I prefer the game and game I've been playing.", "word_count": 14, "label": "", "sentence_structure": "Subject鈥搗erb鈥搊bject", "subjective_objective": {"answer": "Subjective", "rationale": "Because like a subjective attitude.\""}, "sentence_sentiment": {"answer": "Positive", "rationale": "Because the expression \"I prefer\" shows that the interviewees are positive about the game and the game."}, "language_style": {"answer": "Tactful and warm", "rationale": "The entry is more adept and interactive when it is spoken in a gentle and inviting manner, rather than by direct statements or orders."}, "time_dimension": {"answer": "Past", "rationale": "The entries \"showed\" and \"matched and played\" represent past behaviors or events, and therefore judgement is past."}, "sentence_type": "Affirmative declarative sentence", "pos": {"nouns": {"The game": 1, "Game": 1}, "verbs": {"Down": 1, "Bet": 1}, "adverbs": {"More": 1}, "numerals": {"116锛?: 1}, "classifiers": {"individual": 1}, "pronouns": {"I": 1}}}, {"content": "117. Most of the people are honest, mainly because they fear being understood.", "word_count": 22, "label": "", "sentence_structure": "Subject鈥損redicate", "subjective_objective": {"answer": "Subjective", "rationale": "Because the words \"mainstream\" and \"prevents being read\" are described from the subjective point of view of the interviewee."}, "sentence_sentiment": {"answer": "Negative", "rationale": "Because the entry \"to be seen by others\" has negative emotions."}, "language_style": {"answer": "Calm and direct", "rationale": "The entry is based on a direct statement, and the main reason why most people are honest is that they are afraid of being read."}, "time_dimension": {"answer": "Past", "rationale": "\"For fear of being read and understood\" is \"because\" of reasons, and \"for fear of being read and understood\" reflects past behavior or experience. The entry therefore asks whether people have been honest in the past for fear of being read."}, "sentence_type": "Affirmative declarative sentence", "pos": {"nouns": {"Most of them": 1, "Honesty": 1, "Lord": 1, "Yes": 2, "Because": 2, "Don't": 1, "person": 1, "By": 1, "I'm a good friend": 1}, "verbs": {"be": 1, "鈾?And the reason 鈾?: 1, "Because": 1, "Yes": 1, "By": 1, "I'm a good friend": 1}, "adverbs": {"鈾?And the 鈾?: 1, "Lord": 1, "Yes": 1, "That's right": 1, "be": 1, "Because": 1, "Most": 1, "Main": 1}, "numerals": {"117锛?: 1}, "pronouns": {"Large": 1, "Here": 1}}}, {"content": "118. In school, I was sometimes called by the leadership for the sake of nonsense.", "word_count": 18, "label": "", "sentence_structure": "Subject鈥搗erb鈥揷omplement", "subjective_objective": {"answer": "Subjective", "rationale": "Because being called by the leader is the patient's personal experience and feelings, it is a subjective examination."}, "sentence_sentiment": {"answer": "Negative", "rationale": "Because the \"whore called for \" in the entry is negative."}, "language_style": {"answer": "Calm and direct", "rationale": "The entry directly asks whether the person interviewed was called by the leader for his or her nonsense at school, without excessive ado or emotional rendering, and is more objective in tone. The language style of the purpose is therefore \"cool and straight\"."}, "time_dimension": {"answer": "Past", "rationale": "The entry describes past events, i.e., when attending school, once called by the leader for a joke. The temporal dimension is therefore a past."}, "sentence_type": "Affirmative declarative sentence", "pos": {"nouns": {"Time": 1, "School": 1, "Leadership": 1}, "verbs": {"in": 1, "Sometimes": 1, "Because": 1, "- Bullshit": 1, "Called": 1, "Go on": 1}, "adverbs": {"And": 1}, "numerals": {"118": 1}, "pronouns": {"I": 1}}}, {"content": "119. I have always been so slow and slow, and neither vague nor mute.", "word_count": 19, "label": "", "sentence_structure": "Subject鈥搗erb鈥揷omplement", "subjective_objective": {"answer": "Objective", "rationale": "Since the speed, ambiguity and sulfur are objective characteristics of the person ' s linguistic expression, determined by factors such as his or her physical state and personal habits, the patient ' s subjective will or attitude is not required."}, "sentence_sentiment": {"answer": "Neutral", "rationale": "Because the entry does not have any emotional color."}, "language_style": {"answer": "Tactful and warm", "rationale": "The language style of the entry is euphemistic, using the words \"that\" and \"fast\" to describe the speed and sound of speech, and not too directly or strongly express one's views or feelings. At the same time, the whole sentence is more balanced, with little emphasis or excretion, giving a mild sense of moderation."}, "time_dimension": {"answer": "Present", "rationale": "The entry describes the current behaviour and state of the testee, which is \"always speaking like that\" and uses the normal current. So, its temporal dimension is now."}, "sentence_type": "Affirmative declarative sentence", "pos": {"nouns": {"Talk": 1, "That way": 1, "Vague": 1, "Helmet": 1}, "verbs": {"Say it": 1, "Slow": 1, "Come on": 1, "Vague": 1, "Helmet": 1}, "adjectives": {"Not fast": 1, "No ambiguity": 1, "No, he's not": 1}, "adverbs": {"Always": 1}}}, {"content": "120. I eat with my friends outside, much more than at home.", "word_count": 22, "label": "", "sentence_structure": "Subject鈥揷opula鈥損redicative", "subjective_objective": {"answer": "Subjective", "rationale": "Because \"a lot of rules\" is the patient's subjective assessment of his performance in the outside meal."}, "sentence_sentiment": {"answer": "Positive", "rationale": "Because the entry describes a more disciplined way for authors to go out with their friends for dinner, it can be seen as a positive quality of self-control and respect for the behaviour of others."}, "language_style": {"answer": "Tactful and warm", "rationale": "The entry uses a comparative expression to describe the behaviour of the person under investigation, while using a more \"relative rule\" to make language more moderate and euphemistic. Moreover, the contrast between \"at home\" and \"outside\" makes the expression more emotional and more resonant and thought-provoking."}, "time_dimension": {"answer": "Present", "rationale": "\"I eat with my friends outside\" means \"in\" and \"in\" and \"in\" and \"in\" and \"in\" means \"in\" \"in\" it means \"in\" more than home-based.\" It also implies that it is a comparison and an evaluation of current behaviour. So this entry describes the current behaviour and habits of the testee."}, "sentence_type": "Affirmative declarative sentence", "pos": {"nouns": {"Time": 1, "friends": 1, "Rice": 1, "Home": 1}, "verbs": {"in": 1, "Eat": 1, "- Yeah": 1, "More": 1, "Rules": 1}, "adjectives": {"Outside": 1, "Together": 1}, "adverbs": {"very": 1}, "numerals": {"120": 1}, "pronouns": {"I": 1}}}, {"content": "I believe that someone is behind me.", "word_count": 8, "label": "", "sentence_structure": "Subject鈥搗erb鈥搊bject", "subjective_objective": {"answer": "Subjective", "rationale": "Because it is the patient's subjective attitude."}, "sentence_sentiment": {"answer": "Negative", "rationale": "Because the entry expresses a skepticism and mistrust and is a negative emotional expression."}, "language_style": {"answer": "Calm and direct", "rationale": "This language style makes the questionnaire more objective and practical."}, "time_dimension": {"answer": "Present", "rationale": "The entry used to ask the testee ' s views on past beliefs and experiences, whether there had been any hidden experiences, and therefore the temporal dimension was a past."}, "sentence_type": "Affirmative declarative sentence", "pos": {"nouns": {"person": 1, "Undercount": 1}, "verbs": {"Yes": 1, "have": 1}, "numerals": {"121锛?: 1}, "pronouns": {"I": 1}}}, {"content": "122. I seem as smart as the people around me.", "word_count": 15, "label": "", "sentence_structure": "Subject鈥揷opula鈥損redicative", "subjective_objective": {"answer": "Subjective", "rationale": "Because \"I seem\" indicates that it's a self-perception or subjective view, not just an objective description."}, "sentence_sentiment": {"answer": "Neutral", "rationale": "Basis of judgement: The entry does not express positive or negative feelings, attitudes, behaviour, purpose or quality and is therefore neutral."}, "language_style": {"answer": "Calm and direct", "rationale": "The entry is based on a direct statement of the subjective perception of the ability of the subject to know his or her own and those around him, without using excessive rhetorical or emotional rendering, and is more objective in tone. The language style of the article is therefore considered to be straightforward and calm."}, "time_dimension": {"answer": "Present", "rationale": "The entry describes a self-perception of the testee that he feels as smart and capable as the people around him. This perception is now being made, so the temporal dimension is now judged."}, "sentence_type": "Affirmative declarative sentence", "pos": {"nouns": {"Questionnaire": 1, "Entry": 1, "person": 1}, "verbs": {"Yeah": 1, "Smart": 1, "It's working": 1}, "adjectives": {"Same thing": 1}, "adverbs": {"Seems like": 1}, "numerals": {"122锛?: 1}, "pronouns": {"I": 1, "Around": 1}}}, {"content": "123. I believe that someone followed me.", "word_count": 8, "label": "", "sentence_structure": "Subject鈥搗erb鈥搊bject", "subjective_objective": {"answer": "Subjective", "rationale": "Because \"convincing\" is a subjective judgement and perception, which reflects the patient's own mental state and feelings. Although stalking may be objective, the patient's beliefs and feelings are subjective."}, "sentence_sentiment": {"answer": "Negative", "rationale": "Because the expression \"someone's following me\" in the entry is negative."}, "language_style": {"answer": "Calm and direct", "rationale": ""}, "time_dimension": {"answer": "Present", "rationale": "The entry expresses the current conviction or perception of the testee that someone is following him. Although this may have happened in the past, it places greater emphasis on the situation and feelings."}, "sentence_type": "Affirmative declarative sentence", "pos": {"nouns": {"person": 1, "Track": 1}, "verbs": {"Yes": 1, "have": 1}, "adverbs": {"very": 1}, "numerals": {"123": 1}, "pronouns": {"I": 1}}}, {"content": "124. Most people do not want to lose opportunities by improper means to gain.", "word_count": 24, "label": "", "sentence_structure": "Subject鈥搗erb鈥搊bject", "subjective_objective": {"answer": "Subjective", "rationale": "Because \"the majority of people would make a choice\" is the subjective judgement of the investigator."}, "sentence_sentiment": {"answer": "Negative", "rationale": "Because the act of \"improper means of profit\" in the entry is negative."}, "language_style": {"answer": "Calm and direct", "rationale": "The language style of the questionnaire entry is calm. It presents a point of view without any rhetorical or emotional tone, expressing an objective and rational view of humanity. It conveys more directly the subject matter and the intent."}, "time_dimension": {"answer": "Present", "rationale": "The entry expresses an opinion or attitude towards the current social phenomenon, in which many people choose their own means to pursue their own interests, and this behaviour may be continuing. Therefore, the temporal dimension is now judged."}, "sentence_type": "Affirmative declarative sentence", "pos": {"nouns": {"person": 1, "Means": 1, "Interest": 1, "Opportunities": 1}, "verbs": {"Whatever": 1, "- Getting it": 1, "Lose": 1}, "adjectives": {"Irregular": 1}, "numerals": {"Mostly": 1, "124锛?: 1}, "pronouns": {"they": 1}}}, {"content": "125. My stomach has a lot of problems.", "word_count": 8, "label": "", "sentence_structure": "Subject鈥揷opula鈥損redicative", "subjective_objective": {"answer": "Subjective", "rationale": "Because the patient's self-perception and subjective judgment is \"me\" with a lot of stomach problems."}, "sentence_sentiment": {"answer": "Negative", "rationale": "Because the expression \"many things\" is negative, and it means that there's a problem with the stomach."}, "language_style": {"answer": "Calm and direct", "rationale": "The entry directly expresses the question of whether the other party is in a state of stomach discomfort, is not based on excessive rhetorical or emotional rendering, and is more objective in tone."}, "time_dimension": {"answer": "Past", "rationale": "The keyword \"many faults\" indicates that the act has already taken place and may have an impact on the life of the person being tested."}, "sentence_type": "Affirmative declarative sentence", "pos": {"nouns": {"Stomach": 1, "Diseases": 1}, "verbs": {"have": 1}, "adjectives": {"A lot": 1}, "numerals": {"125": 1}}}, {"content": "126. I like drama.", "word_count": 5, "label": "", "sentence_structure": "Subject鈥搗erb鈥搊bject", "subjective_objective": {"answer": "Subjective", "rationale": "Because love is a manifestation of subjective feelings and attitudes, it is a matter of the patient ' s own feelings and perceptions, and therefore it is a subjective issue."}, "sentence_sentiment": {"answer": "Positive", "rationale": "Because the entries \"like drama\" express positive attitudes and emotions."}, "language_style": {"answer": "Tactful and warm", "rationale": "The language style of the entry is more euphemistic. It guides the interviewer to share his/her taste of the theatre rather than direct commands or statements. This expression better triggers the interviewee鈥檚 positive response and interactive engagement."}, "time_dimension": {"answer": "Present", "rationale": "The keyword is \"like\" and it indicates the attitude or feelings of the dominant language to the theatre, without reference to past or future situations. The entry therefore describes the attitudes and behaviour of the subjects."}, "sentence_type": "Affirmative declarative sentence", "pos": {"nouns": {"Theatre": 1}, "verbs": {"Yeah": 1}}}, {"content": "I know who caused my troubles.", "word_count": 12, "label": "", "sentence_structure": "Subject鈥搗erb鈥搊bject", "subjective_objective": {"answer": "Subjective", "rationale": "Because \"know who caused his own trouble\" is the patient's subjective perception and judgment, and it's a subjective view."}, "sentence_sentiment": {"answer": "Neutral", "rationale": "Because the entry does not have a clear emotional orientation or evaluative language, it is presenting a fact."}, "language_style": {"answer": "Calm and direct", "rationale": "The entry is more straightforward in language style, without too much rhetorical or emotional rendering. It opens the door to questions whether the person surveyed knows who caused his or her troubles, and is more objective and clear."}, "time_dimension": {"answer": "Present", "rationale": "The entry describes the testee ' s current perception and understanding of his or her own cause or source of distress, and thus his or her current behaviour or mentality."}, "sentence_type": "Affirmative declarative sentence", "pos": {"nouns": {"Trouble": 1, "Who": 1}, "verbs": {"Yes": 1, "Cause": 1}, "numerals": {"127": 1}, "pronouns": {"I": 1}}}, {"content": "128. When I see blood, I am neither afraid nor miserable.", "word_count": 14, "label": "", "sentence_structure": "Subject鈥揷opula鈥損redicative", "subjective_objective": {"answer": "Objective", "rationale": "Because seeing blood is objective for all people, and not necessarily changing because different people feel differently. Although he says he is neither afraid nor uncomfortable, it is only his subjective attitude and emotional reaction, and it does not change the existence of the objective fact of seeing blood."}, "sentence_sentiment": {"answer": "Neutral", "rationale": "Because the entry merely describes the individual ' s feelings when he sees blood, it does not express positive or negative emotional tendencies."}, "language_style": {"answer": "Calm and direct", "rationale": "The language style of the entry is a calm and straightforward one, because it describes the person's reaction to seeing blood in an objective and concise manner, without too many emotional or rhetorical elements."}, "time_dimension": {"answer": "Present", "rationale": ""}, "sentence_type": "Affirmative declarative sentence", "pos": {"verbs": {"Yeah": 1, "Yes": 1, "It's hard": 1}, "adjectives": {"Neither": 1, "Yeah": 1}, "adverbs": {"Both": 1}, "numerals": {"128锛?: 1}}}, {"content": "129. I often wonder why I am so angry and so angry.", "word_count": 21, "label": "", "sentence_structure": "Subject鈥搗erb鈥搊bject", "subjective_objective": {"answer": "Subjective", "rationale": "Because anger and grumbling are subjective emotional experiences of patients, it is seen from the perspective of the patients themselves."}, "sentence_sentiment": {"answer": "Negative", "rationale": "Because the \"freak and grumble\" in the entry is negative."}, "language_style": {"answer": "Tactful and warm", "rationale": "The entry is more euphemistic, using the expression \"often confused\" to avoid direct accusations or criticism of the other, while expressing concern and support for the other's emotions. Moreover, \"freak and grumbling\" also raises questions in an objective, descriptive way, rather than directly making value judgements."}, "time_dimension": {"answer": "Present", "rationale": "The entry \"self-specific\" denotes a regular act, and the word \"will\" adopts the general current, which means that the act has not only occurred in the past but may still be ongoing. The entry therefore asks about the person's current state and feelings."}, "sentence_type": "Affirmative declarative sentence", "pos": {"nouns": {"self": 1, "Angry": 1, "Scoff": 1}, "verbs": {"Yes": 1, "I know": 1, "Love": 1, "Fan": 1, "Let's go": 1}, "adverbs": {"Often": 1}, "numerals": {"129锛?: 1}}}, {"content": "I never spit blood, or I've been tossed.", "word_count": 12, "label": "", "sentence_structure": "Subject鈥損redicate", "subjective_objective": {"answer": "Objective", "rationale": "Because \" never spitting blood, or getting blood\" is an objective situation for patients, not involving their subjective feelings or ideas."}, "sentence_sentiment": {"answer": "Neutral", "rationale": "As the entry merely describes whether the person surveyed has ever had a certain symptoms, it does not contain any emotional, attitudinal or behavioural tendencies and is therefore neutral."}, "language_style": {"answer": "Calm and direct", "rationale": "The language style of the entry is more objective, accurate and understandable."}, "time_dimension": {"answer": "Present", "rationale": "Now the entry is for the current state of the person being tested, the keywords \"never spitting blood\" and \"bite-bite-ble-ble-bone\" which all indicate the state of the moment. So the temporal dimension of the purpose of the article is now."}, "sentence_type": "Negative declarative sentence", "pos": {"nouns": {"Blood": 2}, "verbs": {"Spit": 1, "Cach": 1}, "adverbs": {"Never": 1, "No, I'm not": 2, "or": 1}, "numerals": {"130": 1}, "pronouns": {"I": 1}}}, {"content": "I am not worried about getting sick.", "word_count": 8, "label": "", "sentence_structure": "Subject鈥揷opula鈥損redicative", "subjective_objective": {"answer": "Objective", "rationale": "Because it is the patient ' s objective condition to be ill or not, fear is the patient ' s subjective feeling."}, "sentence_sentiment": {"answer": "Negative", "rationale": "Because it expresses an attitude of indifference and indifference to one ' s health, it is a negative emotional tendency."}, "language_style": {"answer": "Calm and direct", "rationale": "The language style of the entry is more straightforward, with no excessive rhetorical or emotional rendering, but simply asking if the person under investigation is worried about his or her illness."}, "time_dimension": {"answer": "Present", "rationale": "The temporal dimension of the purpose of the article is therefore now judged."}, "sentence_type": "Negative declarative sentence", "pos": {"verbs": {"not": 1, "Yes": 1, "Okay": 1, "Diseases": 1, "I'm worried": 1}, "adverbs": {"very": 1}}}, {"content": "132. I like to plant flowers or gather flowers.", "word_count": 10, "label": "", "sentence_structure": "Subject鈥搗erb鈥搊bject", "subjective_objective": {"answer": "Subjective", "rationale": "Because \"like to plant flowers or gather flowers\" is a personal preference and interest, and it is the patient's subjective attitude."}, "sentence_sentiment": {"answer": "Positive", "rationale": "Because the \"like to plant flowers or gather flowers\" act is positive."}, "language_style": {"answer": "Tactful and warm", "rationale": "The language style of the entry is more euphemistic, and the invitee is used to guide the interviewee to answer questions, giving a friendly and friendly feeling, which is consistent with the nature of the psychological questionnaire study."}, "time_dimension": {"answer": "Present", "rationale": ""}, "sentence_type": "Affirmative declarative sentence", "pos": {"nouns": {"Yeah": 1, "Flowers": 2, "Collect": 1}, "numerals": {"132": 1}}}, {"content": "I never let myself have any abnormal sexual activity.", "word_count": 21, "label": "", "sentence_structure": "Subject鈥搗erb鈥搊bject", "subjective_objective": {"answer": "Objective", "rationale": "The basis for the determination is that sexual activity is an objective fact experienced by the patient, and that it is the patient's subjective attitude to indulge in it. \""}, "sentence_sentiment": {"answer": "Neutral", "rationale": "As the entry merely stated a fact, it did not express any positive or negative emotional tendencies."}, "language_style": {"answer": "Calm and direct", "rationale": "The language style of the entry is calm and straightforward. It uses simple, plain language to express the questions of the survey, and is not overly emotional or pedigreed, and is objective and neutral."}, "time_dimension": {"answer": "Present", "rationale": ""}, "sentence_type": "Affirmative declarative sentence", "pos": {"nouns": {"Questionnaire Entry": 1, "Sexuality": 1}, "verbs": {"Lend": 1, "Come on": 2}, "adjectives": {"Normal": 1, "Any": 1}, "numerals": {"133": 1, "1": 2}, "pronouns": {"I": 1}}}, {"content": "134. Sometimes my mind runs too fast to express itself.", "word_count": 18, "label": "", "sentence_structure": "Subject鈥搗erb鈥揷omplement", "subjective_objective": {"answer": "Subjective", "rationale": "Because \"thinks run too fast\" and \"failure to express\" are the patient's description of what he feels inside of himself, and it's the patient's subjective thoughts."}, "sentence_sentiment": {"answer": "Neutral", "rationale": "Because the entry does not have any emotional color."}, "language_style": {"answer": "Tactful and warm", "rationale": "The language style of the entry is more euphemistic and warm. It uses the term \"sometimes\" to say that it is not always the case, and it is more gentle and kinder; and the metaphor \"my mind runs too fast\" also gives the author a sense of inner emotion. In general, the entry gives a sense of comfort and proximity to people, and is more suitable for psychological questionnaires."}, "time_dimension": {"answer": "Present", "rationale": "The phrase \"sometimes\" suggests that this is a regular situation, and \"too fast to express\" suggests that this is happening, so time dimensions are now judged."}, "sentence_type": "Affirmative declarative sentence", "pos": {"nouns": {"Ideas": 1, "Expression": 1}, "verbs": {"Too fast": 1, "It's too late": 1}, "adjectives": {"Come on": 1}, "adverbs": {"Sometimes": 1}, "numerals": {"134锛?: 1}}}, {"content": "135. If I could not buy a ticket to a movie and not be detected, I might do it.", "word_count": 25, "label": "", "sentence_structure": "Subject鈥搗erb鈥搊bject", "subjective_objective": {"answer": "Subjective", "rationale": "Since this is a matter of possible action and decision-making by individuals, the patient ' s subjective thoughts and wishes need to be taken into account."}, "sentence_sentiment": {"answer": "Negative", "rationale": "Because the \"no ticket to see a movie\" in the entry is against the law, it has a negative emotional connotation."}, "language_style": {"answer": "Tactful and warm", "rationale": "The entry uses the hypothesis ( \"if I can't buy a ticket to a movie and I won't be discovered\") to guide the interviewee to think and express his thoughts, and also uses the expression of possibility ( \"I may do it\"), which is more euphemism and less pressure or discomfort to the interviewee."}, "time_dimension": {"answer": "Present", "rationale": "The keyword \"can\" and \"will\" indicate that the act is a hypothetical act, that is, that while there may be a chance to do it, it is not certain that it will. Moreover, \"and will not be discovered\" also indicates that it is a future act, because it can only be done without being discovered. In conclusion, the entry looks at the respondent's reaction to a situation that has not occurred but is likely to occur, and the temporal dimension is now judged."}, "sentence_type": "Affirmative declarative sentence", "pos": {"nouns": {"Film": 1, "Tickets": 1, "person": 1, "Yes": 1}, "verbs": {"Yes": 3, "Do it": 1, "No, I'm not buying": 1, "Go on": 1, "I realized": 1}, "adverbs": {"Suppose": 1, "Maybe": 1, "Both": 1}, "numerals": {"135": 1, "One": 1, "III": 1, "Five": 1}, "classifiers": {"Number of times": 1, "individual": 2}, "pronouns": {"I": 1}}}, {"content": "136. If people treat me well, I often doubt that they have an ulterior motive.", "word_count": 18, "label": "", "sentence_structure": "Subject鈥損redicate", "subjective_objective": {"answer": "Subjective", "rationale": "Because \"suspicious\" is a subjective idea or feeling that reflects the patient's personal interpretation and assessment of the behaviour of others."}, "sentence_sentiment": {"answer": "Negative", "rationale": "Because the \"suspect of other people's ulterior motives\" in the entry is negative."}, "language_style": {"answer": "Tactful and warm", "rationale": "The language style of the entry is euphemistic, and it guides the interviewer to think and answer questions by asking questions. Moreover, the phrase \"often\" is used in a regular psychological state, without directly accusing or evaluating the interviewee's behaviour."}, "time_dimension": {"answer": "Present", "rationale": "Now the entry indicates the current mindset or thinking of the testee, \"often\" and \"suspect\" both describe the current situation of the testee. So the time dimension is now."}, "sentence_type": "Negative declarative sentence", "pos": {"nouns": {"person": 1, "I": 2}, "verbs": {"- Wait": 1, "Doubt": 1, "have": 1}, "adjectives": {"Okay": 1, "Often": 1, "Don't be mean": 1}, "numerals": {"136": 1}}}, {"content": "137. I believe that my family life is as happy as many hots I know.", "word_count": 24, "label": "", "sentence_structure": "Subject鈥揷opula鈥損redicative", "subjective_objective": {"answer": "Subjective", "rationale": "Because \"rebel\" and \"happy\" are the personal feelings and perceptions of the patient, and are subjective."}, "sentence_sentiment": {"answer": "Positive", "rationale": "Because the words \"believe me of the happiness of family life\" in the entry express positive emotional attitudes."}, "language_style": {"answer": "Tactful and warm", "rationale": "The language style of this entry is euphemism. It uses a comparative level ( \"happyer than many people I know\") to express its views, and it does not directly compare or evaluate others. It is more relaxed, friendly and respectful of the feelings of others."}, "time_dimension": {"answer": "Present", "rationale": "The entry is for questioning the belief or attitude of the testee, and the time dimension is now judged. The keyword \"convinced\" , \"family life\" and \"happy\" all indicate that it is a view and assessment of the current situation, without reference to past or future situations."}, "sentence_type": "Affirmative declarative sentence", "pos": {"nouns": {"Family life": 1, "Hot": 1}, "verbs": {"Yes": 2, "Happy": 1}, "adjectives": {"Many": 1, "It's hot": 1}, "adverbs": {"Same thing": 1}, "numerals": {"137锛?: 1}, "pronouns": {"I": 1}}}, {"content": "138. I am deeply saddened by criticism and verbal abuse.", "word_count": 12, "label": "", "sentence_structure": "Subject鈥揷opula鈥損redicative", "subjective_objective": {"answer": "Subjective", "rationale": "Because it describes the patient ' s feelings of criticism and abuse and is subjective."}, "sentence_sentiment": {"answer": "Negative", "rationale": "Because \"criticism and scolding\" and \"depressing me\" in the entries are negative emotional attitudes."}, "language_style": {"answer": "Calm and direct", "rationale": "The language style of the entry is calm and straightforward, and it uses a concise and clear language to describe the respondents ' reactions to criticism and abuse, without adding too many emotional colours or rhetorical techniques. At the same time, sentences are simple, easy to understand and meet the basic requirements of questionnaire design."}, "time_dimension": {"answer": "Past", "rationale": ""}, "sentence_type": "Interrogative sentence", "pos": {"nouns": {"Criticism": 1, "Scolding": 1, "Sad": 1}, "verbs": {"鈾?And I'm gonna be 鈾?: 1, "Both": 1}, "adverbs": {"Very": 1}}}, {"content": "139. Sometimes I feel like I have to hurt myself or others.", "word_count": 17, "label": "", "sentence_structure": "Subject鈥搗erb鈥揷omplement", "subjective_objective": {"answer": "Subjective", "rationale": "Because the words \"must\" and \"as if\" imply that it's the feeling and the idea inside of the patient."}, "sentence_sentiment": {"answer": "Negative", "rationale": "Because \"harm yourself or others\" in the entry is a negative behavior and attitude."}, "language_style": {"answer": "Tactful and warm", "rationale": "The entry is more softly phrased, uses metaphorically expressed language (e.g. \"like feeling\") and does not have a direct tone of blame or order. This expression is more acceptable and willing to answer questions."}, "time_dimension": {"answer": "Present", "rationale": "The words \"sometimes\" and \"as if\" in the sentence indicate that the act may be a recurrent occurrence and may be occurring or just ending. Thus, the temporal dimension is now judged."}, "sentence_type": "Affirmative declarative sentence", "pos": {"nouns": {"Time": 1, "I": 2, "others": 1, "self": 1}, "verbs": {"I think": 1, "I have to": 1, "Hurt": 1}, "adverbs": {"It's like": 1}, "numerals": {"139": 1}}}, {"content": "I like cooking and cooking.", "word_count": 7, "label": "", "sentence_structure": "Subject鈥搗erb鈥搊bject", "subjective_objective": {"answer": "Subjective", "rationale": "I like cooking and cooking is patient's subjective attitude."}, "sentence_sentiment": {"answer": "Positive", "rationale": "Because the \"like cooking\" behavior in the entries is positive."}, "language_style": {"answer": "Tactful and warm", "rationale": ""}, "time_dimension": {"answer": "Present", "rationale": ""}, "sentence_type": "Affirmative declarative sentence", "pos": {"nouns": {"140": 1, "I like it": 1, "Cooking": 2}, "numerals": {"140": 1}}}, {"content": "141. Most of my behaviour is governed by the customs of those around me.", "word_count": 17, "label": "", "sentence_structure": "Subject鈥揷opula鈥損redicative", "subjective_objective": {"answer": "Subjective", "rationale": "Because it expresses the self-perception and subjective perception of the patient ' s behaviour, which is influenced by the habits of the surrounding people."}, "sentence_sentiment": {"answer": "Neutral", "rationale": "Because the entry is a mere statement of fact and does not have any obvious emotional colours."}, "language_style": {"answer": "Tactful and warm", "rationale": "The language style of the entry is euphemistic, as it uses an invitation-style tone to question the interviewee, without direct orders or accusations. At the same time, the purpose of the article is expressed in a more moderate manner, avoiding the use of language that is too intense or conflicting to increase the comfort and co-operation of the interviewee."}, "time_dimension": {"answer": "Present", "rationale": "\"My actions\" and \"mostly dominated by...\" indicate that this is a description and interpretation of the current state, without reference to past or future information. Thus, the temporal dimension of this article is now."}, "sentence_type": "Affirmative declarative sentence", "pos": {"nouns": {"Behaviour": 1, "person": 1, "Custom": 1}, "verbs": {"Yes": 1, "Rule": 1}, "adverbs": {"Mostly": 1}, "numerals": {"141锛?: 1}, "pronouns": {"I": 1}}}, {"content": "142. Sometimes I feel I am useless.", "word_count": 12, "label": "", "sentence_structure": "Subject鈥揷opula鈥損redicative", "subjective_objective": {"answer": "Subjective", "rationale": "This is a patient's assessment of himself and is a subjective examination."}, "sentence_sentiment": {"answer": "Negative", "rationale": "Because the expression \"no use\" in the entry is negative."}, "language_style": {"answer": "Tactful and warm", "rationale": "The language style of the entry is more euphemistic, because it asks the other side whether it has ever felt self-denial, in a caring, understanding manner, rather than directly accusing or criticizing. Moreover, the expression \"useless\" is not too emotional and relatively mild."}, "time_dimension": {"answer": "Present", "rationale": "The keyword \"felt\" and \"useless\" all refer to the state of the moment, indicating that the person being tested currently has such feelings or thoughts."}, "sentence_type": "Negative declarative sentence", "pos": {"nouns": {"Time": 1, "I think": 1, "It's useless": 1}, "verbs": {"be": 1, "I think": 1, "have": 1}, "adverbs": {"Sometimes": 1}, "numerals": {"142": 1}, "pronouns": {"I": 1}}}, {"content": "143. When I was a child, I was in a gang, with the same blessings and the same harm.", "word_count": 19, "label": "", "sentence_structure": "Subject鈥搗erb鈥搊bject", "subjective_objective": {"answer": "Objective", "rationale": "Because the experience of childhood is an objective fact and participation in a gang is an objective act. Although it is a recollection and subjective feeling of the patient, it is an objective event that is being examined and is therefore an objective one."}, "sentence_sentiment": {"answer": "Neutral", "rationale": "Because the entry describes a past experience and does not express any positive or negative emotional colours."}, "language_style": {"answer": "Calm and direct", "rationale": ""}, "time_dimension": {"answer": "Past", "rationale": "The entry therefore describes past experiences and behaviours, and the temporal dimension is a matter of the past."}, "sentence_type": "Affirmative declarative sentence", "pos": {"nouns": {"When I was little": 1, "I": 1, "Gang": 1}, "verbs": {"I've been in it": 1, "We'll have a good time": 1, "It's the same as the other": 1}, "adverbs": {"Tsang": 1}, "numerals": {"143锛?: 1}}}, {"content": "144. I like being in the army.", "word_count": 5, "label": "", "sentence_structure": "Subject鈥損redicate", "subjective_objective": {"answer": "Subjective", "rationale": "Because likeness is a subjective feeling and attitude of a person, it reflects individual characteristics such as emotions, values, etc."}, "sentence_sentiment": {"answer": "Positive", "rationale": "Because the entry \"likes to be a soldier\" expresses a positive attitude."}, "language_style": {"answer": "Calm and direct", "rationale": "The language style of the calm direct purpose of the article is simpler, with no excessive adoration or emotional colour, and is a direct expression of the attitude and preferences of the other party towards being a soldier, which is more objective and less."}, "time_dimension": {"answer": "Present", "rationale": "According to the phrase \"like to be a soldier\", it can be seen as a description of the current preferences and attitudes of individuals, which are the current state of behaviour or state."}, "sentence_type": "Exclamatory sentence", "pos": {"nouns": {"Soldiers": 1}, "verbs": {"Yeah": 1, "When": 1}, "numerals": {"144": 1}}}, {"content": "145. Sometimes I want to fight for reasons.", "word_count": 11, "label": "", "sentence_structure": "Subject鈥搗erb鈥搊bject", "subjective_objective": {"answer": "Subjective", "rationale": "Because \"think\" is a state of mind or a desire, it's seen from the patient's subjective perspective."}, "sentence_sentiment": {"answer": "Negative", "rationale": "Because the \"want to fight\" in the entry is negative."}, "language_style": {"answer": "Calm and direct", "rationale": "The entry raises the question in a direct and clear manner, without using a rhetorical or emotional rendering, which is more objective and balanced. This approach is appropriate for serious or more private topics."}, "time_dimension": {"answer": "Present", "rationale": "In the future, the entry contains ideas and expectations for the future, and the keywords are \"sometimes\" and \"ide\" , all of which point to situations that have not yet occurred and that may occur in the future. While the verbs in the sentence are now the usual, the existence of \"sometimes\" suggests that the idea may not be frequent, but it may appear occasionally. Thus, the temporal dimension of the entry is judged by the future."}, "sentence_type": "Imperative sentence", "pos": {"verbs": {"Yes": 1, "I'm sorry": 1, "Fight": 1}, "adverbs": {"Time": 1}, "classifiers": {"145": 1}}}, {"content": "I like to wander around. I'm not happy if I don't.", "word_count": 16, "label": "", "sentence_structure": "Subject鈥搗erb鈥揷omplement", "subjective_objective": {"answer": "Subjective", "rationale": "Because \"like\" and \"dissatisfied\" are both manifestations of patients' subjective feelings."}, "sentence_sentiment": {"answer": "Negative", "rationale": "Because the entries \"not like\" and \"not happy\" express negative attitudes."}, "language_style": {"answer": "Calm and direct", "rationale": "The language style of the entry is more direct, concise, and not too much rhetorical or euphemistic. At the same time, the tone is strong and clear, showing that the speaker is highly inclined and subjective in his or her behaviour and therefore a cold and direct language style."}, "time_dimension": {"answer": "Past", "rationale": "Among them, \"like to wander\" and \"if not, be unhappy\" are descriptions of the situation, which do not relate to past or future points, so the answer is \"now\"."}, "sentence_type": "Affirmative declarative sentence", "pos": {"nouns": {"I": 2, "Yeah": 1, "Everywhere": 1, "I'm just hanging around": 1, "No way": 1, "Just": 1, "I'm not happy": 1}, "verbs": {"Yeah": 1, "I'm just hanging around": 1, "Okay": 1, "Just": 1, "I'm not happy": 1}, "adverbs": {"Everywhere": 1}, "numerals": {"146": 1}}}, {"content": "147. Many opportunities have been missed because I have often been unable to stand up for myself.", "word_count": 19, "label": "", "sentence_structure": "Subject鈥搗erb鈥搊bject", "subjective_objective": {"answer": "Subjective", "rationale": "Because \"often without a chance to decide\" is a subjective view or attitude."}, "sentence_sentiment": {"answer": "Negative", "rationale": "Because \"not in the right place\" and \"lost many good opportunities\" are negative."}, "language_style": {"answer": "Calm and direct", "rationale": "The entry is more directly linguistically, expresses the problem by presenting facts, does not use excessive rhetoric or emotional colours, and therefore can be judged as being straightforward."}, "time_dimension": {"answer": "Past", "rationale": "In it, \"often\" indicates that the act has occurred in the past many times, and \"rejected many opportunities\" indicates that it has led to undesirable results, all of which point to the past."}, "sentence_type": "Interrogative sentence", "pos": {"nouns": {"Opportunities": 2, "Timing": 1}, "verbs": {"Loss": 1, "Yes": 1, "Now, let's get this straight": 1, "Lose": 1}, "adjectives": {"Yes": 1}, "adverbs": {"Regular": 1, "Many": 1}, "numerals": {"147": 1}, "classifiers": {"Number of times": 1}, "pronouns": {"I": 1}}}, {"content": "148. When I am doing something important, I will be impatient if someone asks me to teach or disturb me.", "word_count": 32, "label": "", "sentence_structure": "Subject鈥搗erb鈥揷omplement", "subjective_objective": {"answer": "Subjective", "rationale": "Because the words \"insatisfaction\" in the entry are all about personal emotions and attitudes from the perspective of the patient."}, "sentence_sentiment": {"answer": "Negative", "rationale": "Because the attitude of impatience in the entries is negative."}, "language_style": {"answer": "Calm and direct", "rationale": "The entry is a straightforward expression of a situation and the corresponding reaction in a simple and clear language, without too much rhetorical or emotional."}, "time_dimension": {"answer": "Present", "rationale": "\"Now.\""}, "sentence_type": "Affirmative declarative sentence", "pos": {"nouns": {"Things": 1, "person": 2, "Please": 1, "Excuse me": 1, "I'm impatient": 1}, "verbs": {"Do it": 1, "In the process of": 1, "have": 1, "In": 1, "Please": 1, "Excuse me": 1, "Yes": 1, "I'm impatient": 1}, "adjectives": {"Important": 1}, "numerals": {"148": 1}, "pronouns": {"I": 1}}}, {"content": "149. I have previously written journals.", "word_count": 7, "label": "", "sentence_structure": "Subject鈥搗erb鈥搊bject", "subjective_objective": {"answer": "Subjective", "rationale": "Because \"diary writing\" is a patient's past behavioral experience and therefore is subjective."}, "sentence_sentiment": {"answer": "Neutral", "rationale": "Because the entry does not have any emotional color."}, "language_style": {"answer": "Tactful and warm", "rationale": "The language style of the entry is more euphemistic, using the term \"former\" to guide the other side to remember past behavior rather than directly asking if the other side has written a diary. And the word \"me\" also reflects respect and concern for the other. This expression is more resonant, making the questionnaire more friendly and interactive."}, "time_dimension": {"answer": "Past", "rationale": ""}, "sentence_type": "Affirmative declarative sentence", "pos": {"nouns": {"Questionnaire Entry": 1, "Journal": 1}, "verbs": {"Yes": 1}, "numerals": {"149": 1}, "pronouns": {"I": 1}}}, {"content": "When playing games, I want to win, not lose.", "word_count": 14, "label": "", "sentence_structure": "Subject鈥損redicate", "subjective_objective": {"answer": "Subjective", "rationale": "Because \"yes\" and \"no\" are the patient's personal attitude or feelings."}, "sentence_sentiment": {"answer": "Negative", "rationale": "Because the \"only want to win or not\" attitude in the entry is competitive and competitive, it may have some negative effects."}, "language_style": {"answer": "Calm and direct", "rationale": "The language style of the entry is more concise, and there is no excessive emotional or rhetorical approach, which directly expresses the view that the person interviewed is more likely to win than fail in the game. This expression is more objective and understandable, and is consistent with the characteristics of the psychological questionnaire."}, "time_dimension": {"answer": "Present", "rationale": ""}, "sentence_type": "Affirmative declarative sentence", "pos": {"nouns": {"Time": 1, "Game": 1}, "verbs": {"Do it": 1, "May I": 2, "Win": 1, "Lose": 1}, "adverbs": {"Only": 1}, "numerals": {"150锛?: 1, "1": 3}, "classifiers": {"individual": 1}, "pronouns": {"I": 1}}}, {"content": "151. Someone has been trying to poison me.", "word_count": 8, "label": "", "sentence_structure": "Subject鈥搗erb鈥搊bject", "subjective_objective": {"answer": "Subjective", "rationale": "Because it is a patient ' s perception and interpretation of his or her environment and belongs to the patient ' s subjective idea."}, "sentence_sentiment": {"answer": "Negative", "rationale": "Because the act of \"want to poison me\" in the entry is threatening and offensive, expressing negative emotional attitudes and tendencies."}, "language_style": {"answer": "Calm and direct", "rationale": "The language style of the entry is more calm and straightforward, rather than using excessive rhetoric or emotional rendering, but rather directly describes the problems that the person under investigation may encounter, which is more objective and straightforward."}, "time_dimension": {"answer": "Present", "rationale": "The keyword \"someone has always wanted\" to indicate that this is a past event, but the result may have an impact on the present and is therefore classified as the present."}, "sentence_type": "Negative declarative sentence", "pos": {"nouns": {"person": 1, "Poison": 1, "Die": 1}, "verbs": {"Yes": 1, "Poison": 1}, "numerals": {"151": 1, "Always": 1}, "pronouns": {"I": 1}}}, {"content": "152. Most nights I sleep without any ideological interference.", "word_count": 17, "label": "", "sentence_structure": "Subject鈥搗erb鈥揷omplement", "subjective_objective": {"answer": "Objective", "rationale": "Because \"without any thought\" is the actual experience and feeling of the patient in the night sleep, and it's objective."}, "sentence_sentiment": {"answer": "Neutral", "rationale": "Because the entry does not have any emotional color."}, "language_style": {"answer": "Calm and direct", "rationale": "The language style of the entry is more direct and concise, there is no excessive rhetorical or emotional colour, and the tone is more calm and objective and more in line with the linguistic characteristics of the questionnaire."}, "time_dimension": {"answer": "Present", "rationale": "The keyword \"most nights\" indicates that it is a regular, customary act and emphasizes the continuity and stability of the situation, so that it can be judged as a description of the current situation."}, "sentence_type": "Affirmative declarative sentence", "pos": {"nouns": {"Night": 1, "I": 1, "Ideas": 1, "Interference": 1}, "verbs": {"Sleeping": 1, "Yes": 1, "not": 1, "Impact": 1}, "adjectives": {"Most of them": 1}, "adverbs": {"Time": 1, "What": 1}, "numerals": {"152锛?: 1}, "classifiers": {"individual": 1}}}, {"content": "153. For most of the years, I have been in good health.", "word_count": 16, "label": "", "sentence_structure": "Subject鈥揷opula鈥損redicative", "subjective_objective": {"answer": "Objective", "rationale": "Since the state of health is the physical state of the patient, it is an objective fact that can be observed and measured."}, "sentence_sentiment": {"answer": "Positive", "rationale": "Because the entry is a good health and a positive description."}, "language_style": {"answer": "Tactful and warm", "rationale": "The entry is more polite and thoughtful in the light of the more euphemistic and soft language style, which does not directly describe the physical condition of the person interviewed, but rather asks about his or her physical condition in recent years."}, "time_dimension": {"answer": "Past", "rationale": ""}, "sentence_type": "Affirmative declarative sentence", "pos": {"nouns": {"For years": 1, "Most of the time": 1, "I": 1, "Body": 1}, "verbs": {"Everything's fine": 1}, "adverbs": {"In recent years": 1, "Most of them": 1}, "numerals": {"153锛?: 1, "Both": 1}}}, {"content": "I've never had a twitch.", "word_count": 11, "label": "", "sentence_structure": "Subject鈥搗erb鈥揷omplement", "subjective_objective": {"answer": "Subjective", "rationale": "Because the problem of scrambling is the patient ' s subjective perception and self-assessment."}, "sentence_sentiment": {"answer": "Neutral", "rationale": "Because the entry merely stated a fact, it did not show positive or negative feelings."}, "language_style": {"answer": "Calm and direct", "rationale": "The language style of the entry is more direct, concise and not excessive in rhetoric or emotional rendering, mainly to obtain objective information and data.\""}, "time_dimension": {"answer": "Present", "rationale": "The entry indicates that the testee has never experienced a symptoms or condition of stuporization in the past, and that the keyword \"never\" indicates that the act occurred in the past and was completed for the testee."}, "sentence_type": "Negative declarative sentence", "pos": {"nouns": {"- You're crazy": 1, "Mistake": 1}, "verbs": {"have": 1, "Never": 1}, "numerals": {"154锛?: 1}}}, {"content": "155. I am neither increasing nor decreasing my weight.", "word_count": 16, "label": "", "sentence_structure": "Subject鈥揷opula鈥損redicative", "subjective_objective": {"answer": "Objective", "rationale": "Because weight gain or loss is an objective fact of the patient ' s experience, it is not related to the patient ' s subjective attitude."}, "sentence_sentiment": {"answer": "Neutral", "rationale": "Because the entry does not have any emotional color."}, "language_style": {"answer": "Tactful and warm", "rationale": "The entry uses a more objective statement and does not have any emotional or personal preference for expression, which is a more euphemistic and neutral language."}, "time_dimension": {"answer": "Present", "rationale": ""}, "sentence_type": "Affirmative declarative sentence", "pos": {"nouns": {"Weight": 2, "Increase": 1, "Mitigation": 1}, "verbs": {"have": 1, "Both": 1, "Not yet": 1, "Mitigation": 1, "Increase": 1}, "adverbs": {"Present": 1, "Both": 1, "Not yet": 1}, "numerals": {"155": 1}, "pronouns": {"I": 1}}}, {"content": "156. For some time, I don't remember anything I had done.", "word_count": 18, "label": "", "sentence_structure": "Subject鈥揷opula鈥損redicative", "subjective_objective": {"answer": "Subjective", "rationale": "Because \"for a while, I can't remember anything I've done\" is the patient's subjective feelings and experiences."}, "sentence_sentiment": {"answer": "Negative", "rationale": "Because the \"not remember\" act in the entry is negative."}, "language_style": {"answer": "Tactful and warm", "rationale": ""}, "time_dimension": {"answer": "Past", "rationale": ""}, "sentence_type": "Affirmative declarative sentence", "pos": {"nouns": {"For a while": 1, "Things": 1, "Answer": 1}, "verbs": {"Do it": 1, "Yes": 1}, "adverbs": {"self": 1}, "numerals": {"156": 1, "One": 2}, "classifiers": {"None": 1}}}, {"content": "I feel that I am often punished without any reason.", "word_count": 15, "label": "", "sentence_structure": "Subject鈥揷opula鈥損redicative", "subjective_objective": {"answer": "Subjective", "rationale": "Because it is a subjective feeling and perception of a person who feels punished."}, "sentence_sentiment": {"answer": "Negative", "rationale": "Because the expression \"punishable\" in the entry is negative."}, "language_style": {"answer": "Tactful and warm", "rationale": "The entry uses the subjective expression \"I think\" and asks each other about their feelings in a more moderate and understanding way, rather than directly drawing conclusions or making accusations. This expression is more inclusive and personal."}, "time_dimension": {"answer": "Present", "rationale": "The entry is an inquiry into the present feelings and experiences of the subject, and the keyword \"felt\" and \"frequently\" indicates that the feeling is not a momentous impulse or occasional event, but a state of persistence. Thus, the temporal dimension of the purpose of the article is now judged."}, "sentence_type": "Negative declarative sentence", "pos": {"nouns": {"I": 1, "Punishment": 1}, "verbs": {"I think": 1, "Yes": 1, "Time": 1, "Regular": 1}, "adjectives": {"For no reason": 1}, "adverbs": {"Land": 1, "Often": 1}, "numerals": {"157": 1}}}, {"content": "I'm easy to cry.", "word_count": 4, "label": "", "sentence_structure": "Subject鈥揷opula鈥損redicative", "subjective_objective": {"answer": "Subjective", "rationale": "Because it's an emotional reaction, it's a subjective feeling of the patient."}, "sentence_sentiment": {"answer": "Negative", "rationale": "Because the act of \"easy to cry\" is negative, it is an expression of the negative emotional state of an individual in some cases."}, "language_style": {"answer": "Calm and direct", "rationale": "The entry asks the respondents whether they are easy to cry in direct, concise language, without too many rhetorical or emotional colours, and the language style is straightforward and calm."}, "time_dimension": {"answer": "Present", "rationale": "The entry indicates that the testee describes a current act or state with no clear time reference point and therefore is judged to be \"now\"."}, "sentence_type": "Affirmative declarative sentence", "pos": {"verbs": {"I": 1, "Easy": 1, "Cry": 1}, "adjectives": {"Easy": 1}, "numerals": {"158": 1}, "pronouns": {"I": 1}}}, {"content": "159. I cannot understand what I read as I did before.", "word_count": 17, "label": "", "sentence_structure": "Subject鈥搗erb鈥揷omplement", "subjective_objective": {"answer": "Subjective", "rationale": "Because it's the patient's subjective feelings and experiences."}, "sentence_sentiment": {"answer": "Negative", "rationale": "Because the entry \"I can't understand what I read\" is negative."}, "language_style": {"answer": "Tactful and warm", "rationale": "The entry presents the feelings and experiences of individuals in a more euphemistic way, expressing concerns and concerns about the decline in their cognitive abilities, and the language style that is more resonant and supportive."}, "time_dimension": {"answer": "Present", "rationale": "Now the entry is about the current situation or state, the keywords being \"not as understanding as before\" and \"understanding\" and they are about the current problem of reading and understanding. So the temporal dimension is now."}, "sentence_type": "Negative declarative sentence", "pos": {"nouns": {"Questionnaire": 1, "Entry": 1, "Something": 1}, "verbs": {"Understood": 1, "Read it": 1}, "adjectives": {"No, I can't": 1, "Elephant": 1, "That way": 1}, "adverbs": {"Once upon a time": 1}, "numerals": {"159锛?: 1}}}, {"content": "160. I have never felt better in my life than I do now.", "word_count": 19, "label": "", "sentence_structure": "Subject鈥揷opula鈥損redicative", "subjective_objective": {"answer": "Subjective", "rationale": "Because feeling good is the patient's subjective feeling."}, "sentence_sentiment": {"answer": "Positive", "rationale": "Because the expression \"I've never felt so good\" in the entry is positive."}, "language_style": {"answer": "Calm and direct", "rationale": "Because the language of the entry is simple and not too much adour or emotional, it is a direct statement that asks the other person whether he or she feels best at some stage of life. This language style is more in keeping with the characteristics of the questionnaire and allows direct access to the real feelings and views of the interviewee."}, "time_dimension": {"answer": "Present", "rationale": "The time in this sentence is \"now\", which means the person who speaks is feeling right now, and therefore judging by the behaviour or mentality of the moment."}, "sentence_type": "Affirmative declarative sentence", "pos": {"nouns": {"I": 2, "Fan": 1, "Feel": 1, "Elephant": 1, "Present": 1, "Here": 1}, "verbs": {"I feel": 1, "be": 1}, "adjectives": {"Okay": 1}, "adverbs": {"Never": 1, "Well": 2}, "numerals": {"160": 1}, "pronouns": {"I": 1}}}, {"content": "Sometimes I feel like my head hurts when I touch it.", "word_count": 13, "label": "", "sentence_structure": "Subject鈥揷opula鈥損redicative", "subjective_objective": {"answer": "Objective", "rationale": "Because \"the first touch hurts\" is a phenomenon that can be observed and measured, and is the objective state of the patient's body."}, "sentence_sentiment": {"answer": "Negative", "rationale": "Because the \"head bumps\" in the entry is negative."}, "language_style": {"answer": "Calm and direct", "rationale": "The language style of the entry is calm and straightforward. It expresses the subject of the survey in a concise and straightforward way, without too many emotional adorations or rhetorical techniques, but rather by asking the respondents whether they feel that way. This expression is more objective and direct and suitable for psychological questionnaire research."}, "time_dimension": {"answer": "Present", "rationale": "The sentence describes the current state of the person being tested, uses the general current state and does not address the concept of past or future time. Thus, the temporal dimension is now."}, "sentence_type": "Affirmative declarative sentence", "pos": {"nouns": {"Head": 1}, "verbs": {"Ouch": 1, "I think": 1, "- A bump": 1}, "adverbs": {"Sometimes": 1}, "numerals": {"161锛?: 1, "Just": 1}, "pronouns": {"I": 1}}}, {"content": "162. I hate the fact that I have to be betrayed by being misled by others.", "word_count": 22, "label": "", "sentence_structure": "Subject鈥搗erb鈥揷omplement", "subjective_objective": {"answer": "Subjective", "rationale": "Because \"hate\" is the patient's subjective attitude to the behaviour of others."}, "sentence_sentiment": {"answer": "Negative", "rationale": "Because \"hate\" and \"must lose\" in the entries are all negative emotional acts."}, "language_style": {"answer": "Calm and direct", "rationale": "The entry is based on direct language and explicit emotional expression, without too many qualifiers or euphemisms. At the same time, it clearly expresses the respondent鈥檚 dissatisfaction and aversion to the improper manipulation of others. Thus, the language style of the article can be judged as 鈥渃ooling straight鈥?"}, "time_dimension": {"answer": "Past", "rationale": "The verbs \"hate\" and \"en\" in the entry, which indicate that the act occurred in the past and thus judged to be past."}, "sentence_type": "Negative declarative sentence", "pos": {"nouns": {"Grandpa": 1, "Questionnaire": 1, "Entry": 1, "person": 1, "Means": 1, "- You're kidding me": 1, "I": 2, "Lose": 1}, "verbs": {"Analysis": 1, "Hate": 1, "鈾?And I'm gonna be 鈾?: 1, "I had to": 1, "Recognition": 1}, "adjectives": {"It's not fair": 1}, "numerals": {"162锛?: 1}, "pronouns": {"I": 2}}}, {"content": "163. I am not tired.", "word_count": 6, "label": "", "sentence_structure": "Subject鈥揷opula鈥損redicative", "subjective_objective": {"answer": "Subjective", "rationale": "Because \"hard to get tired\" is the patient's personal feelings and experiences, it is subjective."}, "sentence_sentiment": {"answer": "Neutral", "rationale": ""}, "language_style": {"answer": "Tactful and warm", "rationale": "This is a more euphemistic expression, which is more euphemistic, questions-in-quiries, is more friendly, friendly and does not use direct command or criticism."}, "time_dimension": {"answer": "Present", "rationale": "The keyword is \"hard to wear off\" and it is a state or situation, so the temporal dimension is now judged."}, "sentence_type": "Affirmative declarative sentence", "pos": {"verbs": {"Easy": 1, "Tired": 1}, "adjectives": {"not": 1}}}, {"content": "164. I like to study and read things that are relevant to my current work.", "word_count": 19, "label": "", "sentence_structure": "Subject鈥搗erb鈥搊bject", "subjective_objective": {"answer": "Subjective", "rationale": "Like a description of personal interest is subjective."}, "sentence_sentiment": {"answer": "Positive", "rationale": "Because the words \"like\" and \"research and reading\" in the entries convey a positive, proactive attitude."}, "language_style": {"answer": "Tactful and warm", "rationale": ""}, "time_dimension": {"answer": "Present", "rationale": ""}, "sentence_type": "Affirmative declarative sentence", "pos": {"nouns": {"Research": 1, "Reading": 1, "Work": 1}, "verbs": {"Yeah": 1, "Related": 1, "Something": 1}, "adverbs": {"and": 1, "Current": 1}, "numerals": {"164锛?: 1}}}, {"content": "165. I like to meet some important people, which makes me feel important.", "word_count": 24, "label": "", "sentence_structure": "Subject鈥搗erb鈥搊bject", "subjective_objective": {"answer": "Subjective", "rationale": "Because it is the patient ' s own subjective thoughts and feelings, rather than objective facts or phenomena, that are valued for his or her attachment to other people and his or her social status."}, "sentence_sentiment": {"answer": "Positive", "rationale": "Because the behavior in the entries, \"like to meet some important people\" and the purpose, \"make yourself important,\" have a positive emotional effect."}, "language_style": {"answer": "Tactful and warm", "rationale": "The language style of the entry is more euphemistic, moderate and a positive expression of love for the recognition of a person of importance, and also of the value and expectation of one鈥檚 own. This expression is more personal and interactive."}, "time_dimension": {"answer": "Present", "rationale": "The verbs \"like\" , \"getting to know\" and \"get to feel\" all indicate that the act or attitude is the state of the moment."}, "sentence_type": "Affirmative declarative sentence", "pos": {"nouns": {"People": 1, "self": 1}, "verbs": {"Yeah": 1, "I'm meeting someone": 1, "鈾?And I'm gonna be 鈾?: 1, "I feel": 1}, "adjectives": {"Important": 2}, "numerals": {"165锛?: 1}, "pronouns": {"I": 1}}}, {"content": "I was afraid to look down from the top.", "word_count": 10, "label": "", "sentence_structure": "Subject鈥揷opula鈥損redicative", "subjective_objective": {"answer": "Objective", "rationale": "Fear of being down from the top is a fact and does not require subjective judgement or explanation by the patient."}, "sentence_sentiment": {"answer": "Negative", "rationale": "Because the expression \"I'm scared\" in the entry is negative."}, "language_style": {"answer": "Calm and direct", "rationale": "The entry raises the question in a straightforward and clear manner, without excessive ado or emotional expression, and is more objective and calm."}, "time_dimension": {"answer": "Present", "rationale": "\"I'm afraid to look down from the top\" means a state of emotion or feeling, so the judgment is based on \"now\"."}, "sentence_type": "Affirmative declarative sentence", "pos": {"nouns": {"166": 1, "I": 1, "Scared": 1, "High": 1, "Look down": 1}, "verbs": {"very": 1, "be": 1}, "adverbs": {"From": 1}, "numerals": {"166": 1}}}, {"content": "167. Even if someone broke the law in my family, I would not be nervous.", "word_count": 15, "label": "", "sentence_structure": "Subject鈥損redicate", "subjective_objective": {"answer": "Subjective", "rationale": "Because tension is a subjective feeling and emotional reaction of one. Even if there is a violation of the law in the family, the emotional response of different people will be different, so the subject is viewed from the patient鈥檚 subjective point of view."}, "sentence_sentiment": {"answer": "Negative", "rationale": "Because \"someone in the family broke the law\" was negative, and \"not nervous\" was negative."}, "language_style": {"answer": "Calm and direct", "rationale": "The entry is relatively direct in its language style, with no excessive emotional rendering or euphemism. It clearly presents a point of view and asks the interviewee to answer it, which is more objective and calm."}, "time_dimension": {"answer": "Present", "rationale": "Even \"and\" means a synonym for conditional words, which describe the current situation or attitude. And \"offence\" and \"without tension\" are the expressions of the attitude and behaviour of the person being tested in such a case, so the entry is the current behaviour or mentality of the person being tested, and the temporal dimension is now judged."}, "sentence_type": "Negative declarative sentence", "pos": {"nouns": {"person": 1, "Offences": 1}, "verbs": {"Yes": 1, "Nervous": 1, "have": 1}, "adverbs": {"Even if": 1}, "numerals": {"167": 1}, "pronouns": {"I": 1}}}, {"content": "My head is a little bit out of its mind.", "word_count": 8, "label": "", "sentence_structure": "Subject鈥搗erb鈥揷omplement", "subjective_objective": {"answer": "Subjective", "rationale": "Because \"a little bad\" is a subjective description of self-awareness."}, "sentence_sentiment": {"answer": "Negative", "rationale": "Because the expression \"brain is a bit sick\" in the entry is a negative assessment of his mental or mental state."}, "language_style": {"answer": "Calm and direct", "rationale": "The language style of the entry is more straightforward, concise and not too much rhetorical or emotionally reproducing a direct statement of one鈥檚 own perception."}, "time_dimension": {"answer": "Past", "rationale": "The entry \"something wrong\" represents past experience or symptoms and is therefore judged to be past."}, "sentence_type": "Exclamatory sentence", "pos": {"nouns": {"Brain": 1, "Mistake": 1}, "verbs": {"A little": 1, "be": 1}, "adjectives": {"Mine": 1}, "numerals": {"168": 1}}}, {"content": "I'm not afraid to control the money.", "word_count": 5, "label": "", "sentence_structure": "Subject鈥損redicate", "subjective_objective": {"answer": "Subjective", "rationale": "Because \"not afraid\" is a subjective attitude or feeling that reflects patients' subjective perceptions and emotional reactions to money management."}, "sentence_sentiment": {"answer": "Neutral", "rationale": "Since the entry does not have a clear positive or negative tendency, it merely expresses a person ' s views or attitude."}, "language_style": {"answer": "Calm and direct", "rationale": "The entry describes the subject of the problem in a simple and clear language, i.e., asking if the other party is afraid to manage money."}, "time_dimension": {"answer": "Present", "rationale": "The keyword \"not afraid of money\" shows the testee's attitude and confidence in his current financial management, so the time dimension of this entry is now judged."}, "sentence_type": "Affirmative declarative sentence", "pos": {"verbs": {"Yes": 1, "Pipe": 1}, "adjectives": {"not": 1}, "adverbs": {"not": 1}, "pronouns": {"I": 1, "Money": 1}}}, {"content": "I am not calling people to say what they think of me.", "word_count": 13, "label": "", "sentence_structure": "Subject鈥搗erb鈥揷omplement", "subjective_objective": {"answer": "Subjective", "rationale": "Because \"not quench\" is a term for patient subjective attitudes, which means that patients do not care about others' views of themselves, which is a subjective feeling."}, "sentence_sentiment": {"answer": "Negative", "rationale": "Because \"no one's opinion\" suggests that an individual's neglect of others may have shown some negative emotional attitudes of indifference, arrogance and so on."}, "language_style": {"answer": "Calm and direct", "rationale": "The language style of the entry is relatively calm and straightforward, with no excessive embellishment or emotional rendering, and the attitude of the subject to the views of others is expressed in a concise manner."}, "time_dimension": {"answer": "Present", "rationale": "In the entry, \"No Shout\" indicates current attitudes and behaviour, and \"What do others think of me\" is also a current description. So, the entry is asking the testee about the current thinking and attitude, which is a judgment of the current dimension of time."}, "sentence_type": "Negative declarative sentence", "pos": {"nouns": {"I": 1, "person": 1}, "verbs": {"not": 1, "in": 1, "Whoo": 1, "have": 1}, "adverbs": {"More": 1}, "numerals": {"70": 1}}}, {"content": "171. I would feel uncomfortable and uncomfortable at the parties, despite the fact that some people are doing the best they can.", "word_count": 29, "label": "", "sentence_structure": "Subject鈥搗erb鈥揷omplement", "subjective_objective": {"answer": "Subjective", "rationale": "Because the subject is one of personal feelings and reactions, it is the patient ' s subjective attitude."}, "sentence_sentiment": {"answer": "Negative", "rationale": "Because the attitude of \"fear and uncomfortable\" in the entries is negative."}, "language_style": {"answer": "Tactful and warm", "rationale": "The language style of the entry is more euphemistic and gentle, using expressions such as \"even\" and \"if\" and less verbs, and is not too direct or rigid. Moreover, the entry does not clearly indicate whether the other party's behaviour is problematic, but rather is expressed more in terms of his own feelings, which is more conducive to generating reflection and resonance and enhancing interaction."}, "time_dimension": {"answer": "Present", "rationale": "Now."}, "sentence_type": "Negative declarative sentence", "pos": {"nouns": {"Party": 1, "Windy": 1, "I": 1}, "verbs": {"Out": 1, "Jean": 1, "Do it": 1, "Yes": 1, "I feel": 1, "I'm not feeling well": 1}, "adjectives": {"and": 1}, "numerals": {"171": 1}, "pronouns": {"If": 1, "Yeah": 1}}}, {"content": "172. I often need to try not to be ashamed of myself.", "word_count": 18, "label": "", "sentence_structure": "Subject鈥搗erb鈥揷omplement", "subjective_objective": {"answer": "Subjective", "rationale": "Because feelings and reactions like \"fear\" and \"weak\" are subjective feelings and experiences of patients, the entry is viewed from the patient's subjective perspective."}, "sentence_sentiment": {"answer": "Negative", "rationale": "Because \"weak\" is a negative emotional experience and expression."}, "language_style": {"answer": "Tactful and warm", "rationale": ""}, "time_dimension": {"answer": "Present", "rationale": "The keyword \"often needs\" and \"not to be shy\" suggests that it is a description of the person being tested for the act or the mentality that he is now."}, "sentence_type": "Affirmative declarative sentence", "pos": {"nouns": {"Grandpa": 1, "Questionnaire": 2, "Item": 1, "Problem": 1, "Answer": 1, "Entry": 1, "Look": 1}, "verbs": {"Analysis": 1, "Yes": 2, "Try": 1, "Show": 1, "鈾?And I'm gonna be 鈾?: 1, "not": 1, "Scared": 1, "Shy": 1}, "adjectives": {"Often": 1, "Shameless": 1}, "adverbs": {"Regular": 1}, "numerals": {"172": 1}, "classifiers": {"individual": 1}, "pronouns": {"I": 1}}}, {"content": "173. I used to like school.", "word_count": 7, "label": "", "sentence_structure": "Subject鈥搗erb鈥揷omplement", "subjective_objective": {"answer": "Subjective", "rationale": "Because \"like\" is a subjective feeling and attitude of an individual, which is a manifestation of past preferences or preferences, not an objective fact."}, "sentence_sentiment": {"answer": "Neutral", "rationale": "Because the entry does not have any emotional color."}, "language_style": {"answer": "Tactful and warm", "rationale": ""}, "time_dimension": {"answer": "Past", "rationale": ""}, "sentence_type": "Affirmative declarative sentence", "pos": {"nouns": {"I": 1, "Past": 1, "Learn": 1}, "verbs": {"Yeah": 1, "Go, go, go": 1}, "numerals": {"173": 1}}}, {"content": "I've never fainted.", "word_count": 8, "label": "", "sentence_structure": "Subject鈥損redicate", "subjective_objective": {"answer": "Objective", "rationale": "Because it is an objective fact that a person has fainted, he or she cannot choose or control himself or herself."}, "sentence_sentiment": {"answer": "Neutral", "rationale": "Because the entry does not have any emotional color."}, "language_style": {"answer": "Tactful and warm", "rationale": "The entry is more euphemistic, not directly asking questions or ordering answers, but using a more moderate, friendly tone to guide the interviewee to express his/her views. At the same time, this expression helps to create a good atmosphere of communication and increase the confidence and participation of the interviewee."}, "time_dimension": {"answer": "Past", "rationale": "\"I never fainted\" means that the testee has not fainted in his past experience. So the answer is \"the past.\""}, "sentence_type": "Negative declarative sentence", "pos": {"nouns": {"- I'm fainted": 1}, "verbs": {"have": 1, "Pass": 1}, "adverbs": {"Never": 1, "No, I'm not": 1}, "numerals": {"174": 1, "1": 2}, "classifiers": {"individual": 1}, "pronouns": {"I": 1}}}, {"content": "175. I'm rarely dazzled.", "word_count": 7, "label": "", "sentence_structure": "Subject鈥搗erb鈥揷omplement", "subjective_objective": {"answer": "Subjective", "rationale": "Because the phrase \"I rarely get dizzy\" is a subjective description of the patient."}, "sentence_sentiment": {"answer": "Neutral", "rationale": "Since the entry does not address positive or negative behaviour, attitudes, emotions, etc., it merely describes a fact."}, "language_style": {"answer": "Calm and direct", "rationale": "The entry asks the other side whether the other side is often dizziness, is not using excessive qualifiers or emotional expressions and is more calm and straightforward."}, "time_dimension": {"answer": "Present", "rationale": "The entry is for questioning the testee's past behaviour or state, and the keyword is \"very little dizziness\", indicating that this has occurred less frequently in the past. Therefore, the temporal dimension is judged to be past. Output: past."}, "sentence_type": "Negative declarative sentence", "pos": {"nouns": {"Dizziness": 1, "Eyes": 1}, "verbs": {"Headlight": 1, "Eyes": 1}, "adjectives": {"Less": 1}, "adverbs": {"very": 1}, "numerals": {"175": 1}}}, {"content": "I am not afraid of the snake.", "word_count": 5, "label": "", "sentence_structure": "Subject鈥搗erb鈥揷omplement", "subjective_objective": {"answer": "Objective", "rationale": "Fearing that the snake is an individual experience and emotional reaction, it is subjective. But the subject is described in a form of question, and does not involve personal emotional reactions and experiences, and therefore looks at the issue objectively."}, "sentence_sentiment": {"answer": "Neutral", "rationale": "Because the entry does not have any emotional color."}, "language_style": {"answer": "Calm and direct", "rationale": "The language style of the entry is more straightforward, there is no excessive rhetorical or emotional rendering, but simply asks if the person interviewed is not afraid of the snake."}, "time_dimension": {"answer": "Present", "rationale": "The key word is \"I,\" which means that the subject is the subject, and that \"not afraid\" and \"brave\" form an acclaimed structure, indicating the attitude or conviction of the subject. Because the entry does not have a clear time limit, it can be understood as the current situation."}, "sentence_type": "Affirmative declarative sentence", "pos": {"nouns": {"I": 1, "Snake": 1}, "verbs": {"Yes": 1, "Large": 1}, "adjectives": {"not": 1}, "adverbs": {"very": 1}, "numerals": {"176": 1}}}, {"content": "My mother was a good man.", "word_count": 7, "label": "", "sentence_structure": "Subject鈥揷opula鈥損redicative", "subjective_objective": {"answer": "Subjective", "rationale": "Because this is a subjective assessment and opinion of the patient."}, "sentence_sentiment": {"answer": "Neutral", "rationale": "The entry is not a positive or negative emotional expression."}, "language_style": {"answer": "Tactful and warm", "rationale": "The entry expresses the assessment of the mother in a positive manner, without excessive qualification or criticism, with a warm and friendly language style."}, "time_dimension": {"answer": "Present", "rationale": ""}, "sentence_type": "Affirmative declarative sentence", "pos": {"nouns": {"Mother": 1, "person": 1}, "verbs": {"be": 1}, "adjectives": {"Okay": 1}, "pronouns": {"I": 1}}}, {"content": "178. My memory seems to be good.", "word_count": 10, "label": "", "sentence_structure": "Subject鈥揷opula鈥損redicative", "subjective_objective": {"answer": "Subjective", "rationale": "Because memory is the subjective feeling and cognitive ability of the individual."}, "sentence_sentiment": {"answer": "Neutral", "rationale": "Because the entry is not marked by emotional or attitudinal tendencies."}, "language_style": {"answer": "Tactful and warm", "rationale": "The language style of the entry is more euphemistic, presenting the interviewee ' s perception and assessment of himself in a gentle and modest manner, without over-stated descriptions or direct affirmation of his or her merits, which is more likely to bring the interviewee closer to the respondent."}, "time_dimension": {"answer": "Present", "rationale": "The term \"seemingly\" in the sentence and \"good\" in the main sentence may be judged to describe the current situation and therefore to be part of the current temporal dimension."}, "sentence_type": "Affirmative declarative sentence", "pos": {"nouns": {"Memory": 1}, "verbs": {"Seems like": 1, "Good": 1}, "adverbs": {"And": 1}, "numerals": {"178": 1}}}, {"content": "179. I am troubled by the problems related to sexuality.", "word_count": 12, "label": "", "sentence_structure": "Subject鈥揷opula鈥損redicative", "subjective_objective": {"answer": "Subjective", "rationale": "Because it is a subjective emotional reaction, not an objective fact or situation. While the problem may involve the privacy and sensitivity of the individual, it is a feeling associated with the individual鈥檚 emotional state and therefore subjective."}, "sentence_sentiment": {"answer": "Negative", "rationale": "Because the emotional expression \"fear\" in the entry is negative."}, "language_style": {"answer": "Tactful and warm", "rationale": "The entry is more personal and interactive when it is spoken in a gentle and inviting manner, rather than in direct statements or orders. At the same time, this euphemism also protects the privacy of the interviewees and makes them more comfortable and comfortable in answering questions."}, "time_dimension": {"answer": "Present", "rationale": "The entry asks whether the testee has problems with sex at present, and the time dimension is now judged."}, "sentence_type": "Negative declarative sentence", "pos": {"nouns": {"Problem": 1, "Sex": 1, "Trouble": 1}, "verbs": {"鈾?And I'm gonna be 鈾?: 1}, "numerals": {"179锛?: 1}}}, {"content": "I don't think I know what to say when I meet a living person.", "word_count": 20, "label": "", "sentence_structure": "Subject鈥揷opula鈥損redicative", "subjective_objective": {"answer": "Subjective", "rationale": "Because \"I don't know what to say\" is the patient's subjective feeling and attitude."}, "sentence_sentiment": {"answer": "Negative", "rationale": "Because the entry \"I don't know what to say\" expresses a negative mood and attitude."}, "language_style": {"answer": "Tactful and warm", "rationale": "The entry, which allows respondents to share their feelings in a way that is asked, is not directly evaluated or criticized, but rather questions that lead to reflection and expression, with a more gentle and friendly language style."}, "time_dimension": {"answer": "Present", "rationale": ""}, "sentence_type": "Affirmative declarative sentence", "pos": {"nouns": {"Time": 1, "Livers": 1}, "verbs": {"I think": 1, "I met him": 1, "Say it": 1, "Okay": 1}, "adjectives": {"not": 1, "Okay": 1}, "adverbs": {"Just": 1, "Both": 1, "I don't know": 1}, "numerals": {"180锛?: 1}, "pronouns": {"I": 1}}}, {"content": "181. When bored, I get in trouble and I get happy.", "word_count": 14, "label": "", "sentence_structure": "Subject鈥搗erb鈥揷omplement", "subjective_objective": {"answer": "Subjective", "rationale": "Because \"silent\" and \"problem for happiness\" are the inner experience and behaviour of the patient, and are subjective."}, "sentence_sentiment": {"answer": "Negative", "rationale": "Because \"problem for happiness\" in the entry is negative."}, "language_style": {"answer": "Tactful and warm", "rationale": "Warm and warm."}, "time_dimension": {"answer": "Past", "rationale": ""}, "sentence_type": "Affirmative declarative sentence", "pos": {"verbs": {"Boring": 1, "Yes": 1, "It's a problem": 1, "Seek": 1, "Happy": 1}, "adjectives": {"Time": 1}, "adverbs": {"Just": 1}, "numerals": {"181": 1}}}, {"content": "I'm afraid I'm going crazy.", "word_count": 7, "label": "", "sentence_structure": "Subject鈥搗erb鈥揷omplement", "subjective_objective": {"answer": "Subjective", "rationale": "Because the word \"fear\" represents the patient's subjective feelings and fears."}, "sentence_sentiment": {"answer": "Negative", "rationale": "Because the psychological state of \"fearing to go crazy\" is negative, it suggests that the interviewee may have negative feelings of anxiety or depression."}, "language_style": {"answer": "Tactful and warm", "rationale": "The entry uses a more euphemistic expression that asks the other side whether there are concerns about being mad, rather than directly speaking or ordering. This expression is more personal and interactive, which is better resonance with the interviewees and encourages them to answer questions more frankly."}, "time_dimension": {"answer": "Present", "rationale": "Now the entry describes the experience or anxiety of the testee, \"I'm afraid\" that it expresses the inside experience and emotional reaction of the testee, while \"I'm going crazy\" suggests the psychological stress or distress that the testee may have at present. So the time dimension of the questionnaire should be judged now."}, "sentence_type": "Negative declarative sentence", "pos": {"nouns": {"182": 1, "self": 1, "Crazy": 1}, "verbs": {"Yes": 2, "Crazy": 1}, "numerals": {"182": 1}, "pronouns": {"I": 1}}}, {"content": "183. I object to giving money to beggars.", "word_count": 8, "label": "", "sentence_structure": "Subject鈥搗erb鈥搊bject", "subjective_objective": {"answer": "Subjective", "rationale": "This is the patient ' s personal opinion and attitude and does not involve objective facts."}, "sentence_sentiment": {"answer": "Negative", "rationale": "Because the attitudes in the entries are negative."}, "language_style": {"answer": "Calm and direct", "rationale": "The language style of the entry is more direct, clear and not overly acrimony or emotional, but rather the attitude and views of the author expressed through a simple statement."}, "time_dimension": {"answer": "Present", "rationale": "The entry now expresses the attitude and position of the testee towards giving money to the beggar, describing a current state or view, and thus judging it as now."}, "sentence_type": "Negative declarative sentence", "pos": {"nouns": {"183": 1, "I": 1, "- The beggar": 1, "Money": 1}, "verbs": {"Objection": 1, "Here": 1}, "numerals": {"183": 1}, "pronouns": {"he": 1}}}, {"content": "184. I hear voices often, but I don ' t know where they come from.", "word_count": 21, "label": "", "sentence_structure": "Subject鈥搗erb鈥搊bject", "subjective_objective": {"answer": "Subjective", "rationale": "Because this phrase is subjective in its expression of the feelings and experiences of the patient."}, "sentence_sentiment": {"answer": "Negative", "rationale": "Because the entry describes a constant hearing hallucination (hearing voices), a symptom of mental illness or mental problems, which is usually considered a negative experience."}, "language_style": {"answer": "Tactful and warm", "rationale": "The language style of the entry is more euphemistic, as it uses more moderate terms and expressions, such as \"always hearing\" and \"sound\" and leads a turn with \"but\" to express the doubts and confusion of the speaker, a tone that is more readily accepted and understood by the interviewees."}, "time_dimension": {"answer": "Present", "rationale": "The keyword \"is often heard \" means a regular act, but\" is linked by two sentences that constitute a turning relationship, indicating that the phenomenon is the experience and experience of the moment. The entry therefore asks whether the testee currently has a situation where he/she is often hearing voices but cannot determine the source, and the temporal dimension is now."}, "sentence_type": "Affirmative declarative sentence", "pos": {"nouns": {"Sound": 2, "Local": 1, "Answer": 1}, "verbs": {"Yes": 2}, "adverbs": {"Often": 1}, "numerals": {"184": 1}, "pronouns": {"it": 1, "Where": 1, "Here": 1}}}, {"content": "185. My hearing is clearly as good as most people.", "word_count": 14, "label": "", "sentence_structure": "Subject鈥揷opula鈥損redicative", "subjective_objective": {"answer": "Subjective", "rationale": "Because \"my hearing is obviously as good as most people\" is a person's self-awareness and feelings, a patient's subjective attitude."}, "sentence_sentiment": {"answer": "Neutral", "rationale": "As the entry only objectively describes the individual ' s hearing ability, it does not reflect positive or negative emotional tendencies."}, "language_style": {"answer": "Calm and direct", "rationale": "The entry is presented in a concise and clear language, and is not presented by a speech-making or emotional rendering, so that the language style is relatively calm and straightforward."}, "time_dimension": {"answer": "Past", "rationale": "This entry describes the previous hearing state of the person tested and compares it with that of most people."}, "sentence_type": "Affirmative declarative sentence", "pos": {"nouns": {"Hearing": 1, "person": 2, "Most people": 1}, "verbs": {"be": 1, "Okay": 1}, "adjectives": {"Obviously": 1}, "numerals": {"185": 1, "Same thing": 1}, "pronouns": {"I": 1}}}, {"content": "When I do one thing, I often find my hands shaking.", "word_count": 20, "label": "", "sentence_structure": "Subject鈥揷opula鈥損redicative", "subjective_objective": {"answer": "Subjective", "rationale": "Because the word \"I found\" suggests that it's the patient's subjective feeling."}, "sentence_sentiment": {"answer": "Negative", "rationale": "Because \"hands shaking\" is usually caused by negative emotions such as anxiety or stress."}, "language_style": {"answer": "Tactful and warm", "rationale": "The phrase is more moderate, using the expression \"I found\" to guide the respondents to express their feelings, without too much emphasis or criticism, and to treat each other in an understanding and acceptance. Moreover, the sentence is in the form of a counter-question, allowing the respondents to respond to themselves after thinking, making the questions more open and interactive."}, "time_dimension": {"answer": "Present", "rationale": "Now the keyword \"to find\" and \"to find\" and the verb \"to describe behavior\" point to the state of the current operation. So the entry is now judged."}, "sentence_type": "Affirmative declarative sentence", "pos": {"nouns": {"Yes": 1, "Hands": 1}, "verbs": {"Yes": 1, "Do it": 1, "# Wresh #": 1}, "adverbs": {"Regular": 1}, "numerals": {"186": 1}}}, {"content": "187. My hands are not becoming clumsy.", "word_count": 13, "label": "", "sentence_structure": "Subject鈥揷opula鈥損redicative", "subjective_objective": {"answer": "Objective", "rationale": "Because \"the hands are not clumsy\" is an objective observation that does not require subjective assessment or perception by the patient."}, "sentence_sentiment": {"answer": "Neutral", "rationale": "Because the entry does not have any emotional color."}, "language_style": {"answer": "Tactful and warm", "rationale": ""}, "time_dimension": {"answer": "Present", "rationale": "\"Not becoming\" in the entry suggests that it is a state description of the present situation, that the hands are not being clumsy. So the temporal dimension is now."}, "sentence_type": "Negative declarative sentence", "pos": {"nouns": {"Hands": 1, "Clumsy": 1, "Ling": 1}, "verbs": {"鈾?And become 鈾?: 1, "No, I'm not": 1}, "adjectives": {"Mine": 1}, "numerals": {"187": 1}}}, {"content": "188. I can read for a long time without the eyes feeling tired.", "word_count": 16, "label": "", "sentence_structure": "Subject鈥搗erb鈥揷omplement", "subjective_objective": {"answer": "Subjective", "rationale": "This is because patients feel and evaluate their reading abilities and are subjective."}, "sentence_sentiment": {"answer": "Positive", "rationale": "Because the ability described in the entry is positive, i.e. reading for long periods does not exhaust the eyes."}, "language_style": {"answer": "Tactful and warm", "rationale": "The language style of the entry is more euphemistic and warm. Can you create a friendly atmosphere of communication with the polite words \"under the invitation\" and \"in the gentle manner of asking?\", so that the respondents are comfortable and willing to share their thoughts and experiences? At the same time, the entire sentence expresses a caring and caring attitude, not an order or a charge, which is more reflective of the designers' intentions and humanization."}, "time_dimension": {"answer": "Present", "rationale": ""}, "sentence_type": "Affirmative declarative sentence", "pos": {"nouns": {"Time": 1, "Eyes": 1}, "verbs": {"Reading": 1, "Yes": 1, "Feel": 1}, "adjectives": {"Long": 1, "Tired": 1}, "adverbs": {"Okay": 1}, "numerals": {"188": 1}, "classifiers": {"individual": 1}, "pronouns": {"I": 1}}}, {"content": "189. Many times I feel powerless.", "word_count": 11, "label": "", "sentence_structure": "Subject鈥揷opula鈥損redicative", "subjective_objective": {"answer": "Subjective", "rationale": "Because feeling powerless is a subjective experience of the patient."}, "sentence_sentiment": {"answer": "Negative", "rationale": "Because the mood of feeling weak in the entry is negative."}, "language_style": {"answer": "Tactful and warm", "rationale": "The expression of the entry, which asks questions in a caring and understanding manner, rather than directly judging or blaming the other party for his or her physical condition, is more reflective of the respect and concern of the interviewee."}, "time_dimension": {"answer": "Present", "rationale": "In it, \"many times\" means that this happens often, and \"I feel like I'm incompetent\" describes specific feelings and symptoms, which indicate that this is an observation and description of the current state. Therefore, the time dimension of the article should be now."}, "sentence_type": "Affirmative declarative sentence", "pos": {"nouns": {"Time": 1, "All over": 1, "Strength": 1}, "verbs": {"I think": 1, "have": 1}, "adjectives": {"Many": 1, "None": 1}, "numerals": {"189锛?: 1}, "pronouns": {"I": 1}}}, {"content": "I have little headaches.", "word_count": 5, "label": "", "sentence_structure": "Subject鈥搗erb鈥揷omplement", "subjective_objective": {"answer": "Objective", "rationale": "Because \"headache\" is a physical phenomenon that can be observed, and the subject is about the subjective feeling of the patient. So \"I have little headaches\" is seen from an objective perspective."}, "sentence_sentiment": {"answer": "Neutral", "rationale": "Because the entry does not have any emotional color."}, "language_style": {"answer": "Calm and direct", "rationale": "The entry is more straightforward in language style, is not based on excessive rhetorical or emotional rendering, and is more objective in tone. It is simply a question of whether the person under investigation has a recurring headache."}, "time_dimension": {"answer": "Present", "rationale": ""}, "sentence_type": "Negative declarative sentence", "pos": {"verbs": {"Headache": 1, "Very few": 1}, "numerals": {"190": 1}}}, {"content": "191. Sometimes, when I am embarrassed, I get sweaty, which makes me very upset.", "word_count": 23, "label": "", "sentence_structure": "Subject鈥揷opula鈥損redicative", "subjective_objective": {"answer": "Subjective", "rationale": "Because \"unfortunate\" and \"very bitter\" are subjective feelings and emotional reactions."}, "sentence_sentiment": {"answer": "Negative", "rationale": "Because \"unfortunate\" and \"proficient\" are negative emotional terms, which indicate emotional distress and discomfort."}, "language_style": {"answer": "Tactful and warm", "rationale": "The language style of the entry is more euphemistic and warm. It describes the situation that the other side may encounter in a moderate and caring manner, and expresses its compassion and understanding in the form of \"I'm Worried.\" This is common in psychological questionnaires, which increase the comfort and trust of the interviewee."}, "time_dimension": {"answer": "Present", "rationale": "Now the entry describes the experience and feelings of the testees now, in which the words \"sometimes\" and \"sweet\" and \"freak me\" indicate that this is the current situation."}, "sentence_type": "Affirmative declarative sentence", "pos": {"nouns": {"Time": 2, "I'm embarrassed": 1, "Khan": 1, "Trouble": 1}, "verbs": {"Out": 1, "鈾?And I'm gonna be 鈾?: 1, "I'm embarrassed": 1}, "adjectives": {"A lot": 1, "Very": 1}, "adverbs": {"Sometimes": 1}, "numerals": {"191锛?: 1}, "pronouns": {"I": 1}}}, {"content": "192. It is not difficult to keep my walking steady.", "word_count": 12, "label": "", "sentence_structure": "Subject鈥搗erb鈥揷omplement", "subjective_objective": {"answer": "Objective", "rationale": "This is an objective fact because it is a description of the patient ' s condition in his or her daily life."}, "sentence_sentiment": {"answer": "Neutral", "rationale": "Because the entry does not have any emotional color."}, "language_style": {"answer": "Tactful and warm", "rationale": ""}, "time_dimension": {"answer": "Present", "rationale": "The entry describes the current walk of the testee, and the word \"and\" indicates that both situations exist simultaneously, and \"no difficulty\" indicates that there is no problem with the walk at present, so the temporal dimension is now."}, "sentence_type": "Affirmative declarative sentence", "pos": {"nouns": {"Step": 1, "Road": 1}, "verbs": {"Let's go": 1, "Hold on": 1, "and": 1, "not": 1, "Difficulties": 1}, "adjectives": {"Steady": 1}, "adverbs": {"Time": 1}, "numerals": {"192": 1}}}, {"content": "I have no asthma type.", "word_count": 9, "label": "", "sentence_structure": "Subject鈥搗erb鈥搊bject", "subjective_objective": {"answer": "Objective", "rationale": "Because \"no asthma\" is a state of disease, which is the physical and medical history of the patient, and is an objective fact."}, "sentence_sentiment": {"answer": "Neutral", "rationale": "Because the entry describes a fact and does not involve any emotion, attitude, behaviour, purpose or quality."}, "language_style": {"answer": "Calm and direct", "rationale": "The entry answers questions in a clear and concise language, without too many rhetorical or emotional renderings, and is more objective in tone."}, "time_dimension": {"answer": "Past", "rationale": ""}, "sentence_type": "Negative declarative sentence", "pos": {"nouns": {"Diseases": 1, "Asthma": 1}, "verbs": {"have": 1, "Nope": 1}, "adjectives": {"Category": 1}, "numerals": {"193锛?: 1}}}, {"content": "194. I was in a few instances suddenly unable to control my actions or words, but I was still thinking clearly.", "word_count": 32, "label": "", "sentence_structure": "Subject鈥搗erb鈥揷omplement", "subjective_objective": {"answer": "Subjective", "rationale": "Because \"I can't control my actions or words\" and \"I'm still clear\" are subjective feelings and judgments of patients."}, "sentence_sentiment": {"answer": "Negative", "rationale": "Because the \"uncontrollable\" act in the entry is negative."}, "language_style": {"answer": "Calm and direct", "rationale": "This entry describes a situation that the respondent may have experienced in a direct and concise language, without using excessive rhetorical or emotional techniques."}, "time_dimension": {"answer": "Past", "rationale": "In the entry, \"had \" indicates that this has happened, and therefore the temporal dimension is a past."}, "sentence_type": "Affirmative declarative sentence", "pos": {"nouns": {"Questionnaire Entry": 1, "Number of times": 1, "Action": 1, "Words": 1, "Brain": 1, "self": 2, "Time": 1}, "verbs": {"have": 1, "Yes": 1, "Control": 1, "I can't": 1, "Wake up": 1}, "adjectives": {"Clear": 1}, "adverbs": {"Once": 1, "Suddenly": 1}, "numerals": {"194": 1, "A few times": 1}, "pronouns": {"I": 1}}}, {"content": "I like not every single one of the people I know.", "word_count": 15, "label": "", "sentence_structure": "Subject鈥搗erb鈥搊bject", "subjective_objective": {"answer": "Subjective", "rationale": "Because it's the subjective attitude of the patient that you like or dislike."}, "sentence_sentiment": {"answer": "Neutral", "rationale": "As the entry merely stated a fact, it did not involve any positive or negative emotional aspects."}, "language_style": {"answer": "Tactful and warm", "rationale": "The language style of the entry is more euphemistic. It expresses the view that \"not everyone can get my love\" in a relatively gentle way, avoiding direct denial or offensive rhetoric. It is more friendly and inclusive, and more comfortable and acceptable to the interviewee."}, "time_dimension": {"answer": "Present", "rationale": "The entry is for the testee's opinion of the preferences of the people he knows, and the temporal dimension is now. The keywords \"the people I know\" and \"not everyone I like\" point to the state of consciousness and feeling."}, "sentence_type": "Negative declarative sentence", "pos": {"nouns": {"person": 2, "individual": 1}, "verbs": {"Yes": 1, "Yeah": 1}, "adjectives": {"- A few": 1}, "numerals": {"195": 1}, "pronouns": {"I": 1}}}, {"content": "I like to go on tour where I've never been before.", "word_count": 17, "label": "", "sentence_structure": "Subject鈥搗erb鈥搊bject", "subjective_objective": {"answer": "Subjective", "rationale": "Because it is the patient's own subjective attitude that determines whether or not he likes to go somewhere."}, "sentence_sentiment": {"answer": "Positive", "rationale": "Because \"likes to go to places where you haven't been\" means curiosity and interest in exploring unknown and new things, and it's a positive emotional attitude."}, "language_style": {"answer": "Tactful and warm", "rationale": "The language style of the entry is more euphemistic, and it expands the topic by asking if the other side likes to travel to places that he has not been."}, "time_dimension": {"answer": "Present", "rationale": "The entry is for the present preferences and behaviour of the testee, and the time dimension is now judged."}, "sentence_type": "Affirmative declarative sentence", "pos": {"nouns": {"Local": 1, "Tour": 1}, "verbs": {"Yeah": 1, "Present": 2, "Yes": 1}, "adverbs": {"Never": 1}, "numerals": {"196": 1}, "pronouns": {"I": 1}}}, {"content": "197. Someone's been trying to rob me.", "word_count": 10, "label": "", "sentence_structure": "Subject鈥搗erb鈥搊bject", "subjective_objective": {"answer": "Subjective", "rationale": "Because the idea was created by the patient himself, it was a subjective examination."}, "sentence_sentiment": {"answer": "Negative", "rationale": "Because the \"someone's been trying to rob me\" act in the entry is negative."}, "language_style": {"answer": "Calm and direct", "rationale": "The entry is dialogueable in a direct question-and-answer manner, without excessive rhetorical or emotional rendering, and is more objective in tone. It asks whether the person surveyed has ever experienced attempts to take their belongings, and the question is more practical and direct in design, which helps to obtain more authentic data feedback."}, "time_dimension": {"answer": "Past", "rationale": "The entry describes a past experience, \"Someone's been trying to rob me.\" Although there is no clear time or time frame, it can be inferred that this event has happened and ended by using the old tense \"want to.\""}, "sentence_type": "Affirmative declarative sentence", "pos": {"nouns": {"person": 1, "Something": 1}, "verbs": {"Grab it": 1, "Yes": 1}, "adverbs": {"Always": 1}, "numerals": {"197锛?: 1}}}, {"content": "198. I have little imagination.", "word_count": 5, "label": "", "sentence_structure": "Subject鈥搗erb鈥揷omplement", "subjective_objective": {"answer": "Subjective", "rationale": "Because \"irritive\" is a mind-motion, a patient's subjective experience and feelings."}, "sentence_sentiment": {"answer": "Neutral", "rationale": "The entry is not marked by any emotional or attitudinal bias."}, "language_style": {"answer": "Calm and direct", "rationale": "The entry is in a concise language and asks the respondents whether they are often obscurantist and have no excessive rhetorical or emotional colours, so that the language style is straightforward and calm."}, "time_dimension": {"answer": "Present", "rationale": "In the future, the entry does not have clear time indicators or past or present relevant behaviour or state, so it cannot judge its time dimension. It describes a possible future state of the future - \"very little imagination\" - which suggests that the testees may in the future be more focused on actual action and practice than overthinking."}, "sentence_type": "Negative declarative sentence", "pos": {"verbs": {"Very few": 1, "I'm not sure": 1}, "adjectives": {"very": 1}, "adverbs": {"Less": 1}, "numerals": {"198": 1}}}, {"content": "We should tell the child the main knowledge about 鈥渟ex鈥?", "word_count": 19, "label": "", "sentence_structure": "Subject鈥搗erb鈥搊bject", "subjective_objective": {"answer": "Objective", "rationale": "Because it is a recommendation that should or should not be done, it is an objective observation and evaluation."}, "sentence_sentiment": {"answer": "Positive", "rationale": "Because the purpose of the entry is to educate the children about their main knowledge of sexuality, it is a positive behaviour."}, "language_style": {"answer": "Calm and direct", "rationale": "It does not use excessive rhetoric or emotional colours to express its views, but rather makes recommendations directly in a concise and clear manner."}, "time_dimension": {"answer": "Present", "rationale": "The main words and the title in the entry follow the current state of affairs, indicating a general recommendation or guidance that the child should be informed of the main sexual knowledge at this time."}, "sentence_type": "Imperative sentence", "pos": {"nouns": {"199": 1, "we": 1, "Son": 1, "Knowledge": 1, "Sex": 1}, "verbs": {"I think so": 1, "- Put it on": 1, "Tell": 1}, "adjectives": {"Main": 1}, "numerals": {"199": 1}, "classifiers": {"In terms of": 1, "of": 2}, "pronouns": {"they": 1}}}, {"content": "200. There are people who want to steal my ideas and plans.", "word_count": 12, "label": "", "sentence_structure": "Subject鈥搗erb鈥搊bject", "subjective_objective": {"answer": "Subjective", "rationale": "Because \"I want to steal my thoughts and plans\" is the patient's own idea, so it's a subjective examination."}, "sentence_sentiment": {"answer": "Negative", "rationale": "Because the \"wanted to steal\" act in the entry is negative."}, "language_style": {"answer": "Calm and direct", "rationale": "The entry is based on a straightforward question-and-answer method, without too much qualifier or emotional colour, allowing respondents to express their thoughts and attitudes in a straightforward manner."}, "time_dimension": {"answer": "Present", "rationale": "Keywords include \"someone,\" \"want to steal,\" which suggests that this is a present-day fear or fear. And \"my thoughts and plans\" also highlight the interest and value of the testee in the world."}, "sentence_type": "Negative declarative sentence", "pos": {"nouns": {"person": 1, "Ideas": 1, "Planned": 1}, "verbs": {"Yes": 1, "Steal": 1}, "numerals": {"200锛?: 1}}}, {"content": "201. But I am not as shy as I am now.", "word_count": 12, "label": "", "sentence_structure": "Subject鈥搗erb鈥揷omplement", "subjective_objective": {"answer": "Subjective", "rationale": "Because \"screety\" is a subjective feeling or emotional experience."}, "sentence_sentiment": {"answer": "Negative", "rationale": "Because the quality of shyness in the entries is negative."}, "language_style": {"answer": "Tactful and warm", "rationale": "The language style of the entry is more euphemistic, using the expression \"if you wish\" to express dissatisfaction with your personality and the desire to change your personality, and it is not too direct or impulsive. At the same time, the phrase is also accompanied by some scorn and humility, which increases the degree of kinship and humanization of the language."}, "time_dimension": {"answer": "Present", "rationale": "The entry indicates the current situation, and the \"like it is\" indicates that it is a description of the current situation, and the \"silent\" behaviour is happening, so it is judged to be the present."}, "sentence_type": "Imperative sentence", "pos": {"verbs": {"Original": 1, "Elephant": 1, "Shy": 1}, "adjectives": {"No, it doesn't": 1}, "adverbs": {"of": 2, "Here": 1, "Present": 1}, "pronouns": {"I": 1}}}, {"content": "I believe I am a condemned person.", "word_count": 12, "label": "", "sentence_structure": "Subject鈥搗erb鈥搊bject", "subjective_objective": {"answer": "Subjective", "rationale": "This expression is an expression of self-evaluation and conviction of the patient and is a subjective one."}, "sentence_sentiment": {"answer": "Negative", "rationale": "The entry is an expression of a self-derogation emotional attitude."}, "language_style": {"answer": "Calm and direct", "rationale": "The entry is asked in a direct and clear manner, without any stylistic or emotional rendering, and is more balanced and objective. It is therefore judged that the language style of the article is \"cool and direct\"."}, "time_dimension": {"answer": "Present", "rationale": ""}, "sentence_type": "Affirmative declarative sentence", "pos": {"nouns": {"I": 1, "person": 1}, "verbs": {"Yes": 1, "be": 1, "By": 1, "Condemnation": 1}, "numerals": {"202锛?: 1}}}, {"content": "203. If I were a journalist, I would like to cover the drama.", "word_count": 20, "label": "", "sentence_structure": "Subject鈥搗erb鈥搊bject", "subjective_objective": {"answer": "Subjective", "rationale": "Because \"will like\" is the patient's subjective attitude to the hypothetical situation."}, "sentence_sentiment": {"answer": "Positive", "rationale": "Because the attitude of \"likes to report on the drama\" in the entries is positive."}, "language_style": {"answer": "Tactful and warm", "rationale": "The language style of the entry is more euphemistic and soft, by assuming that you are a journalist and expressing a mild and friendly feeling about reporting on the theatre. This expression is easier to comfort the interviewee and is willing to share his real thoughts."}, "time_dimension": {"answer": "Future", "rationale": "The key words are \"if I were a journalist\" and \"will like\" , which point to situations that have not yet occurred and that may arise in the future."}, "sentence_type": "Imperative sentence", "pos": {"nouns": {"Journalists": 1, "Theatrical world": 1}, "verbs": {"I'll love it": 1, "Reporting": 1}, "adverbs": {"If": 1, "I": 1}, "numerals": {"203": 1}}}, {"content": "204. I like to be a journalist.", "word_count": 10, "label": "", "sentence_structure": "Subject鈥搗erb鈥搊bject", "subjective_objective": {"answer": "Subjective", "rationale": "Because like a subjective feeling and attitude of a person."}, "sentence_sentiment": {"answer": "Positive", "rationale": "Because it expresses a preference and a positive attitude."}, "language_style": {"answer": "Tactful and warm", "rationale": "The language style of the entry is more euphemistic. It does not use direct or strong tone, but rather asks the respondents in a more smooth and friendly way about the degree of preferences for journalists, giving them a friendly and warm feeling. Moreover, the question is expressed in a relatively neutral way, with no excessive subjectivity and easier to get an accurate and realistic answer."}, "time_dimension": {"answer": "Future", "rationale": "The entry indicates that the testee may prefer to be a journalist in the future and that the time dimension is judged to be the future."}, "sentence_type": "Affirmative declarative sentence", "pos": {"nouns": {"Journalists": 1}, "verbs": {"Do it": 1, "Yeah": 1}, "adverbs": {"One": 1}, "numerals": {"204": 1}}}, {"content": "I can't help but try to steal something.", "word_count": 13, "label": "", "sentence_structure": "Subject鈥搗erb鈥揷omplement", "subjective_objective": {"answer": "Subjective", "rationale": "Because \"want to steal\" is a subjective will or impulse. While such behaviour may have an impact on the objective environment (for example, loss of stolen goods), it is a matter of examining the idea itself from a subjective perspective."}, "sentence_sentiment": {"answer": "Negative", "rationale": "Because the steal thing is negative."}, "language_style": {"answer": "Calm and direct", "rationale": "The language style of the entry is more direct, concise and not overly refined, and the expression is more appropriate for psychological survey studies, by asking directly whether the person surveyed had had a history of being able to steal something uncontrollably."}, "time_dimension": {"answer": "Future", "rationale": "The entry is for questioning the behaviour or mentality that the subject may have in the future, and the keywords are \"sometimes\" and \"uncontrollable\", all of which point to situations that have not yet occurred and that may occur in the future."}, "sentence_type": "Affirmative declarative sentence", "pos": {"nouns": {"Time": 1, "I": 1}, "verbs": {"I can't help it": 1, "Yes": 1, "Steal": 1}, "adverbs": {"Sometimes": 1}, "numerals": {"205": 1, "Points": 1}}}, {"content": "206. I believe in God to a greater extent than most.", "word_count": 11, "label": "", "sentence_structure": "Subject鈥搗erb鈥揷omplement", "subjective_objective": {"answer": "Subjective", "rationale": "Because \"very\" and \"more than most\" are personal feelings and views of the patient, and are subjective."}, "sentence_sentiment": {"answer": "Neutral", "rationale": "Because the entry does not address specific emotions, attitudes or behaviours, it is merely a description of a person ' s beliefs and therefore has no apparent positive or negative emotional aspects."}, "language_style": {"answer": "Tactful and warm", "rationale": ""}, "time_dimension": {"answer": "Present", "rationale": "The entry \"I believe in God\" shows the testee's current state of belief, and \"more than majority\" is a description of the strength of his conviction and implies that it exists now. The entry should therefore be classified as an assessment of the present."}, "sentence_type": "Affirmative declarative sentence", "pos": {"nouns": {"God": 1, "Level": 1}, "verbs": {"I believe you": 1, "More than": 1, "Most people": 1}, "adverbs": {"very": 1, "More than": 1}, "numerals": {"206": 1}, "classifiers": {"individual": 1}}}, {"content": "207. I like many different kinds of games and entertainment.", "word_count": 15, "label": "", "sentence_structure": "Subject鈥搗erb鈥搊bject", "subjective_objective": {"answer": "Subjective", "rationale": "Because \"like\" is a subjective attitude or emotional experience."}, "sentence_sentiment": {"answer": "Neutral", "rationale": "Because the entry simply expresses a person ' s preference for many games and entertainments, and does not involve any emotion or attitude."}, "language_style": {"answer": "Tactful and warm", "rationale": "The entry is relatively gentle and friendly, is not too direct or rigid, but rather is a conversation in question, which makes it easier for the interviewees to feel comfortable and acceptable. Moreover, the entry uses positive words such as \"like\" and \"many different kinds\" and helps to create a comfortable and pleasant atmosphere in which the interviewees are more willing to share their thoughts and feelings."}, "time_dimension": {"answer": "Present", "rationale": "The entry asks about the present interests of the subject, which include expressions of \"like\" and \"many different kinds\", which indicate that this is an act or situation describing the current state."}, "sentence_type": "Affirmative declarative sentence", "pos": {"nouns": {"Game": 1, "Recreation": 1}, "verbs": {"Yeah": 1}, "adjectives": {"Many": 1, "Different types": 1}, "numerals": {"207锛?: 1}}}, {"content": "208. I like to joke with the opposite sex.", "word_count": 8, "label": "", "sentence_structure": "Subject鈥搗erb鈥搊bject", "subjective_objective": {"answer": "Subjective", "rationale": "The idea of a person who is a person is not a person, because it is a person's inner feeling or attitude."}, "sentence_sentiment": {"answer": "Positive", "rationale": "Because the \"like and the opposite sex\" act in the entry shows a positive and open personality."}, "language_style": {"answer": "Tactful and warm", "rationale": "This euphemism makes it easier for the interviewee to accept and answer questions."}, "time_dimension": {"answer": "Present", "rationale": "The entry describes a customary sexual act of the testee, \"like\" is psychological, and \"and the opposite sex\" is a specific act, so the temporal dimension is now judged."}, "sentence_type": "Affirmative declarative sentence", "pos": {"nouns": {"Heterosexual": 1, "You're kidding": 1}, "verbs": {"Yeah": 1, "and": 1, "Say it": 1, "Smile": 1}, "adverbs": {"208": 1}, "numerals": {"208": 1}}}, {"content": "209. I believe that my sins are inexcusable.", "word_count": 12, "label": "", "sentence_structure": "Subject鈥搗erb鈥揷omplement", "subjective_objective": {"answer": "Subjective", "rationale": "Because \"convincing\" is a subjective attitude or belief."}, "sentence_sentiment": {"answer": "Negative", "rationale": "Because it expresses negative feelings and attitudes that consider their sins unforgivable."}, "language_style": {"answer": "Calm and direct", "rationale": "The language style of the entry is a straightforward type of calm, which directly expresses the perception and attitude of the other side to their guilt, without excessive ado or emotional rendering. At the same time, this direct expression has some authority and objectivity.\""}, "time_dimension": {"answer": "Past", "rationale": "The entry \"Believe\" and \"Be\" both use the general current, express the current belief, while \"Unforgivable\" implies a certain act or situation of the past and can therefore be judged to be the past."}, "sentence_type": "Negative declarative sentence", "pos": {"nouns": {"Sin": 1, "Forgive me": 1}, "verbs": {"Yes": 1, "be": 1}, "numerals": {"209锛?: 1}, "pronouns": {"I": 1}}}, {"content": "210. Each food tastes different.", "word_count": 14, "label": "", "sentence_structure": "Subject鈥揷opula鈥損redicative", "subjective_objective": {"answer": "Objective", "rationale": "Because \"everything tastes different\" is an objective description, and it does not require subjective attitudes or behaviour. It is an objective experience based on taste and perception."}, "sentence_sentiment": {"answer": "Neutral", "rationale": "Since the entry does not express feelings, attitudes or behaviour, it merely states a fact."}, "language_style": {"answer": "Calm and direct", "rationale": "It's simple, it's straight, it's straight, it's asking if the person under investigation feels that way. So I'll judge it as \"cool and straight.\""}, "time_dimension": {"answer": "Present", "rationale": "The entry describes the taste of the present food taste by the testee, and the temporal dimension is now judged."}, "sentence_type": "Affirmative declarative sentence", "pos": {"nouns": {"Something": 1, "Tastes": 1}, "verbs": {"Eat it": 1, "Both": 1, "It's different": 1}, "adjectives": {"Each one": 1}, "numerals": {"210锛?: 1}}}, {"content": "211. I sleep during the day and I can't sleep at night.", "word_count": 11, "label": "", "sentence_structure": "Subject鈥搗erb鈥揷omplement", "subjective_objective": {"answer": "Subjective", "rationale": "Because it's a subjective statement of the patient, expressing his feelings and experience.\""}, "sentence_sentiment": {"answer": "Negative", "rationale": "Because the behavior in the entries \"sleeping during the day and not at night\" is negative."}, "language_style": {"answer": "Tactful and warm", "rationale": ""}, "time_dimension": {"answer": "Present", "rationale": ""}, "sentence_type": "Affirmative declarative sentence", "pos": {"nouns": {"I": 1, "Daytime": 1, "Sleep": 2, "Night": 1, "I'm not sure": 1}, "verbs": {"Sleep": 3, "not": 1, "Yeah": 1}, "adverbs": {"But": 1}, "numerals": {"211": 1}}}, {"content": "212. My family thinks of me as a child, and I think of me as an adult.", "word_count": 21, "label": "", "sentence_structure": "Subject鈥揷opula鈥損redicative", "subjective_objective": {"answer": "Subjective", "rationale": "Because this sentence relates to patients ' subjective perceptions and perceptions of themselves and their families, it is a reflection of patients ' subjective feelings and attitudes."}, "sentence_sentiment": {"answer": "Neutral", "rationale": "Because the entry is not of a clear emotional nature, it merely presents a fact."}, "language_style": {"answer": "Tactful and warm", "rationale": "The entry uses a soft, invitation-style tone of conversation, in which the speaker does not directly blame or direct the other, but rather directs his answer by allowing the other to reflect on his own. Such expression is more personal and interactive, while avoiding potential conflicts or discomfort."}, "time_dimension": {"answer": "Present", "rationale": "The entry describes the current mindset and feelings of the testee, which are considered children at home, but are considered adults in self-awareness. This mindset and feeling is judged by the time dimension of time."}, "sentence_type": "Affirmative declarative sentence", "pos": {"nouns": {"Home": 1, "person": 2, "Kids": 1, "Sir": 1}, "verbs": {"- Put it on": 4, "I'll take it as a treat": 3, "Look": 1}, "numerals": {"212": 1}, "pronouns": {"I": 2}}}, {"content": "213. When walking, I carefully crossed the line on the sidewalk.", "word_count": 17, "label": "", "sentence_structure": "Subject鈥搗erb鈥揷omplement", "subjective_objective": {"answer": "Objective", "rationale": "Because crossing the seam is an objective behavior of the patient."}, "sentence_sentiment": {"answer": "Neutral", "rationale": "Because the purpose of the article is not expressed in a clear emotional or tendentious way. It simply describes an act (crutinizing through the seams) without involving emotion, attitude or value judgement. We can therefore classify it as neutral."}, "language_style": {"answer": "Tactful and warm", "rationale": "The language style of the entry is euphemistic, because it uses a gentle and inviting tone, rather than a direct statement or order. It is more acrimony and interactive. It also does not use excessive rhetorical or emotional overtones, which are more objective and give people a comfortable and relaxed feeling."}, "time_dimension": {"answer": "Past", "rationale": "The \"very careful cross\" in the entry indicates that the act has occurred in the past and is therefore judged to be past."}, "sentence_type": "Affirmative declarative sentence", "pos": {"nouns": {"Road": 1, "Sidewalk": 1, "Suture": 1}, "verbs": {"Let's go": 1, "Cross": 1, "Very careful": 1}, "adjectives": {"Be careful": 1}, "adverbs": {"Land": 2}, "numerals": {"213": 1}}}, {"content": "214. I've never been bothered by the skin.", "word_count": 15, "label": "", "sentence_structure": "Subject鈥搗erb鈥揷omplement", "subjective_objective": {"answer": "Objective", "rationale": "Because the question of whether a person is troubled by something is the patient's own subjective feelings and experiences, and the question in the title is \"Never before,\" which is an objective fact."}, "sentence_sentiment": {"answer": "Positive", "rationale": "Because the act of \"never getting upset about skin\" in the entry is positive."}, "language_style": {"answer": "Calm and direct", "rationale": "The entry is more direct and objective in its language style, is not accompanied by too much emotional rendering or speech writing and is less fluent. It asks the interviewee whether he has ever been troubled by skin problems in a simple and clear manner, and is intended to obtain an accurate and true answer."}, "time_dimension": {"answer": "Past", "rationale": "The entry describes the testee's state of affairs at some point in the past, i.e. they have never been troubled by skin problems and have therefore been judged \"the past\"."}, "sentence_type": "Affirmative declarative sentence", "pos": {"nouns": {"Skin": 1, "Something": 1}, "verbs": {"Never": 1, "Long": 1, "Trouble": 1}, "adverbs": {"Never": 1}, "numerals": {"214锛?: 1}}}, {"content": "I've been drinking too much.", "word_count": 7, "label": "", "sentence_structure": "Subject鈥損redicate", "subjective_objective": {"answer": "Objective", "rationale": "Because \"had been overdrinking\" is an objective fact that happens to patients, whether they remember or admit it."}, "sentence_sentiment": {"answer": "Negative", "rationale": "Because the \"overdrinking\" act in the entry is negative."}, "language_style": {"answer": "Calm and direct", "rationale": "This expression makes it easier for the respondents to answer questions in a true and accurate manner."}, "time_dimension": {"answer": "Past", "rationale": ""}, "sentence_type": "Affirmative declarative sentence", "pos": {"nouns": {"Questionnaire": 1, "Entry": 1, "Drink": 1, "degrees": 1}, "verbs": {"Once": 1, "Pass": 1}, "numerals": {"215": 1, "One": 1}, "classifiers": {"individual": 1}, "pronouns": {"I": 1}}}, {"content": "216. Compared to other families, my family lacks love and warmth.", "word_count": 18, "label": "", "sentence_structure": "Subject鈥揷opula鈥損redicative", "subjective_objective": {"answer": "Subjective", "rationale": "Since the questionnaire is based on the patient ' s own views and feelings about the family, it is a subjective examination."}, "sentence_sentiment": {"answer": "Negative", "rationale": "Because the description \"lack of love and warmth\" in the entry is negative."}, "language_style": {"answer": "Tactful and warm", "rationale": "The entry expresses grievances or shortcomings about their families in a euphemism, without direct blame or criticism, but rather by suggesting that the problem exists by comparing it with others. Such expression is relatively milder and less likely to provoke resistance among the respondents."}, "time_dimension": {"answer": "Past", "rationale": "Because the \"old\" in this sentence suggests that it is a description of the past."}, "sentence_type": "Negative declarative sentence", "pos": {"nouns": {"others": 1, "Family": 2, "Love": 1, "Warm": 1}, "verbs": {"Comparison": 1, "Lack": 1}, "numerals": {"216": 1}, "pronouns": {"Mine": 1}}}, {"content": "217. I often feel that I am worried about something.", "word_count": 15, "label": "", "sentence_structure": "Subject鈥搗erb鈥搊bject", "subjective_objective": {"answer": "Subjective", "rationale": "Because it's the patient's subjective feeling."}, "sentence_sentiment": {"answer": "Negative", "rationale": "Because the word \"fear\" in the entry is a negative sentiment."}, "language_style": {"answer": "Calm and direct", "rationale": "The entry directly expresses the subject of an emotional query about whether the other party is often concerned, without excessive rhetorical or emotional rendering and with a more objective tone."}, "time_dimension": {"answer": "Past", "rationale": "The entry asks if the testee was often worried in the past and judged by the time dimension."}, "sentence_type": "Affirmative declarative sentence", "pos": {"nouns": {"Entry": 1, "I": 1, "Yes": 1, "Fear": 1}, "verbs": {"I feel": 1, "Yes": 1}, "adverbs": {"Often": 1}, "numerals": {"217": 1}}}, {"content": "218. When I see animals suffering, I am not particularly hard.", "word_count": 19, "label": "", "sentence_structure": "Subject鈥揷opula鈥損redicative", "subjective_objective": {"answer": "Subjective", "rationale": "Because \"not particularly hard\" is a personal subjective feeling and evaluation."}, "sentence_sentiment": {"answer": "Negative", "rationale": "Basis of judgement: because the entry \"is not particularly difficult\" expresses a indifference or indifference that is often seen as a manifestation of negative emotions."}, "language_style": {"answer": "Calm and direct", "rationale": "The language style of the entry is calm, because it directly expresses the feeling of asking the other person when seeing the animal being tortured, without any emotional rendering. This direct, simple way makes it easier for the interviewee to understand the question and answer it."}, "time_dimension": {"answer": "Present", "rationale": "The keyword \"I am not particularly uncomfortable\" indicates that the question is asked about the feelings and experiences of the subjects at the time and is therefore a matter of current times."}, "sentence_type": "Negative declarative sentence", "pos": {"nouns": {"Time": 1, "Animals": 1, "Torture": 1}, "verbs": {"Yeah": 1, "It's hard": 1, "Not special": 1}, "adjectives": {"When": 1, "Special": 1, "Don't": 1}, "adverbs": {"very": 1}, "numerals": {"218": 1}, "pronouns": {"I": 1}}}, {"content": "219. I think I would like to work on construction contracts.", "word_count": 13, "label": "", "sentence_structure": "Subject鈥搗erb鈥搊bject", "subjective_objective": {"answer": "Subjective", "rationale": "Because \"think\" represents the patient's personal will and expectations, which are considered from the patient's perspective."}, "sentence_sentiment": {"answer": "Positive", "rationale": "Because the expression \"likes\" reflects a positive emotional attitude."}, "language_style": {"answer": "Tactful and warm", "rationale": "The language style of the entry is more euphemistic, neutral, and does not show strong emotional tendencies or direct command. This expression makes respondents feel more comfortable and natural. At the same time, the use of the phrase \"I want\" expresses subjectivity and uncertainty, making questions more free and open."}, "time_dimension": {"answer": "Future", "rationale": "We can therefore judge that the temporal dimension of the article is the future."}, "sentence_type": "Affirmative declarative sentence", "pos": {"nouns": {"Work": 1, "Construction contracting": 1}, "verbs": {"Yes": 2, "Yeah": 1}, "adverbs": {"Yes": 1}, "numerals": {"219": 1}, "pronouns": {"I": 1}}}, {"content": "I love my mother.", "word_count": 6, "label": "", "sentence_structure": "Subject鈥搗erb鈥搊bject", "subjective_objective": {"answer": "Subjective", "rationale": "Because love is a subjective emotional attitude."}, "sentence_sentiment": {"answer": "Positive", "rationale": "Because the statement \"I love my mother\" expresses a positive emotional attitude."}, "language_style": {"answer": "Tactful and warm", "rationale": "The language style of the entry is euphemism, a warmer expression of love for the mother, but not a direct and intense depiction or hairing, but rather a gentle way of conveying it. This expression is more likely to resonate with the interviewee and to share his feelings and thoughts."}, "time_dimension": {"answer": "Present", "rationale": ""}, "sentence_type": "Affirmative declarative sentence", "pos": {"nouns": {"Mother": 1, "I": 1}, "verbs": {"Love": 1}, "numerals": {"220": 1}}}, {"content": "221. I like science.", "word_count": 5, "label": "", "sentence_structure": "Subject鈥損redicate", "subjective_objective": {"answer": "Subjective", "rationale": "Like is a subjective attitude."}, "sentence_sentiment": {"answer": "Positive", "rationale": "Because the attitude of \"like science\" in the entries is positive."}, "language_style": {"answer": "Tactful and warm", "rationale": "The entry is more adept and interactive when it is spoken in a gentle and inviting manner, rather than by direct statements or orders."}, "time_dimension": {"answer": "Present", "rationale": ""}, "sentence_type": "Affirmative declarative sentence", "pos": {"nouns": {"Yeah": 1, "Science": 1}, "verbs": {"Yeah": 1}, "numerals": {"221": 1}}}, {"content": "222. Even if I could not repay the favor later, I had sought help from my friends.", "word_count": 19, "label": "", "sentence_structure": "Subject鈥損redicate", "subjective_objective": {"answer": "Subjective", "rationale": "Because \"no reward\" and \"help\" are the thoughts of the patient's own heart, and are the expression of subjective attitudes."}, "sentence_sentiment": {"answer": "Positive", "rationale": "Because the act of \"usual to help friends\" in the entry expresses trust and dependence on friends and is positive."}, "language_style": {"answer": "Tactful and warm", "rationale": "The language style of the entry is relatively euphemistic and gentle. By using the phrase \"even if I cannot repay my grace later\", to express a request for help to a friend, rather than a direct order or request, it is a reflection of the humility and gratitude of the speaker, and is more easily understood and accepted by others. Moreover, the expression \"I also sought help from my friend\" is used to give the listener the sense of sincerity and respect."}, "time_dimension": {"answer": "Present", "rationale": "Now, the \"I can't repay the favors\" and \"I used to ask my friends for help\" in the questionnaire column both indicate the attitude and behavior of the testee, so the time dimension of the entry is now judged."}, "sentence_type": "Affirmative declarative sentence", "pos": {"nouns": {"Grace": 1, "friends": 1}, "verbs": {"Refund": 1, "Help": 1}, "adverbs": {"Later": 1, "Yeah": 2, "Original": 1}, "numerals": {"222锛?: 1}, "pronouns": {"I": 1}}}, {"content": "I like hunting.", "word_count": 6, "label": "", "sentence_structure": "Subject鈥搗erb鈥揷omplement", "subjective_objective": {"answer": "Subjective", "rationale": "Because \"a lot like hunting\" is a human preference and an interest, a manifestation of subjective attitudes."}, "sentence_sentiment": {"answer": "Positive", "rationale": "Because \"like\" expresses positive emotional attitudes."}, "language_style": {"answer": "Tactful and warm", "rationale": "The entry is more adept and interactive when it is spoken in a gentle and inviting manner, rather than by direct statements or orders."}, "time_dimension": {"answer": "Present", "rationale": ""}, "sentence_type": "Affirmative declarative sentence", "pos": {"nouns": {"Hunting": 1}, "verbs": {"Yeah": 1}, "numerals": {"223": 1}}}, {"content": "224. My parents often object to those who are with me.", "word_count": 15, "label": "", "sentence_structure": "Subject鈥搗erb鈥搊bject", "subjective_objective": {"answer": "Subjective", "rationale": "Because \"my parents often object\" is a subjective attitude or view."}, "sentence_sentiment": {"answer": "Negative", "rationale": "Because the \"parents often object to those who are with me\" act is negative."}, "language_style": {"answer": "Calm and direct", "rationale": "The entry is more concise, has less emotional or speechistic styles, and is more direct and objective."}, "time_dimension": {"answer": "Present", "rationale": "\"My parents often object to those who interact with me.\" In \"My parents\" in \"the subject,\" \"often\" means frequency, \"opposed\" means behaviour, \"the person I interact with\" means object or environment. This information is relevant now. So the entry describes the current state and circumstances."}, "sentence_type": "Negative declarative sentence", "pos": {"nouns": {"I": 1, "parents": 1, "person": 1, "- To be with someone": 1}, "verbs": {"Objection": 1, "- To be with someone": 1}, "adverbs": {"Regular": 1}, "numerals": {"224": 1}}}, {"content": "225. Sometimes I can talk about people.", "word_count": 12, "label": "", "sentence_structure": "Subject鈥搗erb鈥揷omplement", "subjective_objective": {"answer": "Subjective", "rationale": "Because it is the subjective act and will of a person to say something about others, it is subjective."}, "sentence_sentiment": {"answer": "Neutral", "rationale": "Since the entry does not express a positive or negative attitude, conduct, purpose or quality, it merely describes a possible act."}, "language_style": {"answer": "Tactful and warm", "rationale": "The entry was dialogueable in a question-and-answer manner and the phrase \"sometimes\" was used to express its views, which was more gentle and kind and had some kind of accompaniment."}, "time_dimension": {"answer": "Present", "rationale": ""}, "sentence_type": "Affirmative declarative sentence", "pos": {"nouns": {"Time": 1, "person": 1, "Baloney": 1}, "verbs": {"Yes": 1, "Tell me": 1}, "adverbs": {"Yeah": 1}, "numerals": {"225锛?: 1}, "pronouns": {"I": 1}}}, {"content": "226. Some of my family ' s habits make me very sick.", "word_count": 15, "label": "", "sentence_structure": "Subject鈥揷opula鈥損redicative", "subjective_objective": {"answer": "Subjective", "rationale": "This is a subjective feeling and attitude of the patient, a reaction to and an evaluation of the behaviour of family members."}, "sentence_sentiment": {"answer": "Negative", "rationale": "Because the entries contain the word \"obsessed\" of negative emotions, which expresses a negative attitude."}, "language_style": {"answer": "Calm and direct", "rationale": "Thus, the language style is considered to be straightforward and calm."}, "time_dimension": {"answer": "Past", "rationale": "\"Somebody's habits\" represent the past, \"I hate them\" means that the feeling of those habits still exists, so the temporal dimension of the article is the past."}, "sentence_type": "Negative declarative sentence", "pos": {"nouns": {"I": 1, "Family": 1, "Custom": 1, "Shit": 1}, "verbs": {"鈾?And I'm gonna be 鈾?: 1}, "adjectives": {"Very": 1}, "numerals": {"226": 1}, "pronouns": {"Some": 1}}}, {"content": "227. I was told that I was up in bed walking (sleeping).", "word_count": 16, "label": "", "sentence_structure": "Subject鈥搗erb鈥搊bject", "subjective_objective": {"answer": "Objective", "rationale": "Because this is what people tell patients, it is an objective perspective."}, "sentence_sentiment": {"answer": "Neutral", "rationale": "Because the entry does not have any emotional color."}, "language_style": {"answer": "Tactful and warm", "rationale": "The language style of this entry is more euphemistic and caring, and by using the expression \"someone tells me\" suggests that the person surveyed may not be aware of his sleepwalking, but rather that they are told. This expression makes the investigation more moderate and humane, and is more likely to obtain real and accurate information."}, "time_dimension": {"answer": "Present", "rationale": ""}, "sentence_type": "Affirmative declarative sentence", "pos": {"nouns": {"He's": 1, "I": 1, "Sleep": 1, "Walk": 1, "Sleepwalking": 1}, "verbs": {"Tell": 1, "in": 2, "Get up": 1, "Let's go": 1, "Dream on": 1}, "adverbs": {"Medium": 1}, "numerals": {"227": 1}}}, {"content": "228. Sometimes I feel that I make decisions very easily.", "word_count": 15, "label": "", "sentence_structure": "Subject鈥揷opula鈥損redicative", "subjective_objective": {"answer": "Subjective", "rationale": "Because \"think\" is a subjective feeling and perception, and making decisions is an objective act. So, \"I think sometimes I'm very easy to make decisions\" is viewed from the patient's subjective point of view."}, "sentence_sentiment": {"answer": "Neutral", "rationale": "The entry is not subject to any apparent emotional orientation or value judgement."}, "language_style": {"answer": "Tactful and warm", "rationale": "The language style of the entry is more euphemistic, engaging in dialogue in a gentle and inviting tone, asking if the other side feels like it, rather than directly speaking or ordering."}, "time_dimension": {"answer": "Present", "rationale": "The keyword \"sometimes\" and \"easy to make a decision\" suggests that it is the person who is being tested for the moment or for the state, and therefore the judgement is now."}, "sentence_type": "Affirmative declarative sentence", "pos": {"nouns": {"Time": 1, "Decision": 1}, "verbs": {"I think": 1, "Do it": 1}, "adjectives": {"Very": 1, "Easy": 1}, "adverbs": {"Land": 1}, "pronouns": {"I": 1}}}, {"content": "229. I like to join several groups at the same time.", "word_count": 11, "label": "", "sentence_structure": "Subject鈥搗erb鈥搊bject", "subjective_objective": {"answer": "Subjective", "rationale": "Because \"like to join multiple groups\" is the patient's subjective attitude and emotional expression."}, "sentence_sentiment": {"answer": "Neutral", "rationale": "Because the entry merely stated a fact and did not express any emotional colour."}, "language_style": {"answer": "Tactful and warm", "rationale": "The language style of the entry is euphemism. It does not use direct or hard language to ask questions, but rather asks the respondents whether they like to join multiple groups at the same time, in a more compassionate and interactive way."}, "time_dimension": {"answer": "Present", "rationale": "The entry describes the current preferences and preferences of the testee, i.e., the preference to join several groups at the same time, and thus the judgement is now."}, "sentence_type": "Affirmative declarative sentence", "pos": {"nouns": {"Groups": 1}, "verbs": {"Yeah": 1, "Participation": 1}, "adverbs": {"Meanwhile": 1}, "numerals": {"How many": 1}, "classifiers": {"individual": 1}}}, {"content": "I've never been in a panic.", "word_count": 11, "label": "", "sentence_structure": "Subject鈥揷opula鈥損redicative", "subjective_objective": {"answer": "Objective", "rationale": "Because a person's nervous condition is a biological feeling, it is an objective description of whether the patient has experienced such an experience."}, "sentence_sentiment": {"answer": "Neutral", "rationale": "Because the entry is not clearly emotional."}, "language_style": {"answer": "Calm and direct", "rationale": "The language style of the entry is calm and straightforward. It does not use excessive rhetorical techniques or emotional rendering, and it is speaking with a sense of objectivity and directness to the subject of asking if the other side had ever felt a panic."}, "time_dimension": {"answer": "Present", "rationale": "The entry \"I never felt\" means that the person tested has not experienced this at the moment and therefore is judged to be in the present state of behaviour or state."}, "sentence_type": "Affirmative declarative sentence", "pos": {"nouns": {"I'm scared": 1, "Short air": 1}, "verbs": {"I feel": 1, "Never": 1, "No, I'm not": 1}, "adverbs": {"Never": 1}, "numerals": {"230": 1}}}, {"content": "231. I like to talk about gender.", "word_count": 11, "label": "", "sentence_structure": "Subject鈥搗erb鈥搊bject", "subjective_objective": {"answer": "Subjective", "rationale": "Like being patient subjective."}, "sentence_sentiment": {"answer": "Positive", "rationale": "Because the behaviour in the entry \"likes to talk about gender\" expresses love and interest in the subject, without any negative or neutral emotional expression."}, "language_style": {"answer": "Tactful and warm", "rationale": "The entry uses a more euphemistic and inviting language style, replacing the words \"like to talk\" with the words \"direct statement or order\" and leads the other side to answer by asking questions, which is more personal and interactive."}, "time_dimension": {"answer": "Present", "rationale": "Now the entry indicates that the testee prefers to talk about gender at the current time, without any reference to the past or future time dimension. So time is judged as \"now\"."}, "sentence_type": "Affirmative declarative sentence", "pos": {"nouns": {"In terms of": 1, "be": 1}, "verbs": {"Yeah": 1, "Talk": 1, "Likes to talk": 1}, "numerals": {"Gender": 1}}}, {"content": "232. I have always been cautious about my desire to lead a life of responsibility.", "word_count": 26, "label": "", "sentence_structure": "Subject鈥搗erb鈥搊bject", "subjective_objective": {"answer": "Subjective", "rationale": "Because \"intentional\" and \"prudence\" are subjective attitudes and behaviours of patients."}, "sentence_sentiment": {"answer": "Positive", "rationale": "It is a positive emotion and quality that is expressed in the article's expression that \"a desire to lead a life of responsibility\" and in its expression that \"a man has a clear understanding of his responsibilities and has always acted with that understanding\"."}, "language_style": {"answer": "Calm and direct", "rationale": "The entry is based on a more objective and direct presentation, without too much emotional rendering or rhetorical. It expresses a person's aims and actions in life, is more plain and direct, and has little euphemism or exaggeration. So we can describe it as a calm and direct style."}, "time_dimension": {"answer": "Past", "rationale": "The words \"had been\" and \"have been so cautious\" in the entry indicate that this is a past experience and present behaviour, and the temporal dimension of the article can therefore be judged to be the past."}, "sentence_type": "Affirmative declarative sentence", "pos": {"nouns": {"Questionnaire": 1, "Life": 1, "Responsibility": 1, "Situation": 1}, "verbs": {"I'm so sorry": 1, "Yes": 1, "Pass": 1, "Here": 1, "To weigh": 1, "And so on": 1, "Be careful": 1}, "adverbs": {"Once": 1, "Always": 1}, "numerals": {"232": 1}, "pronouns": {"I": 1}}}, {"content": "Sometimes I stop people from doing something that has a great impact, and sometimes I should interfere with him 鈥渕orally鈥?", "word_count": 36, "label": "", "sentence_structure": "Subject鈥搗erb鈥搊bject", "subjective_objective": {"answer": "Subjective", "rationale": "It is subjective in view of the moral values and values of the individual."}, "sentence_sentiment": {"answer": "Neutral", "rationale": "Because the expression of the purpose of the article is not clearly emotional. While it refers to \"moral\" interference, it does not necessarily imply a positive or negative attitude, as it does not express a preference or aversion to such conduct."}, "language_style": {"answer": "Tactful and warm", "rationale": "The language style of the entry is more euphemistic, implicit, and implies the author's view of the issue by using the expression \"moral\" and \"should interfere with him\". This expression is more polite and respectful than direct accusations or orders, and it is more resonant and thoughtable with the other side."}, "time_dimension": {"answer": "Present", "rationale": ""}, "sentence_type": "Affirmative declarative sentence", "pos": {"nouns": {"Questionnaire": 1, "Entry": 1, "person": 1, "Yes": 2, "Morality": 1, "he": 1}, "verbs": {"Stop": 1, "Do it": 1, "have": 1, "Intervention": 1, "be": 1}, "adjectives": {"Large": 1}, "adverbs": {"Time": 2}, "numerals": {"233": 1, "That": 1}, "pronouns": {"I": 1}}}, {"content": "234. I was easily angry, but quickly calmed down.", "word_count": 14, "label": "", "sentence_structure": "Subject鈥搗erb鈥揷omplement", "subjective_objective": {"answer": "Subjective", "rationale": "The content of the questionnaire is based on the emotional response and treatment of the individual, the subjective feelings and attitudes of the patient and therefore the subjective examination."}, "sentence_sentiment": {"answer": "Neutral", "rationale": "It is based on the fact that anger is a negative emotional reaction, but it is easy to calm down and it is a positive response. The entry therefore contains both negative and positive elements, without a clear emotional bias, and is neutral."}, "language_style": {"answer": "Calm and direct", "rationale": "This language style is more suitable for psychological questionnaire research and clearly reflects the real situation of the person interviewed."}, "time_dimension": {"answer": "Present", "rationale": "Because the sentence describes the state of the moment, \"I'm easily angry\" and has time to say, \"but calm down very quickly,\" which means that anger and calming is the state of behavior or emotion that is now under way."}, "sentence_type": "Affirmative declarative sentence", "pos": {"nouns": {"Questionnaire": 1, "Entry": 1, "Anger": 1}, "verbs": {"Fan": 1, "Calm down": 1, "Get down": 1}, "adjectives": {"Easy": 1, "Come on": 1}, "adverbs": {"very": 1, "Just": 1}, "numerals": {"234": 1}, "pronouns": {"I": 1}}}, {"content": "235. I am independent and independent of the family.", "word_count": 13, "label": "", "sentence_structure": "Subject鈥搗erb鈥揷omplement", "subjective_objective": {"answer": "Subjective", "rationale": "Because \"I'm independent and independent of my family\" is the patient's subjective perception and assessment of his current situation."}, "sentence_sentiment": {"answer": "Positive", "rationale": "Because the words \"independent\" and \"unbounded by the family\" in the entries are all positive emotional."}, "language_style": {"answer": "Calm and direct", "rationale": "The entry conveys in a direct statement whether the subject has been independent and is no longer subject to family constraints, without the use of rhetorical or emotional representation and with a more objective tone."}, "time_dimension": {"answer": "Present", "rationale": "The word \"has\" in the entry indicates that the actions or state of the past have still had an impact on the present, while the word \"unbound\" represents the present state, so that the temporal dimension of the article is now."}, "sentence_type": "Affirmative declarative sentence", "pos": {"nouns": {"Family": 1, "Constraints": 1}, "verbs": {"Independence and autonomy": 1, "Yes": 1, "Constraints": 1}, "adjectives": {"Autonomy": 1, "Independence": 1}, "adverbs": {"Already": 1}, "numerals": {"235锛?: 1}}}, {"content": "236. I have a lot on my mind.", "word_count": 6, "label": "", "sentence_structure": "Subject鈥搗erb鈥揷omplement", "subjective_objective": {"answer": "Subjective", "rationale": "Because \"many things\" are the feelings and experiences inside the patient, the subjective expression."}, "sentence_sentiment": {"answer": "Negative", "rationale": "Because the entry indicates that there are many things that the interviewees have to deal with and resolve, which is usually seen as a negative emotional state."}, "language_style": {"answer": "Tactful and warm", "rationale": "Warm and warm."}, "time_dimension": {"answer": "Present", "rationale": "In the entry, \"many\" means a large number of things that the testees have at the moment have a lot of heart, and the word \"yes\" uses the usual current state of affairs, which means that this state not only has happened in the past, but may still be ongoing. Thus, the time dimension of the questionnaire entry is now judged."}, "sentence_type": "Affirmative declarative sentence", "pos": {"nouns": {"Something's on the mind": 1}, "verbs": {"have": 1}, "numerals": {"236": 1}, "pronouns": {"I": 1}}}, {"content": "237. Almost all my relatives sympathize with me.", "word_count": 11, "label": "", "sentence_structure": "Subject鈥搗erb鈥搊bject", "subjective_objective": {"answer": "Subjective", "rationale": "Because \"sympathetic\" is an emotional attitude that reflects the degree to which patients feel that their relatives are in agreement with them, and this is the subjective view of the patient."}, "sentence_sentiment": {"answer": "Positive", "rationale": "Because the word \"sympathetic\" is positive."}, "language_style": {"answer": "Tactful and warm", "rationale": "The entry uses a more euphemistic expression, which is \"almost all\" to express the compassion of not all relatives, avoiding a direct denial or an overly absolute expression. This expression is more moderate, respectful and understanding."}, "time_dimension": {"answer": "Present", "rationale": "According to the context, \"my relatives are almost all sympathetic to me\" describes the current situation, indicating that the relatives of the talking person are sympathetic to his situation and therefore judge as the present behaviour or state."}, "sentence_type": "Affirmative declarative sentence", "pos": {"nouns": {"Relatives": 1, "Compassion": 1, "I": 1}, "verbs": {"All": 1, "Both": 1, "Compassion": 1}, "adjectives": {"All": 1, "All of them": 1}, "adverbs": {"Almost": 1}, "numerals": {"237": 1}}}, {"content": "238. Sometimes I am very restless and restless.", "word_count": 11, "label": "", "sentence_structure": "Subject鈥搗erb鈥揷omplement", "subjective_objective": {"answer": "Subjective", "rationale": "Because \"freak, stand up\" is the patient's subjective feeling and experience."}, "sentence_sentiment": {"answer": "Negative", "rationale": "Because the description of \"sweet, restless\" in the entry is negative."}, "language_style": {"answer": "Calm and direct", "rationale": "The relatively calm and straightforward language style of the entry, with no excessive rhetorical or emotional rendering, mainly describes the fact that the person under investigation may be in a situation of irritation and restlessness and that the expression is more objective and balanced."}, "time_dimension": {"answer": "Present", "rationale": "The keywords \"sometimes\" and \"staggered\" and \"staggered\" indicate that this is a description of the current psychological state of the person being tested, so the time dimension of the article is now."}, "sentence_type": "Affirmative declarative sentence", "pos": {"nouns": {"Time": 1, "I": 1}, "verbs": {"Tens": 1, "I'm so upset": 1, "Sit tight": 1}, "adjectives": {"Tens": 1, "I'm so upset": 1}, "numerals": {"238锛?: 1}, "pronouns": {"I": 1}}}, {"content": "I've had a break-up.", "word_count": 6, "label": "", "sentence_structure": "Subject鈥搗erb鈥揷omplement", "subjective_objective": {"answer": "Subjective", "rationale": "Because \"I was\" shows that it's a patient's subjective experience and memory."}, "sentence_sentiment": {"answer": "Neutral", "rationale": "Because the entry does not have any emotional color."}, "language_style": {"answer": "Tactful and warm", "rationale": "The language style of the entry is euphemism. It asks the interviewee, in a gentle and respectful manner, whether he has experienced a loss of love, without directly highlighting or evaluating the good or bad of the experience, but leaving it to the interviewer to decide how to answer it. At the same time, the term is more neutral, objective, and avoids overly emotional expressions."}, "time_dimension": {"answer": "Past", "rationale": ""}, "sentence_type": "Affirmative declarative sentence", "pos": {"nouns": {"Questionnaire": 1, "Entry": 1, "Broken love": 1}, "verbs": {"be": 1, "Tsang": 1, "have": 1}, "adverbs": {"Once": 1}, "numerals": {"239": 1}, "pronouns": {"I": 1}}}, {"content": "I've never been upset about my appearance.", "word_count": 12, "label": "", "sentence_structure": "Subject鈥損redicate", "subjective_objective": {"answer": "Objective", "rationale": "Since the patient ' s attitude and feelings are objective, the view of the patient ' s external appearance is examined, not the actual physical appearance itself."}, "sentence_sentiment": {"answer": "Positive", "rationale": "Because the \"no worries about appearance\" attitude in the entry is positive."}, "language_style": {"answer": "Tactful and warm", "rationale": "The language style of the entry is more euphemistic, using the expression \"never\" to make semantics more demeaning and without any direct denial or affirmation of the value of appearance. This expression makes it easier for respondents to answer with a softer and more realistic idea."}, "time_dimension": {"answer": "Past", "rationale": "The entry expresses the behaviour and mentality of the person who was tested in the past, i.e. never be upset by the appearance. Thus, the temporal dimension is the past."}, "sentence_type": "Affirmative declarative sentence", "pos": {"verbs": {"Sad": 1}, "adjectives": {"Never": 1, "not": 1}, "numerals": {"240": 1}, "pronouns": {"I": 1}}}, {"content": "241. I often dream of something that is not to be seen.", "word_count": 12, "label": "", "sentence_structure": "Subject鈥搗erb鈥搊bject", "subjective_objective": {"answer": "Subjective", "rationale": "Because dreams of something that is not to be seen are the subjective experiences and feelings of a person."}, "sentence_sentiment": {"answer": "Negative", "rationale": "Because the word \"unsaid\" in the entry has a certain negative emotional connotation."}, "language_style": {"answer": "Calm and direct", "rationale": "The entry was asked in a concise and straightforward manner whether the respondents often had certain specific types of dreams, were not over-modified or emotional, and were more objective in tone."}, "time_dimension": {"answer": "Present", "rationale": ""}, "sentence_type": "Affirmative declarative sentence", "pos": {"nouns": {"I": 1, "Yes": 1}, "verbs": {"I dreamt about it": 1, "Say it": 1}, "adjectives": {"No one's ever heard of it": 1}, "numerals": {"241": 1}}}, {"content": "I believe that I am no more sensitive than others.", "word_count": 15, "label": "", "sentence_structure": "Subject鈥搗erb鈥搊bject", "subjective_objective": {"answer": "Subjective", "rationale": "It is believed to be a subjective feeling and attitude, and the entry in the questionnaire is therefore a test of the subjective view of the patient."}, "sentence_sentiment": {"answer": "Positive", "rationale": "Because \"believing that you're no more sensitive than anyone else\" is a confident and positive attitude."}, "language_style": {"answer": "Calm and direct", "rationale": "The language style of the entry is more direct, there is no excessive rhetorical or emotional rendering, and the subject matter is expressed in a concise and clear manner."}, "time_dimension": {"answer": "Present", "rationale": ""}, "sentence_type": "Affirmative declarative sentence", "pos": {"nouns": {"person": 1, "You're a bit of a sane": 1}, "verbs": {"Yes": 2, "- Yeah": 1, "not": 1}, "adjectives": {"You're a bit of a sane": 1}, "adverbs": {"and": 1}, "numerals": {"242": 1}, "pronouns": {"I": 1, "others": 1}}}, {"content": "243. I have little pain anywhere.", "word_count": 11, "label": "", "sentence_structure": "Subject鈥揷opula鈥損redicative", "subjective_objective": {"answer": "Subjective", "rationale": "Because \"I have almost no pain\" is a subjective feeling and self-assessment of the patient, not an objective fact to be observed."}, "sentence_sentiment": {"answer": "Neutral", "rationale": "Because the entry does not show positive or negative feelings, attitudes, behaviour, purpose or quality, it merely describes the physical condition of a person."}, "language_style": {"answer": "Calm and direct", "rationale": "The entry asks the interviewee whether he is physically ill or in pain, is shorter and less verbs, and does not use excessive aphrodisiacs or emotional colours."}, "time_dimension": {"answer": "Past", "rationale": "The entry asks whether the testee has been \"unreserved of pain\" in the past, and therefore the temporal dimension is a matter of the past."}, "sentence_type": "Negative declarative sentence", "pos": {"nouns": {"Pain": 1}, "verbs": {"have": 1, "Almost": 1, "Ouch": 1}, "adjectives": {"What": 1}, "adverbs": {"Almost": 1}, "numerals": {"243": 1}, "pronouns": {"I": 1}}}, {"content": "244. My methods of doing things are easily misunderstood.", "word_count": 13, "label": "", "sentence_structure": "Subject鈥揷opula鈥損redicative", "subjective_objective": {"answer": "Subjective", "rationale": "Because I do things by the patient's subjective perception and practice."}, "sentence_sentiment": {"answer": "Negative", "rationale": "Because the phrase \"doing things in ways that are easily misunderstood\" in the entry is negative."}, "language_style": {"answer": "Tactful and warm", "rationale": "The language style of the entry is more euphemistic and is expressed in a \"facilitatively misleading\" way, rather than directly accusing or complaining, which is more inclusive and understanding."}, "time_dimension": {"answer": "Present", "rationale": "The keyword is \"methods of doing business\", which indicates the conduct currently being carried out; and \"vulnerability to misinterpret\" implies that the act has or is causing some consequence. The temporal dimension of the article should therefore be the present."}, "sentence_type": "Negative declarative sentence", "pos": {"nouns": {"Method of conduct": 1, "others": 1}, "verbs": {"Easy to be subjected to": 1, "Misunderstanding": 1}, "adverbs": {"very": 1}, "numerals": {"244": 1}, "pronouns": {"I": 1}}}, {"content": "245. My parents and family have been scoffing at my past.", "word_count": 14, "label": "", "sentence_structure": "Subject鈥搗erb鈥搊bject", "subjective_objective": {"answer": "Subjective", "rationale": "Because \"choo-hoo\" is an evaluative word and the evaluation is directed at \"I,\" it is a subjective view."}, "sentence_sentiment": {"answer": "Negative", "rationale": "Because the word \"choo-hoo\" usually has a negative emotional connotation."}, "language_style": {"answer": "Calm and direct", "rationale": "The entry is more calm and straightforward, with no excessive emotional or rhetorical style. It directly raises the question of whether the person surveyed feels that his parents and family have been critical of TA in the past."}, "time_dimension": {"answer": "Past", "rationale": "The word \"old\" in the entry indicates a time frame in the past. The keyword \"I used\" indicates that the person tested was talking about his/her past or feelings. The term \"choo-hoo\" is also about past behaviour, not current or future situations. So the time dimension of this questionnaire entry is past."}, "sentence_type": "Negative declarative sentence", "pos": {"nouns": {"parents": 1, "Family": 1, "Past": 1, "Picky": 1}, "verbs": {"to": 1, "I": 2}, "adjectives": {"- Mine": 1}, "numerals": {"245锛?: 1}, "pronouns": {"they": 1}}}, {"content": "246. There are frequent red spots on my neck (caps).", "word_count": 11, "label": "", "sentence_structure": "Subject鈥搗erb鈥搊bject", "subjective_objective": {"answer": "Objective", "rationale": "Because \"red spots are often found on my neck\" is an objective fact."}, "sentence_sentiment": {"answer": "Negative", "rationale": "Because \"red spots\" in entries are usually an abnormal symptom that can cause anxiety and anxiety."}, "language_style": {"answer": "Calm and direct", "rationale": "The entry is more straightforward in its language style, is less mundane or emotional and focuses more on transmitting information and facts. It asks the other person in a simple, clear way about his/her condition, enabling the other person to quickly understand and answer questions."}, "time_dimension": {"answer": "Present", "rationale": ""}, "sentence_type": "Affirmative declarative sentence", "pos": {"nouns": {"Neck": 1, "The neck": 1, "Red spots": 1}, "verbs": {"Come on": 1}, "adverbs": {"Often": 1}, "numerals": {"246": 1}}}, {"content": "247. I have reason to envy some of my family.", "word_count": 13, "label": "", "sentence_structure": "Subject鈥搗erb鈥搊bject", "subjective_objective": {"answer": "Subjective", "rationale": "Because the words \"reasons\" and \"envious\" indicate the patient's subjective perception and emotional reaction to some people in the family."}, "sentence_sentiment": {"answer": "Negative", "rationale": "Because \"jealous\" is a negative sentiment."}, "language_style": {"answer": "Calm and direct", "rationale": "The entry directly asked the interviewees whether they were jealous of their families and did not use excessive rhetorical or emotional rendering, which was more objective."}, "time_dimension": {"answer": "Present", "rationale": "\"I have reason to be jealous\" means \"there is sex,\" and the descriptions of \"enormity\" and \"home\" point to the current family situation, so time dimension is now judged."}, "sentence_type": "Affirmative declarative sentence", "pos": {"nouns": {"Rationale": 1, "Jealousy": 1, "Home": 1, "person": 1}, "verbs": {"have": 1, "Jealousy": 1}, "numerals": {"247": 1}, "pronouns": {"I": 1}}}, {"content": "248. I sometimes feel very happy for no reason, even when things are not going well.", "word_count": 25, "label": "", "sentence_structure": "Subject鈥搗erb鈥揷omplement", "subjective_objective": {"answer": "Subjective", "rationale": "Because \"Feeling very happy\" is the patient's sense of feeling and emotional experience, and it's subjective. And \"Sometimes, even in the event of a bad day\" is an objective description of what happens to these feelings. But, overall, the entry looks at the patient's subjective emotional state."}, "sentence_sentiment": {"answer": "Positive", "rationale": "Because the description \"will feel very happy\" in the entry is positive."}, "language_style": {"answer": "Tactful and warm", "rationale": "The entry is more euphemistic. First, it uses the term \"unprovoked\" to express a sense of happiness, which is relatively soft. Second, the expression \"even in bad times\" implies that happiness is not always relevant and smooth, and that it is relatively euphemism. Finally, the term \"very happy\" also has a positive emotional tone, making the whole subject more warm."}, "time_dimension": {"answer": "Present", "rationale": "The keywords \"sometimes\", \"even in the event of a bad time\" indicate that this feeling is happening now."}, "sentence_type": "Affirmative declarative sentence", "pos": {"nouns": {"248": 1, "Time": 1, "Happy": 1}, "verbs": {"Yes": 1, "I think": 1, "be": 1, "Even if": 1, "Yeah": 1, "For no reason": 1, "in": 1, "Even": 1, "When things went bad": 1}, "adjectives": {"For no reason": 1}, "adverbs": {"Sometimes": 1, "Very": 1, "Even": 1}, "numerals": {"248": 1, "One": 1, "Plant": 1}, "classifiers": {"individual": 1}, "pronouns": {"I": 1}}}, {"content": "249. I believe there is a devil and hell in the shadows.", "word_count": 11, "label": "", "sentence_structure": "Subject鈥損redicate", "subjective_objective": {"answer": "Subjective", "rationale": "Believe is a subjective attitude that reflects the patient鈥檚 degree of acceptance of a certain concept or belief. In such cases, the patient鈥檚 belief that there is a demon and hell in the vagina is based on their personal views and convictions, and is not an objective fact."}, "sentence_sentiment": {"answer": "Negative", "rationale": "Because the idea of \"believing in the shadows there is a devil and hell\" in the entry is negative."}, "language_style": {"answer": "Calm and direct", "rationale": "The entry asks the other side in a direct and clear way about the existence of demons and hell in the vagina, and there is no excessive rhetorical or emotional rendering."}, "time_dimension": {"answer": "Present", "rationale": "The entry is for the testee's belief in \"devil and hell in the shadows\", without information about the past or the future. So the time dimension is now."}, "sentence_type": "Affirmative declarative sentence", "pos": {"nouns": {"The shadow": 1, "Devil": 1, "Hell": 1}, "verbs": {"have": 1, "Yes": 1}, "numerals": {"249": 1}}}, {"content": "I do not blame anyone who wants to take what is available in the world.", "word_count": 25, "label": "", "sentence_structure": "Subject鈥搗erb鈥搊bject", "subjective_objective": {"answer": "Objective", "rationale": "Because this is an author ' s view or attitude, it is not the patient ' s subjective feeling."}, "sentence_sentiment": {"answer": "Neutral", "rationale": "As the entry does not clearly express any positive or negative emotional attitude, behaviour, purpose or quality, it merely describes a phenomenon or view and can therefore be considered neutral."}, "language_style": {"answer": "Tactful and warm", "rationale": "The entry is more personal and interactive when it is spoken in a gentle and inviting manner, rather than in direct statements or orders. Moreover, the content of the entry is not offensive or accusatory, but rather deals with different ideas and behaviours that may exist in a tolerant and understanding manner. Thus, it can be said that the language style of the purpose is euphemistic."}, "time_dimension": {"answer": "Present", "rationale": "\"250. Some people want to take what is available in the world\" to describe what is now, and \"I will not blame him\" to indicate that the attitude or perception of the speaker is also now. So the entry is mainly concerned with the prevailing phenomena and attitudes in modern societies, and the temporal dimension is now."}, "sentence_type": "Affirmative declarative sentence", "pos": {"nouns": {"person": 1, "Something": 1, "Hands": 1}, "verbs": {"Yes": 1, "Take it": 1, "Blame": 1}, "adverbs": {"Never": 1}, "numerals": {"250锛?: 1}, "pronouns": {"he": 1}}}, {"content": "251. I was in a state of panic and I was not aware of what was happening around me.", "word_count": 23, "label": "", "sentence_structure": "Subject鈥損redicate", "subjective_objective": {"answer": "Subjective", "rationale": "Because \"I don't know what's going on around here\" is a subjective feeling and judgment."}, "sentence_sentiment": {"answer": "Negative", "rationale": "Basis of judgement: The entry is a statement of a focusless behaviour that may lead to a decrease in cognitive capacity in the surrounding environment and therefore can be considered negative."}, "language_style": {"answer": "Calm and direct", "rationale": "The entry describes an eventuality in an objective and direct manner, without excessive emotion or rhetorical tactics, and therefore can be judged as being calm and straightforward."}, "time_dimension": {"answer": "Past", "rationale": "The reference to \"had\" in the entry refers to what happened at some point in the past and is therefore judged to be past."}, "sentence_type": "Affirmative declarative sentence", "pos": {"nouns": {"I": 1, "Things": 1}, "verbs": {"Once": 1, "You're so stupid": 1, "Come on": 1, "Yes": 1, "Stop": 1, "Activities": 1, "I don't know": 1, "What": 1}, "adverbs": {"Once": 1, "Whoa": 1, "Stop": 1, "You're so stupid": 1, "I don't know": 1}, "numerals": {"251": 1}}}, {"content": "252. No one cares about what happened to anyone.", "word_count": 9, "label": "", "sentence_structure": "Subject鈥搗erb鈥搊bject", "subjective_objective": {"answer": "Subjective", "rationale": "The subject is the patient's subjective feelings towards the people around him."}, "sentence_sentiment": {"answer": "Negative", "rationale": "Because the act of \"no one cares about what happened to no one\" in the entry is a sign of indifference, indifference and negative."}, "language_style": {"answer": "Tactful and warm", "rationale": "The language style of the entry is euphemism, because it does not directly accuse or criticize others for their indifference to others, but rather raises questions in a moderate and inclusive manner, leaving room for reflection and improvement. Moreover, it is also more balanced and objective, and does not have too much emotional overtones."}, "time_dimension": {"answer": "Past", "rationale": "The entry uses the expression \"no one cares\" in the past and therefore determines its temporal dimension as the past."}, "sentence_type": "Negative declarative sentence", "pos": {"nouns": {"person": 1, "Problems": 1}, "verbs": {"not": 1, "Care": 1}, "adverbs": {"Yeah": 1, "Nobody": 1}, "numerals": {"252": 1}, "pronouns": {"Who": 1}}}, {"content": "253. Some people have done what I think is wrong, but I have been able to treat them amicably.", "word_count": 28, "label": "", "sentence_structure": "Subject鈥搗erb鈥搊bject", "subjective_objective": {"answer": "Subjective", "rationale": "Because \"it's wrong\" is the patient's subjective attitude."}, "sentence_sentiment": {"answer": "Neutral", "rationale": "Because the entry does not express a positive or negative emotional attitude, it merely presents a view."}, "language_style": {"answer": "Tactful and warm", "rationale": "The language style of the entry is more euphemistic. It expresses its views in a polite and respectful manner, emphasizing that even when certain acts are contested, they can be treated in a friendly manner."}, "time_dimension": {"answer": "Past", "rationale": "The entry does not have a clear temporal dimension and does not determine whether the act or mentality is past, present or future."}, "sentence_type": "Affirmative declarative sentence", "pos": {"nouns": {"person": 1, "Yes": 1, "Wrong": 1, "Friendly": 1}, "verbs": {"Do it": 1, "I think": 1, "Treatment": 2}, "adjectives": {"Wrong": 1, "Friendly": 1}, "adverbs": {"But": 1, "Still": 1, "Yes": 1}, "numerals": {"253": 1}, "pronouns": {"they": 1}}}, {"content": "254. I like to be with people who can joke about each other.", "word_count": 17, "label": "", "sentence_structure": "Subject鈥搗erb鈥搊bject", "subjective_objective": {"answer": "Subjective", "rationale": "Because it's the patient's subjective attitude."}, "sentence_sentiment": {"answer": "Positive", "rationale": "Because the entry \"likes to be with someone who can joke about each other\" expresses a positive emotional attitude."}, "language_style": {"answer": "Tactful and warm", "rationale": ""}, "time_dimension": {"answer": "Present", "rationale": "The entry describes the current state or tendency, which is that the words \"like\" and \"together\" point to a constant behaviour, and therefore the judgment is \"now\"."}, "sentence_type": "Affirmative declarative sentence", "pos": {"nouns": {"Questionnaire Entry": 1, "person": 1, "A joke": 1}, "verbs": {"Yeah": 1, "in": 1, "Together": 1, "Open": 1}, "adverbs": {"- Yeah, yeah": 1}, "numerals": {"254": 1}, "classifiers": {"Some": 1}, "pronouns": {"I": 1}}}, {"content": "255. At elections, sometimes I choose someone I do not know.", "word_count": 18, "label": "", "sentence_structure": "Subject鈥搗erb鈥搊bject", "subjective_objective": {"answer": "Subjective", "rationale": "Because \"selecting someone I don't know\" is the patient's choice and idea, and it's subjective."}, "sentence_sentiment": {"answer": "Negative", "rationale": "Because it's negative to choose someone I don't know."}, "language_style": {"answer": "Calm and direct", "rationale": "The language style of the entry is straightforward and directly describes the fact that the person surveyed may choose someone unfamiliar during the election, without excessive ado and emotional."}, "time_dimension": {"answer": "Past", "rationale": "The keyword \"election time\" means that this is an event that has already happened, and the \"I choose someone I'm not familiar\" describes a behavior or attitude, which suggests that the entry asks about the behaviour or attitude of the testee in the past. So the temporal dimension is a past."}, "sentence_type": "Affirmative declarative sentence", "pos": {"nouns": {"Time": 1, "Elections": 1, "person": 1}, "verbs": {"- Yes, I do": 1, "Out": 1}, "adjectives": {"I don't know": 1}, "numerals": {"255锛?: 1}, "pronouns": {"I": 1}}}, {"content": "Only 鈥渃omics鈥?are the most interesting.", "word_count": 10, "label": "", "sentence_structure": "Subject鈥搗erb鈥搊bject", "subjective_objective": {"answer": "Subjective", "rationale": "Because it's the patient's personal perception and assessment of the extent of the comic book in the newspaper."}, "sentence_sentiment": {"answer": "Negative", "rationale": "Since the entry refers only to the interest in the \"comics\" and not to other content, it may indicate a disregard for or lack of interest in other news reports, with some negative emotional connotations."}, "language_style": {"answer": "Tactful and warm", "rationale": "The topic uses euphemism, moderation, and does not express subjective views or criticism directly, but rather asks the interviewee to express his views. At the same time, the \"most interesting\" also has some subjective connotations, which highlight the openness and personalization of the issue. Thus, the language style of the topic can be considered as euphemism."}, "time_dimension": {"answer": "Past", "rationale": "The entry describes the testee's past behaviour or preference and can therefore be judged as \"the past\"."}, "sentence_type": "Affirmative declarative sentence", "pos": {"nouns": {"Newspapers": 1, "Comics": 1}, "verbs": {"have": 1, "It's the funniest": 1}, "adjectives": {"Interesting": 1}, "numerals": {"256": 1}}}, {"content": "257. I expect success in everything I do.", "word_count": 15, "label": "", "sentence_structure": "Subject鈥搗erb鈥搊bject", "subjective_objective": {"answer": "Subjective", "rationale": "Because \"what I do\" and \"what I'm looking for\" are the feelings and expectations of the patient, and they are viewed from a subjective perspective."}, "sentence_sentiment": {"answer": "Positive", "rationale": "Because the entry expresses positive feelings and attitudes, namely optimism, self-confidence and determination."}, "language_style": {"answer": "Tactful and warm", "rationale": "The entry is more adept and interactive when it is spoken in a gentle and inviting manner, rather than by direct statements or orders."}, "time_dimension": {"answer": "Present", "rationale": "According to the \"all I do\" word, it is now being done or done. And \"all I do\" and \"all I can do\" are in the state or the expectation, so the sentence describes the situation."}, "sentence_type": "Affirmative declarative sentence", "pos": {"nouns": {"Things": 1, "I": 1}, "verbs": {"Do it": 1, "Both": 1, "Look": 1, "Yes": 1, "Success": 1}, "adverbs": {"鈾?Where 鈾?: 1}, "numerals": {"257锛?: 1}}}, {"content": "258. I believe in God.", "word_count": 5, "label": "", "sentence_structure": "Subject鈥損redicate", "subjective_objective": {"answer": "Subjective", "rationale": "Because belief in the existence of God is a subjective belief and conviction, it is not based on objective facts."}, "sentence_sentiment": {"answer": "Neutral", "rationale": "Because the entry does not have any emotional color."}, "language_style": {"answer": "Calm and direct", "rationale": ""}, "time_dimension": {"answer": "Future", "rationale": "The entry contains a question about beliefs and perceptions, and uses the word \"convincing,\" which suggests that it is a matter of predicting the future of one's beliefs. At the same time, \"with God\" is a state or event that has not yet happened and may only exist in the imagination of the interviewee, and thus it points to the dimensions of the future."}, "sentence_type": "Affirmative declarative sentence", "pos": {"nouns": {"God": 1, "Questionnaire": 1}, "verbs": {"Yes": 1, "have": 1}, "numerals": {"258": 1}, "pronouns": {"I": 1}}}, {"content": "259. I find it difficult to start anything.", "word_count": 13, "label": "", "sentence_structure": "Subject鈥揷opula鈥損redicative", "subjective_objective": {"answer": "Subjective", "rationale": "Because the hard idea to start is the patient's subjective attitude."}, "sentence_sentiment": {"answer": "Negative", "rationale": "Because the entry indicates that the interviewee will feel difficulties and frustrations in doing his/her work, which is a negative sentiment."}, "language_style": {"answer": "Calm and direct", "rationale": "The language style of the entry is relatively calm and straightforward, rather than using excessive emotional or rhetorical techniques, questions are raised in an objective and concise manner, aimed at collecting the actual situation and feelings of the person interviewed."}, "time_dimension": {"answer": "Past", "rationale": ""}, "sentence_type": "Negative declarative sentence", "pos": {"nouns": {"Things": 1}, "verbs": {"Do it": 1, "I feel": 1, "Start": 1}, "adjectives": {"Hard": 1}, "adverbs": {"Both": 1}, "numerals": {"259": 1}}}, {"content": "260. In school, I'm a stupid student.", "word_count": 10, "label": "", "sentence_structure": "Subject鈥揷opula鈥損redicative", "subjective_objective": {"answer": "Subjective", "rationale": "Because \"smug student\" is a subjective evaluation, not an objective factual description."}, "sentence_sentiment": {"answer": "Negative", "rationale": "Because the expression \"soft student\" is negative."}, "language_style": {"answer": "Calm and direct", "rationale": "This entry is a simple and clear expression of the self-evaluations of the respondents in schools, and there is no excessive rhetorical and emotional rendering."}, "time_dimension": {"answer": "Past", "rationale": "The entry uses the past \"Yes\", indicating that this happened in the past and has ended or not happened again."}, "sentence_type": "Negative declarative sentence", "pos": {"nouns": {"School": 1, "Students": 1}, "verbs": {"be": 1}, "adjectives": {"Stupid": 1}, "numerals": {"260锛?: 1}, "pronouns": {"I": 1}}}, {"content": "If I was a painter, I would like to paint flowers.", "word_count": 12, "label": "", "sentence_structure": "Subject鈥搗erb鈥搊bject", "subjective_objective": {"answer": "Subjective", "rationale": "Because \"like to paint flowers\" is a personal interest and preference of the patient, and it's a subjective description."}, "sentence_sentiment": {"answer": "Neutral", "rationale": "Because the entry does not have any emotional color."}, "language_style": {"answer": "Tactful and warm", "rationale": "The entry is more euphemistic, using virtual \"if\" to express the scenario, while expressing the master language's hobby and interest in painting, and the overall tone is more moderate, without direct command or strong emotional colours."}, "time_dimension": {"answer": "Present", "rationale": "Now the entry describes the idea and interest of the testee, which is, \"If I'm a painter,\" they \"like to paint flowers.\" So, the time dimension is now."}, "sentence_type": "Affirmative declarative sentence", "pos": {"nouns": {"I": 1, "Painter": 1, "Flowers": 1}, "verbs": {"be": 1, "Yeah": 1, "Paint": 1}, "numerals": {"261": 1}}}, {"content": "262. I am not upset by my appearance, although I am not a good-looking person.", "word_count": 15, "label": "", "sentence_structure": "Subject鈥搗erb鈥揷omplement", "subjective_objective": {"answer": "Subjective", "rationale": "Although it is true that the appearance is not good, it is the patient ' s subjective attitude to avoid suffering from it."}, "sentence_sentiment": {"answer": "Neutral", "rationale": "The entry describes the physical features of a person and his psychological state, and does not involve positive or negative emotional aspects."}, "language_style": {"answer": "Tactful and warm", "rationale": "The entry uses a more euphemistic tone, expressing the view of the interviewee about his or her appearance, and does not feel troubled or humbled by his or her shortcomings. Such expression reflects the interviewee鈥檚 feelings in the heart without causing discomfort or resentment."}, "time_dimension": {"answer": "Present", "rationale": "The keywords \"though\" and \"without distress\" indicate that the testees are describing current feelings and attitudes, not past or future situations."}, "sentence_type": "Negative declarative sentence", "pos": {"nouns": {"I": 2, "Trouble": 1, "Look at her": 1}, "verbs": {"Although": 1, "I'm not worried about it": 1}, "adjectives": {"Nice": 1}, "adverbs": {"Yeah": 1}, "numerals": {"262锛?: 1}}}, {"content": "263. Even in cold days, I can sweat easily.", "word_count": 12, "label": "", "sentence_structure": "Subject鈥搗erb鈥揷omplement", "subjective_objective": {"answer": "Subjective", "rationale": "Because \"sweat easily\" is a subjective experience and feeling."}, "sentence_sentiment": {"answer": "Negative", "rationale": "Because the description \"Easier to sweat\" is negative."}, "language_style": {"answer": "Tactful and warm", "rationale": "The entry is more euphemistic and warm, and does not directly blame or criticize the interviewee, but uses a more objective and moderate expression to guide the interviewee to share his/her situation. At the same time, the end-of-the-word symbol gives a sense of affection and makes it easier to accept and respond to the questionnaire."}, "time_dimension": {"answer": "Past", "rationale": "\"Also sweaty\" means that the act has occurred and has a certain frequency and stability, and therefore is judged to be past."}, "sentence_type": "Affirmative declarative sentence", "pos": {"nouns": {"Oh, my God": 1, "Khan": 1}, "verbs": {"Easy": 1, "Sweat": 1}, "adjectives": {"Cold": 1, "Yeah": 1}, "adverbs": {"Even if": 1}, "numerals": {"263锛?: 1}}}, {"content": "264. I am confident.", "word_count": 5, "label": "", "sentence_structure": "Subject鈥揷opula鈥損redicative", "subjective_objective": {"answer": "Subjective", "rationale": "Because confidence is a subjective attitude or feeling of an individual rather than an objective reality."}, "sentence_sentiment": {"answer": "Positive", "rationale": "Because the description of \"very confident\" in the entry is a positive attitude or quality."}, "language_style": {"answer": "Calm and direct", "rationale": "The entry expresses the subject of a more straightforward expression of confidence in the other party, which is more straightforward and does not use excessive rhetoric or emotional rendering."}, "time_dimension": {"answer": "Present", "rationale": "The entry \"I'm very confident\" describes a psychological state or emotional experience, judging the dimensions of time as now."}, "sentence_type": "Affirmative declarative sentence", "pos": {"verbs": {"Confidence": 1}, "adjectives": {"Ten": 1, "min": 1, "From": 1}, "adverbs": {"Extreme": 1}, "numerals": {"264": 1}}}, {"content": "265. Trusting no one is safer.", "word_count": 14, "label": "", "sentence_structure": "Subject鈥揷opula鈥損redicative", "subjective_objective": {"answer": "Subjective", "rationale": "Because it's a subjective idea to think and to think of \"no trust.\""}, "sentence_sentiment": {"answer": "Negative", "rationale": "Because the attitude of \"no trusting anyone\" in the entry is negative."}, "language_style": {"answer": "Calm and direct", "rationale": "The language style of the entry is a calm and direct type. The basis of the judgement is that the entry expresses the view directly in a positive statement, does not use a rhetorical or emotional rendering, is more balanced and objective, and can therefore be judged as a \"cool and direct\" language style."}, "time_dimension": {"answer": "Past", "rationale": "The entry describes a state of behaviour or attitude in which time points to the past, and \"no trust in anyone\" may have existed or occurred in the past."}, "sentence_type": "Negative declarative sentence", "pos": {"nouns": {"person": 2, "Problem": 1, "Trust": 1, "Clear": 1}, "verbs": {"I don't trust you": 1, "be": 1, "Comparison": 1}, "adjectives": {"Any": 1, "Both": 1, "of": 3}, "adverbs": {"Compare": 1}, "numerals": {"265": 1}, "classifiers": {"individual": 1}, "pronouns": {"Here": 1}}}, {"content": "266. I am very excited at least once or twice a week.", "word_count": 14, "label": "", "sentence_structure": "Subject鈥揷opula鈥損redicative", "subjective_objective": {"answer": "Subjective", "rationale": "Because high excitement is a subjective experience, it is the patient ' s personal feelings and perceptions."}, "sentence_sentiment": {"answer": "Positive", "rationale": "Because \"excited\" is a positive emotional experience."}, "language_style": {"answer": "Tactful and warm", "rationale": "The entry is more personal and interactive when it is spoken in a gentle and inviting tone, rather than in direct statements or orders. At the same time, the language style of the article is more positive, encouraging respondents to share their positive emotional experiences by using the term \"very excited\"."}, "time_dimension": {"answer": "Present", "rationale": ""}, "sentence_type": "Affirmative declarative sentence", "pos": {"nouns": {"Week": 1, "Number of times": 1, "Very excited": 1}, "verbs": {"have": 1}, "adverbs": {"At least": 1, "One pair": 1}, "numerals": {"Two": 1, "Six": 1}, "classifiers": {"Number of times": 1}, "pronouns": {"I": 1}}}, {"content": "When there were more people, I didn't know what to say.", "word_count": 15, "label": "", "sentence_structure": "Subject鈥揷opula鈥損redicative", "subjective_objective": {"answer": "Subjective", "rationale": "Because it is a subjective feeling and an idea to know what to say."}, "sentence_sentiment": {"answer": "Negative", "rationale": "There is a sense of confusion and disquiet because of the negative emotional expression \"I don't know what to say\"."}, "language_style": {"answer": "Calm and direct", "rationale": ""}, "time_dimension": {"answer": "Present", "rationale": "The entry asks the testee about the situation in his current state, \"when he's in a crowded state\", and about his behaviour, which he does not know how to speak. So, its temporal dimension is now judged."}, "sentence_type": "Interrogative sentence", "pos": {"nouns": {"Time": 1, "Word": 1}, "verbs": {"Yes": 1, "Say it": 1, "Okay": 1}, "adjectives": {"More": 1}, "adverbs": {"What": 1, "person": 1, "Both": 1, "not": 1, "Land": 1}, "numerals": {"267": 1, "Some": 1}, "classifiers": {"individual": 1}, "pronouns": {"I": 1}}}, {"content": "268. There are always things that cheer me when I'm in a bad mood.", "word_count": 21, "label": "", "sentence_structure": "Subject鈥搗erb鈥揷omplement", "subjective_objective": {"answer": "Subjective", "rationale": "Because \"I'm not feeling well\" and \"I'm happy\" are the feelings and subjective experiences of the patients themselves."}, "sentence_sentiment": {"answer": "Positive", "rationale": "Because the \"something makes me happy\" act is positive."}, "language_style": {"answer": "Tactful and warm", "rationale": "The entry introduces problems by using the expression \"I'm in a bad mood\" to give people a sense of affection and concern. And, in the same way, ask each other if there are things that can bring positive emotions to the face of negative emotions, which are relatively gentle, encouraging and accepting."}, "time_dimension": {"answer": "Present", "rationale": "The sentence expresses a recurrent situation, in which there is always something to be pleased with when the mood is bad. Since this situation is ongoing, it can be judged to be the time dimension of the present."}, "sentence_type": "Affirmative declarative sentence", "pos": {"nouns": {"Time": 1, "The mood": 1, "Things": 1}, "verbs": {"have": 1, "鈾?And I'm gonna be 鈾?: 1, "Rise": 1}, "adjectives": {"It's not good": 1, "Happy": 1}, "adverbs": {"Always": 1}, "numerals": {"268": 1}}}, {"content": "269. I can easily be afraid of me, sometimes I do it on purpose to be happy.", "word_count": 21, "label": "", "sentence_structure": "Subject鈥搗erb鈥搊bject", "subjective_objective": {"answer": "Subjective", "rationale": "Because the sentence describes the patient鈥檚 subjective thoughts and feelings that he/she is capable of fearing and sometimes intentionally doing so to be happy. This reflects a self-perception and emotional state of the patient, which is a subjective perspective."}, "sentence_sentiment": {"answer": "Negative", "rationale": "Because the description \"can easily scare people\" and \"willful to do so to be happy\" in the entries have obvious derogatory and negative feelings."}, "language_style": {"answer": "Calm and direct", "rationale": "First, the question expresses a behaviour and purpose in a direct and clear manner, without too much qualifier or emotional colour; second, with a more neutral tone, without too much subjective emotion or evaluation. It is therefore clear that the language style of the entry is straightforward."}, "time_dimension": {"answer": "Present", "rationale": "The entry describes the present behaviour or attitude of the testee, which \"can\" and \"sometimes\" means that these acts are frequent, and therefore the judgement is now."}, "sentence_type": "Interrogative sentence", "pos": {"nouns": {"person": 1, "Happy": 1}, "verbs": {"鈾?And I'm gonna be 鈾?: 1, "Yes": 1, "Easy": 1, "Search": 1, "Do it": 1, "It scares people": 1}, "adjectives": {"On purpose": 1}, "numerals": {"269": 1}, "pronouns": {"I": 1}}}, {"content": "270. When I left home, I never feared that the doors and windows would be locked.", "word_count": 25, "label": "", "sentence_structure": "Subject鈥搗erb鈥搊bject", "subjective_objective": {"answer": "Objective", "rationale": "Because \"will the door and window be locked in the house\" is the patient's subjective idea and feeling, and the patient's attitude is asked here, and it is therefore subjective. But the result is based on the objective fact that the patient has not feared the problem in practice, and therefore contains some objective factors."}, "sentence_sentiment": {"answer": "Neutral", "rationale": "Because the entry does not have any emotional color."}, "language_style": {"answer": "Tactful and warm", "rationale": "The language style of the entry is euphemism and warmth, which guides the respondents to answer questions in an easy and natural way, without direct orders or demands that they answer. This expression is more friendly and close, helping to create a comfortable and relaxed Question and Answering environment."}, "time_dimension": {"answer": "Present", "rationale": ""}, "sentence_type": "Affirmative declarative sentence", "pos": {"nouns": {"Time": 1, "Home": 1, "Windows and doors": 1, "Lock": 1}, "verbs": {"Leave home": 1, "Out": 1, "I'm worried": 1, "Off": 1, "Okay": 2, "Lock": 1}, "adverbs": {"Never": 1}, "numerals": {"270": 1}, "pronouns": {"I": 1}}}, {"content": "I don't blame a man who has bullied himself and made himself no fun.", "word_count": 15, "label": "", "sentence_structure": "Subject鈥搗erb鈥搊bject", "subjective_objective": {"answer": "Subjective", "rationale": "Because this phrase expresses the personal attitude and the view of the speaker that he will not blame a person who has wronged himself or has lost interest. This attitude is based on the values and beliefs of the speaker and is therefore subjective."}, "sentence_sentiment": {"answer": "Neutral", "rationale": "The entry is not a positive or negative emotional expression."}, "language_style": {"answer": "Calm and direct", "rationale": "The language style of the entry is calm and straightforward. It expresses a view or attitude in a clear and concise manner, without too many rhetorical or emotional renderings, and is more objective in tone."}, "time_dimension": {"answer": "Present", "rationale": ""}, "sentence_type": "Affirmative declarative sentence", "pos": {"nouns": {"Bullying": 2, "person": 2, "It's not funny to find out": 1}, "verbs": {"Blame": 1, "Bullying": 1, "Find": 1}, "adjectives": {"Bullying": 1, "You're looking for yourself": 1}, "numerals": {"271": 1, "One": 3}, "pronouns": {"One": 2}}}, {"content": "272. I am sometimes energetic.", "word_count": 7, "label": "", "sentence_structure": "Subject鈥揷opula鈥損redicative", "subjective_objective": {"answer": "Subjective", "rationale": "Because \"absorbed\" is a personal feeling and experience, subjective."}, "sentence_sentiment": {"answer": "Positive", "rationale": "Because the description of \"energy\" is positive."}, "language_style": {"answer": "Tactful and warm", "rationale": "The entry is more adept and interactive when it is spoken in a gentle and inviting manner, rather than by direct statements or orders."}, "time_dimension": {"answer": "Present", "rationale": "The keyword \"sometimes\" indicates that the testee describes his current state, and \"energy\" is an adjective phrase that indicates that the testee feels dynamic and energyful at some point in time. So, the entry is now in terms of the time dimension of asking the testee about his current state or feelings."}, "sentence_type": "Affirmative declarative sentence", "pos": {"verbs": {"- I'm very energetic": 1}, "adjectives": {"Sometimes": 1}}}, {"content": "273. I have one or two numb spots on my skin.", "word_count": 12, "label": "", "sentence_structure": "Subject鈥揷opula鈥損redicative", "subjective_objective": {"answer": "Objective", "rationale": "As numbing is the physical condition of the patient, it can be observed and measured and it is objective."}, "sentence_sentiment": {"answer": "Negative", "rationale": "Because the \"skin numb\" symptoms in the entries are negative."}, "language_style": {"answer": "Tactful and warm", "rationale": "The entry is more personal and interactive when it is being approached in a gentle and inviting tone, asking whether the other side is insulated from skin rather than directly speaking or ordering."}, "time_dimension": {"answer": "Present", "rationale": "The entry describes the current condition of the subject, which is \"one or two numbs on my skin,\" so the temporal dimension is now."}, "sentence_type": "Affirmative declarative sentence", "pos": {"nouns": {"Skin": 1, "I'm numb": 1}, "verbs": {"have": 1}, "adjectives": {"One or two": 1}, "numerals": {"Twenty-seven three": 1}}}, {"content": "274. My vision is as good as in previous years.", "word_count": 10, "label": "", "sentence_structure": "Subject鈥揷opula鈥損redicative", "subjective_objective": {"answer": "Objective", "rationale": "Since the visual condition is an objective fact, it is irrelevant to the subjective perception of the individual."}, "sentence_sentiment": {"answer": "Neutral", "rationale": "Because the entry describes an objective fact and does not involve any emotional colour."}, "language_style": {"answer": "Calm and direct", "rationale": "The language style of the entry is calm and straightforward. It does not use any rhetorical or emotional rendering, but simply describes the fact that \"the vision is as good as it was in the past.\" This expression is more objective and neutral, less subjective and personal."}, "time_dimension": {"answer": "Present", "rationale": "The entry describes the current visual situation of the person tested, and \"as good as this year\" refers to a situation that has not changed compared to the past, so the temporal dimension is now judged."}, "sentence_type": "Affirmative declarative sentence", "pos": {"nouns": {"Visual": 1, "Year": 1}, "verbs": {"Sample": 1, "Okay": 1}, "adjectives": {"Same as before": 1}, "adverbs": {"and": 1}, "numerals": {"274": 1}}}, {"content": "275. I have control over my mind.", "word_count": 9, "label": "", "sentence_structure": "Subject鈥搗erb鈥搊bject", "subjective_objective": {"answer": "Subjective", "rationale": "Because \"someone controls my mind\" is a subjective feeling or belief, not an objective reality. The subject is the subjective idea or feeling of the patient."}, "sentence_sentiment": {"answer": "Negative", "rationale": "Because the \"controlled mind\" act is negative."}, "language_style": {"answer": "Tactful and warm", "rationale": "The entry asks questions in a more implicit way, namely, whether there is a feeling or experience that others are controlling their own ideas. This way respects the personal privacy of the interviewee and avoids the discomfort that may arise from the use of too direct language."}, "time_dimension": {"answer": "Present", "rationale": "The entry indicates that the respondent is thinking about the issue in his answer and is conducting a self-evaluation."}, "sentence_type": "Negative declarative sentence", "pos": {"nouns": {"person": 1, "Ideas": 1}, "verbs": {"Control": 1}, "numerals": {"275": 1}}}, {"content": "276. I like children.", "word_count": 6, "label": "", "sentence_structure": "Subject鈥搗erb鈥揷omplement", "subjective_objective": {"answer": "Subjective", "rationale": "Like being patient subjective."}, "sentence_sentiment": {"answer": "Neutral", "rationale": "Because the entry does not express positive or negative feelings, attitudes, behaviour, purpose or quality."}, "language_style": {"answer": "Tactful and warm", "rationale": ""}, "time_dimension": {"answer": "Present", "rationale": "The keyword \"likes a child\" indicates that it is a sentence that describes personal preferences, without reference to past or future behaviour or expectations, and therefore the temporal dimension is now judged."}, "sentence_type": "Affirmative declarative sentence", "pos": {"nouns": {"Kids": 1}, "verbs": {"Yeah": 1}}}, {"content": "277. Sometimes I appreciate the tact of the liar, and I even hope he gets over it.", "word_count": 24, "label": "", "sentence_structure": "Subject鈥搗erb鈥搊bject", "subjective_objective": {"answer": "Subjective", "rationale": "Because \"approving the brain of a liar\" and \"and hoping he'll get away with it\" are patients' attitudes and thoughts about the behaviour of a liar, which are subjective perceptions of the patient."}, "sentence_sentiment": {"answer": "Negative", "rationale": "Because the act of \"approving the wits of liars\" is negative."}, "language_style": {"answer": "Tactful and warm", "rationale": "The basis for the judgement: Although the entry expresses a kind of appreciation for the tact of the fraud, it is more mild, implicit, and does not directly criticize or accuse the liar. And the expression \"I hope he even gets mixed up with the past\" reflects a degree of tolerance and understanding, which is more euphemism."}, "time_dimension": {"answer": "Future", "rationale": "The entry \"and even hope he's lucky enough to get past\" expresses his expectations and vision of the future, and the key words are \"and even\" and \"hope,\" all pointing to what has not yet happened and what may happen in the future.\""}, "sentence_type": "Affirmative declarative sentence", "pos": {"nouns": {"I": 2, "Liar": 1, "Smart": 1, "Get over there": 1}, "verbs": {"Very": 1, "Enjoy": 1, "Hope": 1, "Yes": 1, "Lucky": 1, "Fuck": 1}, "adjectives": {"Even": 1}, "adverbs": {"Sometimes": 1}, "numerals": {"277锛?: 1, "One": 1}}}, {"content": "278. I often feel that some strangers stare at me with a picky eye.", "word_count": 19, "label": "", "sentence_structure": "Subject鈥揷opula鈥損redicative", "subjective_objective": {"answer": "Subjective", "rationale": "Because it is a subjective feeling and judgment to feel being watched with a pickle."}, "sentence_sentiment": {"answer": "Negative", "rationale": "Because the expression \"look at me with a pickle\" in the entry is negative emotional."}, "language_style": {"answer": "Tactful and warm", "rationale": "The language style of the entry is euphemistic. It does not directly express whether the other side often feels that it is being singled out by strangers, but rather engages in dialogue in a gentle and inviting manner, which is more friendly and interactive. At the same time, it reduces the pressure or discomfort that may be placed on the interviewee."}, "time_dimension": {"answer": "Present", "rationale": "The keyword \"often\" means regular, customary behavior, \"I think\" and \"unsure\" and \"look at me with a picky eye\" are all descriptions of the state of affairs, so it's a question of the present."}, "sentence_type": "Affirmative declarative sentence", "pos": {"nouns": {"I": 1, "View": 1, "Stranger": 1}, "verbs": {"I think": 1, "Watch": 1, "Use": 1, "Some": 1, "Often": 1}, "adjectives": {"Picky": 1}, "numerals": {"278": 1}, "classifiers": {"individual": 1}}}, {"content": "279. I drink a lot of water every day.", "word_count": 9, "label": "", "sentence_structure": "Subject鈥搗erb鈥揷omplement", "subjective_objective": {"answer": "Objective", "rationale": "Because the amount of water consumed is a fact that can be observed, not just the patient ' s subjective feelings or attitudes."}, "sentence_sentiment": {"answer": "Negative", "rationale": "Because excessive drinking water can have a negative impact on health."}, "language_style": {"answer": "Calm and direct", "rationale": "The entry directly asks the person surveyed about drinking water daily, speaking in a balanced and objective manner, without excessive adoration or emotional colour, and therefore can be judged as being calm and straightforward in language style."}, "time_dimension": {"answer": "Present", "rationale": "The keyword is \"I'm every day\", which means it's a description of the person's current customary behaviour, so the temporal dimension is now."}, "sentence_type": "Exclamatory sentence", "pos": {"nouns": {"I": 1, "Water": 1}, "verbs": {"Drink": 1, "Especially": 1}, "adjectives": {"Chile": 1, "More": 1}, "adverbs": {"Special": 1}, "numerals": {"279锛?: 1, "of": 1}}}, {"content": "280. Most people make friends and are useful to them.", "word_count": 12, "label": "", "sentence_structure": "Subject鈥搗erb鈥揷omplement", "subjective_objective": {"answer": "Subjective", "rationale": "This is a description of the general state of mind and does not directly relate to actual acts or events."}, "sentence_sentiment": {"answer": "Positive", "rationale": "Because the entries \"friending\" and \"useful\" are positive."}, "language_style": {"answer": "Tactful and warm", "rationale": "The language style of the entry is euphemism. It uses the implicit expression \"most people\" to avoid direct attacks or fingering individuals. At the same time, by emphasizing \"friends are useful to them\", respondents are encouraged to share their views and experiences in order to achieve the purpose of the investigation. Such expression is more gentle and friendly, and it can easily generate resonance and participation among the interviewees."}, "time_dimension": {"answer": "Present", "rationale": "The entry describes the benefits or benefits that people receive in their current state (i.e. making friends), and thus the time dimension is now judged."}, "sentence_type": "Affirmative declarative sentence", "pos": {"nouns": {"person": 1, "friends": 1}, "verbs": {"Hand it over": 1, "Use": 1}, "adjectives": {"Large": 1, "have": 1, "Use": 1}, "adverbs": {"Other": 1}, "numerals": {"280锛?: 1, "More": 1}, "classifiers": {"individual": 1}, "pronouns": {"they": 1}}}, {"content": "281. I have paid little attention to my ears.", "word_count": 9, "label": "", "sentence_structure": "Subject鈥搗erb鈥揷omplement", "subjective_objective": {"answer": "Subjective", "rationale": "Because \"I'm not paying much attention\" is the patient's subjective feeling and attitude."}, "sentence_sentiment": {"answer": "Neutral", "rationale": "Because the entry merely stated a fact, it did not contain any positive or negative emotional aspects."}, "language_style": {"answer": "Calm and direct", "rationale": "The language style of the entry is calm and straightforward. It asks the other side directly about the tone of the ear, without too much emotional or rhetorical. It makes the question clearer and more concise and facilitates the achievement of accurate answers."}, "time_dimension": {"answer": "Past", "rationale": "In this entry, \"very little\" indicates past behaviour or state, i.e. the testee has rarely noticed his or her ears ringing for a certain period of time, and has therefore judged to be past."}, "sentence_type": "Negative declarative sentence", "pos": {"nouns": {"# And the ears #": 1}, "verbs": {"Attention": 1, "Less": 1}, "adverbs": {"Very few": 1}, "numerals": {"281": 1}}}, {"content": "I usually love my family and occasionally hate them.", "word_count": 13, "label": "", "sentence_structure": "Subject鈥搗erb鈥搊bject", "subjective_objective": {"answer": "Subjective", "rationale": "Because the emotional experience of love and hate is usually a subjective one."}, "sentence_sentiment": {"answer": "Neutral", "rationale": "Because \"love family\" is positive, and \"hate them\" is negative."}, "language_style": {"answer": "Tactful and warm", "rationale": "The language style of the entry is more euphemistic, reducing the intensity of speech by using expressions like \"normal\" and \"frequent\" and using emotional words like \"love\" and \"hate\" to enable readers to better understand and feel the author's emotional state. Such expressions are more intimate and acceptable."}, "time_dimension": {"answer": "Present", "rationale": "According to the description of \"normal\" and \"frequent\" in the title, the act is a situation that may arise in the past, in the present, in the future, and can therefore be judged as \"now\"."}, "sentence_type": "Affirmative declarative sentence", "pos": {"nouns": {"Questionnaire": 1, "Entry": 1, "Family": 1, "they": 1}, "verbs": {"Love": 1, "Hate": 1, "be": 1}, "adjectives": {"Usually": 1, "Sometimes": 1}, "numerals": {"282": 1}}}, {"content": "283. If I were a journalist, I would be happy to cover sports news.", "word_count": 21, "label": "", "sentence_structure": "Subject鈥搗erb鈥搊bject", "subjective_objective": {"answer": "Subjective", "rationale": "Because \"sport news is a subjective idea of the attitude and desire of the patient to become a journalist.\""}, "sentence_sentiment": {"answer": "Positive", "rationale": "Because the behaviour and attitude in the entries are positive."}, "language_style": {"answer": "Tactful and warm", "rationale": "The entry is a soft and warm language. It is presented in the first person's hypothetical form, and expresses a positive attitude in \"very willing.\" This language is both warm and very friendly and easy to accept, and it is better able to guide the interviewee to open his heart and answer questions with truth."}, "time_dimension": {"answer": "Present", "rationale": ""}, "sentence_type": "Imperative sentence", "pos": {"nouns": {"Journalists": 1, "Sports News": 1}, "verbs": {"Yes": 1, "Reporting": 1}, "adjectives": {"very": 1, "Yes": 2}, "numerals": {"Two, eight, three": 1}, "pronouns": {"I": 1}}}, {"content": "284. I am sure that I am being discussed.", "word_count": 10, "label": "", "sentence_structure": "Subject鈥搗erb鈥揷omplement", "subjective_objective": {"answer": "Subjective", "rationale": "Because \"sure\" is the patient's subjective judgment and feeling."}, "sentence_sentiment": {"answer": "Negative", "rationale": "Because the idea of \"sure that someone is talking about me\" in the entry is negative, implying that the interviewee may have doubts and mistrust towards others."}, "language_style": {"answer": "Tactful and warm", "rationale": "The entry is more personal and interactive when it is presented in a gentle and inviting tone, rather than in direct statements or orders. At the same time, the sentence uses the first person ' s term, which enables respondents to better understand the problem and to feel the respect and interest of researchers in them."}, "time_dimension": {"answer": "Present", "rationale": "The keyword is \"convinced\", indicating that it is a subjective perception of the current situation; and, at the same time, \"is\" indicating that knowledge is in progress, so the entry describes the current mindset or feelings of the subject."}, "sentence_type": "Affirmative declarative sentence", "pos": {"nouns": {"I": 1, "others": 1, "Arguments": 1}, "verbs": {"Sure": 1, "In the process of": 1, "in": 1}, "adverbs": {"Indeed": 1}, "numerals": {"284": 1}}}, {"content": "I sometimes laugh when I hear dirty jokes.", "word_count": 14, "label": "", "sentence_structure": "Subject鈥搗erb鈥揷omplement", "subjective_objective": {"answer": "Subjective", "rationale": "Because it's the patient's subjective feeling and reaction to a joke."}, "sentence_sentiment": {"answer": "Negative", "rationale": "Because \"dirty jokes\" are usually considered indecent or vulgar, hearing them can create uncomfortable feelings and thus negative attitudes."}, "language_style": {"answer": "Tactful and warm", "rationale": "The entry is more personal and interactive when it is spoken in a gentle and inviting tone, rather than in direct statements or orders. It also does not use strong tone or emotional terms to make the respondents comfortable and comfortable and more willing to share their true feelings."}, "time_dimension": {"answer": "Present", "rationale": "The keywords \"frequently\" and \"may\" indicate a customary, sometimes occurring state of affairs, so that time can be judged for now."}, "sentence_type": "Affirmative declarative sentence", "pos": {"nouns": {"A joke": 1, "Smile": 1}, "verbs": {"Listen": 1, "Let's go": 1, "Yes": 1}, "adjectives": {"Naughty": 1, "Sometimes": 1}, "numerals": {"285": 1}, "pronouns": {"I": 1}}}, {"content": "286. I feel happier when I'm alone.", "word_count": 14, "label": "", "sentence_structure": "Subject鈥揷opula鈥損redicative", "subjective_objective": {"answer": "Subjective", "rationale": "Because feeling happy is a subjective experience or feeling. While a person鈥檚 situation is objective, it is a subjective view, because it belongs to the patient鈥檚 own inner experience."}, "sentence_sentiment": {"answer": "Negative", "rationale": "Because the situation of being alone in the entry can create a negative feeling of loneliness, helplessness, etc."}, "language_style": {"answer": "Tactful and warm", "rationale": "The entry is more adept and interactive when it is spoken in a gentle and inviting manner, rather than by direct statements or orders."}, "time_dimension": {"answer": "Present", "rationale": "The words \"when I'm alone\" and \"favorite more\" describe the state or feeling, without any reference to the time dimension of the past or the future. So, the time dimension of the sentence can be judged as \"now\"."}, "sentence_type": "Affirmative declarative sentence", "pos": {"nouns": {"Time": 1, "person": 1}, "verbs": {"I feel": 1}, "adjectives": {"More": 1, "Happy": 1}, "adverbs": {"Alone": 1}, "numerals": {"286": 1}}}, {"content": "287. Much less has scared me than my friends.", "word_count": 15, "label": "", "sentence_structure": "Subject鈥揷opula鈥損redicative", "subjective_objective": {"answer": "Subjective", "rationale": "Because \"what scares me is much less than my friends\" means subjective knowledge and comparison of the number of things that people fear."}, "sentence_sentiment": {"answer": "Positive", "rationale": "Because the entry \"that scares me much less than my friends\" indicates a positive emotional attitude to self-confidence and bravery."}, "language_style": {"answer": "Calm and direct", "rationale": "It directly raises a question and asks respondents to compare, without using any qualifier or emotional description, and with objectivity and straightforwardness."}, "time_dimension": {"answer": "Present", "rationale": ""}, "sentence_type": "Affirmative declarative sentence", "pos": {"nouns": {"Yes": 1, "Friends": 1}, "verbs": {"鈾?And I'm gonna be 鈾?: 1, "Scared": 1, "More": 1, "Less": 1}, "adjectives": {"287锛?: 1, "of": 2, "- Yeah": 1}, "adverbs": {"Land": 1}, "numerals": {"287": 1}, "pronouns": {"I": 1}}}, {"content": "288. I am troubled by the disgusting vomiting.", "word_count": 11, "label": "", "sentence_structure": "Subject鈥搗erb鈥揷omplement", "subjective_objective": {"answer": "Subjective", "rationale": "Because \"I'm upset\" means the patient's subjective feelings and emotional state."}, "sentence_sentiment": {"answer": "Negative", "rationale": "Because \"grudge vomit\" and \"fear\" in the entries are all negative emotional expressions."}, "language_style": {"answer": "Calm and direct", "rationale": "This entry describes the potential problems of the person under investigation in a direct and clear language, without too many emotional or rhetorical styles. Such language styles are usually more common and useful in psychological questionnaires, as it accurately conveys the feelings and experiences of the person under investigation."}, "time_dimension": {"answer": "Present", "rationale": "The entry describes the current state and feeling of the testee, and the \"disgusting sickness\" is the symptom that currently plagues the testee, and thus judges it as now."}, "sentence_type": "Affirmative declarative sentence", "pos": {"nouns": {"Disgusting": 1, "Mistake": 1, "I": 1, "Trouble": 1}, "verbs": {"鈾?And I'm gonna be 鈾?: 1}, "numerals": {"288锛?: 1}}}, {"content": "289. I am tired of the law when a criminal can exonerate himself through a lawyer who can speak with good counsel.", "word_count": 29, "label": "", "sentence_structure": "Subject鈥揷opula鈥損redicative", "subjective_objective": {"answer": "Subjective", "rationale": "Because the feeling of the law is the individual view and attitude of the patient, it is a subjective examination."}, "sentence_sentiment": {"answer": "Negative", "rationale": "Because the emotional attitude of \"tired of the law\" in the entry is negative."}, "language_style": {"answer": "Calm and direct", "rationale": "The language style of the entry is calm and straightforward, and there is no excessive emotional rendering or rhetorical technique. It expresses the views of the interviewee on the law in an objective manner and describes the specific situation (i.e., the fact that lawyers can help the offender to exonerate him)."}, "time_dimension": {"answer": "Present", "rationale": "The entry describes the current feelings and attitudes of the testees, using the current state of affairs. In it, \"when\" indicates events that occur in a given situation, and \"I am tired of the law\" is a reaction to this situation, describing the state of affairs."}, "sentence_type": "Affirmative declarative sentence", "pos": {"nouns": {"Perpetrators": 1, "Counselor": 1, "Responsibility": 1, "Law": 1}, "verbs": {"I feel": 1, "Tired": 1, "Open": 1}, "adjectives": {"It's a good one": 1}, "numerals": {"289锛?: 1}, "pronouns": {"I": 1}}}, {"content": "I always work under stress.", "word_count": 14, "label": "", "sentence_structure": "Subject鈥搗erb鈥揷omplement", "subjective_objective": {"answer": "Subjective", "rationale": "Because \"stretch\" is an emotional state, which is usually subjective, the entry on the questionnaire is a subjective approach to the patient."}, "sentence_sentiment": {"answer": "Negative", "rationale": "Because the \"work under stress\" act in the entry is negative."}, "language_style": {"answer": "Calm and direct", "rationale": "The entry uses a straightforward language style, which expresses the subject of constant tension in the work of the person under investigation, with a clear and concise language, without any reticulation or emotional colour.\""}, "time_dimension": {"answer": "Present", "rationale": "The entry is for the testee ' s emotional state of work, which cannot be determined in terms of time dimension, and is therefore neutral."}, "sentence_type": "Affirmative declarative sentence", "pos": {"nouns": {"Work": 1, "Situation": 1}, "verbs": {"Always": 1, "Nervous": 1, "Work": 1}, "adjectives": {"Nervous": 1}, "adverbs": {"Always": 1}, "numerals": {"290": 1}}}, {"content": "291. In my life, at least once or twice I felt that I had been subjected to some hypnosis.", "word_count": 29, "label": "", "sentence_structure": "Subject鈥搗erb鈥搊bject", "subjective_objective": {"answer": "Subjective", "rationale": "Because it's the patient's subjective feeling and understanding that he feels hypnotized."}, "sentence_sentiment": {"answer": "Negative", "rationale": "Because the entry \"I feel like someone's been doing something with hypnosis\" expresses a feeling of manipulation and threat, and it's negative."}, "language_style": {"answer": "Calm and direct", "rationale": "The entry is more calm and straightforward in language, and does not have too many rhetorical or emotional accents. It clearly presents a point of view and asks the interviewee to comment on it. This type of questionnaire is usually used to collect objective data and information.\""}, "time_dimension": {"answer": "Past", "rationale": "The entry asks if the person tested has had hypnotic experience in his past life. The keywords \"in this life\" and \"at least once or twice\" point to the time dimension of the past. So the answer is the past."}, "sentence_type": "Affirmative declarative sentence", "pos": {"nouns": {"Life": 1, "Once or twice": 1, "I": 1, "person": 1, "Hypnosis": 1, "Yes": 1}, "verbs": {"I think": 1, "- What": 1, "Do it": 1}, "adverbs": {"At least": 1, "in": 1}, "numerals": {"291": 1, "Once or twice": 1}, "pronouns": {"In my life": 1}}}, {"content": "I am not willing to speak to anyone unless he speaks first.", "word_count": 14, "label": "", "sentence_structure": "Subject鈥搗erb鈥搊bject", "subjective_objective": {"answer": "Subjective", "rationale": "This is a subjective attitude of patients who are unwilling to communicate with others, and is not an objective fact."}, "sentence_sentiment": {"answer": "Negative", "rationale": "Because the entry demonstrates a reluctance to engage with others on a proactive basis, it is a negative emotional tendency."}, "language_style": {"answer": "Calm and direct", "rationale": "This entry is a direct expression of reluctance to speak to others on a proactive basis, without too many rhetorical or emotional expressions."}, "time_dimension": {"answer": "Present", "rationale": "The temporal dimension of the article is therefore now."}, "sentence_type": "Negative declarative sentence", "pos": {"nouns": {"I": 1, "person": 1, "he": 1}, "verbs": {"No, I don't": 1, "Same": 1, "Speech": 1, "Unless": 1, "First": 1, "Open up": 1}, "adverbs": {"not": 1, "Only": 1}, "numerals": {"292": 1}}}, {"content": "293. There have been attempts to influence my mind.", "word_count": 11, "label": "", "sentence_structure": "Subject鈥搗erb鈥揷omplement", "subjective_objective": {"answer": "Subjective", "rationale": "Because \"someone has always wanted to influence my mind\" is a personal feeling and perception, a subjective examination."}, "sentence_sentiment": {"answer": "Negative", "rationale": "Because the act of \"someone has always wanted to influence my mind\" in the entry is negative and may mean being interfered with and pressured by others."}, "language_style": {"answer": "Calm and direct", "rationale": "The language style of the entry is more straightforward, there is no excessive rhetorical or emotional overturning, and there is a greater focus on the transmission of facts and information. At the same time, the sentence is simple and easy to understand."}, "time_dimension": {"answer": "Present", "rationale": "The entry is to ask if the testee is in this situation, and \"Always trying to influence my mind\" indicates that it is an act or state of conduct that is currently being performed, so the temporal dimension is now."}, "sentence_type": "Affirmative declarative sentence", "pos": {"nouns": {"person": 1, "Ideas": 1}, "verbs": {"Yes": 1, "Impact": 1}, "adverbs": {"Always": 1}, "numerals": {"293锛?: 1}}}, {"content": "294. I have never broken the law.", "word_count": 8, "label": "", "sentence_structure": "Subject鈥損redicate", "subjective_objective": {"answer": "Subjective", "rationale": "Because \"Never committed law\" is a subjective assessment and perception of a person, not an objective reality. Everyone has their own standards of conduct and values, so different people may answer differently. So the entry \"I never committed a crime\" should be classified as a subjective questionnaire."}, "sentence_sentiment": {"answer": "Neutral", "rationale": "As this entry does not contain any emotional or attitudinal, behavioural or other information."}, "language_style": {"answer": "Calm and direct", "rationale": "The entry is presented in a direct negative form, with a clear language, with no excessive embellishment or emotional rendering, and a more objective and calm tone.\""}, "time_dimension": {"answer": "Past", "rationale": "The entry is a negative view of past criminal conduct by the testee and therefore a temporal dimension is a past."}, "sentence_type": "Negative declarative sentence", "pos": {"verbs": {"Yes": 1, "Pass": 1}, "adjectives": {"Never": 1}, "numerals": {"294": 1}, "pronouns": {"I": 1}}}, {"content": "295. I love novels like \" Dreams of the Red House \" .", "word_count": 13, "label": "", "sentence_structure": "Subject鈥搗erb鈥搊bject", "subjective_objective": {"answer": "Subjective", "rationale": "Because the word \"like\" expresses the patient's personal preference and subjective attitude, the entry on the questionnaire is viewed from a subjective perspective."}, "sentence_sentiment": {"answer": "Positive", "rationale": "Because \"I love to watch Red House Dreams\" is a positive hobby and emotional tendency."}, "language_style": {"answer": "Tactful and warm", "rationale": "The entry is presented in a first person's style, with a more friendly and friendly tone, without excessive emotional colour or direct pressure. At the same time, it also expresses personal preferences in a moderate manner, reflecting the respect and concern of the questionnaire designers."}, "time_dimension": {"answer": "Past", "rationale": "The entry indicates that the testee prefers a novel that he has seen in the past, and the temporal dimension is a past."}, "sentence_type": "Affirmative declarative sentence", "pos": {"nouns": {"295": 1, "Novel": 1, "Red House Dreams": 1}, "verbs": {"Yeah": 1, "Look": 1}, "adjectives": {"Here": 1, "One": 1, "Category": 1}, "numerals": {"Twenty-five": 1}}}, {"content": "Sometimes I feel very happy for no reason.", "word_count": 17, "label": "", "sentence_structure": "Subject鈥揷opula鈥損redicative", "subjective_objective": {"answer": "Subjective", "rationale": "Because \"very happy for no reason\" is the patient's subjective feeling."}, "sentence_sentiment": {"answer": "Positive", "rationale": "Because the entry is about positive emotional experience."}, "language_style": {"answer": "Tactful and warm", "rationale": "The entry is more adept and interactive when it is spoken in a gentle and inviting manner, rather than by direct statements or orders."}, "time_dimension": {"answer": "Past", "rationale": "Therefore, the entry is now."}, "sentence_type": "Affirmative declarative sentence", "pos": {"nouns": {"Time": 1, "person": 1, "Have a nice day": 1}, "verbs": {"Yes": 1, "I think": 1, "For no reason": 1, "Very": 1}, "adverbs": {"Some": 1, "Land": 1}, "numerals": {"296锛?: 1}}}, {"content": "297. I hope that I will not be troubled by ideas that are related to sex.", "word_count": 21, "label": "", "sentence_structure": "Subject鈥搗erb鈥揷omplement", "subjective_objective": {"answer": "Subjective", "rationale": "Because hope is no longer the subject of some thought is the patient's subjective wish."}, "sentence_sentiment": {"answer": "Negative", "rationale": "Because the expectation in the entry that \"that is no longer beset by sexual considerations\" is negative."}, "language_style": {"answer": "Tactful and warm", "rationale": "The entry is more euphemistic and warm, using the first-person pronoun \"I\" to make the sentence more personal and human, and also to express a desire for self-change and growth. Moreover, the more soft language (i.e. \"no longer beset by ideas of that kind and sexual nature\") is used to describe the problem, avoiding the possibility that direct expression may cause discomfort or discomfort."}, "time_dimension": {"answer": "Future", "rationale": "The temporal dimension is therefore judged in future."}, "sentence_type": "Affirmative declarative sentence", "pos": {"nouns": {"Hope": 1, "I": 2, "That": 1, "In terms of": 1, "Thinking": 1, "Trouble": 1}, "verbs": {"鈾?No more 鈾?: 1, "have": 1}, "adjectives": {"Sex-related": 1}, "numerals": {"297锛?: 1}}}, {"content": "298. If a few people have made a mistake, they would have better prepared a false statement without changing it.", "word_count": 24, "label": "", "sentence_structure": "Subject鈥搗erb鈥搊bject", "subjective_objective": {"answer": "Subjective", "rationale": "Because the proposal was based on a certain degree of subjective judgement and values, it was examined in terms of honesty and lies."}, "sentence_sentiment": {"answer": "Negative", "rationale": "Because the suggestion in the entry that \"a set of false words\" and \"no change\" are negative."}, "language_style": {"answer": "Calm and direct", "rationale": "The language style of the entry is more straightforward, and there is no excessive rhetorical or emotional rendering. It expresses the author鈥檚 view in simple and clear language, that in some cases people may choose to lie to avoid responsibility. This expression is more calm and objective, without a strong subjective or emotional tendency, and is more in line with the linguistic characteristics and requirements of the questionnaire."}, "time_dimension": {"answer": "Past", "rationale": ""}, "sentence_type": "Affirmative declarative sentence", "pos": {"nouns": {"person": 1, "Set": 1, "That's a lie": 1, "mouth": 1}, "verbs": {"Break in": 1, "Part": 1, "Change": 1}, "adverbs": {"If some people had made trouble, they would have made up a lie without changing their tongues": 1}, "numerals": {"298": 1}, "classifiers": {"individual": 1}}}, {"content": "299. I think I have more feelings than most people.", "word_count": 13, "label": "", "sentence_structure": "Subject鈥搗erb鈥揷omplement", "subjective_objective": {"answer": "Subjective", "rationale": "Because the word \"considers\" means that it is the patient's personal view and attitude and is therefore subjective."}, "sentence_sentiment": {"answer": "Positive", "rationale": "Because self-evaluations in the entry, \"Assisting I am more emotional than most people,\" express a positive emotional attitude towards myself."}, "language_style": {"answer": "Tactful and warm", "rationale": "The entry uses a more \"more\" expression of one's views, and is based on subjective perceptions, which are more euphemisms of the feelings. This language style is more intimate and moderate, and it can inspire resonance and understanding."}, "time_dimension": {"answer": "Present", "rationale": "The entry expresses the subjective feelings and perceptions of the subject, and the keyword is \"conception\" and \"more than me,\" which represent the current, present state.\""}, "sentence_type": "Affirmative declarative sentence", "pos": {"nouns": {"person": 1, "Emotion": 1}, "verbs": {"I think": 1, "- Yeah": 1, "Heavy": 1}, "adjectives": {"Mostly": 1}, "numerals": {"299": 1}, "pronouns": {"I": 1, "More": 1, "More than most people": 1}}}, {"content": "300. In my life, I've never liked dolls.", "word_count": 17, "label": "", "sentence_structure": "Subject鈥損redicate", "subjective_objective": {"answer": "Subjective", "rationale": "Because \"like\" is a subjective feeling or attitude, the questionnaire entry is a subjective idea to examine patients."}, "sentence_sentiment": {"answer": "Negative", "rationale": "Because the phrase \"never liked dolls\" in the entry has a negative emotional connotation."}, "language_style": {"answer": "Calm and direct", "rationale": "The entry asks the other party in a direct and clear manner whether he has ever liked a doll, without using any rhetorical or emotional rendering, and with a more calm and objective language style."}, "time_dimension": {"answer": "Past", "rationale": "The key words are \"for my life\" and \"for never\" and they all point to the past experience and feelings."}, "sentence_type": "Imperative sentence", "pos": {"nouns": {"Life": 1, "Dolls": 1}, "verbs": {"Yeah": 1, "Never": 1, "in": 1}, "adverbs": {"When": 1, "Medium": 1, "Never": 1}, "numerals": {"300锛?: 1}}}, {"content": "Life is a lot of work for me.", "word_count": 17, "label": "", "sentence_structure": "Subject鈥揷opula鈥損redicative", "subjective_objective": {"answer": "Subjective", "rationale": "This phrase expresses patients' subjective feelings and perceptions of life."}, "sentence_sentiment": {"answer": "Negative", "rationale": "Because the expression \"life is a thing for me\" in the entry is negative."}, "language_style": {"answer": "Tactful and warm", "rationale": "The language style of the entry is euphemism. It uses more moderate language, such as \"many times\" and \"for me,\" to express the feelings of the person under investigation about life and to avoid outright denial or accusations. Moreover, the entire sentence is relatively soft, with no excessive emotional colour or strong emotional expression. Such expression is more intimate, more resonant with the person under investigation and helps to create a good communication atmosphere."}, "time_dimension": {"answer": "Present", "rationale": "The keyword \"many times\" indicates that it is a constant act or state, and \"for me\" emphasizes subjective feelings. Taken together, the entry is about the emotional experience of the present, that is, the situation of being struggling with life."}, "sentence_type": "Affirmative declarative sentence", "pos": {"nouns": {"Time": 2, "Life": 1, "Yes": 1}, "verbs": {"be": 1, "Come on": 1}, "adjectives": {"Many": 1}, "numerals": {"301锛?: 1}, "pronouns": {"I": 1}}}, {"content": "302. I have never suffered from sexual behaviour.", "word_count": 18, "label": "", "sentence_structure": "Subject鈥揷opula鈥損redicative", "subjective_objective": {"answer": "Objective", "rationale": "This is a statement of the patient ' s experience and facts about his sexual behaviour and is an objective examination."}, "sentence_sentiment": {"answer": "Neutral", "rationale": "Because the entry does not show positive or negative attitudes, emotions, behaviour or qualities. It merely presents a fact."}, "language_style": {"answer": "Calm and direct", "rationale": "The entry is based on a simple, objective tone, a statement of the facts, and a clear and straightforward question."}, "time_dimension": {"answer": "Past", "rationale": "\"Never\" denotes a negative result or experience, implying that the person tested never suffered from sexual acts in the past. The temporal dimension of the article is therefore a matter of the past."}, "sentence_type": "Affirmative declarative sentence", "pos": {"nouns": {"Sexual": 1, "Behaviour": 1}, "verbs": {"Never": 1, "Out": 1, "Yes": 1}, "adverbs": {"Never": 1}, "numerals": {"302": 1}, "pronouns": {"I": 1}}}, {"content": "303. I am very sensitive to certain matters so that I cannot mention them.", "word_count": 17, "label": "", "sentence_structure": "Subject鈥揷opula鈥損redicative", "subjective_objective": {"answer": "Subjective", "rationale": "Sensitivity is a subjective emotional experience that involves the patient ' s personal feelings and reactions to events or situations."}, "sentence_sentiment": {"answer": "Negative", "rationale": "Basis of judgement: The expression \"I am sensitive to certain things, so I cannot mention\" indicates that the author has a negative emotional experience (sensitive) and is therefore classified as negative."}, "language_style": {"answer": "Tactful and warm", "rationale": "The entry is more euphemistic, is used in more moderate terms and expressions, is spoken in a question-and-answer manner, and expresses concern and understanding about each other鈥檚 emotions. This expression is easier to share with the interviewees, and to create a good atmosphere of communication."}, "time_dimension": {"answer": "Present", "rationale": "The entry described the testee's past behavior or experience, and the words \"sensitized\" and \"not able to bring it up\" all indicated that this was a matter of what had happened, and therefore judged to be the past."}, "sentence_type": "Negative declarative sentence", "pos": {"nouns": {"Things": 2, "Sensitive": 1, "T": 1}, "verbs": {"be": 1, "No, I can't": 1, "Rise": 1}, "adjectives": {"Some": 1, "very": 1}, "adverbs": {"And so": 1}, "numerals": {"303": 1}, "pronouns": {"I": 1}}}, {"content": "304. In schools, it is very difficult to have me speak at early hours.", "word_count": 17, "label": "", "sentence_structure": "Subject鈥揷opula鈥損redicative", "subjective_objective": {"answer": "Subjective", "rationale": "Because \"very difficult\" is a subjective assessment."}, "sentence_sentiment": {"answer": "Negative", "rationale": "Because the entry \"very hard\" expresses negative emotional attitudes."}, "language_style": {"answer": "Calm and direct", "rationale": "The entry directly expresses the other party ' s speech challenges at school, with no excessive rhetorical or emotional rendering, and a more objective tone."}, "time_dimension": {"answer": "Present", "rationale": "Now the entry describes the experience and feelings of the testees in school, indicating that it is very difficult to speak at early hours. This statement emphasizes the current situation and can therefore be judged as it is."}, "sentence_type": "Negative declarative sentence", "pos": {"nouns": {"In school": 1, "Early shift": 1, "Presentations": 1}, "verbs": {"Yes": 1, "be": 1, "Hard": 1}, "adjectives": {"Very": 1}, "numerals": {"304锛?: 1}}}, {"content": "305. Even with people, I often feel alone.", "word_count": 17, "label": "", "sentence_structure": "Subject鈥揷opula鈥損redicative", "subjective_objective": {"answer": "Subjective", "rationale": "Because the sentence expresses the patient ' s subjective feeling that he/she often feels alone, which is not necessarily related to the surroundings."}, "sentence_sentiment": {"answer": "Negative", "rationale": "Because the feeling of being alone is negative in the entries."}, "language_style": {"answer": "Tactful and warm", "rationale": "The language style of the entry is more euphemistic and warm, using the phrase \"even if I am with people\" instead of \"I am always alone.\" It is more gentle, more friendly, more acceptable and more responsive. And it is not too much to say or to demean the feelings of individuals, more objective and neutral."}, "time_dimension": {"answer": "Present", "rationale": "\"I often feel alone\" describes a state or feeling that does not relate to past or future acts or events and therefore judges current behaviour or mentality."}, "sentence_type": "Negative declarative sentence", "pos": {"nouns": {"Alone": 1, "People": 1}, "verbs": {"Regular": 1, "I feel": 1}, "adjectives": {"Together": 1}, "adverbs": {"Or is it": 1, "Even if": 1}, "numerals": {"305": 1}, "pronouns": {"I": 1}}}, {"content": "306. I've got all the sympathy I deserve.", "word_count": 10, "label": "", "sentence_structure": "Subject鈥搗erb鈥揷omplement", "subjective_objective": {"answer": "Subjective", "rationale": "Because it's the patient's own subjective feeling."}, "sentence_sentiment": {"answer": "Neutral", "rationale": "Because the entry does not address any positive or negative behaviour, attitude or emotional colour. It merely describes the fact that the interviewee feels that he or she has the sympathy he or she deserves."}, "language_style": {"answer": "Tactful and warm", "rationale": "The language style of the entry is euphemism. It expresses in a gentle and positive way the satisfaction and gratitude of the interviewee for having sympathy with others, without using too direct or hard language, and is more resonant."}, "time_dimension": {"answer": "Present", "rationale": "The entry expresses the person's current feelings or mentality, \"I've got it all\" means that the person who was tested feels that he has earned the sympathy that he deserves, based on his current experience and circumstances. Thus, the time dimension of the article is now judged."}, "sentence_type": "Affirmative declarative sentence", "pos": {"nouns": {"Compassion": 1}, "verbs": {"Got it": 2, "You deserve it": 1}, "adverbs": {"All": 1}, "numerals": {"306": 1}, "pronouns": {"I": 1}}}, {"content": "I refuse to play those games I'm not playing well.", "word_count": 14, "label": "", "sentence_structure": "Subject鈥搗erb鈥搊bject", "subjective_objective": {"answer": "Subjective", "rationale": "Because \"good play\" is a subjective judgment and evaluation of the individual."}, "sentence_sentiment": {"answer": "Negative", "rationale": "Because the \"no\" in the entry is negative."}, "language_style": {"answer": "Calm and direct", "rationale": "The language style of the entry is more calm and straightforward, and there is no excessive adoration or emotional colour. It expresses the surveyed's attitude and choice about bad games in a clear language. This language style is suitable for official settings such as questionnaires."}, "time_dimension": {"answer": "Present", "rationale": "The word \"play\" in \"I refuse to play\" uses the current state of affairs, which means that it has not only happened in the past, but may still be. \"The games I played bad\" represent a description of the past, but do not clearly indicate whether it is past, present or future, and the time dimension of the article is uncertain."}, "sentence_type": "Affirmative declarative sentence", "pos": {"nouns": {"Questionnaire Entry": 1, "Game": 2}, "verbs": {"Reject": 1, "Play": 2, "Not good": 1}, "adjectives": {"Okay": 1}, "numerals": {"307": 1}, "pronouns": {"I": 1}}}, {"content": "Sometimes I want to leave home.", "word_count": 9, "label": "", "sentence_structure": "Subject鈥損redicate", "subjective_objective": {"answer": "Subjective", "rationale": "Because \"very much\" reflects the feelings and aspirations of the patient, which is a subjective attitude."}, "sentence_sentiment": {"answer": "Negative", "rationale": "Because the \"very much wanted to leave home\" act in the entry is negative."}, "language_style": {"answer": "Calm and direct", "rationale": "The entry is more concise, direct, and has no excessive emotional accoutrements or rhetorical techniques, and directly expresses the idea of asking if the other party has ever wanted to leave the home. This is more objective and realistic."}, "time_dimension": {"answer": "Present", "rationale": "The entry asks the person who was tested for his or her current mood and feelings, and the time dimension is now judged."}, "sentence_type": "Affirmative declarative sentence", "pos": {"nouns": {"Time": 1, "I": 1, "Home": 1}, "verbs": {"Yes": 1, "Off": 1, "Open": 1}, "adverbs": {"Very": 1}, "numerals": {"308": 1}}}, {"content": "309. It is almost as easy for me to make friends as for others.", "word_count": 15, "label": "", "sentence_structure": "Subject鈥搗erb鈥搊bject", "subjective_objective": {"answer": "Subjective", "rationale": "Subjectivity because it is easy to make friends as a patient's personal feeling and attitude."}, "sentence_sentiment": {"answer": "Neutral", "rationale": "Because the entry does not have any emotional color."}, "language_style": {"answer": "Tactful and warm", "rationale": "The entry is a more euphemistic and easy way of asking each other about their performance in social relations, is more friendly in language and is not too direct or strong."}, "time_dimension": {"answer": "Past", "rationale": "\"I'm as easy as anyone else to be friends\" describes the past experience and feelings, and therefore judges it as the past."}, "sentence_type": "Affirmative declarative sentence", "pos": {"nouns": {"friends": 2, "others": 1}, "verbs": {"Hand it over": 1, "It's just as easy": 1}, "adjectives": {"Easy": 1}, "adverbs": {"Almost": 1}, "numerals": {"309": 1, "Two": 1, "One": 2}, "pronouns": {"I": 1}}}, {"content": "310. My sex life is satisfactory.", "word_count": 9, "label": "", "sentence_structure": "Subject鈥揷opula鈥損redicative", "subjective_objective": {"answer": "Subjective", "rationale": "Since satisfaction is a subjective feeling, the degree of satisfaction of sexual life may vary from one person to another, and therefore it is a subjective entry."}, "sentence_sentiment": {"answer": "Positive", "rationale": "Because it expresses satisfaction and satisfaction, it is a positive emotional attitude."}, "language_style": {"answer": "Calm and direct", "rationale": "The entry is a simple and straightforward way of asking respondents about their satisfaction with their sexual life, without any aesthetic or emotional expression, and with a more calm and straightforward language style."}, "time_dimension": {"answer": "Past", "rationale": "The entry asks about the sexual satisfaction of the person being tested at present, and does not address the time dimension of the past or future, and therefore the judgement is \"now\"."}, "sentence_type": "Affirmative declarative sentence", "pos": {"nouns": {"Sex": 1}, "verbs": {"be": 1, "Satisfied": 1}, "numerals": {"310": 1}}}, {"content": "311. When I was a kid, I did a little bit of petty theft.", "word_count": 19, "label": "", "sentence_structure": "Subject鈥損redicate", "subjective_objective": {"answer": "Subjective", "rationale": "Because \"I did a little bit of the petty theft thing when I was a kid\" is a personal memory and experience, and it is therefore viewed from the subjective perspective of the patient."}, "sentence_sentiment": {"answer": "Negative", "rationale": "Because the behavior of \"the thief\" in the entry is negative."}, "language_style": {"answer": "Tactful and warm", "rationale": "The entry uses a more moderate tone and does not directly accuse or criticize the interviewee, but rather raises the question in a more open and understandable way. Moreover, the beginning of the article implies the interviewee鈥檚 growth experience and increases the resonance between the interviewer and the interviewee."}, "time_dimension": {"answer": "Past", "rationale": "In the entry, \"for a while\" means \"for the past, \"I've done the petty theft\" is also a description of the past, so it's a judgment."}, "sentence_type": "Affirmative declarative sentence", "pos": {"nouns": {"Time": 1, "For a while": 1, "Yes": 1}, "verbs": {"have": 1, "Yes": 1, "Do it": 1}, "adjectives": {"Small": 2}, "adverbs": {"I": 3, "A thief's little hand": 2}, "numerals": {"311": 1, "One": 1}, "classifiers": {"individual": 1}}}, {"content": "I don't like people around me.", "word_count": 10, "label": "", "sentence_structure": "Subject鈥搗erb鈥揷omplement", "subjective_objective": {"answer": "Subjective", "rationale": "Because \"not like\" is a subjective assessment of how people feel when they're around."}, "sentence_sentiment": {"answer": "Negative", "rationale": "Because the expression \"not like someone around me\" in the entry is negative."}, "language_style": {"answer": "Calm and direct", "rationale": "The entry is simple and directly expresses the question of whether the other party does not like the subject of being around him, without too much ado or emotional rendering."}, "time_dimension": {"answer": "Past", "rationale": "The entry is for the purpose of asking the respondent about the memory and description of past behaviour or feelings, and the temporal dimension is a past."}, "sentence_type": "Negative declarative sentence", "pos": {"nouns": {"person": 1, "I": 1, "Side": 1}, "verbs": {"Yeah": 1, "in": 1}, "adjectives": {"not": 1}, "numerals": {"312": 1}}}, {"content": "313. Persons who fail to keep their valuables in safe custody and thereby cause theft are equally responsible.", "word_count": 34, "label": "", "sentence_structure": "Subject鈥搗erb鈥搊bject", "subjective_objective": {"answer": "Objective", "rationale": "Because the sentence describes a particular act, it does not involve subjective attitudes or feelings of individuals."}, "sentence_sentiment": {"answer": "Neutral", "rationale": "Because the entry merely stated a fact, and did not have any emotional colour."}, "language_style": {"answer": "Calm and direct", "rationale": "The entry expresses the author's view in a straightforward and clear manner, without too much rhetorical or euphemism. This expression makes the meaning of the entry clearer.\""}, "time_dimension": {"answer": "Present", "rationale": "The entry describes a current state of conduct or state, namely, \"someone who does not keep his valuables in safe custody and thereby causes others to steal\", and thus judges it as now."}, "sentence_type": "Affirmative declarative sentence", "pos": {"nouns": {"person": 2, "Valuables": 1, "others": 1, "Theft": 1, "Blame": 1, "Thief": 1}, "verbs": {"Not": 1, "Keep it safe": 1, "Cause": 1, "Due": 1}, "adverbs": {"Yeah": 1}, "numerals": {"313": 1}, "pronouns": {"Here": 1}}}, {"content": "314. Occasionally I think of something that is so bad that I can't say.", "word_count": 16, "label": "", "sentence_structure": "Subject鈥搗erb鈥揷omplement", "subjective_objective": {"answer": "Subjective", "rationale": "Because \"Thinking about something bad is the experience and feeling of the patient, it's subjective."}, "sentence_sentiment": {"answer": "Negative", "rationale": "Because the idea of \"bad things\" in the entry is negative."}, "language_style": {"answer": "Calm and direct", "rationale": "The entry expresses in a direct manner the subject of a question about whether the other party has ever done a bad thing, without too many rhetorical or emotional renderings, and with a more objective tone.\""}, "time_dimension": {"answer": "Present", "rationale": "The keywords are \"interim\" and \"chamber\", which means that this is an ongoing, occasional situation, and therefore the temporal dimension is now judged."}, "sentence_type": "Affirmative declarative sentence", "pos": {"nouns": {"Yes": 1}, "verbs": {"Yes": 1, "Say it": 1}, "adjectives": {"Sometimes": 1, "Bad": 1, "I can't say": 1}, "adverbs": {"Okay": 1}, "numerals": {"III": 1, "Fourteen": 1}, "classifiers": {"individual": 1}, "pronouns": {"I": 1}}}, {"content": "315. I am convinced that life is cruel to me.", "word_count": 11, "label": "", "sentence_structure": "Subject鈥揷opula鈥損redicative", "subjective_objective": {"answer": "Subjective", "rationale": "Because belief is subjective."}, "sentence_sentiment": {"answer": "Negative", "rationale": "Because the word \"cruel\" expresses negative emotional attitudes."}, "language_style": {"answer": "Calm and direct", "rationale": "The entry is of a direct, clear, concise type, with no too much accommodative vocabulary or statements, and is cold and firm. It asks whether the person surveyed believes that life is cruel to himself and does not use euphemism or verb to induce or induce the person under investigation to answer."}, "time_dimension": {"answer": "Present", "rationale": ""}, "sentence_type": "Negative declarative sentence", "pos": {"nouns": {"Life": 1, "I": 2, "Cruel": 1}, "verbs": {"I'm sure of that": 1, "be": 1, "to": 1}, "adjectives": {"Cruel": 1}, "numerals": {"315": 1}, "pronouns": {"I": 2}}}, {"content": "316. I think that almost everyone would say something false in order to avoid trouble.", "word_count": 20, "label": "", "sentence_structure": "Subject鈥搗erb鈥搊bject", "subjective_objective": {"answer": "Subjective", "rationale": "Because \"want\" and \"doesn't bother saying a few false words\" are patients' subjective thoughts or attitudes."}, "sentence_sentiment": {"answer": "Negative", "rationale": "Because it expresses an dishonest act."}, "language_style": {"answer": "Tactful and warm", "rationale": "The language style of the entry is more euphemistic, leads the respondents to express their views in a way that is asked, and uses a number of rhetorical techniques to increase kinship and attraction, such as \"almost everyone\" and \"preventing troubles by saying false words.\" These are used in a way that neither directs nor criticizes, nor triggers resonance with the other."}, "time_dimension": {"answer": "Past", "rationale": "The entry is therefore an inquiry into the present behaviour or attitude of the person being tested, and the temporal dimension is now judged."}, "sentence_type": "Affirmative declarative sentence", "pos": {"nouns": {"Everyone": 1, "Trouble": 1, "That's a lie": 1}, "verbs": {"Yes": 2, "Avoid": 1, "Say it": 1}, "adjectives": {"Almost": 1}, "numerals": {"316锛?: 1}}}, {"content": "317. I am more sensitive than most people.", "word_count": 9, "label": "", "sentence_structure": "Subject鈥揷opula鈥損redicative", "subjective_objective": {"answer": "Subjective", "rationale": "Because \"sensitive\" is a subjective feeling and understanding, not an objective fact."}, "sentence_sentiment": {"answer": "Neutral", "rationale": "Because the entry does not have any emotional color."}, "language_style": {"answer": "Tactful and warm", "rationale": "The entry is more euphemistic, soft, neutral and not overly direct or intensely emotional. At the same time, it does not use negative terminology or strong tone to describe the questions that the respondent may have, but rather in a more moderate way to guide the respondent to think and express his feelings. The language style of the entry can therefore be classified as \"precipitous.\""}, "time_dimension": {"answer": "Present", "rationale": "The keyword \"I'm more sensitive than most people\" refers to the quality and performance of the person being tested, without reference to the time dimension of the past or the future, and therefore to the present."}, "sentence_type": "Exclamatory sentence", "pos": {"nouns": {"Questionnaire": 1, "Entry": 1}, "verbs": {"- Yeah": 1, "More": 1, "Sensitive": 1}, "adjectives": {"Most of them": 1, "person": 1}, "numerals": {"317": 1}, "pronouns": {"I": 1}}}, {"content": "318. In my daily life, there are things that interest me.", "word_count": 18, "label": "", "sentence_structure": "Subject鈥揷opula鈥損redicative", "subjective_objective": {"answer": "Subjective", "rationale": "Because \"interest\" is a subjective feeling."}, "sentence_sentiment": {"answer": "Positive", "rationale": "Because the description \"filled with things that interest me\" in the entry is positive."}, "language_style": {"answer": "Tactful and warm", "rationale": "This is a more intimate way of expressing the interest in the daily life of the person surveyed."}, "time_dimension": {"answer": "Present", "rationale": "The entry \"My Daily Life\" means the situation now, \"filled with things that interest me\" is also the act that happens now, so time dimensions are now judged."}, "sentence_type": "Affirmative declarative sentence", "pos": {"nouns": {"Daily life": 1, "Things": 1}, "verbs": {"It's full of": 1}, "adjectives": {"I'm interested": 1}, "numerals": {"318": 1}}}, {"content": "319. Most people are not willing to step forward and help others.", "word_count": 21, "label": "", "sentence_structure": "Subject鈥搗erb鈥搊bject", "subjective_objective": {"answer": "Subjective", "rationale": "Because \"no\" is a psychological state, a patient's subjective feeling."}, "sentence_sentiment": {"answer": "Negative", "rationale": "Because the entry expresses the reluctance to help others and is negative."}, "language_style": {"answer": "Tactful and warm", "rationale": "This is more personal and interactive."}, "time_dimension": {"answer": "Present", "rationale": "The words \"mostly\" , \"inner\" and \"inside\" in the entry indicate that this is a description of the present, so it is possible to judge the dimension of time as it is."}, "sentence_type": "Negative declarative sentence", "pos": {"nouns": {"person": 2, "Most": 1, "Inside": 1, "Help": 1, "others": 1}, "verbs": {"be": 1, "Yes": 1, "Stand up": 1, "Go on": 1}, "adjectives": {"Large": 1, "Most of them": 1, "No, I don't": 1}, "adverbs": {"Both": 1}, "numerals": {"319": 1}, "pronouns": {"they": 1}}}, {"content": "320. My dreams are more about sex.", "word_count": 14, "label": "", "sentence_structure": "Subject鈥搗erb鈥揷omplement", "subjective_objective": {"answer": "Subjective", "rationale": "Because the content of dreams usually reflects the thoughts and desires of the patients, \"My dreams are more about sex\" is a subjective description."}, "sentence_sentiment": {"answer": "Negative", "rationale": "The idea of a sexual dream can lead to discomfort and negative sentiment."}, "language_style": {"answer": "Tactful and warm", "rationale": "The entry expresses the subject of a possible sexual dream in a more subtle and subtle way, avoiding too direct or offensive expressions. Such language styles make it easier for the interviewee to feel comfortable and to answer questions in greater depth."}, "time_dimension": {"answer": "Present", "rationale": ""}, "sentence_type": "Affirmative declarative sentence", "pos": {"nouns": {"Dreams": 1, "In terms of": 1, "Yes": 1}, "verbs": {"have": 1, "Do it": 1}, "adjectives": {"Better": 1}, "adverbs": {"True": 1}, "numerals": {"320": 1}, "pronouns": {"I": 1}}}, {"content": "321. I am easily overwhelmed.", "word_count": 10, "label": "", "sentence_structure": "Subject鈥揷opula鈥損redicative", "subjective_objective": {"answer": "Subjective", "rationale": "It is viewed from a subjective perspective because it is the feelings and experiences of the patient that are easily overwhelmed."}, "sentence_sentiment": {"answer": "Negative", "rationale": "Because the feeling of being \"easy to be confused\" in the entry is negative."}, "language_style": {"answer": "Calm and direct", "rationale": "The language style of the entry is relatively calm, with no excessive rhetorical or emotional rendering, and with a more objective tone, indicating that the subject is easily confused."}, "time_dimension": {"answer": "Present", "rationale": "Now the entry uses the current state of \"easy\" to describe the current state of the person's feeling, without reference to the past or future dimensions of time. At the same time, \"I can easily feel overwhelmed\" to describe a persistent state of personal subjective feeling, and therefore to judge it as now."}, "sentence_type": "Negative declarative sentence", "pos": {"nouns": {"Grandpa": 1, "Questionnaire": 1, "Entry": 1}, "verbs": {"Sensor": 1, "Present": 1, "be": 1, "Sou-san": 1, "Yeah": 1}, "adjectives": {"Yon": 1, "Easy": 1, "I feel": 1, "I don't know": 1, "I'm sure I can": 1}, "numerals": {"321": 1}, "pronouns": {"I": 1}}}, {"content": "I am worried about money and business.", "word_count": 9, "label": "", "sentence_structure": "Subject鈥揷opula鈥損redicative", "subjective_objective": {"answer": "Subjective", "rationale": "Because \"I\" worry about money and business is a subjective or emotional experience."}, "sentence_sentiment": {"answer": "Negative", "rationale": "Because \"fear\" is a negative sentiment."}, "language_style": {"answer": "Calm and direct", "rationale": "The entry uses a direct description of the psychological state that the subject may have, and does not have too many rhetorical or emotional renderings. It therefore tends to be more calm and direct."}, "time_dimension": {"answer": "Present", "rationale": "The keyword \"I'm worried about money and business\" is a sense of anxiety and anxiety about the current situation, and the temporal dimension is now."}, "sentence_type": "Affirmative declarative sentence", "pos": {"nouns": {"Money": 1, "Career": 1}, "verbs": {"Fear": 1}, "numerals": {"322": 1}}}, {"content": "323. I have had a very special and extraordinary experience.", "word_count": 14, "label": "", "sentence_structure": "Subject鈥搗erb鈥揷omplement", "subjective_objective": {"answer": "Subjective", "rationale": "Because \"very special and peculiar\" experiences are usually subjective, the perspective of the questionnaire is subjective."}, "sentence_sentiment": {"answer": "Positive", "rationale": "Because the adjectives \"special\" and \"gentle\" are usually considered positive."}, "language_style": {"answer": "Tactful and warm", "rationale": "The entry is more euphemistic and gentle, using the words \"very special\" and \"unique\" to describe experience, softening the tone, and giving people a sense of affection and affection. At the same time, the entry does not use the direct means of asking questions, but rather uses a more open way of asking questions, encouraging respondents to share their experiences and feelings, which is more personal and interactive."}, "time_dimension": {"answer": "Past", "rationale": "\"At one time\" is a reference to past experience and experience, so the entry is a time dimension of the past."}, "sentence_type": "Affirmative declarative sentence", "pos": {"nouns": {"Experience": 1, "Once": 1}, "verbs": {"Yes": 1, "very": 2, "be": 1}, "adjectives": {"Special": 1, "- Chi": 2}, "adverbs": {"Pass": 1}, "numerals": {"323": 1}}}, {"content": "324. I have never fallen in love with anyone.", "word_count": 11, "label": "", "sentence_structure": "Subject鈥搗erb鈥揷omplement", "subjective_objective": {"answer": "Subjective", "rationale": "Because it is the patient's subjective feeling and judgment that loves someone."}, "sentence_sentiment": {"answer": "Negative", "rationale": "Because the \"no one has fallen in love\" in the entry expresses a negative and rejectionist attitude, which is a negative emotional tendency."}, "language_style": {"answer": "Calm and direct", "rationale": "The language style of the entry is more direct, concise, without any qualifying adjectives or adverbs to enhance emotionality, or any symbolic or metaphorical expression. This tone is usually considered to be more objective, practical and dispassionate, and suitable for use in applications such as questionnaires that require specific information."}, "time_dimension": {"answer": "Past", "rationale": "The entry \"never\" indicates that it is a description or reflection of the past and therefore belongs to the temporal dimension of the past."}, "sentence_type": "Negative declarative sentence", "pos": {"nouns": {"Questionnaire": 1, "person": 1}, "verbs": {"Love": 1, "Go, go, go": 1}, "adverbs": {"Never": 1, "No, I'm not": 2}, "numerals": {"324": 1}, "pronouns": {"I": 1}}}, {"content": "325. I am surprised by what some people in my family have done.", "word_count": 14, "label": "", "sentence_structure": "Subject鈥搗erb鈥揷omplement", "subjective_objective": {"answer": "Subjective", "rationale": "Because \"to surprise\" is a subjective feeling and perception."}, "sentence_sentiment": {"answer": "Neutral", "rationale": "The entry describes the behaviour of family members as surprising, does not express positive or negative emotional tendencies and is therefore neutral."}, "language_style": {"answer": "Tactful and warm", "rationale": "The language style of the entry is euphemism, because it uses a gentle tone, asks questions in an invitation-style manner, and does not speak directly or command each other. This expression is more friendly and interactive, making the interviewee feel more comfortable and comfortable and more willing to answer the questions."}, "time_dimension": {"answer": "Past", "rationale": "The use of the word \"pro\" in the entry suggests that it describes events in the past, that what some people in my family do is a past act, and that the dimensions of time are therefore judged to be past."}, "sentence_type": "Affirmative declarative sentence", "pos": {"nouns": {"Home": 1, "person": 1, "Yes": 1, "Surprise": 1}, "verbs": {"Do it": 1, "鈾?And I'm gonna be 鈾?: 1}, "adjectives": {"Surprise": 1}, "numerals": {"325": 1}, "pronouns": {"I": 1}}}, {"content": "Sometimes I cry and laugh and I can't even control myself.", "word_count": 18, "label": "", "sentence_structure": "Subject鈥搗erb鈥揷omplement", "subjective_objective": {"answer": "Subjective", "rationale": "Because \"cry, laugh\" and \"uncontrollable\" are all described in terms of patients' subjective feelings. This reflects their emotional volatility and inadequate self-regulation, reflecting their subjective experience and emotional state."}, "sentence_sentiment": {"answer": "Negative", "rationale": "Because the \"uncontrollable\" behavior in the entry is negative."}, "language_style": {"answer": "Tactful and warm", "rationale": "The language style of the entry is more euphemistic, using expressions such as \"sometimes\" and \"uncontrollable\" to give people a sense of affection and understanding. At the same time, instead of too much criticism or criticism, the question of the respondents is asked in a caring and receptive manner, which enhances the relevance and effectiveness of the questionnaire."}, "time_dimension": {"answer": "Present", "rationale": "The entry describes the current behaviour or state, and the keywords \"will\" and \"uncontrollable\" all indicate that this is an ongoing situation."}, "sentence_type": "Affirmative declarative sentence", "pos": {"nouns": {"Time": 1, "Yes": 1, "Cry": 1, "Line": 1, "Smile": 1, "Company": 1, "self": 1, "Control": 1}, "verbs": {"have": 1, "No, I can't": 1}, "adverbs": {"Sometimes": 1, "Both": 1}, "numerals": {"326": 1}}}, {"content": "327. My mother or father often asked me to obey him, even if I did not think it was reasonable.", "word_count": 24, "label": "", "sentence_structure": "Subject鈥搗erb鈥搊bject", "subjective_objective": {"answer": "Subjective", "rationale": "Because \"it's not reasonable\" involves the patient's own subjective evaluation and judgment."}, "sentence_sentiment": {"answer": "Negative", "rationale": "Because the expression \"mother or father often asks me to obey him\" in the entry has a feeling of coercion or non-consensualness, implying that a negative family relationship may exist."}, "language_style": {"answer": "Tactful and warm", "rationale": "The language style of the entry is more euphemistic and warm. By using words such as \"and often want\" and \"and even unreasonable\", it expresses a mild attitude of questioning, rather than directly blaming or ordering each other. At the same time, the whole sentence is more apologetic and respectful, facilitating good communication."}, "time_dimension": {"answer": "Past", "rationale": "The entry describes the experience and feelings of the testee in the past, and the keywords \"frequently\" and \"considered unreasonable\" indicate that this is a long-standing situation and has already happened. The temporal dimension is therefore judged to be the past."}, "sentence_type": "Affirmative declarative sentence", "pos": {"nouns": {"Mother": 1, "Father": 1, "I": 1, "Time": 1, "Obey": 1, "he": 1, "I think": 1, "It doesn't make sense": 1}, "verbs": {"Often": 1, "Yes": 1, "Even": 1, "I think": 1}, "adverbs": {"Often": 1, "Even": 1}, "numerals": {"327": 1}, "pronouns": {"I": 1, "he": 1}}}, {"content": "328. I find it difficult to focus on a job.", "word_count": 18, "label": "", "sentence_structure": "Subject鈥搗erb鈥搊bject", "subjective_objective": {"answer": "Subjective", "rationale": "Because \"it's hard to focus on a job\" is a patient's self-perception and experience, and therefore a subjective approach."}, "sentence_sentiment": {"answer": "Negative", "rationale": "Because behavior in the entries \"it's hard to focus on a job\" is negative."}, "language_style": {"answer": "Calm and direct", "rationale": ""}, "time_dimension": {"answer": "Present", "rationale": ""}, "sentence_type": "Negative declarative sentence", "pos": {"nouns": {"Work": 1}, "verbs": {"Found": 1, "Focus": 1}, "adjectives": {"Hard": 1}, "adverbs": {"very": 1}, "numerals": {"328锛?: 1}}}, {"content": "329. I hardly dream.", "word_count": 7, "label": "", "sentence_structure": "Subject鈥揷opula鈥損redicative", "subjective_objective": {"answer": "Objective", "rationale": "Because dreams usually occur in sleep, and are an objective phenomenon, the purpose of the article is to describe them objectively."}, "sentence_sentiment": {"answer": "Neutral", "rationale": "Because the entry does not have any emotional color."}, "language_style": {"answer": "Calm and direct", "rationale": "The entry uses a simple and clear language style, without any rhetorical or emotional rendering, which is more objective and which is often asked whether the other side is dreaming."}, "time_dimension": {"answer": "Past", "rationale": "The entry describes the testee's past behavior or state, which is \"I hardly dream.\""}, "sentence_type": "Negative declarative sentence", "pos": {"verbs": {"Do it": 1, "Dreams": 1}, "adjectives": {"Almost": 1}, "adverbs": {"EMPTY": 1}, "numerals": {"329": 1}}}, {"content": "330. I have never been paralysed.", "word_count": 8, "label": "", "sentence_structure": "Subject鈥揷opula鈥損redicative", "subjective_objective": {"answer": "Objective", "rationale": "Since paralysis is an objective experience of the patient, there is no need to examine subjective ideas."}, "sentence_sentiment": {"answer": "Neutral", "rationale": "Basis of judgement: The entry does not express any positive or negative feelings, attitudes, behaviour, purpose or quality and can therefore be judged neutral."}, "language_style": {"answer": "Calm and direct", "rationale": "The language style of the entry is calm and straightforward. It explicitly asks whether the person surveyed has been paralysed, has not used excessive emotional accoutrements or euphemisms, and is more direct and objective."}, "time_dimension": {"answer": "Past", "rationale": "In the entry, \"never\" indicates that this is a description of the past or experience, and therefore judgement is the past."}, "sentence_type": "Negative declarative sentence", "pos": {"nouns": {"Paralytic": 1, "Entry": 1}, "verbs": {"Never": 1}, "adverbs": {"From": 1, "Come on": 1, "Nope": 1}, "numerals": {"330": 1}, "classifiers": {"individual": 1}, "pronouns": {"I": 1}}}, {"content": "331. I would have achieved much more had it not been for the confrontations with me.", "word_count": 20, "label": "", "sentence_structure": "Subject鈥搗erb鈥揷omplement", "subjective_objective": {"answer": "Subjective", "rationale": "Because \"if it is\" is the patient's imagination, not the objective fact."}, "sentence_sentiment": {"answer": "Negative", "rationale": "Because the expression \"if I don't have anyone against me, I'll be more successful\" in the entry has a self-centred negative attitude."}, "language_style": {"answer": "Calm and direct", "rationale": "The language style of the entry is relatively calm and straightforward, with no excessive rhetorical or emotional rendering, but simply a hypothetical and point of view."}, "time_dimension": {"answer": "Future", "rationale": "The entry contains a hypothetical conditional phrase \"if...\" and relates to what may happen in the future, \"there will be greater achievement\" expresses a desire or desire and is judged as the future."}, "sentence_type": "Negative declarative sentence", "pos": {"nouns": {"person": 1, "- Yes": 1, "Achievements": 1}, "verbs": {"Yes": 1, "have": 1, "I'm sure": 1, "be": 1}, "adverbs": {"Suppose": 1, "not": 1, "Bigger": 1}, "numerals": {"331": 1}, "pronouns": {"I": 2}}}, {"content": "332. Even if I had no cold, I sometimes did not have a voice or a voice to change.", "word_count": 21, "label": "", "sentence_structure": "Subject鈥搗erb鈥揷omplement", "subjective_objective": {"answer": "Subjective", "rationale": "Because the person feels sick in his throat, or cold is judged by the person ' s own feelings and is subjective."}, "sentence_sentiment": {"answer": "Negative", "rationale": "Because the entry \"no cold, no sound or voice change\" is negative."}, "language_style": {"answer": "Tactful and warm", "rationale": "The language style of the entry is more euphemistic. It is a soft, invitation-style conversation, not a direct statement or an order. This expression is more personal and interactive, makes it easier to answer questions and does not make the respondents feel too stressful or uneasy."}, "time_dimension": {"answer": "Present", "rationale": "It is possible to judge by the title 鈥渟ometimes鈥?that the act is present and irregular and therefore is current."}, "sentence_type": "Affirmative declarative sentence", "pos": {"nouns": {"Cold": 1, "Sound": 3}, "verbs": {"No, I'm not": 1, "Yes": 1, "Out": 1, "Change": 1}, "adverbs": {"Sometimes": 1}, "numerals": {"332": 1}, "classifiers": {"individual": 1}, "pronouns": {"I": 2}}}, {"content": "333. It seems that no one knows me.", "word_count": 9, "label": "", "sentence_structure": "Subject鈥揷opula鈥損redicative", "subjective_objective": {"answer": "Subjective", "rationale": "Because the words \"like\" and \"understand\" speak of the feelings and views of the patient, and are subjective."}, "sentence_sentiment": {"answer": "Negative", "rationale": "Because the phrase \"no one can understand me\" in the entry expresses negative feelings and attitudes."}, "language_style": {"answer": "Tactful and warm", "rationale": "The entry leads the respondents to share their thoughts and feelings by describing a feeling and uses moderate and indirect expressions, making the questions more acceptable and answerable."}, "time_dimension": {"answer": "Present", "rationale": ""}, "sentence_type": "Negative declarative sentence", "pos": {"nouns": {"person": 1, "I": 1}, "verbs": {"Yes": 1, "Roger that": 1, "Seems like": 1, "Nobody": 1}, "adjectives": {"Nobody": 1}, "adverbs": {"Both": 1, "not": 1}, "numerals": {"333": 1, "One": 1}, "pronouns": {"Yes": 1}}}, {"content": "334. Sometimes I smell strange.", "word_count": 11, "label": "", "sentence_structure": "Subject鈥搗erb鈥揷omplement", "subjective_objective": {"answer": "Objective", "rationale": "Because \"smelling strange smell\" is an objective fact that patients experience, there is no need to examine subjective ideas."}, "sentence_sentiment": {"answer": "Neutral", "rationale": "Because the entry does not express any emotion, attitude or behaviour, it merely describes a phenomenon."}, "language_style": {"answer": "Calm and direct", "rationale": "The language style of the entry is more direct, there is no over-modified or emotional expression, but rather questions are asked in a concise and clear manner, allowing respondents to understand the meaning of the question and to respond to it."}, "time_dimension": {"answer": "Present", "rationale": "The keywords are \"sometimes\" and \"smelt,\" both of which point to the action or state that is taking place at the moment, so the temporal dimension of the entry is now."}, "sentence_type": "Affirmative declarative sentence", "pos": {"nouns": {"Time": 1, "Smell": 1}, "verbs": {"Yes": 1, "Smell it": 1}, "adjectives": {"Strange": 1}, "numerals": {"334锛?: 1}}}, {"content": "335. I cannot concentrate on one thing.", "word_count": 11, "label": "", "sentence_structure": "Subject鈥搗erb鈥揷omplement", "subjective_objective": {"answer": "Subjective", "rationale": "Because \"focus\" is a subjective feeling or experience that requires the patient to describe and evaluate itself. It is a subjective attitude or feeling of the patient, and it is a subjective examination."}, "sentence_sentiment": {"answer": "Negative", "rationale": "Because \"can't focus on one thing\" is negative."}, "language_style": {"answer": "Calm and direct", "rationale": "The entry presents the potential problems of the subject in a straightforward manner, with no excessive rhetorical or emotional rendering. This cold language allows the person to express his or her feelings and thoughts more accurately, while also reducing the potential for ambiguity and misunderstanding."}, "time_dimension": {"answer": "Past", "rationale": "According to the sentence entered, \"I can't concentrate on one thing\" is a description of the person's current state, so the temporal dimension is the past."}, "sentence_type": "Negative declarative sentence", "pos": {"verbs": {"No, I can't": 1, "Focus": 1, "One thing": 1}, "adjectives": {"Go, go, go": 1}, "numerals": {"335": 1}}}, {"content": "336. I can easily be impatient.", "word_count": 11, "label": "", "sentence_structure": "Subject鈥揷opula鈥損redicative", "subjective_objective": {"answer": "Subjective", "rationale": "Because it is a subjective experience and feeling that people are easily insatiable."}, "sentence_sentiment": {"answer": "Negative", "rationale": "Because the insatiable mood in the entry is negative."}, "language_style": {"answer": "Tactful and warm", "rationale": "The entry is more adept and interactive when it is spoken in a gentle and inviting manner, rather than by direct statements or orders."}, "time_dimension": {"answer": "Present", "rationale": ""}, "sentence_type": "Affirmative declarative sentence", "pos": {"nouns": {"person": 1}, "verbs": {"I feel": 1, "I'm impatient": 1}, "adverbs": {"very": 1, "Easy": 1}, "numerals": {"336锛?: 1}}}, {"content": "337. I've been worried about something or someone almost all day.", "word_count": 18, "label": "", "sentence_structure": "Subject鈥搗erb鈥揷omplement", "subjective_objective": {"answer": "Subjective", "rationale": "Because anxiety is a subjective feeling and experience, it is a description of the patient ' s own state of mind, not an objective fact."}, "sentence_sentiment": {"answer": "Negative", "rationale": "Because \"anxiety\" in the entry is negative."}, "language_style": {"answer": "Tactful and warm", "rationale": "The entry uses a more euphemistic expression, which is more moderate, respectful and understanding of the person鈥檚 feelings by asking whether the person surveyed is often anxious, rather than directly stating his or her possible reasons or extent. At the same time, the issue is not too direct or negative, making it easier for the person surveyed to share his or her true situation."}, "time_dimension": {"answer": "Present", "rationale": ""}, "sentence_type": "Affirmative declarative sentence", "pos": {"nouns": {"Yes": 1, "person": 1, "Anxiety": 1}, "verbs": {"Yes, for": 1}, "adverbs": {"Almost": 1, "All day long": 1}, "numerals": {"337": 1}}}, {"content": "338. I am more concerned than I should be.", "word_count": 18, "label": "", "sentence_structure": "Subject鈥搗erb鈥搊bject", "subjective_objective": {"answer": "Subjective", "rationale": "Because this phrase expresses the subjective feelings and views of the patients."}, "sentence_sentiment": {"answer": "Negative", "rationale": "Because the entries \"mind\" and \"overdue\" have negative emotional connotations."}, "language_style": {"answer": "Tactful and warm", "rationale": "Although the entry expresses the author鈥檚 dissatisfaction with his or her concerns, it is expressed in a relatively soft way, without direct blame or criticism. At the same time, the phrase uses a term such as 鈥渕ore than that鈥? leaving space for reflection and enhancing the orientation and inspiration of the phrase."}, "time_dimension": {"answer": "Present", "rationale": "The entry describes the current feelings and state of the testee, i.e., that they are worried and worried about things that go beyond what is necessary. This concern may be related to their current life, work or personal problems. So the time dimension of the entry is now."}, "sentence_type": "Affirmative declarative sentence", "pos": {"nouns": {"Yes": 2, "Mind your own business": 2}, "verbs": {"More than": 1}, "adjectives": {"Far": 1, "The": 1}, "adverbs": {"Sou-san": 4, "I": 1}, "numerals": {"338锛?: 1}}}, {"content": "339. Most of the time, I think I'm better off dead.", "word_count": 15, "label": "", "sentence_structure": "Subject鈥揷opula鈥損redicative", "subjective_objective": {"answer": "Subjective", "rationale": "Because it is the individual feeling and view of the patient, it is subjective."}, "sentence_sentiment": {"answer": "Negative", "rationale": "Because the purpose of the article expresses a negative and desperate feeling."}, "language_style": {"answer": "Calm and direct", "rationale": "This is a question-and-answer process that is straightforward and clear, is not too much acrimony or metaphoric, and is more language-oriented towards calmness and objectivity."}, "time_dimension": {"answer": "Present", "rationale": "The entry expresses the subjective feeling of the testee in answering the question, that \"most of the time\" feels \"good\" is dead, and that feeling is present, not past or future. The entry therefore describes the status of the testee."}, "sentence_type": "Negative declarative sentence", "pos": {"nouns": {"Time": 1, "I": 2, "Feel": 1, "Okay": 1, "Die": 1}, "verbs": {"I think": 1, "be": 1, "Yes": 1}, "adjectives": {"Large": 1}, "numerals": {"339": 1}, "pronouns": {"Most of them": 1}}}, {"content": "340. Sometimes I get excited and can't sleep.", "word_count": 11, "label": "", "sentence_structure": "Subject鈥搗erb鈥揷omplement", "subjective_objective": {"answer": "Subjective", "rationale": "Because \"sleeping out\" is a subjective feeling or experience."}, "sentence_sentiment": {"answer": "Negative", "rationale": "Because the \"sleeping\" behavior in the entries is negative."}, "language_style": {"answer": "Tactful and warm", "rationale": "The language style of the entry is euphemistic, because it uses the vague expression of \"sometimes\" and expresses the individual's feelings in the way first person calls, rather than directly speaking or ordering. At the same time, the description \"hard to sleep\" has some emotional connotations, making the entry more friendly and accessible to the interviewee."}, "time_dimension": {"answer": "Past", "rationale": "The entry indicates the circumstances that the person was tested, and the keywords are \"sometimes\" and \"excited\" and all point to what has happened and may have happened in the past."}, "sentence_type": "Affirmative declarative sentence", "pos": {"nouns": {"Time": 1, "I": 1}, "verbs": {"Yes": 1, "Excited": 1, "Okay": 1, "Sleep": 1}, "adjectives": {"Hard": 1, "Here": 1}, "adverbs": {"Sometimes": 1}, "numerals": {"340": 1}}}, {"content": "341. Sometimes my hearing is too sharp and it bothers me.", "word_count": 18, "label": "", "sentence_structure": "Subject鈥揷opula鈥損redicative", "subjective_objective": {"answer": "Subjective", "rationale": "Because \"fear\" is the patient's subjective feeling and attitude. While hearing sensitivity may be an objective phenomenon, feelings and judgements of distress are personal subjective."}, "sentence_sentiment": {"answer": "Negative", "rationale": "Because the word \"challenge\" is negative."}, "language_style": {"answer": "Tactful and warm", "rationale": ""}, "time_dimension": {"answer": "Past", "rationale": "In that sentence, \"sometimes\" and \"sometimes\" instead\" indicate that the situation is a past experience or state of affairs and still has an impact on the present. Thus, the judgement is a past."}, "sentence_type": "Affirmative declarative sentence", "pos": {"nouns": {"Hearing": 1, "Smart": 1, "Trouble": 1}, "verbs": {"鈾?And I'm gonna be 鈾?: 1, "I feel": 1}, "adjectives": {"Too much": 1}, "adverbs": {"Sometimes": 1}}}, {"content": "342. I immediately forgot what others said to me.", "word_count": 15, "label": "", "sentence_structure": "Subject鈥損redicate", "subjective_objective": {"answer": "Subjective", "rationale": "Because \"forget it\" is a subjective assessment or feeling, a patient's perception of the ability to remember what others say."}, "sentence_sentiment": {"answer": "Neutral", "rationale": "Because the entry does not have any emotional color."}, "language_style": {"answer": "Tactful and warm", "rationale": "The language style of the entry is more euphemistic and gentle, with the interviewer using a polite phrase like \"please\" and expressing his doubts in a more relaxed way. Such expression neither puts pressure on people, nor reflects respect for and concern for the other."}, "time_dimension": {"answer": "Past", "rationale": "\"I immediately forgot what others said to me\" describes a past experience or behaviour, that is, the testees, in answering this question, need to recall whether they have had a previous experience of forgetting others. Thus, this entry is part of the past."}, "sentence_type": "Affirmative declarative sentence", "pos": {"nouns": {"person": 1, "Word": 1}, "verbs": {"Say it": 1, "Forget it": 1}, "adverbs": {"Now": 1}, "numerals": {"342": 1}, "pronouns": {"I": 1}}}, {"content": "343. Even if it was a trivial matter, I did so after I had considered it.", "word_count": 18, "label": "", "sentence_structure": "Subject鈥搗erb鈥揷omplement", "subjective_objective": {"answer": "Subjective", "rationale": "Since the question is whether the patient will think again before doing what he does, it is subjective to ask the patient about his psychological state and thoughts."}, "sentence_sentiment": {"answer": "Neutral", "rationale": "Because the entry is merely a description of a habit and does not express positive or negative feelings."}, "language_style": {"answer": "Tactful and warm", "rationale": "The language style of the entry is more euphemistic and gentle, expressing concern and concern for the other in a manner that is asked, rather than direct orders or criticism. Moreover, the use of the term \"minus matter\" gives a sense of affection and peace and accessibility."}, "time_dimension": {"answer": "Present", "rationale": ""}, "sentence_type": "Affirmative declarative sentence", "pos": {"nouns": {"It's nothing": 1, "Tiny": 1}, "verbs": {"Do it": 1, "Go on": 1, "Consider": 1}, "adjectives": {"Tiny": 1}, "adverbs": {"Three": 1}, "numerals": {"343": 1}}}, {"content": "344. Sometimes I go around in order to avoid encounters with some people.", "word_count": 18, "label": "", "sentence_structure": "Subject鈥搗erb鈥揷omplement", "subjective_objective": {"answer": "Objective", "rationale": "Because this is the way patients describe their behaviour and avoid encounters with others, it is an objective fact and not the subjective idea or feeling of the patient."}, "sentence_sentiment": {"answer": "Negative", "rationale": "Because the entry \"I will go around to avoid meeting someone\" is negative."}, "language_style": {"answer": "Calm and direct", "rationale": "This language style is usually considered neutral, objective and accurate, and is more common in official settings such as questionnaires."}, "time_dimension": {"answer": "Present", "rationale": "Therefore, the time dimension contained in the entry is now."}, "sentence_type": "Affirmative declarative sentence", "pos": {"nouns": {"Time": 1, "person": 1, "Problems": 1, "Depart": 1, "Okay": 1}, "verbs": {"Avoid": 1, "Yes": 1, "Round": 1, "Let's go": 1}, "adverbs": {"Sometimes": 1}, "numerals": {"344锛?: 1}, "pronouns": {"I": 1}}}, {"content": "I often seem to be the truth about everything.", "word_count": 13, "label": "", "sentence_structure": "Subject鈥搗erb鈥揷omplement", "subjective_objective": {"answer": "Subjective", "rationale": "Because \"often\" and \"as if everything wasn't real\" are subjective feelings of the patient."}, "sentence_sentiment": {"answer": "Negative", "rationale": "Because \"All is not true\" in the entry expresses a skepticism and a negative attitude."}, "language_style": {"answer": "Calm and direct", "rationale": "The entry is simple and not used too much rhetorical or emotional rendering, and directly expresses the subject of asking the other party whether it feels less realistic and objective."}, "time_dimension": {"answer": "Present", "rationale": "The word \"often\" denotes a regular act, and the word \"is\" adopts the general current, meaning that the act not only has occurred in the past, but may still be ongoing. Thus, the temporal dimension of the article is now."}, "sentence_type": "Affirmative declarative sentence", "pos": {"nouns": {"Questionnaire Entry": 1, "Everything": 1}, "verbs": {"be": 1, "It looks like": 1, "Regular": 1, "Do it": 1}, "adjectives": {"Often": 1}, "adverbs": {"Really": 1}, "numerals": {"345锛?: 1}}}, {"content": "346. I have a habit of counting things that are not important, like utility poles on the road.", "word_count": 27, "label": "", "sentence_structure": "Subject鈥搗erb鈥搊bject", "subjective_objective": {"answer": "Subjective", "rationale": "Because \"likes to count something that is not important\" is the patient's personal habits and preferences, and it's subjective."}, "sentence_sentiment": {"answer": "Neutral", "rationale": "The entry is not an emotional one for describing behaviour."}, "language_style": {"answer": "Tactful and warm", "rationale": ""}, "time_dimension": {"answer": "Present", "rationale": ""}, "sentence_type": "Affirmative declarative sentence", "pos": {"nouns": {"Custom": 1, "Yeah": 1, "Points": 1, "It doesn't matter": 1, "Something": 2, "Elephant": 1, "On the way": 1, "Wire poles": 1}, "verbs": {"have": 1, "Yeah": 1, "Points": 1, "Wait": 1}, "adjectives": {"It doesn't matter": 1}, "numerals": {"346": 1, "Some": 1}, "classifiers": {"One": 1, "Wait": 1}, "pronouns": {"I": 1}}}, {"content": "347. I did not really want to hurt my enemies.", "word_count": 12, "label": "", "sentence_structure": "Subject鈥搗erb鈥搊bject", "subjective_objective": {"answer": "Subjective", "rationale": "Because \"no real intention to hurt\" is the patient's subjective view."}, "sentence_sentiment": {"answer": "Neutral", "rationale": "Since the entry merely stated a fact, it did not express any positive or negative emotional tendencies."}, "language_style": {"answer": "Tactful and warm", "rationale": "The language style of this entry is euphemism. It uses the phrase \"no real desire to hurt\" to express respect and understanding of the other and to try to ease tensions. At the same time, the entire sentence structure is relatively easy to reach and resonate with the interviewee."}, "time_dimension": {"answer": "Present", "rationale": "Now the entries \"no,\" \"real want\" and \"my enemy\" are all descriptions of the current situation, so the temporal dimension is now judged."}, "sentence_type": "Negative declarative sentence", "pos": {"nouns": {"The enemy": 1, "I": 1}, "verbs": {"No, I'm not": 1, "Yes": 1, "Hurt": 1, "have": 1}, "adjectives": {"Really": 1}, "adverbs": {"Really": 1}, "numerals": {"347": 1}}}, {"content": "348. I am protected from those who are too close to me.", "word_count": 13, "label": "", "sentence_structure": "Subject鈥搗erb鈥搊bject", "subjective_objective": {"answer": "Subjective", "rationale": "Because \"I'm on the embankment\" is patient's subjective attitude and reaction."}, "sentence_sentiment": {"answer": "Negative", "rationale": "The expression \"defence\" has a negative emotional effect because of the vigilance and vigilance of the term."}, "language_style": {"answer": "Calm and direct", "rationale": "The entry uses a direct language style to ask the respondents about the level of vigilance of the person who is too close, without too much emotional rendering or adoration, and is succinctly expressed."}, "time_dimension": {"answer": "Present", "rationale": "The word \"I\" in \"I'm\" means the first person's number one, meaning that it is a description of his current state, that \"Wire\" means vigilance, caution, that \"those who are too close to me\" is the object, and that the subject's concern is what the subject is concerned with. The entry therefore asks whether the person who was tested is now alert or not to some."}, "sentence_type": "Negative declarative sentence", "pos": {"nouns": {"person": 1, "Quarter": 1}, "verbs": {"to": 1, "Too close": 1, "be": 1}, "adjectives": {"of": 2}, "adverbs": {"Those": 1, "That's too much": 1}, "numerals": {"348": 1}, "pronouns": {"I": 1}}}, {"content": "349. I have some strange and special thoughts.", "word_count": 12, "label": "", "sentence_structure": "Subject鈥搗erb鈥揷omplement", "subjective_objective": {"answer": "Subjective", "rationale": "Because \"unusual and special thoughts\" are a subjective feeling and idea of a human being, they are viewed from a subjective angle."}, "sentence_sentiment": {"answer": "Neutral", "rationale": "Because the entry does not describe the nature or the outcome of these \"unusual and extraordinary thoughts\", nor does it express positive or negative emotional expressions."}, "language_style": {"answer": "Tactful and warm", "rationale": ""}, "time_dimension": {"answer": "Present", "rationale": ""}, "sentence_type": "Affirmative declarative sentence", "pos": {"nouns": {"Questionnaire Entry": 1, "Thinking": 1}, "verbs": {"have": 1}, "adjectives": {"Strange": 1, "Special": 1}, "numerals": {"349锛?: 1}, "classifiers": {"Some": 1}, "pronouns": {"I": 1}}}, {"content": "350. When I was alone, I heard strange voices.", "word_count": 15, "label": "", "sentence_structure": "Subject鈥搗erb鈥揷omplement", "subjective_objective": {"answer": "Subjective", "rationale": "Because hearing strange voices is a personal feeling and experience, a subjective feeling of the patient."}, "sentence_sentiment": {"answer": "Neutral", "rationale": "Because the entry does not have any emotional color."}, "language_style": {"answer": "Calm and direct", "rationale": "The entry raises the question in a direct manner, without excessive ado or emotional, with a flat and objective tone, and is of a calm and direct language style."}, "time_dimension": {"answer": "Past", "rationale": "The entry is an inquiry into the testee's past behaviour or mental state, and therefore is based on the \"old\"."}, "sentence_type": "Affirmative declarative sentence", "pos": {"nouns": {"Time": 1, "Sound": 1}, "verbs": {"Yes": 1, "Alone": 1}, "adjectives": {"Strange": 1}, "numerals": {"350": 1}, "pronouns": {"I": 1}}}, {"content": "351. When I have to leave the house for a short time, I feel a little bit distracted.", "word_count": 21, "label": "", "sentence_structure": "Subject鈥揷opula鈥損redicative", "subjective_objective": {"answer": "Subjective", "rationale": "Because \"frightful\" is the patient's subjective feeling and experience."}, "sentence_sentiment": {"answer": "Negative", "rationale": "Because the mood for \"frightful\" in the entries is negative."}, "language_style": {"answer": "Tactful and warm", "rationale": "Warm and warm."}, "time_dimension": {"answer": "Present", "rationale": "\"When I have to leave the house for a short time\" describes a specific point or period of time, namely, \"short time out of the house,\" which is not spoken in terms of past or future. Therefore, the time dimension of this questionnaire entry should be now."}, "sentence_type": "Affirmative declarative sentence", "pos": {"nouns": {"Questionnaire": 1, "Entry": 1, "Time": 1, "I": 1, "Family": 1}, "verbs": {"Yes": 2, "I feel": 1, "Out": 1, "Short-term": 1}, "adjectives": {"I'm not sure I can": 1}, "numerals": {"351": 1}, "classifiers": {"individual": 1}, "pronouns": {"When": 1, "I have to": 1}}}, {"content": "352. I fear something or someone, although I know they will not harm me.", "word_count": 22, "label": "", "sentence_structure": "Subject鈥揷opula鈥損redicative", "subjective_objective": {"answer": "Subjective", "rationale": "Because \"I'm afraid\" is a subjective feeling and attitude, and even if the other side is not actually threatened, it's still the fear that comes from the patient himself."}, "sentence_sentiment": {"answer": "Negative", "rationale": "Because the word \"fear\" in the entry is an expression of a negative sentiment."}, "language_style": {"answer": "Tactful and warm", "rationale": "This is a more humane way of getting people to resonate with each other and helps to create a good atmosphere of communication."}, "time_dimension": {"answer": "Present", "rationale": "\"I fear\" suggests that it is a state of current feeling, and that \"I know that they will not harm me\" suggests that this fear stems from a cognitive contradiction that the testee realizes that these things or people do not pose a real threat to himself, but is still emotionally afraid. So this entry describes the current psychological state of the testee."}, "sentence_type": "Negative declarative sentence", "pos": {"nouns": {"Something": 1, "person": 1, "Hurt": 1}, "verbs": {"Yes": 2, "I know": 1, "be": 1}, "adverbs": {"Some": 1, "Although": 1}, "numerals": {"352": 1}, "pronouns": {"I": 1}}}, {"content": "If someone already lives in the house and talks together, I am not afraid to go in there alone.", "word_count": 32, "label": "", "sentence_structure": "Subject鈥搗erb鈥搊bject", "subjective_objective": {"answer": "Subjective", "rationale": "It is the patient ' s inner feelings and attitudes that are examined, not the objective circumstances or facts."}, "sentence_sentiment": {"answer": "Positive", "rationale": "Because the entry describes behaviour that does not fear being alone in places where others already gather, there is no negative or neutral emotional colour."}, "language_style": {"answer": "Calm and direct", "rationale": "The entry describes a situation in simple narratives and clearly expresses an attitude that does not fear facing the population alone. It does not use too much qualifier or emotional expression and is therefore a calm and straightforward language style."}, "time_dimension": {"answer": "Present", "rationale": ""}, "sentence_type": "Affirmative declarative sentence", "pos": {"nouns": {"The house": 1, "person": 2, "Talk": 1, "A little": 1, "Not afraid": 1}, "verbs": {"Already": 1, "Get together": 1, "Yes": 1, "Get in": 1}, "adverbs": {"If": 1, "And then": 1, "A little": 1}, "numerals": {"353": 1, "2": 1}, "pronouns": {"I": 1}}}, {"content": "354. I am afraid of using knives or anything sharp.", "word_count": 15, "label": "", "sentence_structure": "Subject鈥搗erb鈥搊bject", "subjective_objective": {"answer": "Subjective", "rationale": "Because fear is the patient's own feelings and experiences, and it's a subjective approach."}, "sentence_sentiment": {"answer": "Negative", "rationale": "Because the word \"fear\" in the entry is an expression of negative emotional attitudes."}, "language_style": {"answer": "Tactful and warm", "rationale": "The basis for the judgement: 1. The question is presented in a more gentle and friendly way, \"you fear\" which is more likely to resonate and respond to the respondents. 2. There is no too direct or hard-line expression, such as \"are you afraid of using knives or sharp objects?\" This expression may put pressure on the respondents. 3. The entries are simple and clear, do not have too much rhetoric or emotional publicity to facilitate their understanding and answer."}, "time_dimension": {"answer": "Past", "rationale": "The word \"fear\" is an emotional fear and anxiety that may have been experienced in the past or a fear for the future. Thus, \"fear\" indicates that the entry is about the past behaviour or feelings of the person being tested, which is part of the past."}, "sentence_type": "Negative declarative sentence", "pos": {"nouns": {"Questionnaire": 1, "Entry": 1, "Something": 1}, "verbs": {"Scared": 1, "Use": 1, "Pointy": 1}, "adjectives": {"354锛?: 1, "Knife": 1}, "adverbs": {"or": 1, "Any": 1}, "numerals": {"354": 1}}}, {"content": "Sometimes I like to torture the people I love.", "word_count": 12, "label": "", "sentence_structure": "Subject鈥搗erb鈥搊bject", "subjective_objective": {"answer": "Subjective", "rationale": "The preference is the patient ' s subjective attitude and torture is also the patient ' s subjective behaviour."}, "sentence_sentiment": {"answer": "Negative", "rationale": "Because the word \"torture\" in the entry is a derogatory term that represents a negative act or attitude."}, "language_style": {"answer": "Tactful and warm", "rationale": "This is a relatively euphemistic way of expressing whether the subject of the inquiry has a relationship with someone he loves."}, "time_dimension": {"answer": "Present", "rationale": "The entry is an inquiry into the behaviour or orientation of the person being tested, the keyword being \"like\" and \"torture\" and both of which indicate an act or behaviour. Although there is no clear time point, given the general current pattern of the verb, it is possible to assume that the act may be present or past customary. Thus, the temporal dimension of the article is to judge past and present."}, "sentence_type": "Affirmative declarative sentence", "pos": {"nouns": {"355": 1, "person": 1, "Love": 1}, "verbs": {"Yeah": 1, "Torture": 1}, "adverbs": {"Sometimes": 1}, "numerals": {"Three-five-five": 1}}}, {"content": "356. I seem to be less able to concentrate than others.", "word_count": 14, "label": "", "sentence_structure": "Subject鈥搗erb鈥揷omplement", "subjective_objective": {"answer": "Subjective", "rationale": "Because \"hard\" is a subjective term, which expresses patients' subjective feelings and self-assessments."}, "sentence_sentiment": {"answer": "Negative", "rationale": "Because the expression \"difficult to concentrate\" is negative."}, "language_style": {"answer": "Calm and direct", "rationale": "The language style of the entry is relatively simple, there is no excessive emotional modification or rhetorical technique, and the tone is more calm and objective, directly expressing the subject of asking the other side whether it is difficult to focus. \""}, "time_dimension": {"answer": "Present", "rationale": "The entry is an inquiry into the subjective perception and assessment of the ability of the testee to concentrate. In terms of time dimension, the sentence does not clearly indicate past, present or future circumstances, and therefore does not allow it to judge the temporal attributes. But the word \"seemingly\" expresses a subjective feeling that may suggest that the testee's perception may have existed for some time, and therefore can be considered an assessment of the past."}, "sentence_type": "Negative declarative sentence", "pos": {"nouns": {"Questionnaire": 1, "Entry": 1, "Focus": 1, "Attention": 1}, "verbs": {"Seems like": 1, "not": 1, "Don't": 1, "on": 1, "It's harder": 1}, "adjectives": {"others": 1}, "adverbs": {"More": 1}, "numerals": {"356锛?: 1}, "pronouns": {"I": 1}}}, {"content": "357. On several occasions I gave up what was being done because I felt too bad about my abilities.", "word_count": 25, "label": "", "sentence_structure": "Subject鈥搗erb鈥揷omplement", "subjective_objective": {"answer": "Subjective", "rationale": "Because feeling that they are less capable is the subjective feeling and evaluation of the patient."}, "sentence_sentiment": {"answer": "Negative", "rationale": "Because the \"release what's going on\" act in the entry is negative."}, "language_style": {"answer": "Tactful and warm", "rationale": "The language style of the entry is euphemism. It expresses the interviewee鈥檚 concerns about his abilities in a first person鈥檚 tone, and it is more apt and understandable to use the word 鈥渂ecause鈥?to channel them. At the same time, the entry is not too much criticism or criticism, but rather encourages the interviewee to look at his problems in a more moderate way."}, "time_dimension": {"answer": "Present", "rationale": "Now, \"a few times\" in the entry suggests that this is a description of what happened in the past, and that \"fearing too bad\" points to the emotional state and feelings of the time. So the time dimension of the article is now."}, "sentence_type": "Affirmative declarative sentence", "pos": {"nouns": {"Number of times": 1, "Yes": 1, "Strength": 1}, "verbs": {"Abandon": 1, "Do it": 1, "Sensor": 1, "be": 1, "have": 1, "Bad": 1}, "adjectives": {"Okay": 1, "Your own": 1, "Too much": 1, "It's not working": 1}, "adverbs": {"very": 1}, "numerals": {"357": 1, "How many": 1}, "classifiers": {"Number of times": 1}, "pronouns": {"I": 1}}}, {"content": "358. I have some bad words in my head that are often terrible, but I cannot get rid of them.", "word_count": 26, "label": "", "sentence_structure": "Subject鈥搗erb鈥揷omplement", "subjective_objective": {"answer": "Subjective", "rationale": "Because the words \"bad\" and \"horrible\" are the subjective feelings and thoughts inside the patient. Although they may be related to objective facts, the patient鈥檚 response and evaluation are subjective."}, "sentence_sentiment": {"answer": "Negative", "rationale": "Because words like \"bad\" and \"horrible\" have negative emotional connotations."}, "language_style": {"answer": "Tactful and warm", "rationale": "The entry asks the interviewees in a more moderate way whether they are experiencing mental distress and tries to create a climate of goodwill and understanding. At the same time, the terms are relatively euphemism and inaccuracy, without directly identifying the problems or symptoms of the interviewees and avoiding discomfort."}, "time_dimension": {"answer": "Present", "rationale": ""}, "sentence_type": "Affirmative declarative sentence", "pos": {"nouns": {"Brain": 1, "Come on": 1, "Some": 1, "Bad": 2, "Words": 1, "Horrible": 1, "They": 1}, "verbs": {"Yes": 1, "be": 1, "Cannot": 1, "Get rid of it": 1}, "adjectives": {"Often": 1}, "adverbs": {"And": 1, "But": 1}, "numerals": {"358": 1, "2": 1}, "pronouns": {"I": 1}}}, {"content": "359. I was sometimes disturbed by the inconsequential thoughts that had been bothering me for many days.", "word_count": 24, "label": "", "sentence_structure": "Subject鈥搗erb鈥揷omplement", "subjective_objective": {"answer": "Subjective", "rationale": "This \"touch\" is a subjective experience and feeling of the patient, and it is not an objective fact or act."}, "sentence_sentiment": {"answer": "Negative", "rationale": "Because \"touch\" and \"disturbing\" in the entries are negative emotional experiences."}, "language_style": {"answer": "Tactful and warm", "rationale": "The language style of the entry is more euphemistic and gentle, using an invitation to ask questions, and not too direct or harsh. This expression is more likely to give the respondents the sense of respect and understanding, thus becoming more willing to participate in the investigation and to answer questions honestly. At the same time, the entry uses a more honest and objective description of the phenomenon, avoiding emotional or exaggerated expressions, making the questionnaire more professional and reliable."}, "time_dimension": {"answer": "Past", "rationale": ""}, "sentence_type": "Affirmative declarative sentence", "pos": {"nouns": {"Thinking": 1, "I": 1, "A few days": 1}, "verbs": {"- A entanglement": 1, "鈾?And I'm gonna be 鈾?: 1, "I feel": 1}, "adjectives": {"It doesn't matter": 1, "For days": 1, "Unsettled": 1}, "adverbs": {"Sometimes": 1}, "numerals": {"359": 1}}}, {"content": "360. Almost every day something happens that scares me.", "word_count": 15, "label": "", "sentence_structure": "Subject鈥揷opula鈥損redicative", "subjective_objective": {"answer": "Subjective", "rationale": "Fear is a subjective feeling and experience, and it is the patient ' s subjective assessment and interpretation of the matter."}, "sentence_sentiment": {"answer": "Negative", "rationale": "Because \"fear\" is negative."}, "language_style": {"answer": "Calm and direct", "rationale": "The entry directly expresses a question about whether the other party is often afraid of the subject, is more objective and is not based on excessive rhetoric or emotional rendering."}, "time_dimension": {"answer": "Present", "rationale": "Now the keywords \"almost every day\" and \"all\" indicate that it is a statement of the present, so it is judged to be the present. And, \"What happens to me is what scares me\" also refers to the present state or experience, not the past or the future."}, "sentence_type": "Affirmative declarative sentence", "pos": {"nouns": {"Things": 1, "Come on": 1}, "verbs": {"have": 1, "鈾?And I'm gonna be 鈾?: 1}, "adjectives": {"Scared": 1}, "adverbs": {"Almost": 1, "Chile": 1}, "numerals": {"360锛?: 1}}}, {"content": "361. I always take things seriously.", "word_count": 11, "label": "", "sentence_structure": "Subject鈥揷opula鈥損redicative", "subjective_objective": {"answer": "Subjective", "rationale": "Because \"always\" and \"seriously\" are the personal feelings and attitudes of the patient, the entry is a subjective thought."}, "sentence_sentiment": {"answer": "Negative", "rationale": "Because \" always takes things seriously\" is negative."}, "language_style": {"answer": "Calm and direct", "rationale": "The language style of the entry is calm and straightforward. It uses a simple and clear language to ask whether the respondents often see things more seriously than they are, without too much emotional or accommodative, and in a more objective way. This language style facilitates the acquisition of accurate data and information."}, "time_dimension": {"answer": "Present", "rationale": "The entry asks the testee about the perception and attitude of the subject, and does not have a clear temporal dimension, and therefore does not allow for a judgement about past, present or future behaviour or mindset."}, "sentence_type": "Affirmative declarative sentence", "pos": {"verbs": {"Yeah": 1, "Look": 1}, "adjectives": {"It's worse": 1}, "adverbs": {"Total": 1}, "numerals": {"361": 1}}}, {"content": "I am more sensitive than most people.", "word_count": 9, "label": "", "sentence_structure": "Subject鈥揷opula鈥損redicative", "subjective_objective": {"answer": "Subjective", "rationale": "Because \"sensitization\" is a subjective feeling and is not objectively measured or validated. It depends on the individual's perception, cognitive and emotional experience."}, "sentence_sentiment": {"answer": "Negative", "rationale": "Because the expression \"more sensitive than most people\" in the entries is negative."}, "language_style": {"answer": "Tactful and warm", "rationale": "The entry is more adept and interactive when it is spoken in a gentle and inviting manner, rather than by direct statements or orders."}, "time_dimension": {"answer": "Present", "rationale": "The entry uses descriptive language like \"I am more sensitive than most people\" and does not specify the time dimension, so it is not possible to judge accurately whether it is past, present or future. But since this comparison is usually based on a person's self-perception and feelings, it can be considered as a representation of the current state."}, "sentence_type": "Affirmative declarative sentence", "pos": {"nouns": {"I": 1, "Most of them": 1, "person": 1}, "verbs": {"- Yeah": 1, "More": 1, "Sensitive": 1}, "adverbs": {"More": 1}, "numerals": {"362": 1, "More": 1}, "classifiers": {"individual": 1}}}, {"content": "Sometimes I like to be tortured by the people I love.", "word_count": 15, "label": "", "sentence_structure": "Subject鈥搗erb鈥搊bject", "subjective_objective": {"answer": "Subjective", "rationale": "Because it's the patient's subjective attitude."}, "sentence_sentiment": {"answer": "Negative", "rationale": "Because \"torture\" is a negative emotional experience."}, "language_style": {"answer": "Tactful and warm", "rationale": "The entry expresses in a more euphemistic way the emotional tendency that a person may in some cases prefer to be tortured by someone he loves. This expression is more implicit and indirect, and does not directly express or order the other person to accept or change his behaviour."}, "time_dimension": {"answer": "Past", "rationale": "The entry describes the individual's past feelings and experiences, and the keyword \"sometimes\" indicates that it is a past act or state, so it is judged to be the past."}, "sentence_type": "Affirmative declarative sentence", "pos": {"nouns": {"Torture": 1, "person": 1}, "verbs": {"Yeah": 1, "Yes": 1}, "adjectives": {"My love": 1}, "adverbs": {"Sometimes": 1}, "numerals": {"363": 1}, "pronouns": {"I": 1}}}, {"content": "364. I was talked about in insulting and indecent terms.", "word_count": 15, "label": "", "sentence_structure": "Subject鈥搗erb鈥搊bject", "subjective_objective": {"answer": "Subjective", "rationale": "Because the sentence describes the patient ' s feelings of others about his or her own words and is a subjective one."}, "sentence_sentiment": {"answer": "Negative", "rationale": "Because the \"Stigmatizing and Demeaning Words\" in the entry is negative."}, "language_style": {"answer": "Calm and direct", "rationale": "The entry is more directly written, without too much rhetoric or emotional overtones, but simply expresses the fact that the other side was attacked by insulting remarks. Such expressions are more appropriate for objective, rational analysis and discussion."}, "time_dimension": {"answer": "Present", "rationale": "The entry describes what is going on, and the keywords are \"persons,\" \"insulting and indecent words\" and \"debate\" which point to the actions or state of the current process."}, "sentence_type": "Negative declarative sentence", "pos": {"nouns": {"person": 1, "Word": 1, "Arguments": 1}, "verbs": {"Use": 1, "Say it": 1, "Insulting": 1, "Naughty": 1}, "numerals": {"364锛?: 1}, "pronouns": {"I": 1}}}, {"content": "365. I'm always upset to be in the house.", "word_count": 10, "label": "", "sentence_structure": "Subject鈥搗erb鈥揷omplement", "subjective_objective": {"answer": "Subjective", "rationale": "Because it's a subjective experience."}, "sentence_sentiment": {"answer": "Negative", "rationale": "Because the \"always troubled\" feeling in the entries is negative."}, "language_style": {"answer": "Tactful and warm", "rationale": ""}, "time_dimension": {"answer": "Present", "rationale": ""}, "sentence_type": "Negative declarative sentence", "pos": {"nouns": {"365": 1, "I": 1, "In the house": 1, "Unsettled": 1}, "verbs": {"Stay": 1, "I feel": 1}, "adverbs": {"Total": 1}}}, {"content": "366. Even with people, I often feel alone.", "word_count": 16, "label": "", "sentence_structure": "Subject鈥揷opula鈥損redicative", "subjective_objective": {"answer": "Subjective", "rationale": "Because feeling alone is a subjective feeling, reflecting the emotional state of the patient."}, "sentence_sentiment": {"answer": "Negative", "rationale": "Because the \"often feeling lonely\" expression of negative emotional attitudes is in the entry."}, "language_style": {"answer": "Calm and direct", "rationale": ""}, "time_dimension": {"answer": "Present", "rationale": "Now the entry describes the current state of the testee's feeling, which is often \"sloof\" 鈥?a recurrent feeling. Although it does not specify the specific point of time, it is the subjective feeling of the testee, so the temporal dimension is now judged."}, "sentence_type": "Affirmative declarative sentence", "pos": {"nouns": {"Questionnaire": 1, "Entry": 1, "person": 1, "Alone": 1}, "verbs": {"in": 1, "Together": 1, "I feel": 1}, "adverbs": {"Regular": 1}, "numerals": {"366": 1}, "pronouns": {"I": 1}}}, {"content": "367. I am not particularly shy.", "word_count": 9, "label": "", "sentence_structure": "Subject鈥揷opula鈥損redicative", "subjective_objective": {"answer": "Subjective", "rationale": "Because \"discretion\" and \"screety\" are subjective feelings and attitudes of patients."}, "sentence_sentiment": {"answer": "Neutral", "rationale": "Because the entry merely stated a fact, it did not express a positive or negative emotional colour."}, "language_style": {"answer": "Calm and direct", "rationale": "It asks the other side simply and clearly whether it has any shyness, and there is no emotional or vague element. Such a formulation usually gives a more objective, professional and direct impression."}, "time_dimension": {"answer": "Present", "rationale": "The keyword \"not particularly shy\" indicates that the testee is not unduly shy or eccentric in his current attitude and behaviour, and that this description occurs now."}, "sentence_type": "Negative declarative sentence", "pos": {"verbs": {"not": 1, "Special": 1, "Shy": 1, "Rule of law": 1}, "adjectives": {"Shy": 1, "Rule of law": 1}, "adverbs": {"and": 1, "not": 1, "Special": 1, "Don't": 1}, "numerals": {"367锛?: 1}}}, {"content": "Sometimes my mind seems more insensitive than usual.", "word_count": 13, "label": "", "sentence_structure": "Subject鈥揷opula鈥損redicative", "subjective_objective": {"answer": "Subjective", "rationale": "Because retardation is a matter of self-perception and subjective observation."}, "sentence_sentiment": {"answer": "Negative", "rationale": "Because the description \"dick mind\" is negative."}, "language_style": {"answer": "Tactful and warm", "rationale": "The entry is based on a soft, inquisitive tone that is not directly stated or ordered. This expression is more personal and interactive, bringing the respondents closer to the respondents, making them more willing to participate and provide real feedback. At the same time, the article is more objective, does not have too many rhetorical or emotional expressions, and makes them comfortable and relaxed, making it easier for the respondents to express their feelings and thoughts in a true way."}, "time_dimension": {"answer": "Past", "rationale": "The entry describes a state that may occur at any time, but is here asking about the testee ' s past experience and feelings, and therefore is part of the temporal dimension of the past."}, "sentence_type": "Affirmative declarative sentence", "pos": {"nouns": {"Brain": 1, "Time": 1, "Normal": 1}, "verbs": {"Seems like": 1, "- Yeah": 1, "Slow": 1}, "adjectives": {"Slow": 1}, "adverbs": {"Sometimes": 1}, "numerals": {"368锛?: 1}}}, {"content": "369. In social settings, I sit alone, or with another person, rather than in the crowd.", "word_count": 33, "label": "", "sentence_structure": "Subject鈥搗erb鈥搊bject", "subjective_objective": {"answer": "Subjective", "rationale": "This is a description of patients ' subjective feelings and behaviour in social settings and examines their social fear tendencies."}, "sentence_sentiment": {"answer": "Negative", "rationale": "Because the entry \"I'm sitting alone, or just sitting with another person\" is negative."}, "language_style": {"answer": "Calm and direct", "rationale": "The entry raises questions in a direct manner, without excessive rhetorical or emotional rendering, and is more objective. At the same time, the sentence clearly indicates the behaviour of the person questioned in the social setting, making it easy to understand and answer questions."}, "time_dimension": {"answer": "Present", "rationale": ""}, "sentence_type": "Affirmative declarative sentence", "pos": {"nouns": {"Socially": 1, "Alone": 2, "The other guy": 1, "Population": 1}, "verbs": {"Mostly": 1, "Sit down": 1, "Call": 1, "Go on": 1}, "adverbs": {"I'm not": 1}, "numerals": {"369": 1}, "classifiers": {"individual": 2}, "pronouns": {"I": 1}}}, {"content": "370. I am often disappointed.", "word_count": 7, "label": "", "sentence_structure": "Subject鈥搗erb鈥揷omplement", "subjective_objective": {"answer": "Subjective", "rationale": "Because the subject concerns people ' s feelings and perceptions, it is examined from the subjective point of view of the patient."}, "sentence_sentiment": {"answer": "Negative", "rationale": "Because the emotional color of \"depression\" is negative."}, "language_style": {"answer": "Tactful and warm", "rationale": ""}, "time_dimension": {"answer": "Present", "rationale": ""}, "sentence_type": "Negative declarative sentence", "pos": {"nouns": {"person": 1, "Disappointment": 1}, "verbs": {"鈾?And I'm gonna be 鈾?: 1, "Disappointment": 1}, "numerals": {"370": 1}, "pronouns": {"I": 1}}}, {"content": "371. I like to go to prom.", "word_count": 8, "label": "", "sentence_structure": "Subject鈥揷opula鈥損redicative", "subjective_objective": {"answer": "Subjective", "rationale": "Because \"I love to go to prom\" is the patient's personal feeling and attitude, and it's subjective."}, "sentence_sentiment": {"answer": "Positive", "rationale": "Because the entry \"I love to go to prom\" expresses a positive attitude."}, "language_style": {"answer": "Tactful and warm", "rationale": "The entry uses a more relaxed, friendly tone to ask if the other party likes to go to the ball, rather than asking \"Do you like to go to the ball\", which is more personal and interactive."}, "time_dimension": {"answer": "Present", "rationale": ""}, "sentence_type": "Affirmative declarative sentence", "pos": {"nouns": {"The ball": 1}, "verbs": {"Yeah": 1, "Participation": 1}, "adjectives": {"very": 1}, "numerals": {"371": 1}}}, {"content": "372. At times, digging is difficult and difficult to overcome.", "word_count": 14, "label": "", "sentence_structure": "Subject鈥搗erb鈥揷omplement", "subjective_objective": {"answer": "Subjective", "rationale": "It is subjective because it is difficult to overcome what is inside and what is in the minds of one."}, "sentence_sentiment": {"answer": "Negative", "rationale": "Because the description \"difficult to overcome\" in the entry is negative."}, "language_style": {"answer": "Tactful and warm", "rationale": ""}, "time_dimension": {"answer": "Past", "rationale": "The words \"sometimes\", \"difficult\" and \"insolvable\" in this entry describe past experiences or feelings, and therefore the dimension of time is the past."}, "sentence_type": "Exclamatory sentence", "pos": {"nouns": {"Time": 1, "Dig": 1, "Regular": 1, "Tired": 1, "Hard": 1, "Heavy": 1, "grams": 1, "- Yeah": 1}, "verbs": {"I feel": 1, "have": 1, "Yes": 1}, "adjectives": {"Thick": 1, "None": 1, "FL": 1}, "adverbs": {"Regular": 1}, "numerals": {"372": 1}}}, {"content": "I often thought, \"I wish I could be a child again.\"", "word_count": 16, "label": "", "sentence_structure": "Subject鈥損redicate", "subjective_objective": {"answer": "Subjective", "rationale": "Because \"I wish I could be a child again\" is the idea and feeling inside the patient, the subjective perspective of the patient."}, "sentence_sentiment": {"answer": "Negative", "rationale": "Because the entries express a feeling of dissatisfaction with the status quo, they want to go back to the past, with a negative emotional tone."}, "language_style": {"answer": "Tactful and warm", "rationale": "The language style of the entry is euphemistic and emotional, and by describing the past memories and aspirations of the respondents, the respondents are guided to their childhood and thus understand their mentalities and needs in greater depth. This expression is more intimate and resonant, and contributes to the effectiveness of the questionnaire."}, "time_dimension": {"answer": "Present", "rationale": ""}, "sentence_type": "Exclamatory sentence", "pos": {"nouns": {"I": 2, "Son": 1}, "verbs": {"I always do": 1, "Yes": 1, "Again": 1}, "adverbs": {"Regular": 1, "Okay": 1}, "numerals": {"373": 1}}}, {"content": "374. If I had the opportunity, I would have done something that would have been of great benefit to the world.", "word_count": 21, "label": "", "sentence_structure": "Subject鈥損redicate", "subjective_objective": {"answer": "Subjective", "rationale": "This is because patients are subjectively assessed and expected of their abilities."}, "sentence_sentiment": {"answer": "Positive", "rationale": "Because the entries express a positive emotional feeling of recognition and confidence in their abilities and future possibilities."}, "language_style": {"answer": "Tactful and warm", "rationale": "The entry is more euphemistic. First, it uses the expression of assumptions and possibilities, without directly asking or ordering each other to act. Second, the phrase \"better for the world\" is also very positive and positive, and it is easier to resonate with the respondents."}, "time_dimension": {"answer": "Future", "rationale": "The entry contains ideas and expectations for the future, and \"if you give me a chance\" means that it is a hypothetical condition that only under certain conditions can happen. And \"must do something that will be of great benefit to the world\" expresses the testee's confidence and expectation for the future, pointing to what has not yet happened and what may happen in the future."}, "sentence_type": "Affirmative declarative sentence", "pos": {"nouns": {"Opportunities": 1, "World": 1}, "verbs": {"Here": 1, "Do it": 1, "Yes": 2}, "adjectives": {"I'm sure": 1, "- Yeah": 1, "Benefits": 1}, "numerals": {"374锛?: 1}, "pronouns": {"I": 1}}}, {"content": "375. I often meet so-called experts, who are not more sophisticated than me.", "word_count": 20, "label": "", "sentence_structure": "Subject鈥搗erb鈥搊bject", "subjective_objective": {"answer": "Subjective", "rationale": "Because the \"smart\" and \"specialist\" evaluations are subjective decisions of patients."}, "sentence_sentiment": {"answer": "Negative", "rationale": "Because the \"so-called expert\" assessment in the entry is demeaning and emotional."}, "language_style": {"answer": "Calm and direct", "rationale": ""}, "time_dimension": {"answer": "Present", "rationale": "The entry described a regular situation, which \"I often meet so-called experts,\" which suggests that it has happened in the past and may continue. The \"not more brilliant\" assessment is the perception and feeling of these experts, and therefore also the behaviour or mentality of the past."}, "sentence_type": "Exclamatory sentence", "pos": {"nouns": {"Experts": 2, "I": 2}, "verbs": {"I met him": 1, "be": 1, "No, it's not like that": 1}, "adjectives": {"So-called": 1, "Smart": 1}, "adverbs": {"Often": 1}, "numerals": {"375": 1}}}, {"content": "When I heard that the people I knew had succeeded, I felt I had failed.", "word_count": 22, "label": "", "sentence_structure": "Subject鈥揷opula鈥損redicative", "subjective_objective": {"answer": "Subjective", "rationale": "Because \"think\" represents the patient's subjective feelings and thoughts."}, "sentence_sentiment": {"answer": "Negative", "rationale": "Because the \"I feel like I'm failing\" attitude in the entry is negative."}, "language_style": {"answer": "Tactful and warm", "rationale": "The entry uses a more euphemistic approach to express a more negative sentiment that when hearing someone else who is familiar with success, it creates a sense of self-failing. It is more emotional, less stressful or uncomfortable for the interviewee, and more conducive to real and objective feedback."}, "time_dimension": {"answer": "Present", "rationale": "I heard that the \"me\" and \"just\" pattern suggests that it is a subjective feeling, while \"hearing\" suggests that this is happening recently. So, the entry is asking about the person who is being tested for behavior or mentalities."}, "sentence_type": "Negative declarative sentence", "pos": {"nouns": {"Questionnaire": 1, "Entry": 1, "person": 2, "Success": 1, "self": 1, "Failed": 1}, "verbs": {"I heard": 1, "I think": 1, "It failed": 1, "It worked": 1}, "adverbs": {"When": 1}, "numerals": {"376锛?: 1}, "pronouns": {"I": 2}}}, {"content": "377. I will certainly be a good leader of the people if I have the opportunity.", "word_count": 19, "label": "", "sentence_structure": "Subject鈥搗erb鈥搊bject", "subjective_objective": {"answer": "Subjective", "rationale": "Because \"must\" and \"can be\" are the subjective judgment and expectations of the patient."}, "sentence_sentiment": {"answer": "Positive", "rationale": "Because the phrase \"must be a good leader of the people\" in the entry expresses positive and positive feelings."}, "language_style": {"answer": "Tactful and warm", "rationale": "The language style of the entry is more euphemistic, using hypothetical expressions like \"if there is a chance\", which do not directly affirm that they will be good leaders, but express their expectations and beliefs in a relatively gentle and modest manner. This is more acceptable and can better trigger the resonance and reflection of interlocutors."}, "time_dimension": {"answer": "Future", "rationale": "The entry expresses the vision and expectations of the testee for the future, and the key words are \"if you have the chance\" and \"if you can.\" They all point to what is not yet happening and what may happen in the future. And \"good leaders of the people\" is also an expectation for the future. So the time dimension of the entry is the future."}, "sentence_type": "Affirmative declarative sentence", "pos": {"nouns": {"Opportunities": 1, "People": 1, "Leader": 1}, "verbs": {"Be": 1, "have": 1}, "adjectives": {"Okay": 1}, "numerals": {"377": 1}, "pronouns": {"I": 1}}}, {"content": "I am embarrassed by the dirty story.", "word_count": 13, "label": "", "sentence_structure": "Subject鈥搗erb鈥揷omplement", "subjective_objective": {"answer": "Subjective", "rationale": "Because this statement reflects the individual sense of shame and discomfort of the patient, it is subjective."}, "sentence_sentiment": {"answer": "Negative", "rationale": "Because \"sorry\" means that emotional reactions to dirty stories are negative."}, "language_style": {"answer": "Tactful and warm", "rationale": "The language style of the entry is euphemistic. It uses an indirect but emotional way to ask each other about the feelings of the dirty story, rather than directly speaking or ordering. This expression is more personal and interactive, making it easier for the interviewee to open his heart and share the real feeling."}, "time_dimension": {"answer": "Present", "rationale": ""}, "sentence_type": "Affirmative declarative sentence", "pos": {"nouns": {"Story": 1, "A dirty story": 1}, "verbs": {"鈾?And I'm gonna be 鈾?: 1, "I feel": 1}, "adjectives": {"Nasty": 1}, "adverbs": {"not": 1, "Okay": 1, "Meaning": 1}, "numerals": {"378锛?: 1, "I": 1}}}, {"content": "379. Generally, people demand that others respect their rights, while they rarely respect others ' rights.", "word_count": 33, "label": "", "sentence_structure": "Subject鈥搗erb鈥搊bject", "subjective_objective": {"answer": "Subjective", "rationale": "Because it expresses the patient ' s view of rights and respect in interpersonal relations, it is the patient ' s subjective attitude."}, "sentence_sentiment": {"answer": "Negative", "rationale": "Because the reference to \"people who demand that others respect their rights, and they rarely respect others' rights\" is selfish and unjust and therefore negative."}, "language_style": {"answer": "Calm and direct", "rationale": "The content of the entry is more straightforward, without excessive rhetorical or emotional rendering. 2. The statement is simple and clear, without complex sentence structure and redundant language. 3. It is more objective in tone and does not deliberately emphasize or denigrate the views of either side."}, "time_dimension": {"answer": "Present", "rationale": ""}, "sentence_type": "Affirmative declarative sentence", "pos": {"nouns": {"person": 2, "Rights": 2, "Powers": 1, "People": 1}, "verbs": {"Request": 1, "Respect": 4, "Comparison": 1, "More": 1, "And": 1, "But": 1, "Very few": 1, "Consider": 1}, "adverbs": {"General": 1, "Comparison": 1, "More": 1, "Very few": 1, "Yes": 1}, "numerals": {"379": 1, "2": 2, "1": 4}, "pronouns": {"they": 1, "others": 2, "of": 5}}}, {"content": "380. I always want to remember the good story and tell it to others.", "word_count": 15, "label": "", "sentence_structure": "Subject鈥搗erb鈥搊bject", "subjective_objective": {"answer": "Subjective", "rationale": "Because \"I always want\" is the patient's own subjective will and attitude."}, "sentence_sentiment": {"answer": "Positive", "rationale": "Because the entry \"I always want to remember the good story, tell the good story,\" is a positive act."}, "language_style": {"answer": "Tactful and warm", "rationale": "Warm and warm."}, "time_dimension": {"answer": "Present", "rationale": "\"Wanted to\" means a constant act and attitude, \"remember good stories, tell others\" is the expression of such behaviour. So the sentence describes the state and behaviour of the present."}, "sentence_type": "Affirmative declarative sentence", "pos": {"nouns": {"Story": 1, "person": 1}, "verbs": {"Yes": 1, "Remember": 1, "Speak": 1, "Here": 1}, "adverbs": {"Total": 1, "Okay": 1}, "numerals": {"380锛?: 1}, "pronouns": {"I": 1, "others": 1}}}, {"content": "381. I like to gamble with little.", "word_count": 11, "label": "", "sentence_structure": "Subject鈥搗erb鈥搊bject", "subjective_objective": {"answer": "Subjective", "rationale": "Because \"like\" is a subjective attitude or emotional expression, and gambling itself is a behavioral choice. So \"likes to win and lose\" is viewed from the patient's subjective perspective."}, "sentence_sentiment": {"answer": "Neutral", "rationale": "Because the entry merely describes a behavioural preference and does not involve emotional colour or moral evaluation."}, "language_style": {"answer": "Calm and direct", "rationale": "The language style of the entry is more straightforward, and it does not use too many rhetorical or emotional techniques. It asks the interviewee whether he likes to play small bets in a simple and direct way. This way makes the problem clearer and easier to understand."}, "time_dimension": {"answer": "Past", "rationale": "It is therefore not possible to classify it as any past, present or future situation."}, "sentence_type": "Affirmative declarative sentence", "pos": {"nouns": {"Win or lose": 1, "Gambling": 1}, "verbs": {"Yeah": 1, "Fuck": 1}, "adverbs": {"Not much": 1}, "numerals": {"381": 1}}}, {"content": "382. To be able to be with people, I like social activities.", "word_count": 17, "label": "", "sentence_structure": "Subject鈥搗erb鈥搊bject", "subjective_objective": {"answer": "Subjective", "rationale": "Because \"like\" is patient's subjective attitude."}, "sentence_sentiment": {"answer": "Positive", "rationale": "Because the \"like social activities\" in the entries is a positive attitude."}, "language_style": {"answer": "Tactful and warm", "rationale": "The language style of the entry is more euphemistic and moderate, and the expression of the other's love for social activities is expressed by using the active term \"like\" without direct orders or criticism, which makes it easier to generate resonance and positive responses from the interviewees."}, "time_dimension": {"answer": "Present", "rationale": "The entry describes a current act or attitude, and the keyword \"likes\" indicates that it is a subjective feeling, while \"can be with people\" suggests that the result of such behaviour or attitude is to interact with people. So \"now\" is the temporal dimension of the article."}, "sentence_type": "Affirmative declarative sentence", "pos": {"nouns": {"For": 1, "person": 1, "Together": 1, "Activities": 1}, "verbs": {"Yeah": 1, "Participation": 1}, "numerals": {"382锛?: 1}, "pronouns": {"Yeah": 1}}}, {"content": "I like the crowds.", "word_count": 10, "label": "", "sentence_structure": "Subject鈥搗erb鈥揷omplement", "subjective_objective": {"answer": "Subjective", "rationale": "Because I like being subjective."}, "sentence_sentiment": {"answer": "Positive", "rationale": "Because this attitude of a hospitable setting is positive."}, "language_style": {"answer": "Calm and direct", "rationale": "The language style of the entry is more direct, not expressed in too many qualifiers or emotional colours, but rather in a simple and clear statement of the interest of the subject in the scene."}, "time_dimension": {"answer": "Present", "rationale": "The keyword \"likes\" and the behavioural verbs that describe personal preferences and preferences indicate that the entry asks the testee about his current attitude and feelings."}, "sentence_type": "Affirmative declarative sentence", "pos": {"nouns": {"Population": 1, "It's hilarious": 1, "Event": 1}, "verbs": {"Yeah": 1}, "numerals": {"383": 1}}}, {"content": "When I was with a group of lively friends, my troubles disappeared.", "word_count": 24, "label": "", "sentence_structure": "Subject鈥揷opula鈥損redicative", "subjective_objective": {"answer": "Subjective", "rationale": "Because my troubles disappear because my own subjective feelings."}, "sentence_sentiment": {"answer": "Positive", "rationale": "Because the behavior in the entry \"with a bunch of lively friends\" is positive and has the positive result \"fears disappear.\""}, "language_style": {"answer": "Tactful and warm", "rationale": "The entry expresses in an invitation tone themes that ease the distress when dealing with friends, is more objective in tone, and is personal and interactive."}, "time_dimension": {"answer": "Present", "rationale": "The keyword \"with a bunch of lively friends\" and \"scattered\" indicate that the act is taking place or is taking place on a regular basis, so it's now."}, "sentence_type": "Affirmative declarative sentence", "pos": {"nouns": {"friends": 1, "Time": 1, "Trouble": 1}, "verbs": {"in": 1, "Disappear": 1}, "adjectives": {"Lively": 1}, "numerals": {"384": 1}, "classifiers": {"A bunch of them": 1}, "pronouns": {"I": 1}}}, {"content": "When people talk about my team, I never get involved.", "word_count": 18, "label": "", "sentence_structure": "Subject鈥搗erb鈥搊bject", "subjective_objective": {"answer": "Subjective", "rationale": "This is because the patient's assessment and description of his/her behaviour is subjective."}, "sentence_sentiment": {"answer": "Neutral", "rationale": "Because the entry merely presents an objective fact, there is no apparent emotional colour."}, "language_style": {"answer": "Tactful and warm", "rationale": "The language style of the entry is more moderate, using the expression \"never get involved\" to avoid direct accusations or criticism of people speaking out. This way reduces the emotions that can trigger conflict or confrontation and encourages each other to think and answer questions more openly."}, "time_dimension": {"answer": "Present", "rationale": "The entry represents a constant act of not participating in the hearing of gossip about one ' s own team, so the temporal dimension is now judged."}, "sentence_type": "Negative declarative sentence", "pos": {"nouns": {"People": 1, "Baloney": 1}, "verbs": {"Say it": 1, "Participation": 1}, "adjectives": {"Never": 1, "not": 2}, "adverbs": {"When": 1}, "numerals": {"385锛?: 1}, "pronouns": {"I": 1}}}, {"content": "386. As long as I start doing one thing, it is difficult to put it down, even temporarily.", "word_count": 20, "label": "", "sentence_structure": "Subject鈥損redicate", "subjective_objective": {"answer": "Subjective", "rationale": "Because words like \"hard to put down\" or \"even temporarily\" indicate that it is a subjective assessment of the patient's behaviour or feelings."}, "sentence_sentiment": {"answer": "Negative", "rationale": "Because the \"hard to put down\" act in the entry is negative."}, "language_style": {"answer": "Calm and direct", "rationale": "This expression is more common in psychological questionnaires, which provide a more accurate picture of the person under investigation鈥檚 thoughts and feelings."}, "time_dimension": {"answer": "Past", "rationale": "The entry describes a pattern of behaviour that is difficult to stop once something has been done and that may have developed in the past and continued to this day."}, "sentence_type": "Affirmative declarative sentence", "pos": {"nouns": {"Yes": 2, "I": 1}, "verbs": {"Start": 1, "Do it": 1, "Put it down": 1}, "adverbs": {"Just": 1, "It's hard": 1, "Even": 1, "For now": 1}, "numerals": {"386锛?: 1}}}, {"content": "387. My defecation is easy and easy to control.", "word_count": 13, "label": "", "sentence_structure": "Subject鈥揷opula鈥損redicative", "subjective_objective": {"answer": "Objective", "rationale": "Because \"crash is not difficult, it is not difficult to control\" is a function of the patient's body and is an objective observation."}, "sentence_sentiment": {"answer": "Neutral", "rationale": "Because the entry describes only the state of a biological function and does not involve positive or negative emotional colours."}, "language_style": {"answer": "Tactful and warm", "rationale": ""}, "time_dimension": {"answer": "Present", "rationale": ""}, "sentence_type": "Affirmative declarative sentence", "pos": {"nouns": {"Shit": 1, "Pee": 1, "Control": 1}, "verbs": {"not": 2, "Yeah": 1, "Hard": 1, "Control": 1, "Difficulties": 1}, "numerals": {"387": 1}}}, {"content": "388. I often find people jealous of my good idea because they did not think of it first.", "word_count": 22, "label": "", "sentence_structure": "Subject鈥搗erb鈥搊bject", "subjective_objective": {"answer": "Subjective", "rationale": "Because \"someone's jealous of my good idea\" is the patient's personal feeling and explanation, and it's subjective."}, "sentence_sentiment": {"answer": "Positive", "rationale": "Because the subjective feeling of the phrase \"I often find people jealous of my good idea\" in the entry is positive, and it is thought that it is a good idea that others have endorsed and taken care of."}, "language_style": {"answer": "Calm and direct", "rationale": "The entry is based on objective factual statements, with no excessive emotional accompaniment or euphemism, and is relatively calm and straightforward. It asks whether the interviewee is often jealous of others and explains the possible reasons for this (i.e., others fail to think of their own good ideas) and is more straightforward."}, "time_dimension": {"answer": "Past", "rationale": "The entry describes the experience and feelings of the testee in the past, and \"often finds\" indicates that this is a continuous act or emotional experience and is therefore a historical state of affairs."}, "sentence_type": "Affirmative declarative sentence", "pos": {"nouns": {"Questionnaire Entry": 1, "person": 2, "Ideas": 1, "Jealousy": 1, "they": 1, "I": 1}, "verbs": {"Found": 1, "be": 1, "Yes": 1, "First thought": 1}, "adjectives": {"Okay": 1}, "adverbs": {"Regular": 1, "Both": 1}, "numerals": {"388锛?: 1, "2": 1}}}, {"content": "389. Whenever possible, I avoid the crowd.", "word_count": 11, "label": "", "sentence_structure": "Subject鈥搗erb鈥揷omplement", "subjective_objective": {"answer": "Subjective", "rationale": "Because avoiding the crowd is the patient's own subjective will and action."}, "sentence_sentiment": {"answer": "Negative", "rationale": "Because the \"scramble\" behavior in the entries is negative."}, "language_style": {"answer": "Calm and direct", "rationale": "The entry is more direct and objective in its language style, is not over-modified or emotionally rendered, and the interviewee is asked whether he or she prefers to avoid crowding and is neutral."}, "time_dimension": {"answer": "Past", "rationale": "The entry \"Screeching the crowd\" denotes past customary behaviour and it can therefore be judged whether the person tested has experienced such past."}, "sentence_type": "Affirmative declarative sentence", "pos": {"verbs": {"Avoid": 1, "Maybe": 1, "Population": 1}, "adjectives": {"Just": 1}, "adverbs": {"Just": 1}, "numerals": {"389": 1}}}, {"content": "I'm not afraid to see anyone.", "word_count": 6, "label": "", "sentence_structure": "Subject鈥搗erb鈥搊bject", "subjective_objective": {"answer": "Subjective", "rationale": "Because \"not afraid of living\" is a personal feeling or attitude, it is subjective."}, "sentence_sentiment": {"answer": "Positive", "rationale": "Because the attitude of \"not afraid of living\" is positive."}, "language_style": {"answer": "Tactful and warm", "rationale": "The language style of the entry is more euphemistic, as it uses the method of questioning, without directly stating or ordering the other party to be afraid of seeing the living person."}, "time_dimension": {"answer": "Present", "rationale": "\"I\" uses the usual current, personal qualities or behaviour of the speaker, while \"no fear\" expresses subjective feelings and attitudes, with no clear time to point, and the entry is therefore a question of describing the current state or character."}, "sentence_type": "Affirmative declarative sentence", "pos": {"verbs": {"Yes": 1, "See": 1}, "adjectives": {"Fan": 1}, "numerals": {"390": 1}}}, {"content": "It is recalled that I had pretended to be sick to avoid doing something.", "word_count": 17, "label": "", "sentence_structure": "Subject鈥搗erb鈥搊bject", "subjective_objective": {"answer": "Subjective", "rationale": "Because \"remember\" and \"had pretended to be sick\" to avoid doing something is a personal experience and a thought of the patient, so it's a subjective angle."}, "sentence_sentiment": {"answer": "Negative", "rationale": "Because the \"do not want to do something but pretend to be sick\" act in the entry is negative."}, "language_style": {"answer": "Tactful and warm", "rationale": "The phrase is more euphemistic, using the expression \"Remember me\" to allow the person under investigation to remember his or her own experience more naturally, without directly indicating whether the other person is lying or dishonest. At the same time, the description \"showing a disease to do something without wanting to do\" is more subtle, avoiding expressions that are too direct or offensive."}, "time_dimension": {"answer": "Present", "rationale": "The entry now describes a past experience or behaviour, but does not specify a specific time period or time reference point. Thus, it concerns acts that occurred in the past and may still exist in the trial memory, but does not indicate whether they are still repeated in the present or in the future."}, "sentence_type": "Affirmative declarative sentence", "pos": {"nouns": {"Questionnaire": 1, "Entry": 1, "Yes": 1, "Diseases": 1}, "verbs": {"Yes": 2, "Once": 1, "Do it": 1, "Load": 1}, "adverbs": {"For": 1, "No, I don't": 1, "Once": 1}, "numerals": {"391锛?: 1}, "pronouns": {"I": 1}}}, {"content": "392. I speak to strangers on trains and buses.", "word_count": 17, "label": "", "sentence_structure": "Subject鈥搗erb鈥搊bject", "subjective_objective": {"answer": "Subjective", "rationale": "Because \"like talking to strangers\" is patient's subjective attitude."}, "sentence_sentiment": {"answer": "Positive", "rationale": "Because the behavior in the entry \"talking to strangers\" is social in nature, it increases the interaction between people."}, "language_style": {"answer": "Tactful and warm", "rationale": "The entry uses the word \"normal\" to describe the frequency of behavior, which is less verb. And \"I talk to strangers on trains and buses\" has the meaning of asking, communicating, not direct orders or statements. This euphemism and accompaniment language can bring people closer to the respondents and increase the effectiveness and acceptance of questionnaires."}, "time_dimension": {"answer": "Present", "rationale": "Now the entry describes the behaviour that the testee is often carrying out in the current day-to-day life, and the word \"normal\" indicates that it is a customary act. And the \"on trains and buses\" also shows specific scene information and points to the current state. So the time dimension of this questionnaire should be now."}, "sentence_type": "Affirmative declarative sentence", "pos": {"nouns": {"Train": 1, "Bus": 1, "Stranger": 1, "Talk": 1}, "verbs": {"in": 1, "Regular": 1, "Call": 1, "Talk": 1}, "adverbs": {"very": 1, "Regular": 1}, "numerals": {"392锛?: 1}}}, {"content": "When things went wrong, I wanted to give up immediately.", "word_count": 16, "label": "", "sentence_structure": "Subject鈥搗erb鈥搊bject", "subjective_objective": {"answer": "Subjective", "rationale": "Because the word \"just\" indicates that it is the patient's personal thoughts and attitudes, not an objective external fact or result."}, "sentence_sentiment": {"answer": "Negative", "rationale": "Because the \"want to give up\" act in the entry is negative."}, "language_style": {"answer": "Calm and direct", "rationale": "The language style of the entry is a cold and direct type. The question is asked in a concise manner about the attitude and behaviour of the subject in the face of difficulties, without too much emotional or accommodative, direct and frank. This type of questionnaire is usually designed to obtain objective and accurate information, not to create an atmosphere or to guide the respondent in his or her thinking."}, "time_dimension": {"answer": "Present", "rationale": "The word 鈥渋n itself鈥?denotes a strong subjective will or sexual preference, which is usually used to describe the current state and feelings, and the sentence therefore describes the present idea."}, "sentence_type": "Negative declarative sentence", "pos": {"nouns": {"Time": 1, "Things": 1, "Abandon": 1}, "verbs": {"Yes": 1, "Just": 1, "Abandon": 1}, "adjectives": {"It's not going well": 1}, "numerals": {"393": 1}, "pronouns": {"I": 1}}}, {"content": "I do not want to let anyone know that I am too much of a person.", "word_count": 17, "label": "", "sentence_structure": "Subject鈥搗erb鈥搊bject", "subjective_objective": {"answer": "Subjective", "rationale": "Because \"not wanting to let anyone know\" is a subjective feeling or attitude."}, "sentence_sentiment": {"answer": "Negative", "rationale": "Because the attitude in the entry is \"unwilling to be known\", it tends to avoid negative evaluations and criticisms and therefore has a certain negative side."}, "language_style": {"answer": "Calm and direct", "rationale": "The language style of the entry is more calm and straightforward, with no excessive emotional accoutrements or rhetorical techniques, but rather with a simple and clear language, which directly expresses the subject of questioning the other person about his or her attitude."}, "time_dimension": {"answer": "Present", "rationale": "Now, \"not willing\" indicates that the person is currently in such a state of mind and attitude, and \"getting people to know\" means that in dealing with others, the person who is tested will try to hide his attitude to things. So the entry is to ask the person who is being tested whether there is a psychological tendency to be reluctant to let others know about his attitude."}, "sentence_type": "Negative declarative sentence", "pos": {"nouns": {"Questionnaire": 1, "Entry": 1, "person": 1, "Attitude": 2}, "verbs": {"Yes": 2, "Jean": 1, "Too much": 1, "Things": 1}, "adjectives": {"Too much": 1}, "numerals": {"394": 1}, "pronouns": {"He's": 1}}}, {"content": "There are times when I feel so strong that I don't need to sleep for days at a time.", "word_count": 24, "label": "", "sentence_structure": "Subject鈥搗erb鈥揷omplement", "subjective_objective": {"answer": "Subjective", "rationale": "Because \"fearing strong\" and \"no sleep\" are patients' subjective feelings and thoughts."}, "sentence_sentiment": {"answer": "Positive", "rationale": "Because \"sweet\" and \"no sleep\" are positive expressions in the entries."}, "language_style": {"answer": "Tactful and warm", "rationale": "The words \"sometimes\" and \"sweetness\" are used in the entry, giving people a sense of ease, pleasure, and a warmer expression. And the phrase \"and for days without sleep\" is also more straightforward, but not too hard or cold."}, "time_dimension": {"answer": "Present", "rationale": "The entry describes a current experience or feeling of the testee, which is \"sometimes strong.\" This feeling can lead to the testee not having to sleep for many days. So the time dimension of the entry is now."}, "sentence_type": "Affirmative declarative sentence", "pos": {"nouns": {"Time": 1, "Powerful": 1, "Oh, my God": 1, "I'm not sure": 1}, "verbs": {"I feel": 1, "Yes": 1, "Sleep": 1}, "adjectives": {"Perfect": 1, "A few days": 1}, "adverbs": {"Some": 1, "And so": 1}, "numerals": {"395锛?: 1}}}, {"content": "I am not ashamed to be in the crowd to take the lead or to comment on things I know.", "word_count": 34, "label": "", "sentence_structure": "Subject鈥搗erb鈥搊bject", "subjective_objective": {"answer": "Subjective", "rationale": "The question is how and how the patient feels inside."}, "sentence_sentiment": {"answer": "Neutral", "rationale": "Because the entry describes a person ' s reaction in a given situation, there is no apparent positive or negative emotional colour."}, "language_style": {"answer": "Calm and direct", "rationale": "The entry directly asks whether the interviewee is able to express his/her views confidently in the crowd, without using excessive rhetorical or emotional rendering."}, "time_dimension": {"answer": "Present", "rationale": ""}, "sentence_type": "Affirmative declarative sentence", "pos": {"nouns": {"Population": 1, "Presentations": 2, "Observations": 1}, "verbs": {"Please": 1, "Lead": 1, "I feel": 1, "not": 1, "Publication": 1}, "adjectives": {"I know you well": 1}, "adverbs": {"and": 1, "not": 1}, "numerals": {"396": 1}, "pronouns": {"I": 2}}}, {"content": "397. I like parties and social events.", "word_count": 10, "label": "", "sentence_structure": "Subject鈥搗erb鈥搊bject", "subjective_objective": {"answer": "Subjective", "rationale": "Because it's the patient's subjective attitude."}, "sentence_sentiment": {"answer": "Positive", "rationale": "Because the \"like party and social activities\" act in the entry expresses a positive attitude."}, "language_style": {"answer": "Tactful and warm", "rationale": "This expression is more personal and interactive and more responsive to the respondents."}, "time_dimension": {"answer": "Present", "rationale": ""}, "sentence_type": "Affirmative declarative sentence", "pos": {"nouns": {"Party": 1, "Social events": 1}, "verbs": {"Yeah": 1}, "numerals": {"397": 1}}}, {"content": "398. I have been unable to move forward in the face of difficulties or dangers.", "word_count": 16, "label": "", "sentence_structure": "Subject鈥損redicate", "subjective_objective": {"answer": "Subjective", "rationale": "This is a subjective examination, because of the patient ' s own psychological reactions and attitudes."}, "sentence_sentiment": {"answer": "Negative", "rationale": "Because \"dumping back\" is a negative act."}, "language_style": {"answer": "Calm and direct", "rationale": "The entry is presented in a direct manner, without any aesthetic or emotional reticulation, and is more calm and objective."}, "time_dimension": {"answer": "Present", "rationale": "The entry asks about the normal behaviour of the person being tested in the face of difficulties or danger, and is therefore described as such."}, "sentence_type": "Negative declarative sentence", "pos": {"nouns": {"Time": 1, "Difficulties": 1, "Danger": 1, "I": 1}, "verbs": {"Face it": 1, "Total": 1, "Retrenched": 1, "I'm not going": 1}, "adverbs": {"Always": 1}, "numerals": {"398": 1}}}, {"content": "399. What I wanted to do was to be easily abandoned if others thought it was not worth it.", "word_count": 23, "label": "", "sentence_structure": "Subject鈥搗erb鈥搊bject", "subjective_objective": {"answer": "Subjective", "rationale": "Because \"easy to give up\" is the patient's own subjective assessment and attitude."}, "sentence_sentiment": {"answer": "Negative", "rationale": "Because the \"easy to give up\" act in the entry is negative."}, "language_style": {"answer": "Calm and direct", "rationale": "The entry is a direct expression of the question about whether the other party is easily influenced by the other party鈥檚 views to abandon what it had planned to do, without using excessive rhetorical or emotional rendering, and with a more balanced and objective tone. Nor is it a euphemism to direct the respondent to further information or expression, but rather a question to be asked to answer his thoughts and behaviour, so the language is more calm and straightforward."}, "time_dimension": {"answer": "Present", "rationale": "\"What I wanted to do\" means past thoughts or behavior, \"if others think it's not worth doing, I can easily give up\" and a attitude that individuals can easily change when others disagree. So this entry asks about the psychological state and behaviour patterns of the person being tested in the present situation."}, "sentence_type": "Affirmative declarative sentence", "pos": {"nouns": {"Yes": 1, "person": 1}, "verbs": {"Yes": 1, "Do it": 2, "Recognition": 1, "Abandon": 1}, "adverbs": {"very": 1, "Easy": 1, "Original": 1}, "numerals": {"399": 1}, "pronouns": {"I": 2, "Here": 1, "Don't": 1}}}, {"content": "I'm not afraid of fire.", "word_count": 4, "label": "", "sentence_structure": "Subject鈥搗erb鈥搊bject", "subjective_objective": {"answer": "Objective", "rationale": "Because fear of fire is a personal emotion and attitude, and \"free from fire\" is the actual experience and reaction of the patient, which is an objective fact."}, "sentence_sentiment": {"answer": "Neutral", "rationale": "The entry is not a positive or negative emotional expression."}, "language_style": {"answer": "Calm and direct", "rationale": "The language style of the entry is a calm and straightforward type, and is intended to allow the interviewee to answer directly whether he is not afraid of fire, without using overly embellished or emotional expressions. This simple language is more accurate in obtaining the interviewee鈥檚 response and avoids possible ambiguity and misunderstanding."}, "time_dimension": {"answer": "Present", "rationale": "The entry describes the current psychological state or tendency of the testee, i.e. that \"I\" is now at a level that is not fire-proof, so the temporal dimension is now."}, "sentence_type": "Affirmative declarative sentence", "pos": {"verbs": {"Not afraid": 1, "Fire": 1}, "numerals": {"400": 1}}}, {"content": "I'm not afraid of water.", "word_count": 4, "label": "", "sentence_structure": "Subject鈥損redicate", "subjective_objective": {"answer": "Objective", "rationale": "The sentence is considered objectively because fear of water is a personal feeling or experience, a subjective description, and the word \"no\" is negative."}, "sentence_sentiment": {"answer": "Neutral", "rationale": "Because the entry does not have any emotional color."}, "language_style": {"answer": "Tactful and warm", "rationale": "The language style of the entry is more euphemistic and warm. It uses a gentle and inviting tone, rather than a direct statement or order, which is more personal and interactive. Moreover, \"no fear of water\" is a more positive expression, with no too many negative emotions or accusations."}, "time_dimension": {"answer": "Present", "rationale": "This entry describes a current attitude or belief that the tested person is not afraid of water."}, "sentence_type": "Affirmative declarative sentence", "pos": {"nouns": {"Water": 1}, "verbs": {"Not afraid": 1}}}, {"content": "402. I often take decisions after careful consideration.", "word_count": 15, "label": "", "sentence_structure": "Subject鈥搗erb鈥揷omplement", "subjective_objective": {"answer": "Subjective", "rationale": "Because \"consider it carefully before making a decision\" is the patient's subjective attitude."}, "sentence_sentiment": {"answer": "Neutral", "rationale": "Because the entry is a description of a person ' s decision-making process and does not reflect positive or negative emotional aspects."}, "language_style": {"answer": "Calm and direct", "rationale": "The entry is more calm and straightforward in language, with no excessive emotional or speech modification, and is expressed in a more concise manner. It is an objective and realistic way of asking questions about whether the interviewee will think carefully before taking a decision."}, "time_dimension": {"answer": "Past", "rationale": "\"Alternally\" and \"after careful consideration\" denotes a pattern of past behaviour, while \"decision\" uses the general current pattern to indicate that the conduct is still in progress. Thus, the entry describes the behaviour of the person tested over time."}, "sentence_type": "Affirmative declarative sentence", "pos": {"nouns": {"Questionnaire Entry": 1, "Decision": 1}, "verbs": {"Analysis": 1, "Make it": 1, "Consider": 1}, "adjectives": {"Often": 1, "Careful": 1}, "numerals": {"402锛?: 1}, "pronouns": {"I": 1}}}, {"content": "403. It is wonderful to live in this rich and diverse age.", "word_count": 18, "label": "", "sentence_structure": "Subject鈥揷opula鈥損redicative", "subjective_objective": {"answer": "Subjective", "rationale": "Because it is a personal feeling and perception, not a fact that can be proved or observed."}, "sentence_sentiment": {"answer": "Positive", "rationale": "Because the expression in the entry \"how wonderful it is to live in this rich and diverse age\" is positive and expresses the appreciation and love of life."}, "language_style": {"answer": "Tactful and warm", "rationale": "The entry is a complimentary and encouraging expression of a positive attitude to life and a positive assessment, which is more likely to attract the participation and resonance of interviewees."}, "time_dimension": {"answer": "Past", "rationale": "In the entry, \"How wonderful it is to live in this rich and diverse age\" is a recollection and praise of the past, and can therefore be judged as the past."}, "sentence_type": "Exclamatory sentence", "pos": {"nouns": {"Life": 1, "Times": 1, "Good": 1}, "verbs": {"Fan": 1, "Live": 1, "in": 1, "be": 1, "More": 1, "Color": 1, "Okay": 1}, "adjectives": {"Rich": 1, "Color": 1, "Beautiful": 1}, "adverbs": {"Here": 1, "How": 1}, "numerals": {"403": 1, "1": 5}, "pronouns": {"This": 1}}}, {"content": "404. When I want to correct others ' mistakes and help them, my good intentions are often misunderstood.", "word_count": 26, "label": "", "sentence_structure": "Subject鈥搗erb鈥搊bject", "subjective_objective": {"answer": "Subjective", "rationale": "Because \"I want to correct others' mistakes and help them\" is the patient's own thoughts and intentions, and \"good intentions are often misunderstood\" is the patient's subjective feelings and experiences, so the questionnaire entry is viewed from a subjective perspective."}, "sentence_sentiment": {"answer": "Negative", "rationale": "Because the \"my good intentions are often misunderstood\" act is negative."}, "language_style": {"answer": "Tactful and warm", "rationale": "The language style of the entry is euphemistic. First, it does not use strong negative or accusatory terms to describe the problem, but rather raises the issue in a more objective and neutral way; second, it also uses an invitation and an interest tone, implying that the other party may encounter the situation and seeking some help or advice. This expression is more easily acceptable to the other party and willing to communicate more deeply with researchers."}, "time_dimension": {"answer": "Present", "rationale": "\"When I want to correct others' mistakes and help them,\" the word \"want\" indicates the current will or intent, and \"often misunderstood\" indicates that this happens often and may continue to the present. So this entry describes the current behaviour or situation."}, "sentence_type": "Affirmative declarative sentence", "pos": {"nouns": {"404": 1, "others": 2, "Error": 1, "Help": 1, "I": 1}, "verbs": {"Yes": 1, "Correct": 1, "Misunderstanded": 1, "Help": 1}, "adverbs": {"Regular": 1}, "numerals": {"404": 1, "2": 1}, "classifiers": {"individual": 1}}}, {"content": "405. I had no difficulty swallowing.", "word_count": 7, "label": "", "sentence_structure": "Subject鈥揷opula鈥損redicative", "subjective_objective": {"answer": "Objective", "rationale": "The objective facts that patients experience because there is no difficulty in swallowing."}, "sentence_sentiment": {"answer": "Neutral", "rationale": "Because the entry merely stated a fact, it did not express any positive or negative feelings."}, "language_style": {"answer": "Calm and direct", "rationale": "The language style of the entry is more direct, concise and does not involve too much reverbs or emotional colours, mainly to describe the feelings or experiences of the person under investigation in a given context in a simple and clear language."}, "time_dimension": {"answer": "Present", "rationale": "The entry asks about the current situation of the person tested, i.e. whether there are difficulties in swallowing. The judgement is therefore now."}, "sentence_type": "Exclamatory sentence", "pos": {"nouns": {"Problem": 1, "Swallow": 1, "Difficulties": 1}, "verbs": {"have": 1, "be": 1, "Swallow": 1, "Difficulties": 1}, "numerals": {"405": 1, "No, I'm not": 1}, "pronouns": {"I": 1}}}, {"content": "I sometimes avoid seeing people because I fear that I will do or will do something that I regret afterwards.", "word_count": 28, "label": "", "sentence_structure": "Subject鈥搗erb鈥搊bject", "subjective_objective": {"answer": "Subjective", "rationale": "Because \"I'm afraid\" and \"may regret\" are patients' subjective feelings and fears."}, "sentence_sentiment": {"answer": "Negative", "rationale": "Because both the entries \"scrambling\" and \"scrambling for something that will happen or will come back to regret.\""}, "language_style": {"answer": "Tactful and warm", "rationale": ""}, "time_dimension": {"answer": "Past", "rationale": ""}, "sentence_type": "Affirmative declarative sentence", "pos": {"nouns": {"Questionnaire": 1, "Entry": 1, "person": 1, "Yes": 2, "Regret": 1}, "verbs": {"Avoidance": 1, "See": 1, "Yes, I will": 1, "Out": 1, "You're": 1}, "adjectives": {"Sometimes": 1, "Back": 1}, "numerals": {"406": 1, "One": 2, "Two": 1}, "classifiers": {"Some": 1}, "pronouns": {"I": 2}}}, {"content": "I'm usually calm and not very excited.", "word_count": 11, "label": "", "sentence_structure": "Subject鈥揷opula鈥損redicative", "subjective_objective": {"answer": "Subjective", "rationale": "Subjectivity, because \"sedit\" and \"uneasy\" are patients' subjective feelings and self-evaluations."}, "sentence_sentiment": {"answer": "Negative", "rationale": "Because the \"uneasy\" act in the entry is negative."}, "language_style": {"answer": "Calm and direct", "rationale": "The entry directly expresses the subject of questioning the other party ' s excavation, does not use excessive rhetorical techniques or emotional rendering, and is more objective in tone."}, "time_dimension": {"answer": "Present", "rationale": ""}, "sentence_type": "Affirmative declarative sentence", "pos": {"nouns": {"I": 1, "Calm down": 1, "Excited": 1}, "verbs": {"Usually": 1, "very": 1, "Easy": 1}, "adjectives": {"Calm down": 1, "Excited": 1}, "adverbs": {"very": 1}, "numerals": {"407": 1}}}, {"content": "I did not easily reveal my feelings so that I was offended, and he did not know it.", "word_count": 27, "label": "", "sentence_structure": "Subject鈥搗erb鈥搊bject", "subjective_objective": {"answer": "Subjective", "rationale": "Because \"not easy to reveal your feelings\" is the patient's subjective attitude or way of acting."}, "sentence_sentiment": {"answer": "Negative", "rationale": "Because \"not easily your feelings\" in the entry is an expression of a negative emotional attitude."}, "language_style": {"answer": "Tactful and warm", "rationale": "The language style of the entry is more euphemistic, implicit, and does not directly express the emotions or attitudes of the other. By describing a phenomenon or behaviour, the other side can understand and reflect on itself. This expression is more likely to resonate and reflect on the other side, and is conducive to good interpersonal relationships."}, "time_dimension": {"answer": "Past", "rationale": "The word \"always\" in \"I hear\" denotes a regular, customary act, while the word \"hear\" uses the usual current pattern, meaning that this act has not only happened in the past, but may still be ongoing."}, "sentence_type": "Negative declarative sentence", "pos": {"nouns": {"Grandpa": 1, "Questionnaire": 1, "Entry": 1, "Emotion": 1, "He's": 1, "I'm sorry": 1}, "verbs": {"Analysis": 1, "The exposed": 1, "Yes": 1}, "adjectives": {"Easy": 1}, "adverbs": {"not": 2}, "numerals": {"408": 1}}}, {"content": "I sometimes get exhausted by taking too much to bear.", "word_count": 18, "label": "", "sentence_structure": "Subject鈥揷opula鈥損redicative", "subjective_objective": {"answer": "Subjective", "rationale": "Because the entry describes the subjective feelings and experiences of patients in the face of stress, such as \"sometimes I'm tired,\" which is viewed from the patient's subjective perspective."}, "sentence_sentiment": {"answer": "Negative", "rationale": "Because the description \"because of the burden, the exhaustion\" in the entry is negative."}, "language_style": {"answer": "Tactful and warm", "rationale": "The entry \"sometimes\" implies that this is an occasional occurrence and uses the expression \"to the point of exhaustion\" with a certain sense of self-protection without directly blaming or criticizing the other. These factors make the language style more euphemismal and gentle."}, "time_dimension": {"answer": "Present", "rationale": "The keywords are \"sometimes\" and \"because\" because they all point to what is happening. \"Too much to bear\" and \"to pay tribute to fatigue\" describe the feelings and experiences of the subjects, indicating that they may be experiencing some kind of stress or fatigue. So the main focus of the article is on the state and emotional experience."}, "sentence_type": "Affirmative declarative sentence", "pos": {"nouns": {"Things": 1, "Cheers": 1, "Tired": 1, "- Oh, shit": 1}, "verbs": {"Undertaking": 1, "to": 1}, "adverbs": {"Sometimes": 1}, "numerals": {"409": 1}, "classifiers": {"Too much": 1}}}, {"content": "I am certainly happy to do it in my own way.", "word_count": 16, "label": "", "sentence_structure": "Subject鈥搗erb鈥搊bject", "subjective_objective": {"answer": "Subjective", "rationale": "Because this phrase expresses the attitude and will of the patient, it is subjective."}, "sentence_sentiment": {"answer": "Positive", "rationale": "Because \"tooth for tooth\" is a positive response."}, "language_style": {"answer": "Tactful and warm", "rationale": "The entry is more personal and interactive in its expression of conversation in a gentle and inviting tone, rather than a direct statement or order. At the same time, the words \"of course\" and \"happy\" convey positive and optimistic emotional expressions that give a warmer sense of feeling."}, "time_dimension": {"answer": "Present", "rationale": "The entry describes an attitude or will that does not involve past or future acts or events, and therefore the temporal dimension is now judged."}, "sentence_type": "Affirmative declarative sentence", "pos": {"nouns": {"Questionnaire": 1, "Entry": 1, "person": 2, "Dow": 1}, "verbs": {"I'm happy": 1, "And": 1, "- Oh, yeah": 1}, "adverbs": {"Sure": 1}, "numerals": {"410锛?: 1}, "pronouns": {"I": 1}}}, {"content": "Religion does not bother me.", "word_count": 7, "label": "", "sentence_structure": "Subject鈥揷opula鈥損redicative", "subjective_objective": {"answer": "Subjective", "rationale": "Whether religion is troubling is the subjective feeling and perception of a patient."}, "sentence_sentiment": {"answer": "Negative", "rationale": "Because religion may cause trouble for some, the sentence expresses a negative attitude."}, "language_style": {"answer": "Calm and direct", "rationale": "This entry directly expresses the question of whether the other party is troubled by religious problems, and is not speaking too much or making emotional presentations, which is more objective. This direct presentation enables the interviewee to answer the questions more honestly and helps researchers to obtain more accurate data."}, "time_dimension": {"answer": "Present", "rationale": "\"Don't worry me\" means that the current situation is that the testees are being plagued by religion when they fill out the questionnaire, and therefore the time dimension is now judged."}, "sentence_type": "Negative declarative sentence", "pos": {"nouns": {"Religion": 1, "I": 1, "Trouble": 1}, "verbs": {"鈾?And I'm gonna be 鈾?: 1}, "adjectives": {"not": 1}, "numerals": {"411": 1}}}, {"content": "412. When I am sick or injured, I am not afraid to seek a doctor.", "word_count": 14, "label": "", "sentence_structure": "Subject鈥搗erb鈥搊bject", "subjective_objective": {"answer": "Objective", "rationale": "It is an objective fact that a doctor is required to assist because of illness or injury, and not related to the patient ' s subjective attitude."}, "sentence_sentiment": {"answer": "Positive", "rationale": "The entry is positive because it expresses an attitude towards seeking medical assistance when the body is not in due course."}, "language_style": {"answer": "Tactful and warm", "rationale": "The entry is more adept and interactive when it is spoken in a gentle and inviting manner, rather than by direct statements or orders."}, "time_dimension": {"answer": "Present", "rationale": "The entry describes the person who was tested for the present state of affairs in the face of the untimely behaviour and attitude of the body, the expression \"in case of illness or injury\" and the expression \"without fear of a doctor\". So the time dimension of the entry is now."}, "sentence_type": "Affirmative declarative sentence", "pos": {"nouns": {"I": 1, "Diseases": 1, "Wounded": 1, "Time": 1, "Doctor": 1}, "verbs": {"Find": 1, "Fan": 1, "Yes": 2}, "adverbs": {"not": 1}, "numerals": {"412": 1}}}, {"content": "413. I am guilty and shall be liable to heavy penalties.", "word_count": 7, "label": "", "sentence_structure": "Subject鈥搗erb鈥搊bject", "subjective_objective": {"answer": "Subjective", "rationale": "Because \"guilty\" and \"serious punishment\" are patients' subjective attitudes and thoughts."}, "sentence_sentiment": {"answer": "Negative", "rationale": "The attitude and view of \"guilty, heavy\" in the entry is negative."}, "language_style": {"answer": "Calm and direct", "rationale": "The entry is relatively calm and straightforward in its language style, not using excessive emotional or rhetorical techniques, but in an objective and neutral manner. At the same time, the entry uses a more traditional questionnaire form to ask the respondents' views on whether they are guilty and what punishment they are liable to.鈥?}, "time_dimension": {"answer": "Past", "rationale": "The entry contains self-evaluations and self-recriminations of the past, such as \"guilty\" and \"serious punishment\", which point to past events."}, "sentence_type": "Affirmative declarative sentence", "pos": {"nouns": {"Sin": 1, "Heavy penalty": 1}, "verbs": {"have": 1, "Due": 2}, "numerals": {"413": 1}}}, {"content": "414. I have taken the disappointment too seriously to be forgotten.", "word_count": 17, "label": "", "sentence_structure": "Subject鈥搗erb鈥搊bject", "subjective_objective": {"answer": "Subjective", "rationale": "The disappointment is viewed from the patient ' s subjective perspective, as it is the patient ' s own feelings and attitudes that are taken too seriously and that are not forgotten."}, "sentence_sentiment": {"answer": "Negative", "rationale": "Because \"depression is too heavy\" and \"remembering\" in the entry is a negative emotional tendency."}, "language_style": {"answer": "Tactful and warm", "rationale": "The language style of the entry is euphemism. It asks the respondents in a gentle and inviting manner whether they often look down on disappointments so much that they can never be forgotten. It is a way of being personal and interactive, and it is easier to resonate and engage the respondents."}, "time_dimension": {"answer": "Present", "rationale": ""}, "sentence_type": "Negative declarative sentence", "pos": {"nouns": {"Yes": 2, "Disappointment": 1}, "verbs": {"- Put it on": 1, "Look": 1, "I can't forget it": 1, "Okay": 1}, "adjectives": {"Too heavy": 1}, "adverbs": {"So much so that": 1}, "numerals": {"414": 1}, "pronouns": {"I": 1}}}, {"content": "415. I do not like rushing to work.", "word_count": 13, "label": "", "sentence_structure": "Subject鈥揷opula鈥損redicative", "subjective_objective": {"answer": "Subjective", "rationale": "Because \"very bad\" means it's a patient's subjective feeling."}, "sentence_sentiment": {"answer": "Negative", "rationale": "Because the entry \"I don't like rushing to work\" expresses a negative mood and attitude."}, "language_style": {"answer": "Calm and direct", "rationale": "The language style of the entry is more direct and concise, and it does not use too many emotional terms or rhetorical techniques to describe the problem, but rather expresses the other side's attitude to \"hurst work\". This expression is more objective and understandable."}, "time_dimension": {"answer": "Present", "rationale": "The entry \"I\" uses the general current, which indicates that it is a feeling or behaviour that is now happening, and \"very bad\" means that subjective attitudes are strong, so it can be judged to be a description of the current situation."}, "sentence_type": "Negative declarative sentence", "pos": {"nouns": {"Work": 1}, "verbs": {"Yeah": 1, "Hurry up": 1}, "adjectives": {"I'm in a hurry": 1}, "numerals": {"415": 1}}}, {"content": "416. Although I know I can do things right, I'm afraid that I'm not gonna be seen to do it.", "word_count": 23, "label": "", "sentence_structure": "Subject鈥損redicate", "subjective_objective": {"answer": "Subjective", "rationale": "Because \"I know I can do it well\" and \"I'm afraid that I'm watching me do it\" are the thoughts and feelings inside the patient, and they're subjective."}, "sentence_sentiment": {"answer": "Negative", "rationale": "Because the expression \"and fear that I will be seen to do\" expresses self-doubt and fear, and is negative."}, "language_style": {"answer": "Tactful and warm", "rationale": "The language style of the entry is more euphemistic and thoughtful, expressing recognition of one's abilities by \"know that you can do well\" and revealing potential insecurity and anxiety by \"but fear that others will look at me\" in a way that preserves the emotional experience of the other, but is not merely a direct irritation or offensive."}, "time_dimension": {"answer": "Present", "rationale": "So the answer is: now."}, "sentence_type": "Affirmative declarative sentence", "pos": {"nouns": {"Yes": 1, "person": 1}, "verbs": {"Do it": 2, "Okay": 1, "Look": 1}, "adjectives": {"Understood": 1, "Don't": 1}, "adverbs": {"Yeah": 2, "Yes": 1, "Just": 1, "Here": 1, "Both": 1}, "numerals": {"416": 1, "One": 2}, "pronouns": {"self": 1, "others": 1}}}, {"content": "417. If someone were to step in front of me in line, I would be angry and blame him.", "word_count": 27, "label": "", "sentence_structure": "Subject鈥搗erb鈥搊bject", "subjective_objective": {"answer": "Subjective", "rationale": "This perception and reaction is the patient ' s personal subjective attitude and emotional experience."}, "sentence_sentiment": {"answer": "Negative", "rationale": "Because \"scold him for being angry\" in the entry is negative."}, "language_style": {"answer": "Calm and direct", "rationale": "The entry is more straightforward, clear-cut, and does not use excessive rhetorical or emotional rendering. It simply expresses attitudes and reactions to queued behaviour, and is more objective and calm."}, "time_dimension": {"answer": "Past", "rationale": "Because \"fear\" and \"scriticism\" are actions or states that have occurred in the past."}, "sentence_type": "Affirmative declarative sentence", "pos": {"nouns": {"Time": 1, "person": 2, "I": 2, "Plug in": 1, "Front": 1, "Blame": 1, "Cut in line": 1}, "verbs": {"If": 1, "I feel": 1, "I'm angry": 1, "Blame": 1, "Plug in": 1, "Go on": 1}, "adjectives": {"Back": 1}, "numerals": {"417": 1}}}, {"content": "418. Sometimes I feel like I'm nothing.", "word_count": 11, "label": "", "sentence_structure": "Subject鈥揷opula鈥損redicative", "subjective_objective": {"answer": "Subjective", "rationale": "Because \"I feel like I'm nothing\" is the patient's subjective feeling and idea."}, "sentence_sentiment": {"answer": "Negative", "rationale": "Because the idea of \"necessity\" in the entry is negative."}, "language_style": {"answer": "Tactful and warm", "rationale": "The entry is more euphemistic and warm. It uses a non-direct way of asking the interviewee whether he has ever had a self-denial feeling, which is easier to express by opening his heart and sharing his real thoughts and feelings. At the same time, the phrase \"sometimes\" also leaves the interviewee some buffer space to answer questions without being too nervous or worried about the outcome."}, "time_dimension": {"answer": "Past", "rationale": ""}, "sentence_type": "Negative declarative sentence", "pos": {"nouns": {"Time": 1, "self": 1}, "verbs": {"I think": 1, "Nothing": 1}, "adverbs": {"Sometimes": 1}, "numerals": {"418锛?: 1}}}, {"content": "419. When I was a child, I often escaped from school.", "word_count": 8, "label": "", "sentence_structure": "Subject鈥損redicate", "subjective_objective": {"answer": "Subjective", "rationale": "Because \"when you're young,\" \"when you're young,\" \"when you're not in school\" are described from the patient's own perspective, it's a subjective attitude and experience."}, "sentence_sentiment": {"answer": "Negative", "rationale": "Because the \"scramble\" in the entry is negative."}, "language_style": {"answer": "Calm and direct", "rationale": "The entry is a direct question-and-answer dialogue, without any pedagogy or emotional colour, and is more balanced and objective."}, "time_dimension": {"answer": "Past", "rationale": "The keyword \"when we were young\" means that the act occurred at some stage in the past, and \"train\" is also a past act. So, judging by the time dimension, this is the past."}, "sentence_type": "Affirmative declarative sentence", "pos": {"nouns": {"Time": 1, "- You're not going to school": 1}, "verbs": {"Often": 1, "I": 1}, "adverbs": {"Small": 1, "Time": 1, "Often": 1}, "numerals": {"419": 1, "One": 1}}}, {"content": "420. I have had a very unusual religious experience.", "word_count": 14, "label": "", "sentence_structure": "Subject鈥損redicate", "subjective_objective": {"answer": "Subjective", "rationale": "Because \"unusual religious experience\" is usually a subjective feeling and understanding of individuals."}, "sentence_sentiment": {"answer": "Neutral", "rationale": "Because the entry describes personal experience, it does not express positive or negative emotional tendencies."}, "language_style": {"answer": "Calm and direct", "rationale": "The entry is more direct and concise, is not used too much rhetorical or emotionally retrofitting, and is more objective. It asks the interviewee in a simple and clear way whether he has had a very special religious experience, giving him a clear sense of clarity."}, "time_dimension": {"answer": "Past", "rationale": ""}, "sentence_type": "Exclamatory sentence", "pos": {"nouns": {"Religion": 1, "Experience": 1}, "verbs": {"have": 1, "Pass": 1, "be": 1}, "adjectives": {"very": 1, "Unusual": 1}, "numerals": {"420": 1}, "pronouns": {"I": 1}}}, {"content": "421. There are people in my family who are very sensitive.", "word_count": 10, "label": "", "sentence_structure": "Subject鈥搗erb鈥搊bject", "subjective_objective": {"answer": "Subjective", "rationale": "As the entry relates to the family situation of the patient, it is the patient ' s own observations and perceptions, and is therefore examined from a subjective perspective."}, "sentence_sentiment": {"answer": "Negative", "rationale": "Because \"sensitization\" is usually seen as a negative emotion or quality."}, "language_style": {"answer": "Tactful and warm", "rationale": "This is more likely to trigger a resonance and reflection among the interviewee, and to avoid the possible negative or counter-spiriting mood."}, "time_dimension": {"answer": "Past", "rationale": "The entry asks if the member of the testee鈥檚 family is neurotic, and the family member is a former member."}, "sentence_type": "Affirmative declarative sentence", "pos": {"nouns": {"Home": 1, "person": 1, "You're a bit of a sane": 1}, "verbs": {"have": 1}, "adjectives": {"very": 1}, "numerals": {"421": 1}, "pronouns": {"I": 1}}}, {"content": "422. I feel embarrassed because of the occupation that is being performed in the family.", "word_count": 20, "label": "", "sentence_structure": "Subject鈥搗erb鈥搊bject", "subjective_objective": {"answer": "Subjective", "rationale": "Because I feel embarrassed about the patient鈥檚 personal feelings and attitudes because of the occupation that someone is in the family. This is the feeling and the idea that is deep inside the patient, and it is therefore subjective."}, "sentence_sentiment": {"answer": "Negative", "rationale": "Because the entry \"excuse me\" expresses a negative emotional attitude."}, "language_style": {"answer": "Tactful and warm", "rationale": "The language style of the entry is relatively euphemistic, using a more euphemistic expression like \"I'm sorry\" rather than direct accusations or criticism. At the same time, it is not too emotional, but rather a matter of a smooth, objective way of asking questions that is more acceptable to the interviewee and willing to answer. Thus, the language style of the questionnaire entry is considered to be euphemism."}, "time_dimension": {"answer": "Present", "rationale": "Now the entry describes the current feelings and thoughts of the testee, and \"I feel embarrassed because of the occupation of the family.\" It is a present emotional experience. So the time dimension of the article is now."}, "sentence_type": "Affirmative declarative sentence", "pos": {"nouns": {"person": 1, "Occupation": 1}, "verbs": {"I feel": 1, "Excuse me": 1}, "adverbs": {"422": 1}, "numerals": {"Because": 1}, "pronouns": {"I": 1}}}, {"content": "423. I like fishing (or I like) very much.", "word_count": 11, "label": "", "sentence_structure": "Subject鈥搗erb鈥揷omplement", "subjective_objective": {"answer": "Subjective", "rationale": "Like being patient subjective."}, "sentence_sentiment": {"answer": "Neutral", "rationale": "Because the entry describes personal preferences or interests, it does not express any positive or negative emotional attitudes."}, "language_style": {"answer": "Tactful and warm", "rationale": "The entry is more adept and interactive when it is spoken in a gentle and inviting manner, rather than by direct statements or orders."}, "time_dimension": {"answer": "Past", "rationale": "At the same time, the words \"like,\" \"like\" or \"like\" also suggest that the act may have ceased or decreased."}, "sentence_type": "Affirmative declarative sentence", "pos": {"nouns": {"Fishing": 1}, "verbs": {"Yeah": 2, "I did": 1}, "adverbs": {"very": 1}, "numerals": {"423": 1}}}, {"content": "424. I almost always feel hungry.", "word_count": 9, "label": "", "sentence_structure": "Subject鈥揷opula鈥損redicative", "subjective_objective": {"answer": "Subjective", "rationale": "Because \"I'm almost always hungry\" is a subjective feeling and experience, not an objective fact."}, "sentence_sentiment": {"answer": "Negative", "rationale": "Because the \"always hungry\" phenomenon in the entry is unhealthy and may suggest a physical problem."}, "language_style": {"answer": "Calm and direct", "rationale": ""}, "time_dimension": {"answer": "Present", "rationale": "The future entry contains the intended feelings and subjective judgement of the testee, the keyword being \"almost always felt\", which suggests that this may not have occurred or is not yet ongoing, but the testee believes that it may be frequent in the future. The time dimension of the entry can therefore be judged as future."}, "sentence_type": "Affirmative declarative sentence", "pos": {"verbs": {"I feel like": 1, "Hungry": 1}, "adjectives": {"Almost": 1}, "numerals": {"424": 1}}}, {"content": "425. I often dream.", "word_count": 5, "label": "", "sentence_structure": "Subject鈥損redicate", "subjective_objective": {"answer": "Subjective", "rationale": "Because dreams are usually a subjective experience and feeling of individuals, the entry is viewed from the subjective perspective of the patient."}, "sentence_sentiment": {"answer": "Neutral", "rationale": "Because the entry does not express a positive and negative emotional expression, it only describes a phenomenon."}, "language_style": {"answer": "Tactful and warm", "rationale": "This expression is more personal and interactive, making it easier for the respondents to answer questions."}, "time_dimension": {"answer": "Present", "rationale": ""}, "sentence_type": "Affirmative declarative sentence", "pos": {"verbs": {"Do it": 1}, "adjectives": {"The long run": 1, "Regular": 1}, "numerals": {"425": 1}, "pronouns": {"I": 2}}}, {"content": "426. I have sometimes had to deal with rude or disgusting people with an unkind attitude.", "word_count": 26, "label": "", "sentence_structure": "Subject鈥搗erb鈥搊bject", "subjective_objective": {"answer": "Objective", "rationale": "Because it describes the circumstances and ways in which a person may be confronted in life, it does not depend on the patient鈥檚 subjective feelings or thoughts. This is an objective statement."}, "sentence_sentiment": {"answer": "Negative", "rationale": "Because \"black or disgusting\" and \"offensive attitude\" are both derogatory words that express a negative emotion."}, "language_style": {"answer": "Tactful and warm", "rationale": "The entry uses the words \"just good\" and \"bold\" and refers to ways to deal with the repugnant, but does not directly criticize or blame each other, but rather expresses their distress and need in a moderate way, so that the language style is more euphemismal."}, "time_dimension": {"answer": "Present", "rationale": "The keywords \"sometimes\" and \"just\" mean that this act may have occurred in the past, but it focuses more on the state of the present. So the answer is \"now\"."}, "sentence_type": "Affirmative declarative sentence", "pos": {"nouns": {"Attitude": 1, "person": 1}, "verbs": {"Use": 1, "Deal": 1}, "adjectives": {"Naughty": 1, "Disgusting": 1}, "adverbs": {"Sometimes": 1, "Just": 1}, "numerals": {"426锛?: 1}, "pronouns": {"I": 1}}}, {"content": "427. I prefer to be interested in different types of hobby rather than to insist on one of them for the long term.", "word_count": 29, "label": "", "sentence_structure": "Subject鈥搗erb鈥搊bject", "subjective_objective": {"answer": "Subjective", "rationale": "Because \"prefer\" and \"unwilling to insist\" are subjective feelings and ideas of the patients themselves, and they look at their interests and attitudes, so it's a subjective view."}, "sentence_sentiment": {"answer": "Neutral", "rationale": "Because the entry does not express any positive or negative emotional aspects, it merely presents a fact."}, "language_style": {"answer": "Tactful and warm", "rationale": ""}, "time_dimension": {"answer": "Present", "rationale": ""}, "sentence_type": "Affirmative declarative sentence", "pos": {"nouns": {"Questionnaire": 1, "Entry": 1, "Various": 1, "Hobbies": 1, "Hold on": 1, "Other": 1, "Medium": 1}, "verbs": {"Prefer": 1, "Come on": 1, "Interest": 1, "No, I don't": 1, "Long term": 1, "Hold on": 1}, "adverbs": {"Different": 1, "Long": 1}, "numerals": {"427": 1}, "classifiers": {"Plant": 1}, "pronouns": {"I": 1}}}, {"content": "428. I like reading editorials in newspapers.", "word_count": 10, "label": "", "sentence_structure": "Subject鈥搗erb鈥搊bject", "subjective_objective": {"answer": "Subjective", "rationale": "Because \"like to read\" is the patient's personal attitude and feelings, and it's subjective."}, "sentence_sentiment": {"answer": "Positive", "rationale": "Because the purpose of the entry is to be positive."}, "language_style": {"answer": "Calm and direct", "rationale": "The entry is in a straightforward way asking the respondents about the attitude to reading editorials in newspapers, which is not overly emotional or rhetorical, and is based on a more calm and straightforward style."}, "time_dimension": {"answer": "Present", "rationale": ""}, "sentence_type": "Affirmative declarative sentence", "pos": {"nouns": {"Newspapers": 1, "editorial": 1}, "verbs": {"Yeah": 1, "Reading": 1}, "numerals": {"428": 1}}}, {"content": "429. I like to hear serious speeches on the subject.", "word_count": 11, "label": "", "sentence_structure": "Subject鈥搗erb鈥搊bject", "subjective_objective": {"answer": "Subjective", "rationale": "Because \"like\" is a subjective feeling and attitude, it's the patient's own feeling and perception, so it's subjective."}, "sentence_sentiment": {"answer": "Positive", "rationale": "Because the \"like to hear\" behavior in the entry is positive."}, "language_style": {"answer": "Calm and direct", "rationale": "The entry is a simple and clear expression of the interest of the subject in listening to serious subject-matter speeches, without too much rhetorical or emotional refinement."}, "time_dimension": {"answer": "Present", "rationale": ""}, "sentence_type": "Affirmative declarative sentence", "pos": {"nouns": {"I like it": 1, "Listen": 1, "Theme": 1, "Seriously": 1, "Speech": 1}, "adjectives": {"Seriously": 1}, "numerals": {"429锛?: 1}}}, {"content": "I'm susceptible to the attraction of the opposite sex.", "word_count": 8, "label": "", "sentence_structure": "Subject鈥搗erb鈥揷omplement", "subjective_objective": {"answer": "Subjective", "rationale": "Because \"vulnerable to the attraction of the opposite sex\" is the patient's own feeling and perception, it's a subjective approach."}, "sentence_sentiment": {"answer": "Positive", "rationale": "/negative/neutral: Positive, because the attitude of \"vulnerable to heterosexual attraction\" in the entries is positive."}, "language_style": {"answer": "Tactful and warm", "rationale": "The entry is more adept and interactive when it is spoken in a gentle and inviting manner, rather than by direct statements or orders."}, "time_dimension": {"answer": "Present", "rationale": "\"Easier to be attracted to opposite sex\" is a representation of personal characteristics or emotional orientation, with a continuing character and mentality, and the entry is therefore the present."}, "sentence_type": "Affirmative declarative sentence", "pos": {"nouns": {"Heterosexual": 1, "Attractive": 1}, "verbs": {"Easy": 1}, "adverbs": {"very": 1}, "numerals": {"430锛?: 1}, "pronouns": {"I": 1}}}, {"content": "I am quite concerned about the unfortunate consequences that may have arisen.", "word_count": 15, "label": "", "sentence_structure": "Subject鈥揷opula鈥損redicative", "subjective_objective": {"answer": "Subjective", "rationale": "Because it describes the feelings and fears of the patient, it is a subjective attitude."}, "sentence_sentiment": {"answer": "Negative", "rationale": "Because the expression \"a fear of what could happen\" in the entry is negative."}, "language_style": {"answer": "Calm and direct", "rationale": "The entry is in an objective and direct tone, asking the respondent whether he is worried about something, and is not over-rendered or incipient, and is more inclement and direct."}, "time_dimension": {"answer": "Past", "rationale": "The keyword \"scared\" is a sign of past anxiety and anxiety, pointing to the past."}, "sentence_type": "Affirmative declarative sentence", "pos": {"nouns": {"431": 1, "I": 1, "Maybe": 1, "Come on": 1, "Unfortunately": 1}, "verbs": {"I'm worried": 1, "Cause": 1}, "adverbs": {"Pretty much": 1}, "numerals": {"431": 1, "1": 2}, "classifiers": {"Maybe": 1, "Come on": 1}, "pronouns": {"Well": 1}}}, {"content": "432. I have a strong political opinion.", "word_count": 10, "label": "", "sentence_structure": "Subject鈥搗erb鈥揷omplement", "subjective_objective": {"answer": "Subjective", "rationale": "For a firm political opinion is one's own idea and is a subjective one."}, "sentence_sentiment": {"answer": "Positive", "rationale": "Because the quality of \"stout political opinion\" in the entry is positive."}, "language_style": {"answer": "Calm and direct", "rationale": "The entry is more straightforward in language style, is not accompanied by too many rhetorical or emotional renderings, and is more calm and objective in tone. It explicitly asks whether the person surveyed has a firm political opinion and thus obtains the relevant data."}, "time_dimension": {"answer": "Present", "rationale": "The entry indicates that the testee has a firm political opinion at the moment he answers the questions. While this view may have developed in the past or have developed over a long period of time, at this particular moment the testee believes that he has such a conviction."}, "sentence_type": "Affirmative declarative sentence", "pos": {"nouns": {"Political opinion": 1}, "verbs": {"have": 1}, "adjectives": {"Firm": 1}, "numerals": {"432": 1}, "pronouns": {"I": 1}}}, {"content": "I had an imaginary companion.", "word_count": 10, "label": "", "sentence_structure": "Subject鈥損redicate", "subjective_objective": {"answer": "Subjective", "rationale": "Because \"a man who thinks of his own\" is a person's subjective perception and feeling, and belongs to the experience and imagination of the inner world."}, "sentence_sentiment": {"answer": "Neutral", "rationale": "Because the entry does not have any emotional color."}, "language_style": {"answer": "Tactful and warm", "rationale": "The more euphemistic, first-person expression and \"had\" expression of the entry implies that the question is not a question to be answered, but rather a question to be asked in a more relaxed and friendly way. At the same time, the term is more gentle and friendly."}, "time_dimension": {"answer": "Past", "rationale": "The term \"had \" denotes a completed action or state, and the entry asks if the person tested had had any imaginary peer experience in the past."}, "sentence_type": "Affirmative declarative sentence", "pos": {"nouns": {"Companion": 1, "Imagine": 1}, "verbs": {"have": 1, "Pass": 1}, "adverbs": {"Once": 1}, "numerals": {"433": 1}}}, {"content": "I wish to become a motorcycle athlete.", "word_count": 14, "label": "", "sentence_structure": "Subject鈥搗erb鈥搊bject", "subjective_objective": {"answer": "Subjective", "rationale": "Because \"I want to be a motorcycle athlete\" is the patient's aspiration and goal, reflecting his subjective attitude and values."}, "sentence_sentiment": {"answer": "Positive", "rationale": "Because \"I want to be\" in this entry is an expression of a person's positive attitude and objective setting."}, "language_style": {"answer": "Tactful and warm", "rationale": "The entry uses a more euphemistic language style, which is not too direct or strong, but rather an invitation to ask the other side whether it wishes to be a motorcycle player."}, "time_dimension": {"answer": "Future", "rationale": "The keyword is \"hope,\" which expresses the individual's wishes and expectations, while \"becoming\" refers to the future act or state. The entry therefore describes the future act or state."}, "sentence_type": "Imperative sentence", "pos": {"nouns": {"Aeroplanes": 1, "Motorcycle": 1, "Car": 1}, "verbs": {"Be": 1, "Hope": 1}, "numerals": {"434": 1}}}, {"content": "I usually prefer to work with women.", "word_count": 12, "label": "", "sentence_structure": "Subject鈥搗erb鈥搊bject", "subjective_objective": {"answer": "Subjective", "rationale": "Because \"like\" is patient's subjective attitude."}, "sentence_sentiment": {"answer": "Neutral", "rationale": "Because the entry merely describes a fact, it does not express a positive or negative emotional colour."}, "language_style": {"answer": "Tactful and warm", "rationale": "The language style of the entry is more euphemistic, expressing preference for female colleagues in a more polite and polite way. Using \"usually\" and \"working together\" instead of \"I like working with women\" directly, it avoids unnecessary misunderstanding or discomfort. This also reflects the attitude of the surveyer to respect and be inclusive."}, "time_dimension": {"answer": "Present", "rationale": "\"I usually like \"normally\" means \"recurringly, habitually\" and \"like\" takes the normal current state of affairs, which means that this behaviour has not only happened in the past, but may still be ongoing. So this questionnaire entry describes the testee's current preferences and preferences, which are now time-dimensional."}, "sentence_type": "Affirmative declarative sentence", "pos": {"nouns": {"I": 1, "Women": 1, "Work": 1}, "verbs": {"Yeah": 1, "Together": 1, "Work": 1}, "adverbs": {"Usually": 1}, "numerals": {"435": 1}}}, {"content": "436. I think there is only one religion.", "word_count": 12, "label": "", "sentence_structure": "Subject鈥揷opula鈥損redicative", "subjective_objective": {"answer": "Subjective", "rationale": "Because \"like\" means the patient's personal perception and feelings is a subjective description."}, "sentence_sentiment": {"answer": "Negative", "rationale": "Because the expression \"only one religion is true\" in the entries expresses a negative attitude towards other religions and has a certain negative tendency."}, "language_style": {"answer": "Tactful and warm", "rationale": "The language style of the entry is more euphemistic and moderate, and does not directly negate or affirm the truth of any religion, but rather allows the interviewee to express his views in an open and respectful manner. Such expression is better able to guide the interviewee鈥檚 thinking and is more acceptable."}, "time_dimension": {"answer": "Past", "rationale": "The entry expresses a belief or attitude of the person tested, does not relate to future conduct or expectations and therefore is judged to be past."}, "sentence_type": "Imperative sentence", "pos": {"nouns": {"Religion": 1}, "verbs": {"I think": 1, "be": 1}, "adjectives": {"Really": 1}, "adverbs": {"Only": 1}, "numerals": {"436锛?: 1}}}, {"content": "437. As long as you do not really break the law, it is possible to take a chance of being caught in a loophole in the law.", "word_count": 23, "label": "", "sentence_structure": "Subject鈥損redicate", "subjective_objective": {"answer": "Subjective", "rationale": "Because it reflects the patient ' s personal views and views, it is subjective."}, "sentence_sentiment": {"answer": "Neutral", "rationale": "As the entry merely presented a point of view, it did not express any positive or negative emotional aspects."}, "language_style": {"answer": "Calm and direct", "rationale": "The wording in this entry appears to be more direct and categorical, even with a certain apathy. It clearly expresses the view that, as long as it is not contrary to criminal law, the use of legal loopholes in some cases is acceptable, a position that is emotionally calm and objective. At the same time, the use of the phrase \"real\" seems to imply that other, more serious violations cannot be tolerated or forgiven."}, "time_dimension": {"answer": "Present", "rationale": ""}, "sentence_type": "Affirmative declarative sentence", "pos": {"nouns": {"Law": 1, "Hole": 1, "Picking up the right": 1}, "verbs": {"be": 1, "Yeah": 1}, "adjectives": {"Really": 1, "Offences": 1}, "numerals": {"437": 1}, "pronouns": {"you": 1}}}, {"content": "438. Some people hate it so much that I would be so happy because they eat their own food.", "word_count": 22, "label": "", "sentence_structure": "Subject鈥搗erb鈥搊bject", "subjective_objective": {"answer": "Subjective", "rationale": "Because it is a subjective feeling and attitude to be happy in the dark."}, "sentence_sentiment": {"answer": "Negative", "rationale": "Because it contains words like \"disguised\" and \"self-eating\" negative emotional expressions."}, "language_style": {"answer": "Tactful and warm", "rationale": "The entry is more personal and interactive when it is spoken in a gentle, rather than direct, statement or order. At the same time, the sentence does not directly display bad faith or hostility towards others, but rather represents a more complex and delicate emotional experience, with a strong subjective and emotional colour, which is in keeping with a soft and warm language style."}, "time_dimension": {"answer": "Present", "rationale": "The entry describes a psychological state and emotional response that may have occurred at some point in the past but is still ongoing. It should therefore be classified as the present state of affairs."}, "sentence_type": "Affirmative declarative sentence", "pos": {"nouns": {"person": 2, "Some people": 1, "they": 1, "self": 1, "Perfect": 1, "In the dark": 1, "Happy": 1}, "verbs": {"Shit": 1, "Yes": 1, "It's a self-inflicted result": 1}, "adjectives": {"Some": 1, "It's disgusting": 1}, "adverbs": {"And": 1, "In the dark": 1}, "numerals": {"438": 1, "1": 2}, "pronouns": {"I": 1}}}, {"content": "439. I'm nervous waiting for me.", "word_count": 8, "label": "", "sentence_structure": "Subject鈥搗erb鈥揷omplement", "subjective_objective": {"answer": "Subjective", "rationale": "Because \"stretch\" is an emotional experience, a subjective feeling of the patient."}, "sentence_sentiment": {"answer": "Negative", "rationale": "Because the entry describes a negative mood - stress."}, "language_style": {"answer": "Tactful and warm", "rationale": "The entry uses metaphoric rhetoric, using \"waiting\" as a metaphor, which vividly expresses the anxiety and anxiety of the waiting period and is more euphemistically tempered."}, "time_dimension": {"answer": "Present", "rationale": "The entry is for the psychological response of the person who is being asked to wait for a certain situation, and the time dimension is now judged. In it, the word \"wait me\" in the phrase \"wait me\" is a conditional relationship, implying that the tension is directly related to the waiting behaviour, so it can be inferred that it is a state of emotion that is now being experienced."}, "sentence_type": "Imperative sentence", "pos": {"verbs": {"Wait": 1, "Nervous": 1}, "adverbs": {"Yes": 1}, "numerals": {"439": 1}}}, {"content": "When I was excited, I was frustrated by the depression.", "word_count": 24, "label": "", "sentence_structure": "Subject鈥搗erb鈥揷omplement", "subjective_objective": {"answer": "Subjective", "rationale": "Because it's the feelings and attitudes of the patients that are being examined."}, "sentence_sentiment": {"answer": "Negative", "rationale": "Because the behavior and emotions of \"damn\" in the entries are negative."}, "language_style": {"answer": "Tactful and warm", "rationale": "The question is one of gentle and inviting responses to the interviewer鈥檚 emotional reactions, rather than direct orders or statements. At the same time, the purpose of the article is expressed in a more intimate and acceptable way, and can therefore be considered as a soft and warm language style."}, "time_dimension": {"answer": "Present", "rationale": "\"When I am excited\" and \"seeing others sad and depressed\" are describing the current state and behaviour of the subjects, and \"I am greatly frustrated\" are subjective reactions to these situations and behaviours. The entry is therefore asking the present feelings and attitudes of the subjects."}, "sentence_type": "Affirmative declarative sentence", "pos": {"nouns": {"Time": 2, "person": 2, "Emotion": 1, "鈾?And the joys are high 鈾?: 1, "It's a bad time": 1}, "verbs": {"Yes": 1, "鈾?And I'm gonna be 鈾?: 1, "Great": 1, "Declining": 1}, "adjectives": {"鈾?And the joys are high 鈾?: 1, "Blue": 1, "Declining": 1}, "numerals": {"440": 1}, "pronouns": {"I": 1}}}, {"content": "441. I like women with high heights.", "word_count": 9, "label": "", "sentence_structure": "Subject鈥搗erb鈥搊bject", "subjective_objective": {"answer": "Subjective", "rationale": "Like being patient subjective."}, "sentence_sentiment": {"answer": "Negative", "rationale": "Because the \"woman who likes to be tall\" in the entry is sexist and negative emotional."}, "language_style": {"answer": "Calm and direct", "rationale": "The entry expresses the subject of male preference for female body in a straightforward manner, without adding too much qualifier or emotional colour, and is more directly objective in tone."}, "time_dimension": {"answer": "Present", "rationale": "The entry describes a person鈥檚 current preferences or attitudes, i.e. the degree of love for a woman with a tall body."}, "sentence_type": "Imperative sentence", "pos": {"nouns": {"Women": 1, "Height": 1}, "verbs": {"Yeah": 1}, "adjectives": {"High": 1}, "numerals": {"441": 1}, "pronouns": {"I": 1}}}, {"content": "442. There have been periods when I was insomniad.", "word_count": 11, "label": "", "sentence_structure": "Subject鈥揷opula鈥損redicative", "subjective_objective": {"answer": "Subjective", "rationale": "Because anxiety is the patient ' s subjective emotion, insomnia is also a subjective experience."}, "sentence_sentiment": {"answer": "Negative", "rationale": "Because the \"sleep out of fear\" act in the entry is negative."}, "language_style": {"answer": "Tactful and warm", "rationale": "The entry uses a more euphemistic approach, beginning with \"sometimes\", implying that this is a common situation and describing the problem in detail as \"I am insomnia\" . This expression avoids direct accusations or criticism, but also generates concern and reflection, more personal and human."}, "time_dimension": {"answer": "Past", "rationale": "The keyword \"sometimes\" indicates that the problem is about the testee's past, and \"insomnia\" is a continuous problem that may occur at different points in time. The entry is therefore an inquiry into whether the testee has experienced insomnia due to anxiety in the past."}, "sentence_type": "Affirmative declarative sentence", "pos": {"nouns": {"Period": 1, "Fear": 1, "Insomnia": 1}, "verbs": {"Some": 1, "And": 1, "be": 1}, "adverbs": {"Because": 1}, "numerals": {"442": 1}}}, {"content": "443. If others think I am inappropriate for certain things, I can easily give up.", "word_count": 25, "label": "", "sentence_structure": "Subject鈥搗erb鈥搊bject", "subjective_objective": {"answer": "Subjective", "rationale": "This is a description of the patient ' s mental state and reflects his/her self-awareness and attitude."}, "sentence_sentiment": {"answer": "Negative", "rationale": "Because the \"easy to give up\" act in the entry is negative."}, "language_style": {"answer": "Tactful and warm", "rationale": "The entry is more adept and interactive when it is spoken in a gentle and inviting manner, rather than by direct statements or orders."}, "time_dimension": {"answer": "Past", "rationale": ""}, "sentence_type": "Negative declarative sentence", "pos": {"nouns": {"Yes": 1, "Approach": 1, "Word": 1}, "verbs": {"I think": 1, "Easy": 1, "Abandon": 1}, "adverbs": {"Suppose": 1, "very": 1}, "numerals": {"443锛?: 1}, "pronouns": {"others": 1, "I": 2}}}, {"content": "444. I do not want to correct those who express their ignorance.", "word_count": 18, "label": "", "sentence_structure": "Subject鈥搗erb鈥搊bject", "subjective_objective": {"answer": "Subjective", "rationale": "Because of the patient's subjective attitude to not wanting to correct others."}, "sentence_sentiment": {"answer": "Negative", "rationale": "Because the \"no correction\" attitude in the entry is negative."}, "language_style": {"answer": "Calm and direct", "rationale": "The language style of the entry is straightforward, because it does not use euphemism or strong emotional expressions, but rather expresses the author鈥檚 view that it is not willing to correct the mistakes of others. This language style is more common in questionnaires, allowing respondents to understand the meaning of the problem more accurately and avoid unnecessary ambiguity."}, "time_dimension": {"answer": "Present", "rationale": "\"I don't want to correct \"no\" means a negative attitude, \"to correct\" is an exercise that is taking place, so the sentence is a state of things and a thought."}, "sentence_type": "Negative declarative sentence", "pos": {"nouns": {"Questionnaire": 1, "Entry": 1, "person": 1, "Observations": 1}, "verbs": {"Go on": 1, "Correct": 1, "Let's go": 1, "Table": 1, "Publication": 1, "Ignorance": 1}, "adjectives": {"Ignorance": 1}, "numerals": {"444锛?: 1, "Well": 1}, "classifiers": {"individual": 1}, "pronouns": {"I": 1}}}, {"content": "445. When I was young, I liked to play.", "word_count": 12, "label": "", "sentence_structure": "Subject鈥揷opula鈥損redicative", "subjective_objective": {"answer": "Subjective", "rationale": "Because \"like to be a hottie\" is the patient's subjective feeling and attitude."}, "sentence_sentiment": {"answer": "Positive", "rationale": "Because the \"like to be a hottie\" in the entry is a positive, optimistic attitude and behavior."}, "language_style": {"answer": "Tactful and warm", "rationale": "The language style of the entry is more euphemistic and implicit, using relatively moderate terms such as \"young\" and \"like to be popular\", and not directly expressing or ordering the interviewee, but rather engaging in dialogue in an invitation-based manner, more personal and interactive."}, "time_dimension": {"answer": "Past", "rationale": "It is judged by \"I was young\" that it is a question of the person who was tested for behaviour or experience at some stage in the past, so the temporal dimension of the article is the past."}, "sentence_type": "Affirmative declarative sentence", "pos": {"nouns": {"Year": 1, "Time": 1, "Children": 1, "Hot": 1}, "verbs": {"Hi, Hi": 1, "- Yes": 1}, "adjectives": {"Young": 1}, "numerals": {"445": 1}}}, {"content": "446. The police are usually honest.", "word_count": 8, "label": "", "sentence_structure": "Subject鈥揷opula鈥損redicative", "subjective_objective": {"answer": "Objective", "rationale": "Since the integrity of the police is an observable phenomenon, it is possible to draw conclusions from research, not just the subjective views of the patient."}, "sentence_sentiment": {"answer": "Neutral", "rationale": "Because the entry merely stated a fact and did not contain any emotional tendencies."}, "language_style": {"answer": "Calm and direct", "rationale": "The language style of the entry is calm and straightforward. It expresses a point in an objective, concise way, without using any rhetorical or emotional colour to strengthen the tone. This style is usually used in research studies to collect data and information."}, "time_dimension": {"answer": "Present", "rationale": "The entry represents a description or perspective of the current situation, does not address past or future situations, and therefore the temporal dimension is now judged."}, "sentence_type": "Affirmative declarative sentence", "pos": {"nouns": {"Police": 1, "Usually": 1, "Honesty": 1}, "adjectives": {"Honest": 1}, "adverbs": {"Usually": 1}, "numerals": {"446锛?: 1}}}, {"content": "When others object to my opinion, I will do whatever it takes to convince him.", "word_count": 19, "label": "", "sentence_structure": "Subject鈥搗erb鈥搊bject", "subjective_objective": {"answer": "Subjective", "rationale": "Because \"do whatever to convince him\" is the patient's subjective attitude and behavior."}, "sentence_sentiment": {"answer": "Positive", "rationale": "Because the behavior in the entry \"do whatever to convince\" is a strong expression of motivation and determination."}, "language_style": {"answer": "Calm and direct", "rationale": "The entry is more directly linguistically, with no excessive emotional rendering or rhetorical techniques, but rather asks the respondent in an objective and clear manner about his attitude and behaviour in the face of objections. Thus, his language style can be judged as \"cool and direct\"."}, "time_dimension": {"answer": "Present", "rationale": "In the past, the entry describes a customary act of the person being tested, i.e. a strong persuasion in the face of objections. It is not possible to determine whether such act has occurred or is continuing in the past, since no specific time period or context is mentioned. But by the expression \"doing whatever to convince him\" it can be inferred that this is a more violent tendency to behave and that a similar situation may have occurred in the past."}, "sentence_type": "Affirmative declarative sentence", "pos": {"nouns": {"others": 1, "Observations": 1, "Convince": 1}, "verbs": {"Objection": 1, "Yes": 1, "Go on": 1, "Convince": 1}, "adverbs": {"Everything": 1, "Whatever": 1}, "numerals": {"447": 1}, "pronouns": {"he": 1}}}, {"content": "448. I feel uncomfortable when I am being watched on the street, in cars or in shops.", "word_count": 23, "label": "", "sentence_structure": "Subject鈥損redicate", "subjective_objective": {"answer": "Subjective", "rationale": "Because \"will be disturbed\" is the patient's subjective feeling."}, "sentence_sentiment": {"answer": "Negative", "rationale": "Because the emotional attitude of \"will be disturbed\" in the entry is negative."}, "language_style": {"answer": "Calm and direct", "rationale": "The article is more straightforward, and there is no excessive rhetorical and emotional rendering. It asks the interviewee whether he or she is disturbed in the streets, cars or shops, if anyone looks at himself. This language is common in psychological questionnaires, because it helps researchers to understand more accurately how the interviewee feels and thinks."}, "time_dimension": {"answer": "Past", "rationale": "The entry is an inquiry into the respondent ' s reaction to others, and the temporal dimension does not clearly point to past, present or future, and therefore cannot be categorized as past, present or future behaviour or mentality."}, "sentence_type": "Affirmative declarative sentence", "pos": {"nouns": {"Street": 1, "Car": 1, "Shop": 1, "person": 1, "Watch": 1, "Unsettled": 1}, "verbs": {"Yes": 2, "I think": 1}, "adverbs": {"very": 1}, "numerals": {"448": 1}}}, {"content": "449. I do not like to see women smoke.", "word_count": 10, "label": "", "sentence_structure": "Subject鈥搗erb鈥搊bject", "subjective_objective": {"answer": "Subjective", "rationale": "Because \"not like seeing\" shows the attitude or feeling of the patient."}, "sentence_sentiment": {"answer": "Negative", "rationale": "Because the \"not like to see\" attitude in the entry is negative."}, "language_style": {"answer": "Tactful and warm", "rationale": "This language style increases the willingness of respondents to listen and respond to the questionnaire and facilitates the effective conduct of the questionnaire."}, "time_dimension": {"answer": "Present", "rationale": ""}, "sentence_type": "Negative declarative sentence", "pos": {"nouns": {"Women": 1, "Smoking": 1}, "verbs": {"I like to see it": 1, "not": 1, "Smoking": 1}, "adjectives": {"I don't like it": 1}, "adverbs": {"I": 1}, "numerals": {"449": 1}}}, {"content": "I'm not much of a depressed person.", "word_count": 9, "label": "", "sentence_structure": "Subject鈥搗erb鈥揷omplement", "subjective_objective": {"answer": "Subjective", "rationale": "Because depression is the patient's own psychological state."}, "sentence_sentiment": {"answer": "Negative", "rationale": "Because the mood of \"depression\" in the entry is negative."}, "language_style": {"answer": "Calm and direct", "rationale": "The entry is more straightforward, concise, and does not use too many rhetorical or emotional colours to directly question whether the person under investigation is often depressed."}, "time_dimension": {"answer": "Present", "rationale": "\"Neither\" denotes the past in frequency, and \"depressiveness\" is a state, so the sentence describes the past."}, "sentence_type": "Negative declarative sentence", "pos": {"nouns": {"Blue": 1, "Mistake": 1}, "verbs": {"have": 1, "Less": 1}, "adjectives": {"very": 1}, "numerals": {"450": 1}, "pronouns": {"I": 1}}}, {"content": "451. If someone has made a foolish and ignorant opinion about what I know, I always try to correct him.", "word_count": 29, "label": "", "sentence_structure": "Subject鈥搗erb鈥搊bject", "subjective_objective": {"answer": "Objective", "rationale": "Because the description of \"someone has a stupid and ignorant opinion of what I know\" is an objective fact, and \"I always try to correct him\" is the result of the act, the entry to the questionnaire is considered from an objective perspective."}, "sentence_sentiment": {"answer": "Positive", "rationale": "Because the actions in the entries are positive, i.e. they attempt to correct the wrong views of others."}, "language_style": {"answer": "Calm and direct", "rationale": "The entry clearly expresses dissatisfaction with the ignorance of others and expresses a corrective attitude."}, "time_dimension": {"answer": "Future", "rationale": "If I always try to correct him, the act has not yet happened, and therefore the future act is judged."}, "sentence_type": "Affirmative declarative sentence", "pos": {"nouns": {"Observations": 1, "Things": 2, "person": 1, "I": 1}, "verbs": {"to": 1, "I know you": 1, "Publication": 1, "Try": 1, "Correct": 1}, "adjectives": {"Stupid": 1, "Ignorance": 1, "Always": 1}, "numerals": {"451": 1, "One": 2, "Two": 1}}}, {"content": "I like to joke about others.", "word_count": 9, "label": "", "sentence_structure": "Subject鈥搗erb鈥搊bject", "subjective_objective": {"answer": "Subjective", "rationale": "Because \"like to joke\" is a personal preference or attitude that is seen from the patient's subjective point of view."}, "sentence_sentiment": {"answer": "Negative", "rationale": "Because \"joke someone else\" could hurt someone's feelings, and it's negative."}, "language_style": {"answer": "Tactful and warm", "rationale": "The language style of the entry is euphemism. It does not ask or order the other person to be a joker, but rather to engage in a gentle, inviting conversation, which is more personal and interactive. Moreover, the statement \"I like to joke about others\" is not much emotional, and it is more objective in tone."}, "time_dimension": {"answer": "Present", "rationale": "Now the entry expresses a personal preference or attitude that does not relate to past or future situations. The keyword is \"like,\" which is the sense and the thought of the present. At the same time, \"joke someone else's joke\" is an act or a customary act, which is generally the current state of affairs, which suggests that the act may have occurred, present or future. Thus, the temporal dimension of the article is now."}, "sentence_type": "Affirmative declarative sentence", "pos": {"nouns": {"Questionnaire": 1, "Entry": 1}, "verbs": {"Yeah": 1, "Open": 1, "Play": 1}, "adjectives": {"others": 1}, "adverbs": {"of": 2, "A joke": 1}, "numerals": {"452锛?: 1}}}, {"content": "453. When I was a child, I had no concern with being part of a gang.", "word_count": 14, "label": "", "sentence_structure": "Subject鈥損redicate", "subjective_objective": {"answer": "Subjective", "rationale": "Because it is not a subjective feeling or attitude to whether or not to join a group."}, "sentence_sentiment": {"answer": "Neutral", "rationale": "Because the entry does not have any emotional color."}, "language_style": {"answer": "Calm and direct", "rationale": ""}, "time_dimension": {"answer": "Past", "rationale": "\"When I was a child\" shows that it is a description of the testee's past, so the temporal dimension is the past."}, "sentence_type": "Negative declarative sentence", "pos": {"nouns": {"When I was little": 1, "Gang": 1}, "verbs": {"to": 1, "Participation": 1, "It doesn't matter": 1}, "adverbs": {"very": 1}, "numerals": {"453.0": 1}, "pronouns": {"I": 1}}}, {"content": "454. I am happy to live alone in a cabin in the mountains or in the old woods.", "word_count": 20, "label": "", "sentence_structure": "Subject鈥搗erb鈥揷omplement", "subjective_objective": {"answer": "Subjective", "rationale": "Because feeling happy is the subjective attitude and feeling of the patient."}, "sentence_sentiment": {"answer": "Positive", "rationale": "Because the entries \"single in the cabins of the mountains or the woods\" and \"get happy\" express positive emotional attitudes."}, "language_style": {"answer": "Tactful and warm", "rationale": "The entry uses indirect questions, by depicting a situation in which the interviewee can imagine and express his views and feelings freely. Moreover, the phrase \"I feel happy\" is used in a language that gives a warm and comfortable atmosphere, which helps to ease the tension of the interviewee. This language style is more friendly, gentle and resonant with the interviewee."}, "time_dimension": {"answer": "Present", "rationale": "In the future, the entry describes and expresses a sense of happiness about the behaviour and feelings that the testee may have in the future, namely, \"living alone in a cabin in the mountains or in the old woods\". The time dimension of the questionnaire is therefore the future."}, "sentence_type": "Affirmative declarative sentence", "pos": {"nouns": {"The cabin": 1, "Deep mountains": 1, "Lin": 1}, "verbs": {"Stay": 1, "I think": 1, "Happy": 1}, "adjectives": {"Alone": 1}, "adverbs": {"Yeah": 1}, "numerals": {"454锛?: 1}}}, {"content": "Many people call me acute.", "word_count": 10, "label": "", "sentence_structure": "Subject鈥搗erb鈥揷omplement", "subjective_objective": {"answer": "Subjective", "rationale": "This is because it is a subjective observation that others evaluate the patient."}, "sentence_sentiment": {"answer": "Neutral", "rationale": "Because the entry merely stated a fact, there was no obvious emotional colour."}, "language_style": {"answer": "Calm and direct", "rationale": "The entry is presented in a direct way, without too much adoration or euphemism, and directly conveys a message asking whether the other side is considered to be acute. This language style is simpler and more clear.\""}, "time_dimension": {"answer": "Past", "rationale": "The entry \"many say \"and \" yes\" is a representation of past actions or states, so it describes past experiences and the temporal dimension is a word of the past."}, "sentence_type": "Affirmative declarative sentence", "pos": {"nouns": {"person": 1, "- Angry": 1}, "verbs": {"I'm telling you": 1, "be": 1}, "adjectives": {"Sex": 1, "Urgent": 1}, "adverbs": {"Both": 1, "Many": 1}, "numerals": {"455锛?: 1}, "pronouns": {"I": 1}}}, {"content": "456. A person who has violated a law which he considers unreasonable should not be punished.", "word_count": 29, "label": "", "sentence_structure": "Subject鈥搗erb鈥搊bject", "subjective_objective": {"answer": "Subjective", "rationale": "Because this entry relates to the individual ' s judgement and attitude to legal reasonableness, it is the patient ' s subjective view."}, "sentence_sentiment": {"answer": "Negative", "rationale": "Because the idea that \"not to be punished\" in the entry is negative."}, "language_style": {"answer": "Tactful and warm", "rationale": "The entry is a question-and-answer dialogue, not a direct expression of views or attitudes towards a person, but a moderate and respectful way of directing the interviewee to think and answer questions. At the same time, the phrase in the sentence is more neutral, without the use of strong rhetoric or emotional colours, and reflects a soft and warm language style."}, "time_dimension": {"answer": "Past", "rationale": "The \"he\" in the questionnaire refers to the person of the past, so it is a judgement and a description of the past. At the same time, the \"no punishment\" has already occurred, and thus points to the past."}, "sentence_type": "Negative declarative sentence", "pos": {"nouns": {"person": 2, "One": 1, "Law": 1, "Punishment": 1}, "verbs": {"Infractions": 1, "I think": 1, "I think so": 1, "Yes": 1}, "adjectives": {"It doesn't make sense": 1}, "numerals": {"456锛?: 1, "One": 2}, "pronouns": {"he": 1}}}, {"content": "457. I think that a person should never drink.", "word_count": 12, "label": "", "sentence_structure": "Subject鈥搗erb鈥搊bject", "subjective_objective": {"answer": "Subjective", "rationale": "Because \"think\" is a subjective attitude or belief of a human being."}, "sentence_sentiment": {"answer": "Negative", "rationale": "Because the expression \"never should\" is used in the purpose of the article, it expresses a negative attitude."}, "language_style": {"answer": "Calm and direct", "rationale": "The language style of the entry is more calm and straightforward, with no excessive rhetorical or emotional rendering, and rather with clear language, the views and positions on alcohol use are expressed."}, "time_dimension": {"answer": "Present", "rationale": "The words \"consider\" and \"should never\" in the entry indicate that it is a personal conviction or attitude and therefore describes the present view and position."}, "sentence_type": "Affirmative declarative sentence", "pos": {"nouns": {"person": 1, "Wine": 1}, "verbs": {"I think": 1, "Never": 1}, "adverbs": {"I think so": 1, "Never": 1}, "numerals": {"457": 1}, "pronouns": {"I": 1}}}, {"content": "458. The person who was close to me when I was a child (father, stepfather, etc.) was very harsh to me.", "word_count": 22, "label": "", "sentence_structure": "Subject鈥搗erb鈥搊bject", "subjective_objective": {"answer": "Subjective", "rationale": "Because this is the memory and experience of the patient with his or her past experience, it involves personal emotional experience and subjective perceptions."}, "sentence_sentiment": {"answer": "Negative", "rationale": "Because the entry describes \"draconian\", it is a negative quality or behaviour that may have a negative impact on interpersonal relations."}, "language_style": {"answer": "Calm and direct", "rationale": "The entry is based on a direct question-and-answer approach, which explicitly asks the respondent about his relationship with his father or stepfather, without using any qualifier language or emotional colours, and in a concise manner."}, "time_dimension": {"answer": "Past", "rationale": ""}, "sentence_type": "Affirmative declarative sentence", "pos": {"nouns": {"When I was little": 1, "person": 1, "Father": 1, "Stepfather": 1, "Hard": 1}, "verbs": {"and": 1, "Closeness": 1, "to": 2}, "adjectives": {"Tens": 1}, "numerals": {"458锛?: 1}}}, {"content": "459. I have several bad habits that are deeply entrenched and difficult to correct.", "word_count": 17, "label": "", "sentence_structure": "Subject鈥搗erb鈥揷omplement", "subjective_objective": {"answer": "Subjective", "rationale": "Because \"I've been deep-rooted\" and \"I've been hard to correct\" are the patient's description of myself, reflecting his subjective feelings and thoughts."}, "sentence_sentiment": {"answer": "Negative", "rationale": "Because \"bad habits\" and \"deepness, hard to correct\" are negative expressions in the entries."}, "language_style": {"answer": "Calm and direct", "rationale": "The language style of the entry is calm and straightforward. It is a direct description of the theme, \"Some bad habits are entrenched and difficult to correct\", and instead of using too many qualifiers or emotional expressions, it is an objective way of asking the interviewee. This style is more common in psychological questionnaires, which avoids triggering the respondent ' s defensive spirit and facilitates a real and effective answer."}, "time_dimension": {"answer": "Past", "rationale": "The words \"already\" and \"hard to correct\" in the entry indicate that these bad habits have existed for some time and are difficult to change, so it can be judged that the entry is describing the past experience and state of affairs."}, "sentence_type": "Negative declarative sentence", "pos": {"nouns": {"Custom": 2, "Bad habits": 1, "Deep and deep": 1, "It's hard to fix": 1}, "verbs": {"have": 1, "Correct": 1}, "adjectives": {"Several": 1, "Bad": 1, "Hard": 1, "Deep and deep": 1}, "numerals": {"459": 1, "Plant": 1}, "classifiers": {"Several": 1}, "pronouns": {"I": 1}}}, {"content": "I only drink a few drinks (or not at all).", "word_count": 16, "label": "", "sentence_structure": "Subject鈥搗erb鈥揷omplement", "subjective_objective": {"answer": "Subjective", "rationale": "Because \"only appropriate\" and \"not at all\" are patients' subjective attitudes to drinking habits."}, "sentence_sentiment": {"answer": "Neutral", "rationale": "This entry only describes the habit and frequency of drinking by the person under investigation, and does not involve positive or negative emotional aspects."}, "language_style": {"answer": "Tactful and warm", "rationale": "The words \"appropriate\" or \"or \"at all\" are used in a more gentle and gentle language, without directly emphasizing or ordering the interviewee not to drink, but rather to ask the interviewee about his drinking habits in a more polite and respectful manner."}, "time_dimension": {"answer": "Present", "rationale": "The entry describes the current behaviour and habits of the testees, i.e. whether they are qualified to drink or not, and therefore judge them as now."}, "sentence_type": "Affirmative declarative sentence", "pos": {"nouns": {"Wine": 1}, "verbs": {"Drink": 2, "Drink it in the right amount": 1, "Not at all": 1}, "adjectives": {"A little": 1, "Appropriate": 1, "not": 1}, "adverbs": {"Only": 1, "Land": 1, "Yeah": 1}, "numerals": {"460": 1}, "classifiers": {"One": 2, "Some": 1}, "pronouns": {"I": 1}}}, {"content": "461. I hope I can avoid the distress caused by the trauma.", "word_count": 19, "label": "", "sentence_structure": "Subject鈥搗erb鈥搊bject", "subjective_objective": {"answer": "Subjective", "rationale": "This is because it is a subjective attitude, which is the patient ' s inner desire and expectations."}, "sentence_sentiment": {"answer": "Positive", "rationale": "Because the action \"to avoid the trauma\" in the entry is positive, so it's to avoid trouble."}, "language_style": {"answer": "Tactful and warm", "rationale": "The entry expresses a desire for idealism by using words such as \"favorite\" and \"avoid\" while encouraging respondents to be positive in their daily lives to be emotional and to reduce conflicts. The overall language is more euphemismal and encouraging, and is in keeping with the warmer language style."}, "time_dimension": {"answer": "Future", "rationale": "The entry is a description of the testee ' s expectations for the future and therefore judged to be future."}, "sentence_type": "Imperative sentence", "pos": {"nouns": {"Hope": 1, "person": 1, "Tetanus": 1, "Trouble": 1}, "verbs": {"Yes": 1, "Avoid": 1, "Cause": 1}, "adverbs": {"Because": 1}, "numerals": {"461": 1}, "pronouns": {"I": 1}}}, {"content": "I feel that I cannot tell anyone everything about myself.", "word_count": 16, "label": "", "sentence_structure": "Subject鈥揷opula鈥損redicative", "subjective_objective": {"answer": "Subjective", "rationale": "Because it is a subjective idea or attitude that patients feel they should do."}, "sentence_sentiment": {"answer": "Negative", "rationale": "Because the entry \"can't tell people everything about yourself\" expresses an emotional tendency to distrust others."}, "language_style": {"answer": "Calm and direct", "rationale": "The entry asks the other side in a direct and clear way about sharing personal information, without too many rhetorical or emotional expressions."}, "time_dimension": {"answer": "Present", "rationale": ""}, "sentence_type": "Negative declarative sentence", "pos": {"nouns": {"self": 1, "Everything": 1, "others": 1}, "verbs": {"I think": 1, "No, I can't": 1, "Tell": 1, "Both": 1}, "adverbs": {"not": 1, "Both": 1}, "numerals": {"462锛?: 1}, "pronouns": {"I": 1}}}, {"content": "I used to play \"jumping house\" (or \"stumping twitching\").", "word_count": 16, "label": "", "sentence_structure": "Subject鈥搗erb鈥搊bject", "subjective_objective": {"answer": "Subjective", "rationale": "Because playing games is a personal experience and feeling of the patient, and it is subjective."}, "sentence_sentiment": {"answer": "Neutral", "rationale": "Because the entry is merely a description of a person ' s past interests and does not express any emotional colours."}, "language_style": {"answer": "Tactful and warm", "rationale": "This entry is presented in a way that is reminiscent of the past, sharing personal experiences, and is spoken in a way that is friendly and has no more direct interview or command tone."}, "time_dimension": {"answer": "Past", "rationale": ""}, "sentence_type": "Imperative sentence", "pos": {"nouns": {"Jumping in the house": 1, "- Jumping": 1, "Game": 1}, "verbs": {"Yeah": 1, "Play": 1}, "numerals": {"463": 1}}}, {"content": "I have never seen a vision.", "word_count": 8, "label": "", "sentence_structure": "Subject鈥搗erb鈥搊bject", "subjective_objective": {"answer": "Objective", "rationale": "Because \"I've seen visions\" are the objective facts that patients have experienced, and \"I've never seen visions\" are the patient's statements, subjective."}, "sentence_sentiment": {"answer": "Neutral", "rationale": "Because the entry does not express any emotion, attitude or behaviour, it merely describes a fact."}, "language_style": {"answer": "Calm and direct", "rationale": "The language style of the entry is more objective and straightforward, without any symmetry or emotional rendering, but simply a simple statement of the facts."}, "time_dimension": {"answer": "Present", "rationale": ""}, "sentence_type": "Negative declarative sentence", "pos": {"nouns": {"Imagination": 1}, "verbs": {"See": 1, "Here": 1}, "adverbs": {"Never": 1}, "numerals": {"464": 1}, "classifiers": {"individual": 1}, "pronouns": {"I": 1}}}, {"content": "465. I have changed my mind several times about my career.", "word_count": 18, "label": "", "sentence_structure": "Subject鈥搗erb鈥搊bject", "subjective_objective": {"answer": "Subjective", "rationale": "Because \"changed mind\" is a description of a person's subjective feelings and attitudes."}, "sentence_sentiment": {"answer": "Negative", "rationale": "Because the \"changed mind\" act in the entry is negative."}, "language_style": {"answer": "Calm and direct", "rationale": "The entry describes changes in the life course of the person surveyed in an objective and direct manner, does not involve excessive rhetorical or emotional rendering, is more balanced and objective, and can therefore be considered as being of a calm and straightforward language style."}, "time_dimension": {"answer": "Present", "rationale": "The entry asks the testee about several changes in his career in his current state, and thus the time dimension is now judged."}, "sentence_type": "Affirmative declarative sentence", "pos": {"nouns": {"I": 1, "Observations": 1, "Lifetime occupation": 1}, "verbs": {"Already": 1, "A few times": 1, "Change": 2, "Yes": 1}, "adjectives": {"For life": 1}, "adverbs": {"Yes": 1}, "numerals": {"465": 1, "A few times": 1}}}, {"content": "I have never taken any medication or sleeping pills other than those prescribed by the doctor.", "word_count": 21, "label": "", "sentence_structure": "Subject鈥搗erb鈥搊bject", "subjective_objective": {"answer": "Objective", "rationale": "Because the use of drugs or sleeping pills is an objective result of behaviour, not the patient ' s own subjective will or ideas."}, "sentence_sentiment": {"answer": "Neutral", "rationale": "Because the entry merely presents a fact, it does not show any positive or negative emotional tendencies."}, "language_style": {"answer": "Calm and direct", "rationale": "The entry uses a straightforward language describing the customary behaviour of the person under investigation, without adding too much emotional colour or rhetorical techniques, so that the language style can be judged as being calm and straightforward.\""}, "time_dimension": {"answer": "Past", "rationale": "The entry covers past actions and commitments to the future, since \" never\" indicates a negative act that has occurred in the past, present and future."}, "sentence_type": "Negative declarative sentence", "pos": {"nouns": {"Doctor": 1, "Order": 1, "Drugs": 1, "Sleeping pills": 1}, "verbs": {"Take it": 2, "Here": 1}, "adverbs": {"Never": 1, "not": 3, "Any": 1}, "numerals": {"466": 1, "2": 1}, "classifiers": {"individual": 1}, "pronouns": {"I": 1}}}, {"content": "I often keep silent notes of numbers (plus car plates, etc.) that are not relevant.", "word_count": 20, "label": "", "sentence_structure": "Subject鈥搗erb鈥揷omplement", "subjective_objective": {"answer": "Subjective", "rationale": "Because \"the usual\" and \"the irrelevant number\" are personal experiences and thoughts of patients, they are subjective."}, "sentence_sentiment": {"answer": "Positive", "rationale": "Because the silent recording of some irrelevant numbers is a manifestation of memory and does not in itself have a negative or negative meaning."}, "language_style": {"answer": "Tactful and warm", "rationale": ""}, "time_dimension": {"answer": "Present", "rationale": "The entry \"I'm often\" means that it is a regular, customary act, and \"mistracting some irrelevant numbers (with car plates, etc.)\" is describing the specific content of the act. So, judging by the dimensions of time and behaviour, we can conclude that the entry describes the person being tested."}, "sentence_type": "Affirmative declarative sentence", "pos": {"nouns": {"Problem": 1, "Questionnaire": 1, "Entry": 1, "No": 2, "Car": 1, "License": 1}, "verbs": {"Silent": 1, "Add": 1}, "adverbs": {"Often": 1}, "numerals": {"467": 1}}}, {"content": "I often feel regret for my temper and complaints.", "word_count": 19, "label": "", "sentence_structure": "Subject鈥搗erb鈥揷omplement", "subjective_objective": {"answer": "Subjective", "rationale": "Because remorse is the patient's subjective feeling."}, "sentence_sentiment": {"answer": "Negative", "rationale": "Because the word \"repentant\" in the entry has negative emotions."}, "language_style": {"answer": "Tactful and warm", "rationale": "The entry is more adept and interactive when it is spoken in a gentle and inviting manner, rather than by direct statements or orders."}, "time_dimension": {"answer": "Present", "rationale": ""}, "sentence_type": "Affirmative declarative sentence", "pos": {"nouns": {"Questionnaire": 1, "Entry": 1, "- Grumpy": 1, "- He's complaining": 1, "Regret": 1}, "verbs": {"Often": 1, "Because": 1, "I feel": 1, "Let's go": 1, "- He's complaining": 1}, "adjectives": {"Love": 1}, "adverbs": {"Often": 1, "Because": 1}, "numerals": {"468锛?: 1}, "pronouns": {"I": 1}}}, {"content": "The lightning is one of the things I fear.", "word_count": 13, "label": "", "sentence_structure": "Subject鈥揷opula鈥損redicative", "subjective_objective": {"answer": "Subjective", "rationale": "Because fear is a subjective sentiment."}, "sentence_sentiment": {"answer": "Negative", "rationale": "Because \"fear\" is negative."}, "language_style": {"answer": "Calm and direct", "rationale": "The language style of the entry is more straightforward, not using too many rhetorical techniques or emotional renderings, but simply stating the fact that lightning is one of the things the interviewees fear. Thus, the language style is a direct, calm expression."}, "time_dimension": {"answer": "Past", "rationale": "The keyword \"I'm afraid\" means that it's an already existing emotion or feeling and that it happens at some point in the past."}, "sentence_type": "Affirmative declarative sentence", "pos": {"nouns": {"Lightning": 1, "Something": 1}, "verbs": {"be": 1, "Scared": 1, "Medium": 1}, "adjectives": {"One": 1}, "numerals": {"469": 1}, "classifiers": {"One": 2, "Plant": 1}, "pronouns": {"I": 1}}}, {"content": "470. The sexual aspect is a source of disgust to me.", "word_count": 11, "label": "", "sentence_structure": "Subject鈥揷opula鈥損redicative", "subjective_objective": {"answer": "Subjective", "rationale": "Because \"aversion\" is a subjective feeling or attitude."}, "sentence_sentiment": {"answer": "Negative", "rationale": "Because the \"detestation\" act in the entry is negative."}, "language_style": {"answer": "Tactful and warm", "rationale": "This language reduces the resistance of the surveyer and increases the rate of survey recovery and the amount of information available."}, "time_dimension": {"answer": "Present", "rationale": "The entry expresses the feeling or attitude of the person who was tested about \"sexual matters\" that may be temporary, instantaneous or persistent. Because of the lack of a clear time frame, it is not certain whether the entry is asking about past or future behaviour or mentality. In terms of language, the sentence follows the general current and can therefore be considered to describe the current feeling or state."}, "sentence_type": "Negative declarative sentence", "pos": {"nouns": {"Sex": 1, "Yes": 1}, "verbs": {"鈾?And I'm gonna be 鈾?: 1, "Disgusting": 1}, "numerals": {"470锛?: 1}}}, {"content": "In schools, teachers always rated my character badly.", "word_count": 18, "label": "", "sentence_structure": "Subject鈥搗erb鈥揷omplement", "subjective_objective": {"answer": "Subjective", "rationale": "Because this is a subjective assessment of the patient ' s perception and perception of his/her character."}, "sentence_sentiment": {"answer": "Negative", "rationale": "Because the expression \"always bad\" in the entry has a negative emotional connotation."}, "language_style": {"answer": "Calm and direct", "rationale": "The entry describes the problems or problems of the interviewee in a direct and clear way, without too many rhetorical or emotional renderings. This way conveys the message more clearly and makes it easier for the reader to understand the problems faced by the interviewee."}, "time_dimension": {"answer": "Past", "rationale": "The entry describes an event in the school, where the teacher evaluates the character of the testee."}, "sentence_type": "Negative declarative sentence", "pos": {"nouns": {"School": 1, "Teacher": 1, "Good conduct": 1, "Assessment": 1}, "verbs": {"to": 1, "Always": 1, "Good": 1}, "adverbs": {"very": 2}, "numerals": {"471锛?: 1, "One": 1}, "pronouns": {"I": 1}}}, {"content": "Fire is an attractive thing to me.", "word_count": 9, "label": "", "sentence_structure": "Subject鈥搗erb鈥揷omplement", "subjective_objective": {"answer": "Subjective", "rationale": "Because \"attractive\" is a subjective feeling or experience."}, "sentence_sentiment": {"answer": "Positive", "rationale": "Because the description \"fire has an appeal to me\" shows a positive emotional attitude or a positive interest in fire."}, "language_style": {"answer": "Tactful and warm", "rationale": "The language style of the entry is more euphemistic and implicit, and by using the expression \"Accept me\" it implies that the other side may have some interest in fire or emotional connection, and does not ask or express it too directly. This expression can better trigger the other side's reflection and self-reflection, while maintaining some respect and understanding."}, "time_dimension": {"answer": "Past", "rationale": "The key word in the entry, \"Fire has an appeal to me\", implies that the person tested has had such an experience or a feeling of attraction to fire, and therefore the temporal dimension is a matter of the past."}, "sentence_type": "Affirmative declarative sentence", "pos": {"nouns": {"Fire": 1, "An attraction": 1}, "verbs": {"have": 1, "Attractive": 1}, "numerals": {"472": 1}}}, {"content": "473. I like to let others guess what I'm going to do next.", "word_count": 17, "label": "", "sentence_structure": "Subject鈥搗erb鈥搊bject", "subjective_objective": {"answer": "Subjective", "rationale": "Because it is a subjective attitude or emotional expression, \"Let's guess what I'm going to do next\" describes the patient's behavioural orientation and personality. So the questionnaire is designed to look at the patient's subjective thoughts and behavioural orientation."}, "sentence_sentiment": {"answer": "Positive", "rationale": "Because \"I like\" is positive."}, "language_style": {"answer": "Tactful and warm", "rationale": ""}, "time_dimension": {"answer": "Present", "rationale": "The keywords \"like\" and \"the next move\" indicate a habit and preference of the person being tested, which is occurring while speaking or is occurring frequently in the past, so the time dimension of the article is now."}, "sentence_type": "Affirmative declarative sentence", "pos": {"nouns": {"473": 1, "Action": 1}, "verbs": {"Yeah": 1, "Jean": 1, "Guess": 1, "Take it": 1}, "adverbs": {"One second": 1}, "numerals": {"473": 1}, "pronouns": {"I": 1}}}, {"content": "474. I have no more pee than others.", "word_count": 11, "label": "", "sentence_structure": "Subject鈥揷opula鈥損redicative", "subjective_objective": {"answer": "Objective", "rationale": "Since the number of urinations is a physical condition of the patient, objective facts can be quantified."}, "sentence_sentiment": {"answer": "Neutral", "rationale": "Because the entry does not have any emotional color."}, "language_style": {"answer": "Calm and direct", "rationale": ""}, "time_dimension": {"answer": "Present", "rationale": "The entry uses a comparative structure of \"me\" as a first person and \"more than others\" which means that it is subjectively felt or observed in its current state of health. It is therefore judged that the entry is in the current state of the person being questioned."}, "sentence_type": "Negative declarative sentence", "pos": {"nouns": {"Number of urination": 1, "others": 1}, "verbs": {"- Yeah": 1, "More": 1}, "numerals": {"474": 1}}}, {"content": "When the last resort was granted, I disclosed only some of the truth that had not been damaged.", "word_count": 24, "label": "", "sentence_structure": "Subject鈥搗erb鈥搊bject", "subjective_objective": {"answer": "Subjective", "rationale": "Because \"only the part of the truth that is not compromised\" is a person's choice and decision, which reflects the patient's attitude and behaviour in a given situation, it is viewed from the patient's subjective point of view."}, "sentence_sentiment": {"answer": "Neutral", "rationale": "Because the entry does not address specific behaviour, attitudes or qualities, nor does it express positive or negative emotional sentiments. It simply describes a possible situation or condition."}, "language_style": {"answer": "Tactful and warm", "rationale": "The language style of the entry is euphemism. First, it uses the polite expression \"everything is necessary\" to express the attitude of revealing the truth in exceptional circumstances. Second, the emphasis on \"no harm to the truth\" shows that the person who talks wants to protect his interests and that the information that he wants to share is selective, not exhaustive. This expression is more personal and interactive, and more acceptable and resonant."}, "time_dimension": {"answer": "Present", "rationale": "In terms of time dimension, the phrase \"only some of the truth that is not damaging to itself\" expresses the act at hand, while the phrase \"when the last resort is necessary\" emphasizes the particular condition under which the act occurs, i.e., only in extreme circumstances. Thus, the article's purpose is to judge the moment."}, "sentence_type": "Affirmative declarative sentence", "pos": {"nouns": {"Time": 1, "I'll have to": 1, "Part of it": 1, "True": 1}, "verbs": {"Show": 1, "Damage": 1, "be": 1}, "adjectives": {"It's not to be harmed by itself": 1}, "numerals": {"475锛?: 1, "When it's time to go": 1, "It's not a loss to your own part of the truth": 1}, "classifiers": {"individual": 1}}}, {"content": "476. I am a special envoy from God.", "word_count": 8, "label": "", "sentence_structure": "Subject鈥揷opula鈥損redicative", "subjective_objective": {"answer": "Subjective", "rationale": "Because \"I am a special envoy from God\" is an expression of self-awareness and belief, a thought and conviction that is deep inside the patient and is subjective."}, "sentence_sentiment": {"answer": "Neutral", "rationale": "The entry is not marked by any emotional or attitudinal bias."}, "language_style": {"answer": "Calm and direct", "rationale": "This entry, which directly identifies itself as a \"God's Envoy\", does not use excessive rhetorical or emotional displays, which are more objective and sound. Such language styles are more direct and clear, and make it easier for respondents to understand and answer questions."}, "time_dimension": {"answer": "Past", "rationale": "Therefore, it is not possible to judge on the basis of the time dimension."}, "sentence_type": "Affirmative declarative sentence", "pos": {"nouns": {"God": 1, "Special Envoy": 1}, "verbs": {"Send it": 1, "be": 1}, "numerals": {"476": 1}, "pronouns": {"I": 1}}}, {"content": "477. If I had the same fault as a few friends, I would have preferred to bear it alone rather than to be in danger.", "word_count": 29, "label": "", "sentence_structure": "Subject鈥搗erb鈥搊bject", "subjective_objective": {"answer": "Subjective", "rationale": "Because this phrase expresses the patient鈥檚 idea and attitude that, in a similar situation, they prefer to take responsibility for themselves rather than to others. It is the patient鈥檚 subjective feeling and thoughts."}, "sentence_sentiment": {"answer": "Negative", "rationale": "The basis for the judgement: the act of \"I prefer to bear it and not to burden others\" in the entry is a negative selfish and irresponsible attitude."}, "language_style": {"answer": "Calm and direct", "rationale": "The language style of the entry is a straightforward and calm type, because it presents a clear view or belief, and does not have too many emotional or rhetorical styles. It is usually used to express strong attitudes or positions, emphasizing objectivity and factuality.\""}, "time_dimension": {"answer": "Present", "rationale": "The keywords \"if\" \"yes\" and \"better\" and so on indicate that it is a hypothetical and current state of thinking, so the entry is now."}, "sentence_type": "Affirmative declarative sentence", "pos": {"nouns": {"Wrong": 1, "friends": 1, "Responsibility": 1}, "verbs": {"Undertaking": 1, "I'm in trouble": 1, "have": 1}, "adverbs": {"I'd rather not": 1, "not": 2, "and": 1, "Here": 1}, "numerals": {"477": 1}, "pronouns": {"I": 1, "others": 1}}}, {"content": "478. I have never been particularly nervous about my family ' s problems.", "word_count": 23, "label": "", "sentence_structure": "Subject鈥搗erb鈥揷omplement", "subjective_objective": {"answer": "Objective", "rationale": "This is a description of the objective facts of the patient ' s past experience."}, "sentence_sentiment": {"answer": "Positive", "rationale": "Because it is a positive attitude and emotion that the entry indicates that a person is not nervous about family problems."}, "language_style": {"answer": "Tactful and warm", "rationale": "The entry is more ludicrous and warm, asking in a gentle, caring tone whether there have been any cases of tension caused by family problems."}, "time_dimension": {"answer": "Past", "rationale": ""}, "sentence_type": "Negative declarative sentence", "pos": {"nouns": {"Home": 2, "person": 1, "Yes": 1, "self": 1}, "verbs": {"Nope": 1, "Fuck": 1, "I feel": 1, "Nervous": 1}, "adjectives": {"Special": 1}, "adverbs": {"Never": 1}, "numerals": {"478": 1}, "pronouns": {"I": 1}}}, {"content": "The only miracle I know is the mutual deception of people.", "word_count": 19, "label": "", "sentence_structure": "Subject鈥揷opula鈥損redicative", "subjective_objective": {"answer": "Subjective", "rationale": "Because the title \"I know\" shows that it's a patient's personal perception and feelings, and it's subjective."}, "sentence_sentiment": {"answer": "Negative", "rationale": "Because the \"fun\" in the entry is negative."}, "language_style": {"answer": "Calm and direct", "rationale": "The entry is more calm and straightforward, is less emotional or isomanic, and is more objective and rational. At the same time, it expresses a clear view that \"the only miracle of human deception is between human beings\" and is more concise."}, "time_dimension": {"answer": "Present", "rationale": "The phrase uses the expression \"all I know\" and \"sole\" to emphasize the perception and feelings of the phenomenon, so it can be judged to be a description of the current state."}, "sentence_type": "Affirmative declarative sentence", "pos": {"nouns": {"person": 2, "STAGE": 2, "And then you cheat each other": 1, "The only miracle": 1}, "verbs": {"be": 1, "Yes": 1, "have": 1}, "adjectives": {"All I know": 1}, "adverbs": {"One": 1}, "numerals": {"479锛?: 1}, "pronouns": {"I": 1}}}, {"content": "I'm often afraid of the dark.", "word_count": 6, "label": "", "sentence_structure": "Subject鈥揷opula鈥損redicative", "subjective_objective": {"answer": "Subjective", "rationale": "Fear of darkness is a subjective feeling and psychological reaction, and the personal experience and perception of the patient is not an objective reality."}, "sentence_sentiment": {"answer": "Negative", "rationale": "Because the \"fear of darkness\" emotion in the entry is negative."}, "language_style": {"answer": "Tactful and warm", "rationale": "The language style of the entry is relatively euphemistic, by asking if the interviewees are often afraid of darkness, and by leading them in a gentle way to share their feelings and experiences. At the same time, the term \"often\" is used to express a universal emotional experience, rather than to directly criticize or criticize, which is more readily accepted and co-opted by the interviewees."}, "time_dimension": {"answer": "Present", "rationale": "The keyword is \"I'm often\" and it means it's a regular, customary situation. And \"fear of darkness\" is an emotional reaction, implying a state of things or feeling that is now in progress. So the time dimension of this article should be now."}, "sentence_type": "Affirmative declarative sentence", "pos": {"nouns": {"Darkness": 1}, "verbs": {"Yes": 1, "Often": 1}, "adverbs": {"Often": 1}, "numerals": {"480": 1}}}, {"content": "481. I am afraid of being alone in the dark.", "word_count": 13, "label": "", "sentence_structure": "Subject鈥搗erb鈥揷omplement", "subjective_objective": {"answer": "Subjective", "rationale": "Because fear is a subjective feeling and emotional reaction."}, "sentence_sentiment": {"answer": "Negative", "rationale": "Because the attitude of \"fear\" in the entries is negative."}, "language_style": {"answer": "Tactful and warm", "rationale": "The entry is more euphemistic, uses first-person expression and asks, in a caring and understanding manner, whether the other side is afraid of being alone in the dark. Such expression is more likely to generate resonance and create a good atmosphere of communication."}, "time_dimension": {"answer": "Past", "rationale": "The entry is about the victims' fear and experience of the past, and the key words are \"fear\" and \"sing out in the dark\" and they point to what has happened."}, "sentence_type": "Negative declarative sentence", "pos": {"nouns": {"Alone": 1, "Darkness": 1}, "verbs": {"Scared": 1, "Stay": 1}, "adverbs": {"Alone": 1}, "numerals": {"481": 1}}}, {"content": "482. My plan always seemed to be difficult, forcing me to give up.", "word_count": 22, "label": "", "sentence_structure": "Subject鈥揷opula鈥損redicative", "subjective_objective": {"answer": "Subjective", "rationale": "Because the difficulties of the \"feel\" plan are the patient's own subjective feelings and experiences."}, "sentence_sentiment": {"answer": "Negative", "rationale": "Because the expression \"the plan always seems difficult\" is negative."}, "language_style": {"answer": "Tactful and warm", "rationale": ""}, "time_dimension": {"answer": "Present", "rationale": "Now the entry describes the current feelings and experiences of the testees, and \"my plan\" suggests that this is an ongoing project. And the expression \"difficult\" and \"have to give up\" further suggests that this is happening or still going on, so the temporal dimension is now."}, "sentence_type": "Negative declarative sentence", "pos": {"nouns": {"Planned": 1, "Difficulties": 1, "Abandon": 1}, "verbs": {"Looks like": 1, "鈾?And I'm gonna be 鈾?: 1, "I had to": 1, "Abandon": 1}, "adjectives": {"Thick": 1}, "adverbs": {"Always": 1}, "numerals": {"482锛?: 1}}}, {"content": "God makes miracles.", "word_count": 5, "label": "", "sentence_structure": "Subject鈥損redicate", "subjective_objective": {"answer": "Subjective", "rationale": "Because \"God's miracle\" is a faith or belief, a patient's subjective attitude."}, "sentence_sentiment": {"answer": "Neutral", "rationale": "As the entry merely presented a point of view, there was no apparent emotional tendency or evaluation."}, "language_style": {"answer": "Tactful and warm", "rationale": "The language style of the entry is more euphemistic, because it uses the term \"God's miracle\" rather than direct statements or orders. This expression is more moderate and inviting, making the respondents more willing to answer questions and participate in the investigation. At the same time, the use of the subject gives the respondents pleasure and appreciation, thus increasing their motivation and cooperation."}, "time_dimension": {"answer": "Future", "rationale": "According to the sentence entered, \"God's miracle\" expresses the hope and faith for the future, and therefore the judgement is the future."}, "sentence_type": "Affirmative declarative sentence", "pos": {"nouns": {"Miracles": 1, "God": 1}, "verbs": {"Create": 1}, "numerals": {"483": 1}}}, {"content": "484. There are shortcomings that I have to acknowledge and try to control, but that cannot be eliminated.", "word_count": 21, "label": "", "sentence_structure": "Subject鈥搗erb鈥搊bject", "subjective_objective": {"answer": "Subjective", "rationale": "Because \"some shortcomings, I have to admit and try to control them, but I cannot eliminate them\" is the attitude and the idea of the patient to his or her shortcomings, which is subjective."}, "sentence_sentiment": {"answer": "Negative", "rationale": "Because the entries \"some shortcomings\" and \"unresolved\" express a negative emotional tone, indicating that respondents have a more negative view of certain aspects of themselves and that they are difficult to change."}, "language_style": {"answer": "Calm and direct", "rationale": "The entry is a direct expression of the attitude and approach of the subject in dealing with certain shortcomings, without excessive rhetorical or emotional rendering, and with a more objective tone."}, "time_dimension": {"answer": "Present", "rationale": ""}, "sentence_type": "Affirmative declarative sentence", "pos": {"nouns": {"Disadvantages": 2, "Control": 1, "Elimination": 1}, "verbs": {"Recognition": 1, "Try": 1, "Add": 1, "Cannot": 1, "Elimination": 1}, "adverbs": {"Just": 1, "and": 1}, "numerals": {"484锛?: 1}, "classifiers": {"Some": 1}, "pronouns": {"I": 1}}}, {"content": "When a man and a woman are together, he usually thinks about her sexual aspects.", "word_count": 30, "label": "", "sentence_structure": "Subject鈥搗erb鈥搊bject", "subjective_objective": {"answer": "Subjective", "rationale": "Because it's a man's idea, it's subjective."}, "sentence_sentiment": {"answer": "Negative", "rationale": "Because the description \"he usually thinks about her sex\" in the entry is tending to be demeaning to women and negative."}, "language_style": {"answer": "Calm and direct", "rationale": "The entry presents a phenomenon or view in a direct and objective manner, without too much emotional rendering or use of rhetoric, and with a calmer and direct tone."}, "time_dimension": {"answer": "Present", "rationale": "This entry describes the general psychological phenomenon that men may be focusing on the sexual aspect of women when they interact with women."}, "sentence_type": "Exclamatory sentence", "pos": {"nouns": {"Men": 1, "Women": 1, "Time": 1, "she": 2, "Sex": 1, "In terms of": 1, "Yes": 1}, "verbs": {"Get along": 1, "I thought": 1, "be": 1}, "numerals": {"485": 1, "One": 2, "He usually": 1}}}, {"content": "I never found blood in my urine.", "word_count": 13, "label": "", "sentence_structure": "Subject鈥搗erb鈥搊bject", "subjective_objective": {"answer": "Objective", "rationale": "It is not subjective or subjective to find blood in urine as an observation of the patient."}, "sentence_sentiment": {"answer": "Neutral", "rationale": "Because the entry is not marked by emotional or attitudinal tendencies."}, "language_style": {"answer": "Calm and direct", "rationale": "The language style of the entry is more straightforward, there is no excessive rhetorical or emotional rendering, but simply asks if the person under investigation has such a situation. This tone makes it feel relatively objective, real and reliable."}, "time_dimension": {"answer": "Past", "rationale": ""}, "sentence_type": "Negative declarative sentence", "pos": {"nouns": {"In the urine": 1, "Blood": 1}, "verbs": {"Found": 1, "have": 1}, "adverbs": {"Never": 1, "No, I'm not": 2}, "numerals": {"486锛?: 1}, "pronouns": {"I": 1}}}, {"content": "When I try to make things wrong and not others get misunderstood, I am often very sad.", "word_count": 31, "label": "", "sentence_structure": "Subject鈥揷opula鈥損redicative", "subjective_objective": {"answer": "Subjective", "rationale": "The reason for the pain is that it is the patient's subjective feeling."}, "sentence_sentiment": {"answer": "Negative", "rationale": "Because the emotional expression \"I'm often very sad\" in the entry is negative."}, "language_style": {"answer": "Tactful and warm", "rationale": "The entry is more personal and interactive when it is spoken in a voice that cares about the feelings of others, rather than directly stating or ordering. At the same time, by using the term \"often\" it expresses the opinion of the surveyer that the emotional experience of the interviewee is not too strong to ask the interviewee to answer."}, "time_dimension": {"answer": "Past", "rationale": "The verbs \"attempted,\" \"made\" and \"felt\" in the entry all indicate events or circumstances of the past. So, judging from the temporal dimension, the entry is about the behaviour and feelings of the past."}, "sentence_type": "Affirmative declarative sentence", "pos": {"nouns": {"Time": 1, "Things": 1, "person": 2, "Error": 1, "Do the job": 1}, "verbs": {"Try": 1, "鈾?And I'm gonna be 鈾?: 1, "Yes": 1, "Misunderstanding": 1, "I feel": 1, "Sorry": 1, "Often": 1, "Do it": 1}, "adverbs": {"Tens": 1}, "numerals": {"487": 1}, "pronouns": {"I": 2}}}, {"content": "488. I pray several times a week.", "word_count": 8, "label": "", "sentence_structure": "Subject鈥損redicate", "subjective_objective": {"answer": "Subjective", "rationale": "Because the frequency of \"pray\" is the patient's subjective perception and behavioral habits, which are subjective in nature."}, "sentence_sentiment": {"answer": "Neutral", "rationale": "Because the entry simply asked the respondents about the frequency of weekly prayers, it did not contain any positive or negative emotional aspects."}, "language_style": {"answer": "Tactful and warm", "rationale": "The entry is more personal and interactive when it is spoken in a gentle, inquisitive manner, rather than in direct statements or orders. At the same time, the term \"pray\" is used to express respect for and tolerance of the religious beliefs of the interviewees."}, "time_dimension": {"answer": "Present", "rationale": "The entry asks about the present behaviour of the subjects, which is the frequency of their weekly prayers, and therefore it is judged to be the present."}, "sentence_type": "Affirmative declarative sentence", "pos": {"nouns": {"Week": 1, "Pray": 1}, "verbs": {"Every time": 1, "be": 1}, "numerals": {"Four hundred eighty-eight": 1, "How many": 1}, "classifiers": {"Number of times": 1}}}, {"content": "489. I sympathize with those who cannot escape their pain and sorrow.", "word_count": 16, "label": "", "sentence_structure": "Subject鈥搗erb鈥搊bject", "subjective_objective": {"answer": "Subjective", "rationale": "Because \"sympathetic\" is a subjective emotional response. While it involves the behaviour or state of others observed (i.e. they are unable to escape their distress and anxiety), emotional reactions are subjective in themselves and not objective facts."}, "sentence_sentiment": {"answer": "Positive", "rationale": "Because the \"sympathetic\" act in the entry is positive."}, "language_style": {"answer": "Tactful and warm", "rationale": "The language style in this entry is more euphemistic and gentle, and it uses the word \"sympathetic\" to express concern and understanding for those who cannot escape their pain and distress. It gives people a sweet, loving feeling, and it can easily resonate and identify."}, "time_dimension": {"answer": "Present", "rationale": "The entry is an inquiry into the respondent's view of \"sympathetic\" and does not have clear time dimension instructions, and therefore it can be considered to be an attitude or view of the present."}, "sentence_type": "Affirmative declarative sentence", "pos": {"nouns": {"489": 1, "I": 1, "person": 2, "Compassion": 1, "Trouble": 1, "Sad": 1}, "verbs": {"Yes": 1, "Get rid of it": 1, "Compassion": 1}, "adjectives": {"No, I can't": 1, "Those": 1, "of": 2, "and": 1, "Sad": 1}, "numerals": {"489": 1, "I": 1, "person": 2, "Compassion": 1, "Trouble": 1, "Sad": 1}, "classifiers": {"individual": 1}, "pronouns": {"Those": 1}}}, {"content": "490. I read the scriptures several times a week.", "word_count": 8, "label": "", "sentence_structure": "Subject鈥搗erb鈥揷omplement", "subjective_objective": {"answer": "Subjective", "rationale": "Because \"twice per week\" is the patient's personal behaviour and habits and is the patient's subjective attitude and behaviour."}, "sentence_sentiment": {"answer": "Neutral", "rationale": "Because the entry does not cover the description of emotion, attitude, behaviour, purpose or quality, it is merely an inquiry into personal habits."}, "language_style": {"answer": "Tactful and warm", "rationale": "The entry is based on a question about the individual beliefs of the other party, which is more gentle, respectful, and less critical or emphasized, so that the interviewee is comfortable and natural in his response. At the same time, the frequency of the \"reading\" is expressed as a freely chosen quantity, not as a criterion to be met, and such a design is conducive to obtaining more realistic and objective data."}, "time_dimension": {"answer": "Past", "rationale": ""}, "sentence_type": "Affirmative declarative sentence", "pos": {"nouns": {"The long run": 1, "Week": 1}, "verbs": {"Read it": 1}, "adverbs": {"How many": 1}, "numerals": {"490": 1}, "classifiers": {"Number of times": 1}}}, {"content": "I am impatient with those who believe that there is only one religion in the world.", "word_count": 25, "label": "", "sentence_structure": "Subject鈥搗erb鈥搊bject", "subjective_objective": {"answer": "Subjective", "rationale": "Because it is the patient ' s own subjective feeling and attitude to be impatient."}, "sentence_sentiment": {"answer": "Negative", "rationale": "Because the emotional reaction of \"insatisfaction\" in the entries is negative."}, "language_style": {"answer": "Calm and direct", "rationale": "The language style of the entry is more direct, without any rhetorical or emotional accents, but simply expresses attitudes towards some people. It is more calm and objective and conveys messages clearly."}, "time_dimension": {"answer": "Present", "rationale": "The keywords \"I'm impatient with thinking,\" \"I'm impatient,\" all of which indicate the feelings and attitudes of the talking person at this moment."}, "sentence_type": "Negative declarative sentence", "pos": {"nouns": {"person": 1, "World": 1, "Religion": 1, "Disgusting": 1}, "verbs": {"I feel": 1, "be": 1, "I think": 1, "There's only one": 1, "I'm tired": 1}, "adverbs": {"Really": 1}, "numerals": {"491": 1}, "pronouns": {"I": 1}}}, {"content": "I was afraid of the earthquake.", "word_count": 8, "label": "", "sentence_structure": "Subject鈥揷opula鈥損redicative", "subjective_objective": {"answer": "Subjective", "rationale": "Because \"I'm afraid of an earthquake\" is the feeling and experience inside the patient, and it's a subjective angle."}, "sentence_sentiment": {"answer": "Negative", "rationale": "Because fear in the entry is a negative emotional color."}, "language_style": {"answer": "Tactful and warm", "rationale": "The language style of the entry is more euphemistic, more accommodative, replacing the directly described \"experience\" with \"remember\" and reducing the language irritation that can cause fear; and the use of invitations to ask questions makes people feel more comfortable and safe."}, "time_dimension": {"answer": "Past", "rationale": "The entry is about past experiences and feelings, and the words \"remember\" and \"fear\" all imply a state of mind or emotion that recalls past events."}, "sentence_type": "Affirmative declarative sentence", "pos": {"nouns": {"Earthquake": 1}, "verbs": {"I remember": 1, "Scared": 1}, "adverbs": {"Just": 1}}}, {"content": "493. I like the kind of work that requires concentration, rather than less (involved) effort.", "word_count": 27, "label": "", "sentence_structure": "Subject鈥搗erb鈥搊bject", "subjective_objective": {"answer": "Subjective", "rationale": "Because \"like\" and \"not like\" are patients' personal emotional attitudes, this is an entry that is subjectively viewed."}, "sentence_sentiment": {"answer": "Negative", "rationale": "Because the expression \"not to be a carefree job\" has a negative emotional connotation."}, "language_style": {"answer": "Tactful and warm", "rationale": "The entry is more adept and interactive when it is spoken in a gentle and inviting manner, rather than by direct statements or orders."}, "time_dimension": {"answer": "Present", "rationale": ""}, "sentence_type": "Affirmative declarative sentence", "pos": {"nouns": {"Work": 2, "Attention": 1}, "verbs": {"Yeah": 2, "Yes": 1, "Focus": 1, "I don't like it": 1, "Save your breath": 1, "It's hard": 1}, "adjectives": {"That": 1, "Focus": 1, "It's not hard": 1}, "adverbs": {"Yeah": 1, "not": 1}, "numerals": {"493": 1}, "classifiers": {"Plant": 1, "Work": 1}, "pronouns": {"I": 1}}}, {"content": "I am afraid of being kept in small rooms or in closed places.", "word_count": 17, "label": "", "sentence_structure": "Subject鈥搗erb鈥搊bject", "subjective_objective": {"answer": "Subjective", "rationale": "Because it's subjective, because it's the feeling and fear inside the patient."}, "sentence_sentiment": {"answer": "Negative", "rationale": "The expression \"fear of being locked in small rooms or closed places\" in the entry is an expression of fear and anxiety about the situation and a negative emotional expression."}, "language_style": {"answer": "Tactful and warm", "rationale": ""}, "time_dimension": {"answer": "Present", "rationale": "The \"fear\" expression of fear, which is based on the current situation, is now judged. And \"I fear being kept in small rooms or closed places\" is a further illustration of what is possible at the moment."}, "sentence_type": "Negative declarative sentence", "pos": {"nouns": {"Room": 1, "Local": 1}, "verbs": {"Yes": 1, "Off": 1, "in": 1}, "adjectives": {"Small": 2}, "numerals": {"494": 1}, "pronouns": {"self": 1}}}, {"content": "For those who want to help them correct or improve, I am always frank.", "word_count": 24, "label": "", "sentence_structure": "Subject鈥搗erb鈥搊bject", "subjective_objective": {"answer": "Subjective", "rationale": "Because \"clarifying\" is what the patient thinks should be done, it's subjective."}, "sentence_sentiment": {"answer": "Positive", "rationale": "Because the entry expresses concern and goodwill towards others, positive action is taken to help others improve."}, "language_style": {"answer": "Calm and direct", "rationale": "This entry directly describes the attitude of the person under investigation in dealing with the person in need of help, without any acrimony or emotional rendering."}, "time_dimension": {"answer": "Present", "rationale": "\"Always\" and \"confidently\" describe current behaviour or attitude, so the temporal dimension of the article is now."}, "sentence_type": "Affirmative declarative sentence", "pos": {"nouns": {"person": 2, "Improvements": 1, "Help": 1}, "verbs": {"Always": 1, "Turn it in": 1, "Yes": 1}, "adjectives": {"- Frank": 1}, "numerals": {"495": 1}, "pronouns": {"they": 1}}}, {"content": "I have never seen a single thing as two pieces (revision phenomenon).", "word_count": 19, "label": "", "sentence_structure": "Subject鈥搗erb鈥搊bject", "subjective_objective": {"answer": "Objective", "rationale": "Because it's an objective symptom."}, "sentence_sentiment": {"answer": "Neutral", "rationale": "Because the entry does not have any emotional color."}, "language_style": {"answer": "Calm and direct", "rationale": "The basis for the determination: 1. The purpose of the article is expressed in a relatively concise and concise manner, without the use of excessive rhetorical techniques or emotional rendering. 2. The words \"never\" in the entry indicate whether the entry has been subjected to specific experiences or feelings of the other party, and is more objective and straightforward."}, "time_dimension": {"answer": "Past", "rationale": "The phrase \"never\" indicates that it is a past experience or behaviour, so it is judged to be the past."}, "sentence_type": "Negative declarative sentence", "pos": {"nouns": {"Move": 1, "One": 1, "Repetition": 1}, "verbs": {"Never": 1, "See": 1}, "adverbs": {"Never": 1}, "numerals": {"496锛?: 1}}}, {"content": "497. I like adventure novels.", "word_count": 7, "label": "", "sentence_structure": "Subject鈥搗erb鈥搊bject", "subjective_objective": {"answer": "Subjective", "rationale": "Because \"like\" is the patient's personal perception and feelings of adventure novels, and it's subjective."}, "sentence_sentiment": {"answer": "Positive", "rationale": "Because the expression \"like adventure novels\" is positive."}, "language_style": {"answer": "Tactful and warm", "rationale": "This language style reduces the resistance of the interviewee and makes him more willing to participate in the investigation."}, "time_dimension": {"answer": "Present", "rationale": ""}, "sentence_type": "Affirmative declarative sentence", "pos": {"nouns": {"Questionnaire": 1, "Entry": 1, "Novel": 1, "Adventure": 1}, "verbs": {"Yeah": 1}, "numerals": {"497": 1}}}, {"content": "498. The frankness is always a good thing.", "word_count": 9, "label": "", "sentence_structure": "Subject鈥揷opula鈥損redicative", "subjective_objective": {"answer": "Subjective", "rationale": "Since frankness is a personal attitude and perception, based on the subjective attitude of the individual based on values, beliefs, etc., the questionnaire is therefore subjective in its purpose."}, "sentence_sentiment": {"answer": "Positive", "rationale": "Because the phrase \"really frank is always a good thing\" in the entry is positive, positive emotional."}, "language_style": {"answer": "Tactful and warm", "rationale": "The entry expresses frankness in a positive tone and conveys positive messages to interviewees in a manner that encourages them, rather than direct orders or criticism, and therefore the language style is more euphemismal."}, "time_dimension": {"answer": "Future", "rationale": "The two key words \"clarification\" and \"always good\" point to an expectation or desire that shows vision and expectations for the future. The temporal dimension of this article is therefore a judgement of the future."}, "sentence_type": "Affirmative declarative sentence", "pos": {"verbs": {"- Frank": 1, "be": 1, "One": 1, "Things": 1}, "adjectives": {"Forever": 1, "Okay": 1}, "numerals": {"498锛?: 1}}}, {"content": "499. I must admit that I sometimes have unreasonable concerns about matters of little importance.", "word_count": 23, "label": "", "sentence_structure": "Subject鈥搗erb鈥搊bject", "subjective_objective": {"answer": "Subjective", "rationale": "Because \"fear\" and \"irrelevance\" are patients' subjective feelings and thoughts."}, "sentence_sentiment": {"answer": "Negative", "rationale": "Because the \"unreasonable fear\" in the entry is negative."}, "language_style": {"answer": "Tactful and warm", "rationale": ""}, "time_dimension": {"answer": "Past", "rationale": "The keyword \"sometimes\" implies a repetition of the situation, while the verb \"acknowledges\" suggests that the act may not be acceptable to society or individuals. The entry therefore asks about the behaviour and attitudes of the person being tested in the past."}, "sentence_type": "Affirmative declarative sentence", "pos": {"nouns": {"Questionnaire": 1, "Entry": 1, "Situation": 1}, "verbs": {"Recognition": 1, "I'm worried": 2, "be": 1}, "adjectives": {"Sometimes": 1, "It doesn't make sense": 1, "It doesn't matter": 1}, "adverbs": {"I have to": 1, "Yes": 1, "Sometimes": 1}, "numerals": {"499": 1}, "pronouns": {"I": 2}}}, {"content": "500. I would be happy to accept 100 per cent of the good advice.", "word_count": 16, "label": "", "sentence_structure": "Subject鈥搗erb鈥搊bject", "subjective_objective": {"answer": "Subjective", "rationale": "Because \"I'd love to\" is a subjective attitude and an expression of will."}, "sentence_sentiment": {"answer": "Positive", "rationale": "Because the entry \"I'd love to accept a 100% good opinion\" expresses a willingness to accept and improve."}, "language_style": {"answer": "Tactful and warm", "rationale": "The entry uses euphemism, which indicates that the other side is open and positive about accepting good suggestions, rather than being tough or command-style. This expression is more acceptable and willing to engage in communication and discussion."}, "time_dimension": {"answer": "Present", "rationale": "Now the entry is an expression of the current attitude and will, that is, the attitude expressed by the testee in reply to this question is \"I would love to.\" While this idea may be applied or applied in the future, the testee is expressing his/her current emotional state and attitude in this question. The time dimension of this purpose should therefore be judged now."}, "sentence_type": "Affirmative declarative sentence", "pos": {"nouns": {"Questionnaire": 1, "Entry": 1, "Percentage": 1, "Observations": 1}, "verbs": {"Accept": 1, "I'd love to": 1}, "adjectives": {"Okay": 1}, "adverbs": {"Total": 1}, "numerals": {"500": 1, "100%": 1}}}, {"content": "I have always been on my own to solve problems and from time to time have asked me to do it.", "word_count": 22, "label": "", "sentence_structure": "Subject鈥搗erb鈥搊bject", "subjective_objective": {"answer": "Subjective", "rationale": "Because it's the patient's subjective attitude and behavior to \"sell your own problems\" and \"get me to do it.\""}, "sentence_sentiment": {"answer": "Positive", "rationale": "Because the \"settle on your own\" act in the entry is positive."}, "language_style": {"answer": "Tactful and warm", "rationale": "The language style of the entry is euphemism. It uses the relatively soft expression \"I've always been\" rather than expressing my views and attitudes too harshly or directly. Moreover, the phrase \"sometimes teach me how to do it\" is more subtle, rather than being too explicit about personal preferences or preferences, but rather asking each other about their habits in a relatively neutral way. These features make the language style of the article more euphemismal."}, "time_dimension": {"answer": "Present", "rationale": "\"I've always\" expressed a normal and customary behaviour, and the word \"general\" emphasized the universality and long-term nature of that behaviour. \"Sometimes\" meant an occasional or intermittent act, \"get me to do it\" was a current move. So this questionnaire entry asks the testee about the way he is doing it."}, "sentence_type": "Affirmative declarative sentence", "pos": {"nouns": {"Problem": 1, "person": 1, "self": 2}, "verbs": {"Shit": 1, "Resolve": 1, "Find": 1, "Teaching": 1, "Do it": 1}, "adverbs": {"Always": 2, "From time to time": 1}, "numerals": {"501": 1}, "classifiers": {"individual": 1}, "pronouns": {"I": 2}}}, {"content": "502. The storm scared me.", "word_count": 6, "label": "", "sentence_structure": "Subject鈥揷opula鈥損redicative", "subjective_objective": {"answer": "Subjective", "rationale": "Because \"making me panic\" is a personal feeling and emotional experience, viewed from the patient's subjective perspective."}, "sentence_sentiment": {"answer": "Negative", "rationale": "Because the description of \"fright\" is usually associated with negative emotions."}, "language_style": {"answer": "Tactful and warm", "rationale": "The entry is more personal and interactive when it is spoken in a gentle and inviting manner, rather than in direct statements or orders. At the same time, the personalized expression of \"storms scare me\" makes the interviewee more comfortable and receptive, facilitating more real and accurate feedback."}, "time_dimension": {"answer": "Past", "rationale": "The \"storms scare me\" in this entry is a description and a sense of the events that have been experienced in the past, so it can be judged that it is an inquiry into the experience and feelings of the subjects."}, "sentence_type": "Affirmative declarative sentence", "pos": {"nouns": {"Storm": 1, "I": 1}, "verbs": {"鈾?And I'm gonna be 鈾?: 1, "Frightened": 1}, "adverbs": {"502锛?: 1}, "numerals": {"502": 1, "鈾?And I'm gonna be 鈾?: 1}, "classifiers": {"锛?: 1}, "pronouns": {"鈾?And I'm gonna be 鈾?: 1}}}, {"content": "I do not often express serious approval or opposition to the actions of others.", "word_count": 20, "label": "", "sentence_structure": "Subject鈥搗erb鈥揷omplement", "subjective_objective": {"answer": "Subjective", "rationale": "Because \"showing a serious yes or a negative\" is a subjective attitude and behaviour of a person."}, "sentence_sentiment": {"answer": "Neutral", "rationale": "Because the entry does not have any emotional color."}, "language_style": {"answer": "Calm and direct", "rationale": "The language style of the entry is a calm and straightforward type. It presents the problem directly, does not use excessive rhetorical or emotional rendering, and is more objective in tone."}, "time_dimension": {"answer": "Past", "rationale": ""}, "sentence_type": "Negative declarative sentence", "pos": {"nouns": {"Action": 1, "Serious": 1, "Aye": 1, "Objection": 1}, "verbs": {"Organisation": 1, "Regular": 1, "to": 1}, "adjectives": {"others": 1}, "adverbs": {"not": 1, "Regular": 1}, "numerals": {"503": 1, "Serious": 1}, "pronouns": {"I": 1}}}, {"content": "I do not wish to hide my bad impression or sympathy for a person so that he does not know what I think of him.", "word_count": 29, "label": "", "sentence_structure": "Subject鈥搗erb鈥搊bject", "subjective_objective": {"answer": "Subjective", "rationale": "The intention not to conceal the personal will and attitude of the patient is a subjective approach to objective situations (bad impressions or sympathy for others)."}, "sentence_sentiment": {"answer": "Negative", "rationale": "Because the emotions in the entry are negative, \"I don't want to hide the bad impression of a person\" and \"and he doesn't know what I think of him\" are negative."}, "language_style": {"answer": "Tactful and warm", "rationale": "The language style of the entry is more euphemistic and implicit, rather than directly stating whether the other side has a negative impression or sympathy, but rather expresses its unwillingness to conceal its views about someone. This expression is more focused on maintaining harmony in relationships and avoiding conflict."}, "time_dimension": {"answer": "Past", "rationale": "\"Don't want to hide\" means a denial of a past act, implying that it has occurred in the past. Moreover, \"I\" is a view of \"a person\" that describes a particular person in the past. So, this entry is an expression of the person who was tested who might have had a bad impression or sympathy for someone in the past and who feared that he had not told his own true thoughts."}, "sentence_type": "Negative declarative sentence", "pos": {"nouns": {"504": 1, "person": 2, "Impression": 1, "Compassion": 1, "Observations": 1}, "verbs": {"I don't want to hide it": 1, "have": 1, "And so": 1, "Yes": 1}, "adjectives": {"Bad": 1}, "adverbs": {"It's for someone": 1, "or": 1}, "numerals": {"504": 1, "2": 1}, "classifiers": {"individual": 1}, "pronouns": {"he": 2, "I": 2}}}, {"content": "I believe that 鈥渁 horse who does not pull a horse should be whipped鈥?", "word_count": 16, "label": "", "sentence_structure": "Subject鈥搗erb鈥搊bject", "subjective_objective": {"answer": "Subjective", "rationale": "This is a manifestation of subjective attitudes, as it is the patient ' s personal and opinion."}, "sentence_sentiment": {"answer": "Negative", "rationale": "Because the view in the entry favours the use of violence to solve problems, there is a clear negative emotional tone."}, "language_style": {"answer": "Calm and direct", "rationale": "The entry is more calm and straightforward in its language, without using too much qualifier or emotionally coloured vocabulary. It presents an objective and neutral view or belief aimed at understanding the views and attitudes of the person under investigation."}, "time_dimension": {"answer": "Past", "rationale": "The key word in the entry is \"thinks\" that it expresses a past view or view, and can therefore be judged to be the past. At the same time, \"shall be whipped\" is also a past vision and expectation, pointing to situations that have not yet occurred and that may arise in the future."}, "sentence_type": "Affirmative declarative sentence", "pos": {"nouns": {"Car": 1, "Horses": 2, "Whipping": 1}, "verbs": {"I think": 1, "I think so": 1, "Pull": 1}, "adverbs": {"Ken": 1}, "numerals": {"505": 1, "One": 1}, "pronouns": {"I": 1}}}, {"content": "I'm a nervously nervous person.", "word_count": 11, "label": "", "sentence_structure": "Subject鈥揷opula鈥損redicative", "subjective_objective": {"answer": "Subjective", "rationale": "Because \"nervous\" is a personal feeling and experience, a subjective assessment of the emotional state of a person. Although this feeling may be linked to objective factors (e.g. life stress, work burden, etc.), it is still a subjective concept in itself. The issue should therefore be classified as subjective."}, "sentence_sentiment": {"answer": "Negative", "rationale": "Because the description of \"high nervous tension\" is usually considered negative and may imply some health or stress problems."}, "language_style": {"answer": "Calm and direct", "rationale": "The language style of the entry is more objective and direct, and there is no excessive rhetorical or emotional rendering, but rather a concise expression of the researcher ' s desire to know whether the subject is nervous or not."}, "time_dimension": {"answer": "Present", "rationale": "The keyword \"I'm a nervously nervous person\" suggests that it's the testee's description of his current state and therefore the time dimension of the present."}, "sentence_type": "Affirmative declarative sentence", "pos": {"nouns": {"person": 1}, "verbs": {"be": 1, "Nervous": 1}, "adjectives": {"Nerves": 1, "Height": 1}, "numerals": {"506": 1}, "pronouns": {"I": 1}}}, {"content": "I often met leaders who attributed their credit to themselves and pushed the mistake to the lower ranks.", "word_count": 26, "label": "", "sentence_structure": "Subject鈥搗erb鈥搊bject", "subjective_objective": {"answer": "Objective", "rationale": "Because this entry describes a phenomenon or behaviour, not a subjective feeling, idea, or emotion. Although this may cause resentment or resentment, it does not itself belong to the patient鈥檚 mental state or experience."}, "sentence_sentiment": {"answer": "Negative", "rationale": "Because the \"deprive your credit, push your mistake to the lower end\" in the entry is negative."}, "language_style": {"answer": "Calm and direct", "rationale": "The entry is a direct expression of the subject matter of questioning whether the other party is often confronted with the subject of attribution of credit to himself, the blame of error to the subordinate leader, the absence of excessive rhetoric or emotional rendering, and the more objective tone of the speech."}, "time_dimension": {"answer": "Past", "rationale": "The entry describes the circumstances that the person was subjected to, and involves a time dimension that is judged to be past."}, "sentence_type": "Affirmative declarative sentence", "pos": {"nouns": {"Leadership": 2, "person": 1, "Error": 1, "Subordinate": 1}, "verbs": {"I met him": 1, "Attribution": 1, "Push it": 1}, "adverbs": {"Regular": 1}, "numerals": {"507": 1, "One": 2}, "pronouns": {"they": 1}}}, {"content": "I believe that my sense of smell is as good as anyone else.", "word_count": 13, "label": "", "sentence_structure": "Subject鈥搗erb鈥搊bject", "subjective_objective": {"answer": "Subjective", "rationale": "This is because of the patient ' s personal convictions and is the expression of subjective attitudes."}, "sentence_sentiment": {"answer": "Neutral", "rationale": "The entry is not a clear expression of positive or negative emotional sentiments."}, "language_style": {"answer": "Tactful and warm", "rationale": "The entry uses the words \"believing\" and \"as good\" to express a positive, optimistic attitude, and is not directly comparable or evaluated, and is more moderate. This makes it easier for respondents to accept the problem and to share their views."}, "time_dimension": {"answer": "Present", "rationale": "The keyword is \"I believe\", implying that it is a subjective statement or belief, not a past experience or a future expectation of behaviour. So the temporal dimension is now."}, "sentence_type": "Affirmative declarative sentence", "pos": {"nouns": {"Smell": 1, "person": 1, "others": 1}, "verbs": {"Yes": 1, "have": 1}, "adverbs": {"Same thing": 1}, "numerals": {"508锛?: 1, "Okay": 1}, "pronouns": {"I": 1}}}, {"content": "I am too formal to be able to maintain my own right sometimes.", "word_count": 22, "label": "", "sentence_structure": "Subject鈥損redicate", "subjective_objective": {"answer": "Subjective", "rationale": "Because the sentence refers to \"I am too rigid,\" which is a self-perception and feeling of the patient, and a subjective description. And the subsequent behavior (which is difficult to maintain) is based on this subjective attitude. So the questionnaire entry looks at the patient from a subjective perspective."}, "sentence_sentiment": {"answer": "Negative", "rationale": "Because the attitude of \"hard to maintain your right opinion\" in the entry is negative."}, "language_style": {"answer": "Tactful and warm", "rationale": "The language style of the entry is euphemism, because it uses the synonym \"because\" and \"so\" and expresses a self-reflectional and self-evaluation attitude in the first person's name. At the same time, it does not directly blame or criticize itself, but rather expresses its own awareness and understanding in a moderate way. This increases the comfort of the person surveyed and is more willing to answer the questions in a genuine manner."}, "time_dimension": {"answer": "Present", "rationale": ""}, "sentence_type": "Affirmative declarative sentence", "pos": {"nouns": {"I": 2, "Rule of law": 1, "Time": 1, "Observations": 1}, "verbs": {"be": 1, "Because": 1, "Too much": 1, "So": 1, "Hold on": 1, "Hard": 1, "Sometimes": 1}, "adjectives": {"Correct": 1}, "adverbs": {"Rule of law": 1, "Sometimes": 1}, "numerals": {"509": 1}, "pronouns": {"self": 1}}}, {"content": "510. Dirty makes me scared or sick.", "word_count": 9, "label": "", "sentence_structure": "Subject鈥揷opula鈥損redicative", "subjective_objective": {"answer": "Subjective", "rationale": "Because \"fearing or disgusting\" is a subjective feeling and emotional reaction. Although it may be associated with the \"dirty\" of the outside world, it is still the inside experience of the patient, not an objective description of the outside world."}, "sentence_sentiment": {"answer": "Negative", "rationale": "Because \"fear\" and \"disgusting\" are negative emotional reactions."}, "language_style": {"answer": "Calm and direct", "rationale": "It asked whether the interviewees were afraid or disgusted by feeling dirty, were not using excessive rhetoric or emotional colours and were speaking in a balanced and objective manner."}, "time_dimension": {"answer": "Present", "rationale": "The sentence describes subjective feelings and reactions to \"dirty\" and indicates the state of the current mood or mental state. It does not refer to specific past or future behaviour or circumstances, and therefore judges now."}, "sentence_type": "Negative declarative sentence", "pos": {"nouns": {"Dirty": 1, "Scared": 1, "Gross": 1}, "verbs": {"鈾?And I'm gonna be 鈾?: 1}, "adjectives": {"Scared": 1, "Gross": 1}, "numerals": {"510": 1}}}, {"content": "I have a dream life that I do not want to tell others.", "word_count": 15, "label": "", "sentence_structure": "Subject鈥搗erb鈥揷omplement", "subjective_objective": {"answer": "Subjective", "rationale": "Because it is an in-depth experience that is not shared, it is the patient ' s subjective feelings and thoughts."}, "sentence_sentiment": {"answer": "Neutral", "rationale": "Because the entry does not have a clear positive, negative or neutral emotional colour. It merely describes a feeling or state of the person under investigation, and does not evaluate such feelings or state or express any emotional preference."}, "language_style": {"answer": "Tactful and warm", "rationale": "The entry is more personal and interactive when it is spoken in a gentle and inviting tone, rather than in direct statements or orders. At the same time, the term \"life of dreams\" is full of mystery and desires, which make people want to know more."}, "time_dimension": {"answer": "Present", "rationale": "\"A dream life that is not willing to tell others\" suggests that the testee is in this situation and can therefore be judged to be the current state of affairs."}, "sentence_type": "Affirmative declarative sentence", "pos": {"nouns": {"Plant": 1, "Dream life": 1}, "verbs": {"have": 1, "Tell": 1}, "adjectives": {"I don't want to": 1, "others": 1}, "numerals": {"Five": 1, "One": 1}, "pronouns": {"I": 1}}}, {"content": "512. I don't like baths.", "word_count": 6, "label": "", "sentence_structure": "Subject鈥搗erb鈥揷omplement", "subjective_objective": {"answer": "Subjective", "rationale": "Because \"not like\" is a subjective attitude."}, "sentence_sentiment": {"answer": "Negative", "rationale": "Because \"not like\" in the entry is a negative emotional attitude."}, "language_style": {"answer": "Calm and direct", "rationale": "The language style of the entry is straightforward. It expresses the attitude of the respondents to bathing in a simple and straightforward manner, without using excessive rhetorical or emotional rendering, and with an objective tone."}, "time_dimension": {"answer": "Present", "rationale": "The entry describes the preferences or habits of the person being tested, and the temporal dimension is now judged."}, "sentence_type": "Negative declarative sentence", "pos": {"nouns": {"512": 1, "Bath": 1}, "verbs": {"Yeah": 1, "Wash": 1}, "adjectives": {"not": 1}, "numerals": {"512": 1}}}, {"content": "513. I believe that the pursuit of happiness for others is greater than the struggle for freedom for others.", "word_count": 21, "label": "", "sentence_structure": "Subject鈥搗erb鈥搊bject", "subjective_objective": {"answer": "Subjective", "rationale": "Because \"concept\" means that it is the patient's personal view and attitude, and it is subjective."}, "sentence_sentiment": {"answer": "Positive", "rationale": "Because the entry expresses a positive value and attitude that is more important to helping others than to pursuing individual freedom, and has a positive sense of social responsibility and humanitarianism."}, "language_style": {"answer": "Tactful and warm", "rationale": "The entry is more proficient and interactive in its expression, in a gentle and inviting tone, rather than in direct statements or orders. At the same time, the article is more neutral in its tone, does not overemphasize personal views or tendentious conclusions, and focuses more on guiding respondents to reflect and express their views."}, "time_dimension": {"answer": "Present", "rationale": "The entry asks the present views and attitudes of the testees, and the words \"considers\" and \"greater\" both indicate that this is a subjective assessment and judgement, and that there is no point in time to the past or the future."}, "sentence_type": "Affirmative declarative sentence", "pos": {"nouns": {"Happiness": 1, "self": 1, "Freedom": 1}, "verbs": {"I think": 1, "Seek": 1, "Fight": 1}, "adjectives": {"Great": 1}, "numerals": {"513": 1}, "pronouns": {"I": 1}}}, {"content": "514. I like women who are manly.", "word_count": 10, "label": "", "sentence_structure": "Subject鈥搗erb鈥揷omplement", "subjective_objective": {"answer": "Subjective", "rationale": "Because it is personal and desirable to like or dislike it is subjective."}, "sentence_sentiment": {"answer": "Negative", "rationale": "Because the gender stereotypes and sexist emotional colours of the term \"a man-like woman\" are negative."}, "language_style": {"answer": "Calm and direct", "rationale": "The entry is simple and clear, directly expressing queries about the preference of female gender roles, with no excessive emotional or rhetorical tact, and with a more objective and calm tone."}, "time_dimension": {"answer": "Present", "rationale": "The keyword \"likes\" indicates that the testee has a positive attitude or preference for a certain quality, and \"a manly woman\" describes the testee's preferred qualities, which point to the current thinking and feeling. So the time dimension of the article is now."}, "sentence_type": "Affirmative declarative sentence", "pos": {"nouns": {"Men": 1, "Women": 2}, "verbs": {"Yeah": 1}, "adjectives": {"Manly": 1}, "numerals": {"514": 1}, "pronouns": {"I": 1}}}, {"content": "515. Our family is always unscathed.", "word_count": 11, "label": "", "sentence_structure": "Subject鈥搗erb鈥揷omplement", "subjective_objective": {"answer": "Objective", "rationale": "Because \"if you're sad\" is an objective situation for the family, not for the patient."}, "sentence_sentiment": {"answer": "Positive", "rationale": "Because the description \"no worries\" means that family is in better economic conditions and is a positive one."}, "language_style": {"answer": "Tactful and warm", "rationale": "The language style of the entry is euphemism. It is more friendly and interactive in a gentle and inviting tone of speech, rather than in direct statements or orders. At the same time, using the expression \"Our family is always free of sorrow\" conveys positive messages while avoiding the feeling of flaunting or boasting, making language more appropriate and friendly."}, "time_dimension": {"answer": "Present", "rationale": ""}, "sentence_type": "Affirmative declarative sentence", "pos": {"nouns": {"Home": 1, "Eat": 1, "Put it on": 1}, "verbs": {"Sad": 2, "It's not sad": 2}, "adverbs": {"Always": 1}, "numerals": {"515": 1}, "pronouns": {"we": 1}}}, {"content": "516. Some of my family is in a state of rage.", "word_count": 10, "label": "", "sentence_structure": "Subject鈥搗erb鈥搊bject", "subjective_objective": {"answer": "Subjective", "rationale": "Because \"grumpy\" is a subjective assessment, reflecting the views and feelings of the interviewees about their families."}, "sentence_sentiment": {"answer": "Negative", "rationale": "Because the term \"grumpy\" is usually considered negative."}, "language_style": {"answer": "Tactful and warm", "rationale": "The entry is more adept and interactive when it is spoken in a gentle and inviting manner, rather than in direct statements or orders. At the same time, the use of the word \"some\" also indicates that the question is not absolute and leaves the respondents some room for reply."}, "time_dimension": {"answer": "Past", "rationale": "The entry asks about the characteristics of the family members of the testee and does not have clear information on the temporal dimensions, so it is not possible to judge whether the behaviour or mentality is past, present or future."}, "sentence_type": "Affirmative declarative sentence", "pos": {"nouns": {"Family": 1, "person": 1, "- Grumpy": 1, "Routine": 1}, "verbs": {"have": 1}, "adjectives": {"Routine": 1}, "numerals": {"516": 1, "Some": 1}, "classifiers": {"individual": 1}, "pronouns": {"I": 1}}}, {"content": "I can't do anything right.", "word_count": 11, "label": "", "sentence_structure": "Subject鈥揷opula鈥損redicative", "subjective_objective": {"answer": "Subjective", "rationale": "Because the sentence is about self-evaluation and subjective feelings of patients, who think they are not good at anything. It is not an objective description of the facts, but a subjective idea or emotional experience of the patient. The questionnaire entry is therefore viewed from the patient鈥檚 subjective perspective."}, "sentence_sentiment": {"answer": "Negative", "rationale": "Because the expression \"does nothing well\" in the entry has negative emotions."}, "language_style": {"answer": "Tactful and warm", "rationale": "The entry uses a more moderate formulation, without too direct or negative expressions, to trigger the perception and feelings of the respondent's abilities in an attitude of concern, understanding and support. At the same time, the use of the word \"both\" emphasizes the low self-confidence of the respondent, a euphemism that better guides the respondent to answer questions in an honest manner and gives them more support and encouragement."}, "time_dimension": {"answer": "Present", "rationale": "The keyword \"neither good\" means that it is a subjective feeling or attitude, not a past or future act or result, and the time dimension of the entry is thus judged to be the present."}, "sentence_type": "Negative declarative sentence", "pos": {"verbs": {"Do it": 1}, "adjectives": {"Okay": 1}, "adverbs": {"Whatever": 1}, "pronouns": {"I": 1}}}, {"content": "I am often ashamed because I think about things differently than I do.", "word_count": 21, "label": "", "sentence_structure": "Subject鈥揷opula鈥損redicative", "subjective_objective": {"answer": "Subjective", "rationale": "Because \"disguised\" and \"inconsistent\" are patients' subjective feelings and thoughts."}, "sentence_sentiment": {"answer": "Negative", "rationale": "Because the \"often ashamed\" emotion in the entry is negative."}, "language_style": {"answer": "Tactful and warm", "rationale": "The language style of the entry is euphemistic. It uses a gentle, understanding tone to ask the interviewees whether they are often ashamed because they think that what they think and do is not consistent. It is more acceptable and willing to share their true feelings."}, "time_dimension": {"answer": "Present", "rationale": ""}, "sentence_type": "Negative declarative sentence", "pos": {"nouns": {"I": 1, "Shame": 1, "Things": 1}, "verbs": {"I feel": 1, "Yes": 1, "Do it": 1, "It's different": 1}, "adverbs": {"Regular": 1}, "numerals": {"518锛?: 1}}}, {"content": "My big organs are a little bit sick.", "word_count": 9, "label": "", "sentence_structure": "Subject鈥搗erb鈥揷omplement", "subjective_objective": {"answer": "Objective", "rationale": "Because the condition of the sexual organs is an objective fact, whether or not the patient is aware or willing to admit it. The question is therefore examined from an objective perspective."}, "sentence_sentiment": {"answer": "Negative", "rationale": "Because this entry involves physical problems, it may cause some negative emotional reactions."}, "language_style": {"answer": "Calm and direct", "rationale": "The entry describes a possible health problem in more straightforward and objective language, with no excessive emotional colour or qualifier, and therefore has a more blunt language style."}, "time_dimension": {"answer": "Present", "rationale": "According to the sentence given, we can see that the entry is asking about the condition of the person being tested, specifically about the \"large sexual organs\". In terms of time dimension, the question is not clear, so we cannot judge precisely the time point of the entry. It may be a state of the past, a state of the present, or a projection of the future. So the answer is \"now/for the future.\""}, "sentence_type": "Affirmative declarative sentence", "pos": {"nouns": {"Sexual organs": 1, "Mistake": 1}, "verbs": {"have": 1}, "numerals": {"Five": 1, "One": 1, "Nine": 1}, "pronouns": {"I": 1}}}, {"content": "520. My principle is to insist on a serious defence of my opinion.", "word_count": 16, "label": "", "sentence_structure": "Subject鈥搗erb鈥搊bject", "subjective_objective": {"answer": "Subjective", "rationale": "Because the words \"principles\" and \"preservation to defend one's opinion\" express the patient's personal views and attitudes."}, "sentence_sentiment": {"answer": "Positive", "rationale": "Because it expresses a positive attitude and quality, namely, the firmness to defend one ' s own point of view."}, "language_style": {"answer": "Calm and direct", "rationale": "The entry expresses the principles of the individual in a direct and clear manner, without excessive adoration or emotional colour, speech temperature and objectivity."}, "time_dimension": {"answer": "Present", "rationale": "The keyword \"My Principles\" expresses personal attitudes or beliefs, while \"maintenance to defend one's opinion\" refers to a form of conduct that persists, so that the article has a temporal dimension."}, "sentence_type": "Affirmative declarative sentence", "pos": {"nouns": {"Principles": 1, "self": 1, "Observations": 1}, "verbs": {"be": 1, "Hold on": 1, "Maintenance": 1}, "adjectives": {"Serious": 1}, "numerals": {"520锛?: 1}}}, {"content": "521. I often consult others.", "word_count": 8, "label": "", "sentence_structure": "Subject鈥搗erb鈥揷omplement", "subjective_objective": {"answer": "Subjective", "rationale": "Subjectivity, because it is a subjective will and action to ask someone else."}, "sentence_sentiment": {"answer": "Positive", "rationale": "Because the purpose of the \"often asking\" act in the entry is positive."}, "language_style": {"answer": "Tactful and warm", "rationale": "The entry is more euphemistic, is used in polite language, \"often\" and \"inquiries\" and is expressed in a more moderate and interactive manner. At the same time, the article is aimed at a warm and friendly language and is easily acceptable to the interviewees."}, "time_dimension": {"answer": "Past", "rationale": "The entry \"always\" denotes a regular, customary act, and the \"please\" action occurred in the past, so the entry describes the testee's past experience."}, "sentence_type": "Affirmative declarative sentence", "pos": {"nouns": {"521": 1, "I": 1, "others": 1, "Please": 1}, "verbs": {"Regular": 1, "In": 1}, "adverbs": {"Often": 1}}}, {"content": "I'm not afraid of spiders.", "word_count": 6, "label": "", "sentence_structure": "Subject鈥損redicate", "subjective_objective": {"answer": "Subjective", "rationale": "Fear or fear of spiders being the patient ' s own emotional experience and attitude is subjective."}, "sentence_sentiment": {"answer": "Positive", "rationale": "Because the expression \"no fear of spiders\" in the entry is positive."}, "language_style": {"answer": "Tactful and warm", "rationale": "The entry is more adept and interactive when it is spoken in a gentle and inviting manner, rather than by direct statements or orders."}, "time_dimension": {"answer": "Present", "rationale": "The entry \"I\" represents the interviewee himself, and the verb \"no fear of spiders\" indicates the current feeling or attitude, so it can be judged as the time dimension of the present."}, "sentence_type": "Negative declarative sentence", "pos": {"nouns": {"Spider": 1}, "verbs": {"No, I'm not": 1}, "numerals": {"522": 1}}}, {"content": "I never had a blush.", "word_count": 6, "label": "", "sentence_structure": "Subject鈥搗erb鈥揷omplement", "subjective_objective": {"answer": "Objective", "rationale": "Because \"silent\" is a physical reaction or external expression of a person, it is not the patient's subjective attitude or behavioural choice."}, "sentence_sentiment": {"answer": "Negative", "rationale": "Because \"silent\" may indicate a lack of shame or guilt, this attitude may be negative in some cases."}, "language_style": {"answer": "Tactful and warm", "rationale": ""}, "time_dimension": {"answer": "Present", "rationale": "\"I never blush\" means a constant state of affairs and behaviour, and it happens at the moment, so it's judged now."}, "sentence_type": "Negative declarative sentence", "pos": {"verbs": {"not": 1, "Red": 1}, "adverbs": {"Never": 1}, "pronouns": {"I": 1}}}, {"content": "524. I am not afraid to spread disease through the door.", "word_count": 12, "label": "", "sentence_structure": "Subject鈥搗erb鈥搊bject", "subjective_objective": {"answer": "Objective", "rationale": "Because fear of infectious diseases is the patient ' s subjective attitude towards them, the infectious nature of the disease is an objective fact."}, "sentence_sentiment": {"answer": "Neutral", "rationale": "Because the entry does not express positive or negative feelings, attitudes, behaviours, aims or qualities. It merely describes the fact or the view that the person interviewed is not afraid to spread disease through the door."}, "language_style": {"answer": "Calm and direct", "rationale": "The basis for the judgement: the entry presents a point in a concise and straightforward way, namely, \"not afraid of the disease from the door. \" This expression is not too much rhetorical or emotional, and is more objective and more in a calm and direct way."}, "time_dimension": {"answer": "Present", "rationale": "\"Not afraid\" means the feeling and attitude of the moment, and \"infectious disease from the door\" is a hypothetical situation, but it does not point to the time dimension of the past or the future. Therefore, the topic should be classified as now."}, "sentence_type": "Negative declarative sentence", "pos": {"nouns": {"I": 1, "Door handle": 1, "Infection": 1, "Diseases": 1}, "verbs": {"Not afraid": 1}, "numerals": {"524锛?: 1}}}, {"content": "525. Some animals make me nervous.", "word_count": 10, "label": "", "sentence_structure": "Subject鈥搗erb鈥搊bject", "subjective_objective": {"answer": "Subjective", "rationale": "Because stress is an emotional experience, it's a subjective feeling of the patient."}, "sentence_sentiment": {"answer": "Negative", "rationale": "The entry is a negative expression of feelings and attitudes."}, "language_style": {"answer": "Tactful and warm", "rationale": "The entry is more euphemistic. First, it uses a softer word \"some\" to express how some animals feel, without making it clear that all animals cause nervous stress. Second, it does not have too much emotional and subjective judgement, but rather expresses in an objective way the problems that the respondents may have. This language helps to make the interviewee comfortable and willing to answer the questions in a real and natural way."}, "time_dimension": {"answer": "Present", "rationale": "In conclusion, the time dimension of this questionnaire entry is \"now\"."}, "sentence_type": "Affirmative declarative sentence", "pos": {"nouns": {"Animals": 1, "Nerves": 1}, "verbs": {"鈾?And I'm gonna be 鈾?: 1, "Nervous": 1}, "numerals": {"525": 1}}}, {"content": "526. My future seems hopeless.", "word_count": 10, "label": "", "sentence_structure": "Subject鈥揷opula鈥損redicative", "subjective_objective": {"answer": "Subjective", "rationale": "Because \"the future seems hopeless\" is the patient's fear and subjective judgment about the future."}, "sentence_sentiment": {"answer": "Negative", "rationale": "Because \"no hope for the future\" in the entry is an expression of a negative emotion and attitude."}, "language_style": {"answer": "Tactful and warm", "rationale": "The language style of the entry is more moderate, leading the respondents to share their thoughts and feelings by raising the question of \"my future seems hopeless.\" This expression is more focused on the inner experience of the respondents and seeks to create an open and comfortable space for the respondents to express their opinions freely."}, "time_dimension": {"answer": "Past", "rationale": "The entry contains a description of the past, which means that the testees are looking back at their past and their feelings at the current point in time. The keyword \"like\" and \"no hope\" point to the time dimension of the past."}, "sentence_type": "Negative declarative sentence", "pos": {"nouns": {"Future": 1, "Hope": 1}, "verbs": {"Seems like": 1, "No, I'm not": 1}, "numerals": {"526": 1}, "classifiers": {"individual": 1}}}, {"content": "527. My family and close relatives live well together.", "word_count": 14, "label": "", "sentence_structure": "Subject鈥搗erb鈥揷omplement", "subjective_objective": {"answer": "Objective", "rationale": "Because \"the family and the next of kin are getting along well\" is an objective description."}, "sentence_sentiment": {"answer": "Positive", "rationale": "Because the \"good with\" behavior in the entry is positive."}, "language_style": {"answer": "Tactful and warm", "rationale": "The entry is more adept and interactive when it is spoken in a gentle and inviting manner, rather than by direct statements or orders."}, "time_dimension": {"answer": "Present", "rationale": "The entry now describes the current situation among family members of the testee, so the temporal dimension is now judged."}, "sentence_type": "Affirmative declarative sentence", "pos": {"nouns": {"Home": 1, "person": 1, "Close relatives": 1, "Guys": 1, "Get along": 1}, "verbs": {"have": 1, "Okay": 1}, "adverbs": {"very": 1}, "numerals": {"527锛?: 1}, "pronouns": {"I": 1}}}, {"content": "528. I am not easy to get more blushed than I am.", "word_count": 9, "label": "", "sentence_structure": "Subject鈥搗erb鈥揷omplement", "subjective_objective": {"answer": "Subjective", "rationale": "Because \"failless blush\" is a subjective assessment or feeling of the patient."}, "sentence_sentiment": {"answer": "Neutral", "rationale": "Because the entry describes a personal character, it does not express positive or negative emotional colours."}, "language_style": {"answer": "Calm and direct", "rationale": "The language style of the entry is calm and straightforward. It asks the respondents in a straightforward way whether they are easy to blush and do not use excessive emotional acrimony or rhetoric to guide the answer. This expression is simple and clear, and it is easier to understand and accept."}, "time_dimension": {"answer": "Present", "rationale": "The keywords are \"not easy\" and \"no\" -- they all point to the state or behaviour of the moment, which means that the testee's situation is not as good as humans."}, "sentence_type": "Negative declarative sentence", "pos": {"nouns": {"Questionnaire": 1, "Entry": 1, "Face": 1}, "verbs": {"and": 1, "- Yeah": 1, "Red": 1}, "adjectives": {"not": 1, "Easy": 1}, "adverbs": {"very": 1}, "numerals": {"528锛?: 1}}}, {"content": "529. I like to wear high-grade clothes.", "word_count": 9, "label": "", "sentence_structure": "Subject鈥搗erb鈥搊bject", "subjective_objective": {"answer": "Subjective", "rationale": "Because the preference for wearing high-grade clothing is the expression of one ' s subjective preferences and values and falls within the realm of individual subjective attitudes."}, "sentence_sentiment": {"answer": "Negative", "rationale": "Because the act of \"like to wear high-grade clothes\" can lead to overconsumption and upwards, and it is negative."}, "language_style": {"answer": "Tactful and warm", "rationale": "Warm and warm."}, "time_dimension": {"answer": "Present", "rationale": ""}, "sentence_type": "Affirmative declarative sentence", "pos": {"nouns": {"Clothes": 1, "High-quality": 1}, "verbs": {"Yeah": 1, "Put it on": 1}, "numerals": {"529锛?: 1}}}, {"content": "I'm often afraid I'll get blushed.", "word_count": 11, "label": "", "sentence_structure": "Subject鈥搗erb鈥揷omplement", "subjective_objective": {"answer": "Subjective", "rationale": "Because \"care\" is subjective."}, "sentence_sentiment": {"answer": "Negative", "rationale": "Because \"fearing to get blushing\" is negative."}, "language_style": {"answer": "Tactful and warm", "rationale": "The entry is more proficient and interactive when it is spoken in a gentle and inviting manner, rather than in direct statements or orders. At the same time, the language style of the article is more euphemistic, using terms such as \"often\" and \"fear\" to give people a sense of caring about each other's feelings and respect for their privacy."}, "time_dimension": {"answer": "Present", "rationale": "\"Assured\" means a regular, customary act, while \"Assisted\" means that the general current, means that this act not only has occurred in the past, but may still be ongoing. \"530. I often fear that I may be blushing.\" The key word is \"I often will.\" They point to the person being tested's current thoughts and feelings."}, "sentence_type": "Affirmative declarative sentence", "pos": {"nouns": {"I'm worried": 1, "self": 1, "Red": 1}, "verbs": {"Yes": 2}, "adverbs": {"Often": 1}, "numerals": {"530锛?: 1}, "pronouns": {"I": 1}}}, {"content": "Even if I thought I had a plan for something, it was easy for others to get me hung up.", "word_count": 28, "label": "", "sentence_structure": "Subject鈥搗erb鈥揷omplement", "subjective_objective": {"answer": "Subjective", "rationale": "Because \"making me believe in changing my mind\" is someone else's subjective behavior, and it's the patient's subjective feelings."}, "sentence_sentiment": {"answer": "Negative", "rationale": "Because the phrase \"others can easily turn me off\" in the entry is negative."}, "language_style": {"answer": "Tactful and warm", "rationale": "The language style of the entry is a euphemism. It uses the implicit expression \"I think I've made a decision about something\" , neither too hard to express my views nor to convey to the other. At the same time, the use of the expression \"I can easily be hung up\" has increased the emphasis on interaction and influence with others, making the questionnaire more personal and interactive."}, "time_dimension": {"answer": "Present", "rationale": ""}, "sentence_type": "Affirmative declarative sentence", "pos": {"nouns": {"Questionnaire": 1, "Entry": 1, "person": 2, "Ideas": 2, "Things": 1}, "verbs": {"I thought": 1, "Hold on": 1, "鈾?And I'm gonna be 鈾?: 1, "Hanging": 1}, "adverbs": {"very": 1, "Yeah": 1}, "numerals": {"531锛?: 1}, "classifiers": {"individual": 1}, "pronouns": {"self": 1, "others": 1}}}, {"content": "I am as capable of suffering as others.", "word_count": 15, "label": "", "sentence_structure": "Subject鈥搗erb鈥搊bject", "subjective_objective": {"answer": "Objective", "rationale": "The basis for the determination: the entry of the questionnaire is to allow the patient to self-evaluate whether he or she believes he or she is capable of suffering the same as others, which is a personal feeling and a subjective assessment. The answer to this question is therefore subjective."}, "sentence_sentiment": {"answer": "Neutral", "rationale": "Because the entry does not have any emotional color."}, "language_style": {"answer": "Calm and direct", "rationale": "The language style of the entry is calm, because it directly expresses the subject of a survey in a concise and straightforward manner, denying that it can suffer the same degree of suffering as others."}, "time_dimension": {"answer": "Present", "rationale": "The entry \"I'm like everyone else\" is a comparison or comparison, and \"can withstand the same pain\" is an expression of ability or behaviour. According to the time statement, it describes the state and capacity of the moment. So the answer is \"now.\""}, "sentence_type": "Affirmative declarative sentence", "pos": {"nouns": {"Pain": 1, "others": 1}, "verbs": {"Experience": 1}, "adjectives": {"Same thing": 1}, "adverbs": {"Yeah": 1}, "numerals": {"532": 1}, "pronouns": {"I": 1}}}, {"content": "533. I am not troubled by the frequent hiccups (Uh tw) that I am.", "word_count": 17, "label": "", "sentence_structure": "Subject鈥揷opula鈥損redicative", "subjective_objective": {"answer": "Subjective", "rationale": "Because it's subjective to feel upset or not."}, "sentence_sentiment": {"answer": "Negative", "rationale": "Because the act of burping in the entry is neutral, but the word \"sort\" in the back is negative emotional."}, "language_style": {"answer": "Tactful and warm", "rationale": "The entry uses the negative word \"no\" to express the notion that hiccuping does not bother the interviewer, and the whole phrase is less ambivalent and objective, is not overly direct or emotional and can therefore be judged as a \"softly warm\" language style."}, "time_dimension": {"answer": "Present", "rationale": "\"Always burping\" indicates that this is a regular act, and that the current state of the sentence means that it has not only happened in the past but may still be ongoing. Thus, the entry asks the testee what it feels like now, and the temporal dimension is now judged."}, "sentence_type": "Negative declarative sentence", "pos": {"nouns": {"533": 1, "Hiccup": 1, "I think": 1, "Trouble": 1}, "verbs": {"No, it's not": 1, "Hiccup": 1, "I think": 1}, "adverbs": {"Often": 1}, "numerals": {"533": 1}, "pronouns": {"I": 1}}}, {"content": "534. On several occasions, I have been alone in the end and finally given up on what I had done.", "word_count": 24, "label": "", "sentence_structure": "Subject鈥搗erb鈥搊bject", "subjective_objective": {"answer": "Subjective", "rationale": "Because \"I'm alone\" is patient's subjective attitude."}, "sentence_sentiment": {"answer": "Negative", "rationale": "Because the \"last thing to do\" act in the entry is negative."}, "language_style": {"answer": "Calm and direct", "rationale": "The entry is based on a simple and clear expression of the situation in which the interviewee will, in some cases, insist on and eventually give up on his own, without excessive rhetoric or emotional accoutrements."}, "time_dimension": {"answer": "Present", "rationale": "The entry \"a few times\" indicates what happened in the past, and \"a man who insists on doing it and finally gives up what he did\" is also a move that was done in the past, so the time dimension is now judged."}, "sentence_type": "Affirmative declarative sentence", "pos": {"nouns": {"Number of times": 2, "person": 1, "Things": 1}, "verbs": {"be": 1, "Abandon": 1, "Do it": 1, "Hold on": 1}, "adjectives": {"Okay": 1, "Finally": 1}, "adverbs": {"Both": 1, "That's right": 1}, "numerals": {"534锛?: 1, "One": 2}, "pronouns": {"I": 1}}}, {"content": "535. I feel dry almost all day.", "word_count": 9, "label": "", "sentence_structure": "Subject鈥揷opula鈥損redicative", "subjective_objective": {"answer": "Subjective", "rationale": "Because the word \"fEE\" shows that it's a patient's own feeling and subjective experience."}, "sentence_sentiment": {"answer": "Negative", "rationale": "Because the feeling of \"sniffing all day\" is negative."}, "language_style": {"answer": "Calm and direct", "rationale": "The entry is in a concise language, and it is more objective and incriminating to ask the interviewees whether they often feel the symptoms of oral drying, without excessive emotional rendering or speech writing."}, "time_dimension": {"answer": "Past", "rationale": "The keyword is \"almost day-to-day feeling,\" which means that the testee has felt that for some time, i.e. \"almost day-to-day\" and therefore judged as the past."}, "sentence_type": "Affirmative declarative sentence", "pos": {"nouns": {"- Mouth dry": 1}, "verbs": {"Sensor": 1, "Present": 1, "All day long": 1, "I feel": 1}, "adjectives": {"Almost": 1}, "numerals": {"535": 1, "One": 1}, "classifiers": {"Oh, my God": 1}}}, {"content": "I'm angry when I'm being pushed.", "word_count": 10, "label": "", "sentence_structure": "Subject鈥損redicate", "subjective_objective": {"answer": "Subjective", "rationale": "Because \"freak\" is an emotional experience, a subjective feeling of the patient."}, "sentence_sentiment": {"answer": "Negative", "rationale": "Because the \"freak\" act in the entry is negative."}, "language_style": {"answer": "Calm and direct", "rationale": "The entry is asked explicitly, by direct question, whether the person under investigation would be angry at being pushed by someone else."}, "time_dimension": {"answer": "Present", "rationale": "The keyword \"just get angry\" suggests that it's an instant emotional reaction, and \"as long as someone pushes me\" is the condition or cause for triggering that emotion, which points to the situation now."}, "sentence_type": "Affirmative declarative sentence", "pos": {"nouns": {"person": 1, "Hurry": 1}, "verbs": {"Angry": 1}, "adjectives": {"Just": 1}, "numerals": {"536": 1}, "pronouns": {"I": 1}}}, {"content": "537. I want to go to the mountains and fight tigers.", "word_count": 11, "label": "", "sentence_structure": "Subject鈥搗erb鈥搊bject", "subjective_objective": {"answer": "Subjective", "rationale": "Because it's the patient's inner thoughts and wishes, and it's a subjective description."}, "sentence_sentiment": {"answer": "Negative", "rationale": "Because the entry is \"to fight tigers in the wild\", it is a dangerous and irresponsible act that may harm humans and animals and therefore has a clear negative emotional colour."}, "language_style": {"answer": "Calm and direct", "rationale": "The entry presents the thoughts and wishes of the person under investigation in a concise statement, without too much ado or emotional."}, "time_dimension": {"answer": "Future", "rationale": "The entry contains visions and expectations for the future, and the keywords are \"want to go\" and \"play\", which point to situations that have not yet occurred and that may arise in the future."}, "sentence_type": "Imperative sentence", "pos": {"nouns": {"Mountain forest": 1, "Tiger": 1}, "verbs": {"Yes": 1, "Go on": 1, "Fight": 1}, "adverbs": {"Deep": 1}, "numerals": {"537锛?: 1}}}, {"content": "I think I'd like to work with the tailor.", "word_count": 11, "label": "", "sentence_structure": "Subject鈥搗erb鈥搊bject", "subjective_objective": {"answer": "Subjective", "rationale": "Because \"like\" is patient's subjective attitude."}, "sentence_sentiment": {"answer": "Positive", "rationale": "Because it expresses the preferences and expectations for the tailor ' s work and is positive in emotional terms."}, "language_style": {"answer": "Tactful and warm", "rationale": "The language style of the entry is more euphemistic, and it is more positive to express the individual鈥檚 preference for tailoring, with a sense of anticipation and vision of the future. At the same time, it is more subtle and intuitive than a statement of certainty."}, "time_dimension": {"answer": "Future", "rationale": "The entry describes the views and expectations of the testees for the future, and the keywords are \"I want\" and \"I like\" and they all point to what may happen in the future and can therefore be judged to be the future."}, "sentence_type": "Affirmative declarative sentence", "pos": {"nouns": {"Work": 1, "A tailor": 1}, "verbs": {"Yes": 2, "Yeah": 1, "Do it": 1}, "adverbs": {"EMPTY": 1}, "numerals": {"538锛?: 1}, "pronouns": {"I": 1}}}, {"content": "I'm not afraid of rats.", "word_count": 5, "label": "", "sentence_structure": "Subject鈥搗erb鈥搊bject", "subjective_objective": {"answer": "Subjective", "rationale": "Because \"no fear\" is a subjective attitude or feeling of a human being, it is an assessment and a description of the degree of fear of a mouse."}, "sentence_sentiment": {"answer": "Neutral", "rationale": "As this entry does not express any positive or negative feelings, attitudes or behaviour."}, "language_style": {"answer": "Calm and direct", "rationale": "The language style of the entry is more direct, without excessive rhetorical or emotional rendering, simply stating a view or feeling. Thus, the language style can be judged as \"cool and direct\"."}, "time_dimension": {"answer": "Present", "rationale": ""}, "sentence_type": "Imperative sentence", "pos": {"nouns": {"Rat": 1}, "verbs": {"Yes": 1, "not": 1, "Say it": 1}, "adverbs": {"not": 1}, "numerals": {"539": 1}}}, {"content": "My face has never been paralyzed.", "word_count": 11, "label": "", "sentence_structure": "Subject鈥揷opula鈥損redicative", "subjective_objective": {"answer": "Objective", "rationale": "Because the facial paralysis is an objective state of the patient, it can be observed and verified."}, "sentence_sentiment": {"answer": "Neutral", "rationale": "Because the entry merely stated a fact, it did not express any positive or negative emotional colour."}, "language_style": {"answer": "Tactful and warm", "rationale": "The language style of the questionnaire entry is euphemistic. It asks questions in a simple, respectful way, without using direct or hard language. Moreover, the issue does not involve any topic that may cause discomfort or distress, and thus makes it easier for the interviewee to feel relaxed and comfortable."}, "time_dimension": {"answer": "Past", "rationale": ""}, "sentence_type": "Affirmative declarative sentence", "pos": {"nouns": {"Face": 1, "Paralytic": 1}, "verbs": {"Never": 1}, "adverbs": {"Pass": 1}, "numerals": {"540": 1}}}, {"content": "541. My skin seems particularly sensitive to touch.", "word_count": 13, "label": "", "sentence_structure": "Subject鈥揷opula鈥損redicative", "subjective_objective": {"answer": "Subjective", "rationale": "Because \"special sensitivity to touch\" is a subjective feeling and self-evaluation, the entry is subjective."}, "sentence_sentiment": {"answer": "Neutral", "rationale": "Basis of judgement: The entry describes the individual ' s state of feeling and physical state, without any apparent emotional tendency."}, "language_style": {"answer": "Tactful and warm", "rationale": "The basis for the determination: the entry is more proficient and interactive by speaking with a gentle, invitationable tone rather than by direct statement or order. At the same time, the entry is not overly rhetorical or emotionally pronouncing, and is more balanced and objective."}, "time_dimension": {"answer": "Present", "rationale": ""}, "sentence_type": "Affirmative declarative sentence", "pos": {"nouns": {"I": 1, "Skin": 1, "Touch": 1}, "verbs": {"to": 1, "Seems like": 1, "Sensitive": 1}, "adjectives": {"Special": 1}, "numerals": {"541锛?: 1}}}, {"content": "I never had any black manure like tar.", "word_count": 15, "label": "", "sentence_structure": "Subject鈥搗erb鈥揷omplement", "subjective_objective": {"answer": "Objective", "rationale": "Because it's an objective fact that patients have experienced when they never had the same black shit as tar."}, "sentence_sentiment": {"answer": "Neutral", "rationale": "Because the entry describes a specific symptom and does not have a human emotional colour."}, "language_style": {"answer": "Calm and direct", "rationale": "The entry describes a physical phenomenon in a straightforward manner, without any aesthetic or emotional rendering, with a speech temperature and objective."}, "time_dimension": {"answer": "Past", "rationale": "The entry is for questioning the testee ' s past behaviour and the temporal dimension is a past."}, "sentence_type": "Negative declarative sentence", "pos": {"nouns": {"Questionnaire": 1, "Entry": 1, "Boil": 1, "Black shit": 1}, "verbs": {"have": 1, "Pass": 1, "Yeah": 1, "Never": 1}, "adjectives": {"Same thing": 1, "Black": 1}, "numerals": {"542锛?: 1}}}, {"content": "543. There are always a few times a week when something terrible seems to be going on.", "word_count": 21, "label": "", "sentence_structure": "Subject鈥搗erb鈥揷omplement", "subjective_objective": {"answer": "Subjective", "rationale": "Because \"the terrible thing is going to happen\" is a subjective feeling or a hunch."}, "sentence_sentiment": {"answer": "Negative", "rationale": "Because the idea of \"horrible things to happen\" in the entry is negative."}, "language_style": {"answer": "Calm and direct", "rationale": "The language style of the entry is calm and straightforward, and it expresses the core of the question in a concise language that is not phrased with any rhetorical or emotional colour. It enables respondents to answer questions more objectively and avoid being influenced by excessive emotional or personal bias."}, "time_dimension": {"answer": "Present", "rationale": "The key words are \"every week has a \"and a \" feeling, which all point to what is not yet happening and what may happen in the future. At the same time, \"some terrible things are happening\" is also a concern and an imagination for future uncertainty, so the temporal dimension of the article is the future."}, "sentence_type": "Affirmative declarative sentence", "pos": {"nouns": {"Week": 1, "Things": 1}, "verbs": {"Total": 1, "I think": 1, "Come on": 1}, "adjectives": {"Horrible": 1}, "adverbs": {"A few times": 1}, "numerals": {"543. Per cent": 1}}}, {"content": "I have been tired for most of my time.", "word_count": 11, "label": "", "sentence_structure": "Subject鈥揷opula鈥損redicative", "subjective_objective": {"answer": "Subjective", "rationale": "Because \"weary\" is a subjective experience and feeling."}, "sentence_sentiment": {"answer": "Negative", "rationale": "Because \"weary\" is a negative emotional expression."}, "language_style": {"answer": "Tactful and warm", "rationale": "The entry does not directly accuse or order the interviewee, but uses a mild, understanding tone, expressing the researcher鈥檚 desire to understand whether the interviewee is tired. Moreover, the vague description, such as 鈥渕ost of the time鈥? avoids overabsolute language and makes the interviewee feel respected and understood."}, "time_dimension": {"answer": "Present", "rationale": "According to the title, \"I'm tired for most of my time\" means the current subjective feeling, so the entry is about the present situation."}, "sentence_type": "Affirmative declarative sentence", "pos": {"nouns": {"Time": 1, "Tired": 1}, "verbs": {"I feel": 1}, "adjectives": {"Large": 1}, "adverbs": {"Both": 1}, "numerals": {"I have been tired for most of my time": 1}}}, {"content": "I sometimes have the same dream time and again.", "word_count": 10, "label": "", "sentence_structure": "Subject鈥搗erb鈥搊bject", "subjective_objective": {"answer": "Subjective", "rationale": "Because this is the patient ' s description and feelings of his or her dream, it is a subjective experience of the individual."}, "sentence_sentiment": {"answer": "Neutral", "rationale": "Because the entry describes an objective phenomenon, it does not express positive or negative emotional sentiments."}, "language_style": {"answer": "Tactful and warm", "rationale": "The entry is relatively gentle and friendly in its language, is a more euphemistic and somewhat inquisitive language, and is not expressed in a way that is too direct or harsh. At the same time, it contains psychological indications that it encourages respondents to open their hearts and share their feelings, and is more interactive and personal."}, "time_dimension": {"answer": "Past", "rationale": "The entry \"repeated\" indicates a pattern of repeated behaviour over a certain period of time, and \"same dream\" is a situation that has already occurred, and the entry is therefore judged to be past."}, "sentence_type": "Interrogative sentence", "pos": {"nouns": {"Dreams": 1}, "verbs": {"Do it": 1, "Repeatedly": 1, "be": 1}, "adverbs": {"Sometimes": 1, "Same": 1}, "numerals": {"545": 1}, "classifiers": {"individual": 1}}}, {"content": "546. I like reading books about history.", "word_count": 12, "label": "", "sentence_structure": "Subject鈥搗erb鈥搊bject", "subjective_objective": {"answer": "Subjective", "rationale": "Like being patient subjective."}, "sentence_sentiment": {"answer": "Positive", "rationale": "Because the \"like to read history\" act is positive."}, "language_style": {"answer": "Tactful and warm", "rationale": "The language style of the entry is more euphemistic and uses polite language to ask if the other person likes to read history-related books."}, "time_dimension": {"answer": "Past", "rationale": ""}, "sentence_type": "Affirmative declarative sentence", "pos": {"nouns": {"546": 1, "Books": 1, "History": 1}, "verbs": {"Yeah": 1, "Reading": 1}, "numerals": {"546": 1}, "classifiers": {"Ben": 1}}}, {"content": "The future is a fluid one, and it is difficult for one person to make serious arrangements.", "word_count": 20, "label": "", "sentence_structure": "Subject鈥揷opula鈥損redicative", "subjective_objective": {"answer": "Objective", "rationale": "Because the future is an objective reality, one that cannot be changed by personal will or will. One can predict and plan for the future, but one cannot control the outcome completely, so it is an objective view."}, "sentence_sentiment": {"answer": "Negative", "rationale": "Because the entry expresses a negative attitude and view that the future is fraught with uncertainty and unpredictable changes, which may create confusion and uncertainty about the development of long-term plans."}, "language_style": {"answer": "Tactful and warm", "rationale": "The language style of the entry is more euphemistic. It does not express a pessimism or mistrust of the future directly, but rather, it leads the interviewees to think by describing facts 鈥?the future is a vagaries, and it implies uncertainty about the future by the statement that it is very difficult for a person to make serious arrangements, which is relatively mild and not strong."}, "time_dimension": {"answer": "Future", "rationale": ""}, "sentence_type": "Affirmative declarative sentence", "pos": {"nouns": {"The future": 1, "Change": 1, "Unusual": 1, "person": 2, "Seriously": 1, "Arrangements": 1}, "verbs": {"Do it": 1, "Out": 1}, "adjectives": {"Seriously": 1, "Unusual": 1}, "numerals": {"547锛?: 1, "Alone": 1}, "classifiers": {"of": 2}, "pronouns": {"be": 1}}}, {"content": "I would never go to a porn show if I could avoid it.", "word_count": 22, "label": "", "sentence_structure": "Subject鈥搗erb鈥搊bject", "subjective_objective": {"answer": "Subjective", "rationale": "Because \"do everything you can to avoid\" and \"never go\" are subjective attitudes and wills of the patient, not objective circumstances."}, "sentence_sentiment": {"answer": "Negative", "rationale": "Because the \"defunct sex show as much as possible\" in the entry is negative."}, "language_style": {"answer": "Calm and direct", "rationale": "The language style of the entry is more direct and clear, and it is not expressed in too many euphemisms or rhetorical ways, and is objective and concise. It simply describes a person鈥檚 attitude to watching pornographic performances, i.e., to avoid it, and to be absolutely sure. It may give a firm and decisive impression, but it may also appear to be a little indifferent or unattractive."}, "time_dimension": {"answer": "Present", "rationale": "The entry will contain visions and expectations for the future, and the keywords \"if avoidable\" and \"never\" point to situations that have not yet occurred and that may occur in the future. At the same time, \"never go to pornographic shows\" indicates that the testee has a clear will and decision in some future circumstances."}, "sentence_type": "Negative declarative sentence", "pos": {"nouns": {"Pornography": 1, "Performing": 1}, "verbs": {"Decides": 1, "Go on": 1, "Look": 1, "Yeah": 1, "Avoid": 2, "- You're not gonna get away with this": 1, "Hundreds": 1, "Like": 1, "Here": 1}, "adverbs": {"Decision": 1, "If": 1}, "numerals": {"548": 1}, "classifiers": {"of": 2, "Yeah": 1}, "pronouns": {"I": 1}}}, {"content": "549. On many occasions, I feel that I have no concern about matters of personality, even if everything goes well.", "word_count": 22, "label": "", "sentence_structure": "Subject鈥搗erb鈥搊bject", "subjective_objective": {"answer": "Subjective", "rationale": "Because \"I feel like I don't care\" is a subjective feeling and attitude, a patient's assessment and perception of personality."}, "sentence_sentiment": {"answer": "Neutral", "rationale": "Because the entry describes the individual ' s feelings and attitudes towards matters of personality, there is no obvious emotional colour."}, "language_style": {"answer": "Tactful and warm", "rationale": "The entry is more euphemistic, and in a gentle way, asks each other about their attitude in the face of important personality issues, without directly expressing feelings of criticism or blame. At the same time, it uses some polite language such as \"many times\" and \"even if everything goes well\" to make the entire questionnaire more friendly and respectful of the interviewees."}, "time_dimension": {"answer": "Present", "rationale": "The entry uses the usual current \"I feel\" to indicate that the feeling is in the current state or view, without making it clear that it occurred at some point in the past. Therefore, the time dimension of the questionnaire entry should be now."}, "sentence_type": "Negative declarative sentence", "pos": {"nouns": {"Time": 1, "Everything": 1, "Good": 1, "Personality": 1, "Things": 1}, "verbs": {"I think": 1}, "adjectives": {"Many": 1, "It doesn't matter": 1}, "adverbs": {"Both": 1}, "numerals": {"549": 1}}}, {"content": "I like fixing the locks.", "word_count": 7, "label": "", "sentence_structure": "Subject鈥搗erb鈥搊bject", "subjective_objective": {"answer": "Subjective", "rationale": "Because \"like\" is a manifestation of personal emotions and attitudes, viewed from the patient's subjective point of view."}, "sentence_sentiment": {"answer": "Neutral", "rationale": "Because the entry does not have any emotional color."}, "language_style": {"answer": "Calm and direct", "rationale": "According to the information provided, \"I like to fix the lock\" is a more straightforward expression, without using too many rhetorical techniques or emotional renderings, so language style is straight and calm."}, "time_dimension": {"answer": "Present", "rationale": "The entry describes the personal preference of the person tested, i.e. whether they prefer to repair the lock, which is a subjective description of the current situation and does not address past or future situations."}, "sentence_type": "Imperative sentence", "pos": {"nouns": {"Door lock": 1}, "verbs": {"Yeah": 1, "Repair": 1}}}, {"content": "At times I can be sure that I'm not.", "word_count": 16, "label": "", "sentence_structure": "Subject鈥搗erb鈥搊bject", "subjective_objective": {"answer": "Subjective", "rationale": "The question is about whether people can feel that they know what they think. This feeling is based on the patient鈥檚 personal experience, thoughts and emotional state, and is therefore subjective."}, "sentence_sentiment": {"answer": "Neutral", "rationale": "Because the entry describes a phenomenon and does not contain any apparent emotional tendencies."}, "language_style": {"answer": "Tactful and warm", "rationale": "The language style of this entry is euphemism, because it uses a neutral term like \"certainty\" rather than an absolute expression, which makes the respondents to the questionnaire more relaxed and comfortable in answering. At the same time, \"I know what I think\" is also a matter of concern, understanding, rather than direct criticism or criticism, which also reflects a warm language."}, "time_dimension": {"answer": "Present", "rationale": "Now the entry describes the experience and experience of a testee, the keyword \"sometimes\", \"certainly\", all pointing to the state of the current process. So, the time dimension of this questionnaire is now."}, "sentence_type": "Affirmative declarative sentence", "pos": {"nouns": {"Time": 1, "I": 1, "others": 1, "What": 1}, "verbs": {"Yeah": 1, "Sure": 1, "Yes": 1, "I'm thinking": 1}, "adverbs": {"Sometimes": 1}, "numerals": {"551": 1}}}, {"content": "552. I love reading books about science.", "word_count": 12, "label": "", "sentence_structure": "Subject鈥搗erb鈥搊bject", "subjective_objective": {"answer": "Subjective", "rationale": "I like reading books about science is patient's subjective attitude."}, "sentence_sentiment": {"answer": "Positive", "rationale": "Because the entries \"like to read\" and \"books about science\" are positive behaviors and attitudes."}, "language_style": {"answer": "Tactful and warm", "rationale": ""}, "time_dimension": {"answer": "Present", "rationale": ""}, "sentence_type": "Affirmative declarative sentence", "pos": {"nouns": {"Books": 1, "Science": 1}, "verbs": {"Yeah": 1, "Reading": 1}, "numerals": {"552锛?: 1}}}, {"content": "I am afraid to be alone in the open.", "word_count": 12, "label": "", "sentence_structure": "Subject鈥搗erb鈥搊bject", "subjective_objective": {"answer": "Subjective", "rationale": "Because \"fear\" is a subjective feeling or emotional reaction."}, "sentence_sentiment": {"answer": "Negative", "rationale": "Because the \"fear\" sentiment in the entry is negative."}, "language_style": {"answer": "Tactful and warm", "rationale": "The entry is more accessible and interactive when it is spoken in question rather than directly speaking or ordering. At the same time, the language style of the entry is more moderate, there is no excessive emotional overturn or negative vocabulary, and it is more acceptable and willing to answer questions."}, "time_dimension": {"answer": "Past", "rationale": "The entry expresses a feeling of fear, which may be long-standing or new. But it does not clearly point to the future or to the present in terms of sentence structure and terminology selection. Therefore, the content of the entry should be in the past, judging by the time dimension."}, "sentence_type": "Negative declarative sentence", "pos": {"nouns": {"Local": 1, "Questionnaire": 1}, "verbs": {"Scared": 1, "Alone": 1, "Stay": 1}, "adjectives": {"Empty": 1}, "numerals": {"553": 1}, "pronouns": {"I": 1}}}, {"content": "554. If I was a painter, I would like to draw children.", "word_count": 14, "label": "", "sentence_structure": "Subject鈥搗erb鈥搊bject", "subjective_objective": {"answer": "Subjective", "rationale": "\"likes is the patient's subjective attitude.\""}, "sentence_sentiment": {"answer": "Positive", "rationale": "Because it expresses a positive emotional attitude towards painting and children."}, "language_style": {"answer": "Tactful and warm", "rationale": "This is a way to make the person surveyed comfortable and comfortable, thus providing better answers to questions."}, "time_dimension": {"answer": "Future", "rationale": "The key word in the entry is \"if\" and \"like,\" which point to future visions and expectations. \"If I'm a painter,\" which suggests that this is a hypothetical situation, \"like to paint a child\" suggests that the person tested may wish to engage in future activities related to paintings and children."}, "sentence_type": "Affirmative declarative sentence", "pos": {"nouns": {"Suppose": 1, "I": 2, "Painter": 1, "Kids": 1}, "verbs": {"be": 1, "Yeah": 1, "Paint": 1}, "adverbs": {"Both": 1}, "numerals": {"554锛?: 1}}}, {"content": "555. Friendly I feel like I'm going to collapse.", "word_count": 10, "label": "", "sentence_structure": "Subject鈥搗erb鈥揷omplement", "subjective_objective": {"answer": "Subjective", "rationale": "Because \"it's about to collapse\" is a subjective feeling and judgement, not an objective fact or event."}, "sentence_sentiment": {"answer": "Negative", "rationale": "Because the entry \"I feel like I'm going to collapse\" expresses negative emotions and attitudes."}, "language_style": {"answer": "Tactful and warm", "rationale": "The entry asks in a more euphemistic and caring way whether the person under investigation is under very high stress, and this expression makes it easier for the person under investigation to open up and to share his or her true feelings."}, "time_dimension": {"answer": "Present", "rationale": "The keyword \"I feel like I'm going down\" indicates the mood and mentality of the subject at this point, so it can be judged that the entry is asking about the present behaviour and mentality of the subject."}, "sentence_type": "Affirmative declarative sentence", "pos": {"nouns": {"Friendly": 1, "I think": 1, "I want it": 1, "Falling": 1}, "verbs": {"I think": 1, "Falling": 1}, "adjectives": {"Friendly": 1}, "numerals": {"555锛?: 1}, "pronouns": {"I": 1}}}, {"content": "556. I'm very sensitive to my dress patterns.", "word_count": 10, "label": "", "sentence_structure": "Subject鈥揷opula鈥損redicative", "subjective_objective": {"answer": "Subjective", "rationale": "Because \"careful\" is a subjective attitude or feeling."}, "sentence_sentiment": {"answer": "Positive", "rationale": "Because the description \"took care\" shows a concern for and appreciation of his image, and a positive attitude."}, "language_style": {"answer": "Tactful and warm", "rationale": "Warm and warm."}, "time_dimension": {"answer": "Present", "rationale": "In this questionnaire entry, \"I\" indicates that the testee's self-statement, \"fully\" is a synphrase of behaviour, which indicates that the act is ongoing or frequently occurring. \"My dress pattern\" indicates that the subject is being looked at and considered by the testee, which is an act of a state of affairs. So the temporal dimension of the article is now judged."}, "sentence_type": "Affirmative declarative sentence", "pos": {"nouns": {"Questionnaire": 1, "Entry": 1, "Clothes": 1, "Pattern": 1}, "verbs": {"Attention": 1}, "adverbs": {"very": 1}, "numerals": {"556": 1}}}, {"content": "557. I like being a private secretary.", "word_count": 10, "label": "", "sentence_structure": "Subject鈥搗erb鈥搊bject", "subjective_objective": {"answer": "Subjective", "rationale": "Like being patient subjective."}, "sentence_sentiment": {"answer": "Positive", "rationale": "Because \"like being a private secretary\" is positive."}, "language_style": {"answer": "Tactful and warm", "rationale": "The entry uses the first-person proxy, \"I,\" which expresses the personal preferences and preferences of the person who speaks, is more acclaimable, natural, is not overly emotional or direct command language. This expression is more resonant and responsive to the respondent."}, "time_dimension": {"answer": "Present", "rationale": "The words \"like,\" \"behind\" and \"private secretary\" all indicate that this is the current preference and attitude of the testees, so the temporal dimension is now judged."}, "sentence_type": "Exclamatory sentence", "pos": {"nouns": {"Private Secretary": 1}, "verbs": {"Yeah": 1, "When": 1}, "numerals": {"557": 1}}}, {"content": "558. Many people are ashamed of having had bad sexual behaviour.", "word_count": 19, "label": "", "sentence_structure": "Subject鈥搗erb鈥搊bject", "subjective_objective": {"answer": "Subjective", "rationale": "The content of this questionnaire item relates to people鈥檚 feelings and attitudes, and is therefore viewed from the patient鈥檚 subjective perspective. Although bad sex is an objective fact, feelings and attitudes that are ashamed are subjective."}, "sentence_sentiment": {"answer": "Negative", "rationale": "Because the attitude of \"smear\" in the entries is negative."}, "language_style": {"answer": "Tactful and warm", "rationale": "This expression is more conducive to building trust and cooperation among the subjects of the inquiry."}, "time_dimension": {"answer": "Present", "rationale": "The description of the keyword \"many people\" and \"disguised\" points to the current state and emotional response, so it can be judged that the entry is asking the subject what it feels now."}, "sentence_type": "Affirmative declarative sentence", "pos": {"nouns": {"person": 2, "Bad": 1, "Sexuality": 1, "Sensor": 1, "Shame": 1}, "verbs": {"have": 1, "Pass": 1, "Because": 1, "I feel": 1}, "adjectives": {"Many": 1, "Bad": 1}, "numerals": {"558": 1, "Both": 1, "One": 1}, "pronouns": {"he": 1}}}, {"content": "I was often frightened in the middle of the night.", "word_count": 11, "label": "", "sentence_structure": "Subject鈥搗erb鈥揷omplement", "subjective_objective": {"answer": "Subjective", "rationale": "Because \"scared\" is a subjective feeling and experience, related to the emotional and psychological state of the patient."}, "sentence_sentiment": {"answer": "Negative", "rationale": "Because \"scared\" is usually considered a negative experience and emotional reaction."}, "language_style": {"answer": "Tactful and warm", "rationale": "The entry is presented in a more euphemistic way, by means of questions, to guide the interviewee to share his experiences and feelings, rather than to make direct statements or orders. Moreover, the use of the term \"often\" also shows a sense of concern and understanding, rather than judgment or criticism. This language style is more appropriate for the purposes of the psychological questionnaire, and can create a climate of ease and security that allows the interviewee to express his thoughts and feelings more openly."}, "time_dimension": {"answer": "Present", "rationale": "So, the entry is basically about asking the testee's current behavior and emotional state. So the output is: \"Now.\""}, "sentence_type": "Affirmative declarative sentence", "pos": {"nouns": {"I": 1, "In the middle of the night": 1, "Surprise": 1}, "verbs": {"Regular": 1, "Received": 1}, "adverbs": {"Lee": 1, "very": 1}, "numerals": {"559": 1}, "classifiers": {"individual": 1}}}, {"content": "I am often troubled by the fact that I cannot remember where I put it.", "word_count": 20, "label": "", "sentence_structure": "Subject鈥揷opula鈥損redicative", "subjective_objective": {"answer": "Subjective", "rationale": "Because it's the feelings and experiences of the patients who can't remember where they put them."}, "sentence_sentiment": {"answer": "Negative", "rationale": "Because \"I can't remember where to put it\" is negative and can be a source of distress."}, "language_style": {"answer": "Tactful and warm", "rationale": ""}, "time_dimension": {"answer": "Present", "rationale": ""}, "sentence_type": "Affirmative declarative sentence", "pos": {"nouns": {"I": 1, "Something": 1, "Trouble": 1}, "verbs": {"Because": 1, "Forget it": 1, "Fire": 1, "Sensor": 1, "Present": 1}, "adjectives": {"Regular": 1, "Clear": 1, "Where": 1}, "numerals": {"560": 1}, "classifiers": {"individual": 1}}}, {"content": "I like riding.", "word_count": 6, "label": "", "sentence_structure": "Subject鈥損redicate", "subjective_objective": {"answer": "Subjective", "rationale": "Because \"like\" is a subjective feeling or emotion that describes the attitude and preferences of the patient towards riding."}, "sentence_sentiment": {"answer": "Positive", "rationale": "Because the expression \"I like horse riding a lot\" in the entry expresses a positive attitude and preference towards riding."}, "language_style": {"answer": "Tactful and warm", "rationale": "The entry is more euphemistic and warm, using the subjective expression \"I like it\" rather than directly speaking or ordering, and more friendly and friendly. At the same time, the subject matter is more comfortable, without serious themes or negative emotions, and more resonant with the interviewee."}, "time_dimension": {"answer": "Present", "rationale": ""}, "sentence_type": "Affirmative declarative sentence", "pos": {"nouns": {"Horses": 1}, "verbs": {"Yeah": 1, "Ride": 1}, "numerals": {"561": 1}}}, {"content": "When I was a child, I was most attached to and admired by a woman (mother, sister, aunt, aunt, aunt, etc.).", "word_count": 24, "label": "", "sentence_structure": "Subject鈥搗erb鈥搊bject", "subjective_objective": {"answer": "Subjective", "rationale": "Because it involves the patient ' s personal memory and emotional reaction, it is a reflection of subjective experience."}, "sentence_sentiment": {"answer": "Neutral", "rationale": "Because the entry does not have any emotional color."}, "language_style": {"answer": "Tactful and warm", "rationale": "The entry is more euphemistic. First, it gives a sense of affection to people, beginning with \"when they were young,\" and uses the expression \"the woman most attached and admired\" to emphasize the emotional identity of the woman rather than an objective assessment. Moreover, the list \"mother, sister, aunt, aunt, aunt, etc.\" is a more subtle one, reflecting the interest and respect of the investigator for the person under investigation."}, "time_dimension": {"answer": "Past", "rationale": ""}, "sentence_type": "Affirmative declarative sentence", "pos": {"nouns": {"Time": 1, "The most devoted and admired": 1, "One": 1, "Women": 1, "Mother, sister, aunt, aunt, aunt, etc": 1}, "verbs": {"be": 1}, "adverbs": {"When I was little": 1}, "numerals": {"562锛?: 1}}}, {"content": "563. I like adventure novels more than love novels.", "word_count": 13, "label": "", "sentence_structure": "Subject鈥搗erb鈥搊bject", "subjective_objective": {"answer": "Subjective", "rationale": "Like being patient subjective."}, "sentence_sentiment": {"answer": "Neutral", "rationale": "Because the entry merely describes a fact, expresses a personal preference and does not display a positive or negative emotional colour."}, "language_style": {"answer": "Calm and direct", "rationale": "This is a straightforward way to reflect the views and attitudes of the interviewee more accurately."}, "time_dimension": {"answer": "Present", "rationale": "In the entry, the words \"like\" and \"over\" express the emotional state and preferences of the testee, and therefore judge now."}, "sentence_type": "Affirmative declarative sentence", "pos": {"nouns": {"Yeah": 1, "Novel": 2, "Adventure": 1, "Love": 1}, "verbs": {"More than anything": 1}, "adjectives": {"Yeah": 1}, "numerals": {"563": 1}}}, {"content": "564. I am not easily angry.", "word_count": 6, "label": "", "sentence_structure": "Subject鈥搗erb鈥揷omplement", "subjective_objective": {"answer": "Subjective", "rationale": "Because \"not easily angry\" is the patient's subjective attitude and emotional experience."}, "sentence_sentiment": {"answer": "Neutral", "rationale": "Because the entry does not have any emotional color."}, "language_style": {"answer": "Calm and direct", "rationale": "The entry expresses a theme in a concise language style, with no excessive rhetorical or emotional rendering, and is more objective and consistent with the immediate and calmness of the tone."}, "time_dimension": {"answer": "Present", "rationale": "The term \"uneasy\" in this entry is the self-presentation and description of the person being tested, referring to the current situation and behaviour patterns, and therefore the temporal dimension is now judged."}, "sentence_type": "Negative declarative sentence", "pos": {"verbs": {"Not easy": 1, "Fan": 1}, "adjectives": {"Easy": 1}, "adverbs": {"not": 1}, "numerals": {"564": 1}}}, {"content": "565. When I stand high, I would love to jump.", "word_count": 16, "label": "", "sentence_structure": "Subject鈥搗erb鈥搊bject", "subjective_objective": {"answer": "Subjective", "rationale": "This is because the patient's description of his/her feelings and thoughts in a given situation is a subjective experience of the individual."}, "sentence_sentiment": {"answer": "Negative", "rationale": "Because the \"I'd love to jump\" act in the entry is negative."}, "language_style": {"answer": "Calm and direct", "rationale": "The entry is in plain and simple language, and it directly expresses the subject of asking the other side whether they have had any experience of jumping down when they stand at a higher level. There are no excessive rhetorical techniques or emotional renderings, and the tone is more objective."}, "time_dimension": {"answer": "Past", "rationale": "Such impulses may be emotions or behaviours that have been experienced in the past, or may be an imaginary or anticipated situation. Thus, the temporal dimension of this entry is likely to be involved in the past, present and future."}, "sentence_type": "Affirmative declarative sentence", "pos": {"nouns": {"Time": 1, "High": 1, "Jump": 1}, "verbs": {"Stop": 1, "Yes": 1, "Go on": 1, "Down": 1, "Jump": 1}, "adverbs": {"Just": 1}, "numerals": {"565": 1}, "pronouns": {"I": 1}}}, {"content": "I love the love scene in the movies.", "word_count": 11, "label": "", "sentence_structure": "Subject鈥搗erb鈥搊bject", "subjective_objective": {"answer": "Subjective", "rationale": "Because \"like\" is a subjective feeling or emotional reaction, describing the patient's personal preferences and attitudes to the scene of love in the film."}, "sentence_sentiment": {"answer": "Positive", "rationale": "Because the word \"like\" expresses a positive emotional attitude."}, "language_style": {"answer": "Tactful and warm", "rationale": "The language style of the entry is euphemistic, because it is a soft, invitation-style conversation, not a direct statement or order. Moreover, the positive term \"like\" is used to give positive feelings and not too much emotional expression, with a greater focus on objective understanding of the views and feelings of the person under investigation."}, "time_dimension": {"answer": "Past", "rationale": ""}, "sentence_type": "Affirmative declarative sentence", "pos": {"nouns": {"I": 1, "Yeah": 1, "Film": 1, "Lee": 1, "Love": 1, "Camera": 1}, "verbs": {"have": 1}, "numerals": {"566锛?: 1}}}]}

{"questionnaire_title": "IPPA questionnaire", "questionnaire_category": "Mental health", "item_count": 75, "emotion_shift_overall": "From positive to negative", "language_style_overall": "Tactful and warm", "items": [{"content": "My mother respects my feelings.", "word_count": 9, "label": "", "sentence_structure": "Subject鈥搗erb鈥搊bject", "subjective_objective": {"answer": "Subjective", "rationale": "Because \"respect\" is a subjective word of evaluation, which expresses the attitude and perception of patients towards their mothers."}, "sentence_sentiment": {"answer": "Positive", "rationale": "Because \"respect\" in the entry means that the mother's attitude is positive."}, "language_style": {"answer": "Tactful and warm", "rationale": "The entry is more acrimony and interactive when it is presented in a gentle and inviting tone, rather than in direct statements or orders. At the same time, it is not overly rhetorical or emotionally retrofitted, and is more objective and suitable for psychological questionnaire research."}, "time_dimension": {"answer": "Present", "rationale": ""}, "sentence_type": "Affirmative declarative sentence", "pos": {"nouns": {"Mother": 1, "Emotion": 1}, "verbs": {"Respect": 1}, "pronouns": {"I": 1}}}, {"content": "I think my mother is a competent mother.", "word_count": 13, "label": "", "sentence_structure": "Subject鈥揷opula鈥損redicative", "subjective_objective": {"answer": "Subjective", "rationale": "As this view is part of the patient ' s personal perception and assessment of the mother, it is examined from a subjective perspective."}, "sentence_sentiment": {"answer": "Positive", "rationale": "Because the entry \"skilled mother\" expresses a positive assessment and recognition of the mother."}, "language_style": {"answer": "Tactful and warm", "rationale": "The entry presents its views in a relatively positive and kind manner, without direct criticism or praise of the other, and in a more moderate manner."}, "time_dimension": {"answer": "Present", "rationale": "The keyword \"considers\" and \"yes\" indicates that the sentence describes the current evaluation of the mother and therefore the judgement is now."}, "sentence_type": "Affirmative declarative sentence", "pos": {"nouns": {"Questionnaire Entry": 1, "Mother": 1, "Work": 1}, "verbs": {"I think": 1, "be": 1}, "adjectives": {"Good": 1}, "pronouns": {"I": 1}}}, {"content": "I wish my mother were someone else.", "word_count": 13, "label": "", "sentence_structure": "Subject鈥搗erb鈥揷omplement", "subjective_objective": {"answer": "Subjective", "rationale": "Because \"hope\" is a subjective emotion and expectation."}, "sentence_sentiment": {"answer": "Negative", "rationale": "Because the emotional expression \"hope for your mother to be someone else\" is negative."}, "language_style": {"answer": "Tactful and warm", "rationale": "The entry expresses the desire and expectation for motherhood in an implicit and euphemistic way, without directly blaming or denying the mother. This expression is more respectful of each other鈥檚 feelings and more resonance and reflection among interlocutors."}, "time_dimension": {"answer": "Present", "rationale": "The sentence expresses the personal feelings and attitudes of the person who speaks to his mother, which are a description or expression of the current state of affairs and which still exist in the present state and can therefore be judged to be the present."}, "sentence_type": "Exclamatory sentence", "pos": {"nouns": {"Mother": 1}, "verbs": {"Hope": 1, "be": 1}, "adjectives": {"Other": 1, "individual": 1}, "numerals": {"3": 1}, "pronouns": {"I": 1}}}, {"content": "My mother recognizes my present state of mind.", "word_count": 12, "label": "", "sentence_structure": "Subject鈥搗erb鈥揷omplement", "subjective_objective": {"answer": "Subjective", "rationale": "Because \"acceptance\" is a subjective feeling, a description of the patient's attitude towards my mother, it is viewed from the patient's subjective perspective."}, "sentence_sentiment": {"answer": "Positive", "rationale": "Because the expression \"I agree\" is positive and is recognized and praised by the mother."}, "language_style": {"answer": "Tactful and warm", "rationale": "The expression \"I'm very good at it\" in the entry does not directly ask the other side about his image, but rather in a more euphemistic and warm way, making it easier for the other side to accept and be willing to answer. And the use of the word \"mother\" also makes the subject of the questionnaire more intimate and sweet."}, "time_dimension": {"answer": "Present", "rationale": ""}, "sentence_type": "Affirmative declarative sentence", "pos": {"nouns": {"I": 1, "Mother": 1, "Look": 1}, "verbs": {"very": 1, "Approval": 1, "be": 1}, "adjectives": {"Present": 1}}}, {"content": "5. I like to hear my mother ' s views on what I care about.", "word_count": 18, "label": "", "sentence_structure": "Subject鈥搗erb鈥搊bject", "subjective_objective": {"answer": "Subjective", "rationale": "Because \"like to hear my mother's opinion\" is the patient's personal attitude and opinion, and it's subjective."}, "sentence_sentiment": {"answer": "Positive", "rationale": "Because this entry expresses the importance that individuals attach to and respect for the views of mothers and has a positive emotional orientation."}, "language_style": {"answer": "Tactful and warm", "rationale": "The language style of the entry is relatively euphemistic and emotional, using the term \"like\" to express the importance and intimacy of the views of the mother. Moreover, the term \"me mother\" emphasizes the importance of family relations. In general, this expression is more natural and resonates with the interviewee."}, "time_dimension": {"answer": "Present", "rationale": ""}, "sentence_type": "Exclamatory sentence", "pos": {"nouns": {"Things": 1, "Observations": 1, "Mother": 1}, "verbs": {"Yeah": 1, "Listen": 1, "in": 1}, "adjectives": {"I care": 1}, "numerals": {"5": 1}, "pronouns": {"I": 2}}}, {"content": "6. I feel that it is useless to show my feelings to my mother.", "word_count": 18, "label": "", "sentence_structure": "Subject鈥搗erb鈥揷omplement", "subjective_objective": {"answer": "Subjective", "rationale": "Because \"think\" expresses the patient's views and opinions on the matter, and it is a manifestation of subjective attitudes."}, "sentence_sentiment": {"answer": "Negative", "rationale": "Because the \"no\" attitude in the entry is negative."}, "language_style": {"answer": "Calm and direct", "rationale": "The language style of the entry is calm, because it expresses the subject clearly and succinctly 鈥?considering that it is ineffective to express feelings to mothers. It does not use excessive emotional qualifiers or complex sentence structures, but rather conveys the message in an objective and clear manner, so that the person surveyed can understand the meaning of the problem clearly."}, "time_dimension": {"answer": "Past", "rationale": "\"I feel that showing my feelings to my mother is useless\" means the testee's past behavior and mentality, and the judgement is based on the \"old.\""}, "sentence_type": "Negative declarative sentence", "pos": {"nouns": {"Feel": 1, "Mother": 1, "Emotion": 1}, "verbs": {"Show": 1, "I think": 1}, "adjectives": {"It works": 1}, "adverbs": {"No, I'm not": 1}, "pronouns": {"self": 1}}}, {"content": "When I feel sorry for something, my mother will see it.", "word_count": 18, "label": "", "sentence_structure": "Subject鈥搗erb鈥揷omplement", "subjective_objective": {"answer": "Subjective", "rationale": "Because \"my mother will see\" is my own feeling and perception, and it's a subjective expression."}, "sentence_sentiment": {"answer": "Positive", "rationale": "According to the entry, \"When I feel sorry for something,\" the expression of motherly love is \"seen,\" which reflects the emotional attitude of mothers to care for their children and to understand them, and is positive."}, "language_style": {"answer": "Tactful and warm", "rationale": "The entry is more adept and interactive when it is spoken in a gentle and inviting manner, rather than by direct statements or orders."}, "time_dimension": {"answer": "Present", "rationale": ""}, "sentence_type": "Affirmative declarative sentence", "pos": {"nouns": {"Time": 1, "Something": 1, "Mother": 1}, "verbs": {"Sorry": 1, "Yeah": 1}, "pronouns": {"I": 1}}}, {"content": "8. It is a shame and a foolish thing to discuss my problems with my mother.", "word_count": 19, "label": "", "sentence_structure": "Subject鈥揷opula鈥損redicative", "subjective_objective": {"answer": "Subjective", "rationale": "Because the feeling of \"smugness\" and \"smugness\" is the patient's subjective experience. It's a description of how he feels when he talks to his mother about his problems, and it's a subjective idea, not an objective result or fact."}, "sentence_sentiment": {"answer": "Negative", "rationale": "Because \"smuggling, stupid\" in the entry is a negative emotional attitude."}, "language_style": {"answer": "Tactful and warm", "rationale": "The language style in the entry is euphemism. Using a description like \"and I feel ashamed and stupid,\" it expresses feelings and feelings, but it is softer, not too direct or harsh, and therefore warm. Moreover, the issue leads the respondents to share their experiences and feelings in a more personal way, with some personality and interaction."}, "time_dimension": {"answer": "Past", "rationale": "The entry describes the behavior or experience of the past, and \"discussed and stupid to discuss my problems with my mother\" is a specific event or situation. So the temporal dimension of the entry is the past."}, "sentence_type": "Negative declarative sentence", "pos": {"nouns": {"Mother": 1, "Trouble": 1, "Shame": 1, "Stupid": 1}, "verbs": {"Discussion": 1, "Jean": 1, "I feel": 1}, "adjectives": {"- I'm not": 1, "very": 2}, "numerals": {"8": 1}}}, {"content": "Mothers expect too much from me.", "word_count": 8, "label": "", "sentence_structure": "Subject鈥揷opula鈥損redicative", "subjective_objective": {"answer": "Subjective", "rationale": "Because \"mothers expect too much of me\" is the patient's own view and feelings, and it is therefore a subjective examination."}, "sentence_sentiment": {"answer": "Negative", "rationale": "Because the expression \"too high expectations\" usually leads to negative emotions and stress."}, "language_style": {"answer": "Tactful and warm", "rationale": "The language style of the entry is more euphemistic, not too straightforward, but rather more subtle, making the subject aware of the problem itself. This expression avoids stimulating the self-esteem of the person under investigation and is more acceptable and understandable."}, "time_dimension": {"answer": "Present", "rationale": "The entry now describes the current feelings or circumstances of the person being tested, and the keyword is \"Mother expects too much of me\", which indicates that there are such feelings or circumstances."}, "sentence_type": "Negative declarative sentence", "pos": {"nouns": {"Mother": 1, "Expectations": 1}, "verbs": {"to": 1, "Too much": 1, "High": 1}, "adjectives": {"Too high": 1}}}, {"content": "I feel very easily for my mother.", "word_count": 11, "label": "", "sentence_structure": "Subject鈥揷opula鈥損redicative", "subjective_objective": {"answer": "Subjective", "rationale": "Because \"sorture\" is a subjective feeling, and it's the patient's description and experience of his feelings."}, "sentence_sentiment": {"answer": "Negative", "rationale": "Because the \"worried for mother\" sentiment in the entry is negative."}, "language_style": {"answer": "Tactful and warm", "rationale": "The entry uses a gentle, caring tone of the feelings of others, asking whether the other is often upset by the mother's story, which is more personal and interactive. At the same time, using the expression \"I am easy\" suggests that it is a relatively common experience, making it easier for the person questioned to open his heart and share his feelings."}, "time_dimension": {"answer": "Past", "rationale": "The entry asks about the testee ' s past behaviour or experience and therefore judges as past."}, "sentence_type": "Affirmative declarative sentence", "pos": {"nouns": {"Mother": 1, "Sorry": 1}, "verbs": {"I feel": 1}, "adjectives": {"Easy": 1}, "numerals": {"10": 1}, "pronouns": {"I": 1}}}, {"content": "I have many unhappy but little that my mother knows.", "word_count": 15, "label": "", "sentence_structure": "Subject鈥搗erb鈥搊bject", "subjective_objective": {"answer": "Subjective", "rationale": "Because \"mother knows very little\" is the patient's subjective idea."}, "sentence_sentiment": {"answer": "Negative", "rationale": "Because the phrase \"a lot of unhappy\" in the entry is negative."}, "language_style": {"answer": "Tactful and warm", "rationale": ""}, "time_dimension": {"answer": "Present", "rationale": ""}, "sentence_type": "Negative declarative sentence", "pos": {"nouns": {"I": 1, "A lot": 1, "I'm not happy": 1, "Mother": 1, "Less": 1}, "verbs": {"have": 1, "Yes": 1}, "adjectives": {"very": 2}, "numerals": {"11": 1, "Very few": 1}, "classifiers": {"individual": 1}}}, {"content": "12. When things are discussed, mothers will take my point very seriously.", "word_count": 18, "label": "", "sentence_structure": "Subject鈥搗erb鈥搊bject", "subjective_objective": {"answer": "Subjective", "rationale": "Because \"care for my point\" is a subjective judgment, a patient's subjective view of the mother's behavior."}, "sentence_sentiment": {"answer": "Positive", "rationale": "Because the \"I care\" in the entry shows that mothers value and respect the views of their children, which is a positive attitude."}, "language_style": {"answer": "Tactful and warm", "rationale": "The entry is more personal and interactive when it is spoken in a gentle and inviting manner, rather than in direct statements or orders. Moreover, the entry uses the term \"careful\" to indicate that mothers take the views of their children very seriously and to convey an emotional atmosphere of love and support."}, "time_dimension": {"answer": "Present", "rationale": "The keyword \"will\" denote a customary act, which is taking place at the moment. So the time dimension of this entry is now."}, "sentence_type": "Affirmative declarative sentence", "pos": {"nouns": {"Things": 1, "Time": 1, "Mother": 1, "Views": 1}, "verbs": {"Discussion": 1, "I'll care": 1}, "numerals": {"12": 1}, "pronouns": {"I": 1}}}, {"content": "My mother trusted my judgement.", "word_count": 9, "label": "", "sentence_structure": "Subject鈥搗erb鈥搊bject", "subjective_objective": {"answer": "Subjective", "rationale": "Because trust is an emotional state, it is the patient's subjective feeling."}, "sentence_sentiment": {"answer": "Positive", "rationale": "Because the expression \"mother trusts my judgment\" in the entry is positive."}, "language_style": {"answer": "Tactful and warm", "rationale": "The language style of the entry is more moderate, and by using the expression \"My mother trusts my judgment\", it expresses both the subject of the survey (the level of confidence of the mother in the interviewee) and avoids direct questioning of the interviewee's own feelings or behaviour, and is more respectful and respectful."}, "time_dimension": {"answer": "Past", "rationale": "The entry \"My mother trusts my judgment\" shows the facts of the past, and therefore the past."}, "sentence_type": "Affirmative declarative sentence", "pos": {"nouns": {"Mother": 1, "Judgement": 1}, "verbs": {"Trust": 1}, "adverbs": {"13": 1}, "numerals": {"13": 1}, "pronouns": {"I": 1}}}, {"content": "14. My mother had her own problems, so I would not bother her with my own.", "word_count": 24, "label": "", "sentence_structure": "Subject鈥搗erb鈥搊bject", "subjective_objective": {"answer": "Subjective", "rationale": "The content of this entry concerns the patient鈥檚 own thoughts and behaviour (i.e., 鈥淚 will not bother her with my own worries鈥?, so it looks at it from the patient鈥檚 subjective point of view. At the same time, while the entry refers to the situation of the patient鈥檚 mother, it focuses primarily on the patient鈥檚 subjective ideas and actions, not on objective facts."}, "sentence_sentiment": {"answer": "Neutral", "rationale": "Since the entry merely describes a fact, it does not involve positive or negative emotional tendencies."}, "language_style": {"answer": "Tactful and warm", "rationale": ""}, "time_dimension": {"answer": "Past", "rationale": ""}, "sentence_type": "Affirmative declarative sentence", "pos": {"nouns": {"Problem": 1, "Mother": 1, "Trouble": 2}, "verbs": {"have": 1, "Take it": 1, "Fuck off": 1}, "adverbs": {"self": 1, "No, I won't": 1, "So": 1, "she": 1}, "numerals": {"14": 1}, "pronouns": {"I": 1, "she": 2}}}, {"content": "My mother helped me to better understand myself.", "word_count": 13, "label": "", "sentence_structure": "Subject鈥搗erb鈥揷omplement", "subjective_objective": {"answer": "Subjective", "rationale": "Because \"mother helps me to know myself better\" is the patient's subjective feeling and experience."}, "sentence_sentiment": {"answer": "Positive", "rationale": "Because the behavior in the entry ( \"Mother helps me to know myself better\") is advantageous and positive."}, "language_style": {"answer": "Tactful and warm", "rationale": "The words \"help\" and \"better understanding\" are used to suggest the importance and positive impact of the mother. At the same time, there is no direct order or request for answers, and the tone is more respectful."}, "time_dimension": {"answer": "Present", "rationale": "Now, the entry describes the situation where the testee is getting help from his mother to better understand himself. Although the person \"my mother\" may have existed in the past, in this sentence it means the situation now. So the time dimension is now."}, "sentence_type": "Affirmative declarative sentence", "pos": {"nouns": {"I": 1, "Mother": 1, "self": 1}, "verbs": {"Help": 1, "Roger that": 1}, "numerals": {"15": 1}}}, {"content": "I told my mother my troubles.", "word_count": 13, "label": "", "sentence_structure": "Subject鈥搗erb鈥搊bject", "subjective_objective": {"answer": "Subjective", "rationale": "Because this is the patient ' s subjective action and decision, it is entirely up to the mother to tell her of her own concerns."}, "sentence_sentiment": {"answer": "Neutral", "rationale": "Because the entry does not have any emotional color."}, "language_style": {"answer": "Tactful and warm", "rationale": "The entry uses a first-person expression and refers to the feeling of \"me\" and the relationship between the mother, giving a warm and warm feeling. At the same time, the language style of the article is more direct, concise, not too much rhetorical or emotional, and more focused on factual description and information transmission."}, "time_dimension": {"answer": "Present", "rationale": "The keyword \"Tell\" and \"mother\" indicates that this is a past, present or potential future. Because there is no clear indication of the time-state of such behaviour (such as \"I'm telling my mother\" or \"I'm going to tell my mother my troubles,\" we cannot be sure whether the act occurred or will, and therefore it can only be classified as now."}, "sentence_type": "Affirmative declarative sentence", "pos": {"nouns": {"鈾?My own troubles 鈾?: 1, "Mother": 1}, "verbs": {"- Put it on": 1, "Tell": 1}, "numerals": {"16": 1}, "classifiers": {"individual": 1}, "pronouns": {"I": 1}}}, {"content": "I'm angry with my mother.", "word_count": 7, "label": "", "sentence_structure": "Subject鈥揷opula鈥損redicative", "subjective_objective": {"answer": "Subjective", "rationale": "Because anger is an emotional experience, it is the patient ' s subjective feeling and attitude."}, "sentence_sentiment": {"answer": "Negative", "rationale": "Because the entry expresses a negative emotion \"smoke\" and a negative assessment of the mother."}, "language_style": {"answer": "Calm and direct", "rationale": "The entry expresses dissatisfaction with the mother in a direct and clear language, without any use of rhetoric or euphemism, and is more serious and apathy."}, "time_dimension": {"answer": "Present", "rationale": "The keyword \"I'm angry\" indicates that the person being tested is experiencing a negative emotion that is related to his mother."}, "sentence_type": "Negative declarative sentence", "pos": {"nouns": {"I": 2, "Mother": 1}, "verbs": {"I'm angry": 1}}}, {"content": "18. I did not receive much attention from my mother.", "word_count": 12, "label": "", "sentence_structure": "Subject鈥搗erb鈥揷omplement", "subjective_objective": {"answer": "Subjective", "rationale": "Because it is the individual feelings and perceptions of the patient that are noticed by the mother, it is subjective."}, "sentence_sentiment": {"answer": "Negative", "rationale": "The entry is a reflection of negative feelings that do not receive the attention of the mother."}, "language_style": {"answer": "Tactful and warm", "rationale": "This is a more euphemistic and implicit expression, avoiding direct accusations or criticism, and allowing the other to understand his/her own situation by expressing his/her own feelings. This language style is more friendly and sweet, reducing each other鈥檚 resistance and increasing the effectiveness of questionnaires."}, "time_dimension": {"answer": "Past", "rationale": "The entry uses \"not receiving\" and \"a lot of attention\", implying that this has happened in the past and has had an impact on the subjects."}, "sentence_type": "Negative declarative sentence", "pos": {"nouns": {"Mother": 1, "Attention": 1}, "verbs": {"Not received": 1, "Here": 1}, "numerals": {"18": 1, "A lot": 1}, "pronouns": {"I": 1}}}, {"content": "My mother helped me discuss my difficulties.", "word_count": 13, "label": "", "sentence_structure": "Subject鈥搗erb鈥搊bject", "subjective_objective": {"answer": "Objective", "rationale": "Because the entry describes the patient ' s interaction with her mother, not her own feelings or thoughts, it is objective."}, "sentence_sentiment": {"answer": "Positive", "rationale": "Because the behaviour in the entry, \"Mother helps to discuss difficulties\", is positive and reflects family care and support, helping to solve problems."}, "language_style": {"answer": "Tactful and warm", "rationale": "The entry conveys a positive, loving emotional atmosphere, with a more compassionate and gentle language style, by describing the behavior that my mother helped."}, "time_dimension": {"answer": "Present", "rationale": "The keyword \"help me discuss\" and the word \"my mother\" show that it's an ongoing or recurrent move, so we can judge the time dimension of this entry is now."}, "sentence_type": "Exclamatory sentence", "pos": {"nouns": {"Mother": 1, "Difficulties": 1}, "verbs": {"Help": 1, "Together": 1, "Discussion": 1}, "adverbs": {"I": 1}, "numerals": {"19": 1}}}, {"content": "My mother understood me.", "word_count": 6, "label": "", "sentence_structure": "Subject鈥搗erb鈥搊bject", "subjective_objective": {"answer": "Subjective", "rationale": "Because \"understanding\" is a subjective word of evaluation that reflects the patient's perception and feelings about his/her relationship with his/her mother."}, "sentence_sentiment": {"answer": "Positive", "rationale": "Because the \"understanding me\" in the entry expresses a positive emotional attitude."}, "language_style": {"answer": "Tactful and warm", "rationale": "The entry expresses in a question-and-answer manner the understanding of the mother of the person surveyed of her concern, the temperature of the language and the sensitivity of the person concerned, and the comfort and willingness of the person interviewed to share the real thoughts."}, "time_dimension": {"answer": "Present", "rationale": "The time indicator in the entry is \"My mother understands me\", which is a statement of the present, describing the current relationship and level of mutual understanding between the testees and their mother. We can therefore classify it as the current dimension of time."}, "sentence_type": "Affirmative declarative sentence", "pos": {"nouns": {"Mother": 1, "Understood": 1, "I": 1}, "verbs": {"Here": 1, "Understood": 1}, "numerals": {"20": 1}, "classifiers": {"individual": 1}}}, {"content": "21. When I was angry about something, my mother tried to understand me.", "word_count": 17, "label": "", "sentence_structure": "Subject鈥搗erb鈥搊bject", "subjective_objective": {"answer": "Subjective", "rationale": "Attempts to understand are subjective attitudes or behaviour, which means that mothers are trying to see the problem from the perspective of the patient."}, "sentence_sentiment": {"answer": "Positive", "rationale": "Because the \"mother tries to understand me\" act is positive."}, "language_style": {"answer": "Tactful and warm", "rationale": "The entry is more personal and interactive when it is spoken in a gentle and inviting tone, rather than in direct statements or orders. At the same time, the tone of the article is filled with elements of understanding and concern, displaying understanding and support for emotions, and leaving a warmer feeling."}, "time_dimension": {"answer": "Present", "rationale": "In conclusion, the answer to this question is \"now\"."}, "sentence_type": "Affirmative declarative sentence", "pos": {"nouns": {"I": 1, "Things": 1, "Mother": 1}, "verbs": {"Angry": 1, "Try": 1, "Understood": 1}, "numerals": {"21": 1}}}, {"content": "I trust my mother.", "word_count": 7, "label": "", "sentence_structure": "Subject鈥搗erb鈥搊bject", "subjective_objective": {"answer": "Subjective", "rationale": "Because \"trust\" is a subjective feeling and attitude."}, "sentence_sentiment": {"answer": "Positive", "rationale": "Because \"trust\" is a positive emotional word that shows that the interviewee has a good emotional basis and relationship with her mother."}, "language_style": {"answer": "Tactful and warm", "rationale": "The entry is in more euphemistic terms, which indicate the degree of confidence that the interviewee has in her mother, without using a direct tone of accusation or order. Moreover, the subject is more friendly, and the interviewee feels respected and understood."}, "time_dimension": {"answer": "Present", "rationale": ""}, "sentence_type": "Affirmative declarative sentence", "pos": {"nouns": {"I": 1, "Mother": 1}, "verbs": {"Trust": 1}, "numerals": {"22": 1}}}, {"content": "My mother did not know what I had to go through on those days.", "word_count": 17, "label": "", "sentence_structure": "Subject鈥搗erb鈥搊bject", "subjective_objective": {"answer": "Subjective", "rationale": "Because the sentence expresses the patient ' s subjective feelings and perceptions that her mother does not know about her experience."}, "sentence_sentiment": {"answer": "Negative", "rationale": "Because the expression \"mother does not know\" in the entry is negative and rebuffive and has a negative emotional tendency."}, "language_style": {"answer": "Calm and direct", "rationale": "The language style of the entry is more straightforward, there is no excessive rhetorical or emotional rendering, and it is more objective in tone.\""}, "time_dimension": {"answer": "Present", "rationale": "The entry to the questionnaire is to ask the testee about the current situation, and the time dimension is now judged. The keyword \"these days\" indicates that it is a review and description of recent experience, and \"know\" that the testee's mother was not aware of it, so it is the state of affairs."}, "sentence_type": "Negative declarative sentence", "pos": {"nouns": {"I": 1, "Mother": 1, "Experience": 1, "What": 1}, "verbs": {"Roger that": 1, "Both": 1, "The long run": 1, "Calendar": 1}, "adverbs": {"Here": 2, "Some": 1, "Oh, my God": 1}, "numerals": {"23": 1}, "pronouns": {"These": 1}}}, {"content": "24. When I need to get rid of something, my mother can count on it.", "word_count": 20, "label": "", "sentence_structure": "Subject鈥揷opula鈥損redicative", "subjective_objective": {"answer": "Subjective", "rationale": "Because the description of \"mothers who can rely on\" is more based on the patient's trust and dependence on the mother, which is a subjective attitude or feeling."}, "sentence_sentiment": {"answer": "Positive", "rationale": "Because the description \"can rely on\" in the entry is a positive emotional attitude."}, "language_style": {"answer": "Tactful and warm", "rationale": "The language style of this entry is more euphemistic, giving people a warm, comforting feeling by describing \"My mother can count on\" . This way encourages respondents to share more real and deeper answers and enhances the effectiveness of the questionnaire."}, "time_dimension": {"answer": "Present", "rationale": "The entry describes the feelings and attitudes of the testee in the present circumstances, which is \"when I need to get rid of something,\" and therefore judges it now."}, "sentence_type": "Affirmative declarative sentence", "pos": {"nouns": {"Time": 1, "Something": 1, "My mother": 1}, "verbs": {"Yeah": 1, "Dependence": 1, "Get rid of it": 1}, "adverbs": {"When": 1}, "numerals": {"24": 1}, "pronouns": {"I": 1}}}, {"content": "25. If my mother knew something was bothering me, she would ask me for details.", "word_count": 24, "label": "", "sentence_structure": "Subject鈥搗erb鈥搊bject", "subjective_objective": {"answer": "Subjective", "rationale": "Since the entry describes the behaviour of the patient as her mother, it is examined from the patient ' s subjective perspective."}, "sentence_sentiment": {"answer": "Positive", "rationale": "Because the \"mother asks for details\" in the entry is positive, it expresses concern and support among families."}, "language_style": {"answer": "Tactful and warm", "rationale": ""}, "time_dimension": {"answer": "Present", "rationale": "The entry describes a hypothetical situation, \"If my mother knows something's bothering me,\" and her reaction is \"to ask me for details.\" Because it is a hypothetical situation, it is impossible to determine its authenticity. But, in grammatical and semantic terms, the temporal dimension of the article is not specific, so it can be considered to be a certain possibility for the present or the future."}, "sentence_type": "Affirmative declarative sentence", "pos": {"nouns": {"I": 1, "Mother": 1, "Things": 1, "Trouble": 1}, "verbs": {"Yes": 3, "Ask": 1, "Details": 1, "Situation": 1}, "adverbs": {"If": 1, "have": 1, "Detailed": 1}, "numerals": {"25": 1}, "pronouns": {"she": 1}}}, {"content": "My father respected my feelings.", "word_count": 9, "label": "", "sentence_structure": "Subject鈥搗erb鈥搊bject", "subjective_objective": {"answer": "Subjective", "rationale": "Since respect is the patient ' s subjective feeling and understanding, it is linked to the father ' s specific behaviour, but remains based primarily on the patient ' s views."}, "sentence_sentiment": {"answer": "Positive", "rationale": "Because the action of \"respect my feelings\" in the entry is positive."}, "language_style": {"answer": "Tactful and warm", "rationale": ""}, "time_dimension": {"answer": "Present", "rationale": ""}, "sentence_type": "Affirmative declarative sentence", "pos": {"nouns": {"I": 1, "Father": 1, "Emotion": 1}, "verbs": {"Respect": 1}, "numerals": {"26": 1}}}, {"content": "I think my father is a good father.", "word_count": 13, "label": "", "sentence_structure": "Subject鈥揷opula鈥損redicative", "subjective_objective": {"answer": "Subjective", "rationale": "Because I think that the patient's own views and opinions are subjective, personal, and emotional experiences."}, "sentence_sentiment": {"answer": "Neutral", "rationale": "Because the entry does not have any emotional color."}, "language_style": {"answer": "Tactful and warm", "rationale": "The entry uses a first-person expression and a positive evaluation as the dominant language, which makes the language style more euphemistic and warmer. At the same time, the tone of the article does not express strong emotions or positions directly, but rather leads the respondents to share their views in a more moderate way."}, "time_dimension": {"answer": "Present", "rationale": "The keyword \"considers\" and \"is competent\" is the general current, which is the current idea and the evaluation, so the entry is judged now."}, "sentence_type": "Affirmative declarative sentence", "pos": {"nouns": {"Questionnaire": 1, "Entry": 1, "Father": 1, "Competency": 1}, "verbs": {"I think": 1, "be": 1}, "numerals": {"27": 1}, "pronouns": {"I": 1}}}, {"content": "I wish my father were someone else.", "word_count": 13, "label": "", "sentence_structure": "Subject鈥搗erb鈥搊bject", "subjective_objective": {"answer": "Subjective", "rationale": "Because hope is the patient's personal subjective emotion and attitude."}, "sentence_sentiment": {"answer": "Negative", "rationale": "The entry is based on a certain negative emotional profile, expressing a desire for a change in the wishes and aspirations of the biological father."}, "language_style": {"answer": "Calm and direct", "rationale": "The entry presents the inner desires or expectations of the person under investigation in a direct presentation, without the use of excessive rhetoric or emotional profiling, and with a more calm and objective language style."}, "time_dimension": {"answer": "Present", "rationale": "In the entry, \"Hope\" expresses current expectations or ideas, and \"My father is someone else\" describes the reality of the situation. The entry is therefore asking about the current mood and mental state of the person being tested."}, "sentence_type": "Exclamatory sentence", "pos": {"nouns": {"Father": 1, "person": 1}, "verbs": {"Hope": 1}, "adjectives": {"Really": 1, "The other one": 1}, "adverbs": {"True": 1}, "numerals": {"28": 1}, "pronouns": {"I": 1}}}, {"content": "My father recognizes the way I am now.", "word_count": 12, "label": "", "sentence_structure": "Subject鈥揷opula鈥損redicative", "subjective_objective": {"answer": "Subjective", "rationale": "Because \"accepting what I am\" is a subjective or emotional expression of what patients think and feel about their father."}, "sentence_sentiment": {"answer": "Positive", "rationale": "Because the phrase \"my father is very supportive of my present appearance\" in the entry is positive."}, "language_style": {"answer": "Calm and direct", "rationale": "This is more objective and neutral, and is appropriate for the needs of the psychological questionnaire."}, "time_dimension": {"answer": "Present", "rationale": "The keyword \"now\" denotes the current state or situation, so the entry is a description of the current state and falls within the present context."}, "sentence_type": "Affirmative declarative sentence", "pos": {"nouns": {"Father": 1, "I": 1, "Present": 1, "Look": 1}, "verbs": {"I'm sure it's true": 1}, "adjectives": {"Present": 1}, "adverbs": {"very": 1}, "numerals": {"29": 1}, "pronouns": {"I": 1}}}, {"content": "30. I like to listen to my father for what I care about.", "word_count": 18, "label": "", "sentence_structure": "Subject鈥搗erb鈥搊bject", "subjective_objective": {"answer": "Subjective", "rationale": "Subjectivity is reflected in the patient ' s subjective attitude, as it is more likely to listen to the father ' s opinion."}, "sentence_sentiment": {"answer": "Positive", "rationale": "Because the \"like to hear my father's opinion\" act in the entry is positive and expresses trust and respect for the father."}, "language_style": {"answer": "Tactful and warm", "rationale": "The entry is more personal and interactive when it is spoken in a gentle and inviting manner, rather than in direct statements or orders. At the same time, it is more verbish by the use of the phrase \"like to hear\"."}, "time_dimension": {"answer": "Present", "rationale": "\"Leave to hear\" suggests that this is a customary act, and \"My father's opinion\" suggests that it is in progress. Thus, the entry indicates that the person tested is listening to his father's advice in the current circumstances."}, "sentence_type": "Exclamatory sentence", "pos": {"nouns": {"Things": 1, "Observations": 1}, "verbs": {"Yeah": 1, "Listen": 1}, "adverbs": {"to": 1, "in": 1, "I care": 1}, "numerals": {"30": 1}, "pronouns": {"I": 2, "he": 1}}}, {"content": "31. I do not think it is useful to show my feelings to my father.", "word_count": 18, "label": "", "sentence_structure": "Subject鈥搗erb鈥揷omplement", "subjective_objective": {"answer": "Subjective", "rationale": "It's a subjective view because it expresses feelings and feelings for the patient."}, "sentence_sentiment": {"answer": "Negative", "rationale": "Because the idea that \"to show your feelings to your father is useless\" in the entry is negative."}, "language_style": {"answer": "Calm and direct", "rationale": "The entry presents a point in a concise and clear manner, without excessive embellishment or emotional colour, and is more direct and objective in tone. \""}, "time_dimension": {"answer": "Past", "rationale": "The entry describes the emotional attitude of the interviewees towards their father, and does not clearly indicate the point of time, and it is therefore not possible to determine whether it is past, present or future."}, "sentence_type": "Negative declarative sentence", "pos": {"nouns": {"Questionnaire Entry": 1, "Father": 1, "self": 2, "Emotion": 1}, "verbs": {"I think": 1, "Show": 1, "It works": 1}, "adjectives": {"Nope": 1, "Use": 1}, "numerals": {"31": 1}, "pronouns": {"I": 1}}}, {"content": "32. When I feel sorry for something, my father will see it.", "word_count": 18, "label": "", "sentence_structure": "Subject鈥搗erb鈥搊bject", "subjective_objective": {"answer": "Subjective", "rationale": "Because \"seen\" requires an interaction between the observer and the person being observed, who is the father of the patient and who is the patient's subjective idea or perception."}, "sentence_sentiment": {"answer": "Positive", "rationale": "Because the expression \"father will see\" conveys a feeling of love and understanding, with a positive emotional color."}, "language_style": {"answer": "Tactful and warm", "rationale": "The language style of the entry is euphemism. It does not directly express criticism or accusations against the person under investigation, but rather gives the person a picture of the situation in question to remember and think. This soft, encouraging way of asking questions makes it easier for the person to feel comfortable and to get accurate and real answers."}, "time_dimension": {"answer": "Present", "rationale": "The word \"when\" and \"will\" in the entry indicate that it is a regular, customary act, and that it occurs when the person talking about it is sad about something. So the time dimension is now."}, "sentence_type": "Affirmative declarative sentence", "pos": {"nouns": {"Time": 1, "Father": 1}, "verbs": {"I can see that": 1}, "adjectives": {"Sorry": 1}, "numerals": {"32": 1}, "pronouns": {"I": 1}}}, {"content": "It was a shame and a foolish thing to discuss my problems with my father.", "word_count": 19, "label": "", "sentence_structure": "Subject鈥搗erb鈥揷omplement", "subjective_objective": {"answer": "Subjective", "rationale": "This is because the patient describes his/her feelings and involves subjective emotions and evaluation."}, "sentence_sentiment": {"answer": "Negative", "rationale": "Because the \"smuggling, stupid\" attitude in the entry is negative."}, "language_style": {"answer": "Calm and direct", "rationale": "The entry uses a direct statement language, does not have too many emotional renderings or speech techniques, expresses the feeling of having a problem with the father and is more objective and incriminating."}, "time_dimension": {"answer": "Present", "rationale": "The entry describes a past act or state, but the expression \"very\" indicates that this feeling still exists and therefore the temporal dimension is now judged."}, "sentence_type": "Negative declarative sentence", "pos": {"nouns": {"Father": 1, "Discussion": 1, "Trouble": 1, "Shame": 1, "Stupid": 1}, "verbs": {"I feel": 1, "Discussion": 1, "Other": 1}, "adjectives": {"very": 2, "Mine": 1}, "numerals": {"33": 1}, "pronouns": {"I": 1}}}, {"content": "Fathers expect too much of me.", "word_count": 8, "label": "", "sentence_structure": "Subject鈥揷opula鈥損redicative", "subjective_objective": {"answer": "Subjective", "rationale": "The expectations of fathers are my own subjective feeling."}, "sentence_sentiment": {"answer": "Negative", "rationale": "Because the description of \"too high expectations\" suggests negative evaluation and pressure."}, "language_style": {"answer": "Tactful and warm", "rationale": ""}, "time_dimension": {"answer": "Past", "rationale": "The entry describes the subjective feelings and views of the testees about the past, and the keyword is \"father expects too much of me\", reflecting the facts and experiences of the past."}, "sentence_type": "Negative declarative sentence", "pos": {"nouns": {"Father": 1, "Expectations": 1}, "verbs": {"to": 1, "Too high": 1}, "numerals": {"34": 1}}}, {"content": "35. I feel very easy for my father.", "word_count": 11, "label": "", "sentence_structure": "Subject鈥揷opula鈥損redicative", "subjective_objective": {"answer": "Subjective", "rationale": "Because \"I feel sorry for my father very easily\" is the subjective feeling and emotional reaction of the patient, which is subjective."}, "sentence_sentiment": {"answer": "Negative", "rationale": "Because the expression \"easy to feel sorry for your father\" in the entry is negative."}, "language_style": {"answer": "Tactful and warm", "rationale": "The entry is more proficient and interactive by engaging in dialogue in a gentle and inviting manner, rather than by direct statements or orders. At the same time, it does not use overly strong rhetorical or emotional renderings, and is more balanced and objective."}, "time_dimension": {"answer": "Present", "rationale": "Now, the entry \"I'm easily sad for my father\" expresses the emotional state and feelings of the subject, so it can be judged now."}, "sentence_type": "Affirmative declarative sentence", "pos": {"nouns": {"I": 1, "Father": 1}, "verbs": {"Easy": 1, "I feel": 1}, "adverbs": {"very": 1}, "numerals": {"35": 1}}}, {"content": "36. I have a lot of unhappy, but my father knows very little.", "word_count": 15, "label": "", "sentence_structure": "Subject鈥搗erb鈥搊bject", "subjective_objective": {"answer": "Subjective", "rationale": "\"I have a lot of unhappy\" is the patient's subjective feeling, \"father knows very little\" and the patient's description of the objective situation."}, "sentence_sentiment": {"answer": "Negative", "rationale": "Because the entry \"a lot of unhappy\" expresses a negative emotional attitude."}, "language_style": {"answer": "Tactful and warm", "rationale": ""}, "time_dimension": {"answer": "Past", "rationale": "The entry is about the mental or emotional state of the person being tested and does not address past, present or future acts or events, and therefore cannot be judged in terms of the temporal dimension."}, "sentence_type": "Negative declarative sentence", "pos": {"nouns": {"36": 1, "I": 2, "A lot": 1, "I'm not happy": 1, "Father": 1, "Yes": 1, "Very few": 1}, "verbs": {"have": 1, "Yes": 1}, "adjectives": {"More": 1, "I'm not happy": 1}, "numerals": {"36": 1, "A lot": 1}, "classifiers": {"individual": 1}, "pronouns": {"I": 2}}}, {"content": "37. When things are discussed, fathers will care about my point of view.", "word_count": 18, "label": "", "sentence_structure": "Subject鈥搗erb鈥搊bject", "subjective_objective": {"answer": "Subjective", "rationale": "Because \"he cares about my point\" is a subjective feeling and an assessment."}, "sentence_sentiment": {"answer": "Positive", "rationale": "This is a positive emotional and attitudinal attitude, as it expresses the view of the father that the child is valued."}, "language_style": {"answer": "Tactful and warm", "rationale": "The entry is more adept and interactive when it is spoken in a gentle and inviting manner, rather than by direct statements or orders."}, "time_dimension": {"answer": "Present", "rationale": "In the entry, \"when things are discussed\" means a situation or behaviour, and \"care\" action takes place in the past, but it has an impact on the results of the present and is therefore a state of affairs."}, "sentence_type": "Affirmative declarative sentence", "pos": {"nouns": {"Time": 1, "Things": 1, "Father": 1, "Views": 1}, "verbs": {"Discussion": 1, "Yes": 1, "I care": 1}, "adjectives": {"very": 1}, "numerals": {"37": 1}, "pronouns": {"I": 1}}}, {"content": "My father trusted my judgment.", "word_count": 9, "label": "", "sentence_structure": "Subject鈥搗erb鈥搊bject", "subjective_objective": {"answer": "Subjective", "rationale": "Because \"my father trusts my judgment\" is a subjective judgment, which means that the patient believes their father trusts their judgment."}, "sentence_sentiment": {"answer": "Positive", "rationale": "Because it expresses \"father trust in me\" and it is a positive attitude and behaviour."}, "language_style": {"answer": "Tactful and warm", "rationale": "The entry uses a more subjective description from the point of view of first person, while expressing a certain degree of certainty and trust in a person, more intimate and cozy language."}, "time_dimension": {"answer": "Present", "rationale": "The entry describes the relationship and communication between the testee and his father, that is, the degree of confidence his father has in the testee."}, "sentence_type": "Affirmative declarative sentence", "pos": {"nouns": {"I": 1, "Father": 1, "Judgement": 1}, "verbs": {"Trust": 1}, "numerals": {"38": 1}}}, {"content": "My father had his own problems, so I would not bother him with my own.", "word_count": 24, "label": "", "sentence_structure": "Subject鈥搗erb鈥搊bject", "subjective_objective": {"answer": "Subjective", "rationale": "Because \"I\" is subjective, and has a sense of feeling, and \"my father\" is objective object, and his existence and behaviour are not affected by \"me.\" So, \"I will not bother him with my own troubles\" is based on subjective judgment of \"me\" rather than objective facts."}, "sentence_sentiment": {"answer": "Negative", "rationale": "Because the behavior in the entry \"to take your own troubles off your father\" is negative, which means a lack of empathy and caring for the emotional color of others."}, "language_style": {"answer": "Tactful and warm", "rationale": "The entry expresses the father鈥檚 own concerns in an understanding and acceptance, and does not want to bring his problems to the other. This is more emotional and caring, and is better able to bring the respondents closer to the questionnaire and make it easier to answer questions."}, "time_dimension": {"answer": "Past", "rationale": "The expression \"my father has his own troubles\" happened in the past, and I will not bother him with my own troubles\" also implies that this is a custom of the past. So the subject describes the behaviour and attitude of the subjects in the past."}, "sentence_type": "Affirmative declarative sentence", "pos": {"nouns": {"I": 2, "Father": 1, "Trouble": 3}, "verbs": {"have": 1, "Take it": 1, "Yes": 1, "Boring": 1, "Fuck off": 1}, "adverbs": {"self": 1, "His own": 1, "So": 1}, "numerals": {"39": 1}, "classifiers": {"individual": 1}, "pronouns": {"he": 1}}}, {"content": "My father helped me to better understand myself.", "word_count": 13, "label": "", "sentence_structure": "Subject鈥搗erb鈥揷omplement", "subjective_objective": {"answer": "Subjective", "rationale": "Because this sentence relates to patients ' subjective perception and feelings about their relationship with their father."}, "sentence_sentiment": {"answer": "Positive", "rationale": "Because the purpose of the action in the entry is to help me better understand myself."}, "language_style": {"answer": "Tactful and warm", "rationale": "The entry uses a positive presentation describing the relationship between the interviewee and the father, and is more moderate, encouraging and positive, and comforting and close. At the same time, the expression \"better understanding\" reflects the interest and support for the growth and development of the interviewee, and therefore the language style is more euphemismal."}, "time_dimension": {"answer": "Past", "rationale": "In this entry, \"help\" and \"better know yourself\" are all actions of the past, so it can be judged that the item describes the past."}, "sentence_type": "Affirmative declarative sentence", "pos": {"nouns": {"Father": 1, "self": 1}, "verbs": {"Help": 1, "Roger that": 1}, "numerals": {"40": 1}}}, {"content": "I told my father my own troubles.", "word_count": 13, "label": "", "sentence_structure": "Subject鈥搗erb鈥搊bject", "subjective_objective": {"answer": "Subjective", "rationale": "Because \"Tell your father your troubles\" is a personal act and decision that reflects patient's subjective attitude and choice."}, "sentence_sentiment": {"answer": "Positive", "rationale": "Because it expresses an act of sharing troubles with others, it has a positive emotional colour."}, "language_style": {"answer": "Tactful and warm", "rationale": "The entry uses a more euphemistic, soft-minded approach to questions, without directly questioning or criticizing the behaviour of the person under investigation. At the same time, the entry uses more intimate language, such as \"me\" and \"you\", which enhances the proximity and communication with the person interviewed."}, "time_dimension": {"answer": "Present", "rationale": ""}, "sentence_type": "Affirmative declarative sentence", "pos": {"nouns": {"Trouble": 1, "Father": 1}, "verbs": {"- Put it on": 1, "Tell": 1, "of": 1}, "numerals": {"41": 1}, "pronouns": {"I": 1}}}, {"content": "I was angry with my father.", "word_count": 7, "label": "", "sentence_structure": "Subject鈥揷opula鈥損redicative", "subjective_objective": {"answer": "Subjective", "rationale": "Because \"I'm angry with my father\" is the subjective description and feeling of my feelings."}, "sentence_sentiment": {"answer": "Negative", "rationale": "Because the \"smuggling\" in the entry is negative."}, "language_style": {"answer": "Calm and direct", "rationale": "The entry expresses the negative feelings of the respondent towards his father in a direct and explicit manner, without excessive ado or euphemism, and is more calm and direct."}, "time_dimension": {"answer": "Past", "rationale": "In conclusion, the entry is the subject of a description of the current mood of the testee, the temporal dimension of which is uncertain."}, "sentence_type": "Negative declarative sentence", "pos": {"nouns": {"I": 1, "Father": 1}, "verbs": {"I'm angry": 1}}}, {"content": "43. I did not receive much attention from my father.", "word_count": 12, "label": "", "sentence_structure": "Subject鈥搗erb鈥揷omplement", "subjective_objective": {"answer": "Subjective", "rationale": "Because \"with much father's attention\" is a subjective feeling and evaluation that involves factors such as individual perception, emotional and other. Although this feeling may be related to objective facts (for example, whether the father really ignores the child), it is subjective in itself and cannot be drawn directly from objective observations."}, "sentence_sentiment": {"answer": "Negative", "rationale": "Because the statement in the entry \"not getting much attention from the father\" is negative."}, "language_style": {"answer": "Tactful and warm", "rationale": "The language style of the entry is more euphemistic, as it expresses a potential problem or difficulty in a more implicit way, rather than a direct accusation or complaint. At the same time, the \"many\" description is used to make the presentation of the issue more objective and neutral, rather than overly emotional."}, "time_dimension": {"answer": "Past", "rationale": ""}, "sentence_type": "Negative declarative sentence", "pos": {"nouns": {"I": 1, "Father": 1, "Attention": 1}, "verbs": {"Not received": 1}, "adverbs": {"very": 1}, "numerals": {"43": 1}}}, {"content": "My father helped me discuss my difficulties.", "word_count": 13, "label": "", "sentence_structure": "Subject鈥搗erb鈥搊bject", "subjective_objective": {"answer": "Objective", "rationale": "The basis for the judgement: This is an objective statement, which describes the fact that the patient鈥檚 father discussed the matter with them. While this may reflect the patient鈥檚 feelings or attitudes (e.g. if they are grateful or understood), the description is itself objective and not based solely on the patient鈥檚 subjective experience."}, "sentence_sentiment": {"answer": "Positive", "rationale": "Because the \"Father helps to discuss difficulties\" in the entry is positive and reflects family support and care."}, "language_style": {"answer": "Tactful and warm", "rationale": "The entry uses a more euphemistic formulation, which, by describing the fact that \"my father helped me discuss my difficulties\", does not ask the other side directly whether it needs help or support, but rather gives the other side the freedom to judge whether it is helped in this situation. This expression is more respectful and caring about the feelings of the interviewee, more personal and warmer."}, "time_dimension": {"answer": "Present", "rationale": "The entry describes the current behavior or state, which is \"my father helped me discuss my difficulties\", and does not address past or future information, so the temporal dimension is now judged."}, "sentence_type": "Exclamatory sentence", "pos": {"nouns": {"Questionnaire Entry": 1, "Difficulties": 1, "Father": 1}, "verbs": {"Help": 1, "We'll discuss it together": 1}, "adverbs": {"Down there": 1}, "numerals": {"44": 1}, "pronouns": {"I": 2}}}, {"content": "My father understood me.", "word_count": 6, "label": "", "sentence_structure": "Subject鈥搗erb鈥搊bject", "subjective_objective": {"answer": "Subjective", "rationale": "Because \"understanding\" is a subjective feeling and evaluation."}, "sentence_sentiment": {"answer": "Neutral", "rationale": "Because the entry does not have any emotional color."}, "language_style": {"answer": "Tactful and warm", "rationale": "The language style of the entry is euphemism, because it is a soft, inviting conversation, expressing understanding and concern about the father of the interviewer, and not too much rhetorical or emotional overtook. This expression is more personal and interactive, and it makes it easier for the interviewee to share his thoughts and feelings."}, "time_dimension": {"answer": "Past", "rationale": "\"My father understands me\" and \"understanding\" are descriptions of past experience or memory, so the temporal dimension of the article is the past."}, "sentence_type": "Affirmative declarative sentence", "pos": {"nouns": {"Father": 1, "Understood": 1, "I": 1}, "verbs": {"Here": 1}, "numerals": {"45": 1}}}, {"content": "46. When I was angry about something, my father tried to understand me.", "word_count": 17, "label": "", "sentence_structure": "Subject鈥搗erb鈥搊bject", "subjective_objective": {"answer": "Objective", "rationale": "* The reference to \"my father tried to understand me\" in the entry sentence is an objective fact, describing a specific act, independent of the subjective feelings of the individual, so the entry to the questionnaire is viewed from an objective perspective."}, "sentence_sentiment": {"answer": "Positive", "rationale": "Because the \"father tries to understand me\" act is positive."}, "language_style": {"answer": "Tactful and warm", "rationale": "The expression is more personal and interactive, in a gentle and inviting tone, rather than in direct statements or orders. At the same time, it shows that the questionnaire designers want participants to share their real feelings and experiences."}, "time_dimension": {"answer": "Present", "rationale": ""}, "sentence_type": "Affirmative declarative sentence", "pos": {"nouns": {"Things": 1, "Father": 1}, "verbs": {"Fan": 1, "Try": 1, "Understood": 1, "Angry": 1}, "adverbs": {"When": 1}, "numerals": {"46": 1}, "classifiers": {"individual": 1}, "pronouns": {"I": 1}}}, {"content": "I trust my father.", "word_count": 7, "label": "", "sentence_structure": "Subject鈥搗erb鈥搊bject", "subjective_objective": {"answer": "Subjective", "rationale": "Because \"trust\" is patient's subjective attitude."}, "sentence_sentiment": {"answer": "Positive", "rationale": "Because trust is a positive emotion."}, "language_style": {"answer": "Tactful and warm", "rationale": "The entry is more adept and interactive when it is spoken in a gentle and inviting manner, rather than by direct statements or orders."}, "time_dimension": {"answer": "Present", "rationale": "The entry uses the now completed state of affairs ( \"I have...\") to indicate that acts that have occurred in the past have affected or resulted in the present. Thus, it can be judged as a description of the present behaviour or attitude."}, "sentence_type": "Affirmative declarative sentence", "pos": {"nouns": {"Questionnaire Entry": 1, "Trust": 1, "Father": 1}, "verbs": {"be": 1}, "numerals": {"47": 1}, "pronouns": {"I": 1}}}, {"content": "My father did not know what I had to go through on those days.", "word_count": 17, "label": "", "sentence_structure": "Subject鈥搗erb鈥搊bject", "subjective_objective": {"answer": "Subjective", "rationale": "Because \"father doesn't know\" is the patient's own view and feelings, and it's subjective."}, "sentence_sentiment": {"answer": "Negative", "rationale": "Because the expression \"father doesn't know\" in the entry is negative."}, "language_style": {"answer": "Tactful and warm", "rationale": "The entry is more personal and interactive when it is spoken in a moderate, sensitive manner, rather than directly speaking or ordering."}, "time_dimension": {"answer": "Past", "rationale": "The entry \"what has been through\" means what happened in the past, so it can be judged as asking the testee what he has been through over time, and the temporal dimension is judged to be the past. At the same time, the expression \"my father does not know\" further highlights the relevance of the issue in the past."}, "sentence_type": "Negative declarative sentence", "pos": {"nouns": {"Father": 1, "Experience": 1}, "verbs": {"Roger that": 1, "Both": 1, "Come on": 1}, "adverbs": {"These days": 1}, "numerals": {"48": 1}, "pronouns": {"I": 2}}}, {"content": "49. My father could count on me when I needed to get rid of something.", "word_count": 20, "label": "", "sentence_structure": "Subject鈥揷opula鈥損redicative", "subjective_objective": {"answer": "Subjective", "rationale": "Because this entry relates to the patient ' s perception of and dependence on his father and is subjective in the patient ' s sense."}, "sentence_sentiment": {"answer": "Positive", "rationale": "Because the purpose of the action of \"father can be relied upon\" in the entry is positive."}, "language_style": {"answer": "Tactful and warm", "rationale": "The entry uses a more euphemistic and warm language style, conveying trust and dependence on fathers by expressing the more emotional term \"we can rely on\" while avoiding overly direct or conflicting expressions that make dialogue more friendly."}, "time_dimension": {"answer": "Present", "rationale": "The keywords are \"need to get rid of something\" and \"what I can count on,\" which point to the current situation."}, "sentence_type": "Affirmative declarative sentence", "pos": {"nouns": {"Time": 1, "Something": 1, "Father": 1}, "verbs": {"Yes": 1, "Get rid of it": 1, "Yeah": 1, "Dependence": 1}, "numerals": {"49": 1}, "pronouns": {"I": 1}}}, {"content": "50. If my father knew something was bothering me, he would ask me for details.", "word_count": 24, "label": "", "sentence_structure": "Subject鈥搗erb鈥搊bject", "subjective_objective": {"answer": "Subjective", "rationale": "Because this statement is an expectation and an assumption of how the interviewees might react to their father, it is examined from the point of view of the interviewees ' subjective perspective."}, "sentence_sentiment": {"answer": "Positive", "rationale": "Because the \"Question me in detail\" act in the entry is positive."}, "language_style": {"answer": "Tactful and warm", "rationale": "The language style of the entry is more euphemistic, and it allows the person to react emotionally by describing the circumstances."}, "time_dimension": {"answer": "Past", "rationale": ""}, "sentence_type": "Affirmative declarative sentence", "pos": {"nouns": {"Father": 1, "Things": 1, "Situation": 1}, "verbs": {"Yes": 3, "have": 1, "Ask": 1, "Details": 1}, "numerals": {"50": 1}, "pronouns": {"I": 1}}}, {"content": "I like to listen to my friends on the issues I value.", "word_count": 19, "label": "", "sentence_structure": "Subject鈥搗erb鈥搊bject", "subjective_objective": {"answer": "Subjective", "rationale": "Like the patient's subjective attitude."}, "sentence_sentiment": {"answer": "Positive", "rationale": "This is because it expresses a positive attitude, i.e., the importance attached to the opinions and opinions of friends."}, "language_style": {"answer": "Tactful and warm", "rationale": "The language style of the entry is more euphemistic, it uses an invitation tone and expresses a respectful and caring attitude that is comforting and pleasant."}, "time_dimension": {"answer": "Present", "rationale": "The time dimension of this questionnaire entry is \"now,\" because it's about personal preferences and behavior. The words \"like,\" \"hearing\" and so on all imply that this is an act that happens now."}, "sentence_type": "Affirmative declarative sentence", "pos": {"nouns": {"Questionnaire": 1, "Entry": 1, "Problem": 2, "Views": 1, "friends": 1}, "verbs": {"Yeah": 1, "Listen": 1}, "adjectives": {"- It's important": 1}, "numerals": {"1": 3}, "pronouns": {"I": 1}}}, {"content": "When I feel sorry for something, my friends can tell.", "word_count": 20, "label": "", "sentence_structure": "Subject鈥搗erb鈥搊bject", "subjective_objective": {"answer": "Subjective", "rationale": "Because \"seen\" reflects the subjective perception of the patient of his feelings and those of others."}, "sentence_sentiment": {"answer": "Positive", "rationale": "Because \"friends can see\" in the entry expresses the emotional attitude of having friends who can support and care in the face of difficulties or setbacks, which is a positive emotional expression."}, "language_style": {"answer": "Tactful and warm", "rationale": "The issue is more euphemistic, not directly asking the other party whether they had had a sad experience or whether they had been detected by friends, but in a more gentle and caring way; and, in the words \"my friend can see it\" it is used to make people feel more friendly and sweet and to be very friendly."}, "time_dimension": {"answer": "Past", "rationale": "Because these may have happened and may have continued. So the answer is \"the past/now\"."}, "sentence_type": "Affirmative declarative sentence", "pos": {"nouns": {"Things": 1, "Sorry": 1, "Time": 1, "friends": 1}, "verbs": {"I can see that": 1}, "numerals": {"Something": 1}, "classifiers": {"individual": 1}, "pronouns": {"I": 1}}}, {"content": "When we discuss things, my friends take my point very seriously.", "word_count": 19, "label": "", "sentence_structure": "Subject鈥搗erb鈥搊bject", "subjective_objective": {"answer": "Subjective", "rationale": "Because \"respect my point\" is a subjective feeling or experience, a patient's subjective attitude and emotional reaction."}, "sentence_sentiment": {"answer": "Positive", "rationale": "Because it expresses good relations and respect for others, it is positive."}, "language_style": {"answer": "Tactful and warm", "rationale": "The entry is more personal and interactive in its expression of conversation in a gentle and inviting tone, rather than in direct statements or orders. At the same time, the sentence also gives respondents a sense of interest and respect by using the first person ' s pronoun, \"I \" , which helps to enhance the credibility and effectiveness of the questionnaire."}, "time_dimension": {"answer": "Present", "rationale": "The keyword \"when we discuss things\" indicates that this is an ongoing scene, and \"to value\" means that it is a tester's expectation and assessment of his own point of view, and therefore the temporal dimension should be now. In conclusion, the temporal dimension of this article should be \"now\"."}, "sentence_type": "Affirmative declarative sentence", "pos": {"nouns": {"we": 1, "Things": 1, "friends": 1, "Views": 1}, "verbs": {"Discussion": 1, "I can take care of it": 1}, "numerals": {"3": 1}, "pronouns": {"I": 1}}}, {"content": "4. It's embarrassing and stupid to discuss my troubles with friends.", "word_count": 19, "label": "", "sentence_structure": "Subject鈥搗erb鈥揷omplement", "subjective_objective": {"answer": "Subjective", "rationale": "Because shame and folly are subjective feelings and judgments, it is the patient ' s own assessment of the bitterness of discussing this with friends."}, "sentence_sentiment": {"answer": "Negative", "rationale": "Because the description of \"smuggling and stupidity\" in the entry is negative."}, "language_style": {"answer": "Calm and direct", "rationale": "The entry directly expresses a more objective and balanced tone in asking if the other party is negative when discussing his or her own distress with his or her friends."}, "time_dimension": {"answer": "Present", "rationale": "The words \"discuss my troubles\" and \"will make me ashamed and stupid\" in the entry all indicate that it is a present or recent experience, and so it is judged now."}, "sentence_type": "Negative declarative sentence", "pos": {"nouns": {"friends": 1, "Trouble": 1, "Shame": 1, "Stupid": 1}, "verbs": {"Discussion": 1, "Jean": 1, "I feel": 1}, "adjectives": {"Mine": 1, "Yes": 1, "Sensor": 1}, "numerals": {"4": 1}, "pronouns": {"I": 1}}}, {"content": "I hope my friend isn't like this.", "word_count": 13, "label": "", "sentence_structure": "Subject鈥損redicate", "subjective_objective": {"answer": "Subjective", "rationale": "Because hope is a subjective idea and expectation."}, "sentence_sentiment": {"answer": "Negative", "rationale": "Because \"I hope that's not the way friends\" in the entry expresses dissatisfaction or disappointment with friends at the moment, and has a negative emotional tone."}, "language_style": {"answer": "Tactful and warm", "rationale": "The language style of the entry is euphemism. It is a gentle and inviting tone of speech, not a direct statement or order. This expression is more personal and interactive. At the same time, the entry is not overly emotional or strongly negative, but rather asks each other about their views in a calm and objective manner. It is therefore judged that the language style of the entry is euphemistic and warm."}, "time_dimension": {"answer": "Present", "rationale": ""}, "sentence_type": "Negative declarative sentence", "pos": {"nouns": {"Hope": 1, "friends": 1}, "verbs": {"be": 1, "Hope": 1, "No, it's not": 1}, "adjectives": {"This way": 1}, "pronouns": {"I": 1}}}, {"content": "My friends understand me.", "word_count": 7, "label": "", "sentence_structure": "Subject鈥搗erb鈥揷omplement", "subjective_objective": {"answer": "Subjective", "rationale": "Because \"my friend understands me\" is the patient's subjective perception and feelings about his relationship with friends."}, "sentence_sentiment": {"answer": "Positive", "rationale": "Because the description of \"friend understands me\" in the entry is positive, and it shows that my friends are very supportive and understanding of me."}, "language_style": {"answer": "Tactful and warm", "rationale": "The entry is more personal and interactive by asking the other side whether it believes that its friends understand themselves in terms of their relationship status rather than directly speaking or ordering."}, "time_dimension": {"answer": "Present", "rationale": "\"My\" friend and \"I\" are in the state of things now, so the entry is in the state or state of things now, and the time dimension is now judged."}, "sentence_type": "Affirmative declarative sentence", "pos": {"nouns": {"friends": 1, "Understood": 1}, "verbs": {"have": 1, "be": 1}, "pronouns": {"I": 1}}}, {"content": "My friends encouraged me to explore my difficulties.", "word_count": 13, "label": "", "sentence_structure": "Subject鈥搗erb鈥搊bject", "subjective_objective": {"answer": "Subjective", "rationale": "Because friends' attitudes and statements are subjective to the patient."}, "sentence_sentiment": {"answer": "Positive", "rationale": "Because the \"friends encouraged me to explore my difficulties\" behavior and attitude in the entry are positive and conducive to solving the problem."}, "language_style": {"answer": "Tactful and warm", "rationale": "The entry gives a positive view of the \"friend\" as the dominant language, gives a sense of affection and \"incentive\" expression. It is more acceptable and willing to answer questions."}, "time_dimension": {"answer": "Past", "rationale": "The entry describes past behaviour, and both the terms \"exploration\" and \"encourage\" indicate that this is an event that has been completed and can therefore be judged to be past."}, "sentence_type": "Imperative sentence", "pos": {"nouns": {"friends": 1, "Encourage": 1, "I": 1, "Difficulties": 1}, "verbs": {"Explore": 1, "Give it to me": 1}, "numerals": {"7": 1}}}, {"content": "My friends accepted me like this.", "word_count": 11, "label": "", "sentence_structure": "Subject鈥搗erb鈥搊bject", "subjective_objective": {"answer": "Subjective", "rationale": "Since admission or non-admission is usually based on individual feelings and perceptions, this option is considered more from the patient ' s subjective perspective."}, "sentence_sentiment": {"answer": "Positive", "rationale": "Because the entry \"Accept me like this\" expresses a positive emotional attitude."}, "language_style": {"answer": "Tactful and warm", "rationale": "The entry is more personal and interactive, speaking in a gentle and inviting tone, rather than directly speaking or ordering. It gives the interviewee a sense of respect and attention and a greater willingness to share his real thoughts. At the same time, it can ease the tension and make the interviewee more relaxed in answering."}, "time_dimension": {"answer": "Present", "rationale": ""}, "sentence_type": "Affirmative declarative sentence", "pos": {"nouns": {"friends": 1, "I": 2, "Look": 1}, "verbs": {"Admission": 1, "be": 1}}}, {"content": "I feel the need to keep in touch with my friends on a regular basis.", "word_count": 16, "label": "", "sentence_structure": "Subject鈥搗erb鈥揷omplement", "subjective_objective": {"answer": "Subjective", "rationale": "Because \"need\" and \"relationship with each other\" are subjective feelings and decisions of patients."}, "sentence_sentiment": {"answer": "Positive", "rationale": "Because the purpose of the entry is to keep in touch with friends on a regular basis."}, "language_style": {"answer": "Tactful and warm", "rationale": "The entry uses a more euphemistic and moderate expression, using the phrase \"needs to be frequent\" to express emotional needs without direct orders or forcing the other to do something, which is more friendly and acceptable."}, "time_dimension": {"answer": "Present", "rationale": "Now, the \"I feel\" and \"often\" in the entry indicate that it is the current feeling and behavior of the testee and therefore is the time dimension of the present."}, "sentence_type": "Affirmative declarative sentence", "pos": {"nouns": {"Questionnaire": 1, "Entry": 1, "friends": 1, "Contact": 1}, "verbs": {"Yes": 1, "Hold on": 1}, "adverbs": {"Regular": 1}, "numerals": {"9": 1}, "pronouns": {"I": 1}}}, {"content": "My friends don't understand what I've been through these days.", "word_count": 17, "label": "", "sentence_structure": "Subject鈥搗erb鈥搊bject", "subjective_objective": {"answer": "Subjective", "rationale": "Because \"friends don't understand what I've been through these days\" is the patient's subjective feelings and thoughts."}, "sentence_sentiment": {"answer": "Negative", "rationale": "Because the \"My friend doesn't understand me\" act is negative, expressing dissatisfaction and loneliness."}, "language_style": {"answer": "Tactful and warm", "rationale": "The language style of the entry is more euphemistic, nuanced and sensitive to each other鈥檚 feelings, using the first person鈥檚 expression, which is more intimate and true. At the same time, questions are not critical of or critical of the behaviour of the other, but rather express concern and help in a way that is understood and supported."}, "time_dimension": {"answer": "Past", "rationale": "The entry \"these days\" refers to the most recent period, which is closer to the past than it is now and the future, and therefore is judged to be the past."}, "sentence_type": "Negative declarative sentence", "pos": {"nouns": {"friends": 1, "Experience": 1, "Oh, my God": 1}, "verbs": {"Understood": 1, "Organisation": 1}, "numerals": {"10": 1, "These": 1}, "pronouns": {"I": 1, "Both": 1, "What": 1}}}, {"content": "I feel isolated when I'm with my friends.", "word_count": 20, "label": "", "sentence_structure": "Subject鈥揷opula鈥損redicative", "subjective_objective": {"answer": "Subjective", "rationale": "The subject is a subjective experience of the patient. The subject is therefore viewed from the perspective of the patient."}, "sentence_sentiment": {"answer": "Negative", "rationale": "Because the \"Isolation Feels\" in the entry is negative."}, "language_style": {"answer": "Tactful and warm", "rationale": ""}, "time_dimension": {"answer": "Present", "rationale": "\"When with my friends\" means the current situation, where the interviewee is or has been with his friends when he answers this question. The time dimension of this article should therefore be now."}, "sentence_type": "Negative declarative sentence", "pos": {"nouns": {"Questionnaire": 1, "friends": 1, "self": 1, "Isolation": 1}, "verbs": {"in": 2, "I feel": 1, "be": 1, "and": 1, "Together": 1}, "adjectives": {"Mine": 1}, "numerals": {"11": 1}, "pronouns": {"I": 1}}}, {"content": "My friend is the one I've been talking to.", "word_count": 11, "label": "", "sentence_structure": "Subject鈥揷opula鈥損redicative", "subjective_objective": {"answer": "Subjective", "rationale": "Because my friend is the one I choose to talk to, and that's subjective, based on personal preferences and emotional needs."}, "sentence_sentiment": {"answer": "Positive", "rationale": "Because the expression \"friends are objects of talk\" in the entry expresses positive attitudes and behaviour."}, "language_style": {"answer": "Tactful and warm", "rationale": "The entry builds a sense of proximity and interaction by using the proxies \"me\" and \"you\" and the descriptive term \"the object of the talk.\" This expression is more euphemism, more gentle, and it allows the respondents to feel understood and accepted. It also guides the respondents to share their thoughts and feelings."}, "time_dimension": {"answer": "Present", "rationale": "The term \"the object of my speech\" indicates a constant, customary situation, which is the general state of affairs, which means that this situation has not only happened in the past, but may still be ongoing. So this entry reflects the behaviour and attitude of the subject."}, "sentence_type": "Affirmative declarative sentence", "pos": {"nouns": {"friends": 1, "Object": 1}, "verbs": {"be": 1, "Speech": 1}, "numerals": {"12": 1}, "pronouns": {"I": 1}}}, {"content": "I think my friend is good.", "word_count": 10, "label": "", "sentence_structure": "Subject鈥揷opula鈥損redicative", "subjective_objective": {"answer": "Subjective", "rationale": "Because it is a subjective feeling, it is the patient's subjective view."}, "sentence_sentiment": {"answer": "Positive", "rationale": "The entry is a positive expression of the opinion of friends."}, "language_style": {"answer": "Tactful and warm", "rationale": "The entry is used in a question-and-answer tone, using the subjective term \"think\" , which allows respondents to express their thoughts and feelings more freely than to be forced to give a clear answer. At the same time, the use of \"me\" in the phrase also increases the interaction with the interviewees."}, "time_dimension": {"answer": "Present", "rationale": "The entry expresses the current feelings and attitudes of the testees and thinks that their friends are good. So the time dimension is now."}, "sentence_type": "Affirmative declarative sentence", "pos": {"nouns": {"friends": 1}, "verbs": {"be": 1, "I think": 1}, "adjectives": {"Okay": 1}, "numerals": {"13": 1}, "pronouns": {"I": 1}}}, {"content": "14. My friends are hard to communicate with.", "word_count": 11, "label": "", "sentence_structure": "Subject鈥搗erb鈥揷omplement", "subjective_objective": {"answer": "Subjective", "rationale": "Because \"easy to communicate\" is a subjective assessment that reflects the subjective view of the interviewees of their ability to communicate with their friends."}, "sentence_sentiment": {"answer": "Negative", "rationale": "Because the \"unable to communicate\" feature in the entry is negative."}, "language_style": {"answer": "Calm and direct", "rationale": "The language style of the entry is more calm and straightforward, and there is no excessive emotional rendering or rhetorical technique, but rather a simple question about the quality of the person."}, "time_dimension": {"answer": "Present", "rationale": "The main words in the entry, \"I\" and \"I\" say \"that it is\" a description of the current subjective feelings or perceptions, and therefore a state of affairs. The keyword \"unable to communicate\" is the tester's view of the current behaviour of friends."}, "sentence_type": "Negative declarative sentence", "pos": {"nouns": {"friends": 1, "person": 1, "Communications": 1}, "verbs": {"Easy": 1}, "numerals": {"14": 1}, "pronouns": {"I": 1}}}, {"content": "When I was angry with something, my friends tried to understand me.", "word_count": 20, "label": "", "sentence_structure": "Subject鈥搗erb鈥搊bject", "subjective_objective": {"answer": "Subjective", "rationale": "Because it is the patient ' s perception of his or her relationship with others that is subjective."}, "sentence_sentiment": {"answer": "Positive", "rationale": "Because the \"friends try to understand me\" act is positive."}, "language_style": {"answer": "Tactful and warm", "rationale": "The entry uses a gentle, friendly language style, and attempts to lead the respondents in a positive way to share their feelings and experiences. At the same time, the first person is called \"I\" and makes the question more personal and intimate, making it more acceptable and more responsive."}, "time_dimension": {"answer": "Present", "rationale": "The entry \"now\" describes the current situation, where a friend of the testee tries to understand him when he is angry. Although this may be a regular act, it does not involve past or future factors. So the temporal dimension is \"now\"."}, "sentence_type": "Affirmative declarative sentence", "pos": {"nouns": {"Things": 1, "Time": 1, "friends": 1, "Understood": 1}, "verbs": {"Angry": 1, "Try": 1, "Understood": 1}, "adverbs": {"When": 1}, "numerals": {"15": 1}, "pronouns": {"I": 1}}}, {"content": "My friends helped me understand myself better.", "word_count": 15, "label": "", "sentence_structure": "Subject鈥搗erb鈥揷omplement", "subjective_objective": {"answer": "Subjective", "rationale": "Because this entry relates to the interaction of patients and friends and the exploration of their own inner world, these are subjective aspects."}, "sentence_sentiment": {"answer": "Positive", "rationale": "According to the article, the purpose of the act \"friends help me to understand myself better\" is positive and can therefore be judged positive."}, "language_style": {"answer": "Tactful and warm", "rationale": "Warm and warm."}, "time_dimension": {"answer": "Present", "rationale": ""}, "sentence_type": "Affirmative declarative sentence", "pos": {"nouns": {"friends": 1, "I": 2, "self": 1}, "verbs": {"Help": 1, "Understood": 1, "Better": 1}, "adverbs": {"Better": 1}}}, {"content": "My friends are concerned about how I'm doing.", "word_count": 12, "label": "", "sentence_structure": "Subject鈥搗erb鈥揷omplement", "subjective_objective": {"answer": "Subjective", "rationale": "Because \"care\" is a subjective description of the patient's attitude and feelings towards his friends."}, "sentence_sentiment": {"answer": "Positive", "rationale": "Because the entry \"Friends are concerned about me\" expresses a positive attitude that friends are very close."}, "language_style": {"answer": "Tactful and warm", "rationale": "The entry uses a more compassionate and moderate language style, rather than expressing the interest of the other, but rather the means of asking questions, which make respondents more willing to share their own situation. At the same time, the use of the word \"very\" has some emotional connotations, adding to the relative sensitivity of the questionnaire."}, "time_dimension": {"answer": "Present", "rationale": "\"My friend's concern\" in \"Careful concern\" suggests that it is a continuous act and is relevant now, so the time dimension of this entry is now."}, "sentence_type": "Interrogative sentence", "pos": {"nouns": {"friends": 1, "Attention": 1, "How's it going": 1}, "verbs": {"very": 1, "Attention": 1, "How's it going": 1}, "adverbs": {"very": 1}, "numerals": {"17": 1}, "pronouns": {"I": 1}}}, {"content": "I'm angry with my friends.", "word_count": 10, "label": "", "sentence_structure": "Subject鈥揷opula鈥損redicative", "subjective_objective": {"answer": "Subjective", "rationale": "Because \"frightful\" is a subjective description."}, "sentence_sentiment": {"answer": "Negative", "rationale": "Because the \"freak\" mood in the entry is negative."}, "language_style": {"answer": "Calm and direct", "rationale": "The entry directly expresses the subject of questioning whether the other party is often angry with friends, is not based on excessive rhetorical or emotional rendering, and is more objective in tone."}, "time_dimension": {"answer": "Present", "rationale": "Now the entry describes the current emotional state of the subject, the key words being \"I feel for my friend\" and \"I'm angry\" and they all point to what's happening."}, "sentence_type": "Negative declarative sentence", "pos": {"nouns": {"I": 1, "friends": 1}, "verbs": {"to": 1, "I feel": 1, "Fan": 1}, "adjectives": {"Gas": 1}}}, {"content": "When I need to get rid of something, I can count on my friends.", "word_count": 20, "label": "", "sentence_structure": "Subject鈥搗erb鈥搊bject", "subjective_objective": {"answer": "Subjective", "rationale": "Because \"friends can be relied on\" is a subjective feeling and attitude of a person, reflecting the individual ' s social abilities and relationships. This involves subjective assessment and emotional experience of the individual, not just the individual ' s behaviour in a particular situation."}, "sentence_sentiment": {"answer": "Positive", "rationale": "Because the purpose of the action of \"can count on my friend\" in the entry is positive."}, "language_style": {"answer": "Tactful and warm", "rationale": "The language style of the entry is more euphemistic, more acclaimed, conveys its importance to the respondents in a positive tone, and uses a query-style phrase to make them more willing to answer. Moreover, the topic is not too direct or emotional, but expressed in a more peaceful way, leaving people feeling comfortable."}, "time_dimension": {"answer": "Present", "rationale": "The terms \"reliable\" and \"need\" both indicate that such conduct is a current, present state or capability and that it is dependent on the presence of others."}, "sentence_type": "Affirmative declarative sentence", "pos": {"nouns": {"Things": 1, "Time": 1, "friends": 1}, "verbs": {"Yes": 3, "Get rid of it": 1, "Dependence": 1}, "adverbs": {"When": 1}, "numerals": {"19": 1}, "pronouns": {"I": 1}}}, {"content": "I trust my friends.", "word_count": 7, "label": "", "sentence_structure": "Subject鈥搗erb鈥揷omplement", "subjective_objective": {"answer": "Subjective", "rationale": "Because \"trust\" is a subjective attitude or emotional experience, it is the perception and feelings of a friend of the interviewee and is therefore subjective."}, "sentence_sentiment": {"answer": "Positive", "rationale": "Because \"trust\" is a positive emotional word, a sign of trust and dependence on friends."}, "language_style": {"answer": "Tactful and warm", "rationale": "The entry is a soft, invited conversation, using the word \"please\" to express respect for and hope for the interviewee. At the same time, the subject of \"I trust my friends\" is more positive, positive, without too strong a negative mood or accusation, and more easily accepted and answered by the interviewee."}, "time_dimension": {"answer": "Present", "rationale": "The entry indicates the current level of trust that the interviewees have in their friends and therefore the present."}, "sentence_type": "Affirmative declarative sentence", "pos": {"nouns": {"I": 1, "friends": 1}, "verbs": {"Trust": 1}}}, {"content": "My friends respect my feelings.", "word_count": 10, "label": "", "sentence_structure": "Subject鈥搗erb鈥揷omplement", "subjective_objective": {"answer": "Subjective", "rationale": "Because respect is an assessment of the attitude towards patients ' subjective feelings."}, "sentence_sentiment": {"answer": "Positive", "rationale": "Because the \"friend respect me\" act is positive."}, "language_style": {"answer": "Tactful and warm", "rationale": "The entry is more personal and interactive when it is presented in a soft, invitation-like tone, rather than in direct statements or orders. At the same time, the entry does not use any negative language or emotional expression, but rather uses a positive and positive way of describing the subject matter, which increases the attractiveness and acceptability of the questionnaire."}, "time_dimension": {"answer": "Present", "rationale": "The keyword is \"My friend respects my feelings\", which means the current state and circumstances, so the temporal dimension of the article is now judged."}, "sentence_type": "Affirmative declarative sentence", "pos": {"nouns": {"friends": 1, "Emotion": 1}, "verbs": {"Respect": 1}, "numerals": {"21": 1}, "pronouns": {"I": 1}}}, {"content": "I'm not a pain in the ass more than my friends know.", "word_count": 15, "label": "", "sentence_structure": "Subject鈥揷opula鈥損redicative", "subjective_objective": {"answer": "Subjective", "rationale": "Because \"more than friends know\" is a comparative expression, involving the patient's perception and assessment of what he feels inside, and is subjective."}, "sentence_sentiment": {"answer": "Negative", "rationale": "Because the phrase \"not much more pain than my friend knows\" in the entry is a certain negative sentiment."}, "language_style": {"answer": "Tactful and warm", "rationale": "The entry uses a more moderate and emotional language, suggesting that the interviewees may have some negative emotions and distress, and inviting them to share more information than direct orders or questions. This language style helps to build good interaction and trust, and is more likely to allow respondents to answer questions in an open manner."}, "time_dimension": {"answer": "Present", "rationale": "In the entry, \"I\" is the dominant word, describing the state and the feeling, the keyword \"not happy\" and \"more than friends know\" are the description of the situation. So, the entry is now."}, "sentence_type": "Negative declarative sentence", "pos": {"nouns": {"I": 1, "friends": 1, "# Happy": 1}, "verbs": {"Yes": 1, "- Yeah": 1}, "adjectives": {"not": 1}, "adverbs": {"Yes": 1}, "numerals": {"22": 1}, "classifiers": {"More": 1}, "pronouns": {"Sou-san": 1}}}, {"content": "It's like my friend can piss me off for no reason.", "word_count": 15, "label": "", "sentence_structure": "Subject鈥搗erb鈥揷omplement", "subjective_objective": {"answer": "Subjective", "rationale": "Because \"can provoke me for no reason\" is the patient's subjective feeling and perception."}, "sentence_sentiment": {"answer": "Negative", "rationale": "Because the \"fated\" act in the entry is negative."}, "language_style": {"answer": "Tactful and warm", "rationale": "The entry uses the term \"like\" to indicate that it is a perceived, not a definite, fact, while it uses a more soft language, which does not overemphasize negative emotions or critical language. Such a formulation makes it easier for the respondents to relax their vigilance and to be more willing to participate in the questionnaire."}, "time_dimension": {"answer": "Present", "rationale": "The keywords \"like\" and \"may\" indicate that this is a general expression of the current state of affairs, involving the current feelings and experiences of the testees, and therefore judgement is now."}, "sentence_type": "Affirmative declarative sentence", "pos": {"nouns": {"friends": 1, "Reason": 1}, "verbs": {"Yes": 1, "Exasperation": 1}, "adjectives": {"Nothing": 1}, "adverbs": {"I think so": 1, "Both": 1}, "numerals": {"23": 1}, "pronouns": {"I": 1}}}, {"content": "I can tell my friends about my problems and my troubles.", "word_count": 17, "label": "", "sentence_structure": "Subject鈥搗erb鈥搊bject", "subjective_objective": {"answer": "Subjective", "rationale": "Because of the willingness to tell friends what troubles they have, it is the subjective will of one."}, "sentence_sentiment": {"answer": "Positive", "rationale": "Because the purpose of the action in the entry is to \"Tell my friends my troubles and worries\" and to seek help and support from others."}, "language_style": {"answer": "Tactful and warm", "rationale": "The entry uses a positive and encouraging language style that encourages respondents to share their worries with friends and express their support and understanding of the interviewee. This language style is more gentle and friendly, and it is easy to feel comfortable and comfortable."}, "time_dimension": {"answer": "Present", "rationale": ""}, "sentence_type": "Imperative sentence", "pos": {"nouns": {"Trouble": 2, "friends": 1}, "verbs": {"Yeah": 1, "- Put it on": 1, "Tell": 1}, "adverbs": {"I": 1}, "numerals": {"24": 1}}}, {"content": "25. If my friend knew something was bothering me, he would ask me for details.", "word_count": 24, "label": "", "sentence_structure": "Subject鈥搗erb鈥搊bject", "subjective_objective": {"answer": "Subjective", "rationale": "This is because of patients' subjective perception or assumptions about the behaviour that their friends may have."}, "sentence_sentiment": {"answer": "Positive", "rationale": "Because the \"Question Me in Details\" act is a positive emotional expression of caring for others and of being willing to help."}, "language_style": {"answer": "Tactful and warm", "rationale": "The language style of the entry is euphemism. It uses a gentle and inviting tone, expresses a hypothetical situation and encourages each other to share their views or experiences. This expression is more personal and interactive, helping to build good relationships and gain more information and feedback."}, "time_dimension": {"answer": "Present", "rationale": ""}, "sentence_type": "Affirmative declarative sentence", "pos": {"nouns": {"friends": 1, "Things": 1, "Trouble": 1, "I will": 1, "he": 1, "Details": 1}, "verbs": {"Yes": 2, "have": 1, "Ask": 1}, "adverbs": {"If": 1}, "numerals": {"25": 1}, "pronouns": {"I": 1}}}]}

{"questionnaire_title": "Russell ' s smoking questionnaire (RSQ)", "questionnaire_category": "Behavioral questionnaire", "item_count": 24, "emotion_shift_overall": "No obvious trend", "language_style_overall": "Neutral", "items": [{"content": "I'll have a cigarette. I'll have a paraplegic.", "word_count": 11, "label": "", "sentence_structure": "Subject鈥搗erb鈥揷omplement", "subjective_objective": {"answer": "Subjective", "rationale": "Because \"no smoking\" and \"smoking addiction\" are the personal feelings and experiences of patients."}, "sentence_sentiment": {"answer": "Negative", "rationale": "Because the expression \"smoking is a habit\" in the entry is negative."}, "language_style": {"answer": "Tactful and warm", "rationale": "The language style of the entry is relatively warm, and in the words \"no smoking\" and \"smoking paraplegic\" it is more subtle to express the effects and harms of smoking on the body, and is balanced and caring, in line with the mild attitude of the psychological questionnaire."}, "time_dimension": {"answer": "Present", "rationale": "According to the sentences given, \"no smoking for a while\" and \"smoking addiction\" are all behaviors and feelings relevant to the present, so the temporal dimension of the entry is now judged."}, "sentence_type": "Affirmative declarative sentence", "pos": {"nouns": {"One": 1, "Yes": 2, "Smoke": 2, "Paraplegic": 1}, "verbs": {"Smoke": 1, "Let's go": 1}, "adverbs": {"not": 1, "Just": 1}, "numerals": {"1": 2}, "pronouns": {"I": 1}}}, {"content": "Sometimes one cigarette is not finished and then another is lit without realizing it.", "word_count": 20, "label": "", "sentence_structure": "Subject鈥搗erb鈥搊bject", "subjective_objective": {"answer": "Subjective", "rationale": "Because \"another one without realizing it\" is the patient's subjective behavior and feelings."}, "sentence_sentiment": {"answer": "Negative", "rationale": "Because the entry \"one cigarette is not finished and another one is lit\" is negative."}, "language_style": {"answer": "Tactful and warm", "rationale": "The entry is more proficient and interactive when it is spoken in a gentle and inviting manner, rather than in direct statements or orders. Moreover, the term \"unintentionally\" is used, which increases the level of detail of the context and makes it easier for readers to resonate."}, "time_dimension": {"answer": "Present", "rationale": "The entry describes a customary act of the subject, using the general current pattern ( \"sometimes\" and \"not finished\") and highlights the repetitive and customary nature of the act. Thus, the temporal dimension of the article can be judged to be the present."}, "sentence_type": "Affirmative declarative sentence", "pos": {"nouns": {"A cigarette": 1, "One": 1}, "verbs": {"No, I'm not": 1, "Finish": 1, "Light it up": 1, "And": 1, "Yes": 1}, "adverbs": {"Sometimes": 1, "I didn't know": 1}, "numerals": {"One": 1}}}, {"content": "I smoke when I'm resting.", "word_count": 8, "label": "", "sentence_structure": "Subject鈥搗erb鈥揷omplement", "subjective_objective": {"answer": "Subjective", "rationale": "Because the expressions \"often\" and \"smoking\" are described from the patient's subjective perspective, reflecting the patient's own habits and behaviour."}, "sentence_sentiment": {"answer": "Negative", "rationale": "Because the \"smoking\" act in the entry is negative."}, "language_style": {"answer": "Tactful and warm", "rationale": "The language style of the entry is more moderate, by asking the other side whether they smoke regularly during their rest, and by directing the interviewee to reflect and answer, while expressing a sense of concern and understanding."}, "time_dimension": {"answer": "Past", "rationale": "The word \"smoking\" in \"I smoke often during rest\" means a regular, customary act, and \"on rest\" means that it occurs at some point in the past, and therefore it is judged to be the past."}, "sentence_type": "Affirmative declarative sentence", "pos": {"verbs": {"I": 1, "Smoking": 1}, "adjectives": {"Regular": 1, "Rest": 1}}}, {"content": "4. Every time I smoke, I think of Shu Yang.", "word_count": 10, "label": "", "sentence_structure": "Subject鈥揷opula鈥損redicative", "subjective_objective": {"answer": "Subjective", "rationale": "Because \"sweet\" is a subjective feeling, not an objective fact."}, "sentence_sentiment": {"answer": "Negative", "rationale": "Because the act of \"smoking\" in the entry is negative, it is always comfortable and it is an indication that smoking has become a habit and not a positive lifestyle."}, "language_style": {"answer": "Tactful and warm", "rationale": "At the same time, emotional terms (such as \"Shu Yang\") are used in the problem, trying to create a friendly and relaxed atmosphere in which the interviewees are more willing to share their real feelings and experiences."}, "time_dimension": {"answer": "Present", "rationale": "According to the context, \"anytime\" means that it is comfortable and pleasant to smoke, whenever it is, so the entry describes the person being tested's current behaviour or mental state."}, "sentence_type": "Affirmative declarative sentence", "pos": {"nouns": {"Time": 1, "Smoking": 2, "Shuu": 1, "Jan": 1}, "verbs": {"Whatever": 1, "Both": 2, "Smoking": 1, "Shuu": 1}, "adverbs": {"What": 1, "Both": 1}, "numerals": {"4": 1}}}, {"content": "5. A cigarette in his hand is a good smoker.", "word_count": 11, "label": "", "sentence_structure": "Subject鈥搗erb鈥揷omplement", "subjective_objective": {"answer": "Subjective", "rationale": "Because \"smoking in the hand is also a smoke pleasure\" is a person's attitude to smoking or feelings, and is subjective."}, "sentence_sentiment": {"answer": "Negative", "rationale": "Because \"smoking\" is a negative health act."}, "language_style": {"answer": "Tactful and warm", "rationale": "The language style of the entry is more euphemistic and warm, and it invites interviewees in an easy and friendly way to share their views on smoking, rather than to make direct statements or orders. At the same time, the expression \"also smokes \" is used, implying that smoking may have its positive side, increasing the openness and inclusiveness of the issue."}, "time_dimension": {"answer": "Past", "rationale": "The entry describes the personal past habits or experiences, the keywords being \"also\" and \"smoking pleasures,\" which point to past behavior. At the same time, the move \"smoking the cigarette in the hand\" also indicates an already developed habit. So the time dimension of this questionnaire item is the past."}, "sentence_type": "Affirmative declarative sentence", "pos": {"nouns": {"In your hand": 1, "Smoke": 1, "Lew": 1}, "verbs": {"Jack": 1, "be": 1, "Suck": 1}, "adjectives": {"Yeah": 1, "Smoker": 1}, "numerals": {"5": 1}}}, {"content": "I think smoking is a good way to go.", "word_count": 9, "label": "", "sentence_structure": "Subject鈥揷opula鈥損redicative", "subjective_objective": {"answer": "Subjective", "rationale": "Because \"the feeling\" expresses the patient's personal views and attitudes and is a subjective examination."}, "sentence_sentiment": {"answer": "Negative", "rationale": "The judgement is based on the fact that the expression \"smoking\" for the purpose of the article is a negative act and that, therefore, the emotional orientation of the purpose of the article is negative."}, "language_style": {"answer": "Tactful and warm", "rationale": "The entry is more personal and interactive in an implicit way, asking the other side about smoking, rather than directly stating or ordering. At the same time, while the phrase contains positive tobacco use, it is not overemphasised or forced, thus giving a warmer feeling of the whole."}, "time_dimension": {"answer": "Present", "rationale": "The keyword \"I feel\" and \"smoking with a sense of style\" indicate the current attitude and perception of the speaker, so the temporal dimension is now judged."}, "sentence_type": "Affirmative declarative sentence", "pos": {"nouns": {"I": 1, "Wind": 1, "Smoke": 1}, "verbs": {"Smoke": 1, "I think": 1}, "adjectives": {"Yeah": 1}}}, {"content": "When things get so annoying, I smoke more.", "word_count": 18, "label": "", "sentence_structure": "Subject鈥搗erb鈥揷omplement", "subjective_objective": {"answer": "Subjective", "rationale": "Because the idea of \"the troublesome thing\" and \"the smoke is more smoke\" is the patient's subjective feeling and judgment."}, "sentence_sentiment": {"answer": "Negative", "rationale": "Because the entry describes the increase in smoking in times of distress, which is a negative situation."}, "language_style": {"answer": "Calm and direct", "rationale": "The entry uses objective representations, without excessive emotional or subjective evaluation, and therefore language styles are straightforward and dispassionate."}, "time_dimension": {"answer": "Present", "rationale": "The entry indicates a customary act ( \"I smoke more\") and it is occurring or has occurred recently. The keyword is \"when something is troubling,\" which suggests that the act occurs in a given situation, so it can be inferred that it is a current act or state."}, "sentence_type": "Affirmative declarative sentence", "pos": {"nouns": {"Things": 1, "Smoke": 2}, "verbs": {"Come on": 1, "Suck": 2, "More": 1}, "adjectives": {"Trouble": 1}, "numerals": {"7": 1}, "pronouns": {"I": 1}}}, {"content": "8. For me, smoking increases emotions and makes me think a little bit.", "word_count": 16, "label": "", "sentence_structure": "Subject鈥揷opula鈥損redicative", "subjective_objective": {"answer": "Subjective", "rationale": "Because this option represents personal feelings and experiences and is a subjective one."}, "sentence_sentiment": {"answer": "Negative", "rationale": "Because the idea that smoking can raise emotions and make thinking agile in the entry is negative, it may mislead respondents in their positive perception of smoking and be detrimental to health."}, "language_style": {"answer": "Tactful and warm", "rationale": "The language style of the entry is more euphemistic. By using \"for me\" to express personal feelings and emphasize the benefits of smoking (e.g., raising emotions, making thinking agile), it makes dialogue more friendly and natural, rather than directly ordering or criticizing each other's smoking behaviour. At the same time, the use of words such as \"can\" and \"make\" makes the tone more positive and optimistic, giving people a sense of encouragement and support."}, "time_dimension": {"answer": "Present", "rationale": "This questionnaire entry uses the term \"for me\" to indicate that it is the individual view and feeling of the testee, not an objective fact. Moreover, the words \"enhanced\" and \"improved thinking\" describe a view of the present, so it can be judged that the question is about the current behaviour or mentality of the testee, and the temporal dimension is about the present."}, "sentence_type": "Affirmative declarative sentence", "pos": {"nouns": {"I": 1, "Emotion": 1, "Thinking": 1, "Smoke": 1}, "verbs": {"Yes": 1, "Increase": 1, "鈾?And I'm gonna be 鈾?: 1, "Suck": 1}, "adjectives": {"Agility": 1}, "numerals": {"8": 1}}}, {"content": "I sometimes don't know how to smoke.", "word_count": 13, "label": "", "sentence_structure": "Subject鈥搗erb鈥揷omplement", "subjective_objective": {"answer": "Subjective", "rationale": "Because \"aware\" is a subjective process of perception and perception, the point of view of the questionnaire is that of the patient."}, "sentence_sentiment": {"answer": "Negative", "rationale": "Because the \"self smoking\" act in the entry is negative."}, "language_style": {"answer": "Tactful and warm", "rationale": "The entry uses the first person's word, \"I\" , which is more natural and leads to answers by asking the other person whether there is a certain situation. This expression is neither too direct nor offensive, but it triggers reflection and self-reflection. It is therefore considered to be a soft language."}, "time_dimension": {"answer": "Present", "rationale": "The keyword \"I\" sometimes indicates that the act is occasional, and \"smoking\" means that it's an ongoing move, so the entry asks about the person's current behaviour."}, "sentence_type": "Negative declarative sentence", "pos": {"nouns": {"I": 2, "self": 1, "Smoking": 1}, "verbs": {"I've known him better": 1, "In the process of": 1}, "adverbs": {"Sometimes": 1}, "numerals": {"9": 1}}}, {"content": "When I didn't smoke, I felt like I was missing something.", "word_count": 19, "label": "", "sentence_structure": "Subject鈥揷opula鈥損redicative", "subjective_objective": {"answer": "Subjective", "rationale": "Because it's a subjective experience, it's the patient's own feelings."}, "sentence_sentiment": {"answer": "Negative", "rationale": "Because the expression \"things are missing\" in the entry conveys an unsatisfactory, uncomfortable emotion, negative."}, "language_style": {"answer": "Tactful and warm", "rationale": "The basis for the judgement: the entry is more personal and interactive, by speaking with a gentle, invitation-like tone, rather than by direct statement or order. At the same time, the entry is not overly emotional, but rather leads the respondents to their answers by giving them a sense of non-smoking, which is more euphemism, warmer and more comfortable."}, "time_dimension": {"answer": "Present", "rationale": "The keyword \"when I don't smoke\" suggests that it is a description of the current situation, while \"something is missing\" expresses a constant feeling or state, and suggests that it may be related to non-smoking. Therefore, the questionnaire entry should be classified as \"present.\""}, "sentence_type": "Negative declarative sentence", "pos": {"nouns": {"Time": 1, "Hands": 1, "Something": 1}, "verbs": {"Total": 1, "I think": 1, "Less": 1, "Missing": 1, "Smoking": 1}, "adverbs": {"not": 1, "in": 1}, "numerals": {"10": 1}, "pronouns": {"I": 1}}}, {"content": "I can't bear to get a cigarette when I'm free.", "word_count": 26, "label": "", "sentence_structure": "Subject鈥搗erb鈥揷omplement", "subjective_objective": {"answer": "Subjective", "rationale": "Because it is unbearable and necessary to get smoke is the subjective feeling and the idea of the patient."}, "sentence_sentiment": {"answer": "Negative", "rationale": "Since the behaviour described in the entry (to get smoke without smoke) is coercive, it can cause health problems and can be addictive, and can therefore be judged as negative."}, "language_style": {"answer": "Calm and direct", "rationale": "The entry is presented directly, indicating that the interviewee has a strong thirst for smoke."}, "time_dimension": {"answer": "Present", "rationale": "Now the entry describes a feeling and behavior that occurs when there is no smoke on hand, using the current \"always\" and the general \"yes\" of the present, which means that the feeling and behaviour are still present. So, the time dimension is now."}, "sentence_type": "Affirmative declarative sentence", "pos": {"nouns": {"Hand": 1, "Smoke": 2, "Feel": 1, "It's unbearable": 1, "I'm sure": 1, "It's just a smoke": 1}, "verbs": {"have": 1, "Got it": 1, "That's right": 1, "Stop": 1}, "adjectives": {"It's unbearable": 1}, "numerals": {"11": 1, "One": 2}, "pronouns": {"I": 1}}}, {"content": "12. When I'm bored, I smoke more.", "word_count": 12, "label": "", "sentence_structure": "Subject鈥搗erb鈥揷omplement", "subjective_objective": {"answer": "Objective", "rationale": "The amount of the withdrawal is determined by the patient ' s behaviour and is not related to the patient ' s subjective feelings."}, "sentence_sentiment": {"answer": "Negative", "rationale": "Because the behavior in the entries is negative."}, "language_style": {"answer": "Calm and direct", "rationale": "The entry directly asks the respondents whether they will increase smoking behaviour in the event of depression, with a more direct and concise language style, and no excessive emotional colour or rhetorical technique."}, "time_dimension": {"answer": "Present", "rationale": "Now the key words are \"when you're bored\" and \"more smoked\" , which describe a customary behavior or state of mind, which increases smoking when you feel depressed. And, \"just more smoked\" uses the usual current pattern, which suggests that it is a regular or customary act, and the temporal dimension is now."}, "sentence_type": "Affirmative declarative sentence", "pos": {"nouns": {"Time": 1, "Questionnaire": 1, "Entry": 1, "Smoke": 1, "It's boring": 1}, "verbs": {"Smoke": 1, "More": 1}, "adverbs": {"Just": 1}, "numerals": {"12": 1}}}, {"content": "Smoking helps me to overcome fatigue.", "word_count": 10, "label": "", "sentence_structure": "Subject鈥搗erb鈥揷omplement", "subjective_objective": {"answer": "Subjective", "rationale": "Because helping me to overcome fatigue is the patient's subjective feeling and perception."}, "sentence_sentiment": {"answer": "Negative", "rationale": "Because the goal of \"elimination\" is good, but smoking is unhealthy."}, "language_style": {"answer": "Calm and direct", "rationale": "The entry directly describes the role of smoking in eliminating fatigue, without adding any rhetorical or emotional colours, and is more calm and straightforward."}, "time_dimension": {"answer": "Present", "rationale": "The use of the word \"capable\" in the entry is a capacity or effect, which suggests that smoking is a meaningful influence on the elimination of fatigue, and that this influence has not only occurred in the past, but may still be ongoing. Thus, we can judge the time dimension of the entry as it is."}, "sentence_type": "Negative declarative sentence", "pos": {"nouns": {"Smoking": 1, "Tired": 1}, "verbs": {"Yes": 1, "Help": 1, "Elimination": 1}, "adverbs": {"Yes": 1, "Help": 1, "Elimination": 1}, "numerals": {"13": 1}}}, {"content": "I found it hard to be an hour away from smoking.", "word_count": 17, "label": "", "sentence_structure": "Subject鈥搗erb鈥揷omplement", "subjective_objective": {"answer": "Subjective", "rationale": "Because \"hard\" is the individual feeling and the assessment of the patient, and it's subjective."}, "sentence_sentiment": {"answer": "Negative", "rationale": "Because the expression \"hard\" in the entry is negative emotional."}, "language_style": {"answer": "Calm and direct", "rationale": "The entry describes the problem in a direct and clear manner, without excessive emotional or rhetorical style, and with a more calm and objective language style."}, "time_dimension": {"answer": "Past", "rationale": "In the entry, \"I find it hard to be free of smoking for an hour\" expresses the experience and feelings of the testee about the past, and therefore judges it as the past."}, "sentence_type": "Negative declarative sentence", "pos": {"nouns": {"Hours": 1, "Smoking": 1}, "verbs": {"Do it": 1, "be": 1, "Found": 1, "Hard": 1}, "adverbs": {"very": 1}, "numerals": {"One": 1, "4": 1}, "classifiers": {"individual": 1}}}, {"content": "I've been on the smoke all the time without knowing.", "word_count": 13, "label": "", "sentence_structure": "Subject鈥搗erb鈥揷omplement", "subjective_objective": {"answer": "Subjective", "rationale": "Because \"smoking the smoke, often unconsciously,\" is a subjective feeling and behaviour description that reflects the habit and inattention of the patient to smoke."}, "sentence_sentiment": {"answer": "Negative", "rationale": "Because the \"smoking on the smoke\" in the entry is negative."}, "language_style": {"answer": "Calm and direct", "rationale": ""}, "time_dimension": {"answer": "Present", "rationale": "In conclusion, the output should read \"Now\"."}, "sentence_type": "Affirmative declarative sentence", "pos": {"nouns": {"Questionnaire": 1, "Entry": 1, "Smoke": 1}, "verbs": {"Light it up": 1}, "adjectives": {"Often": 1, "I didn't know": 1}, "numerals": {"15": 1}, "pronouns": {"I": 1}}}, {"content": "I'm the one who wants to smoke when I'm feeling comfortable and free.", "word_count": 14, "label": "", "sentence_structure": "Subject鈥搗erb鈥揷omplement", "subjective_objective": {"answer": "Subjective", "rationale": "Because \"fine\" and \"freedom\" and \"smoking\" are personal feelings, desires or emotions of the patient, and are subjective."}, "sentence_sentiment": {"answer": "Negative", "rationale": "Because the \"smoking\" act is negative."}, "language_style": {"answer": "Calm and direct", "rationale": "The entry directly expresses the subject of a tendency to smoke when the subject is happy and relaxed, and the language style is more of a calm and straightforward one."}, "time_dimension": {"answer": "Present", "rationale": "The entry describes a state or habit of the moment, where \"I\" is the most interested in smoking when it's in a good mood and free time, so the time dimension is now."}, "sentence_type": "Affirmative declarative sentence", "pos": {"nouns": {"The mood": 1, "鈾?Unsweet 鈾?: 1, "Have a nice time": 1}, "verbs": {"in": 1, "Yes": 1, "Smoke": 1}, "adjectives": {"Most": 1}, "adverbs": {"Time": 1}}}, {"content": "17. Smoking helps me think and concentrate.", "word_count": 16, "label": "", "sentence_structure": "Subject鈥搗erb鈥揷omplement", "subjective_objective": {"answer": "Subjective", "rationale": "Because \"help\" and \"focus\" and so on describe patients' perceptions and feelings about smoking, and are subjective."}, "sentence_sentiment": {"answer": "Negative", "rationale": "Because smoking is harmful to health and long-term inhalation of second-hand smoke can harm others. While the entry expresses a positive intention (i.e., that smoking is considered to be useful for thinking and focusing), it is based on a false perception and ignores the negative effects that smoking can have."}, "language_style": {"answer": "Calm and direct", "rationale": "The entry presents the relationship between smoking and thinking and attention in a direct manner, with no excessive emotional rendering or rhetorical techniques, and a more concise language style."}, "time_dimension": {"answer": "Present", "rationale": "The entry indicates the person's current perception and behaviour, and the keyword is \"help\" and \"now\" and points to the current conduct or state in progress."}, "sentence_type": "Negative declarative sentence", "pos": {"nouns": {"Problem": 1, "Attention": 1}, "verbs": {"Help": 1, "Think": 1, "Focus": 1}, "numerals": {"17": 1}}}, {"content": "I'm not gonna be able to smoke for a while.", "word_count": 11, "label": "", "sentence_structure": "Subject鈥搗erb鈥揷omplement", "subjective_objective": {"answer": "Subjective", "rationale": "Because \"suffering\" is a subjective feeling of patients, reflecting their reactions to non-smoking and emotional experiences. It's not an objective fact or behavioural outcome, but an individual's feelings and experiences."}, "sentence_sentiment": {"answer": "Negative", "rationale": "Because the description of \"distressed\" in the entry is negative."}, "language_style": {"answer": "Calm and direct", "rationale": "The entry uses a direct and clear language style to ask if the interviewee is uncomfortable with smoking, has no excessive rhetoric or emotional colour. Such language style allows the interviewee to understand the problem more clearly and to obtain more precise answers."}, "time_dimension": {"answer": "Present", "rationale": "The term \"all pains\" in the sentence makes it possible to judge the current state and feelings of the sentence, and therefore the current entries."}, "sentence_type": "Negative declarative sentence", "pos": {"verbs": {"Smoking": 1, "It's hard": 1}, "adjectives": {"Just a minute": 1}, "adverbs": {"Both": 1}}}, {"content": "I feel more mature and experienced when smoking.", "word_count": 15, "label": "", "sentence_structure": "Subject鈥揷opula鈥損redicative", "subjective_objective": {"answer": "Subjective", "rationale": "Because \"maturity\" is a subjective assessment, not a fact that can be directly observed."}, "sentence_sentiment": {"answer": "Negative", "rationale": "Because smoking is usually negative, and it's possible that being more mature and sophisticated may be a self-deception or a circumvention."}, "language_style": {"answer": "Calm and direct", "rationale": "The entry asks whether the person interviewed feels more mature and sophisticated when smoking, and the language is clear and simple, without too many embellishments or emotional renderings."}, "time_dimension": {"answer": "Past", "rationale": "The entry asks the testee about the perception and feelings of smoking behaviour, and the temporal dimension is judged to be the past. In it, the word \"think\" indicates that this is the testee's past experience or feeling, not the current or future state."}, "sentence_type": "Affirmative declarative sentence", "pos": {"nouns": {"Smoking": 1, "I": 1, "Feel": 1, "Grown up": 1, "Practice": 1}, "verbs": {"I think": 1, "It seems": 1}, "pronouns": {"self": 1}}}, {"content": "I always thought about smoking when I wasn't smoking.", "word_count": 17, "label": "", "sentence_structure": "Subject鈥搗erb鈥揷omplement", "subjective_objective": {"answer": "Subjective", "rationale": "This is a subjective approach, since it is a personal feeling and reflection of the patient."}, "sentence_sentiment": {"answer": "Negative", "rationale": "Because the \"Smoking about the Tobacco\" act in the entry is negative and expresses the belief that smoking is both a necessity and an option beyond control."}, "language_style": {"answer": "Calm and direct", "rationale": "The entry asks the respondents directly whether they are often reminded of smoking in non-smoking conditions, and the language is more calm and straightforward and does not have too much emotional rendering."}, "time_dimension": {"answer": "Present", "rationale": "The keywords \"when you don't smoke\" and \"just think about smoking\" mean that the testee's thinking behavior is happening and it's now."}, "sentence_type": "Affirmative declarative sentence", "pos": {"nouns": {"20": 1, "Smoking": 2, "Yes": 1}, "verbs": {"be": 1, "in": 1, "Yes": 1, "Smoking": 1}, "adverbs": {"Just": 1, "Old": 1}, "numerals": {"20": 1}, "pronouns": {"I": 1}}}, {"content": "It's hard to get me out of smoking a week.", "word_count": 11, "label": "", "sentence_structure": "Subject鈥搗erb鈥揷omplement", "subjective_objective": {"answer": "Subjective", "rationale": "Because \"I can't do it without smoking\" is a subjective feeling and an idea."}, "sentence_sentiment": {"answer": "Negative", "rationale": "Because \"a week without smoking\" is a negative behavior in the entry, and \"hard to do\" also conveys a negative attitude."}, "language_style": {"answer": "Tactful and warm", "rationale": "The entry uses a more euphemistic and understanding language style, which emphasizes the difficulty of quitting smoking, without directly blaming or criticizing the other party for smoking."}, "time_dimension": {"answer": "Present", "rationale": "The entry describes the current behaviour and feelings of the testee, and \"one week of no smoking\" is an ongoing exercise, so it has a time dimension now."}, "sentence_type": "Negative declarative sentence", "pos": {"nouns": {"Questionnaire Entry": 1, "Week": 1, "Smoking": 1}, "verbs": {"Yes": 1, "Do it": 1}, "adjectives": {"Hard": 1}, "adverbs": {"very": 1}, "numerals": {"21": 1, "One": 2}, "classifiers": {"individual": 1}, "pronouns": {"I": 1}}}, {"content": "I smoke because I have something in my mouth.", "word_count": 14, "label": "", "sentence_structure": "Subject鈥搗erb鈥揷omplement", "subjective_objective": {"answer": "Subjective", "rationale": "Because \"for something in your mouth\" is the patient's subjective motive or cause."}, "sentence_sentiment": {"answer": "Neutral", "rationale": "Because the entry merely describes a motive for smoking, it does not express positive or negative emotional sentiments."}, "language_style": {"answer": "Tactful and warm", "rationale": "This entry uses a milder tone, does not directly criticize or order the other to quit smoking, but rather expresses an invitation to understand and care about smoking."}, "time_dimension": {"answer": "Present", "rationale": "\"Smoking\" and \"for something in your mouth\" are descriptions of behaviour, in which \"often\" means that the act has a certain frequency, and \"yes\" uses the usual current state of affairs, which means that the act is still going on."}, "sentence_type": "Affirmative declarative sentence", "pos": {"nouns": {"Smoking": 1, "Mouth": 1, "Something": 1}, "verbs": {"Always": 1, "have": 1}, "adverbs": {"For": 1}, "numerals": {"22": 1}}}, {"content": "23. Smoking attracts heterosexuals", "word_count": 7, "label": "", "sentence_structure": "Subject鈥搗erb鈥搊bject", "subjective_objective": {"answer": "Subjective", "rationale": "Since this statement involves evaluation and perception of smoking, it is necessary to examine the subjective attitude of the patient."}, "sentence_sentiment": {"answer": "Negative", "rationale": "Because smoking is a negative act."}, "language_style": {"answer": "Calm and direct", "rationale": "The entry describes in a clear and straightforward language one of the possible consequences of smoking, with no excessive rhetoric or emotional rendering, and is expressed in a more objective and calm manner."}, "time_dimension": {"answer": "Present", "rationale": "The keyword is \"smoking,\" which indicates that the person is in the current state or habit of the person being tested; and the word \"capable\" in \"attracting opposite sex\" indicates ability or possibility, which also points to the present. The entry therefore asks whether the person being tested currently has a behaviour or perception that attracts opposite sex by smoking."}, "sentence_type": "Affirmative declarative sentence", "pos": {"nouns": {"Smoking": 1, "Heterosexual": 1}, "verbs": {"Attractive": 1}, "numerals": {"23": 1}}}, {"content": "I smoke when I'm angry.", "word_count": 9, "label": "", "sentence_structure": "Subject鈥搗erb鈥搊bject", "subjective_objective": {"answer": "Subjective", "rationale": "Because smoking is a personal habit and behaviour, it is a reaction of patients in emotional situations and is a subjective attitude of patients."}, "sentence_sentiment": {"answer": "Negative", "rationale": "Because smoking is a negative behaviour in the event of a fire, it is detrimental to physical and mental health."}, "language_style": {"answer": "Tactful and warm", "rationale": "The entry is more personal and interactive when it is presented in a gentle and inviting tone, rather than in direct statements or orders. At the same time, it raises a phenomenon or behaviour and asks whether the other side has a similar behaviour that can trigger reflection and self-reflection by the person under investigation."}, "time_dimension": {"answer": "Present", "rationale": ""}, "sentence_type": "Imperative sentence", "pos": {"nouns": {"I": 1, "Smoke": 1}, "verbs": {"Yes": 1, "Smoke": 1, "- Get mad": 1}, "adverbs": {"Just": 1}, "numerals": {"24": 1}}}]}

{"questionnaire_title": "Self-assessment of sleeping conditions (SRSS)", "questionnaire_category": "Mental health", "item_count": 20, "emotion_shift_overall": "No obvious trend", "language_style_overall": "Neutral", "items": [{"content": "Do you think it's enough to sleep?", "word_count": 10, "label": "", "sentence_structure": "Subject鈥損redicate", "subjective_objective": {"answer": "Subjective", "rationale": "The word \"like\" indicates that it is the patient's subjective feeling, so the entry is subjective."}, "sentence_sentiment": {"answer": "Neutral", "rationale": "Because the entry is asked about objective facts, there is no apparent emotional tendency."}, "language_style": {"answer": "Tactful and warm", "rationale": "The language style of the entry is more euphemistic and gentle, and it is a question-and-answer approach, allowing respondents to express their true feelings rather than coercion or command. Moreover, the term \"normal\" is used to emphasize everyday situations, making questionnaires more practical. Such language styles are more comfortable and relaxed, and are more likely to be more likely to be available for questionnaire surveys."}, "time_dimension": {"answer": "Present", "rationale": "The keyword is \"you feel\" and it is the subject's current subjective perception and perception of his state; the other keyword is \"normal sleep\" , indicating that the problem is related to the situation of the immediate or normal situation. The entry is therefore asking about the current situation."}, "sentence_type": "Interrogative sentence", "pos": {"nouns": {"you": 1, "Sleep": 1, "Enough": 1}, "verbs": {"I think": 1, "Ping": 1, "Time": 1, "That's enough": 1}, "adverbs": {"Normal": 1}}}, {"content": "2. Have you felt sufficiently rested after sleeping?", "word_count": 16, "label": "", "sentence_structure": "Subject鈥搗erb鈥搊bject", "subjective_objective": {"answer": "Subjective", "rationale": "Because \"Feeling well rest\" is a subjective feeling and judgment of the patient, it is a subjective view."}, "sentence_sentiment": {"answer": "Neutral", "rationale": "As the entry does not express any positive or negative emotional aspects, it merely asks the respondents about the effect of sleeping."}, "language_style": {"answer": "Calm and direct", "rationale": "The entry directly asks about the state of the other party ' s sleep, does not have too many speech-making or emotional renderings, and is more objective in tone."}, "time_dimension": {"answer": "Present", "rationale": "The entry asks the current situation of the person being tested, i.e. whether he or she feels sufficiently rest after sleep, and therefore judges it now."}, "sentence_type": "Imperative sentence", "pos": {"nouns": {"Sleep": 1, "Full": 1, "Rest": 1}, "verbs": {"Did you feel it": 1, "Pass": 1}, "adjectives": {"Full": 1}, "adverbs": {"Already": 1, "I'm not sure": 1}, "numerals": {"2": 1}, "pronouns": {"you": 1}}}, {"content": "3. Have you slept at night, do you dozed during the day?", "word_count": 14, "label": "", "sentence_structure": "Subject鈥搗erb鈥搊bject", "subjective_objective": {"answer": "Objective", "rationale": "Because it is an objective fact that can be observed and recorded, i.e. whether the patient is sleeping during the day."}, "sentence_sentiment": {"answer": "Neutral", "rationale": "Since the entry is merely an objective fact, it does not express any positive or negative emotional attitude."}, "language_style": {"answer": "Calm and direct", "rationale": "The entry uses direct and concise language, asking if the person surveyed was sleepy during the day, without excessive rhetoric and emotional colours, and with a more calm and straightforward language style."}, "time_dimension": {"answer": "Present", "rationale": "The entry is for the testee ' s current state, and the time dimension is now judged."}, "sentence_type": "Interrogative sentence", "pos": {"nouns": {"you": 1, "Night": 1, "I'm not sure": 1, "Daytime": 1, "Sleep": 1}, "verbs": {"Already": 1, "Sleep": 1, "Fight": 1, "Is it possible": 1}, "adverbs": {"very": 1, "- You're not": 1}, "numerals": {"3": 1}}}, {"content": "How many hours do you sleep on average every night?", "word_count": 14, "label": "", "sentence_structure": "Subject鈥搗erb鈥搊bject", "subjective_objective": {"answer": "Subjective", "rationale": "The problem is examined from the patient ' s subjective point of view, as it relates to the patient ' s own perception and assessment of his or her sleep."}, "sentence_sentiment": {"answer": "Neutral", "rationale": "Since the entry is merely an objective fact, it does not involve any emotional or attitudinal colour."}, "language_style": {"answer": "Calm and direct", "rationale": "The entry is more straightforward in language, is not based on excessive rhetorical or emotional rendering, is more objective and neutral and is designed to obtain the true sleep of the person under investigation."}, "time_dimension": {"answer": "Present", "rationale": "The entry asks the subject about his current sleep and habits, and the keywords \"average sleep per night\" and \"sleepable hours\" point to the current time dimension."}, "sentence_type": "Interrogative sentence", "pos": {"nouns": {"Questionnaire Entry": 1, "you": 1, "Night": 1, "Hours": 1}, "verbs": {"Yes": 1, "Sleep": 1, "Average": 1, "About": 1}, "adverbs": {"Every": 1, "individual": 1, "Both": 0, "in": 0, "have": 0, "None": 0, "More": 0, "Less": 0, "Okay": 0, "Bad": 0}, "numerals": {"4": 1, "Every one": 1, "Night": 1}, "pronouns": {"you": 1}}}, {"content": "Do you have any sleep difficulties?", "word_count": 8, "label": "", "sentence_structure": "Subject鈥損redicate", "subjective_objective": {"answer": "Subjective", "rationale": "Because sleeping difficulties are a subjective experience and feeling, different people may have different answers and experiences."}, "sentence_sentiment": {"answer": "Negative", "rationale": "Because the act of sleeping difficulties in the entries is negative."}, "language_style": {"answer": "Calm and direct", "rationale": "The entry directly asked the other party whether it had problems with sleeping, without excessive rhetorical or emotional rendering, and with a more balanced and objective tone."}, "time_dimension": {"answer": "Present", "rationale": "The entry asks if the person tested is currently having sleep difficulties, and the time dimension is now judged."}, "sentence_type": "Interrogative sentence", "pos": {"nouns": {"Problem": 1, "Answer": 1}, "verbs": {"have": 1, "Sleep": 1, "Difficulties": 1}, "numerals": {"5": 1}}}, {"content": "6. Are you awake in the middle of your sleep?", "word_count": 10, "label": "", "sentence_structure": "Subject鈥搗erb鈥搊bject", "subjective_objective": {"answer": "Subjective", "rationale": "According to the definition given, it can be seen that the subject is the subject of subjective feelings and experiences of the patient and can therefore be judged as \"subjective\"."}, "sentence_sentiment": {"answer": "Negative", "rationale": "Because the act of waking up is negative."}, "language_style": {"answer": "Calm and direct", "rationale": "The language style of the entry is relatively objective and calm, with no excessive emotional rendering or rhetorical techniques, but simply directs the respondents to ask if they are awakening during their sleep, which is more conducive to obtaining accurate information."}, "time_dimension": {"answer": "Present", "rationale": "The entry is an inquiry into the current behaviour or state of the subject, and the words \"after sleep\", \"in the middle\" and \"awakening\" indicate that this is a sentence describing the current stage, and therefore the judgement is now."}, "sentence_type": "Interrogative sentence", "pos": {"nouns": {"you": 1, "After bed": 1, "Centre": 1, "Wake up": 1}, "numerals": {"6": 1}}}, {"content": "7. Are you having difficulty sleeping again?", "word_count": 11, "label": "", "sentence_structure": "Subject鈥搗erb鈥搊bject", "subjective_objective": {"answer": "Subjective", "rationale": "This is a subjective question, because it concerns the patient鈥檚 personal feelings and experience, i.e. whether it is difficult for the patient to go back to sleep when he wakes up. Although this feeling may be related to certain physical or environmental factors, the problem is mainly concerned with the patient鈥檚 subjective experience."}, "sentence_sentiment": {"answer": "Negative", "rationale": "Because \"hard to sleep\" is negative."}, "language_style": {"answer": "Tactful and warm", "rationale": "The entry is more euphemistic and not used too direct or strong language. It raises questions in a peaceful and inviting manner, making the interviewee comfortable and comfortable, and more willing to share his real feelings. And the use of verbs like \"you\" also increases the sense of affection and respect."}, "time_dimension": {"answer": "Present", "rationale": "The entry asks about the quality of the person's sleep after awakening and the time dimension is now judged."}, "sentence_type": "Interrogative sentence", "pos": {"nouns": {"Wake up": 1, "Sleep": 1}, "verbs": {"Hard": 1, "Re-in": 1}, "adverbs": {"Yes": 1, "Hard": 1, "Again": 1}, "numerals": {"7": 1}}}, {"content": "Do you dream many or are you often awakened by nightmares?", "word_count": 12, "label": "", "sentence_structure": "Subject鈥搗erb鈥搊bject", "subjective_objective": {"answer": "Subjective", "rationale": "Because the dream is the result of subjective experience of individuals, the issue is viewed from the perspective of patients."}, "sentence_sentiment": {"answer": "Negative", "rationale": "Because the entries \"dreaming\" and \"wake by nightmares\" are both negative experiences."}, "language_style": {"answer": "Tactful and warm", "rationale": "The language style of the questionnaire entry is euphemism. Instead of asking the interviewee whether he has sleep problems, it uses a gentle and inviting tone to guide them to think and answer. This expression is more personal and interactive and resonates with the interviewee."}, "time_dimension": {"answer": "Present", "rationale": ""}, "sentence_type": "Interrogative sentence", "pos": {"nouns": {"Questionnaire": 1, "Entry": 1, "Dreams": 2, "Nightmares": 1}, "verbs": {"you": 1, "Is it possible": 1, "Dreams": 1, "Wake up": 1}, "adverbs": {"Regular": 1}, "numerals": {"8": 1}}}, {"content": "Do you take sleeping pills?", "word_count": 11, "label": "", "sentence_structure": "Subject鈥搗erb鈥搊bject", "subjective_objective": {"answer": "Subjective", "rationale": "Because sleeping pills are individual behavioral choices, subjective attitudes and behavior."}, "sentence_sentiment": {"answer": "Negative", "rationale": "Because the behavior in the entry \"sleeping pills\" is usually considered negative."}, "language_style": {"answer": "Calm and direct", "rationale": "The entry is of a calm and straightforward language style. The entry directly asks the interviewee whether he has been given sleeping pills, and there is no excessive emotional or rhetorical approach to express it clearly."}, "time_dimension": {"answer": "Present", "rationale": ""}, "sentence_type": "Interrogative sentence", "pos": {"nouns": {"Sleep": 1, "Sleeping pills": 1}, "verbs": {"Eat": 1, "Is it possible": 1}, "numerals": {"9": 1}}}, {"content": "How are you feeling after you're insomnia?", "word_count": 10, "label": "", "sentence_structure": "Subject鈥搗erb鈥搊bject", "subjective_objective": {"answer": "Subjective", "rationale": "Because insomnia is a mental state, and the mood (heartland) is the patient鈥檚 subjective feeling and experience."}, "sentence_sentiment": {"answer": "Negative", "rationale": "Because insomnia can lead to bad emotions and heart."}, "language_style": {"answer": "Tactful and warm", "rationale": "The entry is more euphemistic, using the words \"memorning\" and \"how\" to ask the respondents about their insomnia, without using direct, hard language to express the problem and give people a sense of affection and peace."}, "time_dimension": {"answer": "Present", "rationale": "The entry asks the testee about the current feeling or mental state of insomnia, and the temporal dimension is now. The keyword \"mature\" and \"how\" point to the emotional state of the testee."}, "sentence_type": "Interrogative sentence", "pos": {"nouns": {"Insomnia": 1, "The mood": 1, "Heartland": 1}, "numerals": {"10": 1}}}, {"content": "Do you think that sleep is enough?", "word_count": 16, "label": "", "sentence_structure": "Subject鈥搗erb鈥搊bject", "subjective_objective": {"answer": "Subjective", "rationale": "Because it is the patient ' s subjective feeling and evaluation of his or her sleep condition."}, "sentence_sentiment": {"answer": "Negative", "rationale": "Because the expression \"sleep time\" in the entries is negative, it expresses a sense of satisfaction with the quality of sleep."}, "language_style": {"answer": "Calm and direct", "rationale": "The entry uses a direct question-and-question phrase to ask the other side about the quality of sleep and to identify clearly possible problems, without excessive rhetorical or emotional rendering, and with more direct and objective tone."}, "time_dimension": {"answer": "Present", "rationale": ""}, "sentence_type": "Interrogative sentence", "pos": {"nouns": {"you": 1, "Normal": 1, "Sleep": 2, "Time": 1}, "verbs": {"I think": 1, "Enough": 2, "Not enough": 1}, "adjectives": {"I don't have enough sleep time": 1}, "numerals": {"1": 4}}}, {"content": "2. Do you feel fully rested after sleep? (Imperate sleep quality)", "word_count": 22, "label": "", "sentence_structure": "Subject鈥搗erb鈥搊bject", "subjective_objective": {"answer": "Subjective", "rationale": "Because this question asks about patients ' subjective feelings and assessment of their sleep, it looks at subjective ideas of patients and is therefore subjective."}, "sentence_sentiment": {"answer": "Negative", "rationale": "Because the description of \"sleep quality\" in the entry is negative."}, "language_style": {"answer": "Tactful and warm", "rationale": "The entry is more euphemistic and sensitive to each other鈥檚 feelings than to point out the problem directly or criticize the other. It leads the interviewee to reflect on the quality of his sleep in a way that leads him to ask questions and gives him an alternative option, 鈥渘ot very good sleep鈥? This expression respects the subject鈥檚 subjective feelings and is effective in collecting useful information."}, "time_dimension": {"answer": "Present", "rationale": ""}, "sentence_type": "Interrogative sentence", "pos": {"nouns": {"Sleep": 2, "Quality": 1}, "verbs": {"I think": 1, "Rest": 1, "Pass": 1}, "adjectives": {"Full": 1, "High": 1}, "numerals": {"2": 1}}}, {"content": "3. You've slept at night, do you dozed during the day? (Inadequate sleep or awakening)", "word_count": 24, "label": "", "sentence_structure": "Subject鈥搗erb鈥搊bject", "subjective_objective": {"answer": "Objective", "rationale": "Because the question is about the patient鈥檚 sleep condition, which can be determined by objective observation and documentation, not by the patient鈥檚 subjective perception. For example, doctors can judge whether the patient is not sleeping or awakening, by examining the patient鈥檚 sleep patterns, sleep time and quality, which are objective data."}, "sentence_sentiment": {"answer": "Negative", "rationale": "Because the behaviour described in the entry \"sleeping\" is usually considered negative, as it may affect the behaviour of a person in his or her daily life and work."}, "language_style": {"answer": "Calm and direct", "rationale": "The entry is based on an objective and clear tone, which is more straightforward, and which asks whether the other party has a problem of daytime nap, without excessive emotional colours or qualifiers."}, "time_dimension": {"answer": "Present", "rationale": "The entry asks about the behaviour of the person tested during the day, i.e. whether he/she will be tired and sleepy during the day."}, "sentence_type": "Interrogative sentence", "pos": {"nouns": {"Night": 1, "I'm not sure": 1, "Oh, my God": 1, "Daytime": 1, "Sleep": 1, "I'm not sleeping enough": 1, "I'm not awake enough": 1}, "verbs": {"Already": 1, "I slept": 1, "Is it possible": 1, "Fight": 1, "Enough": 1, "I'm not sure": 1, "Wake up": 1, "That's enough": 1}, "adverbs": {"Night": 1, "Already": 1, "Daytime": 1, "Enough": 1}, "numerals": {"3": 1}, "classifiers": {"individual": 1}, "pronouns": {"you": 1}}}, {"content": "How many hours do you sleep at night?", "word_count": 18, "label": "", "sentence_structure": "Subject鈥搗erb鈥搊bject", "subjective_objective": {"answer": "Objective", "rationale": "Sleep time is about the objective facts."}, "sentence_sentiment": {"answer": "Neutral", "rationale": "Because the entry merely asks the interviewee about the length of sleep and does not involve any emotional colour."}, "language_style": {"answer": "Calm and direct", "rationale": ""}, "time_dimension": {"answer": "Present", "rationale": "The entry asks about the current daily living habits of the person being tested and can therefore be judged as now."}, "sentence_type": "Interrogative sentence", "pos": {"nouns": {"Night": 1, "Hours": 2, "Sleep": 1, "Time": 1}, "verbs": {"Yes": 1, "Sleep": 1}, "adverbs": {"About": 1}, "numerals": {"How many": 1}, "classifiers": {"individual": 1}, "pronouns": {"you": 1}}}, {"content": "Do you have any sleep difficulties?", "word_count": 12, "label": "", "sentence_structure": "Subject鈥搗erb鈥搊bject", "subjective_objective": {"answer": "Objective", "rationale": "Because the question is a factual description of the patient ' s sleep condition, there is no need for subjective judgement or perception by the patient."}, "sentence_sentiment": {"answer": "Negative", "rationale": "Because the \"sleeping difficulties\" act is negative."}, "language_style": {"answer": "Calm and direct", "rationale": "The language style is usually considered objective, calm and direct, and is more suitable for use in a psychological survey of a formal or scientific nature."}, "time_dimension": {"answer": "Past", "rationale": ""}, "sentence_type": "Interrogative sentence", "pos": {"nouns": {"5": 1, "Questionnaire": 1, "Entry": 1, "Sleeping difficulties": 1}, "adjectives": {"have": 1}, "numerals": {"Do you have sleep difficulties": 1}}}, {"content": "6. Are you awake in the middle of your sleep?", "word_count": 14, "label": "", "sentence_structure": "Subject鈥搗erb鈥搊bject", "subjective_objective": {"answer": "Objective", "rationale": "Because \"wake\" and \"sleep disorder\" are the patient's sleep expression, which is an objective fact. It is subjective to examine how the patient perceives and feels these phenomena."}, "sentence_sentiment": {"answer": "Negative", "rationale": "Because the act of \"wake in the middle of sleep\" in the entry is negative."}, "language_style": {"answer": "Calm and direct", "rationale": "The entry is in a simple and clear language, asking whether the other party is easily awake during sleep, is not using excessive emotional colours or rhetorical techniques and is more objective and calm."}, "time_dimension": {"answer": "Past", "rationale": "The entry asks the person who was tested for past sleep, the keywords being \"intersight\" and \"whether or not to wake up\" point to what has happened and happened in the past. The temporal dimension of the article therefore determines the past."}, "sentence_type": "Interrogative sentence", "pos": {"nouns": {"Questionnaire": 1, "Entry": 1, "Sleep": 2, "Centre": 1}, "verbs": {"Is it possible": 1, "Wake up": 1, "It's not steady": 1}, "numerals": {"6": 1}}}, {"content": "7. Are you having trouble sleeping again?", "word_count": 12, "label": "", "sentence_structure": "Subject鈥搗erb鈥搊bject", "subjective_objective": {"answer": "Subjective", "rationale": "Because it is a subjective feeling that patients feel and experience their sleep."}, "sentence_sentiment": {"answer": "Negative", "rationale": "Because \"hard to sleep\" and \"early awakening\" are both negative conditions associated with the deterioration of sleep quality, the expression in this article is negative."}, "language_style": {"answer": "Tactful and warm", "rationale": "The entry is more personal and interactive in its expression, by a gentle and inviting tone, rather than by direct statement or order. \""}, "time_dimension": {"answer": "Present", "rationale": "The phrases \"awakening\" and \"difficult to sleep\" indicate the experience and feelings of the person being tested in the current state, so the entry should be judged as now."}, "sentence_type": "Interrogative sentence", "pos": {"nouns": {"you": 1, "Wake up": 1, "Hard": 1, "Then go to sleep": 1, "(Awakening early)": 1}}}, {"content": "Do you dream many or are you often awakened by nightmares?", "word_count": 19, "label": "", "sentence_structure": "Subject鈥搗erb鈥搊bject", "subjective_objective": {"answer": "Objective", "rationale": "Because dreams are the objective facts of the patient's experience."}, "sentence_sentiment": {"answer": "Negative", "rationale": "Because \"dreams or nightmares awaken\" in the entry are negative experiences."}, "language_style": {"answer": "Tactful and warm", "rationale": ""}, "time_dimension": {"answer": "Present", "rationale": "The keyword \"is it\" indicates that it is asking what is happening, and the terms \"dreams\", \"dreams\" and \"night terrors\" are described in the general state of affairs. Thus, the temporal dimension of the article is now judged."}, "sentence_type": "Interrogative sentence", "pos": {"nouns": {"Questionnaire": 1, "Entry": 1, "Dreams": 2, "Nightmares": 1, "Night terror": 1}, "verbs": {"Is it possible": 1, "Dreams": 1, "I'm always in the mood": 1, "Wake up": 1}, "adverbs": {"or": 1, "Regular": 1}, "numerals": {"8": 1}}}, {"content": "9. Do you take sleeping pills? (Drinking)", "word_count": 15, "label": "", "sentence_structure": "Subject鈥搗erb鈥搊bject", "subjective_objective": {"answer": "Objective", "rationale": "It is an objective fact because the subject is to ask if the patient has been taking sleeping pills."}, "sentence_sentiment": {"answer": "Negative", "rationale": "Because sleeping pills are a side effect, overdependence can lead to resistance to the body and may have health effects. Therefore, the entry \"Do you take sleeping pills?\" is negative."}, "language_style": {"answer": "Calm and direct", "rationale": ""}, "time_dimension": {"answer": "Present", "rationale": "The temporal dimension of the entry is now judged because the behaviour of sleeping pills is now a state of affairs and is an inquiry into the current state."}, "sentence_type": "Interrogative sentence", "pos": {"nouns": {"Sleep": 1, "Sleeping pills": 1}, "verbs": {"Eat": 1, "Drug use": 1, "Situation": 1}, "numerals": {"9": 1}, "classifiers": {"individual": 1}}}, {"content": "10. What is your state of mind after insomnia? (Sleep attitudes and post-somnia psycho-psychosocial response)", "word_count": 22, "label": "", "sentence_structure": "Subject鈥搗erb鈥搊bject", "subjective_objective": {"answer": "Subjective", "rationale": "The idea is that \"how to sleep in the heart\" is the patient's subjective feeling, so the entry is viewed from the patient's subjective point of view. So it's \"subjective.\""}, "sentence_sentiment": {"answer": "Negative", "rationale": "Because the description of \"how the insomnia is insomnia\" is itself negative and implies that the interviewee may have a negative feeling because of insomnia."}, "language_style": {"answer": "Tactful and warm", "rationale": ""}, "time_dimension": {"answer": "Present", "rationale": "The keywords \"post-sleep\" and \"the state of mind\" point to the current psychological state, indicating that it is an investigation of the psychological response to insomnia."}, "sentence_type": "Interrogative sentence", "pos": {"nouns": {"10": 1, "you": 1, "Insomnia": 2, "Heartland": 1, "Sleeping attitude": 1, "Psychopsychosocial response": 1}, "verbs": {"How's that": 1, "have": 1}, "adverbs": {"Back": 1, "and": 1}, "numerals": {"10": 1, "2": 1}, "classifiers": {"individual": 1}, "pronouns": {"you": 1}}}]}

{"questionnaire_title": "Symptom self-assessment table (SCL-90)", "questionnaire_category": "Mental health", "item_count": 90, "emotion_shift_overall": "From negative to positive", "language_style_overall": "Neutral", "items": [{"content": "1. Headaches.", "word_count": 2, "label": "", "sentence_structure": "Subject鈥揷opula鈥損redicative", "subjective_objective": {"answer": "Objective", "rationale": "Because headaches are a symptom or signs that can be observed, not a subjective feeling or attitude of the patient."}, "sentence_sentiment": {"answer": "Negative", "rationale": "Because the symptoms of \"headache\" are usually considered negative."}, "language_style": {"answer": "Calm and direct", "rationale": "The language style of the entry is relatively calm and objective, and the interviewer is asked whether there are headaches, without using excessive rhetoric or emotional colours, and with a greater focus on access to information and data."}, "time_dimension": {"answer": "Past", "rationale": "The entry describes only the symptoms of headache and does not address the dimensions of time, so it can be judged to be past."}, "sentence_type": "Interrogative sentence", "pos": {"nouns": {"Headache": 1}}}, {"content": "2. Sensitized and insensitive.", "word_count": 9, "label": "", "sentence_structure": "Subject鈥搗erb鈥揷omplement", "subjective_objective": {"answer": "Subjective", "rationale": "Because the sensitivity and inactivity of the patient are their own psychological feelings and experiences, they are subjective."}, "sentence_sentiment": {"answer": "Negative", "rationale": "Because \"sensitization\" and \"insensitive\" are negative descriptions."}, "language_style": {"answer": "Tactful and warm", "rationale": "This is a more moderate expression, asking the interviewee whether he or she is experiencing nervous or uneasy situations, and avoiding direct accusations or evaluation of the other person鈥檚 problems."}, "time_dimension": {"answer": "Present", "rationale": "The entry \"now\" indicates the state or feeling that the person is experiencing, the keyword \"sensitized\" and \"insensitive\" describing the current emotional state of the person being tested."}, "sentence_type": "Interrogative sentence", "pos": {"nouns": {"You're a bit of a sane": 1, "鈾?In the heart 鈾?: 1, "Step on it": 1}, "verbs": {"Yes": 1, "Anxiety": 1}, "adjectives": {"Comparison": 1, "Obviously": 1}}}, {"content": "There are unnecessary ideas or words in the mind.", "word_count": 15, "label": "", "sentence_structure": "Subject鈥搗erb鈥揷omplement", "subjective_objective": {"answer": "Subjective", "rationale": "Because the idea in the mind is a subjective experience and feeling of a person, and belongs to the patient's own psychological state."}, "sentence_sentiment": {"answer": "Negative", "rationale": "Because the act of \"unnecessary thought or words circled\" in the entry is negative."}, "language_style": {"answer": "Tactful and warm", "rationale": "The entry is more adept and interactive when it is spoken in a gentle and inviting manner, rather than by direct statements or orders."}, "time_dimension": {"answer": "Present", "rationale": ""}, "sentence_type": "Affirmative declarative sentence", "pos": {"nouns": {"Brain": 1, "Thoughts": 1, "Words": 1, "Rotate": 1}, "verbs": {"have": 1, "Rotate": 1}, "numerals": {"3": 1}}}, {"content": "4. Dizziness and fainting.", "word_count": 5, "label": "", "sentence_structure": "Subject鈥搗erb鈥揷omplement", "subjective_objective": {"answer": "Subjective", "rationale": "The first is the subjective feeling and experience of people who are dizzy and faint."}, "sentence_sentiment": {"answer": "Negative", "rationale": "Because both dizziness and fainting are symptoms of physical discomfort, they are often associated with negative emotions or state."}, "language_style": {"answer": "Calm and direct", "rationale": "The language style of the entry is relatively calm and straightforward, and there is no excessive emotional qualification, mainly the description and question of symptoms, with the aim of enabling the respondents to answer the questions accurately and provide real data."}, "time_dimension": {"answer": "Present", "rationale": "The entry is for the purpose of asking if the person being tested is currently suffering from dizziness and fainting, and the time dimension is now judged."}, "sentence_type": "Affirmative declarative sentence", "pos": {"nouns": {"Dizziness": 1, "- I'm fainted": 1}}}, {"content": "5. Declining interest in heterosexuality.", "word_count": 8, "label": "", "sentence_structure": "Subject鈥搗erb鈥揷omplement", "subjective_objective": {"answer": "Subjective", "rationale": "Because \"reducing interest\" is a subjective feeling or attitude that reflects the experience and feelings of the patient in a given situation."}, "sentence_sentiment": {"answer": "Negative", "rationale": "Because the \"reducing interest in opposite sex\" in the entry is usually considered negative."}, "language_style": {"answer": "Calm and direct", "rationale": "The language style of the entry is a calm and direct type. It raises a question directly, without adding any rhetoric or emotional colour, or using euphemism to raise problems."}, "time_dimension": {"answer": "Present", "rationale": "The entry asks about the current changes in sexual orientation of the person tested, does not address the past or future time dimension and therefore the judgement is now."}, "sentence_type": "Affirmative declarative sentence", "pos": {"nouns": {"Heterosexual": 1, "Interest": 2, "Declining": 1}, "verbs": {"to": 1, "have": 1, "Declining": 1}, "numerals": {"5": 1}}}, {"content": "6. Full accountability.", "word_count": 7, "label": "", "sentence_structure": "Subject鈥搗erb鈥搊bject", "subjective_objective": {"answer": "Subjective", "rationale": "Because \"compensation\" is a subjective attitude or view that reflects the criticism and expectations of the patient towards others."}, "sentence_sentiment": {"answer": "Negative", "rationale": "Because the \"acquire\" act in the entry is negative."}, "language_style": {"answer": "Tactful and warm", "rationale": "The language style of the entry is more euphemistic and uses polite language to ask the other side whether they are easily dissatisfied or harmed by criticism and accusations. Such expression makes the interviewee more comfortable and respectful, and more willing to share their real thoughts and feelings."}, "time_dimension": {"answer": "Present", "rationale": "The word \"to\" in the entry indicates a reaction to a given situation, and \"compensation for full\" is the content of the description of such a reaction, so it does not clearly point in time to the past, present or future, but rather to a state or behaviour. So the time dimension of the questionnaire entry should be now."}, "sentence_type": "Negative declarative sentence", "pos": {"nouns": {"The others": 1, "Blame": 1, "Full": 1}, "verbs": {"to": 1, "Accountability": 1}}}, {"content": "Feels like someone else can control your mind.", "word_count": 11, "label": "", "sentence_structure": "Subject鈥揷opula鈥損redicative", "subjective_objective": {"answer": "Subjective", "rationale": "Because feeling controlled is the patient's subjective feeling."}, "sentence_sentiment": {"answer": "Negative", "rationale": "Because the feeling in the entry that \"someone else can control your mind\" is negative, it expresses the feeling of being controlled and violated by others, and it can lead to psychological stress and negative emotional reactions."}, "language_style": {"answer": "Tactful and warm", "rationale": "The entry is more euphemistic and respectful, using the term \"fear\" of subjective feelings, which allows respondents to express their thoughts and feelings more freely. At the same time, \"controlling your mind\" is a more sensitive topic, and using euphemism in language styles avoids undue pressure or discomfort on the interviewees."}, "time_dimension": {"answer": "Present", "rationale": "The entry is to ask the testee what he feels now, and the keyword is \"fear\" and \"controllable\", which both represent the current state or feeling and therefore judge it as now."}, "sentence_type": "Negative declarative sentence", "pos": {"nouns": {"you": 1, "Ideas": 1}, "verbs": {"I feel": 1, "Yes": 1, "Control": 1}}}, {"content": "8. Blame others for causing trouble.", "word_count": 8, "label": "", "sentence_structure": "Subject鈥搗erb鈥搊bject", "subjective_objective": {"answer": "Subjective", "rationale": "Because blaming others for their feelings and thoughts is the patient's own subjective attitude."}, "sentence_sentiment": {"answer": "Negative", "rationale": "Because \"criticizing people for making trouble\" is a negative emotional attitude and behavior."}, "language_style": {"answer": "Calm and direct", "rationale": "The language style of the entry is more straightforward, without any reticulation or emotional rendering, and is largely based on simple statements, which are obvious. This expression is very direct, clear and neutral, and suitable for use in, for example, questionnaires."}, "time_dimension": {"answer": "Present", "rationale": "The entry asks if the testee is currently responsible for causing trouble or mentalities, and therefore the temporal dimension is now judged."}, "sentence_type": "Imperative sentence", "pos": {"nouns": {"Blame": 1, "others": 1, "Trouble": 1}, "verbs": {"Manufacturing": 1}}}, {"content": "9. Forgetful is sexual.", "word_count": 4, "label": "", "sentence_structure": "Subject鈥揷opula鈥損redicative", "subjective_objective": {"answer": "Subjective", "rationale": "Because Forget Sex is a subjective assessment, reflecting the patient鈥檚 perception and feelings. While memory can be objectively tested, how it is evaluated is subjective."}, "sentence_sentiment": {"answer": "Negative", "rationale": "Because the expression \"forget sexuality\" in the entry is negative."}, "language_style": {"answer": "Tactful and warm", "rationale": ""}, "time_dimension": {"answer": "Present", "rationale": ""}, "sentence_type": "Negative declarative sentence", "pos": {"verbs": {"Forget it": 1}, "adjectives": {"Large": 1}}}, {"content": "Fear of being dressed and dressed in good manner.", "word_count": 15, "label": "", "sentence_structure": "Subject鈥揷opula鈥損redicative", "subjective_objective": {"answer": "Subjective", "rationale": "Because \"fear\" shows how people feel about their appearance, and it's a subjective attitude and emotional experience."}, "sentence_sentiment": {"answer": "Negative", "rationale": "Because the \"fear\" in the entry indicates a negative emotional state, indicating that the interviewee may be disturbed or unconfident about his or her appearance."}, "language_style": {"answer": "Tactful and warm", "rationale": "The entry is relatively euphemistic. It allows the respondents to share their concerns in a way that they are asked whether they are concerned, rather than directly ordering or asking them to answer. Moreover, the question is not in strong vocabulary or emotional colour, but in a more calm and objective tone. This makes the respondents feel more comfortable and relaxed, and more willing to express their feelings and ideas in a true way."}, "time_dimension": {"answer": "Present", "rationale": ""}, "sentence_type": "Imperative sentence", "pos": {"nouns": {"self": 1, "Clothing": 1, "Instrument": 1}, "verbs": {"I'm worried": 1, "and": 1, "Positive": 1}, "numerals": {"10": 1}}}, {"content": "11. Easier to worry and more excited.", "word_count": 7, "label": "", "sentence_structure": "Subject鈥揷opula鈥損redicative", "subjective_objective": {"answer": "Subjective", "rationale": "Because \"fear and excitement\" is the patient's own psychological state, and it's subjective."}, "sentence_sentiment": {"answer": "Negative", "rationale": "Because \"fear\" and \"excited\" are negative emotions, this emotional orientation is negative."}, "language_style": {"answer": "Calm and direct", "rationale": "The language style of the entry is a calm and straightforward one. It is a simple expression of the subject of asking each other whether they are susceptible to anxiety and excitement, and is not based on too many rhetorical or emotional expressions, which are more objective. Such a question is easier to answer with real answers, and it is also respectful and valued by the interviewee."}, "time_dimension": {"answer": "Present", "rationale": "The keyword is \"fear and excitement\", which refers to the current emotional state or mode of reaction of the person being tested, and therefore to the present."}, "sentence_type": "Affirmative declarative sentence", "pos": {"verbs": {"Easy": 1, "Trouble": 1, "Excited": 1}, "numerals": {"11": 1}}}, {"content": "12. chest pain.", "word_count": 2, "label": "", "sentence_structure": "Subject鈥搗erb鈥揷omplement", "subjective_objective": {"answer": "Objective", "rationale": "Because chest pain is an objective manifestation of the patient ' s state of health."}, "sentence_sentiment": {"answer": "Negative", "rationale": "Because \" chest pain\" is a symptom of disease, it usually causes physical discomfort and pain, and is therefore negative."}, "language_style": {"answer": "Calm and direct", "rationale": "The language style of the entry is more direct and concise, and there is no excessive rhetoric or emotional vocabulary, mainly to convey a clear question or message, and therefore to be a calm, direct style."}, "time_dimension": {"answer": "Present", "rationale": "In the past, the entry asked if the person tested had had experience of chest pain, and the temporal dimension was a past."}, "sentence_type": "Declarative sentence", "pos": {"nouns": {"- Chestaches": 1}}}, {"content": "Fear of vacant places or streets.", "word_count": 10, "label": "", "sentence_structure": "Subject鈥搗erb鈥揷omplement", "subjective_objective": {"answer": "Subjective", "rationale": "Because \"fear\" is an emotional experience and subjective feeling, fear and anxiety of a situation. And an empty place or street is an objective environment, so the entry looks at the patient's subjective feeling and is subjective."}, "sentence_sentiment": {"answer": "Negative", "rationale": "Because the word \"fear\" in the entry is negative."}, "language_style": {"answer": "Tactful and warm", "rationale": "The language style of the entry is more euphemistic. It uses a gentle, invitation-style tone to ask if the interviewee is afraid of an open place or street, which is more personal and interactive and makes it easier for the interviewee to share his feelings."}, "time_dimension": {"answer": "Present", "rationale": ""}, "sentence_type": "Affirmative declarative sentence", "pos": {"nouns": {"Places": 1, "Street": 1}, "verbs": {"Scared": 1, "Empty": 1}}}, {"content": "14. Feels like a decline in energy and activity.", "word_count": 13, "label": "", "sentence_structure": "Subject鈥搗erb鈥揷omplement", "subjective_objective": {"answer": "Subjective", "rationale": "Because the problem involves the feelings and experiences of the patient himself, it is a subjective examination."}, "sentence_sentiment": {"answer": "Negative", "rationale": "Because the descriptions of \"dive\" and \"slow\" in the entries are negative."}, "language_style": {"answer": "Calm and direct", "rationale": "The entry uses a simple and clear language and asks the interviewees whether they have experienced a decline in energy and activity in the recent past, with a more objective and neutral language style, and with no excessive rhetorical or emotional overturn."}, "time_dimension": {"answer": "Past", "rationale": "The entry asks about the current subjective feelings and experiences of the person being tested, and the temporal dimension is a past."}, "sentence_type": "Affirmative declarative sentence", "pos": {"nouns": {"- The energy": 1, "Activities": 1}, "verbs": {"Down": 1, "Slow down": 1}, "numerals": {"14": 1}}}, {"content": "15. To end their lives.", "word_count": 8, "label": "", "sentence_structure": "Subject鈥搗erb鈥搊bject", "subjective_objective": {"answer": "Subjective", "rationale": "Because \"want to end your life\" is the mind and attitude of the patient, and it's subjective."}, "sentence_sentiment": {"answer": "Negative", "rationale": "Because the \"end your life\" act is negative."}, "language_style": {"answer": "Calm and direct", "rationale": "This is a direct and frank way of making certain people uncomfortable, but it helps researchers to understand the mental health of the interviewee and provide the necessary support and assistance."}, "time_dimension": {"answer": "Past", "rationale": "The entry does not have a clear time reference or a landmark verb, but in the context of the context, it is asking the testee whether he or she has any idea or behavior that would want to end his or her life. This idea and behaviour may be a past experience or a state of emotion. Thus, it is judged as \"the past.\""}, "sentence_type": "Imperative sentence", "pos": {"nouns": {"Life": 1}, "verbs": {"I want to end it": 1}, "numerals": {"15": 1}}}, {"content": "16. No one heard the sound.", "word_count": 10, "label": "", "sentence_structure": "Subject鈥搗erb鈥揷omplement", "subjective_objective": {"answer": "Subjective", "rationale": "Because hearing voices that are not heard by others is a personal perception and experience that cannot be directly confirmed or denied by others, it is more concerned with the subjective perspective of the patient."}, "sentence_sentiment": {"answer": "Neutral", "rationale": "As the entry describes a phenomenon or experience, it does not involve any positive or negative emotions, attitudes or behaviour."}, "language_style": {"answer": "Tactful and warm", "rationale": "The entry is more personal and interactive when it is spoken in a gentle and inviting manner, rather than in direct statements or orders. Moreover, the language style of the article is relatively simple and not too much reverb or complex to be understood and answered by the interviewees."}, "time_dimension": {"answer": "Present", "rationale": "The entry is an inquiry into whether the person who was tested has experienced \"hearing voices that are not heard by others.\" The dimension of time, because it is not clear how time period, makes it difficult to judge whether the entry is past, present or future. But, in terms of context, the experience should have taken place, because it involves a sense, not an expectation or a prediction."}, "sentence_type": "Affirmative declarative sentence", "pos": {"nouns": {"Sound": 1, "The others": 1}, "verbs": {"Yes": 1, "Give": 1}, "adverbs": {"not": 1, "Present": 1}, "pronouns": {"Here": 1}}}, {"content": "17. Wright.", "word_count": 2, "label": "", "sentence_structure": "Subject鈥搗erb鈥揷omplement", "subjective_objective": {"answer": "Objective", "rationale": "Because shaking is a physical reaction or a symptom, it is an objective situation."}, "sentence_sentiment": {"answer": "Negative", "rationale": "Because \"shock\" is usually a sign of fear, stress, etc."}, "language_style": {"answer": "Calm and direct", "rationale": "The term \"shocking\" in this title is a very straightforward expression of the behaviour of the person under investigation, without excessive emotional accoutrements or euphemisms, and therefore the language style of the entry on the questionnaire can be judged as being straightforward and straightforward."}, "time_dimension": {"answer": "Present", "rationale": "The keyword is \"shock\" and it means the state or the feeling, so the temporal dimension is now."}, "sentence_type": "Imperative sentence", "pos": {"verbs": {"# Wresh #": 1}}}, {"content": "18. It was felt that most people were not trustworthy.", "word_count": 11, "label": "", "sentence_structure": "Subject鈥揷opula鈥損redicative", "subjective_objective": {"answer": "Subjective", "rationale": "Because it is a subjective feeling and perception that most people cannot be trusted."}, "sentence_sentiment": {"answer": "Negative", "rationale": "Because the \"most people can't trust\" view is negative."}, "language_style": {"answer": "Calm and direct", "rationale": "The entry is simple and clear and the interviewee is asked whether the majority of the respondents consider it untrustworthy and do not have too many emotional acquis or euphemisms, so that the language style is more calm and straightforward."}, "time_dimension": {"answer": "Past", "rationale": "The entry asks the views and feelings of the testees about the majority of the population, without specifying whether it is the past, the present or the future, but the word \"felt\" implies a continuing subjective feeling and can therefore be judged as the past."}, "sentence_type": "Negative declarative sentence", "pos": {"nouns": {"person": 1, "Trust": 1}, "verbs": {"I feel": 1}, "adjectives": {"Most of them": 1, "Both": 1}, "adverbs": {"No way": 1}, "numerals": {"18": 1}}}, {"content": "19. The appetite is bad.", "word_count": 4, "label": "", "sentence_structure": "Subject鈥揷opula鈥損redicative", "subjective_objective": {"answer": "Subjective", "rationale": "Because \"bad appetite\" is a self-perception symptom of a patient, and it's part of the subjective sense."}, "sentence_sentiment": {"answer": "Negative", "rationale": "Because \"bad appetite\" is a negative physical condition."}, "language_style": {"answer": "Tactful and warm", "rationale": "The entry uses a more euphemistic expression to ask the other side whether there is a bad appetite, without direct challenge or order, and gives a sense of affection and concern. At the same time, the term \"bad appetite\" is more subtle and does not cause excessive pain or unnecessary stress."}, "time_dimension": {"answer": "Past", "rationale": "The entry is for the purpose of asking the testee whether he has had a bad appetite at some point in the past and can therefore be judged to be past."}, "sentence_type": "Imperative sentence", "pos": {"verbs": {"-Adulge": 1, "Okay": 1}}}, {"content": "20. Easy to cry.", "word_count": 4, "label": "", "sentence_structure": "Subject鈥揷opula鈥損redicative", "subjective_objective": {"answer": "Subjective", "rationale": "Because the ease of crying is an emotional response, a subjective feeling and experience of the individual."}, "sentence_sentiment": {"answer": "Negative", "rationale": "The basis is that the act of \"cry easy\" is usually considered negative or negative."}, "language_style": {"answer": "Calm and direct", "rationale": "The language style of the entry is relatively simple, with no excessive use of rhetorical techniques or emotional rendering, and is more straightforward and obvious."}, "time_dimension": {"answer": "Present", "rationale": "According to the keyword \"Easier to weep\", the entry describes the current behaviour or state of the person being tested, and the temporal dimension is now judged."}, "sentence_type": "Affirmative declarative sentence", "pos": {"verbs": {"Easy": 1, "Crying": 1}, "adjectives": {"Easy": 1}}}, {"content": "21. Homosexuality is shy and uncomfortable.", "word_count": 13, "label": "", "sentence_structure": "Subject鈥搗erb鈥揷omplement", "subjective_objective": {"answer": "Subjective", "rationale": "Because shyness is a subjective feeling, not an objective fact."}, "sentence_sentiment": {"answer": "Negative", "rationale": "Because the emotional reaction of being shy in the entries is negative."}, "language_style": {"answer": "Tactful and warm", "rationale": "The language style of the entry is more euphemistic, using terms such as \"coherent\" and \"weakness\" and expressing in a gentle and understandable way the subject of asking about the shyness or discomfort of the other in his or her dealings with the opposite sex. This expression is neither stressful nor effective in directing the interviewee to share his or her feelings and experiences."}, "time_dimension": {"answer": "Past", "rationale": ""}, "sentence_type": "Affirmative declarative sentence", "pos": {"nouns": {"Same-sex": 1, "Get along": 1, "Shy": 1, "I'm not comfortable": 1}, "verbs": {"I feel": 1, "Get along": 1}, "adjectives": {"Shy": 1, "I'm not comfortable": 1}, "numerals": {"21": 1}}}, {"content": "22. Feels deceived, trapped or someone wants to catch you.", "word_count": 15, "label": "", "sentence_structure": "Subject鈥搗erb鈥揷omplement", "subjective_objective": {"answer": "Subjective", "rationale": "Because \"fearing to be deceived, being caught in the trap or someone trying to catch you\" is the patient's subjective perception and judgment of the current situation."}, "sentence_sentiment": {"answer": "Negative", "rationale": "Because the expression of the purpose of the article contains negative emotional expressions such as \"fear of being deceived, bemiculated\" and expresses a negative emotion and attitude."}, "language_style": {"answer": "Calm and direct", "rationale": "The language style of the entry is more direct and objective, and there is no excessive rhetorical or emotional rendering, mainly through simple and clear language, so that the interviewee can better understand and answer the questions."}, "time_dimension": {"answer": "Present", "rationale": "Now the entry uses the current state of affairs ( \"fEEL\") and describes a current feeling and experience, so it is judged to be the present."}, "sentence_type": "Affirmative declarative sentence", "pos": {"nouns": {"I feel": 1, "Swindled": 1, "A trap": 1, "person": 1}, "verbs": {"Yes": 1, "Hold on": 1}, "numerals": {"22": 1}}}, {"content": "23. Suddenly, fear was felt without reason.", "word_count": 11, "label": "", "sentence_structure": "Subject鈥搗erb鈥揷omplement", "subjective_objective": {"answer": "Subjective", "rationale": "This is often the emotional experience of the patient, and it is a subjective examination."}, "sentence_sentiment": {"answer": "Negative", "rationale": "Because the \"unnecessary fear\" in the entry is negative."}, "language_style": {"answer": "Tactful and warm", "rationale": "The language style of the entry is more euphemistic. It does not ask whether the respondents are often afraid, but rather whether they have had \"a sudden fear without reason.\" It is more soft and friendly, and it is a way of feeling respected and understood."}, "time_dimension": {"answer": "Present", "rationale": "The entry describes an emotional experience, with no clear time limits, and can therefore be judged as now."}, "sentence_type": "Declarative sentence", "pos": {"nouns": {"Reason": 1, "Yes": 1}, "verbs": {"I feel": 1, "Suddenly": 1, "For no reason": 1}, "adjectives": {"Scared": 1}, "numerals": {"23": 1}}}, {"content": "24. He was not able to control his temper.", "word_count": 10, "label": "", "sentence_structure": "Subject鈥搗erb鈥揷omplement", "subjective_objective": {"answer": "Subjective", "rationale": "Because a temper is a emotional reaction within a person, it cannot exist out of the person's consciousness."}, "sentence_sentiment": {"answer": "Negative", "rationale": "Because the \"uncontrollable temper\" in the entry is negative."}, "language_style": {"answer": "Tactful and warm", "rationale": ""}, "time_dimension": {"answer": "Present", "rationale": ""}, "sentence_type": "Negative declarative sentence", "pos": {"nouns": {"Questionnaire": 1, "Entry": 1, "self": 1, "- Grumpy": 1}, "verbs": {"Control": 1, "Let's go": 1}, "adverbs": {"No, I can't": 1}, "numerals": {"24": 1}}}, {"content": "25. Fear of going out alone.", "word_count": 5, "label": "", "sentence_structure": "Subject鈥揷opula鈥損redicative", "subjective_objective": {"answer": "Subjective", "rationale": "Because the word \"fear\" expresses the fear and fear of being out alone, which is a subjective emotional experience."}, "sentence_sentiment": {"answer": "Negative", "rationale": "Because fear of going out alone is a fear and a sense of unease, and it's negative."}, "language_style": {"answer": "Tactful and warm", "rationale": "The entry is more personal and interactive when it is spoken in a gentle and inviting tone, rather than in direct statements or orders. At the same time, the issue is one of more neutral language styles, avoiding too many emotional words or strong tone, making it easier for respondents to accept and share their real ideas."}, "time_dimension": {"answer": "Present", "rationale": "The keyword \"fearing to go out alone\" is an expression of current fear of emotion and behaviour, so the entry describes the current state of mind and behaviour of the person being tested."}, "sentence_type": "Negative declarative sentence", "pos": {"nouns": {"Questionnaire": 1, "Entry": 1, "Alone": 1, "Out": 1}, "verbs": {"Yes": 1}, "numerals": {"25": 1}}}, {"content": "26. Too often, it was self-inflicted.", "word_count": 6, "label": "", "sentence_structure": "Subject鈥搗erb鈥揷omplement", "subjective_objective": {"answer": "Subjective", "rationale": "Because blame is a subjective emotion and attitude."}, "sentence_sentiment": {"answer": "Negative", "rationale": "Because the \"criticizing\" act in the entry is negative."}, "language_style": {"answer": "Tactful and warm", "rationale": ""}, "time_dimension": {"answer": "Present", "rationale": "The keyword \"always blame yourself\" indicates that it is a customary behaviour or mentality that judge the dimensions of time as now."}, "sentence_type": "Negative declarative sentence", "pos": {"verbs": {"Blame": 1, "self": 1}, "adverbs": {"Regular": 1}, "numerals": {"26": 1}}}, {"content": "27. A back ache.", "word_count": 2, "label": "", "sentence_structure": "Subject鈥搗erb鈥揷omplement", "subjective_objective": {"answer": "Objective", "rationale": "Because of the pain in the back, it's an objective fact that patients experience."}, "sentence_sentiment": {"answer": "Neutral", "rationale": "Because the entry describes only a physical condition and does not express a positive or negative attitude, emotional or behavioural orientation."}, "language_style": {"answer": "Calm and direct", "rationale": ""}, "time_dimension": {"answer": "Past", "rationale": "The entry describes only some symptoms or experiences of the person tested and does not involve future behavioural or mental changes, which can be judged as past entries."}, "sentence_type": "Affirmative declarative sentence", "pos": {"nouns": {"My back hurts": 1}}}, {"content": "28. It was difficult to fulfil the mandate.", "word_count": 8, "label": "", "sentence_structure": "Subject鈥揷opula鈥損redicative", "subjective_objective": {"answer": "Subjective", "rationale": "It is a subjective feeling or attitude to feel that it is difficult to fulfil its mandate."}, "sentence_sentiment": {"answer": "Negative", "rationale": "Because \"hard to do\" is a difficult, frustrated or powerless feeling, and it's negative."}, "language_style": {"answer": "Calm and direct", "rationale": "The entry is a direct indication of the subject matter of the survey, namely, that the respondent would, in certain circumstances, feel the difficulty of completing the task, and that the language style was more direct and objective."}, "time_dimension": {"answer": "Past", "rationale": ""}, "sentence_type": "Negative declarative sentence", "pos": {"nouns": {"Questionnaire": 1, "Entry": 1, "Tasks": 1}, "verbs": {"I feel": 1, "Completed": 1}, "adjectives": {"Hard": 1}, "numerals": {"28": 1}}}, {"content": "29. Feels lonely.", "word_count": 4, "label": "", "sentence_structure": "Subject鈥揷opula鈥損redicative", "subjective_objective": {"answer": "Subjective", "rationale": "Because feeling alone is a subjective emotional experience, it depends on the individual ' s feelings and perceptions."}, "sentence_sentiment": {"answer": "Negative", "rationale": "Because the \"fear alone\" sentiment in the entry is negative."}, "language_style": {"answer": "Tactful and warm", "rationale": "The language style of the entry is euphemistic, and it guides the respondents to think and share their feelings in a question-and-answer manner, rather than directly expressing the demeaning or judgment of loneliness. Moreover, the more specific descriptive term \"single\" is used to help the respondents to better understand the meaning and scope of the problem."}, "time_dimension": {"answer": "Present", "rationale": "The keyword is \"fear\", which indicates the current feelings or state of the subject, and it can therefore be judged that the entry is asking the subject about the current feelings and experiences."}, "sentence_type": "Affirmative declarative sentence", "pos": {"nouns": {"Loneliness": 1}, "verbs": {"I feel": 1}}}, {"content": "30. Disturbing.", "word_count": 4, "label": "", "sentence_structure": "Subject鈥揷opula鈥損redicative", "subjective_objective": {"answer": "Subjective", "rationale": "Because \"suffering\" is an emotional experience, a patient's subjective feeling and emotional reaction."}, "sentence_sentiment": {"answer": "Negative", "rationale": "Because the entry \"suffering\" expresses a negative emotional attitude."}, "language_style": {"answer": "Calm and direct", "rationale": ""}, "time_dimension": {"answer": "Present", "rationale": "The keyword \"distressed\" shows the current emotional state of the subject, and it is therefore judged to be now."}, "sentence_type": "Affirmative declarative sentence", "pos": {"verbs": {"I feel": 1, "I'm bored": 1}}}, {"content": "31. Excessive concern.", "word_count": 4, "label": "", "sentence_structure": "Subject鈥搗erb鈥揷omplement", "subjective_objective": {"answer": "Subjective", "rationale": "Because excessive fear is a subjective feeling and attitude."}, "sentence_sentiment": {"answer": "Negative", "rationale": "Because the \"excessive worry\" act in the entry is negative."}, "language_style": {"answer": "Tactful and warm", "rationale": "The entry is more personal and interactive by speaking in a gentle and inviting manner, rather than by direct statement or order. At the same time, it expresses concern and respect for the person under investigation, avoiding possible negative emotions and resistance."}, "time_dimension": {"answer": "Past", "rationale": ""}, "sentence_type": "Negative declarative sentence", "pos": {"nouns": {"That's too much": 1, "Fear": 1}}}, {"content": "32. There is no interest in things.", "word_count": 7, "label": "", "sentence_structure": "Subject鈥揷opula鈥損redicative", "subjective_objective": {"answer": "Subjective", "rationale": "Because the hobby is the patient ' s personal feeling and attitude, it is viewed from the patient ' s subjective point of view."}, "sentence_sentiment": {"answer": "Negative", "rationale": "Because the \"not interested in things\" attitude in the entry is negative."}, "language_style": {"answer": "Calm and direct", "rationale": "The language style of the entry is straightforward, as it is a simple and straightforward expression of a query about the low interest of the other party and does not add too many rhetorical or emotional colours."}, "time_dimension": {"answer": "Present", "rationale": "The key word is \"not interested in things,\" and that feeling or experience should be in the state of things."}, "sentence_type": "Negative declarative sentence", "pos": {"verbs": {"to": 1, "Sensor": 1, "not": 1, "Hing": 1, "Fun": 1}, "adjectives": {"Yes": 1, "Objects": 1}, "adverbs": {"very": 1}, "numerals": {"32": 1}}}, {"content": "33. Fear.", "word_count": 4, "label": "", "sentence_structure": "Subject鈥揷opula鈥損redicative", "subjective_objective": {"answer": "Subjective", "rationale": "Because fear is a subjective feeling and experience, determined by the patient鈥檚 own emotional state, not by objective facts or circumstances. While fear may be associated with objective factors (such as threats, dangers, etc.), it itself remains a subjective feeling or emotional reaction. So, in this case, \"fearing\" is a subjective questionnaire entry."}, "sentence_sentiment": {"answer": "Negative", "rationale": "Because \"fear\" is usually considered negative."}, "language_style": {"answer": "Calm and direct", "rationale": "The entry describes the possible state of the person ' s feelings in a direct and concise manner, without excessive emotional or rhetorical refinements and renderings, and therefore the language style is more incriminating towards calmness and directness."}, "time_dimension": {"answer": "Past", "rationale": "The entry asks about the testee ' s past feelings, and the temporal dimension is the past."}, "sentence_type": "Affirmative declarative sentence", "pos": {"verbs": {"I feel": 1, "Scared": 1}}}, {"content": "34. My feelings are vulnerable.", "word_count": 10, "label": "", "sentence_structure": "Subject鈥搗erb鈥揷omplement", "subjective_objective": {"answer": "Subjective", "rationale": "Because \"vulnerable\" is an emotional experience or subjective feeling."}, "sentence_sentiment": {"answer": "Negative", "rationale": "Because the expression \"emotional vulnerability\" in the entry is negative."}, "language_style": {"answer": "Tactful and warm", "rationale": "The entry uses a more euphemistic tone, which neither expresses the emotional state of the other too directly nor uses too negative or harsh language. This expression is more comfortable and acceptable to the person under investigation and is more likely to receive a true and accurate answer."}, "time_dimension": {"answer": "Present", "rationale": "The entry describes the current subjective state of feeling and emotion, i.e., the vulnerability of \"me\", and therefore the temporal dimension is now judged."}, "sentence_type": "Affirmative declarative sentence", "pos": {"nouns": {"Emotion": 1, "Hurt": 1}, "verbs": {"Yes": 1, "Present": 1}, "adjectives": {"Easy": 1, "Present": 1}, "numerals": {"34": 1}, "pronouns": {"I": 1}}}, {"content": "35. Your thoughts are known to others.", "word_count": 11, "label": "", "sentence_structure": "Subject鈥搗erb鈥搊bject", "subjective_objective": {"answer": "Subjective", "rationale": "Because it is the patient ' s knowledge and feelings of his own private thoughts that are examined from a subjective perspective."}, "sentence_sentiment": {"answer": "Neutral", "rationale": "Because the entry does not have any emotional color."}, "language_style": {"answer": "Tactful and warm", "rationale": "By asking the other party whether they feel that their private thoughts will be understood, the entry leads the interviewee to think and express his/her views rather than direct orders or statements, and therefore the language style is more euphemismal."}, "time_dimension": {"answer": "Present", "rationale": ""}, "sentence_type": "Negative declarative sentence", "pos": {"nouns": {"The others": 1, "you": 1, "I'm not sure": 1}, "verbs": {"I can tell": 1}, "adverbs": {"Yes": 1}, "numerals": {"35": 1}}}, {"content": "36. Feels that you are not understood by others and do not feel sympathy for you.", "word_count": 12, "label": "", "sentence_structure": "Subject鈥搗erb鈥揷omplement", "subjective_objective": {"answer": "Subjective", "rationale": "It is a subjective feeling and experience to feel that others do not understand themselves and do not feel sympathetic to themselves."}, "sentence_sentiment": {"answer": "Negative", "rationale": "Because the words \"ununderstanding\" and \"unsympathetic\" in the entries have a negative emotional connotation, indicating that the respondents feel the indifference and alienation of others and are therefore negative."}, "language_style": {"answer": "Tactful and warm", "rationale": "The entry is more personal and interactive when it is spoken in a gentle and inviting tone, rather than in direct statements or orders. At the same time, it does not use too harsh or negative language, but rather asks the interviewees in a more active way to share their feelings."}, "time_dimension": {"answer": "Past", "rationale": "The entry is for the testee's feelings or experiences at a certain point in time, and \"fear\" and \"not understand you not to feel sorry for\" are all descriptions of past experiences or feelings. The time dimension of this questionnaire item is therefore judged to be past."}, "sentence_type": "Negative declarative sentence", "pos": {"nouns": {"Questionnaire Entry": 1, "person": 2, "Compassion": 1}, "verbs": {"I feel": 1, "Understood": 1, "I don't understand": 1, "No sympathy": 1}, "adverbs": {"36": 1, "Down there": 1}, "numerals": {"36": 1}, "pronouns": {"you": 2}}}, {"content": "37. It is felt that people are not friendly to you and do not like you.", "word_count": 13, "label": "", "sentence_structure": "Subject鈥搗erb鈥揷omplement", "subjective_objective": {"answer": "Subjective", "rationale": "The first is that the first step is to make the first step towards a better future."}, "sentence_sentiment": {"answer": "Negative", "rationale": "Because it expresses a negative feeling that people are not friendly and do not like themselves."}, "language_style": {"answer": "Tactful and warm", "rationale": "The language style of the entry is relatively warm, because it uses the term \"fear\" to express the problems that the person under investigation may feel. This is more personal and interactive than direct statements or orders."}, "time_dimension": {"answer": "Present", "rationale": "The entry indicates the current feelings and experiences of the testees, the keywords being \"fear\" and \"unfriendly\", which are all terms relevant to the current situation and are therefore judged to be the present."}, "sentence_type": "Interrogative sentence", "pos": {"nouns": {"Questionnaire Entry": 1, "person": 2, "you": 2, "Friendly": 1, "Yeah": 1}, "verbs": {"I feel": 1, "to": 1, "not": 1, "I don't like it": 1}, "adjectives": {"of": 4}, "numerals": {"37": 1}}}, {"content": "38. The slowness of the process must be ensured.", "word_count": 15, "label": "", "sentence_structure": "Subject鈥搗erb鈥揷omplement", "subjective_objective": {"answer": "Subjective", "rationale": "Because \"doing things slowly\" is an individual way of acting and a choice of attitude that reflects patients' subjective perceptions of their behaviour."}, "sentence_sentiment": {"answer": "Negative", "rationale": "Because \"slowly\" is a negative act."}, "language_style": {"answer": "Calm and direct", "rationale": "The language style of the entry is relatively calm and objective, and there is no excessive accrimation or emotional colour. It conveys a point or requirement that things be done slowly to ensure accuracy. This style is usually appropriate for situations where clarity, specificity and rigour are required.\""}, "time_dimension": {"answer": "Present", "rationale": "The entry describes a customary state of conduct, that is, the need to be careful and to ensure accuracy when doing things, which may have occurred in the past but which continues in the present. Thus, the judgement is \"now\"."}, "sentence_type": "Affirmative declarative sentence", "pos": {"nouns": {"Yes": 1, "Promise": 1}, "verbs": {"Do it": 2, "It's slow": 1, "Promised to do it": 1}, "adjectives": {"It's slow": 1, "Correct": 1}, "numerals": {"38": 1}}}, {"content": "39. Heart rate is high.", "word_count": 6, "label": "", "sentence_structure": "Subject鈥揷opula鈥損redicative", "subjective_objective": {"answer": "Subjective", "rationale": "The basis for the judgement: the heart rate is a strong feeling or experience, which is a symptom of subjective experience."}, "sentence_sentiment": {"answer": "Negative", "rationale": "Because \"the heart beats hard\" is a physical discomfort, a negative emotional experience."}, "language_style": {"answer": "Tactful and warm", "rationale": "The entry uses euphemism, not too directly describing the heartbeat of the person under investigation, but rather a question-and-answer expression. This language style is more likely to trigger resonance and motivation among the person under investigation."}, "time_dimension": {"answer": "Present", "rationale": ""}, "sentence_type": "Affirmative declarative sentence", "pos": {"verbs": {"Heart rate": 1, "That's great": 1}, "adverbs": {"very": 1}}}, {"content": "40. Disgusting or stomach problems.", "word_count": 8, "label": "", "sentence_structure": "Subject鈥搗erb鈥揷omplement", "subjective_objective": {"answer": "Subjective", "rationale": "Because nausea or stomach discomfort is usually the subjective feeling and experience of the patient. While this may be an objective situation caused by a disease or external factors, when filling out a questionnaire, the patient needs to answer the question in the light of his or her subjective feelings, and therefore is subjectively viewed."}, "sentence_sentiment": {"answer": "Negative", "rationale": "Because \"disgusting or stomach discomfort\" is a symptom of physical discomfort, it is usually considered negative."}, "language_style": {"answer": "Tactful and warm", "rationale": ""}, "time_dimension": {"answer": "Present", "rationale": ""}, "sentence_type": "Imperative sentence", "pos": {"nouns": {"Gross": 1, "Stomach": 1, "I'm not feeling well": 1}, "numerals": {"40": 1}}}, {"content": "41. It was not as good as others.", "word_count": 7, "label": "", "sentence_structure": "Subject鈥揷opula鈥損redicative", "subjective_objective": {"answer": "Subjective", "rationale": "Because it is not as much as others that it is a subjective assessment and judgement."}, "sentence_sentiment": {"answer": "Negative", "rationale": "Because the entry \"did not feel like anyone else\" expressed a negative emotion and attitude."}, "language_style": {"answer": "Tactful and warm", "rationale": ""}, "time_dimension": {"answer": "Past", "rationale": ""}, "sentence_type": "Negative declarative sentence", "pos": {"nouns": {"Questionnaire": 1, "Entry": 1, "person": 1}, "verbs": {"I feel": 1, "Not as good as it is": 1, "Others": 1}, "numerals": {"41": 1}}}, {"content": "42. Muscle acid.", "word_count": 4, "label": "", "sentence_structure": "Subject鈥揷opula鈥損redicative", "subjective_objective": {"answer": "Objective", "rationale": "As muscle acidity is a physical feeling or symptom that can be observed and measured, it is objective."}, "sentence_sentiment": {"answer": "Negative", "rationale": "Because muscle acidity is a symptom of physical discomfort, it is usually considered negative."}, "language_style": {"answer": "Tactful and warm", "rationale": "The entry is more euphemistic and sensitive to each other鈥檚 feelings, using the term 鈥渕uscle acid鈥?to describe symptoms rather than over-professional or direct language. This approach may make the interviewee more comfortable and willing to share his feelings."}, "time_dimension": {"answer": "Past", "rationale": "The entry is for questioning the testee ' s past symptoms or experiences, and the temporal dimension is a past."}, "sentence_type": "Affirmative declarative sentence", "pos": {"nouns": {"Muscles": 1, "Sour": 1}}}, {"content": "It's like someone's watching you talking about you.", "word_count": 11, "label": "", "sentence_structure": "Subject鈥搗erb鈥揷omplement", "subjective_objective": {"answer": "Subjective", "rationale": "Because being under surveillance is a subjective feeling and experience, not an objective fact or circumstance."}, "sentence_sentiment": {"answer": "Negative", "rationale": "Because the \"Feeling someone's watching\" act in the entry is uncomfortable and therefore negative."}, "language_style": {"answer": "Calm and direct", "rationale": "The purpose of this article is more straightforward, with no over-modified or emotional rendering, and a brief and quick expression of whether the person under investigation would feel the feeling of being watched and listening. This expression is more easily understood by the interviewee and meets the objective and true requirements of the psychological questionnaire."}, "time_dimension": {"answer": "Past", "rationale": "The entry asks the testee about what he had felt, and the keyword \"someone feels watching\" and \"talking\" suggests that these experiences may have passed."}, "sentence_type": "Affirmative declarative sentence", "pos": {"nouns": {"person": 1, "Watch": 1, "Talk": 1, "you": 1}, "verbs": {"I feel": 1, "in": 1, "Watch": 1, "Talk": 1}, "adverbs": {"Someone's here": 1}, "numerals": {"43": 1}}}, {"content": "44. It is difficult to sleep.", "word_count": 4, "label": "", "sentence_structure": "Subject鈥搗erb鈥揷omplement", "subjective_objective": {"answer": "Subjective", "rationale": "Because \"hard to sleep\" is a subjective feeling and experience, and different people have different feelings and assessments of the ease of sleeping, this entry is viewed from the subjective perspective of the patient."}, "sentence_sentiment": {"answer": "Negative", "rationale": "Because the \"sleeping\" behavior in the entries is negative."}, "language_style": {"answer": "Calm and direct", "rationale": "The language style of the entry is relatively simple and not excessive in qualifier or emotional colour, and the expression is more objective and practical by asking whether the person surveyed often has difficulty sleeping at night."}, "time_dimension": {"answer": "Present", "rationale": "The entry describes the current problem of the testee, namely, \"hard to sleep\", and thus judges it now."}, "sentence_type": "Negative declarative sentence", "pos": {"verbs": {"Sleep": 1}, "adjectives": {"Hard": 1}}}, {"content": "45. The conduct of business must be reviewed repeatedly.", "word_count": 8, "label": "", "sentence_structure": "Subject鈥搗erb鈥揷omplement", "subjective_objective": {"answer": "Subjective", "rationale": "Since repeated examinations are a personal habit and attitude, which is determined by the patient himself, they are subjectively viewed."}, "sentence_sentiment": {"answer": "Negative", "rationale": "Because the \"re-check\" act in the entry is negative."}, "language_style": {"answer": "Calm and direct", "rationale": "The entry is presented in a simple and clear language, with no excessive qualifier or emotional colour, and is more direct and objective and therefore of a calm and direct language style."}, "time_dimension": {"answer": "Present", "rationale": "According to the title, \"must be checked repeatedly\", it can be seen that this is a description of current behaviour and therefore the temporal dimension is now judged."}, "sentence_type": "Affirmative declarative sentence", "pos": {"verbs": {"Work": 1, "Inspection": 1}, "adverbs": {"I have to": 1, "Repeatedly": 1}, "numerals": {"45": 1}}}, {"content": "46. It was difficult to make decisions.", "word_count": 6, "label": "", "sentence_structure": "Subject鈥揷opula鈥損redicative", "subjective_objective": {"answer": "Subjective", "rationale": "Because the difficulty of taking decisions reflects the patient ' s inner feelings and experiences, it is a subjective examination."}, "sentence_sentiment": {"answer": "Negative", "rationale": "The basis of the judgment. Because the act of \"hard to make a decision\" is negative."}, "language_style": {"answer": "Tactful and warm", "rationale": "The language style of the entry is euphemistic, because it uses the term \"hard\" to express the problem, without direct accusations or orders."}, "time_dimension": {"answer": "Present", "rationale": "The entry asks if the person tested has encountered difficulties in making decisions and the time dimension is now judged."}, "sentence_type": "Negative declarative sentence", "pos": {"verbs": {"Make it": 1, "Decision": 1}, "adjectives": {"Hard": 1}, "numerals": {"46": 1}}}, {"content": "Fear of being taken by electric train, bus, subway or train.", "word_count": 13, "label": "", "sentence_structure": "Subject鈥搗erb鈥搊bject", "subjective_objective": {"answer": "Subjective", "rationale": "Because fear is a subjective feeling, a patient's inner experience and emotional reaction."}, "sentence_sentiment": {"answer": "Negative", "rationale": "Because the word \"fear\" in the entry expresses a feeling of fear and unease, which is negative."}, "language_style": {"answer": "Tactful and warm", "rationale": "The entry is more personal and interactive when it is spoken in a gentle and inviting tone, rather than in direct statements or orders. At the same time, the issue expresses concern about the psychological state of the interviewee, rather than simply asking whether there is fear of travelling in public transport, and therefore the language style is more euphemismal."}, "time_dimension": {"answer": "Present", "rationale": "The entry indicates that the testee is currently afraid of travelling in public transport and therefore the time dimension is now judged."}, "sentence_type": "Imperative sentence", "pos": {"nouns": {"Train": 2, "Bus": 1, "Subway": 1}, "verbs": {"Yes": 1, "Multiply": 1}, "numerals": {"47": 1}}}, {"content": "48. Respiration is difficult.", "word_count": 5, "label": "", "sentence_structure": "Subject鈥揷opula鈥損redicative", "subjective_objective": {"answer": "Objective", "rationale": "Because breathing difficulties are a phenomenon that can be observed and measured, not subjective feelings or attitudes of the patient."}, "sentence_sentiment": {"answer": "Negative", "rationale": "Because \"breathing is difficult\" is a negative physical condition."}, "language_style": {"answer": "Calm and direct", "rationale": "The entry raises questions in a simple and straightforward manner, without too many rhetorical or emotional colours, with more objective and direct tone, and is more likely to attract the attention and answers of the respondents."}, "time_dimension": {"answer": "Present", "rationale": "The entry is now not clear-cut time reference points, but only describes the current experience or symptoms of the testee and can therefore be judged as now."}, "sentence_type": "Affirmative declarative sentence", "pos": {"nouns": {"Breathe": 1, "Difficulties": 1}}}, {"content": "49. A wave of cold or heat.", "word_count": 8, "label": "", "sentence_structure": "Subject鈥搗erb鈥揷omplement", "subjective_objective": {"answer": "Objective", "rationale": "Since a period of cold or heat is a physical condition of the patient, it can be measured and observed and therefore is objectively assessed."}, "sentence_sentiment": {"answer": "Neutral", "rationale": "Because the entry does not express a positive, negative or neutral attitude/emotional/behaviour, it only describes a symptom."}, "language_style": {"answer": "Calm and direct", "rationale": "The entry is more calm and straightforward, is not over-modified and emotionally retrofitted, and uses simple and clear language to describe the physical symptoms that the subject may experience. This expression is more accurate and clear in communicating the message, making it easier for the respondent to understand and answer the questions."}, "time_dimension": {"answer": "Past", "rationale": "The entry asks whether the person tested had had \"a period of cold or heat\" experience over the past period and therefore is judged on the basis of the past."}, "sentence_type": "Affirmative declarative sentence", "pos": {"nouns": {"Line": 1, "Cold": 1, "Hot": 1}, "verbs": {"Let's go": 1}, "adjectives": {"For a while": 1}, "numerals": {"49": 1}}}, {"content": "50. Certain things, occasions or activities are avoided because of fear.", "word_count": 18, "label": "", "sentence_structure": "Subject鈥搗erb鈥搊bject", "subjective_objective": {"answer": "Subjective", "rationale": "Because \"fear\" is a subjective feeling, the answer to this questionnaire is from the patient's subjective point of view."}, "sentence_sentiment": {"answer": "Negative", "rationale": "Because the entry describes something that is avoided out of fear, which is a negative attitude and behaviour."}, "language_style": {"answer": "Tactful and warm", "rationale": "This entry is a soft, inviting expression that leads to reflection and recollection by asking the interviewees whether they have ever had experience of avoiding something, place or activity because they are afraid."}, "time_dimension": {"answer": "Past", "rationale": "The entry describes the behaviour or feelings of the person who was tested during a certain period of time, and the words \"fear\" and \"shield\" indicate that this is an experience that has already occurred and can therefore be judged as the past."}, "sentence_type": "Affirmative declarative sentence", "pos": {"nouns": {"Scared": 1, "Something": 1, "Event": 1, "Activities": 1}, "verbs": {"Avoid": 1}, "adverbs": {"Because": 1}, "numerals": {"50": 1}}}, {"content": "51. The brain is empty.", "word_count": 5, "label": "", "sentence_structure": "Subject鈥搗erb鈥揷omplement", "subjective_objective": {"answer": "Subjective", "rationale": "Because \"brainless\" is the sense and experience of the patient himself, and it's subjective."}, "sentence_sentiment": {"answer": "Negative", "rationale": "Because the expression \"brainless\" implies negative emotions and attitudes."}, "language_style": {"answer": "Calm and direct", "rationale": "The language style of the entry is calm and straightforward. It expresses concern about the psychological state of the person under investigation in a concise and straightforward manner, with no excessive embellishment or emotional expression, and is more objective and professional."}, "time_dimension": {"answer": "Past", "rationale": ""}, "sentence_type": "Affirmative declarative sentence", "pos": {"nouns": {"Brain": 1, "It's empty": 1}, "verbs": {"Change": 1, "Yes": 1}, "adjectives": {"& & & & & & & & & & & & & & & & & & & & & & & & & & & & & & & & & & & & & & & & & & & & & & & & & & & & & & & & & & & & & & & & & & & & & & & & & & & & & & & & & & & & & & & & & & & & & & & & & & & & & & & & & & & & Z": 1}, "numerals": {"51": 1}}}, {"content": "52. The body is numb or stinging.", "word_count": 7, "label": "", "sentence_structure": "Subject鈥揷opula鈥損redicative", "subjective_objective": {"answer": "Objective", "rationale": "Since the body is numb or sting is an objective expression of the patient ' s state of health, it is irrelevant to the patient ' s subjective will."}, "sentence_sentiment": {"answer": "Negative", "rationale": "Because body atrophy or sting is an uncomfortable physical feeling, it is usually considered negative."}, "language_style": {"answer": "Calm and direct", "rationale": "The language style of the entry is relatively simple, there is no excessive emotional rendering or rhetorical technique, and the person surveyed is asked whether there are any signs of body atrophy or sting, and the tone is objective and direct."}, "time_dimension": {"answer": "Past", "rationale": "The entry asks if the testee has experienced \"body atrophy or sting\" in the past, so the temporal dimension is judged to be past."}, "sentence_type": "Affirmative declarative sentence", "pos": {"nouns": {"Body": 1, "Hair": 1, "Stings": 1}}}, {"content": "53. There is a sense of infarction in the throat.", "word_count": 6, "label": "", "sentence_structure": "Subject鈥揷opula鈥損redicative", "subjective_objective": {"answer": "Objective", "rationale": "Because the feeling of infarction in the throat is an objective fact of the patient ' s body feeling, it is not necessary to be judged subjectively."}, "sentence_sentiment": {"answer": "Negative", "rationale": "Because \"throat infarction\" is an uncomfortable biological feeling, which usually means that there is a problem or discomfort in the body, and is therefore negative."}, "language_style": {"answer": "Calm and direct", "rationale": "The entry is more calm and straightforward in its language, without excessive ado or emotional. It directly asks the interviewee whether there is a problem with the infarction of the throat, and it does not use any euphemism or implied language, which appears to be objective and neutral."}, "time_dimension": {"answer": "Past", "rationale": "The entry describes the current situation and feelings of the person being tested and is therefore a past."}, "sentence_type": "Exclamatory sentence", "pos": {"nouns": {"Throat": 1, "Infarction": 1}}}, {"content": "54. There was no hope for a future.", "word_count": 10, "label": "", "sentence_structure": "Subject鈥揷opula鈥損redicative", "subjective_objective": {"answer": "Subjective", "rationale": "Because \"fear\" is the patient's own emotional experience, which is the subject of subjective feelings."}, "sentence_sentiment": {"answer": "Negative", "rationale": "Because the \"no future no hope\" attitude in the entry is negative."}, "language_style": {"answer": "Tactful and warm", "rationale": "The language style of the entry is relatively euphemistic and sensitive, rather than directly criticizing or denying the other ' s feelings, and this expression is more likely to trigger resonance and positive responses from the interviewees."}, "time_dimension": {"answer": "Present", "rationale": "\"Feeling without future, without hope\" describes an emotion or feeling that occurs at the moment and may be a major problem or distress at the moment. Thus, the temporal dimension of the article is now judged."}, "sentence_type": "Negative declarative sentence", "pos": {"nouns": {"Future": 1, "Hope": 1}, "verbs": {"I feel": 1, "have": 1}, "numerals": {"54": 1}}}, {"content": "55. No focus can be given.", "word_count": 6, "label": "", "sentence_structure": "Subject鈥揷opula鈥損redicative", "subjective_objective": {"answer": "Objective", "rationale": "Because \"not focusing\" is an objective phenomenon or symptom, not a subjective feeling or attitude of the patient. Although patients may feel a sense of subjectivity, such as anxiety, anxiety or helplessness, none of this changes the objective fact that \"not focusing\" is. So, the questionnaire is about objective attention, not subjective feelings or attitudes of the patient."}, "sentence_sentiment": {"answer": "Negative", "rationale": "Because the expression \"not focusing\" implies that there are individual problems of attention deficit, and it is a manifestation of negative emotions or behaviour."}, "language_style": {"answer": "Calm and direct", "rationale": "The entry describes a common psychological problem in a concise language, does not add too many emotional colours or acoustic techniques and is more objective, direct and suitable for psychological measurement and analysis."}, "time_dimension": {"answer": "Present", "rationale": "Now the entry describes the person's current behaviour or state, and \"not focusing\" is the result of the observation that is now, and therefore the judgement is now."}, "sentence_type": "Negative declarative sentence", "pos": {"nouns": {"Attention": 1}, "verbs": {"I can't concentrate": 1}, "numerals": {"55": 1}}}, {"content": "56. A part of the body is felt to be weak.", "word_count": 13, "label": "", "sentence_structure": "Subject鈥揷opula鈥損redicative", "subjective_objective": {"answer": "Objective", "rationale": "Because the body feels objective, not subjective, thinking of the patient."}, "sentence_sentiment": {"answer": "Negative", "rationale": "Because the \"weak\" physical condition in the entry is negative."}, "language_style": {"answer": "Calm and direct", "rationale": "The language style of the entry is calm and direct, and it asks the interviewee whether he or she has ever felt a certain part of the body weak, without any qualifier or emotional rendering."}, "time_dimension": {"answer": "Present", "rationale": "The entry describes the current feelings or experiences of the testee, and the temporal dimensions are judged as now.\""}, "sentence_type": "Affirmative declarative sentence", "pos": {"nouns": {"Part": 1, "Power": 1}, "verbs": {"I feel": 1, "Weakness": 1}, "numerals": {"56": 1}}}, {"content": "57. Tensions or susceptibility to tension.", "word_count": 9, "label": "", "sentence_structure": "Subject鈥揷opula鈥損redicative", "subjective_objective": {"answer": "Subjective", "rationale": "It is a subjective feeling or experience to feel nervous or to be nervous."}, "sentence_sentiment": {"answer": "Negative", "rationale": "Because the emotional state of \"Esperation or Esperation\" in the entry is negative."}, "language_style": {"answer": "Calm and direct", "rationale": "The entry describes the subject in a concise language (i.e., feeling or being nervous), and does not use too much rhetorical or emotional colours. This expression appears to be more direct, objective and suitable for use in, for example, questionnaires."}, "time_dimension": {"answer": "Present", "rationale": "The \"fear\" in the entry indicates that it is a state of present or a feeling, and thus it can be judged as the time dimension of the item."}, "sentence_type": "Affirmative declarative sentence", "pos": {"nouns": {"Nervous": 2, "It's a little nervous": 1}, "verbs": {"I feel": 1}, "adverbs": {"or": 1}, "numerals": {"57": 1}}}, {"content": "58. Feels heavy hands or feet.", "word_count": 7, "label": "", "sentence_structure": "Subject鈥揷opula鈥損redicative", "subjective_objective": {"answer": "Subjective", "rationale": "This is a perception of the patient ' s subjective experience and perception, not an objective fact."}, "sentence_sentiment": {"answer": "Negative", "rationale": "Because feeling heavy hands or feet is a negative emotional experience of physical discomfort."}, "language_style": {"answer": "Tactful and warm", "rationale": "The entry is more adept and interactive when it is spoken in a gentle and inviting manner, rather than by direct statements or orders."}, "time_dimension": {"answer": "Present", "rationale": "The word \"fear\" in the sentence makes it possible to judge that the act is in progress and is therefore in the present state of affairs. At the same time, the \"heavy or heavy\" is a further confirmation of the feeling of the current state of health."}, "sentence_type": "Affirmative declarative sentence", "pos": {"nouns": {"Hands": 1, "Foot": 1, "Heavy": 1}, "verbs": {"Sensor": 1, "Let's go": 1}, "numerals": {"58": 1}}}, {"content": "59. The idea of death.", "word_count": 6, "label": "", "sentence_structure": "Subject鈥搗erb鈥揷omplement", "subjective_objective": {"answer": "Subjective", "rationale": "Because the thought of death is a subjective idea and feeling of a human being, which is part of psychological activity rather than an objective reality."}, "sentence_sentiment": {"answer": "Negative", "rationale": "The expression of this article is negative, as it is a fear and a fear that is felt."}, "language_style": {"answer": "Calm and direct", "rationale": "The entry directly asked whether the interviewees had considered their death, did not use excessive rhetorical techniques or emotional rendering, and the language style was more calm and straightforward."}, "time_dimension": {"answer": "Present", "rationale": "The entry asks whether the testee is often aware of the death, and the time dimension is now judged."}, "sentence_type": "Declarative sentence", "pos": {"nouns": {"Death": 1, "Yes": 1}, "verbs": {"Yes": 1}, "numerals": {"59": 1}}}, {"content": "60. Too much food.", "word_count": 4, "label": "", "sentence_structure": "Subject鈥搗erb鈥揷omplement", "subjective_objective": {"answer": "Subjective", "rationale": "Because \"too much\" is a subjective feeling or evaluation, not an objective fact. Everyone may have different definitions and feelings of \"too much\" and therefore this is a subjective view."}, "sentence_sentiment": {"answer": "Negative", "rationale": "Because \"too much to eat\" is unhealthy, it can be judged negative."}, "language_style": {"answer": "Calm and direct", "rationale": "The entry is simple and straightforward in asking the interviewees whether they often eat too many questions, with objectivity, without excessive rhetorical or emotional rendering.\""}, "time_dimension": {"answer": "Present", "rationale": "The entry is an expression of the person's current state or behaviour, and the keyword is \"too much to eat\", indicating that there are problems or habits now."}, "sentence_type": "Affirmative declarative sentence", "pos": {"nouns": {"Too much": 1}, "verbs": {"Eat": 1}}}, {"content": "61. It is uncomfortable to be seen or talked about.", "word_count": 16, "label": "", "sentence_structure": "Subject鈥搗erb鈥搊bject", "subjective_objective": {"answer": "Subjective", "rationale": "Because the patient is describing his subjective feelings."}, "sentence_sentiment": {"answer": "Negative", "rationale": "Because the feeling of \"frightful\" in the entry is negative."}, "language_style": {"answer": "Calm and direct", "rationale": "The entry raises the question directly in a concise and clear language, without too many rhetorical or emotional renderings, and with a more calm and objective language style."}, "time_dimension": {"answer": "Present", "rationale": ""}, "sentence_type": "Affirmative declarative sentence", "pos": {"nouns": {"others": 1, "Watch": 1, "you": 2, "Talk": 1}, "verbs": {"When": 1, "I feel": 1, "I'm not comfortable": 1}, "numerals": {"61": 1, "One": 1}}}, {"content": "There are ideas that do not belong to you.", "word_count": 12, "label": "", "sentence_structure": "Subject鈥搗erb鈥搊bject", "subjective_objective": {"answer": "Subjective", "rationale": "Because \"some ideas that don't belong to you\" are personal experiences and feelings of patients, subjective considerations."}, "sentence_sentiment": {"answer": "Neutral", "rationale": "Because the entry does not have any emotional color."}, "language_style": {"answer": "Tactful and warm", "rationale": "The entry uses more euphemistic language, and does not directly accuse or criticize the views of the interviewees. At the same time, the expression is more objective and neutral, and gives some certainty and understanding, making the interviewees more willing to answer."}, "time_dimension": {"answer": "Present", "rationale": "The entry describes what the subject is currently going through, and \"some ideas that do not belong to you\" is an ongoing exercise, and therefore the judgement is \"now\"."}, "sentence_type": "Affirmative declarative sentence", "pos": {"nouns": {"Questionnaire": 1, "Entry": 1, "Thoughts": 1}, "verbs": {"have": 1, "belong to": 1}, "numerals": {"62": 1}, "pronouns": {"you": 1}}}, {"content": "63. There is an impulse to beat or hurt people.", "word_count": 12, "label": "", "sentence_structure": "Subject鈥搗erb鈥揷omplement", "subjective_objective": {"answer": "Subjective", "rationale": "Because \"the impulse to beat or hurt\" is the patient's subjective feeling and thoughts, not the objective reality."}, "sentence_sentiment": {"answer": "Negative", "rationale": "Because the entry \"want to hit or hurt someone\" is offensive and negative."}, "language_style": {"answer": "Calm and direct", "rationale": "The entry is in a straightforward manner, asking the person surveyed whether he or she has been motivated to hit or hurt someone, without using excessive rhetoric or emotional rendering."}, "time_dimension": {"answer": "Past", "rationale": "The entry \"with the urge to hit or hurt someone\" indicates the experience and feelings of the past, the dimension of which is the past."}, "sentence_type": "Affirmative declarative sentence", "pos": {"nouns": {"person": 2, "Expedition": 1}, "verbs": {"Fight": 1, "Hurt": 1, "Yes": 1}, "numerals": {"63": 1}}}, {"content": "64. Early wake-up.", "word_count": 4, "label": "", "sentence_structure": "Subject鈥揷opula鈥損redicative", "subjective_objective": {"answer": "Objective", "rationale": "Because it is an objective fact that early awakening occurs whether the patient wishes or likes it."}, "sentence_sentiment": {"answer": "Negative", "rationale": "Because early awakening can affect the quality of human sleep and have negative effects."}, "language_style": {"answer": "Calm and direct", "rationale": "The language style of the entry is calm and straightforward. It describes a question or situation in a concise manner, without too much emotional or rhetorical style. It is usually more easily understood and answered by the interviewee."}, "time_dimension": {"answer": "Past", "rationale": "The keyword \"awaken too early\" indicates that the entry is asked about the testee's past experience and state, and therefore judged to be past."}, "sentence_type": "Negative declarative sentence", "pos": {"verbs": {"Wake up": 1, "Too much": 1}, "adjectives": {"Morning": 1}, "adverbs": {"of": 1, "Too much": 1}, "numerals": {"64": 1}}}, {"content": "65. Hand washing, numbering or touching of something must be repeated.", "word_count": 16, "label": "", "sentence_structure": "Subject鈥搗erb鈥揷omplement", "subjective_objective": {"answer": "Subjective", "rationale": "Since \"repeated hand washing, count or touching something\" is the subjective act and idea of the patient, this act is not an objective fact. While it may be related to objective factors, such as anxiety or coercion, in this questionnaire entry it is primarily the patient who is the subject of such subjective tendencies or behaviour."}, "sentence_sentiment": {"answer": "Negative", "rationale": "Because \"repeated hand washing, count or touch something\" is a coercive act, usually associated with anxiety and coercion, and therefore negative."}, "language_style": {"answer": "Calm and direct", "rationale": ""}, "time_dimension": {"answer": "Present", "rationale": "This questionnaire entry is asking the testee鈥檚 current behavioral habits, i.e. whether it is often necessary to wash hands, dots or touch something repeatedly. Therefore, the time dimension is now judged."}, "sentence_type": "Interrogative sentence", "pos": {"nouns": {"I have to": 1, "Repeatedly": 1, "Wash your hands": 1, "Number of points": 1, "Something": 1}, "adverbs": {"I have to": 1}, "numerals": {"65": 1}, "classifiers": {"individual": 1}}}, {"content": "66. Sleeps are not stable.", "word_count": 6, "label": "", "sentence_structure": "Subject鈥搗erb鈥揷omplement", "subjective_objective": {"answer": "Subjective", "rationale": "Because the quality of sleep is the patient ' s own feeling and experience, it is the patient ' s subjective view."}, "sentence_sentiment": {"answer": "Negative", "rationale": "Because \"sleeping in a bad way\" is a negative description of the poor quality of sleep."}, "language_style": {"answer": "Tactful and warm", "rationale": "The language style of the entry is more euphemistic, using the term \"stable\" to describe the sleep condition of the person under investigation, rather than simply saying that they are insomnia or poor sleep quality. This expression is more moderate and avoids unnecessary stress and discomfort on the person under investigation."}, "time_dimension": {"answer": "Past", "rationale": "The entry describes the behaviour or state of the person tested at a certain point in time (i.e., when the questionnaire is completed) and can therefore be judged to be past."}, "sentence_type": "Negative declarative sentence", "pos": {"verbs": {"Sleep": 1, "Okay": 1, "It's not steady": 1, "Not deep": 1}, "numerals": {"66": 1}}}, {"content": "67. There is an impulse to break or destroy something.", "word_count": 12, "label": "", "sentence_structure": "Subject鈥搗erb鈥揷omplement", "subjective_objective": {"answer": "Subjective", "rationale": "This is a psychological feeling and idea of the patient and is a subjective examination."}, "sentence_sentiment": {"answer": "Negative", "rationale": "Because \"the urge to break or destroy something\" is a destructive act and a negative emotional tendency."}, "language_style": {"answer": "Calm and direct", "rationale": "The entry is of a calm and straightforward language, with no excessive emotional accolades and rhetorical techniques, and is straightforward in asking the testee whether he has any impulse to break or destroy something, and is succinctly expressed."}, "time_dimension": {"answer": "Present", "rationale": ""}, "sentence_type": "Imperative sentence", "pos": {"nouns": {"Questionnaire": 1, "Entry": 1}, "verbs": {"have": 1, "Broken": 1, "Destruction": 1, "Expedition": 1}, "numerals": {"67": 1}}}, {"content": "68. There are ideas or ideas that others do not have.", "word_count": 13, "label": "", "sentence_structure": "Subject鈥搗erb鈥揷omplement", "subjective_objective": {"answer": "Subjective", "rationale": "Because \"somethings or ideas that no one else has\" are the subjective feelings and perceptions of patients."}, "sentence_sentiment": {"answer": "Neutral", "rationale": "Because the entry does not have any emotional color."}, "language_style": {"answer": "Tactful and warm", "rationale": "This entry is more personal and interactive when it is spoken in a conversation with a question and invitation rather than by a direct statement or an order. Moreover, positive language is used to encourage respondents to share their thoughts and experiences, and to demonstrate respect and support for them."}, "time_dimension": {"answer": "Present", "rationale": "The entry asks if the person tested currently has some unique ideas or ideas, so the time dimension is now judged."}, "sentence_type": "Affirmative declarative sentence", "pos": {"nouns": {"Thoughts": 1, "Thinking": 1}, "verbs": {"have": 1}, "numerals": {"68": 1}}}, {"content": "69. People are sensitive to others.", "word_count": 9, "label": "", "sentence_structure": "Subject鈥揷opula鈥損redicative", "subjective_objective": {"answer": "Subjective", "rationale": "Because this is a subjective feeling of the patient, whether he is sensitive requires his own judgement and description."}, "sentence_sentiment": {"answer": "Negative", "rationale": "Because \"sensitization\" is a negative emotional attitude, expressing oversensitivity or anxiety."}, "language_style": {"answer": "Tactful and warm", "rationale": ""}, "time_dimension": {"answer": "Present", "rationale": ""}, "sentence_type": "Affirmative declarative sentence", "pos": {"nouns": {"person": 1, "You're a bit of a sane": 1}, "verbs": {"I feel": 1, "to": 1}, "numerals": {"69": 1}}}, {"content": "70. People are uncomfortable in shops or cinemas, where they are found.", "word_count": 18, "label": "", "sentence_structure": "Subject鈥搗erb鈥揷omplement", "subjective_objective": {"answer": "Subjective", "rationale": "This is an entry that looks at the patient from the subjective point of view. Because \"frightful\" is a personal feeling and experience, a subjective attitude and emotional reaction of the patient, rather than an objective fact. The answer to this questionnaire should therefore be \"subjective.\""}, "sentence_sentiment": {"answer": "Negative", "rationale": "Because the description of \"frightful\" is negative, which means that a person may react in a situation of discomfort, stress, etc."}, "language_style": {"answer": "Calm and direct", "rationale": "The language style of the entry is relatively straightforward and does not involve too much embellishment or emotional expression, mainly to ask if the interviewee is uncomfortable in a given context."}, "time_dimension": {"answer": "Past", "rationale": "The entry describes a feeling or experience of the person being tested, and the \"frightful\" action occurs in shops, cinemas, etc., and these scenes are all in the past. So, judging by the time dimension, the content of the entry is in the past."}, "sentence_type": "Affirmative declarative sentence", "pos": {"nouns": {"Local": 1, "person": 2, "Shop": 1, "Cinema": 1, "I feel": 1, "I'm not comfortable": 1}, "verbs": {"in": 1, "Wait": 1, "I feel": 1}, "adverbs": {"More": 1, "Local": 2}, "numerals": {"70": 1}}}, {"content": "71. Everything was difficult to feel.", "word_count": 10, "label": "", "sentence_structure": "Subject鈥揷opula鈥損redicative", "subjective_objective": {"answer": "Subjective", "rationale": "Because \"fear\" is a verb that expresses the patient's subjective feelings, and \"everything is difficult\" is the patient's subjective view of his own situation."}, "sentence_sentiment": {"answer": "Negative", "rationale": "Because the description \"Esperately difficult\" in the entry is negative and expresses the pessimism and helplessness of the respondents."}, "language_style": {"answer": "Calm and direct", "rationale": ""}, "time_dimension": {"answer": "Present", "rationale": ""}, "sentence_type": "Negative declarative sentence", "pos": {"verbs": {"I feel": 1, "Difficulties": 2}, "adjectives": {"Any": 1, "very": 1}, "numerals": {"71": 1}}}, {"content": "72. A moment of fear or panic.", "word_count": 8, "label": "", "sentence_structure": "Subject鈥搗erb鈥揷omplement", "subjective_objective": {"answer": "Subjective", "rationale": "Because fear or panic is a subjective emotional reaction felt by the patient."}, "sentence_sentiment": {"answer": "Negative", "rationale": "Because the mood of \"a little fear or panic\" in the entry is negative."}, "language_style": {"answer": "Calm and direct", "rationale": "The entry describes the emotional reactions that the person under investigation may have in a concise and clear language, does not use excessive rhetorical techniques or emotional rendering, is more balanced and objective and therefore can be judged as being calm and straightforward."}, "time_dimension": {"answer": "Present", "rationale": "The entry describes the current emotional state of the testee, namely, \"a moment of fear or shock\", and thus judges it as now."}, "sentence_type": "Declarative sentence", "pos": {"nouns": {"Line": 1, "Fear": 1, "Scared": 1}, "verbs": {"For a while": 1}, "numerals": {"72": 1}}}, {"content": "73. It is not comfortable to eat in public.", "word_count": 14, "label": "", "sentence_structure": "Subject鈥揷opula鈥損redicative", "subjective_objective": {"answer": "Subjective", "rationale": "Because this feeling is the patient ' s personal feeling and is subjective."}, "sentence_sentiment": {"answer": "Negative", "rationale": "Because the feeling in the entry \"it's uncomfortable eating in public\" is negative."}, "language_style": {"answer": "Tactful and warm", "rationale": "The language style of the entry is more euphemistic, using the expression \"uncomfort\" rather than directly describing the consequences of the behaviour of the other side, making the issue more relevant and easily resonating with the person under investigation. At the same time, this expression avoids any accusations or attacks against the person under investigation."}, "time_dimension": {"answer": "Present", "rationale": ""}, "sentence_type": "Affirmative declarative sentence", "pos": {"nouns": {"Public": 1, "Eat": 1, "I'm not feeling well": 1}, "verbs": {"I feel": 1, "I'm not feeling well": 1}, "numerals": {"73": 1}}}, {"content": "74. Arguments are often raised.", "word_count": 6, "label": "", "sentence_structure": "Subject鈥搗erb鈥揷omplement", "subjective_objective": {"answer": "Subjective", "rationale": "According to the description given, \"often in dispute\", this is a matter of behaviour and attitude of the patient and is therefore subjective."}, "sentence_sentiment": {"answer": "Negative", "rationale": "Because the practice of \"frequent dispute\" in the entries is usually considered negative, it may lead to quarrels and conflicts."}, "language_style": {"answer": "Calm and direct", "rationale": "The entry is written in a calm and straightforward manner. It asks the respondents whether they are often arguing with people, without using excessive rhetoric or emotional descriptions, and in a fair and objective manner."}, "time_dimension": {"answer": "Present", "rationale": "Time dimension is judged now."}, "sentence_type": "Affirmative declarative sentence", "pos": {"nouns": {"Regular": 1, "person": 1, "Arguments": 1}, "verbs": {"and": 1, "Fight": 1}, "adverbs": {"The long run": 1}, "numerals": {"74": 1}}}, {"content": "75. The individual is nervous.", "word_count": 10, "label": "", "sentence_structure": "Subject鈥揷opula鈥損redicative", "subjective_objective": {"answer": "Subjective", "rationale": "Because it's the patient's subjective feeling."}, "sentence_sentiment": {"answer": "Negative", "rationale": "Because the description of \"nervous\" in the entry is negative."}, "language_style": {"answer": "Calm and direct", "rationale": "The entry directly expresses the subject of questioning the other party ' s vulnerability to stress when being alone, without excessive rhetoric or emotional rendering, and with a more objective tone."}, "time_dimension": {"answer": "Present", "rationale": "The keyword \"one person alone\" means the current state and environment, and \"nerstroke\" is the experience or emotional state of the person being tested at this moment, pointing to the present."}, "sentence_type": "Affirmative declarative sentence", "pos": {"nouns": {"person": 1, "Nerves": 1, "Nervous": 1}, "verbs": {"Time": 1, "Alone": 1, "Yes": 1, "very": 1, "Nervous": 1}, "adverbs": {"very": 1, "Alone": 1}, "numerals": {"75": 1}}}, {"content": "76. Your achievements were not properly evaluated.", "word_count": 16, "label": "", "sentence_structure": "Subject鈥搗erb鈥揷omplement", "subjective_objective": {"answer": "Subjective", "rationale": "Because this is the patient ' s own perception and perception of others ' own performance evaluation, it is subjective."}, "sentence_sentiment": {"answer": "Negative", "rationale": "Because the \"others have not properly evaluated\" act in the entry is negative."}, "language_style": {"answer": "Tactful and warm", "rationale": "This is a more friendly, acceptable and responsive expression."}, "time_dimension": {"answer": "Present", "rationale": "The entry is an inquiry into the past or present experience of the testee, and the description of \"you\" indicates that it is a personal experience. Moreover, \"no proper evaluation\" indicates that it is a negative situation. Therefore, the temporal dimension is now or in the past, and the circumstances need to be determined on the basis of the respondent's response."}, "sentence_type": "Negative declarative sentence", "pos": {"nouns": {"others": 1, "you": 1, "Achievements": 1, "Evaluation": 1}, "verbs": {"Do it": 1, "Out": 1, "No, I'm not": 1, "Make it": 1}, "adjectives": {"Appropriate": 1}, "numerals": {"76": 1}}}, {"content": "77. Even with others, it was felt lonely.", "word_count": 13, "label": "", "sentence_structure": "Subject鈥揷opula鈥損redicative", "subjective_objective": {"answer": "Subjective", "rationale": "Because feeling alone is the subjective feeling and experience of the patient."}, "sentence_sentiment": {"answer": "Negative", "rationale": "Because feeling alone is negative."}, "language_style": {"answer": "Tactful and warm", "rationale": "The language style of the entry tends to be euphemistic, and it uses the expression \"even if with someone else\" to suggest that the person under investigation may often feel alone. This expression is relatively implicit, moderate, and does not directly point to the psychological problems of the person under investigation, but rather leads the person to self-reflect and explore the inner world by asking questions."}, "time_dimension": {"answer": "Present", "rationale": "The entry expresses a subjective feeling or emotional state, and although there is no clear time indicator, the emotional experience of feeling alone usually occurs now and is therefore judged now."}, "sentence_type": "Affirmative declarative sentence", "pos": {"nouns": {"Questionnaire": 1, "Entry": 1, "person": 1, "Alone": 1}, "verbs": {"in": 1, "I feel": 1, "and": 1, "Together": 1, "Yeah": 1, "Alone": 1}, "adverbs": {"Even if": 1, "Yeah": 1}, "numerals": {"77": 1}, "pronouns": {"others": 1, "self": 1}}}, {"content": "78. The feeling of uncertainty is still high.", "word_count": 10, "label": "", "sentence_structure": "Subject鈥揷opula鈥損redicative", "subjective_objective": {"answer": "Subjective", "rationale": "Because feeling restless and unstable is a subjective feeling and experience."}, "sentence_sentiment": {"answer": "Negative", "rationale": "Because the emotional description of \"silentness\" in the entry is negative."}, "language_style": {"answer": "Tactful and warm", "rationale": "The language style of the entry is more euphemistic. It uses descriptive terms such as \"sitting\" and \"deepness\" rather than direct statements or command-style expressions, which are more resonant and understandable to the respondents. And, \"Please analyse below...\", which also means invitation and respect, and more conducive to interaction and communication between the survey researchers and the respondents."}, "time_dimension": {"answer": "Present", "rationale": "The entry describes the current feelings and state of the person being tested and does not address the time dimension of the past or the future."}, "sentence_type": "Affirmative declarative sentence", "pos": {"nouns": {"Sit tight": 1, "I'm not sure I can": 1}, "verbs": {"I feel": 1}, "numerals": {"78": 1}}}, {"content": "79. It was felt that there was little value in it.", "word_count": 10, "label": "", "sentence_structure": "Subject鈥揷opula鈥損redicative", "subjective_objective": {"answer": "Subjective", "rationale": "Because \"fearing yourself worthless\" is a patient's subjective assessment or perception of himself."}, "sentence_sentiment": {"answer": "Negative", "rationale": "Because the expression \"no value\" in the entry is negative emotional."}, "language_style": {"answer": "Tactful and warm", "rationale": "The language style of the entry is euphemistic. It does not directly blame or criticize the interviewee, but rather asks questions in an understanding and supportal manner, hoping that the interviewee will reflect and think more deeply about himself. This expression is more respectful of the interviewee鈥檚 feelings and emotions and more likely to receive positive responses from them."}, "time_dimension": {"answer": "Present", "rationale": "The key word is \"fear,\" which is the current state of immediate feeling and awareness."}, "sentence_type": "Negative declarative sentence", "pos": {"nouns": {"Value": 1}, "verbs": {"I feel": 1, "have": 1}, "adverbs": {"Nothing": 1}, "numerals": {"79": 1}}}, {"content": "80. It is true that things that feel familiar become unknown or unsimilar.", "word_count": 17, "label": "", "sentence_structure": "Subject鈥揷opula鈥損redicative", "subjective_objective": {"answer": "Subjective", "rationale": "Because \"fEssing\" is a subjective expression of perception."}, "sentence_sentiment": {"answer": "Negative", "rationale": "Because the descriptions \"unknown\" and \"not real\" are negative."}, "language_style": {"answer": "Tactful and warm", "rationale": "The language style of the entry is more euphemistic, more moderate, and it leads the respondents to think in a question-and-answer manner, and creates a more cordial and sweet atmosphere by using the words \"fear,\" \"go\" and the words. This way helps to make the respondents more willing to share their feelings and ideas."}, "time_dimension": {"answer": "Present", "rationale": "Now the entry describes the current feelings and experiences of the testee, and the words \"fear\" and \"be\" indicate that it is an instant sense, and the temporal dimension is now."}, "sentence_type": "Affirmative declarative sentence", "pos": {"nouns": {"Something": 1, "I know you": 1, "Strange": 1, "True": 1}, "verbs": {"Change": 1}, "adjectives": {"It doesn't seem like it": 1}, "numerals": {"80": 1}}}, {"content": "Scream or fall.", "word_count": 6, "label": "", "sentence_structure": "Subject鈥搗erb鈥揷omplement", "subjective_objective": {"answer": "Subjective", "rationale": "Because shouting or falling is a person鈥檚 behaviour, a subjective choice and reaction. While these acts may be influenced by external incentives, the decision to take such an act remains in the hands of the individual, so the point of view of the questionnaire is subjective."}, "sentence_sentiment": {"answer": "Negative", "rationale": "Because \"scream\" and \"thumping something\" are negative behavior."}, "language_style": {"answer": "Calm and direct", "rationale": "The language style of the entry is more straightforward, there is no excessive rhetorical or emotional presentation, and it appears to be more balanced and objective, and it tends to raise questions in a calm manner."}, "time_dimension": {"answer": "Past", "rationale": ""}, "sentence_type": "Imperative sentence", "pos": {"nouns": {"Shout out loud": 1, "Drop something": 1}, "verbs": {"Shouting": 1, "Fall": 1}}}, {"content": "Fear of fainting in public.", "word_count": 10, "label": "", "sentence_structure": "Subject鈥揷opula鈥損redicative", "subjective_objective": {"answer": "Subjective", "rationale": "Because fear is a subjective emotional attitude."}, "sentence_sentiment": {"answer": "Negative", "rationale": "Because \"fear\" is negative."}, "language_style": {"answer": "Calm and direct", "rationale": "The entry uses a direct question-and-answer method to explicitly ask the interviewees whether they fear fainting in public, the language style is more calm and objective, and there is no excessive emotional or rhetorical style."}, "time_dimension": {"answer": "Present", "rationale": "The keyword \"fear,\" \"in public,\" \"scramble\" all refers to the current situation. So, the entry is for questioning the person who was tested for the present behavior or state."}, "sentence_type": "Affirmative declarative sentence", "pos": {"nouns": {"Questionnaire": 1, "Entry": 1, "Public": 1, "- I'm fainted": 1}, "verbs": {"Yes": 1, "Scared": 1, "in": 1, "- I'm fainted": 1}, "numerals": {"82": 1}}}, {"content": "Feel like someone else wants to take advantage of you.", "word_count": 10, "label": "", "sentence_structure": "Subject鈥搗erb鈥揷omplement", "subjective_objective": {"answer": "Subjective", "rationale": "Because it's a subjective feeling and judgment to feel that someone else wants to take advantage of you."}, "sentence_sentiment": {"answer": "Negative", "rationale": "Because the emotional tendency of \"Feeling someone else wants to take advantage of you\" in the entry is negative."}, "language_style": {"answer": "Tactful and warm", "rationale": ""}, "time_dimension": {"answer": "Present", "rationale": "The keyword \"fear\" means that it's a feeling or emotional experience, and \"to take advantage of you\" may happen in the past or in the future, but because the title is \"felt,\" the sentence is now time-dimensional."}, "sentence_type": "Imperative sentence", "pos": {"nouns": {"Questionnaire": 1, "Entry": 1, "83": 1, "others": 1, "you": 1, "Cheap": 1}, "verbs": {"I feel": 1, "Yes": 1, "Jim": 1}, "numerals": {"83": 1}, "pronouns": {"others": 1, "you": 1}}}, {"content": "The idea of 鈥渟ex鈥?is troubling.", "word_count": 13, "label": "", "sentence_structure": "Subject鈥搗erb鈥揷omplement", "subjective_objective": {"answer": "Subjective", "rationale": "Because \"suffering\" is the patient's subjective feeling and emotional reaction, and it's subjective. Although the idea of \"sex\" may be objective, it is subjective to the pain and suffering it causes."}, "sentence_sentiment": {"answer": "Negative", "rationale": "Because the word \"torture\" in the entry is negative emotional."}, "language_style": {"answer": "Tactful and warm", "rationale": "The entry is more gentle, is more open to dialogue by means of inquiry, and does not directly present or order, which makes it more comfortable and comfortable. At the same time, the entry uses the emotional term \"torture\" to better resonate with the interviewees and to better understand their inner feelings."}, "time_dimension": {"answer": "Present", "rationale": "The entry is for the current status and feelings of the testee, the keyword is \"suffering\" and the temporal dimension is now."}, "sentence_type": "Affirmative declarative sentence", "pos": {"nouns": {"Sex": 2, "Thoughts": 1, "Trouble": 1}, "verbs": {"Yes": 1, "Trouble": 1}, "numerals": {"84": 1}}}, {"content": "85. You believe that you should be punished for your own fault.", "word_count": 17, "label": "", "sentence_structure": "Subject鈥搗erb鈥搊bject", "subjective_objective": {"answer": "Subjective", "rationale": "As this statement relates to the values and beliefs of individuals, it is examined from a subjective perspective."}, "sentence_sentiment": {"answer": "Positive", "rationale": "Since the entry emphasizes the responsibility and obligation of the individual, it is a positive attitude that he or she should be held accountable for his or her actions."}, "language_style": {"answer": "Calm and direct", "rationale": "The entry raises a question in a direct manner, not in a euphemism or indulging expression, but in a simple and clear language. This language style applies to situations that require a clear and objective expression, such as questionnaires."}, "time_dimension": {"answer": "Present", "rationale": "The entry is an inquiry into the present views and attitudes of the testee, the keyword being \"concepts\" and \"should\" and they all point to the present situation. Moreover, \"fault\" and \"punishment\" are also terms of conduct or events, indicating that the idea relates to past behaviour, but in this context, more attention is given to the current view of the testee."}, "sentence_type": "Affirmative declarative sentence", "pos": {"nouns": {"Wrong": 1, "Punishment": 1}, "verbs": {"I think": 1, "I think so": 1, "Received": 1, "self": 1}, "adverbs": {"Because": 1}, "numerals": {"85": 1}}}, {"content": "86. It was felt that the matter should be completed quickly.", "word_count": 10, "label": "", "sentence_structure": "Subject鈥搗erb鈥揷omplement", "subjective_objective": {"answer": "Subjective", "rationale": "Because \"fear\" is the patient's subjective feeling and attitude."}, "sentence_sentiment": {"answer": "Positive", "rationale": "Because the entry \"Do something quickly\" represents a positive attitude and behaviour, reflecting the positive mindset and the ability of the respondents to accomplish their task."}, "language_style": {"answer": "Tactful and warm", "rationale": "The entry is a more accessible and willing response to questions."}, "time_dimension": {"answer": "Future", "rationale": "The entry expresses a vision or vision of a certain possibility for the future, and \"do it quickly\" means that the person tested will have to complete a task or work at some point in the future. It is therefore a time dimension for the future."}, "sentence_type": "Imperative sentence", "pos": {"verbs": {"I feel": 1, "Do it": 1}, "adjectives": {"Hurry up": 1}, "numerals": {"86": 1}}}, {"content": "87. It is felt that there are serious problems in your body.", "word_count": 12, "label": "", "sentence_structure": "Subject鈥揷opula鈥損redicative", "subjective_objective": {"answer": "Subjective", "rationale": "Because \"fear\" is a patient's subjective feeling, a inner experience, not an objective fact from the outside."}, "sentence_sentiment": {"answer": "Negative", "rationale": "Because the emotional description of \"Esperately serious physical problems\" in the entry is negative."}, "language_style": {"answer": "Tactful and warm", "rationale": "The language of the entry is more moderate, caring and understanding, and the interviewer is guided by the question, without direct accusations or criticism, making it easier for the interviewee to accept and be willing to answer the question. The term \"serious\" is used to express the seriousness of the problem and to give greater attention to the health of the interviewee, reflecting the concern and respect for the interviewee."}, "time_dimension": {"answer": "Past", "rationale": "The entry asks about the feelings and experiences of the person under examination during the survey, which are part of the experience and feelings of the past."}, "sentence_type": "Interrogative sentence", "pos": {"nouns": {"self": 1, "Body": 1, "Problem": 1}, "verbs": {"I feel": 1, "have": 1}, "adjectives": {"Serious": 1}, "numerals": {"87": 1}}}, {"content": "88. No one has ever felt close to anyone else.", "word_count": 11, "label": "", "sentence_structure": "Subject鈥揷opula鈥損redicative", "subjective_objective": {"answer": "Subjective", "rationale": "Because it is a subjective feeling and experience to feel close to others."}, "sentence_sentiment": {"answer": "Negative", "rationale": "Because the expression \"never felt close to anyone else\" conveys a distant, lonely emotional colour, which is negative."}, "language_style": {"answer": "Tactful and warm", "rationale": "The language style of the entry is euphemistic, leading the interviewee to share their feelings in an interview, while avoiding direct questions that may cause discomfort or resistance. This expression helps to build a climate of trust and openness, and makes the interviewee more willing to share his/her real thoughts."}, "time_dimension": {"answer": "Past", "rationale": "In conclusion, for given questionnaire entries, we can judge the time dimension of the questionnaire in terms of the time and context."}, "sentence_type": "Imperative sentence", "pos": {"nouns": {"Questionnaire": 1, "Entry": 1, "person": 1}, "verbs": {"I feel": 1, "and": 1, "very": 1, "Close": 1}, "adjectives": {"Other": 1, "of": 2}, "adverbs": {"Never": 1}, "numerals": {"88": 1}, "pronouns": {"they": 1}}}, {"content": "Feel guilty.", "word_count": 6, "label": "", "sentence_structure": "Subject鈥搗erb鈥揷omplement", "subjective_objective": {"answer": "Subjective", "rationale": "Because feeling guilty is the feeling and experience inside the patient, it is a subjective view."}, "sentence_sentiment": {"answer": "Negative", "rationale": "Because the \"guilty\" feeling in the entry is negative."}, "language_style": {"answer": "Tactful and warm", "rationale": "The entry uses a gentle and invitationful tone of conversation rather than a direct statement or order, which is more personal and interactive. At the same time, it does not have too much emotional overtones, but rather asks each other about their feelings in a more calm and objective way. It is therefore considered to be a softly and warm language."}, "time_dimension": {"answer": "Past", "rationale": "The keyword \"perception of guilt\" indicates a subjective emotional or psychological state, without any specific act or incident, and therefore the entry is more consistent with the investigation of the previous psychological state of the person being tested."}, "sentence_type": "Negative declarative sentence", "pos": {"nouns": {"Sin": 1, "self": 1}, "verbs": {"I feel": 1}, "adverbs": {"89": 1, "have": 1}, "numerals": {"89": 1}, "pronouns": {"self": 1}}}, {"content": "90. Feels like you're out of your mind.", "word_count": 10, "label": "", "sentence_structure": "Subject鈥揷opula鈥損redicative", "subjective_objective": {"answer": "Subjective", "rationale": "Because \"the feeling of a brain out of its head\" is a subjective feeling and perception, not an objective fact or a behavioural outcome."}, "sentence_sentiment": {"answer": "Negative", "rationale": "Because the expression \"brain\" in the entry is negative."}, "language_style": {"answer": "Calm and direct", "rationale": "The entry asks the interviewee in a concise language whether he or she feels that his or her mental ability is in question, that it is not over-modified or emotional, and that it is more direct and objective in tone."}, "time_dimension": {"answer": "Present", "rationale": ""}, "sentence_type": "Negative declarative sentence", "pos": {"nouns": {"Brain": 1, "Diseases": 1}, "verbs": {"I feel": 1}, "adjectives": {"Your own": 1}, "numerals": {"90": 1}}}]}

{"questionnaire_title": "Revised adult attachment scale (AAS)", "questionnaire_category": "Emotion and affect", "item_count": 18, "emotion_shift_overall": "From positive to negative", "language_style_overall": "Tactful and warm", "items": [{"content": "1. I find it easier to be close.", "word_count": 11, "label": "", "sentence_structure": "Subject鈥搗erb鈥搊bject", "subjective_objective": {"answer": "Subjective", "rationale": "Because \"discovery\" and \"relationship is easier\" is the patient's subjective feeling and perception, not the objective fact or result."}, "sentence_sentiment": {"answer": "Positive", "rationale": "Because the phrase \"nearness is easier\" in the entry is positive."}, "language_style": {"answer": "Tactful and warm", "rationale": "The language style of the entry is more euphemistic and warm. By using words like \"discovery,\" the objectivity of a personal experience is not expressed too directly or in a strong manner. At the same time, the expression \"failer to be close to one's person\" is also more intimate and sweet, giving people a positive, open feeling."}, "time_dimension": {"answer": "Present", "rationale": "The keyword \"discovery\" shows that this is a description of the state of the moment, and \"nearness is easier\" is a statement of a fact or experience that has already occurred. So the entry is a description of the person being tested, which is the current feeling and experience, and is the current state."}, "sentence_type": "Affirmative declarative sentence", "pos": {"nouns": {"person": 1, "Close": 1}, "verbs": {"Found": 1, "It's easier": 1}, "adjectives": {"Close": 1}, "adverbs": {"It's easier": 1}, "numerals": {"1": 2}, "pronouns": {"I": 1}}}, {"content": "2.2 I find it difficult to rely on others.", "word_count": 13, "label": "", "sentence_structure": "Subject鈥搗erb鈥搊bject", "subjective_objective": {"answer": "Subjective", "rationale": "Because \"discovery\" and \"difficult to rely on others\" are personal feelings and perspectives that are described from the perspective of the patient."}, "sentence_sentiment": {"answer": "Negative", "rationale": "Because \"dependantly hard\" in the entry expresses a negative emotional attitude."}, "language_style": {"answer": "Tactful and warm", "rationale": "The language style of the entry is more euphemistic, beginning with \"I found\" and expressing my feelings without directly accusing or ordering others. This expression is more respectful of each other's feelings and rights, and more acceptable and responsive."}, "time_dimension": {"answer": "Present", "rationale": "The entry describes the person being tested in his or her current state or feeling, and does not have a clear time point, and therefore the judgement is now."}, "sentence_type": "Exclamatory sentence", "pos": {"nouns": {"Found": 1, "Problem": 1, "Others": 1, "Dependency": 1}, "verbs": {"be": 1, "I'm going": 1, "very": 1, "Difficulties": 1}, "adverbs": {"2.2": 1}}}, {"content": "3. I often worry that couples do not really love me.", "word_count": 13, "label": "", "sentence_structure": "Subject鈥損redicate", "subjective_objective": {"answer": "Subjective", "rationale": "Because \"fear\" is a subjective feeling and attitude of a person, which suggests that the person has doubts and concerns about his relationship. Although this concern may be based on objective facts or behaviour (such as behaviour, means of communication, etc.), the questionnaire entry focuses more on the subjective experience and perception of the concern."}, "sentence_sentiment": {"answer": "Negative", "rationale": "Because the entry \"Fearing that couples don't really love me\" expresses negative emotional attitudes."}, "language_style": {"answer": "Tactful and warm", "rationale": "The language style of the entry is more euphemistic. It asks whether the respondents are often concerned about their lack of sincerity, rather than directly accusing or criticizing them. This expression is more acrimony, gentler, more likely to trigger a resonance and willingness to cooperate among the respondents, and more conducive to a real answer."}, "time_dimension": {"answer": "Present", "rationale": "The entry asks the testee about the emotional state of the current period, and the temporal dimension is now judged."}, "sentence_type": "Negative declarative sentence", "pos": {"nouns": {"Couple": 1, "Really": 1}, "verbs": {"I'm worried": 1}, "adverbs": {"Often": 1}, "numerals": {"3": 1}, "pronouns": {"I": 1}}}, {"content": "I found that others were not willing to approach me as I had hoped.", "word_count": 18, "label": "", "sentence_structure": "Subject鈥搗erb鈥揷omplement", "subjective_objective": {"answer": "Subjective", "rationale": "Because the words \"hope\" and \"think\" indicate that it is a patient's feeling and perception, a subjective examination."}, "sentence_sentiment": {"answer": "Negative", "rationale": "Because the expression \"no one else wants to be as close to me as I want\" in the entry is negative emotional."}, "language_style": {"answer": "Calm and direct", "rationale": "The entry directly expresses the fact that the interviewee has not used excessive rhetorical or emotional renderings in relation to interpersonal relations, and is more objective in terms of tone."}, "time_dimension": {"answer": "Present", "rationale": "The keyword \"discover\" and \"will\" indicate the current state of feeling and awareness of the subjects, that is, that they now realize that their relationship with others is not what they want."}, "sentence_type": "Negative declarative sentence", "pos": {"nouns": {"Questionnaire": 1, "Entry": 1, "Found": 1, "others": 1, "Hope": 1, "Modalities": 1, "Close": 1}, "verbs": {"Let's go": 1, "Current": 1, "May I": 1, "Yeah": 1, "Hope": 1, "That way": 1, "Close": 1}, "adverbs": {"and": 1, "not": 1, "May I": 1, "Yeah": 1, "That way": 1}, "numerals": {"4": 1}, "pronouns": {"I": 1}}}, {"content": "I feel comfortable being able to rely on others.", "word_count": 12, "label": "", "sentence_structure": "Subject鈥揷opula鈥損redicative", "subjective_objective": {"answer": "Subjective", "rationale": "Because the feeling of dependence on others is a feeling inside a person and is subjective."}, "sentence_sentiment": {"answer": "Positive", "rationale": "Because the entry \"can rely on someone else\" indicates a positive emotional need and attitude, and \"make me comfortable\" implies that this emotional need is met, the emotional orientation of the article is positive."}, "language_style": {"answer": "Tactful and warm", "rationale": "The entry uses a more acclaimable and gentle tone to ask if the other is able to rely on others to gain comfort, avoiding the use of too direct or strong language, which makes people feel more comfortable and receptive. At the same time, this expression is easier to open the interviewer ' s box and to share their thoughts and feelings."}, "time_dimension": {"answer": "Present", "rationale": "The entry indicates the current feelings and thoughts of the interviewee, that \"reliable\" is the current behavior or state, that \"comforts me\" is the psychological experience that it brings. The entry is therefore the present state of affairs."}, "sentence_type": "Affirmative declarative sentence", "pos": {"verbs": {"Yes": 1, "Dependency": 1, "Jean": 1}, "adjectives": {"- Yeah": 1}}}, {"content": "I don't care about being too close to me.", "word_count": 10, "label": "", "sentence_structure": "Subject鈥搗erb鈥揷omplement", "subjective_objective": {"answer": "Subjective", "rationale": "Because \"not caring\" and \"too close\" are subjective perceptions and evaluations of the phenomenon, and are subjective attitudes of the patients."}, "sentence_sentiment": {"answer": "Negative", "rationale": "Because \"not caring\" in the entries is a negative attitude."}, "language_style": {"answer": "Tactful and warm", "rationale": "The entry is presented in a more objective and neutral way, rather than directly criticizing or accusing the other. At the same time, the expression \"not caring\" is relatively euphemistic, avoiding the pressure or discomfort that too direct language may bring to the person under investigation."}, "time_dimension": {"answer": "Present", "rationale": ""}, "sentence_type": "Negative declarative sentence", "pos": {"nouns": {"others": 1, "Close": 1}, "verbs": {"I don't care": 1, "Too much": 1, "Close": 1}, "adjectives": {"Someone else's": 1}, "adverbs": {"not": 2, "in": 1, "Yes": 1}, "pronouns": {"I": 1}}}, {"content": "7. I find no one will help me when I need help.", "word_count": 17, "label": "", "sentence_structure": "Subject鈥搗erb鈥搊bject", "subjective_objective": {"answer": "Subjective", "rationale": "The detection of the need for help, the search for help and the availability of others are subjective experiences and observations of the patient."}, "sentence_sentiment": {"answer": "Negative", "rationale": "Because the \"no one will help me\" in the entry is negative."}, "language_style": {"answer": "Tactful and warm", "rationale": "The entry uses subjective expressions of perception, and does not directly blame or criticize others, but rather expresses their emotions and needs in a more euphemistic way. This language style is more likely to generate understanding and resonance among the other side and is more conducive to good interpersonal relationships."}, "time_dimension": {"answer": "Present", "rationale": "The entry expresses the experience and experience of the testee, i.e., the inability to get the support of others when in need of help, and therefore the time dimension is now judged."}, "sentence_type": "Negative declarative sentence", "pos": {"nouns": {"I": 1, "person": 2, "Help": 1}, "verbs": {"Found": 1, "Yes": 2, "Help": 1}, "adverbs": {"When": 1, "Time": 1}, "numerals": {"7": 1}, "classifiers": {"individual": 1}}}, {"content": "8. I feel a little uncomfortable being close to others.", "word_count": 14, "label": "", "sentence_structure": "Subject鈥揷opula鈥損redicative", "subjective_objective": {"answer": "Subjective", "rationale": "Because \"comfort\" and \"incomfort\" are subjective feelings of patients."}, "sentence_sentiment": {"answer": "Negative", "rationale": "Because the attitude of \"uncomfortable\" in the entries is negative."}, "language_style": {"answer": "Calm and direct", "rationale": "The entry expresses the subject of a direct and explicit inquiry into whether the other party would feel uncomfortable in dealing with another person, without too many rhetorical or emotional renderings, and is more objective in tone."}, "time_dimension": {"answer": "Present", "rationale": "\"I feel a little uncomfortable being close to someone else\" describes a feeling in the moment and therefore belongs to the present behavior or mentality."}, "sentence_type": "Negative declarative sentence", "pos": {"nouns": {"person": 2, "Close": 1}, "verbs": {"鈾?And I'm gonna be 鈾?: 1, "I feel": 1}, "adjectives": {"- Yeah": 1, "I'm not feeling well": 1}, "pronouns": {"I": 1}}}, {"content": "I often worry that couples don't want to be with me.", "word_count": 14, "label": "", "sentence_structure": "Subject鈥搗erb鈥搊bject", "subjective_objective": {"answer": "Subjective", "rationale": "Because \"fearing that couples don't want to be with me\" is a patient's subjective idea or concern, not an objective fact. This reflects the patient's uncertainty and anxiety about future relationships."}, "sentence_sentiment": {"answer": "Negative", "rationale": "Because the words \"fear\" and \"not want to be with me\" in the entries are negative emotional expressions of concern and anxiety about the future, and therefore negative."}, "language_style": {"answer": "Tactful and warm", "rationale": "The entry asks each other in a concerned way whether they often have such concerns, in a more moderate and common sense. At the same time, by using the term \"you\" rather than \"you\" it makes the questionnaire more relevant and personal, and better attracts the attention of the interviewees and guides them to answer questions."}, "time_dimension": {"answer": "Present", "rationale": "The keyword \"I'm often worried\" indicates that it is a sentence describing the current state or emotional state of the person being tested, so that the entry is a historical one."}, "sentence_type": "Negative declarative sentence", "pos": {"nouns": {"Questionnaire Entry": 1, "Couple": 1, "I": 2}, "verbs": {"I'm worried": 1, "Yes": 1, "in": 1, "Together": 1}, "adverbs": {"Often": 1}, "numerals": {"9": 1}}}, {"content": "When I express my feelings to others, I fear that they will feel different from me.", "word_count": 26, "label": "", "sentence_structure": "Subject鈥揷opula鈥損redicative", "subjective_objective": {"answer": "Subjective", "rationale": "Because \"fear\" is a subjective feeling and emotional reaction."}, "sentence_sentiment": {"answer": "Negative", "rationale": "Because the emotional expression \"fearing that they will feel different from me\" in the entry is negative."}, "language_style": {"answer": "Tactful and warm", "rationale": "The entry uses a more euphemistic formulation that leads the respondents to share their thoughts and feelings by describing a subjective feeling, which is more likely to generate resonance and support among the respondents and also creates an easy, friendly atmosphere."}, "time_dimension": {"answer": "Past", "rationale": "The act of \"I express my feelings\" has happened in the past, and \"fear\" is an emotional experience in the process of expressing feelings, so it can be judged that the entry describes the past experience or feelings."}, "sentence_type": "Negative declarative sentence", "pos": {"nouns": {"Questionnaire": 1, "Entry": 1, "person": 1, "Emotion": 1, "they": 1, "Feel": 1}, "verbs": {"Expression": 1, "Scared": 1, "It's different": 1}, "adjectives": {"others": 1}, "numerals": {"10": 1}, "pronouns": {"I": 1}}}, {"content": "I often wonder if couples really care about me.", "word_count": 14, "label": "", "sentence_structure": "Subject鈥損redicate", "subjective_objective": {"answer": "Subjective", "rationale": "Because suspicion is a subjective sentiment and attitude."}, "sentence_sentiment": {"answer": "Negative", "rationale": "Because the attitude in the entry \"Suspects if the couple really cares about me\" is negative."}, "language_style": {"answer": "Tactful and warm", "rationale": "The entry is in the form of a question sentence and does not directly accuse or complain about the other, and is expressed in a relatively soft and friendly way, which is more in keeping with the euphemism of the language. At the same time, the entry is not too strongly negative, and is more oriented towards the objective description of its feelings and ideas, which is also a feature of the euphemism of the language style."}, "time_dimension": {"answer": "Present", "rationale": "The words \"suspect\" and \"are really concerned with me\" in the entry indicate that the testee has committed such acts on a regular basis, and the verbs \"suspicion\" and \"are really concerned with me\" are all in the usual current state of affairs, which means that this is the current psychological state of the testee. Thus, the temporal dimension of the article is now."}, "sentence_type": "Interrogative sentence", "pos": {"nouns": {"Couple": 1, "Care": 1}, "verbs": {"Doubt": 1, "be": 1, "Really": 1, "Care": 1}, "adjectives": {"Often": 1, "Really": 1}, "numerals": {"11": 1}, "pronouns": {"I": 2}}}, {"content": "12. I am comfortable with the establishment of close relationships.", "word_count": 16, "label": "", "sentence_structure": "Subject鈥揷opula鈥損redicative", "subjective_objective": {"answer": "Subjective", "rationale": "Because \"sweet\" is a personal feeling and attitude that belongs to the patient's subjective perspective."}, "sentence_sentiment": {"answer": "Positive", "rationale": "Because it expresses a positive attitude, which is \"comfort with intimate relationships\"."}, "language_style": {"answer": "Tactful and warm", "rationale": "The language style of the entry is euphemism. It uses more subjective expressions to ask the interviewee how he feels and to use the more moderate wording."}, "time_dimension": {"answer": "Present", "rationale": "The entry describes a subjective or emotional state of the person being tested, namely, \"comfort with the intimate relationship of others\", which is the current, immediate state. Thus, the temporal dimension of the purpose of the article is the present."}, "sentence_type": "Affirmative declarative sentence", "pos": {"nouns": {"Questionnaire Entry": 1, "others": 1, "Relations": 1, "- Yeah": 1}, "verbs": {"to": 1, "Create": 1, "I feel": 1}, "adjectives": {"very": 1, "Intimacy": 1}, "numerals": {"12": 1}, "pronouns": {"I": 1}}}, {"content": "I feel uncomfortable when someone is too emotionally close to me.", "word_count": 18, "label": "", "sentence_structure": "Subject鈥揷opula鈥損redicative", "subjective_objective": {"answer": "Subjective", "rationale": "Because \"uncomfort\" is a subjective feeling and emotional experience, the problem is considered subjective."}, "sentence_sentiment": {"answer": "Negative", "rationale": "Because the word \"uncomfortable\" in the entry is an expression of negative emotional attitudes."}, "language_style": {"answer": "Tactful and warm", "rationale": "Warm and warm."}, "time_dimension": {"answer": "Present", "rationale": "The \"feel\" in \"when I feel uncomfortable\" uses the current state of affairs, indicating that this sentiment exists at this moment, so the temporal dimension of the article is now."}, "sentence_type": "Interrogative sentence", "pos": {"nouns": {"person": 1, "Emotion": 1, "Close": 1, "I'm not feeling well": 1}, "verbs": {"I feel": 1, "be": 1, "Too much": 1}, "adjectives": {"Close": 1}, "adverbs": {"in": 1, "Go, go, go": 1}, "numerals": {"13": 1}}}, {"content": "I know that when I need help, someone will help me.", "word_count": 18, "label": "", "sentence_structure": "Subject鈥搗erb鈥搊bject", "subjective_objective": {"answer": "Subjective", "rationale": "This is a subjective attitude of the patient, since it is unpredictable or not controlled that assistance is provided when it is needed and cannot be considered as objective."}, "sentence_sentiment": {"answer": "Positive", "rationale": "Because it expresses a positive attitude and quality, i.e. knowing and believing that people will always support it when they need help."}, "language_style": {"answer": "Tactful and warm", "rationale": "The language style of the entry is euphemism. It implies a positive attitude of support and help to the people interviewed in their lives, networks or interpersonal relationships, and encourages them to believe it. This expression is more likely to trigger resonance and positive emotional reactions among the people interviewed."}, "time_dimension": {"answer": "Present", "rationale": "The entry asks the testees about their current beliefs and perceptions, and the key words \"know\" and \"help\" indicate that this is their current perception and attitude."}, "sentence_type": "Affirmative declarative sentence", "pos": {"nouns": {"Problem": 1, "person": 2, "Help": 1}, "verbs": {"Yes": 3, "Help": 1}, "adverbs": {"When": 1, "Total": 1}, "numerals": {"14": 1}, "pronouns": {"I": 1}}}, {"content": "I want to be close to people, but I fear that I will be hurt.", "word_count": 16, "label": "", "sentence_structure": "Subject鈥搗erb鈥搊bject", "subjective_objective": {"answer": "Subjective", "rationale": "Because \"want to be close\" and \"fear to be hurt\" are the thoughts and fears of the patient, which are subjective."}, "sentence_sentiment": {"answer": "Negative", "rationale": "Because the emotional expression \"fearing to hurt\" in the entry is negative."}, "language_style": {"answer": "Tactful and warm", "rationale": ""}, "time_dimension": {"answer": "Present", "rationale": "The keyword \"want\" and \"fear\" are now the usual expressions of the present psychological state or emotional experience of the person being tested. So, the entry describes the current feelings and thoughts."}, "sentence_type": "Affirmative declarative sentence", "pos": {"nouns": {"I": 2, "person": 1, "Close": 1, "Hurt": 1}, "verbs": {"Yes": 4, "- Yes, sir": 1, "Close": 1}, "adjectives": {"Close": 1, "Hurt": 1}, "numerals": {"15": 1, "2": 1, "1": 4}, "pronouns": {"self": 1}}}, {"content": "16. I find it difficult to rely entirely on others.", "word_count": 12, "label": "", "sentence_structure": "Subject鈥搗erb鈥揷omplement", "subjective_objective": {"answer": "Subjective", "rationale": "It is because it is possible to discover whether one can rely entirely on the individual's subjective judgement and feelings."}, "sentence_sentiment": {"answer": "Negative", "rationale": "Because the phrase \"difficult to rely fully on others\" in the entry expresses a negative emotion and attitude."}, "language_style": {"answer": "Tactful and warm", "rationale": ""}, "time_dimension": {"answer": "Present", "rationale": ""}, "sentence_type": "Negative declarative sentence", "pos": {"nouns": {"I": 1, "person": 1, "others": 1}, "verbs": {"Found": 1, "Trust": 1}, "adjectives": {"Hard": 1, "Total": 1}, "numerals": {"16": 1}}}, {"content": "17. Couples want me to be more emotional, which often makes me uncomfortable.", "word_count": 23, "label": "", "sentence_structure": "Subject鈥揷opula鈥損redicative", "subjective_objective": {"answer": "Subjective", "rationale": "This is a subjective description of the patient ' s mental state, expressing their emotional reaction and discomfort."}, "sentence_sentiment": {"answer": "Negative", "rationale": "Because the description \"uncomfortable\" in the entry is negative."}, "language_style": {"answer": "Calm and direct", "rationale": "The entry describes a specific situation in objective and direct language and expresses the feelings of the interviewees, and is not overly emotional or rhetorical, so that the language style is straightforward and calm."}, "time_dimension": {"answer": "Present", "rationale": "Because \"I'm often uncomfortable\" is a description of the current state of feeling and state, the temporal dimension is now judged."}, "sentence_type": "Affirmative declarative sentence", "pos": {"nouns": {"Couple": 1, "Emotion": 1, "Close": 1}, "verbs": {"Yes": 1, "鈾?And I'm gonna be 鈾?: 1, "I feel": 1}, "adjectives": {"I'm not feeling well": 1}, "numerals": {"17": 1}, "pronouns": {"I": 2}}}, {"content": "18. I am not sure that I can always find someone I can rely on when I need them.", "word_count": 20, "label": "", "sentence_structure": "Subject鈥搗erb鈥搊bject", "subjective_objective": {"answer": "Subjective", "rationale": "Because \"can you be sure\" and \"can find\" are patients' subjective feelings and attitudes."}, "sentence_sentiment": {"answer": "Negative", "rationale": "Because the contrast between \"uncertain\" and \"required for someone who can rely on\" in the entry is an expression of a negative emotional ambivalence, suggesting that the interviewee may feel difficulties or anxiety in finding reliable support."}, "language_style": {"answer": "Tactful and warm", "rationale": "The language style of the entry is euphemism. It expresses the theme by using negative tone and highlights the uncertainty that respondents face in finding people they can rely on. It is relatively mild, not too direct or hard, more friendly and inclusive, and helps to create a good atmosphere of communication."}, "time_dimension": {"answer": "Present", "rationale": "So the answer is \"now\"."}, "sentence_type": "Negative declarative sentence", "pos": {"nouns": {"18": 1, "person": 1}, "verbs": {"Found it": 1, "Dependency": 1}, "adjectives": {"Sure": 1}, "adverbs": {"Always": 1, "Yeah": 2}, "pronouns": {"I": 1}}}]}

{"questionnaire_title": "Self-esteem questionnaire", "questionnaire_category": "Self-cognition", "item_count": 58, "emotion_shift_overall": "From positive to negative", "language_style_overall": "Tactful and warm", "items": [{"content": "I spent a lot of time dreaming.", "word_count": 10, "label": "", "sentence_structure": "Subject鈥搗erb鈥搊bject", "subjective_objective": {"answer": "Subjective", "rationale": "Because I'm a person's personal experience and subjective feeling."}, "sentence_sentiment": {"answer": "Neutral", "rationale": "Because the entry is not clearly emotional, it describes an act or habit and does not carry the emotions or attitudes of the person."}, "language_style": {"answer": "Tactful and warm", "rationale": "The entry is more adept and interactive when it is spoken in a gentle and inviting manner, rather than by direct statements or orders."}, "time_dimension": {"answer": "Present", "rationale": ""}, "sentence_type": "Affirmative declarative sentence", "pos": {"nouns": {"Time": 1, "Daydreams": 1}, "verbs": {"Flowers": 1, "Do it": 1}, "adverbs": {"very": 1}, "pronouns": {"I": 1}}}, {"content": "I am sure of myself.", "word_count": 8, "label": "", "sentence_structure": "Subject鈥揷opula鈥損redicative", "subjective_objective": {"answer": "Subjective", "rationale": "Because it is a subjective feeling and a perception of what is in control of one's own."}, "sentence_sentiment": {"answer": "Positive", "rationale": "Because the words in the entries are \"very sure of themselves,\" and they indicate that they have confidence and motivation."}, "language_style": {"answer": "Tactful and warm", "rationale": "This entry is more consistent with the principles of confidence-building and interaction in psychological questionnaires."}, "time_dimension": {"answer": "Present", "rationale": "The entry \"I'm sure of myself\" indicates the mentalities or behaviour of the testee in answering the question, which is a description of the current self-awareness and self-confidence. So the judgment is now."}, "sentence_type": "Affirmative declarative sentence", "pos": {"verbs": {"to": 1, "have": 1}, "adjectives": {"self": 1, "very": 1, "Sure": 1}, "pronouns": {"I": 1}}}, {"content": "I often wish I was someone else.", "word_count": 12, "label": "", "sentence_structure": "Subject鈥搗erb鈥揷omplement", "subjective_objective": {"answer": "Subjective", "rationale": "Because \"fear is his own subjective attitude and desire.\""}, "sentence_sentiment": {"answer": "Negative", "rationale": "Because the attitude of \"whom else you want\" in the entry is an expression of a negative emotion and dissatisfaction with the status quo."}, "language_style": {"answer": "Tactful and warm", "rationale": "The entry is expressed indirectly, asking questions on the theme \"I want to be someone else\" and avoiding touching the other side's personal features or emotions, which is more euphemism and affection. Moreover, the language style is more moderate and friendly, and helps create a comfortable and pleasant investigation atmosphere."}, "time_dimension": {"answer": "Future", "rationale": "Thus, based on the above analysis, the temporal dimension of the entry of the questionnaire is judged as \"now\", as it contains a description of the current self-status and expectations for the future."}, "sentence_type": "Affirmative declarative sentence", "pos": {"nouns": {"self": 1, "others": 1}, "verbs": {"Hope": 1}, "adverbs": {"Regular": 1}, "numerals": {"3": 1}}}, {"content": "I like what I like easily.", "word_count": 8, "label": "", "sentence_structure": "Subject鈥搗erb鈥揷omplement", "subjective_objective": {"answer": "Subjective", "rationale": "Because like is a subjective feeling and attitude."}, "sentence_sentiment": {"answer": "Neutral", "rationale": "Because the entry does not have any emotional color."}, "language_style": {"answer": "Tactful and warm", "rationale": "The language style of the entry is more euphemistic and gentle, and the use of terms is more intimate, and there is no too direct or hard-line expression that gives people a friendly and comfortable feeling, which helps to make respondents more willing to cooperate in completing the questionnaire and sharing their ideas."}, "time_dimension": {"answer": "Present", "rationale": ""}, "sentence_type": "Interrogative sentence", "pos": {"nouns": {"Questionnaire Entry": 1, "I": 1, "What": 1}, "verbs": {"Easy": 1, "I like it": 1}, "adverbs": {"Down there": 1, "Please": 1, "Analysis": 1, "EMPTY": 1}, "numerals": {"4": 1}}}, {"content": "I have had a lot of fun with my parents.", "word_count": 13, "label": "", "sentence_structure": "Subject鈥揷opula鈥損redicative", "subjective_objective": {"answer": "Subjective", "rationale": "Because happiness is a subjective feeling, and different people may feel differently about the same thing, the entry on the questionnaire is subjective."}, "sentence_sentiment": {"answer": "Positive", "rationale": "Because the phrase \"I'm having a lot of fun with my parents\" in the entry reflects positive emotional attitudes."}, "language_style": {"answer": "Tactful and warm", "rationale": "This language style is more resonant and more effective in getting real feedback."}, "time_dimension": {"answer": "Present", "rationale": "The entry \"I'm happy with my parents\" describes the current situation, which is the current state of affairs, so it can be judged now."}, "sentence_type": "Affirmative declarative sentence", "pos": {"nouns": {"I": 2, "and": 1, "parents": 1, "Happy things": 1}, "verbs": {"Together": 1, "have": 1}, "adjectives": {"A lot": 1}, "numerals": {"5": 1}}}, {"content": "I never worry about anything.", "word_count": 9, "label": "", "sentence_structure": "Subject鈥搗erb鈥揷omplement", "subjective_objective": {"answer": "Subjective", "rationale": "Because \"I never worry about anything\" is the patient's subjective assessment and attitude to his emotional state."}, "sentence_sentiment": {"answer": "Negative", "rationale": "Because, while the entry does not express a clear negative sentiment, such behaviour may be considered a negative attitude, as it may imply, to some extent, a lack of concern and concern, which may lead to a failure to address problems or risks in a timely manner."}, "language_style": {"answer": "Calm and direct", "rationale": "The language style of the entry is \"cool and straight.\" It directly expresses whether the subject is always worried about life, without using too many rhetorical or emotional accents. This direct language makes the question more clear and concise."}, "time_dimension": {"answer": "Past", "rationale": "The entry \"never\" denotes something that has never been done before, and therefore is judged as past behaviour."}, "sentence_type": "Interrogative sentence", "pos": {"verbs": {"I": 1, "I'm worried": 1}, "adjectives": {"Any": 1}, "adverbs": {"Never": 1}}}, {"content": "I find it difficult to speak in front of the entire class.", "word_count": 13, "label": "", "sentence_structure": "Subject鈥搗erb鈥搊bject", "subjective_objective": {"answer": "Subjective", "rationale": "Because Discovery shows that it's a patient's personal feeling and perception, a subjective perspective."}, "sentence_sentiment": {"answer": "Negative", "rationale": "Because the phrase \"it's hard to speak in front of the class\" in the entry indicates that there is a certain sense of tension and pressure in the public arena and that it has a negative emotional tone."}, "language_style": {"answer": "Calm and direct", "rationale": "The entry presents a fact or feeling in a straightforward manner, without too much rhetorical or emotional rendering, and is more balanced and objective. The language style of the article is therefore considered to be straightforward and calm."}, "time_dimension": {"answer": "Present", "rationale": "Based on the phrase \"I find\" and \"hard,\" it can be inferred that this is a description of the current situation, indicating that in the current learning environment, \"spoke in front of the class\" is a difficult one for individuals. Thus, the entry is a topic of the current behavioral or mental type."}, "sentence_type": "Affirmative declarative sentence", "pos": {"nouns": {"Questionnaire": 1, "Entry": 1, "All class": 1, "Noodles": 1, "Speech": 1}, "verbs": {"Found": 1, "In front of you": 1, "It's hard": 1}, "numerals": {"7": 1}}}, {"content": "I wish I was younger.", "word_count": 11, "label": "", "sentence_structure": "Subject鈥搗erb鈥揷omplement", "subjective_objective": {"answer": "Subjective", "rationale": "Because hope is the patient's subjective attitude."}, "sentence_sentiment": {"answer": "Negative", "rationale": "Because the idea of \"hope for a younger age\" implies a negative emotion to the dissatisfaction with life and the memory of the past."}, "language_style": {"answer": "Tactful and warm", "rationale": "The language style of the entry is more euphemistic, as it expresses the desire for a young age in a relatively soft way, rather than a direct statement or order."}, "time_dimension": {"answer": "Future", "rationale": "The entry expresses the expectations and hopes of the testees for the future, and the key word is \"want to be younger\", pointing to situations that have not yet occurred and that may arise in the future."}, "sentence_type": "Imperative sentence", "pos": {"nouns": {"Age": 1, "self": 1}, "verbs": {"Hope": 1, "Smaller": 1}, "adverbs": {"Some": 1}, "pronouns": {"I": 1}}}, {"content": "9. I have many things to change, if possible.", "word_count": 16, "label": "", "sentence_structure": "Subject鈥損redicate", "subjective_objective": {"answer": "Subjective", "rationale": "Because \"To change many things about yourself\" is the patient's subjective desire and thought."}, "sentence_sentiment": {"answer": "Negative", "rationale": "Because the phrase \"many things to change\" in the entry implies dissatisfaction with the status quo and negative sentiments."}, "language_style": {"answer": "Tactful and warm", "rationale": "The language style of the entry is more euphemistic and warm, because it uses the means of questioning rather than ordering or making direct statements. This expression is more intimate and interactive, and it is easier to stimulate the thoughts and sharing of the interviewees. Moreover, the phrase \"if possible\" provides the interviewee with a choice and buffer, which makes him feel more comfortable and free."}, "time_dimension": {"answer": "Future", "rationale": "The keyword \"to change\" expresses a subjective desire or intention, while \"if possible\" implies that this change has not yet been achieved. The temporal dimension of the article is therefore the future."}, "sentence_type": "Imperative sentence", "pos": {"nouns": {"Things": 1}, "verbs": {"Yes": 1, "Change": 1, "Do it": 1}, "adjectives": {"Maybe": 1}, "adverbs": {"very": 1}, "numerals": {"9": 1}, "classifiers": {"individual": 1}, "pronouns": {"I": 1}}}, {"content": "10. I need not have too much difficulty in making my own decisions.", "word_count": 13, "label": "", "sentence_structure": "Subject鈥搗erb鈥揷omplement", "subjective_objective": {"answer": "Subjective", "rationale": "Because \"no hard time to make a decision\" is a patient's subjective judgment and feeling."}, "sentence_sentiment": {"answer": "Positive", "rationale": "Because it expresses a self-empowerment and self-confidence attitude."}, "language_style": {"answer": "Tactful and warm", "rationale": "The language style of the entry is more euphemistic and makes the question more comfortable and acceptable by using the expression \"no more difficulties\". Such language style helps to increase the motivation and trust of the interviewee."}, "time_dimension": {"answer": "Present", "rationale": "So output for \"now\"."}, "sentence_type": "Imperative sentence", "pos": {"verbs": {"Yes": 1, "Hold it": 1}, "adjectives": {"Large": 1}, "adverbs": {"Too much": 1}, "numerals": {"10": 1}, "pronouns": {"I": 1}}}, {"content": "I have many happy stories.", "word_count": 7, "label": "", "sentence_structure": "Subject鈥搗erb鈥揷omplement", "subjective_objective": {"answer": "Subjective", "rationale": "Because \"happy\" and \"many happy things\" are subjective feelings and experiences of patients, the view of this questionnaire entry is subjective."}, "sentence_sentiment": {"answer": "Positive", "rationale": "Because the expression \"many happy things\" conveys positive emotions and attitudes."}, "language_style": {"answer": "Tactful and warm", "rationale": ""}, "time_dimension": {"answer": "Present", "rationale": "The keyword is \"many happy things\", which means the state or feeling of the moment, and therefore falls within the realm of the past."}, "sentence_type": "Interrogative sentence", "pos": {"nouns": {"Questionnaire": 1, "Entry": 1, "Many": 1, "Happy things": 1}, "verbs": {"have": 1}, "numerals": {"11": 1}}}, {"content": "12. It is easy to be unhappy at home.", "word_count": 9, "label": "", "sentence_structure": "Subject鈥揷opula鈥損redicative", "subjective_objective": {"answer": "Subjective", "rationale": "Because \"easy to be unhappy\" is an emotional experience, a subjective feeling of the patient."}, "sentence_sentiment": {"answer": "Negative", "rationale": "Because the \"unhappy\" behavior in the entry is negative."}, "language_style": {"answer": "Calm and direct", "rationale": "The entry directly expresses the subject of questioning the other party ' s displeasure at home, without excessive rhetoric or emotional rendering, and with a more balanced and objective tone."}, "time_dimension": {"answer": "Present", "rationale": "The entry describes a subjective feeling or state of affairs, which is \"at home\" indicating that it occurs in a particular place, and \"easy\" means that it is a regular situation, so it can be judged to be a description of the present state of affairs."}, "sentence_type": "Negative declarative sentence", "pos": {"nouns": {"Home": 1, "I'm not happy": 1}, "verbs": {"in": 1, "Easy": 1, "Happy": 1}, "numerals": {"12": 1}, "pronouns": {"I": 1}}}, {"content": "I always do what I have to do.", "word_count": 9, "label": "", "sentence_structure": "Subject鈥搗erb鈥揷omplement", "subjective_objective": {"answer": "Objective", "rationale": "Because \" always do what I have to do\" is an objective description of behaviour, not involving patients' subjective attitudes or feelings."}, "sentence_sentiment": {"answer": "Neutral", "rationale": "Because the entry is not clearly emotional."}, "language_style": {"answer": "Calm and direct", "rationale": "The entry is presented in a simple statement, with no excessive emotional or rhetorical style, and is more straightforward and objective. So I think that its language style is calm and straightforward."}, "time_dimension": {"answer": "Present", "rationale": ""}, "sentence_type": "Affirmative declarative sentence", "pos": {"nouns": {"Questionnaire": 1, "Entry": 1, "Yes": 1}, "verbs": {"Do it": 1, "The": 1, "be": 1}, "adverbs": {"Always": 1}, "numerals": {"13": 1}, "pronouns": {"I": 1}}}, {"content": "I am proud of my work.", "word_count": 11, "label": "", "sentence_structure": "Subject鈥揷opula鈥損redicative", "subjective_objective": {"answer": "Subjective", "rationale": "Because pride is a subjective emotional experience that reflects patients' attitudes and feelings about their homework."}, "sentence_sentiment": {"answer": "Positive", "rationale": "Because in the entry, \"right to self\""}, "language_style": {"answer": "Tactful and warm", "rationale": "The \"I'm proud of myself\" in the entry uses a language style of affirmative, self-identifying, emphasizing the positive attitude and self-confidence of individuals, which is more likely to trigger the echoes of those surveyed and to stimulate their positive feelings."}, "time_dimension": {"answer": "Present", "rationale": "The entry asks the testee about his self-evaluation of his current homework, using the first person, who is proud, and the general current, and thus judging now."}, "sentence_type": "Affirmative declarative sentence", "pos": {"nouns": {"I": 1, "Your own": 1, "Work": 1}, "verbs": {"to": 1, "I feel": 1, "Proud": 1}, "numerals": {"14": 1}}}, {"content": "I always need someone to tell me what to do.", "word_count": 14, "label": "", "sentence_structure": "Subject鈥揷opula鈥損redicative", "subjective_objective": {"answer": "Subjective", "rationale": "Because \"it's always necessary to tell me what to do\" reflects the patient's dependence on his actions and decision-making, and is the patient's subjective feeling."}, "sentence_sentiment": {"answer": "Negative", "rationale": "Because it expresses a lack of self-decision-making capacity and dependence on others, it is a negative emotional attitude."}, "language_style": {"answer": "Calm and direct", "rationale": "The language style of the entry is relatively calm and straightforward, and it raises a question directly, without too much embellishment or emotional rendering, and is expressed in a more concise and concise manner."}, "time_dimension": {"answer": "Present", "rationale": "The keyword \"always need\" and \"tell\" indicates that it is a statement describing the current behaviour or attitude of the interviewees and can therefore be judged as now."}, "sentence_type": "Affirmative declarative sentence", "pos": {"nouns": {"Questionnaire Entry": 1, "person": 1}, "verbs": {"Tell": 1, "Do it": 1}, "adverbs": {"Always": 1, "Yes": 1}, "numerals": {"15": 1}, "classifiers": {"individual": 1}, "pronouns": {"I": 1}}}, {"content": "16. It takes me a long time to get used to new things.", "word_count": 14, "label": "", "sentence_structure": "Subject鈥搗erb鈥搊bject", "subjective_objective": {"answer": "Subjective", "rationale": "Because the habit of new things is the subjective goal of the patient, who needs to invest time and effort to achieve them, it is subjective."}, "sentence_sentiment": {"answer": "Negative", "rationale": "Because it's a \"long time before you get used to\" behavior in the entry is negative."}, "language_style": {"answer": "Tactful and warm", "rationale": "The language style of the entry is more euphemistic, moderate, and it may take longer to adapt to the subject of new things, rather than directly rejecting or criticizing. It makes it easier for the interviewee to feel comfortable and acceptable and to answer questions in a genuine manner."}, "time_dimension": {"answer": "Past", "rationale": "* Enter sentence: \"16. It takes me a long time to get used to new things.\"* Output: \"The Past.\""}, "sentence_type": "Affirmative declarative sentence", "pos": {"nouns": {"Time": 1, "Things": 1}, "verbs": {"Flowers": 1, "Custom": 1, "Yes": 1}, "adjectives": {"Long": 1}, "adverbs": {"very": 1, "That's right": 1}, "numerals": {"16": 1}}}, {"content": "17. I often regret what I have done.", "word_count": 11, "label": "", "sentence_structure": "Subject鈥搗erb鈥揷omplement", "subjective_objective": {"answer": "Subjective", "rationale": "Because \"I often regret what I do\" is the patient's subjective feeling and attitude."}, "sentence_sentiment": {"answer": "Negative", "rationale": "Because the \"often regret\" act in the entry is negative."}, "language_style": {"answer": "Calm and direct", "rationale": "\"Calm down straight.\""}, "time_dimension": {"answer": "Present", "rationale": ""}, "sentence_type": "Affirmative declarative sentence", "pos": {"nouns": {"Yes": 1}, "verbs": {"Do it": 1, "Sensor": 1, "Present": 1, "Regret": 1}, "adverbs": {"Regular": 1}, "numerals": {"17": 1}, "pronouns": {"I": 1}}}, {"content": "18. I am able to eat from among the children of my age.", "word_count": 14, "label": "", "sentence_structure": "Subject鈥搗erb鈥揷omplement", "subjective_objective": {"answer": "Subjective", "rationale": "Because \"eating\" is a subjective assessment and feeling, and it is the patient's perception of his place and popularity among his peers."}, "sentence_sentiment": {"answer": "Positive", "rationale": "Because the entry \"sweetly eats\" shows that you have a high social status and popularity among people of this age group, which is a positive emotion and attitude."}, "language_style": {"answer": "Tactful and warm", "rationale": "The language style of the entry is more euphemistic and friendly, rather than using strong tone or evaluative vocabulary, but rather allowing the respondents to express their views in a question-and-answer manner, giving them a sense of comfort. This euphemism can make the respondents more willing to participate in the questionnaire and answer questions honestly."}, "time_dimension": {"answer": "Present", "rationale": "\"Eats well\" means the person who was tested for feelings and behavior in his current state, so the temporal dimension is now."}, "sentence_type": "Affirmative declarative sentence", "pos": {"nouns": {"Son": 1, "Age": 1, "Same age": 1}, "verbs": {"It's good": 1, "in": 1}, "adverbs": {"very": 1}, "numerals": {"18": 1}, "classifiers": {"individual": 1}, "pronouns": {"I": 1}}}, {"content": "19. My parents usually take my feelings into account.", "word_count": 14, "label": "", "sentence_structure": "Subject鈥搗erb鈥搊bject", "subjective_objective": {"answer": "Objective", "rationale": "Since it is an objective fact to take into account the patient ' s mood, it is not influenced by the patient ' s subjective feelings."}, "sentence_sentiment": {"answer": "Neutral", "rationale": "Because the entry does not express a positive or negative emotional expression."}, "language_style": {"answer": "Tactful and warm", "rationale": "The problem begins with a sense of affection and interaction, as the first person calls it, and the term \"normally takes into account\" is more neutral and objective, not too affirmative or negative, and more in keeping with the warmer expression. 3. The term \"my parents\" is used to make people feel warm and caring."}, "time_dimension": {"answer": "Past", "rationale": "The keyword \"usually\" indicates that the act occurred within a certain period of time in the past and may continue to be so, and therefore falls within the temporal dimension of the past."}, "sentence_type": "Affirmative declarative sentence", "pos": {"nouns": {"I": 1, "parents": 1, "The mood": 1}, "verbs": {"I can take care of it": 1}, "adverbs": {"Usually": 1}}}, {"content": "I've never been unhappy.", "word_count": 8, "label": "", "sentence_structure": "Subject鈥揷opula鈥損redicative", "subjective_objective": {"answer": "Subjective", "rationale": "Because \"unsatisfied\" is a subjective feeling or emotional experience, the question is viewed from the subjective perspective of the patient."}, "sentence_sentiment": {"answer": "Neutral", "rationale": "Because the entry does not display positive or negative emotional colours, it merely describes a person ' s mental state."}, "language_style": {"answer": "Calm and direct", "rationale": "The language style of the entry is calm and straightforward. It expresses a point in a simple and clear language, without using too many emotional colours or rhetorical techniques to emphasize its importance or impact."}, "time_dimension": {"answer": "Present", "rationale": "The keyword \"never\" indicates a customary, normal state of affairs, and \"unsatisfied\" is a term for describing the current emotional state, so the entry asks about the current emotional state of the subject."}, "sentence_type": "Affirmative declarative sentence", "pos": {"verbs": {"Yes": 1, "From": 1, "Come on": 1, "not": 1, "Happy": 1}, "adverbs": {"Never": 1}, "pronouns": {"I": 1}}}, {"content": "21. I am doing the best I can.", "word_count": 13, "label": "", "sentence_structure": "Subject鈥搗erb鈥揷omplement", "subjective_objective": {"answer": "Subjective", "rationale": "Because this is the patient ' s subjective assessment and perception of his current behaviour."}, "sentence_sentiment": {"answer": "Positive", "rationale": "Because the \"doing the best I can\" act is positive."}, "language_style": {"answer": "Tactful and warm", "rationale": "The entry uses a positive and encouraging language style, which expresses the \"doing\" of the current state of play, and uses the positive phrase \"I'm doing the best I can do,\" which in part conveys the author's support and encouragement to the interviewee. This euphemism of language can make the interviewee feel more comfortable and comfortable and more willing to share his thoughts and feelings."}, "time_dimension": {"answer": "Present", "rationale": "The entry uses the verb \"in the process of doing\" in the ongoing process, which indicates the current behaviour or state of the speaker, and therefore judges \"now\"."}, "sentence_type": "Affirmative declarative sentence", "pos": {"verbs": {"Do it": 1, "Yes": 1}, "adjectives": {"I'd better": 1}, "adverbs": {"Most": 1, "Yes": 1}, "pronouns": {"I": 1}}}, {"content": "I can easily submit.", "word_count": 6, "label": "", "sentence_structure": "Subject鈥揷opula鈥損redicative", "subjective_objective": {"answer": "Subjective", "rationale": "Because \"easy to surrender\" is the patient's assessment and perception of himself, and it's in the realm of subjective attitudes."}, "sentence_sentiment": {"answer": "Negative", "rationale": "Because the expression \"easy to surrender\" is negative, it implies a lack of individual determination and perseverance, and may show behaviour that is evasive or compromise when faced with challenges or pressures."}, "language_style": {"answer": "Tactful and warm", "rationale": "The entry uses a more euphemistic tone, by asking questions, in which the respondent answers the question on his/her own initiative, rather than directly stating or ordering him/her to acknowledge his/her shortcomings. At the same time, the question is more closely related to the personal feelings and experiences of the respondent and better guides them to a true self-assessment."}, "time_dimension": {"answer": "Present", "rationale": "The entry asks the testee about his current behaviour or mentality, and \"easy to surrender\" is a customary act, which is based on the prevailing state of affairs and is therefore judged as \"now\"."}, "sentence_type": "Negative declarative sentence", "pos": {"verbs": {"Easy": 1, "Conceal": 1}, "adjectives": {"very": 1}, "numerals": {"22": 1}, "pronouns": {"I": 1}}}, {"content": "I'm usually able to take care of myself.", "word_count": 9, "label": "", "sentence_structure": "Subject鈥揷opula鈥損redicative", "subjective_objective": {"answer": "Subjective", "rationale": "Because \"care for yourself\" is a self-evaluation or attitude, which is a subjective view of the patient."}, "sentence_sentiment": {"answer": "Positive", "rationale": "Because the act of \"care for yourself\" is positive."}, "language_style": {"answer": "Calm and direct", "rationale": "The entry asks the respondent in simple and direct language whether he or she is capable of taking care of himself or herself, is more objective and neutral in tone and does not have too much emotional or rhetorical style."}, "time_dimension": {"answer": "Present", "rationale": "The entry describes the current status of the testees, which means that they can take care of themselves and therefore judge as now."}, "sentence_type": "Affirmative declarative sentence", "pos": {"verbs": {"General": 1, "Care": 1, "self": 1}, "numerals": {"23": 1}}}, {"content": "I'm happy.", "word_count": 4, "label": "", "sentence_structure": "Subject鈥揷opula鈥損redicative", "subjective_objective": {"answer": "Subjective", "rationale": "Because \"happy\" is an emotional experience, a subjective feeling of the patient."}, "sentence_sentiment": {"answer": "Positive", "rationale": "Because the phrase \"I'm happy\" in the entry is a positive emotional attitude."}, "language_style": {"answer": "Tactful and warm", "rationale": "The entry is a positive and encouraging expression of concern and support for the other and a more cordial and sweet tone."}, "time_dimension": {"answer": "Present", "rationale": ""}, "sentence_type": "Affirmative declarative sentence", "pos": {"adjectives": {"Happy": 1}, "adverbs": {"very": 1}}}, {"content": "I would rather play with children younger than me.", "word_count": 13, "label": "", "sentence_structure": "Subject鈥搗erb鈥揷omplement", "subjective_objective": {"answer": "Subjective", "rationale": "This is because it is a tendency or preference of the patient and is part of a subjective attitude."}, "sentence_sentiment": {"answer": "Neutral", "rationale": "As this entry does not express positive or negative tendencies about emotions, attitudes, behaviour, purpose or quality."}, "language_style": {"answer": "Tactful and warm", "rationale": "The entry expresses an individual's preferences or preferences in a first person's name, and uses a comparative expression, rather than a direct statement or order. It is a euphemism, more personal and interactive. At the same time, it is not too emotional in its linguistic style, and therefore tends more to be \"fluent.\""}, "time_dimension": {"answer": "Future", "rationale": "The entry expresses expectations and preferences for the future, and the key words are \"a lot more \" , \"a little younger than me,\" which all point to future behaviour or attitudes."}, "sentence_type": "Affirmative declarative sentence", "pos": {"nouns": {"Son": 1, "Age": 1}, "verbs": {"May I": 1, "and": 1, "Play": 1}, "adjectives": {"- Yeah": 1, "Small": 1}, "numerals": {"25": 1}, "pronouns": {"I": 1}}}, {"content": "26. My parents expect too much from me.", "word_count": 12, "label": "", "sentence_structure": "Subject鈥揷opula鈥損redicative", "subjective_objective": {"answer": "Subjective", "rationale": "Because \"too much to expect\" is a subjective assessment that reflects the patient's feelings and perceptions of his or her parents."}, "sentence_sentiment": {"answer": "Negative", "rationale": "Because the expression \"too much to expect\" in the entry is negative."}, "language_style": {"answer": "Tactful and warm", "rationale": ""}, "time_dimension": {"answer": "Present", "rationale": ""}, "sentence_type": "Negative declarative sentence", "pos": {"nouns": {"parents": 1, "Expectations": 1}, "verbs": {"to": 1, "have": 1}, "adjectives": {"Too much": 1}, "numerals": {"26": 1}, "pronouns": {"I": 1}}}, {"content": "I like everyone I know.", "word_count": 10, "label": "", "sentence_structure": "Subject鈥搗erb鈥搊bject", "subjective_objective": {"answer": "Subjective", "rationale": "Like is the subjective attitude of my patients."}, "sentence_sentiment": {"answer": "Positive", "rationale": "Because the \"like\" in the entry is positive."}, "language_style": {"answer": "Tactful and warm", "rationale": "The language style of the entry is more euphemistic, using the affirmative expression \"I like\" and expressing a positive emotional attitude; and replacing a specific group or range with \"everyone I know\" so that the respondent can feel the respect and acceptance of the investigator without revealing his personal information."}, "time_dimension": {"answer": "Present", "rationale": "The sentence describes the current emotional tendencies and attitudes of the testees, the keywords being \"like\" and \"know\", which both represent the current state or feeling and can therefore be judged as now."}, "sentence_type": "Affirmative declarative sentence", "pos": {"nouns": {"I": 1, "person": 1, "Questionnaire": 1, "Entry": 1}, "verbs": {"Yeah": 1, "Yes": 1}, "numerals": {"27": 1}, "pronouns": {"Everyone": 1}}}, {"content": "I like being in class.", "word_count": 9, "label": "", "sentence_structure": "Subject鈥搗erb鈥揷omplement", "subjective_objective": {"answer": "Subjective", "rationale": "Because \"like\" is an individual's subjective emotional attitude."}, "sentence_sentiment": {"answer": "Positive", "rationale": "Because the word \"like\" in the entry expresses a positive attitude."}, "language_style": {"answer": "Calm and direct", "rationale": "The entry asks the respondent in a direct manner whether he/she prefers to be questioned in the class, the language style is more straightforward and the use of excessive rhetorical or emotional colours does not give a calm and objective impression."}, "time_dimension": {"answer": "Present", "rationale": "The entry is a personal preference or feeling, \"I like\" indicates a subjective attitude and preference, and \"Is asked in class\" is an ongoing move, so it's a current state of affairs."}, "sentence_type": "Affirmative declarative sentence", "pos": {"nouns": {"Class": 1, "STAGE": 1}, "verbs": {"Yeah": 1, "- I'm sorry": 1}, "numerals": {"28": 1}}}, {"content": "I know myself.", "word_count": 6, "label": "", "sentence_structure": "Subject鈥揷opula鈥損redicative", "subjective_objective": {"answer": "Subjective", "rationale": "Because this phrase expresses the patient's subjective perception and attitude."}, "sentence_sentiment": {"answer": "Neutral", "rationale": "Because the entry is not clearly emotional, it is merely asking whether the subject knew himself."}, "language_style": {"answer": "Tactful and warm", "rationale": "The language style of the entry is more euphemistic, using a more open and receptive attitude to guide respondents to reflect on their own understanding of their own self-perception, rather than directly asking whether they understand their own specifics. This expression is more likely to create a positive, friendly and respectful environment for respondents, who are comfortable with answering questions."}, "time_dimension": {"answer": "Present", "rationale": "The keyword is \"know,\" which shows that the testee is describing his/her perception, usually in the present or in the past. At the same time, the use of \"me\" and \"self\" suggests that we are describing the current subjective feelings and perceptions. So we can judge that the entry is primarily about the self-awareness of the testee, and that the temporal dimension is now."}, "sentence_type": "Affirmative declarative sentence", "pos": {"nouns": {"I": 1, "self": 1}, "verbs": {"Roger that": 1}}}, {"content": "I am so unlucky.", "word_count": 8, "label": "", "sentence_structure": "Subject鈥搗erb鈥揷omplement", "subjective_objective": {"answer": "Subjective", "rationale": "Because of the \"too\" and \"unlucky\" words in the sentence, they express the patient's subjective view and assessment of himself. The questionnaire entry is therefore viewed from the patient's subjective point of view."}, "sentence_sentiment": {"answer": "Negative", "rationale": "Because \"too bad\" in the entry is a negative sentiment."}, "language_style": {"answer": "Calm and direct", "rationale": "The entry expresses the other side's feelings in a direct, simple tone, without excessive trimulation or emotional rendering, and with a more calm and objective language style.\""}, "time_dimension": {"answer": "Present", "rationale": "The entry expresses the current feelings and attitudes of the testee, \"unlucky\" is an assessment of past experience, but it is described in the sentence as the current state. The entry is therefore an inquiry into the present behaviour or mentality of the testee."}, "sentence_type": "Exclamatory sentence", "pos": {"verbs": {"Too much": 1, "Bad luck": 1}, "adjectives": {"True": 1}, "adverbs": {"Too much": 1}, "pronouns": {"I": 1}}}, {"content": "31. Everything in my life is a mess.", "word_count": 15, "label": "", "sentence_structure": "Subject鈥揷opula鈥損redicative", "subjective_objective": {"answer": "Subjective", "rationale": "This is because of the patient ' s subjective perception and evaluation of the situation in life."}, "sentence_sentiment": {"answer": "Negative", "rationale": "Because the description of \"discretion\" in the entry is negative."}, "language_style": {"answer": "Calm and direct", "rationale": "The language style of the entry is calm and straightforward. It expresses the confusion of the interviewee in his life in a simple and clear language, without too many rhetorical or emotional expressions. This expression is more objective and straightforward, and it allows for real feedback from the interviewee."}, "time_dimension": {"answer": "Present", "rationale": "The keyword \"in my life\" and \"into chaos\" all indicate that it is a description and evaluation of the current state of life, so the temporal dimension is now."}, "sentence_type": "Affirmative declarative sentence", "pos": {"verbs": {"be": 1, "It's a mess": 1, "鈾?I'm a pack 鈾?: 1}, "adjectives": {"All": 1}, "numerals": {"31": 1}, "pronouns": {"I": 1}}}, {"content": "32. Children usually follow my advice.", "word_count": 12, "label": "", "sentence_structure": "Subject鈥搗erb鈥搊bject", "subjective_objective": {"answer": "Subjective", "rationale": "Because disobedientness is an attitude or choice, it is the subjective perception and behaviour of the patient."}, "sentence_sentiment": {"answer": "Positive", "rationale": "Because the expression \"children usually listen to my mind\" shows the place and influence of the voice in the minds of the children, and it has a positive emotional dimension."}, "language_style": {"answer": "Tactful and warm", "rationale": "The language style of the entry is euphemism. It presents the problem in a way that is asked, without direct orders or accusations, but rather to try to understand the views and feelings of the interviewee."}, "time_dimension": {"answer": "Past", "rationale": "The entry describes a situation that has occurred in the past, namely, that \"the children usually listen to my advice\", and therefore belongs to the temporal dimension of the past."}, "sentence_type": "Exclamatory sentence", "pos": {"nouns": {"Kids": 1, "Ideas": 1}, "verbs": {"Listen": 1, "Yes": 1}, "numerals": {"32": 1}, "pronouns": {"I": 1}}}, {"content": "33. At home, no one pays special attention to me.", "word_count": 11, "label": "", "sentence_structure": "Subject鈥搗erb鈥揷omplement", "subjective_objective": {"answer": "Objective", "rationale": "Because it is an observed phenomenon that is given special attention, not an individual ' s subjective feeling or attitude."}, "sentence_sentiment": {"answer": "Negative", "rationale": "Because the phrase \"no one pays special attention to me\" in the entry conveys a feeling of neglect, neglect and negative emotional colour."}, "language_style": {"answer": "Tactful and warm", "rationale": "The language style of the entry is euphemism, as it uses a more soft expression that gives the interviewee a sense of relative comfort and comfort. At the same time, the sentence does not directly ask the other side whether it is neglected or alone, but rather leads to a more subtle way of sharing its feelings and experiences, with some degree of interaction and personality."}, "time_dimension": {"answer": "Present", "rationale": "\"No one at home has paid special attention to me\" describes the experience and feelings of the subjects in the current family environment, so the temporal dimension is now judged."}, "sentence_type": "Negative declarative sentence", "pos": {"nouns": {"Home": 1, "person": 1, "Watch out": 1}, "verbs": {"in": 1, "Special": 1, "Attention": 1}, "adjectives": {"Special": 1}, "numerals": {"33": 1}, "pronouns": {"I": 1}}}, {"content": "I have never been blamed.", "word_count": 6, "label": "", "sentence_structure": "Subject鈥揷opula鈥損redicative", "subjective_objective": {"answer": "Objective", "rationale": "Since never being blamed is an objective fact, it is verifiable and does not need to rely on a person ' s perception or emotional state."}, "sentence_sentiment": {"answer": "Neutral", "rationale": "Since the entry does not address any emotional colour, attitude, behaviour or purpose, it merely states a fact."}, "language_style": {"answer": "Tactful and warm", "rationale": "The language style of the entry is a euphemismal and warm type. It uses negative expressions and asks in a relatively positive way whether the person under investigation has never been blamed, rather than directly asked or blamed."}, "time_dimension": {"answer": "Present", "rationale": "\"Never being blamed\" suggests that this is a customary experience, using the usual current state of affairs, so the temporal dimension is now."}, "sentence_type": "Negative declarative sentence", "pos": {"nouns": {"Scolding": 1}, "verbs": {"Never": 1, "Scolded": 1}, "numerals": {"34": 1}}}, {"content": "35. I did not perform as well in school as I hoped.", "word_count": 16, "label": "", "sentence_structure": "Subject鈥揷opula鈥損redicative", "subjective_objective": {"answer": "Subjective", "rationale": "This is a self-evaluation of patients and is subjective in nature."}, "sentence_sentiment": {"answer": "Negative", "rationale": "Because the entry \"not as good as I hoped\" expressed a negative attitude and self-evaluation."}, "language_style": {"answer": "Calm and direct", "rationale": "The language style of the entry is more calm and straightforward, and there is no excessive emotional rendering or rhetorical technique, but rather a direct statement of the fact that learning may be less than expected. This expression is more objective and practical."}, "time_dimension": {"answer": "Present", "rationale": ""}, "sentence_type": "Negative declarative sentence", "pos": {"nouns": {"School": 1, "I": 1, "Hope": 1, "Performance": 1}, "verbs": {"Why don't you": 1, "Hope": 1, "Okay": 2}, "adjectives": {"Well": 1}, "adverbs": {"Okay": 1}, "numerals": {"35": 1}, "pronouns": {"I": 1}}}, {"content": "I can make up my mind and hold on to it.", "word_count": 10, "label": "", "sentence_structure": "Subject鈥搗erb鈥揷omplement", "subjective_objective": {"answer": "Subjective", "rationale": "Because \"suspect\" and \"maintenance\" are patient subjective attitudes and actions."}, "sentence_sentiment": {"answer": "Positive", "rationale": "Because the entry is a reflection of the individual ' s will and discretion, and has a positive emotional connotation."}, "language_style": {"answer": "Tactful and warm", "rationale": "The entry uses a positive tone and encouragement to question the views and experiences of the interviewees, without direct questioning or criticism, and therefore the language style is more euphemismal."}, "time_dimension": {"answer": "Present", "rationale": "According to the phrase \"can make up your mind and hold on to it\", it can be seen as describing some of the current capabilities or characteristics of the person being tested, and thus the temporal dimension can be judged to be the present."}, "sentence_type": "Affirmative declarative sentence", "pos": {"verbs": {"Take it": 1, "Freeze": 1, "Ideas": 1, "and": 1, "Hold on": 1, "it": 1}, "adverbs": {"Yes": 1}, "pronouns": {"I": 1}}}, {"content": "I really hate being a boy/girl.", "word_count": 11, "label": "", "sentence_structure": "Subject鈥搗erb鈥搊bject", "subjective_objective": {"answer": "Subjective", "rationale": "Because \"willing or not\" is an individual's subjective feeling and attitude, and it's a subjective angle."}, "sentence_sentiment": {"answer": "Negative", "rationale": "Because \"not willing to do\" is negative."}, "language_style": {"answer": "Tactful and warm", "rationale": "The entry is more moderate and euphemistic, using the term \"unwilling\" to express feelings, without directly accusing or ordering the person under investigation. At the same time, an invitation is used to give the person under investigation more time and space to think about his or her thoughts, avoiding pressure or discomfort."}, "time_dimension": {"answer": "Present", "rationale": "The \"real unwillingness\" in the entry indicates that the testee is not satisfied with his gender, which may be their current thinking or feeling, and thus the temporal dimension is now judged."}, "sentence_type": "Exclamatory sentence", "pos": {"nouns": {"Boys": 1, "Girls": 1, "True": 1}, "verbs": {"Yes": 1, "Do it": 1}, "adverbs": {"True": 1}, "numerals": {"37": 1}}}, {"content": "I have a low rating of myself.", "word_count": 8, "label": "", "sentence_structure": "Subject鈥搗erb鈥揷omplement", "subjective_objective": {"answer": "Subjective", "rationale": "Because \"I'm under-appraising myself\" is a subjective view or feeling that reflects the patient's perception and attitude towards himself. It does not necessarily represent what the reality is, but the patient's own subjective experience and perception."}, "sentence_sentiment": {"answer": "Negative", "rationale": "Because the attitude of \"self-appraising\" in the entries is negative."}, "language_style": {"answer": "Tactful and warm", "rationale": ""}, "time_dimension": {"answer": "Present", "rationale": "The entry indicates that the testee now rated himself less favourably, and therefore the time dimension is now judged."}, "sentence_type": "Negative declarative sentence", "pos": {"verbs": {"You're gonna judge yourself": 1, "Low": 1}, "numerals": {"38": 1}, "pronouns": {"I": 1}}}, {"content": "I do not like being with others.", "word_count": 11, "label": "", "sentence_structure": "Subject鈥損redicate", "subjective_objective": {"answer": "Subjective", "rationale": "Because it's not the patient's subjective feelings and attitudes."}, "sentence_sentiment": {"answer": "Negative", "rationale": "Because the entry \"not like being with others\" expresses negative feelings."}, "language_style": {"answer": "Calm and direct", "rationale": "The entry directly expresses the question whether the other party does not like the subject of being with others, does not use excessive rhetorical or emotional rendering, and is more objective in tone."}, "time_dimension": {"answer": "Past", "rationale": "The entry does not contain clear time indicators, but it expresses a description of an emotional or attitudinal attitude, implying that the person who was tested had had a history of not being with someone else at some point in the past."}, "sentence_type": "Exclamatory sentence", "pos": {"nouns": {"Questionnaire Entry": 1, "person": 1}, "verbs": {"I don't like it": 1, "in": 1, "Together": 1}, "adjectives": {"Other": 1}, "numerals": {"39": 1}}}, {"content": "40. There have been many occasions when I wanted to leave home.", "word_count": 9, "label": "", "sentence_structure": "Subject鈥搗erb鈥揷omplement", "subjective_objective": {"answer": "Subjective", "rationale": "Because \"want to leave home\" is a mind of mind and mind, and it belongs to the subjective feelings and experiences of the individual."}, "sentence_sentiment": {"answer": "Negative", "rationale": "Because \"want to leave home\" expresses a negative emotional attitude."}, "language_style": {"answer": "Calm and direct", "rationale": "The language style of the entry is relatively simple, there is no excessive emotional rendering or euphemism, and the interviewee is asked whether he or she has had any experience of wanting to leave the home, and is more in line with the objective and direct requirements of the psychological questionnaire."}, "time_dimension": {"answer": "Past", "rationale": ""}, "sentence_type": "Affirmative declarative sentence", "pos": {"nouns": {"Number": 1, "Yes": 1, "Leave": 1, "Home": 1}, "verbs": {"have": 1, "be": 1, "Yes": 1, "Leave": 1}, "adverbs": {"Many times": 1}, "numerals": {"40": 1}}}, {"content": "I'm never shy.", "word_count": 5, "label": "", "sentence_structure": "Subject鈥損redicate", "subjective_objective": {"answer": "Subjective", "rationale": "Because \"never shy\" is the patient's subjective attitude."}, "sentence_sentiment": {"answer": "Neutral", "rationale": "Because the entry is not clearly emotional, it is merely a statement of the facts."}, "language_style": {"answer": "Calm and direct", "rationale": "The language style of this entry is relatively simple, with no excessive use of rhetorical techniques or emotional colours, and is calm and direct in tone, so it can be judged as being a blunt and straightforward language style."}, "time_dimension": {"answer": "Present", "rationale": "The keyword \"never\" indicates that such behaviour has been present at the current point in time, so it is judged now."}, "sentence_type": "Affirmative declarative sentence", "pos": {"verbs": {"Shy": 1}, "adverbs": {"Never": 1}}}, {"content": "42. I am often unhappy at school.", "word_count": 10, "label": "", "sentence_structure": "Subject鈥揷opula鈥損redicative", "subjective_objective": {"answer": "Subjective", "rationale": "Because \"unhappiness\" is the personal emotional experience and feeling of the patient, it is subjective."}, "sentence_sentiment": {"answer": "Negative", "rationale": "Because \"unhappiness\" in the entry is an expression of a negative emotional attitude."}, "language_style": {"answer": "Calm and direct", "rationale": "The language style of the entry is calm and straightforward. It clearly expresses the subject of often unhappyness in schools in simple language, without any euphemism or covert language. This direct, simple language style can convey the message directly and is not easy to create ambiguity or misunderstanding."}, "time_dimension": {"answer": "Past", "rationale": "However, the word \"normal\" indicates that it is a constant emotional experience of the testees, which may be a continuing problem and requires further understanding and research."}, "sentence_type": "Negative declarative sentence", "pos": {"nouns": {"School": 1, "Happy": 1}, "verbs": {"Common sense": 1, "I feel": 1}, "adjectives": {"not": 1, "Happy": 1}, "numerals": {"42": 1}, "pronouns": {"I": 1}}}, {"content": "43. I am often ashamed of myself.", "word_count": 9, "label": "", "sentence_structure": "Subject鈥搗erb鈥揷omplement", "subjective_objective": {"answer": "Subjective", "rationale": "Because shame is an emotional experience of a person, it is a subjective feeling of the patient."}, "sentence_sentiment": {"answer": "Negative", "rationale": "Because \"smear\" in the entry is a negative emotional expression."}, "language_style": {"answer": "Tactful and warm", "rationale": "The entry uses a more euphemistic approach, which does not directly criticize or accuse the other, but rather leads the interviewee to think about his or her problems in a more moderate way. Such an expression is more likely to enjoy the trust and cooperation of the interviewee and helps to create a good atmosphere of communication."}, "time_dimension": {"answer": "Present", "rationale": "So, the judgment is \"now\"."}, "sentence_type": "Negative declarative sentence", "pos": {"verbs": {"I feel": 1, "Shame": 1}, "adverbs": {"Regular": 1, "Yes": 1}, "numerals": {"43": 1}, "pronouns": {"I": 1}}}, {"content": "I am not as good-looking as most people.", "word_count": 11, "label": "", "sentence_structure": "Subject鈥揷opula鈥損redicative", "subjective_objective": {"answer": "Subjective", "rationale": "Because \"not as good as most people\" is the patient's assessment of his or her appearance, which is the expression of subjective ideas."}, "sentence_sentiment": {"answer": "Negative", "rationale": "Because the expression \"is not as good as most\" in the entry is a self-denial, negative way of assessing."}, "language_style": {"answer": "Tactful and warm", "rationale": "This item expresses self-evaluation of the person鈥檚 appearance in a more euphemistic way, without using direct attacks or demeaning his/her own language. This way protects the self-esteem and privacy of the person under investigation, while making the questionnaire more humane and acceptable."}, "time_dimension": {"answer": "Present", "rationale": "The keyword is \"extended\" and represents a current state or feature, so the temporal dimension is now judged."}, "sentence_type": "Negative declarative sentence", "pos": {"nouns": {"Questionnaire": 1, "Entry": 1, "person": 1, "Nice": 1}, "verbs": {"- He's a big boy": 1, "Why don't you": 1}, "adjectives": {"Most of them": 1}, "numerals": {"44": 1}, "pronouns": {"I": 1}}}, {"content": "45. If I had something to say, I would normally have said it.", "word_count": 13, "label": "", "sentence_structure": "Subject鈥損redicate", "subjective_objective": {"answer": "Subjective", "rationale": "Because \"if anything\" and \"if anything\" are usually said, are personal preferences and attitudes of the patient and are subjective."}, "sentence_sentiment": {"answer": "Positive", "rationale": "Because the \"something will come out\" act is positive in the entry."}, "language_style": {"answer": "Calm and direct", "rationale": "The language style of the entry is more straightforward, not using too many qualifiers or emotionally strong words, but rather expressing the subject in simple words, asking whether the person surveyed is inclined to express his/her own thoughts when dealing with the matter. This expression is more objective and accurate."}, "time_dimension": {"answer": "Present", "rationale": "\"At times\" suggests that this is a regular practice, and \"says\" and \"speaks\" are acts that have occurred in the past, but are used here to describe current habits or tendencies. Thus, the entry describes the current situation."}, "sentence_type": "Affirmative declarative sentence", "pos": {"verbs": {"Yes": 2, "Speak": 1, "Come out": 1}, "adverbs": {"Usually": 1}, "numerals": {"45": 1}, "pronouns": {"I": 1}}}, {"content": "The children often bully me.", "word_count": 8, "label": "", "sentence_structure": "Subject鈥搗erb鈥搊bject", "subjective_objective": {"answer": "Objective", "rationale": "Because \"the children often bully me\" is an objective fact that reflects the actual experience of the patient in life. Although this may cause negative emotional and psychological stress, it is not a subjective feeling or attitude in itself, but an objective phenomenon that can be observed and measured."}, "sentence_sentiment": {"answer": "Negative", "rationale": "Because the \"bullying\" in the entries is negative."}, "language_style": {"answer": "Tactful and warm", "rationale": "This is a more respectful and understanding expression that helps to create a good communication atmosphere."}, "time_dimension": {"answer": "Present", "rationale": ""}, "sentence_type": "Negative declarative sentence", "pos": {"nouns": {"Son": 1, "Bullying": 1}, "verbs": {"Regular": 1}, "adverbs": {"of": 1}, "numerals": {"46": 1}}}, {"content": "47. My parents know me.", "word_count": 7, "label": "", "sentence_structure": "Subject鈥搗erb鈥搊bject", "subjective_objective": {"answer": "Subjective", "rationale": "Because it is a subjective feeling and assessment of whether parents know about themselves."}, "sentence_sentiment": {"answer": "Neutral", "rationale": "Because the entry does not have a clear emotional tendency, it merely describes the fact that parents know themselves."}, "language_style": {"answer": "Tactful and warm", "rationale": "The entry uses a soft-minded approach, not directly evaluating or judging the person surveyed, but rather leading the other to think and express his/her views. At the same time, personalized proxies such as \"me\" and \"you\" have been used, which have increased the sense of dialogue and interaction, making the questionnaire more friendly and acceptable."}, "time_dimension": {"answer": "Present", "rationale": "The entry asks the testee whether his or her parents know their views and the time dimension is now judged."}, "sentence_type": "Affirmative declarative sentence", "pos": {"nouns": {"parents": 1, "I": 1}, "verbs": {"Roger that": 1}, "numerals": {"47": 1}}}, {"content": "48. I always tell the truth.", "word_count": 6, "label": "", "sentence_structure": "Subject鈥搗erb鈥揷omplement", "subjective_objective": {"answer": "Subjective", "rationale": "Because telling the truth is a personal choice and attitude, it is the patient ' s subjective feeling and behavioural orientation."}, "sentence_sentiment": {"answer": "Neutral", "rationale": "Because the entry does not have any emotional color."}, "language_style": {"answer": "Calm and direct", "rationale": "The language style of the entry is more straightforward, with no excessive embellishment or emotional rendering, and is a more objective presentation."}, "time_dimension": {"answer": "Present", "rationale": ""}, "sentence_type": "Affirmative declarative sentence", "pos": {"verbs": {"Speak": 1}, "adjectives": {"True": 1}, "adverbs": {"Always": 1}, "numerals": {"48": 1}, "pronouns": {"I": 1}}}, {"content": "49. My teacher made me feel bad enough.", "word_count": 13, "label": "", "sentence_structure": "Subject鈥搗erb鈥揷omplement", "subjective_objective": {"answer": "Subjective", "rationale": "Because the word \"thinks\" indicates that it is the patient's own feeling and perception, and it is a subjective attitude."}, "sentence_sentiment": {"answer": "Negative", "rationale": "The entry is a negative assessment of the teacher, who feels that he is not feeling well enough and has some negative feelings."}, "language_style": {"answer": "Tactful and warm", "rationale": "This expression is more pacifying and encouraging, and more easily allows the interviewee to share his real thoughts and feelings."}, "time_dimension": {"answer": "Past", "rationale": "In the entry, \"make me feel\" means experience or feeling of the past, so it can be judged in terms of time dimension."}, "sentence_type": "Negative declarative sentence", "pos": {"nouns": {"Teacher": 1, "self": 1}, "verbs": {"鈾?And I'm gonna be 鈾?: 1, "I think": 1, "Not good enough": 1}, "numerals": {"49": 1}, "pronouns": {"I": 1}}}, {"content": "I don't care what happens to myself.", "word_count": 13, "label": "", "sentence_structure": "Subject鈥搗erb鈥揷omplement", "subjective_objective": {"answer": "Subjective", "rationale": "Because \"not caring\" is a subjective feeling or attitude that reflects the patient's perception of his or her own mind."}, "sentence_sentiment": {"answer": "Negative", "rationale": "Because the attitude of \"not caring\" in the entries is negative."}, "language_style": {"answer": "Calm and direct", "rationale": "The language style of the entry is direct, objective and there is no excessive emotional colour or qualifier, and the question is expressed in a concise manner whether the other party is indifferent to what happens in his life."}, "time_dimension": {"answer": "Present", "rationale": "The entry is an inquiry into the attitudes and values of the subjects to the events encountered, does not address the specific behaviour or mentality of the past, the present and therefore can be judged to be of a general nature."}, "sentence_type": "Negative declarative sentence", "pos": {"verbs": {"self": 1, "I've got it": 1, "What": 1}, "adjectives": {"I don't care": 1}, "numerals": {"50": 1}}}, {"content": "I am a loser.", "word_count": 7, "label": "", "sentence_structure": "Subject鈥揷opula鈥損redicative", "subjective_objective": {"answer": "Subjective", "rationale": "Because \"lost\" is a subjective self-labelling and evaluation that reflects an individual's cognitive and emotional experience of his or her abilities and values."}, "sentence_sentiment": {"answer": "Negative", "rationale": "Because the word \"lost\" is usually considered negative."}, "language_style": {"answer": "Tactful and warm", "rationale": "The entry is more gentle and gentle in its language, using a first-person expression, which does not directly blame or judge the other. This expression avoids offending or injuring the interviewee and contributes to a good atmosphere of communication."}, "time_dimension": {"answer": "Past", "rationale": "So, it's not possible to judge the time dimension of \"the past\", \"now\", \"the future\"."}, "sentence_type": "Negative declarative sentence", "pos": {"nouns": {"Questionnaire Entry": 1, "Loser": 1}, "verbs": {"be": 1}, "numerals": {"51": 1}}}, {"content": "52. When I was being blamed, it became easier to get excited.", "word_count": 15, "label": "", "sentence_structure": "Subject鈥揷opula鈥損redicative", "subjective_objective": {"answer": "Subjective", "rationale": "Because I appear in the sentence as the dominant word, which expresses personal feelings and reactions, it is a subjective examination."}, "sentence_sentiment": {"answer": "Negative", "rationale": "Because the emotional reaction in the entry, \"Easier to get excited,\" is negative."}, "language_style": {"answer": "Calm and direct", "rationale": "Because it directly describes the emotional response when the interviewee is being abused, it is not expressed by any means of rhetoric or emotional rendering. Moreover, the tone is more objective, flat and not too emotional."}, "time_dimension": {"answer": "Present", "rationale": "The term \"easy to become\" and \"scolded\" shows that it describes the reaction of the individual in the current state, so the temporal dimension of the article is now judged."}, "sentence_type": "Affirmative declarative sentence", "pos": {"nouns": {"I": 1, "Scolding": 1, "Easy": 1, "鈾?And become 鈾?: 1, "Excited": 1, "Unsettled": 1}, "verbs": {"When": 1, "By": 1, "When you curse": 1, "鈾?And become 鈾?: 1, "Excited": 1}, "adjectives": {"It's easy": 1}, "numerals": {"52": 1}}}, {"content": "53. Most people are more popular than me.", "word_count": 11, "label": "", "sentence_structure": "Subject鈥揷opula鈥損redicative", "subjective_objective": {"answer": "Subjective", "rationale": "Because \"enjoying\" is a subjective feeling and assessment, not an objective fact or phenomenon. The answer to this question depends on the subjective perception and perception of the individual, not just on objective data or facts."}, "sentence_sentiment": {"answer": "Negative", "rationale": "Because it contains elements that demean themselves, it can easily provoke negative emotions."}, "language_style": {"answer": "Tactful and warm", "rationale": "The entry asks the interviewee in a relatively euphemistic way about his or her popularity, not directly questioning or demeaning the interviewee, but by directing the other to reflect on his or her situation and feelings. This expression is more easily accepted and willing to share real thoughts and experiences."}, "time_dimension": {"answer": "Present", "rationale": "The sentence describes a fact of subjective perceptions, and does not refer to any past or future circumstances, so the temporal dimension is now judged."}, "sentence_type": "Negative declarative sentence", "pos": {"nouns": {"Most people": 1, "I": 1, "person": 1, "Yeah": 1}, "verbs": {"- Yeah": 1, "More": 1, "Move": 1}, "adverbs": {"More": 1}, "numerals": {"53": 1}, "pronouns": {"Here": 1}}}, {"content": "54. I often feel that my parents seem to be punishing me.", "word_count": 16, "label": "", "sentence_structure": "Subject鈥搗erb鈥揷omplement", "subjective_objective": {"answer": "Subjective", "rationale": "It is a subjective perception that it is the individual feeling and understanding of the patient that feels punished."}, "sentence_sentiment": {"answer": "Negative", "rationale": "Because the \"punishment\" in the entry is negative."}, "language_style": {"answer": "Calm and direct", "rationale": "The language style of the entry is relatively calm and straightforward, with no excessive acrimonyms or emotional expressions, and the expression of the other ' s feelings that his or her parents may feel punishing themselves in a concise and clear manner."}, "time_dimension": {"answer": "Past", "rationale": ""}, "sentence_type": "Affirmative declarative sentence", "pos": {"nouns": {"Questionnaire": 1, "Entry": 1, "parents": 1, "Punishment": 1}, "verbs": {"I think": 1, "In the process of": 1, "Seems like": 1}, "adjectives": {"Often": 1}, "adverbs": {"very": 1}, "numerals": {"54": 1}, "pronouns": {"I": 1}}}, {"content": "55. I always know what to say to people.", "word_count": 12, "label": "", "sentence_structure": "Subject鈥搗erb鈥搊bject", "subjective_objective": {"answer": "Subjective", "rationale": "Because knowing and saying what is meant is the patient's own cognitive and subjective feelings."}, "sentence_sentiment": {"answer": "Neutral", "rationale": "Because the entry does not have any emotional color."}, "language_style": {"answer": "Tactful and warm", "rationale": "This is a more gentle and friendly language style, which guides respondents to share their views and experiences in a friendly and encouraging way by asking if they are often able to understand and grasp the topics they communicate with."}, "time_dimension": {"answer": "Present", "rationale": "The \"total\" in the entry is a high frequency or customary behaviour, and \"speaks what\" is an ongoing move, so it's judged now."}, "sentence_type": "Exclamatory sentence", "pos": {"nouns": {"Questionnaire Entry": 1, "People": 1}, "verbs": {"Yes": 2}, "adverbs": {"Total": 1}, "numerals": {"55": 1}, "pronouns": {"I": 1}}}, {"content": "56. I often encounter frustration in schools.", "word_count": 15, "label": "", "sentence_structure": "Subject鈥搗erb鈥搊bject", "subjective_objective": {"answer": "Subjective", "rationale": "Because the sentence is a personal experience and experience of the patient at school, and is a subjective attitude of the patient."}, "sentence_sentiment": {"answer": "Negative", "rationale": "Because the fact that \"often encounters a disheartening thing\" in the entry is negative."}, "language_style": {"answer": "Tactful and warm", "rationale": "The language style of the entry is more euphemistic, not directly negative, or accusing, but rather a more objective and neutral way of describing it."}, "time_dimension": {"answer": "Present", "rationale": "The words \"often\" and \"in school\" in the past indicate that this behaviour has been frequent in the past, while \"depressive things\" describe the specific situation and therefore allow the time to be judged by the past."}, "sentence_type": "Affirmative declarative sentence", "pos": {"nouns": {"School": 1, "Yes": 1}, "verbs": {"I've got it": 1, "You're": 1, "Desperate": 1}, "adverbs": {"Often": 1}, "numerals": {"56": 1}, "pronouns": {"I": 1}}}, {"content": "57. There is usually nothing to bother me about.", "word_count": 11, "label": "", "sentence_structure": "Subject鈥揷opula鈥損redicative", "subjective_objective": {"answer": "Subjective", "rationale": "Because words like \"no\" and \"no\" and \"no\" are subjective descriptions and evaluations of the patient's mental state."}, "sentence_sentiment": {"answer": "Negative", "rationale": "Because the expression \"Nothing can bother me\" suggests that individuals may lack the capacity to respond to the difficulties and challenges of life, which is a negative attitude."}, "language_style": {"answer": "Calm and direct", "rationale": "It is a succinct expression of whether the subject is usually a vexed subject, without too many rhetorical or emotional renderings. Such expressions are more direct and objective."}, "time_dimension": {"answer": "Present", "rationale": ""}, "sentence_type": "Negative declarative sentence", "pos": {"verbs": {"have": 1, "Boring": 1}, "adjectives": {"What": 1, "No, I'm not": 1, "Usually": 1}, "adverbs": {"Yes": 1}, "numerals": {"57": 1}}}, {"content": "I can't be blamed.", "word_count": 7, "label": "", "sentence_structure": "Subject鈥搗erb鈥揷omplement", "subjective_objective": {"answer": "Subjective", "rationale": "Because \"people cannot blame me\" is a personal feeling or opinion, not an objective fact or act, and it is therefore subjective."}, "sentence_sentiment": {"answer": "Negative", "rationale": "Because the entry is a negative act or attitude, i.e., that you cannot be blamed, which may mean that you do not accept criticism or respect others ' views."}, "language_style": {"answer": "Calm and direct", "rationale": "The entry is more straightforward, concise and not subject to too many rhetorical or emotional expressions. It asks the interviewee about a quality of his/her own, and the temperature and objectivity of his/her language."}, "time_dimension": {"answer": "Past", "rationale": "The entry describes the testee ' s past experience and state, and the time dimension is judged to be the past."}, "sentence_type": "Negative declarative sentence", "pos": {"verbs": {"Blame": 1}, "adjectives": {"Cannot": 1}, "adverbs": {"Total": 1, "Cannot": 1}, "pronouns": {"I": 1}}}]}

{"questionnaire_title": "Motive of achievement", "questionnaire_category": "Self-cognition", "item_count": 30, "emotion_shift_overall": "From positive to negative", "language_style_overall": "Neutral", "items": [{"content": "I like novel, difficult tasks, even taking risks.", "word_count": 19, "label": "", "sentence_structure": "Subject鈥搗erb鈥搊bject", "subjective_objective": {"answer": "Subjective", "rationale": "Because it is the patient ' s own subjective feeling and attitude that prefers novelty, difficult tasks and risk taking."}, "sentence_sentiment": {"answer": "Positive", "rationale": "Because the description of \"like novel, difficult tasks, even taking risks\" in the entry shows that individuals have a positive attitude of exploring and challenging themselves and that it is positive emotional."}, "language_style": {"answer": "Tactful and warm", "rationale": "The entry asks in a encouraging and appreciated manner whether the other side likes new and challenging tasks and expresses willingness to take some risks. This expression is more apt and encouraging, giving a positive sense of upward orientation."}, "time_dimension": {"answer": "Future", "rationale": ""}, "sentence_type": "Affirmative declarative sentence", "pos": {"nouns": {"Tasks": 1, "Risk": 1}, "verbs": {"Yeah": 1, "-Put it on": 1}, "adjectives": {"It's new": 1, "It's hard": 1}, "pronouns": {"I": 1}}}, {"content": "I am happy to be able to accomplish my difficult task.", "word_count": 15, "label": "", "sentence_structure": "Subject鈥揷opula鈥損redicative", "subjective_objective": {"answer": "Subjective", "rationale": "Because happiness is the patient ' s own psychological experience and feelings, it is a subjective examination."}, "sentence_sentiment": {"answer": "Positive", "rationale": "Because \"fun\" in the entry is a positive emotional attitude."}, "language_style": {"answer": "Tactful and warm", "rationale": "The language style of the entry is more gentle and friendly, and it uses the means of questioning rather than command or direct statements, which are more likely to resonate and to be open to participation. Moreover, the phrase \"favouring happy\" has a positive tone, making the questionnaire entries more sweet and encouraging.\""}, "time_dimension": {"answer": "Present", "rationale": "The entry used/present/forth is to ask the testee how he feels when he performs his mission, and the time dimension is now. Although \"I am doing a difficult job\" suggests that this feeling may have occurred in the past or in the future, the entire sentence focuses on describing a customary behaviour and feeling (i.e. \"happy\"), so we classify it as now."}, "sentence_type": "Affirmative declarative sentence", "pos": {"nouns": {"Tasks": 1, "Difficulties": 1, "Happy": 1}, "verbs": {"in": 1, "Completed": 1, "I feel": 1}}}, {"content": "I'll be attracted to those who know how talented I am.", "word_count": 21, "label": "", "sentence_structure": "Subject鈥搗erb鈥揷omplement", "subjective_objective": {"answer": "Subjective", "rationale": "Because \"will\" and \"attractive\" are words that express the patient's feelings and attitudes, they are viewed from the patient's subjective perspective."}, "sentence_sentiment": {"answer": "Positive", "rationale": "Because the purpose of the entry is to be positive."}, "language_style": {"answer": "Tactful and warm", "rationale": "The entry uses a more euphemistic tone to express \"I prefer to understand and appreciate my ability\" rather than directly speaking or ordering, which is more personal and interactive."}, "time_dimension": {"answer": "Present", "rationale": "The word \"will\" follows the general future pattern, meaning that it may happen in the future; and \"work that knows how talented it is\" is also a vision of the future. The entry therefore asks the testee about the current and future attitudes and expectations, the time dimension of which is now and in the future."}, "sentence_type": "Affirmative declarative sentence", "pos": {"nouns": {"Work": 1, "Genius": 1}, "verbs": {"Yes": 1, "Attractive": 1, "Roger that": 1}, "adjectives": {"Large": 1}, "adverbs": {"More": 1, "Yes": 1}, "classifiers": {"individual": 1}, "pronouns": {"I": 1}}}, {"content": "I like what I can do with my best.", "word_count": 15, "label": "", "sentence_structure": "Subject鈥搗erb鈥揷omplement", "subjective_objective": {"answer": "Subjective", "rationale": "Because like it or not is a subjective attitude or emotional experience."}, "sentence_sentiment": {"answer": "Positive", "rationale": "Because \"like to work as hard as possible\" is a positive attitude and behavior."}, "language_style": {"answer": "Tactful and warm", "rationale": "The entry encourages respondents to share their views and feelings by using polite language and positive tone, and is not expressed in a too direct or commandive manner, which is comfortable and popular."}, "time_dimension": {"answer": "Present", "rationale": ""}, "sentence_type": "Affirmative declarative sentence", "pos": {"nouns": {"Work": 1}, "verbs": {"Yeah": 1, "End of story": 1, "Completed": 1}, "adjectives": {"Max": 1}, "adverbs": {"Yes": 1}, "numerals": {"4": 1}}}, {"content": "5. I like to work tirelessly on issues that I have not been sure of.", "word_count": 21, "label": "", "sentence_structure": "Subject鈥搗erb鈥揷omplement", "subjective_objective": {"answer": "Subjective", "rationale": "Because \"like\" is a subjective attitude or feeling, which is a preference or preference for things. And whether or not it is a matter of certainty is an objective fact or condition. So, like, \"involved in the problem that I have not been able to solve\" is viewed from the patient's subjective perspective."}, "sentence_sentiment": {"answer": "Positive", "rationale": "Because the \"like to stick to hard work\" act is positive."}, "language_style": {"answer": "Calm and direct", "rationale": "The entry is more calm and straightforward, expresses the core meaning of the problem through a concise and clear statement, and does not use excessive rhetorical or emotional rendering. At the same time, the entry is asked about each other's attitudes and behaviour in an objective and rational manner, which is consistent with the language style of the questionnaire.\""}, "time_dimension": {"answer": "Present", "rationale": "The entry describes the person who was tested for the current act, in which the words \"like\" and \"deeply working\" indicate that it is a current act or attitude."}, "sentence_type": "Affirmative declarative sentence", "pos": {"nouns": {"Problem": 1, "Try": 1}, "verbs": {"Yeah": 1, "have": 1, "Resolve": 1, "Hold on": 1, "Try": 2}, "adjectives": {"I'm not sure": 1}, "adverbs": {"And you keep on": 1}, "pronouns": {"I": 1}}}, {"content": "6. I can easily get involved in difficult tasks, even if they have little meaning.", "word_count": 23, "label": "", "sentence_structure": "Subject鈥搗erb鈥搊bject", "subjective_objective": {"answer": "Subjective", "rationale": "Because the sentence relates to the attitudes and feelings of patients, it expresses their potential for overinvolvement in tasks that are of little significance, a subjective tendency and attitude of patients."}, "sentence_sentiment": {"answer": "Negative", "rationale": "Because the description of \"even if there is no point\" in the entry implies that the meaning of the mandate is negative in its effect on behaviour."}, "language_style": {"answer": "Calm and direct", "rationale": "The entry expresses the attitude or behaviour of the person who is asked about the difficult task, and the language style is more direct and concise."}, "time_dimension": {"answer": "Present", "rationale": "The entry describes the testee's sexual preference in the face of difficult tasks, using the adverb \"so easy\" to indicate that the tendency is frequent. At the same time, the expression \"even if there is no point\" suggests that it is a proactive choice of behaviour rather than a situation of coercion or imperception, and therefore prefers to represent the present state.\""}, "sentence_type": "Affirmative declarative sentence", "pos": {"nouns": {"Tasks": 2, "Difficulties": 1, "Meaning": 1}, "verbs": {"Get in there": 1}, "adjectives": {"Easy": 1}, "adverbs": {"Yeah": 1}}}, {"content": "7. I feel a whipping and a challenge in the face of opportunities to measure my abilities.", "word_count": 22, "label": "", "sentence_structure": "Subject鈥搗erb鈥搊bject", "subjective_objective": {"answer": "Subjective", "rationale": "Because of the perceived subjective attitude of the patient, the attitude and perception of the patient regarding the opportunity are examined."}, "sentence_sentiment": {"answer": "Positive", "rationale": "Because the \"striking and challenge\" in the entry is a positive attitude towards opportunity."}, "language_style": {"answer": "Tactful and warm", "rationale": "The entry expresses an encouraging attitude to opportunity, without directly describing it as a pressure or threat that gives people a more caring and positive feeling. It is also a question of guidance that encourages respondents to think about their attitudes and feelings."}, "time_dimension": {"answer": "Present", "rationale": "The entry describes the emotional response of the testee to the opportunity, and the temporal dimension is now judged. The keyword \"exposed\" and \"percepted\" indicates that it is a description of the current state, and \"opportunities to measure my abilities\" and \"flash and challenge\" are specific situational irritations, which together constitute a description of the subjective perception of the testee in a given situation. So the entry reflects the current psychological state and attitude of the testee."}, "sentence_type": "Affirmative declarative sentence", "pos": {"nouns": {"Opportunities": 1, "I": 2, "Capacity": 1, "Slash": 1, "Challenges": 1}, "verbs": {"Face it": 1, "Measurement": 1, "I feel": 1}, "adjectives": {"Yes": 1, "of": 3}, "classifiers": {"Plant": 1}}}, {"content": "I'll be attracted to difficult tasks.", "word_count": 12, "label": "", "sentence_structure": "Subject鈥揷opula鈥損redicative", "subjective_objective": {"answer": "Subjective", "rationale": "Because \"to be attracted\" is an individual's subjective feeling and attitude."}, "sentence_sentiment": {"answer": "Positive", "rationale": "This is a positive attitude because it represents a personal preference to accept and address challenging tasks."}, "language_style": {"answer": "Tactful and warm", "rationale": "The entry uses an invitation tone, in which a moderate tone is used to ask whether the other person will be attracted to a difficult task, rather than directly speaking or ordering. This expression is more personal and interactive and more resonant with the interviewee and his/her involvement."}, "time_dimension": {"answer": "Past", "rationale": "The keywords \"will\" and \"attractive\" suggest that this is the current situation or tendency of the testee, not past or future behaviour."}, "sentence_type": "Affirmative declarative sentence", "pos": {"verbs": {"Yes": 1, "By": 1, "Attractive": 1}, "adjectives": {"Difficulties": 1}}}, {"content": "9. For those I cannot be sure of success, I am the most attractive.", "word_count": 22, "label": "", "sentence_structure": "Subject鈥搗erb鈥搊bject", "subjective_objective": {"answer": "Subjective", "rationale": "Because \"most attractive to me\" is the patient's subjective attitude and feelings."}, "sentence_sentiment": {"answer": "Positive", "rationale": "Because the \"can attract me\" attitude in the entry is positive."}, "language_style": {"answer": "Tactful and warm", "rationale": "The entry is more personal and interactive in its expression of conversation in a gentle and inviting tone, rather than in direct statements or orders. It also reflects the respect and interest of the questionnaire designers in the respondents, who want to share their real thoughts and feelings."}, "time_dimension": {"answer": "Future", "rationale": "The keywords are \"successful\" and \"most attractive,\" which point to the future and to the expectations. And the expression \"those I can't be sure\" also implies uncertainty and exploration of the future. So, this entry is the future."}, "sentence_type": "Affirmative declarative sentence", "pos": {"nouns": {"Work": 1, "Success": 1, "Most": 1}, "verbs": {"Attractive": 1, "Sure": 1, "Can you": 1}, "adjectives": {"Those": 1}, "adverbs": {"Yes": 3}, "numerals": {"9": 1}, "pronouns": {"I": 1}}}, {"content": "10. Even with sufficient time, I would like to start work immediately.", "word_count": 23, "label": "", "sentence_structure": "Subject鈥搗erb鈥搊bject", "subjective_objective": {"answer": "Subjective", "rationale": "Because \"like to start work immediately\" is the patient's subjective attitude and behavioral tendency."}, "sentence_sentiment": {"answer": "Positive", "rationale": "Because the entry \"likes to start work immediately\" expresses a positive, proactive attitude."}, "language_style": {"answer": "Calm and direct", "rationale": "The entry expresses the attitude of the interviewees in the face of the task, i.e., they tend to start work immediately, whether or not they have enough time. This expression is more straightforward, and there is no excessive emotional or rhetorical use."}, "time_dimension": {"answer": "Present", "rationale": "The entry indicates the person's current behaviour or preference, that is, the immediate reaction and the way he acts in the face of the task. The keyword \"likes to start work immediately\" indicates the person's customary sexual behaviour pattern. Thus, the temporal dimension of the purpose of the article is now judged."}, "sentence_type": "Affirmative declarative sentence", "pos": {"verbs": {"Here": 1, "have": 1, "Yeah": 1, "Start": 1, "Work": 1}, "adjectives": {"Immediately": 1, "Enough": 1}, "numerals": {"10": 1}, "pronouns": {"I": 1}}}, {"content": "The chance to measure my abilities is attractive to me.", "word_count": 18, "label": "", "sentence_structure": "Subject鈥揷opula鈥損redicative", "subjective_objective": {"answer": "Subjective", "rationale": "Because the phrase \"attractive\" is the patient's personal feelings and attitudes and is subjective."}, "sentence_sentiment": {"answer": "Positive", "rationale": "Because the description of \"attractive\" in the entry is positive emotional."}, "language_style": {"answer": "Tactful and warm", "rationale": "Although the entry refers to \"attraction,\" it does not directly express positive effects or benefits for the respondents, but rather uses a more moderate approach to make them more comfortable and natural. Moreover, the use of the term \"opportunities that can measure my abilities\" conveys the need for challenging work to stimulate motivation, without overemphasis on competition or pressure. This language-style questionnaire is more likely to resonate and cooperate with the respondents."}, "time_dimension": {"answer": "Future", "rationale": ""}, "sentence_type": "Affirmative declarative sentence", "pos": {"nouns": {"Opportunities": 1, "I": 2, "Capacity": 1}, "verbs": {"Measurement": 1, "be": 1, "have": 1, "Attractive": 1, "of": 1}, "adverbs": {"Yes": 1}, "numerals": {"11": 1}}}, {"content": "I'm very excited and happy when I'm faced with difficult problems that I can't handle.", "word_count": 21, "label": "", "sentence_structure": "Subject鈥搗erb鈥揷omplement", "subjective_objective": {"answer": "Subjective", "rationale": "Because \"very excited, happy\" is the patient's subjective feeling of emotional state in the face of difficulties."}, "sentence_sentiment": {"answer": "Positive", "rationale": "Because \"excited\" and \"happy\" in the entries are positive emotional expressions that indicate positive attitudes when facing difficult problems."}, "language_style": {"answer": "Tactful and warm", "rationale": "The words of the entry are more euphemistic. By using the words \"very excited, happy,\" they express a positive emotional attitude that makes it easier for respondents to resonate and participate in answering questions. At the same time, using the first person's proxy \"I\" makes the subject's subjective feelings more visible, adding to the relative and interactive nature of the questionnaire."}, "time_dimension": {"answer": "Present", "rationale": ""}, "sentence_type": "Affirmative declarative sentence", "pos": {"nouns": {"Difficulty": 1, "Excited": 1, "Happy": 1}, "verbs": {"Faced": 1, "Overcoming": 1, "Yes": 1}, "adjectives": {"I'm not sure": 1}, "adverbs": {"Very": 1}}}, {"content": "13, if something doesn't get it right away, I'll be interested in it soon.", "word_count": 21, "label": "", "sentence_structure": "Subject鈥搗erb鈥搊bject", "subjective_objective": {"answer": "Subjective", "rationale": "Because the interest in something is a subjective attitude and emotional experience, a feeling and perception within the patient, and is subject to subjective examination."}, "sentence_sentiment": {"answer": "Positive", "rationale": "Because the \"will interest it\" act is positive."}, "language_style": {"answer": "Tactful and warm", "rationale": "The entry is more personal and interactive in terms of encouragement and support, rather than direct orders or criticism. At the same time, positive words are used, positive attitudes and expectations are expressed, attention and respect are felt, and the heart is opened for investigation."}, "time_dimension": {"answer": "Future", "rationale": "According to the sentence entered, \"if something cannot be understood immediately\" is a hypothetical conditional statement, while \"if it will soon be of interest\" is a description of the result of the hypothesis. This description points to future behaviour or circumstances, so the answer is \"the future\"."}, "sentence_type": "Affirmative declarative sentence", "pos": {"nouns": {"Yes": 1, "Understood": 1, "Interest": 1}, "verbs": {"Yes": 1, "Production": 1, "Fan": 1, "to": 1}, "adjectives": {"Now": 1}, "adverbs": {"Soon": 1}, "numerals": {"13": 1}, "pronouns": {"it": 1}}}, {"content": "14 to me, it's important to do something difficult, even if nobody knows.", "word_count": 25, "label": "", "sentence_structure": "Subject鈥揷opula鈥損redicative", "subjective_objective": {"answer": "Subjective", "rationale": "Because \"for me\" means that it's a patient's personal opinion and attitude."}, "sentence_sentiment": {"answer": "Positive", "rationale": "Because the behaviour and attitude of \"doing something difficult\" in the entry is positive, indicating that the interviewee values the challenge to himself. Although the entry refers to \"no one knows and is irrelevant,\" it does not affect the assessment of behaviour, and therefore remains positive."}, "language_style": {"answer": "Calm and direct", "rationale": "The entry is based on a brief and clear statement of the author鈥檚 views and attitudes, without too much rhetorical or emotional overturning. At the same time, it is more balanced and objective, and it is impressive to have a direct account of its values and attitudes."}, "time_dimension": {"answer": "Present", "rationale": "\"To me\" and \"important\" indicate that it is a statement of personal values, and is based on the knowledge and feeling of the present. Moreover, both acts or events are carried out or exist within the current time frame, \"doing difficult things\" or \"even if nobody knows about them.\" Thus, this entry describes the current mindset and behavioral orientation of the subjects."}, "sentence_type": "Affirmative declarative sentence", "pos": {"nouns": {"Yes": 2, "person": 1, "Importance": 1}, "verbs": {"be": 1, "Do it": 1, "Yes": 1, "Related": 1}, "adjectives": {"It's hard": 1, "Nobody": 1}, "adverbs": {"Yeah": 1, "None": 1, "It's important": 1}, "numerals": {"14": 1}, "classifiers": {"individual": 1}, "pronouns": {"I": 1}}}, {"content": "I wish to assign to me the difficult task.", "word_count": 14, "label": "", "sentence_structure": "Subject鈥搗erb鈥搊bject", "subjective_objective": {"answer": "Subjective", "rationale": "Because \"favorite\" is a subjective desire and attitude, which belongs to the individual's feelings and perceptions, it is a subjective view."}, "sentence_sentiment": {"answer": "Negative", "rationale": "The desire to allocate difficult work to itself was an expression of unwillingness to assume responsibility or to escape difficulties."}, "language_style": {"answer": "Tactful and warm", "rationale": "The language style of the entry is more euphemistic. It uses a positive expression of invitation, not direct statements or orders. This expression is more reflective of the respect and interest of the researcher in the tester, and is more likely to receive positive feedback from the testee."}, "time_dimension": {"answer": "Future", "rationale": "The entry describes a desire or desire of the person tested, the keyword being \"hope\" and it refers to a situation that has not yet occurred and that may arise in the future. While the context involves \"work that is difficult\", it simply indicates what type of task the person tested's expectation is related to, and does not clearly indicate whether it has been or will be fulfilled. The entry therefore remains a vision and expectation for the future, and should be judged as the future."}, "sentence_type": "Imperative sentence", "pos": {"nouns": {"Work": 1, "Difficulties": 1}, "verbs": {"Hope": 1, "- Put it on": 1, "Allocation": 1, "Here": 1}, "adverbs": {"have": 1}, "numerals": {"15": 1}, "pronouns": {"I": 1}}}, {"content": "16, I hate working in situations where there is no certainty that it will fail.", "word_count": 21, "label": "", "sentence_structure": "Subject鈥搗erb鈥搊bject", "subjective_objective": {"answer": "Subjective", "rationale": "Because \"obsessed\" is a subjective feeling or emotional reaction, it's the patient's attitude and feelings towards a situation."}, "sentence_sentiment": {"answer": "Negative", "rationale": "Because the word \"disguised\" in the entry expresses a negative attitude to work."}, "language_style": {"answer": "Calm and direct", "rationale": "The entry clearly expresses the attitude of the subject to uncertainty in a direct presentation, and the language style is more calm and objective."}, "time_dimension": {"answer": "Present", "rationale": "\"I hate the \"obsessed\" in \"the current state of attitude or emotion,\" in situations where there is no certainty that failure will be achieved, describes a way of doing things that the testee is currently doing or is used to doing. So, the entry asks what the testee thinks and feels right now."}, "sentence_type": "Negative declarative sentence", "pos": {"nouns": {"I": 1, "Situation": 1, "Work": 1}, "verbs": {"Shit": 1, "in": 1, "Sure": 1, "Yes": 1, "Failed": 1, "Medium": 1, "Work": 1}, "adjectives": {"I'm not sure if I'm gonna fail": 1}, "numerals": {"16": 1}}}, {"content": "17 In the absence of results, I fear failure.", "word_count": 14, "label": "", "sentence_structure": "Subject鈥搗erb鈥搊bject", "subjective_objective": {"answer": "Subjective", "rationale": "Because \"fear\" is patient's subjective attitude."}, "sentence_sentiment": {"answer": "Negative", "rationale": "Because the mood of \"fearing to fail\" in the entry is negative."}, "language_style": {"answer": "Calm and direct", "rationale": "The entry raises a clear question in a concise and clear manner, without too many rhetorical or emotional accents, and with a more direct and objective tone."}, "time_dimension": {"answer": "Present", "rationale": "The entry describes an emotional state or attitude of the testee, namely, \"a fear of failure\", which occurs in circumstances where the outcome is not clear. Thus, the temporal dimension is now judged."}, "sentence_type": "Imperative sentence", "pos": {"nouns": {"Situation": 1, "Result": 1, "Failed": 1}, "verbs": {"in": 1, "I'm worried": 1, "I see": 1}, "adverbs": {"Unknown": 1}, "pronouns": {"I": 1}}}, {"content": "I fear failure in fulfilling what I consider to be a difficult task.", "word_count": 18, "label": "", "sentence_structure": "Subject鈥搗erb鈥搊bject", "subjective_objective": {"answer": "Subjective", "rationale": "Because fear of failure is the patient's subjective perception and concern about the difficulty of the task, not the objective reality or result."}, "sentence_sentiment": {"answer": "Negative", "rationale": "Because the \"fear of failure\" attitude in the entry is negative."}, "language_style": {"answer": "Calm and direct", "rationale": "The language style of the entry is more direct, with no excessive emotional accolades or rhetorical techniques, but rather focused on objective factual statements, expressing concerns and challenges in the accomplishment of difficult tasks, which are consistent with the immediate language characteristics of calmness."}, "time_dimension": {"answer": "Present", "rationale": ""}, "sentence_type": "Negative declarative sentence", "pos": {"nouns": {"Tasks": 1, "Failed": 1}, "verbs": {"I think": 1, "Completed": 1, "I'm worried": 1}, "adjectives": {"Hard": 1}, "numerals": {"18": 1}, "pronouns": {"I": 1}}}, {"content": "I am disturbed by the thought of doing new and difficult work.", "word_count": 23, "label": "", "sentence_structure": "Subject鈥揷opula鈥損redicative", "subjective_objective": {"answer": "Subjective", "rationale": "Because \"uneasy\" is the patient's subjective emotional response. While these may be somewhat challenging or new and curious, patients' feelings for them are subjective and depend on their own feelings and experiences."}, "sentence_sentiment": {"answer": "Negative", "rationale": "Because the \"uneasy\" emotion in the entry is negative."}, "language_style": {"answer": "Tactful and warm", "rationale": "The language style of the entry is euphemism, which describes an emotional response in a gentle and understanding way, rather than a direct accusation or order. It does not use too many strong words or tones, but rather gives the respondents as much affection and understanding as possible. And it does not give too much guidance or hints to the subject to express themselves."}, "time_dimension": {"answer": "Present", "rationale": "The entry describes the person's feelings and psychological state in the current situation, and the key word is \"at first thought\" and \"at first feeling\", which is an immediate emotional reaction."}, "sentence_type": "Affirmative declarative sentence", "pos": {"nouns": {"Work": 2, "Difficulty": 1}, "verbs": {"Do it": 1, "I thought": 1, "I feel": 1}, "adjectives": {"It's new": 1, "It's hard": 1, "Unsettled": 1}, "numerals": {"One": 1}, "pronouns": {"I": 1}}}, {"content": "I don't like those things that measure my abilities.", "word_count": 14, "label": "", "sentence_structure": "Subject鈥搗erb鈥搊bject", "subjective_objective": {"answer": "Subjective", "rationale": "Because \"not like\" is the patient's feelings and attitude to measure his abilities, and it's a subjective view."}, "sentence_sentiment": {"answer": "Negative", "rationale": "Because the \"not like\" attitude in the entries is negative."}, "language_style": {"answer": "Calm and direct", "rationale": "\"Calm down straight.\""}, "time_dimension": {"answer": "Present", "rationale": "The keyword \"not likes\" means the individual's current attitude or feelings, and the judgement is now."}, "sentence_type": "Negative declarative sentence", "pos": {"nouns": {"20": 1, "I like it": 1, "Those": 1, "Measurement": 1, "Capacity": 1, "Show": 1}, "verbs": {"I don't like it": 1}, "numerals": {"20": 1}, "classifiers": {"individual": 1}}}, {"content": "I am concerned about those jobs that are not ready for competence.", "word_count": 18, "label": "", "sentence_structure": "Subject鈥搗erb鈥搊bject", "subjective_objective": {"answer": "Subjective", "rationale": "Because \"fearing\" is a subjective feeling and emotional reaction."}, "sentence_sentiment": {"answer": "Negative", "rationale": "Because the mood of \"fear\" in the entry is negative."}, "language_style": {"answer": "Calm and direct", "rationale": "The entry directly asks the interviewees whether they are worried about the work that may not be competent, and does not use excessive rhetorical techniques or emotional rendering. Its language style is therefore calm and straightforward."}, "time_dimension": {"answer": "Present", "rationale": "The entry describes the current subjective feelings and experiences of the testees, judged by the time dimension of time. The keyword \"I am worried about those jobs that are not sure of being able to do it\" suggests that this is a concern and a disquiet for the future, but it is based on current circumstances and thinking."}, "sentence_type": "Negative declarative sentence", "pos": {"nouns": {"Work": 1, "Hold on": 1, "Anxiety": 1}, "verbs": {"I feel": 1, "Competencies": 1}, "adjectives": {"No, I'm not": 1, "Fear": 1}, "numerals": {"21": 1}, "pronouns": {"I": 1}}}, {"content": "22, I don't like doing things I don't know if I can do, even if others don't.", "word_count": 25, "label": "", "sentence_structure": "Subject鈥搗erb鈥搊bject", "subjective_objective": {"answer": "Subjective", "rationale": "Because \"not like\" is a subjective feeling, and \"can't finish\" is a subjective judgement of the patient."}, "sentence_sentiment": {"answer": "Negative", "rationale": "Because the \"do not like what I don't know whether I can do it\" act is negative."}, "language_style": {"answer": "Calm and direct", "rationale": "The language style of the entry is a calm and direct one. It does not use too many rhetorical or emotional techniques to express its views, but rather to express its thoughts and feelings directly in a concise and clear language. It often gives a clear and objective impression that it applies to situations that require a clear message or rational thinking."}, "time_dimension": {"answer": "Present", "rationale": "The words \"not like\" and \"do not do\" indicate that the testee has a negative emotion or attitude to the current state; while the words \"does not know the same\" and \"does not know the same\" suggest that the testee's behaviour is related to their self-perception. Thus, the entry describes the testee's present state and thoughts."}, "sentence_type": "Negative declarative sentence", "pos": {"nouns": {"Yes": 1, "others": 1}, "verbs": {"I don't like it": 1, "Do it": 1, "Yes": 3, "Completed": 1, "Yeah": 1, "Same thing": 1}, "adjectives": {"I can't tell": 1}, "adverbs": {"not": 1}, "numerals": {"22": 1}, "pronouns": {"I": 1}}}, {"content": "In those situations where my ability is measured, I am disturbed.", "word_count": 17, "label": "", "sentence_structure": "Subject鈥揷opula鈥損redicative", "subjective_objective": {"answer": "Subjective", "rationale": "Because \"disturbing\" is a subjective emotional experience, it is the patient's own perception and assessment of the situation, not the objective fact."}, "sentence_sentiment": {"answer": "Negative", "rationale": "Because the mood of \"frightful\" in the entry is negative."}, "language_style": {"answer": "Calm and direct", "rationale": "The language style of the entry is calm and straightforward, and there is no excessive rhetorical or emotional colour, mainly to convey the problem in an objective and clear manner."}, "time_dimension": {"answer": "Present", "rationale": "The entry asks the situation in which the person tested would be disturbed and the time dimension is now judged."}, "sentence_type": "Negative declarative sentence", "pos": {"nouns": {"Capacity": 1, "Situation": 1, "Unsettled": 1}, "verbs": {"Measurement": 1, "I feel": 1}, "numerals": {"23": 1}, "pronouns": {"I": 1}}}, {"content": "I fear failure in matters that require a specific opportunity to resolve.", "word_count": 20, "label": "", "sentence_structure": "Subject鈥搗erb鈥搊bject", "subjective_objective": {"answer": "Subjective", "rationale": "Fear of failure is a subjective feeling and attitude of one."}, "sentence_sentiment": {"answer": "Negative", "rationale": "Because the attitude of fear of failure in the entries is negative."}, "language_style": {"answer": "Calm and direct", "rationale": "The entry is directly asked to answer the question as to whether the respondent is afraid of failure, the language style is more straightforward and not too much rhetorical or emotional rendering."}, "time_dimension": {"answer": "Present", "rationale": "The entry in the questionnaire is about the person's current attitude or behaviour, and therefore the judgment is \"now.\" The keyword \"will\" indicate a tendentious behaviour, while the main phrase of the sentence \"I\" indicates that it is a description of the person's own situation."}, "sentence_type": "Negative declarative sentence", "pos": {"nouns": {"Yes": 1, "Opportunities": 1, "Failed": 1}, "verbs": {"Yes": 2, "Resolve": 1, "Scared": 1}, "adverbs": {"Specific": 1}, "numerals": {"24": 1}, "classifiers": {"individual": 1}, "pronouns": {"I": 1}}}, {"content": "I'm worried about what seems pretty difficult.", "word_count": 17, "label": "", "sentence_structure": "Subject鈥搗erb鈥搊bject", "subjective_objective": {"answer": "Subjective", "rationale": "Because \"fear\" is a subjective feeling or emotional reaction, it is an expression of individual concern and anxiety about the difficult task."}, "sentence_sentiment": {"answer": "Negative", "rationale": "Because the entry \"scared\" expressed a negative sentiment."}, "language_style": {"answer": "Tactful and warm", "rationale": ""}, "time_dimension": {"answer": "Present", "rationale": "The keyword \"that seems quite difficult\" suggests that it refers to the problems or circumstances that are facing us today, and that \"I am worried when I do\" means that the testees are worried and uneasy about the task, so it can be considered that the entry describes the current state and feelings of the testee."}, "sentence_type": "Affirmative declarative sentence", "pos": {"nouns": {"Yes": 1, "Difficulties": 1, "I'm worried": 1}, "verbs": {"Looks like": 1, "Do it": 1}, "adjectives": {"Pretty much": 1}, "adverbs": {"very": 1}, "numerals": {"25": 1}}}, {"content": "I don't like working in unfamiliar environments, even if nobody knows.", "word_count": 23, "label": "", "sentence_structure": "Subject鈥損redicate", "subjective_objective": {"answer": "Subjective", "rationale": "Because \"not like\" is a subjective attitude or feeling, it reflects the personal perception and emotional reaction of the interviewees to a situation."}, "sentence_sentiment": {"answer": "Negative", "rationale": "Because the words \"not like\" and \"no one knows the same\" in the entries are negative."}, "language_style": {"answer": "Tactful and warm", "rationale": "This entry uses a more euphemistic tone, which expresses the respondent鈥檚 unease and resistance to working in an unfamiliar environment, rather than direct orders or statements."}, "time_dimension": {"answer": "Past", "rationale": "The entry describes attitudes or tendencies of the testees that do not like to work in unfamiliar environments, which are likely to be based on their past experiences and feelings. Thus, judging from the time dimension, the content of the entry can be classified as \"the past\"."}, "sentence_type": "Negative declarative sentence", "pos": {"nouns": {"Work": 1, "Environment": 1}, "verbs": {"I don't like it": 1, "Yes": 1, "Work": 1, "Same thing": 1}, "adjectives": {"I don't know": 1}, "adverbs": {"in": 1, "Yeah": 1, "Nobody": 1}, "pronouns": {"I": 1}}}, {"content": "27 If there is a difficult work to do, I hope not to be allocated to me.", "word_count": 19, "label": "", "sentence_structure": "Subject鈥損redicate", "subjective_objective": {"answer": "Subjective", "rationale": "Because hope is a subjective attitude or desire."}, "sentence_sentiment": {"answer": "Negative", "rationale": "Because the \"I hope not to be assigned\" attitude in the entry is negative."}, "language_style": {"answer": "Calm and direct", "rationale": "The entry is based on the use of a direct negative form of \"unwanted\" to express its attitude towards the distribution of difficult work, with no excessive rhetorical or emotional rendering, and a more objective tone, so it can be judged that its language style is straightforward and calm."}, "time_dimension": {"answer": "Present", "rationale": "Because \"the hard work\" and \"the hope not to be assigned to me\" describe the situation and the expectations, the entry should be judged as now."}, "sentence_type": "Negative declarative sentence", "pos": {"nouns": {"Work": 1, "Difficulties": 1}, "verbs": {"Hope": 1, "Allocation": 1}, "adjectives": {"Hard": 1}, "adverbs": {"not": 2, "Yes": 1}, "numerals": {"27": 1}, "pronouns": {"I": 1}}}, {"content": "I don't want to do things that will make me feel better.", "word_count": 16, "label": "", "sentence_structure": "Subject鈥搗erb鈥搊bject", "subjective_objective": {"answer": "Subjective", "rationale": "Because \"not wanting\" is patient's subjective attitude."}, "sentence_sentiment": {"answer": "Negative", "rationale": "Because the words \"not wanting\" and \"work with my abilities\" in the entries express a negative attitude and emotion."}, "language_style": {"answer": "Tactful and warm", "rationale": "The language style of the entry is more euphemistic and moderate, using negative expressions and questions to guide respondents to reflect on their views, and not directly rejecting or accusing each other. This expression helps the respondents to express their thoughts and feelings more naturally, while also respecting their independence and autonomy."}, "time_dimension": {"answer": "Past", "rationale": "At the same time, the expression \"utilise my powers\" implies that it is describing a general state or tendency, not a particular past or future event, which further confirms that the entry is about the present."}, "sentence_type": "Negative declarative sentence", "pos": {"nouns": {"Work": 1, "Capacity": 1}, "verbs": {"Hope": 1, "Do it": 1, "Play": 1}, "adjectives": {"Yes": 1}, "adverbs": {"not": 1}, "numerals": {"28": 1}, "pronouns": {"I": 1}}}, {"content": "I don't like doing things that I don't know if I can do.", "word_count": 18, "label": "", "sentence_structure": "Subject鈥搗erb鈥搊bject", "subjective_objective": {"answer": "Subjective", "rationale": "Because \"competence\" is a self-assessment and judgement, and it is the patient's subjective feeling."}, "sentence_sentiment": {"answer": "Negative", "rationale": "Because the \"not like\" attitude in the entry is negative."}, "language_style": {"answer": "Tactful and warm", "rationale": "The language style of the entry is more euphemistic, asking questions in a spirit of encouragement and understanding. Using the expression \"not like\" rather than directly rejecting or ordering, it gives people a sense of affection. At the same time, by asking whether the other person does not like to do things that he cannot do, the interviewee is guided to think and express his feelings, so that he can better understand their psychological state."}, "time_dimension": {"answer": "Present", "rationale": "The main phrase of the sentence is \"I\" and the verb \"I don't like\" means that it is a statement describing personal emotions or attitudes. And \"that I don't know if I can do it\" is a further explanation of the reasons for not like it, it's about the perception and feeling of the moment. So the time dimension of this entry is now."}, "sentence_type": "Negative declarative sentence", "pos": {"nouns": {"Yes": 1, "I can do it": 1}, "verbs": {"I don't like it": 1, "Do it": 1}, "adjectives": {"Those": 1, "I don't know": 1, "Can I": 1}, "numerals": {"29": 1}, "pronouns": {"I": 1}}}, {"content": "I'm worried when I'm having problems I can't get right away with.", "word_count": 20, "label": "", "sentence_structure": "Subject鈥搗erb鈥揷omplement", "subjective_objective": {"answer": "Subjective", "rationale": "Because \"crazy\" is a subjective feeling and emotional reaction."}, "sentence_sentiment": {"answer": "Negative", "rationale": "Because the emotional attitude of \"anxiety\" in the entry is negative."}, "language_style": {"answer": "Calm and direct", "rationale": "The language style of the entry is more straightforward, there is no excessive rhetoric or emotional rendering, an objective, rational description of a behaviour characteristic and the use of the expression \"I will\" to indicate the relevance of the problem to the person under investigation. Thus, the language style of the questionnaire entry is more calm and straightforward."}, "time_dimension": {"answer": "Past", "rationale": "The entry asks the subject about the emotional response to the problem and does not clearly describe the time dimension, making it difficult to judge whether it is past, present or future."}, "sentence_type": "Affirmative declarative sentence", "pos": {"nouns": {"Problem": 1, "Anxiety": 1, "Unsettled": 1}, "verbs": {"I met him": 1, "Get it": 1}, "adjectives": {"Immediately": 1}, "adverbs": {"Yes": 1}, "numerals": {"30": 1}}}]}

{"questionnaire_title": "The security test: How much love do you lack?", "questionnaire_category": "Self-cognition", "item_count": 16, "emotion_shift_overall": "No obvious trend", "language_style_overall": "Calm and direct", "items": [{"content": "One never dared to say his own words.", "word_count": 13, "label": "", "sentence_structure": "Subject鈥搗erb鈥搊bject", "subjective_objective": {"answer": "Subjective", "rationale": "Because \"never dared\" or \"provocate your own opinion\" are the subjective attitudes and feelings of the patient."}, "sentence_sentiment": {"answer": "Negative", "rationale": "Because the act of \"determining to speak out\" is negative, it suggests that the interviewee may lack confidence or fear rejection, an attitude that may limit personal growth and development."}, "language_style": {"answer": "Calm and direct", "rationale": "The language style of the entry is more direct, concise and not excessive in rhetorical or emotional rendering, but in objective and neutral expression, which is more suitable for rigorous psychological research."}, "time_dimension": {"answer": "Past", "rationale": "\"Never dare\" is a customary act, while \"never\" is a mental or attitudinal one, so the entry relates to the person who was tested for past and present behaviour or mentalities."}, "sentence_type": "Negative declarative sentence", "pos": {"nouns": {"Questionnaire Entry": 1, "Observations": 1}, "verbs": {"Initiatives": 1, "Say it": 2}, "adjectives": {"Never dared": 1}}}, {"content": "I feel that life is always full of uncertainty and unpredictability.", "word_count": 17, "label": "", "sentence_structure": "Subject鈥揷opula鈥損redicative", "subjective_objective": {"answer": "Subjective", "rationale": "Because \"fear\" is the patient's own feeling and perception, and is subjective. Although there is uncertainty in life, it is the individual's feeling that determines whether or not it is so."}, "sentence_sentiment": {"answer": "Negative", "rationale": "Because the description of \"uncertainty and unpredictability\" in the entry is negative, it means that the interviewee may be disturbed and anxious about life."}, "language_style": {"answer": "Tactful and warm", "rationale": "The language style of the entry is more moderate and friendly, using the term \"always\" to express feelings of life, rather than directly stating or ordering, making the respondents more willing to share their true ideas. At the same time, the language of the question is more open, leaving the respondents enough room for free play, and not too restrictive for their answers."}, "time_dimension": {"answer": "Future", "rationale": "The entry uses the words \"always\" and \"unpredictable and unpredictable\", describing a possibility and expectation, and thus judging it as the future."}, "sentence_type": "Affirmative declarative sentence", "pos": {"nouns": {"Life": 1, "Uncertainty": 1, "Unpredictable": 1}, "verbs": {"I feel": 1}, "adjectives": {"Total": 1, "Full": 1}, "pronouns": {"Here": 1}}}, {"content": "3 used to give up their wishes and demands.", "word_count": 13, "label": "", "sentence_structure": "Subject鈥搗erb鈥揷omplement", "subjective_objective": {"answer": "Subjective", "rationale": "Because custom is an individual ' s tendentious attitude, it reflects the attitude and perception of patients to abandon their aspirations and demands."}, "sentence_sentiment": {"answer": "Negative", "rationale": "Because the \"renounce your wishes and demands\" act in the entry is negative."}, "language_style": {"answer": "Calm and direct", "rationale": "The entry is more straightforward and concise in language, and does not have too many rhetorical or emotional features. It asks whether the interviewee is used to abandoning his wishes and demands, and is more objective and calm."}, "time_dimension": {"answer": "Present", "rationale": "\"Accessive\" suggests a pattern of constant, long-term behaviour that has been developed in the past and continues to the present. The time dimension of this questionnaire entry is therefore now."}, "sentence_type": "Negative declarative sentence", "pos": {"nouns": {"Custom": 1, "Abandon": 1, "Wish": 1, "Request": 1}, "verbs": {"on": 1, "have": 1}, "adverbs": {"Often": 1}, "numerals": {"3": 1}}}, {"content": "Four is always worried about what's gonna happen.", "word_count": 11, "label": "", "sentence_structure": "Subject鈥搗erb鈥搊bject", "subjective_objective": {"answer": "Subjective", "rationale": "Because \"always fear what happens\" is the patient's inner feeling and mind activity, which is subjective."}, "sentence_sentiment": {"answer": "Negative", "rationale": "Because the \"always fear something will happen\" in the entry is negative."}, "language_style": {"answer": "Calm and direct", "rationale": "The language style of the entry is calm and straightforward, and the other party is asked whether the other party is always concerned about the eventuality, is not over-prescriptive or emotional, and is expressed in more objective and direct ways."}, "time_dimension": {"answer": "Future", "rationale": "The keyword \"always worry about it\" is a sign of anxiety and uncertainty about the future, and therefore judgement is the future."}, "sentence_type": "Affirmative declarative sentence", "pos": {"nouns": {"Things": 1, "Not good": 1}, "verbs": {"Come on": 1, "I'm worried": 1}, "adverbs": {"Always": 1}}}, {"content": "Five never refuses a friend's request.", "word_count": 9, "label": "", "sentence_structure": "Subject鈥搗erb鈥搊bject", "subjective_objective": {"answer": "Objective", "rationale": "Because rejection is an objective act, not the patient ' s thoughts or feelings."}, "sentence_sentiment": {"answer": "Neutral", "rationale": "Because the entry does not have any emotional color."}, "language_style": {"answer": "Tactful and warm", "rationale": "This language style is more likely to resonate and engage the respondents, and to facilitate the validity and reliability of the questionnaire."}, "time_dimension": {"answer": "Future", "rationale": "According to the description of the title, \"5 never rejects a friend's request\" is an inquiry into the testee's future conduct and therefore judgement as future conduct."}, "sentence_type": "Negative declarative sentence", "pos": {"nouns": {"Request": 1}, "verbs": {"Reject": 1, "Here": 1, "friends": 1}, "adjectives": {"None": 1, "of": 1}, "adverbs": {"Never": 1}, "numerals": {"5": 1}}}, {"content": "Six of them are unhappy, they're always upset or crying alone.", "word_count": 18, "label": "", "sentence_structure": "Subject鈥搗erb鈥揷omplement", "subjective_objective": {"answer": "Subjective", "rationale": "Because \"smut or weeping alone\" is a description of emotional expression and mental state, subjective feelings and behaviour of the patient."}, "sentence_sentiment": {"answer": "Negative", "rationale": "Because the \"smuggling or crying alone\" in the entry is negative."}, "language_style": {"answer": "Calm and direct", "rationale": "The entry is in a straightforward way to ask the other party whether to be angry or weep alone when it encounters an unhappy situation, without too much embellishment or emotional rendering, and in a more balanced and objective way. It is therefore possible to judge that the language style of the entry is straightforward and calm."}, "time_dimension": {"answer": "Present", "rationale": "The entry asks what kind of response the testee usually takes when he encounters unhappy events, and can therefore be judged to be the present."}, "sentence_type": "Interrogative sentence", "pos": {"nouns": {"Yes": 1, "I'm not happy": 1, "- Oh, shit": 1, "Crying": 1}, "verbs": {"I met him": 1, "Fan": 1, "Alone": 1, "Cry": 1}, "adverbs": {"Always": 1}, "classifiers": {"individual": 1}}}, {"content": "Seven always thinks he's a bad luck.", "word_count": 10, "label": "", "sentence_structure": "Subject鈥搗erb鈥揷omplement", "subjective_objective": {"answer": "Subjective", "rationale": "Because feeling bad is what patients feel and think."}, "sentence_sentiment": {"answer": "Negative", "rationale": "Because the entry \"likes to feel bad\" expresses a negative emotional attitude."}, "language_style": {"answer": "Calm and direct", "rationale": "The language style of the entry is more straightforward, with no excessive rhetorical or emotional rendering, more focused on asking for factual information and more objective in tone."}, "time_dimension": {"answer": "Present", "rationale": ""}, "sentence_type": "Negative declarative sentence", "pos": {"nouns": {"self": 1, "Shit": 1}, "verbs": {"I think": 1, "Yeah": 1, "Always": 1}, "adverbs": {"Yeah": 1, "Always": 1}, "numerals": {"7": 1}, "pronouns": {"self": 1}}}, {"content": "Eight people say you're shy and chicken.", "word_count": 13, "label": "", "sentence_structure": "Subject鈥搗erb鈥揷omplement", "subjective_objective": {"answer": "Objective", "rationale": "Because the descriptions of \"scrambling\" and \"dumping\" are based on the views and assessments of others and reflect objective facts."}, "sentence_sentiment": {"answer": "Negative", "rationale": "Because it contains negative emotional terms, \"weakness, retreat.\""}, "language_style": {"answer": "Tactful and warm", "rationale": "The language style of the entry is more euphemistic, understanding the insider鈥檚 feelings by asking the interviewee鈥檚 opinion about the other person鈥檚 evaluation, without directly accusing or questioning his character. This expression is more likely to create a climate of respect and understanding that will facilitate the interviewee鈥檚 more open expression of his thoughts and feelings."}, "time_dimension": {"answer": "Present", "rationale": "Now the keyword \"someone says \" means that it is a description of what happened in the past, and \"the shy, the retreating\" is an assessment of the character characteristics of the testee, which may be based on past observations and interactions. The entry is therefore an inquiry into the present behaviour or state of the testee."}, "sentence_type": "Negative declarative sentence", "pos": {"nouns": {"others": 1, "person": 1, "Shy": 1, "Retrenched": 1}, "verbs": {"Say it": 1}, "adjectives": {"Shy": 1, "Retrenched": 1}, "numerals": {"8": 1}, "pronouns": {"you": 1}}}, {"content": "Nine always worry about bad friends.", "word_count": 16, "label": "", "sentence_structure": "Subject鈥搗erb鈥搊bject", "subjective_objective": {"answer": "Subjective", "rationale": "Because \"always\" and \"fear\" indicate that it's a patient's personal psychological feeling and subjective concern."}, "sentence_sentiment": {"answer": "Negative", "rationale": "Because the mood and attitude in the entry, \"A fear of being too good for friends\" is negative."}, "language_style": {"answer": "Tactful and warm", "rationale": "The language style of the entry is more euphemistic, and it stimulates discussion by asking if the other side is always worried about bad relationships with friends. This expression is relatively gentle and friendly, making it easier for respondents to share their thoughts and feelings, and helps to communicate and understand the views and mentalities of the interviewees in depth."}, "time_dimension": {"answer": "Present", "rationale": "The two key words \"always\" and \"foretime\" in the sentence can be judged to describe the current thinking and feeling of the testee, and therefore the current state of affairs."}, "sentence_type": "Exclamatory sentence", "pos": {"nouns": {"Always": 1, "I'm worried": 1, "Okay": 1, "friends": 2, "Relations": 1, "Later": 1, "Bad": 1}, "verbs": {"be": 1, "Yes": 1}, "adverbs": {"Too much": 1}, "numerals": {"9": 1}}}, {"content": "Ten pairs of leaders have been far away.", "word_count": 9, "label": "", "sentence_structure": "Subject鈥搗erb鈥搊bject", "subjective_objective": {"answer": "Subjective", "rationale": "For the sake of respect, it is a subjective attitude that examines the attitude and feelings of the patient towards leadership."}, "sentence_sentiment": {"answer": "Negative", "rationale": "Because the \"regardless\" act in the entry is negative."}, "language_style": {"answer": "Calm and direct", "rationale": "The entry is a direct query about the behaviour of the respondent towards the leader, without too much rhetoric or emotional colour, and with a more objective and calm language style."}, "time_dimension": {"answer": "Past", "rationale": ""}, "sentence_type": "Affirmative declarative sentence", "pos": {"nouns": {"Leadership": 2, "To the farthest": 1}, "verbs": {"to": 1}, "adverbs": {"Always": 1, "Ten": 1}, "numerals": {"10": 1}}}, {"content": "11 often worry about losing control of their mind or emotions.", "word_count": 16, "label": "", "sentence_structure": "Subject鈥搗erb鈥揷omplement", "subjective_objective": {"answer": "Subjective", "rationale": "Because \"always worried\" is a subjective emotional experience and attitude."}, "sentence_sentiment": {"answer": "Negative", "rationale": "Because the attitude of \"fearing your mind or emotions out of control\" in the entry is negative."}, "language_style": {"answer": "Calm and direct", "rationale": "The entry is a direct and clear query about whether the interviewee is often concerned about losing control of his mind or emotions, is not over-represented in rhetoric or emotional rendering, and is more balanced and objective. Its language style is therefore straightforward and calm."}, "time_dimension": {"answer": "Present", "rationale": ""}, "sentence_type": "Affirmative declarative sentence", "pos": {"nouns": {"Thinking": 1, "Emotion": 1, "Control": 2}, "verbs": {"I'm worried": 1, "Lose": 1}, "adverbs": {"Often": 1}, "numerals": {"11": 1}}}, {"content": "12 is always... everything is not for anyone.", "word_count": 7, "label": "", "sentence_structure": "Subject鈥搗erb鈥揷omplement", "subjective_objective": {"answer": "Subjective", "rationale": "Because \"always\" is a subjective judgment."}, "sentence_sentiment": {"answer": "Negative", "rationale": "Because the \"doing nothing\" act in the entry is negative and means being too independent and isolated."}, "language_style": {"answer": "Tactful and warm", "rationale": "The language style of the entry is more euphemistic, using the term \"always\" to express the personality of the other, rather than directly describing or accusing, and this is more respectful and understanding."}, "time_dimension": {"answer": "Present", "rationale": "The entry describes the attitude and manner in which the subject is treated, and does not have a clear time point, and therefore is judged to be neutral."}, "sentence_type": "Imperative sentence", "pos": {"nouns": {"12": 1, "Everything": 1, "person": 1}, "verbs": {"Always": 1, "Please": 1}, "numerals": {"1": 1, "2": 1}, "classifiers": {"individual": 1}}}, {"content": "13, afraid of a mess in his life.", "word_count": 12, "label": "", "sentence_structure": "Subject鈥搗erb鈥揷omplement", "subjective_objective": {"answer": "Subjective", "rationale": "Because fear is a subjective state of mind."}, "sentence_sentiment": {"answer": "Negative", "rationale": "Because \"care of your life is a mess\" expresses a negative mood and attitude, that is, fear and anxiety for life."}, "language_style": {"answer": "Calm and direct", "rationale": "The entry is relatively straightforward in its language style, with no excessive rhetorical or emotional rendering, and is more focused on factual statements and objective expressions. For example, the terms \" worry\" and \"fuck\" are more obvious in negative colours, and are presented in a concise manner, without using complex phrase structures or soft language."}, "time_dimension": {"answer": "Present", "rationale": ""}, "sentence_type": "Affirmative declarative sentence", "pos": {"nouns": {"Life": 1, "It's a mess": 1}, "verbs": {"I'm worried": 1}, "numerals": {"13": 1}}}, {"content": "14 Feels powerless to respond to and address sudden dangers", "word_count": 16, "label": "", "sentence_structure": "Subject鈥揷opula鈥損redicative", "subjective_objective": {"answer": "Objective", "rationale": "The objective facts that patients experience are the result of feeling incapable of coping with and dealing with sudden dangers."}, "sentence_sentiment": {"answer": "Negative", "rationale": "Because the description of \"incompetence to cope with and manage sudden dangers\" in the entry is negative."}, "language_style": {"answer": "Calm and direct", "rationale": "The entry is investigated with a specific, practical problem, without adding too many emotional or rhetorical features and with a more direct and objective linguistic style."}, "time_dimension": {"answer": "Present", "rationale": "The \"Esses of inability to cope with and manage sudden dangers\" in the entry to the questionnaire describe the current state or feelings of the person being tested, without reference to the past or future time dimension. It therefore falls within the context of the present state of affairs."}, "sentence_type": "Affirmative declarative sentence", "pos": {"nouns": {"14": 1, "Power": 1, "Break": 1, "Danger": 1}, "verbs": {"Response": 1, "Processing": 1}, "numerals": {"1": 1}}}, {"content": "15 afraid of being close and close to someone else.", "word_count": 14, "label": "", "sentence_structure": "Subject鈥搗erb鈥揷omplement", "subjective_objective": {"answer": "Subjective", "rationale": "Because \"fear\" is a subjective emotion and feeling that means that individuals are afraid or uneasy about establishing and maintaining close relationships."}, "sentence_sentiment": {"answer": "Negative", "rationale": "Because the emotional term \"fear\" in the entry is negative, implying fear and anxiety about human relationships."}, "language_style": {"answer": "Calm and direct", "rationale": "The entry begins with a number, points out the subject matter in a straightforward manner and is more straightforward in language style, with no excessive rhetorical or emotional rendering, expressing an objective inquiry attitude."}, "time_dimension": {"answer": "Present", "rationale": "The term \"fear\" in this entry indicates a subjective sentiment or attitude that the testee immediately felt when completing the questionnaire. The temporal dimension of this article should therefore be judged now."}, "sentence_type": "Negative declarative sentence", "pos": {"nouns": {"15": 1, "person": 1, "Relations": 1, "Fear": 1}, "verbs": {"Create": 1, "Hold on": 1, "Close": 1}, "adjectives": {"Scared": 1}, "numerals": {"15": 1, "1": 1}, "pronouns": {"Others": 1}}}, {"content": "16, no matter what anyone says, they think they're useless.", "word_count": 15, "label": "", "sentence_structure": "Subject鈥搗erb鈥揷omplement", "subjective_objective": {"answer": "Subjective", "rationale": "Because \"I feel useless\" is a patient's subjective feeling and evaluation."}, "sentence_sentiment": {"answer": "Negative", "rationale": "Because self-evaluation in the entry, \"I feel useless,\" is negative."}, "language_style": {"answer": "Calm and direct", "rationale": "The entry directly asks whether the interviewees often consider themselves worthless, with a more straightforward, concise language style, and no euphemism or accoutrements. \""}, "time_dimension": {"answer": "Present", "rationale": "In conclusion, the answer to this question is \"now\"."}, "sentence_type": "Negative declarative sentence", "pos": {"nouns": {"others": 1, "self": 2, "Role": 1}, "verbs": {"Say it": 1, "I think": 1, "have": 1}, "adjectives": {"It's useless": 1}, "numerals": {"16": 1}}}]}

{"questionnaire_title": "Anxiety questionnaire", "questionnaire_category": "Mental health", "item_count": 20, "emotion_shift_overall": "From negative to positive", "language_style_overall": "Tactful and warm", "items": [{"content": "I feel calm.", "word_count": 7, "label": "", "sentence_structure": "Subject鈥揷opula鈥損redicative", "subjective_objective": {"answer": "Subjective", "rationale": "Because \"feeling calm\" is the individual experience and feeling of the patient, and it is a subjective examination."}, "sentence_sentiment": {"answer": "Neutral", "rationale": "Because the entry is not clearly emotional, it is just a description of a state of feeling."}, "language_style": {"answer": "Tactful and warm", "rationale": "The entry is more personal and interactive when it is spoken in a gentle and inviting manner, rather than in direct statements or orders. At the same time, the language style of the article gives people a sense of ease of self-interest, which can trigger the respondents to respond positively to questions."}, "time_dimension": {"answer": "Present", "rationale": "The keyword \"feel\" and \"quiet\" describe the current emotional state of the person being tested, so the entry is in its current state."}, "sentence_type": "Affirmative declarative sentence", "pos": {"nouns": {"I": 1, "The mood": 1}, "verbs": {"Feel": 1, "Calm down": 1}}}, {"content": "I feel safe.", "word_count": 5, "label": "", "sentence_structure": "Subject鈥揷opula鈥損redicative", "subjective_objective": {"answer": "Subjective", "rationale": "Because \"felt safe\" is a subjective feeling and experience based on the individual feelings and judgement of the patient, rather than a phenomenon that can be directly observed or measured."}, "sentence_sentiment": {"answer": "Positive", "rationale": "Because the entry \"I feel safe\" expresses a positive emotional attitude."}, "language_style": {"answer": "Tactful and warm", "rationale": "The entry is based on the following: - The entry is more personal and interactive in terms of a moderate and inviting tone of speech, rather than a direct statement or order. - The article is more balanced, not using too many strong emotional terms, such as \"strong\" and \"extreme\" but neutral and objective, which is consistent with the euphemism of the language. In summary, the language style of the article ' s purpose is considered euphemism."}, "time_dimension": {"answer": "Present", "rationale": "The keyword \"I feel\" means what the testee is feeling, so the entry describes the emotional state of the testee."}, "sentence_type": "Affirmative declarative sentence", "pos": {"nouns": {"Clear": 1}, "verbs": {"I feel": 1}}}, {"content": "I'm nervous.", "word_count": 5, "label": "", "sentence_structure": "Subject鈥揷opula鈥損redicative", "subjective_objective": {"answer": "Subjective", "rationale": "Because \"I'm nervous\" describes the patient's own emotional state, which is the patient's subjective feeling."}, "sentence_sentiment": {"answer": "Negative", "rationale": "Because \"stretch\" in the entry is an expression of a negative emotional state."}, "language_style": {"answer": "Calm and direct", "rationale": "The language style of this entry is more straightforward, not overdoing or emotional, simply asking if the interviewee is nervous and consistent with the \"cool and straight\" feature."}, "time_dimension": {"answer": "Present", "rationale": "\"Yes\" means the state of the moment, so the entry is asking the person who was tested for his present state of mind, \"I\" for the person who was tested, so the entire sentence is describing the subjective feelings and state of the person who was tested, pointing to the present."}, "sentence_type": "Affirmative declarative sentence", "pos": {"verbs": {"be": 1, "Nervous": 1}, "pronouns": {"I": 1}}}, {"content": "I feel restricted.", "word_count": 6, "label": "", "sentence_structure": "Subject鈥揷opula鈥損redicative", "subjective_objective": {"answer": "Subjective", "rationale": "Because it is a subjective feeling or emotional experience, not an objective fact or result, that is limited."}, "sentence_sentiment": {"answer": "Negative", "rationale": "Because \"restricted\" is a negative feeling."}, "language_style": {"answer": "Tactful and warm", "rationale": "The entry uses the more subjective expression \"I feel\" to guide the other side to share personal emotional experiences, to be more gentle, to be inspired and contagious."}, "time_dimension": {"answer": "Present", "rationale": "The key word \"perception of being limited\" would make it possible to judge that it was describing the current subjective feeling or state, so that the temporal dimension of the article was now."}, "sentence_type": "Negative declarative sentence", "pos": {"nouns": {"Questionnaire Entry": 1, "Sensor": 1, "Limit": 1, "Production": 1}, "verbs": {"I feel": 1, "By": 1, "Limits": 1}, "numerals": {"4": 1}}}, {"content": "I feel comfortable.", "word_count": 5, "label": "", "sentence_structure": "Subject鈥揷opula鈥損redicative", "subjective_objective": {"answer": "Subjective", "rationale": "Because \"fear\" is a patient's subjective feeling, the questionnaire entry is viewed from the patient's subjective point of view."}, "sentence_sentiment": {"answer": "Positive", "rationale": "Because \"frightful\" is a positive emotional description, indicating that the interviewee is in a good mood."}, "language_style": {"answer": "Tactful and warm", "rationale": "The entry uses a more moderate and friendly tone to ask the other side about their feelings, and is not expressed in a way that is too direct or hard, which makes it easier for the interviewee to relax and to share real ideas."}, "time_dimension": {"answer": "Present", "rationale": "The entry describes the feelings of the testee at this moment, and the keywords \"I feel\" and \"Easy\" indicate that this is a current sentence, which is the current state of the person speaking or the emotions."}, "sentence_type": "Affirmative declarative sentence", "pos": {"nouns": {"Questionnaire Entry": 1, "Easy": 1}, "verbs": {"I feel": 1}}}, {"content": "I'm so upset.", "word_count": 5, "label": "", "sentence_structure": "Subject鈥揷opula鈥損redicative", "subjective_objective": {"answer": "Subjective", "rationale": "Because disorder is the patient ' s own feelings and experiences, it is a subjective examination."}, "sentence_sentiment": {"answer": "Negative", "rationale": "The basis is that the term \"disturbing\" is usually considered to be a reflection of negative sentiment."}, "language_style": {"answer": "Calm and direct", "rationale": "The entry directly expresses the subject of questioning whether the other party is often troubled, is not based on excessive rhetoric or emotional rendering, and is more objective in tone."}, "time_dimension": {"answer": "Present", "rationale": ""}, "sentence_type": "Negative declarative sentence", "pos": {"nouns": {"Questionnaire": 1, "Entry": 1, "Disturbing": 1}, "verbs": {"I feel": 1}}}, {"content": "I'm worried about the possibility of misfortune.", "word_count": 16, "label": "", "sentence_structure": "Subject鈥搗erb鈥搊bject", "subjective_objective": {"answer": "Subjective", "rationale": "Because \"fear for what may happen\" is a manifestation of subjective emotions and attitudes, which are part of the patient's inner experience."}, "sentence_sentiment": {"answer": "Negative", "rationale": "Because the word \"torture\" itself has negative emotions and is negative."}, "language_style": {"answer": "Calm and direct", "rationale": "The language style of the entry is a calm and direct type. It directly expresses the subject of asking the other party whether it is troubled by what is possible, and is not speaking too much or making emotional presentations, which is more objective. This expression is more appropriate for psychological questionnaires, because it does not affect the emotions of the interviewee and the accuracy of the answers."}, "time_dimension": {"answer": "Present", "rationale": "The keyword \"now\" indicates that it is an ongoing move, and \"unfortunately for what may happen\" is the emotional state that the testee is experiencing, so the entry is a description of the current state of affairs."}, "sentence_type": "Affirmative declarative sentence", "pos": {"nouns": {"Problem": 1, "Trouble": 1}, "verbs": {"Come on": 1, "In the process of": 2}, "adjectives": {"Possible": 1, "Unfortunately": 1}, "numerals": {"7": 1}, "pronouns": {"I": 1}}}, {"content": "8. I'm satisfied.", "word_count": 5, "label": "", "sentence_structure": "Subject鈥揷opula鈥損redicative", "subjective_objective": {"answer": "Subjective", "rationale": "Because \"satisfaction\" is a subjective feeling, an assessment and perception of the patient's own situation."}, "sentence_sentiment": {"answer": "Positive", "rationale": "Because \"satisfaction\" expresses a positive emotional attitude and indicates that the respondents have a positive view of their current state."}, "language_style": {"answer": "Tactful and warm", "rationale": "The entry uses the positive word \"satisfactory\" and gives the interviewee a self-evaluation in a manner that encourages the interviewee to participate actively without being perceived as too direct or aggressive."}, "time_dimension": {"answer": "Present", "rationale": "Now, I'm satisfied that the entry means the current feeling or state, so the temporal dimension is now."}, "sentence_type": "Affirmative declarative sentence", "pos": {"verbs": {"Sensor": 1, "Present": 1, "Satisfied": 1}}}, {"content": "I'm scared.", "word_count": 5, "label": "", "sentence_structure": "Subject鈥揷opula鈥損redicative", "subjective_objective": {"answer": "Subjective", "rationale": "Because fear is a subjective feeling, caused by the individual ' s inner experience and emotional state, not by an objective existence or situation."}, "sentence_sentiment": {"answer": "Negative", "rationale": "Because the \"fear\" sentiment in the entry is negative."}, "language_style": {"answer": "Calm and direct", "rationale": ""}, "time_dimension": {"answer": "Past", "rationale": "The entry describes the emotional feelings of the person who was tested at a certain point in time, and does not clearly indicate the point of time, and therefore does not allow for a judgement as to whether it is a past, present or future act or mentality."}, "sentence_type": "Negative declarative sentence", "pos": {"nouns": {"Questionnaire": 1, "Entry": 1}, "verbs": {"I feel": 1, "Scared": 1}}}, {"content": "I'm comfortable.", "word_count": 5, "label": "", "sentence_structure": "Subject鈥揷opula鈥損redicative", "subjective_objective": {"answer": "Subjective", "rationale": "Because \"favouring\" is a personal experience and feeling, and it's a subjective view of the patient."}, "sentence_sentiment": {"answer": "Neutral", "rationale": "Because the entry merely expresses a feeling and does not involve any emotional or attitudinal bias."}, "language_style": {"answer": "Tactful and warm", "rationale": "This is a more personal and interactive way of expressing the person you are interviewing, and helps the person you are interviewing to be more willing to answer the question in a true and natural way."}, "time_dimension": {"answer": "Present", "rationale": "The entry is for the current feeling or mentality of the testee, and the time dimension is now judged."}, "sentence_type": "Affirmative declarative sentence", "pos": {"verbs": {"I feel": 1, "Comfortable": 1}, "numerals": {"10": 1}}}, {"content": "I'm confident.", "word_count": 5, "label": "", "sentence_structure": "Subject鈥搗erb鈥揷omplement", "subjective_objective": {"answer": "Subjective", "rationale": "Self-confidence is the patient's subjective feeling and attitude."}, "sentence_sentiment": {"answer": "Positive", "rationale": "Because the \"self-confidence\" attitude is positive."}, "language_style": {"answer": "Tactful and warm", "rationale": "The entry is opened in a way that is either in a way of asking and inviting questions, without too direct or harsh a tone, with a more gentle and friendly expression, which is in keeping with the euphemism of the language. At the same time, the purpose of the article is also more closely related to life and more resonance and response by the person under investigation."}, "time_dimension": {"answer": "Present", "rationale": "Now the entry is to ask the testee about his current state and feelings, and the keyword is \"have\" and \"confident\" and they point to the current state and attitude."}, "sentence_type": "Affirmative declarative sentence", "pos": {"nouns": {"Questionnaire Entry": 1, "Self-confidence": 1}, "verbs": {"have": 1}, "numerals": {"11": 1}}}, {"content": "I'm very sensitive.", "word_count": 7, "label": "", "sentence_structure": "Subject鈥揷opula鈥損redicative", "subjective_objective": {"answer": "Subjective", "rationale": "Because \"I think\" means that it's a personal view and feeling of the patient, and it's a subjective examination."}, "sentence_sentiment": {"answer": "Negative", "rationale": "Because \"sensitization\" is a negative emotional experience."}, "language_style": {"answer": "Tactful and warm", "rationale": "The entry, which guides the interviewee in a way of asking questions, does not directly deny or accuse him of his behaviour or emotions, but rather provides a safe space for the interviewee to share freely. Moreover, the expression \"me\" has been used to increase the interviewee's sense of participation and belonging. This has made the language style of the questionnaire more euphemistic."}, "time_dimension": {"answer": "Present", "rationale": ""}, "sentence_type": "Affirmative declarative sentence", "pos": {"nouns": {"You're a bit of a sane": 1}, "adjectives": {"I think": 1}}}, {"content": "I'm extremely nervous.", "word_count": 7, "label": "", "sentence_structure": "Subject鈥揷opula鈥損redicative", "subjective_objective": {"answer": "Subjective", "rationale": "Because extreme stress is an emotional experience of a person and a subjective feeling of a patient."}, "sentence_sentiment": {"answer": "Negative", "rationale": "Because \"extremely nervous\" is a negative emotional experience."}, "language_style": {"answer": "Calm and direct", "rationale": "The entry expresses direct and concise language of questioning about the emotional state of the interviewee, without excessive rhetorical or emotional rendering, and with a more calm and objective language style."}, "time_dimension": {"answer": "Present", "rationale": "So the time dimension is now judged."}, "sentence_type": "Negative declarative sentence", "pos": {"nouns": {"I'm nervous": 1, "Questionnaire Entry": 1}, "verbs": {"Analysis": 1, "be": 1}, "adjectives": {"Extreme": 1}, "numerals": {"13": 1}}}, {"content": "I'm indecisive.", "word_count": 5, "label": "", "sentence_structure": "Subject鈥搗erb鈥揷omplement", "subjective_objective": {"answer": "Subjective", "rationale": "Because \"indecisiveness\" is a character characteristic of a human being, a subjective feeling."}, "sentence_sentiment": {"answer": "Negative", "rationale": "Because the attitude of indecisiveness is negative."}, "language_style": {"answer": "Tactful and warm", "rationale": "The entry uses a more moderate tone, not directly rejecting or accusing the other of character, but rather allowing the other to think about his or her situation in a way that is more resonant and reflective of the other."}, "time_dimension": {"answer": "Present", "rationale": "The keyword now \"I\" suggests that the question is to question the person's own behaviour or characteristics, not past or future behaviour. \"Fructure indecisive\" means a character or tendency that usually lasts for a certain period of time, and therefore the temporal dimension is now judged."}, "sentence_type": "Negative declarative sentence", "pos": {"nouns": {"I": 1, "Indecisiveness": 1}, "adjectives": {"Indecisiveness": 1}}}, {"content": "I'm easy.", "word_count": 5, "label": "", "sentence_structure": "Subject鈥揷opula鈥損redicative", "subjective_objective": {"answer": "Subjective", "rationale": "Because \"easy\" is a term that describes personal feelings and emotions, and it belongs to the patient's subjective attitude."}, "sentence_sentiment": {"answer": "Positive", "rationale": "Because the description of \"easy\" is positive emotional and expresses a positive attitude and good feelings of the interviewees."}, "language_style": {"answer": "Tactful and warm", "rationale": "The entry describes the emotional state of the person under investigation in a positive and positive manner, without using too direct or negative language, which is more likely to resonate and identify the person under investigation. At the same time, this euphemism is more friendly and friendly, and contributes to a good investigation atmosphere."}, "time_dimension": {"answer": "Present", "rationale": "In the future, the entry contains visions and expectations for the future of the testee, the keywords being \"Yes\" and \"Easier\" and all pointing to situations that have not yet occurred and that may arise in the future."}, "sentence_type": "Exclamatory sentence", "pos": {"adjectives": {"Easy": 1}, "pronouns": {"I": 1}}}, {"content": "I'm satisfied.", "word_count": 7, "label": "", "sentence_structure": "Subject鈥揷opula鈥損redicative", "subjective_objective": {"answer": "Subjective", "rationale": "Because \"satisfaction\" is a subjective feeling and experience that cannot be measured or proved by objective facts."}, "sentence_sentiment": {"answer": "Positive", "rationale": "Because \"satisfaction\" means a positive emotional attitude."}, "language_style": {"answer": "Tactful and warm", "rationale": "The entry is more euphemistic. It uses descriptive words of \"satisfaction\" to express the emotional state of the person under investigation, rather than directly asking about the intensity of emotions or feelings, which is more personal and interactive. And \"Please analyse below\" is a more moderate way of guiding the person under investigation, making him more willing to share his views."}, "time_dimension": {"answer": "Present", "rationale": "Now the entry describes the current feelings or state of the subject, \"I feel\" indicating that it is a subjective experience, and \"now\" that it is at the moment of answering the question. So, it can be judged that the time dimension of the entry is now."}, "sentence_type": "Affirmative declarative sentence", "pos": {"verbs": {"I feel": 1, "Satisfied": 1}, "adjectives": {"Heart": 1, "Satisfied": 1}}}, {"content": "I'm a pain in the ass.", "word_count": 5, "label": "", "sentence_structure": "Subject鈥揷opula鈥損redicative", "subjective_objective": {"answer": "Subjective", "rationale": "Because \"I'm a pain\" is the subjective perception and description of the emotional state of the patient."}, "sentence_sentiment": {"answer": "Negative", "rationale": "Because \"fear\" is a negative emotion."}, "language_style": {"answer": "Tactful and warm", "rationale": "The language style of the entry is euphemism, because it uses the first person's proxy \"I\" to express subjective feelings and to make the interviewee feel concerned and understand by asking if the other party agrees or has experienced similar situations. Moreover, the whole issue is more intimate, soft, and the language is not used in too direct or strong a way."}, "time_dimension": {"answer": "Present", "rationale": "Thus, the judgment on the time dimension is now."}, "sentence_type": "Affirmative declarative sentence", "pos": {"nouns": {"Trouble": 1}, "verbs": {"be": 1}}}, {"content": "I was in a panic.", "word_count": 5, "label": "", "sentence_structure": "Subject鈥揷opula鈥損redicative", "subjective_objective": {"answer": "Subjective", "rationale": "Because feeling panic is a subjective feeling of the patient, a feeling of the heart, a subjective examination."}, "sentence_sentiment": {"answer": "Negative", "rationale": "Because \"fright\" in the entry is an expression of a negative emotion."}, "language_style": {"answer": "Calm and direct", "rationale": "This is more objective and direct, allowing the interviewee to understand and answer questions."}, "time_dimension": {"answer": "Present", "rationale": ""}, "sentence_type": "Negative declarative sentence", "pos": {"verbs": {"I feel": 1, "Panic": 1}}}, {"content": "I'm calm.", "word_count": 5, "label": "", "sentence_structure": "Subject鈥揷opula鈥損redicative", "subjective_objective": {"answer": "Subjective", "rationale": "Because it's a psychological state, it's a subjective feeling of the patient."}, "sentence_sentiment": {"answer": "Positive", "rationale": "Because \"fEalse calm\" is positive."}, "language_style": {"answer": "Calm and direct", "rationale": "The entry directly expresses a question about whether the other party often feels calm about the subject, does not use excessive rhetoric or emotional rendering, and is more objective in tone."}, "time_dimension": {"answer": "Present", "rationale": "The \"I feel\" action occurs at the moment, so the entry describes the state or feeling of the moment."}, "sentence_type": "Affirmative declarative sentence", "pos": {"verbs": {"I feel": 1, "Calm down": 1}}}, {"content": "I'm happy.", "word_count": 5, "label": "", "sentence_structure": "Subject鈥揷opula鈥損redicative", "subjective_objective": {"answer": "Subjective", "rationale": "Because feeling happy is a subjective emotional experience."}, "sentence_sentiment": {"answer": "Positive", "rationale": "Because \"happy\" is a positive emotional word."}, "language_style": {"answer": "Tactful and warm", "rationale": "Warm and warm."}, "time_dimension": {"answer": "Present", "rationale": ""}, "sentence_type": "Affirmative declarative sentence", "pos": {"verbs": {"I feel": 1, "Have a nice day": 1}}}]}

{"questionnaire_title": "Fear of negative evaluation (FNE)", "questionnaire_category": "Mental health", "item_count": 29, "emotion_shift_overall": "From negative to positive", "language_style_overall": "Neutral", "items": [{"content": "I fear what people will think of me, although I know it doesn't matter.", "word_count": 21, "label": "", "sentence_structure": "Subject鈥搗erb鈥搊bject", "subjective_objective": {"answer": "Subjective", "rationale": "Because \"I'm worried about what people think about me\" is a subjective feeling and a concern for the patient. Although he knows it may not matter, it's still his personal emotional reaction. So the questionnaire entry is viewed from the patient's subjective point of view."}, "sentence_sentiment": {"answer": "Negative", "rationale": "Because the attitude and emotion of \"a fear of what people will think of me\" in the entry is negative."}, "language_style": {"answer": "Tactful and warm", "rationale": "The language style of the entry is more euphemistic and thoughtful, using the expression \"though I know it doesn't matter\" to ease the tone of the sentence and try to comfort the other side in not being too concerned about the other. Moreover, the sentence is in the form of a question-and-answer sentence, which gives the other side some room for reflection and interaction, and is more in the softer language style."}, "time_dimension": {"answer": "Present", "rationale": "The key words were \"Aware of what people think about me\" and \"I know it doesn't matter,\" which point to the past experience and feelings."}, "sentence_type": "Negative declarative sentence", "pos": {"nouns": {"He's": 1, "I": 1, "Look": 1}, "verbs": {"I'm worried": 1, "Yes": 1, "Nope": 1, "It's important": 1}, "adverbs": {"What": 1, "鈾?Though 鈾?: 1}}}, {"content": "3. I would be very nervous to know that someone is judging me.", "word_count": 22, "label": "", "sentence_structure": "Subject鈥搗erb鈥搊bject", "subjective_objective": {"answer": "Subjective", "rationale": "Because \"it's very nervous\" is the patient's subjective feelings and attitude."}, "sentence_sentiment": {"answer": "Negative", "rationale": "Because the emotional reaction to \"strained\" in the entry is negative."}, "language_style": {"answer": "Tactful and warm", "rationale": ""}, "time_dimension": {"answer": "Present", "rationale": "The word \"hell\" uses a general future pattern, indicating a prediction or scenario of what may occur in the future, and the entry therefore asks the respondent about his or her reaction and state of mind, when he or she learns that someone is judging his or her head, and the temporal dimension is now."}, "sentence_type": "Affirmative declarative sentence", "pos": {"nouns": {"person": 1, "A review of the quality of the body": 1, "I'm nervous": 1}, "verbs": {"Yes": 3, "to": 1, "in": 1}, "adjectives": {"Tens": 1}, "adverbs": {"If": 1}, "pronouns": {"I": 1}}}, {"content": "I don't care if I know people are creating an impression against me.", "word_count": 24, "label": "", "sentence_structure": "Subject鈥搗erb鈥搊bject", "subjective_objective": {"answer": "Subjective", "rationale": "Because \"not caring\" is patient's subjective attitude."}, "sentence_sentiment": {"answer": "Negative", "rationale": "Because the \"not caring\" attitude in the entry is negative."}, "language_style": {"answer": "Calm and direct", "rationale": "The entry is more straightforward, concise, without too many rhetorical or emotional renderings, and is more objective in tone. At the same time, it raises questions in a clear manner and asks the interviewee to answer, which is more direct and calm. It is therefore judged to be the language style of the purpose of the article as being straightforward and straightforward."}, "time_dimension": {"answer": "Present", "rationale": "Even if \"know\" means \"not care\" indicates the emotional attitude of the person being tested, which points to the current psychological state and behavioral reaction of the person being tested in reply, and therefore to the present."}, "sentence_type": "Interrogative sentence", "pos": {"nouns": {"Questionnaire": 1, "Item": 1, "Impression": 1, "person": 1}, "verbs": {"Yes": 1, "Form": 1, "I care": 1}, "adjectives": {"Negative": 1}, "numerals": {"4": 1}, "pronouns": {"I": 2}}}, {"content": "5. When I make a social mistake, I am very unhappy.", "word_count": 17, "label": "", "sentence_structure": "Subject鈥搗erb鈥揷omplement", "subjective_objective": {"answer": "Subjective", "rationale": "Because \"very unhappy\" is a subjective feeling or emotional reaction, which is the subjective angle of the patient."}, "sentence_sentiment": {"answer": "Negative", "rationale": "Because the \"very unhappy\" attitude in the entry is negative."}, "language_style": {"answer": "Calm and direct", "rationale": "The entry expresses in a direct and clear manner the emotional reaction of the person interviewed when he or she makes a mistake in a social setting, without excessive rhetoric or emotional accoutrements, and more in favour of objective and factual descriptions."}, "time_dimension": {"answer": "Present", "rationale": "The entry is asking the testee about his current attitude and feelings, the keywords being \"we've made a social error\" and \"we're very unhappy\" , all pointing to what is happening. So the time dimension of the entry is now."}, "sentence_type": "Negative declarative sentence", "pos": {"nouns": {"I": 1, "Social": 1, "Error": 1}, "verbs": {"Yes": 1, "Out": 1, "It's not good": 1}, "adjectives": {"Very": 1}, "adverbs": {"When": 1, "in": 1}}}, {"content": "I am not much concerned about what important people think of me.", "word_count": 15, "label": "", "sentence_structure": "Subject鈥搗erb鈥搊bject", "subjective_objective": {"answer": "Subjective", "rationale": "The fear is a subjective one because it is the patient ' s own feelings and attitudes."}, "sentence_sentiment": {"answer": "Neutral", "rationale": "Because the entry does not have any emotional color."}, "language_style": {"answer": "Calm and direct", "rationale": "The entry is in a straightforward manner, asking the interviewees whether they are often concerned about others ' views, and are not using any qualifier or emotional colours, and expressing them in a calm and objective manner."}, "time_dimension": {"answer": "Present", "rationale": "The word \"always\" in \"I hear\" indicates a regular, customary act, while the word \"hear\" uses the normal current state, which means that it has not only happened in the past, but may still be ongoing. The word \"no\" and \"how\" in \"not much\" is the current form of denial, indicating that this person is doing this act in answer to the question, so the entry describes the current state."}, "sentence_type": "Negative declarative sentence", "pos": {"nouns": {"Fear": 1, "People": 1, "Observations": 1}, "verbs": {"I'm worried": 1, "What": 1, "to": 1, "be": 1, "not": 1, "have": 1}, "adjectives": {"Important": 1}, "adverbs": {"very": 1, "Both": 1}, "numerals": {"6": 1}, "pronouns": {"I": 1}}}, {"content": "7. I was often afraid that I would look funny or stupid.", "word_count": 17, "label": "", "sentence_structure": "Subject鈥搗erb鈥揷omplement", "subjective_objective": {"answer": "Subjective", "rationale": "I'm often afraid that's what patients feel and think, so the entry is seen from a subjective perspective."}, "sentence_sentiment": {"answer": "Negative", "rationale": "The fear of being ridiculous or stupid is a negative emotional experience."}, "language_style": {"answer": "Tactful and warm", "rationale": ""}, "time_dimension": {"answer": "Present", "rationale": "\"I've often\" expressed regular, customary behaviour, \"fear\" was subjective feeling and thought, at the psychological level of the person tested. So the time dimension of the article is now."}, "sentence_type": "Negative declarative sentence", "pos": {"nouns": {"Questionnaire": 1, "Entry": 1, "self": 2, "Funny": 1, "Ridiculous": 1, "Stupid": 1}, "verbs": {"Often": 1, "Scared": 1, "It seems": 1, "Yes": 1}, "adjectives": {"very": 1, "Stupid": 1}, "adverbs": {"very": 1, "Often": 1}, "classifiers": {"individual": 1}, "pronouns": {"self": 2}}}, {"content": "I have little reaction to others' disagreement with me.", "word_count": 13, "label": "", "sentence_structure": "Subject鈥搗erb鈥揷omplement", "subjective_objective": {"answer": "Subjective", "rationale": "Because \"almost no reaction\" is the personal experience and feeling of the patient, and it is subjective."}, "sentence_sentiment": {"answer": "Negative", "rationale": "This entry is an expression of a lack of emotional feedback and self-reflection and may lead to problems in interpersonal relations."}, "language_style": {"answer": "Calm and direct", "rationale": "The entry describes in a concise manner the emotional reactions of the subject in the face of opposition from others, the more calm and objective language style, and the absence of excessive rhetoric or emotional rendering."}, "time_dimension": {"answer": "Present", "rationale": "The entry is for the testee's reaction to the other person's disagreement, and the time dimension is uncertain whether it is past, present or future. The keyword is \"I'm almost unresponsive to others' disagreement with me\" and expresses a description of an act, not a state of time. Thus, the time dimension of the article is empty."}, "sentence_type": "Negative declarative sentence", "pos": {"nouns": {"I": 1, "Response": 1}, "verbs": {"to": 1, "Yes": 1, "None": 1, "have": 1}, "adjectives": {"Almost": 1}, "adverbs": {"None": 1}, "numerals": {"8": 1}}}, {"content": "I'm afraid people will notice my weaknesses.", "word_count": 15, "label": "", "sentence_structure": "Subject鈥搗erb鈥揷omplement", "subjective_objective": {"answer": "Subjective", "rationale": "Because fear is the psychological feeling of the patient, it is a subjective examination."}, "sentence_sentiment": {"answer": "Negative", "rationale": "Because the \"fear of others to notice my weaknesses\" act in the entry is negative."}, "language_style": {"answer": "Tactful and warm", "rationale": "The language style of the entry is more moderate and euphemistic, using expressions such as \"often\" and \"fear\" to convey the psychological problems that the person under investigation may have, without a direct tone of blame or criticism, giving a sense of affection, understanding and support."}, "time_dimension": {"answer": "Present", "rationale": ""}, "sentence_type": "Negative declarative sentence", "pos": {"nouns": {"Short": 1, "others": 1}, "verbs": {"Regular": 1, "Scared": 1, "Attention": 1}, "adjectives": {"Mine": 1}, "adverbs": {"Yes": 1, "Regular": 1}, "pronouns": {"I": 2}}}, {"content": "10. The disagreement of others has had little effect on me.", "word_count": 14, "label": "", "sentence_structure": "Subject鈥揷opula鈥損redicative", "subjective_objective": {"answer": "Subjective", "rationale": "Because \"others don't agree\" is an external factor, and patients' perceptions and feelings are subjective."}, "sentence_sentiment": {"answer": "Neutral", "rationale": "As this entry does not express positive or negative emotions."}, "language_style": {"answer": "Calm and direct", "rationale": "The entry is more straightforward, concise, has no excessive speech skills or emotional rendering, is more objective and expresses the subject of asking whether the attitude of the other side to the contrary is more affected."}, "time_dimension": {"answer": "Present", "rationale": "The entry \"no impact\" suggests that the testee is less influenced by criticism or denial when expressing his views and behaviour, which is an immediate attitude or feeling. The temporal dimension is therefore now."}, "sentence_type": "Affirmative declarative sentence", "pos": {"nouns": {"others": 1, "Yes": 1, "Impact": 1}, "verbs": {"No, I'm not": 1}, "adjectives": {"Almost": 1}, "numerals": {"10": 1}, "pronouns": {"I": 1}}}, {"content": "If someone were to judge me, I would easily think of the worst.", "word_count": 19, "label": "", "sentence_structure": "Subject鈥搗erb鈥搊bject", "subjective_objective": {"answer": "Subjective", "rationale": "This is because of the patient ' s perception or perception of self-evaluation and is a subjective one."}, "sentence_sentiment": {"answer": "Negative", "rationale": "Basis of judgement: The \"most likely to think of the worst\" in the entry expresses a negative attitude or emotion."}, "language_style": {"answer": "Calm and direct", "rationale": "The entry is more straightforward, concise, and not overly accommodative or emotional. It explicitly asks the other side's psychological response to the evaluation, allowing the respondent to answer the question directly, without having to devote more effort to interpreting and speculating on the meaning of the question."}, "time_dimension": {"answer": "Present", "rationale": "The words \"if\" and \"easy to think\" all suggest that this is a hypothetical or anticipated situation, where the person tested has some concern or imagination about what might happen in the future. The temporal dimension of this article is therefore the future."}, "sentence_type": "Negative declarative sentence", "pos": {"nouns": {"person": 1, "Evaluation": 2, "I": 3}, "verbs": {"have": 1, "Yes": 1}, "adjectives": {"very": 1, "Bad": 1, "Easy": 1}, "adverbs": {"in": 1, "Suppose": 1}, "numerals": {"11": 1, "Most": 1}, "pronouns": {"he": 2}}}, {"content": "12. I hardly care what I am making of others.", "word_count": 19, "label": "", "sentence_structure": "Subject鈥搗erb鈥揷omplement", "subjective_objective": {"answer": "Subjective", "rationale": "Because the entry is asking the patient about his or her self-perception and attitude, i.e. whether the patient is often concerned about whether his or her behaviour is a positive impression for others, it is a subjective reflection."}, "sentence_sentiment": {"answer": "Neutral", "rationale": "Because the entry does not have any emotional color."}, "language_style": {"answer": "Calm and direct", "rationale": "The entry is a direct expression of the concern to ask the other party whether it has made an impression on others, and is not based on excessive rhetoric or emotional rendering, which is more objective and balanced."}, "time_dimension": {"answer": "Present", "rationale": "The keywords are \"near mind\" and \"what impressions\" and they all indicate the person being tested in the present state of behaviour or state, and therefore the judgement is now."}, "sentence_type": "Negative declarative sentence", "pos": {"nouns": {"Questionnaire Entry": 1, "Impression": 1}, "verbs": {"Mind your own business": 1, "Stay": 1, "others": 1}, "adjectives": {"Almost": 1, "not": 1}, "numerals": {"12": 1}, "pronouns": {"I": 1}}}, {"content": "I fear that others will disagree with me.", "word_count": 10, "label": "", "sentence_structure": "Subject鈥搗erb鈥搊bject", "subjective_objective": {"answer": "Subjective", "rationale": "Because fear is a personal feeling and emotional experience, it is considered from the patient ' s subjective perspective."}, "sentence_sentiment": {"answer": "Negative", "rationale": "Because the purpose of the article is negative, it is an expression of fear among individuals that they are rejected or criticized by others."}, "language_style": {"answer": "Tactful and warm", "rationale": "The expression is more personal and interactive, in a gentle and inviting tone, rather than in direct statements or orders. The phrase \"fear\" is used to describe the psychological state of the person under investigation, which is more resonant and does not cause excessive pressure or discomfort on the person under investigation."}, "time_dimension": {"answer": "Present", "rationale": "Now the \"I'm afraid\" in the entry is an expression of the current emotional state of the subject, and \"others will disagree with me\" is the cause of that sentiment, and it is therefore judged as the present behavior or mentality."}, "sentence_type": "Interrogative sentence", "pos": {"nouns": {"I": 1, "person": 1, "Yes": 1}, "verbs": {"Scared": 1, "Yes": 1, "I don't agree": 1}, "numerals": {"13": 1}}}, {"content": "I fear that others will find my mistakes.", "word_count": 12, "label": "", "sentence_structure": "Subject鈥揷opula鈥損redicative", "subjective_objective": {"answer": "Subjective", "rationale": "Because \"fear\" is a subjective emotional experience."}, "sentence_sentiment": {"answer": "Negative", "rationale": "Because the emotional color of fear in the entries is negative."}, "language_style": {"answer": "Tactful and warm", "rationale": "The language style of the entry is more euphemistic and gentle, rather than directly revealing the wrongs or shortcomings of the other side, but rather asking questions in a more thoughtful, understanding and supportive manner. This expression helps to build trust and security and makes the interviewees more open to sharing their feelings."}, "time_dimension": {"answer": "Present", "rationale": "In conclusion, the answer to this question is \"Now\"."}, "sentence_type": "Negative declarative sentence", "pos": {"nouns": {"Questionnaire": 1, "Entry": 1, "others": 1, "Wrong": 1}, "verbs": {"Scared": 1, "Found": 1}, "numerals": {"14": 1}, "pronouns": {"I": 1}}}, {"content": "I am not worried about what others think of me.", "word_count": 14, "label": "", "sentence_structure": "Subject鈥搗erb鈥揷omplement", "subjective_objective": {"answer": "Subjective", "rationale": "Because \"fearing for others' perception of me\" is the patient's subjective attitude and emotional experience."}, "sentence_sentiment": {"answer": "Negative", "rationale": "Because while the entries do not make the impression that others are upset, this is not necessarily positive or may be an attitude to avoid problems."}, "language_style": {"answer": "Calm and direct", "rationale": "\"Calm down straight.\""}, "time_dimension": {"answer": "Past", "rationale": "The word \"no\" is not negative, i.e. there was a situation that was so annoying about what others think of me, but it is not. So the entry is about asking the testee about past behaviors and mentalities, etc., and the dimension of time is a matter of the past."}, "sentence_type": "Negative declarative sentence", "pos": {"nouns": {"Questionnaire": 1, "Entry": 1, "others": 1, "Observations": 1, "I'm worried": 1}, "verbs": {"be": 1, "Yes": 2, "No, it's not": 1, "I'm worried": 1}, "adjectives": {"Down there": 1, "I": 1, "and": 1, "others": 1}, "adverbs": {"And": 1, "of": 3}, "numerals": {"15": 1}}}, {"content": "16. If someone is not happy, I may not care.", "word_count": 16, "label": "", "sentence_structure": "Subject鈥搗erb鈥搊bject", "subjective_objective": {"answer": "Subjective", "rationale": "Because the entry \"may not matter\" reflects the patient's subjective attitude and feelings."}, "sentence_sentiment": {"answer": "Negative", "rationale": "Because the act of \"if you fail to please someone\" in the entry may cause dissatisfaction or disappointment to the other party and is therefore negative."}, "language_style": {"answer": "Tactful and warm", "rationale": ""}, "time_dimension": {"answer": "Present", "rationale": ""}, "sentence_type": "Negative declarative sentence", "pos": {"verbs": {"Jean": 1, "Joy": 1, "Maybe": 1, "It doesn't matter": 1}, "adverbs": {"Suppose": 1, "I'm not letting you": 1, "I might": 1}, "numerals": {"16": 1}, "pronouns": {"Someone": 1}}}, {"content": "When I spoke to others, I was afraid what they would think of me.", "word_count": 18, "label": "", "sentence_structure": "Subject鈥揷opula鈥損redicative", "subjective_objective": {"answer": "Subjective", "rationale": "This is because the fear and anxiety that patients experience in their dealings with others is a manifestation of subjective sentiment."}, "sentence_sentiment": {"answer": "Negative", "rationale": "Because the entry \"worried what they'd think of me\" is negative."}, "language_style": {"answer": "Tactful and warm", "rationale": "The entry uses a more euphemistic and caring tone, without direct accusations or criticism, but rather attempts to understand the situation and feelings of the person under investigation. This expression is more acceptable and may encourage the person under investigation to be more willing to share his or her thoughts and experiences."}, "time_dimension": {"answer": "Present", "rationale": "\"I'm afraid they'll look at me\" and \"fear\" will indicate that this is a constant, customary concern, and that this is happening. So the time dimension of this questionnaire entry is now."}, "sentence_type": "Negative declarative sentence", "pos": {"nouns": {"person": 1, "Talk": 1}, "verbs": {"I'll see": 1, "I'm worried": 1, "Same": 1, "Say it": 1}, "adverbs": {"What": 1}, "numerals": {"17": 1}, "pronouns": {"they": 1, "I": 2}}}, {"content": "18. I think it is inevitable that one should make a mistake in social life, and therefore why should one be upset about it?", "word_count": 27, "label": "", "sentence_structure": "Subject鈥搗erb鈥搊bject", "subjective_objective": {"answer": "Subjective", "rationale": ""}, "sentence_sentiment": {"answer": "Neutral", "rationale": "Because the entry does not have any obvious emotional colours. While it expresses a perception of social errors, it does not express a positive or negative attitude."}, "language_style": {"answer": "Tactful and warm", "rationale": "The language style of the entry is more euphemistic and sweet, and it expresses a sense of comfort and encouragement by using the phrase \"Why be upset about it.\" This is more acceptable, and it also creates good communication between respondents and researchers."}, "time_dimension": {"answer": "Past", "rationale": "The keywords \"thinks\" and \"doesn't have to be indiscreet in social life\" and \"Why should it be in the mood\" describe past behavior or attitude."}, "sentence_type": "Rhetorical question", "pos": {"nouns": {"person": 2, "Social": 1, "Error": 1}, "verbs": {"I think": 1, "Out": 1, "Sad": 1}, "adjectives": {"It's inevitable": 1}, "adverbs": {"Why": 1}, "pronouns": {"Alone": 1}}}, {"content": "I am usually worried about what I am making of others.", "word_count": 21, "label": "", "sentence_structure": "Subject鈥搗erb鈥搊bject", "subjective_objective": {"answer": "Subjective", "rationale": "Because \"always worried\" and \"what I'm thinking\" are the feelings and perceptions of the patient, and they are subjective."}, "sentence_sentiment": {"answer": "Negative", "rationale": "Because \"fear what's left of the entry\" is a negative emotion and attitude."}, "language_style": {"answer": "Tactful and warm", "rationale": "The entry uses the tone of the query and expresses concern and concern for the other in a gentle and understandable way, which is more euphemistic and warm."}, "time_dimension": {"answer": "Past", "rationale": "Past."}, "sentence_type": "Interrogative sentence", "pos": {"nouns": {"Questionnaire Entry": 1, "person": 1, "others": 1, "Impression": 1}, "verbs": {"in": 1, "I'm worried": 1, "Here": 1, "Stay": 1}, "adjectives": {"Always": 1, "What the hell": 1}, "adverbs": {"Both": 1}, "pronouns": {"I": 2}}}, {"content": "I'm worried about how my boss looks at me.", "word_count": 14, "label": "", "sentence_structure": "Subject鈥揷opula鈥損redicative", "subjective_objective": {"answer": "Subjective", "rationale": "Because the idea of \"quietly worried\" and \"how to look at me\" reflects the patient's subjective feelings and attitude."}, "sentence_sentiment": {"answer": "Negative", "rationale": "Because the word \"scared\" is an expression of a negative emotional attitude."}, "language_style": {"answer": "Calm and direct", "rationale": "The language style of the entry is relatively simple, there is no excessive rhetorical or emotional rendering, and it is more balanced and objective and more consistent with the usual style of psychological questionnaire research.\""}, "time_dimension": {"answer": "Present", "rationale": "Now the \"very worried\" in the entry is an expression of a current emotional state, while the \"how to see me\" is an assessment of the current situation and a concern that can be judged as the present behavior or mentality."}, "sentence_type": "Interrogative sentence", "pos": {"nouns": {"I": 1, "Sir": 1, "What": 1}, "verbs": {"very": 1, "I'm worried": 1, "be": 1, "Look": 1}, "adverbs": {"very": 1}, "classifiers": {"20": 1}}}, {"content": "I don't care when I know someone is judging me.", "word_count": 19, "label": "", "sentence_structure": "Subject鈥搗erb鈥搊bject", "subjective_objective": {"answer": "Subjective", "rationale": "Because \"not caring\" is a subjective feeling or attitude, a self-perception and reaction to others' evaluation. It's the patient's subjective idea, not the objective fact."}, "sentence_sentiment": {"answer": "Negative", "rationale": "Because the \"never mind\" attitude in the entry is negative."}, "language_style": {"answer": "Calm and direct", "rationale": "The entry is more calm and straightforward, not using too many qualifiers or emotional expressions, but conveying the main message of the problem in an objective and clear manner."}, "time_dimension": {"answer": "Present", "rationale": "In the future, the entry describes an expectation or attitude of the testee that they want to be able to care when they know that he or she evaluates himself. This suggests that the testee may take such behaviour or mentality in the future and do not rely on past behaviour or experience."}, "sentence_type": "Negative declarative sentence", "pos": {"nouns": {"person": 1, "Evaluation": 1}, "verbs": {"Yes": 1, "I care": 1}, "adjectives": {"A little": 1}, "adverbs": {"not": 1}, "numerals": {"Twenty-one": 1}, "pronouns": {"I": 1}}}, {"content": "I fear that people will think I am useless.", "word_count": 14, "label": "", "sentence_structure": "Subject鈥搗erb鈥搊bject", "subjective_objective": {"answer": "Subjective", "rationale": "This is a subjective concern and idea of the patient, a self-evaluation of the patient ' s heart and therefore a subjective perspective."}, "sentence_sentiment": {"answer": "Negative", "rationale": "Because the idea of \"I'm a useless person\" in the entry is negative."}, "language_style": {"answer": "Tactful and warm", "rationale": "The entry uses the first-person proxy \"I\" to make the problem more direct and personal. At the same time, the words \"concerning\" and \"no-use\" express a more negative mood and self-evaluation, but the whole sentence is less euphemismal and implicit than too intense or offensive."}, "time_dimension": {"answer": "Present", "rationale": "The \"I'm worried\" in the entry means the emotional state and feeling, and \"I'm considered useless\" is the concern and the idea. So the time dimension of this questionnaire should be judged now."}, "sentence_type": "Negative declarative sentence", "pos": {"nouns": {"Questionnaire": 1, "Entry": 1, "person": 1, "A useless man": 1}, "verbs": {"I'm worried": 1, "Recognition": 1, "be": 1}, "adjectives": {"It's useless": 1}, "numerals": {"22": 1}, "pronouns": {"I": 1}}}, {"content": "I'm not really worried about how people think about me.", "word_count": 17, "label": "", "sentence_structure": "Subject鈥搗erb鈥揷omplement", "subjective_objective": {"answer": "Subjective", "rationale": "The concern was a subjective approach to self-evaluation."}, "sentence_sentiment": {"answer": "Neutral", "rationale": "Because the entry does not have any emotional color."}, "language_style": {"answer": "Calm and direct", "rationale": "The entry describes the attitude of the interviewees towards others ' views in a direct and clear manner, without too much rhetorical or emotional projection, and with a more calm and direct language style."}, "time_dimension": {"answer": "Present", "rationale": "The entry describes the attitude and feelings of the testee towards others at the current stage of his or her own view, and thus the judgement is \"now\"."}, "sentence_type": "Negative declarative sentence", "pos": {"nouns": {"I": 1}, "verbs": {"to": 1, "Yes": 1, "I'm worried": 1}, "adjectives": {"Total": 1}, "adverbs": {"Almost": 1}, "numerals": {"23": 1, "not": 1}}}, {"content": "24. Sometimes I think I care too much about what others think of me.", "word_count": 17, "label": "", "sentence_structure": "Subject鈥揷opula鈥損redicative", "subjective_objective": {"answer": "Subjective", "rationale": "Because \"too caring\" is a subjective feeling and an assessment of the state of your mind."}, "sentence_sentiment": {"answer": "Negative", "rationale": "Because the attitude in the entry, \"too much care about what people think about me,\" is negative."}, "language_style": {"answer": "Tactful and warm", "rationale": "The entry is more personal and interactive when it is spoken in a gentle and inviting manner, rather than in direct statements or orders. At the same time, the entry is not based on too many rhetorical or emotional expressions, which are more objective and sound. It is therefore judged to be a euphemism of the language style."}, "time_dimension": {"answer": "Present", "rationale": "\"Sometimes\" means that the act is not always happening, but is an occasional or intermittent situation. And, \"want\" and \"too care\" are a description of a current mentality or feeling, so the entry should be in the current state of affairs."}, "sentence_type": "Affirmative declarative sentence", "pos": {"nouns": {"Time": 1, "I": 1, "Thoughts": 1}, "verbs": {"Yes": 1, "I care": 1, "Look": 1}, "adjectives": {"Too much": 1, "self": 1}, "adverbs": {"Sometimes": 1}, "numerals": {"24": 1}, "pronouns": {"to": 1, "I": 1, "of": 2}}}, {"content": "I often fear that I will say or do something wrong.", "word_count": 14, "label": "", "sentence_structure": "Subject鈥揷opula鈥損redicative", "subjective_objective": {"answer": "Subjective", "rationale": "Because \"fear\" is a subjective feeling and a fear, a psychological experience of the patient."}, "sentence_sentiment": {"answer": "Negative", "rationale": "Because the mood of \"fear\" in the entry is negative."}, "language_style": {"answer": "Tactful and warm", "rationale": ""}, "time_dimension": {"answer": "Present", "rationale": ""}, "sentence_type": "Affirmative declarative sentence", "pos": {"nouns": {"Entry": 1, "I'm worried": 1, "Word": 1, "Yes": 2}, "verbs": {"Often": 1, "Yes": 2, "Say it": 1, "Do it": 1}, "adverbs": {"Often": 1, "Yes": 1}, "numerals": {"25": 1}, "classifiers": {"individual": 1}, "pronouns": {"I": 1}}}, {"content": "26. I am not usually concerned about what others think of me.", "word_count": 14, "label": "", "sentence_structure": "Subject鈥搗erb鈥搊bject", "subjective_objective": {"answer": "Subjective", "rationale": "Because \"I don't usually care\" is a statement of personal attitudes, a subjective examination."}, "sentence_sentiment": {"answer": "Neutral", "rationale": "Because the entry does not address positive or negative behaviour, attitudes, emotions, etc., it merely describes the attitude of one person towards others ' evaluation."}, "language_style": {"answer": "Calm and direct", "rationale": "The entry expresses the person interviewed ' s attitude towards others ' views in a direct manner, without too much rhetoric or emotional rendering, and is more objective in tone."}, "time_dimension": {"answer": "Present", "rationale": "The keyword is \"I am with\" and the attitude and emotion of the subject to its own perception; \"usually unconcerned\" means a constant act or state of affairs, and is now a state of affairs, which means that the act is still ongoing. So the entry is a description of the current emotional attitude of the subject, and the temporal dimension is now."}, "sentence_type": "Negative declarative sentence", "pos": {"verbs": {"have": 1, "Do it": 1}, "adjectives": {"Regular": 1, "not": 1}, "adverbs": {"Usually": 1}, "numerals": {"26": 1}, "pronouns": {"I": 1}}}, {"content": "I am generally confident that others will be impressed with me.", "word_count": 14, "label": "", "sentence_structure": "Subject鈥搗erb鈥揷omplement", "subjective_objective": {"answer": "Subjective", "rationale": "Since self-confidence is a subjective feeling, it is an attitude or belief in self-evaluation."}, "sentence_sentiment": {"answer": "Positive", "rationale": "Because the \"confidence that others will be impressed with me\" attitude in the entry is positive, indicating that individuals have confidence in their external performance and expect others to have a good impression of themselves."}, "language_style": {"answer": "Tactful and warm", "rationale": "The language style of the entry is more euphemistic, and avoids directly confirming or denying the level of confidence of the other. This expression is more friendly and acceptable and helps create a good atmosphere for questionnaire completion."}, "time_dimension": {"answer": "Present", "rationale": "The sentence describes a person who normally has some confidence in his or her image in the eyes of others, which may affect their behaviour and attitude. The term \"general\" is used to indicate that confidence is usually normal or recurrent, without particular emphasis on past or future circumstances."}, "sentence_type": "Affirmative declarative sentence", "pos": {"nouns": {"Questionnaire Entry": 1, "27": 1, "Confidence": 1, "others": 1, "have": 1, "I'm impressed": 1}, "verbs": {"Analysis": 1}, "adverbs": {"General": 1}, "numerals": {"27": 1}, "pronouns": {"I": 1}}}, {"content": "I am always afraid that those who are important to me will not always think of me.", "word_count": 21, "label": "", "sentence_structure": "Subject鈥揷opula鈥損redicative", "subjective_objective": {"answer": "Subjective", "rationale": "Because \"always worried,\" \"the important people don't always think about me\" are the patients' own feelings and thoughts, and therefore are subjective."}, "sentence_sentiment": {"answer": "Negative", "rationale": "Because the word \"fear\" in the entry is negative."}, "language_style": {"answer": "Tactful and warm", "rationale": "The entry raises questions in a way that cares about the other person's feelings, uses \"Will you always?\", a euphemism, rather than direct accusations or orders, which is more acceptable and understandable to the other person. At the same time, uses the expression \"People who are important to me do not always think of me,\" avoids expressing their anxiety directly and is more concerned about each other's feelings and thoughts."}, "time_dimension": {"answer": "Present", "rationale": "In conclusion, I think the time dimension of this questionnaire entry can be summed up as \"now\" and \"for the future.\""}, "sentence_type": "Negative declarative sentence", "pos": {"nouns": {"person": 1, "Fear": 1}, "verbs": {"Yes": 2, "Always": 1, "I'm worried": 1}, "adverbs": {"Always": 1, "not": 1}, "numerals": {"28": 1}, "pronouns": {"I": 1}}}, {"content": "I am unhappy with the way my friends see me.", "word_count": 16, "label": "", "sentence_structure": "Subject鈥揷opula鈥損redicative", "subjective_objective": {"answer": "Subjective", "rationale": "Because the sentence expresses the feelings and thoughts of the patient, i.e., the unhappyness of the person, it is viewed from a subjective perspective."}, "sentence_sentiment": {"answer": "Negative", "rationale": "Because \"unhappiness\" in the entry is an expression of a negative sentiment."}, "language_style": {"answer": "Tactful and warm", "rationale": "The entry uses a more moderate way of asking questions, rather than asking whether the other side is unhappy because of a friend鈥檚 opinion. This way of expressing itself makes it easier for the person under investigation to open his heart and to answer questions in a more honest manner."}, "time_dimension": {"answer": "Present", "rationale": "The keyword \"unhappy\" means that the situation is a state of emotion, and the \"because\" argument is a phrase that uses the general current state of view of friends to suggest that the state of emotion has existed for some time. So the entry looks at the current attitudes and feelings of the person being tested."}, "sentence_type": "Negative declarative sentence", "pos": {"nouns": {"I": 3, "friends": 2, "Observations": 1, "I'm not happy": 1}, "verbs": {"Because": 1, "have": 1, "be": 1, "I'm not happy": 1}, "numerals": {"29": 1}, "classifiers": {"individual": 1}, "pronouns": {"Mine": 1}}}, {"content": "30. I would be nervous to know that my superiors are judging me.", "word_count": 20, "label": "", "sentence_structure": "Subject鈥搗erb鈥揷omplement", "subjective_objective": {"answer": "Subjective", "rationale": "As tension and anxiety in this situation are determined by the patient ' s feelings and are the expression of subjective attitudes."}, "sentence_sentiment": {"answer": "Negative", "rationale": "Because the \"frightful\" mood in the entry is negative."}, "language_style": {"answer": "Calm and direct", "rationale": "\"Calm down straight.\""}, "time_dimension": {"answer": "Present", "rationale": ""}, "sentence_type": "Affirmative declarative sentence", "pos": {"nouns": {"Sir": 1, "Evaluation": 1}, "verbs": {"It's gonna be": 1, "Yes": 1, "I'm nervous": 1}, "adjectives": {"Nervous": 1, "Unsettled": 1}, "numerals": {"30": 1}, "pronouns": {"I": 1}}}]}

{"questionnaire_title": "- I'm not sure.", "questionnaire_category": "Mental health", "item_count": 15, "emotion_shift_overall": "From negative to positive", "language_style_overall": "Tactful and warm", "items": [{"content": "I am often nervous, even at informal gatherings.", "word_count": 17, "label": "", "sentence_structure": "Subject鈥揷opula鈥損redicative", "subjective_objective": {"answer": "Subjective", "rationale": "Because \"often nervous\" is the patient's feelings and judgment about his emotional state, and it's his subjective attitude."}, "sentence_sentiment": {"answer": "Negative", "rationale": "Because the \"often nervous\" act in the entry is negative."}, "language_style": {"answer": "Calm and direct", "rationale": "The entry directly asked about the social anxiety of the person interviewed, did not use excessive emotional qualifiers or expressions, and was more direct and objective."}, "time_dimension": {"answer": "Present", "rationale": "The keyword \"I often feel\" indicates a state of regular, customary feeling, \"even at informal gatherings\" which means that it may not be just in a particular situation, but that it has a certain generality. So the entry describes the current psychological state and feelings of the person being tested, which is the time dimension of the present."}, "sentence_type": "Negative declarative sentence", "pos": {"nouns": {"Party": 1, "Nervous": 1}, "verbs": {"Sensor": 1, "Regular": 1, "be": 1, "I feel": 1, "in": 2, "Informal": 1, "I": 1, "Yeah": 1, "Go, go, go": 1}, "adjectives": {"Nervous": 1, "Informal": 1}, "numerals": {"1": 5}, "classifiers": {"individual": 1}, "pronouns": {"I": 1}}}, {"content": "2. I am usually uncomfortable with a group of people I do not know.", "word_count": 20, "label": "", "sentence_structure": "Subject鈥揷opula鈥損redicative", "subjective_objective": {"answer": "Subjective", "rationale": "The problem is viewed in terms of the feelings and attitudes of the patients in the social sphere and is part of their subjective experience."}, "sentence_sentiment": {"answer": "Negative", "rationale": "Because the entry \"unsatisfied\" expresses a negative emotion or feeling."}, "language_style": {"answer": "Tactful and warm", "rationale": "The entry is more euphemistic. It uses the term \"normally\" to describe the fact that the interviewee may be uncomfortable in some cases, rather than simply saying that they are always uncomfortable. Moreover, it avoids using too negative or offensive language, but rather asks questions in a more understanding and supportive way. These factors make the language style of the article more euphemistic."}, "time_dimension": {"answer": "Present", "rationale": "In the entry, the term \"normally\" denotes a frequent description, implying that it is a normal reaction of the interviewee, while the term \"frightful\" describes the state of the immediate mood, so that it can be judged to describe the present behaviour or feeling."}, "sentence_type": "Affirmative declarative sentence", "pos": {"nouns": {"person": 2, "Group": 1, "Time": 1, "I'm not comfortable": 1}, "verbs": {"Together": 1, "I feel": 1}, "adjectives": {"No, I don't": 1, "Usually": 1}, "numerals": {"2": 1}, "pronouns": {"I": 1}}}, {"content": "3. I usually feel comfortable talking to a heterosexual. (R)", "word_count": 16, "label": "", "sentence_structure": "Subject鈥揷opula鈥損redicative", "subjective_objective": {"answer": "Subjective", "rationale": "Because the subject is the patient's usual subjective feeling, which is \"normally relaxed\", the entry is subjective."}, "sentence_sentiment": {"answer": "Positive", "rationale": "Because it means that talking to opposite sex can bring relief and is a positive emotional experience."}, "language_style": {"answer": "Tactful and warm", "rationale": "The entry is more euphemistic, uses the expression \"usually light\" and provides the answer option (R) in brackets, which is more gentle and inviting, and gives the respondents a more comfortable and free feel."}, "time_dimension": {"answer": "Present", "rationale": "The entry is about the feelings and behavior of the subject in its current state, which is, \"I usually feel relaxed when talking to a heterosexual person.\" So, its time dimension is now."}, "sentence_type": "Affirmative declarative sentence", "pos": {"nouns": {"Heterosexual": 1, "Talk": 1, "One": 1}, "verbs": {"I feel": 1, "Talk": 1}, "adjectives": {"Easy": 1, "Usually": 1}, "numerals": {"3": 1}, "pronouns": {"I": 1}}}, {"content": "4. I was nervous when I had to speak to teachers or supervisors.", "word_count": 17, "label": "", "sentence_structure": "Subject鈥損redicate", "subjective_objective": {"answer": "Subjective", "rationale": "Because feeling nervous is a subjective emotional reaction."}, "sentence_sentiment": {"answer": "Negative", "rationale": "Because \"stretch\" in the entry is a negative emotional experience."}, "language_style": {"answer": "Calm and direct", "rationale": "The entry is a direct question-and-answer dialogue, with no excessive qualifier or emotional colour, and is more neutral and objective in tone."}, "time_dimension": {"answer": "Present", "rationale": "\"Feel\" means the emotional experience of the moment, \"when it has to talk to the teacher or supervisor\" means a situation that may occur in the future, but in this questionnaire it is considered to be a state of the moment. So the time dimension of this entry is now."}, "sentence_type": "Affirmative declarative sentence", "pos": {"nouns": {"Teacher": 1, "Sir": 1, "Talk": 1}, "verbs": {"I feel": 1, "Nervous": 1}, "numerals": {"4": 1}}}, {"content": "5. The parties often cause me anxiety and uncomfortable.", "word_count": 14, "label": "", "sentence_structure": "Subject鈥揷opula鈥損redicative", "subjective_objective": {"answer": "Subjective", "rationale": "Because anxiety and uncomfortable feelings are part of the patient ' s own subjective experience and emotional response."}, "sentence_sentiment": {"answer": "Negative", "rationale": "Because the emotional reaction of \"fear and uncomfortable\" in the entry is negative."}, "language_style": {"answer": "Tactful and warm", "rationale": "The entry is more euphemistic, and it uses \"regular\" rather than \"always\" to make it more relevant and avoids overly absolute expressions. At the same time, \"anxiety and discomfort\" is not a term that directly depicts negative emotions, but rather that allows respondents to express their feelings in an objective manner."}, "time_dimension": {"answer": "Present", "rationale": "Now, in the entry, both \"party\" and \"party\" are expressions of ongoing or customary behaviour, while the \"cause ... anxiety and discomfort\" state of affairs occurs in the present or in the past, so time horizons are now judged."}, "sentence_type": "Negative declarative sentence", "pos": {"nouns": {"Party": 1, "Anxiety": 1, "I'm not comfortable": 1}, "verbs": {"鈾?And I'm gonna be 鈾?: 1, "I feel": 1}}}, {"content": "I may be less shy in social relations than most people.", "word_count": 20, "label": "", "sentence_structure": "Subject鈥揷opula鈥損redicative", "subjective_objective": {"answer": "Subjective", "rationale": "Because the entry is about patients' perception and evaluation of themselves, like \"I may be less shy,\" it is subjectively viewed."}, "sentence_sentiment": {"answer": "Negative", "rationale": "Because the \"less shyness\" attitude in the entries is relatively negative."}, "language_style": {"answer": "Tactful and warm", "rationale": "The entry is more adept and interactive when it is spoken in a gentle and inviting manner, rather than by direct statements or orders."}, "time_dimension": {"answer": "Present", "rationale": ""}, "sentence_type": "Affirmative declarative sentence", "pos": {"nouns": {"Questionnaire Entry": 1, "Most of them": 1, "person": 1, "Social interaction": 1, "Shame": 1}, "verbs": {"in": 1, "and": 1, "Comparison": 1, "Maybe": 1, "Less": 1}, "adverbs": {"Maybe": 1}, "numerals": {"6": 1, "Most of them": 1}, "pronouns": {"I": 1}}}, {"content": "7. I am often nervous when talking to same-sex people that I am not familiar with.", "word_count": 20, "label": "", "sentence_structure": "Subject鈥搗erb鈥搊bject", "subjective_objective": {"answer": "Subjective", "rationale": "As stress is a subjective feeling, it is the patient ' s personal reaction to a conversation with a less familiar person of the same sex."}, "sentence_sentiment": {"answer": "Negative", "rationale": "Because the \"often nervous\" sentiment in the entry is negative."}, "language_style": {"answer": "Tactful and warm", "rationale": "The language style of the entry tends to be euphemistic and moderate, and it does not directly blame or attack the other party's words and actions, but rather asks the other person's feelings and experiences in a more objective and neutral way."}, "time_dimension": {"answer": "Past", "rationale": "The entry asks about the testee's past experience and feelings, and the keyword is \"often feels\", which indicates that this is a regular, past experience."}, "sentence_type": "Affirmative declarative sentence", "pos": {"nouns": {"Questionnaire": 1, "Entry": 1, "Same-sex": 1, "Talk": 1, "Nervous": 1}, "verbs": {"in": 1, "I know you": 1, "I feel": 1, "When we talk": 1}, "adjectives": {"Not really": 1, "I know it": 1}, "numerals": {"7": 1}, "pronouns": {"I": 2}}}, {"content": "I'm nervous about job interviews.", "word_count": 11, "label": "", "sentence_structure": "Subject鈥揷opula鈥損redicative", "subjective_objective": {"answer": "Subjective", "rationale": "Because tension is a subjective feeling, different people may have different manifestations and degrees."}, "sentence_sentiment": {"answer": "Negative", "rationale": "Because the \"stretch\" in the entry is negative."}, "language_style": {"answer": "Tactful and warm", "rationale": "The language style of the entry is more euphemistic and sensitive to the feelings of the interviewee, using the expression \"creative\" and not much criticism or criticism of the interviewee's performance. This sweet, caring language helps to increase the interviewee's trust in the investigation and willingness to cooperate."}, "time_dimension": {"answer": "Past", "rationale": "The entry describes job interviews, but uses the general current pattern to indicate that it is a normal reaction and therefore judgement is the past."}, "sentence_type": "Affirmative declarative sentence", "pos": {"nouns": {"Job interviews": 1, "Nervous": 1}, "verbs": {"Yes": 1, "Answer": 1}}}, {"content": "I want to be more confident in social life.", "word_count": 17, "label": "", "sentence_structure": "Subject鈥搗erb鈥搊bject", "subjective_objective": {"answer": "Subjective", "rationale": "Because \"hope\" and \"confidence\" are subjective feelings and aspirations of patients, so they're subjective."}, "sentence_sentiment": {"answer": "Neutral", "rationale": "Because the entry expresses a personal expectation and does not involve emotional colour."}, "language_style": {"answer": "Tactful and warm", "rationale": "The topic uses a more euphemistic and encouraging language, asking the respondents whether they have a hope of raising their self-confidence, which neither puts pressure or negative emotions on the respondents, nor triggers their reflections and reflections. At the same time, the topic's \"social dimension\" emphasizes that it is a matter of interpersonal interaction, more relevant and practical."}, "time_dimension": {"answer": "Present", "rationale": "The entry represents the respondent ' s dissatisfaction with its current situation and hopes for future improvements, and is therefore current."}, "sentence_type": "Imperative sentence", "pos": {"nouns": {"Questionnaire": 1, "Entry": 1, "self": 1, "Socially": 1, "Confidence": 1}, "verbs": {"Hope": 1, "have": 1, "in": 1, "More": 1, "Some": 1}, "adverbs": {"More": 1, "Some": 1}, "numerals": {"9": 1}, "classifiers": {"individual": 1}, "pronouns": {"self": 1}}}, {"content": "10. I have little anxiety in social settings. (R)", "word_count": 13, "label": "", "sentence_structure": "Subject鈥揷opula鈥損redicative", "subjective_objective": {"answer": "Subjective", "rationale": "Because \"fearing\" is a subjective feeling, not an objective fact."}, "sentence_sentiment": {"answer": "Positive", "rationale": "Because the description \"very rarely gets anxious\" in the entry is positive."}, "language_style": {"answer": "Calm and direct", "rationale": "The language style of the entry is more straightforward and there is no excessive rhetorical or emotional rendering, the main purpose of which is to ask the interviewee about the level of anxiety in the social setting."}, "time_dimension": {"answer": "Present", "rationale": "The entry describes the feelings and experiences of the person being tested in a social setting, which is a description of the behaviour or state of the moment, so the temporal dimension is judged now. At the same time, the English (R) in brackets is usually used to indicate the type of feedback that the respondent receives, such as \"yes\", \"no\" (No), \"partly\" (Yes), etc., and the answer to this question may be useful in understanding the behaviour and psychological condition of the person being tested."}, "sentence_type": "Affirmative declarative sentence", "pos": {"nouns": {"Socially": 1}, "adjectives": {"Very few": 1, "Anxious": 1}, "numerals": {"10": 1}}}, {"content": "I'm generally a shy person.", "word_count": 12, "label": "", "sentence_structure": "Subject鈥揷opula鈥損redicative", "subjective_objective": {"answer": "Subjective", "rationale": "Because \" shyness \" is a personal feeling or perception that reflects the patient ' s subjective attitude or perception."}, "sentence_sentiment": {"answer": "Neutral", "rationale": "Because the entry describes a personal character, it does not involve positive or negative feelings."}, "language_style": {"answer": "Tactful and warm", "rationale": "The entry uses more euphemistic language, avoiding directly highlighting its shortcomings, but instead expresses its tendency to be shy in the form of a relatively neutral expression, \"generally\". Such language style is more proficient, making it easier for the respondents to accept and be willing to provide a true answer."}, "time_dimension": {"answer": "Present", "rationale": "The entry indicates a general assessment or description of the person being tested, i.e. they are usually or often considered shy. The description is about the act or characteristic of the present, and therefore the temporal dimension is now judged."}, "sentence_type": "Imperative sentence", "pos": {"nouns": {"11": 1, "In general": 1, "person": 1}, "verbs": {"be": 1, "Shy": 1}, "adverbs": {"In general": 1}, "numerals": {"11": 1}, "classifiers": {"individual": 1}}}, {"content": "12. I am often nervous when talking to a charming heterosexual.", "word_count": 19, "label": "", "sentence_structure": "Subject鈥揷opula鈥損redicative", "subjective_objective": {"answer": "Subjective", "rationale": "Because feeling nervous is a subjective feeling or experience."}, "sentence_sentiment": {"answer": "Negative", "rationale": "Because the \"often nervous\" behavior in the entries is negative."}, "language_style": {"answer": "Tactful and warm", "rationale": ""}, "time_dimension": {"answer": "Past", "rationale": ""}, "sentence_type": "Affirmative declarative sentence", "pos": {"nouns": {"Heterosexual": 1, "Talk": 1, "Nervous": 1}, "verbs": {"in": 1, "I feel": 1}, "adjectives": {"Fascinating": 1}, "numerals": {"12": 1}}}, {"content": "13. I am usually nervous when calling people who are not familiar.", "word_count": 17, "label": "", "sentence_structure": "Subject鈥揷opula鈥損redicative", "subjective_objective": {"answer": "Subjective", "rationale": "Because feeling stress is a subjective experience or emotional reaction, determined by the patient鈥檚 personal perceptions and feelings. While telephone conversations may bring some stress or discomfort, they are humanly different and depend on many factors, such as communication skills, character characteristics, social anxiety, etc. The question is therefore one of subjective emotions and experiences of the person under investigation, not objective facts."}, "sentence_sentiment": {"answer": "Negative", "rationale": "Because \"stretch\" in the entry is a negative emotional state."}, "language_style": {"answer": "Tactful and warm", "rationale": "The entry is more euphemistic and warm. It uses first-person expressions, and uses \"less familiar\" to replace a specific name for a person or group, avoiding direct accusations or animosity. At the same time, the entry is not too emotional or negative, but is expressed in a relatively neutral tone, more personal and inclusive."}, "time_dimension": {"answer": "Present", "rationale": ""}, "sentence_type": "Affirmative declarative sentence", "pos": {"nouns": {"person": 1, "Telephone": 1, "Nervous": 1}, "verbs": {"Here": 1, "Fight": 1, "I think": 1}, "adjectives": {"Not familiar": 1}, "adverbs": {"Usually": 1}, "numerals": {"13": 1}, "pronouns": {"I": 1}}}, {"content": "14. I was nervous when I spoke to the authorities.", "word_count": 14, "label": "", "sentence_structure": "Subject鈥揷opula鈥損redicative", "subjective_objective": {"answer": "Subjective", "rationale": "The subject is primarily a subjective one, as it examines the feelings and reactions of the interviewees in specific situations."}, "sentence_sentiment": {"answer": "Negative", "rationale": "Because the \"frightful\" sentiment in the entry is negative."}, "language_style": {"answer": "Calm and direct", "rationale": "The language style of the entry is a calm and straightforward one. It opens up a specific question, without adding too many qualifiers or emotional colours, and is objective and direct, and suitable for use in surveys of such situations as needing a clear and concise expression."}, "time_dimension": {"answer": "Present", "rationale": "The entry describes the current behaviour or feelings of the testee, and the expression \"I'm nervous\" indicates that this is an emotional state that is going through and that can therefore be judged as the time dimension of the test."}, "sentence_type": "Affirmative declarative sentence", "pos": {"nouns": {"Questionnaire": 1, "Entry": 1, "Authority": 1, "Talk": 1}, "verbs": {"I feel": 1, "Nervous": 1}, "numerals": {"14": 1}}}, {"content": "15. Even in a group that is quite different from me, I usually feel relaxed. (R)", "word_count": 25, "label": "", "sentence_structure": "Subject鈥揷opula鈥損redicative", "subjective_objective": {"answer": "Subjective", "rationale": "This sentence describes the feelings and attitudes of the patient in a given situation and is a subjective examination."}, "sentence_sentiment": {"answer": "Positive", "rationale": "Because the \"fright to relax\" in the entry is positive."}, "language_style": {"answer": "Calm and direct", "rationale": "The decision-making authority: The article is expressed in relatively simple and clear terms, with no excessive rhetorical or emotional overtones. It asks whether the person under investigation can maintain a relaxed mentality, be objective and direct in the given circumstances."}, "time_dimension": {"answer": "Present", "rationale": "\"even if\" indicates that there is a hypothetical situation of the act, that is, the scene in different groups of people. At the same time, \"the feeling\" suggests the continuity of this feeling, so the entry describes the situation of the person being tested."}, "sentence_type": "Affirmative declarative sentence", "pos": {"nouns": {"Population": 2, "I": 1}, "verbs": {"I feel": 1, "Relax": 1, "Location": 1, "be": 1}, "adjectives": {"Pretty much": 1, "Different": 1}, "adverbs": {"Still": 1}, "numerals": {"15": 1}, "pronouns": {"Here": 1}}}]}

{"questionnaire_title": "Zung Axiety Self-Assessment Table (SAS)", "questionnaire_category": "Mental health", "item_count": 20, "emotion_shift_overall": "From negative to positive", "language_style_overall": "Tactful and warm", "items": [{"content": "I feel more nervous and anxious than usual.", "word_count": 13, "label": "", "sentence_structure": "Subject鈥揷opula鈥損redicative", "subjective_objective": {"answer": "Subjective", "rationale": "Because \"I think\" means that it's a personal feeling and perception of the patient, and it's a subjective perspective."}, "sentence_sentiment": {"answer": "Negative", "rationale": "Because \"stretch and rush\" in the entry is a negative emotional state."}, "language_style": {"answer": "Tactful and warm", "rationale": "This is a more gentle and friendly expression that helps to create a good atmosphere of communication and trust."}, "time_dimension": {"answer": "Present", "rationale": "The entry can be judged to be \"more easily than usual\" in the sentence by the person being questioned about the current feeling or state of the person being tested and therefore the time dimension of the present."}, "sentence_type": "Negative declarative sentence", "pos": {"nouns": {"I": 1, "Nervous": 1, "- It's urgent": 1}, "verbs": {"I think": 1, "- Yeah": 1, "Easy": 1, "Nervous": 1, "I'm in a hurry": 1}, "adjectives": {"Normal": 1}}}, {"content": "I'm afraid for no reason.", "word_count": 10, "label": "", "sentence_structure": "Subject鈥揷opula鈥損redicative", "subjective_objective": {"answer": "Subjective", "rationale": "The fear is the patient's subjective emotional experience and feeling."}, "sentence_sentiment": {"answer": "Negative", "rationale": "Because \"fear without cause\" is negative."}, "language_style": {"answer": "Tactful and warm", "rationale": "The entry is more euphemistic and warm, and guides the respondents to express their feelings in an understanding and supportive tone, rather than directly questioning or criticizing them. At the same time, the article is more open and inclusive, giving the interviewee sufficient time and space to think and answer questions."}, "time_dimension": {"answer": "Present", "rationale": ""}, "sentence_type": "Negative declarative sentence", "pos": {"nouns": {"Questionnaire": 1, "Entry": 1, "Scared": 1}, "verbs": {"have": 1, "Sensor": 1, "I feel": 1, "For no reason": 1}, "adverbs": {"For no reason": 1}, "pronouns": {"I": 1}}}, {"content": "I'm a little upset or scared.", "word_count": 12, "label": "", "sentence_structure": "Subject鈥揷opula鈥損redicative", "subjective_objective": {"answer": "Subjective", "rationale": "Because words like \"easy,\" \"freaky,\" \"frightful\" represent the patient's subjective feelings."}, "sentence_sentiment": {"answer": "Negative", "rationale": "Because \"deep or frightened\" is a negative emotional state in the entry."}, "language_style": {"answer": "Calm and direct", "rationale": "The entry describes the psychological state that the person under investigation may have, in a direct and objective manner, without using excessive rhetorical or emotional rendering, and is more honest and relevant. At the same time, the \"cool straight\" language style better helps the person under investigation to understand the problem and to provide an accurate answer."}, "time_dimension": {"answer": "Present", "rationale": "Now the keywords \"easy to be disturbed\" and \"frightful\" indicate that it is the testee describing his current state of mind, and therefore judging as now."}, "sentence_type": "Negative declarative sentence", "pos": {"nouns": {"I": 1, "In my heart": 1, "Disturbing": 1, "Scared": 1}, "verbs": {"Easy": 1, "I think": 1}, "adverbs": {"or": 1}, "numerals": {"3": 1}}}, {"content": "I think I might be going crazy.", "word_count": 10, "label": "", "sentence_structure": "Subject鈥搗erb鈥揷omplement", "subjective_objective": {"answer": "Subjective", "rationale": "It's a subjective feeling and a perception."}, "sentence_sentiment": {"answer": "Negative", "rationale": "Because the word \"crazy\" is a negative emotional, implying negative emotional and attitudinal."}, "language_style": {"answer": "Tactful and warm", "rationale": "The entry is more euphemistic and caring, not in terms of a too direct or violent expression, but in a more understanding and caring attitude. At the same time, the tone of the entry is more moderate and friendly, which facilitates good communication with the person under investigation."}, "time_dimension": {"answer": "Future", "rationale": "The entry examines the expectations and concerns of the testee for the future, according to the keyword \"may be\" and therefore the temporal dimension is judged to be the future."}, "sentence_type": "Negative declarative sentence", "pos": {"nouns": {"I think": 1, "Crazy": 1}, "verbs": {"Crazy": 1}, "adjectives": {"Maybe": 1, "Yes, I'm going": 1}}}, {"content": "I think everything's fine, and nothing bad will happen.", "word_count": 17, "label": "", "sentence_structure": "Subject鈥揷opula鈥損redicative", "subjective_objective": {"answer": "Subjective", "rationale": "Because \"think\" and \"do not happen\" are patients' subjective attitudes and ideas."}, "sentence_sentiment": {"answer": "Positive", "rationale": "The entry is a positive expression of attitude and opinion."}, "language_style": {"answer": "Calm and direct", "rationale": "The entry expresses the positive expectations of the interviewees for the future in a concise and clear language, and does not use too many rhetorical or emotional terms, so that the language style is more calm and straightforward."}, "time_dimension": {"answer": "Present", "rationale": "The keyword \"sweet\" and \"does not happen\" means the state of the current emotion and the expectations for the future, so the temporal dimension is now."}, "sentence_type": "Affirmative declarative sentence", "pos": {"nouns": {"Everything": 1, "Unfortunately": 1}, "verbs": {"I think": 1, "Come on": 1}, "adjectives": {"Okay": 1}, "adverbs": {"Yeah": 1, "not": 1}, "classifiers": {"very": 1}}}, {"content": "I'm shaking my hands and feet.", "word_count": 7, "label": "", "sentence_structure": "Subject鈥搗erb鈥揷omplement", "subjective_objective": {"answer": "Objective", "rationale": "The basis for the judgement: because \"fright\" is an observed physical reaction or symptom, it is objective. But \"fist\" may be a subjective feeling of the patient and therefore require further investigation."}, "sentence_sentiment": {"answer": "Negative", "rationale": "Since the act of \"fear and tremors\" is usually seen as a manifestation of negative emotions such as tension, fear or anxiety, the emotional orientation of the article's purpose is negative."}, "language_style": {"answer": "Calm and direct", "rationale": "The entry describes a symptom in a simple and straightforward manner (fear and tremors), does not use excessive rhetoric or emotional rendering, and the language style is more calm and objective."}, "time_dimension": {"answer": "Past", "rationale": "The entry contains the expression \"shock\" and \"shock\" of the past, and therefore the judgement is the past."}, "sentence_type": "Affirmative declarative sentence", "pos": {"nouns": {"Hands and feet": 1, "Wreck": 1}, "verbs": {"# Wresh #": 1}, "adjectives": {"# Shatter #": 1}}}, {"content": "I'm upset with headaches, neck pains and back pains.", "word_count": 13, "label": "", "sentence_structure": "Subject鈥搗erb鈥揷omplement", "subjective_objective": {"answer": "Subjective", "rationale": "Because the feeling of headaches, cervical pains and back pains and the response to these diseases are subjective experiences of patients."}, "sentence_sentiment": {"answer": "Negative", "rationale": "Because \"headaches, neck pains and back pains\" in the entry are symptoms of physical discomfort and are usually considered negative."}, "language_style": {"answer": "Tactful and warm", "rationale": "The entry is more moderate in its language, using the word \"torture\" to express sympathy and concern for the testees, and to make them feel understood and respected. At the same time, \"headaches, neck pains and back pains\" are also an objective description, without too much emotional or subjective assessment, to enable the testees to answer questions more honestly."}, "time_dimension": {"answer": "Present", "rationale": "Now the entry asks if the person being tested is currently suffering from headaches, neck pains and back pains, the key word being \"I'm upset\" and \"I'm upset\" and all point to the state of the moment. Thus, the time dimension of the article is now judged."}, "sentence_type": "Negative declarative sentence", "pos": {"nouns": {"Questionnaire": 1, "Entry": 1, "Headache": 1, "Cervical pain": 1, "Backache": 1, "Trouble": 1}, "verbs": {"Analysis": 1, "be": 1}}}, {"content": "I feel weak and tired.", "word_count": 10, "label": "", "sentence_structure": "Subject鈥揷opula鈥損redicative", "subjective_objective": {"answer": "Subjective", "rationale": "Because \"fear is weak and weary\" is a subjective experience or feeling."}, "sentence_sentiment": {"answer": "Negative", "rationale": "Because \"weakness and fatigue\" is a negative physical condition, indicating low moods and lack of energy."}, "language_style": {"answer": "Tactful and warm", "rationale": "This is more accompaniment and caring expression."}, "time_dimension": {"answer": "Present", "rationale": "The keyword \"feel\" means the feeling or state of the moment, so the entry is judged as now."}, "sentence_type": "Negative declarative sentence", "pos": {"nouns": {"Weakness": 1, "Weary": 1}, "verbs": {"Feel": 1}}}, {"content": "I feel calm and easy to sit still.", "word_count": 15, "label": "", "sentence_structure": "Subject鈥揷opula鈥損redicative", "subjective_objective": {"answer": "Subjective", "rationale": "Because \"fear\" and \"cute\" and \"silent\" are the feelings and experiences of the patient, and are subjectively described."}, "sentence_sentiment": {"answer": "Positive", "rationale": "Because the entries \"salent\" and \"silently sit\" are all positive emotional expressions."}, "language_style": {"answer": "Tactful and warm", "rationale": "The entry uses a moderate language, which leads the respondents to questions in an invitation-based manner, and there is no language that is too direct or command-based, which is more adept and interactive."}, "time_dimension": {"answer": "Present", "rationale": "The entry describes the current status or feelings of the respondent in reply to the questionnaire, does not have a clear point of reference for time and therefore is judged to be the present."}, "sentence_type": "Affirmative declarative sentence", "pos": {"nouns": {"Calm down": 1, "Quiet": 1, "Sit down": 1}, "verbs": {"I think": 1, "Easy": 1, "Sit down": 1}, "adjectives": {"Ping": 1, "and": 1, "Quiet": 1}, "adverbs": {"And": 1}}}, {"content": "I think my heart is beating fast.", "word_count": 8, "label": "", "sentence_structure": "Subject鈥揷opula鈥損redicative", "subjective_objective": {"answer": "Subjective", "rationale": "Because it's a subjective feeling to feel a heart beat."}, "sentence_sentiment": {"answer": "Negative", "rationale": "Because it represents an uncomfortable physical reaction and a negative emotional experience."}, "language_style": {"answer": "Tactful and warm", "rationale": "Based on the information provided, it is not possible to determine the language style of the article."}, "time_dimension": {"answer": "Present", "rationale": ""}, "sentence_type": "Affirmative declarative sentence", "pos": {"verbs": {"I think": 1, "- Yeah": 1}, "adjectives": {"Come on": 1}, "numerals": {"10": 1}}}, {"content": "I was upset by a moment of faintness.", "word_count": 11, "label": "", "sentence_structure": "Subject鈥搗erb鈥揷omplement", "subjective_objective": {"answer": "Objective", "rationale": "Because \"headlight\" is a symptom or phenomenon that the patient is experiencing, it is an objective fact, and \"fear\" is the patient's subjective feeling."}, "sentence_sentiment": {"answer": "Negative", "rationale": "The description \"sickness for a while\" in the entry is negative."}, "language_style": {"answer": "Calm and direct", "rationale": "The entry directly expresses the subject of questioning whether the other party is often feeling dizzy, is not being presented in a more balanced and objective manner, using excessive rhetorical or emotional means."}, "time_dimension": {"answer": "Present", "rationale": ""}, "sentence_type": "Affirmative declarative sentence", "pos": {"nouns": {"Dizziness": 1, "Trouble": 1}, "verbs": {"Because": 1, "And": 1, "Okay": 1, "Trouble": 1}, "adverbs": {"For a while": 1}, "numerals": {"11": 1}, "pronouns": {"I": 1}}}, {"content": "I'm having a fainting seizure or feeling like I'm going to faint.", "word_count": 14, "label": "", "sentence_structure": "Subject鈥搗erb鈥揷omplement", "subjective_objective": {"answer": "Subjective", "rationale": "Because \"I have fainted\" and \"I feel like I'm going to faint\" are subjective feelings and experiences of patients. Although these symptoms may be objective, they are perceived and described by the patients themselves, and therefore subjectively."}, "sentence_sentiment": {"answer": "Negative", "rationale": "Because \"stunning\" in the entry is an expression of physical discomfort and is usually considered negative."}, "language_style": {"answer": "Calm and direct", "rationale": "The entry is based on a more objective and balanced tone by asking whether the other party has ever experienced a fainting or similar feeling, without using excessive rhetorical or emotional rendering. The question is designed to gather more accurate information for subsequent analysis and processing."}, "time_dimension": {"answer": "Past", "rationale": ""}, "sentence_type": "Negative declarative sentence", "pos": {"nouns": {"He's fainted": 1, "I'm fainted": 1}, "verbs": {"have": 1, "I think": 1, "Like": 1}, "numerals": {"12": 1}}}, {"content": "I'm so easy to breathe in.", "word_count": 11, "label": "", "sentence_structure": "Subject鈥揷opula鈥損redicative", "subjective_objective": {"answer": "Objective", "rationale": "Because \"exhale easily\" is a feeling or experience, a result of the patient's own perception, a subjective feeling of the patient and therefore objective."}, "sentence_sentiment": {"answer": "Positive", "rationale": "Because \"breathing easy\" is a comfortable, healthy feeling, the emotional orientation of the article is positive."}, "language_style": {"answer": "Tactful and warm", "rationale": "The entry is more personal and interactive when it is presented in a gentle and inviting tone, asking whether the other is comfortable breathing rather than directly speaking or ordering."}, "time_dimension": {"answer": "Present", "rationale": "The entry describes the current breathing feeling of the testee, so the temporal dimension is now judged."}, "sentence_type": "Affirmative declarative sentence", "pos": {"nouns": {"Breathe": 2, "Gas": 2}, "verbs": {"Breathe out": 1, "Breathe in": 1, "I feel": 1, "Both": 1}, "adjectives": {"Easy": 1}, "numerals": {"13": 1}}}, {"content": "My hands and feet are numb and stingy.", "word_count": 9, "label": "", "sentence_structure": "Subject鈥揷opula鈥損redicative", "subjective_objective": {"answer": "Objective", "rationale": "Because numbness and sting are symptoms of the patient and are objective descriptions."}, "sentence_sentiment": {"answer": "Negative", "rationale": "Because \"insensitive hands and feet\" is a feeling of discomfort, usually associated with negative factors such as physical pain, disease, etc."}, "language_style": {"answer": "Calm and direct", "rationale": "The entry uses a concise language describing the problems that researchers want to understand, with no excessive emotional rendering or rhetorical techniques, and a more direct language style.\""}, "time_dimension": {"answer": "Past", "rationale": ""}, "sentence_type": "Interrogative sentence", "pos": {"nouns": {"Hands and feet": 2, "I'm numb": 1, "Stings": 1}, "numerals": {"14": 1}}}, {"content": "I'm upset with stomach pains and indigestion.", "word_count": 13, "label": "", "sentence_structure": "Subject鈥揷opula鈥損redicative", "subjective_objective": {"answer": "Subjective", "rationale": "Because \"torture\" is the patient's subjective feeling, which means that the patient thinks that his stomach pains and digestive disorders are troubling."}, "sentence_sentiment": {"answer": "Negative", "rationale": "Because words like \"tummy pain,\" \"indigestion\", \"fear\" express a uncomfortable, painful feeling, and have a negative emotional color."}, "language_style": {"answer": "Tactful and warm", "rationale": "The expression of concern for the physical discomfort of the person tested in a sympathetic and understanding manner, rather than direct questioning or stigmatization, is more respectful and sensitive."}, "time_dimension": {"answer": "Past", "rationale": "The entry \"I'm upset with stomach pain and indigestion\" describes a condition that has already occurred and has affected the emotional state of the moment, so it can be judged as the past."}, "sentence_type": "Negative declarative sentence", "pos": {"nouns": {"Stomach pain": 1, "Indigestion": 1}, "verbs": {"Trouble": 1}, "numerals": {"15": 1}}}, {"content": "I used to pee.", "word_count": 6, "label": "", "sentence_structure": "Subject鈥搗erb鈥揷omplement", "subjective_objective": {"answer": "Subjective", "rationale": "Because \"often pee\" describes patients' subjective feelings and experiences."}, "sentence_sentiment": {"answer": "Neutral", "rationale": "Because the entry merely describes a biological phenomenon, it does not have any emotional colour."}, "language_style": {"answer": "Calm and direct", "rationale": "The entry is simple and clear in asking whether the person surveyed often feels the need for urine, without using euphemism or accommodative language."}, "time_dimension": {"answer": "Present", "rationale": "The key words in the entry are \"often\" and \"to pee\" , which all indicate a regular, customary behaviour, that is, that the testee may have been and is likely to be so. So the temporal dimension is now."}, "sentence_type": "Affirmative declarative sentence", "pos": {"verbs": {"I": 1, "Always": 1, "Pee": 1}, "adverbs": {"Often": 1}}}, {"content": "My hands and feet are often dry and warm.", "word_count": 12, "label": "", "sentence_structure": "Subject鈥揷opula鈥損redicative", "subjective_objective": {"answer": "Objective", "rationale": "Because dry warmth is an objective observation of the patient's state of health."}, "sentence_sentiment": {"answer": "Neutral", "rationale": "Because the entry describes facts, it does not contain any positive or negative emotional colours."}, "language_style": {"answer": "Tactful and warm", "rationale": "The entry is more euphemistic, in which the person surveyed is asked to answer questions and to resonate and reminisce through the descriptive adjective \"dry warm.\" At the same time, the purpose of the article is relatively gentle and friendly, with no too much emphasis or command language."}, "time_dimension": {"answer": "Present", "rationale": "The description of \"often\" as a constant, habitual state of affairs, \"dry and warm hands\" is also a current state of affairs, so the entry is an expression of the present state of the person being tested."}, "sentence_type": "Affirmative declarative sentence", "pos": {"nouns": {"Hands and feet": 2, "Dry": 1, "Warm": 1}, "verbs": {"Always": 1}, "adjectives": {"Dry": 1, "Warm": 1}, "adverbs": {"Often": 1}, "numerals": {"17": 1}, "classifiers": {"individual": 1}}}, {"content": "I'm blushing.", "word_count": 5, "label": "", "sentence_structure": "Subject鈥揷opula鈥損redicative", "subjective_objective": {"answer": "Objective", "rationale": "Because the red fever is an objective fact of the patient's experience."}, "sentence_sentiment": {"answer": "Negative", "rationale": "Because \"regular fever\" is a symptom of physical discomfort, it is often seen as a negative emotion or experience."}, "language_style": {"answer": "Tactful and warm", "rationale": "The entry is expressed in a more euphemistic way, and does not ask the other side too directly whether there is a red-hot situation. At the same time, the use of the word \"me\" makes the other side feel that they are the object of respect and concern, and the language style is more friendly and sweet."}, "time_dimension": {"answer": "Present", "rationale": "Now, this questionnaire entry, \"My face is red\" describes the current state of feeling or state, and it is therefore judged to be a reflection of the current behavior or mentality."}, "sentence_type": "Affirmative declarative sentence", "pos": {"nouns": {"I": 1, "Face": 1, "Heat": 1}, "verbs": {"Red": 1, "Hot": 1}}}, {"content": "I sleep easily and well overnight.", "word_count": 12, "label": "", "sentence_structure": "Subject鈥搗erb鈥揷omplement", "subjective_objective": {"answer": "Objective", "rationale": "Because \"sleep easy and sleep well overnight\" is a description of the patient's sleep, it is not a subjective perception or feeling of the patient, and it is therefore objective."}, "sentence_sentiment": {"answer": "Positive", "rationale": "Because the description of \"sleep-in-the-sleep-in-the-night\" in the entry is positive, indicating that the individual has a good sleep quality."}, "language_style": {"answer": "Tactful and warm", "rationale": "This expression is more supportive and humane than direct questions."}, "time_dimension": {"answer": "Present", "rationale": "The entry describes the current state of sleep and habits of the person being tested, without reference to the past or future time dimensions. Both terms are \"sleep-easy and sleep-good\" descriptions of the current state."}, "sentence_type": "Affirmative declarative sentence", "pos": {"nouns": {"I": 1, "One night": 1, "Sleep": 1, "Okay": 1}, "verbs": {"Easy": 1, "Enter": 1, "Sleep": 1}, "adjectives": {"Easy": 1, "Okay": 1}, "numerals": {"19": 1, "One": 1}}}, {"content": "I had nightmares.", "word_count": 4, "label": "", "sentence_structure": "Subject鈥搗erb鈥揷omplement", "subjective_objective": {"answer": "Subjective", "rationale": "Because \"dreaming\" is a subjective experience and feeling of a person, a subjective explanation of his or her own dream."}, "sentence_sentiment": {"answer": "Negative", "rationale": "Because \"dreaming\" usually causes fear and anxiety, it has a negative emotional tone."}, "language_style": {"answer": "Tactful and warm", "rationale": "The entry is more euphemistic and sensitive to each other鈥檚 feelings, using a question-and-answer format to ask them if they have had nightmares, without directly denying or confirming. At the same time, the expression is more acclaimed, gentle and sensitive, and it feels that it is cared for and understood."}, "time_dimension": {"answer": "Past", "rationale": "The entry is for questioning the testee's past behaviour, and the keyword is \"dreaming,\" an event that has already occurred and can therefore be judged to be past."}, "sentence_type": "Exclamatory sentence", "pos": {"nouns": {"Nightmares": 1}, "verbs": {"Do it": 1}}}]}

{"questionnaire_title": "Status-specific anxiety questionnaire (STAI)", "questionnaire_category": "Mental health", "item_count": 40, "emotion_shift_overall": "From positive to negative", "language_style_overall": "Tactful and warm", "items": [{"content": "I feel calm.", "word_count": 7, "label": "", "sentence_structure": "Subject鈥揷opula鈥損redicative", "subjective_objective": {"answer": "Subjective", "rationale": "Because the word \"fEE\" shows that it's a patient's own feeling, a subjective observation and description."}, "sentence_sentiment": {"answer": "Neutral", "rationale": "Because the entry does not express positive or negative emotional aspects, it simply describes feelings."}, "language_style": {"answer": "Tactful and warm", "rationale": "The language style of the entry is euphemism. By using the subjective expression \"I feel\" the questionnaire designer gives the interviewer greater freedom to share his feelings and is more compassionate and slow, and less than direct or harsh language. At the same time, the more common psychological term \"settling\" makes the questionnaire more professional and credible."}, "time_dimension": {"answer": "Present", "rationale": "The entry now describes the current state of the person's feelings and emotions, so the time dimension is now judged."}, "sentence_type": "Affirmative declarative sentence", "pos": {"nouns": {"The mood": 1, "Calm down": 1}, "verbs": {"I feel": 1}}}, {"content": "I feel safe.", "word_count": 5, "label": "", "sentence_structure": "Subject鈥揷opula鈥損redicative", "subjective_objective": {"answer": "Subjective", "rationale": "/ Objective. Because \"felt safe\" is an emotion or feeling, it is the patient's own experience and perception, and therefore is subjective. Whether or not such feelings or feelings exist objectively requires further study and research."}, "sentence_sentiment": {"answer": "Positive", "rationale": "Because feeling safe is a positive emotional experience."}, "language_style": {"answer": "Tactful and warm", "rationale": "The entry is more adept and interactive when it is spoken in a gentle and inviting manner, rather than by direct statements or orders."}, "time_dimension": {"answer": "Present", "rationale": "In the future, the entry asks if the person tested will feel safe in the future and therefore the temporal dimension is judged to be the future."}, "sentence_type": "Affirmative declarative sentence", "pos": {"nouns": {"Clear": 1}, "verbs": {"I feel": 1}}}, {"content": "I'm nervous.", "word_count": 5, "label": "", "sentence_structure": "Subject鈥揷opula鈥損redicative", "subjective_objective": {"answer": "Subjective", "rationale": "Because \"stretch\" is a patient's self-perception and experience, and it's subjective."}, "sentence_sentiment": {"answer": "Negative", "rationale": "Because \"stretch\" in the entry is a negative emotion."}, "language_style": {"answer": "Calm and direct", "rationale": "The entry directly expresses the subject of questioning the other party about the existence of tension, without using excessive rhetorical or emotional rendering, and with a more balanced and objective tone. Nor does it use any euphemism or encouraging vocabulary to calm the atmosphere, but rather presents the problem in a direct way, allowing the interviewee to understand the meaning of the problem clearly and clearly."}, "time_dimension": {"answer": "Present", "rationale": "In the phrase \"I am nervous,\" the word \"is\" denotes a state or a situation, while \"strained\" describes the state or characteristics of the dominant language, which point to the current situation. Thus, the temporal dimension of the article is now."}, "sentence_type": "Affirmative declarative sentence", "pos": {"nouns": {"Questionnaire": 1, "Entry": 1}, "verbs": {"be": 1, "Analysis": 1}, "adjectives": {"Nervous": 1}}}, {"content": "I feel nervous and bound.", "word_count": 7, "label": "", "sentence_structure": "Subject鈥揷opula鈥損redicative", "subjective_objective": {"answer": "Subjective", "rationale": "Because it's the patient's subjective feeling."}, "sentence_sentiment": {"answer": "Negative", "rationale": "Because the expression \"stretching\" is negative, indicating emotional depression and discomfort."}, "language_style": {"answer": "Calm and direct", "rationale": "The language style of the entry is more straightforward, not using too many rhetorical or emotional colours, but expressing the emotional reactions that the subject may have in the face of a situation in an objective, concise manner. This expression helps the interviewee to understand the problem accurately and to reflect his feelings and thoughts more honestly."}, "time_dimension": {"answer": "Present", "rationale": "The keyword is \"I feel\", which means that it is a subjective feeling and experience, and that it is being felt, so the temporal dimension is now judged."}, "sentence_type": "Negative declarative sentence", "pos": {"nouns": {"Nervous": 1, "Tie": 1}, "verbs": {"I feel": 1}}}, {"content": "I feel comfortable.", "word_count": 5, "label": "", "sentence_structure": "Subject鈥揷opula鈥損redicative", "subjective_objective": {"answer": "Subjective", "rationale": "Because \"Feel\" is a subjective feeling and experience of the patient, the subject is a subjective one."}, "sentence_sentiment": {"answer": "Positive", "rationale": "Because \"frightful\" is a positive emotional experience, indicating that the person interviewed is in a positive mood."}, "language_style": {"answer": "Tactful and warm", "rationale": "The language style of the entry is more euphemistic and warm. Using the expression \"I feel\" , observers are placed in the role of observers, making them more receptive and answerable, and at the same time showing respect and concern for each other's feelings. Moreover, the term \"easy\" has positive and comfortable meaning, conveying a positive emotional tone in the context, making the questionnaire more accessible and easy to fill."}, "time_dimension": {"answer": "Present", "rationale": "The keyword is \"fear,\" which means the state of the current feeling and emotion, and therefore the judgment is now."}, "sentence_type": "Affirmative declarative sentence", "pos": {"verbs": {"I feel": 1, "Easy": 1}, "adjectives": {"Easy": 1}}}, {"content": "I'm so upset.", "word_count": 5, "label": "", "sentence_structure": "Subject鈥揷opula鈥損redicative", "subjective_objective": {"answer": "Subjective", "rationale": "Because \"disturbing\" is a subjective feeling or emotional reaction."}, "sentence_sentiment": {"answer": "Negative", "rationale": "Because the description of \"disturbing\" in the entry is negative."}, "language_style": {"answer": "Calm and direct", "rationale": "The entry is in plain and simple language, which directly expresses the subject of questioning the other party for feelings of distress, without excessive rhetorical or emotional rendering, and is more objective in tone."}, "time_dimension": {"answer": "Present", "rationale": "The entry describes the current emotional state of the testee, which is judged by the time dimension."}, "sentence_type": "Negative declarative sentence", "pos": {"nouns": {"Questionnaire": 1, "Entry": 1}, "verbs": {"I feel": 1, "Disturbing": 1}}}, {"content": "I'm now in a lot of trouble, and I feel more trouble than I could possibly be.", "word_count": 20, "label": "", "sentence_structure": "Subject鈥揷opula鈥損redicative", "subjective_objective": {"answer": "Subjective", "rationale": "Because \"fear\" and \"unforgettable\" are the feelings and assessments of the patient, subjective attitudes and emotional experiences."}, "sentence_sentiment": {"answer": "Negative", "rationale": "Because \"fear more than possible misfortune\" is a negative emotional attitude."}, "language_style": {"answer": "Tactful and warm", "rationale": "The language style of the entry is more euphemistic, asking the person in question about his or her current emotional state in a caring and understanding manner, and not speaking in too direct or too cold language. Such expression increases the comfort of the person in question and encourages them to share their true feelings."}, "time_dimension": {"answer": "Present", "rationale": "The time word \"now\" contained in the entry represents the situation that the person being tested is experiencing and feeling, and thus judging as the past."}, "sentence_type": "Negative declarative sentence", "pos": {"nouns": {"Questionnaire": 1, "Problem": 1, "Trouble": 1, "Unfortunately": 1}, "verbs": {"I feel": 1, "More than": 1}, "adjectives": {"Possible": 1}, "numerals": {"7": 1}, "pronouns": {"I": 1}}}, {"content": "8. I'm satisfied.", "word_count": 5, "label": "", "sentence_structure": "Subject鈥揷opula鈥損redicative", "subjective_objective": {"answer": "Subjective", "rationale": "Because \"satisfaction\" is a subjective emotional experience and evaluation."}, "sentence_sentiment": {"answer": "Positive", "rationale": "Because the \"satisfaction\" in the entry is a positive attitude."}, "language_style": {"answer": "Tactful and warm", "rationale": "The language style of this entry is euphemistic, because it is a more gentle, inviting tone of speech to ask the interviewee's emotional state, without direct orders or asking the interviewee to answer the question. This expression is more comfortable and relaxed for the interviewee and is more acceptable."}, "time_dimension": {"answer": "Present", "rationale": "The keyword is \"satisfaction\", which means that the respondent answered the question in a way that is representative of the current feelings and experiences and that is therefore judged as the present behaviour or mentality."}, "sentence_type": "Affirmative declarative sentence", "pos": {"verbs": {"I feel": 1, "Satisfied": 1}}}, {"content": "I'm scared.", "word_count": 5, "label": "", "sentence_structure": "Subject鈥揷opula鈥損redicative", "subjective_objective": {"answer": "Subjective", "rationale": "Because fear is a subjective emotional experience of a human being."}, "sentence_sentiment": {"answer": "Negative", "rationale": "Because \"fear\" is a negative emotion."}, "language_style": {"answer": "Calm and direct", "rationale": "The entry directly expresses the question whether the other party is often afraid of the subject, is not using excessive rhetorical or emotional rendering, and is more objective in tone."}, "time_dimension": {"answer": "Past", "rationale": "The entry uses the first-person proxy \"I\" to indicate that it is a personal subjective feeling, while the verb \"Esperate\" and the adjective \"Fear\" suggest that this feeling has occurred in the past."}, "sentence_type": "Negative declarative sentence", "pos": {"nouns": {"Questionnaire Entry": 1, "Scared": 1}, "verbs": {"I feel": 1}}}, {"content": "I'm comfortable.", "word_count": 5, "label": "", "sentence_structure": "Subject鈥揷opula鈥損redicative", "subjective_objective": {"answer": "Subjective", "rationale": "Because comfort is an individual feeling or experience, it is subjective."}, "sentence_sentiment": {"answer": "Positive", "rationale": "Because the emotional attitude of \"comfort\" in the entries is positive."}, "language_style": {"answer": "Tactful and warm", "rationale": "The language style of the entry is more acclaimable and gentle, using the first person's pronoun, \"I,\" and without directly expressing strong feelings or emotions, giving people a sense of ease of comfort, which is easier to open up and give real feelings to the interviewees."}, "time_dimension": {"answer": "Present", "rationale": ""}, "sentence_type": "Affirmative declarative sentence", "pos": {"verbs": {"I feel": 1, "Comfortable": 1}, "adjectives": {"Comfortable": 1}, "numerals": {"10": 1}}}, {"content": "I'm confident.", "word_count": 5, "label": "", "sentence_structure": "Subject鈥搗erb鈥揷omplement", "subjective_objective": {"answer": "Subjective", "rationale": "Because self-confidence is a subjective attitude or feeling."}, "sentence_sentiment": {"answer": "Positive", "rationale": "Because confidence is a positive quality."}, "language_style": {"answer": "Tactful and warm", "rationale": "The entry is a positive and encouraging question, and by emphasizing the positive character of \"confidence\" it encourages respondents to show their strengths and strengths. At the same time, this expression avoids possible negative emotions or discomfort."}, "time_dimension": {"answer": "Present", "rationale": "The keywords are \"I have\" and \"self-confidence\", which both represent the current state and feelings of the testee, so the entry describes the current behavior or mentality."}, "sentence_type": "Affirmative declarative sentence", "pos": {"nouns": {"Questionnaire": 1, "Entry": 1, "Confidence": 1}, "verbs": {"have": 1}, "numerals": {"11": 1}}}, {"content": "I'm very sensitive.", "word_count": 7, "label": "", "sentence_structure": "Subject鈥揷opula鈥損redicative", "subjective_objective": {"answer": "Subjective", "rationale": "Because \"think\" is a subjective adjective, which means that an individual's feelings and perceptions are a subjective description."}, "sentence_sentiment": {"answer": "Negative", "rationale": "Because the description of \"sensitized\" in the entry is negative."}, "language_style": {"answer": "Tactful and warm", "rationale": "The entry is more personal and interactive in a gentle and inviting tone, rather than in direct statements or orders. And \"you feel\" makes it easier for the person under investigation to accept and share his true feelings."}, "time_dimension": {"answer": "Present", "rationale": "The entry asks the testees about their current feelings, i.e. whether they feel sensitive and time dimension is now judged."}, "sentence_type": "Affirmative declarative sentence", "pos": {"nouns": {"You're a bit of a sane": 1}, "verbs": {"I think": 1}}}, {"content": "I'm extremely nervous.", "word_count": 7, "label": "", "sentence_structure": "Subject鈥揷opula鈥損redicative", "subjective_objective": {"answer": "Subjective", "rationale": "Because extreme stress is an emotional experience, it is the subjective feeling of the patient."}, "sentence_sentiment": {"answer": "Negative", "rationale": "Because the emotional state of \"extremely nervous\" in the entries is negative."}, "language_style": {"answer": "Tactful and warm", "rationale": "The word for the \"13. I'm extremely nervous\" entry is more direct, not much rhetorical or emotional, and it's more neutral and objective. So I think it can be described as \"cool and straight.\""}, "time_dimension": {"answer": "Present", "rationale": "In terms of the situation, \"extremely nervous\" is a description of the emotional state of the person being tested at this moment, so the temporal dimension is now."}, "sentence_type": "Negative declarative sentence", "pos": {"nouns": {"Nervous": 1, "Ann": 1}, "verbs": {"I": 1, "Extreme": 1, "Unsettled": 1}, "adverbs": {"Extreme": 1}, "numerals": {"13": 1}}}, {"content": "I'm indecisive.", "word_count": 5, "label": "", "sentence_structure": "Subject鈥搗erb鈥揷omplement", "subjective_objective": {"answer": "Subjective", "rationale": "Because \"indecisive\" is a subjective attitude or character."}, "sentence_sentiment": {"answer": "Negative", "rationale": "Because the act of indecisiveness is negative."}, "language_style": {"answer": "Tactful and warm", "rationale": "The entry is more moderate, euphemistic, and not using too direct or hard-line language to ask the respondents about their situation, but rather to direct them to think and answer questions in a more neutral way. At the same time, this expression is more easily accepted by the respondents and is willing to share their thoughts and feelings."}, "time_dimension": {"answer": "Present", "rationale": "The word \"I am indecisive\" in \"I am indecisive\" indicates that it is an evaluation or description of the person being tested, and the character characteristic of \"indecisiveness\" is usually considered a fixed, long-term pattern of behaviour, so the entry refers to the present behavioural or character."}, "sentence_type": "Negative declarative sentence", "pos": {"verbs": {"be": 1, "Indecisiveness": 1}, "adjectives": {"I": 1}}}, {"content": "I'm easy.", "word_count": 5, "label": "", "sentence_structure": "Subject鈥揷opula鈥損redicative", "subjective_objective": {"answer": "Subjective", "rationale": "Because \"easy\" is a subjective feeling."}, "sentence_sentiment": {"answer": "Positive", "rationale": "Because the description of \"easy\" is positive, it means that people are happy and not nervous."}, "language_style": {"answer": "Tactful and warm", "rationale": "The entry describes itself in a light, self-centered way, a language style that is sensitive and intimate, and that allows the interviewee to feel comfortable and relaxed. At the same time, it does not use overly strong subjective evaluations or emotional expressions, and is more oriented towards the objective presentation of his or her state."}, "time_dimension": {"answer": "Present", "rationale": "According to the sentence, \"it's easy\" it can be seen that the questionnaire entry is asking the subject about the current emotional state and mental state of the person being tested and therefore falls within the scope of \"now\"."}, "sentence_type": "Exclamatory sentence", "pos": {"verbs": {"be": 1, "Easy": 1}, "adjectives": {"Easy": 1}, "numerals": {"15": 1}}}, {"content": "I'm satisfied.", "word_count": 7, "label": "", "sentence_structure": "Subject鈥揷opula鈥損redicative", "subjective_objective": {"answer": "Subjective", "rationale": "Because \"satisfaction\" is a subjective emotional experience."}, "sentence_sentiment": {"answer": "Positive", "rationale": "Because the word \"satisfaction\" is a positive emotional attitude."}, "language_style": {"answer": "Tactful and warm", "rationale": "The entry is more adept and interactive when it is spoken in a gentle and inviting manner, rather than by direct statements or orders."}, "time_dimension": {"answer": "Present", "rationale": "The entry asks the person's current psychological state and feelings, and the time dimension is now judged. The key words are \"fear\" and \"satisfaction\", which all imply that the person who was tested needed to respond to the question in a way that reflected their current emotional state of mind. So we can conclude that this is the question of the present."}, "sentence_type": "Affirmative declarative sentence", "pos": {"verbs": {"I feel": 1, "Satisfied": 1}, "adjectives": {"I'm satisfied": 1}}}, {"content": "I'm a pain in the ass.", "word_count": 5, "label": "", "sentence_structure": "Subject鈥揷opula鈥損redicative", "subjective_objective": {"answer": "Subjective", "rationale": "Because \"fear\" is a subjective state of feeling and emotion, not an objective fact or result of behaviour."}, "sentence_sentiment": {"answer": "Negative", "rationale": "Because the word \"torture\" is usually considered negative."}, "language_style": {"answer": "Calm and direct", "rationale": "The entry expresses the subject in a concise and straightforward manner, without too many rhetorical or emotional renderings, and the language style is a straightforward type of calm."}, "time_dimension": {"answer": "Present", "rationale": "Now the entry is a current state or feeling, and the first person's singular form of \"I am\" indicates that it is the subject's own subjective experience and therefore the judgment is now."}, "sentence_type": "Negative declarative sentence", "pos": {"nouns": {"I": 1, "Trouble": 1}, "verbs": {"be": 1}}}, {"content": "I was in a panic.", "word_count": 5, "label": "", "sentence_structure": "Subject鈥揷opula鈥損redicative", "subjective_objective": {"answer": "Subjective", "rationale": "Because \"fright\" is an emotional experience, viewed from the patient's subjective point of view."}, "sentence_sentiment": {"answer": "Negative", "rationale": "Because the expression \"frightful\" in the article is negative."}, "language_style": {"answer": "Calm and direct", "rationale": "The entry directly expresses the subject of questioning whether the other party is often in a state of panic, does not use excessive rhetorical or emotional rendering, and is more objective in tone."}, "time_dimension": {"answer": "Present", "rationale": "The entry \"Frightened\" describes the current emotional state of the person being tested, so the temporal dimension is now judged."}, "sentence_type": "Affirmative declarative sentence", "pos": {"verbs": {"I feel": 1, "Panic": 1}}}, {"content": "I feel calm.", "word_count": 5, "label": "", "sentence_structure": "Subject鈥揷opula鈥損redicative", "subjective_objective": {"answer": "Subjective", "rationale": "Because \"feel\" is a subjective experience and feeling of the individual."}, "sentence_sentiment": {"answer": "Positive", "rationale": "Because \"feel calm\" expresses a positive attitude and feeling, and there is no negative or neutral emotional colour."}, "language_style": {"answer": "Calm and direct", "rationale": ""}, "time_dimension": {"answer": "Present", "rationale": "The entry is for questioning the current emotional state of the subject, the keywords being \"feel\" and \"settling\" and they all point to the subjective feeling of the subject and therefore judge it as now."}, "sentence_type": "Affirmative declarative sentence", "pos": {"nouns": {"Calm down": 1}, "verbs": {"Feel": 1}}}, {"content": "I'm happy.", "word_count": 5, "label": "", "sentence_structure": "Subject鈥揷opula鈥損redicative", "subjective_objective": {"answer": "Subjective", "rationale": "Because \"happy\" is the patient's subjective emotional experience and attitude."}, "sentence_sentiment": {"answer": "Positive", "rationale": "Because the \"happy\" attitude in the entries is positive."}, "language_style": {"answer": "Tactful and warm", "rationale": "The language style of the entry is euphemism and warmer, and it is used in the descriptive term \"happy\" to resonate with the other side rather than directly asking whether it is happy. This makes it easier for the interviewee to open his heart and participate in the investigation."}, "time_dimension": {"answer": "Present", "rationale": "The keyword \"fEs\" indicates that it is the person who was tested now, so the entry describes the current situation."}, "sentence_type": "Affirmative declarative sentence", "pos": {"nouns": {"Questionnaire": 1, "Entry": 1, "Have a nice day": 1}, "verbs": {"Sensor": 1, "Present": 1}, "numerals": {"20": 1}}}, {"content": "I'm happy.", "word_count": 5, "label": "", "sentence_structure": "Subject鈥揷opula鈥損redicative", "subjective_objective": {"answer": "Subjective", "rationale": "Because \"fEs\" is the patient's own subjective and emotional experience."}, "sentence_sentiment": {"answer": "Positive", "rationale": "Because \"happy\" is positive."}, "language_style": {"answer": "Tactful and warm", "rationale": "The language style of the entry is euphemistic and inviting, presented in the form of a question-and-answer period, which leads the interviewee to express his emotions without being too direct or too rigid. At the same time, the word \"happy\" is soft, non-intrusive, more acceptable and willing to answer."}, "time_dimension": {"answer": "Present", "rationale": "The keyword \"happy\" shows the current emotional state of the testee, so the temporal dimension is now judged."}, "sentence_type": "Affirmative declarative sentence", "pos": {"verbs": {"I feel": 1, "Have a nice day": 1}}}, {"content": "I feel sensitive and uneasy.", "word_count": 10, "label": "", "sentence_structure": "Subject鈥揷opula鈥損redicative", "subjective_objective": {"answer": "Subjective", "rationale": "Because it is a subjective experience, it reflects the psychological state and emotional response of the patient."}, "sentence_sentiment": {"answer": "Negative", "rationale": "Because \"sensitization and discomfort\" is a negative emotional experience."}, "language_style": {"answer": "Calm and direct", "rationale": "The entry directly expresses the subject of questioning the other party for sensitivity and unease, without excessive rhetorical or emotional rendering, and with a more objective tone."}, "time_dimension": {"answer": "Present", "rationale": "The keyword \"fear\" indicates the experience or state of the subject being experienced, and \"sensitized and uneasy\" describes the subjective feelings of the subject, so the entry is asking about the present behaviour and mentality of the subject."}, "sentence_type": "Affirmative declarative sentence", "pos": {"nouns": {"I": 1, "Nerves": 1, "Allergies": 1, "Unsettled": 1}, "verbs": {"I feel": 1}, "numerals": {"22": 1}}}, {"content": "I feel satisfied.", "word_count": 7, "label": "", "sentence_structure": "Subject鈥揷opula鈥損redicative", "subjective_objective": {"answer": "Subjective", "rationale": "Because self-fulfilment is a subjective feeling, it is related to the individual ' s psychological state."}, "sentence_sentiment": {"answer": "Positive", "rationale": "Because self-fulfilment in the entry is a positive emotional experience."}, "language_style": {"answer": "Tactful and warm", "rationale": "Warm and warm."}, "time_dimension": {"answer": "Present", "rationale": ""}, "sentence_type": "Affirmative declarative sentence", "pos": {"verbs": {"I": 1, "I feel": 1}, "adjectives": {"Self": 1, "Satisfied": 1}}}, {"content": "I wish I was as happy as anyone else.", "word_count": 11, "label": "", "sentence_structure": "Subject鈥搗erb鈥揷omplement", "subjective_objective": {"answer": "Subjective", "rationale": "Because hope is a subjective emotion and expectation, it is the feeling and expectation of the patient that is being examined."}, "sentence_sentiment": {"answer": "Neutral", "rationale": "Because the entry does not express positive or negative feelings, it merely expresses a person ' s wish."}, "language_style": {"answer": "Tactful and warm", "rationale": "The entry is more personal and interactive when it is a soft, inviting expression of the researcher ' s interest and understanding of the person being tested, rather than a direct statement or order."}, "time_dimension": {"answer": "Future", "rationale": "The keyword \"hope\" indicates a desire or expectation for the future, and \"can be as happy as others\" suggests that this is not yet the case, so this entry is about the future."}, "sentence_type": "Imperative sentence", "pos": {"nouns": {"I": 1, "others": 1, "Happy": 1}, "verbs": {"Yes": 2, "Yeah": 1, "That way": 1, "Hope": 1}, "numerals": {"24": 1}}}, {"content": "I feel like I'm failing.", "word_count": 9, "label": "", "sentence_structure": "Subject鈥揷opula鈥損redicative", "subjective_objective": {"answer": "Subjective", "rationale": "Because \"feel\" is usually a subjective feeling of a person."}, "sentence_sentiment": {"answer": "Negative", "rationale": "Because \"defeating\" in the entry is a negative emotional state."}, "language_style": {"answer": "Tactful and warm", "rationale": "The entry is in a gentle and understandable way, giving the respondents a sense of care and support and a more euphemism and warmer expression."}, "time_dimension": {"answer": "Past", "rationale": "By the keyword \"felt\" and \"like failure\", it can be assumed that the entry is a question of how the person was feeling at some point or time in the past, and therefore is judged to be past."}, "sentence_type": "Affirmative declarative sentence", "pos": {"nouns": {"Decay": 1}, "verbs": {"Sensor": 1, "Yeah": 1, "Decay": 1}, "numerals": {"25": 1}, "pronouns": {"I": 1}}}, {"content": "I feel calm.", "word_count": 6, "label": "", "sentence_structure": "Subject鈥揷opula鈥損redicative", "subjective_objective": {"answer": "Subjective", "rationale": "Because quiet is a subjective feeling or experience."}, "sentence_sentiment": {"answer": "Positive", "rationale": "Because the entry is about positive emotional experience."}, "language_style": {"answer": "Tactful and warm", "rationale": "The entry uses the more subjective adjective \"quietly\" to describe the feelings of the interviewee, which is more euphemistic and moderate in its language, and is not overly direct or strongly expressed. It is also open to the interviewee to respond freely, depending on his or her actual situation."}, "time_dimension": {"answer": "Present", "rationale": "\"Feeling very calm\" is a description of the emotional experience and state of the subject at this moment, so the answer is \"now\"."}, "sentence_type": "Imperative sentence", "pos": {"verbs": {"I feel": 1, "Peace": 1}, "adverbs": {"very": 1}}}, {"content": "I'm calm, calm and purturistic.", "word_count": 14, "label": "", "sentence_structure": "Subject鈥揷opula鈥損redicative", "subjective_objective": {"answer": "Subjective", "rationale": "Because \"quiet,\" \"cool\", \"cool,\" \"crazy\" are words that describe the patient's inner feelings or state, and that need to be answered in the light of the patient's subjective experience, and therefore subjectively."}, "sentence_sentiment": {"answer": "Positive", "rationale": "Because the emotions and attitudes described in the entries are positive, they represent a personal character of calm, calm and tactful character."}, "language_style": {"answer": "Calm and direct", "rationale": "The language style of the entry is calm and straightforward. It explicitly asks the interviewee whether he has a calm, calm and tactful character, without using too many emotional acrimonyms or euphemisms, and in a more straightforward way."}, "time_dimension": {"answer": "Past", "rationale": "The keywords \"I am calm,\" \"cool\" and \"successful\" are used to describe the psychological state of the subjects, not the past or the future."}, "sentence_type": "Affirmative declarative sentence", "pos": {"nouns": {"I": 1, "Calm down": 2, "I'm glad": 1}, "adjectives": {"be": 1, "of": 3}, "adverbs": {"and": 1}}}, {"content": "I felt a lot of difficulties, so I couldn't overcome it.", "word_count": 17, "label": "", "sentence_structure": "Subject鈥搗erb鈥揷omplement", "subjective_objective": {"answer": "Objective", "rationale": "Because the difficulty is the patient's subjective feeling, \"a collection of things that cannot be overcome\" is an objective description of the dilemma and stress that patients face."}, "sentence_sentiment": {"answer": "Negative", "rationale": "Because the \"unable\" act in the entry is negative."}, "language_style": {"answer": "Calm and direct", "rationale": "The entry is a direct statement in which the respondent is asked whether he or she often feels insurmountable in the face of a difficult situation, with a relatively calm and objective language style, with no excessive rhetoric or emotional colour."}, "time_dimension": {"answer": "Present", "rationale": "The entry uses the words \"sense\" and \"unresolvable\" to express the current psychological state and feelings of the person being tested. Thus, the temporal dimension of the purpose of the article is now judged."}, "sentence_type": "Negative declarative sentence", "pos": {"nouns": {"Difficulties": 1, "Stack": 1, "Overcoming": 1}, "verbs": {"I feel": 1, "Cannot": 1}, "adverbs": {"And so": 1}, "pronouns": {"I": 1}}}, {"content": "I'm worried too much about things that are not really important.", "word_count": 17, "label": "", "sentence_structure": "Subject鈥揷opula鈥損redicative", "subjective_objective": {"answer": "Subjective", "rationale": "Because the descriptions of \"excessive anxiety\" and \"invisible\" are subjective feelings and evaluations of patients."}, "sentence_sentiment": {"answer": "Negative", "rationale": "Because the \"excessive anxiety\" act in the entry is negative."}, "language_style": {"answer": "Calm and direct", "rationale": "The entry expresses the problems that the respondents may have in a straightforward language, with no excessive emotional qualifiers or euphemisms, and is more objective and cold."}, "time_dimension": {"answer": "Present", "rationale": ""}, "sentence_type": "Negative declarative sentence", "pos": {"nouns": {"Yes": 2, "Fear": 1}, "verbs": {"That's too much": 1, "have": 1}, "adjectives": {"It doesn't matter": 1}, "adverbs": {"Too much": 1, "very": 1}, "numerals": {"29": 1}, "pronouns": {"I": 1}}}, {"content": "I'm happy.", "word_count": 5, "label": "", "sentence_structure": "Subject鈥揷opula鈥損redicative", "subjective_objective": {"answer": "Subjective", "rationale": "Because \"happy\" is an emotional experience, a patient's subjective feeling and perception."}, "sentence_sentiment": {"answer": "Positive", "rationale": "Because \"happiness\" is a positive emotional vocabulary."}, "language_style": {"answer": "Tactful and warm", "rationale": "The entry is more adept and interactive when it is spoken in a gentle and inviting manner, rather than by direct statements or orders."}, "time_dimension": {"answer": "Present", "rationale": ""}, "sentence_type": "Affirmative declarative sentence", "pos": {"verbs": {"be": 1, "Happy": 1}, "adjectives": {"Happy": 1}, "numerals": {"30": 1}}}, {"content": "My mind is in disarray.", "word_count": 10, "label": "", "sentence_structure": "Subject鈥揷opula鈥損redicative", "subjective_objective": {"answer": "Subjective", "rationale": "Because \"disorder\" is a personal feeling and subjective experience that reflects the patient's understanding and perception of his or her inner world. It is not objectively seen, and it is therefore viewed from the patient's subjective perspective."}, "sentence_sentiment": {"answer": "Negative", "rationale": "Because the entry \"disturbed mind\" expresses a negative emotional attitude, implying that the interviewees have problems and problems."}, "language_style": {"answer": "Calm and direct", "rationale": "The entry is a simple, non-verbally-prescriptive, direct interview of the person interviewed, expressed in a calmer and straightforward manner, without excessive emotional or rhetorical tact."}, "time_dimension": {"answer": "Present", "rationale": "In the entry, the \"I\" state of mind is a description of the current state, without reference to a specific point of time, past or future. The temporal dimension of the article should therefore be judged as \"now\"."}, "sentence_type": "Affirmative declarative sentence", "pos": {"nouns": {"Ideas": 1, "Disorder Status": 1}, "verbs": {"in": 1}, "numerals": {"31": 1}}}, {"content": "I lack confidence.", "word_count": 6, "label": "", "sentence_structure": "Subject鈥揷opula鈥損redicative", "subjective_objective": {"answer": "Subjective", "rationale": "Because \"lack of self-confidence\" is a subjective feeling and evaluation."}, "sentence_sentiment": {"answer": "Negative", "rationale": "Because the description of \"lack of self-confidence\" in the entry is negative."}, "language_style": {"answer": "Tactful and warm", "rationale": "The entry is more proficient and understanding by asking, in a gentle and caring manner, whether the other side lacks confidence, rather than directly accusing or ordering. At the same time, the language style of the article avoids the use of too negative terms such as \"lack\", \"inconfidence\" and, rather, more objective and neutral descriptions, which are more conducive to the authenticity and accuracy of the questionnaire survey."}, "time_dimension": {"answer": "Present", "rationale": "The keyword \"I'm lacking\" indicates that it is the testee's assessment or perception of himself, without reference to past or future circumstances. The entry therefore asks the testee's current, immediate state and feelings and judges it as \"now\"."}, "sentence_type": "Negative declarative sentence", "pos": {"nouns": {"Self-confidence": 1}, "verbs": {"Lack": 1}, "numerals": {"32": 1}}}, {"content": "I feel safe.", "word_count": 5, "label": "", "sentence_structure": "Subject鈥揷opula鈥損redicative", "subjective_objective": {"answer": "Subjective", "rationale": "Because feeling safe is a subjective feeling of a human being."}, "sentence_sentiment": {"answer": "Positive", "rationale": "Because \"felt safe\" is a positive description, indicating that the interviewees feel protected and safeguarded to some extent."}, "language_style": {"answer": "Tactful and warm", "rationale": "The entry uses a more subjective term, \"I feel\" and guides the respondents to express their feelings in a question-and-answer manner, so it is a euphemism and a warmer language. At the same time, the article is also more balanced, friendly, without too strong or direct expression, and contributes to a good atmosphere of interaction."}, "time_dimension": {"answer": "Present", "rationale": "The \"Feel safe\" in the entry indicates the current state of the testee's feeling, so the temporal dimension is now judged."}, "sentence_type": "Affirmative declarative sentence", "pos": {"nouns": {"I": 1, "Clear": 1}, "verbs": {"I feel": 1}, "numerals": {"33": 1}}}, {"content": "I'm easy to make decisions.", "word_count": 7, "label": "", "sentence_structure": "Subject鈥揷opula鈥損redicative", "subjective_objective": {"answer": "Subjective", "rationale": "Since \"deciding\" is a process of individual thinking and action that reflects the individual's psychological characteristics and preferences, it is viewed from the patient's subjective perspective."}, "sentence_sentiment": {"answer": "Positive", "rationale": "Because \"easy to make a decision\" shows that a person has the capacity to make decisions and is decisive, a positive character and attitude."}, "language_style": {"answer": "Calm and direct", "rationale": "The entry directly asks whether the person surveyed is easy to make decisions, does not use over-modified language or emotional expressions and therefore is a calm and direct language style."}, "time_dimension": {"answer": "Present", "rationale": "\"Easier to make a judgment\" describes a character or a customary state of behaviour, which in this context indicates the character and behaviour of the person being tested at this stage. Thus, it can be judged that the entry is an inquiry into the person being tested."}, "sentence_type": "Affirmative declarative sentence", "pos": {"nouns": {"Decision": 1}, "verbs": {"Make": 1}, "adjectives": {"Easy": 1}, "numerals": {"34": 1}}}, {"content": "I don't feel right.", "word_count": 6, "label": "", "sentence_structure": "Subject鈥揷opula鈥損redicative", "subjective_objective": {"answer": "Subjective", "rationale": "Because \"felt inappropriate\" is the patient's own feelings and perceptions, and it's a subjective description."}, "sentence_sentiment": {"answer": "Negative", "rationale": "Because \"improper\" in the entry conveys a negative emotion, which is a feeling of dissatisfaction or discomfort and is therefore negative."}, "language_style": {"answer": "Tactful and warm", "rationale": "The language style of the entry is more moderate, using the expression \"disappeared inappropriate\" rather than directly expressing dissatisfaction or criticism, giving a relatively relaxed tone. Such language style is more likely to gain the trust of the interviewee and to promote more truthful answers."}, "time_dimension": {"answer": "Present", "rationale": "The keyword \"I feel\" shows that it is the subject's own subjective feeling, and that there is no clear time point, and therefore it is judged as the present behavior or mentality."}, "sentence_type": "Negative declarative sentence", "pos": {"verbs": {"I feel": 1, "Suits": 1}, "numerals": {"35": 1}}}, {"content": "I'm satisfied.", "word_count": 5, "label": "", "sentence_structure": "Subject鈥揷opula鈥損redicative", "subjective_objective": {"answer": "Subjective", "rationale": "Because satisfaction is a subjective experience and feeling of a human being."}, "sentence_sentiment": {"answer": "Positive", "rationale": "Because the \"satisfaction\" attitude in the entry is positive."}, "language_style": {"answer": "Tactful and warm", "rationale": "The entry is asked in a positive and self-identified manner, expressing concern and respect for the satisfaction of the respondents, and is more linguistically friendly and moderate."}, "time_dimension": {"answer": "Past", "rationale": "The entry is an inquiry into the views of the testee on his or her satisfaction and does not address past, present or future time dimensions, and therefore does not allow for time judgement."}, "sentence_type": "Affirmative declarative sentence", "pos": {"verbs": {"be": 1, "Satisfied": 1}, "adjectives": {"Satisfied": 1}}}, {"content": "37. Some of the unimportant thoughts that haunt me and disturb me.", "word_count": 17, "label": "", "sentence_structure": "Subject鈥搗erb鈥搊bject", "subjective_objective": {"answer": "Subjective", "rationale": "Because \"some insignificant thoughts\", \"entangled me\" and \"disturbing me\" are the feelings and experiences of the patient himself, and therefore are subjective."}, "sentence_sentiment": {"answer": "Negative", "rationale": "Because \"involved and disturbed by some insignificant ideas\" is a negative emotional expression in the entries."}, "language_style": {"answer": "Tactful and warm", "rationale": "Warm and warm."}, "time_dimension": {"answer": "Present", "rationale": "\"The sum\" and \"the sum\" indicate that the phenomenon is continuous, while \"entanglement\" and \"disturbing\" indicate that it is the current state. So the time dimension of this questionnaire entry is now."}, "sentence_type": "Negative declarative sentence", "pos": {"nouns": {"Ideas": 1, "I": 2}, "verbs": {"Quest": 1, "Excuse me": 1}, "adjectives": {"Important": 1, "not": 1}, "adverbs": {"Total": 1}, "numerals": {"37": 1}}}, {"content": "38. The frustration I created was so serious that I couldn't exclude them from my mind.", "word_count": 24, "label": "", "sentence_structure": "Subject鈥揷opula鈥損redicative", "subjective_objective": {"answer": "Subjective", "rationale": "Because \"the idea of undesired depression\" is the patient's subjective feeling and idea."}, "sentence_sentiment": {"answer": "Negative", "rationale": "The basis for the judgment: because \"depression\" is a negative sentiment."}, "language_style": {"answer": "Tactful and warm", "rationale": ""}, "time_dimension": {"answer": "Past", "rationale": "Taken together, the entry relates to the description of the past and the impact on the present, so it can be judged as \"the past/now\"."}, "sentence_type": "Negative declarative sentence", "pos": {"nouns": {"Frustration": 1, "Ideas": 1}, "verbs": {"Generate": 1, "Exclude": 1}, "adjectives": {"Serious": 1}, "adverbs": {"I see": 1, "So": 1}, "numerals": {"38": 1, "They": 2}, "pronouns": {"I": 1}}}, {"content": "I'm a calm man.", "word_count": 8, "label": "", "sentence_structure": "Subject鈥揷opula鈥損redicative", "subjective_objective": {"answer": "Subjective", "rationale": "Because I'm a calm person is a self-awareness and evaluation of the patient, a subjective examination."}, "sentence_sentiment": {"answer": "Neutral", "rationale": "Basis of judgement: The entry describes the characteristics of a person and does not express a positive or negative emotional tendency and is therefore neutral."}, "language_style": {"answer": "Calm and direct", "rationale": "The entry directly expresses the subject of questioning whether the other party considers himself a calm person, without excessive rhetorical or emotional rendering, and with a more objective tone."}, "time_dimension": {"answer": "Present", "rationale": "Now, the dominant word in \"I'm a calm man\" -- \"I\" -- is the use of the present state of affairs, which means that the speaker thinks he's calm in his current state. And, \"Yes\" also means a statement or self-perception, which further confirms the present description."}, "sentence_type": "Affirmative declarative sentence", "pos": {"nouns": {"person": 1, "Calm down": 1}, "verbs": {"be": 1}, "numerals": {"39": 1}, "pronouns": {"I": 1}}}, {"content": "I was in a state of tension when I considered my current interests and interests.", "word_count": 22, "label": "", "sentence_structure": "Subject鈥搗erb鈥搊bject", "subjective_objective": {"answer": "Subjective", "rationale": "Because the sentence refers to the feelings and attitudes of me, it is viewed from the patient's subjective perspective."}, "sentence_sentiment": {"answer": "Negative", "rationale": "Because \"stretch\" is a negative emotional experience."}, "language_style": {"answer": "Calm and direct", "rationale": ""}, "time_dimension": {"answer": "Present", "rationale": ""}, "sentence_type": "Affirmative declarative sentence", "pos": {"nouns": {"Questionnaire Entry": 1, "Things": 1, "Interest": 1, "Tension": 1}, "verbs": {"Consider": 1, "Into the": 1}, "numerals": {"40": 1}, "pronouns": {"I": 1}}}]}

{"questionnaire_title": "- I'm not sure.", "questionnaire_category": "Mental health", "item_count": 15, "emotion_shift_overall": "From negative to positive", "language_style_overall": "Tactful and warm", "items": [{"content": "I'm nervous, even at informal gatherings.", "word_count": 17, "label": "", "sentence_structure": "Subject鈥搗erb鈥揷omplement", "subjective_objective": {"answer": "Subjective", "rationale": "Because \"I\" is the patient's subjective feeling and experience."}, "sentence_sentiment": {"answer": "Negative", "rationale": "Because \"frightful\" is a negative emotional experience."}, "language_style": {"answer": "Tactful and warm", "rationale": ""}, "time_dimension": {"answer": "Present", "rationale": "The words \"even at informal gatherings\" and \"often feel\" indicate a constant, customary feeling or state and should therefore fall into the category \"now\"."}, "sentence_type": "Negative declarative sentence", "pos": {"nouns": {"Party": 1, "Nervous": 1}, "verbs": {"I feel": 1, "be": 1, "Regular": 1}, "adjectives": {"Informal": 1}, "adverbs": {"Yeah": 1, "in": 1, "Even if": 1}, "pronouns": {"I": 1}}}, {"content": "I am usually uncomfortable with a group of people I don't know.", "word_count": 20, "label": "", "sentence_structure": "Subject鈥揷opula鈥損redicative", "subjective_objective": {"answer": "Subjective", "rationale": "It's an objective feeling, because it's a feeling in a particular situation, a patient's feelings about seeing a stranger, a subjective experience of the patient."}, "sentence_sentiment": {"answer": "Negative", "rationale": "Because the \"uneasy\" mood in the entries is negative."}, "language_style": {"answer": "Calm and direct", "rationale": "The entry expresses, in a direct statement, the feeling of discomfort that a person may have among strangers, without too much emotional rendering or euphemism, and with a more calm and straightforward language style."}, "time_dimension": {"answer": "Past", "rationale": "At the same time, \"I usually feel uncomfortable\" means \"normally\" that this is a regular situation, which is further confirmation that it is a description of the state of affairs."}, "sentence_type": "Affirmative declarative sentence", "pos": {"nouns": {"person": 2, "Group": 1, "Yes": 1, "Time": 1, "Usually": 1, "I feel": 1, "I'm not comfortable": 1}, "verbs": {"in": 1, "Together": 1, "Time": 1, "Usually": 1, "I feel": 1, "I'm not comfortable": 1}, "adjectives": {"No, I don't": 1, "I'm not comfortable": 1}, "adverbs": {"Usually": 1}, "numerals": {"2": 1}, "classifiers": {"Group": 1}, "pronouns": {"I": 1}}}, {"content": "I usually feel comfortable talking to a heterosexual.", "word_count": 16, "label": "", "sentence_structure": "Subject鈥搗erb鈥揷omplement", "subjective_objective": {"answer": "Subjective", "rationale": "Because feeling relaxed is a subjective emotional experience."}, "sentence_sentiment": {"answer": "Positive", "rationale": "Because \"easiness\" is a positive emotional experience."}, "language_style": {"answer": "Tactful and warm", "rationale": "By describing the individual鈥檚 feelings, rather than directly judging or criticizing the behaviour of the other, the entry appears more euphemistic and moderate, giving a more comfortable and comfortable impression. At the same time, it is more resonant and positive."}, "time_dimension": {"answer": "Present", "rationale": ""}, "sentence_type": "Affirmative declarative sentence", "pos": {"nouns": {"3": 1, "Heterosexual": 1, "Talk": 1}, "verbs": {"I feel": 1, "Easy": 1, "Talk": 1}, "adjectives": {"Easy": 1}, "numerals": {"3": 1}, "pronouns": {"I": 1}}}, {"content": "I was nervous when I had to talk to teachers or supervisors.", "word_count": 17, "label": "", "sentence_structure": "Subject鈥搗erb鈥揷omplement", "subjective_objective": {"answer": "Subjective", "rationale": "Because \"frightful\" is the patient's subjective feeling and attitude."}, "sentence_sentiment": {"answer": "Negative", "rationale": "Because the description of \"strained\" in the entry is negative."}, "language_style": {"answer": "Calm and direct", "rationale": "The language style of the entry is calm, as it is more objective, concise and accurate in obtaining the actual situation and views of the interviewee."}, "time_dimension": {"answer": "Past", "rationale": "The entry asks the testee about his past feelings and experiences and judge the past in terms of the time dimension."}, "sentence_type": "Affirmative declarative sentence", "pos": {"nouns": {"4": 1, "Teacher": 1, "Sir": 1, "Talk": 1}, "verbs": {"I feel": 1, "Nervous": 1}, "numerals": {"One": 1}}}, {"content": "The parties often make me anxious and uncomfortable.", "word_count": 14, "label": "", "sentence_structure": "Subject鈥揷opula鈥損redicative", "subjective_objective": {"answer": "Subjective", "rationale": "Because anxiety and discomfort are subjective emotional reactions of patients, it is the patient ' s subjective perception and perception of what may happen at a gathering."}, "sentence_sentiment": {"answer": "Negative", "rationale": "Because the expression \"anxiety and discomfort\" in the entry is negative."}, "language_style": {"answer": "Calm and direct", "rationale": ""}, "time_dimension": {"answer": "Past", "rationale": "The entry represents a description of what has happened, of past experience or experience, and the keyword is \"Five parties often\" and all point to what has happened."}, "sentence_type": "Negative declarative sentence", "pos": {"nouns": {"Party": 1, "Anxiety": 1, "I'm not comfortable": 1}, "verbs": {"鈾?And I'm gonna be 鈾?: 1, "I feel": 1}, "adjectives": {"Often": 1}, "numerals": {"5": 1}}}, {"content": "I may be less shy in social relations than most people.", "word_count": 20, "label": "", "sentence_structure": "Subject鈥揷opula鈥損redicative", "subjective_objective": {"answer": "Subjective", "rationale": "Because \"less shyness\" is a subjective feeling and self-evaluation that reflects patients' subjective perceptions and perceptions of their social abilities."}, "sentence_sentiment": {"answer": "Positive", "rationale": "Because the \"less shy\" act in the entry is positive."}, "language_style": {"answer": "Tactful and warm", "rationale": "The entry is more personal and interactive when it is spoken in a gentle and inviting manner, rather than directly speaking or ordering. At the same time, it does not clearly identify the shortcomings of the person under investigation, but rather raises a relatively neutral question, which gives the person under investigation the opportunity to share his or her views, which is more euphemism and warmer."}, "time_dimension": {"answer": "Present", "rationale": "According to the keyword \"may be less shy in social relations\", it can be seen that the entry describes the current state of behaviour or mental state of the person being tested in social settings, and therefore the sentence is \"now\"."}, "sentence_type": "Affirmative declarative sentence", "pos": {"nouns": {"person": 1, "Social": 1}, "verbs": {"Shame": 1, "in": 1}, "adjectives": {"Most of them": 1, "Less": 1}, "adverbs": {"and": 1}, "numerals": {"6": 1}}}, {"content": "I was often nervous when I spoke to same-sex people that I was not familiar with.", "word_count": 20, "label": "", "sentence_structure": "Subject鈥搗erb鈥搊bject", "subjective_objective": {"answer": "Subjective", "rationale": "Because \"stretch\" is a subjective feeling, reflecting the individual鈥檚 inner experience and feelings of having a conversation with a less familiar person of the same sex. While this tension may be related to objective factors (such as the subject of dialogue, the context of interaction, etc.), in this questionnaire entry the subject matter is primarily the subject matter of the person interviewed鈥檚 emotional reactions."}, "sentence_sentiment": {"answer": "Negative", "rationale": "Because the \"sweet\" sentiment in the entry is negative."}, "language_style": {"answer": "Calm and direct", "rationale": "The entry directly expresses the subject of questioning the other party in a concise and clear language about whether he or she is nervous when talking to a person of the same sex who is not familiar, without using excessive rhetorical techniques or emotional rendering, and with a more objective tone."}, "time_dimension": {"answer": "Present", "rationale": "The word \"often\" means that it is a regular act, while the word \"strained\" describes the state of emotion, and the entry therefore belongs to the present."}, "sentence_type": "Affirmative declarative sentence", "pos": {"nouns": {"I": 1, "Heterosexual": 1, "Talk": 1}, "verbs": {"Often": 1, "I feel": 1, "Nervous": 1}, "adjectives": {"Not really": 1}, "numerals": {"7": 1}}}, {"content": "I'm nervous about job interviews.", "word_count": 11, "label": "", "sentence_structure": "Subject鈥揷opula鈥損redicative", "subjective_objective": {"answer": "Subjective", "rationale": "Because \"creasure\" is a subjective emotional feeling, it looks at the psychological state that patients may have during job interviews."}, "sentence_sentiment": {"answer": "Negative", "rationale": "Because the \"creature\" behavior in the entry is negative."}, "language_style": {"answer": "Calm and direct", "rationale": "The entry describes in a direct manner an emotional reaction that the respondent may have in the job interview, without excessive adoration or emotional colour, and with a more calm and objective language style."}, "time_dimension": {"answer": "Past", "rationale": ""}, "sentence_type": "Affirmative declarative sentence", "pos": {"nouns": {"Job interviews": 1, "Nervous": 1}, "verbs": {"Yes": 1}, "adjectives": {"8": 1}}}, {"content": "I hope I have more confidence in social life.", "word_count": 17, "label": "", "sentence_structure": "Subject鈥搗erb鈥揷omplement", "subjective_objective": {"answer": "Subjective", "rationale": "Because \"favorite\" represents the mind and expectations of the patient, and is a manifestation of subjective attitudes."}, "sentence_sentiment": {"answer": "Positive", "rationale": "Because the entry \"I want to be more confident in social settings\" expresses a positive attitude and expectations."}, "language_style": {"answer": "Tactful and warm", "rationale": "The entry is more personal and interactive in its expression of conversation in a gentle and encouraging tone, rather than in direct statements or orders. It attempts to make interviewees feel understood and supported, and to encourage them to share their thoughts and feelings."}, "time_dimension": {"answer": "Present", "rationale": "The entry describes the current state of the person's mind and expectations, the key words being \"fear,\" \"self\" and \"confidence\", all pointing to the person's current feelings and expectations."}, "sentence_type": "Imperative sentence", "pos": {"nouns": {"Hope": 1, "self": 1, "Socially": 1, "Confidence": 1}, "verbs": {"in": 1, "have": 1}, "adjectives": {"More": 1, "Just a little bit": 1}, "numerals": {"9": 1}, "pronouns": {"I": 1}}}, {"content": "I'm not very anxious about it in social settings.", "word_count": 13, "label": "", "sentence_structure": "Subject鈥揷opula鈥損redicative", "subjective_objective": {"answer": "Subjective", "rationale": "Because \"very little anxiety\" is a subjective feeling or attitude, not an objective fact. Everyone has different experiences and reactions to social settings, so the question is the psychological state of the individual."}, "sentence_sentiment": {"answer": "Positive", "rationale": "Because \"very rarely anxiety\" is positive."}, "language_style": {"answer": "Tactful and warm", "rationale": "The entry is more personal and interactive when it is spoken in a gentle and inviting manner, rather than in direct statements or orders. At the same time, the language style of the article is more objective and not overly rhetorical or emotional. Thus, the language style of the questionnaire entry is considered to be euphemistic."}, "time_dimension": {"answer": "Present", "rationale": "So, the output is now."}, "sentence_type": "Affirmative declarative sentence", "pos": {"nouns": {"Socially": 1, "Anxiety": 1}, "verbs": {"in": 1, "I feel": 1, "Less": 1}, "adjectives": {"10": 1, "A lot": 1}, "adverbs": {"Very few": 1}, "numerals": {"10": 1}}}, {"content": "I'm generally a shy person.", "word_count": 12, "label": "", "sentence_structure": "Subject鈥揷opula鈥損redicative", "subjective_objective": {"answer": "Subjective", "rationale": "Because the description of \"weakness\" is the patient's subjective perception and evaluation, the questionnaire entry is viewed from the patient's subjective perspective."}, "sentence_sentiment": {"answer": "Neutral", "rationale": "Because the entry does not express a positive or negative emotional tendency. It simply describes a person's personality, and does not evaluate it as good or bad."}, "language_style": {"answer": "Tactful and warm", "rationale": "The language style of the entry is euphemistic, expressed by the use of the words \"generally\" and by the words, which are more euphemistic, less direct, and give people a sense of friendship and respect. At the same time, the description of \"scree\" is not too negative or sharp, but is presented in an objective and self-perception manner, which is more in keeping with the euphemism of the language."}, "time_dimension": {"answer": "Present", "rationale": "In the entry, \"generally\" means that the person who was tested normally behaved, \"a shy person\" is a descriptive term, all pointing to the current state and behaviour. Thus, the temporal dimension is now."}, "sentence_type": "Imperative sentence", "pos": {"nouns": {"In general": 1, "I": 1, "person": 1}, "verbs": {"be": 1, "Shy": 1}, "adjectives": {"In general": 1, "Shy": 1}, "numerals": {"11": 1}, "pronouns": {"I": 1}}}, {"content": "I'm always nervous when I talk to a charming heterosexual.", "word_count": 19, "label": "", "sentence_structure": "Subject鈥揷opula鈥損redicative", "subjective_objective": {"answer": "Subjective", "rationale": "Because it is the patient ' s own feelings and experiences, it is a subjective perspective."}, "sentence_sentiment": {"answer": "Negative", "rationale": "Because the emotional experience of \"often nervous\" in the entries is negative."}, "language_style": {"answer": "Tactful and warm", "rationale": "The entry is described in terms of the first person's name, \"I\" , and it expresses personal feelings and experiences, rather than directly presenting objective facts or ordering others. At the same time, it uses more moderate terms such as \"attractive\" opposite sex, \"often nervous,\" which give people a warm, warm feeling of affection, so it can be judged that the language style of the purpose is euphemism."}, "time_dimension": {"answer": "Present", "rationale": ""}, "sentence_type": "Affirmative declarative sentence", "pos": {"nouns": {"Heterosexual": 1, "Nervous": 1}, "verbs": {"Talk": 1, "I feel": 1}, "adjectives": {"Fascinating": 1}}}, {"content": "I'm usually nervous when I call someone I don't know.", "word_count": 17, "label": "", "sentence_structure": "Subject鈥搗erb鈥搊bject", "subjective_objective": {"answer": "Subjective", "rationale": "Because \"Escrowding\" is a subjective feeling and emotional experience, the entry is viewed from the patient's subjective perspective."}, "sentence_sentiment": {"answer": "Negative", "rationale": "Because the emotional attitude of \"stretch\" in the entries is negative."}, "language_style": {"answer": "Calm and direct", "rationale": "The language style of the entry is more calm and straightforward, with no excessive qualifier or emotional expression, but rather directly describes the phenomenon that the person surveyed usually feels nervous when he or she calls someone less familiar."}, "time_dimension": {"answer": "Present", "rationale": "\"When calling someone who's not familiar,\" \"usually\" means a customary behavior, and \"I feel nervous\" is the emotional state that is now being experienced, so the entry describes the current behaviour and feelings of the subject.\""}, "sentence_type": "Negative declarative sentence", "pos": {"nouns": {"person": 1, "Telephone": 1, "Nervous": 1}, "verbs": {"Here": 1, "Fight": 1, "I think": 1}, "adjectives": {"Not really": 1}, "adverbs": {"Usually": 1}, "numerals": {"13": 1}}}, {"content": "I was nervous when I spoke to the authorities.", "word_count": 14, "label": "", "sentence_structure": "Subject鈥搗erb鈥揷omplement", "subjective_objective": {"answer": "Subjective", "rationale": "Because it's a emotional feeling, it's seen from the patient's subjective perspective."}, "sentence_sentiment": {"answer": "Negative", "rationale": "Because the \"frightful\" mood in the entry is negative."}, "language_style": {"answer": "Tactful and warm", "rationale": "The entry uses a soft, warm language style, with a gentle, inviting tone, asking if the other person is nervous when talking to a person of authority. This expression is not only more personal and interactive, but also gives the interviewee a greater willingness to share his real feelings."}, "time_dimension": {"answer": "Past", "rationale": ""}, "sentence_type": "Affirmative declarative sentence", "pos": {"nouns": {"I": 1, "Authority": 1}, "verbs": {"Talk": 1, "I feel": 1, "Nervous": 1}, "adverbs": {"in": 1}, "numerals": {"14": 1}}}, {"content": "Even in a group quite different from me, I usually feel relaxed.", "word_count": 25, "label": "", "sentence_structure": "Subject鈥搗erb鈥揷omplement", "subjective_objective": {"answer": "Subjective", "rationale": "Because the sentence contains a subjective feeling of \"me\"."}, "sentence_sentiment": {"answer": "Positive", "rationale": "Because the expression \"frightful\" in this article shows that individuals can still maintain a comfortable and calm emotional state in the population, it is a positive attitude or quality."}, "language_style": {"answer": "Tactful and warm", "rationale": "The entry is more euphemistic and gentle, leading each other to think by describing what the interviewees feel in the crowd, avoiding the use of too direct or hard language. Moreover, the entry uses a positive expression ( \"I still feel relaxed \" ), making the issue more friendly and attractive."}, "time_dimension": {"answer": "Past", "rationale": "This entry does not clearly indicate the point of time, and therefore the dimension of time is not certain whether it is past, present or future."}, "sentence_type": "Affirmative declarative sentence", "pos": {"nouns": {"15": 1, "Population": 1, "I": 2}, "verbs": {"Location": 1, "I feel": 1, "Relax": 1}, "adjectives": {"Different": 1, "Pretty much": 1}, "adverbs": {"Even if": 1, "Centre": 1, "Usually": 1}, "numerals": {"15": 1}, "classifiers": {"Group": 1}, "pronouns": {"I": 2}}}]}

{"questionnaire_title": "Questionnaire on simple response modalities", "questionnaire_category": "Behavioral questionnaire", "item_count": 20, "emotion_shift_overall": "From positive to negative", "language_style_overall": "Tactful and warm", "items": [{"content": "1. Relief through work learning or some other activity", "word_count": 15, "label": "", "sentence_structure": "Subject鈥搗erb鈥揷omplement", "subjective_objective": {"answer": "Subjective", "rationale": "Because this answer requires the patient to describe his or her own behaviour and thoughts, it examines the patient ' s subjective attitude and experience."}, "sentence_sentiment": {"answer": "Positive", "rationale": "Because the behaviour \"resolved\" in the entries is positive, it shows that the interviewees want to achieve the objective of reducing stress and relaxing through work learning or other activities."}, "language_style": {"answer": "Calm and direct", "rationale": "The entry directly asks the interviewees whether they are working to relieve stress through their work studies or other activities, without using excessive rhetorical or emotional rendering, and with a more calm and objective language style."}, "time_dimension": {"answer": "Present", "rationale": "According to the sentence entered, \"resolved through work learning or some other activity\" is not expressed in clear time, so it is not possible to judge whether it is past, present or future behavior or mentality. The output is therefore \"undetermined\"."}, "sentence_type": "Imperative sentence", "pos": {"nouns": {"Work": 1, "Learning": 1, "Activities": 1}, "verbs": {"Pass": 1, "Release": 1}}}, {"content": "2. Talk to people, talk to people about their internal concerns", "word_count": 10, "label": "", "sentence_structure": "Subject鈥搗erb鈥揷omplement", "subjective_objective": {"answer": "Subjective", "rationale": "Because it's the patient's subjective attitude and thoughts to talk to someone to ease his trouble."}, "sentence_sentiment": {"answer": "Positive", "rationale": "Because \"talking\" and \"disclaiming internal troubles\" in the entries are positive behaviours that help to relieve stress and solve problems."}, "language_style": {"answer": "Tactful and warm", "rationale": ""}, "time_dimension": {"answer": "Present", "rationale": ""}, "sentence_type": "Imperative sentence", "pos": {"nouns": {"person": 1, "Talk": 1, "Speech": 1, "Inside": 1, "Trouble": 1}, "verbs": {"and": 1, "Talk": 1, "Speech": 1, "Inside": 1}}}, {"content": "3. To see the good side of things as much as possible", "word_count": 10, "label": "", "sentence_structure": "Subject鈥搗erb鈥揷omplement", "subjective_objective": {"answer": "Subjective", "rationale": "Because \"seeing the good side of things as much as possible\" is a personal attitude and choice, which is a subjective view of the patient."}, "sentence_sentiment": {"answer": "Positive", "rationale": "Because the purpose of the entry is to see the good side of things."}, "language_style": {"answer": "Tactful and warm", "rationale": "Warm and warm."}, "time_dimension": {"answer": "Present", "rationale": ""}, "sentence_type": "Imperative sentence", "pos": {"verbs": {"Try": 1, "Look": 1, "Okay": 1}, "adjectives": {"Yes": 1, "Objects": 1, "Noodles": 1}, "adverbs": {"of": 1, "Both": 1}, "pronouns": {"Here": 1}}}, {"content": "4. Change your mind and rediscover what's important in life", "word_count": 18, "label": "", "sentence_structure": "Subject鈥搗erb鈥搊bject", "subjective_objective": {"answer": "Subjective", "rationale": "Because changing ideas and rediscovering the importance of life are the subjective ideas and attitudes of the patients themselves."}, "sentence_sentiment": {"answer": "Positive", "rationale": "Because \"change your mind\" and \"rediscover what matters in life\" in the entries are positive behavior."}, "language_style": {"answer": "Tactful and warm", "rationale": ""}, "time_dimension": {"answer": "Present", "rationale": ""}, "sentence_type": "Imperative sentence", "pos": {"nouns": {"self": 1, "Thoughts": 1, "Life": 1, "Important": 1}, "verbs": {"Change": 1, "Found": 1}, "numerals": {"4": 1}}}, {"content": "5. Not to take the problem too seriously.", "word_count": 9, "label": "", "sentence_structure": "Subject鈥搗erb鈥揷omplement", "subjective_objective": {"answer": "Subjective", "rationale": "Because it is the subjective judgement and attitude of the patient that is taking the issue too seriously."}, "sentence_sentiment": {"answer": "Positive", "rationale": "Because the attitude of \"not looking too seriously\" is positive and reflects optimism and positive mindsets."}, "language_style": {"answer": "Tactful and warm", "rationale": "The more gentle and inviting tone of the language of the entry, rather than direct statements or orders, is more personal and interactive."}, "time_dimension": {"answer": "Present", "rationale": "\"Do not take the issue too seriously\" is an attitude or view that describes the testee, which can be either the testee's customary behaviour or the attitude at this stage. Thus, the temporal dimension of the article is now judged."}, "sentence_type": "Imperative sentence", "pos": {"verbs": {"- Put it on": 1, "Look": 1, "Problem": 1}, "adjectives": {"Serious": 1}, "adverbs": {"Too much": 1}}}, {"content": "6. To stand up for the struggle that you want to get.", "word_count": 16, "label": "", "sentence_structure": "Subject鈥搗erb鈥搊bject", "subjective_objective": {"answer": "Subjective", "rationale": "Because it is the patient ' s subjective attitude and behaviour to stand up for his position and to fight for himself."}, "sentence_sentiment": {"answer": "Positive", "rationale": "Because the actions in the entries \"maintain your position, fight for what you want\" express a positive quality and attitude, that is, a spirit of self-preservation, not easy compromise."}, "language_style": {"answer": "Calm and direct", "rationale": "The entry is relatively calm and direct. It explicitly asks whether the person surveyed has a tendency to fight for his or her ideas or interests in certain circumstances, and is clear, without too much emotional rendering and rhetoric. This direct expression allows the person surveyed to better understand the problem and give a true answer."}, "time_dimension": {"answer": "Future", "rationale": "The keyword \"maintenance of your position\" and \"the struggle for what you want\" represent vision and expectations for the future, and therefore the temporal dimension is the future."}, "sentence_type": "Imperative sentence", "pos": {"nouns": {"Questionnaire": 1, "Entry": 1, "Position": 1, "Fight": 1}, "verbs": {"Hold on": 1, "I can": 1}, "adverbs": {"self": 2, "of": 3}, "numerals": {"6": 1}}}, {"content": "7. Identification of several different ways to solve the problem", "word_count": 14, "label": "", "sentence_structure": "Subject鈥搗erb鈥搊bject", "subjective_objective": {"answer": "Subjective", "rationale": "Because \"find\" is the patient's subjective behaviour and attitude, it requires the patient to proactively discover and try different ways to solve the problem."}, "sentence_sentiment": {"answer": "Neutral", "rationale": "Because the entry does not have any emotional color."}, "language_style": {"answer": "Tactful and warm", "rationale": "The language style of the entry is more moderate, not using too direct or command-type expressions, but rather using a means of asking the interviewer to think freely and answer. This expression respects the subject鈥檚 feelings more fully and encourages their active participation in the solution."}, "time_dimension": {"answer": "Present", "rationale": "The keyword is \"find\" and it means an action or state, and the verb does not clearly indicate whether it is past, present or future, and therefore cannot be accurately judged. However, the subject of \"the way to solve problems\" involves a process of reflection and planning, and the tester is usually asked to answer questions in the current state of mind when conducting a questionnaire, so the entry may be more inclined to ask the testee about his current thoughts and behaviour."}, "sentence_type": "Imperative sentence", "pos": {"nouns": {"Problem": 1, "Methodology": 2}, "verbs": {"Find out": 1, "Resolve": 1}, "numerals": {"How many": 1}, "classifiers": {"Plant": 1}}}, {"content": "8. Seek advice from relatives, friends or classmates", "word_count": 12, "label": "", "sentence_structure": "Subject鈥搗erb鈥搊bject", "subjective_objective": {"answer": "Subjective", "rationale": "The decision is made by the patient himself, although it may be influenced by others."}, "sentence_sentiment": {"answer": "Positive", "rationale": "Because the purpose of the action of \"seeking advice\" in the entry is positive."}, "language_style": {"answer": "Tactful and warm", "rationale": "The entry is more acclaimable and friendly in an interview with the other party about the experience of seeking advice from relatives, friends or classmates, and is expressed in a more moderate manner, which is more acceptable and more responsive."}, "time_dimension": {"answer": "Present", "rationale": "According to the description of the title, \"Seek advice from relatives and friends or classmates\" is an ongoing act and therefore falls within the context of the present state of affairs."}, "sentence_type": "Imperative sentence", "pos": {"nouns": {"Relatives": 1, "friends": 1, "Students": 1, "Recommendations": 1}, "verbs": {"Seek": 1}}}, {"content": "9. Some issues of changing practices or self-standing", "word_count": 17, "label": "", "sentence_structure": "Subject鈥搗erb鈥揷omplement", "subjective_objective": {"answer": "Objective", "rationale": "Because changes in the original practice or some of the problems of the patient are concrete actions in practice, they are objective."}, "sentence_sentiment": {"answer": "Neutral", "rationale": "Because the entry does not have any emotional color."}, "language_style": {"answer": "Tactful and warm", "rationale": "The language style of the entry is more moderate and friendly, using a means of asking questions rather than ordering or making direct statements."}, "time_dimension": {"answer": "Present", "rationale": "The term \"now\" in the sentence makes it possible to judge the time dimension of the questionnaire entry as it is now. At the same time, the description \"some practices or some of their own problems\" also points to the current situation of the subject."}, "sentence_type": "Imperative sentence", "pos": {"nouns": {"Change": 1, "Some": 2, "Approach": 1, "self": 1, "Problem": 1}, "verbs": {"Do it": 1, "have": 1}, "adverbs": {"I see": 1, "of": 4, "Some": 2}, "numerals": {"9": 1}, "pronouns": {"Here": 1}}}, {"content": "10. Approaches to drawing on others to address similar difficult situations", "word_count": 15, "label": "", "sentence_structure": "Subject鈥搗erb鈥搊bject", "subjective_objective": {"answer": "Subjective", "rationale": "Because the entry asks if the patient will be guided by the experience and practices of others, it is subjective."}, "sentence_sentiment": {"answer": "Positive", "rationale": "Because the purpose of the action of \"learning from others\" in the entries is positive and aimed at learning and progress."}, "language_style": {"answer": "Tactful and warm", "rationale": "The language style of the entry is more euphemistic, and the expression \"learning from others\" is used to guide respondents to think and share their experiences and views, rather than to make recommendations or requests directly. At the same time, the term \"like difficult situations\" is more closely related to humanized care, making the whole issue more friendly."}, "time_dimension": {"answer": "Future", "rationale": "The entry \"By Learning from Others\" and \"Applications to Similar Difficult Situations\" point to situations that have not yet occurred and that may arise in the future, and it can therefore be judged to be an inquiry into the behaviour that the subject may be expected to take in the future."}, "sentence_type": "Imperative sentence", "pos": {"nouns": {"Difficulties": 1, "Scenario": 1, "Approach": 1}, "verbs": {"Learning": 1, "Processing": 1}, "numerals": {"10": 1}}}, {"content": "11. Seeking amateurism and actively participating in cultural activities", "word_count": 14, "label": "", "sentence_structure": "Subject鈥損redicate", "subjective_objective": {"answer": "Subjective", "rationale": "Seeking and participating actively are subjective behaviour and attitudes of patients."}, "sentence_sentiment": {"answer": "Positive", "rationale": "Because the entries \"Finding for amateurs\" and \"Actively participating in the arts\" are positive."}, "language_style": {"answer": "Tactful and warm", "rationale": "The entry is more gentle and friendly in language, using an invitation-style tone to encourage the interviewee to participate actively in amateur and cultural activities. This expression is more friendly and interactive, and it makes it easier for the interviewee to feel respected and understood, and thus more willing to participate in the investigation."}, "time_dimension": {"answer": "Present", "rationale": "So the answer is \"now\"."}, "sentence_type": "Imperative sentence", "pos": {"nouns": {"Amateurity": 1, "Physical activities": 1}, "verbs": {"Seek": 1, "Active participation": 1}, "numerals": {"11": 1}}}, {"content": "12. To exercise maximum restraint in their disappointment, remorse, grief and anger.", "word_count": 16, "label": "", "sentence_structure": "Subject鈥搗erb鈥揷omplement", "subjective_objective": {"answer": "Subjective", "rationale": "Because \"restraint,\" \"depression, regret, grief and anger\" are subjective emotions and behaviours of patients."}, "sentence_sentiment": {"answer": "Negative", "rationale": "Because the words \"depression, regret, grief and anger\" are all negative."}, "language_style": {"answer": "Calm and direct", "rationale": "The entry is presented in a more direct language style, with no excessive rhetorical or emotional rendering, and is more objective in tone. It clearly tells the respondents what needs to be done, and does not use too euphemism or exaggerating language to attract attention or create an atmosphere. Thus, its language style is straightforward."}, "time_dimension": {"answer": "Present", "rationale": "The \"best restraint\" in the entry implies that it is an ongoing act or strategy, so the temporal dimension is judged now.\""}, "sentence_type": "Imperative sentence", "pos": {"nouns": {"self": 1, "Disappointment": 1, "Regret": 1, "Sad": 1, "Anger": 1}, "verbs": {"Restraint": 1}, "adverbs": {"Try": 1}, "numerals": {"12": 1}}}, {"content": "13. Attempt to take a break or leave, leaving the problem (fears) aside for a while", "word_count": 16, "label": "", "sentence_structure": "Subject鈥搗erb鈥搊bject", "subjective_objective": {"answer": "Subjective", "rationale": "Attempts to rest or take leave to relieve one ' s emotions and stress are subjective and the patient ' s will."}, "sentence_sentiment": {"answer": "Positive", "rationale": "Because these actions and purposes are positive in terms of \"attempting to rest or take leave\" and \"for the time being to leave the problem behind.\""}, "language_style": {"answer": "Tactful and warm", "rationale": "The language style of the entry is euphemismal, because it uses the words \"try\" and \"leave\" to express attitudes and concerns about the interviewee. Moreover, the \"tried rest or leave\" approach also gives people a comfortable, relaxed feeling, in contrast to the seriousness and stress of the problem."}, "time_dimension": {"answer": "Present", "rationale": "The entry is an inquiry into the behaviour or plan of the person who, in the face of the problem, has attempted to relieve the pressure by rest or leave, the temporal dimension being judged as now."}, "sentence_type": "Imperative sentence", "pos": {"nouns": {"Try": 1, "Rest": 1, "Leave": 1, "Problem": 1, "Trouble": 1}, "verbs": {"Try": 1, "Throw it away": 1}, "numerals": {"13": 1}}}, {"content": "14. To ease the trouble by smoking, drinking alcohol, taking drugs and eating.", "word_count": 17, "label": "", "sentence_structure": "Subject鈥搗erb鈥搊bject", "subjective_objective": {"answer": "Subjective", "rationale": "Since the decision-making power for such acts is vested in the patient, it is his or her own choice and is therefore subjective."}, "sentence_sentiment": {"answer": "Negative", "rationale": "Because the entry \"Smoking, drinking, taking drugs and eating\" is negative. It can cause physical health problems and not really solve them."}, "language_style": {"answer": "Calm and direct", "rationale": "The language style of the entry is calm and straightforward, and it does not use any qualifying words or emotional colours, but rather expresses the content of the problem directly in a concise and clear language."}, "time_dimension": {"answer": "Present", "rationale": "The entry describes the current behaviour or tendency of the subject, and the keywords are \"through\" , \"smoking\", \"drinking\", \"taking drugs\" and \"eating\" all pointing to what is happening or what is happening now or frequently.\""}, "sentence_type": "Imperative sentence", "pos": {"nouns": {"Smoke": 1, "Wine": 1, "Drug use": 1, "Eat": 1, "Trouble": 1}, "verbs": {"Pass": 1, "Undo": 1}, "numerals": {"14": 1}}}, {"content": "15. Considering that time will change the status quo, the only thing to do is wait", "word_count": 18, "label": "", "sentence_structure": "Subject鈥搗erb鈥搊bject", "subjective_objective": {"answer": "Subjective", "rationale": "Because words such as \"think\" and \"do it\" indicate that this is the patient's personal view and attitude and is therefore viewed from a subjective perspective."}, "sentence_sentiment": {"answer": "Neutral", "rationale": "Because the entry does not express any positive or negative emotional aspects, it is merely a statement of one view."}, "language_style": {"answer": "Calm and direct", "rationale": "The entry expresses the view directly, without excessive rhetorical or emotional rendering, is more objective and more consistent with the harsh, direct language style of the questionnaire."}, "time_dimension": {"answer": "Present", "rationale": "Because \"think\" and \"do\" are all the current patterns, and they represent current thinking and behavior. And the expression \"the only thing to do is wait\" also shows that it is a present view and attitude."}, "sentence_type": "Imperative sentence", "pos": {"nouns": {"Time": 1, "Status": 1, "Wait": 1}, "verbs": {"It changes": 1, "Do it": 1, "be": 1}, "adverbs": {"One": 1, "Shit": 1}, "numerals": {"15": 1}, "pronouns": {"self": 1}}}, {"content": "16. Trying to forget the whole thing.", "word_count": 8, "label": "", "sentence_structure": "Subject鈥搗erb鈥揷omplement", "subjective_objective": {"answer": "Subjective", "rationale": "For trying to forget the whole thing is an act of the patient's own initiative, which is his subjective attitude and will."}, "sentence_sentiment": {"answer": "Negative", "rationale": "Because the purpose of the \"tried to forget the whole thing\" in the entry is negative."}, "language_style": {"answer": "Calm and direct", "rationale": "The entry is more directly linguistically, without too much rhetorical or emotional rendering, but simply asks if the person who was tested has ever tried to forget something. This expression is simple, clear, easy to understand and answer."}, "time_dimension": {"answer": "Present", "rationale": ""}, "sentence_type": "Imperative sentence", "pos": {"nouns": {"Things": 1}, "verbs": {"Forget it": 1, "Try": 1}}}, {"content": "17. Reliance on others to solve inter-agency problems", "word_count": 8, "label": "", "sentence_structure": "Subject鈥搗erb鈥搊bject", "subjective_objective": {"answer": "Subjective", "rationale": "Because \"dependance on others\" is the patient's subjective attitude or tendency."}, "sentence_sentiment": {"answer": "Negative", "rationale": "Because the action of relying on others to solve inter-problems in the entries is negative."}, "language_style": {"answer": "Tactful and warm", "rationale": "The entry uses a more moderate and friendly language style, such as \"Leave to others to solve problems\", which is more euphemistic and acceptable, and which also attracts a sense of resonance and participation among respondents, thus improving the rate of recovery and data quality of questionnaires."}, "time_dimension": {"answer": "Present", "rationale": "The keyword \"dependance of others\" means current behavior or state, so the time dimension of this entry is now."}, "sentence_type": "Imperative sentence", "pos": {"nouns": {"Problem": 1, "others": 1}, "verbs": {"Dependence": 1, "Resolve": 1}, "numerals": {"17": 1}, "pronouns": {"you": 1}}}, {"content": "18. Accept reality because there is no other way", "word_count": 12, "label": "", "sentence_structure": "Subject鈥損redicate", "subjective_objective": {"answer": "Objective", "rationale": "Because \"accepting reality\" is an objective act, and the fact is there, whether the patient wishes it or not, and it is therefore an objective perspective."}, "sentence_sentiment": {"answer": "Negative", "rationale": "Because the \"acceptance of reality\" attitude in the entries is negative."}, "language_style": {"answer": "Calm and direct", "rationale": "The language style of the entry is relatively simple and clear, presenting the view in a direct and clear manner, without excessive rhetorical or emotional rendering. It is therefore judged to be \"cool and direct\"."}, "time_dimension": {"answer": "Present", "rationale": "The words \"acceptance\" and \"no other way\" in this entry describe the current situation or attitude of the testee and can therefore be judged as now."}, "sentence_type": "Imperative sentence", "pos": {"nouns": {"Reality": 1, "Approach": 1}, "verbs": {"Accept": 1, "have": 1}, "adverbs": {"Because": 1}, "numerals": {"18": 1}, "pronouns": {"Other": 1}}}, {"content": "19. Imagination could have a miracle to change the situation.", "word_count": 15, "label": "", "sentence_structure": "Subject鈥搗erb鈥搊bject", "subjective_objective": {"answer": "Subjective", "rationale": "Because illusions are usually the subjective imagination and expectation of individuals, not objective facts or circumstances."}, "sentence_sentiment": {"answer": "Neutral", "rationale": "Because the entry merely raised a possibility and did not express any emotional colour."}, "language_style": {"answer": "Tactful and warm", "rationale": "The language style of the entry is mild, using the words \"fanta\", \"possible,\" and gives people a sense of encouragement and hope, not of a strong order or threat. This language style makes it easier for the interviewee to feel comfortable and to be more willing to participate in the questionnaire."}, "time_dimension": {"answer": "Future", "rationale": "The entry contains an imagination and expectations of what may happen in the future, with the key words \"possible\", \"magic\" and \"change the status quo\" pointing to situations that have not yet occurred and that may arise in the future. The temporal dimension of the article is therefore the future."}, "sentence_type": "Imperative sentence", "pos": {"nouns": {"Fantasy": 1, "Miracles": 1, "Status": 1}, "verbs": {"It could happen": 1, "Change": 1}, "numerals": {"19": 1}}}, {"content": "20. Consolate yourself.", "word_count": 6, "label": "", "sentence_structure": "Subject鈥搗erb鈥揷omplement", "subjective_objective": {"answer": "Subjective", "rationale": "Because they are the subjective attitudes and behaviours of the patient."}, "sentence_sentiment": {"answer": "Positive", "rationale": "Because \"self-consolation\" is a positive act of self-regulation and psychological decorum."}, "language_style": {"answer": "Tactful and warm", "rationale": "The language style of the entry is more euphemismal and gentle, and leads the interviewee to answer questions in a way that is caring and understanding, rather than directly ordering or critical. This expression is easier to resonate and comfort with and to get real and effective information."}, "time_dimension": {"answer": "Present", "rationale": "The keyword is \"self-consolation\", and the act occurs at the current point of time or in the near future, so it's now."}, "sentence_type": "Imperative sentence", "pos": {"nouns": {"self": 2, "Consolation": 1}, "verbs": {"Consolation": 1}, "numerals": {"20": 1}}}]}

{"questionnaire_title": "Type A, Type B personality test", "questionnaire_category": "Behavioral questionnaire", "item_count": 25, "emotion_shift_overall": "From negative to positive", "language_style_overall": "Calm and direct", "items": [{"content": "Do you deliberately increase the tone of keywords when you speak?", "word_count": 16, "label": "", "sentence_structure": "Subject鈥搗erb鈥搊bject", "subjective_objective": {"answer": "Subjective", "rationale": "Because the question is whether the patient ' s subjective feelings and attitudes, i.e. whether the tone of the keyword is specifically aggravated when speaking, are not necessarily the same."}, "sentence_sentiment": {"answer": "Negative", "rationale": "Because the act of \"intentionally reinforcing the tone of the keyword\" in the entry may be a source of blame or criticism and have some negative emotional connotations."}, "language_style": {"answer": "Calm and direct", "rationale": "The language style of the entry is relatively calm and objective, and there is no excessive emotional rendering or rhetorical technique to ask the respondents in a straightforward manner about the habits and characteristics of their expression."}, "time_dimension": {"answer": "Present", "rationale": ""}, "sentence_type": "Interrogative sentence", "pos": {"nouns": {"Questionnaire": 1, "Entry": 1, "- You're a good guy": 1}, "verbs": {"Talk": 1, "On purpose": 1, "Increase": 1}, "adverbs": {"I'm doing it": 1}, "numerals": {"1": 2}, "classifiers": {"individual": 1}}}, {"content": "Are you in a hurry to eat and walk?", "word_count": 12, "label": "", "sentence_structure": "Subject鈥搗erb鈥搊bject", "subjective_objective": {"answer": "Subjective", "rationale": "This is a self-described habit of the patient and is observed from a subjective perspective."}, "sentence_sentiment": {"answer": "Negative", "rationale": "Because \"surgery\" is a negative act."}, "language_style": {"answer": "Calm and direct", "rationale": "The entry is an objective and direct question to the respondents about whether they are used to eating and walking in their daily lives, and the tone is not too much rhetorical or emotional, which is more calm and direct language style."}, "time_dimension": {"answer": "Present", "rationale": "The entry asks whether the testee has \"a rush to eat and walk\" in his current behaviour or habits, and therefore the time dimension is now judged."}, "sentence_type": "Imperative sentence", "pos": {"nouns": {"Rice": 1, "Let's go": 1, "Road": 1}, "verbs": {"Eat": 1, "Let's go": 2, "Both": 1, "- It's urgent": 1}, "adjectives": {"- It's urgent": 1}, "numerals": {"2": 1}}}, {"content": "3. Do you think that children should be able to develop a habit of competing with others as children?", "word_count": 19, "label": "", "sentence_structure": "Subject鈥搗erb鈥搊bject", "subjective_objective": {"answer": "Subjective", "rationale": "Because \"think\" represents the patient's subjective view and attitude."}, "sentence_sentiment": {"answer": "Negative", "rationale": "Because it expresses a stronger concept of competition, it may lead to some negative consequences, such as excessive competition, excessive pressure, etc."}, "language_style": {"answer": "Calm and direct", "rationale": "The language style of this entry is of a more direct and explicit type, with no excessive accoutrements or subtle expressions. It raises questions directly and asks the interviewee to give his own opinion, which is more objective in tone.\""}, "time_dimension": {"answer": "Present", "rationale": "So the answer is \"now\"."}, "sentence_type": "Interrogative sentence", "pos": {"nouns": {"Son": 1, "Custom": 1, "Competition": 1}, "verbs": {"I think": 1, "The": 1, "Cultivation": 1, "and": 1, "Just": 1}, "adverbs": {"Since childhood": 1}, "numerals": {"3": 1}, "pronouns": {"you": 1}}}, {"content": "Do you get impatient when others slow down?", "word_count": 18, "label": "", "sentence_structure": "Subject鈥搗erb鈥搊bject", "subjective_objective": {"answer": "Subjective", "rationale": "Because \"deepness\" is the patient's subjective feeling."}, "sentence_sentiment": {"answer": "Negative", "rationale": "Because the emotional reaction of \"deepness\" is negative."}, "language_style": {"answer": "Calm and direct", "rationale": "The entry is based on a direct inquiry, without too much rhetorical or emotional presentation, and is more objective in tone. It asks whether the interviewee is impatient when he or she does something slow, which is a relatively simple and clear expression that is more relevant and operational."}, "time_dimension": {"answer": "Present", "rationale": "The entry asks about the feelings and attitudes of the person being tested at the moment (i.e., at the time of completion of the investigation) and therefore is judged to be \"now\"."}, "sentence_type": "Interrogative sentence", "pos": {"nouns": {"others": 1, "Entry": 1, "Yes": 1, "Slow-Sensey": 1, "I'm tired": 1}, "verbs": {"I feel": 1, "Slow down": 1, "I'm impatient": 1}, "adjectives": {"Entry": 1, "Slow-Sensey": 1}, "adverbs": {"When": 1}, "numerals": {"4": 1}, "pronouns": {"you": 1}}}, {"content": "5. When someone tells you what's going on, will you hurry him to finish?", "word_count": 19, "label": "", "sentence_structure": "Subject鈥搗erb鈥搊bject", "subjective_objective": {"answer": "Subjective", "rationale": "Because this is the subject of examining patients ' subjective feelings and attitudes, questions are asked about the reactions or thoughts that patients may have in certain circumstances."}, "sentence_sentiment": {"answer": "Negative", "rationale": "Because the entry \"pull him\" was a bit rude and hasty, so it was negative."}, "language_style": {"answer": "Calm and direct", "rationale": "The language style of the entry is relatively calm and straightforward, and it uses a clear question form, without any rhetorical or emotional recitation. This language style enables respondents to understand the meaning of the question clearly and to provide accurate answers."}, "time_dimension": {"answer": "Present", "rationale": "The entry describes the behaviour or attitude of the person under examination in the current circumstances, and the keywords are \"will\" and \"trigger\", which indicate that this is a customary and potentially ongoing act."}, "sentence_type": "Interrogative sentence", "pos": {"nouns": {"person": 1, "Things": 1, "Answer": 1}, "verbs": {"Tell you what": 1, "Yes": 1, "- Get him out of here": 1, "Say it": 1}, "adverbs": {"Come on": 1, "Hurry up": 1}, "numerals": {"5": 1}, "pronouns": {"you": 1, "he": 2}}}, {"content": "6. Are you gonna get pissed off when you're on the road, in a car or in a restaurant line?", "word_count": 17, "label": "", "sentence_structure": "Subject鈥搗erb鈥搊bject", "subjective_objective": {"answer": "Subjective", "rationale": "Because the answer to \"yes\" or \"no\" reflects personal emotional reactions and subjective feelings."}, "sentence_sentiment": {"answer": "Negative", "rationale": "Because the \"fated\" sentiment in the entry is negative."}, "language_style": {"answer": "Tactful and warm", "rationale": ""}, "time_dimension": {"answer": "Present", "rationale": "Keywords \"will\", \"crowding\" and \"queuing\" indicate that this behaviour is a current or recent occurrence and therefore is judged to be the present."}, "sentence_type": "Interrogative sentence", "pos": {"nouns": {"On the way": 1, "Car": 1, "Restaurant": 1, "Team": 1, "Yes": 1, "you": 1, "Exasperation": 1}, "verbs": {"Squeeze": 1, "Line": 1}, "numerals": {"6": 1}, "classifiers": {"individual": 1}}}, {"content": "Do you think about your own problems while listening to others?", "word_count": 19, "label": "", "sentence_structure": "Subject鈥搗erb鈥搊bject", "subjective_objective": {"answer": "Subjective", "rationale": "Because this question examines whether the patient, when listening to others, is going to shift his or her attention to thinking, which is an individual subjective feeling and attitude."}, "sentence_sentiment": {"answer": "Negative", "rationale": "Because the \"always think about your problems\" act is negative."}, "language_style": {"answer": "Tactful and warm", "rationale": "The entry is more euphemistic and warm. It is asked in an invitation-based manner, without direct orders or accusations, but rather by asking the questions to guide the interviewees to think and express their thoughts. Moreover, the topic does not use too strong language to emphasize a particular point of view, but is as neutral as possible, so that the interviewees can express their feelings and views more freely."}, "time_dimension": {"answer": "Past", "rationale": "The entry asks whether the person who was tested was ever thinking about his or her own problems while listening to others, and the time dimension is a matter of past."}, "sentence_type": "Interrogative sentence", "pos": {"nouns": {"Listen": 1, "others": 1, "Talk": 1, "Problem": 1}, "verbs": {"Yes": 3}, "adverbs": {"Always": 1}, "numerals": {"7": 1}, "pronouns": {"you": 1}}}, {"content": "Would you shave while you're eating?", "word_count": 22, "label": "", "sentence_structure": "Subject鈥搗erb鈥搊bject", "subjective_objective": {"answer": "Subjective", "rationale": "This is a subjective behaviour habit of the patient and involves the way of life and habits of the individual."}, "sentence_sentiment": {"answer": "Neutral", "rationale": "Because the entry is concerned with a behaviour pattern and there is no apparent positive or negative emotional orientation."}, "language_style": {"answer": "Tactful and warm", "rationale": "The language style of the entry is more euphemistic and friendly, using a question-and-answer approach rather than an order or a direct statement."}, "time_dimension": {"answer": "Present", "rationale": "The entry is therefore asked about the current customary behaviour of the person being tested, and the temporal dimension is now judged."}, "sentence_type": "Interrogative sentence", "pos": {"nouns": {"Questionnaire": 1, "Entry": 1, "Rice": 1, "Notes": 1, "Car": 1, "The beard": 1}, "verbs": {"Yes": 2, "Eat": 1, "Write": 1, "Open": 1, "Scratch": 1}, "adverbs": {"On the side": 3}, "numerals": {"8": 1}}}, {"content": "Will you finish all the work you've been doing before you leave?", "word_count": 17, "label": "", "sentence_structure": "Subject鈥損redicate", "subjective_objective": {"answer": "Objective", "rationale": "The decision is based on the question of whether the patient will take any action (i.e., complete all scheduled work before the leave of absence), and therefore it is an objective question."}, "sentence_sentiment": {"answer": "Negative", "rationale": "Because \"finishing all scheduled jobs\" can lead to overwork and stress, which is a negative behaviour or attitude."}, "language_style": {"answer": "Calm and direct", "rationale": "The entry asked the interviewees in a straightforward and clear manner whether they would complete all work before the leave, and the language style was more objective and calm."}, "time_dimension": {"answer": "Future", "rationale": "The keywords \"before leave\" and \"after all scheduled work\" point to situations that have not yet occurred and that may arise in the future. It is therefore possible to determine that the entry is about the future."}, "sentence_type": "Interrogative sentence", "pos": {"nouns": {"Leave": 1, "Everything": 1}, "verbs": {"Yes": 1, "Finish": 1}, "adverbs": {"Before": 1}, "numerals": {"9": 1}}}, {"content": "Do you always mention your own concerns when you talk to others?", "word_count": 18, "label": "", "sentence_structure": "Subject鈥搗erb鈥搊bject", "subjective_objective": {"answer": "Subjective", "rationale": "This is because the individual attitude and preferences expressed by the patient in answering the questions are subjective."}, "sentence_sentiment": {"answer": "Positive", "rationale": "Because the \"Refer to your own concern\" act is positive."}, "language_style": {"answer": "Tactful and warm", "rationale": "The entry is more adept and interactive when it is spoken in a gentle and inviting manner, rather than by direct statements or orders."}, "time_dimension": {"answer": "Present", "rationale": ""}, "sentence_type": "Interrogative sentence", "pos": {"nouns": {"Talk": 1, "person": 1, "Yes": 1}, "verbs": {"Reference": 1, "Care": 1}, "adverbs": {"Always": 1}, "numerals": {"10": 1}, "pronouns": {"you": 1}}}, {"content": "Did you feel like wasting your time when you stopped working for a while?", "word_count": 22, "label": "", "sentence_structure": "Subject鈥搗erb鈥搊bject", "subjective_objective": {"answer": "Subjective", "rationale": "This is because the patient ' s attitude and feelings about stopping work are subjective."}, "sentence_sentiment": {"answer": "Negative", "rationale": "Because the idea of \"will feel wasted time\" is negative."}, "language_style": {"answer": "Tactful and warm", "rationale": "The entry is more moderate in terms of language style, not directly expressing negative evaluations or criticisms, but rather asking questions in a question-and-answer manner, allowing the interviewee to respond autonomously. At the same time, the expression \"time wasted\" is also neutral, avoiding pressure or discomfort on the interviewee."}, "time_dimension": {"answer": "Present", "rationale": "The word \"let\" in \"let you stop work for a while\" means \"to give up\" is to say what others want or expect, \"stop work for a while\" describes a behavior or state, \"think\" means the subjective feeling of the person being tested. The whole sentence expresses a state or behaviour that is now in place."}, "sentence_type": "Interrogative sentence", "pos": {"nouns": {"you": 2, "Work": 1, "Rest": 1, "Time": 1}, "verbs": {"Stop": 1, "Yes": 2, "I think": 1, "Wasted": 1, "Jean": 1}, "adverbs": {"Just a minute": 1, "- You're not": 1}, "numerals": {"11": 1}, "pronouns": {"Here": 1}}}, {"content": "12. Do you feel like you're working hard to enjoy the beauty of your surroundings?", "word_count": 21, "label": "", "sentence_structure": "Subject鈥搗erb鈥搊bject", "subjective_objective": {"answer": "Subjective", "rationale": "Because \"thinks it\" is a subjective question, it reflects the attitude and perception of the testee towards work."}, "sentence_sentiment": {"answer": "Negative", "rationale": "Because the \"unreceivable view\" in the entry is negative."}, "language_style": {"answer": "Tactful and warm", "rationale": "I therefore consider that the language style of this purpose is euphemism."}, "time_dimension": {"answer": "Present", "rationale": "The terms \"felt\" and \"involved\" both indicate the current perception and state of the subject, so the entry describes the current situation."}, "sentence_type": "Interrogative sentence", "pos": {"nouns": {"Work": 1, "- Oh, beautiful view": 1}, "verbs": {"I think": 1, "I'm all in": 1, "Enjoy": 1}, "adjectives": {"Around": 1, "of": 2, "I don't have time": 1}, "numerals": {"12": 1}, "pronouns": {"you": 1}}}, {"content": "13. Do you feel that you prefer to be pragmatic rather than creative or reformist?", "word_count": 21, "label": "", "sentence_structure": "Subject鈥搗erb鈥搊bject", "subjective_objective": {"answer": "Subjective", "rationale": "Because \"what feels more pragmatic than innovation or change\" is the patient鈥檚 personal thinking and attitude. This is not an objective fact, but a subjective perception and assessment of his situation."}, "sentence_sentiment": {"answer": "Negative", "rationale": "Because the attitude in the entry \"not to do things of innovation or change\" is negative."}, "language_style": {"answer": "Calm and direct", "rationale": "The point is based on the following: the question of the purpose of this article is one of direct questions, with no excessive rhetorical or emotional rendering. It expresses its concern and investigation about whether the interviewee is risk-averse and prefers to act in a robust manner. It is therefore more neutral in its tone and is straightforward in its tone."}, "time_dimension": {"answer": "Present", "rationale": "The entry is to ask the testee about his thoughts and attitude in the current state, the keyword being \"Does he feel\" and it points to the testee's view of an issue and does not refer to the past or future time dimension. Thus, the entry can be judged to be now."}, "sentence_type": "Interrogative sentence", "pos": {"nouns": {"Questionnaire": 1, "Entry": 1, "Innovation": 1, "Reform": 1}, "verbs": {"I think": 1, "I'd rather not": 1, "I don't want to": 1, "Active": 1}, "adjectives": {"Practical": 1, "And": 1}, "numerals": {"13": 1}, "pronouns": {"you": 1}}}, {"content": "14. Have you tried to do more in a limited time?", "word_count": 18, "label": "", "sentence_structure": "Subject鈥搗erb鈥搊bject", "subjective_objective": {"answer": "Subjective", "rationale": "Because \"attempt or not\" is an individual's attitude and choice, depending on the patient's own will and awareness."}, "sentence_sentiment": {"answer": "Positive", "rationale": "Because the purpose of the entry is to \"tried to do more for a limited period of time\"."}, "language_style": {"answer": "Calm and direct", "rationale": "The entry is more straightforward in language style, with no excessive rhetorical or emotional rendering, and asks the interviewee whether he has tried to do more in a limited time. This expression is more objective, concise and easy to understand and answer."}, "time_dimension": {"answer": "Present", "rationale": "The entry asks if the person tested is currently in a habit or preference of \"attempting to do more within a limited time\" and thus judges it as now."}, "sentence_type": "Interrogative sentence", "pos": {"nouns": {"Time": 1, "Things": 1}, "verbs": {"Try": 1, "Do it": 1, "Out": 1}, "adjectives": {"Limited": 1}, "numerals": {"14": 1}}}, {"content": "15. Are you absolutely punctual when you have an appointment with someone else?", "word_count": 13, "label": "", "sentence_structure": "Subject鈥搗erb鈥搊bject", "subjective_objective": {"answer": "Objective", "rationale": "Because the question is an objective examination of the question of whether the interviewees are absolutely punctual, which is a manifestation of their behaviour."}, "sentence_sentiment": {"answer": "Positive", "rationale": "Because punctuality is a positive habit and quality."}, "language_style": {"answer": "Calm and direct", "rationale": "The entry is more straightforward in its language style, is not accompanied by too many emotional or speech-making techniques and is more objective. It is a habit of asking the person surveyed about the observance of the agreed time, which is used to obtain data and information."}, "time_dimension": {"answer": "Present", "rationale": "The entry is to ask the testee whether he is in the current habits of behaviour, whether he is in compliance with time, the keywords being \"when he has made an appointment with someone else\" and \"when he is absolutely on time\", all pointing to the state of affairs that is now under way."}, "sentence_type": "Interrogative sentence", "pos": {"nouns": {"person": 1, "Date": 1, "Timeout": 1}, "verbs": {"have": 1, "Guard": 1}, "adverbs": {"Absolutely": 1}, "numerals": {"15": 1}}}, {"content": "16. Do you hold your fist tight to enhance your tone when expressing your views?", "word_count": 17, "label": "", "sentence_structure": "Subject鈥搗erb鈥搊bject", "subjective_objective": {"answer": "Subjective", "rationale": "Because a tight fist is a physical act and attitude, it is a subjective decision that requires the patient ' s consciousness."}, "sentence_sentiment": {"answer": "Negative", "rationale": "Because holding your fist is a tense, exciting expression that may affect the acceptance of your views."}, "language_style": {"answer": "Tactful and warm", "rationale": "The language style of the article is more direct, and there is no excessive accrimation or rhetorical technique. It simply asks whether the interviewee will be able to strengthen his tone by holding his fists when he expresses his opinion. This type of questionnaire is usually designed to collect objective information without attempting to influence the way the interviewee answers."}, "time_dimension": {"answer": "Present", "rationale": ""}, "sentence_type": "Interrogative sentence", "pos": {"nouns": {"Observations": 1, "Fist": 1, "- You're a good guy": 1}, "verbs": {"Expression": 1, "Hold on tight": 1, "Strengthening": 1}}}, {"content": "Do you have confidence in improving your performance again?", "word_count": 15, "label": "", "sentence_structure": "Subject鈥搗erb鈥搊bject", "subjective_objective": {"answer": "Subjective", "rationale": "Because \"confidence and upgrading\" is a subjective attitude and conviction of individuals and does not represent actual performance."}, "sentence_sentiment": {"answer": "Positive", "rationale": "Because the \"confidence to improve your performance\" attitude in the entry is positive."}, "language_style": {"answer": "Tactful and warm", "rationale": "The language style of the entry is euphemistic, because it uses a gentle and encouraging tone to ask if the other side has confidence in its own efficiency. It does not speak too directly or in a prescriptive manner, but rather in a friendly and supportive way, which makes it easier for respondents to feel comfortable and to share their thoughts and feelings."}, "time_dimension": {"answer": "Present", "rationale": ""}, "sentence_type": "Interrogative sentence", "pos": {"nouns": {"Confidence": 1, "Performance of work": 1}, "verbs": {"have": 1, "T": 1, "_Other Organiser": 1}, "adverbs": {"Again": 1}, "numerals": {"17": 1}, "classifiers": {"you": 1}}}, {"content": "Do you think there's something you're waiting for to do right away?", "word_count": 16, "label": "", "sentence_structure": "Subject鈥搗erb鈥搊bject", "subjective_objective": {"answer": "Subjective", "rationale": "Because it is a subjective feeling or attitude."}, "sentence_sentiment": {"answer": "Positive", "rationale": "Because \"something awaits you to do immediately\" in the entry expresses a positive, proactive attitude and behaviour that implies a sense of responsibility and enforcement on the part of the interviewees."}, "language_style": {"answer": "Calm and direct", "rationale": "The entry is relatively calm and straightforward in its language style, has no excessive speech or emotional rendering, and is more objective in tone. It is conducted in a direct interview mode, allowing respondents to answer questions more intuitively and facilitate data collection and analysis."}, "time_dimension": {"answer": "Present", "rationale": "The entry now asks the testee ' s current feelings and circumstances, 'Does it feel like something awaits you to finish immediately? ' It asks about the situation at this moment, and therefore the time dimension is now."}, "sentence_type": "Interrogative sentence", "pos": {"nouns": {"Yes": 1, "Questionnaire": 1, "Entry": 1}, "verbs": {"I think": 1, "Wait": 1, "Completed": 1}, "adjectives": {"Now": 1}, "numerals": {"18": 1}, "pronouns": {"you": 1}}}, {"content": "19. Have you ever felt dissatisfied with the efficiency of your work?", "word_count": 18, "label": "", "sentence_structure": "Subject鈥搗erb鈥搊bject", "subjective_objective": {"answer": "Subjective", "rationale": "Because the problem involves patients' self-perception and evaluation, it is a subjective approach."}, "sentence_sentiment": {"answer": "Negative", "rationale": "Because the attitude of \"unsatisfactory\" in the entries is negative."}, "language_style": {"answer": "Calm and direct", "rationale": "The entry directly asked whether the respondents were not satisfied with their work efficiency, were clear in language, were not subject to excessive emotional accommodations or euphemism and seemed calmer and straightforward."}, "time_dimension": {"answer": "Present", "rationale": "The entry asks the testee's current feelings and perceptions, the keyword \" whether it feels \" and \"has been dissatisfied\", all of which point to the situation."}, "sentence_type": "Interrogative sentence", "pos": {"nouns": {"you": 1, "Efficiency": 1}, "verbs": {"I think": 1, "Satisfied": 1}, "adjectives": {"Always": 1, "not": 1}, "numerals": {"19": 1}}}, {"content": "Do you feel like you have to win when you compete with someone?", "word_count": 14, "label": "", "sentence_structure": "Subject鈥損redicate", "subjective_objective": {"answer": "Subjective", "rationale": "Because \"like\" is a subjective attitude or feeling that must be won."}, "sentence_sentiment": {"answer": "Negative", "rationale": "Because the \"must win\" attitude in the entries is challenging and aggressive, it is also subject to some stress and anxiety and is therefore considered negative."}, "language_style": {"answer": "Calm and direct", "rationale": "The entry is conducted in a direct question-and-answer manner, with a more direct, clear language style, with no excessive rhetorical or emotional rendering. At the same time, the question is more straightforward about asking the other side whether it must be successful in the competition. So, I think its language style is \"cool and straight.\""}, "time_dimension": {"answer": "Present", "rationale": "The keyword \"now\" contained in the entry indicates that it is a question of the current mindset and thinking of the subjects, i.e. whether they feel this at this stage."}, "sentence_type": "Interrogative sentence", "pos": {"nouns": {"you": 1, "person": 1, "Competition": 1, "Win": 1}, "verbs": {"I think": 1, "be": 1, "Not": 1, "Win": 1}, "adverbs": {"not": 1, "Yeah": 1}}}, {"content": "21. Do you always interrupt people?", "word_count": 11, "label": "", "sentence_structure": "Subject鈥搗erb鈥搊bject", "subjective_objective": {"answer": "Subjective", "rationale": "Since the question is asked about the patient ' s own behaviour, it is based on the patient ' s personal experience and subjective consciousness."}, "sentence_sentiment": {"answer": "Negative", "rationale": "Because \"interrupting someone's words\" is rude and has a negative impact."}, "language_style": {"answer": "Calm and direct", "rationale": "The language style of the entry is straightforward, as it clearly asks whether the person surveyed has frequently interrupted the conversation, has not used too many rhetorical or emotional accents and is more honest and objective."}, "time_dimension": {"answer": "Present", "rationale": ""}, "sentence_type": "Interrogative sentence", "pos": {"nouns": {"Questionnaire": 1, "Entry": 1, "Word": 1}, "verbs": {"be": 1, "Interrupt": 1, "Regular": 1}, "adverbs": {"Regular": 1}, "numerals": {"21": 1}}}, {"content": "Will you be angry when you see someone late?", "word_count": 13, "label": "", "sentence_structure": "Subject鈥搗erb鈥搊bject", "subjective_objective": {"answer": "Subjective", "rationale": "Because anger is an emotional reaction, it is the result of subjective feelings and experiences of individuals."}, "sentence_sentiment": {"answer": "Negative", "rationale": "Because the mood for \"freak\" in the entry is negative."}, "language_style": {"answer": "Calm and direct", "rationale": "The entry is more straightforward, is less speech-making or emotional, and is more objective. It asks whether the person surveyed would be angry when he was late, expressing clear themes and intentions, and therefore more calm and straightforward."}, "time_dimension": {"answer": "Present", "rationale": "The entry is for the emotional reaction of the person who is being questioned when he or she sees another person late, so the time dimension is now judged."}, "sentence_type": "Interrogative sentence", "pos": {"nouns": {"others": 1, "Late": 1, "Angry": 1}, "verbs": {"Yeah": 1, "Yes": 1, "Fan": 1}, "adverbs": {"Yes": 1, "- You're not": 1}, "numerals": {"22": 1}, "pronouns": {"you": 1}}}, {"content": "Did you leave the table as soon as you finished eating?", "word_count": 14, "label": "", "sentence_structure": "Subject鈥損redicate", "subjective_objective": {"answer": "Subjective", "rationale": "Because this behaviour depends on the patient ' s personal habits and choices, it is subjective."}, "sentence_sentiment": {"answer": "Negative", "rationale": "Because the act of \"leave immediately\" is a display of disrespect and disrespect."}, "language_style": {"answer": "Calm and direct", "rationale": "This is a way to make it easier for the person interviewed to understand and accept and to be able to obtain accurate and effective answers."}, "time_dimension": {"answer": "Present", "rationale": "The entry asks the testee about his current behavioral habits, and the keywords \"whether\" and \"immediately\" indicate that this is an instant action, so the temporal dimension is now judged."}, "sentence_type": "Interrogative sentence", "pos": {"nouns": {"Dinner": 1, "Time": 1, "you": 1, "Now": 1, "Dismissed": 1}, "verbs": {"Is it possible": 1, "Eat": 1, "Just": 1, "Off": 1, "Seats": 1}, "adverbs": {"- Finish it": 1}, "numerals": {"23": 1}}}, {"content": "Do you often feel in haste?", "word_count": 13, "label": "", "sentence_structure": "Subject鈥搗erb鈥搊bject", "subjective_objective": {"answer": "Subjective", "rationale": "Because \"fears in haste\" is a person's own subjective experience and feeling."}, "sentence_sentiment": {"answer": "Negative", "rationale": "Because the \"hurry\" feeling in the entry is usually negative."}, "language_style": {"answer": "Calm and direct", "rationale": "The entry is based on direct inquiries, with no excessive adoration and emotional rendering, and is more objective in tone and is in line with the linguistic style of the psychological questionnaire."}, "time_dimension": {"answer": "Present", "rationale": "The entry asks the testee's current state and feelings, and \"there are often\" is a word for frequency, indicating that the question is asking about the current state. The time dimension of the entry is therefore judged to be now."}, "sentence_type": "Interrogative sentence", "pos": {"nouns": {"Feel": 1, "It's a rush": 1}, "verbs": {"have": 1, "Regular": 1}, "adjectives": {"Hurry up": 1}, "adverbs": {"Regular": 1}, "numerals": {"24": 1}}}, {"content": "Are you not satisfied with your recent performance?", "word_count": 14, "label": "", "sentence_structure": "Subject鈥搗erb鈥搊bject", "subjective_objective": {"answer": "Subjective", "rationale": "Because \"satisfaction\" and \"unsatisfactory\" are subjective feelings of the patient himself, and are subjective in nature."}, "sentence_sentiment": {"answer": "Negative", "rationale": "Because \"unsatisfactory\" is negative."}, "language_style": {"answer": "Calm and direct", "rationale": "The entry directly asks the respondents about their satisfaction with their recent performance, which is more direct and objective, and does not appear to be too emotional or rhetorical in tone."}, "time_dimension": {"answer": "Past", "rationale": "The entry asks if the person tested is satisfied with his or her recent performance and the time dimension is a matter of the past."}, "sentence_type": "Interrogative sentence", "pos": {"nouns": {"you": 1, "Recently": 1, "Performance": 1}, "verbs": {"Is it possible": 1, "Satisfied": 1}, "adverbs": {"Recently": 1}, "numerals": {"25": 1}}}]}

{"questionnaire_title": "Individual evaluation questionnaires", "questionnaire_category": "Mental health", "item_count": 54, "emotion_shift_overall": "From positive to negative", "language_style_overall": "Tactful and warm", "items": [{"content": "I am a social person.", "word_count": 8, "label": "", "sentence_structure": "Subject鈥揷opula鈥損redicative", "subjective_objective": {"answer": "Subjective", "rationale": "Because \"coitus\" is a self-perception and evaluation of the individual, and is subjective."}, "sentence_sentiment": {"answer": "Positive", "rationale": "Because the expression \"persons who are able to communicate\" in the entry is positive emotional."}, "language_style": {"answer": "Tactful and warm", "rationale": ""}, "time_dimension": {"answer": "Present", "rationale": "The entry describes the character or attribute of the testee, namely, \"I'm a social person\", a statement of personal qualities that have nothing to do with time, and therefore the judgment is now."}, "sentence_type": "Interrogative sentence", "pos": {"nouns": {"person": 1, "Communication": 1}, "verbs": {"be": 1, "Yes": 1, "Communication": 1}, "adjectives": {"individual": 1}, "classifiers": {"individual": 1}, "pronouns": {"I": 1}}}, {"content": "2. I have been very disappointed in myself on several occasions in recent days.", "word_count": 16, "label": "", "sentence_structure": "Subject鈥搗erb鈥搊bject", "subjective_objective": {"answer": "Subjective", "rationale": "Because \"very disappointed\" is a subjective or emotional experience, reflecting the emotional state and the inner feeling of the patient, not the objective fact or behaviour."}, "sentence_sentiment": {"answer": "Negative", "rationale": "Because the \"very disappointed\" attitude in the entry is negative."}, "language_style": {"answer": "Calm and direct", "rationale": "The type of language that is used is a more objective and practical type. It is not too emotionally qualified or euphemistic, but rather it presents a question in a simple and straightforward way, giving four options for respondents to choose."}, "time_dimension": {"answer": "Present", "rationale": "In the entry, \"for the last few days\" is an indication of the behaviour of the recent period, while \"for several times\" further indicates that this is a recurrent situation, so it can be judged that it is an inquiry into the mental or behavioural state of the person being tested over the past period, and that the temporal dimension is now."}, "sentence_type": "Interrogative sentence", "pos": {"verbs": {"Near": 1, "Come on": 1, "to": 1, "self": 2, "Very": 1, "Disappointment": 1}, "adverbs": {"A few times": 1}, "numerals": {"How many": 1}, "pronouns": {"I": 1}}}, {"content": "3. What bothers me is that I cannot look better.", "word_count": 16, "label": "", "sentence_structure": "Subject鈥揷opula鈥損redicative", "subjective_objective": {"answer": "Subjective", "rationale": "This expression is an expression of the patient ' s dissatisfaction with his or her appearance and desire to change his or her appearance and is part of his or her subjective attitude."}, "sentence_sentiment": {"answer": "Negative", "rationale": "Because the expression \"my appearance is not better seen\" has a negative emotional connotation."}, "language_style": {"answer": "Tactful and warm", "rationale": "The entry uses euphemisms to \"make it\" and then expresses each other's concern about the face in question, with a more gentle and friendly language style."}, "time_dimension": {"answer": "Present", "rationale": "It is therefore possible to judge from this information that the entry is now relevant."}, "sentence_type": "Affirmative declarative sentence", "pos": {"nouns": {"I": 1, "Look at that": 1, "Points": 1}, "verbs": {"鈾?And I'm gonna be 鈾?: 1, "Trouble": 1, "Yes": 1, "It looks better": 1}, "adjectives": {"More": 1, "Nice": 1}, "numerals": {"3": 1}}}, {"content": "4. It is no difficulty for me to maintain a satisfactory relationship of love.", "word_count": 19, "label": "", "sentence_structure": "Subject鈥揷opula鈥損redicative", "subjective_objective": {"answer": "Subjective", "rationale": "Because the entry expresses the patient ' s attitude and opinion towards maintaining a relationship of love, it is a subjective judgement."}, "sentence_sentiment": {"answer": "Neutral", "rationale": "Since the entry does not express positive or negative attitudes, feelings or behaviour, it merely presents a fact."}, "language_style": {"answer": "Tactful and warm", "rationale": "The entry is more personal and interactive in its expression, which is conducted in a gentle and inviting manner, rather than in direct statements or orders. At the same time, it implies a more euphemism in the language of the interviewee鈥檚 inquiry as to whether he or she can maintain a satisfactory relationship with the love."}, "time_dimension": {"answer": "Present", "rationale": "The entry describes the current situation and attitude of the interviewee, using the current state of \"maintained\" and expresses the current state of behaviour or state. The keyword \"satisfactory love\" suggests that this is a question of emotional and interpersonal relationships. It is therefore a question of whether the interviewee has difficulty or experience in maintaining a satisfactory relationship, and the time dimension is now."}, "sentence_type": "Affirmative declarative sentence", "pos": {"nouns": {"Love relationships": 1, "Difficulties": 1}, "verbs": {"Maintenance": 1}, "adjectives": {"Satisfied": 1}, "adverbs": {"No, I'm not": 1}, "numerals": {"4": 1}}}, {"content": "5. I am happier than I have been for weeks.", "word_count": 11, "label": "", "sentence_structure": "Subject鈥揷opula鈥損redicative", "subjective_objective": {"answer": "Subjective", "rationale": "Because this sentence relates to the patient ' s own emotional state and changes, it examines the patient ' s subjective feelings."}, "sentence_sentiment": {"answer": "Positive", "rationale": "Because it expresses a positive emotional attitude, i.e., feeling happier."}, "language_style": {"answer": "Tactful and warm", "rationale": "The language style of the entry is more euphemistic. Instead of asking the interviewee directly about his emotional state, it uses a more moderate and inviting expression, such as \"I'm happier than I have been for weeks,\" which is more friendly and natural, and is easy to accept and answer questions."}, "time_dimension": {"answer": "Present", "rationale": "The keyword \"I'm a few weeks more than I am\" means the state of things and the period of time, so it's \"now\"."}, "sentence_type": "Affirmative declarative sentence", "pos": {"verbs": {"Right now": 1, "- Yeah": 1, "More": 1}, "adjectives": {"Happy": 1}, "adverbs": {"Come on": 1, "Yes": 1, "of": 1}, "numerals": {"How many": 1, "Week": 1}, "pronouns": {"I": 1}}}, {"content": "I am satisfied with my physical appearance.", "word_count": 13, "label": "", "sentence_structure": "Subject鈥揷opula鈥損redicative", "subjective_objective": {"answer": "Subjective", "rationale": "It is the patient ' s subjective assessment of his physical appearance."}, "sentence_sentiment": {"answer": "Positive", "rationale": "Because the attitude of \"satisfaction with your physical appearance\" in the entry is positive."}, "language_style": {"answer": "Tactful and warm", "rationale": ""}, "time_dimension": {"answer": "Present", "rationale": "The keyword \"satisfaction with his physical appearance\" indicates the current subjective perception and state of the subject, and thus the judgement is now."}, "sentence_type": "Affirmative declarative sentence", "pos": {"nouns": {"self": 1, "Body": 1, "Looks": 1}, "verbs": {"I feel": 1, "Satisfied": 1}}}, {"content": "I sometimes do not go to ball and non-formal sports because I do not think I will search for it for long.", "word_count": 30, "label": "", "sentence_structure": "Subject鈥搗erb鈥搊bject", "subjective_objective": {"answer": "Subjective", "rationale": "\"Assumption\" is patient's subjective attitude."}, "sentence_sentiment": {"answer": "Negative", "rationale": "Because the entry \"No more ball and non-formal sports\" is negative."}, "language_style": {"answer": "Calm and direct", "rationale": "The language style of the entry is more calm and straightforward, with no excessive rhetoric or emotional expression, but simply presents a fact and point of view. Such style is usually more appropriate for such a more rigorous application of the questionnaire."}, "time_dimension": {"answer": "Present", "rationale": "The keyword \"sometimes without participating\" and \"thinking that he's not good at it\" means that it's the person being tested for the act and the idea."}, "sentence_type": "Negative declarative sentence", "pos": {"nouns": {"Activities": 2, "Balls": 1, "Sports": 1, "self": 1}, "verbs": {"Participation": 1, "I think": 1, "to": 1, "No, no, no, no, no, no, no, no, no, no": 1}, "adjectives": {"Sometimes": 1, "Informal": 1}, "numerals": {"7": 1}, "pronouns": {"I": 1}}}, {"content": "8. Public statements make me uncomfortable.", "word_count": 10, "label": "", "sentence_structure": "Subject鈥揷opula鈥損redicative", "subjective_objective": {"answer": "Subjective", "rationale": "Because \"uncomfort\" is a subjective feeling, and it's a personal view of the person who speaks in public."}, "sentence_sentiment": {"answer": "Negative", "rationale": "Because the emotional reaction of \"uncomfortable\" in the entry is negative."}, "language_style": {"answer": "Tactful and warm", "rationale": "The language style of the entry is more euphemistic, and it asks the interviewee how he feels about speaking in public, rather than directly ordering or criticizing."}, "time_dimension": {"answer": "Present", "rationale": "The entry describes the current feelings and experiences of the testee, and the expression \"I'm not comfortable\" suggests that it is a subjective feeling or emotional state, and therefore it is now judged."}, "sentence_type": "Negative declarative sentence", "pos": {"nouns": {"Public speaking": 1, "I": 1, "I'm not feeling well": 1}, "verbs": {"鈾?And I'm gonna be 鈾?: 1}}}, {"content": "I am willing to meet more people, but I am not willing to go out and meet them.", "word_count": 22, "label": "", "sentence_structure": "Subject鈥搗erb鈥搊bject", "subjective_objective": {"answer": "Subjective", "rationale": "Because the idea of \"willing to know more people\" and \"willing not go out and meet them\" is the patient's subjective attitude."}, "sentence_sentiment": {"answer": "Neutral", "rationale": "This item is an expression of a person ' s willingness to expand the social circle and his concern to go out and meet with others at the same time, but does not show a positive or negative emotional profile."}, "language_style": {"answer": "Tactful and warm", "rationale": "The language style of the entry is euphemistic, asking questions in an invitation-style manner, encouraging the respondents to express their thoughts and feelings. At the same time, the use of the \"no...\" format expresses a contradictory view, making the questionnaire more open and inclusive and more able to guide the respondents to reflect and express themselves."}, "time_dimension": {"answer": "Present", "rationale": "\"I'm willing to know more people\" expresses a desire or desire of the testees, and what happens in the future, and \"I'm not willing to go out and meet them,\" and what happens in the current situation or behaviour of the testees, is what it is. So the entry contains both visions of the future and current behaviour, and it is judged to be present and future."}, "sentence_type": "Affirmative declarative sentence", "pos": {"nouns": {"Questionnaire Entry": 1, "person": 2, "Meet me": 1}, "verbs": {"May I": 2, "Recognition": 1, "Out": 1, "and": 1, "See": 1}, "adjectives": {"More": 1, "External": 1, "Same": 1}, "adverbs": {"More": 1, "And": 1}, "numerals": {"9": 1}, "pronouns": {"I": 1}}}, {"content": "10. Sport and physical education are among my long searches.", "word_count": 11, "label": "", "sentence_structure": "Subject鈥揷opula鈥損redicative", "subjective_objective": {"answer": "Subjective", "rationale": "Because the desire to sport is the personal feeling and perception of the patient, it is a subjective examination."}, "sentence_sentiment": {"answer": "Positive", "rationale": "Because the entry \"Sport is one of my searchers\" expresses a positive attitude and ability of the individual."}, "language_style": {"answer": "Tactful and warm", "rationale": "The entry uses euphemism to describe the fact that the interviewee is good at sports and avoids being too direct or flaunted."}, "time_dimension": {"answer": "Present", "rationale": "The entry is for the purpose of asking the testee about the degree of preferences and good practices of sport, and does not clearly indicate the time point of sexual orientation, and therefore the judgement is now."}, "sentence_type": "Affirmative declarative sentence", "pos": {"nouns": {"Sports": 1, "Chief": 1}, "verbs": {"be": 1}, "adjectives": {"Mine": 1}, "numerals": {"Ten": 1}, "pronouns": {"I": 1}}}, {"content": "11. Academic performance is one aspect of showing my ability to make my achievements known.", "word_count": 25, "label": "", "sentence_structure": "Subject鈥揷opula鈥損redicative", "subjective_objective": {"answer": "Subjective", "rationale": "Because the sentence expresses the patient ' s perception and evaluation of his or her academic performance, it is subjective."}, "sentence_sentiment": {"answer": "Positive", "rationale": "Because the entries \"students\" and \"shows my abilities\" and \"gets people to know my grades\" are positive expressions."}, "language_style": {"answer": "Calm and direct", "rationale": "The language style of the entry is calm and straightforward. It clearly sets out the view that academic performance is one of the criteria for measuring individual abilities and achievement. It is not expressed in any qualifier or emotionally coloured language, but rather in simple, objective language. The entry can therefore be classified as \"cool and direct.\""}, "time_dimension": {"answer": "Present", "rationale": "The entry will express visions and expectations for the future, and the keyword \"the future\" will mean what is not yet happening and what may be possible in the future. At the same time, the descriptions of \"academic performance\" and \"advising\" will also imply that the testee may have the motivation and willingness to improve his or her academic performance in the future."}, "sentence_type": "Affirmative declarative sentence", "pos": {"nouns": {"Performance of studies": 1, "Capacity": 1, "others": 1, "Achievements": 1, "In terms of": 1}, "verbs": {"Show": 1, "Jean": 1, "Yes": 1}, "adverbs": {"One": 1}, "numerals": {"11": 1}, "pronouns": {"Mine": 2, "I": 1}}}, {"content": "I'm better-looking than the average person.", "word_count": 9, "label": "", "sentence_structure": "Subject鈥揷opula鈥損redicative", "subjective_objective": {"answer": "Subjective", "rationale": "Because the \"good\" standard varies from person to person and there is a self-awareness and evaluation of the individual, this is a subjective approach."}, "sentence_sentiment": {"answer": "Neutral", "rationale": "Because the entry does not have any emotional color."}, "language_style": {"answer": "Calm and direct", "rationale": "\"Calm down straight.\""}, "time_dimension": {"answer": "Present", "rationale": "\"Be good-looking\" is a self-evaluation, which is based on subjective perceptions and judgements of the external appearance of the individual. So, the expression \"I look better than the average person\" is a description of the self-perception and attitude that exists today."}, "sentence_type": "Affirmative declarative sentence", "pos": {"verbs": {"- Yeah": 1, "- He's a big boy": 1}, "adjectives": {"General": 1, "Nice": 1}, "numerals": {"12": 1}, "pronouns": {"I": 1, "Normal": 1}}}, {"content": "13. I am afraid to think about the performance of programmes and speeches in public.", "word_count": 17, "label": "", "sentence_structure": "Subject鈥搗erb鈥搊bject", "subjective_objective": {"answer": "Subjective", "rationale": "Because \"never mind\" expresses the patient's inner feelings and attitudes, and it's subjective."}, "sentence_sentiment": {"answer": "Negative", "rationale": "Because the expression \"never dared to think\" in the entry is full of negative emotions."}, "language_style": {"answer": "Tactful and warm", "rationale": "The entry uses the expression \"I can't even think about it\" and emphasizes the sense of fear among respondents of performing in public, without being too direct or harsh language styles, reflecting a kind and understanding attitude."}, "time_dimension": {"answer": "Present", "rationale": ""}, "sentence_type": "Negative declarative sentence", "pos": {"nouns": {"Public": 1, "Programs": 1, "Speech": 1}, "verbs": {"Play": 1, "Speak": 1}, "adverbs": {"I'm afraid": 1}, "numerals": {"13": 1}}}, {"content": "When I think of most sports activities, I am filled with passion and desire, not fear and anxiety.", "word_count": 27, "label": "", "sentence_structure": "Subject鈥揷opula鈥損redicative", "subjective_objective": {"answer": "Subjective", "rationale": "This positive emotional reaction is the feeling and attitude of the patient inside and is subjective."}, "sentence_sentiment": {"answer": "Positive", "rationale": "Because the expression \"sent and eager\" is positive and indicates a positive attitude towards sports activities."}, "language_style": {"answer": "Tactful and warm", "rationale": "The entry uses a gentle and encouraging tone of questioning, without directly questioning or accusing the person under investigation. It attempts to create a friendly and positive atmosphere in which the person under investigation is more willing to express his thoughts and feelings in a genuine way. This language is more appropriate for psychological questionnaire research and can help build good mutual trust."}, "time_dimension": {"answer": "Present", "rationale": ""}, "sentence_type": "Affirmative declarative sentence", "pos": {"nouns": {"Sports activities": 1, "鈾?Hot": 1, "Desire": 1, "Fear": 1, "Anxiety": 1}, "verbs": {"I thought": 1, "Fill": 1}, "adjectives": {"Most of them": 1}, "numerals": {"14": 1}}}, {"content": "15. Even in those occasions where I have been able to cope well in the past, I have often been uncertain about myself.", "word_count": 29, "label": "", "sentence_structure": "Subject鈥搗erb鈥揷omplement", "subjective_objective": {"answer": "Subjective", "rationale": "This is a subjective attitude of patients who feel self-inflicted and lack confidence and uncertainty."}, "sentence_sentiment": {"answer": "Negative", "rationale": "Because the expression \"un certain\" in the entry is negative."}, "language_style": {"answer": "Tactful and warm", "rationale": ""}, "time_dimension": {"answer": "Present", "rationale": ""}, "sentence_type": "Negative declarative sentence", "pos": {"nouns": {"Questionnaire Entry": 1, "Event": 2, "self": 2}, "verbs": {"- Where": 1, "Payable": 1, "Regular": 1, "have": 1}, "adjectives": {"Okay": 2, "Those": 1, "Tsang": 1}, "adverbs": {"Still": 1, "Often": 1}, "numerals": {"15": 1, "2": 3}, "pronouns": {"I": 2}}}, {"content": "I often doubt that I have the talent to successfully achieve my professional and professional goals.", "word_count": 28, "label": "", "sentence_structure": "Subject鈥搗erb鈥搊bject", "subjective_objective": {"answer": "Subjective", "rationale": "Because suspicion is a subjective assessment of the patient ' s ability."}, "sentence_sentiment": {"answer": "Negative", "rationale": "Because the entry expresses a self-suspicion, which is negative."}, "language_style": {"answer": "Tactful and warm", "rationale": "The language style of the entry is euphemismal. Instead of directly asking the person interviewed about his or her talents or abilities, it uses an implicit and encouraging way to allow the person to think about himself and express his or her feelings. This expression reduces the pressure on the person interviewed and makes them more willing to participate in the questionnaire and share real ideas and feelings."}, "time_dimension": {"answer": "Past", "rationale": "\"At times\" is a constant act, and \"suspicion\" is an act of reflection or retroactivity of the past. The temporal dimension of this article is therefore the past."}, "sentence_type": "Interrogative sentence", "pos": {"nouns": {"self": 1, "Natural talent": 1, "Success": 1, "Achieved": 1, "Occupation": 1, "Professional objectives": 2}, "verbs": {"Doubt": 1, "have": 1, "Yes": 1, "Do it": 1}, "adverbs": {"Regular": 1}, "numerals": {"16": 1}, "pronouns": {"Here": 1}}}, {"content": "17. I am better at sports than most people of the same age and sex.", "word_count": 20, "label": "", "sentence_structure": "Subject鈥揷opula鈥損redicative", "subjective_objective": {"answer": "Subjective", "rationale": "Because \"sports\" is a self-evaluation and comparison of an individual, a subjective feeling of the patient."}, "sentence_sentiment": {"answer": "Positive", "rationale": "Because the expression \"most people of the same age and sex are better at sports\" expresses a self-assertion and self-confidence, with positive emotional aspects."}, "language_style": {"answer": "Calm and direct", "rationale": "The language style of the entry is relatively simple and the interviewee is asked whether he/she considers himself better than his/her peers in sports, and does not have too many emotional or rhetorical styles."}, "time_dimension": {"answer": "Present", "rationale": "The keyword \"I\" is a reference to the ability of the testee to compare his abilities and to the current state of the art, which is a description of the current situation. So, the time dimension of this entry is now."}, "sentence_type": "Affirmative declarative sentence", "pos": {"nouns": {"Questionnaire Entry": 1, "person": 2, "Sports": 1, "Age": 1, "Gender": 1}, "verbs": {"- Yeah": 1, "Good at it": 1, "Same": 1, "More": 1, "Do it": 1}, "adjectives": {"Mostly": 1}, "adverbs": {"Both": 1}, "numerals": {"17": 1, "2": 1}, "pronouns": {"I": 2, "Most people of the same age, sex": 1}}}, {"content": "18. I lack some of the key competencies that have made me successful.", "word_count": 14, "label": "", "sentence_structure": "Subject鈥揷opula鈥損redicative", "subjective_objective": {"answer": "Subjective", "rationale": "Because \"some important competencies\" are a subjective criterion of evaluation, which depends on the values and objectives of the individual. Everyone has his or her own perspective and perspective on what skills are important and what these importance is assessed as subjective."}, "sentence_sentiment": {"answer": "Negative", "rationale": "Because the phrase \"lack of some important abilities that make me successful\" in the entry expresses negative emotions and attitudes."}, "language_style": {"answer": "Tactful and warm", "rationale": "The language style of the entry is a soft and warm one. It asks the interviewee whether he lacks the skills needed to succeed, rather than pointing out the problem directly or exerting pressure. This expression is more likely to generate resonance and positive feedback from the other side and helps to create a good investigation atmosphere."}, "time_dimension": {"answer": "Present", "rationale": ""}, "sentence_type": "Interrogative sentence", "pos": {"nouns": {"18": 1, "Success": 1, "Capacity": 1}, "verbs": {"Missing": 1, "鈾?And I'm gonna be 鈾?: 1}, "adverbs": {"EMPTY": 3}, "numerals": {"18": 1}, "classifiers": {"Some": 1}, "pronouns": {"EMPTY": 1}}}, {"content": "When I speak in public, I am often confident that I will express my views clearly and effectively.", "word_count": 27, "label": "", "sentence_structure": "Subject鈥揷opula鈥損redicative", "subjective_objective": {"answer": "Subjective", "rationale": "The question is addressed from a subjective perspective, as it is about the individual's feelings and experience. The question is whether people can express their thoughts with confidence in public statements. It is a question of personal feelings and beliefs that cannot be answered by objective facts alone."}, "sentence_sentiment": {"answer": "Positive", "rationale": "Because the behavior in the entry \"show your views clearly and effectively\" is positive."}, "language_style": {"answer": "Tactful and warm", "rationale": "The entry is more personal and interactive when it is spoken in a gentle and inviting manner, rather than directly speaking or ordering. At the same time, the entry does not impose too many demands or pressures on the respondent, but rather creates a dialogue by asking about the other side鈥檚 feelings, giving one a sense of respect and understanding."}, "time_dimension": {"answer": "Present", "rationale": ""}, "sentence_type": "Affirmative declarative sentence", "pos": {"nouns": {"Questionnaire Entry": 1, "person": 1, "Public speaking": 1, "Hold on": 1, "self": 1, "Observations": 1}, "verbs": {"Often": 1, "Do it": 1, "Clear": 1, "Effective": 1, "Expression": 1}, "adjectives": {"It works": 1, "Clear": 1}, "adverbs": {"Often": 1}, "classifiers": {"One": 2}, "pronouns": {"I": 1}}}, {"content": "I am so glad I'm pretty.", "word_count": 10, "label": "", "sentence_structure": "Subject鈥揷opula鈥損redicative", "subjective_objective": {"answer": "Subjective", "rationale": "Because \"happiness\" and \"pretty\" are subjective feelings and evaluations of patients."}, "sentence_sentiment": {"answer": "Positive", "rationale": "Because the \"grateful\" in the entry is a positive emotional attitude."}, "language_style": {"answer": "Tactful and warm", "rationale": "The words of the entry are more euphemistic. It uses the word \"grateful\" to express a positive and self-affirming feeling. Moreover, \"pretty\" is a measure of praise for its physical features, but it is not overemphasis or boasting, but rather in a more moderate way."}, "time_dimension": {"answer": "Present", "rationale": "The keywords are \"I am so glad\" and \"pretty\" and they express self-evaluation and feelings of the subjects, both current and immediate emotional states, and the issue is therefore an investigation of current behaviour or mentality."}, "sentence_type": "Exclamatory sentence", "pos": {"nouns": {"I": 1, "I'm glad": 1}, "verbs": {"Long": 1, "Okay": 1, "Nice": 1}, "adjectives": {"self": 1, "Really": 1, "Nice": 1}, "adverbs": {"True": 1}}}, {"content": "I've realized that I'm not a good student compared to most of the people I compete with.", "word_count": 26, "label": "", "sentence_structure": "Subject鈥揷opula鈥損redicative", "subjective_objective": {"answer": "Subjective", "rationale": "This is a subjective assessment of the patient ' s ability to learn."}, "sentence_sentiment": {"answer": "Negative", "rationale": "Because the expression \"I'm not a good student\" in the entry is a negative assessment with negative emotions."}, "language_style": {"answer": "Calm and direct", "rationale": "The language style of the entry is calm and straightforward, as it directly expresses the interviewee鈥檚 awareness and assessment of his academic performance. While this assessment may be negative, it does not have too much emotional or rhetorical accommodation."}, "time_dimension": {"answer": "Present", "rationale": "The keyword \"aware\" indicates that it is a description of the past, while \"good student\" is a state of judgement, so the entry is primarily about the present self-awareness and attitude of the testee."}, "sentence_type": "Negative declarative sentence", "pos": {"nouns": {"Questionnaire Entry": 1, "person": 2, "Competition": 1, "Students": 1}, "verbs": {"Already": 1, "Awareness": 1, "be": 1, "- Yeah": 1, "Competition": 1, "Learn": 1}, "adjectives": {"Okay": 1}, "adverbs": {"Mostly": 1, "individual": 1}, "numerals": {"21": 1}, "pronouns": {"I": 1}}}, {"content": "22. In recent days, I have been more dissatisfied than ever with myself.", "word_count": 19, "label": "", "sentence_structure": "Subject鈥揷opula鈥損redicative", "subjective_objective": {"answer": "Subjective", "rationale": "Because \"unsatisfied with the place\" is the patient's own feelings and evaluations, it's subjective."}, "sentence_sentiment": {"answer": "Negative", "rationale": "Because the word \"unsatisfactory\" in the entry expresses a negative attitude."}, "language_style": {"answer": "Tactful and warm", "rationale": "The entry is more softly phrased, not too direct or intensely chosen, but rather in a more peaceful way to guide respondents to share their feelings. At the same time, the question is asked more about the emotional state and self-perception of individuals, and is more likely to receive in-depth and real answers."}, "time_dimension": {"answer": "Present", "rationale": "\"Recently\" in \"the last few days\" means behaviour in the past, and \"dissatisfaction\" may have occurred in this time, so the sentence describes a feeling now."}, "sentence_type": "Exclamatory sentence", "pos": {"nouns": {"Oh, my God": 1, "self": 1}, "verbs": {"Satisfied": 1, "Local": 1, "More": 1}, "adjectives": {"Most recently": 1, "Dissatisfied": 1}, "adverbs": {"More than ever": 1}, "numerals": {"22": 1}, "classifiers": {"individual": 1}, "pronouns": {"I": 1}}}, {"content": "23. The lack of long searches for sports is a major drawback for me.", "word_count": 18, "label": "", "sentence_structure": "Subject鈥揷opula鈥損redicative", "subjective_objective": {"answer": "Subjective", "rationale": "Because \"sport is not good\" is a personal perception and self-evaluation, and is a subjective attitude of patients."}, "sentence_sentiment": {"answer": "Negative", "rationale": "Because the word \"defect\" in the entry is negative."}, "language_style": {"answer": "Calm and direct", "rationale": "\" I think its language style is calm and straightforward. The entry presents an objective fact in a concise and clear way, that the interviewees are not good at sports, are not too emotional or are not used in rhetoric. This expression conveys both a clear message and avoids emotional effects that may cause discomfort."}, "time_dimension": {"answer": "Past", "rationale": ""}, "sentence_type": "Negative declarative sentence", "pos": {"nouns": {"Sports": 1, "Disadvantages": 1}, "verbs": {"Not good at it": 1, "be": 1, "Chief": 1}, "adjectives": {"It's big": 1}, "numerals": {"23": 1}, "pronouns": {"I": 1}}}, {"content": "24. For me, it is a pleasure to have a new friend.", "word_count": 21, "label": "", "sentence_structure": "Subject鈥搗erb鈥搊bject", "subjective_objective": {"answer": "Subjective", "rationale": "Because \"hope\" and \"favorite\" are patients' subjective thoughts and feelings."}, "sentence_sentiment": {"answer": "Positive", "rationale": "Because the phrase \"to meet a new friend is the pleasure I want\" in the entry is positive emotional."}, "language_style": {"answer": "Tactful and warm", "rationale": "The entry is more personal and interactive when it is spoken in a gentle and inviting tone, rather than in direct statements or orders. At the same time, the \"happy feeling I want\" in the entry implies concern and respect for each other's emotional experience."}, "time_dimension": {"answer": "Present", "rationale": "The entry expresses the person's mental state and expectations, and the keyword \"is\" and \"is\" points to the current situation."}, "sentence_type": "Affirmative declarative sentence", "pos": {"nouns": {"I": 1, "friends": 1, "Feel": 1}, "verbs": {"be": 1, "Hope": 1, "I'm meeting someone": 1, "Have a nice day": 1}, "adjectives": {"New": 1, "Happy": 2}, "numerals": {"24": 1}, "classifiers": {"individual": 1}, "pronouns": {"I": 1}}}, {"content": "25. Many times I feel less capable than many around me.", "word_count": 21, "label": "", "sentence_structure": "Subject鈥搗erb鈥搊bject", "subjective_objective": {"answer": "Subjective", "rationale": "Because \"showing yourself like many people around\" is a subjective feeling and self-evaluation of the patient. It is a subjective question of examining the patient's perception and attitude to his or her abilities."}, "sentence_sentiment": {"answer": "Negative", "rationale": "Because the entry \"not as talented as many around you\" expresses a negative and disgruntled feeling about yourself, and it is a negative feeling."}, "language_style": {"answer": "Calm and direct", "rationale": "The entry directly expresses the subject of questioning the other party about self-denial or inferiority, does not use excessive rhetoric or emotional rendering, and is more objective in tone."}, "time_dimension": {"answer": "Present", "rationale": ""}, "sentence_type": "Negative declarative sentence", "pos": {"nouns": {"Time": 1, "person": 2, "self": 1, "It's a thing": 1}, "verbs": {"I feel": 1, "Yeah": 1, "have": 1}, "adjectives": {"Many": 1}, "adverbs": {"That way": 1}, "numerals": {"25": 1}, "pronouns": {"self": 1}}}, {"content": "26. I have almost never felt comfortable at parties or other social gatherings.", "word_count": 22, "label": "", "sentence_structure": "Subject鈥搗erb鈥揷omplement", "subjective_objective": {"answer": "Subjective", "rationale": "Because \"almost never feeling uncomfortable\" is the patient's subjective feeling and attitude."}, "sentence_sentiment": {"answer": "Positive", "rationale": "It is a positive experience that the entry indicates that the author has hardly been comfortable at the party."}, "language_style": {"answer": "Tactful and warm", "rationale": "The entry is more adept and interactive when it is spoken in a gentle and inviting manner, rather than by direct statements or orders."}, "time_dimension": {"answer": "Present", "rationale": ""}, "sentence_type": "Affirmative declarative sentence", "pos": {"nouns": {"Party": 1, "Social parties": 1}, "verbs": {"Never": 1, "I feel": 1, "I'm not feeling well": 1}, "adverbs": {"Almost": 1}, "numerals": {"26": 1}}}, {"content": "27. I doubt my abilities less than most people.", "word_count": 17, "label": "", "sentence_structure": "Subject鈥揷opula鈥損redicative", "subjective_objective": {"answer": "Subjective", "rationale": "Because \"suspecting your abilities\" is a subjective idea or feeling, related to patient self-evaluation."}, "sentence_sentiment": {"answer": "Positive", "rationale": "Because the expression \"less doubting your abilities\" in the entry is positive, expressing confidence and motivation."}, "language_style": {"answer": "Tactful and warm", "rationale": "This entry uses a more euphemistic approach to asking the interviewee about the level of confidence in himself, without directly using a positive or negative tone."}, "time_dimension": {"answer": "Present", "rationale": "The entry asks about the testee's perception and assessment of his/her self-capacity and confidence, and the temporal dimension does not clearly point to the past, present or future. Therefore, it cannot be described as \"the past\", \"now\" or \"the future\"."}, "sentence_type": "Affirmative declarative sentence", "pos": {"nouns": {"self": 1, "Capacity": 1}, "verbs": {"Doubt": 1, "- Yeah": 1, "Come on": 1}, "adjectives": {"Mostly": 1, "Less": 1, "More": 1}, "numerals": {"27": 1}, "classifiers": {"individual": 1}, "pronouns": {"I": 1}}}, {"content": "28. I have had more difficulties in building relationships than most people.", "word_count": 18, "label": "", "sentence_structure": "Subject鈥揷opula鈥損redicative", "subjective_objective": {"answer": "Subjective", "rationale": "This is because patients are assessed and perceived as they are subjective."}, "sentence_sentiment": {"answer": "Negative", "rationale": "Because the entry is about the difficulty of establishing a relationship of love, which has a certain negative emotional connotation."}, "language_style": {"answer": "Calm and direct", "rationale": "The entry expresses in a direct, concise language the challenge of the person under investigation in establishing a relationship of love, without too much ado or emotional rendering, and is more objective and neutral in tone.\""}, "time_dimension": {"answer": "Present", "rationale": "\"In building a relationship of love\" indicates that the behaviour is currently being performed, and \"more difficult than most people\" expresses a subjective feeling or evaluation, both of which point to the current situation. Thus, the temporal dimension of the article is now."}, "sentence_type": "Negative declarative sentence", "pos": {"nouns": {"Love relationships": 1, "person": 1}, "verbs": {"Create": 1, "Difficulties": 1}, "adjectives": {"Most of them": 1, "More": 1}, "numerals": {"28": 1}, "pronouns": {"I": 1}}}, {"content": "Today I'm more uncertain than I normally am.", "word_count": 16, "label": "", "sentence_structure": "Subject鈥揷opula鈥損redicative", "subjective_objective": {"answer": "Subjective", "rationale": "Because the assessment of one ' s abilities is the patient ' s subjective idea."}, "sentence_sentiment": {"answer": "Negative", "rationale": "Because the description \"more uncertain about his abilities\" in the entry is negative and expresses mistrust and suspicion about his abilities."}, "language_style": {"answer": "Calm and direct", "rationale": "This expression is more capable of conveying the information that the surveyer would like to obtain, while also making it easier for the respondents to understand the implications of the problem."}, "time_dimension": {"answer": "Present", "rationale": "\"Today\" in the entry indicates time constraints, implying that it is a description of the current state of feeling and state. \"I am more uncertain about my abilities\" is a subjective feeling or belief, and it is in the present state. So the answer is \"Now.\""}, "sentence_type": "Negative declarative sentence", "pos": {"nouns": {"Today": 1, "I": 1, "Normal": 1, "self": 1, "Capacity": 1}, "verbs": {"- Yeah": 1, "have": 1}, "adjectives": {"Normal": 1, "I don't know": 1}, "numerals": {"29": 1}, "pronouns": {"I": 1}}}, {"content": "30. I am troubled by the fact that I am not intellectually comparable to others.", "word_count": 17, "label": "", "sentence_structure": "Subject鈥揷opula鈥損redicative", "subjective_objective": {"answer": "Subjective", "rationale": "This is a subjective examination of patients ' perception and evaluation of themselves."}, "sentence_sentiment": {"answer": "Negative", "rationale": "Because the expression \"incompetently as intellectually as others\" in the entry is a negative self-evaluation."}, "language_style": {"answer": "Calm and direct", "rationale": "The language style of the entry is relatively straightforward, and it is not too much rhetorical or emotional to express the question in an objective and clear way, allowing the interviewee to understand the meaning and purpose of the question more clearly. At the same time, it is more likely to trigger the interviewee's reflection and answers.\""}, "time_dimension": {"answer": "Present", "rationale": "The entry is about the person's current thoughts and feelings, and the keyword is \"I'm upset with it,\" which shows the person's current emotional state. So the judgment is based on the present."}, "sentence_type": "Negative declarative sentence", "pos": {"nouns": {"Intellectual": 1, "person": 2, "Trouble": 1}, "verbs": {"be": 1, "Not as good as it is": 1}, "adverbs": {"I'm": 1, "Other": 1}, "numerals": {"30": 1}, "pronouns": {"I": 1}}}, {"content": "When things get worse, I usually believe I can handle them properly.", "word_count": 23, "label": "", "sentence_structure": "Subject鈥揷opula鈥損redicative", "subjective_objective": {"answer": "Subjective", "rationale": "Because \"believing in themselves to handle them properly\" is the patient's personal conviction and attitude, and is subjective."}, "sentence_sentiment": {"answer": "Positive", "rationale": "Because the \"convinced that you can handle them properly\" attitude in the entry is positive."}, "language_style": {"answer": "Calm and direct", "rationale": "The entry is brief and clear in asking the respondents about their attitudes and beliefs in the face of difficulties, without too many rhetorical or emotional renderings, and with a more calm and objective language style.\""}, "time_dimension": {"answer": "Present", "rationale": "In the entry, \"usually\" denotes regular, customary behaviour, and \"be sure that you can handle them properly\" is a current attitude or belief, so it is possible to judge the temporal dimension of the article as it is."}, "sentence_type": "Affirmative declarative sentence", "pos": {"nouns": {"Things": 1, "Shit": 1, "Yes": 1, "self": 1, "Processing": 1}, "verbs": {"鈾?And become 鈾?: 1, "Usually": 1, "Yes": 1, "It's a good thing": 1, "Processing": 1}, "adjectives": {"Shit": 1}, "adverbs": {"Usually": 1, "Well done": 1}, "numerals": {"31": 1}, "pronouns": {"self": 1}}}, {"content": "32. I am more concerned than most about my ability to speak in public.", "word_count": 22, "label": "", "sentence_structure": "Subject鈥搗erb鈥揷omplement", "subjective_objective": {"answer": "Subjective", "rationale": "Because \"more fear\" is a subjective feeling or attitude that reflects a patient's self-evaluation of his ability to speak in public."}, "sentence_sentiment": {"answer": "Negative", "rationale": "Because the \"more worried\" attitude in the entries is negative."}, "language_style": {"answer": "Tactful and warm", "rationale": "The language style of the entry is more euphemistic. By using the comparison, \"I am more worried than most people\" the respondents are more receptive and willing to answer the question. Nor is it too direct or negative to use the phrase, which makes people feel more friendly and friendly."}, "time_dimension": {"answer": "Present", "rationale": ""}, "sentence_type": "Affirmative declarative sentence", "pos": {"nouns": {"Capacity": 1, "Most of them": 1, "person": 1, "Speech": 1}, "verbs": {"I'm worried": 1, "in": 1, "self": 1, "- Yeah": 1, "More": 1}, "adjectives": {"Public": 1, "Event": 1}, "adverbs": {"- Yeah": 1}, "numerals": {"32": 1, "Most of them": 1}, "pronouns": {"I": 1}}}, {"content": "I am more confident than most of the people I know.", "word_count": 12, "label": "", "sentence_structure": "Subject鈥揷opula鈥損redicative", "subjective_objective": {"answer": "Subjective", "rationale": "Because confidence is a subjective feeling, not an objective fact. Although the phrase refers to \"most people,\" it is still a description of what patients feel about themselves."}, "sentence_sentiment": {"answer": "Positive", "rationale": "Because the quality of \"more confident\" in the entry is positive."}, "language_style": {"answer": "Tactful and warm", "rationale": "The entry uses a comparative and adjective \"confidence\" to describe the situation of the interviewee, and is not directly expressing an affirmative or negative attitude, but is guided in a more moderate and encouraging manner, in keeping with the euphemism of the language."}, "time_dimension": {"answer": "Present", "rationale": "The entry indicates a current state of mind, where the person tested feels more confident than others. Since the sentence does not mention past or future points, the temporal dimension is now judged."}, "sentence_type": "Affirmative declarative sentence", "pos": {"nouns": {"Questionnaire": 1, "Entry": 1, "person": 2, "Most people": 1}, "verbs": {"- Yeah": 1, "Yes": 1, "More": 1, "Confidence": 1}, "adjectives": {"More": 1}, "adverbs": {"Most": 1}, "numerals": {"33": 1}, "pronouns": {"I": 1}}}, {"content": "34. When I considered continuing my date, I was nervous or uncertain.", "word_count": 18, "label": "", "sentence_structure": "Subject鈥揷opula鈥損redicative", "subjective_objective": {"answer": "Subjective", "rationale": "Because \"I'm nervous or unsure\" is the patient's subjective emotions and feelings."}, "sentence_sentiment": {"answer": "Negative", "rationale": "Because the emotional attitude of \"frightful or uncertain\" in the entries is negative."}, "language_style": {"answer": "Calm and direct", "rationale": "The language style of the entry is more calm and objective, and there is no excessive emotional rendering or rhetorical technique to express the subject matter in a direct way. At the same time, the terms \"stress\" and \"uncertainty\" make the issue more straightforward and clear."}, "time_dimension": {"answer": "Present", "rationale": "The entry describes the current mindset and feelings of the testee, the key words being \"I think\" and \"I feel\" , which point to the state of the current process. At the same time, \"when going on a date\" indicates that this feeling is related to a particular situation, i.e., when considering going on a date, it is tense or uncertain. Therefore, the time dimension of this questionnaire entry should be now."}, "sentence_type": "Affirmative declarative sentence", "pos": {"nouns": {"Date": 1, "Nervous": 1, "Hold on": 1}, "verbs": {"Consider": 1, "Go on": 1, "I feel": 1}, "adjectives": {"When": 1}, "adverbs": {"or": 1}, "numerals": {"34": 1}, "pronouns": {"I": 1}}}, {"content": "35. Most people may find my appearance unattractive.", "word_count": 18, "label": "", "sentence_structure": "Subject鈥揷opula鈥損redicative", "subjective_objective": {"answer": "Subjective", "rationale": "This is because patients have subjective knowledge and evaluation of the perception of others about their appearance."}, "sentence_sentiment": {"answer": "Negative", "rationale": "Because the word \"concealed that I'm not attractive\" in the entry is negative."}, "language_style": {"answer": "Calm and direct", "rationale": "The basis for the determination: the entry expresses the possible views of the other party in a direct statement, without any rhetorical or emotional rendering, and is more objective. Moreover, the expression \"most people may think\" also shows a common view, without a direct assessment of the individual ' s appearance. Thus, the language style of the article can be considered as being calm and straightforward."}, "time_dimension": {"answer": "Present", "rationale": "The entry asks the testee's opinion of the attraction of his or her appearance, which is a description of the current situation, and therefore is judged to be \"now\"."}, "sentence_type": "Negative declarative sentence", "pos": {"nouns": {"Most people": 1, "Appearance": 1, "Attractive": 1}, "verbs": {"I think": 1}, "adjectives": {"It's not attractive": 1}, "numerals": {"35": 1}, "pronouns": {"I": 1}}}, {"content": "When I take a new course, I usually can confirm that I am in the first quarter of my class at the end of the class.", "word_count": 29, "label": "", "sentence_structure": "Subject鈥搗erb鈥搊bject", "subjective_objective": {"answer": "Subjective", "rationale": "Since this questionnaire entry deals with self-assessment and expectations of patients, it examines subjective factors such as their self-confidence and self-awareness."}, "sentence_sentiment": {"answer": "Positive", "rationale": "Because the behavior in the entries is self-affirming and has a positive objective."}, "language_style": {"answer": "Tactful and warm", "rationale": "The language style of the entry is relatively moderate, and the respondents are guided by questions to self-evaluate their learning and performance, and is more acclaimable and acceptable."}, "time_dimension": {"answer": "Future", "rationale": "The entry expresses the vision and expectations of the testee for the future, and the keywords \"usually certain\" and \"at the end of the period in the first quarter of the class\" point to situations that have not yet occurred and that may arise in the future."}, "sentence_type": "Affirmative declarative sentence", "pos": {"nouns": {"Questionnaire Entry": 1, "New class": 1, "End": 1, "Achievements": 1, "Class": 1, "Front": 1, "A quarter": 1}, "verbs": {"Yeah": 2, "Sure": 1, "Learn": 1, "in": 1, "Internal": 1}, "adjectives": {"Usually": 1, "Different": 1, "Okay": 1}, "numerals": {"36": 1, "Four": 1}, "classifiers": {"Door": 1, "individual": 1}, "pronouns": {"I": 1}}}, {"content": "I am as capable as most of us of speaking in public.", "word_count": 15, "label": "", "sentence_structure": "Subject鈥搗erb鈥搊bject", "subjective_objective": {"answer": "Subjective", "rationale": "Because \"most people are just like able\" is a subjective view or judgment, not a description of objective facts."}, "sentence_sentiment": {"answer": "Positive", "rationale": "Because the entry expresses a sense of confidence and self-esteem, and the ability to speak in public is a positive emotional tendency."}, "language_style": {"answer": "Tactful and warm", "rationale": "The entry is more neutral in asking the respondents whether they are capable of speaking in public, are not subject to excessive judgement or emotional colours and do not direct the answer, and therefore the language style is more euphemism and moderation."}, "time_dimension": {"answer": "Present", "rationale": ""}, "sentence_type": "Affirmative declarative sentence", "pos": {"nouns": {"Capacity": 1, "person": 2, "Public speaking": 1}, "verbs": {"have": 1, "Speak": 1}, "adjectives": {"Most of them": 1}, "adverbs": {"Same thing": 1}, "numerals": {"37": 1}, "pronouns": {"I": 1, "they": 1}}}, {"content": "38. When I attend social gatherings, I often feel clumsy and uncomfortable.", "word_count": 19, "label": "", "sentence_structure": "Subject鈥揷opula鈥損redicative", "subjective_objective": {"answer": "Subjective", "rationale": "Because \"frightful and uncomfortable\" is a subjective feeling."}, "sentence_sentiment": {"answer": "Negative", "rationale": "Because the emotional attitude of feeling clumsy and uncomfortable in the entry is negative."}, "language_style": {"answer": "Tactful and warm", "rationale": "The language style of the entry is more euphemistic and warm, and expresses the discomfort of the other in social settings by using the word \"me\" and the descriptive term \"mute and uncomfortable.\" At the same time, the way in which questions are asked is more gentle and friendly."}, "time_dimension": {"answer": "Past", "rationale": "\"I\" in \"I'm at a social meeting\" indicates that it's a description of the person who was tested, \"at a social meeting\" means that it's a past behavior or state, \"often feeling\" means that it's a constant, customary feeling, \"very clumsy and uncomfortable\" means that it's a subjective feeling of the person who was tested, so the entry can be judged to describe the experience and feelings of the past."}, "sentence_type": "Negative declarative sentence", "pos": {"nouns": {"Social parties": 1, "Clumsy": 1, "I'm not comfortable": 1}, "verbs": {"Participation": 1, "I feel": 1}, "adjectives": {"very": 1}, "numerals": {"38": 1}}}, {"content": "39. Usually my love life seems to be better than most.", "word_count": 16, "label": "", "sentence_structure": "Subject鈥揷opula鈥損redicative", "subjective_objective": {"answer": "Subjective", "rationale": "Because \"better\" than most people is a patient's self-evaluation and subjective feeling."}, "sentence_sentiment": {"answer": "Positive", "rationale": "Because it says \"Love seems to be better than most people\" and has a positive emotional color."}, "language_style": {"answer": "Tactful and warm", "rationale": "The language style of the entry is euphemism, as it uses a positive and encouraging expression to ask each other about their love life. At the same time, it avoids negative or critical comments that may exist, making the person under investigation more comfortable and relaxed."}, "time_dimension": {"answer": "Present", "rationale": "The entry describes the current situation of the person being tested for love and uses a comparative \"better\" than most people, indicating that the situation is not past or future, but current. Thus, the time dimension of the article is now."}, "sentence_type": "Affirmative declarative sentence", "pos": {"nouns": {"Love life": 1, "Most people": 1}, "verbs": {"Seems like": 1, "- Yeah": 1, "Okay": 1}, "adjectives": {"Most of them": 1}, "numerals": {"39": 1}, "pronouns": {"I": 1}}}, {"content": "Sometimes I avoid classes or other things because I don't want to speak in public.", "word_count": 21, "label": "", "sentence_structure": "Subject鈥搗erb鈥搊bject", "subjective_objective": {"answer": "Subjective", "rationale": "This is seen from the patient's subjective point of view. The phrases \"sometimes\" and \"because they don't want to speak in public\" are all part of the patient's subjective feelings and ideas, and are therefore part of the subjective examination."}, "sentence_sentiment": {"answer": "Negative", "rationale": "Because the entries \"do not want to speak in public\" and \"leave from classes or do something else\" are negative."}, "language_style": {"answer": "Calm and direct", "rationale": "The entry asks the respondents in a simple and straightforward manner whether they have refrained from participating in certain activities because they were afraid to speak in public, and the language style is more objective and calm."}, "time_dimension": {"answer": "Present", "rationale": "The keyword \"sometimes\" indicates a customary situation, while the verb \"avoidance\" indicates that the act is taking place or has already taken place. Thus, the temporal dimension of the purpose of the article is now judged."}, "sentence_type": "Affirmative declarative sentence", "pos": {"nouns": {"Time": 1, "I": 2, "No, I don't": 1, "Public statements": 1, "Classes": 1, "Other things": 1}, "verbs": {"Avoidance": 1, "Go, go, go": 1, "Do it": 1}, "adverbs": {"Sometimes": 1}, "numerals": {"40": 1}}}, {"content": "41. When I have to pass a major exam or other professional assignment, I know I can do it.", "word_count": 26, "label": "", "sentence_structure": "Subject鈥揷opula鈥損redicative", "subjective_objective": {"answer": "Subjective", "rationale": "This is a patient ' s subjective attitude, as it is an assessment of his or her abilities and a manifestation of confidence in his or her abilities."}, "sentence_sentiment": {"answer": "Positive", "rationale": "Because the entries express confidence and faith in their abilities and are positive emotional attitudes."}, "language_style": {"answer": "Tactful and warm", "rationale": "The language style of the entry is euphemism. First, the questioner uses the structure of the \"Do you...\" query, which is more polite and respectful of the views of the interviewee and more likely to provoke a positive response from the other side; second, the expression \"I know I can do it\" has the effect of encouraging and stimulating the interviewee to feel accepted and supported, while also conveying an optimistic attitude."}, "time_dimension": {"answer": "Present", "rationale": "Now, in the entry, \"I know I can do it\" indicates that it is a present belief and attitude, so it is possible to judge the time dimension of the article."}, "sentence_type": "Affirmative declarative sentence", "pos": {"nouns": {"Questionnaire": 1, "Entry": 1, "Examinations": 1, "Professional": 1, "Tasks": 1}, "verbs": {"Yes": 1, "I have to": 1, "Pass": 1, "Yes, it can": 2}, "adjectives": {"Important": 1}, "numerals": {"41": 1}, "pronouns": {"I": 1}}}, {"content": "I seem to have been looking for new friends longer than most.", "word_count": 16, "label": "", "sentence_structure": "Subject鈥揷opula鈥損redicative", "subjective_objective": {"answer": "Subjective", "rationale": "Because \"more than most people\" is a comparative description, involving the evaluation and judgement of individual abilities and performance, and is the subjective perception and perception of the patient."}, "sentence_sentiment": {"answer": "Positive", "rationale": "Because the emotional attitude of the phrase \"more than most people know new friends\" is positive."}, "language_style": {"answer": "Tactful and warm", "rationale": "The language style of the entry is euphemistic, because the questioner uses the word \"seemingly\" to express his views, without directly asserting that he is better than most people to have new friends. At the same time, the questioner does not use strong tone or emotional vocabulary, but rather asks questions in a gentle and friendly manner, giving people a sense of affection."}, "time_dimension": {"answer": "Past", "rationale": ""}, "sentence_type": "Affirmative declarative sentence", "pos": {"nouns": {"person": 1, "friends": 1}, "verbs": {"Chief": 1, "I'm meeting someone": 1}, "adjectives": {"More": 1, "Large": 1}, "adverbs": {"Seems like": 1, "More": 1}, "numerals": {"42": 1}}}, {"content": "43. I am more confident today than usual.", "word_count": 10, "label": "", "sentence_structure": "Subject鈥揷opula鈥損redicative", "subjective_objective": {"answer": "Subjective", "rationale": "Because \"more self-confidence than usual\" is an expression of subjective feelings or attitudes, reflecting subjective assessments of the patient's abilities, performance, etc."}, "sentence_sentiment": {"answer": "Positive", "rationale": "Because the expression \"more confident than usual\" in the entry is positive and expresses a good self-perception and mental state."}, "language_style": {"answer": "Tactful and warm", "rationale": "The moderate way in which the entry expresses its self-confidence today by using a comparative level without directly expressing whether it is very high or very low makes it easier for each other to accept and share their feelings. At the same time, the comparison between \"Today\" and \"At times\" highlights the rise in confidence, which is more inspiring and encouraging."}, "time_dimension": {"answer": "Present", "rationale": ""}, "sentence_type": "Affirmative declarative sentence", "pos": {"nouns": {"Questionnaire": 1, "Entry": 1, "Confidence": 1}, "verbs": {"be": 1, "- Yeah": 1, "More": 1}, "adjectives": {"Normal": 1, "Today": 1}, "adverbs": {"Yes": 1}, "numerals": {"43": 1}}}, {"content": "I avoid those with whom I may have a relationship, because I feel too nervous around them.", "word_count": 36, "label": "", "sentence_structure": "Subject鈥搗erb鈥搊bject", "subjective_objective": {"answer": "Subjective", "rationale": "This is the patient ' s description and interpretation of his or her emotional state, which is subjective."}, "sentence_sentiment": {"answer": "Negative", "rationale": "Because the words \"screw out possible lovers\" and \"strained\" in the entries all have negative emotional aspects."}, "language_style": {"answer": "Calm and direct", "rationale": "The language style of the entry is calm and straightforward, and it directly describes the behaviour and feelings of the subject, without any use of rhetorical or emotional rendering. It is simple, objective and suitable for use in psychological questionnaire studies.\""}, "time_dimension": {"answer": "Present", "rationale": ""}, "sentence_type": "Affirmative declarative sentence", "pos": {"nouns": {"I": 2, "Those": 1, "person": 2, "Love relationships": 1, "Nervous": 1}, "verbs": {"Avoid": 1, "Generate": 1, "Yes": 2, "I feel": 1}, "adjectives": {"Maybe": 1, "Too much": 1}, "adverbs": {"Time": 1, "Yes": 1}, "numerals": {"44": 1}, "classifiers": {"individual": 1}}}, {"content": "45. I hope I can change my face.", "word_count": 12, "label": "", "sentence_structure": "Subject鈥搗erb鈥搊bject", "subjective_objective": {"answer": "Subjective", "rationale": "Because hope is a subjective idea or aspiration of the patient, not an objective reality."}, "sentence_sentiment": {"answer": "Negative", "rationale": "Because the idea of \"wanted to change your face\" in the entry may be due to external disaffection or anxiety, and is a negative emotional tendency."}, "language_style": {"answer": "Tactful and warm", "rationale": "The entry is more personal and interactive when it is presented in a gentle and inviting tone, rather than in direct statements or orders. At the same time, the content of the article is more personal and sensitive, and therefore the euphemism is more protective of the privacy and self-esteem of the person under investigation."}, "time_dimension": {"answer": "Future", "rationale": "The words \"hope,\" \"can\" and \"change\" all imply the expectations and aspirations for the future. So the answer is the future."}, "sentence_type": "Imperative sentence", "pos": {"nouns": {"Looks like a": 1}, "verbs": {"Hope": 1, "Change": 1}, "numerals": {"45": 1}}}, {"content": "46. I am less afraid than the older people to speak in public.", "word_count": 16, "label": "", "sentence_structure": "Subject鈥揷opula鈥損redicative", "subjective_objective": {"answer": "Subjective", "rationale": "Because \"fear\" is a subjective feeling or emotion, it's seen from the patient's subjective perspective."}, "sentence_sentiment": {"answer": "Positive", "rationale": "Because the position expressed in this entry is one of confidence and optimism."}, "language_style": {"answer": "Calm and direct", "rationale": "The language style of the entry is a cool and straightforward type. It asks whether the interviewees are less concerned than most people about speaking in public, and do not use excessive rhetorical or emotional colours to render and reprove. This expression is clear and easier to understand and answer."}, "time_dimension": {"answer": "Present", "rationale": "\"Better\" means a comparison with others, and \"less worry\" indicates a lower degree of anxiety about public speeches. Both terms point to the current psychological state and emotional response of the person being tested, so the entry should be \"now\"."}, "sentence_type": "Affirmative declarative sentence", "pos": {"nouns": {"Questionnaire Entry": 1, "person": 1, "Speech": 1}, "verbs": {"- Yeah": 1, "I'm worried": 1, "in": 1, "Less": 1, "Speak": 1}, "adjectives": {"Large": 1}, "numerals": {"46": 1}, "pronouns": {"I": 1}}}, {"content": "47. I feel more optimistic and positive now than usual.", "word_count": 14, "label": "", "sentence_structure": "Subject鈥揷opula鈥損redicative", "subjective_objective": {"answer": "Subjective", "rationale": "Because positive optimism is a subjective emotional experience of one person."}, "sentence_sentiment": {"answer": "Positive", "rationale": "Because \"more optimistic and positive\" in the entry is positive."}, "language_style": {"answer": "Tactful and warm", "rationale": "The entry uses an encouraging language style, which is more personal and interactive, by means of a gentle and inviting tone of questioning about the other party ' s current emotional state, rather than by direct statements or orders."}, "time_dimension": {"answer": "Present", "rationale": "The entry describes the current emotional state of the subject, and the point of time \"now\" clearly points to the situation that is now under way."}, "sentence_type": "Interrogative sentence", "pos": {"nouns": {"Present": 1, "I": 1, "Optimistic": 1, "Positive": 1}, "verbs": {"I feel": 1, "- Yeah": 1, "More": 2}, "adjectives": {"Optimistic": 1, "Positive": 1}, "adverbs": {"Present": 1, "More": 2}, "numerals": {"47": 1}, "pronouns": {"I": 1}}}, {"content": "48. For me, attracting a eager boyfriend or girlfriend is never a problem.", "word_count": 26, "label": "", "sentence_structure": "Subject鈥搗erb鈥搊bject", "subjective_objective": {"answer": "Subjective", "rationale": "Because the descriptions of \"required\" and \"attractive\" are based on the patient's subjective perspective. Although this is a patient's observation and assessment of his or her own situation, it is based on the patient's personal experience, feelings and expectations and is therefore considered subjective."}, "sentence_sentiment": {"answer": "Positive", "rationale": "Because the entry describes the individual ' s abilities and self-confidence and has a positive emotional connotation."}, "language_style": {"answer": "Tactful and warm", "rationale": "The language style of the entry is more euphemism and complacency than direct presentation or command, giving people a sense of love and strength, confidence and charm. At the same time, the topic is not much emotional, but simply expresses their self-confidence in attracting heterosexuals."}, "time_dimension": {"answer": "Present", "rationale": "So the time dimension is now judged."}, "sentence_type": "Affirmative declarative sentence", "pos": {"nouns": {"I": 1, "Problem": 1, "Boyfriend": 1, "Girlfriend": 1}, "verbs": {"Attractive": 1, "Be": 1}, "adjectives": {"I'm thirsty": 1}, "numerals": {"48": 1}}}, {"content": "49. If I had been more confident, my life would have been better.", "word_count": 17, "label": "", "sentence_structure": "Subject鈥揷opula鈥損redicative", "subjective_objective": {"answer": "Subjective", "rationale": "Because \"be more confident\" and \"my life will be better\" are the subjective views and expectations of the patients."}, "sentence_sentiment": {"answer": "Positive", "rationale": "Because \"a little more confident\" and \"a little better life\" in the entries are positive expressions."}, "language_style": {"answer": "Tactful and warm", "rationale": "The language style of the entry is more moderate, friendly and full of encouragement and support. It does not directly criticize or dictate the interviewee, but rather expresses the author鈥檚 views and expectations in a positive and constructive way that is more likely to generate resonance and reflection among the interviewee and is more conducive to good interaction."}, "time_dimension": {"answer": "Past", "rationale": "The temporal dimension of the purpose of the article is therefore judged to be past."}, "sentence_type": "Affirmative declarative sentence", "pos": {"nouns": {"Confidence": 1, "Life": 1}, "verbs": {"More": 1, "Yes": 2, "Okay": 1}, "adjectives": {"Some": 1}, "adverbs": {"A little": 1}, "numerals": {"49": 1}, "pronouns": {"I": 1}}}, {"content": "I pursue those intellectually challenging activities, because I know I can do better than most people.", "word_count": 32, "label": "", "sentence_structure": "Subject鈥搗erb鈥搊bject", "subjective_objective": {"answer": "Subjective", "rationale": "This is because the patient ' s personal thoughts and convictions are subjective in that he or she is intellectually superior to others."}, "sentence_sentiment": {"answer": "Positive", "rationale": "Because the position of the entry is positive, it expresses recognition of self-capacity and self-confidence in value."}, "language_style": {"answer": "Tactful and warm", "rationale": ""}, "time_dimension": {"answer": "Present", "rationale": ""}, "sentence_type": "Affirmative declarative sentence", "pos": {"nouns": {"Activities": 1, "Intellectual": 1, "Challenge": 1}, "verbs": {"Seek": 1, "Yes": 3, "Do it": 1}, "adjectives": {"Rich": 1, "More": 1}, "adverbs": {"Go, go, go": 1}, "numerals": {"50": 1, "Most of them": 1}, "pronouns": {"I": 1}}}, {"content": "I can get many dates without difficulty.", "word_count": 13, "label": "", "sentence_structure": "Subject鈥搗erb鈥揷omplement", "subjective_objective": {"answer": "Subjective", "rationale": "Because the entry describes the patient鈥檚 own feelings and experiences, rather than the outside facts or objective circumstances. For example, patients may feel that they can easily contact people or have access to social activities, but it does not necessarily mean that they have actually successfully arranged multiple dates."}, "sentence_sentiment": {"answer": "Positive", "rationale": "Because the \"many dates without difficulty\" act is positive in the entries."}, "language_style": {"answer": "Tactful and warm", "rationale": "The language style of the entry is more euphemistic, it uses a more intimate expression, avoiding the use of too direct or hard language. In addition, the entry uses the first person ' s verb 'I', making the questionnaire more personal and interactive."}, "time_dimension": {"answer": "Present", "rationale": "The entry now represents the current status and ability of the testee, i.e., under the present circumstances, the testee believes that he or she can get many dates without any effort."}, "sentence_type": "Affirmative declarative sentence", "pos": {"nouns": {"I": 1, "Date": 1}, "verbs": {"Yes": 1, "Got it": 1, "It's no problem": 1}, "adjectives": {"Many": 1}, "numerals": {"51": 1}}}, {"content": "52. I cannot be as comfortable in the crowd as most people.", "word_count": 18, "label": "", "sentence_structure": "Subject鈥揷opula鈥損redicative", "subjective_objective": {"answer": "Subjective", "rationale": "Because feeling uncomfortable is subjective."}, "sentence_sentiment": {"answer": "Negative", "rationale": "Because \"not as comfortable as most people\" in the entry indicates an incomprehensible and uncomfortable mood, which is negative."}, "language_style": {"answer": "Tactful and warm", "rationale": "By describing the feelings of the interviewees in the crowd, the entry leads the other to reflect on their social and psychological situation, and to express their language in a more moderate manner, without direct denial or stigmatization, with a greater focus on communication and understanding with the interviewees."}, "time_dimension": {"answer": "Past", "rationale": "\"I can't feel comfortable like most people\" is a sense or state of the past, and therefore judgement is the past."}, "sentence_type": "Negative declarative sentence", "pos": {"nouns": {"Population": 1, "person": 1, "Comfortable": 1}, "verbs": {"Yes": 1, "I feel": 1, "Yeah": 1, "That way": 1}, "adjectives": {"Most of them": 1, "- Yeah": 1}, "numerals": {"52": 1}, "pronouns": {"I": 1}}}, {"content": "Today I am more uncertain than I am normally.", "word_count": 13, "label": "", "sentence_structure": "Subject鈥搗erb鈥揷omplement", "subjective_objective": {"answer": "Subjective", "rationale": "Because \"uncertain\" is a personal emotion and feeling, a subjective view of the patient."}, "sentence_sentiment": {"answer": "Negative", "rationale": "Because \"uncertain\" is a negative attitude or emotion."}, "language_style": {"answer": "Calm and direct", "rationale": "The language style of the entry is a calm and straightforward type. It describes the fact that the person surveyed today has less control over himself than usual. There is no excessive emotional colour or accoutrements, and the language is simple and straightforward."}, "time_dimension": {"answer": "Present", "rationale": ""}, "sentence_type": "Negative declarative sentence", "pos": {"verbs": {"Today": 1, "- Yeah": 1, "Normal": 1, "To yourself": 1, "More": 1, "I don't know": 1}, "adjectives": {"Today": 1, "Normal": 1, "self": 1}, "adverbs": {"More": 1, "I don't know": 1}, "numerals": {"53": 1}, "pronouns": {"I": 1}}}, {"content": "54. If I look better, I'll be more successful on a date.", "word_count": 19, "label": "", "sentence_structure": "Subject鈥搗erb鈥搊bject", "subjective_objective": {"answer": "Subjective", "rationale": "This is because the patient ' s subjective view of the relationship between his or her appearance and the outcome of the date does not involve the occurrence of objective facts or acts."}, "sentence_sentiment": {"answer": "Negative", "rationale": "Because the entries express a dependency and a desire for external profiling to succeed, they may affect individual self-esteem and values."}, "language_style": {"answer": "Tactful and warm", "rationale": "The language style of the entry is more euphemistic, subtle, using hypothetical language, and does not directly present a clear conclusion. This expression can make the respondents more comfortable and comfortable, while encouraging them to provide more realistic and accurate answers."}, "time_dimension": {"answer": "Future", "rationale": "Moreover, the \"better look\" also indicates that it is a potential, unfulfilled state or condition, which further supports its judgement as a future conclusion."}, "sentence_type": "Affirmative declarative sentence", "pos": {"nouns": {"I": 1, "Date": 1, "Success": 1}, "verbs": {"Long": 1, "Yes": 2, "It looks better": 1, "Okay": 1, "in": 1, "More": 2}, "adjectives": {"Nice": 1}, "adverbs": {"A little": 1, "Both": 1}, "numerals": {"54": 1}}}]}

{"questionnaire_title": "Table of sources of control of marriage", "questionnaire_category": "Self-cognition", "item_count": 44, "emotion_shift_overall": "From positive to negative", "language_style_overall": "Tactful and warm", "items": [{"content": "8. I know what I am doing in our marriage when it starts to get in trouble.", "word_count": 25, "label": "", "sentence_structure": "Subject鈥搗erb鈥搊bject", "subjective_objective": {"answer": "Subjective", "rationale": "This is because patients are subjective in their perception and self-evaluation."}, "sentence_sentiment": {"answer": "Positive", "rationale": "Because the \"know what I'm playing in\" act in the entry is positive."}, "language_style": {"answer": "Calm and direct", "rationale": "The type of question is usually presented in question form and does not have too many emotional adorations or rhetorical techniques."}, "time_dimension": {"answer": "Present", "rationale": ""}, "sentence_type": "Affirmative declarative sentence", "pos": {"nouns": {"Marriage": 1, "Trouble": 1, "Role": 1}, "verbs": {"Start": 1, "Come on": 1, "Yes": 1, "Of which": 1, "It's a play": 1}, "adverbs": {"When": 1}, "pronouns": {"I": 1}}}, {"content": "11. When my spouse argued with me, I always made a settlement.", "word_count": 17, "label": "", "sentence_structure": "Subject鈥搗erb鈥搊bject", "subjective_objective": {"answer": "Subjective", "rationale": "Because reconciliation is the patient ' s subjective feeling and attitude."}, "sentence_sentiment": {"answer": "Positive", "rationale": "Because the \"conciliation\" in the entry is positive."}, "language_style": {"answer": "Tactful and warm", "rationale": "The entry is more personal and interactive when it is presented in a gentle and inviting tone, rather than in direct statements or orders. At the same time, it does not overemphasize personal views, but rather allows the interviewee to self-evaluate by asking questions, which is more euphemismal and friendly."}, "time_dimension": {"answer": "Present", "rationale": "The entry describes the behaviour or mentality of the person being tested in the current state, i.e. whether the spouse can successfully reconcile with himself or herself when he or she is arguing."}, "sentence_type": "Affirmative declarative sentence", "pos": {"nouns": {"Questionnaire": 1, "Entry": 1, "Spouse": 1, "Argument": 1, "Reconciliation": 1}, "verbs": {"Achieved": 1, "Argument": 1}, "numerals": {"11": 1}, "pronouns": {"I": 1}}}, {"content": "16. When our marriage is unhappy, I always know how I do it will help to solve the problem.", "word_count": 29, "label": "", "sentence_structure": "Subject鈥搗erb鈥搊bject", "subjective_objective": {"answer": "Subjective", "rationale": "Because the title says \"I know how I do it,\" which shows the subjective views and attitudes of the patient."}, "sentence_sentiment": {"answer": "Positive", "rationale": "Because the entry expresses a problem-solving attitude and ability, and a positive emotional attitude."}, "language_style": {"answer": "Calm and direct", "rationale": "This language style helps the interviewee to understand the substance of the question and the content of the questions that need to be answered."}, "time_dimension": {"answer": "Present", "rationale": "The keyword \"always\" indicates that it is a customary act, while the verb \"knows how I do it to help solve the problem\" suggests that it is being done. So, the entry is about the present capacity of the testee to resolve the marriage problem."}, "sentence_type": "Affirmative declarative sentence", "pos": {"nouns": {"Marriage": 1, "It's not good": 1, "Problem": 1}, "verbs": {"Come on": 1, "Yes": 1, "Do it": 1, "It helps solve problems": 1}, "adverbs": {"Always": 1}, "numerals": {"16": 1}, "classifiers": {"individual": 1}, "pronouns": {"I": 2}}}, {"content": "19. Parents are good when they teach their children with due diligence.", "word_count": 19, "label": "", "sentence_structure": "Subject鈥損redicate", "subjective_objective": {"answer": "Subjective", "rationale": "Because \"if parents do their best to teach their children\" is the patient's subjective view and expectation."}, "sentence_sentiment": {"answer": "Positive", "rationale": "Because the words \"parents are doing their best to teach their children\" and \"good conduct\" in the entries are positive expressions."}, "language_style": {"answer": "Calm and direct", "rationale": "The language style of the entry is relatively calm and straightforward, and it presents assumptions and conclusions in a clear and concise manner, without excessive rhetorical or emotional colours and with a more balanced and objective tone."}, "time_dimension": {"answer": "Present", "rationale": "In this entry, \"If\" means \"supposed\" the \"good-faith\" and \"good-faith\" are both descriptions or evaluations of the status quo, so the temporal dimension is now judged."}, "sentence_type": "Affirmative declarative sentence", "pos": {"nouns": {"Questionnaire Entry": 1, "Children": 1, "parents": 2, "Good conduct": 1}, "verbs": {"Teaching": 1, "Yes": 1, "Good conduct": 1}, "adverbs": {"Suppose": 1, "I'm sure": 1}, "numerals": {"19": 1}, "pronouns": {"they": 1}}}, {"content": "20. If my spouse and I may have difficulties in sexual life, we can certainly overcome them.", "word_count": 26, "label": "", "sentence_structure": "Subject鈥搗erb鈥搊bject", "subjective_objective": {"answer": "Subjective", "rationale": "This is because of the patient ' s subjective belief and attitude that he or she can cope with sexual difficulties together with his or her spouse."}, "sentence_sentiment": {"answer": "Positive", "rationale": "This entry is an expression of confidence and positive attitude towards the solution of the problem."}, "language_style": {"answer": "Tactful and warm", "rationale": "The language style of the entry is more moderate, using positive words such as \"if\" and \"if it can be overcome\" to express confidence in the possible sexual problems between spouses, and avoiding direct criticism or accusations."}, "time_dimension": {"answer": "Present", "rationale": "The entry expresses the views of the person tested on the difficulties that he or she and his spouse may face in future, emphasizes the possibility of future and includes the hypothetical context, and therefore judges the future."}, "sentence_type": "Affirmative declarative sentence", "pos": {"nouns": {"Questionnaire": 1, "Entry": 1, "Spouse": 1, "Sex": 1, "Difficulties": 1, "Problem": 1}, "verbs": {"I met him": 1, "Maybe": 1, "Overcoming": 1}, "adjectives": {"Sure": 1}, "numerals": {"20": 1}, "pronouns": {"we": 1}}}, {"content": "25. In my view, maintaining a full marriage is just a skill; something like luck does not work.", "word_count": 34, "label": "", "sentence_structure": "Subject鈥揷opula鈥損redicative", "subjective_objective": {"answer": "Subjective", "rationale": "Because \"in my opinion\" means that it's a personal opinion and opinion of the patient."}, "sentence_sentiment": {"answer": "Neutral", "rationale": "Because the entry expresses the view of maintenance of marriage, it does not involve emotional colour."}, "language_style": {"answer": "Calm and direct", "rationale": "The entry is expressed in a direct presentation, without the use of rhetorical techniques or excessive emotional rendering, and with a relatively calm and objective language style."}, "time_dimension": {"answer": "Present", "rationale": "The entry expresses the views and views of the interviewees on the maintenance of marriage, which have been developed at this point in time and are therefore judged to be the present."}, "sentence_type": "Affirmative declarative sentence", "pos": {"nouns": {"In my opinion": 1, "Maintain": 1, "Plant": 2, "- Oh, it's nice": 1, "Fill": 1, "Marriage": 1, "Skills": 1, "Lucky": 1, "Something": 1, "It works": 1}, "verbs": {"Look": 1, "be": 2, "Maintenance": 1, "have": 1, "Play": 1}, "adjectives": {"Plant": 2, "- Oh, it's nice": 1, "Fill": 1}, "adverbs": {"Just": 1, "Here": 1}, "numerals": {"25": 1}, "classifiers": {"Plant": 2}, "pronouns": {"I": 1}}}, {"content": "26. Good communication between husband and wife is nothing more than learning and applying such skills; nothing really disturbs this good communication.", "word_count": 40, "label": "", "sentence_structure": "Subject鈥損redicate", "subjective_objective": {"answer": "Subjective", "rationale": "Because this sentence is an expression of a view and a view that good communication between husband and wife is only a skill that needs to be learned and applied and that is not interfered with in any way. It is a subjective judgement and interpretation."}, "sentence_sentiment": {"answer": "Positive", "rationale": "Because the entries \"good communication between husband and wife\" and \"nothing really interferes with this good communication\" are positive."}, "language_style": {"answer": "Tactful and warm", "rationale": "This is a more personal and interactive expression that helps to increase the participation of the interviewees and the reliability of the answers."}, "time_dimension": {"answer": "Present", "rationale": "In this entry, \"just a thing to learn and apply this technique\" and \"nothing really disturbs this good exchange\" are describing the current situation, emphasizing the practical and lasting nature of good communication between couples and therefore judging it as now."}, "sentence_type": "Affirmative declarative sentence", "pos": {"nouns": {"Couples": 1, "Communications": 2, "Yes": 1, "Skills": 1, "Good": 1, "- What": 1}, "verbs": {"be": 1, "Just": 1, "Yes": 2, "Learning": 1, "Apply": 1, "Really": 1, "Interference": 1, "This one": 2}, "adjectives": {"Good": 1, "- What": 1}, "adverbs": {"Just": 1, "Nothing": 1, "Really": 1}, "numerals": {"26": 1}, "classifiers": {"Annex": 1}, "pronouns": {"Here": 2}}}, {"content": "33. A few programmes on parenting would eliminate most of the conflicts between spouses.", "word_count": 29, "label": "", "sentence_structure": "Subject鈥搗erb鈥揷omplement", "subjective_objective": {"answer": "Subjective", "rationale": "Because \"a little plan can eliminate most conflicts between spouses\" is the researcher's perception or assumption of the issue, not an objective fact. It is based on a certain theory and assumption and is therefore subjective."}, "sentence_sentiment": {"answer": "Positive", "rationale": "The description in the entry shows that the programmes in child-rearing can help to eliminate most of the conflicts between spouses, which is a positive advice and attitude."}, "language_style": {"answer": "Tactful and warm", "rationale": "Warm and warm."}, "time_dimension": {"answer": "Present", "rationale": "\"In the field of parenting\", \"just a little planning\" and \"all that will eliminate most of the conflict between husband and wife\" suggest that the sentence describes the current situation or recommendation. So the time dimension of this questionnaire entry is now."}, "sentence_type": "Affirmative declarative sentence", "pos": {"nouns": {"Child-rearing": 1, "Conflict": 1}, "verbs": {"Planned": 1, "Elimination": 1, "Just a little": 1}, "adverbs": {"Just": 1, "I'll be fine": 1}, "numerals": {"33": 1}}}, {"content": "34. It seems that the problems of our marriage never disappear over time; what we usually do to resolve them.", "word_count": 37, "label": "", "sentence_structure": "Subject鈥搗erb鈥搊bject", "subjective_objective": {"answer": "Subjective", "rationale": "Since the entry expresses the views and feelings of patients on the issues in their marriage, it examines subjective ideas."}, "sentence_sentiment": {"answer": "Negative", "rationale": "Because the reference to the negative situation that \"the problem does not seem to disappear on its own\" in the entry is negative, it raises the need for measures to be taken to address it, and therefore has a negative side."}, "language_style": {"answer": "Tactful and warm", "rationale": "This is a more gentle, thoughtful and supportive expression that the interviewee feels understood and supported."}, "time_dimension": {"answer": "Present", "rationale": "The entry is an inquiry into the current situation and feelings of the testee, where the words \"seemingly\" and \"never\" imply a long-standing phenomenon, and the expression \"what to do to solve\" suggests that the problem needs to be addressed. Thus, the time dimension of the entry can be judged as it is now."}, "sentence_type": "Affirmative declarative sentence", "pos": {"nouns": {"Marriage": 1, "Problem": 2, "Time": 1, "Resolve": 1}, "verbs": {"Yes": 2, "Disappear": 1, "Do it": 1, "Resolve": 1}, "adjectives": {"Never": 1, "Self": 1, "Usually": 1}, "numerals": {"34": 1}, "pronouns": {"we": 1}}}, {"content": "36. My spouse and I live well together because we know the skills of people; not about luck, integrity, etc.", "word_count": 34, "label": "", "sentence_structure": "Subject鈥揷opula鈥損redicative", "subjective_objective": {"answer": "Subjective", "rationale": "This is a subjective entry, as it relates to the patient ' s perception and explanation of the reasons for his/her good relations with his/her spouse, which is the patient ' s subjective attitude and perception."}, "sentence_sentiment": {"answer": "Positive", "rationale": "Because the result of the entry \"Spouse and I get along well\" is positive and for reasons described as \"know-how-to-people\" without any negative factors."}, "language_style": {"answer": "Tactful and warm", "rationale": "The language style of the questionnaire entry is euphemism. It does not directly criticize or judge the other side's views, but rather expresses itself in a moderate tone and presents a point that can be discussed. This expression is more easily accepted by the respondents and offers real ideas and feelings."}, "time_dimension": {"answer": "Present", "rationale": ""}, "sentence_type": "Affirmative declarative sentence", "pos": {"nouns": {"Questionnaire": 1, "Entry": 1, "Spouse": 1, "Interpersonal skills": 1, "Lucky": 1, "Validity": 1, "Something": 1}, "verbs": {"Get along": 1, "be": 2, "I know you well": 1, "Not relevant": 1}, "adverbs": {"Okay": 1, "Wait": 1}, "numerals": {"36": 1}, "pronouns": {"we": 1}}}, {"content": "38. I always find ways to help end the controversy between me and my spouse and to make both sides happy.", "word_count": 30, "label": "", "sentence_structure": "Subject鈥搗erb鈥搊bject", "subjective_objective": {"answer": "Subjective", "rationale": "Because the descriptions of \" always find a way\" and \"happy\" are all expressions of the patient's own feelings and attitudes and are subjective."}, "sentence_sentiment": {"answer": "Positive", "rationale": "Because the entry \"Find a way to end the debate and make both sides happy\" is positive."}, "language_style": {"answer": "Tactful and warm", "rationale": "The language style of this questionnaire entry is more euphemistic. First, the term \"always\" expresses a positive attitude and determination, emphasizes the possibility of solving the problem and optimistic expectations, and gives readers a sense of goodwill and concern on the part of the designers. Second, the description \"helps to end the controversy between me and my spouse, and makes both sides happy\" expresses the goal of solving the problem in a moderate way, and instead of directly blaming or criticizing each other's behaviour, expresses the expectation and desire for harmonious relations. This language style creates a friendly and positive atmosphere, making respondents more willing to participate in the questionnaire."}, "time_dimension": {"answer": "Present", "rationale": "The entry describes the current behaviour or attitude of the testee, the keyword \" always find a way\", and the result is \"end of the debate\" and \"make both parties happy\", so it can be judged that the dimension of time is now."}, "sentence_type": "Affirmative declarative sentence", "pos": {"nouns": {"Approach": 1, "End": 1, "I": 2, "Spouse": 1, "Between": 1, "Arguments": 1, "Both sides": 1, "The mood": 1, "Have a nice day": 1}, "verbs": {"Found it": 1, "It helps": 1, "Jean": 1}, "adverbs": {"Always": 1}, "numerals": {"38": 1}}}, {"content": "43. The good times of our marriage are not just opportunities; they are usually also planned.", "word_count": 26, "label": "", "sentence_structure": "Subject鈥搗erb鈥搊bject", "subjective_objective": {"answer": "Subjective", "rationale": "The purpose of the questionnaire is subjective. It expresses the researchers鈥?perception and understanding of the good times of marriage, which is based on their experience and views, and does not reflect the experiences and views of all. At the same time, it also contains subjective language, such as \"not only by opportunity; it usually needs to be planned.\" This suggests the researchers鈥?personal perspective."}, "sentence_sentiment": {"answer": "Positive", "rationale": "Because the expression \"good times\" in the entry is not just an opportunity, but a positive expression, through \"planning\", emphasizing proactive attitudes and behaviour."}, "language_style": {"answer": "Tactful and warm", "rationale": ""}, "time_dimension": {"answer": "Present", "rationale": ""}, "sentence_type": "Affirmative declarative sentence", "pos": {"nouns": {"we": 1, "Marriage": 1, "Good times": 1, "Come on": 1, "Opportunities": 1, "Planning": 1}, "verbs": {"be": 1, "Shit": 1, "Yes": 1}, "adjectives": {"Just": 1, "Usually": 1}, "numerals": {"43": 1}, "pronouns": {"we": 1}}}, {"content": "3. Efforts to work for the relationship between the two parties are in fact guarantees of a successful marriage.", "word_count": 21, "label": "", "sentence_structure": "Subject鈥搗erb鈥搊bject", "subjective_objective": {"answer": "Objective", "rationale": "Because \"work for a relationship\" and \"successful marriage\" are observable acts and outcomes, and need not be determined by the patient ' s subjective feelings or explanations. This is an objective view of marriage."}, "sentence_sentiment": {"answer": "Positive", "rationale": "Because the entries \"work for the relationship\" and \"assurance of a successful marriage\" are both positive emotional expressions of the value of successful pursuit and relationship."}, "language_style": {"answer": "Tactful and warm", "rationale": "The language style of the entry is more euphemistic and moderate. It does not direct orders or threats, but influences the reader鈥檚 views by presenting ideas and expressing beliefs. At the same time, it uses some positive language terms (such as 鈥渟uccess鈥? 鈥渁ssurance鈥? that make it more inspiring and attractive."}, "time_dimension": {"answer": "Present", "rationale": "The words \"actually\", \"is\" and \"guarantee\" in that sentence indicate that this is an objective fact or view, and does not address the temporal dimension of the past or the future. The sentence should therefore be judged as now."}, "sentence_type": "Affirmative declarative sentence", "pos": {"nouns": {"Both sides": 1, "Relations": 1, "Try": 1, "Success": 1, "Marriage": 1, "Promise": 1}, "verbs": {"Give": 1, "be": 1, "Promise": 1}, "adjectives": {"Actually": 1}, "numerals": {"3": 1}}}, {"content": "5. If my marriage is likely to end with a divorce, I suspect that I am not giving enough attention to its preservation.", "word_count": 34, "label": "", "sentence_structure": "Subject鈥損redicate", "subjective_objective": {"answer": "Subjective", "rationale": "This is because it involves patients' assessment of themselves and subjective ideas."}, "sentence_sentiment": {"answer": "Negative", "rationale": "Because the expression \"suspect yourself insufficiently committed\" in the entry is a self-critical and negative emotional expression, a negative attitude and emotional reaction."}, "language_style": {"answer": "Calm and direct", "rationale": "The entry uses a more straightforward language expression, without qualification, which presents assumptions and views, and does not use euphemism, emotional terms to adjust the emotional response of the reader, and therefore belongs to the \"cool-direct\" language style."}, "time_dimension": {"answer": "Present", "rationale": "Now, the entry \"if my marriage ends with a divorce\" is a hypothetical scenario, which suggests that the issue is to explore the attitudes and views of the testee, now or in the near future. And the latter, \"I'll doubt that I'm not investing enough energy to preserve it,\" is a speculation and expectation for the future, that if a marriage is in question, the testee will feel guilty or regret. So the time horizon of this article is now."}, "sentence_type": "Negative declarative sentence", "pos": {"nouns": {"Marriage": 1, "Divorce": 2, "- The energy": 1}, "verbs": {"Yes": 1, "Doubt": 1, "Input": 1}, "adjectives": {"Enough": 1}, "numerals": {"5": 1}, "pronouns": {"I": 1}}}, {"content": "Among the factors that generate marital satisfaction, the role of the environment is very limited; it is mainly due to efforts and the importance attached to it.", "word_count": 36, "label": "", "sentence_structure": "Subject鈥搗erb鈥搊bject", "subjective_objective": {"answer": "Subjective", "rationale": "The reason for examining marital satisfaction is subjective attitudes, i.e. \"mainly by effort and the importance attached to it\", rather than objective environmental factors. The answer to this article is therefore \"subjective\"."}, "sentence_sentiment": {"answer": "Neutral", "rationale": "Because the entry describes the less important role of the environment in generating a sense of marital satisfaction, it emphasizes the importance of personal effort and importance, without any obvious emotional colour."}, "language_style": {"answer": "Calm and direct", "rationale": "The language style of the entry is relatively calm and straightforward, and the objective, concise language is used to express the view that \"the environment is of little use in marriage satisfaction, and depends primarily on effort and attention\" and that there is no excessive emotional modification or rhetorical technique."}, "time_dimension": {"answer": "Present", "rationale": ""}, "sentence_type": "Affirmative declarative sentence", "pos": {"nouns": {"Marriage satisfaction": 1, "Factors": 2, "Role": 1, "Environment": 1, "Try": 1, "Value": 1}, "verbs": {"Generate": 1, "Rise": 1, "Limited": 1, "Shit": 1, "Value": 1}, "adjectives": {"Very": 1, "Main": 1}, "adverbs": {"Mostly": 1}, "classifiers": {"individual": 1}}}, {"content": "9. The success of educating children depends entirely on the ability of a person to do his or her best, and the opportunity is irrelevant.", "word_count": 27, "label": "", "sentence_structure": "Subject鈥搗erb鈥搊bject", "subjective_objective": {"answer": "Subjective", "rationale": "Because this view suggests that success depends more on individual efforts than on the external environment and opportunities, it is a subjective view."}, "sentence_sentiment": {"answer": "Negative", "rationale": "Because the emphasis in the entry on \"the only condition for success is to work\" negates the role of opportunity and has a certain negative emotional tone."}, "language_style": {"answer": "Calm and direct", "rationale": "The language style of the entry is straightforward. It expresses the view that the success of educating a child depends primarily on the individual鈥檚 level of effort and that opportunities do not play a decisive role in this regard. This expression does not use any euphemism or ambiguity, but rather conveys the researchers鈥?ideas in a clear way."}, "time_dimension": {"answer": "Present", "rationale": "The entry is about a view or belief, and there is no clear temporal dimension. It expresses a vision of how successful it is to educate a child, which includes the concepts of \"best efforts\" and \"opportunities\" but does not specifically address a certain period of time, past, present or future. So we can judge the time dimension of the entry as it is."}, "sentence_type": "Interrogative sentence", "pos": {"nouns": {"Education": 1, "Son": 1, "Success": 1, "Alone": 1, "Opportunities": 1, "Relations": 1}, "verbs": {"It depends": 1, "Try": 1, "have": 1, "This": 1, "Nothing": 1}, "adjectives": {"Total": 1, "Is it possible": 2}, "adverbs": {"I'm sure": 1}, "numerals": {"One": 3}, "classifiers": {"individual": 1}, "pronouns": {"This": 1}}}, {"content": "14. All I was asked to do to make our marriage life a happy one was to make some effort.", "word_count": 26, "label": "", "sentence_structure": "Subject鈥搗erb鈥搊bject", "subjective_objective": {"answer": "Subjective", "rationale": "Because the changes requested are judged by the patient's expectations and needs for marital life, the patient's subjective opinion is examined."}, "sentence_sentiment": {"answer": "Positive", "rationale": "Because the entry \"for the sake of a happy marriage\" expresses positive intentions and expectations and the effort required is also to achieve this goal."}, "language_style": {"answer": "Calm and direct", "rationale": "This is a direct and explicit way of expressing a view, without too much rhetorical or emotional overtook, and with a more objective and calm tone, without too much emotional. This way of speaking directly and clearly helps the interviewees to understand and answer the questions more quickly."}, "time_dimension": {"answer": "Present", "rationale": "The entry describes the current or current situation, and the keywords are \"what I am asked to do\" and \"what is\" which point to the current conduct or state in progress. Moreover, \"marriage life\" is also a concept that is relevant to the present, because it needs to be operated and maintained in reality. Thus, the temporal dimension of the article can be judged as it is."}, "sentence_type": "Affirmative declarative sentence", "pos": {"nouns": {"Marriage": 1, "All": 1, "Try": 1}, "verbs": {"Request": 1, "Do it": 1, "Pay": 1, "Out": 1}, "adjectives": {"Have a nice day": 1, "Some": 1}, "numerals": {"14": 1}, "classifiers": {"Only": 1}}}, {"content": "18. No matter how difficult the situation is, my spouse and I will be happy as long as we have decided.", "word_count": 27, "label": "", "sentence_structure": "Subject鈥搗erb鈥搊bject", "subjective_objective": {"answer": "Subjective", "rationale": "Because \"happiness\" is the patient's subjective expectation and belief, it is the patient's subjective perception of his/her relationship with his/her spouse."}, "sentence_sentiment": {"answer": "Positive", "rationale": "Because the purpose of the entry is to be positive, \"Happiness is always in the best of circumstances.\""}, "language_style": {"answer": "Tactful and warm", "rationale": "The language style of the entry is euphemism, tenderness, initiative, and expression of hope and confidence in happiness. Moreover, \"if we have decided\" emphasizes the role of individual will, it also seems relatively calm and straightforward."}, "time_dimension": {"answer": "Future", "rationale": "The entry contains visions and expectations for the future, and the key words are \"as long as we have decided\" and \"as long as we've decided\" and \"as long as we've lived together\" and they all point to situations that have not yet occurred and that may occur in the future."}, "sentence_type": "Affirmative declarative sentence", "pos": {"nouns": {"Situation": 1, "Hard": 1, "Decision": 1, "Spouse": 1, "Happiness": 1, "Get along": 1}, "verbs": {"Yes": 2, "Whatever": 1, "鈾?To live happily ever after 鈾?: 1}, "adjectives": {"Hard": 1}, "adverbs": {"Just": 1}, "pronouns": {"we": 1}}}, {"content": "23. Even among the most beloved couples, the emotional relationship of satisfaction is not a coincidence, but rather the result of the efforts of both parties.", "word_count": 34, "label": "", "sentence_structure": "Subject鈥揷opula鈥損redicative", "subjective_objective": {"answer": "Subjective", "rationale": "Because the phrase \"not by coincidence\" is the result of both parties' efforts, it shows that it is the patient's perception and evaluation of emotional relations, and it is subjective."}, "sentence_sentiment": {"answer": "Positive", "rationale": "Because the phrase \"the outcome of both parties' efforts\" in the entry is positive."}, "language_style": {"answer": "Calm and direct", "rationale": "The entry expresses a view or observation in a direct presentation, does not use excessive rhetorical or emotional rendering, and is more objective in tone."}, "time_dimension": {"answer": "Present", "rationale": "The phrase \"not by coincidence,\" but by the result of efforts by both parties, can be judged to be a universal understanding of the establishment and maintenance of emotional relations, that is, of the emotional relationships that require effort to be satisfied. This understanding is not limited to past or future situations, but rather to the current state, and is therefore current."}, "sentence_type": "Imperative sentence", "pos": {"nouns": {"Questionnaire Entry": 1, "Emotional relations": 1, "Couple": 1, "Result": 1, "Coincidence": 1, "Both sides": 1, "Try": 1}, "verbs": {"be": 2, "in": 1, "Analysis": 1, "Even if": 1, "In love": 1, "Satisfied": 1, "Access": 1}, "adverbs": {"Most": 1, "Yeah": 1, "No, it's not": 1, "It's": 1}, "numerals": {"23": 1}, "pronouns": {"鈾?And the 鈾?: 1, "Other": 1, "Here": 1}}}, {"content": "29. If my sexual life with my spouse is not as good as I would have liked, I would have thought that I was not investing enough energy in it.", "word_count": 35, "label": "", "sentence_structure": "Subject鈥搗erb鈥搊bject", "subjective_objective": {"answer": "Subjective", "rationale": "Because this statement relates to the patient ' s judgement of self-awareness and self-responsibility, it is the patient ' s subjective attitude."}, "sentence_sentiment": {"answer": "Positive", "rationale": "Because of the positive behaviour and attitude of the entries, it is felt that more effort is needed to improve sexual life."}, "language_style": {"answer": "Tactful and warm", "rationale": "The entry is more euphemistic. It does not ask the interviewee whether he or she has devoted enough energy to his or her sex life, but rather leads the interviewee to think about the issue in a hypothetical way and to ask questions in a caring and understanding manner."}, "time_dimension": {"answer": "Present", "rationale": "The keyword \"will\" say \"is a subjective judgement, not a past fact or future expectation. Besides, the assumption \"if my sexual life with my spouse is not as good as it would have been\" implies that this is the current situation, not the past or the future. So the time dimension of this questionnaire entry is now."}, "sentence_type": "Affirmative declarative sentence", "pos": {"nouns": {"I": 1, "Spouse": 1, "Sex": 1, "- The energy": 1}, "verbs": {"Yes": 2, "I think": 1, "be": 1, "For that reason": 1, "- The pitch": 1, "No, I'm not": 1}, "adjectives": {"Not exactly what I expected": 1}, "adverbs": {"Suppose": 1}, "numerals": {"29": 1}, "pronouns": {"self": 1}}}, {"content": "31. Good and clear communication between couples is not based on factors such as compatibility or character, but on ongoing practice.", "word_count": 31, "label": "", "sentence_structure": "Subject鈥搗erb鈥搊bject", "subjective_objective": {"answer": "Subjective", "rationale": "Because \"good, clear communication\", \"compatibility or character\" and \"continuous practice\" are all described from the point of view and experience of the patient."}, "sentence_sentiment": {"answer": "Positive", "rationale": "Because the words \"good communication\" and \"continuous practice\" in the entries are positive."}, "language_style": {"answer": "Tactful and warm", "rationale": "The language style of the questionnaire entry is euphemism. While it directly indicates that the subject matter - good communication between couples is not dependent on certain factors, but is built and maintained through continuous practice, it does not convey this view too strongly or directly, but rather uses a soft, invitee language that makes respondents more willing to participate in the questionnaire and share their views and experiences. In addition, it emphasizes the need for good communication between couples to be \"performing\" , which shows that authors are very action-oriented and practice-oriented, and that language style is more positive and encouraging."}, "time_dimension": {"answer": "Present", "rationale": "The entry describes the current situation, where good couple relationships need to be achieved through continuous practice. The keyword \"is not\" but\" is \"is\" and they all imply that it is a current view or belief."}, "sentence_type": "Affirmative declarative sentence", "pos": {"nouns": {"Couple": 1, "Communications": 2, "Factors": 1, "Practice": 1}, "verbs": {"It's": 1, "Compatibility": 1, "Character": 1, "Constantly": 1, "Practice": 1}, "adjectives": {"Good": 1, "Clear": 1}, "numerals": {"31": 1}}}, {"content": "40. Couples with satisfactory emotional relationships focus on continuing efforts to improve their relationships; good relationships cannot be spontaneous.", "word_count": 39, "label": "", "sentence_structure": "Subject鈥搗erb鈥搊bject", "subjective_objective": {"answer": "Subjective", "rationale": "Because it expresses the views and opinions of researchers on emotional relations, it is subjective."}, "sentence_sentiment": {"answer": "Positive", "rationale": "Since the wording of the article emphasizes \"continuing efforts to improve their relations\", such an attitude is positive."}, "language_style": {"answer": "Calm and direct", "rationale": "The language style of the entry is a calm and straightforward one, which clearly describes a fact, without excessive rhetorical and emotional rendering. At the same time, it also highlights the fact that good emotional relations between couples require constant effort to improve, which is more objective and rational.\""}, "time_dimension": {"answer": "Present", "rationale": "In the future, the entry refers to \"continuing efforts to improve their relationship\" and \"good relations cannot be spontaneous\" , both of which imply that efforts are needed to improve emotional relations in the future. Moreover, \"a couple with satisfactory emotional relations\" also indicates a state of non-occurrence and future, and therefore judgement is the future."}, "sentence_type": "Affirmative declarative sentence", "pos": {"nouns": {"Questionnaire Entry": 1, "Emotional relations": 2, "Couple": 1, "Relations": 3, "Try": 1, "Auto": 1}, "verbs": {"Possession": 1, "The point is": 1, "I'm getting better": 1, "Generate": 1}, "adjectives": {"Satisfactory": 1, "Good": 1, "Impossible": 1}, "adverbs": {"in": 1, "Auto": 1}, "numerals": {"40": 1, "2": 1, "1": 3}, "pronouns": {"they": 1}}}, {"content": "I was often overwhelmed when I had a disagreement with my spouse.", "word_count": 18, "label": "", "sentence_structure": "Subject鈥搗erb鈥揷omplement", "subjective_objective": {"answer": "Subjective", "rationale": "Because \"often unconscious\" is a personal psychological experience and feeling, and it is the patient's subjective attitude."}, "sentence_sentiment": {"answer": "Negative", "rationale": "Because the act of \"discovery\" is negative."}, "language_style": {"answer": "Tactful and warm", "rationale": "The entry is more euphemistic in language style, which leads the interviewee to think and express his feelings and experiences by describing a common life scenario, and is also more gentle, friendly, friendly and interactive."}, "time_dimension": {"answer": "Present", "rationale": ""}, "sentence_type": "Negative declarative sentence", "pos": {"nouns": {"Questionnaire Entry": 1, "Disagreement": 1, "I": 2, "Spouse": 1, "Response": 1}, "verbs": {"be": 1, "Regular": 1, "I know what I'm doing": 1}, "adverbs": {"Often": 1}, "numerals": {"1": 4}}}, {"content": "2. Sexual harmony is something that is incredible to me; it is a mere coincidence.", "word_count": 22, "label": "", "sentence_structure": "Subject鈥揷opula鈥損redicative", "subjective_objective": {"answer": "Subjective", "rationale": "This sentence is an expression of patients ' subjective views and evaluations of sexual life."}, "sentence_sentiment": {"answer": "Negative", "rationale": "Because the expression \"sexual harmony is a little weird to me\" is negative."}, "language_style": {"answer": "Tactful and warm", "rationale": "The language style of the entry is more euphemistic and implicit, and the author does not express his thoughts or feelings directly, but rather presents his views in a more objective and calm way. Moreover, the term \"some wonder\" and \"just coincidence\" is used to express doubts and uncertainty about sexual life, which is more moderate and not overly emotional."}, "time_dimension": {"answer": "Present", "rationale": "\"Sexual harmony is a bit weird to me\" and \"it's just a coincidence\" are the words that describe the current thinking or feeling of the testee, so it can be judged that the entry is an inquiry into the present behavior or mentality."}, "sentence_type": "Negative declarative sentence", "pos": {"nouns": {"Sex": 1, "Harmony": 1, "I": 1, "Coincidence": 1}, "verbs": {"Tell me": 1, "Some": 1, "Just": 1, "be": 1, "Plant": 1, "Existence": 1}, "adjectives": {"Unbelievable": 1}, "adverbs": {"A little": 1}, "pronouns": {"it": 1}}}, {"content": "6. Unhappy experiences in our marriage can happen regardless of what I do.", "word_count": 25, "label": "", "sentence_structure": "Subject鈥搗erb鈥搊bject", "subjective_objective": {"answer": "Subjective", "rationale": "Because \"unhappy experience can happen no matter what I do\" is a personal subjective perception and feeling."}, "sentence_sentiment": {"answer": "Negative", "rationale": "Because the entry \"unhappy experience can happen no matter what I do\" expresses a negative, pessimistic attitude and emotion."}, "language_style": {"answer": "Calm and direct", "rationale": "The entry is based on a direct and concise statement of views, without too much acrimonym or emotional language. It clearly expresses the fact that unpleasant experiences of marriage may occur at any time, and that no matter what action is taken by itself, it is not immune. This expression is calm and objective, straightforward and more direct in language style."}, "time_dimension": {"answer": "Present", "rationale": "The sentence \"I can do anything with an unhappy experience\" suggests that the entry asks about problems and challenges in the current marital status, and therefore the time dimension is the present."}, "sentence_type": "Negative declarative sentence", "pos": {"nouns": {"Marriage": 1, "Experience": 1}, "verbs": {"Come on": 1}, "adjectives": {"Have a nice day": 1, "Whatever": 1, "Both": 1}, "adverbs": {"Whatever": 1, "I": 1, "Do it": 1, "What": 1, "have": 1, "Maybe": 1}, "numerals": {"6": 1}, "classifiers": {"individual": 1}}}, {"content": "I found how the external environment, such as in everyday life, has had a significant impact on our relationship.", "word_count": 30, "label": "", "sentence_structure": "Subject鈥搗erb鈥搊bject", "subjective_objective": {"answer": "Subjective", "rationale": "Because the descriptions of \"discovery\" and \"impact\" are based on the patient's subjective view, which indicates that the patient believes that the outside environment has an impact on their marital relationship."}, "sentence_sentiment": {"answer": "Neutral", "rationale": "Because the entry does not have any emotional color."}, "language_style": {"answer": "Tactful and warm", "rationale": "The entry is more euphemistic, using the expression \"How we get along\" to avoid direct criticism or criticism of each other. It also expresses the interest and concern of investigators about the relationship between the couple, giving people a sweet, loving feeling."}, "time_dimension": {"answer": "Present", "rationale": "The entry describes the current situation and observations, the keyword \"discover\" and \"how we get along\" and \"how we get along\" point to the behavior and phenomena that are happening now. So the temporal dimension is now."}, "sentence_type": "Exclamatory sentence", "pos": {"nouns": {"Found": 1, "External environment": 1, "Daily life": 1, "Things": 1, "We're married": 1, "Get along": 1, "Impact": 1}, "verbs": {"have": 1, "to": 1, "very": 1, "Large": 1}, "adjectives": {"External": 1, "Day": 1, "A lot": 1}, "numerals": {"10": 1, "One": 2, "very": 1}, "classifiers": {"individual": 1}, "pronouns": {"we": 1, "Couple": 1}}}, {"content": "12. It is often difficult to draw my thoughts from my spouse when I want to do something that is not part of his/her plan.", "word_count": 33, "label": "", "sentence_structure": "Subject鈥搗erb鈥搊bject", "subjective_objective": {"answer": "Subjective", "rationale": "The subject is the patient ' s subjective feelings and perceptions of the way he/she communicates with his/her spouse."}, "sentence_sentiment": {"answer": "Negative", "rationale": "Because the entry \"It's hard to draw the spouse's own mind\" is negative."}, "language_style": {"answer": "Tactful and warm", "rationale": "The language style of the entry is euphemism. It uses more moderate language, without direct orders or accusations. It also does not use overly radical emotional terms, but rather allows the respondents to think and answer questions themselves by asking and directing. This expression is more acceptable and better able to promote effective communication and communication."}, "time_dimension": {"answer": "Present", "rationale": "The entry is an inquiry into the present behaviour and mentality of the person being tested, and the keyword is \"often difficult\", indicating that this is often the case and that the temporal dimension is now judged."}, "sentence_type": "Negative declarative sentence", "pos": {"nouns": {"Questionnaire": 1, "Entry": 1, "Spouse": 1, "Yes": 1, "Thinking": 1}, "verbs": {"I want to": 1, "Do it": 1, "No, I'm not": 1, "- Oh, that's sweet": 1}, "adjectives": {"It's hard": 1}, "numerals": {"12": 1}, "pronouns": {"he": 1, "she": 1}}}, {"content": "Misunderstanding between our couples is usually caused by purely environmental causes.", "word_count": 20, "label": "", "sentence_structure": "Subject鈥揷opula鈥損redicative", "subjective_objective": {"answer": "Objective", "rationale": "Because this sentence describes the misunderstanding between the spouses, which is usually caused by the environment, it is a statement of objective facts and does not include subjective attitudes or feelings of the patient."}, "sentence_sentiment": {"answer": "Neutral", "rationale": "The entry is not clearly emotional or tendentious."}, "language_style": {"answer": "Tactful and warm", "rationale": "The entry describes the reasons for misunderstandings between couples in a more euphemistic way, with a sense of understanding and tolerance, and attempts to reduce the negative feelings of respondents."}, "time_dimension": {"answer": "Present", "rationale": "The entry describes the misunderstanding of the relationship between the spouses and points out that this is usually due to environmental factors. In terms of time dimension, this description is made within the current or near-term time frame and is therefore judged to be the present."}, "sentence_type": "Affirmative declarative sentence", "pos": {"nouns": {"Misunderstanding": 1, "Couple": 1, "Environment": 1}, "verbs": {"Come on": 1, "Cause": 1}, "adjectives": {"Pure": 1}, "numerals": {"13": 1}, "pronouns": {"we": 1}}}, {"content": "17. One or another environment plays a major role in determining whether our marriage is perfect.", "word_count": 26, "label": "", "sentence_structure": "Subject鈥揷opula鈥損redicative", "subjective_objective": {"answer": "Subjective", "rationale": "Because the sentence relates to the assessment and view of \"determinating the gratification of our marriage\" and is the patient's subjective view of one or another environment."}, "sentence_sentiment": {"answer": "Neutral", "rationale": "Because the entry merely presented a point of view, it did not involve positive or negative emotional aspects."}, "language_style": {"answer": "Tactful and warm", "rationale": "The entry is more personal and interactive when it is spoken in a gentle and inviting manner, rather than in direct statements or orders. Moreover, the article is more objective and has no excessive rhetorical or emotional overtones, which give a calmer sense of calm."}, "time_dimension": {"answer": "Present", "rationale": "The keywords \"environment\" and \"decision\" indicate the degree and importance of this factor, while \"compensation\" implies the outcome and scope of the problem. The purpose of the article can therefore be classified as \"now\"."}, "sentence_type": "Affirmative declarative sentence", "pos": {"nouns": {"Environment": 2, "Marriage": 1, "Role": 1}, "verbs": {"Decision": 1, "Rise": 1}, "adjectives": {"- Oh, it's nice": 1, "Some or some other kind": 1}, "numerals": {"17": 1}}}, {"content": "24. I sometimes seem to have no way of resolving the discord between me and my spouse.", "word_count": 21, "label": "", "sentence_structure": "Subject鈥揷opula鈥損redicative", "subjective_objective": {"answer": "Subjective", "rationale": "Because the idea that I sometimes seem to have no way of resolving the discord between me and my spouse is a subjective feeling for the patient."}, "sentence_sentiment": {"answer": "Negative", "rationale": "Because the entry \"no way to resolve the discord between me and my spouse\" is negative."}, "language_style": {"answer": "Tactful and warm", "rationale": ""}, "time_dimension": {"answer": "Present", "rationale": "The words \"seemingly \" , \" innocuous\" indicate that the person who was tested was filling out the questionnaire in a way that described his current emotional state and distress, so that the entry could be judged to be about the present behaviour or mentality."}, "sentence_type": "Negative declarative sentence", "pos": {"nouns": {"Questionnaire": 1, "Entry": 1, "I": 2, "Time": 1, "Resolve": 1, "Problem": 1, "Spouse": 1, "Between": 1, "- No, no, no": 1, "Approach": 1}, "verbs": {"Sometimes": 1, "Seems like": 1, "Nothing": 1, "Approach": 1}, "adverbs": {"I": 2}, "numerals": {"24": 1}, "classifiers": {"individual": 1}}}, {"content": "It is often my spouse who properly ends a dispute.", "word_count": 17, "label": "", "sentence_structure": "Subject鈥搗erb鈥揷omplement", "subjective_objective": {"answer": "Objective", "rationale": "It is an objective situation to have the dispute properly concluded by the spouse, and not to involve the patient ' s subjective attitude or feelings."}, "sentence_sentiment": {"answer": "Positive", "rationale": "Because the entry \"to end a dispute properly by the spouse\" is positive."}, "language_style": {"answer": "Tactful and warm", "rationale": "The entry uses a more euphemistic tone, in which the respondent is guided by a question-and-answerer, and is friendly and gentle."}, "time_dimension": {"answer": "Past", "rationale": ""}, "sentence_type": "Affirmative declarative sentence", "pos": {"nouns": {"Arguments": 1, "Spouse": 1}, "verbs": {"End": 1}, "adjectives": {"Often": 1}, "numerals": {"27": 1}, "pronouns": {"I": 1}}}, {"content": "28. The manner in which your children grow up depends to a great extent on external factors, such as the kind of person who lives in your neighbourhood.", "word_count": 38, "label": "", "sentence_structure": "Subject鈥搗erb鈥搊bject", "subjective_objective": {"answer": "Objective", "rationale": "Because good behaviour depends largely on the fact that external factors are objective, such as the neighbourhood environment. While parents鈥?education and guidance are important, the end result is objectively influenced by the surrounding environment."}, "sentence_sentiment": {"answer": "Neutral", "rationale": "Because the entry merely expresses a point of view and does not involve any emotional colour."}, "language_style": {"answer": "Tactful and warm", "rationale": "The entry is more proficient and interactive when it is spoken in a gentle and inviting manner, rather than in direct statements or orders. It also implies the important role of the interviewees in the education of their children, rather than all the responsibility being attributed to the outside environment or others."}, "time_dimension": {"answer": "Present", "rationale": "The keyword \"old\" and \"dependant\" all indicate that it is a description or view of the status quo, without any reference to future or past events. So the temporal dimension is now."}, "sentence_type": "Affirmative declarative sentence", "pos": {"nouns": {"Children": 1, "Good conduct": 1, "It's big": 1, "Level": 1, "External factors": 1, "Neighbors": 2, "What kind of person": 1}, "verbs": {"When I grew up": 1, "It depends": 1, "Stay": 1}, "adjectives": {"How's that": 1, "What": 1}, "adverbs": {"To a large extent": 1}, "classifiers": {"individual": 1}, "pronouns": {"you": 1}}}, {"content": "35. I do not seem to have much influence on when we have a moment of intimate relations in our marriage; they appear to have taken place according to our own pattern.", "word_count": 40, "label": "", "sentence_structure": "Subject鈥揷opula鈥損redicative", "subjective_objective": {"answer": "Objective", "rationale": "The objective feelings and experiences of patients, i.e., perceptions of the intimate moment of marriage, are not examined as involving the influence of subjective attitudes or wishes."}, "sentence_sentiment": {"answer": "Negative", "rationale": "Because the emotions and attitudes expressed in the entries are negative, that is, the author is not satisfied or dissatisfied with them."}, "language_style": {"answer": "Calm and direct", "rationale": ""}, "time_dimension": {"answer": "Present", "rationale": "The entry describes the views and feelings of the testees about the moment of intimateness in marriage and uses the general currents to express this view."}, "sentence_type": "Affirmative declarative sentence", "pos": {"nouns": {"Marriage": 1, "Intimacy": 2, "Time": 2, "Pattern": 1}, "verbs": {"Come on": 2, "have": 1}, "adjectives": {"How old": 1, "As you wish": 1}, "numerals": {"35": 1}, "pronouns": {"I": 1}}}, {"content": "37. My spouse ' s emotions are often as my mystery as to what causes me to change my mind.", "word_count": 32, "label": "", "sentence_structure": "Subject鈥揷opula鈥損redicative", "subjective_objective": {"answer": "Subjective", "rationale": "Because \"the mind of the patient\" is the patient's subjective feeling and thinking. Although the emotional state of the spouse is objective, the patient's interpretation and understanding of it is subjective."}, "sentence_sentiment": {"answer": "Negative", "rationale": "Because the phrase \"emotionality is often a mystery to me\" in the entry indicates that the interviewee has a low level of knowledge of the emotional state of the spouse, and that there may be problems of communication or understanding, and therefore has a certain negative emotional colour."}, "language_style": {"answer": "Tactful and warm", "rationale": "The entry is more softly phrased, uses the metaphor \"emotional like a mystery\" and expresses confusion and impatience about changes in the emotional behaviour of the spouse, and is more sensitive and sensitive to each other's feelings."}, "time_dimension": {"answer": "Present", "rationale": "The entry is for the current situation of the subject, and the keywords \"often\" and \"in my mind\" indicate that this feeling is still going on and is therefore judged to be now."}, "sentence_type": "Interrogative sentence", "pos": {"nouns": {"Questionnaire": 1, "Entry": 1, "Emotion": 2, "Change": 1, "Heart": 1}, "verbs": {"to": 1, "Tell me": 2, "Yeah": 1, "Cause": 1, "鈾?In the heart 鈾?: 1, "Countless": 1}, "adjectives": {"Often": 1, "It's a mystery": 1}, "numerals": {"37": 1}, "pronouns": {"I": 2}}}, {"content": "39. No matter how hard parents were educated, some children were hopeless.", "word_count": 24, "label": "", "sentence_structure": "Subject鈥搗erb鈥搊bject", "subjective_objective": {"answer": "Subjective", "rationale": "Because the sense and the assessment of \"no way\" is the patient鈥檚 own subjective feeling and judgement, not the objective reality. Although parents鈥?education methods may be problematic, it cannot simply be considered that all children are uneducated, which requires deeper exploration and understanding."}, "sentence_sentiment": {"answer": "Negative", "rationale": "Because the description of \"some children are hopeless\" in the entry gives people a sense of frustration and frustration."}, "language_style": {"answer": "Calm and direct", "rationale": ""}, "time_dimension": {"answer": "Past", "rationale": ""}, "sentence_type": "Affirmative declarative sentence", "pos": {"nouns": {"Parents": 1, "Kung Fu": 1, "Son": 1, "Approach": 1}, "verbs": {"Down": 1, "Education": 1, "You're": 1, "have": 1}, "adjectives": {"None": 1, "The way": 1}, "numerals": {"39": 1}, "pronouns": {"they": 1}}}, {"content": "41. When I had a sincere exchange with my spouse, we had done nothing special to that end.", "word_count": 25, "label": "", "sentence_structure": "Subject鈥搗erb鈥搊bject", "subjective_objective": {"answer": "Objective", "rationale": "This is a description of the communication between the spouses, an objective fact and does not involve subjective attitudes or feelings of the patient."}, "sentence_sentiment": {"answer": "Neutral", "rationale": ""}, "language_style": {"answer": "Tactful and warm", "rationale": "The language style of the entry is euphemistic, because it uses a more moderate tone and presentation, avoiding direct, hard language. Moreover, it does not provide too specific or immediate answers or suggestions, but rather raises a question for the interviewee to think and answer, which is different from the calm and direct language style."}, "time_dimension": {"answer": "Present", "rationale": "\"When I speak with my spouse in good faith,\" which means \"when...\" means \"what is going on, and \"does nothing special\" suggests that it is a normal practice, not a recent event. So the entry is to ask the testee what is going on and to judge the dimensions of the moment."}, "sentence_type": "Affirmative declarative sentence", "pos": {"nouns": {"Questionnaire": 1, "Entry": 1, "Me and": 2, "I": 1, "Spouse": 1, "Truth": 1, "Communications": 1}, "verbs": {"Do it": 2, "I didn't do it": 1}, "adjectives": {"Special": 1}, "adverbs": {"For that reason": 1, "and": 1}, "numerals": {"41": 1, "What": 1}, "classifiers": {"Yes": 1}, "pronouns": {"we": 1}}}, {"content": "42. How well I get along with my spouse is largely up to how he/she felt that day.", "word_count": 28, "label": "", "sentence_structure": "Subject鈥搗erb鈥搊bject", "subjective_objective": {"answer": "Subjective", "rationale": "Because \"getting along well\" depends on the other person's feelings, which are the patient's personal and subjective feelings."}, "sentence_sentiment": {"answer": "Negative", "rationale": "Because the expression \"depending on his/her mood that day\" in the entry is negative emotional."}, "language_style": {"answer": "Tactful and warm", "rationale": "The question was posed in a euphemism, without directly pointing out the questions of the respondents, but rather in an interesting and understandable tone, asking about their relationship with their spouses and highlighting the emotional impact of the relationship, which could better trigger the reflection and answers of the respondents."}, "time_dimension": {"answer": "Present", "rationale": "Now, the words \"how does he get along\" and \"well depends on how he/she feels on the day\" are all the words describing the current situation, so it is possible to judge the present. And, while I am the main word, the guest language of \"how do I get along with my spouse\" is he/she, and the phrase \"a great deal depends on how he/she feels that day\" indicates that the entry is a subjective description rather than an objective statement."}, "sentence_type": "Affirmative declarative sentence", "pos": {"nouns": {"Spouse": 1, "Get along": 1, "Level": 1, "he": 1, "she": 1, "Oh, my God": 1, "The mood": 1}, "verbs": {"Same": 1, "Limit to": 1, "How's it going": 1}, "adjectives": {"A lot": 1}, "adverbs": {"How's that": 1}, "numerals": {"42": 1}, "classifiers": {"individual": 1}, "pronouns": {"I": 1}}}, {"content": "4. Most of the troubles I had with my spouse were due to the occasional conversation.", "word_count": 20, "label": "", "sentence_structure": "Subject鈥搗erb鈥搊bject", "subjective_objective": {"answer": "Subjective", "rationale": "Because \"most of the trouble between me and my spouse is the occasional problem\" is the subjective perception and feeling of the patient."}, "sentence_sentiment": {"answer": "Neutral", "rationale": "Because the entry is only a description of a phenomenon and does not have any emotional colour."}, "language_style": {"answer": "Tactful and warm", "rationale": "The language style of the entry is euphemistic, as it is expressed in a more gentle, polite and sweet manner than in direct statements or orders. It also attempts to direct respondents to share their experiences and feelings in order to better understand their marital relationship."}, "time_dimension": {"answer": "Present", "rationale": "The keywords \"problem\" and \"mostly\" and the verb \"cause\" all indicate that it is a statement describing the current or recent situation. Moreover, the emergence of the topic suggests that it is a matter of interpersonal relations, not of past experience. So we can judge that the entry is an inquiry into the current situation of the subject, and the temporal dimension is now."}, "sentence_type": "Affirmative declarative sentence", "pos": {"nouns": {"Questionnaire": 1, "Entry": 1, "I": 1, "Spouse": 1, "Trouble": 1, "Topic": 1}, "verbs": {"Out": 1, "Cause of": 1, "Analysis": 1}, "adjectives": {"It's a coincidence": 1}, "numerals": {"4": 1}}}, {"content": "15. It is a matter of luck to have satisfactory sexual relations with their partners.", "word_count": 27, "label": "", "sentence_structure": "Subject鈥揷opula鈥損redicative", "subjective_objective": {"answer": "Subjective", "rationale": "This is because the patient ' s views and evaluation of sexual relations are subjective."}, "sentence_sentiment": {"answer": "Negative", "rationale": "Because the words \"sexual relations\" and \"luck\" in the entry are negative, implying that such conduct or circumstances may not be determined by personal efforts."}, "language_style": {"answer": "Tactful and warm", "rationale": "The entry is more euphemistic and implicit, is softer, is expressed in relatively indirect terms, does not directly touch sensitive topics or is described in strong language. Moreover, the sentence is also characterized by an objective and rational emotional tone, showing respect and caution on the part of the questionnaire designers."}, "time_dimension": {"answer": "Present", "rationale": "The entry asks whether the testee ' s satisfaction with the relationship with its pair is considered to be of luck-related, and therefore it does not have a clear temporal dimension, past, present and future."}, "sentence_type": "Affirmative declarative sentence", "pos": {"nouns": {"Questionnaire Entry": 1, "person": 1, "Sexual relations": 1, "Yes": 1}, "verbs": {"Hold on": 1, "be": 2, "Related": 1, "It's a piece": 1}, "adjectives": {"Satisfied": 1, "Lucky": 1}, "numerals": {"How much": 1, "15": 1}, "pronouns": {"self": 1}}}, {"content": "21. Success in child-rearing is a result of the luck of this.", "word_count": 17, "label": "", "sentence_structure": "Subject鈥揷opula鈥損redicative", "subjective_objective": {"answer": "Objective", "rationale": "Because successful parenting is an objective fact, not a matter of luck."}, "sentence_sentiment": {"answer": "Negative", "rationale": "Because the phrase \"success in raising children is the result of luck in this regard\" in the entry devalues personal effort and talent and has a negative emotional connotation."}, "language_style": {"answer": "Tactful and warm", "rationale": "The entry is a softer, more inviting expression, where the other person is asked about his/her views in the education of the child, rather than directly speaking or ordering. At the same time, the term \"lucky\" is used, which expresses a more modest and objective attitude, without emphasizing his/her views or achievements, but rather a focus on listening to and understanding the other person's thoughts."}, "time_dimension": {"answer": "Past", "rationale": "The entry describes what has happened or events, and the keyword \"successful parenting\" and \"results of luck in this regard\" indicate a reflection or a reflection of the past."}, "sentence_type": "Affirmative declarative sentence", "pos": {"nouns": {"Son": 1, "Corrections": 1, "Success": 1, "In terms of": 1, "Result": 1}, "verbs": {"be": 1, "Lucky": 1, "in": 1}, "adjectives": {"Here": 1}, "numerals": {"21": 1}, "pronouns": {"Well": 1}}}, {"content": "22. If my marriage was long and happy, I would say I was lucky.", "word_count": 21, "label": "", "sentence_structure": "Subject鈥損redicate", "subjective_objective": {"answer": "Subjective", "rationale": "Because \"lucky\" is a subjective view and evaluation, it expresses the subjective sense of the patient's enduring and happy marriage."}, "sentence_sentiment": {"answer": "Negative", "rationale": "Because the attitude in the entries is negative, the expression \"just lucky\" implies a denial of self-preservation."}, "language_style": {"answer": "Tactful and warm", "rationale": "The language style of the entry is euphemistic, because it does not directly express \"I\" strong confidence or pride in marriage happiness, but rather demonstrates an emotional attitude that is internal and implicit through the hypothetical phrase \"if you are lucky\" and the modest expression \"if you are lucky\" (as in the case of \"just lucky.\" At the same time, it makes the interviewee more relaxed in answering questions and is willing to share his views and feelings about marriage."}, "time_dimension": {"answer": "Future", "rationale": "The temporal dimension of the article is therefore future."}, "sentence_type": "Affirmative declarative sentence", "pos": {"nouns": {"Marriage": 1, "Happiness": 1, "Lucky": 1}, "verbs": {"Persistence": 1, "Yes": 1, "Sure": 1}, "adjectives": {"Lucky": 1}, "adverbs": {"It's just": 1}, "numerals": {"22": 1}, "pronouns": {"I": 1}}}, {"content": "When I look back at my marriage, I wonder if it is not meant to be.", "word_count": 27, "label": "", "sentence_structure": "Subject鈥搗erb鈥搊bject", "subjective_objective": {"answer": "Subjective", "rationale": "Because \"I wonder if everything is not meant to be this way\" is the patient's subjective feeling and thinking."}, "sentence_sentiment": {"answer": "Negative", "rationale": "Because the question \"is everything not meant to be so\" in the entry is an emotional expression of uncertainty and doubt."}, "language_style": {"answer": "Tactful and warm", "rationale": "The entry, in a recollection, brings out perceptions and reflections of marriage, a relatively moderate, euphemistic style, and no overly direct or emotional expression. At the same time, the phrase \"is everything not meant to be so\" leaves room for the interviewee to reflect and respond to himself."}, "time_dimension": {"answer": "Present", "rationale": "The term \"recall\" in this entry indicates reflection and reflection on the past, and \"just think\" of the act takes place now, so the temporal dimension of the article is now."}, "sentence_type": "Interrogative sentence", "pos": {"nouns": {"Marriage": 1, "Experience": 1}, "verbs": {"Recalling": 1, "Yes": 1, "be": 1, "Not really": 1, "Destiny": 1}, "adjectives": {"Your own": 1}, "adverbs": {"That's right": 1, "Here": 1}, "numerals": {"30": 1, "Everything": 1}, "pronouns": {"I": 1}}}, {"content": "32. It was fortunate that couples who had not experienced problems in all aspects of marriage had been able to do so.", "word_count": 26, "label": "", "sentence_structure": "Subject鈥揷opula鈥損redicative", "subjective_objective": {"answer": "Subjective", "rationale": "Because \"no trouble\" and \"thankfully\" are based on subjective perceptions and judgment of the interviewees."}, "sentence_sentiment": {"answer": "Positive", "rationale": "Because this entry expresses appreciation and blessing for the successful marriage."}, "language_style": {"answer": "Tactful and warm", "rationale": "The entry expresses a view in a praising and blessing, rather than criticism or denigration, which is more respectful and encouraging. At the same time, it does not use too direct or strong a tone, but rather is expressed in a more moderate way."}, "time_dimension": {"answer": "Past", "rationale": "In the entry, the description \"without trouble\" is about past experiences and circumstances of \"all aspects of marriage\" and therefore the past is judged."}, "sentence_type": "Affirmative declarative sentence", "pos": {"nouns": {"Couple": 1, "Marriage": 1, "In terms of": 1, "Trouble": 1, "Lucky": 1}, "verbs": {"I've got it": 1, "No, I'm not": 1, "be": 1, "I met him": 1, "Lucky": 1}, "adjectives": {"Each": 1, "In terms of": 1, "very": 1}, "adverbs": {"Not even": 1}, "numerals": {"32": 1}, "pronouns": {"Those": 1, "No, I'm not": 1}}}, {"content": "44. Achieving a relationship of affection that is satisfactory to both parties requires something other than the will and ability of the couple; this is a special magic that may or may not exist.", "word_count": 50, "label": "", "sentence_structure": "Subject鈥搗erb鈥搊bject", "subjective_objective": {"answer": "Subjective", "rationale": "Because \"special magic\" is a subjective view or interpretation, not an objective thing. And other parts like \"emotional relationships\" and \"the will and ability of couples\" are objective facts."}, "sentence_sentiment": {"answer": "Neutral", "rationale": "Because the entry does not have any emotional color."}, "language_style": {"answer": "Tactful and warm", "rationale": "The entry is more personal and interactive in terms of dialogue in a way that explores and asks questions and expresses views in some poetic language."}, "time_dimension": {"answer": "Present", "rationale": "The entry describes an emotional relationship, using terms such as \"need\", \"presence\" and \"possibly non-existence\" that indicate a situation that is, or may occur in the future."}, "sentence_type": "Interrogative sentence", "pos": {"nouns": {"Both sides": 1, "Emotional relations": 1, "Will": 2, "Capacity": 1, "Something": 1, "Magic": 1}, "verbs": {"Achieved": 1, "Yes": 1, "Existence": 1, "Maybe": 3, "Cannot initialise Evolution's mail component": 1, "Special": 1, "be": 1, "Give": 1}, "adjectives": {"Satisfied": 1, "Couple of them": 1, "Special": 1}, "adverbs": {"Yeah": 2}, "numerals": {"44": 1}, "classifiers": {"Plant": 1}, "pronouns": {"Here": 1}}}]}

{"questionnaire_title": "Social anxiety table", "questionnaire_category": "Mental health", "item_count": 12, "emotion_shift_overall": "From positive to negative", "language_style_overall": "Neutral", "items": [{"content": "1. In the new environment, I have spent a lot of time overcoming shame.", "word_count": 18, "label": "", "sentence_structure": "Subject鈥搗erb鈥搊bject", "subjective_objective": {"answer": "Subjective", "rationale": "Because the entry is a description of the patient's feelings and perceptions, which take time to overcome shyness. This feeling is subjective, so the answer is \"subjective.\""}, "sentence_sentiment": {"answer": "Negative", "rationale": "Because the entry \"to take a lot of time to overcome shyness\" is negative."}, "language_style": {"answer": "Tactful and warm", "rationale": "The entry is more adept and interactive when it is spoken in a gentle and inviting manner, rather than by direct statements or orders."}, "time_dimension": {"answer": "Present", "rationale": "This entry indicates that the testee is facing a new environment and that it requires time and effort to overcome his own shyness. So the time dimension is now judged. And, while \"to spend\" indicates that it is a future goal or plan, in the context of this issue, the main emphasis is on the current state and challenges."}, "sentence_type": "Affirmative declarative sentence", "pos": {"nouns": {"New Environment": 1, "Time": 1, "Overcoming": 1, "Shame": 1}, "verbs": {"- Yes, it's a flower": 1, "Go, go, go": 1}, "adjectives": {"A lot": 1}, "numerals": {"One": 1}, "classifiers": {"individual": 1}}}, {"content": "When I was looked at, I was always in a mess.", "word_count": 15, "label": "", "sentence_structure": "Subject鈥搗erb鈥搊bject", "subjective_objective": {"answer": "Subjective", "rationale": "Because \"someone looks at me\" is a psychological state, which is shaped by the patient's own feelings and observations, and is not an objective fact. So the question is about the patient's subjective feelings and attitudes."}, "sentence_sentiment": {"answer": "Negative", "rationale": "Because the \"doing the house is always wrong\" in the entry is negative."}, "language_style": {"answer": "Tactful and warm", "rationale": "The entry does not directly express accusations or criticism, but rather uses a more moderate tone to ask questions. At the same time, the entry uses the term \"always\" to express concern and concern about the issue, giving people a warm and warm feeling of affection. The language style of the article is therefore more euphemismal."}, "time_dimension": {"answer": "Present", "rationale": ""}, "sentence_type": "Negative declarative sentence", "pos": {"nouns": {"person": 1, "Work": 1, "- What a mess": 1}, "verbs": {"Watch": 1, "Total": 1, "Fuck": 1, "Out": 1}, "adverbs": {"Always": 1}, "pronouns": {"I": 1}}}, {"content": "I am very vulnerable.", "word_count": 7, "label": "", "sentence_structure": "Subject鈥揷opula鈥損redicative", "subjective_objective": {"answer": "Subjective", "rationale": "Because \"very vulnerable\" is an emotional experience and subjective feeling that reflects the subjective reaction of individuals to an awkward, uncomfortable social situation."}, "sentence_sentiment": {"answer": "Negative", "rationale": "Because \"easy\" is negative."}, "language_style": {"answer": "Tactful and warm", "rationale": ""}, "time_dimension": {"answer": "Present", "rationale": "The entry uses the state or behaviour of the dominant language in its present-day state, indicating the current situation and thus judging it as the present."}, "sentence_type": "Affirmative declarative sentence", "pos": {"verbs": {"I'm embarrassed": 1}, "adjectives": {"Very": 1}}}, {"content": "I find it not difficult to talk to strangers.", "word_count": 13, "label": "", "sentence_structure": "Subject鈥搗erb鈥搊bject", "subjective_objective": {"answer": "Subjective", "rationale": "Because \"discovery\" is the patient's subjective feeling, and whether it is difficult to talk to a stranger is the patient's subjective view of the situation."}, "sentence_sentiment": {"answer": "Positive", "rationale": "Because it is not difficult to discover and communicate with strangers, it is a positive quality of self-confidence, social skills, etc."}, "language_style": {"answer": "Tactful and warm", "rationale": "The language style of the entry is euphemistic. It does not ask the interviewee whether he is afraid to talk to strangers, but rather it stimulates the resonance and participation of the other by stating the fact that he finds it easy to talk to strangers, making it more natural and easier to open his heart. And, \"I find\" is a way to share personal experience and feelings, enhancing the interaction and authenticity of communication."}, "time_dimension": {"answer": "Present", "rationale": "The keyword \"discovery\" denotes past conduct or state, while \"no difficulty\" implies that the act or state continues to this point, so the entry describes the present situation."}, "sentence_type": "Affirmative declarative sentence", "pos": {"nouns": {"I found out": 1, "Stranger": 1, "Talk": 1, "Difficulties": 1}, "verbs": {"Found": 1, "Talk": 1, "not": 1, "Difficulties": 1}, "adjectives": {"Strange": 1}}}, {"content": "I was anxious when I spoke in front of the crowd.", "word_count": 12, "label": "", "sentence_structure": "Subject鈥揷opula鈥損redicative", "subjective_objective": {"answer": "Subjective", "rationale": "Because anxiety is a subjective emotional experience."}, "sentence_sentiment": {"answer": "Negative", "rationale": "Because \"fear\" is a negative act."}, "language_style": {"answer": "Tactful and warm", "rationale": "The entry is more personal and interactive when it is spoken in a gentle and inviting manner, rather than in direct statements or orders. At the same time, the use of the word \"I\" to guide the respondents to share their feelings reflects respect for and protection of the privacy of the interviewees."}, "time_dimension": {"answer": "Present", "rationale": ""}, "sentence_type": "Affirmative declarative sentence", "pos": {"nouns": {"Questionnaire Entry": 1, "Population": 1, "Talk": 1, "Anxiety": 1}, "verbs": {"I feel": 1}, "adverbs": {"in": 1, "Front": 1}, "pronouns": {"I": 1}}}, {"content": "A large group of people makes me nervous.", "word_count": 9, "label": "", "sentence_structure": "Subject鈥揷opula鈥損redicative", "subjective_objective": {"answer": "Subjective", "rationale": "Because feeling nervous is a subjective experience and emotional response."}, "sentence_sentiment": {"answer": "Negative", "rationale": "Because the emotional reaction to \"stretch\" in the entry is negative."}, "language_style": {"answer": "Calm and direct", "rationale": "The entry expresses a clear view in a concise manner, without too much emotional accoutrements or rhetorical techniques, and is more calm and objective in tone.\""}, "time_dimension": {"answer": "Present", "rationale": "The entry expresses a feeling or emotional reaction to the current situation, does not mention past or future changes in behaviour or mindset, and therefore determines \"now\"."}, "sentence_type": "Affirmative declarative sentence", "pos": {"nouns": {"Population": 1, "Nervous": 1}, "verbs": {"鈾?And I'm gonna be 鈾?: 1}, "adjectives": {"Big group": 1}}}, {"content": "I have to spend a lot of time overcoming my shyness in the new environment.", "word_count": 17, "label": "", "sentence_structure": "Subject鈥搗erb鈥搊bject", "subjective_objective": {"answer": "Subjective", "rationale": "Because \"overcoming shyness\" is an individual's inner reaction and feeling, and it's a subjective angle."}, "sentence_sentiment": {"answer": "Negative", "rationale": "Because the act of \"overcoming shyness\" in the entry is negative."}, "language_style": {"answer": "Calm and direct", "rationale": "The entry describes a specific act or situation in simple language, without too much emotional rendering or rhetorical techniques, and therefore can be judged as \"cool and direct \" language style."}, "time_dimension": {"answer": "Present", "rationale": "Because the sentence describes a current situation, in which it takes time to overcome shy behaviour or mentalities in the new environment."}, "sentence_type": "Affirmative declarative sentence", "pos": {"nouns": {"Environment": 1, "Time": 1, "Shame": 1}, "verbs": {"It's a cost": 1, "Overcoming": 1}, "adverbs": {"A lot": 1}, "numerals": {"1": 2}, "pronouns": {"I": 1}}}, {"content": "I was working hard when I was looked at.", "word_count": 12, "label": "", "sentence_structure": "Subject鈥搗erb鈥揷omplement", "subjective_objective": {"answer": "Subjective", "rationale": "Because \"look at me\" is the subjective feeling of the person being observed, and \"working hard\" is the subjective experience of the person being observed."}, "sentence_sentiment": {"answer": "Negative", "rationale": "Because the \"work hard\" act in the entry is negative."}, "language_style": {"answer": "Calm and direct", "rationale": "This question is more straightforward and concise, and it is difficult to ask the respondents whether they are looking at the work. There are no excessive rhetorical techniques or emotional rendering, and it is more objective in tone and is consistent with the calm and direct language style."}, "time_dimension": {"answer": "Past", "rationale": "The keywords \"look at me\" and \"work hard\" describe past experiences or behaviours, and therefore judge as past."}, "sentence_type": "Affirmative declarative sentence", "pos": {"nouns": {"person": 1, "I": 1, "Live": 1, "Powerful": 1}, "verbs": {"Watch": 1, "Fuck": 1, "Come on": 1}, "adjectives": {"very": 1}, "numerals": {"2": 1}}}, {"content": "3. I am very vulnerable.", "word_count": 7, "label": "", "sentence_structure": "Subject鈥搗erb鈥揷omplement", "subjective_objective": {"answer": "Subjective", "rationale": "Because \"damn\" is a subjective feeling and experience, different people may feel and react differently."}, "sentence_sentiment": {"answer": "Negative", "rationale": "Because the word \"dressed\" in the entry has some negative emotional connotations."}, "language_style": {"answer": "Tactful and warm", "rationale": "Warm and warm."}, "time_dimension": {"answer": "Present", "rationale": ""}, "sentence_type": "Negative declarative sentence", "pos": {"verbs": {"I'm embarrassed": 1}, "adjectives": {"Very": 1}}}, {"content": "4. It is easy for me to talk to strangers.", "word_count": 10, "label": "", "sentence_structure": "Subject鈥搗erb鈥搊bject", "subjective_objective": {"answer": "Subjective", "rationale": "Because the ease of talking to strangers is a subjective feeling and attitude, it is not an objective fact or experience."}, "sentence_sentiment": {"answer": "Neutral", "rationale": "Because the entry does not have any emotional color."}, "language_style": {"answer": "Tactful and warm", "rationale": "The language style of the entry is more euphemistic and warm, and the information that the surveyer would like to know if the interviewee would be willing to deal with strangers is conveyed by using the more objective presentation \"I speak with strangers\" and is not too direct or hard to speak, with some degree of affection and interaction."}, "time_dimension": {"answer": "Present", "rationale": "The entry describes the current behaviour of the subjects, that is, their feelings when talking to strangers, and thus the temporal dimension is now judged."}, "sentence_type": "Affirmative declarative sentence", "pos": {"verbs": {"Talk": 1, "Easy": 1}, "adjectives": {"Strange": 1}, "adverbs": {"very": 1}, "pronouns": {"I": 1}}}, {"content": "5. I was nervous when I spoke in front of the crowd.", "word_count": 12, "label": "", "sentence_structure": "Subject鈥揷opula鈥損redicative", "subjective_objective": {"answer": "Subjective", "rationale": "The answer to this questionnaire is \"subjective\"."}, "sentence_sentiment": {"answer": "Negative", "rationale": "Because the \"frightful\" sentiment in the entry is negative."}, "language_style": {"answer": "Calm and direct", "rationale": "The entry is more direct and concise, is not based on excessive rhetorical or emotional rendering, and is more objective. It is straightforward in asking whether the respondents are nervous in front of the crowd, and expresses the information that researchers want to get, without excessive emotional or personal subjective judgement."}, "time_dimension": {"answer": "Present", "rationale": "The entry indicates that the person who was tested experienced an emotional reaction (strained) in his current life and that this emotional experience occurred in a particular situation (talking in front of a crowd). Thus, we can judge that the entry describes the person's current behaviour or mental state."}, "sentence_type": "Affirmative declarative sentence", "pos": {"nouns": {"Population": 1, "Talk": 1}, "verbs": {"in": 1, "Sensor": 1, "Present": 1, "Nervous": 1}}}, {"content": "A large group of people makes me nervous.", "word_count": 9, "label": "", "sentence_structure": "Subject鈥揷opula鈥損redicative", "subjective_objective": {"answer": "Subjective", "rationale": "Because it is the patient's own subjective feeling that is nervous."}, "sentence_sentiment": {"answer": "Negative", "rationale": "Because the emotional description of \"would make me nervous\" in the entry is negative."}, "language_style": {"answer": "Calm and direct", "rationale": "The entry asks the interviewee whether he or she is nervous in the crowd, and does not use any qualifier language or expression to calm the atmosphere or guide the answer."}, "time_dimension": {"answer": "Present", "rationale": "The keyword is \"will,\" which means that it is a customary emotional reaction, usually in the current state. At the same time, the situation of \"a large group of people\" is a specific trigger, further proof of the description of the current emotional state."}, "sentence_type": "Affirmative declarative sentence", "pos": {"nouns": {"Population": 1, "I": 1}, "verbs": {"Yes": 1, "鈾?And I'm gonna be 鈾?: 1, "Nervous": 1}, "adjectives": {"Large": 1}, "adverbs": {"very": 1}, "numerals": {"One": 1}}}]}

{"questionnaire_title": "OLSON Qualification of Marriage", "questionnaire_category": "Emotion and affect", "item_count": 124, "emotion_shift_overall": "From negative to positive", "language_style_overall": "Tactful and warm", "items": [{"content": "We all like the same kind of social activity.", "word_count": 13, "label": "", "sentence_structure": "Subject鈥搗erb鈥搊bject", "subjective_objective": {"answer": "Subjective", "rationale": "The subjective attitude and preference of the patients are examined, namely, whether they all like the same type of social activity. This subjective feeling and view is not influenced by the external context and objective facts, but is determined by the individual鈥檚 inner experience."}, "sentence_sentiment": {"answer": "Positive", "rationale": "Because the \"like the same kind of social activity\" in the entry is positive."}, "language_style": {"answer": "Tactful and warm", "rationale": "The entry is more personal and interactive in its tone of conversation in a gentle and inviting manner, expressing curiosity and expectations about what the respondents have in common with themselves."}, "time_dimension": {"answer": "Present", "rationale": "The keywords \"like\" and \"social activity\" both indicate current behaviour or state, so the judgement is now."}, "sentence_type": "Affirmative declarative sentence", "pos": {"nouns": {"we": 1, "Social activities": 1}, "verbs": {"Both": 1, "Yeah": 1}, "adverbs": {"Same type": 1}}}, {"content": "It is very easy to express my true feelings to a spouse.", "word_count": 17, "label": "", "sentence_structure": "Subject鈥揷opula鈥損redicative", "subjective_objective": {"answer": "Subjective", "rationale": "Because \"it's very easy to express my true feelings\" is a subjective assessment or opinion that reflects the patient's perception of his ability to communicate in a marriage."}, "sentence_sentiment": {"answer": "Positive", "rationale": "Because \"expressing true feelings\" in the entry is positive."}, "language_style": {"answer": "Tactful and warm", "rationale": "This entry uses more euphemistic and sweet language, which, by emphasizing \"real feelings\" and \"very easy\", makes each other feel comfortable and supportive in their emotional expression and encourages them to communicate more openly with their spouses. Overall, this language style helps to create a positive and harmonious atmosphere that will facilitate the successful conduct of the questionnaire."}, "time_dimension": {"answer": "Present", "rationale": "The entry \"is\" uses the general current, indicating that the act has not only occurred in the past, but may still be ongoing. The description \"very easy\" is also about the current situation. So the time dimension of the questionnaire entry is now judged."}, "sentence_type": "Affirmative declarative sentence", "pos": {"nouns": {"Spouse": 1, "Feel": 1}, "verbs": {"Expression": 1, "be": 1, "Very": 1, "Easy": 1}, "adjectives": {"Real": 1, "of": 2}, "numerals": {"2": 1}, "pronouns": {"I": 1}}}, {"content": "3. Education of him/her about his/her religious beliefs", "word_count": 16, "label": "", "sentence_structure": "Subject鈥搗erb鈥揷omplement", "subjective_objective": {"answer": "Subjective", "rationale": "Because \"he/she is taught about religious beliefs\" is an education that the patient has received himself, and it looks at the patient's subjective thoughts and experiences."}, "sentence_sentiment": {"answer": "Neutral", "rationale": "Because the entry describes the fact that a person has been educated on religious beliefs, it does not relate to emotions, attitudes or behaviour."}, "language_style": {"answer": "Calm and direct", "rationale": "The decision is based on the following: the purpose of the article is simple and clear, not to use too much qualifier or rhetoric to describe the situation of the other party, but to ask the other side what religion or belief is taught."}, "time_dimension": {"answer": "Present", "rationale": "The entry asks the testee about the level of knowledge of the education he receives about religious beliefs, and the temporal dimension is now judged."}, "sentence_type": "Declarative sentence", "pos": {"nouns": {"he": 1, "she": 1, "Religious beliefs": 1, "Education": 1}, "verbs": {"Received": 1}, "numerals": {"3": 1}, "pronouns": {"EMPTY": 1}}}, {"content": "4. In order to put an end to the dispute as soon as possible, I have often given in.", "word_count": 14, "label": "", "sentence_structure": "Subject鈥搗erb鈥搊bject", "subjective_objective": {"answer": "Subjective", "rationale": "Because \"immediate concessions\" are patient subjective behavior and attitude."}, "sentence_sentiment": {"answer": "Negative", "rationale": "Because the purpose of the act of \"concession\" in the entry is negative."}, "language_style": {"answer": "Calm and direct", "rationale": "This language style is usually used in official settings such as questionnaires to ensure that the respondents understand the problem accurately and avoid ambiguity."}, "time_dimension": {"answer": "Present", "rationale": "Now, the word \"I often give in\" means \"recurring, customary\" and \"immediate\" means \"immediate\" is a move to complete the current state of affairs, which means that it has already happened in the past and may have an impact on the present. So, this entry asks the tester about the way he usually behaves in the face of a quarrel, and the time dimension is now."}, "sentence_type": "Affirmative declarative sentence", "pos": {"verbs": {"End": 1, "Argument": 1, "Compliment": 1}, "adverbs": {"As soon as possible": 1, "Immediately": 1}, "pronouns": {"I": 1}}}, {"content": "5. In our home, fathers spend not enough time with their children.", "word_count": 21, "label": "", "sentence_structure": "Subject鈥搗erb鈥搊bject", "subjective_objective": {"answer": "Subjective", "rationale": "Because \"the time spent with the child by the father\" is a subjective feeling and an assessment of the patient, not an objective reality."}, "sentence_sentiment": {"answer": "Negative", "rationale": "Because the entry states that the father is not spent enough time with the child, this may have a negative impact on the emotional health and development of the child and therefore be negative."}, "language_style": {"answer": "Calm and direct", "rationale": "The basis for the determination: The entry is structured in a simple and clear sentence, which directly raises the question of whether the father and the child spends sufficient time at home. This expression is not too emotional or rhetorical, but presents the problem in an objective and direct way, so it can be judged that the language style of the entry is straightforward and straightforward."}, "time_dimension": {"answer": "Present", "rationale": "So the time dimension is now judged."}, "sentence_type": "Negative declarative sentence", "pos": {"nouns": {"Time": 1, "Home": 1, "Father": 1, "Son": 2}, "verbs": {"Stay": 1, "Flowers": 1, "That's enough": 1}, "numerals": {"5": 1}, "classifiers": {"One": 1, "Sou-san": 1}, "pronouns": {"we": 1}}}, {"content": "When a couple asks a conflict, my spouse is silent.", "word_count": 18, "label": "", "sentence_structure": "Subject鈥搗erb鈥揷omplement", "subjective_objective": {"answer": "Subjective", "rationale": "However, in the above-mentioned questionnaire entries, the question is asked about the specific fact that \"my spouse is often silent when a couple asks a contradiction.\" So the question is considered from an objective perspective. The answer to this question depends on the actual situation, not on the patient's subjective perception or feelings."}, "sentence_sentiment": {"answer": "Negative", "rationale": "Because the act of \"spouses often silence\" in the entry is negative."}, "language_style": {"answer": "Calm and direct", "rationale": "Thus, the language style is considered to be straightforward and calm."}, "time_dimension": {"answer": "Present", "rationale": "\"When\" in \"when\" means conditions or circumstances, \"when\" in \"in\" is a verb describing a state, and \"in\" in \"in silence\" is a \"in a regular, customary act. The above-mentioned information is combined with the behaviour of the spouse when asking the testee about the conflict between the couple, and the act may still be occurring at this time. Thus, the time frame of this questionnaire is now."}, "sentence_type": "Negative declarative sentence", "pos": {"nouns": {"Paradoxious": 1, "Couples": 1, "Spouse": 2}, "verbs": {"Come on": 1, "Question": 1, "Silence": 1}, "numerals": {"6": 1}, "pronouns": {"I": 1}}}, {"content": "Some of our relatives have made our marriage nervous.", "word_count": 16, "label": "", "sentence_structure": "Subject鈥搗erb鈥搊bject", "subjective_objective": {"answer": "Subjective", "rationale": "This perception and perception is subjectively responsive and evaluated because the behaviour of the relatives and friends has an impact on the patient ' s marital relationship."}, "sentence_sentiment": {"answer": "Negative", "rationale": "Because the \"making our marriage nervous\" act in the entry is negative."}, "language_style": {"answer": "Tactful and warm", "rationale": "The entry is more euphemistic and warm, using the more subjective term \"some people\" to describe the problem, and is not too direct or offensive, but rather to guide the interviewee to express his feelings and experiences in an understanding and caring manner."}, "time_dimension": {"answer": "Present", "rationale": ""}, "sentence_type": "Affirmative declarative sentence", "pos": {"nouns": {"Friends": 1, "Marriage": 1, "Nervous": 1}, "verbs": {"鈾?And I'm gonna be 鈾?: 1}}}, {"content": "My spouse is too picky or often negative.", "word_count": 17, "label": "", "sentence_structure": "Subject鈥揷opula鈥損redicative", "subjective_objective": {"answer": "Subjective", "rationale": "Since the description of the purpose of the questionnaire mainly concerns the patient's perception and feelings regarding his/her spouse's attitude, which are part of the patient's subjective experience and opinion, the answer is \"subjective\"."}, "sentence_sentiment": {"answer": "Negative", "rationale": "Because \"too picky\" and \"often negative\" in the entries are negative emotional tendencies, describing the negative character characteristics that may exist in the other side."}, "language_style": {"answer": "Calm and direct", "rationale": ""}, "time_dimension": {"answer": "Past", "rationale": "The entry asks whether the testee has encountered spousal pick-up or negative views in his or her past experience, and the temporal dimension is judged to be past."}, "sentence_type": "Negative declarative sentence", "pos": {"nouns": {"Spouse": 1, "Views": 1}, "verbs": {"Too much": 1, "Picky": 1, "Hold": 1, "Negative": 1}}}, {"content": "I'm completely satisfied with my spouse's feelings for me.", "word_count": 12, "label": "", "sentence_structure": "Subject鈥搗erb鈥揷omplement", "subjective_objective": {"answer": "Subjective", "rationale": "Because \"satisfaction\" is a subjective feeling that belongs to the patient's assessment and perception of his/her feelings."}, "sentence_sentiment": {"answer": "Positive", "rationale": "Because the description of \"satisfaction\" is positive and indicates a positive attitude and feelings towards his spouse."}, "language_style": {"answer": "Tactful and warm", "rationale": "The entry uses a gentle, invitation-style tone to ask the other person about the satisfaction of his/her spouse ' s feelings, rather than direct orders or statements, which are more intimate and interactive."}, "time_dimension": {"answer": "Present", "rationale": "\"I'm completely satisfied\" means the current feelings and attitudes, so the judgment is now."}, "sentence_type": "Affirmative declarative sentence", "pos": {"nouns": {"Spouse": 1, "Emotion": 1}, "verbs": {"Satisfied": 1}, "adjectives": {"Total": 1}, "numerals": {"9": 1}, "pronouns": {"I": 1}}}, {"content": "10. There is often disagreement between me and the spouse on how best to resolve the conflict.", "word_count": 24, "label": "", "sentence_structure": "Subject鈥搗erb鈥揷omplement", "subjective_objective": {"answer": "Subjective", "rationale": "Because \"unsatisfactory\" is a subjective assessment, it reflects patients' perceptions and feelings of differences with their spouses in resolving their problems."}, "sentence_sentiment": {"answer": "Negative", "rationale": "Because the \"often inconsistent\" act in the entry is negative, implying conflict and disagreement between spouses."}, "language_style": {"answer": "Calm and direct", "rationale": "The entry directly describes the discrepancy between the subject and the spouse in resolving the conflict, with a more concise language style, with no excessive rhetoric or emotional colour."}, "time_dimension": {"answer": "Past", "rationale": "The entry \"often\" indicates that such acts have occurred many times in the past and may continue to occur, and therefore the temporal dimension is a matter of the past."}, "sentence_type": "Affirmative declarative sentence", "pos": {"nouns": {"I": 1, "Spouse": 1, "Paradoxious": 1, "Methodology": 1, "Resolve": 1}, "verbs": {"Just": 1, "Adoption": 1, "Best": 1, "Often": 1, "Observations": 1, "Inconsistencies": 1}, "adverbs": {"How's that": 1}, "numerals": {"10锛?: 1}}}, {"content": "I think that both spouses should have the same understanding of religion.", "word_count": 17, "label": "", "sentence_structure": "Subject鈥搗erb鈥搊bject", "subjective_objective": {"answer": "Subjective", "rationale": "Because \"think\" represents the patient's personal view and opinion, it's a subjective examination."}, "sentence_sentiment": {"answer": "Neutral", "rationale": "Because the entry describes the views or beliefs of the individual and does not address emotional, attitudinal or behavioural aspects, it is therefore neutral."}, "language_style": {"answer": "Tactful and warm", "rationale": "The language style of the entry is euphemistic and sweet, leading the conversation by asking each other鈥檚 views, without using too direct or strong a tone."}, "time_dimension": {"answer": "Present", "rationale": "The entry shows the views and views of the testee at present, without reference to past or future circumstances. Therefore, the temporal dimension is now judged."}, "sentence_type": "Affirmative declarative sentence", "pos": {"nouns": {"Couple": 1, "Religion": 1, "Understood": 1}, "verbs": {"I think": 1, "have": 1, "Reactions": 1, "Same": 1}, "numerals": {"11锛?: 1, "to": 1, "Couple": 1}, "pronouns": {"I": 1}}}, {"content": "I think women should stay mainly at home.", "word_count": 12, "label": "", "sentence_structure": "Subject鈥搗erb鈥搊bject", "subjective_objective": {"answer": "Subjective", "rationale": "This is a personal view of the patient and is a subjective examination."}, "sentence_sentiment": {"answer": "Negative", "rationale": "The view that \"women should stay mainly at home\" in the entry is sexist and therefore negative emotional."}, "language_style": {"answer": "Calm and direct", "rationale": "The entry is presented in a direct way, without excessive rhetorical or emotional rendering and is more objective in tone, and therefore can be judged as \" calm and direct \" language style."}, "time_dimension": {"answer": "Present", "rationale": "The entry is an inquiry into the views and perceptions of the testee on the role of women, and the temporal dimension is not clearly defined by past, present or future references."}, "sentence_type": "Affirmative declarative sentence", "pos": {"nouns": {"Women": 1, "Home": 1}, "verbs": {"I think": 1, "Stay": 1}, "numerals": {"12": 1}, "pronouns": {"I": 1}}}, {"content": "Sometimes I'm concerned about the temper of my spouse.", "word_count": 12, "label": "", "sentence_structure": "Subject鈥揷opula鈥損redicative", "subjective_objective": {"answer": "Subjective", "rationale": "Because \"I care\" expresses the patient's own subjective feelings."}, "sentence_sentiment": {"answer": "Negative", "rationale": "Because the \"spouses are very sensitive\" act in the entry is negative."}, "language_style": {"answer": "Tactful and warm", "rationale": "The entry is more personal and interactive when it is spoken in a gentle and inviting tone, rather than in direct statements or orders. At the same time, the word \"sometimes\" implies that this is a relatively neutral issue and does not make people feel too accusing or attacking."}, "time_dimension": {"answer": "Present", "rationale": "\"Specificly\" means a state of current psychology and feeling, and the time dimension of the entry is therefore judged to be now."}, "sentence_type": "Affirmative declarative sentence", "pos": {"nouns": {"Time": 1, "I": 1, "Spouse": 1, "- Grumpy": 1}, "verbs": {"to": 1, "I care": 1}, "adverbs": {"very": 1}, "numerals": {"13": 1}, "classifiers": {"individual": 1}}}, {"content": "I don't like the personal habits of a spouse.", "word_count": 14, "label": "", "sentence_structure": "Subject鈥搗erb鈥揷omplement", "subjective_objective": {"answer": "Subjective", "rationale": "Because \"not like\" is a subjective attitude, and the personality and habits of a spouse are objective facts."}, "sentence_sentiment": {"answer": "Negative", "rationale": "Because the expression \"not likes\" in the entry is negative."}, "language_style": {"answer": "Calm and direct", "rationale": "The entry directly expresses the fact that the other party does not use excessive rhetorical or emotional renderings to ask the other party whether the other party prefers the subject in terms of the spouse ' s personality and personal habits, and is more objective in terms of tone."}, "time_dimension": {"answer": "Present", "rationale": "The entry \"not likes\" means the emotional attitude at hand, while \"personal habits\" is the object or context of the description, which points to the current situation."}, "sentence_type": "Negative declarative sentence", "pos": {"nouns": {"Spouse": 1, "Character": 1, "person": 1, "Custom": 1}, "verbs": {"Yeah": 1, "not": 1}, "adjectives": {"individual": 1}, "numerals": {"14": 1}, "pronouns": {"I": 1}}}, {"content": "15. To keep sex fun, we're trying to find new ways to do it.", "word_count": 21, "label": "", "sentence_structure": "Subject鈥搗erb鈥搊bject", "subjective_objective": {"answer": "Subjective", "rationale": "Because \"seeing new ways\" is a subjective idea and behavior of a human being."}, "sentence_sentiment": {"answer": "Positive", "rationale": "Because the behavior in the entry \"tried to find some new way\" is positive."}, "language_style": {"answer": "Tactful and warm", "rationale": "The entry is more euphemistic and gentle, uses the words \"tried\" and \"fun\" and does not directly address specific sexual behaviours or descriptions, giving people a lighter, friendly feeling. At the same time, the entry shows a more positive attitude of attention and exploration towards sexual life."}, "time_dimension": {"answer": "Present", "rationale": "\"Making sex fun\" is the goal or the end, and it is a relatively abstract concept that does not directly concern the temporal dimension of the act. Thus, the entry contains both a vision of the future and a description of the current behaviour, which can be considered as a questionnaire that spans the temporal dimension."}, "sentence_type": "Affirmative declarative sentence", "pos": {"nouns": {"Sexual relations": 1, "Fun": 1, "Approach": 1}, "verbs": {"鈾?And I'm gonna be 鈾?: 1, "Hold on": 1, "Try": 1, "Find": 1, "New": 1}, "adverbs": {"鈾?And I'm gonna be 鈾?: 1, "Some": 1}, "numerals": {"15": 1}}}, {"content": "Sometimes, I wish my spouse would not spend money.", "word_count": 11, "label": "", "sentence_structure": "Subject鈥搗erb鈥搊bject", "subjective_objective": {"answer": "Subjective", "rationale": "Because \"favorite\" is a subjective term that shows the expectations and aspirations of patients."}, "sentence_sentiment": {"answer": "Negative", "rationale": "Because the attitude in the entry \"I hope that spouses don't spend money\" is negative."}, "language_style": {"answer": "Calm and direct", "rationale": "The language style of the entry is more straightforward, with no excessive rhetorical or emotional rendering, but rather a direct expression of the perception and expectations of the person who asks about the spouse鈥檚 ilk. This expression is more objective and practical.\""}, "time_dimension": {"answer": "Present", "rationale": "The entry asks about the feelings and thoughts of the testee in the current circumstances, and the time dimension is judged to be now. In it, \"sometimes\" indicates that this is an occasional feeling or thought, \"hope\" may not be a frequent occurrence, and \"do not spend money\" is an expectation or a suggestion of the testee's behaviour towards the spouse, and is now needed. Thus, the entry points to the current situation in terms of time and content."}, "sentence_type": "Negative declarative sentence", "pos": {"nouns": {"Time": 1, "Spouse": 1, "Money": 1}, "verbs": {"Hope": 1, "Flowers": 1}, "adjectives": {"Chaos": 1}, "adverbs": {"Don't": 1, "Sometimes": 1}, "numerals": {"16": 1}, "classifiers": {"individual": 1}, "pronouns": {"I": 1}}}, {"content": "My spouse seems to lack the time and energy to entertain me.", "word_count": 19, "label": "", "sentence_structure": "Subject鈥揷opula鈥損redicative", "subjective_objective": {"answer": "Subjective", "rationale": "Because the lack of time and energy is the subjective feeling and judgement of the patient, this entry is viewed from a subjective perspective."}, "sentence_sentiment": {"answer": "Negative", "rationale": "Because the entry \"Low time and energy to entertain with me\" expresses a negative emotional attitude."}, "language_style": {"answer": "Tactful and warm", "rationale": "The more moderate and euphemistic style of the entry, which does not directly accuse or criticize the spouse, but expresses its desire and desire for the companionship of the spouse, is more acceptable and a source of reflection."}, "time_dimension": {"answer": "Present", "rationale": "\"It seems\" indicates that the speaker is not sure whether the spouses really lack time and energy, but it is their current perception and observation and therefore the judgement is now."}, "sentence_type": "Negative declarative sentence", "pos": {"nouns": {"Spouse": 1, "Time": 1, "- The energy": 1, "I": 1, "Recreation": 1}, "verbs": {"Missing": 1, "and": 2, "Together": 1}, "numerals": {"17": 1}}}, {"content": "I would rather do anything else than spend one night alone.", "word_count": 20, "label": "", "sentence_structure": "Subject鈥搗erb鈥搊bject", "subjective_objective": {"answer": "Subjective", "rationale": "Because it's the patient's subjective attitude and feelings."}, "sentence_sentiment": {"answer": "Negative", "rationale": "Because the phrase \"will do anything else, rather than spend one night alone\" in the entry expresses a negative attitude."}, "language_style": {"answer": "Tactful and warm", "rationale": "The entry is more euphemistic, presented in a question-and-answer manner, rather than in direct command or statement."}, "time_dimension": {"answer": "Present", "rationale": ""}, "sentence_type": "Negative declarative sentence", "pos": {"verbs": {"I'd rather": 1, "Do it": 1, "Other": 1, "Things": 1, "I don't want to": 1, "Single": 1, "From": 1, "Stay": 1, "One": 2, "Night": 1}, "adverbs": {"I'd rather not": 1, "Single": 1, "From": 1}, "numerals": {"One": 2}, "pronouns": {"I": 1}}}, {"content": "I am very pleased with the responsibility of both spouses in marriage.", "word_count": 18, "label": "", "sentence_structure": "Subject鈥揷opula鈥損redicative", "subjective_objective": {"answer": "Subjective", "rationale": "Since satisfaction is the individual ' s feeling and assessment, it is a subjective attitude."}, "sentence_sentiment": {"answer": "Positive", "rationale": "Because the entry \"very satisfied\" expresses a positive attitude and satisfaction, the respondents are satisfied with the responsibilities of both spouses in marriage."}, "language_style": {"answer": "Tactful and warm", "rationale": "The entry is more personal and interactive when it is spoken in a gentle and inviting manner, rather than in direct statements or orders. At the same time, the language style of the article is more euphemistic, using the term \"very satisfied\" to express an attitude towards the responsibilities of both spouses in marriage, which gives people a warmer sense."}, "time_dimension": {"answer": "Present", "rationale": "The entries use expressions such as \"very satisfactory\" and \"responsibility in marriage\", which refer to current feelings and attitudes and therefore judge them as now."}, "sentence_type": "Affirmative declarative sentence", "pos": {"nouns": {"Responsibility": 1, "Couple": 1, "Marriage": 1}, "verbs": {"Undertaking": 1, "Satisfied": 1}, "adjectives": {"Very": 1}, "numerals": {"19锛?: 1}, "pronouns": {"I": 1}}}, {"content": "20. My spouse and I always agree on how to spend money.", "word_count": 15, "label": "", "sentence_structure": "Subject鈥搗erb鈥揷omplement", "subjective_objective": {"answer": "Subjective", "rationale": "This expression is an expression of the patient ' s and the spouse ' s views, which are a manifestation of subjective feelings and attitudes."}, "sentence_sentiment": {"answer": "Positive", "rationale": "Since the entry describes the consensus and coordination of the spouses in the area of money consumption, this behaviour and attitude are positive."}, "language_style": {"answer": "Tactful and warm", "rationale": "Because it does not ask directly whether the other spouse is often in agreement with how the other spouse spends money, but rather uses the word \"always\" to say that this is a normal situation. Moreover, the expression \"me and the spouse\" makes the sentence sound more intimate and sweet."}, "time_dimension": {"answer": "Present", "rationale": "The keyword is \"always,\" which means that this situation continues to this day and is likely to continue. So it's now."}, "sentence_type": "Affirmative declarative sentence", "pos": {"nouns": {"Spouse": 1, "Money": 1, "Flowers": 1}, "verbs": {"be": 1, "Observations": 1, "Unanimously": 1}, "numerals": {"20": 1}, "classifiers": {"individual": 1}, "pronouns": {"I": 1}}}, {"content": "I'm pleased with the division of responsibilities for the upbringing of children.", "word_count": 16, "label": "", "sentence_structure": "Subject鈥揷opula鈥損redicative", "subjective_objective": {"answer": "Subjective", "rationale": "Because the evaluations of \"satisfaction\" and \"responsibility\" are viewed from the subjective point of view of the patient."}, "sentence_sentiment": {"answer": "Positive", "rationale": "Because the \"satisfactory\" in this entry expresses a positive emotional attitude, indicating that respondents have a positive attitude and assessment of this division of responsibilities."}, "language_style": {"answer": "Calm and direct", "rationale": "The entry expresses a fact or view in a concise and clear language, which is not overly emotional or qualifier, and is more direct and objective."}, "time_dimension": {"answer": "Present", "rationale": "The entry describes the current feelings and attitudes of the testee, namely, satisfaction with the division of responsibilities for child-rearing, and thus the time dimension is now judged."}, "sentence_type": "Affirmative declarative sentence", "pos": {"nouns": {"Division of responsibilities": 1, "Children": 1}, "verbs": {"I'm satisfied": 1, "to": 1, "Maintenance": 1}, "adverbs": {"very": 1}, "numerals": {"31锛?: 1}}}, {"content": "22. Common faiths help to develop our relationship", "word_count": 15, "label": "", "sentence_structure": "Subject鈥搗erb鈥揷omplement", "subjective_objective": {"answer": "Subjective", "rationale": "Because \"common faith helps to develop our relationship\" is a subjective view or view."}, "sentence_sentiment": {"answer": "Positive", "rationale": "Because the entry \"Common faith helps to develop our relationship\" expresses a positive attitude and purpose."}, "language_style": {"answer": "Tactful and warm", "rationale": "The language style of the entry is euphemistic. It expresses concern about the development of relationships among the interviewees in a moderate way and implies the role of common faith in it, without direct or direct orders to the interviewees to follow certain rules."}, "time_dimension": {"answer": "Future", "rationale": "The entry describes a future expectation and vision that \"common faith\" can promote the development of interpersonal relations, and that such relationships may occur or occur on a future date. Therefore, judging by the time dimension, the content of the questionnaire should be future."}, "sentence_type": "Affirmative declarative sentence", "pos": {"nouns": {"Faith": 1, "Relations": 1, "Development": 1}, "verbs": {"have": 1, "Helping": 1}, "numerals": {"22": 1}}}, {"content": "23. If both spouses have a job, the husband should have the same domestic duties as the wife.", "word_count": 27, "label": "", "sentence_structure": "Subject鈥損redicate", "subjective_objective": {"answer": "Subjective", "rationale": "This is because it expresses the author's subjective view that \"the spouses should share the household equally\"."}, "sentence_sentiment": {"answer": "Neutral", "rationale": "Because the entry describes a factual situation, it does not express positive or negative emotional sentiments."}, "language_style": {"answer": "Calm and direct", "rationale": "The entry raises a question in a direct statement and clearly indicates what needs to be answered. There is no rhetorical or emotional rendering, which is more objective, and it has some guidance and regulation."}, "time_dimension": {"answer": "Present", "rationale": ""}, "sentence_type": "Negative declarative sentence", "pos": {"nouns": {"Couples": 2, "Work": 2, "Husband": 1, "Wife": 1, "Domestic work": 1}, "verbs": {"I think so": 1, "Undertaking": 1, "Same thing": 1, "More": 1}, "adverbs": {"Both": 1}, "numerals": {"23锛?: 1, "2": 2, "1": 6}, "pronouns": {"If": 1}}}, {"content": "24. I am concerned about the unhappiness and isolation of spouses.", "word_count": 18, "label": "", "sentence_structure": "Subject鈥搗erb鈥搊bject", "subjective_objective": {"answer": "Subjective", "rationale": "Because my fear of a spouse is a subjective feeling and an assessment."}, "sentence_sentiment": {"answer": "Negative", "rationale": "Because the attitude \"regarding the unsatisfied and isolated spouse\" in the entry is negative."}, "language_style": {"answer": "Tactful and warm", "rationale": "The entry uses more euphemistic language, such as \"unsatisfied and isolated with the spouse\" rather than \"I am often angry with or alienated from the spouse,\" which avoids creating resistance, makes the other party more willing to participate in the questionnaire and provide real feedback. At the same time, \"fearing\" also conveys the author's concern and concern about the situation more mildly."}, "time_dimension": {"answer": "Past", "rationale": "\"I am worried about the unsatisfied and isolated spouses\" describes the concerns and concerns of the tested over the behaviour and state of the spouses over time. The temporal dimension is therefore a matter of the past."}, "sentence_type": "Affirmative declarative sentence", "pos": {"nouns": {"Spouse": 1, "I'm alone": 1}, "verbs": {"It seems": 1, "I'm worried": 1}, "adjectives": {"It's not good": 1}, "numerals": {"24锛?: 1}, "pronouns": {"I": 1}}}, {"content": "I fear that spouses may not be sexually attractive to me.", "word_count": 17, "label": "", "sentence_structure": "Subject鈥搗erb鈥搊bject", "subjective_objective": {"answer": "Subjective", "rationale": "Because \"fear\" and \"not excited\" are patients' subjective feelings and attitudes."}, "sentence_sentiment": {"answer": "Negative", "rationale": "Because it expresses feelings of anxiety and anxiety and has a negative emotional connotation."}, "language_style": {"answer": "Calm and direct", "rationale": "The entry directly expresses the subject of questioning the sexual concerns of the other spouse, which is not accompanied by excessive rhetorical or emotional rendering, and is more objective in tone."}, "time_dimension": {"answer": "Present", "rationale": "In the sentence, \"I\" is the dominant expression of the person who speaks, the \"fear\" is the expression of a psychological state or emotional experience, usually used to describe the current situation, and \"spouse may be... less excited\" is about the possibility of the future. So this entry asks about the person who is being questioned about his current fears and emotional feelings, which are the current state of affairs."}, "sentence_type": "Imperative sentence", "pos": {"nouns": {"Problem": 1, "Spouse": 1, "Sex": 1, "In terms of": 1, "not": 1, "鈾?The big bang 鈾?: 1}, "verbs": {"I'm worried": 1, "Maybe": 1}, "adjectives": {"25锛?: 1}, "numerals": {"25": 1}, "pronouns": {"I": 1}}}, {"content": "26. It's hard to make decisions about economic arrangements.", "word_count": 14, "label": "", "sentence_structure": "Subject鈥搗erb鈥搊bject", "subjective_objective": {"answer": "Subjective", "rationale": "Because \"decisions on economic arrangements\" involve the will and choice of the individual, they are subjective. While they may be influenced by objective circumstances, the final decision depends on the individual鈥檚 thoughts and judgement."}, "sentence_sentiment": {"answer": "Negative", "rationale": "Because the \"hard to make a decision\" act in the entry is negative."}, "language_style": {"answer": "Calm and direct", "rationale": "The entry describes in a concise and clear language the difficulties that the subject may face, the lack of excessive rhetorical or emotional rendering, and the directness and objectivity of the language."}, "time_dimension": {"answer": "Past", "rationale": "The entry describes past experience and feelings, and the word \"hard\" indicates the degree of difficulty in taking decisions. Thus, the temporal dimension is a past."}, "sentence_type": "Negative declarative sentence", "pos": {"nouns": {"Economic arrangements": 1, "Decision": 1}, "verbs": {"Make it": 1, "It's hard": 1}, "adverbs": {"very": 1}, "numerals": {"26锛?: 1}}}, {"content": "27. The time we spend on our family and friends is the right one.", "word_count": 13, "label": "", "sentence_structure": "Subject鈥揷opula鈥損redicative", "subjective_objective": {"answer": "Subjective", "rationale": "Because the idea of spending time is patient subjectivity."}, "sentence_sentiment": {"answer": "Negative", "rationale": "Because the act of \"the time we spend for our relatives and friends\" may be considered positive without specific circumstances, but in this case the expression \"the root is right\" is negative, implying that there may be an imbalance or inappropriate distribution of time between relatives and friends, and the entry is therefore in a negative emotional way."}, "language_style": {"answer": "Tactful and warm", "rationale": "The language style of the entry is more euphemistic and friendly, expressing attitudes and views towards the interviewee in a question-based manner, without direct orders or evaluation of the behaviour of the other party. At the same time, the expression \"root\" is more moderate, avoiding the use of too direct or negative language."}, "time_dimension": {"answer": "Present", "rationale": "The entry is for the testee's opinion or attitude to \"the appropriateness of the time we spend for our relatives and friends\", the time dimension of which is now judged."}, "sentence_type": "Interrogative sentence", "pos": {"nouns": {"Time": 1, "Friends": 1}, "verbs": {"Cost": 1}, "adjectives": {"Appropriate": 1}, "numerals": {"27": 1}}}, {"content": "I care about the fact that there is too little interest or hobby in the spouse.", "word_count": 14, "label": "", "sentence_structure": "Subject鈥搗erb鈥揷omplement", "subjective_objective": {"answer": "Subjective", "rationale": "Because \"careful\" reflects the patient's personal feelings and subjective attitudes."}, "sentence_sentiment": {"answer": "Negative", "rationale": "Because the entry \"sweetly\" expresses negative emotional attitudes."}, "language_style": {"answer": "Tactful and warm", "rationale": "The entry uses the first person's name \"I\" to express what you feel and to describe the expectations of the interest or hobby of a spouse, the more moderate and euphemismal language. This expression is more resonant with the other person and makes the dialogue more friendly and natural."}, "time_dimension": {"answer": "Present", "rationale": "The entry \"are concerned\" indicates the current emotional state and attitude of the testee, and therefore the judgement is now."}, "sentence_type": "Negative declarative sentence", "pos": {"nouns": {"Spouse": 1, "Interest": 1, "Hobbies": 1}, "verbs": {"to": 1, "I care": 1}, "adjectives": {"Too little": 1}, "numerals": {"28": 1}}}, {"content": "My wife should not be out of town until it's economically necessary.", "word_count": 17, "label": "", "sentence_structure": "Subject鈥搗erb鈥搊bject", "subjective_objective": {"answer": "Subjective", "rationale": "Because it is the patient's subjective view and expectations of what his/her spouse should do.\""}, "sentence_sentiment": {"answer": "Negative", "rationale": "The practice of \"not to work outside the home\" in the entry is a restriction on women's freedom and development, has a sexist orientation and therefore has a negative emotional connotation."}, "language_style": {"answer": "Calm and direct", "rationale": "The language of the entry is more calm and straightforward, expressing the view that women should be out of work, and that there are no excessive rhetorical or emotional expressions."}, "time_dimension": {"answer": "Past", "rationale": "The word \"should\" in the entry indicates what should and should be, which is a presumption or expectation of the past. And \"unless economic need\" suggests that it is a conditional phrase, that is, that a situation is not expected to occur under certain conditions. Thus, the entire sentence expresses an expectation or view that existed in the past."}, "sentence_type": "Interrogative sentence", "pos": {"nouns": {"Questionnaire": 1, "Entry": 1, "Economy": 1, "Yes": 1, "Wife": 1, "Out": 1, "Work": 1}, "verbs": {"Reactions": 1, "Out": 1, "in": 1, "Yes": 1}, "adjectives": {"Unless": 1, "Go, go, go": 1}, "adverbs": {"not": 1}, "pronouns": {"I": 1}}}, {"content": "30. My spouse has problems smoking and/or drinking alcohol.", "word_count": 12, "label": "", "sentence_structure": "Subject鈥搗erb鈥搊bject", "subjective_objective": {"answer": "Subjective", "rationale": "\"My spouse smokes and/or drinks is a problem\" is a patient's observation and evaluation of his or her behaviour and is therefore subjective."}, "sentence_sentiment": {"answer": "Negative", "rationale": "Because the act of \"spouse smoking and/or drinking\" is negative in the entry."}, "language_style": {"answer": "Calm and direct", "rationale": ""}, "time_dimension": {"answer": "Present", "rationale": ""}, "sentence_type": "Negative declarative sentence", "pos": {"nouns": {"I": 1, "Spouse": 1}, "verbs": {"Smoking": 1, "Drink": 1, "Problem": 1}, "adverbs": {"/": 1, "or": 1}, "numerals": {"30": 1}}}, {"content": "31. I have little pressure to engage in social activities with my spouse.", "word_count": 16, "label": "", "sentence_structure": "Subject鈥揷opula鈥損redicative", "subjective_objective": {"answer": "Subjective", "rationale": "Because it is the subjective feeling of the patient, and participation in social activities is an act, the view of the purpose of the questionnaire is more oriented towards the subjective perception."}, "sentence_sentiment": {"answer": "Positive", "rationale": "Because the behavior in the entries \"to participate in social activities with spouses\" and the feeling \"very little pressure\" are positive."}, "language_style": {"answer": "Tactful and warm", "rationale": "The entry is more euphemistic and warm, and asks the other side, in a more moderate and encouraging manner, whether it feels pressured to participate in social events with the spouse."}, "time_dimension": {"answer": "Present", "rationale": "\"Participation\" in \"Participation with spouses\" indicates that it is an ongoing act. And \"very little pressure\" indicates that this feeling is in the state of the moment, not in the past or in the future. So, this entry is now asking the person who was tested. In conclusion, it is asking about the person's current behaviour or mental state."}, "sentence_type": "Negative declarative sentence", "pos": {"nouns": {"Questionnaire Entry": 1, "Social events": 1, "Spouse": 1, "Pressure": 1}, "verbs": {"Participation": 1, "I feel": 1}, "adjectives": {"Very few": 1}, "numerals": {"31锛?: 1}, "pronouns": {"I": 2}}}, {"content": "I'm not happy with the couple. My spouse doesn't understand me.", "word_count": 18, "label": "", "sentence_structure": "Subject鈥搗erb鈥搊bject", "subjective_objective": {"answer": "Subjective", "rationale": "This is because patients are dissatisfied with their communication with their spouses and they feel that the spouses do not understand their subjective feelings."}, "sentence_sentiment": {"answer": "Negative", "rationale": "Because the expression \"unsatisfactory\" and \"unsense\" in the entries are negative terms, expressing dissatisfaction and confusion, the emotional colour of the article is negative."}, "language_style": {"answer": "Calm and direct", "rationale": "The entry directly expresses the complaints of the subject of the survey about the communication between the spouses and the lack of understanding of the spouses, without any euphemism or suggestion."}, "time_dimension": {"answer": "Present", "rationale": ""}, "sentence_type": "Negative declarative sentence", "pos": {"nouns": {"Communications": 1, "Couples": 1, "Spouse": 1, "I": 2}, "verbs": {"Unsatisfactory": 1, "Understood": 1}, "numerals": {"32": 1}}}, {"content": "I'm always happy about how and where our family is on vacation.", "word_count": 20, "label": "", "sentence_structure": "Subject鈥揷opula鈥損redicative", "subjective_objective": {"answer": "Subjective", "rationale": "Because this is the patient ' s attitude and feelings towards family vacations and is a subjective view of the individual."}, "sentence_sentiment": {"answer": "Positive", "rationale": "Because the \"I'm always satisfied\" attitude in the entry is positive."}, "language_style": {"answer": "Tactful and warm", "rationale": "The entry uses the subjective feeling of \"I feel satisfied\" to describe the attitude to family vacations, which is relatively gentle and friendly, and is not expressed too directly or strongly. Moreover, the use of the first person's term \"I\" also enhances the personality and affection of expression. So I think the language of the questionnaire is more euphemistic."}, "time_dimension": {"answer": "Present", "rationale": "The entry uses the word \"always\" to indicate that it has been maintained, and therefore is a description of the current state. At the same time, the feeling of \"satisfaction\" is also in the current context, and can therefore be judged as the present state."}, "sentence_type": "Affirmative declarative sentence", "pos": {"nouns": {"Our home": 1, "Holiday": 2}, "verbs": {"Total": 1, "I think": 1, "Satisfied": 1}, "adverbs": {"Always": 1}, "numerals": {"33": 1}, "pronouns": {"I": 1}}}, {"content": "34. We have a complete understanding of each other.", "word_count": 11, "label": "", "sentence_structure": "Subject鈥揷opula鈥損redicative", "subjective_objective": {"answer": "Subjective", "rationale": "Because \"mutual understanding\" is a subjective feeling and perception."}, "sentence_sentiment": {"answer": "Positive", "rationale": "Because the \"total understanding\" attitude in the entry is positive."}, "language_style": {"answer": "Tactful and warm", "rationale": "The language style of the entry is euphemism. It expresses in a gentle and inviting tone the expectations of complete understanding between the spouses, rather than direct statements or orders, which are more personal and interactive."}, "time_dimension": {"answer": "Present", "rationale": "The entry describes the current situation, where \"we have a complete understanding\" is the state or behaviour of the present, so it's judged to be \"now\"."}, "sentence_type": "Affirmative declarative sentence", "pos": {"nouns": {"In our marriage": 1, "Understood": 1}, "verbs": {"Total": 1, "To each other": 1}, "numerals": {"34锛?: 1}}}, {"content": "35. The spouses agree on the discipline of their children.", "word_count": 13, "label": "", "sentence_structure": "Subject鈥揷opula鈥損redicative", "subjective_objective": {"answer": "Subjective", "rationale": "Since the subject is primarily about views and attitudes between spouses, it is a subjective one."}, "sentence_sentiment": {"answer": "Neutral", "rationale": "Because the entry describes the unity of views of the couple in the discipline of their children, it does not express positive or negative feelings."}, "language_style": {"answer": "Calm and direct", "rationale": "The entry is a direct query to the respondent to answer questions about the discipline of children in the family, without excessive emotional colours or qualifiers."}, "time_dimension": {"answer": "Present", "rationale": "The keyword \"in the discipline of children\" means that it is a particular behavior or situation, and \"consensual husband and wife\" is the attitude or view of the act or situation. Since this information is a description of the current situation, the temporal dimension is now."}, "sentence_type": "Affirmative declarative sentence", "pos": {"nouns": {"Corrections": 1, "Children": 1, "Couples": 1}, "verbs": {"Concurring": 1}, "adverbs": {"in": 1}, "numerals": {"35": 1}}}, {"content": "36. I am very pleased with the way we make decisions and resolve conflicts.", "word_count": 18, "label": "", "sentence_structure": "Subject鈥揷opula鈥損redicative", "subjective_objective": {"answer": "Subjective", "rationale": "Because \"very satisfied\" is a phrase that expresses emotional attitudes and is subjectively felt and evaluated by patients."}, "sentence_sentiment": {"answer": "Positive", "rationale": "Because the \"satisfaction\" attitude in the entry is positive."}, "language_style": {"answer": "Tactful and warm", "rationale": "The language style of the entry is more euphemistic and moderate, using positive terms such as \"very satisfactory\" and \"the way in which decisions and conflicts are resolved\" and not directly expressing accusations or criticisms of the other side. This expression enhances the relevance and interaction of the questionnaire, making it easier for respondents to accept and participate in the survey."}, "time_dimension": {"answer": "Present", "rationale": "The keywords \"very satisfied\", \"decision-making\" and \"resolution of conflict\" are describing the current situation, so it can be judged that this is the evaluation of decision-making and conflict management by the testees."}, "sentence_type": "Affirmative declarative sentence", "pos": {"nouns": {"Questionnaire Entry": 1, "Decision": 1, "Conflict": 1}, "verbs": {"I'm very satisfied": 1, "Make a decision": 1, "Resolve": 1}, "adjectives": {"Satisfied": 1}, "adverbs": {"Very": 1}, "numerals": {"36": 1}}}, {"content": "37. Sometimes, my spouse does not depend on me, and it is not always the same.", "word_count": 17, "label": "", "sentence_structure": "Subject鈥搗erb鈥揷omplement", "subjective_objective": {"answer": "Subjective", "rationale": "The reason for this is that people are dependent, and the cloud is the patient's subjective feeling."}, "sentence_sentiment": {"answer": "Neutral", "rationale": "Because it does not clearly express positive or negative feelings, attitudes, behaviours, aims or qualities. It simply states the fact that \"my spouse does not depend on me, not always the same.\""}, "language_style": {"answer": "Calm and direct", "rationale": "The entry is presented in fact, with no excessive emotional rendering or rhetorical style, and is more objective in tone. It is also not expressed in euphemism, invitation, but rather in a straightforward manner, so that the interviewee can answer the questions clearly."}, "time_dimension": {"answer": "Past", "rationale": "So it cannot simply be classified as \"the past\", \"now\" or \"the future\"."}, "sentence_type": "Affirmative declarative sentence", "pos": {"nouns": {"Time": 1, "Spouse": 1, "I": 2, "person": 1, "Clouds": 1, "Yeah": 1}, "verbs": {"No dependent": 1, "Not always": 1, "Man and woman": 1}, "adjectives": {"Sometimes": 1}, "adverbs": {"Always": 1, "Yeah": 1}, "classifiers": {"individual": 1}}}, {"content": "38. I am satisfied with the decision on how much money a family should save.", "word_count": 18, "label": "", "sentence_structure": "Subject鈥揷opula鈥損redicative", "subjective_objective": {"answer": "Subjective", "rationale": "Because it is the patient's subjective feeling to decide whether to be satisfied."}, "sentence_sentiment": {"answer": "Positive", "rationale": "Because the entry expresses the respondent ' s satisfaction with his or her decision to save in the home, it is a positive emotional expression."}, "language_style": {"answer": "Tactful and warm", "rationale": "The entry is more personal and interactive in terms of speaking in a gentle and inviting tone, rather than directly speaking or ordering. At the same time, the expression of the subjective feeling of \"I am satisfied\" makes the language style more euphemism and affectionate."}, "time_dimension": {"answer": "Present", "rationale": "The questionnaire entry asks the testee about the feelings and attitudes of the present moment, and the time dimension is therefore judged to be the present."}, "sentence_type": "Affirmative declarative sentence", "pos": {"nouns": {"Family": 1, "Money": 1, "Decision": 1, "Satisfied": 1}, "verbs": {"Savings": 1, "I feel": 1}, "numerals": {"How much": 1}, "pronouns": {"I": 1}}}, {"content": "When discussing a particular issue, I usually feel that the spouse understands me.", "word_count": 20, "label": "", "sentence_structure": "Subject鈥搗erb鈥搊bject", "subjective_objective": {"answer": "Subjective", "rationale": "Because \"Feel\" and \"understanding\" are the feelings and thoughts of the patients themselves, this questionnaire is viewed from the patient's subjective perspective."}, "sentence_sentiment": {"answer": "Positive", "rationale": "Because the attitude and behavior of \"understanding me\" in the entry is positive."}, "language_style": {"answer": "Tactful and warm", "rationale": "The entry uses a more euphemistic expression to ask the other party whether, in discussing a question, the spouse understands himself or herself as the subject and to increase the moderation of tone by using the term \"normally\". At the same time, the expression \"spouse understands me\" makes it easier to comfort and accommodate the interviewee and thus to answer questions in a true and frank manner."}, "time_dimension": {"answer": "Present", "rationale": "The entry now describes the feelings of the testee in discussing a problem, a reaction to the current, ongoing situation, and therefore the judgement is \"now\". At the same time, \"spouse is understanding me\" indicates that the testee's spouse may have shown an understanding in the past, but this does not affect the temporal dimension of the article."}, "sentence_type": "Affirmative declarative sentence", "pos": {"nouns": {"Problem": 1, "Understood": 1, "Spouse": 1}, "verbs": {"I feel": 1, "be": 1, "Discussion": 1}, "adverbs": {"Usually": 1}, "numerals": {"39": 1}, "pronouns": {"I": 1}}}, {"content": "My spouse sometimes blames me for demeaning my opinion.", "word_count": 16, "label": "", "sentence_structure": "Subject鈥搗erb鈥搊bject", "subjective_objective": {"answer": "Subjective", "rationale": "Because this entry describes patients ' subjective feelings and views, i.e. their perception of the spousal ' s speech and emotional reactions."}, "sentence_sentiment": {"answer": "Negative", "rationale": "Because the \"scratch of some demeaning opinion\" in the entry is negative."}, "language_style": {"answer": "Tactful and warm", "rationale": "The entry uses more euphemistic terms, such as \"sometimes\" and \"some\" that reduce the intensity of the tone, while avoiding direct attacks on the other. Moreover, the article ' s entry is more simple, easily understandable and meets the linguistic style requirements of the questionnaire."}, "time_dimension": {"answer": "Present", "rationale": "The keyword \"sometimes\" indicates that the act took place within a certain time period and that it may be repeated, so the temporal dimension is now."}, "sentence_type": "Negative declarative sentence", "pos": {"nouns": {"Spouse": 1, "Observations": 1}, "verbs": {"Blaming": 1, "Demeaning": 1}, "adverbs": {"Sometimes": 1}, "numerals": {"40": 1}, "pronouns": {"I": 1}}}, {"content": "41. It was easy and easy for me to talk about sex with a spouse.", "word_count": 20, "label": "", "sentence_structure": "Subject鈥揷opula鈥損redicative", "subjective_objective": {"answer": "Subjective", "rationale": "Because it's personal feelings and perceptions that are \"easy and easy for me\" and it's a subjective judgment."}, "sentence_sentiment": {"answer": "Positive", "rationale": "Because the description of \"easy and easy\" in the entry suggests that the attitude of the speaker to sexual issues is positive."}, "language_style": {"answer": "Tactful and warm", "rationale": "This is a more natural expression that makes it easier for respondents to share their ideas and to use them more easily."}, "time_dimension": {"answer": "Present", "rationale": ""}, "sentence_type": "Affirmative declarative sentence", "pos": {"nouns": {"Spouse": 1, "Sexual issues": 1, "I": 1}, "verbs": {"Talk": 1, "be": 1, "Easy": 2}, "adverbs": {"very": 1}, "numerals": {"41": 1}}}, {"content": "My spouse understands and feels the same every emotional change I have.", "word_count": 27, "label": "", "sentence_structure": "Subject鈥搗erb鈥揷omplement", "subjective_objective": {"answer": "Subjective", "rationale": "Because patients are examined whether their spouses fully understand and share their emotional changes, this is the patient ' s subjective view."}, "sentence_sentiment": {"answer": "Positive", "rationale": "Because the expression \"every emotional change is fully understood and feels the same\" expresses the support and understanding of the spouses, which is a positive quality or behaviour."}, "language_style": {"answer": "Tactful and warm", "rationale": "The language style of this entry is more euphemistic, more compassionate and positive, and it is a matter of concern and respect, and of courtesy and affection."}, "time_dimension": {"answer": "Present", "rationale": ""}, "sentence_type": "Affirmative declarative sentence", "pos": {"nouns": {"Spouse": 1, "Emotional change": 1}, "verbs": {"Understood": 1, "Feel": 1}, "adverbs": {"Every time": 1, "Both": 1, "Total": 1}, "numerals": {"42": 1}, "classifiers": {"One": 1}, "pronouns": {"I": 1, "we": 1}}}, {"content": "43. In our marriage, wives should be more obedient to their husbands ' wishes.", "word_count": 19, "label": "", "sentence_structure": "Subject鈥搗erb鈥搊bject", "subjective_objective": {"answer": "Subjective", "rationale": "Because \"the wife should be more obedient to her husband's wishes\" is a subjective value and expectation that reflects the personal views and attitudes of the researcher or the person under investigation, rather than the objective facts."}, "sentence_sentiment": {"answer": "Negative", "rationale": "The phrase \"the wife should be more obedient to her husband's wishes\" in the entry is sexist and unequal and has negative emotional tendencies."}, "language_style": {"answer": "Tactful and warm", "rationale": "The expression in this entry is based on the following: 1. The expression is more moderate, polite and not overly strong or direct; 2. While in some traditional cultures it is considered to be a woman's expression of due conduct, in modern society it may be considered gender-discriminatory and unfair. Thus, a more euphemistic expression can avoid such misunderstandings and be more readily accepted by the interviewees. 3. The language of this article is relatively polite and respectful, which is consistent with the linguistic style that a good psychological survey should have."}, "time_dimension": {"answer": "Present", "rationale": "The entry now asks the respondents about the role that they expect from the current marriage, namely, that \"the wife should be more obedient to the wishes of her husband\"."}, "sentence_type": "Affirmative declarative sentence", "pos": {"nouns": {"Marriage": 1, "Wife": 1, "Husband": 2, "Wish": 1}, "verbs": {"I think so": 1, "Submissive": 1, "Wish": 1}, "numerals": {"43": 1, "One": 1}, "pronouns": {"we": 1, "More": 1}}}, {"content": "When we're with someone, sometimes I get upset about the behavior of my spouse.", "word_count": 22, "label": "", "sentence_structure": "Subject鈥揷opula鈥損redicative", "subjective_objective": {"answer": "Subjective", "rationale": "Because \"uneasy\" is the patient's subjective emotional experience and judgment."}, "sentence_sentiment": {"answer": "Negative", "rationale": "Because the \"uneasy\" act in the entry is negative."}, "language_style": {"answer": "Tactful and warm", "rationale": "The language style of the entry is euphemistic. It expresses a possible problem in a mild and understanding way, without directly blaming or ordering each other. At the same time, the term \"sometimes\" is used to suggest that it is an occasional situation, but rather a very different one, which makes the phrase more mild and inclusive."}, "time_dimension": {"answer": "Present", "rationale": "\"We are living with others\" and \"I am sometimes disturbed by the behaviour of my spouse\" all point to a situation that is now under way, so it is the current feelings and circumstances of the person being tested that can be judged."}, "sentence_type": "Affirmative declarative sentence", "pos": {"nouns": {"we": 1, "others": 1, "Time": 1, "Behaviour": 1, "Spouse": 1, "Feel": 1, "Unsettled": 1}, "verbs": {"Together": 1, "I feel": 1}, "adverbs": {"Sometimes": 1}, "numerals": {"44": 1}, "pronouns": {"I": 1}}}, {"content": "We all know the debt we owe... and it's not a problem.", "word_count": 19, "label": "", "sentence_structure": "Subject鈥搗erb鈥揷omplement", "subjective_objective": {"answer": "Subjective", "rationale": "Because \"know\" and \"not a problem\" are subjective attitudes and evaluations of patients."}, "sentence_sentiment": {"answer": "Negative", "rationale": "Because the \"liabilities\" act is negative in the entries."}, "language_style": {"answer": "Tactful and warm", "rationale": "The language style of the entry is more euphemistic, soft, using expressions like \"We know\" and \"It's not a problem,\" and there is no too strong or direct tone that gives people a friendly, relaxed feeling. This moderate expression helps respondents to be more willing to cooperate in answering questions."}, "time_dimension": {"answer": "Present", "rationale": "Since both terms describe the current situation and state of perception, the time dimension of this questionnaire entry can be judged to be the present."}, "sentence_type": "Affirmative declarative sentence", "pos": {"nouns": {"Debt": 1, "Problem": 1}, "verbs": {"Yes": 1, "Own": 1, "Done": 1, "be": 1}, "adjectives": {"Both": 1}, "numerals": {"45": 1}, "pronouns": {"we": 2}}}, {"content": "46. My religious beliefs are an important part of the influence of our marriage.", "word_count": 20, "label": "", "sentence_structure": "Subject鈥揷opula鈥損redicative", "subjective_objective": {"answer": "Subjective", "rationale": "Because religion is a belief and value of one's own, and belongs to the subjective experience of the individual."}, "sentence_sentiment": {"answer": "Neutral", "rationale": "Since the entry describes the role of religious belief in marriage, it does not reflect positive or negative emotional aspects."}, "language_style": {"answer": "Tactful and warm", "rationale": "This is because the entry uses a more moderate and euphemistic language, expressing respect for and understanding of the role of religious belief in marriage, and is not too strong or straightforward. This expression is more likely to resonate with the respondents and is more conducive to a good questionnaire relationship."}, "time_dimension": {"answer": "Present", "rationale": "The entry for the questionnaire is to ask the present views and circumstances of the subject, and the keyword \"is\" and \"impact\" all indicate that this is the general current and does not address past or future situations."}, "sentence_type": "Affirmative declarative sentence", "pos": {"nouns": {"Faith": 1, "Religion": 1, "Marriage": 2, "Part": 1}, "verbs": {"be": 1, "Shadow": 1, "Ring": 1, "we": 1, "Marriage": 1, "One": 1, "individual": 1}, "adjectives": {"Heavy": 1}, "adverbs": {"I": 1}, "numerals": {"46": 1}}}, {"content": "47. Sometimes I'm afraid that spouses have the idea of seeking extramarital sex.", "word_count": 19, "label": "", "sentence_structure": "Subject鈥搗erb鈥搊bject", "subjective_objective": {"answer": "Subjective", "rationale": "The fear that the spouses would seek sexual relations outside marriage is a subjective assumption and a fear of the patient, not an objective fact."}, "sentence_sentiment": {"answer": "Negative", "rationale": "Because the idea of \"a fear that a spouse will seek extramarital sexual relations\" in the entry is negative."}, "language_style": {"answer": "Tactful and warm", "rationale": "This is a way to make it easier for the respondents to resonate and respond honestly and to facilitate data collection and analysis."}, "time_dimension": {"answer": "Present", "rationale": ""}, "sentence_type": "Negative declarative sentence", "pos": {"nouns": {"Time": 1, "I": 1, "Spouse": 1, "Thoughts": 1}, "verbs": {"Fear": 1, "have": 1, "Yes": 1, "Seek": 1, "Sexual relations outside marriage": 1}, "adverbs": {"Sometimes": 1}, "numerals": {"47锛?: 1}}}, {"content": "I think the spouse is too close to his/her family or too affected by it.", "word_count": 22, "label": "", "sentence_structure": "Subject鈥搗erb鈥搊bject", "subjective_objective": {"answer": "Subjective", "rationale": "Because the word \"considers\" indicates that it is a personal view and feeling of the patient and is a subjective examination."}, "sentence_sentiment": {"answer": "Negative", "rationale": "Because the phrase \"spouse is too close to or too affected by his/her home\" in the entry indicates some dissatisfaction or problems, which are negative emotional."}, "language_style": {"answer": "Calm and direct", "rationale": "The entry expresses, in a direct manner, concerns and inquiries about possible problems in the marital relationship of the respondent, does not use excessive rhetorical or emotional rendering, and is more objective in tone."}, "time_dimension": {"answer": "Present", "rationale": "Now, the entry \"I think\" indicates that this is the subjective view of the subject at hand and does not involve past or future acts or events. So the temporal dimension is now."}, "sentence_type": "Negative declarative sentence", "pos": {"nouns": {"Spouse": 1, "Home": 1, "Lee": 1, "he": 1, "she": 1, "Impact": 1, "Large": 1}, "verbs": {"I think": 1, "and": 1, "Too much": 1, "Close": 1, "or": 1, "To hell with it": 1, "Too big": 1}, "adverbs": {"Too much": 2}, "numerals": {"48锛?: 1}, "classifiers": {"individual": 1}, "pronouns": {"he": 1, "she": 1}}}, {"content": "49. Children seem to be a major source of conflict in our marriage.", "word_count": 19, "label": "", "sentence_structure": "Subject鈥揷opula鈥損redicative", "subjective_objective": {"answer": "Subjective", "rationale": "The problem is more characterized by the patient ' s perception and feelings of the child as the main source of conflict in marriage and is therefore subjective."}, "sentence_sentiment": {"answer": "Negative", "rationale": "Because the expression \"the child appears to be the main source of conflict\" in the entry implies that the child has a negative impact on marriage and is a negative sentiment."}, "language_style": {"answer": "Tactful and warm", "rationale": "This is a more acceptable expression for respondents and can trigger their reflection and reflection."}, "time_dimension": {"answer": "Present", "rationale": ""}, "sentence_type": "Affirmative declarative sentence", "pos": {"nouns": {"Children": 1, "Marriage": 1, "Paradoxious": 1, "Source": 1}, "verbs": {"Seems like": 1, "be": 1, "One": 1, "Main": 1, "From": 1}, "numerals": {"49锛?: 1, "One": 2}, "classifiers": {"individual": 1}}}, {"content": "We agree on the number of children needed.", "word_count": 14, "label": "", "sentence_structure": "Subject鈥搗erb鈥揷omplement", "subjective_objective": {"answer": "Subjective", "rationale": "Because \"consensus\" is the patient's subjective feeling and attitude, it means they have a common understanding of how many children are needed."}, "sentence_sentiment": {"answer": "Neutral", "rationale": "Because the entry does not have any emotional color."}, "language_style": {"answer": "Tactful and warm", "rationale": "This is a more moderate and friendly expression, which helps to increase the responsiveness of the interviewee and the reliability of his or her answers."}, "time_dimension": {"answer": "Present", "rationale": "The entry asks whether the spouses are currently in agreement on the number of children and therefore falls within the description of the current temporal dimension."}, "sentence_type": "Affirmative declarative sentence", "pos": {"nouns": {"Number": 1, "Children": 1, "Observations": 1, "Unanimously": 1}, "verbs": {"to": 1, "Yes": 1, "have": 1}, "adverbs": {"Unanimously": 1}, "numerals": {"50": 1}, "pronouns": {"we": 1}}}, {"content": "We pay regularly according to our economic power.", "word_count": 16, "label": "", "sentence_structure": "Subject鈥搗erb鈥搊bject", "subjective_objective": {"answer": "Objective", "rationale": "It is an objective fact that spending is a consumption of individuals, in accordance with economic power."}, "sentence_sentiment": {"answer": "Neutral", "rationale": "Because the entry does not have any emotional color."}, "language_style": {"answer": "Tactful and warm", "rationale": "The entry is more euphemistic, using a question-and-answer tone, without direct orders or criticism of the other side's spending habits. At the same time, \"regularly paying\" is also a more neutral term, without overemphasis on the demands of economic power, and therefore the whole expression is more gentle and friendly."}, "time_dimension": {"answer": "Present", "rationale": "The keywords are \"by\" and \"by order\", which all indicate an ongoing behavior or state of affairs, in which the tested are now spending money in a regular manner according to their economic power."}, "sentence_type": "Affirmative declarative sentence", "pos": {"nouns": {"we": 2, "Economic power": 1, "Money": 1}, "verbs": {"Press": 1, "Regular flowers": 1}, "adverbs": {"There's a regular pattern": 1}, "numerals": {"51锛?: 1}}}, {"content": "I'm not satisfied with the way our economic position determines economic affairs.", "word_count": 21, "label": "", "sentence_structure": "Subject鈥搗erb鈥搊bject", "subjective_objective": {"answer": "Subjective", "rationale": "This statement is a subjective attitude of patients who express their dissatisfaction with and appreciation of the way in which economic affairs are decided."}, "sentence_sentiment": {"answer": "Negative", "rationale": "Because the attitude of \"unsatisfactory\" in the entries is negative."}, "language_style": {"answer": "Calm and direct", "rationale": "The entry expresses, in a calm and direct manner, dissatisfaction with the way economic status determines economic affairs, without excessive ado or emotional, and with a more calm and direct language style."}, "time_dimension": {"answer": "Past", "rationale": "The entry is for the purpose of asking the views and attitudes of the testee on the current economic situation, without clearly identifying past or future behaviour or mentality, and therefore can be judged as the present."}, "sentence_type": "Negative declarative sentence", "pos": {"nouns": {"we": 1, "Economic status": 1, "Economic affairs": 1}, "verbs": {"Unsatisfactory": 1, "Decision": 1}, "numerals": {"52": 1}}}, {"content": "I'm very pleased with our amateur activities and the time we spent together.", "word_count": 22, "label": "", "sentence_structure": "Subject鈥搗erb鈥揷omplement", "subjective_objective": {"answer": "Subjective", "rationale": "Because \"very satisfied\" and \"time spent together\" are personal feelings and attitudes of patients, subjectively."}, "sentence_sentiment": {"answer": "Positive", "rationale": "Because the \"very satisfied\" assessment is positive and expresses positive attitudes and feelings."}, "language_style": {"answer": "Tactful and warm", "rationale": "The entry describes, in a positive and positive tone, the feelings of the amateur activity that couples share, emphasizes the sense of satisfaction and uses a number of rhetorical techniques (such as \"very\") to further strengthen this positive sentiment. The language style of the purpose is therefore more euphemismal."}, "time_dimension": {"answer": "Present", "rationale": ""}, "sentence_type": "Affirmative declarative sentence", "pos": {"nouns": {"we": 2, "Amateur activities": 1, "Time": 1}, "verbs": {"I'm very satisfied": 1, "Pass": 1}, "adjectives": {"Satisfied": 1}, "adverbs": {"Very": 1}, "numerals": {"53": 1}, "pronouns": {"I": 1, "Couples": 1}}}, {"content": "Sometimes I can't look for my spouse to ask for what I need.", "word_count": 15, "label": "", "sentence_structure": "Subject鈥搗erb鈥搊bject", "subjective_objective": {"answer": "Subjective", "rationale": "Because \"never\" is a subjective attitude or emotional reaction."}, "sentence_sentiment": {"answer": "Negative", "rationale": "Because \"I dare not find my spouse what I need\" is negative."}, "language_style": {"answer": "Tactful and warm", "rationale": ""}, "time_dimension": {"answer": "Present", "rationale": "The keywords are \"sometimes\" and \"no\" , which indicate that it is a regular act and that it may be a state of current or emotional expression, and should therefore be classified as \"now\"."}, "sentence_type": "Negative declarative sentence", "pos": {"nouns": {"Questionnaire": 1, "Entry": 1, "Spouse": 1, "Something": 1}, "verbs": {"I'm afraid": 1, "Find": 1, "Yes": 2}, "adverbs": {"Sometimes": 1}, "numerals": {"54": 1}, "pronouns": {"I": 1}}}, {"content": "55. Even if the wife is employed, she should be responsible for managing the household.", "word_count": 19, "label": "", "sentence_structure": "Subject鈥損redicate", "subjective_objective": {"answer": "Subjective", "rationale": "Because this sentence emphasizes the views and attitudes of individuals with respect to family responsibilities as subjective elements."}, "sentence_sentiment": {"answer": "Negative", "rationale": "Because of the gender bias in the entry, the \"responsibility to manage the household\" is not fair to women and is therefore negative."}, "language_style": {"answer": "Calm and direct", "rationale": "It implies male dominance in the family and considers domestic work as a female responsibility. This expression reflects society鈥檚 traditional perception of gender roles and may have a discriminatory impact on some."}, "time_dimension": {"answer": "Present", "rationale": "The view expressed in this entry is a traditional one that still exists in modern society, with men assuming more family responsibilities, including domestic work. Although this view may be criticized and challenged in some respects, it is still a prevailing situation in the society today."}, "sentence_type": "Exclamatory sentence", "pos": {"nouns": {"Wife": 1, "Work": 1, "Responsibility": 1}, "verbs": {"I think so": 1, "Burden": 1, "Management": 1}, "adverbs": {"Even if": 1}, "numerals": {"55锛?: 1}, "pronouns": {"EMPTY": 1, "she": 1}}}, {"content": "56. Disagreements between spouses in matters relating to religious beliefs", "word_count": 20, "label": "", "sentence_structure": "Subject鈥搗erb鈥搊bject", "subjective_objective": {"answer": "Subjective", "rationale": "Because \"discretion\" involves patients' subjective attitudes and perceptions."}, "sentence_sentiment": {"answer": "Negative", "rationale": "Because the \"dissenting\" act is negative."}, "language_style": {"answer": "Tactful and warm", "rationale": "The article is more euphemistic and inclusive, and does not directly accuse or criticize the spouses for their differences in their religious beliefs."}, "time_dimension": {"answer": "Present", "rationale": "The keywords \"both husband and wife\" and \"relative activities\" point to the current situation."}, "sentence_type": "Affirmative declarative sentence", "pos": {"nouns": {"Couples": 2, "Both sides": 1, "Religious beliefs": 1, "Activities": 1, "Observations": 1}, "verbs": {"have": 1, "No, no, no": 1}, "numerals": {"56锛?: 1}}}, {"content": "57. I am unhappy with my relatives or those of my spouse.", "word_count": 19, "label": "", "sentence_structure": "Subject鈥揷opula鈥損redicative", "subjective_objective": {"answer": "Subjective", "rationale": "Because this is the patient ' s sense and attitude, it is subjective."}, "sentence_sentiment": {"answer": "Negative", "rationale": "Because the emotional attitude of \"discomfort\" in the entry is negative."}, "language_style": {"answer": "Calm and direct", "rationale": "The entry expresses the respondents鈥?concern directly about whether the interviewee is unhappy with his family."}, "time_dimension": {"answer": "Past", "rationale": "The entry is about the behaviour or feelings of the testee, the key word being \"with my relatives or the spouse's family,\" which refers to the experience and feelings of the past. And \"I feel unhappy\" also shows that this is a state of emotion in the past. So we can judge that this entry is about the past."}, "sentence_type": "Imperative sentence", "pos": {"nouns": {"Mine": 1, "Spouse": 1, "Relatives": 1}, "verbs": {"Together": 1, "I feel": 1, "It's not good": 1}, "numerals": {"57锛?: 1}, "pronouns": {"I": 1}}}, {"content": "When I get into trouble, I always tell my spouse.", "word_count": 14, "label": "", "sentence_structure": "Subject鈥搗erb鈥揷omplement", "subjective_objective": {"answer": "Subjective", "rationale": "Because \"I always tell my spouse\" is patient's subjective attitude and behavioral choice."}, "sentence_sentiment": {"answer": "Neutral", "rationale": "Because the entry does not express a positive or negative emotional expression."}, "language_style": {"answer": "Tactful and warm", "rationale": "The entry is more personal and interactive when it is spoken in a gentle and inviting tone, rather than in direct statements or orders. It also implies that investigators want to know whether they are trying to communicate with their spouses in the face of a difficult situation, and use polite language to \"always tell\" to make it more euphemistic and warm."}, "time_dimension": {"answer": "Past", "rationale": "The inclusion of \"always tell the spouse\" in the entry indicates that it is an already customary practice and repeats it when faced with difficulties, and therefore is judged as a past experience."}, "sentence_type": "Affirmative declarative sentence", "pos": {"nouns": {"Difficulties": 1, "Spouse": 1}, "verbs": {"I met him": 1, "Tell": 1}, "adverbs": {"Always": 1}, "pronouns": {"I": 1}}}, {"content": "My spouse's more concerned about our children than about our marriage, which makes me uncomfortable.", "word_count": 24, "label": "", "sentence_structure": "Subject鈥揷opula鈥損redicative", "subjective_objective": {"answer": "Subjective", "rationale": "The sentence expresses the patient ' s subjective feelings and perception that the behaviour of the spouse makes the patient uncomfortable."}, "sentence_sentiment": {"answer": "Negative", "rationale": "It is also a negative description because the entry \"uncomfortable\" conveys a negative emotional connotation and refers to the spousal concern for too many children."}, "language_style": {"answer": "Calm and direct", "rationale": "The language style of the entry is more direct, objective, and there is no excessive emotional or rhetorical approach, which expresses, in a concise manner, the dissatisfaction of the interviewees with the over-attractiveness of the spouses to their children and to their neglect of their marriage."}, "time_dimension": {"answer": "Present", "rationale": "The keyword \"I'm not comfortable\" and the verb \"I'm more concerned with our children than with our marriage\" all indicate that the entry is describing the current feelings and experiences. So the judgment is based on the present."}, "sentence_type": "Negative declarative sentence", "pos": {"nouns": {"Spouse": 1, "Children": 2, "Marriage": 1, "I'm not feeling well": 1}, "verbs": {"Attention": 1, "鈾?And I'm gonna be 鈾?: 1}, "adjectives": {"More than": 1}, "numerals": {"59锛?: 1}, "pronouns": {"Here": 1, "I": 1}}}, {"content": "60. I think we have had a good time on vacation and travel.", "word_count": 15, "label": "", "sentence_structure": "Subject鈥揷opula鈥損redicative", "subjective_objective": {"answer": "Subjective", "rationale": "Because \"think\" is the patient's subjective feeling."}, "sentence_sentiment": {"answer": "Positive", "rationale": "Because the expression \"I think we've had a good time on vacation and travel\" is positive."}, "language_style": {"answer": "Tactful and warm", "rationale": "The words \"sweet\" and \"good\" are used to express satisfaction and enjoyment of holidays and travel. This is more acclaimable, and better to guide respondents to express their views and feelings."}, "time_dimension": {"answer": "Past", "rationale": "The entry describes the testee's assessment of the experience of a certain period of time, and can therefore be judged as \"the past\"."}, "sentence_type": "Affirmative declarative sentence", "pos": {"nouns": {"Holiday": 1, "Tourism": 1}, "verbs": {"I think": 1, "Pass": 1}, "adjectives": {"Okay": 1}, "numerals": {"60": 1}, "pronouns": {"we": 1}}}, {"content": "Our husband is the head of the family.", "word_count": 10, "label": "", "sentence_structure": "Subject鈥搗erb鈥搊bject", "subjective_objective": {"answer": "Subjective", "rationale": "Because \"the head of the family\" is a symbol of social roles and power, it usually takes personal will, attitudes and behaviour to achieve it. Thus, the phrase \"we husband is the head of the family\" reflects the patient's perception and expectations of his role in the family and is subjective in its approach."}, "sentence_sentiment": {"answer": "Negative", "rationale": "Because the \"household\" in the entry implies male dominance in the family, it may have a negative impact on the concept of gender equality."}, "language_style": {"answer": "Tactful and warm", "rationale": "The entry is more tingious in language by asking about the division of roles within the respondent's family and expressing concern and respect for each other's family life in the words \"our family\" and \"the head of the family\"."}, "time_dimension": {"answer": "Present", "rationale": "The entry now describes the role and status of a husband in a family, which is a current, objective and factual description, and does not address past or future behaviour or mentalities."}, "sentence_type": "Affirmative declarative sentence", "pos": {"nouns": {"Our home": 1, "Husband": 1, "Head of the family": 1}, "numerals": {"61": 1}}}, {"content": "62. For me, our sexual relations are satisfactory and perfect.", "word_count": 17, "label": "", "sentence_structure": "Subject鈥揷opula鈥損redicative", "subjective_objective": {"answer": "Subjective", "rationale": "Because \"satisfaction and perfection\" is the patient's subjective perception and evaluation of sexual relations."}, "sentence_sentiment": {"answer": "Positive", "rationale": "Because the entry describes personal satisfaction and satisfaction, it is a positive emotional attitude."}, "language_style": {"answer": "Tactful and warm", "rationale": "The entry is more intimate and interactive when it is spoken in a gentle and inviting manner, rather than in direct statements or orders. At the same time, the formulation of the article is more implicit, avoiding language that is too direct or offensive and gives people a comfortable and pleasant feeling."}, "time_dimension": {"answer": "Present", "rationale": "The entry describes the current feelings and state of the subject, the word \"yes\" in the words \"to be\" indicates that it is about the feelings of the person, and the description \"is satisfactory and perfect\" is the current state."}, "sentence_type": "Affirmative declarative sentence", "pos": {"nouns": {"Sexual relations": 1, "Satisfied": 1, "Perfect": 1}, "verbs": {"Tell me": 1, "be": 1}, "adverbs": {"I": 1, "and": 1}, "numerals": {"62锛?: 1}, "classifiers": {"individual": 1}}}, {"content": "Sometimes, my spouse is too stubborn.", "word_count": 9, "label": "", "sentence_structure": "Subject鈥揷opula鈥損redicative", "subjective_objective": {"answer": "Subjective", "rationale": "Because stubbornness is a subjective adjective, describing the views of observers rather than the behaviour or state of the persons being observed."}, "sentence_sentiment": {"answer": "Negative", "rationale": "Because the term \"too stubborn\" in the entry is derogatory and negative."}, "language_style": {"answer": "Calm and direct", "rationale": "The entry describes in a direct and objective manner a possible character characteristic of the spouse of the person under investigation, without the use of excessive rhetorical techniques or emotional rendering, and with a calmer tone."}, "time_dimension": {"answer": "Past", "rationale": "The time dimension of the article is \"the past\" as it is judged by the principle of time dimension in the title, \"sometimes\" as an expression of an unrecurring event or state in the past."}, "sentence_type": "Negative declarative sentence", "pos": {"nouns": {"Spouse": 1, "Obstinacy": 1}, "verbs": {"Sometimes": 1, "be": 1}, "adjectives": {"Too much": 1}, "numerals": {"63": 1}, "pronouns": {"I": 1}}}, {"content": "64. Our marriage was a very successful one.", "word_count": 11, "label": "", "sentence_structure": "Subject鈥揷opula鈥損redicative", "subjective_objective": {"answer": "Subjective", "rationale": "Because \"very successful\" is a subjective evaluation, which reflects the satisfaction and perception of the respondents about marriage."}, "sentence_sentiment": {"answer": "Positive", "rationale": "Because the description of \"very successful\" is usually considered a positive assessment, indicating a good and satisfactory state of marriage."}, "language_style": {"answer": "Tactful and warm", "rationale": "This is a positive expression of the degree of success of the marriage, and a more euphemistic, soft language style is used to avoid being too direct or offensive in questioning."}, "time_dimension": {"answer": "Future", "rationale": "The entry expresses expectations and visions for the future, and the \"very successful\" evaluation points to marital status that has not yet occurred in the future."}, "sentence_type": "Affirmative declarative sentence", "pos": {"nouns": {"Marriage": 1, "Success": 1}, "verbs": {"be": 1, "Very": 1}, "adjectives": {"Success": 1}, "adverbs": {"Very": 1}, "numerals": {"64": 1}}}, {"content": "65. Pray with your spouse. It's important to me.", "word_count": 12, "label": "", "sentence_structure": "Subject鈥揷opula鈥損redicative", "subjective_objective": {"answer": "Subjective", "rationale": "Because the sentence relates to the feelings and perceptions of individuals, namely, \"important to me\", it is a subjective examination."}, "sentence_sentiment": {"answer": "Positive", "rationale": "Because the entry expresses a positive emotion and attitude that it is important to pray with the spouse."}, "language_style": {"answer": "Tactful and warm", "rationale": "The entry is more personal and interactive when it is presented in a gentle and inviting tone, rather than in direct statements or orders. At the same time, the expression \"I'm important to me\" is used, highlighting individual feelings and subjective experiences, reflecting the respect and concern of the questionnaire designers for the interviewees."}, "time_dimension": {"answer": "Present", "rationale": "The entry \"I'm important to me\" indicates that it's the person's current mentality and feelings, and therefore the judgment is \"now.\" And, \"Pray with your spouse\" is a certain act or habit of the person, and therefore can be understood from this perspective as the act of the moment."}, "sentence_type": "Affirmative declarative sentence", "pos": {"nouns": {"Questionnaire": 1, "Entry": 1, "Spouse": 1, "Pray": 1}, "verbs": {"to": 1, "It's important": 1}, "numerals": {"65": 1}}}, {"content": "I hope that the spouse would be more willing to share his/her feelings with me.", "word_count": 17, "label": "", "sentence_structure": "Subject鈥搗erb鈥搊bject", "subjective_objective": {"answer": "Subjective", "rationale": "Because hope represents the patient's expectations and wishes, it's his subjective attitude."}, "sentence_sentiment": {"answer": "Positive", "rationale": "Because the desire to \"like a spouse to share his/her feelings\" expresses the expectation of a deeper relationship, which is positive emotional."}, "language_style": {"answer": "Tactful and warm", "rationale": "The language style of the entry is expressed in a euphemism, gentler way, with the words \"hope\" and \"better\" used to ask questions, leaving more room and space for the interviewees to think and answer. This expression is more personal and interactive."}, "time_dimension": {"answer": "Present", "rationale": "It can be inferred from the sentence that this is the expectation and expectation of the present behavior. At the same time, sharing his/her feelings is also about the present or future behavior, not what has happened in the past. The entry is therefore a description of the behavior or mentality of the present dimension of time."}, "sentence_type": "Imperative sentence", "pos": {"nouns": {"Spouse": 1, "Feel": 1}, "verbs": {"Hope": 1, "Yes": 1, "Share": 1}, "adjectives": {"More": 1}, "numerals": {"66锛?: 1}, "pronouns": {"he": 1, "she": 1}}}, {"content": "67. Having children makes our marriage closer.", "word_count": 15, "label": "", "sentence_structure": "Subject鈥搗erb鈥揷omplement", "subjective_objective": {"answer": "Subjective", "rationale": "Because \"the closer marriage\" is a subjective feeling or evaluation."}, "sentence_sentiment": {"answer": "Positive", "rationale": "Because the result of the entry \"making our marriage closer\" is positive."}, "language_style": {"answer": "Tactful and warm", "rationale": "The term is more euphemistic. Using the neutral description of \"with a child\" instead of directly describing the change in marital relations, the interviewer is asked to think and express his opinion, which is more accompanied and respectful of the views of the interviewee. At the same time, \"making our marriage closer\" expresses a positive attitude that avoids stress or discomfort on the interviewee."}, "time_dimension": {"answer": "Present", "rationale": "The words \"with children\" and \"better marriage\" in the entry both indicate the state of the present and thus the temporal dimension of the article can be judged to be the present."}, "sentence_type": "Affirmative declarative sentence", "pos": {"nouns": {"Son": 1, "Marriage": 1}, "verbs": {"have": 1, "鈾?And I'm gonna be 鈾?: 1, "Closer": 1}, "numerals": {"67锛?: 1}}}, {"content": "My spouse likes all my friends.", "word_count": 12, "label": "", "sentence_structure": "Subject鈥搗erb鈥搊bject", "subjective_objective": {"answer": "Subjective", "rationale": "Because of the subjective view that the friend of the other is the spouse."}, "sentence_sentiment": {"answer": "Positive", "rationale": "The basis for the judgment: because the expression \"my spouse likes all my friends\" in the entry conveys a positive emotion, that is, the acceptance of and respect for all friends by the spouse."}, "language_style": {"answer": "Tactful and warm", "rationale": "The entry is more personal and interactive when asked in an invitation tone whether the respondent considers his spouse to like all his friends rather than directly speaking or ordering. At the same time, the entry uses a more positive phrase to encourage the interviewee to express his views, and the language is more euphemismal."}, "time_dimension": {"answer": "Present", "rationale": "The entry describes the relationship between a testee and his spouse, and the keyword \"likes\" represents the current feeling or behaviour and therefore the judgement is now."}, "sentence_type": "Interrogative sentence", "pos": {"nouns": {"Spouse": 1, "friends": 2}, "verbs": {"Yeah": 1}, "numerals": {"68": 1}, "pronouns": {"I": 1}}}, {"content": "I'm not going to be gentle with my spouse because it's often mistaken for a sexual expression.", "word_count": 28, "label": "", "sentence_structure": "Subject鈥搗erb鈥搊bject", "subjective_objective": {"answer": "Subjective", "rationale": "Because the attitude of \"unwilling\" and \"misunderstanding\" is the patient's subjective feeling and perception."}, "sentence_sentiment": {"answer": "Negative", "rationale": "The basis for the judgment: \"no\" in the entry expresses negative emotional attitudes."}, "language_style": {"answer": "Tactful and warm", "rationale": "The entry is more euphemistic, using expressions such as \"unwilling\" or \"mispered\" to avoid direct conflict or negative emotional triggers. At the same time, the entry provides a thought-provoking question, inviting interviewees to think and reflect, rather than simply to make judgements or answers. This soft and inspiring language style helps to create a positive and harmonious atmosphere of dialogue and to increase the participation and feedback of questionnaires."}, "time_dimension": {"answer": "Present", "rationale": "The use of the keywords \"unwilling\" and \"often misconstrued as \"is\" suggests that it is a description of the current situation or behaviour, and thus the temporal dimension is now judged."}, "sentence_type": "Negative declarative sentence", "pos": {"nouns": {"Spouse": 1, "Sex": 2, "Organisation": 3}, "verbs": {"I don't want to": 1, "Mistake": 1, "鈾?Mistaked 鈾?: 1}, "adjectives": {"very": 1, "Be gentle": 1}, "numerals": {"69": 1, "One": 2, "Regular": 1}, "pronouns": {"it": 1, "One": 1, "Here": 1}}}, {"content": "I think there's something missing from our marriage.", "word_count": 16, "label": "", "sentence_structure": "Subject鈥揷opula鈥損redicative", "subjective_objective": {"answer": "Subjective", "rationale": "It's a subjective feeling and a perception."}, "sentence_sentiment": {"answer": "Negative", "rationale": "The expression \"scarce of something\" in the entry implies dissatisfaction and disappointment with the marriage, and is therefore negative."}, "language_style": {"answer": "Tactful and warm", "rationale": ""}, "time_dimension": {"answer": "Present", "rationale": ""}, "sentence_type": "Negative declarative sentence", "pos": {"nouns": {"Marriage": 1, "Something": 1}, "verbs": {"I think": 1, "Missing": 1}, "adverbs": {"I": 1}, "numerals": {"70锛?: 1}}}, {"content": "We sometimes have serious disputes about issues that are not important.", "word_count": 22, "label": "", "sentence_structure": "Subject鈥搗erb鈥搊bject", "subjective_objective": {"answer": "Subjective", "rationale": "Because it is a subjective view of the patient to describe his own thoughts and feelings about whether there is a serious dispute."}, "sentence_sentiment": {"answer": "Negative", "rationale": "Because \"serious dispute\" in the entry is negative."}, "language_style": {"answer": "Tactful and warm", "rationale": "The entry is more adept and interactive when it is spoken in a question-and-answer tone rather than directly speaking or ordering. At the same time, words such as \"sometimes\" and \"not important\" are used to temper tone and to show concern and respect for the other."}, "time_dimension": {"answer": "Present", "rationale": "The keyword \"sometimes\" indicates that the dispute is not continuous, but rather an occasional, intermittent phenomenon; the word \"serious\" suggests that it may have a negative impact on the relationship between the parties. The combination of the words \"sometimes\" and \"serious\" point to the current situation."}, "sentence_type": "Negative declarative sentence", "pos": {"nouns": {"Problem": 2, "Arguments": 1}, "verbs": {"Generate": 1, "Serious": 1, "Regular": 1, "in": 1, "Some": 1, "Go, go, go": 1, "we": 1, "Sometimes": 1}, "adjectives": {"It's not important": 1}, "adverbs": {"Sometimes": 1}, "numerals": {"71": 1}, "classifiers": {"Some": 1}, "pronouns": {"EMPTY": 1}}}, {"content": "I feel that the couple didn't spend enough time together to spend their leisure time.", "word_count": 24, "label": "", "sentence_structure": "Subject鈥揷opula鈥損redicative", "subjective_objective": {"answer": "Subjective", "rationale": "Because \"fear\" represents the patient's own subjective feelings and perceptions."}, "sentence_sentiment": {"answer": "Negative", "rationale": "Because the \"not enough time to spend leisure time together\" in the entry is negative."}, "language_style": {"answer": "Tactful and warm", "rationale": ""}, "time_dimension": {"answer": "Present", "rationale": "According to the keyword \"felt\" and \"does not spend enough time with leisure time\", it can be seen that this phrase is describing the current state and feeling, and therefore the entry is now."}, "sentence_type": "Negative declarative sentence", "pos": {"nouns": {"Couple": 1, "Time": 1, "Amateurity": 1}, "verbs": {"I feel": 1, "Cost": 1, "Pass": 1}, "adverbs": {"Enough": 1, "Together": 1}, "numerals": {"72锛?: 1}, "pronouns": {"I": 1}}}, {"content": "Sometimes, I can't believe everything my spouse tells me.", "word_count": 17, "label": "", "sentence_structure": "Subject鈥揷opula鈥損redicative", "subjective_objective": {"answer": "Subjective", "rationale": "Because \"unbelievable\" is a subjective attitude and feeling."}, "sentence_sentiment": {"answer": "Negative", "rationale": "Because the expression \"hard to believe\" in the entry is negative."}, "language_style": {"answer": "Tactful and warm", "rationale": "The language style of the entry tends to be euphemistic and sweet, and is more polite and emotional, expressing respect and concern for the person under investigation. At the same time, the entry also takes the form of a question and question sentence, making it easier for the person under investigation to accept and answer questions."}, "time_dimension": {"answer": "Present", "rationale": "In conclusion, the time dimension of the questionnaire entry is now judged."}, "sentence_type": "Negative declarative sentence", "pos": {"nouns": {"Spouse": 1, "Annex": 1}, "verbs": {"Tell": 1, "Letter": 1}, "adjectives": {"Hard": 1, "Every one": 1}, "adverbs": {"Sometimes": 1}, "numerals": {"73": 1}, "pronouns": {"I": 2}}}, {"content": "I try to avoid conflict with my spouse.", "word_count": 12, "label": "", "sentence_structure": "Subject鈥搗erb鈥搊bject", "subjective_objective": {"answer": "Subjective", "rationale": "Because it is the patient ' s subjective attitude and behaviour that is avoided as much as possible."}, "sentence_sentiment": {"answer": "Negative", "rationale": "Because the act of \"as little as possible avoids conflict\" in the entry is negative."}, "language_style": {"answer": "Tactful and warm", "rationale": "The language style of the entry is more euphemistic and sensitive to the other's feelings, and it starts with \"I try\" and asks if the other side has the same situation. This expression is more personal and interactive, and it attracts resonance and interest in answering questions."}, "time_dimension": {"answer": "Past", "rationale": "The keywords \"as far as possible avoid\" and \"conflict\" both imply that this is an act or attitude that has been taken, and therefore that act is a past one."}, "sentence_type": "Negative declarative sentence", "pos": {"nouns": {"Questionnaire": 1, "Entry": 1, "Spouse": 1, "Conflict": 1}, "verbs": {"Avoid": 1, "Come on": 1}, "adjectives": {"Try": 1}, "numerals": {"74锛?: 1}}}, {"content": "75. For us, the husband ' s profession is more important than the wife ' s occupation.", "word_count": 20, "label": "", "sentence_structure": "Subject鈥搗erb鈥揷omplement", "subjective_objective": {"answer": "Subjective", "rationale": "This is because of patients ' subjective perception and attitude to the importance of the husband ' s profession and that of his wife."}, "sentence_sentiment": {"answer": "Negative", "rationale": "This sentence is an expression of a gender-based and occupational bias, which considers that male occupations are more important than female occupations and a negative emotional aspect."}, "language_style": {"answer": "Calm and direct", "rationale": "The entry expresses the importance attached to the professional position of men in a concise and clear language, which is straightforward and not overly acrimony or emotional."}, "time_dimension": {"answer": "Past", "rationale": "The entry asks the respondents about their views and attitudes towards the past, and whether the husband鈥檚 profession is more important than the wife鈥檚 occupation."}, "sentence_type": "Affirmative declarative sentence", "pos": {"nouns": {"we": 1, "Husband": 1, "Occupation": 2, "Wife": 1}, "verbs": {"Compare": 1, "Heavy": 1}, "adjectives": {"Important": 1}, "numerals": {"75": 1}, "classifiers": {"锛?: 1}}}, {"content": "I think our marriage is influenced by religious beliefs.", "word_count": 16, "label": "", "sentence_structure": "Subject鈥揷opula鈥損redicative", "subjective_objective": {"answer": "Subjective", "rationale": "Because \"the feeling\" expresses the patient's personal feelings and views, it's a subjective description."}, "sentence_sentiment": {"answer": "Negative", "rationale": "Because the fact that marriage is \"affected by religious beliefs\" in the entry may have a negative impact on marriage."}, "language_style": {"answer": "Tactful and warm", "rationale": "The entry uses a more moderate and understanding tone to ask the respondents whether they believe that their marriage is influenced by religious beliefs, and that this expression is better able to generate resonance and reflection among the other, without creating pressure or discomfort."}, "time_dimension": {"answer": "Present", "rationale": "The keywords are \"like\" and \"take\", which show the subjective perception and feelings of the testee about the current marital status. Moreover, the emergence of \"religious beliefs\" also shows that the marriage is influenced by external factors."}, "sentence_type": "Affirmative declarative sentence", "pos": {"nouns": {"Marriage": 1, "Religious beliefs": 1}, "verbs": {"I think": 1, "Yes": 1, "Impact": 1}, "adverbs": {"I": 1}, "numerals": {"76": 1}}}, {"content": "Our economy has become tense, like too much credit.", "word_count": 15, "label": "", "sentence_structure": "Subject鈥搗erb鈥揷omplement", "subjective_objective": {"answer": "Objective", "rationale": "The title is an objective fact describing the economic situation."}, "sentence_sentiment": {"answer": "Negative", "rationale": "Because the words \"economic stress\" and \"over credit\" in the entries are negative, indicating a poor economic situation."}, "language_style": {"answer": "Calm and direct", "rationale": "This language style is more consistent with the requirements of the questionnaire and the accuracy of the data."}, "time_dimension": {"answer": "Past", "rationale": "According to the description of \"have become\" and \"too much\" in the sentence, the entry is about the economic situation of the past and therefore the temporal dimension can be judged to be the past."}, "sentence_type": "Affirmative declarative sentence", "pos": {"nouns": {"Economy": 1, "Nervous": 1, "Credit": 1, "More": 1}, "verbs": {"Has become": 1}, "numerals": {"77锛?: 1}}}, {"content": "78. I am troubled by the frequent delays in the marriage of spouses.", "word_count": 13, "label": "", "sentence_structure": "Subject鈥揷opula鈥損redicative", "subjective_objective": {"answer": "Subjective", "rationale": "Because the word \"make me\" means that it's the patient's subjective feeling and experience."}, "sentence_sentiment": {"answer": "Negative", "rationale": "Because the emotional tendency of the description \"spouse drags me down\" in the entry is negative."}, "language_style": {"answer": "Calm and direct", "rationale": "Since the entry does not use excessive rhetorical or emotional overtones, it is a direct statement of a person ' s dissatisfaction and distress at the spousal drag, which is more objective and concise."}, "time_dimension": {"answer": "Present", "rationale": ""}, "sentence_type": "Affirmative declarative sentence", "pos": {"nouns": {"Spouse": 1, "Trouble": 1}, "verbs": {"- I'm sorry": 1, "鈾?And I'm gonna be 鈾?: 1}, "adjectives": {"Regular": 1}, "numerals": {"78": 1}}}, {"content": "Sometimes I feel like the couple's dispute is over and I don't think it's over.", "word_count": 22, "label": "", "sentence_structure": "Subject鈥搗erb鈥搊bject", "subjective_objective": {"answer": "Subjective", "rationale": "Because the sentence uses the subjective term \"I think\" to express the patient's feelings and views. It's from the patient's subjective point of view."}, "sentence_sentiment": {"answer": "Negative", "rationale": "Because the \"debate is endless\" in the entry is negative."}, "language_style": {"answer": "Tactful and warm", "rationale": "The phrase is more euphemistic and implicit, using words such as \"sometimes\" and \"I think\" to express a view, and not directly accusing or attacking the other. At the same time, it contains emotional elements such as the disturbing and helpless nature of the dispute between the couple, which also makes the language style of the purpose more warm and human."}, "time_dimension": {"answer": "Present", "rationale": "Now, both \"Feel\" and \"Recognition Unsolved\" in the entry point indicate that it is a current feeling or state, and therefore judgement is now."}, "sentence_type": "Negative declarative sentence", "pos": {"nouns": {"Couples": 1, "Arguments": 1, "Resolve": 1}, "verbs": {"I think": 1, "Okay": 1, "Do you recognize me": 1, "Present": 1, "Yes": 1, "It's not over": 1}, "adverbs": {"Sometimes": 1}, "numerals": {"79锛?: 1}}}, {"content": "80. A wife should not work outside the home if she has a small child.", "word_count": 18, "label": "", "sentence_structure": "Subject鈥搗erb鈥搊bject", "subjective_objective": {"answer": "Subjective", "rationale": "Because the entry in the questionnaire expresses a subjective opinion or view that a wife should not be out of work if she has a small child."}, "sentence_sentiment": {"answer": "Negative", "rationale": "The view in the entry that women should abandon their career development to take care of their families is a manifestation of gender discrimination and has a negative emotional connotation."}, "language_style": {"answer": "Calm and direct", "rationale": "This language style is more straightforward and clearly indicates the author鈥檚 attitude and opinion."}, "time_dimension": {"answer": "Past", "rationale": "The entry contains past events and circumstances, and the word \"yes\" indicates that this was the case in the past, while the word \"should not\" indicates that an act was taken in the circumstances. The title therefore describes the past as a view and a proposal."}, "sentence_type": "Negative declarative sentence", "pos": {"nouns": {"Home": 1, "Children": 1, "Wife": 1, "Work": 1}, "verbs": {"have": 1, "Reactions": 1, "Out": 1, "Do it": 1}, "adjectives": {"Small": 1, "very": 1}, "numerals": {"80": 1, "One": 2}, "classifiers": {"individual": 1}, "pronouns": {"he": 1}}}, {"content": "I often don't tell my spouse how I feel, because he/she should know.", "word_count": 23, "label": "", "sentence_structure": "Subject鈥搗erb鈥搊bject", "subjective_objective": {"answer": "Subjective", "rationale": "Because \"not telling your spouse what you feel\" is a patient's subjective decision and behavior."}, "sentence_sentiment": {"answer": "Negative", "rationale": "Because the behavior in the entry is \"not to tell the spouse how he feels\", it expresses a negative emotional attitude."}, "language_style": {"answer": "Tactful and warm", "rationale": "The language style of the entry is euphemism. It expresses its feelings by using the more euphemism \"often without telling my feelings to my spouse\" and, after the passage, by saying \"because he/she should have felt\" it gave the other side a certain expectation and hope, rather than direct accusations or criticism, and thus shows a gentle and considerate tone."}, "time_dimension": {"answer": "Present", "rationale": ""}, "sentence_type": "Negative declarative sentence", "pos": {"nouns": {"Questionnaire": 1, "Entry": 1, "Feel": 1, "Spouse": 1}, "verbs": {"Regular": 1, "No, I'm not": 2, "Tell": 1, "I can see it": 1}, "adverbs": {"He/she should": 1}, "numerals": {"81锛?: 1}, "pronouns": {"I": 1}}}, {"content": "82. I am satisfied with the emotional and sexual aspects of our couples ' expression.", "word_count": 25, "label": "", "sentence_structure": "Subject鈥揷opula鈥損redicative", "subjective_objective": {"answer": "Subjective", "rationale": "As satisfaction is a subjective feeling or attitude, it depends on individual experience and perspectives."}, "sentence_sentiment": {"answer": "Positive", "rationale": "The basis for the judgment: \"satisfaction\" in the entry expresses positive attitudes and emotions."}, "language_style": {"answer": "Tactful and warm", "rationale": "The language style of the entry is relatively euphemistic and implicit, using a more subjective description ( \"satisfactory\") and not directly related to specific acts or scenes, and is more inclusive and proximate."}, "time_dimension": {"answer": "Present", "rationale": ""}, "sentence_type": "Imperative sentence", "pos": {"nouns": {"we": 1, "Couples": 1, "Emotion": 1, "Sex": 1, "Yes": 1}, "verbs": {"Yes": 1, "Expression": 1, "very": 1, "Satisfied": 1}, "adjectives": {"What": 1}, "numerals": {"82锛?: 1}}}, {"content": "83. When there is disagreement between spouses, we communicate our feelings openly and decide how to deal with it.", "word_count": 31, "label": "", "sentence_structure": "Subject鈥搗erb鈥搊bject", "subjective_objective": {"answer": "Subjective", "rationale": "Since the questionnaire entries relate to the means of communication and decision-making between spouses, these are more of a subjective examination of the views and attitudes of the patients themselves."}, "sentence_sentiment": {"answer": "Positive", "rationale": "Because the entries \"open and open to the feeling\" and \"problem.\""}, "language_style": {"answer": "Tactful and warm", "rationale": "The language style of the entry is more euphemistic and gentle, and the interviewer is asked and invited to answer the questions. The words \"open and open\" and \"communicating with feelings\" are used in the expression, emphasizing the importance of communication and understanding, and conveying a harmonious and positive atmosphere. Thus, the language style of the entry can be judged as being a warmer type."}, "time_dimension": {"answer": "Present", "rationale": ""}, "sentence_type": "Affirmative declarative sentence", "pos": {"nouns": {"Couples": 2, "Observations": 1, "Time": 1, "Communications": 1, "Feel": 1, "Decision": 1, "Problem": 1, "Resolve": 1}, "verbs": {"Come on": 2, "Open and honest": 1, "Communications": 1, "Feel": 1, "What": 1, "Resolve": 2}, "adverbs": {"Open and honest": 1, "What": 1}, "numerals": {"83锛?: 1}, "pronouns": {"we": 1}}}, {"content": "84. I rarely joke unless I am with my spouse.", "word_count": 16, "label": "", "sentence_structure": "Subject鈥搗erb鈥搊bject", "subjective_objective": {"answer": "Subjective", "rationale": "Because this phrase reflects the patient ' s subjective attitude and feelings that patients rarely joke about in the absence of their spouse."}, "sentence_sentiment": {"answer": "Negative", "rationale": "Because the expression \"very rarely joke\" in the entry is negative."}, "language_style": {"answer": "Tactful and warm", "rationale": "The language style of the entry is more euphemistic and more polite. Moreover, the term \"very rarely\" is used, rather than outright denial, to avoid situations that are too direct or offensive to the other, while maintaining a certain openness and flexibility. The language style of the article is therefore euphemistic and warm."}, "time_dimension": {"answer": "Present", "rationale": "The \"now\" in the entry to the questionnaire refers to the present behaviour and attitude of the testee, and \"less jokes\" is the current status of the testee."}, "sentence_type": "Negative declarative sentence", "pos": {"nouns": {"Questionnaire": 1, "Entry": 1, "Spouse": 1}, "verbs": {"Analysis": 1, "Together": 1, "You're kidding": 1}, "adjectives": {"Unless": 1, "Very few": 1, "Together": 1}, "adverbs": {"Otherwise": 1}, "pronouns": {"I": 1}}}, {"content": "85. We are very focused on deciding how to spend our money on the most important things.", "word_count": 20, "label": "", "sentence_structure": "Subject鈥搗erb鈥搊bject", "subjective_objective": {"answer": "Subjective", "rationale": "Because the entry is an inquiry into patients ' subjective perceptions and behaviour towards spending, rather than an objective expenditure of money."}, "sentence_sentiment": {"answer": "Positive", "rationale": "Because the attitude in the entry, \"Clinding to decide how to spend money on the most important thing,\" is positive."}, "language_style": {"answer": "Tactful and warm", "rationale": "The questionnaire entry is more euphemistic and personal, and guides respondents to express their thoughts and attitudes in a way that is both informative and informative. It also uses more active language expressions such as \"high-profile\" and \"most important\" language, emphasizing the importance and expectations of financial management, which makes the language warm and powerful."}, "time_dimension": {"answer": "Present", "rationale": "The phrase \"we focus\" indicates that the act is currently being done, that the \"money spent on the most important thing\" is happening now, and so it's now."}, "sentence_type": "Affirmative declarative sentence", "pos": {"nouns": {"Decision": 1, "Money": 1, "Things": 1}, "verbs": {"Flowers": 1, "Notes": 1, "in": 1}, "adjectives": {"Most important": 1}, "numerals": {"85": 1}, "pronouns": {"we": 1}}}, {"content": "86. Sometimes my spouse spends too much time with friends.", "word_count": 17, "label": "", "sentence_structure": "Subject鈥搗erb鈥搊bject", "subjective_objective": {"answer": "Subjective", "rationale": "Because the entry describes the patient ' s perception of the time spent with his spouse and friends and is a subjective feeling of the patient."}, "sentence_sentiment": {"answer": "Negative", "rationale": "Because the \"spouse and friends\" in the entry are too long, it can create negative feelings of jealousy, displeasure, etc."}, "language_style": {"answer": "Tactful and warm", "rationale": "The language style of the entry is more euphemistic and gentle. It is not directly critical or reproached, but rather expresses concern and understanding in a way that allows the person under investigation to express his thoughts and feelings more freely. At the same time, this soft language style is more readily accepted and co-opted by the person under investigation."}, "time_dimension": {"answer": "Present", "rationale": "The entry \"sometimes\" indicates that this phenomenon is not frequent, but still exists. And the entry \"over time\" indicates that it is a state of current relevance and is happening, so it can be judged that the content of the questionnaire is about the current relationship between the testee's spouse and friends."}, "sentence_type": "Negative declarative sentence", "pos": {"nouns": {"Time": 3, "Spouse": 1, "friends": 1}, "verbs": {"Together": 2}, "adverbs": {"Sometimes": 1}, "numerals": {"86锛?: 1}, "pronouns": {"I": 1}}}, {"content": "87. My husband and I have different views on the religious education of children.", "word_count": 22, "label": "", "sentence_structure": "Subject鈥損redicate", "subjective_objective": {"answer": "Subjective", "rationale": "This is the subjective view of the patient of the difference of opinion between himself and his spouse in religious education."}, "sentence_sentiment": {"answer": "Negative", "rationale": "Because the description of \"dissenting\" in the entry implies that there may be contradictions or conflicts and is negative."}, "language_style": {"answer": "Calm and direct", "rationale": "The entry directly illustrates the different opinions of the subjects in the religious education of their children, and the language style is relatively objective and direct."}, "time_dimension": {"answer": "Present", "rationale": "Now in the entry, \"I and my spouse have different views on the religious education of their children\" describes the current situation and does not clearly point to the past or the future. It is therefore an entry for this period."}, "sentence_type": "Affirmative declarative sentence", "pos": {"nouns": {"Questionnaire Entry": 1, "Spouse": 1, "Children": 1, "Religious education": 1}, "verbs": {"Conduct": 1, "have": 1, "Different": 1, "Observations": 1}, "adverbs": {"87锛?: 1}, "numerals": {"87锛?: 1, "I": 1, "Spouse": 1, "Children": 1, "Religious education": 1}}}, {"content": "I'm not satisfied with the division of blame for parents.", "word_count": 16, "label": "", "sentence_structure": "Subject鈥搗erb鈥揷omplement", "subjective_objective": {"answer": "Subjective", "rationale": "Because this entry addresses patients ' perceptions and feelings regarding the distribution of family responsibilities, it is a subjective attitude and emotional response of the patients."}, "sentence_sentiment": {"answer": "Negative", "rationale": "Because the word \"unsatisfactory\" in the entry is an expression of negative emotional attitudes."}, "language_style": {"answer": "Calm and direct", "rationale": "The entry expresses, in a direct and clear language, dissatisfaction with the current distribution of family responsibilities, is not overly rhetorical or emotional and is more objective and firm."}, "time_dimension": {"answer": "Present", "rationale": "The entry now indicates the respondent ' s dissatisfaction with the current division of blame for responsibility, so the time dimension is now judged."}, "sentence_type": "Negative declarative sentence", "pos": {"nouns": {"Blame": 1, "Division of labour": 1}, "verbs": {"Undertaking": 1, "Unsatisfactory": 1}, "adjectives": {"Parents": 1, "of": 2}, "numerals": {"88锛?: 1}}}, {"content": "89. Love for a spouse, so I can feel it even more deeply; God is kind.", "word_count": 17, "label": "", "sentence_structure": "Subject鈥揷opula鈥損redicative", "subjective_objective": {"answer": "Subjective", "rationale": "Because \"love\" and \"know God is kind\" are the views and feelings of patients about these things and are therefore subjectively viewed."}, "sentence_sentiment": {"answer": "Positive", "rationale": "Because the words \"love your spouse\" and \"know God is kind\" both express positive emotional attitudes."}, "language_style": {"answer": "Tactful and warm", "rationale": "The entry uses descriptive language styles and expresses the subject in a positive emotional way, giving people a warm and warm feeling. Moreover, it also gives a metaphor of how God鈥檚 loving kindness is expressed through his spouse, allowing respondents to understand the meaning more deeply when thinking about the issue."}, "time_dimension": {"answer": "Past", "rationale": ""}, "sentence_type": "Affirmative declarative sentence", "pos": {"nouns": {"Spouse": 1, "Feel it": 1, "Jesus": 2, "Mercy": 1}, "verbs": {"Love": 1, "鈾?And I'm gonna be 鈾?: 1, "Deep": 1, "I'm aware of it": 1, "be": 1}, "adverbs": {"More": 1, "Land": 1}, "numerals": {"89": 1}, "pronouns": {"I": 1}}}, {"content": "I think both parents are looking too much for our attention and help.", "word_count": 23, "label": "", "sentence_structure": "Subject鈥搗erb鈥揷omplement", "subjective_objective": {"answer": "Subjective", "rationale": "The first is the fact that the patient's attitude and feelings are subjective."}, "sentence_sentiment": {"answer": "Negative", "rationale": "Because the description of \"high expectations\" in the entry implies an excessive pressure and dissatisfaction, a negative emotion."}, "language_style": {"answer": "Calm and direct", "rationale": "The entry presents in a straightforward manner the feelings of the respondents regarding the high expectations of both parents, with no excessive emotional rendering or euphemism, and with a more objective tone."}, "time_dimension": {"answer": "Present", "rationale": "According to the questionnaire entry, \"I think that both parents expect too much from us\" describes the feelings and perceptions of the current testees, so it is possible to judge the time dimension of the article as it is now."}, "sentence_type": "Affirmative declarative sentence", "pos": {"nouns": {"Both sides": 1, "parents": 2, "Care": 1, "Help": 1}, "verbs": {"I think": 1, "I'm looking too much": 1, "Got it": 1, "Give it to me": 1}, "adjectives": {"Too high": 1, "of": 3}, "numerals": {"90锛?: 1}, "pronouns": {"we": 1}}}, {"content": "I'm very pleased with the way couples talk to each other.", "word_count": 16, "label": "", "sentence_structure": "Subject鈥揷opula鈥損redicative", "subjective_objective": {"answer": "Subjective", "rationale": "Because \"satisfaction\" and \"mutual conversation\" are personal feelings and attitudes of the patient and are subjective."}, "sentence_sentiment": {"answer": "Positive", "rationale": "Because the entry indicates a very high level of satisfaction with the way in which couples talk to each other, it is a positive emotional attitude."}, "language_style": {"answer": "Tactful and warm", "rationale": ""}, "time_dimension": {"answer": "Present", "rationale": "The entry describes the satisfaction of the interviewees with the current mode of communication between themselves and their spouses and therefore the judgement is now."}, "sentence_type": "Affirmative declarative sentence", "pos": {"nouns": {"Couples": 1, "Modalities": 1}, "verbs": {"Satisfied": 1, "Talk": 1, "be": 1}, "adjectives": {"Very": 1}, "numerals": {"91锛?: 1}}}, {"content": "92. I think our parents are causing problems with our marriage.", "word_count": 18, "label": "", "sentence_structure": "Subject鈥搗erb鈥搊bject", "subjective_objective": {"answer": "Subjective", "rationale": "Because \"the feeling\" represents the patient's personal perception and feelings, and it is viewed from a subjective perspective."}, "sentence_sentiment": {"answer": "Negative", "rationale": "Because the expression \"the marriage of the parents causes problems\" in the entry is negative."}, "language_style": {"answer": "Tactful and warm", "rationale": "The entry is based on the first person's \"I\" perspective and expresses a subjective feeling, not a direct accusation or evaluation of others. Moreover, it uses more moderate language styles, such as \"think\" and \"we\" that make language expression more kinetic and emotional. These factors tend to favour this language style for this purpose."}, "time_dimension": {"answer": "Past", "rationale": "Therefore, depending on the content of the input, it is possible to judge that the time dimension of the article is now."}, "sentence_type": "Negative declarative sentence", "pos": {"nouns": {"parents": 1, "we": 1, "Marriage": 1, "Problem": 1}, "verbs": {"Here": 1, "Cause": 1}, "adverbs": {"I think": 1}, "numerals": {"92": 1}}}, {"content": "I'm worried. I can't spend without a spouse's permission.", "word_count": 16, "label": "", "sentence_structure": "Subject鈥損redicate", "subjective_objective": {"answer": "Subjective", "rationale": "Because \"sorture\" and \"I can't spend without a spouse\" are subjective thoughts and emotional reactions."}, "sentence_sentiment": {"answer": "Negative", "rationale": "Because \"unsatisfied\" and \"not paying without the permission of a spouse\" in the entries indicate negative emotions and restrictive behaviour."}, "language_style": {"answer": "Tactful and warm", "rationale": ""}, "time_dimension": {"answer": "Present", "rationale": ""}, "sentence_type": "Exclamatory sentence", "pos": {"nouns": {"Trouble": 1, "Spouse": 1, "Money": 1}, "verbs": {"No, I'm not": 1, "Allow": 1, "Flowers": 1}, "numerals": {"93": 1}, "pronouns": {"I": 2}}}, {"content": "94. Since having children, couples rarely ask to be alone.", "word_count": 19, "label": "", "sentence_structure": "Subject鈥搗erb鈥揷omplement", "subjective_objective": {"answer": "Subjective", "rationale": "Because \"we have little time alone\" is the patient's subjective feeling and experience."}, "sentence_sentiment": {"answer": "Negative", "rationale": "Because the entries describe the negative phenomenon of \"very little time for couples to be alone\"."}, "language_style": {"answer": "Tactful and warm", "rationale": "The entry is more personal and interactive when it is presented in a gentle and inviting tone, rather than in direct statements or orders. At the same time, the article is not too strong or aggressive, but rather calm and objective, helping to increase the respondents ' willingness to answer questions."}, "time_dimension": {"answer": "Present", "rationale": ""}, "sentence_type": "Negative declarative sentence", "pos": {"nouns": {"Son": 1, "Couples": 2, "Time": 1, "Alone": 1, "Together": 1}, "verbs": {"Got it": 1, "Very few": 1, "have": 1, "Question": 1, "There's time": 1, "鈾?Have time alone 鈾?: 1, "Together": 1}, "adverbs": {"Since": 1, "Very few": 1}, "numerals": {"94锛?: 1, "2": 1}}}, {"content": "95. I feel like I'm in a state of uncertainty about my spouse.", "word_count": 18, "label": "", "sentence_structure": "Subject鈥搗erb鈥搊bject", "subjective_objective": {"answer": "Subjective", "rationale": "Because \"fearless\" is the patient's subjective feeling and attitude."}, "sentence_sentiment": {"answer": "Negative", "rationale": "Because the \"frightful\" attitude in the entry is negative."}, "language_style": {"answer": "Calm and direct", "rationale": "The entry uses a simple language to ask the interviewee directly about the feelings of the spouse when he or she is experiencing a spousal mood swing, without too much embellishment or emotional expression."}, "time_dimension": {"answer": "Present", "rationale": "Now, the words \"sometimes\" and \"fear\" in the entry are both expressions of the current feelings and experiences of the speaker, and therefore the judgment is now. And, \"frightfulness towards the spouse\" is a description of the status quo, and it is the current state of affairs."}, "sentence_type": "Negative declarative sentence", "pos": {"nouns": {"Spouse": 1, "鈾?The mood is unchanging 鈾?: 1, "Sometimes": 1, "Nothing": 1}, "verbs": {"Yes": 1, "I feel": 1, "be": 1}, "adverbs": {"Unusual": 1, "Sometimes": 1}, "numerals": {"95锛?: 1}, "pronouns": {"I": 2}}}, {"content": "I often feel that our spouses are not taking our differences seriously.", "word_count": 18, "label": "", "sentence_structure": "Subject鈥搗erb鈥搊bject", "subjective_objective": {"answer": "Subjective", "rationale": "The blogger says that the problem is that the patient is not a patient, because it is the patient's own subjective feeling that is not taken seriously."}, "sentence_sentiment": {"answer": "Negative", "rationale": "Because the phrase \"the feeling that the spouses are not taking our differences seriously\" in the entry is negative."}, "language_style": {"answer": "Calm and direct", "rationale": "The entry is more direct in language style, is calm and objective, expresses questions and grievances about the behaviour of the other, is not overly emotional or rhetorical and is more capable of transmitting information."}, "time_dimension": {"answer": "Present", "rationale": ""}, "sentence_type": "Negative declarative sentence", "pos": {"nouns": {"Spouse": 1, "Difference": 1}, "verbs": {"I feel": 1, "Seriously": 1}, "adjectives": {"Regular": 1}, "adverbs": {"No, I'm not": 1}, "numerals": {"96锛?: 1}, "pronouns": {"we": 1}}}, {"content": "97. In our home, the husband has the final say on most important matters.", "word_count": 25, "label": "", "sentence_structure": "Subject鈥揷opula鈥損redicative", "subjective_objective": {"answer": "Subjective", "rationale": "Since the title referred to \"the husband should have final decision\" as the opinion and opinion of the interviewees themselves, it looked at the subjective attitude of the interviewees."}, "sentence_sentiment": {"answer": "Negative", "rationale": "This is because it emphasizes the dominant position of men in the family, neglects the rights and opinions of women and has a discriminatory gender dimension."}, "language_style": {"answer": "Calm and direct", "rationale": "This language style is suitable for the entry of a questionnaire where the views or positions are expressed."}, "time_dimension": {"answer": "Past", "rationale": "The entry describes a traditional or customary act in the family, namely, \"the husband shall have the final decision on most important matters\", which is usually considered as a past act or state of affairs."}, "sentence_type": "Affirmative declarative sentence", "pos": {"nouns": {"Our home": 1, "Husband": 2, "Things": 1, "Finally": 1, "Decision-making authority": 1}, "verbs": {"You should": 1, "have": 1, "Decision": 1}, "adverbs": {"In most cases, it's important": 1}, "numerals": {"97": 1}, "pronouns": {"we": 1}}}, {"content": "98. Fearing that the spouse is angry, I do not always tell him/her some of the worries that are in his/her heart.", "word_count": 27, "label": "", "sentence_structure": "Subject鈥搗erb鈥搊bject", "subjective_objective": {"answer": "Subjective", "rationale": "It is the patient ' s subjective idea to express his or her fear about the spousal temper and whether he or she is told of the choice."}, "sentence_sentiment": {"answer": "Negative", "rationale": "Because the words \"fear of the spouse's temper\" and \"not always tell him/her some of his/her worries\" are negative."}, "language_style": {"answer": "Tactful and warm", "rationale": "The entry is more personal and interactive by speaking in a gentle, invitation-like manner, rather than by direct statement or order. At the same time, by using the more euphemismal language \"because of fear\" it avoids criticism or accusations of the other party's behaviour and is more likely to receive a positive response from the other party."}, "time_dimension": {"answer": "Past", "rationale": "The entry is for the purpose of asking the person who was tested for a certain past experience or behaviour, and the keyword \"fear of the spouse to be angry\" and \"not always to tell\" all point to the past behaviour or state."}, "sentence_type": "Affirmative declarative sentence", "pos": {"nouns": {"Spouse": 1, "The mood": 1, "Some": 1, "Trouble": 1}, "verbs": {"I'm worried": 1, "- You're losing your temper": 1, "Tell": 2, "Always": 1}, "adjectives": {"In my heart": 1}, "adverbs": {"not": 2, "Total": 1}, "numerals": {"98": 1, "Some": 1}, "classifiers": {"individual": 1}, "pronouns": {"He/she": 1}}}, {"content": "I'm not happy with our relationship with our parents, our friends.", "word_count": 16, "label": "", "sentence_structure": "Subject鈥搗erb鈥揷omplement", "subjective_objective": {"answer": "Subjective", "rationale": "Because dissatisfaction is a subjective feeling."}, "sentence_sentiment": {"answer": "Negative", "rationale": "Because the expression of dissatisfaction in the entries is negative."}, "language_style": {"answer": "Calm and direct", "rationale": "The entry directly points to the problem in the negative, with a more straightforward and concise language style, with no excessive accoutrements and emotional colours, giving people a calm, sharp feeling."}, "time_dimension": {"answer": "Present", "rationale": "The entry is to ask the testee about his satisfaction with his relationship with his parents, friends, the temporal dimension of which is now. The key words are \"unsatisfactory\" and \"relationship,\" which point to the current state and feeling."}, "sentence_type": "Negative declarative sentence", "pos": {"nouns": {"we": 1, "Parents": 1, "friends": 1, "Relations": 1}, "verbs": {"Unsatisfactory": 1, "and": 1, "of": 3}, "adverbs": {"99锛?: 1}, "numerals": {"99": 1}, "classifiers": {"individual": 1}, "pronouns": {"I": 1}}}, {"content": "100. My husband and I disagree with regard to the religious education I received.", "word_count": 20, "label": "", "sentence_structure": "Subject鈥揷opula鈥損redicative", "subjective_objective": {"answer": "Subjective", "rationale": "Because \"discretion\" is the patient's subjective feeling or opinion."}, "sentence_sentiment": {"answer": "Negative", "rationale": "The entry refers to differences between spouses, suggesting that problems may exist."}, "language_style": {"answer": "Calm and direct", "rationale": "The entry asks the respondents and spouses in an objective and direct manner whether there are differences in religious education, and it is more calm and direct than it is in the form of excessive emotional overtones or euphemisms."}, "time_dimension": {"answer": "Present", "rationale": "The entry of the questionnaire asked about the current situation of respondents, i.e. whether they and their spouses had differences in religious education, and the time dimension was now judged."}, "sentence_type": "Negative declarative sentence", "pos": {"nouns": {"Questionnaire": 1, "Entry": 1, "individual": 1, "Spouse": 1, "Religion": 1, "In terms of": 1, "Education": 2, "Observations": 1}, "verbs": {"Here": 1, "Yes": 1, "No, no, no": 1}, "adverbs": {"very": 1}, "numerals": {"100锛?: 1, "One": 2}, "pronouns": {"I": 1, "Sou-san": 1}}}, {"content": "I never regretted my parents' relationship, even for a moment.", "word_count": 18, "label": "", "sentence_structure": "Subject鈥搗erb鈥搊bject", "subjective_objective": {"answer": "Subjective", "rationale": "Because regret is a subjective emotion and feeling, \"I never regret my parents' relationship\" is the patient's subjective attitude."}, "sentence_sentiment": {"answer": "Positive", "rationale": "The position expressed in this entry is positive, i.e. the interviewee never regretted his or her relationship with his or her parents."}, "language_style": {"answer": "Tactful and warm", "rationale": ""}, "time_dimension": {"answer": "Present", "rationale": ""}, "sentence_type": "Affirmative declarative sentence", "pos": {"nouns": {"101": 1, "Relations": 1, "I": 2, "parents": 1, "鈾?And in a moment 鈾?: 1}, "verbs": {"Never regret it": 1, "have": 1}, "adverbs": {"not": 1}, "numerals": {"One": 1, "Ten": 1, "100": 1}, "classifiers": {"individual": 1}}}, {"content": "I02. How much should we do for our children is one of the reasons for our conflict.", "word_count": 21, "label": "", "sentence_structure": "Subject鈥揷opula鈥損redicative", "subjective_objective": {"answer": "Subjective", "rationale": "Because \"should\" and \"conflict\" are patients' subjective feelings or attitudes."}, "sentence_sentiment": {"answer": "Negative", "rationale": "Because the \"conflict\" in the entry is negative."}, "language_style": {"answer": "Calm and direct", "rationale": "The language style of the entry is calm and straightforward. It clearly raises a question, not using excessive rhetorical techniques or emotional rendering. At the same time, the question is not subject to any conditions or assumptions, but rather directly asks the views of the interviewee, which is more objective and neutral."}, "time_dimension": {"answer": "Present", "rationale": "\"Yes\" means that it is an ongoing event or state, \"a cause of our conflict\" means that the child problem is a current and possibly continuing one. So, the entry is about the current situation and state."}, "sentence_type": "Interrogative sentence", "pos": {"nouns": {"Children": 1, "Conflict": 1, "Reason": 1}, "verbs": {"I think so": 1, "Do it": 1, "Come on": 1}, "adverbs": {"How much": 1}, "numerals": {"One": 2}, "pronouns": {"we": 1}}}, {"content": "I'm really happy to be with all my friends.", "word_count": 16, "label": "", "sentence_structure": "Subject鈥搗erb鈥揷omplement", "subjective_objective": {"answer": "Subjective", "rationale": "Because \"happy\" is a subjective emotional experience."}, "sentence_sentiment": {"answer": "Positive", "rationale": "Because the expression \"surely happy\" in the entry is a positive emotional expression."}, "language_style": {"answer": "Tactful and warm", "rationale": "The entry expresses in a positive and complimentary manner the attitude of the other party to dealing with the spouse ' s friends, in a more cordial and sweet manner, and is more acceptable and resonant."}, "time_dimension": {"answer": "Present", "rationale": "The sentence given makes it possible to judge that the entry is an inquiry into the attitude and feelings of the person who was tested with his or her spouse's friends, and that there is no apparent point in the time dimension, so the answer is \"now\"."}, "sentence_type": "Affirmative declarative sentence", "pos": {"nouns": {"Questionnaire Entry": 1, "I": 1, "Spouse": 1, "All": 1, "friends": 1, "Travel": 1}, "verbs": {"Happy": 1, "and": 1, "- To be with someone": 1}, "adjectives": {"Indeed": 1}, "numerals": {"l03": 1}}}, {"content": "I'm close to my spouse because of our religious beliefs.", "word_count": 20, "label": "", "sentence_structure": "Subject鈥揷opula鈥損redicative", "subjective_objective": {"answer": "Subjective", "rationale": "The question is examined in relation to the relationship between the patient and the spouse, as well as in relation to the feelings based on religious belief, and is therefore a subjective feeling of the patient."}, "sentence_sentiment": {"answer": "Positive", "rationale": "Because the description of \"felt close\" in the entry is positive."}, "language_style": {"answer": "Tactful and warm", "rationale": "The entry is more moderate in its language style, where dialogue takes place in an invitation-style tone rather than directly speaking or ordering. Furthermore, the entry refers to the relationship between religious belief and intimacy, which is more intimate and warmer."}, "time_dimension": {"answer": "Present", "rationale": "The entry uses the verb \"I feel\" in the general current state of the day, which means that the current feeling or state is so time-dimensional that it is now. And, \"Me and my spouse\" refers to the present situation and state."}, "sentence_type": "Interrogative sentence", "pos": {"nouns": {"Religious beliefs": 1, "Me and my wife": 1, "Intimacy": 1}, "verbs": {"I think": 1, "very": 1, "be": 1}, "adjectives": {"Ours": 1}, "numerals": {"l04锛?: 1}, "pronouns": {"Because": 1}}}, {"content": "105. The wife must believe and accept her husband ' s judgment on important issues.", "word_count": 20, "label": "", "sentence_structure": "Subject鈥搗erb鈥搊bject", "subjective_objective": {"answer": "Subjective", "rationale": "Because the content of \"believable and accept\" is subjective, and the wife and husband are both individual, the subjective idea is being examined."}, "sentence_sentiment": {"answer": "Neutral", "rationale": "Because the entry does not address any emotional or attitudinal aspects. It merely refers to the constructive view that the wife should believe and accept the husband ' s judgment on important issues."}, "language_style": {"answer": "Tactful and warm", "rationale": "The language style of the questionnaire entry is euphemism. It uses a gentle, invitation-style tone, implying that the interviewee should trust and accept the judgement of his partner on important issues, rather than directly speaking or ordering. This expression is more personal and interactive, making the interviewee more willing to participate and answer the question."}, "time_dimension": {"answer": "Present", "rationale": "This sentence is a question of a perception or expectation of a marital relationship in the current society, and thus the temporal dimension is now judged."}, "sentence_type": "Affirmative declarative sentence", "pos": {"nouns": {"Wife": 1, "Problem": 1, "Judgement": 2}, "verbs": {"I think so": 1, "Yes": 1, "Accept": 1}, "adjectives": {"Important": 1}, "numerals": {"105": 1}, "pronouns": {"Husband": 1}}}, {"content": "Sometimes, I care about the sexual interests of my spouse.", "word_count": 18, "label": "", "sentence_structure": "Subject鈥揷opula鈥損redicative", "subjective_objective": {"answer": "Subjective", "rationale": "Because the perception and reaction of others is usually a manifestation of subjective attitudes."}, "sentence_sentiment": {"answer": "Negative", "rationale": "Because the \"careful\" attitude in the entry is negative, it is indicative of some concern and dissatisfaction with the interest of spouses."}, "language_style": {"answer": "Tactful and warm", "rationale": ""}, "time_dimension": {"answer": "Present", "rationale": "The word \"very\" in \"careful\" indicates the strength or degree of concern of the testee with regard to this issue. And \"careful\" is used in the current state of affairs, which means that it is an ongoing and ongoing state. So the entry to the questionnaire asks about the current feelings and state of the testee, judging by the present."}, "sentence_type": "Affirmative declarative sentence", "pos": {"nouns": {"Time": 1, "very": 1, "Meaning": 1, "I": 1, "Spouse": 1, "Sexual interest": 1, "Inconsistencies": 1}, "verbs": {"I care": 1, "and": 1}, "adverbs": {"very": 1}, "numerals": {"l06锛?: 1}}}, {"content": "107. I am satisfied with the decision on family planning and the number of children to be born.", "word_count": 19, "label": "", "sentence_structure": "Subject鈥揷opula鈥損redicative", "subjective_objective": {"answer": "Subjective", "rationale": "Because \"I'm satisfied\" is a subjective description of the patient's positive and positive feelings about their family planning and the decision to have children."}, "sentence_sentiment": {"answer": "Positive", "rationale": "Because the \"satisfactory\" in the entry is a positive attitude."}, "language_style": {"answer": "Tactful and warm", "rationale": "The language style of the entry is more euphemistic and gentle, using relatively neutral terms and expressions such as \"satisfaction\", \"decisions on family planning and number of children\", and not too emotional or subjectively evaluated, and more objective and sensitive."}, "time_dimension": {"answer": "Present", "rationale": "\"I\" shows that the entry describes the feelings of the testee himself, and \"satisfying with the decision on family planning and the number of children to be born\" indicates the current attitude and decision-making, so the temporal dimension is now."}, "sentence_type": "Affirmative declarative sentence", "pos": {"nouns": {"Questionnaire": 1, "Entry": 1, "Family": 1, "Planned": 1, "Childbirth": 1, "Decision": 1}, "verbs": {"Satisfied": 1, "About": 1}, "numerals": {"107锛?: 1, "very": 1}}}, {"content": "I don't care if a spouse and a heterosexual friend are there.", "word_count": 13, "label": "", "sentence_structure": "Subject鈥搗erb鈥揷omplement", "subjective_objective": {"answer": "Subjective", "rationale": "Because it's the patient's subjective attitude."}, "sentence_sentiment": {"answer": "Negative", "rationale": "Because the \"not mind\" attitude in the entries is negative."}, "language_style": {"answer": "Calm and direct", "rationale": "The language style of the entry is calm and straightforward, as it directly asks whether the respondent does not care about the spouse being with a heterosexual friend."}, "time_dimension": {"answer": "Past", "rationale": "The entry is an inquiry into the attitude or perception of the testee regarding the spouse with a heterosexual friend, and does not address past, present or future conduct, and thus the temporal dimension can be judged to be general."}, "sentence_type": "Negative declarative sentence", "pos": {"nouns": {"Spouse": 1, "Fae friend": 1, "Together": 1}, "verbs": {"I don't care": 1}, "numerals": {"108": 1}, "pronouns": {"I": 1}}}, {"content": "When I speak, the couple listens carefully.", "word_count": 12, "label": "", "sentence_structure": "Subject鈥搗erb鈥揷omplement", "subjective_objective": {"answer": "Subjective", "rationale": "Because \"when speaking, spouses always listen carefully\" requires judgement based on the subjective feelings of the interviewee."}, "sentence_sentiment": {"answer": "Positive", "rationale": "Because the \"spouse always listens\" in the entry is positive."}, "language_style": {"answer": "Tactful and warm", "rationale": "This is a more personal and interactive expression, which is conducted in a gentle and inviting tone, rather than in direct statements or orders. At the same time, by describing the \"me\" feeling, it leads to reflection and reflection on the behaviour of each other in communicating with their spouses. This way avoids direct criticism or accusations, is more acceptable and understood, and helps to promote communication and harmony between the two sides."}, "time_dimension": {"answer": "Present", "rationale": ""}, "sentence_type": "Affirmative declarative sentence", "pos": {"nouns": {"Talk": 1, "Spouse": 1}, "verbs": {"Total": 1, "Listen": 1, "Here": 1}, "adjectives": {"Seriously": 1}, "adverbs": {"Always": 1}, "numerals": {"109": 1}, "pronouns": {"I": 1}}}, {"content": "I care who cares about the money.", "word_count": 7, "label": "", "sentence_structure": "Subject鈥搗erb鈥搊bject", "subjective_objective": {"answer": "Subjective", "rationale": "Because \"care\" is a subjective feeling and attitude, it's the patient's subjective idea."}, "sentence_sentiment": {"answer": "Negative", "rationale": "Because the attitude and behaviour of \"who cares about money\" has a certain negative tendency to imply a desire for money control or a sense of mistrust."}, "language_style": {"answer": "Tactful and warm", "rationale": "The entry is more personal and interactive when it is spoken in a gentle and inviting tone, rather than in direct statements or orders. At the same time, the use of the word \"I\" makes the issue more personal and relevant, and makes it easier to generate resonance and reflection among the respondents."}, "time_dimension": {"answer": "Present", "rationale": "Now the verb \"intention\" and \"incarceration\" in the entry indicates that this is a normal current-day act, a current state or situation, and therefore the temporal dimension is now judged."}, "sentence_type": "Interrogative sentence", "pos": {"nouns": {"I": 1, "Who": 1, "Money": 1}, "verbs": {"I care": 1, "Pipe": 1}, "adverbs": {"very": 1}, "numerals": {"110": 1}}}, {"content": "I was troubled by the unfair way in which 111 couples agreed or refused to have sex.", "word_count": 23, "label": "", "sentence_structure": "Subject鈥搗erb鈥搊bject", "subjective_objective": {"answer": "Subjective", "rationale": "The question is more subjective, since the problem is subjective, and \"spouses who use unfair means to consent or refuse sex\" is an objective fact that patients experience."}, "sentence_sentiment": {"answer": "Negative", "rationale": "Because the acts described in the entries (unfairly consenting or refusing to have sex) and the resulting results (which bother me) are negative."}, "language_style": {"answer": "Calm and direct", "rationale": "The entry directly asked the respondents whether they were troubled by the unfair manner in which the spouses used their sex life, and the language style was objective and not too much rhetorical or emotional overtook."}, "time_dimension": {"answer": "Present", "rationale": "In the entry, the words \"applying unfair ways\", \"consent or reject\" and \"problem me\" indicate that such acts are occurring or have affected the subjects, and therefore the judgement is now."}, "sentence_type": "Affirmative declarative sentence", "pos": {"nouns": {"Spouse": 1, "Modalities": 1, "Sex": 1}, "verbs": {"Apply": 1, "Agreed": 1, "Reject": 1, "鈾?And I'm gonna be 鈾?: 1, "very": 1, "Trouble": 1}, "adjectives": {"It's not fair": 1}, "numerals": {"111": 1}}}, {"content": "112. When we argue, I usually do not think that it was my fault.", "word_count": 18, "label": "", "sentence_structure": "Subject鈥揷opula鈥損redicative", "subjective_objective": {"answer": "Subjective", "rationale": "Because \"not to think it's my fault\" is a subjective attitude or opinion."}, "sentence_sentiment": {"answer": "Negative", "rationale": "Because the entry \"Don't think it's my fault\" is negative."}, "language_style": {"answer": "Tactful and warm", "rationale": "The entry is more adept and interactive when it is spoken in a gentle and inviting manner, rather than by direct statements or orders."}, "time_dimension": {"answer": "Past", "rationale": "The entry \"When we argue\" indicates that this is an event that has already happened, and \"it is usually my fault\" indicates a past tendency or habit. So, judging by the time dimension, the entry describes the past behaviour or attitude."}, "sentence_type": "Exclamatory sentence", "pos": {"nouns": {"Argument": 1, "Wrong": 1}, "verbs": {"Go on": 1, "Yes": 1, "be": 1, "Louder": 1}, "adverbs": {"Usually": 1}, "numerals": {"112": 1}, "pronouns": {"I": 1}}}, {"content": "113. I feel good about our religious beliefs and values.", "word_count": 18, "label": "", "sentence_structure": "Subject鈥揷opula鈥損redicative", "subjective_objective": {"answer": "Subjective", "rationale": "Because \"think\" is a term that describes the patient's subjective feelings, the question entry is subjective."}, "sentence_sentiment": {"answer": "Positive", "rationale": "Because the entry \"felt good\" expresses a positive emotion and attitude."}, "language_style": {"answer": "Tactful and warm", "rationale": "The \"Feeling well\" in the entry is a subjective assessment, and does not express the specific feelings and attitudes of the other party to religious beliefs and values, giving a relatively moderate and neutral tone. And \"I feel well\" is a more euphemism, avoiding direct accusations or criticism of the other, and more respectful and understanding."}, "time_dimension": {"answer": "Present", "rationale": "The word \"I feel good\" indicates a subjective feeling or attitude that usually occurs and changes at a given point in time, so it can be assumed that the entry expresses the subjective perception of the person being tested, i.e. the state of the religion or belief."}, "sentence_type": "Affirmative declarative sentence", "pos": {"nouns": {"Religious beliefs": 1, "Values": 1}, "verbs": {"I think": 1, "Okay": 1}, "adjectives": {"Ours": 1, "Good": 1}, "numerals": {"113锛?: 1}, "pronouns": {"I": 1}}}, {"content": "l14. My spouses spend their free time together and separately fairly.", "word_count": 22, "label": "", "sentence_structure": "Subject鈥揷opula鈥損redicative", "subjective_objective": {"answer": "Subjective", "rationale": "The issue is examined in terms of the perception and perception of the patient that his or her spouse spends his or her leisure time with him or her and that he or she spends his or her leisure time alone, and is therefore subjective."}, "sentence_sentiment": {"answer": "Positive", "rationale": "The \"fair distribution of leisure time\" in the entry is positive, expressing recognition and affirmation of fairness and equality in the relationship between spouses, and therefore positive feelings can be judged."}, "language_style": {"answer": "Tactful and warm", "rationale": "The entry is more acceptable and willing to answer questions by asking the respondent's spouse whether the time spent in the spare time was fairly distributed, using a more moderate question method, without directly judging or evaluating the situation of the other."}, "time_dimension": {"answer": "Present", "rationale": ""}, "sentence_type": "Affirmative declarative sentence", "pos": {"nouns": {"Spouse": 1, "Amateur Time": 1, "Fair": 1}, "verbs": {"Together": 1, "Separate": 1, "Pass": 1, "Allocation": 1}, "adverbs": {"very": 1}, "pronouns": {"I": 1}}}, {"content": "Sometimes I think a couple is too aggressive.", "word_count": 13, "label": "", "sentence_structure": "Subject鈥揷opula鈥損redicative", "subjective_objective": {"answer": "Subjective", "rationale": "Because \"think\" is a subjective feeling and judgment."}, "sentence_sentiment": {"answer": "Negative", "rationale": "Because the description of \"too aggressive\" in the entry is negative."}, "language_style": {"answer": "Tactful and warm", "rationale": "The entry asks the respondents in a more moderate and respectful way whether they feel like a spouse is abusive, avoiding the possibility of direct accusations or attacks. At the same time, the first person ' s pronoun, \"I,\" is used to enhance the participants ' sense of participation and identity."}, "time_dimension": {"answer": "Present", "rationale": "The entry indicates that the testee felt that the spouse was being overly aggressive in the current marriage and could therefore be judged to describe the current situation."}, "sentence_type": "Negative declarative sentence", "pos": {"nouns": {"115": 1, "Time": 1, "Spouse": 1, "You're a badass": 1}, "verbs": {"I think": 1, "Too much": 1, "You're a badass": 1}, "adjectives": {"Too much": 1}, "numerals": {"115": 1, "Time": 1}, "pronouns": {"I": 1}}}, {"content": "I don't think any couple living together has a good relationship.", "word_count": 22, "label": "", "sentence_structure": "Subject鈥搗erb鈥搊bject", "subjective_objective": {"answer": "Subjective", "rationale": "Because the title \"I think\" indicates that it's a patient's own view and that it's subjective."}, "sentence_sentiment": {"answer": "Negative", "rationale": "Because the expression \"without harmony between us\" in the entry is a negative and negative emotional expression of our marital life."}, "language_style": {"answer": "Tactful and warm", "rationale": "The language style of the entry is more euphemistic, and dialogue is conducted in an invitation and friendly language, trying to understand the views of the interviewee on his/her relationship with his/her spouse."}, "time_dimension": {"answer": "Present", "rationale": "The entry is for questioning the attitude and perception of the testee in relation to the marriage relationship, which is now judged in terms of time dimension. In it, \"I think\" expresses subjective attitudes or beliefs, \"no spouse living together has a bond of arms\" is the testee's view or evaluation, and does not have a clear point of time, and is therefore a current state of affairs."}, "sentence_type": "Affirmative declarative sentence", "pos": {"nouns": {"Life": 2, "Together": 1, "Spouse": 2, "Couples": 2, "Harmony": 2}, "verbs": {"I think": 1, "No, I'm not": 1}, "numerals": {"116锛?: 1, "Any": 1}, "pronouns": {"we": 1}}}, {"content": "Sometimes I feel like I'm not in love with my spouse.", "word_count": 16, "label": "", "sentence_structure": "Subject鈥揷opula鈥損redicative", "subjective_objective": {"answer": "Subjective", "rationale": "This is a subjective feeling of the patient, a sense of the spouse, and a subjective view."}, "sentence_sentiment": {"answer": "Negative", "rationale": "Because the attitude of \"not feeling love\" in the entry is negative."}, "language_style": {"answer": "Calm and direct", "rationale": "The entry is in a straightforward manner to ask the respondents whether they may at some point feel free of love or affection, have a calmer and objective language style and have no excessive emotional rendering or rhetorical skills."}, "time_dimension": {"answer": "Present", "rationale": "The entry indicates the current state and feeling of the testee, i.e. that they sometimes feel that they are not feeling love or affection for their spouse. This emotional state may be a continuing problem, not just an event in the past. So the time dimension of the entry is now."}, "sentence_type": "Negative declarative sentence", "pos": {"nouns": {"Spouse": 2, "Love": 2, "Emotion": 2}, "verbs": {"I think": 1, "I feel": 1, "have": 1}, "adverbs": {"Sometimes": 1, "Marriage": 1}, "numerals": {"117锛?: 1}, "pronouns": {"I": 1}}}, {"content": "118. Sometimes, a spouse does something that makes me unhappy.", "word_count": 14, "label": "", "sentence_structure": "Subject鈥搗erb鈥搊bject", "subjective_objective": {"answer": "Subjective", "rationale": "Because \"I'm not happy with it\" is a subjective feeling and evaluation, a patient's subjective attitude and perception of the behaviour of his spouse."}, "sentence_sentiment": {"answer": "Negative", "rationale": "Because the entry \"doing something that makes me unhappy\" is negative."}, "language_style": {"answer": "Calm and direct", "rationale": "The entry describes a specific scene or behaviour in a concise manner, does not use excessive rhetorical or emotional colours and is more calm and objective."}, "time_dimension": {"answer": "Present", "rationale": ""}, "sentence_type": "Affirmative declarative sentence", "pos": {"nouns": {"Time": 1, "Spouse": 1, "Yes": 1}, "verbs": {"Do it": 1, "鈾?And I'm gonna be 鈾?: 1, "It's not good": 1}, "adjectives": {"Have a nice day": 1}, "numerals": {"118锛?: 1}, "pronouns": {"I": 1}}}, {"content": "If there's anything wrong with the spouse, I don't know.", "word_count": 14, "label": "", "sentence_structure": "Subject鈥搗erb鈥搊bject", "subjective_objective": {"answer": "Subjective", "rationale": "Because \"not knowing\" is the patient's subjective attitude and perception."}, "sentence_sentiment": {"answer": "Neutral", "rationale": "Because the entry merely describes a person ' s attitude and perception, it does not contain any apparent emotional tendencies."}, "language_style": {"answer": "Tactful and warm", "rationale": "The entry is more proficient and interactive in its expression of a moderate and inviting tone of speech, rather than a direct statement or order. At the same time, the article is more balanced, without obvious accusations or criticism, and more like an attempt to explore and understand."}, "time_dimension": {"answer": "Present", "rationale": "According to the description of \"not aware\" the entry asks the testee whether he or she is aware of the fault of the spouse in the current marriage and the temporal dimension is now judged."}, "sentence_type": "Negative declarative sentence", "pos": {"nouns": {"Spouse": 1, "Wrong": 1, "Awareness": 1}, "verbs": {"have": 1, "I didn't realize": 1}, "numerals": {"119": 1}, "pronouns": {"I": 1}}}, {"content": "120. Even if every person in the world who is opposite sex wishes to marry me, I can't make a better choice than the one I'm married to.", "word_count": 32, "label": "", "sentence_structure": "Subject鈥揷opula鈥損redicative", "subjective_objective": {"answer": "Subjective", "rationale": "Because this is the patient ' s personal thoughts and feelings, not an objective fact or an external environment judgement."}, "sentence_sentiment": {"answer": "Negative", "rationale": "Because the expression in the entry is \"no better choice than to make a marriage now\" is a negative attitude and evaluation."}, "language_style": {"answer": "Calm and direct", "rationale": "The language style of the entry is calm and straightforward. It clearly expresses the attitude and position of the person surveyed when facing marriage options, without any euphemism or exaggeration. It allows the person to express his thoughts and feelings more accurately and honestly, and thus obtain more reliable research data."}, "time_dimension": {"answer": "Present", "rationale": ""}, "sentence_type": "Negative declarative sentence", "pos": {"nouns": {"World": 1, "Heterosexual": 1, "Marriage": 3}, "verbs": {"May it be with you": 1, "Marriage": 2, "Do it": 1, "- Yeah": 1, "Selection": 1}, "adjectives": {"Better": 1}, "adverbs": {"Both": 1, "Yeah": 1}, "numerals": {"120锛?: 1, "Every one of them": 1}, "pronouns": {"I": 2, "Present": 1}}}, {"content": "121. Our husband and wife are better suited to each other than anyone else in the world.", "word_count": 18, "label": "", "sentence_structure": "Subject鈥揷opula鈥損redicative", "subjective_objective": {"answer": "Subjective", "rationale": "Because \"goodly fit\" is a subjective feeling and an evaluation."}, "sentence_sentiment": {"answer": "Positive", "rationale": "This entry is a positive emotional expression of the harmony and adaptability of the relationship between the spouses."}, "language_style": {"answer": "Tactful and warm", "rationale": "The entry is more personal and interactive in its expression of self-acclaimment and affirmation than direct statements or orders. At the same time, it is used to emphasize that the degree of harmony between one's husband and wife is higher than that of others, and it is also expressed in a certain narcissistic manner."}, "time_dimension": {"answer": "Present", "rationale": "The keywords \"we husband and wife\" and \"goodly fit\" point to the current situation, so this entry describes the present situation."}, "sentence_type": "Affirmative declarative sentence", "pos": {"nouns": {"Couples": 1, "World": 1, "person": 1}, "verbs": {"Adaptation": 1, "- Yeah": 1}, "adjectives": {"Okay": 1}, "adverbs": {"Both": 1, "Most": 1}, "numerals": {"121": 1}, "pronouns": {"we": 1, "Anyone": 1}}}, {"content": "122. Every new thing about a spouse makes me happy.", "word_count": 16, "label": "", "sentence_structure": "Subject鈥揷opula鈥損redicative", "subjective_objective": {"answer": "Subjective", "rationale": "Because pleasure is a subjective feeling, not an objective fact or phenomenon."}, "sentence_sentiment": {"answer": "Positive", "rationale": "Because the entry \"everything new makes me happy\" is positive."}, "language_style": {"answer": "Tactful and warm", "rationale": ""}, "time_dimension": {"answer": "Present", "rationale": "The keywords \"every new thing about a spouse\" and \"make me happy\" express the current feelings and perceptions, and therefore judge now."}, "sentence_type": "Affirmative declarative sentence", "pos": {"nouns": {"Spouse": 1, "Something new": 1}, "verbs": {"鈾?And I'm gonna be 鈾?: 1, "Happy": 1}, "adjectives": {"Every piece": 1}, "numerals": {"122": 1, "Both": 1}, "pronouns": {"I": 1}}}, {"content": "123. Our relationship is better than it should be.", "word_count": 14, "label": "", "sentence_structure": "Subject鈥揷opula鈥損redicative", "subjective_objective": {"answer": "Subjective", "rationale": "Because it's \"our relationship,\" it's a subjective feeling or perception."}, "sentence_sentiment": {"answer": "Positive", "rationale": "Because the expression \"relationship is better than it deserves\" in the entry is positive and indicates that the evaluation of the state of relationship is positive."}, "language_style": {"answer": "Tactful and warm", "rationale": "The entry is more adept and interactive when it is spoken in a gentle and inviting manner, rather than by direct statements or orders."}, "time_dimension": {"answer": "Present", "rationale": "The entry asks the testee for his subjective assessment of the state of our relationship, and therefore the judgment is \"now.\" The keyword \"better than it should\" suggests that the testee believes that the relationship is better than it should be."}, "sentence_type": "Affirmative declarative sentence", "pos": {"nouns": {"Relations": 1, "Status": 1}, "verbs": {"- Yeah": 1, "have": 1}, "adjectives": {"Better": 1}, "numerals": {"123锛?: 1, "It's what it deserves": 1}, "pronouns": {"we": 1}}}, {"content": "When I'm with my spouse, I don't think anyone can be happier than us.", "word_count": 24, "label": "", "sentence_structure": "Subject鈥揷opula鈥損redicative", "subjective_objective": {"answer": "Subjective", "rationale": "Because it is not possible for anyone to be happier than himself or his spouse is the patient's subjective feeling."}, "sentence_sentiment": {"answer": "Positive", "rationale": "Because the expression \"I don't think that anyone can be happier than us\" in the entry expresses a positive emotional attitude to the love of the spouse and the satisfaction of his or her life."}, "language_style": {"answer": "Tactful and warm", "rationale": "The entry is in a comparative form that expresses the value and satisfaction of the voice in his relationship with his spouse. At the same time, the expression \"I think\" also shows a certain subjectivity, which makes readers feel that the voice is sharing its own feelings, not directly speaking the truth. Thus, the language style of the article is more euphemismal."}, "time_dimension": {"answer": "Present", "rationale": "According to the sentence, \"when I'm with my spouse,\" it is a description of the situation, and it is now. At the same time, \"I don't think anyone can be happier than us\" is a statement that expresses subjective feelings and attitudes, emphasizing current feelings and perceptions. So, in sum, the questionnaire entry should be judged as now."}, "sentence_type": "Exclamatory sentence", "pos": {"nouns": {"Questionnaire": 1, "Entry": 1, "Time": 1, "person": 1, "Spouse": 1, "Anyone": 1, "Happiness": 2}, "verbs": {"I think": 1, "Impossible": 1, "- Yeah": 1, "Together": 1}, "adjectives": {"Any": 1}, "adverbs": {"Both": 1}, "numerals": {"124": 1, "2": 1}, "classifiers": {"individual": 1}, "pronouns": {"I": 1}}}]}

{"questionnaire_title": "Mental health diagnostic test (MHT)", "questionnaire_category": "Mental health", "item_count": 99, "emotion_shift_overall": "From negative to positive", "language_style_overall": "Neutral", "items": [{"content": "(1) Do you always think about tomorrow's homework when you sleep at night?", "word_count": 16, "label": "", "sentence_structure": "Subject鈥損redicate", "subjective_objective": {"answer": "Subjective", "rationale": "Because \"for tomorrow's homework\" is a subjective feeling and attitude, a patient's observation and description of his own inner state."}, "sentence_sentiment": {"answer": "Negative", "rationale": "Because the \"want to think about tomorrow's homework\" thing in the entry is negative."}, "language_style": {"answer": "Calm and direct", "rationale": "The language style of the entry is calm and straightforward. It asks the respondents directly, through simple and clear questions, whether they are always thinking about tomorrow's homework at night, without too much emotional rendering or modification. This direct and objective language style facilitates accurate data.\""}, "time_dimension": {"answer": "Present", "rationale": ""}, "sentence_type": "Interrogative sentence", "pos": {"nouns": {"Night": 1, "Sleep": 1, "Work": 1}, "verbs": {"Think about it": 1, "Do it": 1}}}, {"content": "(2) When a teacher asks a class-wide question, do you feel disturbed by asking yourself?", "word_count": 26, "label": "", "sentence_structure": "Subject鈥搗erb鈥搊bject", "subjective_objective": {"answer": "Subjective", "rationale": "Because it's the patient's own subjective feelings and perceptions."}, "sentence_sentiment": {"answer": "Negative", "rationale": "Because the mood of \"frightful\" in the entry is negative."}, "language_style": {"answer": "Tactful and warm", "rationale": "The entry is more personal and interactive in its expression of a gentle and respectful tone, rather than a direct statement or order. At the same time, the question is in the subjective language of \"you feel\" and reflects the individual differences in the respondents, making the questionnaire more inclusive and human."}, "time_dimension": {"answer": "Present", "rationale": ""}, "sentence_type": "Interrogative sentence", "pos": {"nouns": {"Teacher": 1, "All class": 1, "Problem": 1, "Answer": 1}, "verbs": {"Question": 1, "Yes": 1, "I think": 1, "I feel": 1, "Unsettled": 1}, "adjectives": {"Your own": 1}, "adverbs": {"Is it possible": 1}, "numerals": {"2": 1}, "pronouns": {"you": 1}}}, {"content": "(3) Are you nervous when you hear that you want to take a test?", "word_count": 14, "label": "", "sentence_structure": "Subject鈥搗erb鈥搊bject", "subjective_objective": {"answer": "Subjective", "rationale": "Because \"psychological stress\" is a subjective feeling and emotional reaction, it's the patient's psychological state."}, "sentence_sentiment": {"answer": "Negative", "rationale": "Because the behavior in the entries is negative as soon as you hear that you're nervous about taking the test."}, "language_style": {"answer": "Tactful and warm", "rationale": "The entry uses a soft, invitation-style tone for dialogue, rather than direct statements or orders, which is more personal and interactive. At the same time, the question \"Do you have to test to be nervous\" is also asking the interviewee in a more friendly way, avoiding undue pressure or insensitiveness to the interviewee."}, "time_dimension": {"answer": "Past", "rationale": ""}, "sentence_type": "Interrogative sentence", "pos": {"nouns": {"Questionnaire": 1, "Item": 1, "Examinations": 1}, "verbs": {"I heard": 1, "Psychological": 1, "Nervous": 1}, "numerals": {"One": 1}}}, {"content": "(4) Is your test not good for your mind?", "word_count": 16, "label": "", "sentence_structure": "Subject鈥搗erb鈥揷omplement", "subjective_objective": {"answer": "Subjective", "rationale": "Because \"frightful\" is the subjective reaction and feeling of patients with poor grades."}, "sentence_sentiment": {"answer": "Negative", "rationale": "Because the attitude of \"psychologically unhappy\" in the entries is negative."}, "language_style": {"answer": "Tactful and warm", "rationale": "The language style of the entry is more euphemistic, using the interview tone, and not directly expressing negative feelings. This way reduces the resistance of the other side and makes it more willing to participate in the investigation."}, "time_dimension": {"answer": "Present", "rationale": "\"When the test is not good\" describes the current behavioural situation, and \"psychological discomfort\" is an inquiry into the psychological state of the person tested in this situation, so the time dimension of the purpose is now."}, "sentence_type": "Interrogative sentence", "pos": {"nouns": {"Achievements": 1, "Psychological": 1, "Not fast": 1}, "verbs": {"Examinations": 1, "I feel": 1, "Not fast": 1}, "adverbs": {"Yes": 1}, "numerals": {"4": 1}}}, {"content": "(5) Are you always afraid when you have a bad academic achievement?", "word_count": 16, "label": "", "sentence_structure": "Subject鈥搗erb鈥搊bject", "subjective_objective": {"answer": "Subjective", "rationale": "Because \"not good at learning\" and \"crazy\" are the subjective feelings and experiences of patients."}, "sentence_sentiment": {"answer": "Negative", "rationale": "Because the attitude and behaviour of \"crazy\" in the entries is negative."}, "language_style": {"answer": "Tactful and warm", "rationale": "The entry is softly phrased, dialogue is conducted in an inquisitorial manner, seeking to understand the mindset and feelings of the respondents in the event of poor learning performance, and is expressed in a more personal and interactive manner."}, "time_dimension": {"answer": "Past", "rationale": ""}, "sentence_type": "Interrogative sentence", "pos": {"nouns": {"Achievements": 1, "Learning": 1}, "verbs": {"Not good": 1, "I'm afraid": 2}, "adjectives": {"Not good": 1}, "adverbs": {"Always": 1}, "numerals": {"5": 1}}}, {"content": "(6) When you take the test, when you can't remember what you know, do you feel anxious?", "word_count": 25, "label": "", "sentence_structure": "Subject鈥搗erb鈥搊bject", "subjective_objective": {"answer": "Subjective", "rationale": "Because \"fearing\" is the patient's subjective feeling."}, "sentence_sentiment": {"answer": "Negative", "rationale": "Because the emotional reaction of \"fearing\" in the entry is negative."}, "language_style": {"answer": "Tactful and warm", "rationale": "The words are more euphemistic, more verb-like, less direct accusations or orders. The use of words such as \"whether or not,\" \"fear\" makes the question more flexible, gives the other side some space to answer questions and psychological comfort. Moreover, the issue is not one of too technical a professional term, but rather of a language that is easy to understand, which helps the respondents to better understand the problem and to express their true feelings."}, "time_dimension": {"answer": "Past", "rationale": "This entry asks about the feelings and reactions of the person being tested in a given situation, i.e., the anxiety of the examination when difficulties are encountered."}, "sentence_type": "Interrogative sentence", "pos": {"nouns": {"Examinations": 1, "Knowledge": 1, "Anxiety": 1}, "verbs": {"Yes": 3, "I feel": 1, "Got it": 1}, "adverbs": {"When": 1, "Time": 1, "Original": 1}, "numerals": {"6": 1}, "pronouns": {"you": 2}}}, {"content": "(7) After your exams, do you always feel relieved until you know your grades.", "word_count": 21, "label": "", "sentence_structure": "Subject鈥搗erb鈥搊bject", "subjective_objective": {"answer": "Subjective", "rationale": "Because \"no worries\" is an expression of subjective emotions and attitudes."}, "sentence_sentiment": {"answer": "Negative", "rationale": "Because the \"reward\" act in the entry is negative."}, "language_style": {"answer": "Calm and direct", "rationale": "The entry uses simple and clear language and asks the respondents whether they are worried or uneasy after the examination, without any rhetorical or emotional rendering, and with a more objective and calm tone."}, "time_dimension": {"answer": "Present", "rationale": "\"Unknown\" means a specific point or period of time, usually between the end of a given examination and the time of learning about the results. And \"always resting\" is a customary, recurring psychological state that may have existed in the past or may be repeated in the future, and therefore more appropriate to describe it in its present-day state."}, "sentence_type": "Interrogative sentence", "pos": {"nouns": {"Examinations": 1, "Achievements": 1, "Before": 1, "Problem": 1, "Answer": 1}, "verbs": {"be": 1, "Always": 1, "Don't worry": 1}, "adverbs": {"No, I'm not": 1, "in": 1, "After": 1}, "numerals": {"7": 1}, "pronouns": {"you": 1}}}, {"content": "(8) Whether you've met the test, you're afraid it'll fail.", "word_count": 14, "label": "", "sentence_structure": "Subject鈥搗erb鈥搊bject", "subjective_objective": {"answer": "Subjective", "rationale": "The fear is the patient's subjective feeling and thoughts."}, "sentence_sentiment": {"answer": "Negative", "rationale": "Because the mood of \"fears to fail\" in the entry is negative."}, "language_style": {"answer": "Calm and direct", "rationale": "The entry is more straightforward in language style, is not subject to much rhetorical or emotional rendering, and is more objective in tone. It asks if the other person has concerns at the test, is simple, and clear, so that the respondent can understand and express his/her views clearly."}, "time_dimension": {"answer": "Past", "rationale": ""}, "sentence_type": "Interrogative sentence", "pos": {"nouns": {"Examinations": 1, "Bad": 1}, "verbs": {"I met him": 1, "I'm worried": 1, "Examination": 1}, "adverbs": {"Just": 1}, "numerals": {"VIII": 1}, "pronouns": {"you": 1}}}, {"content": "(9) Do you wish the examination to pass successfully?", "word_count": 12, "label": "", "sentence_structure": "Subject鈥損redicate", "subjective_objective": {"answer": "Subjective", "rationale": "Because \"favorite\" is an individual's expectation and desire, a subjective feeling and attitude."}, "sentence_sentiment": {"answer": "Positive", "rationale": "Because the \"I hope the exam will pass\" attitude in the entry is positive."}, "language_style": {"answer": "Tactful and warm", "rationale": "The language style of the entry is more euphemistic, as it uses a question-and-answer form to ask the other side about the test, and does not directly indicate his or her position or pressure."}, "time_dimension": {"answer": "Future", "rationale": "Based on the information provided in the title, the entry asks the testee about the future test results and therefore determines the future."}, "sentence_type": "Interrogative sentence", "pos": {"nouns": {"Examinations": 1, "Good": 1, "Pass": 1}, "verbs": {"Hope": 1}, "numerals": {"9": 1}}}, {"content": "(10) Do you always worry about the mission being completed before it is completed?", "word_count": 20, "label": "", "sentence_structure": "Subject鈥搗erb鈥搊bject", "subjective_objective": {"answer": "Subjective", "rationale": "This is a question of subjective feelings of anxiety and anxiety among patients."}, "sentence_sentiment": {"answer": "Negative", "rationale": "Because the entry \"always worried about not doing the job\" expresses a negative emotional attitude."}, "language_style": {"answer": "Calm and direct", "rationale": "The entry raises the question in a direct manner, without too much emotional rendering or euphemism, and is more objective and calm in tone. \""}, "time_dimension": {"answer": "Present", "rationale": "The keyword \"whether\" and \"always fear\" indicates that the act took place in the person's current psychological state, so the entry describes the present situation."}, "sentence_type": "Interrogative sentence", "pos": {"nouns": {"Questionnaire Entry": 1, "Tasks": 2}, "verbs": {"Completed": 2, "I'm worried": 1, "I'm gonna finish my job": 1}, "adverbs": {"Is it possible": 1, "Always": 1}, "numerals": {"10": 1}, "classifiers": {"individual": 1}}}, {"content": "(11) Are you always afraid to read the script in front of everyone?", "word_count": 19, "label": "", "sentence_structure": "Subject鈥搗erb鈥搊bject", "subjective_objective": {"answer": "Subjective", "rationale": "Because the problem is viewed in terms of the patient ' s psychological response and feelings in a given situation, it is the patient ' s subjective perspective."}, "sentence_sentiment": {"answer": "Negative", "rationale": "Because the \"fear of reading\" in the entry is negative."}, "language_style": {"answer": "Tactful and warm", "rationale": "The language style of the entry is euphemistic, using the expression \"Whether or not to always\" to avoid direct accusations or commanding. At the same time, the term \"fear of reading wrong\" suggests that there may be some self-denial, and that there is a need for more gentle questioning and guidance."}, "time_dimension": {"answer": "Present", "rationale": "The entry describes the person who was tested in the current state of behavior, that is, the feeling of reading the text in front of everyone. So the time dimension is now."}, "sentence_type": "Interrogative sentence", "pos": {"nouns": {"Everyone": 1, "Noodles": 1, "Text": 1}, "verbs": {"Speak": 2, "Total": 1, "Yes": 1, "Error": 1}, "adverbs": {"Always": 1, "In front of you": 1, "of": 3}, "numerals": {"11": 1}, "pronouns": {"you": 1, "Is it possible": 1}}}, {"content": "(12) Do you think that school results are always unreliable?", "word_count": 22, "label": "", "sentence_structure": "Subject鈥搗erb鈥搊bject", "subjective_objective": {"answer": "Subjective", "rationale": "Because \"think\" expresses the views of the patient or the views of the patient."}, "sentence_sentiment": {"answer": "Negative", "rationale": "Because the idea that learning achievement is not always reliable in the entry is negative."}, "language_style": {"answer": "Calm and direct", "rationale": "The entry raises questions in a direct and unamended language, without using euphemism or excessive emotional rendering, and is more objective and calm."}, "time_dimension": {"answer": "Present", "rationale": "The entry asks the testee about his current views or attitudes, and the keywords are \"whether or not he thinks \" and \"learning achievement is always unreliable\", all pointing to the current situation."}, "sentence_type": "Interrogative sentence", "pos": {"nouns": {"School": 1, "Learning achievement": 1, "Reliability": 1}, "verbs": {"I think": 1, "Got it": 1, "Always": 1, "It's not reliable": 1}, "adjectives": {"Reliable": 1}, "adverbs": {"Always": 1, "Not much": 1}, "numerals": {"12": 1}, "classifiers": {"individual": 1}, "pronouns": {"you": 1}}}, {"content": "(13) Do you think you're more worried about learning than anyone else?", "word_count": 14, "label": "", "sentence_structure": "Subject鈥搗erb鈥搊bject", "subjective_objective": {"answer": "Subjective", "rationale": "Because \"think\" is the patient's subjective attitude."}, "sentence_sentiment": {"answer": "Negative", "rationale": "Because the entry expresses feelings that are negative, and that they are more concerned than others that learning can bring stress and anxiety."}, "language_style": {"answer": "Calm and direct", "rationale": "The entry directly asks whether the respondents have over-self-evaluations, the language style is more objective and cold, and there are no excessive emotional qualifiers or rhetorical techniques, so that they can be judged as \"cool and direct\"."}, "time_dimension": {"answer": "Present", "rationale": "The keyword \"Do you think\" indicates that the tester's subjective assessment of his current mentality, and therefore the temporal dimension is now judged."}, "sentence_type": "Interrogative sentence", "pos": {"nouns": {"Questionnaire": 1, "Entry": 1, "person": 2, "Learning": 1}, "verbs": {"I think": 1, "- Yeah": 1, "More": 1, "I'm worried": 1}, "numerals": {"13": 1}, "pronouns": {"you": 1, "others": 1}}}, {"content": "(14) Have you ever had a bad dream with a bad test?", "word_count": 12, "label": "", "sentence_structure": "Subject鈥搗erb鈥搊bject", "subjective_objective": {"answer": "Subjective", "rationale": "Because bad exams are a manifestation of negative emotions, dreams are usually the inner feeling of the patient and are subjective."}, "sentence_sentiment": {"answer": "Negative", "rationale": "Because the \"dicks are bad\" in the entry is negative."}, "language_style": {"answer": "Tactful and warm", "rationale": ""}, "time_dimension": {"answer": "Past", "rationale": "The entry asks whether the person tested had experienced the \"bad test\" dream, which occurred at some point in the past and was therefore judged to be the past."}, "sentence_type": "Interrogative sentence", "pos": {"nouns": {"Questionnaire": 1, "Entry": 1, "Dreams": 1}, "verbs": {"Do it": 1, "Bad": 1, "Examination": 1, "Bad test": 1}, "numerals": {"14": 1}}}, {"content": "(15) Have you ever had a dream of being reprimanded by a mother or a mother or a teacher when you had a bad academic achievement?", "word_count": 25, "label": "", "sentence_structure": "Subject鈥搗erb鈥搊bject", "subjective_objective": {"answer": "Objective", "rationale": "Because this is an objective fact that patients have experienced, not a subjective feeling."}, "sentence_sentiment": {"answer": "Negative", "rationale": "Because the experience of being reprimanded by parents or teachers in the entries usually leads to negative emotional and psychological stress."}, "language_style": {"answer": "Tactful and warm", "rationale": "The entry is more euphemistic and warm, using a query rather than a direct statement or order."}, "time_dimension": {"answer": "Past", "rationale": "The keyword \"do\" indicates that it is an inquiry into past behaviour and therefore belongs to the temporal dimension of the past."}, "sentence_type": "Interrogative sentence", "pos": {"nouns": {"you": 1, "Learning achievement": 1, "Mom and Dad": 2, "Teacher": 1, "Censorship": 1, "Dreams": 1}, "verbs": {"Do it": 1, "Yes": 1, "Censorship": 1}, "adverbs": {"not": 1}, "numerals": {"15": 1}, "classifiers": {"individual": 1}, "pronouns": {"you": 1, "Learning achievement": 1, "Mom and Dad": 2, "Teacher": 1}}}, {"content": "(16) Do you often feel that there are people who say bad things about you behind your back?", "word_count": 17, "label": "", "sentence_structure": "Subject鈥搗erb鈥搊bject", "subjective_objective": {"answer": "Subjective", "rationale": "Because it is a subjective feeling and judgment to feel that you are being told bad."}, "sentence_sentiment": {"answer": "Negative", "rationale": "Because the \"said you bad\" act in the entry is negative."}, "language_style": {"answer": "Calm and direct", "rationale": "The entry is based on a direct interview, without too much rhetorical or emotional rendering. It is simple to ask if the person surveyed often feels that he or she has a bad story behind his or her back, is more objective in tone and is more straightforward in language."}, "time_dimension": {"answer": "Present", "rationale": "In conclusion, the analysis of the input sentence shows \"now\"."}, "sentence_type": "Interrogative sentence", "pos": {"nouns": {"Students": 1, "Behind": 1, "Bad word": 1}, "verbs": {"I think": 1, "Say it": 1}, "adjectives": {"Often": 1}, "numerals": {"16": 1}}}, {"content": "(17) Are you always over-minded and concerned when you are judged by your parents?", "word_count": 19, "label": "", "sentence_structure": "Subject鈥搗erb鈥搊bject", "subjective_objective": {"answer": "Subjective", "rationale": "This is a question of how patients feel and react to their parents鈥?judgment, such as whether they are always insatiable and caring. This requires that the patient respond to the questions and express his feelings and thoughts."}, "sentence_sentiment": {"answer": "Negative", "rationale": "Because \"inconvenient\" and \"intentional\" are negative words in the entries."}, "language_style": {"answer": "Tactful and warm", "rationale": "The language style of the entry tends to be euphemistic. It uses a question-and-answer tone, not direct orders or statements, and starts with the phrase \"are you always\" that is more moderate and friendly, and that can trigger positive responses and reflection by the respondents. Moreover, the term \"care\" is used to describe the situation of the respondents after they have been judged by their parents, which shows the researchers' interest in and understanding of the respondents."}, "time_dimension": {"answer": "Past", "rationale": "\"Between\" and \"have to think about it?\" means \"receiving\" and \"receiving.\""}, "sentence_type": "Interrogative sentence", "pos": {"nouns": {"Parental judgement": 1, "Heart": 1}, "verbs": {"Received": 1, "I can't": 1, "Fire": 1}, "adjectives": {"Always": 1}, "adverbs": {"Is it possible": 1}, "numerals": {"17": 1}}}, {"content": "(18) Do you want to quit if you lose to each other in games or competitions?", "word_count": 24, "label": "", "sentence_structure": "Subject鈥搗erb鈥搊bject", "subjective_objective": {"answer": "Subjective", "rationale": "Because it is the patient ' s subjective attitude and reaction."}, "sentence_sentiment": {"answer": "Negative", "rationale": "Because \"lost to the other side, I don't want to do it anymore\" is negative."}, "language_style": {"answer": "Calm and direct", "rationale": "The language style of the entry is more direct, with no excessive rhetorical and emotional rendering, but simply asks whether the other party is willing to continue to participate after losing the competition or competing. This tone is more in line with the professional and objective requirements of the questionnaire."}, "time_dimension": {"answer": "Present", "rationale": "The entry asks the testee's reaction and feelings in a given situation, and the temporal dimension is now. The keyword \"does not want to do it\" suggests that it is a topic of immediate reaction, not directly related to the past or the future."}, "sentence_type": "Interrogative sentence", "pos": {"nouns": {"you": 1, "Game": 1, "others": 1, "Competition": 1, "The other side": 1, "Wins and losses": 1}, "verbs": {"Lose": 1, "No, I don't": 1, "Fuck": 1}, "adjectives": {"Again": 1}, "adverbs": {"Is it possible": 1}, "pronouns": {"you": 1}}}, {"content": "(19) Are you sick of people talking about you behind your back?", "word_count": 15, "label": "", "sentence_structure": "Subject鈥搗erb鈥搊bject", "subjective_objective": {"answer": "Subjective", "rationale": "The reason for this is that it is the patient's subjective emotional reaction."}, "sentence_sentiment": {"answer": "Negative", "rationale": "Because the behavior in the entry \"someone talks about you behind your back\" is negative, and the respondents are negative about whether they hate it or not."}, "language_style": {"answer": "Calm and direct", "rationale": "It raises questions in a concise manner, without too much accommodative terminology or expression, or attempts to channel the mood of the respondents. This calm, objective language style helps to ensure that the responses to the questionnaire are more accurate and true."}, "time_dimension": {"answer": "Present", "rationale": "\"Does it bother you?\" \"Do you hate it?\" and ask the testee how or how he feels about it, which is the current context. So the time dimension of the article is now."}, "sentence_type": "Interrogative sentence", "pos": {"nouns": {"He's": 1, "Behind": 1, "Arguments": 1, "you": 2, "Shit": 1}, "verbs": {"Is it possible": 1, "I feel": 1}, "adverbs": {"very": 1, "in": 1}, "numerals": {"19": 1}}}, {"content": "(20) Do you have a red face in front of everyone or when you are questioned by your teacher?", "word_count": 18, "label": "", "sentence_structure": "Subject鈥搗erb鈥搊bject", "subjective_objective": {"answer": "Subjective", "rationale": "Because it's an emotional reaction, it's a subjective experience of the patient."}, "sentence_sentiment": {"answer": "Negative", "rationale": "Because the \"Hama\" act in the entry is negative."}, "language_style": {"answer": "Calm and direct", "rationale": "The language style of the entry is more objective and straightforward, and there is no excessive emotional qualification or rhetorical technique, mainly in the form of questions and answers to the respondents."}, "time_dimension": {"answer": "Past", "rationale": "The \"in front of you\" and \"in response to the teacher's questions\" and \"in the face of the red\" are past experiences and behaviour, so the temporal dimension of the purpose is the past."}, "sentence_type": "Interrogative sentence", "pos": {"nouns": {"you": 1, "Everyone": 1, "In front of you": 1, "Teacher": 1, "Problem": 1, "Time": 1, "Is it possible": 2, "Red": 1}, "verbs": {"Yes": 2, "Question": 1}, "adverbs": {"in": 1, "By": 1}, "classifiers": {"individual": 1}}}, {"content": "(21) Are you worried about being a Ben's cadre?", "word_count": 13, "label": "", "sentence_structure": "Subject鈥搗erb鈥搊bject", "subjective_objective": {"answer": "Subjective", "rationale": "Because \"a lot of worry\" is a personal psychological feeling and attitude, a subjective view of the patient."}, "sentence_sentiment": {"answer": "Negative", "rationale": "The term \"fear\" in this entry is negative and indicates that the interviewees have some concerns or concerns about being in the class."}, "language_style": {"answer": "Tactful and warm", "rationale": "The entry is more personal and interactive in terms of euphemistic questions, a soft, inviting tone of conversation, and an inquiry into the level of concern of the other party about the staff member ' s position, rather than a direct statement or order."}, "time_dimension": {"answer": "Present", "rationale": "The keyword \"Are you\" means that it is a question of the current state of the person, and the description \"are worried\" is also about the current situation, so it can be judged that the entry is an inquiry into the current emotional state of the person being tested."}, "sentence_type": "Interrogative sentence", "pos": {"nouns": {"Questionnaire Entry": 1, "Ben's cadre": 1}, "verbs": {"Appointments": 1, "Please": 1, "I'm worried": 1}, "adverbs": {"very": 1}, "numerals": {"21": 1}}}, {"content": "(22) Do you always feel like someone's watching you?", "word_count": 15, "label": "", "sentence_structure": "Subject鈥損redicate", "subjective_objective": {"answer": "Subjective", "rationale": "Because it's the subjective feeling that patients feel."}, "sentence_sentiment": {"answer": "Negative", "rationale": "Because the \"like someone's watching you\" attitude in the entry is negative."}, "language_style": {"answer": "Tactful and warm", "rationale": "The entry is more personal and interactive in its expression of conversation in a gentle and inviting tone, rather than direct statements or orders. It also uses the phrase \"you think\" to make it easier for respondents to share their feelings."}, "time_dimension": {"answer": "Present", "rationale": "The entry asks the testee's current feelings and experiences, whether \"always\" means \"always\" means \"always\" is a constant feeling, and \"like someone's watching you\" is a current-day move. So the time dimension of this entry is now."}, "sentence_type": "Interrogative sentence", "pos": {"nouns": {"person": 1, "Attention": 1}, "verbs": {"Always": 1, "I think": 1, "in": 1}, "adjectives": {"Okay": 1}, "adverbs": {"Yeah": 1, "Always": 1}, "numerals": {"22": 1, "One": 1}, "pronouns": {"you": 1}}}, {"content": "(23) Are you nervous when you work or study, if someone pays attention to you?", "word_count": 21, "label": "", "sentence_structure": "Subject鈥搗erb鈥搊bject", "subjective_objective": {"answer": "Subjective", "rationale": "Because stress is a psychological feeling and subjective attitude of the patient."}, "sentence_sentiment": {"answer": "Negative", "rationale": "Because the \"stretch\" in the entry is negative."}, "language_style": {"answer": "Tactful and warm", "rationale": "The entry is more personal and interactive when it is spoken in a gentle and inviting manner, rather than in direct statements or orders. Moreover, the question is one of \"whether or not\" and allows respondents to express their views and feelings more freely than to be subject to excessive guidance or restriction."}, "time_dimension": {"answer": "Past", "rationale": "The entry enquired about the testee ' s past and judged the time dimension as the past."}, "sentence_type": "Interrogative sentence", "pos": {"nouns": {"person": 1, "Work": 1, "Learning": 1, "Time": 1, "In my heart": 1, "Nervous": 1}, "verbs": {"Attention": 1, "Is it possible": 1, "Nervous": 1}, "adverbs": {"If": 1}, "numerals": {"23": 1}, "pronouns": {"you": 1}}}, {"content": "(24) Are you always unhappy when you are judged?", "word_count": 15, "label": "", "sentence_structure": "Subject鈥搗erb鈥搊bject", "subjective_objective": {"answer": "Subjective", "rationale": "Because psychological feelings are a subjective experience of the individual."}, "sentence_sentiment": {"answer": "Negative", "rationale": "Because the \"unhappy mind\" act in the entry is negative."}, "language_style": {"answer": "Tactful and warm", "rationale": "The entry is more adept and interactive when it is spoken in a gentle and inviting manner, rather than by direct statements or orders."}, "time_dimension": {"answer": "Present", "rationale": ""}, "sentence_type": "Interrogative sentence", "pos": {"nouns": {"The judges": 1, "Heart": 1}, "verbs": {"Received": 1, "Is it possible": 1, "Always": 1, "It's not good": 1}, "adverbs": {"Always": 1}, "numerals": {"24": 1}}}, {"content": "(25) Were you always upset when you were judged by your teacher?", "word_count": 16, "label": "", "sentence_structure": "Subject鈥搗erb鈥搊bject", "subjective_objective": {"answer": "Subjective", "rationale": "Because it is a description of the state of mind, reflecting the emotional reaction of the patient, it is subjective."}, "sentence_sentiment": {"answer": "Negative", "rationale": "Because the mental state of \"always uneasy\" in the entries is negative."}, "language_style": {"answer": "Calm and direct", "rationale": ""}, "time_dimension": {"answer": "Past", "rationale": ""}, "sentence_type": "Interrogative sentence", "pos": {"nouns": {"Teacher": 1, "The judges": 1, "In my heart": 1, "Unsettled": 1}, "verbs": {"Received": 1, "The judges": 1, "Always": 1, "I feel": 1}, "adjectives": {"Ann": 1}, "adverbs": {"Total": 1, "be": 1}, "numerals": {"25": 1}, "pronouns": {"you": 1}}}, {"content": "(26) Are you not laughing when the students laugh?", "word_count": 13, "label": "", "sentence_structure": "Subject鈥搗erb鈥搊bject", "subjective_objective": {"answer": "Subjective", "rationale": "This is because of the patient ' s personal feelings and attitudes and is subjective."}, "sentence_sentiment": {"answer": "Negative", "rationale": "Because the behavior in the entry \"never laughs\" is negative."}, "language_style": {"answer": "Tactful and warm", "rationale": "The language style of the entry is euphemism. It is more friendly and interactive in a gentle and inviting tone, rather than in direct statements or orders. The phrase in the entry is \"Doesn't your classmates laugh?\" is a question, and is made in the form of inquiries, not orders or criticisms. Also, \"Do you not laugh?\" It is not a negative emotional expression in itself, but a question of the other side, which is more gentle and friendly."}, "time_dimension": {"answer": "Past", "rationale": "The entry contains a description of the testee's past behavior, the \"sympiads laugh.\" Whether\" indicates that this is a selective question, asking what happened in the past. So the time dimension of the entry is the past."}, "sentence_type": "Interrogative sentence", "pos": {"nouns": {"Students": 1, "Smile": 1}, "verbs": {"have": 1, "No, I won't": 1}, "adverbs": {"Yeah": 1}, "numerals": {"26": 1, "you": 1}, "pronouns": {"Is it possible": 1}}}, {"content": "(27) Do you think it's better to play at your own home?", "word_count": 19, "label": "", "sentence_structure": "Subject鈥搗erb鈥搊bject", "subjective_objective": {"answer": "Subjective", "rationale": "The subject is the patient's subjective feelings."}, "sentence_sentiment": {"answer": "Negative", "rationale": "Because the attitude in the entry \"I think it's better to play at my schoolmates' house\" is negative."}, "language_style": {"answer": "Tactful and warm", "rationale": "The entry uses a question-and-answer format, which is a soft, open and respectful expression of the other. This expression helps to encourage respondents to share their real thoughts rather than being forced to make a specific answer."}, "time_dimension": {"answer": "Present", "rationale": "The keyword \"you feel\" indicates that it is an inquiry into the present feelings or attitudes of the testees, so the temporal dimension is now judged."}, "sentence_type": "Interrogative sentence", "pos": {"nouns": {"Questionnaire": 1, "Entry": 1, "Students": 1, "Home": 2, "Play": 2}, "verbs": {"I think": 1, "in": 1, "Home": 1, "Why don't you": 1}, "adverbs": {"Present": 1}, "numerals": {"27": 1}, "pronouns": {"you": 1}}}, {"content": "(28) Do you feel alone when you're with everyone?", "word_count": 22, "label": "", "sentence_structure": "Subject鈥搗erb鈥搊bject", "subjective_objective": {"answer": "Subjective", "rationale": "Because the feeling of being alone is a subjective experience of a human being."}, "sentence_sentiment": {"answer": "Negative", "rationale": "Because the emotional color of \"slelhood\" in the entries is negative."}, "language_style": {"answer": "Tactful and warm", "rationale": "The entry is more personal and interactive in a gentle and inviting tone of speech, rather than in direct statements or orders. At the same time, the language style of the article is euphemism, which is a soft sentence, allowing the interviewee to think and answer the question, rather than to give the answer directly. This language style is more respectful of the feelings and ideas of the interviewee and better able to inspire their enthusiasm and honesty in participating."}, "time_dimension": {"answer": "Present", "rationale": ""}, "sentence_type": "Interrogative sentence", "pos": {"nouns": {"you": 1, "Everyone": 1, "self": 1, "person": 1}, "verbs": {"I think": 1, "be": 1, "Alone": 1, "Together": 1}, "numerals": {"28": 1}, "classifiers": {"individual": 1}, "pronouns": {"Well": 1}}}, {"content": "(29) Do you think you should play with your classmates instead of playing alone?", "word_count": 19, "label": "", "sentence_structure": "Subject鈥搗erb鈥搊bject", "subjective_objective": {"answer": "Subjective", "rationale": "Because the subject is about the patient's own feelings, which are \"like,\" it's subjective."}, "sentence_sentiment": {"answer": "Negative", "rationale": "Because the attitude in the entry \"I feel like playing with my classmates, I think it's better to play alone\" is negative."}, "language_style": {"answer": "Calm and direct", "rationale": "The language style of the entry is relatively simple and not too much emotional or euphemism, but rather more direct and clear to ask the other side about the personal feelings of playing with the fellow students and playing alone."}, "time_dimension": {"answer": "Present", "rationale": "The entry asks the testee what he feels and what he thinks, and the key word is \"do you feel\" and \"do you feel better than you play alone,\" all pointing to the current, ongoing situation."}, "sentence_type": "Interrogative sentence", "pos": {"nouns": {"Students": 1, "Problem": 1}, "verbs": {"I think": 1, "Play": 2, "Why don't you": 1}, "adverbs": {"One": 2}, "numerals": {"29": 1}}}, {"content": "(30) Do you not want to join the class while they talk?", "word_count": 14, "label": "", "sentence_structure": "Subject鈥損redicate", "subjective_objective": {"answer": "Subjective", "rationale": "The sentence is based on the patient鈥檚 subjective feelings and attitudes, i.e. whether they feel they do not want to be involved in the communication. Although it is an observed act (i.e. whether others notice that the patient is not involved in the communication), its causes and motivations remain the patient鈥檚 subjective thoughts and feelings."}, "sentence_sentiment": {"answer": "Negative", "rationale": "Because it expresses a negative behaviour, \"not wanting to join\" suggests that the respondents may have social fears or disliked participation in group discussions."}, "language_style": {"answer": "Tactful and warm", "rationale": "The entry is more euphemistic in language style and takes a soft investigative approach to asking each other about their feelings and views."}, "time_dimension": {"answer": "Present", "rationale": "The entry is for the present situation of the testee, and the keywords \"when talking\" and \"if you do not want to join\" point to the current behaviour or state. Thus, the time dimension of the article is now judged in terms of the time dimension."}, "sentence_type": "Interrogative sentence", "pos": {"nouns": {"Class": 1, "Talk": 1, "Add": 1}, "verbs": {"Yes": 1, "Add": 1}, "adverbs": {"not": 1}, "numerals": {"30": 1}, "pronouns": {"you": 1}}}, {"content": "(31) Do you feel like you're redundant when you're with everyone?", "word_count": 19, "label": "", "sentence_structure": "Subject鈥搗erb鈥搊bject", "subjective_objective": {"answer": "Subjective", "rationale": "Since the question is about the patient ' s self-perception in a social setting, the patient ' s subjective attitude and feelings need to be taken into account."}, "sentence_sentiment": {"answer": "Negative", "rationale": "Because the mental state of the entry, \"I feel like I'm redundant,\" is negative."}, "language_style": {"answer": "Tactful and warm", "rationale": "It uses the phrase \"when you're with everyone\" to ask the respondents in an indirect way how they feel in social settings, so that they feel understood and respected, not questioned or accused. And there is no overly emotional tone in the sentence, which is more moderate, moderate and consistent with the euphemism of the language."}, "time_dimension": {"answer": "Present", "rationale": "The entry can be judged by the title \"Does it feel redundant?\", and the entry is asked about the current feelings or mindset of the testee. Therefore, the time dimension is now."}, "sentence_type": "Interrogative sentence", "pos": {"nouns": {"self": 1, "person": 2, "Everyone": 1}, "verbs": {"I think": 1, "be": 1, "Overload": 1}, "adjectives": {"It's redundant": 1}, "adverbs": {"Is it possible": 1, "Together": 1}, "numerals": {"31": 1}, "pronouns": {"you": 1}}}, {"content": "(32) Do you hate sports and arts?", "word_count": 16, "label": "", "sentence_structure": "Subject鈥搗erb鈥搊bject", "subjective_objective": {"answer": "Subjective", "rationale": "Because \"obsessed\" is a subjective emotional attitude."}, "sentence_sentiment": {"answer": "Negative", "rationale": "Because the attitude in the entry \"I hate to go to sports and art shows\" is negative."}, "language_style": {"answer": "Calm and direct", "rationale": "The language style of the entry is more straightforward and concise, and there is no excessive rhetorical or emotional colour to express the interest of the interviewees in sports and arts performances in a question-and-answer manner."}, "time_dimension": {"answer": "Present", "rationale": "The entry uses the word \"whether or not\" to indicate that the person being questioned is the view or attitude of the person being questioned about the situation and therefore judge it as the present. At the same time, participation in sports and arts events is also a question of the present situation, which is further confirmed by the answer."}, "sentence_type": "Interrogative sentence", "pos": {"nouns": {"Games": 1, "Art fair": 1}, "verbs": {"Participation": 1, "Shit": 1}, "numerals": {"32": 1}}}, {"content": "(33) Are your friends very few?", "word_count": 8, "label": "", "sentence_structure": "Subject鈥揷opula鈥損redicative", "subjective_objective": {"answer": "Subjective", "rationale": "Because \"friends are few\" is a subjective feeling and an evaluation."}, "sentence_sentiment": {"answer": "Negative", "rationale": "Because the description of \"very few friends\" in the entry is negative."}, "language_style": {"answer": "Tactful and warm", "rationale": "The entry uses a soft, inquisitive language style, whereby interviewees are invited to share their situation rather than directly speaking or ordering."}, "time_dimension": {"answer": "Present", "rationale": "The entry asks about the current social status of the testee, which is judged by the time dimension. The keyword \"your friend\" and \"are few\" point to the current state or situation."}, "sentence_type": "Interrogative sentence", "pos": {"nouns": {"friends": 1, "Less": 1}, "verbs": {"Is it possible": 1, "be": 1}, "adjectives": {"A lot": 1}, "numerals": {"33": 1}}}, {"content": "(34) Do you not like talking to others?", "word_count": 11, "label": "", "sentence_structure": "Subject鈥搗erb鈥搊bject", "subjective_objective": {"answer": "Subjective", "rationale": "Because the desire to talk to someone is a subjective attitude and feeling of a person, not an objective fact or result."}, "sentence_sentiment": {"answer": "Negative", "rationale": "Because the \"not like talking to other people\" act is negative."}, "language_style": {"answer": "Calm and direct", "rationale": "The language style of the entry is more direct, concise, and does not have too many adorations and emotional colours, and it is more focused on the collection of facts and data, which facilitates subsequent analysis and research."}, "time_dimension": {"answer": "Present", "rationale": "In the expression \"does not like\" the word \"no\" indicates that it is an inquiry into the current state, so the temporal dimension is now judged."}, "sentence_type": "Interrogative sentence", "pos": {"nouns": {"Questionnaire": 1, "Entry": 1, "person": 2, "Talk": 1}, "verbs": {"be": 1, "Yeah": 1, "Talk": 1}, "adjectives": {"With someone else": 1, "of": 3, "I don't like it": 1}, "numerals": {"34": 1}, "pronouns": {"you": 1}}}, {"content": "(35) Are you afraid of being in a crowded place?", "word_count": 13, "label": "", "sentence_structure": "Subject鈥搗erb鈥搊bject", "subjective_objective": {"answer": "Subjective", "rationale": "The fear is a subjective feeling of a person, a subjective reaction of the individual to the surrounding environment and circumstances."}, "sentence_sentiment": {"answer": "Negative", "rationale": "Because the \"fear\" in the entry is negative."}, "language_style": {"answer": "Calm and direct", "rationale": "The entry is presented in a direct and concise question, is not overly accommodative or emotional, is more objective and air-sensitive and is of a straightforward language style."}, "time_dimension": {"answer": "Present", "rationale": "In the entry, \"in a crowded place\" means the current situation or state, and \"you feel afraid\" reflects the person being tested. So, the entry describes the current situation."}, "sentence_type": "Interrogative sentence", "pos": {"nouns": {"Local": 1, "person": 1, "Yes": 1}, "verbs": {"I think": 1, "very": 1, "be": 1, "Yes": 1}, "adjectives": {"More": 1, "Land": 1}, "adverbs": {"in": 1}, "numerals": {"35": 1}}}, {"content": "(36) Did you always think you were bad when you lost sports games like volleyball, basketball, football, tugley, radio?", "word_count": 31, "label": "", "sentence_structure": "Subject鈥搗erb鈥搊bject", "subjective_objective": {"answer": "Subjective", "rationale": "Because \"whether or not you always think you're bad\" is the patient's subjective idea and attitude."}, "sentence_sentiment": {"answer": "Negative", "rationale": "Because the entries \"lost the sports game\" and \"thinks you're bad\" have negative emotional aspects."}, "language_style": {"answer": "Tactful and warm", "rationale": "The entry is a conversation that takes place in a conversation with a voice of inquiry and concern, not in a direct denial or blame, but in an individual鈥檚 feelings and thoughts."}, "time_dimension": {"answer": "Present", "rationale": ""}, "sentence_type": "Interrogative sentence", "pos": {"nouns": {"Volleyball": 1, "Basketball": 1, "Football": 1, "To the river": 1, "Radio": 1, "Sports competitions": 1, "Lose": 1, "self": 1}, "verbs": {"I think": 1}, "adjectives": {"Not good": 1}, "adverbs": {"Always": 1}, "numerals": {"36": 1}}}, {"content": "(37) Do you always think you're bad after being criticized?", "word_count": 16, "label": "", "sentence_structure": "Subject鈥搗erb鈥搊bject", "subjective_objective": {"answer": "Subjective", "rationale": "Because this question relates to the patient ' s perception and interpretation of what he feels inside of him, it is a subjective examination."}, "sentence_sentiment": {"answer": "Negative", "rationale": "Because the attitude of \"always feeling bad\" in the entry is negative."}, "language_style": {"answer": "Calm and direct", "rationale": ""}, "time_dimension": {"answer": "Past", "rationale": "The entry asks the testee's reaction and feelings in the face of criticism, and the temporal dimension is the past. In it, \"the criticism indicates\" that this is an event that has already taken place, and \"always thinks that you're not good\"? It reflects the psychological state or behavior of the testee."}, "sentence_type": "Interrogative sentence", "pos": {"nouns": {"Criticism": 1, "self": 1}, "verbs": {"Received": 1, "Is it possible": 2, "I think": 1, "Okay": 1}, "adverbs": {"Always": 1}, "numerals": {"37": 1}}}, {"content": "(38) When people laugh at you, do you think it's because you don't have to work?", "word_count": 22, "label": "", "sentence_structure": "Subject鈥搗erb鈥搊bject", "subjective_objective": {"answer": "Subjective", "rationale": "The question is about the views and attitudes of the patients themselves."}, "sentence_sentiment": {"answer": "Negative", "rationale": "Because \"When people laugh at you\" in the entry is a negative and degrading experience, and \"do you think it's because you don't have to do it?\" can trigger self-criticalness and anxiety, which are negative emotional experiences."}, "language_style": {"answer": "Calm and direct", "rationale": "The entry is asked in a direct and clear manner, without excessive rhetorical or emotional rendering, and is more objective and incriminating. This way applies to situations requiring specific and clear answers, which helps to reduce subjective judgement and misleading behaviour of the person under investigation."}, "time_dimension": {"answer": "Present", "rationale": ""}, "sentence_type": "Interrogative sentence", "pos": {"nouns": {"Time": 1, "person": 1, "Reason": 1, "you": 2, "Smile": 1, "Yes": 1, "self": 1, "Don't be hard": 1}, "verbs": {"I think": 1, "be": 1, "others": 1, "Smile": 1, "Yes": 1, "- You're good": 1, "For reasons of": 1}, "adverbs": {"Yes": 2}, "numerals": {"38": 1}}}, {"content": "(39) Do you always think you're not doing your job because you're not doing well?", "word_count": 23, "label": "", "sentence_structure": "Subject鈥搗erb鈥搊bject", "subjective_objective": {"answer": "Subjective", "rationale": "Because good or bad results are the subjective feeling of the patient, it is also based on his own subjective judgement that the use of his or her work is done."}, "sentence_sentiment": {"answer": "Negative", "rationale": "Because the idea of \"thinking you're not doing your job\" in the entry is negative."}, "language_style": {"answer": "Calm and direct", "rationale": "This is a more objective expression. This expression better captures the actual thoughts and attitudes of the interviewees."}, "time_dimension": {"answer": "Present", "rationale": ""}, "sentence_type": "Interrogative sentence", "pos": {"nouns": {"Learning achievement": 1, "- You're good": 1}, "verbs": {"Not good": 1, "Always": 1, "I think": 1}, "adverbs": {"not": 2, "self": 1}, "numerals": {"39": 1}}}, {"content": "(40) Do you always feel responsible when you fail?", "word_count": 18, "label": "", "sentence_structure": "Subject鈥損redicate", "subjective_objective": {"answer": "Subjective", "rationale": "Because \"thinks it's his own responsibility\" is a subjective assessment or perception."}, "sentence_sentiment": {"answer": "Negative", "rationale": "Because the act and attitude in the entry, \"always think it's his own responsibility\", shows a negative emotional semblance."}, "language_style": {"answer": "Calm and direct", "rationale": "The entry is a direct question to whether respondents will be held responsible for failure, with a more concise language style, without too many rhetorical and emotional features."}, "time_dimension": {"answer": "Past", "rationale": "- Past: Past experience may have affected the testee鈥檚 current self-reliance. If the testee has often attributed responsibility to himself in past failures, he may be inclined to answer the question as to whether failure is always his own responsibility, depending on the way they think and the emotional state they are currently thinking. If they are demanding and often anxious or stressed, they may prefer to blame failure. - Future experience may affect the testee鈥檚 self-reliance."}, "sentence_type": "Interrogative sentence", "pos": {"nouns": {"Time": 2, "Failed": 1, "Responsibility": 1}, "verbs": {"be": 1, "I think": 1, "Total": 1}, "adjectives": {"self": 1}, "adverbs": {"Always": 1}, "numerals": {"40": 1}}}, {"content": "(41) When everyone is blamed, do you think it is primarily your fault?", "word_count": 20, "label": "", "sentence_structure": "Subject鈥搗erb鈥搊bject", "subjective_objective": {"answer": "Subjective", "rationale": "This is because patients have subjective knowledge and evaluation of their own responsibilities."}, "sentence_sentiment": {"answer": "Negative", "rationale": "Because the attitude of \"priorly blame yourself\" in the entry is negative."}, "language_style": {"answer": "Calm and direct", "rationale": "The entry is more directly linguistically, without too much rhetoric or emotional rendering, and directly asks the other party whether it considers itself wrong when blamed. \""}, "time_dimension": {"answer": "Present", "rationale": ""}, "sentence_type": "Interrogative sentence", "pos": {"nouns": {"Everyone": 1, "Blame": 1, "Wrong": 1}, "verbs": {"Yes": 1, "I think": 1, "be": 1, "Main": 1, "of": 2, "Pass": 1, "Accountability": 1}, "adverbs": {"Time": 1, "Yes": 1, "Is it possible": 1}, "numerals": {"41": 1}, "pronouns": {"you": 1, "self": 1}}}, {"content": "(42) Were you particularly careful when you made a mistake at sports games like ping-pong, badminton, basketball, football, tug-ha, radio?", "word_count": 33, "label": "", "sentence_structure": "Subject鈥搗erb鈥搊bject", "subjective_objective": {"answer": "Subjective", "rationale": "Because it examines the psychological response of the patient after the error in the competition, which is the patient ' s subjective attitude."}, "sentence_sentiment": {"answer": "Neutral", "rationale": "The purpose of this article is not expressed in a clear emotional manner, but in a way that asks the person interviewed about the behaviour of the person."}, "language_style": {"answer": "Calm and direct", "rationale": "The entry is written in a calm and straightforward manner. It asks whether the respondents are particularly careful about their mistakes when they are participating in sports, and is not over-prescriptive or emotional, and is more objective in terms of tone."}, "time_dimension": {"answer": "Present", "rationale": "The questionnaire entry asked if the person tested was particularly concerned about his or her mistakes in the current sports competition and thus judged it to be now."}, "sentence_type": "Interrogative sentence", "pos": {"nouns": {"Questionnaire": 1, "Entry": 1, "Ping-pong ball": 1, "Badminton": 1, "Basketball": 1, "Football": 1, "To the river": 1, "Radio": 1, "Sports": 1, "The game": 1, "Wrong": 1, "Special": 1, "Watch out": 1}, "verbs": {"in": 1, "Is it possible": 2, "Error": 1, "It's special": 1, "Watch out": 1}, "adverbs": {"Both": 1, "One": 2}, "numerals": {"42": 1}, "classifiers": {"Wait": 1}, "pronouns": {"you": 1}}}, {"content": "(43) Do you think you can handle the difficult things you have to do?", "word_count": 19, "label": "", "sentence_structure": "Subject鈥搗erb鈥搊bject", "subjective_objective": {"answer": "Subjective", "rationale": "The problem is a subjective one because it involves the feelings and perceptions of the patient himself."}, "sentence_sentiment": {"answer": "Negative", "rationale": "Because the attitude of \"unmanageable\" in the entries is negative."}, "language_style": {"answer": "Calm and direct", "rationale": "The language style of the entry is more direct and objective, and there is no excessive emotional rendering or euphemism, but rather a direct question as to whether the other party feels unresponsive when it encounters difficulties. \""}, "time_dimension": {"answer": "Past", "rationale": ""}, "sentence_type": "Interrogative sentence", "pos": {"nouns": {"Things": 1, "Problem": 1, "Hard": 1, "- It's hard": 1}, "verbs": {"I think": 1, "self": 1, "Payable": 1, "I've got it": 1, "Can you": 1}, "adjectives": {"Hard": 1}, "numerals": {"43": 1}, "pronouns": {"you": 1}}}, {"content": "Do you regret it sometimes?", "word_count": 15, "label": "", "sentence_structure": "Subject鈥搗erb鈥搊bject", "subjective_objective": {"answer": "Subjective", "rationale": "Because it is a subjective examination of the patient ' s inner thoughts and feelings."}, "sentence_sentiment": {"answer": "Negative", "rationale": "Because the mood of regret in the entry is negative."}, "language_style": {"answer": "Calm and direct", "rationale": "The entry is in direct, concise language, without too much rhetoric or emotional rendering. It asks whether the person surveyed has ever had a moment of regret and clearly states that this time is about \"that thing.\" This language style is more objective and calm, and suitable for psychological questionnaire research."}, "time_dimension": {"answer": "Past", "rationale": "The key words in the entry, \"regrets\" and \"doesn't do it\" all indicate that it is a reflection of a decision or act of the past and that it regrets that no action was taken at that time. The entry is therefore a past."}, "sentence_type": "Interrogative sentence", "pos": {"nouns": {"Yes": 2, "Regret": 1}, "verbs": {"Do it": 1, "Okay": 1, "Time": 1, "Yes": 1, "Regret": 1}, "adverbs": {"Sometimes": 1}, "numerals": {"44": 1}}}, {"content": "(45) Do you always think it's your fault after you and your classmates have a fight?", "word_count": 19, "label": "", "sentence_structure": "Subject鈥搗erb鈥搊bject", "subjective_objective": {"answer": "Subjective", "rationale": "Because \"thinking it's your fault\" is what patients think about themselves and how they feel about themselves, and it's subjective."}, "sentence_sentiment": {"answer": "Negative", "rationale": "Because the attitude in the entry that \"always thinks it's his fault\" is negative."}, "language_style": {"answer": "Calm and direct", "rationale": "The entry is more direct, objective, and does not have too much emotional or rhetorical style. It is inspired by a specific scene and then guides the question-and-answer process in question form, which is more calm and direct."}, "time_dimension": {"answer": "Past", "rationale": "In this entry, \"always\" means \"acts or ideas that have existed in some circumstances in the past, and the time description of \"after a schoolboy's quarrel\" indicates that such act or idea occurred after a specific event in the past. The temporal dimension of this questionnaire item is therefore judged to be \"the past\"."}, "sentence_type": "Interrogative sentence", "pos": {"nouns": {"Students": 1, "Fight": 1}, "verbs": {"I think": 1, "Louder": 1}, "adjectives": {"Always": 1}, "numerals": {"45": 1}, "pronouns": {"you": 1}}}, {"content": "Do you always want to do good for your class?", "word_count": 14, "label": "", "sentence_structure": "Subject鈥損redicate", "subjective_objective": {"answer": "Subjective", "rationale": "Because \"want\" is patient's subjective attitude."}, "sentence_sentiment": {"answer": "Positive", "rationale": "Because the entry \"wanted to do good for the class\" expresses a positive emotional attitude."}, "language_style": {"answer": "Tactful and warm", "rationale": "The entry uses euphemism and raises questions with encouragement and support, rather than direct orders or questioning. This expression makes it easier for the interviewee to feel comfortable and to share his thoughts."}, "time_dimension": {"answer": "Present", "rationale": ""}, "sentence_type": "Interrogative sentence", "pos": {"nouns": {"Psychological": 1, "Classes": 2, "Good news": 1}, "verbs": {"Yes": 1, "Do it": 1}, "numerals": {"46": 1}}}, {"content": "(47) Do you learn to distance your mind from one another?", "word_count": 15, "label": "", "sentence_structure": "Subject鈥搗erb鈥搊bject", "subjective_objective": {"answer": "Subjective", "rationale": "Because \"Does the mind often leave?\" is a question of asking patients about subjective feelings and experiences."}, "sentence_sentiment": {"answer": "Negative", "rationale": "Because the \"done thinking\" in the entry is negative."}, "language_style": {"answer": "Calm and direct", "rationale": "The entry is in direct, concise language, and the interviewer ' s learning habits are questioned, and there is no excessive emotional or oral style, and the tone is more objective and calm."}, "time_dimension": {"answer": "Present", "rationale": "The entry asks whether the testee has been constantly distracted and thoughtless in his current learning behaviour. \""}, "sentence_type": "Interrogative sentence", "pos": {"nouns": {"Time": 1, "Ideas": 1, "- Hey, you": 1}, "verbs": {"Learning": 1, "Regular": 1, "Open": 1, "Is it possible": 1}, "adverbs": {"very": 1, "Very": 1}, "numerals": {"47": 1}}}, {"content": "(48) When you lend something to someone, do you worry that someone will break it?", "word_count": 21, "label": "", "sentence_structure": "Subject鈥搗erb鈥搊bject", "subjective_objective": {"answer": "Subjective", "rationale": "Because \"fearing someone else to break things\" is a subjective feeling and a fear for a human being."}, "sentence_sentiment": {"answer": "Negative", "rationale": "Because the attitude in the entry, \"A fear that someone will break something,\" is negative."}, "language_style": {"answer": "Tactful and warm", "rationale": "The entry is more accessible and interactive by speaking in a question-and-answer manner rather than by direct statement or order. At the same time, the language style of the entry is more euphemismal, not in strong or critical language, and is more acceptable and willing to answer questions."}, "time_dimension": {"answer": "Present", "rationale": "The keyword \"are afraid\" suggests that this is a description of the current situation. And, \"when lending something to someone else\" also highlights that it is an act of interaction with others, which involves a general problem that people may face in their daily lives. So the content of this questionnaire can be classified as:"}, "sentence_type": "Interrogative sentence", "pos": {"nouns": {"you": 1, "Something": 2, "others": 1}, "verbs": {"- Put it on": 2, "Loan": 1, "I'm worried": 1, "Broken": 1}, "adverbs": {"Is it possible": 1}, "numerals": {"48": 1}, "classifiers": {"individual": 1}}}, {"content": "(49) Were you upset when things went wrong?", "word_count": 17, "label": "", "sentence_structure": "Subject鈥損redicate", "subjective_objective": {"answer": "Subjective", "rationale": "The subject is subjective because it asks about the patient ' s own feelings and mental state."}, "sentence_sentiment": {"answer": "Negative", "rationale": "Because the \"when things go wrong\" thing in the entry is negative, \"Are you upset?\" The question is emotional and negative."}, "language_style": {"answer": "Tactful and warm", "rationale": "Warm and warm."}, "time_dimension": {"answer": "Past", "rationale": "The entry is a psychological response to the testee ' s past difficult or unsettling circumstances, and the temporal dimension is a past."}, "sentence_type": "Interrogative sentence", "pos": {"nouns": {"Things": 1, "Psychological": 1, "I'm so upset": 1}, "verbs": {"Pong": 1, "In my heart": 1}, "numerals": {"49": 1}}}, {"content": "(50) Are you very worried about people in your family who are sick or dead?", "word_count": 16, "label": "", "sentence_structure": "Subject鈥搗erb鈥搊bject", "subjective_objective": {"answer": "Subjective", "rationale": "Because \"fear\" is a subjective emotional attitude that reflects the feelings and thoughts inside the patient."}, "sentence_sentiment": {"answer": "Negative", "rationale": "Because the \"very worried\" attitude in the entry is negative."}, "language_style": {"answer": "Tactful and warm", "rationale": "The entry is more gentle, caring and considerate by asking the other party about the extent of the concern about the illness or death of a family member, rather than directly expressing or ordering. At the same time, using a euphemism would also make the interviewee more willing to share his/her true feelings and thoughts, and would help to obtain more accurate data results."}, "time_dimension": {"answer": "Present", "rationale": ""}, "sentence_type": "Interrogative sentence", "pos": {"nouns": {"Questionnaire": 1, "Problem": 1, "person": 2, "Home": 1, "Sick": 1, "Die": 1}, "verbs": {"I'm worried": 1, "have": 1, "be": 1, "Give": 1}, "adjectives": {"Very": 1}, "numerals": {"50": 1}}}, {"content": "(52) Are you particularly sensitive to the sound of radios and cars?", "word_count": 17, "label": "", "sentence_structure": "Subject鈥搗erb鈥搊bject", "subjective_objective": {"answer": "Objective", "rationale": "Because sensitivity to sound is a fact that can be measured, not a subjective feeling of the patient."}, "sentence_sentiment": {"answer": "Neutral", "rationale": "Because the entry does not have any emotional color."}, "language_style": {"answer": "Calm and direct", "rationale": "The language style of the entry is clear, there is no excessive rhetorical or emotional rendering, and the tone of the interview is more objective and correct in the sense of the radio and car sound."}, "time_dimension": {"answer": "Present", "rationale": "The keyword \"is\" or \"is\" \"is\" a particularly sensitive word is all about the state or feeling."}, "sentence_type": "Interrogative sentence", "pos": {"nouns": {"you": 1, "Radio": 1, "Sound": 2, "Car": 1, "Sensitivity": 1}, "verbs": {"be": 1, "Special": 1, "Sensitive": 1}, "numerals": {"52": 1}}}, {"content": "(53) Do you always feel like something's not right?", "word_count": 18, "label": "", "sentence_structure": "Subject鈥損redicate", "subjective_objective": {"answer": "Subjective", "rationale": "Because it is a feeling or emotion, a feeling and experience inside the patient, and a subjective perspective."}, "sentence_sentiment": {"answer": "Negative", "rationale": "Because \"it's like something's not right\" is usually a negative, anxiety situation."}, "language_style": {"answer": "Tactful and warm", "rationale": ""}, "time_dimension": {"answer": "Present", "rationale": ""}, "sentence_type": "Interrogative sentence", "pos": {"nouns": {"In my heart": 1, "Yes": 2}, "verbs": {"Total": 1, "Do it": 1, "have": 1}, "adjectives": {"Okay": 1}, "adverbs": {"I think so": 1}, "numerals": {"53": 1, "One": 1, "Two": 1}, "pronouns": {"you": 1}}}, {"content": "(54) Are you worried about what's going to happen?", "word_count": 14, "label": "", "sentence_structure": "Subject鈥搗erb鈥搊bject", "subjective_objective": {"answer": "Subjective", "rationale": "Fear is a subjective emotional experience and belongs to the patient ' s personal psychological state."}, "sentence_sentiment": {"answer": "Negative", "rationale": "Because the attitude of \"fear of an accident\" in the entry is negative."}, "language_style": {"answer": "Calm and direct", "rationale": "\"Calm down straight.\""}, "time_dimension": {"answer": "Present", "rationale": "The keyword \"fear\" and \"will\" mean that it is the person who is being tested now feeling and attitude, so the entry asks about the situation now."}, "sentence_type": "Interrogative sentence", "pos": {"nouns": {"Yes": 1, "Accident": 1}, "verbs": {"Come on": 1, "I'm worried": 1}, "numerals": {"54": 1}}}, {"content": "(55) Do you always hesitate to decide what to do?", "word_count": 18, "label": "", "sentence_structure": "Subject鈥損redicate", "subjective_objective": {"answer": "Subjective", "rationale": "This is because of the patient ' s own subjective perception and feelings about whether he will hesitate to make decisions."}, "sentence_sentiment": {"answer": "Negative", "rationale": "Because \"rescientity\" is often considered a negative behaviour or attitude, it is indicative of hesitation and uncertainty in the decision-making process."}, "language_style": {"answer": "Tactful and warm", "rationale": "The entry is more adept and interactive when it is spoken in a gentle and inviting manner, rather than by direct statements or orders."}, "time_dimension": {"answer": "Present", "rationale": "The keywords are \"in doing something\" and \"inwards\" and \"invariably indecisive,\" which are both current behaviors or state. So, the time dimension of this questionnaire entry is now."}, "sentence_type": "Interrogative sentence", "pos": {"nouns": {"Yes": 2, "Relentless": 1}, "verbs": {"Do it": 1, "Decision": 1, "be": 1}, "adverbs": {"Total": 1, "Is it possible": 1}, "numerals": {"55": 1}}}, {"content": "(56) Do you sweat often?", "word_count": 9, "label": "", "sentence_structure": "Subject鈥損redicate", "subjective_objective": {"answer": "Objective", "rationale": "Because it is an objective fact to examine the physical condition of the patient, whether or not he sweats regularly, and does not require subjective evaluation by the patient."}, "sentence_sentiment": {"answer": "Neutral", "rationale": "Because the entry merely asks about a physical phenomenon, it does not have any emotional colour."}, "language_style": {"answer": "Tactful and warm", "rationale": "The entry is more adept and interactive when it is spoken in a gentle and inviting manner, rather than by direct statements or orders."}, "time_dimension": {"answer": "Past", "rationale": "The entry asks the testee about past customary behaviour and therefore belongs to the past."}, "sentence_type": "Interrogative sentence", "pos": {"nouns": {"Questionnaire": 1, "Entry": 1, "Khan": 1}, "verbs": {"Is it possible": 1, "Sweat": 1}, "numerals": {"56": 1}}}, {"content": "(57) Will you be blushing when you're shy?", "word_count": 9, "label": "", "sentence_structure": "Subject鈥損redicate", "subjective_objective": {"answer": "Subjective", "rationale": "Because the face is a physical reaction and shyness is a psychological state, the problem is viewed from the patient ' s subjective perspective."}, "sentence_sentiment": {"answer": "Neutral", "rationale": "Because the entry does not have any emotional color."}, "language_style": {"answer": "Tactful and warm", "rationale": "The language style of the entry is more euphemistic, using a question-and-answer sentence, giving people a sense of kindness and moderation; it also avoids the use of language of direct orders or evaluation, which is more respectful of the feelings and privacy of the person under investigation."}, "time_dimension": {"answer": "Past", "rationale": "The entry asks if the person tested has been shy and blushing at some point in the past and therefore belongs to a description and recollection of past behaviour or state."}, "sentence_type": "Interrogative sentence", "pos": {"nouns": {"Shy": 1, "Red": 1}, "verbs": {"Yes": 1}, "adjectives": {"Humiliated": 1}, "numerals": {"57": 1}}}, {"content": "(58) Do you have a lot of headaches?", "word_count": 7, "label": "", "sentence_structure": "Subject鈥損redicate", "subjective_objective": {"answer": "Subjective", "rationale": "Since headaches are often a matter of personal feelings and experiences, and different human experiences may differ, they are subjective in their approach."}, "sentence_sentiment": {"answer": "Neutral", "rationale": "Because the entry is concerned with a physical condition and does not involve any emotion, attitude or behaviour."}, "language_style": {"answer": "Calm and direct", "rationale": ""}, "time_dimension": {"answer": "Present", "rationale": "The keyword \"often\" in the entry indicates that it is a question of the current state and asks if the person tested has a regular headache, which is now judged in terms of time dimension."}, "sentence_type": "Interrogative sentence", "pos": {"nouns": {"Headache": 1}, "verbs": {"be": 1, "The long run": 1, "Regular": 1, "have": 1}, "adverbs": {"not": 1, "Yes": 1}, "numerals": {"58": 1}}}, {"content": "(59) When you were asked by your teacher, did you always get nervous?", "word_count": 16, "label": "", "sentence_structure": "Subject鈥搗erb鈥搊bject", "subjective_objective": {"answer": "Subjective", "rationale": "Because \"Does the mind always get nervous\" is the patient's subjective feeling and attitude."}, "sentence_sentiment": {"answer": "Negative", "rationale": "Because the \"always nervous\" mood in the entry is negative."}, "language_style": {"answer": "Calm and direct", "rationale": "The entry is a direct query about the feelings of the person questioned by the teacher, without excessive ado or emotional rendering, and is more objective and calm."}, "time_dimension": {"answer": "Present", "rationale": "The \"always\" in the entry indicates a normal psychological state, while the \"when questioned by the teacher\" temporal expression indicates that this is usually the case at a particular point in time or in circumstances, so it can be judged to be an investigation of the present psychological condition of the person being tested."}, "sentence_type": "Interrogative sentence", "pos": {"nouns": {"you": 1, "Teacher": 1, "Question": 1, "In my heart": 1, "Nervous": 1}, "verbs": {"By": 1, "Is it possible": 1, "Always": 1, "very": 1, "be": 1}, "adjectives": {"Nervous": 1}, "adverbs": {"very": 1, "Always": 1}, "numerals": {"59": 1}, "pronouns": {"you": 1}}}, {"content": "(60) You're not in sports, do you often jump through your heart?", "word_count": 19, "label": "", "sentence_structure": "Subject鈥損redicate", "subjective_objective": {"answer": "Subjective", "rationale": "Because this question examines whether the patient ' s heart is often beating quickly, it is a matter of perception and experience and therefore of subjective perspective."}, "sentence_sentiment": {"answer": "Negative", "rationale": "Because the expression \"hearts are constantly beating through the ground\" in the entry suggests that there may be physical health problems and some negative colours."}, "language_style": {"answer": "Calm and direct", "rationale": "The style of the entry is more calm and straightforward, and there is no excessive emotional colour or rhetorical technique, mainly to ask the person's heart status in simple questions."}, "time_dimension": {"answer": "Present", "rationale": "The entry asks if the person tested has a heart condition, and the time dimension is now judged."}, "sentence_type": "Interrogative sentence", "pos": {"nouns": {"Sports": 1, "Heart": 1, "Jump": 1}, "verbs": {"Participation": 1, "Regular": 1, "Pumping up, puffing up, puffing up, puffing up, puffing up, puffing up, puffing up, puffing up, puffing up, puffing up, puffing up, puffing up, puffing up, puffing up, puffing up, puffing up, puffing up, puffing up, puffing up, puffing up, puffing up, puffing up, puffing up, puffing up, puffing up, puffing up, puffing up, puffing, puffing, puffing, puffing up, puffing, puffing, puffing, puffing, puffing, puffing, puffing, puffing, puffing, puffing, puffing": 1, "Jump": 1}, "adverbs": {"No, I'm not": 1, "Regular": 1}, "numerals": {"60": 1}}}, {"content": "(61) Are you tired easily?", "word_count": 8, "label": "", "sentence_structure": "Subject鈥損redicate", "subjective_objective": {"answer": "Subjective", "rationale": "Because fatigue is the patient's own subjective feeling and experience."}, "sentence_sentiment": {"answer": "Negative", "rationale": "Because the expression \"easy fatigue\" in the entry is negative and indicates poor health."}, "language_style": {"answer": "Calm and direct", "rationale": "The entry directly asks the other side whether there is a situation of fatigue, there is no excessive rhetorical or emotional rendering, is more objective and is of a calm and direct linguistic style."}, "time_dimension": {"answer": "Past", "rationale": "The entry is for the testee ' s past physical condition, and the time dimension is judged to be past."}, "sentence_type": "Interrogative sentence", "pos": {"nouns": {"Tired": 1}, "verbs": {"Is it possible": 1, "Easy": 1}, "adjectives": {"very": 1}, "numerals": {"61": 1}}}, {"content": "(62) Are you very reluctant to take your medication?", "word_count": 8, "label": "", "sentence_structure": "Subject鈥搗erb鈥搊bject", "subjective_objective": {"answer": "Subjective", "rationale": "Because \"no\" is a subjective emotional attitude, it's a subjective view."}, "sentence_sentiment": {"answer": "Negative", "rationale": "Because the attitude of \"not taking pills\" in the entry is negative."}, "language_style": {"answer": "Calm and direct", "rationale": "The entry asks the other side in a direct and clear manner about the attitude to the drug, does not have too many rhetorical or emotional renderings, and is more objective in tone."}, "time_dimension": {"answer": "Present", "rationale": "The entry \"Does not\" indicate a question about the current situation, and \"very reluctant\" indicates the attitude or feelings of the testee, so the subject is asking about the current attitude and feelings of the testee and about the present."}, "sentence_type": "Interrogative sentence", "pos": {"nouns": {"Questionnaire": 1, "Entry": 1}, "verbs": {"Analysis": 1}, "adverbs": {"very": 1, "not": 1}, "numerals": {"62": 1}}}, {"content": "(63) Is it hard for you to dream at night?", "word_count": 9, "label": "", "sentence_structure": "Subject鈥搗erb鈥搊bject", "subjective_objective": {"answer": "Objective", "rationale": "This is a factual question of whether the patient is sleeping and there is no interference with personal subjective attitudes or emotions."}, "sentence_sentiment": {"answer": "Negative", "rationale": "Because \"hard to dream\" usually means poor sleep quality, which is a negative experience."}, "language_style": {"answer": "Tactful and warm", "rationale": "The entry is more personal and interactive in its expression of a soft, invitation-like tone of conversation, rather than a direct statement or order. At the same time, questions are used to guide respondents to questions and to obtain their active participation and real feedback."}, "time_dimension": {"answer": "Present", "rationale": "The entry asks whether the person tested is currently having difficulty sleeping and does not address past or future time dimensions, and therefore is judged as \"now\"."}, "sentence_type": "Interrogative sentence", "pos": {"nouns": {"Lee": 1, "Night": 1, "Dreams": 1}, "verbs": {"very": 1, "Hard": 1, "Enter": 1}, "numerals": {"63": 1}}}, {"content": "(64) Do you feel like you're having a problem with your body?", "word_count": 14, "label": "", "sentence_structure": "Subject鈥搗erb鈥搊bject", "subjective_objective": {"answer": "Subjective", "rationale": "Because it's the patient's subjective feeling that you feel that you have a problem with your body."}, "sentence_sentiment": {"answer": "Negative", "rationale": "Because the description \"what's wrong with your body\" in the entry is negative."}, "language_style": {"answer": "Tactful and warm", "rationale": "The entry is more personal and interactive when it is spoken in a gentle and inviting manner, rather than in direct statements or orders. At the same time, it avoids the use of too direct or negative language, making it easier for the respondents to respond with less psychological care and more real feedback."}, "time_dimension": {"answer": "Past", "rationale": "The entry asks the testee whether there has been a feeling or experience before, and the temporal dimension is judged to be past."}, "sentence_type": "Interrogative sentence", "pos": {"nouns": {"Questionnaire": 1, "Entry": 1, "Body": 1, "Mistake": 1}, "verbs": {"I think": 1}, "adverbs": {"I think so": 1}, "numerals": {"64": 1}}}, {"content": "(65) Do you often think you're worse-looking than others?", "word_count": 20, "label": "", "sentence_structure": "Subject鈥損redicate", "subjective_objective": {"answer": "Subjective", "rationale": "Since this is an issue involving patient self-evaluation, the patient ' s subjective attitude needs to be taken into account."}, "sentence_sentiment": {"answer": "Negative", "rationale": "Because the description of \"your own body and face are worse than others\" in the entry is a negative emotional expression that demeans and negates self-worth."}, "language_style": {"answer": "Tactful and warm", "rationale": "The entry uses a more euphemistic form of question, avoiding direct attacks or harm to the self-esteem of the person interviewed, and is also asked about the person ' s own self-perception of body and face, with greater respect."}, "time_dimension": {"answer": "Present", "rationale": "The entry asks the testee's current feelings and perceptions, and does not address past or future behaviour or events. The answer is therefore \"now\"."}, "sentence_type": "Interrogative sentence", "pos": {"nouns": {"Questionnaire Entry": 1, "Body": 1, "Face": 1, "others": 1}, "verbs": {"I think": 1, "- Yeah": 1, "It's ugly": 1}, "adverbs": {"Regular": 1}, "numerals": {"65": 1}, "pronouns": {"Your own": 1}}}, {"content": "(66) Do you often feel bad about the stomach?", "word_count": 11, "label": "", "sentence_structure": "Subject鈥損redicate", "subjective_objective": {"answer": "Subjective", "rationale": "The basis for the judgment: because \"you feel bad about the stomach\" is the patient's subjective feeling and experience."}, "sentence_sentiment": {"answer": "Negative", "rationale": "Because \"bone stomach\" is a negative physical condition."}, "language_style": {"answer": "Tactful and warm", "rationale": ""}, "time_dimension": {"answer": "Present", "rationale": "The keyword \"often feeling bad stomach\" suggests that it is a question of asking the current state of the interviewee, so the temporal dimension is now judged."}, "sentence_type": "Interrogative sentence", "pos": {"nouns": {"- The stomach": 1, "Not good": 1}, "verbs": {"I think": 1, "Is it possible": 1, "Regular": 1, "Yes": 1}, "adverbs": {"very": 1}, "numerals": {"66": 1}}}, {"content": "(67) Do you bite your nails often?", "word_count": 8, "label": "", "sentence_structure": "Subject鈥損redicate", "subjective_objective": {"answer": "Subjective", "rationale": "Because \"often biting your fingernails\" is a patient's subjective habit or behaviour, determined by factors such as the individual's mental state and emotional state."}, "sentence_sentiment": {"answer": "Negative", "rationale": "Because the \"bit your nails\" act is negative in the entry."}, "language_style": {"answer": "Calm and direct", "rationale": "The entry asked in a direct and clear manner whether the respondents were often used to bite their fingernails, were not subject to excessive rhetoric or emotional colours, and were more calm and objective in their language style."}, "time_dimension": {"answer": "Present", "rationale": ""}, "sentence_type": "Interrogative sentence", "pos": {"nouns": {"- Nail": 1, "Questionnaire Entry": 1}, "verbs": {"Bite": 1, "be": 1, "Regular": 1, "have": 1}, "adverbs": {"very": 1}, "numerals": {"67": 1}, "pronouns": {"you": 1}}}, {"content": "(68) Do you lick your fingers?", "word_count": 7, "label": "", "sentence_structure": "Subject鈥損redicate", "subjective_objective": {"answer": "Subjective", "rationale": "Because licking a finger is a personal habit or preference and is a subjective act of the patient."}, "sentence_sentiment": {"answer": "Neutral", "rationale": "Because the entry merely asked about an act, it did not involve any emotion or attitude."}, "language_style": {"answer": "Calm and direct", "rationale": "The language style of the entry is more calm and straightforward, presenting problems in a question-and-answer manner, and does not use excessive rhetorical or emotional rendering."}, "time_dimension": {"answer": "Present", "rationale": "The entry now asks the testee for his current behaviour, \"Is it licking his finger?\" is a clear description of current-day behaviour, and therefore judges now."}, "sentence_type": "Interrogative sentence", "pos": {"nouns": {"Questionnaire": 1, "Entry": 1, "Finger": 1}, "verbs": {"Lick it": 1}, "numerals": {"68": 1}}}, {"content": "(69) Do you often feel difficulty breathing?", "word_count": 11, "label": "", "sentence_structure": "Subject鈥損redicate", "subjective_objective": {"answer": "Objective", "rationale": "Because respiratory difficulties are a physical condition that can be observed and measured, they are examined from the perspective of the patient ' s objectivity."}, "sentence_sentiment": {"answer": "Negative", "rationale": "As the entry describes symptoms of physical discomfort, it is usually considered negative."}, "language_style": {"answer": "Calm and direct", "rationale": "The entry expresses in a direct, concise language the question of the person ' s breathing, is more objective and does not involve excessive rhetorical or emotional rendering."}, "time_dimension": {"answer": "Present", "rationale": "The keyword \"often\" indicates that it is a question of the state of the moment, and that \"fearing breathing difficulties\" is the feeling that the person being tested is experiencing, and therefore judging as now."}, "sentence_type": "Interrogative sentence", "pos": {"nouns": {"Breathe": 1, "Difficulties": 1}, "verbs": {"I feel": 1, "Breathe": 1, "Difficulties": 1}, "numerals": {"69": 1}}}, {"content": "(70) Did you go to the bathroom more often than anyone else?", "word_count": 13, "label": "", "sentence_structure": "Subject鈥搗erb鈥搊bject", "subjective_objective": {"answer": "Subjective", "rationale": "The basis for the determination is the patient ' s subjective perception and comparison of his or her own defecation, not the objective fact."}, "sentence_sentiment": {"answer": "Negative", "rationale": "Because the entry involves personal privacy and implies a dual opposition between \"normal\" and \"unusual\" that may create unnecessary stress and anxiety."}, "language_style": {"answer": "Calm and direct", "rationale": "The entry is dialogueable in a direct question-and-answer manner, and is not based on excessive rhetorical or emotional rendering, and is more objective in tone. \""}, "time_dimension": {"answer": "Present", "rationale": "The keyword \"now\" indicates that it is a question of the person's current behaviour or state, and that \"if more than anyone else\" suggests that it is a difference between a particular act or situation of the person being tested and the situation of others."}, "sentence_type": "Interrogative sentence", "pos": {"nouns": {"Number": 1, "Toilet": 1, "person": 1}, "verbs": {"Is it possible": 1, "More": 1}, "numerals": {"70": 1}}}, {"content": "(71) Are you afraid to go to higher places?", "word_count": 12, "label": "", "sentence_structure": "Subject鈥搗erb鈥搊bject", "subjective_objective": {"answer": "Subjective", "rationale": "Because fear is a subjective emotional reaction."}, "sentence_sentiment": {"answer": "Negative", "rationale": "Because the \"fear to go high\" mood in the entry is negative."}, "language_style": {"answer": "Calm and direct", "rationale": "The language style of the entry is more direct and clear, it is not over-modified or emotionally rendered, and it is more factual and objective.\""}, "time_dimension": {"answer": "Present", "rationale": "The reference to the \"are afraid\" and \"now\" in the sentence makes it possible to judge that the entry in the questionnaire is to ask the person who was tested about his or her current feelings and behaviour."}, "sentence_type": "Interrogative sentence", "pos": {"nouns": {"Questionnaire": 1, "Entry": 1, "Local": 1}, "verbs": {"Is it possible": 1, "Scared": 1, "Go on": 1}, "adjectives": {"very": 1, "High": 1}, "adverbs": {"Land": 1}, "numerals": {"71": 1}}}, {"content": "(72) Are you afraid of a lot?", "word_count": 9, "label": "", "sentence_structure": "Subject鈥損redicate", "subjective_objective": {"answer": "Subjective", "rationale": "Because fear is a subjective feeling or emotional reaction, different individuals may have different objects and degrees of fear. Thus, the problem is viewed from the perspective of patients ' subjective views."}, "sentence_sentiment": {"answer": "Negative", "rationale": "Because \"fearing a lot\" in the entry is an expression of a negative emotion."}, "language_style": {"answer": "Calm and direct", "rationale": "Calm down straight."}, "time_dimension": {"answer": "Present", "rationale": "The entry asks the testee's current feelings and state, and \"are afraid\" is a question of describing the testee's current emotional or psychological state, and therefore it is judged \"now\"."}, "sentence_type": "Interrogative sentence", "pos": {"nouns": {"Questionnaire": 1, "Entry": 1, "Something": 1}, "verbs": {"Is it possible": 1, "Scared": 1, "More": 1}, "adverbs": {"very": 1}, "numerals": {"72": 1}}}, {"content": "(73) Do you often have nightmares?", "word_count": 8, "label": "", "sentence_structure": "Subject鈥損redicate", "subjective_objective": {"answer": "Subjective", "rationale": "This is a subjective examination of the experience, feelings and perceptions of the patients themselves."}, "sentence_sentiment": {"answer": "Negative", "rationale": "Because nightmares are often an unpleasant experience, the purpose of this questionnaire is negative."}, "language_style": {"answer": "Calm and direct", "rationale": "The entry is in a straightforward manner asking the other party whether they often have nightmares, without excessive modifications or emotional rendering, and with more objective and calm tone."}, "time_dimension": {"answer": "Present", "rationale": "The keyword \"often\" and the verb \"doing\" indicates that the entry asks about the current state of conduct of the subject, i.e. whether there are now frequent nightmares."}, "sentence_type": "Interrogative sentence", "pos": {"nouns": {"Nightmare": 1}, "verbs": {"Do it": 1, "Regular": 1}, "adverbs": {"The long run": 1}, "numerals": {"73": 1}}}, {"content": "(74) Are you a little bit of a coward?", "word_count": 7, "label": "", "sentence_structure": "Subject鈥搗erb鈥揷omplement", "subjective_objective": {"answer": "Subjective", "rationale": "Because the size of the guts is a subjective experience and feeling of the patient."}, "sentence_sentiment": {"answer": "Negative", "rationale": "Because the description of \"coward\" in the entry is negative."}, "language_style": {"answer": "Tactful and warm", "rationale": "The entry is more proficient and interactive when it is spoken in a gentle and inviting manner, rather than in direct statements or orders. At the same time, the issue is couched in more neutral terms, without negative terminology or strong tone, and is more readily accepted and answered by the interviewees."}, "time_dimension": {"answer": "Past", "rationale": ""}, "sentence_type": "Interrogative sentence", "pos": {"nouns": {"you": 1, "- You're a coward": 1}, "verbs": {"Is it possible": 1, "Very small": 1}, "numerals": {"74": 1}}}, {"content": "(75) Are you afraid to sleep alone in your room at night?", "word_count": 16, "label": "", "sentence_structure": "Subject鈥搗erb鈥搊bject", "subjective_objective": {"answer": "Subjective", "rationale": "The subject is the fear of the patient, the subjective attitude and the feeling of the patient."}, "sentence_sentiment": {"answer": "Negative", "rationale": "Because \"I'm afraid of sleeping in a room\" is negative."}, "language_style": {"answer": "Tactful and warm", "rationale": "The entry is more friendly and interactive when it is presented in a gentle and inviting tone, rather than in direct statements or orders. At the same time, the article is more acclaimed, understood, and does not directly provoke fear among the interviewees, but rather asks if they are afraid in specific circumstances, which is more likely to enjoy the trust and cooperation of the interviewees."}, "time_dimension": {"answer": "Past", "rationale": ""}, "sentence_type": "Interrogative sentence", "pos": {"nouns": {"Night": 1, "person": 2, "Room": 1, "Sleep": 1}, "verbs": {"Do you": 1, "Yes": 1, "in": 1, "Lee": 1, "Sleep": 1}, "adverbs": {"very": 1}, "numerals": {"75": 1}, "pronouns": {"you": 1}}}, {"content": "(76) Were you afraid when you drove through tunnels or high bridges?", "word_count": 15, "label": "", "sentence_structure": "Subject鈥搗erb鈥搊bject", "subjective_objective": {"answer": "Subjective", "rationale": "The fear of the passengers is subjective."}, "sentence_sentiment": {"answer": "Negative", "rationale": "Because the \"scared\" mood in the entry is negative."}, "language_style": {"answer": "Calm and direct", "rationale": "The entry directly asked whether the person surveyed was afraid to cross a tunnel or high bridge, was speaking with a clear tone and did not have too many rhetorical or emotional renderings."}, "time_dimension": {"answer": "Present", "rationale": "The keyword \"are afraid\" suggests that it is an inquiry into the current state of the subjects, i.e. how they feel when they cross tunnels or high bridges by car. Thus, the temporal dimension of the article is now judged."}, "sentence_type": "Interrogative sentence", "pos": {"nouns": {"Tunnel": 1, "Takahashi": 1, "Car": 1}, "verbs": {"Multiply": 1, "Put it on": 1, "Pass": 1, "I'm scared": 1}, "adverbs": {"Is it possible": 2}, "numerals": {"76": 1}}}, {"content": "(77) Do you like sleeping with lights on at night?", "word_count": 12, "label": "", "sentence_structure": "Subject鈥損redicate", "subjective_objective": {"answer": "Subjective", "rationale": "Because \"like\" is a subjective attitude or emotional reaction."}, "sentence_sentiment": {"answer": "Neutral", "rationale": "Because the entry does not have any emotional color."}, "language_style": {"answer": "Tactful and warm", "rationale": "The entry is more personal and interactive when it is spoken in a gentle, inquisitive manner, rather than in direct statements or orders. At the same time, the expression \"likes\" is more euphemismal and avoids unnecessary pressure or discomfort on the person under investigation."}, "time_dimension": {"answer": "Present", "rationale": "The entry asks the testee's current state of behaviour, i.e. whether they are used to sleeping at night with lights on. Therefore, the time dimension is now judged."}, "sentence_type": "Interrogative sentence", "pos": {"nouns": {"Questionnaire": 1, "Entry": 1, "Night": 1, "Lights": 1, "Sleep": 1}, "verbs": {"Open": 1, "Sleep": 1}, "adverbs": {"Is it possible": 2}, "numerals": {"77": 1}, "classifiers": {"individual": 1}}}, {"content": "(78) Are you afraid to hear thunder?", "word_count": 12, "label": "", "sentence_structure": "Subject鈥搗erb鈥搊bject", "subjective_objective": {"answer": "Subjective", "rationale": "Because fear is a subjective feeling and experience."}, "sentence_sentiment": {"answer": "Negative", "rationale": "Because \"fear\" is a negative mood and attitude."}, "language_style": {"answer": "Tactful and warm", "rationale": "The entry is more adept and interactive when it is spoken in a gentle and inviting manner, rather than by direct statements or orders."}, "time_dimension": {"answer": "Present", "rationale": "\"You heard\" and \"hear\" means \"is it now\" is \"are you very scared?\" Asks how it feels now, so the temporal dimension of the article is now."}, "sentence_type": "Interrogative sentence", "pos": {"nouns": {"you": 1, "Thunder": 1, "Scared": 1}, "verbs": {"Yes": 1, "Very": 1, "Is it possible": 1}, "adverbs": {"Very": 1}, "numerals": {"78": 1}}}, {"content": "(79) Are you very afraid of the dark?", "word_count": 9, "label": "", "sentence_structure": "Subject鈥損redicate", "subjective_objective": {"answer": "Subjective", "rationale": "Because fear is a subjective feeling."}, "sentence_sentiment": {"answer": "Negative", "rationale": "Because the expression \"very afraid\" has a negative emotional connotation."}, "language_style": {"answer": "Tactful and warm", "rationale": "The entry uses a moderate and encouraging language style, which leads the respondents to share their feelings and experiences, rather than directly asking questions or orders. In addition, the entry expresses interest and understanding of the issue by using the word \"extraordinary\"."}, "time_dimension": {"answer": "Past", "rationale": "The entry asks about the testee's past experience and feelings, and the key words are \"Are you\" and \"are you very afraid\", all pointing to the past."}, "sentence_type": "Interrogative sentence", "pos": {"nouns": {"you": 1, "Darkness": 1}, "verbs": {"Scared": 1}, "adjectives": {"Very": 1}, "numerals": {"79": 1}}}, {"content": "(80) Do you often feel that someone is following you?", "word_count": 14, "label": "", "sentence_structure": "Subject鈥損redicate", "subjective_objective": {"answer": "Subjective", "rationale": "Because \"I feel people following you\" is an individual feeling and perception, and it's part of subjective experience."}, "sentence_sentiment": {"answer": "Negative", "rationale": "Because it describes an emotional feeling of anxiety and fear, which is a negative emotional expression."}, "language_style": {"answer": "Calm and direct", "rationale": ""}, "time_dimension": {"answer": "Present", "rationale": ""}, "sentence_type": "Interrogative sentence", "pos": {"nouns": {"person": 1, "Questionnaire": 1, "Entry": 1}, "verbs": {"I feel": 1, "Call": 1, "Here": 1}, "adverbs": {"Regular": 1}, "numerals": {"80": 1}}}, {"content": "(81) Do you get angry often?", "word_count": 7, "label": "", "sentence_structure": "Subject鈥損redicate", "subjective_objective": {"answer": "Subjective", "rationale": "Because it is a subjective feeling and experience to be angry often, it requires a patient to answer it himself."}, "sentence_sentiment": {"answer": "Negative", "rationale": "Because \"freak\" is often seen as a negative emotional state, which suggests that the questionnaire designer wanted to know whether the testees were experiencing negative emotions on a regular basis."}, "language_style": {"answer": "Calm and direct", "rationale": "The entry directly asks whether the interviewees are often angry and have not used any qualifier or emotionally strong expressions, and is more concise and clear."}, "time_dimension": {"answer": "Present", "rationale": ""}, "sentence_type": "Interrogative sentence", "pos": {"nouns": {"Questionnaire Entry": 1, "Angry": 1}, "verbs": {"Is it possible": 2, "Regular": 1}, "numerals": {"81": 1}}}, {"content": "(82) Don't you want to get good grades?", "word_count": 11, "label": "", "sentence_structure": "Subject鈥損redicate", "subjective_objective": {"answer": "Subjective", "rationale": "The mental state and attitude of the patient are subjective in nature, since the desire to achieve good results is a subjective one."}, "sentence_sentiment": {"answer": "Negative", "rationale": "Because the behavior in the entry \"does not want good grades\" is negative."}, "language_style": {"answer": "Calm and direct", "rationale": "The language style of the entry is relatively simple, it is not based on excessive rhetorical or emotional techniques, and it is more straightforward in tone.\""}, "time_dimension": {"answer": "Past", "rationale": "The entry \"does not want to get good grades\" expresses the testee's attitude and thoughts about a certain point in the past and therefore judges as the past."}, "sentence_type": "Interrogative sentence", "pos": {"nouns": {"Achievements": 1}, "verbs": {"Yes": 1, "Got it": 1}, "numerals": {"82": 1}, "pronouns": {"you": 1}}}, {"content": "(83) Will you suddenly cry?", "word_count": 8, "label": "", "sentence_structure": "Subject鈥損redicate", "subjective_objective": {"answer": "Subjective", "rationale": "Because it's a subjective feeling or emotional experience to cry."}, "sentence_sentiment": {"answer": "Negative", "rationale": "Because the \"breathing\" act in the entry is negative."}, "language_style": {"answer": "Tactful and warm", "rationale": "The entry is more adept and interactive when it is spoken in a gentle and inviting manner, rather than in direct statements or orders. At the same time, the language style of the article is more concise, not too much rhetorical or emotional, and is more objective in tone."}, "time_dimension": {"answer": "Present", "rationale": "The entry asks if the person who was tested was suddenly trying to cry, and the time dimension is judged by past and present. Because the word \"will\" indicates that it is a customary or frequent act, implying that such emotional reactions may have occurred in the past or now."}, "sentence_type": "Interrogative sentence", "pos": {"nouns": {"Questionnaire": 1, "Entry": 1, "Yes": 1}, "verbs": {"Suddenly": 1, "Yes": 1, "Cry": 1}, "numerals": {"83": 1}, "pronouns": {"you": 1}}}, {"content": "(84) Have you ever lied before?", "word_count": 9, "label": "", "sentence_structure": "Subject鈥損redicate", "subjective_objective": {"answer": "Objective", "rationale": "Because lying is an objective act, whether or not the patient admits it, it is a fact that has already happened. The question is therefore examined from the perspective of the patient ' s objectivity."}, "sentence_sentiment": {"answer": "Neutral", "rationale": "Because the entry does not have any emotional color."}, "language_style": {"answer": "Calm and direct", "rationale": "The entry directly asks the other side whether there has been a lie in the past, without adding excessive qualifiers or emotional colours, and with a more direct and objective language style."}, "time_dimension": {"answer": "Past", "rationale": ""}, "sentence_type": "Interrogative sentence", "pos": {"nouns": {"Questionnaire": 1, "Entry": 1}, "verbs": {"Say it": 1, "Is it possible": 1, "Yes": 1}, "adverbs": {"Before": 1, "Is it possible": 1}, "numerals": {"84": 1}}}, {"content": "(85) Do you sometimes feel better or die?", "word_count": 12, "label": "", "sentence_structure": "Subject鈥損redicate", "subjective_objective": {"answer": "Subjective", "rationale": "Because it's the patient's subjective feeling."}, "sentence_sentiment": {"answer": "Negative", "rationale": "Because the idea of \"good dead\" in the entry is negative."}, "language_style": {"answer": "Tactful and warm", "rationale": "The entry is more personal and interactive when it is spoken in a gentle and inviting manner, rather than in direct statements or orders. At the same time, the purpose of the article is set more euphemistic, avoiding feelings of resentment or resistance on the part of the interviewees, and helping them to better answer questions and open themselves up."}, "time_dimension": {"answer": "Present", "rationale": "The future entry contains visions and expectations for the future, \"sometimes feeling\" indicating that the testee may have such an idea in the future and can therefore be judged to be the future."}, "sentence_type": "Interrogative sentence", "pos": {"nouns": {"Time": 1, "Die": 1}, "verbs": {"I think": 1}, "adverbs": {"Sometimes": 1}, "numerals": {"85": 1}}}, {"content": "(86) Have you never missed an appointment?", "word_count": 11, "label": "", "sentence_structure": "Subject鈥損redicate", "subjective_objective": {"answer": "Subjective", "rationale": "Because \"no\" or \"no\" answers depend on the patient's subjective judgement and behaviour, and are subjectively viewed."}, "sentence_sentiment": {"answer": "Neutral", "rationale": "Because the entry is an act that does not involve positive or negative emotional attitudes."}, "language_style": {"answer": "Calm and direct", "rationale": "The entry directly asks whether the interviewee has never breached the law, does not use any acculturation language or emotional colour, language temperature and objectivity, and is consistent with the style of the direct question."}, "time_dimension": {"answer": "Present", "rationale": "The keyword \"not at all\" implies that it is a question of behaviour over a certain period of time, but that there is a lack of time-frame, and therefore a need to further confirm the specific time frame through context or other questions."}, "sentence_type": "Interrogative sentence", "pos": {"nouns": {"Failure to make a deal": 1, "Number of times": 1}, "verbs": {"have": 1, "Nope": 1, "Failure to make a deal": 1}, "adverbs": {"From": 1, "Not": 1}, "numerals": {"86": 1, "Once": 1, "No, I'm not": 1}}}, {"content": "(87) Do you often want to shout?", "word_count": 10, "label": "", "sentence_structure": "Subject鈥損redicate", "subjective_objective": {"answer": "Subjective", "rationale": "Because \"want to shout\" is the patient's inner feeling and thoughts, and it's a subjective angle."}, "sentence_sentiment": {"answer": "Negative", "rationale": "Because the \"scream out\" in the entry is negative."}, "language_style": {"answer": "Tactful and warm", "rationale": "The language style of the entry is more euphemistic. It is asked in an invitation tone whether the other side has ever had an experience of shouting, without directly giving an evaluation or an order. This expression is more personal and interactive."}, "time_dimension": {"answer": "Past", "rationale": ""}, "sentence_type": "Interrogative sentence", "pos": {"nouns": {"you": 1, "Sound": 1, "Shout": 1}, "verbs": {"Yes": 1, "Shout": 1, "Louder": 1, "# Hoo-ooo #": 1}, "adjectives": {"Large": 1, "Sound": 1}, "numerals": {"87": 1}}}, {"content": "(88) Are you unwilling to tell what others are not allowed to tell you?", "word_count": 14, "label": "", "sentence_structure": "Subject鈥搗erb鈥搊bject", "subjective_objective": {"answer": "Subjective", "rationale": "The reason for this is that it is not the patient's own subjective attitude and ideas."}, "sentence_sentiment": {"answer": "Negative", "rationale": "Because the attitude of \"not wanting to say what others are not going to say\" in the entry is negative."}, "language_style": {"answer": "Tactful and warm", "rationale": "The entry is more adept and interactive when it is spoken in a gentle and inviting manner, rather than by direct statements or orders."}, "time_dimension": {"answer": "Past", "rationale": "\"Leave\" means that it is a past or present attitude, tendency or behaviour, and that \"speak what others are not allowed to say\" concerns the attitude and reaction of the person being tested to a situation or event, so the entry covers both the past and the present."}, "sentence_type": "Interrogative sentence", "pos": {"nouns": {"Questionnaire Entry": 1, "person": 1, "Yes": 1}, "verbs": {"Say it": 2, "Jean": 1}, "numerals": {"88": 1}, "pronouns": {"you": 1, "others": 1}}}, {"content": "(89) Do you sometimes think you're going to go far away alone?", "word_count": 19, "label": "", "sentence_structure": "Subject鈥搗erb鈥搊bject", "subjective_objective": {"answer": "Subjective", "rationale": "Because \"thinking\" is the patient's personal thought and feeling, and it's a subjective angle."}, "sentence_sentiment": {"answer": "Neutral", "rationale": "Because the entry does not have any emotional color."}, "language_style": {"answer": "Tactful and warm", "rationale": "The entry is more personal and interactive, using a soft, invitation-style tone of dialogue rather than direct statements or orders. At the same time, by asking the other side whether they have had similar ideas, the interviewee can feel respected and understood, which will facilitate the smooth running of the questionnaire."}, "time_dimension": {"answer": "Future", "rationale": "\"Sometimes\" means that there has been or is now such an idea, \"thinking\" is a verb for the future, so the temporal dimension of the article is the future."}, "sentence_type": "Interrogative sentence", "pos": {"nouns": {"Local": 1, "Far away": 1}, "verbs": {"Yes": 1, "Pass": 1, "Go on": 1}, "adverbs": {"Sometimes": 1}, "numerals": {"89": 1}}}, {"content": "(90) Are you always polite?", "word_count": 9, "label": "", "sentence_structure": "Subject鈥損redicate", "subjective_objective": {"answer": "Subjective", "rationale": "Because being polite or not is a subjective expression of the individual, it is not an objective fact."}, "sentence_sentiment": {"answer": "Positive", "rationale": "Because \"behavedly\" in the entry is a positive quality."}, "language_style": {"answer": "Tactful and warm", "rationale": "The entry is more adept and interactive when it is spoken in a gentle and inviting manner, rather than by direct statements or orders."}, "time_dimension": {"answer": "Present", "rationale": "The entry is a question about the current behaviour of the subject and uses the word \"always\" to indicate a high frequency, so it is now judged."}, "sentence_type": "Interrogative sentence", "pos": {"numerals": {"90": 1}, "classifiers": {"individual": 1}, "pronouns": {"you": 1}}}, {"content": "(91) Are you being misspoked and want to act with ambition immediately?", "word_count": 18, "label": "", "sentence_structure": "Subject鈥搗erb鈥搊bject", "subjective_objective": {"answer": "Subjective", "rationale": "Because the problem involves patients鈥?reactions and ideas, it needs to be examined from a subjective perspective. For example, some patients may want immediate revenge, while others may not be so impulsive, depending on their personality, emotional state and perception of things."}, "sentence_sentiment": {"answer": "Negative", "rationale": "Because the entry \"want immediate retaliation\" is negative."}, "language_style": {"answer": "Tactful and warm", "rationale": ""}, "time_dimension": {"answer": "Present", "rationale": "The temporal dimension of the article is therefore now."}, "sentence_type": "Interrogative sentence", "pos": {"nouns": {"person": 2, "Bad word": 1, "Action": 1}, "verbs": {"Say it": 1, "Yes": 1, "Adoption": 1}, "adverbs": {"Immediately": 1}, "numerals": {"91": 1}, "pronouns": {"you": 1}}}, {"content": "(92) Do you do everything you say, either by your teacher or your parents?", "word_count": 14, "label": "", "sentence_structure": "Subject鈥搗erb鈥搊bject", "subjective_objective": {"answer": "Subjective", "rationale": "Because the entry asks about the patient鈥檚 personal attitudes and behaviour choices, rather than the objective reality."}, "sentence_sentiment": {"answer": "Negative", "rationale": "Because the \"do as\" in the entry is negative."}, "language_style": {"answer": "Calm and direct", "rationale": "The language style of the entry is more calm and straightforward, and the question is simple, and it does not use too many rhetorical or emotional terms. It is intended to understand whether the interviewee will comply fully with the advice and requests of the teacher or parent, and does not imply any answers, but rather to allow the interviewee to make his own judgement. This language style makes the questionnaire more objective and scientific."}, "time_dimension": {"answer": "Past", "rationale": ""}, "sentence_type": "Interrogative sentence", "pos": {"nouns": {"Teacher": 1, "parents": 1, "Word": 1}, "verbs": {"Say it": 2, "Do it": 1}, "numerals": {"92": 1}}}, {"content": "(93) Are you unhappy, throwing things away?", "word_count": 15, "label": "", "sentence_structure": "Subject鈥搗erb鈥搊bject", "subjective_objective": {"answer": "Subjective", "rationale": "Because the answer to \"will\" or \"no\" is the patient's subjective attitude and behaviour."}, "sentence_sentiment": {"answer": "Negative", "rationale": "Because \"throwing, smashing\" is destructive and is often seen as a sign of negative emotions."}, "language_style": {"answer": "Tactful and warm", "rationale": "The entry uses a more euphemistic approach to questions, trying to understand the potential behaviour of the respondent in the event of a low level of mood. By using a more moderate and respectful language style, the interviewee is guided to think about issues and does not feel challenged or attacked, thus being more friendly and interactive."}, "time_dimension": {"answer": "Present", "rationale": "\"will\" or \"no\"."}, "sentence_type": "Interrogative sentence", "pos": {"nouns": {"Psychological": 1, "Happy": 1, "Something": 1}, "verbs": {"Yes": 1, "Throw it away": 1, "Smash it": 1}, "numerals": {"93": 1}}}, {"content": "Have you ever been angry?", "word_count": 6, "label": "", "sentence_structure": "Subject鈥損redicate", "subjective_objective": {"answer": "Subjective", "rationale": "Because the existence of anger is a psychological state of a person, it is a subjective examination."}, "sentence_sentiment": {"answer": "Negative", "rationale": "Because \"gream\" is usually considered negative."}, "language_style": {"answer": "Calm and direct", "rationale": "The entry directly asks whether the person surveyed had had an emotional experience of anger, a more concise language style, and not too much accommodative or emotional colour, so that the language style can be judged as \"cool and direct\"."}, "time_dimension": {"answer": "Past", "rationale": "The verb \"run\" contained in the entry uses the usual past pattern, indicating that the act occurred at a certain point in the past. The answer is therefore the past."}, "sentence_type": "Interrogative sentence", "pos": {"nouns": {"Anger": 1, "Questionnaire": 1, "Entry": 1}, "verbs": {"Let's go": 1, "be": 1}, "adverbs": {"Yes": 1, "Pass": 1, "Is it possible": 1}, "numerals": {"94": 1}}}, {"content": "(95) Is that what you want?", "word_count": 15, "label": "", "sentence_structure": "Subject鈥搗erb鈥搊bject", "subjective_objective": {"answer": "Subjective", "rationale": "Because it is the subjective desire and attitude of one."}, "sentence_sentiment": {"answer": "Neutral", "rationale": "Basis of judgement: The entry does not express a positive or negative emotional colour, but rather describes a problem or situation."}, "language_style": {"answer": "Tactful and warm", "rationale": "The language style of the entry is relatively euphemistic and moderate, and by asking the views of the other side, it attempts to guide the interviewee to reflect on his thoughts and behaviour."}, "time_dimension": {"answer": "Past", "rationale": ""}, "sentence_type": "Interrogative sentence", "pos": {"nouns": {"Something": 1, "Yes": 1}, "verbs": {"Yes": 3, "Got it": 1}, "adverbs": {"I'm sure": 1}, "numerals": {"95": 1}}}, {"content": "(96) Are you particularly pleased that the teacher dropped out early in the course you don't like?", "word_count": 21, "label": "", "sentence_structure": "Subject鈥搗erb鈥搊bject", "subjective_objective": {"answer": "Subjective", "rationale": "Because it is particularly happy to be a subjective feeling and emotional reaction of one person. While this is an objective situation (teachers drop out early), it is an individual subjective experience to feel and react to it."}, "sentence_sentiment": {"answer": "Negative", "rationale": "Because the descriptions of \"not like class\" and \"teacher early discharge\" in the entries are negative, and the question \"are you particularly happy?\" indicates that it is a negative emotion."}, "language_style": {"answer": "Calm and direct", "rationale": "The language style of the entry is more direct and objective, and there is no excessive emotional rendering or rhetorical technique. It asks the interviewees in a direct question how they feel about a particular situation.\""}, "time_dimension": {"answer": "Present", "rationale": "The keyword \"Is it particularly happy\" suggests that the question is asked whether the person tested has changed in the emotional reaction or attitude of the moment. Moreover, the situation of \"not like, teachers drop early\" is also a question of the present situation. The entry therefore meets the current time dimension test."}, "sentence_type": "Interrogative sentence", "pos": {"nouns": {"Classes": 1, "Teacher": 1, "Happy": 1}, "verbs": {"Early": 1, "Class dismissed": 1, "I feel": 1}, "adjectives": {"Special": 1}, "numerals": {"96": 1}}}, {"content": "(97) Do you often want to jump off high ground?", "word_count": 14, "label": "", "sentence_structure": "Subject鈥搗erb鈥搊bject", "subjective_objective": {"answer": "Subjective", "rationale": "Because \"often think\" is a subjective idea or feeling."}, "sentence_sentiment": {"answer": "Negative", "rationale": "Because \"jumping from high ground\" is a dangerous act, prone to physical injury and threats to life, with obvious negative emotional colours."}, "language_style": {"answer": "Calm and direct", "rationale": "The entry raises questions in a more direct and concise language style, with no excessive accoutrements or rhetorical techniques, and is expressed in a calm and objective manner, suitable for investigation and research."}, "time_dimension": {"answer": "Past", "rationale": "\"Recurring\" means frequency, and \"intending to jump from high ground\" occurs at a point or time in the past and can therefore be judged as the past."}, "sentence_type": "Interrogative sentence", "pos": {"nouns": {"Questionnaire": 1, "Entry": 1, "person": 1, "High places": 1}, "verbs": {"Yes": 1, "Jump": 1}, "adverbs": {"Regular": 1}, "numerals": {"97": 1}}}, {"content": "(98) Are you close to anyone?", "word_count": 10, "label": "", "sentence_structure": "Subject鈥搗erb鈥搊bject", "subjective_objective": {"answer": "Subjective", "rationale": "Because the degree of alienation is a subjective feeling and attitude."}, "sentence_sentiment": {"answer": "Neutral", "rationale": "As the entry does not address any positive or negative behaviour, attitude, feelings, etc., it is neutral."}, "language_style": {"answer": "Tactful and warm", "rationale": "The language style of the entry is euphemistic and warm. It uses a gentle and inviting tone, rather than a direct statement or order, which is more acrimony and interactive. Moreover, the issue does not contain any negative or critical language, but rather asks the respondents in a positive way about their attitudes and behaviour."}, "time_dimension": {"answer": "Present", "rationale": "The entry \"Whether or not\" indicates that this is a selective question, and that \"whoever is close to\" indicates that it is the behaviour of the testee in answering the question that needs to be thought and chosen, so the temporal dimension is now judged."}, "sentence_type": "Interrogative sentence", "pos": {"nouns": {"Questionnaire Entry": 1, "you": 2, "person": 2, "Who": 1}, "verbs": {"Is it possible": 1, "Both": 1, "very": 1, "Kiss": 1}, "numerals": {"98": 1}}}, {"content": "(99) Do you often get too anxious to sit?", "word_count": 13, "label": "", "sentence_structure": "Subject鈥搗erb鈥搊bject", "subjective_objective": {"answer": "Subjective", "rationale": "Because irritation and irritation are subjective experiences and feelings of patients."}, "sentence_sentiment": {"answer": "Negative", "rationale": "Because \"surgery\" and \"staggering\" in the entries are negative behavioral expressions."}, "language_style": {"answer": "Calm and direct", "rationale": "The language style of the entry is calm and straightforward. It uses simple and clear language to ask the respondents whether they are in a state of impatience, or have no excessive emotional or rhetorical style. This language style is common in the questionnaire, avoiding unnecessary pressure or misleading the respondents."}, "time_dimension": {"answer": "Present", "rationale": "The word \"will\" indicates the state of affairs, and \"suspensiveness\" is a behaviour, so it can be judged to be a description of the situation of the person being tested."}, "sentence_type": "Interrogative sentence", "pos": {"nouns": {"you": 1, "Routine": 1, "Sit tight": 1}, "verbs": {"Yes": 1, "Regular": 1}, "adjectives": {"Routine": 1, "Unsettled": 1}, "adverbs": {"Regular": 1, "Land": 1}, "numerals": {"99": 1}}}, {"content": "(100) Would you like to know someone you don't know?", "word_count": 13, "label": "", "sentence_structure": "Subject鈥搗erb鈥搊bject", "subjective_objective": {"answer": "Subjective", "rationale": "The perception and perception of the patient is subjective because it is the individual feeling or perception of the patient."}, "sentence_sentiment": {"answer": "Neutral", "rationale": "Because the entry does not have any emotional color."}, "language_style": {"answer": "Calm and direct", "rationale": "The entry is based on a direct question-and-answer approach, without using a rhetorical technique or excessive emotional colour, and with a more objective and calm tone."}, "time_dimension": {"answer": "Past", "rationale": "The word \"will\" in the entry indicates that it was a past act or attitude, and \"all\" means that it is the same for all, and it therefore relates to the general situation of the past."}, "sentence_type": "Interrogative sentence", "pos": {"nouns": {"person": 2, "Questionnaire Entry": 1, "Yes": 1}, "verbs": {"Yes": 2, "Yeah": 1, "Both": 1}, "adverbs": {"to": 1, "not": 1, "Is it possible": 1}, "numerals": {"100": 1, "2": 1, "1": 4}, "pronouns": {"you": 1}}}]}

{"questionnaire_title": "Hospital Anger and Depression Scale (HAD)", "questionnaire_category": "Mental health", "item_count": 14, "emotion_shift_overall": "From negative to positive", "language_style_overall": "Neutral", "items": [{"content": "1. I feel nervous (or painful) (A)", "word_count": 8, "label": "", "sentence_structure": "Subject鈥揷opula鈥損redicative", "subjective_objective": {"answer": "Subjective", "rationale": "Because \"I'm nervous\" describes the subjective feelings of the patient, which are subjective."}, "sentence_sentiment": {"answer": "Negative", "rationale": "Because \"stretch\" and \"suffer\" are negative emotional terms in the entries."}, "language_style": {"answer": "Calm and direct", "rationale": "The entry is in plain and clear language, and it is more direct and objective to ask the interviewees whether they are feeling nervous or painful at the moment, without too many emotional renderings or speech-writing techniques."}, "time_dimension": {"answer": "Past", "rationale": "The entry asks the testee about past feelings, uses the verb \"fear\" and the bracketed term \"A\" to indicate that this feeling has occurred, so the temporal dimension is a past."}, "sentence_type": "Interrogative sentence", "pos": {"nouns": {"Questionnaire": 1, "Entry": 1, "Nervous": 1, "Pain": 1, "A": 1}, "verbs": {"I feel": 1}}}, {"content": "2. I remain interested in what has been an interest in the past (D)", "word_count": 15, "label": "", "sentence_structure": "Subject鈥揷opula鈥損redicative", "subjective_objective": {"answer": "Subjective", "rationale": "Because \"interest\" is a personal feeling and attitude that is seen from the patient's subjective perspective."}, "sentence_sentiment": {"answer": "Neutral", "rationale": "Because the entry does not have any emotional color."}, "language_style": {"answer": "Tactful and warm", "rationale": "The language style of the entry is more euphemistic and gentle, allowing the respondent to express his or her views without pressure by using the questionword and the selection. At the same time, this tone is more able to guide the respondent to open his or her heart and answer the questions."}, "time_dimension": {"answer": "Past", "rationale": "The \"old\" in the entry shows that there is no change in interest in \"old\" or \"old\" and is therefore judged as asking about the testee's past."}, "sentence_type": "Interrogative sentence", "pos": {"nouns": {"Things": 1, "Interest": 2}, "verbs": {"to": 1, "And": 1, "have": 1}, "adjectives": {"Previous": 1, "of": 1}, "adverbs": {"Or is it": 1, "D": 1}, "pronouns": {"I": 1}}}, {"content": "I'm a little scared, like I'm expecting something terrible to happen.", "word_count": 22, "label": "", "sentence_structure": "Subject鈥揷opula鈥損redicative", "subjective_objective": {"answer": "Subjective", "rationale": "Because fear is a subjective emotional experience, it may be influenced by external events, but it is the patient ' s own perception and reaction."}, "sentence_sentiment": {"answer": "Negative", "rationale": "Because \"fear\" in the entries, \"foresee something terrible to happen\" are all negative emotions and feelings."}, "language_style": {"answer": "Tactful and warm", "rationale": "The entry uses a euphemism to collect information by asking if the other side feels that something bad is about to happen. This expression is more moderate and friendly, and it makes it easier for the interviewee to open his heart and answer questions. It also avoids the pressure or discomfort that may arise from the direct use of negative terms such as \"fear\"."}, "time_dimension": {"answer": "Present", "rationale": "The entry indicates the current feelings and emotions of the testees, and the keywords are \"a little scared,\" \"like a hunch\", all pointing to the behaviour and feelings that are currently being carried out. So the judgment is now."}, "sentence_type": "Affirmative declarative sentence", "pos": {"nouns": {"Questionnaire": 1, "Answer": 2, "Problem": 1, "Entry": 1, "Options": 1, "Terrible things": 1}, "verbs": {"I feel": 1, "Foreground": 1, "Come on": 1}, "adjectives": {"Scared": 1, "Yes": 1}, "adverbs": {"A little": 1, "I think so": 1}, "numerals": {"3": 1}, "pronouns": {"I": 1}}}, {"content": "I can laugh and see the good side of things.", "word_count": 16, "label": "", "sentence_structure": "Subject鈥搗erb鈥搊bject", "subjective_objective": {"answer": "Subjective", "rationale": "Because it's a man's subjective attitude to laugh and see things as good as they are."}, "sentence_sentiment": {"answer": "Positive", "rationale": "Because the attitude of seeing things on the good side in the entry is positive."}, "language_style": {"answer": "Tactful and warm", "rationale": "The entry is more adept and interactive when it is spoken in a gentle and inviting manner, rather than by direct statements or orders."}, "time_dimension": {"answer": "Present", "rationale": "The entry describes the current ability and attitude of the testee to laugh and see the good side of things at this moment. It is therefore the time dimension of the present."}, "sentence_type": "Affirmative declarative sentence", "pos": {"nouns": {"I": 1, "# Ha ha ha ha ha ha ha ha ha ha ha ha ha ha ha ha ha ha ha ha ha ha ha ha ha ha ha ha ha ha ha ha ha ha ha ha ha ha ha ha ha ha ha ha ha ha ha ha ha ha ha ha ha ha ha ha ha ha ha ha ha ha ha ha ha ha ha ha ha ha ha ha ha ha ha ha ha ha ha ha ha ha ha ha ha ha ha ha ha ha ha ha ha ha ha ha ha ha ha ha ha ha ha ha ha ha ha ha ha ha ha ha ha ha ha ha ha ha ha ha ha ha ha ha ha ha ha ha ha ha ha ha ha ha ha ha ha ha ha ha ha ha ha ha ha ha ha ha ha ha ha ha ha ha ha ha ha ha ha ha ha ha ha ha ha ha ha ha ha ha ha ha ha ha ha ha ha ha ha ha ha ha ha ha ha ha ha ha ha ha ha ha ha ha ha ha ha ha ha ha ha ha ha ha ha ha ha ha ha ha ha ha ha ha ha ha ha ha ha ha ha ha ha ha ha ha ha ha ha ha ha ha ha ha ha ha ha ha ha ha ha ha ha ha ha ha ha ha ha ha ha ha ha ha": 1, "Things": 1, "One side": 1}, "verbs": {"Yeah": 1, "Yes": 1, "and": 1, "Smile": 1}, "adjectives": {"Okay": 1}, "adverbs": {"Large": 1, "Ha ha": 1}}}, {"content": "5. My heart is full of troubles (A)", "word_count": 8, "label": "", "sentence_structure": "Subject鈥揷opula鈥損redicative", "subjective_objective": {"answer": "Subjective", "rationale": "Because \"scarce\" is the patient's subjective feeling and psychological state, it's a subjective view."}, "sentence_sentiment": {"answer": "Negative", "rationale": "Because the word \"fear\" in the entry is negative."}, "language_style": {"answer": "Calm and direct", "rationale": "The language style of the entry is calm and straightforward, as it does not use any rhetorical or emotional language, but rather directs the question of whether the person under investigation has any problems. At the same time, \"filled\" is also a very direct and clear statement, without ambiguity."}, "time_dimension": {"answer": "Present", "rationale": "The entry asks about the current inner feeling and state of the person being tested, so the time dimension is now judged."}, "sentence_type": "Affirmative declarative sentence", "pos": {"nouns": {"Heart": 1, "Trouble": 1}, "verbs": {"Fill": 1}, "numerals": {"5": 1}, "pronouns": {"I": 1}}}, {"content": "I'm happy (D)", "word_count": 5, "label": "", "sentence_structure": "Subject鈥揷opula鈥損redicative", "subjective_objective": {"answer": "Subjective", "rationale": "Because it is the patient's subjective feeling and attitude to be happy."}, "sentence_sentiment": {"answer": "Positive", "rationale": "Because the feeling of \"happy\" is positive."}, "language_style": {"answer": "Calm and direct", "rationale": "The language style of the entry is relatively simple and clear, with no excessive rhetoric or emotional rendering, and the person surveyed is asked whether he or she is happy at the moment and therefore falls within the \"cool and straight\" language style."}, "time_dimension": {"answer": "Present", "rationale": "This entry describes the current feelings of the testee, \"favourable\" is a term for emotional state, and the letter \"D\" in brackets may represent a scoring method or option number, but it does not affect our judgement that its time dimension is now."}, "sentence_type": "Affirmative declarative sentence", "pos": {"adjectives": {"Have a nice day": 1}, "numerals": {"6": 1}, "classifiers": {"individual": 1}, "pronouns": {"I": 1}}}, {"content": "I can sit down in a free and easy manner (A)", "word_count": 11, "label": "", "sentence_structure": "Subject鈥搗erb鈥揷omplement", "subjective_objective": {"answer": "Subjective", "rationale": "The basis for the judgement: the description of \"sitting free and easy\" in this questionnaire entry is the subjective feeling and attitude of the patient, and it is therefore subjective."}, "sentence_sentiment": {"answer": "Positive", "rationale": "Because the \"sitting free and easy\" act in the entry is positive."}, "language_style": {"answer": "Tactful and warm", "rationale": "The entry is more euphemistic and warm in language, and uses adjectives such as \"relax\" and \"easy\" to describe the state, making the question more friendly. In addition, the entry uses question sentences to guide respondents in a more natural way to express their thoughts and feelings, and to enhance the relevance and interaction of the questionnaire."}, "time_dimension": {"answer": "Present", "rationale": "Now, the \"I can\" expression that the dominant language has some kind of ability or character, and \"sit in peace\" is the way to describe that ability, which does not relate to past or future acts or events, so the entry in this questionnaire is asking about the situation of the person being tested."}, "sentence_type": "Affirmative declarative sentence", "pos": {"nouns": {"I": 1, "Sit down": 1}, "verbs": {"Yes": 1, "Sitting down in a little bit": 1}, "adjectives": {"It's all right": 1, "Easy": 1}, "adverbs": {"And": 1, "Land": 1}, "classifiers": {"individual": 1}}}, {"content": "I'm losing interest in my own appearance (D).", "word_count": 15, "label": "", "sentence_structure": "Subject鈥搗erb鈥搊bject", "subjective_objective": {"answer": "Subjective", "rationale": "The entry is based on the patient ' s level of interest in his or her own manner and on his or her subjective feelings and attitudes."}, "sentence_sentiment": {"answer": "Negative", "rationale": "Because the \"lost interest\" act is negative."}, "language_style": {"answer": "Calm and direct", "rationale": "The language style of the entry is straightforward, and it is a simple question to ask the interviewees whether they have lost interest in their own dress, without using too many rhetorical or emotional colours to render it, and with a calmer and objective tone."}, "time_dimension": {"answer": "Present", "rationale": ""}, "sentence_type": "Negative declarative sentence", "pos": {"nouns": {"Questionnaire Entry": 1, "self": 1, "Yum": 1, "Get dressed": 1, "Interest": 1}, "verbs": {"to": 1, "Lose": 1}, "adverbs": {"very": 1}, "numerals": {"8": 1}}}, {"content": "I'm a little restless, and I feel like I have to move.", "word_count": 17, "label": "", "sentence_structure": "Subject鈥搗erb鈥揷omplement", "subjective_objective": {"answer": "Subjective", "rationale": "Because \"fright to move\" is the personal feeling and experience of the patient."}, "sentence_sentiment": {"answer": "Positive", "rationale": "Because the purpose of \"must do something\" is positive."}, "language_style": {"answer": "Calm and direct", "rationale": ""}, "time_dimension": {"answer": "Present", "rationale": "The entry describes the current behaviour or state, \"a little restless\" and \"a feeling of necessity\" are all ongoing acts, and therefore the temporal dimension is now judged."}, "sentence_type": "Affirmative declarative sentence", "pos": {"verbs": {"have": 1, "I feel": 1, "Not": 1, "Yes": 1, "Live": 1, "Move": 1, "not": 1, "Yeah": 1}, "adjectives": {"Points": 1, "Sit tight": 1}, "adverbs": {"Elephant": 1, "Yes": 1}, "pronouns": {"I": 1}}}, {"content": "I'm optimistic about everything going forward (D)", "word_count": 12, "label": "", "sentence_structure": "Subject鈥揷opula鈥損redicative", "subjective_objective": {"answer": "Subjective", "rationale": "Because \"I am optimistic about everything going forward\" is an expression of subjective attitudes or views."}, "sentence_sentiment": {"answer": "Positive", "rationale": "Because \"optimistic looking forward\" expresses positive attitudes and emotions."}, "language_style": {"answer": "Calm and direct", "rationale": "The entry is more calm and straightforward in language style, and there is no excessive emotional or rhetorical style. It asks the interviewees in an objective and concise manner about their attitude to the matter, and expresses it in a clear and direct manner."}, "time_dimension": {"answer": "Future", "rationale": "According to the keyword \"optimistically forward\", it can be seen that the entry is an inquiry into the future's perception and attitude of the subjects and is therefore a matter of future time dimensions."}, "sentence_type": "Affirmative declarative sentence", "pos": {"verbs": {"be": 1, "I'm optimistic": 1, "Look forward": 1}, "adjectives": {"Optimistic": 1}, "adverbs": {"Land": 1}, "numerals": {"10": 1, "Everything": 1}}}, {"content": "I suddenly found panic.", "word_count": 8, "label": "", "sentence_structure": "Subject鈥搗erb鈥揷omplement", "subjective_objective": {"answer": "Subjective", "rationale": "Because \"fright\" is a patient's own feeling, a subjective experience. It may be connected to outside stimulation, but it is still subjective. So, \"A\" suddenly comes from the patient's subjective perspective."}, "sentence_sentiment": {"answer": "Negative", "rationale": "Because \"fright\" is usually seen as a negative sentiment, the expression of this purpose is negative."}, "language_style": {"answer": "Calm and direct", "rationale": "The entry is more straightforward and concise in language style, with no excessive rhetorical or emotional rendering. It starts with a specific act (discovering a sense of panic), then asks questions in short language (whether or not this has been experienced), which is more objective and pragmatic."}, "time_dimension": {"answer": "Present", "rationale": "The keyword is \"I suddenly found\" , which means it's a description of the current experience or feelings of the person being tested, and it's therefore judged now."}, "sentence_type": "Affirmative declarative sentence", "pos": {"nouns": {"I found out": 1, "Panic": 1, "A": 1}, "verbs": {"Suddenly": 1}, "numerals": {"11": 1}}}, {"content": "I feel like I'm getting lower.", "word_count": 12, "label": "", "sentence_structure": "Subject鈥揷opula鈥損redicative", "subjective_objective": {"answer": "Subjective", "rationale": "Because I feel emotionally as a subjective experience of a human being."}, "sentence_sentiment": {"answer": "Negative", "rationale": "Because \"frightful\" is a negative emotional experience."}, "language_style": {"answer": "Tactful and warm", "rationale": "The language style of the entry is euphemism. Although it involves negative emotions (i.e. emotional \"down\" ), it uses ambiguous language expressions (e.g. \"like feeling\") rather than direct statements or orders, which are more moderate and friendly and encourage respondents to share their true feelings."}, "time_dimension": {"answer": "Present", "rationale": "The keyword \"frights are falling\" indicates that the person being tested is experiencing an emotional experience that may be ongoing or temporary, but is now in a state of progress."}, "sentence_type": "Negative declarative sentence", "pos": {"nouns": {"Emotion": 1, "Down": 1}, "verbs": {"I feel": 1}, "adjectives": {"Gradient": 1, "D": 1}}}, {"content": "I'm a little scared, like some internal organ is broken.", "word_count": 18, "label": "", "sentence_structure": "Subject鈥揷opula鈥損redicative", "subjective_objective": {"answer": "Subjective", "rationale": "Fear and fear are determined by the patient ' s own subjective feelings, not by objective facts."}, "sentence_sentiment": {"answer": "Negative", "rationale": "Because the entries \"fear\" and \"a part of the internal organs has broken\" have negative emotional aspects."}, "language_style": {"answer": "Calm and direct", "rationale": "The entry presents the problem in a direct manner, without too much embellishment or emotional overture, and conveys more objectively and accurately the message that needs to be answered by the person surveyed. At the same time, the question is also more neutral, avoiding language that could trigger the emotional fluctuations of the person under investigation."}, "time_dimension": {"answer": "Present", "rationale": ""}, "sentence_type": "Affirmative declarative sentence", "pos": {"nouns": {"Problem": 1, "Questionnaire": 1, "Entry": 1, "Points": 1, "Scared": 1, "Internal organs": 1, "Bad": 1}, "verbs": {"I feel": 1, "It looks like": 1, "Change": 1}, "adjectives": {"A little": 1}, "numerals": {"13": 1}}}, {"content": "I can appreciate a good book or a good radio or television show (D)", "word_count": 20, "label": "", "sentence_structure": "Subject鈥搗erb鈥搊bject", "subjective_objective": {"answer": "Subjective", "rationale": "Because \"appear\" is the patient's subjective attitude and feelings."}, "sentence_sentiment": {"answer": "Positive", "rationale": "Because the \"can enjoy a good book or a good radio or television show\" is positive."}, "language_style": {"answer": "Tactful and warm", "rationale": "The language style of the entry is more euphemistic, because it uses the words \"can\" and \"or\" to give people a sense of choice. And the expressions \"a good book\", \"a good radio\" and \"television\" are also more neutral and objective, without excessive emotional or personal subjectivity. The whole sentence is more balanced and gives people a positive upward feeling."}, "time_dimension": {"answer": "Present", "rationale": "The keyword \"can\" uses the general current, which means that it is a question of the current capabilities of the testee. So the temporal dimension is now."}, "sentence_type": "Interrogative sentence", "pos": {"nouns": {"Books": 1, "Radio": 1, "Television programmes": 1}, "verbs": {"Yes": 1, "Enjoy": 1}, "adjectives": {"Okay": 2}, "numerals": {"14": 1}, "pronouns": {"I": 1}}}]}

{"questionnaire_title": "Multidimensional Health Lotus of Health Scales", "questionnaire_category": "Mental health", "item_count": 29, "emotion_shift_overall": "From negative to positive", "language_style_overall": "Neutral", "items": [{"content": "1. Children are in trouble because their parents blame them too much.", "word_count": 22, "label": "", "sentence_structure": "Subject鈥搗erb鈥搊bject", "subjective_objective": {"answer": "Objective", "rationale": "Because the title refers to the fact that \"the children are having problems\" and believes that this is due to the parents' culpability of the children. These descriptions are based on observations and data, not on personal feelings or beliefs, and are therefore objective."}, "sentence_sentiment": {"answer": "Negative", "rationale": "Because the expression \"too much blame\" in the entry contains negative emotional tendencies."}, "language_style": {"answer": "Calm and direct", "rationale": "The entry is a direct statement of views, does not use excessive rhetorical or emotional rendering, and is more objective in tone."}, "time_dimension": {"answer": "Present", "rationale": "The entry describes a current situation or view that \"the children are having problems because their parents blame them too much\". It does not address any past or future events or events. So the temporal dimension is now."}, "sentence_type": "Affirmative declarative sentence", "pos": {"nouns": {"Kids": 1, "Problem": 1, "Parents": 1, "they": 2}, "verbs": {"Out": 1, "be": 1, "Blame": 1, "Too much": 1}, "adjectives": {"of": 4}, "numerals": {"One": 2}, "pronouns": {"Here": 1}}}, {"content": "2. Many unfortunate events in people ' s lives have had some bearing on bad luck.", "word_count": 22, "label": "", "sentence_structure": "Subject鈥搗erb鈥搊bject", "subjective_objective": {"answer": "Objective", "rationale": "Because \"bad luck\" is an objective concept, the sentence is about whether this objective fact is connected to people's misfortune."}, "sentence_sentiment": {"answer": "Negative", "rationale": "Because it expresses the unfortunate and somewhat pessimistic nature of life that bad luck can lead to."}, "language_style": {"answer": "Tactful and warm", "rationale": "The entry is relatively euphemistic, in that it guides the respondents to express their views in an interview, without direct judgement or criticism. At the same time, the expression \"certain\" is used, adding ambiguity to the phrase and greater respect for the views and feelings of the respondents."}, "time_dimension": {"answer": "Present", "rationale": "Now the entry is a description of a general phenomenon or view, and there is no clear time pointing to sex, so it's \"now\"."}, "sentence_type": "Affirmative declarative sentence", "pos": {"nouns": {"People": 1, "Life": 1, "Yes": 1, "Lucky": 1}, "verbs": {"Come on": 1}, "adjectives": {"Many": 1, "Unfortunately": 1}, "adverbs": {"Both": 1, "I'm sure": 1}, "pronouns": {"and": 1}}}, {"content": "3. One of the main reasons for the war was the lack of political interest.", "word_count": 24, "label": "", "sentence_structure": "Subject鈥揷opula鈥損redicative", "subjective_objective": {"answer": "Subjective", "rationale": "Because \"insensitive\" is the view and opinion of the individual, and is subjective."}, "sentence_sentiment": {"answer": "Negative", "rationale": "Because the result of the \"war\" in the entry is negative and it is believed that the lack of political interest in people has led to such an outcome."}, "language_style": {"answer": "Calm and direct", "rationale": "The entry indicates in direct and clear language one of the main causes of war, without any rhetorical or emotional tact, and with a more calm and direct language style."}, "time_dimension": {"answer": "Past", "rationale": ""}, "sentence_type": "Affirmative declarative sentence", "pos": {"nouns": {"War": 1, "Reason": 1, "Care": 1, "Political": 1}, "verbs": {"Generate": 1, "It's": 1, "Not enough": 1}, "adverbs": {"Main": 1, "Not enough": 1}, "numerals": {"3": 1}}}, {"content": "4. Ultimately, people will be given the respect he deserves in this world.", "word_count": 18, "label": "", "sentence_structure": "Subject鈥搗erb鈥搊bject", "subjective_objective": {"answer": "Subjective", "rationale": "Because \"due respect\" is a subjective assessment of a situation or an individual's expectations."}, "sentence_sentiment": {"answer": "Positive", "rationale": "The entry is an expression of respect for and impartiality of the human person."}, "language_style": {"answer": "Tactful and warm", "rationale": "Warm and warm."}, "time_dimension": {"answer": "Future", "rationale": "The keyword \"finally\" indicates that the entry is at some point in the discussion of the future, while the dominant word \"people\" refers to a group of people in the future. Thus, it can be judged to be about the vision and expectations of the future, in which people will be given the respect they deserve."}, "sentence_type": "Affirmative declarative sentence", "pos": {"nouns": {"People": 1, "World": 1, "Respect": 1}, "verbs": {"Got it": 1, "be": 1}, "adjectives": {"Eventually": 1, "Here": 1}, "numerals": {"4": 1}, "pronouns": {"he": 1}}}, {"content": "5. The perception of teachers as being unfair to students is unfounded.", "word_count": 21, "label": "", "sentence_structure": "Subject鈥揷opula鈥損redicative", "subjective_objective": {"answer": "Objective", "rationale": "This is because it is a judgement of a prevailing view and does not involve subjective feelings and experiences of the individual patient."}, "sentence_sentiment": {"answer": "Negative", "rationale": "Because the expression \"teachers' perception of students' unfairness is absurd\" in the entry is derogatory and negative, emotional tendencies are negative."}, "language_style": {"answer": "Calm and direct", "rationale": "The entry was asked in a more direct and explicit language, and was not used in excessive terms or euphemism, which was more intruding and straightforward."}, "time_dimension": {"answer": "Present", "rationale": "The entry now expresses a view or view that does not address past behaviour or future expectations. In it, the words \"concepts\" and \"views\" represent a description and judgement of the current state."}, "sentence_type": "Negative declarative sentence", "pos": {"nouns": {"Teachers": 1, "Students": 2, "Observations": 1, "Nonsense": 1}, "verbs": {"I think": 1, "Not enough": 1, "Fair": 1, "be": 1, "It's nonsense": 1}, "adjectives": {"That": 1, "Not enough": 1}, "adverbs": {"to": 1, "of": 3}, "numerals": {"5": 1, "2": 1}, "pronouns": {"Teachers to students": 1}}}, {"content": "6. Without the right opportunities, one cannot be a good leader.", "word_count": 23, "label": "", "sentence_structure": "Subject鈥揷opula鈥損redicative", "subjective_objective": {"answer": "Objective", "rationale": "Because the ability to be a good leader is determined by a number of factors, not only by the availability of suitable opportunities."}, "sentence_sentiment": {"answer": "Neutral", "rationale": "Because the entry does not have any emotional color."}, "language_style": {"answer": "Calm and direct", "rationale": ""}, "time_dimension": {"answer": "Present", "rationale": ""}, "sentence_type": "Negative declarative sentence", "pos": {"nouns": {"person": 1, "Leader": 1, "Opportunities": 1}, "verbs": {"Maybe": 1, "Be": 1, "No, I'm not": 1}, "adjectives": {"Suitable": 1, "Excellent": 1}, "adverbs": {"Impossible": 1}, "numerals": {"6": 1}, "classifiers": {"One": 2}, "pronouns": {"he": 1}}}, {"content": "7. Whatever you do, some people just don ' t like you.", "word_count": 16, "label": "", "sentence_structure": "Subject鈥搗erb鈥搊bject", "subjective_objective": {"answer": "Subjective", "rationale": "The basis for the judgment: because \"like\" is the patient's emotional attitude, it's subjective."}, "sentence_sentiment": {"answer": "Negative", "rationale": "Because the expression \"some people don't like you\" in the entry is emotional and negative, and expresses a negative attitude."}, "language_style": {"answer": "Calm and direct", "rationale": "The entry directly describes the possible existence of a situation in which some people, even if you try so hard, cannot change their perception of you, and the language style is more calm and objective."}, "time_dimension": {"answer": "Present", "rationale": "The entry describes a current phenomenon or situation, which is that \"some people just don't like you\", and emphasizes that no matter how hard they try, they can change that fact. So the time dimension is now."}, "sentence_type": "Affirmative declarative sentence", "pos": {"nouns": {"Questionnaire": 1, "Entry": 1, "person": 1}, "verbs": {"Yeah": 1, "Try": 1}, "adjectives": {"Whatever": 1, "What": 1}, "adverbs": {"Both": 1}, "numerals": {"7": 1}, "pronouns": {"you": 1}}}, {"content": "8. Genetics play a major decisive role in a person ' s personality.", "word_count": 17, "label": "", "sentence_structure": "Subject鈥損redicate", "subjective_objective": {"answer": "Objective", "rationale": "Since genetics is a natural characteristic of a human being, it is not determined by subjective will or behaviour."}, "sentence_sentiment": {"answer": "Neutral", "rationale": "Since the entry expresses a fact or view, there is no apparent emotional tendency."}, "language_style": {"answer": "Tactful and warm", "rationale": "The entry is more proficient and interactive in its expression, in a gentle and inviting tone, rather than in direct statements or orders. At the same time, the language style of the issue is neutral and objective, is not overly emotional and is more in line with the attitude and requirements of scientific research."}, "time_dimension": {"answer": "Present", "rationale": ""}, "sentence_type": "Affirmative declarative sentence", "pos": {"nouns": {"Genetic": 1, "Personality": 1, "Role": 1}, "verbs": {"to": 1, "Rise": 1, "Decision": 1}, "adjectives": {"Main": 1}, "numerals": {"8": 1}}}, {"content": "I've often found out that what's going to happen is happening.", "word_count": 18, "label": "", "sentence_structure": "Subject鈥搗erb鈥揷omplement", "subjective_objective": {"answer": "Subjective", "rationale": "This is because patients' predictions and expectations about the future are a reflection of their subjective attitude."}, "sentence_sentiment": {"answer": "Positive", "rationale": "Because the \"often discovering that what is about to happen is real\" is positive, indicating that the interviewees have a sense of predictability and insight."}, "language_style": {"answer": "Tactful and warm", "rationale": "The entry is more personal and interactive when it is spoken in a gentle and inviting tone, rather than in direct statements or orders. At the same time, the article is relatively easy to use, without too much emotional and serious. It is therefore a warm judgement that its language style is euphemism."}, "time_dimension": {"answer": "Present", "rationale": "The word \"I'm often\" denotes a regular, customary act, while both \"discovery\" and \"occupying\" use the normal current state of affairs, which means that this act has not only occurred in the past, but may still be ongoing. So the entry primarily asks the testee about the experience and experience of the present, and the temporal dimension is now."}, "sentence_type": "Affirmative declarative sentence", "pos": {"nouns": {"Yes": 2, "Come on": 1}, "verbs": {"Found": 1, "Yes": 1, "True": 1}, "adjectives": {"Often": 1}, "adverbs": {"True": 1}, "pronouns": {"Those": 1, "Will": 1, "Here": 1}}}, {"content": "10. For a fully prepared student, unfair examinations and other things do not exist.", "word_count": 28, "label": "", "sentence_structure": "Subject鈥揷opula鈥損redicative", "subjective_objective": {"answer": "Subjective", "rationale": "Since this entry relates to the subjective view and attitude of the student, i.e. whether the student believes that he or she is well prepared and that the examination is unfair, there is no such thing."}, "sentence_sentiment": {"answer": "Positive", "rationale": "Because the words \"students who are well prepared\" and \"unfair exams don't exist\" in the entries are positive."}, "language_style": {"answer": "Calm and direct", "rationale": "The article expresses the view in a single statement, without any qualifying or emotional expression. At the same time, it appears to be very categorical and leaves no room for any. This language style is more direct, clear, and it is not too soft or vague and is consistent with the immediate and calmness of the person."}, "time_dimension": {"answer": "Present", "rationale": "The entry for the questionnaire is a description of the current view or belief that unfair examinations do not exist for adequately prepared students. Thus, the time dimension is now judged."}, "sentence_type": "Negative declarative sentence", "pos": {"nouns": {"Students": 1, "Examinations": 2, "Yes": 1, "Existence": 1}, "verbs": {"Ready": 1, "Come on": 1, "I think": 1, "Fair": 1, "be": 3}, "adjectives": {"Full": 1, "It's not fair": 1}, "numerals": {"10": 1, "One": 2}, "pronouns": {"One": 2}}}, {"content": "Success is hard work, and luck is almost totally irrelevant.", "word_count": 24, "label": "", "sentence_structure": "Subject鈥揷opula鈥損redicative", "subjective_objective": {"answer": "Subjective", "rationale": "This is a subjective perspective, as success requires hard work, and whether luck has an impact on the patient ' s personal perception and conviction."}, "sentence_sentiment": {"answer": "Positive", "rationale": "This is a positive attitude, since it expresses the hard work necessary for success, while ignoring the role of luck."}, "language_style": {"answer": "Calm and direct", "rationale": "The language style of the entry is more calm and straightforward, without too much emotional and rhetorical, but simply presents the idea that success requires hard work and that luck is less. This expression is more objective and rational and suitable for official settings such as questionnaires."}, "time_dimension": {"answer": "Present", "rationale": "The entry describes a general view or belief, and does not clearly indicate the point of time. Therefore, the dimension of time is now judged."}, "sentence_type": "Affirmative declarative sentence", "pos": {"nouns": {"Success": 1, "Give": 1, "Hard work": 1, "Lucky": 2, "Total": 1}, "verbs": {"Access": 1, "be": 1, "Yes": 1, "Give": 1, "Hard work": 1, "Almost": 1, "Even": 1, "It's not relevant": 1}, "adjectives": {"Hard": 1, "Even": 1, "Total": 1}, "adverbs": {"Almost": 1, "Even": 1}, "numerals": {"11": 1, "2": 1}, "classifiers": {"individual": 1}, "pronouns": {"Here": 1}}}, {"content": "12. The general population also has an impact on government decision-making.", "word_count": 16, "label": "", "sentence_structure": "Subject鈥搗erb鈥搊bject", "subjective_objective": {"answer": "Subjective", "rationale": "Because \"the general population will influence government decision-making\" is a view or belief, and it is the individual opinion of the interviewee, it is subjective."}, "sentence_sentiment": {"answer": "Neutral", "rationale": "As the entry merely stated a fact, it did not express any positive or negative feelings, attitudes or views."}, "language_style": {"answer": "Tactful and warm", "rationale": "The entry presents the point in a more objective and open manner, without overemphasising or undermining the view of one party, so that the interviewee feels respected and encouraged to express his views. At the same time, the dictum makes the tone more friendly."}, "time_dimension": {"answer": "Present", "rationale": "The keywords \"common people\" and \"government decision-making\" suggest that this is a description of current social phenomena, not past or future. At the same time, the word \"will\" implies that this is a constant behaviour, in which ordinary people often influence government decision-making."}, "sentence_type": "Affirmative declarative sentence", "pos": {"nouns": {"People": 1, "Government": 1, "Decision-making": 1}, "verbs": {"Yes": 1, "Generate": 1, "Impact": 1}, "adverbs": {"Normal": 1}, "numerals": {"12": 1}}}, {"content": "When I made my plans, I was almost certain that I could implement them.", "word_count": 20, "label": "", "sentence_structure": "Subject鈥搗erb鈥揷omplement", "subjective_objective": {"answer": "Subjective", "rationale": "Because \"I can almost be sure I can practice them\" is the patient's personal conviction and attitude, and it is subjective."}, "sentence_sentiment": {"answer": "Positive", "rationale": "Because the phrase \"almost sure I can do them\" in the entry conveys a sense of confidence and optimism, and is positive."}, "language_style": {"answer": "Tactful and warm", "rationale": ""}, "time_dimension": {"answer": "Present", "rationale": ""}, "sentence_type": "Affirmative declarative sentence", "pos": {"nouns": {"Planned": 1}, "verbs": {"Staple": 1, "Yeah": 2, "Implementation": 1}, "adjectives": {"I'm sure it is": 1}, "adverbs": {"Almost": 1, "Total": 1}, "numerals": {"13": 1}, "classifiers": {"Some": 1}, "pronouns": {"They": 1}}}, {"content": "There is a lack of merit in a person.", "word_count": 9, "label": "", "sentence_structure": "Subject鈥揷opula鈥損redicative", "subjective_objective": {"answer": "Subjective", "rationale": "Because it's a personal judgment to assess whether a person is useless."}, "sentence_sentiment": {"answer": "Negative", "rationale": "Because \"never good\" is a negative way of saying that this person has no merit or value."}, "language_style": {"answer": "Calm and direct", "rationale": "The language style of the entry uses the ironic phrase \"nothing\" and expresses derogatory and critical attitudes towards some, which are offensive and provocative and may give rise to feelings of repulsion or resistance on the part of the person being evaluated."}, "time_dimension": {"answer": "Past", "rationale": "The entry describes a subjective judgement or view, which does not involve the conduct or feelings of the person being tested, and therefore is judged by the temporal dimension of the past."}, "sentence_type": "Negative declarative sentence", "pos": {"nouns": {"person": 1}, "verbs": {"have": 1, "be": 1}, "adjectives": {"None": 1, "Plant": 1}, "adverbs": {"One": 1}, "numerals": {"XIV": 1}, "classifiers": {"individual": 1}}}, {"content": "15. For me, it is not luck to get what I want.", "word_count": 18, "label": "", "sentence_structure": "Subject鈥揷opula鈥損redicative", "subjective_objective": {"answer": "Subjective", "rationale": "Because \"I can get what I want\" and \"luck has nothing to do with\" are subjective views and opinions of patients."}, "sentence_sentiment": {"answer": "Neutral", "rationale": "Because the entry does not have any emotional color."}, "language_style": {"answer": "Tactful and warm", "rationale": "The entry is a first-person expression of personal views and is more balanced, rational, and not overly emotional or strongly expressed. This language style is more personal and interactive, allowing respondents to be more willing to participate in the questionnaire and to express their views more honestly."}, "time_dimension": {"answer": "Present", "rationale": "\"What I want is not luck\" is the personal view and attitude of the testee, which emphasizes the importance of individual efforts in achieving the goal, and therefore the time dimension is now judged."}, "sentence_type": "Affirmative declarative sentence", "pos": {"nouns": {"I": 1, "Something": 1, "Lucky": 1}, "verbs": {"Yes": 1, "Got it": 1, "and": 1, "Not relevant": 1}, "adverbs": {"Just": 1}, "numerals": {"15": 1}}}, {"content": "16. The ability to be a boss often depends on his having taken a favourable position first.", "word_count": 25, "label": "", "sentence_structure": "Subject鈥搗erb鈥搊bject", "subjective_objective": {"answer": "Subjective", "rationale": "Because \"we're lucky to have a good seat first\" is a subjective view or an explanation, not an objective fact."}, "sentence_sentiment": {"answer": "Negative", "rationale": "This is a negative perception, since it implies that success depends primarily on luck, while ignoring factors such as individual effort and capacity."}, "language_style": {"answer": "Tactful and warm", "rationale": "The language style of the entry is more euphemistic and implicit, using the words \"often\" and \"very fortunate\" to express a subjective view rather than a direct assertion or order. Such expression is more moderate and personal, and better able to guide respondents to reflect and answer questions."}, "time_dimension": {"answer": "Past", "rationale": "The sentence describes the factors (luck and location) that have succeeded in becoming the boss, and does not clearly point to the future or the current time dimension. Thus, the past can be judged."}, "sentence_type": "Affirmative declarative sentence", "pos": {"nouns": {"Boss": 1, "bit": 1}, "verbs": {"When": 1, "Occupation": 1, "Lucky": 1, "Decision": 1}, "adjectives": {"very": 1, "Good": 1}, "numerals": {"16": 1}, "pronouns": {"Who": 1, "he": 2}}}, {"content": "17. In terms of world affairs, most of us are victims of efforts that we neither understand nor control.", "word_count": 35, "label": "", "sentence_structure": "Subject鈥揷opula鈥損redicative", "subjective_objective": {"answer": "Subjective", "rationale": "This sentence explores human power and confusion in world affairs."}, "sentence_sentiment": {"answer": "Neutral", "rationale": "The entry is not a positive or negative emotional expression."}, "language_style": {"answer": "Tactful and warm", "rationale": "The language style of the entry is euphemistic, and it stimulates resonance and reflection by describing the powerlessness and impotence of the majority in world affairs. This expression is more emotional and humane, and better attracts the attention and participation of the respondents."}, "time_dimension": {"answer": "Present", "rationale": "The entry describes how people feel and experience when dealing with world affairs, using the point of time \"now\" and does not address future or past events or situations. It therefore belongs to this time frame."}, "sentence_type": "Negative declarative sentence", "pos": {"nouns": {"World affairs": 1, "we": 2, "Most people": 1, "Try": 1, "The victim": 1}, "verbs": {"be": 1, "Understood": 1, "Control": 1, "Give": 1}, "adjectives": {"not": 3, "Cannot": 1, "of": 2, "Both": 1}, "numerals": {"17": 1, "1": 4}, "pronouns": {"鈾?And the 鈾?: 1, "Other": 1}}}, {"content": "18. Most people are unaware that their lives are somewhat influenced by accidents.", "word_count": 29, "label": "", "sentence_structure": "Subject鈥搗erb鈥搊bject", "subjective_objective": {"answer": "Objective", "rationale": "Because the sentence describes a widespread fact, it does not involve the patient ' s personal experience or feelings and is therefore objective."}, "sentence_sentiment": {"answer": "Neutral", "rationale": "Because the entry does not have any emotional color."}, "language_style": {"answer": "Tactful and warm", "rationale": "The entry is more personal and interactive in its expression of conversation in a gentle and inviting tone, rather than in direct statements or orders. It also offers an observation or reflection perspective that leads respondents to reflect on their lives and, in this way, increases the appeal and participation of questionnaires."}, "time_dimension": {"answer": "Present", "rationale": "The keyword \"mostly unaware\" and \"in part influenced by an accident\" suggests that it is a description of the current situation, so the answer is \"now\"."}, "sentence_type": "Affirmative declarative sentence", "pos": {"nouns": {"person": 1, "Life": 1, "Accident": 1}, "verbs": {"I didn't realize": 1, "Left and left": 1}, "adjectives": {"Most of them": 1, "In a way": 1}, "numerals": {"18": 1}, "pronouns": {"they": 1}}}, {"content": "19. A person should be ready to admit mistakes.", "word_count": 13, "label": "", "sentence_structure": "Subject鈥損redicate", "subjective_objective": {"answer": "Subjective", "rationale": "Because it is believed that \"a person should be ready to admit wrong\" is the expression of a subjective attitude and values of a human being."}, "sentence_sentiment": {"answer": "Positive", "rationale": "Because \"acknowledged error\" in the entry is positive behavior and attitude."}, "language_style": {"answer": "Tactful and warm", "rationale": "The entry is more softly phrased, using the words \"should\" and \"ready\" to guide and advise the respondents on their attitudes and behaviour, rather than direct orders or criticism. At the same time, the theme \"acknowledged error\" is expressed more subtlely, without overemphasising the seriousness of the error, but rather on promoting self-reflection and corrective behaviour. Such language styles help to create a harmonious atmosphere of dialogue and encourage respondents to participate more actively in the questionnaire."}, "time_dimension": {"answer": "Present", "rationale": "The keyword \"should be ready\" indicates that it is a normal and constant act, and the entry therefore asks about current attitudes or behaviour."}, "sentence_type": "Affirmative declarative sentence", "pos": {"nouns": {"person": 1, "Error": 1}, "verbs": {"I think so": 1, "Ready": 1, "Recognition": 1}, "adverbs": {"Anytime": 1}}}, {"content": "It is difficult to know whether a person really likes you.", "word_count": 16, "label": "", "sentence_structure": "Subject鈥揷opula鈥損redicative", "subjective_objective": {"answer": "Subjective", "rationale": "Because \"want to know\" is a subjective idea or feeling, and \"do you really like\" is a subjective view that needs to be judged by the behaviour and attitude of the other party."}, "sentence_sentiment": {"answer": "Negative", "rationale": "Because the entry expresses an emotion of difficulty and challenge, implying that it is not easy to understand the feelings of another person, with some negative emotional colour."}, "language_style": {"answer": "Tactful and warm", "rationale": "The language style of the entry is euphemistic, as it uses a more subjective expression and does not directly present or order. The entry is a soft, invitation-based conversation, asking if the interviewees agree with it, and this expression is more personal and interactive."}, "time_dimension": {"answer": "Present", "rationale": ""}, "sentence_type": "Affirmative declarative sentence", "pos": {"nouns": {"person": 1, "Yeah": 1, "you": 1}, "verbs": {"Yes": 2, "Hard": 1}, "adjectives": {"Really": 1}, "numerals": {"20": 1}}}, {"content": "21. Ultimately, we will be faced with the same bad and good things.", "word_count": 15, "label": "", "sentence_structure": "Subject鈥揷opula鈥損redicative", "subjective_objective": {"answer": "Subjective", "rationale": "Since parity is a subjective view, it depends on the individual ' s definition and assessment of good and bad."}, "sentence_sentiment": {"answer": "Neutral", "rationale": "Because the entry merely stated a point of view, it did not involve any positive or negative emotional aspects."}, "language_style": {"answer": "Tactful and warm", "rationale": "The language style of the entry is euphemism. It is more personal and interactive in a more gentle and encouraging way to ask questions, rather than direct orders or statements. Moreover, the word \"parity\" reflects the tolerance of the questionnaire designer to the respondents."}, "time_dimension": {"answer": "Past", "rationale": "The entry does not contain any vision or expectations for the future, but rather a description of the past, so the temporal dimension is judged to be the past."}, "sentence_type": "Affirmative declarative sentence", "pos": {"nouns": {"Bad things": 1, "Good news": 1, "Equality": 1}, "verbs": {"I've got it": 1, "be": 1, "Yes": 1}, "adverbs": {"Eventually": 1}, "numerals": {"21": 1}, "pronouns": {"we": 1}}}, {"content": "22. We can eradicate political corruption with sufficient effort.", "word_count": 19, "label": "", "sentence_structure": "Subject鈥搗erb鈥搊bject", "subjective_objective": {"answer": "Subjective", "rationale": "Because the eradication of political corruption is a subjective judgement, depending on the values and beliefs of the individual. While the effort is an objective act, the successful eradication of political corruption is a subjective conclusion."}, "sentence_sentiment": {"answer": "Positive", "rationale": "Because the purpose of the \"Eradicate Political Corruption\" in the entry is positive, it is believed that efforts can be made."}, "language_style": {"answer": "Calm and direct", "rationale": "The language style of the entry is more calm and straightforward, and it clearly expresses the view that, with sufficient effort, we can eradicate political corruption. This expression is not too rhetorical or emotional, but rather conveys the message in an objective and clear manner."}, "time_dimension": {"answer": "Future", "rationale": "The keyword \"We can eradicate political corruption with enough effort\" shows a vision and expectation for the future that efforts can change the current political situation. So, the entry deals with visions and expectations for the future, and the time dimension is judged to be the future."}, "sentence_type": "Affirmative declarative sentence", "pos": {"nouns": {"Try": 1, "Political corruption": 1}, "verbs": {"Give": 1, "Eradication": 1}, "adverbs": {"Enough": 1}, "numerals": {"22": 1}}}, {"content": "23. Sometimes I don ' t understand how teachers are able to score on the side of a scroll.", "word_count": 22, "label": "", "sentence_structure": "Subject鈥搗erb鈥搊bject", "subjective_objective": {"answer": "Subjective", "rationale": "Because \"not understanding\" is the patient's subjective feeling."}, "sentence_sentiment": {"answer": "Negative", "rationale": "Because the word \"ununderstand\" in the entry expresses a sense of confusion and dissatisfaction."}, "language_style": {"answer": "Tactful and warm", "rationale": "This expression is more comfortable and acceptable to the interviewees."}, "time_dimension": {"answer": "Present", "rationale": "In that sentence, \"sometimes\" and \"but I don't get it\" indicate that it is a state of the moment or a feeling, and therefore it is judged as now."}, "sentence_type": "Interrogative sentence", "pos": {"nouns": {"Time": 1, "Teachers": 1, "Rolling face": 1, "Score": 1}, "verbs": {"Understood": 1, "Hit": 1}, "adjectives": {"Really": 1, "What": 1}, "adverbs": {"Sometimes": 1}, "numerals": {"23": 1}, "pronouns": {"I": 1}}}, {"content": "A good leader would encourage people to make their own choices about what to do.", "word_count": 23, "label": "", "sentence_structure": "Subject鈥搗erb鈥搊bject", "subjective_objective": {"answer": "Subjective", "rationale": "Because this is more the expression of a subjective view or value that a good leader should encourage people to make decisions on their own. This is not an objective fact, but a subjective judgement based on personal experience, views and convictions."}, "sentence_sentiment": {"answer": "Positive", "rationale": "Because the words \"good leader\" and \"encourage people to make decisions\" in the entries are all positive."}, "language_style": {"answer": "Tactful and warm", "rationale": "The language style of the entry is euphemismal. It does not direct orders or impose opinions, but rather directs the respondents to think and express their views in an invitation and inspirational way that is more acceptable and willing to participate."}, "time_dimension": {"answer": "Present", "rationale": "The entry now describes a general style of leadership or management, and does not address past or future behavior or mentality. Thus, the time dimension is now."}, "sentence_type": "Affirmative declarative sentence", "pos": {"nouns": {"bit": 1, "Leadership": 1, "person": 1, "Ideas": 1}, "verbs": {"Yes": 1, "Encourage": 1, "Do it": 1, "Take it": 1, "self": 1}, "adjectives": {"Okay": 1}, "numerals": {"24": 1}}}, {"content": "25. I have felt too often that I am powerless to deal with my own experiences.", "word_count": 19, "label": "", "sentence_structure": "Subject鈥揷opula鈥損redicative", "subjective_objective": {"answer": "Subjective", "rationale": "Because this feeling is usually an experience and attitude within the individual, it is subjective."}, "sentence_sentiment": {"answer": "Negative", "rationale": "Because the attitude of \"incompetently affected\" in the entry is negative."}, "language_style": {"answer": "Calm and direct", "rationale": "The entry \"Calm down straight\" directly expresses the subject of asking the other party whether it is unable to respond to the situation it is experiencing, without excessive rhetorical or emotional rendering, and is more objective in tone."}, "time_dimension": {"answer": "Present", "rationale": "Among them, \"many times\" indicates that this feeling is not sporadic, but recurrent; and \"all feel powerless\" expresses the strong sense of weakness and helplessness of the testee. Thus, the article's purpose is to be judged by the description of the time dimension and emotional state of the person concerned as to the feelings and mental state of the person."}, "sentence_type": "Interrogative sentence", "pos": {"nouns": {"Time": 1, "I": 2, "Problems": 1, "There's nothing I can do": 1}, "verbs": {"Both": 1, "Sensor": 1, "Present": 1, "be": 1, "I feel": 1, "to": 2, "self": 1, "of": 4, "Problems": 1, "There's nothing I can do": 1}, "adverbs": {"Many times": 1, "Both": 1}, "classifiers": {"individual": 1}, "pronouns": {"I": 2}}}, {"content": "26. The people were alone because they did not try to be friendly.", "word_count": 21, "label": "", "sentence_structure": "Subject鈥揷opula鈥損redicative", "subjective_objective": {"answer": "Subjective", "rationale": "Because it is the patient's perception and explanation of why people feel alone."}, "sentence_sentiment": {"answer": "Negative", "rationale": "Because the sentence negatively evaluates and blames those \"people\" who feel that their isolation is due to their own problems, which is a negative attitude."}, "language_style": {"answer": "Tactful and warm", "rationale": "The language style of the entry is more euphemistic, and it does not directly blame or criticize, but rather uses a guiding tone, which attempts to give the respondents a sense of whether their actions may lead to isolation. This expression is more moderate, inclusive, and tends to resonate and respond positively to the respondents."}, "time_dimension": {"answer": "Present", "rationale": "The \"they\" in the entry refers to some people now, and the \"no attempt to be friendly\" act is a description of the current behaviour and attitude of these people. So, the time dimension of the entry is now."}, "sentence_type": "Negative declarative sentence", "pos": {"nouns": {"person": 1, "Loneliness": 1, "Friendly": 1}, "verbs": {"be": 1, "Because": 1, "Try": 1, "It seems": 1}, "adverbs": {"Some": 1}, "numerals": {"26": 1}, "pronouns": {"Those": 1, "they": 1}}}, {"content": "27. The emphasis on sport in secondary schools has been too much.", "word_count": 13, "label": "", "sentence_structure": "Subject鈥搗erb鈥揷omplement", "subjective_objective": {"answer": "Subjective", "rationale": "Because \"too much\" is a subjective judgement and evaluation."}, "sentence_sentiment": {"answer": "Negative", "rationale": "Because the phrase \"the importance attached to sports in secondary schools is too much\" in the entry expresses negative attitudes."}, "language_style": {"answer": "Calm and direct", "rationale": "The language style of the entry is more direct and clear, and there is no excessive emotional or rhetorical style, but rather a representation of the facts, giving a calm and clear impression."}, "time_dimension": {"answer": "Present", "rationale": "According to the keyword and sentence structure, the entry is describing the current situation and thus judged as \"now\"."}, "sentence_type": "Negative declarative sentence", "pos": {"nouns": {"Secondary": 1, "Sports": 1, "Value": 1}, "verbs": {"Lee": 1, "That's too much": 1, "be": 1}, "adjectives": {"Too much": 1}, "numerals": {"27": 1}}}, {"content": "28. The outcome depends entirely on what I do.", "word_count": 16, "label": "", "sentence_structure": "Subject鈥搗erb鈥搊bject", "subjective_objective": {"answer": "Subjective", "rationale": "Because \"how things end\" is a subjective judgement that requires the patient to assess and evaluate itself. And \"depending on what I do\" is also the patient's subjective attitude and perception."}, "sentence_sentiment": {"answer": "Positive", "rationale": "Because the attitude in the entries is one of self-assertion and belief that decisions can influence the development of things. This positive attitude usually inspires motivation and self-confidence and contributes to the desired goal."}, "language_style": {"answer": "Tactful and warm", "rationale": "The entry uses a more subjective and positive language style, which leads each other to think by affirming their abilities, is more moderate and encouraging."}, "time_dimension": {"answer": "Present", "rationale": "The entry expresses the view or belief that it is in control of the outcome of the matter. This belief may have an impact on the behaviour of the person being tested and may reflect his current mentalities and attitudes."}, "sentence_type": "Affirmative declarative sentence", "pos": {"verbs": {"End of story": 1, "It's up to you": 1, "Do it": 1}, "adverbs": {"How's that": 1, "Total": 1}, "numerals": {"28": 1}, "pronouns": {"I": 1}}}, {"content": "29. I do not understand why politicians do so for most of the time.", "word_count": 21, "label": "", "sentence_structure": "Subject鈥搗erb鈥搊bject", "subjective_objective": {"answer": "Subjective", "rationale": "The patient ' s thoughts and understanding are examined and therefore subjectively."}, "sentence_sentiment": {"answer": "Negative", "rationale": "Because the \"ununderstandable\" attitude in the entry is negative."}, "language_style": {"answer": "Calm and direct", "rationale": "Because the article is neutral and objective, it does not use excessive rhetorical or emotional terms. It directly asks the respondents about the level of understanding of politicians鈥?behaviour and asks questions in a direct and non-ambitious manner."}, "time_dimension": {"answer": "Present", "rationale": "The words \"mostly\" and \"not understandable\" and \"doing so\" suggest that the entry is describing the current feelings or attitudes and therefore judging as the present."}, "sentence_type": "Negative declarative sentence", "pos": {"nouns": {"Time": 1, "Both": 1, "I": 1, "Reason": 1, "Politicians": 1, "Behaviour": 1}, "verbs": {"Understood": 1, "Yes": 1, "I see": 1, "Act": 1}, "adjectives": {"Most of them": 1}, "adverbs": {"Both": 1}, "numerals": {"29": 1}}}]}

{"questionnaire_title": "UCLA Lonely Measure", "questionnaire_category": "Mental health", "item_count": 20, "emotion_shift_overall": "From positive to negative", "language_style_overall": "Tactful and warm", "items": [{"content": "Do you often feel in harmony with the people around you?", "word_count": 14, "label": "", "sentence_structure": "Subject鈥損redicate", "subjective_objective": {"answer": "Subjective", "rationale": "Because the subject looks at the patient鈥檚 self-perception, i.e. whether they often feel in harmony with the people around them. This is a subjective assessment based on personal experience."}, "sentence_sentiment": {"answer": "Positive", "rationale": "Because the entry expresses a positive feeling and attitude as to whether individuals often feel comfortable with the people around them."}, "language_style": {"answer": "Tactful and warm", "rationale": "The entry is more adept and interactive when it is spoken in a gentle and inviting manner, rather than by direct statements or orders."}, "time_dimension": {"answer": "Present", "rationale": "The keyword \"often feels\" indicates that it is a description of the current feeling, and the temporal dimension is now."}, "sentence_type": "Interrogative sentence", "pos": {"nouns": {"person": 1, "Relations": 1, "Harmony": 1}, "verbs": {"Sensor": 1, "Regular": 1, "and": 1, "I feel": 1, "have": 1}, "adjectives": {"Around": 1}, "adverbs": {"- You're not": 1}, "pronouns": {"you": 1}}}, {"content": "Do you often feel the lack of partners?", "word_count": 9, "label": "", "sentence_structure": "Subject鈥搗erb鈥揷omplement", "subjective_objective": {"answer": "Subjective", "rationale": "Because \"lack of partners\" is a subjective feeling and experience, it cannot be measured or validated by objective criteria."}, "sentence_sentiment": {"answer": "Neutral", "rationale": "Because the entry does not have any emotional color."}, "language_style": {"answer": "Tactful and warm", "rationale": "The entry is more adept and interactive when it is spoken in a gentle and inviting manner, rather than by direct statements or orders."}, "time_dimension": {"answer": "Past", "rationale": "In this entry, \"often feeling\" indicates a constant state of affairs or emotion that has occurred in the past and may continue. The temporal dimension of the questionnaire is therefore in the past."}, "sentence_type": "Interrogative sentence", "pos": {"nouns": {"Partners": 1}, "verbs": {"I always feel": 1, "Missing": 1, "- You're not": 1}, "adverbs": {"Regular": 1, "Sensor": 1, "Missing": 1, "Less": 1}}}, {"content": "Do you often feel that no one can trust?", "word_count": 11, "label": "", "sentence_structure": "Subject鈥搗erb鈥搊bject", "subjective_objective": {"answer": "Subjective", "rationale": "Because \"fear\" is a term for the patient's feelings, and it's subjective."}, "sentence_sentiment": {"answer": "Negative", "rationale": "Because \"no one can trust\" in the entry expresses negative emotions and attitudes."}, "language_style": {"answer": "Tactful and warm", "rationale": "The entry uses a question-and-answer language style, allowing interviewees to express their feelings in a soft and inviting manner and avoiding direct statements or orders, which are more personal and interactive."}, "time_dimension": {"answer": "Present", "rationale": "In this entry, \"often\" means regular, customary behaviour, \"like\" takes the present state of affairs, which means that this feeling may be occurring or occurring frequently. So, judging from the temporal dimension, the entry describes the psychological state and emotional experience of the person being tested."}, "sentence_type": "Interrogative sentence", "pos": {"nouns": {"person": 1, "Trust": 1}, "verbs": {"I feel": 1, "Yeah": 1}, "adverbs": {"Regular": 1, "- You're not": 1}, "numerals": {"3": 1}, "pronouns": {"you": 1}}}, {"content": "Do you often feel lonely?", "word_count": 7, "label": "", "sentence_structure": "Subject鈥損redicate", "subjective_objective": {"answer": "Subjective", "rationale": "Because loneliness is a subjective feeling, it looks at the emotional state and psychological experience of the patient."}, "sentence_sentiment": {"answer": "Neutral", "rationale": "Because the entry does not have any emotional color."}, "language_style": {"answer": "Tactful and warm", "rationale": ""}, "time_dimension": {"answer": "Present", "rationale": ""}, "sentence_type": "Interrogative sentence", "pos": {"nouns": {"Questionnaire Entry": 1, "Loneliness": 1}, "verbs": {"I always feel": 1, "- You're not": 1}, "adverbs": {"Regular": 1}, "numerals": {"4": 1}}}, {"content": "Do you often feel like one of your friends?", "word_count": 14, "label": "", "sentence_structure": "Subject鈥搗erb鈥搊bject", "subjective_objective": {"answer": "Subjective", "rationale": "The question is whether the patient feels that he or she is a member of a group of friends."}, "sentence_sentiment": {"answer": "Positive", "rationale": "Because it expresses an emotion of desire to belong to social groups, it is positive."}, "language_style": {"answer": "Tactful and warm", "rationale": "The basis for the judgement: the entry is more personal and interactive when it is spoken in a gentle and inquisitive manner, rather than by direct statements or orders. At the same time, \"5. Do you often feel like you are one of your friends?\" The phrase is not too strong or emotional in terms, but rather expresses concern for social relations among the interviewees in a balanced and objective way, reflecting a soft and warm language style."}, "time_dimension": {"answer": "Present", "rationale": "The entry uses the word \"normal\" to denote regular, customary behaviour, while the word \"sense\" and \"to\" to adopt the general current, which means that this feeling has existed not only in the past, but may still be present. Thus, the entry is a question of the present situation of the person being tested, which is a judgment of the present behaviour or mentality."}, "sentence_type": "Interrogative sentence", "pos": {"nouns": {"Questionnaire": 1, "Entry": 1, "Friends": 1, "One": 1}, "verbs": {"I always feel": 1, "belong to": 1, "- You're not": 1}, "adverbs": {"Regular": 1}, "numerals": {"5": 1}, "pronouns": {"you": 1}}}, {"content": "Do you often feel that you have much in common with the people around you?", "word_count": 16, "label": "", "sentence_structure": "Subject鈥搗erb鈥搊bject", "subjective_objective": {"answer": "Subjective", "rationale": "Because this question asks the patient about his personal feelings and subjective views, namely whether they often feel they have something in common with the people around them. This is not a description or observation based on objective facts, and therefore is a subjective issue."}, "sentence_sentiment": {"answer": "Neutral", "rationale": "Because the entry does not have any emotional color."}, "language_style": {"answer": "Tactful and warm", "rationale": "The entry is more personal and interactive when it is spoken in a gentle and inviting manner, rather than in direct statements or orders. At the same time, the issue uses the term \"many\" to suggest that the subject of the investigation has many things in common with others, and is therefore more positive in tone."}, "time_dimension": {"answer": "Present", "rationale": "The entry asks what the person is feeling and what he thinks is now, and the keyword is \"You often feel\" , which indicates that it is a customary act or state. Moreover, \"many things in common\" imply that it is a relatively long-term, continuous state, and it is therefore possible to judge the time dimension of the questionnaire entry."}, "sentence_type": "Interrogative sentence", "pos": {"nouns": {"Questionnaire": 1, "Entry": 1, "person": 2, "Common ground": 1}, "verbs": {"I feel": 1, "have": 1, "Regular": 1, "Many": 1}, "adverbs": {"Regular": 1, "Many": 1}, "numerals": {"6": 1}, "classifiers": {"individual": 1}, "pronouns": {"you": 1, "Around": 1}}}, {"content": "Do you often feel intimacy to anyone?", "word_count": 14, "label": "", "sentence_structure": "Subject鈥損redicate", "subjective_objective": {"answer": "Subjective", "rationale": "Because it is a subjective feeling, not an objective fact."}, "sentence_sentiment": {"answer": "Negative", "rationale": "Because the expression \"fear not intimacy\" in the entry is negative."}, "language_style": {"answer": "Tactful and warm", "rationale": "The entry is in the form of a question and question sentence, which expresses a moderate and topical concern, without direct blame or criticism of the other, and is more acrimony and affectionate."}, "time_dimension": {"answer": "Present", "rationale": ""}, "sentence_type": "Interrogative sentence", "pos": {"nouns": {"you": 1, "person": 1, "Feel": 1, "With anyone": 1}, "verbs": {"I always feel": 1, "not": 1, "Intimacy": 1}, "adverbs": {"Regular": 1}, "numerals": {"7": 1}}}, {"content": "Do you often feel that your interest and thoughts are different from those of the people around you?", "word_count": 20, "label": "", "sentence_structure": "Subject鈥搗erb鈥搊bject", "subjective_objective": {"answer": "Subjective", "rationale": "Because \"fear,\" \"interest\" and \"ideology\" are all words that relate to the patient's inner feelings and subjective knowledge, the entry on the questionnaire is therefore viewed from a subjective perspective."}, "sentence_sentiment": {"answer": "Negative", "rationale": "Because the expression \"I feel your interest and thoughts are different from those around you\" is negative."}, "language_style": {"answer": "Tactful and warm", "rationale": "The language style of the entry is euphemistic and uses the question phrase to guide the interviewee to think and express his thoughts. At the same time, the phrase \"often feels\" gives a sense of affection and concern, making the interviewee more willing to participate in answering questions."}, "time_dimension": {"answer": "Present", "rationale": "The entry \"You often feel\" means it's a regular or customary act and uses the usual current state of affairs, so it can be judged to be the time dimension of the entry \"now\"."}, "sentence_type": "Interrogative sentence", "pos": {"nouns": {"Interest": 1, "Thoughts": 1, "person": 1}, "verbs": {"I always feel": 1, "Same thing": 1, "It's different": 1}, "adverbs": {"Regular": 1}, "numerals": {"8": 1}}}, {"content": "Do you often feel like having friends and friends?", "word_count": 15, "label": "", "sentence_structure": "Subject鈥搗erb鈥搊bject", "subjective_objective": {"answer": "Subjective", "rationale": "The subject is the patient's subjective feelings."}, "sentence_sentiment": {"answer": "Positive", "rationale": "Because the attitude of \"wanting to be friends and to be friends\" is positive."}, "language_style": {"answer": "Tactful and warm", "rationale": "The language style of the entry is more euphemistic and moderate, with the subject being expressed in question, while the word \"often felt\" is introduced to be positive, making the questionnaire more personal and interactive."}, "time_dimension": {"answer": "Present", "rationale": "And the word \"often\" in \"often feels\" indicates that the behaviour is frequent, habitual, and that this feeling may persist. So, judging from the time dimension, the entry is now."}, "sentence_type": "Interrogative sentence", "pos": {"nouns": {"Questionnaire": 1, "Entry": 1, "friends": 1}, "verbs": {"Regular": 1, "I feel": 1, "Yes": 1, "Travel": 1, "Deal": 1}, "adverbs": {"Regular": 1}, "numerals": {"9": 1}, "pronouns": {"you": 1}}}, {"content": "Do you often feel close to others?", "word_count": 9, "label": "", "sentence_structure": "Subject鈥損redicate", "subjective_objective": {"answer": "Subjective", "rationale": "Because it is a subjective feeling to feel close to someone."}, "sentence_sentiment": {"answer": "Neutral", "rationale": "Because the entry does not have any emotional color."}, "language_style": {"answer": "Tactful and warm", "rationale": ""}, "time_dimension": {"answer": "Present", "rationale": "In this entry, \"often\" means more frequent behaviour, while \"like\" uses the general current, which means that this feeling may be present at present. Thus, the time dimension of this questionnaire entry can be judged as it is."}, "sentence_type": "Interrogative sentence", "pos": {"nouns": {"Questionnaire": 1, "Entry": 1, "person": 1}, "verbs": {"I feel": 1, "Close": 1}, "adverbs": {"Regular": 1}, "numerals": {"10": 1}, "classifiers": {"individual": 1}}}, {"content": "Do you often feel left alone?", "word_count": 9, "label": "", "sentence_structure": "Subject鈥損redicate", "subjective_objective": {"answer": "Subjective", "rationale": "Because \"often feeling left alone\" is the patient's subjective feeling and experience."}, "sentence_sentiment": {"answer": "Negative", "rationale": "Because being left alone usually leads to negative emotional experiences, like loneliness, helplessness, etc."}, "language_style": {"answer": "Tactful and warm", "rationale": "The entry is a conversation in a question-and-answer format and uses a more euphemistic language to express the questions, avoiding direct accusations or feelings."}, "time_dimension": {"answer": "Present", "rationale": "You often feel that the word \"always\" means \"recurring, \"likely\" is \"in a normal state of affairs,\" which means that it not only happened in the past, but may still be going on. So the entry to the questionnaire is to ask the person who was tested about what it feels now."}, "sentence_type": "Interrogative sentence", "pos": {"nouns": {"person": 1, "Cold": 1}, "verbs": {"I always feel": 1}, "adverbs": {"- You're not": 1}, "numerals": {"11": 1}}}, {"content": "Do you often feel that you have no sense in being with someone else?", "word_count": 15, "label": "", "sentence_structure": "Subject鈥搗erb鈥搊bject", "subjective_objective": {"answer": "Subjective", "rationale": "Because feeling is a patient's subjective experience."}, "sentence_sentiment": {"answer": "Negative", "rationale": "Because the expression \"distant and meaningless\" is negative."}, "language_style": {"answer": "Calm and direct", "rationale": "The language style of the entry is relatively calm and straightforward, rather than using excessive emotional colours or rhetorical techniques, it is presented in a concise question form, asking the other side more objectively and directly whether it has ever felt this way."}, "time_dimension": {"answer": "Present", "rationale": ""}, "sentence_type": "Interrogative sentence", "pos": {"nouns": {"Questionnaire": 1, "Entry": 1, "Travel": 1, "Meaning": 1}, "verbs": {"I feel": 1, "have": 1, "Nothing": 1}, "adverbs": {"Regular": 1, "Both": 1, "- You're not": 1}, "numerals": {"12": 1}}}, {"content": "13. Do you often feel that no one knows you well?", "word_count": 12, "label": "", "sentence_structure": "Subject鈥損redicate", "subjective_objective": {"answer": "Subjective", "rationale": "The question is asked about the feelings and experiences of the patient, and the mental and emotional state of the patient is examined and therefore subjectively."}, "sentence_sentiment": {"answer": "Negative", "rationale": "Because the \"Feeling no one knows you\" emotion in the entry is a negative expression of emotion."}, "language_style": {"answer": "Tactful and warm", "rationale": "The entry uses a more euphemistic and moderate approach to questions, avoiding direct accusations or negative evaluations, and enhancing the comfort and co-operation of the person under investigation. At the same time, the space and space for the person under investigation to express his or her true feelings is also left."}, "time_dimension": {"answer": "Present", "rationale": "The entry contains the verb \"common\" which indicates the current situation and does not have a clear past or future time indicator, and therefore the judgement is now."}, "sentence_type": "Interrogative sentence", "pos": {"nouns": {"person": 1, "you": 2, "Questionnaire": 1, "Entry": 1}, "verbs": {"I always feel": 1, "Roger that": 1}, "adjectives": {"very": 1}, "numerals": {"13": 1}}}, {"content": "Do you often feel isolated from others?", "word_count": 11, "label": "", "sentence_structure": "Subject鈥損redicate", "subjective_objective": {"answer": "Subjective", "rationale": "Because it is a subjective experience and feeling, it is not an objective fact."}, "sentence_sentiment": {"answer": "Negative", "rationale": "Because the \"fear separate\" attitude is negative."}, "language_style": {"answer": "Tactful and warm", "rationale": "The entry is more euphemistic, using subjective expressions like \"You often feel\" to guide the interviewee to express his feelings while avoiding a direct assessment or accusing tone. Moreover, the question mark at the end of the sentence shows the expectation and respect for the interviewee's response."}, "time_dimension": {"answer": "Present", "rationale": ""}, "sentence_type": "Interrogative sentence", "pos": {"nouns": {"you": 1, "others": 1, "Interval": 1}, "verbs": {"I always feel": 1, "Separate": 1}, "adverbs": {"Regular": 1}, "numerals": {"14": 1}}}, {"content": "Do you often feel like you can find your partner when you want?", "word_count": 16, "label": "", "sentence_structure": "Subject鈥損redicate", "subjective_objective": {"answer": "Subjective", "rationale": "The feeling that it is the patient's subjective perception and idea to find a partner or not."}, "sentence_sentiment": {"answer": "Positive", "rationale": "Because the \"Find a Partner\" act in the entry is positive."}, "language_style": {"answer": "Tactful and warm", "rationale": "The language style of the entry is euphemism. It uses the form of questioning whether the interviewee is often able to find a partner and the expression of positive language, \"Find when you want to\" , gives people a sense of encouragement and support, thus making it easier to get the interviewee ' s cooperation and real answers."}, "time_dimension": {"answer": "Present", "rationale": "The phrase \"common sense\" and \"capable\" suggests that the behaviour is recurrent and that the main words are in a state where partners can be found, so the question is asking the person who was tested how he feels."}, "sentence_type": "Interrogative sentence", "pos": {"nouns": {"Partners": 1, "Entry": 1}, "verbs": {"I feel": 1, "Yes": 2, "Find": 1}, "adjectives": {"Regular": 1}, "adverbs": {"When": 1, "Time": 1, "Just": 1, "Yes": 1}, "numerals": {"15": 1}, "classifiers": {"individual": 1}, "pronouns": {"you": 1, "When you": 1}}}, {"content": "Do you often feel that someone really knows you?", "word_count": 12, "label": "", "sentence_structure": "Subject鈥搗erb鈥搊bject", "subjective_objective": {"answer": "Subjective", "rationale": "Since the problem involves the feelings and subjective perceptions of the patients, it is a subjective examination that they often feel that someone really knows themselves."}, "sentence_sentiment": {"answer": "Neutral", "rationale": "Because the entry does not have any emotional color."}, "language_style": {"answer": "Tactful and warm", "rationale": "The language style of the entry is euphemism. First, it uses an invitation type of starting to guide respondents to think and answer questions, rather than direct orders or statements of facts; second, it uses more friendly and moderate language, such as \"You often feel\" and \"Do You really know you?\", making the questionnaire more friendly and acceptable. This language style helps to increase respondents' response rates and to obtain their true feelings and opinions more effectively."}, "time_dimension": {"answer": "Past", "rationale": ""}, "sentence_type": "Interrogative sentence", "pos": {"nouns": {"person": 1, "you": 2, "Really": 1, "Roger that": 1}, "verbs": {"Sensor": 1, "Regular": 1, "Present": 1, "have": 1}, "adjectives": {"Regular": 1, "True": 1}, "adverbs": {"Regular": 1}, "numerals": {"16": 1}, "pronouns": {"you": 2}}}, {"content": "Do you often feel shy?", "word_count": 7, "label": "", "sentence_structure": "Subject鈥搗erb鈥揷omplement", "subjective_objective": {"answer": "Subjective", "rationale": "Because being shy is a subjective feeling and emotional experience."}, "sentence_sentiment": {"answer": "Negative", "rationale": "Because \"weak\" is a negative emotional attitude."}, "language_style": {"answer": "Tactful and warm", "rationale": ""}, "time_dimension": {"answer": "Present", "rationale": ""}, "sentence_type": "Interrogative sentence", "pos": {"nouns": {"Shame": 1}, "verbs": {"I always feel": 1, "- You're not": 1}, "adverbs": {"Regular": 1, "- You're not": 1}}}, {"content": "Do you often feel like people are around you, but they don't care about you?", "word_count": 16, "label": "", "sentence_structure": "Subject鈥搗erb鈥搊bject", "subjective_objective": {"answer": "Subjective", "rationale": "Because \"often feeling\" and \"care about you\" are the patient's personal subjective feelings and experiences."}, "sentence_sentiment": {"answer": "Negative", "rationale": "Because the \"often feeling around you, but not caring about you\" sentiment in the entry is negative."}, "language_style": {"answer": "Tactful and warm", "rationale": "The entry is more personal and interactive when it is spoken in a gentle and inviting tone, rather than in direct statements or orders. At the same time, the words in the entry do not use any strong emotional vocabulary or critical language, but rather express more concern and concern for the emotional situation of the person under investigation in a more balanced and objective manner, and therefore the language style tends to be more euphemistic and warm."}, "time_dimension": {"answer": "Present", "rationale": ""}, "sentence_type": "Interrogative sentence", "pos": {"nouns": {"you": 2, "person": 1, "Care": 1}, "verbs": {"Common sense": 1, "Round": 1, "- No, it's not": 1}, "adverbs": {"But": 1, "No, it's not": 1, "- You're not": 1}, "numerals": {"18": 1}}}, {"content": "Do you often feel like someone wants to talk to you?", "word_count": 13, "label": "", "sentence_structure": "Subject鈥損redicate", "subjective_objective": {"answer": "Subjective", "rationale": "Because \"often someone feels like talking to you\" is the patient's subjective feeling and experience."}, "sentence_sentiment": {"answer": "Neutral", "rationale": "It does not express any positive or negative feelings, attitudes, behaviour, purpose or quality, but merely a feeling of the interviewee."}, "language_style": {"answer": "Tactful and warm", "rationale": "The language style of the entry is relatively euphemistic, and the question is asked to guide the interviewee to think and express his/her thoughts, without directly evaluating or ordering the interviewee."}, "time_dimension": {"answer": "Present", "rationale": "The word \"normal\" means regular, customary behavior, and \"you often feel\" happens now, so the entry describes the state or feeling."}, "sentence_type": "Interrogative sentence", "pos": {"nouns": {"person": 1, "Talk": 1}, "verbs": {"May I": 1, "Sensor": 1, "and": 1, "Talk": 1}, "adverbs": {"Regular": 1}, "numerals": {"19": 1}, "pronouns": {"you": 1}}}, {"content": "Do you often feel that someone deserves your trust?", "word_count": 12, "label": "", "sentence_structure": "Subject鈥損redicate", "subjective_objective": {"answer": "Subjective", "rationale": "Because it is a subjective feeling and experience of one who feels that there is someone to trust."}, "sentence_sentiment": {"answer": "Neutral", "rationale": "Because the entry does not have any emotional color."}, "language_style": {"answer": "Tactful and warm", "rationale": "The language style of the entry is mild and encouraging, using the expression \"common sense\" to avoid direct questioning or accusations, and using the active term \"trustworthy\" to guide respondents to share their feelings and experiences. Such language styles are more likely to make respondents feel respected and supported and to get real feedback."}, "time_dimension": {"answer": "Present", "rationale": "The \"You often feel\" in the entry indicates a constant subjective feeling that is closely related to the current situation and therefore judged as the present behavior or mentality."}, "sentence_type": "Interrogative sentence", "pos": {"nouns": {"person": 1, "It's worth it": 1, "Trust": 1}, "verbs": {"I feel": 1, "have": 1}, "adverbs": {"Regular": 1, "you": 1}, "numerals": {"20": 1}}}]}

{"questionnaire_title": "Self-assessment depression tables and depression questionnaires", "questionnaire_category": "Mental health", "item_count": 40, "emotion_shift_overall": "From negative to positive", "language_style_overall": "Tactful and warm", "items": [{"content": "I feel bored and depressed.", "word_count": 11, "label": "", "sentence_structure": "Subject鈥揷opula鈥損redicative", "subjective_objective": {"answer": "Subjective", "rationale": "Because \"silent\" and \"silent\" are the psychological feelings of the patient, which are viewed from the patient's subjective point of view."}, "sentence_sentiment": {"answer": "Negative", "rationale": "Because the entries \"silent\" and \"silent\" express a negative emotional state."}, "language_style": {"answer": "Tactful and warm", "rationale": "\"I feel bored and depressed\" is a phrase that directly describes what I feel inside, without too much rhetorical or emotional rendering. So my judgment is: calm and straightforward. The sentence is based on: 1. The expression is a straightforward expression of the mood of the speaker, and the language style is more objective. 2. Although negative sentiments are expressed, it does not use too harsh a vocabulary or tone to maintain a degree of calm and restraint. 3. It may be expressed in a concise language, without too much embellishment or emotional reverence."}, "time_dimension": {"answer": "Past", "rationale": "The entry indicates the current emotional state of the testee, \"frightful\" and \"silent\" and \"insensitive\" are all words that describe what is being felt, so the temporal dimension is a past."}, "sentence_type": "Interrogative sentence", "pos": {"nouns": {"I": 1, "It's boring": 1, "Emotion": 1, "Low": 1}, "verbs": {"I think": 1}}}, {"content": "I think it's best to have a morning.", "word_count": 11, "label": "", "sentence_structure": "Subject鈥揷opula鈥損redicative", "subjective_objective": {"answer": "Subjective", "rationale": "Because \"think\" is the patient's subjective feeling and opinion, and it's a subjective view."}, "sentence_sentiment": {"answer": "Positive", "rationale": "Because the expression \"best morning\" in the entry is positive."}, "language_style": {"answer": "Tactful and warm", "rationale": "The entry is more adept and interactive when it is spoken in a gentle and inviting manner, rather than by direct statements or orders."}, "time_dimension": {"answer": "Present", "rationale": "In conclusion, the entry for the questionnaire should read that, at present, the person tested felt it would be preferable to have a subjective view and a preference for which period of the day."}, "sentence_type": "Interrogative sentence", "pos": {"nouns": {"In the sky": 1, "Morning": 1}, "verbs": {"I think": 1, "Okay": 1}, "adverbs": {"鈾?And the 鈾?: 1, "Medium": 1}, "numerals": {"2": 1}}}, {"content": "I cried for a while or I felt like I was crying.", "word_count": 12, "label": "", "sentence_structure": "Subject鈥搗erb鈥揷omplement", "subjective_objective": {"answer": "Subjective", "rationale": "Because \"like to cry\" is a subjective or emotional experience."}, "sentence_sentiment": {"answer": "Negative", "rationale": "Because the entries \"cry out\" and \"like to cry\" express a negative sentiment."}, "language_style": {"answer": "Tactful and warm", "rationale": "The entry uses a soft, invitation-style tone, which asks the interviewee questions in an understanding and acceptance, rather than directly speaking or ordering. This expression is more friendly, friendly, and creates a good sense of interaction and trust. At the same time, the entry does not overemphasize the negative feelings of the interviewee, but expresses the emotional concerns and understanding of the interviewee in a euphemism, making it easier for the interviewee to accept and answer questions."}, "time_dimension": {"answer": "Present", "rationale": "The keywords are \"for a while,\" \"cry out\" and \"like to cry\", all of which imply the current emotional state of the person being tested, so the time dimension of the article is now."}, "sentence_type": "Affirmative declarative sentence", "pos": {"nouns": {"Form": 1, "Cry": 2, "I'm crying": 1}, "verbs": {"Cry": 1, "I think": 1}, "adverbs": {"For a while": 1}}}, {"content": "I'm not sleeping well at night.", "word_count": 7, "label": "", "sentence_structure": "Subject鈥揷opula鈥損redicative", "subjective_objective": {"answer": "Subjective", "rationale": "Because sleep problems are caused by the patient ' s own feelings and experiences, they are observed from a subjective perspective."}, "sentence_sentiment": {"answer": "Negative", "rationale": "Because the expression \"sleeping bad\" is negative."}, "language_style": {"answer": "Tactful and warm", "rationale": "The entry is presented in a more euphemistic way, in a way that is concerned, asks, not accusing, or directly speaking, and reflects the respect and concern of researchers with the testees. At the same time, the \"fructuous\" language style is more acceptable and willing to participate in the survey, which will help to increase the effectiveness of the questionnaire and the rate of recovery."}, "time_dimension": {"answer": "Present", "rationale": "According to the sentence entered, \"I'm sleeping at night\" and \"not at night\" both mean the situation, so the time dimension of the questionnaire entry is now judged."}, "sentence_type": "Negative declarative sentence", "pos": {"nouns": {"Night": 1, "Sleep": 1}, "verbs": {"I": 1, "Sleep": 1, "Okay": 1}, "numerals": {"4": 1}}}, {"content": "I ate as much as I normally did.", "word_count": 9, "label": "", "sentence_structure": "Subject鈥搗erb鈥揷omplement", "subjective_objective": {"answer": "Objective", "rationale": "Because the amount of food that is eaten is a fact that can be observed and measured, not the patient's subjective attitude or feelings, the content of this questionnaire entry is objective."}, "sentence_sentiment": {"answer": "Neutral", "rationale": "Because the entry does not have any emotional color."}, "language_style": {"answer": "Tactful and warm", "rationale": "The language style of the entry is more gentle and friendly, and there is no overly direct tone or command-style expression, but rather a more friendly and understanding way of asking about the other person鈥檚 eating habits."}, "time_dimension": {"answer": "Present", "rationale": ""}, "sentence_type": "Affirmative declarative sentence", "pos": {"nouns": {"Questionnaire": 1, "Entry": 1, "I": 2, "Normal": 1, "More": 1}, "verbs": {"Eat": 1, "Okay": 1, "Yeah": 1, "Same thing": 1}, "adverbs": {"- It's normal": 1}, "numerals": {"5锛?: 1}, "pronouns": {"I": 2}}}, {"content": "I was as happy in my close contact with the opposite sex as ever.", "word_count": 18, "label": "", "sentence_structure": "Subject鈥揷opula鈥損redicative", "subjective_objective": {"answer": "Subjective", "rationale": "Because it is the patient ' s personal feeling, it is a subjective examination."}, "sentence_sentiment": {"answer": "Neutral", "rationale": "Because the entry does not express any positive or negative feelings, it merely describes the feeling of close contact with the opposite sex."}, "language_style": {"answer": "Tactful and warm", "rationale": "The language style of the entry is euphemistic. It is more intimate and interactive when speaking in a gentle and inviting manner, asking whether the other person is as happy in intimate contact with the opposite sex as before, without a direct statement or order."}, "time_dimension": {"answer": "Present", "rationale": "The entry describes the feelings of the testee in close heterosexual contact and uses the term \"as usual\" as a comparison, so it is now judged."}, "sentence_type": "Affirmative declarative sentence", "pos": {"nouns": {"Heterosexual": 1, "Exposure": 1, "Time": 1, "Previous": 1, "Have a nice day": 1}, "verbs": {"I feel": 1, "Close": 1, "Exposure": 1}, "adjectives": {"Have a nice day": 1}, "numerals": {"6锛?: 1}, "classifiers": {"One": 2}, "pronouns": {"I": 1}}}, {"content": "I found my weight falling.", "word_count": 10, "label": "", "sentence_structure": "Subject鈥搗erb鈥揷omplement", "subjective_objective": {"answer": "Subjective", "rationale": "Because weight loss is a fact, it is subjective to feel and recognize it."}, "sentence_sentiment": {"answer": "Negative", "rationale": "Because \"weight loss\" is usually a negative situation, which may mean health problems or malnutrition."}, "language_style": {"answer": "Calm and direct", "rationale": "The entry is a simple and straightforward way of asking the interviewees whether they have experienced a loss of weight, a more objective and neutral language style, and no excessive rhetoric or emotional colour."}, "time_dimension": {"answer": "Present", "rationale": "The entry describes the current state of the person being tested, using the two terms \"discovery\" and \"deduction\" to indicate the key now, and it can therefore be judged to be a question of the behaviour or state of the moment."}, "sentence_type": "Affirmative declarative sentence", "pos": {"nouns": {"Weight": 1, "Down": 1}, "verbs": {"I realized": 1}}}, {"content": "I have a problem with constipation.", "word_count": 7, "label": "", "sentence_structure": "Subject鈥搗erb鈥揷omplement", "subjective_objective": {"answer": "Subjective", "rationale": "Because constipation is the patient ' s personal experience and experience, it is subjective."}, "sentence_sentiment": {"answer": "Negative", "rationale": "Because the expression \"conciliation pains\" in the entry is negative."}, "language_style": {"answer": "Calm and direct", "rationale": "The entry directly expresses the subject of asking the other party whether it has a problem of constipation, without using excessive rhetoric or emotional rendering, and with a more balanced and objective tone."}, "time_dimension": {"answer": "Past", "rationale": ""}, "sentence_type": "Negative declarative sentence", "pos": {"nouns": {"Decency": 1, "Trouble": 1}, "verbs": {"have": 1}}}, {"content": "My heart beats faster than usual.", "word_count": 7, "label": "", "sentence_structure": "Subject鈥揷opula鈥損redicative", "subjective_objective": {"answer": "Subjective", "rationale": "Because the heart rate is slow is the patient's own feeling, and therefore the subjective angle."}, "sentence_sentiment": {"answer": "Negative", "rationale": "Because \"heart beats faster than usual\" are often considered to be a manifestation of physical discomfort or health problems, with some negative emotional colour."}, "language_style": {"answer": "Calm and direct", "rationale": "The entry is directly available to the respondents on the heartbeat, and is not used in euphemism or rhetoric, and is expressed in a more objective and straightforward manner."}, "time_dimension": {"answer": "Present", "rationale": ""}, "sentence_type": "Affirmative declarative sentence", "pos": {"nouns": {"Heart rate": 1, "Normal": 1}, "verbs": {"- Yeah": 1, "Come on": 1}, "numerals": {"9": 1}}}, {"content": "I'm tired for no reason.", "word_count": 10, "label": "", "sentence_structure": "Subject鈥揷opula鈥損redicative", "subjective_objective": {"answer": "Subjective", "rationale": "Because fatigue is a subjective experience, the causes and extent of fatigue vary from one person to another."}, "sentence_sentiment": {"answer": "Negative", "rationale": "Because the word \"tired\" is an expression of a negative emotional state."}, "language_style": {"answer": "Tactful and warm", "rationale": "The entry is more adept and interactive when it is spoken in a gentle and inviting manner, rather than by direct statements or orders."}, "time_dimension": {"answer": "Present", "rationale": "The entry describes the current physical feelings of the testee, using the general current state of the moment ( \"the feeling\" ), so the temporal dimension is now."}, "sentence_type": "Negative declarative sentence", "pos": {"nouns": {"Weary": 1}, "verbs": {"I feel": 1, "For no reason": 1}, "adverbs": {"For no reason": 1}}}, {"content": "I'm as clear as I normally am.", "word_count": 11, "label": "", "sentence_structure": "Subject鈥揷opula鈥損redicative", "subjective_objective": {"answer": "Subjective", "rationale": "Because the sobering of the mind is the subjective perception and cognitive assessment of the individual."}, "sentence_sentiment": {"answer": "Neutral", "rationale": "Because the entry does not have any emotional color."}, "language_style": {"answer": "Calm and direct", "rationale": "The entry is a direct statement of the person ' s state of mind at a given point in time, without too much rhetoric or emotional colour, and with a more calm and objective language style."}, "time_dimension": {"answer": "Present", "rationale": "\"My mind is as clear as usual\" means that the situation is the same as it was or will be, and therefore the judgment is now."}, "sentence_type": "Affirmative declarative sentence", "pos": {"nouns": {"Brain": 1, "Normal": 1}, "verbs": {"Same thing": 1, "Clear": 1}, "numerals": {"11锛?: 1}}}, {"content": "I don't think it's hard to do things all the time.", "word_count": 14, "label": "", "sentence_structure": "Subject鈥揷opula鈥損redicative", "subjective_objective": {"answer": "Subjective", "rationale": "Because \"the feeling\" is about the patient's subjective feelings and perceptions."}, "sentence_sentiment": {"answer": "Negative", "rationale": "Because the entry expresses a lack of challenge and growth attitude, which may be a lack of motivation or an overly easy expression, it is negative."}, "language_style": {"answer": "Calm and direct", "rationale": "The entry directly expresses the subject of questioning the other party about the difficulty of doing things, without excessive rhetorical or emotional rendering, and is more objective in tone."}, "time_dimension": {"answer": "Present", "rationale": ""}, "sentence_type": "Affirmative declarative sentence", "pos": {"nouns": {"Things": 1, "Difficulties": 1}, "verbs": {"I think": 1, "Do it": 1, "have": 1}, "adjectives": {"Regular": 1}, "adverbs": {"The long run": 1}, "numerals": {"12": 1}, "classifiers": {"individual": 1}, "pronouns": {"I": 1}}}, {"content": "I feel uneasy and uneasy.", "word_count": 11, "label": "", "sentence_structure": "Subject鈥揷opula鈥損redicative", "subjective_objective": {"answer": "Subjective", "rationale": "Because \"frightful and uneasy\" is the experience of feelings and emotions inside the patient, which is a subjective examination."}, "sentence_sentiment": {"answer": "Negative", "rationale": "Because the mood of \"unease\" is negative and the expression \"unsettling\" implies a negative mood."}, "language_style": {"answer": "Tactful and warm", "rationale": "Warm and warm."}, "time_dimension": {"answer": "Present", "rationale": "The \"I feel\" in the questionnaire entry is a reference to subjective feelings and experiences, which are the present state of affairs, and \"uneasy and unsettled\" states of which the current feeling and experience is also the present. So, the entry describes the current state of the subject, judging by \"now\"."}, "sentence_type": "Affirmative declarative sentence", "pos": {"nouns": {"Unsettled": 1, "Calm down": 1}, "verbs": {"I think": 1, "I'm not coming down": 1}, "adverbs": {"And": 1, "I": 1}, "numerals": {"13": 1}}}, {"content": "I have hope for the future.", "word_count": 8, "label": "", "sentence_structure": "Subject鈥搗erb鈥揷omplement", "subjective_objective": {"answer": "Subjective", "rationale": "Because hope is a subjective attitude of one person."}, "sentence_sentiment": {"answer": "Positive", "rationale": "Because the \"hope for the future\" approach in the entry is positive."}, "language_style": {"answer": "Tactful and warm", "rationale": ""}, "time_dimension": {"answer": "Future", "rationale": "The entry contains visions and expectations for the future, with the key words \"the future\" and \"the hope\" pointing to situations that have not yet occurred and that may arise in the future."}, "sentence_type": "Affirmative declarative sentence", "pos": {"nouns": {"Future": 1, "Hope": 1}, "verbs": {"Have": 1}}}, {"content": "I'm more angry than usual.", "word_count": 10, "label": "", "sentence_structure": "Subject鈥揷opula鈥損redicative", "subjective_objective": {"answer": "Subjective", "rationale": "Because \"freaky\" is the patient's own feelings and experiences, and it's a subjective examination."}, "sentence_sentiment": {"answer": "Negative", "rationale": "Because \"fun\" in the entry is a negative behavior or emotional state."}, "language_style": {"answer": "Tactful and warm", "rationale": ""}, "time_dimension": {"answer": "Present", "rationale": "\"Before usual\" implies a comparison with past or future, but in this sentence it is used to describe the current state, so the temporal dimension of the article is now."}, "sentence_type": "Affirmative declarative sentence", "pos": {"nouns": {"Questionnaire Entry": 1, "I": 1, "Normal": 1, "Angry": 1, "Excited": 1}, "verbs": {"- Yeah": 1, "Easy": 1, "Angry": 1, "Excited": 1}, "adjectives": {"Normal": 1, "Easy": 1}, "numerals": {"15": 1}}}, {"content": "I think it's easy to make decisions.", "word_count": 11, "label": "", "sentence_structure": "Subject鈥揷opula鈥損redicative", "subjective_objective": {"answer": "Subjective", "rationale": "Because it's the patient's subjective attitude."}, "sentence_sentiment": {"answer": "Positive", "rationale": "Because it expresses the attitude of respondents that they find it easy to make decisions, it is an expression of certainty and confidence."}, "language_style": {"answer": "Calm and direct", "rationale": "The entry is straightforward, concise, not too much adoration and emotional, and is more objective and neutral. It asks the interviewee whether it is easy to make a decision in a more calm way, allowing the interviewee to quickly understand the question and give a clear answer."}, "time_dimension": {"answer": "Present", "rationale": "The entry describes the current feelings and perceptions of the testees, and the keyword \"felt\" and \"easy\" are all descriptions of the current state and can therefore be judged as now."}, "sentence_type": "Affirmative declarative sentence", "pos": {"nouns": {"Decision": 1}, "verbs": {"I think": 1, "Do it": 1, "be": 1, "Easy": 1}, "adverbs": {"very": 1}, "pronouns": {"I": 1}}}, {"content": "I feel like I'm a useful person. Someone needs me.", "word_count": 16, "label": "", "sentence_structure": "Subject鈥揷opula鈥損redicative", "subjective_objective": {"answer": "Subjective", "rationale": "Because \"think\" is the patient's subjective feeling and attitude, reflecting his self-perception and value orientation."}, "sentence_sentiment": {"answer": "Positive", "rationale": "Because the expression in the entry, \"I feel useful, someone needs me,\" is positive and expresses confidence and acceptance."}, "language_style": {"answer": "Tactful and warm", "rationale": "\"Slow and warm.\""}, "time_dimension": {"answer": "Present", "rationale": "The entry describes the current feelings and self-perception of the testee and therefore the judgement is now."}, "sentence_type": "Affirmative declarative sentence", "pos": {"nouns": {"person": 2, "Questionnaire": 1, "Entry": 1}, "verbs": {"I think": 1, "be": 1, "Yes": 1}, "adjectives": {"It works": 1}, "numerals": {"17": 1}, "pronouns": {"I": 1}}}, {"content": "I've had a really interesting life.", "word_count": 10, "label": "", "sentence_structure": "Subject鈥揷opula鈥損redicative", "subjective_objective": {"answer": "Subjective", "rationale": "Because \"interest\" is a subjective feeling and an evaluation, not an objective fact or description."}, "sentence_sentiment": {"answer": "Positive", "rationale": "Because the entry \"Life's fun\" expresses a positive emotional attitude."}, "language_style": {"answer": "Tactful and warm", "rationale": "The language style of the entry is euphemismal and warm, and it uses a positive and positive expression that encourages the other to share their own views and feelings about life. At the same time, it gives people a sense of kindness and kindness, and makes it easier to attract the other side to the investigation and provide real answers."}, "time_dimension": {"answer": "Present", "rationale": "The entry is to ask the testee what it feels like to be in the time dimension. The keyword \"my life\" and \"very interesting\" suggests that it is a descriptive statement, not a past or future event. So, \"now\" is the most accurate answer."}, "sentence_type": "Affirmative declarative sentence", "pos": {"verbs": {"Pass": 1, "Okay": 1, "Interesting": 1}, "adjectives": {"very": 1}, "numerals": {"18": 1}}}, {"content": "I think if I die, someone else will live better.", "word_count": 16, "label": "", "sentence_structure": "Subject鈥搗erb鈥搊bject", "subjective_objective": {"answer": "Subjective", "rationale": "Because \"think\" expresses patients' subjective thoughts and attitudes, it is viewed from the perspective of patients."}, "sentence_sentiment": {"answer": "Negative", "rationale": "Because \"others will live better\" in the entry is a negative perception of others, who are likely to benefit from their own death. Such negative emotions and attitudes can have a negative impact on an individual's mental health."}, "language_style": {"answer": "Calm and direct", "rationale": "The language style of the entry is a calm and straightforward one. It expresses a view or belief in a concise and clear way, without too many rhetorical or emotional expressions. At the same time, it is more balanced and objective. It is more appropriate for data collection and analysis to obtain a more accurate view of the views and attitudes of the interviewees."}, "time_dimension": {"answer": "Future", "rationale": "The entry contains visions and expectations for the future, and the assumption that \"if I die\" indicates that this scenario has not yet been realized, but the tester believes that it may occur in the future. The temporal dimension therefore determines the future."}, "sentence_type": "Affirmative declarative sentence", "pos": {"nouns": {"person": 2, "Life": 1}, "verbs": {"I think": 1, "Die": 1, "Yes": 2, "Live": 1}, "adverbs": {"If": 1, "Some": 1}, "numerals": {"19": 1}, "pronouns": {"I": 1, "others": 1}}}, {"content": "I'm still interested in things that are usually of interest.", "word_count": 15, "label": "", "sentence_structure": "Subject鈥揷opula鈥損redicative", "subjective_objective": {"answer": "Objective", "rationale": "Because the descriptions of \"intributive things\" and \"still interested\" are observations and records of the patient鈥檚 actual situation, and are objective facts. While these situations may be influenced by patient subjective perceptions, in this case researchers are more concerned with the patient鈥檚 actual interest than their thoughts or feelings, and therefore the issue is considered objective."}, "sentence_sentiment": {"answer": "Neutral", "rationale": "Because the entry does not have any emotional color."}, "language_style": {"answer": "Tactful and warm", "rationale": ""}, "time_dimension": {"answer": "Present", "rationale": "The entry \"Attractive Matters\" indicates that it is a question of describing the current state of the subject, and \"I remain interested\" indicates that it is a description of current behaviour or attitude, and therefore the entry should be classified as \"now\"."}, "sentence_type": "Affirmative declarative sentence", "pos": {"nouns": {"Yes": 1, "Interest": 1, "Same thing": 1}, "verbs": {"Normal": 1, "Interested": 1, "Still": 1, "Same thing": 1, "Do it": 1}, "adjectives": {"I like it": 1, "Original": 1}, "adverbs": {"Still": 1}, "classifiers": {"individual": 1}, "pronouns": {"I": 1}}}, {"content": "Are you depressed?", "word_count": 10, "label": "", "sentence_structure": "Subject鈥損redicate", "subjective_objective": {"answer": "Subjective", "rationale": "Because this is the subjective sense and perception of the patient of his or her emotional state, it is subjective."}, "sentence_sentiment": {"answer": "Negative", "rationale": "Because the entry \"Feeling depressed, depressed\" expresses a negative emotional state."}, "language_style": {"answer": "Calm and direct", "rationale": "The language style of the entry is relatively simple, not over-modified or emotionally rendered, and the person concerned is asked whether he or she is in emotional distress and speaking relatively directly."}, "time_dimension": {"answer": "Present", "rationale": "The entry asks about the current emotional state of the person being tested, and therefore the temporal dimension is now judged."}, "sentence_type": "Interrogative sentence", "pos": {"nouns": {"Emotion": 1, "Frustration": 1, "I'm depressed": 1}, "verbs": {"I feel": 1, "Depression": 1}}}, {"content": "Are you going to cry or want to cry?", "word_count": 7, "label": "", "sentence_structure": "Subject鈥搗erb鈥搊bject", "subjective_objective": {"answer": "Subjective", "rationale": "Because \"to cry or want to cry\" reflects the emotional state of the patient, a subjective feeling and emotional reaction."}, "sentence_sentiment": {"answer": "Negative", "rationale": "Because the act of \"to cry or to cry\" is negative, indicating a low mood or a negative feeling."}, "language_style": {"answer": "Calm and direct", "rationale": "The entry is in plain and simple language and directly asks the respondents whether they have a desire or emotion to cry, and is more direct and objective."}, "time_dimension": {"answer": "Present", "rationale": "The entry is for the current emotional state of the subject, and the keywords \"will\" and \"cry or want\" both indicate the movement under way, and the time dimension is now judged."}, "sentence_type": "Interrogative sentence", "pos": {"nouns": {"Questionnaire": 1, "Entry": 1, "you": 1}, "verbs": {"Yes": 2, "Cry": 1}, "numerals": {"2": 1}}}, {"content": "3. Do you feel the best in the morning?", "word_count": 10, "label": "", "sentence_structure": "Subject鈥搗erb鈥搊bject", "subjective_objective": {"answer": "Subjective", "rationale": "Because the word \"fear\" indicates that it's a personal feeling or subjective experience of the patient."}, "sentence_sentiment": {"answer": "Positive", "rationale": "Because \"I feel the best in the morning\" expresses a positive emotional attitude."}, "language_style": {"answer": "Tactful and warm", "rationale": "The language style of the entry is euphemistic, because it asks the interviewee whether he feels best in the morning in a gentle and inviting manner. This expression is more personal and interactive than a direct statement or order, and helps to create a good atmosphere of communication and to get a more realistic answer."}, "time_dimension": {"answer": "Present", "rationale": "The keyword \"Feeling the best mood in the morning\" describes the subjective feeling and state of the subject, so the temporal dimension is now judged."}, "sentence_type": "Interrogative sentence", "pos": {"nouns": {"Morning": 1, "The mood": 1}, "verbs": {"I feel": 1, "I'd better": 1}, "numerals": {"3": 1}}}, {"content": "4. Is it not good for you to sleep at night? Do you wake up early?", "word_count": 13, "label": "", "sentence_structure": "Subject鈥搗erb鈥搊bject", "subjective_objective": {"answer": "Subjective", "rationale": "This questionnaire entry is viewed from the patient's subjective perspective. Because \"no sleep\" and \"frequent early awakening\" are the conditions that patients feel themselves and need to answer through their own observations and experiences."}, "sentence_sentiment": {"answer": "Negative", "rationale": "Because \"sleep bad\" and \"always awake\" are terms that describe a negative state, with negative emotional colours."}, "language_style": {"answer": "Calm and direct", "rationale": "The entry is in a concise language and directly asks the interviewees about the quality of their night sleep and whether they often wake up early, without excessive adoration or emotional colour, and the language style is more incriminating towards calm and direct."}, "time_dimension": {"answer": "Present", "rationale": "The entry asks about the current situation of the subjects, i.e. whether they sleep poorly or often wake up early at night."}, "sentence_type": "Interrogative sentence", "pos": {"nouns": {"Night": 1, "Sleep": 1, "Wake up": 1}, "verbs": {"Not good": 1, "Regular": 1, "- You're not": 2}, "numerals": {"4": 1}}}, {"content": "Do you eat as much as you normally eat? How is your appetite?", "word_count": 14, "label": "", "sentence_structure": "Subject鈥搗erb鈥搊bject", "subjective_objective": {"answer": "Objective", "rationale": "The problem is considered from an objective perspective, as it is asking about the patient ' s physical condition and behaviour habits, not the patient ' s subjective feelings or attitudes."}, "sentence_sentiment": {"answer": "Neutral", "rationale": "As the entry is concerned with the individual ' s diet, there is no apparent positive or negative tendency."}, "language_style": {"answer": "Tactful and warm", "rationale": "The language style of the entry is more euphemistic and gentle. It guides the interviewee to answer in a quiz, not directly ordering or forcing him to answer. This expression is more intimate and interactive. It also attempts to make the interviewee comfortable and not offended or pressured."}, "time_dimension": {"answer": "Present", "rationale": "The entry asks about the current status of the person being tested, and about the behaviour of \"eating\" and \"eating.\" While this may cover a certain time frame (i.e., asking about the recent situation), the most appropriate answer is \"now\" because the point of reference for past or future time is not clearly identified."}, "sentence_type": "Interrogative sentence", "pos": {"nouns": {"Rice": 1, "Normal": 1, "A appetite": 1}, "verbs": {"Eat": 1, "Elephant": 1}, "adverbs": {"Same thing": 1}, "pronouns": {"you": 1}}}, {"content": "6. Did you feel less weight?", "word_count": 9, "label": "", "sentence_structure": "Subject鈥損redicate", "subjective_objective": {"answer": "Subjective", "rationale": "Because weight loss is a physical feeling and belongs to the patient ' s subjective experience."}, "sentence_sentiment": {"answer": "Neutral", "rationale": "The entry was merely an inquiry into whether the interviewees felt that they were losing weight and did not contain any emotional colour."}, "language_style": {"answer": "Calm and direct", "rationale": ""}, "time_dimension": {"answer": "Present", "rationale": "The entry is for the present feeling or state of the testee, and the time dimension is now judged."}, "sentence_type": "Interrogative sentence", "pos": {"nouns": {"Weight": 1, "Mitigation": 1}, "verbs": {"I feel": 1, "It's a little light": 1}, "adverbs": {"- You're not": 1}}}, {"content": "Are you sexually functioning? Are you happy to be attractive to the opposite sex and to be with him/her and to speak?", "word_count": 31, "label": "", "sentence_structure": "Subject鈥搗erb鈥搊bject", "subjective_objective": {"answer": "Subjective", "rationale": "Because this question is about patients鈥?perceptions and feelings about their sexual functions, including their self-evaluation and desire. This involves subjective experience and attitudes of individuals, rather than objective facts or behavioural outcomes."}, "sentence_sentiment": {"answer": "Neutral", "rationale": "The entry does not address specific emotions, attitudes or behaviour, but merely asks the interviewees about some basic factual circumstances and is therefore neutral."}, "language_style": {"answer": "Calm and direct", "rationale": ""}, "time_dimension": {"answer": "Present", "rationale": "The words \"sexual function\", \"care\" and \"willing\" point to the state of the moment, so the entry is to ask the testee what the situation is, and the time dimension is now."}, "sentence_type": "Interrogative sentence", "pos": {"nouns": {"Questionnaire": 1, "Sex function": 1, "Heterosexual": 2, "Attractive": 1, "he": 1, "she": 1, "Together": 1, "Talk": 1}, "verbs": {"Attention": 1, "Okay": 1, "and": 1, "Together": 1, "Say it": 1}, "numerals": {"7": 1, "2": 1}, "pronouns": {"you": 1}}}, {"content": "- Are you worried about constipation?", "word_count": 7, "label": "", "sentence_structure": "Subject鈥損redicate", "subjective_objective": {"answer": "Subjective", "rationale": "The test is based on: constipation is a physical condition or a symptom, a subjective experience and feeling of the patient, and therefore the problem is subjective. Although it may be caused by objective physical causes, the patient鈥檚 perception and reaction to it is subjective."}, "sentence_sentiment": {"answer": "Negative", "rationale": "Because constipation is a physical discomfort, it is often seen as a negative experience or symptom."}, "language_style": {"answer": "Tactful and warm", "rationale": "The entry is more personal and interactive in its expression of a gentle and caring tone rather than a direct statement or order. At the same time, it is used in a question-and-answer format to make respondents more willing to share their feelings, thus enhancing the effectiveness and reliability of the questionnaire."}, "time_dimension": {"answer": "Past", "rationale": ""}, "sentence_type": "Interrogative sentence", "pos": {"nouns": {"Decency": 1}, "verbs": {"Trouble": 1, "Yes": 1}}}, {"content": "Is your heart beating faster than usual?", "word_count": 9, "label": "", "sentence_structure": "Subject鈥搗erb鈥搊bject", "subjective_objective": {"answer": "Objective", "rationale": "The test is based on: heart rate is a physical reaction, which is an objective fact. Although patients may feel their heart rate is accelerating, this is itself caused by objective physical conditions."}, "sentence_sentiment": {"answer": "Negative", "rationale": "Because heart rate acceleration is usually a physical reaction to stress or stress, it is a negative emotional or state."}, "language_style": {"answer": "Calm and direct", "rationale": "The entry is based on a simple and clear question about whether the person interviewed has a faster heart rate than usual, does not use any rhetorical or emotionally colourful language to promote the atmosphere or to guide emotions, and is expressed in a very direct manner, consistent with the calm and direct language style."}, "time_dimension": {"answer": "Present", "rationale": "The entry asks the testee's current heart rate, which is not about time, so it's \"now\"."}, "sentence_type": "Interrogative sentence", "pos": {"nouns": {"Heart rate": 1, "Normal": 1}, "verbs": {"- Yeah": 1, "Come on": 1}}}, {"content": "Are you tired of being out of your mind?", "word_count": 8, "label": "", "sentence_structure": "Subject鈥搗erb鈥搊bject", "subjective_objective": {"answer": "Subjective", "rationale": "Because fatigue is a subjective experience, and although it may be related to certain health conditions, the answer depends more on the patient鈥檚 feelings and understanding. For example, some patients may be exhausted by stress, anxiety, or depression, which is subjective."}, "sentence_sentiment": {"answer": "Negative", "rationale": "Because the emotional reaction of \"undue fatigue\" in the entry is negative."}, "language_style": {"answer": "Tactful and warm", "rationale": "Warm and warm."}, "time_dimension": {"answer": "Present", "rationale": ""}, "sentence_type": "Interrogative sentence", "pos": {"nouns": {"Tired": 1, "For no reason": 1}, "verbs": {"I feel": 1}, "adverbs": {"For no reason": 1}, "numerals": {"10": 1}}}, {"content": "Are you restless and difficult to keep calm?", "word_count": 12, "label": "", "sentence_structure": "Subject鈥搗erb鈥揷omplement", "subjective_objective": {"answer": "Subjective", "rationale": "Because \"sitting\" and \"unusual to keep calm\" are subjective feelings of patients."}, "sentence_sentiment": {"answer": "Negative", "rationale": "Because the entries \"sitting\" and \"hard to keep quiet\" have some negative feelings."}, "language_style": {"answer": "Tactful and warm", "rationale": ""}, "time_dimension": {"answer": "Present", "rationale": "The entry asks about the current behaviour of the subject, the keywords \"sitting\" and \"hard to keep calm\" indicate the feeling that is now going through, and therefore the judgement is now."}, "sentence_type": "Interrogative sentence", "pos": {"nouns": {"you": 1, "Sit tight": 1, "Calm down": 1}, "verbs": {"Hold on": 1, "Sit tight": 1}, "adverbs": {"Hard": 1}, "numerals": {"11": 1}}}, {"content": "12. Are you doing things slower than usual?", "word_count": 9, "label": "", "sentence_structure": "Subject鈥損redicate", "subjective_objective": {"answer": "Objective", "rationale": "Because the speed of doing things is usually an objective fact, independent of the subjective sense of the individual. The problem is therefore to examine the behaviour of patients in a given situation, not their subjective feelings or attitudes."}, "sentence_sentiment": {"answer": "Negative", "rationale": "Because \"doing things slower than usual\" is often considered a negative description, which means inefficiency or slowness."}, "language_style": {"answer": "Tactful and warm", "rationale": ""}, "time_dimension": {"answer": "Present", "rationale": "The keywords are \"lower than usual\" and \"now\" and they all point to the current state of conduct or state."}, "sentence_type": "Interrogative sentence", "pos": {"nouns": {"Things": 1, "Normal": 1}, "verbs": {"Do it": 1, "Slow": 1}, "adverbs": {"- Yeah": 1, "Normal": 1}, "numerals": {"12": 1}}}, {"content": "Is your mind as clear as usual?", "word_count": 12, "label": "", "sentence_structure": "Subject鈥損redicate", "subjective_objective": {"answer": "Objective", "rationale": "Because it is an objective fact that a patient ' s judgment is not required."}, "sentence_sentiment": {"answer": "Positive", "rationale": "Because the adjective \"clear mind\" in the entry is positive."}, "language_style": {"answer": "Calm and direct", "rationale": "The language style of the entry is calm and straightforward. It is a short, clear-cut query, without any qualifier or accommodative, which is more objective and neutral, and is intended to obtain an accurate answer from the person under investigation."}, "time_dimension": {"answer": "Present", "rationale": "The entry asks the testee's current state, \"Does your mind seem as clear as usual?\", does not address the time dimension of the past or the future, and therefore judges now."}, "sentence_type": "Interrogative sentence", "pos": {"nouns": {"Brain": 1, "As usual": 1}, "verbs": {"Elephant": 1, "Clear": 1}, "adverbs": {"Same thing": 1}, "numerals": {"13": 1}}}, {"content": "Do you feel like life is empty?", "word_count": 9, "label": "", "sentence_structure": "Subject鈥損redicate", "subjective_objective": {"answer": "Subjective", "rationale": "Because feeling the void of life is the patient's subjective feeling and thoughts."}, "sentence_sentiment": {"answer": "Negative", "rationale": "Because the emotional expression \"frightful life\" is negative."}, "language_style": {"answer": "Tactful and warm", "rationale": "The entry uses a soft question, using the \"Are you\" formula, to ask each other in a more moderate way about their feelings, and to avoid directly pointing out or highlighting the emptiness of their lives. At the same time, the question \"Establishing?\" is also more respectful of each other's emotional experiences and personal privacy, making them more comfortable and natural in their answers."}, "time_dimension": {"answer": "Present", "rationale": ""}, "sentence_type": "Interrogative sentence", "pos": {"nouns": {"Life": 1, "Empty": 1}, "verbs": {"Sensor": 1, "Present": 1}, "adjectives": {"Empty": 1}}}, {"content": "Do you have any hope for the future?", "word_count": 10, "label": "", "sentence_structure": "Subject鈥損redicate", "subjective_objective": {"answer": "Subjective", "rationale": "Because \"there's hope\" is the patient's subjective feeling and expectation for the future."}, "sentence_sentiment": {"answer": "Positive", "rationale": "Because \"gets hope\" is positive."}, "language_style": {"answer": "Tactful and warm", "rationale": "The entry is more personal and interactive when it is presented in a gentle and inviting tone, asking the other side about the future, rather than directly speaking or ordering. At the same time, the term \"promising\" is not too emotional, making the tone of the problem more neutral and objective and meets the linguistic requirements of the psychological questionnaire."}, "time_dimension": {"answer": "Future", "rationale": ""}, "sentence_type": "Interrogative sentence", "pos": {"nouns": {"The future": 1, "Hope": 1}, "verbs": {"I feel": 1}, "numerals": {"15": 1}}}, {"content": "Do you think it's easy to decide what's going on?", "word_count": 12, "label": "", "sentence_structure": "Subject鈥搗erb鈥搊bject", "subjective_objective": {"answer": "Subjective", "rationale": "This is a subject of subjectivity, because it is a question of how people think and feel about things. It is a subjective experience, and the experience of patients who feel that doing something depends on their personal experience and feelings. So the answer is \"subjective.\""}, "sentence_sentiment": {"answer": "Neutral", "rationale": "Since the entry does not address positive or negative behaviour, attitudes, emotions, etc., it is merely an inquiry into the subjective perception of the respondent in taking decisions."}, "language_style": {"answer": "Tactful and warm", "rationale": "The entry uses a gentle, invitee tone to guide respondents to answer questions, is more friendly and close, avoids the use of orders or direct statements, and makes it easier for respondents to answer. The language style of this purpose is therefore euphemism."}, "time_dimension": {"answer": "Present", "rationale": "The keywords \"felt\" and \"easy\" both indicate that the entry is subjectively felt and assessed in the current state, and can therefore be judged to be current."}, "sentence_type": "Interrogative sentence", "pos": {"nouns": {"Decision": 1, "Yes": 1}, "verbs": {"I think": 1, "Easy": 1}, "adverbs": {"very": 1}, "numerals": {"16": 1}, "classifiers": {"individual": 1}}}, {"content": "Are you more angry than usual?", "word_count": 10, "label": "", "sentence_structure": "Subject鈥搗erb鈥搊bject", "subjective_objective": {"answer": "Subjective", "rationale": "Because \"facilitation\" is a subjective feeling and emotional experience, depending on the patient's own internal state and emotional response. Although this sentiment may be influenced by external stimulation, it is still subjectively assessed."}, "sentence_sentiment": {"answer": "Negative", "rationale": "Because the \"facilitation\" in the entry is negative."}, "language_style": {"answer": "Calm and direct", "rationale": "The entry directly asks whether the interviewees are in some cases more angry than usual, without any acrimony or emotional colour, and therefore the language style is straightforward and calm."}, "time_dimension": {"answer": "Present", "rationale": "The entry asks about the person's current behaviour or state, and the keyword \"more irritating than usual\" indicates that this is an ongoing state and therefore judgement is now."}, "sentence_type": "Interrogative sentence", "pos": {"nouns": {"17": 1, "you": 1, "Normal": 1, "Exasperation": 1}, "verbs": {"- Yeah": 1, "Easy": 1, "Exasperation": 1}, "adjectives": {"Normal": 1, "Easy": 1}, "numerals": {"17": 1, "1": 2}, "pronouns": {"you": 1, "Here": 1}}}, {"content": "Do you still love what you like?", "word_count": 15, "label": "", "sentence_structure": "Subject鈥搗erb鈥搊bject", "subjective_objective": {"answer": "Subjective", "rationale": "Because it's the patient's subjective attitude to like or not."}, "sentence_sentiment": {"answer": "Positive", "rationale": "Because \"love\" is a positive word."}, "language_style": {"answer": "Tactful and warm", "rationale": "The entry follows the inquiry approach and emphasizes the hobby of the other side in the word \"remain\" 鈥?a gesture that is both polite and friendly. At the same time, the word \"still\" conveys a desire to receive a positive answer. So the language style of the purpose is euphemism."}, "time_dimension": {"answer": "Present", "rationale": "The entry asks about the current situation of the person being tested, i.e. whether he/she still likes things that are normal, and therefore the time dimension is now judged."}, "sentence_type": "Interrogative sentence", "pos": {"nouns": {"Things": 1, "Yeah": 1}, "verbs": {"Still": 1, "Love": 1}, "adverbs": {"From": 1, "Ping": 1, "Normal": 1}, "numerals": {"18": 1}, "pronouns": {"you": 1}}}, {"content": "19. Do you feel useful and indispensable?", "word_count": 17, "label": "", "sentence_structure": "Subject鈥搗erb鈥搊bject", "subjective_objective": {"answer": "Subjective", "rationale": "Because the question is about the patient ' s self-awareness and feelings, it is subjective."}, "sentence_sentiment": {"answer": "Positive", "rationale": "Because the expression \"useful and indispensable person\" in the entry is positive and positive."}, "language_style": {"answer": "Tactful and warm", "rationale": "The entry is more adept and interactive when it is spoken in a gentle and inviting manner, rather than by direct statements or orders."}, "time_dimension": {"answer": "Present", "rationale": "The entry is for the testee's current self-awareness, and the keyword is \"fear\" and \"now\", so the judgment is now."}, "sentence_type": "Interrogative sentence", "pos": {"nouns": {"Questionnaire": 1, "Entry": 1, "person": 1}, "verbs": {"I feel": 1, "be": 2, "It works": 1, "and": 1, "No way": 1, "Missing": 1}, "adjectives": {"self": 1, "have": 1, "None": 1}, "numerals": {"19": 1}, "classifiers": {"Grandpa": 1}, "pronouns": {"you": 1}}}, {"content": "Have you ever thought about suicide?", "word_count": 8, "label": "", "sentence_structure": "Subject鈥搗erb鈥搊bject", "subjective_objective": {"answer": "Subjective", "rationale": "Because \"want\" is a subjective feeling and an idea."}, "sentence_sentiment": {"answer": "Negative", "rationale": "Because the act of \"suicide\" in the entry is negative and may have adverse emotional and psychological effects, it requires careful handling and questioning."}, "language_style": {"answer": "Calm and direct", "rationale": "The entry asks the other person in a direct and clear language whether he or she has committed suicide, is calm and objective, is not overly pedantic or emotional and is more suitable for serious mental health investigations."}, "time_dimension": {"answer": "Past", "rationale": "The keyword \"had thought about\" indicates that this idea or event occurred at some point in the past, and the temporal dimension of the article is therefore the past."}, "sentence_type": "Interrogative sentence", "pos": {"nouns": {"Suicide": 1}, "verbs": {"Yes": 1, "Pass": 1}, "numerals": {"20": 1}}}]}

{"questionnaire_title": "UCLA Lonely Count (UCLC-LS)", "questionnaire_category": "Mental health", "item_count": 20, "emotion_shift_overall": "From positive to negative", "language_style_overall": "Tactful and warm", "items": [{"content": "Do you often feel in harmony with the people around you?", "word_count": 13, "label": "", "sentence_structure": "Subject鈥損redicate", "subjective_objective": {"answer": "Subjective", "rationale": "The question is examined from a subjective point of view because it relates to the feelings and perceptions of the patient himself."}, "sentence_sentiment": {"answer": "Positive", "rationale": "Because the expression \"often feels harmonious with the people around you\" expresses a positive attitude or quality."}, "language_style": {"answer": "Tactful and warm", "rationale": "The entry is more adept and interactive when it is spoken in a gentle and inviting manner, rather than by direct statements or orders."}, "time_dimension": {"answer": "Present", "rationale": ""}, "sentence_type": "Interrogative sentence", "pos": {"nouns": {"you": 1, "People around": 1, "Relations": 1, "Harmony": 1}, "verbs": {"I always feel": 1, "and": 1}, "adjectives": {"Harmony": 1}, "numerals": {"1": 2}, "pronouns": {"you": 1}}}, {"content": "Do you often feel the lack of partners?", "word_count": 9, "label": "", "sentence_structure": "Subject鈥搗erb鈥搊bject", "subjective_objective": {"answer": "Subjective", "rationale": "Because feeling the lack of partners is a subjective feeling and emotional experience."}, "sentence_sentiment": {"answer": "Neutral", "rationale": "Because the entry does not have any emotional color."}, "language_style": {"answer": "Tactful and warm", "rationale": "\"Slow and warm.\""}, "time_dimension": {"answer": "Past", "rationale": "It was, is and will be impossible to judge, as the sentence does not provide sufficient context to determine the temporal dimension."}, "sentence_type": "Interrogative sentence", "pos": {"nouns": {"Partners": 1}, "verbs": {"I always feel": 1, "Missing": 1}}}, {"content": "Do you often feel that no one can trust?", "word_count": 11, "label": "", "sentence_structure": "Subject鈥損redicate", "subjective_objective": {"answer": "Subjective", "rationale": "Because it feels like it can be assessed subjectively."}, "sentence_sentiment": {"answer": "Negative", "rationale": "Because the \"no one can trust\" attitude in the entry is negative."}, "language_style": {"answer": "Tactful and warm", "rationale": "The language style of the entry is more moderate, sensitive and questions-based, rather than direct statements or orders, which are more personal and interactive and more accessible to respondents."}, "time_dimension": {"answer": "Present", "rationale": ""}, "sentence_type": "Interrogative sentence", "pos": {"nouns": {"person": 1, "Trust": 1}, "verbs": {"Common sense": 1, "Yeah": 1, "Trust": 1}, "adverbs": {"Regular": 1}, "numerals": {"3": 1}, "pronouns": {"you": 1}}}, {"content": "Do you often feel the silence?", "word_count": 7, "label": "", "sentence_structure": "Subject鈥搗erb鈥揷omplement", "subjective_objective": {"answer": "Subjective", "rationale": "Because feeling lonely is a subjective emotional experience."}, "sentence_sentiment": {"answer": "Negative", "rationale": "Because \"loneliness\" in this entry is usually considered a negative emotional experience."}, "language_style": {"answer": "Tactful and warm", "rationale": "The entry is more adept and interactive when it is spoken in a gentle and inviting manner, rather than by direct statements or orders."}, "time_dimension": {"answer": "Present", "rationale": "The entry is to ask the testee what he feels now, the key words are \"Do you often feel\" and \"Do you feel the silence\" , which point to the state in progress and now, and therefore judge it as now."}, "sentence_type": "Interrogative sentence", "pos": {"nouns": {"Silent": 1}, "verbs": {"I feel": 1, "Regular": 1}, "adverbs": {"Regular": 1, "- You're not": 1}, "numerals": {"4": 1}}}, {"content": "Do you often feel like one of your friends?", "word_count": 13, "label": "", "sentence_structure": "Subject鈥搗erb鈥搊bject", "subjective_objective": {"answer": "Subjective", "rationale": "Because the question relates to the feelings and attitudes of the patient, i.e., whether he feels like a friend or not, it is a subjective examination."}, "sentence_sentiment": {"answer": "Positive", "rationale": "Because \"often feeling like a friend\" expresses a positive emotional attitude."}, "language_style": {"answer": "Tactful and warm", "rationale": ""}, "time_dimension": {"answer": "Present", "rationale": "The entry asks the testee about the feelings and experiences at this stage, and the keywords are \"often feeling\" and \"belonging to a friend\", which denotes a continuous state or experience and therefore judge as now."}, "sentence_type": "Interrogative sentence", "pos": {"nouns": {"friends": 1, "One": 1}, "verbs": {"Sensor": 1, "belong to": 1}, "adverbs": {"Regular": 1}, "numerals": {"5": 1}}}, {"content": "6. Do you often feel much in common with the people around you?", "word_count": 15, "label": "", "sentence_structure": "Subject鈥搗erb鈥搊bject", "subjective_objective": {"answer": "Subjective", "rationale": "Since the issue involves the perception and perception of individuals, it is examined from a subjective perspective."}, "sentence_sentiment": {"answer": "Neutral", "rationale": "Because the entry does not have any emotional color."}, "language_style": {"answer": "Tactful and warm", "rationale": "The entry is more personal and interactive when it is spoken in a gentle and inviting manner, rather than in direct statements or orders. At the same time, the purpose of the article is more relaxed, friendly and gives people a comfortable feeling."}, "time_dimension": {"answer": "Present", "rationale": "The keywords are \"you often feel\" and \"many things in common\", which represent a current state of conduct or state, so the temporal dimension of the article should be the present."}, "sentence_type": "Interrogative sentence", "pos": {"nouns": {"Questionnaire": 1, "Entry": 1, "person": 2, "Common ground": 1}, "verbs": {"you": 1, "I always feel": 1, "have": 1, "and": 1}, "adjectives": {"Many": 1}, "adverbs": {"Regular": 1, "- You're not": 1}, "classifiers": {"individual": 2}}}, {"content": "Do you often feel intimacy to anyone?", "word_count": 14, "label": "", "sentence_structure": "Subject鈥損redicate", "subjective_objective": {"answer": "Subjective", "rationale": "Because feeling close or alienated is a subjective feeling, the question is about the subjective emotional state of the patient."}, "sentence_sentiment": {"answer": "Negative", "rationale": "Because the entry \"often feels intimacy with anyone\" expresses negative feelings, which means that the person under investigation often feels alone and alienated."}, "language_style": {"answer": "Tactful and warm", "rationale": "The entry uses a question-and-answer approach, in a gentle and inviting manner, to the respondents, avoiding the use of direct or command language, making it easier for the respondents to accept and be willing to answer questions. At the same time, the article is more apt and caring language, conveying the interest and understanding felt by the respondents, and contributing to the effectiveness and accuracy of the questionnaire."}, "time_dimension": {"answer": "Present", "rationale": "\"You often feel that the word \"always\" means \"recurring, customary\" and \"fear\" means \"normally\" in the current state of affairs, which means that this behaviour has not only happened in the past, but may still be ongoing. So the time dimension of this questionnaire is now."}, "sentence_type": "Interrogative sentence", "pos": {"nouns": {"Questionnaire": 1, "Entry": 1, "person": 1, "Intimacy": 1}, "verbs": {"I feel": 1, "Both": 1, "- You're not": 1}, "adjectives": {"Regular": 1}, "numerals": {"7": 1}}}, {"content": "Do you often feel that your interest and thoughts are different from those of the people around you?", "word_count": 20, "label": "", "sentence_structure": "Subject鈥搗erb鈥搊bject", "subjective_objective": {"answer": "Subjective", "rationale": "Because this is a subjective question about the patient ' s own feelings and views."}, "sentence_sentiment": {"answer": "Neutral", "rationale": "Because the entry does not have any emotional color."}, "language_style": {"answer": "Tactful and warm", "rationale": ""}, "time_dimension": {"answer": "Present", "rationale": ""}, "sentence_type": "Interrogative sentence", "pos": {"nouns": {"Interest": 1, "Thoughts": 1, "person": 1}, "verbs": {"I feel": 1, "Same thing": 1}, "adjectives": {"Regular": 1}, "adverbs": {"not": 1}, "numerals": {"8": 1}, "pronouns": {"you": 1}}}, {"content": "Do you often feel like having friends and friends?", "word_count": 15, "label": "", "sentence_structure": "Subject鈥搗erb鈥搊bject", "subjective_objective": {"answer": "Subjective", "rationale": "Because \"I feel like I want to be with someone and have friends\" is the patient's subjective feeling and attitude."}, "sentence_sentiment": {"answer": "Positive", "rationale": "Because the act of \"wanting to have contact with someone, making friends\" in the entry is a positive expression of attitude."}, "language_style": {"answer": "Tactful and warm", "rationale": "The entry uses a soft-minded approach, without direct orders or a request for answers, but rather a caring and understanding attitude to ask questions, which makes it easier to accept and share their feelings. At the same time, the article is more accompanied and sweet, and more in line with the characteristics of the psychological questionnaire, which helps to create a good investigation atmosphere."}, "time_dimension": {"answer": "Present", "rationale": "\"Now\"."}, "sentence_type": "Interrogative sentence", "pos": {"nouns": {"Questionnaire": 1, "Entry": 1, "person": 2, "friends": 1}, "verbs": {"Yes": 1, "I feel": 1, "Travel": 1, "Deal": 1}, "adverbs": {"Regular": 1}, "numerals": {"9": 1}}}, {"content": "Do you often feel close to others?", "word_count": 9, "label": "", "sentence_structure": "Subject鈥搗erb鈥搊bject", "subjective_objective": {"answer": "Subjective", "rationale": "Because \"fear\" is a subjective expression of feeling, reflecting the feelings and experiences of the patient."}, "sentence_sentiment": {"answer": "Neutral", "rationale": "Since the entry did not express a positive or negative emotional profile, the respondents were asked whether they often felt close to each other."}, "language_style": {"answer": "Tactful and warm", "rationale": "The entry is more adept and interactive when it is spoken in a gentle and inviting manner, rather than by direct statements or orders."}, "time_dimension": {"answer": "Present", "rationale": "This is a topic that is now ( \"often\") done, which is a regular or customary act. And the main phrase \"you\" implies that the question is to ask about the current personal feelings and experiences, so it describes the present state and experience."}, "sentence_type": "Interrogative sentence", "pos": {"nouns": {"Questionnaire": 1, "Entry": 1, "Close": 1}, "verbs": {"I feel": 1, "Regular": 1, "person": 1}, "adverbs": {"very": 1}, "numerals": {"10": 1}, "pronouns": {"you": 1}}}, {"content": "Do you often feel left alone?", "word_count": 9, "label": "", "sentence_structure": "Subject鈥搗erb鈥搊bject", "subjective_objective": {"answer": "Subjective", "rationale": "Because \"often feeling left alone\" is a subjective description that requires the patient to answer it himself."}, "sentence_sentiment": {"answer": "Negative", "rationale": "Because the feeling of being left behind in the entries is negative."}, "language_style": {"answer": "Tactful and warm", "rationale": "The more euphemistic and warm language of the entry, the less direct or tough language used in asking the respondents in a gentle and caring tone, can better create a friendly and comfortable atmosphere and facilitate the positive collaboration of the respondents."}, "time_dimension": {"answer": "Present", "rationale": "The keywords are \"you often feel\" and \"closer\", which all imply that the act exists in the life of the person being tested, so it can be judged to be a question of asking what it feels like."}, "sentence_type": "Interrogative sentence", "pos": {"nouns": {"you": 1, "person": 1}, "verbs": {"I feel": 1, "Cold": 1, "Regular": 1}, "adjectives": {"By": 1}, "adverbs": {"Regular": 1}, "numerals": {"11": 1}, "classifiers": {"individual": 1}, "pronouns": {"you": 1}}}, {"content": "12. Do you often feel that your dealings with others are meaningless?", "word_count": 16, "label": "", "sentence_structure": "Subject鈥搗erb鈥搊bject", "subjective_objective": {"answer": "Subjective", "rationale": "Since the issue concerns patients ' perception and perception of interpersonal relationships and is based on their own subjective experience, it can be judged as a subjective examination."}, "sentence_sentiment": {"answer": "Negative", "rationale": "Because the emotional attitude of \"feeling meaningless with other people\" in the entry is negative."}, "language_style": {"answer": "Calm and direct", "rationale": "The basis for the determination: The entry raises a question in a direct way, i.e. whether the person surveyed often feels that his/her social interaction is of no value. This question is more direct and simple, without the use of too much accommodative or emotional language, and with a more calm and objective tone, and therefore can be judged as being of a calm and straightforward language style."}, "time_dimension": {"answer": "Present", "rationale": "\"At times\" means a regular act, \"You feel\" uses the usual current state of affairs, which means that this feeling has not only happened in the past, but may still be ongoing. So the entry is asking the person who was tested about what it feels now."}, "sentence_type": "Interrogative sentence", "pos": {"nouns": {"Questionnaire": 1, "Entry": 1, "others": 1, "Travel": 1, "Meaning": 1}, "verbs": {"I feel": 1, "None": 1, "Regular": 1}, "adverbs": {"Nothing": 1}, "numerals": {"12": 1}, "classifiers": {"you": 1}}}, {"content": "Do you often feel that no one knows you well?", "word_count": 12, "label": "", "sentence_structure": "Subject鈥搗erb鈥搊bject", "subjective_objective": {"answer": "Subjective", "rationale": "Because \"fear\" is a subjective experience and feeling, and \"no one knows you well\" is a personal feeling and perception. So, it's a question of looking at patients' subjective attitudes."}, "sentence_sentiment": {"answer": "Negative", "rationale": "Because the \"no one understands\" feeling in the entries is usually negative."}, "language_style": {"answer": "Tactful and warm", "rationale": "The entry raises questions in a question-and-answer tone, while being gentle and friendly, without directly denying or confirming the other ' s feelings, which is more personal and interactive."}, "time_dimension": {"answer": "Present", "rationale": "\"You often feel that the word \"always\" means a regular, customary act, and \"likely\" uses the normal current, which means that this act has not only happened in the past, but may still be ongoing."}, "sentence_type": "Interrogative sentence", "pos": {"nouns": {"person": 2, "you": 1}, "verbs": {"Regular": 1, "I feel": 1, "Roger that": 1}, "adjectives": {"Nobody knows you well": 1}, "adverbs": {"Regular": 1}, "numerals": {"13": 1}, "pronouns": {"you": 1}}}, {"content": "Do you often feel isolated from others?", "word_count": 11, "label": "", "sentence_structure": "Subject鈥損redicate", "subjective_objective": {"answer": "Subjective", "rationale": "Because it is a subjective experience and feeling that separates people from others."}, "sentence_sentiment": {"answer": "Negative", "rationale": "Because the emotional experience of \"separating from others\" in the entries is negative."}, "language_style": {"answer": "Tactful and warm", "rationale": ""}, "time_dimension": {"answer": "Present", "rationale": "The keywords are \"common sense\" and \"separate\", which both indicate regular behaviour or feelings and do not refer to past or future time references, and therefore judge now."}, "sentence_type": "Interrogative sentence", "pos": {"nouns": {"you": 2, "person": 1, "Entry": 1, "Questionnaire": 1, "Sensor": 1, "others": 1, "Separate": 1}, "verbs": {"Regular": 1, "I feel": 1, "and": 1, "Separate": 1}, "adverbs": {"- You're not": 1}, "numerals": {"14": 1}}}, {"content": "Do you often feel like you can find your partner when you want?", "word_count": 16, "label": "", "sentence_structure": "Subject鈥搗erb鈥搊bject", "subjective_objective": {"answer": "Subjective", "rationale": "Because it is a subjective feeling and a perception that partners can be found."}, "sentence_sentiment": {"answer": "Positive", "rationale": "Since the entry describes the ability of individuals to find partners when needed, such behaviour and attitudes are often considered positive."}, "language_style": {"answer": "Tactful and warm", "rationale": "The language style of the entry is more euphemistic and friendly, and dialogue is conducted in a question-and-answer manner, rather than directly speaking or ordering. The term \"you\" and \"partner\" are used to make the questionnaire more personal and interactive, making it easier for the respondents to accept and answer questions."}, "time_dimension": {"answer": "Present", "rationale": ""}, "sentence_type": "Interrogative sentence", "pos": {"nouns": {"15": 1, "you": 2, "I always feel": 1, "Time": 1, "Yes": 1, "Found it": 1, "Partners": 1}, "verbs": {"Yes": 1, "I can": 1, "I feel": 1, "Found it": 1}, "adverbs": {"Regular": 1, "When": 1}, "numerals": {"15": 1}, "classifiers": {"individual": 1}, "pronouns": {"you": 2}}}, {"content": "Do you often feel that someone really knows you?", "word_count": 13, "label": "", "sentence_structure": "Subject鈥搗erb鈥搊bject", "subjective_objective": {"answer": "Subjective", "rationale": "Because there is a sense that someone really understands your subjective feelings and perceptions as a patient."}, "sentence_sentiment": {"answer": "Neutral", "rationale": "Because the entry does not have any emotional color."}, "language_style": {"answer": "Tactful and warm", "rationale": "The language style of the entry is more euphemistic, expressing the inner care of the interviewee in a way that is both a matter of inquiry and concern."}, "time_dimension": {"answer": "Present", "rationale": "The phrase \"you often feel\" makes it possible to judge the way the subject is feeling and the circumstances of the trial."}, "sentence_type": "Interrogative sentence", "pos": {"nouns": {"you": 2, "person": 2, "Real": 1, "Roger that": 1}, "verbs": {"I always feel": 1}, "adjectives": {"Really": 1}, "numerals": {"16": 1}}}, {"content": "Do you often feel shy?", "word_count": 7, "label": "", "sentence_structure": "Subject鈥損redicate", "subjective_objective": {"answer": "Subjective", "rationale": "Because being shy is a subjective emotional experience."}, "sentence_sentiment": {"answer": "Negative", "rationale": "Because \"often shy\" is a negative sentiment, which indicates a degree of self-confidence and disquiet in their abilities and behaviour."}, "language_style": {"answer": "Tactful and warm", "rationale": "The entry is more adept and interactive when it is spoken in a gentle and inviting manner, rather than by direct statements or orders."}, "time_dimension": {"answer": "Present", "rationale": "The keyword \"common sense\" means a constant, customary act, and \"weakness\" is a subjective feeling, and this behaviour or state of mind usually occurs now. So, this entry relates to the current time dimension."}, "sentence_type": "Interrogative sentence", "pos": {"nouns": {"Questionnaire": 1, "Entry": 1, "Shame": 1}, "verbs": {"Common sense": 1, "I feel": 1}, "adverbs": {"- You're not": 1}, "numerals": {"17": 1}}}, {"content": "Do you often feel like people are around you, but they don't care about you?", "word_count": 16, "label": "", "sentence_structure": "Subject鈥搗erb鈥搊bject", "subjective_objective": {"answer": "Subjective", "rationale": "Because \"care for you\" is a subjective feeling."}, "sentence_sentiment": {"answer": "Negative", "rationale": "Because the \"not caring\" attitude in the entries is negative."}, "language_style": {"answer": "Tactful and warm", "rationale": "The entry uses a soft, but not direct, approach to asking questions, which is more acceptable and willing to answer questions. It also implies that there may be disturbing situations and that the respondents are being guided in a moderate way to reflect and reflect."}, "time_dimension": {"answer": "Past", "rationale": "\"18.\" is the title number, \"You often feel\" means \"recurrent, customary\" behaviour, while \"fEalse\" uses the general current, which means that the behaviour has not only occurred in the past, but may still be ongoing. The entry therefore asks the testee about his past behaviour and feelings, and the temporal dimension is a past."}, "sentence_type": "Interrogative sentence", "pos": {"nouns": {"you": 2, "People": 1, "Round": 1, "Care": 1}, "verbs": {"I always feel": 1, "Round and round": 1, "not": 1, "Care": 1}, "adjectives": {"I always feel": 1, "Round and round": 1, "not": 1, "Care": 1}, "adverbs": {"I always feel": 1, "Round and round": 1, "not": 1}, "numerals": {"18": 1}, "pronouns": {"you": 2, "People": 1}}}, {"content": "Do you often feel like someone wants to talk to you?", "word_count": 13, "label": "", "sentence_structure": "Subject鈥搗erb鈥搊bject", "subjective_objective": {"answer": "Subjective", "rationale": "Because this is a subjective view of the patient ' s own feelings and perceptions."}, "sentence_sentiment": {"answer": "Neutral", "rationale": "Because the entry does not have any emotional color."}, "language_style": {"answer": "Tactful and warm", "rationale": "The entry is more euphemistic, expressing a more positive and friendly attitude by using the term \"willing\". At the same time, the form of the question is more open, not directly asking the other side to answer, but rather to allow the other side to answer according to its own feelings, which are more likely to resonate and share with the other side."}, "time_dimension": {"answer": "Present", "rationale": "In this entry, the word \"normal\" denotes regular, customary behaviour, while the words \"fear,\" \"will\" and \"talk\" take the current state of affairs, which means that this feeling has not only happened in the past, but may still be ongoing. So the time dimension of this questionnaire is now."}, "sentence_type": "Interrogative sentence", "pos": {"nouns": {"person": 1, "Talk": 1}, "verbs": {"May I": 1, "Sensor": 1, "and": 1, "Talk": 1}, "numerals": {"19": 1}, "pronouns": {"you": 1}}}, {"content": "Do you often feel that someone deserves your trust?", "word_count": 12, "label": "", "sentence_structure": "Subject鈥搗erb鈥搊bject", "subjective_objective": {"answer": "Subjective", "rationale": "Because it is a subjective feeling and perception of a person that someone can be trusted, it is not an objective fact."}, "sentence_sentiment": {"answer": "Positive", "rationale": "Because the attitude of \"someone deserves your trust\" in the entry is positive."}, "language_style": {"answer": "Tactful and warm", "rationale": "The language style of the entry is more euphemistic, as it allows respondents to express their feelings and ideas by means of questioning, without direct pressure or order. At the same time, the question is one of positive words, \"often felt\" , which can be perceived as being of interest and respect and which can help to create a good investigation atmosphere."}, "time_dimension": {"answer": "Present", "rationale": "\"Now.\""}, "sentence_type": "Interrogative sentence", "pos": {"nouns": {"Trust": 1, "person": 1}, "verbs": {"I feel": 1, "It's worth it": 1, "Trust": 1}, "adverbs": {"Regular": 1}, "numerals": {"20": 1}, "pronouns": {"you": 1}}}]}

{"questionnaire_title": "Hospital Anger and Depression Scale (HAD)", "questionnaire_category": "Mental health", "item_count": 14, "emotion_shift_overall": "From negative to positive", "language_style_overall": "Tactful and warm", "items": [{"content": "I feel nervous (or painful)", "word_count": 8, "label": "", "sentence_structure": "Subject鈥揷opula鈥損redicative", "subjective_objective": {"answer": "Subjective", "rationale": "Because stress and pain are the feelings and experiences of the patient himself, they are subjective observations."}, "sentence_sentiment": {"answer": "Negative", "rationale": "Because the entry \"Esperate (or painful)\" expresses a negative emotional attitude."}, "language_style": {"answer": "Tactful and warm", "rationale": "The entry uses descriptive language, which is not too direct or strong."}, "time_dimension": {"answer": "Present", "rationale": ""}, "sentence_type": "Interrogative sentence", "pos": {"nouns": {"Nervous": 1, "Pain": 1}, "verbs": {"Sensor": 1, "Present": 1}}}, {"content": "I'm still interested in things that have been of interest to me.", "word_count": 15, "label": "", "sentence_structure": "Subject鈥揷opula鈥損redicative", "subjective_objective": {"answer": "Subjective", "rationale": "Because \"interest\" is a subjective feeling or attitude that requires the patient to describe and evaluate itself."}, "sentence_sentiment": {"answer": "Positive", "rationale": "Because the entry \"are interested in things that have been of interest to the past\" expresses a continuing interest and enthusiasm, and is a positive emotional attitude."}, "language_style": {"answer": "Tactful and warm", "rationale": ""}, "time_dimension": {"answer": "Present", "rationale": ""}, "sentence_type": "Interrogative sentence", "pos": {"nouns": {"Things": 1, "Interest": 2}, "verbs": {"be": 1, "have": 1}, "adjectives": {"Previous": 1}, "numerals": {"2": 1}, "pronouns": {"I": 1}}}, {"content": "I'm a little scared, like I'm expecting something terrible to happen.", "word_count": 22, "label": "", "sentence_structure": "Subject鈥揷opula鈥損redicative", "subjective_objective": {"answer": "Subjective", "rationale": "Because \"a little fear\" and \"a little fear\" and \"a feeling of something terrible going on\" are the subjective feelings and thoughts of the patients themselves."}, "sentence_sentiment": {"answer": "Negative", "rationale": "Because the descriptions \"a little scared\" and \"a little scary thing to happen\" in the entries are negative."}, "language_style": {"answer": "Calm and direct", "rationale": "The entry expresses in a direct manner the feelings that the subject may have been afraid or premonitioned, does not have too many rhetorical techniques or emotional renderings, and is more calm and objective in its language style."}, "time_dimension": {"answer": "Present", "rationale": "The sentence expresses the feelings and thoughts of the testees now, and \"fEE\" and \"Emperate\" are all actions or states in progress, so it can be judged as now."}, "sentence_type": "Affirmative declarative sentence", "pos": {"nouns": {"Questionnaire Entry": 1, "Scared": 1, "Foreground": 1, "Things": 1}, "verbs": {"I feel": 1, "Come on": 1}, "adjectives": {"Horrible": 1}, "adverbs": {"A little": 1}, "numerals": {"3": 1}, "pronouns": {"I": 1}}}, {"content": "I can laugh and see the good side of things.", "word_count": 16, "label": "", "sentence_structure": "Subject鈥揷opula鈥損redicative", "subjective_objective": {"answer": "Subjective", "rationale": "Because the descriptions of \"I can\" and \"seeing the good side of things\" are all subjective feelings and attitudes of patients."}, "sentence_sentiment": {"answer": "Positive", "rationale": "Because \"Ha ha ha ha ha ha ha ha\" and \"seeing the good side of things\" are positive emotional attitudes and behavior."}, "language_style": {"answer": "Tactful and warm", "rationale": "The entry expresses, in a positive tone, the expectation of personal optimism, in a more cordial and encouraging manner, and gives respondents a greater willingness to answer."}, "time_dimension": {"answer": "Present", "rationale": "The entry describes the current emotional state and attitude of the testees, using the current state of the day ( \"can\", \"see\") to describe their current abilities and views. At the same time, \"Haha laugh\" and \"seeing the good side\" are two specific behavioural or mental descriptions, and are now manifestations. So the time dimension of the article is now."}, "sentence_type": "Affirmative declarative sentence", "pos": {"nouns": {"In terms of": 1, "Things": 1}, "verbs": {"Yeah": 1, "# Ha ha ha ha ha ha ha ha ha ha ha ha ha ha ha ha ha ha ha ha ha ha ha ha ha ha ha ha ha ha ha ha ha ha ha ha ha ha ha ha ha ha ha ha ha ha ha ha ha ha ha ha ha ha ha ha ha ha ha ha ha ha ha ha ha ha ha ha ha ha ha ha ha ha ha ha ha ha ha ha ha ha ha ha ha ha ha ha ha ha ha ha ha ha ha ha ha ha ha ha ha ha ha ha ha ha ha ha ha ha ha ha ha ha ha ha ha ha ha ha ha ha ha ha ha ha ha ha ha ha ha ha ha ha ha ha ha ha ha ha ha ha ha ha ha ha ha ha ha ha ha ha ha ha ha ha ha ha ha ha ha ha ha ha ha ha ha ha ha ha ha ha ha ha ha ha ha ha ha ha ha ha ha ha ha ha ha ha ha ha ha ha ha ha ha ha ha ha ha ha ha ha ha ha ha ha ha ha ha ha ha ha ha ha ha ha ha ha ha ha ha ha ha ha ha ha ha ha ha ha ha ha ha ha ha ha ha ha ha ha ha ha ha ha ha ha ha ha ha ha ha ha ha ha": 2}, "adjectives": {"Okay": 1}, "numerals": {"4": 1}, "pronouns": {"I": 1}}}, {"content": "5. My heart is full of trouble.", "word_count": 8, "label": "", "sentence_structure": "Subject鈥揷opula鈥損redicative", "subjective_objective": {"answer": "Subjective", "rationale": "Because the pain in the heart is a human experience and feeling, it is a subjective examination."}, "sentence_sentiment": {"answer": "Negative", "rationale": "Because \"in the heart\" of the entry is a negative emotion and attitude."}, "language_style": {"answer": "Calm and direct", "rationale": "The topic was presented in a direct, concise manner, without excessive emotional or rhetorical overtones, and asked whether respondents were often troubled."}, "time_dimension": {"answer": "Present", "rationale": "The keyword \"in the heart\" expresses the current state of the person's heart and feelings, and \"in the heart\" further highlights the extent of that feeling, so the entry asks about the current emotional state of the person's being tested. The temporal dimension is now."}, "sentence_type": "Affirmative declarative sentence", "pos": {"nouns": {"鈾?In the heart 鈾?: 1, "Trouble": 1}, "verbs": {"Fill": 1}}}, {"content": "I'm happy.", "word_count": 5, "label": "", "sentence_structure": "Subject鈥揷opula鈥損redicative", "subjective_objective": {"answer": "Subjective", "rationale": "Because \"I'm happy\" is the patient's subjective feeling of his own emotions."}, "sentence_sentiment": {"answer": "Positive", "rationale": "Because \"happy\" is a positive emotional word."}, "language_style": {"answer": "Tactful and warm", "rationale": "The entry uses a more euphemistic and gentle language style, which allows dialogue by asking about the other's feelings, rather than direct statements or orders, which is more personal and interactive. At the same time, \"happy\" is also a more subjective description, requiring respondents to respond to their own reality, so that this soft-minded approach to questions also encourages respondents to express their feelings more honestly."}, "time_dimension": {"answer": "Present", "rationale": "Because \"I'm happy\" is the expression of the current emotional feeling, so it's now."}, "sentence_type": "Affirmative declarative sentence", "pos": {"verbs": {"I feel": 1, "Have a nice day": 1}}}, {"content": "I can sit in a free and easy place.", "word_count": 11, "label": "", "sentence_structure": "Subject鈥揷opula鈥損redicative", "subjective_objective": {"answer": "Subjective", "rationale": "This is because of the patient ' s subjective feelings about his or her sitting position and physical and mental state."}, "sentence_sentiment": {"answer": "Neutral", "rationale": "Because the entry describes only the state of a person sitting in a sitting and does not involve any information such as emotion, attitude or behaviour."}, "language_style": {"answer": "Calm and direct", "rationale": "The language of the article is simple and clear, there is no excessive rhetorical or emotional rendering, and it is more objective. It asks the respondents in a direct way whether they are able to sit and sit and do not use euphemism. The language style of the article is therefore straightforward."}, "time_dimension": {"answer": "Present", "rationale": "\"Can sit free and easy\" means a current capability or state, and can therefore be judged as now."}, "sentence_type": "Exclamatory sentence", "pos": {"nouns": {"I": 1, "- No, no, no": 1, "Easy": 1, "Seats": 1}, "verbs": {"Yes": 1, "Sit down": 1}, "adjectives": {"- No, no, no": 1, "Easy": 1}, "adverbs": {"And": 1, "Land": 1}, "classifiers": {"individual": 1}, "pronouns": {"I": 1}}}, {"content": "I lost interest in my appearance.", "word_count": 15, "label": "", "sentence_structure": "Subject鈥搗erb鈥搊bject", "subjective_objective": {"answer": "Subjective", "rationale": "Because it is self-awareness and feelings of the patient, it is subjective observation."}, "sentence_sentiment": {"answer": "Negative", "rationale": "Because the act of \"lost interest in his own appearance\" is negative."}, "language_style": {"answer": "Tactful and warm", "rationale": "The words of the entry are more gentle and friendly, using the expression \"I\" to make you feel more close and understandable. Asking if the other person is not interested in self-dressing, and showing a caring attitude. Such expression makes it easier for the interviewee to open his heart and answer the questions."}, "time_dimension": {"answer": "Present", "rationale": "\"I lost interest in my appearance,\" means \"lost interest\" in the state of the present, so it can be judged that the time dimension of the article is now."}, "sentence_type": "Negative declarative sentence", "pos": {"nouns": {"I": 1, "Yum": 1, "Get yourself dressed": 1}, "verbs": {"to": 1, "Lose": 1, "Interest": 1}, "numerals": {"8": 1}}}, {"content": "I'm a little restless. I feel like I have to move.", "word_count": 17, "label": "", "sentence_structure": "Subject鈥揷opula鈥損redicative", "subjective_objective": {"answer": "Subjective", "rationale": "Because being restless is a subjective feeling, which reflects the emotional state and needs of the patient. While this feeling may be related to objective circumstances (for example, if the patient works in an environment that requires a long sitting time), the patient鈥檚 feeling is subjective and not entirely controlled by objective factors."}, "sentence_sentiment": {"answer": "Negative", "rationale": "Because the act of \"sitting\" in the entry is negative."}, "language_style": {"answer": "Tactful and warm", "rationale": ""}, "time_dimension": {"answer": "Past", "rationale": "The entry describes the current feelings and state of the person being tested, does not address past or future time dimensions, and therefore the judgement is now."}, "sentence_type": "Affirmative declarative sentence", "pos": {"nouns": {"Sit tight": 1, "Activities": 1}, "verbs": {"I feel": 1, "You have to": 1, "Yes": 1, "No way": 1}, "adjectives": {"A little": 1}}}, {"content": "I'm optimistic about everything.", "word_count": 12, "label": "", "sentence_structure": "Subject鈥揷opula鈥損redicative", "subjective_objective": {"answer": "Subjective", "rationale": "This is because of the patient ' s personal views and attitudes and is subjective."}, "sentence_sentiment": {"answer": "Positive", "rationale": "Because the entry \"optimistically looking forward\" expresses a positive and positive attitude."}, "language_style": {"answer": "Tactful and warm", "rationale": "The language style of the entry is more euphemistic, using positive and encouraging expressions like \"optimistic looking forward\" to guide respondents, comforting and comforting. At the same time, the absence of direct questions or command-style tone makes the respondents ' answers more free and natural."}, "time_dimension": {"answer": "Present", "rationale": "The keyword \"I am optimistic about everything going forward\" expresses the current attitude and conviction of the testees that the vision of the future is positive, and the entry therefore describes the current behavior or mentality."}, "sentence_type": "Affirmative declarative sentence", "pos": {"nouns": {"Everything": 1, "Optimistic": 1, "Front": 1}, "verbs": {"Look": 1}, "adverbs": {"Both": 1, "Land": 2}, "numerals": {"10锛?: 1}}}, {"content": "I suddenly found out I was in a panic.", "word_count": 8, "label": "", "sentence_structure": "Subject鈥搗erb鈥揷omplement", "subjective_objective": {"answer": "Subjective", "rationale": "Because panic is a subjective feeling."}, "sentence_sentiment": {"answer": "Negative", "rationale": "Because \"fright\" in the entry is a negative emotional experience."}, "language_style": {"answer": "Calm and direct", "rationale": "The entry is very straightforward in expressing the subject, without any rhetorical or emotional overture. It does not attempt to hide the seriousness of the problem in euphemism, but rather directly describes the situation in which the subject may feel frightened. This direct and clear language style helps the person surveyed to express his feelings more accurately and truthfully."}, "time_dimension": {"answer": "Future", "rationale": "The entry contains \"accidental discovery\" and \"frightfulness\" 鈥?both terms suggest that the current feeling has not yet appeared, so the judgement is the future. Moreover, the act of \"discovery\" implies a perception of the state of the unknown, and further supports the future of time judgement."}, "sentence_type": "Affirmative declarative sentence", "pos": {"nouns": {"Panic": 1, "Suddenly": 1}, "verbs": {"Found": 1}, "adverbs": {"very": 1}, "numerals": {"11": 1, "1": 2}, "pronouns": {"I": 1}}}, {"content": "I feel like I'm getting low.", "word_count": 12, "label": "", "sentence_structure": "Subject鈥揷opula鈥損redicative", "subjective_objective": {"answer": "Subjective", "rationale": "Because it is the patient's subjective feeling and judgment of his emotional state."}, "sentence_sentiment": {"answer": "Negative", "rationale": "Because it expresses a negative emotional attitude, which is \"the mood is falling.\""}, "language_style": {"answer": "Tactful and warm", "rationale": "The entry is more euphemistic, is more friendly and friendly by asking the interviewee whether he or she feels depressed, and avoids direct criticism or accusations. Moreover, this expression is more likely to trigger resonance and open responses from the interviewee, which will help to improve the effectiveness and accuracy of the questionnaire."}, "time_dimension": {"answer": "Past", "rationale": ""}, "sentence_type": "Affirmative declarative sentence", "pos": {"nouns": {"Emotion": 1, "Down": 1}, "verbs": {"I feel": 1, "Gradient": 1}, "adverbs": {"in": 1}, "numerals": {"12": 1}, "classifiers": {"individual": 1}, "pronouns": {"I": 1}}}, {"content": "I'm a little scared. It looks like some internal organ has broken down.", "word_count": 18, "label": "", "sentence_structure": "Subject鈥揷opula鈥損redicative", "subjective_objective": {"answer": "Subjective", "rationale": "Because \"fear\" and \"a broken internal organ\" are the psychological feelings and self-evaluations of patients, and they are subjective thoughts of patients."}, "sentence_sentiment": {"answer": "Negative", "rationale": "Because the \"fear\" sentiment in the entry is negative."}, "language_style": {"answer": "Calm and direct", "rationale": "The entry directly expresses concerns about the health of the person under investigation in a concise language, without too much rhetorical or emotional rendering, and is more objective. The language style of the entry should therefore be \"cool and direct\"."}, "time_dimension": {"answer": "Present", "rationale": "Now, according to the description of \"I feel\" and \"like a part of the internal organs has broken,\" it can be seen that it's the current feeling and experience of the subject, so the time dimension of the article is now."}, "sentence_type": "Affirmative declarative sentence", "pos": {"nouns": {"Questionnaire": 1, "Entry": 1, "Internal organs": 1}, "verbs": {"I feel": 1, "Bad": 1}, "adjectives": {"A little": 1, "Scared": 1}, "numerals": {"13": 1}, "pronouns": {"I": 1}}}, {"content": "I can appreciate a good book or a good radio or television show.", "word_count": 20, "label": "", "sentence_structure": "Subject鈥搗erb鈥搊bject", "subjective_objective": {"answer": "Subjective", "rationale": "Because appreciation is the patient's subjective attitude."}, "sentence_sentiment": {"answer": "Positive", "rationale": "Because the word \"applause\" in the entries is positive and expresses appreciation for and appreciation for good books, radio and television programmes."}, "language_style": {"answer": "Tactful and warm", "rationale": "The entry is more proficient and interactive in its expression of dialogue in a question-and-answer tone rather than direct statements or orders. At the same time, the use of the term \"capable\" also shows that the issue is not mandatory but rather expects a positive response from the interviewees."}, "time_dimension": {"answer": "Present", "rationale": "The keyword \"can\" means that it is capable of doing so, and \"appease\" means that it is an ongoing act, so the entry is to ask the testee what the situation is."}, "sentence_type": "Affirmative declarative sentence", "pos": {"nouns": {"I": 1, "Books": 1, "Radio": 1, "Television programmes": 1}, "verbs": {"Yes": 1, "Enjoy": 1}, "adjectives": {"Okay": 2}, "numerals": {"14": 1}}}]}

{"questionnaire_title": "Self-harmonized Scale", "questionnaire_category": "Self-cognition", "item_count": 35, "emotion_shift_overall": "From negative to positive", "language_style_overall": "Neutral", "items": [{"content": "I'm a little bit of a ambivalent about myself.", "word_count": 20, "label": "", "sentence_structure": "Subject鈥搗erb鈥揷omplement", "subjective_objective": {"answer": "Subjective", "rationale": "This is because patients describe their views and reflect their subjective feelings and thoughts."}, "sentence_sentiment": {"answer": "Negative", "rationale": "Because the description of \"some contradiction of one's own views\" in the entry is negative and may indicate self-doubt, self-confidence, etc. of the interviewees."}, "language_style": {"answer": "Calm and direct", "rationale": "The entry, which is more directly linguistically, expresses more succinctly some of the doubts about the self-perception of the respondents."}, "time_dimension": {"answer": "Present", "rationale": "The words \"often\" and \"likely\" in the sentence make it clear that the entry describes the person being tested in his current behaviour or state and is therefore a historical state."}, "sentence_type": "Affirmative declarative sentence", "pos": {"nouns": {"person": 1, "Observations": 1, "Paradoxious": 1}, "verbs": {"I think": 1, "to": 2}, "adverbs": {"Often": 1}, "pronouns": {"I": 1}}}, {"content": "I sometimes feel dissatisfied with my own performance in some areas.", "word_count": 17, "label": "", "sentence_structure": "Subject鈥搗erb鈥揷omplement", "subjective_objective": {"answer": "Subjective", "rationale": "Because it is a subjective attitude and emotional attitude to the patient ' s own perception and feelings."}, "sentence_sentiment": {"answer": "Negative", "rationale": "Because the attitude of \"unsatisfactory\" in the entries is negative."}, "language_style": {"answer": "Tactful and warm", "rationale": "The language style of the entry is more euphemistic. It does not directly blame or criticize the other, but rather asks in an understanding and supportive tone, which reduces the other鈥檚 resistance and makes him more willing to participate in the questionnaire. At the same time, the word 鈥渟ometimes鈥?implies that this is a more general situation, rather than a specific individual or particular situation, adding further euphemism to the euphemism of the statement."}, "time_dimension": {"answer": "Present", "rationale": "The words \"sometimes\" and \"will\" in the entry indicate a regular, customary act, which is occurring or may be continuing. Thus, the temporal dimension is now judged."}, "sentence_type": "Negative declarative sentence", "pos": {"nouns": {"In terms of": 1, "Performance": 1}, "verbs": {"Yes": 1, "Unsatisfactory": 1}, "adjectives": {"self": 1}, "adverbs": {"Sometimes": 1}}}, {"content": "3. Whenever difficulties are encountered, I always first analyse the causes of the difficulties.", "word_count": 20, "label": "", "sentence_structure": "Subject鈥搗erb鈥搊bject", "subjective_objective": {"answer": "Objective", "rationale": "This is the way patients act and think in the face of difficulties and is an objective perspective."}, "sentence_sentiment": {"answer": "Neutral", "rationale": "Since the entry describes only an act, i.e., the first analysis of the cause in case of difficulty, does not express any emotional colour and is therefore neutral."}, "language_style": {"answer": "Calm and direct", "rationale": "The language style of the entry is straightforward. It describes a behavioral habit in plain and clear language, without using too many rhetorical or emotional techniques to make the atmosphere more visible. This expression is more objective, straightforward, easy to understand and accept."}, "time_dimension": {"answer": "Past", "rationale": ""}, "sentence_type": "Affirmative declarative sentence", "pos": {"nouns": {"Difficulties": 1, "Reason": 1}, "verbs": {"I met him": 1, "First of all": 1, "Analysis": 2, "Cause": 1}, "adverbs": {"Always": 1}, "numerals": {"3": 1}}}, {"content": "4. It is difficult for me to properly express my emotional response to others.", "word_count": 17, "label": "", "sentence_structure": "Subject鈥搗erb鈥揷omplement", "subjective_objective": {"answer": "Subjective", "rationale": "Because \"hard\" and \"rightly expressed\" reflect patients' own subjective feelings and perceptions."}, "sentence_sentiment": {"answer": "Negative", "rationale": "Because it's a \"difficult to express\" behavior in the entry is negative."}, "language_style": {"answer": "Tactful and warm", "rationale": "The entry is more personal and interactive when it is spoken in a question-and-answer tone, rather than directly speaking or ordering. It also expresses the interest and respect of researchers for the subjects, and seeks to understand their inner feelings rather than simply acquiring data."}, "time_dimension": {"answer": "Present", "rationale": "The keywords are \"hard\" and \"rightly expressed\" and indicate that the subject has problems in the current state of conduct, and therefore the judgement is now."}, "sentence_type": "Negative declarative sentence", "pos": {"nouns": {"Questionnaire Entry": 1, "I": 2, "Emotional response": 1, "others": 1}, "verbs": {"Expression": 1, "Correct": 1, "to": 1}, "adjectives": {"Hard": 1, "Appropriate": 1}, "numerals": {"4": 1}}}, {"content": "I have my own views on many things, but I don't ask anyone to do the same.", "word_count": 26, "label": "", "sentence_structure": "Subject鈥搗erb鈥搊bject", "subjective_objective": {"answer": "Subjective", "rationale": "Because it is the patient's subjective feeling and attitude."}, "sentence_sentiment": {"answer": "Positive", "rationale": "Because the attitude in the entry is positive, which is, \"I have my own perspective on a lot of things, but I don't need anyone else to agree with me.\""}, "language_style": {"answer": "Calm and direct", "rationale": "The article is based on a judgment: its purpose is to be honest and objective, without too much rhetorical or emotional overtones. It expresses the view in a straightforward and clear manner that the interviewee has his own unique point of view and does not press for consent. This expression is more calm and straightforward and meets the objective requirements of the questionnaire."}, "time_dimension": {"answer": "Present", "rationale": ""}, "sentence_type": "Affirmative declarative sentence", "pos": {"nouns": {"Things": 1, "Views": 1, "others": 1}, "verbs": {"have": 1, "Request": 1, "Same thing": 1}, "adjectives": {"A lot": 1, "Different": 1}, "adverbs": {"Both": 1, "But": 1, "and": 1}, "pronouns": {"I": 1}}}, {"content": "6. Once I have developed a vision of the matter, I will not change.", "word_count": 17, "label": "", "sentence_structure": "Subject鈥損redicate", "subjective_objective": {"answer": "Subjective", "rationale": "Because the formation of an opinion is a subjective perception and attitude of one person, it does not change the subjective will and decision of another."}, "sentence_sentiment": {"answer": "Negative", "rationale": "Because the attitude in the entry \"if you have a vision of things, you will not change\" is conservative and unwilling to accept new ideas, and is therefore negative."}, "language_style": {"answer": "Calm and direct", "rationale": "The language style of the entry is more cold and objective, and there is no excessive emotional or oral linguistic style, and it is more direct and firm."}, "time_dimension": {"answer": "Present", "rationale": "The entry expresses a state or behaviour that is \"if you have a view of the matter\" and will not change, so it can be judged as \"now\"."}, "sentence_type": "Affirmative declarative sentence", "pos": {"verbs": {"Form": 1, "Observations": 1, "Change": 1}, "adverbs": {"鈾?Once 鈾?: 1}, "pronouns": {"I": 1}}}, {"content": "7. I am often dissatisfied with my actions.", "word_count": 12, "label": "", "sentence_structure": "Subject鈥揷opula鈥損redicative", "subjective_objective": {"answer": "Subjective", "rationale": "Because this is a subjective observation of the patient ' s perception and feelings of his or her behaviour."}, "sentence_sentiment": {"answer": "Negative", "rationale": "Because \"unsatisfactory\" is negative."}, "language_style": {"answer": "Calm and direct", "rationale": "The entry is in plain and simple language and asks whether the person surveyed is often dissatisfied with his or her behaviour and does not use excessive rhetoric or emotional colours."}, "time_dimension": {"answer": "Past", "rationale": "The term \"always\" in the entry indicates a customary, long-standing state, and therefore a sense of time dimension. At the same time, the description of \"self\" indicates that it is an individual feeling or assessment, not an act that is being performed, so it can also be classified as a past."}, "sentence_type": "Negative declarative sentence", "pos": {"nouns": {"Behaviour": 1, "self": 1}, "verbs": {"Regular": 1, "Satisfied": 1}, "adverbs": {"Regular": 1, "to": 1}, "classifiers": {"One": 1}, "pronouns": {"I": 1}}}, {"content": "8. While sometimes I have to do something I do not want to do, I have to do it largely as I wish.", "word_count": 28, "label": "", "sentence_structure": "Subject鈥搗erb鈥揷omplement", "subjective_objective": {"answer": "Subjective", "rationale": "Because \"doing what you want\" is a personal feeling and attitude that reflects the patient's self-perception and values. This is viewed from the patient's subjective perspective."}, "sentence_sentiment": {"answer": "Positive", "rationale": "Because the entry \"doing essentially as it wants\" expresses respect and satisfaction of the individual's will, it is a positive attitude."}, "language_style": {"answer": "Tactful and warm", "rationale": "The language style of the entry is mild, using the term \"basically\" to make it more euphemistic, not too direct or hard to express. At the same time, the entry uses positive and positive language expressions, such as \"do as you wish,\" which also makes the language style more warm and encouraging."}, "time_dimension": {"answer": "Present", "rationale": "According to the sentence entered, \"I'm basically doing what I want\" means that it's the person being tested in the act or state, and therefore it's \"now\"."}, "sentence_type": "Affirmative declarative sentence", "pos": {"nouns": {"Time": 1, "Yes": 1, "Wish": 1}, "verbs": {"Okay": 1, "Do it": 2, "Press": 1, "OPERATIONS": 1}, "adjectives": {"I don't want to": 1, "Your own": 1}, "adverbs": {"Basic": 1, "Go, go, go": 1, "be": 1, "of": 2, "Both": 1}, "classifiers": {"Some": 1, "individual": 1}, "pronouns": {"I": 1}}}, {"content": "9. One thing is good, one thing is bad, nothing can be ambiguous.", "word_count": 23, "label": "", "sentence_structure": "Subject鈥揷opula鈥損redicative", "subjective_objective": {"answer": "Objective", "rationale": "Because this sentence is a description of certainty and an objective fact."}, "sentence_sentiment": {"answer": "Neutral", "rationale": "Because the entry describes an attitude or view and does not contain a clear emotional colour, it is judged neutral."}, "language_style": {"answer": "Calm and direct", "rationale": "This expression is more appropriate for formal psychological questionnaires."}, "time_dimension": {"answer": "Present", "rationale": "The keywords \"good is good,\" \"bad is bad\" and \"no one can be vague\" are all descriptions and evaluations of the present, both of which are present or relevant. So, the entry describes the present mentality or view."}, "sentence_type": "Affirmative declarative sentence", "pos": {"verbs": {"be": 2, "have": 1}, "adjectives": {"Okay": 2, "Not good": 1, "Yeah": 1, "Vague": 1}, "adverbs": {"Just": 2}, "pronouns": {"One thing": 1, "What": 1}}}, {"content": "If I don't get along with something, I'm always suspicious of my abilities.", "word_count": 23, "label": "", "sentence_structure": "Subject鈥搗erb鈥搊bject", "subjective_objective": {"answer": "Subjective", "rationale": "Because it is the subjective perception and perception of the patient of his or her own situation."}, "sentence_sentiment": {"answer": "Negative", "rationale": "Because the act of \"suspecting your abilities\" in the entry is negative."}, "language_style": {"answer": "Tactful and warm", "rationale": "The entry uses a mild and invitational tone to guide the testees to answer questions, not to speak or order directly. At the same time, it implies understanding and support for the testees, implying that they are not alone in the face of difficulties and challenges. This expression is more personal and interactive, contributing to a good research climate and making the testees more willing to share their thoughts and feelings."}, "time_dimension": {"answer": "Present", "rationale": "The entry describes a mode of thinking or belief of the person being tested, which exists at some point in time and influences the person ' s thinking and behaviour, and is therefore present."}, "sentence_type": "Affirmative declarative sentence", "pos": {"nouns": {"Yes": 1, "Capacity": 1}, "verbs": {"Yes": 2, "Doubt": 1, "in": 1, "Often": 1, "It's not going well": 1, "I": 1, "Something": 1}, "adjectives": {"self": 1}, "adverbs": {"Often": 1}, "numerals": {"10": 1}, "pronouns": {"If": 1}}}, {"content": "I have at least a few friends who know each other.", "word_count": 11, "label": "", "sentence_structure": "Subject鈥搗erb鈥揷omplement", "subjective_objective": {"answer": "Subjective", "rationale": "Because the number of friends who know is a subjective feeling and perception of a person, it is not an objective fact."}, "sentence_sentiment": {"answer": "Neutral", "rationale": "Because the entry was not of any emotional colour, it was merely an inquiry into the number of friends of the person surveyed."}, "language_style": {"answer": "Tactful and warm", "rationale": "The language style of the entry is euphemism, which leads the respondents to share their thoughts and feelings by asking questions and using relatively soft and polite language. This way makes the respondents comfortable and relaxed, and is more open to sharing real ideas and experiences."}, "time_dimension": {"answer": "Present", "rationale": "The entry asks about the current situation of the subjects, i.e. how much of their knowing friends they have, and therefore the time dimension is now judged."}, "sentence_type": "Affirmative declarative sentence", "pos": {"nouns": {"friends": 2, "I know what you're doing": 1}, "verbs": {"have": 1, "to": 1}, "adjectives": {"At least": 1}, "numerals": {"How many": 1, "individual": 1}, "pronouns": {"I": 1}}}, {"content": "12. I feel that much of what I have done is wrong.", "word_count": 17, "label": "", "sentence_structure": "Subject鈥揷opula鈥損redicative", "subjective_objective": {"answer": "Subjective", "rationale": "This expression is an expression of the patient ' s assessment and perception of his or her behaviour and is part of his or her subjective feelings and views."}, "sentence_sentiment": {"answer": "Negative", "rationale": "Because the expression of the article contains a negative and self-responsible emotional connotation, it is negative."}, "language_style": {"answer": "Calm and direct", "rationale": "The entry is directly linguistically expressed without any qualifier or emotional colour, and is very objective in asking the person under investigation about his or her perception and evaluation of his or her behaviour."}, "time_dimension": {"answer": "Present", "rationale": "The \"Feel\" and \"Do\" in the entry indicate the current psychological state and behaviour of the subject, and therefore the judgement is \"now\"."}, "sentence_type": "Negative declarative sentence", "pos": {"nouns": {"A lot": 1, "Things": 1}, "verbs": {"Do it": 1, "I think": 1, "be": 1}, "adjectives": {"I shouldn't": 1}, "adverbs": {"very": 1, "Both": 1}, "numerals": {"12": 1}, "pronouns": {"I": 1}}}, {"content": "13. Whatever others say, my views remain unchanged.", "word_count": 15, "label": "", "sentence_structure": "Subject鈥損redicate", "subjective_objective": {"answer": "Subjective", "rationale": "Because \"whatever you say\" represents the views or opinions of others, \"by never changing my views\" reflects the subjective attitude of the patient."}, "sentence_sentiment": {"answer": "Negative", "rationale": "Because the article is formulated too firmly and forcefully, it may lead to problems in communicating with others."}, "language_style": {"answer": "Calm and direct", "rationale": "The language style of the entry is relatively simple, there is no excessive emotional rendering or rhetorical style, and the tone is more direct and decisive, expressing the attitude and expectations of the questioners towards the respondents ' adherence to their own views."}, "time_dimension": {"answer": "Future", "rationale": "The keywords \"no matter what others say\" and \"no change\" point to the circumstances that may arise in the future, indicating that the testees will remain in the position of their own view in the face of different views or pressures. The judgement is therefore the future."}, "sentence_type": "Affirmative declarative sentence", "pos": {"verbs": {"Say it": 1, "Change": 1}, "adjectives": {"others": 1}, "adverbs": {"Never": 1}, "pronouns": {"I": 1}}}, {"content": "14. People often misconstrue my bad taste for them.", "word_count": 14, "label": "", "sentence_structure": "Subject鈥搗erb鈥搊bject", "subjective_objective": {"answer": "Subjective", "rationale": "The purpose of the questionnaire is to determine the patient鈥檚 subjective attitude and views."}, "sentence_sentiment": {"answer": "Negative", "rationale": "Because the phrase \"others often misread\" in the entry is negative."}, "language_style": {"answer": "Tactful and warm", "rationale": "The entry is more euphemistic, is more moderate, does not directly blame or criticize the other, but raises questions in a more understanding and respectful tone, thus avoiding the risk of infusing resistance and negative reactions from the other side. At the same time, the entry uses a first person's perspective (\"I\"), making the questionnaire more personal and interactive."}, "time_dimension": {"answer": "Present", "rationale": "The entry uses the expression \"others often\" , in which \"often\" is a regular, customary act, while the word \"will\" adopts the general current pattern, which means that the act not only has occurred in the past but may continue to be so. Thus, the temporal dimension of the article is now judged."}, "sentence_type": "Negative declarative sentence", "pos": {"nouns": {"others": 1, "Misunderstanding": 1, "That's disgusting": 1}, "verbs": {"Yes": 1, "to": 1}, "adverbs": {"Often": 1}, "numerals": {"14": 1}, "pronouns": {"I": 1}}}, {"content": "15. In many cases, I have had to express my doubts about my capabilities.", "word_count": 19, "label": "", "sentence_structure": "Subject鈥揷opula鈥損redicative", "subjective_objective": {"answer": "Subjective", "rationale": "Because \"suspecting one's abilities\" is a subjective emotion and attitude, it is the perception and perception of one's abilities that is being examined."}, "sentence_sentiment": {"answer": "Negative", "rationale": "Because the attitude of \"suspecting your abilities\" in the entry is negative."}, "language_style": {"answer": "Calm and direct", "rationale": "The entry is based on direct questions, is not based on excessive rhetoric or emotional rendering and is more objective in tone."}, "time_dimension": {"answer": "Past", "rationale": ""}, "sentence_type": "Negative declarative sentence", "pos": {"nouns": {"Situation": 1, "self": 2, "Capacity": 1, "Doubt": 1}, "verbs": {"Organisation": 1, "I had to": 1}, "adjectives": {"A lot": 1}, "adverbs": {"very": 1}, "numerals": {"15": 1}}}, {"content": "Some of my friends are different from me, which does not affect our relationship.", "word_count": 25, "label": "", "sentence_structure": "Subject鈥揷opula鈥損redicative", "subjective_objective": {"answer": "Subjective", "rationale": "Because it's about the patients' thoughts and attitudes, whether they think \"the differences between friends don't affect their relationship.\""}, "sentence_sentiment": {"answer": "Positive", "rationale": "Because the idea that \"the person who is different from me doesn't affect our relationship\" in the entry expresses a positive, open and inclusive attitude."}, "language_style": {"answer": "Tactful and warm", "rationale": "The language style of the entry is relatively euphemistic, friendly and friendly. Using words such as \"some,\" \"no impact\" and \"insensitive\" to express tolerance and acceptance of people who are different from their own personality, which is more readily accepted by the interviewees and can make them comfortable and relaxed."}, "time_dimension": {"answer": "Present", "rationale": ""}, "sentence_type": "Affirmative declarative sentence", "pos": {"nouns": {"friends": 2, "Relations": 1, "person": 1}, "verbs": {"have": 1, "Impact": 1, "It's quite different": 1}, "adjectives": {"A completely different person": 1}, "adverbs": {"not": 1}, "numerals": {"16": 1, "Some": 1}, "pronouns": {"I": 2}}}, {"content": "17. Too much interaction with others can easily expose their privacy.", "word_count": 16, "label": "", "sentence_structure": "Subject鈥搗erb鈥搊bject", "subjective_objective": {"answer": "Subjective", "rationale": "Because \"vulnerability\" is the patient's subjective concern and perception, the point of view of the questionnaire is subjective."}, "sentence_sentiment": {"answer": "Negative", "rationale": "Because the \"exposure of privacy\" in the entry is negative."}, "language_style": {"answer": "Calm and direct", "rationale": "The entry presents a fact in an objective and direct manner - the problem of excessive interaction with others that could lead to the disclosure of personal information, and is not based on excessive rhetorical or emotional rendering, which is more objective and objective."}, "time_dimension": {"answer": "Present", "rationale": "The keyword \"exposed\" indicates that it is a constant situation, which is the current state of affairs. At the same time, the article ' s purpose is related to the current psychological state, behaviour habits of the person being tested and can therefore be considered as a description of the present problem."}, "sentence_type": "Affirmative declarative sentence", "pos": {"nouns": {"- To be with someone": 1, "Privacy": 1}, "verbs": {"Easy": 1, "Exposure": 1, "self": 1}, "adjectives": {"More": 1}, "adverbs": {"and": 1}, "numerals": {"17": 1}}}, {"content": "18. I know my feelings for the people around me.", "word_count": 13, "label": "", "sentence_structure": "Subject鈥揷opula鈥損redicative", "subjective_objective": {"answer": "Subjective", "rationale": "Because knowledge of one ' s feelings is a subjective perception and understanding of the patient and a subjective assessment of his or her psychological state."}, "sentence_sentiment": {"answer": "Positive", "rationale": "Because it expresses a self-confidence and self-awareness attitude that it is well aware of its emotions and motivated."}, "language_style": {"answer": "Calm and direct", "rationale": "The purpose of the article is expressed in direct representations, without the use of euphemism or vague language, which is more objective and clear, and which gives a clear picture of the subject of its inquiry as to how they feel about the people around them."}, "time_dimension": {"answer": "Present", "rationale": ""}, "sentence_type": "Affirmative declarative sentence", "pos": {"nouns": {"self": 1, "Emotion": 1, "person": 1}, "verbs": {"Roger that": 1}, "adverbs": {"very": 1}, "numerals": {"18": 1}}}, {"content": "19. I feel that my current situation is far from my requirements.", "word_count": 19, "label": "", "sentence_structure": "Subject鈥搗erb鈥揷omplement", "subjective_objective": {"answer": "Subjective", "rationale": "Because the sentence relates to the patient ' s comparison and evaluation of his or her status and expectations, it is the patient ' s subjective feeling and attitude."}, "sentence_sentiment": {"answer": "Negative", "rationale": "Because it expresses dissatisfaction and disappointment with the status quo, it is a negative emotional attitude."}, "language_style": {"answer": "Calm and direct", "rationale": "The language style of the entry is of a more direct, concise and clear type, with no euphemism or ambiguity. It shows in a clear way that the respondents have a clear understanding of the gap between their status and expectations.\""}, "time_dimension": {"answer": "Present", "rationale": "The term \"currently\" indicates that the question is to ask the present feelings and perceptions of the testees, so the time dimension of the article is now."}, "sentence_type": "Negative declarative sentence", "pos": {"nouns": {"Situation": 2, "Request": 1}, "verbs": {"I think": 1, "Distance": 1, "Far": 1}, "adjectives": {"Current": 1, "Too much": 1}, "numerals": {"19": 1}, "pronouns": {"I": 1}}}, {"content": "I rarely wondered whether what I was doing was right.", "word_count": 15, "label": "", "sentence_structure": "Subject鈥搗erb鈥揷omplement", "subjective_objective": {"answer": "Subjective", "rationale": "This is a self-assessment and reflection of patients and is a subjective observation."}, "sentence_sentiment": {"answer": "Neutral", "rationale": "Because the entry is merely a description of a person ' s behaviour and does not express positive or negative feelings."}, "language_style": {"answer": "Calm and direct", "rationale": "The language style of the entry is more straightforward, there is no excessive rhetoric or emotional rendering, and the respondents are asked whether they considered the correctness of what they do."}, "time_dimension": {"answer": "Present", "rationale": "In the future, the entry \" hardly wants\" to indicate past behaviour and habits, while \"should\" is a reflection and decision-making about future behaviour. Thus, the temporal dimension of the article can be judged to be future."}, "sentence_type": "Negative declarative sentence", "pos": {"nouns": {"Questionnaire": 1, "Entry": 1, "Yes": 1}, "verbs": {"Do it": 1, "Yes": 1, "I think so": 1}, "adverbs": {"Very few": 1, "self": 1}, "numerals": {"20": 1}, "pronouns": {"I": 1}}}, {"content": "21. Many of the problems I have encountered cannot be solved by myself.", "word_count": 16, "label": "", "sentence_structure": "Subject鈥揷opula鈥損redicative", "subjective_objective": {"answer": "Subjective", "rationale": "Because this is the patient ' s own subjective feeling and judgment, the perception of whether or not he or she can solve the problem is subjective."}, "sentence_sentiment": {"answer": "Negative", "rationale": "Because the \"many problems cannot solve\" act in the entry is negative."}, "language_style": {"answer": "Tactful and warm", "rationale": "The entry is more proficient and interactive when it is presented in a gentle and inviting tone, rather than in direct statements or orders. At the same time, the article is more neutral, has no excessive emotional or biased bias, and is more in line with the rigour and objectivity of the psychological questionnaire study."}, "time_dimension": {"answer": "Present", "rationale": "The entry indicates that the testee has encountered many problems that he cannot solve himself in his current life, and thus the time dimension is now judged."}, "sentence_type": "Negative declarative sentence", "pos": {"nouns": {"Problem": 1, "self": 1}, "verbs": {"I met him": 1, "Resolve": 1}, "adjectives": {"A lot": 1}, "numerals": {"21": 1}, "pronouns": {"I": 1}}}, {"content": "22. I know exactly what I am.", "word_count": 12, "label": "", "sentence_structure": "Subject鈥揷opula鈥損redicative", "subjective_objective": {"answer": "Subjective", "rationale": "Because \"what I am\" is a self-awareness and self-evaluation, a patient's subjective attitude."}, "sentence_sentiment": {"answer": "Positive", "rationale": "Because the entries express a clear perception of and confidence in themselves, and are positive emotional attitudes."}, "language_style": {"answer": "Tactful and warm", "rationale": "The entry is a positive phrase, a positive, confident attitude and a more euphemistic or emotional tone, which makes the language more euphemistic. At the same time, the question of the entry is more direct, allowing the respondent to clearly answer his real thoughts."}, "time_dimension": {"answer": "Present", "rationale": "The \"I\" and \"clear\" in the entry indicate that this is a description of the current state, that is, the testee has a clear idea of himself. So the time dimension of the entry is now."}, "sentence_type": "Affirmative declarative sentence", "pos": {"nouns": {"Questionnaire": 1, "Entry": 1}, "verbs": {"Analysis": 1}, "adjectives": {"Clear": 1}, "numerals": {"22": 1}, "pronouns": {"self": 1}}}, {"content": "I can express myself in a very comfortable manner.", "word_count": 15, "label": "", "sentence_structure": "Subject鈥搗erb鈥揷omplement", "subjective_objective": {"answer": "Subjective", "rationale": "Because \"showing what I want to say\" is the patient's subjective feeling and attitude."}, "sentence_sentiment": {"answer": "Positive", "rationale": "Because the act of \"expressing my own words\" is positive."}, "language_style": {"answer": "Calm and direct", "rationale": "The entry presents the capacity of the respondent to express his or her views in a concise and clear manner, with no excessive rhetoric or emotional colour, and with a more objective and direct tone."}, "time_dimension": {"answer": "Present", "rationale": ""}, "sentence_type": "Affirmative declarative sentence", "pos": {"verbs": {"Yes": 1, "Expression": 2}, "adjectives": {"It's a good thing": 1}, "numerals": {"23": 1}}}, {"content": "24. If there is enough evidence, I can also change my own point of view.", "word_count": 20, "label": "", "sentence_structure": "Subject鈥搗erb鈥搊bject", "subjective_objective": {"answer": "Subjective", "rationale": "Because the problem involves a change in the attitude and perception of the patient, it is the subjective perception and will of the individual."}, "sentence_sentiment": {"answer": "Positive", "rationale": "Because the behaviour in the entry is proactive and is intended to be open and adaptable to changing attitudes, this is usually positive."}, "language_style": {"answer": "Tactful and warm", "rationale": "The language style of the entry is more euphemistic, using assumptions and possibilities, without directly emphasizing or ordering what the other party must do. This expression makes the interviewee more comfortable and comfortable and more willing to participate in the questionnaire."}, "time_dimension": {"answer": "Present", "rationale": "The keywords \"also\" and \"may\" indicate that the sentence describes a current attitude or behaviour that is willing to change its own view with sufficient evidence."}, "sentence_type": "Affirmative declarative sentence", "pos": {"nouns": {"Evidence": 1, "Views": 1}, "verbs": {"have": 1, "Change": 1}, "adverbs": {"Yeah": 1, "Enough": 1}, "numerals": {"24": 1}, "pronouns": {"I": 1}}}, {"content": "I rarely think of what I am.", "word_count": 15, "label": "", "sentence_structure": "Subject鈥搗erb鈥揷omplement", "subjective_objective": {"answer": "Subjective", "rationale": "The objective is to examine the patient ' s subjective attitude, i.e. the degree of reflection on his/her identity."}, "sentence_sentiment": {"answer": "Neutral", "rationale": "Because the entry does not have any emotional color."}, "language_style": {"answer": "Calm and direct", "rationale": "The entry is based on a straightforward approach to asking each other about their identity, without too many rhetorical or emotional expressions, and in a more objective and objective manner. At the same time, the question does not show a clear euphemism or affection, and is more focused on obtaining the real thoughts and feelings of the interviewee."}, "time_dimension": {"answer": "Present", "rationale": "The entry \"less thought\" indicates that it is a customary act and is describing the current state of the person being tested, and thus judging as now."}, "sentence_type": "Negative declarative sentence", "pos": {"nouns": {"Questionnaire": 1, "Entry": 1, "person": 1}, "verbs": {"Consider": 1, "be": 1}, "adverbs": {"Very few": 1}, "numerals": {"25": 1}, "pronouns": {"self": 1}}}, {"content": "26. Telling people what they think can cause trouble, not only without help.", "word_count": 22, "label": "", "sentence_structure": "Subject鈥搗erb鈥搊bject", "subjective_objective": {"answer": "Subjective", "rationale": "Because it is a subjective choice and attitude of the individual to put it in the heart."}, "sentence_sentiment": {"answer": "Negative", "rationale": "Because the phrase \"not only is it not helpful to tell others the truth, but it may cause trouble\" in the entry is negative and expresses a negative attitude towards the practice of verbalization."}, "language_style": {"answer": "Calm and direct", "rationale": "The entry is based on a more direct description of the negative effects that the person who told him of his heart may have, without using excessive rhetoric or emotionalization. It is more objective and balanced, giving the impression of calm and rational."}, "time_dimension": {"answer": "Past", "rationale": ""}, "sentence_type": "Negative declarative sentence", "pos": {"nouns": {"In my heart": 1, "others": 1, "Help": 1, "Trouble": 1}, "verbs": {"- Put it on": 1, "Tell": 1, "Got it": 1, "Present": 1}, "adjectives": {"Not only": 1, "Maybe": 1}, "numerals": {"26": 1}, "pronouns": {"you": 1}}}, {"content": "27. When confronted with problems, I felt that others were far away from me.", "word_count": 17, "label": "", "sentence_structure": "Subject鈥揷opula鈥損redicative", "subjective_objective": {"answer": "Subjective", "rationale": "Because it is a subjective emotion and experience, it is an objective fact that others are close to themselves."}, "sentence_sentiment": {"answer": "Negative", "rationale": "Because the expression \"likes someone to be far away\" in the entry expresses negative emotions and attitudes, such as loneliness, helplessness, etc."}, "language_style": {"answer": "Calm and direct", "rationale": "The entry is a direct question-and-answer dialogue, without the use of too much qualifier or emotional terms, and is more balanced and objective and of a calm and direct language style."}, "time_dimension": {"answer": "Present", "rationale": "The entry is more reflective of the current mindset and emotional state of the subjects and is a time dimension, as it is not likely to persist in a given situation."}, "sentence_type": "Negative declarative sentence", "pos": {"nouns": {"Problem": 1, "person": 1}, "verbs": {"I think": 1, "Off": 1, "very": 1}, "adjectives": {"Far": 1}, "adverbs": {"Both": 1}, "numerals": {"27": 1}, "pronouns": {"I": 1}}}, {"content": "I find it difficult to achieve the level I deserve.", "word_count": 15, "label": "", "sentence_structure": "Subject鈥搗erb鈥揷omplement", "subjective_objective": {"answer": "Subjective", "rationale": "This entry examines the patient ' s own views and feelings and is subjective."}, "sentence_sentiment": {"answer": "Negative", "rationale": "Because the expression \"difficulties to achieve the level of self-esteem\" in the entry is negative, indicating some doubt or lack of confidence in their abilities."}, "language_style": {"answer": "Tactful and warm", "rationale": "The entry leads to the other side to express their feelings in a way that encourages and understands, rather than directly criticizes or accuses. This way reduces the psychological pressure on the other side and makes them more willing to share their real thoughts."}, "time_dimension": {"answer": "Present", "rationale": "\"I feel very difficult to play at my own level\" is both the current verbs, which indicate that the voice is feeling when it answers the questionnaire."}, "sentence_type": "Negative declarative sentence", "pos": {"nouns": {"Horizontal": 1}, "verbs": {"I think": 1, "Play": 1}, "adjectives": {"Hard": 1, "You should": 1}, "numerals": {"28": 1}, "pronouns": {"self": 1}}}, {"content": "I am concerned that what I have done can be misunderstood.", "word_count": 19, "label": "", "sentence_structure": "Subject鈥揷opula鈥損redicative", "subjective_objective": {"answer": "Subjective", "rationale": "Because it's the feelings and the worries inside of the patient."}, "sentence_sentiment": {"answer": "Negative", "rationale": "Because \"required\" and \"misunderstanding\" in the entries are words with negative emotional connotations."}, "language_style": {"answer": "Tactful and warm", "rationale": "The language style of the entry is euphemistic. It conveys the feelings and inner feelings of the voice by using the expression \"very worried\" and emphasizes that their concerns are caused by the reaction or behaviour of others. Moreover, the expression \"doing what you do\" is more modest, avoiding the possibility of directly blaming others or boasting about yourself. In general, the language style of the entry reflects the concern and respect for the feelings of others."}, "time_dimension": {"answer": "Present", "rationale": "The entry expresses the current anxiety and anxiety of the subject, and the keywords \"are worried\" and \"misunderstanding\" point to the present situation."}, "sentence_type": "Negative declarative sentence", "pos": {"nouns": {"I'm worried": 1, "self": 1, "What I did": 1, "others": 1, "Misunderstanding": 1}, "verbs": {"Cause": 1, "Fear": 1}, "adverbs": {"very": 1}, "numerals": {"29": 1}}}, {"content": "30. If I found myself underperforming in some areas, I would like to make up for it as soon as possible.", "word_count": 23, "label": "", "sentence_structure": "Subject鈥搗erb鈥搊bject", "subjective_objective": {"answer": "Subjective", "rationale": "Because \"I hope to make it up soon\" is the patient's subjective attitude and expectations."}, "sentence_sentiment": {"answer": "Positive", "rationale": "Because it expresses a self-improvement attitude, it is positive."}, "language_style": {"answer": "Calm and direct", "rationale": "The sentence is based on the following: the entry presents a certain psychological tendency on the other side in a direct manner, without too much rhetorical or emotional rendering, and is more objective in tone. At the same time, the expression \"in the hope of making it up soon\" is more straightforward, and is not expressed in euphemism or vague language."}, "time_dimension": {"answer": "Present", "rationale": ""}, "sentence_type": "Affirmative declarative sentence", "pos": {"nouns": {"self": 1, "In terms of": 1, "Compensatory": 1}, "verbs": {"Found": 1, "Performance": 1, "Hope": 1, "Compensatory": 1}, "adverbs": {"As soon as possible": 1}, "numerals": {"30": 1}, "pronouns": {"I": 1}}}, {"content": "31. Everyone was busy with his own business and had difficulty communicating with them.", "word_count": 18, "label": "", "sentence_structure": "Subject鈥搗erb鈥搊bject", "subjective_objective": {"answer": "Objective", "rationale": "Because the sentence describes an objective fact that people are busy with their own things, making effective communication difficult. While it contains subjective perceptions and feelings (such as \"hard\"), in general it is a statement based on observed situations and is therefore considered objective."}, "sentence_sentiment": {"answer": "Negative", "rationale": "Because the behavior and the result of \"hard to communicate\" in the entry is negative."}, "language_style": {"answer": "Tactful and warm", "rationale": "The language style of the entry is more euphemistic, using the expression \"Everyone is busy with his own business\" to convey the problem rather than directly accusing or complaining. At the same time, by asking each other's views and suggestions, the interviewee is given the opportunity to express his views and feelings, thus enhancing the interactive and equal sense of communication."}, "time_dimension": {"answer": "Past", "rationale": ""}, "sentence_type": "Negative declarative sentence", "pos": {"nouns": {"person": 2, "Things": 1, "Communications": 1}, "verbs": {"in": 2, "Busy": 1, "self": 1, "Both": 1, "very": 1, "and": 1}, "adjectives": {"Hard": 1, "Every": 1}, "adverbs": {"Both": 1, "very": 1}, "numerals": {"31": 1}, "pronouns": {"they": 1}}}, {"content": "32. I believe that those who are better equipped may also encounter difficulties.", "word_count": 17, "label": "", "sentence_structure": "Subject鈥搗erb鈥搊bject", "subjective_objective": {"answer": "Subjective", "rationale": "\"and the patient's subjective attitude and opinion.\""}, "sentence_sentiment": {"answer": "Neutral", "rationale": "The basis for the determination is that the entry is merely a narrative sentence and that there is no apparent emotional tendency or attitude."}, "language_style": {"answer": "Tactful and warm", "rationale": "The entry is more adept and interactive when it is spoken in a gentle and inviting manner, rather than by direct statements or orders."}, "time_dimension": {"answer": "Present", "rationale": "The entry expresses the current view and conviction of the testee that even a person with greater capacity may face difficulties or challenges. The temporal dimension is therefore judged as \"now\"."}, "sentence_type": "Affirmative declarative sentence", "pos": {"nouns": {"Problem": 1, "person": 2, "Capacity": 1, "Strong": 1, "Difficulty": 1}, "verbs": {"I think": 1, "Go, go, go": 1, "Yes": 1}, "adjectives": {"Stronger": 1}, "numerals": {"32": 1}, "pronouns": {"he": 1}}}, {"content": "I often feel I am alone.", "word_count": 13, "label": "", "sentence_structure": "Subject鈥揷opula鈥損redicative", "subjective_objective": {"answer": "Subjective", "rationale": "Because \"fEs\" is the patient's subjective feeling."}, "sentence_sentiment": {"answer": "Negative", "rationale": "Because the entry is an individual's negative emotional experience, and \"Isolation\" is a negative emotional state."}, "language_style": {"answer": "Calm and direct", "rationale": "The language style of the entry is more calm and straightforward, and there is no excessive emotional or rhetorical style, but rather a simple and clear statement of the feeling of the person under investigation. This expression makes the answer more objective and realistic."}, "time_dimension": {"answer": "Present", "rationale": "\"Recurring\" and \"fear\" indicate that it is a normal feeling of the testee, so the temporal dimension is now judged."}, "sentence_type": "Affirmative declarative sentence", "pos": {"nouns": {"Questionnaire": 1, "Entry": 1, "self": 1, "Isolation": 1, "No help": 1}, "verbs": {"I feel": 1, "Regular": 1, "be": 1, "of": 1}, "adjectives": {"Isolation": 1, "No help": 1}, "adverbs": {"Regular": 1}, "numerals": {"33": 1}, "pronouns": {"I": 1}}}, {"content": "34. If there was trouble, it would not help.", "word_count": 16, "label": "", "sentence_structure": "Subject鈥損redicate", "subjective_objective": {"answer": "Subjective", "rationale": "Because \"no good\" is a subjective feeling and judgment."}, "sentence_sentiment": {"answer": "Negative", "rationale": "Because the entry \"doing nothing\" is a negative attitude."}, "language_style": {"answer": "Calm and direct", "rationale": "The entry asks whether the other party feels helpless when it is in trouble, does not use excessive rhetoric or emotional rendering, and is more objective. Its language style is therefore more calm and straightforward."}, "time_dimension": {"answer": "Present", "rationale": "The keywords \"if in trouble\" and \"if nothing\" describe a possible psychological state or behavioural orientation of the person being tested, which can help researchers understand the relevant characteristics of the person's coping strategies or psychological resilience."}, "sentence_type": "Negative declarative sentence", "pos": {"nouns": {"Trouble": 1}, "verbs": {"I met him": 1, "Do it": 1}, "adjectives": {"What": 1, "It's not gonna help": 1}, "adverbs": {"Whatever": 1}, "numerals": {"34": 1}}}, {"content": "35. I always have a clear understanding of my feelings.", "word_count": 13, "label": "", "sentence_structure": "Subject鈥搗erb鈥揷omplement", "subjective_objective": {"answer": "Subjective", "rationale": "Because \"to know your feelings clearly\" is what patients know and understand about themselves, and it's in the context of individual subjective experience."}, "sentence_sentiment": {"answer": "Positive", "rationale": "Because \"clearly understanding your feelings\" is a positive expression."}, "language_style": {"answer": "Tactful and warm", "rationale": "The entry is more adept and interactive when it is spoken in a gentle and inviting manner, rather than by direct statements or orders."}, "time_dimension": {"answer": "Present", "rationale": ""}, "sentence_type": "Affirmative declarative sentence", "pos": {"verbs": {"Always": 1, "Roger that": 1}, "adjectives": {"Clear": 1}, "adverbs": {"Land": 1}, "numerals": {"35": 1}, "pronouns": {"I": 1}}}]}

{"questionnaire_title": "Self-measurement of student relationship", "questionnaire_category": "Mental health", "item_count": 28, "emotion_shift_overall": "From negative to positive", "language_style_overall": "Tactful and warm", "items": [{"content": "1. There is some confusion about their own concerns.", "word_count": 11, "label": "", "sentence_structure": "Subject鈥搗erb鈥揷omplement", "subjective_objective": {"answer": "Subjective", "rationale": "Because \"speech\" is a subjective or emotional experience."}, "sentence_sentiment": {"answer": "Negative", "rationale": "Because the \"speech\" act in the entry is negative."}, "language_style": {"answer": "Tactful and warm", "rationale": "The entry uses euphemism to ask the other side whether it has any obscurantious problems and does not directly pursue or blame, which is more compassionate and comprehensible."}, "time_dimension": {"answer": "Past", "rationale": "The \"old\" in the entry indicates that the question is about the experience and feelings of the testee."}, "sentence_type": "Negative declarative sentence", "pos": {"nouns": {"self": 1, "Trouble": 1}, "verbs": {"have": 1, "Say it": 1, "Statements": 1}, "adverbs": {"That's a good idea": 1}}}, {"content": "2. It is not natural to meet with a living person.", "word_count": 10, "label": "", "sentence_structure": "Subject鈥搗erb鈥揷omplement", "subjective_objective": {"answer": "Subjective", "rationale": "Because \"feel\" is the patient's subjective feeling."}, "sentence_sentiment": {"answer": "Negative", "rationale": "Because the description of \"frightful\" in the entry is negative."}, "language_style": {"answer": "Calm and direct", "rationale": "Although the entry asks about a subjective feeling, it does not use excessive rhetoric or emotional rendering, but rather asks the interviewee in a more objective, straightforward way. It is therefore judged that the language style of the entry is \"cool and straight\"."}, "time_dimension": {"answer": "Present", "rationale": ""}, "sentence_type": "Negative declarative sentence", "pos": {"nouns": {"Livers": 1, "Feel": 1, "Meet me": 1}, "verbs": {"and": 1, "Meet me": 1, "Feel": 1}, "adjectives": {"It's not natural": 1}}}, {"content": "3. Exalt envy and envy.", "word_count": 10, "label": "", "sentence_structure": "Subject鈥搗erb鈥搊bject", "subjective_objective": {"answer": "Subjective", "rationale": "Because \"excessive,\" \"envious and jealous\" are all words that describe patients' subjective feelings."}, "sentence_sentiment": {"answer": "Negative", "rationale": "Because \"envious and jealous\" in the entry is negative."}, "language_style": {"answer": "Calm and direct", "rationale": "The language style of the entry is more direct, concise, with no excessive qualifier or emotional colour, and is typical of the calm, direct language style."}, "time_dimension": {"answer": "Future", "rationale": "The keyword is \"excessive envy and envy\", which describes a mental state or behaviour that may arise in the future and is therefore judged to be future."}, "sentence_type": "Negative declarative sentence", "pos": {"nouns": {"Grandpa": 1, "Investigations": 1, "Questionnaire": 1, "Entry": 1, "person": 1, "others": 1}, "verbs": {"Envious": 1, "Jealousy": 1, "It's too much": 1}, "adjectives": {"That's too much": 1}, "adverbs": {"Land": 1}, "numerals": {"3锛?: 1}, "pronouns": {"you": 1}}}, {"content": "4. Too little is being done with heterosexuals.", "word_count": 7, "label": "", "sentence_structure": "Subject鈥搗erb鈥揷omplement", "subjective_objective": {"answer": "Subjective", "rationale": "Because less interaction is a subjective assessment, it may be influenced by factors such as the values, experiences and the patient ' s own experience."}, "sentence_sentiment": {"answer": "Negative", "rationale": "Because the \"too little\" act in the entry is negative."}, "language_style": {"answer": "Tactful and warm", "rationale": "The entry is more proficient and interactive, using a gentle and inviting tone of speech rather than a direct statement or order. Moreover, the article is more objective and does not have too much rhetorical or emotional accent. The language style of the entry can therefore be considered as warm."}, "time_dimension": {"answer": "Future", "rationale": "The entry describes a possible future scenario or trend in which the testee may have fewer heterosexual experiences in the future. The judgement is therefore future."}, "sentence_type": "Negative declarative sentence", "pos": {"nouns": {"Heterosexual": 1, "- To be with someone": 1}, "verbs": {"Too much": 1, "Less": 1}, "adverbs": {"not": 1, "and": 1}, "numerals": {"4": 1}}}, {"content": "5. The ongoing talks were difficult.", "word_count": 12, "label": "", "sentence_structure": "Subject鈥搗erb鈥揷omplement", "subjective_objective": {"answer": "Subjective", "rationale": "Because the difficulty of having continuous talks is the patient ' s personal subjective feelings and experiences."}, "sentence_sentiment": {"answer": "Negative", "rationale": "Because \"difficult\" in the entry is negative."}, "language_style": {"answer": "Calm and direct", "rationale": "The language style of the item is more direct and clear. It is not used in any qualifier or emotional rendering, but is presented in a simple question that makes it difficult for the answeree to be able to talk constantly. Thus, the language style of the item can be described as being straightforward."}, "time_dimension": {"answer": "Present", "rationale": "The entry describes a current symptom or experience, i.e. the testee will feel difficult when talking on a continuous basis. The time dimension is therefore now judged."}, "sentence_type": "Negative declarative sentence", "pos": {"nouns": {"Meetings": 1, "Difficulties": 1}, "verbs": {"to": 1, "Continuous": 1, "I feel": 1, "Conduct": 1}, "adjectives": {"Always": 1}, "numerals": {"5锛?: 1}}}, {"content": "6. Tensions are felt in social settings.", "word_count": 9, "label": "", "sentence_structure": "Subject鈥揷opula鈥損redicative", "subjective_objective": {"answer": "Subjective", "rationale": "Because feeling nervous is a subjective emotional experience and feeling."}, "sentence_sentiment": {"answer": "Negative", "rationale": "Because the \"frightful\" sentiment in the entry is negative."}, "language_style": {"answer": "Calm and direct", "rationale": "The language style of the entry is more direct, concise, and there is no excessive qualifier or emotional rendering, and the person is asked whether he or she is nervous in a social setting."}, "time_dimension": {"answer": "Present", "rationale": "The entry indicates a emotional reaction that is often present in social settings and is now the norm."}, "sentence_type": "Affirmative declarative sentence", "pos": {"nouns": {"Socially": 1, "Nervous": 1}, "verbs": {"I feel": 1}}}, {"content": "7. People are often hurt.", "word_count": 6, "label": "", "sentence_structure": "Subject鈥搗erb鈥揷omplement", "subjective_objective": {"answer": "Subjective", "rationale": "Because \"often hurting\" is a subjective description of behaviour and attitude that needs to be assessed in the light of the patient's own feelings and behaviour."}, "sentence_sentiment": {"answer": "Negative", "rationale": "Because the \"harming\" act is negative, it has a negative emotional tone."}, "language_style": {"answer": "Calm and direct", "rationale": "The entry uses a simple and clear language, asking directly whether the other party is often hurting others, without excessive acrimonyms or emotional colours, and is more calm and straightforward."}, "time_dimension": {"answer": "Present", "rationale": "In this entry, \"frequently\" means frequency and custom, while \"harming someone\" is an ongoing, uncompleted act, and it can be judged that the act continues in the life of the person being tested. Thus, the temporal dimension of the article is now."}, "sentence_type": "Negative declarative sentence", "pos": {"nouns": {"Hurt": 1, "others": 1}, "verbs": {"Often": 1, "Hurt": 1}}}, {"content": "8. It is not natural to have sexual relations with opposite sex.", "word_count": 10, "label": "", "sentence_structure": "Subject鈥搗erb鈥揷omplement", "subjective_objective": {"answer": "Subjective", "rationale": "Because \"feel\" is a subjective feeling and experience of a human being, a subjective perspective."}, "sentence_sentiment": {"answer": "Negative", "rationale": "Because the attitude of \"frightful\" in the entries is negative."}, "language_style": {"answer": "Tactful and warm", "rationale": "The language style of the entry is more euphemistic and moderate, using the form of a question and question phrase, expressing a sense of concern and understanding without direct criticism or criticism. At the same time, the tone is easier and friendly, and it is easier to resonate and engage the respondents."}, "time_dimension": {"answer": "Present", "rationale": "The entry asks about the current feelings and state of the person being tested, and does not address past or future situations, so it is judged as \"now\"."}, "sentence_type": "Negative declarative sentence", "pos": {"nouns": {"Heterosexual": 1, "- To be with someone": 1}, "verbs": {"Travel": 1, "Feel": 1, "It's not natural": 1}, "numerals": {"8锛?: 1}}}, {"content": "9. With a large group of friends, it is common to feel lonely or lost.", "word_count": 17, "label": "", "sentence_structure": "Subject鈥揷opula鈥損redicative", "subjective_objective": {"answer": "Subjective", "rationale": "Because it's a subjective experience."}, "sentence_sentiment": {"answer": "Negative", "rationale": "Because \"silent\" and \"lost\" in the entries are negative emotional states."}, "language_style": {"answer": "Tactful and warm", "rationale": ""}, "time_dimension": {"answer": "Present", "rationale": ""}, "sentence_type": "Negative declarative sentence", "pos": {"nouns": {"friends": 1, "Group": 1, "Feel": 1, "Loneliness": 1, "Lost": 1}, "verbs": {"and": 1, "in": 1, "Together": 1, "I always feel": 1}, "adverbs": {"Large": 1, "Regular": 1}, "numerals": {"One": 2}, "classifiers": {"Group": 1, "individual": 1}}}, {"content": "10. Extreme vulnerability.", "word_count": 4, "label": "", "sentence_structure": "Subject鈥揷opula鈥損redicative", "subjective_objective": {"answer": "Subjective", "rationale": "Because vulnerability is the result of a subjective feeling."}, "sentence_sentiment": {"answer": "Negative", "rationale": "Because \"vulnerable\" is a negative attitude and feeling."}, "language_style": {"answer": "Tactful and warm", "rationale": "The language style of the entry is euphemism. Instead of being too direct, it asks the interviewee whether he is embarrassed or embarrassed in a gentle and respectful manner, which makes it easier for the interviewee to share his real feelings."}, "time_dimension": {"answer": "Present", "rationale": ""}, "sentence_type": "Negative declarative sentence", "pos": {"verbs": {"I'm embarrassed": 1}, "adjectives": {"Very easy": 1}}}, {"content": "I am not in harmony with people.", "word_count": 9, "label": "", "sentence_structure": "Subject鈥搗erb鈥揷omplement", "subjective_objective": {"answer": "Subjective", "rationale": "Because it is a self-evaluation of the feelings and thoughts of the patients themselves, namely, \"not living in harmony with the human being\", it is subjective."}, "sentence_sentiment": {"answer": "Negative", "rationale": "Because the \"not living together\" act in the entry is negative."}, "language_style": {"answer": "Tactful and warm", "rationale": "The entry is more euphemistic and moderate, not directly accusing or criticizing the person surveyed, but rather allowing the person to share his/her feelings and experiences in an invitation manner. This expression is more respectful of the interviewee and more likely to receive real answers and feedback."}, "time_dimension": {"answer": "Present", "rationale": ""}, "sentence_type": "Negative declarative sentence", "pos": {"nouns": {"Questionnaire": 1, "Entry": 1}, "verbs": {"Analysis": 1, "Harmony": 1}, "numerals": {"11锛?: 1}}}, {"content": "12. It was not clear how it was possible to stop living with opposite sex.", "word_count": 14, "label": "", "sentence_structure": "Subject鈥搗erb鈥揷omplement", "subjective_objective": {"answer": "Subjective", "rationale": "Because \"how to stop\" requires the patient's personal experience and judgment to answer, it is subjective."}, "sentence_sentiment": {"answer": "Neutral", "rationale": "Because the entry does not have any emotional color."}, "language_style": {"answer": "Tactful and warm", "rationale": "The entry asks each other about the confusion about dealing with heterosexual relationships in a gentle and respectful manner, without direct accusations or criticism, but rather with care and support. This expression is more likely to gain the trust of the interviewee and to be motivated by a willingness to share real ideas."}, "time_dimension": {"answer": "Present", "rationale": "The future of the entry implies that the testee needs to acquire some new social skills in the future, and therefore it involves future actions and attitudes. At the same time, \"unsure\" suggests that the testee may not yet have a full grasp of the skills to be able to stop in the face of the opposite sex, which can also be seen as a future goal or demand."}, "sentence_type": "Interrogative sentence", "pos": {"nouns": {"Heterosexual": 1, "Get along": 1, "Stop it": 1}, "verbs": {"Yes": 1, "How's that": 1, "Get along": 1}, "adverbs": {"Stop it": 1}, "numerals": {"12": 1}}}, {"content": "13. Unacquainted people often feel uncomfortable when they tell themselves about their lives and their experiences in order to gain sympathy.", "word_count": 31, "label": "", "sentence_structure": "Subject鈥搗erb鈥搊bject", "subjective_objective": {"answer": "Subjective", "rationale": "Because feeling uncomfortable is a personal, subjective feeling and experience."}, "sentence_sentiment": {"answer": "Negative", "rationale": "Because the \"frightful\" behavior and emotional reaction in the entry is negative."}, "language_style": {"answer": "Tactful and warm", "rationale": "The basis for the judgement: The article is more moderate, does not direct the order or criticism of the other, but asks the respondent in an understanding and care about the feelings of a certain situation. At the same time, the words \"speak\" and \"sympathize\" are used to highlight the emotional colour of care and understanding."}, "time_dimension": {"answer": "Past", "rationale": "In combination, the temporal dimension of the article is mainly the past and present."}, "sentence_type": "Affirmative declarative sentence", "pos": {"nouns": {"person": 1, "Problems": 1, "Compassion": 1, "self": 2}, "verbs": {"to": 1, "Speech": 1, "I feel": 1}, "adjectives": {"I don't know": 1, "Life": 1, "Regular": 1, "I'm not comfortable": 1}, "numerals": {"13": 1}}}, {"content": "Fear of what bad impressions others have of themselves.", "word_count": 13, "label": "", "sentence_structure": "Subject鈥搗erb鈥揷omplement", "subjective_objective": {"answer": "Subjective", "rationale": "Because \"fear\" is a subjective feeling or attitude."}, "sentence_sentiment": {"answer": "Negative", "rationale": "Because the mood for \"fearing someone to have a bad impression of themselves\" in the entry is negative."}, "language_style": {"answer": "Tactful and warm", "rationale": "The entry is more adept and interactive when it is spoken in a gentle and inviting manner, rather than by direct statements or orders."}, "time_dimension": {"answer": "Present", "rationale": "The entry describes an emotional occurrence, in which the testee is worried about the problem when he fills out the questionnaire, and the temporal dimension is now judged."}, "sentence_type": "Interrogative sentence", "pos": {"nouns": {"person": 1, "self": 1, "Impression": 1}, "verbs": {"I'm worried": 1, "have": 1, "Bad": 1}, "adjectives": {"Bad": 1}, "numerals": {"14": 1}, "pronouns": {"What": 1}}}, {"content": "15. Every effort was made to impress themselves.", "word_count": 11, "label": "", "sentence_structure": "Subject鈥搗erb鈥揷omplement", "subjective_objective": {"answer": "Subjective", "rationale": "Because the \"always try\" in the title indicates that it is a self-evaluation and effort of the patient and a subjective attitude."}, "sentence_sentiment": {"answer": "Positive", "rationale": "Because the actions in the entries \"do your best to make others appreciate themselves\" express a positive individual mentality and a desire to seek approval from others."}, "language_style": {"answer": "Tactful and warm", "rationale": "The entry uses a more moderate and encouraging language style to resonate and actively participate. It does not direct orders or accusations, but rather makes the other person more willing to share his/her thoughts and feelings, thereby better understanding his/her motivation and mental state. This expression is more personal and interactive, stimulating each other鈥檚 motivation and self-reflection, and helps the questionnaire researchers to better collect data and understand the behaviour and ideas of the interviewee."}, "time_dimension": {"answer": "Present", "rationale": "The keyword \"always try to make\" indicates that it is a sentence describing the current behaviour, that the testee is doing it on a regular basis at the present time. So the time dimension of this questionnaire entry is now."}, "sentence_type": "Imperative sentence", "pos": {"nouns": {"Grandpa": 1, "Entry": 1}, "verbs": {"Down": 1, "Please": 1, "Analysis": 1, "Admirable": 1, "鈾?And I'm gonna be 鈾?: 1, "Try": 1}, "adjectives": {"Down there": 1, "self": 1}, "numerals": {"15": 1}, "classifiers": {"锛?: 1}}}, {"content": "16. Self-consciousness is a thing of the opposite sex.", "word_count": 6, "label": "", "sentence_structure": "Subject鈥損redicate", "subjective_objective": {"answer": "Subjective", "rationale": "Because it's the patient's personal psychological state."}, "sentence_sentiment": {"answer": "Negative", "rationale": "Because the act of \"mixing yourself with opposite sex\" in the entry can cause negative emotional and psychological stress."}, "language_style": {"answer": "Tactful and warm", "rationale": ""}, "time_dimension": {"answer": "Present", "rationale": ""}, "sentence_type": "Interrogative sentence", "pos": {"nouns": {"From the shadows": 1, "Smooze": 1, "Heterosexual": 1}}}, {"content": "17. It is often avoided to express their feelings.", "word_count": 11, "label": "", "sentence_structure": "Subject鈥搗erb鈥揷omplement", "subjective_objective": {"answer": "Subjective", "rationale": "Because \"resisting from expressing your feelings\" is a manifestation of a person's psychological activity and attitude and is subjective."}, "sentence_sentiment": {"answer": "Negative", "rationale": "Because the \"avoid expressing your feelings\" act in the entry is negative."}, "language_style": {"answer": "Calm and direct", "rationale": "The entry directly expresses the subject of asking the other party whether it is often avoided expressing its feelings, without excessive rhetoric or emotional rendering, and with a more balanced and objective tone."}, "time_dimension": {"answer": "Present", "rationale": "The keyword \"often avoids expressing his feelings\" refers to the current behaviour and state, and thus the present is judged."}, "sentence_type": "Imperative sentence", "pos": {"nouns": {"Feel": 1}, "verbs": {"Avoid": 1, "Expression": 1}, "adverbs": {"Often": 1}}}, {"content": "18. Lack of confidence in the way you behave.", "word_count": 12, "label": "", "sentence_structure": "Subject鈥搗erb鈥揷omplement", "subjective_objective": {"answer": "Subjective", "rationale": "Because lack of confidence in one ' s own morphology (morphology) is a subjective feeling or assessment, not an objective fact."}, "sentence_sentiment": {"answer": "Negative", "rationale": "Because the \"lack of confidence\" attitude in the entries is negative."}, "language_style": {"answer": "Tactful and warm", "rationale": ""}, "time_dimension": {"answer": "Present", "rationale": ""}, "sentence_type": "Negative declarative sentence", "pos": {"nouns": {"self": 1, "Ceremonial": 1, "Looks like a": 1}, "verbs": {"Lack": 1, "Confidence": 1}}}, {"content": "19. Disgusting someone or being hated by someone.", "word_count": 11, "label": "", "sentence_structure": "Subject鈥搗erb鈥揷omplement", "subjective_objective": {"answer": "Subjective", "rationale": "Because \"hate\" and \"repulsed\" are subjective feelings and attitudes of a person."}, "sentence_sentiment": {"answer": "Negative", "rationale": "Because the \"hot or hated\" act in the entry is negative."}, "language_style": {"answer": "Tactful and warm", "rationale": "The entry raises questions in a more moderate way, avoiding language that is too direct or offensive, and makes it easier for respondents to accept and share their views by using the term \"hot or hated\". This expression is more appropriate for psychological questionnaire research."}, "time_dimension": {"answer": "Past", "rationale": "The term \"disguised\" and \"obsessed\" in the entry are all descriptions of interpersonal relationships, but they do not clearly indicate whether they are past, present or future. Thus, by context and common sense, the temporal dimension of the entry can be the state of the relationship, past or present."}, "sentence_type": "Affirmative declarative sentence", "pos": {"nouns": {"person": 2}, "verbs": {"Shit": 2, "Sou-san": 1}, "numerals": {"19": 1, "Some": 1}, "pronouns": {"Someone": 1}}}, {"content": "20. The opposite sex is not to be seen.", "word_count": 5, "label": "", "sentence_structure": "Subject鈥搗erb鈥揷omplement", "subjective_objective": {"answer": "Subjective", "rationale": "Because the contempt for opposite sex is a subjective attitude or perception, it reflects the assessment and attitude of the patient towards the opposite sex and is the expression of the person ' s inner feelings and thoughts."}, "sentence_sentiment": {"answer": "Negative", "rationale": "Because the attitude of \"deserving opposite sex\" is negative, it expresses disrespect for and discrimination against opposite sex."}, "language_style": {"answer": "Tactful and warm", "rationale": "Warm and warm."}, "time_dimension": {"answer": "Past", "rationale": ""}, "sentence_type": "Affirmative declarative sentence", "pos": {"nouns": {"Heterosexual": 1}, "verbs": {"I'm not looking down": 1}}}, {"content": "21. There is no focus on listening.", "word_count": 7, "label": "", "sentence_structure": "Subject鈥搗erb鈥揷omplement", "subjective_objective": {"answer": "Subjective", "rationale": "Because the inability to listen is a subjective experience and feeling."}, "sentence_sentiment": {"answer": "Negative", "rationale": "Because the \"not listening\" in the entry is negative."}, "language_style": {"answer": "Tactful and warm", "rationale": ""}, "time_dimension": {"answer": "Present", "rationale": ""}, "sentence_type": "Negative declarative sentence", "pos": {"nouns": {"Focus": 1, "Listen": 1}, "verbs": {"Yes": 1, "Focus": 1, "Listen": 1}, "adjectives": {"No, I can't": 1}, "adverbs": {"Land": 1}}}, {"content": "22. No one can complain about their own problems.", "word_count": 10, "label": "", "sentence_structure": "Subject鈥搗erb鈥揷omplement", "subjective_objective": {"answer": "Subjective", "rationale": "Because \"no one can complain\" is a personal subjective feeling and attitude that reflects the subjective experience of patients who are unable to understand and support their own worries."}, "sentence_sentiment": {"answer": "Negative", "rationale": "Because the \"no one can complain about their troubles\" in the entry is negative."}, "language_style": {"answer": "Tactful and warm", "rationale": "The language style of the entry is more euphemistic, not directly expressing the meaning of each other鈥檚 distress or need for help, but rather, in a spirit of encouragement and understanding, to guide the interviewee to express his thoughts. This moderate presentation is more comfortable and willing to share his troubles, which is very beneficial for the researchers of the psychological questionnaire."}, "time_dimension": {"answer": "Present", "rationale": "The entry asks whether the person tested is currently in a state of distress that prevents him from being heard by others, and therefore the basis of the judgement is now."}, "sentence_type": "Negative declarative sentence", "pos": {"nouns": {"Trouble": 1, "The complaint": 1}, "verbs": {"have": 1, "No one": 1}, "adverbs": {"self": 1}, "numerals": {"22": 1}}}, {"content": "23. Exclusion and indifference.", "word_count": 8, "label": "", "sentence_structure": "Subject鈥搗erb鈥揷omplement", "subjective_objective": {"answer": "Subjective", "rationale": "Because being excluded and apathy is the patient ' s subjective feelings and experience and is viewed from a subjective perspective."}, "sentence_sentiment": {"answer": "Negative", "rationale": "Because \"exclusion and indifference\" in the entries is a negative emotional color."}, "language_style": {"answer": "Tactful and warm", "rationale": "The entry uses a more euphemistic term, \"exclusion and indifference\", without the use of negative terms such as \"repulsion\" and \"neglect\" directly, which are more acceptable to the interviewees and avoid triggering their resistance. At the same time, this language style conveys a sense of concern and understanding that can create better communication."}, "time_dimension": {"answer": "Past", "rationale": ""}, "sentence_type": "Negative declarative sentence", "pos": {"nouns": {"Exclusion": 1, "Coldness": 1}, "verbs": {"Yes": 1, "Exclusion": 1, "Coldness": 1}, "numerals": {"23": 1}}}, {"content": "24. Discretion from the opposite sex.", "word_count": 6, "label": "", "sentence_structure": "Subject鈥搗erb鈥揷omplement", "subjective_objective": {"answer": "Subjective", "rationale": "Because being \"sighted at the opposite sex\" is a subjective feeling and evaluation, different people may have different views and experiences. This subjective perception and evaluation is considered to be a subjective perspective of psychological questionnaires."}, "sentence_sentiment": {"answer": "Negative", "rationale": "Because the act of being despised by opposite sex is negative."}, "language_style": {"answer": "Tactful and warm", "rationale": ""}, "time_dimension": {"answer": "Past", "rationale": "In the entry, \"sighted by the opposite sex\" is a past experience or feeling, and therefore the temporal dimension is a past."}, "sentence_type": "Negative declarative sentence", "pos": {"nouns": {"Heterosexual": 1, "I'm not looking down": 1}, "verbs": {"By": 1, "I'm not looking down": 1}}}, {"content": "25. Views and opinions cannot be widely heard.", "word_count": 13, "label": "", "sentence_structure": "Subject鈥搗erb鈥揷omplement", "subjective_objective": {"answer": "Subjective", "rationale": "The attitude and opinion of the patient, i.e. whether or not they can be heard widely, is considered subjective."}, "sentence_sentiment": {"answer": "Negative", "rationale": "Because the act of \"not hearing a wide range of opinions and opinions\" in the entry is negative."}, "language_style": {"answer": "Calm and direct", "rationale": "The entry uses a concise language, which directly describes the problems that the person under investigation has in hearing from others, is not over-modified or emotional and is more calm and objective."}, "time_dimension": {"answer": "Present", "rationale": "The entry describes a state of capacity or behaviour, using the general current \"no\", which indicates that this phenomenon is taking place."}, "sentence_type": "Negative declarative sentence", "pos": {"nouns": {"Observations": 2}, "verbs": {"Listen": 1, "Hiro": 1, "Remove": 1}, "adjectives": {"Wide": 1}, "adverbs": {"Land": 2}, "numerals": {"25": 1}}}, {"content": "26. He was often in deep pain because of his injuries.", "word_count": 11, "label": "", "sentence_structure": "Subject鈥搗erb鈥揷omplement", "subjective_objective": {"answer": "Subjective", "rationale": "Since self-inflicted self-pity is an emotional experience and feeling of an individual, it is a subjective approach."}, "sentence_sentiment": {"answer": "Negative", "rationale": "Because the \"silent self-depressing\" act in the entry is negative."}, "language_style": {"answer": "Tactful and warm", "rationale": "The entry is a more gentle and friendly interview with the interviewee about the fact that he/she is often sad about being hurt, and the term is more euphemistic and more personal and interactive."}, "time_dimension": {"answer": "Present", "rationale": "The keyword \"always\" indicates that it is a customary act, that both the actions of \"injured\" and \"intimately sad\" are the result of past, present, so the subject should be classified as now."}, "sentence_type": "Affirmative declarative sentence", "pos": {"nouns": {"self": 1, "Wounded": 1, "Sad": 1}, "verbs": {"Regular": 1, "Because": 1, "From the shadows": 1, "Sad": 1}, "adverbs": {"Regular": 1, "From the shadows": 1}, "numerals": {"26": 1}}}, {"content": "27. People often talk about it and fool around.", "word_count": 8, "label": "", "sentence_structure": "Subject鈥搗erb鈥揷omplement", "subjective_objective": {"answer": "Subjective", "rationale": "The first is the fact that the people are often talked about, and are duped about, the subjective feelings and experiences of patients."}, "sentence_sentiment": {"answer": "Negative", "rationale": "Because the \"often talked about and fooled\" in the entry is negative."}, "language_style": {"answer": "Tactful and warm", "rationale": "The language style of the entry is more euphemistic, it is described in a more moderate way, it is not too direct or rigid language, and it is more acceptable and understandable. At the same time, \"to be talked about\" and \"smuggling\" are relatively negative elements, and in this way, it is more reflective of the interest and respect shown to the interviewee."}, "time_dimension": {"answer": "Present", "rationale": ""}, "sentence_type": "Negative declarative sentence", "pos": {"nouns": {"person": 1, "Talk": 1, "Smuggle": 1}, "verbs": {"I'm always in the mood": 1}, "adverbs": {"very": 1}, "numerals": {"27锛?: 1}}}, {"content": "28. It was not clear how heterosexual relationships could be better managed.", "word_count": 14, "label": "", "sentence_structure": "Subject鈥搗erb鈥搊bject", "subjective_objective": {"answer": "Subjective", "rationale": "This sentence is about patients鈥?subjective feelings and thoughts about their situation, which is that they feel difficult and troubled when they have sex with opposite sex."}, "sentence_sentiment": {"answer": "Negative", "rationale": "Because the fact that \"I don't know how to get along\" in the entry implies that the interviewee has difficulties and shortcomings in heterosexual relations, is a negative emotional colour."}, "language_style": {"answer": "Tactful and warm", "rationale": "The entry is more euphemistic and warm, not directly accusing or criticizing the other party for its shortcomings in heterosexual relations, but rather engaging in a dialogue in a sense of inquiry and assistance, so that the interviewee feels more friendly and supportive. At the same time, the question gives the interviewee sufficient space to reflect and reflect on his behaviour and to seek ways to improve it."}, "time_dimension": {"answer": "Present", "rationale": ""}, "sentence_type": "Interrogative sentence", "pos": {"nouns": {"Heterosexual": 1, "- To be with someone": 1, "Get along": 1}, "verbs": {"I don't know": 1, "How's that": 1, "Better": 1, "Land": 1, "Hand it over": 1, "Location": 1}, "adverbs": {"More": 1, "Land": 1}, "numerals": {"28锛?: 1}}}]}

{"questionnaire_title": "Drinking Questionnaire (ADS)", "questionnaire_category": "Behavioral questionnaire", "item_count": 25, "emotion_shift_overall": "No obvious trend", "language_style_overall": "Calm and direct", "items": [{"content": "1. How many did you drink last time?", "word_count": 12, "label": "", "sentence_structure": "Subject鈥搗erb鈥搊bject", "subjective_objective": {"answer": "Objective", "rationale": "The basis for the judgement: because \"how much is drunk\" is an objective fact, it can be measured and recorded. Although individual drinking habits and preferences may differ subjectively, the fact of asking is, in itself, objective in this particular question."}, "sentence_sentiment": {"answer": "Neutral", "rationale": "Because the entry does not have any emotional color."}, "language_style": {"answer": "Calm and direct", "rationale": "The language style of the entry is more concise, and there is no excessive emotional or rhetorical style, but rather a direct way of asking questions, which enables the respondents to answer the questions accurately."}, "time_dimension": {"answer": "Past", "rationale": "The entry \"last\" suggests that this is an event of the past, and that \"drinking\" is a specific act of action, so it is judged to be the past."}, "sentence_type": "Interrogative sentence", "pos": {"nouns": {"Most recently": 1, "Number of times": 1, "Wine": 1, "Time": 1}, "verbs": {"Drink": 1, "How much": 1}, "adverbs": {"Most": 1, "Near": 1}, "numerals": {"One": 1}, "classifiers": {"Number of times": 1, "Cups": 1}, "pronouns": {"you": 1}}}, {"content": "2. Do you often get drunk on holidays or weekends and have headaches, nausea, and all kinds of discomfort the next morning?", "word_count": 27, "label": "", "sentence_structure": "Subject鈥搗erb鈥搊bject", "subjective_objective": {"answer": "Objective", "rationale": "Because \"the following morning, often after a holiday or weekend of intoxication, is an objective fact experienced by the patient, and although it is related to his behaviour and feelings, it is not the patient's subjective thoughts or attitudes."}, "sentence_sentiment": {"answer": "Negative", "rationale": "Because the symptoms described in the entry as \"headaches, nausea, discomfort\" are negative."}, "language_style": {"answer": "Calm and direct", "rationale": "The language style of the entry is more calm and straightforward, without too much embellishment and emotional rendering. It raises the question in an objective and straightforward way of asking whether the person surveyed often suffers from hangovers after getting drunk on holidays or weekends."}, "time_dimension": {"answer": "Present", "rationale": ""}, "sentence_type": "Interrogative sentence", "pos": {"nouns": {"Holidays": 1, "Weekend": 1, "Drunk": 1, "Next": 1, "Morning": 1, "Head": 1, "Gross": 1, "All over": 1, "I'm not well": 1, "Hangover": 1}, "verbs": {"Drink": 1, "Still": 1, "Headache": 1, "Gross": 1, "I'm not well": 1, "Hangover": 1}, "adverbs": {"Regular": 1, "Still": 1}, "numerals": {"2": 1}}}, {"content": "Are you shaking your hands after you wake up?", "word_count": 8, "label": "", "sentence_structure": "Subject鈥搗erb鈥搊bject", "subjective_objective": {"answer": "Objective", "rationale": "It is an objective question whether the handshake is a real expression of alcohol consumption without taking into account the patient鈥檚 subjective attitude or emotional orientation."}, "sentence_sentiment": {"answer": "Negative", "rationale": "Because the phenomenon of hand shaking is often seen as an abnormal physical reaction, it may imply bad habits such as alcohol or health problems."}, "language_style": {"answer": "Calm and direct", "rationale": "The entry is more calmly and straightforwardly, and in a concise and clear manner, the person surveyed is asked whether he or she is awake to the symptoms of hand shaking, and there is no excessive emotional colour or acrimony."}, "time_dimension": {"answer": "Present", "rationale": ""}, "sentence_type": "Interrogative sentence", "pos": {"nouns": {"Wake up": 1, "Shake your hands": 1}, "verbs": {"have": 1, "Whim": 1}, "numerals": {"3": 1}}}, {"content": "4. Have you ever felt uncomfortable (e.g., disgusting, stomach ache) because of drinking?", "word_count": 19, "label": "", "sentence_structure": "Subject鈥搗erb鈥搊bject", "subjective_objective": {"answer": "Subjective", "rationale": "Because \"incommodity\" is the patient's subjective experience and feeling. While drinking may be an objective factor, self-incommitment is the patient's own subjective reaction and evaluation."}, "sentence_sentiment": {"answer": "Negative", "rationale": "Because the behavior in the entries \"drinking discomfort\" is negative."}, "language_style": {"answer": "Tactful and warm", "rationale": ""}, "time_dimension": {"answer": "Past", "rationale": "4. You've been feeling uncomfortable for drinking (e.g., disgusting, stomach pain)? [input sentence]"}, "sentence_type": "Interrogative sentence", "pos": {"nouns": {"Reason": 1, "Wine": 2, "you": 2, "I'm not well": 1, "Feel": 1, "Gross": 1, "Stomach pain": 1}, "verbs": {"Self-conscious": 1, "Drink": 1, "I'm not feeling well": 1}, "numerals": {"4": 1}, "pronouns": {"Tsang": 1}}}, {"content": "Have you ever had a vibrating moment? (The vibrating means hearing a voice that does not exist, seeing something that does not exist, feeling very uneasy, anxious, excited, shaking hands, etc.)", "word_count": 42, "label": "", "sentence_structure": "Subject鈥搗erb鈥搊bject", "subjective_objective": {"answer": "Objective", "rationale": "Because tremors are a pathological symptom and an objective condition."}, "sentence_sentiment": {"answer": "Negative", "rationale": "Because the entry describes an abnormal state of mind that may lead to negative emotions and behaviour."}, "language_style": {"answer": "Calm and direct", "rationale": "The entry describes the symptoms of tremors in a concise and clear language and asks the interviewees whether they have ever been."}, "time_dimension": {"answer": "Present", "rationale": "The questionnaire entry asks if the person tested had had a history of tremors, which is a description of the past. But, because the symptoms can persist, they can also be considered as current. Thus, the time dimension of the article is both historical and present."}, "sentence_type": "Interrogative sentence", "pos": {"nouns": {"鈾?The tremors 鈾?: 1, "You're a good man": 1, "Sound": 1, "Something": 1, "Feel": 1, "Unsettled": 1, "Anxiety": 1, "Excited": 1, "Shake your hands": 1}, "verbs": {"There was": 1, "Finger": 1, "Yes": 1, "Yeah": 1, "I feel": 1, "Very": 1, "Unsettled": 1, "Anxiety": 1, "Excited": 1, "Shake your hands": 1}, "adverbs": {"There was": 1, "Very": 1, "Anxiety": 1, "Excited": 1}, "numerals": {"5": 1}, "pronouns": {"you": 1}}}, {"content": "When you were drinking, there was a time when you were in a state of instability, you were in a state of discomfort, you couldn't say anything?", "word_count": 21, "label": "", "sentence_structure": "Subject鈥損redicate", "subjective_objective": {"answer": "Objective", "rationale": "Because \"stable\" and \"sliding\" and \"sniffing\" are the objective manifestations that can be expected from drinking."}, "sentence_sentiment": {"answer": "Negative", "rationale": "Because the entries describe \"stable, tumbled, incoherent\" and so on as \"overdoured, and so on, negative effects."}, "language_style": {"answer": "Calm and direct", "rationale": "The entry is of a calm and straightforward language style, and it explicitly asks the respondents whether they have had any instability, difficulty walking, and lack of mouth."}, "time_dimension": {"answer": "Past", "rationale": "In the entry, \"had \" indicates that this was a past experience that had taken place, and it describes the past behaviour or state."}, "sentence_type": "Interrogative sentence", "pos": {"nouns": {"Questionnaire": 1, "Entry": 1, "Wine": 1, "Actions": 1, "Step": 2, "Talk": 1}, "verbs": {"I did": 1, "Drink": 1, "Steady": 1, "Boom": 1, "Clear": 1}, "adverbs": {"When": 1}, "numerals": {"6": 1}, "classifiers": {"individual": 1}}}, {"content": "Have you ever felt all hot and sweaty for drinking?", "word_count": 18, "label": "", "sentence_structure": "Subject鈥搗erb鈥搊bject", "subjective_objective": {"answer": "Subjective", "rationale": "The perception of a body reaction is subjective, though drinking may be an objective factor."}, "sentence_sentiment": {"answer": "Negative", "rationale": "Because the consequences of the act \"You've been feeling all hot and sweaty because of drinking\" in the entry are negative."}, "language_style": {"answer": "Calm and direct", "rationale": "The entry uses direct and objective language describing the physical reactions that alcohol consumption may bring, without excessive emotional colours or qualifiers, and can therefore be judged as \"cooling straight\"."}, "time_dimension": {"answer": "Past", "rationale": "Thus, the temporal dimension is judged to be the past."}, "sentence_type": "Interrogative sentence", "pos": {"nouns": {"Reason": 1, "Drink": 1, "All over": 1, "Tide fever": 1, "Sweat": 1}, "verbs": {"I'm feeling it": 1, "Present": 1, "Drink": 1, "have": 1}, "adjectives": {"Because": 1}, "adverbs": {"- You're not": 1}, "classifiers": {"Tsang": 1}}}, {"content": "8. Have you ever seen anything that does not exist for drinking?", "word_count": 20, "label": "", "sentence_structure": "Subject鈥搗erb鈥搊bject", "subjective_objective": {"answer": "Subjective", "rationale": "Because it is the behaviour of the patient to drink, whether or not what is actually not available is the perception and perception of the patient, which is viewed from the patient ' s subjective perspective."}, "sentence_sentiment": {"answer": "Negative", "rationale": "Because \"seeing what actually doesn't exist\" in the entry is a negative consequence of drinking."}, "language_style": {"answer": "Tactful and warm", "rationale": "The entry uses a quiz to guide respondents to answer questions and avoids the language of direct accusations or judgment that the other party should drink. This approach makes it easier for respondents to accept and share their experiences, thus better completing the questionnaire."}, "time_dimension": {"answer": "Past", "rationale": "The entry asks if the person tested has seen something that does not actually exist after drinking, and this experience usually occurs at some point in the past. The temporal dimension of the purpose of the article is therefore judged by the past."}, "sentence_type": "Interrogative sentence", "pos": {"nouns": {"Reason": 1, "Drink": 1, "Something": 1}, "verbs": {"I saw it": 1, "Existence": 1}, "numerals": {"8": 1}}}, {"content": "Have you ever been alarmed by the need to drink, but not by the fact that you are not getting to drink?", "word_count": 20, "label": "", "sentence_structure": "Subject鈥搗erb鈥搊bject", "subjective_objective": {"answer": "Subjective", "rationale": "The issue is based on the theory that the patient鈥檚 emotional reaction and feelings (frightful) are considered from the patient鈥檚 subjective point of view. Although the question refers to the objective fact of drinking, it is primarily used to induce the patient鈥檚 emotional reaction and therefore remains subject to subjective scrutiny."}, "sentence_sentiment": {"answer": "Negative", "rationale": "Because the mood of \"fright\" in the entry is negative."}, "language_style": {"answer": "Calm and direct", "rationale": "The question of the entry is simple, not using too many rhetorical techniques or emotional renderings, but simply asking if the person surveyed had been alarmed by the lack of access to alcohol. This straightforward language style makes the questionnaire clearer and easier to understand, while reducing possible misunderstandings and ambiguities."}, "time_dimension": {"answer": "Past", "rationale": "The words \"had\" and \"and panic\" expressed what had happened in the past, so the entry was a past act or mentality."}, "sentence_type": "Interrogative sentence", "pos": {"nouns": {"you": 1, "Wine": 2, "Frightened": 1, "Unsettled": 1}, "verbs": {"Zeng Zhen": 1, "Yes": 1, "Drink": 3, "Got it": 1, "And": 1, "Frightened": 1, "Unsettled": 1, "- You're not": 1}, "adverbs": {"Zeng Zhen": 1, "Yes": 1, "Drink": 3, "Got it": 1, "And": 1, "Frightened": 1, "Unsettled": 1, "- You're not": 1}, "numerals": {"9": 1, "2": 1}, "pronouns": {"you": 1}}}, {"content": "Have you ever had a memory of a drink (not so drunk at the time)?", "word_count": 29, "label": "", "sentence_structure": "Subject鈥搗erb鈥搊bject", "subjective_objective": {"answer": "Objective", "rationale": "Because the entry is asking whether the patient has experienced some physical symptoms or experience, which are objective. Even if the patient鈥檚 subjective feelings may influence their answers, the answer to the article is based on objective facts."}, "sentence_sentiment": {"answer": "Negative", "rationale": "Because the act of \"lost a memory\" in the entry is negative."}, "language_style": {"answer": "Tactful and warm", "rationale": "The language style of the entry is relatively euphemistic, using the means of questioning rather than direct statements or orders. At the same time, the question is not too strongly emotional or negative, but rather is asked in a fair and objective manner. This expression is better able to guide respondents to respond positively and honestly to questions and to the results of the questionnaire."}, "time_dimension": {"answer": "Past", "rationale": "So it's decided: \"The Past.\""}, "sentence_type": "Interrogative sentence", "pos": {"nouns": {"Alcohol": 1, "Memory": 1, "One": 1}, "verbs": {"Loss": 1, "have": 1, "Drink": 2, "Drunk and intoxicated": 1}, "adverbs": {"Once": 1, "No, I'm not": 1}, "numerals": {"10": 1}, "classifiers": {"Paragraph": 1}, "pronouns": {"you": 1}}}, {"content": "Were you out with a bottle or put it near you?", "word_count": 19, "label": "", "sentence_structure": "Subject鈥搗erb鈥搊bject", "subjective_objective": {"answer": "Subjective", "rationale": "The subject is the patient ' s personal habits and behaviour choices, which are based on his own wishes and decisions. The subject is therefore considered subjectively in terms of his drinking behaviour."}, "sentence_sentiment": {"answer": "Negative", "rationale": "Because the behavior in the entry \"with a bottle or place it near you\" could have adverse consequences, such as driving with alcohol."}, "language_style": {"answer": "Calm and direct", "rationale": "The entry is more straightforward, concise and does not use excessive accommodations or rhetorical techniques. It specifically asks whether the person surveyed will carry a bottle or place it in the vicinity while he/she is away, which is more objective and practical."}, "time_dimension": {"answer": "Present", "rationale": ""}, "sentence_type": "Interrogative sentence", "pos": {"nouns": {"Questionnaire": 1, "Entry": 1, "you": 2, "Time": 1, "A bottle": 1, "Wine": 1, "Near": 1}, "verbs": {"Take it": 1, "Fire": 1}, "adverbs": {"or": 1, "EMPTY": 1}, "numerals": {"11": 1}}}, {"content": "12. Have you failed to drink again with a great amount of alcohol after you quit drinking?", "word_count": 20, "label": "", "sentence_structure": "Subject鈥搗erb鈥搊bject", "subjective_objective": {"answer": "Subjective", "rationale": "The question is asked about the subjective feelings and experiences of the patient with his or her own absortment experience, which can be judged as \"subjective\"."}, "sentence_sentiment": {"answer": "Negative", "rationale": "The \"failure of drinking again\" in the entry is negative."}, "language_style": {"answer": "Calm and direct", "rationale": "The language style of the entry is calm and straightforward, and it explicitly asks the interviewee about the situation after the alcohol has been given and uses a negative expression, which is both air temperature and straightforward."}, "time_dimension": {"answer": "Past", "rationale": "The entry asked the testee about his or her experience after drinking, and the temporal dimension was therefore judged to be past."}, "sentence_type": "Interrogative sentence", "pos": {"nouns": {"Wine": 3, "A clean drink": 1, "Again": 1, "Failed": 1, "End of story": 1}, "verbs": {"Here": 1, "A lot of wine": 1, "It's over": 1}, "numerals": {"12": 1, "3": 1}}}, {"content": "Have you ever been drunk in the last 12 months?", "word_count": 17, "label": "", "sentence_structure": "Subject鈥搗erb鈥搊bject", "subjective_objective": {"answer": "Objective", "rationale": "Because \"drinking drunk\" is an objective fact that patients have experienced."}, "sentence_sentiment": {"answer": "Negative", "rationale": "Because the \"drinking drunk\" act in the entry is negative."}, "language_style": {"answer": "Calm and direct", "rationale": "The entry is harshly language-clause, asking directly whether the other person has had a very drunk experience in the past 12 months, and is not speaking too much or making it emotionally sound, and is more rigid and serious. This style may be more suitable for serious research or investigative settings."}, "time_dimension": {"answer": "Past", "rationale": "The entry is for the purpose of questioning the behaviour of the person tested over the past 12 months, and the temporal dimension is judged to be the past."}, "sentence_type": "Interrogative sentence", "pos": {"nouns": {"Past": 1, "12 months": 1, "Month": 1, "Year": 1, "you": 1, "Entry": 1, "Drunk": 1}, "verbs": {"Yes": 1}, "adjectives": {"No. No": 1}, "numerals": {"12": 1}}}, {"content": "Have you ever had a pick after drinking?", "word_count": 9, "label": "", "sentence_structure": "Subject鈥損redicate", "subjective_objective": {"answer": "Subjective", "rationale": "Because \"had there ever been a pick-up\" is a question of asking about the patient's personal experience and needs to be examined from the patient's subjective point of view."}, "sentence_sentiment": {"answer": "Negative", "rationale": "Because \"smoking\" is a negative act."}, "language_style": {"answer": "Calm and direct", "rationale": "The language style of the entry is more direct and concise, with no excessive emotional rendering or rhetorical techniques, but rather a straightforward inquiry into the behaviour of the person surveyed in smoking after drinking, which is more consistent with the seriousness and objectivity of the questionnaire."}, "time_dimension": {"answer": "Present", "rationale": "The entry \"after drinking\" means behaviour at some point in the past, but the context of the question is asking about the state or habits of the present. So the answer is \"now\"."}, "sentence_type": "Interrogative sentence", "pos": {"nouns": {"Drink": 1, "Scrap": 1}, "verbs": {"Yes": 1}, "adverbs": {"in": 1, "Back": 1}, "numerals": {"14": 1}}}, {"content": "- You want to drink all the time?", "word_count": 10, "label": "", "sentence_structure": "Subject鈥損redicate", "subjective_objective": {"answer": "Subjective", "rationale": "Because it's a question of whether the patient has a mental state, \"Do you want to drink?\" is the patient's inner thought and is therefore subjectively viewed."}, "sentence_sentiment": {"answer": "Negative", "rationale": "Because the act of \"drinking at all times\" is unhealthy and may cause physical and mental health problems."}, "language_style": {"answer": "Calm and direct", "rationale": "The entry is of a calm and direct type. The basis of the judgement is that it directly expresses the question whether the other party often wishes to drink, does not use excessive rhetorical or emotional rendering, and is more objective. At the same time, it is used in the form of a questionword, which is more straightforward and more explicit to the interviewee, and is usually considered to be straightforward and straightforward."}, "time_dimension": {"answer": "Present", "rationale": "The entry asks if the person tested is currently interested in drinking, has no clear time limit and therefore is judged to be now."}, "sentence_type": "Interrogative sentence", "pos": {"nouns": {"Time and time": 1, "Wine": 1}, "verbs": {"Yes": 1, "Drink": 1}, "adverbs": {"Both": 1}, "numerals": {"15": 1}}}, {"content": "16. After a lot of alcohol, is your mind confused?", "word_count": 23, "label": "", "sentence_structure": "Subject鈥損redicate", "subjective_objective": {"answer": "Subjective", "rationale": "The question is about the subjective feelings and experiences of patients following the high amount of alcohol they drink, and is a subjective examination."}, "sentence_sentiment": {"answer": "Negative", "rationale": "Because the entry \"brain is confused and unclear\" is negative."}, "language_style": {"answer": "Calm and direct", "rationale": ""}, "time_dimension": {"answer": "Present", "rationale": ""}, "sentence_type": "Interrogative sentence", "pos": {"nouns": {"A lot of wine": 1, "Brain": 1, "Thinking": 1, "I'm confused": 1, "I don't know": 1}, "verbs": {"鈾?Turn into 鈾?: 1}, "adjectives": {"Blur": 1, "Clear": 1}, "numerals": {"Mass": 1, "16": 1}}}, {"content": "17. Do you feel your heart beat fast because of drinking?", "word_count": 16, "label": "", "sentence_structure": "Subject鈥搗erb鈥搊bject", "subjective_objective": {"answer": "Subjective", "rationale": "Because heart rate acceleration may be a patient鈥檚 self-perception rather than an objective phenomenon. Moreover, the question of 鈥渂ecause of drinking鈥?suggests that this may be associated with drinking, further suggesting that it is the patient鈥檚 subjective response to the effect of drinking."}, "sentence_sentiment": {"answer": "Negative", "rationale": "Because the \"drinking causes a heart rate\" act in the entry is negative."}, "language_style": {"answer": "Calm and direct", "rationale": "The entry asks the interviewee whether he or she is suffering from an acceleration of his or her heart rate due to alcohol consumption, is simple and has no excessive accommodative or emotional colour. The language style of the purpose is therefore \"cool and straight\"."}, "time_dimension": {"answer": "Past", "rationale": "The entry asks if the person tested had experienced an acceleration of the heart rate at a certain point in time (i.e. after drinking), and the dimension of time was judged to be past."}, "sentence_type": "Interrogative sentence", "pos": {"nouns": {"Reason": 1, "Wine": 1, "Heart rate": 1}, "verbs": {"I think": 1, "Drink": 1, "Come on": 1}, "adverbs": {"very": 1}, "numerals": {"17": 1, "you": 1}}}, {"content": "Do you think about drinking?", "word_count": 14, "label": "", "sentence_structure": "Subject鈥搗erb鈥搊bject", "subjective_objective": {"answer": "Subjective", "rationale": "Because it's a process of examining patients' own subjective feelings and ideas."}, "sentence_sentiment": {"answer": "Negative", "rationale": "Because the \"continuous thought of drinking\" act in the entry is negative."}, "language_style": {"answer": "Calm and direct", "rationale": "The language of the entry is relatively simple, there is no excessive rhetorical or emotional rendering, and the person is asked whether he or she has been thinking frequently about drinking, and the tone is more objective and objective."}, "time_dimension": {"answer": "Present", "rationale": ""}, "sentence_type": "Interrogative sentence", "pos": {"nouns": {"you": 1, "Brain": 1, "Drink": 1}, "verbs": {"Yes": 1, "Drink": 1}, "adverbs": {"Constantly": 1}, "numerals": {"18": 1}}}, {"content": "Have you 鈥渉eard鈥?any voices that do not exist because of alcohol?", "word_count": 18, "label": "", "sentence_structure": "Subject鈥搗erb鈥搊bject", "subjective_objective": {"answer": "Subjective", "rationale": "Because hearing sounds is the patient's subjective experience and feeling."}, "sentence_sentiment": {"answer": "Negative", "rationale": "Because the entry describes a possible alcohol-related symptom, i.e. a hearing, which is a negative behaviour or state of affairs."}, "language_style": {"answer": "Calm and direct", "rationale": "The entry is based on a straightforward presentation, without too much rhetorical or emotional rendering. It raises questions in an objective, neutral way, so that the respondents can understand and answer them clearly. This language style is appropriate for a rigorous scientific research and psychological questionnaire."}, "time_dimension": {"answer": "Present", "rationale": "The word \"had\" means that the movement that happened in the past, \"hear\" is a continuous act, and the time of this entry is now."}, "sentence_type": "Interrogative sentence", "pos": {"nouns": {"Drink": 1, "Sound": 2, "you": 1, "Questionnaire Entry": 1}, "verbs": {"Yes": 1, "Existence": 1, "Drink": 1}, "adverbs": {"Because": 1}, "numerals": {"19": 1}, "classifiers": {"Tsang": 1, "individual": 1}, "pronouns": {"Here": 1}}}, {"content": "When you drink, do you feel surprised and frightened?", "word_count": 18, "label": "", "sentence_structure": "Subject鈥搗erb鈥搊bject", "subjective_objective": {"answer": "Subjective", "rationale": "This is a subjective topic, because \"fright and panic\" is a description based on personal feelings and requires patients to judge themselves. While drinking may affect some people in this way, it is a human-specific feeling, and therefore requires questioning patients about their subjective experiences."}, "sentence_sentiment": {"answer": "Negative", "rationale": "Because it involves negative physical reactions (surprising and panic), it may imply overdrinking or alcohol dependence."}, "language_style": {"answer": "Calm and direct", "rationale": "The language style of the entry is more direct and objective, and there is no excessive emotional rendering or rhetorical technique. It asks the respondents in a concise and clear way whether they have ever had a sense of surprise or fear during the drinking process, and is intended to obtain real data and information. Therefore, the entry is a \"cool and direct\" language style."}, "time_dimension": {"answer": "Present", "rationale": "In the entry, the word \"yes\" uses the usual current pattern, asking what the testee feels now. Besides, \"drinking\" means that it's happening and it's happening. So, the time dimension of this questionnaire is now."}, "sentence_type": "Interrogative sentence", "pos": {"nouns": {"you": 1, "Wine": 1, "Feel": 1}, "verbs": {"Yes": 1, "Drink": 1, "Surprised": 1, "Scared": 1}, "adverbs": {"When": 1}, "numerals": {"20": 1}}}, {"content": "21. Have you ever felt like you were crawling on your mother or a little bug for drinking?", "word_count": 27, "label": "", "sentence_structure": "Subject鈥搗erb鈥搊bject", "subjective_objective": {"answer": "Objective", "rationale": "Because it is the patient's subjective experience, but it is the objective thing that produces it 鈥?the drinking. So it is objective to look at whether it exists."}, "sentence_sentiment": {"answer": "Neutral", "rationale": "Because the entry is merely asking the interviewees whether they have had any feelings and not expressing any emotional colours."}, "language_style": {"answer": "Calm and direct", "rationale": "The entry is relatively direct and objective in its language style, and does not use too many emotional terms or euphemisms. It explicitly asks whether the person tested had ever felt like an ant or a worm crawling over his body because of alcohol, and is more cold and direct, and more suitable for use in a questionnaire."}, "time_dimension": {"answer": "Past", "rationale": "In combination, the entry is a query about the testee's past behaviour and should therefore be judged as \"the past\"."}, "sentence_type": "Interrogative sentence", "pos": {"nouns": {"Reason": 1, "Wine": 1, "Mom": 1, "Bugs": 1, "Feel": 1, "you": 2}, "verbs": {"Drink": 1, "Climb": 1}, "adverbs": {"Tsang": 1, "have": 1, "Because": 1}, "numerals": {"21": 1}, "classifiers": {"individual": 1}}}, {"content": "22. About the loss of memory after drinking?", "word_count": 10, "label": "", "sentence_structure": "Subject鈥搗erb鈥搊bject", "subjective_objective": {"answer": "Objective", "rationale": "Because the loss of memory is an objective observation of the patient's drinking."}, "sentence_sentiment": {"answer": "Neutral", "rationale": "Because the entry does not express any emotional colour."}, "language_style": {"answer": "Tactful and warm", "rationale": "The entry is in the form of a question sentence, which does not clearly indicate the accusations or doubts against the person interviewed, but rather leads the interviewer in a gentle way to share his experiences and views, a language that is more friendly, respectful and understandable."}, "time_dimension": {"answer": "Present", "rationale": "The entry asks if the person tested will lose memory for a certain period of time after drinking, and does not mention specific events, past or future, and therefore the temporal dimension is now judged."}, "sentence_type": "Interrogative sentence", "pos": {"nouns": {"After the wine": 1, "Memory": 1, "Paragraph": 1}, "verbs": {"Loss": 1, "About": 1}, "numerals": {"22": 1}, "classifiers": {"One": 1}}}, {"content": "Have you ever had any experience of trying to reduce alcohol consumption but ending up failing?", "word_count": 22, "label": "", "sentence_structure": "Subject鈥搗erb鈥搊bject", "subjective_objective": {"answer": "Objective", "rationale": "Because \"had tried to reduce the amount of alcohol\" and \"failed experience\" are the objective facts and behavioral results of the patient."}, "sentence_sentiment": {"answer": "Negative", "rationale": "Because the experience in the entry \"tried to reduce the amount of alcohol, but ended in failure\" is negative."}, "language_style": {"answer": "Calm and direct", "rationale": "The entry is a direct question, without any rhetorical or emotional recitation, and is more objective in tone. The expression \"the experience of failure\" is also more straightforward and not much reverb."}, "time_dimension": {"answer": "Present", "rationale": "The entry used to ask the testee about his past behaviour and experience, and the keyword \"had ever \" indicated that this was something that had happened in the past."}, "sentence_type": "Interrogative sentence", "pos": {"nouns": {"Investigations": 1, "Questionnaire": 1, "Drinking": 1, "Experience": 1}, "verbs": {"have": 1, "Try": 1, "Lower": 1, "End of story": 1}, "adverbs": {"Tsang": 1, "But": 1, "Here": 1, "And": 1}, "numerals": {"23": 1}, "pronouns": {"you": 1}}}, {"content": "Did you drink fast, and drink a few drinks in a row?", "word_count": 18, "label": "", "sentence_structure": "Subject鈥搗erb鈥搊bject", "subjective_objective": {"answer": "Subjective", "rationale": "Because \"drinking quickly, drinking in consecutive cups\" is the patient's drinking habit and behaviour, and it is his subjective feeling and attitude."}, "sentence_sentiment": {"answer": "Negative", "rationale": "Because it describes a health-impairment behaviour, \"drinking fast\" and \"drinking over a few drinks in a row\" are a bad habit of overdrinking."}, "language_style": {"answer": "Calm and direct", "rationale": ""}, "time_dimension": {"answer": "Present", "rationale": ""}, "sentence_type": "Interrogative sentence", "pos": {"nouns": {"you": 1, "Wine": 1, "Cups": 1}, "verbs": {"Drink": 2, "Yes": 1, "Continuous": 1, "A few": 1, "Drink it up": 1}, "adverbs": {"Soon": 1, "Continuous": 1}, "numerals": {"24": 1, "How many": 1}, "classifiers": {"Cups": 1}}}, {"content": "25. After a few drinks, can you stop drinking?", "word_count": 13, "label": "", "sentence_structure": "Subject鈥搗erb鈥搊bject", "subjective_objective": {"answer": "Objective", "rationale": "This is an objective observation and inquiry into the alcohol consumption of the patient."}, "sentence_sentiment": {"answer": "Negative", "rationale": "Because the behaviour in the entry \"stop\" may involve overdrinking and have some negative connotations."}, "language_style": {"answer": "Tactful and warm", "rationale": "The question was simple and straightforward, and the interviewee was asked about his control capacity after drinking."}, "time_dimension": {"answer": "Present", "rationale": "The entry is for self-control after the testee has been asked to drink, and the time dimension is now judged."}, "sentence_type": "Interrogative sentence", "pos": {"nouns": {"Cups": 1, "Drink": 3, "Stop": 1}, "verbs": {"Yes": 1, "Stop": 1, "Drink": 1}, "adverbs": {"in": 1, "Back": 1}, "numerals": {"How many": 1}, "classifiers": {"Cups": 1}}}]}

{"questionnaire_title": "PDQ-4", "questionnaire_category": "Mental health", "item_count": 104, "emotion_shift_overall": "From positive to negative", "language_style_overall": "Neutral", "items": [{"content": "I avoid working with people who might criticize myself.", "word_count": 16, "label": "", "sentence_structure": "Subject鈥搗erb鈥搊bject", "subjective_objective": {"answer": "Subjective", "rationale": "Because the entry contains subjective feelings and feelings of \"self \" that patients may be afraid or uneasy about criticism from others, which is viewed from the patient's subjective perspective."}, "sentence_sentiment": {"answer": "Negative", "rationale": "Because the words \"avoid\" and \"criticize\" in the entries are both negative emotional."}, "language_style": {"answer": "Calm and direct", "rationale": "The entry presents a point in a straightforward way, namely, \"I avoid working with people who might criticize me.\" It is simple, powerful, without too much rhetorical or emotional overtook, and it is more objective in tone."}, "time_dimension": {"answer": "Past", "rationale": "The words \"avoid\" and \"work together\" in that sentence all indicate the movement or state of the past, and therefore judge as the past."}, "sentence_type": "Interrogative sentence", "pos": {"nouns": {"person": 1, "Work": 1}, "verbs": {"I": 1, "Avoid": 1, "and": 1, "Together": 1, "Criticism": 1}, "adverbs": {"Maybe": 1}, "numerals": {"1": 2}}}, {"content": "2. I cannot take a decision without advice or repeated assurances from others.", "word_count": 21, "label": "", "sentence_structure": "Subject鈥揷opula鈥損redicative", "subjective_objective": {"answer": "Subjective", "rationale": "Because \"advisory advice or repeated assurances from others\" have an impact on patients' decisions, which reflects patients' subjective attitudes and beliefs."}, "sentence_sentiment": {"answer": "Negative", "rationale": "Because the phrase \"I cannot take a decision without the advice or repeated assurances of others\" in the entry expresses a negative attitude of overdependence on others."}, "language_style": {"answer": "Calm and direct", "rationale": "The language style of the entry is more straightforward, is not overly emotional or rhetorical, and is mainly expressed in concise language, asking the other side whether it needs advice and assurance from others in making a decision."}, "time_dimension": {"answer": "Present", "rationale": "The entry \"just\" indicates that if there is no condition (i.e. \"advisory advice or repeated assurances from others\") then the result will occur (i.e. \"no decision\"). This situation is going through right now, and it is now."}, "sentence_type": "Interrogative sentence", "pos": {"nouns": {"others": 1, "- I'm not sure": 1, "Repeatedly": 1, "Promise": 1, "Decision": 1}, "verbs": {"No, I'm not": 1, "Yes": 1, "Make": 1}, "adverbs": {"Just": 2}, "pronouns": {"I": 1}}}, {"content": "I've often focused too much on detail and neglected the larger aspects.", "word_count": 16, "label": "", "sentence_structure": "Subject鈥揷opula鈥損redicative", "subjective_objective": {"answer": "Subjective", "rationale": "Because \"too much attention is given to detail, and the big aspects are ignored\" are the patient's description and perception of his or her psychological state, which is subjective."}, "sentence_sentiment": {"answer": "Negative", "rationale": "Because the entry \"too much attention to detail, and neglecting the larger aspect\" is negative."}, "language_style": {"answer": "Tactful and warm", "rationale": "The entry is more euphemistic and warm, and in a question-and-answer tone, guides the interviewee to reflect on his/her behaviour rather than directly speaking or ordering. At the same time, the expression \"too much attention to detail\" and \"too little to be seen\" is more mild and less stressful to the interviewee."}, "time_dimension": {"answer": "Present", "rationale": "The keyword \"I'm often\" indicates that it's a sentence describing the current behaviour of the subject, which is a constant habit."}, "sentence_type": "Negative declarative sentence", "pos": {"nouns": {"Details": 1, "In general": 1}, "verbs": {"Focus": 1, "Neglect": 1}, "adjectives": {"That's too much": 1}, "pronouns": {"I": 1}}}, {"content": "I need to be the center of attention.", "word_count": 12, "label": "", "sentence_structure": "Subject鈥搗erb鈥揷omplement", "subjective_objective": {"answer": "Subjective", "rationale": "Because \"need\" is a person's mental state, which is viewed from the patient's subjective point of view."}, "sentence_sentiment": {"answer": "Negative", "rationale": "Because the \"need to be the focus of attention\" in the entry expresses a strong demand and desire, often arising out of low self-esteem, lack of self-confidence, etc. The expression is therefore somewhat negative."}, "language_style": {"answer": "Tactful and warm", "rationale": "The entry is more adept and interactive when it is spoken in a gentle and inviting manner, rather than by direct statements or orders."}, "time_dimension": {"answer": "Present", "rationale": "In conclusion, the time dimension of this questionnaire entry can be judged as the future."}, "sentence_type": "Imperative sentence", "pos": {"nouns": {"Centre": 1, "People": 1}, "verbs": {"Yes": 1, "Be": 1, "Attention": 1}, "pronouns": {"I": 1}}}, {"content": "I've achieved more than others have praised me for.", "word_count": 17, "label": "", "sentence_structure": "Subject鈥搗erb鈥揷omplement", "subjective_objective": {"answer": "Subjective", "rationale": "Because \"I've accomplished\" and \"I've been praised\" are subjective views of patients or self-evaluations, and therefore subjective approaches."}, "sentence_sentiment": {"answer": "Positive", "rationale": "Because the phrase \"achieving more than others praise me\" in the entry expresses a sense of confidence, complacency and positive emotionality."}, "language_style": {"answer": "Calm and direct", "rationale": "The entry uses a relatively straightforward language style, which is not over-modified or emotional, but rather a concise expression of recognition and affirmation of the achievements of the interviewees. \""}, "time_dimension": {"answer": "Past", "rationale": "The words \"I've accomplished\" and \"others' praise\" in this entry refer to acts that have already taken place, so the dimension of time is the past."}, "sentence_type": "Affirmative declarative sentence", "pos": {"nouns": {"Achievements": 1, "- I'm impressed": 1, "others": 1}, "verbs": {"Access": 1, "Beyond": 1}, "adjectives": {"Far": 1}, "numerals": {"5": 1}, "pronouns": {"I": 1}}}, {"content": "I'll use excessive means to stop the people I love from leaving me.", "word_count": 18, "label": "", "sentence_structure": "Subject鈥搗erb鈥搊bject", "subjective_objective": {"answer": "Subjective", "rationale": "Because \"it's too much to stop\" is a subjective will and behaviour of a person."}, "sentence_sentiment": {"answer": "Negative", "rationale": "Because the \"over-the-counter\" act in the entry is negative."}, "language_style": {"answer": "Calm and direct", "rationale": "The entry is based on a straightforward question about whether the person interviewed would be too aggressive to prevent the person he loves from leaving, without using a rhetorical or emotional presentation, and in a more objective manner. This expression allows the interviewee to answer questions clearly and clearly, without ambiguity or misunderstanding."}, "time_dimension": {"answer": "Past", "rationale": "The entry uses the word \"will\" to indicate future behaviour, but the word \"will\" here refers to behaviour that may occur in the future for some time, not to a particular future moment. Moreover, \"the person I love leaves me\" also indicates that the act is a reaction to a situation in the past. The time dimension of this questionnaire should therefore be judged as past."}, "sentence_type": "Imperative sentence", "pos": {"nouns": {"Questionnaire": 1, "Entry": 1, "Means": 1, "person": 1, "Love": 1, "Leave": 1}, "verbs": {"Yes": 1, "Stop": 1, "Sou-san": 1}, "adjectives": {"It's too much": 1}, "numerals": {"6": 1}, "pronouns": {"I": 2, "He/she": 1}}}, {"content": "I've had several legal problems (e.g. detention, prosecution or re-education through labour)", "word_count": 22, "label": "", "sentence_structure": "Subject鈥搗erb鈥搊bject", "subjective_objective": {"answer": "Objective", "rationale": "Since it is an objective fact to encounter legal problems, the patient is not required to ask for his own subjective attitude."}, "sentence_sentiment": {"answer": "Negative", "rationale": "Because \"detention,\" \"prosecuted\" and \"re-education through labour\" are negative incidents."}, "language_style": {"answer": "Calm and direct", "rationale": "The language style of this entry is relatively harsh, and it uses a clear question form to ask if the interviewee has ever encountered a legal problem. This tone does not try to alleviate or dilute the seriousness of the problem, but rather uses a clear and concise language to express it. Moreover, the terms \"detention\", \"indicted\" and \"re-education through labour\" are more formal and legal terms, which also strengthens the directness of the purpose."}, "time_dimension": {"answer": "Past", "rationale": "The entry is for questioning the testee's past, and the keywords \"has encountered several times\" and \"detained, prosecuted or re-education through labour\" point to the past."}, "sentence_type": "Negative declarative sentence", "pos": {"nouns": {"Law": 1, "Trouble": 2, "Detention": 1, "Prosecution": 1, "Reeducation through labour": 1}, "verbs": {"I met him": 1, "By": 3}, "numerals": {"A few times": 1}, "pronouns": {"I": 1}}}, {"content": "I'm not interested in spending time with family or friends.", "word_count": 17, "label": "", "sentence_structure": "Subject鈥揷opula鈥損redicative", "subjective_objective": {"answer": "Subjective", "rationale": "The investigator understands the emotional state, values, etc. of the person surveyed by asking him or her about the level of interest in certain activities, which is seen from a subjective perspective."}, "sentence_sentiment": {"answer": "Negative", "rationale": "Because the attitude in the entry \"uninterested in spending time with family or friends\" is negative."}, "language_style": {"answer": "Calm and direct", "rationale": "The entry directly expresses the subject of an inquiry about the interest in spending time with family or friends, and does not use excessive rhetorical or emotional rendering, which is more objective and balanced."}, "time_dimension": {"answer": "Past", "rationale": ""}, "sentence_type": "Negative declarative sentence", "pos": {"nouns": {"Family": 1, "friends": 1, "Time": 1}, "verbs": {"Common": 1, "Not interested": 1}, "adjectives": {"not": 1, "very": 1, "Interested": 1}, "numerals": {"8": 1}}}, {"content": "I can get special information about what's happening around here.", "word_count": 18, "label": "", "sentence_structure": "Subject鈥搗erb鈥揷omplement", "subjective_objective": {"answer": "Subjective", "rationale": "This sentence describes the patient ' s cognitive and interpretive ability to understand the surrounding environment, which is the patient ' s subjective feeling and perception."}, "sentence_sentiment": {"answer": "Positive", "rationale": "Because the entry is about a capacity and the word \"can\" is about motivation."}, "language_style": {"answer": "Tactful and warm", "rationale": "The entry is more adept and interactive when it is spoken in a gentle and inviting manner, rather than by direct statements or orders."}, "time_dimension": {"answer": "Present", "rationale": "The entry asks whether the testee can currently obtain special information from what happens around it, and the time dimension is now judged. \""}, "sentence_type": "Interrogative sentence", "pos": {"nouns": {"Things": 1, "Information": 1}, "verbs": {"Yes": 1, "From": 1, "Got it": 1, "Come on": 1, "Special": 1}, "adjectives": {"Special": 1}, "adverbs": {"Around": 1}}}, {"content": "I know that if I don't take care of myself, someone else will take advantage of me or try to cheat me.", "word_count": 25, "label": "", "sentence_structure": "Subject鈥搗erb鈥搊bject", "subjective_objective": {"answer": "Subjective", "rationale": "This is the subjective idea and belief of the patients and reflects their perception and attitude to self-protection."}, "sentence_sentiment": {"answer": "Negative", "rationale": "Because \"take me for a price\" and \"try to cheat me\" in the entries are negative."}, "language_style": {"answer": "Calm and direct", "rationale": "The article is more objective and straightforward, with no excessive rhetorical or emotional rendering. It expresses the subject directly, and does not contain much personal or emotional, but rather a more calm and rational way. So I think the language style of this entry is straightforward and calm."}, "time_dimension": {"answer": "Present", "rationale": ""}, "sentence_type": "Affirmative declarative sentence", "pos": {"nouns": {"Questionnaire Entry": 1, "self": 1, "Watch out": 1, "others": 2, "Cheap": 1, "Try": 1, "- Liar": 1}, "verbs": {"Yes": 2, "Jim": 1, "I was just": 1, "Try": 1}, "adjectives": {"of": 4}, "adverbs": {"If": 1, "Just": 1}, "numerals": {"10": 1, "1": 2, "2": 1}, "classifiers": {"Grandpa": 1}, "pronouns": {"I": 1}}}, {"content": "I get upset sometimes.", "word_count": 7, "label": "", "sentence_structure": "Subject鈥搗erb鈥揷omplement", "subjective_objective": {"answer": "Subjective", "rationale": "Because \"disturbed\" is a subjective feeling or emotional experience, it looks at the patient's subjective feelings and thoughts."}, "sentence_sentiment": {"answer": "Negative", "rationale": "Because \"drilling\" in the entries is an expression of a negative emotional state."}, "language_style": {"answer": "Tactful and warm", "rationale": ""}, "time_dimension": {"answer": "Present", "rationale": "Now the entry indicates the feelings or state of the respondent in reply to the questionnaire, in which both \"sometimes\" and \"disturbed\" suggest that this is a statement describing the current feeling. Thus, the temporal dimension is now."}, "sentence_type": "Affirmative declarative sentence", "pos": {"nouns": {"I'm upset": 1}, "verbs": {"Sometimes": 1}}}, {"content": "I only make friends with people when I'm sure they like me.", "word_count": 19, "label": "", "sentence_structure": "Subject鈥搗erb鈥搊bject", "subjective_objective": {"answer": "Subjective", "rationale": "Because \"when people like me\" is the patient's subjective feeling and attitude."}, "sentence_sentiment": {"answer": "Negative", "rationale": "Because the \"Friends of People Only When They Sure They Like Me\" act in the entry demonstrates a lack of trust and self-worth."}, "language_style": {"answer": "Calm and direct", "rationale": "The entry is more direct, not using too many qualifiers or emotional colours, but rather presenting a point in a concise manner, asking whether the person surveyed is influenced by the degree of preferences of others when establishing a relationship."}, "time_dimension": {"answer": "Past", "rationale": "\"Only if there is certainty\" indicates that this is a past experience or habit, and the temporal dimension of the article is therefore the past."}, "sentence_type": "Affirmative declarative sentence", "pos": {"nouns": {"Questionnaire": 1, "Entry": 1, "person": 1, "Make friends": 1}, "verbs": {"Sure": 1, "Yeah": 1, "That's right": 1, "and": 1, "Hand it over": 1}, "adverbs": {"Only": 1, "When": 1}, "numerals": {"12": 1}, "pronouns": {"I": 1}}}, {"content": "I'd rather have others take responsibility for myself.", "word_count": 14, "label": "", "sentence_structure": "Subject鈥搗erb鈥揷omplement", "subjective_objective": {"answer": "Subjective", "rationale": "Because \"will\" is patient's subjective attitude."}, "sentence_sentiment": {"answer": "Negative", "rationale": "Because it expresses a evading of responsibility towards itself, tends to shirk responsibility from others. This may reflect a negative attitude or quality."}, "language_style": {"answer": "Tactful and warm", "rationale": "The language style of the entry is more euphemistic and gentle, using the expression \"more willing\" rather than directly ordering or imposing a duty on the other party. This is more friendly and acceptable, and better able to guide the person under investigation to express his or her true thoughts."}, "time_dimension": {"answer": "Present", "rationale": "The keywords \"better\" and \"place responsibility on others\" suggest that this is a statement of personal preference and involves a decision or attitude in the moment."}, "sentence_type": "Affirmative declarative sentence", "pos": {"nouns": {"Responsibility": 1, "Undertaking": 1}, "verbs": {"Yes": 1, "Jean": 1}, "adverbs": {"More": 1}, "numerals": {"13": 1}, "pronouns": {"self": 1}}}, {"content": "I've spent time making things perfect.", "word_count": 14, "label": "", "sentence_structure": "Subject鈥搗erb鈥揷omplement", "subjective_objective": {"answer": "Subjective", "rationale": "Because I'm here to express my feelings and attitudes, which are viewed from the subjective perspective of the patient."}, "sentence_sentiment": {"answer": "Negative", "rationale": "Because the goal of \"excessive perfection\" is an overly pro-optimal goal, it can lead to excessive anxiety and dissatisfaction."}, "language_style": {"answer": "Tactful and warm", "rationale": "The words \"I've taken time\" suggest that it is personal experience and feelings, not direct statements or orders. Moreover, \"excessively perfect\" also conveys in a more moderate way the level of attention given to the details, avoiding undue pressure or discomfort on the respondents."}, "time_dimension": {"answer": "Present", "rationale": "The keyword \"now spent\" means that the act is in progress."}, "sentence_type": "Affirmative declarative sentence", "pos": {"nouns": {"Time": 1, "Things": 1, "Perfect": 1}, "verbs": {"Cost": 1, "鈾?And I'm gonna be 鈾?: 1}, "adjectives": {"That's too much": 1}, "numerals": {"14": 1}, "pronouns": {"I": 1}}}, {"content": "I'm more sexy than most people.", "word_count": 8, "label": "", "sentence_structure": "Subject鈥揷opula鈥損redicative", "subjective_objective": {"answer": "Subjective", "rationale": "Because \"I'm more sexy than most people\" is a self-evaluation, a subjective attitude and feelings of patients."}, "sentence_sentiment": {"answer": "Positive", "rationale": "Because it expresses a self-acceptance and self-confidence and expresses a positive emotional attitude."}, "language_style": {"answer": "Calm and direct", "rationale": "The entry uses a language of subjective judgement, evaluates the external characteristics of the individual and is more straightforward in tone, and does not use too much euphemism or emotional expressions."}, "time_dimension": {"answer": "Present", "rationale": "The entry uses the now completed fashion \"more sexy than most people\", indicating that the speaker believes that he is more sexy than most and that this belief may be in the current state or feeling, so it is judged now."}, "sentence_type": "Affirmative declarative sentence", "pos": {"verbs": {"- Yeah": 1, "More": 1}, "adjectives": {"Most": 1, "Sexy": 2}, "numerals": {"15": 1}, "pronouns": {"I": 1}}}, {"content": "I've often thought that I was or would be a great man.", "word_count": 19, "label": "", "sentence_structure": "Subject鈥揷opula鈥損redicative", "subjective_objective": {"answer": "Subjective", "rationale": "Because \"often thinking\" and \"great\" are the personal feelings and thoughts of the patient, and they are subjective."}, "sentence_sentiment": {"answer": "Positive", "rationale": "Because the entry expresses a recognition of one ' s abilities and self-confidence, which is a positive emotion."}, "language_style": {"answer": "Calm and direct", "rationale": "This language style gives people a direct and strong sense of personal engagement by using the first person's pronoun, \"I\". At the same time, the expression \"how great\" is filled with positive and positive sentiments, encouraging and inspiring. Moreover, the entry does not use any negative or negative terminology or expression, but rather emphasizes the growth and development of the individual, which further strengthens its language style of self-confidence and self-exploration."}, "time_dimension": {"answer": "Present", "rationale": "In conclusion, the temporal dimension of the questionnaire entry: \"I often think I'm a man or what I'm going to be.\""}, "sentence_type": "Affirmative declarative sentence", "pos": {"nouns": {"person": 2, "Great": 1}, "verbs": {"Yes": 1, "Be": 1}, "adjectives": {"More": 1, "How": 1}, "adverbs": {"Regular": 1}, "numerals": {"16": 1}, "pronouns": {"I": 2}}}, {"content": "I love someone or hate them, there's no middle.", "word_count": 18, "label": "", "sentence_structure": "Subject鈥損redicate", "subjective_objective": {"answer": "Subjective", "rationale": "Because \"love someone\" and \"hate them\" are expressions of personal emotional attitudes, subjective."}, "sentence_sentiment": {"answer": "Negative", "rationale": "Because the expression \"love or hate someone, without middle\" in the entry indicates an extremely negative, neutral or positive attitude towards others."}, "language_style": {"answer": "Calm and direct", "rationale": "The entry uses plain language to describe emotional state, which is more calm and direct than too much adoration or euphemism."}, "time_dimension": {"answer": "Present", "rationale": "\"I love or hate someone, there is no middle ground\" describes the current emotional state and perception of the person being tested, and there is no past or future of time dimension. So, now is the judgement."}, "sentence_type": "Negative declarative sentence", "pos": {"nouns": {"Situation": 1, "they": 1, "Someone": 1}, "verbs": {"be": 1, "Love": 1, "Hate": 2, "have": 1, "No, I'm not": 1}, "adverbs": {"Just": 1, "not": 1, "Centre": 1}, "numerals": {"17": 1}, "pronouns": {"I": 1}}}, {"content": "I was involved in a lot of fights.", "word_count": 9, "label": "", "sentence_structure": "Subject鈥搗erb鈥揷omplement", "subjective_objective": {"answer": "Objective", "rationale": "Because \"fight\" is an objective fact of behaviour, and \"multiple\" can be a subjective description. So, \"I've been involved in fights many times\" contains both objective and subjective aspects."}, "sentence_sentiment": {"answer": "Negative", "rationale": "Because the \"getting involved in a fight\" act is negative."}, "language_style": {"answer": "Calm and direct", "rationale": "The entry asks the respondents directly and objectively whether they have been involved in numerous fights, without excessive emotional reticulation or guidance, and the language style is a straightforward type of calm."}, "time_dimension": {"answer": "Past", "rationale": ""}, "sentence_type": "Negative declarative sentence", "pos": {"nouns": {"I": 1, "Fighting": 2}, "verbs": {"Intrusion": 1, "Multiple": 1}, "adverbs": {"Multiple": 1}, "numerals": {"18": 1, "Multiple": 1}}}, {"content": "I'd rather do things myself than with others.", "word_count": 16, "label": "", "sentence_structure": "Subject鈥搗erb鈥搊bject", "subjective_objective": {"answer": "Subjective", "rationale": "Because \"better\" means that it's the patient's personal will and preferences, and it's the expression of subjective attitudes."}, "sentence_sentiment": {"answer": "Negative", "rationale": "Because the tendency in the entry to \"do things on its own rather than with others\" is to be lonely, to be self-centered, which can lead to social disorder or interpersonal problems, and is a negative act."}, "language_style": {"answer": "Calm and direct", "rationale": "The entry directly expresses the subject of asking the other party whether it prefers to do things alone rather than to cooperate with others, without using excessive rhetorical or emotional rendering, and with a more balanced and objective tone."}, "time_dimension": {"answer": "Present", "rationale": "Based on the terms \"willing to do\" and \"doing things without doing with others\", the entry asks about the present behavioural tendencies of the subject."}, "sentence_type": "Affirmative declarative sentence", "pos": {"nouns": {"Questionnaire Entry": 1, "Things": 2, "Others": 1}, "verbs": {"I'd rather": 1, "Do it": 3, "and": 1}, "adverbs": {"self": 1}, "pronouns": {"I": 1}}}, {"content": "I have the ability to premonition before something actually happens.", "word_count": 20, "label": "", "sentence_structure": "Subject鈥搗erb鈥揷omplement", "subjective_objective": {"answer": "Subjective", "rationale": "Because \"presupplied ability\" is a personal feeling or experience, not an objective fact. It is a subjective view to examine it."}, "sentence_sentiment": {"answer": "Positive", "rationale": "Because the expression \"presuppositional ability\" in the entry is positive."}, "language_style": {"answer": "Tactful and warm", "rationale": ""}, "time_dimension": {"answer": "Future", "rationale": ""}, "sentence_type": "Affirmative declarative sentence", "pos": {"nouns": {"Capacity": 1, "Things": 1}, "verbs": {"have": 1, "Yes": 1, "I'm not sure": 1, "Come on": 1}, "adjectives": {"Actual": 1}, "adverbs": {"Presupposition": 1}}}, {"content": "I've often wondered if all the people I know really deserve to be trusted.", "word_count": 20, "label": "", "sentence_structure": "Subject鈥揷opula鈥損redicative", "subjective_objective": {"answer": "Subjective", "rationale": "This is because the patient ' s perception and perception of trust in interpersonal relationships is subjective."}, "sentence_sentiment": {"answer": "Negative", "rationale": "Because the entry \"often doubts\" and \"are they really trustworthy\" have a negative emotional connotation, showing mistrust and uncertainty about human relationships."}, "language_style": {"answer": "Tactful and warm", "rationale": ""}, "time_dimension": {"answer": "Past", "rationale": "\"Are people really trustworthy\" is a subjective assessment of current behaviour, and the entry is therefore to ask the testee what he thinks and feels."}, "sentence_type": "Interrogative sentence", "pos": {"nouns": {"Questionnaire": 1, "Entry": 1, "person": 1, "Trust": 1}, "verbs": {"Regular": 1, "Doubts": 1, "Yes": 1, "It's worth it": 1}, "adjectives": {"Really": 1}, "numerals": {"21": 1}, "pronouns": {"I": 2, "Guys": 1}}}, {"content": "22. I sometimes talk about people behind my back.", "word_count": 10, "label": "", "sentence_structure": "Subject鈥搗erb鈥揷omplement", "subjective_objective": {"answer": "Subjective", "rationale": "Because \"sometimes speaking behind people\" is an individual act, a patient's subjective attitude and behavioral habits."}, "sentence_sentiment": {"answer": "Negative", "rationale": "Because the behavior in the entry is derogatory and therefore negative."}, "language_style": {"answer": "Calm and direct", "rationale": "The entry directly asks the respondents whether they are talking about the behaviour of others behind their backs, the language style is relatively simple and clear, there is no excessive emotional colour and rhetoric, and there is more objective description and investigation needs."}, "time_dimension": {"answer": "Present", "rationale": "The entry is for questioning the present behaviour or orientation of the subject, the keywords being \"frequently\" and \"persons behind the debate\", which indicate that this is an act that can occur in the past, in the present and in the future. Thus, the temporal dimension is now."}, "sentence_type": "Affirmative declarative sentence", "pos": {"nouns": {"Questionnaire Entry": 1, "Behind": 1, "People": 1}, "verbs": {"in": 1, "Arguments": 1}, "adverbs": {"Sometimes": 1}, "numerals": {"22": 1}}}, {"content": "I'm limiting myself to being intimate with others because I'm afraid I'm being mocked.", "word_count": 25, "label": "", "sentence_structure": "Subject鈥搗erb鈥搊bject", "subjective_objective": {"answer": "Subjective", "rationale": "Fear of being mocked is a subjective feeling and thought."}, "sentence_sentiment": {"answer": "Negative", "rationale": "Because the behavior in the entry, \"I'm limiting my intimate relationship with others,\" and the reason I'm afraid of being mocked, are negative emotional expressions that the interviewee may have some degree of social fear or low self-esteem."}, "language_style": {"answer": "Tactful and warm", "rationale": ""}, "time_dimension": {"answer": "Present", "rationale": "\"I limit my intimate relations with others\" indicates the conduct that is being carried out, and \"because I fear being mocked\" indicates the motive or cause of such behaviour. The entry therefore describes the attitudes and behaviour of the present."}, "sentence_type": "Interrogative sentence", "pos": {"nouns": {"Questionnaire": 1, "Entry": 1, "Relations": 1, "person": 1, "Scoffing": 1}, "verbs": {"Limits": 1, "and": 1, "have": 1, "Scared": 1, "Received": 1}, "adverbs": {"very": 1}, "numerals": {"23": 1, "One": 1}, "classifiers": {"individual": 1}, "pronouns": {"I": 1}}}, {"content": "I'm afraid if I don't approve of others, I'm afraid I'll lose them.", "word_count": 21, "label": "", "sentence_structure": "Subject鈥搗erb鈥搊bject", "subjective_objective": {"answer": "Subjective", "rationale": "Because fear is a manifestation of personal feelings and attitudes, it is a subjective approach."}, "sentence_sentiment": {"answer": "Negative", "rationale": "Because the \"fear of losing support\" in the entry is negative."}, "language_style": {"answer": "Tactful and warm", "rationale": "The language style of the entry tends to be euphemistic. It uses a more moderate way to channel the interviewer to think, not to direct orders or statements, by asking questions. This expression is usually more intimate, friendly and acceptable, and helps to establish good investigative relationships. Moreover, the entry contains some hint that the interviewee may lose his support by not agreeing to others, a hint that can prompt the interviewee to reflect more deeply and provide more valuable feedback."}, "time_dimension": {"answer": "Present", "rationale": "According to \"24. I'm afraid,\" I can judge that this is the current psychological state or feeling of the subject, so the answer is now."}, "sentence_type": "Negative declarative sentence", "pos": {"nouns": {"24": 1, "I": 1, "others": 1, "Lose": 1, "they": 1, "Support": 1}, "verbs": {"Yes": 1, "If": 1, "Aye": 1, "Lose": 1, "Got it": 0, "Hold on": 0, "Access": 0, "Enjoyment": 0, "Possession": 0, "Achieved": 0, "Success": 0, "Completed": 0}, "adjectives": {"Scared": 1}, "numerals": {"24": 1, "If": 1}, "classifiers": {"individual": 1}, "pronouns": {"I": 1, "they": 1}}}, {"content": "I see work as more important than spending time or entertainment with my family or friends.", "word_count": 24, "label": "", "sentence_structure": "Subject鈥搗erb鈥搊bject", "subjective_objective": {"answer": "Subjective", "rationale": "Because this is the patient ' s personal opinion and attitude and is subjective."}, "sentence_sentiment": {"answer": "Negative", "rationale": "Because the behaviour in the entry \"to see work more important than spending time or entertainment with family or friends\" is too work-oriented, neglecting family and social life, with some negative impact."}, "language_style": {"answer": "Calm and direct", "rationale": "The language style of the entry is straightforward, it raises the question in a concise and clear manner and does not use excessive rhetoric or emotional colours to qualify the question, so that the interviewee can clearly understand the meaning of the question."}, "time_dimension": {"answer": "Present", "rationale": "The entry describes the current attitude and perception of the testee, which is still present, as the keyword \"work for work\" adopts the general current state of affairs."}, "sentence_type": "Affirmative declarative sentence", "pos": {"nouns": {"Work": 1, "Family": 1, "friends": 1, "Time": 1, "Recreation": 1}, "verbs": {"Look": 1, "- Put it on": 1, "Common": 1, "More important": 1}, "adverbs": {"- Yeah": 1, "or": 1}, "numerals": {"25": 1}, "pronouns": {"I": 1}}}, {"content": "I'm easy to express my feelings.", "word_count": 9, "label": "", "sentence_structure": "Subject鈥揷opula鈥損redicative", "subjective_objective": {"answer": "Subjective", "rationale": "Because \"expression\" is the mind and attitude of a person, and it is subjective."}, "sentence_sentiment": {"answer": "Positive", "rationale": "Because the act of \"expression\" in the entry is positive and indicates that the individual is more capable of expressing emotions."}, "language_style": {"answer": "Tactful and warm", "rationale": "The language style of the entry is more euphemistic, as it uses invitations to ask if the other side is often expressing its feelings, without direct orders or statements."}, "time_dimension": {"answer": "Present", "rationale": ""}, "sentence_type": "Affirmative declarative sentence", "pos": {"nouns": {"Questionnaire Entry": 1, "Emotion": 1}, "verbs": {"Performance": 1, "Easy": 1}, "numerals": {"26": 1}}}, {"content": "There are only certain special people who really appreciate me and understand me.", "word_count": 19, "label": "", "sentence_structure": "Subject鈥搗erb鈥搊bject", "subjective_objective": {"answer": "Subjective", "rationale": "Because \"to really appreciate and understand me\" is a subjective feeling, only the patient can judge exactly whether or not he really is appreciated and understood."}, "sentence_sentiment": {"answer": "Negative", "rationale": "Because the entry expresses a feeling of loneliness and misunderstanding, which is negative."}, "language_style": {"answer": "Tactful and warm", "rationale": "This entry is expressed in an invitation and gentle tone, emphasizing that only certain individuals can understand and appreciate themselves. This language style is more personal and interactive, making respondents more willing to share their thoughts and feelings."}, "time_dimension": {"answer": "Past", "rationale": "The entry contains memories of the past and observations of the present, so it can be judged as the past/now. The keyword \"Be able to really appreciate and understand me\" shows the expectations and feelings of the subjects about relationships, which relate to the emotional state and social needs of individuals, and is one of the important elements of psychological questionnaire research."}, "sentence_type": "Negative declarative sentence", "pos": {"nouns": {"person": 2, "Enjoy": 1, "Understood": 1}, "verbs": {"have": 1, "True": 1}, "adjectives": {"Special": 1, "Some": 1}, "numerals": {"27": 1}, "pronouns": {"I": 1}}}, {"content": "I've always wondered what kind of person I am.", "word_count": 15, "label": "", "sentence_structure": "Subject鈥揷opula鈥損redicative", "subjective_objective": {"answer": "Subjective", "rationale": "The issue is subjective because it involves the patient ' s self-awareness and self-understanding."}, "sentence_sentiment": {"answer": "Negative", "rationale": "Because the entry \"Don't know who he is\" expresses a confused, confused mood."}, "language_style": {"answer": "Tactful and warm", "rationale": "The entry is more personal and interactive when it is spoken in a gentle and inviting manner, rather than in direct statements or orders. At the same time, the first person ' s questionable sentence is used, making it easier for the person surveyed to resonate and be willing to participate in the answer."}, "time_dimension": {"answer": "Present", "rationale": "The words \"often think\" and \"what's the person\" in the entry indicate that this is a constant psychological state or a process of thinking, which occurs now or in the past."}, "sentence_type": "Interrogative sentence", "pos": {"nouns": {"Questionnaire Entry": 1, "self": 1, "person": 1}, "verbs": {"Yes": 1, "be": 1, "I always do": 1}, "adverbs": {"What the hell": 1}, "numerals": {"28": 1}, "pronouns": {"I": 1}}}, {"content": "I didn't pay the dues on time because I didn't have a long-term, stable job.", "word_count": 23, "label": "", "sentence_structure": "Subject鈥搗erb鈥搊bject", "subjective_objective": {"answer": "Subjective", "rationale": "The basis for the judgment: \"I don't pay the dues on time\" and \"I don't have a long-term, stable job\" are the patient's description and explanation of myself, and are subjective."}, "sentence_sentiment": {"answer": "Negative", "rationale": "Because the behavior in the entry \"failure to pay the dues\" is negative."}, "language_style": {"answer": "Calm and direct", "rationale": "The language style of this entry is more straightforward, with no over-modification or emotional expression, but rather a direct description of the status and behaviour of the other party, with a more objective and cold tone."}, "time_dimension": {"answer": "Past", "rationale": "The word \"yes\" in it indicates that there was a past, a past experience, and \"no long-term, stable work\" suggests that this occurred in the past. So the entry to the questionnaire is to ask the testee about the past behaviour, which is the dimension of the past."}, "sentence_type": "Negative declarative sentence", "pos": {"nouns": {"Work": 1}, "verbs": {"not": 1, "Pay": 1, "have": 1, "It's time": 1}, "adjectives": {"Long-term stability": 1}, "adverbs": {"Because": 1}, "numerals": {"29": 1, "One": 1}, "pronouns": {"I": 1}}}, {"content": "The sex content can't really interest me.", "word_count": 16, "label": "", "sentence_structure": "Subject鈥揷opula鈥損redicative", "subjective_objective": {"answer": "Subjective", "rationale": "Because interest is a subjective feeling and attitude of one, the answer to the question \"Is the content of sex really interesting to me?\" depends on the preferences and thoughts of the individual."}, "sentence_sentiment": {"answer": "Negative", "rationale": "Because the expression \"sexual content cannot really interest me\" is negative."}, "language_style": {"answer": "Tactful and warm", "rationale": "The entry uses a soft expression to ask the other side whether it is interested in content in sexual terms, rather than using a negative term directly, but rather engaging in a more moderate and moderate way. Such expression is more personal and interactive, and contributes to a good communication climate."}, "time_dimension": {"answer": "Present", "rationale": "The entry describes the degree of interest of the testee in sexual content, using the general current \"not really causing\" that this feeling still exists. So it is judged now."}, "sentence_type": "Interrogative sentence", "pos": {"nouns": {"In terms of": 1, "Contents": 1, "Sex": 1, "Interest": 1}, "verbs": {"Yes": 1, "Really": 1, "Cause": 1}, "adjectives": {"Sex": 1, "No, I can't": 1}, "numerals": {"30": 1}}}, {"content": "I'm always aware of or feeling things that people don't know.", "word_count": 19, "label": "", "sentence_structure": "Subject鈥搗erb鈥揷omplement", "subjective_objective": {"answer": "Subjective", "rationale": "Because \"aware or feel something that others do not know\" is a subjective experience and feeling that reflects the patient's perception and understanding of the world inside. This perception may be real or illusory, but it is, in any case, determined by the patient's subjective experience. So, the view of this questionnaire is subjective."}, "sentence_sentiment": {"answer": "Positive", "rationale": "Because the act of \"aware or feel something that others do not know\" in the entry is positive and may indicate that the person under investigation has a high degree of insight and observation."}, "language_style": {"answer": "Tactful and warm", "rationale": "The language style of the entry is euphemism. It is a soft, inviting tone of conversation, asking whether respondents are often able to recognize or feel things that others do not know, rather than directly speaking or ordering, which is more personal and interactive. At the same time, the entry uses words such as \"aware,\" \"favorite,\" which makes language more subtle and emotional, thus increasing the attractiveness and readability of questionnaires."}, "time_dimension": {"answer": "Present", "rationale": "I often say \"I\" is a regular act, and \"I'm conscious\" and \"I'm feeling\" is a general current, which means that this act has not only happened in the past, but may still be going on. So this entry asks the testee about the current behavior or state, and it's based on the time dimension of the present."}, "sentence_type": "Affirmative declarative sentence", "pos": {"nouns": {"Things": 1, "person": 1}, "verbs": {"Yes": 2, "I'm aware of that": 1, "Feel": 1}, "adjectives": {"Regular": 1, "Don't": 1, "not": 1, "of": 2}, "numerals": {"31": 1}, "pronouns": {"I": 1}}}, {"content": "32. People will use what I tell them or what I tell them to oppose me.", "word_count": 20, "label": "", "sentence_structure": "Subject鈥搗erb鈥搊bject", "subjective_objective": {"answer": "Subjective", "rationale": "Because \"someone will use what I tell them to oppose me\" is the patient's subjective feeling and thoughts."}, "sentence_sentiment": {"answer": "Negative", "rationale": "Because the entry says \"someone uses what I tell them to oppose me\" is negative."}, "language_style": {"answer": "Tactful and warm", "rationale": "The language style of the entry is euphemistic, using the means of questioning, and without direct criticism or criticism of the other. This is more comfortable and open to the respondents, and thus more willing to share their real thoughts and feelings."}, "time_dimension": {"answer": "Present", "rationale": "The keywords are \"others will\" and \"use\" and they all point to what is happening or what is going on, so the entry describes what the person is feeling or experiencing."}, "sentence_type": "Negative declarative sentence", "pos": {"nouns": {"others": 1, "Word": 1, "Things": 1, "self": 1}, "verbs": {"Tell": 1, "Utilization": 1, "Objection": 1}, "numerals": {"32": 1}, "classifiers": {"individual": 1}, "pronouns": {"I": 1}}}, {"content": "There are some people I don't like.", "word_count": 8, "label": "", "sentence_structure": "Subject鈥搗erb鈥搊bject", "subjective_objective": {"answer": "Subjective", "rationale": "Because \"not like\" is a subjective emotional attitude, not just a description of the objective facts."}, "sentence_sentiment": {"answer": "Negative", "rationale": "Because the \"not like\" attitude in the entry is negative."}, "language_style": {"answer": "Calm and direct", "rationale": "The entry expresses in a straightforward manner the question of whether the other party does not like the subject, does not use excessive rhetoric or emotional rendering, and is more objective in tone."}, "time_dimension": {"answer": "Present", "rationale": "In conclusion, the time dimension of the questionnaire can be determined by reference to the time information involved in the survey. Generally, current patterns usually indicate current behaviour or state; past patterns usually indicate what has happened; and future patterns are used to describe what may occur in the future."}, "sentence_type": "Negative declarative sentence", "pos": {"nouns": {"person": 1, "Yeah": 1}, "verbs": {"have": 1, "not": 1}, "numerals": {"33": 1}, "pronouns": {"Some": 1}}}, {"content": "I'm more sensitive to criticism or rejection than most people.", "word_count": 14, "label": "", "sentence_structure": "Subject鈥揷opula鈥損redicative", "subjective_objective": {"answer": "Subjective", "rationale": "This is because the patient describes his or her feelings, and it relates to the psychological state of the individual and is subjective."}, "sentence_sentiment": {"answer": "Negative", "rationale": "Because the term \"sensitive\" in the entry is usually considered to be a negative description, implying that the person may be frustrated, upset or hurt, and therefore is a negative emotional colour."}, "language_style": {"answer": "Calm and direct", "rationale": "The entry is asked in a clear statement, and is expressed more directly without a rhetorical or emotional language, so that it can be judged as \"cool and direct\"."}, "time_dimension": {"answer": "Present", "rationale": "It is judged by the phrase \"I am more sensitive to criticism or rejection\" in the sentence to describe the present behaviour or attitude of the testee. The answer is therefore \"now\"."}, "sentence_type": "Negative declarative sentence", "pos": {"nouns": {"Questionnaire Entry": 1, "Batch": 1, "Reject": 1}, "verbs": {"- Yeah": 1, "to": 1, "Min": 1}, "adjectives": {"Most": 1, "Sensitive": 1}, "adverbs": {"More": 1}, "numerals": {"34": 1}, "pronouns": {"I": 1}}}, {"content": "If I had to do something myself, I found it hard to start.", "word_count": 18, "label": "", "sentence_structure": "Subject鈥揷opula鈥損redicative", "subjective_objective": {"answer": "Subjective", "rationale": "It is difficult to find that it is the patient ' s subjective feelings and attitudes."}, "sentence_sentiment": {"answer": "Negative", "rationale": "Because the expression \"hard to start\" in the entry is negative."}, "language_style": {"answer": "Tactful and warm", "rationale": "The language style of the entry is more euphemistic and gentle, asking questions in a question-and-answer manner, and giving the interviewees more room for reflection and expression. At the same time, using the phrase \"if I have to do something myself\" makes the questionnaire more friendly, more acceptable and more responsive to the respondents."}, "time_dimension": {"answer": "Present", "rationale": "The keyword \"discovery difficult to begin\" indicates that the testee may have a difficult start when faced with a task that he or she has to perform."}, "sentence_type": "Negative declarative sentence", "pos": {"nouns": {"Questionnaire Entry": 1, "Something": 1}, "verbs": {"Found": 1, "Do it": 1, "Start": 1}, "adjectives": {"Hard": 1, "self": 1}, "adverbs": {"If": 1, "I had to": 1}, "numerals": {"35": 1}, "pronouns": {"I": 1}}}, {"content": "I have a higher moral standard than anyone else.", "word_count": 12, "label": "", "sentence_structure": "Subject鈥搗erb鈥揷omplement", "subjective_objective": {"answer": "Subjective", "rationale": "Because this is a statement of patients' assessment and perception of their moral standards, it is a manifestation of subjective attitudes."}, "sentence_sentiment": {"answer": "Neutral", "rationale": "Because the entry is not emotional, it is merely a statement of a view or a fact."}, "language_style": {"answer": "Calm and direct", "rationale": "The entry asks the other in a straightforward and clear manner whether the other party has a higher moral standard than others, is not over-represented in rhetoric or emotional colours, and is speaking in a fair and objective manner."}, "time_dimension": {"answer": "Present", "rationale": "The keyword \"more than\" indicates that it is a comparison with others, that \"a higher moral standard\" is a description of one's own behaviour or attitude, and \"me\" indicates that the speaker is doing something. So the entry is mainly about the present situation."}, "sentence_type": "Affirmative declarative sentence", "pos": {"nouns": {"Questionnaire Entry": 1, "Ethical standards": 1, "others": 1}, "verbs": {"Analysis": 1, "- Yeah": 1, "have": 1}, "adjectives": {"Higher": 1}, "numerals": {"36": 1}, "pronouns": {"I": 1, "others": 1}}}, {"content": "When I want to be noticed, I use my own appearance to attract them.", "word_count": 21, "label": "", "sentence_structure": "Subject鈥損redicate", "subjective_objective": {"answer": "Subjective", "rationale": "Because \"wanted to be noticed\" and \"inflicted to them with their own appearance\" are the inner thoughts and attitudes of patients, and they are subjective."}, "sentence_sentiment": {"answer": "Positive", "rationale": "Because the \"incentive them with their own appearance\" behavior in the entry is positive."}, "language_style": {"answer": "Calm and direct", "rationale": ""}, "time_dimension": {"answer": "Present", "rationale": "The words \"I want\" and \"I use\" are both the present-day expressions of the first-person singular, indicating that the act is taking place. So the temporal dimension of this entry is now."}, "sentence_type": "Affirmative declarative sentence", "pos": {"nouns": {"Problem": 1, "person": 2, "Attention": 2, "Appearance": 2, "Attractive": 2}, "verbs": {"Yes": 1, "Use": 1, "By": 2, "Attractive": 1, "Come on": 1, "they": 1}, "adjectives": {"Your own": 1, "others": 1}, "adverbs": {"When": 1, "Time": 1}, "numerals": {"37": 1}, "pronouns": {"I": 1}}}, {"content": "I need to be noticed or complimented.", "word_count": 14, "label": "", "sentence_structure": "Subject鈥搗erb鈥揷omplement", "subjective_objective": {"answer": "Subjective", "rationale": "Because \"I need to be noticed or praised\" is an expression of subjective emotion and need."}, "sentence_sentiment": {"answer": "Positive", "rationale": "Because the \"very much needed\" in the entry expresses a strong desire and demand, the attention and praise of others are often considered positive."}, "language_style": {"answer": "Tactful and warm", "rationale": "The language style of the entry is more euphemistic, expressing the emotional needs of the person under investigation by using the descriptive term \"very much needed\" while avoiding direct criticism or accusations. This soft language is better able to facilitate an open and honest response from the subject."}, "time_dimension": {"answer": "Present", "rationale": "\"I really need to\" express the strongness of \"very\" and \"need\" the state of mind, so this entry describes the behavior or mentality of the present."}, "sentence_type": "Imperative sentence", "pos": {"nouns": {"person": 1, "Praise": 1}, "verbs": {"Yes": 1, "Attention": 1}, "adjectives": {"Very": 1}, "numerals": {"38": 1}, "pronouns": {"I": 1}}}, {"content": "I tried to hurt myself or kill myself.", "word_count": 9, "label": "", "sentence_structure": "Subject鈥搗erb鈥搊bject", "subjective_objective": {"answer": "Subjective", "rationale": "Because self-inflicted and suicide are self-defeating and subjective behaviour of patients."}, "sentence_sentiment": {"answer": "Negative", "rationale": "Because the act of \"self-inflicted or suicide\" in the entry is negative."}, "language_style": {"answer": "Calm and direct", "rationale": "The entry is more calm and straightforward in its language, and it explicitly asks whether the other party has tried self-inflicted or suicide, without too many rhetorical or emotional renderings."}, "time_dimension": {"answer": "Present", "rationale": "The entry is for questioning the testee's past behavior and experience, the keywords being \"tried\" and \"self-injured or suicide\", all of which point to events that have occurred and that have occurred in the past. Thus, the temporal dimension is a past."}, "sentence_type": "Negative declarative sentence", "pos": {"nouns": {"Questionnaire": 1, "Entry": 1}, "verbs": {"Try": 1, "Pass": 1, "Self-inflicted": 1, "or": 1, "Suicide": 1}, "numerals": {"39": 1}}}, {"content": "I do a lot of things without taking consequences into account.", "word_count": 12, "label": "", "sentence_structure": "Subject鈥搗erb鈥揷omplement", "subjective_objective": {"answer": "Subjective", "rationale": "Because \"without consequences\" is an individual decision-making and attitude, it is subjective."}, "sentence_sentiment": {"answer": "Negative", "rationale": "Because the act of \"without consequences\" in the entry is negative and may pose unnecessary risks and problems."}, "language_style": {"answer": "Tactful and warm", "rationale": "The entry is more personal and interactive when it is spoken in a soft, invitation-like tone, rather than in direct statements or orders. Moreover, it is not too negative or critical, but rather raises a question that leads the respondents to self-reflection, reflecting the interest and support of the questionnaire researchers."}, "time_dimension": {"answer": "Present", "rationale": "Now, \"doing many things without taking consequences\" describes a customary form of behaviour, and it happens in the state in which the questionnaire was answered, so the temporal dimension is now judged."}, "sentence_type": "Exclamatory sentence", "pos": {"nouns": {"Things": 1, "Consequences": 1}, "verbs": {"Do it": 1, "Consider": 1}, "adverbs": {"very": 1, "not": 1}, "numerals": {"40": 1}, "pronouns": {"I": 1}}}, {"content": "There's hardly any activity that would interest me.", "word_count": 14, "label": "", "sentence_structure": "Subject鈥揷opula鈥損redicative", "subjective_objective": {"answer": "Subjective", "rationale": "Because interest is a personal feeling, it is viewed from a subjective perspective."}, "sentence_sentiment": {"answer": "Negative", "rationale": "Because the emotions expressed in the entries are negative, that is, the feelings of not interested activities are negative."}, "language_style": {"answer": "Tactful and warm", "rationale": "The entry is more personal and interactive when it is spoken in a gentle and inviting manner, rather than directly speaking or ordering. At the same time, it is not too emotional, but rather, it is understood by asking questions that trigger the respondents to think and answer, in order to understand their attitudes and perceptions of the activities of interest."}, "time_dimension": {"answer": "Present", "rationale": "The entry \"near anything that interests me\" expresses a current feeling and state, and therefore the judgement is now."}, "sentence_type": "Negative declarative sentence", "pos": {"nouns": {"Interest": 1, "Activities": 1}, "verbs": {"鈾?And I'm gonna be 鈾?: 1, "Interested": 1}, "adjectives": {"How many": 1}, "numerals": {"41": 1}, "pronouns": {"I": 1}}}, {"content": "It's often hard to understand what I'm saying.", "word_count": 13, "label": "", "sentence_structure": "Subject鈥揷opula鈥損redicative", "subjective_objective": {"answer": "Subjective", "rationale": "Because \"ununderstandable\" is a subjective feeling that represents a patient who thinks that others cannot understand their expression."}, "sentence_sentiment": {"answer": "Negative", "rationale": "Because the \"hard to understand\" act in the entry is negative."}, "language_style": {"answer": "Tactful and warm", "rationale": "The entry is more personal and interactive when it is spoken in a gentle and inviting tone, rather than in direct statements or orders. At the same time, the term \"hard to understand\" is not too emotional, but rather a more objective description of the problem."}, "time_dimension": {"answer": "Present", "rationale": "It's hard to understand what I'm saying, \"As usual\" means regular, customary behaviour, \"It's hard to understand\" is the state of things, so it's a case of what's happening."}, "sentence_type": "Negative declarative sentence", "pos": {"nouns": {"People": 1, "Contents": 1}, "verbs": {"Regular": 1, "Understood": 1, "Say it": 1}, "adjectives": {"It's hard": 1}, "adverbs": {"very": 1}, "numerals": {"42": 1}, "pronouns": {"I": 1}}}, {"content": "I'm keeping an eye on what people say.", "word_count": 17, "label": "", "sentence_structure": "Subject鈥搗erb鈥搊bject", "subjective_objective": {"answer": "Subjective", "rationale": "Because the examination is about the patient ' s understanding and judgement of the language of others, it is about the subjective thinking and attitude of the individual."}, "sentence_sentiment": {"answer": "Neutral", "rationale": "Because the entry does not have any obvious emotional colours. It is a description of the act, not a sentence that expresses positive or negative feelings or attitudes."}, "language_style": {"answer": "Calm and direct", "rationale": "The entry is in the form of a direct statement that makes clear the subject of asking the other side whether it has a habit of being alert to understand what others mean. It is more objective and balanced without using too many emotions or rhetorical techniques. So I think the language style of the entry is calm and straightforward."}, "time_dimension": {"answer": "Present", "rationale": "Now \"Keep\" is an ongoing move, and \"Say\" is an exercise that is now being done, so the entry is an attempt to understand what the other person is saying."}, "sentence_type": "Affirmative declarative sentence", "pos": {"nouns": {"Watch out": 1, "People": 1, "Meaning": 1}, "verbs": {"Hold on": 1, "I know": 1, "Say it": 1}, "adverbs": {"Really": 1}, "numerals": {"43": 1}, "pronouns": {"I": 1}}}, {"content": "I never lie.", "word_count": 7, "label": "", "sentence_structure": "Subject鈥搗erb鈥揷omplement", "subjective_objective": {"answer": "Subjective", "rationale": "Because I never lie is a subjective statement that reflects the attitude and beliefs of the patient."}, "sentence_sentiment": {"answer": "Positive", "rationale": "Because the \"never lie\" act is positive, indicating that the interviewee has the qualities of honesty and trust."}, "language_style": {"answer": "Calm and direct", "rationale": "The entry is simple and straightforward, has no overly reductive language, expresses the code of conduct \"Never lie\" and is objective and clear."}, "time_dimension": {"answer": "Past", "rationale": "In the context of \"I never lie\", \"Never\" is a negative act of the past, so the temporal dimension of the article is the past."}, "sentence_type": "Negative declarative sentence", "pos": {"nouns": {"Lies": 1}, "verbs": {"Speak": 1, "Never": 1}, "numerals": {"44": 1}, "pronouns": {"I": 1}}}, {"content": "I'm afraid of meeting someone else because I'm uncomfortable.", "word_count": 15, "label": "", "sentence_structure": "Subject鈥搗erb鈥揷omplement", "subjective_objective": {"answer": "Subjective", "rationale": "Because fear, feeling uncomfortable are the psychological feelings and perceptions of the patient, and are subjective."}, "sentence_sentiment": {"answer": "Negative", "rationale": "Because the entries \"fear\" and \"discomfort\" express negative feelings."}, "language_style": {"answer": "Tactful and warm", "rationale": "The entry is a soft, understanding conversation, expressing concern and understanding about what the interviewer feels inside, rather than directly criticizing or rejecting. This expression is more personal and mutual, helping to create a good investigation atmosphere."}, "time_dimension": {"answer": "Present", "rationale": "Based on the two key words \"I'm afraid\" and \"I'm uncomfortable,\" we can tell that this is what's happening at the moment that we're talking, and therefore in the state that we are."}, "sentence_type": "Negative declarative sentence", "pos": {"nouns": {"Questionnaire Entry": 1, "Scared": 1, "I met him": 1, "Livers": 1, "Because": 1, "Feel": 1, "I'm not comfortable": 1}, "verbs": {"I": 2, "Scared": 1, "I met him": 1, "I feel": 1, "Answer": 1}, "adverbs": {"Down there": 1}, "numerals": {"45": 1}, "pronouns": {"I": 1}}}, {"content": "I'm so desperate to make people like me, so I'm willing to do things I don't want to do.", "word_count": 24, "label": "", "sentence_structure": "Subject鈥搗erb鈥搊bject", "subjective_objective": {"answer": "Subjective", "rationale": "Because \"too much to make people like me\" and \"doing what I don't want to do\" are the thoughts and behaviours of the patients themselves, and they are subjective."}, "sentence_sentiment": {"answer": "Negative", "rationale": "Because the act of \"voluntaryly doing what I don't want to do\" in the entry is negative."}, "language_style": {"answer": "Tactful and warm", "rationale": "The language style in this entry is euphemistic, because it uses expressions such as \"too\" and \"volunteer,\" which emphasize emotions and will, rather than direct orders or assertions. It is more gentle, friendly, and easily resonates with the audience."}, "time_dimension": {"answer": "Past", "rationale": "\"Too much to make people like me\" and \"to do things I don't want to.\" means the behavior or mentality that happened in the past, so the time dimension of the entry is the past."}, "sentence_type": "Affirmative declarative sentence", "pos": {"nouns": {"I": 2, "People": 1, "Things": 2}, "verbs": {"鈾?And I'm gonna be 鈾?: 1, "Yeah": 1, "Do it": 3, "Go on": 1}, "adjectives": {"Too much": 1, "I'd love to": 1}, "adverbs": {"Too much": 1}, "numerals": {"46": 1}, "pronouns": {"I": 1}}}, {"content": "I am saving up a lot of things I don't need, but I can't afford to throw them away.", "word_count": 22, "label": "", "sentence_structure": "Subject鈥搗erb鈥搊bject", "subjective_objective": {"answer": "Subjective", "rationale": "Because \"spare to throw away\" is a subjective attitude and emotional experience. While the problem describes objective facts (possessing much of what is not needed), it focuses on the feelings and attitudes of the patient with regard to these items, and it is therefore the subjective perspective of the patient that is being examined."}, "sentence_sentiment": {"answer": "Negative", "rationale": "Because \"still throw them away\" means negative attitudes and behavior about what is not needed."}, "language_style": {"answer": "Tactful and warm", "rationale": "The entry uses a first person's expression and leads the interviewee to share their feelings and ideas in a way that is asked. At the same time, by emphasizing the \"but\"-led turn-around, the expression highlights the contradictions and problems within the interviewee, which are more euphemismal, friendly and can better trigger the echoes and engagement of the interviewee."}, "time_dimension": {"answer": "Present", "rationale": ""}, "sentence_type": "Negative declarative sentence", "pos": {"nouns": {"I": 2, "A lot": 1, "No, I don't": 1, "Something": 1, "But": 1, "I'm not going to leave you alone": 1, "Throw it away": 1, "They": 1}, "verbs": {"Storage": 1, "Throw it away": 1}, "adjectives": {"No, I don't": 1}, "adverbs": {"A lot": 1, "But": 1, "very": 1}, "numerals": {"47": 1}, "pronouns": {"I": 2}}}, {"content": "Even though I've said a lot, people say I haven't caught the point.", "word_count": 19, "label": "", "sentence_structure": "Subject鈥損redicate", "subjective_objective": {"answer": "Subjective", "rationale": "This is a subjective assessment of the views or feelings of others as expressed by the patient."}, "sentence_sentiment": {"answer": "Negative", "rationale": "Because the description of \"not catching the point\" in the entry is negative and indicates that there are problems in communication."}, "language_style": {"answer": "Tactful and warm", "rationale": "The language style of the entry is a gentle and warm type. It raises questions in a gentle and friendly manner and invites respondents to answer instead of direct orders or criticisms of the interviewee. Moreover, the entry uses some polite language (e.g. \"even if I have spoken a lot\" ), which further underscores its euphemism and respectable language style."}, "time_dimension": {"answer": "Present", "rationale": ""}, "sentence_type": "Negative declarative sentence", "pos": {"nouns": {"Questionnaire": 1, "Points": 1}, "verbs": {"Yes": 1, "Hold on": 1, "Say it": 1}, "adverbs": {"Many": 1, "And": 1}, "numerals": {"48": 1}, "pronouns": {"I": 1}}}, {"content": "I expect favors from others, even if I don't usually give them.", "word_count": 21, "label": "", "sentence_structure": "Subject鈥搗erb鈥搊bject", "subjective_objective": {"answer": "Subjective", "rationale": "Because \"expectation\" and \"generally not\" are subjective ideas and attitudes of patients."}, "sentence_sentiment": {"answer": "Negative", "rationale": "Because the \"seeing someone to give me mercy\" act in the entry is negative."}, "language_style": {"answer": "Calm and direct", "rationale": "The language style of the entry can be described as cynical, as it implies selfish and immoral behaviour and expresses an ironic attitude towards those who accept the favours of others. On the other hand, it can be seen as a neutral language style, as it directly presents an observation without involving personal positions or values."}, "time_dimension": {"answer": "Present", "rationale": "The entry for this questionnaire therefore relates to the current behaviour or state."}, "sentence_type": "Affirmative declarative sentence", "pos": {"nouns": {"others": 1, "Grace": 2, "I": 3}, "verbs": {"Expectations": 1, "Here": 1, "I'm not giving it to you": 1}, "adverbs": {"Usually": 1}, "numerals": {"49": 1}}}, {"content": "I'm a man of rage.", "word_count": 8, "label": "", "sentence_structure": "Subject鈥揷opula鈥損redicative", "subjective_objective": {"answer": "Subjective", "rationale": "Because \"freakless\" is an emotional experience and self-awareness that is part of the subjective sense of the patient."}, "sentence_sentiment": {"answer": "Negative", "rationale": "Because the term \"freakless\" is usually considered negative."}, "language_style": {"answer": "Tactful and warm", "rationale": "The language style of the entry is euphemistic, as it asks the interviewer in an invitation tone, not in a direct statement or order. This expression is more personal and interactive and is more acceptable to the interviewee and is willing to answer the question."}, "time_dimension": {"answer": "Present", "rationale": "The entry is for the testee ' s own character characteristics, and the time dimension is now judged."}, "sentence_type": "Affirmative declarative sentence", "pos": {"nouns": {"person": 1, "鈾?The mood is unchanging 鈾?: 1}, "adjectives": {"Unusual": 1}, "numerals": {"50": 1}, "pronouns": {"I": 1}}}, {"content": "It's easy for me to lie. I do that all the time.", "word_count": 14, "label": "", "sentence_structure": "Subject鈥搗erb鈥搊bject", "subjective_objective": {"answer": "Subjective", "rationale": "The idea and behaviour of lying are subjective attitudes and behaviour of the patient and are therefore subjective."}, "sentence_sentiment": {"answer": "Negative", "rationale": "Because the description of \"false\" and \"often do\" in the entries is all negative emotional."}, "language_style": {"answer": "Calm and direct", "rationale": "The entry expresses the frequency and difficulty of the person under investigation lying in a direct and clear manner, and is expressed in a more blunt and straightforward manner without a euphemism or implicit language."}, "time_dimension": {"answer": "Present", "rationale": ""}, "sentence_type": "Affirmative declarative sentence", "pos": {"nouns": {"Liar": 1, "I": 2}, "verbs": {"Do it": 1}, "adjectives": {"Easy": 1}, "adverbs": {"very": 1, "Regular": 1}, "numerals": {"51": 1, "Here": 1}}}, {"content": "I don't care if there's any close friends.", "word_count": 13, "label": "", "sentence_structure": "Subject鈥揷opula鈥損redicative", "subjective_objective": {"answer": "Subjective", "rationale": "Because \"not concerned with the existence of a close friend\" is a personal attitude or perception, and it is subjective."}, "sentence_sentiment": {"answer": "Neutral", "rationale": "The entry is not a positive or negative emotional expression."}, "language_style": {"answer": "Calm and direct", "rationale": "The entry is presented in a concise and direct language, with no excessive adoration or emotional rendering, and in a calm and objective manner, easy to understand and answer."}, "time_dimension": {"answer": "Present", "rationale": ""}, "sentence_type": "Negative declarative sentence", "pos": {"nouns": {"friends": 1}, "verbs": {"have": 1, "be": 1, "It doesn't matter": 1}, "adverbs": {"to": 1}, "numerals": {"52": 1}}}, {"content": "I'm always on guard against the benefits being taken.", "word_count": 12, "label": "", "sentence_structure": "Subject鈥搗erb鈥搊bject", "subjective_objective": {"answer": "Subjective", "rationale": "Because vigilance is for the protection of oneself, it involves the individual feelings and attitudes of the patient."}, "sentence_sentiment": {"answer": "Negative", "rationale": "Because the entry \"Alert to prevent the benefits from being taken away\" expresses an emotion of vigilance and preparedness, which is somewhat negative."}, "language_style": {"answer": "Calm and direct", "rationale": "The entry uses a more direct language style, does not have too many rhetorical or emotional renderings and simply expresses the interest of the subject in protecting his or her rights and interests."}, "time_dimension": {"answer": "Present", "rationale": "In the future, the entry contains visions and expectations for the future, with the key words \"prevent\" and \"take away\" pointing to situations that have not yet occurred and that may arise in the future."}, "sentence_type": "Affirmative declarative sentence", "pos": {"nouns": {"I": 1, "Benefits": 1, "Watch out": 1, "Take it away": 1}, "verbs": {"Regular": 1, "have": 1}, "adverbs": {"Regular": 1}, "numerals": {"53": 1}}}, {"content": "I never forget or forgive anyone who did something wrong to me.", "word_count": 16, "label": "", "sentence_structure": "Subject鈥搗erb鈥搊bject", "subjective_objective": {"answer": "Subjective", "rationale": "Because \"forgetting or forgiving\" is the patient's subjective attitude and behavioral choice."}, "sentence_sentiment": {"answer": "Negative", "rationale": "Because \"forgetting\" and \"forgive\" are neutral words, but the premise of \"for those who have done wrong\" in combination makes this object a negative emotional aspect."}, "language_style": {"answer": "Tactful and warm", "rationale": "The entry uses more euphemistic language, expressing the subject of questioning each other about their attitudes and behaviour in dealing with others. Rather than using a direct command or criticism of language, it communicates in a more moderate and friendly way, helping to create a good communication atmosphere."}, "time_dimension": {"answer": "Past", "rationale": "The entry asks about the attitudes and behaviour of the testee towards \"forgotten\" or \"forgivable\" behaviour based on personal experience and memory, and can therefore be judged as past."}, "sentence_type": "Interrogative sentence", "pos": {"nouns": {"I": 1, "person": 1, "Wrong": 1}, "verbs": {"Forget it": 1, "Forgive me": 1}, "adverbs": {"Never": 1}, "numerals": {"54": 1}}}, {"content": "A nuclear war may not be a bad idea.", "word_count": 15, "label": "", "sentence_structure": "Subject鈥搗erb鈥揷omplement", "subjective_objective": {"answer": "Subjective", "rationale": "Because it is a view or opinion, not an objective fact."}, "sentence_sentiment": {"answer": "Negative", "rationale": "Because the concept of \"nuclear war\" in the entry is devastating and \"may not be a bad idea\" expresses an incorrect perception of nuclear war, the whole emotional color is negative."}, "language_style": {"answer": "Calm and direct", "rationale": "The entry is straightforward, without any qualifier or expression, and is impressive in its objective and concise expression of views on nuclear war."}, "time_dimension": {"answer": "Present", "rationale": "The entry expresses the view that \"a nuclear war may not be a bad idea.\" This view is now, because it is formed at the current point of time. While it may involve future situations (if there is a nuclear war), it is the current point of view in the discussion at this point."}, "sentence_type": "Negative declarative sentence", "pos": {"nouns": {"Nuclear war": 1}, "verbs": {"be": 1, "Maybe": 1, "not": 1, "Bad": 1, "Ideas": 1}, "adjectives": {"very": 1, "Bad": 1}, "adverbs": {"very": 1}, "numerals": {"55": 1}}}, {"content": "When I was alone, I felt helpless and unable to take care of myself.", "word_count": 20, "label": "", "sentence_structure": "Subject鈥揷opula鈥損redicative", "subjective_objective": {"answer": "Subjective", "rationale": "It is the patient ' s subjective feeling that feels helpless and unable to take care of himself."}, "sentence_sentiment": {"answer": "Negative", "rationale": "Because the emotional \"incompetent and unable to take care of yourself\" in the entry is negative."}, "language_style": {"answer": "Calm and direct", "rationale": "This entry is a direct interview with the interviewee鈥檚 feelings when he is alone, and is not packaged with any euphemism or rhetoric. This cold and plain expression is more suitable for the interviewee to truly reflect his or her inner feelings than to attempt to influence his or her answers by modifying his or her language."}, "time_dimension": {"answer": "Present", "rationale": ""}, "sentence_type": "Negative declarative sentence", "pos": {"nouns": {"person": 1, "Helpless": 1, "Self": 1, "Care": 1}, "verbs": {"I feel": 1, "No, I can't": 1}, "adjectives": {"Alone": 1}, "adverbs": {"Time": 1}, "numerals": {"56": 1}, "pronouns": {"I": 1}}}, {"content": "If someone else can't do something right, I'd rather do it myself.", "word_count": 21, "label": "", "sentence_structure": "Subject鈥搗erb鈥搊bject", "subjective_objective": {"answer": "Subjective", "rationale": "Because \"I'd rather do it myself\" is the patient's subjective idea and attitude."}, "sentence_sentiment": {"answer": "Positive", "rationale": "Because the actions in the entries are voluntary and have a positive emotional character."}, "language_style": {"answer": "Calm and direct", "rationale": "Although the phrase expresses the individual鈥檚 willingness to solve the problem, it does not use too much euphemism or warm words, which appear to be calm and objective."}, "time_dimension": {"answer": "Past", "rationale": "So the entry is both about the past (because something \"can't be done correctly\" may have happened) and about the future (because \"if someone else can't do it myself\" is a prediction or a preference for the future). So the time dimension of the questionnaire is past and future."}, "sentence_type": "Interrogative sentence", "pos": {"nouns": {"others": 1, "Things": 2, "self": 1}, "verbs": {"Yes": 1, "Do it": 3, "Come on": 1}, "adverbs": {"Correct": 1, "If": 1, "I'd rather not": 1}, "numerals": {"57": 1}, "pronouns": {"I": 1}}}, {"content": "I have a talent for acting.", "word_count": 6, "label": "", "sentence_structure": "Subject鈥搗erb鈥揷omplement", "subjective_objective": {"answer": "Subjective", "rationale": "Because \"a talent for acting\" is a self-awareness and evaluation of the patient, and it's a subjective perspective."}, "sentence_sentiment": {"answer": "Positive", "rationale": "Because \"Performance gift\" in the entry is a positive quality."}, "language_style": {"answer": "Tactful and warm", "rationale": "The language style of the entry is more euphemistic. The term \"natural\" is used to describe itself, to make people feel encouraged and appreciated, and not to express too directly their self-confidence and superiority, reflecting a more modest and respectful mindset."}, "time_dimension": {"answer": "Present", "rationale": "The entry describes self-evaluation by the testee, with no clear time-pointing, but is generally considered to be a description of the current state."}, "sentence_type": "Affirmative declarative sentence", "pos": {"nouns": {"Performing": 1, "Talent": 1}, "verbs": {"have": 1}, "numerals": {"58": 1}, "pronouns": {"I": 1}}}, {"content": "Some people think I take advantage of others.", "word_count": 12, "label": "", "sentence_structure": "Subject鈥搗erb鈥搊bject", "subjective_objective": {"answer": "Subjective", "rationale": "Because it is perceived as subjective."}, "sentence_sentiment": {"answer": "Negative", "rationale": "Because the \"bringing off\" act in the entry is considered negative."}, "language_style": {"answer": "Calm and direct", "rationale": "The entry directly asks the interviewees whether they are perceived to be cheap, is neutral and does not have too many rhetorical or emotional accents. Thus, the language style is \"cool and direct\"."}, "time_dimension": {"answer": "Present", "rationale": "The keyword \"some people think \"and \"taking advantage of others\" suggests that this is a description of the testee's current behaviour or attitude, and therefore the judgement is now."}, "sentence_type": "Negative declarative sentence", "pos": {"nouns": {"person": 1, "Cheap": 1}, "verbs": {"I think": 1, "Jim": 1}, "adjectives": {"Some": 1}, "numerals": {"59": 1}, "pronouns": {"I": 1}}}, {"content": "I feel that my life is monolithic and meaningless.", "word_count": 15, "label": "", "sentence_structure": "Subject鈥揷opula鈥損redicative", "subjective_objective": {"answer": "Subjective", "rationale": "Because it is the patient's subjective feeling and evaluation."}, "sentence_sentiment": {"answer": "Negative", "rationale": "Because \"uniformity and meaningless\" is a negative description, it is an expression of dissatisfaction or confusion about life."}, "language_style": {"answer": "Tactful and warm", "rationale": "The entry uses a relatively euphemistic description, by asking if the other side has such feelings about his life, to guide the other to share their own inner world. At the same time, it uses emotional words like \"uniform\" and \"unmeaning\" to make the words emotional. Such language does not make the interviewee feel too stingy or offensive, but it can effectively capture their real thoughts and feelings."}, "time_dimension": {"answer": "Present", "rationale": "The term \"sweet\" denotes the person being tested's immediate feelings, so the temporal dimension of the article is now."}, "sentence_type": "Negative declarative sentence", "pos": {"nouns": {"Life": 1, "Meaning": 1}, "verbs": {"I feel": 1, "Undo": 1, "No, I'm not": 1}, "numerals": {"60": 1}, "pronouns": {"self": 1}}}, {"content": "I don't care what people say about me.", "word_count": 10, "label": "", "sentence_structure": "Subject鈥搗erb鈥揷omplement", "subjective_objective": {"answer": "Subjective", "rationale": "Because it doesn't matter what people think is the patient's subjective attitude and emotional experience."}, "sentence_sentiment": {"answer": "Negative", "rationale": "Because the attitude in the entries is one that does not care about others' evaluation, it is a negative attitude."}, "language_style": {"answer": "Calm and direct", "rationale": "The entry directly asked whether the interviewee did not care about the other person ' s evaluation, was clear and clear in language and was more calm and objective in tone."}, "time_dimension": {"answer": "Present", "rationale": "Thus, the entry describes the current state of behaviour or mental state of the person being tested, which should be judged as \"now\"."}, "sentence_type": "Negative declarative sentence", "pos": {"verbs": {"I care": 1, "Say it": 1}, "adjectives": {"Don't": 1, "I": 1}, "adverbs": {"not": 1}, "numerals": {"61": 1}, "pronouns": {"What": 1}}}, {"content": "62. In one-on-one situations, I have difficulty dealing with people.", "word_count": 18, "label": "", "sentence_structure": "Subject鈥搗erb鈥搊bject", "subjective_objective": {"answer": "Subjective", "rationale": "Because the title refers to \"me,\" which means that it is a personal feeling and perception of the patient and is a subjective examination."}, "sentence_sentiment": {"answer": "Negative", "rationale": "Because the \"difficult\" act in the entry is negative."}, "language_style": {"answer": "Calm and direct", "rationale": "The entry expresses the subject matter directly in a concise and clear language, without using excessive rhetorical or emotional rendering, and is more objective in tone."}, "time_dimension": {"answer": "Present", "rationale": ""}, "sentence_type": "Negative declarative sentence", "pos": {"nouns": {"Situation": 1, "person": 1, "- To be with someone": 1}, "verbs": {"have": 1, "Fight": 1, "Hand it over": 1, "Hard": 1}, "adjectives": {"One": 2, "One on one": 1}, "adverbs": {"in": 1, "Down there": 1}, "numerals": {"62": 1}, "pronouns": {"I": 1, "others": 1}}}, {"content": "People often complain that I don't feel the troubles in their hearts.", "word_count": 17, "label": "", "sentence_structure": "Subject鈥搗erb鈥搊bject", "subjective_objective": {"answer": "Subjective", "rationale": "Because \"not to feel their worries\" is the patient's subjective feeling and understanding. Although it's about other people's thoughts, it's still subjective."}, "sentence_sentiment": {"answer": "Negative", "rationale": "Because the \"complaint\" act is negative, expressing dissatisfaction or frustration. And \"failed to feel their worries\" means that the other person feels neglected or ununderstanded, and it is also negative emotional."}, "language_style": {"answer": "Tactful and warm", "rationale": "The entry uses a euphemismal and thoughtful approach, which attempts to make the interviewees feel understood and cared for, rather than blamed or criticized. Such an expression reduces the resistance of the interviewees and is more likely to receive their true feedback."}, "time_dimension": {"answer": "Present", "rationale": ""}, "sentence_type": "Negative declarative sentence", "pos": {"nouns": {"People": 1, "- He's complaining": 1, "I": 2, "they": 1, "Psychological": 1, "Trouble": 1}, "verbs": {"Regular": 1, "Yes": 1, "Feel it": 1, "- He's complaining": 1}, "adverbs": {"Regular": 1}, "numerals": {"63": 1}}}, {"content": "When people look at me, they think I'm weird, weird or weird.", "word_count": 21, "label": "", "sentence_structure": "Subject鈥揷opula鈥損redicative", "subjective_objective": {"answer": "Subjective", "rationale": "Because \"I think what people think\" is a subjective idea."}, "sentence_sentiment": {"answer": "Negative", "rationale": "Because the expressions \"eccentric\", \"unsurprising\" and \"uncharacteristic\" in the entries are derogatory and reflect negative emotional expressions."}, "language_style": {"answer": "Tactful and warm", "rationale": "Warm and warm."}, "time_dimension": {"answer": "Present", "rationale": "The entry describes a situation that may occur in people's daily lives 鈥?when others stare at them, they may feel strange. This can happen now or at some point in the past, but in any case, it is a description of current or past observations and perceptions, and is therefore classified as now."}, "sentence_type": "Negative declarative sentence", "pos": {"nouns": {"People": 1, "Problem": 1, "Weird": 2, "Strange": 1}, "verbs": {"Watch": 1, "I think": 1, "Yes": 1}, "adjectives": {"Very": 1}, "adverbs": {"Maybe": 1}, "numerals": {"64": 1}, "pronouns": {"I": 1}}}, {"content": "I like to do dangerous things for fun.", "word_count": 11, "label": "", "sentence_structure": "Subject鈥搗erb鈥搊bject", "subjective_objective": {"answer": "Subjective", "rationale": "Because it's the patient's subjective attitude."}, "sentence_sentiment": {"answer": "Negative", "rationale": "Because the \"likes to do dangerous things\" act negatively in the entry."}, "language_style": {"answer": "Calm and direct", "rationale": "The language style of the entry is more straightforward, without any symmetry or emotional rendering, and directly expresses the subject of asking if the other party likes to do dangerous things to get happy. So, its language style is \"cool straight.\""}, "time_dimension": {"answer": "Present", "rationale": "Now, because the title uses \"like\" and \"do\" to express the current state of interest and behaviour, not past or future situations. So the temporal dimension of the topic is now."}, "sentence_type": "Affirmative declarative sentence", "pos": {"nouns": {"Things": 1, "Danger": 1, "Enjoy": 1}, "verbs": {"Yeah": 1, "Do it": 1}, "adverbs": {"Yeah": 1}, "numerals": {"65": 1}}}, {"content": "I lied a lot in this questionnaire.", "word_count": 12, "label": "", "sentence_structure": "Subject鈥搗erb鈥揷omplement", "subjective_objective": {"answer": "Subjective", "rationale": "Because lying is a subjective act, whether or not the respondent decides to do so, and the outcome of such acts is assessed by the interviewee itself. The answer to this questionnaire should therefore be considered subjective."}, "sentence_sentiment": {"answer": "Negative", "rationale": "Because the behavior of the entry \"fake\" is negative."}, "language_style": {"answer": "Tactful and warm", "rationale": "This is a more euphemistic and acceptable expression that helps maintain a good atmosphere of communication."}, "time_dimension": {"answer": "Past", "rationale": "The \"many lies\" in the entry are a description and evaluation of the past, and therefore judgement is the past."}, "sentence_type": "Negative declarative sentence", "pos": {"nouns": {"Questionnaire": 1, "Lies": 1}, "verbs": {"Scram": 1, "in": 1, "be": 1}, "adverbs": {"A lot": 1}, "numerals": {"66": 1}, "pronouns": {"I": 1}}}, {"content": "I have difficulty controlling my anger and temper.", "word_count": 13, "label": "", "sentence_structure": "Subject鈥揷opula鈥損redicative", "subjective_objective": {"answer": "Subjective", "rationale": "Because \"hard to control your anger and temper\" is a subjective feeling and experience, a patient's description of his emotional state."}, "sentence_sentiment": {"answer": "Negative", "rationale": "Because the \"hard to control your anger and temper\" act in the entry is negative."}, "language_style": {"answer": "Calm and direct", "rationale": "The entry directly expresses the subject of questioning the other party about the difficulty of emotional management, and is not based on excessive rhetorical or emotional rendering, which is more objective and sound."}, "time_dimension": {"answer": "Present", "rationale": "The keyword \"I have difficulty controlling\" and \"my anger and temper\" all imply that this is a state or behaviour of the moment, so it's judged to be the present."}, "sentence_type": "Negative declarative sentence", "pos": {"nouns": {"self": 1, "Anger": 1, "- Grumpy": 1}, "verbs": {"Control": 1, "be": 1}, "adverbs": {"It's hard": 1}, "numerals": {"67": 1}}}, {"content": "There are some people who envy me.", "word_count": 7, "label": "", "sentence_structure": "Subject鈥搗erb鈥揷omplement", "subjective_objective": {"answer": "Subjective", "rationale": "Because jealousy is an emotional experience, it is felt and experienced by the patient, not an objective fact. The answer to this should therefore be based on the patient鈥檚 subjective feelings."}, "sentence_sentiment": {"answer": "Negative", "rationale": "Because the \"envious\" act in the entry is negative."}, "language_style": {"answer": "Calm and direct", "rationale": "The entry is based on direct inquiries, with no excessive use of rhetorical techniques, and is sound and objective, consistent with the calm and direct language style."}, "time_dimension": {"answer": "Past", "rationale": "At the same time, \"envious\" is an emotional state that requires consideration of the duration of time, i.e. whether jealousy persists over a certain period of time, and therefore can also be considered as the present situation."}, "sentence_type": "Affirmative declarative sentence", "pos": {"nouns": {"Some people": 1, "Jealousy": 1}, "verbs": {"have": 1}, "numerals": {"68": 1}, "classifiers": {"individual": 1}, "pronouns": {"I": 1}}}, {"content": "I'm vulnerable to others.", "word_count": 9, "label": "", "sentence_structure": "Subject鈥揷opula鈥損redicative", "subjective_objective": {"answer": "Subjective", "rationale": "Because being affected is a subjective feeling and perception of a person."}, "sentence_sentiment": {"answer": "Negative", "rationale": "Because the act of being \"vulnerable to others\" in the entry is negative."}, "language_style": {"answer": "Tactful and warm", "rationale": "The entry is more personal and interactive in its expression of a gentle and inviting tone, rather than a direct statement or order. At the same time, it uses the word \"I\" to enable respondents to better participate in the answers to questions and to be more closely related to humanized communication."}, "time_dimension": {"answer": "Present", "rationale": "The entry describes the degree to which the person tested is vulnerable to exposure in his/her dealings with others, without clear time-pointing. Thus, it can be judged that the entry does not belong to any of the past, present or future situations, but rather to a general statement."}, "sentence_type": "Negative declarative sentence", "pos": {"nouns": {"Impact": 1}, "verbs": {"Easy": 1, "Yes": 1}, "adjectives": {"Others": 1}, "numerals": {"69": 1}, "pronouns": {"I": 1}}}, {"content": "I think I'm a very timid person, but I'm considered a little timid.", "word_count": 16, "label": "", "sentence_structure": "Subject鈥揷opula鈥損redicative", "subjective_objective": {"answer": "Subjective", "rationale": "Because \"think\" is a personal thought and opinion, subjective."}, "sentence_sentiment": {"answer": "Neutral", "rationale": "Since the entry merely describes the self-evaluation of one person and the views of others, it is not clearly emotional."}, "language_style": {"answer": "Tactful and warm", "rationale": "The entry is more personal and interactive when it is presented in a gentle and inviting tone, rather than in direct statements or orders. At the same time, the entry is more euphemistic in its content, which is also asked about the views of the respondent in relation to those of others."}, "time_dimension": {"answer": "Present", "rationale": ""}, "sentence_type": "Negative declarative sentence", "pos": {"nouns": {"Questionnaire": 1, "Entry": 1, "person": 1, "self": 1, "Reveal": 1, "others": 1, "- You're a little bit of a jerk": 1}, "verbs": {"I think": 2, "very": 1, "But": 1, "Both": 1, "Yeah": 1, "Call": 1, "Chat": 1, "Analysis": 1}, "adjectives": {"Reveal": 1, "- You're a little bit of a jerk": 1}, "adverbs": {"very": 1}, "numerals": {"70": 1, "One": 2}, "pronouns": {"I": 1, "self": 1}}}, {"content": "When one relationship ends, I need to establish a close relationship with another immediately.", "word_count": 27, "label": "", "sentence_structure": "Subject鈥搗erb鈥搊bject", "subjective_objective": {"answer": "Subjective", "rationale": "Because the need to establish close relationships with another person immediately is a subjective attitude or need of the patient."}, "sentence_sentiment": {"answer": "Positive", "rationale": "Because the purpose of the act in the entry is to \"need to establish a close relationship with another person immediately\"."}, "language_style": {"answer": "Calm and direct", "rationale": "The entry is based on a straightforward approach to the response of the person surveyed after the close relationship has been processed, without excessive rhetorical or emotional rendering, and is more objective in tone and therefore of a calm and direct language style."}, "time_dimension": {"answer": "Present", "rationale": "The keywords \"need\" and \"immediately\" both indicate that this is an act or state that is now taking place. \"Construction of a close relationship\" is also a verb that is now taking place, implying that the act is in progress. The entry therefore describes the current attitude or orientation of the person being tested."}, "sentence_type": "Affirmative declarative sentence", "pos": {"nouns": {"Plant": 1, "Intimacy": 2, "person": 2}, "verbs": {"Create": 1, "Yes": 1, "and": 1, "End": 1}, "adjectives": {"When": 1, "Immediately": 1, "The other one": 1, "Close": 2}, "numerals": {"71": 1}, "pronouns": {"I": 1}}}, {"content": "I'm suffering from a low self-evaluation.", "word_count": 11, "label": "", "sentence_structure": "Subject鈥揷opula鈥損redicative", "subjective_objective": {"answer": "Subjective", "rationale": "Because self-evaluation of low feelings and suffering is the patient ' s own subjective experience and feelings."}, "sentence_sentiment": {"answer": "Negative", "rationale": "The basis for the judgment. It is negative because the description of \"self-evaluation is too low and painful\" in the entry indicates that the individual feels negative emotional experience."}, "language_style": {"answer": "Tactful and warm", "rationale": "The entry is more personal and interactive when it is spoken in a gentle and inviting manner, rather than in direct statements or orders. At the same time, it implies compassion and understanding of the feelings of the interviewees, by using the structure \"by reason...\"."}, "time_dimension": {"answer": "Present", "rationale": "According to the keyword \"I am suffering from my own low self-evaluation\", it can be seen that the entry to the questionnaire describes the behaviour and feelings of the subjects at present, and therefore the temporal dimension is now judged."}, "sentence_type": "Negative declarative sentence", "pos": {"nouns": {"Pain": 1, "Evaluation": 1}, "verbs": {"Because": 1, "Self": 1, "And": 1, "Pain": 1}, "adjectives": {"Low": 1}, "numerals": {"72": 1}, "pronouns": {"I": 1}}}, {"content": "I don't spend time reproducing to those who insult me.", "word_count": 15, "label": "", "sentence_structure": "Subject鈥搗erb鈥搊bject", "subjective_objective": {"answer": "Subjective", "rationale": "Because \"regard\" is a behaviour that requires subjective decision-making, the decision-making process for which is based on the patient ' s own attitudes and values."}, "sentence_sentiment": {"answer": "Negative", "rationale": "Because the entry expresses tolerance and inaction towards insults, it is a negative attitude."}, "language_style": {"answer": "Calm and direct", "rationale": "The language style of the entry is more calm and objective, and there is no excessive emotional or rhetorical style, which directly describes the attitudes and behaviour of the person speaking. This expression gives people a sense of reason and reality, and is less likely to produce strong resonance or emotional reactions."}, "time_dimension": {"answer": "Past", "rationale": "The entry is therefore an inquiry into the manner in which the person tested treated his or her own insults and attitudes over a period of time."}, "sentence_type": "Negative declarative sentence", "pos": {"nouns": {"Time": 1, "Insulting": 1, "person": 1}, "verbs": {"Flowers": 1, "- To you": 1, "Insulting": 1}, "adverbs": {"not": 1, "Go on": 1}, "numerals": {"73": 1}, "pronouns": {"I": 1}}}, {"content": "I'm nervous about someone else around.", "word_count": 12, "label": "", "sentence_structure": "Subject鈥揷opula鈥損redicative", "subjective_objective": {"answer": "Subjective", "rationale": "Because tension is a subjective emotional experience and reaction, it is the result of factors such as the individual ' s own emotional, psychological state."}, "sentence_sentiment": {"answer": "Negative", "rationale": "Because the \"frightful\" mood in the entry is negative."}, "language_style": {"answer": "Calm and direct", "rationale": "The entry directly expresses the subject of questioning the other party about the stress in a given situation in a concise and clear language, with no excessive rhetorical or emotional rendering, and is more objective in tone."}, "time_dimension": {"answer": "Present", "rationale": "\"I feel nervous when there are others around me\" means that it's an immediate, immediate reaction or feeling, and so it's now."}, "sentence_type": "Negative declarative sentence", "pos": {"nouns": {"Around": 1, "person": 1, "Nervous": 1}, "verbs": {"have": 1, "Sensor": 1, "Present": 1}, "adverbs": {"Just": 1}, "numerals": {"74": 1}}}, {"content": "75. I'm afraid of being embarrassed or embarrassed in a new occasion.", "word_count": 14, "label": "", "sentence_structure": "Subject鈥揷opula鈥損redicative", "subjective_objective": {"answer": "Subjective", "rationale": "Fear of embarrassment or humiliation is a feeling and attitude within a person and is subjective."}, "sentence_sentiment": {"answer": "Negative", "rationale": "Because it's a negative emotion, which is \"regarding embarrassment or shame\"."}, "language_style": {"answer": "Calm and direct", "rationale": "The language style of the entry is more concise, and it does not use too much qualifier or emotional vocabulary, but rather directly presents a situation that the person surveyed may face and asks about his or her feelings."}, "time_dimension": {"answer": "Future", "rationale": "The entry is for the purpose of asking the subject about the fear and fear of what might happen in the future, and the keywords are \"fear\" and \"in the new context\", all of which point to situations that have not yet occurred and that may arise in the future."}, "sentence_type": "Negative declarative sentence", "pos": {"nouns": {"Event": 1, "Yes": 1, "I'm embarrassed": 1, "Shame": 1}, "verbs": {"in": 1, "Scared": 1}, "adverbs": {"New": 1}, "numerals": {"75": 1}}}, {"content": "I'm afraid of being alone.", "word_count": 9, "label": "", "sentence_structure": "Subject鈥揷opula鈥損redicative", "subjective_objective": {"answer": "Subjective", "rationale": "Because \"fear\" is a subjective emotion and feeling."}, "sentence_sentiment": {"answer": "Negative", "rationale": "Because fear of being alone is a negative emotional reaction."}, "language_style": {"answer": "Tactful and warm", "rationale": "The entry uses a mild and caring tone to ask the other side whether there is a sense of fear, rather than a direct statement or order, which is more personal and interactive. At the same time, the more euphemism of \"singing alone\" is used to avoid unnecessary tension and discomfort."}, "time_dimension": {"answer": "Present", "rationale": ""}, "sentence_type": "Negative declarative sentence", "pos": {"nouns": {"Questionnaire Entry": 1, "I": 1, "Alone": 1, "individual": 1}, "verbs": {"Scared": 1, "Stay": 1}, "numerals": {"76": 1}}}, {"content": "People complain I'm stubborn like a bull.", "word_count": 12, "label": "", "sentence_structure": "Subject鈥搗erb鈥揷omplement", "subjective_objective": {"answer": "Objective", "rationale": "Because \"damn like a cow\" is an objective description of the patient."}, "sentence_sentiment": {"answer": "Negative", "rationale": "Because the term \"dilemma\" in the entry is negative."}, "language_style": {"answer": "Calm and direct", "rationale": "The language style of the entry is more calm and straightforward, with no excessive rhetorical or emotional rendering, and it is clear and objective. At the same time, the term \"complaint\" is used to imply negative perceptions of the character characteristics of the person being evaluated."}, "time_dimension": {"answer": "Past", "rationale": "The \"people complain\" move happened in the past, and \"hard as a cow\" is a description of the past, and therefore judgement is the past."}, "sentence_type": "Negative declarative sentence", "pos": {"nouns": {"People": 1, "- He's complaining": 1, "I": 2, "Yeah": 1, "Snail": 1, "Obstinate": 1}, "verbs": {"- He's complaining": 1, "Yeah": 1, "Obstinate": 1}, "adjectives": {"Obstinate": 1}, "numerals": {"77": 1}, "pronouns": {"I": 2}}}, {"content": "I'm more serious about each other than the people around me.", "word_count": 16, "label": "", "sentence_structure": "Subject鈥搗erb鈥揷omplement", "subjective_objective": {"answer": "Subjective", "rationale": "Because it is a personal assessment and subjective feeling of the patient."}, "sentence_sentiment": {"answer": "Positive", "rationale": "Because the \"dealing with each other more seriously\" in the entry is positive."}, "language_style": {"answer": "Calm and direct", "rationale": "The entry is based on a straightforward approach to the subject of questioning whether the other party is more serious than others in dealing with human relationships, without too much rhetorical or emotional overtook, and with a more balanced and objective tone. At the same time, the question is not worded in too euphemism or ambiguity, but in a clear and straightforward way, which expresses the subject and purpose of the investigation."}, "time_dimension": {"answer": "Past", "rationale": "In conclusion, the output answer is: \"Now/Before\"."}, "sentence_type": "Affirmative declarative sentence", "pos": {"nouns": {"Questionnaire": 1, "Entry": 1, "Relations": 2, "person": 1}, "verbs": {"Processing": 1, "Seriously": 1}, "adjectives": {"- A little bit": 1, "Around": 1}, "numerals": {"78": 1}, "pronouns": {"I": 1}}}, {"content": "I'm considered a bit of a fool.", "word_count": 13, "label": "", "sentence_structure": "Subject鈥揷opula鈥損redicative", "subjective_objective": {"answer": "Subjective", "rationale": "Because \"others think\" involves subjective views of others, and \"very unthinking\" is also the patient's own subjective feeling."}, "sentence_sentiment": {"answer": "Negative", "rationale": "Because the \"very unthinking\" assessment is negative."}, "language_style": {"answer": "Calm and direct", "rationale": "The entry is more straightforward, concise, less talkative or emotional, and more objective. It is asked whether the respondents find them to be incontrovertible and more clearly expressed."}, "time_dimension": {"answer": "Present", "rationale": "Now the key words are \"I'm thought\" and \"I'm very insensitive,\" which all represent current behaviour, state or feeling, and therefore judgmental."}, "sentence_type": "Negative declarative sentence", "pos": {"nouns": {"person": 1, "Brain": 1}, "verbs": {"I think": 1, "I can't touch it": 1}, "adjectives": {"very": 1, "You're": 1, "person": 1}, "numerals": {"79": 1}, "pronouns": {"I": 1}}}, {"content": "When I'm under great stress, something happens to me, like delusional or temporary memory loss.", "word_count": 30, "label": "", "sentence_structure": "Subject鈥搗erb鈥搊bject", "subjective_objective": {"answer": "Subjective", "rationale": "Because the appearance of delusional or temporary memory loss is the patient ' s subjective experience and feelings."}, "sentence_sentiment": {"answer": "Negative", "rationale": "Because the act of \"a delusional or temporary memory loss\" in the entry is negative."}, "language_style": {"answer": "Calm and direct", "rationale": "Basis of judgement: The entry describes in a direct and objective manner the symptoms that the person under investigation may have in the face of stress, without excessive emotional rendering or euphemism."}, "time_dimension": {"answer": "Present", "rationale": "The entry uses the word \"will\" to denote customary behaviour, and it also indicates that this phenomenon exists at the moment. The temporal dimension of the entry is therefore now judged."}, "sentence_type": "Negative declarative sentence", "pos": {"nouns": {"Pressure": 1, "Things": 1, "Paranoia": 1, "Memory loss": 1}, "verbs": {"Come on": 2, "Yes": 1, "Like": 1, "For now": 1}, "adjectives": {"Significant": 1, "Some": 1}, "numerals": {"80": 1}, "classifiers": {"individual": 1}}}, {"content": "I don't care if anyone else gets hurt because I want to.", "word_count": 20, "label": "", "sentence_structure": "Subject鈥損redicate", "subjective_objective": {"answer": "Subjective", "rationale": "Because the attitude of \"not knowing whether or not others are hurt\" is the patient's own subjective judgment and choice."}, "sentence_sentiment": {"answer": "Negative", "rationale": "Because the entry is \"not concerned about the harm done to others\" and it is irresponsible and selfish, disrespectful to others and therefore negative."}, "language_style": {"answer": "Tactful and warm", "rationale": "The language style of the entry is more euphemistic. It uses a question-and-answer approach to ask questions, express concerns and understanding about the feelings of the person under investigation, and avoids direct criticism or language."}, "time_dimension": {"answer": "Present", "rationale": "The words \"only\" and \"just\" indicate that this is a conditional phrase, and that both \"not care\" and \"whether or not it is hurt\" in the sentence are at the moment when the testee decides, so it is now."}, "sentence_type": "Affirmative declarative sentence", "pos": {"nouns": {"Grandpa": 1, "Questionnaire": 1, "Entry": 1, "others": 1, "Hurt": 1}, "verbs": {"Analysis": 1, "Yes": 3, "I don't care": 1, "And so": 1}, "adverbs": {"Only": 1, "Just": 2, "not": 1, "Here": 1}, "numerals": {"81": 1}, "pronouns": {"I": 2}}}, {"content": "I'm away from other people.", "word_count": 8, "label": "", "sentence_structure": "Subject鈥搗erb鈥揷omplement", "subjective_objective": {"answer": "Subjective", "rationale": "Because \"me\" and \"other\" are subjective perspectives, and distance is personal attitudes and choices."}, "sentence_sentiment": {"answer": "Negative", "rationale": "Because the \"show distance\" behavior in the entry is negative."}, "language_style": {"answer": "Calm and direct", "rationale": "The entry starts with a number and clearly expresses the theme \"I am far away from others\" with a relatively simple, direct language style, without too many rhetorical or emotional renderings. It is therefore clear that the language style of the questionnaire entry is straightforward and calm."}, "time_dimension": {"answer": "Present", "rationale": ""}, "sentence_type": "Affirmative declarative sentence", "pos": {"nouns": {"I": 1, "Others": 1, "Distance": 1}, "verbs": {"Hold on": 1}, "numerals": {"82": 1}}}, {"content": "I've often wondered if my wife was being faithful to me.", "word_count": 22, "label": "", "sentence_structure": "Subject鈥搗erb鈥搊bject", "subjective_objective": {"answer": "Subjective", "rationale": "Because \"suspicious\" is the patient's subjective speculation and apprehension about the behaviour of his partner, the fact is objective."}, "sentence_sentiment": {"answer": "Negative", "rationale": "Because the entry expresses an emotion of suspicion and mistrust, it is a negative sentiment."}, "language_style": {"answer": "Calm and direct", "rationale": "The sentence is based on the following: 1. The entry directly expresses doubts about the existence of infidelity of the other spouse, does not use excessive rhetorical or emotional rendering, and is more objective in tone. 2. In terms of expression, the entry appears in a form of direct questioning, rather than using euphemism, implication or speculative language, making the question more clear and direct."}, "time_dimension": {"answer": "Present", "rationale": "The temporal dimension of this purpose is therefore judged now."}, "sentence_type": "Interrogative sentence", "pos": {"nouns": {"I": 2, "Wife": 1, "Husband": 1, "Girlfriend": 1, "Boyfriend": 1}, "verbs": {"Doubts": 1, "Is it possible": 1, "- Not faithful": 1, "Regular": 1, "be": 1}, "adjectives": {"Faithful": 1}, "adverbs": {"to": 1}, "numerals": {"83": 1}, "classifiers": {"individual": 1}, "pronouns": {"I": 1}}}, {"content": "a) Excess of expenses", "word_count": 4, "label": "", "sentence_structure": "Subject鈥搗erb鈥揷omplement", "subjective_objective": {"answer": "Objective", "rationale": "Because \"expension\" is a reality or fact, an objective description."}, "sentence_sentiment": {"answer": "Negative", "rationale": "Because \"costs are more than\" is negative."}, "language_style": {"answer": "Calm and direct", "rationale": "The language style of the questionnaire entry is calm and straightforward. It directly asks the respondents whether they have problems with economic expenditure or if they exceed the budget, without using excessive rhetorical techniques or emotional rendering. This way allows the respondents to answer questions clearly and clearly, while reducing possible subjective bias and misunderstanding."}, "time_dimension": {"answer": "Present", "rationale": "In the future, the entry is to ask if the testee will experience an over-expenditure in future potential, and therefore to judge it as future. The keyword \"will\" indicates an expectation or possibility and is likely to occur in future."}, "sentence_type": "Negative declarative sentence", "pos": {"nouns": {"Entry": 1, "Questionnaire": 1, "Cost": 1}, "verbs": {"Over": 1}}}, {"content": "(b) Sexual relations with people I hardly know", "word_count": 13, "label": "", "sentence_structure": "Subject鈥搗erb鈥搊bject", "subjective_objective": {"answer": "Subjective", "rationale": "Since sexual intercourse is a subjective act and will of the patient, it is a subjective one."}, "sentence_sentiment": {"answer": "Negative", "rationale": "Because the actions in the entries are immoral and irresponsible, they can cause harm to both parties."}, "language_style": {"answer": "Calm and direct", "rationale": "The entry uses direct and clear language, does not use excessive rhetorical or emotional rendering, is more neutral and objective in tone, and is consistent with the rigour and scientific nature of the psychological questionnaire survey."}, "time_dimension": {"answer": "Past", "rationale": "The keyword \"near known person\" indicates that the act has occurred or is completed, and \"sexuality\" is a completed action or state. Thus, the temporal dimension of the article is past."}, "sentence_type": "Interrogative sentence", "pos": {"nouns": {"I": 1, "person": 2}, "verbs": {"have": 1, "Sexual relations": 1, "Yes": 1}, "adjectives": {"Almost": 1}, "numerals": {"b)": 1}}}, {"content": "c) Overdrinking", "word_count": 4, "label": "", "sentence_structure": "Subject鈥搗erb鈥揷omplement", "subjective_objective": {"answer": "Objective", "rationale": "Because overdouring is an objective fact of the patient's experience."}, "sentence_sentiment": {"answer": "Negative", "rationale": "Because the \"overdrink\" act is negative."}, "language_style": {"answer": "Calm and direct", "rationale": "The entry is more straightforward in language style, not using excessive rhetorical or emotional colours, but rather asking questions in an objective, concise manner. This expression is easier for respondents to understand and answer, and does not trigger emotional fluctuations."}, "time_dimension": {"answer": "Present", "rationale": "The entry now asks the testee whether there is currently a problem of overdrinking, and therefore the temporal dimension is now judged."}, "sentence_type": "Interrogative sentence", "pos": {"nouns": {"Drink": 1, "Excess": 1}}}, {"content": "d) Use of drugs (specifically addiction-dependent drugs)", "word_count": 13, "label": "", "sentence_structure": "Subject鈥搗erb鈥搊bject", "subjective_objective": {"answer": "Subjective", "rationale": "Since the entry examines whether the patient uses a specific drug, it is the patient ' s subjective choice and behaviour and is therefore viewed from a subjective perspective."}, "sentence_sentiment": {"answer": "Negative", "rationale": "Because the use of drugs in the entries is negative, especially for addiction-dependent drugs."}, "language_style": {"answer": "Calm and direct", "rationale": "The language style of the entry is more objective, with no excessive rhetorical or emotional rendering, and is more flat, and is intended to ask the other party whether they have used an addictive drug."}, "time_dimension": {"answer": "Past", "rationale": "\"Use of drugs\" is a practice that encompasses both past (if the person tested has abused drugs) and present and future (if the person tested continues to use drugs or is planning to use drugs)."}, "sentence_type": "Interrogative sentence", "pos": {"nouns": {"Drugs": 1, "Special finger": 1, "Dependence dependency": 1}, "verbs": {"鈾?And I'm gonna be 鈾?: 1, "Use": 1}, "adverbs": {"Down there": 1, "Please": 1, "Analysis": 1}, "numerals": {"d": 1}}}, {"content": "(e) Drinking and drinking and driving me crazy", "word_count": 7, "label": "", "sentence_structure": "Subject鈥搗erb鈥搊bject", "subjective_objective": {"answer": "Subjective", "rationale": "Because \"drinking, drinking and crazy\" are psychological activities and subjective experiences that patients may experience."}, "sentence_sentiment": {"answer": "Negative", "rationale": "Because the \"drinking, demented\" act in the entry is negative."}, "language_style": {"answer": "Tactful and warm", "rationale": "The entry uses a more euphemistic language style, beginning with \"drinking and playing\", avoiding the use of words that may cause unwelcome feelings, such as \"drinking\" and \"drinking\" and adding the description of \"drinking\" to express the subject without being too direct or stimulating. This expression is more mild and easily accepted and answered by the interviewees."}, "time_dimension": {"answer": "Present", "rationale": "The entry describes the present behaviour or habits of the testee, \"drinking\" and \"drinking mad\" are all ongoing acts, so it's now judged."}, "sentence_type": "Imperative sentence", "pos": {"nouns": {"Drinking and playing": 1, "Drunk": 1}}}, {"content": "f) Routine driving or riding", "word_count": 7, "label": "", "sentence_structure": "Subject鈥搗erb鈥搊bject", "subjective_objective": {"answer": "Subjective", "rationale": "Because \"a reckless driving or riding\" is a personal choice and attitude, determined by the patient himself, it is subjective."}, "sentence_sentiment": {"answer": "Negative", "rationale": "Because the word \"reckless\" is a negative attitude."}, "language_style": {"answer": "Calm and direct", "rationale": "The entry is based on a simple and straightforward question about whether the person interviewed has been recklessly driving or riding, has not used excessive rhetorical or emotional rendering, and is more objective. This expression conveys the purpose and content of the investigation, while avoiding the resistance of the person interviewed."}, "time_dimension": {"answer": "Past", "rationale": "The keyword \"frequent driving or riding\" points to what has happened and what may have happened in the past."}, "sentence_type": "Imperative sentence", "pos": {"nouns": {"You're reckless": 1, "Drive": 1, "Crawling": 1}, "verbs": {"Open": 1, "Ride": 1}}}, {"content": "(a) I'm thought to be a bullying student", "word_count": 15, "label": "", "sentence_structure": "Subject鈥搗erb鈥搊bject", "subjective_objective": {"answer": "Subjective", "rationale": "Because \"the perception\" is based on the opinion and opinion of others to assess the character characteristics of the student, which is a subjective view."}, "sentence_sentiment": {"answer": "Negative", "rationale": "Because the description of \"bullying with the weak\" in the entry is negative, which means that the student is of poor quality."}, "language_style": {"answer": "Tactful and warm", "rationale": "Although it is asking the views of the other party, it is not speaking directly against or criticizing the other party, but rather in a more moderate and understanding manner, showing respect and concern for the person under investigation."}, "time_dimension": {"answer": "Past", "rationale": "The term \"perception\" in this entry refers to the evaluation of the person tested over a period of time, and therefore the temporal dimension is judged to be past."}, "sentence_type": "Imperative sentence", "pos": {"nouns": {"People": 1, "Students": 1}, "verbs": {"I think": 1, "be": 1, "Bullying": 1}, "adjectives": {"Strong": 1, "Weak": 1}, "pronouns": {"I": 1}}}, {"content": "(b) I used to start a fight with other kids.", "word_count": 15, "label": "", "sentence_structure": "Subject鈥搗erb鈥揷omplement", "subjective_objective": {"answer": "Subjective", "rationale": "Because \"intribution\" and \"fighting\" are based on patient's subjective intent and behaviour."}, "sentence_sentiment": {"answer": "Negative", "rationale": "Because the entry \"to provoke a fight with another child\" is negative."}, "language_style": {"answer": "Tactful and warm", "rationale": "The language style of the entry is a euphemism. By using the expression \"often in the past\" it avoids direct accusations or criticism of each other's behaviour, while also conveying an attitude of understanding and support. Moreover, the expression \"inducing a fight with another child\" is relatively flat, is not overly emotional and is more conducive to good interaction."}, "time_dimension": {"answer": "Past", "rationale": "The entry is for the testee's past experience and behaviour, and the temporal dimension is the past.\""}, "sentence_type": "Imperative sentence", "pos": {"nouns": {"Matters": 1, "Son": 1}, "verbs": {"Start": 1, "Fight": 1}, "adverbs": {"Past": 1, "Regular": 1}, "classifiers": {"individual": 1}, "pronouns": {"I": 1}}}, {"content": "(c) I used a murder weapon in a fight.", "word_count": 10, "label": "", "sentence_structure": "Subject鈥搗erb鈥搊bject", "subjective_objective": {"answer": "Objective", "rationale": "Because the murder weapon was used as an objective fact of the patient's fight."}, "sentence_sentiment": {"answer": "Negative", "rationale": "Because the act was offensive and dangerous, it could cause harm to others."}, "language_style": {"answer": "Calm and direct", "rationale": "The entry describes an act in a direct and clear language, without excessive rhetoric or emotional rendering, and is more objective in tone."}, "time_dimension": {"answer": "Present", "rationale": "The entry is for questioning the testee's past experience and behaviour, and therefore is based on \"the past.\" The keyword \"in a fight\" and \"used\" all indicate that this is a description of what happened in the past."}, "sentence_type": "Affirmative declarative sentence", "pos": {"nouns": {"Fight": 1, "The murder weapon": 1}, "verbs": {"Use": 1}}}, {"content": "(d) I've robbed or killed someone.", "word_count": 10, "label": "", "sentence_structure": "Subject鈥搗erb鈥搊bject", "subjective_objective": {"answer": "Subjective", "rationale": "Because robbery or assault is a subjective fact of the patient's experience."}, "sentence_sentiment": {"answer": "Negative", "rationale": "Because the \"robbery or murder\" in the entry is negative."}, "language_style": {"answer": "Calm and direct", "rationale": "The entry is more direct in language style, with no excessive adoration or emotional colour. It is more calm and direct in its tone by asking in an objective and clear manner whether the other party has committed robbery or murder."}, "time_dimension": {"answer": "Past", "rationale": "The entry contains a description of the events of the past, and \"had\" indicated that the action had been completed, and the act had therefore occurred in the past period."}, "sentence_type": "Affirmative declarative sentence", "pos": {"nouns": {"I": 1, "others": 1, "Robbery": 1, "Murderer": 1}, "verbs": {"Tsang": 1, "to": 1}}}, {"content": "I've been violent to others.", "word_count": 12, "label": "", "sentence_structure": "Subject鈥搗erb鈥搊bject", "subjective_objective": {"answer": "Subjective", "rationale": "Because \"the violence that was brutal to others\" is a subjective act and attitude of a human being."}, "sentence_sentiment": {"answer": "Negative", "rationale": "Because the entry describes acts of cruel violence, which is a negative and negative emotion and behaviour."}, "language_style": {"answer": "Calm and direct", "rationale": "The language style of the entry is more direct and objective, not to use excessive rhetorical or emotional colours to describe behaviour, but to present it in a more austere and neutral way. This expression helps to avoid leading the respondents to emotional reactions, thereby obtaining more realistic and accurate data."}, "time_dimension": {"answer": "Past", "rationale": "The keyword \"difficult violence against others\" refers to actions or events that occurred in the past and are therefore judged to be past."}, "sentence_type": "Negative declarative sentence", "pos": {"nouns": {"Questionnaire": 1, "Entry": 1, "others": 1, "Violence": 1}, "verbs": {"Implementation": 1, "Cruel": 1}}}, {"content": "(f) I'm violent against animals.", "word_count": 12, "label": "", "sentence_structure": "Subject鈥搗erb鈥搊bject", "subjective_objective": {"answer": "Objective", "rationale": "Since acts of violence against animals are objective, even if the patient is aware that he or she has committed such acts."}, "sentence_sentiment": {"answer": "Negative", "rationale": "Because \"violence against animals\" is a negative act and attitude that can cause harm and suffering to animals."}, "language_style": {"answer": "Calm and direct", "rationale": "The language style of the entry is calm, because it clearly asks the interviewee whether he has committed acts of cruel violence against animals, and not in any euphemism or euphemism. This straightforward question-and-answerer can make the interviewee more truthful and precise."}, "time_dimension": {"answer": "Past", "rationale": "The keyword \"act\" indicates the act of the past, and the entire sentence describes an event or situation that has already occurred, so the temporal dimension of this entry is the past."}, "sentence_type": "Negative declarative sentence", "pos": {"nouns": {"Animals": 1, "Violence": 1}, "verbs": {"Implementation": 1, "to": 1}, "adverbs": {"In cold blood": 1}}}, {"content": "(g) I forced him to have sex with me.", "word_count": 14, "label": "", "sentence_structure": "Subject鈥搗erb鈥搊bject", "subjective_objective": {"answer": "Objective", "rationale": "This is a patient ' s description and does not involve subjective feelings or attitudes of the patient."}, "sentence_sentiment": {"answer": "Negative", "rationale": "Because the act of \"coercion\" in the entry is negative and involves sexual assault, it has serious moral and legal implications."}, "language_style": {"answer": "Tactful and warm", "rationale": "The language style of the entry is more euphemistic, and by using a more neutral expression, \"I forced him/her,\" it avoids direct description of personal behaviour while maintaining the objectivity and relevance of the issue. Moreover, the description \"he/her had sexual relations with me\" also has greater respect for the person under investigation and minimizes the possible negative impact and discomfort."}, "time_dimension": {"answer": "Present", "rationale": "The word \"I forced\" in \"I forced\" to indicate the past, and \"he/she had sexual relations with me\" was the specific content of the description of that behaviour. The entry therefore asked whether the person tested had forced someone to have sexual intercourse with him in the past, and the temporal dimension judged it now."}, "sentence_type": "Negative declarative sentence", "pos": {"nouns": {"Grandpa": 2, "I": 3, "person": 2, "he": 1, "she": 1, "Sexual relations": 1}, "verbs": {"Come on": 1, "Forced": 1}, "numerals": {"g": 1, "Two": 1}, "classifiers": {"individual": 2}, "pronouns": {"he": 1, "she": 1}}}, {"content": "鈾?I've lied many times 鈾?, "word_count": 5, "label": "", "sentence_structure": "Subject鈥搗erb鈥揷omplement", "subjective_objective": {"answer": "Subjective", "rationale": "Because lying is a subjective attitude and behaviour, which may be intended to conceal objective facts or achieve certain objectives, it is still a matter of personal determination. The question is therefore one of subjective thinking and behaviour of the patient."}, "sentence_sentiment": {"answer": "Negative", "rationale": "Because \"false\" is a negative act."}, "language_style": {"answer": "Calm and direct", "rationale": "The language style of the entry is more straightforward, with no qualifying words or strong emotional expressions, but rather a straightforward question about whether the person under investigation is often lying."}, "time_dimension": {"answer": "Past", "rationale": "Because the verb \"multi-false\" is used in the title, and it is not clear whether it is past, present or future, but we can see it in the word \"multi-place\" as a description of past behaviour. Because people do not usually lie all the time, but rather do so within a specific time period. So the entry asks whether the subjects have often lied in the past."}, "sentence_type": "Negative declarative sentence", "pos": {"nouns": {"I": 1, "Lies": 1}, "verbs": {"Scram": 1, "Multiple": 1}, "adverbs": {"Multiple": 1}}}, {"content": "I'm not allowed to spend the night without my parents' permission.", "word_count": 14, "label": "", "sentence_structure": "Subject鈥搗erb鈥揷omplement", "subjective_objective": {"answer": "Subjective", "rationale": "Because \"overnight without parental permission\" is a decision and action made by the patient himself and is a subjective attitude."}, "sentence_sentiment": {"answer": "Negative", "rationale": "Because the behavior in the entry \"sleeping out without parental permission\" is negative."}, "language_style": {"answer": "Calm and direct", "rationale": "The entry is more flat in terms of the need to ask the other party whether he or she has spent the night outside without parental permission in an objective and direct language."}, "time_dimension": {"answer": "Past", "rationale": ""}, "sentence_type": "Negative declarative sentence", "pos": {"nouns": {"parents": 1, "Outside": 1, "Stay the night": 1}, "verbs": {"I didn't get it": 1, "Jean": 1, "in": 1, "Pass": 1}, "adverbs": {"Just": 1}, "pronouns": {"I": 1}}}, {"content": "I've stolen from someone else.", "word_count": 8, "label": "", "sentence_structure": "Subject鈥搗erb鈥揷omplement", "subjective_objective": {"answer": "Subjective", "rationale": "Because theft is the subjective act of others."}, "sentence_sentiment": {"answer": "Negative", "rationale": "Because the \"snatching someone's things\" act in the entry is negative."}, "language_style": {"answer": "Calm and direct", "rationale": "The entry directly asked the sensitive question of whether the person under investigation had stolen the property of another person, without too much embellishment or covert language, and in a more straightforward manner."}, "time_dimension": {"answer": "Present", "rationale": "The entry asks if the person tested had ever committed theft, and the dimension of time is the past. The keyword is \"snatching\" and \"things of others,\" which point to a certain act of the past, so the answer should be the past."}, "sentence_type": "Negative declarative sentence", "pos": {"nouns": {"Questionnaire": 1, "Entry": 1, "person": 1, "Something": 1}, "verbs": {"- I did": 1}, "numerals": {"j": 1}}}, {"content": "鈾?I've been on fire 鈾?, "word_count": 4, "label": "", "sentence_structure": "Subject鈥損redicate", "subjective_objective": {"answer": "Subjective", "rationale": "Because I set fire to the patient's subjective statement."}, "sentence_sentiment": {"answer": "Negative", "rationale": "Because arson is illegal, it is destructive and harmful."}, "language_style": {"answer": "Calm and direct", "rationale": "The entry directly asks the other side whether it has ever set fire to a sensitive question, the language style is more direct and clear, and there is no excessive rhetorical or emotional rendering. This tone is more conducive to obtaining a true and accurate answer."}, "time_dimension": {"answer": "Past", "rationale": "The term \"over-fire\" in this entry is a past event and should therefore be classified as a past event."}, "sentence_type": "Affirmative declarative sentence", "pos": {"nouns": {"Questionnaire": 1, "Entry": 1, "Fire": 1}, "verbs": {"iv": 1, "Pass": 1, "Analysis": 1}, "pronouns": {"I": 1}}}, {"content": "(l) I've deliberately broken windows or destroyed property", "word_count": 13, "label": "", "sentence_structure": "Subject鈥搗erb鈥搊bject", "subjective_objective": {"answer": "Subjective", "rationale": "Because the descriptions of \"intentional\" or \"break-out of windows or destruction of property\" are based on the patient's subjective perspective, and the patient's motivation and attitude are examined."}, "sentence_sentiment": {"answer": "Negative", "rationale": "Because \"break windows or destroy property\" is a negative act."}, "language_style": {"answer": "Calm and direct", "rationale": "The entry directly asked the interviewees whether there had been any deliberate break-ups or destruction of property, without any use of rhetorical techniques or emotional rendering, and with a more objective and cold tone."}, "time_dimension": {"answer": "Past", "rationale": "The keyword \"break the window\" and \"damaged property\" describes a certain past act, so the temporal dimension of the article is the past."}, "sentence_type": "Negative declarative sentence", "pos": {"nouns": {"Window": 1, "Property": 1}, "verbs": {"Break it up": 1, "Destruction": 1, "On purpose": 1}}}, {"content": "(m) I've been out for the night not only once.", "word_count": 13, "label": "", "sentence_structure": "Subject鈥搗erb鈥揷omplement", "subjective_objective": {"answer": "Subjective", "rationale": "Because the terms \"scrambling\" and \"overnight\" are subjective behaviour and experience of patients, looking at subjective attitudes in terms of their emotions, will and decision-making."}, "sentence_sentiment": {"answer": "Negative", "rationale": "Because \"scrambling home\" and \"overnight\" in the entries are all negative."}, "language_style": {"answer": "Tactful and warm", "rationale": "The entry uses a first-person expression, and emphasizes the frequency of behaviour by using the term \"more than once\" without overstateing the specific circumstances or feelings. Such expression can lead respondents to self-reflection without being too offensive or stimulating their sensitivities."}, "time_dimension": {"answer": "Past", "rationale": "The words \"more than once\" and \"overnight\" in the entry indicate that this is a description of past behaviour and can therefore be judged as past."}, "sentence_type": "Negative declarative sentence", "pos": {"nouns": {"Questionnaire Entry": 1, "m": 1, "Run away from home": 1, "Night": 1}, "verbs": {"in": 1, "Here": 1, "Analysis": 1, "Give": 1, "Leave home": 1, "Run away": 1, "Outside": 1, "Stay the night": 1}, "adverbs": {"Not just once": 1}, "numerals": {"One": 2, "Number of times": 1}}}, {"content": "I've been avoiding school a lot since I was 13.", "word_count": 11, "label": "", "sentence_structure": "Subject鈥損redicate", "subjective_objective": {"answer": "Subjective", "rationale": "Because \"start\" and \"repeated\" are descriptions based on the memory and experience of the patient, and are subjective."}, "sentence_sentiment": {"answer": "Negative", "rationale": "Because the \"trunny\" act in the entry is negative."}, "language_style": {"answer": "Calm and direct", "rationale": "This entry presents the subject of repeated truancy before the age of 13 in a straightforward and concise way, without excessive qualifier language or emotional colour."}, "time_dimension": {"answer": "Past", "rationale": "The description of \"start\" and \"precedent\" in the entry refers to the actions or state of the past, and therefore the past is judged."}, "sentence_type": "Affirmative declarative sentence", "pos": {"nouns": {"I": 1, "Age": 1, "Before": 1, "Multiple": 1, "- You're not going to school": 1}, "verbs": {"Start": 1, "Run": 1}, "adverbs": {"Before": 1, "Multiple": 1}, "numerals": {"13": 1}, "pronouns": {"I": 1}}}, {"content": "I broke into people's homes and buildings without permission.", "word_count": 14, "label": "", "sentence_structure": "Subject鈥搗erb鈥搊bject", "subjective_objective": {"answer": "Subjective", "rationale": "This is because patients evaluate and interpret their behaviour and is subjective."}, "sentence_sentiment": {"answer": "Negative", "rationale": "Because the actions in the entries \"breach into the home and buildings of others\" are negative and not in accordance with social ethics and legal requirements."}, "language_style": {"answer": "Calm and direct", "rationale": "The entry is a straightforward expression of the subject of questioning whether the other party has entered the home or building of another person without authorization, and is more concise and plain, without any rhetorical or emotional rendering."}, "time_dimension": {"answer": "Past", "rationale": "The keywords \"unauthorized entry\" and \"into another's house, building\" indicate an event that has already occurred, so we can infer that the act occurred in the past. At the same time, the act \"unauthorized entry into another's home, building\" is usually considered to be criminal, which means that it may lead to legal consequences or social punishment. So the temporal dimension of this entry is the past. In conclusion, the temporal dimension of this article is the past."}, "sentence_type": "Negative declarative sentence", "pos": {"nouns": {"Questionnaire": 1, "Entry": 1, "I": 1, "Allow": 1, "others": 1, "Home": 1, "Buildings": 1}, "verbs": {"Break in": 1, "Not yet": 1}, "adverbs": {"Please": 1, "Down there": 1}}}]}

{"questionnaire_title": "Solitude test", "questionnaire_category": "Mental health", "item_count": 20, "emotion_shift_overall": "From positive to negative", "language_style_overall": "Tactful and warm", "items": [{"content": "You feel comfortable with the people around you, and you feel like you're a group.", "word_count": 19, "label": "", "sentence_structure": "Subject鈥搗erb鈥揷omplement", "subjective_objective": {"answer": "Subjective", "rationale": "It is viewed from a subjective perspective because it is perceived as the patient ' s personal feelings and views."}, "sentence_sentiment": {"answer": "Positive", "rationale": "Because the words \"concrete\" and \"the sense of convergence\" in the entries are positive."}, "language_style": {"answer": "Tactful and warm", "rationale": ""}, "time_dimension": {"answer": "Present", "rationale": "The entry is for the current feelings of the testee, and the keyword \"felt\" and \"have\" are now known as the current perception and experience, and therefore belong to the present."}, "sentence_type": "Affirmative declarative sentence", "pos": {"nouns": {"person": 2, "Get along": 1, "Gathering of objects in groups": 1}, "verbs": {"I think": 1, "have": 1, "It's nice": 1}, "numerals": {"1": 3}, "pronouns": {"you": 1}}}, {"content": "You feel like you don't have a date.", "word_count": 7, "label": "", "sentence_structure": "Subject鈥搗erb鈥揷omplement", "subjective_objective": {"answer": "Subjective", "rationale": "This is viewed from a subjective perspective because of the subjective sense and needs of a person to feel the need for company."}, "sentence_sentiment": {"answer": "Neutral", "rationale": "As the entry expresses a fact or situation, it does not involve feelings or attitudes."}, "language_style": {"answer": "Calm and direct", "rationale": "The entry is a direct narrative, with no too much emotional or rhetorical style, which expresses the theme \"You feel you lack a partner.\" This language is more calm and straightforward, and it makes it easier for the interviewee to understand the meaning of the question and to give a true answer."}, "time_dimension": {"answer": "Present", "rationale": "\"2 You feel like you don't have a partner\" and \"you don't have a partner\" are all descriptions of the current state of mind or the feelings of the person being tested, so the temporal dimension is now."}, "sentence_type": "Declarative sentence", "pos": {"nouns": {"- Hey, buddy": 1}, "verbs": {"I think": 1, "Missing": 1, "individual": 1}, "adverbs": {"2": 1}}}, {"content": "You think no one can turn to, share or depend on.", "word_count": 14, "label": "", "sentence_structure": "Subject鈥搗erb鈥搊bject", "subjective_objective": {"answer": "Subjective", "rationale": "Because \"think\" is a subjective assessment of the patient's perception and feelings about whether or not there is anyone who can turn to, share or depend on. This is not an objective fact, but a personal experience and emotional response."}, "sentence_sentiment": {"answer": "Negative", "rationale": "Because the \"no one can turn to, share or rely on\" in the entry is negative, expressing a feeling of loneliness and helplessness."}, "language_style": {"answer": "Tactful and warm", "rationale": "The entry uses a soft, invitation-style tone for dialogue, asking if the other party is alone in the face of difficulties and is not over-represented or emotionally retrofitted, which is more acrimony and interactive."}, "time_dimension": {"answer": "Present", "rationale": "The entry is to ask if the person tested felt that no one could turn to, share or rely on, in the current situation, and the time dimension is now judged."}, "sentence_type": "Interrogative sentence", "pos": {"nouns": {"person": 2, "Help": 1, "Share": 1, "Dependence": 1}, "verbs": {"I think": 1, "Yeah": 1}, "numerals": {"3": 1}}}, {"content": "Four you feel alone.", "word_count": 5, "label": "", "sentence_structure": "Subject鈥搗erb鈥揷omplement", "subjective_objective": {"answer": "Subjective", "rationale": "Because feeling alone is the patient's own feeling and subjective experience, it is an emotional state inherent in the individual and therefore a subjective view."}, "sentence_sentiment": {"answer": "Negative", "rationale": "Because being alone is often considered a negative emotion."}, "language_style": {"answer": "Tactful and warm", "rationale": "The entry is more euphemistic, using implicit expressions that avoid direct or overly strong emotional rendering. It also gives people a sense of care and understanding that can better trigger the resonance and collaboration of the person under investigation."}, "time_dimension": {"answer": "Present", "rationale": "The entry asks the testee about his state of affairs, \"you\" and \"think\" both indicate that it is subjective, personal, or experience, and does not address the time dimension of the past or the future. So, judging now."}, "sentence_type": "Interrogative sentence", "pos": {"nouns": {"Alone": 1}, "verbs": {"I think": 1}, "adverbs": {"4": 1}}}, {"content": "You think you're one of the friends.", "word_count": 11, "label": "", "sentence_structure": "Subject鈥揷opula鈥損redicative", "subjective_objective": {"answer": "Subjective", "rationale": "Because \"think\" is a subjective feeling and attitude. Although this may be based on objective facts (for example, you may interact with these people much), perceptions of identity are usually subjective and are influenced by personal perceptions."}, "sentence_sentiment": {"answer": "Neutral", "rationale": "Since the entry does not address words that have a clear positive or negative orientation, such as emotions, attitudes, behaviour, purpose or quality, it merely describes the perception and feelings of the person under investigation about his or her role in the circle of friends and can therefore be judged neutral."}, "language_style": {"answer": "Tactful and warm", "rationale": "The language style of the entry is more euphemistic, and it makes the interviewee feel cared for and understood by asking him about his place in the circle of friends. At the same time, the expression \"you feel\" is more natural and less stressful or comfortable."}, "time_dimension": {"answer": "Present", "rationale": "The key word in the entry is \"you think\" and it expresses the current feelings and perceptions of the subject, so it can be judged that the item describes the current state."}, "sentence_type": "Affirmative declarative sentence", "pos": {"nouns": {"Friends": 1, "One": 1}, "verbs": {"I think": 1, "be": 1}, "numerals": {"5": 1}}}, {"content": "You feel like you're outward and friendly.", "word_count": 10, "label": "", "sentence_structure": "Subject鈥搗erb鈥揷omplement", "subjective_objective": {"answer": "Subjective", "rationale": "Because it's a subjective feeling and an assessment of a person."}, "sentence_sentiment": {"answer": "Positive", "rationale": "Because \"outside friendly\" is a positive description in the entry."}, "language_style": {"answer": "Tactful and warm", "rationale": "The article's purpose is more moderate and friendly, using the first person's alias, \"self\", and using positive adjectives, \"extra\" and \"friendly\", to make the respondents feel respected and accepted. Nor is it too strongly directed or implied to give the respondents some room for autonomous choice, reflecting the consideration and respect of the questionnaire designers."}, "time_dimension": {"answer": "Present", "rationale": ""}, "sentence_type": "Interrogative sentence", "pos": {"nouns": {"Questionnaire": 1, "Entry": 1, "self": 1, "Outward": 1, "Friendly": 1}, "verbs": {"I think": 1}, "adjectives": {"Outward": 1, "Friendly": 1}, "numerals": {"9": 1}, "pronouns": {"you": 1}}}, {"content": "You don't think you can share your interests and ideas with people around you.", "word_count": 21, "label": "", "sentence_structure": "Subject鈥搗erb鈥搊bject", "subjective_objective": {"answer": "Subjective", "rationale": "The idea of sharing one's interests and ideas with others is a subjective feeling for patients."}, "sentence_sentiment": {"answer": "Negative", "rationale": "Because the entry \"can't share your interests and ideas with those around you\" is negative."}, "language_style": {"answer": "Tactful and warm", "rationale": "The entry is more euphemistic, uses subjective expressions like \"you feel\" and does not directly negate the behaviour or opinion of the other. At the same time, it is also somewhat guided by the desire of the person surveyed to express his or her views and feelings on his or her own initiative. These factors make the language style of the article more euphemistic and moderate."}, "time_dimension": {"answer": "Present", "rationale": "\"You feel\" is an expression of a psychological feeling or emotional state, and \"you cannot share your interests and thoughts with the people around you\" is an act or state that is now being performed, so the entry is primarily about the current psychological state and behaviour of the person being tested."}, "sentence_type": "Interrogative sentence", "pos": {"nouns": {"Questionnaire Entry": 1, "self": 2, "person": 1, "Interest": 1, "Thoughts": 1}, "verbs": {"I think": 1, "Yes": 1, "Share": 1, "and": 1, "Around": 1}, "adverbs": {"8": 1, "No, I can't": 1}, "classifiers": {"individual": 1}}}, {"content": "You don't feel close to anyone anymore.", "word_count": 13, "label": "", "sentence_structure": "Subject鈥搗erb鈥揷omplement", "subjective_objective": {"answer": "Subjective", "rationale": "Because the word \"think\" means that it is a subjective feeling or perception of a patient, not just an objective fact. While this feeling may be related to objective situations, such as social isolation or interpersonal relations, the specific feeling is subjective in itself."}, "sentence_sentiment": {"answer": "Negative", "rationale": "Because \"no closer\" is negative."}, "language_style": {"answer": "Calm and direct", "rationale": "The entry expresses in a direct presentation the question of whether the other party feels no closer to anyone, and does not use excessive rhetorical or emotional rendering, which is more objective and balanced."}, "time_dimension": {"answer": "Present", "rationale": "The keyword \"Feeling no longer close to anyone\" suggests that it is a description of the person being tested at the moment and therefore falls within the scope of the present."}, "sentence_type": "Interrogative sentence", "pos": {"nouns": {"person": 1, "Close": 1}, "verbs": {"I think": 1, "Not anymore": 1, "Yes": 1}, "adverbs": {"7": 1, "Both": 1}}}, {"content": "You feel a lot in common with the people around you.", "word_count": 14, "label": "", "sentence_structure": "Subject鈥搗erb鈥揷omplement", "subjective_objective": {"answer": "Subjective", "rationale": "Because it is the patient's own subjective perception of his relationship with the people around him."}, "sentence_sentiment": {"answer": "Positive", "rationale": "Because \"there's a lot in common\" in the entry expresses a positive emotional attitude."}, "language_style": {"answer": "Tactful and warm", "rationale": "The language style of the entry is more euphemistic and friendly, using a query-style language with a certain sense of care and affection, which allows respondents to feel respected and valued."}, "time_dimension": {"answer": "Present", "rationale": "The entry describes the current feelings or views of the testees, the keywords being \"like\" and \"many\", which indicate that this is the current subjective experience."}, "sentence_type": "Affirmative declarative sentence", "pos": {"nouns": {"you": 1, "Around": 1, "person": 1, "Common ground": 1}, "verbs": {"I think": 1, "have": 1, "A lot": 1}, "adverbs": {"very": 1}, "numerals": {"6": 1}}}, {"content": "You feel close to someone else.", "word_count": 9, "label": "", "sentence_structure": "Subject鈥搗erb鈥揷omplement", "subjective_objective": {"answer": "Subjective", "rationale": "Because it is a subjective feeling or perception that is close to others, it is a subjective perspective of the patient."}, "sentence_sentiment": {"answer": "Positive", "rationale": "Because the attitude of \"like to feel close to someone else\" in the entry is positive."}, "language_style": {"answer": "Tactful and warm", "rationale": "In a more moderate way, the entry asks about the degree of proximity between the person interviewed and others, and is expressed in a way that is not directly stated or ordered, but rather in a more personal and interactive way."}, "time_dimension": {"answer": "Present", "rationale": "The entry asks the testee how he feels about his relationship with others, and the time dimension is now judged."}, "sentence_type": "Interrogative sentence", "pos": {"nouns": {"you": 1, "others": 1}, "verbs": {"I think": 1, "Close": 1}, "adjectives": {"very": 1}, "numerals": {"10": 1}}}, {"content": "You feel like you're being left alone.", "word_count": 9, "label": "", "sentence_structure": "Subject鈥搗erb鈥揷omplement", "subjective_objective": {"answer": "Subjective", "rationale": "Because \"the feeling of being left alone\" is the personal feeling and perception of the patient, and it is a subjective examination."}, "sentence_sentiment": {"answer": "Negative", "rationale": "Because the emotional attitude expressed in this entry is negative, and \"frustrated\" is a feeling of isolation and exclusion, with some negative sentiment."}, "language_style": {"answer": "Tactful and warm", "rationale": ""}, "time_dimension": {"answer": "Present", "rationale": ""}, "sentence_type": "Interrogative sentence", "pos": {"nouns": {"self": 1, "person": 1, "Cold": 1}, "verbs": {"I think": 1, "Shit": 1}, "numerals": {"11": 1}}}, {"content": "You don't think your relationship with someone is meaningful.", "word_count": 15, "label": "", "sentence_structure": "Subject鈥搗erb鈥搊bject", "subjective_objective": {"answer": "Subjective", "rationale": "Because it is a subjective feeling or perception that it makes no sense to interact with others."}, "sentence_sentiment": {"answer": "Negative", "rationale": "Because the expression in the entry is negative, i.e., that it is meaningless to think that it is in contact with someone else."}, "language_style": {"answer": "Calm and direct", "rationale": "This is a statement that may be uncomfortable or frustrated."}, "time_dimension": {"answer": "Past", "rationale": "The entry asks about the subjective perception of the past by the person being tested, and the keywords are \"self\", \"other person\" and \"disconnection has no meaning\" , all of which point to what happened in the past. Thus, it can be judged to be about the behaviour or mentality of the past."}, "sentence_type": "Interrogative sentence", "pos": {"nouns": {"self": 1, "others": 1, "- To be with someone": 1, "Meaning": 1}, "verbs": {"I think": 1, "have": 1, "- To be with someone": 1}, "numerals": {"12": 1}, "classifiers": {"individual": 1}, "pronouns": {"you": 1}}}, {"content": "You think nobody really knows you.", "word_count": 10, "label": "", "sentence_structure": "Subject鈥搗erb鈥搊bject", "subjective_objective": {"answer": "Subjective", "rationale": "Because the word \"like\" expresses the patient's emotional and subjective feelings, the sentence is viewed from the patient's subjective perspective."}, "sentence_sentiment": {"answer": "Negative", "rationale": "Because it expresses a negative sentiment and attitude, i.e. feeling alone, misconstrued, etc."}, "language_style": {"answer": "Tactful and warm", "rationale": "The entry expressed in a more implicit way the subject of asking the other party whether it felt that it was understood less, without directly accusing or attacking the other party, which was more inclusive and respectful."}, "time_dimension": {"answer": "Present", "rationale": "The entry asks the testee about his current feelings and the time dimension is now judged."}, "sentence_type": "Interrogative sentence", "pos": {"nouns": {"13": 1, "person": 1, "Roger that": 1}, "verbs": {"I think": 1, "True": 1, "of": 2}, "adjectives": {"Nope": 1, "Really": 1}, "numerals": {"13": 1}, "pronouns": {"you": 1}}}, {"content": "You feel isolated.", "word_count": 11, "label": "", "sentence_structure": "Subject鈥搗erb鈥揷omplement", "subjective_objective": {"answer": "Subjective", "rationale": "Because \"think\" is a subjective feeling and perception that means that it's the subjective view of the patient to describe his situation."}, "sentence_sentiment": {"answer": "Negative", "rationale": "Because the word \"separation\" in the entries has a negative emotional color."}, "language_style": {"answer": "Calm and direct", "rationale": "This expression makes the questionnaire more objective and clear, and also facilitates the respondent鈥檚 understanding and answers."}, "time_dimension": {"answer": "Past", "rationale": "The keyword \"thinks\" means that the testee remembers and describes his past, and \"segregates himself from others\" is a sense or experience of the testee over time."}, "sentence_type": "Negative declarative sentence", "pos": {"nouns": {"self": 1, "Others": 1}, "verbs": {"I think": 1, "Isolation": 1}, "adverbs": {"14": 1}, "numerals": {"1": 2}}}, {"content": "You think you'll find someone if you want.", "word_count": 15, "label": "", "sentence_structure": "Subject鈥搗erb鈥搊bject", "subjective_objective": {"answer": "Subjective", "rationale": "Because finding a partner depends primarily on the patient's will and action, and \"think\" and \"think\" and \"think\" are subjective feelings and ideas."}, "sentence_sentiment": {"answer": "Positive", "rationale": "Because the entry \"If you want, you'll find a companion\" expresses a positive and optimistic attitude."}, "language_style": {"answer": "Tactful and warm", "rationale": "The entry is more personal and interactive in its expression of a gentle and inviting tone, rather than a direct statement or order. It also attempts to convey a positive message that it is possible to find a partner and to encourage respondents to believe in their abilities."}, "time_dimension": {"answer": "Future", "rationale": "The keyword \"if\" indicates that it is a statement of assumptions and possibilities, and both \"to find\" and \"to find it\" point to future intentions and expectations. Thus, the temporal dimension of the article is judged to be future."}, "sentence_type": "Interrogative sentence", "pos": {"nouns": {"you": 1, "- Hey, buddy": 1}, "verbs": {"Yes": 2, "Find": 1}, "adverbs": {"I'm sure": 1, "Just": 1}, "numerals": {"15": 1}, "pronouns": {"Here": 1}}}, {"content": "You think someone really understands you.", "word_count": 12, "label": "", "sentence_structure": "Subject鈥搗erb鈥揷omplement", "subjective_objective": {"answer": "Subjective", "rationale": "Because \"think\" is the patient's subjective perception of his own feelings."}, "sentence_sentiment": {"answer": "Positive", "rationale": "Because the entry expresses an emotional feeling of understanding and acceptance, it is positive."}, "language_style": {"answer": "Tactful and warm", "rationale": "The entry uses a more moderate, inviting tone, which attempts to understand whether the interviewees feel that they can really understand. This expression is neither direct nor prescriptive, but rather communicates with the interviewees in a more personal and interactive manner, making it easier to gain the trust and cooperation of the interviewees."}, "time_dimension": {"answer": "Present", "rationale": "The words \"and feel\" and \"someone really understands you\" both indicate that this is a question about the current feelings of the testees, so it is possible to judge the time dimension of the article as it is."}, "sentence_type": "Affirmative declarative sentence", "pos": {"nouns": {"person": 1, "Really": 1, "Understood": 1}, "verbs": {"I think": 1, "be": 1, "have": 1}, "adverbs": {"And": 1}, "numerals": {"16": 1}, "pronouns": {"you": 1}}}, {"content": "You're shy.", "word_count": 5, "label": "", "sentence_structure": "Subject鈥搗erb鈥搊bject", "subjective_objective": {"answer": "Subjective", "rationale": "Because shyness is a subjective emotional experience and psychological state."}, "sentence_sentiment": {"answer": "Negative", "rationale": "Because \"screety\" is a negative emotion or attitude."}, "language_style": {"answer": "Calm and direct", "rationale": "The entry asks the interviewees whether they are shy, are not using euphemism or rhetoric, and is more straightforward and clear-cut."}, "time_dimension": {"answer": "Past", "rationale": ""}, "sentence_type": "Affirmative declarative sentence", "pos": {"nouns": {"you": 1, "Shy": 1}, "verbs": {"I think": 1}}}, {"content": "You think you're around someone, but they're not really with you.", "word_count": 22, "label": "", "sentence_structure": "Subject鈥搗erb鈥搊bject", "subjective_objective": {"answer": "Subjective", "rationale": "Because it's subjective for the patient to feel and feel the people around him. So it's: subjective."}, "sentence_sentiment": {"answer": "Negative", "rationale": "Because the entry describes a feeling of loneliness, with some negative emotions."}, "language_style": {"answer": "Tactful and warm", "rationale": "The language style of the entry is more euphemistic, expressing a relatively soft, indirect sentiment by using words such as \"think\" and \"though\". At the same time, the entry uses descriptive language (such as \"person,\" \"not really with you\") that allows respondents to understand the problem more deeply and to provide their views."}, "time_dimension": {"answer": "Present", "rationale": "The word \"you think\" means \"the feeling\" means \"the feeling\" and \"18\" means it's a problem on the self-reporting scale. So, this entry is asking the testee's current status and feelings."}, "sentence_type": "Interrogative sentence", "pos": {"nouns": {"person": 2, "Around": 1}, "verbs": {"I think": 1, "have": 1, "in": 1}, "adjectives": {"True": 1}, "adverbs": {"Nope": 1}, "numerals": {"18": 1}, "pronouns": {"you": 2, "they": 1}}}, {"content": "You think someone can still talk.", "word_count": 12, "label": "", "sentence_structure": "Subject鈥搗erb鈥搊bject", "subjective_objective": {"answer": "Subjective", "rationale": "Because \"think\" is a subjective term, which means that it is a personal feeling and perception of the patient, not an objective fact."}, "sentence_sentiment": {"answer": "Neutral", "rationale": "Because the entry is a mere statement of fact and does not express any emotional colours."}, "language_style": {"answer": "Tactful and warm", "rationale": "The language style of the entry is euphemistic and warm, using the words \"think\" and \"may\" to express an attitude of openness and willingness to communicate with others, and without being too direct or hard-line. This makes the questionnaire more acceptable and accessible."}, "time_dimension": {"answer": "Present", "rationale": "The keywords \"think,\" \"also\" and \"can\" indicate that it is a subjective feeling and attitude, while the main word \"you\" points to the subject itself. So the entry is primarily looking at the social needs and status of the testee."}, "sentence_type": "Interrogative sentence", "pos": {"nouns": {"person": 2, "Word": 1}, "verbs": {"I think": 1, "Say it": 1}, "adverbs": {"And": 1}, "numerals": {"19": 1}, "pronouns": {"you": 1, "he": 1}}}, {"content": "You think there's someone who can help, share, or depend.", "word_count": 16, "label": "", "sentence_structure": "Subject鈥搗erb鈥搊bject", "subjective_objective": {"answer": "Subjective", "rationale": "Because \"the feeling\" represents the subjective attitude and feelings of the patient."}, "sentence_sentiment": {"answer": "Positive", "rationale": "Because the purpose of the entry is to \"help, share, or rely on\" the act."}, "language_style": {"answer": "Tactful and warm", "rationale": "The language style of the entry is more moderate and encouraging, using the means of asking for the other to express their views and give some recognition and support."}, "time_dimension": {"answer": "Present", "rationale": "The entry uses the words \"like\" and \"or \" as a description of a subjective feeling, without specifying the past or future. It should therefore be judged as an act or mentality of the present."}, "sentence_type": "Affirmative declarative sentence", "pos": {"nouns": {"person": 1, "Help": 1, "Share": 1, "Dependence": 1}, "verbs": {"I think": 1, "Yeah": 1, "Help": 1, "Share": 1, "Dependence": 1}, "adverbs": {"20": 1}, "numerals": {"20": 1}}}]}

{"questionnaire_title": "Alcohol-dependent screening table (MAST)", "questionnaire_category": "Behavioral questionnaire", "item_count": 25, "emotion_shift_overall": "From positive to negative", "language_style_overall": "Calm and direct", "items": [{"content": "Do you like drinking a lot?", "word_count": 7, "label": "", "sentence_structure": "Subject鈥損redicate", "subjective_objective": {"answer": "Subjective", "rationale": "The basis for the judgement: the examination of the patient ' s personal habits and preferences is therefore subjective."}, "sentence_sentiment": {"answer": "Negative", "rationale": "Because the \"often drinking\" act is negative."}, "language_style": {"answer": "Calm and direct", "rationale": "This entry is presented in a direct, concise interview, without any syllable or emotional colour, and is more objective and neutral."}, "time_dimension": {"answer": "Present", "rationale": ""}, "sentence_type": "Interrogative sentence", "pos": {"nouns": {"you": 1, "Wine": 1}, "verbs": {"Drink": 1}, "adverbs": {"The long run": 1, "Regular": 1}, "classifiers": {"0锛?: 1}}}, {"content": "Do you drink the same or less than most people?", "word_count": 14, "label": "", "sentence_structure": "Subject鈥搗erb鈥搊bject", "subjective_objective": {"answer": "Subjective", "rationale": "Because \"drink quantity\" is a physical characteristic or a human ability, it needs to be considered subjectively."}, "sentence_sentiment": {"answer": "Neutral", "rationale": "As this entry does not address any positive or negative emotional, attitudinal, behavioural, purpose or quality."}, "language_style": {"answer": "Calm and direct", "rationale": "The language style of the entry is more neutral and objective, and there is no excessive emotional or rhetorical approach, which is a direct expression of calmness."}, "time_dimension": {"answer": "Present", "rationale": "The entry is an inquiry into the testee ' s comparison and evaluation of his or her alcohol volume, with no clear indication of the temporal dimension, and it can be considered as a neutral, time-consuming issue."}, "sentence_type": "Interrogative sentence", "pos": {"nouns": {"Wine": 1, "Most people": 1}, "verbs": {"Same thing": 1, "Less": 1, "- You're not": 1}, "adjectives": {"of": 2}, "numerals": {"One": 1}, "classifiers": {"individual": 1}}}, {"content": "Did you drink the following night and wake up the next morning and not remember some of the things you went through?", "word_count": 27, "label": "", "sentence_structure": "Subject鈥搗erb鈥搊bject", "subjective_objective": {"answer": "Objective", "rationale": "It is an objective fact that the overnight experience is the result of the patient ' s physical reaction and memory."}, "sentence_sentiment": {"answer": "Negative", "rationale": "Because the entry \"Drinking the next night, waking up the next morning without thinking about part of the night\" is negative."}, "language_style": {"answer": "Tactful and warm", "rationale": "The entry is more gentle, using the means of inquiry, without direct orders or statements. At the same time, it is not too emotional, but rather a conversation in a balanced and objective tone."}, "time_dimension": {"answer": "Present", "rationale": ""}, "sentence_type": "Interrogative sentence", "pos": {"nouns": {"you": 1, "The next day": 1, "Night": 1, "Drink": 1, "The morning after that": 1, "Wake up": 1, "Part of it": 1, "Things": 1}, "verbs": {"There was": 1, "I can't remember": 1, "Experience": 1}, "adverbs": {"Separatism": 1, "Number of times": 1, "Wake up": 1}, "numerals": {"2锛?: 1, "of": 1, "One": 3}}}, {"content": "Have your spouses, parents or other close relatives ever been concerned or complained about your drinking?", "word_count": 24, "label": "", "sentence_structure": "Subject鈥搗erb鈥搊bject", "subjective_objective": {"answer": "Subjective", "rationale": "The question is about how the patient feels about his or her spouse, parents or other close relatives, and about his or her drinking."}, "sentence_sentiment": {"answer": "Negative", "rationale": "Because the \"fear or complain\" act in the entry is negative."}, "language_style": {"answer": "Calm and direct", "rationale": "The entry is a direct question to the respondent, asking if the person concerned has any concerns or complaints about his drinking, and is of a more objective and guiding language style."}, "time_dimension": {"answer": "Present", "rationale": ""}, "sentence_type": "Interrogative sentence", "pos": {"nouns": {"Spouse": 1, "parents": 1, "Close relatives": 1, "Drink": 1}, "verbs": {"to": 1, "I feel": 1, "- He's complaining": 1}, "adjectives": {"I'm worried": 1}, "numerals": {"3锛?: 1}, "pronouns": {"you": 2}}}, {"content": "When you've had one or two drinks, you can stop drinking without any effort.", "word_count": 18, "label": "", "sentence_structure": "Subject鈥搗erb鈥搊bject", "subjective_objective": {"answer": "Subjective", "rationale": "Because the entry is concerned with the patient ' s drinking behaviour and self-control, which are the expression of subjective experience and attitudes."}, "sentence_sentiment": {"answer": "Positive", "rationale": "Because the behaviour of \"many control over no more drinking\" in the entry represents individual self-control and self-management capacity, and is positive quality and attitude."}, "language_style": {"answer": "Tactful and warm", "rationale": "The language style of the entry is more euphemistic and implicit, and does not directly express criticism or praise for alcohol consumption. Instead, the question is asked in a more objective and neutral way, allowing the person surveyed to think and answer. This language is more acceptable and can reduce the negative emotional reactions that may arise."}, "time_dimension": {"answer": "Present", "rationale": "So the answer is \"now\"."}, "sentence_type": "Interrogative sentence", "pos": {"nouns": {"Wine": 1, "Cups": 1}, "verbs": {"Drink": 2, "Control": 1, "Drink up": 1}, "adverbs": {"It's not hard": 1}, "numerals": {"1锝?": 1}, "classifiers": {"Cups": 1}}}, {"content": "Have you ever felt guilty about drinking?", "word_count": 10, "label": "", "sentence_structure": "Subject鈥搗erb鈥搊bject", "subjective_objective": {"answer": "Subjective", "rationale": "Because guilt is a subjective emotional experience of the patient, and it's a subjective perspective."}, "sentence_sentiment": {"answer": "Negative", "rationale": "Because the emotional attitude of guilt in the entries is negative."}, "language_style": {"answer": "Calm and direct", "rationale": "The entry is more straightforward in its language style, is not over-modified or istrophized, but asks the interviewee in an objective and clear manner whether he or she has ever felt guilty about drinking. \""}, "time_dimension": {"answer": "Past", "rationale": "The entry is for questioning the testee ' s past behaviour or mental state, and the time dimension is judged to be past."}, "sentence_type": "Interrogative sentence", "pos": {"nouns": {"Drink": 1, "Guilt": 1}, "verbs": {"I feel": 1}, "adverbs": {"Tsang": 1, "- You're not": 1}}}, {"content": "Do your relatives and friends think you're drinking like the usual?", "word_count": 20, "label": "", "sentence_structure": "Subject鈥搗erb鈥搊bject", "subjective_objective": {"answer": "Objective", "rationale": "This is an objective fact because it is an assessment of the drinking habits of the patients by the relatives and friends who are asked about them."}, "sentence_sentiment": {"answer": "Neutral", "rationale": "As the entry does not express any emotion, attitude or behaviour, it merely asks the relatives and friends of the interviewee about the alcohol habits of the interviewee."}, "language_style": {"answer": "Calm and direct", "rationale": "The entry is directly asked, without excessive ado or emotional rendering, and is more calm, objective and of a calm and direct language style."}, "time_dimension": {"answer": "Present", "rationale": "Now the entry asks if the testee's current drinking habits are similar to those of the average person, so the temporal dimension is now judged. And \"the normal habits\" also suggest that this is a general phenomenon, not a particular situation in the past."}, "sentence_type": "Imperative sentence", "pos": {"nouns": {"you": 1, "Friends": 1, "Drink": 1, "Custom": 1, "General": 1}, "verbs": {"I think": 1, "Almost": 1}, "adjectives": {"Normal": 1}, "classifiers": {"individual": 1}}}, {"content": "When you're going to stop drinking, can you do it?", "word_count": 17, "label": "", "sentence_structure": "Subject鈥搗erb鈥搊bject", "subjective_objective": {"answer": "Subjective", "rationale": "The basis for the determination is that the question is primarily one of whether the patient can control his or her drinking desire and behaviour, and therefore from the patient ' s subjective point of view."}, "sentence_sentiment": {"answer": "Negative", "rationale": "Because the behavior in the entry \"intending not to drink\" is negative."}, "language_style": {"answer": "Calm and direct", "rationale": "The entry asks the respondents directly about their ability to stop drinking, does not use excessive rhetoric or emotional colours to depict the situation, and the language style is more calm and objective."}, "time_dimension": {"answer": "Present", "rationale": "The keywords \"intending\" and \"can do\" indicate that the entry is asking the testee whether he or she can currently quit drinking, and thus judging now."}, "sentence_type": "Interrogative sentence", "pos": {"nouns": {"Time": 1, "Wine": 1}, "verbs": {"I'm going to": 1, "Do it": 1}, "numerals": {"7": 1}}}, {"content": "Have you ever been to an AA?", "word_count": 10, "label": "", "sentence_structure": "Subject鈥搗erb鈥搊bject", "subjective_objective": {"answer": "Subjective", "rationale": "Because participation in alcohol-revenging activities is a person ' s experience and behaviour choices and is a patient ' s subjective attitude."}, "sentence_sentiment": {"answer": "Neutral", "rationale": "Because the entry was not of any emotional nature, it was merely a question of whether the person interviewed had ever been involved in alcohol-revenging activities."}, "language_style": {"answer": "Calm and direct", "rationale": "The answer is based on the following: The subject raises questions in a direct, concise manner, without using any qualifier or expression to project the atmosphere or guide the respondents. This direct question is common in the questionnaire design, which aims to obtain the most immediate and realistic answers."}, "time_dimension": {"answer": "Present", "rationale": "The entry asks if the person tested has ever participated in a specific activity (the alcohol-revenging event) and therefore the temporal dimension is judged to be \"now\"."}, "sentence_type": "Interrogative sentence", "pos": {"nouns": {"Activities": 1, "A clean drink": 1}, "verbs": {"Participation": 1}}}, {"content": "Did you ever fight after drinking?", "word_count": 11, "label": "", "sentence_structure": "Subject鈥搗erb鈥搊bject", "subjective_objective": {"answer": "Objective", "rationale": "Because the fact that a fight has occurred is an objective fact and does not depend on the subjective feelings or ideas of the individual."}, "sentence_sentiment": {"answer": "Negative", "rationale": "Because the fight in the entry is negative."}, "language_style": {"answer": "Calm and direct", "rationale": "The language style of the entry is more calm and straightforward, and does not use any qualifying language or expression to alleviate the seriousness of the problem, but rather uses a simple and straightforward question form, which directly asks the interviewee whether he has had a history of fighting after drinking."}, "time_dimension": {"answer": "Present", "rationale": "The entry asked the respondent whether at any point in time there had been any act, namely, \"had been a fight with a person after drinking\", which had occurred for some time in the past and was now over or continued. The time dimension of the entry was therefore now."}, "sentence_type": "Interrogative sentence", "pos": {"nouns": {"Drink": 1, "person": 1, "Fighting": 1}, "verbs": {"in": 1, "Tsang": 1, "and": 1, "Fighting": 1}, "adverbs": {"Back": 1}, "numerals": {"9": 1}}}, {"content": "Have you ever had any problems with your spouse, parents or other close relatives as a result of drinking?", "word_count": 24, "label": "", "sentence_structure": "Subject鈥搗erb鈥搊bject", "subjective_objective": {"answer": "Subjective", "rationale": "The question is about whether the patient has problems with his spouse, parents or other close relatives because of alcohol."}, "sentence_sentiment": {"answer": "Negative", "rationale": "Because the entry \"on the contrary with relatives due to alcohol problems\" is negative."}, "language_style": {"answer": "Calm and direct", "rationale": "The entry is in the form of a direct question, without too much rhetorical or emotional overtones, and questions are asked in an objective and neutral manner, allowing the interviewee to answer the question clearly and clearly, reflecting a calm and direct language style."}, "time_dimension": {"answer": "Past", "rationale": "The entry asks whether the person tested had in the past had had problems with his spouse, parents or other close relatives because of alcohol consumption problems and therefore was part of the temporal dimension of the past."}, "sentence_type": "Interrogative sentence", "pos": {"nouns": {"Problem": 1, "Drink": 1, "Paradoxious": 1, "Spouse": 1, "parents": 1, "Close relatives": 1}, "verbs": {"Tsang": 1, "Because": 1, "Generate": 1}, "numerals": {"10": 1}, "pronouns": {"you": 1}}}, {"content": "Has your spouse (or other family members) ever sought help from others for your drinking?", "word_count": 24, "label": "", "sentence_structure": "Subject鈥搗erb鈥搊bject", "subjective_objective": {"answer": "Subjective", "rationale": "Because the subject is the patient's own views and experiences, the question is whether your spouse has sought help in your drinking problems and is therefore subjective."}, "sentence_sentiment": {"answer": "Negative", "rationale": "Because the act of \"had asked for help for your drink\" in the entry implies the problem of drinking or overdrinking, which is a negative act."}, "language_style": {"answer": "Tactful and warm", "rationale": "The entry is more gentle, does not directly identify the wrongs of the other party or uses blameful language."}, "time_dimension": {"answer": "Present", "rationale": "The entry asks about the current situation of the person tested, i.e. whether the spouse or other family member has sought help from others because of the person tested for alcohol. The keyword \"had\" indicates that the act occurred in the past, but the question remains in its present state."}, "sentence_type": "Interrogative sentence", "pos": {"nouns": {"Spouse": 1, "Other": 1, "Family": 1, "Members": 1, "Drink": 1, "Yes": 1, "Others": 1}, "verbs": {"Tsang": 1, "Yes": 2, "Help": 2}, "numerals": {"11": 1, "One": 1, "individual": 2}, "pronouns": {"you": 1, "of": 2, "Here": 1}}}, {"content": "Have you ever broken up with a friend for drinking?", "word_count": 14, "label": "", "sentence_structure": "Subject鈥搗erb鈥搊bject", "subjective_objective": {"answer": "Subjective", "rationale": "Because the result of \"caused break-up with a friend\" is the combination of the patient's behaviour (drinking) and subjective judgement (which is believed to lead to break-up) and is subjective in nature."}, "sentence_sentiment": {"answer": "Negative", "rationale": "Because the \"breeding of a friend\" in the entry is negative."}, "language_style": {"answer": "Calm and direct", "rationale": "The entry explicitly asked the interviewees whether they had broken up with friends because of alcohol consumption, which was more objective, straightforward and not excessive in terms of rhetorical or emotional overtones."}, "time_dimension": {"answer": "Past", "rationale": "And \"breakup\" is the result, the description of the past. So, the judgment is based on the past."}, "sentence_type": "Interrogative sentence", "pos": {"nouns": {"Problem": 1, "Entry": 1, "Drink": 1, "Friends": 1, "Break up": 1}, "verbs": {"Result": 1, "Tsang": 1, "Because": 1, "min": 1, "and": 1, "you": 1, "And": 1, "- You're not": 1}, "numerals": {"12锛?: 1}}}, {"content": "Have you had problems at work and in your studies because you drank?", "word_count": 16, "label": "", "sentence_structure": "Subject鈥搗erb鈥搊bject", "subjective_objective": {"answer": "Subjective", "rationale": "Because it is a subjective feeling to ask if the patient has had such experience."}, "sentence_sentiment": {"answer": "Negative", "rationale": "Because \"problems at work, learning\" in the entry is negative."}, "language_style": {"answer": "Tactful and warm", "rationale": "The language style of the entry is more euphemistic and gentle, and it is easier to be accepted by the interviewees to ask them, in a caring and understanding manner, whether they have had problems at work or in their studies because of alcohol consumption, and have no language of direct accusation or criticism."}, "time_dimension": {"answer": "Past", "rationale": "The entry asks if the testee has had experience of problems at work or learning due to alcohol consumption, and therefore the temporal dimension is judged to be past."}, "sentence_type": "Interrogative sentence", "pos": {"nouns": {"Drink": 1, "Work": 1, "Learning": 1, "Problem": 1}, "verbs": {"Zeng Zhen": 1, "in": 1, "Out": 1}, "adverbs": {"Tsang": 1, "in": 1}, "numerals": {"13": 1}, "classifiers": {"individual": 1}}}, {"content": "Did you ever get fired for drinking?", "word_count": 9, "label": "", "sentence_structure": "Subject鈥搗erb鈥搊bject", "subjective_objective": {"answer": "Objective", "rationale": "Since the dismissal is an objective fact, it does not need to be judged from the patient ' s subjective point of view."}, "sentence_sentiment": {"answer": "Negative", "rationale": "Because the entry was alcohol-drinked and resulted in dismissal, there was a clear negative emotional profile."}, "language_style": {"answer": "Calm and direct", "rationale": "The entry is more straightforward, has less acrimony or emotional colour, and is more practical in its factual approach. At the same time, the issue is sensitive, and a less nuanced and neutral approach reduces each other鈥檚 resistance and facilitates access to real and effective information."}, "time_dimension": {"answer": "Past", "rationale": ""}, "sentence_type": "Interrogative sentence", "pos": {"nouns": {"Drink": 1, "Termination": 1}, "verbs": {"By": 1, "Tsang": 1}, "adverbs": {"- You're not": 1}, "numerals": {"14": 1}}}, {"content": "Have you been drinking for more than two days in a row, and have you failed to work or to leave your family behind?", "word_count": 28, "label": "", "sentence_structure": "Subject鈥搗erb鈥搊bject", "subjective_objective": {"answer": "Objective", "rationale": "Because \"had been drinking for more than two days in a row\" and \"relationship, failure to work or neglecting the family\" are the actual acts and consequences of the patient, they are considered from an objective perspective."}, "sentence_sentiment": {"answer": "Negative", "rationale": "Because the entry \"Little duty, failure to work or disregard for the family\" is negative."}, "language_style": {"answer": "Calm and direct", "rationale": "It explicitly asks whether the person surveyed has been guilty of misbehaviour, failure to work or lack of responsibility for the family because of drinking for more than two days in a row."}, "time_dimension": {"answer": "Present", "rationale": "The entry used to ask the testee whether past alcohol consumption had led to consequences such as excessive liability, failure to work or neglect of the family, and the temporal dimension was a matter of past."}, "sentence_type": "Interrogative sentence", "pos": {"nouns": {"Oh, my God": 1, "Drink": 2, "Responsibility": 1, "Work": 1, "Family": 1}, "verbs": {"Tsang": 1, "Continuous": 1, "Always": 1, "Mistake": 1, "Go on": 1, "Set": 1, "I'll give up": 1}, "adverbs": {"Above": 1, "Two": 1}, "numerals": {"15": 1, "One": 2, "Two": 1}}}, {"content": "Do you drink often in the morning?", "word_count": 9, "label": "", "sentence_structure": "Subject鈥搗erb鈥搊bject", "subjective_objective": {"answer": "Objective", "rationale": "Because \"drinking in the morning\" is an objective fact that can be observed and measured, not the patient's subjective attitude or feeling."}, "sentence_sentiment": {"answer": "Negative", "rationale": "Because \"drinking\" is usually negative."}, "language_style": {"answer": "Calm and direct", "rationale": "The entry asks the respondents in a concise and clear language whether they often drink alcohol in the morning, in an objective, direct and non-pleasant manner or in a emotional way."}, "time_dimension": {"answer": "Present", "rationale": ""}, "sentence_type": "Interrogative sentence", "pos": {"nouns": {"a.m": 1, "Drink": 1}, "verbs": {"Regular": 1, "in": 1}, "adverbs": {"- You're not": 1}, "numerals": {"16": 1}}}, {"content": "Did the doctor say you had liver problems or cirrhosis?", "word_count": 17, "label": "", "sentence_structure": "Subject鈥搗erb鈥搊bject", "subjective_objective": {"answer": "Objective", "rationale": "This is an objective fact, as it is the patient ' s medical history and medical diagnosis."}, "sentence_sentiment": {"answer": "Negative", "rationale": "Because the fact that the entry \"hepatic is in a bad condition or hepatic cirrhosis\" is negative."}, "language_style": {"answer": "Calm and direct", "rationale": "This language style helps ensure that the interviewee understands the intentions of the issue and is able to provide real and specific information."}, "time_dimension": {"answer": "Past", "rationale": "The entry asks whether the person tested had been diagnosed by a doctor with liver problems or cirrhosis, and the temporal dimension was in the past."}, "sentence_type": "Interrogative sentence", "pos": {"nouns": {"Doctor": 1, "Problem": 1, "Hepatic cirrhosis": 1}, "verbs": {"I said": 1, "have": 2}, "adverbs": {"Tsang": 1}, "numerals": {"17": 1}, "classifiers": {"individual": 1}, "pronouns": {"you": 1}}}, {"content": "18. After a lot of alcohol, you've had a tremor or heard the truth.", "word_count": 19, "label": "", "sentence_structure": "Subject鈥搗erb鈥搊bject", "subjective_objective": {"answer": "Objective", "rationale": "Since the problem concerns the drinking behaviour of the patient and its possible objective consequences (i.e. tremors or hearing the actual) it is examined from an objective perspective."}, "sentence_sentiment": {"answer": "Negative", "rationale": "Because the description of the act \"a great amount of alcohol and a tremors or actual hearing\" in the entry is negative."}, "language_style": {"answer": "Calm and direct", "rationale": ""}, "time_dimension": {"answer": "Present", "rationale": "The entry describes the person's current behaviour or state, and \"a presence\" means that the act has occurred in the past, but not necessarily continues. At the same time, \"hearing\" is also the expression of a current state of affairs. The time dimension of the entry should therefore be judged as now."}, "sentence_type": "Interrogative sentence", "pos": {"nouns": {"Drink": 1, "鈾?The tremors 鈾?: 1, "You're a good man": 1, "Yes": 1, "Actual": 1}, "verbs": {"Come on": 1, "Mass": 1}, "adverbs": {"in": 1, "Tsang": 1}, "numerals": {"18锛?: 1}}}, {"content": "Have you ever sought help from anyone for problems caused by drinking?", "word_count": 17, "label": "", "sentence_structure": "Subject鈥搗erb鈥搊bject", "subjective_objective": {"answer": "Subjective", "rationale": "Because it is a subjective attitude and behaviour of the patient to ask him about personal problems arising from drinking and to seek help from others."}, "sentence_sentiment": {"answer": "Negative", "rationale": "Because the \"problems of drinking to help others\" in the entry are negative."}, "language_style": {"answer": "Calm and direct", "rationale": "The language style of the entry is calm and straightforward. It asks the interviewee whether he has had problems with alcohol and has sought help from others in an objective and clear manner, without using any emotional aesthetic or euphemism."}, "time_dimension": {"answer": "Past", "rationale": ""}, "sentence_type": "Interrogative sentence", "pos": {"nouns": {"Drink": 1, "Problem": 1, "Others": 1}, "verbs": {"Tsang": 1, "Cause": 1, "Help": 1}, "adverbs": {"Go on": 1}, "classifiers": {"19锛?: 1}}}, {"content": "Have you ever been hospitalized for problems caused by drinking?", "word_count": 15, "label": "", "sentence_structure": "Subject鈥搗erb鈥搊bject", "subjective_objective": {"answer": "Subjective", "rationale": "It is a subjective experience and feeling of the patient who is examined for his or her experience in hospital because of problems caused by drinking."}, "sentence_sentiment": {"answer": "Negative", "rationale": "Because the entry describes a misdemeanour (hospital hospitalization due to problems caused by alcohol consumption), it expresses a negative emotional attitude."}, "language_style": {"answer": "Calm and direct", "rationale": "The language style of the entry is straightforward. It is simple to ask whether the person surveyed has ever been admitted to hospital for problems caused by alcohol consumption, without too much rhetoric or emotional colour, and with an objective and straightforward tone, which is easily understandable."}, "time_dimension": {"answer": "Present", "rationale": "The entry used to ask the testee whether he had been admitted to hospital for drinking and the time dimension was a matter of the past."}, "sentence_type": "Interrogative sentence", "pos": {"nouns": {"Problem": 1, "Hospitalization": 1, "Drink": 1}, "verbs": {"Cause": 1, "Hospitalization": 1}, "adverbs": {"Tsang": 1}, "numerals": {"20": 1}}}, {"content": "Have you been hospitalized in psychiatric hospitals or general hospitals for problems caused by drinking?", "word_count": 28, "label": "", "sentence_structure": "Subject鈥搗erb鈥搊bject", "subjective_objective": {"answer": "Objective", "rationale": "It is an objective fact that the hospital is hospitalized for drinking."}, "sentence_sentiment": {"answer": "Negative", "rationale": "The fact that \"inpatients in psychiatric hospitals or general hospitals due to alcohol problems\" are found in the entry is negative."}, "language_style": {"answer": "Calm and direct", "rationale": "The language of the entry is relatively calm and straightforward, with no excessive adoration or emotional colour, and the main purpose is to ask the other party clearly whether he or she has been hospitalized in psychiatric hospitals or general hospitals for problems caused by alcohol consumption."}, "time_dimension": {"answer": "Past", "rationale": "The entry asks whether the person tested has had a history of alcohol problems that require psychiatric hospitalization in a psychiatric hospital or general hospital."}, "sentence_type": "Interrogative sentence", "pos": {"nouns": {"Problem": 1, "Mental hospital": 1, "General hospital": 1, "Psychiatric": 1}, "verbs": {"Tsang": 1, "Cause": 1, "in": 1, "Hospitalization": 1}, "adverbs": {"Tsang": 1, "in": 1}, "numerals": {"21锛?: 1, "you": 1}, "pronouns": {"you": 1}}}, {"content": "22. Have you ever sought help from psychiatrists, other medical doctors, social workers, psychologists, for emotional problems caused in part by alcohol consumption?", "word_count": 41, "label": "", "sentence_structure": "Subject鈥搗erb鈥搊bject", "subjective_objective": {"answer": "Objective", "rationale": "Since recourse is an objective fact of the patient ' s experience, it is subjective to examine subjective ideas."}, "sentence_sentiment": {"answer": "Negative", "rationale": "The entry \"Drinking-related emotional problems\" and \"Recourse to psychiatric, other medical, social, psychological\" are negative."}, "language_style": {"answer": "Calm and direct", "rationale": "The entry asks questions in a straightforward manner, without using excessive rhetorical or emotional rendering, and in a more objective manner."}, "time_dimension": {"answer": "Past", "rationale": "The entry is for questioning the testee ' s past behaviour and the temporal dimension is a past."}, "sentence_type": "Interrogative sentence", "pos": {"nouns": {"Reason": 1, "Drink": 1, "Emotional problems": 1, "Help": 1, "Psychiatric": 1, "Other sections": 1, "Doctor": 2, "Social workers": 1, "Psychological": 1}, "verbs": {"Zeng Zhen": 1, "Resulted": 1, "And": 1, "Appeal": 1, "Consultants": 1}, "adverbs": {"Part": 1}, "numerals": {"22": 1}}}, {"content": "Have you ever been detained for drinking or driving?", "word_count": 22, "label": "", "sentence_structure": "Subject鈥搗erb鈥搊bject", "subjective_objective": {"answer": "Objective", "rationale": "This question is asked whether the patient has ever done so, not what they feel or feel. It is examined from an objective perspective."}, "sentence_sentiment": {"answer": "Negative", "rationale": "Because the behavior in the entry \"drinking or driving drunk\" is negative."}, "language_style": {"answer": "Calm and direct", "rationale": ""}, "time_dimension": {"answer": "Present", "rationale": "The keyword \"you\" and \"you\" have been used several times together to indicate that this is an inquiry into the testee's past behaviour, but that this behaviour has been detained and therefore the temporal dimension is now judged."}, "sentence_type": "Interrogative sentence", "pos": {"nouns": {"you": 1, "Drink": 1, "Drunk": 1, "Drive": 1, "Detention": 1, "Number of times": 1}, "verbs": {"Zeng Zhen": 1, "After drinking": 1, "Drunk": 1, "Drive": 1, "By": 1, "Detention": 1, "If there is": 1, "Total": 1}, "adverbs": {"How much": 2, "Zeng Zhen": 1, "Drunk": 1}, "numerals": {"23": 1, "Total": 1}, "classifiers": {"Number of times": 1}, "pronouns": {"you": 1}}}, {"content": "Have you been detained for more than a few hours for other alcohol-related activities?", "word_count": 27, "label": "", "sentence_structure": "Subject鈥搗erb鈥搊bject", "subjective_objective": {"answer": "Objective", "rationale": "Because this entry is asking whether a patient has been detained for drinking, it does not examine the patient鈥檚 subjective thoughts or feelings. This is based on objective facts of the past and is therefore objective."}, "sentence_sentiment": {"answer": "Negative", "rationale": "As the entry describes a possible negative act (detention for alcohol use), it can be considered negative."}, "language_style": {"answer": "Calm and direct", "rationale": "The entry is more objective and direct in language style, and does not have too many emotional or rhetorical techniques. It asks the interviewee in a concise manner whether he has had any experience of detention for other alcohol-drinking practices, which is calm and professional."}, "time_dimension": {"answer": "Past", "rationale": "The entry asks whether the person tested was detained for drinking in the past and therefore judged as \"the past\"."}, "sentence_type": "Interrogative sentence", "pos": {"nouns": {"Alcohol behaviour": 1, "Detention": 1, "Hours": 1, "Number": 1}, "verbs": {"Yes": 1, "Detention": 1, "Total": 1}, "adverbs": {"Tsang": 1, "Above": 1}, "numerals": {"24": 1, "How many": 1, "A few times": 1}, "classifiers": {"Number of times": 1}}}]}

{"questionnaire_title": "Torrance's creative personality self-examining.", "questionnaire_category": "Self-cognition", "item_count": 20, "emotion_shift_overall": "No obvious trend", "language_style_overall": "Tactful and warm", "items": [{"content": "(1) Be able to concentrate when doing things, observing things or listening to people.", "word_count": 18, "label": "", "sentence_structure": "Subject鈥搗erb鈥揷omplement", "subjective_objective": {"answer": "Objective", "rationale": "Because \"recentness\" is behaviour that can be assessed by observation or testing, without the patient's subjective attitude or feelings."}, "sentence_sentiment": {"answer": "Positive", "rationale": "Because the \"focus\" act in the entry is positive."}, "language_style": {"answer": "Tactful and warm", "rationale": "The language style of the entry is relatively euphemistic and gentle. It leads the interviewee to answer questions by using the invitee word \"please\" and does not use negative, critical or other coercive language, which makes people comfortable and conducive to a good atmosphere of communication. At the same time, \"focusing\" is a descriptive term that inspires the interviewee to be positive and to make him more willing to share his ideas and experiences."}, "time_dimension": {"answer": "Past", "rationale": "If the testee has demonstrated this behaviour in the past and has referred to past experience in the answer to the question, the entry may point to the past; the reverse may point to the present."}, "sentence_type": "Interrogative sentence", "pos": {"nouns": {"Things": 2, "person": 1}, "verbs": {"OPERATIONS": 1, "Observation": 1, "Listen": 1, "Say it": 1, "Yes": 1, "Focus": 1}, "numerals": {"One": 2}, "classifiers": {"individual": 1}, "pronouns": {"it": 1}}}, {"content": "(2) The method of speaking and writing is often by analogy.", "word_count": 13, "label": "", "sentence_structure": "Subject鈥搗erb鈥揷omplement", "subjective_objective": {"answer": "Subjective", "rationale": "Because the analogy is an expression and a habit, a personal thought and expression habit, and a subjective view."}, "sentence_sentiment": {"answer": "Neutral", "rationale": "The entry is not a positive or negative emotional trend."}, "language_style": {"answer": "Tactful and warm", "rationale": "The language style of the entry is more euphemistic, as the question is asked whether the respondents often use analogues in their speech and writing, and this tone does not put the respondents under stress or discomfort. Moreover, the language structure of the question is relatively simple and understandable."}, "time_dimension": {"answer": "Present", "rationale": "The entry describes the current behaviour or habits of the subject, the keywords being \"frequently\" and \"use\", which point to the present behaviour or state. Thus, the temporal dimension is now judged."}, "sentence_type": "Interrogative sentence", "pos": {"nouns": {"Talk": 1, "Writing": 1, "Methodology": 1}, "verbs": {"Use": 1, "Similarity": 1}, "adverbs": {"Regular": 1}, "numerals": {"2": 1}}}, {"content": "(3) Be able to read, write and paint with full attention.", "word_count": 13, "label": "", "sentence_structure": "Subject鈥搗erb鈥揷omplement", "subjective_objective": {"answer": "Objective", "rationale": "Since the ability to read, write and paint with all due attention is the actual expression or capacity of the patient, objective facts are examined."}, "sentence_sentiment": {"answer": "Positive", "rationale": "Because the act of \"reading, writing and painting with all your attention\" described in the entry is positive."}, "language_style": {"answer": "Calm and direct", "rationale": "While the phrase \"careful\" in the entry indicates the need for focused activities, the sentence is not overly prescriptive or emotional, and is more objective. Such an expression is easier to understand the questions and does not have an excessive emotional impact on their answers."}, "time_dimension": {"answer": "Present", "rationale": "The entry is for the testee ' s current state of behaviour or ability, and the time dimension is now judged."}, "sentence_type": "Affirmative declarative sentence", "pos": {"nouns": {"Books": 1, "Writing": 1, "Painting": 1}, "verbs": {"Yes": 1, "Read it with all your attention": 1, "Read": 1, "Writing": 1, "Painting": 1}, "adverbs": {"鈾?With all your attention 鈾?: 1}, "numerals": {"3": 1}}}, {"content": "(4) There is always a sense of excitement after the completion of the work assigned by the teacher.", "word_count": 17, "label": "", "sentence_structure": "Subject鈥搗erb鈥揷omplement", "subjective_objective": {"answer": "Subjective", "rationale": "Because the experience after the job is done is the patient's subjective experience."}, "sentence_sentiment": {"answer": "Positive", "rationale": "Because the expression of the purpose of the article contains a positive emotional term of \"excited\" that is a sign of satisfaction and pleasure after the completion of the mission."}, "language_style": {"answer": "Tactful and warm", "rationale": "The entry uses descriptive language, emphasizing the feelings of completing the work that the teacher has set up, rather than asking the other side whether he or she is excited. This expression is more easily accepted and willing to share his or her true feelings, and is of good affection and interaction."}, "time_dimension": {"answer": "Present", "rationale": ""}, "sentence_type": "Affirmative declarative sentence", "pos": {"nouns": {"Teacher": 1, "Set": 1, "Operations": 1, "Excited": 1}, "verbs": {"Completed": 1, "There's always one": 1}, "adverbs": {"Back": 1, "Total": 1}, "numerals": {"4": 1}}}, {"content": "(5) Dare to challenge authority.", "word_count": 7, "label": "", "sentence_structure": "Subject鈥損redicate", "subjective_objective": {"answer": "Subjective", "rationale": "Because the entry describes the patient ' s courage to challenge authority, which is his subjective attitude and willingness."}, "sentence_sentiment": {"answer": "Positive", "rationale": "Because the \"bold to challenge the authority\" in the entry is a positive expression of courage and perseverance."}, "language_style": {"answer": "Calm and direct", "rationale": "The language style of the entry is relatively calm and straightforward, and the simple words clearly indicate whether the interviewee dares challenge the authority. Instead of using excessive rhetorical or emotional colours, the dialogue is conducted in an objective and direct manner, allowing the respondents to the questionnaire to understand the questions clearly and clearly."}, "time_dimension": {"answer": "Future", "rationale": ""}, "sentence_type": "Imperative sentence", "pos": {"verbs": {"How dare you": 1, "In": 1, "Challenges": 1}, "adjectives": {"Authority": 1}, "adverbs": {"How dare you": 1}, "numerals": {"5": 1}}}, {"content": "(6) The reasons for being used to looking for things.", "word_count": 12, "label": "", "sentence_structure": "Subject鈥搗erb鈥搊bject", "subjective_objective": {"answer": "Objective", "rationale": "Because the reasons for looking for things are a behaviour that is objective and not the subjective attitude or feelings of the patient."}, "sentence_sentiment": {"answer": "Neutral", "rationale": "Because the entry does not have any emotional color."}, "language_style": {"answer": "Calm and direct", "rationale": "The entry is directly asked in a concise and clear language, without a rhetorical or emotional language, which is more calm and objective and easier to understand and answer by the interviewees."}, "time_dimension": {"answer": "Present", "rationale": "The term \"customary\" in the entry to the questionnaire indicates that it is a constant and persistent act, and that \"seeking for reasons\" is an ongoing action, so the entry describes a current state or behaviour."}, "sentence_type": "Affirmative declarative sentence", "pos": {"nouns": {"Custom": 1, "Search": 1, "Things": 1, "Various": 1, "Reason": 1}, "verbs": {"Search": 1, "Form": 1}, "adverbs": {"on": 1}, "numerals": {"6": 1}}}, {"content": "(7) can observe things carefully.", "word_count": 8, "label": "", "sentence_structure": "Subject鈥搗erb鈥揷omplement", "subjective_objective": {"answer": "Objective", "rationale": "Because it is the patient鈥檚 ability or skill to observe things carefully, it exists objectively, free from the subjective will of the individual. It is therefore seen from an objective perspective."}, "sentence_sentiment": {"answer": "Neutral", "rationale": "The entry does not express positive or negative emotional expressions, but rather describes a capability or behavioural characteristic and is therefore neutral."}, "language_style": {"answer": "Tactful and warm", "rationale": "The entry is more euphemistic, friendly and uses an invitation-style tone to try to share their views and experiences in a light atmosphere. At the same time, the word \"caution\" is more objective, without too much emotional or subjective assessment, and reflects the neutrality of the surveyer."}, "time_dimension": {"answer": "Present", "rationale": "The entry asks the testee about the current behaviour or attitude, and the word \"capable\" indicates that it is a capability, which is an ongoing move, and therefore the time dimension is now judged."}, "sentence_type": "Imperative sentence", "pos": {"verbs": {"Yes": 1, "Careful": 1, "Observation": 1, "Things": 1}, "adjectives": {"Careful": 1}, "adverbs": {"Land": 1}, "numerals": {"7": 1}}}, {"content": "(8) The problems can be identified from other people ' s conversations.", "word_count": 11, "label": "", "sentence_structure": "Subject鈥搗erb鈥揷omplement", "subjective_objective": {"answer": "Subjective", "rationale": "Because finding the problem is a subjective process of perception and judgment."}, "sentence_sentiment": {"answer": "Positive", "rationale": "Because the act of \"can find problems in someone's conversation\" in the entry is positive, indicating that the person has a strong ability to observe and think."}, "language_style": {"answer": "Calm and direct", "rationale": "The entry directly raises the question of whether the person surveyed has the ability to discover problems from someone else ' s conversations, is not over-modified or emotional and belongs to a more calm and direct language style."}, "time_dimension": {"answer": "Present", "rationale": ""}, "sentence_type": "Affirmative declarative sentence", "pos": {"nouns": {"others": 1, "Talk": 1, "Problem": 1}, "verbs": {"Yes": 1, "Found": 1}, "numerals": {"8": 1}}}, {"content": "(9) Time is often forgotten when creative thinking is being carried out.", "word_count": 17, "label": "", "sentence_structure": "Subject鈥搗erb鈥搊bject", "subjective_objective": {"answer": "Subjective", "rationale": "Because \"often forget time\" is the subjective experience and feeling of the patient and is the subject of a subjective examination."}, "sentence_sentiment": {"answer": "Positive", "rationale": "Because the act of \"creative thinking\" in the entry is positive. While \"forgetting time\" may be negative, in this context it expresses a high degree of individual commitment to and focus on creative activity and can therefore be considered positive emotional tendencies."}, "language_style": {"answer": "Tactful and warm", "rationale": ""}, "time_dimension": {"answer": "Present", "rationale": ""}, "sentence_type": "Affirmative declarative sentence", "pos": {"nouns": {"Creative thinking activities": 1, "Time": 1}, "verbs": {"Conduct": 1, "Forget it": 1}, "adverbs": {"Regular": 1}, "numerals": {"9": 1}}}, {"content": "(10) The ability to identify problems on a proactive basis and to identify relationships related to them.", "word_count": 20, "label": "", "sentence_structure": "Subject鈥損redicate", "subjective_objective": {"answer": "Subjective", "rationale": "Because \"can find problems\" and \"discover relationships\" are the expression of the patient's abilities and attitudes, and are part of his subjective feelings."}, "sentence_sentiment": {"answer": "Positive", "rationale": "Because the act of \"proactive detection of problems and the ability to identify relationships associated with them\" in the entry is positive."}, "language_style": {"answer": "Tactful and warm", "rationale": "The entry is more euphemistic, using the words \"can\" and \"and\" to express the request without direct orders or toughness. At the same time, it contains an affirmation of individual capabilities and encouragement, such as \"proactive detection of problems\", which implies that the respondent is more willing to answer the questions, thus improving the effectiveness of the questionnaire."}, "time_dimension": {"answer": "Present", "rationale": "The entry describes the ability of the testee to act, i.e. whether they can proactively identify and identify the relevance of the problem. This is an entry to assess the existing behaviour characteristics of the testee, so the temporal dimension is now judged."}, "sentence_type": "Affirmative declarative sentence", "pos": {"nouns": {"Problem": 1, "Relations": 1}, "verbs": {"Found": 1, "Find out": 1, "Yes": 2}, "adjectives": {"Initiatives": 1}, "numerals": {"10": 1}}}, {"content": "(11) Except for everyday life, most of the time is spent in school.", "word_count": 19, "label": "", "sentence_structure": "Subject鈥搗erb鈥搊bject", "subjective_objective": {"answer": "Objective", "rationale": "This is an objective fact, as it is a case of activities outside the daily life of the patient."}, "sentence_sentiment": {"answer": "Positive", "rationale": "Because this entry expresses positive behaviour and attitudes of the individual."}, "language_style": {"answer": "Tactful and warm", "rationale": "The language style of the entry is more euphemistic, less direct or command-style, but expressed in an invitation and inquiry, which makes it more friendly."}, "time_dimension": {"answer": "Present", "rationale": "The entries \"in time\" and \"in time of study\" describe the current situation of the person being tested or the customary behaviour, and can therefore be judged as questioning the current situation of the person being tested."}, "sentence_type": "Affirmative declarative sentence", "pos": {"nouns": {"Life": 1, "Book studies": 1}, "verbs": {"in": 1, "Read it": 1, "Learn": 1}, "adverbs": {"Normal": 1, "Most of the time": 1}, "numerals": {"11": 1}}}, {"content": "(12) Always curious about what's around.", "word_count": 11, "label": "", "sentence_structure": "Subject鈥搗erb鈥揷omplement", "subjective_objective": {"answer": "Subjective", "rationale": "Because \"acceptance\" is the patient's personal subjective attitude and feelings."}, "sentence_sentiment": {"answer": "Positive", "rationale": "Because the \"always curious\" attitude in the entries is positive."}, "language_style": {"answer": "Tactful and warm", "rationale": "The reasons for this are as follows: - The entry is more personal and interactive in its expression of a moderate and inviting tone rather than a direct statement or order; - \"Sustainful curiosity\" is a positive and positive term in describing the characteristics of the subject under investigation, without any negative terminology or judgement of the subject, which is in keeping with the euphemism and warm language style."}, "time_dimension": {"answer": "Present", "rationale": "The entry describes the current behaviour or attitude of the testee, the word \"always\" indicating that it is a customary act, while \"acceptance\" is a state or psychological characteristic that indicates the condition of the testee at the moment. The entry should therefore be classified as the present behaviour or attitude."}, "sentence_type": "Affirmative declarative sentence", "pos": {"nouns": {"Around things": 1, "Curiosity": 1}, "adverbs": {"Total": 1}, "numerals": {"12": 1}}}, {"content": "(13) When a problem is discovered, there is always a great deal of excitement.", "word_count": 20, "label": "", "sentence_structure": "Subject鈥搗erb鈥揷omplement", "subjective_objective": {"answer": "Subjective", "rationale": "Because \"new discovery\" and \"excited\" are the feelings and experiences inside the patient, and they are subjective."}, "sentence_sentiment": {"answer": "Positive", "rationale": "Because the words \"new discovery\" and \"abnormal excitement\" in the entries are positive, expressing a positive attitude and emotion."}, "language_style": {"answer": "Tactful and warm", "rationale": "The entry is more personal and interactive, by speaking in a gentle and inviting manner, rather than by direct statement or order. At the same time, the entry uses the term \"mental\" to give people a deeper, more personal feeling and to be more resonant with the person under investigation."}, "time_dimension": {"answer": "Present", "rationale": "The keywords \"when you find out\" and \"abnormally excited\" indicate that the act is happening, so it's now."}, "sentence_type": "Affirmative declarative sentence", "pos": {"nouns": {"Problem": 1, "Found": 1, "Spirit": 1, "Excited": 1}, "verbs": {"have": 1, "I feel": 1}, "adjectives": {"Unusual": 1}, "numerals": {"13": 1}}}, {"content": "(14) It is usually possible to predict the outcome of the event and to correctly validate it.", "word_count": 20, "label": "", "sentence_structure": "Subject鈥搗erb鈥搊bject", "subjective_objective": {"answer": "Subjective", "rationale": "This is because it is self-evaluation and cognitive competence of the patient and is subjective in nature."}, "sentence_sentiment": {"answer": "Positive", "rationale": "Because the expression \"can predict the results of something and can be correctly verified\" is a positive description of capability."}, "language_style": {"answer": "Tactful and warm", "rationale": "The entry is more softly phrased, dialogues are conducted in a question-and-answer manner, rather than direct statements or orders. This expression is more personal and interactive. At the same time, the entry does not use too complex or professional terms, making it easier for the person surveyed to understand."}, "time_dimension": {"answer": "Present", "rationale": ""}, "sentence_type": "Affirmative declarative sentence", "pos": {"nouns": {"Yes": 1, "Objects": 1, "Result": 1, "Authentication": 1}, "verbs": {"Projections": 1, "Yes": 2, "Correct": 1, "and": 1, "Here": 1}, "adverbs": {"Usually": 1}, "numerals": {"14": 1}}}, {"content": "(15) Even if there are difficulties and setbacks, there is no discouragement.", "word_count": 13, "label": "", "sentence_structure": "Subject鈥損redicate", "subjective_objective": {"answer": "Objective", "rationale": "Because it describes a person ' s character or belief, it is an intrinsic attribute of a person and is therefore objective."}, "sentence_sentiment": {"answer": "Positive", "rationale": "Because the entry \"problems and frustrations, and neither discourages\" is positive."}, "language_style": {"answer": "Calm and direct", "rationale": "The entry is more calm and objective in its language, and does not have too much emotional or rhetorical style. It clearly asks the respondents about their attitude in the face of difficulties and frustrations, and is very flat."}, "time_dimension": {"answer": "Future", "rationale": "The entry describes a state of expectation or of purpose that may occur in the future and therefore is judged to be future behaviour or mentality."}, "sentence_type": "Exclamatory sentence", "pos": {"nouns": {"Difficulties": 1, "Frustration": 1}, "verbs": {"I met him": 1, "I'm not discouraged": 1}, "adverbs": {"Even if": 1}}}, {"content": "(16) Thinking about new answers and new results.", "word_count": 14, "label": "", "sentence_structure": "Subject鈥搗erb鈥搊bject", "subjective_objective": {"answer": "Subjective", "rationale": "Because it's the patient's subjective attitude."}, "sentence_sentiment": {"answer": "Positive", "rationale": "Because the entry \"New answers and new results to think about things\" is positive."}, "language_style": {"answer": "Calm and direct", "rationale": "The entry directly expresses the subject of new answers and new results that ask each other whether they often think about things, without too much rhetoric or emotional rendering, and with a more objective tone."}, "time_dimension": {"answer": "Present", "rationale": "The entry uses the current \"thinking\" as it is, indicating that the act is in progress, so the temporal dimension is now judged."}, "sentence_type": "Affirmative declarative sentence", "pos": {"nouns": {"Think": 1, "Things": 1, "Answer": 1, "Result": 1}, "verbs": {"Regular": 1, "Think": 1}, "adverbs": {"Regular": 1}, "numerals": {"16": 1}}}, {"content": "(17) A keen observational and problem-solving capability.", "word_count": 17, "label": "", "sentence_structure": "Subject鈥搗erb鈥揷omplement", "subjective_objective": {"answer": "Subjective", "rationale": "Because a keen observation and the ability to ask questions are subjective assessments of the patient's ability to do so."}, "sentence_sentiment": {"answer": "Positive", "rationale": "According to the sentence, \"sensitivity\" and \"the ability to ask questions\" are positive qualities. The purpose of this questionnaire is therefore positive."}, "language_style": {"answer": "Tactful and warm", "rationale": "The entry is more personal and interactive when it is spoken in a gentle and inviting manner, rather than in direct statements or orders. At the same time, the purpose of the article is more modest and respectful of the other, without too much subjective assessment or emphasis on its own strengths, and makes it more comfortable and comfortable."}, "time_dimension": {"answer": "Present", "rationale": "The entry describes a capability or quality, does not clearly indicate the point of time, and can therefore be judged as now."}, "sentence_type": "Affirmative declarative sentence", "pos": {"nouns": {"Sensitivity": 1, "Observation": 1, "Questions raised": 1, "Capacity": 1}, "verbs": {"Yes": 1}, "adverbs": {"and": 1}, "numerals": {"17": 1}}}, {"content": "(18) Learning with a unique subject of study of its choice and with a unique method of discovery and research.", "word_count": 34, "label": "", "sentence_structure": "Subject鈥搗erb鈥揷omplement", "subjective_objective": {"answer": "Subjective", "rationale": "Because the questionnaire entry asks about the individual attitudes and behaviour of patients in the course of their studies, including their interests, choices, uniqueness and innovation, all of which are viewed from the patient ' s subjective perspective."}, "sentence_sentiment": {"answer": "Positive", "rationale": "Because the entries \"unique research subject\" and \"unique discovery and research method\" have positive emotional aspects."}, "language_style": {"answer": "Tactful and warm", "rationale": "The basis for the judgement: - The article is expressed in a relatively mild way, without too direct or command-style tone, but in an invitation-based way, which is more personal and interactive. - The descriptions of \"with a unique research subject of one's choice\" and \"with a unique method of discovery and research of one's own making\" are all designed to guide the respondents to share their experiences and views rather than to impose a requirement on them to answer questions."}, "time_dimension": {"answer": "Present", "rationale": "The entry describes the behaviour or state of the person currently being studied, i.e., whether they have their own research topics and unique research methods, and therefore the temporal dimension is now judged."}, "sentence_type": "Imperative sentence", "pos": {"nouns": {"Learning": 1, "Unique research topics": 1, "Find Methods": 1, "Research methodology": 1}, "verbs": {"have": 1, "Select": 1, "Adoption": 1}, "adjectives": {"It's unique to you": 1}, "numerals": {"18": 1}}}, {"content": "(19) When confronted with problems, possibilities are often explored in many ways, rather than being fixed in one line of thought or limited in one aspect.", "word_count": 34, "label": "", "sentence_structure": "Subject鈥損redicate", "subjective_objective": {"answer": "Subjective", "rationale": "Because the words \"often\" and \"not\" indicate that it's the way people think and the attitude they have."}, "sentence_sentiment": {"answer": "Positive", "rationale": "Because the \"diverse possibilities\" act in the entry is positive."}, "language_style": {"answer": "Tactful and warm", "rationale": "The entry is an encouraging and positive way of asking questions, expressing appreciation and acceptance of the interviewee鈥檚 approach to the problem. Such language styles enhance the interviewee鈥檚 positive mood, allow him to share his experience and views, and contribute to a good investigation atmosphere."}, "time_dimension": {"answer": "Present", "rationale": "The words \"in the face of problems\" and \"often in many ways\" in this entry, rather than being fixed in one line or limited in one aspect, indicate a description of the present behaviour and are therefore judged to be the present."}, "sentence_type": "Affirmative declarative sentence", "pos": {"nouns": {"Problem": 1, "In terms of": 2, "Possibilities": 1, "Thinking": 1, "Limits": 1, "In a certain way": 1}, "verbs": {"I met him": 1, "- I'm not sure": 1, "Explore": 1, "Fixed": 1}, "adverbs": {"More": 1}, "numerals": {"19": 1}}}, {"content": "(20) There are always new ideas that emerge in the brain and that can emerge even when playing.", "word_count": 24, "label": "", "sentence_structure": "Subject鈥搗erb鈥揷omplement", "subjective_objective": {"answer": "Subjective", "rationale": "Because this entry describes the experience and the idea inside the patient, that \"there is always a new idea in the brain,\" which needs to be examined through the patient's subjective perception. Although this idea may have arisen during a trip, it does not change its subjective nature. So, the view of this questionnaire entry is subjective."}, "sentence_sentiment": {"answer": "Positive", "rationale": "Because the expression \"there is always a new vision in the brain\" shows an emotional tendency towards innovative thinking and motivation."}, "language_style": {"answer": "Tactful and warm", "rationale": ""}, "time_dimension": {"answer": "Present", "rationale": ""}, "sentence_type": "Affirmative declarative sentence", "pos": {"nouns": {"Brain": 1, "Ideas": 1}, "verbs": {"Out of the water": 1, "Generate": 1, "Play": 1}, "adverbs": {"There's always": 1, "In my head": 1, "Even if": 1, "And I can": 1, "New": 2}, "numerals": {"20": 1}}}]}

{"questionnaire_title": "State-of-the-art anxiety questionnaire.", "questionnaire_category": "Self-cognition", "item_count": 37, "emotion_shift_overall": "From positive to negative", "language_style_overall": "Neutral", "items": [{"content": "I feel calm.", "word_count": 7, "label": "", "sentence_structure": "Subject鈥揷opula鈥損redicative", "subjective_objective": {"answer": "Subjective", "rationale": "Because the mood is the patient's subjective feeling."}, "sentence_sentiment": {"answer": "Positive", "rationale": "Because \"single\" is a positive emotional experience."}, "language_style": {"answer": "Tactful and warm", "rationale": "The entry is more adept and interactive when it is spoken in a gentle and inviting manner, rather than by direct statements or orders."}, "time_dimension": {"answer": "Present", "rationale": ""}, "sentence_type": "Affirmative declarative sentence", "pos": {"nouns": {"The mood": 1}, "verbs": {"I feel": 1, "Calm down": 1}}}, {"content": "I feel safe.", "word_count": 5, "label": "", "sentence_structure": "Subject鈥揷opula鈥損redicative", "subjective_objective": {"answer": "Subjective", "rationale": "Because feeling safe is a subjective feeling."}, "sentence_sentiment": {"answer": "Positive", "rationale": "Because the description of \"fear safety\" expresses a positive emotional attitude."}, "language_style": {"answer": "Tactful and warm", "rationale": "The entry is more euphemistic, is more nuanced, uses a more active term, \"safe,\" and is presented in questionwords that are very nice and comfortable. This expression is better able to bring people closer to the respondents, improve their cooperation and their real willingness to answer."}, "time_dimension": {"answer": "Present", "rationale": ""}, "sentence_type": "Affirmative declarative sentence", "pos": {"nouns": {"Questionnaire": 1, "Entry": 1, "Clear": 1}, "verbs": {"I feel": 1}}}, {"content": "I'm nervous.", "word_count": 5, "label": "", "sentence_structure": "Subject鈥揷opula鈥損redicative", "subjective_objective": {"answer": "Subjective", "rationale": "Because \"stretch\" is a subjective feeling or attitude."}, "sentence_sentiment": {"answer": "Negative", "rationale": "Because \"stretch\" is a negative emotional word, which means an unpleasant emotional state."}, "language_style": {"answer": "Calm and direct", "rationale": "The entry directly expresses the subject of questioning the other party for stress, without excessive rhetoric or emotional rendering, and is more objective in tone."}, "time_dimension": {"answer": "Present", "rationale": "Based on the expression \"I am\" in the sentence, and the absence of clear time indicators (such as \"the past\", \"the future\", etc.), it is possible to judge that the question in the entry is the feeling of the subject at the current point in time, so the answer is \"now\"."}, "sentence_type": "Affirmative declarative sentence", "pos": {"verbs": {"be": 1}, "adjectives": {"Nervous": 1}}}, {"content": "I feel nervous and bound.", "word_count": 7, "label": "", "sentence_structure": "Subject鈥揷opula鈥損redicative", "subjective_objective": {"answer": "Subjective", "rationale": "Because \"fear\" is the patient's own subjective feelings and experiences."}, "sentence_sentiment": {"answer": "Negative", "rationale": "Because the feeling of \"stretching\" is negative."}, "language_style": {"answer": "Tactful and warm", "rationale": "But this is only a preliminary analysis, and further evaluation needs to be made in conjunction with specific questionnaire design and research objectives. For example, if this is a psychological questionnaire to assess people鈥檚 ability to cope with stress, then a calm and direct language style may be more appropriate; on the contrary, if this questionnaire is intended to help people alleviate stress or anxiety, then a soft and warmer language style may be more likely to attract the participants."}, "time_dimension": {"answer": "Present", "rationale": "The keyword \"I feel\" indicates that it's the subjective feeling of the testee now, and \"stretching\" is the state or characteristic of the feeling, which is judged by the temporal dimension of this entry as now."}, "sentence_type": "Negative declarative sentence", "pos": {"nouns": {"I": 1, "Nervous": 1, "Tie": 1}, "verbs": {"I feel": 1}, "adjectives": {"Nervous": 1, "Tie": 1}}}, {"content": "I feel comfortable.", "word_count": 5, "label": "", "sentence_structure": "Subject鈥揷opula鈥損redicative", "subjective_objective": {"answer": "Subjective", "rationale": "Because \"fEs\" is a subjective feeling of the patient, the entry is viewed from the patient's subjective perspective."}, "sentence_sentiment": {"answer": "Positive", "rationale": "Because \"easy\" is a positive word, satisfaction, comfort."}, "language_style": {"answer": "Tactful and warm", "rationale": "The language style of this questionnaire entry is relatively euphemistic and implicit. It does not directly question or order the interviewee, but rather expresses its concern and understanding of the interviewee's emotional state in a more moderate way. Moreover, it does not use excessive rhetorical techniques or strong emotional colours to give people a relatively calm and comfortable feeling."}, "time_dimension": {"answer": "Present", "rationale": "The keyword is \"fear,\" which means that the state of affairs is now being or is experiencing. At the same time, \"easy\" also implies the current emotional state of the subject, so the temporal dimension of the article is now judged."}, "sentence_type": "Affirmative declarative sentence", "pos": {"verbs": {"I feel": 1, "Easy": 1}, "adjectives": {"Easy": 1}}}, {"content": "I'm so upset.", "word_count": 5, "label": "", "sentence_structure": "Subject鈥揷opula鈥損redicative", "subjective_objective": {"answer": "Subjective", "rationale": "Because \"disturbing\" is an emotional reaction or subjective feeling."}, "sentence_sentiment": {"answer": "Negative", "rationale": "Because \"disturbing\" is a negative emotional experience, a discomfortful state."}, "language_style": {"answer": "Calm and direct", "rationale": "The entry uses a direct, concise language format to ask the respondents about their emotional state, without excessive emotional modification or rhetorical techniques, and is more calm and straightforward."}, "time_dimension": {"answer": "Present", "rationale": ""}, "sentence_type": "Negative declarative sentence", "pos": {"verbs": {"I feel": 1, "Disturbing": 1}, "adjectives": {"Disturbing": 1}}}, {"content": "I'm now in a lot of trouble, and I feel more trouble than I could possibly be.", "word_count": 20, "label": "", "sentence_structure": "Subject鈥搗erb鈥揷omplement", "subjective_objective": {"answer": "Subjective", "rationale": "Because it is more unfortunate than possible that the patient is subjectively felt and evaluated."}, "sentence_sentiment": {"answer": "Negative", "rationale": "Because \"fear\" and \"more than possible misfortune\" both express negative feelings."}, "language_style": {"answer": "Calm and direct", "rationale": "This entry expresses directly whether the person under investigation is experiencing trouble and clearly indicates that it is more than likely. This expression is more objective and direct, and does not have too much emotional or rhetorical style."}, "time_dimension": {"answer": "Present", "rationale": "And \"the feeling of this distress is more unfortunate than possible\" is also a description of the current feelings or emotions, which are also the current state of affairs. So the answer is \"now\"."}, "sentence_type": "Negative declarative sentence", "pos": {"nouns": {"Questionnaire": 1, "Entry": 1, "I'm here": 1, "Trouble": 2, "Unfortunately": 1}, "verbs": {"Positive": 1, "More than": 1, "I feel": 1}, "adjectives": {"Possible": 1}, "numerals": {"7": 1}}}, {"content": "8. I'm satisfied.", "word_count": 5, "label": "", "sentence_structure": "Subject鈥揷opula鈥損redicative", "subjective_objective": {"answer": "Subjective", "rationale": "Because \"satisfaction\" is a subjective feeling and evaluation of the patient, and a subjective observation."}, "sentence_sentiment": {"answer": "Positive", "rationale": "Because the \"satisfaction\" attitude is positive."}, "language_style": {"answer": "Calm and direct", "rationale": "The entry is simple and simple, and the person surveyed is asked whether he or she is satisfied with something, is not over-exposed or euphemisms, and therefore can be judged to be \"cool and direct\"."}, "time_dimension": {"answer": "Present", "rationale": "The entry indicates the person who was tested at the moment of the current response to the questionnaire and therefore is judged to be \"now\"."}, "sentence_type": "Affirmative declarative sentence", "pos": {"verbs": {"I feel": 1, "Satisfied": 1}}}, {"content": "I'm scared.", "word_count": 5, "label": "", "sentence_structure": "Subject鈥揷opula鈥損redicative", "subjective_objective": {"answer": "Subjective", "rationale": "/Objective: Subjective. Because \"fear\" is a subjective emotional experience, reflecting patients' subjective feelings and reactions to a situation or thing. Although fear may be related to reality, it is subjective in itself, and cannot be determined by objective observation."}, "sentence_sentiment": {"answer": "Negative", "rationale": "Because \"fear\" is negative."}, "language_style": {"answer": "Calm and direct", "rationale": "The entry directly asked the respondents whether they were afraid, and not using excessive acrimonyms or emotional expressions, which were more objective and direct."}, "time_dimension": {"answer": "Present", "rationale": "The main word \"I'm afraid\" is \"I\" and it means that the verb \"I feel\" is the general current, meaning that it not only exists in the past but may still be present. Thus, the entry is in the current state of inquiry into the subject, judging by the present."}, "sentence_type": "Negative declarative sentence", "pos": {"nouns": {"Scared": 1}, "verbs": {"Sensor": 1, "Present": 1}}}, {"content": "I'm comfortable.", "word_count": 5, "label": "", "sentence_structure": "Subject鈥揷opula鈥損redicative", "subjective_objective": {"answer": "Subjective", "rationale": "Because \"comfort\" is the patient's subjective feeling."}, "sentence_sentiment": {"answer": "Neutral", "rationale": "Because the entry is not emotional, it is a description of a fact."}, "language_style": {"answer": "Tactful and warm", "rationale": "The language style of this entry is euphemism. It uses an invitation tone to ask if the other side is comfortable in certain circumstances, is not in a direct command or criticism, is more friendly and interactive. This gentle and friendly way makes it easier for the interviewee to open his heart and express his feelings."}, "time_dimension": {"answer": "Present", "rationale": "According to the sentence entered, \"comfort\" is a description of the person's current feelings, which does not relate to the past or the future time dimension, and therefore is judged as \"now\"."}, "sentence_type": "Affirmative declarative sentence", "pos": {"verbs": {"I feel": 1, "Comfortable": 1}}}, {"content": "I'm confident.", "word_count": 5, "label": "", "sentence_structure": "Subject鈥搗erb鈥揷omplement", "subjective_objective": {"answer": "Subjective", "rationale": "Because self-confidence is a subjective feeling and attitude of the individual."}, "sentence_sentiment": {"answer": "Positive", "rationale": "Because self-confidence is usually seen as a positive quality or attitude."}, "language_style": {"answer": "Tactful and warm", "rationale": "The language style of the entry is more gentle and friendly, without the use of too cold or direct language, giving people a friendly and comfortable feeling. At the same time, \"confidence\" is a positive and positive term that is encouraging and encouraging, further reflecting the emotional tendencies expressed in the entry."}, "time_dimension": {"answer": "Present", "rationale": "The entry expresses the person's current subjective perception or state of affairs, and the keyword \"there\" indicates existence, possession, and is the present state of affairs."}, "sentence_type": "Affirmative declarative sentence", "pos": {"nouns": {"Questionnaire": 1, "Entry": 1, "Confidence": 1}, "verbs": {"have": 1, "Here": 1}, "numerals": {"11": 1}, "classifiers": {"individual": 1}, "pronouns": {"I": 2}}}, {"content": "I'm very sensitive.", "word_count": 7, "label": "", "sentence_structure": "Subject鈥揷opula鈥損redicative", "subjective_objective": {"answer": "Subjective", "rationale": "Because the word \"thinks\" suggests that it's a subjective feeling or perception of the patient."}, "sentence_sentiment": {"answer": "Negative", "rationale": "Because \"sensitization\" in the entries is usually considered a negative emotional or psychological state."}, "language_style": {"answer": "Calm and direct", "rationale": "The entry directly expresses the subject of questioning the other party in a concise language, without excessive rhetorical or emotional rendering, and with a more objective tone."}, "time_dimension": {"answer": "Past", "rationale": ""}, "sentence_type": "Affirmative declarative sentence", "pos": {"nouns": {"You're a bit of a sane": 1}, "verbs": {"I think": 1}, "numerals": {"12": 1}}}, {"content": "I'm extremely nervous.", "word_count": 7, "label": "", "sentence_structure": "Subject鈥揷opula鈥損redicative", "subjective_objective": {"answer": "Subjective", "rationale": "Because \"extremely nervous\" describes the feelings and experiences inside the patient, and it is a subjective emotional state."}, "sentence_sentiment": {"answer": "Negative", "rationale": "Because the \"extremely nervous\" act in the entry is negative."}, "language_style": {"answer": "Calm and direct", "rationale": "The entry expresses the question of the psychological state of the person under investigation in a direct and concise language, without excessive rhetorical or emotional rendering, so that the language style is \"cool and direct\"."}, "time_dimension": {"answer": "Present", "rationale": "\"Extra-heavy stress\" describes the current feelings or state of the testee, and thus judges it now."}, "sentence_type": "Negative declarative sentence", "pos": {"nouns": {"I": 1, "Nervous": 1, "Unsettled": 1}, "verbs": {"Extreme": 1, "be": 1}, "adverbs": {"Extreme": 1}, "numerals": {"13": 1}}}, {"content": "I'm indecisive.", "word_count": 5, "label": "", "sentence_structure": "Subject鈥搗erb鈥揷omplement", "subjective_objective": {"answer": "Subjective", "rationale": "Because \"indecisiveness\" is a description of subjective attitudes or character characteristics that reflect individual feelings and perceptions."}, "sentence_sentiment": {"answer": "Negative", "rationale": "Basis of judgement: \"Empower indecisiveness\" in this entry is often considered to be negative character because it indicates that individuals may hesitate to make decisions or lack decisiveness."}, "language_style": {"answer": "Tactful and warm", "rationale": "The language style of the entry is more moderate and friendly, and rather than directly accusing or criticizing the person under investigation, questions are asked in an understanding and supportive manner, which makes it easier to gain the resonance and trust of the person under investigation."}, "time_dimension": {"answer": "Present", "rationale": "The main word in the entry, \"I\" and the verb \"Empowerful Indecisive\", indicate that this act or state exists at the moment and is therefore judged to be the present."}, "sentence_type": "Negative declarative sentence", "pos": {"nouns": {"I": 1, "Indecisiveness": 1}, "adjectives": {"Indecisiveness": 1}}}, {"content": "I'm easy.", "word_count": 5, "label": "", "sentence_structure": "Subject鈥揷opula鈥損redicative", "subjective_objective": {"answer": "Subjective", "rationale": "Because \"Easy\" is a subjective feeling or attitude, it describes the patient's own feelings and mental state. This feeling can be influenced by many factors, including the individual's personality, experience, environment, etc. So, looking at the entry \"I'm Easy\" requires understanding the patient's subjective perspective of what they feel about themselves and how they feel about them."}, "sentence_sentiment": {"answer": "Positive", "rationale": "Because \"easy\" is a positive, positive emotional description."}, "language_style": {"answer": "Tactful and warm", "rationale": "The entry is more personal and interactive in its expression of a gentle and inviting tone, rather than a direct statement or order. At the same time, the expression \"I am easy\" is more subjective, may require the respondent to self-assess his emotional state and is therefore more appropriate to raise questions in a soft and warm language style."}, "time_dimension": {"answer": "Present", "rationale": "The entry describes the person who was tested in his current state or feeling, which is \"I am easy.\" So the time dimension is now."}, "sentence_type": "Affirmative declarative sentence", "pos": {"verbs": {"be": 1}, "adjectives": {"Easy": 1}}}, {"content": "I'm satisfied.", "word_count": 7, "label": "", "sentence_structure": "Subject鈥揷opula鈥損redicative", "subjective_objective": {"answer": "Subjective", "rationale": "Because \"satisfaction\" is a subjective feeling, and it is the patient's assessment and perception of his or her status, not an objective fact or behaviour."}, "sentence_sentiment": {"answer": "Positive", "rationale": "Because \"satisfaction\" expresses a positive emotional attitude."}, "language_style": {"answer": "Tactful and warm", "rationale": "The entry is more proficient and interactive when it is spoken in a gentle and inviting manner, rather than in direct statements or orders. At the same time, \"satisfaction\" is a subjective assessment that is more able to channel the interviewee's natural feelings into reality and thus to obtain more accurate information."}, "time_dimension": {"answer": "Present", "rationale": "The keyword \"I feel\" indicates that it is the person who was tested, and \"satisfaction\" indicates that it is a psychological experience, so the entry asks about the person's current state when he fills out the questionnaire."}, "sentence_type": "Affirmative declarative sentence", "pos": {"verbs": {"I feel": 1, "Satisfied": 1}, "adjectives": {"I'm satisfied": 1}}}, {"content": "I'm a pain in the ass.", "word_count": 5, "label": "", "sentence_structure": "Subject鈥揷opula鈥損redicative", "subjective_objective": {"answer": "Subjective", "rationale": "Because trouble is a subjective feeling and experience of one."}, "sentence_sentiment": {"answer": "Negative", "rationale": "Because the word \"fear\" in the entry is a negative emotional word."}, "language_style": {"answer": "Tactful and warm", "rationale": ""}, "time_dimension": {"answer": "Present", "rationale": ""}, "sentence_type": "Exclamatory sentence", "pos": {"nouns": {"I'm a pain in the ass": 1}}}, {"content": "I was in a panic.", "word_count": 5, "label": "", "sentence_structure": "Subject鈥揷opula鈥損redicative", "subjective_objective": {"answer": "Subjective", "rationale": "Because \"fEs\" is the patient's subjective feeling."}, "sentence_sentiment": {"answer": "Negative", "rationale": "Because \"frightful\" expresses a negative emotional state."}, "language_style": {"answer": "Calm and direct", "rationale": ""}, "time_dimension": {"answer": "Present", "rationale": ""}, "sentence_type": "Negative declarative sentence", "pos": {"nouns": {"Panic": 1}, "verbs": {"I feel": 1}}}, {"content": "I feel calm.", "word_count": 5, "label": "", "sentence_structure": "Subject鈥揷opula鈥損redicative", "subjective_objective": {"answer": "Subjective", "rationale": "Because \"feel\" is a subjective feeling, it's the individual perception and description of the emotional state of the patient."}, "sentence_sentiment": {"answer": "Neutral", "rationale": "Because the entry is merely an expression of feelings, without any apparent emotional tendency."}, "language_style": {"answer": "Tactful and warm", "rationale": "The entry uses a more euphemistic and moderate language style, using descriptive terms such as \"feel\" to express the subjective feelings of the person under investigation, which are more focused on emotional communication and resonance with the person under investigation. At the same time, the entire sentence is more relaxed and comfortable, without strong guidance and stress, and more respectful of the person under investigation's right to free reply and privacy protection."}, "time_dimension": {"answer": "Present", "rationale": "The keyword \"feel\" and \"settling\" both indicate the current psychological state and feelings of the person being tested, so the entry is to ask the person who was being tested about his or her current psychological condition."}, "sentence_type": "Affirmative declarative sentence", "pos": {"verbs": {"Feel": 1, "Calm down": 1}}}, {"content": "I'm happy.", "word_count": 5, "label": "", "sentence_structure": "Subject鈥揷opula鈥損redicative", "subjective_objective": {"answer": "Subjective", "rationale": "Because happiness is a subjective or emotional experience of the patient."}, "sentence_sentiment": {"answer": "Positive", "rationale": "Because the entry \"happy\" expresses a positive sentiment."}, "language_style": {"answer": "Tactful and warm", "rationale": "The language style of the entry is a warm and euphemistic type. It raises questions in a positive and encouraging way about the pleasure of the interviewee. This expression makes the interviewee more willing to share his/her feelings and does not make them feel stressed or uncomfortable."}, "time_dimension": {"answer": "Past", "rationale": "The word \"feel\" in \"I am happy\" indicates that the person being tested is experiencing an emotional state that may be a feeling of the past, the present or the future. So, \"favorite\" may be a sense of past, or of present, or a expectation of future."}, "sentence_type": "Affirmative declarative sentence", "pos": {"verbs": {"I feel": 1, "Have a nice day": 1}}}, {"content": "I'm happy. I'm sensitive and uneasy.", "word_count": 14, "label": "", "sentence_structure": "Subject鈥揷opula鈥損redicative", "subjective_objective": {"answer": "Subjective", "rationale": "None"}, "sentence_sentiment": {"answer": "Positive", "rationale": "I was happy and positive, indicating that the interviewees had a pleasant emotional experience."}, "language_style": {"answer": "Tactful and warm", "rationale": "The language style of the two entries is more euphemistic and gentle, not so much direct or hard-line as to lead a conversation by asking each other about their emotions, and being more friendly and friendly. Such expression makes it easier for the interviewee to feel relaxed and comfortable, and thus more willing to share his real ideas."}, "time_dimension": {"answer": "Past", "rationale": "Both sentences describe the current emotional state of the testee, without clearly indicating whether it is the past or the present state, and therefore cannot determine the time dimension. The first sentence expresses the happy state of the testee, while the second sentence expresses the sensitive and uneasy state of the testee."}, "sentence_type": "Affirmative declarative sentence", "pos": {"verbs": {"I feel": 2, "Have a nice day": 1, "You're a bit of a sane": 1, "Unsettled": 1}, "adjectives": {"Have a nice day": 1, "You're a bit of a sane": 1, "Unsettled": 1}, "numerals": {"21": 1, "22": 1}}}, {"content": "I feel satisfied. I wish I was as happy as anyone else.", "word_count": 18, "label": "", "sentence_structure": "Subject鈥搗erb鈥搊bject", "subjective_objective": {"answer": "Subjective", "rationale": "Because \"fulfilment\" and \"hope to be as happy as anyone\" are subjective feelings and expectations of patients, these two questions are examined from a subjective perspective."}, "sentence_sentiment": {"answer": "Positive", "rationale": "Because it expresses satisfaction with the status quo."}, "language_style": {"answer": "Calm and direct", "rationale": "23. I am satisfied that this sentence is a straightforward expression of the satisfaction of the interviewees with their status quo, without too much rhetoric or emotional overtones; I hope to be as happy as others, and as an expression of the expectations and objectives of the interviewees, with more objective and peaceful rhetoric."}, "time_dimension": {"answer": "Present", "rationale": "The time dimensions of the entries \"I'm content\" and \"I'm happy as others\" are in the present state, so they all belong to the present."}, "sentence_type": "[Affirmative declarative sentence, Imperative sentence]", "pos": {"nouns": {"I": 2, "Self-satisfaction": 1, "others": 1, "Happy": 1}, "verbs": {"I feel": 1, "Hope": 1}, "adverbs": {"Elephant": 1}, "numerals": {"23": 1, "24": 1}}}, {"content": "I feel like I'm failing.", "word_count": 9, "label": "", "sentence_structure": "Subject鈥揷opula鈥損redicative", "subjective_objective": {"answer": "Subjective", "rationale": "Because \"fEat\" is a subjective feeling."}, "sentence_sentiment": {"answer": "Negative", "rationale": "Because the term \"defeating\" expresses a negative, weak feeling, the emotional colour of the purpose of the article is negative."}, "language_style": {"answer": "Calm and direct", "rationale": "The entry expresses a sense or situation in a concise manner, without excessive rhetoric or emotional colours, and is more objective and direct."}, "time_dimension": {"answer": "Present", "rationale": "The entry describes the current feelings and state of the person being tested, does not address past or future behaviour or mentalities, etc., and therefore the temporal dimension is now judged."}, "sentence_type": "Affirmative declarative sentence", "pos": {"nouns": {"Questionnaire": 1, "Entry": 1, "25": 1, "I": 1, "Decay": 1}, "verbs": {"I feel": 1, "Elephant": 1, "Sample": 1}, "adjectives": {"Decay": 1}, "numerals": {"25": 1}, "pronouns": {"I": 1}}}, {"content": "I feel calm.", "word_count": 6, "label": "", "sentence_structure": "Subject鈥揷opula鈥損redicative", "subjective_objective": {"answer": "Subjective", "rationale": "Because \"settling\" is a subjective feeling."}, "sentence_sentiment": {"answer": "Positive", "rationale": "Because \"settling\" is a positive emotional experience."}, "language_style": {"answer": "Tactful and warm", "rationale": "The entry is a soft and inviting tone, which allows respondents to feel respected and understood and encourages them to share their feelings. Moreover, the entry does not use the tone of a direct statement or order, but rather communicates with the interviewee in a more intimate and interactive way, making it more acceptable and willing to participate in the questionnaire."}, "time_dimension": {"answer": "Present", "rationale": ""}, "sentence_type": "Affirmative declarative sentence", "pos": {"nouns": {"Questionnaire": 1, "Entry": 1, "Peace": 1}, "verbs": {"I feel": 1}, "adjectives": {"very": 1}, "numerals": {"26": 1}}}, {"content": "I'm calm, calm and purturistic.", "word_count": 14, "label": "", "sentence_structure": "Subject鈥搗erb鈥揷omplement", "subjective_objective": {"answer": "Subjective", "rationale": "The reason for this is that calm, calm and tact are subjective assessments of the patient."}, "sentence_sentiment": {"answer": "Neutral", "rationale": "Because the entry does not have any emotional color."}, "language_style": {"answer": "Calm and direct", "rationale": "The entry expresses a character character in a positive statement, with no excessive emotional colour or qualifier, and a more concise language style."}, "time_dimension": {"answer": "Present", "rationale": ""}, "sentence_type": "Affirmative declarative sentence", "pos": {"nouns": {"I": 1, "Calm down": 2, "I'm glad": 1}, "adjectives": {"be": 1}, "adverbs": {"of": 4}, "numerals": {"27": 1}}}, {"content": "I felt a lot of difficulties, so I couldn't overcome it.", "word_count": 17, "label": "", "sentence_structure": "Subject鈥搗erb鈥揷omplement", "subjective_objective": {"answer": "Subjective", "rationale": "Because I feel difficult, it's the patient's own subjective feelings and experiences."}, "sentence_sentiment": {"answer": "Negative", "rationale": "The basis for the judgment is that the entry \"unresolved\" expresses negative attitudes and emotions."}, "language_style": {"answer": "Calm and direct", "rationale": "The language style of the entry is more straightforward, with no excessive embellishment or emotional rendering, and it is more empirical and objective in expressing the feelings and challenges of the subject in the face of the difficulties."}, "time_dimension": {"answer": "Present", "rationale": "The term \"sense\" and \"insuperable\" in this questionnaire entry indicates the current state of feelings and behaviour of the subjects, and thus the temporal dimension of the article can be judged to be the present."}, "sentence_type": "Negative declarative sentence", "pos": {"nouns": {"Difficulties": 1, "Stack": 1, "Overcoming": 1}, "verbs": {"I feel": 1, "Jack": 1, "And so": 1, "Cannot": 1}, "adverbs": {"One": 1, "Stack": 1, "Get up": 1, "And so": 1, "Cannot": 1}, "numerals": {"28": 1}, "pronouns": {"I": 1}}}, {"content": "I've been over-considered. It's not really important.", "word_count": 17, "label": "", "sentence_structure": "Subject鈥揷opula鈥損redicative", "subjective_objective": {"answer": "Subjective", "rationale": "Because \"excessive care\" and \"innocent\" are subjective evaluations and judgements of the patient."}, "sentence_sentiment": {"answer": "Negative", "rationale": "Because both the descriptions of \"excessive care\" and \"insensitive\" in the entries are negative emotional."}, "language_style": {"answer": "Calm and direct", "rationale": "The whole expression is more moderate and acceptable, giving a sense of concern and understanding."}, "time_dimension": {"answer": "Present", "rationale": ""}, "sentence_type": "Negative declarative sentence", "pos": {"nouns": {"29": 1, "Things": 1}, "verbs": {"I'm too careful": 1, "Actual": 1, "It doesn't matter": 1}, "adjectives": {"Some": 1, "It's important": 1}, "adverbs": {"Too much": 1}, "pronouns": {"These": 1}}}, {"content": "I'm happy.", "word_count": 5, "label": "", "sentence_structure": "Subject鈥揷opula鈥損redicative", "subjective_objective": {"answer": "Subjective", "rationale": "Because \"happiness\" is a subjective feeling or attitude."}, "sentence_sentiment": {"answer": "Positive", "rationale": "Because the \"happiness\" in the entry is a positive emotional attitude."}, "language_style": {"answer": "Tactful and warm", "rationale": "The language style of the entry is more euphemistic, more compassionate, and in a light, more positive way, it is more friendly and friendly to direct the respondents to the questions."}, "time_dimension": {"answer": "Present", "rationale": "The entry shows the status or feelings of the respondent in reply to the questionnaire, and the time judgement is now."}, "sentence_type": "Affirmative declarative sentence", "pos": {"verbs": {"be": 1, "Happy": 1}, "adjectives": {"Happy": 1}}}, {"content": "My mind is in disarray.", "word_count": 10, "label": "", "sentence_structure": "Subject鈥揷opula鈥損redicative", "subjective_objective": {"answer": "Subjective", "rationale": "Because \"my mind is in disarray\" is a patient's description of his subjective feelings."}, "sentence_sentiment": {"answer": "Negative", "rationale": "Because \"dreaming\" is often considered a negative state of mind, indicating that there are problems with the ability of individuals to think, understand or make decisions."}, "language_style": {"answer": "Tactful and warm", "rationale": "The language style of the entry is more euphemistic and moderate, with little use of too direct or harsh a vocabulary. By using a description like \"disorder\" it is possible to express the feelings of the respondents more objectively than to directly blame or criticize their problems. This expression is more respectful of the respondents and helps to create a good atmosphere of dialogue."}, "time_dimension": {"answer": "Present", "rationale": "In terms of time dimension, \"my mind is in disarray\" is a description of the current state of mind of the testee, so it's now."}, "sentence_type": "Negative declarative sentence", "pos": {"nouns": {"Ideas": 1, "Status": 1}, "verbs": {"in": 1, "Chaos": 1}, "numerals": {"31": 1}}}, {"content": "I lack confidence.", "word_count": 6, "label": "", "sentence_structure": "Subject鈥揷opula鈥損redicative", "subjective_objective": {"answer": "Subjective", "rationale": "Because lack of self-confidence is a subjective feeling and assessment within a person."}, "sentence_sentiment": {"answer": "Negative", "rationale": "Because \"lack of self-confidence\" is a negative attitude or quality."}, "language_style": {"answer": "Tactful and warm", "rationale": "The language style of the entry is more euphemistic, making the questionnaire more proficient and inclusive by asking if the other side felt \"lack of confidence\" rather than directly accusing or evaluating. At the same time, the more common question-and-answer style is used, avoiding too rigid a narrative, making the questionnaire more acceptable to the respondents."}, "time_dimension": {"answer": "Present", "rationale": "The entry describes the current state or feeling of the testee, the keywords being \"I lack\" and \"I have confidence\" which point to the actual situation."}, "sentence_type": "Negative declarative sentence", "pos": {"nouns": {"Confidence": 1, "Heart": 1}, "verbs": {"Lack": 1}, "numerals": {"32": 1}}}, {"content": "I feel safe.", "word_count": 5, "label": "", "sentence_structure": "Subject鈥揷opula鈥損redicative", "subjective_objective": {"answer": "Subjective", "rationale": "Because feeling safe is a subjective feeling and experience."}, "sentence_sentiment": {"answer": "Positive", "rationale": "Because \"felt safe\" in the entry is positive emotional."}, "language_style": {"answer": "Calm and direct", "rationale": "\"Calm down straight.\""}, "time_dimension": {"answer": "Present", "rationale": "The entry describes the current emotional state and feelings of the testee, and therefore the temporal dimension is now judged."}, "sentence_type": "Affirmative declarative sentence", "pos": {"nouns": {"Clear": 1}, "verbs": {"I feel": 1}}}, {"content": "I'm easy to make decisions.", "word_count": 7, "label": "", "sentence_structure": "Subject鈥搗erb鈥揷omplement", "subjective_objective": {"answer": "Subjective", "rationale": "Because \"I'm easy to make a decision\" is a patient's self-awareness and self-evaluation, reflecting his subjective attitude and opinion."}, "sentence_sentiment": {"answer": "Positive", "rationale": "Because \"deciding\" is positive."}, "language_style": {"answer": "Calm and direct", "rationale": "The entry expresses the content of the question in an objective and clear tone, without excessive rhetorical or emotional rendering, and with a more calm and straightforward language style."}, "time_dimension": {"answer": "Present", "rationale": "The entry asks the behaviour or characteristics of the person being tested at the moment, \"easy\" means frequent behaviour, and \"deciding\" is a current, ongoing exercise and therefore judgement is now."}, "sentence_type": "Affirmative declarative sentence", "pos": {"nouns": {"Decision": 1}, "verbs": {"Make": 1, "Easy": 1}, "adverbs": {"Easy": 1}, "numerals": {"34": 1}}}, {"content": "I don't feel right.", "word_count": 6, "label": "", "sentence_structure": "Subject鈥揷opula鈥損redicative", "subjective_objective": {"answer": "Subjective", "rationale": "Because the word \"fear\" reflects the feelings and feelings of the patient, and it's a subjective angle."}, "sentence_sentiment": {"answer": "Negative", "rationale": "Because \"unsuitable\" is usually considered as negative emotion or evaluation."}, "language_style": {"answer": "Calm and direct", "rationale": "The language style of the entry is straightforward, and it asks the other side whether it is often inappropriate, without excessive rhetorical or emotional rendering, and with a more balanced and objective tone."}, "time_dimension": {"answer": "Present", "rationale": "The key word in the entry, \"I feel\" means that the dominant language is feeling or experiencing an emotion or situation, and it is now judged in time."}, "sentence_type": "Negative declarative sentence", "pos": {"verbs": {"I feel": 1, "Suits": 1}, "adjectives": {"not": 1}}}, {"content": "I'm satisfied.", "word_count": 5, "label": "", "sentence_structure": "Subject鈥揷opula鈥損redicative", "subjective_objective": {"answer": "Subjective", "rationale": "\"I am satisfied\" is patient opinion and perception."}, "sentence_sentiment": {"answer": "Positive", "rationale": "Because \"fulfilment\" is a positive emotional word for individual satisfaction with life, work, etc."}, "language_style": {"answer": "Tactful and warm", "rationale": "The entry is more personal and interactive by using positive statements to express the satisfaction of the other party with life, being more moderate and active and not directly speaking or ordering."}, "time_dimension": {"answer": "Present", "rationale": "The entry uses the general current pattern of the first-person singular, which indicates that it is a subjective feeling or state description of the person who was tested when completing the questionnaire, and therefore is judged as \"now\"."}, "sentence_type": "Affirmative declarative sentence", "pos": {"verbs": {"be": 1, "Satisfied": 1}, "adjectives": {"Satisfied": 1}}}, {"content": "37. Some of the unimportant thoughts that haunt me and disturb me.", "word_count": 17, "label": "", "sentence_structure": "Subject鈥搗erb鈥揷omplement", "subjective_objective": {"answer": "Subjective", "rationale": "Because it's the patient's subjective feelings and experiences."}, "sentence_sentiment": {"answer": "Negative", "rationale": "Because the entry \"entangled me and disturbed me\" is negative."}, "language_style": {"answer": "Tactful and warm", "rationale": "The entry uses a gentle and caring tone, asking whether the interviewees are often troubled by irrelevant ideas, which is more easily comfortable and accepted, and can encourage them to share their true feelings."}, "time_dimension": {"answer": "Past", "rationale": "\"37\" indicates that it's a number or serial number, \"some of the insignificant ideas that haunt me and disturb me\" is the problem or feeling that the testee is experiencing, and the temporal dimension is the past."}, "sentence_type": "Negative declarative sentence", "pos": {"nouns": {"Ideas": 1, "I": 1}, "verbs": {"鈾?Crushing around 鈾?: 1, "Excuse me": 1}, "adjectives": {"It doesn't matter": 1}, "numerals": {"37": 1}}}, {"content": "38. The frustration I created was so great that I couldn't exclude them from my mind. I was a calm man.", "word_count": 32, "label": "", "sentence_structure": "Subject鈥揷opula鈥損redicative", "subjective_objective": {"answer": "Subjective", "rationale": "Because the descriptions of \"depression\" and \"can't be excluded from thought\" reflect the emotional experience of the patient."}, "sentence_sentiment": {"answer": "Negative", "rationale": "\"I am so depressed that I cannot exclude them from my mind\" is a negative emotional expression, a negative expression. \"I am a calm person\" is a positive expression of attitude and quality."}, "language_style": {"answer": "Calm and direct", "rationale": "According to the two entries entered, \"38. I create frustration so serious that I cannot exclude them from my mind\" and \"39. I am a calm person\" that both items are directly expressing the subject. The 38th entry expresses the degree of frustration of the interviewee, while the 39th entry expresses the calm character that the interviewee thinks it is. The language of both entries is flat and objective, without too many rhetoric or emotional accents, so it can be judged that the language style of the questionnaire is straightforward and calm."}, "time_dimension": {"answer": "Present", "rationale": "38. In the past, the entry described negative feelings that the subject had generated over time and thus judged the past.39 The entry now describes the present personality characteristics of the testee, namely, that he is a \"settled\" person, without clearly identifying past or future circumstances."}, "sentence_type": "[Negative declarative sentence]", "pos": {"nouns": {"Frustration": 1, "person": 1, "Ideas": 1}, "verbs": {"Generate": 1, "be": 2, "Yes": 1, "Exclude": 1}, "adjectives": {"Serious": 1, "Calm down": 1}, "numerals": {"38": 1, "39": 1}, "pronouns": {"I": 2}}}, {"content": "I was nervous when I thought about my current situation and interests.", "word_count": 22, "label": "", "sentence_structure": "Subject鈥搗erb鈥揷omplement", "subjective_objective": {"answer": "Subjective", "rationale": "Because \"stretch\" is a subjective feeling, it's the patient's psychological state and experience."}, "sentence_sentiment": {"answer": "Negative", "rationale": "Because the act of \"stretching into tension\" in the entry is negative."}, "language_style": {"answer": "Calm and direct", "rationale": "The language style of the entry is more straightforward, and there is no excessive emotional rendering or rhetorical technique. It asks whether the person surveyed is nervous when considering his current circumstances and interests, and whether the language is simple and straightforward."}, "time_dimension": {"answer": "Present", "rationale": ""}, "sentence_type": "Affirmative declarative sentence", "pos": {"nouns": {"Things": 1, "Interest": 1, "Status": 1}, "verbs": {"Consider": 1, "Into the": 1, "Nervous": 1}, "numerals": {"40": 1}, "pronouns": {"I": 1}}}]}

{"questionnaire_title": "Defense Mode Questionnaire (DSQ)", "questionnaire_category": "Behavioral questionnaire", "item_count": 88, "emotion_shift_overall": "From positive to negative", "language_style_overall": "Neutral", "items": [{"content": "I am satisfied by helping others, and if I do not do so I will become depressed.", "word_count": 26, "label": "", "sentence_structure": "Subject鈥搗erb鈥搊bject", "subjective_objective": {"answer": "Subjective", "rationale": "The entry is based on subjective perceptions and attitudes of the patient."}, "sentence_sentiment": {"answer": "Positive", "rationale": "Because the behavior in the entries \"help others\" and \"resatisfaction\" are positive."}, "language_style": {"answer": "Tactful and warm", "rationale": "Warm and warm."}, "time_dimension": {"answer": "Present", "rationale": "The entry describes an emotional state and behaviour of the person tested, and does not address a particular point of time in the future or in the past. Therefore, in terms of time dimension, the content of the entry should be in the current state of affairs."}, "sentence_type": "Affirmative declarative sentence", "pos": {"nouns": {"I": 2, "Others": 1, "Satisfied": 1, "not": 1, "Here": 1, "Just": 1, "Yes": 1, "鈾?And become 鈾?: 1, "Emotional depression": 1}, "verbs": {"Help": 1, "Access": 1, "Yes": 1, "鈾?And become 鈾?: 1, "If": 1, "Do it": 1, "Depression": 1}, "adjectives": {"Satisfied": 1, "Emotion": 1}, "adverbs": {"Because": 1, "not": 1, "Just": 1}, "pronouns": {"This": 1}}}, {"content": "I am often called a man of temper.", "word_count": 13, "label": "", "sentence_structure": "Subject鈥搗erb鈥揷omplement", "subjective_objective": {"answer": "Objective", "rationale": "Because `people often say' is an observed fact, not a subjective feeling of the patient."}, "sentence_sentiment": {"answer": "Negative", "rationale": "Because \"grumpy\" in the entry is a negative assessment."}, "language_style": {"answer": "Tactful and warm", "rationale": "The entry uses a euphemism that avoids directly evaluating each other鈥檚 personality characteristics, while not expressing overly strong negative sentiments. It can be more comfortable and respectful and help create a more positive and constructive atmosphere of dialogue."}, "time_dimension": {"answer": "Present", "rationale": "Now, the word \"always\" in \"I'm a grumpy man\" is a regular, customary act, while the word \"speak\" is a general current, meaning that it has not only happened in the past, but may still be ongoing. So, this entry describes a current act or state."}, "sentence_type": "Affirmative declarative sentence", "pos": {"nouns": {"person": 1, "- Grumpy": 1, "- Routine": 1}, "verbs": {"Say it": 1, "be": 1, "Regular": 1, "have": 1}, "adjectives": {"- He's a bitch": 1}, "numerals": {"2": 1}, "pronouns": {"I": 1}}}, {"content": "3. When I have no time to deal with a difficult matter, I can set it aside.", "word_count": 25, "label": "", "sentence_structure": "Subject鈥搗erb鈥搊bject", "subjective_objective": {"answer": "Subjective", "rationale": "This is the patient鈥檚 subjective attitude, because 鈥渃an set it aside鈥?is the way the patient decides to deal with it. Although this ability may be affected by objective conditions (such as whether there is enough time), in this case, 鈥渘o time鈥?is assessed by the person questioned and is therefore subjective."}, "sentence_sentiment": {"answer": "Neutral", "rationale": "Because the entry describes an act or state, there is no obvious emotional colour."}, "language_style": {"answer": "Calm and direct", "rationale": "The basis for the determination: the entry expresses the core of the problem in a straightforward and clear way, i.e., whether it is capable of temporarily dropping it in the face of problems that cannot be solved immediately. This is a simple formulation, which is not accompanied by too many rhetorical or emotional colours, and is more objective and calm."}, "time_dimension": {"answer": "Present", "rationale": "The entry is an inquiry into the manner in which the person is acting in the face of problems that cannot be addressed immediately, and therefore the temporal dimension is now judged."}, "sentence_type": "Affirmative declarative sentence", "pos": {"nouns": {"I": 1, "Time": 1, "Things": 1}, "verbs": {"No, I'm not": 1, "Processing": 1, "Hold it": 1, "On the side": 1}, "adjectives": {"It's a problem": 1}, "numerals": {"3": 1}}}, {"content": "4. I am always treated unfairly.", "word_count": 11, "label": "", "sentence_structure": "Subject鈥搗erb鈥搊bject", "subjective_objective": {"answer": "Subjective", "rationale": "This is the result of patients ' subjective evaluation and perception of themselves and of their own observations and experience."}, "sentence_sentiment": {"answer": "Negative", "rationale": "Because \"unfairly\" in the entries is a negative emotional tendency."}, "language_style": {"answer": "Tactful and warm", "rationale": "The language style of the entry is euphemistic and warm. Although it expresses a negative view (which is unfair to me), it does not use a strong tone or emotional vocabulary, but rather raises the question in a more moderate way, which is more likely to trigger resonance and reflection."}, "time_dimension": {"answer": "Present", "rationale": "The entry describes the current feelings and experiences of the testee, and therefore the time dimension is now judged."}, "sentence_type": "Negative declarative sentence", "pos": {"nouns": {"person": 1, "It's not fair": 1, "Treatment": 1, "I": 1}, "verbs": {"Always": 1, "Treatment": 1}, "adjectives": {"Unfair": 1, "Fair": 1}, "adverbs": {"Land": 1, "Always": 1}, "numerals": {"4": 1}}}, {"content": "I have been able to escape my anxiety by doing some positive or creative things, such as painting, carpentry, etc.", "word_count": 34, "label": "", "sentence_structure": "Subject鈥搗erb鈥搊bject", "subjective_objective": {"answer": "Subjective", "rationale": "Because this is a subjective statement describing the actions taken by the patient to ease anxiety. While these actions may have the objective result (e.g., a painting completed or a woodwork produced), the purpose of this article is focused on the patient鈥檚 subjective motivation and behavioural choices, and is therefore subjective."}, "sentence_sentiment": {"answer": "Positive", "rationale": "Because the behavior described in the entry - \"doing something positive or creative\" - is positive and can help people to escape anxiety, it can be judged positive emotional."}, "language_style": {"answer": "Tactful and warm", "rationale": "The language style of the entry is relatively euphemistic and warm. Some descriptive terms (such as \"positive\" and \"innovation\") are used to describe these actions, and there is no direct order or question about whether the interviewee has taken similar action. This expression can make the interviewee more willing to share his ideas and experiences and make it easier to build a good atmosphere of communication."}, "time_dimension": {"answer": "Present", "rationale": "The entry describes the current behaviour of the testee and the way he is responding to anxiety, in which \"I am\" means the behaviour that is currently being performed, so the temporal dimension is now judged."}, "sentence_type": "Affirmative declarative sentence", "pos": {"nouns": {"Questionnaire": 1, "Entry": 1, "Things": 2, "Anxiety": 1, "Painting": 1, "Carpentry": 1}, "verbs": {"Pass": 1, "Do it": 3, "Get rid of it": 1, "Like": 1}, "adjectives": {"Positive": 1, "Ideas": 1}, "numerals": {"5": 1}, "classifiers": {"individual": 1}, "pronouns": {"self": 1}}}, {"content": "I occasionally defer some of the things that should be done today to tomorrow.", "word_count": 20, "label": "", "sentence_structure": "Subject鈥搗erb鈥搊bject", "subjective_objective": {"answer": "Subjective", "rationale": "Because \"deferring to tomorrow something that is to be done today\" is a choice of behaviour and attitude, a subjective idea and behaviour of the patient."}, "sentence_sentiment": {"answer": "Negative", "rationale": "Because postponing what is to be done until tomorrow is a form of delay and has some negative character."}, "language_style": {"answer": "Tactful and warm", "rationale": "The entry is more euphemistic and sweet, using the word \"frequently\" to describe the frequency of behaviour, without any sense of blame or criticism. At the same time, it is also more modest, without too strong or direct demands, and gives people a sense of relative comfort."}, "time_dimension": {"answer": "Present", "rationale": "This entry describes a behaviour that the subject may have in some cases -- to postpone what should be done today until tomorrow. It happens \"on occasion,\" and the move \"to postpone something to tomorrow\" is described in the current state of affairs. So we can judge the time dimension of this purpose is now."}, "sentence_type": "Affirmative declarative sentence", "pos": {"verbs": {"- Put it on": 1, "Do it": 2, "Postponement": 1}, "adverbs": {"Sometimes": 1, "Today": 1, "Tomorrow": 1}, "numerals": {"6": 1}, "pronouns": {"I": 1}}}, {"content": "I do not know why the same frustrations were encountered.", "word_count": 17, "label": "", "sentence_structure": "Subject鈥搗erb鈥搊bject", "subjective_objective": {"answer": "Objective", "rationale": "Because it is the objective fact of the patient鈥檚 experience that the same situation is encountered, it is not the patient鈥檚 subjective attitude or emotional experience. It is the patient鈥檚 perception and understanding of the same situation that is part of the objective examination."}, "sentence_sentiment": {"answer": "Neutral", "rationale": "Because the entry merely presents a fact, it does not express a positive or negative emotional expression."}, "language_style": {"answer": "Tactful and warm", "rationale": "The language style of the entry is more euphemistic, warm, and not too direct or emotional. It uses an inquisitive tone to stimulate the interviewee to think and share his experiences and feelings, which are comfortable and welcome."}, "time_dimension": {"answer": "Past", "rationale": "The entry is a time-scaled past because it is confused and overwhelmed by the fact that the same situation was encountered before the person was asked."}, "sentence_type": "Interrogative sentence", "pos": {"nouns": {"Questionnaire Entry": 1, "- Inconvenient": 1}, "verbs": {"Yes": 1, "I'll meet you": 1}, "adverbs": {"I don't know": 1, "Why": 1, "Same": 1}, "numerals": {"7": 1}, "pronouns": {"I": 1}}}, {"content": "I can laugh at myself quite easily.", "word_count": 13, "label": "", "sentence_structure": "Subject鈥搗erb鈥揷omplement", "subjective_objective": {"answer": "Subjective", "rationale": "Because it's a subjective attitude and behaviour of one."}, "sentence_sentiment": {"answer": "Positive", "rationale": "Because the act of \"scoffing yourself\" in the entry is a self-scoffing expression, which is usually considered a reflection of humor and self-confidence."}, "language_style": {"answer": "Tactful and warm", "rationale": "The language style of the entry is euphemism. By using \"slightly\" and \"smuggling myself,\" it expresses a self-acceptance and self-scoffing attitude, and it is positive. It makes it more acceptable and accessible to people, and is willing to answer the question."}, "time_dimension": {"answer": "Present", "rationale": "Now the entry describes the current behaviour or state of the subject, which is \"I can laugh at myself quite easily.\" It does not relate to past or future behaviour or state."}, "sentence_type": "Affirmative declarative sentence", "pos": {"verbs": {"Yes": 1, "Smile": 1, "Scoffing": 1}, "adjectives": {"Easy": 1}, "adverbs": {"Pretty much": 1}, "pronouns": {"I": 1}}}, {"content": "When I was frustrated, I was acting like a child.", "word_count": 13, "label": "", "sentence_structure": "Subject鈥揷opula鈥損redicative", "subjective_objective": {"answer": "Subjective", "rationale": "Because \"act like a child\" describes the patient's self-perception and emotional reaction, which is a subjective experience of the individual."}, "sentence_sentiment": {"answer": "Negative", "rationale": "Because the \"act like a child when you're frustrated\" act in the entry is negative."}, "language_style": {"answer": "Tactful and warm", "rationale": "The entry uses a metaphor, describing its state of affairs in the event of frustration as a child-like act, with a more euphemism and implicit expression, without direct attack or blame. At the same time, this expression is more akin to nature and can bring the person under investigation closer.\""}, "time_dimension": {"answer": "Present", "rationale": "The keyword \"I get frustration\" refers to the current situation and state of affairs, and \"expression is like a child\" describes the way the person tested behaves or reacts at this time. So, the entry is in the current context."}, "sentence_type": "Affirmative declarative sentence", "pos": {"nouns": {"Frustration": 1, "Son": 1}, "verbs": {"I": 1, "Yes": 1, "Performance": 1, "Elephant": 1}, "adjectives": {"Just": 1}}}, {"content": "10. I am ashamed to be in the interests of others.", "word_count": 16, "label": "", "sentence_structure": "Subject鈥搗erb鈥搊bject", "subjective_objective": {"answer": "Subjective", "rationale": "This sentence expresses the patient ' s subjective view as to whether he should be in conflict with others for his own benefit."}, "sentence_sentiment": {"answer": "Negative", "rationale": "Because the act of \"smuggling against others\" is negative and emotional, implying a lack of confidence or hesitation in the struggle for their own rights."}, "language_style": {"answer": "Tactful and warm", "rationale": "The entry is presented in a more moderate way, \"smuggling\" to express an emotional tendency that the other party may hesitate or be less confident in defending its interests. This expression is more humane, more friendly and more powerful, and better attracts respondents to answer."}, "time_dimension": {"answer": "Present", "rationale": "According to the \"smuggling\" in \"smuggling\" we can assume that it is a description of personal emotions and attitudes, which happens in the present state. So the time dimension of this questionnaire entry is now."}, "sentence_type": "Negative declarative sentence", "pos": {"nouns": {"Interest": 1, "In terms of": 1}, "verbs": {"Maintenance": 1, "Shame": 1, "Count": 1}, "adverbs": {"Shame": 1}, "numerals": {"10": 1}, "pronouns": {"I": 1}}}, {"content": "I am better than most of those I know.", "word_count": 13, "label": "", "sentence_structure": "Subject鈥揷opula鈥損redicative", "subjective_objective": {"answer": "Subjective", "rationale": "Because \"greater than most people I know\" is self-evaluation and subjective feelings of patients."}, "sentence_sentiment": {"answer": "Positive", "rationale": "Because the expression \"more than most people\" in the entry is a sign of self-assertion and self-confidence."}, "language_style": {"answer": "Tactful and warm", "rationale": "The basis for the judgement: the wording of the entry is soft, not too direct or hard-line, but rather a euphemism and a gentle, gentle presentation of individual feelings and assessments. Moreover, the expression \"more\" makes the expression more objective, neutral, less subjective and more euphemisms."}, "time_dimension": {"answer": "Present", "rationale": "The entry is a statement by the testee that he or she compares himself or herself with others, and is not clearly time-directed, and thus is judged to be now."}, "sentence_type": "Affirmative declarative sentence", "pos": {"verbs": {"- Yeah": 1, "Both": 1, "Strong": 1}, "adjectives": {"More": 1}, "adverbs": {"Medium": 1}, "numerals": {"11": 1, "Most of them": 1}, "pronouns": {"I": 1, "person": 1}}}, {"content": "12. People often abuse me.", "word_count": 7, "label": "", "sentence_structure": "Subject鈥搗erb鈥搊bject", "subjective_objective": {"answer": "Subjective", "rationale": "Because \"people often abuse me\" is the subjective way in which patients are treated, and it's seen and evaluated from the subjective point of view of the patients."}, "sentence_sentiment": {"answer": "Negative", "rationale": "Because the \"abuse\" in the entry is negative."}, "language_style": {"answer": "Tactful and warm", "rationale": "The entry uses the expression \"people often\" to suggest that the problem may not be entirely true, while avoiding direct accusations of abuse, which would make the person questioned more willing to share his/her true feelings. At the same time, the appearance of the word \"me\" also shows that it is a personal issue, not a group problem, and that this expression is more targeted and personal."}, "time_dimension": {"answer": "Present", "rationale": "The keyword \"often\" means that it is a regular, customary act, and the action of \"abuse\" has occurred in the past, so it can be judged that the entry describes the current or recent experience and feelings of the person being tested."}, "sentence_type": "Negative declarative sentence", "pos": {"nouns": {"People": 1, "Abuse": 1}, "verbs": {"Often": 1, "Abuse": 1}, "adverbs": {"Often": 1}, "numerals": {"12": 1}, "pronouns": {"I": 1}}}, {"content": "13. If someone had lied to me or stolen my money, I would have preferred that he be helped rather than punished.", "word_count": 27, "label": "", "sentence_structure": "Subject鈥搗erb鈥搊bject", "subjective_objective": {"answer": "Subjective", "rationale": "Because it is the patient's subjective idea and attitude."}, "sentence_sentiment": {"answer": "Positive", "rationale": "Because the article is formulated out of compassion and understanding, it is prepared to help the other to correct the error and has a positive emotional tone."}, "language_style": {"answer": "Tactful and warm", "rationale": "The language style of the entry is more euphemistic and gentle, expressing a more tolerant and sympathetic attitude by asking questions. At the same time, the contrast between \"helped\" and \"punished\" gives a sense of balance, without strong negative emotions or acrimony."}, "time_dimension": {"answer": "Present", "rationale": "The entry to the questionnaire is an inquiry into the attitude and behaviour choices of the subjects in certain circumstances, i.e., when someone cheats or steals them, what is their priority. In such cases, \"if someone cheats me or steals my money\" is a hypothetical situation, but it relates to what is happening now or recently (i.e., the act of deception or theft), and therefore is judged on the basis of \"now\"."}, "sentence_type": "Affirmative declarative sentence", "pos": {"nouns": {"Someone": 1, "Money": 1, "Help": 1, "Punishment": 1}, "verbs": {"Liar": 1, "Steal": 1, "Okay": 1, "Yes": 1}, "adverbs": {"I'd rather not": 1, "Not": 1}, "numerals": {"13": 1}, "pronouns": {"I": 1}}}, {"content": "14. Occasionally, I think there are bad things that I cannot say.", "word_count": 16, "label": "", "sentence_structure": "Subject鈥搗erb鈥揷omplement", "subjective_objective": {"answer": "Subjective", "rationale": "Because it's the patient's subjective judgment to think of bad things."}, "sentence_sentiment": {"answer": "Negative", "rationale": "Because the \"thinking of something so bad that you can't say it\" act in the entry is negative, expressing negative feelings and attitudes."}, "language_style": {"answer": "Calm and direct", "rationale": "The entry is more direct and concise in language style, without too much embellishment or emotional rendering. It asks the respondents questions in an objective and neutral manner, with the aim of obtaining their real thoughts and feelings."}, "time_dimension": {"answer": "Present", "rationale": "The word \"want\" follows the general current, meaning that the act has not only occurred in the past but may still be ongoing. The word \"occupying\" means that the act is not occurring at a high frequency, but does not exclude the fact that it is now occurring. The entry is therefore asking about the current situation of the person being tested."}, "sentence_type": "Affirmative declarative sentence", "pos": {"nouns": {"Things": 2, "mouth": 1}, "verbs": {"Yes": 1, "Say it": 1, "Out": 1, "Go on": 1, "Okay": 1}, "adjectives": {"Bad": 1, "No, I can't": 1, "Sometimes": 1}, "adverbs": {"mouth": 1}, "numerals": {"14": 1}, "pronouns": {"I": 1}}}, {"content": "15. Occasionally, I laugh at some dirty jokes.", "word_count": 14, "label": "", "sentence_structure": "Subject鈥搗erb鈥揷omplement", "subjective_objective": {"answer": "Subjective", "rationale": "Because \"smoke\" is a subjective feeling and reaction of a person, not an objective fact. Although the joke itself may be an objective element, whether or not it causes a laugh to someone is the result of his subjective feeling. So, the entry is a test of the patient's subjective attitude."}, "sentence_sentiment": {"answer": "Neutral", "rationale": "Because the entry does not have any emotional color."}, "language_style": {"answer": "Calm and direct", "rationale": "The entry uses direct, concise language, is not too emotional or rhetorical, and asks the other side whether it laughs because of some dirty jokes. The language style is neutral and objective, and meets the needs of the questionnaire."}, "time_dimension": {"answer": "Present", "rationale": ""}, "sentence_type": "Affirmative declarative sentence", "pos": {"nouns": {"Questionnaire": 1, "Entry": 1}, "verbs": {"Analysis": 1, "Because": 1, "Smile": 1}, "adjectives": {"Nasty": 1}, "adverbs": {"Sometimes": 1}, "numerals": {"15": 1}, "pronouns": {"I": 1}}}, {"content": "16. I was said to be a camel bird, burying my head in the sand, in other words, I tended to ignore unpleasant things.", "word_count": 37, "label": "", "sentence_structure": "Subject鈥搗erb鈥搊bject", "subjective_objective": {"answer": "Subjective", "rationale": "Because this phrase expresses the patient鈥檚 subjective feelings and thoughts that he or she deliberately ignores unpleasant things. This is the patient鈥檚 interpretation and understanding of his or her behaviour, and therefore is a subjective examination."}, "sentence_sentiment": {"answer": "Negative", "rationale": "Because the act of \"intentionally ignoring something unpleasant\" in the entry is negative."}, "language_style": {"answer": "Calm and direct", "rationale": "The style of the entry is relatively calm and straightforward, using metaphors and concise expressions, and not too much emotional or euphemism."}, "time_dimension": {"answer": "Present", "rationale": "The word \"always\" in the keyword \"always\" denotes a regular, customary act, while the word \"listen\" adopts the general current, meaning that the act has not only occurred in the past but may still be ongoing. So the entry is about asking the person who was tested for the present behavior or mentality."}, "sentence_type": "Affirmative declarative sentence", "pos": {"nouns": {"People": 1, "A camel bird": 1, "Head": 1, "Sand": 1, "Things": 1}, "verbs": {"Say it": 1, "Elephant": 1, "Bury it": 1, "Neglect": 1, "Intentionally": 1, "Come on": 1}, "adjectives": {"It's not good": 1}, "adverbs": {"Often": 1, "In some cases": 1}, "classifiers": {"Only": 1, "Some": 1, "One": 2}, "pronouns": {"I": 1}}}, {"content": "I often cannot compete with people as much as I can.", "word_count": 14, "label": "", "sentence_structure": "Subject鈥搗erb鈥揷omplement", "subjective_objective": {"answer": "Subjective", "rationale": "Because \"can't do everything\" is a subjective feeling and attitude of a person that reflects an individual's perception of his or her performance in competition."}, "sentence_sentiment": {"answer": "Negative", "rationale": "Because the \"can't compete with people\" act in the entry is negative."}, "language_style": {"answer": "Tactful and warm", "rationale": "The entry uses more euphemistic language to ask if the other party often fails to show full strength in competition, and is not using too direct or hard language, making the respondent feel more comfortable and comfortable in answering the questions."}, "time_dimension": {"answer": "Present", "rationale": "The entry \"I'm often\" denotes a regular act, using the current state of affairs, which suggests that the act may still be ongoing and can therefore be judged to be the present."}, "sentence_type": "Negative declarative sentence", "pos": {"nouns": {"Entry": 1, "Questionnaire": 1, "person": 1, "Strength": 2}, "verbs": {"- All of it": 1, "Competition": 1}, "adverbs": {"Often": 1, "Land": 1}, "numerals": {"17": 1}}}, {"content": "I often feel better than the people I am with.", "word_count": 14, "label": "", "sentence_structure": "Subject鈥揷opula鈥損redicative", "subjective_objective": {"answer": "Subjective", "rationale": "Because \"I'm better than the people I'm with\" is a subjective assessment or feeling of a person."}, "sentence_sentiment": {"answer": "Positive", "rationale": "Because the expression \"more than me\" in the entry expresses a sense of self-confidence and self-assertion."}, "language_style": {"answer": "Tactful and warm", "rationale": "The entry asks the other side whether it feels this way, in a more euphemistic and encouraging way, rather than directly questioning or criticizing, in a way that is more respectful and understanding of the interviewee鈥檚 feelings. At the same time, the language is more warm and friendly, and is easy to accept and share in its thoughts and experiences."}, "time_dimension": {"answer": "Present", "rationale": ""}, "sentence_type": "Affirmative declarative sentence", "pos": {"nouns": {"person": 1}, "verbs": {"Sensor": 1, "in": 1, "Strong": 1}, "adjectives": {"Regular": 1}, "adverbs": {"- Yeah": 1, "I": 2, "Together": 1}, "classifiers": {"individual": 1}}}, {"content": "19. A person is trying to deprive me of everything I have.", "word_count": 14, "label": "", "sentence_structure": "Subject鈥搗erb鈥揷omplement", "subjective_objective": {"answer": "Subjective", "rationale": "Because \"want to take away everything I've got\" is a subjective idea and feeling of a person. Although this person may act or act to realize his ideas, the idea itself is subjective. So, the entry is primarily a review of patients' subjective feelings and ideas, not objective facts."}, "sentence_sentiment": {"answer": "Negative", "rationale": "Because the behavior in the entry is \"to deprive me of everything I have\" and that attitude and behavior is negative."}, "language_style": {"answer": "Calm and direct", "rationale": "The language style of the entry is straightforward, without any rhetorical or emotional recitation, and allows the respondents to express their feelings clearly and clearly through simple statements."}, "time_dimension": {"answer": "Present", "rationale": ""}, "sentence_type": "Negative declarative sentence", "pos": {"nouns": {"Someone": 1, "Everything": 1}, "verbs": {"Yes": 1, "Deprivation": 1, "Okay": 1}, "pronouns": {"I": 1}}}, {"content": "I sometimes get angry.", "word_count": 5, "label": "", "sentence_structure": "Subject鈥搗erb鈥揷omplement", "subjective_objective": {"answer": "Subjective", "rationale": "Because \"greed\" is an emotional reaction, a subjective feeling and experience of the patient."}, "sentence_sentiment": {"answer": "Negative", "rationale": "Because \"gream\" is a negative emotion."}, "language_style": {"answer": "Tactful and warm", "rationale": "The entry is more personal and interactive when it is spoken in a gentle and inviting manner, rather than in direct statements or orders. While it describes a state of mood that may be less satisfactory, the term is relatively soft and does not have overly harsh denials or criticisms, which put psychological pressure on the interviewees."}, "time_dimension": {"answer": "Present", "rationale": "\"I get angry sometimes.\" \"Sometimes\" means occasional, not regular behaviour, \"excited\" is an action that, in the usual current state of affairs, may be taking place at this moment. So the entry asks if the person tested is now angry."}, "sentence_type": "Affirmative declarative sentence", "pos": {"verbs": {"- Get angry": 1}, "adverbs": {"Time": 1, "Sometimes": 1}}}, {"content": "21. I have often acted autonomously, driven by some internal force.", "word_count": 24, "label": "", "sentence_structure": "Subject鈥搗erb鈥揷omplement", "subjective_objective": {"answer": "Subjective", "rationale": "Because it describes the experience and feelings inside the patient, it is the patient ' s subjective attitude."}, "sentence_sentiment": {"answer": "Positive", "rationale": "Because the entry \"driven by the inner force\" shows some motivation."}, "language_style": {"answer": "Tactful and warm", "rationale": "The language style of the entry is more euphemistic, warm, and the expression is more personal and interactive, with a gentle and inviting tone, rather than a direct statement or order."}, "time_dimension": {"answer": "Present", "rationale": "In the entry, the words \"frequently\" denotes a regular act, \"driven by inner force\", \"involved\" and \"made\" indicate that the act is taking place now. So, time dimensions are now judged."}, "sentence_type": "Affirmative declarative sentence", "pos": {"nouns": {"Questionnaire Entry": 1, "Inside power": 1, "Driver": 1, "Behaviour": 1}, "verbs": {"Often": 1, "Make": 1, "I can't help it": 1}, "adverbs": {"Often": 1, "I can't help it": 1}, "numerals": {"21": 1}}}, {"content": "I would rather starve to death than be forced to eat.", "word_count": 12, "label": "", "sentence_structure": "Subject鈥搗erb鈥揷omplement", "subjective_objective": {"answer": "Subjective", "rationale": "This is because the patient ' s personal choice and attitude to a given situation is a subjective view of the patient."}, "sentence_sentiment": {"answer": "Negative", "rationale": "Because the phrase \"I'd rather starve than be forced to eat\" in the entry is negative."}, "language_style": {"answer": "Calm and direct", "rationale": "The language style of the entry is calm, because it expresses the idea of not being forced to eat, and there is no excessive emotional or accoutrements. It conveys the attitudes and positions of the respondents in a clear and categorical manner."}, "time_dimension": {"answer": "Present", "rationale": "The entry expresses the views of the testee on hunger and eating in the current state, where the words \"better\" and \"no\" imply that this is the testee's current attitude and choice, and therefore the judgement is now."}, "sentence_type": "Negative declarative sentence", "pos": {"verbs": {"I'd rather": 1, "Starving": 1, "By": 1, "- Oh, shit": 1, "Eat": 1}, "adverbs": {"I'd rather not": 1, "And": 1}, "numerals": {"22": 1}, "pronouns": {"I": 1}}}, {"content": "I have often deliberately ignored some of the dangers, as if I were a Superman.", "word_count": 18, "label": "", "sentence_structure": "Subject鈥搗erb鈥搊bject", "subjective_objective": {"answer": "Subjective", "rationale": "Because the descriptions of \"intentional neglect\" and \"superhuman\" are the patient's personal feelings and subjective attitudes."}, "sentence_sentiment": {"answer": "Negative", "rationale": "Because the act of \"intentional neglect of some danger\" is negative."}, "language_style": {"answer": "Calm and direct", "rationale": "The decision is based on the fact that the purpose of this article is expressed more directly, without excessive rhetorical or emotional overtones. It clearly asks whether the interviewees often ignore danger and uses more intuitive language to describe the situation, which seems to be calm and straightforward."}, "time_dimension": {"answer": "Past", "rationale": "The word \"always\" in \"I'm often\" denotes a regular, customary act, while the verbs \"neglect\" and \"seemingly\" adopt the general current, meaning that the act not only has occurred in the past, but may still continue. Thus, the entry is a question of the testee's past behaviour, the temporal dimension being judged by the past."}, "sentence_type": "Affirmative declarative sentence", "pos": {"nouns": {"Questionnaire Entry": 1, "Danger": 1, "Superman": 1}, "verbs": {"Neglect": 1, "be": 1, "Often": 1, "On purpose": 1}, "adverbs": {"Often": 1, "I think so": 1}, "numerals": {"23": 1}, "pronouns": {"I": 1}}}, {"content": "24. I am proud of my ability to denigrate others.", "word_count": 15, "label": "", "sentence_structure": "Subject鈥搗erb鈥揷omplement", "subjective_objective": {"answer": "Subjective", "rationale": "Because pride is a subjective emotion and attitude."}, "sentence_sentiment": {"answer": "Negative", "rationale": "Because the act of \"demeaning others' prestige\" in the entry is negative and indicates pride in what they do and negative feelings."}, "language_style": {"answer": "Calm and direct", "rationale": "The entry is more directly quiet, has no excessive emotional rendering or euphemism, and asks whether the person surveyed is proud of some of his or her abilities. This directness helps researchers to understand more accurately the views and attitudes of the person under investigation."}, "time_dimension": {"answer": "Past", "rationale": ""}, "sentence_type": "Affirmative declarative sentence", "pos": {"verbs": {"have": 1, "Demeaning": 1, "Capacity": 1, "Proud": 1}, "adjectives": {"Someone else's": 1, "Honorable": 1}, "numerals": {"24": 1}, "pronouns": {"I": 1}}}, {"content": "25. I was told that I had always felt killed.", "word_count": 13, "label": "", "sentence_structure": "Subject鈥搗erb鈥搊bject", "subjective_objective": {"answer": "Subjective", "rationale": "Because \"there's always a sense of being killed\" is a subjective feeling of a person, shaped by his emotions, experience, beliefs, etc. While others may tell him that they feel that, it doesn't mean that it must be an objective fact. So, the entry looks at the patient's subjective feelings and cognitive processes."}, "sentence_sentiment": {"answer": "Negative", "rationale": "Because it expresses a negative sentiment and attitude, namely, the \"fear of being killed\", which implies the possibility of being harmed or unfairly treated."}, "language_style": {"answer": "Tactful and warm", "rationale": "The language style of the entry is euphemistic, because it uses the second person's alias \"you\" and asks the other person in a gentle way whether he or she has ever felt killed, rather than directly speaking or ordering. This expression is more personal and interactive, and it can more naturally lead the interviewee to share his or her experiences and feelings."}, "time_dimension": {"answer": "Present", "rationale": "The content of this questionnaire entry is a description of a person's feelings and experiences, that's \"people tell me,\" which means that the testee is going through, not past or future. And, \"I always feel killed\" is also a description of the current feeling, so the entry describes the present situation."}, "sentence_type": "Affirmative declarative sentence", "pos": {"nouns": {"Feel": 1, "person": 1}, "verbs": {"Tell": 1, "have": 1, "Total": 1, "be": 1}, "adjectives": {"Shit": 1}, "adverbs": {"Too much": 1}, "numerals": {"25": 1}, "pronouns": {"I": 1}}}, {"content": "26. Sometimes I get angry when I feel bad.", "word_count": 12, "label": "", "sentence_structure": "Subject鈥搗erb鈥搊bject", "subjective_objective": {"answer": "Subjective", "rationale": "Because anger is the patient's subjective behaviour and emotional reaction."}, "sentence_sentiment": {"answer": "Negative", "rationale": "Because it's negative in the entry."}, "language_style": {"answer": "Tactful and warm", "rationale": "The entry uses a euphemism, without directly blaming or evaluating the behaviour of the other, but rather in a moderate way leading the other to think and reflect on the management of their emotions, with greater love and interaction. At the same time, the entry provides a strategy for solving the problem: \"26. Sometimes I get angry when I feel bad.\""}, "time_dimension": {"answer": "Present", "rationale": "The keyword \"frightful\" and \"crazy\" means the person's current behavior or state. So the time dimension of the entry is now judged."}, "sentence_type": "Affirmative declarative sentence", "pos": {"nouns": {"Time": 1, "Feel": 1, "- Grumpy": 1}, "verbs": {"Let's go": 1, "have": 1}, "adjectives": {"Not good": 1}, "adverbs": {"Just": 1}, "numerals": {"26": 1}, "pronouns": {"I": 1}}}, {"content": "When something bothers me, I often do things on my own.", "word_count": 23, "label": "", "sentence_structure": "Subject鈥搗erb鈥搊bject", "subjective_objective": {"answer": "Subjective", "rationale": "Because \"somethin's free\" is the patient's subjective feeling and reaction."}, "sentence_sentiment": {"answer": "Negative", "rationale": "Because the act of \"some act of an autonomous nature\" in the entry is negative."}, "language_style": {"answer": "Tactful and warm", "rationale": ""}, "time_dimension": {"answer": "Past", "rationale": "The entry describes the customary sexual behaviour of a person who was tested, \"often by doing something of his own free will\" indicating that such behaviour is frequent and may be unconscious. Thus, the temporal dimension of the purpose of the article is past."}, "sentence_type": "Affirmative declarative sentence", "pos": {"nouns": {"Things": 1, "I": 1, "Behaviour": 1}, "verbs": {"鈾?And I'm gonna be 鈾?: 1, "Do it": 1, "- You're a pain in the ass": 1}, "adverbs": {"Often": 1, "I can't help it": 1}, "numerals": {"27": 1}, "pronouns": {"Some": 1}}}, {"content": "28. When things go wrong, I get sick.", "word_count": 12, "label": "", "sentence_structure": "Subject鈥揷opula鈥損redicative", "subjective_objective": {"answer": "Subjective", "rationale": "Because it is not an objective fact that the patient ' s own subjective feelings determine whether or not he or she is sick."}, "sentence_sentiment": {"answer": "Negative", "rationale": "Because \"sick\" in the entry is a negative result, expressing a negative attitude or emotion."}, "language_style": {"answer": "Tactful and warm", "rationale": "The language style of the entry is more euphemistic and implicit, using the expression \"when things go wrong\", which does not directly state that \"infirmity\" is caused by emotional problems. This expression is more flexible and acceptable, and it is felt that the questionnaire designer is concerned with the feelings of the interviewee, not just with data collection."}, "time_dimension": {"answer": "Present", "rationale": "The word \"when\" indicates that an action occurs at a given point in time, \"when you get sick\" expresses a situation that leads to a physical condition when something goes wrong. The entry is therefore concerned primarily with the current situation, asking what reactions and results the person tested usually has in the event of a situation of hardship."}, "sentence_type": "Affirmative declarative sentence", "pos": {"nouns": {"Yes": 1, "Diseases": 1}, "verbs": {"Yes": 2, "On your own": 1, "Fan": 1}, "adjectives": {"not": 1, "Sunshine": 1}, "adverbs": {"Just": 1}, "numerals": {"28": 1}}}, {"content": "I am a very self-sufficient man.", "word_count": 11, "label": "", "sentence_structure": "Subject鈥揷opula鈥損redicative", "subjective_objective": {"answer": "Subjective", "rationale": "Because \"have control\" is self-evaluation and subjective feeling of the patient."}, "sentence_sentiment": {"answer": "Positive", "rationale": "Because the entry is about personal qualities being \"self-contained,\" which is a positive assessment."}, "language_style": {"answer": "Tactful and warm", "rationale": "This is more euphemistic and gentle, and it avoids direct evaluation or command by asking if the other side agrees with a self-sufficient person."}, "time_dimension": {"answer": "Present", "rationale": "The entry shows a self-evaluation of the subjects, who consider themselves \"self-contained.\" This self-assessment is about the current beliefs or attitudes, so we can consider it as the present."}, "sentence_type": "Affirmative declarative sentence", "pos": {"nouns": {"I": 1, "person": 1, "Self-restraint": 1}, "verbs": {"be": 1}, "adjectives": {"very": 1, "have": 1}, "numerals": {"29": 1}}}, {"content": "I'm like an undesired artist.", "word_count": 16, "label": "", "sentence_structure": "Subject鈥搗erb鈥揷omplement", "subjective_objective": {"answer": "Subjective", "rationale": "Because \"like an undesired artist\" is a subjective view of the interviewee's own evaluation and description."}, "sentence_sentiment": {"answer": "Negative", "rationale": "Because the \"unwillful\" in the entry is an expression of a negative emotion and attitude."}, "language_style": {"answer": "Tactful and warm", "rationale": "The words \"like\" and \"unwillful\" in the entry are somewhat metaphorically symmetrical, and the first person's word \"I\" is used to make the language style more friendly and self-presentive. Overall, the language style of the purpose is more of a softer type."}, "time_dimension": {"answer": "Present", "rationale": "The sentence expresses the subjective feeling of the speaker, without reference to past or future situations. \"It's just like...\" is an exaggeration to emphasize the current emotional state of the speaker, so the temporal dimension is now judged."}, "sentence_type": "Affirmative declarative sentence", "pos": {"nouns": {"Artist": 1}, "verbs": {"Yeah": 1, "You're right": 1}, "adjectives": {"It's just": 1}, "pronouns": {"I": 1}}}, {"content": "31. I do not always tell the truth.", "word_count": 7, "label": "", "sentence_structure": "Subject鈥損redicate", "subjective_objective": {"answer": "Subjective", "rationale": "Because whether \"I\" always speak the truth is a subjective judgement, depends on the self-perception and values of the person who speaks. This does not involve objective facts or behaviour."}, "sentence_sentiment": {"answer": "Negative", "rationale": "Because the \"not always telling the truth\" act is negative."}, "language_style": {"answer": "Calm and direct", "rationale": ""}, "time_dimension": {"answer": "Present", "rationale": "\"Not always\" denotes a frequent act, and \"truth\" is a general current act that suggests that the habit may be a current and continuing act. The entry therefore contains a description of the present act or attitude."}, "sentence_type": "Negative declarative sentence", "pos": {"verbs": {"Always": 1, "Say it": 1, "The truth": 1}, "adverbs": {"not": 1}}}, {"content": "32. When I feel my self-esteem is hurt, I will avoid it.", "word_count": 16, "label": "", "sentence_structure": "Subject鈥搗erb鈥搊bject", "subjective_objective": {"answer": "Subjective", "rationale": "Because \"fearing self-esteem is hurt\" and \"divide\" is the patient's subjective experience and choice."}, "sentence_sentiment": {"answer": "Negative", "rationale": "Because the \"divide\" act in the entry is negative."}, "language_style": {"answer": "Calm and direct", "rationale": "The language style of this entry is calm and straightforward. It presents a point or situation in a concise manner, without using too many emotional terms or rhetorical techniques, or attempting to influence the respondents' answers through emotional guidance. This style is more in line with the objective and neutral requirements of psychological questionnaire research."}, "time_dimension": {"answer": "Future", "rationale": "Moreover, the \"when self-esteem is injured\" implies that the act may be a reaction caused by some kind of irritation or circumstance, so the entry relates to the situation that the person tested may encounter in the future and the way in which they may respond."}, "sentence_type": "Exclamatory sentence", "pos": {"nouns": {"Self-esteem": 1, "Hurt": 1}, "verbs": {"Yes": 1, "Avoidance": 1}, "adverbs": {"Just": 1}, "pronouns": {"I": 1}}}, {"content": "I have often been compelled to do things so much more than I can do, that others have to limit me.", "word_count": 31, "label": "", "sentence_structure": "Subject鈥搗erb鈥搊bject", "subjective_objective": {"answer": "Subjective", "rationale": "Because it describes the patient鈥檚 own feelings and behaviour, it is a subjective experience and perception. While this may affect others, it is more described from the patient鈥檚 subjective perspective."}, "sentence_sentiment": {"answer": "Negative", "rationale": "The expression of the purpose of the article contained negative terms such as coercion, excesses and so forth, indicating that the tendency of the speaker was negative."}, "language_style": {"answer": "Tactful and warm", "rationale": "The entry is more moderate and implicit, using a more euphemistic approach to express the potential excesses of the respondent and inviting the other to reflect and respond. Such a formulation is more acceptable to the respondent and is willing to respond honestly."}, "time_dimension": {"answer": "Present", "rationale": "Now, in the entry, \"often\" means \"a regular, customary act, \"a self-absorbed to force itself to do something too much\" indicates that the act is a psychological tendency or habit of the person being tested, and therefore the time can be judged to be the present."}, "sentence_type": "Affirmative declarative sentence", "pos": {"nouns": {"Questionnaire": 1, "Entry": 1, "Things": 1, "person": 1, "Limits": 1}, "verbs": {"Forced": 2, "I can't do it on my own": 2, "We have to limit it": 1}, "adverbs": {"Often": 1, "Too far": 1, "Other": 1}, "numerals": {"33": 1}, "pronouns": {"I": 1, "self": 1, "Others": 1}}}, {"content": "My friends see me as a country boy.", "word_count": 12, "label": "", "sentence_structure": "Subject鈥揷opula鈥損redicative", "subjective_objective": {"answer": "Subjective", "rationale": "This is because the patient himself is subjectively aware and evaluates his role in society."}, "sentence_sentiment": {"answer": "Negative", "rationale": "Because the description \"becoming a country bumpkin\" in the entry has the meaning of derogatory, disrespectful, and negative emotional."}, "language_style": {"answer": "Calm and direct", "rationale": "The entry asks in a direct and clear manner whether the person interviewed is considered a \" hick\" by his friends, which is more calm and straightforward and does not have too many rhetorical or emotional expressions.\""}, "time_dimension": {"answer": "Present", "rationale": "According to the context, \"My friends see me as a country boy\" describes the current situation or state, and therefore judges \"now\"."}, "sentence_type": "Negative declarative sentence", "pos": {"nouns": {"friends": 1, "I": 1, "Country boy": 1}, "verbs": {"- Put it on": 1, "Look": 1, "Do it": 1}, "adjectives": {"Country": 1}, "numerals": {"34": 1}, "pronouns": {"Guys": 1}}}, {"content": "35. I often avoid when I am angry.", "word_count": 12, "label": "", "sentence_structure": "Subject鈥搗erb鈥揷omplement", "subjective_objective": {"answer": "Subjective", "rationale": "Because I respond to anger on the basis of personal emotions and choices, which reflect patients' subjective attitudes."}, "sentence_sentiment": {"answer": "Negative", "rationale": "Because the \"divide\" act in the entry is negative."}, "language_style": {"answer": "Calm and direct", "rationale": "The language style of the entry is more calm, and there is no excessive rhetorical or emotional rendering, and the subject matter of the study is expressed through simple statements. This is more objective and practical.\""}, "time_dimension": {"answer": "Present", "rationale": ""}, "sentence_type": "Negative declarative sentence", "pos": {"nouns": {"Time": 1, "Anger": 1, "Avoidance": 1}, "verbs": {"in": 1, "Often": 1, "I": 2}, "adverbs": {"Often": 1}, "numerals": {"35": 1}, "pronouns": {"I": 1}}}, {"content": "I am often more vigilant than I should have suspected of being truly kind to me.", "word_count": 30, "label": "", "sentence_structure": "Subject鈥搗erb鈥揷omplement", "subjective_objective": {"answer": "Subjective", "rationale": "Because I'm the subject of the questionnaire, the sentence describes the subjective ideas and attitudes of me."}, "sentence_sentiment": {"answer": "Negative", "rationale": "Because the \"Better Vigilance\" act in the entry is negative."}, "language_style": {"answer": "Calm and direct", "rationale": "Because the entry is presented in a more direct way, without excessive rhetorical or emotional rendering, and is based on factual statements, expressing the vigilance and friendship of the subject in the interpersonal contacts."}, "time_dimension": {"answer": "Past", "rationale": "The entry describes a customary act, \"I am often\" , which may have occurred many times in the past and may still be ongoing. Moreover, \"to be more vigilant towards those who are really friendly to me than I should doubt\" is also an act based on experience, observation and judgement and is therefore relevant to the past."}, "sentence_type": "Affirmative declarative sentence", "pos": {"nouns": {"Questionnaire": 1, "Entry": 1, "person": 2, "Vigilance": 1}, "verbs": {"Often": 1, "to": 1, "Hold on": 1, "Doubt": 1}, "adjectives": {"Friendly": 1, "High": 1}, "adverbs": {"More": 1, "I think so": 1}, "numerals": {"36": 1}, "pronouns": {"I": 2, "Those": 1, "self": 1}}}, {"content": "37. I have learned a special talent that will enable me to live a life without problems.", "word_count": 22, "label": "", "sentence_structure": "Subject鈥搗erb鈥搊bject", "subjective_objective": {"answer": "Subjective", "rationale": "Because \"I have learned a special talent\" is the self-awareness and subjective feeling of the patient."}, "sentence_sentiment": {"answer": "Positive", "rationale": "Because the entry expresses a sense of confidence and optimism that there are special abilities to successfully meet the challenges of life, positive feelings and attitudes."}, "language_style": {"answer": "Tactful and warm", "rationale": "The language style of the entry is more euphemistic, implicit, and expresses in a gentle manner speculation or advice that the other party may possess some special talents or abilities, rather than a direct statement or order."}, "time_dimension": {"answer": "Present", "rationale": "The word \"recognised\" in \"accomplished\" denotes an act that has been completed, while \"recognised\" means that the act has taken place in the past. The keyword is \"special ability\" and it points to the particular talent that the tester believes he has. So, the entry asks the testee what he thinks and what he thinks of his particular talents."}, "sentence_type": "Affirmative declarative sentence", "pos": {"nouns": {"Capacity": 1, "Special": 1, "Talent": 1, "Problem": 1, "Fan": 1}, "verbs": {"Learn": 1, "鈾?And I'm gonna be 鈾?: 2, "Pass": 1}, "adjectives": {"Special": 1}, "adverbs": {"It's no problem": 1}, "numerals": {"37": 1}, "pronouns": {"I": 1}}}, {"content": "38. Sometimes, at the time of the elections, I choose those I hardly know.", "word_count": 22, "label": "", "sentence_structure": "Subject鈥搗erb鈥搊bject", "subjective_objective": {"answer": "Subjective", "rationale": "Because the choice of who to run is a subjective attitude and decision of one person. While there may be objective factors that influence the decision-making of voters (e.g. the candidate鈥檚 policies, performance, etc.), in this particular case the degree of 鈥渦nunderstanding鈥?depends on the individual鈥檚 perception and assessment, so the entry looks at subjective ideas."}, "sentence_sentiment": {"answer": "Negative", "rationale": "Because the \"selection of those I hardly know\" in the entry is negative, suggesting that the respondents may be irresponsible, impulsive or irrational in voting."}, "language_style": {"answer": "Tactful and warm", "rationale": "The language style of the questionnaire entry is euphemistic. It does not use direct language or command tone to question the other party's ideas, but rather it incorporates soft and inviting elements to make the issue more friendly and acceptable. For example, the phrase \"sometimes\" implies a more general phenomenon than criticism or criticism of individuals; and the statement \"I often choose those I hardly know\" does not speak too directly about the other party's problems, but rather in a more euphemismal way, making it more acceptable and more understandable."}, "time_dimension": {"answer": "Present", "rationale": "The keyword \"sometimes\" means that such behaviour does not always occur, but there is a certain frequency, and \"often\" means that it is a common way of doing things, and \"selection of those I hardly know\" describes the specific content of the act."}, "sentence_type": "Affirmative declarative sentence", "pos": {"nouns": {"Time": 2, "person": 2, "Elections": 1, "Roger that": 1}, "verbs": {"Choose": 1, "Often": 1, "I don't know": 1}, "adjectives": {"Those": 1, "Almost": 1}, "adverbs": {"Sometimes": 1}, "numerals": {"38": 1}, "pronouns": {"I": 1}}}, {"content": "I have often been unable to meet on time.", "word_count": 9, "label": "", "sentence_structure": "Subject鈥搗erb鈥揷omplement", "subjective_objective": {"answer": "Subjective", "rationale": "Because the timely arrival of the patient depends on his or her own will and action, this is the patient ' s subjective attitude and behaviour."}, "sentence_sentiment": {"answer": "Negative", "rationale": "The expression \"failure to make an appointment\" has a negative meaning, indicating delays and non-compliance in the conduct of individuals."}, "language_style": {"answer": "Tactful and warm", "rationale": "The language style of this entry is more euphemistic, because it uses words like \"often\" to express the frequency and universality of the phenomenon, rather than to direct accusations against each other. At the same time, the act of \"failure to come on time\" is not portrayed as a negative act, but as an area that needs to be improved. This expression is more readily acceptable to respondents and willing to provide real answers."}, "time_dimension": {"answer": "Present", "rationale": ""}, "sentence_type": "Negative declarative sentence", "pos": {"verbs": {"Often": 1, "Date": 1}, "adverbs": {"No, I can't": 1, "On time": 1}, "numerals": {"39": 1}}}, {"content": "I have many illusions and can do less in real life.", "word_count": 15, "label": "", "sentence_structure": "Subject鈥搗erb鈥揷omplement", "subjective_objective": {"answer": "Subjective", "rationale": "Because \"fanta\" and \"less\" in real life are subjective feelings and attitudes of patients. Although these may be based on objective facts (such as the fact that the patient has not actually acted to realize their ideas), the assessment itself is based on the subjective perspective of the patient. So the answer to this questionnaire is subjective."}, "sentence_sentiment": {"answer": "Negative", "rationale": "Because the \"Imagination, Less in Real Life\" act is negative."}, "language_style": {"answer": "Tactful and warm", "rationale": ""}, "time_dimension": {"answer": "Present", "rationale": ""}, "sentence_type": "Affirmative declarative sentence", "pos": {"nouns": {"Fantasy": 1, "Real life": 1}, "verbs": {"Fantasy": 1, "I'm working on it": 1}, "adverbs": {"More": 1, "Yeah": 1, "Less": 1}, "numerals": {"40": 1}}}, {"content": "I am ashamed to deal with people.", "word_count": 8, "label": "", "sentence_structure": "Subject鈥揷opula鈥損redicative", "subjective_objective": {"answer": "Subjective", "rationale": "Because shame is a subjective feeling and attitude of the individual, which reflects the psychological experience and emotional response of the patient to interpersonal interactions."}, "sentence_sentiment": {"answer": "Negative", "rationale": "Because the attitude of \"smuggling to deal with people\" in the entry is negative."}, "language_style": {"answer": "Tactful and warm", "rationale": "The language style of the entry is euphemistic, because it uses the \"smuggling\" description to express situations in which people may feel difficult or uncomfortable in their interpersonal relationships, which is more moderate and does not cause discomfort among the person questioned. At the same time, \"smuggling\" implies that the issue may require more attention and help to encourage the interviewee to open his or her own voicebox to share his or her feelings."}, "time_dimension": {"answer": "Present", "rationale": ""}, "sentence_type": "Negative declarative sentence", "pos": {"nouns": {"I": 1, "person": 1, "Dealing": 1}, "verbs": {"Shame": 1, "and": 1}, "adverbs": {"Shame": 1}, "numerals": {"41": 1}}}, {"content": "42. I am not afraid of anything.", "word_count": 6, "label": "", "sentence_structure": "Subject鈥搗erb鈥揷omplement", "subjective_objective": {"answer": "Subjective", "rationale": "Because \"not afraid\" is subjective."}, "sentence_sentiment": {"answer": "Positive", "rationale": "Because \"not afraid\" is positive."}, "language_style": {"answer": "Tactful and warm", "rationale": "The entry is more euphemistic, not too hard or directly asking the other side whether it is fearless, but rather expresses in a relatively soft tone an understanding of and support for the person under investigation. At the same time, the entry has an encouraging and positive implication that it can resonate and actively engage the person under investigation."}, "time_dimension": {"answer": "Present", "rationale": "According to the sentence given in the title, \"I'm not afraid of anything\" suggests that it is the current mindset or belief of the testee, and therefore the time dimension can be judged as it is now."}, "sentence_type": "Affirmative declarative sentence", "pos": {"verbs": {"Yes": 1}, "adjectives": {"Not even": 1}, "numerals": {"42": 1}}}, {"content": "Sometimes I think I'm an angel, sometimes I think I'm a demon.", "word_count": 20, "label": "", "sentence_structure": "Subject鈥搗erb鈥搊bject", "subjective_objective": {"answer": "Subjective", "rationale": "Because the title contains \"I think\" which shows that it's a subjective view and self-perception of the patient."}, "sentence_sentiment": {"answer": "Negative", "rationale": "Because it contains \"thinking that you're the devil,\" this expression has a certain negative emotional connotation."}, "language_style": {"answer": "Tactful and warm", "rationale": "The entry uses a metaphoric approach to describe the complexity and multidimensional nature of an individual ' s self-perception, while also being more peaceful in tone, not too direct or indifferent, and therefore can be judged as a soft and warm language style."}, "time_dimension": {"answer": "Present", "rationale": "The entry indicates the person interviewed ' s self-awareness and feelings at a certain point in time, and therefore the judgement is now."}, "sentence_type": "Affirmative declarative sentence", "pos": {"nouns": {"Questionnaire": 1, "Entry": 1, "Angel": 1, "Demon": 1}, "verbs": {"I think": 2, "be": 2, "Time": 2}, "adverbs": {"Sometimes": 2}, "numerals": {"43": 1}}}, {"content": "44. In the competition, I prefer to win to lose.", "word_count": 12, "label": "", "sentence_structure": "Subject鈥損redicate", "subjective_objective": {"answer": "Subjective", "rationale": "Because \"winner than lose\" is the mind and attitude of the patient, and it is a subjective exercise."}, "sentence_sentiment": {"answer": "Positive", "rationale": "Because the \"winner than lose\" attitude in the entries is positive."}, "language_style": {"answer": "Calm and direct", "rationale": "The entry expresses the view of the interviewees in a straightforward manner that the competition is more about victory than failure, without too many rhetorical or emotional features, and that it can therefore be judged as \"cool and direct\"."}, "time_dimension": {"answer": "Present", "rationale": "According to the keyword \"at the time of the competition\" it means that the act occurred at a certain point in the past or present and may be repeated in the future. And the phrase is generally speaking now, indicating that it is a description of a normal or customary act, and therefore the entry should be classified as an act or mentality of the present."}, "sentence_type": "Affirmative declarative sentence", "pos": {"verbs": {"The game": 1, "I'd rather": 1, "Win": 2, "I don't want to": 1, "Lose": 1}, "numerals": {"44": 1}}}, {"content": "45. When I was angry, I became eager to be bitter.", "word_count": 15, "label": "", "sentence_structure": "Subject鈥揷opula鈥損redicative", "subjective_objective": {"answer": "Subjective", "rationale": "Because \"getting ready to squawk\" is the patient's feeling and attitude in anger, and it's the patient's subjective idea."}, "sentence_sentiment": {"answer": "Negative", "rationale": "Because the \"becoming willing to squawk\" act in the entry is negative."}, "language_style": {"answer": "Calm and direct", "rationale": "The entry describes the behaviour of the subject in anger in a direct and concise language, with no excessive emotional rendering or rhetorical techniques. This language style is generally more acceptable and understandable and more accurately reflects the actual situation of the interviewee."}, "time_dimension": {"answer": "Past", "rationale": "\"I'm angry\" and \"I'm willing to be bitter\" both occurred at some point in the past, so the temporal dimension of this questionnaire entry is the past."}, "sentence_type": "Exclamatory sentence", "pos": {"nouns": {"Time": 1, "person": 1, "I": 2}, "verbs": {"鈾?And become 鈾?: 1, "Scratch": 1}, "adjectives": {"Anger": 1}, "numerals": {"45": 1}}}, {"content": "46. I publicly responded when my self-esteem was harmed.", "word_count": 15, "label": "", "sentence_structure": "Subject鈥搗erb鈥搊bject", "subjective_objective": {"answer": "Subjective", "rationale": "Because the behaviour and the idea of a return are subjective in nature."}, "sentence_sentiment": {"answer": "Negative", "rationale": "Because the \"publicly\" action in the entry is negative."}, "language_style": {"answer": "Calm and direct", "rationale": "The entry is in plain and simple language, which expresses the actions that are taken when self-esteem is harmed, without any euphemism, consolation or encouragement. This language style is more direct and strong, and does not have too much emotional overturn."}, "time_dimension": {"answer": "Present", "rationale": "Now the entry indicates that when the testee's self-esteem is hurt, they will choose to publicly respond to the act or reaction. Because the word \"just\" in the sentence indicates that it is an instant, conditional reaction, not a future or past consideration. So, the time dimension of the entry is now."}, "sentence_type": "Affirmative declarative sentence", "pos": {"verbs": {"in": 1, "Just": 1, "Public": 1, "Fight back": 1}, "adjectives": {"I": 1, "Self-esteem": 1}, "adverbs": {"Yes": 1, "Hurt": 1, "Time": 1}}}, {"content": "47. I think I should turn my back when I get hurt.", "word_count": 15, "label": "", "sentence_structure": "Subject鈥搗erb鈥搊bject", "subjective_objective": {"answer": "Subjective", "rationale": "Because \"think\" means that the patient's personal opinion and conviction are subjectively viewed."}, "sentence_sentiment": {"answer": "Negative", "rationale": "Because the act of \"shattering\" is a negative emotion and attitude."}, "language_style": {"answer": "Calm and direct", "rationale": "The language style of the entry is more direct, not overly pedestal or emotional, and is more objective and clear, expressing a view of the way in which harm is handled."}, "time_dimension": {"answer": "Future", "rationale": "The keyword \"I think\" and \"should turn back\" indicates that it is a vision and a vision for the future, that is, if the person being tested is hurt one day, he/she thinks he/she should strike back immediately. So this entry is about future behavior or mentality."}, "sentence_type": "Affirmative declarative sentence", "pos": {"nouns": {"Hurt": 1, "Face": 1}, "verbs": {"I think": 1, "Yes": 1, "Just": 2, "I think so": 1, "Flip": 1}, "adverbs": {"When": 1}, "numerals": {"47": 1}, "pronouns": {"I": 1}}}, {"content": "48. Not every page of my daily newspaper.", "word_count": 14, "label": "", "sentence_structure": "Subject鈥搗erb鈥搊bject", "subjective_objective": {"answer": "Subjective", "rationale": "Because \"I read every day\" and \"not every page\" are patient subjective experiences and attitudes."}, "sentence_sentiment": {"answer": "Neutral", "rationale": "Because the entry describes a person ' s reading habits and does not express positive or negative emotional tendencies."}, "language_style": {"answer": "Tactful and warm", "rationale": "This entry presents the habits and preferences of the respondents in reading newspapers in a concise and clear manner, without using excessive emotional or rhetorical techniques. This is more appropriate for objective data collection and analysis."}, "time_dimension": {"answer": "Present", "rationale": ""}, "sentence_type": "Negative declarative sentence", "pos": {"nouns": {"I": 1, "Oh, my God": 1, "Report": 2, "Board": 2}, "verbs": {"Read it": 1, "be": 1, "Every": 1, "Both": 1}, "adverbs": {"Chile": 1, "No, it's not": 1}, "numerals": {"48": 1}, "classifiers": {"individual": 2}}}, {"content": "49. When I am depressed, I avoid.", "word_count": 8, "label": "", "sentence_structure": "Subject鈥搗erb鈥揷omplement", "subjective_objective": {"answer": "Subjective", "rationale": "This is a manifestation of subjective attitudes and behaviour, as patients describe their emotional responses and coping strategies."}, "sentence_sentiment": {"answer": "Negative", "rationale": "Because the \"shield\" act in the entry is negative."}, "language_style": {"answer": "Calm and direct", "rationale": "The entry is more concise in language style, and asks the interviewee what to do when facing frustration. There is no over-modified or emotional rendering that gives people an objective, direct sense."}, "time_dimension": {"answer": "Present", "rationale": ""}, "sentence_type": "Affirmative declarative sentence", "pos": {"nouns": {"Frustration": 1, "Avoidance": 1}, "verbs": {"Yes": 1, "Avoid": 1}, "adverbs": {"Time": 1}, "numerals": {"49": 1}}}, {"content": "I am shy about the issue of sexuality.", "word_count": 9, "label": "", "sentence_structure": "Subject鈥揷opula鈥損redicative", "subjective_objective": {"answer": "Subjective", "rationale": "Because being shy is the patient's subjective feeling and attitude."}, "sentence_sentiment": {"answer": "Negative", "rationale": "Because shyness is a negative emotional word that means uncomfortable feeling and reaction to sexual problems."}, "language_style": {"answer": "Tactful and warm", "rationale": "The entry uses a gentle and respectful tone to ask the other side about their sexual feelings, without directly denying or accusing them, but rather engaging in a caring and supportive dialogue that makes it easier for the interviewees to open their minds."}, "time_dimension": {"answer": "Present", "rationale": "The entry indicates that the testee feels shy about the issue of sexuality at this time."}, "sentence_type": "Affirmative declarative sentence", "pos": {"nouns": {"Problem": 1, "Sex": 1, "Shame": 1, "Answer": 1}, "verbs": {"to": 1, "Sensor": 1, "Present": 1, "I feel": 1, "Shame": 1, "Back": 1, "Reactions": 1}, "adjectives": {"Shy": 1, "Sex": 1}, "numerals": {"50": 1}}}, {"content": "51. I always feel like someone I know is a protector.", "word_count": 18, "label": "", "sentence_structure": "Subject鈥揷opula鈥損redicative", "subjective_objective": {"answer": "Subjective", "rationale": "Because \"always feeling\" means that it's a subjective feeling and a perception."}, "sentence_sentiment": {"answer": "Positive", "rationale": "Because the expression \"like a protector\" in the entry is positive, indicating that the person questioned has a strong sense of trust and dependence on the person."}, "language_style": {"answer": "Tactful and warm", "rationale": "The language style of the entry is euphemism. It uses metaphors, it uses the term \"someone\" as a term of \"protector,\" and it uses the expression \"always feeling\" to make the expression positive and emotional. This euphemism, warm expression, allows the person under investigation to feel concerned and supportive and to answer the questions more honestly."}, "time_dimension": {"answer": "Present", "rationale": "I always felt that the word \"general\" meant a regular, customary act, and \"sweet\" used the general current, meaning that it had not only happened in the past, but might still be going on. So the entry asks the person who was tested about his or her current feelings and mental state."}, "sentence_type": "Affirmative declarative sentence", "pos": {"nouns": {"person": 1, "Protect the gods": 1}, "verbs": {"I feel": 1, "Yes": 1, "Elephant": 1}, "adjectives": {"Always": 1}, "numerals": {"51": 1}, "pronouns": {"I": 1}}}, {"content": "My philosophies are: 鈥淏elieve, do not do, do not do鈥?", "word_count": 19, "label": "", "sentence_structure": "Subject鈥損redicate", "subjective_objective": {"answer": "Subjective", "rationale": "Because \"no reason, no reason, no reason\" is a personal conviction and attitude that belongs to the patient's subjective idea."}, "sentence_sentiment": {"answer": "Negative", "rationale": "Because the purpose of the act is negative."}, "language_style": {"answer": "Calm and direct", "rationale": "The language style of the entry is more calm and straightforward, and it does not use too many rhetorical techniques or emotional renderings. It expresses the philosophies of the subject in a concise manner 鈥?only what is reasonable is worth believing, doing and seeing. This language style is more conducive to ensuring the authenticity and objectivity of the questionnaire."}, "time_dimension": {"answer": "Present", "rationale": "The word \"is\" in the entry denotes the current state or characteristics of the dominant language, so the sentence describes the testee's current philosophies and is now the norm."}, "sentence_type": "Affirmative declarative sentence", "pos": {"nouns": {"Philosophy of the world": 1, "I'm not": 3, "Letter": 1, "Do it": 1, "View": 1}, "verbs": {"be": 1, "I don't believe it": 1, "Do not do it": 1, "Do not look": 1}, "adverbs": {"I'm not": 3}}}, {"content": "53. I think that there are good and bad people.", "word_count": 9, "label": "", "sentence_structure": "Subject鈥搗erb鈥搊bject", "subjective_objective": {"answer": "Subjective", "rationale": "Because \"think\" is the patient's own idea and opinion, and it's seen from the patient's subjective perspective."}, "sentence_sentiment": {"answer": "Neutral", "rationale": "Because the entry does not have a clear positive or negative sentiment. It simply expresses a person ' s basic understanding and view of humanity, without reference to specific behaviours, attitudes or emotional colours."}, "language_style": {"answer": "Calm and direct", "rationale": "The language style of the entry is straightforward, it is not based on excessive rhetorical or emotional rendering, it is more objective and it expresses a judgement of humanity."}, "time_dimension": {"answer": "Present", "rationale": "The keyword is \"conception,\" which indicates that it is the present view and attitude of the testee. Although there is no clear time term in this sentence, it should be judged now in terms of the dimension of time, because it expresses a personal conviction or opinion."}, "sentence_type": "Affirmative declarative sentence", "pos": {"nouns": {"person": 2, "Good and bad": 1}, "verbs": {"have": 1, "min": 1}, "adjectives": {"Bad": 1}, "numerals": {"53": 1}, "pronouns": {"I": 1}}}, {"content": "54. If my superiors upset me, I may find myself in trouble at work or in a seamstress to avenge him.", "word_count": 29, "label": "", "sentence_structure": "Subject鈥搗erb鈥搊bject", "subjective_objective": {"answer": "Subjective", "rationale": "This is a subjective attitude and behavioural projection of patients and reflects their negative emotional reactions to their superiors."}, "sentence_sentiment": {"answer": "Negative", "rationale": "Because the entry's \"probable or mower\" is negative."}, "language_style": {"answer": "Calm and direct", "rationale": "The language style of the entry is a calm and straightforward one, which expresses predictions and expectations of behavioural outcomes mainly by way of presentation and description. This style is usually more concise, avoiding excessive emotional colours and rhetorical techniques, and more objective and accurate."}, "time_dimension": {"answer": "Future", "rationale": "The entry contains visions and expectations for the future, with the keyword \"possible\" and \"reprisal\" pointing to situations that have not yet occurred and that may arise in the future."}, "sentence_type": "Affirmative declarative sentence", "pos": {"nouns": {"Sir": 1, "I": 2, "Work": 1, "Trouble": 1, "A seaman": 1, "Revenge": 1}, "verbs": {"Fuck": 1, "Maybe": 1, "Find": 1, "Yes": 1, "in": 1, "Work": 1, "Medium": 1, "Grind": 1, "Revenge": 1}, "adjectives": {"Angry": 1, "of": 2}, "adverbs": {"Maybe": 1}, "numerals": {"54": 1}, "classifiers": {"individual": 1}, "pronouns": {"he": 1}}}, {"content": "Everyone is against me.", "word_count": 9, "label": "", "sentence_structure": "Subject鈥搗erb鈥搊bject", "subjective_objective": {"answer": "Subjective", "rationale": "Because it is the patient ' s subjective feeling, not the objective fact."}, "sentence_sentiment": {"answer": "Negative", "rationale": "Because the expression \"everyone\" in the entry conveys a negative emotional color with the expression \"I'm against you\"."}, "language_style": {"answer": "Tactful and warm", "rationale": "The language style of the entry is relatively gentle, using the more neutral expression \"everyone\" to avoid direct accusations or attacks on the other. At the same time, the term \"to the ends\" is used to convey the meaning of contradictions and conflicts rather than to describe hostile behaviour directly, making the problem more implicit and acceptable."}, "time_dimension": {"answer": "Future", "rationale": "The key words for the sentence, which indicates a description of what may happen in the future, are \"both\" and \"hand-on\" and point to situations that have not yet occurred and that may arise in the future."}, "sentence_type": "Negative declarative sentence", "pos": {"nouns": {"person": 1, "I": 1}, "verbs": {"- To the front": 1}, "numerals": {"55": 1}}}, {"content": "I am often friendly to those I hate.", "word_count": 16, "label": "", "sentence_structure": "Subject鈥搗erb鈥揷omplement", "subjective_objective": {"answer": "Subjective", "rationale": "This is a subjective attitude and behaviour of the patient, reflecting his attitude and behaviour choices towards the hateful."}, "sentence_sentiment": {"answer": "Negative", "rationale": "Because the expression \"the hateful person\" in the entry is negative, and it's not like or hate."}, "language_style": {"answer": "Tactful and warm", "rationale": "The language style of this entry is more euphemistic, and in a gentle way, asks whether the respondents often express their feelings of friendship to the person they hate. This expression is more friendly and acceptable, reducing the resistance that direct questions may generate."}, "time_dimension": {"answer": "Past", "rationale": "The keyword \"often\" indicates that it is a regular act, and \"I hate people\" indicates that it is an act of choice of the testee, so it can be inferred that the entry is asking about the testee's past behaviour."}, "sentence_type": "Negative declarative sentence", "pos": {"nouns": {"Entry": 1, "person": 2, "Friendly": 1}, "verbs": {"I": 2, "Organisation": 1, "Shit": 1, "to": 1}, "adjectives": {"Those": 1, "Often": 1, "It's disgusting": 1}, "adverbs": {"And": 1}, "numerals": {"56": 1}}}, {"content": "57. If I had a engine failure on the plane, I would have been very nervous.", "word_count": 22, "label": "", "sentence_structure": "Subject鈥搗erb鈥搊bject", "subjective_objective": {"answer": "Subjective", "rationale": "Because \"very nervous\" is the patient's subjective feeling and reaction."}, "sentence_sentiment": {"answer": "Negative", "rationale": "Because the \"very nervous\" mood in the entry is negative."}, "language_style": {"answer": "Calm and direct", "rationale": "The entry uses a direct presentation, indicating a hypothetical situation and the reaction, with a more calm and objective language style, without excessive ado or emotional colour."}, "time_dimension": {"answer": "Past", "rationale": "The keyword \"if\" and \"will\" indicate that it is a question of a reaction in a given situation in the past, and the temporal dimension of the article is therefore judged to be the past."}, "sentence_type": "Affirmative declarative sentence", "pos": {"nouns": {"Aircraft": 1, "Engines": 1}, "verbs": {"Take it": 1, "It's not working": 1, "Yes": 1, "Nervous": 1}, "adjectives": {"Very": 1}, "numerals": {"57": 1}, "pronouns": {"I": 1}}}, {"content": "I know a man who can do anything and does it with integrity.", "word_count": 22, "label": "", "sentence_structure": "Subject鈥搗erb鈥搊bject", "subjective_objective": {"answer": "Subjective", "rationale": "Because it's the patient's perception and evaluation of such a person that is subjective."}, "sentence_sentiment": {"answer": "Positive", "rationale": "Because \"reasonable integrity\" is a positive quality."}, "language_style": {"answer": "Tactful and warm", "rationale": "The entry is more personal and interactive in its expression of a gentle and inviting tone, rather than a direct statement or order. At the same time, the article ' s purpose is more positive and appreciated, giving a positive impression."}, "time_dimension": {"answer": "Present", "rationale": "The entry describes the current state or situation of the person being tested, in which \"I know such a person\" indicates that it is the subjective feeling or view of the person being tested. The latter description is an assessment of the person ' s abilities and qualities, and is an observation and judgement of the current state. The entry should therefore be placed in the present context."}, "sentence_type": "Affirmative declarative sentence", "pos": {"nouns": {"person": 1, "Yes": 2, "Both": 1, "Reasonable": 1, "Integrity": 1}, "verbs": {"Yes": 2, "Do it": 2}, "adjectives": {"Here": 1, "Everything": 1}, "adverbs": {"And": 1}, "numerals": {"One": 3}, "classifiers": {"individual": 1}, "pronouns": {"he": 1}}}, {"content": "59. If my emotional outburst prevents me from doing what I am doing, I can control it.", "word_count": 27, "label": "", "sentence_structure": "Subject鈥損redicate", "subjective_objective": {"answer": "Subjective", "rationale": "Because \"controllable\" is the patient's subjective attitude and ability."}, "sentence_sentiment": {"answer": "Positive", "rationale": "Because the attitude that the entries express to control emotions is positive."}, "language_style": {"answer": "Calm and direct", "rationale": "The entry is more calm and straightforward, and does not have too much rhetorical or emotional overtook. It expresses its opinion of emotional management in an objective and rational way, i.e., if the loss of a relationship is a hindrance to the cause, it should learn to control it. This expression is more logical and practical."}, "time_dimension": {"answer": "Present", "rationale": "The \"if\" in this questionnaire entry indicates a scenario, \"I can control it\" means that it is the attitude and perception of the testee, so the time dimension of the article is now."}, "sentence_type": "Affirmative declarative sentence", "pos": {"nouns": {"Emotion": 1, "- Out": 1, "Career": 1}, "verbs": {"- Intrusive": 1, "Control": 1}, "adverbs": {"If": 1, "Just": 1}, "numerals": {"59": 1, "I": 2}}}, {"content": "60. Some people are plotting to kill me.", "word_count": 10, "label": "", "sentence_structure": "Subject鈥搗erb鈥搊bject", "subjective_objective": {"answer": "Objective", "rationale": "Because this is an external information that the patient senses, it is a description of the objective facts."}, "sentence_sentiment": {"answer": "Negative", "rationale": "Because the \"scheming to harm me\" in the entry is threatening and offensive, it's negative."}, "language_style": {"answer": "Calm and direct", "rationale": "The entry uses a more direct expression to ask the other party whether he has ever been subjected to a conspiracy against himself, which is more straightforward, objective and not overly emotional or rhetorical.\""}, "time_dimension": {"answer": "Present", "rationale": "The keyword is \"is,\" \"harm,\" which means that the behavior is currently under way, so the judgment is now."}, "sentence_type": "Affirmative declarative sentence", "pos": {"nouns": {"person": 1, "Shit": 1, "I": 1}, "verbs": {"In the process of": 1, "Conspiracy": 1, "Shit": 1}, "adverbs": {"Some": 1}, "numerals": {"60": 1}}}, {"content": "61. I usually see the positive side of the bad.", "word_count": 16, "label": "", "sentence_structure": "Subject鈥搗erb鈥揷omplement", "subjective_objective": {"answer": "Subjective", "rationale": "Because it is the patient's personal views and attitudes that are good to see in the worst of the situation."}, "sentence_sentiment": {"answer": "Positive", "rationale": "Because the act of \"seeing the good side of the bad\" in the entry is a positive attitude."}, "language_style": {"answer": "Tactful and warm", "rationale": ""}, "time_dimension": {"answer": "Present", "rationale": "The entry uses the current state of affairs and describes the person ' s current behaviour or state, and thus the present is judged."}, "sentence_type": "Affirmative declarative sentence", "pos": {"nouns": {"I": 1, "Bad times": 1, "Noodles": 1}, "verbs": {"Yeah": 2, "Medium": 1, "When": 1, "Okay": 1, "of": 2, "One side": 1}, "adjectives": {"Okay": 1}, "adverbs": {"Usually": 1}}}, {"content": "62. When I had to do something I did not want to do, I had a headache.", "word_count": 20, "label": "", "sentence_structure": "Subject鈥揷opula鈥損redicative", "subjective_objective": {"answer": "Subjective", "rationale": "Because headaches are a subjective symptom, probably caused by the patient鈥檚 emotional or psychological state. While the sentence refers to objective events (i.e., 鈥渢he need to do something I do not want to do鈥?, they do not in themselves directly cause headaches, so headaches are subjective experiences of patients."}, "sentence_sentiment": {"answer": "Negative", "rationale": "Because the \"headache\" act in the entry is negative."}, "language_style": {"answer": "Calm and direct", "rationale": "The language style of the entry is more direct and concise, and there is no excessive rhetorical or emotional rendering, presenting the problem in an objective manner, which enables the person surveyed to understand the meaning of the problem accurately."}, "time_dimension": {"answer": "Past", "rationale": "\"I have to do something I don't want to do,\" which means it's a situation that has happened in the past and that cannot be changed, so the time dimension of this questionnaire entry is the past."}, "sentence_type": "Affirmative declarative sentence", "pos": {"nouns": {"Headache": 1, "Things": 1, "I don't want to": 1}, "verbs": {"Do it": 1, "Go on": 1, "Okay": 1, "Jean": 1}, "adjectives": {"I had to": 1, "Some": 1, "of": 4}, "numerals": {"62": 1}, "classifiers": {"individual": 1}, "pronouns": {"I": 1}}}, {"content": "63. I have often found myself very friendly to those who deserve to be hated.", "word_count": 20, "label": "", "sentence_structure": "Subject鈥搗erb鈥搊bject", "subjective_objective": {"answer": "Subjective", "rationale": "Because \"I often find myself very friendly to those who deserve to hate\" is the patient's subjective attitude."}, "sentence_sentiment": {"answer": "Negative", "rationale": "Because \"to show friendship to those who deserve it\" is not normal and may cause harm to their interests, it is a negative emotional colour."}, "language_style": {"answer": "Tactful and warm", "rationale": "The entry is more adept and interactive when it is spoken in a gentle and inviting manner, rather than by direct statements or orders."}, "time_dimension": {"answer": "Past", "rationale": "\"Always\" in the entry indicates that the act is a past custom, and \"I find that I am friendly to those who deserve to be hated\" means a description of self-awareness and experience over time, and therefore the temporal dimension is a past."}, "sentence_type": "Affirmative declarative sentence", "pos": {"nouns": {"Questionnaire Entry": 1, "person": 2}, "verbs": {"Found": 1, "Organisation": 1, "Hate": 1, "Friendly": 1}, "adjectives": {"Often": 1}, "numerals": {"63": 1}, "pronouns": {"I": 1, "Those": 1}}}, {"content": "I think that 鈥渆veryone has good will鈥?does not exist, and if you are not, you are not well.", "word_count": 27, "label": "", "sentence_structure": "Subject鈥搗erb鈥搊bject", "subjective_objective": {"answer": "Subjective", "rationale": "Because the entry in the questionnaire expresses the opinion and opinion of the interviewees that \"everyone has good will\", it is a subjective judgement."}, "sentence_sentiment": {"answer": "Negative", "rationale": "According to the words in the entry, \"everyone has good will\" is judged to be \"no\" and if a person is not good, everything is bad. It is a very negative, negative attitude or view, and it is therefore possible to judge that the emotional orientation of the purpose is negative."}, "language_style": {"answer": "Calm and direct", "rationale": "The language style of the entry is more straightforward, concise, and not too much rhetorical or emotionally strong, but rather an objective and rational approach to the author鈥檚 views. Thus, the language style can be described as being straightforward and calm."}, "time_dimension": {"answer": "Present", "rationale": "\"Between,\" \"Be exists\" and \"If you're not good, then you're not good\" are all describing the views and attitudes of the interviewees today, so the entry is judged now."}, "sentence_type": "Negative declarative sentence", "pos": {"nouns": {"Questionnaire": 1, "Entry": 1, "Good will": 1, "Existence": 1, "you": 2, "Everything": 1, "It's not good": 1}, "verbs": {"I think": 1, "be": 1, "Existence": 1, "have": 1}, "adjectives": {"Everyone": 1, "Not good": 1}, "numerals": {"64": 1}, "pronouns": {"I": 1}}}, {"content": "I will never be angry with those I hate.", "word_count": 16, "label": "", "sentence_structure": "Subject鈥搗erb鈥搊bject", "subjective_objective": {"answer": "Objective", "rationale": "Because \"I will never be angry with those I hate\" is determined by the patient's objective attitude and behaviour. This is not based on the patient's feelings, thoughts or feelings, but on their beliefs or values. So this entry is based on objective observations."}, "sentence_sentiment": {"answer": "Negative", "rationale": "Because the word \"obsessed\" in the entry indicates a negative emotional attitude."}, "language_style": {"answer": "Calm and direct", "rationale": "The entry expresses in a straightforward manner the subject of an angry response to an undesirable person, which is more objective and not too much rhetorical or emotional."}, "time_dimension": {"answer": "Present", "rationale": "The entry asks the testee about his current attitude and behaviour, and the time dimension is now judged. The keyword \"never\" expresses a strong, positive attitude, and \"expressed anger\" is a specific description of behaviour. The questionnaire entry is therefore asking if the testee often displays anger towards those they hate and the testee is strongly opposed to doing so."}, "sentence_type": "Negative declarative sentence", "pos": {"nouns": {"Questionnaire": 1, "Entry": 1, "person": 1}, "verbs": {"Never": 1, "Organisation": 1, "to": 1, "Disgusting": 1}, "adjectives": {"Shit": 1}, "numerals": {"65": 1}}}, {"content": "66. I am convinced that life is unjust to me.", "word_count": 12, "label": "", "sentence_structure": "Subject鈥揷opula鈥損redicative", "subjective_objective": {"answer": "Subjective", "rationale": "It is a subjective attitude towards the perception of life."}, "sentence_sentiment": {"answer": "Negative", "rationale": "Because the entry \"life is unjust to me\" expresses a negative emotional attitude."}, "language_style": {"answer": "Calm and direct", "rationale": "The language style of the entry is more direct, objective, and there is no excessive emotional and rhetorical approach to convey the attitude of the subject to life in a simple and clear manner."}, "time_dimension": {"answer": "Present", "rationale": "The entry shows the current attitude and conviction of the testee that life is unfair to him and that the time dimension is now judged."}, "sentence_type": "Negative declarative sentence", "pos": {"nouns": {"Life": 1, "I": 2, "Injustice": 1}, "verbs": {"Sure": 1, "to": 1, "be": 1}, "adjectives": {"Justice": 1}, "numerals": {"66": 1}}}, {"content": "67. I will fall under severe blow.", "word_count": 13, "label": "", "sentence_structure": "Subject鈥揷opula鈥損redicative", "subjective_objective": {"answer": "Subjective", "rationale": "Because it is a description of the patient ' s psychological feelings, it reflects the self-awareness and attitude of the patient in the face of stress or shock."}, "sentence_sentiment": {"answer": "Negative", "rationale": "Because \"falling\" in the entries is a negative emotion and attitude."}, "language_style": {"answer": "Calm and direct", "rationale": "The language style of the entry is more calm and straightforward, and it does not use too much qualifier or emotional expression, but rather expresses the problem in an objective, concise way. This expression is clearer and easier to understand."}, "time_dimension": {"answer": "Present", "rationale": "The keyword \"will fall\" indicates a negative mental or behavioural reaction."}, "sentence_type": "Negative declarative sentence", "pos": {"nouns": {"Serious": 1, "Strike": 1, "Falling down": 1}, "verbs": {"Yes": 1, "Falling down": 1}, "adjectives": {"Serious": 1}, "numerals": {"67": 1}}}, {"content": "68. When I realized that I had to face a dilemma, such as the examination, and the trade union, I tried to imagine what it would do and to plan ways to deal with it.", "word_count": 46, "label": "", "sentence_structure": "Subject鈥搗erb鈥搊bject", "subjective_objective": {"answer": "Subjective", "rationale": "This entry deals with patients鈥?subjective ideas and behavioural plans, which are to deal with the situation through imagination and planning."}, "sentence_sentiment": {"answer": "Positive", "rationale": "Because the behavior and attitude in the entries are positive, like \"aware,\" \"tries to imagine,\" \"plans to deal with it.\""}, "language_style": {"answer": "Calm and direct", "rationale": "The entry describes in a calm and direct tone the methods usually employed by the interviewees in the event of a situation, without excessive emotional or rhetorical style, and with a more concise language style.\""}, "time_dimension": {"answer": "Present", "rationale": ""}, "sentence_type": "Affirmative declarative sentence", "pos": {"nouns": {"I": 1, "The dilemma": 1, "Examinations": 1, "Recruitment fair": 1}, "verbs": {"I'm aware of that": 1, "Faced": 1, "Imagine": 1, "Plan": 1, "Payable": 1}, "adverbs": {"Just": 2}, "numerals": {"68": 1}}}, {"content": "The doctors will never really figure out what I am suffering from.", "word_count": 17, "label": "", "sentence_structure": "Subject鈥搗erb鈥揷omplement", "subjective_objective": {"answer": "Subjective", "rationale": "This is because patients view and evaluate doctors and is a manifestation of subjective attitudes."}, "sentence_sentiment": {"answer": "Negative", "rationale": "Because the phrase \"doctors will never really know what my disease is\" in the entry, which expresses mistrust of doctors and concerns that they cannot be accurately diagnosed, is a negative emotional color."}, "language_style": {"answer": "Calm and direct", "rationale": "The language style of the entry is more straightforward, with no excessive rhetoric or emotional rendering, and is more objective and straightforward."}, "time_dimension": {"answer": "Past", "rationale": "The term \"never\" is a word of determination and decision, implying that this belief has been formed in the past and continues to this day, and the temporal dimension of the article is therefore judged by the past."}, "sentence_type": "Negative declarative sentence", "pos": {"nouns": {"Doctor": 1, "Diseases": 1}, "verbs": {"Never": 1, "I know": 1, "Patient": 1}, "adverbs": {"True": 1}, "numerals": {"69": 1}, "pronouns": {"I": 1}}}, {"content": "70. I am not sad when someone close to me dies.", "word_count": 18, "label": "", "sentence_structure": "Subject鈥搗erb鈥揷omplement", "subjective_objective": {"answer": "Subjective", "rationale": "Because \"sorrow\" is an emotional response, it is subjective to look at whether the individual feels sad when facing the death of his or her loved ones. Although the death of a loved one is an objective fact, the emotional response to that fact is subjective."}, "sentence_sentiment": {"answer": "Negative", "rationale": "Because the entry \"When someone close to me dies, not sad\" is negative and indicates a lack of care and compassion for others."}, "language_style": {"answer": "Calm and direct", "rationale": "The entry is presented in a straightforward manner, without too much rhetoric or emotional rendering, and is more objective and quiet. It is designed to understand the emotional reactions of the interviewees in the face of the death of their relatives and friends, and does not attempt to induce or influence their answers."}, "time_dimension": {"answer": "Past", "rationale": "The temporal dimension of the purpose of the article is therefore past."}, "sentence_type": "Negative declarative sentence", "pos": {"nouns": {"person": 1, "Die": 1}, "verbs": {"When": 1, "very": 1, "Close": 1, "Sad": 1, "No, it's not": 1, "Die": 1}, "adjectives": {"I": 1}, "adverbs": {"not": 1}, "numerals": {"70": 1}}}, {"content": "71. After my struggles for my interests and for others, I often apologize for my rudeness.", "word_count": 26, "label": "", "sentence_structure": "Subject鈥搗erb鈥搊bject", "subjective_objective": {"answer": "Subjective", "rationale": "It can be inferred from the sentence that this is the patient ' s subjective feeling."}, "sentence_sentiment": {"answer": "Negative", "rationale": "Because words like \"fight\" and \"bold\" all have negative emotional connotations, which indicate that the act is negative."}, "language_style": {"answer": "Calm and direct", "rationale": "The entry is presented directly, without excessive rhetorical or emotional rendering. It describes a situation in which, after a dispute over interests, people are apologized for rudeness and the language is more objective."}, "time_dimension": {"answer": "Past", "rationale": "The entry refers to past behaviour, \"after\"... and \"often\" indicates that it is a regular, customary sexual act. Thus, the temporal dimension of the entry is the past."}, "sentence_type": "Affirmative declarative sentence", "pos": {"nouns": {"I": 3, "person": 2, "Interest": 1, "Fighting": 1, "Naughty": 1, "Apologize": 1}, "verbs": {"For": 1, "Fighting": 1, "Often": 1, "Because": 1, "In": 1, "Apologize": 1}, "adjectives": {"Naughty": 1}, "adverbs": {"After": 1, "Often": 1}, "numerals": {"71": 1}, "pronouns": {"I": 2}}}, {"content": "Most of what happened to me was not my responsibility.", "word_count": 19, "label": "", "sentence_structure": "Subject鈥揷opula鈥損redicative", "subjective_objective": {"answer": "Subjective", "rationale": "Because \"most things\" and \"not my responsibility\" are the subjective perceptions and attitudes of the patients themselves."}, "sentence_sentiment": {"answer": "Negative", "rationale": "Because the phrase \"most things are not my responsibility\" in the entry is negative."}, "language_style": {"answer": "Calm and direct", "rationale": "The entry presents a point of view in a direct, concise manner, without too many rhetorical or emotional features, and with a more calm and direct language style."}, "time_dimension": {"answer": "Past", "rationale": ""}, "sentence_type": "Negative declarative sentence", "pos": {"nouns": {"Things": 1, "Responsibility": 1}, "verbs": {"Come on": 1, "be": 1}, "adjectives": {"Mostly": 1, "Related": 1}, "adverbs": {"No, it's not": 1}, "numerals": {"72": 1}, "pronouns": {"I": 1, "of": 1}}}, {"content": "73. When I feel depressed or anxious, I feel better at eating something.", "word_count": 26, "label": "", "sentence_structure": "Subject鈥搗erb鈥揷omplement", "subjective_objective": {"answer": "Subjective", "rationale": "Because it is a subjective feeling and idea of the patient, not an objective fact. The patient thinks that eating something makes him feel better, which is a subjective judgment and interpretation."}, "sentence_sentiment": {"answer": "Negative", "rationale": "Because eating can have some negative health effects as a way to cope with stress. While it may be better in the short term, it may lead to weight gain, health problems, etc. in the long run."}, "language_style": {"answer": "Tactful and warm", "rationale": "The entry describes a possible response in a mild and understanding way, without directly evaluating or criticizing the behaviour. At the same time, using the expression \"can make me feel better\" conveys a sense of encouragement and support, giving people a sense of kindness and kindness."}, "time_dimension": {"answer": "Present", "rationale": "\"Feeling emotional depression or anxiety,\" \"Eat something,\" \"Eat better\" are all descriptions of current, ongoing behaviour or circumstances, so this questionnaire entry should be judged as now."}, "sentence_type": "Affirmative declarative sentence", "pos": {"nouns": {"Time": 1, "Emotion": 1, "Repression": 1, "Anxiety": 1, "Unsettled": 1, "Eat": 1, "Something": 1, "鈾?Make me 鈾?: 1, "Feel": 2, "Better": 1}, "verbs": {"be": 1, "It can do": 1}, "adjectives": {"It's depressing": 1, "I'm worried": 1}, "numerals": {"73": 1}, "classifiers": {"individual": 1}, "pronouns": {"I": 1}}}, {"content": "74. I feel better working hard.", "word_count": 10, "label": "", "sentence_structure": "Subject鈥揷opula鈥損redicative", "subjective_objective": {"answer": "Subjective", "rationale": "Because \"fear better\" is the patient's subjective feeling and attitude."}, "sentence_sentiment": {"answer": "Positive", "rationale": "Because the expression \"working hard makes me feel better\" has a positive emotional effect."}, "language_style": {"answer": "Tactful and warm", "rationale": ""}, "time_dimension": {"answer": "Present", "rationale": ""}, "sentence_type": "Affirmative declarative sentence", "pos": {"nouns": {"I'm working hard": 1, "Work": 1, "Feel": 1}, "verbs": {"鈾?And I'm gonna be 鈾?: 1, "Better": 1}, "adverbs": {"More": 1}, "numerals": {"74": 1}}}, {"content": "75. Doctors cannot really help me with this.", "word_count": 12, "label": "", "sentence_structure": "Subject鈥搗erb鈥搊bject", "subjective_objective": {"answer": "Subjective", "rationale": "This is a question that reflects the patient ' s perception of and attitude towards the doctor and is subjective."}, "sentence_sentiment": {"answer": "Negative", "rationale": "Because it expresses a negative and disappointing attitude that doctors cannot really solve their problems."}, "language_style": {"answer": "Calm and direct", "rationale": "The language style of the entry is more straightforward, there is no excessive rhetorical or emotional rendering, and it is more objective and incriminating. It expresses the other side鈥檚 view of whether the doctor can really help him or her to solve the problem in a direct way, which is more realistic and realistic."}, "time_dimension": {"answer": "Present", "rationale": "The entry is for the purpose of asking the person ' s views and attitude towards the doctor, and the time dimension is now judged."}, "sentence_type": "Negative declarative sentence", "pos": {"nouns": {"Doctor": 1, "Problem": 1}, "verbs": {"Yes": 1, "Help": 1, "Resolve": 1}, "adjectives": {"True": 1}, "numerals": {"75": 1}}}, {"content": "I have heard a lot about my feelings.", "word_count": 15, "label": "", "sentence_structure": "Subject鈥搗erb鈥搊bject", "subjective_objective": {"answer": "Subjective", "rationale": "Because \"not to reveal your feelings\" is a personal feeling or attitude of the patient, not an objective fact. This reflects the patient's subjective perception and evaluation of how he or she expresses his or her feelings."}, "sentence_sentiment": {"answer": "Neutral", "rationale": "As this entry does not express any positive or negative feelings, attitudes, behaviour, purpose or quality."}, "language_style": {"answer": "Calm and direct", "rationale": "The language style of the entry is relatively calm and straightforward, and there is no excessive emotional rendering or rhetorical technique to express the information that the investigator wants to understand in a simple and clear manner. At the same time, the use of the word \"often\" also shows that this is a relatively common phenomenon and does not only target a person or a particular situation."}, "time_dimension": {"answer": "Past", "rationale": "The entry is therefore an inquiry into the current situation and behaviour of the person being tested, and the temporal dimension is now judged."}, "sentence_type": "Negative declarative sentence", "pos": {"nouns": {"Questionnaire": 1, "Entry": 1, "People": 1, "Emotion": 1}, "verbs": {"Regular": 1, "Listen": 1, "Say it": 2, "Exposure": 1}, "adjectives": {"self": 1, "of": 2}, "adverbs": {"not": 2, "Regular": 1, "Land": 1}, "numerals": {"76": 1}, "pronouns": {"I": 1}}}, {"content": "77. I believe that people are watching movies, plays or books for more than they are meant to express.", "word_count": 35, "label": "", "sentence_structure": "Subject鈥搗erb鈥搊bject", "subjective_objective": {"answer": "Subjective", "rationale": "Because \"thinks\" is a subjective term for judgment."}, "sentence_sentiment": {"answer": "Positive", "rationale": "Because the entry expresses the subjective view of the author and is positive, namely that people can gain a deeper understanding and meaning from artistic works."}, "language_style": {"answer": "Tactful and warm", "rationale": "The language style is more acrimony and gentler than that of the entry, which uses a comparative and subjective expression of opinion and does not directly challenge or dictate to the other."}, "time_dimension": {"answer": "Past", "rationale": "The entry describes a view or belief of the person tested, which was formed in the past, possibly in the context of watching films, theatres or books. Thus, judging from the time dimension, it is the past."}, "sentence_type": "Affirmative declarative sentence", "pos": {"nouns": {"person": 1, "Film": 1, "Theatre": 1, "Books": 1, "Meaning": 2, "Works": 1}, "verbs": {"I think": 1, "I'm watching a movie": 1, "Theatre": 1, "Reading books": 1, "Learn": 1, "Expression": 1, "More than that": 1}, "adverbs": {"More": 1}, "numerals": {"77": 1}, "pronouns": {"These": 1}}}, {"content": "I feel that I have some customary or ritual behaviour that I have to do on my own and that I have caused me a lot of trouble.", "word_count": 32, "label": "", "sentence_structure": "Subject鈥揷opula鈥損redicative", "subjective_objective": {"answer": "Subjective", "rationale": "Because \"no autonomy\", \"customary or ritual behaviour\" and \"problems\" are the feelings and experiences of the patient, the question is the subjective feelings of the patient."}, "sentence_sentiment": {"answer": "Negative", "rationale": "Because the words \"involved custom or ritual\" and \"involved in a lot of trouble\" in the entries are negative descriptions."}, "language_style": {"answer": "Tactful and warm", "rationale": "The language style of the entry is relatively euphemistic and gentle. It leads the interviewee to wonder in a way whether he or she has some custom or ritual behavior, and whether it has caused himself or herself trouble. It is a less expression that puts too much pressure or discomfort on the interviewee and helps him or her to answer the questions more honestly."}, "time_dimension": {"answer": "Present", "rationale": "Now the \"fear\" and \"yes\" actions in the entry are in the current state of the speaker, and therefore are judged to be current. At the same time, \"problems a lot of trouble\" also imply that this custom or ritual act has an impact on the daily life of the speaker, further proving that this is a current problem."}, "sentence_type": "Affirmative declarative sentence", "pos": {"nouns": {"I": 1, "Some": 1, "Custom": 1, "Ceremonial acts": 1, "A lot": 1, "Trouble": 1}, "verbs": {"Feel it": 1, "have": 1, "Go on": 1, "Do it": 1, "Bring it": 1}, "adjectives": {"It's not up to you": 1, "Lots": 1}, "numerals": {"78": 1}, "classifiers": {"Some": 1, "A lot": 1}, "pronouns": {"I": 2}}}, {"content": "When I was nervous, I drank or took pills.", "word_count": 11, "label": "", "sentence_structure": "Subject鈥搗erb鈥搊bject", "subjective_objective": {"answer": "Subjective", "rationale": "The entry describes the patient鈥檚 behaviour in the event of stress, i.e. drinking alcohol or taking medication. It is a subjective attitude and behaviour choice of the patient, not an objective fact. Therefore, the entry is subjective."}, "sentence_sentiment": {"answer": "Negative", "rationale": "Because the \"drinking or taking medication\" in the entry is negative."}, "language_style": {"answer": "Calm and direct", "rationale": "The entry expresses a habit in a direct and concise manner, with no excessive rhetorical or emotional rendering, and is more objective in tone. Thus, the language style is \"cool and straight\"."}, "time_dimension": {"answer": "Present", "rationale": ""}, "sentence_type": "Affirmative declarative sentence", "pos": {"nouns": {"Time": 1, "Wine": 1, "Medicine": 1}, "verbs": {"Just drink": 1, "Eat": 1, "Nervous": 1}, "adverbs": {"When": 1}, "numerals": {"79": 1}}}, {"content": "80. When I was in a bad mood, I wanted to be with someone else.", "word_count": 17, "label": "", "sentence_structure": "Subject鈥搗erb鈥揷omplement", "subjective_objective": {"answer": "Subjective", "rationale": "Because \"unsatisfied\" and \"want to be with someone else\" are the subjective feelings and thoughts of the patients themselves."}, "sentence_sentiment": {"answer": "Positive", "rationale": "Because the purpose of the entry, \"Inconveniently in a mood to be with someone else\", is to alleviate negative emotions, to seek social support and to be positive."}, "language_style": {"answer": "Tactful and warm", "rationale": "The language style of the entry is euphemistic, because it is asked in an invitation-style way to try to understand whether the other person has a need to be with others when he/she is depressed, rather than to direct orders or statements."}, "time_dimension": {"answer": "Present", "rationale": "The keyword \"want to be with someone else\" represents a current will or need, so the entry describes the person being tested in the present, present act or state."}, "sentence_type": "Affirmative declarative sentence", "pos": {"nouns": {"The mood": 1, "Have a nice day": 1}, "verbs": {"Yes": 1, "Stay": 1}, "adjectives": {"not": 1}, "adverbs": {"Just": 1}, "numerals": {"80": 1}, "pronouns": {"I": 1, "others": 1}}}, {"content": "I can cope better with it if I can feel frustration in advance.", "word_count": 23, "label": "", "sentence_structure": "Subject鈥搗erb鈥搊bject", "subjective_objective": {"answer": "Subjective", "rationale": "This reflects the patient ' s personal attitude and convictions."}, "sentence_sentiment": {"answer": "Positive", "rationale": "Because the descriptions of \"can feel frustrated\" and \"can better deal with it\" in the entries are positive."}, "language_style": {"answer": "Tactful and warm", "rationale": "The entry is more personal and interactive when it is spoken in a gentle and inviting manner, rather than in direct statements or orders. At the same time, the article is more objective and has no excessive rhetorical or emotional accents, which are consistent with the \"sweet\" language style."}, "time_dimension": {"answer": "Present", "rationale": "The key words for the future are \"can feel\" and \"can better deal with it\", which show that the testees have expectations of the frustration that may occur in the future and hope that their negative effects can be mitigated by prediction and response. The entry therefore describes future behaviour or mindset."}, "sentence_type": "Affirmative declarative sentence", "pos": {"nouns": {"Questionnaire items": 1, "Capacity": 1, "Frustration": 2, "Time": 1}, "verbs": {"Yeah": 1, "Foreground": 1, "Payable": 1}, "adjectives": {"Better": 1}, "numerals": {"81": 1, "2": 1}, "pronouns": {"I": 1}}}, {"content": "82. No matter how much I complain, I have never had a satisfactory outcome.", "word_count": 18, "label": "", "sentence_structure": "Subject鈥損redicate", "subjective_objective": {"answer": "Subjective", "rationale": "Because \"shut\" and \"unsatisfied\" are subjective feelings and judgments of patients, this entry is viewed from the patient's subjective perspective."}, "sentence_sentiment": {"answer": "Negative", "rationale": "Because the \"unsatisfactory outcome\" in the entry is negative."}, "language_style": {"answer": "Calm and direct", "rationale": "The entry uses a direct negative statement and describes a negative situation, which is more calm and objective, and does not involve too much emotional and rhetorical. It is therefore clear that the language style is straightforward."}, "time_dimension": {"answer": "Past", "rationale": "In the entry, \"a never satisfactory outcome\" is a description and reflection of the past, indicating that the testee has complained at some point in the past and has not had satisfactory results. The time dimension of the entry is therefore the past."}, "sentence_type": "Negative declarative sentence", "pos": {"nouns": {"Scoff": 1, "Result": 1}, "verbs": {"Got it": 1, "Let's go": 1}, "adjectives": {"Satisfied": 1, "Never": 1}, "numerals": {"82": 1}, "pronouns": {"I": 1}}}, {"content": "83. I often find myself insensitive when the environment is about to provoke a serious emotional reaction.", "word_count": 27, "label": "", "sentence_structure": "Subject鈥揷opula鈥損redicative", "subjective_objective": {"answer": "Subjective", "rationale": "Because it's the patient's subjective feeling and perception."}, "sentence_sentiment": {"answer": "Negative", "rationale": "Because \"insensitive\" in the entry is a negative emotional attitude and behavior."}, "language_style": {"answer": "Calm and direct", "rationale": "The entry is presented in a direct presentation, without too much rhetorical or emotional rendering. It is more objective in tone, and it is designed to understand the actual feelings and psychological state of the person under investigation. So I think the language style for this purpose is \"cool and direct.\""}, "time_dimension": {"answer": "Present", "rationale": "\"Always\" and \"I'm going to be insensitive\" means that this act is often occurring and has a certain frequency and stability. Thus, the entry is a description of the person being tested and of the circumstances and behaviour of the person."}, "sentence_type": "Affirmative declarative sentence", "pos": {"nouns": {"Environment": 1, "Emotional response": 1, "Insensitive": 1}, "verbs": {"Found": 1, "Cause": 1, "Yes": 1, "Insensitive": 1}, "adjectives": {"Serious": 1}, "adverbs": {"Often": 1, "Just": 1}, "numerals": {"83": 1}, "pronouns": {"I": 2}}}, {"content": "84. Working in isolation will free me from emotional depression and anxiety.", "word_count": 19, "label": "", "sentence_structure": "Subject鈥揷opula鈥損redicative", "subjective_objective": {"answer": "Subjective", "rationale": "Because \"forgetting to work\" and \" getting rid of emotional depression and anxiety\" are all patients' observations and evaluations of themselves, it's a subjective examination."}, "sentence_sentiment": {"answer": "Positive", "rationale": "Because the \"forget work\" act in the entry helps people to ease negative emotions, and it is therefore positive."}, "language_style": {"answer": "Tactful and warm", "rationale": "The language style of the entry is euphemism. It asks questions in an invitation and understanding manner, without direct orders or criticism, and focuses more on good communication with the interviewees. At the same time, the entry uses positive words, such as \"forget work\" and \"get rid of emotional depression and anxiety,\" which convey an encouragement and positive upward attitude."}, "time_dimension": {"answer": "Past", "rationale": "The entry \"Unforgetting to work\" shows that it is an experience or behavior that has taken place, a past. And the entry \"to free me from emotional depression and anxiety\" also implies that it has a positive impact on the state of the mood. So the time dimension of this questionnaire is a past."}, "sentence_type": "Affirmative declarative sentence", "pos": {"nouns": {"Work": 1, "Emotion": 1, "Blue": 1, "Anxiety": 1}, "verbs": {"Forget about me": 1, "鈾?And I'm gonna be 鈾?: 1, "Get rid of it": 1}, "adjectives": {"84": 1, "Forget about me": 1}, "adverbs": {"EMPTY": 2}, "numerals": {"84": 1}}}, {"content": "85. When you're nervous, I smoke.", "word_count": 9, "label": "", "sentence_structure": "Subject鈥搗erb鈥揷omplement", "subjective_objective": {"answer": "Subjective", "rationale": "Because I smoke is a matter of subjective thinking and behavior, initiated by the patient himself. While smoking is widely regarded as a way of dealing with stress, the way everyone reacts to stress is different, so it is primarily a matter of the patient鈥檚 subjective attitude."}, "sentence_sentiment": {"answer": "Negative", "rationale": "Because smoking is a health hazard and can cause addiction."}, "language_style": {"answer": "Tactful and warm", "rationale": "The entry is presented in a more subtle way, not by asking the other side whether they smoke when they are nervous, but by describing their behaviour as leading the conversation."}, "time_dimension": {"answer": "Past", "rationale": "The entry is for the purpose of questioning the responses and behaviour of the testee in the face of stress, and the temporal dimension is a matter of the past."}, "sentence_type": "Affirmative declarative sentence", "pos": {"verbs": {"Just": 1, "Smoking": 1}, "adjectives": {"Nervous": 1}, "numerals": {"85": 1}}}, {"content": "86. If I was in some kind of crisis, I would look for someone else who had the same fate as me.", "word_count": 28, "label": "", "sentence_structure": "Subject鈥搗erb鈥搊bject", "subjective_objective": {"answer": "Subjective", "rationale": "Because \"I\" is the patient's subjective view."}, "sentence_sentiment": {"answer": "Negative", "rationale": "Because the entries \"in crisis\" and \"in search of someone with the same fate\" have negative emotional aspects."}, "language_style": {"answer": "Tactful and warm", "rationale": "This entry uses a more moderate and friendly presentation, without directly expressing judgment or criticism of the behaviour of the other. At the same time, the use of the phrase \"another\" and \"a similar fate\" gives the other the sense of companionship and support, making the language more intimate and emotional."}, "time_dimension": {"answer": "Present", "rationale": "The entry describes the person's current behaviour or attitude, \"if I'm in some crisis\" suggests that it is a hypothetical situation, and \"who will find another person of the same fate as me\" is a statement of the behaviour that the person being tested may have in such a situation, so the temporal dimension of the entry is now judged."}, "sentence_type": "Affirmative declarative sentence", "pos": {"nouns": {"Crisis": 1, "person": 2, "Destiny": 1}, "verbs": {"Yes": 2, "Search": 1, "Into the": 1}, "adjectives": {"Same thing": 1, "Another one": 1}, "numerals": {"86": 1}, "pronouns": {"I": 2}}}, {"content": "87. If I do something wrong, I cannot be blamed.", "word_count": 12, "label": "", "sentence_structure": "Subject鈥揷opula鈥損redicative", "subjective_objective": {"answer": "Subjective", "rationale": "Because \"not to be blamed\" is a subjective feeling and perception that reflects the fear and anxiety of the patient at being criticized or accused."}, "sentence_sentiment": {"answer": "Negative", "rationale": "Because the \"not to be blamed\" attitude in the entry is negative."}, "language_style": {"answer": "Tactful and warm", "rationale": "This is a more acceptable and open answer to questions."}, "time_dimension": {"answer": "Present", "rationale": "And in conclusion, the output is: \"Now.\""}, "sentence_type": "Negative declarative sentence", "pos": {"verbs": {"Wrong": 1, "Blame": 1}, "adjectives": {"Like": 1}, "numerals": {"87": 1}, "pronouns": {"I": 1}}}, {"content": "88. If I had the idea of attacking others, I felt the need to do something to divert that idea.", "word_count": 31, "label": "", "sentence_structure": "Subject鈥搗erb鈥搊bject", "subjective_objective": {"answer": "Subjective", "rationale": "Because \"I have an idea of attacking people\" and \"I feel like I need to do something\" are subjective attitudes or feelings of patients."}, "sentence_sentiment": {"answer": "Negative", "rationale": "Because the idea and behavior of \"attacking someone\" in the entry is negative."}, "language_style": {"answer": "Tactful and warm", "rationale": "The language style of the entry is euphemism. It uses a gentle and inviting tone of speech to engage in dialogue, rather than a direct statement or order, which is more personal and interactive. At the same time, it does not overemphasize or demean the idea of attacking others, but rather raises questions in a balanced and objective manner, which helps the interviewee to feel comfortable and to share his real ideas."}, "time_dimension": {"answer": "Present", "rationale": "Now the entry describes the experience and feelings of the testees at this moment, that is, when they have an idea of attacking others, they feel that there is a need to do something to divert that idea. So, time dimensions are now judged."}, "sentence_type": "Affirmative declarative sentence", "pos": {"nouns": {"Thoughts": 2, "Others": 1, "Approach": 1, "Transfer": 1}, "verbs": {"have": 1, "Attack": 1, "Feel": 1, "Do it": 1, "Yes": 1, "Transfer": 1, "This one": 1}, "adverbs": {"If": 1, "Just": 2}, "numerals": {"88": 1, "Plant": 1}, "pronouns": {"I": 1}}}]}

{"questionnaire_title": "Control Circle", "questionnaire_category": "Self-cognition", "item_count": 30, "emotion_shift_overall": "From positive to negative", "language_style_overall": "Tactful and warm", "items": [{"content": "I got what I wanted, mainly because I worked hard for it.", "word_count": 22, "label": "", "sentence_structure": "Subject鈥搗erb鈥搊bject", "subjective_objective": {"answer": "Subjective", "rationale": "This is the subjective interpretation and evaluation of the results of the patient ' s behaviour."}, "sentence_sentiment": {"answer": "Positive", "rationale": "Because \"get what you want\" and \"do what you want\" in the entries are positive behavioral."}, "language_style": {"answer": "Tactful and warm", "rationale": "The entry is based on the following: 1. The entry is presented in the first person's name, giving a sense of affection and nature. 2. The language of positive affirmation is \"what I want\" and emphasizes that it is the result of personal efforts. This language style is positive in its encouragement and support, giving positive psychological intuitive effect and motivation. 3. No criticism or negative evaluation terms are used to avoid causing negative or counter-spiriting feelings in the person under investigation."}, "time_dimension": {"answer": "Present", "rationale": "The keywords \"getting\" and \"mostly\" and \"working hard for it\" indicate that the entry describes the current feelings and experiences of the testee and therefore the time dimension can be judged to be the present."}, "sentence_type": "Affirmative declarative sentence", "pos": {"nouns": {"I": 2, "Yes": 1, "Something": 1, "It's mostly because of": 1, "self": 1, "Once": 1, "Give": 1, "Try": 1}, "verbs": {"Got it": 1, "Yes": 1, "I'm working hard": 1}, "adverbs": {"Main locations": 1}, "numerals": {"1": 2}, "pronouns": {"Here": 1}}}, {"content": "2. I'm almost certain I can do it when I make a plan.", "word_count": 15, "label": "", "sentence_structure": "Subject鈥搗erb鈥搊bject", "subjective_objective": {"answer": "Subjective", "rationale": "Because \"almost certain\" is a subjective evaluation, reflecting the faith and faith of the patient."}, "sentence_sentiment": {"answer": "Positive", "rationale": "Because the \"I can plan\" act is positive."}, "language_style": {"answer": "Tactful and warm", "rationale": "The language style of the questionnaire entry is more euphemistic. It uses the term \"almost certain\" to express confidence in the realization of the plan, rather than simply claiming that it will be possible to achieve it. This expression is more moderate, avoids the feeling of over-hardness or arrogance, is more acceptable and is willing to fill out the questionnaire."}, "time_dimension": {"answer": "Past", "rationale": "The time dimension of the article is therefore the present."}, "sentence_type": "Affirmative declarative sentence", "pos": {"nouns": {"Planned": 1}, "verbs": {"Staple": 1, "Implementation": 1}, "adjectives": {"Almost": 1, "Sure": 1}, "numerals": {"2": 1, "it": 1}, "pronouns": {"I": 1}}}, {"content": "I prefer games that rely on luck rather than purely on technology.", "word_count": 22, "label": "", "sentence_structure": "Subject鈥搗erb鈥搊bject", "subjective_objective": {"answer": "Subjective", "rationale": "Because \"more partial\" is a subjective feeling and attitude, and it's the personal preference of the interviewee for the type of game."}, "sentence_sentiment": {"answer": "Neutral", "rationale": "Because the entry does not have any emotional color."}, "language_style": {"answer": "Tactful and warm", "rationale": "The entry is more proficient and interactive when it is spoken in a gentle and inviting manner, rather than in direct statements or orders. At the same time, the language style of the entry is relatively euphemistic, not overly strong or emotional, and is more biased towards a balanced and objective description."}, "time_dimension": {"answer": "Present", "rationale": ""}, "sentence_type": "Affirmative declarative sentence", "pos": {"nouns": {"Game": 1, "Lucky": 1, "Technology": 2}, "verbs": {"Prejudiced": 1}, "adjectives": {"Pure": 1}, "adverbs": {"More": 1}, "numerals": {"3": 1}, "pronouns": {"I": 1}}}, {"content": "4. I can learn almost anything if I make my mind up.", "word_count": 16, "label": "", "sentence_structure": "Subject鈥損redicate", "subjective_objective": {"answer": "Subjective", "rationale": "Because I'm making up my mind and learning that things are patients' own subjective attitudes and behavior."}, "sentence_sentiment": {"answer": "Positive", "rationale": "Because the expression in the entry is positive, it is positive to think that a lot can be learned with determination."}, "language_style": {"answer": "Tactful and warm", "rationale": "The entry is asked in an encouraging and supportive manner to express the researcher鈥檚 trust and expectations of the testee. At the same time, this euphemism is more personal and interactive, making it easier for the testee to feel comfortable and to cooperate with the questionnaire."}, "time_dimension": {"answer": "Future", "rationale": "The key words in the entry are \"suspect\" and \"almost learn\" , which all imply possible future behavior or attitude. And the expression \"know anything\" also shows expectations and confidence in the future. So I think the time dimension of this goal should be the future."}, "sentence_type": "Affirmative declarative sentence", "pos": {"verbs": {"Yes": 1, "Learn": 1}, "adjectives": {"Just": 1, "Almost": 1, "Any": 1}, "numerals": {"4": 1}, "pronouns": {"I": 2}}}, {"content": "5. Most of my major achievements have come from hard work and talent.", "word_count": 19, "label": "", "sentence_structure": "Subject鈥揷opula鈥損redicative", "subjective_objective": {"answer": "Subjective", "rationale": "Because achievements are the result of hard work and talent as patients' subjective assessment of their own experience."}, "sentence_sentiment": {"answer": "Positive", "rationale": "Because the entries \"work hard\" and \"achievements from talent\" are positive."}, "language_style": {"answer": "Tactful and warm", "rationale": "The entry is more euphemistic. It uses the term \"main achievements\" and emphasizes that they come from \"working hard and talented\", without directly indicating what is more important, or demeaning or emphasizing any party. This expression is more moderate and respectful of the interviewees, and is more acceptable."}, "time_dimension": {"answer": "Present", "rationale": "\"Most\" and \"from\" means \"this is the present, not the past or the future.\" So the time dimension of this entry is now."}, "sentence_type": "Affirmative declarative sentence", "pos": {"nouns": {"Achievements": 1, "Work": 1, "Natural talent": 1}, "verbs": {"Main": 1, "From": 2, "I'm working hard": 1}, "adjectives": {"Mostly": 1}, "numerals": {"5": 1}, "pronouns": {"I": 1}}}, {"content": "6. I do not normally make plans because I have difficulty in keeping it up.", "word_count": 16, "label": "", "sentence_structure": "Subject鈥搗erb鈥搊bject", "subjective_objective": {"answer": "Subjective", "rationale": "Because \"hard to stick with\" reflects the patient's own subjective feelings and attitudes."}, "sentence_sentiment": {"answer": "Negative", "rationale": "Because the practice of \"hard to keep \" in the entries is negative, it shows the difficulties encountered in designing and implementing the plan."}, "language_style": {"answer": "Tactful and warm", "rationale": "The language style of the entry is more euphemistic and warm, and it expresses a common psychological state and experience in a non-critical way, i.e., that some find it difficult to stick to the plan. Such expression is more likely to make the interviewee feel understood and accepted."}, "time_dimension": {"answer": "Past", "rationale": "The phrase \"generally not\" and \"hard to insist\" shows that it is a description of past behaviour and therefore the entry is judged to be \"old\"."}, "sentence_type": "Negative declarative sentence", "pos": {"nouns": {"Planned": 1, "Hold on": 1}, "verbs": {"Staple": 1, "Implementation": 1}, "adjectives": {"General": 1, "Hard": 1}, "numerals": {"6": 1}, "pronouns": {"I": 1}}}, {"content": "7. Competition for excellence.", "word_count": 6, "label": "", "sentence_structure": "Subject鈥搗erb鈥揷omplement", "subjective_objective": {"answer": "Subjective", "rationale": "Because \"competition creates excellence\" is a view or belief that reflects the subjective attitude and perception of the investigator, not just an account of objective facts."}, "sentence_sentiment": {"answer": "Positive", "rationale": "Because the idea of \"competition for excellence\" in the entry is positive."}, "language_style": {"answer": "Calm and direct", "rationale": "The language style of the entry is more direct and concise, and instead of using rhetorical techniques or excessive emotional rendering, the view that \"competition contributes to performance\" is expressed in a clear and unambiguous manner."}, "time_dimension": {"answer": "Future", "rationale": ""}, "sentence_type": "Affirmative declarative sentence", "pos": {"nouns": {"Competition": 1, "Excellent": 1}, "verbs": {"Create": 1}}}, {"content": "8. The extent to which individuals can achieve their achievements is often determined by opportunities.", "word_count": 16, "label": "", "sentence_structure": "Subject鈥搗erb鈥搊bject", "subjective_objective": {"answer": "Objective", "rationale": "Since the achievement of results is not within the control of the patient or foreseen by him/herself, it is dependent on external factors and opportunities and is therefore objectively viewed."}, "sentence_sentiment": {"answer": "Negative", "rationale": "Because it expresses \"the individual's achievements are largely dependent on opportunities\", this attitude may weaken the individual's sense of responsibility and motivation."}, "language_style": {"answer": "Tactful and warm", "rationale": "The entry is more adept and interactive when it is spoken in a gentle and inviting manner, rather than by direct statements or orders."}, "time_dimension": {"answer": "Future", "rationale": "The entry contains ideas and expectations for the future, \"often\" expressing the possibility of repetition, and \"how much can be achieved\" is a projection or expectation for the future. It can therefore be judged as a description of the future."}, "sentence_type": "Affirmative declarative sentence", "pos": {"nouns": {"Achievements": 1, "Opportunities": 1}, "verbs": {"Access": 1, "Decision": 1}, "pronouns": {"Personal": 1}}}, {"content": "9. Whatever the examination or competition, I would like to know how I compare with others.", "word_count": 27, "label": "", "sentence_structure": "Subject鈥搗erb鈥搊bject", "subjective_objective": {"answer": "Subjective", "rationale": "Because \"I want to know how I compare to other people\" is the patient's idea and attitude."}, "sentence_sentiment": {"answer": "Positive", "rationale": "Because the entry expresses a desire for knowledge and a desire for competition, it is a positive attitude."}, "language_style": {"answer": "Calm and direct", "rationale": "The entry is a direct expression of the subject of questioning the other party ' s performance in the examination or competition compared to others, without excessive rhetorical or emotional rendering and with more objective tone."}, "time_dimension": {"answer": "Present", "rationale": "The entry describes the current mindset and behaviour of the testee, i.e., they are concerned about their comparison with others when taking an examination or competition. Therefore, the time dimension is now judged."}, "sentence_type": "Interrogative sentence", "pos": {"nouns": {"Examinations": 1, "Competition": 1, "person": 1}, "verbs": {"Yes": 1, "Do it": 1, "- Yeah": 1}, "adverbs": {"What": 1}, "numerals": {"Nine": 1}, "pronouns": {"I": 2, "Other": 1}}}, {"content": "10. Despite my best efforts, little has been achieved.", "word_count": 14, "label": "", "sentence_structure": "Subject鈥搗erb鈥揷omplement", "subjective_objective": {"answer": "Subjective", "rationale": "Because \"do your best\" and \"almost nothing\" are subjective feelings of the patient."}, "sentence_sentiment": {"answer": "Negative", "rationale": "Because the result of \"almost nothing\" in the entry is negative."}, "language_style": {"answer": "Calm and direct", "rationale": "The entry uses a direct tone of statement, does not use excessive rhetorical or emotional rendering, and is expressed in a more objective and cold manner.\""}, "time_dimension": {"answer": "Present", "rationale": "The keywords \"best efforts\" and \"nearly nothing\" indicate that this is the description of the testee of his current situation, so it can be judged that the entry is an inquiry into the present behaviour or mentality."}, "sentence_type": "Negative declarative sentence", "pos": {"nouns": {"Try": 1, "Yes": 1}, "verbs": {"- All of it": 1, "Done": 1}, "adjectives": {"How many": 1, "Almost": 1, "All": 1}, "adverbs": {"But": 1}, "numerals": {"Ten": 1, "One": 2}}}, {"content": "Although I am confident in most things, I still lack the capacity to control the human condition.", "word_count": 28, "label": "", "sentence_structure": "Subject鈥搗erb鈥揷omplement", "subjective_objective": {"answer": "Subjective", "rationale": "Because \"confident in most things\" and \"lack of control of people\" are subjective feelings of patients."}, "sentence_sentiment": {"answer": "Negative", "rationale": "Because the description of the act \"lack of control of the human condition\" in the entry is negative."}, "language_style": {"answer": "Tactful and warm", "rationale": "The language style of the entry is euphemistic, and it is a sign of confidence in most things by expressing \"I\" but lack the capacity to control the human condition to convey indirectly the psychological state of the person, and to avoid directly exposing the individual's weaknesses or weaknesses, which can reduce the feelings of resentment and rejection. At the same time, the use of the \"though...\" turn-on also shows that it is trying to change and promote its own side, and is more positive."}, "time_dimension": {"answer": "Present", "rationale": "Although there are \"me\" and \"but\" in the sentence, there are no clear points of time for these words, so the sentence is mainly about the present state and feelings of the speaker. At the same time, the \"still lack\" indicates that the problem is not sudden, but is a continuing one. So, the questionnaire entry is about the present."}, "sentence_type": "Affirmative declarative sentence", "pos": {"nouns": {"Things": 1, "Capacity": 1}, "verbs": {"Have": 1, "Lack": 1, "Control": 1, "Human": 1, "Status": 1}, "adjectives": {"Most of them": 1, "Confidence": 1}, "adverbs": {"Both": 1, "Still": 1}, "classifiers": {"individual": 1}, "pronouns": {"I": 2}}}, {"content": "I have no problems in making friends and maintaining friendship.", "word_count": 15, "label": "", "sentence_structure": "Subject鈥搗erb鈥搊bject", "subjective_objective": {"answer": "Objective", "rationale": "This is because it is a subjective feeling of self-evaluation of one ' s own social aspect."}, "sentence_sentiment": {"answer": "Neutral", "rationale": "Because the entry does not have any emotional color."}, "language_style": {"answer": "Calm and direct", "rationale": "The language style of the entry is calm and straightforward, as it presents a fact in a concise and clear manner, with no excessive emotional rendering or rhetorical techniques and with more objective and direct expression."}, "time_dimension": {"answer": "Present", "rationale": "The description of \"I have no problems\" and \"remaining friendship\" in the sentence suggests that the act is a state of present or experience, so it is exported as \"now\"."}, "sentence_type": "Affirmative declarative sentence", "pos": {"nouns": {"Make friends": 1, "Friendship": 1, "Problem": 1}, "verbs": {"in": 1, "Hold on": 1, "have": 1}, "adverbs": {"No, I'm not": 1}, "pronouns": {"I": 1}}}, {"content": "3. I was not very sure of the process of talking to some.", "word_count": 19, "label": "", "sentence_structure": "Subject鈥搗erb鈥搊bject", "subjective_objective": {"answer": "Subjective", "rationale": "Because \"the process of conversation is less likely\" is the patient's subjective assessment and perception of his abilities. Although this is the case with other people, it is more the result of the individual's own feelings and experiences, and therefore the subjective perspective."}, "sentence_sentiment": {"answer": "Negative", "rationale": "Because the \"not very sure about the conversation\" behavior in the entry is negative."}, "language_style": {"answer": "Tactful and warm", "rationale": "The language style of the entry is euphemistic. It uses a milder and more inviting tone, which leads the respondents to share their views and experiences in a relatively accessible way, rather than to direct orders or tough attitudes. At the same time, the entry uses the expression \"less able to grasp the conversational process\" to make the issue more specific and understandable, enhancing the effectiveness and reliability of the investigation."}, "time_dimension": {"answer": "Present", "rationale": ""}, "sentence_type": "Negative declarative sentence", "pos": {"nouns": {"person": 1, "Talk": 2, "Process": 1}, "verbs": {"Not sure": 1, "Talk": 1}, "adjectives": {"Some": 1}, "adverbs": {"In the same": 1}, "numerals": {"3": 1}, "pronouns": {"I": 1}}}, {"content": "I usually have a closer personal relationship with those who I find attractive.", "word_count": 26, "label": "", "sentence_structure": "Subject鈥搗erb鈥搊bject", "subjective_objective": {"answer": "Subjective", "rationale": "Because \"Feeling sexual charm\" is a subjective assessment of the patient."}, "sentence_sentiment": {"answer": "Positive", "rationale": "Because the entry \"can establish a closer personal relationship with those who I find attractive\" is positive."}, "language_style": {"answer": "Tactful and warm", "rationale": "The entry is a softer, more invited language, and asks if the interviewees are usually able to establish a closer relationship with someone they think are attractive. This expression is neither too direct nor offensive, but it also triggers the interviewee's reflections and answers."}, "time_dimension": {"answer": "Present", "rationale": "The keywords are \"normally\" and \"capable\", which all indicate that it is a customary act, and that it is likely that it is occurring when the person who was tested answers the questionnaire. The entry should therefore be classified as an act or attitude of the moment."}, "sentence_type": "Affirmative declarative sentence", "pos": {"nouns": {"Questionnaire": 1, "Entry": 1, "person": 2, "Sex charm": 1, "Private relations": 1}, "verbs": {"Yes": 1, "Same": 1, "Create": 1, "have": 1}, "adjectives": {"Usually": 1, "More intimate": 1}, "numerals": {"4": 1}, "pronouns": {"I": 1}}}, {"content": "5. When I do interviews, I can generally direct the questioners to the subjects I want to talk about and avoid the topics I do not want to talk about.", "word_count": 34, "label": "", "sentence_structure": "Subject鈥搗erb鈥搊bject", "subjective_objective": {"answer": "Subjective", "rationale": "Because \"leading the questioner\" and \"leave the non-talking topic\" are the thoughts and behaviours of the patient, and the subjective attitudes and abilities of the patient are examined."}, "sentence_sentiment": {"answer": "Positive", "rationale": "Because it's positive that the entry \"leads the questioner to ask me what I want to talk about, to avoid what I don't want to talk about.\""}, "language_style": {"answer": "Calm and direct", "rationale": "The language style of the entry is more straightforward, with no excessive adoration and emotional rendering, with objective factual statements as the main expression of reason."}, "time_dimension": {"answer": "Present", "rationale": "\"In interview\" refers to an ongoing act or state, so the temporal dimension of the article is now."}, "sentence_type": "Affirmative declarative sentence", "pos": {"nouns": {"Interview": 1, "Questioner": 1, "Problem": 1, "Topic": 2, "Avoidance": 1}, "verbs": {"Participation": 1, "Yes": 1, "Guide": 1, "Talk": 2, "Avoid": 1}, "adverbs": {"General": 1}, "numerals": {"5": 1}, "pronouns": {"I": 1}}}, {"content": "6. If I need help in implementing my plan, it is often difficult for me to find someone who can help.", "word_count": 27, "label": "", "sentence_structure": "Subject鈥損redicate", "subjective_objective": {"answer": "Subjective", "rationale": "Because \"hard to find someone who can help\" is the patient's own feeling and opinion, and it's subjective."}, "sentence_sentiment": {"answer": "Negative", "rationale": "Because the entry \"It's hard to find someone who can help\" expresses a negative emotion or attitude."}, "language_style": {"answer": "Tactful and warm", "rationale": "Warm and warm."}, "time_dimension": {"answer": "Present", "rationale": "And the expression \"I usually have difficulty finding someone who can help\" suggests that it is a recurring issue and has an impact on the plan being implemented. So the answer is \"now.\""}, "sentence_type": "Negative declarative sentence", "pos": {"nouns": {"Questionnaire Entry": 1, "person": 1}, "verbs": {"Implementation": 1, "Yes": 1, "Find": 1, "Help": 1}, "adjectives": {"Hard": 1}, "adverbs": {"Usually": 1}, "numerals": {"6": 1, "I": 1}, "classifiers": {"individual": 1}}}, {"content": "7. If I wanted to agree to a meeting with someone, I would normally be able to make arrangements. (x)", "word_count": 20, "label": "", "sentence_structure": "Subject鈥搗erb鈥搊bject", "subjective_objective": {"answer": "Subjective", "rationale": "Because it involves the will and capacity of the individual, it is a subjective view."}, "sentence_sentiment": {"answer": "Positive", "rationale": "Because the purpose of the entry is to \"if I want to agree to meet someone, I can usually arrange.\""}, "language_style": {"answer": "Tactful and warm", "rationale": "The language style in this entry is euphemismal. It answers questions with a positive tone and provides additional information by adding the number in brackets to increase the confidence and satisfaction of the respondents. Moreover, the more positive and encouraging expression of the tone reflects its mild and cordial character."}, "time_dimension": {"answer": "Present", "rationale": "\"If I want to\" indicate a possibility or hypothetical situation, \"generally I can\" indicates normal behaviour or habits, \"formulate\" occurs now. So the sentence means \"if I decide to meet someone in the usual situation, I will make the arrangements immediately.\" It is the current situation."}, "sentence_type": "Affirmative declarative sentence", "pos": {"nouns": {"I": 2, "Arrangements": 1, "Meet me": 1}, "verbs": {"Yes": 2, "Do it": 1, "Agreed": 1, "Arrangements": 1}, "adjectives": {"General": 1}, "adverbs": {"Both": 1}, "numerals": {"Ten": 1}}}, {"content": "I often find it difficult to make my point acceptable to others.", "word_count": 17, "label": "", "sentence_structure": "Subject鈥搗erb鈥搊bject", "subjective_objective": {"answer": "Subjective", "rationale": "Because \"hard to get accepted\" is the patient's subjective feeling and perception."}, "sentence_sentiment": {"answer": "Negative", "rationale": "Because the entry \"It's hard to get my opinion\" is negative."}, "language_style": {"answer": "Tactful and warm", "rationale": "The language style of the entry is more euphemistic and respectful of each other鈥檚 feelings. It uses an inquisitorial tone, and it does not directly blame or criticize others, but rather expresses a troubled and helpless mood that is more resonant and understandable. Such expression reduces each other鈥檚 resistance and makes the questionnaire more smooth."}, "time_dimension": {"answer": "Present", "rationale": ""}, "sentence_type": "Negative declarative sentence", "pos": {"nouns": {"Questionnaire": 1, "Entry": 1, "person": 1, "Views": 1}, "verbs": {"Found": 1, "Jean": 1, "Accept": 1, "Often": 1}, "adjectives": {"Hard": 1}, "adverbs": {"very": 1}, "pronouns": {"I": 2, "others": 1}}}, {"content": "9. I had intended to calm the debate, but it was often made worse.", "word_count": 16, "label": "", "sentence_structure": "Subject鈥搗erb鈥揷omplement", "subjective_objective": {"answer": "Subjective", "rationale": "Because the part of \"intended\" and \"often spoiled\" is the patient's inner thought, reflecting the patient's subjective perception and evaluation of his behaviour."}, "sentence_sentiment": {"answer": "Negative", "rationale": "Because the \"making the debate worse\" act in the entry is negative."}, "language_style": {"answer": "Calm and direct", "rationale": "The entry directly expresses the fact that the subject of the survey often makes the situation worse when dealing with the dispute, and that the language style is more objective and direct."}, "time_dimension": {"answer": "Past", "rationale": "Although the sentence does not specify the specific time period of the act, the tone and context of the sentence suggests that the act may have occurred in the nearest or earlier time. Thus, the temporal dimension of the purpose of the article is judged to be the past.鈥?}, "sentence_type": "Negative declarative sentence", "pos": {"nouns": {"Arguments": 1, "it": 1}, "verbs": {"I'm going to": 1, "Quiet": 1, "Do it": 1, "Okay": 1}, "adverbs": {"Regular": 1, "But": 1}, "numerals": {"9": 1}, "pronouns": {"I": 1}}}, {"content": "I find that I can play an important role in most gatherings very easily.", "word_count": 23, "label": "", "sentence_structure": "Subject鈥搗erb鈥搊bject", "subjective_objective": {"answer": "Subjective", "rationale": "Because \"it's easy to play a role\" is the subjective perception and evaluation of what people do in social settings."}, "sentence_sentiment": {"answer": "Positive", "rationale": "Since the entry describes the ability to play an important role in a meeting setting, this ability is usually considered positive."}, "language_style": {"answer": "Tactful and warm", "rationale": "The topic uses a gentle and encouraging tone to question the respondents about their performance at a gathering, without too direct or hard language, giving them a sense of affection. At the same time, it avoids negative language that could have negative effects (such as \"I find myself incapable of working\"), but instead leads the respondents to reflect and share their experiences on a positive basis, which is more conducive to gathering real and useful information."}, "time_dimension": {"answer": "Present", "rationale": "The keyword \"discovery\" and \"can\" indicate that it is a description of the state of affairs, while \"in most gatherings\" gives a picture of the behaviour that occurs, and therefore judges it now."}, "sentence_type": "Affirmative declarative sentence", "pos": {"nouns": {"Questionnaire Entry": 1, "Most of them": 1, "Party time": 1, "Important role": 1}, "verbs": {"Found": 1, "Yes": 2, "Play": 1, "Easy": 1}, "adjectives": {"Important": 1, "Easy": 1}, "adverbs": {"very": 1}, "numerals": {"10": 1, "Most of them": 1}, "pronouns": {"I": 1}}}, {"content": "1. We can control the world by playing an active role in political and social affairs.", "word_count": 29, "label": "", "sentence_structure": "Subject鈥搗erb鈥搊bject", "subjective_objective": {"answer": "Subjective", "rationale": "Because this view expresses the ideas and attitudes of individuals who believe that their own actions can have an impact on society and are subjective.\""}, "sentence_sentiment": {"answer": "Positive", "rationale": "According to the phrase \"by playing an active role in controlling things in the world\", this is a positive, positive attitude and behaviour."}, "language_style": {"answer": "Calm and direct", "rationale": "The entry presents the point in a more direct and explicit way, without too many rhetorical or emotional renderings. At the same time, the terminology used is relatively objective and formal. Thus, its language style can be described as \"cool and direct\"."}, "time_dimension": {"answer": "Future", "rationale": "The keyword \"can\" , \"by making a difference\" and \"by controlling things in the world\" are all expressions of a desire or of a goal that is intended to be achieved in the future. Thus, the entry describes future behaviour or mentality."}, "sentence_type": "Imperative sentence", "pos": {"nouns": {"World": 1, "Political": 1, "Social": 2, "Role": 1}, "verbs": {"Play": 1, "Control": 1, "be": 1, "in": 1}, "adverbs": {"Pass": 1, "Yeah": 1}, "numerals": {"1": 4}, "pronouns": {"we": 1}}}, {"content": "2. The general public can also influence government decision-making.", "word_count": 16, "label": "", "sentence_structure": "Subject鈥搗erb鈥搊bject", "subjective_objective": {"answer": "Subjective", "rationale": "Since this sentence relates to the extent to which ordinary people influence government decision-making, it is a fact that cannot be directly observed and therefore needs to be judged on the basis of the subjective views of the interviewees."}, "sentence_sentiment": {"answer": "Positive", "rationale": "The entry is a positive attitude, since it expresses the possibility and value of the general public being able to participate in the decision-making process of the Government."}, "language_style": {"answer": "Tactful and warm", "rationale": "The language style of the entry is euphemism. It is more personal and interactive by using a gentle, invitation-style tone, emphasizing that ordinary people can also influence government decisions, not directly speaking or ordering."}, "time_dimension": {"answer": "Present", "rationale": "The keyword \"can also\" and \"act on\" suggests that it is a statement describing the situation, pointing to the current phenomenon or fact and thus judging as \"now\"."}, "sentence_type": "Affirmative declarative sentence", "pos": {"nouns": {"People": 1, "Government": 2, "Decision-making": 1}, "verbs": {"Yes": 1, "Play": 1, "Impact": 1}, "adverbs": {"Normal": 1}, "pronouns": {"Yeah": 1, "to": 2}}}, {"content": "3. It is difficult to control what those politicians do in their offices.", "word_count": 22, "label": "", "sentence_structure": "Subject鈥搗erb鈥搊bject", "subjective_objective": {"answer": "Subjective", "rationale": "Because the sentence expresses the opinion and evaluation of the politicians in the office, it does not directly examine the subjective feelings or behaviour of the patient. It is therefore subjective."}, "sentence_sentiment": {"answer": "Negative", "rationale": "Because the entry \"hard to control\" expresses a negative and impotent emotional tone, implying that people are weak or dissatisfied with it."}, "language_style": {"answer": "Tactful and warm", "rationale": "The entry raises questions in a more objective and neutral way, without directly attacking or criticizing any person or group. At the same time, more euphemistic terms and tone are used, making the question more personal and interactive and more likely to trigger the reflections and answers of the person under investigation."}, "time_dimension": {"answer": "Past", "rationale": "The entry does not have clear time indicators, but it describes a general phenomenon or problem and can therefore be judged as past."}, "sentence_type": "Negative declarative sentence", "pos": {"nouns": {"People": 1, "Yes": 2, "Politicians": 1, "Office": 1}, "verbs": {"Hard": 1, "Do it": 1, "have": 1, "Control": 1}, "adjectives": {"It's hard": 1, "To those": 1}, "adverbs": {"in": 1, "Lee": 1}, "pronouns": {"they": 1}}}, {"content": "4. The world was run by a small number of powerful people, and little people could do much about it.", "word_count": 29, "label": "", "sentence_structure": "Subject鈥揷opula鈥損redicative", "subjective_objective": {"answer": "Subjective", "rationale": "This view is subjective because it expresses the views and beliefs of the interviewees about the current situation in society."}, "sentence_sentiment": {"answer": "Negative", "rationale": "Because the entry expresses a negative view of the world order, the view that the status quo cannot be changed by the small person."}, "language_style": {"answer": "Calm and direct", "rationale": "This language style is more consistent with the serious and objective nature of the questionnaire."}, "time_dimension": {"answer": "Present", "rationale": "In the future, the entry expresses expectations and views for the future that in the future, \"a few powerful people\" will continue to hold power and that it will be difficult for ordinary people to change the situation. The time dimension of the issue is therefore directed to the future."}, "sentence_type": "Affirmative declarative sentence", "pos": {"nouns": {"World": 1, "person": 2, "Things": 1}, "verbs": {"By": 1, "Control": 1, "Do it": 1}, "adjectives": {"have": 1, "Small": 1, "Too much": 1}, "numerals": {"How many": 1}, "pronouns": {"Here": 1, "This": 1}}}, {"content": "5. We can eradicate political corruption if we do our best.", "word_count": 14, "label": "", "sentence_structure": "Subject鈥搗erb鈥搊bject", "subjective_objective": {"answer": "Subjective", "rationale": "Because \"do your best\" and \"do your best to clear the political corruption\" are subjective views and expectations of the patient about the development of things."}, "sentence_sentiment": {"answer": "Positive", "rationale": "Because the purpose of the entry is to \"we can clear the political corruption\" with all our best efforts."}, "language_style": {"answer": "Tactful and warm", "rationale": "The entry is more forthcoming and interactive by encouraging and positive comments that the respondents believe that the goal can be achieved through efforts that do not directly present or order."}, "time_dimension": {"answer": "Present", "rationale": "The entry in the future expresses hope and confidence in the future that by doing our best we can eliminate political corruption. The keywords \"as long as you can\" and \"as much as you can\" point to future actions and results. The temporal dimension of the entry can therefore be judged to be the future."}, "sentence_type": "Affirmative declarative sentence", "pos": {"nouns": {"we": 1, "Political corruption": 1}, "verbs": {"Yes": 1, "Clear": 1, "Try": 1}, "adverbs": {"Just": 1}}}, {"content": "6. A major cause of the outbreak of war was the lack of political interest.", "word_count": 24, "label": "", "sentence_structure": "Subject鈥揷opula鈥損redicative", "subjective_objective": {"answer": "Subjective", "rationale": "Because it is a subjective view of researchers' perception and assessment of whether people are interested in politics and the causes of war."}, "sentence_sentiment": {"answer": "Negative", "rationale": "The article ' s formulation, which implies criticism and accusations of a lack of political participation, has a derogatory meaning and therefore has a negative emotional connotation."}, "language_style": {"answer": "Calm and direct", "rationale": "The language style of the entry is more straightforward, it is not over-modified or emotionally rendered, it leads to dialogue by stating a fact, and it is more persuasive and objective."}, "time_dimension": {"answer": "Present", "rationale": "The keywords \"people\" and \"political interest\" both indicate that this is a statement describing the state or phenomenon, so the temporal dimension is now."}, "sentence_type": "Negative declarative sentence", "pos": {"nouns": {"War": 1, "Reason": 1, "People": 1, "Political": 1, "Care": 1}, "verbs": {"Breaking out": 1, "be": 1, "Too little": 1}, "adjectives": {"Main": 1, "Too much": 1}, "numerals": {"Six": 1}, "pronouns": {"it": 1}}}, {"content": "7. We, the consumers, are almost powerless to face the rising cost of living.", "word_count": 22, "label": "", "sentence_structure": "Subject鈥搗erb鈥搊bject", "subjective_objective": {"answer": "Subjective", "rationale": "Because \"incompetence\" is a subjective feeling or attitude that reflects the sense of weakness and helplessness of the patient in terms of rising cost of living."}, "sentence_sentiment": {"answer": "Negative", "rationale": "Because the attitude and behaviour of \"incompetence\" in the entries is negative."}, "language_style": {"answer": "Calm and direct", "rationale": "The language style of the entry is a calm, straightforward, simple and unspoken one, with too many rhetorical or emotional terms. It presents the fact in an objective and rational tone that consumers have difficulty coping with rising costs of living."}, "time_dimension": {"answer": "Present", "rationale": "This questionnaire entry describes the current situation, where \"we consumers\" are experiencing \"a rise in the cost of living\" and feeling \"incompetent\" at the current point of time. So the time dimension of this article is now."}, "sentence_type": "Negative declarative sentence", "pos": {"nouns": {"we": 1, "Consumers": 1, "Cost of living": 1, "Rise": 1, "Almost": 1, "Incompetent": 1, "- Fortune": 1}, "verbs": {"Face it": 1, "Rise": 1}, "adjectives": {"These": 1, "Life": 1, "Costs": 1, "Rise": 1}, "adverbs": {"Almost": 1}, "classifiers": {"individual": 1}, "pronouns": {"we": 1}}}, {"content": "8. I have carefully observed that it is impossible to make a real difference to what politicians do.", "word_count": 32, "label": "", "sentence_structure": "Subject鈥揷opula鈥損redicative", "subjective_objective": {"answer": "Objective", "rationale": "Because the insights gained are based on actual observations and analyses, not on subjective attitudes or feelings of patients."}, "sentence_sentiment": {"answer": "Negative", "rationale": "Because the expression \"can't\" in the entry is negative emotional and expresses a negative attitude."}, "language_style": {"answer": "Tactful and warm", "rationale": "The expression is more personal and interactive, in a gentle and inviting tone, rather than in direct statements or orders. At the same time, the language style of the article is more objective, neutral, without too much emotional or subjective evaluation, and more in line with the characteristics of the psychological questionnaire study."}, "time_dimension": {"answer": "Present", "rationale": "The entry expresses the current perception or attitude of the subject, and the keywords \"recognizing\" that \"real impact is impossible\" are all expressions of the present state of affairs, so the temporal dimension is now judged."}, "sentence_type": "Negative declarative sentence", "pos": {"nouns": {"Observation": 1, "Politicians": 1, "Yes": 1, "Significant impact": 1}, "verbs": {"Yes": 1, "Do it": 1, "Play": 1, "be": 1, "Possible": 1}, "adjectives": {"Careful": 1, "Really": 1}, "numerals": {"8": 1, "Maybe": 1}, "pronouns": {"I": 1}}}, {"content": "I would prefer to concentrate on other things rather than on solving world problems.", "word_count": 27, "label": "", "sentence_structure": "Subject鈥搗erb鈥搊bject", "subjective_objective": {"answer": "Subjective", "rationale": "It is a subjective approach to be taken because it is a personal attitude and choice to concentrate more on other things."}, "sentence_sentiment": {"answer": "Negative", "rationale": "Because it expresses a negative attitude that is not willing to engage in action to solve world problems."}, "language_style": {"answer": "Calm and direct", "rationale": "The language style of the entry is more straightforward, with no excessive rhetorical or emotional rendering. It simply expresses the willingness of the person surveyed to devote his or her energies to solving world problems, and the language is well structured and logical. This language style is more common in psychological questionnaires, because it conveys the intent of the investigator more accurately and facilitates his or her understanding and response."}, "time_dimension": {"answer": "Future", "rationale": "The entry expresses the expectation that there may be behaviors or ideas in the future, that \"I would rather concentrate on other things than on solving world problems.\" This expectation is about a scenario and vision of the future, not necessarily what is happening now. So the time dimension is the future."}, "sentence_type": "Affirmative declarative sentence", "pos": {"nouns": {"Problem": 1, "World": 1}, "verbs": {"Yes": 1, "Flowers": 1, "Resolve": 1, "Focus": 1, "On the... on the": 2}, "adverbs": {"More": 1}, "pronouns": {"I": 1}}}, {"content": "10. We are ultimately responsible as voters for the poor state and local government sectors.", "word_count": 28, "label": "", "sentence_structure": "Subject鈥搗erb鈥搊bject", "subjective_objective": {"answer": "Subjective", "rationale": "Because the words \"we\" and \"responsible\" in the sentence indicate that the questionnaire looked at the perception of the attitude and responsibility of the electorate and was subjective."}, "sentence_sentiment": {"answer": "Neutral", "rationale": "Because the entry describes a statement of fact and does not express any emotional colour."}, "language_style": {"answer": "Tactful and warm", "rationale": "The language style of the questionnaire entry is euphemism. It does not use direct orders or language of blame, but rather expresses the view that voters are responsible for their actions in a gentle and inviting manner. This is more acceptable and resonant."}, "time_dimension": {"answer": "Present", "rationale": "The entry describes a perception or attitude that voters are responsible for the conduct of national and local governments. This expression does not relate to past or future situations, but rather to an opinion or assessment of the current situation, so the temporal dimension is now judged."}, "sentence_type": "Affirmative declarative sentence", "pos": {"nouns": {"Voters": 1, "Country": 1, "Local government sector": 1, "Bad": 1}, "verbs": {"Responsible": 1}, "adverbs": {"Whatever": 1, "Eventually": 1}, "numerals": {"10": 1}}}]}

{"questionnaire_title": "Comprehensive diagnostic table on interpersonal relations (complete)", "questionnaire_category": "Behavioral questionnaire", "item_count": 28, "emotion_shift_overall": "From negative to positive", "language_style_overall": "Tactful and warm", "items": [{"content": "1. I can't tell you what's wrong with me.", "word_count": 11, "label": "", "sentence_structure": "Subject鈥搗erb鈥揷omplement", "subjective_objective": {"answer": "Subjective", "rationale": "Because \"a hard word\" is a patient's subjective assessment and perception of himself, and a subjective perspective."}, "sentence_sentiment": {"answer": "Negative", "rationale": "Because the \"speech\" act in the entry is negative."}, "language_style": {"answer": "Tactful and warm", "rationale": "The entry, which is popular by describing a phenomenon (i.e., some of the concerns cannot be expressed in words), resonates with readers, is relatively gentle and sympathetic. At the same time, the sentence does not specify specific problems or solutions, but rather, it guides the respondents to reflect on their feelings and experiences in an open and receptive manner."}, "time_dimension": {"answer": "Past", "rationale": "The entry asks the testee about his perception and feelings of past experience, and the keyword is \"about himself,\" which implies a subjective and personal process. The phrase \"with words\" is an adjective phrase that indicates that he or she cannot express his or her thoughts or feelings through words. Therefore, based on time dimensions and content analysis, the questionnaire entry should be judged as past."}, "sentence_type": "Negative declarative sentence", "pos": {"nouns": {"self": 1, "Trouble": 1, "mouth": 1, "Statements": 1}, "verbs": {"have": 1, "That's a good idea": 1}, "adverbs": {"About": 1}}}, {"content": "2. It is not natural to meet with a living person.", "word_count": 10, "label": "", "sentence_structure": "Subject鈥搗erb鈥揷omplement", "subjective_objective": {"answer": "Subjective", "rationale": "Because \"feel\" is a subjective feeling that reflects the experience of patients in their inner lives when they face strangers."}, "sentence_sentiment": {"answer": "Negative", "rationale": "Because \"irregular\" is a negative experience of uncomfortable, uncomfortable emotional experience."}, "language_style": {"answer": "Tactful and warm", "rationale": "The language style of the entry is more euphemistic, because it asks the other side in a more gentle way about how it feels when meeting with the living, and does not directly express criticism or questioning of the situation. Moreover, the use of the term \"irresistance\" is relatively implicit, avoiding language that might be offensive to the other side."}, "time_dimension": {"answer": "Present", "rationale": "The entry describes the feelings of dealing with strangers, with no clear time frame and can therefore be judged as now."}, "sentence_type": "Negative declarative sentence", "pos": {"nouns": {"Livers": 1, "Meet me": 1, "Feel": 1, "It's not natural": 1}, "verbs": {"and": 1, "See": 1, "Sensor": 1, "Present": 1}, "adjectives": {"From": 1, "Yeah": 1}, "numerals": {"2锛?: 1}}}, {"content": "3. Exalt envy and envy.", "word_count": 10, "label": "", "sentence_structure": "Subject鈥搗erb鈥揷omplement", "subjective_objective": {"answer": "Subjective", "rationale": "Because envy and jealousy are emotional reactions and subjective perceptions of patients."}, "sentence_sentiment": {"answer": "Negative", "rationale": "Because \"envious and jealous\" is a negative emotional reaction."}, "language_style": {"answer": "Tactful and warm", "rationale": "The entry is more adept and interactive when it is spoken in a gentle and inviting manner, rather than by direct statements or orders."}, "time_dimension": {"answer": "Present", "rationale": "The entry describes a mental or emotional state of the person being tested, and the keywords \"excessive\" and \"envious\" point to the emotional experience of the moment. Thus, the temporal dimension of the purpose of the article is now."}, "sentence_type": "Negative declarative sentence", "pos": {"nouns": {"That's too much": 1, "Envious": 1, "Jealousy": 1, "others": 1}, "adjectives": {"of": 2, "That's too much": 1, "Envious": 1, "Jealousy": 1}, "numerals": {"3锛?: 1}}}, {"content": "4. Too little is being done with heterosexuals.", "word_count": 7, "label": "", "sentence_structure": "Subject鈥搗erb鈥揷omplement", "subjective_objective": {"answer": "Objective", "rationale": "Because the extent of heterosexual interaction is an objective fact that can be observed and measured, not the subjective attitude or feeling of the patient."}, "sentence_sentiment": {"answer": "Negative", "rationale": "Because the entry is an expression of a person ' s possible social impairment or impairment, which has a certain negative emotional connotation."}, "language_style": {"answer": "Tactful and warm", "rationale": "The language style of the entry is more moderate, more compassionate, and not too direct or cold language, which is more acceptable and more open to questions. At the same time, the subject does not cause too many negative emotions and pressures, which is a more friendly way of asking questions."}, "time_dimension": {"answer": "Present", "rationale": "According to the sentence, \"now\", the entry is asking the testee about his current behaviour or state, and thus judging as now."}, "sentence_type": "Negative declarative sentence", "pos": {"nouns": {"Heterosexual": 1, "- To be with someone": 1}, "verbs": {"Too much": 1, "Less": 1}, "adverbs": {"and": 1}, "numerals": {"4锛?: 1}}}, {"content": "5. The continuous talks were difficult.", "word_count": 12, "label": "", "sentence_structure": "Subject鈥搗erb鈥揷omplement", "subjective_objective": {"answer": "Subjective", "rationale": "Because it is a subjective view that patients describe their feelings and experiences."}, "sentence_sentiment": {"answer": "Negative", "rationale": "Because the attitude of \"difficult\" in the entry is negative."}, "language_style": {"answer": "Calm and direct", "rationale": ""}, "time_dimension": {"answer": "Present", "rationale": "The entry describes whether the testee has encountered problems in his current state, namely, \"difficult to continuous talks\". The temporal dimension therefore is now judged."}, "sentence_type": "Negative declarative sentence", "pos": {"nouns": {"Difficulties": 1, "Meetings": 2}, "verbs": {"to": 1, "I feel": 1, "It's been going on and on": 1}, "adverbs": {"Land": 1, "It's been going on and on": 1}, "numerals": {"5锛?: 1, "Two": 1}}}, {"content": "6. Tensions are felt in social settings.", "word_count": 9, "label": "", "sentence_structure": "Subject鈥揷opula鈥損redicative", "subjective_objective": {"answer": "Subjective", "rationale": "Because feeling nervous is a subjective emotional experience."}, "sentence_sentiment": {"answer": "Negative", "rationale": "Because the \"frightful\" mood in the entry is negative."}, "language_style": {"answer": "Tactful and warm", "rationale": "The language style of the entry is more euphemistic, and it does not use direct criticism or strong negative emotional terminology to describe the situation of the other, but rather asks the respondents, in a concerned and understanding, whether they are nervous in the social setting. Such expression is easier to accept and are willing to participate in the responses to the questionnaire."}, "time_dimension": {"answer": "Present", "rationale": "The keywords \"in social settings\" and \"in a state of tension\" indicate that the question is asked about the current, ongoing feelings of the subject, so the judgment is \"now\"."}, "sentence_type": "Affirmative declarative sentence", "pos": {"nouns": {"Socially": 1, "Nervous": 1}, "verbs": {"I feel": 1}}}, {"content": "7. People are often hurt.", "word_count": 6, "label": "", "sentence_structure": "Subject鈥搗erb鈥揷omplement", "subjective_objective": {"answer": "Subjective", "rationale": "Because \"often hurting\" is the patient's subjective behaviour and attitude, reflecting the patient's perception of and treatment of interpersonal relationships, and subjective."}, "sentence_sentiment": {"answer": "Negative", "rationale": "Because \"too often hurting\" is a negative act, a sign of disrespect and responsibility."}, "language_style": {"answer": "Tactful and warm", "rationale": "The entry is more personal and interactive when it is spoken in a gentle and inviting manner, rather than in direct statements or orders. At the same time, it does not have too much blame or criticism, but rather leads the interviewee to reflect on his behaviour by asking questions."}, "time_dimension": {"answer": "Present", "rationale": ""}, "sentence_type": "Negative declarative sentence", "pos": {"nouns": {"Hurt": 1, "others": 1}, "verbs": {"Often": 1}}}, {"content": "8. It is not natural to have sexual relations with opposite sex.", "word_count": 10, "label": "", "sentence_structure": "Subject鈥揷opula鈥損redicative", "subjective_objective": {"answer": "Subjective", "rationale": "Because it is natural that a feeling is a subjective experience and feeling, and it is the patient ' s subjective assessment of his or her sexual relations with the opposite sex."}, "sentence_sentiment": {"answer": "Negative", "rationale": "The expression of the purpose of the article is negative, indicating that the individual is uncomfortable and uncomfortable with the opposite sex."}, "language_style": {"answer": "Tactful and warm", "rationale": "The entry uses a more euphemistic approach, not asking whether the other party is unnatural about opposite sex, but rather leading the other party to think by expressing a feeling. At the same time, the way in which the question is asked is mild and does not make the person under investigation uncomfortable or awkward."}, "time_dimension": {"answer": "Present", "rationale": ""}, "sentence_type": "Negative declarative sentence", "pos": {"nouns": {"Heterosexual": 1, "Travel": 1, "Feel": 1, "Naturally": 1}, "verbs": {"and": 1, "- To be with someone": 1}, "adjectives": {"It's not natural": 1}}}, {"content": "9. With a large group of friends, it is common to feel lonely or lost.", "word_count": 17, "label": "", "sentence_structure": "Subject鈥揷opula鈥損redicative", "subjective_objective": {"answer": "Subjective", "rationale": "Because feeling lonely or lost is the subjective feeling and experience of the patient."}, "sentence_sentiment": {"answer": "Negative", "rationale": "Because the \"sorting or falling\" sentiment in the entry is negative."}, "language_style": {"answer": "Tactful and warm", "rationale": "So the language style of this questionnaire entry can be considered \"precipitous.\""}, "time_dimension": {"answer": "Past", "rationale": "While the time dimension of past, present and future is not clearly identified, usually such questions are more concerned with the emotional experience of individuals at this moment, so the answer should be \"now\"."}, "sentence_type": "Affirmative declarative sentence", "pos": {"nouns": {"A bunch of them": 1, "friends": 1, "Loneliness": 1, "Lost": 1}, "verbs": {"in": 1, "and": 1, "I feel": 1}, "adjectives": {"Big": 1}, "numerals": {"9": 1}}}, {"content": "10. Extreme vulnerability.", "word_count": 4, "label": "", "sentence_structure": "Subject鈥揷opula鈥損redicative", "subjective_objective": {"answer": "Subjective", "rationale": "Because vulnerability is the subjective feeling and experience of the patient."}, "sentence_sentiment": {"answer": "Negative", "rationale": "Because the act of \"vulnerability\" is negative."}, "language_style": {"answer": "Calm and direct", "rationale": "The entry directly asks the respondents about the vulnerability, the lack of excessive emotional accommodations or euphemisms, and the relatively calm and direct language style."}, "time_dimension": {"answer": "Present", "rationale": "The entry is not clearly timed, but describes the person's current feelings and state, and thus the present."}, "sentence_type": "Negative declarative sentence", "pos": {"adverbs": {"Very easy": 1, "I'm embarrassed": 1}}}, {"content": "11. There is no harmony with others.", "word_count": 9, "label": "", "sentence_structure": "Subject鈥搗erb鈥揷omplement", "subjective_objective": {"answer": "Subjective", "rationale": "Because \"not living together\" is a subjective assessment and feeling."}, "sentence_sentiment": {"answer": "Negative", "rationale": "Because the behavior in the entry \"not living together\" is negative."}, "language_style": {"answer": "Tactful and warm", "rationale": "The entry is more adept and interactive when it is spoken in a gentle and inviting manner, rather than by direct statements or orders."}, "time_dimension": {"answer": "Present", "rationale": "The entry is to ask the testee whether there is a problem of being in harmony with others at this stage, and therefore the temporal dimension is now judged."}, "sentence_type": "Negative declarative sentence", "pos": {"nouns": {"person": 1, "Harmony": 1, "Get along": 1}, "verbs": {"Yes": 1}, "numerals": {"11": 1}}}, {"content": "12. No idea how to end a relationship with a heterosexual.", "word_count": 14, "label": "", "sentence_structure": "Subject鈥搗erb鈥揷omplement", "subjective_objective": {"answer": "Subjective", "rationale": "Because the criteria for \"dependability\" vary from person to person, the question requires a subjective examination of the patient's personal thoughts and perceptions."}, "sentence_sentiment": {"answer": "Negative", "rationale": "Because the question of \"know how to live with opposite sex\" may lead to discomfort or offensive behaviour in the context of heterosexual relations, and is therefore negative."}, "language_style": {"answer": "Tactful and warm", "rationale": "The entry is more personal and interactive when it is being approached in a gentle and inviting manner, asking whether the other party has encountered problems in having an unknown moment to stop, rather than directly speaking or ordering."}, "time_dimension": {"answer": "Present", "rationale": "The entry does not contain clear time indications or past experience, but rather questions the views and understanding of the interviewees in the current circumstances, and therefore determines it as \"now\"."}, "sentence_type": "Interrogative sentence", "pos": {"nouns": {"Heterosexual": 1, "Get along": 1, "Stop it": 1}, "verbs": {"Yes": 1, "How's that": 1, "Get along": 1}, "adverbs": {"I don't know": 1}, "numerals": {"12": 1}}}, {"content": "13. When someone who is not familiar tells himself about his life and his experiences in an attempt to feel sympathy, he often feels he is not.", "word_count": 30, "label": "", "sentence_structure": "Subject鈥搗erb鈥揷omplement", "subjective_objective": {"answer": "Subjective", "rationale": "Because \"often feeling overwhelmed\" is the patient's own feelings and experiences. It's the patient's subjective attitude and reaction."}, "sentence_sentiment": {"answer": "Negative", "rationale": "Because the entry describes the feeling of being unconscious, which is a negative emotional reaction."}, "language_style": {"answer": "Tactful and warm", "rationale": "The entry is more euphemistic and gentle, asking the interviewee how he feels when he talks about personal encounters and seeks sympathy from strangers, rather than direct orders or criticism. At the same time, the subjective description of \"self-perception\" is used to guide the interviewee's thinking and enhance communication with the interviewee."}, "time_dimension": {"answer": "Present", "rationale": "The word \"when\" indicates a condition or a situation, and the word \"normal\" indicates that this situation often happens. Moreover, the act of \"frightful\" occurs now, and it is now judged."}, "sentence_type": "Negative declarative sentence", "pos": {"nouns": {"person": 2, "Compassion": 1, "Lifetime": 1, "self": 1}, "verbs": {"to": 1, "Speech": 1, "Please": 1, "I always feel": 1}, "adjectives": {"I don't know": 2}, "adverbs": {"Regular": 1, "Here": 1}, "numerals": {"13": 1}, "pronouns": {"self": 2}}}, {"content": "14. Fear of what people might think of themselves.", "word_count": 13, "label": "", "sentence_structure": "Subject鈥搗erb鈥揷omplement", "subjective_objective": {"answer": "Subjective", "rationale": "Because \"fear\" is a subjective feeling or emotion."}, "sentence_sentiment": {"answer": "Negative", "rationale": "Because the mental state of \"fear\" in the entry is negative."}, "language_style": {"answer": "Tactful and warm", "rationale": "The entry leads the interviewee to wonder whether he has any concerns about the image of others, which is relatively gentle, friendly, and without direct accusations or criticism. Moreover, the expression \"bad impressions of himself\" is more euphemismal, avoiding the use of too sharp or negative language. Thus, it is considered a warm language."}, "time_dimension": {"answer": "Present", "rationale": "The word \"fear\" in the entry indicates a feeling or emotional experience, while \"no\" and \"no\" involve an assessment or prediction of past or future circumstances. The entry therefore primarily looks at the testee's concerns about his or her appearance, behaviour, etc. in his or her current state."}, "sentence_type": "Interrogative sentence", "pos": {"nouns": {"Questionnaire": 1, "Entry": 1, "person": 1, "Bad impression": 1}, "verbs": {"I'm worried": 1, "have": 1}, "numerals": {"14": 1}, "pronouns": {"self": 1}}}, {"content": "15. Always try to be appreciated.", "word_count": 11, "label": "", "sentence_structure": "Subject鈥搗erb鈥揷omplement", "subjective_objective": {"answer": "Subjective", "rationale": "Because it is always the subjective attitude and direction of others who try to appreciate themselves as patients."}, "sentence_sentiment": {"answer": "Positive", "rationale": "Because the purpose of \"always try to be appreciated\" is positive."}, "language_style": {"answer": "Calm and direct", "rationale": "Although it expresses a desire to be accepted by others, it does not use excessive rhetorical or emotional rendering, but rather is expressed in simple and clear language. At the same time, its tone is neutral and peaceful, with no apparent tendency to express a preference for praise. So, the language of the questionnaire entry is closer to \"cooling straight.\""}, "time_dimension": {"answer": "Present", "rationale": "The entry \"always try\" means a continuous state of behaviour, while \"behave yourself\" is an expectation or expected outcome, pointing to current or recent conditions."}, "sentence_type": "Interrogative sentence", "pos": {"nouns": {"person": 1, "Admirable": 1}, "verbs": {"Always": 1, "Try": 1, "be": 1, "self": 1}, "adverbs": {"Total": 1}, "numerals": {"15": 1}}}, {"content": "16. Concealed the opposite sex . 12", "word_count": 6, "label": "", "sentence_structure": "Subject鈥搗erb鈥揷omplement", "subjective_objective": {"answer": "Subjective", "rationale": "Because \"suspect\" is a subjective emotional experience and idea, it looks at the emotional tendencies and attitudes inside the individual."}, "sentence_sentiment": {"answer": "Neutral", "rationale": "Because the entry merely stated a fact, there was no apparent emotional tendency."}, "language_style": {"answer": "Tactful and warm", "rationale": "The language style of the entry is a kind of euphemism. First, the term \"simplified and different\" does not directly blame or evaluate each other, although it relates to personal emotions and behaviour. Second, the phrase is expressed in a gentle and understanding way that makes people feel respected and concerned. This language style increases the willingness of respondents to share their true thoughts and feelings, and is more conducive to the accuracy and reliability of questionnaires."}, "time_dimension": {"answer": "Present", "rationale": "The entry asks whether the person currently in the test is secretly interested in a particular sex, and the time dimension is now judged."}, "sentence_type": "Interrogative sentence", "pos": {"nouns": {"Questionnaire": 1, "Entry": 1, "Heterosexual": 1}, "verbs": {"I'm thinking about myself": 1}}}, {"content": "17. It is often avoided to express their feelings.", "word_count": 11, "label": "", "sentence_structure": "Subject鈥搗erb鈥揷omplement", "subjective_objective": {"answer": "Subjective", "rationale": "Because \"often avoiding expressing one's feelings\" is a subjective attitude or behaviour that reflects the patient's choice of his or her emotional experience and expression."}, "sentence_sentiment": {"answer": "Negative", "rationale": "Because the \"avoid expressing your feelings\" act in the entry is negative."}, "language_style": {"answer": "Calm and direct", "rationale": "The language style of the entry is relatively simple, it is not based on excessive rhetorical or emotional rendering, and it is more objective and straightforward in the form of statements. I therefore think that the language style of the purpose is straightforward and calm."}, "time_dimension": {"answer": "Present", "rationale": "The keyword \"frequently\" denotes a regular, customary act, and the act \"avoiding to express one's feelings\" is not clear whether it was in the past or in the future, so the temporal dimension of the article should be judged as now."}, "sentence_type": "Imperative sentence", "pos": {"nouns": {"Feel": 1, "Entry": 1}, "verbs": {"Often": 1, "Avoid": 1, "Expression": 1}, "adverbs": {"Often": 1}, "numerals": {"17": 1}}}, {"content": "18. Lack of confidence in their own (physical) appearance.", "word_count": 12, "label": "", "sentence_structure": "Subject鈥揷opula鈥損redicative", "subjective_objective": {"answer": "Subjective", "rationale": "Because lack of confidence is a subjective feeling."}, "sentence_sentiment": {"answer": "Negative", "rationale": "Because the attitude of \"lack of confidence in your own instrument\" in the entry is negative."}, "language_style": {"answer": "Tactful and warm", "rationale": "The entry is a euphemism, which leads the interviewee to reflect on his or her appearance and avoid direct accusations or criticism of the interviewee."}, "time_dimension": {"answer": "Present", "rationale": "The entry describes the current behaviour or state of the person being tested, and the expression \"to himself\" indicates that it is an individual's subjective feeling or evaluation, while \"lack of confidence\" is a negative assessment of his own instrument, without reference to past or future events."}, "sentence_type": "Negative declarative sentence", "pos": {"nouns": {"Instrument": 1, "Looks like a": 1}, "verbs": {"Lack": 1, "Confidence": 1}, "numerals": {"18": 1}}}, {"content": "19. Disgusting someone or being hated by someone.", "word_count": 11, "label": "", "sentence_structure": "Subject鈥搗erb鈥搊bject", "subjective_objective": {"answer": "Subjective", "rationale": "Because \"hate\" and \"repulsed\" are personal subjective emotional experiences."}, "sentence_sentiment": {"answer": "Negative", "rationale": "Because the \"hot or hated\" in the entry is negative."}, "language_style": {"answer": "Calm and direct", "rationale": "The language style of the entry is straightforward, because it explicitly asks whether the person interviewed hates someone or is not. This direct question is usually used in psychological questionnaires to understand the emotional and emotional state of the person interviewed."}, "time_dimension": {"answer": "Present", "rationale": "The entry is a time dimension that judges the moment when asking the person who was tested about his or her emotional state of \"disguised or hated.\""}, "sentence_type": "Negative declarative sentence", "pos": {"nouns": {"person": 2, "Shit": 2}, "verbs": {"By": 1, "Sou-san": 1}}}, {"content": "20. The opposite sex is not to be seen.", "word_count": 5, "label": "", "sentence_structure": "Subject鈥搗erb鈥搊bject", "subjective_objective": {"answer": "Subjective", "rationale": "It is a subjective sentiment and attitude to look down on."}, "sentence_sentiment": {"answer": "Negative", "rationale": "Because the act of \"defeating opposite sex\" in the entry is negative."}, "language_style": {"answer": "Tactful and warm", "rationale": "The entry is more adept and interactive when it is spoken in a gentle and inviting manner, rather than by direct statements or orders."}, "time_dimension": {"answer": "Present", "rationale": "The entry can be judged by the sentence 's \"ignoble ' to question the current behaviour or attitude of the person being tested, so the temporal dimension is now."}, "sentence_type": "Negative declarative sentence", "pos": {"nouns": {"Heterosexual": 1}, "verbs": {"I'm not looking down": 1}}}, {"content": "21. Not to listen with a focus.", "word_count": 7, "label": "", "sentence_structure": "Subject鈥搗erb鈥揷omplement", "subjective_objective": {"answer": "Subjective", "rationale": "Because the lack of attention is the subjective feeling and experience of the patient, it is a subjective self-evaluation or subjective judgement."}, "sentence_sentiment": {"answer": "Negative", "rationale": "Because the \"not listening\" in the entry is negative."}, "language_style": {"answer": "Tactful and warm", "rationale": "The term \"listen\" is also positive, leading the person to think about his or her ability to listen in everyday life, rather than simply criticize or criticize."}, "time_dimension": {"answer": "Present", "rationale": "The entry describes a capacity or state, i.e. whether the person tested is able to focus on listening."}, "sentence_type": "Negative declarative sentence", "pos": {"nouns": {"Focus": 1, "Listen": 1}, "verbs": {"No, I can't": 1, "Land": 1}}}, {"content": "22. No one can talk about their own problems.", "word_count": 10, "label": "", "sentence_structure": "Subject鈥搗erb鈥揷omplement", "subjective_objective": {"answer": "Subjective", "rationale": "Because \"no one can talk\" is a subjective feeling and experience, a subjective perception of the difficulties and problems that patients face in their lives that they cannot be properly addressed and supported."}, "sentence_sentiment": {"answer": "Negative", "rationale": "Because \"no one can talk about trouble\" in the entry expresses a negative emotion and attitude."}, "language_style": {"answer": "Tactful and warm", "rationale": "The language style of the entry is more euphemistic and warm, and dialogue is conducted in a way that cares about the other side and encourages them to share their feelings. At the same time, the expression \"no one can talk\" implies that the interviewee may feel emotionally lonely and helpless, which can resonate with the interviewee and promote open-minded conversations."}, "time_dimension": {"answer": "Past", "rationale": "It describes a subjective feeling or state of affairs of the person being tested, i.e., that at some point it is troubled and that no one can speak. This feeling may be a past experience, or a present experience, and may also indicate that similar situations may occur in the future. The temporal dimension of the article is therefore uncertain."}, "sentence_type": "Negative declarative sentence", "pos": {"nouns": {"self": 1, "Trouble": 1, "person": 1, "Speech": 1}, "verbs": {"have": 1}, "numerals": {"22": 1}}}, {"content": "23. Exclusion and indifference . 12", "word_count": 8, "label": "", "sentence_structure": "Subject鈥搗erb鈥揷omplement", "subjective_objective": {"answer": "Subjective", "rationale": "Because the sense of exclusion and indifference is usually a subjective experience of individuals, different people may feel different and react differently. While some may indeed be capable of doing something that others feel excluded or apathy, such an act itself is still an objective fact, while the sense of exclusion and indifference that is felt is often seen as subjective."}, "sentence_sentiment": {"answer": "Negative", "rationale": "Because the act of \"exclusion and indifference\" in the entry is negative."}, "language_style": {"answer": "Calm and direct", "rationale": "The entry directly asks whether the person surveyed has experienced rejection and indifference from others, has a more concise and rapid language style, and has not been over-represented in rhetoric and emotionalization.\""}, "time_dimension": {"answer": "Present", "rationale": "The term \"exclusion and indifference\" in this entry describes a current feeling and experience, without reference to the time dimension of the past or the future. Thus, the basis of the judgement is now."}, "sentence_type": "Negative declarative sentence", "pos": {"nouns": {"others": 1, "Exclusion": 1, "Coldness": 1}, "verbs": {"Yes": 1}, "numerals": {"23": 1}}}, {"content": "24. Disrespected by the opposite sex.", "word_count": 6, "label": "", "sentence_structure": "Subject鈥搗erb鈥揷omplement", "subjective_objective": {"answer": "Subjective", "rationale": "Because \"understanding\" or \"underlooking\" is usually a subjective feeling and judgement of a person, not an objective fact."}, "sentence_sentiment": {"answer": "Negative", "rationale": "Because the emotional experience of being despised by opposite sex in the entry is negative."}, "language_style": {"answer": "Calm and direct", "rationale": "This entry expresses the core of the problem in direct and concise language, without too many qualifiers or emotional colours to influence the other. This language style is usually more appropriate for serious, formal psychological questionnaire studies."}, "time_dimension": {"answer": "Present", "rationale": "The act of \"neglect\" is happening now, so it's judged now."}, "sentence_type": "Negative declarative sentence", "pos": {"nouns": {"Heterosexual": 1, "I'm not looking down": 1}, "numerals": {"24": 1}}}, {"content": "25. The diversity of views and opinions is not widely heard.", "word_count": 15, "label": "", "sentence_structure": "Subject鈥搗erb鈥揷omplement", "subjective_objective": {"answer": "Subjective", "rationale": "Because it is the patient's subjective attitude and thoughts that are examined."}, "sentence_sentiment": {"answer": "Negative", "rationale": "Because the act of \"not hearing a wide range of views and opinions\" in the entry is negative."}, "language_style": {"answer": "Calm and direct", "rationale": "The entry is relatively calm and straightforward in its language style, is not overly pedestic or emotional, and focuses more on expressing the objectivity and accuracy of the information itself. It asks the respondents about their performance or preferences in a certain area, and asks the respondents to respond on the basis of facts rather than expressing their views by describing emotions or feelings."}, "time_dimension": {"answer": "Present", "rationale": "The entry \"can't\" denote the current state of conduct or capacity, while \"hearing the views and opinions\" is an exercise that is now under way, so the entry describes the present behaviour and attitude of the person being tested."}, "sentence_type": "Negative declarative sentence", "pos": {"nouns": {"Questionnaire": 1, "Entry": 1, "Observations": 2}, "verbs": {"No, I can't": 1, "Listen": 2, "Hiro": 1}, "adjectives": {"A lot of things": 1}, "numerals": {"25": 1}}}, {"content": "26. He often felt hurt and felt sad.", "word_count": 12, "label": "", "sentence_structure": "Subject鈥揷opula鈥損redicative", "subjective_objective": {"answer": "Subjective", "rationale": "Because injury and grief are emotional experiences that reflect patients ' subjective feelings and emotional state."}, "sentence_sentiment": {"answer": "Negative", "rationale": "Because the \"injured and heartbroken\" act in the entry is negative."}, "language_style": {"answer": "Tactful and warm", "rationale": "The entry is more personal and humane by speaking in a gentle and caring manner, by asking if the other party is often sad about the injury, and by looking at and understanding the emotional state of the person under investigation."}, "time_dimension": {"answer": "Present", "rationale": "The words \"normally\" and \"silently sad\" in the entry indicate that it is a sentence describing the current situation of the testees and should therefore be classified as the past."}, "sentence_type": "Affirmative declarative sentence", "pos": {"nouns": {"self": 1, "Hurt": 1, "Sad": 1}, "verbs": {"Common cause": 1, "Yes": 1, "And": 1, "From the shadows": 1, "Sad": 1}, "adverbs": {"Common cause": 1, "From the shadows": 1}, "numerals": {"26": 1}}}, {"content": "27. People often talk about it and fool around.", "word_count": 8, "label": "", "sentence_structure": "Subject鈥搗erb鈥揷omplement", "subjective_objective": {"answer": "Subjective", "rationale": "Because the feeling that people talk about and fool about is the subjective experience and feeling of the patient."}, "sentence_sentiment": {"answer": "Negative", "rationale": "Because the \"screw, fool\" in the entry is negative."}, "language_style": {"answer": "Tactful and warm", "rationale": "The entry is more soft, neutral, and does not directly accuse or attack each other. It also uses \"often\" and \"smuggling\" to express a more objective statement of fact. It is more acceptable and more conducive to the questionnaire."}, "time_dimension": {"answer": "Present", "rationale": "So the entry looks at whether people are talking about it or making fun of it. So the output is: \"Now.\""}, "sentence_type": "Negative declarative sentence", "pos": {"nouns": {"others": 1, "Talk": 1, "Smuggle": 1}, "verbs": {"By": 1, "Talk": 1, "Smuggle": 1}, "adverbs": {"Regular": 1}, "numerals": {"27": 1}}}, {"content": "28. It was not clear how heterosexual relationships could be better.", "word_count": 13, "label": "", "sentence_structure": "Subject鈥搗erb鈥揷omplement", "subjective_objective": {"answer": "Subjective", "rationale": "Because it is the attitude and the idea of the patient regarding heterosexual relations that is examined from a subjective perspective."}, "sentence_sentiment": {"answer": "Negative", "rationale": "Because the \"I don't know how to get along\" in the entry is negative."}, "language_style": {"answer": "Tactful and warm", "rationale": "The language style of the entry is more euphemistic, and it does not directly blame or criticize the other party for his or her problems in dealing with opposite sex, but rather raises questions in a caring and understanding tone, leading the other party to think and explore better ways of living together. This expression is more acceptable and stimulates the other party鈥檚 motivation and participation."}, "time_dimension": {"answer": "Past", "rationale": "The entry asks whether the person tested has experienced difficulties in dealing with heterosexuals and the temporal dimension is a matter of past."}, "sentence_type": "Interrogative sentence", "pos": {"nouns": {"Heterosexual": 1, "- To be with someone": 1, "Get along": 1}, "verbs": {"I don't know": 1, "How's that": 1, "Better": 1, "More": 1}, "adverbs": {"28锛?: 1}, "numerals": {"28": 1}}}]}

{"questionnaire_title": "Self-assessment of anxiety (SAS)", "questionnaire_category": "Mental health", "item_count": 20, "emotion_shift_overall": "From negative to positive", "language_style_overall": "Calm and direct", "items": [{"content": "I feel more agitated than I always do.", "word_count": 15, "label": "", "sentence_structure": "Subject鈥揷opula鈥損redicative", "subjective_objective": {"answer": "Subjective", "rationale": "Because \"fEsperate\" is the patient's subjective feeling."}, "sentence_sentiment": {"answer": "Negative", "rationale": "Because the description \"sensitized anxiety\" is negative emotion."}, "language_style": {"answer": "Tactful and warm", "rationale": ""}, "time_dimension": {"answer": "Present", "rationale": "The entry expresses the current feelings of the testees, and the keywords \"fear\" and \"more sensitive anxiety than usual\" describe the present situation."}, "sentence_type": "Affirmative declarative sentence", "pos": {"nouns": {"I": 1, "Feel": 1, "Normal": 1, "You're a bit of a sane": 1, "Anxiety": 1}, "verbs": {"I feel": 1, "- Yeah": 1, "More": 1}, "adjectives": {"He's a sensitive man": 1}, "adverbs": {"As usual": 1, "Land": 1}}}, {"content": "I'm worried for no reason.", "word_count": 9, "label": "", "sentence_structure": "Subject鈥搗erb鈥揷omplement", "subjective_objective": {"answer": "Subjective", "rationale": "Because the word \"for no reason\" reflects the patient's own feelings and thoughts, and it is viewed from a subjective perspective."}, "sentence_sentiment": {"answer": "Negative", "rationale": "Because \"fear\" in the entries is negative, and worries and worries are negative emotional."}, "language_style": {"answer": "Calm and direct", "rationale": ""}, "time_dimension": {"answer": "Present", "rationale": "According to the description of \"feared\" and \"for no reason\", the entry is to ask the testee about the current state of feeling or emotion, and therefore the temporal dimension is now judged."}, "sentence_type": "Interrogative sentence", "pos": {"nouns": {"Grandpa": 1, "Questionnaire": 1}, "verbs": {"Please": 1, "Analysis": 1, "I feel": 1}, "adjectives": {"For no reason": 1}, "numerals": {"2": 1}, "pronouns": {"I": 1}}}, {"content": "I'm upset or scared.", "word_count": 12, "label": "", "sentence_structure": "Subject鈥揷opula鈥損redicative", "subjective_objective": {"answer": "Subjective", "rationale": "Because \"disturbed\" and \"frightful\" are feelings inside the patient, and they are viewed from the patient's subjective point of view."}, "sentence_sentiment": {"answer": "Negative", "rationale": "Because \"drilling\" and \"frightful\" are negative emotions, the purpose of this article is negative."}, "language_style": {"answer": "Calm and direct", "rationale": "The entry expresses in plain and simple language the question of whether the other party is angry or panic-ridden, is not accompanied by excessive rhetorical or emotional rendering, and is more objective in tone."}, "time_dimension": {"answer": "Present", "rationale": "The main phrase in \"3 I am distraught or panicned\" is \"I\" and it is a personal subjective feeling, while the verb \"easy or panic\" uses the usual current state of affairs, indicating that this feeling may be occurring or occurring frequently. Thus, the entry is a description of the present situation."}, "sentence_type": "Negative declarative sentence", "pos": {"nouns": {"I": 1, "I'm upset": 1, "Panic": 1}, "verbs": {"Easy": 1}, "numerals": {"3": 1}}}, {"content": "I feel like my body is broken up and broken.", "word_count": 18, "label": "", "sentence_structure": "Subject鈥搗erb鈥揷omplement", "subjective_objective": {"answer": "Subjective", "rationale": "It is a subjective feeling or experience to feel that the body is broken into pieces and broken."}, "sentence_sentiment": {"answer": "Negative", "rationale": "Because the expression \"break-up\" has a painful and uncomfortable emotional colour and is a negative description."}, "language_style": {"answer": "Calm and direct", "rationale": "Because the entry does not use excessive rhetorical or emotional rendering, it is more balanced and objective in tone, and it directly expresses the subject of asking the other party whether it feels physically divided."}, "time_dimension": {"answer": "Past", "rationale": "\"I feel\" means a past feeling or state, and \"like being divided into pieces and broken\" is a descriptive language, implying that this feeling has been in existence for some time. So the entry is mainly concerned with whether the person tested has experienced physical separation and incompleteness in the past."}, "sentence_type": "Affirmative declarative sentence", "pos": {"nouns": {"Body": 1, "A few": 1, "鈾?Broken 鈾?: 1}, "verbs": {"I feel": 1, "Split": 1}, "adjectives": {"I think so": 1}, "numerals": {"4": 1}, "pronouns": {"I": 1}}}, {"content": "I feel like everything's going well. Nothing bad will happen.", "word_count": 19, "label": "", "sentence_structure": "Subject鈥揷opula鈥損redicative", "subjective_objective": {"answer": "Subjective", "rationale": "This sentence is an expression of the patient ' s personal feelings and attitudes, not of objective facts or phenomena."}, "sentence_sentiment": {"answer": "Positive", "rationale": "Because the entries are \"everything is going well\", \"nothing bad will happen,\" and they express positive optimism and emotion."}, "language_style": {"answer": "Tactful and warm", "rationale": "The entry uses a more positive and optimistic expression, which is more moderate in tone and attempts to convey a positive sentiment and attitude. At the same time, it is more friendly and more likely to arouse the resonance and participation of the person under investigation.\""}, "time_dimension": {"answer": "Present", "rationale": "The keywords are \"fear\" and \"do not.\" They both indicate the current feeling and the expectations for the future, so the time dimension is now."}, "sentence_type": "Affirmative declarative sentence", "pos": {"nouns": {"Things": 2, "Shit": 1}, "verbs": {"Come on": 1, "I feel": 1, "Good": 1}, "adjectives": {"Good": 1}, "adverbs": {"very": 1}, "numerals": {"5": 1}, "pronouns": {"I": 1}}}, {"content": "Six of my limbs twitched and tremor.", "word_count": 9, "label": "", "sentence_structure": "Subject鈥搗erb鈥揷omplement", "subjective_objective": {"answer": "Objective", "rationale": "Because the entanglement and tremor are objective facts of the patient ' s experience."}, "sentence_sentiment": {"answer": "Negative", "rationale": "Because \"twirl and tremors\" are a symptom of physical discomfort and are usually considered negative."}, "language_style": {"answer": "Calm and direct", "rationale": "The entry describes physical symptoms that may exist in the person under investigation in a direct, concise language, without adding excessive emotional accoutrements or rhetorical techniques, and with a more objective and calm language style."}, "time_dimension": {"answer": "Past", "rationale": ""}, "sentence_type": "Affirmative declarative sentence", "pos": {"nouns": {"Four limbs": 1, "鈾?The tremors 鈾?: 1}, "verbs": {"Blend": 1}, "numerals": {"6": 1}}}, {"content": "I'm upset about headaches, neck pains and back pains.", "word_count": 12, "label": "", "sentence_structure": "Subject鈥揷opula鈥損redicative", "subjective_objective": {"answer": "Subjective", "rationale": "It's the patient's subjective feeling that's upset because I feel these pains."}, "sentence_sentiment": {"answer": "Negative", "rationale": "Because the entry \"headaches, neck pains and back pains\" are all manifestations of physical discomfort, they express a negative emotional attitude."}, "language_style": {"answer": "Calm and direct", "rationale": "The entry directly expresses the subject of questioning whether the other party is troubled by headaches, neck pains and back pains, and is not using excessive rhetoric or emotional rendering, which is more objective and balanced."}, "time_dimension": {"answer": "Past", "rationale": ""}, "sentence_type": "Negative declarative sentence", "pos": {"nouns": {"Headache": 1, "Cervical pain": 1, "Backache": 1, "Trouble": 1}, "verbs": {"Because": 1, "And": 1, "I'm upset": 1}, "numerals": {"7": 1}}}, {"content": "I feel weak and tired.", "word_count": 11, "label": "", "sentence_structure": "Subject鈥揷opula鈥損redicative", "subjective_objective": {"answer": "Subjective", "rationale": "Because feeling weak and vulnerable to fatigue is a subjective feeling of the patient."}, "sentence_sentiment": {"answer": "Negative", "rationale": "Because \"fearing weak and vulnerable\" is a physical discomfort, and is often considered a negative emotional state."}, "language_style": {"answer": "Calm and direct", "rationale": "The language style of the entry is more direct, not using too many qualifiers or emotional terms, but rather describing the feelings of the person under investigation in a concise and concise manner, reflecting objective, factual linguistic characteristics."}, "time_dimension": {"answer": "Past", "rationale": "The entry describes the feelings of the testee, which is judged by the time dimension."}, "sentence_type": "Negative declarative sentence", "pos": {"nouns": {"- I'm weak": 1, "Tired": 1}, "verbs": {"I feel": 1, "Easy": 1}, "adjectives": {"- I'm weak": 1, "Easy": 1}, "numerals": {"8": 1}, "pronouns": {"I": 1}}}, {"content": "I feel calm. I can sit down.", "word_count": 11, "label": "", "sentence_structure": "Subject鈥搗erb鈥揷omplement", "subjective_objective": {"answer": "Subjective", "rationale": "Because \"settling\" and \"sitting\" are the feelings and experiences of the patients themselves, and they are subjective."}, "sentence_sentiment": {"answer": "Positive", "rationale": "Because the descriptions of \"feeling calm\" and \"sitting quietly\" are positive and express a comfortable, relaxed mood."}, "language_style": {"answer": "Tactful and warm", "rationale": "The entry is more adept and interactive when it is spoken in a gentle and inviting manner, rather than by direct statements or orders."}, "time_dimension": {"answer": "Present", "rationale": "According to the words \"I feel calm, I sit quietly\" and \"I feel\" and \"I can\" it is a sentence that expresses the state of the moment, the psychological feeling. So the time dimension of the questionnaire entry is now."}, "sentence_type": "Affirmative declarative sentence", "pos": {"nouns": {"Questionnaire Entry": 1, "Calm down": 1, "Quiet": 1}, "verbs": {"I feel": 1, "Sit down": 1}, "adjectives": {"I": 1}, "numerals": {"9": 1}}}, {"content": "I feel my heart beating faster.", "word_count": 9, "label": "", "sentence_structure": "Subject鈥揷opula鈥損redicative", "subjective_objective": {"answer": "Subjective", "rationale": "Because heart beats are usually subjective experiences, each person may feel different about the pace of heart beating. Although the rate of heart beating can be measured by medical equipment, the rate of heart beating that patients feel is also an important factor in assessing their psychological state."}, "sentence_sentiment": {"answer": "Negative", "rationale": "Because the physical reaction of \"heart beats faster\" in the entry is usually associated with negative emotions such as stress, anxiety, etc."}, "language_style": {"answer": "Calm and direct", "rationale": ""}, "time_dimension": {"answer": "Past", "rationale": ""}, "sentence_type": "Affirmative declarative sentence", "pos": {"nouns": {"Heart rate": 1}, "verbs": {"I feel": 1, "Compare": 1}, "adjectives": {"Come on": 1}, "numerals": {"10": 1}, "pronouns": {"I": 1}}}, {"content": "I'm not feeling well because of the dizziness of the formation.", "word_count": 11, "label": "", "sentence_structure": "Subject鈥揷opula鈥損redicative", "subjective_objective": {"answer": "Subjective", "rationale": "Because it's the patient's subjective feeling and experience."}, "sentence_sentiment": {"answer": "Negative", "rationale": "Because \"stunning\" is a uncomfortable symptom, the description has some negative emotions."}, "language_style": {"answer": "Calm and direct", "rationale": "The entry directly asked whether the person surveyed was uncomfortable because of dizziness, was not using euphemism or exaggerating language and was more direct."}, "time_dimension": {"answer": "Present", "rationale": "The entry is to ask the testee's current state and feelings, \"because\" is the reason, \"incommodity\" describes the current feeling, and therefore judges it now."}, "sentence_type": "Negative declarative sentence", "pos": {"nouns": {"I": 1, "Stifler": 1, "I'm not feeling well": 1}, "verbs": {"Because": 1, "Form": 1, "And": 1, "not": 1, "- Yeah": 1}, "adjectives": {"Form": 1}, "numerals": {"11": 1}}}, {"content": "I feel like I'm gonna faint for a while.", "word_count": 10, "label": "", "sentence_structure": "Subject鈥搗erb鈥揷omplement", "subjective_objective": {"answer": "Subjective", "rationale": "The first one is the \"scene\" of the \"scene\" of the disease, because it is the subjective feeling and experience of the patient."}, "sentence_sentiment": {"answer": "Negative", "rationale": "Because the physical symptoms of \"frightful fainting\" in the entry are negative."}, "language_style": {"answer": "Tactful and warm", "rationale": "The entry uses a more moderate and caring expression, rather than direct questioning or accusations, which can more easily inspire the confidence and resonance of the respondents. At the same time, the description of \"fright to faint\" is relatively euphemism, avoiding excessive intrusion on personal privacy."}, "time_dimension": {"answer": "Present", "rationale": "The keyword \"formation\" indicates that the intensity, frequency and duration of the feeling may vary, suggesting that the person tested describes his or her symptoms in as much detail as possible."}, "sentence_type": "Exclamatory sentence", "pos": {"nouns": {"Feel": 1}, "verbs": {"I'm fainted": 1, "have": 1, "Form": 1}, "adverbs": {"Down there": 1, "you": 1}, "numerals": {"12": 1}, "classifiers": {"individual": 1}}}, {"content": "I can't breathe and breathe.", "word_count": 13, "label": "", "sentence_structure": "Subject鈥揷opula鈥損redicative", "subjective_objective": {"answer": "Objective", "rationale": "This is an objective description of the patient ' s state of health and does not require subjective judgement."}, "sentence_sentiment": {"answer": "Neutral", "rationale": "Because the entry describes the physical state of the body, there is no obvious emotional colour."}, "language_style": {"answer": "Tactful and warm", "rationale": "The entry is more gentle and caring, and the person is not directly evaluated or criticized for his or her health."}, "time_dimension": {"answer": "Present", "rationale": ""}, "sentence_type": "Affirmative declarative sentence", "pos": {"nouns": {"Breathe": 1, "Breathe in": 1, "Take it out": 1, "- It's hard": 1}, "verbs": {"Breathe": 1, "Breathe in": 1, "Take it out": 1, "Fees": 1}, "adjectives": {"It's not hard": 1}, "numerals": {"13": 1}}}, {"content": "I'm numb and irritated by my fingers and toes.", "word_count": 14, "label": "", "sentence_structure": "Subject鈥搗erb鈥揷omplement", "subjective_objective": {"answer": "Subjective", "rationale": "Because feeling numb and irritating is the patient ' s own feeling and is subjective."}, "sentence_sentiment": {"answer": "Negative", "rationale": "Because the \"finger and toe feeling numb and irritated\" symptoms of physical discomfort are negative."}, "language_style": {"answer": "Calm and direct", "rationale": "The entry describes the conditions that the person tested may encounter in a direct and concise language, without adding too many emotional colours or rhetorical techniques and with a more calm and straightforward language style."}, "time_dimension": {"answer": "Present", "rationale": "The keyword \"fear\" means the action that is being done, and the entire sentence is \"now,\" so the entry describes the physical feeling of the person being tested."}, "sentence_type": "Exclamatory sentence", "pos": {"nouns": {"Finger": 2, "Toes": 2, "I'm numb": 1, "Exciting": 1}, "verbs": {"I feel": 1}, "numerals": {"14": 1}}}, {"content": "I'm upset about stomach pain and indigestion.", "word_count": 12, "label": "", "sentence_structure": "Subject鈥搗erb鈥揷omplement", "subjective_objective": {"answer": "Subjective", "rationale": "The pain caused by stomach pain and indigestion is a subjective feeling."}, "sentence_sentiment": {"answer": "Negative", "rationale": "Because the expression \"problems with stomach pain and indigestion\" in the entry is negative."}, "language_style": {"answer": "Calm and direct", "rationale": "The entry is a direct indication of the physical condition of the person under investigation, which is not over-modified or emotionally rendered, and is more objective in tone."}, "time_dimension": {"answer": "Past", "rationale": "The entry \"problems caused by...\" indicates the distress that has been in existence for some time, and therefore is judged to be a description of the past."}, "sentence_type": "Affirmative declarative sentence", "pos": {"nouns": {"Stomach pain": 1, "Indigestion": 1, "Trouble": 1}, "verbs": {"Because": 1, "And": 1, "Trouble": 1}, "numerals": {"15": 1}, "pronouns": {"I": 1}}}, {"content": "Sixteen. I have to pee a lot.", "word_count": 7, "label": "", "sentence_structure": "Subject鈥搗erb鈥揷omplement", "subjective_objective": {"answer": "Subjective", "rationale": "Because the descriptions of \"must\" and \"frequent\" are subjective feelings and judgments from the point of view of the patient. Although this may be related to objective physical condition, the perception and feelings of the patient are still subjective."}, "sentence_sentiment": {"answer": "Negative", "rationale": "Because the act of \"high-intensity urine\" is negative and may indicate that there are problems in the body."}, "language_style": {"answer": "Calm and direct", "rationale": "The language style of the entry is calm and straightforward, as it directly enquired about the physical condition of the person interviewed, and did not use excessive qualifier or emotional expressions, but rather used a more objective and concise approach."}, "time_dimension": {"answer": "Present", "rationale": "The key word is \"must pee frequently,\" which means that the testee needs to look back at the past when he answers the question, i.e. whether he or she often felt the need to pee frequently in the past. So the time dimension of this entry is the past."}, "sentence_type": "Imperative sentence", "pos": {"nouns": {"Pee": 1, "Frequency": 1}, "verbs": {"Line": 1, "I have to": 1}, "numerals": {"16": 1}}}, {"content": "17, my hands are always warm and dry.", "word_count": 10, "label": "", "sentence_structure": "Subject鈥揷opula鈥損redicative", "subjective_objective": {"answer": "Objective", "rationale": "Because \"the temperature and humidity of the hand\" is an objective fact of the patient's body, it can be directly observed."}, "sentence_sentiment": {"answer": "Neutral", "rationale": "Because the entry is a description of a fact and does not have any emotional colour."}, "language_style": {"answer": "Tactful and warm", "rationale": "The language style of the entry is more euphemistic and warm. It uses graphic descriptions, such as \"hot\" and \"dry,\" to describe the characteristics of the subject. Besides, the use of \"always\" gives people a sense of affection and care."}, "time_dimension": {"answer": "Present", "rationale": "The time expression in the entry is \"Always\", indicating that the state of affairs has been in place and does not involve the time dimension of the past or the future, and therefore the judgement is now."}, "sentence_type": "Affirmative declarative sentence", "pos": {"nouns": {"Hands": 1, "Warm": 1, "Dry": 1}, "verbs": {"be": 1}, "adjectives": {"Mine": 1, "Always": 1}, "numerals": {"17": 1}}}, {"content": "I feel a red-haired face.", "word_count": 8, "label": "", "sentence_structure": "Subject鈥揷opula鈥損redicative", "subjective_objective": {"answer": "Objective", "rationale": "The entry is therefore based on objective considerations."}, "sentence_sentiment": {"answer": "Negative", "rationale": "Because \"face fever red\" is usually seen as a symptom of physical discomfort, related to negative health-related emotions."}, "language_style": {"answer": "Calm and direct", "rationale": "The language style of the entry is a calm and straightforward type. It is a simple and straightforward statement of a feeling that is more objective and direct than that of an excessive amount of emotional aesthetic or speechual. This type of language style is appropriate for describing information about facts, feelings or experiences."}, "time_dimension": {"answer": "Present", "rationale": "The entry now indicates that the testee felt fever and red in the face at the time of the questionnaire, and thus the temporal dimension is now judged."}, "sentence_type": "Affirmative declarative sentence", "pos": {"nouns": {"18": 1, "I think": 1, "Face": 1, "Fever": 1, "Red": 1}, "verbs": {"I think": 1}, "adjectives": {"Fever": 1, "Red": 1}, "numerals": {"18": 1}, "classifiers": {"individual": 1}, "pronouns": {"I": 1}}}, {"content": "I sleep easy. I rest well at night.", "word_count": 11, "label": "", "sentence_structure": "Subject鈥搗erb鈥揷omplement", "subjective_objective": {"answer": "Subjective", "rationale": "Because it's easy to sleep and good night rest that patients feel subjectively."}, "sentence_sentiment": {"answer": "Positive", "rationale": "Because \"sleep easy\" and \"sleep well\" are positive, positive emotions and attitudes."}, "language_style": {"answer": "Tactful and warm", "rationale": "The entry uses a positive term for \"sleep-easy\" and \"sleep-in-night\" and a kind and encouraging tone to describe the sleep of the person under investigation, which is more protruding and motivated."}, "time_dimension": {"answer": "Past", "rationale": ""}, "sentence_type": "Affirmative declarative sentence", "pos": {"nouns": {"I": 1, "Night": 1, "Sleep": 1, "Rest": 1}, "verbs": {"Sleep": 1, "Okay": 2}, "adjectives": {"Easy": 1}, "numerals": {"19": 1}, "classifiers": {"One": 1}}}, {"content": "Twenty I had nightmares.", "word_count": 4, "label": "", "sentence_structure": "Subject鈥搗erb鈥揷omplement", "subjective_objective": {"answer": "Subjective", "rationale": "Because nightmares are usually the subjective feelings and experiences of patients."}, "sentence_sentiment": {"answer": "Negative", "rationale": "Because \"dreaming\" is negative."}, "language_style": {"answer": "Calm and direct", "rationale": "The entry directly asks whether the subject of nightmares is a common subject for the respondents, and the language style is more straightforward and is not modified by excessive rhetoric or emotional colours. \""}, "time_dimension": {"answer": "Past", "rationale": "The entry is for the purpose of asking the testee whether he has had nightmares in the past and the temporal dimension is the past."}, "sentence_type": "Exclamatory sentence", "pos": {"nouns": {"Nightmares": 1, "20": 1}, "verbs": {"Do it": 1}, "numerals": {"20": 1}}}]}

{"questionnaire_title": "PGC Confidence Scale for the Philadelphia Center for the Elderly", "questionnaire_category": "Self-cognition", "item_count": 23, "emotion_shift_overall": "From negative to positive", "language_style_overall": "Neutral", "items": [{"content": "1. This year has been marked by more trifling matters.", "word_count": 11, "label": "", "sentence_structure": "Subject鈥搗erb鈥揷omplement", "subjective_objective": {"answer": "Subjective", "rationale": "Because this is the personal feeling and perception of the patient, it is considered from a subjective perspective."}, "sentence_sentiment": {"answer": "Negative", "rationale": "Because \"more trivial\" is negative."}, "language_style": {"answer": "Calm and direct", "rationale": "The entry is a simple and clear expression of the fact that there is no excessive emotional modification or rhetorical technique, which is more objective and direct in tone."}, "time_dimension": {"answer": "Present", "rationale": ""}, "sentence_type": "Negative declarative sentence", "pos": {"nouns": {"Year": 1, "It's a little bit of a matter": 1}, "verbs": {"have": 1, "I'm sorry": 1}, "adjectives": {"More": 1, "of": 2}, "numerals": {"1": 2}}}, {"content": "I sometimes become too worried to sleep.", "word_count": 14, "label": "", "sentence_structure": "Subject鈥搗erb鈥搊bject", "subjective_objective": {"answer": "Subjective", "rationale": "The inability to sleep because of excessive fear is a subjective feeling and experience of the patient."}, "sentence_sentiment": {"answer": "Negative", "rationale": "Because the mental state of \"excessive fear\" in the entries is negative."}, "language_style": {"answer": "Tactful and warm", "rationale": "The entry, which is asked in a gentle and caring manner, expresses concern about the sleep conditions of the interviewee, without the use of language that is too direct or commandive, giving the impression of affection and understanding."}, "time_dimension": {"answer": "Present", "rationale": ""}, "sentence_type": "Affirmative declarative sentence", "pos": {"nouns": {"I": 1, "That's too much": 1, "I'm worried": 1, "Sleep": 1}, "verbs": {"Sometimes": 1, "Because": 1, "And": 1, "No, I can't": 1, "Sleep": 1}, "adjectives": {"That's too much": 1}, "adverbs": {"Sometimes": 1}, "classifiers": {"individual": 1}, "pronouns": {"I": 1}}}, {"content": "I am sorry for many things.", "word_count": 10, "label": "", "sentence_structure": "Subject鈥揷opula鈥損redicative", "subjective_objective": {"answer": "Subjective", "rationale": "Because it's an emotional experience, it's a subjective feeling of the patient."}, "sentence_sentiment": {"answer": "Negative", "rationale": "Because the entry \"suffering\" expresses a negative emotional attitude."}, "language_style": {"answer": "Tactful and warm", "rationale": "The language style of the entry is euphemistic. It uses softer language, such as \"worried,\" and does not directly express negative feelings or blame each other. This expression is more acceptable to the interviewees and is willing to share their real thoughts and feelings."}, "time_dimension": {"answer": "Present", "rationale": ""}, "sentence_type": "Affirmative declarative sentence", "pos": {"nouns": {"Yes": 1, "Hard": 1}, "verbs": {"Sensor": 1, "Present": 1}, "adjectives": {"More": 1}, "numerals": {"3": 1}, "pronouns": {"I": 1}}}, {"content": "4. I am afraid of many things.", "word_count": 10, "label": "", "sentence_structure": "Subject鈥揷opula鈥損redicative", "subjective_objective": {"answer": "Subjective", "rationale": "Because fear is an emotional experience, it is a subjective and cognitive response of the individual, not an objective reality or external matter."}, "sentence_sentiment": {"answer": "Negative", "rationale": "Because \"fear\" is a negative emotion, which means it's negative about the thing."}, "language_style": {"answer": "Tactful and warm", "rationale": "The language style of the entry is more euphemistic, not so much as to express the questions of the subject, but rather to direct the other to express their thoughts and feelings by asking. Moreover, the description of \"fear\" is relatively mild and does not use too strong negative terms. The language style of the questionnaire entry can therefore be judged as \"fluent.\""}, "time_dimension": {"answer": "Past", "rationale": "There is no clear time indicator in the entry, but \"fear\" is an emotional reaction, usually arising from experience of an event or situation. So, \"I'm afraid of many things.\" In the entry, \"I\" may have experienced something that scares him or her in the past, or is experiencing something that scares him or her in the present."}, "sentence_type": "Affirmative declarative sentence", "pos": {"nouns": {"Things": 1, "Fear": 1}, "verbs": {"to": 1, "I feel": 1}, "numerals": {"Four": 1}}}, {"content": "5. I have lost control of my senses more than ever before.", "word_count": 17, "label": "", "sentence_structure": "Subject鈥揷opula鈥損redicative", "subjective_objective": {"answer": "Subjective", "rationale": "This is because the patient ' s self-observation and description of his or her situation is subjective."}, "sentence_sentiment": {"answer": "Negative", "rationale": "Because \"lost control\" is a negative description of behavior."}, "language_style": {"answer": "Calm and direct", "rationale": "It asked the interviewees in an objective manner whether they had more reason to lose control of the situation, and had not used too many emotional terms or rhetorical techniques, which were more verbically correct."}, "time_dimension": {"answer": "Present", "rationale": "The entry \"more than ever\" indicates a comparison between past and present, so the temporal dimension is now judged.\""}, "sentence_type": "Negative declarative sentence", "pos": {"nouns": {"I": 1, "Reason": 1, "Number": 2, "Before": 1, "Control": 1}, "verbs": {"Lose": 1, "- Yeah": 1, "More": 1}, "adjectives": {"In a rational way": 1}, "numerals": {"5": 1, "Yes": 1}, "classifiers": {"of": 3}}}, {"content": "6. For most of the time, I felt that life was difficult.", "word_count": 12, "label": "", "sentence_structure": "Subject鈥揷opula鈥損redicative", "subjective_objective": {"answer": "Subjective", "rationale": "Because \"life is difficult\" is a subjective emotion and experience, not an objective fact. It is a feeling inside the patient, linked to objective circumstances, circumstances, etc., but it is not the same as an objective reality."}, "sentence_sentiment": {"answer": "Negative", "rationale": "Because \"life hard\" in the entry is a negative emotional attitude."}, "language_style": {"answer": "Calm and direct", "rationale": "The entry uses a direct, concise language to ask the person ' s state of life, is not rendered in excessive speech or emotional colours and is therefore considered to be of a cold and straightforward language style."}, "time_dimension": {"answer": "Past", "rationale": ""}, "sentence_type": "Negative declarative sentence", "pos": {"nouns": {"Time": 1, "Internal": 1, "Sensor": 1, "Life": 1, "Hard": 1}, "verbs": {"Most": 1, "I feel": 1, "Time": 1}, "adjectives": {"Hard": 1}, "adverbs": {"Hard": 1, "Internal": 1}, "numerals": {"More": 1, "Time": 1}, "classifiers": {"Number of times": 1, "Internal": 1}, "pronouns": {"I": 1}}}, {"content": "I'm nervous about my work.", "word_count": 7, "label": "", "sentence_structure": "Subject鈥揷opula鈥損redicative", "subjective_objective": {"answer": "Subjective", "rationale": "Because \"strained\" is the patient's subjective perception and assessment of his or her psychological state, not the objective fact or result."}, "sentence_sentiment": {"answer": "Negative", "rationale": "Because \"strained\" in the entries describes a negative emotional state."}, "language_style": {"answer": "Calm and direct", "rationale": "The entry raises questions in a simple statement, without too much emotional or rhetorical, and in a calmer and more direct way.\""}, "time_dimension": {"answer": "Present", "rationale": "Now, the keyword \"I'm doing something\" means it's a move that's happening, and the overall context of the sentence shows it's a state or a feeling. So, the time dimension of the article is now."}, "sentence_type": "Affirmative declarative sentence", "pos": {"verbs": {"Do it": 1, "Yes": 1, "Nervous": 1}, "adjectives": {"very": 1}}}, {"content": "I am easily uneasy.", "word_count": 8, "label": "", "sentence_structure": "Subject鈥揷opula鈥損redicative", "subjective_objective": {"answer": "Subjective", "rationale": "Because \"sitting\" is a subjective feeling and emotional state."}, "sentence_sentiment": {"answer": "Negative", "rationale": "Because the act of \"easy to sit up\" is negative."}, "language_style": {"answer": "Tactful and warm", "rationale": ""}, "time_dimension": {"answer": "Present", "rationale": "According to the \"Please analyse the following \"and please\" in the next \"and \"I'm easily uneasy.\" The word \"very\" in the \"extremely\" is a good way to judge the time dimension of this \"questionnaire\" as it is now."}, "sentence_type": "Affirmative declarative sentence", "pos": {"verbs": {"Sit tight": 1}, "adjectives": {"Easy": 1}}}, {"content": "1. Things are getting worse and worse as I get older.", "word_count": 13, "label": "", "sentence_structure": "Subject鈥搗erb鈥揷omplement", "subjective_objective": {"answer": "Subjective", "rationale": "Because \"old and old\" and \"bad and worse\" are the feelings and subjective judgements of patients, not the objective facts."}, "sentence_sentiment": {"answer": "Negative", "rationale": "Because the expression \"the things are getting worse\" in the entry is negative."}, "language_style": {"answer": "Calm and direct", "rationale": "The entry is more direct and concise in language style, without too many rhetorical or emotional renderings. It is a straightforward expression of the subject that can get worse with age, and is more objective and realistic, suitable for use in, for example, questionnaires."}, "time_dimension": {"answer": "Present", "rationale": "The entry describes the personal experience and feelings of the person being tested, and the time dimension is not clear about the past, present or future. It therefore does not allow for a clear determination of the time period of the generation it refers to. However, depending on the context of the sentence, it can be inferred that the entry may be the subjective view and perception of the person being tested of the current situation."}, "sentence_type": "Affirmative declarative sentence", "pos": {"nouns": {"Age": 1, "Things": 1}, "verbs": {"Change": 1, "Shit": 1}, "adjectives": {"Old": 1}, "adverbs": {"Tsk": 1, "Come on": 1}, "numerals": {"One": 2}, "pronouns": {"I": 1}}}, {"content": "1. My energy is as strong as last year.", "word_count": 11, "label": "", "sentence_structure": "Subject鈥揷opula鈥損redicative", "subjective_objective": {"answer": "Subjective", "rationale": "Because energy is a subjective feeling or experience."}, "sentence_sentiment": {"answer": "Neutral", "rationale": "As this entry simply describes the individual ' s state of energy and does not address positive or negative attitudes, behaviour, purpose or quality."}, "language_style": {"answer": "Calm and direct", "rationale": "The entry is not based on too much rhetorical or emotional overtook, and is more objective in tone. It directly expresses the subject of asking if the other side feels that its level of energy has changed compared to last year. It can therefore be considered as being calm and straightforward in its language style."}, "time_dimension": {"answer": "Past", "rationale": "The entry is a reference to the past in terms of the time dimension, compared with the current energy status of the testee and the situation in the past year."}, "sentence_type": "Affirmative declarative sentence", "pos": {"nouns": {"- The energy": 1, "Year": 1}, "verbs": {"and": 1, "Same thing": 1, "Wang-seok": 1}, "adjectives": {"Last year": 1}, "numerals": {"1": 2}, "pronouns": {"I": 1}}}, {"content": "I've had more trouble this year.", "word_count": 11, "label": "", "sentence_structure": "Subject鈥揷opula鈥損redicative", "subjective_objective": {"answer": "Subjective", "rationale": "Because \"more trivial matters bother me\" is the subjective perception and evaluation of this year's situation, which is subjective."}, "sentence_sentiment": {"answer": "Negative", "rationale": "Because the \"more and more\" in the entry is negative."}, "language_style": {"answer": "Calm and direct", "rationale": "The language style of the entry is more calm and straightforward, rather than using excessive emotional or rhetorical techniques, it is a direct statement of the facts of the question, allowing the interviewees to understand and answer the questions more clearly."}, "time_dimension": {"answer": "Present", "rationale": "The keyword \"this year\" means the current year, the \"yes\" action is in progress, and \"more triviality\" is an ongoing event, so this entry describes the current situation."}, "sentence_type": "Negative declarative sentence", "pos": {"nouns": {"This year": 1, "It's a little bit of a matter": 1}, "verbs": {"have": 1, "Trouble": 1}, "numerals": {"More": 2}, "classifiers": {"Annex": 1}, "pronouns": {"I": 1}}}, {"content": "3. The old man is no longer useful.", "word_count": 10, "label": "", "sentence_structure": "Subject鈥損redicate", "subjective_objective": {"answer": "Subjective", "rationale": "Because \"unutilized\" is a subjective judgement that reflects expectations and the assessment of the capacities of older persons."}, "sentence_sentiment": {"answer": "Negative", "rationale": "Because the expression \"incompetent\" in the entry is negative, it implies that older persons are powerless and demeaning."}, "language_style": {"answer": "Calm and direct", "rationale": "The entry is more straightforward and concise, is not over-modified or emotionally rendered and is more consistent with the description of objective facts."}, "time_dimension": {"answer": "Present", "rationale": "The entry describes a stereotyped image and prejudice of older persons, and the temporal dimension is now judged."}, "sentence_type": "Negative declarative sentence", "pos": {"nouns": {"person": 1, "Old": 1, "Medium": 1}, "verbs": {"Naturally": 1, "not": 1, "Just": 1, "Yes": 1}, "adverbs": {"Just": 1}, "numerals": {"3": 1}}}, {"content": "4. When I was old, I found out better than I had thought.", "word_count": 16, "label": "", "sentence_structure": "Subject鈥搗erb鈥揷omplement", "subjective_objective": {"answer": "Subjective", "rationale": "Because the words \"feel\" and \"think\" express the subjective feelings and perceptions of the patient."}, "sentence_sentiment": {"answer": "Positive", "rationale": "Because of the positive attitude and the perception expressed in the entries that older persons may find themselves in fact better off."}, "language_style": {"answer": "Tactful and warm", "rationale": "The entry uses positive tone and descriptive language (such as \"older\" and \"gooder than originally thought\"), which can make the interviewee feel understood and supported. At the same time, it does not directly ask the interviewee about his feelings or circumstances, but rather leads them to think and answer questions in a more moderate way. The language style of the article can therefore be considered as euphemism."}, "time_dimension": {"answer": "Future", "rationale": "The entry \"older\" indicates that it is a vision and a expectation for the future, and the keyword \"older\" points to situations that have not yet occurred and that may arise in the future. The temporal dimension therefore is the future."}, "sentence_type": "Affirmative declarative sentence", "pos": {"nouns": {"Things": 1, "Okay": 1}, "verbs": {"Found": 1, "- Yeah": 1, "Imagine": 1}, "adjectives": {"Old": 1}, "numerals": {"4": 1}, "pronouns": {"I": 1, "Original": 1}}}, {"content": "5. I sometimes feel that life is worthless.", "word_count": 11, "label": "", "sentence_structure": "Subject鈥搗erb鈥搊bject", "subjective_objective": {"answer": "Subjective", "rationale": "Because \"fearing life is worthless\" is an emotional experience, a subjective feeling and attitude of the patient."}, "sentence_sentiment": {"answer": "Negative", "rationale": "Because the attitude of \"fearing life for nothing\" in the entry is negative."}, "language_style": {"answer": "Calm and direct", "rationale": "The entry directly asks the respondents whether they have ever had doubts or feelings of denial about the value of life in their daily lives and the language style is more direct and concise.\""}, "time_dimension": {"answer": "Past", "rationale": "The keyword \"sometimes feel\" means that it is a past or present feeling, but does not specify the time period in which it occurred. It is therefore not possible to determine whether it is the past or the present, but only to judge it as past or the present."}, "sentence_type": "Negative declarative sentence", "pos": {"nouns": {"Life": 1, "Value": 1}, "verbs": {"I feel": 1}, "adjectives": {"Sometimes": 1}, "pronouns": {"I": 1}}}, {"content": "6. I am as happy as I was when I was young.", "word_count": 11, "label": "", "sentence_structure": "Subject鈥揷opula鈥損redicative", "subjective_objective": {"answer": "Subjective", "rationale": "Because happiness is a subjective feeling, different people may feel different."}, "sentence_sentiment": {"answer": "Neutral", "rationale": "Because the entry is merely a statement of facts and does not express any positive or negative feelings."}, "language_style": {"answer": "Tactful and warm", "rationale": "The language style of the entry is euphemistic and warm, using a more personal questionword, \"Are you as happy as you were young?\" instead of asking if the other person was happy. This expression is more intimate and caring, and it attracts the attention of the interviewee and makes him willing to answer the questions."}, "time_dimension": {"answer": "Present", "rationale": "According to the sentence, \"I'm right now,\" it can be seen that the entry is for questioning the feelings of the subject in the current state, and therefore for judging \"now\"."}, "sentence_type": "Affirmative declarative sentence", "pos": {"nouns": {"Questionnaire": 1, "Entry": 1, "Young": 1, "Happiness": 1}, "verbs": {"Present": 1, "and": 1, "Same thing": 1}, "adjectives": {"Happiness": 1}, "adverbs": {"Present": 1, "Same thing": 1}, "numerals": {"6": 1}, "pronouns": {"I": 1}}}, {"content": "Have you been alone for a long time?", "word_count": 13, "label": "", "sentence_structure": "Subject鈥損redicate", "subjective_objective": {"answer": "Subjective", "rationale": "Because feeling alone is a subjective emotional experience, which reflects the person's own inner feelings and emotional state."}, "sentence_sentiment": {"answer": "Negative", "rationale": "Because the emotional experience of feeling alone is usually negative."}, "language_style": {"answer": "Tactful and warm", "rationale": "The language style of the entry is more moderate, allowing the interviewee to share his/her feelings in a way that does not directly express criticism or accusations."}, "time_dimension": {"answer": "Present", "rationale": "The keyword \"Whether or not you feel alone\" indicates that the entry is asking the person who was tested for his or her current state and feelings and therefore judging as it is now."}, "sentence_type": "Interrogative sentence", "pos": {"nouns": {"Time": 1, "Loneliness": 1}, "verbs": {"I feel": 1, "be": 1}, "adverbs": {"very": 1}, "numerals": {"A lot": 1}, "pronouns": {"you": 1}}}, {"content": "I meet with friends and relatives on a regular basis.", "word_count": 10, "label": "", "sentence_structure": "Subject鈥搗erb鈥搊bject", "subjective_objective": {"answer": "Subjective", "rationale": "Because meeting friends and relatives is a personal choice and action of the patient, reflecting their subjective will and social behaviour."}, "sentence_sentiment": {"answer": "Positive", "rationale": "Because the \"meeting with friends and relatives\" act is positive."}, "language_style": {"answer": "Tactful and warm", "rationale": ""}, "time_dimension": {"answer": "Present", "rationale": "\"I'm often\" means a regular, customary act, and \"Meet\" also uses the usual current state of affairs, which means that this act has not only occurred in the past, but may still be ongoing. The entry is therefore asking the person who was tested about his current behaviour or state."}, "sentence_type": "Affirmative declarative sentence", "pos": {"nouns": {"friends": 1, "Relatives": 1, "Meet me": 1}, "verbs": {"Constant with": 1}, "adverbs": {"Constant with": 1}, "classifiers": {"individual": 1}, "pronouns": {"I": 1}}}, {"content": "I sometimes feel that there is no point in living.", "word_count": 12, "label": "", "sentence_structure": "Subject鈥揷opula鈥損redicative", "subjective_objective": {"answer": "Subjective", "rationale": "Because \"I sometimes feel that life is meaningless\" is a personal emotion and experience, a subjective perspective of the patient."}, "sentence_sentiment": {"answer": "Negative", "rationale": "Because the attitude in the entry \"Esperate that life is meaningless\" is negative."}, "language_style": {"answer": "Tactful and warm", "rationale": "The entry uses the words \"sometimes,\" \"no sense,\" which are more apologetic in expressing confusion and confusion about life than directly criticizing or denying it. And the term \"you\" also makes the interviewee feel more close and concerned."}, "time_dimension": {"answer": "Present", "rationale": "The entries were, are and will be asked about the current feelings and experiences of the subjects, so the temporal dimension is judged to be \"now\"."}, "sentence_type": "Interrogative sentence", "pos": {"nouns": {"Alive": 1, "Meaning": 1}, "verbs": {"I feel": 1, "have": 1, "Nope": 1, "Meaning": 1}, "adverbs": {"Sometimes": 1}, "numerals": {"3": 1}}}, {"content": "4. Most of the time, my life was hard.", "word_count": 12, "label": "", "sentence_structure": "Subject鈥搗erb鈥揷omplement", "subjective_objective": {"answer": "Subjective", "rationale": "Because \"my life is hard\" is a personal experience and experience of the patient, a subjective judgment."}, "sentence_sentiment": {"answer": "Negative", "rationale": "Because the description of \"life is hard\" is negative and expresses the dissatisfaction and distress of the interviewees."}, "language_style": {"answer": "Calm and direct", "rationale": "The entry is presented in a direct way, describing the living conditions of the respondents for most of their time, without using too many euphemisms or encouraging language, and with a more calm and objective tone."}, "time_dimension": {"answer": "Past", "rationale": "The entry describes the current life of the person being tested, using terms such as \"most time\" and \"very difficult\" 鈥?all of which relate to the past. The entry should therefore be attributed to the past."}, "sentence_type": "Negative declarative sentence", "pos": {"nouns": {"Time": 1, "Life": 1, "Problem": 1}, "verbs": {"have": 1, "very": 1, "Hard": 1}, "adjectives": {"Most of them": 1, "Hard": 1}, "numerals": {"4": 1}, "pronouns": {"I": 1}}}, {"content": "5. Are you satisfied with your current life?", "word_count": 15, "label": "", "sentence_structure": "Subject鈥搗erb鈥搊bject", "subjective_objective": {"answer": "Subjective", "rationale": "This is a subjective assessment of the patient ' s satisfaction with his or her life."}, "sentence_sentiment": {"answer": "Positive", "rationale": "Because the word \"satisfactory\" in the entry is positive, it is an indication that the interviewee has a positive attitude and an assessment of his or her life."}, "language_style": {"answer": "Calm and direct", "rationale": "The entry is based on a direct question-and-answer approach, with no excessive rhetorical or emotional rendering, and a more calm and objective language style."}, "time_dimension": {"answer": "Present", "rationale": ""}, "sentence_type": "Interrogative sentence", "pos": {"nouns": {"Life": 1, "Satisfied": 1}, "verbs": {"I feel": 1}, "adverbs": {"Is it possible": 1, "Current": 1}, "numerals": {"5": 1}}}, {"content": "I am sorry for many things.", "word_count": 10, "label": "", "sentence_structure": "Subject鈥揷opula鈥損redicative", "subjective_objective": {"answer": "Subjective", "rationale": "The problem is subjective, and it is subjective, as it looks at their emotional state and experience."}, "sentence_sentiment": {"answer": "Negative", "rationale": "Because \"sorture\" expresses a negative emotional attitude."}, "language_style": {"answer": "Tactful and warm", "rationale": ""}, "time_dimension": {"answer": "Present", "rationale": "The entry is for the current feelings and state of the testee, and the time dimension is now judged."}, "sentence_type": "Affirmative declarative sentence", "pos": {"nouns": {"Things": 1, "Sorry": 1}, "verbs": {"I feel": 1, "Do it": 0}, "adjectives": {"Many": 1}, "numerals": {"6": 1}, "pronouns": {"I": 1}}}, {"content": "7. People were happier than they were.", "word_count": 10, "label": "", "sentence_structure": "Subject鈥揷opula鈥損redicative", "subjective_objective": {"answer": "Subjective", "rationale": "Because happiness is a subjective feeling and assessment of the patient, it looks at the subjective perception and feelings of the past."}, "sentence_sentiment": {"answer": "Negative", "rationale": "Because it expresses a greater sense of happiness than it is, it is a negative comparison and evaluation."}, "language_style": {"answer": "Calm and direct", "rationale": "The entry, which is presented directly, explicitly asks the respondents whether people were happier in the past, without too many rhetorical or emotional renderings, and with a more concise language style.\""}, "time_dimension": {"answer": "Past", "rationale": "The emergence of the key word \"the past\" in the entry suggests that the issue is one of perceptions and feelings of the past and is therefore judged to be the past."}, "sentence_type": "Interrogative sentence", "pos": {"nouns": {"People": 1, "Past": 1, "Present": 1, "Happiness": 1}, "verbs": {"- Yeah": 1, "More": 1}}}, {"content": "8. Human beings should live today without being rewarded for tomorrow.", "word_count": 16, "label": "", "sentence_structure": "Subject鈥損redicate", "subjective_objective": {"answer": "Subjective", "rationale": "Because fear of the future is patient's subjective attitude, and \"life for today\" is patient's subjective view of life."}, "sentence_sentiment": {"answer": "Positive", "rationale": "Because the words \"for today's life\" and \"not for tomorrow\" are positive, they express the view that the present is to be valued and that the future is not to be feared too much."}, "language_style": {"answer": "Calm and direct", "rationale": "The entry is clear and does not use euphemism, rendering or invitation, but rather directly sets out the point of view."}, "time_dimension": {"answer": "Present", "rationale": "The entry is mainly an expression of a view and attitude to life today, and the expression \"for today\" and \"not for tomorrow\" are all describing current behaviour or ideas and are therefore in the current state of affairs."}, "sentence_type": "Affirmative declarative sentence", "pos": {"nouns": {"person": 2, "Life": 1, "Tomorrow": 1}, "verbs": {"I think so": 1, "Yes": 3, "You're a good man": 1}, "adverbs": {"not": 1}, "numerals": {"8": 1}}}]}

{"questionnaire_title": "Anxious self-assessment form", "questionnaire_category": "Mental health", "item_count": 20, "emotion_shift_overall": "From negative to positive", "language_style_overall": "Neutral", "items": [{"content": "I feel more nervous and anxious than usual.", "word_count": 13, "label": "", "sentence_structure": "Subject鈥揷opula鈥損redicative", "subjective_objective": {"answer": "Subjective", "rationale": "Because \"think\" is the patient's subjective feeling and attitude."}, "sentence_sentiment": {"answer": "Negative", "rationale": "Because the mood of \"fright and anxious\" in the entry is negative."}, "language_style": {"answer": "Calm and direct", "rationale": "The entry is a simple, straightforward expression of whether the other party is asked whether it feels more nervous and anxious than usual, without too many rhetorical or emotional renderings."}, "time_dimension": {"answer": "Present", "rationale": "\"Esperately more nervous and anxious than usual\" describes the experience and experience of the person being tested, so the time dimension of the article is now."}, "sentence_type": "Negative declarative sentence", "pos": {"nouns": {"I": 1, "Nervous": 1, "I'm in a hurry": 1, "Normal": 1}, "verbs": {"I think": 1, "- Yeah": 1, "Easy": 1}, "adverbs": {"More than usual": 1}}}, {"content": "I was afraid for no reason.", "word_count": 10, "label": "", "sentence_structure": "Subject鈥揷opula鈥損redicative", "subjective_objective": {"answer": "Subjective", "rationale": "For \"no reason\" suggests that fear is not motivated by outside stimulation or logic, but rather by the patient's inner feeling and subjective judgment."}, "sentence_sentiment": {"answer": "Negative", "rationale": "Because \"fear without cause\" in the entry is a negative emotional experience."}, "language_style": {"answer": "Calm and direct", "rationale": "The entry directly expresses a sense of fear that the subject is often questioned without reason, and is not interpreted in a manner that is more objective and balanced."}, "time_dimension": {"answer": "Present", "rationale": "The keyword \"I am afraid without cause\" suggests that it is a subjective feeling, with no clear time limits or specific context, so that it can be judged as the present behavior or mentality."}, "sentence_type": "Negative declarative sentence", "pos": {"nouns": {"Reason": 1, "Scared": 1}, "verbs": {"I feel": 1, "For no reason": 1}, "adverbs": {"For no reason": 1}}}, {"content": "I'm a little upset or scared.", "word_count": 12, "label": "", "sentence_structure": "Subject鈥揷opula鈥損redicative", "subjective_objective": {"answer": "Subjective", "rationale": "Because the mental state is the individual ' s own perception and experience, and it is subjective."}, "sentence_sentiment": {"answer": "Negative", "rationale": "Because \"deeply disturbed\" and \"frightful\" are both negative emotional experiences."}, "language_style": {"answer": "Tactful and warm", "rationale": "The entry is more personal and interactive when it is spoken in a gentle and inviting manner, rather than in direct statements or orders. Moreover, the wording of the entry is neutral, objective, without too much emotional vocabulary or rhetorical techniques, and is more acceptable and willing to answer."}, "time_dimension": {"answer": "Present", "rationale": "The keyword \"easy,\" \"like\" indicates that it is a state of mind, and therefore judgment is now."}, "sentence_type": "Negative declarative sentence", "pos": {"nouns": {"I": 1, "In my heart": 1, "Disturbing": 1, "Scared": 1}, "verbs": {"Easy": 1, "I think": 1}, "numerals": {"3": 1}}}, {"content": "I think I might be going crazy.", "word_count": 10, "label": "", "sentence_structure": "Subject鈥揷opula鈥損redicative", "subjective_objective": {"answer": "Subjective", "rationale": "Because \"may go crazy\" is a subjective concern and expectation."}, "sentence_sentiment": {"answer": "Negative", "rationale": "Because the mood for \"may go crazy\" in the entry is negative."}, "language_style": {"answer": "Tactful and warm", "rationale": "The entry is more gentle and has an invitation-style tone, does not use too direct or strong a tone, giving people a sense of kindness and inclusion. At the same time, the entry leaves some space for reflection and self-evaluation, rather than mandatory asking questions of yes or no."}, "time_dimension": {"answer": "Future", "rationale": "The entry contains visions and expectations for the future, \"possible\" indicating a possibility and uncertainty, and it therefore points to situations that have not yet occurred and that may arise in the future."}, "sentence_type": "Negative declarative sentence", "pos": {"nouns": {"I": 1, "Crazy": 1}, "verbs": {"I think": 1, "Let's go": 1}, "adjectives": {"Maybe": 1}, "numerals": {"4锛?: 1}}}, {"content": "I think everything's fine, and nothing bad will happen.", "word_count": 17, "label": "", "sentence_structure": "Subject鈥揷opula鈥損redicative", "subjective_objective": {"answer": "Subjective", "rationale": "Because it is the feelings and perceptions of the patients themselves that feel that everything is going well and that no misfortune will happen, it is subjective."}, "sentence_sentiment": {"answer": "Negative", "rationale": "This entry is an expression of an overly optimistic attitude that may lead to neglect and inadequate response."}, "language_style": {"answer": "Tactful and warm", "rationale": "In a optimistic way, the entry uses the positive words \"all is well\" and \"no misfortune\" to leave a more relaxed and pleasant feeling. At the same time, it is more euphemismal and does not express its views or attitudes too directly."}, "time_dimension": {"answer": "Future", "rationale": "The words \"think\" and \"will\" in the entry indicate expectations and perceptions of the future, that is, that everything will be good and nothing will be unfortunate. So the time dimension is the future."}, "sentence_type": "Affirmative declarative sentence", "pos": {"nouns": {"Everything": 1, "Unfortunately": 1}, "verbs": {"I think": 1, "Yes": 1, "Come on": 1}, "adjectives": {"Okay": 1}, "adverbs": {"Yeah": 1}, "pronouns": {"I": 1}}}, {"content": "I'm shaking my hands and feet.", "word_count": 7, "label": "", "sentence_structure": "Subject鈥搗erb鈥揷omplement", "subjective_objective": {"answer": "Subjective", "rationale": "Because shaking is a subjective feeling, it is a reaction to the individual鈥檚 own state of health, not an external environment or other person鈥檚 influence. So the tremors are subjective."}, "sentence_sentiment": {"answer": "Negative", "rationale": "Because \"fear and shaking\" is a physical reaction, usually associated with negative emotions such as anxiety, fear, etc."}, "language_style": {"answer": "Calm and direct", "rationale": "This style helps to obtain real feedback from the person under investigation, but may lack a sense of affection and interaction."}, "time_dimension": {"answer": "Present", "rationale": "According to the sentence, \"I'm shaking my hands and feet\", it can be judged that the entry is asking the testee about his current state of health and is therefore in the current state of affairs."}, "sentence_type": "Affirmative declarative sentence", "pos": {"nouns": {"Hands and feet": 1, "# Wresh #": 1, "# Shatter #": 1}, "verbs": {"Wreck": 1}}}, {"content": "I'm suffering from headaches, headaches and back pains.", "word_count": 14, "label": "", "sentence_structure": "Subject鈥揷opula鈥損redicative", "subjective_objective": {"answer": "Subjective", "rationale": "The question is subjective because it is an inquiry into the feelings and experiences of the patient."}, "sentence_sentiment": {"answer": "Negative", "rationale": "Because the term \"headaches, neck pains and back pains\" described in the entry are symptoms of physical discomfort, which usually cause pain and distress, the emotional colour of the purpose of the article is negative."}, "language_style": {"answer": "Calm and direct", "rationale": "The language style of the entry is a straightforward type of calm. It directly illustrates the possible health problems of the person under investigation, and does not use excessive emotional accolades or rhetorical techniques, which are simple and clear.\""}, "time_dimension": {"answer": "Present", "rationale": "The entry is for the interview of the present situation of the subject, the key words being \"me,\" \"headache\", \"headache\" and \"backache\" and all of them relate to the current state of the subject, so it can be judged as now."}, "sentence_type": "Affirmative declarative sentence", "pos": {"nouns": {"Headache": 2, "Backache": 1, "Trouble": 1}, "verbs": {"Because": 1, "And": 1, "Trouble": 1}, "numerals": {"7": 1}}}, {"content": "I feel weak and tired.", "word_count": 10, "label": "", "sentence_structure": "Subject鈥揷opula鈥損redicative", "subjective_objective": {"answer": "Subjective", "rationale": "Because \"feel\" is the patient's subjective feeling."}, "sentence_sentiment": {"answer": "Negative", "rationale": "Because the description of \"vulnerability and fatigue\" expresses poor health and is a negative emotional attitude."}, "language_style": {"answer": "Tactful and warm", "rationale": "This is a more subjective expression, which is to ask the respondents about their own feelings, rather than to make a judgement or evaluation directly. It is to be more respectful of the respondents and makes them more willing to share their real feelings and experiences."}, "time_dimension": {"answer": "Present", "rationale": ""}, "sentence_type": "Negative declarative sentence", "pos": {"nouns": {"I": 1, "Weakness": 1, "Weary": 1}, "verbs": {"Feel": 1, "Easy": 1, "Come on": 1}, "adjectives": {"Easy": 1, "Tired": 1}, "numerals": {"8锛?: 1}}}, {"content": "I feel calm and easy to sit still.", "word_count": 15, "label": "", "sentence_structure": "Subject鈥揷opula鈥損redicative", "subjective_objective": {"answer": "Subjective", "rationale": "Because \"fear\" and \"cute\" and \"silent\" are the subjective feelings and experiences of the patient, and it looks at the subjective emotional state of the patient."}, "sentence_sentiment": {"answer": "Positive", "rationale": "Because \"creature\" and \"silently sitting\" are positive descriptions."}, "language_style": {"answer": "Tactful and warm", "rationale": "This is a more gentle and friendly language, and it is a question-and-answer process that guides the interviewee to reflect on his/her feelings, rather than direct orders or statements."}, "time_dimension": {"answer": "Present", "rationale": "The key words \"felt\" and \"salent\" and \"silently sit\" all indicate that this is a subjective feeling of the state of affairs, and therefore the judgement is now."}, "sentence_type": "Affirmative declarative sentence", "pos": {"nouns": {"Calm down": 1, "Sit still": 1}, "verbs": {"I think": 1, "Easy": 1, "Sit down": 1}, "adjectives": {"Ping": 1, "Quiet": 1}, "adverbs": {"and": 2}, "pronouns": {"I": 1}}}, {"content": "I think my heart is beating fast.", "word_count": 8, "label": "", "sentence_structure": "Subject鈥揷opula鈥損redicative", "subjective_objective": {"answer": "Subjective", "rationale": "Because of the subjective sense of heart beating, different people have different feelings and experiences of the same situation."}, "sentence_sentiment": {"answer": "Negative", "rationale": "It's based on the fact that \"the heart beats fast\" is a physical reaction, usually associated with negative emotions such as stress, fear, etc."}, "language_style": {"answer": "Calm and direct", "rationale": "The entry gives a concise description of the person ' s heart rate, is not overly accommodative or emotional, and is more direct and objective."}, "time_dimension": {"answer": "Present", "rationale": ""}, "sentence_type": "Affirmative declarative sentence", "pos": {"nouns": {"Heart rate": 1}, "verbs": {"I think": 1, "Come on": 1}, "adjectives": {"very": 1}, "numerals": {"10": 1}, "pronouns": {"I": 1}}}, {"content": "I was upset by a moment of faintness.", "word_count": 11, "label": "", "sentence_structure": "Subject鈥揷opula鈥損redicative", "subjective_objective": {"answer": "Subjective", "rationale": "Because the suffering of dizziness is the subjective feeling and emotional reaction of the patient, the subject is examined from a subjective perspective."}, "sentence_sentiment": {"answer": "Negative", "rationale": "Because the entry \"suffering\" expresses negative emotional attitudes."}, "language_style": {"answer": "Calm and direct", "rationale": "The language style of the entry is more objective and calm, and there is no excessive emotional rendering or euphemism, which directly describes the problems faced by the person under investigation."}, "time_dimension": {"answer": "Present", "rationale": "The entry describes the current behaviour and mentality of the testee, the keywords \"swipe\" and \"torture\" which indicate that the situation is either ongoing or present. So the answer is:"}, "sentence_type": "Negative declarative sentence", "pos": {"nouns": {"Dizziness": 1, "Trouble": 1}, "verbs": {"Because": 1, "have": 1, "And": 1, "Trouble": 1}, "adverbs": {"For a while": 1}, "numerals": {"11": 1}, "pronouns": {"I": 1}}}, {"content": "I'm having a fainting seizure or feeling like I'm going to faint.", "word_count": 14, "label": "", "sentence_structure": "Subject鈥搗erb鈥揷omplement", "subjective_objective": {"answer": "Subjective", "rationale": "This feeling is the patient ' s own subjective experience and judgement and is not an objective reality."}, "sentence_sentiment": {"answer": "Negative", "rationale": "Since fainting is usually a symptom of physical discomfort and may represent a health problem, the emotional colour of the purpose of the article is negative."}, "language_style": {"answer": "Calm and direct", "rationale": "This is more consistent with the requirements of the questionnaire鈥檚 professionalism and rigour."}, "time_dimension": {"answer": "Past", "rationale": "\"Be\" and \"be\" both indicate what happened in the past, and \"Embassy\" and \"Scoff\" describe the past. So the entry asks if the subject has experienced similar symptoms in the past."}, "sentence_type": "Exclamatory sentence", "pos": {"nouns": {"鈾?And the attacks 鈾?: 1, "I'm fainted": 1}, "verbs": {"have": 1, "I think": 1, "I'm fainting": 1}, "numerals": {"12": 1}}}, {"content": "I'm so easy to breathe in.", "word_count": 11, "label": "", "sentence_structure": "Subject鈥揷opula鈥損redicative", "subjective_objective": {"answer": "Subjective", "rationale": "Because \"easy\" is a subjective expression of perception, reflecting the feelings and experiences of the interviewee in his own breath."}, "sentence_sentiment": {"answer": "Neutral", "rationale": "Because the entry describes a biological feeling of the body, there is no apparent emotional tendency."}, "language_style": {"answer": "Tactful and warm", "rationale": "The language style of the entry is more euphemistic and friendly, making the respondents feel more comfortable in answering the questions. At the same time, the phrase is in the form of a question sentence, which is easier to generate reflection and answers from the respondents."}, "time_dimension": {"answer": "Present", "rationale": "The keywords are \"I'm now\" and \"Easy,\" which indicate that the subject, in answering the question, is describing his current situation, namely, whether he has difficulty breathing."}, "sentence_type": "Affirmative declarative sentence", "pos": {"nouns": {"Questionnaire": 1, "Entry": 1, "Breathe": 2}, "verbs": {"I feel": 1, "Both": 1, "Easy": 1}, "adjectives": {"very": 1}, "numerals": {"13锛?: 1, "1": 1}, "pronouns": {"I": 1}}}, {"content": "My hands and feet are numb and stingy.", "word_count": 9, "label": "", "sentence_structure": "Subject鈥揷opula鈥損redicative", "subjective_objective": {"answer": "Objective", "rationale": "Because numbness and stings are symptoms of the patient and are objective physiological phenomena."}, "sentence_sentiment": {"answer": "Negative", "rationale": "Because \"smoke and sting\" is a negative physical condition."}, "language_style": {"answer": "Calm and direct", "rationale": "The language style of the entry is more direct and concise, with no excessive qualifier or emotional colour, and more emphasis on communicating facts and information."}, "time_dimension": {"answer": "Past", "rationale": "The entry describes the symptoms of the testee, judging by the time dimension as the past. The keywords \"silent\" and \"sting\" indicate that this is a physical condition that has already occurred and that may still exist."}, "sentence_type": "Affirmative declarative sentence", "pos": {"nouns": {"Hands and feet": 2, "I'm numb": 1, "Stings": 1}, "numerals": {"14": 1}}}, {"content": "I'm upset with stomach pains and indigestion.", "word_count": 13, "label": "", "sentence_structure": "Subject鈥揷opula鈥損redicative", "subjective_objective": {"answer": "Objective", "rationale": "The stomach pain and ingestion are objective facts that patients experience and are therefore objective."}, "sentence_sentiment": {"answer": "Negative", "rationale": "Because \" stomach pain and indigestion\" in the entry is a symptom of physical discomfort, which is usually uncomfortable or painful, and therefore carries a certain negative emotional colour."}, "language_style": {"answer": "Tactful and warm", "rationale": "The language style of the entry is euphemism. It uses the term \"because of stomach pain and digestive indigestion\" to avoid direct involvement in the health of the person under investigation, but also to convey the attention of the investigator to their health problems. Moreover, the entry uses the adjective \"proficient\" to give people a sense of care and understanding. This language style is easier for the person under investigation to accept and be willing to participate in the questionnaire."}, "time_dimension": {"answer": "Past", "rationale": "The entry \"I'm upset with stomach pain and indigestion\" describes the behaviour or state of the past and therefore is a dimension of the past."}, "sentence_type": "Affirmative declarative sentence", "pos": {"nouns": {"Stomach pain": 1, "Indigestion": 1, "Trouble": 1}, "verbs": {"Because": 1, "And": 1, "Trouble": 1}, "numerals": {"15": 1}, "pronouns": {"I": 1}}}, {"content": "I used to pee.", "word_count": 6, "label": "", "sentence_structure": "Subject鈥損redicate", "subjective_objective": {"answer": "Objective", "rationale": "Because \"often pee\" is the patient's biological need, it does not require subjective judgement and interpretation."}, "sentence_sentiment": {"answer": "Negative", "rationale": "Because the act of \"often peeing\" is negative."}, "language_style": {"answer": "Calm and direct", "rationale": "The language style of the entry is more straightforward, it is not used for any rhetorical or emotional rendering, and it is more objective and suitable for psychological investigation."}, "time_dimension": {"answer": "Present", "rationale": "The time dimension of this purpose is therefore now."}, "sentence_type": "Affirmative declarative sentence", "pos": {"nouns": {"Pee": 1}, "verbs": {"Yes": 1}, "adverbs": {"Often": 1}}}, {"content": "My hands are often dry and warm.", "word_count": 11, "label": "", "sentence_structure": "Subject鈥揷opula鈥損redicative", "subjective_objective": {"answer": "Objective", "rationale": "As drying and warmth are the real state of the hand, there is no need to look at the patient from a subjective perspective."}, "sentence_sentiment": {"answer": "Neutral", "rationale": "Because the entry only describes a fact, and there is no obvious emotional colour."}, "language_style": {"answer": "Tactful and warm", "rationale": "The entry is more personal and interactive when it is spoken in a gentle and inviting tone, rather than in direct statements or orders. At the same time, the purpose of the article is to describe it as more specific, objective, and not overly emotional or intensely emotional, so it can be judged that its language style is euphemistic and warm."}, "time_dimension": {"answer": "Present", "rationale": ""}, "sentence_type": "Affirmative declarative sentence", "pos": {"nouns": {"Hands": 1, "Dry": 1, "Wandering": 1}, "verbs": {"be": 1, "Often": 1}, "adjectives": {"Dry": 1, "Wandering": 1}, "adverbs": {"Often": 1}, "classifiers": {"individual": 1}, "pronouns": {"I": 1}}}, {"content": "I'm blushing.", "word_count": 5, "label": "", "sentence_structure": "Subject鈥揷opula鈥損redicative", "subjective_objective": {"answer": "Objective", "rationale": "Because of the red fever that patients experience."}, "sentence_sentiment": {"answer": "Negative", "rationale": "Because the \"refruit fever\" in the entry is a negative physical symptom."}, "language_style": {"answer": "Tactful and warm", "rationale": "The entry is more personal and interactive when it is spoken in a conversation, rather than directly speaking or ordering. At the same time, the expression \"redface hot\" is not too straightforward to reveal the emotional state of the other side, but rather leaves the interviewee to think, remember and answer, which better protects the privacy and dignity of the interviewee."}, "time_dimension": {"answer": "Past", "rationale": "The entry asks if the testee has had any experience, i.e. whether the act or circumstance of \"my face is red\" has occurred in the past and therefore the temporal dimension has been judged to be past."}, "sentence_type": "Affirmative declarative sentence", "pos": {"nouns": {"Face": 1, "Hot": 1}, "verbs": {"Red": 1, "Heat": 1}}}, {"content": "I sleep easily and I sleep well overnight.", "word_count": 13, "label": "", "sentence_structure": "Subject鈥搗erb鈥揷omplement", "subjective_objective": {"answer": "Subjective", "rationale": "Because \"easy to sleep and good to sleep one night\" is the patient's subjective feeling and experience."}, "sentence_sentiment": {"answer": "Positive", "rationale": "Because \"sleep-easy\" and \"sleep-in-the-night\" in the entries are positive words for good sleep quality."}, "language_style": {"answer": "Tactful and warm", "rationale": "The entry is more personal and interactive when it is spoken in a gentle, inquisitive manner rather than directly speaking or ordering."}, "time_dimension": {"answer": "Present", "rationale": "The entry describes the current situation of the person being tested, and the keywords \"sleep easy\" and \"sleep well\" indicate that this is an observation or evaluation of the current state of sleep."}, "sentence_type": "Affirmative declarative sentence", "pos": {"nouns": {"I": 2, "One night": 1}, "verbs": {"It's easy to sleep": 1, "Sleep well": 1}, "adjectives": {"Good": 1}, "adverbs": {"And": 1}, "numerals": {"19": 1}}}, {"content": "I had nightmares.", "word_count": 4, "label": "", "sentence_structure": "Subject鈥搗erb鈥揷omplement", "subjective_objective": {"answer": "Subjective", "rationale": "Because nightmares are often the expression of fear or anxiety within the patient, they are subjective feelings and experiences."}, "sentence_sentiment": {"answer": "Negative", "rationale": "Because \"dreaming\" usually breeds negative feelings of fear, unease, etc., it expresses a negative emotional attitude."}, "language_style": {"answer": "Calm and direct", "rationale": "The \"Cool Direct\" language style is more straightforward, with no excessive qualifier or emotional rendering, and is a cool direct language style."}, "time_dimension": {"answer": "Present", "rationale": "The keyword is \"dreaming,\" which means that the person tested is experiencing or has experienced a state of nightmare. Time dimensions are now judged."}, "sentence_type": "Affirmative declarative sentence", "pos": {"nouns": {"Nightmares": 1}, "verbs": {"Do it": 1}}}]}

{"questionnaire_title": "Young people's depression self-censorship.", "questionnaire_category": "Mental health", "item_count": 20, "emotion_shift_overall": "No obvious trend", "language_style_overall": "Neutral", "items": [{"content": "I feel more patient now than ever.", "word_count": 14, "label": "", "sentence_structure": "Subject鈥揷opula鈥損redicative", "subjective_objective": {"answer": "Subjective", "rationale": "Because \"think\" is a subjective feeling and experience, reflecting the patient's assessment of his or her self-status, and it's a subjective perspective."}, "sentence_sentiment": {"answer": "Negative", "rationale": "Because the attitude of \"desatisfaction\" in the entries is negative."}, "language_style": {"answer": "Calm and direct", "rationale": "The entry is in a straightforward manner, asking the other side about the current state of affairs, without excessive rhetoric or emotional colours, and with a more objective and calm language style."}, "time_dimension": {"answer": "Present", "rationale": "The keywords are \"I feel more impatient now than ever\" and \"I feel less patient,\" which point to the current feelings and state of the subjects, so the time dimension is now."}, "sentence_type": "Affirmative declarative sentence", "pos": {"nouns": {"I'm here": 1, "Patience": 1}, "verbs": {"I think": 1, "- Yeah": 1, "Easy": 1, "Lose": 1}, "adjectives": {"Lose": 1}, "numerals": {"01": 1, "Before": 1}, "pronouns": {"I": 1}}}, {"content": "I'm a little more riled than usual.", "word_count": 9, "label": "", "sentence_structure": "Subject鈥揷opula鈥損redicative", "subjective_objective": {"answer": "Subjective", "rationale": "Because \"freak\" is an emotional state, a subjective feeling of the patient."}, "sentence_sentiment": {"answer": "Negative", "rationale": "Because the mood of \"easiness\" in the entries is negative."}, "language_style": {"answer": "Calm and direct", "rationale": "The entry directly expresses the subject of asking the other party whether in some cases it would feel more ecstatic than usual, without too much rhetorical or emotional rendering, and with a more objective tone."}, "time_dimension": {"answer": "Present", "rationale": "So the time dimension is now judged."}, "sentence_type": "Affirmative declarative sentence", "pos": {"nouns": {"Questionnaire": 1, "Entry": 1, "I'm so upset": 1}, "verbs": {"be": 1, "Easy": 1, "- Yeah": 1}, "adjectives": {"Normal": 1, "More": 1}, "pronouns": {"I": 1}}}, {"content": "I want to leave the current environment.", "word_count": 11, "label": "", "sentence_structure": "Subject鈥搗erb鈥搊bject", "subjective_objective": {"answer": "Subjective", "rationale": "Because it is the patient ' s inner thought and will, it is a subjective observation."}, "sentence_sentiment": {"answer": "Negative", "rationale": "Because \"want to leave the current environment\" is a negative emotional attitude."}, "language_style": {"answer": "Tactful and warm", "rationale": "The entry uses a more euphemistic approach to asking the other side whether it is an idea to leave the current environment, giving the other side more space and possibilities to answer questions by using the expression \"I want\" and not directly imposing personal opinions or emotions, which is more thoughtful and respectful."}, "time_dimension": {"answer": "Future", "rationale": "The keyword \"wanted to leave the current living environment\" expresses the testee's expectations and plans for the future, namely, to change the current living environment and therefore to judge the future."}, "sentence_type": "Imperative sentence", "pos": {"nouns": {"Life": 1, "Environment": 1}, "verbs": {"Yes": 1, "Leave": 1}, "numerals": {"03": 1}}}, {"content": "I'm getting a little more angry than I used to be.", "word_count": 10, "label": "", "sentence_structure": "Subject鈥揷opula鈥損redicative", "subjective_objective": {"answer": "Subjective", "rationale": "Because \"freaker\" is the result of patient subjective perceptions and self-evaluations, not an objective fact or observation."}, "sentence_sentiment": {"answer": "Negative", "rationale": "Because the entry \"getting more angry than ever\" expresses a negative emotional attitude, which is a diminished emotional control."}, "language_style": {"answer": "Calm and direct", "rationale": "The entry directly asked whether the interviewees were more angry, were not over-spoken or emotionally rendered, and were more direct and concise."}, "time_dimension": {"answer": "Past", "rationale": "The keyword \"more than before\" indicates that it is a comparison of the past, while the entire sentence describes the current state and behaviour of the testee, and therefore the temporal dimension is a past."}, "sentence_type": "Negative declarative sentence", "pos": {"nouns": {"I": 1, "Gas": 1}, "verbs": {"Change": 1, "Fan": 1}, "adjectives": {"- Yeah": 1, "Before": 1, "Easy": 1}, "numerals": {"04": 1}}}, {"content": "I'm in a bad mood.", "word_count": 8, "label": "", "sentence_structure": "Subject鈥揷opula鈥損redicative", "subjective_objective": {"answer": "Subjective", "rationale": "Because change of mood is a subjective feeling and experience of a person, it is not an objective reality or phenomenon."}, "sentence_sentiment": {"answer": "Negative", "rationale": "Because the entry \"failed\" expresses negative emotions."}, "language_style": {"answer": "Tactful and warm", "rationale": "The language style of the entry is more euphemistic, because it uses more moderate language (e.g. \"failed\"), and is not expressed in too direct or harsh a language. This expression is more respectful of each other's feelings and emotions, and helps to create a good atmosphere of communication."}, "time_dimension": {"answer": "Present", "rationale": "The entry describes the current situation of the testee, the key word being \"I'm getting very upset\", which means that the testee is currently in a state of negative emotion, so the temporal dimension is now judged."}, "sentence_type": "Negative declarative sentence", "pos": {"nouns": {"I": 1, "The mood": 1, "Not good": 1}, "verbs": {"鈾?And become 鈾?: 1}, "adverbs": {"very": 1}}}, {"content": "I've become lazy and incompetent all day.", "word_count": 12, "label": "", "sentence_structure": "Subject鈥搗erb鈥揷omplement", "subjective_objective": {"answer": "Subjective", "rationale": "Because \"getting\" means it's the patient's subjective feeling."}, "sentence_sentiment": {"answer": "Negative", "rationale": "Because the \"Lack and No Scratch\" status in the entry is negative."}, "language_style": {"answer": "Calm and direct", "rationale": "The language style of the entry is more straightforward, without any rhetoric or emotional rendering, but rather with a simple language that conveys the symptoms of \"sluggering and insinuating\" in a way that allows respondents to understand the questions more clearly and to facilitate their answers."}, "time_dimension": {"answer": "Present", "rationale": "The entry describes the current symptoms or state, and \"getting\" is a term for change, implying that the symptoms begin in the past and may continue into the future. Thus, the temporal dimension is judged as \"now\"."}, "sentence_type": "Affirmative declarative sentence", "pos": {"nouns": {"Oh, my God": 1, "person": 1, "Fine": 1, "Pick": 1}, "verbs": {"Change": 1, "Tight": 1, "Lazy": 1, "I'm not good at it": 1}, "adjectives": {"All day long": 1, "Lazy": 1, "I'm not good at it": 1}, "classifiers": {"Oh, my God": 1}}}, {"content": "I'm not feeling well.", "word_count": 8, "label": "", "sentence_structure": "Subject鈥揷opula鈥損redicative", "subjective_objective": {"answer": "Subjective", "rationale": "Because it is the patient's subjective feeling, not the objective fact."}, "sentence_sentiment": {"answer": "Negative", "rationale": "Because the physical discomfort expressed in the entry is negative emotion or feeling."}, "language_style": {"answer": "Calm and direct", "rationale": "The entry is based on a direct, clear language style, which directly asks the interviewee whether he or she feels ill, does not have too many adorations or emotional renderings and is expressed in a simpler and more concise manner."}, "time_dimension": {"answer": "Present", "rationale": "Now the entry expresses the current physical feeling of the testee, and the word \"I\" uses the first person 's number, which means that it's a statement of the usual current state of affairs, so the temporal dimension is now."}, "sentence_type": "Affirmative declarative sentence", "pos": {"nouns": {"Body": 1, "I'm not feeling well": 1}, "verbs": {"I think": 1}, "numerals": {"07": 1}}}, {"content": "I'm always bored with breasts.", "word_count": 6, "label": "", "sentence_structure": "Subject鈥揷opula鈥損redicative", "subjective_objective": {"answer": "Subjective", "rationale": "Because \"often feeling bored\" is the patient's feeling and subjective experience."}, "sentence_sentiment": {"answer": "Negative", "rationale": "Because \"breathing\" is a negative physical discomfort."}, "language_style": {"answer": "Tactful and warm", "rationale": ""}, "time_dimension": {"answer": "Past", "rationale": "So the entry is about asking the person who was tested for his or her current behavior or state."}, "sentence_type": "Affirmative declarative sentence", "pos": {"nouns": {"- Chesty": 1}, "verbs": {"Regular": 1, "I'm not sure": 1}, "numerals": {"08": 1}}}, {"content": "I've been feeling really weak lately.", "word_count": 14, "label": "", "sentence_structure": "Subject鈥揷opula鈥損redicative", "subjective_objective": {"answer": "Subjective", "rationale": "Because feeling incompetent is a subjective feeling."}, "sentence_sentiment": {"answer": "Negative", "rationale": "Because \"whole body infirmity\" is usually associated with negative state of physical incomprehensibleness, fatigue, etc."}, "language_style": {"answer": "Tactful and warm", "rationale": "The entry is more personal and interactive when it is spoken in a gentle and sensitive manner, rather than by direct questioning or command."}, "time_dimension": {"answer": "Past", "rationale": "The keyword \"most recently\" indicates that this feeling has often occurred or persisted over time and is therefore a past act or state."}, "sentence_type": "Affirmative declarative sentence", "pos": {"nouns": {"Time": 1, "All over": 1, "Strength": 1}, "verbs": {"I think": 1, "- I'm weak": 1}, "adverbs": {"Near": 1, "Mostly": 1, "Time": 1}, "classifiers": {"individual": 1}}}, {"content": "I'm getting sleepless, and I can easily sleep or wake up.", "word_count": 16, "label": "", "sentence_structure": "Subject鈥搗erb鈥揷omplement", "subjective_objective": {"answer": "Subjective", "rationale": "Because insomnia, awakening is the subjective feeling and experience of the patient, it is a subjective approach."}, "sentence_sentiment": {"answer": "Negative", "rationale": "Because \"sleepless\" and \"sleepless\" and \"woke\" in the entries are negative, indicating negative emotions."}, "language_style": {"answer": "Tactful and warm", "rationale": "The language style of the entry is relatively euphemistic, and the interviewer is guided by the way he/she is asked to express his/her feelings and experiences, and is more gentle and friendly, making it easier for the interviewee to open his/her heart. At the same time, the vocabulary and sentence structure of the entry is simpler and less complicated, avoiding the use of too complex terms or professional language to facilitate understanding and response by the wider audience."}, "time_dimension": {"answer": "Present", "rationale": "The entry describes a state of present or situation, i.e., \"I became sleepless\" and further explains the specific manifestation of this state, \"so easily insomnia or waking up.\" Thus, the temporal dimension of the purpose of the article is now."}, "sentence_type": "Negative declarative sentence", "pos": {"nouns": {"I": 2, "Sleep": 1, "Peace": 1, "Insomnia": 1, "Wake up": 1}, "verbs": {"鈾?And become 鈾?: 1, "Easy": 1, "Insomnia or waking up": 1}, "adjectives": {"Unsettled": 1}, "numerals": {"10": 1}, "classifiers": {"individual": 1}}}, {"content": "I've become so tired of going to school.", "word_count": 8, "label": "", "sentence_structure": "Subject鈥搗erb鈥揷omplement", "subjective_objective": {"answer": "Subjective", "rationale": "Because \"I don't want to go to school\" is the patient's personal feeling and attitude, and it's subjective."}, "sentence_sentiment": {"answer": "Negative", "rationale": "Because the act of \"getting really tired of going to school\" is negative and expresses dissatisfaction and resistance to school life."}, "language_style": {"answer": "Tactful and warm", "rationale": "The entry is euphemistic, not so directly revealing the problems or problems that the other side may have, but in a more moderate way, leading the respondents to answer. This language style makes it easier for the respondents to feel comfortable and to share their thoughts and feelings."}, "time_dimension": {"answer": "Present", "rationale": "The entry is for the testee's current behaviour or feelings, and \"getting\" and \"not going to school\" means it's describing the current state, and therefore judging it as now."}, "sentence_type": "Negative declarative sentence", "pos": {"nouns": {"I": 1, "School": 1}, "verbs": {"鈾?And become 鈾?: 1, "Yes": 1, "School": 1}, "adjectives": {"very": 1}, "numerals": {"11": 1}}}, {"content": "I've become disinterested in many things.", "word_count": 12, "label": "", "sentence_structure": "Subject鈥搗erb鈥揷omplement", "subjective_objective": {"answer": "Subjective", "rationale": "Because \"lost interest\" is a personal feeling and experience, and it is subjective observation and judgment."}, "sentence_sentiment": {"answer": "Negative", "rationale": "Because the \"lost interest\" act in the entry is negative."}, "language_style": {"answer": "Tactful and warm", "rationale": "The entry is more personal and interactive when it is spoken in a gentle and inviting manner, rather than in direct statements or orders. Moreover, the tone of the article is more neutral and objective, and does not have a strong emotional or tendentious tone, which helps respondents to express their situation more naturally."}, "time_dimension": {"answer": "Present", "rationale": "The term \"changed\" is a process of change under way, and \"many things are lost in interest\" describes the current situation, so the entry is about the current behaviour or state."}, "sentence_type": "Negative declarative sentence", "pos": {"nouns": {"Yes": 1, "Interest": 1}, "verbs": {"鈾?And become 鈾?: 1, "Lose": 1, "Both": 1}, "numerals": {"12": 1}, "pronouns": {"I": 1}}}, {"content": "I'm getting restless.", "word_count": 11, "label": "", "sentence_structure": "Subject鈥搗erb鈥揷omplement", "subjective_objective": {"answer": "Subjective", "rationale": "Because the word \"becoming\" means that it's a patient's own feeling and experience, and it's a subjective observation."}, "sentence_sentiment": {"answer": "Negative", "rationale": "Because the words \"silent\" and \"silent\" in the entries are negative."}, "language_style": {"answer": "Calm and direct", "rationale": "The entry describes the symptoms or feelings that the interviewees may have in a concise and objective language style, without excessive emotional rendering or rhetorical techniques. \""}, "time_dimension": {"answer": "Past", "rationale": "The entry enquired about the emotional state of the person being tested during the most recent period, and the temporal dimension was judged to be past."}, "sentence_type": "Negative declarative sentence", "pos": {"nouns": {"Sit tight": 1, "I can't stay": 1}, "verbs": {"鈾?And become 鈾?: 1, "Come on": 1}, "adverbs": {"very": 1, "Down": 1, "not": 2}, "numerals": {"13": 1}, "pronouns": {"I": 1}}}, {"content": "I've become so lonely.", "word_count": 10, "label": "", "sentence_structure": "Subject鈥搗erb鈥揷omplement", "subjective_objective": {"answer": "Subjective", "rationale": "Because \"getting\" means a change or a change, which is the patient's subjective perception and description of his condition."}, "sentence_sentiment": {"answer": "Negative", "rationale": "Because the \"only want to be alone\" act in the entry is negative."}, "language_style": {"answer": "Calm and direct", "rationale": "The entry is more calm and straightforward, without excessive rhetoric or emotional colours, and directly asks whether the interviewees would in some cases prefer to be alone."}, "time_dimension": {"answer": "Present", "rationale": ""}, "sentence_type": "Affirmative declarative sentence", "pos": {"nouns": {"person": 1, "Alone": 1}, "verbs": {"鈾?And become 鈾?: 1, "Yes": 1, "Alone": 1}, "pronouns": {"I": 1}}}, {"content": "I've become nothing I want to do.", "word_count": 10, "label": "", "sentence_structure": "Subject鈥揷opula鈥損redicative", "subjective_objective": {"answer": "Subjective", "rationale": "Because \"doing nothing\" is a subjective feeling or attitude."}, "sentence_sentiment": {"answer": "Negative", "rationale": "Because \"doesn't want to do anything\" in the entry is negative."}, "language_style": {"answer": "Calm and direct", "rationale": "The entry directly expresses the subject of an inquiry into the recent appearance of a situation in which the other party has no interest in doing anything, without excessive rhetoric or emotional rendering, and with a more objective tone."}, "time_dimension": {"answer": "Present", "rationale": "The entry is a question about the current status and feelings of the testee, \"15.\" is a number, not a hint of the temporal dimension, but the word \"to become\" indicates that it is a move of the past to a state of time, but it is not possible to determine the specific temporal dimension because there is no clear point or reference. Therefore, the questionnaire entry describes the current situation in the context."}, "sentence_type": "Negative declarative sentence", "pos": {"nouns": {"Yes": 1, "Questionnaire": 1, "Entry": 1}, "verbs": {"Change": 1, "Yes": 2, "Do it": 1}, "adverbs": {"Both": 1}, "numerals": {"15": 1}, "pronouns": {"I": 1}}}, {"content": "I'm not going to make me better, no matter what I do.", "word_count": 15, "label": "", "sentence_structure": "Subject鈥揷opula鈥損redicative", "subjective_objective": {"answer": "Subjective", "rationale": "This is a personal and subjective perception of the patient, and is not an objective fact or event."}, "sentence_sentiment": {"answer": "Negative", "rationale": "Because the entry expresses a negative attitude that its efforts will not bear fruit."}, "language_style": {"answer": "Calm and direct", "rationale": "The entry is more straightforward in language style and does not have too many rhetorical or emotional renderings."}, "time_dimension": {"answer": "Present", "rationale": "The keywords \"do\" and \"let\" mean that the act is or has been committed, not in relation to time, and therefore the judgment is \"now\"."}, "sentence_type": "Negative declarative sentence", "pos": {"nouns": {"Questionnaire": 1, "Item": 1, "Entry": 1}, "verbs": {"Do it": 1, "Jean": 2, "Change": 1}, "adjectives": {"Okay": 2}, "adverbs": {"Whatever": 1}, "pronouns": {"I": 1}}}, {"content": "I feel like I'm a bad person.", "word_count": 8, "label": "", "sentence_structure": "Subject鈥揷opula鈥損redicative", "subjective_objective": {"answer": "Subjective", "rationale": "It is the patient's subjective feeling and evaluation."}, "sentence_sentiment": {"answer": "Negative", "rationale": "Basis: The expression \"likes to feel bad\" in the entry expresses negative emotions and attitudes."}, "language_style": {"answer": "Calm and direct", "rationale": "The language style of the entry is a calm and straightforward one. The question is asked of the interviewees in a concise and clear manner about their assessment of themselves, without excessive emotional or rhetorical style, and directly and objectively expressing the subject matter, which meets the general requirements of the questionnaire."}, "time_dimension": {"answer": "Present", "rationale": "The entry is a subjective feeling and self-evaluation of the interviewee, and does not address the time dimension of the past or future. Thus, the time dimension of the article can be judged as \"now\"."}, "sentence_type": "Negative declarative sentence", "pos": {"nouns": {"Investigations": 1, "Entry": 1}, "verbs": {"I think": 1, "It's bad": 1}, "adjectives": {"Bad": 1}, "numerals": {"17": 1}}}, {"content": "I'm losing my focus.", "word_count": 12, "label": "", "sentence_structure": "Subject鈥揷opula鈥損redicative", "subjective_objective": {"answer": "Subjective", "rationale": "Because it is the patient ' s subjective feeling that he/she feels he/she cannot concentrate."}, "sentence_sentiment": {"answer": "Negative", "rationale": "Because the \"unfocused\" behavior in the entries is negative."}, "language_style": {"answer": "Calm and direct", "rationale": "The entry directly expresses a question about whether the other party may be unable to focus in certain circumstances, without excessive rhetoric or emotional rendering and with a more objective tone."}, "time_dimension": {"answer": "Past", "rationale": "The keyword \"getting\" represents the process of change, and the \"no way to focus\" is a description of the current state, which shows that the problem has happened in the past and continues to this day, and is therefore judged to be the past."}, "sentence_type": "Negative declarative sentence", "pos": {"nouns": {"Attention": 1}, "verbs": {"鈾?And become 鈾?: 1, "Cannot": 1, "Focus": 1}, "adverbs": {"There's no way": 1}}}, {"content": "I'm disappointed in myself.", "word_count": 7, "label": "", "sentence_structure": "Subject鈥搗erb鈥揷omplement", "subjective_objective": {"answer": "Subjective", "rationale": "Because disappointment is an emotional experience, determined by the subjective feeling of the individual, not an objective fact."}, "sentence_sentiment": {"answer": "Negative", "rationale": "Because \"disappointment\" is often seen as a negative emotion or attitude that expresses dissatisfaction or dissatisfaction with something."}, "language_style": {"answer": "Tactful and warm", "rationale": "The entry is asked in a caring and understanding manner, trying to make the interviewee comfortable and supportive in answering questions and directing them to share their feelings and ideas. At the same time, language style is more gentle and friendly, avoiding the possible negative effects of direct accusations or criticism."}, "time_dimension": {"answer": "Present", "rationale": "Now the entry \"now\" indicates a description or feeling of the current state, that is, the feeling of the testee in replying to a question is disappointment to himself."}, "sentence_type": "Exclamatory sentence", "pos": {"nouns": {"self": 1}, "verbs": {"to": 1, "very": 1, "Disappointment": 1}, "adverbs": {"very": 1}, "pronouns": {"I": 1}}}, {"content": "I want to disappear.", "word_count": 7, "label": "", "sentence_structure": "Subject鈥搗erb鈥揷omplement", "subjective_objective": {"answer": "Subjective", "rationale": "Because \"want\" is usually a subjective idea or wish."}, "sentence_sentiment": {"answer": "Negative", "rationale": "Because the idea of \"want to disappear\" in the entry is negative and may indicate negative feelings and attitudes of individuals, such as running away from reality, self-denial."}, "language_style": {"answer": "Tactful and warm", "rationale": "Warm and warm."}, "time_dimension": {"answer": "Future", "rationale": "The keyword is \"I want to disappear\", which expresses expectations or visions of the future, and therefore judgement is future."}, "sentence_type": "Imperative sentence", "pos": {"nouns": {"I": 1, "Gone": 1}, "verbs": {"Yes": 1}}}]}

{"questionnaire_title": "Self-confidence test.", "questionnaire_category": "Mental health", "item_count": 40, "emotion_shift_overall": "From positive to negative", "language_style_overall": "Tactful and warm", "items": [{"content": "1. Once you have made up your mind, will you insist on doing it after all, even if no one agrees?", "word_count": 23, "label": "", "sentence_structure": "Subject鈥損redicate", "subjective_objective": {"answer": "Subjective", "rationale": "Because this is a question of questioning the patient ' s personal attitude and decision-making process, rather than a description or observation of objective facts."}, "sentence_sentiment": {"answer": "Positive", "rationale": "Because it expresses a person ' s firm belief and indomitable spirit, it is a positive emotional attitude."}, "language_style": {"answer": "Tactful and warm", "rationale": "Warm and warm."}, "time_dimension": {"answer": "Present", "rationale": "In the future, the entry asks the testee whether he has a certain determination and a certain capacity for action in future possible behaviour or decision-making, and the keyword \"if it will\" indicates a scenario for the future."}, "sentence_type": "Interrogative sentence", "pos": {"nouns": {"Resolve": 1, "person": 1}, "verbs": {"Down": 1, "Hold on": 1, "Do it": 1}, "adjectives": {"Nobody": 1, "Yes": 1, "What the hell": 1}, "adverbs": {"鈾?Once 鈾?: 1}, "pronouns": {"you": 2}}}, {"content": "- Will you stand up until the party ends, even if you want to go to the bathroom?", "word_count": 25, "label": "", "sentence_structure": "Subject鈥搗erb鈥搊bject", "subjective_objective": {"answer": "Subjective", "rationale": "The problem involves the patient鈥檚 behaviour and ideas, which need to be viewed from the point of view of the patient鈥檚 subjectivity. While this is an objective situation (i.e., whether or not to stop the toilet at dinners), the focus of the problem is on the decision-making and the perception of the patient, and therefore the analysis needs to be made from a subjective point of view."}, "sentence_sentiment": {"answer": "Negative", "rationale": "Because the behavior in the entry \"not having to hold the bathroom\" is unhealthy and negative."}, "language_style": {"answer": "Calm and direct", "rationale": "The entry raises a question in a concise manner, without too much emotional rendering or speech-writing, and is more objective and cold. This style is more suitable for use in questionnaires, tests, etc., and allows more accurate access to the answers that have been tested."}, "time_dimension": {"answer": "Present", "rationale": "The entry asks the testee about the behaviour of the person in the specific circumstances (at dinners), and the temporal dimension is now judged."}, "sentence_type": "Imperative sentence", "pos": {"nouns": {"Dinner": 1, "Bathroom": 1}, "verbs": {"Participation": 1, "Hold on": 1, "End": 1}, "adverbs": {"Even if": 1, "Yeah": 1}}}, {"content": "If you want to buy sexy underwear, would you try to mail it instead of going to the store yourself?", "word_count": 23, "label": "", "sentence_structure": "Subject鈥搗erb鈥搊bject", "subjective_objective": {"answer": "Subjective", "rationale": "This is a reflection of the patient ' s personal purchasing behaviour and psychological orientation and is viewed from a subjective perspective."}, "sentence_sentiment": {"answer": "Positive", "rationale": "Because the \"mail order\" in the entry can be understood as a simple shopping process with some positive emotional color. And the term \"sexy lingerie\" is not clearly emotional."}, "language_style": {"answer": "Tactful and warm", "rationale": "The entry is more euphemistic and guiding, rather than asking the other side whether it would avoid buying sexy lingerie in person. The question is expressed in the words \"if and \" if you try to\" to make the issue more open and flexible, while avoiding unduly strong pressure or discomfort."}, "time_dimension": {"answer": "Past", "rationale": "Output: Now"}, "sentence_type": "Interrogative sentence", "pos": {"nouns": {"Sexy underwear": 1, "Shop": 1}, "verbs": {"Yes": 2, "Try": 1, "Buy": 1, "Mail order": 1, "not": 1, "Personally": 1, "Go on": 1}, "adjectives": {"Sexy": 1}, "adverbs": {"If": 1, "And": 1}, "classifiers": {"One": 1}, "pronouns": {"you": 1}}}, {"content": "- You think you're a better person?", "word_count": 13, "label": "", "sentence_structure": "Subject鈥搗erb鈥搊bject", "subjective_objective": {"answer": "Subjective", "rationale": "Because this is the patient ' s perception of self-awareness and is subjective."}, "sentence_sentiment": {"answer": "Neutral", "rationale": "Because the entry asks about an individual ' s perception of himself, it does not involve specific behavioural or emotional colours."}, "language_style": {"answer": "Calm and direct", "rationale": "The reasons for this are as follows: 1. The question is expressed in a direct statement, is not expressed in terms of rhetoric or emotional rendering, and is more objective in terms of tone. 2. The subject of the question is more clear, and the interviewee is asked whether he considers himself to be a better person, without ambiguity or simplicity."}, "time_dimension": {"answer": "Present", "rationale": "The entry asks the testee about a subjective assessment of himself, namely, \"Does he think he is the perfect person\" , based on the present perception and self-perception. So the temporal dimension is now."}, "sentence_type": "Interrogative sentence", "pos": {"nouns": {"person": 1, "Perfect": 1}, "verbs": {"I think": 1, "be": 1}, "adjectives": {"Perfect": 1}, "numerals": {"4": 1, "individual": 1}, "classifiers": {"individual": 1}, "pronouns": {"you": 1}}}, {"content": "Five. If the shopkeeper's service is not good, will you tell their manager?", "word_count": 21, "label": "", "sentence_structure": "Subject鈥搗erb鈥搊bject", "subjective_objective": {"answer": "Subjective", "rationale": "Because good and bad service is a subjective feeling and evaluation, the issue is one of subjective attitudes in examining patients."}, "sentence_sentiment": {"answer": "Positive", "rationale": "This entry is positive in emotional terms because it encourages consumers to provide feedback and complaints about poor services."}, "language_style": {"answer": "Calm and direct", "rationale": "The entry is more concise, with no excessive emotional or rhetorical style. It asks the interviewee, in a direct question, whether he will inform the manager of the shop about the poor attitude of the service, and is speaking objectively and calmly.\""}, "time_dimension": {"answer": "Present", "rationale": "\"will tell\" indicates that this is a future will in a hypothetical situation, but in this hypothetical situation, \"to tell\" is an action to be taken, so the temporal dimension is now."}, "sentence_type": "Interrogative sentence", "pos": {"nouns": {"Shop clerk": 1, "Service attitude": 1, "Manager": 1}, "verbs": {"Yes": 1, "Tell": 1, "Tell them": 1}, "adjectives": {"Not good": 1}, "numerals": {"5": 1}, "pronouns": {"you": 1}}}, {"content": "- Don't you like your pictures?", "word_count": 11, "label": "", "sentence_structure": "Subject鈥搗erb鈥揷omplement", "subjective_objective": {"answer": "Subjective", "rationale": "Because the ideas of \"unusual\" and \"approval\" are all about patients' subjective attitudes."}, "sentence_sentiment": {"answer": "Neutral", "rationale": "Because the entry does not have any emotional color."}, "language_style": {"answer": "Tactful and warm", "rationale": "The words of the entry are more euphemistic, more warm, using the term \"unusual\" and in the form of a question-word, which neither makes people too direct and offensive, nor causes the other to think and answer. Moreover, the topic \"appearing one's own picture\" is more personal, and it is easier to use moderate language to open the door to the interviewer and share real ideas."}, "time_dimension": {"answer": "Present", "rationale": "The keywords are \"frequent\" and \"approval\", which both indicate a frequency or attitude of behaviour, suggest that the person tested may have some dissatisfaction with his or her appearance or lack confidence in himself."}, "sentence_type": "Interrogative sentence", "pos": {"nouns": {"you": 1, "self": 1, "Photos": 1}, "verbs": {"Not often": 1, "Enjoy": 1, "- You're not": 1}, "adverbs": {"Regular": 1}, "numerals": {"6": 1}}}, {"content": "Do you feel bad about being criticized?", "word_count": 12, "label": "", "sentence_structure": "Subject鈥搗erb鈥搊bject", "subjective_objective": {"answer": "Subjective", "rationale": "Because feeling sad is the patient's subjective experience."}, "sentence_sentiment": {"answer": "Negative", "rationale": "Because the emotional reaction of \"worried\" in the entry is negative."}, "language_style": {"answer": "Tactful and warm", "rationale": "The entry is more personal and interactive when it is spoken in a gentle and inviting manner, asking the other side about the reaction to criticisms of others rather than directly speaking or ordering. \""}, "time_dimension": {"answer": "Present", "rationale": ""}, "sentence_type": "Interrogative sentence", "pos": {"nouns": {"others": 1, "Criticism": 1, "you": 1, "Sorry": 1}, "verbs": {"Yes": 2, "I think": 1}, "adjectives": {"Sorry": 1}, "numerals": {"7": 1}, "pronouns": {"you": 1}}}, {"content": "- You don't say anything to people?", "word_count": 14, "label": "", "sentence_structure": "Subject鈥搗erb鈥搊bject", "subjective_objective": {"answer": "Subjective", "rationale": "Because \"very few\" and \"real opinion\" are subjective views and attitudes of patients."}, "sentence_sentiment": {"answer": "Negative", "rationale": "Because \"You rarely say what you really think\" in the entry expresses negative attitudes and behaviours, that is, a reluctance to express real personal views."}, "language_style": {"answer": "Calm and direct", "rationale": "The entry raises questions directly and does not use excessive rhetorical or emotional colours, and is relatively calm and straightforward in language style, aimed at obtaining the true situation of the person under investigation."}, "time_dimension": {"answer": "Past", "rationale": "The keywords \"very few\", \"your real opinion\" and \"said\" indicate that the entry asks if the person who was tested has ever been in the past without expressing his true opinion, and therefore judges the past."}, "sentence_type": "Interrogative sentence", "pos": {"nouns": {"Questionnaire": 1, "Entry": 1, "person": 1, "Observations": 1}, "verbs": {"Say it": 2, "Very few": 1, "to": 1, "Give": 1}, "adjectives": {"Real": 1}, "adverbs": {"very": 1}, "numerals": {"8": 1}, "pronouns": {"you": 2}}}, {"content": "- You're skeptical about the praises of others?", "word_count": 14, "label": "", "sentence_structure": "Subject鈥搗erb鈥搊bject", "subjective_objective": {"answer": "Subjective", "rationale": "Because doubt is a subjective attitude, a judgement and a view of something."}, "sentence_sentiment": {"answer": "Negative", "rationale": "Because the attitude of \"suspicion\" in the entry is negative."}, "language_style": {"answer": "Tactful and warm", "rationale": "The language style of this entry is euphemismal and warm, depending on the presentation and tone of the question. It appears in a way that asks and invites, without using a soft or mandatory language. This expression is more likely to trigger reflection and communication, and to make people feel more friendly and comfortable."}, "time_dimension": {"answer": "Present", "rationale": "The entry asks the testee about the complimentary attitude of others, which is the case now."}, "sentence_type": "Interrogative sentence", "pos": {"nouns": {"others": 1, "Praise": 1}, "verbs": {"Hold": 1, "Doubt": 1, "Attitude": 1}, "numerals": {"9": 1}, "classifiers": {"individual": 1}, "pronouns": {"you": 1}}}, {"content": "Do you always feel worse than others?", "word_count": 12, "label": "", "sentence_structure": "Subject鈥搗erb鈥搊bject", "subjective_objective": {"answer": "Subjective", "rationale": "Because this is what patients are subjective about themselves."}, "sentence_sentiment": {"answer": "Negative", "rationale": "Because it expresses a low-level feeling, which is negative."}, "language_style": {"answer": "Calm and direct", "rationale": "The entry is more directly linguistically, is less verbically or is less verbically used, and is more calm and objective. It explicitly asks whether the interviewee often feels heath of being demeaning, allows the interviewee to answer the question with precision and is not affected by excessive emotion."}, "time_dimension": {"answer": "Present", "rationale": "The entry asks the testee about the current behaviour or mentality, so the time dimension is now judged."}, "sentence_type": "Interrogative sentence", "pos": {"nouns": {"You": 1, "others": 1}, "verbs": {"I think": 1, "- Yeah": 1, "Bad": 1}, "adjectives": {"Total": 1}, "numerals": {"10": 1}}}, {"content": "Are you satisfied with your appearance?", "word_count": 10, "label": "", "sentence_structure": "Subject鈥損redicate", "subjective_objective": {"answer": "Subjective", "rationale": "Because it is subjective to see the patient as he or she looks at his or her appearance."}, "sentence_sentiment": {"answer": "Neutral", "rationale": "Because the entry does not have any emotional color."}, "language_style": {"answer": "Tactful and warm", "rationale": "While directly asking the other side whether he/she is satisfied with his/her appearance, the expression is more personal and interactive by using a gentle and inviting tone of speech rather than by direct statements or orders. At the same time, \"satisfaction\" is a more subjective term for evaluation, and by euphemism questions can avoid unnecessary pressure and discomfort."}, "time_dimension": {"answer": "Present", "rationale": "The entry is for the subjective evaluation of the person ' s appearance, and the time dimension is now judged."}, "sentence_type": "Interrogative sentence", "pos": {"nouns": {"self": 1, "Appearance": 1}, "verbs": {"Satisfied": 1}, "numerals": {"11": 1}}}, {"content": "- Do you think you're better than anyone else?", "word_count": 13, "label": "", "sentence_structure": "Subject鈥搗erb鈥搊bject", "subjective_objective": {"answer": "Subjective", "rationale": "Because the word \"thinks\" indicates that it's a subjective view or attitude of the patient."}, "sentence_sentiment": {"answer": "Negative", "rationale": "Because the expression \"failer\" is negative."}, "language_style": {"answer": "Calm and direct", "rationale": "The language style of the entry is so calm that it opens up questions and does not use excessive rhetorical or emotional publicity to allow respondents to answer questions directly.\""}, "time_dimension": {"answer": "Present", "rationale": "The entry asks about the current views and views of the subject, the keyword being \"conceptive\" and \"bad\", both of which point to the current subjective perception. Thus, the temporal dimension is now judged."}, "sentence_type": "Interrogative sentence", "pos": {"nouns": {"Capacity": 1, "others": 1}, "verbs": {"I think": 1, "Bad": 1}, "numerals": {"12": 1}, "pronouns": {"you": 1}}}, {"content": "At the party, you're the only one who's not dressed properly.", "word_count": 23, "label": "", "sentence_structure": "Subject鈥搗erb鈥搊bject", "subjective_objective": {"answer": "Subjective", "rationale": "Because it's a subjective experience and feeling of a human being."}, "sentence_sentiment": {"answer": "Negative", "rationale": "Because the \"frightful\" attitude in the entry is negative."}, "language_style": {"answer": "Calm and direct", "rationale": "This way to communicate the questions clearly and clearly, making it easier for the respondents to understand and answer the questions.\""}, "time_dimension": {"answer": "Present", "rationale": ""}, "sentence_type": "Interrogative sentence", "pos": {"nouns": {"Party": 1, "person": 2, "Not Formal": 1, "I feel": 1, "I'm not comfortable": 1}, "verbs": {"Put it on": 1, "Yes": 2}, "adjectives": {"At the party": 1}, "numerals": {"XIII": 1}, "classifiers": {"individual": 1}, "pronouns": {"you": 1}}}, {"content": "Are you a popular man?", "word_count": 9, "label": "", "sentence_structure": "Subject鈥搗erb鈥搊bject", "subjective_objective": {"answer": "Subjective", "rationale": "Because \"popular\" is a subjective assessment, different people may have different opinions about the same person, so it is viewed from the patient's subjective perspective."}, "sentence_sentiment": {"answer": "Neutral", "rationale": "As the entry does not address specific emotions, attitudes, behaviour, purpose or quality, it merely asks whether a person is popular and is therefore neutral."}, "language_style": {"answer": "Tactful and warm", "rationale": "The language style of the entry is euphemistic. The basis of the judgement is that the entry is more personal and interactive in its expression, which is conducted in a gentle and inviting manner, rather than in direct statements or orders."}, "time_dimension": {"answer": "Present", "rationale": "The entry asks the testee ' s current state and characteristics, and therefore the time dimension is now judged."}, "sentence_type": "Interrogative sentence", "pos": {"nouns": {"person": 1, "Questionnaire": 1, "Entry": 1}, "verbs": {"be": 1, "Welcome": 1}, "numerals": {"14": 1}, "pronouns": {"you": 1}}}, {"content": "- Do you think you're attractive?", "word_count": 10, "label": "", "sentence_structure": "Subject鈥損redicate", "subjective_objective": {"answer": "Subjective", "rationale": "The problem is examined by self-evaluation and subjective perceptions of the patient."}, "sentence_sentiment": {"answer": "Neutral", "rationale": "Because the entry does not have any emotional color."}, "language_style": {"answer": "Tactful and warm", "rationale": "The entry uses a soft and invitation-style tone of dialogue rather than direct statements or orders, which is more personal and interactive. At the same time, the \"you think\" form of questioning also makes respondents feel more comfortable and comfortable in answering, and increases the availability and acceptance of questionnaires."}, "time_dimension": {"answer": "Present", "rationale": "The key words are \"Do you think\" and \"Do you find it attractive,\" which point to subjective perceptions and perceptions of the subject."}, "sentence_type": "Interrogative sentence", "pos": {"nouns": {"15": 1, "Charm": 1}, "verbs": {"I think": 1, "have": 1}, "numerals": {"15": 1}, "pronouns": {"you": 1}}}, {"content": "Do you have a sense of humor?", "word_count": 6, "label": "", "sentence_structure": "Subject鈥損redicate", "subjective_objective": {"answer": "Subjective", "rationale": "Based on the information provided in the title, I consider the purpose of the questionnaire to be based on subjectivity, since \"honorousness\" is the characteristic and behaviour of a person, which is the expression of a subjective individual's perception and evaluation and is therefore subjective."}, "sentence_sentiment": {"answer": "Positive", "rationale": "Because \"honoriness\" is usually considered a positive quality."}, "language_style": {"answer": "Tactful and warm", "rationale": "The language style of the entry is euphemistic, because it uses a query-style sentence, not a direct statement or a command statement. It also does not use any negative or offensive vocabulary, but asks the interviewee in a friendly and open way. Such expression is more comfortable for the interviewee and more likely to receive a positive response from him."}, "time_dimension": {"answer": "Present", "rationale": "The entry asks the testee about the current situation or character, i.e. whether he has a sense of humor and the time dimension is now judged."}, "sentence_type": "Interrogative sentence", "pos": {"nouns": {"Humor": 1}, "verbs": {"have": 1}}}, {"content": "Is your job your specialty?", "word_count": 11, "label": "", "sentence_structure": "Subject鈥揷opula鈥損redicative", "subjective_objective": {"answer": "Subjective", "rationale": "This is because patients are aware of and evaluate their work and fall within the realm of subjective perceptions and attitudes."}, "sentence_sentiment": {"answer": "Neutral", "rationale": "Because the entry asks whether a person is good at the current job and does not express positive or negative emotional tendencies."}, "language_style": {"answer": "Calm and direct", "rationale": "The entry is asked in a direct and clear manner, without any rhetoric or emotional qualification, so that the respondent can answer his or her actual situation."}, "time_dimension": {"answer": "Present", "rationale": "The entry asks about the current state and feelings of the testee, so the time dimension is now judged."}, "sentence_type": "Interrogative sentence", "pos": {"nouns": {"Work": 1, "Expertise": 1}, "verbs": {"be": 1, "of": 1}, "numerals": {"17": 1}, "pronouns": {"you": 1}}}, {"content": "- 18, you know how to fit?", "word_count": 8, "label": "", "sentence_structure": "Subject鈥損redicate", "subjective_objective": {"answer": "Subjective", "rationale": "Because \"know how to fit clothes\" is a subjective question that requires patients to answer on the basis of their own knowledge and experience."}, "sentence_sentiment": {"answer": "Neutral", "rationale": "Because the entry does not have any emotional color."}, "language_style": {"answer": "Tactful and warm", "rationale": "The language style of the entry is more euphemismal and warm, and it asks the interviewee in a more friendly and friendly way whether he understands how to fit his clothes, rather than direct orders or questions. This expression can trigger a positive response from the interviewee and make people feel more comfortable and natural."}, "time_dimension": {"answer": "Present", "rationale": "The entry asks if the person tested currently has some ability or knowledge, the keyword being \"you know\" and \"you\" and they all indicate the state of the moment."}, "sentence_type": "Interrogative sentence", "pos": {"nouns": {"Match": 1, "Clothes": 1}, "verbs": {"Yes": 1, "- You're not": 1}, "numerals": {"18": 1}}}, {"content": "Are you calm when you're in a crisis?", "word_count": 8, "label": "", "sentence_structure": "Subject鈥損redicate", "subjective_objective": {"answer": "Subjective", "rationale": "This is a subjective examination because it involves questioning the patient ' s self-perception and attitude in the event of risk."}, "sentence_sentiment": {"answer": "Positive", "rationale": "Because it is positive to stay calm at times of crisis."}, "language_style": {"answer": "Calm and direct", "rationale": "The language style of the entry is relatively straightforward, with no excessive rhetorical or emotional colours, and the question is whether the other party can remain calm and express itself in a more objective manner in times of crisis."}, "time_dimension": {"answer": "Present", "rationale": "The entry asks the person tested for psychological response and response in a dangerous or emergency situation, and the time dimension determines the state or behaviour of the present."}, "sentence_type": "Interrogative sentence", "pos": {"nouns": {"Emergency": 1, "you": 1}, "verbs": {"It's cool": 1}, "adverbs": {"Time": 1}}}, {"content": "Are you working with anyone else?", "word_count": 9, "label": "", "sentence_structure": "Subject鈥損redicate", "subjective_objective": {"answer": "Subjective", "rationale": "This is because it involves patients ' own subjective attitude to their own performance and evaluation in the team."}, "sentence_sentiment": {"answer": "Positive", "rationale": "Because the \"cooperative\" act is positive."}, "language_style": {"answer": "Tactful and warm", "rationale": "The language style of the entry is more euphemistic, and it is more apologetic and slow to ask questions about how you work with others. At the same time, the adjective \"uninterrupted\" is used to describe partnerships, implying high demands and expectations for the quality of cooperation. Such expressions are more likely to resonate with the interviewee and to facilitate the collection of real and accurate information."}, "time_dimension": {"answer": "Present", "rationale": "The entry asks the testee ' s current situation, i.e. whether there is no room for cooperation, and therefore the temporal dimension is now judged."}, "sentence_type": "Interrogative sentence", "pos": {"nouns": {"Questionnaire": 1, "Entry": 1, "person": 1, "Cooperation": 1, "No space": 1}, "verbs": {"and": 1, "have": 1}, "adverbs": {"- You're not": 1}, "numerals": {"20": 1}, "classifiers": {"individual": 1}}}, {"content": "Do you think you're just an ordinary person?", "word_count": 12, "label": "", "sentence_structure": "Subject鈥搗erb鈥搊bject", "subjective_objective": {"answer": "Subjective", "rationale": "Because \"think\" expresses the patient's subjective attitude."}, "sentence_sentiment": {"answer": "Neutral", "rationale": "Because the entry does not contain a clear emotional colour."}, "language_style": {"answer": "Tactful and warm", "rationale": "This soft question and answer approach is more likely to trigger a resonance and real response from the interviewee and to help collect more valuable data."}, "time_dimension": {"answer": "Present", "rationale": "The entry asks the testee about his current perception and self-awareness, so the temporal dimension is now judged."}, "sentence_type": "Interrogative sentence", "pos": {"nouns": {"Questionnaire": 1, "Entry": 1, "self": 1, "Normal people": 1}, "verbs": {"I think": 1, "be": 1}, "numerals": {"21": 1}, "pronouns": {"you": 1}}}, {"content": "22, do you often wish you looked like someone else?", "word_count": 14, "label": "", "sentence_structure": "Subject鈥搗erb鈥搊bject", "subjective_objective": {"answer": "Subjective", "rationale": "Because hope looks like the subjective desire and attitude of a patient."}, "sentence_sentiment": {"answer": "Negative", "rationale": "Because the idea of \"I want to look like someone else\" may have feelings of dissatisfaction with my appearance, and it's negative."}, "language_style": {"answer": "Tactful and warm", "rationale": "The language style of the entry is a euphemism. First, it expresses concern and respect for the inner thoughts of the person under investigation by using the word \"hope.\" Second, \"looks like someone\" is a more sensitive subject, but the entry does not ask the question directly, but rather uses an indirect approach that avoids offending or unnecessary discomfort. Finally, the whole sentence is more peaceful, natural, giving people a sense of affection and goodwill."}, "time_dimension": {"answer": "Present", "rationale": "The word \"often\" in the entry indicates that it is a question of the current situation, so the temporal dimension is now. And, \"Do you often want to look like someone else?\" is asking the interviewee about his current psychological state and expectations, not about the past or the future. So the answer is \"now.\""}, "sentence_type": "Interrogative sentence", "pos": {"nouns": {"self": 1, "Someone": 1}, "verbs": {"Hope": 1, "Long": 1}, "adverbs": {"Regular": 1}, "numerals": {"22": 1}}}, {"content": "- Are you always envious of what people have done?", "word_count": 11, "label": "", "sentence_structure": "Subject鈥搗erb鈥搊bject", "subjective_objective": {"answer": "Subjective", "rationale": "For envy is a subjective feeling, driven by the gap between the position and expectations of individuals vis-脿-vis others."}, "sentence_sentiment": {"answer": "Negative", "rationale": "Because the \"enjoyed for achievement\" act in the entry is negative."}, "language_style": {"answer": "Tactful and warm", "rationale": "The language style of the entry is relatively euphemistic and friendly, and instead of asking the other side whether they are jealous or envious, it is directing the interviewee to express his feelings in a way that is caring and understanding."}, "time_dimension": {"answer": "Present", "rationale": "The keyword is the description of \"often envious\" and it emphasizes the current situation."}, "sentence_type": "Interrogative sentence", "pos": {"nouns": {"you": 1, "others": 1, "Achievements": 1}, "verbs": {"Envious": 1}, "adjectives": {"Regular": 1}, "numerals": {"23": 1}}}, {"content": "- You give up what you like to make others feel better?", "word_count": 20, "label": "", "sentence_structure": "Subject鈥搗erb鈥搊bject", "subjective_objective": {"answer": "Subjective", "rationale": "The question is subjective because it concerns whether the patient changes his behaviour and preferences for the sake of the emotions of others."}, "sentence_sentiment": {"answer": "Negative", "rationale": "Because the \"release\" in the entry is negative."}, "language_style": {"answer": "Tactful and warm", "rationale": "The entry is more personal and interactive when it is spoken in a gentle, inquisitive manner, rather than in direct statements or orders. At the same time, it avoids the use of too direct language to ask questions, but encourages the interviewee to freely share his thoughts and feelings through an open question."}, "time_dimension": {"answer": "Present", "rationale": "Now the entry asks whether the testee will give up what he likes to make others feel bad. The keywords \"you,\" \"to make others feel better\" and \"to give up what he likes\" all point to an investigation of the behaviour or attitude of the present."}, "sentence_type": "Interrogative sentence", "pos": {"nouns": {"person": 1, "Yes": 1}, "verbs": {"Abandon": 1, "Do it": 1}, "adjectives": {"Yeah": 1}, "adverbs": {"not": 1}, "numerals": {"24": 1}, "pronouns": {"you": 1}}}, {"content": "- Will you dress up to please others?", "word_count": 12, "label": "", "sentence_structure": "Subject鈥搗erb鈥搊bject", "subjective_objective": {"answer": "Subjective", "rationale": "Because the motivation for \"love\" is the patient's subjective idea, the issue is considered subjective."}, "sentence_sentiment": {"answer": "Negative", "rationale": "Because the \"hot\" behaviour in the entries is based on a desire to accommodate the needs of others, a tendency to demean themselves and to over-appreciate others' evaluation."}, "language_style": {"answer": "Tactful and warm", "rationale": "The entry uses a question-and-answer language style, which guides the interviewee to think and answer questions in a gentle and inviting manner, and is expressed in a more personal and interactive manner. It also does not directly evaluate or criticize the interviewee鈥檚 behaviour, but rather attempts to understand the views and feelings of the interviewee, which are more euphemistic and warmer."}, "time_dimension": {"answer": "Present", "rationale": "The entry asks about the behaviour or attitude of the subject, the keywords \"will\" and \"dress\" are the verbs of the current state. So it's now."}, "sentence_type": "Interrogative sentence", "pos": {"nouns": {"person": 1, "Beloved": 1, "Get dressed": 1}, "verbs": {"Yes": 1, "For": 1, "Beloved": 1}, "numerals": {"25": 1}}}, {"content": "- You're not doing much of the things you're not willing to do?", "word_count": 15, "label": "", "sentence_structure": "Subject鈥搗erb鈥搊bject", "subjective_objective": {"answer": "Subjective", "rationale": "Because \"yes\" and \"no\" are subjective feelings of individuals, the questionnaire entry is viewed from the subjective point of view of patients."}, "sentence_sentiment": {"answer": "Negative", "rationale": "Because \"sustained to do many things that they were unwilling to do\" expressed a negative emotion and attitude, implying that the respondents lacked initiative or choice in facing a non-lovable task."}, "language_style": {"answer": "Calm and direct", "rationale": "The language style of the entry is more calm and straightforward, and there is no excessive emotional or rhetorical touch, but rather a dialogue in a direct interview, which makes it easier for the interviewees to provide a true and accurate answer."}, "time_dimension": {"answer": "Present", "rationale": "In conclusion, the answer to this question is \"now\"."}, "sentence_type": "Interrogative sentence", "pos": {"nouns": {"you": 1, "self": 1, "Yes": 1}, "verbs": {"Do it": 1, "Yes": 1, "I barely": 1}, "adverbs": {"Many": 1, "not": 1}, "numerals": {"26": 1}}}, {"content": "27, you let others control your life?", "word_count": 13, "label": "", "sentence_structure": "Subject鈥搗erb鈥搊bject", "subjective_objective": {"answer": "Subjective", "rationale": "The question is whether the individual is willing to have his or her life controlled, and it is examined from the point of view of the patient."}, "sentence_sentiment": {"answer": "Negative", "rationale": "Because the \"let others control your life\" behavior in the entries is negative."}, "language_style": {"answer": "Calm and direct", "rationale": "The language style of the entry is calm and straightforward. It asks the respondents whether they are willing to let others control their lives, without excessive accoutrements or emotional colours."}, "time_dimension": {"answer": "Present", "rationale": "The entry asks the testee's current state and customary sexual behaviour, which is \"Is it left to others to control your life\", so the answer is \"now\"."}, "sentence_type": "Interrogative sentence", "pos": {"nouns": {"Life": 1, "Questionnaire Entry": 1}, "verbs": {"I'm not going anywhere": 1, "Rule": 1}, "adverbs": {"27, you": 1, "Come on": 1}, "numerals": {"27": 1}, "pronouns": {"you": 1}}}, {"content": "Do you think you have more merit than disadvantage?", "word_count": 12, "label": "", "sentence_structure": "Subject鈥搗erb鈥搊bject", "subjective_objective": {"answer": "Subjective", "rationale": "As this entry requires the respondent to evaluate his/her own ratio of advantage or disadvantage, it is subjective."}, "sentence_sentiment": {"answer": "Positive", "rationale": "Because \"more than \" \" is an active self-evaluation."}, "language_style": {"answer": "Tactful and warm", "rationale": "The entry allows respondents to reflect on their strengths and weaknesses in a way that is more respectful of the subject ' s subjective feelings and self-perception than of direct judgement or comparison, and is expressed in a more moderate manner and more readily acceptable to the interviewee."}, "time_dimension": {"answer": "Present", "rationale": "The issue is to ask the testee about the perception of self-evaluation, and the time dimension is now judged."}, "sentence_type": "Interrogative sentence", "pos": {"nouns": {"Advantages": 1, "Disadvantages": 1}, "verbs": {"I think": 1, "More": 1}, "numerals": {"28": 1}}}, {"content": "29, do you always say sorry to people, even if it's not your fault?", "word_count": 20, "label": "", "sentence_structure": "Subject鈥搗erb鈥搊bject", "subjective_objective": {"answer": "Subjective", "rationale": "Because \"often\" and \"even if it's not your fault\" are subjective attitudes or behaviours of patients."}, "sentence_sentiment": {"answer": "Negative", "rationale": "Because \"showing sorry\" is a polite act, but in this situation it's asked, \"Will you apologize even if you're not wrong?\" It's a certain negative emotional color."}, "language_style": {"answer": "Tactful and warm", "rationale": "The expression in which the entry is used is more personal and interactive, in a gentle and inviting tone, rather than in direct statements or orders. Moreover, the expression of concern and understanding that seeks to understand the feelings and experiences of the person under investigation also makes the questionnaire more humane and acceptable."}, "time_dimension": {"answer": "Present", "rationale": "Now the keyword \"you\" means that it's a customary act, and the description \"even if you're not wrong\" means that it's happening. So the time dimension of this questionnaire is now."}, "sentence_type": "Interrogative sentence", "pos": {"nouns": {"you": 1, "person": 1, "Situation": 1}, "verbs": {"Call": 1, "Say it": 1, "Sorry": 1, "be": 1, "Wrong": 1}, "adverbs": {"Regular": 1, "Even if": 1, "It's not your fault": 1}, "numerals": {"29": 1}, "classifiers": {"individual": 1}}}, {"content": "Would it upset you if you broke someone's heart without any intention?", "word_count": 21, "label": "", "sentence_structure": "Subject鈥搗erb鈥搊bject", "subjective_objective": {"answer": "Subjective", "rationale": "Because it is an emotional reaction, a personal experience and feelings of the patient, and a subjective perspective."}, "sentence_sentiment": {"answer": "Positive", "rationale": "Because the \"worried\" attitude in the entry is positive."}, "language_style": {"answer": "Tactful and warm", "rationale": "The entry asks questions in a moderate and caring manner, avoiding the use of direct or strong language to stimulate the mood of the person under investigation. At the same time, using the term \"intentional\" shows that the concern is not about the deliberate harm done to others, but rather about the care and reflection of the suffering caused by the unintended suffering of others."}, "time_dimension": {"answer": "Present", "rationale": "The question is whether the person who was tested is guilty or suffering from his or her actions when he or she harms the feelings of others by accident. Thus, the temporal dimension of the article is not a clear past, present or future, but rather a description of a possible situation and emotional reaction, so the answer is \"yes\" or \"no\"."}, "sentence_type": "Interrogative sentence", "pos": {"nouns": {"Situation": 1, "person": 1, "Heart": 1}, "verbs": {"Yes": 2, "Wounded": 1, "Sorry": 1, "Unintentionally": 1}, "adjectives": {"Unintentional": 1}, "adverbs": {"In... in": 1, "If": 1, "- You're not": 1}, "numerals": {"30": 1}, "classifiers": {"individual": 1}, "pronouns": {"you": 1}}}, {"content": "31 Do you want to have more talent and talent?", "word_count": 16, "label": "", "sentence_structure": "Subject鈥搗erb鈥搊bject", "subjective_objective": {"answer": "Subjective", "rationale": "The desire for more talent and talent is a subjective aspiration and expectation of one person, and is therefore viewed from a subjective perspective."}, "sentence_sentiment": {"answer": "Positive", "rationale": "Because the attitude of \"wanting more talent and talent\" in the entry is positive."}, "language_style": {"answer": "Tactful and warm", "rationale": "The language style of the entry is euphemism. It does not ask the other side directly whether it wants more talent and talent, but rather guides the other side to think about it in a gentle and inviting tone, which is more personal and interactive."}, "time_dimension": {"answer": "Present", "rationale": "\"Hope\" is a verb that expresses will or expectation, and \"have more talent and talent\" describes the wishes or expectations of individuals, which indicate that the entry is asking about the current mindset or thinking of the subject. The entry can therefore be classified as \"now\"."}, "sentence_type": "Interrogative sentence", "pos": {"nouns": {"Capacity": 1, "Talent": 1}, "verbs": {"Hope": 1, "Yes": 1}, "numerals": {"More": 1}, "classifiers": {"More": 1, "Some": 1}, "pronouns": {"you": 1}}}, {"content": "32 - Do you listen to people often?", "word_count": 11, "label": "", "sentence_structure": "Subject鈥搗erb鈥揷omplement", "subjective_objective": {"answer": "Subjective", "rationale": "Because listening to others is the subjective will and choice of the individual."}, "sentence_sentiment": {"answer": "Neutral", "rationale": "Because the entry is about personal behaviour and does not have a human emotional colour."}, "language_style": {"answer": "Tactful and warm", "rationale": "The language style of the entry is more euphemistic, polite and of an interview nature, without direct orders or criticism of the other, and this expression is more acceptable to the interviewees and is willing to answer questions."}, "time_dimension": {"answer": "Present", "rationale": ""}, "sentence_type": "Interrogative sentence", "pos": {"nouns": {"Observations": 1, "others": 1}, "verbs": {"Listen": 1}, "adverbs": {"Regular": 1}, "numerals": {"32": 1, "you": 1}}}, {"content": "At the party, did you wait for someone to say hello first?", "word_count": 17, "label": "", "sentence_structure": "Subject鈥搗erb鈥搊bject", "subjective_objective": {"answer": "Subjective", "rationale": "Because \"when someone says hello first\" is a waiting act, a patient's subjective attitude."}, "sentence_sentiment": {"answer": "Neutral", "rationale": "Because the entry merely asks the interviewees about their behaviour at the meeting, it does not express positive or negative emotional sentiments."}, "language_style": {"answer": "Tactful and warm", "rationale": "The entry is more adept and interactive when it is spoken in a gentle and inviting manner, rather than by direct statements or orders."}, "time_dimension": {"answer": "Present", "rationale": "\"Always\" means that such acts are customary and frequently occurring, and the basis of the judgement is now."}, "sentence_type": "Interrogative sentence", "pos": {"nouns": {"Party": 1, "person": 2, "Say hello": 1}, "verbs": {"Wait": 1, "Call": 1, "Say hello": 1}, "adverbs": {"Regular": 1}, "numerals": {"33": 1}, "pronouns": {"you": 1}}}, {"content": "34 - You look in the mirror more than three times a day?", "word_count": 11, "label": "", "sentence_structure": "Subject鈥損redicate", "subjective_objective": {"answer": "Subjective", "rationale": "Because the frequency of looking in the mirror is the patient ' s subjective behaviour and attitude, not the objective fact."}, "sentence_sentiment": {"answer": "Neutral", "rationale": "Because the entry does not cover a description of emotions, attitudes, behaviour, purpose or quality, it merely asks for a factual situation."}, "language_style": {"answer": "Calm and direct", "rationale": "The language style of the entry is relatively simple and not expressed in too much qualifier or emotional terms, with a greater focus on obtaining objective information. \""}, "time_dimension": {"answer": "Present", "rationale": "The key word \"periscope\" and \"over three\" indicate that it is a question of the current behaviour of the person being tested and therefore is of the current temporal dimension."}, "sentence_type": "Interrogative sentence", "pos": {"nouns": {"Mirror": 1, "Son": 1}, "verbs": {"Photo": 1, "Super": 1, "Pass": 1}, "adjectives": {"Every": 1, "Oh, my God": 1}, "adverbs": {"III": 1, "Number of times": 1}, "numerals": {"34": 1}}}, {"content": "- You have a strong personality?", "word_count": 7, "label": "", "sentence_structure": "Subject鈥搗erb鈥揷omplement", "subjective_objective": {"answer": "Subjective", "rationale": "Because a person is a person's identity and identity, it is the individual's creation in the course of a long life, and it is the individual's subjective feeling and perception."}, "sentence_sentiment": {"answer": "Neutral", "rationale": "Because the entry merely asks about the strength of the personality and does not express any emotional colour."}, "language_style": {"answer": "Calm and direct", "rationale": "Because it asked the person under investigation \"Are you very strong?\" The question was not expressed in too many terms or in a way that was more objective and direct."}, "time_dimension": {"answer": "Present", "rationale": "The entry now asks the testee about his current character characteristics and does not address past or future circumstances, so it is now judged."}, "sentence_type": "Interrogative sentence", "pos": {"nouns": {"Personality": 1}, "verbs": {"very": 1, "Strong": 1}, "numerals": {"35": 1}}}, {"content": "- You're a good leader?", "word_count": 10, "label": "", "sentence_structure": "Subject鈥損redicate", "subjective_objective": {"answer": "Subjective", "rationale": "It is a subjective examination because it is a personal view or feeling that one considers himself a good leader."}, "sentence_sentiment": {"answer": "Positive", "rationale": "Because the expression \"good leader\" is a positive one."}, "language_style": {"answer": "Calm and direct", "rationale": "The entry is more directly linguistically, not using too much qualifier or emotionally strong terminology to describe the problem, but rather presenting it in a concise question form to the respondents, enabling them to respond directly to their views."}, "time_dimension": {"answer": "Future", "rationale": "The entry asks the testee about his/her self-evaluation and expectations for the future, and the keywords are \"excellent\" and \"leader\" and they point to situations that have not yet occurred and that may arise in the future."}, "sentence_type": "Interrogative sentence", "pos": {"nouns": {"Excellent": 1, "Leader": 1}, "verbs": {"be": 1}, "adjectives": {"individual": 1}, "numerals": {"36": 1}}}, {"content": "- How's your memory?", "word_count": 7, "label": "", "sentence_structure": "Subject鈥損redicate", "subjective_objective": {"answer": "Subjective", "rationale": "Because good and bad memories are the subjective feelings and cognitive abilities of individuals, they are viewed from the subjective perspective of the patient."}, "sentence_sentiment": {"answer": "Positive", "rationale": "Because the expression \"good memory\" is usually seen as a positive feature or capability."}, "language_style": {"answer": "Tactful and warm", "rationale": "The language style of the entry is more euphemistic and friendly, and instead of asking directly whether the other party has a good memory or no memory problem, it leads to a question about the level of memory. Moreover, the expression \"your memory\" makes the questionnaire more human and friendly."}, "time_dimension": {"answer": "Present", "rationale": "The entry asks the testee about the current memory, does not address past or future time dimensions, and therefore the judgement is now."}, "sentence_type": "Interrogative sentence", "pos": {"nouns": {"Memory": 1, "Questionnaire": 1, "Entry": 1}, "verbs": {"Okay": 1, "be": 1}, "adjectives": {"very": 1}, "numerals": {"37": 1}}}, {"content": "- Are you attracted to opposite sex?", "word_count": 9, "label": "", "sentence_structure": "Subject鈥損redicate", "subjective_objective": {"answer": "Subjective", "rationale": "Because the issue involves subjective perceptions and perceptions of individuals."}, "sentence_sentiment": {"answer": "Negative", "rationale": "Because the concept of \"attraction\" in this entry is usually subjective and evaluative, it can trigger emotional reactions from the person questioned and can generate negative emotions."}, "language_style": {"answer": "Calm and direct", "rationale": "The entry asks the respondents in a straightforward manner whether they are attractive to the opposite sex, with a concise language style, and without too much ado or emotional colour.\""}, "time_dimension": {"answer": "Past", "rationale": "It is therefore not possible to classify it as any category past, present or future."}, "sentence_type": "Interrogative sentence", "pos": {"nouns": {"Heterosexual": 1, "Attractive": 1, "you": 1}, "verbs": {"have": 1}, "numerals": {"38": 1}}}, {"content": "- You know how to handle money?", "word_count": 6, "label": "", "sentence_structure": "Subject鈥損redicate", "subjective_objective": {"answer": "Objective", "rationale": "Because knowledge of financial management is an objective skill or knowledge, not a patient ' s own feelings or attitudes."}, "sentence_sentiment": {"answer": "Neutral", "rationale": "Because the entry does not have any emotional color."}, "language_style": {"answer": "Tactful and warm", "rationale": "The entry takes the form of a question and question sentence, and a soft, invitation-based conversation allows the interviewee to feel comfortable and to answer questions. At the same time, the entry does not directly evaluate or accuse the interviewee of his financial competence, but rather leads the other to share his views and experience in a question-and-answer manner."}, "time_dimension": {"answer": "Present", "rationale": "The \"know\" action occurs now and asks if the person tested has the current knowledge or ability to manage money."}, "sentence_type": "Interrogative sentence", "pos": {"nouns": {"39": 1, "Questionnaire": 1, "Entry": 1, "Financial management": 1}, "verbs": {"Yes": 1}, "numerals": {"39": 1}, "classifiers": {"individual": 1}}}, {"content": "Do you usually listen to others before buying clothes?", "word_count": 16, "label": "", "sentence_structure": "Subject鈥搗erb鈥搊bject", "subjective_objective": {"answer": "Subjective", "rationale": "Because listening to others is a subjective choice and attitude of one."}, "sentence_sentiment": {"answer": "Neutral", "rationale": "The entry does not describe whether the hearing of others is positive or negative, nor does it involve any emotional colour."}, "language_style": {"answer": "Tactful and warm", "rationale": "The entry is a conversation in question, using polite language, and does not directly order or describe the behaviour of the other party. At the same time, the use of the subjective question phrase \"you usually\" makes the questionnaire more open and free, leaving the interviewee with enough space for reflection and expression. Such a language style is more gentle and friendly, and better able to direct the interviewee to the investigation and provide a true answer."}, "time_dimension": {"answer": "Present", "rationale": ""}, "sentence_type": "Interrogative sentence", "pos": {"nouns": {"Clothes": 1, "Observations": 1}, "verbs": {"Buy": 1, "Listen": 1, "First": 1, "Do it": 1}, "adverbs": {"Usually": 1}, "numerals": {"40": 1}, "pronouns": {"you": 1}}}]}

{"questionnaire_title": "DSS", "questionnaire_category": "Mental health", "item_count": 20, "emotion_shift_overall": "From negative to positive", "language_style_overall": "Tactful and warm", "items": [{"content": "One, I feel bored and depressed.", "word_count": 11, "label": "", "sentence_structure": "Subject鈥揷opula鈥損redicative", "subjective_objective": {"answer": "Subjective", "rationale": "Because \"fright,\" \"silent\" and \"silent\" are the feelings and experiences of patients themselves, and are viewed from a subjective perspective."}, "sentence_sentiment": {"answer": "Negative", "rationale": "Because the words \"silent\" and \"silent\" in the entries are the words of negative emotions."}, "language_style": {"answer": "Tactful and warm", "rationale": "This is a more personal and interactive expression that helps to create a good atmosphere of communication."}, "time_dimension": {"answer": "Present", "rationale": ""}, "sentence_type": "Interrogative sentence", "pos": {"nouns": {"I": 1, "It's boring": 1, "Emotion": 1, "Low": 1}, "verbs": {"I think": 1}}}, {"content": "2 I think it's best in the morning.", "word_count": 11, "label": "", "sentence_structure": "Subject鈥揷opula鈥損redicative", "subjective_objective": {"answer": "Subjective", "rationale": "This is a subjective feeling and view of the patient personally, rather than an objective fact or situation."}, "sentence_sentiment": {"answer": "Positive", "rationale": "Because the expression \"thinks it's best\" is positive, indicating that the interviewee has a positive view and feeling of the morning."}, "language_style": {"answer": "Tactful and warm", "rationale": "The entry uses a euphemism and invitation language style, which is \"you think\" to express the inquiry, gives a sense of affection and openness, while avoiding direct denial or affirmation of the other. Such expression is more appropriate for psychological questionnaire research, which better facilitates communication and reflection among the subjects."}, "time_dimension": {"answer": "Present", "rationale": "The keyword \"I think\" and \"the morning is best\" is the expression of what the speaker feels and what he thinks is now. \"I think \"using the general current, which means that it is a regular or customary act, and \"the morning best\" indicates that it is a statement of the current situation, so the entry describes the present situation of the speaker."}, "sentence_type": "Interrogative sentence", "pos": {"nouns": {"One day": 1, "Morning": 1}, "verbs": {"I think": 1, "Okay": 1}, "adverbs": {"鈾?And the 鈾?: 1, "Most": 1}, "numerals": {"2": 1}}}, {"content": "Three, you're crying or you're crying.", "word_count": 13, "label": "", "sentence_structure": "Subject鈥搗erb鈥揷omplement", "subjective_objective": {"answer": "Subjective", "rationale": "Because emotional reactions are often subjective feelings and experiences."}, "sentence_sentiment": {"answer": "Negative", "rationale": "Because \"screeching\" and \"wanting to cry\" in the entries are negative emotional expressions."}, "language_style": {"answer": "Calm and direct", "rationale": "The entry asks directly whether the interviewees often cry without cause, without excessive ado or emotional colour, and in a more calm and objective manner."}, "time_dimension": {"answer": "Present", "rationale": ""}, "sentence_type": "Affirmative declarative sentence", "pos": {"nouns": {"Cry": 2, "What's wrong": 1}, "verbs": {"Come out": 1, "I think": 1}, "adjectives": {"Always": 1}, "adverbs": {"Land": 1}}}, {"content": "I don't sleep well at night.", "word_count": 7, "label": "", "sentence_structure": "Subject鈥搗erb鈥揷omplement", "subjective_objective": {"answer": "Subjective", "rationale": "Subjectivity because \"sleep bad\" is the patient's subjective experience or feeling."}, "sentence_sentiment": {"answer": "Negative", "rationale": "Because \"sleeping bad\" is often seen as a negative health condition, it can lead to fatigue, low concentration, etc."}, "language_style": {"answer": "Calm and direct", "rationale": "The topic was directly asked, not using excessive rhetoric or emotional rendering, and was more balanced and objective, and therefore was of a calm and direct linguistic nature."}, "time_dimension": {"answer": "Present", "rationale": "The keyword is \"I sleep badly at night,\" which means that the testee, in answering this question, describes his/her present situation, which is the problem at hand. So the temporal dimension is now."}, "sentence_type": "Negative declarative sentence", "pos": {"nouns": {"Sleep": 1, "Night": 1}, "verbs": {"Okay": 1}, "numerals": {"4": 1}, "pronouns": {"I": 1}}}, {"content": "Five, I eat as much as I normally do.", "word_count": 9, "label": "", "sentence_structure": "Subject鈥搗erb鈥揷omplement", "subjective_objective": {"answer": "Objective", "rationale": "Because the amount of \"eating\" and \"eating\" is an objective fact, not influenced by personal emotions, etc."}, "sentence_sentiment": {"answer": "Neutral", "rationale": "Because the entry describes only one act, it does not express a positive or negative attitude."}, "language_style": {"answer": "Tactful and warm", "rationale": "The entry is more adept and interactive when it is spoken in a gentle and inviting manner, rather than by direct statements or orders."}, "time_dimension": {"answer": "Present", "rationale": "\"I eat as much as I normally do,\" which means \"like normal, customary\" behaviour, while \"eating\" action takes the normal current state of affairs, which means that it not only has occurred in the past, but may still be ongoing. So, the entry describes the person who was tested in his present state and behaviour."}, "sentence_type": "Interrogative sentence", "pos": {"nouns": {"Rice": 1, "Normal": 1}, "verbs": {"Eat": 1, "Yeah": 1, "Same thing": 1}, "adverbs": {"More": 1}, "numerals": {"5": 1}, "classifiers": {"individual": 1}}}, {"content": "I was as happy in my close contact with the opposite sex as I ever was.", "word_count": 18, "label": "", "sentence_structure": "Subject鈥揷opula鈥損redicative", "subjective_objective": {"answer": "Subjective", "rationale": "Because feeling happy is a subjective emotional experience."}, "sentence_sentiment": {"answer": "Positive", "rationale": "Because the behavior in the entry \"in close contact with the opposite sex\" and \"in the pleasure\" are positive emotional."}, "language_style": {"answer": "Calm and direct", "rationale": "The language style of the entry is more calm and objective, and there is no excessive amount of embellishment or emotional rendering, and the interviewee is asked whether he/she is still happy when he/she has had contact with the opposite sex. This expression makes the issue clearer and more concise."}, "time_dimension": {"answer": "Present", "rationale": "The keyword is \"now,\" because the title describes a feeling in the moment, \"when I have close contact with the opposite sex,\" and whether it happens in the present as before. And at the same time, the act does not involve any past or future situation, but rather describes the current state of feeling and state."}, "sentence_type": "Affirmative declarative sentence", "pos": {"nouns": {"Questionnaire Entry": 1, "Heterosexual": 1, "Close contact": 1, "Have a nice day": 1}, "verbs": {"I feel": 1, "With": 1, "Same thing": 1}, "adverbs": {"As always": 1}, "pronouns": {"I": 1}}}, {"content": "Seven. I feel my weight is falling.", "word_count": 11, "label": "", "sentence_structure": "Subject鈥搗erb鈥揷omplement", "subjective_objective": {"answer": "Subjective", "rationale": "Because \"feel\" is usually subjective experience or perception, not objective fact. Although changes in weight may be objective, individual feelings about changes in weight are subjective, so the problem is viewed from the patient's subjective perspective."}, "sentence_sentiment": {"answer": "Negative", "rationale": "Because the expression \"weight loss\" in the entry is negative."}, "language_style": {"answer": "Calm and direct", "rationale": "The entry asks the other side in a direct manner about how he/she is feeling about his/her weight changes, without too much ado or emotional colour, and with a more calm and objective language style."}, "time_dimension": {"answer": "Present", "rationale": ""}, "sentence_type": "Affirmative declarative sentence", "pos": {"nouns": {"Weight": 1, "Down": 1}, "verbs": {"Feel": 1, "in": 1, "Down": 1}, "adverbs": {"self": 1}, "numerals": {"7": 1}}}, {"content": "I have constipation problems.", "word_count": 7, "label": "", "sentence_structure": "Subject鈥搗erb鈥揷omplement", "subjective_objective": {"answer": "Subjective", "rationale": "Because constipation is a personal and subjective experience of the patient, not an objective fact or event."}, "sentence_sentiment": {"answer": "Negative", "rationale": "Because constipation is a symptom of physical discomfort, it has a certain negative emotional color."}, "language_style": {"answer": "Tactful and warm", "rationale": "The entry is a soft, understanding way of asking the interviewees whether they have questions about constipation, are more sensitive and respect the personal privacy of the interviewees. At the same time, it does not focus too much on or blame constipation, but rather uses a euphemism to guide the interviewees to share their feelings and experiences, which is more in line with the principles of psychological investigation."}, "time_dimension": {"answer": "Present", "rationale": "The entry is asking the testee's current state and feelings, \"I have\" indicating that the action or state is now in progress, so the time dimension is now judged."}, "sentence_type": "Negative declarative sentence", "pos": {"nouns": {"Decency": 1, "Trouble": 1}, "verbs": {"have": 1}, "numerals": {"8": 1}}}, {"content": "Nine. I think my heart beats faster than usual.", "word_count": 10, "label": "", "sentence_structure": "Subject鈥揷opula鈥損redicative", "subjective_objective": {"answer": "Subjective", "rationale": "Because \"heart beat faster\" is a subjective feeling and experience, not an objective fact or behaviour. Although heart beat is indeed a measurable biological indicator, the rate of heart beats that patients feel is based on their subjective feeling, not on objective measurements. So this questionnaire entry is viewed from the patient's subjective perspective."}, "sentence_sentiment": {"answer": "Negative", "rationale": "Because heart rate is usually a physical reaction, it can be caused by stress, anxiety, or disease. While this does not necessarily represent a serious health problem, it may still be uncomfortable and agitated and therefore be considered negative."}, "language_style": {"answer": "Tactful and warm", "rationale": "This is more personal and interactive when it is spoken in a conversation with an interest in the interview rather than in a direct statement or order. At the same time, euphemism avoids undue psychological pressure on the interviewee and helps to better collect real feedback."}, "time_dimension": {"answer": "Present", "rationale": "The entry describes the experience and experience of the testee, \"I think\" which indicates that it is subjective, or observational, of the testee, and is present, not past. The keyword \"heart beats faster than usual\" indicates that this feeling is happening. So the temporal dimension of the article is now."}, "sentence_type": "Affirmative declarative sentence", "pos": {"nouns": {"Heart rate": 1, "Normal": 1}, "verbs": {"I think": 1, "Almost there": 1}}}, {"content": "I'm tired for no reason.", "word_count": 9, "label": "", "sentence_structure": "Subject鈥搗erb鈥揷omplement", "subjective_objective": {"answer": "Subjective", "rationale": "For fatigue is a subjective feeling, not an objective fact."}, "sentence_sentiment": {"answer": "Negative", "rationale": "Because the feeling of \"difficult without cause\" in the entries is negative."}, "language_style": {"answer": "Tactful and warm", "rationale": "Warm and warm."}, "time_dimension": {"answer": "Present", "rationale": "The keyword \"difficult\" means the person being tested in his current state or feeling, and the temporal dimension is now."}, "sentence_type": "Negative declarative sentence", "pos": {"nouns": {"Questionnaire": 1, "Entry": 1, "Weary": 1}, "verbs": {"I feel": 1, "For no reason": 2}, "adverbs": {"For no reason": 1}, "numerals": {"10": 1}, "pronouns": {"I": 1}}}, {"content": "I'm as clear as I normally am.", "word_count": 11, "label": "", "sentence_structure": "Subject鈥揷opula鈥損redicative", "subjective_objective": {"answer": "Subjective", "rationale": "Because it is the subjective assessment of the patient's self-perception that is clear to the mind."}, "sentence_sentiment": {"answer": "Neutral", "rationale": "Since the entry does not express positive or negative emotional expressions, it merely describes an objective fact."}, "language_style": {"answer": "Calm and direct", "rationale": "Because the entry does not use too much rhetorical or emotional overture, it is a direct question of the subject鈥檚 subjective perception in a given situation. It is more objective, not too emotional or personal. So I think the language style of the entry is 鈥渃ool and direct鈥?"}, "time_dimension": {"answer": "Present", "rationale": "The key words in the sentence are \"as usual\" and \"clear\" , which describe the current state and do not address the time dimension of the past or future. Therefore, the time dimension of this questionnaire entry should be judged as now."}, "sentence_type": "Affirmative declarative sentence", "pos": {"nouns": {"Brain": 1, "Normal": 1}, "verbs": {"Call": 1, "Same thing": 1, "Clear": 1}, "numerals": {"11": 1}, "pronouns": {"I": 1}}}, {"content": "I'm not as hard as I normally feel.", "word_count": 17, "label": "", "sentence_structure": "Subject鈥搗erb鈥揷omplement", "subjective_objective": {"answer": "Subjective", "rationale": "This is because the subject is asked whether a patient feels difficult to do something, which requires that the patient assess his/her own feelings and experiences. The answer to this question should therefore be based on his/her own perception and thoughts, not on objective facts."}, "sentence_sentiment": {"answer": "Neutral", "rationale": "Because the entry describes what a person feels when doing things, it does not involve emotional colour and is therefore neutral."}, "language_style": {"answer": "Calm and direct", "rationale": "The language style of the entry is calm and straightforward. It expresses in a concise manner the subject of questioning whether the other party has encountered difficulties in fulfilling its mission, without excessive emotional or rhetorical styles, and with a more objective tone."}, "time_dimension": {"answer": "Present", "rationale": "Now the entry indicates the feelings and experiences of the person who was tested in reply to the questionnaire, and \"no difficulties\" describes the current state and therefore the present."}, "sentence_type": "Affirmative declarative sentence", "pos": {"nouns": {"Things": 1, "Normal": 1, "Difficulties": 1}, "verbs": {"Do it": 1, "I feel": 1, "have": 1}, "adverbs": {"Yeah": 1, "Same thing": 1}, "numerals": {"12": 1}, "pronouns": {"I": 1}}}, {"content": "13, I'm restless, I can't keep my peace.", "word_count": 11, "label": "", "sentence_structure": "Subject鈥搗erb鈥揷omplement", "subjective_objective": {"answer": "Subjective", "rationale": "Because \"sitting\" and \"unusual to keep calm\" are subjective feelings of patients."}, "sentence_sentiment": {"answer": "Negative", "rationale": "Because the description \"sitting, difficult to keep calm\" in the entry is negative."}, "language_style": {"answer": "Tactful and warm", "rationale": ""}, "time_dimension": {"answer": "Present", "rationale": ""}, "sentence_type": "Negative declarative sentence", "pos": {"nouns": {"I": 1, "Ann": 1, "Calm down": 1}, "verbs": {"Sit down": 1, "Get down": 1, "Hold on": 1}, "adjectives": {"Hard": 1, "Hold on": 1}, "adverbs": {"not": 1, "Ann": 1}}}, {"content": "14. I have hope for the future.", "word_count": 9, "label": "", "sentence_structure": "Subject鈥揷opula鈥損redicative", "subjective_objective": {"answer": "Subjective", "rationale": "For hope for the future is an individual feeling and attitude, and a subjective perspective."}, "sentence_sentiment": {"answer": "Positive", "rationale": "Because \"to hope for the future\" expresses a positive attitude and emotion."}, "language_style": {"answer": "Tactful and warm", "rationale": "The entry uses a moderate and encouraging language style, expresses positive expectations about the future of the interviewees and is not accompanied by direct statements or orders, and is more personal and interactive."}, "time_dimension": {"answer": "Future", "rationale": "The keyword \"the future\" indicates that the entry relates to the future's vision and expectations, while the verb \"felt\" indicates the current emotional state of the testee, but the entire sentence places greater emphasis on the feelings and expectations for the future. So, judging by the time dimension, the article is intended for the future."}, "sentence_type": "Affirmative declarative sentence", "pos": {"nouns": {"The future": 1, "Hope": 1}, "verbs": {"I feel": 1}, "numerals": {"14": 1}}}, {"content": "I'm a little more angry than usual.", "word_count": 10, "label": "", "sentence_structure": "Subject鈥搗erb鈥揷omplement", "subjective_objective": {"answer": "Subjective", "rationale": "Because \"freaky\" is a subjective description of the patient's own feelings and experiences."}, "sentence_sentiment": {"answer": "Negative", "rationale": "Because \"freak and excitement\" in the entry is a sign of negative emotion."}, "language_style": {"answer": "Tactful and warm", "rationale": "The language style of the entry is relatively euphemistic and gentle, asking the other side whether it is more angry or excited than usual, rather than directly accusing or critical. This expression is more likely to bring the investigators closer to the respondents and help to establish good interaction."}, "time_dimension": {"answer": "Present", "rationale": "\"I'm more angry than usual\" means \"more than usual\" means compared to the past or the future, but in general, the phrase describes a normal state of affairs, so the temporal dimension is now."}, "sentence_type": "Affirmative declarative sentence", "pos": {"nouns": {"Grandpa": 1, "Questionnaire": 1, "Entry": 1}, "verbs": {"Analysis": 1, "Easy": 1, "Angry": 1, "Excited": 1}, "adverbs": {"Normal": 1, "- Yeah": 1}, "numerals": {"15": 1}, "pronouns": {"I": 1}}}, {"content": "16, I think it's easy to make a decision.", "word_count": 12, "label": "", "sentence_structure": "Subject鈥揷opula鈥損redicative", "subjective_objective": {"answer": "Subjective", "rationale": "Because \"think\" is a term for expressing personal feelings or views, usually described from a subjective perspective."}, "sentence_sentiment": {"answer": "Positive", "rationale": "Since the expression of the purpose of the article expressed positive feelings and attitudes, it was considered that it was easy to take decisions."}, "language_style": {"answer": "Calm and direct", "rationale": "The entry is more directly written, with no excessive emotional or speech modification, and is more objective. It simply and clearly indicates whether the subject of the inquiry believes that it is easy to make a decision."}, "time_dimension": {"answer": "Present", "rationale": ""}, "sentence_type": "Affirmative declarative sentence", "pos": {"nouns": {"Decision": 1, "Yes": 1}, "verbs": {"Do it": 1, "I think": 1, "be": 1, "Easy": 1}, "adjectives": {"of": 2}, "numerals": {"16": 1, "1": 2}, "pronouns": {"I": 1}}}, {"content": "I feel like I'm useful. People need me.", "word_count": 15, "label": "", "sentence_structure": "Subject鈥揷opula鈥損redicative", "subjective_objective": {"answer": "Subjective", "rationale": "It's the patient's subjective feeling."}, "sentence_sentiment": {"answer": "Positive", "rationale": "Because self-evaluation and the needs of others in the entries are positive emotional."}, "language_style": {"answer": "Tactful and warm", "rationale": "The article is expressed in a more euphemistic manner, without direct statements or orders, but in a gentle and inviting tone. Moreover, it reflects a positive emotional focus on self-worth and the needs of others, and adds to the relative and interactive nature of the questionnaire. In short, the language of the article is \"precipitous\" and aims to guide respondents to reflect on their roles and values in life and how to meet the needs of others, with a certain psychological inspiration."}, "time_dimension": {"answer": "Present", "rationale": ""}, "sentence_type": "Affirmative declarative sentence", "pos": {"nouns": {"self": 1, "person": 2, "others": 1}, "verbs": {"I think": 1, "It works": 1, "Yes": 1}, "numerals": {"17": 1}, "pronouns": {"I": 1}}}, {"content": "I've had a very interesting life.", "word_count": 10, "label": "", "sentence_structure": "Subject鈥揷opula鈥損redicative", "subjective_objective": {"answer": "Subjective", "rationale": "Because meaning is a subjective feeling or an evaluation."}, "sentence_sentiment": {"answer": "Positive", "rationale": "Because it expresses the relevance of the lives of the interviewees and their positive emotional orientation."}, "language_style": {"answer": "Tactful and warm", "rationale": ""}, "time_dimension": {"answer": "Present", "rationale": ""}, "sentence_type": "Affirmative declarative sentence", "pos": {"nouns": {"Life": 1, "Meaning": 1}, "verbs": {"Pass": 1}, "adjectives": {"Very": 1}, "numerals": {"18": 1}}}, {"content": "19 I think if I die, I'll live better.", "word_count": 16, "label": "", "sentence_structure": "Subject鈥搗erb鈥揷omplement", "subjective_objective": {"answer": "Subjective", "rationale": "Because it is perceived as a subjective judgement and idea."}, "sentence_sentiment": {"answer": "Negative", "rationale": "Because the emotions and attitudes expressed in the entries are negative, it is possible to reflect a pessimistic or negative mindset that believes that one ' s own death will make others ' lives better."}, "language_style": {"answer": "Tactful and warm", "rationale": "The entry is more euphemistic and warm, expresses a subjective view in the first person's name, and uses the expression \"if I die, I'll live better\", which is more subtle and incisive. Moreover, the entry does not directly blame or criticize anyone or anything, but explores the feelings and views of the individual in a gentle way, which is more personal and interactive."}, "time_dimension": {"answer": "Present", "rationale": "In that sentence, \"consider\" expresses the present view or belief, so the temporal dimension is now judged."}, "sentence_type": "Negative declarative sentence", "pos": {"nouns": {"I think": 1, "I": 1, "Die": 1, "Don't": 1, "person": 1, "Fan": 1, "Live": 1}, "verbs": {"I think": 1, "Yes": 2, "Better": 2}, "adverbs": {"If": 1, "Just": 1}}}, {"content": "I'm still interested in things that are usually of interest.", "word_count": 15, "label": "", "sentence_structure": "Subject鈥揷opula鈥損redicative", "subjective_objective": {"answer": "Subjective", "rationale": "Because interest is a subjective feeling or attitude."}, "sentence_sentiment": {"answer": "Positive", "rationale": "Because the entry is a positive attitude and quality, \"I remain interested in things that are of ordinary interest\" means that the person has the ability and attitude to keep his interest in the matter."}, "language_style": {"answer": "Tactful and warm", "rationale": "Because the entry uses positive language, emphasizing that the other party ' s preferences still exist, it does not use too direct or incorrupt language for questioning or evaluation, giving a mild and caring feeling."}, "time_dimension": {"answer": "Present", "rationale": "The \"remain\" in the entry indicates the continuity and stability of the action, implying that it has existed until now. At the same time, the expression \"for things of common interest\" indicates that interest exists in the past. So the time dimension of the sentence as a whole is now."}, "sentence_type": "Affirmative declarative sentence", "pos": {"nouns": {"Yes": 1, "Interest": 1}, "verbs": {"to": 1, "Still": 1, "Interested": 2}, "adverbs": {"Normal": 1}, "numerals": {"20": 1}, "pronouns": {"I": 1}}}]}

{"questionnaire_title": "Creative Measurement Table", "questionnaire_category": "Mental health", "item_count": 50, "emotion_shift_overall": "From positive to negative", "language_style_overall": "Tactful and warm", "items": [{"content": "1. In schools, I like to try to guess things or problems, even if they are not necessarily true.", "word_count": 29, "label": "", "sentence_structure": "Subject鈥搗erb鈥搊bject", "subjective_objective": {"answer": "Subjective", "rationale": "Because \"like\" is the patient's subjective attitude, \"specify\" is also the patient's own thoughts and behaviour, and this is an entry that is examined from a subjective point of view."}, "sentence_sentiment": {"answer": "Positive", "rationale": "Because the action of \"likes to guess things or problems\" in the entry is positive. Although the result may not be entirely correct, this attempt and exploration is encouraging."}, "language_style": {"answer": "Tactful and warm", "rationale": "The language style of the entry is a warm and euphemistic type. First, the positive words and tone of the title, \"I like it,\" shows a kind, encouraging and receptive attitude. Second, the expression \"even if not right\" reflects the surveyed's respect for and understanding of the personality of the person being tested, making it comfortable and free to answer questions. Such language helps to create a pleasant atmosphere of investigation and improves the effectiveness and reliability of the questionnaire."}, "time_dimension": {"answer": "Present", "rationale": "The entry describes the testee ' s customary behaviour and attitudes in the learning environment, and the temporal dimension is now judged."}, "sentence_type": "Affirmative declarative sentence", "pos": {"nouns": {"School": 1, "Yes": 1, "Problem": 1, "Guess": 1}, "verbs": {"Yeah": 1, "Try": 1, "Do it": 1, "Guess": 1, "not": 1, "It doesn't matter": 1}, "adverbs": {"I'm sure": 1, "Both": 1}, "numerals": {"One": 2}, "pronouns": {"I": 1}}}, {"content": "I would like to look closely at what I have not seen in order to get a detailed picture.", "word_count": 23, "label": "", "sentence_structure": "Subject鈥搗erb鈥搊bject", "subjective_objective": {"answer": "Subjective", "rationale": "Because it is a mindless act, it is the personal preference and tendency of the patient."}, "sentence_sentiment": {"answer": "Positive", "rationale": "Because the behavior in the entries, \"like to look closely at what I have never seen,\" is a manifestation of curiosity and exploration, with positive emotional aspects."}, "language_style": {"answer": "Tactful and warm", "rationale": "The entry is more personal and interactive when it is presented in a soft, invitation-like tone, rather than in direct statements or orders. At the same time, the positive emotional expression of \"like\" is used to convey the respect and concern of the questionnaire designers for the testees, which helps to create a good questionnaire climate and to increase motivation for completing the questionnaire."}, "time_dimension": {"answer": "Present", "rationale": "The entry describes a customary behaviour of the testee, which likes to look closely at things that have not been seen. This behaviour is currently being carried out, so it is judged as \"now\"."}, "sentence_type": "Affirmative declarative sentence", "pos": {"nouns": {"I": 1, "Something": 1, "Situation": 1}, "verbs": {"Yeah": 1, "Observation": 1, "Roger that": 1, "See": 1}, "adjectives": {"Careful": 1, "Details": 1}, "numerals": {"2": 1}}}, {"content": "I like the story of change and imagination.", "word_count": 16, "label": "", "sentence_structure": "Subject鈥搗erb鈥揷omplement", "subjective_objective": {"answer": "Subjective", "rationale": "Like being patient subjective."}, "sentence_sentiment": {"answer": "Positive", "rationale": "Because \"a story that likes to change and to be imaginative\" is a positive attitude or quality."}, "language_style": {"answer": "Tactful and warm", "rationale": "The language style of the entry is euphemistic, because it does not directly ask or order the other to answer questions, but rather to share their views in an invitation. At the same time, the words \"changed\" and \"imaginative\" convey positive, curious feelings that are more likely to resonate with the interviewee and to engage."}, "time_dimension": {"answer": "Present", "rationale": "The entry describes the current preferences of the testees, which are multi-dimensional and imaginative. The \"like\" in it suggests that it is a subjective feeling or attitude, while \"changed and imaginative stories\" are its specific manifestations. So the time dimension of the article is now."}, "sentence_type": "Affirmative declarative sentence", "pos": {"nouns": {"I": 1, "Story": 1}, "verbs": {"Yeah": 1}, "adjectives": {"Change Multiple": 1, "Imagination": 1}}}, {"content": "I like to draw pictures of other people's works.", "word_count": 13, "label": "", "sentence_structure": "Subject鈥搗erb鈥搊bject", "subjective_objective": {"answer": "Subjective", "rationale": "Because it's a subjective idea and a tendency to draw people."}, "sentence_sentiment": {"answer": "Negative", "rationale": "Because the \"Present Works\" act in the entry may indicate that the interviewees lack innovative thinking and independence."}, "language_style": {"answer": "Calm and direct", "rationale": "The language style of the entry is relatively calm and straightforward, and it clearly expresses the fact that the person surveyed likes to draw the work of others."}, "time_dimension": {"answer": "Past", "rationale": "The time dimension needs to be determined in conjunction with context and other issues."}, "sentence_type": "Affirmative declarative sentence", "pos": {"nouns": {"Draw": 1, "Works": 1}, "verbs": {"Yeah": 1, "Not bad": 1}, "adverbs": {"Time": 1, "I": 1}, "classifiers": {"individual": 1}}}, {"content": "I like to make fun of all kinds of waste, such as old newspapers, old calendars and old canisters.", "word_count": 29, "label": "", "sentence_structure": "Subject鈥搗erb鈥搊bject", "subjective_objective": {"answer": "Subjective", "rationale": "Because \"like\" is the emotional attitude and will of individuals and is subjective. While waste use is also a form of behaviour, the focus on this issue is the degree of preference of the respondents for such behaviour, so the subjective element is more important."}, "sentence_sentiment": {"answer": "Positive", "rationale": "Because it describes an innovative and environmentally friendly behaviour that can bring about positive emotional experiences."}, "language_style": {"answer": "Tactful and warm", "rationale": "The language style of the entry is more euphemistic, not directly expressing the preference for waste or encouraging people to do it, but rather raising an interesting question in a moderate way that gives the other side space for reflection and participation. This way makes people more comfortable and more open to dialogue."}, "time_dimension": {"answer": "Present", "rationale": ""}, "sentence_type": "Affirmative declarative sentence", "pos": {"nouns": {"Yeah": 1, "Old paper": 1, "Old Calendar": 1, "Old canton box": 1, "Waste": 1, "Something": 1}, "verbs": {"Utilization": 1, "Do it": 1, "Done": 1}, "numerals": {"5": 1}}}, {"content": "I like to fantasize about something I want to know or want to do.", "word_count": 16, "label": "", "sentence_structure": "Subject鈥搗erb鈥搊bject", "subjective_objective": {"answer": "Subjective", "rationale": "Because fantasies are the personal feelings and attitudes of the patient, they are subjective."}, "sentence_sentiment": {"answer": "Neutral", "rationale": "Because the entry describes a person ' s hobby and does not contain any emotional colour, it is therefore neutral."}, "language_style": {"answer": "Tactful and warm", "rationale": "The language style of the entry is more euphemistic and gentle, and it asks the other side whether it likes to imagine things, without direct orders or strong words. This expression is more likely to trigger resonance and positive responses from the respondents."}, "time_dimension": {"answer": "Present", "rationale": ""}, "sentence_type": "Affirmative declarative sentence", "pos": {"nouns": {"Fantasy": 1, "Yes": 2}, "verbs": {"Yeah": 1, "Yes": 2, "Do it": 1}, "numerals": {"Six": 1}, "pronouns": {"I": 1}}}, {"content": "If things cannot be finished at once, I will continue to try until they are completed.", "word_count": 22, "label": "", "sentence_structure": "Subject鈥搗erb鈥搊bject", "subjective_objective": {"answer": "Subjective", "rationale": "Because this is the patient ' s subjective attitude and conviction."}, "sentence_sentiment": {"answer": "Positive", "rationale": "Because the act of \"continuing to finish\" shows a positive tenacity and determination."}, "language_style": {"answer": "Calm and direct", "rationale": "The entry presents in a direct and clear manner the attitudes and behaviour of the interviewees in the face of unfinished business, with no excessive emotional rendering or rhetorical use, and with a more calm and straightforward language style."}, "time_dimension": {"answer": "Present", "rationale": "\"will continue to try until completion\" means a customary act or attitude which may continue into the future. The temporal dimension of this article should therefore be the present."}, "sentence_type": "Affirmative declarative sentence", "pos": {"nouns": {"Things": 1, "Once": 1, "Completed": 2, "Try": 1}, "verbs": {"Yes": 1, "Go on": 1, "Completed": 1, "Try": 1, "Until": 1, "Until then": 1}, "adverbs": {"No, I can't": 1}, "numerals": {"One": 2}, "classifiers": {"Number of times": 1}, "pronouns": {"I": 1}}}, {"content": "8. I would like to refer to a variety of sources in order to gain a wide range of insights in my work.", "word_count": 26, "label": "", "sentence_structure": "Subject鈥搗erb鈥搊bject", "subjective_objective": {"answer": "Subjective", "rationale": "Because \"likes to refer to different data\" is the patient's subjective attitude."}, "sentence_sentiment": {"answer": "Positive", "rationale": "Because the purpose of the action of \"like to refer to different data\" in the entries is to obtain more knowledge and information, and it is positive."}, "language_style": {"answer": "Tactful and warm", "rationale": "This expression creates a positive, friendly atmosphere that is more easily accessible to the interviewee and trusts."}, "time_dimension": {"answer": "Present", "rationale": "The word \"time\" in \"worktime\" denotes a specific time, usually referring to the current or the latest period, and the entry is therefore a description of the current situation."}, "sentence_type": "Affirmative declarative sentence", "pos": {"nouns": {"Work": 1, "Information": 1, "Roger that": 1}, "verbs": {"Yeah": 1, "References": 1, "Okay": 1, "Do it": 1}, "adverbs": {"Different": 1, "Various aspects": 1}, "classifiers": {"Plant": 1}, "pronouns": {"I": 1}}}, {"content": "I like doing things the same way and not looking for new ways.", "word_count": 23, "label": "", "sentence_structure": "Subject鈥搗erb鈥搊bject", "subjective_objective": {"answer": "Subjective", "rationale": "Because \"like\" is a personal emotional attitude, a subjective approach."}, "sentence_sentiment": {"answer": "Negative", "rationale": "Because the attitude in the entry \"not to look for other new ways\" is negative."}, "language_style": {"answer": "Tactful and warm", "rationale": ""}, "time_dimension": {"answer": "Present", "rationale": ""}, "sentence_type": "Affirmative declarative sentence", "pos": {"nouns": {"Questionnaire": 1, "Entry": 1, "Methodology": 2, "Work": 1}, "verbs": {"Yeah": 2, "Use": 1, "Find": 1, "Do it": 1}, "adjectives": {"Same": 1, "New": 1}, "numerals": {"9": 1}, "pronouns": {"I": 1}}}, {"content": "I like to explore the truth.", "word_count": 10, "label": "", "sentence_structure": "Subject鈥搗erb鈥揷omplement", "subjective_objective": {"answer": "Subjective", "rationale": "Because \"like\" is a subjective attitude or emotion, and the search for the truth about things is the act that this attitude triggers."}, "sentence_sentiment": {"answer": "Positive", "rationale": "Because the \"like to explore the truth\" act in the entry is positive."}, "language_style": {"answer": "Tactful and warm", "rationale": "The language style of the entry is relatively euphemistic, turning the issue into an invitation-style inquiry, giving a friendly and friendly feeling, using the positive expression \"like to explore.\" At the same time, the term \"the truth of the matter\" is relatively neutral, with no overly emotional tone, making the questionnaire survey more objective and professional."}, "time_dimension": {"answer": "Present", "rationale": "The entry is a question of subjective attitudes or values of the person being tested, and the temporal dimension is now judged."}, "sentence_type": "Affirmative declarative sentence", "pos": {"nouns": {"Things": 1, "The truth": 1}, "verbs": {"Yeah": 1, "Explore": 1}, "numerals": {"10": 1}}}, {"content": "11. I like to do a lot of new things.", "word_count": 10, "label": "", "sentence_structure": "Subject鈥搗erb鈥揷omplement", "subjective_objective": {"answer": "Subjective", "rationale": "Because it is a subjective feeling and attitude to like doing many new things."}, "sentence_sentiment": {"answer": "Positive", "rationale": "Because the entry \"likes to do many new things\" expresses a positive emotional mentality of active exploration and experimentation."}, "language_style": {"answer": "Tactful and warm", "rationale": "The language style of the entry is euphemism. It raises questions in a gentle and positive way, without direct orders or a request for answers from the interviewee. Using the word \"like\" suggests that it is an optional question, which is intended to understand the personal interest and attitude of the interviewee, rather than to force them to answer specific answers. This language style usually resonates with the interviewee and makes him more willing to participate in the questionnaire."}, "time_dimension": {"answer": "Present", "rationale": "The term \"likes to do\" in this entry uses the present-day pattern and does not refer to past or future circumstances, so it can be judged as the present behaviour or state."}, "sentence_type": "Affirmative declarative sentence", "pos": {"verbs": {"Yeah": 1, "Do it": 1, "Many": 1, "Fresh": 1}, "adjectives": {"Yes": 1}, "numerals": {"11": 1}}}, {"content": "I don't like making new friends.", "word_count": 8, "label": "", "sentence_structure": "Subject鈥損redicate", "subjective_objective": {"answer": "Subjective", "rationale": "Because \"not like\" is a subjective emotional attitude."}, "sentence_sentiment": {"answer": "Negative", "rationale": "Because the entry \"not like new friends\" expresses a negative attitude."}, "language_style": {"answer": "Calm and direct", "rationale": "The entry directly asks the respondents about their attitude towards the establishment of new friendships, is not overly adour or emotional, and is more objective and neutral."}, "time_dimension": {"answer": "Present", "rationale": "Now."}, "sentence_type": "Negative declarative sentence", "pos": {"nouns": {"Questionnaire": 1, "Entry": 1, "New friends": 1}, "verbs": {"Yeah": 1, "Hand it over": 1}, "adverbs": {"not": 1}, "numerals": {"12": 1}}}, {"content": "I like to think about things that don't happen to me.", "word_count": 16, "label": "", "sentence_structure": "Subject鈥搗erb鈥揷omplement", "subjective_objective": {"answer": "Subjective", "rationale": "Because it's the patient's own subjective attitude that you like to think about."}, "sentence_sentiment": {"answer": "Neutral", "rationale": "Because the entry merely describes an act and does not express any emotional colour."}, "language_style": {"answer": "Tactful and warm", "rationale": "The language style of the entry is more euphemistic and implicit, and it does not use strong language or express views directly, but rather raises questions in a moderate and understanding manner. It makes it easier for the interviewee to feel comfortable and to share their thoughts and feelings."}, "time_dimension": {"answer": "Present", "rationale": "The entry will describe the views and expectations of the testees for the future, and the key words are \"like to think\" and \"does not happen to me\", all pointing to situations that have not yet occurred and that may occur in the future."}, "sentence_type": "Affirmative declarative sentence", "pos": {"nouns": {"Yeah": 1, "Yes": 2}, "verbs": {"Yes": 2, "Come on": 1}, "adverbs": {"Some": 1, "On me": 1}, "numerals": {"13": 1}, "classifiers": {"individual": 1}}}, {"content": "14. I like to imagine one day becoming an artist, a musician or a poet.", "word_count": 20, "label": "", "sentence_structure": "Subject鈥搗erb鈥搊bject", "subjective_objective": {"answer": "Subjective", "rationale": "Because \"like\" is a subjective expression of the patient's desire and expectation to become an artist, a musician or a poet."}, "sentence_sentiment": {"answer": "Positive", "rationale": "Because the words \"like to imagine\" and \"can be an artist, a musician or a poet\" are positive."}, "language_style": {"answer": "Tactful and warm", "rationale": "The language style of the entry is euphemism. It uses the term \"like to imagine\" to express a positive mindset and vision of the future, rather than direct orders or criticism. This expression is more likely to resonate with the respondents and to be interested in participating in the questionnaire."}, "time_dimension": {"answer": "Future", "rationale": ""}, "sentence_type": "Affirmative declarative sentence", "pos": {"nouns": {"Oh, my God": 1, "Artist": 1, "Musicians": 1, "Poet": 1}, "verbs": {"Yeah": 1, "Done": 1}, "numerals": {"14": 1}, "pronouns": {"I": 1}}}, {"content": "15. I will forget about other things because of some exciting thoughts.", "word_count": 20, "label": "", "sentence_structure": "Subject鈥搗erb鈥揷omplement", "subjective_objective": {"answer": "Subjective", "rationale": "Because excitement is a subjective emotional experience, the subject is examined from the patient ' s subjective perspective."}, "sentence_sentiment": {"answer": "Positive", "rationale": "Because the \"excited thoughts\" in the entries usually lead to positive emotions and attitudes."}, "language_style": {"answer": "Tactful and warm", "rationale": ""}, "time_dimension": {"answer": "Present", "rationale": "Keywords \"will\" and \"forgetting\" suggest that this is a current-day description of behavior, which refers to what the testee may have done in his current state. Moreover, \"some exciting thoughts\" suggest that this is a temporary, related diversion of attention in the current situation. So, the entry is mainly about the ability of the testee to control his attention and the current emotional state."}, "sentence_type": "Affirmative declarative sentence", "pos": {"nouns": {"Thinking": 1, "Yes": 1}, "verbs": {"Yes": 2, "Forget it": 1}, "adjectives": {"Excited": 1}, "numerals": {"15": 1}, "pronouns": {"I": 1}}}, {"content": "I would rather live on a space station than on Earth.", "word_count": 18, "label": "", "sentence_structure": "Subject鈥搗erb鈥搊bject", "subjective_objective": {"answer": "Subjective", "rationale": "Because the expression \"better than\" reveals the subjective attitude and preferences of the person under investigation."}, "sentence_sentiment": {"answer": "Negative", "rationale": "Because it expresses dissatisfaction with and rejection of the Earth, it is a negative emotional attitude."}, "language_style": {"answer": "Tactful and warm", "rationale": "The language style of the entry is more euphemistic, because it uses the term \"better\" to express the individual preferences and choices of the other, without directly criticizing or denying the value of the planet. At the same time, by asking the other side about his thoughts and wishes, the subject creates an open, respectful and supportive attitude that makes the interviewees more comfortable and comfortable."}, "time_dimension": {"answer": "Present", "rationale": "The entry \"I prefer\" and \"I don't want\" to indicate that it is a statement of subjective will, while the entries \"Live on the Space Station\" and \"Live on Earth\" refer to the state and environment of the day. So the time dimension of the questionnaire item is now judged."}, "sentence_type": "Affirmative declarative sentence", "pos": {"nouns": {"Space Station": 1, "Earth": 1}, "verbs": {"Life": 2, "I'd rather": 1, "I don't want to": 1}, "numerals": {"16": 1}, "pronouns": {"I": 1}}}, {"content": "17. I believe that all questions have fixed answers.", "word_count": 13, "label": "", "sentence_structure": "Subject鈥揷opula鈥損redicative", "subjective_objective": {"answer": "Subjective", "rationale": "Because \"think\" is the patient's subjective attitude."}, "sentence_sentiment": {"answer": "Negative", "rationale": "Because the idea in the entry that \"all questions have fixed answers\" is too absolute and negative."}, "language_style": {"answer": "Calm and direct", "rationale": "The entry is more calm and objective in its language style, with no excessive emotional or rhetorical style. It directly expresses the interviewer鈥檚 query about whether there are fixed answers to all questions, and is more direct and less emotional."}, "time_dimension": {"answer": "Present", "rationale": "The keyword \"thinks\" is the subjective feeling and the idea of the testee, which is in the context of the present. Moreover, the word \"yes\" indicates that the idea exists and may be of a continuing nature. The entry thus expresses the view of the testee as it is."}, "sentence_type": "Affirmative declarative sentence", "pos": {"nouns": {"Problem": 1, "Answer": 1}, "verbs": {"I think": 1, "have": 1, "Fixed": 1}, "numerals": {"17": 1}}}, {"content": "I like things different.", "word_count": 10, "label": "", "sentence_structure": "Subject鈥搗erb鈥搊bject", "subjective_objective": {"answer": "Subjective", "rationale": "Like being patient subjective."}, "sentence_sentiment": {"answer": "Neutral", "rationale": "Because the entry did not express any emotional colour, it merely stated an objective fact."}, "language_style": {"answer": "Tactful and warm", "rationale": "The entry uses a mild and encouraging expression that allows dialogue to take place by asking if the other side likes something different, rather than directly speaking or ordering. This is more personal and interactive, and can better induce the interviewee to actively participate and think."}, "time_dimension": {"answer": "Present", "rationale": ""}, "sentence_type": "Affirmative declarative sentence", "pos": {"nouns": {"Questionnaire": 1, "Entry": 1, "Things": 1}, "verbs": {"Yeah": 1, "It's different": 1}, "adjectives": {"of": 2}}}, {"content": "I often want to know what people are thinking.", "word_count": 13, "label": "", "sentence_structure": "Subject鈥搗erb鈥搊bject", "subjective_objective": {"answer": "Subjective", "rationale": "Because \"to know what other people are thinking\" is a personal will and expectation, reflects the mind of the patient, and is subjective."}, "sentence_sentiment": {"answer": "Negative", "rationale": "Because the act of \"wanting to know what other people are thinking\" in the entry may indicate that there is an excessive concern for the emotions and ideas of others, which is a negative attitude or quality."}, "language_style": {"answer": "Calm and direct", "rationale": "The entry directly expresses the subject of an idea that the other party often has an idea to know what others think, does not use excessive rhetorical or emotional rendering, and is more objective in tone."}, "time_dimension": {"answer": "Present", "rationale": ""}, "sentence_type": "Interrogative sentence", "pos": {"nouns": {"Questionnaire Entry": 1, "person": 1, "others": 1, "Thoughts": 1}, "verbs": {"I always do": 1, "Yes": 1, "I'm thinking": 1}, "adverbs": {"I always do": 1}, "numerals": {"19": 1}, "pronouns": {"I": 1}}}, {"content": "I like stories or television programmes.", "word_count": 15, "label": "", "sentence_structure": "Subject鈥搗erb鈥搊bject", "subjective_objective": {"answer": "Subjective", "rationale": "Because \"like\" is a subjective attitude that reflects the patient's level of preferences for stories or events on television."}, "sentence_sentiment": {"answer": "Positive", "rationale": "Because the \"like\" attitude in the entries is positive."}, "language_style": {"answer": "Tactful and warm", "rationale": "The entry is more euphemistic, uses an invitation tone and avoids too direct a presentation."}, "time_dimension": {"answer": "Present", "rationale": "The words \"like\" and \"typography\" in the entry are verbs expressing current attitudes and feelings, and therefore judging as now."}, "sentence_type": "Affirmative declarative sentence", "pos": {"nouns": {"Story": 1, "Television programmes": 1, "Yes": 1}, "verbs": {"Yeah": 1, "Writing": 1}, "numerals": {"20": 1}}}, {"content": "21. I like to be with friends and share my thoughts with them.", "word_count": 18, "label": "", "sentence_structure": "Subject鈥搗erb鈥搊bject", "subjective_objective": {"answer": "Subjective", "rationale": "Because \"like\" is the patient's subjective emotional attitude."}, "sentence_sentiment": {"answer": "Positive", "rationale": "The entry is a positive expression of a preference for engaging with friends and sharing ideas."}, "language_style": {"answer": "Tactful and warm", "rationale": "The words \"I like\" and \"sharing\" are used in the entries, and are positive and positive, giving people a feeling of warmth and pleasure."}, "time_dimension": {"answer": "Present", "rationale": ""}, "sentence_type": "Affirmative declarative sentence", "pos": {"nouns": {"friends": 1, "Thoughts": 1}, "verbs": {"Yeah": 1, "in": 1, "Together": 1, "Share": 1}, "numerals": {"21": 1}, "pronouns": {"I": 1}}}, {"content": "22. If the last page of a story book is torn, I will make up a story myself and add the results.", "word_count": 32, "label": "", "sentence_structure": "Subject鈥搗erb鈥搊bject", "subjective_objective": {"answer": "Subjective", "rationale": "This reflects patients ' subjective attitudes and behaviour."}, "sentence_sentiment": {"answer": "Positive", "rationale": "Because entries are proactive story-making, creative thinking, and not negative emotional."}, "language_style": {"answer": "Tactful and warm", "rationale": "The language style of the entry is more euphemistic and lively, as it does not directly ask questions or order answers, but expresses its thoughts in an invitation and sharing format and uses a number of visualized language to describe its behaviour (such as \"precognizing a story\" and \"compensating results\" )."}, "time_dimension": {"answer": "Present", "rationale": "The entry is an inquiry into the victim's understanding of the storybook and his ability to create, and the temporal dimension is judged to be the present. In it, the phrase \"if it is for...\" indicates that it is hypothetical, that is, that the tester may do so in the real world. And the key words \"tore the last page\" and \"to make a story\" point to the present context, which is a description and expression of the present situation."}, "sentence_type": "Affirmative declarative sentence", "pos": {"nouns": {"Storybook": 1, "Last Page": 1, "I": 1, "Story": 1, "Result": 1}, "verbs": {"Rip it off": 1, "Fiction": 1, "Fill": 1}, "adverbs": {"Just": 1}, "numerals": {"22": 1}, "classifiers": {"Ben": 1}}}, {"content": "23. When I grew up, I wanted to do something that nobody else had ever thought about.", "word_count": 16, "label": "", "sentence_structure": "Subject鈥搗erb鈥搊bject", "subjective_objective": {"answer": "Subjective", "rationale": "Because \"want to do something that no one else ever thought about\" is the patient's inner desire and desire, which is an expression of subjective attitudes and emotions."}, "sentence_sentiment": {"answer": "Positive", "rationale": "Because the entry \"want to do something that no one else had ever thought of\" expressed a positive, innovative, exploratory attitude."}, "language_style": {"answer": "Tactful and warm", "rationale": "This entry is an encouraging expression that guides respondents to share their aspirations and aspirations, rather than directly questioning or criticizing them. This expression is more stimulating and attractive."}, "time_dimension": {"answer": "Future", "rationale": "The words \"older\" and \"want to do\" are all about the future, and the key words are \"older\" and \"want,\" which point to what is not yet happening and what may happen in the future. The time dimension of this questionnaire is therefore judged to be the future."}, "sentence_type": "Affirmative declarative sentence", "pos": {"nouns": {"When I grew up": 1, "person": 1, "Yes": 1}, "verbs": {"Yes": 1, "Do it": 1}, "numerals": {"23": 1}, "pronouns": {"I": 1}}}, {"content": "24. The attempt at new games and activities is an interesting one.", "word_count": 16, "label": "", "sentence_structure": "Subject鈥揷opula鈥損redicative", "subjective_objective": {"answer": "Subjective", "rationale": "Because \"interesting\" is a feeling of new games and activities, and it's an individual subjective experience."}, "sentence_sentiment": {"answer": "Positive", "rationale": "Because \"Trying a new game and activity\" in the entry is an interesting thing, and it's a positive attitude."}, "language_style": {"answer": "Tactful and warm", "rationale": "The language style of the entry is more euphemistic, because it uses a tone of inquiry rather than direct orders or statements. Moreover, the term \"fun\" is used to give people a positive and positive feeling."}, "time_dimension": {"answer": "Present", "rationale": "The entry expresses a description of the current behaviour, and the keywords \"tried new games and activities\" and \"an interesting thing\" indicate that this is an ongoing act or state. Therefore, the entry should be classified as now."}, "sentence_type": "Affirmative declarative sentence", "pos": {"nouns": {"Questionnaire": 1, "Game": 1, "Activities": 1, "Yes": 1}, "verbs": {"Try": 1, "be": 1}, "adjectives": {"New": 1, "Interesting": 1}, "adverbs": {"One": 1}, "numerals": {"24": 1}}}, {"content": "I do not like being subject to too many rules.", "word_count": 11, "label": "", "sentence_structure": "Subject鈥損redicate", "subjective_objective": {"answer": "Subjective", "rationale": "Because \"not like\" is the patient's subjective feeling or attitude."}, "sentence_sentiment": {"answer": "Negative", "rationale": "Because the attitude of \"not too much rule-bound\" in the entries is negative."}, "language_style": {"answer": "Calm and direct", "rationale": "The entry presents the views of the respondents in a straightforward and clear manner, without too many rhetorical or emotional expressions, and is more objective in tone. It does not attempt to arouse the emotional resonance of the respondents or to induce them to provide more detailed or in-depth answers, but rather expresses the attitudes and positions of the respondents in a simple and clear manner. This calm and direct language style is common in psychological questionnaire studies, because it conveys the implications of the problem accurately and clearly, and helps researchers to obtain real and effective data."}, "time_dimension": {"answer": "Present", "rationale": "The entry is for the purpose of asking the respondent about the attitude and perception of the rules, and does not have information on the specific temporal dimension, and is therefore judged to be general."}, "sentence_type": "Negative declarative sentence", "pos": {"verbs": {"I don't like it": 1, "Yes": 1, "Limits": 1}, "adjectives": {"Too much": 1}, "adverbs": {"Too much": 1}}}, {"content": "26. I like to solve problems, even without the right answer.", "word_count": 19, "label": "", "sentence_structure": "Subject鈥搗erb鈥搊bject", "subjective_objective": {"answer": "Subjective", "rationale": "Because \"like\" is a subjective expression."}, "sentence_sentiment": {"answer": "Positive", "rationale": "Because the act of \"like to solve problems\" is positive, it is okay, even without the right answer, and it shows the spirit of exploration and attempt on the part of the interviewees."}, "language_style": {"answer": "Tactful and warm", "rationale": "The entry raises questions in a way that encourages and supports them, expressing appreciation and recognition of the respondent ' s ability and attitude to solve problems, and is more gentle and friendly."}, "time_dimension": {"answer": "Present", "rationale": "The entry describes a personal tendency and attitude, which is \"I like to solve the problem,\" and which is present and ongoing. Although it mentions \"even if there is no correct answer,\" it does not mean that it is a possible act in the future or an expectation of the future. So the temporal dimension is now."}, "sentence_type": "Affirmative declarative sentence", "pos": {"nouns": {"Problem": 1, "Answer": 1}, "verbs": {"Yeah": 1, "Resolve": 1, "have": 1}, "adjectives": {"Correct": 1}, "adverbs": {"Yeah": 1, "Nope": 1}, "numerals": {"26": 1}, "pronouns": {"I": 1}}}, {"content": "There are many things I would like to try in person.", "word_count": 14, "label": "", "sentence_structure": "Subject鈥搗erb鈥揷omplement", "subjective_objective": {"answer": "Subjective", "rationale": "Because \"there are many things I would like to try personally\" is a patient's description of what is inside me and what is inside me is a personal attitude and thinking, and therefore a subjective perspective."}, "sentence_sentiment": {"answer": "Positive", "rationale": "Because the phrase \"many things I would like to try\" in the entry is a positive attitude to new things and a spirit of courage to challenge."}, "language_style": {"answer": "Tactful and warm", "rationale": "The entry leads the other side to think and share its thoughts and experiences in a way that is more gentle and friendly and does not give answers or instructions directly, and that is more likely to trigger the resonance and participation of the other side."}, "time_dimension": {"answer": "Present", "rationale": "The word \"both\" in the entry indicates that the testee has the same idea of all the times past, present and future, and that there is no clear time limit for the action \"I would like to try it personally\" and therefore it can be considered as describing the current thinking and status of the testee."}, "sentence_type": "Affirmative declarative sentence", "pos": {"nouns": {"Things": 1, "Try": 1}, "verbs": {"have": 1, "Yes": 1, "Go on": 1}, "adjectives": {"Many": 1}, "numerals": {"27": 1}, "pronouns": {"I": 1}}}, {"content": "I like to sing new songs that nobody knows.", "word_count": 12, "label": "", "sentence_structure": "Subject鈥搗erb鈥搊bject", "subjective_objective": {"answer": "Subjective", "rationale": "Like singing is a subjective attitude or preference."}, "sentence_sentiment": {"answer": "Neutral", "rationale": "Because the entry merely stated a fact, it did not express any emotional tendency."}, "language_style": {"answer": "Tactful and warm", "rationale": "The entry is more personal and interactive when it is presented in a gentle and inviting tone rather than in direct statements or orders. At the same time, the article has a more relaxed and less serious or tense tone, which makes it easier to resonate with and engage the respondents."}, "time_dimension": {"answer": "Present", "rationale": "Now both \"like\" and \"sing\" in the entry are in the current state of the person being tested, in the current state of his or her behavior or state. At the same time, \"new song\" refers to a song that exists, and does not point to past or future situations. So the time dimension of this article is now."}, "sentence_type": "Affirmative declarative sentence", "pos": {"nouns": {"Yeah": 1, "Sing it": 1, "Song": 1, "person": 1, "New song": 1}, "verbs": {"Yeah": 1, "Sing it": 1}, "adjectives": {"No, I'm not": 1, "People know": 2}, "numerals": {"28": 1}}}, {"content": "29. I do not like to be heard in front of my class.", "word_count": 15, "label": "", "sentence_structure": "Subject鈥搗erb鈥揷omplement", "subjective_objective": {"answer": "Subjective", "rationale": "Because \"not like\" is a subjective feeling or attitude, not a description of objective facts. It's a subjective view of the patient's thoughts and feelings."}, "sentence_sentiment": {"answer": "Negative", "rationale": "Because the \"not like to speak in front of class\" act in the entry is negative."}, "language_style": {"answer": "Tactful and warm", "rationale": "The entry is presented in a first person's form to the interviewees, and is described in a negative way, \"not like\", which is relatively gentle and does not cause undue pressure or discomfort. At the same time, the topic \"Assertation in front of class class\" is also more common and easily resonated with the interviewees and their reflections."}, "time_dimension": {"answer": "Present", "rationale": ""}, "sentence_type": "Negative declarative sentence", "pos": {"nouns": {"Questionnaire Entry": 1, "Class": 1, "In front of the students": 1, "Observations": 1}, "verbs": {"Yeah": 1, "Publication": 1}, "adverbs": {"not": 1}, "numerals": {"29": 1}}}, {"content": "When I read novels or watch television, I like to think of myself as a character in stories.", "word_count": 24, "label": "", "sentence_structure": "Subject鈥搗erb鈥搊bject", "subjective_objective": {"answer": "Subjective", "rationale": "Because \"likes to think of yourself as a character in a story\" is a personal feeling and attitude, a patient's subjective experience."}, "sentence_sentiment": {"answer": "Positive", "rationale": "Because the behavior in the entries, \"I think of myself as the person in the story,\" has a certain imagination and creativity, and it's a more positive behavior."}, "language_style": {"answer": "Tactful and warm", "rationale": "The entry is more euphemistic and gentle, without using direct or command tone. It is more interactive and personal by asking if the other side likes to think of itself as a role in a story."}, "time_dimension": {"answer": "Present", "rationale": ""}, "sentence_type": "Affirmative declarative sentence", "pos": {"nouns": {"Novel": 1, "Television": 1, "People": 1}, "verbs": {"Read it": 1, "Yeah": 1, "Think of yourself as": 1}, "numerals": {"30": 1}, "pronouns": {"I": 1}}}, {"content": "31. I like to imagine what human life was 200 years ago.", "word_count": 14, "label": "", "sentence_structure": "Subject鈥搗erb鈥搊bject", "subjective_objective": {"answer": "Subjective", "rationale": "Because it's the patient's subjective attitude."}, "sentence_sentiment": {"answer": "Neutral", "rationale": "Because the entry does not have any emotional color."}, "language_style": {"answer": "Tactful and warm", "rationale": "The language style of the entry is more euphemistic, with the words \"I like\" beginning with the expression of personal interests and \"the situation of human life 200 years ago\" instead of \"the situation of human life\" as a direct questioner, and more gentle."}, "time_dimension": {"answer": "Past", "rationale": "The entry describes the past (200 years ago) human life in a way that is both a hobby and an imagination and therefore can be judged as \"the past\"."}, "sentence_type": "Affirmative declarative sentence", "pos": {"nouns": {"Fantasy": 1, "200 years ago": 1, "Humans": 1, "Situation": 1}, "verbs": {"Yeah": 1, "Life": 1}, "numerals": {"31": 1, "200": 1}}}, {"content": "32. I often wanted to write a new song for myself.", "word_count": 10, "label": "", "sentence_structure": "Subject鈥搗erb鈥揷omplement", "subjective_objective": {"answer": "Subjective", "rationale": "Because \"want\" is the patient's subjective idea."}, "sentence_sentiment": {"answer": "Positive", "rationale": "Because the \"often wanting to make a new song\" in the entry is positive."}, "language_style": {"answer": "Tactful and warm", "rationale": "The entry uses a more euphemistic formulation that opens the subject by asking if the other party has ever had an idea of creating a song, without directly expressing one鈥檚 views or attitudes. This tone is more intimate and interactive, making it easier for the interviewees to accept and share their ideas."}, "time_dimension": {"answer": "Present", "rationale": ""}, "sentence_type": "Affirmative declarative sentence", "pos": {"nouns": {"Questionnaire Entry": 1, "First": 1, "New song": 1}, "verbs": {"Yes": 1, "Part": 1}, "adverbs": {"Regular": 1}, "numerals": {"32": 1}, "pronouns": {"self": 1}}}, {"content": "I like to flip over and rewind. Look what's in there.", "word_count": 18, "label": "", "sentence_structure": "Subject鈥搗erb鈥搊bject", "subjective_objective": {"answer": "Subjective", "rationale": "Because it's the patient's subjective attitude and behavior that likes to go over and over."}, "sentence_sentiment": {"answer": "Neutral", "rationale": "Because the entry is merely a description of the individual ' s behaviour and does not express positive or negative emotional sentiments."}, "language_style": {"answer": "Tactful and warm", "rationale": "The entry is more adept and interactive when it is spoken in a gentle and inviting manner, rather than by direct statements or orders."}, "time_dimension": {"answer": "Present", "rationale": "According to the sentence, \"like to turn over the cabinet\" and \"see it\" it can be seen as a description of the behaviour or habit of the person being tested, with no clear time dimension. So it can be judged now."}, "sentence_type": "Affirmative declarative sentence", "pos": {"nouns": {"Box": 1, "The cabinet": 1, "Something": 1}, "verbs": {"Yeah": 1, "Flip": 1, "Look": 2, "have": 1, "in": 1, "Inside": 1}, "adverbs": {"Down there": 1, "Some": 1}, "numerals": {"33": 1}}}, {"content": "34. I like to change the colour and shape of things when drawing.", "word_count": 19, "label": "", "sentence_structure": "Subject鈥搗erb鈥揷omplement", "subjective_objective": {"answer": "Subjective", "rationale": "Because \"like\" is patient's subjective attitude."}, "sentence_sentiment": {"answer": "Positive", "rationale": "Because the \"I love changing colours and shapes\" act in the entries is positive and shows a love for and creativity in drawing work."}, "language_style": {"answer": "Tactful and warm", "rationale": "The entry uses the first person's pronoun, \"I\" , which expresses personal feelings and preferences, and a more friendly and moderate language style. In addition, descriptive terms are used to emphasize the extent of the problem, making it easier for respondents to understand the intentions of the question. This expression is more interactive and personal and appropriate for psychological questionnaire research."}, "time_dimension": {"answer": "Present", "rationale": "This questionnaire entry asks the testee about his behaviour or preferences in the context of his drawing activities, and the keyword \"I like\" means that it is a state of the moment or a customary behaviour, and it is therefore judged as now."}, "sentence_type": "Affirmative declarative sentence", "pos": {"nouns": {"Draw": 1, "Colour": 1, "Shape": 1}, "verbs": {"Yeah": 1, "Change": 1}, "adjectives": {"Various": 1, "of": 2}, "numerals": {"34": 1}, "pronouns": {"I": 1}}}, {"content": "I am not sure that I am right about everything.", "word_count": 16, "label": "", "sentence_structure": "Subject鈥搗erb鈥揷omplement", "subjective_objective": {"answer": "Subjective", "rationale": "Because \"I'm not sure\" is a subjective feeling and attitude that indicates that patients have doubts and uncertainties about what they think about themselves. In this case, it is the subjective thinking of the patients."}, "sentence_sentiment": {"answer": "Negative", "rationale": "Because the \"uncertain\" in the entry indicates the author's hesitation and unease, which is a negative emotional attitude."}, "language_style": {"answer": "Calm and direct", "rationale": "The language style of the entry is more calm and straightforward, and there is no excessive emotional or rhetorical style. It asks the other side whether it has doubts about itself and is not confident. This expression helps the person under investigation to express his thoughts and feelings more honestly.\""}, "time_dimension": {"answer": "Present", "rationale": "The description of the keyword \"determinate\" and \"view\" indicates the current feelings and mindset of the subject and therefore the present is judged."}, "sentence_type": "Negative declarative sentence", "pos": {"nouns": {"Questionnaire Entry": 1, "The way things are going": 1}, "verbs": {"Sure": 1, "to": 2}, "adverbs": {"not": 1, "Both": 1}, "numerals": {"35": 1}, "pronouns": {"I": 1}}}, {"content": "36. This is an interesting approach to guessing one thing and then to see if it is right.", "word_count": 27, "label": "", "sentence_structure": "Subject鈥揷opula鈥損redicative", "subjective_objective": {"answer": "Subjective", "rationale": "This is because the patient ' s perception and feelings of such behaviour are subjective."}, "sentence_sentiment": {"answer": "Positive", "rationale": "Because the expression \"close\" in the entry is positive."}, "language_style": {"answer": "Tactful and warm", "rationale": "The entry presents the \"guess\" method in a light-speaker way, and it says it's interesting. It's a language style that makes people feel more friendly and close, more acceptable and willing to participate."}, "time_dimension": {"answer": "Present", "rationale": "The keyword \"guess\" and \"then guesses\" and then whether it's right\" describes the person being tested for the current act or habit. Thus, the temporal dimension of the article is now judged."}, "sentence_type": "Affirmative declarative sentence", "pos": {"nouns": {"Things": 1, "Methodology": 1, "Fun": 1}, "verbs": {"Guess": 2, "Look": 2, "to": 1, "be": 1, "First": 1, "Again": 1, "Interesting": 1}, "adjectives": {"very": 1}, "numerals": {"36": 1}}}, {"content": "37. Play games like guess puzzles was interesting, because I wanted to know what the results were.", "word_count": 21, "label": "", "sentence_structure": "Subject鈥揷opula鈥損redicative", "subjective_objective": {"answer": "Subjective", "rationale": "Plays like guesswork and wants to know what the result is is subjective."}, "sentence_sentiment": {"answer": "Positive", "rationale": "Because \"uninterested\" and \"wanting to know what you're doing\" are words that express positive feelings."}, "language_style": {"answer": "Tactful and warm", "rationale": "The entry raises questions in a simple and friendly manner and expresses curiosity about the outcome of the game."}, "time_dimension": {"answer": "Present", "rationale": ""}, "sentence_type": "Affirmative declarative sentence", "pos": {"nouns": {"Game": 1, "A guess": 1, "Result": 1}, "verbs": {"Play": 1, "Yes": 2}, "adjectives": {"Interesting": 1}, "numerals": {"37": 1}, "pronouns": {"I": 1}}}, {"content": "I'm interested in the machine, and I want to know what it looks like and how it's spinning.", "word_count": 30, "label": "", "sentence_structure": "Subject鈥搗erb鈥搊bject", "subjective_objective": {"answer": "Subjective", "rationale": "Because the sentence is a subjective attitude, which expresses the patient's interest in and curiosity about the machine."}, "sentence_sentiment": {"answer": "Positive", "rationale": "Because the entries express interest and curiosity in machines, and are positive emotional attitudes."}, "language_style": {"answer": "Calm and direct", "rationale": "The entry is presented in a direct way, without excessive rhetorical or emotional colours. It directly expresses the interest and curiosity of the subject in the machine, is more concise and is of a calm and direct language style."}, "time_dimension": {"answer": "Present", "rationale": "In the entry, \"I\" shows the attitude and feelings of the subject, and the words \"interested\" and \"wanted to know\" indicate the current state and behaviour of the subject. Thus, the temporal dimension of the purpose of the article is now judged."}, "sentence_type": "Interrogative sentence", "pos": {"nouns": {"Interest": 1, "Machine": 2, "Inside": 1, "Look": 1, "Turn": 1}, "verbs": {"to": 1, "Yes": 1, "Interested": 1, "be": 2}, "adjectives": {"very": 1}, "numerals": {"38": 1}, "pronouns": {"it": 2}}}, {"content": "I like toys that can be broken.", "word_count": 12, "label": "", "sentence_structure": "Subject鈥搗erb鈥揷omplement", "subjective_objective": {"answer": "Subjective", "rationale": "Because \"like\" is the patient's personal feeling and attitude, and it's the subjective aspect of the examination."}, "sentence_sentiment": {"answer": "Positive", "rationale": "Because the entries \"like\" and \"toys that can be broken apart\" express a positive attitude and appreciation."}, "language_style": {"answer": "Tactful and warm", "rationale": "The entry uses a more compassionate and gentle language style, which leads the respondents to share their preferences in a question-and-answer manner, rather than to direct statements or orders. Moreover, \"discovery\" makes the issue more interesting and attractive and more likely to attract the interest and participation of the respondents."}, "time_dimension": {"answer": "Present", "rationale": "By the point of time and presentation given under the title, \"I\" uses the first person's singular and expresses a preference or preference, so the phrase describes the current situation and is the current state of affairs."}, "sentence_type": "Affirmative declarative sentence", "pos": {"nouns": {"I like it": 1, "Toys": 1}, "verbs": {"Open it up and play": 1}, "numerals": {"39": 1}}}, {"content": "I like to think of new ideas, that is, it doesn't matter if they are not used.", "word_count": 18, "label": "", "sentence_structure": "Subject鈥搗erb鈥搊bject", "subjective_objective": {"answer": "Subjective", "rationale": "Because \"like to think of new ideas\" is patient's subjective attitude."}, "sentence_sentiment": {"answer": "Positive", "rationale": "Because \"likes to think of new ideas\" in the entry is a positive attitude of active exploration and innovation."}, "language_style": {"answer": "Tactful and warm", "rationale": "The entry raises questions in a question-and-answer tone and adds a personal emotional colour to the expression, and the word \"likes\" gives a positive and optimistic feeling. Moreover, the entire sentence does not appear to be too direct or hard, and is more balanced in tone, so it can be considered as a euphemism."}, "time_dimension": {"answer": "Present", "rationale": "The keywords \"like\" and \"want\" mean the act that is now being performed, while the phrase \"that is, it doesn't matter whether it is being used\" indicates that it is not for practical purposes, but for personal interests or preferences. So, the entry is asking the person who is being tested about what he thinks and feels."}, "sentence_type": "Affirmative declarative sentence", "pos": {"nouns": {"Ideas": 1}, "verbs": {"Yeah": 1, "Yes": 1, "Use": 1, "Here": 1, "None": 1, "So-called": 1}, "adjectives": {"New": 1}, "adverbs": {"That's": 1, "Yeah": 1}, "numerals": {"40": 1}, "pronouns": {"I": 1}}}, {"content": "41. A good article should contain many different views or views.", "word_count": 20, "label": "", "sentence_structure": "Subject鈥搗erb鈥搊bject", "subjective_objective": {"answer": "Subjective", "rationale": "The content of this questionnaire entry is a subjective view and understanding of the individual of a \"good article\" and is therefore a subjective examination."}, "sentence_sentiment": {"answer": "Neutral", "rationale": "As the entry contains a factual statement, it does not involve any emotional colour."}, "language_style": {"answer": "Tactful and warm", "rationale": "The entry uses suggested language to ask if the interviewees believe that a good article should contain many different opinions or views."}, "time_dimension": {"answer": "Present", "rationale": ""}, "sentence_type": "Affirmative declarative sentence", "pos": {"nouns": {"Title": 1, "Articles": 1, "Observations": 1, "Views": 1}, "verbs": {"Reactions": 1, "Ham": 1, "have": 1}, "adjectives": {"Okay": 1, "Different": 1}, "numerals": {"41": 1}}}, {"content": "42. It was an exciting thing to find answers to questions that might arise in the future.", "word_count": 22, "label": "", "sentence_structure": "Subject鈥揷opula鈥損redicative", "subjective_objective": {"answer": "Subjective", "rationale": "Because \"excited\" is a subjective feeling or emotional reaction, it is a personal experience and feeling, and therefore a subjective perspective."}, "sentence_sentiment": {"answer": "Positive", "rationale": "Because the \"excited\" description is positive emotional."}, "language_style": {"answer": "Tactful and warm", "rationale": "The expression \"excited things\" in this entry gives a positive and emotional sense of encouragement and inspiration. Moreover, the question takes the form of a question sentence, which is more stimulating to the respondents' thinking and expression, and is more euphemismal."}, "time_dimension": {"answer": "Future", "rationale": "The entry contains visions and expectations for the future and refers to \"problems that may arise in the future\", which point to situations that have not yet occurred and that may arise in the future. The temporal dimension of this purpose is therefore judged to be the future."}, "sentence_type": "Affirmative declarative sentence", "pos": {"nouns": {"Problem": 1, "Answer": 1, "Things": 1}, "verbs": {"Find": 1, "be": 1, "You're": 1}, "adjectives": {"Maybe": 1, "Future": 1, "Excited": 1}, "numerals": {"42": 1}}}, {"content": "43. I like to try new things, just to know what will happen.", "word_count": 24, "label": "", "sentence_structure": "Subject鈥搗erb鈥揷omplement", "subjective_objective": {"answer": "Subjective", "rationale": "Because the ideas of \"like to try something new\" and \"to know what will happen\" are subjective."}, "sentence_sentiment": {"answer": "Positive", "rationale": "Because the \"like to try something new\" act in the entry is positive, and the \"to know what's going to happen\" purpose is positive."}, "language_style": {"answer": "Calm and direct", "rationale": "The basis for the judgement: the language of the article is very simple, and it does not use too many rhetorical or emotional expressions. It asks the interviewee whether he likes to try something new and explains its purpose simply to know what the result is. This language style gives people a sense of being realistic, unrefuted, and therefore of being a straightforward type of calm."}, "time_dimension": {"answer": "Present", "rationale": "The keywords \"like to try something new\" and \"to know what's going to happen\" indicate that the testee is acting or habitually, and therefore judging now."}, "sentence_type": "Affirmative declarative sentence", "pos": {"nouns": {"Things": 2, "Purpose": 1, "Result": 1}, "verbs": {"Yeah": 1, "Try": 1, "Yes": 1}, "adverbs": {"Just": 1}, "numerals": {"43": 1}, "pronouns": {"I": 1}}}, {"content": "44. When playing games, I am usually interested in participating, not in winning or losing.", "word_count": 19, "label": "", "sentence_structure": "Subject鈥搗erb鈥搊bject", "subjective_objective": {"answer": "Subjective", "rationale": "Because \"interested\" and \"not caring about winning\" are patients' thoughts or attitudes, subjective."}, "sentence_sentiment": {"answer": "Positive", "rationale": "Because the attitude and behaviour of \"intribution\" in the entry is positive."}, "language_style": {"answer": "Tactful and warm", "rationale": "The language style of the entry is more moderate, not directly indicating the attitude and motivation of the person surveyed in playing the game, but by asking questions to express himself. This expression is more respectful of the person interviewed and more accessible to real and effective information."}, "time_dimension": {"answer": "Present", "rationale": "The phrase \"usually\" denotes a normal or customary behaviour, while \"playing games\" indicates that this is an ongoing scene. It therefore makes it clear that the sentence describes the current situation."}, "sentence_type": "Affirmative declarative sentence", "pos": {"nouns": {"Game": 1, "Win or lose": 1}, "verbs": {"Play": 1, "Participation": 1, "I care": 1}, "adjectives": {"have": 1, "Usually": 1}, "adverbs": {"And": 1, "not": 1}, "pronouns": {"I": 1}}}, {"content": "I like to think about things that others often talk about.", "word_count": 15, "label": "", "sentence_structure": "Subject鈥搗erb鈥搊bject", "subjective_objective": {"answer": "Subjective", "rationale": "Because it is the patient ' s attitude to thinking or emotional experience of something, it is the patient ' s subjective feeling and perspective."}, "sentence_sentiment": {"answer": "Neutral", "rationale": "Because the entry only describes an act and does not express positive or negative emotional colours."}, "language_style": {"answer": "Tactful and warm", "rationale": "The language style of the entry is more moderate. It is a conversation in a way that is asked, not a direct statement or an order. This expression is more personal and interactive, and it triggers positive responses and reflections from the respondents. And \"like to think about things that others often talk about\" is also a neutral issue, and does not put pressure or discomfort on the respondents."}, "time_dimension": {"answer": "Past", "rationale": "The words \"like\" and \"often talk about\" in the entry are descriptions of past memories or experiences, and it can be judged whether the subjects were considered in the past."}, "sentence_type": "Affirmative declarative sentence", "pos": {"nouns": {"Things": 1, "Yeah": 1, "person": 1}, "verbs": {"Yes": 2}, "adverbs": {"Often": 1}, "numerals": {"45": 1}}}, {"content": "When I saw a picture of a stranger, I liked to guess what kind of person he was.", "word_count": 28, "label": "", "sentence_structure": "Subject鈥搗erb鈥搊bject", "subjective_objective": {"answer": "Subjective", "rationale": "Because it's like guessing that strangers are a subjective attitude and feeling of a human being."}, "sentence_sentiment": {"answer": "Neutral", "rationale": "Because the entry describes only a habit of the person under investigation and does not involve emotional colour."}, "language_style": {"answer": "Tactful and warm", "rationale": "The language style of the entry is euphemistic, because it uses a way of asking and understanding, not directly speaking or ordering. By using the word \"like\" to express interest and curiosity about the act, instead of explicitly asking or directing the interviewee to do so, the interviewee feels more comfortable and respectful. At the same time, the article is more gentle, without using too strong or harsh language, making the overall atmosphere of the questionnaire more friendly and friendly."}, "time_dimension": {"answer": "Present", "rationale": "The entry indicates that the testee is \"like to guess what kind of person he is\" at the moment, and thus judges now."}, "sentence_type": "Affirmative declarative sentence", "pos": {"nouns": {"Questionnaire": 1, "Entry": 1, "Chang": 1, "Stranger": 2, "Photos": 1, "I": 1, "person": 1}, "verbs": {"Yeah": 2, "Go on": 1, "Guess": 1, "be": 1, "How's it going": 1}, "adverbs": {"When": 1, "Strange": 1}, "numerals": {"46": 1}, "classifiers": {"individual": 1}}}, {"content": "47. I like to read books and magazines, but just for a brief overview.", "word_count": 19, "label": "", "sentence_structure": "Subject鈥搗erb鈥搊bject", "subjective_objective": {"answer": "Subjective", "rationale": "It's subjective. Because it's the patient's attitude to read books and magazines. They just want to get a general idea, but it's still their personal preference and preference."}, "sentence_sentiment": {"answer": "Neutral", "rationale": "Because the entry does not have any emotional color."}, "language_style": {"answer": "Tactful and warm", "rationale": "The entry is more euphemistic in language style and with some courtesy and respect, expresses the other side's interests by using the words \"like\" and \"generally understand\" and does not directly order or compel the other side to read in some way, so it can be judged as a soft language style."}, "time_dimension": {"answer": "Present", "rationale": "The entry uses the terms \"like\" and \"intention\" , both of which indicate the current state of the person being tested, and do not address past or future behaviour. Thus, the temporal dimension of the article is now."}, "sentence_type": "Affirmative declarative sentence", "pos": {"nouns": {"I like it": 1, "Books": 1, "Magazines": 1, "General": 1, "Roger that": 1}, "verbs": {"Browse": 1, "Yes": 1, "have": 1}, "adverbs": {"and": 1, "Just": 2}, "numerals": {"47": 1}}}, {"content": "48. I do not like to look into the various reasons why things happen.", "word_count": 15, "label": "", "sentence_structure": "Subject鈥搗erb鈥揷omplement", "subjective_objective": {"answer": "Subjective", "rationale": "Because words like \"like\" and \"not like\" represent the individual feelings, attitudes and views of the patient, and are subjective."}, "sentence_sentiment": {"answer": "Negative", "rationale": "Because the attitude of the entry is \"not like,\" and it expresses a negative emotion."}, "language_style": {"answer": "Calm and direct", "rationale": "The entry is more directly written, is not over-modified or emotionally retrofitted, and expresses the questioner鈥檚 attitude to the search for the reason. Thus, its language style can be described as 鈥渃ooling straight鈥?"}, "time_dimension": {"answer": "Present", "rationale": "Now the key words are \"not like\" and \"prospecting,\" which shows that the person tested has no interest in or does not like to explore the various reasons for what happened, and that is an act that describes the current emotional attitude."}, "sentence_type": "Negative declarative sentence", "pos": {"nouns": {"Things": 1, "Come on": 1}, "verbs": {"I don't like it": 1, "Search": 1}, "adjectives": {"Various": 1}, "adverbs": {"not": 1}, "numerals": {"48": 1}}}, {"content": "49. I like to ask questions that others do not think of.", "word_count": 15, "label": "", "sentence_structure": "Subject鈥搗erb鈥搊bject", "subjective_objective": {"answer": "Subjective", "rationale": "Because \"like\" is a subjective attitude or feeling, it is objective to ask whether the idea of others is taken into account and to draw conclusions through observation and analysis."}, "sentence_sentiment": {"answer": "Neutral", "rationale": "Basis of judgement: The entry does not express a positive or negative emotional tendency, but rather describes an act."}, "language_style": {"answer": "Tactful and warm", "rationale": "The entry is more personal and interactive when it is presented in a gentle and inviting tone, asking whether the counterparty is interested in asking questions that others do not think of, and not directly speaking or ordering."}, "time_dimension": {"answer": "Present", "rationale": ""}, "sentence_type": "Affirmative declarative sentence", "pos": {"nouns": {"Problem": 2, "person": 1}, "verbs": {"Yeah": 1, "Question": 1, "I thought": 1}, "adjectives": {"others": 1}, "numerals": {"49": 1}, "pronouns": {"Some": 1}}}, {"content": "50. I always like to do many interesting things, both at home and in school.", "word_count": 22, "label": "", "sentence_structure": "Subject鈥搗erb鈥搊bject", "subjective_objective": {"answer": "Subjective", "rationale": "Because \"likes to do many interesting things\" is the patient's subjective attitude. Although what happens at home or in school is objective, the patient's preferences and feelings are subjective."}, "sentence_sentiment": {"answer": "Positive", "rationale": "Because the act of \"like to do many interesting things\" expresses positive attitudes and emotions."}, "language_style": {"answer": "Tactful and warm", "rationale": "The entry is more euphemistic and friendly, using more positive language, and without strong direct statements or orders. At the same time, the issue is an invitation to the researcher to express his interest and concern for the interviewee鈥檚 preferences, which enhances the interaction of the questionnaire with the interviewee and facilitates the receipt of real and effective feedback."}, "time_dimension": {"answer": "Present", "rationale": "The word \"always\" denotes a regular act, and \"likes to do many interesting things\" is a customary or normal description, so the entry asks about the current state and orientation of the testee."}, "sentence_type": "Affirmative declarative sentence", "pos": {"nouns": {"Home": 1, "School": 1, "Yes": 1}, "verbs": {"Yeah": 1, "Do it": 1, "Total": 1}, "adjectives": {"Many": 1, "Interesting": 1}, "adverbs": {"Always": 1, "Whatever": 1}, "numerals": {"50": 1}}}]}

{"questionnaire_title": "Emotional Social Solo Survey (ESLI)", "questionnaire_category": "Mental health", "item_count": 30, "emotion_shift_overall": "No obvious trend", "language_style_overall": "Calm and direct", "items": [{"content": "1. The situation in life, I have no best friend.", "word_count": 11, "label": "", "sentence_structure": "Subject鈥揷opula鈥損redicative", "subjective_objective": {"answer": "Subjective", "rationale": "Because \"no best friend\" is the patient's subjective assessment and perception of his personal relationship."}, "sentence_sentiment": {"answer": "Neutral", "rationale": "Because the entry does not have any emotional color."}, "language_style": {"answer": "Calm and direct", "rationale": "The entry uses straightforward language expressions, which do not use too much qualifying or emotional language, but rather directly raises a clear question of whether the interviewee can answer directly whether he or she has a good friend in his or her life. This expression reduces the psychological burden on the interviewee and makes him or her more willing to respond honestly. At the same time, the calmer and direct language style makes the questionnaire more objective and scientific, and allows for better data collection."}, "time_dimension": {"answer": "Present", "rationale": ""}, "sentence_type": "Negative declarative sentence", "pos": {"nouns": {"Life": 1, "Status": 1, "Friend": 1}, "numerals": {"I": 1}}}, {"content": "2. The situation in life, with others, is that they want to take advantage of me.", "word_count": 20, "label": "", "sentence_structure": "Subject鈥搗erb鈥揷omplement", "subjective_objective": {"answer": "Objective", "rationale": "This is because the patient is describing a situation or fact that has occurred in him/her. Even if the patient feels that situation, it is still an objective description, as it is not determined by the patient鈥檚 subjective opinion."}, "sentence_sentiment": {"answer": "Negative", "rationale": "Because the description of \"who wants to take advantage of me\" in the entry is negative."}, "language_style": {"answer": "Tactful and warm", "rationale": "The entry is more personal and interactive when it is spoken in a gentle and inviting manner, rather than in direct statements or orders. And the expression \"takes advantage of me\" is more euphemism and avoids too direct acrimony."}, "time_dimension": {"answer": "Present", "rationale": "The \"life situation\" indicates the current living conditions of the subject, \"with others\" and \"with others to take advantage of me\" are all describing what is happening now. So the time dimension of this article is now."}, "sentence_type": "Interrogative sentence", "pos": {"nouns": {"Status": 1, "person": 1, "Dow": 1, "Jim": 1, "I": 1, "Cheap": 1}, "verbs": {"Call": 1, "Together": 1, "Yes": 1, "Jim": 1}, "adverbs": {"Medium": 1, "Call": 1}, "pronouns": {"He's": 1, "of": 2}}}, {"content": "3. Life conditions, I have no partner (or male/girlfriend)", "word_count": 16, "label": "", "sentence_structure": "Subject鈥揷opula鈥損redicative", "subjective_objective": {"answer": "Objective", "rationale": "Because the existence of a partner is an objective fact, not a subjective idea of the patient."}, "sentence_sentiment": {"answer": "Neutral", "rationale": "Because the entry merely describes a fact and does not express any emotional colour."}, "language_style": {"answer": "Tactful and warm", "rationale": "The language style of the entry is more euphemistic and moderate, by using the more objective description of \"no partner\" to avoid directly involving the emotional state of the other, leaving the person surveyed less embarrassed or unwell in answering the question. At the same time, the expression \"status in life\" is more neutral and open, providing the person surveyed with a relatively free space to answer."}, "time_dimension": {"answer": "Past", "rationale": "The entry asks the testee about his past life, and the time dimension is the past. The keyword \"no partner\" indicates that this is a description of the past, not the future expectations or the present state."}, "sentence_type": "Negative declarative sentence", "pos": {"nouns": {"Status": 1, "Partner": 1, "Men": 1, "Girlfriend": 1}, "adverbs": {"No, I'm not": 1}, "numerals": {"3": 1}, "pronouns": {"I": 1}}}, {"content": "I don't want to burden others with my own difficulties.", "word_count": 24, "label": "", "sentence_structure": "Subject鈥搗erb鈥搊bject", "subjective_objective": {"answer": "Subjective", "rationale": "It is the subjective attitude of the patient to be reluctant to make others feel burdened by their own difficulties."}, "sentence_sentiment": {"answer": "Neutral", "rationale": "Since the entry does not express positive or negative feelings, attitudes, behaviour, purpose or quality, it merely describes a situation."}, "language_style": {"answer": "Tactful and warm", "rationale": "The entry expresses a gentle and understanding expression of concern about what others feel, rather than a direct accusation or order. This expression is more friendly and interactive, and is more acceptable and willing to respond to the problem."}, "time_dimension": {"answer": "Present", "rationale": ""}, "sentence_type": "Negative declarative sentence", "pos": {"nouns": {"Life": 1, "Status": 1, "self": 2, "Difficulties": 1, "others": 1, "Burden": 1}, "verbs": {"I don't want to": 1, "Because": 1, "Jean": 1}, "numerals": {"4": 1}, "pronouns": {"I": 1}}}, {"content": "The situation in life, no one in my life is dependent on me.", "word_count": 18, "label": "", "sentence_structure": "Subject鈥揷opula鈥損redicative", "subjective_objective": {"answer": "Objective", "rationale": "Because it's about the objective fact that there's no one in life who depends on you."}, "sentence_sentiment": {"answer": "Neutral", "rationale": "Because the entry does not express a clear emotional colour, it is merely a description of a fact."}, "language_style": {"answer": "Calm and direct", "rationale": "The entry directly asked the respondents about their living conditions, whether they were relied upon, in a concise and clear manner, without too much rhetoric or emotional publicity."}, "time_dimension": {"answer": "Present", "rationale": "Based on the phrase \"in my life\" and \"innocent,\" it can be inferred that the entry is to ask the testee whether he needs to rely on them in his current life, and the time dimension is now."}, "sentence_type": "Negative declarative sentence", "pos": {"nouns": {"Life": 1, "Status": 1, "person": 1, "Dependency": 1}, "verbs": {"in": 1, "be": 1}, "adverbs": {"Medium": 1}, "numerals": {"5": 1}, "pronouns": {"I": 1}}}, {"content": "I don't know what's going on in life.", "word_count": 15, "label": "", "sentence_structure": "Subject鈥搗erb鈥搊bject", "subjective_objective": {"answer": "Objective", "rationale": "Because this is a description of the past, what has happened, and is objective. Although it involves the feelings and attitudes of the patient, it is still part of the objective description."}, "sentence_sentiment": {"answer": "Negative", "rationale": "Because the phrase \"no one has a relationship with me\" in the entry is negative and indicates that there are obstacles or difficulties in socialization."}, "language_style": {"answer": "Calm and direct", "rationale": "The entry is based on a direct and clear inquiry into whether the person surveyed feels unable to establish in-depth contact with others in his daily life."}, "time_dimension": {"answer": "Present", "rationale": ""}, "sentence_type": "Negative declarative sentence", "pos": {"nouns": {"Life": 1, "Status": 1, "person": 1, "Heart": 1}, "verbs": {"Call": 2, "I'm not": 1}, "numerals": {"Any": 1, "6": 1}, "pronouns": {"I": 1}}}, {"content": "The situation in life, no one wants to know me.", "word_count": 17, "label": "", "sentence_structure": "Subject鈥搗erb鈥搊bject", "subjective_objective": {"answer": "Subjective", "rationale": "Because this phrase expresses the subjective feelings and perceptions of patients, who feel that others do not know themselves. This is not an objective fact, but a manifestation of the patient鈥檚 subjective emotions or attitudes."}, "sentence_sentiment": {"answer": "Negative", "rationale": "Because the phrase \"no one wants to know me\" in the entry is negative."}, "language_style": {"answer": "Calm and direct", "rationale": "The language style of the entry is more direct, there is no excessive rhetoric or emotional rendering, and the tone is more calm and objective, and directs the concerns of the interviewee regarding his living conditions."}, "time_dimension": {"answer": "Present", "rationale": ""}, "sentence_type": "Negative declarative sentence", "pos": {"nouns": {"Life": 2, "Status": 1, "person": 1}, "verbs": {"Yes": 1, "Roger that": 1}, "numerals": {"7": 1}, "pronouns": {"I": 1}}}, {"content": "8. The situation in life, no one wants to be affected by me.", "word_count": 20, "label": "", "sentence_structure": "Subject鈥搗erb鈥搊bject", "subjective_objective": {"answer": "Subjective", "rationale": "This is a subjective judgement of the patient about what others think of themselves."}, "sentence_sentiment": {"answer": "Negative", "rationale": "Because the description \"no one wants to be caught in my way\" in the entry expresses negative feelings and attitudes that they can put a burden on others."}, "language_style": {"answer": "Calm and direct", "rationale": "The entry is a direct and explicit question-and-answer dialogue, is not based on too much rhetorical or emotional presentation, and is more objective in tone. It is a succinct expression of concern and inquiry about whether the subject is being associated with the life of the interviewee."}, "time_dimension": {"answer": "Present", "rationale": "The keywords \"the state of life\" and \"no one wants to be burdened with me\" point to the situation that the testee is currently facing, so the entry describes the current behaviour or state."}, "sentence_type": "Negative declarative sentence", "pos": {"nouns": {"Status": 1, "Life": 1, "person": 1, "I'm in trouble": 1}, "verbs": {"Yes": 2, "Present": 1, "Copy that": 1}, "adverbs": {"No, I'm not": 1, "Medium": 1}, "numerals": {"8": 1}, "pronouns": {"I": 1}}}, {"content": "I've had a lot of time alone in my life.", "word_count": 16, "label": "", "sentence_structure": "Subject鈥搗erb鈥揷omplement", "subjective_objective": {"answer": "Subjective", "rationale": "Because \"I have a lot of time alone\" is a subjective feeling and experience of a person that reflects the patient's self-status and emotional orientation in his everyday life."}, "sentence_sentiment": {"answer": "Neutral", "rationale": "Because the entry does not express positive or negative feelings, attitudes, behaviours, aims or qualities. It describes the individual ' s unique time."}, "language_style": {"answer": "Calm and direct", "rationale": "The entry is more directly written, not using excessive emotional accoutrements or rhetorical techniques, but rather asking the interviewee how often they spend their lives alone."}, "time_dimension": {"answer": "Past", "rationale": "The entry uses \"many hours alone\" to describe the living conditions of the subjects, which is a way of expressing the past and therefore judging it as the past."}, "sentence_type": "Affirmative declarative sentence", "pos": {"nouns": {"Status": 1, "Time": 2, "Alone": 1}, "verbs": {"have": 1, "Stay": 1}, "numerals": {"9": 1}}}, {"content": "10. The situation in life, I am not a member of any association or organization", "word_count": 17, "label": "", "sentence_structure": "Subject鈥搗erb鈥揷omplement", "subjective_objective": {"answer": "Objective", "rationale": "Since the absence of membership in an association or organization is an objective fact, it is not necessary to examine it from the perspective of the patient."}, "sentence_sentiment": {"answer": "Neutral", "rationale": "Since the entry describes only a fact, it does not contain any apparent feelings, attitudes or evaluations."}, "language_style": {"answer": "Calm and direct", "rationale": "This language style is of a direct, concise type, with no excessive rhetorical and accommodative language, and a greater focus on the transmission of information and the presentation of facts. It can therefore be concluded that the language style of the entry is \"cool and direct\"."}, "time_dimension": {"answer": "Present", "rationale": "The entry is for the current living conditions of the person being tested, the keywords being \"the living conditions\" and \"no association or organization\" which are the terms describing the current state. The time dimension of the article is therefore now."}, "sentence_type": "Negative declarative sentence", "pos": {"nouns": {"Status": 1, "Associations": 1, "Organizations": 1}, "verbs": {"Add": 1, "Not": 1}, "adverbs": {"Medium": 1, "Life": 1}, "numerals": {"10": 1}, "pronouns": {"I": 1}}}, {"content": "I'm not talking to anyone today.", "word_count": 17, "label": "", "sentence_structure": "Subject鈥搗erb鈥揷omplement", "subjective_objective": {"answer": "Objective", "rationale": "Today it is over, so it is the objective result. The act is done with objectivity, and the subjective idea is examined with subjective intent."}, "sentence_sentiment": {"answer": "Negative", "rationale": "Because the entries describe situations where there is no communication with others, they express a lonely, helpless emotion, which is negative."}, "language_style": {"answer": "Calm and direct", "rationale": "The entry is in a straightforward manner asking the respondent whether he or she spoke to anyone at a given time, whether the temperature of the speech and the implications of the inquiry were excessive or not overly qualified or emotional expressions."}, "time_dimension": {"answer": "Present", "rationale": "The entry uses the time keyword \"Today\" to indicate the current behaviour or state, and therefore to judge it as now. At the same time, the description \"no one speaks\" is also given in response to the circumstances of the day, and further supports this."}, "sentence_type": "Negative declarative sentence", "pos": {"nouns": {"Life": 1, "Status": 1, "person": 2, "Word": 2}, "verbs": {"Talk": 2, "I didn't say": 1}, "adverbs": {"Both": 1, "Any": 1}, "numerals": {"Today": 1, "11": 1}, "pronouns": {"I": 1}}}, {"content": "I have no common ground with the people around me.", "word_count": 20, "label": "", "sentence_structure": "Subject鈥搗erb鈥揷omplement", "subjective_objective": {"answer": "Objective", "rationale": "Because \"I have no common subject with people around me\" is an objective fact that describes the current state of life, not the subjective factors of the patient's mind, emotions, etc."}, "sentence_sentiment": {"answer": "Negative", "rationale": "Because the description \"there is no common subject\" in the entry is negative and indicates a lack of communication and understanding with the people around it."}, "language_style": {"answer": "Calm and direct", "rationale": "The entry is more objective and straightforward in its language style, without using too many rhetorical techniques or emotional rendering. It is simple to ask questions about the relationship of the interviewee with the people around it, and whether there are communication barriers."}, "time_dimension": {"answer": "Present", "rationale": ""}, "sentence_type": "Negative declarative sentence", "pos": {"nouns": {"Status": 1, "person": 1, "Talk": 1}, "verbs": {"have": 1, "Call": 1, "Talk": 1}, "adverbs": {"Nope": 1}, "numerals": {"12": 1}, "classifiers": {"individual": 1}, "pronouns": {"I": 1}}}, {"content": "I don't tell myself much more about myself when I'm with someone else.", "word_count": 22, "label": "", "sentence_structure": "Subject鈥揷opula鈥損redicative", "subjective_objective": {"answer": "Objective", "rationale": "Because \"I don't show myself much more when I'm with someone else\" is an observation, not a patient's subjective attitude or thoughts."}, "sentence_sentiment": {"answer": "Negative", "rationale": "Because the act of \"not more revealing yourself\" in the entry is negative, it suggests that there may be some gap or guard in human relations."}, "language_style": {"answer": "Calm and direct", "rationale": "The language style of this entry is relatively cold and straightforward, and the attitude to self-expression is expressed by using words that are \"not much more \" in a clear and negative way. At the same time, the sentence does not use any rhetorical or emotional description, but rather is presented directly in a concise and clear manner, so it can be judged that the language style is cold and straightforward."}, "time_dimension": {"answer": "Present", "rationale": "The term \"status\" in \"status in life\" indicates that the problem is a description of the behaviour or mentality of the person being tested in everyday life, so that the time dimension can be judged as it is now. And the description \"I don't tell myself much more when I'm with someone else\" also shows that this is a phenomenon that may exist in the past and now, so the time dimension of this entry can be understood as past and present."}, "sentence_type": "Negative declarative sentence", "pos": {"nouns": {"Status": 1, "Life": 1, "person": 1, "Get along": 1}, "verbs": {"be": 1, "and": 1, "More": 1, "Tanlu": 1}, "adjectives": {"self": 1}, "adverbs": {"not": 1}, "numerals": {"13": 1, "1": 2}, "classifiers": {"individual": 1}, "pronouns": {"I": 1}}}, {"content": "I'm not taking any chances with social life.", "word_count": 13, "label": "", "sentence_structure": "Subject鈥搗erb鈥搊bject", "subjective_objective": {"answer": "Subjective", "rationale": "Because \"no social risk\" is a personal choice and attitude, a patient's subjective idea."}, "sentence_sentiment": {"answer": "Negative", "rationale": "Because the entry is an expression of a social evasion, which is negative."}, "language_style": {"answer": "Calm and direct", "rationale": "The entry uses a simple and clear language, asking the interviewee whether he or she is willing to take risks in certain circumstances of life."}, "time_dimension": {"answer": "Present", "rationale": ""}, "sentence_type": "Negative declarative sentence", "pos": {"nouns": {"Status": 1, "Social": 1}, "verbs": {"No, I'm not": 1}, "adverbs": {"Life": 1}, "pronouns": {"I": 1}}}, {"content": "I'm not very interesting in my life.", "word_count": 14, "label": "", "sentence_structure": "Subject鈥揷opula鈥損redicative", "subjective_objective": {"answer": "Objective", "rationale": "Because it is an observed phenomenon or fact, i.e., the perception of me by others, and not the subjective feelings or attitudes of the patient."}, "sentence_sentiment": {"answer": "Negative", "rationale": "Because the phrase \"I don't feel interesting\" in the entry is negative, which means that the person being evaluated is not attractive and interesting to others."}, "language_style": {"answer": "Tactful and warm", "rationale": "The entry is dialogueable in a question-and-answer manner and does not directly express whether the other side is interesting, gentle and friendly."}, "time_dimension": {"answer": "Past", "rationale": "\"Don't think I'm funny\" also shows that this may have been going on for some time, or it's a state of continuity. So, the entry is asking the testee about his feelings and experiences in some aspects of his past life."}, "sentence_type": "Negative declarative sentence", "pos": {"nouns": {"Status": 1, "Life": 1, "People": 1, "Fun": 1}, "verbs": {"I think": 1, "Interesting": 1}, "numerals": {"15": 1}, "pronouns": {"I": 1}}}, {"content": "I don't feel like I have a good friend.", "word_count": 13, "label": "", "sentence_structure": "Subject鈥損redicate", "subjective_objective": {"answer": "Subjective", "rationale": "Because \"no one who feels good\" is a subjective feeling and perception."}, "sentence_sentiment": {"answer": "Negative", "rationale": "Because the expression \"no one who feels good\" expresses a negative emotion and attitude that thinks that there are no real friends in life."}, "language_style": {"answer": "Calm and direct", "rationale": "The entry is based on a simple and clear query about whether the interviewees have no close friends in their lives, which is more direct and objective, without any rhetorical or emotional rendering. Moreover, the sentence is simple, easy to understand, and is in line with the language style of calm and straightforward."}, "time_dimension": {"answer": "Present", "rationale": "The keyword \"not feeling subjectively\" and \"with a good friend\" is an objective phenomenon that requires respondents to respond in the light of their own experience."}, "sentence_type": "Negative declarative sentence", "pos": {"nouns": {"Life": 1, "Experience": 1, "Friend": 1}, "verbs": {"I don't think so": 1}, "adverbs": {"have": 1}, "pronouns": {"I": 1}}}, {"content": "17. I'm afraid of believing in people.", "word_count": 13, "label": "", "sentence_structure": "Subject鈥搗erb鈥搊bject", "subjective_objective": {"answer": "Subjective", "rationale": "Because \"believing in others\" is a subjective attitude or belief. While this attitude may be influenced by past experience and circumstances, it is based on individual perceptions and feelings and can therefore be considered a subjective experience."}, "sentence_sentiment": {"answer": "Negative", "rationale": "Because fear of believing in someone else expresses a mistrust and fear, and it is a negative emotional attitude."}, "language_style": {"answer": "Calm and direct", "rationale": "The language style of the entry is more direct and objective, and there is no excessive emotional or rhetorical use. It simply raises concerns about the possible existence of trust in people in life, which is more calm and rational."}, "time_dimension": {"answer": "Past", "rationale": "In conclusion, the answer to this question is: \"The past/now.\""}, "sentence_type": "Negative declarative sentence", "pos": {"nouns": {"Life": 1, "Experience": 1, "person": 1}, "verbs": {"Scared": 1, "Yes": 1}, "numerals": {"17": 1}, "pronouns": {"I": 1}}}, {"content": "I don't think I have a partner in life.", "word_count": 19, "label": "", "sentence_structure": "Subject鈥揷opula鈥損redicative", "subjective_objective": {"answer": "Objective", "rationale": "Because this is a statement of what the patient feels, it is a subjective attitude of the patient. But the life experience it describes is an objective fact and can therefore be considered an objective result."}, "sentence_sentiment": {"answer": "Negative", "rationale": "The phrase \"I don't feel like I have a partner\" in the entry shows that the respondents have a negative view of their emotional state."}, "language_style": {"answer": "Calm and direct", "rationale": "The entry is based on a direct, simple question about whether the person interviewed feels he has a partner, does not use excessive rhetorical or emotional rendering, and is more objective in tone. It is therefore clear that the language style of the article is straightforward and calm."}, "time_dimension": {"answer": "Past", "rationale": "The entry asks the testee about the views and feelings of the past life experience, and therefore the temporal dimension is a past."}, "sentence_type": "Negative declarative sentence", "pos": {"nouns": {"Life": 1, "Experience": 1, "Partner": 2, "Men": 1, "Women": 1, "friends": 1}, "verbs": {"I think": 1, "have": 1}, "numerals": {"18": 1}, "pronouns": {"I": 1}}}, {"content": "My best friend felt a burden when he shared my difficulties.", "word_count": 24, "label": "", "sentence_structure": "Subject鈥搗erb鈥搊bject", "subjective_objective": {"answer": "Subjective", "rationale": "Because \"felt a burden\" is the subjective feeling and attitude of the patient's best friend."}, "sentence_sentiment": {"answer": "Negative", "rationale": "Because the phrase \"good friends feel a burden\" in the entry is negative."}, "language_style": {"answer": "Tactful and warm", "rationale": ""}, "time_dimension": {"answer": "Present", "rationale": ""}, "sentence_type": "Negative declarative sentence", "pos": {"nouns": {"Life": 1, "Experience": 1, "Share": 2, "Difficulties": 1, "Friends": 1, "Burden": 2}, "verbs": {"I think": 1, "be": 1, "When": 1, "Share": 1}, "adverbs": {"Medium": 1, "When": 1}, "numerals": {"19": 1}, "pronouns": {"Mine": 2}}}, {"content": "I feel like I'm not dependent on anyone, and I don't matter.", "word_count": 22, "label": "", "sentence_structure": "Subject鈥搗erb鈥揷omplement", "subjective_objective": {"answer": "Subjective", "rationale": "Because the words \"thinking\" and \"important\" are subjective perceptions and evaluations of the patient, they are viewed in the patient's inner experience and thoughts and are therefore subjective in nature."}, "sentence_sentiment": {"answer": "Negative", "rationale": "Because the expression \"others do not rely on me, nor do I feel important\" in the entry is negative, suggesting that the interviewee may feel neglected or unimportant."}, "language_style": {"answer": "Calm and direct", "rationale": ""}, "time_dimension": {"answer": "Present", "rationale": ""}, "sentence_type": "Negative declarative sentence", "pos": {"nouns": {"Life": 1, "Experience": 1, "person": 1, "Important": 1}, "verbs": {"I think": 2, "Dependency": 1, "Not really": 1}, "adverbs": {"I": 2}, "numerals": {"20": 1}}}, {"content": "21. I can't feel like I can't talk to anyone.", "word_count": 18, "label": "", "sentence_structure": "Subject鈥揷opula鈥損redicative", "subjective_objective": {"answer": "Subjective", "rationale": "I think it's the patient's subjective attitude."}, "sentence_sentiment": {"answer": "Negative", "rationale": "Because the emotional attitude of \"not having a heart\" in the entry is negative."}, "language_style": {"answer": "Tactful and warm", "rationale": "The language style of the entry is euphemism. Although it expresses the obsession with social interaction, it does not directly blame or blame others, but rather expresses their feelings in a moderate, understanding and inclusive manner."}, "time_dimension": {"answer": "Present", "rationale": "The entry expresses the feelings and thoughts of the testee, both \"felt\" and \"failure\" indicate that this is a subjective assessment of the state of the moment. Moreover, there is no reference to past or future situations in the sentence, so the temporal dimension is now."}, "sentence_type": "Negative declarative sentence", "pos": {"nouns": {"Life": 1, "Experience": 1, "person": 1, "Heart": 1}, "verbs": {"I think": 1, "Cannot": 1, "- A relationship": 1}, "adverbs": {"Any": 1}, "numerals": {"21": 1}, "pronouns": {"I": 1}}}, {"content": "I don't think I understand the experience of life.", "word_count": 13, "label": "", "sentence_structure": "Subject鈥搗erb鈥揷omplement", "subjective_objective": {"answer": "Subjective", "rationale": "Because \"fearing incomprehensible\" is a subjective feeling and assessment of what people experience."}, "sentence_sentiment": {"answer": "Negative", "rationale": "Because the \"ununderstandable\" feeling in the entry is negative."}, "language_style": {"answer": "Tactful and warm", "rationale": "The entry is more personal and interactive in terms of a more euphemistic, invitation-like tone of dialogue than direct statements or orders."}, "time_dimension": {"answer": "Past", "rationale": "So, \"I've heard people say I'm not understanding,\" and this entry is about the experience and feelings of the testees now, and the time dimension is now."}, "sentence_type": "Negative declarative sentence", "pos": {"nouns": {"Life": 1, "Experience": 1}, "verbs": {"I think": 1, "I don't understand": 1}, "pronouns": {"I": 1}}}, {"content": "I don't feel safe to ask for someone else.", "word_count": 16, "label": "", "sentence_structure": "Subject鈥揷opula鈥損redicative", "subjective_objective": {"answer": "Subjective", "rationale": "Life experiences are personal and subjective. It is also the patient鈥檚 own feeling and view that feels unsafe."}, "sentence_sentiment": {"answer": "Negative", "rationale": "Because the idea of \"no one else is safe\" in the entry is a negative attitude."}, "language_style": {"answer": "Calm and direct", "rationale": "The language style of the entry is more calm and objective, with no excessive emotional or rhetorical approach, and a greater focus on the direct transmission of information and facts. At the same time, the entry expresses the view that it may be disturbing to seek help in life. \""}, "time_dimension": {"answer": "Present", "rationale": "The entry indicates the current feelings and views of the testee, \"Feel\" and \"not safe\" are descriptions of the current situation and can therefore be judged as now."}, "sentence_type": "Affirmative declarative sentence", "pos": {"nouns": {"Life": 1, "Experience": 1, "I think": 1, "Please": 1, "others": 1, "Clear": 1}, "verbs": {"I think": 1, "Please": 1, "It's not safe": 1}, "numerals": {"23": 1}, "pronouns": {"I": 1}}}, {"content": "24. I'm alone in my life.", "word_count": 11, "label": "", "sentence_structure": "Subject鈥揷opula鈥損redicative", "subjective_objective": {"answer": "Subjective", "rationale": "Because feeling alone is the subjective feeling of the patient."}, "sentence_sentiment": {"answer": "Negative", "rationale": "Because \"sloneliness\" is a negative emotional experience."}, "language_style": {"answer": "Tactful and warm", "rationale": "This entry uses a more moderate, caring tone to guide respondents to express their true feelings, rather than direct orders or questioning. Moreover, this expression is more likely to generate the feelings and trust of the interviewee."}, "time_dimension": {"answer": "Present", "rationale": "The entry describes the feelings that the person who was tested has felt in his life, and \"fear\" is an ongoing exercise that shows that this feeling still exists. So we can judge the time dimension of the article as it is."}, "sentence_type": "Affirmative declarative sentence", "pos": {"nouns": {"Life": 1, "Experience": 1, "Loneliness": 1}, "verbs": {"I feel": 1}, "numerals": {"24": 1}}}, {"content": "25. I don't think I'm a member of any association or organization.", "word_count": 22, "label": "", "sentence_structure": "Subject鈥揷opula鈥損redicative", "subjective_objective": {"answer": "Subjective", "rationale": "The patient ' s subjective feelings and attitudes are not perceived as being part of any association or organization."}, "sentence_sentiment": {"answer": "Neutral", "rationale": "Because the entry does not have a clear positive or negative emotional tendency, it is a description of life experience."}, "language_style": {"answer": "Tactful and warm", "rationale": "The language style of the entry is more euphemistic, and by using the subjective sense and perspective of \"me\" to guide the interviewee to answer questions, giving a friendly and friendly feeling. At the same time, the use of the negative word \"do not feel\" also shows that the investigator does not force the interviewee to be a member of any association or organization, but rather respects the individual choice and freedom of each individual."}, "time_dimension": {"answer": "Present", "rationale": "\"Not to be a member of any association or organization\" is the current feeling and perception of the testee, so the temporal dimension is now judged."}, "sentence_type": "Negative declarative sentence", "pos": {"nouns": {"Life": 1, "Experience": 1, "Associations": 1, "Organizations": 1, "Members": 1}, "verbs": {"Not really": 1}, "numerals": {"25": 1}, "pronouns": {"I": 1}}}, {"content": "I don't think I've had any contact with anyone today.", "word_count": 19, "label": "", "sentence_structure": "Subject鈥揷opula鈥損redicative", "subjective_objective": {"answer": "Objective", "rationale": "It's over today, so it's objective. It's objective. It's subjective."}, "sentence_sentiment": {"answer": "Negative", "rationale": "Because the entry describes no contact with anyone, it is a negative social experience."}, "language_style": {"answer": "Calm and direct", "rationale": "The language style of the entry is more calm and straightforward, with no excessive rhetorical or emotional rendering, simply asking a question and asking the other side's views. This expression is more objective and clear and suitable for collecting data and information in psychological questionnaires."}, "time_dimension": {"answer": "Present", "rationale": "The keywords \"thinks\" and \"day\" indicate that this is a description of the current state of affairs, in which the person who was tested was expressing his/her current feelings and experiences, and the temporal dimension of the article is now."}, "sentence_type": "Affirmative declarative sentence", "pos": {"nouns": {"Life": 1, "Experience": 1, "person": 1}, "verbs": {"I think": 1, "Exposure": 1, "Today": 1}, "adverbs": {"Any": 1}, "numerals": {"26": 1}, "pronouns": {"I": 1}}}, {"content": "27. The experience of life, I feel like I have nothing to say to anyone else.", "word_count": 16, "label": "", "sentence_structure": "Subject鈥揷opula鈥損redicative", "subjective_objective": {"answer": "Objective", "rationale": "Because what has happened is objective and the patient's subjective feeling is subjective."}, "sentence_sentiment": {"answer": "Negative", "rationale": "Because the \"no words\" in the entry is negative."}, "language_style": {"answer": "Tactful and warm", "rationale": "The language style of the entry is more euphemistic and moderate, expressed in a sense of enquiry and concern, and attempts to understand the feelings and experiences of the person under investigation in life. This way enhances the relevance and interaction of the questionnaire and makes the person under investigation more willing to share his or her thoughts and experiences."}, "time_dimension": {"answer": "Past", "rationale": "The entry asks about the feelings and experiences of the subject at a certain point in time, that is, the experience of \"life\", and uses the old-temporal \"think\" to indicate that the problem is something that has happened."}, "sentence_type": "Negative declarative sentence", "pos": {"nouns": {"Life": 1, "Experience": 1, "others": 1, "Word": 1}, "verbs": {"I think": 1, "Nothing to say": 1}, "numerals": {"27": 1}, "pronouns": {"I": 1}}}, {"content": "I feel like I'm not the same person I am when I'm with someone else.", "word_count": 22, "label": "", "sentence_structure": "Subject鈥搗erb鈥揷omplement", "subjective_objective": {"answer": "Subjective", "rationale": "Because it is not the person who is originally the person who is the person who is the person who is the person who is the person who is the person who is the person who is the person who is the person who is the person who is the person who is the person who is the person who is the person who is the person who is the person who is the person who is the person who is the person who is the person who is the person who is the person who is the person who is the person who is the person who is the person who is the person who is the person who is the person who is the person who is."}, "sentence_sentiment": {"answer": "Negative", "rationale": "Because \"I feel like I'm not the same person anymore\" expresses a loss of self and a loss of real sense, which is a negative feeling."}, "language_style": {"answer": "Tactful and warm", "rationale": "The entry is more personal and interactive when it is spoken in a gentle and inviting tone, rather than in direct statements or orders. At the same time, it is not too emotional or strong, but rather raises questions in an objective, neutral way that makes people feel comfortable and relaxed."}, "time_dimension": {"answer": "Present", "rationale": "The expression \"thinks\" and \"never\" both indicates that it is the subjective feeling of the person being tested about the current living conditions and is therefore judged as the present behaviour or mentality."}, "sentence_type": "Affirmative declarative sentence", "pos": {"nouns": {"Life": 1, "Experience": 1, "person": 1, "Get along": 1}, "verbs": {"I think": 1, "be": 1, "Again": 1, "No, it's not": 1}, "adjectives": {"It was": 1}, "numerals": {"28": 1}, "pronouns": {"I": 1}}}, {"content": "29. I'm embarrassed about living with others.", "word_count": 17, "label": "", "sentence_structure": "Subject鈥搗erb鈥揷omplement", "subjective_objective": {"answer": "Subjective", "rationale": "Because being embarrassed is a subjective feeling and experience within a person."}, "sentence_sentiment": {"answer": "Negative", "rationale": "Because the emotional state of \"disturbing\" in the entry is negative."}, "language_style": {"answer": "Calm and direct", "rationale": "The entry is a direct question-and-answer dialogue, with no excessive emotional or rhetorical touch and a more objective tone."}, "time_dimension": {"answer": "Present", "rationale": "The entry describes the experience of the testee in his current life, and the keyword \"life experience\" and \"now\" point to what is happening now."}, "sentence_type": "Affirmative declarative sentence", "pos": {"nouns": {"Life": 1, "Experience": 1, "others": 1, "Get along": 1, "I'm embarrassed": 1}, "verbs": {"I feel": 1}, "numerals": {"29": 1}}}, {"content": "I don't feel like I'm funny about the experience of life.", "word_count": 14, "label": "", "sentence_structure": "Subject鈥揷opula鈥損redicative", "subjective_objective": {"answer": "Subjective", "rationale": "Because \"interest\" is a subjective criterion for evaluation, and different people may have different views and feelings, such self-evaluation is an examination of individual subjectivity."}, "sentence_sentiment": {"answer": "Negative", "rationale": "Because the attitude of \"not feeling interesting\" in the entry is negative."}, "language_style": {"answer": "Calm and direct", "rationale": "The entry directly asks the respondents about their feelings, without too much embellishment or emotional rendering, and the language style is more calm and straightforward."}, "time_dimension": {"answer": "Present", "rationale": "\"Don't feel funny\" suggests that the testee's feelings about the current life experience are what he is experiencing and that the temporal dimension is now."}, "sentence_type": "Negative declarative sentence", "pos": {"nouns": {"Life": 1, "Experience": 1, "Fun": 1}, "verbs": {"Not really": 1, "self": 1, "Interesting": 1}, "numerals": {"30": 1}, "pronouns": {"I": 1}}}]}

{"questionnaire_title": "Personal maturity test", "questionnaire_category": "Self-cognition", "item_count": 25, "emotion_shift_overall": "From positive to negative", "language_style_overall": "Tactful and warm", "items": [{"content": "The attitude of the head of my unit (or teacher at school) towards me is:", "word_count": 21, "label": "", "sentence_structure": "Subject鈥搗erb鈥搊bject", "subjective_objective": {"answer": "Subjective", "rationale": "Because \"leader attitude\" is the patient's perception and feeling of leadership, it's a subjective perspective."}, "sentence_sentiment": {"answer": "Neutral", "rationale": "As the entry describes an objective fact, it does not express any positive or negative emotional tendencies."}, "language_style": {"answer": "Tactful and warm", "rationale": "The language style of the entry is more euphemistic, because questions are expressed in question, without direct criticism or criticism, and the polite language is \"the head of my unit\" and \"the attitude towards me\". This way of asking questions is more likely to resonate and give positive feedback to the interviewees."}, "time_dimension": {"answer": "Present", "rationale": "The word \"in\" in \"my unit\" indicates the state of things, and the \"treatment\" is also happening now, so the entry describes the current situation and judges it now."}, "sentence_type": "Interrogative sentence", "pos": {"nouns": {"Units": 1, "Leadership": 1, "School": 1, "Teacher": 1}, "verbs": {"Treatment": 1}, "adjectives": {"I": 1}, "classifiers": {"individual": 1}}}, {"content": "If I lose in the game, my usual practice is:", "word_count": 16, "label": "", "sentence_structure": "Subject鈥損redicate", "subjective_objective": {"answer": "Subjective", "rationale": "This is because the patient describes how he or she reacts after losing the game, reflects his or her personal attitude and feelings and is subjective in nature."}, "sentence_sentiment": {"answer": "Negative", "rationale": "Because the expression \"lost\" in the entry is negative."}, "language_style": {"answer": "Calm and direct", "rationale": "The entry uses a direct question-and-answer method, without any qualification or implication, and is more calm and objective and more in line with the professional requirements of the questionnaire."}, "time_dimension": {"answer": "Present", "rationale": "The entry now asks the testee ' s usual practice in the current race failure, and therefore the time dimension is now judged."}, "sentence_type": "Affirmative declarative sentence", "pos": {"nouns": {"The game": 1, "Lose": 1, "Approach": 1}, "verbs": {"be": 1, "have": 1, "Usually": 1}, "adverbs": {"If": 1, "in": 1, "I": 1}, "numerals": {"2": 1}, "pronouns": {"I": 1}}}, {"content": "When life is in a state of great frustration (e.g., high school, love loss), I feel:", "word_count": 23, "label": "", "sentence_structure": "Subject鈥搗erb鈥搊bject", "subjective_objective": {"answer": "Subjective", "rationale": "This is because of the subjective feelings of the patient in certain circumstances."}, "sentence_sentiment": {"answer": "Negative", "rationale": "Because words like \"significant setback\" and \"fear\" in the entries have a clear negative emotional color."}, "language_style": {"answer": "Calm and direct", "rationale": "The style of the entry is more calm and straightforward, and it opens up questions in the mountains, without too many adorations and emotional expressions."}, "time_dimension": {"answer": "Present", "rationale": "The description of \"Strategic Frustration\" and \"Esses\" suggests an emotional response that may occur in the current life situation. Thus, it can be judged by asking the testee for immediate emotional reactions in the face of similar situations, and the temporal dimension is now."}, "sentence_type": "Interrogative sentence", "pos": {"nouns": {"Life": 1, "Medium": 1, "Significant": 1, "Frustration": 1, "Like": 1, "High examination": 1, "Down with the list": 1, "Broken love": 1}, "verbs": {"I met him": 1, "Yes": 1, "I feel": 1, "EMPTY": 1}, "adjectives": {"Significant": 1}, "numerals": {"3": 1}}}, {"content": "I'm the one who likes me.", "word_count": 9, "label": "", "sentence_structure": "Subject鈥搗erb鈥揷omplement", "subjective_objective": {"answer": "Subjective", "rationale": "Because this is the patient ' s subjective view of his or her own evaluation in the eyes of others."}, "sentence_sentiment": {"answer": "Neutral", "rationale": "Because the entry is merely asking about the degree of preferences of others and does not involve any emotional colour."}, "language_style": {"answer": "Tactful and warm", "rationale": "The entry is more personal and interactive when it is spoken in a gentle and inviting manner, rather than in direct statements or orders. At the same time, it does not ask the other side too directly about sensitive issues such as self-worth or social status, but rather by asking the respondents themselves to think and evaluate others鈥?own assessments, which are more acceptable and willing to participate."}, "time_dimension": {"answer": "Present", "rationale": "The entry asks the testee about his current state and feelings, and the time dimension is now judged."}, "sentence_type": "Imperative sentence", "pos": {"nouns": {"Level": 1, "Questionnaire": 1, "Entry": 1, "others": 1}, "verbs": {"Yeah": 1, "be": 1}, "numerals": {"4": 1}, "pronouns": {"I": 1}}}, {"content": "My attitude to talking about my failures is:", "word_count": 14, "label": "", "sentence_structure": "Subject鈥揷opula鈥損redicative", "subjective_objective": {"answer": "Subjective", "rationale": "Because attitudes are subjective ideas or emotions, they reflect individual perceptions and perceptions of a given situation."}, "sentence_sentiment": {"answer": "Negative", "rationale": "Because the \"talking about your failure\" act in the entry is usually considered negative and may give rise to uncomfortable emotional experiences."}, "language_style": {"answer": "Tactful and warm", "rationale": "The language style of this entry is more euphemistic, not too direct or hard-line, but rather in a more peaceful way to ask the respondents. At the same time, the subjective proxy \"I\" is used to guide the answer, making the conversation more intimate and natural."}, "time_dimension": {"answer": "Present", "rationale": "\"Attitudes to\" and \"talking about\" your own failures are all questions about the situation, so it's judged to be the present."}, "sentence_type": "Affirmative declarative sentence", "pos": {"nouns": {"Attitude": 1, "Experience": 1, "Talk": 1, "self": 1, "Failed": 1}, "verbs": {"to": 1, "be": 1}, "adverbs": {"5": 1}}}, {"content": "In general, all people who disagree with me are", "word_count": 17, "label": "", "sentence_structure": "Subject鈥搗erb鈥揷omplement", "subjective_objective": {"answer": "Subjective", "rationale": "Because it is a question based on patient subjective views."}, "sentence_sentiment": {"answer": "Neutral", "rationale": "Because the entry does not express an emotional colour, it is a description of a situation."}, "language_style": {"answer": "Tactful and warm", "rationale": "The style of the entry is more euphemistic, implicit, and does not directly attack or criticize people who disagree with their views, but rather uses more polite and respectful language."}, "time_dimension": {"answer": "Present", "rationale": "The term \"general\" in this entry refers to the current, prevailing situation, while \"all\" indicates that this situation persists. Thus, the temporal dimension of this entry is now."}, "sentence_type": "Interrogative sentence", "pos": {"nouns": {"person": 1, "Observations": 1, "Situation": 1}, "verbs": {"be": 1, "Different": 1}, "adverbs": {"Both": 1, "General": 1}, "numerals": {"6": 1}, "pronouns": {"I": 1}}}, {"content": "I like to meet the competitions in games and competitions:", "word_count": 16, "label": "", "sentence_structure": "Subject鈥搗erb鈥搊bject", "subjective_objective": {"answer": "Subjective", "rationale": "The question is asked about the subjective views and preferences of individuals about the type of opponent they prefer in the game or in the competition, and is therefore subjective."}, "sentence_sentiment": {"answer": "Neutral", "rationale": "Because the entry is not clearly emotional, it is simply asking what kind of opponent it likes."}, "language_style": {"answer": "Tactful and warm", "rationale": "The language style of the entry is mild and somewhat inviting, giving the respondents a sense of the goodwill and respect of the researchers. By using active words such as \"like\" and \"for encounters\" the questionnaire makes it more attractive and easier to induce the respondents to participate. At the same time, the expression \"in games or competitions\" implies that the questionnaire is linked to entertainment, competition, etc., and may be more suitable for young groups to participate."}, "time_dimension": {"answer": "Present", "rationale": "The keywords are \"like\" and \"accede\", which indicate that they are a description of the current situation and do not address the temporal dimension of the past or the future. Thus, the article's purpose is time to judge \"now\"."}, "sentence_type": "Interrogative sentence", "pos": {"nouns": {"Game": 1, "Competition": 1, "Opponent": 1}, "verbs": {"Yeah": 1, "I met him": 1}}}, {"content": "My favorite social environment is:", "word_count": 9, "label": "", "sentence_structure": "Subject鈥搗erb鈥搊bject", "subjective_objective": {"answer": "Subjective", "rationale": "Because love is an emotional experience and attitude of an individual, it is subjective."}, "sentence_sentiment": {"answer": "Neutral", "rationale": "Because the entry is merely an inquiry into the preferences of the individual for the social environment, it does not involve any emotional or attitudinal tendencies."}, "language_style": {"answer": "Tactful and warm", "rationale": "This is a more euphemistic expression that makes the person surveyed comfortable and willing to participate actively in the questionnaire."}, "time_dimension": {"answer": "Present", "rationale": "The entry asks what social circumstances the person is currently in favour of, which is a description of the status quo and a manifestation of preference, and therefore the temporal dimension is now judged."}, "sentence_type": "Declarative sentence", "pos": {"nouns": {"Social environment": 1, "Yeah": 1}, "verbs": {"be": 1}, "adverbs": {"very": 1}, "numerals": {"8": 1}}}, {"content": "I'm dealing with the argument:", "word_count": 9, "label": "", "sentence_structure": "Subject鈥搗erb鈥搊bject", "subjective_objective": {"answer": "Subjective", "rationale": "Because attitudes are usually subjective views and feelings of individuals."}, "sentence_sentiment": {"answer": "Neutral", "rationale": "The entry is not an emotional expression."}, "language_style": {"answer": "Calm and direct", "rationale": "The entry directly asks the respondents about their attitude to the debate, without excessive qualifiers and emotional colours, and is more concise in language style, and therefore judged as \"cool and direct\"."}, "time_dimension": {"answer": "Present", "rationale": "The entry asks the testee's current attitude or perception, and the temporal dimension is judged to be the present. The keyword \"attitude to argument is\" means that it is a question of describing the testee's current orientation or habits, without reference to past or future situations."}, "sentence_type": "Interrogative sentence", "pos": {"nouns": {"Arguments": 1, "Attitude": 1}, "verbs": {"Treatment": 1}, "numerals": {"9": 1}}}, {"content": "When accused, I usually react:", "word_count": 14, "label": "", "sentence_structure": "Subject鈥搗erb鈥搊bject", "subjective_objective": {"answer": "Subjective", "rationale": "Because responses are usually subjective feelings and emotional expressions of individuals."}, "sentence_sentiment": {"answer": "Negative", "rationale": "Because being accused is usually a negative emotional experience, \"reaction\" should also be negative."}, "language_style": {"answer": "Tactful and warm", "rationale": "The article is based on a more neutral and objective language style, with no excessive emotional overtones or subjective evaluations, and therefore can be considered as \"cool and direct.\" At the same time, the entry does not use too complex a vocabulary or phrase, which is easy to understand, more pro-people and more acceptable."}, "time_dimension": {"answer": "Present", "rationale": "Now, the keyword \"normal reaction\" suggests that the entry is asking the subject about the current customary behaviour or reaction. At the same time, the premise \"when someone else blames\" implies that it is a matter of individual emotions or behaviour, not a retrospect of the past or a prediction of the future. So the time horizon of the article is now."}, "sentence_type": "Interrogative sentence", "pos": {"nouns": {"Blame": 1, "Response": 1}, "verbs": {"Yes": 1, "Usually": 1, "be": 1, "have": 1}, "adverbs": {"10": 1}, "numerals": {"10": 1}}}, {"content": "I think that the impact of help from relatives and friends on a person's success is:", "word_count": 20, "label": "", "sentence_structure": "Subject鈥搗erb鈥搊bject", "subjective_objective": {"answer": "Subjective", "rationale": "Because \"concept\" indicates that this is the patient's personal opinion and opinion, and it is subjective."}, "sentence_sentiment": {"answer": "Neutral", "rationale": "Because the entry merely presents a point of view, it does not express a positive or negative emotional colour."}, "language_style": {"answer": "Tactful and warm", "rationale": "The entry is more adept and interactive when it is spoken in a gentle and inviting manner, rather than by direct statements or orders."}, "time_dimension": {"answer": "Present", "rationale": "The entry asks the testee's opinion on the \"Impact of help for the success of a person's career\" and does not specify the time frame, and therefore the judgement is now."}, "sentence_type": "Interrogative sentence", "pos": {"nouns": {"Help": 1, "person": 2, "Career": 1, "Success": 1, "Impact": 1}, "verbs": {"I think": 1, "be": 1, "Here": 1}, "adverbs": {"to": 1}, "numerals": {"11": 1}, "pronouns": {"I": 1}}}, {"content": "I think the right attitude to the environment of social life is:", "word_count": 17, "label": "", "sentence_structure": "Subject鈥搗erb鈥搊bject", "subjective_objective": {"answer": "Subjective", "rationale": "Because the subject is \"I think\" attitude, the examination is about the patient's personal views and attitudes, and it is subjective."}, "sentence_sentiment": {"answer": "Neutral", "rationale": "It does not express any apparent feelings, attitudes or behaviour, but rather a point of view."}, "language_style": {"answer": "Tactful and warm", "rationale": "The entry is more personal and interactive when it is spoken in a gentle and inviting tone, rather than in direct statements or orders. At the same time, it does not use too much emotional terms or rhetorical techniques, but rather directs the interviewee to the correct attitude to the social environment, making the language more objective."}, "time_dimension": {"answer": "Present", "rationale": ""}, "sentence_type": "Interrogative sentence", "pos": {"nouns": {"Attitude": 1, "Social": 1, "Living environment": 1}, "verbs": {"I think": 1, "Treatment": 1}, "adjectives": {"Correct": 1}, "numerals": {"12": 1}}}, {"content": "My attitude to death is:", "word_count": 8, "label": "", "sentence_structure": "Subject鈥搗erb鈥揷omplement", "subjective_objective": {"answer": "Subjective", "rationale": "Because attitudes are personal subjective perceptions and feelings, \"my attitude to death is\" is subjective."}, "sentence_sentiment": {"answer": "Neutral", "rationale": "Because the entry does not address any positive or negative emotional, attitude, behaviour, purpose or quality. It is merely asking about the views of individuals on death, and therefore it is neutral."}, "language_style": {"answer": "Tactful and warm", "rationale": "This is a language that is more emotional and personal and more easily triggers the active participation and deep reflection of the respondents."}, "time_dimension": {"answer": "Present", "rationale": "The entry asks the testee about the perception or perception of the current attitude to death, so the temporal dimension is now judged."}, "sentence_type": "Interrogative sentence", "pos": {"nouns": {"Attitude": 1, "Death": 1}, "verbs": {"be": 1}, "numerals": {"13": 1}}}, {"content": "In order to give others a good impression of themselves, my approach was:", "word_count": 18, "label": "", "sentence_structure": "Subject鈥損redicate", "subjective_objective": {"answer": "Subjective", "rationale": "This is an approach that is initiated by the patient himself in order to expect the approval and approval of others and is subjective in nature."}, "sentence_sentiment": {"answer": "Positive", "rationale": "Because the practice in the entry \"to give others a good impression of themselves\" is a positive act with a clear purpose."}, "language_style": {"answer": "Tactful and warm", "rationale": "The entry uses an invitation-style tone to guide respondents to answer questions on the topic \"Let others have a good impression of themselves.\" This expression is more personal and interactive, making it easier for respondents to share their thoughts. At the same time, the tone of the article is more moderate, with no direct orders or feelings of blame, and is more conducive to a good survey."}, "time_dimension": {"answer": "Present", "rationale": "The entry asks about the testee's current behavior or strategy, and therefore judges it as \"now.\" The keyword \"for\" and \"do\" is the normal current, which means that it is a continuous act or state."}, "sentence_type": "Imperative sentence", "pos": {"nouns": {"others": 1, "self": 1, "Impression": 1}, "verbs": {"Jean": 1, "Do it": 1}, "adverbs": {"very": 1}, "numerals": {"14": 1}, "pronouns": {"I": 1}}}, {"content": "I think that if I want to live happily and meaningfully, I must live:", "word_count": 22, "label": "", "sentence_structure": "Subject鈥揷opula鈥損redicative", "subjective_objective": {"answer": "Subjective", "rationale": "Because the questionnaire entry is asking patients about their ideas and views, which I think is \"I think...\", it is a subjective examination."}, "sentence_sentiment": {"answer": "Neutral", "rationale": "Since the purpose of the article was not expressed in a clear emotional or tendentious manner, it merely presented a point of view or proposal."}, "language_style": {"answer": "Tactful and warm", "rationale": "Warm and warm."}, "time_dimension": {"answer": "Present", "rationale": ""}, "sentence_type": "Interrogative sentence", "pos": {"nouns": {"self": 1, "Life": 2, "Have a nice day": 1, "Meaning": 1}, "verbs": {"I think": 1, "I have to": 1, "Pass": 1}, "adjectives": {"Happy and meaningful": 1}, "numerals": {"15": 1}, "classifiers": {"individual": 1}, "pronouns": {"I": 1}}}, {"content": "When difficulties are encountered in working or learning, I usually:", "word_count": 16, "label": "", "sentence_structure": "Subject鈥搗erb鈥搊bject", "subjective_objective": {"answer": "Subjective", "rationale": "Because \"difficulties at work or learning\" and \"usually\" are personal experiences and feelings of patients and are subjective in nature."}, "sentence_sentiment": {"answer": "Neutral", "rationale": "The entry merely questions the way the person under investigation responds to the difficulties encountered, and does not express a positive or negative emotional profile."}, "language_style": {"answer": "Calm and direct", "rationale": "The language style of the entry is relatively calm and straightforward, rather than using excessive rhetorical or emotional rendering, it is a simple and clear way of asking each other about the general response to the difficulties faced."}, "time_dimension": {"answer": "Present", "rationale": ""}, "sentence_type": "Imperative sentence", "pos": {"nouns": {"Work": 1, "Learning": 1, "Difficulties": 1, "I": 1}, "verbs": {"be": 1}, "numerals": {"16": 1}}}, {"content": "When my own family wrongly blames me, I usually:", "word_count": 17, "label": "", "sentence_structure": "Subject鈥搗erb鈥搊bject", "subjective_objective": {"answer": "Subjective", "rationale": "Because the question is one of the patient ' s reaction or feelings, it is subjective."}, "sentence_sentiment": {"answer": "Negative", "rationale": "Because the \"wrongly blamed\" in the entries is negative, and the latter reactions are usually emotional, such as anger, grief, etc., so the whole subject is negative."}, "language_style": {"answer": "Calm and direct", "rationale": "The entry is based on a direct question-and-answer approach, asking the respondents how to respond to the wrong blame of their loved ones, which is more calm and objective, and which does not involve too much emotional or rhetorical."}, "time_dimension": {"answer": "Present", "rationale": "The entry asks about the reaction of the person under examination in the immediate circumstances, and the keyword \"usually\" indicates customary, recurrent behaviour. The temporal dimension of the article should therefore be judged as now.\""}, "sentence_type": "Interrogative sentence", "pos": {"nouns": {"Family": 1, "Error": 1, "Blame": 1}, "verbs": {"Usually": 1, "When": 1}, "adverbs": {"self": 1}, "numerals": {"17": 1}, "pronouns": {"I": 1}}}, {"content": "In my dealings with others, I usually:", "word_count": 12, "label": "", "sentence_structure": "Subject鈥揷opula鈥損redicative", "subjective_objective": {"answer": "Subjective", "rationale": "Because \"usually\" is an individual's custom or preference, which involves the patient's own subjective attitude and behaviour habits, this is a subjective view."}, "sentence_sentiment": {"answer": "Neutral", "rationale": "Because the entry does not have any emotional color."}, "language_style": {"answer": "Calm and direct", "rationale": "The language style of the entry is calm, because it directly asks the interviewees about normality in interpersonal relationships."}, "time_dimension": {"answer": "Present", "rationale": "The entry of the questionnaire asks about the current behaviour or state of the person being tested, i.e., \"in dealing with someone else\", and therefore the time dimension is now judged."}, "sentence_type": "Interrogative sentence", "pos": {"nouns": {"- To be with someone": 1, "others": 1}, "verbs": {"Usually": 1}, "numerals": {"18": 1}}}, {"content": "19 Appearances to me:", "word_count": 6, "label": "", "sentence_structure": "Subject鈥搗erb鈥揷omplement", "subjective_objective": {"answer": "Subjective", "rationale": "This is the view and evaluation of the patient ' s external watch and falls within the realm of subjective perceptions and attitudes of individuals."}, "sentence_sentiment": {"answer": "Neutral", "rationale": "The entry is based on the opinion of the individual external watch and there is no apparent emotional tendency."}, "language_style": {"answer": "Calm and direct", "rationale": "The question is more straightforward, concise, not overly accommodative or emotional, and appears to be calm and objective and suitable for use in research or research."}, "time_dimension": {"answer": "Present", "rationale": "The entry was used to judge the past in terms of time dimension when it asked the respondent about his or her appearance and attitude."}, "sentence_type": "Interrogative sentence", "pos": {"verbs": {"Come on": 1}, "adjectives": {"19": 1, "Appearance": 1}}}, {"content": "I like to date people who are:", "word_count": 12, "label": "", "sentence_structure": "Subject鈥揷opula鈥損redicative", "subjective_objective": {"answer": "Subjective", "rationale": "Because it is the patient ' s choice and preferences that prefer to be in contact that are the expression of subjective attitudes."}, "sentence_sentiment": {"answer": "Neutral", "rationale": "Because the entry merely asks who you like to be with, it does not express positive or negative emotional tendencies."}, "language_style": {"answer": "Tactful and warm", "rationale": ""}, "time_dimension": {"answer": "Present", "rationale": ""}, "sentence_type": "Interrogative sentence", "pos": {"nouns": {"I": 1, "person": 1}, "verbs": {"Yeah": 1, "- To be with someone": 1}, "numerals": {"20": 1}}}, {"content": "When I had to speak in public, I always:", "word_count": 16, "label": "", "sentence_structure": "Subject鈥搗erb鈥揷omplement", "subjective_objective": {"answer": "Subjective", "rationale": "Since the examination is of the feelings and reactions of the patient in a given situation, it is of a subjective nature."}, "sentence_sentiment": {"answer": "Negative", "rationale": "Because \"always\" means that there are some difficulties and problems in this situation, which is a negative description."}, "language_style": {"answer": "Calm and direct", "rationale": ""}, "time_dimension": {"answer": "Present", "rationale": ""}, "sentence_type": "Interrogative sentence", "pos": {"nouns": {"It's a public place": 1, "Speech": 1}, "verbs": {"I": 1, "I have to": 1, "be": 1, "Total": 1, "in": 1, "Speak": 1}, "adverbs": {"Always": 1}, "numerals": {"21": 1}, "pronouns": {"I": 1}}}, {"content": "My opinion on the use of palm-reading, eight-word readings is that:", "word_count": 16, "label": "", "sentence_structure": "Subject鈥搗erb鈥搊bject", "subjective_objective": {"answer": "Subjective", "rationale": "Because \"view\" is usually a subjective view or attitude of the individual."}, "sentence_sentiment": {"answer": "Negative", "rationale": "Because the words \"reading palms, measuring eight words to tell fortune\" in this entry are usually considered superstitions and therefore have a negative emotional colour."}, "language_style": {"answer": "Tactful and warm", "rationale": "- Warmly: This is a question of exploring your views on astronomy and fortune tellers, and leading the interviewee to express his views in some moderate ways. This style may make the interviewee feel more relaxed and comfortable. - Calm down: on the other hand, the entry may be considered a direct questionnaire design, which does not have too much emotional or rhetorical style, but rather directs the interviewee鈥檚 views. This style may place more emphasis on data accuracy and objectivity. It is important to note that this is only a possible judgement of the language style for the purpose of the article, and that the specific style needs to be considered in a comprehensive manner, based on the overall design and context of the questionnaire."}, "time_dimension": {"answer": "Present", "rationale": "The keyword \"view\" indicates that the question is asked about the current attitude or view of the subject, and that the description \"reading the palm, measuring the eight words to tell the fate\" refers to the current situation, so that the temporal dimension of the article should be the present."}, "sentence_type": "Affirmative declarative sentence", "pos": {"nouns": {"Observations": 1, "Watch your palms": 1, "Eight words": 1}, "verbs": {"be": 1, "Use": 1}, "adverbs": {"22": 1}, "numerals": {"22": 1}}}, {"content": "I usually attend several seminars:", "word_count": 15, "label": "", "sentence_structure": "Subject鈥搗erb鈥搊bject", "subjective_objective": {"answer": "Subjective", "rationale": "Since the problem concerns the behaviour and feelings of the patient himself, it is necessary to look at it from the patient ' s subjective perspective."}, "sentence_sentiment": {"answer": "Neutral", "rationale": "Since the entry describes only the usual behaviour of the respondent in a discussion session, it does not involve any emotional or attitudinal colours."}, "language_style": {"answer": "Tactful and warm", "rationale": "The entry is more adept and interactive when it is spoken in a gentle and inviting manner, rather than by direct statements or orders."}, "time_dimension": {"answer": "Present", "rationale": "The term \"normally\" in the entry indicates that it is a customary act or preference and refers to the current situation. Thus, the temporal dimension is now judged."}, "sentence_type": "Interrogative sentence", "pos": {"nouns": {"Seminar": 1, "person": 1}, "verbs": {"Participation": 1, "Usually": 1}, "numerals": {"23": 1}, "pronouns": {"I": 1}}}, {"content": "My view of society is:", "word_count": 8, "label": "", "sentence_structure": "Subject鈥搗erb鈥搊bject", "subjective_objective": {"answer": "Subjective", "rationale": "Since the perception of society is a subjective one and the view of the individual, the entry is viewed from the subjective perspective of the patient."}, "sentence_sentiment": {"answer": "Neutral", "rationale": "Because the entry does not have any emotional color."}, "language_style": {"answer": "Calm and direct", "rationale": "The entry directly enquires about the views of the respondent on society, without using any qualifier or emotionally strong vocabulary, which is relatively neutral and direct."}, "time_dimension": {"answer": "Present", "rationale": "According to the title, \"24 I think of society as: \"The word \"for\" expresses subjective views, attitudes or views without specifying whether it is past, present or future behaviour, and therefore the temporal dimension can be judged as it is now."}, "sentence_type": "Affirmative declarative sentence", "pos": {"nouns": {"Social": 1, "Observations": 1}, "verbs": {"be": 1}, "adverbs": {"to": 1, "24": 1}}}, {"content": "When I'm on the road to life, I always have a test of personal maturity.", "word_count": 32, "label": "", "sentence_structure": "Subject鈥搗erb鈥搊bject", "subjective_objective": {"answer": "Subjective", "rationale": "Because \"always\" and \"personal maturity tests\" are all evaluated and described from the patient's subjective point of view. The subject is the patient's self-perception and mental state in the face of a life test, and therefore subjective."}, "sentence_sentiment": {"answer": "Neutral", "rationale": "Because the entry describes only an act or situation and does not express any emotional colour, it is neutral."}, "language_style": {"answer": "Calm and direct", "rationale": "The entry is more straightforward and concise in its language style, with no excessive rhetorical or emotional rendering. It asks the interviewee about the degree of maturity he has shown in facing the challenges of life, which usually require clear and objective answers, and therefore the language style is more calm and straightforward."}, "time_dimension": {"answer": "Present", "rationale": ""}, "sentence_type": "Interrogative sentence", "pos": {"nouns": {"Test": 1, "Life path": 1, "High examination": 1, "Candidatures": 1, "Personal maturity test": 1}, "verbs": {"I met him": 1, "Always": 1}, "adjectives": {"Mature": 1}, "numerals": {"25": 1}}}]}

{"questionnaire_title": "Self-measurement table for SCL-90 symptoms", "questionnaire_category": "Mental health", "item_count": 90, "emotion_shift_overall": "From negative to positive", "language_style_overall": "Neutral", "items": [{"content": "1. Headaches", "word_count": 2, "label": "", "sentence_structure": "Subject鈥揷opula鈥損redicative", "subjective_objective": {"answer": "Objective", "rationale": "Because headaches are a symptom or a physical sign, are a physical reaction of the patient and are described as objective facts."}, "sentence_sentiment": {"answer": "Negative", "rationale": "Because \"headache\" is an uncomfortable physical feeling, which is usually seen as a negative emotion or experience."}, "language_style": {"answer": "Calm and direct", "rationale": "The entry directly asked the question of whether the person under investigation had headaches, was simple in language, was not too much rhetorical or emotional, and was more objective and practical."}, "time_dimension": {"answer": "Past", "rationale": "The entry is for the purpose of asking the person tested for past headaches, which are judged by the time dimension."}, "sentence_type": "Negative declarative sentence", "pos": {"nouns": {"Headache": 1}}}, {"content": "2. He's a sensitive man, he's not a real one.", "word_count": 9, "label": "", "sentence_structure": "Subject鈥揷opula鈥損redicative", "subjective_objective": {"answer": "Subjective", "rationale": "Because \"sensitized\" and \"insensitive\" are the feelings and experiences inside the patient, and are subjective."}, "sentence_sentiment": {"answer": "Negative", "rationale": "Because the sensitivity of the mind and the inactivity are negative emotional states."}, "language_style": {"answer": "Tactful and warm", "rationale": "Warm and warm."}, "time_dimension": {"answer": "Present", "rationale": ""}, "sentence_type": "Interrogative sentence", "pos": {"nouns": {"You're a bit of a sane": 1, "鈾?In the heart 鈾?: 1, "Step on it": 1}, "verbs": {"Allergies": 1, "鈾?In the heart 鈾?: 1, "Step on it": 1}, "adjectives": {"Nerves": 1, "Unsettled": 1}}}, {"content": "3. There are unnecessary ideas or words in the mind", "word_count": 15, "label": "", "sentence_structure": "Subject鈥搗erb鈥搊bject", "subjective_objective": {"answer": "Subjective", "rationale": "Because the idea or word of the mind is the subjective feeling and experience of the patient, not the objective fact. This is psychological activity within the patient, and therefore subjective."}, "sentence_sentiment": {"answer": "Negative", "rationale": "Because \"unnecessary ideas or words\" in the entries are usually negative. This state of affairs can affect individual emotions and thinking, causing anxiety, depression, etc. Therefore, the purpose is negative."}, "language_style": {"answer": "Tactful and warm", "rationale": ""}, "time_dimension": {"answer": "Present", "rationale": "The entry asks if the testee currently has \"unnecessary thought or word-turned\" in his mind, so the temporal dimension is now judged."}, "sentence_type": "Affirmative declarative sentence", "pos": {"nouns": {"Brain": 1, "Thoughts": 1, "Words": 1, "Rotate": 1}, "verbs": {"have": 1, "Come on": 1}, "adjectives": {"Not necessary": 1}}}, {"content": "4. Dim or fainting", "word_count": 5, "label": "", "sentence_structure": "Subject鈥搗erb鈥揷omplement", "subjective_objective": {"answer": "Objective", "rationale": "Because of the objective fact that the person is unconscious or unconscious, the person is not required to describe or judge himself."}, "sentence_sentiment": {"answer": "Negative", "rationale": "Because \"heads faint or faint\" are symptoms of physical discomfort, they are usually associated with negative physical health."}, "language_style": {"answer": "Calm and direct", "rationale": "The entry is a straightforward question of whether the person interviewed has been unconscious or fainted, without any use of rhetorical or other means of expression, and with a relatively calm and direct language style.\""}, "time_dimension": {"answer": "Past", "rationale": "The entry is for the purpose of asking the testee whether there have been past symptoms of overscrowding or fainting, and the temporal dimension is judged to be past."}, "sentence_type": "Negative declarative sentence", "pos": {"nouns": {"Headlight": 1, "- I'm fainted": 1}}}, {"content": "5. Declining interest in heterosexuality", "word_count": 8, "label": "", "sentence_structure": "Subject鈥搗erb鈥揷omplement", "subjective_objective": {"answer": "Subjective", "rationale": "Because interest is a subjective emotion and attitude."}, "sentence_sentiment": {"answer": "Negative", "rationale": "Because the description of the act of \"lost interest\" is negative."}, "language_style": {"answer": "Tactful and warm", "rationale": ""}, "time_dimension": {"answer": "Present", "rationale": "The term \"reducing\" in \"v. interest in heterosexuality\" represents a change and does not have a clear time reference, so it can be judged that it is the current state."}, "sentence_type": "Negative declarative sentence", "pos": {"nouns": {"Heterosexual": 1, "Interest": 1}, "verbs": {"Declining": 1}}}, {"content": "6. Accountability for others", "word_count": 7, "label": "", "sentence_structure": "Subject鈥搗erb鈥揷omplement", "subjective_objective": {"answer": "Subjective", "rationale": "Because \"compensation\" is a subjective assessment and attitude that reflects patients' reaction to criticisms."}, "sentence_sentiment": {"answer": "Negative", "rationale": "Because the \"compelling for the whole\" act is negative."}, "language_style": {"answer": "Tactful and warm", "rationale": ""}, "time_dimension": {"answer": "Present", "rationale": ""}, "sentence_type": "Imperative sentence", "pos": {"nouns": {"The others": 1, "Blame": 1, "Full": 1}, "verbs": {"to": 1, "Blame": 1, "Full": 1}}}, {"content": "Feels like someone else can control your mind.", "word_count": 11, "label": "", "sentence_structure": "Subject鈥揷opula鈥損redicative", "subjective_objective": {"answer": "Subjective", "rationale": "Because the perception of other people ' s mind control is a subjective feeling and idea that reflects the feelings and perceptions inside the patient, rather than an objective reality."}, "sentence_sentiment": {"answer": "Negative", "rationale": "Because the emotional expression \"I feel like someone can control your mind\" in the entry is negative."}, "language_style": {"answer": "Calm and direct", "rationale": ""}, "time_dimension": {"answer": "Present", "rationale": "Past"}, "sentence_type": "Negative declarative sentence", "pos": {"nouns": {"person": 1, "Ideas": 1}, "verbs": {"I feel": 1, "Control": 1}}}, {"content": "- You're not gonna blame anyone for the trouble.", "word_count": 8, "label": "", "sentence_structure": "Subject鈥搗erb鈥搊bject", "subjective_objective": {"answer": "Subjective", "rationale": "Because blame is a subjective emotion and attitude of the patient."}, "sentence_sentiment": {"answer": "Negative", "rationale": "Because the \"criticism\" in the entry is negative."}, "language_style": {"answer": "Calm and direct", "rationale": "The language style of the entry is more straightforward, and it does not use euphemism or rhetoric, but rather expresses the subject of a simple and clear inquiry into whether the other party is often blaming others for causing trouble."}, "time_dimension": {"answer": "Present", "rationale": ""}, "sentence_type": "Imperative sentence", "pos": {"nouns": {"Blame": 1, "others": 1, "Trouble": 1}, "verbs": {"Manufacturing": 1}}}, {"content": "9. Forget sex.", "word_count": 4, "label": "", "sentence_structure": "Subject鈥揷opula鈥損redicative", "subjective_objective": {"answer": "Subjective", "rationale": "For forgetting sex is an expression of the individual ' s own subjective sense and cognitive ability."}, "sentence_sentiment": {"answer": "Negative", "rationale": "Basis for judgement: Forgetting sex is often considered a negative quality because it means having bad memory or often forgetting things."}, "language_style": {"answer": "Calm and direct", "rationale": "The entry directly asked the respondents whether they had poor memory, the language style was more objective and straightforward, and not too much speech or emotional colour was used for the refinement."}, "time_dimension": {"answer": "Present", "rationale": "The keyword is \"forget sexual\" and it refers to the person's current state or feelings, not to past or future behaviour or mentality. Thus, the temporal dimension of the article is now."}, "sentence_type": "Negative declarative sentence", "pos": {"nouns": {"Forget sex": 1, "Large": 1}}}, {"content": "Fear of being dressed and dressed properly.", "word_count": 15, "label": "", "sentence_structure": "Subject鈥搗erb鈥揷omplement", "subjective_objective": {"answer": "Subjective", "rationale": "Because \"fear\" is a subjective psychological state, the way you look at your own instrument is also from a personal perspective. So, \"fear of your own dress and the proper way of doing it\" is the patient's subjective attitude and thoughts."}, "sentence_sentiment": {"answer": "Negative", "rationale": "Because \"regarded\" is negative."}, "language_style": {"answer": "Tactful and warm", "rationale": "The language style of the entry is more euphemistic and friendly, leading them to think and answer questions by asking them whether they are worried about their own clothes and their manners. This expression brings the questionnaire closer to the emotional and psychological state of the interviewee, and increases the sensitivity and interaction of the questionnaire."}, "time_dimension": {"answer": "Present", "rationale": "The keyword \"fear\" suggests that this is a concern or a reflection that exists, while \"dressing and proper\" is a description of the current state. Thus, the time dimension of the entry is now judged."}, "sentence_type": "Imperative sentence", "pos": {"nouns": {"Fear": 1, "self": 1, "Clothing": 1, "Clean": 1, "Ceremonial": 1, "Positive": 1}, "verbs": {"I'm worried": 1, "and": 1}, "numerals": {"10": 1}, "pronouns": {"self": 1}}}, {"content": "11. Easier to worry and more excited.", "word_count": 7, "label": "", "sentence_structure": "Subject鈥搗erb鈥揷omplement", "subjective_objective": {"answer": "Subjective", "rationale": "Because pain and excitement are feelings inside the patient, they are subjective."}, "sentence_sentiment": {"answer": "Negative", "rationale": "Because \"fear\" and \"excited\" in the entries are negative emotional experiences."}, "language_style": {"answer": "Tactful and warm", "rationale": ""}, "time_dimension": {"answer": "Present", "rationale": "The entry asks about the current situation of the person being tested, the keywords being \"fear-prone\" and \"excited\", which indicate an emotional state, which is usually used to describe the psychological characteristics of the individual in the current situation. Thus, the temporal dimension is now judged."}, "sentence_type": "Imperative sentence", "pos": {"nouns": {"Trouble": 1, "Excited": 1}, "verbs": {"Easy": 1}, "numerals": {"11": 1}}}, {"content": "12. Heartaches", "word_count": 2, "label": "", "sentence_structure": "Subject鈥搗erb鈥揷omplement", "subjective_objective": {"answer": "Objective", "rationale": "Since chest pain is a physical condition of the patient, it is an objective fact."}, "sentence_sentiment": {"answer": "Negative", "rationale": "Because \" chest pain\" is usually considered a disease or a manifestation of physical discomfort, with a negative connotation."}, "language_style": {"answer": "Calm and direct", "rationale": "The entry uses a more direct, concise language style, which clearly asks the other side whether there has been chest pain, is not overly adoctractive or emotional and is a calm and direct language style."}, "time_dimension": {"answer": "Present", "rationale": "The entry is to ask the testee whether he is currently suffering from chest pain and therefore the time dimension is now judged."}, "sentence_type": "Interrogative sentence", "pos": {"nouns": {"- Chestaches": 1}, "numerals": {"12": 1}}}, {"content": "13. Fear of vacant places or streets", "word_count": 10, "label": "", "sentence_structure": "Subject鈥搗erb鈥揷omplement", "subjective_objective": {"answer": "Subjective", "rationale": "Because fear is an emotional reaction and experience of an individual, it is based on the individual ' s psychological state and experience and is therefore subjective in nature."}, "sentence_sentiment": {"answer": "Negative", "rationale": "Because the word \"fear\" in the entry is negative."}, "language_style": {"answer": "Tactful and warm", "rationale": "It is more friendly and interactive in its expression of a gentle and inviting tone, rather than a direct statement or order. Moreover, the article is more sensitive in its content, and the use of euphemism better protects the privacy and self-esteem of the person under investigation."}, "time_dimension": {"answer": "Past", "rationale": "The expression \"fear of an empty place or street\" is a description of the person's current psychological state or emotional response, and can therefore be judged as now."}, "sentence_type": "Negative declarative sentence", "pos": {"nouns": {"Places": 1, "Street": 1}, "verbs": {"Scared": 1}, "numerals": {"13": 1}}}, {"content": "14. Feels less energy and slower activity", "word_count": 13, "label": "", "sentence_structure": "Subject鈥揷opula鈥損redicative", "subjective_objective": {"answer": "Subjective", "rationale": "This questionnaire entry examines the patient鈥檚 subjective feelings, and therefore it is exported to subjective people."}, "sentence_sentiment": {"answer": "Negative", "rationale": "Because \"dreak\" and \"slow\" are negative descriptions."}, "language_style": {"answer": "Calm and direct", "rationale": "The entry asks the respondents in a direct and clear manner whether there is a decline in energy, a slowdown in activity, no excessive embellishment or emotional rendering, and a calm and straightforward language style."}, "time_dimension": {"answer": "Past", "rationale": ""}, "sentence_type": "Negative declarative sentence", "pos": {"nouns": {"- The energy": 1, "Activities": 1}, "verbs": {"Down": 1, "Slow down": 1}, "numerals": {"14": 1}}}, {"content": "15. To end your life.", "word_count": 8, "label": "", "sentence_structure": "Subject鈥搗erb鈥揷omplement", "subjective_objective": {"answer": "Subjective", "rationale": "Because \"want to end your life\" is a subjective idea and will that reflects patients鈥?subjective feelings and attitudes to life, relationships, etc. While this idea may be influenced by objective factors, it is more accurate to look at the patient from the subjective point of view."}, "sentence_sentiment": {"answer": "Negative", "rationale": "Because the entry relates to the idea of suicide, it is a very negative and dangerous idea."}, "language_style": {"answer": "Calm and direct", "rationale": "The entry uses direct and clear language to ask the other party whether it is suicidal and does not attempt to glorify or conceal the seriousness of the problem, so that the language style is straightforward and calm."}, "time_dimension": {"answer": "Future", "rationale": "The entry asks if the person tested intends to end his or her life in the future, and the temporal dimension is judged to be the future."}, "sentence_type": "Imperative sentence", "pos": {"nouns": {"Life": 3}, "verbs": {"I want to end it": 1}, "numerals": {"15": 1}}}, {"content": "16. Hearing voices that no one else heard", "word_count": 10, "label": "", "sentence_structure": "Subject鈥搗erb鈥揷omplement", "subjective_objective": {"answer": "Subjective", "rationale": "Because hearing voices that are not heard is a subjective feeling and experience, and it reflects the sense and state of consciousness that the patient has inside. While sometimes such voices may be an objective presence (such as the effects of certain diseases or medicines), in this case, they are more of a subjective perception of the patient."}, "sentence_sentiment": {"answer": "Negative", "rationale": "This is a negative expression, as hearing voices that are not heard by others may indicate that individuals are suffering from mental illness."}, "language_style": {"answer": "Tactful and warm", "rationale": "The entry is more personal and interactive when it is spoken in a gentle and inviting tone, rather than in direct statements or orders. At the same time, the \"sweet\" language style makes the interviewee feel respected and understood, and more willing to share his real thoughts and feelings."}, "time_dimension": {"answer": "Future", "rationale": "The keyword \"hearing voices that no one else can hear\" describes a situation that may arise in the future and is therefore judged to be the future."}, "sentence_type": "Interrogative sentence", "pos": {"nouns": {"Questionnaire": 1, "Entry": 1, "Sound": 1}, "verbs": {"Yes": 1, "The others": 1}, "numerals": {"16": 1}}}, {"content": "17. Wrecking.", "word_count": 2, "label": "", "sentence_structure": "Subject鈥搗erb鈥揷omplement", "subjective_objective": {"answer": "Objective", "rationale": "Since shaking is a physical reaction of a person, not controlled by the individual ' s will, it is an objective view."}, "sentence_sentiment": {"answer": "Negative", "rationale": "Because \"shock\" is usually considered a negative physical reaction."}, "language_style": {"answer": "Tactful and warm", "rationale": "For the title \"17. Wrestle,\" I will judge the language style according to the following criteria: 1. The use of the vocabulary: if the term used in the questionnaire is soft, close, and the term \"shock\" is used instead of \"diverse,\" it may give a warmer sense of euphemism. 2. The sentence structure: If the sentence is simple, not too much phrasing and rhetorical, and is sound and objective, it may be a calm and direct expression. In combination, I will judge the language style of the questionnaire entry as \"cool and direct.\" The basis of the judgement is that the questionnaire entry uses relatively neutral terms \"shock\" without excessive emotional thawing. At the same time, the sentence is simple, without excessive stair or emotional sensibility, and the tone is more objective, so I think its language style is \"cool and direct.\""}, "time_dimension": {"answer": "Future", "rationale": "The entry describes future behaviour or state, and the keyword is \"shocking\" , which means that this is a possible situation and has nothing to do with past and present experience."}, "sentence_type": "Interrogative sentence", "pos": {"verbs": {"# Wresh #": 1}}}, {"content": "18. It felt that most people were not trustworthy.", "word_count": 11, "label": "", "sentence_structure": "Subject鈥揷opula鈥損redicative", "subjective_objective": {"answer": "Subjective", "rationale": "Because \"fear\" is the patient's own sense and experience, and it's a subjective feeling, not an objective fact."}, "sentence_sentiment": {"answer": "Negative", "rationale": "Because the \"Feeling Most Untrustable\" attitude in the entry is negative."}, "language_style": {"answer": "Calm and direct", "rationale": "The entry expresses, in a direct and concise manner, the opinion of the subject of the survey about the prevailing sense of mistrust in society, which is not accompanied by too many emotional or rhetorical techniques, and is more objective in tone. The language style of the purpose is therefore straightforward and calm."}, "time_dimension": {"answer": "Present", "rationale": "The \"feel\" in the entry shows what the testee is experiencing, and the description \"most people cannot trust\" is based on the testee's present experience. So the entry should be classified as \"now\"."}, "sentence_type": "Negative declarative sentence", "pos": {"nouns": {"Questionnaire": 1, "Entry": 1, "person": 1, "Most of them": 1}, "verbs": {"I feel": 1, "Both": 1, "Trustable": 1}, "adverbs": {"18": 1}, "numerals": {"18": 1}}}, {"content": "19. The appetite is bad.", "word_count": 4, "label": "", "sentence_structure": "Subject鈥搗erb鈥揷omplement", "subjective_objective": {"answer": "Subjective", "rationale": "Because \"a bad appetite\" is a subjective feeling and experience, reflecting the patient's desire for food and the satisfaction of the person. It is not influenced by the outside world, but depends on the patient's physical and mental state."}, "sentence_sentiment": {"answer": "Negative", "rationale": "Because \"bad appetite\" is often considered a manifestation of physical discomfort or low mood, it has a negative emotional connotation."}, "language_style": {"answer": "Tactful and warm", "rationale": "The entry raises questions in a sense of concern and understanding of the other party ' s feelings, without direct accusations or criticism, and is more gentle and friendly and more likely to trigger a positive response from the other party."}, "time_dimension": {"answer": "Past", "rationale": "The entry is judged to be past, as it is known from the time indicator \"19.\" The entry is for questioning the conduct or circumstances of the person who was tested over time."}, "sentence_type": "Imperative sentence", "pos": {"nouns": {"-Adulge": 1}, "verbs": {"Okay": 1}}}, {"content": "20. Weep easily.", "word_count": 4, "label": "", "sentence_structure": "Subject鈥揷opula鈥損redicative", "subjective_objective": {"answer": "Subjective", "rationale": "Because the ease of crying is an emotional response, a subjective feeling and experience of the patient."}, "sentence_sentiment": {"answer": "Negative", "rationale": "Because the \"Easier to Cry\" act in the entry is negative."}, "language_style": {"answer": "Calm and direct", "rationale": "The language style of the entry is calm and straightforward, without excessive embellishment or emotional rendering, and instead directly asks the interviewee whether he/she is easily weeping with a view to obtaining objective and accurate answers."}, "time_dimension": {"answer": "Present", "rationale": "According to the sentence entered, \"20. Easy to cry\" is a question of the current state of the testee, which does not address the time dimension of the past or the future, and therefore is now judged."}, "sentence_type": "Affirmative declarative sentence", "pos": {"nouns": {"Crying": 1}, "verbs": {"Easy": 1}}}, {"content": "21. Relent when you're dealing with opposite sex.", "word_count": 13, "label": "", "sentence_structure": "Subject鈥揷opula鈥損redicative", "subjective_objective": {"answer": "Subjective", "rationale": "Being shy is a subjective feeling and experience associated with individual emotions, cognitive factors, etc."}, "sentence_sentiment": {"answer": "Negative", "rationale": "Because the \"smuggling\" act in the entry is negative."}, "language_style": {"answer": "Tactful and warm", "rationale": ""}, "time_dimension": {"answer": "Present", "rationale": ""}, "sentence_type": "Affirmative declarative sentence", "pos": {"nouns": {"Heterosexual": 1, "Get along": 1, "Shy": 1, "I'm not comfortable": 1}, "verbs": {"I feel": 1, "Get along": 1}, "adjectives": {"Shy": 1, "I'm not comfortable": 1}, "numerals": {"21": 1}}}, {"content": "22. I feel cheated, caught in the trap or someone trying to catch you.", "word_count": 14, "label": "", "sentence_structure": "Subject鈥搗erb鈥揷omplement", "subjective_objective": {"answer": "Subjective", "rationale": "Because \"fearing to be deceived, being in a trap or wanting to arrest you\" is a subjective feeling and idea of something. Although this may be related to some objective fact, it is subjective in itself."}, "sentence_sentiment": {"answer": "Negative", "rationale": "Because the entries are all negative, like \"smuggling, trapping\"."}, "language_style": {"answer": "Calm and direct", "rationale": "The entry is more directly and explicitly asked whether the person surveyed had been deceived, tricked or mala fided, was not using excessive rhetorical techniques or emotional rendering, and was more objective."}, "time_dimension": {"answer": "Past", "rationale": ""}, "sentence_type": "Exclamatory sentence", "pos": {"nouns": {"- Liar": 1, "A trap": 1, "person": 1}, "verbs": {"I feel": 1, "Swindled": 1, "Medium": 1, "Grab him": 1}, "adverbs": {"Yes": 1}, "numerals": {"22": 1}, "classifiers": {"individual": 1}}}, {"content": "23. Suddenly, for no reason, it was a fear.", "word_count": 11, "label": "", "sentence_structure": "Subject鈥搗erb鈥揷omplement", "subjective_objective": {"answer": "Subjective", "rationale": "Because \"a sudden fear without cause\" is a psychological experience or emotional reaction that can be experienced by a patient and is seen from a subjective perspective."}, "sentence_sentiment": {"answer": "Negative", "rationale": "Because the \"sudden fear without reason\" in the entry is negative."}, "language_style": {"answer": "Calm and direct", "rationale": "The entry directly expresses the subject of an inquiry into the existence of unwarranted fear, and is not based on excessive rhetorical or emotional rendering, and is more objective in tone."}, "time_dimension": {"answer": "Past", "rationale": ""}, "sentence_type": "Affirmative declarative sentence", "pos": {"nouns": {"For no reason": 1, "Scared": 1}, "verbs": {"Suddenly, I felt": 1}, "adverbs": {"For no reason": 1}, "numerals": {"23": 1}}}, {"content": "24. I can't control myself.", "word_count": 11, "label": "", "sentence_structure": "Subject鈥搗erb鈥揷omplement", "subjective_objective": {"answer": "Subjective", "rationale": "Because \"incontrol\" means it's a subjective act caused by the patient's inner feelings."}, "sentence_sentiment": {"answer": "Negative", "rationale": "Because the \"grumpy\" in the entry is a negative act, which means that emotions are out of control and not conducive to the physical and mental health of individuals and social interaction."}, "language_style": {"answer": "Tactful and warm", "rationale": "The entry uses a more euphemistic description than simply asking the other side whether this is happening, for example, by replacing the expression \"uncontrolled\" with the expression \"often\" to make the problem more objective and neutral. Such language styles are usually more acceptable to the respondents and more likely to receive real feedback."}, "time_dimension": {"answer": "Present", "rationale": "The keyword \"uncontrollable anger\" describes the current state of emotion and behaviour, so the entry asks about the person being tested's current behaviour or mentality."}, "sentence_type": "Negative declarative sentence", "pos": {"nouns": {"self": 1, "- Grumpy": 1}, "verbs": {"No, I can't": 1, "Control": 1, "Big hair": 1}, "adverbs": {"Land": 1}, "numerals": {"24": 1}}}, {"content": "25. Fear of leaving alone.", "word_count": 5, "label": "", "sentence_structure": "Subject鈥搗erb鈥揷omplement", "subjective_objective": {"answer": "Subjective", "rationale": "Fear of going out alone is a subjective feeling and attitude, which reflects the fear of patients in public alone."}, "sentence_sentiment": {"answer": "Negative", "rationale": "Because the act of \"fearing to go out alone\" in the entry is negative."}, "language_style": {"answer": "Tactful and warm", "rationale": "The entry is more adept and interactive when it is spoken in a gentle and inviting manner, rather than by direct statements or orders."}, "time_dimension": {"answer": "Future", "rationale": "The keyword \"fearing to leave alone\" expresses a fear that may occur in the future, so the temporal dimension of the article is the future."}, "sentence_type": "Negative declarative sentence", "pos": {"nouns": {"Questionnaire": 1, "Entry": 1, "Alone": 1, "Out": 1}, "verbs": {"Yes": 1, "Analysis": 1}, "numerals": {"25": 1}}}, {"content": "26. Too often blame yourself.", "word_count": 6, "label": "", "sentence_structure": "Subject鈥搗erb鈥揷omplement", "subjective_objective": {"answer": "Subjective", "rationale": "Because blame is the patient's subjective attitude."}, "sentence_sentiment": {"answer": "Negative", "rationale": "Because the \"criticizing\" act is negative."}, "language_style": {"answer": "Calm and direct", "rationale": "The entry \"Calm down straight\" asks, in the form of direct inquiries, whether the other party often blames himself, that the language style is more calm and objective, and that there is no excessive emotional modification or rhetorical technique."}, "time_dimension": {"answer": "Present", "rationale": "The term \"often\" means that the act is a frequent one, usually a customary one. At the same time, \"criticizing yourself\" is a psychological activity that occurs in a person, so the entry is asking about the current customary psychological state of the person being tested. On the basis of these judgements, we can conclude that the questionnaire entries reflect the current situation."}, "sentence_type": "Negative declarative sentence", "pos": {"nouns": {"Blame": 1, "self": 1}, "verbs": {"Regular": 1}, "numerals": {"26": 1}}}, {"content": "27. A backache.", "word_count": 2, "label": "", "sentence_structure": "Subject鈥搗erb鈥揷omplement", "subjective_objective": {"answer": "Objective", "rationale": "Because a back pain is a physical condition that can be observed and measured, it is not a subjective feeling or idea of the patient."}, "sentence_sentiment": {"answer": "Negative", "rationale": "Because \"penal pain\" is a physical discomfort, which is usually considered negative."}, "language_style": {"answer": "Calm and direct", "rationale": "The subject is a direct question of the question of whether the interviewee has a backache, and does not use euphemism or emotional language, so it is clear that the language style is straightforward and calm."}, "time_dimension": {"answer": "Past", "rationale": ""}, "sentence_type": "Affirmative declarative sentence", "pos": {"nouns": {"My back hurts": 1}}}, {"content": "28. Difficulties in fulfilling the mandate", "word_count": 8, "label": "", "sentence_structure": "Subject鈥揷opula鈥損redicative", "subjective_objective": {"answer": "Subjective", "rationale": "Because the word \"fear\" represents the emotional and subjective feelings of the patient."}, "sentence_sentiment": {"answer": "Negative", "rationale": "Because the feeling of \"hard to do\" in the entry is negative."}, "language_style": {"answer": "Calm and direct", "rationale": "The entry asked the respondent whether he or she was having difficulty in completing his or her mission, without excessive ado or emotional, and therefore the language style was \"cool and straight\"."}, "time_dimension": {"answer": "Present", "rationale": "It is judged by the phrase \"felt\" and \"difficult to accomplish\" that this is a status quo or current feeling expressed by the testees in their responses to the survey questions, and therefore the time dimension should be the present."}, "sentence_type": "Interrogative sentence", "pos": {"nouns": {"Tasks": 1}, "verbs": {"I feel": 1, "Completed": 1}, "numerals": {"28": 1}}}, {"content": "29. Feeling alone.", "word_count": 4, "label": "", "sentence_structure": "Subject鈥揷opula鈥損redicative", "subjective_objective": {"answer": "Subjective", "rationale": "Because feeling alone is the individual feeling and experience of the patient, and it is in the realm of subjective emotions."}, "sentence_sentiment": {"answer": "Negative", "rationale": "Because feeling alone is an expression of a negative emotion, implying a negative psychological state of the individual."}, "language_style": {"answer": "Tactful and warm", "rationale": "The language style of the entry is more euphemistic, asking each other whether they are often alone in a caring and understanding tone, and this expression is more accommodative and warm, reducing the resistance of the respondents and increasing the rate of questionnaire recovery."}, "time_dimension": {"answer": "Present", "rationale": "The expression \"fearing alone\" means that the dominant language feels or experiences at the moment, and the entry is therefore an inquiry into the current emotional state of the person being tested, and the temporal dimension is now judged."}, "sentence_type": "Affirmative declarative sentence", "pos": {"nouns": {"Loneliness": 1}}}, {"content": "30. Disturbing", "word_count": 4, "label": "", "sentence_structure": "Subject鈥揷opula鈥損redicative", "subjective_objective": {"answer": "Subjective", "rationale": "Because feeling bored is a subjective emotional experience, determined by the individual ' s feelings and perceptions."}, "sentence_sentiment": {"answer": "Negative", "rationale": "Because \"distressed\" is a negative emotional state."}, "language_style": {"answer": "Calm and direct", "rationale": "The entry asks the interviewees whether they are often bored in a straightforward and clear manner, with a more calm and straightforward language style, without too much ado or emotional colour.\""}, "time_dimension": {"answer": "Present", "rationale": ""}, "sentence_type": "Interrogative sentence", "pos": {"nouns": {"I'm bored": 1}, "verbs": {"I feel": 1}, "numerals": {"30": 1}}}, {"content": "31. Excessive concern", "word_count": 4, "label": "", "sentence_structure": "Subject鈥搗erb鈥揷omplement", "subjective_objective": {"answer": "Subjective", "rationale": "Because \"excessive worry\" is the patient's assessment and perception of his emotional state, and it's subjective."}, "sentence_sentiment": {"answer": "Negative", "rationale": "Because the entry \"too worried\" expresses a negative emotional attitude."}, "language_style": {"answer": "Calm and direct", "rationale": "The entry directly asks whether the respondent is over-concerned about a problem, with a more straightforward, concise and unsure language style, with no excessive aesthetic or emotional colour, which is typical of a calm and direct language style."}, "time_dimension": {"answer": "Present", "rationale": ""}, "sentence_type": "Interrogative sentence", "pos": {"nouns": {"That's too much": 1, "Fear": 1}}}, {"content": "32. Not interested in things.", "word_count": 7, "label": "", "sentence_structure": "Subject鈥搗erb鈥揷omplement", "subjective_objective": {"answer": "Subjective", "rationale": "Because \"not interested in things\" is a subjective feeling or perception that reflects the patient's inner state and emotional response. This subjective experience is not measured by objective facts, but is obtained through subjective reports or psychological tests. Thus, the question is viewed from the perspective of the patient."}, "sentence_sentiment": {"answer": "Negative", "rationale": "Because the attitude of \"not interested in things\" is negative."}, "language_style": {"answer": "Calm and direct", "rationale": "The entry asks the respondents directly whether they are not interested in the matter, are not using excessive rhetoric or emotional colours, and are more objective and neutral."}, "time_dimension": {"answer": "Present", "rationale": "According to the phrase \"not interested in things\", the entry is a question of whether the person tested has no interest in things at the current attitude or state, and thus can be judged as now."}, "sentence_type": "Negative declarative sentence", "pos": {"nouns": {"Things": 1, "Interest": 1}, "verbs": {"to": 1, "Not interested": 1}, "numerals": {"32": 1}}}, {"content": "33. Fear", "word_count": 4, "label": "", "sentence_structure": "Subject鈥揷opula鈥損redicative", "subjective_objective": {"answer": "Subjective", "rationale": "It's the patient's subjective feeling to be afraid."}, "sentence_sentiment": {"answer": "Negative", "rationale": "Because \"fear\" is a negative emotional experience."}, "language_style": {"answer": "Calm and direct", "rationale": "The theme of \"fear\" is not over-repeated, but is presented in a more objective, straightforward way, with more calm tone. This expression allows respondents to understand and answer questions more intuitively."}, "time_dimension": {"answer": "Past", "rationale": "The keyword \"fear\" means that the person who was tested has experienced it at some point in the past."}, "sentence_type": "Affirmative declarative sentence", "pos": {"verbs": {"I feel": 1, "Scared": 1}}}, {"content": "My feelings are vulnerable.", "word_count": 10, "label": "", "sentence_structure": "Subject鈥揷opula鈥損redicative", "subjective_objective": {"answer": "Subjective", "rationale": "Because \"vulnerable\" is a personal feeling and attitude that belongs to the subjective views and experiences of patients."}, "sentence_sentiment": {"answer": "Negative", "rationale": "Because the description of \"emotional vulnerability\" in the entry is negative."}, "language_style": {"answer": "Tactful and warm", "rationale": ""}, "time_dimension": {"answer": "Present", "rationale": ""}, "sentence_type": "Negative declarative sentence", "pos": {"nouns": {"Emotion": 1, "Hurt": 1}, "verbs": {"Easy": 1, "Yes": 1}, "adverbs": {"Regular": 1}, "numerals": {"34": 1}}}, {"content": "35. The others know your thoughts.", "word_count": 11, "label": "", "sentence_structure": "Subject鈥搗erb鈥揷omplement", "subjective_objective": {"answer": "Subjective", "rationale": "Because it is possible to know whether your private thoughts are based on observations and inferences from others, which are subjective in nature."}, "sentence_sentiment": {"answer": "Negative", "rationale": "Because the act of \"someone else knows your private thoughts\" in the entry may give individuals a sense of insecurity and risk of disclosure of privacy, and it is in a negative emotional state."}, "language_style": {"answer": "Calm and direct", "rationale": "The entry uses a direct, clear language to describe the problem, without too many rhetorical or emotional colours, and with a more objective and calm tone. This language style is suitable for official settings such as questionnaires, which can be effective in avoiding misunderstanding or ambiguity and in improving data accuracy and effectiveness."}, "time_dimension": {"answer": "Present", "rationale": "The keywords \"know\" and \"private thoughts\" indicate that this is a description of the person's current behaviour, so the time dimension of the article is now."}, "sentence_type": "Exclamatory sentence", "pos": {"nouns": {"The others": 1, "you": 1, "Private": 1, "Thoughts": 1}, "verbs": {"Yes": 2, "EMPTY": 1}, "adverbs": {"35": 1}, "numerals": {"35": 1}}}, {"content": "36. It's not like you're being understood.", "word_count": 12, "label": "", "sentence_structure": "Subject鈥搗erb鈥揷omplement", "subjective_objective": {"answer": "Subjective", "rationale": "Because \"fear\" is a subjective feeling that reflects the patient's perception of the surrounding environment and of others."}, "sentence_sentiment": {"answer": "Negative", "rationale": "Because the words \"ununderstanding\" and \"unsympathetic\" in the entries are negative, expressing a sense of misperception and indifference."}, "language_style": {"answer": "Calm and direct", "rationale": "The entry is a direct question to the respondents about whether they have been misconstrued or not understood in their social contacts, and the language style is relatively calm and objective."}, "time_dimension": {"answer": "Present", "rationale": ""}, "sentence_type": "Negative declarative sentence", "pos": {"nouns": {"you": 1, "Compassion": 1}, "verbs": {"I feel": 1, "I don't understand": 1}, "numerals": {"36": 1}}}, {"content": "I feel like you're not friendly. I don't like you.", "word_count": 13, "label": "", "sentence_structure": "Subject鈥揷opula鈥損redicative", "subjective_objective": {"answer": "Subjective", "rationale": "Because words like \"fear\" and \"like\" are the feelings and attitudes inside the patient, and they are subjective."}, "sentence_sentiment": {"answer": "Negative", "rationale": "Because the \"Feeling that you're not friendly, don't like you\" act is negative."}, "language_style": {"answer": "Calm and direct", "rationale": "The entry is more direct and clear in language style, is not subject to excessive rhetoric or emotional rendering, and is more objective and neutral in tone. It asks whether the person surveyed feels negative feelings about them, which are more easily accessible to real answers and data."}, "time_dimension": {"answer": "Present", "rationale": "The entry in the past asked whether the testee had ever felt unfriendly or unfriendly in his past experience and feelings. The key words were \"fear\" and \"unfriendly\" , all of which were descriptions of past experiences, so they were judged to be past."}, "sentence_type": "Interrogative sentence", "pos": {"nouns": {"Questionnaire Entry": 1, "person": 2}, "verbs": {"I feel": 1, "For you": 1, "Unfriendly": 1, "Yeah": 1}, "adjectives": {"Friendly": 1}, "adverbs": {"not": 1}, "numerals": {"37": 1}, "pronouns": {"you": 1}}}, {"content": "38. Doing things very slowly to make sure it's done right.", "word_count": 15, "label": "", "sentence_structure": "Subject鈥搗erb鈥揷omplement", "subjective_objective": {"answer": "Subjective", "rationale": "The purpose of the questionnaire, as it is supposed to be, is \"to do things very slowly to ensure that they are done correctly\", which is the patient's own subjective feeling and idea. Therefore, the entry is subjective in nature."}, "sentence_sentiment": {"answer": "Negative", "rationale": "Because the \"doing things very slowly\" act in the entry is negative."}, "language_style": {"answer": "Calm and direct", "rationale": "The entry is simple, is not too emotionally or orally expressive and is more direct. The aim is to understand how the person surveyed is about speed and quality when doing things, and therefore is more linguistically oriented towards objectivity, calmness and directness."}, "time_dimension": {"answer": "Present", "rationale": "The entry asks about the current behaviour or habits of the person being tested, and the key words \"doing business\" and \"must be done very slowly to ensure that it is done correctly\" indicate that this is a state or habit that is now in progress."}, "sentence_type": "Interrogative sentence", "pos": {"nouns": {"Yes": 2, "Slow": 1, "Promise": 1, "Correct": 1}, "verbs": {"Do it": 1, "I have to": 1, "Okay": 2, "very": 1, "Here": 1}, "adverbs": {"Slow": 1, "very": 1}, "numerals": {"38": 1}, "classifiers": {"individual": 1}}}, {"content": "39. Heart rate is high.", "word_count": 6, "label": "", "sentence_structure": "Subject鈥揷opula鈥損redicative", "subjective_objective": {"answer": "Subjective", "rationale": "Because a heart beating is a subjective feeling of the patient."}, "sentence_sentiment": {"answer": "Negative", "rationale": "Because the expression \"heart beating\" has a negative emotional effect."}, "language_style": {"answer": "Calm and direct", "rationale": "The entry directly asks the respondents whether they experience an acceleration of the heart rate in a given situation, does not use excessive rhetoric or emotional colours, and is more calm and straightforward in language style."}, "time_dimension": {"answer": "Present", "rationale": "The entry now asks about the current heartbeat of the person being tested, does not address past or future behaviour or mentalities, etc., and therefore determines the current behaviour or state."}, "sentence_type": "Affirmative declarative sentence", "pos": {"nouns": {"Heart rate": 1, "That's great": 1}, "verbs": {"Okay": 1, "very": 1}, "adjectives": {"That's great": 1}, "adverbs": {"very": 1}, "numerals": {"39": 1}}}, {"content": "40. Disgusting or stomach.", "word_count": 8, "label": "", "sentence_structure": "Subject鈥搗erb鈥揷omplement", "subjective_objective": {"answer": "Subjective", "rationale": "Because nausea or stomach discomfort is a subjective feeling or experience, it is a description of the patient ' s own body and is therefore subjective in nature."}, "sentence_sentiment": {"answer": "Negative", "rationale": "Because it is an expression of physical discomfort and negative emotional colour."}, "language_style": {"answer": "Tactful and warm", "rationale": "The entry is interactive and questions with the use of terms such as \"obsessed\" and \"infirmated stomach\" to describe possible symptoms, which are relatively mild and not too negative. Moreover, the tone of the problem is relatively soft, and the sense of the person is more accommodative and understanding, reflecting the concern and respect for the person under investigation."}, "time_dimension": {"answer": "Present", "rationale": ""}, "sentence_type": "Imperative sentence", "pos": {"nouns": {"Gross": 1, "Stomach": 1, "I'm not feeling well": 1}, "numerals": {"40": 1}}}, {"content": "41. I feel like I'm not as good as anyone else.", "word_count": 7, "label": "", "sentence_structure": "Subject鈥揷opula鈥損redicative", "subjective_objective": {"answer": "Subjective", "rationale": "Because \"fEss\" is patient subjective and self-evaluation."}, "sentence_sentiment": {"answer": "Negative", "rationale": "Because the \"near as good as other\" sentiment in the entry is negative."}, "language_style": {"answer": "Calm and direct", "rationale": "The entry is more straightforward in language style, with no too many pedestals or euphemisms, but rather a straightforward question about whether the interviewee feels like he or she is inferior to others. This simple and clear expression is more conducive to obtaining the real thoughts and feelings of the interviewee."}, "time_dimension": {"answer": "Present", "rationale": "\"Feel\" is the current state of affairs, \"not as much as other people\" describes a state of present or emotional experience, so the entry asks about the current feelings and perceptions of the subjects."}, "sentence_type": "Exclamatory sentence", "pos": {"nouns": {"41": 1, "Others": 1}, "verbs": {"I feel": 1, "Not as good as it is": 1}, "numerals": {"41": 1}}}, {"content": "42. Muscle acidic pain", "word_count": 4, "label": "", "sentence_structure": "Subject鈥揷opula鈥損redicative", "subjective_objective": {"answer": "Objective", "rationale": "As muscle acidity is a physical symptom of physical experience, it is not the patient ' s idea or feeling, and is therefore objectively viewed."}, "sentence_sentiment": {"answer": "Neutral", "rationale": "Because the entry is only a description of a symptom and does not involve any emotional or attitudinal tendencies."}, "language_style": {"answer": "Calm and direct", "rationale": "The language style of the entry is clear, there is no oral or emotional rendering, and the interviewee is simply asked whether there was a muscular sore in the exercise."}, "time_dimension": {"answer": "Past", "rationale": "The entry asks if the person tested had past muscle acidity and therefore judged \"the past\"."}, "sentence_type": "Affirmative declarative sentence", "pos": {"nouns": {"Muscles": 1, "Sour": 1}, "numerals": {"42": 1}}}, {"content": "I feel someone is watching you talking about you.", "word_count": 11, "label": "", "sentence_structure": "Subject鈥搗erb鈥揷omplement", "subjective_objective": {"answer": "Subjective", "rationale": "The perception of being under surveillance is a subjective feeling and experience of the patient and a subjective judgement of the surrounding environment."}, "sentence_sentiment": {"answer": "Negative", "rationale": "Because the expression \"someone is watching you\" in the entry is a feeling of aggression, mistrust, and negative."}, "language_style": {"answer": "Tactful and warm", "rationale": "The entry is more adept and interactive when it is spoken in a gentle and inviting manner, rather than by direct statements or orders."}, "time_dimension": {"answer": "Past", "rationale": "The keyword \"seems someone watching you talk about you\" describes an experience or a feeling that may be past experience or current experience, and the temporal dimension of this article is therefore past."}, "sentence_type": "Interrogative sentence", "pos": {"nouns": {"person": 1, "Watch": 1, "Talk": 1, "you": 2}, "verbs": {"Sensor": 1, "in": 1, "Watch": 1, "Talk": 1}, "numerals": {"43": 1}, "pronouns": {"you": 2}}}, {"content": "44. Hard to sleep", "word_count": 4, "label": "", "sentence_structure": "Subject鈥搗erb鈥揷omplement", "subjective_objective": {"answer": "Subjective", "rationale": "The questionnaire entry \"44. The difficulty of sleeping\" is subjective. Because it is a subjective problem for the patient, not an objective fact. Although insomnia is an objective problem, whether or not a person feels difficult to sleep requires an assessment by the patient's subjective feeling."}, "sentence_sentiment": {"answer": "Negative", "rationale": "Because the \"sleeping\" behavior in the entries is negative."}, "language_style": {"answer": "Calm and direct", "rationale": ""}, "time_dimension": {"answer": "Present", "rationale": "The entry is now judged on the time dimension of the testee ' s current difficulty in sleeping."}, "sentence_type": "Exclamatory sentence", "pos": {"nouns": {"Investigations": 1, "Sleep disorder": 1, "Insomnia": 1, "Difficulties": 1}, "verbs": {"Come on": 1, "Result": 1, "Impact": 1}, "adverbs": {"Serious": 1, "Hard": 1}, "numerals": {"44": 1}}}, {"content": "45. Business has to be checked over and over again.", "word_count": 8, "label": "", "sentence_structure": "Subject鈥搗erb鈥揷omplement", "subjective_objective": {"answer": "Subjective", "rationale": "Because repeated inspections are the subjective behaviour and habit of a person."}, "sentence_sentiment": {"answer": "Negative", "rationale": "Because the behaviour of \"must be checked repeatedly\" in the entry is negative, implying a lack of confidence or excessive caution."}, "language_style": {"answer": "Calm and direct", "rationale": "The entry describes in a concise and concise language whether the subject needs to be examined repeatedly when the person is doing his or her business, without too many rhetorical or emotional renderings, and with a more straightforward language style."}, "time_dimension": {"answer": "Present", "rationale": ""}, "sentence_type": "Affirmative declarative sentence", "pos": {"nouns": {"Things": 1, "Inspection": 1}, "verbs": {"Do it": 1, "Repeatedly": 1, "I have to": 1}, "numerals": {"45": 1}}}, {"content": "46. Difficulties in taking decisions", "word_count": 6, "label": "", "sentence_structure": "Subject鈥揷opula鈥損redicative", "subjective_objective": {"answer": "Subjective", "rationale": "Because \"hard to make a decision\" is a subjective description of the patient's own feelings and experiences."}, "sentence_sentiment": {"answer": "Negative", "rationale": "Because the \"hard to make a decision\" in the entry represents a difficulty and a challenge, with a negative emotional tone."}, "language_style": {"answer": "Tactful and warm", "rationale": ""}, "time_dimension": {"answer": "Past", "rationale": ""}, "sentence_type": "Negative declarative sentence", "pos": {"nouns": {"Decision": 1}, "verbs": {"Do it": 1, "Hard": 1, "Out": 1}, "adjectives": {"Hard": 1}, "numerals": {"46": 1}}}, {"content": "47. Fear of trams, buses, subways or trains.", "word_count": 13, "label": "", "sentence_structure": "Subject鈥搗erb鈥揷omplement", "subjective_objective": {"answer": "Subjective", "rationale": "Because \"fear\" is a subjective sentiment, not an objective fact."}, "sentence_sentiment": {"answer": "Negative", "rationale": "Because the word \"fear\" in the entry is an expression of a negative emotion."}, "language_style": {"answer": "Calm and direct", "rationale": "The entry asks the counterparty in a direct manner whether they fear travelling in public transport, without excessive adoration or emotional rendering, and in a more objective and neutral manner."}, "time_dimension": {"answer": "Present", "rationale": "The entry uses the word \"fear\" to indicate the feelings and feelings of the subjects, implying that this is an act or state that is happening now."}, "sentence_type": "Imperative sentence", "pos": {"nouns": {"Yes": 1, "Train": 2, "Bus": 1, "Subway": 1}, "verbs": {"Multiply": 1}, "adverbs": {"Very": 1, "very": 1}, "numerals": {"47": 1}}}, {"content": "48. Respiration difficulties", "word_count": 5, "label": "", "sentence_structure": "Subject鈥揷opula鈥損redicative", "subjective_objective": {"answer": "Objective", "rationale": "Because respiratory difficulties are an objective manifestation of the patient ' s state of health."}, "sentence_sentiment": {"answer": "Negative", "rationale": "Because \"respiration difficulties\" are usually considered as an uncomfortable and even life-threatening symptom, it has a negative meaning."}, "language_style": {"answer": "Calm and direct", "rationale": "The entry is simple, is not too much rhetorical or emotional, is more objective and directs the respondents to the questions."}, "time_dimension": {"answer": "Present", "rationale": "Based on the phrase \"respiration difficulties\", we can judge that this is a question of describing the symptoms at hand, so the entry is a description of the behaviour or state at hand and should be classified as now."}, "sentence_type": "Affirmative declarative sentence", "pos": {"nouns": {"Breathe": 1, "Difficulties": 1}, "verbs": {"have": 1}, "numerals": {"48": 1}}}, {"content": "49. A little cold or heat.", "word_count": 8, "label": "", "sentence_structure": "Subject鈥搗erb鈥揷omplement", "subjective_objective": {"answer": "Objective", "rationale": "The fact that a moment of cold or heat is a matter of objective fact is a case of patients."}, "sentence_sentiment": {"answer": "Negative", "rationale": "Because \"spills of cold or heat\" are a negative sign of discomfort."}, "language_style": {"answer": "Tactful and warm", "rationale": "The entry uses a gentle and caring tone, asking the respondents whether they have experienced a period of cold or heat, a more peaceful tone, and not a direct expression of strong emotions. Such language styles are more acceptable and willing to share their experiences."}, "time_dimension": {"answer": "Past", "rationale": ""}, "sentence_type": "Affirmative declarative sentence", "pos": {"nouns": {"For a while": 1, "Cold": 1, "Heat": 1}, "numerals": {"49": 1}}}, {"content": "50. Avoiding certain things, occasions or activities because of fear", "word_count": 18, "label": "", "sentence_structure": "Subject鈥搗erb鈥搊bject", "subjective_objective": {"answer": "Subjective", "rationale": "Avoiding something, occasion or activity because of fear is the patient ' s subjective feeling and behavioural choice."}, "sentence_sentiment": {"answer": "Negative", "rationale": "Because the entry \"to avoid something, to avoid it, to be seen or to be active\" is negative."}, "language_style": {"answer": "Calm and direct", "rationale": "The entry directly asks whether the interviewee avoids certain things, events or activities because of fear, and the language style is more straightforward. There is no excessive adoration or emotional rendering, and more emphasis is placed on the transmission of information and the clarity of the issue. It is therefore judged to be \"cool and direct\"."}, "time_dimension": {"answer": "Present", "rationale": "In the future, the entry contains visions and expectations for the future, and the keyword \"negatively, because of fear\" points to situations that have not yet occurred and that may arise in the future."}, "sentence_type": "Affirmative declarative sentence", "pos": {"nouns": {"Scared": 1, "Something": 1, "Event": 1, "Activities": 1}, "verbs": {"Avoid": 1}, "adverbs": {"Because": 1}, "numerals": {"50": 1}}}, {"content": "51. The brain is empty.", "word_count": 5, "label": "", "sentence_structure": "Subject鈥揷opula鈥損redicative", "subjective_objective": {"answer": "Subjective", "rationale": "Because \"brainbrain is empty\" is a subjective feeling or experience that reflects patients' subjective perception and perception of their reduced cognitive abilities."}, "sentence_sentiment": {"answer": "Negative", "rationale": "Because the expression \"brainless\" has a negative emotional connotation, expressing dissatisfaction and concern about itself."}, "language_style": {"answer": "Tactful and warm", "rationale": "The entry uses an implicit and moderate formulation, without directly accusing or criticizing the other party's \"brainless.\" At the same time, it avoids using too strong negative terms, such as \"smugness\" and \"mute\" and is expressed in relatively neutral and acceptable language. This approach helps to create a more friendly and positive atmosphere of communication and enhances the willingness and satisfaction of the other party to respond."}, "time_dimension": {"answer": "Past", "rationale": "The entry is to ask the subject about his current state of affairs, the keywords being \"brainless\" and \"please\" and all point to what is happening now."}, "sentence_type": "Affirmative declarative sentence", "pos": {"nouns": {"Brain": 1, "Empty": 1}, "verbs": {"Change": 1}, "numerals": {"51": 1}}}, {"content": "52. Atrophy or sting.", "word_count": 7, "label": "", "sentence_structure": "Subject鈥搗erb鈥揷omplement", "subjective_objective": {"answer": "Objective", "rationale": "Because body atrophy or sting is the experience and feeling of the patient, it is an objective situation."}, "sentence_sentiment": {"answer": "Negative", "rationale": "Because \"body numbs or stings\" is an uncomfortable feeling, which is usually considered negative."}, "language_style": {"answer": "Tactful and warm", "rationale": "The entry uses a more euphemistic and respectful language style, and when asking about the other person ' s feelings of discomfort, it does not directly deny or confirm, but rather in a mild tone, which allows the person surveyed to answer the question more comfortably and naturally."}, "time_dimension": {"answer": "Past", "rationale": "The entry asks the testee whether he has experienced a physical atrophy or a sting in the past, and the temporal dimension is a past."}, "sentence_type": "Affirmative declarative sentence", "pos": {"nouns": {"Body": 1, "Hair": 1, "Stings": 1}, "numerals": {"52": 1}}}, {"content": "53. There's an infarction in the throat.", "word_count": 6, "label": "", "sentence_structure": "Subject鈥揷opula鈥損redicative", "subjective_objective": {"answer": "Objective", "rationale": "Because the infarction of the throat is an objective fact of the patient's physical experience."}, "sentence_sentiment": {"answer": "Negative", "rationale": "Because the entry \"throat infarction\" is a negative biological feeling."}, "language_style": {"answer": "Tactful and warm", "rationale": "The entry asks the respondents in a more euphemistic way whether they have had an infarction of their throat, rather than directly making or ordering, which is more respectful of the feelings of the respondents and helps to create a good atmosphere for questionnaire filling."}, "time_dimension": {"answer": "Present", "rationale": "Now the entry describes the symptoms of the testee, and the temporal dimension is now judged. The keyword \"sniffed throat\" suggests that it is a questionnaire on body feelings that may be used to collect health information about the testee."}, "sentence_type": "Exclamatory sentence", "pos": {"nouns": {"Throat": 1, "Infarction": 1}, "verbs": {"have": 1}}}, {"content": "54. Feels hopeless about the future.", "word_count": 9, "label": "", "sentence_structure": "Subject鈥揷opula鈥損redicative", "subjective_objective": {"answer": "Subjective", "rationale": "Because of the subjective feeling, the patient ' s emotional and psychological state is examined."}, "sentence_sentiment": {"answer": "Negative", "rationale": "Because the \"no hope for future\" attitude in the entry is negative."}, "language_style": {"answer": "Calm and direct", "rationale": "The language style of the entry is calm and straightforward. It clearly sets out a theme and uses a simple and clear language to describe it without any emotional or rhetorical interference.\""}, "time_dimension": {"answer": "Present", "rationale": "\"Feel\" means the subjective feeling of the testee, \"No hope for the future\" means the testee's attitude and expectations for the future. The answer, therefore, is \"now\"."}, "sentence_type": "Negative declarative sentence", "pos": {"nouns": {"Future": 1, "Hope": 1}, "verbs": {"I feel": 1}, "numerals": {"54": 1}}}, {"content": "55. I can't concentrate.", "word_count": 7, "label": "", "sentence_structure": "Subject鈥揷opula鈥損redicative", "subjective_objective": {"answer": "Subjective", "rationale": "Because \"not concentrating\" is a subjective feeling or experience of a person, reflecting a patient鈥檚 self-assessment and description of his or her state of attention. While this may be related to objective factors (e.g. fatigue, lack of sleep, etc.), this subjective experience itself is still viewed from the patient鈥檚 personal perspective."}, "sentence_sentiment": {"answer": "Negative", "rationale": "Because the \"not focusing\" act is negative."}, "language_style": {"answer": "Tactful and warm", "rationale": "The entry is more euphemistic, and the words \"not to focus\" are used as a direct but not harsh expression to make the respondents feel that their feelings are being cared for and understood, rather than criticized or criticized. At the same time, the following is a more accommodative guide, which helps to increase the rate and efficiency of the questionnaire."}, "time_dimension": {"answer": "Present", "rationale": "The entry describes the current status or performance of the testee, and the keyword is \"not to focus\" and points to the current situation. The temporal dimension therefore is now judged."}, "sentence_type": "Negative declarative sentence", "pos": {"nouns": {"Attention": 1, "Focus": 1}, "verbs": {"No, I can't": 1}, "numerals": {"55": 1}}}, {"content": "56. A part of the body feels weak.", "word_count": 13, "label": "", "sentence_structure": "Subject鈥揷opula鈥損redicative", "subjective_objective": {"answer": "Subjective", "rationale": "Because \"the feeling of a part of the body is a subjective feeling or experience, a subjective judgement and a sense of the patient's own physical condition."}, "sentence_sentiment": {"answer": "Negative", "rationale": "Because the physical condition of \"Esperating a part of the body to be weak\" in the entry is negative."}, "language_style": {"answer": "Tactful and warm", "rationale": "The language style of the entry is euphemism, because it uses a gentle and inviting tone of speech to engage in dialogue rather than direct statements or orders, which is more personal and interactive. At the same time, it does not overemphasize or blame each other for their body problems, but rather asks each other about their feelings in a caring and supportive manner, which makes them more comfortable and relaxed."}, "time_dimension": {"answer": "Past", "rationale": ""}, "sentence_type": "Affirmative declarative sentence", "pos": {"nouns": {"Body": 1, "Part": 1, "Weakness": 1}, "adjectives": {"Part": 1}, "numerals": {"56": 1}}}, {"content": "57. Tension or susceptibility to stress", "word_count": 9, "label": "", "sentence_structure": "Subject鈥揷opula鈥損redicative", "subjective_objective": {"answer": "Subjective", "rationale": "Because feeling nervous or being nervous is a subjective or emotional experience."}, "sentence_sentiment": {"answer": "Negative", "rationale": "Because \"stretch\" is a negative emotional attitude."}, "language_style": {"answer": "Calm and direct", "rationale": "The entry directly expresses the subject of questioning whether the other party is often or easily nervous, is not based on excessive rhetoric or emotional rendering and is more objective in tone."}, "time_dimension": {"answer": "Past", "rationale": ""}, "sentence_type": "Affirmative declarative sentence", "pos": {"nouns": {"Nervous": 2, "Easy": 1}, "verbs": {"I feel": 1}, "numerals": {"57": 1}}}, {"content": "58. Feel the weight of hands or feet", "word_count": 7, "label": "", "sentence_structure": "Subject鈥揷opula鈥損redicative", "subjective_objective": {"answer": "Objective", "rationale": "Because the feeling of sinking hands or feet is a subjective feeling of the patient, it can be observed and measured and therefore also considered an objective result."}, "sentence_sentiment": {"answer": "Negative", "rationale": "Because the feeling of feeling a hand or a foot is negative."}, "language_style": {"answer": "Tactful and warm", "rationale": "The entry is more euphemistic. It does not directly ask the interviewee whether he is ill, but rather guides the other to self-assessment by describing a possible symptom."}, "time_dimension": {"answer": "Present", "rationale": "In the entry, \"Feel\" and \"sink\" means the experience that the testee is currently experiencing, and therefore judgement is now."}, "sentence_type": "Affirmative declarative sentence", "pos": {"nouns": {"Hands": 1, "Foot": 1, "Heavy feeling": 1}, "verbs": {"I feel": 1, "Let's go": 1}, "adjectives": {"Sink": 1}, "numerals": {"58": 1}}}, {"content": "59. Think about death.", "word_count": 8, "label": "", "sentence_structure": "Subject鈥搗erb鈥揷omplement", "subjective_objective": {"answer": "Subjective", "rationale": "Because \"think\" is a subjective psychological activity of a person, indicating interest in and reflection on a subject, the subject is examined from the subjective perspective of the patient."}, "sentence_sentiment": {"answer": "Negative", "rationale": "Because \"death\" is a topic of negative emotions, which can trigger negative emotions such as fear and grief."}, "language_style": {"answer": "Tactful and warm", "rationale": "The entry, in a more euphemistic way, asked the respondents about their thoughts about death, avoiding the use of language that was too direct or shocking to make them more comfortable and comfortable in answering their questions."}, "time_dimension": {"answer": "Present", "rationale": "The main word in the entry, \"you\", indicates that it is an ongoing action or state, that is, that you are thinking about death right now, and therefore is part of the time dimension."}, "sentence_type": "Interrogative sentence", "pos": {"nouns": {"Yes": 1, "Death": 1}, "verbs": {"Yes": 1}, "numerals": {"59": 1}}}, {"content": "- I ate too much.", "word_count": 4, "label": "", "sentence_structure": "Subject鈥搗erb鈥揷omplement", "subjective_objective": {"answer": "Objective", "rationale": "Because the amount of food is objective."}, "sentence_sentiment": {"answer": "Negative", "rationale": "Because \"too much\" is unhealthy and negative."}, "language_style": {"answer": "Calm and direct", "rationale": "The language style of the entry is more concise, it is not too much rhetorical or emotional, it is more objective in tone, and it is more easily understood and answered by the interviewee."}, "time_dimension": {"answer": "Present", "rationale": "The entry describes a constant, present behavior or state, which is \"too much to eat\", and therefore is judged \"now\"."}, "sentence_type": "Negative declarative sentence", "pos": {"nouns": {"Too much": 1, "More": 1, "Eat": 1}, "numerals": {"60": 1}}}, {"content": "It's uncomfortable when people look at you or talk about you.", "word_count": 16, "label": "", "sentence_structure": "Subject鈥揷opula鈥損redicative", "subjective_objective": {"answer": "Subjective", "rationale": "Because feeling uncomfortable is a personal feeling or emotional experience, it is subjective."}, "sentence_sentiment": {"answer": "Negative", "rationale": "Because the emotional description of \"frightful\" in the entry is negative."}, "language_style": {"answer": "Tactful and warm", "rationale": "The basis for the judgment: - The expression of the article is relatively euphemistic, moderate, and does not ask the other side too directly. - To replace \"you\" with \"you\" with \"you\" with \"you\" with more respect and more affectionate. - \"Insatisfaction\" is a more sensitive topic, using this euphemism to avoid uncomfortableness."}, "time_dimension": {"answer": "Present", "rationale": "The word \"when\" indicates the action that occurs in a given situation, \"when others look at you or talk about you\" is a specific description of the situation, and \"frightful\" is a reaction to the testee in this situation. The entry therefore asks about the current feelings and state of the testee."}, "sentence_type": "Affirmative declarative sentence", "pos": {"nouns": {"others": 1, "you": 2, "Talk": 1}, "verbs": {"Watch": 1, "Talk": 1, "I feel": 1, "I'm not comfortable": 1}, "adverbs": {"When": 1}, "numerals": {"61": 1}}}, {"content": "There are some ideas that don't belong to you.", "word_count": 12, "label": "", "sentence_structure": "Subject鈥搗erb鈥搊bject", "subjective_objective": {"answer": "Subjective", "rationale": "Because \"some things that don't belong to you\" are the inner feelings and perceptions of a person, and they are part of a subjective experience."}, "sentence_sentiment": {"answer": "Neutral", "rationale": "Since the entry does not show positive or negative feelings, attitudes, behaviour, purpose or quality, it simply states a fact."}, "language_style": {"answer": "Tactful and warm", "rationale": "The entry is more adept and interactive when it is spoken in a gentle and inviting manner, rather than by direct statements or orders."}, "time_dimension": {"answer": "Past", "rationale": "The entry contains the term \"Yes\", which indicates what happened at some point in time in the past and therefore is judged to be the past."}, "sentence_type": "Negative declarative sentence", "pos": {"nouns": {"Questionnaire": 1, "Entry": 1, "Thoughts": 1}, "verbs": {"belong to": 1, "have": 1}, "numerals": {"62": 1}}}, {"content": "63. An impulse to hit or hurt someone.", "word_count": 12, "label": "", "sentence_structure": "Subject鈥搗erb鈥揷omplement", "subjective_objective": {"answer": "Subjective", "rationale": "Because \"want to hit or hurt someone\" is a subjective emotion and thought in the heart. Although this impulse may be influenced by external stimulus, it is essentially the patient's own psychological state."}, "sentence_sentiment": {"answer": "Negative", "rationale": "Because the \"beat or hurt\" in the entry is negative."}, "language_style": {"answer": "Calm and direct", "rationale": "The language style of the entry is relatively calm and straightforward, and it clearly asks whether the person surveyed has any impulse to attack the ideas and behaviour of others, without too many rhetorical or emotional renderings. This expression can send a message clearly and accurately, and avoids some terminologies that could lead to misunderstanding or create unnecessary tension."}, "time_dimension": {"answer": "Past", "rationale": "The keywords \"intentional\" and \"exulent\" indicate that the question is asking the testee about his past behaviour and can therefore be judged as past."}, "sentence_type": "Affirmative declarative sentence", "pos": {"nouns": {"person": 1, "Expedition": 1}, "verbs": {"Fight": 1, "Hurt": 1, "Yes": 1}, "numerals": {"63": 1}}}, {"content": "Early wake-up call.", "word_count": 4, "label": "", "sentence_structure": "Subject鈥搗erb鈥揷omplement", "subjective_objective": {"answer": "Subjective", "rationale": "Because \"break up too early\" is the patient's subjective feeling and experience."}, "sentence_sentiment": {"answer": "Negative", "rationale": "Because \"awakening too early\" is usually considered a uncomfortable and uncomfortable situation, it has a negative emotional color."}, "language_style": {"answer": "Calm and direct", "rationale": "The language style of the entry is relatively simple and direct, asking the interviewee whether he/she is often too early to be too agglomerated or emotional."}, "time_dimension": {"answer": "Present", "rationale": "The keyword is \" too early to wake up\", which indicates the situation that the person being tested is experiencing, without reference to past or future factors, and therefore the temporal dimension is now judged."}, "sentence_type": "Negative declarative sentence", "pos": {"nouns": {"Wake up": 1, "Too early": 1}, "verbs": {"Morning": 1}, "adverbs": {"Too much": 1}, "numerals": {"64": 1}}}, {"content": "65. Must wash hands, count or touch something repeatedly.", "word_count": 16, "label": "", "sentence_structure": "Subject鈥搗erb鈥揷omplement", "subjective_objective": {"answer": "Objective", "rationale": "Because repeated hand washing, count or touching of certain things are the manifestation of the patient ' s coercion and are objective acts."}, "sentence_sentiment": {"answer": "Negative", "rationale": "Because these acts, \"repeated hand washing, count or touch something\", are manifestations of forced thinking and coercion and are a negative emotional problem."}, "language_style": {"answer": "Calm and direct", "rationale": "The entry describes the behaviour of the subject in objectively clear terms, does not involve excessive rhetorical or emotional rendering and is more objective in tone."}, "time_dimension": {"answer": "Present", "rationale": ""}, "sentence_type": "Interrogative sentence", "pos": {"nouns": {"Hands": 2, "Number": 1, "Something": 1}, "verbs": {"Repeatedly": 1, "Wash": 1, "Points": 1, "Touch": 1}, "adverbs": {"I have to": 1}, "numerals": {"65": 1}}}, {"content": "66. Sleepy.", "word_count": 6, "label": "", "sentence_structure": "Subject鈥搗erb鈥揷omplement", "subjective_objective": {"answer": "Subjective", "rationale": "Because \"sleep in a state of insomnia\" is a patient's self-perception and subjective experience, which reflects patient's subjective assessment of the quality of sleep."}, "sentence_sentiment": {"answer": "Negative", "rationale": "Because the entry \"sleeping in a state of insomnia\" describes a negative situation."}, "language_style": {"answer": "Tactful and warm", "rationale": "The entry uses descriptive language to express the problem, does not direct the order or blame the quality of the sleep, but rather directs the other to think in a way that is concerned and understanding. Moreover, the article is more modest, does not have too much emotional or intense emotional orientation and can therefore be considered as a \"fructual\" language style."}, "time_dimension": {"answer": "Present", "rationale": "The entry describes the person ' s sleep and does not contain information about past or future, and therefore the judgement is now."}, "sentence_type": "Exclamatory sentence", "pos": {"nouns": {"Questionnaire": 1, "Entry": 1, "Sleep": 1}, "verbs": {"Sleep": 1, "Steady": 1, "Deep": 1}, "adjectives": {"It's not steady": 1, "Not deep": 1}, "numerals": {"66": 1}}}, {"content": "67. An impulse to break or destroy something.", "word_count": 12, "label": "", "sentence_structure": "Subject鈥搗erb鈥揷omplement", "subjective_objective": {"answer": "Subjective", "rationale": "Because \"the urge to break or destroy something\" is a person's inner feeling and thought, and it's a subjective view."}, "sentence_sentiment": {"answer": "Negative", "rationale": "Because the idea and impulse in the entry, \"I want to break or destroy the impulse to break things,\" is a negative emotional state."}, "language_style": {"answer": "Calm and direct", "rationale": "The entry directly asks the respondents whether there is an impulse, which is more objective and direct in language style, is not overly rhetorical or emotional and makes it easier to obtain accurate information and data."}, "time_dimension": {"answer": "Present", "rationale": ""}, "sentence_type": "Imperative sentence", "pos": {"nouns": {"Expedition": 1, "Something": 1}, "verbs": {"Yes": 1, "Broken": 1, "Destruction": 1}, "numerals": {"67": 1}}}, {"content": "There are ideas or ideas that no one else has.", "word_count": 13, "label": "", "sentence_structure": "Subject鈥搗erb鈥揷omplement", "subjective_objective": {"answer": "Subjective", "rationale": "Because of the fact that there is or does not have ideas or thoughts, this experience is only felt by the patient himself and is subjective."}, "sentence_sentiment": {"answer": "Neutral", "rationale": "Because the entry is not emotional, it is a description of the behaviour of some people."}, "language_style": {"answer": "Tactful and warm", "rationale": "The entry is more adept and interactive when it is spoken in a gentle and inviting manner, rather than by direct statements or orders."}, "time_dimension": {"answer": "Present", "rationale": "The entry is for the purpose of asking the testee whether he currently has some unique ideas or ideas, and therefore the judgement is \"now\"."}, "sentence_type": "Affirmative declarative sentence", "pos": {"nouns": {"Thoughts": 2, "Thinking": 1, "others": 1}, "verbs": {"have": 1, "No, I'm not": 1}, "adverbs": {"Some": 1}, "numerals": {"68": 1}, "pronouns": {"or": 1}}}, {"content": "69. He's a little paranoid.", "word_count": 9, "label": "", "sentence_structure": "Subject鈥揷opula鈥損redicative", "subjective_objective": {"answer": "Subjective", "rationale": "Because \"sensitization\" is an emotion or a feeling, a subjective experience and an assessment of the patient."}, "sentence_sentiment": {"answer": "Negative", "rationale": "Because the attitude of being \"sensitized to others\" in the entry is negative."}, "language_style": {"answer": "Tactful and warm", "rationale": "This is a more friendly and interactive expression that helps to increase the participation of respondents and the completion rate of questionnaires."}, "time_dimension": {"answer": "Present", "rationale": "The entry is for the testee ' s current state of feeling, so the time dimension is now judged."}, "sentence_type": "Interrogative sentence", "pos": {"nouns": {"person": 2, "You're a bit of a sane": 1, "Grandpa": 1, "Questionnaire": 1, "Entry": 1, "I feel": 1}, "verbs": {"to": 1, "be": 1, "Pass": 1, "Min": 1, "I feel": 1}, "adjectives": {"others": 1, "You're a bit of a sane": 1}, "numerals": {"69": 1}, "classifiers": {"individual": 1}}}, {"content": "70. Disturbed in places with many people in shops or cinemas", "word_count": 18, "label": "", "sentence_structure": "Subject鈥搗erb鈥揷omplement", "subjective_objective": {"answer": "Subjective", "rationale": "Because feeling uncomfortable is a person ' s inner feeling and experience, it is a subjective examination."}, "sentence_sentiment": {"answer": "Negative", "rationale": "Because the feeling of being uncomfortable in the entry is negative."}, "language_style": {"answer": "Calm and direct", "rationale": "The entry directly asked whether the person surveyed would feel in a given context, without using any rhetorical or emotional rendering, and with a more objective and direct linguistic style."}, "time_dimension": {"answer": "Present", "rationale": "The entry asks about the current behaviour and feelings of the subject, the keywords being \"in a store or cinema with a lot of people\" and \"inconvenient\" and all pointing to the current state."}, "sentence_type": "Negative declarative sentence", "pos": {"nouns": {"Shop": 1, "Cinema": 1, "Local": 1, "There's a lot of people": 1}, "verbs": {"I feel": 1, "I'm not comfortable": 1}, "numerals": {"70": 1}}}, {"content": "71. It's hard to do anything.", "word_count": 10, "label": "", "sentence_structure": "Subject鈥揷opula鈥損redicative", "subjective_objective": {"answer": "Subjective", "rationale": "Because \"Esperate that everything is hard to do\" is the patient's subjective feeling and attitude, and it's a subjective view."}, "sentence_sentiment": {"answer": "Negative", "rationale": "Because the expression \"Esperate that anything is difficult to do\" in this entry expresses a negative sentiment that the interviewees may be in a difficult and frustrated state."}, "language_style": {"answer": "Calm and direct", "rationale": "The language style of the entry is more straightforward, with no too many emotional or rhetorical moves, and an open-ended expression of the subject of the inquiry 鈥?whether it is difficult to feel much of life. This is a simple, clear expression that makes it easier for the interviewee to understand and respond."}, "time_dimension": {"answer": "Past", "rationale": "In the entry, the main word \"fear\" is \"you\" and it means the person who was tested, and it points to the experience and feelings of the past in time, so it is judged as the past."}, "sentence_type": "Negative declarative sentence", "pos": {"nouns": {"Questionnaire": 1, "Entry": 1, "Things": 2}, "verbs": {"I feel": 1, "Do it": 1}, "adjectives": {"Any": 1, "Hard": 1}, "numerals": {"71": 1}}}, {"content": "72. A moment of fear or panic.", "word_count": 8, "label": "", "sentence_structure": "Subject鈥搗erb鈥揷omplement", "subjective_objective": {"answer": "Subjective", "rationale": "Fear or panic is the subjective feeling and experience of the patient."}, "sentence_sentiment": {"answer": "Negative", "rationale": "Because the mood of \"fear or panic\" in the entry is negative."}, "language_style": {"answer": "Tactful and warm", "rationale": "The entry is more euphemistic, implicit, and not directly using strong negative terms, but rather, it guides the interviewer to think and answer by depicting feelings. Moreover, \"for a while\" gives people a gradual, uncertain sense of the word, which is more in line with the euphemism of asking questions."}, "time_dimension": {"answer": "Past", "rationale": "The entry describes the psychological state experienced by the person tested, and the temporal dimension is a past."}, "sentence_type": "Declarative sentence", "pos": {"nouns": {"Fear": 1, "Scared": 1}, "verbs": {"Form": 1}, "numerals": {"72": 1}}}, {"content": "It's not easy to eat in public.", "word_count": 14, "label": "", "sentence_structure": "Subject鈥揷opula鈥損redicative", "subjective_objective": {"answer": "Subjective", "rationale": "Because \"fear\" and \"incommodity\" are the feelings and experiences of the patient, so it's subjective."}, "sentence_sentiment": {"answer": "Negative", "rationale": "Because the behaviour in the entry \"eat in public\" is considered to be rude or insensitive, it has a negative emotional colour."}, "language_style": {"answer": "Calm and direct", "rationale": "The entry describes a possible problem in a concise manner, which is not overly adorational or emotional and is more in line with the needs of actual investigations."}, "time_dimension": {"answer": "Past", "rationale": "In the entry, the words \"fear\" and \"discomfort\" indicate the person who was tested for the past, so the temporal dimension is the past."}, "sentence_type": "Negative declarative sentence", "pos": {"nouns": {"Questionnaire Entry": 1, "Public": 1, "Something": 1}, "verbs": {"I feel": 1, "Eat": 1, "I'm not feeling well": 1}, "numerals": {"73": 1}, "classifiers": {"very": 1}}}, {"content": "74. Frequent disputes", "word_count": 6, "label": "", "sentence_structure": "Subject鈥搗erb鈥揷omplement", "subjective_objective": {"answer": "Subjective", "rationale": "Because the word \"often\" is the patient's subjective feeling and attitude, which is a description of his behaviour from the patient's perspective."}, "sentence_sentiment": {"answer": "Negative", "rationale": "Because the \"always arguing\" act in the entry is negative."}, "language_style": {"answer": "Calm and direct", "rationale": "The language style of the entry is more direct, with no excessive qualifier or emotional colour, more than a statement of facts, which gives people a chilling and direct sense of being."}, "time_dimension": {"answer": "Present", "rationale": "The term \"often\" shows that such behaviour is very frequent, while the term \"disputing\" is an action that is possible in the past, present or future, but is being done in this particular time period (i.e. during the questionnaire). The time dimension of this entry can therefore be judged as now."}, "sentence_type": "Imperative sentence", "pos": {"nouns": {"74": 1, "person": 1, "Arguments": 1}, "verbs": {"Regular": 1}, "numerals": {"74": 1}}}, {"content": "75. Nervousness when left alone.", "word_count": 10, "label": "", "sentence_structure": "Subject鈥揷opula鈥損redicative", "subjective_objective": {"answer": "Subjective", "rationale": "Because \"nervous\" is the patient's own feeling, a subjective description."}, "sentence_sentiment": {"answer": "Negative", "rationale": "Because the \"nervous\" mood in the entry is negative."}, "language_style": {"answer": "Tactful and warm", "rationale": "The entry uses a euphemism to guide the question by \"Do you feel\" rather than directly stating whether the other side is in existence, which is more intimate and concerned. At the same time, it is more mildly worded, and \"nerstroke when one is alone\" is not too strong in negative emotional aspects, and it is easier for the respondents to accept and share their true feelings."}, "time_dimension": {"answer": "Present", "rationale": ""}, "sentence_type": "Negative declarative sentence", "pos": {"nouns": {"person": 1, "Nerves": 1}, "verbs": {"Nervous": 1, "Alone": 1}, "adverbs": {"very": 1}, "numerals": {"75": 1}}}, {"content": "76. Your performance has not been properly evaluated.", "word_count": 16, "label": "", "sentence_structure": "Subject鈥搗erb鈥揷omplement", "subjective_objective": {"answer": "Objective", "rationale": "Because \"someone has not properly evaluated your performance\" is a fact that patients experience. Although it may affect their self-perception, it is an objective fact that is not influenced by individual subjective attitudes."}, "sentence_sentiment": {"answer": "Negative", "rationale": "Basis of judgement: The entry \"failure to make a proper assessment\" expresses negative feelings and attitudes."}, "language_style": {"answer": "Calm and direct", "rationale": "The language style of the entry is more straightforward, not using too many qualifiers or rhetorical techniques, but rather a simple expression of the question that the surveyer wants to know about the opinion of the person surveyed about the other person鈥檚 evaluation of his or her academic performance."}, "time_dimension": {"answer": "Present", "rationale": "The keywords are \"others\" and \"made,\" which point to what is happening now. The term \"appropriate evaluation\" is an evaluation of past behaviour or events, so the time dimension of the article is now."}, "sentence_type": "Interrogative sentence", "pos": {"nouns": {"others": 1, "you": 1, "Achievements": 2, "Evaluation": 1}, "verbs": {"Do it": 1, "to": 1, "have": 1, "Make it": 1}, "adjectives": {"Appropriate": 1}, "numerals": {"76": 1}, "pronouns": {"you": 1}}}, {"content": "77. I feel lonely even with someone else.", "word_count": 13, "label": "", "sentence_structure": "Subject鈥搗erb鈥揷omplement", "subjective_objective": {"answer": "Subjective", "rationale": "Because feeling alone is a subjective feeling."}, "sentence_sentiment": {"answer": "Negative", "rationale": "Because the feeling of being alone in this entry is negative."}, "language_style": {"answer": "Tactful and warm", "rationale": "The entry is more personal and interactive in terms of a moderate and inviting tone of dialogue, rather than a direct statement or order. At the same time, the issue is designed in a less explicit manner, avoiding the discomfort that too direct and sharp language may cause to the respondents, thereby increasing the acceptance of the questionnaire."}, "time_dimension": {"answer": "Present", "rationale": "The keyword \"even if with someone else\" describes a state or feeling that is not clearly time-bound in the query and can therefore be judged as now."}, "sentence_type": "Negative declarative sentence", "pos": {"nouns": {"Questionnaire": 1, "Entry": 1, "person": 1, "Alone": 1}, "verbs": {"in": 1, "Together": 1, "I feel": 1}, "adverbs": {"Yeah": 1, "Even if": 1}, "numerals": {"77": 1}, "pronouns": {"and": 1, "others": 1}}}, {"content": "I'm not sure I'm comfortable with it.", "word_count": 10, "label": "", "sentence_structure": "Subject鈥揷opula鈥損redicative", "subjective_objective": {"answer": "Subjective", "rationale": "Because of the uncertainty of being a subjective feeling and experience, it is determined by the patient ' s state of mind."}, "sentence_sentiment": {"answer": "Negative", "rationale": "Because the description of \"silentness\" in the entry is negative and expresses an uncomfortable, uneasy state of mind."}, "language_style": {"answer": "Calm and direct", "rationale": "The entry is based on the following criteria: it expresses the feelings of the respondents directly, does not use excessive rhetorical or emotional rendering, and is more objective in tone. At the same time, this simple and clear presentation is more in line with the language style of the questionnaire survey and helps to obtain accurate data feedback."}, "time_dimension": {"answer": "Past", "rationale": ""}, "sentence_type": "Affirmative declarative sentence", "pos": {"nouns": {"78": 1, "Mind": 1}, "verbs": {"I feel": 1, "Sit tight": 1, "Not cool": 1}, "numerals": {"78": 1}, "classifiers": {"individual": 1}}}, {"content": "79. Feels like they're of little value.", "word_count": 10, "label": "", "sentence_structure": "Subject鈥揷opula鈥損redicative", "subjective_objective": {"answer": "Subjective", "rationale": "Because feeling that you have nothing to gain is a subjective feeling."}, "sentence_sentiment": {"answer": "Negative", "rationale": "Because it expresses the individual ' s rejection of his or her value and is a negative emotional attitude."}, "language_style": {"answer": "Tactful and warm", "rationale": "This expression enhances the comfort and trust of the person interviewed and makes them more open to questions."}, "time_dimension": {"answer": "Past", "rationale": "The entry \"Esperately of little value\" describes the emotions and feelings of the subjects over time and therefore judge them as past."}, "sentence_type": "Negative declarative sentence", "pos": {"nouns": {"self": 1, "Value": 1}, "verbs": {"I feel": 1}, "numerals": {"79": 1}}}, {"content": "80. It's true that things that are familiar become strangers or not.", "word_count": 17, "label": "", "sentence_structure": "Subject鈥搗erb鈥揷omplement", "subjective_objective": {"answer": "Subjective", "rationale": "This description is more of a patient ' s sense and perception and is a subjective examination."}, "sentence_sentiment": {"answer": "Negative", "rationale": "Because the words \"unknown\" and \"not true\" in the entries give people a negative feeling."}, "language_style": {"answer": "Tactful and warm", "rationale": "The language style of the entry is more euphemistic, using the words \"reversible\" and \"unknown\" to make the issue more detailed and friendly. At the same time, the entry is presented in question, not directly, but by direct statement or order, expressing a concern for and respect for the interviewee."}, "time_dimension": {"answer": "Past", "rationale": ""}, "sentence_type": "Negative declarative sentence", "pos": {"nouns": {"Something": 2, "I know you": 1, "Strange": 1, "True": 1}, "verbs": {"Change": 1, "I feel": 1, "Yeah": 1}, "adjectives": {"Strange": 1, "Really": 1}, "numerals": {"80": 1}, "classifiers": {"individual": 2}}}, {"content": "Scream or fall something.", "word_count": 6, "label": "", "sentence_structure": "Subject鈥損redicate", "subjective_objective": {"answer": "Objective", "rationale": "Because screaming or falling is a patient's behaviour and is an objective fact."}, "sentence_sentiment": {"answer": "Negative", "rationale": "Because yelling or falling something is a negative act."}, "language_style": {"answer": "Calm and direct", "rationale": "The language style of the entry is straightforward, as it describes the possible behaviour of the person under investigation in a clear and concise manner, without any emotional or rhetorical approach. This approach is more appropriate for the collection of objective data and information."}, "time_dimension": {"answer": "Present", "rationale": "The term \"scream or falling\" describes the person's immediate state or habitual behaviour at the time of completing the questionnaire, so that the temporal dimension can be judged as it is now."}, "sentence_type": "Imperative sentence", "pos": {"nouns": {"Shouting": 1, "Something": 1}, "verbs": {"Fall": 1}}}, {"content": "Fear of fainting in public.", "word_count": 10, "label": "", "sentence_structure": "Subject鈥搗erb鈥揷omplement", "subjective_objective": {"answer": "Subjective", "rationale": "Fear of fainting in public is a subjective emotion and fear of patients, reflecting their feelings of possible discomfort or embarrassment. While this may happen, it depends on many factors, and not all people experience it."}, "sentence_sentiment": {"answer": "Negative", "rationale": "Because the expression \"fear of fainting in public\" in the entry expresses negative emotions and attitudes."}, "language_style": {"answer": "Tactful and warm", "rationale": "The language style of the entry is more euphemistic and warm. By asking the respondents whether they fear fainting in public, they express concern and support, avoiding any possible discomfort or negative sentiments that may arise from the direct expression of the words."}, "time_dimension": {"answer": "Present", "rationale": "The time dimension of the questionnaire entry is now judged by the two key words \"82.\" and \"fear\" in the sentence. \"82.\" It may be a score or ranking, indicating the degree of concern of the testee on the issue, and \"fear\" indicates a state of mind, not past or future behaviour or events. Thus, the entry is the current time dimension."}, "sentence_type": "Exclamatory sentence", "pos": {"nouns": {"Questionnaire": 1, "Entry": 1, "Public": 1, "- I'm fainted": 1}, "verbs": {"Yes": 2}, "adverbs": {"Fear": 1}, "numerals": {"82": 1}}}, {"content": "Feels like someone else wants to take advantage of you.", "word_count": 10, "label": "", "sentence_structure": "Subject鈥搗erb鈥揷omplement", "subjective_objective": {"answer": "Subjective", "rationale": "Because feeling the cost of occupation is a subjective feeling."}, "sentence_sentiment": {"answer": "Negative", "rationale": "Because the phrase \"like someone else wants to take advantage of you\" in the entry is negative."}, "language_style": {"answer": "Calm and direct", "rationale": "This entry, which starts with the number \"83\", appears objective and cold, without any qualifier language. It directly raises the question of whether the interviewee often feels that others are trying to take advantage of themselves. This tone is more direct, is less nuanced or emotional, and is more in keeping with the calm and direct language style."}, "time_dimension": {"answer": "Present", "rationale": "The entry asks the testee about the feelings and experiences of the person at the time, and therefore judges it as \"now\"."}, "sentence_type": "Imperative sentence", "pos": {"nouns": {"person": 1, "Cheap": 1}, "verbs": {"I feel": 1, "Yes": 1, "Jim": 1}, "adverbs": {"Down there": 1, "Please": 1, "you": 1}, "numerals": {"83": 1}}}, {"content": "I'm upset about some ideas about sex.", "word_count": 13, "label": "", "sentence_structure": "Subject鈥搗erb鈥揷omplement", "subjective_objective": {"answer": "Subjective", "rationale": "Because \"fear\" is the patient's subjective feeling, it is the patient's distress and distress at these thoughts."}, "sentence_sentiment": {"answer": "Negative", "rationale": "Because it describes a disturbing and negative emotional experience, and the idea of \"sex\" can lead to negative emotions such as shame, unease, etc."}, "language_style": {"answer": "Tactful and warm", "rationale": "This is a more acceptable expression and can protect the privacy and dignity of the subject."}, "time_dimension": {"answer": "Past", "rationale": "It is therefore possible to judge whether the entry was an inquiry into whether the person tested had experienced such distress in the past."}, "sentence_type": "Interrogative sentence", "pos": {"nouns": {"84": 1, "Some": 1, "Off": 1, "Sex": 1, "Thoughts": 1, "Trouble": 1}, "adverbs": {"EMPTY": 2}, "numerals": {"84": 1}, "classifiers": {"Some": 1, "Related": 1}, "pronouns": {"EMPTY": 3}}}, {"content": "85. Considers it appropriate to punish for its own fault.", "word_count": 16, "label": "", "sentence_structure": "Subject鈥搗erb鈥搊bject", "subjective_objective": {"answer": "Subjective", "rationale": "Because of the patient ' s awareness of and attitude towards self-responsibility."}, "sentence_sentiment": {"answer": "Negative", "rationale": "Because the entry expresses a view of punishment, which tends to be negative."}, "language_style": {"answer": "Tactful and warm", "rationale": "The entry is more personal and interactive when it is spoken in a gentle and inviting manner, rather than in direct statements or orders. At the same time, the phrase is more accessible and easily accepted and understood by the interviewees, and does not make people too serious or too cold."}, "time_dimension": {"answer": "Present", "rationale": "The keywords are \"thinks\" and \"should\" and they all indicate that this is the current attitude and view of the testee, so the entry describes the current behavior or mentality."}, "sentence_type": "Affirmative declarative sentence", "pos": {"nouns": {"Wrong": 1, "Punishment": 1}, "verbs": {"I think": 1, "I think so": 1, "Received": 1}, "adverbs": {"self": 1, "Because": 1}, "numerals": {"85": 1}}}, {"content": "86. Feels like we need to finish this.", "word_count": 10, "label": "", "sentence_structure": "Subject鈥搗erb鈥揷omplement", "subjective_objective": {"answer": "Subjective", "rationale": "Because it is the subjective experience of the patient, it is not an objective situation."}, "sentence_sentiment": {"answer": "Positive", "rationale": "The sense of urgency in getting things done shows some enthusiasm and initiative."}, "language_style": {"answer": "Calm and direct", "rationale": ""}, "time_dimension": {"answer": "Present", "rationale": "The entry \"Feeling to finish\" is about the current feelings and attitudes of the testees, and the time dimension is now judged."}, "sentence_type": "Imperative sentence", "pos": {"verbs": {"I feel": 1, "Yes": 1, "- Put it on": 1, "Things": 1, "Do it": 1}, "adverbs": {"Hurry up": 1}, "numerals": {"86": 1}}}, {"content": "87. Feels like a serious problem with your body.", "word_count": 12, "label": "", "sentence_structure": "Subject鈥揷opula鈥損redicative", "subjective_objective": {"answer": "Subjective", "rationale": "Because this is the physical state of the patient ' s self-perception, it is subjective."}, "sentence_sentiment": {"answer": "Negative", "rationale": "Because the description \"Esperately serious problems with his body\" is negative, implying that the interviewees are concerned and disturbed about his health."}, "language_style": {"answer": "Calm and direct", "rationale": "The language style of the entry is a calm and straightforward one. The basis of the judgement is: - The entry directly describes the physical problems that the person under investigation may have, does not have too many rhetorical or emotional renderings, and is expressed in an objective and calm manner. - The entry is based on direct questioning, avoiding too many guidance and assumptions to enable the person under investigation to express his or her feelings more accurately."}, "time_dimension": {"answer": "Past", "rationale": "According to the title, \"Esses of serious physical problems\" means the physical discomfort experienced by the person tested at a point or time in the past, and therefore judgement is the past."}, "sentence_type": "Affirmative declarative sentence", "pos": {"nouns": {"self": 1, "Body": 1, "Problem": 1}, "verbs": {"I feel": 1, "have": 1, "Serious": 1, "Problem": 1}, "adjectives": {"Serious": 1}, "numerals": {"87": 1}, "pronouns": {"self": 1}}}, {"content": "88. Never felt close to anyone else.", "word_count": 11, "label": "", "sentence_structure": "Subject鈥搗erb鈥揷omplement", "subjective_objective": {"answer": "Objective", "rationale": "Because feeling close or alienated is a subjective emotional experience, \"never feeling close to others\" is an objective fact."}, "sentence_sentiment": {"answer": "Negative", "rationale": "Because the description \"never felt close to anyone else\" expresses social isolation and is negative."}, "language_style": {"answer": "Tactful and warm", "rationale": "The entry is more adept and interactive when it is spoken in a gentle and inviting manner, rather than by direct statements or orders."}, "time_dimension": {"answer": "Past", "rationale": "The entry asks if the person tested had ever had a \"fearing close to others\" or the keyword \"never\" and it represents a denial or state of affairs at a point in time in the past. It is therefore a judgement that the entry is a description of the past."}, "sentence_type": "Imperative sentence", "pos": {"nouns": {"Questionnaire": 1, "Entry": 1, "person": 1, "Close": 1}, "verbs": {"I feel": 1, "Other": 1}, "adjectives": {"very": 1}, "numerals": {"88": 1}}}, {"content": "89. Feel guilty.", "word_count": 6, "label": "", "sentence_structure": "Subject鈥揷opula鈥損redicative", "subjective_objective": {"answer": "Subjective", "rationale": "Because \"felt guilty\" is a person's inner feeling and self-evaluation, and it is the patient's subjective view in evaluating his behaviour and attitude."}, "sentence_sentiment": {"answer": "Negative", "rationale": "Because the attitude of feeling guilty in the entry is negative."}, "language_style": {"answer": "Calm and direct", "rationale": "The entry starts with a specific number, which is more objective and straightforward, without too many rhetorical or emotional renderings, and therefore language style is straightforward and calm."}, "time_dimension": {"answer": "Present", "rationale": "At the same time, \"guilty\" is a description of an emotional experience, which further confirms that it is asking what it is now. So the time dimension of the article is now."}, "sentence_type": "Negative declarative sentence", "pos": {"nouns": {"Sin": 1}, "verbs": {"Sensor": 1, "have": 1}, "numerals": {"89": 1}}}, {"content": "90. Feels like you're out of your mind.", "word_count": 10, "label": "", "sentence_structure": "Subject鈥揷opula鈥損redicative", "subjective_objective": {"answer": "Subjective", "rationale": "Because feeling a brain in its own way is a subjective feeling or perception, not an objective fact."}, "sentence_sentiment": {"answer": "Negative", "rationale": "Because the description of \"brain\" in the entry is negative."}, "language_style": {"answer": "Calm and direct", "rationale": "The language style of the entry is more direct, concise, and there is no excessive rhetorical or emotional rendering, but rather direct questions are asked to ask the respondents whether they feel that they have problems with their brain. This expression is more in keeping with the nature and characteristics of the psychological questionnaire, which can effectively capture the actual thoughts and feelings of the respondents."}, "time_dimension": {"answer": "Past", "rationale": "The entry is for the purpose of asking if the person tested had ever been \"perceptive of his brain\" and therefore could be judged as being past."}, "sentence_type": "Negative declarative sentence", "pos": {"nouns": {"Brain": 1, "Mistake": 1}, "verbs": {"I feel": 1, "have": 1}, "numerals": {"90": 1}}}]}

{"questionnaire_title": "ESS sleep table", "questionnaire_category": "Mental health", "item_count": 8, "emotion_shift_overall": "No obvious trend", "language_style_overall": "Calm and direct", "items": [{"content": "1. Sitting while reading", "word_count": 5, "label": "", "sentence_structure": "Subject鈥搗erb鈥揷omplement", "subjective_objective": {"answer": "Objective", "rationale": "The fact that the patient is living in a school is an objective fact."}, "sentence_sentiment": {"answer": "Neutral", "rationale": "Because the entry does not have any emotional color."}, "language_style": {"answer": "Calm and direct", "rationale": "The language style of the entry is relatively simple, with no excessive rhetoric or emotional colour, and a direct presentation of the scene and questions, with a more objective and calm tone."}, "time_dimension": {"answer": "Present", "rationale": "The keyword \"sitting\" describes the conduct in progress, so it can be judged to be asking what the subject is doing right now."}, "sentence_type": "Exclamatory sentence", "pos": {"nouns": {"Sit down": 1, "Read": 1}, "verbs": {"Look": 1}}}, {"content": "2. When watching television", "word_count": 4, "label": "", "sentence_structure": "Subject鈥損redicate", "subjective_objective": {"answer": "Subjective", "rationale": "Because \"watch TV\" is a voluntary choice of behaviour, and it is up to the patient to decide whether or not to proceed, it is a question of the patient's subjective perspective."}, "sentence_sentiment": {"answer": "Neutral", "rationale": "Because the entry does not have any emotional color."}, "language_style": {"answer": "Calm and direct", "rationale": "The entry is in a concise language, directly asking the person under investigation about certain behaviour or experience while watching television, without excessive emotional modification or rhetorical techniques, and with a more calm and objective language style."}, "time_dimension": {"answer": "Present", "rationale": "\"Television\" refers to the act or state in progress and thus the temporal dimension is now judged."}, "sentence_type": "Exclamatory sentence", "pos": {"nouns": {"Television": 1}, "verbs": {"Look": 1}, "adverbs": {"Time": 1}}}, {"content": "3. Sitting in public places (e.g. theatres and conferences)", "word_count": 15, "label": "", "sentence_structure": "Subject鈥損redicate", "subjective_objective": {"answer": "Objective", "rationale": "Because it is an objective description of behaviour in a public place, it does not involve subjective attitudes or feelings of the patient."}, "sentence_sentiment": {"answer": "Neutral", "rationale": "Because the entry only describes a scene of behaviour, there is no apparent emotional orientation or attitude."}, "language_style": {"answer": "Calm and direct", "rationale": "The entry presents the behaviour of the person questioned in a direct and concise manner in a given context, without adding too many rhetorical or emotional colours and with a more calm and straightforward language style."}, "time_dimension": {"answer": "Present", "rationale": "\"Sitting in public\" describes a current act or state, so the temporal dimension of the article is now."}, "sentence_type": "Imperative sentence", "pos": {"nouns": {"Public places": 1, "Sit down": 1, "Time": 1, "Theater": 1, "Meetings": 1}, "verbs": {"in": 1, "Sit down": 1}, "numerals": {"3": 1}}}, {"content": "4. No rest for one hour in a row", "word_count": 11, "label": "", "sentence_structure": "Subject鈥搗erb鈥揷omplement", "subjective_objective": {"answer": "Subjective", "rationale": "Because the behaviour of a continuous car and the absence of rest are personal experiences and perceptions of the patient, it is subjective."}, "sentence_sentiment": {"answer": "Negative", "rationale": "Because the continuous absence of rest in a car can have adverse effects on the body, it is a negative act or habit."}, "language_style": {"answer": "Tactful and warm", "rationale": ""}, "time_dimension": {"answer": "Present", "rationale": "In the future, the entry describes a situation that may arise in the future, i.e., the person tested needs to travel for one consecutive hour without rest for the next period. The time dimension of the purpose is therefore judged to be the future."}, "sentence_type": "Interrogative sentence", "pos": {"nouns": {"Hours": 1, "Rest": 1}, "verbs": {"Continuous": 1, "- By car": 1}, "adverbs": {"No, I'm not": 1}, "numerals": {"One": 1}}}, {"content": "5. When the environment allows, lie down after noon.", "word_count": 15, "label": "", "sentence_structure": "Subject鈥搗erb鈥搊bject", "subjective_objective": {"answer": "Subjective", "rationale": "Because \"the environment allows\" and \"the rest of the bed\" are the patient's own will and choice, and it's a subjective judgment."}, "sentence_sentiment": {"answer": "Neutral", "rationale": "Because the entry does not have any emotional color."}, "language_style": {"answer": "Tactful and warm", "rationale": "The language style of the entry is more euphemistic. It starts with \"if circumstances permit\" and it is a hypothetical scene, and the questions are more casual, without excessive pressure or command, and it feels more close and friendly. At the same time, the entry does not use too complex or professional terms, which make it easy for the respondents to understand and answer."}, "time_dimension": {"answer": "Past", "rationale": "The temporal dimension is therefore now judged. At the same time, the entry does not address the description of past or future behaviour or attitude, so it does not address the temporal dimension of the past and future."}, "sentence_type": "Imperative sentence", "pos": {"nouns": {"Environment": 1, "Afternoon": 1, "Rest": 1}, "verbs": {"Allow": 1, "Lie down": 1, "Time": 1}, "adverbs": {"If": 1, "If you": 1}}}, {"content": "While sitting with others", "word_count": 8, "label": "", "sentence_structure": "Subject鈥搗erb鈥揷omplement", "subjective_objective": {"answer": "Objective", "rationale": "Because this is a descriptive question, the person surveyed is asked about the situation when he or she sits and talks to others, without involving the subjective feelings or attitudes of the person under investigation."}, "sentence_sentiment": {"answer": "Neutral", "rationale": "Because the entry describes only the situation of sitting in conversation with others, it does not contain any positive or negative emotional colours."}, "language_style": {"answer": "Tactful and warm", "rationale": "The language style of the entry is relatively euphemistic and warm, and it does not ask the respondent directly about the feelings or behaviour of the person who is sitting in a conversation with someone else, but rather provides a situation and leads the other to self-reflection and expression. Such expression is more respectful of each other鈥檚 feelings and wishes, and is conducive to a good atmosphere of communication and to the effective conduct of the questionnaire."}, "time_dimension": {"answer": "Present", "rationale": "The entry asks about the situation of the person who was being tested while sitting with others, and the time dimension is now judged."}, "sentence_type": "Imperative sentence", "pos": {"nouns": {"person": 1, "Talk": 1}, "verbs": {"Sit down": 1, "Talk": 1}, "classifiers": {"individual": 1}}}, {"content": "7. Sitting after lunch (no alcohol)", "word_count": 9, "label": "", "sentence_structure": "Subject鈥搗erb鈥揷omplement", "subjective_objective": {"answer": "Subjective", "rationale": "This is because the patient's description of his activities and feelings after lunch is subjective or empirical."}, "sentence_sentiment": {"answer": "Neutral", "rationale": "As the entry is simply a statement of the facts, it does not express any positive or negative emotional tendencies."}, "language_style": {"answer": "Calm and direct", "rationale": "The entry describes in an objective and concise language the behavioural requirement of not drinking and sitting silent after lunch, without excessive emotional rendering or rhetorical techniques, and therefore more linguistically incriminating and straightforward."}, "time_dimension": {"answer": "Present", "rationale": "The entry asks whether the person tested will choose the sedentary behaviour or habit after lunch, and the time dimension will be now."}, "sentence_type": "Imperative sentence", "pos": {"nouns": {"Lunch": 1, "Drink": 1, "Sit down": 1}, "verbs": {"Back": 1, "be": 1}, "numerals": {"7": 1}}}, {"content": "8. Traffic jams in taxis.", "word_count": 17, "label": "", "sentence_structure": "Subject鈥搗erb鈥揷omplement", "subjective_objective": {"answer": "Objective", "rationale": "It is an objective fact that taxis are forced to stop because of traffic jams encountered during their journeys, regardless of the subjective feelings of the passengers. This is therefore an objective question to be examined."}, "sentence_sentiment": {"answer": "Neutral", "rationale": "The entry describes an objective fact and does not express any emotion or position."}, "language_style": {"answer": "Calm and direct", "rationale": "The language style of the entry is more objective and calm, with no excessive rhetorical or emotional rendering, but simply describes a situation and asks the respondents how they might react in such a situation. Thus, its language style can be described as \"cool and straight\"."}, "time_dimension": {"answer": "Present", "rationale": "The entry describes a current or recent experience or situation, which is \"a taxi is blocked and stopped for a few minutes.\" So the dimension of time is now."}, "sentence_type": "Interrogative sentence", "pos": {"nouns": {"Taxi": 1, "Traffic jam": 1, "Stop the car": 1, "min": 1}, "verbs": {"Take it": 1, "I met him": 1, "Stop the car": 1}, "adverbs": {"A few minutes": 1}, "numerals": {"8": 1, "A few minutes": 1}}}]}

{"questionnaire_title": "A self-esteem scale.", "questionnaire_category": "Self-cognition", "item_count": 10, "emotion_shift_overall": "From positive to negative", "language_style_overall": "Tactful and warm", "items": [{"content": "I feel that I am a valuable person, at least on the same level as others.", "word_count": 24, "label": "", "sentence_structure": "Subject鈥揷opula鈥損redicative", "subjective_objective": {"answer": "Subjective", "rationale": "Because this phrase expresses patients ' subjective views and self-evaluation of their own values."}, "sentence_sentiment": {"answer": "Positive", "rationale": "Because it expresses a positive attitude of \"value\" and \"at the same point of departure\"."}, "language_style": {"answer": "Tactful and warm", "rationale": "The language style of the entry is euphemism. It uses a positive, inspiring language to make the interviewee feel accepted and supported, and to encourage them to think about their own values. Moreover, the word \"at least\" gives the interviewee the space to feel free from undue pressure."}, "time_dimension": {"answer": "Present", "rationale": "The entry describes the present feelings and self-evaluation of the testee, and the keyword \"felt\" and \"yes\" point to the current state, so it can be judged to be now.\""}, "sentence_type": "Imperative sentence", "pos": {"nouns": {"person": 2, "Value": 1, "Horizontal": 1}, "verbs": {"I feel": 1, "be": 1, "in": 1}, "adjectives": {"have": 1, "Same": 1}, "numerals": {"One": 3}, "pronouns": {"I": 2}}}, {"content": "I feel that I have many good qualities.", "word_count": 11, "label": "", "sentence_structure": "Subject鈥揷opula鈥損redicative", "subjective_objective": {"answer": "Subjective", "rationale": "Because \"I feel I have many good qualities\" is a personal subjective feeling and assessment, not an objective fact or behaviour."}, "sentence_sentiment": {"answer": "Positive", "rationale": "Because the expression \"I have many good qualities\" is a positive assessment of myself, a sense of self-confidence and self-worth."}, "language_style": {"answer": "Tactful and warm", "rationale": "The language style of the entry is more euphemistic, not in the form of strong emotional colours or direct orders, but in the form of a voice of inquiry and invitation to direct respondents to the questions."}, "time_dimension": {"answer": "Present", "rationale": "The key word is \"I feel,\" which means that the testee is feeling and experiencing in the moment; and \"many good qualities\" describes a state or feeling, and it also occurs in the time dimension of the moment. So the purpose of the article is primarily to relate to the self-awareness and emotional experience of the testee, judging by the present."}, "sentence_type": "Affirmative declarative sentence", "pos": {"nouns": {"Questionnaire": 1, "Entry": 1, "Quality": 1}, "verbs": {"I feel": 1, "have": 1}, "adjectives": {"Many": 1, "Okay": 1}, "pronouns": {"I": 2}}}, {"content": "In the final analysis, I tend to feel like a loser.", "word_count": 18, "label": "", "sentence_structure": "Subject鈥揷opula鈥損redicative", "subjective_objective": {"answer": "Subjective", "rationale": "Because the words \"like\" and \"think\" imply that it is a personal view and attitude of the patient, and it is subjective."}, "sentence_sentiment": {"answer": "Negative", "rationale": "Because the expression \"thinks you're a loser\" is negative."}, "language_style": {"answer": "Calm and direct", "rationale": "The entry asks the respondent ' s views on his/her identity in a direct and clear manner, without using too much glorified or obscure expressions, and is more calm and objective."}, "time_dimension": {"answer": "Present", "rationale": "According to the phrase \"prefer\" and \"thinking that he is a loser,\" it can be seen as a description of the current mindset of the individual, and therefore judgment is now."}, "sentence_type": "Negative declarative sentence", "pos": {"nouns": {"In the end": 1, "self": 1, "Loser": 1}, "verbs": {"Prefer": 1, "I think": 1, "be": 1}, "adverbs": {"Most": 1}, "pronouns": {"I": 2}}}, {"content": "I can do what most people do.", "word_count": 14, "label": "", "sentence_structure": "Subject鈥搗erb鈥揷omplement", "subjective_objective": {"answer": "Subjective", "rationale": "Because this is the patient ' s assessment and perception, and because it involves personal feelings and attitudes, it is subjective."}, "sentence_sentiment": {"answer": "Positive", "rationale": "Because the \"can do it\" thing is positive."}, "language_style": {"answer": "Tactful and warm", "rationale": "The language style of the entry is euphemism. It is more friendly and interactive in an invitation-style tone, rather than in a direct statement or order, which makes the interviewee more comfortable and comfortable, willing to share his real thoughts and feelings. At the same time, the expression \"can be like most people\" is more modest, does not overemphasize himself, and is more reflective of an attitude of equal treatment and respect for others."}, "time_dimension": {"answer": "Present", "rationale": "The entry describes the current state of the person's behaviour or capability, and the word \"capable\" indicates the possession of such capability, does not clearly indicate the point of time, and therefore the judgement is now."}, "sentence_type": "Affirmative declarative sentence", "pos": {"nouns": {"Problem": 1, "person": 1, "Yes": 1}, "verbs": {"Yes": 1, "Do it": 1, "Yeah": 1}, "adjectives": {"Large": 1, "Okay": 1}, "numerals": {"Four": 1, "One": 1}, "pronouns": {"I": 1}}}, {"content": "I feel that I have few places to be proud of.", "word_count": 14, "label": "", "sentence_structure": "Subject鈥揷opula鈥損redicative", "subjective_objective": {"answer": "Subjective", "rationale": "Because \"no more pride\" is the patient's personal subjective feeling and evaluation."}, "sentence_sentiment": {"answer": "Negative", "rationale": "Because the word \"not much\" in the entry is negative."}, "language_style": {"answer": "Tactful and warm", "rationale": "The entry uses a more euphemistic and thoughtful language style, which conveys concern and understanding by asking how much one is proud of one another, without directly criticizing or rejecting the other. This expression makes it easier for the interviewee to share the real thoughts inside."}, "time_dimension": {"answer": "Present", "rationale": "The entry indicates the current feelings and thoughts of the testee, and the word \"felt\" uses the general current, which means that this feeling may be continuing. At the same time, the entry does not address past or future situations. So the answer is \"now\"."}, "sentence_type": "Negative declarative sentence", "pos": {"nouns": {"Local": 1}, "verbs": {"I feel": 1, "It's worth it": 1, "Proud": 1, "Not much": 1}, "numerals": {"5": 1}, "pronouns": {"I": 1}}}, {"content": "I am positive about myself.", "word_count": 9, "label": "", "sentence_structure": "Subject鈥揷opula鈥損redicative", "subjective_objective": {"answer": "Subjective", "rationale": "Because a positive perception of self is the patient ' s own inner feeling and assessment, it is a subjective approach."}, "sentence_sentiment": {"answer": "Positive", "rationale": "Because the phrase \"a positive attitude towards yourself\" in the entry is positive."}, "language_style": {"answer": "Tactful and warm", "rationale": "The entry is more euphemistic, expresses a message of encouragement and support in order to express confidence in the subject, without direct orders or accusations. At the same time, the first person ' s pronoun, \"I\", is used to bring the person ' s feelings and psychological state closer to the person ' s psychological state, making the questionnaire more friendly and interactive."}, "time_dimension": {"answer": "Present", "rationale": "The entry uses \"self\" to indicate that it is a description or evaluation of the person; \"yes\" and \"attitude\" are descriptions of the person's inner feelings and behavioural orientation. Since these descriptions are made in the context of the current situation, it can be judged that the entry is an inquiry into the person's current attitude."}, "sentence_type": "Affirmative declarative sentence", "pos": {"verbs": {"Hold": 1, "Sure": 1}, "adjectives": {"self": 1}}}, {"content": "7. Overall, I am satisfied with myself.", "word_count": 12, "label": "", "sentence_structure": "Subject鈥揷opula鈥損redicative", "subjective_objective": {"answer": "Subjective", "rationale": "Because satisfaction with oneself is a subjective assessment or feeling. While this satisfaction may be influenced by objective factors, it is based on the individual ' s inner feeling and assessment and is therefore subjective."}, "sentence_sentiment": {"answer": "Positive", "rationale": "This is because it expresses a positive assessment of self-awareness, i.e. satisfaction with its performance or state."}, "language_style": {"answer": "Tactful and warm", "rationale": "The entry uses a positive word of \"satisfaction\" and answers in an encouraging tone, so that people feel accepted and accepted. At the same time, the expression is relatively moderate, and there is no too direct or hard language style. So I think the language style of the questionnaire entry is euphemism."}, "time_dimension": {"answer": "Present", "rationale": ""}, "sentence_type": "Affirmative declarative sentence", "pos": {"verbs": {"be": 1, "Satisfied": 1}, "adverbs": {"Overall": 1}, "pronouns": {"I": 1}}}, {"content": "I hope I can earn more respect for myself.", "word_count": 14, "label": "", "sentence_structure": "Subject鈥搗erb鈥搊bject", "subjective_objective": {"answer": "Subjective", "rationale": "Because the word \"hope\" expresses a patient's expectation and expectation for the future, which is his own subjective feeling."}, "sentence_sentiment": {"answer": "Positive", "rationale": "Because the attitude and purpose of \"want to be respected\" in the entry are positive."}, "language_style": {"answer": "Tactful and warm", "rationale": "The entry is more personal and interactive when it is spoken in a gentle and inviting tone, rather than in direct statements or orders. At the same time, it is not too emotional, but rather leads each other to think and share their thoughts through questioning, giving people a sense of goodwill."}, "time_dimension": {"answer": "Present", "rationale": "The entry describes a desire or hope of the testee that there be greater respect in the life of the present, and therefore the time dimension is now judged."}, "sentence_type": "Imperative sentence", "pos": {"nouns": {"Hope": 1, "self": 1, "Respect": 1}, "verbs": {"Yes": 2, "Got it": 1}, "adverbs": {"More": 1}, "numerals": {"8": 1}, "pronouns": {"I": 1}}}, {"content": "9. I do often feel useless.", "word_count": 11, "label": "", "sentence_structure": "Subject鈥揷opula鈥損redicative", "subjective_objective": {"answer": "Subjective", "rationale": "Because \"fearing useless\" is a personal feeling or attitude, not an objective observation or fact."}, "sentence_sentiment": {"answer": "Negative", "rationale": "Because the emotional expression \"felt useless\" in the entry is negative."}, "language_style": {"answer": "Tactful and warm", "rationale": "The entry uses a more moderate approach to asking respondents whether they have experienced feeling useless rather than asking them directly or ordering them. At the same time, positive sentences are used to increase the acceptance and openness of the respondents and to increase the likelihood of receiving real feedback."}, "time_dimension": {"answer": "Present", "rationale": "\"I do feel useless all the time\" means that it's true, \"often\" means that it happens, both words indicate that it's a feeling that is now going on."}, "sentence_type": "Negative declarative sentence", "pos": {"verbs": {"I feel": 1, "Nothing": 1, "Useful": 1}, "adverbs": {"Indeed": 1, "Often": 1}}}, {"content": "I often think I am useless.", "word_count": 11, "label": "", "sentence_structure": "Subject鈥揷opula鈥損redicative", "subjective_objective": {"answer": "Subjective", "rationale": "Because it is a subjective description of the patient ' s own thoughts and opinions."}, "sentence_sentiment": {"answer": "Negative", "rationale": "Because the description \"nothing\" in the entry is negative."}, "language_style": {"answer": "Calm and direct", "rationale": "The entry asks the other side in a straightforward manner whether there is a self-denial, and the language style is more straightforward and objective."}, "time_dimension": {"answer": "Present", "rationale": ""}, "sentence_type": "Imperative sentence", "pos": {"verbs": {"I think": 1, "be": 1, "Nothing": 1}, "adverbs": {"Often": 1}, "pronouns": {"I": 1}}}]}

{"questionnaire_title": "Fear of negative evaluation (FNE)", "questionnaire_category": "Mental health", "item_count": 30, "emotion_shift_overall": "From positive to negative", "language_style_overall": "Neutral", "items": [{"content": "I'm rarely afraid of being stupid in other eyes.", "word_count": 16, "label": "", "sentence_structure": "Subject鈥搗erb鈥揷omplement", "subjective_objective": {"answer": "Subjective", "rationale": "Because \"care about yourself seems silly\" is a subjective feeling and an idea."}, "sentence_sentiment": {"answer": "Neutral", "rationale": "Because the entry is not clearly emotional."}, "language_style": {"answer": "Tactful and warm", "rationale": "The language style of the entry is more euphemistic and encouraging, encouraging respondents to express their thoughts and feelings and to engage in dialogue in a gentle and inviting manner."}, "time_dimension": {"answer": "Present", "rationale": "The key words \"very little worries\" and \"self-appearing silly\" point to the future state and outcome, so the entry is judged to be the future."}, "sentence_type": "Negative declarative sentence", "pos": {"nouns": {"Questionnaire": 1, "Entry": 1, "Eye": 1, "self": 1, "Stupid": 1}, "verbs": {"I'm worried": 1, "It seems": 1, "Look": 1}, "adjectives": {"Very few": 1}, "numerals": {"1": 2}, "pronouns": {"I": 1}}}, {"content": "I fear what people will think of me, although I know it doesn't matter.", "word_count": 21, "label": "", "sentence_structure": "Subject鈥搗erb鈥搊bject", "subjective_objective": {"answer": "Subjective", "rationale": "I am concerned about the subjective feelings and thoughts of the patients."}, "sentence_sentiment": {"answer": "Negative", "rationale": "Because \"I'm afraid people will look at me\" is negative."}, "language_style": {"answer": "Tactful and warm", "rationale": "The basis for the judgement: The entry, while expressing the concerns and concerns of the other party, uses the expression \"though I know it doesn't matter\" to try to comfort the other party and to ease its anxiety, which is relatively gentle and friendly. The language style of the article can therefore be considered as euphemism."}, "time_dimension": {"answer": "Past", "rationale": "The entry describes a process of anxiety and reflection of the person being tested, which, although it may be derived from past experience or events, is itself a state of mind. Thus, in terms of time dimension, the entry should be classified as \"the past\"."}, "sentence_type": "Negative declarative sentence", "pos": {"nouns": {"Questionnaire": 1, "Entry": 1, "person": 1, "He's": 1, "Sample": 1}, "verbs": {"I'm worried": 1, "Look": 2, "Yes": 1, "have": 1, "It's important": 1}, "adjectives": {"Here": 1, "What": 1}, "adverbs": {"鈾?Though 鈾?: 1}, "pronouns": {"I": 1}}}, {"content": "3. I would be very nervous to know that someone is judging me.", "word_count": 22, "label": "", "sentence_structure": "Subject鈥搗erb鈥搊bject", "subjective_objective": {"answer": "Subjective", "rationale": "Because \"very nervous\" is a subjective emotional reaction."}, "sentence_sentiment": {"answer": "Negative", "rationale": "Because \"sweet\" in the entry is a negative emotional reaction."}, "language_style": {"answer": "Tactful and warm", "rationale": "The language style of the entry is more euphemistic and caring, using hypothetical words like \"if you know\", and expressing the subject by describing emotional reactions, rather than telling the facts directly. This language style makes it easier for respondents to accept and share their thoughts and feelings."}, "time_dimension": {"answer": "Present", "rationale": "The entry describes the feelings and reactions of the person who is being evaluated when the person is being assessed, which are present and therefore present."}, "sentence_type": "Affirmative declarative sentence", "pos": {"nouns": {"person": 1, "- The headline": 1, "I'm nervous": 1}, "verbs": {"Yes": 2, "to": 1, "Conduct": 1, "A review of the quality of the body": 1}, "adjectives": {"Tens": 1}, "adverbs": {"If": 1}, "pronouns": {"I": 1}}}, {"content": "I don't care if I know people are creating an impression against me.", "word_count": 24, "label": "", "sentence_structure": "Subject鈥損redicate", "subjective_objective": {"answer": "Subjective", "rationale": "Because \"not caring\" is the patient's subjective attitude and emotional reaction."}, "sentence_sentiment": {"answer": "Negative", "rationale": "Because the attitude of \"not caring\" in the entries is negative."}, "language_style": {"answer": "Calm and direct", "rationale": "The entry expresses the subject of the attitude and reaction of the person asking the other in the face of adverse impressions in a direct and concise language, without excessive rhetorical or emotional rendering, and is more objective in tone."}, "time_dimension": {"answer": "Present", "rationale": "The combination of \"even\" and \"even\" expresses a hypothetical situation, while \"not care\" expresses the current attitude and behaviour, so the entry asks about the current mindset and behaviour of the testee."}, "sentence_type": "Negative declarative sentence", "pos": {"nouns": {"Questionnaire": 1, "Entry": 1, "People": 1, "Impression": 1}, "verbs": {"Yes": 1, "Form": 1, "I care": 1}, "adjectives": {"Negative": 1}, "adverbs": {"Even if": 1}, "pronouns": {"I": 1}}}, {"content": "5. When I make a social mistake, I am very unhappy.", "word_count": 17, "label": "", "sentence_structure": "Subject鈥揷opula鈥損redicative", "subjective_objective": {"answer": "Subjective", "rationale": "Because this is a description of the patient ' s emotional response and is subjective."}, "sentence_sentiment": {"answer": "Negative", "rationale": "Because the \"very unhappy\" mood in the entry is negative."}, "language_style": {"answer": "Tactful and warm", "rationale": "The entry presents its emotional experience in terms of first person's perspective, expressed in a relatively moderate and euphemistic way rather than in direct statements or orders, which is more personal and interactive. At the same time, the use of emotional terms such as \"very unpleasant\" increases emotional colours and makes it easier for respondents to resonate."}, "time_dimension": {"answer": "Present", "rationale": "The phrase \"will\" in this sentence indicates a tendentious or regular behaviour, usually associated with the general current state of affairs, which means that this situation is present at times. The entry therefore expresses the person's current behaviour or state."}, "sentence_type": "Negative declarative sentence", "pos": {"nouns": {"Questionnaire": 1, "Entry": 1, "Error": 1}, "verbs": {"Yes": 1, "Out": 1, "Very": 1, "Have a nice day": 1}, "adjectives": {"Social": 1, "It's not good": 1}, "numerals": {"5": 1}}}, {"content": "I am not much concerned about what important people think of me.", "word_count": 15, "label": "", "sentence_structure": "Subject鈥搗erb鈥搊bject", "subjective_objective": {"answer": "Subjective", "rationale": "Because it involves the patient ' s personal feelings and thoughts, i.e. whether or not there is a fear that the person in question will be perceived as such, is a subjective assessment."}, "sentence_sentiment": {"answer": "Negative", "rationale": "The basis for the determination is that the entry expresses an attitude of indifference or indifference towards the views of others, which in some cases may lead to self-closure, isolation and so benevolence."}, "language_style": {"answer": "Calm and direct", "rationale": "The language style of the entry is relatively calm and objective, and there is no excessive emotional rendering or rhetorical technique, and the subject of questioning whether the other party is not concerned about the views of the important person is directly presented."}, "time_dimension": {"answer": "Present", "rationale": "The keywords are \"not much to worry\" and \"the opinion of the person who's important to me,\" which describe the person's current attitude or behaviour."}, "sentence_type": "Negative declarative sentence", "pos": {"nouns": {"Problem": 1, "People": 1}, "verbs": {"Analysis": 1, "I'm worried": 1, "to": 1, "Observations": 1}, "adjectives": {"Important": 1, "Not much": 1}, "adverbs": {"very": 1}, "numerals": {"6": 1}}}, {"content": "7. I was often afraid that I would look funny or stupid.", "word_count": 17, "label": "", "sentence_structure": "Subject鈥揷opula鈥損redicative", "subjective_objective": {"answer": "Subjective", "rationale": "Because fear is an emotional response, it is the patient ' s subjective attitude and feelings."}, "sentence_sentiment": {"answer": "Negative", "rationale": "Because the sentiment in the entry, \"fearing that you'll look funny or stupid,\" is a negative expression of emotion."}, "language_style": {"answer": "Tactful and warm", "rationale": ""}, "time_dimension": {"answer": "Present", "rationale": "The word \"I'm often\" means regular, customary behaviour, and the word \"fear\" uses the usual current state of affairs, which means that this sentiment has not only happened in the past, but may still be present."}, "sentence_type": "Negative declarative sentence", "pos": {"nouns": {"Questionnaire": 1, "Entry": 1}, "verbs": {"Analysis": 1}, "adjectives": {"Often": 1, "Scared": 1, "self": 1, "Funny": 1, "Ridiculous": 1, "Stupid": 1}, "adverbs": {"Yes": 1, "very": 1}, "pronouns": {"I": 1}}}, {"content": "I have little reaction to others' disagreement with me.", "word_count": 13, "label": "", "sentence_structure": "Subject鈥搗erb鈥揷omplement", "subjective_objective": {"answer": "Subjective", "rationale": "Because \"no response\" is the patient's subjective feeling and attitude."}, "sentence_sentiment": {"answer": "Negative", "rationale": "Because the \"near to agree with me\" act in the entry is negative."}, "language_style": {"answer": "Calm and direct", "rationale": "The entry uses a concise narrative that describes a theme that is whether the interviewee is emotionally responding to someone else鈥檚 disapproval of himself. The tone is more objective, and there is no excessive emotional or pedigree. It is therefore judged to be 鈥渃ool and direct鈥?"}, "time_dimension": {"answer": "Present", "rationale": "The sentence describes the situation and state of the person being tested, in which \"unapproval of me\" is expressed or criticisms of others do not have a significant impact on him, and \"near reaction\" is an indication of his less volatile mood. The entry is therefore used to understand the resilience and psychological resilience of the person being tested and is applied to the current state of affairs."}, "sentence_type": "Affirmative declarative sentence", "pos": {"nouns": {"Response": 1, "others": 1}, "verbs": {"to": 1, "Yes": 1, "None": 1}, "adjectives": {"Almost": 1}, "pronouns": {"I": 1}}}, {"content": "9. I am often afraid that others will notice my shortcomings.", "word_count": 15, "label": "", "sentence_structure": "Subject鈥搗erb鈥揷omplement", "subjective_objective": {"answer": "Subjective", "rationale": "Because the words \"fear\" and \"fear\" indicate that it is a patient's own feeling or fear, and it is subjective."}, "sentence_sentiment": {"answer": "Negative", "rationale": "Because the words \"fear\" and \"not notice the shortness\" in the entries are all negative emotional expressions of anxiety and anxiety."}, "language_style": {"answer": "Tactful and warm", "rationale": "The entry uses a first-person expression and a soft, invitation-style tone of dialogue rather than direct statements or orders, which is more personal and interactive. At the same time, it does not overemphasize or criticize the other side鈥檚 weaknesses, but provides an opportunity to express their feelings freely and to make the interviewee feel understood and respected."}, "time_dimension": {"answer": "Present", "rationale": "The entry uses the phrase \"I'm often\" to suggest that it's a sentence describing current behaviour, so it's judged as \"now.\" And the words \"fear\" and \"notice\" also suggest that this is an emotional problem."}, "sentence_type": "Negative declarative sentence", "pos": {"nouns": {"I": 1, "others": 1, "Short": 1}, "verbs": {"Scared": 1, "Attention": 1, "Present": 1, "have": 1}, "adjectives": {"Regular": 1, "People will notice my weaknesses": 1}, "numerals": {"9": 1}}}, {"content": "10. The disagreement of others has had little effect on me.", "word_count": 14, "label": "", "sentence_structure": "Subject鈥搗erb鈥揷omplement", "subjective_objective": {"answer": "Subjective", "rationale": "Because \"adoption\" is an external factor that has an impact on patients' feelings and thoughts, it is a subjective examination."}, "sentence_sentiment": {"answer": "Neutral", "rationale": "Because the entry does not have any emotional color."}, "language_style": {"answer": "Calm and direct", "rationale": "This is a more calm and direct language style, which does not use too many embellishments or emotional renderings, but simply asks whether the interviewee is influenced by opposition. This language style can be more accurate and more effective in gathering information, without creating excessive psychological pressure on the interviewee."}, "time_dimension": {"answer": "Future", "rationale": "The entry addresses situations that the testee may face in the future, which are related to the views of others, and the keywords \"others\" and \"dissenting\" point to situations that have not yet occurred and that may arise in the future."}, "sentence_type": "Affirmative declarative sentence", "pos": {"verbs": {"to": 1, "have": 1}, "adjectives": {"Almost": 1, "No, I'm not": 1}, "numerals": {"10": 1}, "pronouns": {"I": 1}}}, {"content": "If someone were to judge me, I would easily think of the worst.", "word_count": 19, "label": "", "sentence_structure": "Subject鈥損redicate", "subjective_objective": {"answer": "Subjective", "rationale": "Because this sentence concerns patients鈥?subjective thoughts and concerns about themselves, that when others evaluate themselves, they tend to think that others may give the worst assessment. This is what patients feel in their heart, and it is therefore subjective."}, "sentence_sentiment": {"answer": "Negative", "rationale": "Because the mental state of \"the worst is thought to\" in the entry is negative."}, "language_style": {"answer": "Calm and direct", "rationale": "The entry uses a hypothetical tone, presents a situation and asks the interviewee how he feels. This description is more direct and objective, and there is no excessive emotional rendering or rhetorical technique, so the language style is more calm and straightforward."}, "time_dimension": {"answer": "Future", "rationale": "\"The future.\""}, "sentence_type": "Negative declarative sentence", "pos": {"nouns": {"person": 1, "Evaluation": 2, "I": 1}, "verbs": {"Yes": 1, "be": 1}, "adjectives": {"The worst": 1}, "adverbs": {"Easy": 1, "in": 1, "Suppose": 1}, "numerals": {"11": 1}}}, {"content": "12. I hardly care what I am making of others.", "word_count": 19, "label": "", "sentence_structure": "Subject鈥搗erb鈥搊bject", "subjective_objective": {"answer": "Subjective", "rationale": "Because this is the patient ' s own feeling and attitude and is subjective."}, "sentence_sentiment": {"answer": "Neutral", "rationale": "Because the entry describes the manner of conduct, it does not express positive or negative emotional tendencies."}, "language_style": {"answer": "Calm and direct", "rationale": "The entry directly enquired about the psychological condition of the person interviewed, was not packaged using excessive rhetorical or emotional colours, and was more calm and direct in language style."}, "time_dimension": {"answer": "Present", "rationale": "The keyword \"I'm hardly concerned\" means the current state and behaviour, and it is therefore judged now."}, "sentence_type": "Negative declarative sentence", "pos": {"nouns": {"Questionnaire Entry": 1, "I": 2, "person": 1, "Impression": 1}, "verbs": {"Mind your own business": 1, "Stay": 1}, "adjectives": {"Almost": 1}, "adverbs": {"Here": 1}, "numerals": {"12": 1}, "pronouns": {"Don't": 1}}}, {"content": "I fear that others will disagree with me.", "word_count": 10, "label": "", "sentence_structure": "Subject鈥搗erb鈥揷omplement", "subjective_objective": {"answer": "Subjective", "rationale": "Because \"fear\" is the patient's subjective feeling and thoughts."}, "sentence_sentiment": {"answer": "Negative", "rationale": "Because the \"fear\" in the entry is negative."}, "language_style": {"answer": "Tactful and warm", "rationale": ""}, "time_dimension": {"answer": "Present", "rationale": "The keyword \"fear\" and \"will not agree with me\" means what the subject thinks and feels now, so the entry is about the person's current state."}, "sentence_type": "Interrogative sentence", "pos": {"nouns": {"13": 1, "I": 2, "others": 1, "Yes": 1}, "verbs": {"Yes": 1, "I don't agree": 1}, "numerals": {"13": 1}, "pronouns": {"I": 2}}}, {"content": "I fear that others will find my mistakes.", "word_count": 12, "label": "", "sentence_structure": "Subject鈥搗erb鈥揷omplement", "subjective_objective": {"answer": "Subjective", "rationale": "Because \"I'm afraid\" is the patient's subjective feeling and fear."}, "sentence_sentiment": {"answer": "Negative", "rationale": "Because \"fear\" is negative."}, "language_style": {"answer": "Calm and direct", "rationale": "The language style of the entry is calm and straightforward, and it asks the interviewee whether he fears that he or she will be found to have made a mistake. Instead of using too many rhetorical techniques or emotional rendering, he or she asks questions in a balanced and objective manner, making them clearer and more understandable."}, "time_dimension": {"answer": "Present", "rationale": "Now the entry indicates the feelings and thoughts of the testee, and the word \"I'm afraid\" in it indicates that it's the subject matter of the testee, and that \"someone will find\" and \"my fault\" suggests that it is an emotion of fear or fear that is likely to influence the conduct and decision-making of the testee."}, "sentence_type": "Negative declarative sentence", "pos": {"nouns": {"I": 1, "others": 1, "Wrong": 1}, "verbs": {"Scared": 1, "Found": 1}, "numerals": {"14": 1}}}, {"content": "I'm not worried about what people think of me.", "word_count": 14, "label": "", "sentence_structure": "Subject鈥搗erb鈥搊bject", "subjective_objective": {"answer": "Subjective", "rationale": "Because \"not to worry about...\" is the patient's subjective attitude."}, "sentence_sentiment": {"answer": "Neutral", "rationale": "Because the entry describes only a fact, there is no apparent emotional tendency."}, "language_style": {"answer": "Calm and direct", "rationale": "The entry is directly, succinctly, asking the respondent whether he or she is troubled by his or her reputation or evaluation, without any rhetorical or emotional rendering, and relatively calm and straightforward in his or her language style. \""}, "time_dimension": {"answer": "Present", "rationale": "\"Not for\" expresses a state of present and attitude that the testee is not currently troubled by the perception of himself. The entry therefore describes the current emotional state of the testee, and is judged by the time dimension of \"now\"."}, "sentence_type": "Negative declarative sentence", "pos": {"nouns": {"Grandpa": 1, "Questionnaire": 1, "Entry": 1, "Observations": 1}, "verbs": {"be": 1, "I'm worried": 1}, "adjectives": {"he": 1}, "adverbs": {"No, it's not": 1}, "numerals": {"15": 1}, "pronouns": {"I": 1, "others": 1}}}, {"content": "16. If someone is not happy, I may not care.", "word_count": 16, "label": "", "sentence_structure": "Subject鈥搗erb鈥揷omplement", "subjective_objective": {"answer": "Subjective", "rationale": "Since this entry relates to the patient ' s emotional reaction and attitude to failure to please a person, it is examined from the patient ' s subjective perspective."}, "sentence_sentiment": {"answer": "Negative", "rationale": "Because the attitude of \"doesn't matter\" in the entry is negative."}, "language_style": {"answer": "Tactful and warm", "rationale": "The entry is more personal and interactive when it is spoken in a gentle and inviting manner, rather than in direct statements or orders. At the same time, the sentence also expresses the attitude that the other side may have towards others, and can therefore be considered a soft and warm language style."}, "time_dimension": {"answer": "Present", "rationale": ""}, "sentence_type": "Negative declarative sentence", "pos": {"verbs": {"Jean": 1, "Joy": 1, "Maybe": 1, "It doesn't matter": 1}, "adverbs": {"Suppose": 1, "Nope": 1, "Someone": 1}, "numerals": {"16": 1}, "pronouns": {"I": 1}}}, {"content": "When I spoke to others, I was afraid what they would think of me.", "word_count": 18, "label": "", "sentence_structure": "Subject鈥揷opula鈥損redicative", "subjective_objective": {"answer": "Subjective", "rationale": "Because it is the patient ' s inner feeling and thoughts, it is a subjective examination."}, "sentence_sentiment": {"answer": "Negative", "rationale": "Because the \"worried they'd look at me\" attitude in the entry is negative."}, "language_style": {"answer": "Tactful and warm", "rationale": "The entry is a conversation in a question-and-answer manner and expresses the anxiety that might exist in the person under investigation by using the word \"concerned\" in a more moderate, euphemistic and sensitive way to the person's feelings."}, "time_dimension": {"answer": "Present", "rationale": "Now the keyword \"when I talk to someone\" says it's an ongoing scene, and \"I'm afraid they'll look at me\" is a sentence that expresses emotional or psychological state, so it can be judged that the entry primarily asks the person who was tested about the mood and mental state of the person who was talking to someone else at the moment."}, "sentence_type": "Negative declarative sentence", "pos": {"nouns": {"When": 1, "I": 2, "others": 1, "Talk": 1, "they": 1, "Look": 1}, "verbs": {"Yes": 1, "I'm worried": 1, "Same": 1, "Say it": 1, "How do you think": 1}, "adverbs": {"very": 1}, "numerals": {"17": 1}, "pronouns": {"I": 2, "they": 1}}}, {"content": "18. I think it is inevitable that one should make a mistake in social life, and therefore why should one be upset about it?", "word_count": 27, "label": "", "sentence_structure": "Subject鈥搗erb鈥搊bject", "subjective_objective": {"answer": "Subjective", "rationale": "Because words like \"think\" and \"Why worry about it\" are all words that describe patients' subjective feelings and attitudes. The topic is a subjective one, considering patients' attitude to social error."}, "sentence_sentiment": {"answer": "Positive", "rationale": "Because the attitude in the entry is positive and the social error is inevitable, there is no need to be grieved about it."}, "language_style": {"answer": "Tactful and warm", "rationale": "The entry is more euphemistic, less directly critical or commanding that one should not be troubled by minor social errors. Instead, it uses a tone of encouragement and comfort, which makes the interviewee comfortable and willing to share their views. This expression is more acceptable and helps create a good questionnaire atmosphere."}, "time_dimension": {"answer": "Present", "rationale": ""}, "sentence_type": "Affirmative declarative sentence", "pos": {"nouns": {"Alone": 1, "Socially": 1, "Error": 1, "Required": 1, "For": 1, "Sad": 1}, "verbs": {"I think": 1, "Out": 1, "It's inevitable": 1, "And so": 1, "Why": 1, "Sad": 1, "And": 1}, "adverbs": {"in": 1, "Medium": 1, "Points": 1, "No way": 1, "Yes": 1, "For that reason": 1}, "numerals": {"18": 1, "1": 4}, "pronouns": {"I": 1}}}, {"content": "I am usually worried about what I am making of others.", "word_count": 21, "label": "", "sentence_structure": "Subject鈥搗erb鈥搊bject", "subjective_objective": {"answer": "Subjective", "rationale": "Because it expresses the feelings and thoughts of the patient himself and is a subjective observation."}, "sentence_sentiment": {"answer": "Negative", "rationale": "Because the \"always worry\" attitude in the entries is negative."}, "language_style": {"answer": "Tactful and warm", "rationale": "The language style of the entry is more euphemistic, expressing the fears and anxiety that might exist in the person under investigation by using the words \"always\" and \"what is left of the impressions.\" At the same time, the phrase \"whatever I am\" implies uncertainty and self-doubt about self-evaluation, which is more acrimony and emotional."}, "time_dimension": {"answer": "Present", "rationale": "The entry asks about the current behaviour and mentality of the subject, which is \"always\" and \"in\" and \"in the end\" key words, which indicate a state of continuity and habit. So, judging from the temporal dimension, the entry is now."}, "sentence_type": "Interrogative sentence", "pos": {"nouns": {"I": 2, "I'm worried": 1, "others": 1, "Impression": 1}, "verbs": {"Always": 1, "Stay": 1}, "adverbs": {"What the hell": 1}, "numerals": {"19": 1}}}, {"content": "I'm worried about how my boss looks at me.", "word_count": 14, "label": "", "sentence_structure": "Subject鈥揷opula鈥損redicative", "subjective_objective": {"answer": "Subjective", "rationale": "Because \"I'm worried\" is a word for patient subjective feelings and fears, the questionnaire entry is viewed from the patient's subjective perspective."}, "sentence_sentiment": {"answer": "Negative", "rationale": "The entry was a negative sentiment because it expressed concern and disquiet over the views of the supervisor."}, "language_style": {"answer": "Calm and direct", "rationale": "The entry is of a direct, clear language style, does not use any rhetorical or emotional rendering, and focuses more on the transmission of facts and information than on attempts to provoke emotional reactions or emotional fluctuations among the person under investigation. \""}, "time_dimension": {"answer": "Present", "rationale": ""}, "sentence_type": "Interrogative sentence", "pos": {"nouns": {"Sir": 1, "Observations": 1}, "verbs": {"I'm worried": 1}, "adverbs": {"What": 1}, "pronouns": {"I": 1}}}, {"content": "I don't care when I know someone is judging me.", "word_count": 19, "label": "", "sentence_structure": "Subject鈥搗erb鈥搊bject", "subjective_objective": {"answer": "Subjective", "rationale": "Because \"not caring\" is the patient's subjective feeling and attitude."}, "sentence_sentiment": {"answer": "Negative", "rationale": "Because the \"not caring\" attitude in the entry is negative."}, "language_style": {"answer": "Calm and direct", "rationale": "The language style of the entry is more straightforward, with no excessive rhetorical or emotional rendering, and a calm attitude that does not care about others ' evaluation."}, "time_dimension": {"answer": "Past", "rationale": "It is therefore not possible to determine precisely its time frame."}, "sentence_type": "Negative declarative sentence", "pos": {"nouns": {"person": 1, "Evaluation": 1}, "verbs": {"Yes": 1, "I care": 1}, "adjectives": {"Not at all": 1}, "numerals": {"21": 1}, "pronouns": {"I": 1}}}, {"content": "I fear that people will think I am useless.", "word_count": 14, "label": "", "sentence_structure": "Subject鈥搗erb鈥搊bject", "subjective_objective": {"answer": "Subjective", "rationale": "Because \"fear\" is the patient's subjective feeling and thought."}, "sentence_sentiment": {"answer": "Negative", "rationale": "Because the entry expresses negative assessments and concerns about itself."}, "language_style": {"answer": "Tactful and warm", "rationale": "The entry uses a first person's expression and has a certain subjective emotional aspect, and the interrogator is worried about what he thinks is \"no use.\" This is a warmer tone, more concerned with the feelings and thoughts of the other."}, "time_dimension": {"answer": "Past", "rationale": "The entry asks the current feelings and mindset of the testee, and the keywords \"concern\" and \"think\" indicate that this is a subjective feeling and idea, and that there are no clear time indicators of the temporal dimension, and it is therefore not possible to judge clearly whether it is past, present or future behaviour or mentality."}, "sentence_type": "Negative declarative sentence", "pos": {"nouns": {"Questionnaire": 1, "Entry": 1, "person": 1, "A useless man": 1}, "verbs": {"I'm worried": 1, "I think": 1, "be": 2, "Yes": 1, "Fear": 1}, "adjectives": {"It's useless": 1}, "numerals": {"22": 1}, "pronouns": {"I": 1}}}, {"content": "I'm not really worried about how people think about me.", "word_count": 17, "label": "", "sentence_structure": "Subject鈥揷opula鈥損redicative", "subjective_objective": {"answer": "Subjective", "rationale": "Because fear of others' perceptions is a subjective feeling and emotion."}, "sentence_sentiment": {"answer": "Neutral", "rationale": "Because the entry describes the degree of individual concern about others ' views, it does not clearly express positive or negative emotional tendencies."}, "language_style": {"answer": "Calm and direct", "rationale": "The language style of the entry is a calm and direct type. It directly describes the subject matter of the investigation (i.e. the degree of personal concern for the views of others), without using excessive rhetorical or emotional rendering, and with a fair and objective tone.\""}, "time_dimension": {"answer": "Present", "rationale": "The keyword \"I\" and \"almost unafraid\" point to the current tested attitude and emotional state, so the entry describes the current situation."}, "sentence_type": "Negative declarative sentence", "pos": {"nouns": {"He's": 1, "What": 1, "I": 1}, "verbs": {"to": 1, "be": 1, "Yes": 1, "I'm worried": 1}, "adjectives": {"Total": 1}, "adverbs": {"Almost": 1}, "numerals": {"23": 1}}}, {"content": "24. Sometimes I think I care too much about what others think of me.", "word_count": 17, "label": "", "sentence_structure": "Subject鈥揷opula鈥損redicative", "subjective_objective": {"answer": "Subjective", "rationale": "Because \"too much care for what people think of me\" is a subjective feeling and attitude."}, "sentence_sentiment": {"answer": "Negative", "rationale": "Because the \"too much care\" in the entry is negative."}, "language_style": {"answer": "Tactful and warm", "rationale": "The entry uses a more moderate tone, so that the respondents are not stressed or uncomfortable in their responses. At the same time, it is expressed in terms of \"sometimes\" and gives people a more open and receptive attitude."}, "time_dimension": {"answer": "Present", "rationale": ""}, "sentence_type": "Interrogative sentence", "pos": {"nouns": {"Time": 1, "Yes": 1, "self": 1, "Too much": 1, "I care": 1, "others": 1, "Observations": 1}, "verbs": {"Yes": 1, "I care": 1}, "adjectives": {"Too much": 1}, "adverbs": {"Sometimes": 1}, "numerals": {"24": 1}, "pronouns": {"I": 1}}}, {"content": "I often fear that I will say or do something wrong.", "word_count": 14, "label": "", "sentence_structure": "Subject鈥搗erb鈥揷omplement", "subjective_objective": {"answer": "Subjective", "rationale": "Because \"fear\" is a feeling and attitude that is both inner and subjective."}, "sentence_sentiment": {"answer": "Negative", "rationale": "Because the attitude in the entry that I'm afraid I'm going to say or do wrong is negative."}, "language_style": {"answer": "Calm and direct", "rationale": "The entry is based on a direct question, with a more objective tone, with no excessive rhetorical or emotional rendering, and a language style of a straightforward type of calm.\""}, "time_dimension": {"answer": "Present", "rationale": "\"I've often\" expressed that fear as a regular, customary act, while the word \"will\" has adopted the usual future pattern, meaning that it may occur in the future. So, this entry is now."}, "sentence_type": "Affirmative declarative sentence", "pos": {"nouns": {"Questionnaire": 1, "Entry": 1, "I'm worried": 1, "I": 1, "Word": 1, "Yes": 1}, "verbs": {"Often": 1, "Say it": 1, "Do it": 1, "Yes": 2}, "adverbs": {"Wrong": 2}, "numerals": {"25": 1}}}, {"content": "26. I am not usually concerned about what others think of me.", "word_count": 14, "label": "", "sentence_structure": "Subject鈥揷opula鈥損redicative", "subjective_objective": {"answer": "Subjective", "rationale": "Because \"care\" is the subjective feeling and attitude of the individual, the attitude of the other person."}, "sentence_sentiment": {"answer": "Neutral", "rationale": "Because the entry describes the attitude of one person towards others ' views of one another, there is no apparent positive or negative tendency."}, "language_style": {"answer": "Calm and direct", "rationale": "The language of the entry is more straightforward, without any symmetry or emotional rendering, and is more objective in tone, and directly asks the respondents about the attitude of others to their own views."}, "time_dimension": {"answer": "Present", "rationale": "And by the word \"normal\", it can be judged to describe the current state and trends."}, "sentence_type": "Negative declarative sentence", "pos": {"nouns": {"I": 1, "others": 1, "Observations": 1}, "verbs": {"to": 2, "I don't care": 1}, "adverbs": {"Usually": 1}, "numerals": {"26": 1}}}, {"content": "I am generally confident that others will be impressed with me.", "word_count": 14, "label": "", "sentence_structure": "Subject鈥搗erb鈥揷omplement", "subjective_objective": {"answer": "Subjective", "rationale": "Because confidence is a subjective emotion and attitude."}, "sentence_sentiment": {"answer": "Positive", "rationale": "Because the \"assure that someone else will be impressed with me\" attitude in the entry is positive."}, "language_style": {"answer": "Tactful and warm", "rationale": "The entry uses a language style that is active, encouraging and supportive, raises questions and leads respondents to reflect on their attitudes and levels of self-confidence in others' views, and is soft in language, does not directly deny or confirm the answers of the interviewees. Such language styles are more personal and interactive and help to create an atmosphere of comfort and free expression."}, "time_dimension": {"answer": "Past", "rationale": "The entry describes the past subjective feelings and beliefs of the testee and therefore judges the past."}, "sentence_type": "Affirmative declarative sentence", "pos": {"nouns": {"Questionnaire": 1, "Entry": 1, "Confidence": 1, "person": 1, "Impression": 1}, "verbs": {"Yes": 1, "have": 1, "Here": 1}, "adverbs": {"General": 1}, "numerals": {"27": 1}, "pronouns": {"I": 1}}}, {"content": "I am always afraid that those who are important to me will not always think of me.", "word_count": 21, "label": "", "sentence_structure": "Subject鈥搗erb鈥搊bject", "subjective_objective": {"answer": "Subjective", "rationale": "It is the patient ' s own feelings and perceptions that are always concerned about whether others will always think about themselves and are subjective in their approach."}, "sentence_sentiment": {"answer": "Negative", "rationale": "Because it expresses an emotional feeling of anxiety and anxiety and is negative."}, "language_style": {"answer": "Tactful and warm", "rationale": "The entry is more softly phrased, and resonates by expressing feelings of concern and concern to others. It does not directly blame or order, but engages in dialogue in a kind invitation, which is more likely to elicit positive responses and engagement."}, "time_dimension": {"answer": "Present", "rationale": "The entry expresses a current concern and feeling of the interviewee, \"Always worried,\" which is occurring at the moment. And, \"those who are important to me\" shows that this is a theme of relationships with others, not a future or past event. So, the time dimension of this questionnaire is now."}, "sentence_type": "Negative declarative sentence", "pos": {"nouns": {"person": 2, "Important": 1, "I": 2, "Always": 1}, "verbs": {"Yes": 2, "Present": 1, "I'm worried": 1}, "adjectives": {"It's important": 1}, "adverbs": {"Total": 1}, "numerals": {"28": 1}, "pronouns": {"Those": 1, "Guys": 1}}}, {"content": "I am unhappy with the way my friends see me.", "word_count": 16, "label": "", "sentence_structure": "Subject鈥揷opula鈥損redicative", "subjective_objective": {"answer": "Subjective", "rationale": "Because the perception of friends is a subjective feeling of the patient, the question is one of the subjective feelings and thoughts of the patient."}, "sentence_sentiment": {"answer": "Negative", "rationale": "Because the emotional expression \"unhappiness\" in the entry is negative."}, "language_style": {"answer": "Calm and direct", "rationale": "The entry is in short, direct language and asks the interviewee whether he is unhappy because of his friends ' views, is objective and clear, and does not have too much emotional adornment or euphemism."}, "time_dimension": {"answer": "Past", "rationale": "The entry describes the emotional state and experience of the testee, the key words being \"my views of my friends\" and \"unhappiness\" and points to what happened in the past."}, "sentence_type": "Negative declarative sentence", "pos": {"nouns": {"Questionnaire": 1, "Entry": 1, "friends": 1, "Observations": 1, "I'm not happy": 1}, "verbs": {"Because": 1, "have": 1, "to": 1, "And": 1, "Here": 1}, "adverbs": {"Mine": 1, "And so": 1}, "numerals": {"29": 1}, "pronouns": {"I": 1}}}, {"content": "30. I would be nervous to know that my superiors are judging me.", "word_count": 20, "label": "", "sentence_structure": "Subject鈥揷opula鈥損redicative", "subjective_objective": {"answer": "Subjective", "rationale": "Because stress is a subjective feeling or emotional reaction, it is the perception and expectations of the patient鈥檚 superiors. This is a subjective view of the patient鈥檚 mental state."}, "sentence_sentiment": {"answer": "Negative", "rationale": "Because the \"get nervous\" act in the entry is negative."}, "language_style": {"answer": "Calm and direct", "rationale": "The entry is more objective and direct in its direct way by asking the other party about the emotional reaction of the supervisor when he learned that he was evaluating himself, and is clear in language, without too many rhetorical or emotional colours."}, "time_dimension": {"answer": "Future", "rationale": "The keyword \"will\" and \"will\" indicates that this is an expected and possible future scenario, i.e., one that is tense when it comes to knowing that the superior is evaluating himself."}, "sentence_type": "Affirmative declarative sentence", "pos": {"nouns": {"Questionnaire Entry": 1, "Sir": 1, "Evaluation": 1}, "verbs": {"Yes": 1, "It's gonna be": 1, "I'm nervous": 1}, "adjectives": {"30": 1, "of": 2}, "numerals": {"30": 1}, "pronouns": {"I": 1}}}]}

{"questionnaire_title": "Pittsburgh sleep quality index (PSQI)", "questionnaire_category": "Emotion and affect", "item_count": 9, "emotion_shift_overall": "No obvious trend", "language_style_overall": "Calm and direct", "items": [{"content": "1. For almost one month, bedtime is usually at _ o ' clock.", "word_count": 14, "label": "", "sentence_structure": "Subject鈥搗erb鈥搊bject", "subjective_objective": {"answer": "Objective", "rationale": "Because the title requires the last night of bedtime, it is an objective description of the facts and does not involve subjective feelings or attitudes of the patient."}, "sentence_sentiment": {"answer": "Neutral", "rationale": "Because the entry does not have any emotional color."}, "language_style": {"answer": "Tactful and warm", "rationale": "The language style of the entry is more euphemistic, and by using the expression \"usually\" it is neither mandatory for the interviewee to answer the specific time, nor can it be respected and understood. Moreover, the description of \"nighttime bed\" is neutral and does not have too many negative emotional aspects."}, "time_dimension": {"answer": "Present", "rationale": "The entry asked the testee about sleep habits during the last month, which were part of the investigation of current behaviour or state, and therefore judged \"now\"."}, "sentence_type": "Interrogative sentence", "pos": {"nouns": {"Nearly 1 month": 1, "Night": 1, "Go to bed": 1, "Points": 1}, "verbs": {"be": 1}, "adverbs": {"Usually": 1}, "numerals": {"1": 2, "Month": 1}}}, {"content": "2. For almost one month, it usually takes _ minutes from bedtime to sleep.", "word_count": 15, "label": "", "sentence_structure": "Subject鈥搗erb鈥揷omplement", "subjective_objective": {"answer": "Objective", "rationale": "This is an objective fact that asks how long it takes for the patient to sleep."}, "sentence_sentiment": {"answer": "Neutral", "rationale": "Because the entry is only a description of a time period and does not involve any emotional colour."}, "language_style": {"answer": "Tactful and warm", "rationale": "The language style of the entry is relatively euphemistic and warm. It allows the respondents to ask questions about personal information rather than direct orders or statements. At the same time, more friendly words and words, such as \"near a month\" and \"usually needed\" are used to make the questionnaire more interactive and friendly."}, "time_dimension": {"answer": "Present", "rationale": ""}, "sentence_type": "Interrogative sentence", "pos": {"nouns": {"Near": 1, "Month": 1, "min": 1}, "verbs": {"Yes": 1, "Get in bed": 1, "People sleep": 1}, "adverbs": {"Usually": 1}, "numerals": {"1": 2}}}, {"content": "3. Almost a month, usually wake up at _ o'clock.", "word_count": 10, "label": "", "sentence_structure": "Subject鈥搗erb鈥揷omplement", "subjective_objective": {"answer": "Objective", "rationale": "It is an objective fact that the question is a regular activity for the patient to be questioned for a certain period of time in the past."}, "sentence_sentiment": {"answer": "Neutral", "rationale": "As the entry describes a fact or behaviour, it does not contain any emotional tendencies."}, "language_style": {"answer": "Calm and direct", "rationale": "The basis for the judgement is that the article is designed to ask questions in a more concise language, without any qualifier or emotional colour, and to be objective and objective. It directly asks the interviewee about the number of times he has been up in the morning of the past month, using no euphemism or ambiguity."}, "time_dimension": {"answer": "Past", "rationale": "The entry is for the testee ' s life habits over the past month and the time dimension is judged to be past."}, "sentence_type": "Interrogative sentence", "pos": {"nouns": {"Month": 1, "Points": 2, "The bed": 1}, "verbs": {"Rise": 1, "Sleep": 1, "Wake up": 1}, "adverbs": {"Near": 1, "Usually": 2}, "numerals": {"3": 1, "1": 3}}}, {"content": "4. For nearly one month, sleep per night is usually actual _ hours (not equal to bedtime).", "word_count": 20, "label": "", "sentence_structure": "Subject鈥搗erb鈥揷omplement", "subjective_objective": {"answer": "Objective", "rationale": "Because sleep time is an objective and visible fact."}, "sentence_sentiment": {"answer": "Neutral", "rationale": "Because the entry is merely an inquiry into the basic sleep of the person under investigation and is not emotional."}, "language_style": {"answer": "Calm and direct", "rationale": "The entry is relatively calm and straightforward in language, and does not have too much adoration or emotional colour. It asks the respondents in a simple and clear manner about the average actual sleep time in the last month, and is of high information accessibility and objectivity."}, "time_dimension": {"answer": "Present", "rationale": "The entry asks the person who was tested for the actual sleep time per night during the past month, emphasizing the recent situation and thus judging as \"now\"."}, "sentence_type": "Interrogative sentence", "pos": {"nouns": {"Month": 1, "Night": 1, "Time": 1}, "verbs": {"Near": 1, "Usually": 1, "Sleep": 1}, "adjectives": {"Actual": 1, "It's not like bedtime": 1}, "adverbs": {"Every": 1, "Wait": 1}, "numerals": {"4": 1, "Hours": 1}, "classifiers": {"individual": 1}, "pronouns": {"EMPTY": 1}}}, {"content": "5. For almost one month, the following conditions have been disturbing because of sleep-related effects:", "word_count": 15, "label": "", "sentence_structure": "Subject鈥搗erb鈥搊bject", "subjective_objective": {"answer": "Subjective", "rationale": "This question is examined from the patient鈥檚 subjective point of view, because it asks whether the patient has been troubled by certain conditions and has affected their sleep in the last month. This subjective feeling and experience is not possible by objective observation or measurement, but only by the patient himself."}, "sentence_sentiment": {"answer": "Neutral", "rationale": "According to the given entry, \"fearing because the following affect sleep\" is an objective statement, which does not express any emotional colour. The expression of this article is therefore neutral."}, "language_style": {"answer": "Calm and direct", "rationale": ""}, "time_dimension": {"answer": "Present", "rationale": "The keyword \"almost a month\" indicates the range of time in the last month, which is a time-description of the current state. And \"fearful because of the following: sleep-drowsting\" is also a description of the current state. So, the entry is now."}, "sentence_type": "Interrogative sentence", "pos": {"nouns": {"Month": 1, "Situation": 1, "Sleep": 1}, "verbs": {"Impact": 1, "Trouble": 1}, "numerals": {"Near": 1}, "classifiers": {"Below": 1, "And": 1}}}, {"content": "6. For almost one month, you generally consider yourself sleep quality (1) to be good (2) to be better (3) to be poor (4);", "word_count": 25, "label": "", "sentence_structure": "Subject鈥搗erb鈥搊bject", "subjective_objective": {"answer": "Subjective", "rationale": "The question is asked about the patient ' s subjective assessment of the quality of his sleep."}, "sentence_sentiment": {"answer": "Neutral", "rationale": "Because the entry does not have any emotional color."}, "language_style": {"answer": "Calm and direct", "rationale": "The language style of the entry is calm and objective. It directly asks the interviewee about the quality of his sleep in the last month and offers a set of options for the interviewee to choose, without excessive emotional colours or rhetorical techniques. This expression allows the interviewee to reflect his feelings and state more accurately and truthfully."}, "time_dimension": {"answer": "Present", "rationale": "The keyword \"nearly a month\" indicates that the entry is asked about the condition or behaviour of the person being tested in the last month, and the temporal dimension is judged to be now. Moreover, \"In general, you think that your sleep quality is...?\" is also asked about the current self-perception, which further confirms its current state of affairs."}, "sentence_type": "Interrogative sentence", "pos": {"nouns": {"January": 1, "Sleep quality": 1, "self": 1}, "verbs": {"I think": 1}, "adjectives": {"very": 1, "Better": 1, "Poor": 1, "Bad": 1}, "numerals": {"6. Near": 1, "1": 2, "2": 1, "3": 1, "4": 1}, "classifiers": {"individual": 1}}}, {"content": "7. For almost one month, you have been using drug hypnotics (1) (not) < 1 per week (3) 1-2 per week (4) 鈮?3 per week;", "word_count": 19, "label": "", "sentence_structure": "Subject鈥搗erb鈥搊bject", "subjective_objective": {"answer": "Objective", "rationale": "Since the entry of the questionnaire asks about the frequency of drug hypnosis use by patients over the past month, it is a fact that can be objectively measured and therefore it is objective."}, "sentence_sentiment": {"answer": "Neutral", "rationale": "Because the entry does not have any emotional color."}, "language_style": {"answer": "Calm and direct", "rationale": "The entry uses a more objective and explicit language style, quantifys the frequency of drug hypnotic use by the subjects and provides specific options for their choice. This expression is relatively simple and clear, does not have too many emotional colours or accommodative techniques and is consistent with the basic characteristics of the questionnaire."}, "time_dimension": {"answer": "Present", "rationale": "The entry asked about the frequency of drug hypnosis in the victim ' s use within the last month, and the temporal dimension is now judged."}, "sentence_type": "Interrogative sentence", "pos": {"nouns": {"Month": 1, "Situation": 1, "Drugs": 1, "Hypnotic": 1}, "verbs": {"Use": 1, "Hypnotic": 1}, "adjectives": {"Near": 1}, "numerals": {"1": 2, "7": 1}, "classifiers": {"individual": 1}}}, {"content": "8. In the last month, are you often tired of (1) (ii) < 1 / 3 < 3 / weeks) < 1 / 2 / 3 / weeks > ;", "word_count": 17, "label": "", "sentence_structure": "Subject鈥搗erb鈥搊bject", "subjective_objective": {"answer": "Subjective", "rationale": "Because \"Do you get tired of being a patient for almost a month\" is a question of asking the patient about what it feels like to be subjective. The question is whether the patient has been tired of being in the last month, rather than observing or recording the facts."}, "sentence_sentiment": {"answer": "Neutral", "rationale": "Because the entry does not have any emotional color."}, "language_style": {"answer": "Calm and direct", "rationale": "The language style of the entry is relatively calm, and the number and specific time frequency are used to express it, and there are no excessive emotional qualifiers or rhetorical techniques to make the questionnaire presentation more objective, accurate and understandable."}, "time_dimension": {"answer": "Present", "rationale": "The entry asks about the behaviour and feelings of the person under examination in the last month, \"near a month\" indicating that the dimension of time is now and that the act has continued in the last month."}, "sentence_type": "Interrogative sentence", "pos": {"nouns": {"Month": 1, "Tired": 1}, "verbs": {"Common sense": 1, "I feel": 1, "Tired": 1}, "adjectives": {"Near": 1, "None": 1, "< 1 per week": 1, "1-2 meetings/weeks": 1, "3 times/week": 1}, "numerals": {"1": 2, "2": 1, "3": 1, "4": 1, "Week": 2}, "classifiers": {"Number of times": 2}, "pronouns": {"you": 1}}}, {"content": "9. In the last month, you have been under-respiring to do things?", "word_count": 24, "label": "", "sentence_structure": "Subject鈥搗erb鈥搊bject", "subjective_objective": {"answer": "Objective", "rationale": "This is an objective fact that the patient has experienced, as it is to examine whether he has been under-inspiring in the last month."}, "sentence_sentiment": {"answer": "Neutral", "rationale": "Because the entry does not have any emotional color."}, "language_style": {"answer": "Calm and direct", "rationale": "The entry is a factual statement that directly asks the respondents whether they have been under-resourced in the last month, and that the language is simple, non-emotional or rhetorical, and is a more calm and direct expression."}, "time_dimension": {"answer": "Present", "rationale": "The entry asks about the energy of the person who was tested for the last month to do things, and the time dimension is now judged."}, "sentence_type": "Interrogative sentence", "pos": {"nouns": {"you": 1, "Near": 1, "Month": 1, "Do something": 1, "- The energy": 1}, "verbs": {"Insufficient": 1, "Do it": 1}, "adverbs": {"Sometimes": 2, "Regular": 1}, "numerals": {"1": 4, "2": 1, "3": 1, "4": 1}}}]}

{"questionnaire_title": "Baker Anxiety Scale (BAI)", "questionnaire_category": "Mental health", "item_count": 21, "emotion_shift_overall": "No obvious trend", "language_style_overall": "Calm and direct", "items": [{"content": "1. Insemination or sting", "word_count": 5, "label": "", "sentence_structure": "Subject鈥搗erb鈥揷omplement", "subjective_objective": {"answer": "Objective", "rationale": "Because numbness or sting is the actual experience of the patient and a description of the objective facts."}, "sentence_sentiment": {"answer": "Negative", "rationale": "Because \"smoke or sting\" in the entry is usually considered as a physical discomfort, with negative emotional colours."}, "language_style": {"answer": "Tactful and warm", "rationale": "The entry is more profane and interactive by engaging in dialogue in a gentle and inviting tone, rather than by direct statements or orders. At the same time, terms such as \"smut\" and \"sting\" describe symptoms of physical discomfort, are more objective and neutral in tone and do not use too much emotional colour."}, "time_dimension": {"answer": "Present", "rationale": "The entry asks whether the person tested is often numb or stinging, and has no specific temporal dimensions, and therefore cannot be clearly judged as past, present or future."}, "sentence_type": "Interrogative sentence", "pos": {"nouns": {"I'm numb": 1, "Stings": 1}}}, {"content": "2. Fever.", "word_count": 4, "label": "", "sentence_structure": "Subject鈥揷opula鈥損redicative", "subjective_objective": {"answer": "Objective", "rationale": "Because \"heat\" is a physical symptom of the patient, an objective fact."}, "sentence_sentiment": {"answer": "Negative", "rationale": "Because the \"fever\" is unhealthy, and it has some negative emotions."}, "language_style": {"answer": "Calm and direct", "rationale": "The entry describes the symptoms of the subjects in a concise and clear language, does not have too many emotional and rhetorical features and is more directly objective in tone."}, "time_dimension": {"answer": "Present", "rationale": "\"Feel hot\" describes the person who was tested in his current state or feeling, so the judgment is based on \"now\"."}, "sentence_type": "Interrogative sentence", "pos": {"nouns": {"Heat": 1}, "verbs": {"I feel": 1}}}, {"content": "3. Leg tremors", "word_count": 4, "label": "", "sentence_structure": "Subject鈥搗erb鈥揷omplement", "subjective_objective": {"answer": "Objective", "rationale": "\"Stand-twitch\" is a symptom or manifestation that can be observed and is therefore an objective angle."}, "sentence_sentiment": {"answer": "Negative", "rationale": "Because the expression \"legs shaking\" is usually associated with negative emotions such as stress, fear, etc."}, "language_style": {"answer": "Calm and direct", "rationale": "The questionnaire entry directly asks the respondents whether they have problems with leg tremors, do not have too many rhetorical or emotional renderings, and is more objective in tone, so it can be judged that the language style of the questionnaire entry is calm and straightforward."}, "time_dimension": {"answer": "Past", "rationale": "Therefore, I suggest that you use specific time reference points (e.g. \"for the past week\", \"for the present day\" and so on) in designing the questionnaire, so that data can be collected more accurately."}, "sentence_type": "Affirmative declarative sentence", "pos": {"nouns": {"Legs": 1, "Wreck": 1}}}, {"content": "- No relaxing.", "word_count": 4, "label": "", "sentence_structure": "Subject鈥損redicate", "subjective_objective": {"answer": "Objective", "rationale": "Because of the possibility of relaxing the objective facts that patients experience in their lives, not the subjective idea."}, "sentence_sentiment": {"answer": "Negative", "rationale": "Because \"not relax\" in the entry is a coercive, restrictive attitude, negative emotional."}, "language_style": {"answer": "Calm and direct", "rationale": "The entry is in plain and simple language, is not subject to too many rhetorical or emotional accents, and is more directly objective. At the same time, \"not relax\" is a strong command, is more persuasive and instructive, and is better suited to psychological counselling, health surveys, etc."}, "time_dimension": {"answer": "Past", "rationale": "The entry is to ask whether the testee has often felt uneasy over a certain period of time and the dimension of time is judged to be past."}, "sentence_type": "Negative declarative sentence", "pos": {"nouns": {"Questionnaire Entry": 1, "Relax": 1}, "verbs": {"Analysis": 1, "No, I can't": 1}}}, {"content": "Fear of bad things", "word_count": 9, "label": "", "sentence_structure": "Subject鈥搗erb鈥揷omplement", "subjective_objective": {"answer": "Subjective", "rationale": "Because fear is the patient's subjective emotional reaction."}, "sentence_sentiment": {"answer": "Negative", "rationale": "Because \"fear\" is negative."}, "language_style": {"answer": "Tactful and warm", "rationale": "The entry is more personal and interactive when it is spoken in a gentle and inviting manner, rather than in direct statements or orders. At the same time, this euphemism makes the interviewee more comfortable and willing to share his real feelings."}, "time_dimension": {"answer": "Present", "rationale": "The entry asks if the testee has any idea or emotion of fear of bad things, and the time dimension is now judged."}, "sentence_type": "Negative declarative sentence", "pos": {"nouns": {"Things": 1, "Come on": 1, "Bad": 1}, "verbs": {"Scared": 1}, "adjectives": {"It's not good": 1}}}, {"content": "6. Dizziness", "word_count": 2, "label": "", "sentence_structure": "Subject鈥揷opula鈥損redicative", "subjective_objective": {"answer": "Objective", "rationale": "Because dizziness is a symptom of the patient and an objective fact."}, "sentence_sentiment": {"answer": "Negative", "rationale": "Because \"headlight\" is usually considered a physical discomfort or disease, with negative emotional aspects."}, "language_style": {"answer": "Calm and direct", "rationale": ""}, "time_dimension": {"answer": "Past", "rationale": "The entry is for the testee ' s past symptoms, and the temporal dimension is judged to be past."}, "sentence_type": "Negative declarative sentence", "pos": {"nouns": {"Dizziness": 1}}}, {"content": "7. Heart loss or acceleration of heart rate", "word_count": 7, "label": "", "sentence_structure": "Subject鈥搗erb鈥揷omplement", "subjective_objective": {"answer": "Objective", "rationale": "Because heart palpitation or acceleration is a physical condition of the patient, it is an objective fact."}, "sentence_sentiment": {"answer": "Negative", "rationale": "Because \"heart palpitation\" and \"acceleration\" are symptoms of physical discomfort, which are often considered negative experiences."}, "language_style": {"answer": "Tactful and warm", "rationale": "Warm and warm."}, "time_dimension": {"answer": "Past", "rationale": "The entry asks if the person tested has symptoms of heart palpitation or acceleration, and does not address the time dimension of past, present and future, and therefore cannot be judged by time."}, "sentence_type": "Affirmative declarative sentence", "pos": {"nouns": {"Heartbreak": 1, "Heart rate": 1}, "verbs": {"Faster": 1}}}, {"content": "8. Unsure.", "word_count": 4, "label": "", "sentence_structure": "Subject鈥搗erb鈥揷omplement", "subjective_objective": {"answer": "Subjective", "rationale": "Because \"dreaming\" is the patient's subjective feeling and experience."}, "sentence_sentiment": {"answer": "Negative", "rationale": "Because \"deep\" usually means emotional instability or anxiety, it's negative."}, "language_style": {"answer": "Calm and direct", "rationale": "The subject was directly asked in short language whether the respondents were often distraught, were not using excessive rhetorical techniques or emotional rendering, and were more direct and objective."}, "time_dimension": {"answer": "Past", "rationale": "The entry represents the person's state of mind at a certain point in time (i.e., when the questionnaire is completed) and therefore is judged to be \"the past\"."}, "sentence_type": "Negative declarative sentence", "pos": {"nouns": {"Mind": 1, "Uncertainty": 1}}}, {"content": "9. Frightening", "word_count": 2, "label": "", "sentence_structure": "Subject鈥搗erb鈥揷omplement", "subjective_objective": {"answer": "Subjective", "rationale": "According to the description of the subject, \"Scare\" is a psychological response that a patient produces in a given situation, and it can therefore be judged from the patient's subjective perspective. The output is therefore \"subjective\"."}, "sentence_sentiment": {"answer": "Negative", "rationale": "Because \"fright\" is usually a negative emotional experience."}, "language_style": {"answer": "Calm and direct", "rationale": "The subject of the article is to ask the interviewee whether he was ever frightened, to express himself more directly, to speak more objectively without excessive emotional accoutrements or speech. Moreover, the entry does not use any choreography, but rather raises the question in a direct statement, making it more concise. Thus, the language style of the entry \"Scare\" is straightforward."}, "time_dimension": {"answer": "Present", "rationale": "\"Scare\" is a term that describes the emotions or experiences that the person being tested may encounter in the current time frame, so the temporal dimension is now judged."}, "sentence_type": "Imperative sentence", "pos": {"nouns": {"Surprise": 1}}}, {"content": "10. Tension", "word_count": 2, "label": "", "sentence_structure": "Subject鈥搗erb鈥揷omplement", "subjective_objective": {"answer": "Subjective", "rationale": "/Objective: Subjective. Because stress is usually a psychological reaction and feeling of the individual, it is not an objective state or fact."}, "sentence_sentiment": {"answer": "Negative", "rationale": "Because \"stretch\" is usually considered a negative mood or state, not about being positive or neutral."}, "language_style": {"answer": "Tactful and warm", "rationale": ""}, "time_dimension": {"answer": "Future", "rationale": "The keyword is \"stretching,\" which describes an emotional state, usually related to what might happen in the future. The purpose of this article is to ask the testee whether he or she has emotional reactions to the pressures and challenges that may arise in the future. The temporal dimension therefore determines the future."}, "sentence_type": "Imperative sentence", "pos": {"nouns": {"Nervous": 1}}}, {"content": "11. Stiflerism", "word_count": 3, "label": "", "sentence_structure": "Subject鈥搗erb鈥揷omplement", "subjective_objective": {"answer": "Subjective", "rationale": "Because \"suffocation\" is a subjective experience and feeling of the patient."}, "sentence_sentiment": {"answer": "Negative", "rationale": "Because \"suffocation\" usually means an unpleasant, negative feeling."}, "language_style": {"answer": "Calm and direct", "rationale": "The language style of this entry is calm and straightforward, because it directly asks whether the person surveyed has experienced a \"suffocation\" experience, has no excessive aesthetic or emotional colour, and has a flat and objective tone. Such a design would focus the respondents more on providing true and accurate information than on adding unnecessary personal bias or emotional colours to the description of emotions or experiences."}, "time_dimension": {"answer": "Present", "rationale": ""}, "sentence_type": "Interrogative sentence", "pos": {"nouns": {"Suffocation": 1}}}, {"content": "12. Hand shaking", "word_count": 3, "label": "", "sentence_structure": "Subject鈥搗erb鈥揷omplement", "subjective_objective": {"answer": "Objective", "rationale": "The entry is therefore based on objective observations."}, "sentence_sentiment": {"answer": "Negative", "rationale": "Because hand shaking is usually a negative physical condition."}, "language_style": {"answer": "Calm and direct", "rationale": "This is a more objective and professional expression that allows for accurate data."}, "time_dimension": {"answer": "Past", "rationale": "The keyword is \"hand shaking\", which asks the person who was tested whether there were signs of hand shaking in the past and therefore determines the past."}, "sentence_type": "Exclamatory sentence", "pos": {"nouns": {"Hands": 1, "Whim": 1}, "verbs": {"# Wresh #": 1}, "numerals": {"12": 1}}}, {"content": "-Swaying.", "word_count": 2, "label": "", "sentence_structure": "Subject鈥搗erb鈥揷omplement", "subjective_objective": {"answer": "Subjective", "rationale": "Because \"scrambling\" is a subjective feeling or experience, different people may experience it differently. While shaking may be related to an objective physical environment (e.g. earthquakes, wind, etc.), the focus of the topic is to examine the subjective feelings of the person under investigation and should therefore be considered as a subjective questionnaire entry."}, "sentence_sentiment": {"answer": "Neutral", "rationale": "Since the entry did not express any positive or negative feelings, it merely described a phenomenon."}, "language_style": {"answer": "Tactful and warm", "rationale": "The issue is tempered, not directly asking the other side whether it has experienced anything bad, but rather directing it to share its experiences and feelings in an invitation way, allowing the interviewee to express his thoughts and feelings more easily and naturally. At the same time, the word \"sway\" itself has some symbolic meaning, implying uncertainty and instability in life, and this metaphor adds to the temperature and acrimony."}, "time_dimension": {"answer": "Present", "rationale": ""}, "sentence_type": "Imperative sentence", "pos": {"verbs": {"Shake": 1}}}, {"content": "14. Fear of losing control.", "word_count": 4, "label": "", "sentence_structure": "Subject鈥搗erb鈥揷omplement", "subjective_objective": {"answer": "Subjective", "rationale": "Fear of losing control is a patient ' s inner feeling and experience, and is a subjective examination."}, "sentence_sentiment": {"answer": "Negative", "rationale": "Because \"fears to lose control\" is negative."}, "language_style": {"answer": "Calm and direct", "rationale": "The entry is in a concise language and asks the interviewees whether they are afraid of losing control, are not using excessive rhetoric or emotional rendering and are speaking with a more objective and calm tone."}, "time_dimension": {"answer": "Present", "rationale": "It is therefore possible to judge that the title is now."}, "sentence_type": "Negative declarative sentence", "pos": {"nouns": {"Out of control": 1}, "verbs": {"Scared": 1}}}, {"content": "15. Respiration difficulties", "word_count": 4, "label": "", "sentence_structure": "Subject鈥搗erb鈥揷omplement", "subjective_objective": {"answer": "Objective", "rationale": "Because respiratory difficulties are a physical condition that can be observed and measured, they are objective."}, "sentence_sentiment": {"answer": "Negative", "rationale": "Because \"respiration difficulties\" are symptoms of a disease, which usually means that there is a physical problem and therefore negative."}, "language_style": {"answer": "Calm and direct", "rationale": "The entry describes a common physical reaction in a concise and clear language, with no excessive adoration or emotional colour, and a more calm and straightforward language style."}, "time_dimension": {"answer": "Past", "rationale": "The entry is for the purpose of asking the testee whether he has had past experience of breathing difficulties and the temporal dimension is a past."}, "sentence_type": "Imperative sentence", "pos": {"nouns": {"I can't breathe": 1}}}, {"content": "16. Fear of dying.", "word_count": 6, "label": "", "sentence_structure": "Subject鈥搗erb鈥揷omplement", "subjective_objective": {"answer": "Subjective", "rationale": "Because \"fear\" is a subjective emotional reaction."}, "sentence_sentiment": {"answer": "Negative", "rationale": "Because the attitude of \"fear\" in the entries is negative."}, "language_style": {"answer": "Calm and direct", "rationale": "The entry asks the testee whether he fears death in a direct and clear manner, does not use excessive rhetorical or emotional rendering, and is more objective in tone. \""}, "time_dimension": {"answer": "Future", "rationale": "The keyword \"fearing to die\" expresses the fear and fear of the testees for the future, pointing to situations that have not yet occurred and that may arise in the future, and thus judge as the future."}, "sentence_type": "Negative declarative sentence", "pos": {"nouns": {"Scared": 1, "Die": 1}, "verbs": {"Come on": 1, "Yes": 1}}}, {"content": "17. Panic", "word_count": 2, "label": "", "sentence_structure": "Subject鈥損redicate", "subjective_objective": {"answer": "Subjective", "rationale": "Because panic is a subjective feeling and experience of one person, different individuals may have different degrees of panic over the same thing."}, "sentence_sentiment": {"answer": "Negative", "rationale": "According to the title, \"fright\" is usually considered a negative emotional state, so the expression of the purpose of the questionnaire can be judged to be negative."}, "language_style": {"answer": "Calm and direct", "rationale": "The language style of the entry is more direct, not using too many rhetorical techniques or more emotional terms, but rather using a simple and clear language to express what researchers want to understand. At the same time, this cold, objective expression can make respondents feel more real and credible."}, "time_dimension": {"answer": "Present", "rationale": "The entry asks if the person tested is currently in a state of panic, and therefore the time dimension is now judged."}, "sentence_type": "Affirmative declarative sentence", "pos": {"nouns": {"Panic": 1}}}, {"content": "18. Indigestion or abdominal disorders", "word_count": 9, "label": "", "sentence_structure": "Subject鈥揷opula鈥損redicative", "subjective_objective": {"answer": "Objective", "rationale": "Since indigestion or abdominal infirmity is the symptom of the patient, it is an objective fact that does not need to be determined by subjective judgement."}, "sentence_sentiment": {"answer": "Negative", "rationale": "Because indigestion and abdominal discomfort are negative symptoms of physical discomfort, the emotional color of the article is negative."}, "language_style": {"answer": "Tactful and warm", "rationale": "The entry is more adept and interactive when it is spoken in a gentle and inviting manner, rather than by direct statements or orders."}, "time_dimension": {"answer": "Past", "rationale": "The entry asks if the person tested has experienced \"indigestion or abdominal discomfort\" and therefore determines the past."}, "sentence_type": "Interrogative sentence", "pos": {"nouns": {"Indigestion": 1, "Abdominal": 1, "I'm not well": 1}}}, {"content": "19. Stunning", "word_count": 2, "label": "", "sentence_structure": "Subject鈥損redicate", "subjective_objective": {"answer": "Objective", "rationale": "Because fainting is a symptom or condition that actually occurs, it can be observed and diagnosed, for example, through a medical examination, without depending on the patient鈥檚 sense or subjective consciousness."}, "sentence_sentiment": {"answer": "Negative", "rationale": "Because fainting is usually a negative situation, it means that serious problems have arisen."}, "language_style": {"answer": "Calm and direct", "rationale": "The entry was succinctly presented as a subject of investigation, without excessive rhetoric or emotional rendering, and with a more objective and calm language style."}, "time_dimension": {"answer": "Past", "rationale": "The entry describes past experiences or symptoms, and \"19\" is also a numerical number, implying that it is a description of past behaviour. Thus, the judgement is the past."}, "sentence_type": "Exclamatory sentence", "pos": {"nouns": {"Stupefy": 1}}}, {"content": "20-face red.", "word_count": 3, "label": "", "sentence_structure": "Subject鈥搗erb鈥揷omplement", "subjective_objective": {"answer": "Objective", "rationale": "Because the red face is an objective biological phenomenon, it has nothing to do with the patient ' s own mental state."}, "sentence_sentiment": {"answer": "Neutral", "rationale": "Because the entry does not express any positive or negative emotional colour, it is only an objective description."}, "language_style": {"answer": "Tactful and warm", "rationale": "The entry uses an informal and more friendly tone rather than directly asking or ordering the other party to be blushing, which makes it easier for the interviewee to relax and to share his real thoughts and feelings."}, "time_dimension": {"answer": "Past", "rationale": "The entry is for the purpose of asking the testee about past symptoms or behaviour, and the temporal dimension is a past."}, "sentence_type": "Affirmative declarative sentence", "pos": {"nouns": {"Face": 1, "Red": 1}, "verbs": {"Let's go": 1}}}, {"content": "21. Sweat.", "word_count": 8, "label": "", "sentence_structure": "Subject鈥搗erb鈥揷omplement", "subjective_objective": {"answer": "Subjective", "rationale": "This is a subjective feeling and experience of the patient, not an objective physical phenomenon."}, "sentence_sentiment": {"answer": "Neutral", "rationale": ""}, "language_style": {"answer": "Tactful and warm", "rationale": "The language style of the entry is more euphemistic and moderate, rather than directly expressing negative sentiments or strong negative attitudes, but rather questions in a gentle and inviting manner, which makes it easier for the interviewees to feel comfortable and open."}, "time_dimension": {"answer": "Present", "rationale": "The entry asks if the testee is easily sweaty without heat, and can therefore be judged as now."}, "sentence_type": "Negative declarative sentence", "pos": {"nouns": {"Sweat": 1, "Summer": 1}, "verbs": {"Out": 1}, "adverbs": {"/": 1, "(Not because of the heat)": 1}, "numerals": {"21锛?: 1}}}]}

{"questionnaire_title": "Emotional and social isolation tables", "questionnaire_category": "Behavioral questionnaire", "item_count": 10, "emotion_shift_overall": "From negative to positive", "language_style_overall": "Tactful and warm", "items": [{"content": "1. Everyone around me seems like a stranger.", "word_count": 14, "label": "", "sentence_structure": "Subject鈥搗erb鈥揷omplement", "subjective_objective": {"answer": "Subjective", "rationale": "Because the sentence expresses the patient ' s subjective feelings and perceptions, not the objective facts."}, "sentence_sentiment": {"answer": "Negative", "rationale": "Because the expression \"everyone like a stranger\" in the entry gives people a lonely, helpless and passive emotion."}, "language_style": {"answer": "Calm and direct", "rationale": "The language style of the entry is a calm and straightforward type. It is a straightforward language that expresses the information that the investigator wants to know, without using excessive emotional acrimony and rhetorical techniques.\""}, "time_dimension": {"answer": "Present", "rationale": "The entry is to ask the testee about the feeling of the people around him, and the time dimension cannot be judged by past, present or future. But, \"Everyone seems like a stranger\" expresses a subjective feeling, so it can be assumed that this is a description of the current situation. So, the answer is \"now.\""}, "sentence_type": "Affirmative declarative sentence", "pos": {"nouns": {"Around": 1, "person": 2, "Stranger": 1}, "verbs": {"Elephant": 1}, "adjectives": {"Every one": 1}, "adverbs": {"Both": 1}, "pronouns": {"I": 1}}}, {"content": "I cannot get much satisfaction from the collective in which I am involved.", "word_count": 18, "label": "", "sentence_structure": "Subject鈥搗erb鈥搊bject", "subjective_objective": {"answer": "Subjective", "rationale": "The subject is subjective because it is about asking the patient his or her own feelings and views."}, "sentence_sentiment": {"answer": "Negative", "rationale": "Because the result of \"not getting much satisfaction\" in the entry is negative."}, "language_style": {"answer": "Calm and direct", "rationale": "The language style of the entry is more straightforward, there is no excessive emotional qualification or euphemism, but rather a concise and clear description of the problem of participation in a collective inability to satisfy, which is more consistent with the rigour and objectivity of the questionnaire."}, "time_dimension": {"answer": "Present", "rationale": "The phrase \"the collective I'm part of\" and \"the inability to get much satisfaction\" can be judged to describe the current feelings or experiences of the testee, and the temporal dimensions are now."}, "sentence_type": "Negative declarative sentence", "pos": {"nouns": {"I": 1, "Collective": 1, "Satisfied": 1}, "verbs": {"Participation": 1, "Access": 1}, "adjectives": {"A lot": 1}}}, {"content": "3. Many around me understand my views and beliefs.", "word_count": 16, "label": "", "sentence_structure": "Subject鈥搗erb鈥搊bject", "subjective_objective": {"answer": "Subjective", "rationale": "Because anyone understands that their views and beliefs are subjective feelings and perceptions of the patients."}, "sentence_sentiment": {"answer": "Positive", "rationale": "Because the purpose of the action \"someone understands my opinion and belief\" in the entry is positive."}, "language_style": {"answer": "Tactful and warm", "rationale": "This entry uses a more euphemistic, inviting language style, without direct orders or accusations, but rather raises questions in a mild way that is easier for respondents to accept and be willing to cooperate in the completion of the questionnaire."}, "time_dimension": {"answer": "Present", "rationale": ""}, "sentence_type": "Affirmative declarative sentence", "pos": {"nouns": {"person": 1, "Observations": 1, "Faith": 1}, "verbs": {"Understood": 1, "have": 1}, "numerals": {"not": 1, "Less": 1}, "classifiers": {"individual": 1}, "pronouns": {"I": 1}}}, {"content": "No one has ever been able to close me for a long time.", "word_count": 20, "label": "", "sentence_structure": "Subject鈥搗erb鈥揷omplement", "subjective_objective": {"answer": "Objective", "rationale": "This is a subjective examination of patients ' perceptions and feelings of interpersonal relationships."}, "sentence_sentiment": {"answer": "Negative", "rationale": "Because the phrase \"no one can feel close enough for a long time\" in the entry reflects negative emotions and attitudes."}, "language_style": {"answer": "Calm and direct", "rationale": "The entry expresses the views of people in relation to relationships in a direct way, with no excessive rhetorical or emotional rendering, and a calmer and objective language style."}, "time_dimension": {"answer": "Present", "rationale": "The keyword \"for a long time\" indicates that it is a statement describing the current situation and contains a time frame and is therefore in the current state of affairs."}, "sentence_type": "Negative declarative sentence", "pos": {"nouns": {"person": 1, "Time": 1}, "verbs": {"Yes": 1, "Jean": 1, "I feel": 1, "鈾?Intimacy 鈾?: 1}, "adverbs": {"Any": 1, "Yes": 1, "For a long time": 1}, "numerals": {"4": 1}, "classifiers": {"Lee": 1, "individual": 1}, "pronouns": {"I": 1}}}, {"content": "I have a lover (lover, lover, lover) who can give me support and encouragement.", "word_count": 21, "label": "", "sentence_structure": "Subject鈥搗erb鈥搊bject", "subjective_objective": {"answer": "Subjective", "rationale": "It is the patient's own subjective feeling and thoughts that are the existence of a person of interest and the support and encouragement that the other person gives to himself."}, "sentence_sentiment": {"answer": "Positive", "rationale": "Because the \"Send me and encourage\" in the entry is positive."}, "language_style": {"answer": "Tactful and warm", "rationale": "The entry expresses in a gentle and caring tone the subject of asking if the other side has a heart-wrenching theme that gives them support and encouragement. This language style is more likely to create a friendly and friendly atmosphere, which makes people comfortable and willing to share their thoughts and feelings."}, "time_dimension": {"answer": "Present", "rationale": ""}, "sentence_type": "Affirmative declarative sentence", "pos": {"nouns": {"Questionnaire Entry": 1, "My love": 1, "Lover": 1, "Lovers": 2, "he": 1, "she": 1, "Support": 1, "Encourage": 1}, "verbs": {"have": 1, "Yes": 1, "Here": 1}, "numerals": {"5": 1}, "classifiers": {"individual": 1}, "pronouns": {"I": 1}}}, {"content": "I am one of my friends.", "word_count": 10, "label": "", "sentence_structure": "Subject鈥揷opula鈥損redicative", "subjective_objective": {"answer": "Subjective", "rationale": "Because \"a friend of a friend\" is a patient's self-evaluation and subjective feeling, it is a subjective examination."}, "sentence_sentiment": {"answer": "Positive", "rationale": "Because the expression \"belonging to one of our friends\" in the entry expresses a sense of belonging and social satisfaction, and is a positive emotional tendency."}, "language_style": {"answer": "Tactful and warm", "rationale": "The entry uses a euphemism and warmth, describing itself as a member of a friend ' s circle, expressing a sense of belonging and self-worth, and also leading the interviewee to reflect on his/her social relations."}, "time_dimension": {"answer": "Present", "rationale": "The entry is therefore a question of the social relations of the person being tested, and the time dimension is now judged."}, "sentence_type": "Affirmative declarative sentence", "pos": {"nouns": {"friends": 1, "One": 1}, "verbs": {"belong to": 1}}}, {"content": "I can be accompanied.", "word_count": 8, "label": "", "sentence_structure": "Subject鈥損redicate", "subjective_objective": {"answer": "Subjective", "rationale": "Because \"someone can be with me\" reflects the patient's need and expectations for company, and it's a subjective feeling and attitude."}, "sentence_sentiment": {"answer": "Positive", "rationale": "Because the act of \"someone can be with me\" is positive, expressing the need for people to be company and socialize."}, "language_style": {"answer": "Tactful and warm", "rationale": "The entry is more personal and interactive in its expression, in a gentle and inviting tone, rather than in direct statements or orders. At the same time, \"someone can be with me\" is a relatively subjective feeling, and the euphemism is more reflective of the understanding and respect of the questionnaire researchers."}, "time_dimension": {"answer": "Past", "rationale": "The entry does not have a clear time indicator, but \"someone can be with me\" suggests that a person has experienced isolation or lack of company at some point in the past, so the entry tends to ask if the person tested has experienced such experience in the past."}, "sentence_type": "Interrogative sentence", "pos": {"nouns": {"person": 1, "Together": 1}, "verbs": {"have": 1, "Partners": 1}, "pronouns": {"I": 1}}}, {"content": "No one has a special relationship with me that makes me feel as understanding as I am.", "word_count": 20, "label": "", "sentence_structure": "Subject鈥揷opula鈥損redicative", "subjective_objective": {"answer": "Subjective", "rationale": "Because \"special relationships that make people feel understand each other\" are subjective perceptions and evaluations of relationships. Although they may not exist in objective reality, the perceptions and experiences of patients are real and subjective."}, "sentence_sentiment": {"answer": "Negative", "rationale": "Because the \"no one understands\" attitude in the entry is negative."}, "language_style": {"answer": "Calm and direct", "rationale": "This language style helps the interviewee to understand and answer questions more accurately and clearly."}, "time_dimension": {"answer": "Present", "rationale": "The keyword \"No one with me\" shows that this is a question of describing the state of affairs, and \"maintaining a special relationship that makes me feel as understanding\" underlines the importance of this relationship and the impact on the person being tested. Therefore, the entry should be judged as it is now."}, "sentence_type": "Negative declarative sentence", "pos": {"nouns": {"person": 1, "Special relations": 1, "Mutual understanding": 1}, "verbs": {"Hold on": 1, "鈾?Make me 鈾?: 2, "I feel": 1, "Understood": 1}, "numerals": {"8": 1}}}, {"content": "9. I have a major influence on the feelings of another person.", "word_count": 17, "label": "", "sentence_structure": "Subject鈥搗erb鈥搊bject", "subjective_objective": {"answer": "Subjective", "rationale": "Because \"I have a significant influence on another person's feelings\" is a personal conviction or opinion, a patient's subjective attitude and opinion, and not an objective fact or result of behaviour."}, "sentence_sentiment": {"answer": "Positive", "rationale": "Because it expresses a sense of confidence and certainty in its abilities."}, "language_style": {"answer": "Tactful and warm", "rationale": "The entry is more gentle and inspiring by asking questions about the impact of their personal interaction, rather than directly making statements or making judgements. At the same time, the content of the article is more personal and sensitive, and therefore euphemism is more likely to protect the privacy and personal feelings of the interviewee."}, "time_dimension": {"answer": "Future", "rationale": ""}, "sentence_type": "Interrogative sentence", "pos": {"nouns": {"Emotion": 2, "The other guy": 1, "Impact": 1}, "verbs": {"It's a product": 1}, "adverbs": {"Heavy": 1, "Large": 1}, "numerals": {"Nine": 1}, "pronouns": {"I": 1}}}, {"content": "I have no special relationship with love.", "word_count": 10, "label": "", "sentence_structure": "Subject鈥揷opula鈥損redicative", "subjective_objective": {"answer": "Subjective", "rationale": "Because \"no special relationship of love\" is a subjective statement, reflecting the view and perception of the respondent as to whether he or she has a special relationship of love. Although this relationship may not exist in objective reality, its existence or non-existence is determined by the subjective feeling of the person under investigation."}, "sentence_sentiment": {"answer": "Neutral", "rationale": "Because the entry merely stated a fact, it did not express any positive or negative emotional colour."}, "language_style": {"answer": "Tactful and warm", "rationale": "The entry is more personal and interactive when it is spoken in a gentle and inviting manner, rather than in direct statements or orders. At the same time, this euphemism does not make the interviewee uncomfortable or under pressure."}, "time_dimension": {"answer": "Present", "rationale": "The keyword \"no\" means that the testee is currently single and that this situation may have been maintained for some time. The subject is therefore asking about the present emotional situation of the testee, which is the current state."}, "sentence_type": "Negative declarative sentence", "pos": {"nouns": {"Love relationships": 1, "Special": 1}, "verbs": {"No, I'm not": 1}, "numerals": {"10": 1}}}]}

{"questionnaire_title": "Response questionnaire", "questionnaire_category": "Behavioral questionnaire", "item_count": 62, "emotion_shift_overall": "From positive to negative", "language_style_overall": "Tactful and warm", "items": [{"content": "1. Be able to manage the plight rationally", "word_count": 8, "label": "", "sentence_structure": "Subject鈥搗erb鈥揷omplement", "subjective_objective": {"answer": "Objective", "rationale": "Because \"sure to deal with a dilemma\" is a human ability or behaviour, an objective fact, not a subjective feeling or attitude of the individual. The entry is therefore viewed from an objective perspective."}, "sentence_sentiment": {"answer": "Positive", "rationale": "Because the purpose of the entry is to deal with the dilemma rationally."}, "language_style": {"answer": "Tactful and warm", "rationale": "The entry is more adept and interactive when it is spoken in a gentle and inviting manner, rather than by direct statements or orders."}, "time_dimension": {"answer": "Present", "rationale": "The entry asks if the person tested is currently capable and rational in dealing with the dilemma, and the time dimension is now judged."}, "sentence_type": "Affirmative declarative sentence", "pos": {"nouns": {"The dilemma": 1, "Reason": 1}, "verbs": {"Payable": 1}, "adverbs": {"Land": 1}}}, {"content": "2. Learning from failures", "word_count": 10, "label": "", "sentence_structure": "Subject鈥搗erb鈥揷omplement", "subjective_objective": {"answer": "Subjective", "rationale": "Because \"good\" is a subjective assessment or attitude, indicating whether the patient is conscious to learn and grow from failure. And \"learning from failure\" is the patient's actions or behaviour, and it is an objective result. The questionnaire therefore looks at both the patient's subjective attitudes and their objective behaviour."}, "sentence_sentiment": {"answer": "Positive", "rationale": "Because the \"good to learn from failure\" approach in the entry is positive."}, "language_style": {"answer": "Tactful and warm", "rationale": "The entry uses a positive, encouraging language style that leads the respondents to reflect on their strengths and abilities, and to give them recognition and support by asking questions. This language style is more moderate, friendly, and it helps to increase the participants鈥?sense of participation and self-confidence and their willingness to share real ideas and feelings."}, "time_dimension": {"answer": "Present", "rationale": "The keyword \"good\" and \"learning from experience\" suggests that it is a habit or attitude of the testee that is more in line with the present description than it has been and will be. The entry should therefore be classified as \"now\"."}, "sentence_type": "Affirmative declarative sentence", "pos": {"nouns": {"Failed": 1, "Experience": 1}, "verbs": {"Good at it": 1, "Draw": 1}}}, {"content": "3. Develop and plan to overcome difficulties", "word_count": 17, "label": "", "sentence_structure": "Subject鈥搗erb鈥搊bject", "subjective_objective": {"answer": "Subjective", "rationale": "Because the patient ' s active involvement is required for design and planning, it is the patient ' s subjective action."}, "sentence_sentiment": {"answer": "Positive", "rationale": "Because the entries \"developing plans to overcome difficulties\" and \"doing as planned\" are positive."}, "language_style": {"answer": "Tactful and warm", "rationale": "The language style of the entry is relatively euphemistic and encouraging, helping people overcome difficulties by proposing the steps of \"preparation\" and \"doing as planned.\" This expression is more acceptable to the interviewees and stimulates their positive mood and willingness to act."}, "time_dimension": {"answer": "Present", "rationale": "The entry is on the future action plan and implementation of the testee, with the key words \"developing\", \"plan to overcome difficulties\" and \"do as planned.\" They all point to situations that have not yet occurred and that may occur in the future. The judgement is therefore based on the future."}, "sentence_type": "Imperative sentence", "pos": {"nouns": {"Difficulties": 1, "Planned": 1, "Do it": 1}, "verbs": {"Development": 1, "Overcoming": 1, "Go as planned": 1}, "numerals": {"Some": 1}}}, {"content": "4. It is hoped that the difficulties encountered will be resolved.", "word_count": 15, "label": "", "sentence_structure": "Subject鈥搗erb鈥搊bject", "subjective_objective": {"answer": "Subjective", "rationale": "Because hope is a subjective term, representing the patient鈥檚 expectations and aspirations, and a subjective attitude. Although the problem involves an objective difficulty, the patient鈥檚 attitude to solving it is subjective, so the entry is viewed from the patient鈥檚 subjective point of view."}, "sentence_sentiment": {"answer": "Positive", "rationale": "Because the attitude of \"often hoping to solve the difficulties\" in the entry is positive.\""}, "language_style": {"answer": "Calm and direct", "rationale": "The entry expresses in a more objective and direct way the question of whether the other party has the hope of solving the problem in the face of difficulties, without too much emotional accoutrements or rhetorical techniques."}, "time_dimension": {"answer": "Future", "rationale": "The expression of hope and desire is usually linked to the future, and the expression of \"worked out\" indicates that the result has been or is now achieved, and the entry therefore expresses the vision and expectations for the future."}, "sentence_type": "Imperative sentence", "pos": {"nouns": {"Questionnaire": 1, "Entry": 1, "self": 1, "Difficulties": 1}, "verbs": {"Resolve": 1, "Hope": 1}, "adverbs": {"Regular": 1}, "numerals": {"4": 1}}}, {"content": "5. Confidence in their ability to succeed", "word_count": 14, "label": "", "sentence_structure": "Subject鈥搗erb鈥搊bject", "subjective_objective": {"answer": "Subjective", "rationale": "This is because the patient ' s belief in and attitude towards his or her abilities are part of his or her subjective feelings and thoughts."}, "sentence_sentiment": {"answer": "Positive", "rationale": "Because the \"confident\" attitude in the entries is positive."}, "language_style": {"answer": "Tactful and warm", "rationale": "This language style better inspires the surveyed people鈥檚 motivation and self-confidence and makes them more willing to share their real ideas."}, "time_dimension": {"answer": "Present", "rationale": "Please provide more context information."}, "sentence_type": "Imperative sentence", "pos": {"nouns": {"self": 1, "Capacity": 1, "Success": 1}, "verbs": {"Access": 1, "Fill": 1, "Confidence": 1}}}, {"content": "Considers that 鈥渓ife experience is a bitterness鈥?", "word_count": 10, "label": "", "sentence_structure": "Subject鈥搗erb鈥搊bject", "subjective_objective": {"answer": "Subjective", "rationale": "This is because of the patient ' s personal convictions or opinions and is subjective."}, "sentence_sentiment": {"answer": "Negative", "rationale": "Because the attitudes in the entries are negative, life is just a difficult one."}, "language_style": {"answer": "Calm and direct", "rationale": "The language style of the entry is more calm and straightforward, and there is no excessive rhetorical or emotional rendering, but rather a direct expression of the view that asks if the other side agrees with \"life experience is pain.\" This expression is objective and clear, and it helps the interviewee to understand and answer the questions accurately."}, "time_dimension": {"answer": "Past", "rationale": "The entry describes a concept or view, without specifying whether the testee is being asked about past, present or future behaviour or mentality, but the notion that \"life experience is a bitterness\" should be developed in the past and continue to this day. Thus, the temporal dimension of the article is a matter of the past."}, "sentence_type": "Affirmative declarative sentence", "pos": {"nouns": {"Life experience": 1, "The trials": 1}, "verbs": {"I think": 1}, "numerals": {"6": 1}}}, {"content": "7. The difficulties of life are often lamented.", "word_count": 8, "label": "", "sentence_structure": "Subject鈥損redicate", "subjective_objective": {"answer": "Subjective", "rationale": "Because it is the patient ' s personal feelings and attitudes, it is subjective."}, "sentence_sentiment": {"answer": "Negative", "rationale": "Because the behavior and attitude of \"exclaiming the hard times of life\" in the entries is negative."}, "language_style": {"answer": "Calm and direct", "rationale": "The entry is more straightforward in language style, does not have too many rhetorical or emotional renderings, but rather expresses the emotional state of the person being investigated in an objective and concise manner."}, "time_dimension": {"answer": "Past", "rationale": "The entry indicates that the testees have experienced the difficult life they have often experienced in the past and can therefore be judged as the past."}, "sentence_type": "Exclamatory sentence", "pos": {"nouns": {"Life": 1, "Hard": 1}, "verbs": {"I'm sorry": 1}, "adverbs": {"Regular": 1}}}, {"content": "8. Focusing on work or learning to forget it.", "word_count": 13, "label": "", "sentence_structure": "Subject鈥搗erb鈥揷omplement", "subjective_objective": {"answer": "Subjective", "rationale": "Because focusing on work or learning is a subjective attitude or willingness, neglect is the result."}, "sentence_sentiment": {"answer": "Positive", "rationale": "Because the behavior in the entry \"to work or learn\" is to achieve the purpose of forgetting and is positive emotional."}, "language_style": {"answer": "Tactful and warm", "rationale": "The language style of the entry is more moderate, using the words \"focus\" and \"forgetting out\" and not directly expressing negative feelings or forcing the interviewee to perform something. This language style helps to create a comfortable and comfortable Question and Answer atmosphere and makes it easier to get the real answers from the interviewee."}, "time_dimension": {"answer": "Present", "rationale": "The entry is a question of whether the person tested is currently focusing on work or learning to forget the unpleasant experiences and behaviours, and the time dimension is now judged."}, "sentence_type": "Imperative sentence", "pos": {"nouns": {"Focus": 1, "Work": 1, "Learning": 1, "Not fast": 1}, "verbs": {"Forget it": 1}}}, {"content": "It is often believed that 鈥渓ife is a life and death, and wealth is in heaven鈥?, "word_count": 11, "label": "", "sentence_structure": "Subject鈥搗erb鈥搊bject", "subjective_objective": {"answer": "Subjective", "rationale": "This is because patients ' perception of life and wealth is a reflection of their subjective attitude."}, "sentence_sentiment": {"answer": "Negative", "rationale": "Because the view in the entry \"Life and death, wealth and fortune\" is often seen as a negative fatalism, expressing an attitude of impotence."}, "language_style": {"answer": "Calm and direct", "rationale": "The entry is in plain language and asks whether the interviewees often think that \"life is a thing and wealth is in the world\" and that they do not use any rhetorical or emotional rendering."}, "time_dimension": {"answer": "Past", "rationale": "The word \"normal\" in the sentence denotes a regular, customary act, \"considers\" that the action has been completed or is in progress in the past, and the entry therefore asks about the behaviour and mentality of the person who was tested."}, "sentence_type": "Exclamatory sentence", "pos": {"nouns": {"Live or die": 1, "Life": 1, "Rich": 1, "Oh, my God": 1}, "adjectives": {"Regular": 1}}}, {"content": "10. Often, people talk to people to ease their troubles.", "word_count": 13, "label": "", "sentence_structure": "Subject鈥搗erb鈥揷omplement", "subjective_objective": {"answer": "Subjective", "rationale": "Like being patient subjective."}, "sentence_sentiment": {"answer": "Positive", "rationale": "Because the purpose of the entry is to find someone to talk to to ease trouble."}, "language_style": {"answer": "Tactful and warm", "rationale": "The entry is more personal and interactive when it is presented in a gentle and inviting tone, asking whether the other party has a preference for a less troubled person, without a direct statement or order."}, "time_dimension": {"answer": "Present", "rationale": ""}, "sentence_type": "Imperative sentence", "pos": {"nouns": {"Questionnaire Entry": 1, "person": 1, "Chat": 1, "Trouble": 1}, "verbs": {"Often": 1, "Yeah": 1, "Find": 1, "Mitigation": 1}, "adverbs": {"Here": 1}, "numerals": {"10": 1}}}, {"content": "Requesting help to overcome difficulties", "word_count": 12, "label": "", "sentence_structure": "Subject鈥搗erb鈥搊bject", "subjective_objective": {"answer": "Subjective", "rationale": "Because \"requesting help\" is a subjective act, reflecting the patient's spirit of recourse and need, and is a manifestation of the patient's initiative to seek support from others."}, "sentence_sentiment": {"answer": "Positive", "rationale": "Because the purpose and attitude of the act of asking for help to overcome the difficulties in the entry are positive."}, "language_style": {"answer": "Tactful and warm", "rationale": "The entry is more euphemistic, more compassionate, and more modest and polite by using the words \"request\" and \"help\" to express the idea of needing the help of others to solve the problem. At the same time, the entry does not speak too directly about its plight, but rather conveys the message of recourse to the interviewees in a more positive and positive way."}, "time_dimension": {"answer": "Present", "rationale": "The term \"request\" and \"help\" in this entry both indicate an act or state currently being performed, and the temporal dimension of the purpose of the article is therefore judged to be the present."}, "sentence_type": "Imperative sentence", "pos": {"nouns": {"others": 1, "self": 1, "Difficulties": 1}, "verbs": {"Request": 1, "Help": 1, "Overcoming": 1}, "numerals": {"11": 1}}}, {"content": "12. Often only as they see fit, without taking into account the consequences.", "word_count": 14, "label": "", "sentence_structure": "Subject鈥搗erb鈥揷omplement", "subjective_objective": {"answer": "Subjective", "rationale": "Because \"just do what you want\" and \"no consequences\" are subjective behaviour and attitude of the patient."}, "sentence_sentiment": {"answer": "Negative", "rationale": "Because the act \"do as you wish\" in the entry is a self-centered act, and without regard to its consequences, it may have a negative impact on itself or others and therefore has a certain negative emotional connotation."}, "language_style": {"answer": "Tactful and warm", "rationale": "The entry is a soft and warm language. It guides the respondents to think and answer questions in a question-and-answer manner, without using a direct command or a language of accusation. It also uses affirmative sentences to make the respondents more responsive and proactive."}, "time_dimension": {"answer": "Present", "rationale": ""}, "sentence_type": "Negative declarative sentence", "pos": {"nouns": {"Questionnaire": 1, "Entry": 1}, "verbs": {"Analysis": 1, "Press": 1, "Do it": 1, "Consider": 1}, "adjectives": {"Regular": 1, "Only": 1, "and": 1}, "adverbs": {"not": 1}, "numerals": {"12": 1}}}, {"content": "13. Reluctance to reflect too much on the issues that affect their emotions", "word_count": 16, "label": "", "sentence_structure": "Subject鈥搗erb鈥揷omplement", "subjective_objective": {"answer": "Subjective", "rationale": "Because this is something that patients are unwilling to do, it is their subjective attitude and psychological orientation, and it is not an objective fact."}, "sentence_sentiment": {"answer": "Negative", "rationale": "Because the act of \"not thinking too much\" in the entry is negative."}, "language_style": {"answer": "Calm and direct", "rationale": "The language style of the entry is calm and straightforward. It raises the question in a concise and straightforward manner, without any rhetorical or emotional expressions."}, "time_dimension": {"answer": "Present", "rationale": "The entry describes the person's current behaviour or state, the keywords being \"unwilling\" and \"too much thinking,\" which all indicate that this is happening. And \"now\" confirms this from the time dimension."}, "sentence_type": "Imperative sentence", "pos": {"nouns": {"Problem": 1, "Emotion": 1}, "verbs": {"Think": 1, "Impact": 1}, "adjectives": {"self": 1, "Pass": 1, "More": 1}, "adverbs": {"I don't want to": 1}, "numerals": {"13": 1}}}, {"content": "14. To engage in other social activities and find new ones", "word_count": 13, "label": "", "sentence_structure": "Subject鈥搗erb鈥搊bject", "subjective_objective": {"answer": "Subjective", "rationale": "Because it reflects the social activities of the patient in search of psychological comfort and care, it is the patient ' s subjective will and behaviour."}, "sentence_sentiment": {"answer": "Positive", "rationale": "Because the behavior in the entries, \"into other social activities\" and the aim of \"in search of new ones,\" are positive."}, "language_style": {"answer": "Calm and direct", "rationale": "\"Calm down straight.\""}, "time_dimension": {"answer": "Present", "rationale": "The entry asks whether the person tested is involved in other social activities to seek new placements, and there is no clear indication of direction in time, and therefore it is not possible to judge accurately whether it is past, present or future."}, "sentence_type": "Imperative sentence", "pos": {"nouns": {"Social activities": 1, "Mailing": 1}, "verbs": {"- Get in": 1, "Search": 1}, "numerals": {"14": 1}}}, {"content": "15. Often self-absorption", "word_count": 5, "label": "", "sentence_structure": "Subject鈥搗erb鈥揷omplement", "subjective_objective": {"answer": "Subjective", "rationale": "Because self-abuse is a subjective attitude and emotional experience that reflects the feelings and perceptions of the patient, rather than the objective facts or behavioural outcomes."}, "sentence_sentiment": {"answer": "Negative", "rationale": "Because \"suggested\" is a negative act."}, "language_style": {"answer": "Tactful and warm", "rationale": "The entry uses a more euphemistic approach to questions, without directly blaming or criticizing the behaviour of the interviewees, but rather a more moderate and caring attitude to guide the other party to thinking and expressing their feelings, which is more acrimony and friendly."}, "time_dimension": {"answer": "Present", "rationale": "\"At times\" means a regular, customary act, \"a self-absorption\" means a renunciation of effort and hope, a term that describes the emotional state and psychological characteristics of the individual. Thus, the temporal dimension of this entry is now judged."}, "sentence_type": "Negative declarative sentence", "pos": {"nouns": {"Regular": 1, "Riot": 1, "Quit": 1}, "numerals": {"15": 1}}}, {"content": "16. Often, it's a little unsettling to hide what's inside.", "word_count": 16, "label": "", "sentence_structure": "Subject鈥搗erb鈥揷omplement", "subjective_objective": {"answer": "Subjective", "rationale": "Because \"always indifferently conceals the feeling of the person\" is the patient's personal subjective feeling and behaviour, it is a subjective examination."}, "sentence_sentiment": {"answer": "Negative", "rationale": "Because \"apologetic attitude\" in the entry is a negative emotional expression."}, "language_style": {"answer": "Calm and direct", "rationale": "The purpose of this article is to ask more directly, without too many rhetorical or emotional expressions, and to be more objective. It asks the interviewee whether he is used to conceal his feelings with \"a careless attitude\" in a simple and straightforward way, which makes it easier for the interviewee to answer."}, "time_dimension": {"answer": "Present", "rationale": "The word \"always\" in \"16.\" often hides feelings in the mind, means a regular, customary act, and the word \"securing\" not only in the past but may still be ongoing, so time horizons are now judged."}, "sentence_type": "Imperative sentence", "pos": {"nouns": {"Questionnaire": 1, "Entry": 1, "Attitude": 1, "Feel": 1}, "verbs": {"Regular": 1, "Here": 1, "Come on": 1, "Cover it up": 1, "Inside": 1}, "adjectives": {"It doesn't matter": 1, "of": 2, "Regular": 1, "Inside": 1}, "adverbs": {"Regular": 1}, "numerals": {"16": 1}, "pronouns": {"Here": 1}}}, {"content": "I always thought, \"This is not true.\"", "word_count": 10, "label": "", "sentence_structure": "Subject鈥搗erb鈥揷omplement", "subjective_objective": {"answer": "Subjective", "rationale": "This is the patient ' s inner feeling and thoughts, and it is a subjective examination."}, "sentence_sentiment": {"answer": "Negative", "rationale": "Because the idea of \"not true is good\" in the entry is an expression of dissatisfaction with reality and evasion, and is a negative emotional attitude."}, "language_style": {"answer": "Tactful and warm", "rationale": ""}, "time_dimension": {"answer": "Present", "rationale": "The keyword is \"often thought\" and it means that it is a customary behaviour or a way of thinking, and the temporal dimension is now judged."}, "sentence_type": "Exclamatory sentence", "pos": {"nouns": {"Regular": 1, "True": 1, "Okay": 1, "Yes": 1}, "verbs": {"Yes": 1, "be": 1}, "adverbs": {"not": 1}, "numerals": {"17": 1}}}, {"content": "18. Considers that many of their failures are due to external causes", "word_count": 13, "label": "", "sentence_structure": "Subject鈥搗erb鈥搊bject", "subjective_objective": {"answer": "Subjective", "rationale": "Because \"think\" is a subjective idea or belief."}, "sentence_sentiment": {"answer": "Negative", "rationale": "Because \"failure\" and \"cause of external causes\" in the entries are negative terms, expressing a negative emotional attitude."}, "language_style": {"answer": "Calm and direct", "rationale": "The entry is a direct question-and-answer exercise, which clearly asks the respondents about their perception of the reasons for their failure, is more calm and objective in language and does not involve excessive emotional modification or rhetoric."}, "time_dimension": {"answer": "Past", "rationale": "\""}, "sentence_type": "Negative declarative sentence", "pos": {"nouns": {"self": 1, "Failed": 1, "External factors": 1, "Result": 1}, "verbs": {"I think": 1, "Yes": 1}, "numerals": {"18": 1}}}, {"content": "19. A wait-and-see attitude to difficulties", "word_count": 16, "label": "", "sentence_structure": "Subject鈥搗erb鈥搊bject", "subjective_objective": {"answer": "Subjective", "rationale": "Because attitudes towards difficulties are the patient ' s personal perception and attitude."}, "sentence_sentiment": {"answer": "Negative", "rationale": "Because \"waiting for its development\" is a negative attitude, indicating a lack of positive response to the difficulties."}, "language_style": {"answer": "Calm and direct", "rationale": "The language style of the entry is more straightforward, with no excessive rhetoric or emotional colour, but rather a clear expression of the attitude of asking the other side whether to wait and see when facing difficulties. This expression is more objective and professional and helps to obtain accurate answers."}, "time_dimension": {"answer": "Present", "rationale": "The entry describes the attitude and choice of a testee when facing difficulties, namely, \"waiting to see it develop.\" It is a manifestation of behavior or mentality, and can therefore be judged as now."}, "sentence_type": "Exclamatory sentence", "pos": {"nouns": {"Difficulties": 1, "Attitude": 1}, "verbs": {"Adoption": 1, "Development": 1, "Wait": 1, "Watch": 1}, "adverbs": {"Other": 1}, "numerals": {"19": 1}}}, {"content": "20. Conflict with people is often caused by the strangeness of the other.", "word_count": 14, "label": "", "sentence_structure": "Subject鈥搗erb鈥揷omplement", "subjective_objective": {"answer": "Subjective", "rationale": "Because personality weirdness is a subjective assessment and is the subjective perception and perception of the personality of others by the interviewees."}, "sentence_sentiment": {"answer": "Negative", "rationale": "Because the expression \"others' personality is strange\" in the entry is negative and emotional, implying that the conflict is caused by the other party's problems, not by his own problems."}, "language_style": {"answer": "Calm and direct", "rationale": "The entry presents the causes of the possible conflict in a direct manner, without excessive rhetoric or emotional rendering and with more objective tone."}, "time_dimension": {"answer": "Present", "rationale": "The word \"normal\" denotes a regular, customary act, while the word \"yes\" adopts the usual current pattern, meaning that it has not only happened in the past, but may still be ongoing. The entry is therefore an inquiry into whether the person tested currently has a conflict with a person, and whether these conflicts are usually caused by the personality of the other party. The temporal dimension is now."}, "sentence_type": "Interrogative sentence", "pos": {"nouns": {"person": 1, "Conflict": 1, "The other side": 1, "Character": 1, "Weird": 1}, "verbs": {"Regular": 1, "Cause": 1}, "adjectives": {"Weird": 1}, "adverbs": {"Regular": 1}, "numerals": {"20": 1}}}, {"content": "21. The usual anger towards the people and events that cause the problem", "word_count": 13, "label": "", "sentence_structure": "Subject鈥搗erb鈥搊bject", "subjective_objective": {"answer": "Subjective", "rationale": "Because anger is the patient's subjective attitude and behaviour."}, "sentence_sentiment": {"answer": "Negative", "rationale": "Because the behavior in the entry is usually considered a negative act."}, "language_style": {"answer": "Calm and direct", "rationale": "The language style of the entry is more direct and concise, without too many emotional accommodations or euphemisms, but rather a simple and straightforward expression of the subject of asking the other party whether it will often be offensive to the person or events that cause the problem."}, "time_dimension": {"answer": "Present", "rationale": "Because \"normal\" means constant, customary behaviour, and \"fatal\" is an ongoing act or state, it can be concluded that this is an inquiry into the present behaviour of the person being tested."}, "sentence_type": "Imperative sentence", "pos": {"nouns": {"person": 1, "Yes": 1, "- Grumpy": 1}, "verbs": {"Regular": 1, "In": 1, "Let's go": 1}, "adverbs": {"Regular": 1}, "numerals": {"21": 1}, "classifiers": {"individual": 1}}}, {"content": "22. I've always imagined that I had the ability to overcome a difficult superhuman.", "word_count": 15, "label": "", "sentence_structure": "Subject鈥搗erb鈥揷omplement", "subjective_objective": {"answer": "Subjective", "rationale": "Because the illusion is the patient's subjective imagination and attitude."}, "sentence_sentiment": {"answer": "Positive", "rationale": "Because the idea of \"overcoming the difficult superhuman talent\" in the entry is positive, encouraging individuals to face challenges and seek solutions."}, "language_style": {"answer": "Calm and direct", "rationale": "Because the entry is in plain language and asks whether the interviewees often fantasize about their ability to overcome difficulties, and is direct and non-emotional."}, "time_dimension": {"answer": "Future", "rationale": ""}, "sentence_type": "Affirmative declarative sentence", "pos": {"nouns": {"Difficulties": 1, "Superman": 1, "Your Majesty": 1}, "verbs": {"Fantasy": 1, "Overcoming": 1}, "numerals": {"22": 1}}}, {"content": "23. Frequent self-pity", "word_count": 5, "label": "", "sentence_structure": "Subject鈥搗erb鈥揷omplement", "subjective_objective": {"answer": "Subjective", "rationale": "Because \"self-responsible\" is a mental activity and emotional experience that reflects the individual's assessment and reflection of his or her behaviour, attitude and attitude, and is subjective in its perspective."}, "sentence_sentiment": {"answer": "Negative", "rationale": "Because the \"self-pity\" act in the entry is negative."}, "language_style": {"answer": "Tactful and warm", "rationale": "It is not a direct order or challenge, but rather a request and invitation to ask questions that are comfortable and respected. Moreover, the sentence does not use too harsh or negative terms, but rather expresses the subject in a relatively mild way, which is more acceptable and understandable."}, "time_dimension": {"answer": "Present", "rationale": "The keyword \"always\" denotes a regular, customary act, while the word \"self-responsible\" indicates the person's current attitude and behaviour, so the entry reflects the current situation."}, "sentence_type": "Imperative sentence", "pos": {"verbs": {"Blame": 1, "Regular": 1}, "adverbs": {"Self": 1}, "numerals": {"23": 1}}}, {"content": "24. The usual way to sleep escapes pain.", "word_count": 11, "label": "", "sentence_structure": "Subject鈥搗erb鈥揷omplement", "subjective_objective": {"answer": "Subjective", "rationale": "Because the words \"usual\" and \"evading\" are described from the point of view of the patient, emphasizing his subjective feelings and behaviour."}, "sentence_sentiment": {"answer": "Negative", "rationale": "Because the act of \"evading pain\" in the entry is negative."}, "language_style": {"answer": "Tactful and warm", "rationale": "The entry uses a gentle tone and a friendly language style, which is not a direct question about whether the other side is sleeping to escape pain, but rather a euphemism and respectable way of guiding the other side to think about its own behaviour. This language style is more personal and interactive."}, "time_dimension": {"answer": "Present", "rationale": "\"Attempted\" suggests a customary act, and the word \"sleeping out of pain\" uses the word \"sleeping out\" to use the word \"normally\" to mean that the act has not only occurred in the past, but may still be ongoing. Thus, the entry describes the person being tested for the current act or habit."}, "sentence_type": "Affirmative declarative sentence", "pos": {"nouns": {"Modalities": 1, "Pain": 1}, "verbs": {"Common": 1, "Sleep": 1, "Run away": 1}, "numerals": {"24": 1}}}, {"content": "25. Recreational activities are often used to overcome the troubles.", "word_count": 11, "label": "", "sentence_structure": "Subject鈥搗erb鈥揷omplement", "subjective_objective": {"answer": "Subjective", "rationale": "Because recreational activities are the way that patients choose to ease their emotions, they are a subjective exercise."}, "sentence_sentiment": {"answer": "Positive", "rationale": "Because the purpose of the entry is to \"severe the troubles with entertainment.\""}, "language_style": {"answer": "Tactful and warm", "rationale": "The language style of the entry is mild, and it does not ask questions or orders directly, but rather, in an invitation tone, guides the interviewee to answer questions, giving a sense of affection and affection. At the same time, the entry does not use too serious or professional terms, which are more in line with everyday speech. So I think the language style for this purpose is \"false.\""}, "time_dimension": {"answer": "Present", "rationale": ""}, "sentence_type": "Affirmative declarative sentence", "pos": {"nouns": {"Recreational activities": 1, "Trouble": 1}, "verbs": {"Borrow": 1, "Elimination": 1}, "adverbs": {"Regular": 1}, "numerals": {"25": 1}}}, {"content": "26. I always thought of something happy to comfort myself.", "word_count": 12, "label": "", "sentence_structure": "Subject鈥搗erb鈥揷omplement", "subjective_objective": {"answer": "Subjective", "rationale": "Because it's the patient's subjective attitude."}, "sentence_sentiment": {"answer": "Positive", "rationale": "Because the \"I've always thought of something happy\" act is positive."}, "language_style": {"answer": "Tactful and warm", "rationale": "The entry is more euphemistic, warm, and uses more friendly language and tone, trying to create a friendly and comfortable atmosphere in which the respondents can feel relaxed and comfortable. At the same time, the question is not asked about each other too directly or harshly, but rather leads the respondents to think and answer in a more moderate way."}, "time_dimension": {"answer": "Present", "rationale": "So the article's purpose is time to judge \"now\"."}, "sentence_type": "Affirmative declarative sentence", "pos": {"nouns": {"Things": 1, "Self": 1, "Consolation": 1}, "verbs": {"Yes": 1, "Love": 1, "Do it": 1}, "adjectives": {"Happy": 1}, "numerals": {"26": 1}}}, {"content": "27. Avoiding difficulties to find peace in your heart.", "word_count": 10, "label": "", "sentence_structure": "Subject鈥搗erb鈥揷omplement", "subjective_objective": {"answer": "Subjective", "rationale": "This is a behaviour that the patient chooses to avoid, and is a subjective attitude and idea of the patient."}, "sentence_sentiment": {"answer": "Negative", "rationale": "Because the action of avoiding difficulties in the entry is negative."}, "language_style": {"answer": "Tactful and warm", "rationale": ""}, "time_dimension": {"answer": "Present", "rationale": ""}, "sentence_type": "Imperative sentence", "pos": {"nouns": {"Difficulties": 1, "Heart": 1, "Peace": 1}, "verbs": {"Avoid": 1, "Please": 1}, "numerals": {"27": 1}}}, {"content": "28. Worried about not being able to avoid difficulties", "word_count": 10, "label": "", "sentence_structure": "Subject鈥搗erb鈥揷omplement", "subjective_objective": {"answer": "Subjective", "rationale": "Because \"satisfaction\" is a subjective emotional reaction that reflects the attitude and feelings of patients who cannot avoid difficulties."}, "sentence_sentiment": {"answer": "Negative", "rationale": "Because the mood of \"freak\" in the entry is negative."}, "language_style": {"answer": "Calm and direct", "rationale": "The language style of the entry is more calm and objective, and there is no excessive emotional rendering or rhetorical technique, but rather direct and concise questioning of whether the interviewee is upset about the difficulties. This language style helps to ensure the accuracy and effectiveness of the questionnaire and avoids the influence of subjective sentiment on the answer."}, "time_dimension": {"answer": "Present", "rationale": "The keyword is \"to avoid difficulties\", it describes a state of emotion that is going through right now, so the entry asks about the emotional reaction of the person being tested."}, "sentence_type": "Negative declarative sentence", "pos": {"nouns": {"Difficulties": 1, "I'm upset": 1}, "verbs": {"Avoidance": 1, "Yes": 1}, "adverbs": {"No, I can't": 1}}}, {"content": "29. More than two common solutions to difficulties", "word_count": 13, "label": "", "sentence_structure": "Subject鈥搗erb鈥搊bject", "subjective_objective": {"answer": "Subjective", "rationale": "This is because the behaviour and habits reported by the patient itself are subjective."}, "sentence_sentiment": {"answer": "Positive", "rationale": "Because the behaviour in the entry \"relatively more than two solutions to difficulties\" is positive, it shows that the respondents have the flexibility to solve problems when faced with difficulties."}, "language_style": {"answer": "Tactful and warm", "rationale": "The entry is more liberal in its language style. It uses the means of questioning, not direct orders or statements, which make the respondents more comfortable and comfortable. Moreover, it does not use too professional or complex terms to enable respondents to better understand and answer questions. These are the warmer language styles of the questionnaire."}, "time_dimension": {"answer": "Present", "rationale": "The entry asks whether the testees often use multiple methods to resolve difficulties, so it is possible to judge that the time dimension of the article is now."}, "sentence_type": "Imperative sentence", "pos": {"nouns": {"Questionnaire": 1, "Entry": 1, "Difficulties": 1}, "verbs": {"Use": 1, "Resolve": 1, "Approach": 1}, "numerals": {"Two": 1, "More than one type": 1, "of": 3}, "classifiers": {"individual": 2}}}, {"content": "It's often felt unnecessary to fight for success.", "word_count": 15, "label": "", "sentence_structure": "Subject鈥搗erb鈥搊bject", "subjective_objective": {"answer": "Subjective", "rationale": "Because \"it is not necessary to fight for success or failure\" is a personal view and attitude, and it is subjective."}, "sentence_sentiment": {"answer": "Negative", "rationale": "Because the entry expresses a negative attitude to success or failure, it is a negative sentiment."}, "language_style": {"answer": "Tactful and warm", "rationale": "The language style of the entry is more euphemistic and moderate, using an invitation to speak rather than to speak or order directly. This expression is more personal and interactive, and it enables the person surveyed to feel more comfortable and natural to answer the questions."}, "time_dimension": {"answer": "Present", "rationale": "The phrase \"often believes\" in the entry to be a constant thought or view, while \"there is no need to struggle to win or lose\" indicates the respondent's attitude and perception of the matter. The entire sentence therefore describes a continuing state of affairs that should be in the current state of affairs."}, "sentence_type": "Negative declarative sentence", "pos": {"nouns": {"Required": 1, "Strength": 1, "Fight": 1, "Success or failure": 2}, "verbs": {"I think it's a good idea": 1, "Fight": 1, "have": 1}, "adverbs": {"Well": 1}, "numerals": {"30": 1}}}, {"content": "31. Efforts to change the status quo and turn the situation to the positive side", "word_count": 17, "label": "", "sentence_structure": "Subject鈥搗erb鈥揷omplement", "subjective_objective": {"answer": "Subjective", "rationale": "Because this entry is a subjective view of the patient in terms of his or her attitude and behaviour towards himself or herself, and his or her willingness to seek change on his or her own initiative."}, "sentence_sentiment": {"answer": "Positive", "rationale": "Because the words \"to try to change the status quo\" and \"to change the situation to the better\" in this entry are positive acts and purposes."}, "language_style": {"answer": "Calm and direct", "rationale": "The language style of the entry is more straightforward, not too much retrofitting or emotional rendering, but rather a simple expression of the questionnaire, in which the interviewee is expected to reflect and answer the question."}, "time_dimension": {"answer": "Present", "rationale": "The verbs \"to try\" and \"to change the status quo\" and the goal \"to turn the situation to the better\" describe the efforts and the expected future results of the testees, so the time dimension is now judged."}, "sentence_type": "Imperative sentence", "pos": {"nouns": {"Try": 1, "Status": 1, "Conversion": 1}, "verbs": {"Go on": 1, "Change": 1, "鈾?And I'm gonna be 鈾?: 1}, "adjectives": {"Okay": 1}, "numerals": {"31": 1}}}, {"content": "32. Reluctance by tobacco or alcohol", "word_count": 6, "label": "", "sentence_structure": "Subject鈥搗erb鈥揷omplement", "subjective_objective": {"answer": "Subjective", "rationale": "This is an act of the patient ' s choice and is a subjective attitude and behaviour of the individual."}, "sentence_sentiment": {"answer": "Negative", "rationale": "Because the act of \"smoking or drinking\" is negative and may cause bodily harm."}, "language_style": {"answer": "Tactful and warm", "rationale": "The entry is more personal and interactive when it is spoken in a gentle and inviting manner, rather than in direct statements or orders. Moreover, \"remuneration\" is a more implicit term, which does not explicitly require the use of tobacco or alcohol to address the troubles, but rather uses the word \"remuneration\" to draw attention to and reflect on the emotional situation of the other party."}, "time_dimension": {"answer": "Present", "rationale": "The entry asks the testee whether he or she has acted to relieve stress and to remove distress by smoking or drinking, and the time dimension is not clear, present or future. Therefore, it cannot be judged on the time dimension."}, "sentence_type": "Imperative sentence", "pos": {"nouns": {"Borrow": 1, "Smoke": 1, "Wine": 1, "Sad": 1}, "verbs": {"Dismissed": 1}, "numerals": {"32": 1}}}, {"content": "33. Too often to blame.", "word_count": 5, "label": "", "sentence_structure": "Subject鈥搗erb鈥揷omplement", "subjective_objective": {"answer": "Subjective", "rationale": "Because \"criticizing others\" is a subjective attitude or perception that depends on the way individuals think and feel. This view may be influenced by individual emotions, values, beliefs, etc., which are subjective. Thus, the entry \"commonly blaming others\" is a survey of patients' subjective tendencies or patterns of behaviour."}, "sentence_sentiment": {"answer": "Negative", "rationale": "Because the \"criticizing others\" act in the entry is negative."}, "language_style": {"answer": "Calm and direct", "rationale": "The entry is more objective and straightforward in terms of direct questioning of the respondents about the frequent blame, the lack of use of rhetorical techniques or emotional rendering."}, "time_dimension": {"answer": "Past", "rationale": ""}, "sentence_type": "Imperative sentence", "pos": {"nouns": {"Others": 1, "Responsibility": 1}, "verbs": {"Blame": 1}, "numerals": {"33": 1}}}, {"content": "34. The usual evasive attitude to difficulties", "word_count": 11, "label": "", "sentence_structure": "Subject鈥搗erb鈥揷omplement", "subjective_objective": {"answer": "Subjective", "rationale": "The attitude taken to deal with difficulties is a subjective choice and preference of one."}, "sentence_sentiment": {"answer": "Negative", "rationale": "Because \"aversion\" is often seen as a passive act of avoiding problems, contrary to a positive attitude towards, and solving, problems."}, "language_style": {"answer": "Tactful and warm", "rationale": "The entry is more personal and interactive when it is spoken in a gentle and inviting tone, rather than in direct statements or orders. Moreover, it does not have too strong emotional accents or rhetorical techniques, but rather describes a behavioural characteristic in an objective and neutral language."}, "time_dimension": {"answer": "Present", "rationale": ""}, "sentence_type": "Imperative sentence", "pos": {"nouns": {"Difficulties": 1, "Attitude": 1}, "verbs": {"Adopt": 1, "Avoidance": 1, "Regular": 1}, "adverbs": {"Regular": 1}, "numerals": {"34": 1}}}, {"content": "Considers that 鈥渁 step back is natural鈥?, "word_count": 9, "label": "", "sentence_structure": "Subject鈥損redicate", "subjective_objective": {"answer": "Subjective", "rationale": "Because this view is one person ' s perception and understanding of things and is subjective."}, "sentence_sentiment": {"answer": "Positive", "rationale": "Because the view in the entry is positive, it is suggested that an appropriate subordination in the face of difficulties could lead to better results."}, "language_style": {"answer": "Tactful and warm", "rationale": "The language style of the entry is more euphemistic, using the popular phrase \"reverse one step of natural breadth\" to guide the interviewee to think, and to express it in a more moderate manner, without direct command or a strong tone. This expression is more readily acceptable to the interviewee and is willing to answer questions."}, "time_dimension": {"answer": "Past", "rationale": "In the entry, the \"concept\" action occurred in the past and was related to the testee's experience or observation of the past, and thus judged to be the past."}, "sentence_type": "Affirmative declarative sentence", "pos": {"nouns": {"Step": 1, "Kwang": 1}, "verbs": {"Recognition": 1, "Back": 1, "Kwang": 1}, "adjectives": {"From": 1, "Yeah": 1}, "numerals": {"35": 1}}}, {"content": "36. Bury the unpleasant.", "word_count": 10, "label": "", "sentence_structure": "Subject鈥搗erb鈥揷omplement", "subjective_objective": {"answer": "Subjective", "rationale": "Because it is a personal approach and attitude to bury unpleasant things in the heart, reflecting the emotional management strategies and coping mechanisms of the patient. This involves subjective factors such as emotional experience, cognitive evaluation, and so it is subjective."}, "sentence_sentiment": {"answer": "Negative", "rationale": "Because the entry \"Playing Inconvenient Things in the Heart\" is negative and detrimental to physical and mental health."}, "language_style": {"answer": "Tactful and warm", "rationale": "The entry is more euphemistic. It does not ask whether the respondents always suppress negative emotions, but rather directs them to think and express their feelings in a more moderate and attractive way, which is more accessible to the respondents and encourages them to express their thoughts more honestly."}, "time_dimension": {"answer": "Present", "rationale": ""}, "sentence_type": "Imperative sentence", "pos": {"nouns": {"Yes": 1, "Heart": 1}, "verbs": {"- Put it on": 1, "Buried": 1}, "adjectives": {"It's not good": 1}, "numerals": {"36": 1}}}, {"content": "37. Always humbled.", "word_count": 5, "label": "", "sentence_structure": "Subject鈥搗erb鈥揷omplement", "subjective_objective": {"answer": "Subjective", "rationale": "The first is the fact that the first step is to make the most of the people who are sick."}, "sentence_sentiment": {"answer": "Negative", "rationale": "Because \"self-pity\" is a negative emotion and attitude."}, "language_style": {"answer": "Tactful and warm", "rationale": "The language style of the entry is relatively euphemistic, not directly denying or attacking the self-esteem of the other, but directing the other to thinking in a caring and understanding way. This moderate language makes it easier for the interviewee to accept and share his thoughts."}, "time_dimension": {"answer": "Past", "rationale": "According to the keyword and sentence structure, the entry expresses the person ' s feelings or state at a certain point in time, does not specify past, present or future behavioural or mental changes, and therefore cannot determine the time dimension."}, "sentence_type": "Negative declarative sentence", "pos": {"verbs": {"Regular": 1, "You're a low man": 1, "Self-pity": 1}, "numerals": {"37": 1}}}, {"content": "38. It's often thought of as a form of injustice to one ' s own life.", "word_count": 16, "label": "", "sentence_structure": "Subject鈥搗erb鈥搊bject", "subjective_objective": {"answer": "Subjective", "rationale": "Because \"think\" is the patient's subjective attitude."}, "sentence_sentiment": {"answer": "Negative", "rationale": "Because the expression \"often considered it an expression of injustice to itself\" expresses negative emotions and attitudes."}, "language_style": {"answer": "Calm and direct", "rationale": "The entry is presented in a direct question-and-answer manner and is more calm and straightforward."}, "time_dimension": {"answer": "Present", "rationale": "The temporal dimension of this entry can therefore be judged as now."}, "sentence_type": "Imperative sentence", "pos": {"nouns": {"Life": 1, "self": 1, "Performance": 1}, "verbs": {"I think": 1, "be": 1, "to": 1, "Fair": 1, "of": 2, "Regular": 1, "not": 1, "Table": 1, "Icon": 1}, "adjectives": {"It's not fair": 1}, "adverbs": {"Regular": 1}, "numerals": {"38": 1, "Here": 1}, "pronouns": {"Here": 1, "to": 1, "self": 1, "of": 2, "Regular": 1, "not": 1}}}, {"content": "39. The anger and discontent that often repressed the heart", "word_count": 11, "label": "", "sentence_structure": "Subject鈥搗erb鈥揷omplement", "subjective_objective": {"answer": "Subjective", "rationale": "The idea of depression and attitudes are the subjective psychological state of the patient."}, "sentence_sentiment": {"answer": "Negative", "rationale": "Because the act of \"pressing inner anger and discontent\" in the entries is negative."}, "language_style": {"answer": "Tactful and warm", "rationale": "The language style of the article is \"precipitous.\" Although it involves a negative emotion (irregation and dissatisfaction), it is not expressed in a strong negative or critical language, but rather in a less intrusive tone. It makes the interviewee feel understood and respected, and more willing to share their feelings. At the same time, the entry does not direct the person to answer the questions, but rather leads them to think and answer in a way that is more accessible to the interviewee, and that gives them more valuable information."}, "time_dimension": {"answer": "Past", "rationale": ""}, "sentence_type": "Negative declarative sentence", "pos": {"nouns": {"Repression": 1, "Inside": 1, "Anger": 1, "Dissatisfied": 1}, "verbs": {"Regular": 1}, "adverbs": {"and": 1, "not": 1, "Regular": 1}, "numerals": {"39": 1}}}, {"content": "40. Learning from one ' s own or others ' experience to cope with difficulties", "word_count": 15, "label": "", "sentence_structure": "Subject鈥搗erb鈥揷omplement", "subjective_objective": {"answer": "Objective", "rationale": "This is the result of the actual actions taken by the patient in the face of difficulties. Although it may contain subjective ideas and judgements, the whole process of behaviour itself is objective."}, "sentence_sentiment": {"answer": "Positive", "rationale": "Because the act of \"learning from your own or others' experience to cope with difficulties\" is positive and shows determination and ability to solve problems."}, "language_style": {"answer": "Tactful and warm", "rationale": "The language style of the entry is euphemism. It raises questions in a gentle, encouraging way, not directly ordering or accusing the interviewee. It is more acceptable and stimulates the interviewee鈥檚 positive feelings and participation. Moreover, the issue uses the term 鈥渓earning from his own or others鈥?experiences鈥?to give a warm and caring feeling, which helps to create a good atmosphere of communication."}, "time_dimension": {"answer": "Past", "rationale": ""}, "sentence_type": "Imperative sentence", "pos": {"nouns": {"Difficulties": 1, "Experience": 2}, "verbs": {"Draw": 1, "Payable": 1}, "numerals": {"40": 1}}}, {"content": "I don't believe in things that are bad for me.", "word_count": 13, "label": "", "sentence_structure": "Subject鈥搗erb鈥搊bject", "subjective_objective": {"answer": "Subjective", "rationale": "Because \"untrusted\" is a word of personal mental state, emphasizing the patient's own attitudes and perceptions."}, "sentence_sentiment": {"answer": "Negative", "rationale": "Because \"not believing in something bad for yourself\" may mean avoiding and ignoring negative information, which is a negative attitude."}, "language_style": {"answer": "Calm and direct", "rationale": "The entry is based on a direct question-and-answer approach and a positive negative-formation sentence structure (i.e. \"Is it?\"), a more concise language style, with no excessive emotional colour or rhetorical techniques, and therefore can be judged as a calm and straightforward language style."}, "time_dimension": {"answer": "Present", "rationale": ""}, "sentence_type": "Negative declarative sentence", "pos": {"nouns": {"Yes": 1}, "verbs": {"Letter": 1, "have": 1}, "adjectives": {"Regular": 1, "Negative": 1}, "adverbs": {"Those": 1}, "numerals": {"41": 1}}}, {"content": "42. For self-esteem, people often do not want to know what they're going through.", "word_count": 16, "label": "", "sentence_structure": "Subject鈥揷opula鈥損redicative", "subjective_objective": {"answer": "Subjective", "rationale": "The reluctance to let people know that their experiences are subjective attitudes or feelings reflects the importance and protection of self-esteem."}, "sentence_sentiment": {"answer": "Negative", "rationale": "Because the purpose of the entry, \"always unwilling to let people know what they are suffering\", is to protect themselves and preserve self-esteem, but to some extent may also lead to a sense of loneliness and difficulty in obtaining help from others, thus bearing a certain negative emotional colour."}, "language_style": {"answer": "Calm and direct", "rationale": "The entry is simple and straightforward. The language style is therefore more calm and straightforward."}, "time_dimension": {"answer": "Past", "rationale": "\"As a result of self-esteem, the reluctance to let people know what happened to them\" contains a description of the past and such behaviour continues, so that the temporal dimension of the article can be judged to be past and present."}, "sentence_type": "Imperative sentence", "pos": {"nouns": {"Self-esteem": 1, "person": 1, "Problems": 1}, "verbs": {"May I": 1, "Jean": 1, "Yes": 1, "Here": 1, "Do it": 1, "Regular": 1, "I don't want to": 1}, "adjectives": {"Your own": 1}, "adverbs": {"Regular": 1, "not": 1}, "numerals": {"42": 1}, "pronouns": {"self": 1}}}, {"content": "43. Often discussing solutions with colleagues and friends.", "word_count": 17, "label": "", "sentence_structure": "Subject鈥搗erb鈥搊bject", "subjective_objective": {"answer": "Objective", "rationale": "Because it is an act of social activity of the patient and an objective fact."}, "sentence_sentiment": {"answer": "Positive", "rationale": "Because the purpose and outcome of the action \"discussing the solution\" in the entry is positive."}, "language_style": {"answer": "Tactful and warm", "rationale": "The entry leads the interviewee to review his behaviour in an interview and raises questions with encouragement and support rather than with orders or criticism, so the language style is euphemismal."}, "time_dimension": {"answer": "Past", "rationale": ""}, "sentence_type": "Affirmative declarative sentence", "pos": {"nouns": {"Co-workers": 1, "friends": 1, "Problem": 1, "Approach": 1}, "verbs": {"Constant with": 1, "Discussion": 1, "Resolve": 1}, "adverbs": {"Together": 1}, "numerals": {"43": 1}}}, {"content": "He often warns himself 鈥渢o be safe for the ninja鈥?", "word_count": 10, "label": "", "sentence_structure": "Subject鈥搗erb鈥揷omplement", "subjective_objective": {"answer": "Subjective", "rationale": "Because it is the patient who warns himself, it is a manifestation of subjective ideas or attitudes."}, "sentence_sentiment": {"answer": "Positive", "rationale": "Because the attitude and behavior of the Ninja are positive."}, "language_style": {"answer": "Tactful and warm", "rationale": "The language style of the entry is euphemistic and warm. It uses a recommendatory tone, not direct orders or statements, which is more personal and interactive. At the same time, the phrase \"Enable Ninja\" gives a warm and inspiring feeling of encouragement to the interviewee to remain patient and calm in life."}, "time_dimension": {"answer": "Present", "rationale": "Now, the keyword \"always warn yourself\" indicates that it is a constant act of self-alarming, and both \"caution\" and \"self\" adopt the general current, meaning that this act has not only happened in the past, but may still be ongoing. So, the entry is the current dimension of time."}, "sentence_type": "Affirmative declarative sentence", "pos": {"nouns": {"self": 1}, "verbs": {"Warning": 1, "You can't be a ninja": 1}, "adverbs": {"Regular": 1}, "numerals": {"44": 1}}}, {"content": "45. I pray to God.", "word_count": 7, "label": "", "sentence_structure": "Subject鈥搗erb鈥揷omplement", "subjective_objective": {"answer": "Subjective", "rationale": "Because prayer is a personal belief and attitude and is the subjective experience and perception of the patient, the entry to the questionnaire is subjective."}, "sentence_sentiment": {"answer": "Positive", "rationale": "Because the act of \"pity and blessings\" in the entry expresses a positive, faith-based feeling."}, "language_style": {"answer": "Tactful and warm", "rationale": "The language style of the entry is more moderate, using a prayer sentence to guide the interviewees to their thinking, while the religious vocabulary of \"godly blessing\" is used in the sentence, making the expression more friendly and respectful."}, "time_dimension": {"answer": "Present", "rationale": ""}, "sentence_type": "Imperative sentence", "pos": {"nouns": {"Pray": 1, "Spirit": 1, "Bless you": 1}, "verbs": {"Regular": 1}, "numerals": {"45": 1}}}, {"content": "46. Common humor or joke-based ways to mitigate conflict or delay", "word_count": 17, "label": "", "sentence_structure": "Subject鈥搗erb鈥搊bject", "subjective_objective": {"answer": "Subjective", "rationale": "Because the usual way of de-escalating conflict or inefficiency is the strategy and attitude of patients, it is their subjective choice and behaviour."}, "sentence_sentiment": {"answer": "Positive", "rationale": "Because the purpose of the entry is to mitigate conflict or inefficiency by humor or joke, which helps to reduce tension and improve relationships."}, "language_style": {"answer": "Tactful and warm", "rationale": "The entry uses a gentle, inviting tone to ask the other side whether the way humor or jokes is commonly used to mitigate conflict or infrequently, which is more proficient and interactive. At the same time, it is not based on too many rhetorical or emotional expressions, and is more objective in tone."}, "time_dimension": {"answer": "Present", "rationale": "The entry asks whether the testees are now often using humor or jokes to mitigate conflicts or improvities, and therefore judge them as current behaviors or mentalities."}, "sentence_type": "Imperative sentence", "pos": {"nouns": {"Questionnaire": 1, "Entry": 1, "Modalities": 1, "Mitigation": 1, "Conflict": 1, "Not fast": 1}, "verbs": {"Use": 1, "Regular": 1, "Humor": 1, "A joke": 1, "Mitigation": 1}, "adverbs": {"or": 1, "Common": 1, "Down there": 1, "of": 2, "Not fast": 1}, "numerals": {"46": 1}, "pronouns": {"Here": 1}}}, {"content": "47. Limited capacity, patience.", "word_count": 10, "label": "", "sentence_structure": "Subject鈥搗erb鈥揷omplement", "subjective_objective": {"answer": "Subjective", "rationale": "Because patience is a subjective feeling and attitude."}, "sentence_sentiment": {"answer": "Negative", "rationale": "Because the entries \"self-limited\" and \"resistence\" express a sense of impatience and negative sentiment."}, "language_style": {"answer": "Tactful and warm", "rationale": "The entry is a moderate and understanding-based dialogue, highlighting the limitations that individuals may encounter in some cases and trying to create an atmosphere of tolerance and understanding. Such expressions are more personal and interactive and are more able to attract the active participation of interviewees."}, "time_dimension": {"answer": "Present", "rationale": "The entries, \"Hand of my own power\" and \"Stand up\" describe the current state of mind and behaviour of the testee, and the temporal dimension is now judged."}, "sentence_type": "Imperative sentence", "pos": {"nouns": {"self": 1, "Capacity": 1, "Hold on": 1}, "verbs": {"Limited": 1, "have": 1, "Hold on": 1}, "adverbs": {"Only": 1}, "numerals": {"47": 1}, "pronouns": {"self": 1}}}, {"content": "The reason I'm not good enough is that I'm not good enough.", "word_count": 7, "label": "", "sentence_structure": "Subject鈥搗erb鈥揷omplement", "subjective_objective": {"answer": "Subjective", "rationale": "Because feeling like you're not paying off is a subjective assessment or feeling."}, "sentence_sentiment": {"answer": "Negative", "rationale": "Because the word \"failure\" has a negative emotional connotation."}, "language_style": {"answer": "Tactful and warm", "rationale": "The entry is more adept and interactive when it is spoken in a gentle and inviting manner, rather than by direct statements or orders."}, "time_dimension": {"answer": "Past", "rationale": "The keywords \"often odd\" and \"unprofitable\" both indicate that the entry is a description of the person's past behaviour or mental state, and therefore a judgement of the past."}, "sentence_type": "Negative declarative sentence", "pos": {"nouns": {"self": 1, "- It's good": 1}, "verbs": {"It's weird": 1}, "numerals": {"48": 1}}}, {"content": "49. I always fantasize about things that are unrealistic to remove the trouble.", "word_count": 16, "label": "", "sentence_structure": "Subject鈥搗erb鈥揷omplement", "subjective_objective": {"answer": "Subjective", "rationale": "Because fantasy is the patient's subjective attitude and behaviour."}, "sentence_sentiment": {"answer": "Negative", "rationale": "Because the \"fantasy of something unrealistic\" in the entry is negative."}, "language_style": {"answer": "Calm and direct", "rationale": "The basis for the determination: The entry presents in a concise and clear manner the question of whether the interviewees often alleviate their troubles by fantasizing unrealistic things. This expression is not too much rhetorical or emotional, is more objective and meets the linguistic requirements of the questionnaire."}, "time_dimension": {"answer": "Present", "rationale": ""}, "sentence_type": "Affirmative declarative sentence", "pos": {"nouns": {"Fantasy": 1, "Yes": 1, "Trouble": 1}, "verbs": {"Always": 1, "Elimination": 1}, "adverbs": {"not": 1, "Reality": 1}, "numerals": {"49": 1}, "classifiers": {"Some": 1}}}, {"content": "50. I've complained about my incompetence.", "word_count": 7, "label": "", "sentence_structure": "Subject鈥搗erb鈥揷omplement", "subjective_objective": {"answer": "Subjective", "rationale": "Because \"always complaining\" is an individual's behaviour and attitude, which is the patient's subjective perception and feelings."}, "sentence_sentiment": {"answer": "Negative", "rationale": "Because the \"always complaining about incompetence\" act in the entry is negative."}, "language_style": {"answer": "Tactful and warm", "rationale": "The language style of the entry is more euphemistic, not directly critical or accusing the interviewee, but rather a caring attitude to ask questions. It is easier to express the interviewer鈥檚 feelings and thoughts, and to share them. At the same time, this moderate tone helps to create a good atmosphere of communication that allows the interviewee to feel respected and supported."}, "time_dimension": {"answer": "Present", "rationale": ""}, "sentence_type": "Exclamatory sentence", "pos": {"nouns": {"Questionnaire": 1, "Entry": 1, "Capacity": 1, "- He's complaining": 1}, "verbs": {"Regular": 1, "be": 1, "- He's complaining": 2}, "adjectives": {"Incompetent": 1, "Your own": 1}, "numerals": {"50": 1}, "classifiers": {"individual": 1}}}, {"content": "51. There are good aspects to bad things that are often seen.", "word_count": 12, "label": "", "sentence_structure": "Subject鈥搗erb鈥揷omplement", "subjective_objective": {"answer": "Objective", "rationale": "Since it is objective to see good and bad, subjective attitudes are subjective."}, "sentence_sentiment": {"answer": "Positive", "rationale": "Because the \"seeing the good side of bad things\" in the entry is positive."}, "language_style": {"answer": "Tactful and warm", "rationale": "The entry is more pro-active and interactive in its expression of a positive tone of speech rather than a direct statement or order. At the same time, it encourages respondents to view the issue positively and convey a sense of optimism."}, "time_dimension": {"answer": "Present", "rationale": "\"Attractive\" indicates that this is a customary act, while \"seen\" uses the general current, indicating that the act not only has occurred in the past, but may still be ongoing. The entry therefore asks whether the testees are often able to see the good side of bad things in their current lives."}, "sentence_type": "Affirmative declarative sentence", "pos": {"nouns": {"Bad things": 1, "Good news": 1}, "verbs": {"Yeah": 1, "have": 1, "of": 2, "One side": 1}, "adjectives": {"Bad": 1, "Okay": 1}, "adverbs": {"Regular": 1, "Yes": 1}, "numerals": {"51": 1}}}, {"content": "52. Self-depression is a test of self.", "word_count": 11, "label": "", "sentence_structure": "Subject鈥揷opula鈥損redicative", "subjective_objective": {"answer": "Subjective", "rationale": "Because \"self-retardation is a test of self\" is a subjective view or belief. It reflects patients' attitude and response to frustration, not objective facts."}, "sentence_sentiment": {"answer": "Positive", "rationale": "Because the \"depression\" in the entry is objective, and it's a positive attitude to view it as \"a test of your own\"."}, "language_style": {"answer": "Tactful and warm", "rationale": "The entry is more personal and interactive when it is spoken in a gentle, inviting manner, rather than in direct statements or orders. At the same time, the entry does not use too hard or professional language, but rather uses a more intimate and understandable way of making it easier for respondents to accept and be willing to participate in the answer."}, "time_dimension": {"answer": "Future", "rationale": "The entry expresses the views and attitudes of the testees regarding possible future setbacks and can therefore be judged as future."}, "sentence_type": "Affirmative declarative sentence", "pos": {"nouns": {"Frustration": 1, "self": 1, "Test": 1}, "verbs": {"Self-conscious": 1, "be": 1, "to": 1, "of": 3}, "numerals": {"52": 1}}}, {"content": "53. Approach to solving problems with experienced relatives and teachers", "word_count": 18, "label": "", "sentence_structure": "Subject鈥搗erb鈥搊bject", "subjective_objective": {"answer": "Subjective", "rationale": "Because it is the patient ' s subjective behaviour and willingness to turn to experienced people for help."}, "sentence_sentiment": {"answer": "Positive", "rationale": "Because the entry \"Teaching experienced friends and friends to solve problems\" is positive."}, "language_style": {"answer": "Tactful and warm", "rationale": "The language style of the entry is more euphemistic and gentle, expressing respect and appreciation for each other by using the terms \"please\" and \"teaching\". At the same time, more objective and neutral language is used, with no excessive emotional or personal preference."}, "time_dimension": {"answer": "Present", "rationale": "The practice of \"teaching experienced relatives and friends, teachers\" is the present state and may continue and therefore falls within the present context."}, "sentence_type": "Exclamatory sentence", "pos": {"nouns": {"Experience": 1, "Friends": 1, "Sir": 1, "Problem": 1, "Methodology": 1}, "verbs": {"In": 1, "- You're a teacher": 1, "Resolve": 1}, "numerals": {"53": 1}, "pronouns": {"Experienced": 1}}}, {"content": "54. Calm down and lighten up the troubles.", "word_count": 8, "label": "", "sentence_structure": "Subject鈥搗erb鈥揷omplement", "subjective_objective": {"answer": "Subjective", "rationale": "This is the subjective desire and action of the patient to try to adjust and control his or her emotional and psychological state."}, "sentence_sentiment": {"answer": "Neutral", "rationale": "Because the entry describes an attitude and approach, it does not express positive or negative emotional sentiments."}, "language_style": {"answer": "Tactful and warm", "rationale": "The entry is designed to guide the interviewee in a manner that encourages and advises him to remain calm and to face the difficulties of life, with a more moderate and friendly language, which is more resonant and acceptable to the interviewee."}, "time_dimension": {"answer": "Present", "rationale": ""}, "sentence_type": "Imperative sentence", "pos": {"nouns": {"Calm down": 1, "Trouble": 1}, "verbs": {"Dilution": 1}, "numerals": {"54": 1}}}, {"content": "55. Efforts to find inter-agency solutions", "word_count": 11, "label": "", "sentence_structure": "Subject鈥搗erb鈥揷omplement", "subjective_objective": {"answer": "Subjective", "rationale": "Because \"working to find solutions to inter-problems\" reflects the attitudes and behaviour of the patient and is part of his or her subjective feelings and wishes."}, "sentence_sentiment": {"answer": "Positive", "rationale": "Because the entries \"tough to find\" and \"solve\" are positive, expressing a proactive and responsible attitude."}, "language_style": {"answer": "Calm and direct", "rationale": "The entry is based on a direct question about the attitude of the person surveyed to actively seek solutions to problems, without using excessive emotional aesthetic or euphemism. This straightforward question is more objective and clear."}, "time_dimension": {"answer": "Future", "rationale": ""}, "sentence_type": "Imperative sentence", "pos": {"nouns": {"Approach": 1, "Problem": 1}, "verbs": {"Search": 1, "Resolve": 1}, "adverbs": {"Try": 1}, "numerals": {"55": 1}}}, {"content": "56. Inadequate career choices are the main cause of frequent setbacks.", "word_count": 18, "label": "", "sentence_structure": "Subject鈥揷opula鈥損redicative", "subjective_objective": {"answer": "Objective", "rationale": "Because the choice of occupation is determined by the subjective will of the individual, not by objective facts. Thus, the main reason for the improper choice of occupation is that it is often viewed from the patient鈥檚 subjective perspective."}, "sentence_sentiment": {"answer": "Negative", "rationale": "Because the \"opt-in\" behaviour in the entry is negative."}, "language_style": {"answer": "Calm and direct", "rationale": "The entry is a direct indicator of the relationship between inappropriate career choices and frequent setbacks to individuals, with no excessive rhetoric and emotional colours, and a more objective and incipient language style.\""}, "time_dimension": {"answer": "Present", "rationale": "The key words of the questionnaire entry are \"choice of profession\" and \"recurring frustrations,\" which indicate the problems or challenges that the testee is facing. At the same time, \"mainly because of himself,\" which highlights the impact of the subjectivity of the testee on the problem, and is also an expression of the current state of affairs. Thus, the entry can be judged by the current situation, and the time dimension by the present."}, "sentence_type": "Imperative sentence", "pos": {"nouns": {"Occupation": 1, "Frustration": 2, "Main reasons": 1}, "verbs": {"Selection": 1, "Yes": 1}, "adjectives": {"Not right": 1, "Main": 1}, "numerals": {"56": 1}}}, {"content": "57. It's my fault.", "word_count": 6, "label": "", "sentence_structure": "Subject鈥揷opula鈥損redicative", "subjective_objective": {"answer": "Subjective", "rationale": "Because \"I blame myself\" is an expression of subjective emotions and attitudes."}, "sentence_sentiment": {"answer": "Negative", "rationale": "Because the attitude of \"don't blame yourself\" in the entries is negative."}, "language_style": {"answer": "Tactful and warm", "rationale": "The entry is more euphemistic, using the implicit term \"the Mansion,\" which does not directly point to the behaviour of the other party in terms of self-responsibility or self-denial, but rather leads to reflection in a moderate way. At the same time, the expression \"not good\" is more subtle, avoiding too much negative emotion and more personal and interactive."}, "time_dimension": {"answer": "Present", "rationale": ""}, "sentence_type": "Imperative sentence", "pos": {"nouns": {"self": 1}, "verbs": {"Weird": 1, "Okay": 1}, "adverbs": {"Total": 1}, "numerals": {"57": 1}}}, {"content": "58. Often, you see the dust, you don't care about your own misfortunes.\"", "word_count": 17, "label": "", "sentence_structure": "Subject鈥搗erb鈥揷omplement", "subjective_objective": {"answer": "Subjective", "rationale": "Because \"seeing the dust, not caring about your own misfortune\" is a subjective attitude or perception, not an objective fact or result. This subjective view is viewed from the patient's subjective perspective."}, "sentence_sentiment": {"answer": "Negative", "rationale": "Because the act and attitude of \"seeing the dust, not caring about your own misfortune\" is negative."}, "language_style": {"answer": "Calm and direct", "rationale": "The entry describes a phenomenon or behaviour in more objective language, does not use excessive emotional or rhetorical techniques and therefore is more language-oriented and straightforward."}, "time_dimension": {"answer": "Present", "rationale": "So the answer is \"now\"."}, "sentence_type": "Affirmative declarative sentence", "pos": {"nouns": {"58": 1, "Reddust": 1, "Problems": 1}, "verbs": {"be": 1, "- Look at that": 1, "I care": 1, "I met him": 1}, "adjectives": {"Regular": 1}, "adverbs": {"not": 2}, "numerals": {"58": 1, "1": 4}, "pronouns": {"self": 1}}}, {"content": "59. Too often, bad luck.", "word_count": 7, "label": "", "sentence_structure": "Subject鈥搗erb鈥揷omplement", "subjective_objective": {"answer": "Subjective", "rationale": "Because bad luck is a subjective assessment."}, "sentence_sentiment": {"answer": "Negative", "rationale": "Because the expression \"frightful\" in the entry is negative."}, "language_style": {"answer": "Tactful and warm", "rationale": "The entry is more adept and interactive when it is spoken in a gentle and inviting manner, rather than by direct statements or orders."}, "time_dimension": {"answer": "Present", "rationale": "The word \"normally\" in \"commonly self-conscious\" denotes a regular, customary behaviour, while \"self-conscious\" adopts the general current, meaning that this behaviour has not only occurred in the past, but may still be ongoing."}, "sentence_type": "Exclamatory sentence", "pos": {"nouns": {"Lucky": 1, "Not good": 1}, "verbs": {"Common sense": 1}, "numerals": {"59": 1}}}, {"content": "60. Tell people what's in your heart.", "word_count": 10, "label": "", "sentence_structure": "Subject鈥損redicate", "subjective_objective": {"answer": "Subjective", "rationale": "Because the complaint is the patient ' s subjective attitude."}, "sentence_sentiment": {"answer": "Positive", "rationale": "Because it is positive to tell others about the distress in their hearts, it can alleviate negative sentiments."}, "language_style": {"answer": "Tactful and warm", "rationale": "The language style of the entry is euphemism, which is a gentle and inviting tone of conversation, rather than a direct statement or order. This expression is more personal and interactive, encouraging respondents to participate more actively in the questionnaire and share their real feelings and experiences."}, "time_dimension": {"answer": "Present", "rationale": "The keyword \"to say to others\" indicates that it is an ongoing act, while \"the distress in the heart\" indicates that it is the current emotional state of the subject. So, the time dimension of this entry is now."}, "sentence_type": "Exclamatory sentence", "pos": {"nouns": {"Others": 1, "鈾?In the heart 鈾?: 1, "Trouble": 1}, "verbs": {"Tell me": 1, "In": 1}, "numerals": {"60": 1}}}, {"content": "61. I'm always indifferent to what I'm doing.", "word_count": 12, "label": "", "sentence_structure": "Subject鈥搗erb鈥搊bject", "subjective_objective": {"answer": "Subjective", "rationale": "Because \"always inert and natural\" is a sense and attitude of the person's own heart, a psychological state and emotional experience, it is a subjective examination."}, "sentence_sentiment": {"answer": "Negative", "rationale": "Because the attitude of \"inaction\" and \"leave it to nature\" in the entries is negative."}, "language_style": {"answer": "Calm and direct", "rationale": "The language style of the entry is more objective and calm, and it directly expresses whether the investigator wants to know whether the other party often feels that it has nothing to do with it. There is no excessive rhetorical or emotional overtookness that gives people a sense of reason and pragmatism."}, "time_dimension": {"answer": "Present", "rationale": "The keyword \"always self-conscious\" denotes a regular, customary act that uses the usual current state of affairs, so that the act has not only occurred in the past but may still continue."}, "sentence_type": "Exclamatory sentence", "pos": {"nouns": {"I'm not doing anything": 1, "Naturally": 1}, "verbs": {"Regular": 1, "Sensor": 1, "Ren": 1, "Other": 1}, "adjectives": {"From": 1, "Sensor": 1, "None": 1}, "adverbs": {"Regular": 1}, "numerals": {"61": 1}, "pronouns": {"And": 1}}}, {"content": "62. Seeking understanding and compassion from others", "word_count": 10, "label": "", "sentence_structure": "Subject鈥搗erb鈥搊bject", "subjective_objective": {"answer": "Subjective", "rationale": "Seeking understanding and compassion is the patient ' s subjective attitude and expectations."}, "sentence_sentiment": {"answer": "Positive", "rationale": "Because seeking understanding and compassion is an act that people often adopt when facing a dilemma, it expresses the positive attitude and emotional need of people to seek help in facing problems."}, "language_style": {"answer": "Tactful and warm", "rationale": "The entry is expressed by using the words \"seek\" and \"understanding and compassion\" and conveys a message that needs help and support, with a more euphemism and a more euphemism of language, with a meaning of request, and a feeling of warmth and compassion."}, "time_dimension": {"answer": "Present", "rationale": "The keyword \"seeking understanding and compassion from others\" indicates the conduct or attitude being followed by the person being tested, so the temporal dimension of the article is now."}, "sentence_type": "Imperative sentence", "pos": {"nouns": {"Understood": 1, "Compassion": 1}, "verbs": {"Seek": 1}, "numerals": {"62": 1}}}]}

{"questionnaire_title": "Patient depression self-assessment tool (PHQ-9)", "questionnaire_category": "Mental health", "item_count": 9, "emotion_shift_overall": "No obvious trend", "language_style_overall": "Neutral", "items": [{"content": "One is not strong or interested in doing things.", "word_count": 12, "label": "", "sentence_structure": "Subject鈥搗erb鈥揷omplement", "subjective_objective": {"answer": "Subjective", "rationale": "Because words like \"uninterested\" and \"not interested\" express a subjective feeling and attitude."}, "sentence_sentiment": {"answer": "Negative", "rationale": "Because the entry \"uninterested or uninterested\" is a manifestation of negative emotions and attitudes."}, "language_style": {"answer": "Tactful and warm", "rationale": ""}, "time_dimension": {"answer": "Present", "rationale": ""}, "sentence_type": "Negative declarative sentence", "pos": {"nouns": {"Yes": 2, "Powerful": 1, "Fun": 1}, "verbs": {"Do it": 1, "T": 1}, "numerals": {"One": 1}}}, {"content": "2 feeling depressed, depressed, or desperate.", "word_count": 11, "label": "", "sentence_structure": "Subject鈥揷opula鈥損redicative", "subjective_objective": {"answer": "Subjective", "rationale": "Because this emotional feeling is an inner experience of a human being, it is a subjective examination."}, "sentence_sentiment": {"answer": "Negative", "rationale": "Because \"depressed,\" \"depressed\" and \"depressed\" are negative emotional terms."}, "language_style": {"answer": "Tactful and warm", "rationale": ""}, "time_dimension": {"answer": "Present", "rationale": "The \"feel\" in the entry indicates that it is a subjective feeling that is being made and that it can therefore be judged to describe the current emotional state."}, "sentence_type": "Imperative sentence", "pos": {"nouns": {"The mood": 1, "Frustration": 1, "Despair": 1}, "verbs": {"I feel": 1, "Down": 1}, "adjectives": {"Despair": 1}, "classifiers": {"2": 1}}}, {"content": "3 sleep difficulties, sleep disorders or sleep problems", "word_count": 12, "label": "", "sentence_structure": "Subject鈥搗erb鈥揷omplement", "subjective_objective": {"answer": "Subjective", "rationale": "Because of the difficulty of sleeping, the lack of sleep or the greater sleep, it is likely that the patient has subjective feelings and experiences."}, "sentence_sentiment": {"answer": "Negative", "rationale": "Because the entries are \"sleeping difficulties, sleep disorders\" and so on are negative emotions and attitudes."}, "language_style": {"answer": "Calm and direct", "rationale": "The entry is more concise, is not subject to excessive rhetoric or emotional rendering, and directly expresses the subject of asking the other person whether he or she has sleeping difficulties, sleep difficulties or sleep overdoses."}, "time_dimension": {"answer": "Present", "rationale": "Now the entry asks if the person tested has a \"sleeping difficulty\" or \"sleeping\" or \"sleeping more\" situation, which is all about the state of the moment. So the time dimension is now."}, "sentence_type": "Affirmative declarative sentence", "pos": {"nouns": {"3": 1, "Sleeping difficulties": 1, "I can't sleep": 1, "Sleeping more": 1}, "numerals": {"More": 1}, "classifiers": {"individual": 1}}}, {"content": "Four is tired or not alive.", "word_count": 9, "label": "", "sentence_structure": "Subject鈥揷opula鈥損redicative", "subjective_objective": {"answer": "Subjective", "rationale": "For fatigue and lack of dynamism are subjective feelings, not objective facts or acts."}, "sentence_sentiment": {"answer": "Negative", "rationale": "Because \"tired\" and \"not alive\" are negative emotional experiences, the expression is negative."}, "language_style": {"answer": "Tactful and warm", "rationale": "The entry, in a mild and caring tone, asks whether the interviewees are often tired or insufficiently dynamic, rather than directly accusing or ordering, is more personal and interactive. At the same time, the term \"feel\" is used more euphemistically to express the subject of the problem and avoids too direct or strong language."}, "time_dimension": {"answer": "Present", "rationale": "The entry is about the current feelings and state of the subject, the keywords being \"flashed\" and \"not alive\", all of which are descriptions of the current state of mind and mind, and therefore the judgement is now."}, "sentence_type": "Affirmative declarative sentence", "pos": {"nouns": {"Feel": 1, "Tired": 1, "Dynamic": 1}, "verbs": {"have": 1}, "adjectives": {"No, I'm not": 1}, "numerals": {"4": 1}}}, {"content": "Five is either hungry or eating too much.", "word_count": 8, "label": "", "sentence_structure": "Subject鈥損redicate", "subjective_objective": {"answer": "Subjective", "rationale": "Because \"failure or eating too much\" is the patient's own subjective feelings and behaviour, it looks at the patient's inner experience and behaviour. While these feelings and behaviours may be influenced by objective circumstances, they are still subjective in themselves."}, "sentence_sentiment": {"answer": "Negative", "rationale": "Because \"failed appetite\" and \"too much eating\" are negative physiological conditions."}, "language_style": {"answer": "Tactful and warm", "rationale": "The entry leads the interviewee to reflect on his eating habits in a question-and-answer manner, without directly evaluating or criticizing the other, but rather asking questions in a caring and understanding manner, with a relatively euphemism and moderation of language style."}, "time_dimension": {"answer": "Past", "rationale": ""}, "sentence_type": "Negative declarative sentence", "pos": {"nouns": {"A appetite": 1, "More": 1}, "verbs": {"It's not working": 1, "Eat": 2}, "adverbs": {"Too much": 1}, "numerals": {"5": 1}}}, {"content": "Six thinks they're bad -- or they think they're bad, or they let themselves or their families down.", "word_count": 23, "label": "", "sentence_structure": "Subject鈥搗erb鈥搊bject", "subjective_objective": {"answer": "Subjective", "rationale": "Because the words \"fear\" and \"disappointed\" are the expression of the patient's own feelings and emotions, it is viewed from the patient's subjective perspective."}, "sentence_sentiment": {"answer": "Negative", "rationale": "Because the words \"felt bad\" in the entries, \"felt failed\" and \"defeating yourself or family\" all express negative feelings and attitudes, the purpose of the article is negative."}, "language_style": {"answer": "Calm and direct", "rationale": "The language style of the entry is calm and straightforward, because it clearly indicates whether the subject of the inquiry often feels bad, fails, or disappoints others, without using excessive rhetorical or emotional techniques. This expression is both concise and objective, and helps the subject to understand and answer the questions accurately."}, "time_dimension": {"answer": "Present", "rationale": "The entry asks the testee about his current feelings and mental state, and the time dimension is now judged."}, "sentence_type": "Negative declarative sentence", "pos": {"nouns": {"self": 2, "Family": 1, "Failed": 1, "Disappointment": 1}, "verbs": {"I think": 3, "Jean": 1}, "adjectives": {"Shit": 1}, "numerals": {"6": 1}}}, {"content": "7 has difficulty focusing on things, like reading newspapers or watching TV", "word_count": 19, "label": "", "sentence_structure": "Subject鈥搗erb鈥揷omplement", "subjective_objective": {"answer": "Subjective", "rationale": "Because \"it's difficult to focus on things\" is a patient's self-perception and subjective feeling. While reading newspapers or watching television is objective, whether the patient can concentrate on doing these things is subjective experience and feeling. So, the questionnaire entry is viewed from the patient's subjective point of view."}, "sentence_sentiment": {"answer": "Negative", "rationale": "Because the action of \"difficult to focus on things\" in the entry is negative."}, "language_style": {"answer": "Calm and direct", "rationale": "The entry is based on a simple and straightforward language that directly expresses the subject matter, is not based on excessive rhetorical or emotional rendering, and is more objective in tone. At the same time, the purpose of the article is clearly structured, is simple to understand and meets the scientific and objective requirements of psychological questionnaires."}, "time_dimension": {"answer": "Present", "rationale": "The entry \"7\" indicates a frequency level, while the description \"focusing on difficult\" or \"reading newspapers or watching television\" is for current behaviour or circumstances. Thus, the time dimension of the entry can be judged as now."}, "sentence_type": "Interrogative sentence", "pos": {"nouns": {"Things": 1, "Focus": 1, "Difficulties": 1, "Reading": 1, "Newspapers": 1, "Television": 1}, "verbs": {"have": 1, "For example": 1, "Look": 1}, "numerals": {"7": 1, "or": 1}}}, {"content": "Eight moves or talking slow enough to be seen? Or the opposite -- more agitated or restless, moving than usual.", "word_count": 40, "label": "", "sentence_structure": "Subject鈥搗erb鈥揷omplement", "subjective_objective": {"answer": "Subjective", "rationale": "Because \"slow motion or speech\", \"freak or restless, moving more than usual\" is the patient's subjective feeling and observation, not the objective fact."}, "sentence_sentiment": {"answer": "Neutral", "rationale": "Because the entry does not have any emotional color."}, "language_style": {"answer": "Calm and direct", "rationale": "The language style of the entry is more objective, with no excessive emotional colours or rhetorical techniques. It describes the possible behaviour problems of the person under investigation in a straightforward manner and provides two extremes for reference. This language style is more of a calm, rational and practical one, suitable for official settings such as psychological questionnaires."}, "time_dimension": {"answer": "Present", "rationale": "The entry can be judged by the time characteristics of \"emerging\" and \"ongoing\" in terms of the person's current behaviour and state. The keyword \"slow or speaking\" or \"tired or restless\" or \"scrambling\" indicates that this is a description of the person's current behaviour and is a state of affairs."}, "sentence_type": "Exclamatory sentence", "pos": {"nouns": {"Actions": 2, "Talk speed": 1, "person": 1, "Entry": 1, "I'm so upset": 1, "Sit tight": 1, "Move around": 1, "Situation": 1, "Normal": 1}, "verbs": {"Observe": 1, "On the contrary": 1, "Better than": 1}, "adverbs": {"More": 1}, "numerals": {"8": 1}}}, {"content": "It's better to die or to hurt yourself in some way.", "word_count": 18, "label": "", "sentence_structure": "Subject鈥搗erb鈥搊bject", "subjective_objective": {"answer": "Subjective", "rationale": "Because the entry asks patients about subjective ideas, about \"not as dead as dead.\""}, "sentence_sentiment": {"answer": "Negative", "rationale": "Because the behavior and idea of \"not as good as death or harming yourself in some way\" in the entry is negative."}, "language_style": {"answer": "Tactful and warm", "rationale": "The entry is more personal and interactive in its expression of a moderate and sensitive approach to the other鈥檚 feelings, rather than a direct statement or order. It also expresses the importance of caring about the mental health of the interviewee, with a certain temperature in the language style."}, "time_dimension": {"answer": "Future", "rationale": "The word \"yes\" in the entry uses the current state of affairs, indicating that the act occurs at the moment of speaking, while the idea \"not to die or to harm yourself in some way\" is to imagine and expect the future, the key words being \"not to die\" and \"harm yourself,\" all pointing to situations that have not yet occurred and that may occur in the future. Thus, the entry concerns the temporal dimension of the future."}, "sentence_type": "Negative declarative sentence", "pos": {"nouns": {"Thinking": 1, "Die": 1}, "verbs": {"have": 1, "Hurt": 1}, "adjectives": {"Some": 1}, "adverbs": {"not": 1}, "numerals": {"9": 1}}}]}

{"questionnaire_title": "Health status tables", "questionnaire_category": "Mental health", "item_count": 34, "emotion_shift_overall": "From positive to negative", "language_style_overall": "Neutral", "items": [{"content": "2 and 1 year ago, you felt your health condition was as follows:", "word_count": 16, "label": "", "sentence_structure": "Subject鈥搗erb鈥揷omplement", "subjective_objective": {"answer": "Objective", "rationale": "As a comparison of the patient ' s current and past health status is made, objective facts are examined."}, "sentence_sentiment": {"answer": "Neutral", "rationale": "Since the entry merely asked the respondents about their health relative to a year ago, it did not involve emotional attitudes or behavioural evaluations and was therefore neutral."}, "language_style": {"answer": "Calm and direct", "rationale": "The entry is based on a direct inquiry, without excessive rhetorical or emotional rendering. The subject and purpose of the investigation are communicated to the person surveyed in a simple and clear language, which is objective and airtight."}, "time_dimension": {"answer": "Past", "rationale": "The \"symmetry\" in this entry represents a comparison with the past, so the temporal dimension is the past."}, "sentence_type": "Interrogative sentence", "pos": {"nouns": {"you": 1, "self": 1, "Health status": 1}, "verbs": {"I think": 1, "be": 1, "- Yeah": 1, "1 year ago": 1}, "adjectives": {"Call": 1}, "numerals": {"2": 1, "1": 2}, "classifiers": {"Before": 1}}}, {"content": "(1) Heavy physical activity.", "word_count": 17, "label": "", "sentence_structure": "Subject鈥搗erb鈥搊bject", "subjective_objective": {"answer": "Objective", "rationale": "Because \"heavy activity\" is an objective act and activity that the patient needs to perform, it is not necessary for the patient to make subjective judgements."}, "sentence_sentiment": {"answer": "Neutral", "rationale": "As the entry describes only a type of activity, it does not involve any positive or negative emotional or behavioural tendencies and is therefore neutral."}, "language_style": {"answer": "Calm and direct", "rationale": "The entry clearly indicates whether the person under investigation was involved in heavy physical activity, without using excessive rhetorical or emotional colours and with a clear tone."}, "time_dimension": {"answer": "Past", "rationale": "The entry is for the purpose of asking the testee about past physical exercise and the temporal dimension is a past."}, "sentence_type": "Declarative sentence", "pos": {"nouns": {"Physical activity": 1, "Run": 1, "Lift": 1, "Sports": 2}, "verbs": {"Like": 1, "Participation": 1, "Wait": 1}, "numerals": {"1": 2, "2": 1}, "classifiers": {"individual": 1}}}, {"content": "(2) Moderate activity.", "word_count": 24, "label": "", "sentence_structure": "Subject鈥搗erb鈥搊bject", "subjective_objective": {"answer": "Objective", "rationale": "This is because it is an objective examination of whether the patient has performed some specific physical activity."}, "sentence_sentiment": {"answer": "Positive", "rationale": "Because \"proper activity\" can bring physical and psychological benefits, and it's a positive act."}, "language_style": {"answer": "Tactful and warm", "rationale": "The language style of the entry is more moderate, with a recommendatory tone to guide respondents to appropriate activities and without too direct or command-style tone of expression, which is more readily acceptable to the respondents and willing to cooperate."}, "time_dimension": {"answer": "Present", "rationale": "The entry describes some of the physical activities that the person being tested can carry out at present, which do not require too much physical strength and skill, and therefore is judged to be present."}, "sentence_type": "Interrogative sentence", "pos": {"nouns": {"Activities": 2, "A table": 1, "Sweep": 1, "Tai Chi Boxing": 1, "Gymnastics": 1}, "verbs": {"Like": 1, "Move": 1, "Sweep": 1, "Fight": 1, "Do it": 1}, "adjectives": {"Moderate": 1}, "numerals": {"2": 1}, "classifiers": {"Chang": 1}}}, {"content": "(3) Hand-held items.", "word_count": 11, "label": "", "sentence_structure": "Subject鈥搗erb鈥揷omplement", "subjective_objective": {"answer": "Objective", "rationale": "The handheld items are a specific activity and are observed from an objective perspective."}, "sentence_sentiment": {"answer": "Neutral", "rationale": "Because the entry describes only a routine act, it does not involve any positive or negative emotional colour."}, "language_style": {"answer": "Tactful and warm", "rationale": "The language style of the entry is more euphemistic. By asking the other side whether it needs to buy daily goods in everyday life, rather than directly making statements or orders, this expression is more personal and interactive, making it easier for the interviewees to accept and share their own reality."}, "time_dimension": {"answer": "Present", "rationale": "This entry describes a common activity in daily life, namely, the use of hand-held goods for the purchase of food and daily items."}, "sentence_type": "Imperative sentence", "pos": {"nouns": {"Handheld": 1, "Daily supplies": 1, "Buying food": 1, "Shopping": 1}, "verbs": {"Like": 1, "Buy": 1, "Wait": 1}, "numerals": {"3": 1}, "classifiers": {"individual": 1}}}, {"content": "(4) Upstairs:", "word_count": 5, "label": "", "sentence_structure": "Subject鈥搗erb鈥搊bject", "subjective_objective": {"answer": "Objective", "rationale": "Because the stairs above are objective acts that are not influenced by subjective will."}, "sentence_sentiment": {"answer": "Neutral", "rationale": "The entry was merely a question of the person ' s floor information and did not address any emotional or attitudinal tendencies."}, "language_style": {"answer": "Calm and direct", "rationale": "The entry is simple and clear in asking the respondents how many stairs they normally have to climb, without too many rhetorical or emotional renderings, and in a more objective and objective manner."}, "time_dimension": {"answer": "Past", "rationale": "The entry asks about the physical activity of the person tested in the past, and the temporal dimension is the past."}, "sentence_type": "Imperative sentence", "pos": {"nouns": {"Stairs": 1, "Layer": 1}, "verbs": {"Go, go, go": 1}, "adverbs": {"How many": 1}, "numerals": {"4": 1}, "classifiers": {"individual": 1}}}, {"content": "(5) Upstairs:", "word_count": 5, "label": "", "sentence_structure": "Subject鈥搗erb鈥搊bject", "subjective_objective": {"answer": "Objective", "rationale": "Because the behaviour on the stairs is objective and not related to the patient ' s subjective attitude."}, "sentence_sentiment": {"answer": "Neutral", "rationale": "Because the entry is not clearly emotional."}, "language_style": {"answer": "Tactful and warm", "rationale": "But from the point of view of the problem itself, the \"upstairs\" are a more direct and objective issue, and there is no excessive emotional overturn or rhetorical use. So the language style of the problem can be considered more calm and straightforward."}, "time_dimension": {"answer": "Present", "rationale": "The entry now describes the current behaviour or experience of the testee, and the keyword \"upstairs\" means that this is an ongoing move and therefore the judgement is now."}, "sentence_type": "Imperative sentence", "pos": {"nouns": {"Stairs": 1}, "verbs": {"Go, go, go": 1}, "adverbs": {"One": 1}, "numerals": {"Five": 1}}}, {"content": "(6) Bend over, knees down, knees down:", "word_count": 6, "label": "", "sentence_structure": "Subject鈥搗erb鈥揷omplement", "subjective_objective": {"answer": "Subjective", "rationale": "Since the patient ' s perception and subjective evaluation are examined, it is difficult or painful to feel these movements."}, "sentence_sentiment": {"answer": "Neutral", "rationale": "Because it's not clearly emotional."}, "language_style": {"answer": "Calm and direct", "rationale": "The entry is in direct, concise language, which asks the respondents whether they often do the \"toughing, kneeing, crouching\" in their daily lives, and the language style is more objective and calm. There is no excessive emotional overturn or rhetorical use, and more emphasis is placed on the accuracy and efficiency of information transmission."}, "time_dimension": {"answer": "Present", "rationale": "The entry asks about the current state of the person ' s health and abilities, and therefore the temporal dimension is now judged."}, "sentence_type": "Imperative sentence", "pos": {"nouns": {"Bend over": 1, "Knees": 1, "Down": 1}, "numerals": {"6": 1}}}, {"content": "(7) Walking over 1,500 metres:", "word_count": 8, "label": "", "sentence_structure": "Subject鈥搗erb鈥揷omplement", "subjective_objective": {"answer": "Objective", "rationale": "Because walking over 1,500 metres is a specific distance and action, it is not determined by the patient ' s subjective judgement or perception."}, "sentence_sentiment": {"answer": "Neutral", "rationale": "Because the entry merely describes a fact and does not address positive or negative attitudes, behaviour, etc."}, "language_style": {"answer": "Calm and direct", "rationale": "The entry is based on a direct and explicit query about whether the person interviewed can walk at a distance of 1,500 metres or more, without using excessive accompaniment or emotional colour, and is more objective and direct. This language style is more suitable for scientific research or practical investigation to ensure the accuracy and effectiveness of the problem."}, "time_dimension": {"answer": "Present", "rationale": "The entry asks if the person tested is currently able to walk more than 1,500 metres, and the time dimension is now judged."}, "sentence_type": "Imperative sentence", "pos": {"nouns": {"Walk": 1, "1,500 meters": 1, "Road": 1}, "verbs": {"EMPTY": 1}, "adverbs": {"EMPTY": 1}, "numerals": {"1500": 1}, "classifiers": {"Rice": 1, "of": 1}}}, {"content": "(8) 1,000 metres walk:", "word_count": 6, "label": "", "sentence_structure": "Subject鈥搗erb鈥揷omplement", "subjective_objective": {"answer": "Objective", "rationale": "Because the 1,000-metre walk is an objective fact that can be measured and observed in real terms."}, "sentence_sentiment": {"answer": "Neutral", "rationale": "Because the entry does not contain any clear information about emotion, attitude, behaviour, etc., it merely describes an objective fact."}, "language_style": {"answer": "Calm and direct", "rationale": "The language style of the entry is more blunt, it is not expressed with too much adoration or emotional colour, it is more like a clear instruction or regulation, which gives a sense of indifference and distance."}, "time_dimension": {"answer": "Future", "rationale": "The entry asks if the person tested will be able to perform a future mission 鈥?walking 1,000 metres 鈥?and therefore the temporal dimension is the future."}, "sentence_type": "Imperative sentence", "pos": {"nouns": {"Walk": 1, "1000 meters": 1, "Road": 1}, "numerals": {"8": 1, "1000": 1}}}, {"content": "(9) 100 m walk:", "word_count": 6, "label": "", "sentence_structure": "Subject鈥搗erb鈥揷omplement", "subjective_objective": {"answer": "Subjective", "rationale": "Since walking a 100-metre distance is the patient ' s subjective experience and feelings, the physical condition and mobility of the patient are examined."}, "sentence_sentiment": {"answer": "Neutral", "rationale": "The entry does not express any emotional colour but rather describes an objective act or situation."}, "language_style": {"answer": "Tactful and warm", "rationale": "The entry is more euphemistic and warm. First, it uses a simple language expression, without too complex or difficult to understand professional terms. Second, it is also soft and friendly, as it is in a friendly dialogue with the interviewee. Finally, the term for the purpose of the article is not accompanied by any mandatory or command-style tone, which makes the interviewee feel more comfortable and comfortable."}, "time_dimension": {"answer": "Past", "rationale": ""}, "sentence_type": "Imperative sentence", "pos": {"nouns": {"Walk": 1, "Road": 1, "100 meters": 1}, "numerals": {"100": 1}, "classifiers": {"Rice": 1}}}, {"content": "(10) Taking a bath and dressing themselves:", "word_count": 6, "label": "", "sentence_structure": "Subject鈥搗erb鈥揷omplement", "subjective_objective": {"answer": "Subjective", "rationale": "Because \"self bathing, dressing\" is a personal custom and will of a person and belongs to the patient's subjective attitude and behaviour."}, "sentence_sentiment": {"answer": "Neutral", "rationale": "The entry is merely an interview with the interviewee about his bathing and clothes and does not involve any positive or negative emotional aspects."}, "language_style": {"answer": "Calm and direct", "rationale": "The entry directly asked whether the person surveyed could perform the basic life skills of bathing and dressing on his own, with objectivity and without excessive rhetorical modifications. The language style was therefore \"cool and straight\"."}, "time_dimension": {"answer": "Present", "rationale": "The entry asks whether the person being tested is currently able to complete the basic life skills of bathing and clothing on his own, and the time dimension is now judged."}, "sentence_type": "Imperative sentence", "pos": {"nouns": {"self": 1, "Bath": 1, "Clothes": 1}, "verbs": {"Wash": 1, "Put it on": 1}, "numerals": {"10": 1}}}, {"content": "(1) Reduction of working or other activity time:", "word_count": 12, "label": "", "sentence_structure": "Subject鈥搗erb鈥揷omplement", "subjective_objective": {"answer": "Objective", "rationale": "Because reducing working or working time is the objective behaviour of patients, not their subjective thoughts or feelings."}, "sentence_sentiment": {"answer": "Negative", "rationale": "As reducing the time spent working or other activities is usually considered as a negative act."}, "language_style": {"answer": "Calm and direct", "rationale": "The entry is in a concise language and asks the respondent whether the time spent on work or activities has been reduced for some reason, without using excessive emotional acrimony or rhetoric, and in a more direct and objective manner."}, "time_dimension": {"answer": "Present", "rationale": "The entry asks the testee whether he has reduced his work or activity time over the current period, and the time dimension is now judged."}, "sentence_type": "Imperative sentence", "pos": {"nouns": {"Work": 1, "Activities": 1, "Time": 1}, "verbs": {"Reduction": 1}}}, {"content": "(2) The only thing that would have been possible had been to accomplish only partially:", "word_count": 15, "label": "", "sentence_structure": "Subject鈥搗erb鈥揷omplement", "subjective_objective": {"answer": "Subjective", "rationale": "Because \"what would have been done\" is a subjective idea or plan, and \"only part of it\" is also based on personal circumstances and environmental factors, and it is therefore a subjective perspective."}, "sentence_sentiment": {"answer": "Negative", "rationale": "Because the result of \"only part of the finish\" in the entry is negative."}, "language_style": {"answer": "Tactful and warm", "rationale": "Warm and warm."}, "time_dimension": {"answer": "Present", "rationale": ""}, "sentence_type": "Imperative sentence", "pos": {"nouns": {"Things": 1, "Part": 1}, "verbs": {"Completed": 1, "Yes": 1}, "adverbs": {"Only": 1, "It was": 1}, "numerals": {"2": 1}}}, {"content": "(3) The type of work or activity desired is restricted:", "word_count": 15, "label": "", "sentence_structure": "Subject鈥搗erb鈥搊bject", "subjective_objective": {"answer": "Subjective", "rationale": "Because \"the type of work or activity that you want to do\" belongs to the patient's inner thoughts and expectations, and it's subjective."}, "sentence_sentiment": {"answer": "Negative", "rationale": "Because the expression \"restricted\" in the entry is negative."}, "language_style": {"answer": "Tactful and warm", "rationale": "The entry is interactive and more personal by means of inquiry, without direct statements or orders. At the same time, \"type of work or activity desired\" is a more sensitive topic, and the use of euphemisms can better protect the privacy and self-esteem of the person under investigation."}, "time_dimension": {"answer": "Present", "rationale": "The entry asks if the person tested has been restricted in the choice of work and activities for physical reasons, and the time dimension is now judged."}, "sentence_type": "Declarative sentence", "pos": {"nouns": {"Work": 2, "Activities": 2, "Category": 1, "Limits": 1}, "verbs": {"EMPTY": 4, "Yes": 1, "Received": 1}, "adjectives": {"of": 3, "or": 1}, "numerals": {"锛?锛?: 1, "2": 1, "1": 3}, "pronouns": {"EMPTY": 4, "Yes": 1}}}, {"content": "(4) Increased difficulties in completing work or other activities (e.g., additional efforts required):", "word_count": 22, "label": "", "sentence_structure": "Subject鈥搗erb鈥揷omplement", "subjective_objective": {"answer": "Subjective", "rationale": "It is a subjective feeling and experience that patients are required to assess and judge themselves for the difficulty of completing their work or other activities."}, "sentence_sentiment": {"answer": "Negative", "rationale": "The phenomenon of \"increasing difficulties in completing work or other activities\" in the entries is negative and implies that respondents may encounter problems and challenges in their work or activities."}, "language_style": {"answer": "Calm and direct", "rationale": "The language style of the entry is more direct, with no excessive rhetoric or emotional rendering, but rather a simple and clear language. At the same time, it is more objective, not bringing personal emotions or subjective judgements, and more in line with the professional requirements of the questionnaire."}, "time_dimension": {"answer": "Present", "rationale": "The entry asks about the current status and feelings of the testee, and the time dimension is now judged."}, "sentence_type": "Affirmative declarative sentence", "pos": {"nouns": {"Work": 1, "Activities": 1}, "verbs": {"Completed": 1, "Increase": 1}, "adjectives": {"Difficulties": 1, "Extra": 1}, "numerals": {"4": 1, "Other": 1}}}, {"content": "(1) Reduction of working or activity time:", "word_count": 10, "label": "", "sentence_structure": "Subject鈥搗erb鈥揷omplement", "subjective_objective": {"answer": "Objective", "rationale": "Since reducing working or working hours is an objective fact of the patient ' s experience, whether the patient is subjective or not."}, "sentence_sentiment": {"answer": "Negative", "rationale": "This behaviour is negative because of the risk of inefficiencies and adverse consequences of reducing the time spent on work or activities."}, "language_style": {"answer": "Calm and direct", "rationale": ""}, "time_dimension": {"answer": "Present", "rationale": ""}, "sentence_type": "Declarative sentence", "pos": {"nouns": {"Questionnaire Entry": 1, "Work": 1, "Activity Time": 1}, "verbs": {"Decreased": 1}}}, {"content": "(2) The only thing that would have been possible had been to accomplish only partially:", "word_count": 15, "label": "", "sentence_structure": "Subject鈥搗erb鈥揷omplement", "subjective_objective": {"answer": "Subjective", "rationale": "Because \"what would have been done was done only in part\" is a patient's subjective feeling or experience, not an objective fact."}, "sentence_sentiment": {"answer": "Negative", "rationale": "Because the \"only part of the\" in the entry is negative."}, "language_style": {"answer": "Calm and direct", "rationale": "The entry is simple and simple, and it is more objective and insensitive to ask if the other party has had experience of doing only part of what was planned for various reasons, without using too much qualifier or emotional expressions."}, "time_dimension": {"answer": "Present", "rationale": "The keyword \"the only thing that would have done\" meant the act or state of conduct that was currently being carried out, and the temporal dimension of the article was therefore judged to be the present."}, "sentence_type": "Negative declarative sentence", "pos": {"nouns": {"Things": 1, "Part of": 1}, "verbs": {"Yes": 1, "Completed": 1}, "numerals": {"Two": 1}}}, {"content": "(3) The officers should not be as careful as they normally are:", "word_count": 9, "label": "", "sentence_structure": "Subject鈥搗erb鈥揷omplement", "subjective_objective": {"answer": "Subjective", "rationale": "Because \"officers are less careful than usual\" is a subjective feeling or an evaluation that reflects patients鈥?attitudes and perceptions of doing things. While this may be related to objective factors (e.g. fatigue, stress, etc.), it is essentially a subjective experience or attitude."}, "sentence_sentiment": {"answer": "Negative", "rationale": "Because the \"not as careful as usual\" act in the entry is negative."}, "language_style": {"answer": "Calm and direct", "rationale": "The entry is expressed in straightforward language, with no excessive qualifier or emotional expression, which is more objective and less intuitive and more in line with the psychological questionnaire."}, "time_dimension": {"answer": "Present", "rationale": "The entry indicates that the testee has a \"not as careful as usual\" situation in his current state and therefore the test is now judged."}, "sentence_type": "Negative declarative sentence", "pos": {"nouns": {"Things": 1, "Normal": 1, "Careful": 1}, "verbs": {"Fuck": 1}, "adverbs": {"Why don't you": 1, "Normal": 1}}}, {"content": "7 - Have you had any physical pain in the last 4 weeks?", "word_count": 14, "label": "", "sentence_structure": "Subject鈥搗erb鈥搊bject", "subjective_objective": {"answer": "Subjective", "rationale": "Because physical pain is the patient ' s own perception and experience and is subjective."}, "sentence_sentiment": {"answer": "Neutral", "rationale": "Since the entry merely asks whether there has been physical pain in the past four weeks, it does not involve any emotional attitudes or behavioural tendencies and is therefore neutral."}, "language_style": {"answer": "Calm and direct", "rationale": "The language style of the entry is more calm and objective, and there is no excessive emotional rendering or rhetorical technique. It is simple and clear to ask if the person under investigation has experienced physical pain in the past four weeks.\""}, "time_dimension": {"answer": "Past", "rationale": ""}, "sentence_type": "Interrogative sentence", "pos": {"nouns": {"Past": 1, "Week": 1, "Body": 1, "Pain": 1}, "verbs": {"have": 1}, "adverbs": {"Four": 1}, "numerals": {"7": 1, "4": 1}, "classifiers": {"Week": 1, "individual": 1}}}, {"content": "Have your physical pains affected your work and your housework in the last four weeks?", "word_count": 24, "label": "", "sentence_structure": "Subject鈥搗erb鈥搊bject", "subjective_objective": {"answer": "Objective", "rationale": "It can be demonstrated by data or observations whether physical pain affects work and household chores as an objective fact of the patient ' s experience."}, "sentence_sentiment": {"answer": "Negative", "rationale": "Because \"physical pain affects your work and your housework\" in the entry is a negative expression, meaning that physical incomprehension has a negative impact on life."}, "language_style": {"answer": "Tactful and warm", "rationale": "The entry asks the other side, in a caring and understanding manner, whether the physical pain of the past four weeks has affected daily life and work, rather than direct orders or accusations, which is more easily expressed by the interviewee to open his heart and provide a true answer."}, "time_dimension": {"answer": "Past", "rationale": ""}, "sentence_type": "Interrogative sentence", "pos": {"nouns": {"Past": 1, "4 weeks": 1, "you": 2, "Body": 1, "Pain": 1, "Impact": 1, "Work": 1, "Domestic work": 1}, "verbs": {"Lee": 1, "have": 1, "- You're not": 1, "It's affecting": 1}, "numerals": {"Four": 1}, "pronouns": {"Past": 1}}}, {"content": "(1) You feel that life is full:", "word_count": 7, "label": "", "sentence_structure": "Subject鈥揷opula鈥損redicative", "subjective_objective": {"answer": "Subjective", "rationale": "Because \"fear\" and \"full\" are subjective feelings and evaluations of patients."}, "sentence_sentiment": {"answer": "Neutral", "rationale": "Because the entry merely asked the respondents whether they had a good life, there was no apparent positive or negative tendency."}, "language_style": {"answer": "Calm and direct", "rationale": "The entry directly asks the respondents about their own lives, without any rhetorical or emotional rendering, and is more linguistically sound and straightforward."}, "time_dimension": {"answer": "Present", "rationale": "The entry is for the purpose of asking the testee about his or her own current state of life, and the temporal dimension is now judged."}, "sentence_type": "Interrogative sentence", "pos": {"nouns": {"Life": 1, "Fill": 1}, "verbs": {"I think": 1, "Fill": 1}}}, {"content": "(2) You are a sensitive person:", "word_count": 8, "label": "", "sentence_structure": "Subject鈥揷opula鈥損redicative", "subjective_objective": {"answer": "Subjective", "rationale": "Because \"sensitivity\" is a subjective feeling and evaluation, different people may have different views."}, "sentence_sentiment": {"answer": "Neutral", "rationale": "Because the entry does not express any apparent emotional tendency, it is merely asking whether the other party has some kind of quality."}, "language_style": {"answer": "Tactful and warm", "rationale": "The entry, which guides the interviewee to self-awareness in a question-and-answer manner, does not directly evaluate or point fingers, is relatively gentle and friendly, and has a certain degree of kinship and interaction."}, "time_dimension": {"answer": "Present", "rationale": "The keyword \"is\" and \"a sensitive person\" indicates that it is a description of the current characteristics of the subject and is of the current temporal dimension."}, "sentence_type": "Interrogative sentence", "pos": {"nouns": {"person": 1, "Questionnaire": 1, "Entry": 1}, "verbs": {"be": 1, "Answer": 1}, "adjectives": {"Sensitive": 1}, "numerals": {"2": 1}}}, {"content": "(3) Your emotions are very bad, and nothing can please you:", "word_count": 20, "label": "", "sentence_structure": "Subject鈥揷opula鈥損redicative", "subjective_objective": {"answer": "Subjective", "rationale": "Because \"very bad mood\" and \"not happy\" are the emotional state that patients feel themselves, and it's a subjective examination."}, "sentence_sentiment": {"answer": "Negative", "rationale": "Because the emotional \"very bad\" in the entry is negative."}, "language_style": {"answer": "Calm and direct", "rationale": "The entry is presented in a direct way, clearly asking the interviewee about his emotional state, without adding too much adoration or emotional colour, and thus judging his language style as being straightforward."}, "time_dimension": {"answer": "Present", "rationale": "The entry describes the current emotional state of the testee, which is \"very bad\" and \"nothing will please you,\" so the temporal dimension is now."}, "sentence_type": "Interrogative sentence", "pos": {"nouns": {"Emotion": 1, "Yes": 1, "Happy": 1}, "verbs": {"鈾?And I'm gonna be 鈾?: 1}, "adjectives": {"Very": 1, "Okay": 1, "not": 1, "No, I can't": 1}, "adverbs": {"Both": 1, "Rise": 1}, "numerals": {"3": 1}}}, {"content": "(4) Your mind is calm:", "word_count": 7, "label": "", "sentence_structure": "Subject鈥揷opula鈥損redicative", "subjective_objective": {"answer": "Subjective", "rationale": "Because mental state is the patient's subjective feeling and experience."}, "sentence_sentiment": {"answer": "Positive", "rationale": "Because \"psychiatric peace\" is a positive emotional state."}, "language_style": {"answer": "Tactful and warm", "rationale": "The language style of the entry is relatively euphemistic and warm. First, it does not use the negative word directly to describe the situation of the person surveyed, but rather to direct the interviewer to answer the question with a positive positive tone, making it easier for the interviewee to accept and be willing to participate in the investigation. Second, the expression \"Your mind is calm\" is also more friendly and friendly, which reduces the tension of the interviewee and makes it more natural to answer the question."}, "time_dimension": {"answer": "Present", "rationale": "The entry describes the current psychological state of the subject, and the keyword \"silent\" indicates that it is a subjective feeling, which is an expression of the emotional experience of the moment."}, "sentence_type": "Declarative sentence", "pos": {"verbs": {"have": 1, "very": 1}, "adjectives": {"Calm down": 1}, "numerals": {"4": 1}}}, {"content": "(5) You are energetic:", "word_count": 7, "label": "", "sentence_structure": "Subject鈥搗erb鈥揷omplement", "subjective_objective": {"answer": "Subjective", "rationale": "Because \"absorbed\" is a subjective attitude or state of mind that patients feel or self-evaluate."}, "sentence_sentiment": {"answer": "Neutral", "rationale": "Because the entry does not have any emotional color."}, "language_style": {"answer": "Tactful and warm", "rationale": "The language style of the entry is more euphemistic, leading the respondents to answer questions by asking questions and avoiding to some extent the use of direct or compulsory expressions, thereby increasing the comfort and participation of the interviewees."}, "time_dimension": {"answer": "Present", "rationale": ""}, "sentence_type": "Affirmative declarative sentence", "pos": {"verbs": {"Work": 1, "- I'm very energetic": 1}, "adjectives": {"鈾?Full of 鈾?: 1}}}, {"content": "(6) Your mood is low:", "word_count": 6, "label": "", "sentence_structure": "Subject鈥搗erb鈥揷omplement", "subjective_objective": {"answer": "Subjective", "rationale": "Because depression is the patient's subjective judgement and description of his or her current emotional state."}, "sentence_sentiment": {"answer": "Negative", "rationale": "Because the term \"moral depression\" is usually associated with negative emotions."}, "language_style": {"answer": "Calm and direct", "rationale": "The reason is as follows: the article is simple and plain, without too many rhetorical and emotional expressions. At the same time, it directly raises questions about whether the respondents were in a low mood, were straightforward and did not turn the corner, and therefore could be considered as a calm and straightforward language style."}, "time_dimension": {"answer": "Past", "rationale": "The entry is for the purpose of asking the testee whether he has had a low-temporal experience, and the time dimension is judged by past and present."}, "sentence_type": "Interrogative sentence", "pos": {"nouns": {"Emotion": 1, "Down": 1}, "adverbs": {"EMPTY": 2}, "numerals": {"6": 1}}}, {"content": "(7) You feel exhausted:", "word_count": 7, "label": "", "sentence_structure": "Subject鈥揷opula鈥損redicative", "subjective_objective": {"answer": "Subjective", "rationale": "Because the problem involves the feelings and perceptions of the patient, it is a subjective examination."}, "sentence_sentiment": {"answer": "Negative", "rationale": "Because \"tired\" is a negative emotional experience, the entry is negative."}, "language_style": {"answer": "Calm and direct", "rationale": "This is a more neutral and calm expression. This expression helps the interviewees to understand and answer questions more accurately."}, "time_dimension": {"answer": "Present", "rationale": "The entry asks the testee ' s current perception in answering the question, and the time dimension is now judged."}, "sentence_type": "Interrogative sentence", "pos": {"nouns": {"I'm exhausted": 1}, "verbs": {"I think": 1}}}, {"content": "(8) You are a happy man:", "word_count": 7, "label": "", "sentence_structure": "Subject鈥揷opula鈥損redicative", "subjective_objective": {"answer": "Subjective", "rationale": "Because \"happy\" is a subjective emotional state."}, "sentence_sentiment": {"answer": "Neutral", "rationale": "Because the entry does not have any emotional color."}, "language_style": {"answer": "Tactful and warm", "rationale": "The entry is more adept and interactive when it is spoken in a gentle and inviting manner, rather than by direct statements or orders."}, "time_dimension": {"answer": "Present", "rationale": "The entry is for questioning the personality or self-perception of the person being tested and belongs to the present."}, "sentence_type": "Interrogative sentence", "pos": {"nouns": {"person": 1, "Happy": 1}, "adjectives": {"individual": 1}, "numerals": {"8": 1}}}, {"content": "(9) You are bored:", "word_count": 5, "label": "", "sentence_structure": "Subject鈥搗erb鈥揷omplement", "subjective_objective": {"answer": "Subjective", "rationale": "Because boredom is a subjective feeling."}, "sentence_sentiment": {"answer": "Negative", "rationale": "Because \"incontinent\" is a negative emotional attitude, which is a sign of dissatisfaction or displeasure with something or circumstances."}, "language_style": {"answer": "Calm and direct", "rationale": "According to the information provided, the issue is expressed in a relatively simple and straightforward manner, without the use of excessive rhetorical or emotional language, and therefore can be judged as being \"cool and direct\"."}, "time_dimension": {"answer": "Present", "rationale": "Based on the phrase \"feeling\" and \"happy,\" we can deduce that this is a question of what it is, and therefore is the present."}, "sentence_type": "Interrogative sentence", "pos": {"nouns": {"Boring": 1}, "verbs": {"Feel": 1, "Boring": 1}, "numerals": {"9": 1}}}, {"content": "10: Inhealth affects your social activities (e.g., visiting friends and family):", "word_count": 17, "label": "", "sentence_structure": "Subject鈥搗erb鈥搊bject", "subjective_objective": {"answer": "Subjective", "rationale": "This is because it concerns the subjective perception and perception of the patient of the impact of his or her own health in terms of his or her participation in society."}, "sentence_sentiment": {"answer": "Negative", "rationale": "Because the entries \"inhealth\" and \"infecting your social activities\" are both negative emotional terms, implying that the person's poor state of health has a negative impact on his or her social life."}, "language_style": {"answer": "Calm and direct", "rationale": "The entry is more concise in language, and the interviewee is asked whether the effects of ill health have reduced social activity."}, "time_dimension": {"answer": "Past", "rationale": "The entry asks whether the person tested was unable to perform some social activities in the past because of his health. The temporal dimension is therefore judged as the past.\""}, "sentence_type": "Negative declarative sentence", "pos": {"nouns": {"Questionnaire": 1, "Entry": 1, "Ill health": 1, "Impact": 1, "Social activities": 1, "Go visit your friends": 1}, "verbs": {"Yes": 1}, "numerals": {"10": 1}}}, {"content": "I'm more vulnerable to illness than anyone else:", "word_count": 10, "label": "", "sentence_structure": "Subject鈥揷opula鈥損redicative", "subjective_objective": {"answer": "Subjective", "rationale": "Because \"freaker than others\" is a self-perception or self-evaluation, a subjective view of the patient."}, "sentence_sentiment": {"answer": "Negative", "rationale": "Because the expression \"predisposition to disease\" is negative and indicates poor health."}, "language_style": {"answer": "Tactful and warm", "rationale": "The language style of this entry is euphemism. It asks questions in a more moderate and encouraging way, making the interviewee feel more comfortable and respectful, and thus more willing to share his real feelings. At the same time, the language is more neutral, without too strong a negative or blame tone, and more conducive to collecting accurate and true questionnaire results."}, "time_dimension": {"answer": "Past", "rationale": "The phrase \"I seem to be more vulnerable to illness than others\" shows that the testee's subjective perception and comparison of his health status belongs to the past, and is therefore judged to be the past."}, "sentence_type": "Interrogative sentence", "pos": {"nouns": {"Diseases": 1}, "verbs": {"It looks like": 1, "- Yeah": 1, "Sick": 1}, "adjectives": {"Don't": 1, "Easy": 1}, "numerals": {"1": 2}, "pronouns": {"I": 1, "others": 1}}}, {"content": "(2) I am as healthy as anyone else:", "word_count": 9, "label": "", "sentence_structure": "Subject鈥揷opula鈥損redicative", "subjective_objective": {"answer": "Subjective", "rationale": "Because \"I'm as healthy as anyone else\" is the patient's subjective perception and assessment of his health."}, "sentence_sentiment": {"answer": "Neutral", "rationale": "Because the entry is not marked by a positive or negative tendency, it is merely a description of a fact."}, "language_style": {"answer": "Tactful and warm", "rationale": "The language style of the entry is more euphemistic and warm, and instead of asking or ordering the other to answer the question, it guides the other to think about his situation in a friendly and inclusive way."}, "time_dimension": {"answer": "Present", "rationale": ""}, "sentence_type": "Interrogative sentence", "pos": {"nouns": {"Questionnaire": 1, "Entry": 1, "Around": 1, "person": 1, "Health": 1}, "verbs": {"Call": 1, "Same thing": 1, "Health": 1}, "numerals": {"2": 1}, "pronouns": {"I": 1}}}, {"content": "(3) I think my health is getting worse:", "word_count": 12, "label": "", "sentence_structure": "Subject鈥搗erb鈥搊bject", "subjective_objective": {"answer": "Subjective", "rationale": "Because \"consider\" is the patient's subjective perception and judgement of his or her health, it is subjective in its perspective."}, "sentence_sentiment": {"answer": "Negative", "rationale": "Because the expression \"my health is getting worse\" in the entry is negative."}, "language_style": {"answer": "Calm and direct", "rationale": "The entry presents a point in a concise manner that the interviewee considers his health to be deteriorating."}, "time_dimension": {"answer": "Present", "rationale": "In the future, the entry asks the testee about the expectations and projections of future health status, the keywords being \"conceptive\" and \"in the process of deterioration\", which point to situations that have not yet occurred and that may arise in the future."}, "sentence_type": "Negative declarative sentence", "pos": {"nouns": {"Health status": 1, "Bad": 1}, "verbs": {"I think": 1, "It's getting worse": 1}, "numerals": {"3": 1}}}, {"content": "(4) I am in a very good state of health:", "word_count": 9, "label": "", "sentence_structure": "Subject鈥揷opula鈥損redicative", "subjective_objective": {"answer": "Subjective", "rationale": "Because \"I'm in a very good state of health\" is a patient's self-evaluation and subjective feeling, not an objective fact or observation."}, "sentence_sentiment": {"answer": "Positive", "rationale": "Because \"very good health\" is a positive expression, a good physical state."}, "language_style": {"answer": "Tactful and warm", "rationale": "The language of the entry is relatively euphemistic and sweet, and the way in which the interviewee is asked to make a positive assessment of his health and self-awareness, without direct advice or instructions, appears to be more personal and human."}, "time_dimension": {"answer": "Present", "rationale": "The entry describes the current health of the person tested, so the time dimension is now judged."}, "sentence_type": "Affirmative declarative sentence", "pos": {"adjectives": {"Very": 1}, "adverbs": {"Okay": 1}}}]}

{"questionnaire_title": "Medical response questionnaire (MCMQ)", "questionnaire_category": "Behavioral questionnaire", "item_count": 20, "emotion_shift_overall": "From positive to negative", "language_style_overall": "Calm and direct", "items": [{"content": "To what extent do you wish to participate in making treatment decisions?", "word_count": 21, "label": "", "sentence_structure": "Subject鈥搗erb鈥搊bject", "subjective_objective": {"answer": "Subjective", "rationale": "The entry to the questionnaire is viewed from the patient鈥檚 subjective point of view, as it asks the patient about the extent of his or her desire to participate in treatment decision-making."}, "sentence_sentiment": {"answer": "Positive", "rationale": "Because the attitude of \"want to participate in all kinds of therapeutic decisions\" in the entry is positive."}, "language_style": {"answer": "Calm and direct", "rationale": "The entry is based on a direct question-and-answer approach, without any rhetorical or emotional rendering, but simply asking the respondent about the extent of his participation in the decision-making process."}, "time_dimension": {"answer": "Present", "rationale": "The questionnaire entry asks the extent to which the person tested expects himself in the current or near future (i.e. now) and therefore the time dimension is now judged."}, "sentence_type": "Interrogative sentence", "pos": {"nouns": {"Level": 1, "self": 2, "Various": 1, "Treatment": 1, "Decision": 1}, "verbs": {"Hope": 1, "Participation": 1, "Do it": 1, "Out": 1}, "adjectives": {"Large": 1}, "adverbs": {"in": 1}, "numerals": {"How old": 1}, "pronouns": {"you": 1}}}, {"content": "Do you often want to talk about your illness with relatives and friends?", "word_count": 17, "label": "", "sentence_structure": "Subject鈥搗erb鈥搊bject", "subjective_objective": {"answer": "Subjective", "rationale": "Because \"think\" is the patient's subjective attitude, and discussing illness with relatives and friends is a possible act. So the entry looks at the patient's subjective idea, which is subjective."}, "sentence_sentiment": {"answer": "Negative", "rationale": "Since the question was \"disease talk\", the topic was usually a source of negative emotions and concerns, and the emotional colour of the article tended to be negative."}, "language_style": {"answer": "Calm and direct", "rationale": "The language style of the entry is more straightforward, with no excessive adoration or emotional colour, and the interviewee鈥檚 opinion about whether he or she is willing to share the disease with his or her family or friends."}, "time_dimension": {"answer": "Present", "rationale": ""}, "sentence_type": "Interrogative sentence", "pos": {"nouns": {"Relatives and friends": 1, "Diseases": 1}, "verbs": {"Yes": 1, "Talk": 1, "Comment": 1}, "adverbs": {"The long run": 1, "Regular": 1}, "numerals": {"2": 1}, "pronouns": {"you": 1}}}, {"content": "3. When discussing your illness, do you often find yourself thinking about something else?", "word_count": 27, "label": "", "sentence_structure": "Subject鈥搗erb鈥搊bject", "subjective_objective": {"answer": "Subjective", "rationale": "The problem is subjective because it involves the patient ' s observation and description of his or her psychological state."}, "sentence_sentiment": {"answer": "Negative", "rationale": "Because the \"thinking other things\" act in the entry is negative."}, "language_style": {"answer": "Tactful and warm", "rationale": "The entry is presented in a question-and-answer format rather than in direct orders or statements, which are more balanced and caring, and which makes it easier for the person surveyed to feel comfortable and to cooperate in answering the questions."}, "time_dimension": {"answer": "Present", "rationale": ""}, "sentence_type": "Interrogative sentence", "pos": {"nouns": {"Diseases": 1, "Time": 2, "self": 1}, "verbs": {"Discussion": 1, "Found": 1, "Consider": 1}, "adverbs": {"Regular": 1}, "pronouns": {"you": 1}}}, {"content": "4. Do you often feel that you have no hope for a full recovery?", "word_count": 22, "label": "", "sentence_structure": "Subject鈥搗erb鈥搊bject", "subjective_objective": {"answer": "Subjective", "rationale": "This requires an examination of the patient鈥檚 inner thoughts and emotional experience, and therefore is subjective."}, "sentence_sentiment": {"answer": "Negative", "rationale": "Because the expression \"no hope\" in the entry is negative."}, "language_style": {"answer": "Calm and direct", "rationale": "The entry is based on a straightforward approach: the person surveyed has not used excessive rhetorical or emotional renderings to ask if he or she has any idea that there is no hope for a complete recovery."}, "time_dimension": {"answer": "Past", "rationale": "The entry asks if the testee had ever felt that he or she could not fully recover from health, and the temporal dimension was a matter of past."}, "sentence_type": "Interrogative sentence", "pos": {"nouns": {"Health": 1, "self": 2, "Hope": 1, "Restore": 1}, "verbs": {"I think": 1, "be": 1, "No, I'm not": 1, "have": 1, "Restore": 1}, "adjectives": {"Total": 1, "Health": 1}, "adverbs": {"Regular": 1, "No, I'm not": 1}, "numerals": {"4": 1}, "classifiers": {"individual": 1}, "pronouns": {"you": 1}}}, {"content": "How much knowledge have you received from doctors, nurses and others over the past few months about diseases?", "word_count": 27, "label": "", "sentence_structure": "Subject鈥搗erb鈥搊bject", "subjective_objective": {"answer": "Objective", "rationale": "It is an objective fact that the entry is asking how much information about the disease has been obtained from doctors, nurses, etc. over the past few months."}, "sentence_sentiment": {"answer": "Neutral", "rationale": "Because the entry does not have any emotional color."}, "language_style": {"answer": "Calm and direct", "rationale": "The decision-making basis: the article is neutral and objective, and does not use too many rhetorical or emotional terms. It simply asks the respondents how much knowledge they have gained from professionals about disease, highlighting the subject of access to information, rather than trying to mobilize the emotions of the reader or to arouse resonance."}, "time_dimension": {"answer": "Past", "rationale": "And in conclusion, the output is: \"The Past.\""}, "sentence_type": "Interrogative sentence", "pos": {"nouns": {"Month": 1, "Doctor": 1, "Nurse": 2, "Yes, I do": 1, "person": 1, "Diseases": 1, "Knowledge": 1}, "verbs": {"Come on": 1, "Got it": 1, "Related": 1}, "adverbs": {"How much": 1, "From": 1}, "numerals": {"How many": 1}, "classifiers": {"individual": 1}, "pronouns": {"you": 1}}}, {"content": "6. Do you often feel that you are not concerned about the future because of illness?", "word_count": 25, "label": "", "sentence_structure": "Subject鈥搗erb鈥搊bject", "subjective_objective": {"answer": "Subjective", "rationale": "The questionnaire entry examines patients鈥?subjective views of the disease鈥檚 impact on their future."}, "sentence_sentiment": {"answer": "Negative", "rationale": "Because the attitude of \"not caring about the future\" in the entry is negative."}, "language_style": {"answer": "Tactful and warm", "rationale": "The entry is more adept and interactive when it is spoken in a gentle and inviting manner, rather than by direct statements or orders."}, "time_dimension": {"answer": "Past", "rationale": "The entry is to ask the testee whether he or she has been in the past without attention to the future because of illness, and therefore the temporal dimension is a matter of the past."}, "sentence_type": "Interrogative sentence", "pos": {"nouns": {"Diseases": 1, "Yes": 1, "Future": 1}, "verbs": {"I think": 1, "I don't care": 1}, "adverbs": {"Regular": 1, "self": 1}, "numerals": {"6": 1}}}, {"content": "7. To what extent would you like to talk to your relatives and friends about other things, since you do not have to think about disease?", "word_count": 29, "label": "", "sentence_structure": "Subject鈥搗erb鈥搊bject", "subjective_objective": {"answer": "Subjective", "rationale": "This is because it involves the patient ' s subjective will and attitude."}, "sentence_sentiment": {"answer": "Neutral", "rationale": "Because the entry does not have any emotional color."}, "language_style": {"answer": "Tactful and warm", "rationale": "The words are more personal and interactive. At the same time, the words are euphemistic, avoiding the use of language that is too direct, intense or could provoke negative feelings. Such language helps create a relaxed, positive atmosphere and encourages respondents to answer questions more frankly."}, "time_dimension": {"answer": "Present", "rationale": "The entry is an inquiry into the current attitude or behaviour of the subject, the keywords being \"to what extent\" and \"because there is no need to think about disease\" and all point to the state of the present."}, "sentence_type": "Interrogative sentence", "pos": {"nouns": {"Level": 1, "In terms of": 1, "you": 2, "Friends": 1, "Things": 1, "Diseases": 1}, "verbs": {"Yes": 1, "Talk": 1, "Think about it": 1}, "adjectives": {"How old": 1, "Required": 1, "Old": 1}, "adverbs": {"very": 1}, "numerals": {"7": 1}, "classifiers": {"individual": 1}}}, {"content": "8. To what extent does your illness make you think more positively about things in life?", "word_count": 29, "label": "", "sentence_structure": "Subject鈥搗erb鈥搊bject", "subjective_objective": {"answer": "Subjective", "rationale": "The question is about subjective attitudes and behaviour changes in the patient ' s condition."}, "sentence_sentiment": {"answer": "Neutral", "rationale": "Because the entry asks about the impact of disease on individual attitudes, there is no clear positive or negative emotional colour."}, "language_style": {"answer": "Tactful and warm", "rationale": "The entry uses the word \"level\" to guide respondents to reflect on how their disease affects their attitude, without direct orders or evaluation. At the same time, the more active description is asking whether the interviewee has changed positively, rather than directly telling them whether or not he has made progress. This expression is more moderate and respectful and helps to build good interaction."}, "time_dimension": {"answer": "Present", "rationale": "So the time dimension is now judged."}, "sentence_type": "Interrogative sentence", "pos": {"nouns": {"Level": 1, "Diseases": 1, "Attitude": 1, "Life": 1, "Something": 1}, "verbs": {"鈾?And I'm gonna be 鈾?: 1, "Consider": 1}, "adjectives": {"More positive": 1}, "numerals": {"How old": 1}}}, {"content": "When you think about your disease, do you do something else to distract yourself?", "word_count": 28, "label": "", "sentence_structure": "Subject鈥搗erb鈥搊bject", "subjective_objective": {"answer": "Subjective", "rationale": "Because this is the patient鈥檚 subjective attitude and behaviour. Although the question is to examine the patient鈥檚 thoughts, it is to ask whether they choose to do something to distract them from their concerns, which requires the patient鈥檚 subjective will and action."}, "sentence_sentiment": {"answer": "Negative", "rationale": "Because the \"severe\" act in the entry is negative."}, "language_style": {"answer": "Calm and direct", "rationale": "The entry asks the respondent in a direct and clear language whether he or she will be able to divert attention in a certain way in the face of a disease."}, "time_dimension": {"answer": "Present", "rationale": "The entry asks the testee for his current behaviour, and the word \"whether or not\" indicates that this is a selective question that requires the testee to judge and answer, and therefore the temporal dimension is now."}, "sentence_type": "Interrogative sentence", "pos": {"nouns": {"Diseases": 1, "Attention": 1}, "verbs": {"Yes": 2, "Do it": 1, "Spread out": 1}, "adverbs": {"When": 1, "Time": 1}, "numerals": {"9": 1}, "pronouns": {"you": 2}}}, {"content": "Have you often asked doctors what you should do about your illness?", "word_count": 22, "label": "", "sentence_structure": "Subject鈥搗erb鈥搊bject", "subjective_objective": {"answer": "Subjective", "rationale": "Because the words \"often\" and \"how to do\" are the expression of patients' subjective attitudes and actions."}, "sentence_sentiment": {"answer": "Positive", "rationale": "Because the \"quiet the doctor how to handle his disease\" in the entry is positive."}, "language_style": {"answer": "Calm and direct", "rationale": "The language style of the entry is a calm and straightforward one. It uses a direct language expression and a simple speech structure, with no excessive emotional accoutrements or rhetorical techniques. The style is characterized by clarity, directness, clarity, and greater understanding and acceptance by the interviewee."}, "time_dimension": {"answer": "Present", "rationale": "The entry is a question about the present behaviour of the testee, and the keywords \"whether often\", \"questioning\" and \"how to do\" suggest that this is an investigation of the current situation."}, "sentence_type": "Interrogative sentence", "pos": {"nouns": {"Questionnaire": 1, "Entry": 1, "Diseases": 1, "you": 2}, "verbs": {"Regular": 1, "In": 1, "Ask": 1, "Do it": 1}, "adverbs": {"How's that": 1}, "numerals": {"10": 1}}}, {"content": "Do you often try to change the subject when relatives and friends talk to you about your illness?", "word_count": 25, "label": "", "sentence_structure": "Subject鈥搗erb鈥搊bject", "subjective_objective": {"answer": "Subjective", "rationale": "The attempt to change the subject is a psychological response and behaviour of the patient and is a subjective attitude of the patient."}, "sentence_sentiment": {"answer": "Negative", "rationale": "Because the \"torture to change the subject\" act in the entry is negative."}, "language_style": {"answer": "Calm and direct", "rationale": "This language style is more suitable for psychological surveying to ensure that the interviewee understands the meaning of the question and is more truthful and accurate in answering the question."}, "time_dimension": {"answer": "Past", "rationale": "In the entry, \"often\" means that there has been a higher frequency in the past, and the act of \"torture to change the subject\" has occurred in the past. So we can judge that entry by asking the testee about past behavior, and by the time dimension, by way of passing."}, "sentence_type": "Interrogative sentence", "pos": {"nouns": {"Relatives": 1, "friends": 1, "Diseases": 1, "Topic": 1}, "verbs": {"Talk": 1, "Try": 1, "Convert": 1}, "adverbs": {"Regular": 1}, "numerals": {"11": 1}, "pronouns": {"you": 2}}}, {"content": "12. In recent months, what do you know about your illness in books, magazines, newspapers?", "word_count": 26, "label": "", "sentence_structure": "Subject鈥搗erb鈥搊bject", "subjective_objective": {"answer": "Objective", "rationale": "Since the amount of information actually reached by the patient is known, it is part of the objective examination."}, "sentence_sentiment": {"answer": "Neutral", "rationale": "Because the entry is only a question of the amount of information the respondents have on the disease, it is not clearly emotional."}, "language_style": {"answer": "Calm and direct", "rationale": "Such language styles are more objective and clear, and allow respondents to give precise answers."}, "time_dimension": {"answer": "Past", "rationale": "The time dimension is judged as past."}, "sentence_type": "Interrogative sentence", "pos": {"nouns": {"A few months": 1, "Books": 1, "Magazines": 1, "Newspapers": 1, "Information": 1, "Diseases": 1}, "verbs": {"Roger that": 1}, "adverbs": {"Near": 1}, "numerals": {"12": 1}}}, {"content": "Do you often feel like you're going to give in to a disease?", "word_count": 16, "label": "", "sentence_structure": "Subject鈥搗erb鈥搊bject", "subjective_objective": {"answer": "Subjective", "rationale": "Because it is a subjective feeling and attitude to feel that you want to submit to disease."}, "sentence_sentiment": {"answer": "Negative", "rationale": "Because the attitude in the entry, \"I feel like I'm going to give in to disease,\" is negative."}, "language_style": {"answer": "Calm and direct", "rationale": "The entry is presented in a direct, concise interview, without too much emotional or rhetorical overtones to give people a sense of the seriousness and importance of the problem."}, "time_dimension": {"answer": "Present", "rationale": ""}, "sentence_type": "Interrogative sentence", "pos": {"nouns": {"self": 1, "Diseases": 1}, "verbs": {"I think": 1, "Yes": 1, "Conceal": 1}, "adverbs": {"Regular": 1}, "numerals": {"13": 1}, "pronouns": {"you": 1}}}, {"content": "To what extent do you want to forget your illness?", "word_count": 14, "label": "", "sentence_structure": "Subject鈥搗erb鈥搊bject", "subjective_objective": {"answer": "Subjective", "rationale": "Because \"want to forget\" is a subjective attitude or will that is related to the patient's inner feelings and thoughts."}, "sentence_sentiment": {"answer": "Negative", "rationale": "Because the \"want to forget your disease\" act in the entry is positive, but the \"level\" in the sentence indicates that it has a negative emotional color, namely negative."}, "language_style": {"answer": "Tactful and warm", "rationale": "The expression is more personal and interactive, in a gentle and inviting tone, rather than in direct statements or orders. At the same time, using the \"to what extent\" form of open questions allows respondents to express their thoughts and feelings more freely than to be confined to a fixed choice."}, "time_dimension": {"answer": "Present", "rationale": "The entry asks the testee about his current thoughts and feelings, and the time dimension is now judged."}, "sentence_type": "Interrogative sentence", "pos": {"nouns": {"Level": 1, "Problem": 1}, "verbs": {"Yes": 1, "Forget it": 1, "have": 1}, "adjectives": {"Large": 1, "you": 1}, "adverbs": {"More": 1}, "numerals": {"14": 1}, "pronouns": {"Yours": 1}}}, {"content": "15. How many questions did you ask the doctor about the disease?", "word_count": 14, "label": "", "sentence_structure": "Subject鈥搗erb鈥搊bject", "subjective_objective": {"answer": "Subjective", "rationale": "This is a question of the patient ' s initiative and level of concern in the face of the disease, and of his/her subjective attitude and behaviour."}, "sentence_sentiment": {"answer": "Neutral", "rationale": "Because the entry does not have any emotional color."}, "language_style": {"answer": "Calm and direct", "rationale": "The language style of the entry is more straightforward, with no excessive rhetoric or emotional rendering, more factual presentation and purpose of the investigation, and more objective and calm tone."}, "time_dimension": {"answer": "Past", "rationale": "The entry is for medical history and behaviour of the person tested, and the keyword is \"on illness\" and \"on how many questions have been asked to the doctor\" and all point to what has happened and what has happened in the past."}, "sentence_type": "Interrogative sentence", "pos": {"nouns": {"Diseases": 1, "Doctor": 1, "Problem": 1}, "verbs": {"In": 1, "Question": 1}, "adverbs": {"How much": 1}, "numerals": {"15": 1}, "classifiers": {"individual": 1}}}, {"content": "How much details do you usually talk to a person with the same disease?", "word_count": 27, "label": "", "sentence_structure": "Subject鈥搗erb鈥搊bject", "subjective_objective": {"answer": "Subjective", "rationale": "Since the problem concerns the views and behaviour of the patients themselves, they are asked how much details of the disease they prefer to share with people with the same disease and are therefore subjective in nature."}, "sentence_sentiment": {"answer": "Neutral", "rationale": "Because the entry does not have any emotional color."}, "language_style": {"answer": "Tactful and warm", "rationale": ""}, "time_dimension": {"answer": "Present", "rationale": "The entry asks about the situation of the person tested when he or she encounters a sick person in his or her current life, and therefore is now."}, "sentence_type": "Interrogative sentence", "pos": {"nouns": {"Questionnaire": 1, "Entry": 1, "person": 2, "Diseases": 2, "Details": 1}, "verbs": {"I met him": 1, "Yes": 1, "Talk": 1, "have": 1}, "adverbs": {"Usually": 1}, "numerals": {"How much": 1, "16": 1}, "pronouns": {"he": 1}}}, {"content": "Do you often distract yourself from disease by watching movies, television, etc.?", "word_count": 25, "label": "", "sentence_structure": "Subject鈥搗erb鈥搊bject", "subjective_objective": {"answer": "Objective", "rationale": "Because it is an objective question to ask patients whether they will distract from the disease by watching films, television, etc."}, "sentence_sentiment": {"answer": "Negative", "rationale": "Because the \"dispersion of your own attention\" in the entry is negative."}, "language_style": {"answer": "Tactful and warm", "rationale": "The entry is more adept and interactive when it is spoken in a gentle and inviting manner, rather than by direct statements or orders."}, "time_dimension": {"answer": "Present", "rationale": ""}, "sentence_type": "Interrogative sentence", "pos": {"nouns": {"Problem": 1, "Modalities": 2, "Attention": 1, "Film": 1, "Television": 1, "Diseases": 1}, "verbs": {"be": 1, "Regular": 1, "Here": 1, "Let's spread out": 1, "self": 1}, "adjectives": {"Wait": 1}, "adverbs": {"to": 1, "Come on": 1, "Regular": 1}, "classifiers": {"individual": 1}, "pronouns": {"self": 1}}}, {"content": "18. Do you often feel powerless about disease?", "word_count": 16, "label": "", "sentence_structure": "Subject鈥損redicate", "subjective_objective": {"answer": "Subjective", "rationale": "Because the word \"thinks\" shows that it's the patient's subjective feeling and opinion."}, "sentence_sentiment": {"answer": "Negative", "rationale": "Because the attitude of feeling powerless about disease in the entry is negative."}, "language_style": {"answer": "Calm and direct", "rationale": "The entry was asked in a direct and clear manner whether they felt unable to cope with the disease, without excessive adoration and emotional rendering, and with a more calm and objective language style."}, "time_dimension": {"answer": "Present", "rationale": "\"The entry now asks about the person who is being tested for his current behaviour or feelings, that is, \"Does he often feel he is incapable of disease?\""}, "sentence_type": "Interrogative sentence", "pos": {"nouns": {"self": 1, "Diseases": 1}, "verbs": {"I think": 1, "There's nothing I can do": 1}, "adverbs": {"Regular": 1}, "numerals": {"18": 1}, "pronouns": {"you": 1}}}, {"content": "19. Do you often share with you many details of your illness when you are asked by family and friends?", "word_count": 25, "label": "", "sentence_structure": "Subject鈥搗erb鈥搊bject", "subjective_objective": {"answer": "Subjective", "rationale": "Because the patient ' s response is based on his or her own feelings and behaviour, it is the patient ' s subjective attitude that is examined."}, "sentence_sentiment": {"answer": "Negative", "rationale": "Because \"Do you often talk to your family and friends about the details of your illness?\" This behaviour may cause anxiety and concern, and is therefore negative."}, "language_style": {"answer": "Calm and direct", "rationale": "The entry expresses the subject of a direct, simple statement asking the other party whether it will disclose the details of the illness when the person concerned is a relative, without excessive rhetorical or emotional rendering, and is more objective in tone."}, "time_dimension": {"answer": "Present", "rationale": "The entry, \"When you're asked by your family and friends about the condition\" and \"Do you often talk to him about the details of the condition,\" suggests that this is a question of the current behaviour of the testee, and therefore the temporal dimension is now judged."}, "sentence_type": "Interrogative sentence", "pos": {"nouns": {"Family and friends": 1, "Medical conditions": 1, "Details of the condition": 1}, "verbs": {"Ask": 1, "Talk": 1, "Regular": 1}, "adverbs": {"Many": 1}, "classifiers": {"individual": 1}, "pronouns": {"you": 1}}}, {"content": "20. Do you feel that you are only in the hands of God with regard to your illness?", "word_count": 21, "label": "", "sentence_structure": "Subject鈥搗erb鈥搊bject", "subjective_objective": {"answer": "Subjective", "rationale": "This is a description of what the patient thinks and feels about himself, and is viewed from a subjective perspective."}, "sentence_sentiment": {"answer": "Negative", "rationale": "Because the \"go to God\" attitude in the entry is negative."}, "language_style": {"answer": "Calm and direct", "rationale": "The subject is based on the following criteria: the subject is directly asked, without too many emotional amenity and rhetorical techniques, and is more objective in tone. Moreover, the entry does not attempt to induce or suggest how the interviewee will answer, but is presented in a factual question, which is more calm and straightforward."}, "time_dimension": {"answer": "Past", "rationale": "But \"often feeling\" suggests that it's a habit, so it can be considered a problem of the present."}, "sentence_type": "Interrogative sentence", "pos": {"nouns": {"Diseases": 1, "self": 1}, "verbs": {"I feel": 1, "I'm going to pay you a reward": 1, "It's up to you": 1}, "adverbs": {"Only": 1}, "numerals": {"20": 1}}}]}

{"questionnaire_title": "The timidity gauge.", "questionnaire_category": "Self-cognition", "item_count": 13, "emotion_shift_overall": "From positive to negative", "language_style_overall": "Neutral", "items": [{"content": "I was nervous when I was with someone I wasn't sure about.", "word_count": 17, "label": "", "sentence_structure": "Subject鈥揷opula鈥損redicative", "subjective_objective": {"answer": "Subjective", "rationale": "Because \"frightful\" is the feeling and experience inside the patient, and it's a subjective observation."}, "sentence_sentiment": {"answer": "Negative", "rationale": "Because the \"frightful\" in the entry is an expression of a negative sentiment."}, "language_style": {"answer": "Tactful and warm", "rationale": "The language style of the entry is more euphemistic and warm, inviting interviewees to share their feelings in a kind and gentle way. Using a description like \"unfamiliar,\" avoiding direct naming or accusing each other, the expression \"I feel nervous\" shows respect and understanding of the interviewee. At the same time, the expression \"I feel nervous\" is more objective and neutral, and there is no excessive emotional overtones or subjective evaluation, making the interviewee more willing to express his thoughts and feelings."}, "time_dimension": {"answer": "Present", "rationale": "The entry describes the experience and feelings of the person being tested at the moment, that is, the situation \"when it is\" and thus the present."}, "sentence_type": "Interrogative sentence", "pos": {"nouns": {"person": 2, "Time": 1, "Nervous": 1}, "verbs": {"in": 1, "Together": 1, "I feel": 1}, "adjectives": {"Not really": 1, "I know you": 1}, "numerals": {"1": 3}, "classifiers": {"individual": 1}, "pronouns": {"I": 1}}}, {"content": "2. I am very poor in social terms.", "word_count": 10, "label": "", "sentence_structure": "Subject鈥揷opula鈥損redicative", "subjective_objective": {"answer": "Subjective", "rationale": "This is a self-evaluation of the patient and a subjective description."}, "sentence_sentiment": {"answer": "Negative", "rationale": "Because the expression \"slightly bad\" in the entry is negative, implying that the testee is not performing well in social terms."}, "language_style": {"answer": "Calm and direct", "rationale": "The entry presents the problem in a direct presentation, with a more concise language style, with no excessive emotional colour or rhetoric, and with direct and objective expression."}, "time_dimension": {"answer": "Present", "rationale": ""}, "sentence_type": "Interrogative sentence", "pos": {"nouns": {"I": 1, "Social": 1, "In terms of": 1, "Bad": 1}, "verbs": {"in": 1, "Pretty much": 1}, "adjectives": {"Bad": 1}, "adverbs": {"Pretty much": 1}, "pronouns": {"I": 1}}}, {"content": "I don't feel like it's hard to ask people about something.", "word_count": 15, "label": "", "sentence_structure": "Subject鈥搗erb鈥揷omplement", "subjective_objective": {"answer": "Subjective", "rationale": "Because \"Esperate\" is a subjective feeling or perception, the entry is subjective."}, "sentence_sentiment": {"answer": "Positive", "rationale": "Because the \"not feeling difficult\" attitude in the entry is positive."}, "language_style": {"answer": "Calm and direct", "rationale": "The entry is based on the following: - The entry uses the general question phrase to express the subject matter of the inquiry into the matter, is direct, is not expressed in a euphemism or vague manner. - The sentence is simple and is clearly structured, has no qualifying language or rhetorical techniques, and makes people feel calm and objective."}, "time_dimension": {"answer": "Present", "rationale": "The key word \"unreceived difficulties\" now indicates that the act is a subjective feeling that may be temporary or long-term, and that the temporal dimension of the article should therefore be the present."}, "sentence_type": "Negative declarative sentence", "pos": {"nouns": {"Things": 1, "To someone else": 1}, "verbs": {"- I'm asking": 1, "Not really": 1, "Difficulties": 1}, "adverbs": {"to": 1}, "numerals": {"3": 1}}}, {"content": "I am often uncomfortable with meetings or other social events.", "word_count": 19, "label": "", "sentence_structure": "Subject鈥揷opula鈥損redicative", "subjective_objective": {"answer": "Subjective", "rationale": "Because \"often feeling uncomfortable\" is the patient's subjective and emotional experience."}, "sentence_sentiment": {"answer": "Negative", "rationale": "Because the entry \"often feels uncomfortable at a party or other social events\" is negative."}, "language_style": {"answer": "Calm and direct", "rationale": "As the language style of the entry is more straightforward, there is no excessive qualifier or emotionally strong vocabulary, but rather objective, concise language."}, "time_dimension": {"answer": "Present", "rationale": "Now the entry indicates that the testee has a feeling of discomfort in the current social event, and that the temporal dimension is now. The keywords \"often\" and \"fearless\" all indicate that it is a state of habit, continuity, not past or future."}, "sentence_type": "Affirmative declarative sentence", "pos": {"nouns": {"I": 1, "Party": 1, "Other": 1, "Social events": 1, "Activities": 1}, "verbs": {"in": 1, "I feel": 1, "I'm not comfortable": 1}, "adjectives": {"Regular": 1}, "numerals": {"4": 1}}}, {"content": "When I'm in a group, I can hardly find the right conversation.", "word_count": 21, "label": "", "sentence_structure": "Subject鈥揷opula鈥損redicative", "subjective_objective": {"answer": "Subjective", "rationale": "Because \"it's difficult to find the right conversation\" is a subjective feeling and experience. Although this may be influenced by objective factors (such as the nature of the population, the interest in the topic, etc.), ultimately the feeling is unique to the individual. Thus, the entry is primarily about subjective ideas and feelings."}, "sentence_sentiment": {"answer": "Negative", "rationale": "Because the \"hard to find the right conversation\" in the entry is negative."}, "language_style": {"answer": "Tactful and warm", "rationale": ""}, "time_dimension": {"answer": "Past", "rationale": "The entry asks about the testee ' s past experience and feelings, and the temporal dimension is the past."}, "sentence_type": "Negative declarative sentence", "pos": {"nouns": {"Population": 1, "Talk": 1}, "verbs": {"Location": 1, "Find": 1}, "adjectives": {"Suits": 1}, "numerals": {"One": 2, "Hard": 1}, "pronouns": {"I": 1}}}, {"content": "6. I do not need to spend a long time overcoming my shyness in the new environment.", "word_count": 22, "label": "", "sentence_structure": "Subject鈥搗erb鈥搊bject", "subjective_objective": {"answer": "Subjective", "rationale": "Because \"Wasn't it take long to overcome shyness\" is the patient's self-perception and subjective judgment."}, "sentence_sentiment": {"answer": "Negative", "rationale": "Because \"overcoming the shyness in the new environment\" usually requires positive attitudes and behaviour, and the expression \"not taking a long time\" in the entry gives a negative hint that the process seems to be considered either easy or irrelevant."}, "language_style": {"answer": "Tactful and warm", "rationale": "The language style of the entry is euphemistic. It uses the negative form \"no need\" and the word \"weakness,\" but it is not a matter of too much emotional or negative rhetoric in general, but rather of raising questions in a mild way that allows respondents to be more willing to share their feelings and ideas. This expression is more personal and interactive."}, "time_dimension": {"answer": "Present", "rationale": "The keyword \"does not take a long time\" suggests that this is a description of the state of affairs."}, "sentence_type": "Negative declarative sentence", "pos": {"nouns": {"Time": 1, "Environment": 1, "Shame": 1}, "verbs": {"Overcoming": 1, "Required": 1, "In": 1}, "adjectives": {"Long": 1, "New": 1}, "adverbs": {"No, it's not": 1, "very": 1}, "pronouns": {"I": 1}}}, {"content": "7. It is difficult for me to act naturally with the living.", "word_count": 15, "label": "", "sentence_structure": "Subject鈥揷opula鈥損redicative", "subjective_objective": {"answer": "Subjective", "rationale": "Because \"hard\" is naturally a reflection of the patient's own feelings and subjective views."}, "sentence_sentiment": {"answer": "Negative", "rationale": "Because the \"I'm Hard to act naturally\" act in the entry is negative."}, "language_style": {"answer": "Calm and direct", "rationale": "The entry is a direct and explicit inquiry into the behaviour of the other party in a social setting, without excessive rhetorical or emotional rendering, and is more objective in tone."}, "time_dimension": {"answer": "Present", "rationale": "Now the entry describes the feelings and behaviour of the subject in the current circumstances, \"when with the living\" means that it's in the present scene, and \"I'm hard to act naturally\" is also a description of the present situation."}, "sentence_type": "Negative declarative sentence", "pos": {"nouns": {"Livers": 1, "Time": 1, "Naturally": 1}, "verbs": {"in": 1, "Okay": 1, "Performance": 1}, "adjectives": {"It's hard": 1, "Together": 1}, "classifiers": {"One": 2, "individual": 1}, "pronouns": {"I": 1}}}, {"content": "8. I was nervous when I spoke to people with authority.", "word_count": 15, "label": "", "sentence_structure": "Subject鈥揷opula鈥損redicative", "subjective_objective": {"answer": "Subjective", "rationale": "Because feeling nervous is a subjective emotional experience."}, "sentence_sentiment": {"answer": "Negative", "rationale": "Because the emotional attitude of \"strained\" in the entry is negative."}, "language_style": {"answer": "Calm and direct", "rationale": "The entry is more straightforward, concise, has no excessive symmetry or emotional rendering, and is more calm and objective. It expresses in a direct way the subject of questioning whether the other party is nervous when talking to an authority."}, "time_dimension": {"answer": "Present", "rationale": "\"Escrowding\" is a verb that is now in a state of time, indicating the sense that the person who is talking is experiencing when filling out the questionnaire, so the entry describes the current behaviour or mentality."}, "sentence_type": "Affirmative declarative sentence", "pos": {"nouns": {"Questionnaire": 1, "Entry": 1, "Authority": 1, "person": 1, "Talk": 1}, "verbs": {"I feel": 1, "Nervous": 1}, "numerals": {"8": 1}, "pronouns": {"I": 1}}}, {"content": "I have no doubt about my social skills.", "word_count": 12, "label": "", "sentence_structure": "Subject鈥揷opula鈥損redicative", "subjective_objective": {"answer": "Subjective", "rationale": "Because \"no doubt\" is a subjective attitude and perception, an assessment and judgement of their social abilities."}, "sentence_sentiment": {"answer": "Positive", "rationale": "Because the expression \"no doubt about my social skills\" shows confidence and positive attitude."}, "language_style": {"answer": "Tactful and warm", "rationale": "The entry is more encouraging and confident by asking the other side, in a moderate and positive way, about his/her social skills, rather than directly questioning or denying them. At the same time, it avoids answering that could provoke negative emotions."}, "time_dimension": {"answer": "Present", "rationale": "The entry, which expresses self-evaluation of the person's ability to socialize, \"unquestioned\" indicates that it is a customary, recurrent act and conviction, and therefore the temporal dimension is now judged."}, "sentence_type": "Affirmative declarative sentence", "pos": {"nouns": {"Social skills": 1}, "verbs": {"No doubt": 1, "to": 1}, "numerals": {"9": 1}}}, {"content": "I have difficulty facing the people before me.", "word_count": 9, "label": "", "sentence_structure": "Subject鈥搗erb鈥揷omplement", "subjective_objective": {"answer": "Subjective", "rationale": "Because \"right-wing\" is a subjective perception and attitude that expresses an individual's attitude and perception of people being in contact with them."}, "sentence_sentiment": {"answer": "Negative", "rationale": "Because the \"unremarkable\" behavior in the entry is negative."}, "language_style": {"answer": "Calm and direct", "rationale": "The language style of the entry is straightforward. It is straightforward in a simple and straightforward way, raising a question, without any rhetorical or emotional rendering, and is more objective and calm."}, "time_dimension": {"answer": "Present", "rationale": "According to the title, \"persons who are hard to face\" are an ongoing, ongoing act or state and should therefore be classified as \"now\"."}, "sentence_type": "Negative declarative sentence", "pos": {"nouns": {"Questionnaire": 1, "Entry": 1, "person": 1}, "verbs": {"Analysis": 1, "Face-to-face": 1}, "adjectives": {"Hard": 1}, "adverbs": {"Down there": 1}}}, {"content": "11. I feel very limited in social settings.", "word_count": 13, "label": "", "sentence_structure": "Subject鈥揷opula鈥損redicative", "subjective_objective": {"answer": "Subjective", "rationale": "Because \"frightfully limited\" is a personal feeling and experience, a subjective description."}, "sentence_sentiment": {"answer": "Negative", "rationale": "Because the \"frightfully restricted\" behavior in the entries is negative."}, "language_style": {"answer": "Tactful and warm", "rationale": "The entry uses relatively euphemistic language to make the interviewee feel respected and understood. At the same time, the interviewee is given the opportunity to express his/her feelings, and to increase his/her interaction and participation by asking."}, "time_dimension": {"answer": "Present", "rationale": ""}, "sentence_type": "Negative declarative sentence", "pos": {"nouns": {"Socially": 1, "Limits": 1}, "verbs": {"I feel": 1, "Limited": 1}, "numerals": {"11": 1}, "pronouns": {"I": 1}}}, {"content": "I do not see any difficulty in talking to strangers.", "word_count": 16, "label": "", "sentence_structure": "Subject鈥搗erb鈥搊bject", "subjective_objective": {"answer": "Subjective", "rationale": "Because \"I think\" is the patient's subjective attitude."}, "sentence_sentiment": {"answer": "Neutral", "rationale": "Because the entry does not have any emotional color."}, "language_style": {"answer": "Tactful and warm", "rationale": "The language style of the entry is a warmer type. The use of \"not feeling\" a less subtle negative word, which is not directly expressed as \"I find it difficult\", reduces the resistance of the person under investigation and is more conducive to his or her real and free expression of his or her views and feelings. At the same time, the relatively neutral way in which the subject is described is used, avoiding too subjective or negative a vocabulary that makes the entire questionnaire more friendly and intimate."}, "time_dimension": {"answer": "Present", "rationale": ""}, "sentence_type": "Negative declarative sentence", "pos": {"nouns": {"I": 1, "Stranger": 1, "Word": 1, "Difficulties": 1}, "verbs": {"I think": 1, "have": 1, "Talk": 1}, "adjectives": {"not": 1, "What": 1}, "adverbs": {"and": 1, "Okay": 1, "More": 1}, "numerals": {"12": 1, "individual": 1}}}, {"content": "I am even more shy when dealing with heterosexuals.", "word_count": 12, "label": "", "sentence_structure": "Subject鈥揷opula鈥損redicative", "subjective_objective": {"answer": "Subjective", "rationale": "Because shyness is a psychological state of a human being, it is a manifestation of subjective feelings."}, "sentence_sentiment": {"answer": "Negative", "rationale": "Because \"weak\" is a negative emotional attitude."}, "language_style": {"answer": "Tactful and warm", "rationale": "The language style of the entry is relatively euphemistic and moderate, and it is not too much direct criticism or criticism, but rather a question and concern to guide the interviewee to self-reflection, which is more acceptable and willing to share his thoughts and feelings."}, "time_dimension": {"answer": "Present", "rationale": "Now the entry describes a manifestation or feeling of the testee in the current social context, which is \"I'm more shy.\" So the judgment is now based on the judgment."}, "sentence_type": "Affirmative declarative sentence", "pos": {"nouns": {"Heterosexual": 1, "- To be with someone": 1}, "verbs": {"in": 1, "More": 2, "Shame": 1}, "numerals": {"13": 1, "1": 1}, "pronouns": {"I": 1}}}]}

{"questionnaire_title": "A social solo questionnaire.", "questionnaire_category": "Behavioral questionnaire", "item_count": 30, "emotion_shift_overall": "From positive to negative", "language_style_overall": "Neutral", "items": [{"content": "I have no best friend.", "word_count": 5, "label": "", "sentence_structure": "Subject鈥損redicate", "subjective_objective": {"answer": "Subjective", "rationale": "Because \"no best friend\" is the patient's subjective feeling and perception, it's not an objective fact."}, "sentence_sentiment": {"answer": "Negative", "rationale": "Because the \"no best friend\" status in the entry is negative."}, "language_style": {"answer": "Calm and direct", "rationale": ""}, "time_dimension": {"answer": "Present", "rationale": "The entry in the past described the current state of the subject, and \"no friend\" was an existing situation and thus judged to be the past."}, "sentence_type": "Negative declarative sentence", "pos": {"nouns": {"Friend": 1}, "verbs": {"Nope": 1, "have": 1}}}, {"content": "2. When they are with others, they want to take advantage of me.", "word_count": 14, "label": "", "sentence_structure": "Subject鈥搗erb鈥搊bject", "subjective_objective": {"answer": "Subjective", "rationale": "Because this is the patient ' s perception and perception of the behaviour of others, it is subjective."}, "sentence_sentiment": {"answer": "Negative", "rationale": "Because the \"want to take advantage of me\" act is negative."}, "language_style": {"answer": "Tactful and warm", "rationale": "The language style of the entry is more euphemistic, rather than directly accusing or attacking the other side, but rather raising the issue in a relatively less lax way, which is more resonant and thought-provoking for the person under investigation. At the same time, the use of \"other\" to refer to others makes the tone of the sentence more natural and more acceptable."}, "time_dimension": {"answer": "Present", "rationale": "Now the entry describes the subject's current subjective feelings and experience, and the keyword \"who wants to take advantage of me\" is an act or state that is now in progress. So the judgment is now based on the present."}, "sentence_type": "Affirmative declarative sentence", "pos": {"nouns": {"others": 1, "Dow": 1, "Time": 1, "person": 1, "Jim": 1, "Cheap": 1}, "verbs": {"Call": 1, "Together": 1}, "adverbs": {"One": 1}, "pronouns": {"I": 1}}}, {"content": "3. I have no partner (or male/girlfriend).", "word_count": 10, "label": "", "sentence_structure": "Subject鈥損redicate", "subjective_objective": {"answer": "Objective", "rationale": "Since the existence of a partner is an objective situation, the existence of a partner is not controlled by the individual ' s will."}, "sentence_sentiment": {"answer": "Neutral", "rationale": "Because the entry does not have any emotional color."}, "language_style": {"answer": "Calm and direct", "rationale": "The entry is more straightforward in language style, with no excessive rhetorical or emotional rendering, but rather a concise presentation of the facts. This way allows respondents to understand and answer questions more quickly and accurately."}, "time_dimension": {"answer": "Present", "rationale": "The sentence contains a general negative statement, which is now, that is, the current situation, where the person tested had no partner when completing the questionnaire. The time dimension is therefore now judged."}, "sentence_type": "Negative declarative sentence", "pos": {"nouns": {"Partner": 1, "Men/girlfriends": 1}, "verbs": {"have": 1}, "adverbs": {"No, I'm not": 1}, "numerals": {"3": 1, "I": 1}, "pronouns": {"or": 1}}}, {"content": "I do not want to burden others with my own difficulties.", "word_count": 17, "label": "", "sentence_structure": "Subject鈥搗erb鈥搊bject", "subjective_objective": {"answer": "Subjective", "rationale": "Because this is a subjective examination of the patient ' s own thoughts and attitudes to the unwillingness to burden others."}, "sentence_sentiment": {"answer": "Positive", "rationale": "This is positive because the entry expresses an attitude of unwillingness to cause trouble and burden to others."}, "language_style": {"answer": "Tactful and warm", "rationale": "The language style of the entry is more euphemistic, as it uses softer language when asking the other side whether it is willing to burden others because of its own difficulties, without direct orders or accusations. This expression is more acceptable and responsive and facilitates good communication."}, "time_dimension": {"answer": "Present", "rationale": "According to the entry, \"resistance\" expresses a behavioural tendency, which arises at the moment. Moreover, \"burdening others\" is also a current-day act, indicating that it is an ongoing, immediate act. Thus, the entry mainly reflects the present thinking and attitude of the testee, not past or future."}, "sentence_type": "Negative declarative sentence", "pos": {"nouns": {"Difficulties": 1, "Burden": 1}, "verbs": {"Jean": 1, "I feel": 1, "have": 1, "I don't want to": 1}, "adverbs": {"Because": 1, "Your own": 1}, "pronouns": {"others": 1, "self": 1}}}, {"content": "5. No one in my life is dependent on me.", "word_count": 12, "label": "", "sentence_structure": "Subject鈥搗erb鈥搊bject", "subjective_objective": {"answer": "Subjective", "rationale": "Because \"someone depends on me\" is an objective fact, and the patient thinks that \"no one depends on me\" is their subjective feeling or belief."}, "sentence_sentiment": {"answer": "Neutral", "rationale": "Because the entry does not express any positive or negative emotional aspects, it merely presents a fact."}, "language_style": {"answer": "Calm and direct", "rationale": "The entry presents a problem in a concise and clear statement, with no excessive emotional or rhetorical style, and a more direct and calm language style."}, "time_dimension": {"answer": "Present", "rationale": "The entry is to ask the testee about the current situation, and the words \"in my life\" and \"no one depends on me\" all imply that this is a description of the current situation, so the time dimension is now."}, "sentence_type": "Negative declarative sentence", "pos": {"nouns": {"Life": 1, "person": 1}, "verbs": {"Dependency": 1, "I": 2}, "numerals": {"5": 1}}}, {"content": "No one has any contact with me.", "word_count": 9, "label": "", "sentence_structure": "Subject鈥搗erb鈥揷omplement", "subjective_objective": {"answer": "Subjective", "rationale": "Because \"not with anyone\" is a subjective feeling and attitude."}, "sentence_sentiment": {"answer": "Negative", "rationale": "Because the \"untouched\" act in the entry is negative."}, "language_style": {"answer": "Calm and direct", "rationale": "The language style of the entry is relatively calm and straightforward, and there is no excessive rhetorical or emotional rendering, but rather a direct statement of the person鈥檚 attitude to communicating with others."}, "time_dimension": {"answer": "Present", "rationale": "The keyword \"not with anyone\" denotes a status quo or current attitude, thought or behaviour, so the time dimension of the entry is now judged."}, "sentence_type": "Negative declarative sentence", "pos": {"nouns": {"person": 1, "I": 2, "- A relationship": 1}, "verbs": {"Call": 2, "I don't know": 1}, "adverbs": {"Any": 1}}}, {"content": "No one in life wants to know me.", "word_count": 11, "label": "", "sentence_structure": "Subject鈥搗erb鈥搊bject", "subjective_objective": {"answer": "Subjective", "rationale": "Because the entry asks about patients ' subjective feelings and attitudes, i.e. whether they are wanted to know, it is subjective."}, "sentence_sentiment": {"answer": "Negative", "rationale": "Because the entry expresses a neglected, lonely feeling and is negative."}, "language_style": {"answer": "Tactful and warm", "rationale": "Warm and warm."}, "time_dimension": {"answer": "Present", "rationale": ""}, "sentence_type": "Negative declarative sentence", "pos": {"nouns": {"Life": 1, "person": 1, "Roger that": 1}, "verbs": {"Yes": 3}, "numerals": {"7": 1}, "pronouns": {"I": 1}}}, {"content": "No one in life wants to be burdened by me.", "word_count": 14, "label": "", "sentence_structure": "Subject鈥搗erb鈥揷omplement", "subjective_objective": {"answer": "Subjective", "rationale": "Because \"no one wants to be my burden\" is the patient's subjective view or feeling."}, "sentence_sentiment": {"answer": "Negative", "rationale": "Because the expression \"resistance to be my burden\" in the entry is negative, it implies negative perceptions and attitudes of others towards the person under investigation."}, "language_style": {"answer": "Tactful and warm", "rationale": "The entry uses a more euphemistic tone, a gentle, inviting tone to ask if the other side has experience in life that is associated with others. This expression is more personal and interactive, while avoiding direct verbal attacks that may provoke resistance."}, "time_dimension": {"answer": "Present", "rationale": ""}, "sentence_type": "Negative declarative sentence", "pos": {"nouns": {"Life": 1, "person": 2, "I'm in trouble": 1}, "verbs": {"Yes": 2, "Present": 1, "of": 3, "Received": 1}, "adverbs": {"No, I'm not": 1, "Both": 1}, "numerals": {"8": 1}, "pronouns": {"I": 1}}}, {"content": "I have spent a lot of time alone.", "word_count": 10, "label": "", "sentence_structure": "Subject鈥搗erb鈥揷omplement", "subjective_objective": {"answer": "Subjective", "rationale": "Because \"many hours alone\" is a personal feeling and experience, and belongs to the patient's own subjective attitude and feelings."}, "sentence_sentiment": {"answer": "Neutral", "rationale": "Because the entry does not have any emotional color."}, "language_style": {"answer": "Tactful and warm", "rationale": ""}, "time_dimension": {"answer": "Present", "rationale": ""}, "sentence_type": "Affirmative declarative sentence", "pos": {"nouns": {"Time": 1, "Alone": 1}, "verbs": {"have": 1, "Stay": 1}, "adjectives": {"Many": 1}, "numerals": {"9": 1}}}, {"content": "10. I have no associations or organizations.", "word_count": 11, "label": "", "sentence_structure": "Subject鈥損redicate", "subjective_objective": {"answer": "Objective", "rationale": "It is objective to consider membership in an association or organization as a real manifestation of the patient ' s behaviour."}, "sentence_sentiment": {"answer": "Neutral", "rationale": "Because the entry merely stated a fact and did not involve any emotional colour."}, "language_style": {"answer": "Calm and direct", "rationale": "The language style of the entry is calm. It clearly asks whether the person surveyed is a member of any association or organization, and is not expressed in a rhetorical or emotional language."}, "time_dimension": {"answer": "Present", "rationale": "The keywords are \"not joining\" and \"no association or organization\", indicating that the person under examination is in a state of non-participation in any association or organization, and that the act is described in the conduct of the time, and therefore is judged to be the present act."}, "sentence_type": "Negative declarative sentence", "pos": {"nouns": {"Questionnaire": 1, "Entry": 1, "Associations": 1, "Organizations": 1}, "verbs": {"Add": 2}, "adverbs": {"Any": 1}, "numerals": {"10": 1}, "classifiers": {"individual": 1}, "pronouns": {"I": 1}}}, {"content": "I have not spoken to anyone today.", "word_count": 11, "label": "", "sentence_structure": "Subject鈥搗erb鈥揷omplement", "subjective_objective": {"answer": "Objective", "rationale": "Because \"No one talks to you today\" is an objective fact, which describes the specific behaviour of the patient in the day. Although it may be related to the patient ' s subjective emotional state, the behaviour itself is objective. Thus, the entry is considered from an objective perspective."}, "sentence_sentiment": {"answer": "Negative", "rationale": "Because the \"no talk\" act in the entry is negative."}, "language_style": {"answer": "Calm and direct", "rationale": "The entry describes the social behaviour of the person under investigation in direct and concise language, without excessive emotional accoutrements or subjective evaluation, and with a more calm and objective language style."}, "time_dimension": {"answer": "Present", "rationale": ""}, "sentence_type": "Affirmative declarative sentence", "pos": {"nouns": {"person": 2, "Word": 1}, "verbs": {"Say it": 1, "Call": 1}, "adverbs": {"Any": 1, "Today": 1}, "numerals": {"11": 1}, "pronouns": {"I": 1}}}, {"content": "12. I have no common themes with those around me.", "word_count": 14, "label": "", "sentence_structure": "Subject鈥揷opula鈥損redicative", "subjective_objective": {"answer": "Subjective", "rationale": "Because \"the common theme\" is the feeling and experience of the patient, and it's a subjective judgment."}, "sentence_sentiment": {"answer": "Neutral", "rationale": "Because the entry merely stated a fact, it did not express any emotional colour."}, "language_style": {"answer": "Tactful and warm", "rationale": "The entry is more euphemistic, conveys feelings and attitudes in a first person鈥檚 way of expression, and expresses the individual鈥檚 social distress and desires. At the same time, the subject is not spoken in too direct or negative terms, but rather in a more peaceful way."}, "time_dimension": {"answer": "Present", "rationale": "In that sentence, the relationship between me and the people around us is the state of things, and the \"no common subject\" is the current situation. So, the entry describes the current situation."}, "sentence_type": "Negative declarative sentence", "pos": {"nouns": {"Problem": 1, "person": 1, "Topic": 1, "Discussion": 1}, "verbs": {"Call": 1, "have": 1, "No, I'm not": 1, "Yeah, I can talk about it": 1}, "numerals": {"12": 1}, "classifiers": {"individual": 1}, "pronouns": {"I": 1}}}, {"content": "I do not show myself more when I'm with others.", "word_count": 16, "label": "", "sentence_structure": "Subject鈥搗erb鈥揷omplement", "subjective_objective": {"answer": "Subjective", "rationale": "Because \"more disclosure\" is a subjective attitude and behaviour of an individual."}, "sentence_sentiment": {"answer": "Negative", "rationale": "Because the \"no more self-dissemination\" in the entry is negative."}, "language_style": {"answer": "Calm and direct", "rationale": "The entry is more calm and straightforward, without using too much qualifier or emotionally strong expressions. It simply raises the question of whether individuals tend to show themselves more in their relations with others, in an objective and impartial manner."}, "time_dimension": {"answer": "Present", "rationale": "\"When you're with someone else\" means \"to be with someone else\" or \"to be with someone else,\" \"to be less open\" means \"not negative,\" and \"to be more open\" is an ongoing move. So the entry asks the testee about his behavioral orientation in dealing with someone else, and the temporal dimension is now."}, "sentence_type": "Negative declarative sentence", "pos": {"verbs": {"Get along": 1, "Tanlu": 1}, "adjectives": {"self": 1}, "adverbs": {"No, it's not": 1}, "numerals": {"13": 1}}}, {"content": "I do not take the risk of socialization.", "word_count": 7, "label": "", "sentence_structure": "Subject鈥搗erb鈥揷omplement", "subjective_objective": {"answer": "Subjective", "rationale": "Because \"I\" is patient's subjective attitude."}, "sentence_sentiment": {"answer": "Negative", "rationale": "Because \"no social risk\" suggests that the person may be reluctant to engage and establish contact with others on his own initiative, this may be negative."}, "language_style": {"answer": "Calm and direct", "rationale": "The entry is simple and direct in asking the other party whether it is risk-taking, is not too muzzled or emotional, and is more objective and calm."}, "time_dimension": {"answer": "Present", "rationale": "I mean, \"I\" is the first person's number, \"no\" is the negative, \"social risk\" is a moving phrase, which means a behavior or state. So, \"I don't risk social life\" is the current situation, where the testee does not now choose to take risks in social activities."}, "sentence_type": "Negative declarative sentence", "pos": {"nouns": {"Social": 1, "Risk": 1}, "verbs": {"-Put it on": 1}, "adverbs": {"not": 1}}}, {"content": "I was not seen as interesting.", "word_count": 8, "label": "", "sentence_structure": "Subject鈥搗erb鈥揷omplement", "subjective_objective": {"answer": "Subjective", "rationale": "Because \"Does I seem interesting to people\" is based on the views and opinions of others and is subjective."}, "sentence_sentiment": {"answer": "Negative", "rationale": "Because the entry indicates that there is no interest or disinterest in the interviewee, and it is somewhat derogatory."}, "language_style": {"answer": "Tactful and warm", "rationale": "The entry uses an indirect and moderate expression that avoids direct accusations or attacks on the other, making it more comfortable and comfortable to answer. At the same time, it also increases the willingness of respondents to share real ideas and feelings, thereby increasing the effectiveness and accuracy of questionnaires."}, "time_dimension": {"answer": "Present", "rationale": "\"Don't feel\" expresses a sense and a view of the present, so the temporal dimension of the article is now."}, "sentence_type": "Negative declarative sentence", "pos": {"nouns": {"person": 1, "I": 1}, "verbs": {"I think": 1, "Interesting": 1}, "adverbs": {"not": 1}, "numerals": {"15": 1, "1": 2}, "classifiers": {"individual": 1}}}, {"content": "I do not feel like a close friend.", "word_count": 7, "label": "", "sentence_structure": "Subject鈥搗erb鈥揷omplement", "subjective_objective": {"answer": "Subjective", "rationale": "Because \"think\" is a subjective feeling or judgment, reflects the patient's subjective assessment of his/her social relations and is a subjective view."}, "sentence_sentiment": {"answer": "Neutral", "rationale": "The entry is not a positive or negative emotional expression."}, "language_style": {"answer": "Tactful and warm", "rationale": "By using the negative word \"no feeling\" instead of simply saying that there is no friend, the entry avoids being too direct in language style, making respondents more comfortable and natural. Moreover, the use of the word \"good friend\" gives people a warmer, more caring feeling, and more willingness to share their true thoughts."}, "time_dimension": {"answer": "Present", "rationale": "\"No feeling\" is a negative assessment of the current state, while \"have a friend\" is a reference to the current situation of having a friend, so the entry is a question of whether the person tested currently has a friend, and the time dimension is now."}, "sentence_type": "Negative declarative sentence", "pos": {"nouns": {"Friend": 1}, "verbs": {"I think": 1, "have": 1}, "adverbs": {"Nope": 1}}}, {"content": "I'm afraid to believe in others.", "word_count": 7, "label": "", "sentence_structure": "Subject鈥搗erb鈥揷omplement", "subjective_objective": {"answer": "Subjective", "rationale": "Because the words \"fear\" and \"convinced\" are all words that describe the patient's own feelings and attitudes, they are examined from a subjective point of view."}, "sentence_sentiment": {"answer": "Negative", "rationale": "Because the word \"fear\" has a negative emotional connotation."}, "language_style": {"answer": "Calm and direct", "rationale": "The entry directly expresses the subject of asking the other party whether they are often afraid to believe that others are feeling, and is not based on excessive rhetorical or emotional rendering, which is more objective in tone."}, "time_dimension": {"answer": "Present", "rationale": ""}, "sentence_type": "Negative declarative sentence", "pos": {"verbs": {"I": 1, "Scared": 1, "Yes": 1}, "adjectives": {"Don't": 1}, "adverbs": {"very": 1}, "pronouns": {"others": 1}}}, {"content": "I don't think I have a partner (or a man/girlfriend).", "word_count": 13, "label": "", "sentence_structure": "Subject鈥搗erb鈥揷omplement", "subjective_objective": {"answer": "Subjective", "rationale": "Because this sentence describes the subjective attitude of the patient to self-perception and cognitive knowledge, that is, they think they have no partner. This is not an objective fact, but rather a subjective feeling of the patient."}, "sentence_sentiment": {"answer": "Negative", "rationale": "The expression of this article is negative because it expresses the loneliness of a person and the lack of a partner."}, "language_style": {"answer": "Calm and direct", "rationale": "The subject is based on a direct and clear question, without any qualifier or emotional term. It asks the person surveyed whether he or she has a fixed partner, is clear in language, is non-subjective and is consistent with the straight language style of calmness."}, "time_dimension": {"answer": "Present", "rationale": "The entry is the subjective assessment of the present partner of the testee, with no clear time limit, and therefore is judged as \"now\"."}, "sentence_type": "Negative declarative sentence", "pos": {"nouns": {"Partner": 1, "Men": 1, "Girlfriend": 1}, "verbs": {"I think": 1, "have": 1}, "adverbs": {"Nope": 1}, "numerals": {"3": 1}, "pronouns": {"I": 1}}}, {"content": "4. When sharing my difficulties, my friends felt a burden.", "word_count": 18, "label": "", "sentence_structure": "Subject鈥搗erb鈥搊bject", "subjective_objective": {"answer": "Subjective", "rationale": "Because feeling a burden is a subjective feeling of the patient's best friend."}, "sentence_sentiment": {"answer": "Negative", "rationale": "Because the attitude of feeling burden in the entry is negative."}, "language_style": {"answer": "Tactful and warm", "rationale": "The entry uses a more euphemistic expression to describe the fact that \"a good friend feels that sharing difficulties is a burden.\" Although the expression is somewhat negative, it is not too direct or critical of the other, but rather presents it in a peaceful way. It is more friendly, more acceptable and able to effectively guide the respondents to reflect and answer questions."}, "time_dimension": {"answer": "Past", "rationale": "The entry describes the experience of the testee鈥檚 best friends with the past, i.e., at some point in the past, they consider sharing the testee鈥檚 difficulties to be a burden. The time dimension is therefore a matter of the past."}, "sentence_type": "Negative declarative sentence", "pos": {"nouns": {"Share": 1, "Difficulties": 1, "Friends": 1, "Burden": 1}, "verbs": {"I think": 1, "be": 1, "Share": 1}, "numerals": {"Four": 1}, "pronouns": {"I": 1}}}, {"content": "5. I feel that others do not rely on me, nor do I feel important.", "word_count": 16, "label": "", "sentence_structure": "Subject鈥搗erb鈥揷omplement", "subjective_objective": {"answer": "Subjective", "rationale": "Because this is a subjective examination of the patient ' s own views and feelings."}, "sentence_sentiment": {"answer": "Negative", "rationale": "Because the words \"not depending on me\" and \"not important to me\" in the entries are all negative emotional."}, "language_style": {"answer": "Calm and direct", "rationale": "The entry is more concise, has no excessive rhetorical or emotional rendering, and is more objective. It asks whether the person surveyed feels that he or she is in the eyes of others and that the question is generally sensitive or uncomfortable, but the direct expression is more accurate in the person under investigation."}, "time_dimension": {"answer": "Present", "rationale": "The term \"the feeling\" in the entry uses the general current, which is the current state of feeling and awareness, and therefore the judgement is now."}, "sentence_type": "Negative declarative sentence", "pos": {"nouns": {"others": 1, "Dependency": 1, "I": 2, "Important": 1}, "verbs": {"I think": 2}, "adverbs": {"not": 2}}}, {"content": "I don't think I can talk to anyone.", "word_count": 12, "label": "", "sentence_structure": "Subject鈥揷opula鈥損redicative", "subjective_objective": {"answer": "Subjective", "rationale": "I think it's the patient's subjective idea."}, "sentence_sentiment": {"answer": "Negative", "rationale": "Because the \"not having a heart of anyone\" in the entry expresses a feeling of loneliness and helplessness, a negative emotional attitude."}, "language_style": {"answer": "Tactful and warm", "rationale": "The entry is more adept and interactive when it is spoken in a gentle and inviting manner, rather than by direct statements or orders."}, "time_dimension": {"answer": "Present", "rationale": "The keyword \"thinks\" and \"can't\" and \"conceals with anyone\" all indicate the subjective perception of the testee of the current state, so the temporal dimension of the article is now."}, "sentence_type": "Negative declarative sentence", "pos": {"nouns": {"Questionnaire Entry": 1, "person": 1, "- A relationship": 1}, "verbs": {"I think": 1, "Cannot": 1, "Call": 2}, "adverbs": {"Any": 1}, "classifiers": {"individual": 1}, "pronouns": {"I": 1}}}, {"content": "I don't think it's understandable.", "word_count": 7, "label": "", "sentence_structure": "Subject鈥揷opula鈥損redicative", "subjective_objective": {"answer": "Subjective", "rationale": "Because \"think\" is a verb of subjective feelings, which means that the patient does not think that he is understood and accepted. It is a personal experience and judgment of the patient, and therefore is subjective."}, "sentence_sentiment": {"answer": "Negative", "rationale": "Because the attitude of \"not understood\" in the entry is negative."}, "language_style": {"answer": "Calm and direct", "rationale": "The language style of the entry is relatively calm, not too much rhetorical or emotional, but rather a direct statement of what the interviewee may feel, i.e., feeling ununderstandable. This simple and clear language style helps the interviewee to express his thoughts and feelings more accurately."}, "time_dimension": {"answer": "Present", "rationale": "The keyword \"I feel\" and \"I don't understand\" means the current feelings and thoughts of the speaker, so the time dimension of the article is now."}, "sentence_type": "Negative declarative sentence", "pos": {"nouns": {"Understood": 1}, "verbs": {"I think": 1, "By": 1}, "pronouns": {"I": 1}}}, {"content": "I don't think it's safe to beg.", "word_count": 10, "label": "", "sentence_structure": "Subject鈥揷opula鈥損redicative", "subjective_objective": {"answer": "Subjective", "rationale": "Because \"I feel\" is a subjective verb, which indicates that it is a patient's personal feeling or belief, not a proven fact. So, \"I don't feel safe to ask for someone else\" is viewed from the patient's subjective perspective."}, "sentence_sentiment": {"answer": "Negative", "rationale": "Because the word \"unsafe\" in the entry is negative, it expresses concern and anxiety about seeking help from others."}, "language_style": {"answer": "Calm and direct", "rationale": "The language style of the entry is more straightforward, with no excessive embellishment or emotional rendering, and is more objective in tone, and can therefore be judged as \"cool straight\"."}, "time_dimension": {"answer": "Present", "rationale": "In the future, the entry indicates that the testee may feel this in the future, and the keyword \"felt\" and \"unsafe\" points to situations that have not yet occurred and that may occur in the future. The judgement is therefore for the future."}, "sentence_type": "Negative declarative sentence", "pos": {"nouns": {"Questionnaire Entry": 1, "person": 2, "Clear": 1}, "verbs": {"I think": 1, "Please": 1, "and": 1, "not": 1}, "adjectives": {"others": 1, "self": 1}, "adverbs": {"No, it's not": 1}, "numerals": {"8": 1}, "pronouns": {"I": 1}}}, {"content": "I feel so alone.", "word_count": 5, "label": "", "sentence_structure": "Subject鈥揷opula鈥損redicative", "subjective_objective": {"answer": "Subjective", "rationale": "Because loneliness is a subjective emotional experience, a feeling and perception within a person, not an objective reality or behaviour. While it may be influenced by external factors (such as changes in social circumstances, changes in relationships, etc.), it ultimately depends on the individual鈥檚 psychological state and subjective feelings."}, "sentence_sentiment": {"answer": "Negative", "rationale": "Because the \"lonely\" emotion is negative."}, "language_style": {"answer": "Tactful and warm", "rationale": "The entry is more personal and interactive in a gentle and inviting tone of speech, rather than in direct statements or orders. At the same time, the theme \"I feel alone\" is relatively sensitive and personal, and the euphemism is more protective of the feelings and privacy of the interviewees."}, "time_dimension": {"answer": "Present", "rationale": "The entry does not refer to a particular point or event of the past or future, but rather expresses a current emotional state or feeling, so that it can be judged as \"now\"."}, "sentence_type": "Affirmative declarative sentence", "pos": {"nouns": {"Loneliness": 1}, "verbs": {"I feel": 1}}}, {"content": "I feel like a member of any association or organization.", "word_count": 15, "label": "", "sentence_structure": "Subject鈥揷opula鈥損redicative", "subjective_objective": {"answer": "Subjective", "rationale": "The patient's subjective feelings and attitudes are felt as part of any association or organization."}, "sentence_sentiment": {"answer": "Neutral", "rationale": "Since the entry does not express positive or negative feelings or attitudes, it merely describes a feeling or view."}, "language_style": {"answer": "Tactful and warm", "rationale": "The entry is more euphemistic and warm. It asks the other side whether it wishes to be a member of an association or organization in an invitation-based manner, without being too direct or hard-lined. Moreover, the use of the subjective term \"I think it\" is more reflective of the investigative 's desire to get personal feelings and views from the person under investigation."}, "time_dimension": {"answer": "Present", "rationale": "The entry expresses the present sense of self-identification and belonging of the person being tested and is a description of the behaviour in its present state."}, "sentence_type": "Affirmative declarative sentence", "pos": {"nouns": {"Associations": 1, "Organizations": 1, "Members": 1}, "verbs": {"I think": 1, "be": 1, "One": 1, "Members": 1}, "numerals": {"Any": 1, "10": 1}}}, {"content": "I feel like I have no contact with anyone today.", "word_count": 13, "label": "", "sentence_structure": "Subject鈥搗erb鈥揷omplement", "subjective_objective": {"answer": "Objective", "rationale": "Because today is over, it is the objective result. The act is done with objectivity, and the subjective idea is examined with subjectiveness."}, "sentence_sentiment": {"answer": "Negative", "rationale": "Because the \"no contact\" act in the entry is negative."}, "language_style": {"answer": "Calm and direct", "rationale": "This language style is usually used in psychological questionnaires to collect basic information about the person interviewed or to view phenomena, behaviours and behaviours, to facilitate the researcher鈥檚 data analysis and further research."}, "time_dimension": {"answer": "Present", "rationale": "The keywords \"like\" and \"today\" indicate that the entry is asking about the current state of feeling and state and is therefore a matter of past times."}, "sentence_type": "Affirmative declarative sentence", "pos": {"verbs": {"I think": 1, "Exposure": 1, "Anyone": 1}, "adjectives": {"Today": 1}, "adverbs": {"Both": 1}, "numerals": {"11": 1}, "pronouns": {"I": 1}}}, {"content": "I feel like I have nothing to say to anyone else.", "word_count": 10, "label": "", "sentence_structure": "Subject鈥揷opula鈥損redicative", "subjective_objective": {"answer": "Subjective", "rationale": "Because the word \"thinks\" suggests that it's a subjective expression of what patients feel about themselves."}, "sentence_sentiment": {"answer": "Negative", "rationale": "Because the \"necessary feeling\" attitude in the entry is negative."}, "language_style": {"answer": "Tactful and warm", "rationale": "The language style of the entry is more euphemistic and implicit, using the expression of a subjective individual's perception, without directly accusing or criticizing others, making the respondents more comfortable and natural in answering questions. At the same time, \"no words\" is also a more common social experience, which is more resonant and understandable."}, "time_dimension": {"answer": "Present", "rationale": "The keyword \"necessary to say\" means the current feeling and state, and the time is now."}, "sentence_type": "Negative declarative sentence", "pos": {"nouns": {"Questionnaire": 1, "Entry": 1, "others": 1}, "verbs": {"I think": 1, "and": 1, "Nothing to say": 1}, "adverbs": {"I think": 1}, "numerals": {"12": 1}}}, {"content": "I feel that I am no longer the person I am when I am with.", "word_count": 16, "label": "", "sentence_structure": "Subject鈥揷opula鈥損redicative", "subjective_objective": {"answer": "Subjective", "rationale": "Because \"not me,\" is the patient's own subjective feeling and perception."}, "sentence_sentiment": {"answer": "Negative", "rationale": "Because the entry expresses a negative emotion and attitude, which means feeling lost to yourself."}, "language_style": {"answer": "Tactful and warm", "rationale": "The entry is more personal and interactive when it is spoken in a gentle and inviting manner, expressing feelings of dealing with people and not directly speaking or ordering."}, "time_dimension": {"answer": "Present", "rationale": "The entry is for the purpose of asking the testee about his/her own perception and feelings during his/her dealings with others, and the time dimension is now judged."}, "sentence_type": "Affirmative declarative sentence", "pos": {"nouns": {"Questionnaire Entry": 1, "Get along": 1, "I": 2, "others": 1}, "verbs": {"I think": 1, "be": 1, "Not anymore": 1}, "adverbs": {"It was": 1}, "numerals": {"13": 1}}}, {"content": "14. I am embarrassed to be with others.", "word_count": 11, "label": "", "sentence_structure": "Subject鈥揷opula鈥損redicative", "subjective_objective": {"answer": "Subjective", "rationale": "Because embarrassment is a subjective emotion and feeling, it is a description of the feelings that patients experience in their interpersonal relationships and is therefore a subjective examination."}, "sentence_sentiment": {"answer": "Negative", "rationale": "Because the \"smuggling\" in the entry is negative."}, "language_style": {"answer": "Calm and direct", "rationale": "The entry uses a simple and clear language style, asking the other person whether he or she feels embarrassed in his or her dealings with another person, without using excessive qualifiers or emotional colours. Thus, his or her language style can be described as \"cooling straight\"."}, "time_dimension": {"answer": "Present", "rationale": ""}, "sentence_type": "Negative declarative sentence", "pos": {"nouns": {"others": 1, "Get along": 1}, "verbs": {"I feel": 1, "I'm embarrassed": 1}}}, {"content": "I don't feel like I'm funny.", "word_count": 8, "label": "", "sentence_structure": "Subject鈥搗erb鈥揷omplement", "subjective_objective": {"answer": "Subjective", "rationale": "Because \"not feeling interesting\" is what patients say about themselves and feel about themselves."}, "sentence_sentiment": {"answer": "Negative", "rationale": "Because the expression \"does not find yourself interesting\" in the entry is an expression of a negative attitude."}, "language_style": {"answer": "Tactful and warm", "rationale": "The entry appears to be more gentle and euphemistic by asking the other side about himself rather than directly evaluating whether the other side is interesting, and also provides the interviewee with an opportunity to express his feelings."}, "time_dimension": {"answer": "Present", "rationale": ""}, "sentence_type": "Negative declarative sentence", "pos": {"nouns": {"self": 1, "Fun": 1}, "verbs": {"Not really": 1}, "adjectives": {"Interesting": 1}, "numerals": {"15": 1}, "pronouns": {"I": 1}}}]}

{"questionnaire_title": "Examination anxiety table (score-counting rules and evaluation)", "questionnaire_category": "Mental health", "item_count": 37, "emotion_shift_overall": "From positive to negative", "language_style_overall": "Neutral", "items": [{"content": "One, when a big test comes, I always think people are smarter than me.", "word_count": 25, "label": "", "sentence_structure": "Subject鈥揷opula鈥損redicative", "subjective_objective": {"answer": "Subjective", "rationale": "Because \"better than anyone else\" is the patient's perception and evaluation of himself, and it's a subjective view."}, "sentence_sentiment": {"answer": "Negative", "rationale": "Because the attitude in the entry, \"I'm always thinking that people are much smarter than me,\" is negative."}, "language_style": {"answer": "Calm and direct", "rationale": "The language style of the entry is relatively calm and straightforward, and it raises questions in a simple and clear presentation, without too many emotional or rhetorical techniques. This expression makes it easier to understand and answer questions and avoids subjective assumptions and misleading situations."}, "time_dimension": {"answer": "Present", "rationale": "The entry describes a psychological state or behaviour that often occurs in a given situation (i.e., the forthcoming test) (i.e., the idea that someone is always smarter than himself). Since this mental state or behaviour has already occurred in the past, we can judge that it is a description of the past."}, "sentence_type": "Negative declarative sentence", "pos": {"nouns": {"Number of times": 1, "Major examinations": 1, "person": 1}, "verbs": {"Yes": 2, "Come on": 2, "- Yeah": 1, "More": 1}, "adjectives": {"Smart": 1}, "adverbs": {"Always": 1, "Just": 1}, "numerals": {"1": 3, "2": 1}, "classifiers": {"When": 1}, "pronouns": {"I": 1, "others": 1}}}, {"content": "If I'm going to do a smart test, I'll be very anxious before I do it.", "word_count": 22, "label": "", "sentence_structure": "Subject鈥損redicate", "subjective_objective": {"answer": "Subjective", "rationale": "Because anxiety is a subjective emotional experience, it's the feeling that comes from the heart of the individual, so \"I'll be very anxious before I do it\" is the patient's subjective attitude."}, "sentence_sentiment": {"answer": "Negative", "rationale": "Because the description of \"very anxious\" in the entry is negative."}, "language_style": {"answer": "Calm and direct", "rationale": "The language style of the entry is more direct and objective, and there is no excessive emotional or rhetorical use, but simply a statement of the fact that there was anxiety about the test."}, "time_dimension": {"answer": "Present", "rationale": ""}, "sentence_type": "Affirmative declarative sentence", "pos": {"nouns": {"Number of times": 1, "Smart Test": 1, "Do it": 1, "Front": 1, "Anxiety": 1}, "verbs": {"Yes, I'm going": 1, "Do it": 2, "Yes": 1}, "adjectives": {"Very": 1}, "adverbs": {"Before": 1}, "numerals": {"2": 1}, "pronouns": {"I": 1}}}, {"content": "Three, if I knew there would be a smart test, I'd feel confident and relaxed before that.", "word_count": 27, "label": "", "sentence_structure": "Subject鈥搗erb鈥揷omplement", "subjective_objective": {"answer": "Subjective", "rationale": "Because \"fears confident, relaxing\" is the patient's subjective feeling and attitude."}, "sentence_sentiment": {"answer": "Positive", "rationale": "Because the entry \"felts confident and easy\" expresses positive emotional attitudes."}, "language_style": {"answer": "Tactful and warm", "rationale": "The entry is more personal and interactive when it is spoken in a gentle and inviting tone, rather than in direct statements or orders. At the same time, the word \"you\" is used to reach out to the interviewees, to make them feel that they are being cared for and respected, and therefore the language style is more euphemistic and warm."}, "time_dimension": {"answer": "Present", "rationale": ""}, "sentence_type": "Exclamatory sentence", "pos": {"nouns": {"Smart Test": 1, "Confidence": 1, "Easy": 1}, "verbs": {"Yes": 2, "have": 1, "I feel": 1, "be": 1, "Yes, I will": 1, "in": 1, "Before": 1, "very": 2}, "adjectives": {"Smart": 1, "Self-confident": 1, "Easy": 1}, "adverbs": {"Before": 1, "very": 2}, "numerals": {"3": 1}, "pronouns": {"I": 1}}}, {"content": "I'll sweat a lot when I take a big test.", "word_count": 13, "label": "", "sentence_structure": "Subject鈥搗erb鈥揷omplement", "subjective_objective": {"answer": "Objective", "rationale": "Because sweating is a natural reaction to stress, not a subjective attitude of the patient."}, "sentence_sentiment": {"answer": "Negative", "rationale": "Because sweating is usually seen as a form of tension or anxiety, in this situation, sweating can be a negative emotional reaction."}, "language_style": {"answer": "Calm and direct", "rationale": "The entry is directly asked whether the person under investigation is sweating when taking a major examination, is more objective and does not use excessive emotional colours or rhetorical techniques."}, "time_dimension": {"answer": "Present", "rationale": "The keyword \"at the time of the major exam\" indicates that this is an ongoing situation, and that's what's going to sweat, and the time dimension of this questionnaire entry is now."}, "sentence_type": "Affirmative declarative sentence", "pos": {"nouns": {"Examinations": 1, "Khan": 1}, "verbs": {"Participation": 1, "Out": 1}, "adverbs": {"Significant": 1}, "numerals": {"4": 1}}}, {"content": "During the exam, I found myself thinking about things that have nothing to do with the exam.", "word_count": 24, "label": "", "sentence_structure": "Subject鈥搗erb鈥搊bject", "subjective_objective": {"answer": "Subjective", "rationale": "Because \"thinking something that has nothing to do with the test\" is the patient's subjective idea."}, "sentence_sentiment": {"answer": "Negative", "rationale": "Because the \"always thinking of something that has nothing to do with the test\" act in the entry is negative."}, "language_style": {"answer": "Calm and direct", "rationale": "The basis for the judgement: The entry is expressed in a concise and clear way, i.e., it is distracted during the examination by other things that are not relevant to the examination. It is described in simple and straightforward language, without too much emotional or euphemism, and can therefore be judged as a straightforward style of calm."}, "time_dimension": {"answer": "Present", "rationale": "\"Step of examination\" refers to a specific period of time, i.e., the examination process under way or in the future, and the entry therefore describes the current behaviour."}, "sentence_type": "Affirmative declarative sentence", "pos": {"nouns": {"Examinations": 1, "Period": 1, "self": 1, "Contents": 1, "Yes": 1}, "verbs": {"Found": 1, "Always": 1, "Yes": 1, "Not relevant": 1, "Examination": 1}, "adverbs": {"and": 1, "Some": 1, "It's not about that": 1}, "numerals": {"5": 1}, "pronouns": {"I": 1}}}, {"content": "Six, I was scared when a sudden attack test came.", "word_count": 19, "label": "", "sentence_structure": "Subject鈥搗erb鈥搊bject", "subjective_objective": {"answer": "Subjective", "rationale": "Because \"fear\" is a subjective feeling or emotional reaction, a self-perception of the patient's feelings in the face of a sudden examination."}, "sentence_sentiment": {"answer": "Negative", "rationale": "Because the word \"fear\" in the entry is an expression of a negative emotion."}, "language_style": {"answer": "Calm and direct", "rationale": "The entry is asked in a direct, concise manner, without using euphemism or rhetoric, but rather directly expressing the information that the interrogator wants to know, in a more honest and objective manner."}, "time_dimension": {"answer": "Present", "rationale": "The keyword \"when a sudden attack comes\" means a sudden situation, in which case the \"I'm afraid\" reaction is also immediate, and therefore judgment is now."}, "sentence_type": "Negative declarative sentence", "pos": {"nouns": {"Examinations": 1, "Attack": 1, "Yes": 1}, "verbs": {"Come on": 1, "I feel": 1}, "adjectives": {"Suddenly": 1}, "adverbs": {"When": 1, "Time": 1, "very": 1}, "numerals": {"6": 1}}}, {"content": "Seven. I always thought I'd fail during the exam.", "word_count": 12, "label": "", "sentence_structure": "Subject鈥搗erb鈥揷omplement", "subjective_objective": {"answer": "Subjective", "rationale": "Because \"often\" indicates that it's a patient's own subjective idea or concern."}, "sentence_sentiment": {"answer": "Negative", "rationale": "Because the idea of \"thinking it's gonna fail\" in the entry is negative."}, "language_style": {"answer": "Calm and direct", "rationale": ""}, "time_dimension": {"answer": "Present", "rationale": "The entry asks whether the testee will often fail during the examination, and the time dimension is judged by past and present. The keyword \"testing\" indicates a particular situation, and \"often\" highlights the frequency and persistence of this idea. Thus, the entry relates to both past behaviour (the previous examination experience) and the current psychological state (the present feelings of fear, anxiety, etc.)."}, "sentence_type": "Negative declarative sentence", "pos": {"nouns": {"7": 1, "Examination period": 1, "Failed": 1}, "verbs": {"Yes": 2, "Present": 1}, "adverbs": {"Regular": 1}, "numerals": {"7": 1}, "pronouns": {"I": 1}}}, {"content": "After the big test, I get nervous and I get sick.", "word_count": 18, "label": "", "sentence_structure": "Subject鈥搗erb鈥揷omplement", "subjective_objective": {"answer": "Subjective", "rationale": "It's the patient's subjective feeling and experience."}, "sentence_sentiment": {"answer": "Negative", "rationale": "Because the description of \"frightful\" and \"incontestine\" in the entries is negative."}, "language_style": {"answer": "Tactful and warm", "rationale": "The language style of the entry is more euphemistic and concerned with the individual's feelings and experiences, making the issue more humane and close to the mood of the person under investigation by using descriptive terms such as \"often\" and \"so\" . At the same time, there is no too direct or command-style tone, which makes it more acceptable and willing to answer the question."}, "time_dimension": {"answer": "Present", "rationale": ""}, "sentence_type": "Affirmative declarative sentence", "pos": {"nouns": {"Major examinations": 1, "Nervous": 1, "Stomach": 1, "I'm not feeling well": 1}, "verbs": {"I feel": 1, "鈾?And I'm gonna be 鈾?: 1}, "adverbs": {"Regular": 1, "So": 1}, "numerals": {"8": 1}, "pronouns": {"I": 1}}}, {"content": "Nine, I'm always getting a little bit confused about things like smart and finals.", "word_count": 20, "label": "", "sentence_structure": "Subject鈥搗erb鈥搊bject", "subjective_objective": {"answer": "Subjective", "rationale": "Because \"fun\" is the patient's subjective feeling and attitude to things."}, "sentence_sentiment": {"answer": "Negative", "rationale": "Because the mood of \"fun\" in the entry is negative."}, "language_style": {"answer": "Calm and direct", "rationale": "The entry directly expresses the subject of questioning whether the other party is afraid of similar things, such as smart or final exams, and is not using excessive rhetorical or emotional rendering, which is more objective and balanced."}, "time_dimension": {"answer": "Past", "rationale": "The word \"for the whole feeling\" in \"for the whole feeling\" indicates a constant state of mind or habit, implying that the feeling has been going on for some time. And the \"frightful feeling\" movement has taken place over the past period, so it can be judged that the entry describes the feelings and experiences of the subject."}, "sentence_type": "Negative declarative sentence", "pos": {"nouns": {"Smart test": 1, "Final examination": 1, "Yes": 1}, "verbs": {"to": 1, "I feel like": 1, "- What": 1}, "adverbs": {"Total": 1}, "numerals": {"9": 1}}}, {"content": "10, a good score in one test doesn't seem to increase my confidence in the second.", "word_count": 29, "label": "", "sentence_structure": "Subject鈥搗erb鈥搊bject", "subjective_objective": {"answer": "Subjective", "rationale": "Because it is the patient ' s own subjective view and feeling that increases confidence if good results are achieved."}, "sentence_sentiment": {"answer": "Negative", "rationale": "Because the idea that \"good results don't increase my confidence in the second exam\" in the entry is negative."}, "language_style": {"answer": "Tactful and warm", "rationale": ""}, "time_dimension": {"answer": "Past", "rationale": "The entry describes reflections and summations of past examination experience, with the keywords \"achievement of good results\" and \"not increasing my confidence in the second exam.\" It can therefore be judged as questioning the testee's past behavior or mentality."}, "sentence_type": "Negative declarative sentence", "pos": {"nouns": {"Achievements": 2, "Examinations": 2, "Confidence": 1}, "verbs": {"Access": 1, "Increase": 1, "Yes": 1, "in": 1}, "adjectives": {"Okay": 1}, "adverbs": {"Seems like": 1, "No, I can't": 1}, "numerals": {"10": 1, "Once": 1, "Second": 1}, "pronouns": {"I": 1}}}, {"content": "Eleven, during the big test, I sometimes felt my heart beat fast.", "word_count": 16, "label": "", "sentence_structure": "Subject鈥搗erb鈥揷omplement", "subjective_objective": {"answer": "Subjective", "rationale": "Because \"heart beats fast\" is a subjective feeling. Although the pace of heart beats is caused by a physical reaction, it is a personal subjective experience and explanation."}, "sentence_sentiment": {"answer": "Negative", "rationale": "Because \"fast heartbeat\" is usually associated with anxiety or stress, which is a negative emotional reaction."}, "language_style": {"answer": "Tactful and warm", "rationale": ""}, "time_dimension": {"answer": "Present", "rationale": "The entry is for the testee ' s heart rate during the major examination, and the time dimension is now judged."}, "sentence_type": "Affirmative declarative sentence", "pos": {"nouns": {"Major examination period": 1, "Heart rate": 1}, "verbs": {"I feel": 1}, "adverbs": {"Sometimes": 1}, "numerals": {"11": 1}}}, {"content": "I always thought I'd do better after the exam.", "word_count": 20, "label": "", "sentence_structure": "Subject鈥揷opula鈥損redicative", "subjective_objective": {"answer": "Subjective", "rationale": "Because this feeling is an individual's subjective judgement and experience."}, "sentence_sentiment": {"answer": "Positive", "rationale": "Because the entry \"thinks it can do better than it actually does\" expresses a positive attitude, that is, a sense of confidence and expectation."}, "language_style": {"answer": "Calm and direct", "rationale": "The basis for the decision: The article is formulated in plain language, without any rhetorical or emotional recitation, and the interviewee is asked whether he or she always feels better than he or she actually does after the examination. Such expression is more objective, direct and understandable."}, "time_dimension": {"answer": "Past", "rationale": ""}, "sentence_type": "Affirmative declarative sentence", "pos": {"nouns": {"Examinations": 1, "End": 1, "I think": 1}, "verbs": {"Over": 1, "Do it": 1, "- Yeah": 1}, "adjectives": {"Actual": 1}, "adverbs": {"Always": 1}, "numerals": {"12": 1}, "pronouns": {"I": 1}}}, {"content": "I'm always depressed after the exams.", "word_count": 13, "label": "", "sentence_structure": "Subject鈥揷opula鈥損redicative", "subjective_objective": {"answer": "Subjective", "rationale": "Because \"suffering from depression\" is a subjective feeling."}, "sentence_sentiment": {"answer": "Negative", "rationale": "Because the attitude of \"distressed\" in the entry is negative."}, "language_style": {"answer": "Tactful and warm", "rationale": "The language style of the entry is euphemistic, because it uses a gentle and inviting tone of speech to engage in dialogue, does not use excessive rhetorical or emotional accents, and is more objective. It expresses the feelings that the other party may feel depressed after the examination in a kind and interactive manner, avoids direct accusations or orders and makes it easier for the interviewees to accept and respond."}, "time_dimension": {"answer": "Present", "rationale": ""}, "sentence_type": "Affirmative declarative sentence", "pos": {"nouns": {"Examinations": 1, "Depression": 1}, "verbs": {"Over": 1, "I feel": 1, "be": 1}, "adjectives": {"very": 1}, "numerals": {"13": 1}, "pronouns": {"I": 1}}}, {"content": "I've always had a nervous feeling before every final exam.", "word_count": 20, "label": "", "sentence_structure": "Subject鈥揷opula鈥損redicative", "subjective_objective": {"answer": "Subjective", "rationale": "Because \"frightful feeling\" is a subjective experience and feeling that patients describe their psychological state. Although this feeling may be related to the upcoming final exam, it is itself generated by the patient's subjective consciousness."}, "sentence_sentiment": {"answer": "Negative", "rationale": "Because \"sweet\" is usually a negative emotional experience."}, "language_style": {"answer": "Calm and direct", "rationale": "The language style of the entry is relatively simple, with no excessive rhetoric or emotional rendering, but simply asked the interviewee whether he felt nervous before the final exam and was more objective and calm."}, "time_dimension": {"answer": "Present", "rationale": "Now, \"before each final exam\" in the entry indicates that it is a customary act, while \"always there\" emphasizes the frequency of this feeling. So the time dimension of this article is now."}, "sentence_type": "Affirmative declarative sentence", "pos": {"nouns": {"Final examination": 1, "Feel": 1}, "verbs": {"have": 1, "There's always one": 1, "Here": 1}, "adjectives": {"I'm nervous": 1}, "adverbs": {"Every time": 1}, "numerals": {"14": 1}, "pronouns": {"I": 1}}}, {"content": "I'm not gonna be disturbed by my emotional reaction at the test.", "word_count": 16, "label": "", "sentence_structure": "Subject鈥搗erb鈥揷omplement", "subjective_objective": {"answer": "Objective", "rationale": "Because it is an objective fact to interfere with the examination, emotional reactions can be measured and observed."}, "sentence_sentiment": {"answer": "Neutral", "rationale": "Because the entry is only a description of a situation and does not involve positive or negative emotional aspects."}, "language_style": {"answer": "Calm and direct", "rationale": "The language style of the entry is a calm and direct one, because it clearly indicates whether the emotional reaction of the subject during the examination will have a negative impact on it. There are no excessive rhetorical or emotional renderings, and the tone is more flat and objective.\""}, "time_dimension": {"answer": "Past", "rationale": ""}, "sentence_type": "Negative declarative sentence", "pos": {"nouns": {"I": 1, "Emotional response": 1, "Examinations": 2}, "verbs": {"Yes": 1, "Interference": 1}, "adverbs": {"not": 1, "Time": 1}, "numerals": {"15": 1}}}, {"content": "Sixteen, I was so nervous during the exam that I forgot what I knew.", "word_count": 22, "label": "", "sentence_structure": "Subject鈥搗erb鈥揷omplement", "subjective_objective": {"answer": "Subjective", "rationale": "Because \"often nervous\" and \"what was known was forgotten\" is the patient's subjective feelings and perceptions of his state during the exam."}, "sentence_sentiment": {"answer": "Negative", "rationale": "Because the entries \"sweet\" and \"what was known was forgotten\" are negative."}, "language_style": {"answer": "Tactful and warm", "rationale": "This language style helps create a positive, supportive and understanding environment, encouraging the interviewee to open his heart and share his feelings and experiences."}, "time_dimension": {"answer": "Past", "rationale": "The entry describes the behaviour and mentality of the person tested at a certain point in time (during the examination) and can therefore be judged to be past."}, "sentence_type": "Affirmative declarative sentence", "pos": {"nouns": {"16": 1, "Examination period": 1, "Nervous": 1, "Something": 1}, "verbs": {"Yes": 2, "Forget it": 1}, "adjectives": {"very": 1}, "adverbs": {"Regular": 1, "So": 1}, "numerals": {"16": 1}, "classifiers": {"individual": 1}, "pronouns": {"I": 1}}}, {"content": "17, a review of the important exam seems to be a big challenge for me.", "word_count": 21, "label": "", "sentence_structure": "Subject鈥揷opula鈥損redicative", "subjective_objective": {"answer": "Subjective", "rationale": "Because \"it seems to me\" is the patient's subjective feeling and evaluation."}, "sentence_sentiment": {"answer": "Negative", "rationale": "Because the Great Challenge expresses a difficult and unpleasant feeling."}, "language_style": {"answer": "Tactful and warm", "rationale": "The entry uses a more euphemistic approach to describe the challenges faced by the other party in the review of important examinations, and is not expressed too directly or strongly. Such language styles reduce each other鈥檚 stress and resistance and make it easier to get their cooperation and support."}, "time_dimension": {"answer": "Present", "rationale": "The words \"retrospect\" and \"as if\" in the sentence were a great challenge meant the current situation and feelings, and thus the judgement was now."}, "sentence_type": "Affirmative declarative sentence", "pos": {"nouns": {"17": 1, "Review": 1, "Important": 1, "Examinations": 1, "I": 1, "Seems like": 1, "One": 2, "Big": 1, "Challenges": 1}, "verbs": {"be": 1, "I think so": 1, "Review": 1}, "adjectives": {"Important": 1, "Large": 1}, "adverbs": {"very": 2}, "numerals": {"17": 1, "One": 3}, "classifiers": {"individual": 1}, "pronouns": {"I": 1}}}, {"content": "I'm confused by the more I try to review one of the exams.", "word_count": 17, "label": "", "sentence_structure": "Subject鈥搗erb鈥揷omplement", "subjective_objective": {"answer": "Subjective", "rationale": "Because \"sweet\" is a subjective experience, influenced by personal feelings and attitudes."}, "sentence_sentiment": {"answer": "Negative", "rationale": "Because the \"I'm getting confused\" behavior and feeling in the entry is negative."}, "language_style": {"answer": "Calm and direct", "rationale": ""}, "time_dimension": {"answer": "Present", "rationale": "The entry describes a current feeling and state of mind, that is, the sense that the testee is confused while trying to review a particular examination. The feeling is now going through, not past or future.\""}, "sentence_type": "Affirmative declarative sentence", "pos": {"nouns": {"Examinations": 1, "Confused": 1}, "verbs": {"Review": 1, "I feel": 1}, "adjectives": {"Tsk": 1}, "adverbs": {"The more you try": 1}, "numerals": {"18": 1}}}, {"content": "I tried to stop worrying about it, but I couldn't.", "word_count": 20, "label": "", "sentence_structure": "Subject鈥搗erb鈥搊bject", "subjective_objective": {"answer": "Subjective", "rationale": "Because \"trying to stop\" and \"failure\" are patients' psychological state, patients' subjective feelings."}, "sentence_sentiment": {"answer": "Negative", "rationale": "Because the entry \"tried to stop the fear but failed\" is negative."}, "language_style": {"answer": "Tactful and warm", "rationale": "The language style of the entry is more euphemistic, using softer expressions such as \"tempting\" and \"failing to do\" and avoiding too direct or hard language. This expression is more likely to resonate and accept the respondents and is conducive to a good investigation atmosphere."}, "time_dimension": {"answer": "Past", "rationale": ""}, "sentence_type": "Negative declarative sentence", "pos": {"nouns": {"19": 1, "Examinations": 1, "One": 1, "End": 1, "I": 2, "Stop": 1, "Related": 1, "Fear": 1, "Do it": 1}, "verbs": {"Try": 1, "Stop": 1, "Fear": 1, "Do it": 1}, "adverbs": {"Some": 1, "But": 1}, "numerals": {"19": 1, "One": 1}, "pronouns": {"I": 2}}}, {"content": "Twenty, during the exam, I sometimes wonder if I can finish my studies.", "word_count": 17, "label": "", "sentence_structure": "Subject鈥搗erb鈥搊bject", "subjective_objective": {"answer": "Subjective", "rationale": "Because sometimes I wonder if I can finish school is the patient's personal psychological activity and thoughts, and it's a subjective examination."}, "sentence_sentiment": {"answer": "Negative", "rationale": "Because the expression \"sometimes I want to finish school\" in the entry expresses a sense of fear and uncertainty about the future of students' studies, and is a negative emotion."}, "language_style": {"answer": "Calm and direct", "rationale": "The entry is of a calm and direct type. It asks the testee whether he or she is anxious or uneasy during the examination, without using excessive speech techniques or emotional rendering, and with a clear and objective tone."}, "time_dimension": {"answer": "Present", "rationale": "The entry now indicates that during the examination the testee sometimes has the idea of whether he or she will finish his or her studies. This idea is part of the activity currently under way and is therefore in the current state of affairs."}, "sentence_type": "Interrogative sentence", "pos": {"nouns": {"Examination period": 1, "I": 1, "Studies": 1}, "verbs": {"Yes": 1, "Completed": 1}, "adverbs": {"Sometimes": 1}, "numerals": {"20": 1}}}, {"content": "I'd rather write a paper than take an exam as a result of a course.", "word_count": 26, "label": "", "sentence_structure": "Subject鈥搗erb鈥搊bject", "subjective_objective": {"answer": "Subjective", "rationale": "Because this phrase describes patients ' preferences and choices for writing papers and taking exams as subjective attitudes."}, "sentence_sentiment": {"answer": "Negative", "rationale": "Because the \"Better Write a Paper than Take a Test\" act in the entry has negative feelings."}, "language_style": {"answer": "Calm and direct", "rationale": "The language style of the entry is relatively straightforward, and there is no excessive rhetorical or emotional rendering. It clearly expresses the resistance of the other party to taking the examination and the preference for writing a paper to evaluate its performance in a given course. This expression is more objective and rational, and is less subjective."}, "time_dimension": {"answer": "Present", "rationale": ""}, "sentence_type": "Affirmative declarative sentence", "pos": {"nouns": {"21": 1, "Paper": 1, "Examinations": 1, "Courses": 1, "Achievements": 1}, "verbs": {"I'd rather": 1, "Write": 1, "Participation": 1, "As": 1}, "numerals": {"21": 1}, "classifiers": {"Title": 1, "Number of times": 1, "Door": 1}, "pronouns": {"I": 1}}}, {"content": "I wish I could stop being so annoying about the exam.", "word_count": 12, "label": "", "sentence_structure": "Subject鈥搗erb鈥揷omplement", "subjective_objective": {"answer": "Subjective", "rationale": "Because hope is a subjective expression of the view and feeling of the interviewee about the exam."}, "sentence_sentiment": {"answer": "Negative", "rationale": "Because the word \"fun\" in the entry is a negative expression of sentiment."}, "language_style": {"answer": "Tactful and warm", "rationale": "The entry expresses dissatisfaction with the examination in a simple and cordial way, rather than attacking or complaining directly. This expression is more personal and interactive and can trigger resonance among the respondents."}, "time_dimension": {"answer": "Future", "rationale": "The purpose of the article was therefore to be to act or to think in the future."}, "sentence_type": "Interrogative sentence", "pos": {"nouns": {"Examinations": 1, "Trouble": 1}, "verbs": {"Hope": 1, "Don't": 1, "Well": 1, "It's annoying": 1}, "adjectives": {"Boring": 1}, "adverbs": {"True": 1}, "numerals": {"22": 1}, "pronouns": {"I": 1}}}, {"content": "23, I'm sure I'd be better off if I took the exam alone and without a time limit.", "word_count": 28, "label": "", "sentence_structure": "Subject鈥搗erb鈥搊bject", "subjective_objective": {"answer": "Subjective", "rationale": "This expression is a subjective attitude, as it expresses the belief and expectations of patients about their performance in a given situation."}, "sentence_sentiment": {"answer": "Positive", "rationale": "Because the words \"convinced\" in the entry, \"separated examination\" and \"better\" are positive. Although there are hypothetical situations (e.g. \"no time limit\", there is no overall motivation."}, "language_style": {"answer": "Tactful and warm", "rationale": "By using the expression \"I believe\" the entry conveys confidence and optimism about one another's abilities, while portraying itself in a hypothetical manner in the desired context, making language style more euphemism and goodwill."}, "time_dimension": {"answer": "Past", "rationale": "In terms of time dimension, the issue does not relate to what happened in the past, nor does it have a clear future plan or expectations, and it is therefore now judged."}, "sentence_type": "Affirmative declarative sentence", "pos": {"nouns": {"23": 1, "Examinations": 1, "Time": 1, "Limits": 1}, "verbs": {"Yes": 1, "Participation": 1, "- You're a good student": 1}, "adverbs": {"Alone": 1, "If": 1, "No, I'm not": 1, "If you": 1, "Better": 1}, "numerals": {"23": 1}, "classifiers": {"individual": 1}, "pronouns": {"I": 1}}}, {"content": "Think how much I can score in the exam has affected my review and the exam.", "word_count": 22, "label": "", "sentence_structure": "Subject鈥搗erb鈥搊bject", "subjective_objective": {"answer": "Subjective", "rationale": "The idea of what to score in the examination is the patient ' s subjective thinking, which affects his or her review and examination behaviour."}, "sentence_sentiment": {"answer": "Negative", "rationale": "Because the entry \"Thinking of how much I can score in the exam\" can cause the tester to get too anxious, and thus affect his or her review and examination performance."}, "language_style": {"answer": "Tactful and warm", "rationale": ""}, "time_dimension": {"answer": "Present", "rationale": "Now the entry \"think\" indicates that it's a current behavior or state, that \"how much can be scored in the test\" is the matter of thinking, and \"impact\" is the result of the current behavior or state. So, the time dimension of this questionnaire is now judged."}, "sentence_type": "Exclamatory sentence", "pos": {"nouns": {"min": 1, "Examinations": 2, "Review": 1}, "verbs": {"Okay": 1, "Yes": 4, "Impact": 1, "I": 1}, "adverbs": {"More": 1}, "numerals": {"24": 1}, "classifiers": {"individual": 1}}}, {"content": "25, if the exam can be abolished, I think I can learn more.", "word_count": 17, "label": "", "sentence_structure": "Subject鈥搗erb鈥搊bject", "subjective_objective": {"answer": "Subjective", "rationale": "Because \"I think I can learn more\" is about the patient's personal expectations and desires, and it's about the patient's subjective attitude."}, "sentence_sentiment": {"answer": "Positive", "rationale": "Because the entries \"Absolute the exam\" and \"I think I can learn more\" express positive emotions and attitudes."}, "language_style": {"answer": "Tactful and warm", "rationale": "The entry presents a hypothetical view and expresses personal feelings and expectations through \"I want\" that is more euphemistic, without expressing strong emotions or demands directly. At the same time, it uses a more verbal and easy way of expressing, which enhances kinship and interaction and is in keeping with the euphemism of the language."}, "time_dimension": {"answer": "Future", "rationale": "The phrase \"if the exam can be abolished\" contains ideas and expectations for the future, and \"I think I can learn more\" implies a situation that has not yet happened and that may occur in the future. So this questionnaire entry is about the future."}, "sentence_type": "Affirmative declarative sentence", "pos": {"nouns": {"Examinations": 1, "Word": 1}, "verbs": {"Abolition": 1, "Yes": 4, "Learn": 2}, "adverbs": {"More": 2}, "numerals": {"25": 1}, "pronouns": {"I": 1}}}, {"content": "I'm taking this attitude to the exam: \"I don't understand it right now, but I'm not worried.\"", "word_count": 23, "label": "", "sentence_structure": "Subject鈥搗erb鈥搊bject", "subjective_objective": {"answer": "Subjective", "rationale": "Because attitudes are perceptions or feelings of something, and they are subjective cognitive and emotional experiences. The phrase \"I'm taking this attitude towards the exam\" shows that the voice has his own perception and feelings about the exam, and is therefore subjective."}, "sentence_sentiment": {"answer": "Positive", "rationale": "Because the approach in the entry is positive, that is, to have sufficient confidence and optimism in facing the difficulties and challenges."}, "language_style": {"answer": "Tactful and warm", "rationale": "Warm and warm."}, "time_dimension": {"answer": "Present", "rationale": ""}, "sentence_type": "Affirmative declarative sentence", "pos": {"nouns": {"Attitude": 1, "Examinations": 1}, "verbs": {"Hug": 1, "Yes": 1, "I'm worried": 1}, "adjectives": {"Here's the thing": 1}, "adverbs": {"But": 1, "No, it's not": 1}, "numerals": {"26": 1}, "pronouns": {"I": 1}}}, {"content": "I don't understand why some people are so nervous about the exams.", "word_count": 18, "label": "", "sentence_structure": "Subject鈥搗erb鈥搊bject", "subjective_objective": {"answer": "Subjective", "rationale": "Because words like \"not understanding\" and \"stretch\" are the subjective feelings and attitudes of the patient."}, "sentence_sentiment": {"answer": "Negative", "rationale": "Because the phrase \"some people are so nervous about the exam\" in the entry is negative."}, "language_style": {"answer": "Calm and direct", "rationale": "The entry expresses, in a direct tone, the confusion about the reasons for which some people are nervous about the examination, and is more objective and direct than it is when it is not over-exposed to emotional colours or acrimonyms."}, "time_dimension": {"answer": "Present", "rationale": "The entry \"I really don't understand\" expresses the current mood and feelings of the testee, and \"why are some people so nervous about the test\" is a process of thinking and understanding, both elements of which indicate that this is a description of the current situation. So, the time dimension of this questionnaire entry is now."}, "sentence_type": "Exclamatory sentence", "pos": {"nouns": {"27": 1, "person": 1, "Examinations": 1}, "verbs": {"Understood": 1, "to": 1, "Well": 1, "Nervous": 1}, "adjectives": {"True": 1}, "numerals": {"27": 1, "One": 1}, "pronouns": {"I": 1}}}, {"content": "My bad ideas are gonna get me in the wrong way.", "word_count": 18, "label": "", "sentence_structure": "Subject鈥揷opula鈥損redicative", "subjective_objective": {"answer": "Subjective", "rationale": "Because \"bad ideas\" and \"disturbing performance in the exam\" are subjective feelings and assessments of patients."}, "sentence_sentiment": {"answer": "Negative", "rationale": "Because the \"bad idea\" in the entry is negative."}, "language_style": {"answer": "Calm and direct", "rationale": "The language style of the entry is more straightforward, and it is not used in excessive terms or in euphemism, but rather in an objective and calm tone."}, "time_dimension": {"answer": "Present", "rationale": "In the entry, \"I\" means the first person's odd number, and \"the bad idea will interfere with my performance in the examination\" is a statement describing the current state or feeling, so the time dimension of the article is now."}, "sentence_type": "Negative declarative sentence", "pos": {"nouns": {"28": 1, "Thoughts": 1, "Interference": 1, "Examinations": 1, "Performance": 1}, "verbs": {"very": 1, "Bad": 1, "Yes": 1, "in": 1, "Medium": 1}, "adverbs": {"Bad": 1, "very": 1}, "numerals": {"28": 1}}}, {"content": "I'm not much more effective at the final exam than at the usual exam.", "word_count": 19, "label": "", "sentence_structure": "Subject鈥搗erb鈥揷omplement", "subjective_objective": {"answer": "Subjective", "rationale": "Because \"the final exam is no more laborious than the re-reading examination\" is a subjective judgement and perception of the conduct of the re-study."}, "sentence_sentiment": {"answer": "Negative", "rationale": "Because the behavior in the entries \"is not more powerful than the retinal exams\" expresses a negative attitude."}, "language_style": {"answer": "Tactful and warm", "rationale": "The entry is relatively euphemistic, is couched in a more plain language, with no too much negative emotion or direct criticism."}, "time_dimension": {"answer": "Past", "rationale": "The entry expresses the behaviour and attitude of the person tested at some point in the past (final examination) and therefore is judged to be \"the past\"."}, "sentence_type": "Exclamatory sentence", "pos": {"nouns": {"Final examination": 1, "-A usual exam": 1}, "verbs": {"Review": 2, "No, it's not like that": 1, "More": 1, "Work hard": 1}, "adverbs": {"More": 1}, "numerals": {"29": 1}, "pronouns": {"I": 1}}}, {"content": "I'm still worried, though I'm doing a good job of studying some exams.", "word_count": 20, "label": "", "sentence_structure": "Subject鈥搗erb鈥揷omplement", "subjective_objective": {"answer": "Subjective", "rationale": "Because it describes the patient ' s personal feelings and emotional state, it is a subjective examination."}, "sentence_sentiment": {"answer": "Negative", "rationale": "Because the emotional attitude of \"fearing\" in the entries is negative."}, "language_style": {"answer": "Calm and direct", "rationale": "The language style of the entry is more direct, concise, and not too much speech-making or emotional rendering, but rather a direct expression of the other's review and psychological feelings about a given examination. This description is more consistent with the statement of facts and does not put additional psychological pressure on the interviewee."}, "time_dimension": {"answer": "Present", "rationale": "\"I've studied well\" and \"fearful\" all at the moment, so the time dimension of the article is now."}, "sentence_type": "Negative declarative sentence", "pos": {"nouns": {"30": 1, "Examinations": 1, "Review": 1, "Anxiety": 1}, "verbs": {"to": 1, "I feel": 1}, "adjectives": {"Okay": 1, "Anxious": 1}, "adverbs": {"鈾?Though 鈾?: 1, "Still": 1}, "numerals": {"30": 1}, "pronouns": {"I": 1}}}, {"content": "I'm not good before the big test.", "word_count": 11, "label": "", "sentence_structure": "Subject鈥搗erb鈥揷omplement", "subjective_objective": {"answer": "Objective", "rationale": "This is the physical reaction of the patient, described as being an objective observation."}, "sentence_sentiment": {"answer": "Negative", "rationale": "Because \"eating bad\" is a description of a person's physical reaction to stress, and is usually considered a negative expression."}, "language_style": {"answer": "Calm and direct", "rationale": "The entry is more concise, has no excessive rhetorical or emotional rendering, and directly expresses the subject of asking the other party whether there will be a decline in appetite before a major examination. It is therefore judged that the language style is calm and straightforward."}, "time_dimension": {"answer": "Present", "rationale": "The sentence \"before major examinations\" can tell that this is a description of what happened over a certain period of time, not what happened or will happen in the past or in the future. So the time dimension is now."}, "sentence_type": "Negative declarative sentence", "pos": {"nouns": {"31": 1, "Examinations": 1}, "verbs": {"Before": 1, "Eat": 2, "Hong": 1}, "numerals": {"31": 1, "One": 2}, "pronouns": {"I": 1}}}, {"content": "I found my arm shaking before the big test.", "word_count": 16, "label": "", "sentence_structure": "Subject鈥搗erb鈥揷omplement", "subjective_objective": {"answer": "Subjective", "rationale": "This is a description of the patient ' s self-perception and experience, which is subjective."}, "sentence_sentiment": {"answer": "Negative", "rationale": "Because the \"arms will shake\" in the entry is negative."}, "language_style": {"answer": "Calm and direct", "rationale": "The entry describes in a concise and direct manner the physical reactions that an individual may have before a major examination, without too much qualifier or emotional colour.\""}, "time_dimension": {"answer": "Past", "rationale": "The keywords \"discovery\" and \"dithered\" indicate that this is an experience or state that has already occurred, and that is a judgement of the past in time."}, "sentence_type": "Affirmative declarative sentence", "pos": {"nouns": {"32": 1, "Examinations": 1, "Arms": 1}, "verbs": {"Found": 1, "Wreck": 1}, "adverbs": {"In the big one": 1}, "numerals": {"Mine": 1}, "pronouns": {"Yes": 1}}}, {"content": "33 I rarely need a \"suspension\" before I take the exam.", "word_count": 16, "label": "", "sentence_structure": "Subject鈥搗erb鈥揷omplement", "subjective_objective": {"answer": "Objective", "rationale": "Since the act of \"temporary paganism\" is a specific act taken by the patient prior to the examination, the act is an objective fact and is not related to the patient's subjective attitude."}, "sentence_sentiment": {"answer": "Negative", "rationale": "Because the behavior in the entry is negative."}, "language_style": {"answer": "Calm and direct", "rationale": "The language style of the entry is straightforward and it clearly describes a behaviour that is not based on excessive rhetorical or emotional rendering, and is more objective in tone."}, "time_dimension": {"answer": "Present", "rationale": "\"Pre-test\" is a point of time, and \"I have very few\" is a description of frequency and quantity, so the entry is asking about the behaviour or state of the person tested in the past."}, "sentence_type": "Negative declarative sentence", "pos": {"nouns": {"Examinations": 1, "I'm holding my feet": 1}, "verbs": {"have": 1, "Yes": 1}, "adjectives": {"Less": 1}, "adverbs": {"in": 1, "Front": 1, "Very few": 1}, "numerals": {"33": 1, "One": 1}, "pronouns": {"I": 1}}}, {"content": "34 - The school should realize that some students are anxious about the exam, which affects their grades.", "word_count": 30, "label": "", "sentence_structure": "Subject鈥搗erb鈥搊bject", "subjective_objective": {"answer": "Subjective", "rationale": "Because it expresses the author鈥檚 personal view and opinion that school authorities should recognize and value student anxiety about examinations, and that it can have an impact on student performance. This is not an objective factual description, but a subjective view based on the author鈥檚 views and experience."}, "sentence_sentiment": {"answer": "Negative", "rationale": "Because the entries \"retired about the exam\" and \"improving their test results\" have a negative emotional effect."}, "language_style": {"answer": "Calm and direct", "rationale": ""}, "time_dimension": {"answer": "Present", "rationale": "So the time dimension is now. And, \"know\" shows the attitude and the way the author views the issue, and it is now. So the time dimension of this questionnaire is mainly a description of the current phenomenon and the recommendations made, and an analysis of the current situation."}, "sentence_type": "Affirmative declarative sentence", "pos": {"nouns": {"34": 1, "School": 1, "Students": 1, "Examinations": 2, "Anxiety": 1, "Impact": 1, "Achievements": 1}, "verbs": {"I should have known": 1, "Some": 1, "to": 1, "Yes": 1, "And": 1, "Here": 1}, "adverbs": {"Compare": 1}, "numerals": {"34": 1}, "pronouns": {"they": 1}}}, {"content": "I don't think it's gonna be that tight during the exam.", "word_count": 18, "label": "", "sentence_structure": "Subject鈥搗erb鈥揷omplement", "subjective_objective": {"answer": "Subjective", "rationale": "Because it's a \"concept\" that shows the patient's personal views and attitudes."}, "sentence_sentiment": {"answer": "Negative", "rationale": "Because the entry \"should not make it so tense\" expresses a negative attitude and emotion."}, "language_style": {"answer": "Tactful and warm", "rationale": "The language style of the entry is more moderate, expressing personal views in a first person鈥檚 name, and does not use strong emotional or direct criticism."}, "time_dimension": {"answer": "Present", "rationale": "The entry uses subjective judgement terms like \"think\" and \"should\" and describes the observation and perception of the status quo in \"seen.\" These terms and expressions all indicate the current attitude and perception of the interviewees."}, "sentence_type": "Affirmative declarative sentence", "pos": {"nouns": {"Examination period": 1, "Nervous": 1}, "verbs": {"I think": 1, "I think so": 1, "Fuck": 1, "Okay": 1}, "adjectives": {"Well": 1}, "adverbs": {"Seems like": 1}, "numerals": {"35": 1}}}, {"content": "I feel uncomfortable when I get into the papers that were sent.", "word_count": 17, "label": "", "sentence_structure": "Subject鈥搗erb鈥揷omplement", "subjective_objective": {"answer": "Subjective", "rationale": "Because \"frightful\" is the patient's subjective feeling and emotional reaction."}, "sentence_sentiment": {"answer": "Negative", "rationale": "Because the \"frightful\" sentiment in the entry is negative."}, "language_style": {"answer": "Calm and direct", "rationale": "The entry uses a simple and clear language describing the responses of the testee in the test context, and does not contain any qualifying words or emotional colours. Thus, its language style is of the \"cool and direct\" type."}, "time_dimension": {"answer": "Past", "rationale": "The entry \"as soon as you get ahold of it...\" indicates that it is a past experience and feeling, and that it is a description of what happened in the past. So the temporal dimension of the entry is the past."}, "sentence_type": "Affirmative declarative sentence", "pos": {"nouns": {"36": 1, "Tests": 1}, "verbs": {"Exposure": 1, "I think": 1, "Send": 1, "Here": 1}, "adjectives": {"I'm not comfortable": 1}, "adverbs": {"Just": 1}, "numerals": {"36": 1}}}, {"content": "I hate teachers who like to take \"sudden attack\" exams.", "word_count": 18, "label": "", "sentence_structure": "Subject鈥搗erb鈥搊bject", "subjective_objective": {"answer": "Subjective", "rationale": "Because the words \"happy\" and \"like\" in the title are the feelings and attitudes of the patient, and are viewed from the patient's subjective perspective."}, "sentence_sentiment": {"answer": "Negative", "rationale": "Because the words \"obsessed\" and \"brash attack\" in the entry all have negative emotional connotations."}, "language_style": {"answer": "Calm and direct", "rationale": "The entry is in plain and simple language, which directly expresses the other鈥檚 displeasure with the teacher of the course, does not add too many rhetorical techniques or emotional renderings, and is more objective. It is therefore a calm and straightforward language style."}, "time_dimension": {"answer": "Present", "rationale": "Now."}, "sentence_type": "Negative declarative sentence", "pos": {"nouns": {"37": 1, "Teacher": 1, "Courses": 1}, "verbs": {"Shit": 1, "Yeah": 1, "Fuck": 1, "Examinations": 1, "Attack": 1}, "adjectives": {"Suddenly": 1, "Pattern": 1}, "numerals": {"37": 1}, "classifiers": {"individual": 1}, "pronouns": {"I": 1}}}]}

{"questionnaire_title": "Chinese version of the Family Environment Sheet (FES-CV)", "questionnaire_category": "Emotion and affect", "item_count": 90, "emotion_shift_overall": "From positive to negative", "language_style_overall": "Tactful and warm", "items": [{"content": "1. We, the family members, always offer each other the greatest help and support.", "word_count": 21, "label": "", "sentence_structure": "Subject鈥搗erb鈥搊bject", "subjective_objective": {"answer": "Objective", "rationale": "This is a description of the family, an objective fact.\""}, "sentence_sentiment": {"answer": "Positive", "rationale": "Because the behaviour and attitude of \"best help and support to each other\" in the entry is positive."}, "language_style": {"answer": "Tactful and warm", "rationale": ""}, "time_dimension": {"answer": "Present", "rationale": "The entry \"Always giving each other the greatest help and support\" describes the current situation and state of family members and therefore the present."}, "sentence_type": "Affirmative declarative sentence", "pos": {"nouns": {"we": 1, "Family": 1, "Members": 2, "Max": 1, "Help": 1, "Support": 1}, "verbs": {"Always": 1, "Give it to me": 1}, "adverbs": {"- Yeah, yeah": 1, "Both": 1}, "numerals": {"1": 2}, "pronouns": {"we": 1, "Family": 1, "Members": 2, "Max": 1, "Help": 1, "Support": 1}}}, {"content": "2. Family members always hide their feelings from other family members.", "word_count": 26, "label": "", "sentence_structure": "Subject鈥搗erb鈥揷omplement", "subjective_objective": {"answer": "Subjective", "rationale": "The ideas and behaviour of family members are examined from a subjective perspective."}, "sentence_sentiment": {"answer": "Negative", "rationale": "Because the entry \"hidden your feelings in your heart without telling the rest of your family\" is negative."}, "language_style": {"answer": "Tactful and warm", "rationale": "The entry is more euphemistic, does not directly point to the seriousness of the problem or blames family members. It asks about the emotional expression of family members in a moderate and caring manner, and is more friendly and friendly. This expression better enables the respondents to feel comfortable and comfortable, and is more likely to get real and effective feedback."}, "time_dimension": {"answer": "Present", "rationale": "\"Always\" and \"hidden in the heart without revealing to other family members\" indicate that this is a customary act and that it is continuing, so the entry is now."}, "sentence_type": "Exclamatory sentence", "pos": {"nouns": {"Family members": 2, "Emotion": 1, "Psychological": 1, "Other": 1}, "verbs": {"Total": 1, "- Put it on": 1, "Hide": 1, "Disclosure": 1}, "adjectives": {"Your own": 1, "not": 1}, "numerals": {"2": 1}, "pronouns": {"Other family members": 1}}}, {"content": "3. Fighting is common in the home.", "word_count": 6, "label": "", "sentence_structure": "Subject鈥揷opula鈥損redicative", "subjective_objective": {"answer": "Objective", "rationale": "Because the fact that there are often disputes at home is an objective observation rather than a subjective feeling or attitude of the patient."}, "sentence_sentiment": {"answer": "Negative", "rationale": "Because the \"challenge\" in the entry is negative."}, "language_style": {"answer": "Calm and direct", "rationale": "The entry directly asked whether there were frequent altercations in the family of the interviewee, which was relatively simple and clear, and which did not have too many adorations or emotional colours.\""}, "time_dimension": {"answer": "Present", "rationale": "The entry now describes the phenomenon in the current family situation, which shows that at this moment, the families of the interviewees often have a fight."}, "sentence_type": "Affirmative declarative sentence", "pos": {"nouns": {"Home": 1, "Louder": 1, "Array": 1}, "verbs": {"Regular": 1, "have": 1, "in": 1}, "adverbs": {"very": 1, "Regular": 1}, "numerals": {"III": 1}}}, {"content": "4. In our homes, we rarely act alone.", "word_count": 13, "label": "", "sentence_structure": "Subject鈥搗erb鈥揷omplement", "subjective_objective": {"answer": "Subjective", "rationale": "Because \"very few activities are carried out individually\" is an expression of a subjective feeling, it is the patient's perception of his or her behaviour and experience in the family."}, "sentence_sentiment": {"answer": "Negative", "rationale": "Because the \"very few activities on their own\" act in the entry is negative."}, "language_style": {"answer": "Tactful and warm", "rationale": "This is a more euphemistic expression, which is not directly critical or critical, but rather a more moderate way of asking about the respondents."}, "time_dimension": {"answer": "Present", "rationale": "The words \"at home\", \"very few\" and \"single activities\" in the entry all denote a status quo or customary sexual behaviour, and therefore the judgement is now."}, "sentence_type": "Negative declarative sentence", "pos": {"nouns": {"Family": 1, "Activities": 1}, "verbs": {"Very few": 1, "From": 1, "Alone": 1, "Activities": 1}, "adverbs": {"very": 1, "Home": 1}, "numerals": {"4": 1}}}, {"content": "5. Family members do whatever they can.", "word_count": 17, "label": "", "sentence_structure": "Subject鈥搗erb鈥揷omplement", "subjective_objective": {"answer": "Subjective", "rationale": "This is because of the patient ' s perception and evaluation of the behaviour of family members and because it is a subjective attitude of the patient."}, "sentence_sentiment": {"answer": "Neutral", "rationale": "Because the entry is not clearly positive or negative, it is merely a description of a fact."}, "language_style": {"answer": "Tactful and warm", "rationale": "This expression is more likely to resonate and identify the respondents and thus to be more willing to participate in the follow-up questionnaire."}, "time_dimension": {"answer": "Present", "rationale": "\"5. Family members do whatever they can\" means \"in every case,\" which is a generic description, and therefore the temporal dimension is now judged."}, "sentence_type": "Affirmative declarative sentence", "pos": {"nouns": {"Family members": 1, "Yes": 1}, "verbs": {"Do it": 1, "Try": 1, "And it's": 1}, "adjectives": {"Try": 1}, "adverbs": {"Both": 1}, "classifiers": {"individual": 1}}}, {"content": "6. Our family often talks about politics and social affairs.", "word_count": 14, "label": "", "sentence_structure": "Subject鈥搗erb鈥搊bject", "subjective_objective": {"answer": "Subjective", "rationale": "It's about our family, and it looks at patients' subjective attitudes and perceptions."}, "sentence_sentiment": {"answer": "Positive", "rationale": "Because the entry expresses a positive attitude of concern and discussion about political and social issues."}, "language_style": {"answer": "Calm and direct", "rationale": "The entry is a simple and straightforward way of asking the families of the interviewees whether they often discuss political and social issues, without too much ado or emotional expression."}, "time_dimension": {"answer": "Present", "rationale": "The keyword \"often talks\" means that it is a customary act, which occurs now. Thus, the time dimension of the article is now."}, "sentence_type": "Affirmative declarative sentence", "pos": {"nouns": {"Our home": 1, "Political": 1, "Social issues": 1}, "verbs": {"I've been talking about it a lot": 1}, "adverbs": {"Regular": 1}, "numerals": {"6": 1}}}, {"content": "7. Most weekends and evenings are spent at home by family members, without going out to social or recreational events.", "word_count": 32, "label": "", "sentence_structure": "Subject鈥搗erb鈥搊bject", "subjective_objective": {"answer": "Objective", "rationale": "This is a description of the patient ' s daily life and does not involve subjective feelings or ideas."}, "sentence_sentiment": {"answer": "Neutral", "rationale": "Since the entry does not address a description of emotion, attitude, behaviour, purpose or quality, it merely presents a fact."}, "language_style": {"answer": "Tactful and warm", "rationale": "The entry is more personal and interactive when it is spoken in a gentle and inviting manner, asking whether the interviewees choose to spend weekends and nights at home with their families rather than directly speaking or ordering for the most part. At the same time, this way of asking questions avoids the use of language styles that may be strongly negative or questionable to the interviewee."}, "time_dimension": {"answer": "Present", "rationale": ""}, "sentence_type": "Affirmative declarative sentence", "pos": {"nouns": {"Weekend": 1, "Night": 1, "Family members": 1, "Home": 1, "Social": 1, "Recreational activities": 1}, "verbs": {"Pass": 1, "Participation": 1, "be": 1}, "adverbs": {"Most of them": 1, "Both": 1, "not": 1}, "numerals": {"7": 1}, "pronouns": {"Here": 1}}}, {"content": "8. We all believe that, no matter how difficult, children should be the first to meet the needs of the elderly.", "word_count": 28, "label": "", "sentence_structure": "Subject鈥搗erb鈥搊bject", "subjective_objective": {"answer": "Subjective", "rationale": "Because \"we think\" is patient's subjective attitude and ideas."}, "sentence_sentiment": {"answer": "Positive", "rationale": "Because it expresses respect and concern for the elderly, it is a positive emotional attitude."}, "language_style": {"answer": "Tactful and warm", "rationale": "This expression is more gentle and friendly, and is easily accepted and understood by the interviewee."}, "time_dimension": {"answer": "Present", "rationale": "The entry is about perceptions and perceptions of current social phenomena, and the temporal dimension is now judged. In it, \"concepts\" , \"no matter how difficult\" and \"should satisfy\" are terms that describe the current state or situation, indicating that it is an expression of prevailing views and attitudes."}, "sentence_type": "Affirmative declarative sentence", "pos": {"nouns": {"Difficulties": 1, "Children": 1, "Old man": 1, "Requirements": 1}, "verbs": {"Both": 1, "I think": 1, "have": 1, "Satisfied": 1}, "adjectives": {"Large": 1}, "adverbs": {"Both": 1}, "numerals": {"How old": 1, "Various": 1}, "pronouns": {"we": 1, "First of all": 1}}}, {"content": "9. The larger activities in the home are carefully organized.", "word_count": 16, "label": "", "sentence_structure": "Subject鈥揷opula鈥損redicative", "subjective_objective": {"answer": "Subjective", "rationale": "The sentence entered suggests that it is viewed from the patient鈥檚 subjective perspective. The reason is that 鈥渟cholarly鈥?is the patient鈥檚 attitude and perception of family activities, which is subjective. The problem is therefore to examine the patient鈥檚 subjective thoughts and attitudes, not just the objective facts."}, "sentence_sentiment": {"answer": "Positive", "rationale": "Because the \"scareful arrangement\" is positive."}, "language_style": {"answer": "Tactful and warm", "rationale": "The entry is more euphemistic, moderate, and does not use too direct or hard language. It leads the respondents to questions by asking questions, and uses a positive phrase like \"all carefully arranged\" to express respect and trust for the person under investigation, rather than to stimulate the other by denying or questioning."}, "time_dimension": {"answer": "Present", "rationale": "According to the description of \"all\" and \"all carefully arranged\" in the sentence, it can be seen as a statement of the current state of family life. The article therefore determines the time dimension of the purpose as now."}, "sentence_type": "Affirmative declarative sentence", "pos": {"nouns": {"Activities": 1, "Home": 1, "Medium": 1}, "verbs": {"Both": 1, "Yes": 1, "Careful": 1, "Arrangements": 1}, "adverbs": {"Bigger": 1}, "numerals": {"9": 1}}}, {"content": "10. Family members rarely force other family members to comply with family rules.", "word_count": 17, "label": "", "sentence_structure": "Subject鈥搗erb鈥搊bject", "subjective_objective": {"answer": "Objective", "rationale": "Because \"the family rarely demands that other family members comply with the family rules\" is an objective fact that can be proved or perjured, rather than the patient's personal subjective feelings or ideas."}, "sentence_sentiment": {"answer": "Neutral", "rationale": "Because the entry is a mere statement of fact and does not express positive or negative emotional tendencies."}, "language_style": {"answer": "Tactful and warm", "rationale": "The entry is presented in a more moderate way, without too strong or straightforward language. It also gives a sense of comfort and inclusion, indicating that the questionnaire designer wants the interviewee to feel comfortable and relaxed in answering the questions."}, "time_dimension": {"answer": "Present", "rationale": ""}, "sentence_type": "Affirmative declarative sentence", "pos": {"nouns": {"Family": 1, "Family rules": 1}, "verbs": {"Force": 1, "Compliance": 1}, "adjectives": {"Less": 1, "Other": 1}, "numerals": {"10": 1}, "classifiers": {"individual": 1}, "pronouns": {"Family": 1}}}, {"content": "11. At home, we are bored.", "word_count": 10, "label": "", "sentence_structure": "Subject鈥揷opula鈥損redicative", "subjective_objective": {"answer": "Subjective", "rationale": "Because \"we're bored\" reflects patients' subjective feelings and emotions."}, "sentence_sentiment": {"answer": "Negative", "rationale": "Because the entry \"sick\" expresses negative emotions."}, "language_style": {"answer": "Tactful and warm", "rationale": "This is a soft and caring expression, asking if the interviewee is often bored at home and does not use too direct or sharp language."}, "time_dimension": {"answer": "Present", "rationale": "The entry is for the current feelings and state of the testee, and the time dimension is now judged."}, "sentence_type": "Affirmative declarative sentence", "pos": {"nouns": {"Home": 1}, "verbs": {"I feel": 1, "Boring": 1}, "adverbs": {"very": 1}}}, {"content": "12. At home, we can say what we want.", "word_count": 15, "label": "", "sentence_structure": "Subject鈥損redicate", "subjective_objective": {"answer": "Subjective", "rationale": "Because this entry describes the patient ' s subjective feelings and attitudes, i.e., the freedom to express his thoughts and opinions at home."}, "sentence_sentiment": {"answer": "Neutral", "rationale": "The entry describes a situation that does not involve any positive or negative emotional attitude, behaviour or purpose and is therefore neutral."}, "language_style": {"answer": "Tactful and warm", "rationale": "The language style of the entry tends to be euphemistic, warm, and attempts to create a climate of easy, friendly conversation by asking about the family atmosphere of the other party and their feelings in this environment. At the same time, the phrase uses statements rather than questions, which are more gentle and friendly."}, "time_dimension": {"answer": "Present", "rationale": "\"In the home\" means the place, \"whatever you can say\" is the views and behaviour of the person being tested, pointing to the current situation and customary sexual behaviour, and the entry is now."}, "sentence_type": "Affirmative declarative sentence", "pos": {"nouns": {"Home": 1, "Say it": 1}, "verbs": {"Yes": 1, "Yeah": 1, "Just": 1}, "adverbs": {"What": 1, "Both": 1}, "numerals": {"12": 1}, "pronouns": {"we": 1}}}, {"content": "Family members rarely publicly anger each other.", "word_count": 14, "label": "", "sentence_structure": "Subject鈥揷opula鈥損redicative", "subjective_objective": {"answer": "Objective", "rationale": "Because \"family members are rarely openly angry with each other\" is an observed family phenomenon, not a subjective feeling or attitude of the patient. It can be determined by asking family members or observing their behaviour."}, "sentence_sentiment": {"answer": "Neutral", "rationale": "Because the entry does not express a positive or negative emotional tendency, it merely describes an objective fact."}, "language_style": {"answer": "Tactful and warm", "rationale": "The entry is more personal and interactive when it is spoken in a gentle and inviting manner, rather than in direct statements or orders. At the same time, the expression \"very rarely publicly angry\" is more objective and neutral, is not overly emotional and is in keeping with the euphemism of the language."}, "time_dimension": {"answer": "Present", "rationale": ""}, "sentence_type": "Affirmative declarative sentence", "pos": {"nouns": {"Family members": 1, "You too": 1, "Anger": 1}, "verbs": {"Very few": 1, "Let's go": 1, "Public": 1, "Anger": 1}, "adverbs": {"Very few": 1, "Public": 1}, "numerals": {"13": 1}, "classifiers": {"individual": 1}}}, {"content": "14. We all strongly encourage the spirit of independence of our families.", "word_count": 16, "label": "", "sentence_structure": "Subject鈥搗erb鈥搊bject", "subjective_objective": {"answer": "Subjective", "rationale": "Because the attitude of \"very encouraging\" and \"independent\" is the patient's subjective idea."}, "sentence_sentiment": {"answer": "Positive", "rationale": "Because the \"very encouraged\" attitude in the entry is positive."}, "language_style": {"answer": "Tactful and warm", "rationale": "The entry is more personal and interactive when it is spoken in a gentle and inviting tone, rather than in direct statements or orders. At the same time, the term \"very encouraged\" conveys recognition and praise of the spirit of independence of family members, which gives people support and approval in answering questions."}, "time_dimension": {"answer": "Future", "rationale": "The words \"are very encouraging\" and \"have an independent spirit\" appear in the entry, which indicate expectations and visions for the future and can therefore be judged as the future."}, "sentence_type": "Affirmative declarative sentence", "pos": {"nouns": {"we": 1, "Family": 1, "Independence": 1}, "verbs": {"Encourage": 1, "Yes": 1}, "numerals": {"14": 1}}}, {"content": "15. Family members have devoted almost all their energy to a promising future.", "word_count": 21, "label": "", "sentence_structure": "Subject鈥搗erb鈥搊bject", "subjective_objective": {"answer": "Objective", "rationale": "Because \"good future\" and \"family members have spent almost all their energy\" are factual statements that do not involve patients' own subjective attitudes or feelings. So the questionnaire entry is an objective examination."}, "sentence_sentiment": {"answer": "Positive", "rationale": "Because the entries \"for a good future\" and \"for almost all energy\" express a positive, upward attitude and behaviour, indicating that family members are hopeful and willing to work for their children's future."}, "language_style": {"answer": "Calm and direct", "rationale": "The entry describes in an objective and straightforward manner the considerable efforts made by family members for the future of their children, without excessive rhetorical or emotional rendering, and in a more balanced and direct manner."}, "time_dimension": {"answer": "Present", "rationale": "Now the entry describes the behaviour and circumstances of family members at present, \"for a good future\" indicating that they are working hard and that this situation is likely to continue. So the time dimension is now."}, "sentence_type": "Affirmative declarative sentence", "pos": {"nouns": {"Future": 1, "Family members": 1, "- The energy": 1}, "verbs": {"Flowers": 1, "have": 1}, "adjectives": {"Okay": 1}, "adverbs": {"Almost": 1}, "numerals": {"15": 1}, "pronouns": {"Both": 1}}}, {"content": "16. We rarely go out to listen to lectures, to watch plays or to museums and to watch exhibitions.", "word_count": 21, "label": "", "sentence_structure": "Subject鈥搗erb鈥搊bject", "subjective_objective": {"answer": "Objective", "rationale": "This is an objective condition of the patient ' s life and a fact that has already occurred."}, "sentence_sentiment": {"answer": "Negative", "rationale": "Because the behavior in the entry \"very rarely out\" is negative."}, "language_style": {"answer": "Calm and direct", "rationale": "The language style of the entry is more objective, direct, and there is no excessive rhetorical or emotional colour, and the tone is more flat."}, "time_dimension": {"answer": "Past", "rationale": "The entry is a test case of past judgement by asking whether the person who was tested has been out for cultural activities on a regular basis for some time."}, "sentence_type": "Negative declarative sentence", "pos": {"nouns": {"Lectures": 1, "The play": 1, "Museums": 1, "Exhibition": 1}, "verbs": {"Out": 1, "Listen": 1, "Look": 3}, "adjectives": {"Very few": 1}, "numerals": {"16": 1}, "classifiers": {"individual": 1}}}, {"content": "17. Family members often go out to friends ' homes to play and eat together.", "word_count": 19, "label": "", "sentence_structure": "Subject鈥搗erb鈥搊bject", "subjective_objective": {"answer": "Objective", "rationale": "Because the sentence describes the behaviour and circumstances of family members, it does not relate to subjective attitudes or feelings of the patient."}, "sentence_sentiment": {"answer": "Neutral", "rationale": "Because the entry describes the behaviour of a family member, it does not express any positive or negative emotional colours."}, "language_style": {"answer": "Tactful and warm", "rationale": "The entry uses friendly, friendly words like \"showing out\" and \"playing together\" and \"eating together\" and is more gentle and euphemistic, giving people a comfortable and relaxed feeling, so language style can be considered \"fluent.\""}, "time_dimension": {"answer": "Present", "rationale": "The entry describes events in the past, \"often\" as the customary past, \"dinner together\" and \"showing out\" all the same."}, "sentence_type": "Affirmative declarative sentence", "pos": {"nouns": {"Family members": 1, "friends": 1, "Rice": 1}, "verbs": {"Regular": 1, "External": 1, "Go play": 1, "in": 1, "Together": 1, "Eat": 1}, "adverbs": {"and": 1, "Regular": 1}, "numerals": {"17": 1}, "classifiers": {"Home": 1, "Number of times": 1}}}, {"content": "18. Family members agreed that they should act in a manner that was socially appropriate.", "word_count": 16, "label": "", "sentence_structure": "Subject鈥搗erb鈥搊bject", "subjective_objective": {"answer": "Subjective", "rationale": "The views of family members are subjective and are personal or belief rather than objective facts."}, "sentence_sentiment": {"answer": "Neutral", "rationale": "Because the entry does not have any emotional color."}, "language_style": {"answer": "Calm and direct", "rationale": ""}, "time_dimension": {"answer": "Present", "rationale": "The entry asks the views and opinions of the family members of the testee, indicating that the inquiry is conducted at the current point of time and is therefore judged to be \"now\"."}, "sentence_type": "Affirmative declarative sentence", "pos": {"nouns": {"Family members": 1, "Yes": 1, "Social climate": 1}, "verbs": {"I think": 1, "Reactions": 1, "Complementarity": 1}, "numerals": {"18": 1}}}, {"content": "19. Generally speaking, we are all concerned about keeping our homes in order.", "word_count": 20, "label": "", "sentence_structure": "Subject鈥搗erb鈥揷omplement", "subjective_objective": {"answer": "Subjective", "rationale": "Because the answer depends on the patient鈥檚 personal perception and attitude. If the patient believes that this is an important value and works hard to practice, they may answer yes, and no."}, "sentence_sentiment": {"answer": "Neutral", "rationale": "Because the entry does not have any emotional color."}, "language_style": {"answer": "Tactful and warm", "rationale": "The entry is more intimate and interactive when it is spoken in a question-and-answer tone rather than directly speaking or ordering. At the same time, the term \"situation\" has positive and positive connotations that make people feel comfortable."}, "time_dimension": {"answer": "Past", "rationale": "The keywords \"general\" and \"general\" both indicate that this is a regular, customary act, which has existed in the past. Thus, the temporal dimension is a past."}, "sentence_type": "Affirmative declarative sentence", "pos": {"nouns": {"General": 1, "Everyone": 1, "Home": 1, "Pack up": 1, "The well is organized": 1}, "verbs": {"Attention": 1, "- Put it on": 1, "Okay": 1}, "adjectives": {"Both": 1}, "adverbs": {"Yes": 1}, "pronouns": {"we": 1}}}, {"content": "20. There are few regular patterns of living and family norms in the family.", "word_count": 15, "label": "", "sentence_structure": "Subject鈥搗erb鈥揷omplement", "subjective_objective": {"answer": "Objective", "rationale": "This is a description of the actual situation of family life and is not a subjective attitude or feeling of the patient."}, "sentence_sentiment": {"answer": "Negative", "rationale": "Because the description \"very few fixed living patterns and family rules\" in the entry implies a chaotic and disorderly family environment, which may lead to contradictions and conflicts between family members."}, "language_style": {"answer": "Calm and direct", "rationale": "The language style of the entry is a calm and direct type. It presents a fact or situation in an objective, concise manner, without excessive rhetorical or emotional language."}, "time_dimension": {"answer": "Present", "rationale": "Because the sentence describes the current situation, the general current is used. \"There are few fixed living patterns and family rules in the family\" suggests that there are no fixed patterns and rules in family life."}, "sentence_type": "Negative declarative sentence", "pos": {"nouns": {"Family": 1, "Life": 1, "Pattern": 1, "Family rules": 1}, "verbs": {"Very few": 1, "Fixed": 1}, "adjectives": {"20": 1}, "numerals": {"20": 1}}}, {"content": "21. Family members are willing to devote a great deal of their energy to their family.", "word_count": 17, "label": "", "sentence_structure": "Subject鈥揷opula鈥損redicative", "subjective_objective": {"answer": "Subjective", "rationale": "It is the patient ' s subjective attitude to be willing to devote a great deal of effort to family matters, which cannot be judged by objective observations."}, "sentence_sentiment": {"answer": "Positive", "rationale": "Because the act of \"willing to devote a lot of energy to the family\" in the entry reflects the love and responsibility of family members and is a positive attitude and emotion."}, "language_style": {"answer": "Tactful and warm", "rationale": "The language style of the entry is more euphemistic, using a question-and-answer tone rather than a direct statement or order. It uses a more moderate term, \"a willingness to devote a lot of effort,\" which shows that the investigator is concerned about the family situation of the person under investigation and wants to know in this way the family situation and attitude of the person under investigation. Moreover, the entry does not use overly strong or emotional emotional terms or rhetorical techniques, which are more objective and have a certain degree of sensitivity and interaction."}, "time_dimension": {"answer": "Present", "rationale": "The entry describes the current situation, i.e. whether family members are willing to devote a lot of effort to doing their family business. The keyword \"will\" and \"do\" point to the current behavior or state."}, "sentence_type": "Affirmative declarative sentence", "pos": {"nouns": {"Family members": 1, "- The energy": 1, "Home": 1, "Yes": 1}, "verbs": {"Yes": 1, "Flowers": 1, "Do it": 1}, "adjectives": {"Big": 1}, "numerals": {"21": 1}}}, {"content": "22. It is easy to bore families with complaints at home.", "word_count": 13, "label": "", "sentence_structure": "Subject鈥搗erb鈥搊bject", "subjective_objective": {"answer": "Subjective", "rationale": "Because \"easiness to irritate family\" is a subjective view or judgment, not an objective description of fact."}, "sentence_sentiment": {"answer": "Negative", "rationale": "Because the \"fun\" act in the entry is negative."}, "language_style": {"answer": "Calm and direct", "rationale": "The entry is more straightforward in language style, with no excessive rhetorical or emotional rendering, but rather conveys the message by means of simple statements."}, "time_dimension": {"answer": "Present", "rationale": "The keywords \"sorture at home\" and \"happy at family\" exist in the current family environment, so the temporal dimension is now judged."}, "sentence_type": "Negative declarative sentence", "pos": {"nouns": {"Home": 1, "Complaints": 1, "Family": 1, "Boring": 1}, "verbs": {"鈾?And I'm gonna be 鈾?: 1, "in": 1}, "adverbs": {"It's easy": 1}, "numerals": {"22": 1}}}, {"content": "23. Sometimes family members fall when they are angry.", "word_count": 12, "label": "", "sentence_structure": "Subject鈥搗erb鈥揷omplement", "subjective_objective": {"answer": "Objective", "rationale": "Because \"sometimes a family member falls in anger\" is an event that is objective. Although there may be subjective reasons for family members to get angry and fall, the act itself is objective."}, "sentence_sentiment": {"answer": "Negative", "rationale": "Because the act of \"thumping something when angry\" in the entry is negative."}, "language_style": {"answer": "Calm and direct", "rationale": "This is more objective and concise, helping the interviewees to understand and answer questions accurately."}, "time_dimension": {"answer": "Past", "rationale": "The entry describes the behaviour of the family members of the person being tested, does not have clear time limits and a basis for judgement on the dimensions of time past, present and future, and therefore does not allow for precise classification of time."}, "sentence_type": "Affirmative declarative sentence", "pos": {"nouns": {"Time": 1, "Family members": 1, "Anger": 1, "Something": 1}, "verbs": {"- Get angry": 1, "Fall": 1}, "adverbs": {"Sometimes": 1}, "numerals": {"23": 1}}}, {"content": "24. Family members are independent in their thinking.", "word_count": 11, "label": "", "sentence_structure": "Subject鈥揷opula鈥損redicative", "subjective_objective": {"answer": "Subjective", "rationale": "Because \"independent thinking\" is a subjective, individual-based attitude and perception, not an objective fact or behaviour."}, "sentence_sentiment": {"answer": "Positive", "rationale": "Because \"independent thinking\" is positive behavior."}, "language_style": {"answer": "Tactful and warm", "rationale": "The language style of the entry is more euphemistic, and it guides the interviewee to reflect on the current situation of his or her family rather than to make a direct judgement. This expression is more acceptable to the interviewee and is willing to share his or her thoughts and experiences."}, "time_dimension": {"answer": "Present", "rationale": "The keyword \"family members think independently\" suggests that it is a description or assessment of the current state of the family. While the situation may have existed at some point in the past, the title does not clearly point to the past, but rather to the current state of the family, and should therefore be judged as \"now\"."}, "sentence_type": "Affirmative declarative sentence", "pos": {"nouns": {"Family members": 1, "Problem": 1}, "verbs": {"Independent thinking": 1}, "numerals": {"24": 1}}}, {"content": "25. Family members agree that it is more important to raise living standards than anything else.", "word_count": 24, "label": "", "sentence_structure": "Subject鈥搗erb鈥搊bject", "subjective_objective": {"answer": "Subjective", "rationale": "The views of family members are personal and subjective."}, "sentence_sentiment": {"answer": "Positive", "rationale": "Because the attitude in the entry, \"Assuring that improving living standards is more important than anything else\", is positive and expresses the desire to pursue quality of life."}, "language_style": {"answer": "Tactful and warm", "rationale": "The entry is more softly spoken, in which the interviewee answers questions in a question-and-answer manner, and does not use too direct or hard language. It also expresses a more general view that family members consider it important to improve their standard of living, and that this view may resonate and foster acceptance among the interviewee."}, "time_dimension": {"answer": "Present", "rationale": "The word \"considers\" in the entry indicates that this is a subjective view and evaluation, while \"family members\" suggests that this view is being made in the immediate future. Thus, the temporal dimension of the entry is now judged."}, "sentence_type": "Affirmative declarative sentence", "pos": {"nouns": {"Family members": 1, "Standard of living": 1, "Things": 1}, "verbs": {"I think": 1, "鈾?And I'm gonna be 鈾?: 1, "Increase": 1, "Important": 1}, "adverbs": {"Both": 2}, "numerals": {"25": 1}}}, {"content": "26. We all believe that learning new knowledge is more important than anything else.", "word_count": 20, "label": "", "sentence_structure": "Subject鈥搗erb鈥搊bject", "subjective_objective": {"answer": "Subjective", "rationale": "Because \"think\" expresses the patient's subjective attitude."}, "sentence_sentiment": {"answer": "Positive", "rationale": "Because the views in the entries are positive, the importance and recognition of learning new knowledge is expressed."}, "language_style": {"answer": "Tactful and warm", "rationale": "The entry uses an invitation expression, which is a \"we all think\" expression to guide respondents to think and answer questions. It is relatively mild and easy to accept and respond."}, "time_dimension": {"answer": "Present", "rationale": ""}, "sentence_type": "Affirmative declarative sentence", "pos": {"nouns": {"we": 1, "Learning": 1, "Knowledge": 1, "Yes": 1}, "verbs": {"I think": 1, "Learn": 1, "- Yeah": 1, "Heavy": 1}, "adjectives": {"New": 1, "Other": 1}, "numerals": {"26": 1}, "pronouns": {"Both": 1}}}, {"content": "27. No one in the family participates in various sporting activities.", "word_count": 12, "label": "", "sentence_structure": "Subject鈥揷opula鈥損redicative", "subjective_objective": {"answer": "Objective", "rationale": "Because it is an objective fact that participation in sports is not influenced by individual subjective attitudes."}, "sentence_sentiment": {"answer": "Negative", "rationale": "Because this entry expresses a negative situation, where no family member is involved in various sporting activities."}, "language_style": {"answer": "Tactful and warm", "rationale": "The entry is more euphemistic. It does not directly criticize or accuse family members, but rather raises questions by asking questions, and uses more objective terms such as \"to participate in sports activities\" rather than subjective evaluations."}, "time_dimension": {"answer": "Present", "rationale": "The entry describes the current status and circumstances of the person being tested, does not address past or future time dimensions, and therefore is judged to be \"now\"."}, "sentence_type": "Negative declarative sentence", "pos": {"nouns": {"Home": 1, "person": 1, "Sports activities": 1}, "verbs": {"Participation": 1}, "adverbs": {"Nope": 1}, "numerals": {"27": 1}}}, {"content": "28. Family members often help the elderly and disabled around them.", "word_count": 22, "label": "", "sentence_structure": "Subject鈥搗erb鈥搊bject", "subjective_objective": {"answer": "Objective", "rationale": "Since it is an objective fact that family members often help the elderly and disabled around them, it can be ascertained by observation or enquiry."}, "sentence_sentiment": {"answer": "Positive", "rationale": "Because the entry describes family members as caring for and helping groups in need, it is a positive behaviour and attitude."}, "language_style": {"answer": "Tactful and warm", "rationale": "The entry, by asking the respondents about their behaviour in the family, emphasizes mutual assistance and care among family members, and does not indicate directly what should be done or criticized those who do not, which is a more moderate and encouraging expression."}, "time_dimension": {"answer": "Present", "rationale": "In the entry, \"Assisting in life\" indicates that the act is taking place or is going on in the near future, so the temporal dimension is \"now\"."}, "sentence_type": "Affirmative declarative sentence", "pos": {"nouns": {"Family members": 1, "Life": 1, "Around": 1, "Older persons": 1, "Persons with disabilities": 1}, "verbs": {"Helping often": 1}, "adverbs": {"in": 1, "Go, go, go": 1, "of": 2}, "numerals": {"28": 1}}}, {"content": "29. In our home, it is often not found when something needs to be used.", "word_count": 19, "label": "", "sentence_structure": "Subject鈥搗erb鈥搊bject", "subjective_objective": {"answer": "Subjective", "rationale": "Because \"when something is needed, it is often not found\" is the patient's subjective feeling and experience. Although it is an objective situation, in this questionnaire researchers would like to know how patients feel and feel about it, and should therefore be classified as subjective."}, "sentence_sentiment": {"answer": "Negative", "rationale": "Because the \"nil\" act in the entry is negative."}, "language_style": {"answer": "Tactful and warm", "rationale": ""}, "time_dimension": {"answer": "Present", "rationale": "The entry describes a common situation in family life at present, where certain things are often not found when they are needed, suggesting that the testee may be experiencing this situation. The temporal dimension is therefore now judged."}, "sentence_type": "Negative declarative sentence", "pos": {"nouns": {"Questionnaire Entry": 1, "Home": 1, "Something": 1}, "verbs": {"Use": 1, "Found it": 1}, "adverbs": {"Often": 1}, "numerals": {"29": 1, "Some": 1}, "pronouns": {"we": 1}}}, {"content": "30. The time spent eating and sleeping in our home is constant.", "word_count": 19, "label": "", "sentence_structure": "Subject鈥搗erb鈥揷omplement", "subjective_objective": {"answer": "Objective", "rationale": "Because the timetable is an objective fact that is developed by the family together."}, "sentence_sentiment": {"answer": "Neutral", "rationale": "Because the entry merely describes a fact, it does not express a positive or negative emotional tendency."}, "language_style": {"answer": "Tactful and warm", "rationale": ""}, "time_dimension": {"answer": "Present", "rationale": ""}, "sentence_type": "Affirmative declarative sentence", "pos": {"nouns": {"Home": 1, "Time": 1, "Rice": 1, "Sleep": 1}, "verbs": {"be": 1, "Change": 1, "Eat": 1, "Sleep": 1}, "adjectives": {"It's the same": 1}, "numerals": {"30": 1}, "pronouns": {"we": 1}}}, {"content": "31. There is a climate of harmony and harmony in our home.", "word_count": 14, "label": "", "sentence_structure": "Subject鈥揷opula鈥損redicative", "subjective_objective": {"answer": "Subjective", "rationale": "This is because patients have a subjective sense and assessment of the family climate and is subjective in nature."}, "sentence_sentiment": {"answer": "Positive", "rationale": "Because the \"climate of harmony\" in the entry is positive."}, "language_style": {"answer": "Tactful and warm", "rationale": "This euphemism often creates a friendly and relaxed atmosphere and encourages respondents to participate more actively in the questionnaire."}, "time_dimension": {"answer": "Present", "rationale": ""}, "sentence_type": "Affirmative declarative sentence", "pos": {"nouns": {"The atmosphere": 1, "Home": 1, "One": 1, "Harmony": 1}, "verbs": {"have": 1, "in": 1}, "adjectives": {"Unanimously": 1, "Harmony": 1}, "numerals": {"31": 1}, "classifiers": {"Plant": 1}, "pronouns": {"we": 1}}}, {"content": "32. Everyone in the family can speak of their difficulties and problems.", "word_count": 18, "label": "", "sentence_structure": "Subject鈥搗erb鈥搊bject", "subjective_objective": {"answer": "Subjective", "rationale": "The thoughts and feelings of each individual in the family are subjective, so the perspective of this questionnaire entry is subjective."}, "sentence_sentiment": {"answer": "Neutral", "rationale": "Because the entry does not have any emotional color."}, "language_style": {"answer": "Tactful and warm", "rationale": "The entry is more euphemistic and warm, and does not directly order or request the interviewee to perform a particular task, but rather asks the interviewee in an invitation and support manner about the existence of a supportive and communication atmosphere in the family."}, "time_dimension": {"answer": "Present", "rationale": "The entry in the questionnaire is for a description of the current family situation, including the key words \"everyone\" and \"may say\" which indicate that this is an act in progress and therefore is judged to be the present."}, "sentence_type": "Affirmative declarative sentence", "pos": {"nouns": {"Family": 1, "person": 1, "Difficulties": 1, "Trouble": 1}, "verbs": {"Yeah": 1, "Tell me": 1}, "adverbs": {"Every one": 1}, "numerals": {"32": 1}, "pronouns": {"self": 1}}}, {"content": "33. Little temper among family members.", "word_count": 10, "label": "", "sentence_structure": "Subject鈥搗erb鈥揷omplement", "subjective_objective": {"answer": "Subjective", "rationale": "The way in which family members interact is a subjective description rather than an objective fact."}, "sentence_sentiment": {"answer": "Neutral", "rationale": "As this entry does not express positive or negative emotions."}, "language_style": {"answer": "Tactful and warm", "rationale": "The language style of the entry is more euphemistic. It uses a more positive expression that emphasizes harmony and harmony among family members, without directly pointing to the existence of contradictions or quarrels, which are more acceptable and comfortable."}, "time_dimension": {"answer": "Present", "rationale": "The entry describes the emotional communication between family members, which is now judged in time dimension. In it, the term \"very little\" indicates that this has occurred for some time and may continue to be so, so it can be considered to be the present state."}, "sentence_type": "Affirmative declarative sentence", "pos": {"nouns": {"Family members": 1, "- Grumpy": 2}, "verbs": {"Very few": 1, "have": 1}, "adverbs": {"Between": 1, "Very few": 1, "EMPTY": 1}, "numerals": {"33": 1}}}, {"content": "34. Everyone in our family is completely free.", "word_count": 15, "label": "", "sentence_structure": "Subject鈥搗erb鈥揷omplement", "subjective_objective": {"answer": "Subjective", "rationale": "Because \"everyone in our family is completely free\" is a personal conviction or attitude, not an objective fact. This reflects the subjective expression of the views and values of the family members in terms of decision-making and action."}, "sentence_sentiment": {"answer": "Neutral", "rationale": "Because the entry describes an objective fact and does not address emotional, attitudinal, behavioural, etc."}, "language_style": {"answer": "Tactful and warm", "rationale": "The language style of the entry is more moderate, friendly and open, is relatively soft, and does not speak too directly or strongly. At the same time, it uses positive sentences that give positive positive impression. This expression helps create a comfortable atmosphere of communication that is more acceptable to respondents and willing to share their ideas."}, "time_dimension": {"answer": "Present", "rationale": "The content of the questionnaire entries relates to the current family situation, with no clear time frame or past, future or future circumstances, and can therefore be judged as \"now\"."}, "sentence_type": "Affirmative declarative sentence", "pos": {"nouns": {"person": 2, "Home": 1, "Total": 1, "Freedom": 1}, "verbs": {"Out": 1, "be": 1}, "adverbs": {"Every one": 1}, "numerals": {"34": 1}, "pronouns": {"we": 1}}}, {"content": "35. We all believe that competition is good in any case.", "word_count": 16, "label": "", "sentence_structure": "Subject鈥搗erb鈥搊bject", "subjective_objective": {"answer": "Subjective", "rationale": "Because the words \"confident\" and \"anything\" indicate that this is the patient's subjective attitude and belief. Although competition may be an objective situation, whether it is considered good is the result of the patient's subjective judgement."}, "sentence_sentiment": {"answer": "Positive", "rationale": "Because the attitude in the entries is \"both believe\" and \"good\" and shows a positive view of competition."}, "language_style": {"answer": "Calm and direct", "rationale": "This type of subject is usually suitable for objective data collection and analysis."}, "time_dimension": {"answer": "Present", "rationale": "The entry expresses a belief or attitude and does not clearly indicate a time-oriented nature, and therefore the judgement is now."}, "sentence_type": "Affirmative declarative sentence", "pos": {"nouns": {"Situation": 1, "Competition": 1, "Good news": 1}, "verbs": {"Yes": 1, "be": 1, "have": 1}, "adverbs": {"In any case": 1}, "numerals": {"35": 1}, "pronouns": {"we": 1}}}, {"content": "We are not so interested in cultural activities.", "word_count": 13, "label": "", "sentence_structure": "Subject鈥搗erb鈥搊bject", "subjective_objective": {"answer": "Subjective", "rationale": "Because \"less interested\" is the attitude or feeling of the patient about cultural activities, which is subjective."}, "sentence_sentiment": {"answer": "Negative", "rationale": "Because \"not so interested\" expresses a negative attitude."}, "language_style": {"answer": "Calm and direct", "rationale": "The language style of the entry is more straightforward, with no excessive qualifier or emotional colour, and directly expresses the question of whether the other party is not interested in cultural activities."}, "time_dimension": {"answer": "Past", "rationale": "The \"less interested\" in the entry shows the degree of interest that the testee has in the past, and the time dimension is a past."}, "sentence_type": "Negative declarative sentence", "pos": {"nouns": {"Cultural activities": 1}, "verbs": {"Interested": 1, "Not so much": 1, "to": 1}, "adverbs": {"Well": 1}, "numerals": {"36": 1}}}, {"content": "37. We watch movies or sports games, go out on a field trip, etc.", "word_count": 16, "label": "", "sentence_structure": "Subject鈥搗erb鈥搊bject", "subjective_objective": {"answer": "Objective", "rationale": "The following is a video of the event:"}, "sentence_sentiment": {"answer": "Positive", "rationale": "Because the entry describes people ' s frequent involvement in interesting activities and socialization, this behaviour is often considered positive."}, "language_style": {"answer": "Tactful and warm", "rationale": "The entry uses a more euphemistic, inviting language style, which does not require direct orders or mandatory answers, but rather leads to a gentle way of sharing experiences and interests, which is more easily expressed to the respondents to feel comfortable and willing to participate."}, "time_dimension": {"answer": "Present", "rationale": "The phrase \"we\" in that sentence indicates that the person who speaks and listens is involved in this act, \"often watching movies or sports games, going out on the field, etc.\" means that the act is recurrent, customary and therefore can be judged to have a time dimension."}, "sentence_type": "Affirmative declarative sentence", "pos": {"nouns": {"Film": 1, "Sports competitions": 1, "A field trip": 1}, "verbs": {"Look": 1, "Out": 1}, "adverbs": {"Regular": 1, "Wait": 1}, "numerals": {"37": 1}}}, {"content": "38. We consider active and passive bribery to be an acceptable phenomenon.", "word_count": 18, "label": "", "sentence_structure": "Subject鈥搗erb鈥搊bject", "subjective_objective": {"answer": "Subjective", "rationale": "Because \"think\" indicates that it is a patient's subjective attitude or belief, not an objective fact or observation."}, "sentence_sentiment": {"answer": "Negative", "rationale": "Because the \"bribery\" in the entry is negative."}, "language_style": {"answer": "Tactful and warm", "rationale": "Warm and warm."}, "time_dimension": {"answer": "Past", "rationale": ""}, "sentence_type": "Negative declarative sentence", "pos": {"nouns": {"phenomena": 1, "Bribery": 2}, "verbs": {"I think": 1, "be": 1, "It's acceptable": 1, "Receive": 1}, "adjectives": {"Accepted": 1}, "numerals": {"38": 1}, "pronouns": {"we": 1}}}, {"content": "39. In our home, the importance of punctuality is paramount.", "word_count": 12, "label": "", "sentence_structure": "Subject鈥搗erb鈥揷omplement", "subjective_objective": {"answer": "Objective", "rationale": "Because \"Be punctual\" is a fact, a family's values or rules. It is an observed external act or event, not a patient's subjective feeling or idea, and it is therefore an objective look at."}, "sentence_sentiment": {"answer": "Positive", "rationale": "Because the \"Early time for doing things\" attitude in the entry is positive."}, "language_style": {"answer": "Tactful and warm", "rationale": "The language style of the entry is more euphemistic, implicit, and the requirement that the other party must be punctual, without direct orders or coercion, is to express in a more moderate way the value and expectation of what is done on time. At the same time, the term \"our home\" is used, which brings personal behaviour to the family level, increases affection and cohesion."}, "time_dimension": {"answer": "Present", "rationale": "\"Earning\" is the current verb of behaviour, and \"doing as it is\" implies that the act or attitude is being implemented or exists. The entry therefore describes the current situation or attitude."}, "sentence_type": "Affirmative declarative sentence", "pos": {"nouns": {"Our home": 1, "Things": 1, "On time": 1}, "verbs": {"Value": 1, "Do it": 1}, "adverbs": {"very": 1}, "numerals": {"39": 1}}}, {"content": "There is a fixed way in which everything in our home is done.", "word_count": 14, "label": "", "sentence_structure": "Subject鈥搗erb鈥搊bject", "subjective_objective": {"answer": "Objective", "rationale": "Because \"there is a fixed way\" is an objective fact of family behaviour, it is subjective to look at subjective views."}, "sentence_sentiment": {"answer": "Neutral", "rationale": "Because the entry does not have any emotional color."}, "language_style": {"answer": "Tactful and warm", "rationale": "The language style of the entry is more euphemistic, and it is more acceptable and willing to answer questions by asking the family whether they have a regular way of doing things."}, "time_dimension": {"answer": "Present", "rationale": "The entry, which indicates the present family situation described by the testee, does not address past or future concepts and therefore is judged to be \"now\"."}, "sentence_type": "Affirmative declarative sentence", "pos": {"nouns": {"Home": 1, "Yes": 1, "Modalities": 1}, "verbs": {"Do it": 1, "have": 1}, "adverbs": {"Fixed": 1}, "numerals": {"40": 1}}}, {"content": "When there's a family problem, few people volunteer.", "word_count": 13, "label": "", "sentence_structure": "Subject鈥損redicate", "subjective_objective": {"answer": "Objective", "rationale": "Since the description of the purpose of this questionnaire is a factual statement, it reflects the fact that very few people in family matters have offered help."}, "sentence_sentiment": {"answer": "Negative", "rationale": "Because the description of \"very few people volunteer\" in the entry is negative."}, "language_style": {"answer": "Tactful and warm", "rationale": "It is more personal and interactive in its expression of a gentle and inviting tone, rather than of direct statements or orders. At the same time, the sentence implies the impatience and compassion of the authors who are unwilling to help others, demonstrating the care and understanding of the authors."}, "time_dimension": {"answer": "Past", "rationale": ""}, "sentence_type": "Negative declarative sentence", "pos": {"nouns": {"Home": 1, "Yes": 1, "person": 2}, "verbs": {"have": 1, "Do it": 1}, "adverbs": {"Very few": 1}, "numerals": {"41": 1}, "classifiers": {"individual": 1}}}, {"content": "42. Family members often express their feelings in public.", "word_count": 18, "label": "", "sentence_structure": "Subject鈥搗erb鈥揷omplement", "subjective_objective": {"answer": "Objective", "rationale": "Because \"family members often express their feelings in public\" is an objective phenomenon or fact that does not require subjective assessment or assessment by the patient."}, "sentence_sentiment": {"answer": "Positive", "rationale": "Because the behaviour of \"always expressing feelings among themselves in public\" in the entry is positive and expresses emotional and communication between family members."}, "language_style": {"answer": "Tactful and warm", "rationale": "The language style of the entry tends to be euphemistic and sweet. First, it uses the term \"often\" to describe the frequency of emotional expression among family members, indicating that it is a normal phenomenon; secondly, the \"public expression of mutual feelings\" conveys a message of openness and good faith; and finally, the \"relationship between them\" expresses a warm feeling of love and understanding among family members. These words and expressions are both euphemism and a warm, warm-mouthed tone of speech."}, "time_dimension": {"answer": "Present", "rationale": "The keyword \"often\" indicates that it is a customary act, and that it is also an act of a state of affairs. Thus, the entry describes a situation of the present."}, "sentence_type": "Affirmative declarative sentence", "pos": {"nouns": {"Family members": 1, "Emotion": 1}, "verbs": {"Publicly": 1}, "adverbs": {"Regular": 1}, "numerals": {"42": 1}}}, {"content": "43. Family members often blame and criticize each other.", "word_count": 14, "label": "", "sentence_structure": "Subject鈥搗erb鈥揷omplement", "subjective_objective": {"answer": "Subjective", "rationale": "Mutual blame and criticism among family members is the result of patients ' subjective feelings and observations."}, "sentence_sentiment": {"answer": "Negative", "rationale": "Because the \"mutual blame and criticism\" in the entry is negative."}, "language_style": {"answer": "Tactful and warm", "rationale": "The language style of the entry is more euphemistic, because it uses neutral terms such as \"common blame and criticism\" to avoid direct accusations or attacks on the other side, and is not overly emotional. This way helps the respondents to the questionnaire to accept and answer questions more easily."}, "time_dimension": {"answer": "Present", "rationale": "The entry describes a frequent, customary phenomenon, i.e. mutual blame and criticism among family members, and thus the temporal dimension is now judged."}, "sentence_type": "Negative declarative sentence", "pos": {"nouns": {"Family members": 1, "Blame": 1, "Criticism": 1}, "verbs": {"Between": 1, "Regular": 1, "- Yeah, yeah": 1}, "numerals": {"43": 1}}}, {"content": "44. Family members rarely take into account the views of other family members.", "word_count": 19, "label": "", "sentence_structure": "Subject鈥搗erb鈥揷omplement", "subjective_objective": {"answer": "Subjective", "rationale": "Because the subject of the questionnaire, \"Low consideration for the views of other family members\", is the patient's perception and assessment of family members and is a subjective attitude of the patient."}, "sentence_sentiment": {"answer": "Negative", "rationale": "Because the \"very little consideration for the views of the rest of the family\" in the entry is negative."}, "language_style": {"answer": "Tactful and warm", "rationale": "The entry is more personal and interactive when it is spoken in a gentle, inquisitive manner rather than directly speaking or ordering."}, "time_dimension": {"answer": "Present", "rationale": "The entry describes a phenomenon or situation that exists in the family at present, i.e., that family members rarely take into account other family members when they do things."}, "sentence_type": "Negative declarative sentence", "pos": {"nouns": {"Family members": 1, "Observations": 1}, "verbs": {"Do it": 1, "Consider": 1}, "adjectives": {"Very few": 1, "Home": 1, "Other": 1}, "numerals": {"44": 1}, "pronouns": {"they": 1}}}, {"content": "We have been constantly reflecting on ourselves and forcing ourselves to do what we can to do better than once.", "word_count": 27, "label": "", "sentence_structure": "Subject鈥損redicate", "subjective_objective": {"answer": "Subjective", "rationale": "Because \"retroactive\" and \"forced yourself to do better than once\" are subjective acts and attitudes of patients."}, "sentence_sentiment": {"answer": "Positive", "rationale": "Because the position of the entry is positive, it is manifested in a desire for self-promoting and improvement."}, "language_style": {"answer": "Tactful and warm", "rationale": "This is a more euphemistic and warm entry. It encourages respondents to reflect and promote themselves in a positive manner, without excessive pressure or order."}, "time_dimension": {"answer": "Present", "rationale": "Now the entry describes the current behaviour and attitude of the testee, the keywords being \"we always\" , \"retrogressive\" and \"compulsive,\" which point to the situation that is now under way. Moreover, the expression \"better than once\" also indicates that the act is continuing. Thus, the temporal dimension of the article can be judged as now."}, "sentence_type": "Affirmative declarative sentence", "pos": {"nouns": {"Retrospect": 1, "self": 2, "Things": 1, "Strength": 1}, "verbs": {"Total": 1, "Do it": 1, "Okay": 1, "Fuck": 1, "be": 1, "- Put it on": 1}, "adverbs": {"Constantly": 1, "Better once than once": 1}, "numerals": {"45": 1, "One": 2}, "pronouns": {"we": 1}}}, {"content": "46. We rarely discuss issues related to scientific and technological knowledge.", "word_count": 17, "label": "", "sentence_structure": "Subject鈥搗erb鈥搊bject", "subjective_objective": {"answer": "Objective", "rationale": "Because \"we rarely discuss issues related to scientific and technological knowledge\" is a description of what happened in the past at a certain point in time, and is an objective look."}, "sentence_sentiment": {"answer": "Negative", "rationale": "Because \"very rarely talk\" is negative."}, "language_style": {"answer": "Tactful and warm", "rationale": "The language style of the entry is more euphemistic, not so direct or harsh as to be expressed, but rather to guide respondents to share their ideas and experiences in a question and look. At the same time, the sentence does not use too many professional terms or complex language structures, making it easier for respondents to understand and answer questions."}, "time_dimension": {"answer": "Present", "rationale": ""}, "sentence_type": "Negative declarative sentence", "pos": {"nouns": {"Problem": 1, "Scientific and technical knowledge": 1}, "verbs": {"Discussion": 1, "be": 1, "Very few": 1}, "adjectives": {"Related": 1}, "adverbs": {"In terms of": 1, "Very few": 1}, "numerals": {"46": 1}}}, {"content": "47. Each of us has a special interest in one or two recreational activities.", "word_count": 18, "label": "", "sentence_structure": "Subject鈥搗erb鈥搊bject", "subjective_objective": {"answer": "Subjective", "rationale": "Because \"we all\" is a subjective expression, emphasizing the individual feelings and perceptions of family members. And the interest in recreational activities is also subjective, and different people may have different preferences and interests. So, \"Everyone in our family is particularly interested in one or two entertainments\" is viewed from the patient's subjective point of view."}, "sentence_sentiment": {"answer": "Positive", "rationale": "Because the entry \"special interest\" expresses a positive attitude and interest."}, "language_style": {"answer": "Tactful and warm", "rationale": "The language style of the entry is more euphemistic and sweet, and the respondents are introduced to the questionnaire in a friendly manner and are guided to share their interests, which will improve the rate and accuracy of the questionnaire."}, "time_dimension": {"answer": "Present", "rationale": "According to the phrase \"everyone in our family\" and \"special interest in...\" it can be seen that this is a description of the current state of interest of family members. The time dimension of the questionnaire entry should therefore be now."}, "sentence_type": "Affirmative declarative sentence", "pos": {"nouns": {"Our home": 1, "person": 1, "Recreational activities": 1, "Interest": 1}, "verbs": {"Both": 1, "to": 1, "Very interested": 1}, "adjectives": {"Every": 1}, "adverbs": {"Special": 1}, "numerals": {"47": 1, "1-2 items": 1}, "classifiers": {"individual": 1}}}, {"content": "48. We believe that, in any case, the elders should be persuaded by the elders.", "word_count": 21, "label": "", "sentence_structure": "Subject鈥搗erb鈥搊bject", "subjective_objective": {"answer": "Subjective", "rationale": "Because the words \"think\" and \"should\" reflect the subjectivity and values of the interviewee."}, "sentence_sentiment": {"answer": "Positive", "rationale": "Because the position of the entry is positive, it is believed that the older generation should be advised by the older generation."}, "language_style": {"answer": "Calm and direct", "rationale": "It clearly sets out the view that, in any case, the elders should be advised by their elders, without any rhetorical or emotional rendering, and be more honest and objective. This expression is more straightforward and helps ensure that the interviewee understands and answers the questions accurately."}, "time_dimension": {"answer": "Present", "rationale": ""}, "sentence_type": "Affirmative declarative sentence", "pos": {"nouns": {"we": 1, "Sir": 1, "Senior": 1, "Construction": 1}, "verbs": {"I think": 1, "I think so": 1, "Accept": 1}, "adverbs": {"How's it going": 1}, "numerals": {"48": 1}}}, {"content": "49. Our family often changes their plans.", "word_count": 14, "label": "", "sentence_structure": "Subject鈥搗erb鈥搊bject", "subjective_objective": {"answer": "Subjective", "rationale": "Because the description of \"often\" and \"change plan\" relates to the thoughts and behaviour of the patient's family members, and is the patient's subjective perception."}, "sentence_sentiment": {"answer": "Neutral", "rationale": "Because the entry does not have any emotional color."}, "language_style": {"answer": "Calm and direct", "rationale": "The entry is simple and not too simplistic, and it is asked directly whether the family members of the person under investigation have changed their plans regularly and expressed in a more direct and objective manner."}, "time_dimension": {"answer": "Past", "rationale": "\"Always\" means that such acts are frequent, and \"change their plans\" refers to past actions or states, so the temporal dimension of the article is the past."}, "sentence_type": "Affirmative declarative sentence", "pos": {"nouns": {"person": 1, "Planned": 1}, "verbs": {"Often": 1, "Change": 1}, "adverbs": {"Often": 1}, "numerals": {"49": 1}, "pronouns": {"they": 1}}}, {"content": "50. We have a strong emphasis on the observance of regular living patterns and family rules.", "word_count": 20, "label": "", "sentence_structure": "Subject鈥搗erb鈥搊bject", "subjective_objective": {"answer": "Objective", "rationale": "Because compliance with regular living rules and family rules is a rule of the family and is not the patient ' s own subjective feeling or behaviour."}, "sentence_sentiment": {"answer": "Positive", "rationale": "Because the attitude in the entry, which emphasizes the observance of fixed living rules and family rules, is positive, it expresses a value that values family order and discipline."}, "language_style": {"answer": "Calm and direct", "rationale": "The entry is a direct illustration of the importance that families attach to the observance of living standards and family rules, and is not based on excessive rhetoric or emotional rendering, which is more objective and balanced. It is therefore considered that the language style of the purpose is straightforward and calm."}, "time_dimension": {"answer": "Present", "rationale": "According to the phrase \"Our family is very focused\" and \"to comply\", it can be seen that this behaviour is a current family policy and expectation, and so it is judged now."}, "sentence_type": "Affirmative declarative sentence", "pos": {"nouns": {"Family": 1, "Lifestyle": 1, "Family rules": 1}, "verbs": {"Emphasizing": 1, "Compliance": 1, "Fixed": 1}, "adjectives": {"Very": 1}, "numerals": {"50": 1}}}, {"content": "51. Family members are always supportive of each other.", "word_count": 14, "label": "", "sentence_structure": "Subject鈥搗erb鈥搊bject", "subjective_objective": {"answer": "Objective", "rationale": "Because support between family members is an objective relationship, it is not a subjective attitude of the patient."}, "sentence_sentiment": {"answer": "Positive", "rationale": "Because the \"good support\" in the entry is a positive emotion and attitude."}, "language_style": {"answer": "Tactful and warm", "rationale": "The words are more euphemistic and natural, and do not express directly the expectations or demands of relationships between family members. At the same time, the term \"intimate\" is used to describe the degree of mutual support and to make it more mild."}, "time_dimension": {"answer": "Present", "rationale": "The entry now describes the current level of support among family members, and the temporal dimension is now judged."}, "sentence_type": "Affirmative declarative sentence", "pos": {"nouns": {"Family members": 1, "- Yeah, yeah": 1, "Support": 1}, "verbs": {"Both": 1, "Always": 1, "I'm not sure": 1, "Give": 1}, "adjectives": {"Heart": 1}, "adverbs": {"Both": 1, "Always": 1}, "numerals": {"51": 1}}}, {"content": "52. If complaints about family matters were to be made at home, it would be uncomfortable.", "word_count": 21, "label": "", "sentence_structure": "Subject鈥搗erb鈥搊bject", "subjective_objective": {"answer": "Subjective", "rationale": "Because this phrase expresses the views and feelings of the patient regarding expressing dissatisfaction at home, it is the patient ' s subjective attitude."}, "sentence_sentiment": {"answer": "Negative", "rationale": "Because the emotional color of \"uncomfortable\" in the entries is negative."}, "language_style": {"answer": "Tactful and warm", "rationale": "The language style of the entry is euphemism. It does not directly accuse or order the interviewee to refrain from complaining at home, but uses a gentle, understanding tone, conveying a message that \"may be uncomfortable.\" It is easier for the interviewee to accept this expression and more likely to trigger their real answer."}, "time_dimension": {"answer": "Present", "rationale": ""}, "sentence_type": "Affirmative declarative sentence", "pos": {"nouns": {"Home": 1, "Dissatisfied": 1, "Family matters": 1, "person": 1, "- Yeah": 1}, "verbs": {"Say it": 1, "I think": 1}, "adverbs": {"If": 1, "Yes": 1}, "numerals": {"52": 1}, "classifiers": {"individual": 1}, "pronouns": {"you": 1}}}, {"content": "53. Family members sometimes fight each other.", "word_count": 10, "label": "", "sentence_structure": "Subject鈥搗erb鈥揷omplement", "subjective_objective": {"answer": "Objective", "rationale": "Because \"family members sometimes fight each other\" is an event that happens, and it is an objective fact. Although it may bring subjective emotional experience and perceptions, it is objective in itself."}, "sentence_sentiment": {"answer": "Negative", "rationale": "Because the \"fights\" act is negative."}, "language_style": {"answer": "Tactful and warm", "rationale": "The language style of the entry is more euphemistic, and it does not directly blame or criticize behaviour between family members, but rather leads the interviewee to reflect and express his/her views in a gentle way."}, "time_dimension": {"answer": "Past", "rationale": ""}, "sentence_type": "Affirmative declarative sentence", "pos": {"nouns": {"Family members": 1, "Fight": 1}, "verbs": {"Sometimes": 1, "- Yeah, yeah": 1, "Fight": 1}, "adverbs": {"Sometimes": 1}, "numerals": {"53": 1}}}, {"content": "54. Family members rely on their families ' help to resolve their difficulties.", "word_count": 22, "label": "", "sentence_structure": "Subject鈥搗erb鈥搊bject", "subjective_objective": {"answer": "Objective", "rationale": "Because relying on family help to solve difficulties is a statement of fact, not the patient ' s own subjective feelings or attitudes."}, "sentence_sentiment": {"answer": "Negative", "rationale": "Because the behaviour of \"family members rely on family help\" in the entry is negative, it shows a lack of capacity and self-confidence on the part of family members to solve their problems independently."}, "language_style": {"answer": "Tactful and warm", "rationale": "The language style of the entry is more euphemistic, using the terms \"help\" and \"solve problems\" to express it relatively mildly. At the same time, the expression \"both relying on\" is more subtle and not too straightforward. So the language style of the purpose can be described as euphemism."}, "time_dimension": {"answer": "Past", "rationale": "The entry asks about the past behaviour and circumstances of the \"family member\" - whether, for a certain period of time, family members have been able to resolve difficulties by relying on family help."}, "sentence_type": "Affirmative declarative sentence", "pos": {"nouns": {"Family members": 1, "Family": 1, "Problem": 1, "Difficulties": 1}, "verbs": {"Dependency": 1, "Resolve": 1, "I met him": 1}, "numerals": {"54": 1}, "pronouns": {"they": 1}}}, {"content": "Family members are less concerned about promotions, learning achievement, etc.", "word_count": 19, "label": "", "sentence_structure": "Subject鈥搗erb鈥搊bject", "subjective_objective": {"answer": "Subjective", "rationale": "Because the entry describes, inter alia, the patient ' s attitude and perception of family members, which is the patient ' s subjective feeling."}, "sentence_sentiment": {"answer": "Negative", "rationale": "Because the expression \"not very concerned\" in the entry is negative."}, "language_style": {"answer": "Tactful and warm", "rationale": "The entry is more personal and interactive when it is spoken in a gentle and inviting manner, rather than in direct statements or orders. Moreover, the question does not contain negative emotions or a word of evaluation, but rather leads the respondents to questions in a question-and-answer manner, reflecting the fairness and objectivity of the questionnaire design."}, "time_dimension": {"answer": "Present", "rationale": "This entry shows the attitude and perceptions of family members on issues such as promotion and academic achievement, and does not clearly indicate whether this phenomenon has occurred or will occur in the past."}, "sentence_type": "Negative declarative sentence", "pos": {"nouns": {"Family members": 1, "Functions": 1, "Learning achievement": 1}, "verbs": {"Not really": 1, "Upgrade": 1}, "adverbs": {"Too much": 1, "not": 1, "Wait": 1}, "numerals": {"55": 1}}}, {"content": "56. There are people playing musical instruments in their homes.", "word_count": 7, "label": "", "sentence_structure": "Subject鈥搗erb鈥揷omplement", "subjective_objective": {"answer": "Objective", "rationale": "This type of entry is usually used to collect information about the family environment or living conditions and is therefore considered to be objective."}, "sentence_sentiment": {"answer": "Neutral", "rationale": "The entry merely describes a fact and does not express any positive or negative emotional colours."}, "language_style": {"answer": "Tactful and warm", "rationale": "The language style of the entry is more euphemistic. It raises the question in a descriptive phrase, not directly asking or ordering, but in a gentle, invitation-style conversation, which is more personal and interactive. Nor is it using overly emotional terms or tone, which gives people a sense of comfort and calm."}, "time_dimension": {"answer": "Past", "rationale": ""}, "sentence_type": "Affirmative declarative sentence", "pos": {"nouns": {"Home": 1, "person": 1, "Instrument": 1}, "verbs": {"have": 1, "Play": 1}, "numerals": {"56": 1}}}, {"content": "57. Family members do not often engage in recreational activities except for work studies.", "word_count": 18, "label": "", "sentence_structure": "Subject鈥搗erb鈥搊bject", "subjective_objective": {"answer": "Objective", "rationale": "Since the subject is about the recreational activities of family members, which are objective facts, it is about the behaviour of family members, not their subjective ideas."}, "sentence_sentiment": {"answer": "Negative", "rationale": "This entry is an expression of the lack of recreational activities of family members and implies a negative state of life or attitude."}, "language_style": {"answer": "Calm and direct", "rationale": "The language style of the entry is more direct, without excessive rhetorical or emotional colours, but simply describes the fact that family members of the person under investigation regularly engage in recreational activities outside work and study, which gives an objective and rational sense of expression. \""}, "time_dimension": {"answer": "Present", "rationale": "According to \"unusual\" and \"now\", it can be judged that the act is in the state of the act in progress."}, "sentence_type": "Negative declarative sentence", "pos": {"nouns": {"Family members": 1, "Work studies": 2, "Recreational activities": 1}, "verbs": {"Divide": 1, "Regular": 1}, "adverbs": {"not": 1}, "numerals": {"57": 1}, "classifiers": {"External": 1}}}, {"content": "Family members volunteer for public sanitation.", "word_count": 15, "label": "", "sentence_structure": "Subject鈥搗erb鈥揷omplement", "subjective_objective": {"answer": "Objective", "rationale": "Because the title describes the objective behaviour of a family member, i.e. whether they are willing to participate in the work of public sanitation. This is an objective fact that does not require a patient鈥檚 subjective attitude or ideas to interpret or evaluate."}, "sentence_sentiment": {"answer": "Positive", "rationale": "Because the act of \"all of us voluntarily go to public sanitation\" is positive, it expresses the interest and love of families in helping each other and in the community environment."}, "language_style": {"answer": "Tactful and warm", "rationale": "The entry, which leads respondents to share information on the willingness of family members to participate in public sanitation by asking questions, is more gentle and respectful of the will of the interviewee and more natural."}, "time_dimension": {"answer": "Past", "rationale": "In this entry, \"family members voluntarily go to public sanitation\" describes past behaviour or state and therefore falls within the realm of the past."}, "sentence_type": "Affirmative declarative sentence", "pos": {"nouns": {"Family members": 1, "Environmental health": 1}, "verbs": {"Do it": 1, "Go on": 1, "Yes": 1}, "adjectives": {"Public": 1}, "numerals": {"58": 1}}}, {"content": "Family members carefully maintain the cleaning of their rooms.", "word_count": 16, "label": "", "sentence_structure": "Subject鈥搗erb鈥搊bject", "subjective_objective": {"answer": "Objective", "rationale": "Since this sentence describes the conduct of a family member, which can be verified by observation, it is an objective description."}, "sentence_sentiment": {"answer": "Positive", "rationale": "Because the entry describes the positive behaviour of family members - to keep their rooms clean."}, "language_style": {"answer": "Tactful and warm", "rationale": "The topic, in an encouraging tone, expressed a recognition and praise for the behaviour of the family members of the interviewee, while also providing a positive pattern of behaviour, which made the language more euphemismal."}, "time_dimension": {"answer": "Present", "rationale": ""}, "sentence_type": "Affirmative declarative sentence", "pos": {"nouns": {"Family members": 1, "Room": 1, "Clean": 1}, "verbs": {"Hold on": 1}, "adjectives": {"Seriously": 1}, "adverbs": {"Land": 1}, "numerals": {"59": 1}}}, {"content": "Family members may leave at night at random without prior consultation with their families.", "word_count": 21, "label": "", "sentence_structure": "Subject鈥損redicate", "subjective_objective": {"answer": "Subjective", "rationale": "The possibility that the patient's thoughts and attitudes need to be discussed with his or her family in advance is subjective. The fact that \"family members may leave at night at random\" may be justified by objective conditions or by the subjective will of the individual, and the entry is therefore primarily viewed from the patient's subjective perspective."}, "sentence_sentiment": {"answer": "Negative", "rationale": "Since the act of \"without prior consultation with family\" in this entry may result in a lack of communication and understanding between family members and may have a negative impact on family relations."}, "language_style": {"answer": "Calm and direct", "rationale": ""}, "time_dimension": {"answer": "Present", "rationale": ""}, "sentence_type": "Negative declarative sentence", "pos": {"nouns": {"Family members": 1, "Night": 1, "Before": 1, "Family": 1}, "verbs": {"Yeah": 1, "Out": 1, "- Let's talk": 1}, "adverbs": {"No, I don't": 1}, "numerals": {"60": 1, "1": 4}}}, {"content": "61. The collective spirit of our family is very low.", "word_count": 10, "label": "", "sentence_structure": "Subject鈥揷opula鈥損redicative", "subjective_objective": {"answer": "Subjective", "rationale": "Because \"collective spirit\" is a subjective feeling that reflects the patient's perception of relationships and interactions among his or her family members."}, "sentence_sentiment": {"answer": "Negative", "rationale": "Because \"very few\" in the entries means that the family's collective spirit is not good, and it's negative."}, "language_style": {"answer": "Tactful and warm", "rationale": "The language style of the entry is relatively euphemistic, using \"very few\" negative forms to describe the collective mental state of the family, without direct accusations or criticism. At the same time, it is not too harsh or insensitive to be more inclined to lead the interviewee to express his thoughts and feelings in a gentle and understanding manner."}, "time_dimension": {"answer": "Present", "rationale": "The keywords \"the collective spirit of our family\" and \"very few\" indicate that the respondents are dissatisfied with the interaction and solidarity of their family members, and this may be continuing."}, "sentence_type": "Negative declarative sentence", "pos": {"nouns": {"Home": 1, "Collective spirit": 1}, "verbs": {"Very few": 1}, "numerals": {"61": 1}}}, {"content": "62. Our family can speak openly about the economic issues of the family.", "word_count": 17, "label": "", "sentence_structure": "Subject鈥搗erb鈥搊bject", "subjective_objective": {"answer": "Subjective", "rationale": "Because \"can talk openly\" is a subjective attitude or feeling, not just an objective fact about family economy. This reflects patients' perception of family atmosphere and communication, and is therefore subjective."}, "sentence_sentiment": {"answer": "Positive", "rationale": "Because the act of \"open talk about the economy of the family\" is positive and indicates good trust and communication among family members."}, "language_style": {"answer": "Tactful and warm", "rationale": "The language style of the entry is more euphemistic, using the words \"may\" and \"publicly speaking\" and neither the direct nor the negative or negative emotional tone, which appears to be more moderate and open and more conducive to positive responses and real expression by the respondents to the questionnaire."}, "time_dimension": {"answer": "Present", "rationale": "The \"can\" and \"publicly talk\" in the questionnaire entries indicate the current situation, so it's now. And, at the same time, \"our house\" shows that it's a scene within the family, not a point in time, past or future."}, "sentence_type": "Affirmative declarative sentence", "pos": {"nouns": {"Our home": 1, "Family": 1, "Economy": 1, "Problem": 1}, "verbs": {"Yeah": 1, "Talk openly": 1}, "adjectives": {"Public": 1}, "adverbs": {"Land": 1, "Yeah": 1}, "numerals": {"62": 1}, "pronouns": {"Home": 1}}}, {"content": "63. When the views of family members diverged, we avoided them to maintain peace.", "word_count": 25, "label": "", "sentence_structure": "Subject鈥搗erb鈥搊bject", "subjective_objective": {"answer": "Subjective", "rationale": "Whether or not the views of family members are divided and avoid it is the patient ' s own idea of peace and is therefore subjective."}, "sentence_sentiment": {"answer": "Negative", "rationale": "Because the \"scramble\" act in the entry is negative."}, "language_style": {"answer": "Tactful and warm", "rationale": "The expression \"we all avoid it\" is used in this title, which is euphemism, avoiding direct conflict or criticism. Moreover, the phrase \"in order to maintain peace\" also shows that the entry seeks to resolve the issue of differences of opinion among family members in a harmonious and warm way. The language of the topic is therefore more euphemistic and warm."}, "time_dimension": {"answer": "Past", "rationale": "The entry \"has always avoided it\" means a past customary act, and it therefore judges the past."}, "sentence_type": "Negative declarative sentence", "pos": {"nouns": {"Family members": 1, "Observations": 1, "Difference": 1, "# And the twilight #": 1}, "verbs": {"Generate": 1, "Avoidance": 1, "Hold on": 1}, "adverbs": {"Both": 1, "Always": 1}, "numerals": {"63": 1}, "pronouns": {"we": 1}}}, {"content": "64. Family members want their families to solve their problems independently.", "word_count": 15, "label": "", "sentence_structure": "Subject鈥搗erb鈥搊bject", "subjective_objective": {"answer": "Subjective", "rationale": "The degree of hope of family members is the patient ' s subjective feeling."}, "sentence_sentiment": {"answer": "Positive", "rationale": "Because the \"hope for family independence\" approach in the entry is positive, it is an expression that family members encourage each and every one of them to be able to deal with the problem and grow up independently."}, "language_style": {"answer": "Calm and direct", "rationale": "The entry is a straightforward and clear-cut narrative of the core of the problem, which is not retrofitted with excessive rhetoric or emotional colours and is more in line with a calm and objective presentation."}, "time_dimension": {"answer": "Present", "rationale": "In the future, the entry describes the expectations or vision of family members, who want to be able to solve the problem independently."}, "sentence_type": "Affirmative declarative sentence", "pos": {"nouns": {"Family members": 1, "Problem": 1, "Family": 1}, "verbs": {"Hope": 1, "Independent settlement": 2}, "numerals": {"64": 1}}}, {"content": "65. Our family is less active in achieving achievements.", "word_count": 16, "label": "", "sentence_structure": "Subject鈥損redicate", "subjective_objective": {"answer": "Subjective", "rationale": "Because \"positive\" is a term for subjective attitudes, which describe the attitude of family members towards achievement."}, "sentence_sentiment": {"answer": "Negative", "rationale": "Because the \"not so positive about what's going on\" approach in the entry is negative."}, "language_style": {"answer": "Calm and direct", "rationale": "The entry is in an objective and direct manner, and the language is relatively straightforward inquiring about the attitude of the family members of the interviewee to the achievement of the achievement."}, "time_dimension": {"answer": "Present", "rationale": "The phrase \"our family\" refers to family members, while the phrase \"not so active in achieving achievements\" expresses the current attitude and perception. Thus, the sentence describes the current behaviour or mentality."}, "sentence_type": "Negative declarative sentence", "pos": {"nouns": {"Our home": 1, "person": 2, "Achievements": 1}, "verbs": {"to": 1, "Access": 1, "Not so much": 1, "Positive": 1}, "adverbs": {"Not so much": 1}, "numerals": {"65": 1}, "pronouns": {"Our home": 1}}}, {"content": "66. Family members often go to the library.", "word_count": 9, "label": "", "sentence_structure": "Subject鈥搗erb鈥搊bject", "subjective_objective": {"answer": "Objective", "rationale": "Because \"family members go to libraries often\" is an objective fact."}, "sentence_sentiment": {"answer": "Neutral", "rationale": "Because the entry does not express any positive or negative emotional colours, it is merely a description of a fact."}, "language_style": {"answer": "Tactful and warm", "rationale": "The entry uses the tone of the inquiry and the term \"frequently\" to avoid the expression of direct orders or judgements, and is expressed in a more moderate manner and more easily elicited positive responses from the respondents."}, "time_dimension": {"answer": "Present", "rationale": "The word \"normal\" in \"family members often go to the library\" indicates a regular behaviour, and the word \"go\" adopts the usual current pattern, which means that the behaviour has not only occurred in the past, but may still be ongoing. The entry thus describes the current activity of family members, which is the current period."}, "sentence_type": "Affirmative declarative sentence", "pos": {"nouns": {"Family members": 1, "Library": 1}, "verbs": {"Always": 1}, "numerals": {"66": 1}}}, {"content": "67. Family members sometimes take recreational studies according to their preferences or interests.", "word_count": 21, "label": "", "sentence_structure": "Subject鈥搗erb鈥搊bject", "subjective_objective": {"answer": "Objective", "rationale": "This is an objective description of the behaviour of the patient ' s family members and does not involve subjective feelings or attitudes of the patient."}, "sentence_sentiment": {"answer": "Positive", "rationale": "Because the entry \"Participate in recreational studies according to personal preferences or interests\" is positive."}, "language_style": {"answer": "Tactful and warm", "rationale": "The entry is more euphemistic and has a certain degree of intimacy. Instead of asking the interviewee whether he or she is involved in family recreational studies, it is directing the interviewee to think about whether or not he or she is doing something similar by describing the behaviour of family members, and using it as an opportunity to further communicate and explore."}, "time_dimension": {"answer": "Past", "rationale": "The description of \"family member behaviour sometimes\" in the entry indicates that it is an act that has already occurred and is therefore judged as a past."}, "sentence_type": "Affirmative declarative sentence", "pos": {"nouns": {"Family members": 1, "Learning": 1, "Entertainment studies": 1}, "verbs": {"Press": 1, "Participation": 1, "Sometimes": 1}, "adjectives": {"Personal hobby": 1, "Interest": 1}, "adverbs": {"Sometimes": 1}, "numerals": {"67": 1}, "classifiers": {"individual": 1}}}, {"content": "68. Family members agreed to act in a moral capacity.", "word_count": 17, "label": "", "sentence_structure": "Subject鈥搗erb鈥搊bject", "subjective_objective": {"answer": "Subjective", "rationale": "Because the word \"thinks\" indicates that it is the views and ideas of the patient or the interviewee, not an objective description. The view of this questionnaire entry is subjective."}, "sentence_sentiment": {"answer": "Negative", "rationale": "Because the act of \"damn morality\" in the entries may be too conservative and inflexible, it can be considered a negative attitude."}, "language_style": {"answer": "Calm and direct", "rationale": "The entry expresses the view, expressed directly by the family members of the person under investigation, that there is a need to follow ethical norms in dealing with matters, and that the language style is calm and objective, and there is no excessive rhetoric or emotional overturn."}, "time_dimension": {"answer": "Past", "rationale": "In the past, family members had thought that the practice of doing what was ethical was described in the sentence as a historical situation."}, "sentence_type": "Affirmative declarative sentence", "pos": {"nouns": {"Family members": 1, "Ethical precepts": 1, "Operations": 1}, "verbs": {"I think": 1, "Stay put": 1}, "numerals": {"68": 1}}}, {"content": "69. The division of labour among individuals is clear in our home.", "word_count": 14, "label": "", "sentence_structure": "Subject鈥揷opula鈥損redicative", "subjective_objective": {"answer": "Objective", "rationale": "This is a description of the family structure and does not involve subjective attitudes or feelings of the patient."}, "sentence_sentiment": {"answer": "Neutral", "rationale": "As the entry merely stated a fact, it did not express any positive or negative feelings or attitudes."}, "language_style": {"answer": "Calm and direct", "rationale": "The language style of the entry is more straightforward, not using too many rhetorical or emotional techniques, but simply presenting a fact."}, "time_dimension": {"answer": "Present", "rationale": "The keyword \"division of labour is clear\" expresses a state of reality, while \"our home\" refers to the current family situation. So the time dimension of this entry should be now."}, "sentence_type": "Affirmative declarative sentence", "pos": {"nouns": {"Our home": 1, "Everyone": 2, "Division of labour": 1}, "verbs": {"be": 1, "Clear": 1}, "adjectives": {"Clear": 1}, "numerals": {"69": 1}, "pronouns": {"Each": 1}}}, {"content": "70. In our home, there are no strict rules to bind us.", "word_count": 16, "label": "", "sentence_structure": "Subject鈥搗erb鈥揷omplement", "subjective_objective": {"answer": "Subjective", "rationale": "Because this sentence relates to the patient ' s perception and feelings of family rules and is a subjective attitude of the patient."}, "sentence_sentiment": {"answer": "Neutral", "rationale": "Because the entry does not have any emotional color."}, "language_style": {"answer": "Tactful and warm", "rationale": "The language style of the entry is more euphemistic, using the more acclaimed name \"Our Home\" and expressing the imprescriptible state of family rules in a descriptive way. This expression is more likely to resonate with the interviewee and to be willing to share his views and experiences."}, "time_dimension": {"answer": "Present", "rationale": ""}, "sentence_type": "Negative declarative sentence", "pos": {"nouns": {"Our home": 1, "Rule": 1}, "verbs": {"No, I'm not": 1, "Constraints": 1}, "adjectives": {"Strict": 1}, "numerals": {"70": 1}, "pronouns": {"we": 1}}}, {"content": "71. Family members have always been compatible with each other.", "word_count": 14, "label": "", "sentence_structure": "Subject鈥揷opula鈥損redicative", "subjective_objective": {"answer": "Subjective", "rationale": "The relationship between family members is a subjective view of the patient."}, "sentence_sentiment": {"answer": "Positive", "rationale": "Because the description \"family members always fit in with each other\" in the entry is positive and reflects the harmony of family relations."}, "language_style": {"answer": "Tactful and warm", "rationale": ""}, "time_dimension": {"answer": "Present", "rationale": "The entry asks about current family relationships, indicating whether the relationship between the testee and his family members has been good and therefore falls under the current time dimension."}, "sentence_type": "Affirmative declarative sentence", "pos": {"nouns": {"Family members": 1, "You too": 1, "Between": 1}, "verbs": {"Always": 1, "It's a good fit": 1}, "adverbs": {"Both": 1}, "numerals": {"71": 1}}}, {"content": "Family members speak to each other with great care to avoid harming each other's feelings.", "word_count": 22, "label": "", "sentence_structure": "Subject鈥搗erb鈥揷omplement", "subjective_objective": {"answer": "Objective", "rationale": "This is an objective description, as it relates to the way in which family members communicate, and it is a practical situation that does not involve the patient鈥檚 subjective feelings or thoughts."}, "sentence_sentiment": {"answer": "Positive", "rationale": "Because the behavior in the entry is positive \"to avoid hurting the other person's feelings.\""}, "language_style": {"answer": "Tactful and warm", "rationale": "The entry is a soft, invitation-based dialogue that emphasizes mutual respect and understanding among family members, and is more personal and interactive in its expression, which can better resonate with and engage the respondents."}, "time_dimension": {"answer": "Past", "rationale": ""}, "sentence_type": "Affirmative declarative sentence", "pos": {"nouns": {"Family members": 1, "Emotion": 1}, "verbs": {"Attention": 1, "Avoid": 1, "Hurt": 1, "Speak": 1}, "adverbs": {"Both": 1}, "numerals": {"72": 1}}}, {"content": "Family members often want to out-think each other.", "word_count": 12, "label": "", "sentence_structure": "Subject鈥搗erb鈥揷omplement", "subjective_objective": {"answer": "Subjective", "rationale": "The idea of family members is subjective, and this competing relationship is also subjective in its perception and attitude."}, "sentence_sentiment": {"answer": "Negative", "rationale": "Because the \"often wants to be better than the other\" act in the entry is negative."}, "language_style": {"answer": "Calm and direct", "rationale": "The entry is written in a simple and clear language, which is a subject of competition between family members, does not involve too much emotional or rhetorical style, and is therefore more objective and neutral. The language style of the purpose is therefore \"cool and direct\"."}, "time_dimension": {"answer": "Present", "rationale": ""}, "sentence_type": "Affirmative declarative sentence", "pos": {"nouns": {"Family members": 1, "The other side": 1}, "verbs": {"Yes": 1, "More than anything": 1}, "numerals": {"73": 1}}}, {"content": "74. If family members often act alone, they can hurt the feelings of others in the family.", "word_count": 22, "label": "", "sentence_structure": "Subject鈥搗erb鈥搊bject", "subjective_objective": {"answer": "Objective", "rationale": "This result/impact is based on the objective fact that family members are often acting alone, and does not depend on the views or experience of the interviewee. The perspective of the entry is therefore objective."}, "sentence_sentiment": {"answer": "Negative", "rationale": "The basis for the determination: the entry expresses \"harming the feelings of the rest of the family\", which is a negative result of behaviour. The emotional colour of the purpose of the article is therefore negative."}, "language_style": {"answer": "Tactful and warm", "rationale": "This language style is more euphemistic, moderate, and does not directly accuse or order the person under investigation, and is more personal and interactive, helping to increase the acceptance and effectiveness of the questionnaire."}, "time_dimension": {"answer": "Present", "rationale": "In this entry, the fact that \"family members often act alone\" is present, and the result \"that would hurt the feelings of the rest of the family\" is described in terms of the present or the recent situation, and thus judged to be present."}, "sentence_type": "Affirmative declarative sentence", "pos": {"nouns": {"Family members": 1, "Activities": 1, "Wounded": 1, "Home": 1, "Others": 1, "Emotion": 1}, "verbs": {"If": 1, "Regular": 1, "Alone": 1, "Yes": 1, "Wounded": 1, "have": 1}, "adverbs": {"Yes": 1}, "numerals": {"74": 1}, "pronouns": {"he": 1, "Guys": 1}}}, {"content": "75. It is the old habits of our family to work and enjoy.", "word_count": 14, "label": "", "sentence_structure": "Subject鈥揷opula鈥損redicative", "subjective_objective": {"answer": "Subjective", "rationale": "Because \"work and enjoy our old habits\" are patients' perceptions and evaluations of their way of life, and they are subjective feelings and attitudes."}, "sentence_sentiment": {"answer": "Neutral", "rationale": "Because the entry merely stated a fact, did not express any emotional colour and was therefore neutral."}, "language_style": {"answer": "Tactful and warm", "rationale": "The entry is more euphemistic and gentle, using a narrative tone rather than a direct inquiry or order giving people a sense of affection and nature. At the same time, the entry is not overly emotional or rhetorical, and is more focused on objective, true presentation."}, "time_dimension": {"answer": "Present", "rationale": "The entry indicates a habit or notion that work is done before life is enjoyed, and that the habit already exists in the family of the testee. Thus, the temporal dimension is now judged. \""}, "sentence_type": "Affirmative declarative sentence", "pos": {"nouns": {"Work": 1, "Enjoy": 1, "we": 1, "Family": 1, "Custom": 1}, "verbs": {"be": 1, "Work first": 1, "Enjoy the rest": 1, "Be": 1}, "adjectives": {"Old": 1}, "numerals": {"75": 1}, "pronouns": {"we": 1}}}, {"content": "76. Television is more important in our home than in reading.", "word_count": 13, "label": "", "sentence_structure": "Subject鈥揷opula鈥損redicative", "subjective_objective": {"answer": "Subjective", "rationale": "The idea that watching television is more important than reading is the patient's own subjective judgement and attitude."}, "sentence_sentiment": {"answer": "Negative", "rationale": "Because the idea that watching television is more important than reading is downplaying, and therefore negative."}, "language_style": {"answer": "Calm and direct", "rationale": "The language style of the inquiry is neutral and objective, with no apparent verbishness or emotional tendency, and is straightforward and straightforward. The problem itself is not subject to any subjective judgement or value orientation, but simply asks the respondents what is more important about watching television and reading, and can therefore be considered as a neutral issue."}, "time_dimension": {"answer": "Present", "rationale": "Now the entry describes a current situation or attitude, which is \"We watch TV in our house more than we read.\" It does not address the time dimension of the past or the future."}, "sentence_type": "Affirmative declarative sentence", "pos": {"nouns": {"Home": 1, "Television": 1, "Books": 1}, "verbs": {"Look": 1, "Read it": 1, "Important": 1}, "adjectives": {"More": 1}, "numerals": {"76": 1}, "pronouns": {"we": 1}}}, {"content": "77. Family members often participate in social events outside the home during their leisure time.", "word_count": 21, "label": "", "sentence_structure": "Subject鈥損redicate", "subjective_objective": {"answer": "Objective", "rationale": "Because it is a descriptive statement describing the behaviour and habits of family members, not their subjective feelings or ideas."}, "sentence_sentiment": {"answer": "Positive", "rationale": "Because the behaviour of \"family members often participate in social activities outside the home during their leisure time\" in the entry can have positive effects, such as enhancing interaction and communication among family members."}, "language_style": {"answer": "Tactful and warm", "rationale": "The entry is more personal and interactive when it is spoken in a gentle and inviting manner, rather than in direct statements or orders. At the same time, the language style of the article is more objective, neutral, and not overly emotional, consistent with the characteristics of the questionnaire."}, "time_dimension": {"answer": "Present", "rationale": "\"Relationship\" indicates that it is a customary, frequent movement, \"participating\" and \"social activity outside the home\" are all possible past, present or future situations. Taken together, this entry describes customary behaviour in the current context."}, "sentence_type": "Affirmative declarative sentence", "pos": {"nouns": {"Family members": 2, "Amateur Time": 1, "Family": 2, "Social events": 1}, "verbs": {"Participation": 1, "Always": 1}, "adverbs": {"Outside": 1}, "numerals": {"77": 1}}}, {"content": "78. We consider divorce, in any case, immoral.", "word_count": 16, "label": "", "sentence_structure": "Subject鈥搗erb鈥揷omplement", "subjective_objective": {"answer": "Subjective", "rationale": "Because \"think\" indicates that this is the patient's personal view or belief, and there is no objective evidence in the context to support this view. It is therefore subjective."}, "sentence_sentiment": {"answer": "Negative", "rationale": "This may affect the respondent鈥檚 response and create prejudice."}, "language_style": {"answer": "Calm and direct", "rationale": "The entry expresses the views on divorce in a direct statement, does not use a rhetorical or emotional rendering, and the language is more calm and objective."}, "time_dimension": {"answer": "Present", "rationale": "The entry is a reflection of the view and opinion of divorce, which may be the current view or belief of the testee and may affect their behaviour."}, "sentence_type": "Negative declarative sentence", "pos": {"nouns": {"we": 1, "Divorce": 1, "Ethics": 1}, "verbs": {"I think": 1, "be": 1}, "adverbs": {"What": 1, "Both": 1}, "numerals": {"78": 1}, "pronouns": {"Well": 1}}}, {"content": "79. There are no plans for spending money in our home.", "word_count": 9, "label": "", "sentence_structure": "Subject鈥搗erb鈥搊bject", "subjective_objective": {"answer": "Subjective", "rationale": "Because \"scheme\" or \"unplanned\" are judged by the actual situation of the patient's family and are subjective views and attitudes of the patient."}, "sentence_sentiment": {"answer": "Negative", "rationale": "Because the \"no plan\" in the entry is an expression of a negative behaviour or attitude."}, "language_style": {"answer": "Tactful and warm", "rationale": ""}, "time_dimension": {"answer": "Present", "rationale": ""}, "sentence_type": "Negative declarative sentence", "pos": {"nouns": {"Home": 1, "Money": 1, "Planned": 1}, "verbs": {"have": 1, "Flowers": 1, "Do it": 1}, "numerals": {"79": 1}}}, {"content": "80. The family rules and family rules are not changed.", "word_count": 17, "label": "", "sentence_structure": "Subject鈥搗erb鈥搊bject", "subjective_objective": {"answer": "Subjective", "rationale": "Because \"not to change\" is the patient's subjective attitude or belief, it is the individual's subjective feeling and perception."}, "sentence_sentiment": {"answer": "Negative", "rationale": "Because the expression \"can't change\" in the entry has negative emotional connotations."}, "language_style": {"answer": "Calm and direct", "rationale": "The language style of the entry is more calm and straightforward, and it does not use excessive emotional descriptions or rhetorical techniques, and is more balanced and objective in tone.\""}, "time_dimension": {"answer": "Present", "rationale": "The entry is to judge the time dimension of the testee's attitude to family life patterns or family rules as now. At the same time, the expression 鈥渋s inflexible鈥?implies that the attitude is firm and stable, and therefore may reflect, to some extent, the character characteristics and values of the testee."}, "sentence_type": "Affirmative declarative sentence", "pos": {"nouns": {"Home": 1, "Life": 1, "Pattern": 1, "Family rules": 1}, "verbs": {"be": 2, "Change": 1}, "adverbs": {"not": 1}, "numerals": {"80": 1}, "pronouns": {"we": 1}}}, {"content": "81. Every member of the family has always received adequate attention.", "word_count": 17, "label": "", "sentence_structure": "Subject鈥揷opula鈥損redicative", "subjective_objective": {"answer": "Objective", "rationale": "Because \"every member has always been given sufficient attention\" is an objective fact that can be observed and measured. While this assessment may be based on the views or feelings of the patient, it is itself verifiable and does not involve subjective attitudes or emotions of the patient."}, "sentence_sentiment": {"answer": "Positive", "rationale": "Because the entry \"Every member has always been given the full attention\" is positive."}, "language_style": {"answer": "Tactful and warm", "rationale": "The entry is more acclaimed and inspired by the fact that it is conducted in a way that recognizes and praises family members in a way that is more moderate, positive and free from too many criticisms or accusations."}, "time_dimension": {"answer": "Present", "rationale": "The sentence describes the current situation, where every member of the family is given full attention. The keyword \"has always been given sufficient attention\" indicates that this is a regular act and is still ongoing."}, "sentence_type": "Affirmative declarative sentence", "pos": {"nouns": {"Family": 1, "Members": 1, "Full": 1, "Care": 1}, "verbs": {"Got it": 1, "be": 1}, "adjectives": {"Every one": 1, "Always": 1}, "numerals": {"81": 1}}}, {"content": "82. Our family often talks spontaneously about sensitive family issues.", "word_count": 18, "label": "", "sentence_structure": "Subject鈥搗erb鈥揷omplement", "subjective_objective": {"answer": "Subjective", "rationale": "Because the title refers to \"Our family is often spontaneous,\" it is a manifestation of subjective behaviour and attitudes, looking at the patient's family situation and the way family members interact, and therefore at a subjective point of view."}, "sentence_sentiment": {"answer": "Neutral", "rationale": "Because the entry describes an act or state and does not involve positive or negative emotional aspects."}, "language_style": {"answer": "Tactful and warm", "rationale": ""}, "time_dimension": {"answer": "Present", "rationale": ""}, "sentence_type": "Affirmative declarative sentence", "pos": {"nouns": {"we": 1, "Home": 1, "Problem": 1}, "verbs": {"Lecture": 1, "Auto-symmetrical": 1}, "adjectives": {"Sensitive": 1}, "adverbs": {"Regular": 1}, "numerals": {"82": 1}}}, {"content": "83. Family members sometimes argue loudly when there are contradictions.", "word_count": 13, "label": "", "sentence_structure": "Subject鈥搗erb鈥搊bject", "subjective_objective": {"answer": "Objective", "rationale": "Because this statement describes the behaviour and behaviour of patients in family conflicts, it is an objective observation. While it may be caused by subjective feelings or attitudes of patients, it is itself a description of the objective situation."}, "sentence_sentiment": {"answer": "Negative", "rationale": "Because the \"screaming\" in the entries is offensive and destructive, and negative."}, "language_style": {"answer": "Tactful and warm", "rationale": "The entry is more personal and interactive when it is spoken in a gentle and inviting manner, rather than in direct statements or orders. At the same time, it does not use too strong or emotional language to describe family conflicts, but rather to ask questions in an objective and neutral manner, which will lead the interviewee to think calmly and to answer honestly."}, "time_dimension": {"answer": "Present", "rationale": "The entry describes a common reaction of the testee when a family is in conflict. \"Sometimes loudly quarrel\" suggests that this is a frequent act, and the temporal dimension is now."}, "sentence_type": "Affirmative declarative sentence", "pos": {"nouns": {"Family": 1, "Paradoxious": 1, "Argument": 1}, "verbs": {"have": 1, "- Louder fights": 2}, "adverbs": {"Sometimes": 1}, "numerals": {"83": 1}}}, {"content": "84. It is true that in our home members are encouraged to move freely.", "word_count": 15, "label": "", "sentence_structure": "Subject鈥損redicate", "subjective_objective": {"answer": "Subjective", "rationale": "Since encouraging members to move freely is a family attitude or view, it is a subjective examination."}, "sentence_sentiment": {"answer": "Positive", "rationale": "Because the \"encouragement of members to move freely\" is positive."}, "language_style": {"answer": "Tactful and warm", "rationale": "The language style of the entry tends to be moderate and friendly, in order to better communicate with the interviewees and to get their sincere answers. At the same time, this euphemism can also give respondents a sense of respect and understanding, thereby increasing the motivation and authenticity of the questionnaire."}, "time_dimension": {"answer": "Present", "rationale": "It is possible to judge from the time words \"do\" and \"both\" in the sentence that this act has occurred in the past and is still going on, and is therefore the present."}, "sentence_type": "Affirmative declarative sentence", "pos": {"nouns": {"Our home": 1, "Members": 1, "Activities": 1}, "verbs": {"Encourage": 1, "Freedom": 1}, "adverbs": {"Both": 1, "Indeed": 1}, "numerals": {"84": 1}}}, {"content": "85. Family members often compare with others, depending on who is studying well at work.", "word_count": 19, "label": "", "sentence_structure": "Subject鈥搗erb鈥揷omplement", "subjective_objective": {"answer": "Subjective", "rationale": "Because \"often comparing\" is a subjective attitude and behaviour."}, "sentence_sentiment": {"answer": "Negative", "rationale": "Because the \"often comparing\" behavior in the entry is negative."}, "language_style": {"answer": "Tactful and warm", "rationale": "The entry uses a relatively euphemistic approach to ask respondents whether family members prefer to compare with each other, so as to avoid giving rise to overly direct or negative reactions. At the same time, the phrases \"often\" and \"who is well-educated at work\" also make the tone more mild."}, "time_dimension": {"answer": "Present", "rationale": "The entry describes the current behaviour and attitude of the family members of the testee, the keyword being \"often\" and \"comparison\", which all indicate that this is a regular, present act."}, "sentence_type": "Affirmative declarative sentence", "pos": {"nouns": {"Family members": 1, "others": 1, "Work studies": 1}, "verbs": {"Often": 1, "and": 1, "Comparison": 1, "Look": 1, "Who": 1, "Okay": 1}, "adverbs": {"Regular": 1}, "numerals": {"85": 1}}}, {"content": "86. Family members are very fond of music, art and literature.", "word_count": 14, "label": "", "sentence_structure": "Subject鈥搗erb鈥搊bject", "subjective_objective": {"answer": "Objective", "rationale": "As this statement is an objective fact describing the preferences of family members, it does not involve subjective attitudes or ideas of the patient."}, "sentence_sentiment": {"answer": "Positive", "rationale": "Because the entry \"a lot of music, art and literature\" expresses a positive attitude or emotion."}, "language_style": {"answer": "Tactful and warm", "rationale": "The entry is more proficient and interactive by engaging in dialogue in a gentle and inviting manner, rather than by direct statement or order. At the same time, the article is more neutral, is not overly emotional or strongly attitudinal, and is more in line with the objectivity and professionalism of psychological questionnaires."}, "time_dimension": {"answer": "Present", "rationale": "The entry describes the current situation, describing the preferences of family members, without reference to past or future time reference points. The word \"very\" indicates the high level of such preferences, while \"like music, art and literature\" is a specific description of the interests of family members."}, "sentence_type": "Affirmative declarative sentence", "pos": {"nouns": {"Family members": 1, "Music": 1, "Arts": 1, "Literature": 1}, "verbs": {"Yeah": 1}, "adverbs": {"very": 1, "and": 1}, "numerals": {"86": 1}}}, {"content": "87. Our main way of entertainment is to watch television, listen to radio, not go out.", "word_count": 25, "label": "", "sentence_structure": "Subject鈥搗erb鈥搊bject", "subjective_objective": {"answer": "Subjective", "rationale": "Because it describes the recreational habits of the person interviewed, it is viewed from the patient鈥檚 subjective perspective. The answer is:"}, "sentence_sentiment": {"answer": "Negative", "rationale": "Because the description of the behaviour \"mainly by watching television, listening to radio\" in the entry implies a certain negative aspect of the lack of a proactive attitude to life and the habit of outdoor exercise."}, "language_style": {"answer": "Calm and direct", "rationale": "The entry begins with data \"87,\" which identifies the main theme of the discussion - the main way of entertainment. In the next sentence, \"the main way to watch TV, listen to radio, and not go out of the house\" is to present the facts and results directly, without using rhetorical or emotional means of rendering them up, and is more objective in tone, so it can be judged that the language style of the article is calm and straightforward."}, "time_dimension": {"answer": "Present", "rationale": "The entry describes the current state of behaviour, \"the main way we entertain\" means it's their current major behaviour, and \"the TV, the radio, not the outside,\" it describes their specific behavior, so the time dimension of the entry is now."}, "sentence_type": "Affirmative declarative sentence", "pos": {"nouns": {"we": 1, "Recreational activities": 1, "Modalities": 1, "Television": 1, "Radio": 1, "Outgoing activities": 1}, "verbs": {"be": 1, "Look": 1, "Listen": 1, "Not": 1, "Out": 1}, "adjectives": {"Main": 1}, "adverbs": {"More": 1}, "numerals": {"87": 1}}}, {"content": "88. We believe that it is more important to improve the standard of living in the family than to uphold ethical standards.", "word_count": 24, "label": "", "sentence_structure": "Subject鈥搗erb鈥搊bject", "subjective_objective": {"answer": "Subjective", "rationale": "Because \"think\" represents the views and attitudes of the patient, the entry is subjective."}, "sentence_sentiment": {"answer": "Neutral", "rationale": "Because the entry merely stated a point of view, it did not express a positive or negative emotional tendency."}, "language_style": {"answer": "Tactful and warm", "rationale": "The entry is more personal and interactive when it is spoken in a gentle and inviting manner, rather than in direct statements or orders. Moreover, euphemism is used to ask each other's views, giving them a more open and inclusive impression."}, "time_dimension": {"answer": "Present", "rationale": "Based on the phrase \"thinks\" and \"will\" it can be inferred that this is a current view or attitude, and therefore judgement is now."}, "sentence_type": "Affirmative declarative sentence", "pos": {"nouns": {"Life": 1, "Horizontal": 1, "Family": 1, "Ethics": 2, "Standard": 1}, "verbs": {"Increase": 1, "I think": 1, "Hold tight": 1}, "adverbs": {"And": 1}, "numerals": {"88": 1}}}, {"content": "89. The dishes must be washed immediately after our meals.", "word_count": 14, "label": "", "sentence_structure": "Subject鈥損redicate", "subjective_objective": {"answer": "Subjective", "rationale": "Since it is the subjective decision and action of the family members to wash dishes immediately after the meal."}, "sentence_sentiment": {"answer": "Negative", "rationale": "Because the requirement that \"you must have someone washing the dishes immediately after dinner\" is mandatory, may be stressful and dissatisfied and therefore negative."}, "language_style": {"answer": "Calm and direct", "rationale": "The language style of the entry is straightforward and it clearly describes the fact that after meals, someone must be washing dishes."}, "time_dimension": {"answer": "Present", "rationale": "The keyword \"must be immediately\" means that it is a requirement or requirement that it be executed after dinner, and the temporal dimension of the purpose is therefore judged to be now."}, "sentence_type": "Imperative sentence", "pos": {"nouns": {"After dinner": 1, "person": 1, "Wash dishes": 1}, "verbs": {"I have to": 1, "Go on": 1, "Wash": 1}, "adverbs": {"Immediately": 1}, "numerals": {"89": 1}, "pronouns": {"we": 1, "Home": 1}}}, {"content": "90. Those who violate family rules at home are severely criticized.", "word_count": 16, "label": "", "sentence_structure": "Subject鈥搗erb鈥搊bject", "subjective_objective": {"answer": "Objective", "rationale": "Because breaking the rules of the family is an objective fact, and accepting criticism is an objective decision made by other people, such as parents. Although this may affect patients鈥?feelings and attitudes, it is not a subjective factor that patients can control."}, "sentence_sentiment": {"answer": "Negative", "rationale": "Because it describes a negative act and result, i.e. that violators will be severely criticized."}, "language_style": {"answer": "Calm and direct", "rationale": "The entry clearly describes the penalties that are to be imposed on violators of family rules in objective and concise language, without adding too many emotional or rhetorical features, and with a more direct and calm language style."}, "time_dimension": {"answer": "Present", "rationale": "The main phrase in the sentence, \"family violator\" and the word \"blackhouse violator\" are harshly criticized, referring to the current situation or state. Therefore, the time dimension of this questionnaire entry is now."}, "sentence_type": "Affirmative declarative sentence", "pos": {"nouns": {"Home": 1, "Family rules": 1, "Criticism": 1}, "verbs": {"Violations": 1, "Yes": 1, "Received": 1, "Hard": 1}, "adjectives": {"Hard": 1}, "numerals": {"90": 1}, "classifiers": {"individual": 1}}}]}

{"questionnaire_title": "Eisenke personality questionnaire.", "questionnaire_category": "Self-cognition", "item_count": 85, "emotion_shift_overall": "From negative to positive", "language_style_overall": "Neutral", "items": [{"content": "Do you have a wide range of interests?", "word_count": 9, "label": "", "sentence_structure": "Subject鈥搗erb鈥搊bject", "subjective_objective": {"answer": "Subjective", "rationale": "Because a wide range of preferences is an individual ' s choice and preference, it is subjective."}, "sentence_sentiment": {"answer": "Neutral", "rationale": "Because the entry does not have any emotional color."}, "language_style": {"answer": "Tactful and warm", "rationale": "The entry is more personal and interactive in its expression, in a gentle and inviting tone, rather than in direct statements or orders. Moreover, it leaves the interviewees some space for reflection and gives them the opportunity to express their true thoughts and feelings."}, "time_dimension": {"answer": "Present", "rationale": "The entry asks the testee about his current behaviour or state, i.e. whether he or she has a wide range of preferences and the temporal dimension is now judged."}, "sentence_type": "Imperative sentence", "pos": {"nouns": {"you": 1, "Wide": 1, "Hobbies": 1}, "verbs": {"have": 1}, "adverbs": {"Is it possible": 1}, "numerals": {"One": 1}}}, {"content": "Do you have to think about anything before you do anything else?", "word_count": 18, "label": "", "sentence_structure": "Subject鈥搗erb鈥搊bject", "subjective_objective": {"answer": "Subjective", "rationale": "This is a description of the patient ' s way of thinking and behaviour and is a subjective examination."}, "sentence_sentiment": {"answer": "Negative", "rationale": "Because \"first think\" may mean too much caution and hesitation, which may limit mobility and speed of decision-making."}, "language_style": {"answer": "Calm and direct", "rationale": "The entry was in a concise manner asked the interviewees whether they would think sufficiently before the action, with a more objective and calm language style, with no excessive emotional rendering or rhetorical techniques, and with a \"cool and direct\" judgement."}, "time_dimension": {"answer": "Present", "rationale": ""}, "sentence_type": "Interrogative sentence", "pos": {"nouns": {"Things": 1, "A little bit": 1}, "verbs": {"Do it": 1, "Yes": 1, "Consider": 1}, "adverbs": {"Both": 1, "Again": 1}, "pronouns": {"you": 1}}}, {"content": "Do you have a constant mood swing?", "word_count": 9, "label": "", "sentence_structure": "Subject鈥搗erb鈥搊bject", "subjective_objective": {"answer": "Subjective", "rationale": "Because emotional fluctuations are subjective feelings of a person, related to the individual ' s psychological state, they are viewed from a subjective perspective."}, "sentence_sentiment": {"answer": "Negative", "rationale": "Because \"emotional fluctuations\" in the entries are negative emotional states, negative expressions."}, "language_style": {"answer": "Tactful and warm", "rationale": "The entry is more personal and interactive when it is spoken in a question-and-answer tone, without direct statements or orders. It also does not use too direct and strong language to ask about personal sensitivities, but rather uses a gentle and euphemism that makes people comfortable and respected."}, "time_dimension": {"answer": "Present", "rationale": ""}, "sentence_type": "Interrogative sentence", "pos": {"nouns": {"Emotion": 1, "Volatility": 1, "What": 1}, "verbs": {"Often": 1}}}, {"content": "4. Do you feel proud when others do good things and people around think you do?", "word_count": 26, "label": "", "sentence_structure": "Subject鈥搗erb鈥搊bject", "subjective_objective": {"answer": "Subjective", "rationale": "Because the question is about the patient ' s feelings and reactions in a given situation, it is the patient ' s subjective attitude."}, "sentence_sentiment": {"answer": "Negative", "rationale": "Because the attitude of \"exulent\" in the entries is negative."}, "language_style": {"answer": "Tactful and warm", "rationale": "The entry is more adept and interactive when it is spoken in a gentle and inviting manner, rather than by direct statements or orders."}, "time_dimension": {"answer": "Present", "rationale": "\"The good people around think you did\" happened right now, and the description of the tester of this feeling also shows that it is an immediate reaction, so the entry is the current dimension of time."}, "sentence_type": "Interrogative sentence", "pos": {"nouns": {"Good news": 1, "People around": 1}, "verbs": {"Do it": 2, "I think": 1, "I feel": 1, "I'm proud of you": 1}, "adverbs": {"But": 1}, "numerals": {"4": 1}, "classifiers": {"individual": 1}}}, {"content": "Are you a talker?", "word_count": 9, "label": "", "sentence_structure": "Subject鈥揷opula鈥損redicative", "subjective_objective": {"answer": "Subjective", "rationale": "Because \"talking\" is a subjective feeling and self-perception of individuals, the definition of talkative is different from one person to another. The entry is therefore viewed from the patient ' s subjective perspective."}, "sentence_sentiment": {"answer": "Neutral", "rationale": "Because the entry is personal and does not have a clear emotional orientation or behavioural orientation."}, "language_style": {"answer": "Tactful and warm", "rationale": "The entry uses a more euphemistic and gentle way of asking questions, and invites the interviewee to answer questions in a more friendly way, giving him a comfortable and relaxed feeling."}, "time_dimension": {"answer": "Present", "rationale": ""}, "sentence_type": "Interrogative sentence", "pos": {"nouns": {"person": 1, "Talkative": 1}, "verbs": {"be": 1}, "adjectives": {"One": 1}, "numerals": {"5": 1}}}, {"content": "Have you ever felt sorry for yourself for no reason?", "word_count": 15, "label": "", "sentence_structure": "Subject鈥搗erb鈥搊bject", "subjective_objective": {"answer": "Subjective", "rationale": "Because feeling sorry is a subjective or emotional experience."}, "sentence_sentiment": {"answer": "Negative", "rationale": "Because the \"fear yourself\" sentiment in the entry is negative."}, "language_style": {"answer": "Calm and direct", "rationale": "The entry is a direct, simple question of whether the other party has had a poor and unprovoked experience, and is more calm and objective in its language, without excessive embellishment or emotional rendering."}, "time_dimension": {"answer": "Past", "rationale": "The entry asks about the experience and feelings of the testee, and the term \"had\" indicates that this was an event that occurred in the past and it is possible to judge that the temporal dimension of the entry is the past."}, "sentence_type": "Interrogative sentence", "pos": {"nouns": {"Questionnaire": 1, "Entry": 1, "Sensor": 1, "Poor thing": 1}, "verbs": {"Tsang": 1, "I feel": 1, "have": 1, "For no reason": 1, "self": 1}, "adjectives": {"For no reason": 1, "of": 2}, "adverbs": {"Once": 1, "For no reason": 1}, "classifiers": {"individual": 1}, "pronouns": {"you": 1}}}, {"content": "Have you ever had an extra-material benefit from greed?", "word_count": 20, "label": "", "sentence_structure": "Subject鈥搗erb鈥搊bject", "subjective_objective": {"answer": "Subjective", "rationale": "The acquisition of extra-material benefits for greed is the patient ' s subjective behaviour and attitude, and it is the patient ' s inner thoughts and motivations that are examined."}, "sentence_sentiment": {"answer": "Negative", "rationale": "Because the \"growth\" act in the entry is negative."}, "language_style": {"answer": "Calm and direct", "rationale": "The language style of the entry is calm, because it asks the respondents whether they have ever gained additional material benefits because of greed. The question is not using euphemism or indirect language to induce emotional reactions or to direct the respondents to their answers. Rather, it asks the respondents in a simple and clear way."}, "time_dimension": {"answer": "Past", "rationale": "The entry \"was\" indicates past behaviour or experience, and \"gret\" and \"material interest\" also indicates that the issue relates to past events. Thus, the entry is a time dimension of the past."}, "sentence_type": "Interrogative sentence", "pos": {"nouns": {"Questionnaire Entry": 1, "Greedy": 1, "Material benefits": 1}, "verbs": {"Yes": 1, "Access": 1}, "adverbs": {"Once": 1}, "numerals": {"7": 1}}}, {"content": "Will you be careful locking the door at night?", "word_count": 13, "label": "", "sentence_structure": "Subject鈥損redicate", "subjective_objective": {"answer": "Objective", "rationale": "Because it is the patient ' s objective behaviour to lock the door carefully."}, "sentence_sentiment": {"answer": "Neutral", "rationale": "Because the entry does not have any emotional color."}, "language_style": {"answer": "Calm and direct", "rationale": "The entry is a direct question-and-answer dialogue, is not based on too many rhetorical or emotional expressions and is more objective in tone. It asks if the interviewee will carefully lock the door at night, be clear in language, be easily understood and answerable."}, "time_dimension": {"answer": "Present", "rationale": "The entry asks about the current behaviour or tendency of the subject, and the words \"night\" and \"will\" indicate that this is a regular, customary act and therefore the judgement is now."}, "sentence_type": "Interrogative sentence", "pos": {"nouns": {"Night": 1, "you": 1, "Door": 1, "Lock": 1}, "verbs": {"Yes": 1, "Watch out": 1, "- Put it on": 1, "Lock it up": 1}, "adverbs": {"very": 1}, "pronouns": {"Here": 1}}}, {"content": "Do you think you're alive?", "word_count": 8, "label": "", "sentence_structure": "Subject鈥損redicate", "subjective_objective": {"answer": "Subjective", "rationale": "Because \"vio-violation\" is a subjective adjective, the criteria for evaluation vary from person to person and require the patient to answer according to his or her own feelings."}, "sentence_sentiment": {"answer": "Positive", "rationale": "Because the word \"vioper\" usually has a positive meaning."}, "language_style": {"answer": "Tactful and warm", "rationale": "The entry uses a more objective and neutral question form, beginning with \"You think\" and leaves open space for reflection, while the language style is more moderate and does not directly evaluate or judge the character characteristics of the other."}, "time_dimension": {"answer": "Present", "rationale": ""}, "sentence_type": "Interrogative sentence", "pos": {"nouns": {"you": 1, "self": 1, "Lively": 1}, "verbs": {"I think": 1, "Lively": 1}, "numerals": {"9": 1}}}, {"content": "10. Does it feel bad when you see a child or (animal) tortured?", "word_count": 21, "label": "", "sentence_structure": "Subject鈥搗erb鈥搊bject", "subjective_objective": {"answer": "Subjective", "rationale": "Because this question is examined by the subjective feelings and reactions of patients in certain situations, rather than by objective facts. For example, even if some see the torture of children or animals, they do not feel so bad, while others have strong feelings of compassion and discomfort, which is a manifestation of personal subjective feelings."}, "sentence_sentiment": {"answer": "Positive", "rationale": "Because the \"worried\" in the entry is positive."}, "language_style": {"answer": "Tactful and warm", "rationale": "The language style of the entry is euphemism, because it is expressed in a mild way, using the words \"whether or not\" to guide the person surveyed to reflect on his or her feelings, without direct orders or statements."}, "time_dimension": {"answer": "Past", "rationale": "The entry asks about the emotional reactions of the person who is tested when seeing a particular situation (child or animal being tortured), which may be based on past experience and emotional memory."}, "sentence_type": "Interrogative sentence", "pos": {"nouns": {"Kids": 1, "Animals": 1, "Torture": 1}, "verbs": {"Yes": 1, "I feel": 1, "It's hard": 1}, "adverbs": {"very": 1}, "numerals": {"10": 1}, "pronouns": {"you": 1}}}, {"content": "Do you often worry that you will say (or do) what you should not say (or do)?", "word_count": 23, "label": "", "sentence_structure": "Subject鈥搗erb鈥搊bject", "subjective_objective": {"answer": "Subjective", "rationale": "Because \"fear\" is a subjective feeling."}, "sentence_sentiment": {"answer": "Negative", "rationale": "Because the emotional expression \"or do what is not right\" in the entry is negative."}, "language_style": {"answer": "Tactful and warm", "rationale": "The entry is more euphemistic, uses the form of a query and asks the other side whether it has such concerns, does not mean direct orders or criticism. At the same time, it gives a sense of affection, understanding and support, which helps to establish a good survey relationship."}, "time_dimension": {"answer": "Past", "rationale": "\"At times worried\" suggests that it is a constant emotional reaction, and that \"speaking (or doing) what should not be said (or done)\" is a statement of behaviour, so the entry to the questionnaire covers both the current state of emotion and past behaviour."}, "sentence_type": "Interrogative sentence", "pos": {"nouns": {"you": 2, "Things": 2}, "verbs": {"Yes": 2, "Say it": 1, "Make": 1, "I'm worried": 1, "I think so": 1}, "adverbs": {"Often": 1, "not": 1}, "numerals": {"11": 1}, "pronouns": {"Here": 1}}}, {"content": "12. If you say you have to do something, do you have to do it whatever difficulties you may encounter?", "word_count": 25, "label": "", "sentence_structure": "Subject鈥搗erb鈥搊bject", "subjective_objective": {"answer": "Subjective", "rationale": "Since this is a matter of self-evaluation and attitude, it is a subjective examination to ask whether the patient will continue to fulfil his or her mandate in the face of difficulties."}, "sentence_sentiment": {"answer": "Positive", "rationale": "Because the entry \"do it whatever you have to.\" is positive."}, "language_style": {"answer": "Tactful and warm", "rationale": "The language style of the entry is euphemistic, it is a question-and-answer process, without direct orders or a strong demand that the interviewee must answer. At the same time, the question is not too strongly couched or emotionally recast, making the questionnaire more friendly and friendly."}, "time_dimension": {"answer": "Present", "rationale": "The entry now asks the testee whether he or she will insist on doing and overcome any difficulties when he or she says he or she is going to do something. The keyword \"to do\" and \"to do it\" point to the testee's current behaviour or attitude. Thus, the time dimension of the entry can be judged as now."}, "sentence_type": "Interrogative sentence", "pos": {"nouns": {"Yes": 1, "Difficulties": 1}, "verbs": {"Say it": 1, "Do it": 1, "I met him": 1}, "adverbs": {"Both": 1}, "numerals": {"12": 1}, "pronouns": {"you": 1}}}, {"content": "Did you enjoy your party?", "word_count": 14, "label": "", "sentence_structure": "Subject鈥搗erb鈥搊bject", "subjective_objective": {"answer": "Subjective", "rationale": "Because \"Enjoy\" is the patient's feelings and attitude to attending a meeting, and it's subjective."}, "sentence_sentiment": {"answer": "Positive", "rationale": "Because the \"Enjoy\" act in the entry is positive."}, "language_style": {"answer": "Tactful and warm", "rationale": "The language style of the entry is more euphemistic, leads the question with the words \"if\" and uses the words \"happy party\" and \"leave\" to express the subject. This is more friendly and soft, and it is easy to feel comfortable and to answer questions."}, "time_dimension": {"answer": "Present", "rationale": "The entry uses the word \"whether or not\" for questioning and describes a current or recent scene (happy party) and therefore the judgement is now."}, "sentence_type": "Interrogative sentence", "pos": {"nouns": {"Party": 1, "Have a nice day": 1}, "verbs": {"Is it possible": 1, "Enjoy": 1}, "adjectives": {"Have a nice day": 1}, "numerals": {"13": 1}}}, {"content": "Are you an angry man?", "word_count": 10, "label": "", "sentence_structure": "Subject鈥損redicate", "subjective_objective": {"answer": "Subjective", "rationale": "Because irritation is a subjective feeling and self-awareness."}, "sentence_sentiment": {"answer": "Negative", "rationale": "Because the character of \"freaky\" is usually considered negative."}, "language_style": {"answer": "Calm and direct", "rationale": "The entry is written in a calm and straightforward manner. It asks the respondent in a straightforward and clear manner whether he is easily angry, does not use excessive rhetorical or emotional colours, is more balanced and objective, and is intended to obtain the actual answers of the respondent."}, "time_dimension": {"answer": "Present", "rationale": "\"Are you an angry man?\" The description of \"a\" and \"irresponsible\" in it indicates that it is an assessment of the individual character, namely, whether it exists at this time. So the time frame of the article is now."}, "sentence_type": "Interrogative sentence", "pos": {"nouns": {"person": 1, "It's so irritating": 1}, "verbs": {"be": 1}}}, {"content": "Have you ever been to blame anyone for doing something wrong?", "word_count": 20, "label": "", "sentence_structure": "Subject鈥搗erb鈥搊bject", "subjective_objective": {"answer": "Subjective", "rationale": "Because blame for others ' feelings and ideas is generated by the patient himself, it is subjective."}, "sentence_sentiment": {"answer": "Negative", "rationale": "Because the entry describes a person who has done the wrong thing and has abdicated responsibility, it is a negative act."}, "language_style": {"answer": "Calm and direct", "rationale": "The entry is asked in a direct and clear manner, without too many emotional terms or rhetorical techniques, and is more calm and objective in tone."}, "time_dimension": {"answer": "Present", "rationale": "The entry asks the testee for his current behaviour or experience, and the words \"are there\" and \"have\" mean that this is a past but still ongoing state and therefore the judgement is now."}, "sentence_type": "Interrogative sentence", "pos": {"nouns": {"Yes": 1, "Blame": 1, "person": 1}, "verbs": {"have": 1, "Wrong": 1, "Here": 1, "Blame": 1}, "adverbs": {"very": 1}, "numerals": {"15": 1}, "pronouns": {"self": 1}}}, {"content": "Do you like meeting strangers?", "word_count": 9, "label": "", "sentence_structure": "Subject鈥搗erb鈥搊bject", "subjective_objective": {"answer": "Subjective", "rationale": "Because it is a subjective feeling and attitude of a person to like or dislike seeing strangers."}, "sentence_sentiment": {"answer": "Neutral", "rationale": "Because the entry does not have any emotional color."}, "language_style": {"answer": "Tactful and warm", "rationale": "The language style of the entry is more euphemistic, because it is a soft, invitation-style conversation, rather than a direct statement or order. This expression is more personal and interactive, making the interviewee more comfortable and relaxed. Moreover, the question itself is more open and inclusive, without predefined answers or preferences, and encourages the interviewee to express his thoughts and feelings freely."}, "time_dimension": {"answer": "Present", "rationale": ""}, "sentence_type": "Interrogative sentence", "pos": {"nouns": {"Questionnaire": 1, "Entry": 1, "Stranger": 1}, "verbs": {"Yeah": 1, "Yes": 1, "See": 1}, "adverbs": {"What": 1}}}, {"content": "17. Do you believe that participation in savings is a good way to go?", "word_count": 15, "label": "", "sentence_structure": "Subject鈥損redicate", "subjective_objective": {"answer": "Subjective", "rationale": "The belief that participating in savings is a good way to do so is the patient ' s personal perception and attitude."}, "sentence_sentiment": {"answer": "Positive", "rationale": "Because \"convincing to be a good way to save\" expresses positive, positive emotions and attitudes."}, "language_style": {"answer": "Calm and direct", "rationale": "This language style is more objective, neutral and more consistent with the characteristics and purposes of the questionnaire."}, "time_dimension": {"answer": "Present", "rationale": "The entry asks the testee's current attitude or belief, \"Be sure\" suggests that this is a subjective assessment, so the temporal dimension is now judged."}, "sentence_type": "Interrogative sentence", "pos": {"nouns": {"Savings": 1, "Approach": 1}, "verbs": {"Participation": 1, "Yes": 1, "be": 1}, "adverbs": {"One": 1, "Okay": 1}, "numerals": {"7": 1}, "pronouns": {"you": 1}}}, {"content": "Are your feelings vulnerable?", "word_count": 12, "label": "", "sentence_structure": "Subject鈥損redicate", "subjective_objective": {"answer": "Subjective", "rationale": "Because emotional vulnerability is an individual ' s feelings and experience, and is viewed from a subjective perspective."}, "sentence_sentiment": {"answer": "Negative", "rationale": "Because the description of \"removability of feelings\" in this entry is usually negative emotional."}, "language_style": {"answer": "Tactful and warm", "rationale": ""}, "time_dimension": {"answer": "Present", "rationale": "The entry asks the person who was tested about his current situation or state, i.e. whether he or she feels vulnerable to emotional harm at the moment of answering the question."}, "sentence_type": "Interrogative sentence", "pos": {"nouns": {"Emotion": 1, "Hurt": 1}, "verbs": {"Yes": 1, "Present": 1, "Impact": 1}, "adjectives": {"Easy": 1}, "numerals": {"18": 1}}}, {"content": "You want to take drugs with a strange effect or dangerous?", "word_count": 19, "label": "", "sentence_structure": "Subject鈥搗erb鈥搊bject", "subjective_objective": {"answer": "Subjective", "rationale": "Because \"want\" is a subjective will or idea of a person."}, "sentence_sentiment": {"answer": "Negative", "rationale": "Because the expression \"drugs with unusual effects or dangerous effects\" in the entry itself has a negative connotation, indicating potential risks and side effects."}, "language_style": {"answer": "Calm and direct", "rationale": "The entry is a direct and clear query to the interviewees about their willingness to use drugs that may have a strange effect or are dangerous, and the language temperature and not excessive emotional colours, which are in a calm and objective language style."}, "time_dimension": {"answer": "Future", "rationale": "The entry asks if the person tested is prepared to take a drug in the future, and therefore the temporal dimension is judged to be the future. At the same time, the description of \"unique effects\" and \"risk\" also implies that the person tested is thinking about the possibility and risk of the drug, not just past experience or current state."}, "sentence_type": "Interrogative sentence", "pos": {"nouns": {"Questionnaire": 1, "Entry": 1, "Drugs": 2}, "verbs": {"Yes": 1, "Take it": 1, "have": 3, "Kit": 1, "Effects": 1, "Or": 1, "Risk": 1}, "adjectives": {"Kit": 1, "Risk": 1}, "numerals": {"19": 1, "2": 1}, "classifiers": {"individual": 1}}}, {"content": "Do you often feel 鈥渁nd bored鈥?", "word_count": 11, "label": "", "sentence_structure": "Subject鈥搗erb鈥搊bject", "subjective_objective": {"answer": "Subjective", "rationale": "Because feeling \"and bored\" is the emotional state that patients experience subjectively."}, "sentence_sentiment": {"answer": "Negative", "rationale": "Because the emotional state of \"extremely bored\" in the entries is negative."}, "language_style": {"answer": "Calm and direct", "rationale": "The entry is a \"cool and direct\" language style, as it is a direct question-and-answer dialogue, without any rhetorical or emotional accent, and is more objective in tone."}, "time_dimension": {"answer": "Present", "rationale": "The entry asks the subject's current subjective feelings, and the temporal dimension is now judged.\""}, "sentence_type": "Interrogative sentence", "pos": {"nouns": {"you": 1, "Time": 1, "Sensor": 1, "Boring": 1}, "verbs": {"Regular": 1, "I feel": 1, "Boring": 1}, "adjectives": {"Other": 1}, "adverbs": {"Often": 1}}}, {"content": "Have you ever had more than one person's things?", "word_count": 19, "label": "", "sentence_structure": "Subject鈥搗erb鈥搊bject", "subjective_objective": {"answer": "Subjective", "rationale": "Because \"the things that are too much and more than others\" are subjective acts of patients, the attitude or tendency to look at them is subjective."}, "sentence_sentiment": {"answer": "Negative", "rationale": "Because the \"over-possession, over-the-mass\" act is immoral and has a negative emotional connotation."}, "language_style": {"answer": "Calm and direct", "rationale": "The entry asks the other party in a direct and clear manner whether or not he or she has taken more or less of what he or she has done, is more objective and neutral in tone, and does not have too much emotional or rhetorical style."}, "time_dimension": {"answer": "Past", "rationale": "The keywords \"had\" and \"haves\" all indicate that the act has happened and that the dimensions of time are in the past."}, "sentence_type": "Interrogative sentence", "pos": {"nouns": {"Questionnaire": 1, "Entry": 1, "you": 2, "Something": 1, "One shot, one shot": 1}, "verbs": {"Zenith": 1, "- A lot": 1, "others": 1, "- You're not": 1}, "adverbs": {"Even": 1}, "numerals": {"21": 1, "One shot, one shot": 1}, "classifiers": {"individual": 1}}}, {"content": "22. Do you like to travel often, if conditions permit?", "word_count": 16, "label": "", "sentence_structure": "Subject鈥損redicate", "subjective_objective": {"answer": "Subjective", "rationale": "Because it's the patient's subjective attitude."}, "sentence_sentiment": {"answer": "Positive", "rationale": "Because the entry \"likes to travel often\" expresses a positive emotional attitude."}, "language_style": {"answer": "Tactful and warm", "rationale": "The entry is interactive and is used in polite language \"if conditions permit\" which is more friendly and respectful of the choices of the person under investigation, and is not direct or rigid."}, "time_dimension": {"answer": "Present", "rationale": "The entry asks if the person tested prefers to travel frequently under the current circumstances, and does not address past or future situations. The answer is therefore \"now\"."}, "sentence_type": "Interrogative sentence", "pos": {"nouns": {"Conditions": 1, "Allow": 1, "Yeah": 1, "Regular": 1, "Out": 1, "Travel": 1}, "verbs": {"Yeah": 1, "Out": 1, "Travel": 1}, "adverbs": {"Regular": 1}, "numerals": {"22": 1}}}, {"content": "Do you have a joke about people you like?", "word_count": 21, "label": "", "sentence_structure": "Subject鈥損redicate", "subjective_objective": {"answer": "Subjective", "rationale": "The attitude and perception of the patient regarding his or her behaviour are examined from a subjective perspective."}, "sentence_sentiment": {"answer": "Negative", "rationale": "Because the \"overplaying joke\" in the entry is an act that can cause displeasure or even harm to the other party, and is a negative emotional color."}, "language_style": {"answer": "Tactful and warm", "rationale": "The entry is a soft, inviting conversation, asking if the interviewee has been too much of a joke when he or she treats someone he or she likes. This expression is more personal and interactive than a direct statement or order, making the interviewee more willing to share his or her real thoughts and feelings."}, "time_dimension": {"answer": "Present", "rationale": "The question for the entry to the questionnaire is whether the key word \"is\" indicates that it is a selective question, while \"now\" indicates that it is a question of the current situation. So the time dimension of the entry is now."}, "sentence_type": "Interrogative sentence", "pos": {"nouns": {"person": 1, "Enjoy": 1, "A joke": 1}, "verbs": {"to": 1, "Yeah": 1, "be": 1, "Yes": 1, "What": 1}, "adjectives": {"of": 2, "you": 1}, "numerals": {"23": 1}, "pronouns": {"Here": 1}}}, {"content": "Do you often get upset by the sense of 鈥渃rime鈥?", "word_count": 11, "label": "", "sentence_structure": "Subject鈥搗erb鈥搊bject", "subjective_objective": {"answer": "Subjective", "rationale": "Because \"self-incrimination\" is a subjective emotional experience and feeling, it is a subjective angle."}, "sentence_sentiment": {"answer": "Negative", "rationale": "Because \"self-incrimination\" is usually associated with a negative emotional and psychological state, the entry may express negative emotions and attitudes."}, "language_style": {"answer": "Calm and direct", "rationale": "The entry is based on a determination that the person interviewed is often troubled by guilt or guilt, is speaking objectively and without any qualifier or emotional colour. Thus, its language style is classified as being calm and straightforward."}, "time_dimension": {"answer": "Present", "rationale": "The entry contains a description of an act or state ( \"Is there a sinful problem?\") and uses the general current, so we can judge that this is a description of the state.\""}, "sentence_type": "Interrogative sentence", "pos": {"nouns": {"Self-incrimination": 1, "Trouble": 1}, "verbs": {"Is it possible": 1, "Common cause": 1, "You're gonna blame yourself": 1}, "numerals": {"24": 1}}}, {"content": "Are you talking about something you don't know?", "word_count": 17, "label": "", "sentence_structure": "Subject鈥搗erb鈥搊bject", "subjective_objective": {"answer": "Subjective", "rationale": "This is a question of the behaviour or orientation of the patient and requires an understanding of the patient ' s thoughts and feelings."}, "sentence_sentiment": {"answer": "Neutral", "rationale": "As the entry does not express feelings, attitudes, behaviour, purpose or quality that are clearly positive or negative."}, "language_style": {"answer": "Calm and direct", "rationale": "The reasoning is that the article is more objective and has not been used in too many terms or emotionally in order to ask the interviewee whether he or she has ever spoken about topics he or she does not understand. Thus, its language style can be described as \"cool and straight\"."}, "time_dimension": {"answer": "Present", "rationale": "The entry asks if the testee is talking about something he does not understand. The keywords \"talking\" and \"understanding\" are both current, indicating that this is a description of the current behaviour, so it's now."}, "sentence_type": "Interrogative sentence", "pos": {"nouns": {"Questionnaire Entry": 1, "Things": 1}, "verbs": {"Talk": 1, "Nothing": 1}, "numerals": {"25": 1}}}, {"content": "Would you rather read books than want to meet someone else?", "word_count": 16, "label": "", "sentence_structure": "Subject鈥搗erb鈥搊bject", "subjective_objective": {"answer": "Subjective", "rationale": "Because \"better\" is patient's subjective attitude."}, "sentence_sentiment": {"answer": "Negative", "rationale": "Because the behaviour and attitude of \"I prefer to read books rather than want to meet with others\" in the entry is negative, indicating that there may be problems of social fear or unwillingness to interact with others."}, "language_style": {"answer": "Tactful and warm", "rationale": "The entry is a soft, respectful question, using a phrase \"better than...\", which is more euphemistic, friendly, and does not force acceptance or rejection. At the same time, the language of the article is more in line with the psychological questionnaire, with emphasis on protecting the privacy and interests of the person under investigation."}, "time_dimension": {"answer": "Present", "rationale": "The key words of the questionnaire entries are \"better\" and \"not\" , which express the attitude and choice of the testees with regard to the current situation and can therefore be judged as now."}, "sentence_type": "Interrogative sentence", "pos": {"nouns": {"Questionnaire": 1, "Entry": 1, "person": 1, "Books": 1, "Meeting": 1}, "verbs": {"be": 1, "I'd rather": 1, "Look at this": 1, "No, I don't": 1, "Go on": 1}, "adverbs": {"very": 2}, "numerals": {"26": 1}, "pronouns": {"you": 1}}}, {"content": "Is there a bad guy trying to hurt you?", "word_count": 8, "label": "", "sentence_structure": "Subject鈥搗erb鈥搊bject", "subjective_objective": {"answer": "Subjective", "rationale": "This is a question of asking patients whether they fear that they may become victims."}, "sentence_sentiment": {"answer": "Negative", "rationale": "Because the phrase \"some bad guy wants to hurt you\" in the entry is negative."}, "language_style": {"answer": "Calm and direct", "rationale": "The language style of the entry is more straightforward, and there is no excessive rhetorical or emotional rendering, mainly to obtain the real perception and perception of the potential threat."}, "time_dimension": {"answer": "Present", "rationale": "So output is \"now\"."}, "sentence_type": "Interrogative sentence", "pos": {"nouns": {"Bad guy": 1, "Shit": 1, "you": 1}, "verbs": {"Yes": 1}, "adverbs": {"What": 1}, "numerals": {"27": 1}}}, {"content": "Do you think you're \"sensitized\"?", "word_count": 10, "label": "", "sentence_structure": "Subject鈥搗erb鈥搊bject", "subjective_objective": {"answer": "Subjective", "rationale": "Because it's the patient's subjective view that thinks he's \"sensitized.\""}, "sentence_sentiment": {"answer": "Negative", "rationale": "Because the entry is often considered \"sensitized\" as a negative emotion or state, indicating emotional instability or oversensitivity."}, "language_style": {"answer": "Tactful and warm", "rationale": "It uses mild words, such as \"Do you think?\" rather than asking the other side whether it is. Moreover, the term \"sensitized\" is used, not as a direct offensive or negative term, but as a more neutral expression. Overall, the language style of the article is more friendly and understanding."}, "time_dimension": {"answer": "Present", "rationale": "The keyword \"thinks himself\" indicates the person's perception or assessment of his state; it is a present behavior and mentality; \"sensitized\" describes a subjective feeling or experience of the person being tested, and is relevant to the moment. The entry to the questionnaire therefore asks about the person's current psychological condition."}, "sentence_type": "Interrogative sentence", "pos": {"nouns": {"You're a bit of a sane": 1}, "verbs": {"I think": 1, "have": 1}, "numerals": {"28": 1}, "pronouns": {"self": 1}}}, {"content": "Do you have many friends?", "word_count": 6, "label": "", "sentence_structure": "Subject鈥損redicate", "subjective_objective": {"answer": "Objective", "rationale": "Because \"friends\" are a quantifiable fact that can be observed and measured, not just the patient's subjective feelings. For example, it is possible to judge whether the patient has more friends than the number of friends. So, \"friends are many?\" The question is considered objectively."}, "sentence_sentiment": {"answer": "Neutral", "rationale": "Because the entry does not have any emotional color."}, "language_style": {"answer": "Tactful and warm", "rationale": "The language style of the entry is more euphemistic and personal, raising questions in the form of a question-and-answer period without directly assessing or criticizing the relationship status of the other. Moreover, the question of \"friends?\" is relatively neutral, does not involve too much emotional or personal privacy and is therefore more suitable for use in the questionnaire."}, "time_dimension": {"answer": "Present", "rationale": "The entry asks about the current status of the subject, the keyword is \"you\" and \"now\"."}, "sentence_type": "Interrogative sentence", "pos": {"nouns": {"friends": 1, "More": 1}, "verbs": {"have": 1}, "adverbs": {"not": 1, "- You're not": 1}, "numerals": {"29": 1}}}, {"content": "Are you a man of great concern?", "word_count": 10, "label": "", "sentence_structure": "Subject鈥損redicate", "subjective_objective": {"answer": "Subjective", "rationale": "Because the fear exists in the individual's heart, it is a subjective attitude and feeling of one person and is a subjective one."}, "sentence_sentiment": {"answer": "Negative", "rationale": "Because the description \"scared\" is negative."}, "language_style": {"answer": "Calm and direct", "rationale": "The entry is straightforward in asking the other party whether it has an excessive fear or anxiety, without using excessive rhetorical techniques or emotional rendering, and with a more objective and calm tone."}, "time_dimension": {"answer": "Present", "rationale": "The word \"are you a man of great concern?\" The word \"is\" is a description of the current situation, while the word \"person of great concern\" is an adjective of an evaluative nature, pointing to the present state and character. The entry therefore asks whether the testee is in his current state of preference for emotional reactions such as anxiety, anxiety, etc., and is now a problem."}, "sentence_type": "Interrogative sentence", "pos": {"nouns": {"person": 1, "Fear": 2, "Thick": 1}, "verbs": {"be": 1}, "adjectives": {"individual": 1, "I'm worried": 1}, "numerals": {"30": 1}}}, {"content": "31. Were you at the mercy of adults in childhood without complaint?", "word_count": 23, "label": "", "sentence_structure": "Subject鈥搗erb鈥搊bject", "subjective_objective": {"answer": "Subjective", "rationale": "The subject is subjective in that it asks about the individual鈥檚 childhood experiences and attitudes. It looks at the individual鈥檚 attitudes and behaviour, not the objective facts."}, "sentence_sentiment": {"answer": "Positive", "rationale": "Because the behavior in the entry, \"As your lords command\" and \"absolutely\" is positive."}, "language_style": {"answer": "Calm and direct", "rationale": "The entry is in a direct presentation, and the questioner is not used too much emotionally or in a rhetorical way to speak in a balanced and objective manner. The language style of the entry can therefore be described as \"cool and straight\"."}, "time_dimension": {"answer": "Past", "rationale": "The entry is for the testee's experiences and behaviours in childhood, and the temporal dimension is the past. Key words: \"Children's age\", \"As you will immediately obey your Lord\" and \"without any grievance\" point to past experiences or behaviour."}, "sentence_type": "Interrogative sentence", "pos": {"nouns": {"you": 1, "Children": 1, "Sir": 1, "Yes": 1, "Grievances": 1}, "verbs": {"Is it possible": 1, "Immediately": 1, "Listen": 1, "Nothing": 1, "have": 1}, "adverbs": {"And": 1, "None": 1, "Is it possible": 1}, "numerals": {"31": 1}, "pronouns": {"Here": 1}}}, {"content": "Are you a carefree man?", "word_count": 15, "label": "", "sentence_structure": "Subject鈥揷opula鈥損redicative", "subjective_objective": {"answer": "Subjective", "rationale": "The question is whether you are a carefree, free person who needs to be assessed by the respondent according to his or her actual situation and therefore is subjective."}, "sentence_sentiment": {"answer": "Neutral", "rationale": "Because the entry does not have any emotional color."}, "language_style": {"answer": "Tactful and warm", "rationale": "The entry is more euphemistic, in a more pleasant way, to guide the interviewee to answer questions and avoid a direct criticism or evaluation of the interviewee鈥檚 attitude. This language style is personal and interactive, making it easier for the interviewee to open his heart.\""}, "time_dimension": {"answer": "Present", "rationale": ""}, "sentence_type": "Interrogative sentence", "pos": {"nouns": {"you": 1, "person": 1, "No worries": 1, "I'm free": 1}, "verbs": {"be": 1}, "adjectives": {"No worries": 1, "I'm free": 1}, "numerals": {"32": 1}}}, {"content": "Is it important for you to be polite and clean?", "word_count": 12, "label": "", "sentence_structure": "Subject鈥搗erb鈥搊bject", "subjective_objective": {"answer": "Subjective", "rationale": "Because it's important or not is a subjective assessment."}, "sentence_sentiment": {"answer": "Positive", "rationale": "Because \"be polite, be nice and clean\" is a positive quality."}, "language_style": {"answer": "Tactful and warm", "rationale": "The language style of the entry is more gentle and friendly, allowing the interviewee to think about the issue in a question-and-answer manner, without directly expressing his/her views or orders."}, "time_dimension": {"answer": "Present", "rationale": "The entry asks the respondent about a view of the current view, and therefore the temporal dimension is now judged."}, "sentence_type": "Interrogative sentence", "pos": {"nouns": {"Courage": 1, "Clean": 1, "Important": 1}, "verbs": {"have": 1, "Love": 1, "to": 1}, "adjectives": {"very": 2}, "numerals": {"33": 1}}}, {"content": "34. Are you worried about something terrible going to happen?", "word_count": 14, "label": "", "sentence_structure": "Subject鈥搗erb鈥搊bject", "subjective_objective": {"answer": "Subjective", "rationale": "There may be elements of subjective concern, but the circumstances need to be judged in relation to other issues and patient statements."}, "sentence_sentiment": {"answer": "Negative", "rationale": "Because the entries contain the negative emotional term \"fear,\" they express a negative attitude."}, "language_style": {"answer": "Calm and direct", "rationale": "The language style of the entry is a calm and straightforward type. It directly asks the respondents about the level of concern about possible terrible events, and does not use excessive emotional accommodations or rhetoric techniques to express it clearly and succinctly."}, "time_dimension": {"answer": "Present", "rationale": "The word \"no\" in \"are you concerned\" is a future concern and can be considered as asking the present thoughts and feelings of the subjects."}, "sentence_type": "Interrogative sentence", "pos": {"nouns": {"Things": 1, "Horrible": 1}, "verbs": {"Come on": 1, "I'm worried": 1}, "numerals": {"34": 1}}}, {"content": "35. Are you usually proactive when you meet new friends?", "word_count": 15, "label": "", "sentence_structure": "Subject鈥搗erb鈥搊bject", "subjective_objective": {"answer": "Subjective", "rationale": "Because the title \"usually\" shows that it's a patient's subjective feeling or attitude."}, "sentence_sentiment": {"answer": "Positive", "rationale": "Because the \"proactive meeting of new friends\" in the entry is positive."}, "language_style": {"answer": "Calm and direct", "rationale": "This language style is more easily understood and answered by the interviewee."}, "time_dimension": {"answer": "Present", "rationale": "\"At times\" indicates that this is a description of customary conduct, which does not specify a specific point in the past or in the future. We can therefore assume that the entry is asking the testee what is happening now."}, "sentence_type": "Interrogative sentence", "pos": {"nouns": {"New friends": 1, "Initiatives": 1}, "verbs": {"I'm meeting someone": 1, "be": 1, "Usually": 1}, "adverbs": {"in": 1, "Time": 1, "- You're not": 1}, "numerals": {"35": 1}}}, {"content": "Do you think you're a very sensitive person?", "word_count": 15, "label": "", "sentence_structure": "Subject鈥搗erb鈥搊bject", "subjective_objective": {"answer": "Subjective", "rationale": "Because the subject questions the patient ' s personal feelings and self-perception, subjective attitudes are examined."}, "sentence_sentiment": {"answer": "Negative", "rationale": "Because the description of \"very sensitive\" in the entry is negative."}, "language_style": {"answer": "Tactful and warm", "rationale": "The language style of the entry is euphemistic. It is more personal and interactive when it talks in a gentle and inviting tone, asking the person under investigation about his or her perception, rather than directly speaking or ordering."}, "time_dimension": {"answer": "Present", "rationale": "The entry \"Feeling yourself\" and \"are very sensitive?\" is asking the testee about his current self-awareness and feelings, and judging now."}, "sentence_type": "Interrogative sentence", "pos": {"nouns": {"Questionnaire": 1, "person": 1}, "verbs": {"I think": 1, "be": 1, "self": 1}, "adjectives": {"Very": 1, "Sensitive": 1}, "numerals": {"36": 1}, "pronouns": {"you": 1}}}, {"content": "37. Don't you talk much when you're with someone else?", "word_count": 16, "label": "", "sentence_structure": "Subject鈥搗erb鈥搊bject", "subjective_objective": {"answer": "Subjective", "rationale": "Because the question is whether an individual ' s feelings or attitudes, i.e., the tendency to speak less in social settings, are subjective in nature."}, "sentence_sentiment": {"answer": "Negative", "rationale": "Because the \"not often speaking\" act in the entry is negative."}, "language_style": {"answer": "Calm and direct", "rationale": "The entry is presented in a concise manner, without excessive emotional or rhetorical overtones, and more directly asks the person under investigation about his or her behaviour in dealings with others."}, "time_dimension": {"answer": "Present", "rationale": "The entry asks the testee about his current state or behaviour, and the keywords are \"when with someone else\" and \"if he doesn't talk much?\" all point to the current situation."}, "sentence_type": "Interrogative sentence", "pos": {"nouns": {"Time": 1, "others": 1}, "verbs": {"Is it possible": 2, "Not much": 1}, "adjectives": {"Together": 1}, "numerals": {"37": 1}, "pronouns": {"you": 1}}}, {"content": "38. Do you think marriage is a frame and should be abolished?", "word_count": 15, "label": "", "sentence_structure": "Subject鈥搗erb鈥搊bject", "subjective_objective": {"answer": "Subjective", "rationale": "Because the question is asked about the views and attitudes of the patient, it is subjective."}, "sentence_sentiment": {"answer": "Negative", "rationale": "Because the word \"revocation\" in the entry is negative emotional."}, "language_style": {"answer": "Tactful and warm", "rationale": "The language style of the entry is euphemistic, using the term \"revocation\" but not directly denying or attacking the marriage system. At the same time, the form \"you feel...\" also makes the questionnaire more open and interactive, and allows easy access to the real thoughts and feelings of the interviewees."}, "time_dimension": {"answer": "Present", "rationale": "The \"Feel\" in the entry, which uses the general current, means that the idea still exists and may still affect the behaviour and decision-making of the testee. So the judgement is \"now\"."}, "sentence_type": "Interrogative sentence", "pos": {"nouns": {"Marriage": 1, "Box": 1}, "verbs": {"I think": 1, "Abolition": 1}, "numerals": {"38": 1}}}, {"content": "Do you sometimes brag about yourself?", "word_count": 10, "label": "", "sentence_structure": "Subject鈥搗erb鈥搊bject", "subjective_objective": {"answer": "Subjective", "rationale": "Because \"showing himself\" is a subjective attitude or behaviour that patients themselves evaluate."}, "sentence_sentiment": {"answer": "Negative", "rationale": "Because the act of \"blowing himself\" is usually considered self-exclaiming and a negative act."}, "language_style": {"answer": "Calm and direct", "rationale": "The language style of the entry is calm and straightforward. It asks if the person surveyed is able to boast about himself, is not over-exposed or pampered, is honest and objective and is easy to understand."}, "time_dimension": {"answer": "Present", "rationale": "The entry asks about the current behaviour or state of the subject, and the keywords are \"sometimes\" and \"blowing himself up\" and all point to situations that are currently under way or exist.\""}, "sentence_type": "Interrogative sentence", "pos": {"nouns": {"you": 1, "Time": 1, "Points": 1, "鈾?Blowing your own battles 鈾?: 1}, "verbs": {"have": 1, "be": 1}, "adjectives": {"A little": 1}, "numerals": {"39": 1}}}, {"content": "In a dreary situation, can you make us angry?", "word_count": 18, "label": "", "sentence_structure": "Subject鈥搗erb鈥搊bject", "subjective_objective": {"answer": "Subjective", "rationale": "This question is examined from the subjective perspective of the patient, as it asks about the attitudes and behaviour of the individual in a given situation."}, "sentence_sentiment": {"answer": "Positive", "rationale": "Because the article is formulated in the hope of bringing life and dynamism to the scene of boredom, it is a positive, positive attitude and behaviour."}, "language_style": {"answer": "Tactful and warm", "rationale": "The language style of the entry is relatively euphemistic and gentle, and it does not directly ask the other side whether it can be active in a dreary setting, but rather raises the question in a more positive and encouraging way, which is more acceptable.\""}, "time_dimension": {"answer": "Present", "rationale": ""}, "sentence_type": "Interrogative sentence", "pos": {"nouns": {"Event": 1, "Everyone": 1, "Angry": 1}, "verbs": {"Yes": 1, "Tim": 1}, "adjectives": {"Boring": 1}, "numerals": {"40": 1}}}, {"content": "Does the slow-drive driver make you sick?", "word_count": 14, "label": "", "sentence_structure": "Subject鈥搗erb鈥搊bject", "subjective_objective": {"answer": "Subjective", "rationale": "Because it is a subjective view, different people may have different assessments of it."}, "sentence_sentiment": {"answer": "Negative", "rationale": "Because the phrase \"does the slow-move drivers make you sick?\" in the entry expresses a negative attitude, i.e., dissatisfaction or irritation with the drivers."}, "language_style": {"answer": "Calm and direct", "rationale": ""}, "time_dimension": {"answer": "Present", "rationale": "The entry asks the current feelings and attitudes of the testee and does not address past or future situations, so the temporal dimension is now judged."}, "sentence_type": "Interrogative sentence", "pos": {"nouns": {"Driver": 1, "Shit": 1}, "verbs": {"Drive": 1, "鈾?And I'm gonna be 鈾?: 1, "Slow down": 1, "Shit": 1}, "adjectives": {"Slow down": 1}, "numerals": {"41": 1}}}, {"content": "Are you worried about your health?", "word_count": 9, "label": "", "sentence_structure": "Subject鈥搗erb鈥搊bject", "subjective_objective": {"answer": "Subjective", "rationale": "Because \"fear\" is a subjective emotional experience, it examines the extent to which patients are concerned about their health."}, "sentence_sentiment": {"answer": "Negative", "rationale": "Because \"fear\" is negative."}, "language_style": {"answer": "Tactful and warm", "rationale": "The language style of the entry is more euphemistic and warm, leading the conversation by asking if the other side is concerned about its health, and not directly expressing anxiety or stress. This tone makes it easier for the interviewee to open his heart and share his real thoughts and feelings."}, "time_dimension": {"answer": "Present", "rationale": "The entry asks the testee whether he is currently concerned about his health and therefore the temporal dimension is judged to be \"now\"."}, "sentence_type": "Interrogative sentence", "pos": {"nouns": {"Health": 1, "self": 1}, "verbs": {"I'm worried": 1}, "numerals": {"42": 1}}}, {"content": "Do you like to joke and talk about funny things?", "word_count": 16, "label": "", "sentence_structure": "Subject鈥搗erb鈥搊bject", "subjective_objective": {"answer": "Subjective", "rationale": "Because \"like\" is a subjective assessment or emotional, which is the patient's own feelings or attitudes."}, "sentence_sentiment": {"answer": "Positive", "rationale": "Because the action of \"likes to say jokes and talk about interesting things\" in the entry is positive."}, "language_style": {"answer": "Tactful and warm", "rationale": "The language style of the entry is more euphemistic and warm, and it asks the respondents whether they like to make jokes and talk about interesting things in a simple and sweet tone, which is more likely to resonate with the respondents and thus enhance the effectiveness of the questionnaire."}, "time_dimension": {"answer": "Present", "rationale": ""}, "sentence_type": "Interrogative sentence", "pos": {"nouns": {"A joke": 1, "Talk": 1, "Things": 1}, "verbs": {"Yeah": 1, "Say it": 1, "Talk": 1}, "numerals": {"43": 1}}}, {"content": "Do you think most things don't matter to you?", "word_count": 20, "label": "", "sentence_structure": "Subject鈥搗erb鈥搊bject", "subjective_objective": {"answer": "Subjective", "rationale": "Because the word \"thinks\" indicates that it is a personal opinion or feeling of the patient."}, "sentence_sentiment": {"answer": "Negative", "rationale": "Because the attitude in the entry \"does not matter for most things\" is negative."}, "language_style": {"answer": "Calm and direct", "rationale": "The entry is asked in a direct and clear manner, without too much embellishment or emotional rendering, and in a more calm and objective manner."}, "time_dimension": {"answer": "Present", "rationale": "The entry is for the testee ' s current behaviour and mentality, and the time dimension is now judged."}, "sentence_type": "Interrogative sentence", "pos": {"nouns": {"Things": 2, "you": 3, "Most of them": 1}, "verbs": {"I think": 1, "be": 1, "Both": 1, "It doesn't matter": 1}, "adjectives": {"It doesn't matter": 1}, "numerals": {"44": 1}, "pronouns": {"Here": 1}}}, {"content": "Have you ever treated parents recklessly and disrespectfully as a child?", "word_count": 20, "label": "", "sentence_structure": "Subject鈥搗erb鈥搊bject", "subjective_objective": {"answer": "Subjective", "rationale": "Because \"Whether or not to act recklessly or rudely\" is a patient's self-evaluation of his or her behaviour, it is his or her subjective attitude and experience that is examined."}, "sentence_sentiment": {"answer": "Negative", "rationale": "Because the \"brash, rude behaviour of parents\" in the entry is negative."}, "language_style": {"answer": "Calm and direct", "rationale": "The entry is more objective, direct, and does not use excessive rhetorical or emotional colours. It explicitly asks whether the person surveyed has ever acted recklessly and disrespectfully against his or her parents, is more neutral and aims to obtain real data."}, "time_dimension": {"answer": "Past", "rationale": ""}, "sentence_type": "Interrogative sentence", "pos": {"nouns": {"When I was little": 1, "You're reckless": 1, "Behaviour": 1, "parents": 2}, "verbs": {"have": 1, "Treatment": 1, "Pass": 1, "- You're not": 1}, "adjectives": {"Insolent": 1}, "numerals": {"45": 1}}}, {"content": "Do you like to be with others all day long?", "word_count": 18, "label": "", "sentence_structure": "Subject鈥搗erb鈥搊bject", "subjective_objective": {"answer": "Subjective", "rationale": "Because \"like\" is patient's subjective attitude."}, "sentence_sentiment": {"answer": "Positive", "rationale": "Because the entry \"likes to be in a relationship with others all day long\" expresses a positive interpersonal attitude."}, "language_style": {"answer": "Tactful and warm", "rationale": "The entry is more adept and interactive when it is spoken in a gentle and inviting manner, rather than by direct statements or orders."}, "time_dimension": {"answer": "Present", "rationale": "Based on the phrase \"likes\" and \"getting together all day,\" it can be inferred that the entry is asked about the current attitude and habits of the testee, and therefore the temporal dimension is now judged."}, "sentence_type": "Interrogative sentence", "pos": {"nouns": {"Questionnaire": 1, "Entry": 1, "person": 1, "Oh, my God": 1, "Snippets": 1}, "verbs": {"Yeah": 1, "Done": 1, "Get along": 1, "in": 2, "Together": 1}, "adverbs": {"With someone else": 1, "All day long": 1}, "numerals": {"46": 1}, "classifiers": {"individual": 1, "Blocks": 1}, "pronouns": {"you": 1}}}, {"content": "Are you insomniac?", "word_count": 4, "label": "", "sentence_structure": "Subject鈥損redicate", "subjective_objective": {"answer": "Subjective", "rationale": "Because insomnia is a subjective experience and feeling."}, "sentence_sentiment": {"answer": "Neutral", "rationale": "Because the entry is merely an insomnia query, it does not contain any emotional or evaluative language."}, "language_style": {"answer": "Calm and direct", "rationale": "The language style of the entry is a calm, straightforward one, and the interviewee is asked directly in a concise and clear manner whether there is insomnia, without excessive rhetoric or emotional rendering.\""}, "time_dimension": {"answer": "Present", "rationale": "The entry asks if the person tested is currently insomnia and the time dimension is now judged."}, "sentence_type": "Interrogative sentence", "pos": {"nouns": {"Insomnia": 1}, "verbs": {"have": 1}, "numerals": {"47": 1}}}, {"content": "Do you wash your hands before dinner?", "word_count": 9, "label": "", "sentence_structure": "Subject鈥損redicate", "subjective_objective": {"answer": "Subjective", "rationale": "Because the hand wash is a human ' s life and hygiene habits, it is subjective attitudes and behaviour."}, "sentence_sentiment": {"answer": "Positive", "rationale": "Because \"brain your hands before dinner\" is a good hygiene habit, a positive behavior."}, "language_style": {"answer": "Calm and direct", "rationale": "The basis for the determination is as follows: 1. The question is formulated in a very specific and clear manner without using vague or euphemistic language; 2. The tone of the question is neutral and objective, and does not favour any answer; 3. The question does not contain an element of encouragement or inducement, but is presented to the interviewee in a purely factual manner."}, "time_dimension": {"answer": "Present", "rationale": "Now the entry asks the testee's current behavior, which is, \"Do you have to wash your hands before eating?\" is a matter of current-day behaviour."}, "sentence_type": "Interrogative sentence", "pos": {"nouns": {"Before dinner": 1, "Hands": 1}, "verbs": {"Yes": 1, "First": 1, "Wash": 1, "Eat": 1}, "adverbs": {"I'm sure": 1}, "numerals": {"48": 1}}}, {"content": "When people ask you, do you answer the question?", "word_count": 14, "label": "", "sentence_structure": "Subject鈥搗erb鈥搊bject", "subjective_objective": {"answer": "Subjective", "rationale": "This is a question of patients ' subjective feelings and self-evaluation, i.e. their performance and reactions to questions from others."}, "sentence_sentiment": {"answer": "Positive", "rationale": "Because the \"reactions\" in the entry are positive."}, "language_style": {"answer": "Calm and direct", "rationale": "The entry is presented in a direct question-and-answer manner, with no excessive adoration or emotional colour, and is more neutral and objective in tone, which is in keeping with the calm and direct language style."}, "time_dimension": {"answer": "Present", "rationale": ""}, "sentence_type": "Interrogative sentence", "pos": {"nouns": {"others": 1, "Word": 2, "Problem": 1, "It's a good answer": 1}, "verbs": {"Question": 1, "Answer": 1, "Is it possible": 1}, "adverbs": {"When": 1}, "numerals": {"49": 1}, "pronouns": {"you": 1}}}, {"content": "Would you rather have some time to go on a date earlier?", "word_count": 20, "label": "", "sentence_structure": "Subject鈥損redicate", "subjective_objective": {"answer": "Subjective", "rationale": "The questionnaire is intended to address the personal preferences and attitudes of the patients."}, "sentence_sentiment": {"answer": "Positive", "rationale": "Because the entries \"Better time than time\" and \"Being on a date\" are positive."}, "language_style": {"answer": "Tactful and warm", "rationale": "The entry uses a question-and-answer tone, and by the words \"better\" and \"early\", allows the person under investigation to express his or her views freely, rather than simply tell them what to do. Such euphemism reduces the resistance of the person under investigation and makes them more willing to answer the questions."}, "time_dimension": {"answer": "Present", "rationale": "The entry is to ask the testee about the current dating situation, and the keywords \"better time\" and \"early departure\" point to the current situation."}, "sentence_type": "Interrogative sentence", "pos": {"nouns": {"Rich time": 1, "Date": 1}, "verbs": {"I'd rather": 1, "have": 1, "Morning": 1, "Get out of here": 1}, "adverbs": {"More": 1}, "numerals": {"50": 1}, "classifiers": {"individual": 1}}}, {"content": "Do you often get tired or incompetent for no reason?", "word_count": 17, "label": "", "sentence_structure": "Subject鈥搗erb鈥搊bject", "subjective_objective": {"answer": "Subjective", "rationale": "It is the patient's subjective feeling that he is tired or insinuated."}, "sentence_sentiment": {"answer": "Negative", "rationale": "Because \"tired\" and \"incompetent\" are negative emotional descriptions."}, "language_style": {"answer": "Calm and direct", "rationale": "The entry is a direct question to whether the respondents are often tired or insufficiently trained, without excessive rhetorical and emotional rendering, and more calm and straightforward language styles."}, "time_dimension": {"answer": "Present", "rationale": "\"Worried or insufficiently beaten\" describes the emotional state of the person being tested. The entry therefore asks whether the person being tested is often tired or insufficiently satisfied in his current state of mood and in his daily life, which is now."}, "sentence_type": "Interrogative sentence", "pos": {"nouns": {"Questionnaire": 1, "Entry": 1, "Regular": 1, "For no reason": 1, "I feel": 1, "Tired": 1, "I'm not good at it": 1, "you": 2}, "verbs": {"have": 1, "Sensor": 1, "Present": 1, "be": 1}, "adjectives": {"For no reason": 1, "Tired": 1, "I'm not good at it": 1}, "adverbs": {"Regular": 1}, "numerals": {"51": 1, "you": 2}, "pronouns": {"you": 2}}}, {"content": "Do you cheat often in games or cards?", "word_count": 13, "label": "", "sentence_structure": "Subject鈥搗erb鈥搊bject", "subjective_objective": {"answer": "Objective", "rationale": "Because \"false\" is an objective act, not a patient's subjective attitude or experience."}, "sentence_sentiment": {"answer": "Negative", "rationale": "Because the \"cheating\" act is negative."}, "language_style": {"answer": "Calm and direct", "rationale": "The entry is more straightforward and clear in language style, not using euphemism or vague expression, but rather asking the respondent whether he or she would cheat in a game or a card game. This straightforward way of asking questions helps researchers to get more accurate and realistic answers."}, "time_dimension": {"answer": "Past", "rationale": "The entry asks if the person tested had cheated in the past and thus judged the past."}, "sentence_type": "Interrogative sentence", "pos": {"nouns": {"Game": 1, "Cards": 1, "Cheating": 1}, "verbs": {"in": 1, "Play": 1, "Regular": 1, "Do it": 1}, "adverbs": {"- You're not": 1}, "numerals": {"52": 1}}}, {"content": "Do you like to work in a tight place?", "word_count": 9, "label": "", "sentence_structure": "Subject鈥搗erb鈥搊bject", "subjective_objective": {"answer": "Subjective", "rationale": "Because it is the patient ' s subjective attitude to like or not like intense work."}, "sentence_sentiment": {"answer": "Negative", "rationale": "Because \"stretching work\" is a work environment that is usually considered negative."}, "language_style": {"answer": "Tactful and warm", "rationale": "The entry is more adept and interactive when it is spoken in a gentle and inviting manner, rather than by direct statements or orders."}, "time_dimension": {"answer": "Present", "rationale": "The entry asks about the testee's preferences or preferences at the current point in time, so it's \"now\"."}, "sentence_type": "Interrogative sentence", "pos": {"nouns": {"Work": 1, "Yeah": 1, "Nervous": 1}, "verbs": {"Yeah": 1, "Nervous": 1}, "numerals": {"53": 1}}}, {"content": "You always feel your life is so unwieldy?", "word_count": 14, "label": "", "sentence_structure": "Subject鈥損redicate", "subjective_objective": {"answer": "Subjective", "rationale": "Because it's the subjective feeling that the patient feels."}, "sentence_sentiment": {"answer": "Negative", "rationale": "Because the expression \"likes your life to be so monotonous\" in the entry is negative."}, "language_style": {"answer": "Tactful and warm", "rationale": ""}, "time_dimension": {"answer": "Present", "rationale": ""}, "sentence_type": "Interrogative sentence", "pos": {"nouns": {"Life": 1, "Undo": 1}, "verbs": {"I think": 1}, "adverbs": {"Often": 1}, "numerals": {"54": 1}}}, {"content": "Have you ever used anyone else for your own sake?", "word_count": 14, "label": "", "sentence_structure": "Subject鈥搗erb鈥搊bject", "subjective_objective": {"answer": "Subjective", "rationale": "Because \"use\" is a subjective behavioural assessment, it requires patient self-evaluation and reflection."}, "sentence_sentiment": {"answer": "Negative", "rationale": "Because the \"exploitation\" is negative and immoral."}, "language_style": {"answer": "Calm and direct", "rationale": "The entry is in a straightforward language style, is not overtly rhetorical or emotionally recast, and is more objective in tone. \""}, "time_dimension": {"answer": "Present", "rationale": "In the past, the entry asked the testee whether he had used the other person in the past and the dimension of time was judged to be past."}, "sentence_type": "Interrogative sentence", "pos": {"nouns": {"you": 1, "self": 1, "others": 1}, "verbs": {"Once": 1, "Used": 2}, "adverbs": {"For": 1}, "numerals": {"55": 1}}}, {"content": "Have you participated in too many activities and exceeded the time you might allocate?", "word_count": 22, "label": "", "sentence_structure": "Subject鈥搗erb鈥搊bject", "subjective_objective": {"answer": "Subjective", "rationale": "Because the idea and decision to participate in the event is the patient's own subjective attitude."}, "sentence_sentiment": {"answer": "Negative", "rationale": "Because it expresses a possible problem or distress, i.e., too many activities involved, which result in a time-frame that cannot be reasonably scheduled and which has a certain negative emotional connotation."}, "language_style": {"answer": "Calm and direct", "rationale": "The language style of the entry is more straightforward, and there is no excessive emotional or rhetorical approach, mainly to ask questions in an objective and neutral manner, which makes them easier to understand and answer."}, "time_dimension": {"answer": "Present", "rationale": "The entry asks the testee about the current behaviour or circumstances, in which the \"whether you are involved in too much activity and are more than you may allocate\" is the \"whether\" and \"participation\" pattern, which indicates that the act is in progress or just being completed, and therefore the temporal dimension is now judged."}, "sentence_type": "Interrogative sentence", "pos": {"nouns": {"Activities": 1, "Time": 1}, "verbs": {"Participation": 1, "Allocation": 1, "Too much": 1}, "adjectives": {"Maybe": 1}, "adverbs": {"Too much": 1}, "numerals": {"56": 1}}}, {"content": "Do you have a few people who hide from you?", "word_count": 14, "label": "", "sentence_structure": "Subject鈥搗erb鈥搊bject", "subjective_objective": {"answer": "Subjective", "rationale": "Because \"often\" is a subjective feeling and attitude of a person, it is viewed from the patient's subjective perspective."}, "sentence_sentiment": {"answer": "Negative", "rationale": "Because the act of hiding in the entry is negative."}, "language_style": {"answer": "Calm and direct", "rationale": "The language style of the entry is more straightforward, and there is no over-doping or emotionalization. It is dialogueable in simple question form and is very clear in itself, allowing respondents to quickly understand and answer questions. This expression is more appropriate for questionnaires, because it reduces misunderstanding and confusion and improves the accuracy and effectiveness of data."}, "time_dimension": {"answer": "Present", "rationale": "The entry asks if the person being tested is currently in the position of being often hidden from him/her and therefore judged to be now."}, "sentence_type": "Interrogative sentence", "pos": {"nouns": {"person": 2, "I'm avoiding you": 1}, "verbs": {"have": 1, "Often": 1}, "adverbs": {"Well": 1}, "numerals": {"57": 1}}}, {"content": "Do you think people spend too much time trying to figure out how to protect their future?", "word_count": 27, "label": "", "sentence_structure": "Subject鈥搗erb鈥搊bject", "subjective_objective": {"answer": "Subjective", "rationale": "Because \"thinking\" expresses the views and opinions of the patient is a subjective judgement."}, "sentence_sentiment": {"answer": "Negative", "rationale": "Because the \"too much time for careful and diligent\" behavior in the entries is negative."}, "language_style": {"answer": "Calm and direct", "rationale": "The entry is in the form of a question-and-answer sentence. The respondents are asked about the over-calculated, time-consuming phenomenon of people over-calculating their future."}, "time_dimension": {"answer": "Present", "rationale": "The entry is to ask the respondent ' s views on current behaviour and phenomena, and the keyword \"is considered to\" points to a judgement that is now under way."}, "sentence_type": "Interrogative sentence", "pos": {"nouns": {"you": 1, "People": 1, "Future": 1, "Fine calculations": 1, "Fancy": 1, "Time": 1}, "verbs": {"I think": 1, "Safeguards": 1, "Too much": 1, "Too many": 1}, "adjectives": {"Guaranteed": 1, "Future": 1}, "adverbs": {"I'm gonna be very careful": 1, "Fancy": 1}, "numerals": {"58": 1}}}, {"content": "Have you ever thought about dying?", "word_count": 10, "label": "", "sentence_structure": "Subject鈥搗erb鈥搊bject", "subjective_objective": {"answer": "Subjective", "rationale": "Because \"Thinking of Death\" is the patient's subjective feeling and thought, the answer is \"subjective.\""}, "sentence_sentiment": {"answer": "Negative", "rationale": "Because the \"want to die\" act in the entry is negative."}, "language_style": {"answer": "Calm and direct", "rationale": "The entry is a direct question to the respondents about whether they have had a suicide thought, the language is simple and not too much rhetorical or emotional to facilitate a true and objective answer."}, "time_dimension": {"answer": "Past", "rationale": "The keyword \"had \" and \"wanted to pass\" means that the act or state of conduct occurred over time and was completed or terminated, and can therefore be judged to have passed."}, "sentence_type": "Interrogative sentence", "pos": {"nouns": {"you": 1, "Die": 1}, "verbs": {"I'm going to go over there": 1, "Go on": 1}, "numerals": {"59": 1}}}, {"content": "Would you pay less if you knew you wouldn't be found?", "word_count": 19, "label": "", "sentence_structure": "Subject鈥搗erb鈥搊bject", "subjective_objective": {"answer": "Subjective", "rationale": "Because it is a matter of personal ethics and values, the level of honesty of each individual is different, the issue is examined from the subjective point of view of the patient."}, "sentence_sentiment": {"answer": "Negative", "rationale": "Because the act was dishonest and negative."}, "language_style": {"answer": "Calm and direct", "rationale": "The entry, in the form of a direct inquiry, clearly raises a question and is not expressed in a more objective and calm manner using any rhetorical or emotional colours. \""}, "time_dimension": {"answer": "Present", "rationale": "The \"will\" in the entry indicates the present will or attitude, and \"when it is known that it will not be discovered\" suggests that this is a hypothetical situation, but the effect on the behaviour still occurs at this moment. So the time dimension of the entry is now."}, "sentence_type": "Interrogative sentence", "pos": {"nouns": {"I know": 1, "No, I won't": 1, "Found": 1, "He's": 1, "Money": 1}, "verbs": {"Yes": 2, "Less than paid": 1}, "adverbs": {"If": 1}, "numerals": {"60": 1}, "pronouns": {"you": 1}}}, {"content": "Can you make a party work?", "word_count": 12, "label": "", "sentence_structure": "Subject鈥搗erb鈥搊bject", "subjective_objective": {"answer": "Subjective", "rationale": "Because making the party a success is the patient's subjective goal or expectation."}, "sentence_sentiment": {"answer": "Neutral", "rationale": "Because the entry is not emotional, it is a question of ability."}, "language_style": {"answer": "Tactful and warm", "rationale": "The language style of the entry is a soft and warm type. It asks questions in a gentle and gentle way, which makes the respondent feel respected and valued, and which does not require excessive pressure or responsibility."}, "time_dimension": {"answer": "Present", "rationale": ""}, "sentence_type": "Interrogative sentence", "pos": {"nouns": {"- We're having a party": 1, "Success": 1}, "verbs": {"鈾?And I'm gonna be 鈾?: 1}, "numerals": {"61": 1}}}, {"content": "62. Do you try to be less rude?", "word_count": 11, "label": "", "sentence_structure": "Subject鈥搗erb鈥搊bject", "subjective_objective": {"answer": "Subjective", "rationale": "Since the best of effort is an assessment of the subjective attitude or level of effort, it requires patient self-evaluation and judgement."}, "sentence_sentiment": {"answer": "Positive", "rationale": "Because the action in the entry \"do your best not to be rude\" is positive."}, "language_style": {"answer": "Calm and direct", "rationale": "The entry uses a clear, concise language style, which is direct to the other party, asking whether the other party has done its best to avoid rude behaviour, without using excessive rhetoric or emotional rendering."}, "time_dimension": {"answer": "Present", "rationale": "The keyword \"Whether or not to try\" suggests that this is a question of the current state of the person being tested, so the temporal dimension is now judged."}, "sentence_type": "Interrogative sentence", "pos": {"nouns": {"Questionnaire Entry": 1, "self": 1}, "verbs": {"Try": 1, "鈾?And I'm gonna be 鈾?: 1, "Naughty": 1}, "adjectives": {"not": 1}, "numerals": {"62": 1}}}, {"content": "Will something that has made you uncomfortable be a long time after it has been over?", "word_count": 22, "label": "", "sentence_structure": "Subject鈥搗erb鈥搊bject", "subjective_objective": {"answer": "Subjective", "rationale": "Because it was the emotional reaction of the patient that was examined, it was subjective."}, "sentence_sentiment": {"answer": "Negative", "rationale": "Because the \"long trouble\" act in the entry is negative."}, "language_style": {"answer": "Calm and direct", "rationale": "The entry raises the question in a straightforward manner and is clear in language, without too much embellishment or emotional rendering, so it can be judged that the language style of the questionnaire is straightforward and calm."}, "time_dimension": {"answer": "Past", "rationale": "In the entry, the term \"old\" indicates that this has happened and has an impact on the person being tested, and is therefore a part of the past behaviour or mentality."}, "sentence_type": "Interrogative sentence", "pos": {"nouns": {"Annex": 1, "Things": 1, "鈾?Make you 鈾?: 2, "- It's hard": 1, "After": 1, "Yes": 1, "you": 2, "Trouble": 1, "It's been a long time": 1}, "verbs": {"Past": 1, "鈾?And I'm gonna be 鈾?: 1, "It'll make you feel better": 1}, "adjectives": {"Difficulties": 1, "It's been a long time": 1}, "numerals": {"63": 1}}}, {"content": "64. Are you insisting on following your lead?", "word_count": 14, "label": "", "sentence_structure": "Subject鈥搗erb鈥搊bject", "subjective_objective": {"answer": "Subjective", "rationale": "Because it is the patient ' s thoughts and attitudes that are examined and subjective."}, "sentence_sentiment": {"answer": "Positive", "rationale": "Because the words \"continue to do what you think\" in the entry represent a self-acceptance and self-confidence, and are positive emotional tendencies."}, "language_style": {"answer": "Calm and direct", "rationale": "The entry is in a clear and straightforward language and asks the interviewees whether they insist on acting on their own ideas, without too many rhetorical or emotional renderings, and in a more calm and straightforward way."}, "time_dimension": {"answer": "Present", "rationale": "The entry asks the testee about his current attitude and behaviour, the keywords being \"or not to insist\" and \"do what you think.\" Both parts describe the current state, and therefore judge \"now\"."}, "sentence_type": "Interrogative sentence", "pos": {"nouns": {"Questionnaire Entry": 1, "Thoughts": 1}, "verbs": {"Hold on": 1, "According to": 1, "Operations": 1}, "adverbs": {"Is it possible": 1, "Yes": 1}, "numerals": {"64": 1}}}, {"content": "Will you arrive at the last minute when you get on the train?", "word_count": 18, "label": "", "sentence_structure": "Subject鈥搗erb鈥搊bject", "subjective_objective": {"answer": "Subjective", "rationale": "The last minute of arrival is a subjective view, as it is an individual ' s habit or choice."}, "sentence_sentiment": {"answer": "Negative", "rationale": "Because the last minute of the entry is negative."}, "language_style": {"answer": "Calm and direct", "rationale": "The language style of the entry is more straightforward, and there is no excessive emotional or rhetorical approach, mainly by means of brief inquiries to ask the interviewees about their customary behaviour."}, "time_dimension": {"answer": "Past", "rationale": "And the last minute to arrive, the description of the behavior, also points to the past. So the answer is \"the past.\""}, "sentence_type": "Interrogative sentence", "pos": {"nouns": {"you": 2, "Train": 1, "min": 1}, "verbs": {"Yes": 1, "Here we are": 1}, "adverbs": {"Finally": 1, "One": 1}, "numerals": {"65": 1}}}, {"content": "Are you nervous?", "word_count": 6, "label": "", "sentence_structure": "Subject鈥搗erb鈥搊bject", "subjective_objective": {"answer": "Subjective", "rationale": "Because \"nervous\" is a psychological or character characteristic that belongs to the subjective perception and feelings of the patient."}, "sentence_sentiment": {"answer": "Negative", "rationale": "Because \"nervous\" is usually considered a negative quality."}, "language_style": {"answer": "Tactful and warm", "rationale": "The language style of the entry is more euphemistic and caring, rather than directly using negative terms or highlighting personal defects, but rather asking the person under investigation whether he or she has a neurotic condition in a more moderate way."}, "time_dimension": {"answer": "Present", "rationale": ""}, "sentence_type": "Interrogative sentence", "pos": {"nouns": {"Questionnaire Entry": 1, "you": 1, "Nerves": 1}, "verbs": {"be": 1}, "numerals": {"66": 1}}}, {"content": "Do you often feel lonely?", "word_count": 7, "label": "", "sentence_structure": "Subject鈥損redicate", "subjective_objective": {"answer": "Subjective", "rationale": "Because the feeling of loneliness is an individual subjective experience."}, "sentence_sentiment": {"answer": "Negative", "rationale": "Because the \"sloking\" act in the entry is negative."}, "language_style": {"answer": "Tactful and warm", "rationale": "The language style of the entry is more euphemistic, and it asks each other, in a sensitive and understanding tone, whether they are often lonely, rather than expressing the subject directly and in a blunt manner. This expression is more personal and interactive, and it makes the interviewees more willing to share their true feelings."}, "time_dimension": {"answer": "Present", "rationale": "The keywords are \"you often feel\" and \"slept\" , which point to current, recurrent feelings. And the description of \"slouse\" also shows that it is a subjective experience, not a past or future. So the entry is now."}, "sentence_type": "Interrogative sentence", "pos": {"nouns": {"Questionnaire": 1, "Entry": 1, "Loneliness": 1}, "verbs": {"I feel": 1, "Regular": 1, "Loneliness": 2}, "adverbs": {"Regular": 1}, "numerals": {"67": 1}}}, {"content": "Do you always say and do what you do?", "word_count": 10, "label": "", "sentence_structure": "Subject鈥損redicate", "subjective_objective": {"answer": "Subjective", "rationale": "Because the same words and deeds are the expression of a subjective attitude and behaviour of a person."}, "sentence_sentiment": {"answer": "Neutral", "rationale": "Because the entry asks about the consistency of individual behaviour, it does not have any emotional colour."}, "language_style": {"answer": "Calm and direct", "rationale": "The basis for the judgement is as follows: 1. The problem itself is simple, not too much acrimonym, and the language is direct; 2. The article's purpose is more objective, without any emotional or subjective judgement, and is merely an inquiry about a particular act; 3. The \"fluent\" language style is usually more focused on interaction and communication with the other than the first entry in the example, which places greater emphasis on the question of the facts. Thus, according to the above analysis, the article's purpose is \"cool and direct\"."}, "time_dimension": {"answer": "Present", "rationale": "The entry is for the present behaviour or habits of the testee, and the keyword \"always consistent\" indicates that it is an evaluation and description of the present situation. The temporal dimension therefore is now judged."}, "sentence_type": "Interrogative sentence", "pos": {"nouns": {"Words and lines": 2, "Unanimously": 1}, "verbs": {"be": 1, "Total": 1, "have": 1}, "adverbs": {"Always": 1}, "numerals": {"68": 1}, "pronouns": {"you": 1}}}, {"content": "Do you sometimes like to play with animals?", "word_count": 10, "label": "", "sentence_structure": "Subject鈥搗erb鈥搊bject", "subjective_objective": {"answer": "Subjective", "rationale": "Because \"like to play with animals\" is a subjective attitude or tendency."}, "sentence_sentiment": {"answer": "Negative", "rationale": "Because of the irresponsible and cruel behaviour of playing animals, it can cause harm to animals."}, "language_style": {"answer": "Tactful and warm", "rationale": "This is more personal and interactive."}, "time_dimension": {"answer": "Present", "rationale": "The entry is an inquiry into the current behaviour or attitude of the subject, and the temporal dimension is judged to be the present. The keyword \"sometimes likes\" means that it is a situation that is somewhat manageable and sometimes occurring, and can therefore be considered part of the present behaviour."}, "sentence_type": "Interrogative sentence", "pos": {"nouns": {"you": 1, "Animals": 1}, "verbs": {"Yeah": 1, "Play": 1}, "adverbs": {"Sometimes": 1}, "numerals": {"69": 1}}}, {"content": "70. Are you easily discouraged by a man who is asking questions about you or your work?", "word_count": 25, "label": "", "sentence_structure": "Subject鈥搗erb鈥搊bject", "subjective_objective": {"answer": "Subjective", "rationale": "This is a subjective assessment of the patient ' s emotional response to criticism and accusations."}, "sentence_sentiment": {"answer": "Negative", "rationale": "Because the expression \"always harms your motivation\" is negative."}, "language_style": {"answer": "Tactful and warm", "rationale": "The entry is based on a judgment that begins with a soft, invitation-style tone of dialogue rather than a direct statement or order, which is more personal and interactive. At the same time, the entry does not ask the other side too directly about the vulnerability of others to the risk of being harmed, but rather uses a more euphemism to ask questions, showing respect and understanding of the interviewee."}, "time_dimension": {"answer": "Present", "rationale": "The entry \"is it\" indicates a subjective judgement and feeling that requires the testee to evaluate the current situation. Moreover, the \"blowing\" and \"harming motivation\" behaviour is also an ongoing one, so the time dimension of the entry is now."}, "sentence_type": "Interrogative sentence", "pos": {"nouns": {"person": 1, "Active": 1}, "verbs": {"to": 1, "- You're a liar": 1, "Hurt": 1, "Is it possible": 1, "Easy": 1}, "adjectives": {"of": 2, "Yours": 2}, "numerals": {"70": 1}, "pronouns": {"you": 1, "or": 1}}}, {"content": "Were you late for a date or work?", "word_count": 13, "label": "", "sentence_structure": "Subject鈥搗erb鈥搊bject", "subjective_objective": {"answer": "Objective", "rationale": "The fact that lateness is an objective fact is irrelevant to the patient ' s subjective attitude."}, "sentence_sentiment": {"answer": "Negative", "rationale": "Because lateness is a negative act."}, "language_style": {"answer": "Calm and direct", "rationale": "The entry is more straightforward in language style, with no excessive emotional rendering or rhetorical techniques, and asks whether the person surveyed has been late for a date or work. The question is simple, easy to understand, and quickly to obtain the information that is required."}, "time_dimension": {"answer": "Present", "rationale": "Because the entry asked whether the person tested was currently acting or used to be doing so, the temporal dimension was now judged."}, "sentence_type": "Interrogative sentence", "pos": {"nouns": {"Date": 1, "Work": 1}, "verbs": {"Go on": 2, "Late": 1, "Present": 1, "Go ahead": 1}, "numerals": {"71": 1}}}, {"content": "Do you like to have a lot of fun and joy around you?", "word_count": 21, "label": "", "sentence_structure": "Subject鈥搗erb鈥搊bject", "subjective_objective": {"answer": "Subjective", "rationale": "Because \"like\" is patient's subjective attitude."}, "sentence_sentiment": {"answer": "Positive", "rationale": "Because \"like\" in the entries expresses a positive attitude, and \"many things are fun and happy\" is a positive description."}, "language_style": {"answer": "Tactful and warm", "rationale": "The language style of the entry is more euphemistic, using an invitation-like tone rather than a direct statement or order, which is more personal and interactive. At the same time, by using the description \"many things of joy and pleasure\" it makes each other more comfortable and relaxed and encourages them to actively participate and share their thoughts and feelings."}, "time_dimension": {"answer": "Present", "rationale": "The entry asks the testee about his current attitude or preferences, i.e. whether he/she enjoys a virulent and pleasant atmosphere around him, and therefore the temporal dimension is now judged."}, "sentence_type": "Interrogative sentence", "pos": {"nouns": {"Questionnaire": 1, "Entry": 1, "Around": 1, "Things": 1}, "verbs": {"Yeah": 1, "have": 1}, "adjectives": {"Many": 1, "It's hilarious": 1, "Happy": 1}, "numerals": {"72": 1}}}, {"content": "Do you want to scare people about you?", "word_count": 9, "label": "", "sentence_structure": "Subject鈥損redicate", "subjective_objective": {"answer": "Subjective", "rationale": "Since the entry concerned the will and attitude of the individual, it was subjective."}, "sentence_sentiment": {"answer": "Negative", "rationale": "Because \"fearing\" is a negative emotional attitude, it involves a relationship of control and power, often associated with negative quality."}, "language_style": {"answer": "Calm and direct", "rationale": "The entry uses a direct and clear-cut approach, without too many rhetorical or emotional colours to render the problem, and is less formal and straightforward."}, "time_dimension": {"answer": "Present", "rationale": "The entry asks the testee about his current attitude and will, and the key words are \"will you\" and \"will you make others afraid of you?\" It indicates that this is an entry that asks questions about the situation."}, "sentence_type": "Interrogative sentence", "pos": {"nouns": {"Questionnaire": 1, "Entry": 1}, "verbs": {"Yes": 2, "Jean": 1}, "numerals": {"73": 1}, "pronouns": {"you": 1}}}, {"content": "Do you have a good time, but sometimes you're lazy and you don't want to move?", "word_count": 19, "label": "", "sentence_structure": "Subject鈥搗erb鈥搊bject", "subjective_objective": {"answer": "Subjective", "rationale": "Because \"Gangy and Lazy\" are the feelings and thoughts inside the patient, it's an entry that looks at from a subjective angle."}, "sentence_sentiment": {"answer": "Negative", "rationale": "Because the \"slugger doesn't want to move\" act in the entry is negative."}, "language_style": {"answer": "Tactful and warm", "rationale": "The entry is a conversation in a question-and-answer language, and uses a more verbal and routine expression that gives people a sense of kindness and moderation, making it easier for respondents to respond. At the same time, some direct, hard-line formulations are avoided that may provoke resistance among respondents."}, "time_dimension": {"answer": "Present", "rationale": ""}, "sentence_type": "Interrogative sentence", "pos": {"nouns": {"you": 2, "Time": 2, "鈾?And the joy 鈾?: 1, "Lazy": 1, "No, I don't": 1, "Move": 1}, "verbs": {"have": 1, "be": 1, "Sometimes he's lazy": 1, "Yes": 1}, "adjectives": {"Bob": 1, "Lazy": 1}, "adverbs": {"Sometimes": 2, "But it's": 1}, "numerals": {"74": 1}}}, {"content": "Do you sometimes drag up what you should do today to tomorrow?", "word_count": 17, "label": "", "sentence_structure": "Subject鈥搗erb鈥搊bject", "subjective_objective": {"answer": "Subjective", "rationale": "This is because it is self-observation and assessment of the patient and is subjective or behavioural."}, "sentence_sentiment": {"answer": "Negative", "rationale": "Because the entry \"Tow to tomorrow what should be done today\" is negative."}, "language_style": {"answer": "Calm and direct", "rationale": "The entry is in a direct, concise language, asking the other side whether it is often delaying the task that should be performed today, without using too many rhetorical techniques or emotional renderings, and with a more objective tone."}, "time_dimension": {"answer": "Present", "rationale": "In this entry, the words \"sometimes\", \"will\", \"today\", \"today\" and \"tomorrow\" are all words describing current behaviour or state, without reference to the time dimension of the past or the future, and therefore judgement is now."}, "sentence_type": "Interrogative sentence", "pos": {"nouns": {"Things": 1, "Oh, my God": 1, "Tomorrow": 1}, "verbs": {"Drag": 1, "Do it": 1, "Sometimes": 1}, "adverbs": {"Sometimes": 1}, "numerals": {"75": 1}}}, {"content": "Do people think you're alive?", "word_count": 13, "label": "", "sentence_structure": "Subject鈥搗erb鈥搊bject", "subjective_objective": {"answer": "Objective", "rationale": "Since the question is asked about the views and opinions of others, it is examined from an objective perspective."}, "sentence_sentiment": {"answer": "Positive", "rationale": "Because \"violation\" is usually a positive adjective, which means energy and energy."}, "language_style": {"answer": "Tactful and warm", "rationale": "The entry is more adept and interactive when it is spoken in a gentle and inviting manner, rather than by direct statements or orders."}, "time_dimension": {"answer": "Past", "rationale": "The entry used to ask the impression that the subjects had in the eyes of others that they had been identified with the dynamic features they had displayed in the past."}, "sentence_type": "Interrogative sentence", "pos": {"nouns": {"others": 1, "- Yes, he's very lively": 1}, "verbs": {"I think": 1}, "adjectives": {"- Yes, he's very lively": 1}, "numerals": {"76": 1}, "pronouns": {"you": 1}}}, {"content": "Have you been told many lies?", "word_count": 12, "label": "", "sentence_structure": "Subject鈥搗erb鈥搊bject", "subjective_objective": {"answer": "Subjective", "rationale": "Because \"if you have been told many lies\" is a subjective judgement that needs to be assessed on the basis of the patient's own experience, feelings and understanding."}, "sentence_sentiment": {"answer": "Negative", "rationale": "Because the \"false\" act in the entry is negative."}, "language_style": {"answer": "Calm and direct", "rationale": "The language style of the entry is one of calm and straightforward. It opens up questions without too much trim and rendering, but rather asks respondents in plain and clear language whether they have ever been told lies, which is more direct and objective, and which allows the interviewee to understand and answer with precision."}, "time_dimension": {"answer": "Past", "rationale": "The word \"speak\" in \"does anyone else have told you many lies?\" uses the usual past pattern, meaning that the act occurred in the past and the temporal dimension is therefore judged in the past."}, "sentence_type": "Interrogative sentence", "pos": {"nouns": {"person": 1, "Lies": 1, "Questionnaire": 1}, "verbs": {"Say it": 1}, "adverbs": {"Many": 1}, "numerals": {"77": 1}}}, {"content": "Are you angry about something that's easy?", "word_count": 13, "label": "", "sentence_structure": "Subject鈥搗erb鈥搊bject", "subjective_objective": {"answer": "Subjective", "rationale": "Because the emotional haste is the patient's own psychological state and is subjective."}, "sentence_sentiment": {"answer": "Negative", "rationale": "Because \"Easy-Easy-Eat\" is negative."}, "language_style": {"answer": "Calm and direct", "rationale": "The entry directly raises the question of whether the interviewee is made more impatient or angry because of certain things, is not using excessive rhetoric or emotional rendering, and is more objective. The language style of the entry is therefore straightforward and calm."}, "time_dimension": {"answer": "Present", "rationale": ""}, "sentence_type": "Interrogative sentence", "pos": {"nouns": {"Things": 1, "Sexual rush": 1, "Gas": 1}, "verbs": {"be": 1, "Easy": 1}, "adjectives": {"Some": 1}, "numerals": {"78": 1}}}, {"content": "If you were wrong, would you be willing to admit it?", "word_count": 11, "label": "", "sentence_structure": "Subject鈥搗erb鈥搊bject", "subjective_objective": {"answer": "Subjective", "rationale": "Because the problem is examined by the patient ' s subjective attitude and thoughts in the face of the error."}, "sentence_sentiment": {"answer": "Positive", "rationale": "Because the \"acknowledged error\" act in the entry is positive."}, "language_style": {"answer": "Calm and direct", "rationale": "The entry is in a straightforward manner, asking the other party whether it has the courage to admit its mistakes, and is less verbically and objectively speaking, without using euphemism or acrimony."}, "time_dimension": {"answer": "Present", "rationale": "The entry asks about the current attitude and behaviour of the testee, \"Is there\" indicating that this is an act that may exist in the past, present or future? \"Whether or not to admit\" suggests that this is an ongoing selective act. So the focus of the issue is now."}, "sentence_type": "Interrogative sentence", "pos": {"nouns": {"Error": 1, "Recognition": 1}, "verbs": {"have": 1, "May I": 1, "Meaning": 1, "I'm sorry": 1, "Recognition": 1}, "adverbs": {"Yes": 1}, "numerals": {"79": 1}}}, {"content": "You're a neat, neat man?", "word_count": 15, "label": "", "sentence_structure": "Subject鈥揷opula鈥損redicative", "subjective_objective": {"answer": "Subjective", "rationale": "The question is subjective because it is asking the views or attitudes of the interviewees themselves."}, "sentence_sentiment": {"answer": "Positive", "rationale": "Because \"clean and neat\" and \"sequenced\" are positive adjectives, which mean that this person has good habits and organizational skills."}, "language_style": {"answer": "Tactful and warm", "rationale": "The language style of the entry is more moderate, and the person is asked in an invitation manner whether he or she is a neat, orderly person."}, "time_dimension": {"answer": "Present", "rationale": "The words \"intensely and neatly\" and \"are you a person?\" are \"is\" which indicate that the act or character is present at the time of self-evaluation and therefore can be judged to be the present state."}, "sentence_type": "Interrogative sentence", "pos": {"nouns": {"Questionnaire Entry": 1, "person": 1}, "verbs": {"be": 2, "Clean": 1, "Strict": 1, "It's a little organized": 1}, "adjectives": {"Clean": 1, "Strict": 1, "It's a little organized": 1}, "numerals": {"80": 1}, "pronouns": {"you": 1}}}, {"content": "81. Do you always throw fruit or paper in the trash in parks or on the road?", "word_count": 24, "label": "", "sentence_structure": "Subject鈥搗erb鈥搊bject", "subjective_objective": {"answer": "Objective", "rationale": "This is a question of the de facto nature of the act. While an individual鈥檚 moral and environmental awareness may affect his or her answer, the question is primarily about objective behaviour."}, "sentence_sentiment": {"answer": "Positive", "rationale": "Because the purpose of the entry is to \"slam the fruit or paper in the trash.\""}, "language_style": {"answer": "Tactful and warm", "rationale": "The language style of the entry is more euphemistic and gentle, using the word \"whether or not\" to guide the interviewee to answer questions, and there is no excessive emotional or command tone in the context."}, "time_dimension": {"answer": "Present", "rationale": "\"In the park or on the road\" shows that this is the current place of behavior, and \"Do you always throw the bark or paper in the trash?\" and asks the testee about the current customary behaviour. So the time dimension of the entry is now."}, "sentence_type": "Interrogative sentence", "pos": {"nouns": {"Park": 1, "Road": 1, "- Goose": 1, "Waste Paper": 1, "Garbage cans": 1}, "verbs": {"Throw it": 1, "Total": 1}, "adverbs": {"Is it possible": 1}, "numerals": {"81": 1}}}, {"content": "Do you have any idea what's going on?", "word_count": 15, "label": "", "sentence_structure": "Subject鈥搗erb鈥搊bject", "subjective_objective": {"answer": "Subjective", "rationale": "This is a description of the patient ' s attitude and a subjective view of his or her ability to decide when he or she faces difficulties."}, "sentence_sentiment": {"answer": "Negative", "rationale": "Because the \"uncertainty\" act in the entry is negative."}, "language_style": {"answer": "Tactful and warm", "rationale": "The language style of the entry is a soft and warm one. It leads the interviewee to think and answer questions, rather than to direct statements or orders."}, "time_dimension": {"answer": "Present", "rationale": "The entry is for questioning the behaviour or mentality of the person under examination in the event of difficulties, and the time dimension is now judged."}, "sentence_type": "Interrogative sentence", "pos": {"nouns": {"Things": 1, "Ideas": 1}, "verbs": {"I met him": 1, "I can't hold it": 1, "Is it possible": 1}, "adverbs": {"- It's hard": 1}, "numerals": {"82": 1}}}, {"content": "Have you ever been a scolder?", "word_count": 12, "label": "", "sentence_structure": "Subject鈥損redicate", "subjective_objective": {"answer": "Subjective", "rationale": "Because \"scolding people at will\" is the patient's subjective attitude and behaviour."}, "sentence_sentiment": {"answer": "Negative", "rationale": "Because \"scolding\" is negative in the entries."}, "language_style": {"answer": "Calm and direct", "rationale": "The entry is a direct and explicit question-and-answer dialogue, without excessive rhetoric or emotional rendering and is more objective in tone."}, "time_dimension": {"answer": "Present", "rationale": "The keyword \"yes\" means that it is a question of the past or the present state, and \"when you say \"it is a question of whether the person who was tested has done or is not. So the time dimension of the problem is now."}, "sentence_type": "Interrogative sentence", "pos": {"nouns": {"Questionnaire": 1, "Entry": 1, "Time": 1}, "verbs": {"have": 1, "Scolded": 1, "Anytime": 1}, "adjectives": {"Have you ever been a scolder": 1}, "numerals": {"83": 1}}}, {"content": "84. Do you fear collisions or accidents when you travel by car or by plane?", "word_count": 23, "label": "", "sentence_structure": "Subject鈥搗erb鈥搊bject", "subjective_objective": {"answer": "Subjective", "rationale": "The fear of collision or accident is a personal feeling and fear of the patient and is subjective."}, "sentence_sentiment": {"answer": "Negative", "rationale": "Because the mood of \"a fear of collision or accident\" in the entry is negative."}, "language_style": {"answer": "Tactful and warm", "rationale": "The language style of the entry is euphemistic. Although asking the other side whether it has concerns, it is expressed in a gentle, polite manner, without direct statements or orders, making the questionnaire more friendly and interactive. At the same time, the question-taking approach, \"Will it crash or happen by accident?\", also gives the interviewee a sense of respect and understanding rather than being blamed or criticized."}, "time_dimension": {"answer": "Past", "rationale": "The entry asks if the person tested was afraid of a traffic accident or accident in his past experience and therefore determines the past."}, "sentence_type": "Interrogative sentence", "pos": {"nouns": {"Questionnaire": 1, "Entry": 1, "Time": 2, "you": 3, "- By car": 1, "On a plane": 1, "Out": 1, "I'm worried": 1, "It's gonna crash": 1, "or": 1, "Accident": 1}, "verbs": {"If": 1, "Yes": 2, "I'm worried": 1, "Collision": 1, "Out": 1, "Accident": 1}, "adverbs": {"Is it possible": 1}, "numerals": {"84": 1}, "classifiers": {"individual": 1}}}, {"content": "Are you a lover?", "word_count": 10, "label": "", "sentence_structure": "Subject鈥損redicate", "subjective_objective": {"answer": "Subjective", "rationale": "Because \"like to be in a relationship\" is an individual's subjective feeling and attitude, the problem is viewed from the patient's subjective perspective."}, "sentence_sentiment": {"answer": "Positive", "rationale": "Because the purpose of the article is positive, it is a positive expression of the quality of the human person."}, "language_style": {"answer": "Tactful and warm", "rationale": "The language style of the entry is more euphemistic, and it leads the interviewee to think and answer questions in a question-and-answer manner, rather than directly speaking or ordering."}, "time_dimension": {"answer": "Present", "rationale": "The description of \"a person in love\" also emphasizes the current character or tendency, further confirming the current attributes of the purpose of the article."}, "sentence_type": "Interrogative sentence", "pos": {"nouns": {"person": 1, "- To be with someone": 1}, "verbs": {"be": 1, "Love": 1}, "adjectives": {"I'm a lover": 1}, "numerals": {"85": 1}}}]}

{"questionnaire_title": "Summary screening scale for dementia (BSSD)", "questionnaire_category": "Behavioral questionnaire", "item_count": 30, "emotion_shift_overall": "No obvious trend", "language_style_overall": "Calm and direct", "items": [{"content": "1. What year is it?", "word_count": 6, "label": "", "sentence_structure": "Subject鈥損redicate", "subjective_objective": {"answer": "Objective", "rationale": "Because \"what year\" is a specific year, an objective fact."}, "sentence_sentiment": {"answer": "Neutral", "rationale": "Because the entry is merely a question of fact and does not involve any emotion, attitude or behaviour."}, "language_style": {"answer": "Calm and direct", "rationale": "The language style of the entry is relatively simple and straightforward, it is not used too often, nor does it display a strong emotional tone, which tends to present the problem in a factual and objective manner."}, "time_dimension": {"answer": "Present", "rationale": "The entry is asked about the current time, i.e. the year the person was responding was now or was the year of the past, and the judgement is therefore \"now\"."}, "sentence_type": "Interrogative sentence", "pos": {"nouns": {"Year": 1, "Questionnaire": 1}, "verbs": {"be": 1, "Analysis": 1, "Answer": 1}, "numerals": {"One": 1}, "pronouns": {"you": 1}}}, {"content": "2. What month is it now?", "word_count": 6, "label": "", "sentence_structure": "Subject鈥揷opula鈥損redicative", "subjective_objective": {"answer": "Objective", "rationale": "Because \"It's months now\" is an objective fact that does not require subjective judgement by the patient."}, "sentence_sentiment": {"answer": "Neutral", "rationale": "Since the entry is merely a question of the current month, it does not express any feelings, attitudes or behaviour and is therefore neutral."}, "language_style": {"answer": "Calm and direct", "rationale": "The language style of the entry is more direct and concise, and it does not use excessive rhetorical or emotional colours to express the problem, but is presented in an objective and clear manner."}, "time_dimension": {"answer": "Present", "rationale": "The entry is asked about the current time and is in the current time range."}, "sentence_type": "Interrogative sentence", "pos": {"nouns": {"Month": 1}, "verbs": {"be": 1}, "numerals": {"How many": 1}, "classifiers": {"individual": 1}}}, {"content": "3. What days are we in?", "word_count": 5, "label": "", "sentence_structure": "Subject鈥搗erb鈥揷omplement", "subjective_objective": {"answer": "Objective", "rationale": "Since time is an objective fact, it is not diverted by the subjective will of the human person."}, "sentence_sentiment": {"answer": "Neutral", "rationale": "Because the entry is asking about a fact, without any emotional colour."}, "language_style": {"answer": "Tactful and warm", "rationale": "The language style of the entry is euphemistic, as it uses a polite inquiry phrase rather than direct orders or statements, making the respondents more comfortable and willing to participate. At the same time, the issue is not overly emotional or subjective, but simply objectively asks the respondents about their current time and makes it easier to answer."}, "time_dimension": {"answer": "Present", "rationale": "By the phrase, \"it's a few days\" means the current time situation, so the basis of the judgement is \"now\"."}, "sentence_type": "Interrogative sentence", "pos": {"nouns": {"Questionnaire": 1, "Entry": 1, "Day": 1}, "verbs": {"be": 1, "have": 1, "Analysis": 1}, "numerals": {"How many": 1}, "pronouns": {"Present": 1}}}, {"content": "What day is it?", "word_count": 6, "label": "", "sentence_structure": "Subject鈥搗erb鈥揷omplement", "subjective_objective": {"answer": "Objective", "rationale": "Because the Sundays are an objective fact that does not depend on the will of the human being, there is no need to examine the subjective ideas of the patient."}, "sentence_sentiment": {"answer": "Neutral", "rationale": "Because the entry does not have any emotional color."}, "language_style": {"answer": "Calm and direct", "rationale": "The entry uses a direct, non-emotional language style, which is simple and clear enough to ask the interviewee for information that is currently on the week, without any qualifying language or emotional expression. Therefore, its language style should be described as \"cool and straight\"."}, "time_dimension": {"answer": "Present", "rationale": "The entry is asked about the current time, the current time when the person was responding, and is therefore described in the current time period."}, "sentence_type": "Interrogative sentence", "pos": {"nouns": {"Week": 1, "How many": 1}, "verbs": {"be": 1}}}, {"content": "What city is this?", "word_count": 7, "label": "", "sentence_structure": "Subject鈥損redicate", "subjective_objective": {"answer": "Objective", "rationale": "Since this is a matter of specific information, there is no subjective judgement or attitude on the part of the patient."}, "sentence_sentiment": {"answer": "Neutral", "rationale": "The entry is not an expression of any emotion, attitude or behaviour, but a question of the municipality or province in which the person interviewed is located."}, "language_style": {"answer": "Calm and direct", "rationale": "The language style of the entry is calm and straightforward. It asks the respondent in a concise and straightforward manner about the city or province, without any qualifier or emotional colour, and with a more neutral and objective tone."}, "time_dimension": {"answer": "Present", "rationale": "The entry used to be for information about the city or province where the person was tested, and did not address future or present behaviour or mentalities."}, "sentence_type": "Interrogative sentence", "pos": {"nouns": {"City": 1, "Province": 1}, "verbs": {"be": 1}, "numerals": {"5": 1}, "classifiers": {"individual": 1}, "pronouns": {"Here": 1}}}, {"content": "What area is this?", "word_count": 7, "label": "", "sentence_structure": "Subject鈥損redicate", "subjective_objective": {"answer": "Objective", "rationale": "This is a question of geographical location, which does not require subjective judgement or attitude on the part of the patient, but simply objective facts."}, "sentence_sentiment": {"answer": "Neutral", "rationale": "Because the entry is merely a question of geographic location and does not have any emotional colour."}, "language_style": {"answer": "Calm and direct", "rationale": "The entry is simple and clear, does not have too many emotional renderings or rhetorical techniques and is a more objective and calm expression."}, "time_dimension": {"answer": "Present", "rationale": "The entry asks for information about the location of the person currently tested, the keywords \"here\" and \"is\" indicating the behaviour being performed, and the \"area (county)\" is a specific question that requires the person tested to answer. So the temporal dimension is now judged."}, "sentence_type": "Interrogative sentence", "pos": {"nouns": {"Sector": 1, "County": 1}, "verbs": {"be": 1}, "numerals": {"6": 1}}}, {"content": "What street is this?", "word_count": 9, "label": "", "sentence_structure": "Subject鈥揷opula鈥損redicative", "subjective_objective": {"answer": "Objective", "rationale": "Because it is an objective fact of the street, not a subjective attitude or experience of the patient."}, "sentence_sentiment": {"answer": "Neutral", "rationale": "Because the entry is merely asking for names of streets or villages, it does not contain any emotional colours."}, "language_style": {"answer": "Calm and direct", "rationale": "The language style of the entry is relatively straightforward, without excessive rhetorical or emotional rendering, and with a sound and objective tone, with the main purpose of obtaining the required information."}, "time_dimension": {"answer": "Present", "rationale": "So the answer is \"now\"."}, "sentence_type": "Interrogative sentence", "pos": {"nouns": {"Street": 1, "Country": 1, "Town": 1}, "verbs": {"be": 1}, "numerals": {"What": 1}, "classifiers": {"individual": 1}}}, {"content": "What is this road?", "word_count": 7, "label": "", "sentence_structure": "Subject鈥搗erb鈥揷omplement", "subjective_objective": {"answer": "Objective", "rationale": "Because roads and villages are objective things, not subjective thoughts or feelings of patients."}, "sentence_sentiment": {"answer": "Neutral", "rationale": "Because the entry is merely asking for information about the location, it does not express any emotional colour."}, "language_style": {"answer": "Calm and direct", "rationale": "According to the information provided on the subject, the language style of the entry is objective and neutral, and there is no excessive emotional or rhetorical approach, and it can be judged as \"cool and direct\"."}, "time_dimension": {"answer": "Present", "rationale": "The entry is asked about the current location and the time dimension is judged to be now."}, "sentence_type": "Interrogative sentence", "pos": {"nouns": {"Road": 1, "Village": 1}, "verbs": {"be": 1}}}, {"content": "9. Take out five cents, please name it.", "word_count": 12, "label": "", "sentence_structure": "Subject鈥搗erb鈥搊bject", "subjective_objective": {"answer": "Subjective", "rationale": "Because the name of a coin is a subjective process of perception and expression, not an objective fact or behaviour. While the nickel is an objective thing, its perception and description depend on factors such as the experience, knowledge and language skills of individuals, which are subjective."}, "sentence_sentiment": {"answer": "Neutral", "rationale": "Because the entry is asked about the name of the coin and does not address positive or negative elements such as emotions, attitudes, behaviour, purpose or quality."}, "language_style": {"answer": "Calm and direct", "rationale": "The entry clearly requires the interviewee to perform specific operations in a concise manner, and the content of the questions is objective, not overly emotional or rhetorical."}, "time_dimension": {"answer": "Present", "rationale": "The entry now requires the respondent to perform specific acts in answering the question, i.e., observe and name a five-point coin, so the temporal dimension is now judged."}, "sentence_type": "Interrogative sentence", "pos": {"nouns": {"Five cents": 1, "Name": 1}, "verbs": {"Checkout": 1, "Say it": 1}, "numerals": {"Nine": 1}}}, {"content": "10. Remove the pen, please name it.", "word_count": 11, "label": "", "sentence_structure": "Subject鈥搗erb鈥揷omplement", "subjective_objective": {"answer": "Subjective", "rationale": "Because the problem is the patient ' s ability to name a pen, which is a subjective perception and expression."}, "sentence_sentiment": {"answer": "Neutral", "rationale": "Because the entry simply describes an act and does not express any emotion, attitude or evaluation."}, "language_style": {"answer": "Tactful and warm", "rationale": "The language style of the entry is more euphemistic, as it asks the interviewee in a polite, respectful and cooperative manner, rather than directly ordering or criticizing. Using the term \"please\" increases the comfort of the interviewee and encourages him to participate in the investigation. In addition, the questions in the entry are relatively simple and clear, avoiding complex terms or wording that may lead to misunderstandings."}, "time_dimension": {"answer": "Present", "rationale": "The entry requires the person tested to perform a task immediately and describe its results, so the time dimension is now judged."}, "sentence_type": "Interrogative sentence", "pos": {"nouns": {"Plugs": 1}, "verbs": {"Checkout": 1, "Please say it": 1}, "numerals": {"10": 1}}}, {"content": "11. Take out the key ring, please name it.", "word_count": 11, "label": "", "sentence_structure": "Subject鈥搗erb鈥揷omplement", "subjective_objective": {"answer": "Objective", "rationale": "Because it is a question of objective factuality that the person tested is required to describe the name of a specific object."}, "sentence_sentiment": {"answer": "Neutral", "rationale": "As the entry simply required the respondent to name an object, it did not express any emotional colour."}, "language_style": {"answer": "Calm and direct", "rationale": "The entry is made by specific action to guide respondents to the questions, without the use of excessive rhetoric or emotional language, which can be considered as a more direct language style."}, "time_dimension": {"answer": "Present", "rationale": "The entry asks the subject about the behaviour currently being performed, \"take out the key ring\" and \"name it\" are verbs of the current state of affairs, so the entry is asked about the present situation."}, "sentence_type": "Imperative sentence", "pos": {"nouns": {"Keyring": 1}, "verbs": {"Checkout": 1, "Please say it": 2, "Other": 1}, "numerals": {"11": 1, "Other": 1}}}, {"content": "Remove the items and ask 鈥渨hat you saw just now鈥?(pents).", "word_count": 18, "label": "", "sentence_structure": "Subject鈥搗erb鈥搊bject", "subjective_objective": {"answer": "Objective", "rationale": "Because it is an objective way of observing and asking patients what they see after they remove the items."}, "sentence_sentiment": {"answer": "Neutral", "rationale": ""}, "language_style": {"answer": "Calm and direct", "rationale": "The language style of the entry is relatively calm and objective, and it asks the interviewee directly, without too many rhetorical or emotional renderings."}, "time_dimension": {"answer": "Present", "rationale": "The keyword \"just\" and \"read\" have happened at some point in the past, but the question is now. So the time dimension of this article is now."}, "sentence_type": "Imperative sentence", "pos": {"nouns": {"Remove": 1, "Items": 1, "Just now": 1, "you": 1, "Yes": 1, "Which ones": 1, "Something": 2, "Five cents": 1}, "verbs": {"Remove": 1, "Question": 1, "Yes": 1}, "adverbs": {"Just now": 1}, "numerals": {"XII": 1, "Five points": 1}, "classifiers": {"individual": 1}, "pronouns": {"you": 1}}}, {"content": "Remove the items and ask 鈥渨hat have you seen just now鈥?(plugs)", "word_count": 17, "label": "", "sentence_structure": "Subject鈥搗erb鈥搊bject", "subjective_objective": {"answer": "Objective", "rationale": "Because it is an observational issue, it does not involve subjective feelings or attitudes of the patient."}, "sentence_sentiment": {"answer": "Neutral", "rationale": "Because the entry simply describes an act, it does not have a clear emotional, attitudinal or sexual orientation."}, "language_style": {"answer": "Calm and direct", "rationale": "This language style is more direct and clear, and there is no excessive emotional rendering or euphemism, so that the language style of the entry on the questionnaire can be judged as being straightforward and straightforward."}, "time_dimension": {"answer": "Present", "rationale": "The entry describes an operation conducted during the experiment and asks the respondent about the operation. As this is part of the ongoing experiment, the time dimension of the article is now."}, "sentence_type": "Imperative sentence", "pos": {"nouns": {"Items": 1, "Just now": 1, "Something": 1, "Plugs": 1}, "verbs": {"Remove": 1, "Look": 1, "Pass": 1}, "adverbs": {"Which ones": 1}, "numerals": {"13": 1}}}, {"content": "Remove the items and ask, \"What have you seen just now?\" (Keyring)", "word_count": 17, "label": "", "sentence_structure": "Subject鈥搗erb鈥搊bject", "subjective_objective": {"answer": "Objective", "rationale": "The problem is an objective one, as it is an examination of the memory of the patient of the objects he has seen before, not the subjective feelings or thoughts of the patient."}, "sentence_sentiment": {"answer": "Neutral", "rationale": "Because the entry was only a matter of sight before the person was questioned, it did not involve any emotional colour."}, "language_style": {"answer": "Calm and direct", "rationale": "The article is very concise and not used too much rhetorical or emotional rendering. It asks the interviewees whether they have noticed certain items and asks them for information in a direct and objective manner."}, "time_dimension": {"answer": "Present", "rationale": "The entry is used to operate the testee, i.e. to require the testee to remove some items and ask what they have seen. The time dimension is therefore now judged."}, "sentence_type": "Interrogative sentence", "pos": {"nouns": {"Items": 2, "Problem": 1, "Keyring": 1}, "verbs": {"Remove": 1, "Question": 1, "Yes": 1}, "numerals": {"14": 1}}}, {"content": "How much money do we have left?", "word_count": 10, "label": "", "sentence_structure": "Subject鈥搗erb鈥搊bject", "subjective_objective": {"answer": "Objective", "rationale": "Because the answer to this question requires mathematical calculations, it is considered from an objective perspective."}, "sentence_sentiment": {"answer": "Neutral", "rationale": "Because the entry is merely a description of a fact and does not involve any emotion, attitude or behaviour, etc."}, "language_style": {"answer": "Calm and direct", "rationale": "The entry is a specific mathematical calculation that does not involve emotions, attitudes or subjective judgements, but rather raises questions in a straightforward manner and awaits answers.\""}, "time_dimension": {"answer": "Past", "rationale": ""}, "sentence_type": "Interrogative sentence", "pos": {"nouns": {"A dollar": 1, "Seven minutes": 1, "How much": 1}, "verbs": {"Use it": 1, "Left": 1}, "numerals": {"15": 1, "1": 2, "7": 1}}}, {"content": "16. Plus seven points, equals what?", "word_count": 7, "label": "", "sentence_structure": "Subject鈥搗erb鈥搊bject", "subjective_objective": {"answer": "Objective", "rationale": "Because it's a mathematical question, not about patients' subjective attitudes or feelings. The answer is objective, regardless of how the patient answers it."}, "sentence_sentiment": {"answer": "Neutral", "rationale": "Because the entry is asking about a mathematical problem and has no obvious emotional colour."}, "language_style": {"answer": "Calm and direct", "rationale": "The entry uses a very direct, concise language style, without any symmetry or emotional modifications, and is straightforward in asking each other a mathematical question, which is more objective and cold."}, "time_dimension": {"answer": "Present", "rationale": "The questionnaire entry does not contain information about past, present or future time, and therefore does not allow for a time dimension to be judged. It simply raises a mathematical question about how the testee calculates the answer to \"16 + 7 =?\" So the answer should read: \"This entry is not a time dimension.\""}, "sentence_type": "Interrogative sentence", "pos": {"nouns": {"Grandpa": 2, "Add": 1, "Equal": 1, "How much": 1}, "verbs": {"And then": 1}, "numerals": {"16": 1, "7": 1}}}, {"content": "How much is the seven?", "word_count": 7, "label": "", "sentence_structure": "Subject鈥搗erb鈥揷omplement", "subjective_objective": {"answer": "Objective", "rationale": "Because it's mathematical, and it doesn't require a patient's subjective judgment."}, "sentence_sentiment": {"answer": "Neutral", "rationale": "Because the entry is a simple mathematical problem and does not have any emotional color."}, "language_style": {"answer": "Tactful and warm", "rationale": "The language style of the entry is more moderate and oriented. By using the expression \"plus\" it implies that the interviewees need to calculate or think and encourage them to give answers. This way of asking questions is not clearly prescriptive, but rather attempts to involve the interviewees, making them more comfortable and willing to answer questions."}, "time_dimension": {"answer": "Past", "rationale": "The entry is a simple mathematical calculation that is asked of the person tested, does not address the dimensions of time and therefore does not belong to any of the past, present or future."}, "sentence_type": "Interrogative sentence", "pos": {"nouns": {"min": 2, "Questionnaire Entry": 1}, "verbs": {"Add": 1, "Equal": 1}, "numerals": {"17": 1, "7": 1}}}, {"content": "18. Please use your right hand to get paper.", "word_count": 8, "label": "", "sentence_structure": "Subject鈥搗erb鈥揷omplement", "subjective_objective": {"answer": "Objective", "rationale": "Because the title requires that hand-held paper be an objective instruction of behaviour, it is not the patient ' s own subjective attitude or feelings."}, "sentence_sentiment": {"answer": "Neutral", "rationale": "Because the entry does not have any emotional color."}, "language_style": {"answer": "Calm and direct", "rationale": "The entry is in plain and simple language, is not subject to excessive rhetorical or emotional rendering, is more objective and direct. It simply sets out a motion requirement and expects the interviewee to operate as instructed, and the language style is a calm and straightforward type."}, "time_dimension": {"answer": "Present", "rationale": "Keywords include \"Please\" and \"Take Paper with Right Hand\" which indicate the behaviour instructions that are now being implemented."}, "sentence_type": "Interrogative sentence", "pos": {"nouns": {"Right hand": 1, "Paper": 1, "Remove": 1}, "verbs": {"Take it": 1, "Please": 1}, "adverbs": {"Please": 1}, "numerals": {"18": 1}}}, {"content": "19. Retangible paper", "word_count": 6, "label": "", "sentence_structure": "Subject鈥搗erb鈥搊bject", "subjective_objective": {"answer": "Subjective", "rationale": "Because it is a subjective act, it requires the patient to execute it and describe its process."}, "sentence_sentiment": {"answer": "Neutral", "rationale": "Because the entry does not have any emotional color."}, "language_style": {"answer": "Calm and direct", "rationale": "The entry clearly and clearly sets out a specific operational task, with no excessive emotional or rhetorical modifications, and is more straightforward and objective."}, "time_dimension": {"answer": "Present", "rationale": "The entry is a specific action that the testee is required to perform, and therefore it is a current state of affairs."}, "sentence_type": "Interrogative sentence", "pos": {"nouns": {"Paper": 1, "Debit": 2}, "verbs": {"Compose": 1}, "numerals": {"19": 1}}}, {"content": "20. Please place the paper on the table.", "word_count": 9, "label": "", "sentence_structure": "Subject鈥搗erb鈥揷omplement", "subjective_objective": {"answer": "Objective", "rationale": "Because \"Foot\" is an action, an act that can be seen directly by observers, an objective description."}, "sentence_sentiment": {"answer": "Neutral", "rationale": "Because the entry simply describes an action and does not involve any emotion or attitude."}, "language_style": {"answer": "Calm and direct", "rationale": "The language style of the entry is calm and straightforward, and it gives a clear and concise action order without any superfluous word-refuting or emotional color.\""}, "time_dimension": {"answer": "Present", "rationale": "In the past, the entry asked the testee whether he had done anything in the past to place paper on the table, and the time dimension was judged to be past."}, "sentence_type": "Imperative sentence", "pos": {"nouns": {"Paper": 1, "Table": 1}, "verbs": {"Fire": 1}}}, {"content": "21. Think again. What do you see?", "word_count": 17, "label": "", "sentence_structure": "Subject鈥搗erb鈥搊bject", "subjective_objective": {"answer": "Objective", "rationale": "Since a nickel is an objective object, it does not require subjective evaluation or interpretation by the patient."}, "sentence_sentiment": {"answer": "Neutral", "rationale": "Because the entry does not have any emotional color."}, "language_style": {"answer": "Tactful and warm", "rationale": "The entry is more personal and interactive when it is spoken in a gentle and inviting tone, rather than in direct statements or orders. At the same time, the tone of the questionnaire does not overemphasize or induce the subject to answer a particular answer, but provides an open and exploratory space for the respondent to think and answer."}, "time_dimension": {"answer": "Past", "rationale": "In conclusion, the time dimension of this questionnaire entry is the past."}, "sentence_type": "Imperative sentence", "pos": {"nouns": {"you": 1, "Something": 1, "Five cents": 1}, "verbs": {"Look": 1, "Yes": 1, "Again": 1}, "adverbs": {"Again": 1}, "numerals": {"21": 1}, "pronouns": {"What": 1}}}, {"content": "Just think again, what do you see?", "word_count": 16, "label": "", "sentence_structure": "Subject鈥搗erb鈥搊bject", "subjective_objective": {"answer": "Subjective", "rationale": "Because the pen that gives the patient a recollection of the pen is the patient's subjective experience and feelings."}, "sentence_sentiment": {"answer": "Neutral", "rationale": "Because the entry does not have any emotional color."}, "language_style": {"answer": "Calm and direct", "rationale": "The entry directly asks whether the person interviewed has been concerned or impressed with a particular object (plug). The language is simple and clear, and there are no excessive rhetorical or emotional renderings."}, "time_dimension": {"answer": "Present", "rationale": "The keyword \"think again\" indicates that the person tested is reminiscent of the past, while the verb \"sees\" uses the usual past pattern to indicate that the act occurred in the past. The entry therefore primarily asks what objects the person tested has seen in the past (plugs)."}, "sentence_type": "Imperative sentence", "pos": {"nouns": {"Something": 1, "Plugs": 1}, "verbs": {"Look": 1, "Yes": 1, "Jean": 1}, "numerals": {"22": 1}, "pronouns": {"you": 1}}}, {"content": "Think again. What do you see?", "word_count": 16, "label": "", "sentence_structure": "Subject鈥搗erb鈥搊bject", "subjective_objective": {"answer": "Objective", "rationale": "Because this is a question of asking the patient to recall what happened in the past, it does not involve the patient ' s subjective attitude or feelings."}, "sentence_sentiment": {"answer": "Neutral", "rationale": "Because the entry merely asked whether the person surveyed had ever seen an object, it did not express positive or negative emotional colours."}, "language_style": {"answer": "Tactful and warm", "rationale": "The language style of the entry is more euphemistic. It asks the respondents, in a gentle and inviting manner, whether they have seen a particular object at some point 鈥?the key ring 鈥?which makes the questionnaire more friendly and acceptable. At the same time, the expression is more personal and interactive."}, "time_dimension": {"answer": "Past", "rationale": "The entry asks the respondent about what he saw in the past (keyring), and the time dimension is therefore judged to be past."}, "sentence_type": "Imperative sentence", "pos": {"nouns": {"Questionnaire": 1, "Entry": 1, "Something": 1, "Keyring": 1}, "verbs": {"Jean": 1, "Look": 1, "Yes": 2, "Again": 1, "Pass": 1}, "adverbs": {"Please": 1, "One second": 1}, "numerals": {"23": 1}, "pronouns": {"you": 1}}}, {"content": "Take out the picture (Sun Nakayama or other celebrity) and ask, \"Whose picture is this?\"", "word_count": 21, "label": "", "sentence_structure": "Subject鈥搗erb鈥搊bject", "subjective_objective": {"answer": "Subjective", "rationale": "The subject is to examine the cognitive and judgement abilities of patients and to ask them about the person in the picture, which is based on his or her own experience, knowledge and observation, and is therefore viewed from a subjective perspective."}, "sentence_sentiment": {"answer": "Neutral", "rationale": "Because the purpose of the article is not expressed in terms of any positive or negative emotion, attitude, behaviour, purpose or quality. It is merely an inquiry into the person ' s perception and perception of a person."}, "language_style": {"answer": "Calm and direct", "rationale": "The entry is not based on excessive rhetorical or emotional rendering, and is more objective in tone. It asks the person in question about the identity of the person in the picture in a direct way, without attempting to direct the person in question鈥檚 response direction or emotional orientation through language. It is therefore considered to be a calm and straightforward language style."}, "time_dimension": {"answer": "Present", "rationale": "The entry is for the testee to determine the time dimension of his ability to identify Sun or other celebrities."}, "sentence_type": "Interrogative sentence", "pos": {"nouns": {"Picture": 1, "Sun Zhongshan": 1, "Other celebrities": 1, "- A celebrity picture": 1}, "verbs": {"Checkout": 1, "Look": 1, "be": 1, "Question": 1, "Please": 1}, "numerals": {"24": 1}, "classifiers": {"Chang": 1, "Like a piece": 1}, "pronouns": {"Who": 1}}}, {"content": "Take out the pictures (Mao Zedong or other celebrity) and ask, 鈥淲hose picture is this?鈥?, "word_count": 21, "label": "", "sentence_structure": "Subject鈥搗erb鈥搊bject", "subjective_objective": {"answer": "Subjective", "rationale": "Because the questions were about the perception and attitude of the person on the picture, the subject ' s subjective thoughts and experiences were examined."}, "sentence_sentiment": {"answer": "Neutral", "rationale": "Because the entry describes only an objective act and does not express any emotional colour."}, "language_style": {"answer": "Tactful and warm", "rationale": "When asking if the person interviewed knew the person in the picture, the entry used a more gentle, inviting tone, avoiding direct orders or forced answers. At the same time, the expressions \"Question\" and \"See\" also appeared more polite and respectful. These factors brought language styles closer to the warmer type."}, "time_dimension": {"answer": "Present", "rationale": "The entry is for the testee ' s cognitive and cognitive ability to view the picture, and the time dimension is now judged."}, "sentence_type": "Imperative sentence", "pos": {"nouns": {"Picture": 1, "Mao Zedong": 1, "Celebrity": 1, "Like a piece": 1}, "verbs": {"Checkout": 1, "Look": 1, "Question": 1}, "numerals": {"25": 1}, "classifiers": {"Chang": 1}}}, {"content": "26. Take out the pictures and let the subjects of the images be presented.", "word_count": 16, "label": "", "sentence_structure": "Subject鈥搗erb鈥搊bject", "subjective_objective": {"answer": "Objective", "rationale": "Because the subject is about the content of the responses of the subjects, which are based on their observation and understanding of the pictures, this is an objective examination."}, "sentence_sentiment": {"answer": "Neutral", "rationale": "Because the entry does not have any emotional color."}, "language_style": {"answer": "Calm and direct", "rationale": "The entry is relatively straightforward, concise and not over-modified or emotionally retrofitted, and is primarily designed to allow the subject to be described by the testee. Thus, the language style can be summarized as \"cool and direct\"."}, "time_dimension": {"answer": "Present", "rationale": "The entry describes the behaviour or state in progress, and the keywords \"take out\" and \"speak\" both indicate the action in progress and therefore judge it as now."}, "sentence_type": "Interrogative sentence", "pos": {"nouns": {"Picture": 1, "Tested": 1, "Theme": 1, "- An umbrella": 1}, "verbs": {"Checkout": 1, "Say it": 1}, "numerals": {"26": 1}}}, {"content": "27. Take out the pictures and let the subjects of the images be known.", "word_count": 16, "label": "", "sentence_structure": "Subject鈥搗erb鈥搊bject", "subjective_objective": {"answer": "Subjective", "rationale": "Because the theme of the map needs to be answered in the light of the patient's understanding and perception, it is subjective."}, "sentence_sentiment": {"answer": "Neutral", "rationale": "As the entry describes only an act or an operation, it does not contain any apparent emotion, attitude or evaluation."}, "language_style": {"answer": "Calm and direct", "rationale": "The entry describes the steps of the study in a concise and clear language, without using a rhetorical or emotional rendering, and with a more objective and calm tone."}, "time_dimension": {"answer": "Present", "rationale": "Depending on the time nature and behaviour description of the entry itself, it can be judged to be the current state of affairs. The output is therefore: \"Now\"."}, "sentence_type": "Interrogative sentence", "pos": {"nouns": {"Picture": 1, "Tested": 1, "Theme": 1, "Oil": 1}, "verbs": {"Checkout": 1, "Say it": 1, "Buy": 1}, "numerals": {"27": 1}, "classifiers": {"individual": 1}}}, {"content": "Who is our Prime Minister?", "word_count": 7, "label": "", "sentence_structure": "Subject鈥揷opula鈥損redicative", "subjective_objective": {"answer": "Objective", "rationale": "This is an objective fact and does not require examination from the patient ' s subjective point of view."}, "sentence_sentiment": {"answer": "Neutral", "rationale": "As the entry is asked about an objective fact, it does not contain any emotion, attitude or preference and is therefore neutral."}, "language_style": {"answer": "Calm and direct", "rationale": ""}, "time_dimension": {"answer": "Present", "rationale": "The keyword \"Who is the Prime Minister of our country\" is a question of current information and it is therefore possible to judge that the entry belongs to the present."}, "sentence_type": "Imperative sentence", "pos": {"nouns": {"My country": 1, "Prime Minister": 1}, "verbs": {"be": 1}, "numerals": {"28": 1}}}, {"content": "29. How many days a year?", "word_count": 6, "label": "", "sentence_structure": "Subject鈥損redicate", "subjective_objective": {"answer": "Objective", "rationale": "Because \"how many days a year\" is an objective fact, it does not depend on patient's subjective attitude or experience."}, "sentence_sentiment": {"answer": "Neutral", "rationale": "As the entry is an objective description, it does not express any feelings or attitudes."}, "language_style": {"answer": "Tactful and warm", "rationale": "The language style of the questionnaire entry is a warm and gentle type. It asks respondents in a gentle and inviting manner about the number of days in the year, avoiding the use of cold, direct or command language, which enhances interaction and personality."}, "time_dimension": {"answer": "Present", "rationale": "The entry is for the current perception of the testee and does not address past or future behaviour or mentality, so the temporal dimension is now judged."}, "sentence_type": "Imperative sentence", "pos": {"nouns": {"Year": 1, "Oh, my God": 1}, "verbs": {"have": 1}, "numerals": {"Twenty-nine": 1}}}, {"content": "30. New China was founded that year.", "word_count": 10, "label": "", "sentence_structure": "Subject鈥搗erb鈥搊bject", "subjective_objective": {"answer": "Objective", "rationale": "Because the title is asking about an objective historical fact."}, "sentence_sentiment": {"answer": "Neutral", "rationale": "Because the entry does not have any emotional color."}, "language_style": {"answer": "Calm and direct", "rationale": "The language style of the entry is more objective and direct, and there is no excessive emotional or rhetorical approach. It is of greater relevance and practical value to the respondents in a concise and clear manner."}, "time_dimension": {"answer": "Past", "rationale": "The entry is a question about the time when the testee was aware of a historical event (new China was founded) and the dimension of time is judged to be the past."}, "sentence_type": "Interrogative sentence", "pos": {"nouns": {"China": 1, "Year": 1, "Establishment": 1}, "verbs": {"be": 1}, "numerals": {"III": 1, "Ten": 1}, "classifiers": {"individual": 1}}}]}

{"questionnaire_title": "Anxious behaviour questionnaire", "questionnaire_category": "Mental health", "item_count": 20, "emotion_shift_overall": "From positive to negative", "language_style_overall": "Tactful and warm", "items": [{"content": "I'm happy.", "word_count": 5, "label": "", "sentence_structure": "Subject鈥揷opula鈥損redicative", "subjective_objective": {"answer": "Subjective", "rationale": "Because \"I am happy\" is the patient's personal feelings and attitude, which is subjective."}, "sentence_sentiment": {"answer": "Positive", "rationale": "Because \"happy\" is a positive emotional attitude."}, "language_style": {"answer": "Tactful and warm", "rationale": "The entry creates a warm and warm atmosphere, with a softer and warmer language style, by describing subjective feelings."}, "time_dimension": {"answer": "Present", "rationale": "The key word \"is\" indicates that it is a positive statement, and \"happy\" is an adjective that describes the feeling or emotional state of the dominant language, so it can be concluded that the main element of the entry is to ask about the current emotional feeling."}, "sentence_type": "Interrogative sentence", "pos": {"adjectives": {"Have a nice day": 1}, "pronouns": {"I": 1}}}, {"content": "I feel sensitive and uneasy.", "word_count": 10, "label": "", "sentence_structure": "Subject鈥揷opula鈥損redicative", "subjective_objective": {"answer": "Subjective", "rationale": "Because words like \"feel\" and \"felt\" indicate that it's the patient's subjective feeling."}, "sentence_sentiment": {"answer": "Negative", "rationale": "Because the emotional description of \"sensitized and uneasy\" is negative."}, "language_style": {"answer": "Calm and direct", "rationale": "The language style of the entry is more direct, with no excessive adoration or emotional rendering, but rather a direct statement of the psychological state that the person under investigation may have."}, "time_dimension": {"answer": "Present", "rationale": "The \"feel\" in the entry indicates the current experience, and \"sensitized and uneasy\" is also a description of the current state, and therefore is judged to be the present behavior or mentality.\""}, "sentence_type": "Interrogative sentence", "pos": {"nouns": {"You're a bit of a sane": 1, "Unsettled": 1}, "verbs": {"I feel": 1, "Allergies": 1, "Unsettled": 1}}}, {"content": "I feel satisfied.", "word_count": 7, "label": "", "sentence_structure": "Subject鈥揷opula鈥損redicative", "subjective_objective": {"answer": "Subjective", "rationale": "Because \"self-pleasure\" is a personal feeling or emotional experience that reflects patients' subjective perceptions and feelings."}, "sentence_sentiment": {"answer": "Positive", "rationale": "Because \"self-fulfilment\" is a positive emotional attitude, expressing personal satisfaction and satisfaction with their state."}, "language_style": {"answer": "Tactful and warm", "rationale": "Warm and warm."}, "time_dimension": {"answer": "Present", "rationale": "The entry describes the current self-perception of the testee, \"I feel\" which means that it is the present, so it can be judged by the time."}, "sentence_type": "Affirmative declarative sentence", "pos": {"nouns": {"Self": 1, "Satisfied": 1}, "verbs": {"I feel": 1}}}, {"content": "I wish I was as happy as anyone else.", "word_count": 11, "label": "", "sentence_structure": "Subject鈥搗erb鈥揷omplement", "subjective_objective": {"answer": "Subjective", "rationale": "Because hope is a term of subjectivity."}, "sentence_sentiment": {"answer": "Positive", "rationale": "Because the entry \"I hope to be as happy as anyone\" expresses a positive emotional attitude towards individual desires and pursuits of happiness."}, "language_style": {"answer": "Tactful and warm", "rationale": "The language style of the entry is more euphemistic, friendly and inclusive, expressing the desire and expectation for a certain emotion in the dominant language of \" hope\", while using the expression \"like others\" to reduce the psychological burden on the other and to avoid the use of too direct or harsh language, making it easier for the person under investigation to accept and be willing to participate in the investigation."}, "time_dimension": {"answer": "Present", "rationale": "The words \"hope\" and \"happiness like others\" in the entry suggest the views and expectations of the subjects now, so its time dimension is now."}, "sentence_type": "Imperative sentence", "pos": {"nouns": {"Questionnaire": 1, "Entry": 1, "Hope": 1, "others": 1, "Happy": 1}, "verbs": {"Yeah": 1, "That way": 1, "Hope": 1}, "adverbs": {"That's it": 1}, "numerals": {"4": 1}, "pronouns": {"I": 1}}}, {"content": "I feel like a loser.", "word_count": 8, "label": "", "sentence_structure": "Subject鈥揷opula鈥損redicative", "subjective_objective": {"answer": "Subjective", "rationale": "Because \"fear\" is a subjective feeling, and whether or not it is a failure is judged by objective circumstances. The entry therefore primarily looks at the patient's subjective feelings."}, "sentence_sentiment": {"answer": "Negative", "rationale": "Because the expression \"fear like a loser\" is negative, and it means negative assessment and low feelings."}, "language_style": {"answer": "Tactful and warm", "rationale": "The language style of the entry is euphemism. It asks each other, in a caring and understanding manner, whether they feel like losers, rather than directly blaming or criticizing each other. This expression is more caring and supportive and helps create a good atmosphere of communication."}, "time_dimension": {"answer": "Present", "rationale": "The entry uses the now completed time-style \"feel\" and represents the current emotional state and feelings of the person being tested, which is the judgement of the current time dimension."}, "sentence_type": "Negative declarative sentence", "pos": {"nouns": {"Questionnaire": 1, "Entry": 1, "person": 1, "Loser": 1}, "verbs": {"I feel": 1, "Yeah": 1, "be": 1}, "adjectives": {"individual": 1}, "numerals": {"5": 1}, "pronouns": {"I": 1}}}, {"content": "I feel calm.", "word_count": 6, "label": "", "sentence_structure": "Subject鈥揷opula鈥損redicative", "subjective_objective": {"answer": "Subjective", "rationale": "Because the word \"fear\" suggests that it is a subjective feeling or experience."}, "sentence_sentiment": {"answer": "Neutral", "rationale": "Because the entry does not have any emotional color."}, "language_style": {"answer": "Tactful and warm", "rationale": "The language style of the entry is more euphemistic and sensitive, not to use too many direct expressions, but to ask questions by directing the interviewee to remember his own feelings, which is more gentle and friendly. At the same time, the phrase \"I feel calm\" implies attention to the inner world of the interviewee, adding to the relative and interactive nature of the questionnaire."}, "time_dimension": {"answer": "Present", "rationale": "In the entry, both \"fear\" and \"quiet\" are terms describing the current state, with no clear indication of past or future. Thus, it can be judged that the entry is asking the testee about the current feeling, which is the current temporal dimension."}, "sentence_type": "Imperative sentence", "pos": {"nouns": {"Peace": 1}, "verbs": {"I feel": 1}}}, {"content": "I am 鈥渜uite, calm, calm鈥?", "word_count": 12, "label": "", "sentence_structure": "Subject鈥揷opula鈥損redicative", "subjective_objective": {"answer": "Subjective", "rationale": "Because emotional state is usually a subjective feeling and assessment of the individual."}, "sentence_sentiment": {"answer": "Positive", "rationale": "Because the words \"quiet, calm, calm\" in this entry are positive emotional descriptions."}, "language_style": {"answer": "Tactful and warm", "rationale": "The entry uses a more moderate description, which does not directly evaluate or judge the personality characteristics of the other party, but rather asks the respondent about the issue in a more objective and neutral way. Such expression is more acceptable to the interviewee and is willing to share his/her own thoughts."}, "time_dimension": {"answer": "Present", "rationale": "I hear \"I don't reveal my feelings\" often, which means that it's a description of the current state of the person, and \"quiet, calm, and calm\" that it's a description of the current state of affairs. So, this entry is an inquiry into the current character or emotional state of the person being tested, and the temporal dimension is now."}, "sentence_type": "Affirmative declarative sentence", "pos": {"verbs": {"be": 1}, "adjectives": {"Calm down": 2, "And calm": 1}}}, {"content": "I feel like I'm having a hard time over this.", "word_count": 11, "label": "", "sentence_structure": "Subject鈥揷opula鈥損redicative", "subjective_objective": {"answer": "Subjective", "rationale": "Because it is a subjective feeling or emotional experience, the ability to overcome it depends on the individual capacity and action of the patient. Thus, the purpose of this questionnaire is more to look at the patient鈥檚 psychological state and feelings from the subjective perspective."}, "sentence_sentiment": {"answer": "Negative", "rationale": "Because the attitude of \"difficultness is impossible\" in the entries is negative."}, "language_style": {"answer": "Tactful and warm", "rationale": "The language style of the entry is more euphemistic and warm. It uses the word \"fear\" to convey the emotional state of the other side without directly accusing or denying. This expression is more readily acceptable to the interviewee and is willing to answer the questions."}, "time_dimension": {"answer": "Present", "rationale": "Keywords \"fear,\" \"hard\" and \"unresolved\" point to the current psychological state of the subjects, reflecting the stress and distress they are experiencing."}, "sentence_type": "Negative declarative sentence", "pos": {"nouns": {"Difficulties": 1, "Stack": 1, "Cannot": 1, "Overcoming": 1}, "verbs": {"I feel": 1, "Done": 1, "be": 1}, "adverbs": {"Cannot": 1}, "numerals": {"8": 1}}}, {"content": "I'm worried too much about the irrelevant.", "word_count": 13, "label": "", "sentence_structure": "Subject鈥搗erb鈥搊bject", "subjective_objective": {"answer": "Subjective", "rationale": "Because excessive anxiety is the patient's own subjective feeling and thoughts."}, "sentence_sentiment": {"answer": "Negative", "rationale": "Because the \"excessive anxiety\" act in the entry is negative."}, "language_style": {"answer": "Calm and direct", "rationale": "The entry is a straightforward expression of the subject of asking the other party whether it is too concerned about matters of no importance, and is not based on too much rhetorical or emotional presentation, which is more objective and straightforward."}, "time_dimension": {"answer": "Present", "rationale": "The entry can be judged by the words \"excessive anxiety\" and \"involved matters\" in the sentence to be the current behaviour or mentality of the subject. Thus, the temporal dimension of the article is now."}, "sentence_type": "Negative declarative sentence", "pos": {"nouns": {"9": 1, "I": 1, "Yes": 1}, "verbs": {"I'm worried too much": 1}, "adjectives": {"It doesn't matter": 1}}}, {"content": "I'm happy.", "word_count": 5, "label": "", "sentence_structure": "Subject鈥揷opula鈥損redicative", "subjective_objective": {"answer": "Subjective", "rationale": "Because happiness is a subjective feeling."}, "sentence_sentiment": {"answer": "Positive", "rationale": "Because \"happy\" is a positive emotional word."}, "language_style": {"answer": "Tactful and warm", "rationale": "The language style of the entry is euphemism and warm. By asking the person in question about his emotional state at a given moment, he/she is given a light, friendly atmosphere in which to answer questions for better information and more realistic data. This mild and attractive expression increases the willingness of the person in question to participate in the questionnaire and to reflect their real feelings."}, "time_dimension": {"answer": "Present", "rationale": "The entry indicates the current emotional state of the testee, and therefore the temporal dimension is now judged."}, "sentence_type": "Affirmative declarative sentence", "pos": {"verbs": {"be": 1, "Happy": 1}, "adjectives": {"Happy": 1}, "pronouns": {"I": 1}}}, {"content": "I'm confused.", "word_count": 6, "label": "", "sentence_structure": "Subject鈥揷opula鈥損redicative", "subjective_objective": {"answer": "Subjective", "rationale": "Because \"disorder\" is a subjective feeling and experience, reflecting the mental and emotional state of the patient. Although this feeling may be caused by an objective situation (e.g. stress, disease, etc.), individual perceptions and reactions to it are subjective, so the entry in this questionnaire looks at the patient鈥檚 subjective feelings."}, "sentence_sentiment": {"answer": "Negative", "rationale": "Because \"disorder\" is a negative description, which means that the respondents are not in a good state of mind."}, "language_style": {"answer": "Tactful and warm", "rationale": "The entry leads the interviewee to reflect on his or her situation in a more moderate language style and avoids direct evaluation or stigmatization, expressing in a way that is more respectful of the interviewee ' s personal feelings and privacy."}, "time_dimension": {"answer": "Present", "rationale": "The keyword is \"me\" in the state of mind, which means what you feel and think now, so the temporal dimension is now."}, "sentence_type": "Negative declarative sentence", "pos": {"nouns": {"Ideas": 1, "Chaos": 1}, "verbs": {"be": 1}, "numerals": {"11": 1}}}, {"content": "I lack confidence.", "word_count": 5, "label": "", "sentence_structure": "Subject鈥揷opula鈥損redicative", "subjective_objective": {"answer": "Subjective", "rationale": "Because \"lack of confidence\" is a subjective feeling or perception, it is a patient's assessment and perception of his or her own and therefore is a subjective examination."}, "sentence_sentiment": {"answer": "Negative", "rationale": "Because the quality of \"lack of confidence\" in the entry is negative."}, "language_style": {"answer": "Tactful and warm", "rationale": "The entry uses euphemism to ask if the interviewee lacks confidence and does not directly criticize or criticize."}, "time_dimension": {"answer": "Present", "rationale": "So the answer is \"now\"."}, "sentence_type": "Negative declarative sentence", "pos": {"nouns": {"Confidence": 1}, "verbs": {"Lack": 1}}}, {"content": "I feel safe.", "word_count": 5, "label": "", "sentence_structure": "Subject鈥揷opula鈥損redicative", "subjective_objective": {"answer": "Subjective", "rationale": "Because feeling security is a subjective emotional experience of a human being."}, "sentence_sentiment": {"answer": "Positive", "rationale": "Because it expresses a positive feeling for the individual."}, "language_style": {"answer": "Tactful and warm", "rationale": "The entry uses a more moderate, intimate language style to ask the respondents about their security, without a strong language or command tone, making them more acceptable and willing to answer questions. At the same time, by using the expression \"I feel\" the person surveyed is given the initiative to give them more opportunities to participate and express themselves."}, "time_dimension": {"answer": "Present", "rationale": ""}, "sentence_type": "Affirmative declarative sentence", "pos": {"nouns": {"Questionnaire Entry": 1, "Clear": 1}, "verbs": {"I feel": 1}, "numerals": {"13": 1}}}, {"content": "I'm a quick judge.", "word_count": 7, "label": "", "sentence_structure": "Subject鈥揷opula鈥損redicative", "subjective_objective": {"answer": "Subjective", "rationale": "Because a decision is a subjective act and attitude."}, "sentence_sentiment": {"answer": "Positive", "rationale": "Because the \"decisive\" act in the entry is positive."}, "language_style": {"answer": "Calm and direct", "rationale": "The entry is based on a direct question, without too much qualifier or emotional colour, and is more relevant and objective.\""}, "time_dimension": {"answer": "Present", "rationale": "The entry indicates whether the testee is easy to make decisions in the current state, and therefore the time dimension is now judged."}, "sentence_type": "Affirmative declarative sentence", "pos": {"verbs": {"I": 1, "Make it": 1, "Decision": 1}, "adjectives": {"Easy": 1}}}, {"content": "I don't feel so good.", "word_count": 6, "label": "", "sentence_structure": "Subject鈥揷opula鈥損redicative", "subjective_objective": {"answer": "Subjective", "rationale": "Because \"feel\" is usually a subjective feeling and experience of the individual, not an objective fact."}, "sentence_sentiment": {"answer": "Negative", "rationale": "The article is a negative expression of sentiment."}, "language_style": {"answer": "Calm and direct", "rationale": "The entry uses a concise language to express the subject of the question whether the other party is not feeling well, does not use excessive rhetorical or emotional rendering, and is more objective in tone."}, "time_dimension": {"answer": "Present", "rationale": "According to the sentence entered, \"Esperate\" describes the current feelings or state of the person being tested, so it can be judged that the subject is to ask about the current behavior or mentality."}, "sentence_type": "Negative declarative sentence", "pos": {"nouns": {"I": 1, "Not good": 1}, "verbs": {"I feel": 1}, "numerals": {"15": 1}}}, {"content": "I'm satisfied.", "word_count": 5, "label": "", "sentence_structure": "Subject鈥揷opula鈥損redicative", "subjective_objective": {"answer": "Subjective", "rationale": "Because satisfaction is a subjective assessment, not an objective fact."}, "sentence_sentiment": {"answer": "Positive", "rationale": "Because \"satisfaction\" expresses a positive emotional attitude."}, "language_style": {"answer": "Tactful and warm", "rationale": ""}, "time_dimension": {"answer": "Present", "rationale": "The word in the entry is \"I'm content,\" which describes the current emotional state and feeling, so it's judged now."}, "sentence_type": "Affirmative declarative sentence", "pos": {"nouns": {"Questionnaire": 1, "Entry": 1, "Satisfied": 1}, "verbs": {"be": 1}}}, {"content": "I'm obsessed with some unimportant ideas.", "word_count": 14, "label": "", "sentence_structure": "Subject鈥搗erb鈥搊bject", "subjective_objective": {"answer": "Subjective", "rationale": "This is because it is the pain and distress that the patient feels and because it is the subjective feeling of the patient."}, "sentence_sentiment": {"answer": "Negative", "rationale": "Because the \"unimportant thought\" in the entry is negative."}, "language_style": {"answer": "Tactful and warm", "rationale": "The language style of the entry is relatively euphemism, caring, using words such as \"problem,\" giving people a sense of affection, without direct accusations or criticism. This expression makes the interviewee more comfortable and more easily promotes their real answers."}, "time_dimension": {"answer": "Present", "rationale": "The keyword \"I'm entangled by some unimportant ideas\" suggests that this is what the tester is going through right now, so the time dimension of the article is now."}, "sentence_type": "Negative declarative sentence", "pos": {"nouns": {"Problem": 2, "Questionnaire": 1, "Item": 1, "Thoughts": 1, "Trouble": 1}, "verbs": {"be": 1, "have": 1}, "adjectives": {"Important": 1}, "numerals": {"17": 1}, "pronouns": {"I": 1}}}, {"content": "I'm so depressed I can't get out.", "word_count": 9, "label": "", "sentence_structure": "Subject鈥揷opula鈥損redicative", "subjective_objective": {"answer": "Subjective", "rationale": "Because \"showing so depressed\" is patient subjective feeling and self-evaluation, and it is a subjective examination of patient emotional state."}, "sentence_sentiment": {"answer": "Negative", "rationale": "Because \"depression\" and \"inexact\" in the entries are both expressions of negative emotional state."}, "language_style": {"answer": "Calm and direct", "rationale": "The article is straightforward, not using too many rhetorical techniques or emotional renderings, and is more objective in tone. It asks whether the person surveyed feels helpless and difficult to escape when confronted with negative emotions, and does not attempt to alleviate the pain of the person under investigation in a moderate or comforting language."}, "time_dimension": {"answer": "Present", "rationale": "\"Now.\""}, "sentence_type": "Negative declarative sentence", "pos": {"nouns": {"18": 1, "Frustration": 1}, "verbs": {"Cannot": 1, "Get rid of it": 1}, "adjectives": {"I see": 1}}}, {"content": "I'm a stable man.", "word_count": 8, "label": "", "sentence_structure": "Subject鈥揷opula鈥損redicative", "subjective_objective": {"answer": "Subjective", "rationale": "Because \"stability\" is a personal characteristic of a human being, a subjective perception and self-evaluation."}, "sentence_sentiment": {"answer": "Positive", "rationale": "Because the quality of stability is usually considered positive."}, "language_style": {"answer": "Tactful and warm", "rationale": "The entry is more personal and interactive in a more euphemistic way in which the respondents are asked about their personality characteristics and are not directly judged or evaluated."}, "time_dimension": {"answer": "Present", "rationale": "The sentence contains a description of the character of the individual, \"I am a very stable person\" indicating that the tester thinks he is stable, and that description occurs at this moment or in the recent past."}, "sentence_type": "Affirmative declarative sentence", "pos": {"nouns": {"person": 1, "Steady": 1}, "verbs": {"be": 1}, "adjectives": {"very": 1, "Steady": 1}, "pronouns": {"I": 1}}}, {"content": "I think of things and interests that are now, and I get distracted and nervous.", "word_count": 23, "label": "", "sentence_structure": "Subject鈥揷opula鈥損redicative", "subjective_objective": {"answer": "Subjective", "rationale": "Because \"dry and nervous\" is the patient's subjective feeling."}, "sentence_sentiment": {"answer": "Negative", "rationale": "Because the words \"disturbed\" and \"stretch\" in the entries express negative emotions and attitudes."}, "language_style": {"answer": "Tactful and warm", "rationale": "Because the entry uses a soft, invitation-style tone for dialogue, it does not use excessive rhetorical or emotional rendering. At the same time, it is more objective in tone, aimed at understanding the emotional responses that respondents may have in the face of current events and interests, and it is more personal and interactive."}, "time_dimension": {"answer": "Present", "rationale": "The entry describes the person's immediate feelings and state, and the \"I think\" in it indicates that this is an ongoing act, and the \"will become\" suggests that the psychological reaction is immediate. So the time dimension of the entry is now."}, "sentence_type": "Exclamatory sentence", "pos": {"nouns": {"Things": 1, "Interest": 1}, "verbs": {"It's gonna be": 1, "I'm upset": 1, "Nervous": 1, "I thought": 1}, "adjectives": {"Current": 1}, "adverbs": {"One": 1}, "numerals": {"20": 1}, "pronouns": {"I": 1}}}]}

{"questionnaire_title": "Self-esteem scale (SES)", "questionnaire_category": "Self-cognition", "item_count": 10, "emotion_shift_overall": "From positive to negative", "language_style_overall": "Tactful and warm", "items": [{"content": "I feel like I'm worth it, at least on the same level as everyone else.", "word_count": 25, "label": "", "sentence_structure": "Subject鈥揷opula鈥損redicative", "subjective_objective": {"answer": "Subjective", "rationale": "The sentence expresses the patient's subjective feelings and self-evaluation. The patient considers himself \"worthy\" and at the same level as others, a subjective state of self-awareness and emotion, and therefore falls within the scope of subjective examination."}, "sentence_sentiment": {"answer": "Positive", "rationale": "Because it expresses the recognition of the individual ' s self-worth and is a positive emotional attitude."}, "language_style": {"answer": "Tactful and warm", "rationale": "The language style of the entry is euphemistic. It uses positive expressions to ask respondents about their sense of value and to engage in a gentle and inviting tone of dialogue rather than direct statements or orders, which are more personal and interactive."}, "time_dimension": {"answer": "Present", "rationale": "The keyword \"felt\" and \"is\" means that it is a description of the current self-awareness, so the time dimension is now."}, "sentence_type": "Affirmative declarative sentence", "pos": {"nouns": {"Questionnaire Entry": 1, "person": 2, "Horizontal": 1}, "verbs": {"I feel": 1, "be": 1, "have": 1, "in": 1, "Same": 1}, "adjectives": {"It's worth it": 1}, "numerals": {"One": 1}, "classifiers": {"individual": 2, "person": 1}, "pronouns": {"I": 1, "Other": 1}}}, {"content": "I feel like I have a lot of good qualities.", "word_count": 12, "label": "", "sentence_structure": "Subject鈥揷opula鈥損redicative", "subjective_objective": {"answer": "Subjective", "rationale": "Because \"fear\" is the patient's subjective feeling and perception, and \"have many good qualities\" is part of the patient's self-evaluation, it is viewed from the patient's subjective point of view."}, "sentence_sentiment": {"answer": "Positive", "rationale": "Because the \"many good quality\" in the entry is a positive, positive emotional attitude."}, "language_style": {"answer": "Tactful and warm", "rationale": "The entry is in an encouraging language to guide the person under investigation to reflect on his or her strengths and strengths, is more accommodative and helps to stimulate the positive feelings of the person under investigation. At the same time, the purpose of the article is expressed in a more open and inclusive manner, without placing too much burden or pressure on the person under investigation."}, "time_dimension": {"answer": "Present", "rationale": "The keyword \"fear\" indicates that it is a description of the state of the moment, and \"there are many good qualities\" is the testee's assessment of himself, so the entry relates to the present behavior or mentality."}, "sentence_type": "Affirmative declarative sentence", "pos": {"nouns": {"2": 1, "self": 1, "Quality": 1}, "verbs": {"I feel": 1}, "adjectives": {"Many": 1, "Okay": 1}}}, {"content": "In the end, I tend to think of myself as a loser.", "word_count": 18, "label": "", "sentence_structure": "Subject鈥搗erb鈥搊bject", "subjective_objective": {"answer": "Subjective", "rationale": "Since the \"prefers to be\" is a subjective view or view of a person, the answer to the article is subjective."}, "sentence_sentiment": {"answer": "Negative", "rationale": "Because the idea in the entry that I think I'm a loser is negative."}, "language_style": {"answer": "Calm and direct", "rationale": "The entry is straightforward, without too much rhetoric or emotional rendering, and directly presents the views and self-perceptions of the subjects surveyed, with language styles that tend to be calm and direct."}, "time_dimension": {"answer": "Present", "rationale": "According to the phrase \"prefers to\" and \"yes\", it can be judged as the subjective perception and attitude of the subject at present, and thus the temporal dimension is now."}, "sentence_type": "Affirmative declarative sentence", "pos": {"nouns": {"In the end": 1, "self": 1, "Loser": 1}, "verbs": {"Preferative": 1, "I think": 1, "be": 1}, "adjectives": {"Successor": 1}, "numerals": {"3": 1}}}, {"content": "I can do things like most people.", "word_count": 14, "label": "", "sentence_structure": "Subject鈥搗erb鈥揷omplement", "subjective_objective": {"answer": "Subjective", "rationale": "This sentence is intended to convey self-evaluation and subjective feelings of patients and is therefore subjective."}, "sentence_sentiment": {"answer": "Positive", "rationale": "Because \"can do things like most people\" in the entry is a positive attitude that is a recognition of their abilities."}, "language_style": {"answer": "Tactful and warm", "rationale": "The language style of the entry is more euphemistic, because it uses positive language and expresses a positive upward attitude. Moreover, it is more friendly and friendly, with no excessive criticism or criticism, but rather leads the respondents to the questions in a way that encourages and supports them."}, "time_dimension": {"answer": "Present", "rationale": "The terms \"can\" and \"like most people\" indicate the current capabilities and state of the person being tested, so the temporal dimension of the article is now judged."}, "sentence_type": "Affirmative declarative sentence", "pos": {"nouns": {"Yes": 1, "person": 1}, "verbs": {"Do it": 1}, "adjectives": {"More": 1}, "adverbs": {"Yeah": 1, "Yes": 2}, "numerals": {"4": 1}, "pronouns": {"I": 1, "Large": 1}}}, {"content": "I don't feel so much pride in myself.", "word_count": 14, "label": "", "sentence_structure": "Subject鈥揷opula鈥損redicative", "subjective_objective": {"answer": "Subjective", "rationale": "Because there is little room for pride in self-evaluation by patients, it is in the realm of subjective perceptions."}, "sentence_sentiment": {"answer": "Negative", "rationale": "Because the expression \"no place to be proud of\" in the entry is negative."}, "language_style": {"answer": "Tactful and warm", "rationale": "This euphemism helps to create a good atmosphere of interaction and trust."}, "time_dimension": {"answer": "Past", "rationale": "The entry uses the first-person proxy \"me\" and \"me\" of the past, which expresses the testee's assessment of his situation over the past period, and therefore the temporal dimension is a matter of the past."}, "sentence_type": "Negative declarative sentence", "pos": {"nouns": {"Local": 1, "Proud": 1}, "verbs": {"I feel": 1, "It's worth it": 1, "More": 1}, "adverbs": {"not": 1}, "numerals": {"5": 1}, "pronouns": {"I": 1}}}, {"content": "I'm positive about myself.", "word_count": 9, "label": "", "sentence_structure": "Subject鈥搗erb鈥揷omplement", "subjective_objective": {"answer": "Subjective", "rationale": "For it is a subjective attitude or belief."}, "sentence_sentiment": {"answer": "Positive", "rationale": "Because the \"yes\" attitude in the entry is positive."}, "language_style": {"answer": "Tactful and warm", "rationale": "The expression in which the entry is used in an encouraging and supportive manner to ask the respondent about his or her own views rather than directly pointing to his or her shortcomings or shortcomings is more conducive to stimulating the person ' s motivation and self-confidence."}, "time_dimension": {"answer": "Present", "rationale": "The entry expresses the current attitude and conviction of the testee that he/she believes he/she is positive, that this attitude may be long-standing and may affect his/her behaviour and decision-making. The temporal dimension of the entry is therefore judged as \"now\"."}, "sentence_type": "Affirmative declarative sentence", "pos": {"nouns": {"self": 1, "Positive": 1}, "verbs": {"Hold": 1}, "adverbs": {"to": 1, "Land": 1}, "numerals": {"6": 1}}}, {"content": "I'm satisfied with myself, in general.", "word_count": 12, "label": "", "sentence_structure": "Subject鈥揷opula鈥損redicative", "subjective_objective": {"answer": "Subjective", "rationale": "This phrase describes patients' subjective assessment and perception of themselves."}, "sentence_sentiment": {"answer": "Positive", "rationale": "Because \"satisfaction\" in the entry is a positive emotional attitude."}, "language_style": {"answer": "Tactful and warm", "rationale": "The language style of the entry is more euphemistic and gentle, and gives a warm and comfortable feeling by using the terms \"general\" and \"satisfaction\" to express concern and respect for the person under investigation, without directly giving an assessment or criticism."}, "time_dimension": {"answer": "Present", "rationale": "The sentence describes the current self-perception, and the keyword \"generally\" and \"satisfaction\" indicates that it is an assessment of the current situation, and therefore the time dimension is now judged."}, "sentence_type": "Affirmative declarative sentence", "pos": {"verbs": {"Satisfied": 1, "be": 1}, "adjectives": {"Overall": 1}, "adverbs": {"7": 1}, "pronouns": {"I": 1}}}, {"content": "I wish I could earn more respect for myself.", "word_count": 14, "label": "", "sentence_structure": "Subject鈥搗erb鈥搊bject", "subjective_objective": {"answer": "Subjective", "rationale": "Because hope and winning more respect are subjective attitudes and aspirations of patients."}, "sentence_sentiment": {"answer": "Positive", "rationale": "Because the attitude of \"want to win respect\" in the entry is positive."}, "language_style": {"answer": "Tactful and warm", "rationale": "The entry uses the words that are so emotional, the language style is more euphemistic, and attempts to guide the interviewee to express his inner feelings and needs rather than to direct orders or assessments. At the same time, the theme \"Better respect for himself\" is more emotional and personal, and requires a more moderate dialogue. So I think the language style of the questionnaire entry is more euphemistic."}, "time_dimension": {"answer": "Future", "rationale": "The keywords are \"hope\" and \"cap\" and they all point to situations that have not yet occurred and that may arise in the future, so the entry is a judgement on the time dimension of the future."}, "sentence_type": "Imperative sentence", "pos": {"nouns": {"8": 1, "Hope": 1, "I": 2, "self": 1, "Winning": 1, "Respect": 1}, "adjectives": {"Yes": 1, "More": 1}, "adverbs": {"More": 1}, "numerals": {"8": 1}, "pronouns": {"I": 2}}}, {"content": "I do feel useless all the time.", "word_count": 14, "label": "", "sentence_structure": "Subject鈥揷opula鈥損redicative", "subjective_objective": {"answer": "Subjective", "rationale": "Because \"I feel useless\" is the patient's subjective feeling and evaluation."}, "sentence_sentiment": {"answer": "Negative", "rationale": "Because the expression \"no use\" in the entry is negative."}, "language_style": {"answer": "Calm and direct", "rationale": "The entry asks the interviewees in a direct manner whether they often feel useless, with a calm and objective tone, and without excessive rhetorical or emotional publicity."}, "time_dimension": {"answer": "Present", "rationale": "The entry describes the current state of the person being tested, and \"I do feel often\" is \"if often\" indicating that this emotional experience is happening regularly."}, "sentence_type": "Negative declarative sentence", "pos": {"nouns": {"9": 1, "I": 2, "self": 1, "Useful": 1}, "verbs": {"be": 1, "I feel": 1, "No, I don't": 1}, "adverbs": {"Often": 1}, "numerals": {"9": 1}, "pronouns": {"self": 1}}}, {"content": "I always think I'm nothing.", "word_count": 11, "label": "", "sentence_structure": "Subject鈥搗erb鈥揷omplement", "subjective_objective": {"answer": "Subjective", "rationale": "The question entry is viewed from a subjective perspective because it is the patient's personal perception and attitude."}, "sentence_sentiment": {"answer": "Negative", "rationale": "Because the idea and attitude of feeling useless is negative."}, "language_style": {"answer": "Calm and direct", "rationale": "The entry asks the interviewees in a direct and clear manner whether they often feel they are self-denial, with objectivity and without excessive rhetoric.鈥?}, "time_dimension": {"answer": "Past", "rationale": ""}, "sentence_type": "Imperative sentence", "pos": {"nouns": {"Questionnaire": 1, "Entry": 1}, "verbs": {"I think": 1, "have": 1, "be": 1, "Location": 1}, "adjectives": {"Nothing": 1}, "adverbs": {"Often": 1}, "numerals": {"10": 1}}}]}

{"questionnaire_title": "Cannot initialise Evolution's mail component.", "questionnaire_category": "Mental health", "item_count": 25, "emotion_shift_overall": "From negative to positive", "language_style_overall": "Tactful and warm", "items": [{"content": "1. People are too easily directed.", "word_count": 9, "label": "", "sentence_structure": "Subject鈥搗erb鈥揷omplement", "subjective_objective": {"answer": "Subjective", "rationale": "Because \"too easy to be directed\" is a subjective assessment or view, describing people's perception of their own behaviour or abilities. It is not an objective reality, but a subjective judgement based on individual experience, perceptions and feelings. The entry is therefore viewed from a subjective perspective."}, "sentence_sentiment": {"answer": "Negative", "rationale": "Because the expression \"too easy to command\" in the entry has negative emotional connotations."}, "language_style": {"answer": "Tactful and warm", "rationale": "This language is more appropriate for psychological counselling or exploratory dialogue, and can better guide the other to the discussion."}, "time_dimension": {"answer": "Present", "rationale": "The entry does not refer to the past time reference, nor does it contain a clear future time reference, so it can be judged to describe the current phenomenon or state, and is now."}, "sentence_type": "Negative declarative sentence", "pos": {"nouns": {"People": 1, "Command": 1}, "verbs": {"It's too easy": 1, "By": 1}, "adjectives": {"Easy": 1}, "adverbs": {"Too much": 1}}}, {"content": "I like people I know.", "word_count": 10, "label": "", "sentence_structure": "Subject鈥搗erb鈥搊bject", "subjective_objective": {"answer": "Subjective", "rationale": "Like being patient subjective."}, "sentence_sentiment": {"answer": "Positive", "rationale": "Because the \"like\" attitude in the entry is positive."}, "language_style": {"answer": "Tactful and warm", "rationale": "The positive expression of the title, which uses positive tone to describe the subject of people who like to know themselves, makes it easier to resonate and to identify and therefore to be classified as a soft and warm language style."}, "time_dimension": {"answer": "Past", "rationale": "The entry describes the degree of interest of the person being tested in the people they know, does not address the time dimension of the past, present or future, and is therefore judged neutral."}, "sentence_type": "Affirmative declarative sentence", "pos": {"nouns": {"People": 1, "person": 1}, "verbs": {"Yeah": 1, "Roger that": 1}, "pronouns": {"I": 1}}}, {"content": "3. Today ' s moral standards are too low.", "word_count": 12, "label": "", "sentence_structure": "Subject鈥揷opula鈥損redicative", "subjective_objective": {"answer": "Subjective", "rationale": "Because \"the moral standards of people today are too low\" is a subjective assessment that reflects the view and assessment of the present social moral climate by the interviewees."}, "sentence_sentiment": {"answer": "Negative", "rationale": "Because the idea of \"moral low\" in the entries is negative."}, "language_style": {"answer": "Calm and direct", "rationale": "The language style of the entry is more straightforward, with no excessive accoutrements and euphemisms, and it is more serious and harsh. It is a direct expression of the current moral standards of society, which is a strong sense of shock and criticism."}, "time_dimension": {"answer": "Present", "rationale": "The entry \"Today\" means that in this age, \"the moral standards of people are too low\" are a description and an assessment of the current situation, so the temporal dimension is now."}, "sentence_type": "Negative declarative sentence", "pos": {"nouns": {"People": 1, "Moral standards": 1, "Too low": 1}, "verbs": {"Today": 1, "I think": 1, "Horizontal": 1}, "adjectives": {"Low": 1, "Too high": 1}, "adverbs": {"Yes": 1, "Too much": 1}}}, {"content": "4. Most people are quite arrogant and never face their shortcomings.", "word_count": 18, "label": "", "sentence_structure": "Subject鈥搗erb鈥搊bject", "subjective_objective": {"answer": "Subjective", "rationale": "Because the sentence describes subjective attitudes and perceptions of the people, it is not an objective fact or result of the conduct."}, "sentence_sentiment": {"answer": "Negative", "rationale": "Because \"self-defeating\" and \"not looking at your own shortcomings\" in the entries are negative emotional."}, "language_style": {"answer": "Calm and direct", "rationale": "The language style of the entry is more straightforward, with no excessive rhetorical or emotional colours. It directly expresses the view of the investigator that most people are self-righteous and unwilling to admit their shortcomings. Thus, the language style is \"cool and straight.\""}, "time_dimension": {"answer": "Present", "rationale": "The entry describes a general phenomenon or view that many people consider themselves superior and ignore their own shortcomings, a view that is common in present-day societies and does not clearly point to past or future situations. Thus, the temporal dimension is now."}, "sentence_type": "Affirmative declarative sentence", "pos": {"nouns": {"Most people": 1, "self": 1, "Disadvantages": 1}, "verbs": {"Pretty much": 1, "He's a great man": 1, "Face-to-face": 1}, "adjectives": {"He's a great man": 1}, "adverbs": {"Pretty much": 1}, "numerals": {"4": 1}, "pronouns": {"self": 1}}}, {"content": "I can live with almost all kinds of people.", "word_count": 15, "label": "", "sentence_structure": "Subject鈥揷opula鈥損redicative", "subjective_objective": {"answer": "Subjective", "rationale": "Because \"favourable with all types of people\" is a personal subjective feeling and attitude. Although it is a description of an act or ability, it is based on the individual's attitude and emotional experience. So, it is a subjective entry."}, "sentence_sentiment": {"answer": "Positive", "rationale": "Because the expression \"almost having a pleasant time with all types of people\" in the entry is positive and indicates that the person under investigation has a better social ability."}, "language_style": {"answer": "Tactful and warm", "rationale": "The entry is more personal and interactive by speaking in a gentle and inviting manner, rather than by direct statement or order. At the same time, by using the term \"almost\" it avoids overconfidence or complacency, which is more modest and objective."}, "time_dimension": {"answer": "Present", "rationale": "The keyword \"almost\" and \"happy living\" suggests that it is a description or evaluation of the current state, and therefore the judgement is now."}, "sentence_type": "Exclamatory sentence", "pos": {"nouns": {"person": 1, "Type": 1, "Have a nice day": 1}, "verbs": {"Get along": 1}, "adjectives": {"Almost": 1}}}, {"content": "6. Today, people seem to talk about movies and television.", "word_count": 21, "label": "", "sentence_structure": "Subject鈥搗erb鈥搊bject", "subjective_objective": {"answer": "Subjective", "rationale": "This is a subjective feeling and view of the patient, not an objective fact."}, "sentence_sentiment": {"answer": "Neutral", "rationale": "Because the entry does not express positive or negative emotional sentiments. It simply describes a phenomenon and does not involve perceptions or evaluations of it."}, "language_style": {"answer": "Calm and direct", "rationale": "The basis for the judgement: the article is expressed in a more concise and clear manner, without too much rhetorical or emotional presentation, and in a more objective tone. At the same time, the entry directly describes the fact that \"the people are talking about things like movies and television today.\" There are no too many questions or leading language, which is a calm and direct language style."}, "time_dimension": {"answer": "Present", "rationale": "The keywords \"Today\" and \"Looks like\" indicate that it is a statement describing current social phenomena or trends. And, \"Saying\" is an ongoing move, pointing to the present. So the sentence should be judged as \"now\"."}, "sentence_type": "Affirmative declarative sentence", "pos": {"nouns": {"People": 1, "Film": 1, "Television": 1, "Yes": 1}, "verbs": {"Talk": 1, "be": 1, "Category": 1}, "adjectives": {"Today": 1}, "numerals": {"6": 1, "One": 2}, "pronouns": {"Here": 1}}}, {"content": "7. Success depends on the door rather than on knowledge.", "word_count": 16, "label": "", "sentence_structure": "Subject鈥搗erb鈥搊bject", "subjective_objective": {"answer": "Subjective", "rationale": "Because \"doorway\" and \"knowledge\" are terms that describe successful ways, and the individual values \"doorway\" and \"knowledge\" differently, the entry looks at the subjective views of the interviewee."}, "sentence_sentiment": {"answer": "Negative", "rationale": "Because the word \"doorway\" in this entry is derogatory and implies that success depends on interpersonal relationships and contexts, while neglecting individual efforts and capabilities."}, "language_style": {"answer": "Calm and direct", "rationale": "The entry is plain and simple, does not have too many emotional renderings or rhetorical techniques, and conveys the view in the form of a statement of facts."}, "time_dimension": {"answer": "Present", "rationale": "The sentence describes a general view or belief that people rely more on \"door\" than \"knowledge\" for success. It is a view of social phenomena, so the time dimension is now judged."}, "sentence_type": "Affirmative declarative sentence", "pos": {"nouns": {"People": 1, "Success": 1, "The doorway": 1, "Knowledge": 1}, "verbs": {"Access": 1, "Shit": 1, "be": 2, "And": 1, "not": 1, "of": 3, "No, it's not": 1, "It's": 1, "Knowledge": 1}, "adverbs": {"Both": 1}, "numerals": {"7": 1}, "pronouns": {"People": 1, "of": 3}}}, {"content": "8. Once you start helping someone, he will despise you.", "word_count": 15, "label": "", "sentence_structure": "Subject鈥搗erb鈥搊bject", "subjective_objective": {"answer": "Subjective", "rationale": "This statement, which expresses the subjective feelings and views of the patient, does not involve specific objective facts or behaviour."}, "sentence_sentiment": {"answer": "Negative", "rationale": "Because the act of \"negative\" in the entry is negative."}, "language_style": {"answer": "Tactful and warm", "rationale": "The entry is more personal and interactive when it is presented in a gentle and inviting tone, rather than in direct statements or orders. At the same time, the conditional phrase \"as soon as\" is used to allow respondents to think about issues from multiple perspectives and avoid an extreme view."}, "time_dimension": {"answer": "Present", "rationale": "The entry is an enquiry into the testee ' s expectations of the other ' s perception and attitude towards himself, and therefore there is no specific time reference point for the temporal dimension and it is not possible to determine whether the past, present or future behaviour or mentality is present."}, "sentence_type": "Negative declarative sentence", "pos": {"nouns": {"Help": 1, "Someone": 1}, "verbs": {"Start": 1, "Yes": 1, "Disgrace": 1}, "adverbs": {"鈾?Once 鈾?: 1}, "numerals": {"8": 1}, "pronouns": {"you": 1}}}, {"content": "People are too self-centred.", "word_count": 8, "label": "", "sentence_structure": "Subject鈥揷opula鈥損redicative", "subjective_objective": {"answer": "Subjective", "rationale": "Because \"self-centred\" is subjective."}, "sentence_sentiment": {"answer": "Negative", "rationale": "Because \"self-centre\" is usually seen as a negative personality."}, "language_style": {"answer": "Tactful and warm", "rationale": ""}, "time_dimension": {"answer": "Present", "rationale": ""}, "sentence_type": "Negative declarative sentence", "pos": {"nouns": {"People": 1, "Self-centred": 1}, "adjectives": {"Too much": 1}}}, {"content": "10. People are always unhappy and constantly looking for new things.", "word_count": 16, "label": "", "sentence_structure": "Subject鈥損redicate", "subjective_objective": {"answer": "Subjective", "rationale": "Because \"unsatisfied and constantly searching for new things\" is a subjective idea and attitude, not an objective reality or fact."}, "sentence_sentiment": {"answer": "Positive", "rationale": "Because the \"Remaining Search\" act in the entry is positive."}, "language_style": {"answer": "Tactful and warm", "rationale": "The language style of this questionnaire entry is euphemism. It is more intimate and interactive in a gentle and inviting tone, rather than in a direct statement or order. At the same time, the sentence contains a certain emotional element, implying a psychological state of constant pursuit of new things, making the whole question-and-answer process more natural."}, "time_dimension": {"answer": "Present", "rationale": "The entry now describes a widespread phenomenon, namely, the pursuit of new things, which does not have a clear temporal dimension, and therefore it is now judged."}, "sentence_type": "Affirmative declarative sentence", "pos": {"nouns": {"person": 2, "Something new": 1}, "verbs": {"Always": 1, "Unsatisfactory": 1, "Search": 1}, "adverbs": {"and": 1, "Land": 1}, "numerals": {"10": 1}, "pronouns": {"they": 1}}}, {"content": "11. Many people are intolerable.", "word_count": 10, "label": "", "sentence_structure": "Subject鈥搗erb鈥揷omplement", "subjective_objective": {"answer": "Subjective", "rationale": "The subject is to ask patients about their attitudes towards others, that is, subjective feelings and perceptions."}, "sentence_sentiment": {"answer": "Negative", "rationale": "Because the entry describes \"many people are intolerable\" and that sentiment is negative."}, "language_style": {"answer": "Calm and direct", "rationale": "The language style of the entry is harsh, it portrays differences and unacceptable behaviour in a negative manner, and it is more direct, sharp and less euphemistic."}, "time_dimension": {"answer": "Present", "rationale": "The entry does not have a clear time reference, but describes a current feeling or experience, so the temporal dimension is now judged."}, "sentence_type": "Negative declarative sentence", "pos": {"nouns": {"person": 1}, "verbs": {"have": 1, "You're": 1, "Cannot": 1, "Tolerance": 1}, "adjectives": {"Many": 1}, "numerals": {"11": 1}}}, {"content": "12. If you do something of your own will, you can hurt some people.", "word_count": 22, "label": "", "sentence_structure": "Subject鈥搗erb鈥搊bject", "subjective_objective": {"answer": "Subjective", "rationale": "Because this is based on the patient鈥檚 subjective perception and understanding that it is a subjective judgement and concern that others may be harmed if they act on their own will. While this concern may be based on reality, it is still subjective in itself."}, "sentence_sentiment": {"answer": "Negative", "rationale": "Because the \"harming someone\" act in the entry is negative."}, "language_style": {"answer": "Calm and direct", "rationale": "The entry is presented directly to the respondent, asking him how much consideration he or she has for the possible effects of his or her actions."}, "time_dimension": {"answer": "Past", "rationale": "The entry describes a scenario and asks how the testee may act in such a case, judging by the time dimension of the past."}, "sentence_type": "Affirmative declarative sentence", "pos": {"nouns": {"Yes": 1, "person": 1}, "verbs": {"Do it": 1, "have": 1, "Hurt": 1}, "adverbs": {"If": 1, "Just": 1, "Maybe": 1}, "numerals": {"12": 1}, "pronouns": {"you": 1, "self": 1}}}, {"content": "13. One really needs a strong, intelligent leader.", "word_count": 16, "label": "", "sentence_structure": "Subject鈥揷opula鈥損redicative", "subjective_objective": {"answer": "Subjective", "rationale": "Because this is the opinion and opinion of the interviewees that \"people need tough, smart leaders.\""}, "sentence_sentiment": {"answer": "Positive", "rationale": "Because \"hard\" and \"smart\" are positive adjectives, which describe the characteristics of leaders and express a positive attitude."}, "language_style": {"answer": "Calm and direct", "rationale": "The entry is a direct indication of whether the respondents want to know whether they think people need a strong, intelligent leader, and the language style is more concise, and there is no excessive rhetorical or emotional overtook. So I think the language style of the purpose is straightforward and calm."}, "time_dimension": {"answer": "Future", "rationale": "The keyword \"do need\" indicates that it is a description of the future vision or ideal state, while \"hard, smart leaders\" also refers to what may happen to the future. Thus, the time dimension of the entry is judged to be the future."}, "sentence_type": "Affirmative declarative sentence", "pos": {"nouns": {"People": 1, "Leader": 2}, "verbs": {"Yes": 1, "be": 1}, "adjectives": {"Hard": 1, "Smart": 1}, "numerals": {"13": 1}, "pronouns": {"One": 2}}}, {"content": "14. When I was alone and away from the crowd, I appreciated myself most.", "word_count": 18, "label": "", "sentence_structure": "Subject鈥揷opula鈥損redicative", "subjective_objective": {"answer": "Subjective", "rationale": "This is a self-evaluation and subjective feeling of the patient."}, "sentence_sentiment": {"answer": "Neutral", "rationale": "Because the entry does not have any obvious emotional colour."}, "language_style": {"answer": "Tactful and warm", "rationale": "This entry uses a soft language style, does not direct the other party to order or ask for answers, but rather directs the other party to express its views and feelings in a way that is more personal and interactive and that encourages and affirms."}, "time_dimension": {"answer": "Present", "rationale": "The entry asks the testees about their current or recent behaviour and feelings, especially when they are alone. Although it does not explicitly refer to past or future time indicators, its content is more in favour of current state and emotional experience. Thus, in terms of time dimension, the subject of the article is \"now\"."}, "sentence_type": "Affirmative declarative sentence", "pos": {"nouns": {"Population": 1, "self": 2}, "verbs": {"I like it": 1, "Off": 1, "Single": 1, "Far": 1}, "adjectives": {"One": 1, "Lonely": 1}, "numerals": {"14": 1}, "pronouns": {"I": 2}}}, {"content": "I wish people were more honest with you.", "word_count": 13, "label": "", "sentence_structure": "Subject鈥搗erb鈥揷omplement", "subjective_objective": {"answer": "Subjective", "rationale": "Because \"favorite\" is a subjective emotion and expectation that reflects the individual feelings of the patient about the honesty of others."}, "sentence_sentiment": {"answer": "Negative", "rationale": "Because the attitude of \"wanting someone to be honest\" is based on a feeling of dissatisfaction with the current level of honesty and a desire to change, with some negative emotional colour."}, "language_style": {"answer": "Tactful and warm", "rationale": "The language style of the entry is more euphemistic, and it resonates with the other side by expressing a desire or desire, rather than directly accusing or ordering. This expression is more likely to bring the person under investigation closer, and helps to create a good atmosphere of communication. At the same time, the expression \"more honest\" is relatively mild, avoiding an overly emotional tone."}, "time_dimension": {"answer": "Future", "rationale": "The entry expresses a desire or desire for the future, and the keyword is \"hope\", which points to situations that have not yet occurred and that may arise in the future."}, "sentence_type": "Exclamatory sentence", "pos": {"nouns": {"person": 1, "Honesty": 1}, "verbs": {"Hope": 1, "to": 1, "More": 1, "Some": 1}, "adjectives": {"True": 1}, "adverbs": {"Some": 1}, "numerals": {"15": 1}, "pronouns": {"you": 1}}}, {"content": "I like to be with a lot of people.", "word_count": 10, "label": "", "sentence_structure": "Subject鈥搗erb鈥揷omplement", "subjective_objective": {"answer": "Subjective", "rationale": "I like being patient subjective."}, "sentence_sentiment": {"answer": "Positive", "rationale": "Because the attitude of \"likes to be with a lot of people\" in the entry is positive."}, "language_style": {"answer": "Tactful and warm", "rationale": "Warm and warm."}, "time_dimension": {"answer": "Present", "rationale": ""}, "sentence_type": "Affirmative declarative sentence", "pos": {"nouns": {"I": 1, "person": 1}, "verbs": {"Yeah": 1, "in": 1, "Together": 1}, "adverbs": {"very": 1}, "numerals": {"A lot": 1}}}, {"content": "17. In my experience, human beings are rather stubborn and irrational animals.", "word_count": 20, "label": "", "sentence_structure": "Subject鈥揷opula鈥損redicative", "subjective_objective": {"answer": "Subjective", "rationale": "Because \"human stubbornness and lack of reason\" is a view or assessment that reflects the author's own subjective views and perceptions."}, "sentence_sentiment": {"answer": "Negative", "rationale": "Because the words \"dulgent\" and \"incomprehensible\" in the entries are negative."}, "language_style": {"answer": "Calm and direct", "rationale": "The entry presents its views in an objective and calm manner, does not add excessive emotional colours or rhetorical techniques, and therefore it can be judged as being straightforward in its language style."}, "time_dimension": {"answer": "Past", "rationale": "The phrase \"based on my experience\" shows that this is a description of past observations or experiences, and therefore the temporal dimension is a past."}, "sentence_type": "Exclamatory sentence", "pos": {"nouns": {"Experience": 1, "person": 2, "Animals": 1}, "verbs": {"be": 1, "Obstinate": 1, "Lack": 1, "Reason": 1, "have": 1}, "adjectives": {"Pretty much": 1, "Obstinate": 1, "Lack": 1, "Reason": 1}, "numerals": {"17": 1}, "pronouns": {"I": 1}}}, {"content": "I can be happy with people who have different values from myself.", "word_count": 22, "label": "", "sentence_structure": "Subject鈥揷opula鈥損redicative", "subjective_objective": {"answer": "Subjective", "rationale": "Because \"happy\" is a subjective experience and feeling, the questionnaire entry is viewed from the patient's subjective perspective."}, "sentence_sentiment": {"answer": "Positive", "rationale": "Because the \"can have fun\" behavior and attitude in the entry is positive."}, "language_style": {"answer": "Tactful and warm", "rationale": "The entry is more personal and interactive in its expression of a soft, invitation-style conversation rather than a direct statement or order. At the same time, it implies an implication that the interviewee would answer yes, making the questionnaire more friendly and attractive."}, "time_dimension": {"answer": "Future", "rationale": "The entry deals with visions and expectations for the future, and the keywords are \"with\" and \"with pleasure\" , all pointing to situations that have not yet happened and that may occur in the future. Moreover, \"with people who are different from their own values\" suggests that they may happen in the future."}, "sentence_type": "Affirmative declarative sentence", "pos": {"nouns": {"Questionnaire": 1, "Entry": 1, "person": 1, "Values": 1, "Have a nice day": 1}, "verbs": {"Call": 1, "in": 1, "Together": 1, "Time": 1, "Yes": 1, "I feel": 1}, "adjectives": {"Different": 1}, "pronouns": {"I": 1}}}, {"content": "19. Everyone wanted to be good.", "word_count": 7, "label": "", "sentence_structure": "Subject鈥揷opula鈥損redicative", "subjective_objective": {"answer": "Subjective", "rationale": "Because \"want to be good\" is the patient's personal conviction and expectation, reflecting their subjective attitude and values."}, "sentence_sentiment": {"answer": "Positive", "rationale": "Because the purpose of the article is positive, it expresses expectations and aspirations for good."}, "language_style": {"answer": "Tactful and warm", "rationale": "The language style of the entry is more euphemistic and implicit, and it uses an encouraging tone to express universal desires and expectations. At the same time, it is not expressed in a strong tone or in a command style that makes people feel friendly and comfortable and can easily trigger the affection and resonance of the other."}, "time_dimension": {"answer": "Present", "rationale": "The entry expresses a general desire or view that good deeds are widely desired. It does not address the time dimension of the past or the future, but rather reflects current societal attitudes or values."}, "sentence_type": "Affirmative declarative sentence", "pos": {"nouns": {"Everyone": 1, "Good man": 1}, "verbs": {"Yes": 1, "Do it": 1}}}, {"content": "20. The general population is not very satisfied with itself.", "word_count": 12, "label": "", "sentence_structure": "Subject鈥揷opula鈥損redicative", "subjective_objective": {"answer": "Subjective", "rationale": "This is a self-evaluation of patients and a reflection of subjective perceptions."}, "sentence_sentiment": {"answer": "Negative", "rationale": "Because the \"not very satisfied\" attitude in the entry is negative."}, "language_style": {"answer": "Tactful and warm", "rationale": "The entry uses implicit, euphemistic expressions rather than outright denial or accusations, so that respondents can avoid harm or over-responsible in answering questions. At the same time, it gives people a space for reflection and self-reflection, encouraging them to face their shortcomings honestly, rather than simply avoiding or denying them. Such language styles are more humane and warm."}, "time_dimension": {"answer": "Present", "rationale": "The keywords \"general person\" and \"not very satisfied with himself\" all point to a question of describing the current state, and therefore the judgement is \"now\"."}, "sentence_type": "Negative declarative sentence", "pos": {"nouns": {"General": 1, "person": 1, "self": 1}, "verbs": {"to": 1, "Not": 1, "Full": 1, "Meaning": 1}, "adjectives": {"Tens": 1}, "adverbs": {"and": 1}}}, {"content": "I am being harsh.", "word_count": 8, "label": "", "sentence_structure": "Subject鈥揷opula鈥損redicative", "subjective_objective": {"answer": "Subjective", "rationale": "The attitude towards the interviewees is a subjective experience felt by the interviewees."}, "sentence_sentiment": {"answer": "Negative", "rationale": "Because the phrase \"people are very hard on me\" in the entry is derogatory and expresses a negative emotional attitude."}, "language_style": {"answer": "Calm and direct", "rationale": "The entry expresses the views of the other side in a direct tone of presentation, is not overly pedantic or emotional and is of a calm and direct language style."}, "time_dimension": {"answer": "Past", "rationale": "The entry describes a fact or state that has happened, namely, \"I'm being very hard on people\", and it can be judged to be in the past."}, "sentence_type": "Negative declarative sentence", "pos": {"verbs": {"People": 1, "to": 1, "I": 2, "Pretty much": 1, "I'm tough": 1}, "adjectives": {"I'm tough": 1}, "pronouns": {"I": 2}}}, {"content": "I feel 鈥渞emaining鈥?as if people were not willing to keep me by their side.", "word_count": 21, "label": "", "sentence_structure": "Subject鈥揷opula鈥損redicative", "subjective_objective": {"answer": "Subjective", "rationale": "Because feeling left behind and people don't want me to be around them is the patient's subjective feeling and idea."}, "sentence_sentiment": {"answer": "Negative", "rationale": "Because the expression \"felt left over\" in the entry indicates that the interviewee feels alone and excluded, which is a negative emotion. And \"people don't want me to be around them\" is a negative expression that implies that others don't like themselves. So the emotional color of the questionnaire is negative."}, "language_style": {"answer": "Tactful and warm", "rationale": ""}, "time_dimension": {"answer": "Present", "rationale": ""}, "sentence_type": "Negative declarative sentence", "pos": {"nouns": {"Questionnaire": 1, "Entry": 1, "person": 1, "they": 1}, "verbs": {"I feel": 1, "Remaining": 1, "Yes": 1, "- Wait": 1}, "adjectives": {"Remaining": 1, "not": 1, "Yes": 1}, "adverbs": {"It looks like": 1}, "classifiers": {"individual": 1}, "pronouns": {"I": 1}}}, {"content": "3. There seems to be respect for my views on the matter.", "word_count": 13, "label": "", "sentence_structure": "Subject鈥揷opula鈥損redicative", "subjective_objective": {"answer": "Subjective", "rationale": "Because respect is a subjective perception and evaluation of what the patient sees in the eyes of others."}, "sentence_sentiment": {"answer": "Positive", "rationale": "Because the word \"respect\" in the entry is a positive emotional expression of the positive attitude towards the author."}, "language_style": {"answer": "Tactful and warm", "rationale": "The entry uses a more subjective expression, \"people seem to respect\" , which is more euphemismal and euphemistic. At the same time, \"view\" is a relatively vague term, which does not clearly indicate what is at stake, and is therefore more in keeping with the euphemism of the language."}, "time_dimension": {"answer": "Present", "rationale": ""}, "sentence_type": "Affirmative declarative sentence", "pos": {"nouns": {"People": 1, "Observations": 1}, "verbs": {"Seems like": 1, "Respect": 1, "to": 1, "Do it": 1}, "adjectives": {"of": 2, "I": 1, "Things": 1}, "numerals": {"3": 1}, "pronouns": {"Here": 1}}}, {"content": "I'm a great fan of people.", "word_count": 8, "label": "", "sentence_structure": "Subject鈥揷opula鈥損redicative", "subjective_objective": {"answer": "Subjective", "rationale": "Because it is a subjective feeling and assessment, different people may have different views and feelings about the same thing. The description of the questionnaire is subjective."}, "sentence_sentiment": {"answer": "Positive", "rationale": "Because the word \"likes\" in the entries has a positive emotional tendency."}, "language_style": {"answer": "Tactful and warm", "rationale": "The entry describes in a positive and positive manner the characteristics or behaviour that the person under investigation may have, the temperature of the language and the absence of too direct or personal opinions. At the same time, the word \"seemingly\" also reflects a euphemism that avoids stress or discomfort."}, "time_dimension": {"answer": "Present", "rationale": ""}, "sentence_type": "Affirmative declarative sentence", "pos": {"verbs": {"Yeah": 1}, "adjectives": {"very": 1}, "adverbs": {"Seems like": 1}, "pronouns": {"I": 1}}}, {"content": "5. Most seem to understand how I feel about things.", "word_count": 14, "label": "", "sentence_structure": "Subject鈥揷opula鈥損redicative", "subjective_objective": {"answer": "Subjective", "rationale": "Because \"know your feelings\" is a subjective judgement that involves patient's subjective assessment and perception of the views of others."}, "sentence_sentiment": {"answer": "Neutral", "rationale": "Because the entry does not have any emotional color."}, "language_style": {"answer": "Tactful and warm", "rationale": "The language style of the entry is more euphemistic, not too direct or too strong, but rather a gentle, question-and-answer approach. At the same time, the term \"most people\" is also inclusive and understanding, which makes people feel more friendly and comfortable."}, "time_dimension": {"answer": "Present", "rationale": "The entry asks the testee's perception and understanding of his emotional expression, the keywords being \"most\" , \"seemingly understanding\" and \"favouring about things\", which indicate that the testee has a degree of awareness and understanding of his emotional expression, and therefore the temporal dimension is now judged."}, "sentence_type": "Affirmative declarative sentence", "pos": {"nouns": {"person": 1, "Things": 1}, "verbs": {"Roger that": 1}, "adjectives": {"Most": 1, "to": 1}, "adverbs": {"Seems like": 1}, "numerals": {"5": 1}, "classifiers": {"individual": 1}, "pronouns": {"I": 1}}}]}

{"questionnaire_title": "Table of mutually adjusted quantities", "questionnaire_category": "Mental health", "item_count": 11, "emotion_shift_overall": "From positive to negative", "language_style_overall": "Tactful and warm", "items": [{"content": "I have often taken the initiative in sexual activities.", "word_count": 11, "label": "", "sentence_structure": "Subject鈥搗erb鈥搊bject", "subjective_objective": {"answer": "Subjective", "rationale": "Because \"active action often takes place in sexual activity\" is an expression of personal behaviour and attitudes, reflecting the subjective feelings and wishes of the patient."}, "sentence_sentiment": {"answer": "Positive", "rationale": "Because the \"sexual activity takes the initiative\" in the entry is positive."}, "language_style": {"answer": "Calm and direct", "rationale": "The language style of the entry is so calm and straightforward that it explicitly asks the respondents whether they are often active in their sexual activities, without using excessive rhetorical or emotional accents and being more objective. The advantage of this style is to avoid making the interviewee feel offended or uncomfortable, and to be more in line with the professionalism and objectivity of the questionnaire survey."}, "time_dimension": {"answer": "Past", "rationale": "\"At times\" is a customary act, and \"active\" acts are, or will be, possible in the past, now or in the future, so the entry relates to both past, present and future acts."}, "sentence_type": "Affirmative declarative sentence", "pos": {"nouns": {"Sexual activity": 1, "Initiatives": 1}, "verbs": {"Adoption": 1, "be": 1}, "adverbs": {"Often": 1}, "pronouns": {"I": 1}}}, {"content": "2. If my sexual relations are not satisfactory, I have no power to improve the situation.", "word_count": 24, "label": "", "sentence_structure": "Subject鈥搗erb鈥搊bject", "subjective_objective": {"answer": "Subjective", "rationale": "The sentence is an expression of the patient's subjective feelings and views."}, "sentence_sentiment": {"answer": "Negative", "rationale": "Because the attitude in the entry \"to do nothing to improve the situation\" is negative."}, "language_style": {"answer": "Calm and direct", "rationale": "The entry is more neutral and straightforward in its language, using more objective expressions, without excessive emotional modification or rendering."}, "time_dimension": {"answer": "Present", "rationale": "Basis for judgement: The keyword \"if\" and \"if there is nothing to do to improve the situation\" expresses hypothetical scenarios and possible outcomes. This suggests that the issue is not just about past experience, but about the testee's perception of what might happen in the future and his ability to anticipate."}, "sentence_type": "Negative declarative sentence", "pos": {"nouns": {"Sexual relations": 1, "Status": 1}, "verbs": {"Satisfied": 1, "Improvement": 1, "Yes, there's nothing I can do": 1}, "adjectives": {"- I'm sorry": 1}, "adverbs": {"not": 1}, "pronouns": {"I": 1}}}, {"content": "3. I have maintained the frequency of my sexual relations with partners that I wish to have.", "word_count": 17, "label": "", "sentence_structure": "Subject鈥搗erb鈥揷omplement", "subjective_objective": {"answer": "Subjective", "rationale": "This is because it involves the personal expectations and feelings of the patient and is subjective."}, "sentence_sentiment": {"answer": "Positive", "rationale": "Because the description of \"maintain the frequency I want\" in the entry is positive."}, "language_style": {"answer": "Calm and direct", "rationale": "The entry describes the sexual relationship of the subject with his partner in a concise and direct language, with no excessive emotional or rhetorical modification, and with a more calm and direct language style."}, "time_dimension": {"answer": "Present", "rationale": "The word \"maintain\" in this entry indicates that it is a description of the current state, while \"the frequency I want\" indicates that it involves personal expectations or targets. The question is therefore whether the present sexual relationship of the testee with his partner is in line with the expected frequency, and the time dimension is now judged."}, "sentence_type": "Affirmative declarative sentence", "pos": {"nouns": {"Questionnaire": 1, "Entry": 1, "I": 2, "Partner": 1, "Sexual relations": 1, "Hope": 1, "Frequency": 1}, "verbs": {"Hold on": 1, "I'm hoping": 1}, "adverbs": {"Keep it": 1, "I wish": 1}, "numerals": {"3": 1}}}, {"content": "4. I have a sexual intention, which enables me ... to have a pleasant sexual experience with my partner.", "word_count": 27, "label": "", "sentence_structure": "Subject鈥搗erb鈥搊bject", "subjective_objective": {"answer": "Subjective", "rationale": "Because \"I have a intention to have sex\" and \"it makes me have a good sexual experience with my partner\" are subjective thoughts and wishes of patients."}, "sentence_sentiment": {"answer": "Negative", "rationale": "Since this entry implies sexual intent, which may be considered immoral or irresponsible, the emotional colour of expression is negative."}, "language_style": {"answer": "Tactful and warm", "rationale": "The language style of the entry is more euphemistic and sensitive to each other鈥檚 feelings and needs. Using a presentation like 鈥淚 have鈥?is more proactive and open, and it emphasizes sharing a good sexual experience with a partner rather than directly pursuing personal satisfaction, which reflects respect for and appreciation of the relationship and a more moderate language style."}, "time_dimension": {"answer": "Present", "rationale": ""}, "sentence_type": "Affirmative declarative sentence", "pos": {"nouns": {"Entry": 1, "Sexual exposure": 1, "I'm going to": 1, "Partner": 1, "Happy sex": 1}, "verbs": {"have": 1, "鈾?And I'm gonna be 鈾?: 1, "Generate": 1, "Sex": 1}, "adjectives": {"Sex is good": 1}, "numerals": {"4": 1, "One": 2}, "pronouns": {"I": 1}}}, {"content": "I find it difficult for my partner to do what makes me feel good in sexual intercourse.", "word_count": 25, "label": "", "sentence_structure": "Subject鈥搗erb鈥搊bject", "subjective_objective": {"answer": "Subjective", "rationale": "Because \"I feel very hard to make my partner do what makes me feel good in sex\" is a patient's description of his feelings, and it's about his subjective experience and emotional reaction."}, "sentence_sentiment": {"answer": "Negative", "rationale": "Because it expresses a sense of difficulty and frustration, it is a negative emotional tendency."}, "language_style": {"answer": "Tactful and warm", "rationale": "The entry is more euphemistic and respectful of each other鈥檚 feelings, using subjective expressions like 鈥淚 feel鈥?to give people a sense of calmer, better understanding and support, rather than hard-line accusations or direct criticism. At the same time, the issue is expressed more implicitly and privately, avoiding topics that could give rise to embarrassment or conflict."}, "time_dimension": {"answer": "Present", "rationale": "The entry describes the experience and experience of the subjects at present, and concerns their emotional and sexual life, so the temporal dimension is now judged."}, "sentence_type": "Negative declarative sentence", "pos": {"nouns": {"I": 1, "Partner": 1, "Fuck": 1, "Yes": 1}, "verbs": {"I feel": 1, "Jean": 1, "Do it": 1, "鈾?And I'm gonna be 鈾?: 1, "Feel": 1}, "adjectives": {"Wonderful": 1}, "numerals": {"5": 1}}}, {"content": "6. I feel that sexual contact between me and my partner is usually earlier than I wish.", "word_count": 24, "label": "", "sentence_structure": "Subject鈥搗erb鈥搊bject", "subjective_objective": {"answer": "Subjective", "rationale": "\"I think\" means it's a subjective description, that is, the patient's personal feeling. So the question entry is subjective."}, "sentence_sentiment": {"answer": "Negative", "rationale": "Because the \"sexual contact is usually sooner than I want to end\" behavior and attitude in the entry are negative."}, "language_style": {"answer": "Tactful and warm", "rationale": "The entry is more personal and interactive in its expression of a soft, invitation-like tone of dialogue rather than a direct statement or order. At the same time, by using the euphemism of \"I hope\" it avoids making the respondents feel blamed or criticized, thereby increasing the acceptance of the questionnaire."}, "time_dimension": {"answer": "Present", "rationale": "Now the entry describes the experience and feelings of the testee, \"Feeling\" and \"sexual contact is usually sooner than I want to end\" are the expression of the current state of affairs, and therefore the judgment is now."}, "sentence_type": "Negative declarative sentence", "pos": {"nouns": {"I": 2, "Partner": 1, "Sex": 1, "Exposure": 1, "End": 1}, "verbs": {"I think": 1, "have": 1, "Earlier than": 1, "Hope": 1}, "adverbs": {"Usually": 1}, "numerals": {"6": 1, "One": 2}, "classifiers": {"individual": 1}}}, {"content": "7. When I have no interest in sexual activity, I can easily refuse my partner ' s sexual invitation.", "word_count": 25, "label": "", "sentence_structure": "Subject鈥搗erb鈥搊bject", "subjective_objective": {"answer": "Subjective", "rationale": "Because the ideas and attitudes of \"not interested in me\" and \"not able to say it\" are subjective views and feelings of patients."}, "sentence_sentiment": {"answer": "Positive", "rationale": "Because the act of \"receiving my partner's sexual invitation\" in the entry is positive as it means that individuals have the right to choose and reject."}, "language_style": {"answer": "Calm and direct", "rationale": "The entry is presented in direct and concise language, and is not used in over-modified or implied language, so that its language style can be considered as being straightforward and calm."}, "time_dimension": {"answer": "Present", "rationale": "The word \"capable\" in the sentence denotes the ability or state of the person speaking, in a given situation, the act or psychological state, i.e., the ability to refuse a partner ' s sexual invitation when there is no sexual interest. Since there is no explicit time word or other keyword in the sentence to indicate the dimension of time, it is possible to judge that the time of the entry is present."}, "sentence_type": "Affirmative declarative sentence", "pos": {"nouns": {"Sexual activity": 1, "Interest": 1, "Partner": 1, "Sex invitation": 1}, "verbs": {"When": 1, "Interested": 1, "Yes": 2, "Self-dejectedly refused": 1, "It's easy to get along": 1}, "adjectives": {"Nope": 1}, "adverbs": {"Land": 2}, "numerals": {"7": 1, "1": 3}}}, {"content": "I want my partner to lead our sexual contact.", "word_count": 15, "label": "", "sentence_structure": "Subject鈥搗erb鈥搊bject", "subjective_objective": {"answer": "Subjective", "rationale": "This is because of the patient ' s personal wishes and expectations and because it is subject to subjective attitudes."}, "sentence_sentiment": {"answer": "Negative", "rationale": "Because the entry expresses the need for control over sexual contact with the partner, it may lead to a passive position and some negative emotional ambivalence in the relationship."}, "language_style": {"answer": "Tactful and warm", "rationale": "The entry is more proficient and interactive when it is spoken in a gentle and inviting manner, rather than in direct statements or orders. At the same time, it is not too much rhetorical or emotionally rendered, and is more objective in tone and more in line with the language style of the psychological questionnaire."}, "time_dimension": {"answer": "Present", "rationale": "In that sentence, both \"wanted\" and \"dominant\" actions expressed a desire or a desire, and that state could be either a current or a continuing state, so the temporal dimension would be the present."}, "sentence_type": "Imperative sentence", "pos": {"nouns": {"Partner": 1, "Sexual exposure": 1}, "verbs": {"Yes": 1, "Jean": 1}, "adverbs": {"I": 1}}}, {"content": "I found it fun to have sex sometimes when I'm a proactive actor and keep my partner in a passive position.", "word_count": 33, "label": "", "sentence_structure": "Subject鈥搗erb鈥搊bject", "subjective_objective": {"answer": "Subjective", "rationale": "This is a subjective feeling and experience of the patient and reflects his or her attitude and preferences towards the role of the sexual activity."}, "sentence_sentiment": {"answer": "Negative", "rationale": "Because the act of \"putting the other side in a passive position\" may in some cases be considered unequal or disrespectful, it has a certain negative emotional connotation."}, "language_style": {"answer": "Tactful and warm", "rationale": "The language style of the questionnaire entry is euphemistic. The basis of the judgement is that it uses more neutral terms and sentences, without directly describing sexual or emotional reactions. At the same time, the question is presented in a more moderate manner, with respect for the individual鈥檚 will, and reflects a certain level of care and understanding."}, "time_dimension": {"answer": "Present", "rationale": "Now, the \"I find\" in the entry indicates that it's a personal discovery or experience, not a past experience. And, \"Sometimes I'm a proactive actor and I'm having a very fun partner's passive position\" is a current feeling and experience, and it's now, and it's judged."}, "sentence_type": "Affirmative declarative sentence", "pos": {"nouns": {"Sexual activity": 2, "Role": 1, "Initiatives": 1, "Partner": 1, "Passive": 1, "Status": 1, "Fun": 1}, "verbs": {"Found": 1, "have": 1, "Act as": 1, "Jean": 1, "in": 1, "very": 1, "be": 1}, "adverbs": {"Sometimes": 1, "Passive": 1}, "numerals": {"9": 1}, "classifiers": {"individual": 1}, "pronouns": {"I": 1}}}, {"content": "10. If the sexual stimulation given by my partner is not sufficient, I feel that I am not comfortable with orgasm.", "word_count": 32, "label": "", "sentence_structure": "Subject鈥搗erb鈥揷omplement", "subjective_objective": {"answer": "Subjective", "rationale": "The question is based on the patient鈥檚 personal feelings and experiences, and it looks at the patient鈥檚 subjective assessment and expectations of sexual behaviour."}, "sentence_sentiment": {"answer": "Negative", "rationale": "Because the phrase \"not getting high\" in the entry implies negative emotions and dissatisfaction."}, "language_style": {"answer": "Tactful and warm", "rationale": ""}, "time_dimension": {"answer": "Present", "rationale": "The entry describes the feelings and perceptions of the testee in the current state of sexual irritation and orgasm, does not relate to past or future behaviour or mentality and therefore is judged to be present."}, "sentence_type": "Imperative sentence", "pos": {"nouns": {"Partner": 1, "Sexual stimulation": 1, "Climax": 1, "I'm not feeling well": 1}, "verbs": {"Give it to me": 1, "Achieved": 1}, "adverbs": {"Suppose": 1, "Not enough": 1, "and": 1}, "numerals": {"10": 1}, "pronouns": {"I": 1}}}, {"content": "11. In some sexual contacts, I found my partner active and passive would be fun for me.", "word_count": 24, "label": "", "sentence_structure": "Subject鈥搗erb鈥搊bject", "subjective_objective": {"answer": "Subjective", "rationale": "This question describes the feelings and experiences of individuals and is a subjective examination."}, "sentence_sentiment": {"answer": "Neutral", "rationale": "Because the entry describes only one aspect of sexual behaviour and does not express positive or negative emotional expressions."}, "language_style": {"answer": "Tactful and warm", "rationale": "This is more moderate, respectful and understanding of the other鈥檚 feelings and needs."}, "time_dimension": {"answer": "Present", "rationale": "Now the words \"discovery\", \"active\" and \"passive\" in the entry are all indicative of current behaviour or state, so it is judged to be now."}, "sentence_type": "Affirmative declarative sentence", "pos": {"nouns": {"Sexual exposure": 1, "Partner": 1, "Fun": 1}, "verbs": {"Found": 1, "Yes": 1, "have": 1, "Passive": 1, "Initiatives": 1, "I": 2}, "adjectives": {"very": 1}, "numerals": {"11": 1}, "pronouns": {"I": 1}}}]}

{"questionnaire_title": "Lonely Classification", "questionnaire_category": "Mental health", "item_count": 60, "emotion_shift_overall": "From negative to positive", "language_style_overall": "Tactful and warm", "items": [{"content": "I found it easy to express feelings to my family.", "word_count": 13, "label": "", "sentence_structure": "Subject鈥搗erb鈥搊bject", "subjective_objective": {"answer": "Subjective", "rationale": "Because \"easy\" is a subjective sense of feeling, meaning that the patient's own feelings of expressing feelings to his family are a subjective description."}, "sentence_sentiment": {"answer": "Positive", "rationale": "Because the expression \"discovering it easy to express feelings to family\" expresses positive emotions and attitudes, that is, it is a relatively easy thing to communicate with family."}, "language_style": {"answer": "Tactful and warm", "rationale": "The entry uses a soft, invitation-style tone for dialogue, rather than a direct statement or order, which is more personal and interactive. At the same time, the word \"discovery\" also indicates that the expression of the article is objective and not subjective in its emotional nature."}, "time_dimension": {"answer": "Present", "rationale": "The description of \"discovery\" and \"expression of emotion\" point to the current state or situation, so the temporal dimension of the article is now."}, "sentence_type": "Affirmative declarative sentence", "pos": {"nouns": {"Found": 1, "Family": 1, "Emotion": 1}, "verbs": {"Expression": 1, "Easy": 1}, "numerals": {"1": 2}}}, {"content": "Almost everyone around me is strange.", "word_count": 12, "label": "", "sentence_structure": "Subject鈥揷opula鈥損redicative", "subjective_objective": {"answer": "Subjective", "rationale": "Because \"unknown\" is the patient's subjective feeling and perception, not the objective fact."}, "sentence_sentiment": {"answer": "Negative", "rationale": "Because the expression \"unknown\" in the entries is negative."}, "language_style": {"answer": "Calm and direct", "rationale": "The entry uses objective descriptions to interview the social environment around the interviewee, does not use excessive rhetoric or emotional colours and is more calm and direct."}, "time_dimension": {"answer": "Present", "rationale": "The entry represents the current status or perception and does not address the past or future time dimension."}, "sentence_type": "Negative declarative sentence", "pos": {"nouns": {"Around": 1, "Everyone": 1, "Stranger": 1}, "verbs": {"very": 1, "Strange": 1}, "adverbs": {"Almost": 1}, "pronouns": {"I": 1}}}, {"content": "I usually have to wait for my friends to call me and invite me to plan my trip.", "word_count": 19, "label": "", "sentence_structure": "Subject鈥搗erb鈥搊bject", "subjective_objective": {"answer": "Subjective", "rationale": "Because \"waiting for friends to call me, inviting me\" is the personal feeling and attitude of the patients, reflecting their inner experience."}, "sentence_sentiment": {"answer": "Negative", "rationale": "Because the entry says, \"Wait for my friends to call me and invite me to plan out,\" and it's negative."}, "language_style": {"answer": "Calm and direct", "rationale": "The entry asks the other party in a straightforward manner whether it needs to wait for an invitation from a friend to plan to leave, without excessive rhetorical or emotional colours, and is more objective in tone."}, "time_dimension": {"answer": "Present", "rationale": "The entry indicates that the testee is in the current state of behaviour, i.e., needs to wait for an invitation to start planning to leave, and thus is judged to be \"now\"."}, "sentence_type": "Affirmative declarative sentence", "pos": {"nouns": {"friends": 2, "Invitations": 1, "Planned": 1, "Out": 1}, "verbs": {"Wait": 1, "Please": 1, "Yes": 1, "Planned": 1, "Go on": 1}, "adverbs": {"Usually": 1}, "numerals": {"3": 1}, "pronouns": {"I": 1}}}, {"content": "4. Most friends have an understanding of my motives and reasons for doing things.", "word_count": 19, "label": "", "sentence_structure": "Subject鈥搗erb鈥搊bject", "subjective_objective": {"answer": "Subjective", "rationale": "The sentence is based on the patient ' s own understanding of the motivation and justification of others, which is a subjective feeling of the patient."}, "sentence_sentiment": {"answer": "Neutral", "rationale": "Because the entry describes the degree of understanding of your behaviour by the majority, and there is no obvious emotional colour."}, "language_style": {"answer": "Tactful and warm", "rationale": "The words \"most friends\" and \"to understand\" are more gentle and polite. Moreover, the expression \"motives and reasons for my actions\" is more sensitive to each other's feelings and understanding than direct demands or orders, so the language style of the article can be considered as euphemism."}, "time_dimension": {"answer": "Present", "rationale": "This questionnaire entry asks the testee about the current time point (i.e., when completing the questionnaire) and judges it now. The keyword \"most\" and \"understanding\" all indicate that it is a description and observation of the current situation, not a projection or a recollection of the future or the past."}, "sentence_type": "Affirmative declarative sentence", "pos": {"nouns": {"friends": 1, "Motive": 1, "Rationale": 1}, "verbs": {"Do it": 1, "Organisation": 1, "Understood": 1}, "adjectives": {"Most of them": 1}, "numerals": {"4": 1}, "pronouns": {"I": 1}}}, {"content": "There is no lover who is so important to me at this time.", "word_count": 15, "label": "", "sentence_structure": "Subject鈥搗erb鈥揷omplement", "subjective_objective": {"answer": "Subjective", "rationale": "Because the problem relates to the patient ' s personal feelings and subjective belief that there is a significant lover."}, "sentence_sentiment": {"answer": "Neutral", "rationale": "Since the entry merely objectively presents a fact, it does not contain any emotional tendencies."}, "language_style": {"answer": "Calm and direct", "rationale": "According to the content of the question, \"Is there a lover of my great interest?\" The purpose of the question is to know if the person under investigation has a significant partner in love, so the language style can be described as being straightforward."}, "time_dimension": {"answer": "Present", "rationale": "The entry asks the testee whether he or she currently has a lover or partner who has a significant influence on their relationship and therefore judge the time dimension as now."}, "sentence_type": "Negative declarative sentence", "pos": {"nouns": {"Lovers": 2, "Lover": 1}, "adjectives": {"Significant": 1}, "adverbs": {"Current": 1}, "numerals": {"5": 1}, "classifiers": {"individual": 1}, "pronouns": {"I": 1}}}, {"content": "I'm not very good with my family.", "word_count": 8, "label": "", "sentence_structure": "Subject鈥揷opula鈥損redicative", "subjective_objective": {"answer": "Subjective", "rationale": "Because \"failure\" is a subjective assessment of a patient's relationship with his family."}, "sentence_sentiment": {"answer": "Negative", "rationale": "Because the statement in the entry \"failure to get along with his family\" is negative."}, "language_style": {"answer": "Calm and direct", "rationale": ""}, "time_dimension": {"answer": "Present", "rationale": "The entry is about the feelings and experiences of the testee in family relations, and the time dimension is now judged. The keyword \"I'm not well with my family\" shows the subjective feelings of the testee, implying the current predicament or distress."}, "sentence_type": "Negative declarative sentence", "pos": {"nouns": {"I": 1, "Family": 1}, "verbs": {"Location": 1}, "adjectives": {"Okay": 1}, "numerals": {"6": 1}}}, {"content": "7. I have at least one good friend of the same sex.", "word_count": 10, "label": "", "sentence_structure": "Subject鈥揷opula鈥損redicative", "subjective_objective": {"answer": "Subjective", "rationale": "The idea is that the people who have a good friend of the same sex are subjective and experienced."}, "sentence_sentiment": {"answer": "Neutral", "rationale": "Because the entry does not have any emotional color."}, "language_style": {"answer": "Tactful and warm", "rationale": "The entry is more euphemistic and friendly, by using \"at least one\" to ask the interviewee whether he has a friend of the same sex and to avoid asking questions about his sexual orientation directly."}, "time_dimension": {"answer": "Present", "rationale": "The keyword is \"I\" and refers to the interviewee himself, while \"Be\" and \"at least one of the same-sex friends\" are describing the status or status of the interviewee, so the subject is asking about the current situation of the interviewee and is a current state of affairs."}, "sentence_type": "Affirmative declarative sentence", "pos": {"nouns": {"Questionnaire": 1, "Entry": 1, "Same-sex friends": 1}, "verbs": {"have": 1, "be": 1}, "adverbs": {"At least": 1}, "numerals": {"One": 1}, "classifiers": {"individual": 1}, "pronouns": {"I": 1}}}, {"content": "8: I rely on any collective or organizational support for moral or financial support in case of trouble.", "word_count": 29, "label": "", "sentence_structure": "Subject鈥搗erb鈥搊bject", "subjective_objective": {"answer": "Subjective", "rationale": "This is the subjective idea and attitude of the patient and a description of the way in which he or she has managed the difficulties."}, "sentence_sentiment": {"answer": "Negative", "rationale": "For the act of \"reliance on any collective or organizational group\" is in most cases considered negative, as it usually involves dependency and lack of independence."}, "language_style": {"answer": "Calm and direct", "rationale": "The language style of the entry is more straightforward, and there is no excessive rhetorical or emotional rendering. It clearly indicates whether the subject of the inquiry will seek collective or organizational support in case of trouble and will be considered morally and economically. This expression is more objective, neutral and more accurate in understanding the views and attitudes of the subject."}, "time_dimension": {"answer": "Present", "rationale": "So the time dimension is now judged."}, "sentence_type": "Negative declarative sentence", "pos": {"nouns": {"Trouble": 1, "Collective": 1, "Organizations": 1, "Morality": 1, "Money": 1, "Support": 1}, "verbs": {"I met him": 1, "Oh, shit": 1, "Dependence": 1, "Got it": 1}, "numerals": {"8": 1}, "pronouns": {"I": 1}}}, {"content": "I have a lover or lover, and we can work together in good faith.", "word_count": 19, "label": "", "sentence_structure": "Subject鈥搗erb鈥揷omplement", "subjective_objective": {"answer": "Subjective", "rationale": "This requires the patient to answer his own thoughts, so it is subjective."}, "sentence_sentiment": {"answer": "Positive", "rationale": "Because the \"can work together in good faith\" act in the entry is positive."}, "language_style": {"answer": "Tactful and warm", "rationale": "The language style of the entry is more euphemistic, raises questions in a question-and-answer manner, and emphasizes the importance of \"work in good faith.\" This expression makes the respondents feel more comfortable and free to answer questions, while also better communicating the intentions and concerns of researchers."}, "time_dimension": {"answer": "Present", "rationale": "The keyword \"now\" shows that the entry is discussed in the current relationship status of the subjects and their relationship with their lovers. So, the time dimension of the purpose is now."}, "sentence_type": "Affirmative declarative sentence", "pos": {"nouns": {"Present": 1, "Yes": 1, "person": 1, "we": 1, "Two": 1, "Try": 1, "Truth": 1, "Cooperation": 1}, "verbs": {"have": 1, "Yes": 1, "Cooperation": 1}, "adjectives": {"Truth": 1}, "numerals": {"Nine": 1}, "pronouns": {"I": 1}}}, {"content": "I've often become shy and passive in being with my family.", "word_count": 17, "label": "", "sentence_structure": "Subject鈥搗erb鈥揷omplement", "subjective_objective": {"answer": "Subjective", "rationale": "Because \"weakness and passivity\" is a subjective feeling or attitude that reflects the mental state of the patient in his or her relationship with his or her family."}, "sentence_sentiment": {"answer": "Negative", "rationale": "Because \"weak\" and \"passive\" are negative emotional attitudes, expressing self-confidence and lack of initiative towards people close to us."}, "language_style": {"answer": "Tactful and warm", "rationale": "Because the entries use terms such as \"often\" and \"weakness\" to indicate that the questioner prefers to ask the person in question about his or her emotional state, rather than directly expressing his or her views or making criticisms. This expression is more acclaimed and understood, and more likely to trigger the echoes and active participation of the person in the inquiry."}, "time_dimension": {"answer": "Past", "rationale": "The time dimension of this questionnaire entry is therefore judged now."}, "sentence_type": "Affirmative declarative sentence", "pos": {"nouns": {"Family": 1, "Shame": 1, "Passive": 1}, "verbs": {"Together": 1, "鈾?And become 鈾?: 1}, "adjectives": {"Regular": 1, "Often": 1}, "numerals": {"10": 1}, "pronouns": {"I": 1}}}, {"content": "Some friends are my back when I'm having any trouble.", "word_count": 20, "label": "", "sentence_structure": "Subject鈥搗erb鈥揷omplement", "subjective_objective": {"answer": "Subjective", "rationale": "Because the entry relates to patients ' attitudes and perceptions towards friends, it is the patient ' s subjective feeling and perception."}, "sentence_sentiment": {"answer": "Positive", "rationale": "Because the \"friends as my back\" act is positive."}, "language_style": {"answer": "Tactful and warm", "rationale": "This entry is softly phrased and spoken in a friendly manner, and the interrogators express their gratitude to their friends and try to understand whether they are always willing to help."}, "time_dimension": {"answer": "Present", "rationale": "The entry describes the current situation, so the time dimension is now judged. The keywords \"some friends\" and \"any difficulty will be my back,\" which suggests that this is an ongoing act or state, implying that the presence of these friends has some positive impact on and support for the testee."}, "sentence_type": "Affirmative declarative sentence", "pos": {"nouns": {"friends": 2, "I": 2, "Difficulties": 1, "Back up": 1}, "verbs": {"I met him": 1, "Do it": 1}, "adverbs": {"Both": 1}, "numerals": {"11": 1}}}, {"content": "The people in the unit don't really appreciate my thoughts and feelings.", "word_count": 19, "label": "", "sentence_structure": "Subject鈥搗erb鈥揷omplement", "subjective_objective": {"answer": "Subjective", "rationale": "Because \"the people in the unit don't really value my thoughts and feelings\" is a subjective view or feeling that reflects the views of the interviewees about their position in the unit and their relationships, not the objective facts."}, "sentence_sentiment": {"answer": "Negative", "rationale": "Because the description \"not really important\" in the entry is negative and expresses negative emotional attitudes."}, "language_style": {"answer": "Calm and direct", "rationale": "The entry expresses concern about the respect that the other party receives in the working environment, is balanced and objective, does not use excessive rhetorical or emotional rendering, and is of a calm and direct linguistic style."}, "time_dimension": {"answer": "Past", "rationale": "At the same time, the description of \"my thoughts and feelings\" points to the feelings and experiences of individuals, which further confirms this. The article should be the present in a holistic sense."}, "sentence_type": "Negative declarative sentence", "pos": {"nouns": {"- In the unit": 1, "person": 1, "Thoughts": 1, "Feel": 1}, "verbs": {"Value": 1}, "adjectives": {"True": 1}, "adverbs": {"and": 1, "Not": 1}, "numerals": {"12": 1}, "pronouns": {"I": 1}}}, {"content": "I tried to make friends, I tried to make people like me, but I rarely did.", "word_count": 20, "label": "", "sentence_structure": "Subject鈥搗erb鈥搊bject", "subjective_objective": {"answer": "Subjective", "rationale": "It is subjective to look at the subjective behaviour and attitude of people trying to make friends. Although the results are rarely what they want, they are based on the patient鈥檚 own will and efforts."}, "sentence_sentiment": {"answer": "Negative", "rationale": "Because the result of \"very rarely gets what you want\" in the entry is negative."}, "language_style": {"answer": "Tactful and warm", "rationale": ""}, "time_dimension": {"answer": "Past", "rationale": "The use of the old-temporal \"attempt\" and \"very rarely get what you want\" in the entry suggests that it is describing the testee's past experience or behaviour. So the time dimension of this questionnaire entry is the past."}, "sentence_type": "Affirmative declarative sentence", "pos": {"nouns": {"friends": 1, "person": 2, "Wish": 1}, "verbs": {"Hand it over": 1, "Try": 2, "It's so sweet": 1, "Yes": 1, "I'll do it": 1}, "adverbs": {"Very few": 1}, "numerals": {"13": 1}, "pronouns": {"I": 1}}}, {"content": "14. I took time to speak to everyone in the family alone.", "word_count": 17, "label": "", "sentence_structure": "Subject鈥搗erb鈥揷omplement", "subjective_objective": {"answer": "Objective", "rationale": "This is because the behaviour described by the patient in the questionnaire is an objective one."}, "sentence_sentiment": {"answer": "Positive", "rationale": "The behaviour in the entry \"leaves time to speak to everyone in the family alone\" expresses a positive attitude and willingness, with the aim of promoting communication and relationships between family members."}, "language_style": {"answer": "Tactful and warm", "rationale": "The phrase \"show a certain amount of time\" also shows that this is an achievable goal, without setting too high standards or requirements to give people a sense of affection and care."}, "time_dimension": {"answer": "Present", "rationale": "The entry describes the behaviour that the subject is doing, and the keyword \"screw\" and \"talk\" all indicate that this is an ongoing move.\""}, "sentence_type": "Affirmative declarative sentence", "pos": {"nouns": {"Time": 1, "Home": 1, "Everyone": 1, "Talk": 1}, "verbs": {"Smoke": 1, "Out": 1, "and": 1, "Talk": 1}, "adverbs": {"I'm sure": 1}, "numerals": {"14": 1}, "classifiers": {"individual": 1}, "pronouns": {"I": 1}}}, {"content": "I find it difficult to show love to others.", "word_count": 12, "label": "", "sentence_structure": "Subject鈥搗erb鈥搊bject", "subjective_objective": {"answer": "Subjective", "rationale": "Because it's hard to express love to others, it's a human feeling and a thought. It's subjective."}, "sentence_sentiment": {"answer": "Negative", "rationale": "Because the \"difficult to express love\" in the entry is negative."}, "language_style": {"answer": "Tactful and warm", "rationale": "The entry uses a soft, invitee language style, which does not directly deny or criticize the other, but rather directs the interviewee to share his/her feelings, which is more personal and interactive."}, "time_dimension": {"answer": "Past", "rationale": "By way of interpretation, \"I realize\" indicates that the experience and feelings of the past, and \"difficult to express love\" can be considered as the present state or tendency, so the temporal dimension of the article can be seen as either the past or the present."}, "sentence_type": "Negative declarative sentence", "pos": {"nouns": {"I": 1, "person": 1, "Love": 1}, "verbs": {"Found": 1, "In": 1, "Expression": 1}, "adjectives": {"Hard": 1}, "numerals": {"15": 1}}}, {"content": "I don't have many friends in my city.", "word_count": 12, "label": "", "sentence_structure": "Subject鈥揷opula鈥損redicative", "subjective_objective": {"answer": "Subjective", "rationale": "Because \"no friends\" are the patient's personal feelings and subjective evaluations, not the objective facts."}, "sentence_sentiment": {"answer": "Negative", "rationale": "Because \"no friends\" in the entries express a negative emotion or attitude."}, "language_style": {"answer": "Tactful and warm", "rationale": "The entry is more personal and interactive when it is spoken in a gentle and inviting tone, rather than in direct statements or orders. At the same time, the expression \"no friends\" is not too much stressed or criticized, but rather uses a relatively neutral phrase to keep respondents from feeling pressured when they answer."}, "time_dimension": {"answer": "Present", "rationale": "The keywords \"My City\" and \"My Friends are not many\" indicate the current situation or state, so the time dimension of this questionnaire entry is now judged."}, "sentence_type": "Negative declarative sentence", "pos": {"nouns": {"Urban": 1, "friends": 1}, "verbs": {"Stay": 1, "More": 1}, "adjectives": {"I": 1}, "numerals": {"16": 1}, "classifiers": {"individual": 1}, "pronouns": {"I": 1}}}, {"content": "17. I worked well with others in a collective.", "word_count": 15, "label": "", "sentence_structure": "Subject鈥搗erb鈥揷omplement", "subjective_objective": {"answer": "Subjective", "rationale": "Because it is a description of a subjective feeling, it involves subjective judgement of one ' s own performance and of others ' evaluation."}, "sentence_sentiment": {"answer": "Positive", "rationale": "Because \"good cooperation\" in the entry is a positive attitude and behaviour."}, "language_style": {"answer": "Tactful and warm", "rationale": "This is a more gentle and polite expression, which does not directly question or order, but rather asks the interviewee whether he or she can work well with others. This expression is more friendly and can create a more comfortable environment for communication, which will facilitate the smooth running of the questionnaire survey."}, "time_dimension": {"answer": "Past", "rationale": "The entry describes the behaviour or state of the person tested at a certain point in time in the past and therefore determines the past."}, "sentence_type": "Affirmative declarative sentence", "pos": {"nouns": {"Collective": 1, "person": 1, "Cooperation": 1}, "verbs": {"in": 1, "Do it": 1}, "adjectives": {"Okay": 1}, "numerals": {"17": 1}, "pronouns": {"I": 1, "others": 1}}}, {"content": "I am important to the physical and mental state of lovers or spouses.", "word_count": 16, "label": "", "sentence_structure": "Subject鈥揷opula鈥損redicative", "subjective_objective": {"answer": "Subjective", "rationale": "Because \"important\" is a subjective term for evaluation, which expresses the attitude and feelings of the individual, it is a subjective view."}, "sentence_sentiment": {"answer": "Positive", "rationale": "Because the behavior in the entry \"is important to the physical and mental state of the lover\" expresses a positive attitude and concern."}, "language_style": {"answer": "Tactful and warm", "rationale": "This is a more gentle, friendly and understandable expression."}, "time_dimension": {"answer": "Present", "rationale": "The entry indicates the current situation, and \"I'm important to the physical and mental state of a lover\" is a current statement. So the time dimension of the article is now."}, "sentence_type": "Affirmative declarative sentence", "pos": {"nouns": {"Lovers": 1, "Spouse": 1, "Physical and mental state": 1}, "verbs": {"Yes": 1, "Important": 1}, "numerals": {"18": 1}, "pronouns": {"I": 1}}}, {"content": "I don't think I can count on my friends around when I need help.", "word_count": 19, "label": "", "sentence_structure": "Subject鈥揷opula鈥損redicative", "subjective_objective": {"answer": "Subjective", "rationale": "This is a subjective approach, because the words \"thinking\" and \"can't rely on\" express the subjective feelings and the assessment of the patient that he cannot get help from friends."}, "sentence_sentiment": {"answer": "Negative", "rationale": "Because the entry \"can't rely on friends\" expresses negative feelings, implying no-one's recourse or helplessness."}, "language_style": {"answer": "Tactful and warm", "rationale": "The language style of the entry is more euphemistic and mild. It uses the word \"think\" to express a subjective feeling rather than to directly accuse or criticize others. Moreover, \"I cannot rely on friends around me when I need help\" is a more subtle phrase, not to point directly to the problem, but to allow the interviewee to feel and reflect on it. So the language style of the questionnaire is considered to be euphemism."}, "time_dimension": {"answer": "Present", "rationale": "Now the time dimension of the keyword \"felt\" and \"unable to rely on\" is the current feeling or state, so the entry is to ask the subject what it feels like today."}, "sentence_type": "Negative declarative sentence", "pos": {"nouns": {"I": 1, "friends": 1, "Help": 1}, "verbs": {"I think": 1, "Yeah": 1, "Dependence": 1}, "adverbs": {"When": 1, "Yes": 1, "Time": 1}}}, {"content": "I don't think my family really knows me.", "word_count": 13, "label": "", "sentence_structure": "Subject鈥揷opula鈥損redicative", "subjective_objective": {"answer": "Subjective", "rationale": "The family is aware of my subjective view as a patient."}, "sentence_sentiment": {"answer": "Negative", "rationale": "Because it expresses a negative feeling of being misunderstood and alone."}, "language_style": {"answer": "Tactful and warm", "rationale": "The entry is more adept and interactive when it is spoken in a gentle and inviting manner, rather than by direct statements or orders."}, "time_dimension": {"answer": "Past", "rationale": "The key word is \"I don't think that my family really knows me\", which shows that the testees have felt and experienced this in the past and can therefore be judged as a description of the past."}, "sentence_type": "Negative declarative sentence", "pos": {"nouns": {"Family": 1, "I": 1}, "verbs": {"I think": 1, "Roger that": 1}, "adjectives": {"Really": 1}, "numerals": {"20": 1}}}, {"content": "I have a lover or a spouse who can meet my emotional needs.", "word_count": 18, "label": "", "sentence_structure": "Subject鈥揷opula鈥損redicative", "subjective_objective": {"answer": "Subjective", "rationale": "Since it is a personal feeling and experience to satisfy emotional needs, this is viewed from the patient ' s subjective perspective."}, "sentence_sentiment": {"answer": "Positive", "rationale": "Because the entry expresses the desire of a person who meets his or her emotional needs, it is positive."}, "language_style": {"answer": "Tactful and warm", "rationale": "The entry is more adept and interactive when it is spoken in a gentle and inviting manner, rather than by direct statements or orders."}, "time_dimension": {"answer": "Present", "rationale": "The sentence \"I have one\" indicates the current state of affairs, which is the actual situation, and \"the lover or spouse who meets my emotional needs\" describes the current situation. It is therefore possible to judge that the entry is the present in terms of the emotional status of the testee, and the time dimension is the present."}, "sentence_type": "Affirmative declarative sentence", "pos": {"nouns": {"Emotion": 1, "Yes": 1, "Lovers": 1, "Spouse": 1}, "verbs": {"have": 1, "Yes": 1, "Satisfied": 1}, "adjectives": {"I": 2, "One": 1}, "numerals": {"21": 1}}}, {"content": "22. Although not exhaustive, friends are generally concerned about what I do.", "word_count": 22, "label": "", "sentence_structure": "Subject鈥搗erb鈥揷omplement", "subjective_objective": {"answer": "Subjective", "rationale": "Because it's about \"the level of interest of friends in me,\" which is seen from the patient's subjective perspective."}, "sentence_sentiment": {"answer": "Positive", "rationale": "Because it expresses the attitude of friends to what they do and is a positive emotional color."}, "language_style": {"answer": "Tactful and warm", "rationale": "The entry uses a gentle and inviting tone to express the attitude and level of interest of the interviewees, without direct statements or orders, which is more personal and interactive. Moreover, the expression \"though not an issue of question\" implies that the interviewees do not need to report to their friends without great detail, which is a more euphemism."}, "time_dimension": {"answer": "Present", "rationale": ""}, "sentence_type": "Affirmative declarative sentence", "pos": {"nouns": {"- The thing": 1, "Friends": 1, "I": 1, "Do it": 1, "Yes": 1}, "verbs": {"Ask": 1, "General": 1, "very": 1, "Care": 1, "be": 1}, "adjectives": {"No, it's not": 1}, "adverbs": {"Both": 1}, "numerals": {"22": 1}, "pronouns": {"they": 1}}}, {"content": "Family would like to see my friends.", "word_count": 9, "label": "", "sentence_structure": "Subject鈥搗erb鈥搊bject", "subjective_objective": {"answer": "Subjective", "rationale": "This is the subjective feeling or perception of the patients that they believe their families are willing to meet their friends."}, "sentence_sentiment": {"answer": "Neutral", "rationale": "Because the entry does not express any emotion or attitude, it simply describes a fact."}, "language_style": {"answer": "Tactful and warm", "rationale": "The entry is a soft, inviting conversation, expressing concern and curiosity about the willingness of the family to meet their friends. This expression is more personal and interactive, making it easier for the interviewee to feel comfortable and to share his thoughts."}, "time_dimension": {"answer": "Past", "rationale": "The term \"willing to meet\" in the entry refers to the situation in the past in a certain period of time and is therefore judged to be the past."}, "sentence_type": "Affirmative declarative sentence", "pos": {"nouns": {"Family": 1, "friends": 1}, "verbs": {"Yes": 1}, "adverbs": {"very": 1}, "numerals": {"23": 1}}}, {"content": "24. I have allowed myself to interact closely with friends.", "word_count": 12, "label": "", "sentence_structure": "Subject鈥搗erb鈥搊bject", "subjective_objective": {"answer": "Subjective", "rationale": "Because \"I allow myself\" is the patient's subjective attitude or choice."}, "sentence_sentiment": {"answer": "Positive", "rationale": "Because the act of \"permissing himself to have a close relationship with friends\" in the entry is positive and indicates the willingness of the interviewees to establish and maintain good interpersonal relations."}, "language_style": {"answer": "Tactful and warm", "rationale": "The entry uses a more euphemistic and encouraging language to express support and recognition for the close interaction of the interviewee with his friends, rather than direct orders or criticism. This language style creates a more friendly and positive atmosphere, which increases the willingness of the interviewee to participate in the questionnaire and share his real ideas."}, "time_dimension": {"answer": "Present", "rationale": ""}, "sentence_type": "Affirmative declarative sentence", "pos": {"nouns": {"self": 1, "friends": 1}, "verbs": {"Allow": 1, "Travel": 1}, "numerals": {"24": 1}}}, {"content": "Families are usually busy with their own business and do not get me wrong.", "word_count": 19, "label": "", "sentence_structure": "Subject鈥搗erb鈥搊bject", "subjective_objective": {"answer": "Objective", "rationale": "Because \"family is busy with their own business\" and \"not to be in my head\" are objective facts that need not be judged by patients' subjective opinions."}, "sentence_sentiment": {"answer": "Negative", "rationale": "Because the expression \"working on my own, not hurting my mind\" in the entry gives people a feeling of neglect and loneliness, and it's a negative emotional color."}, "language_style": {"answer": "Tactful and warm", "rationale": "The entry describes the attitude of the family in a more positive and positive way, and does not directly express the other鈥檚 concern or concern for himself, but rather indirectly asks the person surveyed about the family鈥檚 feelings about their own affairs."}, "time_dimension": {"answer": "Present", "rationale": "The entry describes the current situation of the testee, which is that \"family is usually busy with their own business, and does not hurt my mind.\" Although this situation may be long-standing, it has nothing to do with the past or the future."}, "sentence_type": "Interrogative sentence", "pos": {"nouns": {"Family": 1, "Yes": 2}, "verbs": {"Busy with": 1, "Yes": 1, "You're a badass": 1}, "adjectives": {"General": 1}, "numerals": {"25": 1}, "pronouns": {"I": 1}}}, {"content": "26. Almost no one among my friends understands me as I would have liked.", "word_count": 17, "label": "", "sentence_structure": "Subject鈥搗erb鈥揷omplement", "subjective_objective": {"answer": "Subjective", "rationale": "Because \"friend understanding\" is the patient's expectation and desire, and it's subjective."}, "sentence_sentiment": {"answer": "Negative", "rationale": "Because \"near\" in the entries is an expression of a negative emotion, implying disappointment and dissatisfaction in social relations."}, "language_style": {"answer": "Tactful and warm", "rationale": "The entry expresses an implicit and caring expression of an individual's feelings about relationships, without directly criticizing or accusing others, but rather by presenting their own feelings to guide each other to thinking and resonance. At the same time, the term 鈥渁lmost鈥?also reflects subjectivity and uncertainty, avoiding over-prescriptive language."}, "time_dimension": {"answer": "Past", "rationale": "The entry describes the experience and feelings of the testee, the key words being \"near anyone in friends\" and \"understanding as I would have liked\" and all pointing to what has happened."}, "sentence_type": "Negative declarative sentence", "pos": {"nouns": {"friends": 1, "person": 1, "Understood": 1}, "verbs": {"Elephant": 1, "Expectations": 1, "That way": 1, "Understood": 1}, "adjectives": {"Almost": 1}, "numerals": {"26": 1}, "classifiers": {"individual": 1}, "pronouns": {"I": 1}}}, {"content": "No one around is concerned about me.", "word_count": 8, "label": "", "sentence_structure": "Subject鈥搗erb鈥揷omplement", "subjective_objective": {"answer": "Subjective", "rationale": "Because \"care\" is a subjective feeling or evaluation, not an objective fact."}, "sentence_sentiment": {"answer": "Negative", "rationale": "Because the expression \"no one cares about me\" in the entry expresses the feeling of loneliness and helplessness of the person under investigation, and is negative."}, "language_style": {"answer": "Tactful and warm", "rationale": "The entry is more proficient and interactive when it is spoken in a gentle and inviting manner, rather than in direct statements or orders. At the same time, the article has a certain emotional tone in its tone, allowing the interviewee to better understand and feel the meaning of the problem and the emotional needs behind it."}, "time_dimension": {"answer": "Present", "rationale": ""}, "sentence_type": "Negative declarative sentence", "pos": {"nouns": {"Around": 1, "person": 1, "Care": 1}, "verbs": {"Nope": 1, "be": 1}, "adjectives": {"very": 1}, "numerals": {"27": 1}, "pronouns": {"I": 1}}}, {"content": "There is no real harmony in my relationship or marriage.", "word_count": 22, "label": "", "sentence_structure": "Subject鈥揷opula鈥損redicative", "subjective_objective": {"answer": "Subjective", "rationale": "Because \"real harmony\" is a subjective feeling and evaluation, it requires a subjective attitude on the part of the patient to determine whether it exists."}, "sentence_sentiment": {"answer": "Negative", "rationale": "Because the description \"no real harmony\" in the entry is negative and implies contradictions and inconsistencies in the current relationship."}, "language_style": {"answer": "Tactful and warm", "rationale": "The entry uses a more euphemistic approach to questions, avoiding a direct blame or attack, making the interviewee more comfortable and natural in his response. At the same time, this expression is more likely to resonate and reflect on the interviewee鈥檚 feelings and provide him with an opportunity to reflect further on whether his relationship or relationship is really a problem."}, "time_dimension": {"answer": "Present", "rationale": "The entry \"currently\" denotes the time dimension of the present state, and \"there is no real harmony in love or marriage\" describes the status of the person tested and thus judges the present."}, "sentence_type": "Negative declarative sentence", "pos": {"nouns": {"Love": 1, "Marriage": 1, "Relations": 1}, "verbs": {"have": 1, "in": 1, "No, I'm not": 1, "Really": 1, "Harmony": 1, "- I'm sorry": 1}, "adjectives": {"Current": 1}, "numerals": {"28": 1}, "pronouns": {"I": 1}}}, {"content": "29. Family can give me the support I need.", "word_count": 12, "label": "", "sentence_structure": "Subject鈥搗erb鈥揷omplement", "subjective_objective": {"answer": "Subjective", "rationale": "Because it examines patients鈥?subjective feelings and perceptions. While \"family support\" is an objective fact, the patient鈥檚 perception and evaluation of such support is subjective."}, "sentence_sentiment": {"answer": "Positive", "rationale": "Because \"to give me the support I need\" is a positive emotional attitude, a sign of family concern and support for the individual."}, "language_style": {"answer": "Tactful and warm", "rationale": "The entry is more adept and interactive when it is spoken in a gentle and inviting manner, rather than by direct statements or orders."}, "time_dimension": {"answer": "Present", "rationale": "The entry asks if the person tested has the support of his family in the current circumstances, and therefore the time dimension is now judged."}, "sentence_type": "Affirmative declarative sentence", "pos": {"nouns": {"Family": 1, "Support": 1}, "verbs": {"I can give you that": 1}, "numerals": {"29": 1}}}, {"content": "I've had a lot of bad relations.", "word_count": 15, "label": "", "sentence_structure": "Subject鈥搗erb鈥揷omplement", "subjective_objective": {"answer": "Subjective", "rationale": "Because disappointment is a subjective feeling and evaluation."}, "sentence_sentiment": {"answer": "Negative", "rationale": "Because the result of \"Friendly Disappointment\" in the entry is negative."}, "language_style": {"answer": "Tactful and warm", "rationale": "The entry uses a more euphemistic and friendly tone, asking questions about the interviewee. By using the terms \"many\" and \"final\" the question does not directly blame or criticize anyone, but rather provides an idea of the interviewee's friend relationship. This expression is more acceptable to the interviewee and helps to build trust."}, "time_dimension": {"answer": "Present", "rationale": "From a temporal point of view, \"some of my friendships eventually become disappointing\" is a description of the current situation or state, and therefore the judgement is \"now\"."}, "sentence_type": "Negative declarative sentence", "pos": {"nouns": {"Friendship": 1, "Disappointment": 1}, "verbs": {"have": 1, "Change": 1, "Okay": 1, "You're": 1}, "adjectives": {"A lot": 1, "Eventually": 1}, "adverbs": {"None": 1}, "numerals": {"30": 1}, "pronouns": {"I": 1}}}, {"content": "I have many support and encouragements for lovers or spouses.", "word_count": 14, "label": "", "sentence_structure": "Subject鈥搗erb鈥揷omplement", "subjective_objective": {"answer": "Subjective", "rationale": "The support and encouragement are a test of the patient ' s subjective feelings."}, "sentence_sentiment": {"answer": "Positive", "rationale": "Because the \"showing support and encouragement\" in the entry is positive."}, "language_style": {"answer": "Tactful and warm", "rationale": ""}, "time_dimension": {"answer": "Present", "rationale": "The keyword \"lovers or spouses give me many support and encouragement\" means the state or behaviour of the moment, so the temporal dimension is now judged."}, "sentence_type": "Affirmative declarative sentence", "pos": {"nouns": {"Lovers": 1, "Spouse": 1, "Support": 1, "Encourage": 1}, "verbs": {"Here": 1}, "numerals": {"31": 1}}}, {"content": "I can't open my heart to my family completely.", "word_count": 12, "label": "", "sentence_structure": "Subject鈥搗erb鈥揷omplement", "subjective_objective": {"answer": "Subjective", "rationale": "Because \"not completely open to family\" is a person's inner feeling and attitude, a subjective examination."}, "sentence_sentiment": {"answer": "Negative", "rationale": "Because the act of \"not being completely open to family\" in the entry indicates some psychological impairment or gap, which is a negative emotional expression."}, "language_style": {"answer": "Calm and direct", "rationale": "\"Calm down straight.\""}, "time_dimension": {"answer": "Present", "rationale": "Because \"not completely open to family\" is a state of the art phrase describing the state or behaviour, indicating that the person currently tested has such a feeling or condition."}, "sentence_type": "Negative declarative sentence", "pos": {"nouns": {"Family": 1, "鈾?Heart to heart 鈾?: 1}, "verbs": {"Open your heart": 1, "Yes": 1}, "adverbs": {"Total": 1}, "numerals": {"32": 1}, "pronouns": {"I": 1}}}, {"content": "I often resent some of my friends' behavior.", "word_count": 13, "label": "", "sentence_structure": "Subject鈥搗erb鈥揷omplement", "subjective_objective": {"answer": "Subjective", "rationale": "Because resentment is an emotional response, it is the subjective feeling and attitude of the patient."}, "sentence_sentiment": {"answer": "Negative", "rationale": "Because the \"hate\" attitude is negative."}, "language_style": {"answer": "Calm and direct", "rationale": "The entry is simple and clear, and the other is asked whether the other party regularly complains about the behaviour of friends, without using too many qualifiers or strong emotional expressions. The entry can therefore be classified as a calm and straightforward language style."}, "time_dimension": {"answer": "Present", "rationale": ""}, "sentence_type": "Negative declarative sentence", "pos": {"nouns": {"friends": 2, "Behaviour": 1}, "verbs": {"Hate": 1, "Often": 1, "have": 1}, "adverbs": {"Some": 1}, "numerals": {"33": 1}, "pronouns": {"I": 1}}}, {"content": "I'm embarrassed by the behaviour of my family.", "word_count": 12, "label": "", "sentence_structure": "Subject鈥揷opula鈥損redicative", "subjective_objective": {"answer": "Subjective", "rationale": "Because \"smuggling\" is a subjective feeling that reflects patients' perceptions and feelings of family behaviour."}, "sentence_sentiment": {"answer": "Negative", "rationale": "Because the \"smuggling\" in the entry is negative."}, "language_style": {"answer": "Calm and direct", "rationale": "The entry is more calm and straightforward, does not use too many qualifiers or emotional terms, but rather asks the interviewee whether he or she is embarrassed at home in a concise manner. Such expression is more objective and neutral and conveys the purpose of the problem clearly."}, "time_dimension": {"answer": "Present", "rationale": "Now the keywords \"family behavior\" and \"I'm embarrassed\" point to the current situation, indicating that the person being tested is going through it, and judging it is now."}, "sentence_type": "Negative declarative sentence", "pos": {"nouns": {"Home": 1, "person": 2, "Behaviour": 1, "I": 1, "I'm embarrassed": 1}, "verbs": {"鈾?And I'm gonna be 鈾?: 1, "I feel": 1}, "numerals": {"34": 1}}}, {"content": "35. People who love me are usually just used more rationally to achieve their own ends.", "word_count": 30, "label": "", "sentence_structure": "Subject鈥搗erb鈥搊bject", "subjective_objective": {"answer": "Subjective", "rationale": "This sentence, which relates to the feelings and perceptions of the individual, expresses the subjective attitude of the person under investigation towards the person whom he has shown love."}, "sentence_sentiment": {"answer": "Negative", "rationale": "Because the purpose and emotional colour of the act \"to use me more rationally to achieve their own ends\" in the entry is negative."}, "language_style": {"answer": "Calm and direct", "rationale": "The entry presents the author鈥檚 views in a direct and clear manner, without using a euphemism or implied language. This language style is intended to provide clear information and enable readers to quickly understand the author鈥檚 meaning."}, "time_dimension": {"answer": "Present", "rationale": "The entry for this questionnaire is asking the present feelings and perceptions of the subjects, the keywords being \"normally\" and \"for their purposes\", all pointing to what is happening now."}, "sentence_type": "Negative declarative sentence", "pos": {"nouns": {"person": 1, "Purpose": 1}, "verbs": {"A love": 1, "Usually": 1, "Utilization": 1, "Achieved": 1, "be": 1}, "adjectives": {"It makes sense": 1, "Your own": 1}, "numerals": {"35": 1}, "classifiers": {"individual": 1}, "pronouns": {"they": 1}}}, {"content": "36. I am well connected to most members of my own small family.", "word_count": 16, "label": "", "sentence_structure": "Subject鈥揷opula鈥損redicative", "subjective_objective": {"answer": "Objective", "rationale": "This is a self-evaluation of the patient ' s relationship with the family and is subjective."}, "sentence_sentiment": {"answer": "Positive", "rationale": "Because the entry is about the positive emotion of being in good relationships."}, "language_style": {"answer": "Calm and direct", "rationale": "The entry directly enquired about the status of the respondent ' s relationship with most members of his small family, and did not have too many rhetorical or emotional expressions, which were more objective and balanced."}, "time_dimension": {"answer": "Present", "rationale": "The temporal dimension of the purpose of the article is therefore now judged."}, "sentence_type": "Affirmative declarative sentence", "pos": {"nouns": {"Questionnaire": 1, "Entry": 1, "Family": 1, "Members": 1}, "verbs": {"Relations": 1, "Okay": 1}, "adverbs": {"self": 1, "Most": 1}, "numerals": {"36": 1}}}, {"content": "37. Both positive and negative feelings are generally expressed in my relationships.", "word_count": 21, "label": "", "sentence_structure": "Subject鈥揷opula鈥損redicative", "subjective_objective": {"answer": "Subjective", "rationale": "Because \"positive and negative\" are the subjective experiences and feelings of the patient, and therefore the subjective perspective."}, "sentence_sentiment": {"answer": "Positive", "rationale": "The expression \"both positive and negative feelings of one's own are generally expressed\" in the entry indicates that the person has confidence and self-awareness in his/her dealings and can express his/her feelings in a frank manner, which is a positive quality."}, "language_style": {"answer": "Tactful and warm", "rationale": "The entry uses a more moderate, query-style language style, dialogue in a more intimate, friendly and understandable manner, and is more acceptable and cooperative to the interviewees. At the same time, it avoids the use of overly direct or command language, reduces the resistance of the interviewees and helps to increase the rate of recovery of questionnaires."}, "time_dimension": {"answer": "Present", "rationale": "Now the entry describes the behavior and attitude of the subject in the current relationship, the use of \"me,\" \"self\" and \"expressive\" actions, all of which indicate that this is happening and it is happening. So the time dimension is now."}, "sentence_type": "Affirmative declarative sentence", "pos": {"nouns": {"- To be with someone": 1, "Emotion": 2}, "verbs": {"in": 1, "Yes": 2, "Expression": 1}, "adjectives": {"Positive and negative": 2}, "adverbs": {"General": 1}, "numerals": {"37": 1}, "pronouns": {"I": 1}}}, {"content": "I have not been able to get much satisfaction from the group I'm part of.", "word_count": 18, "label": "", "sentence_structure": "Subject鈥搗erb鈥揷omplement", "subjective_objective": {"answer": "Subjective", "rationale": "The questionnaire entry is a subjective examination of the patient ' s own feelings and views."}, "sentence_sentiment": {"answer": "Negative", "rationale": "Because \"unsatisfactory\" in the entry is an expression of a negative emotional attitude and result."}, "language_style": {"answer": "Calm and direct", "rationale": "The entry is a direct query about the feelings of the respondents in their collective activities, does not use any rhetorical or emotional rendering, and the temperature of the words and their objectivity make it possible to judge the language style as \"cool and direct\"."}, "time_dimension": {"answer": "Present", "rationale": "The entry describes the current feelings and state of the testee, and the words \"failure\", \"gets a lot of satisfaction\" suggest that this is a situation that is going through, so the temporal dimension is now judged."}, "sentence_type": "Negative declarative sentence", "pos": {"nouns": {"38": 1, "I": 2, "Collective": 1, "Satisfied": 1}, "verbs": {"Participation": 1, "Got it": 1, "Not": 1}, "adjectives": {"A lot": 1}, "adverbs": {"very": 1}, "classifiers": {"individual": 1}}}, {"content": "I've got a lot of help and support from friends.", "word_count": 15, "label": "", "sentence_structure": "Subject鈥搗erb鈥搊bject", "subjective_objective": {"answer": "Subjective", "rationale": "Because \"getting a lot of help and support from friends\" is the patient's subjective sense and perception, and it's a subjective view."}, "sentence_sentiment": {"answer": "Positive", "rationale": "Because the \"with a lot of help and support\" act is positive."}, "language_style": {"answer": "Tactful and warm", "rationale": "The language style of the entry is euphemismal, because it uses the expression \"getting from friends\" to give people a positive, positive feeling and to emphasize the help and support of friends. It is usually comfortable and interesting, more open to questions and willingness to participate in investigations."}, "time_dimension": {"answer": "Present", "rationale": "The phrase \"I\" and \"with a lot of help and support\" suggests that the entry is asking about the current behaviour or state of the subject. So the temporal dimension is now."}, "sentence_type": "Affirmative declarative sentence", "pos": {"nouns": {"39": 1, "I": 1, "friends": 1, "Help": 1, "Support": 1}, "verbs": {"Got it": 1, "Here": 1}, "numerals": {"39": 1}, "pronouns": {"There": 1}}}, {"content": "I don't think I have much to say to my family.", "word_count": 12, "label": "", "sentence_structure": "Subject鈥揷opula鈥損redicative", "subjective_objective": {"answer": "Subjective", "rationale": "Because \"think\" is a subjective adjective, which represents the patient's personal views and feelings, the entry is viewed from the patient's subjective perspective."}, "sentence_sentiment": {"answer": "Negative", "rationale": "Because the \"I don't feel like I have much to say to my family\" in the entry is negative."}, "language_style": {"answer": "Calm and direct", "rationale": "The entry is more directly linguistically, not using too many qualifiers or emotionally strong words, but expressing the views of the interviewee in an objective and concise manner."}, "time_dimension": {"answer": "Present", "rationale": "The entry describes the current state of feeling and feeling, and the key words \"like,\" \"not much to say to family\" indicate that the act or feeling is happening. So the temporal dimension is now."}, "sentence_type": "Negative declarative sentence", "pos": {"nouns": {"I think": 1, "Family": 1, "Word": 1}, "verbs": {"Call": 1, "Yeah": 1}, "numerals": {"40": 1}}}, {"content": "I don't have that kind of love that really understands each other.", "word_count": 14, "label": "", "sentence_structure": "Subject鈥損redicate", "subjective_objective": {"answer": "Subjective", "rationale": "Because \"no true understanding of love\" is a description of subjective feelings and experiences."}, "sentence_sentiment": {"answer": "Negative", "rationale": "Because the entry \"no true understanding of love\" expresses a negative emotion and attitude."}, "language_style": {"answer": "Tactful and warm", "rationale": "The language style of the entry is more euphemistic and sweet, using the expression \"no true understanding of love\" and without too many negative emotions or criticisms, giving people a more peaceful and comfortable feeling. At the same time, it can trigger resonance and reflection among the respondents and make it easier to access effective information."}, "time_dimension": {"answer": "Present", "rationale": "The keyword \"no true understanding of love\" expresses the feelings and perceptions of the subjects now, so the temporal dimension is now."}, "sentence_type": "Negative declarative sentence", "pos": {"nouns": {"Love": 1}, "verbs": {"have": 1, "Understood": 1}, "adjectives": {"Really": 1, "To each other": 1}, "numerals": {"41": 1}, "pronouns": {"I": 1}}}, {"content": "42. I do feel like a family.", "word_count": 12, "label": "", "sentence_structure": "Subject鈥搗erb鈥揷omplement", "subjective_objective": {"answer": "Subjective", "rationale": "Because \"I feel\" expresses the patient's subjective feelings and attitudes."}, "sentence_sentiment": {"answer": "Positive", "rationale": "Because the expression \"belonging to a family\" in the entry expresses a sense of belonging and identity, and is a positive emotional attitude."}, "language_style": {"answer": "Tactful and warm", "rationale": "The language style of the entry is more euphemistic, because it uses a positive tone ( \"I do feel...\") and expresses a sense of belonging and affection, which makes the entire sentence more intimate and sweet."}, "time_dimension": {"answer": "Present", "rationale": "The entry describes the person who was tested now in his or her feelings and views, and thus the judgement is now."}, "sentence_type": "Affirmative declarative sentence", "pos": {"nouns": {"Family": 1}, "verbs": {"I think": 1, "belong to": 1}, "adverbs": {"Indeed": 1}}}, {"content": "I have few friends who can talk frankly.", "word_count": 14, "label": "", "sentence_structure": "Subject鈥搗erb鈥揷omplement", "subjective_objective": {"answer": "Subjective", "rationale": "Because I'm the patient's subjective view of a friend who doesn't have a frank conversation, it's the patient's personal feelings and evaluations."}, "sentence_sentiment": {"answer": "Negative", "rationale": "Because the phrase \"near friends who can talk frankly\" in the entry is negative."}, "language_style": {"answer": "Calm and direct", "rationale": "The entry asks whether the person surveyed has few friends who can communicate honestly, is speaking in an objective and cold manner, and does not have too many speech techniques or emotional renderings."}, "time_dimension": {"answer": "Present", "rationale": "The keyword \"almost\" represents a degree of denial, while the verb \"can speak frankly\" is the general current, so the entry refers to the current state and circumstances. The temporal dimension of the entry can therefore be judged as the present."}, "sentence_type": "Negative declarative sentence", "pos": {"nouns": {"friends": 1, "Talk to me frankly": 1}, "verbs": {"have": 1, "Almost": 1, "No, I'm not": 1, "Yes": 1, "Talk to me frankly": 1}, "adverbs": {"Very few": 1}, "numerals": {"43": 1}, "pronouns": {"I": 1}}}, {"content": "Family is the key to me.", "word_count": 7, "label": "", "sentence_structure": "Subject鈥揷opula鈥損redicative", "subjective_objective": {"answer": "Subjective", "rationale": "Family is important to me as a subjective description of the patient ' s personal feelings and as a subjective perspective."}, "sentence_sentiment": {"answer": "Positive", "rationale": "Because the words \"family is key to me\" in the entry express the importance of family, and it is a positive emotional attitude."}, "language_style": {"answer": "Tactful and warm", "rationale": "The entry uses subjective terms \"critical\" and is expressed in a first person's way, giving people a sense of being close and concerned about the individual's feelings, and is relatively gentle in tone. It can therefore be judged as being euphemismal in its language style."}, "time_dimension": {"answer": "Present", "rationale": ""}, "sentence_type": "Affirmative declarative sentence", "pos": {"nouns": {"Family": 1, "Chile": 1}, "verbs": {"to": 1}, "numerals": {"44": 1}}}, {"content": "My love life is rich.", "word_count": 9, "label": "", "sentence_structure": "Subject鈥揷opula鈥損redicative", "subjective_objective": {"answer": "Subjective", "rationale": "Because \"rich\" is a subjective feeling and an assessment, the understanding of love life may differ from one person to another, so the entry to the questionnaire is to examine the subjective views of the respondents."}, "sentence_sentiment": {"answer": "Positive", "rationale": "Because \"love life is rich\" expresses positive emotional attitudes and experiences, and it is a positive expression."}, "language_style": {"answer": "Tactful and warm", "rationale": "The language style of the entry is more euphemistic and gentle, asking each other whether they are rich in love in an invitation-style expression, which is more intimate and interactive. At the same time, the expression \"very rich\" has a certain emotional connotation, implying positive attitudes and desires to love life."}, "time_dimension": {"answer": "Present", "rationale": "And judging by the keywords and the context, \"my love life is rich\" describes a state of affairs or a state of affairs, so the time dimension is now."}, "sentence_type": "Affirmative declarative sentence", "pos": {"nouns": {"Love life": 1, "Rich": 1}, "adjectives": {"very": 1}, "numerals": {"45": 1}}}, {"content": "46. Few friends can trust and fail to meet the mutual commitments.", "word_count": 22, "label": "", "sentence_structure": "Subject鈥搗erb鈥搊bject", "subjective_objective": {"answer": "Subjective", "rationale": "Because this entry relates to patients鈥?subjective feelings and perceptions that they feel that they have little trust in their friends and are unable to meet the mutual commitments. This is viewed from the perspective of the patients themselves, and therefore is subjective."}, "sentence_sentiment": {"answer": "Negative", "rationale": "Because the behaviour and attitude of the entry, \"almost untrustworthy, unable to meet the mutually committed objectives\", is negative and indicates poor interpersonal relations and a lack of trust."}, "language_style": {"answer": "Calm and direct", "rationale": ""}, "time_dimension": {"answer": "Present", "rationale": "The entry now describes a state of affairs or situation in which virtually no one of the tested friends can trust to meet the mutual commitments. Keywords \"near anyone can trust\" and \"unable to meet the mutual commitments\" indicate that the state is a state of affairs, not a past or future. So the time dimension of this questionnaire item is now judged."}, "sentence_type": "Negative declarative sentence", "pos": {"nouns": {"friends": 1, "person": 1, "Objective": 1}, "verbs": {"Yeah": 1, "Trust": 1, "Satisfied": 1, "Commitments": 1, "Achieved": 1}, "adjectives": {"Almost": 1, "Nobody": 1, "To each other": 1}, "numerals": {"46": 1}}}, {"content": "47. Generally speaking, family members understand my strengths and strengths.", "word_count": 15, "label": "", "sentence_structure": "Subject鈥搗erb鈥搊bject", "subjective_objective": {"answer": "Objective", "rationale": "This is an objective description of family relations and does not involve subjective attitudes or feelings of the patient."}, "sentence_sentiment": {"answer": "Positive", "rationale": "Because the behaviour of \"family knows my strengths and strengths\" in the entry is positive."}, "language_style": {"answer": "Tactful and warm", "rationale": "The language style of the entry is more euphemistic, and the respondents are asked in a more soft way whether they feel that their families know their merits."}, "time_dimension": {"answer": "Present", "rationale": "\"Generally\" indicates that family members of the tested are usually aware of their strengths and advantages, a description of which is the current situation. The answer is therefore \"now\"."}, "sentence_type": "Affirmative declarative sentence", "pos": {"nouns": {"Home": 1, "person": 2, "Strengths": 1, "Advantages": 1}, "verbs": {"Roger that": 1, "In general": 1}, "adverbs": {"Land": 1}, "numerals": {"47": 1}, "pronouns": {"Mine": 1}}}, {"content": "I have at least one real friend.", "word_count": 11, "label": "", "sentence_structure": "Subject鈥搗erb鈥揷omplement", "subjective_objective": {"answer": "Subjective", "rationale": "Because \"real friends\" are a subjective criterion for evaluation, different people may have different definitions and understandings, and therefore need to be viewed from the patient's subjective perspective."}, "sentence_sentiment": {"answer": "Positive", "rationale": "Because having a true friend is a positive human relationship and social state."}, "language_style": {"answer": "Tactful and warm", "rationale": "The language style of the entry is more euphemismal. It asks the interviewee in a more friendly and friendly way whether he has a real friend, without direct orders or mandatory language. This expression is more likely to guide the interviewee to open his heart and answer the questions honestly."}, "time_dimension": {"answer": "Present", "rationale": "The keyword \"yes\" means that the time is now complete, and it means that the person who was tested has at least one friend at the moment of answering the question."}, "sentence_type": "Affirmative declarative sentence", "pos": {"nouns": {"friends": 1, "Grandpa": 1}, "verbs": {"have": 1, "Up to": 1, "One": 2}, "adjectives": {"Really": 1}, "adverbs": {"At least": 1}, "numerals": {"48": 1}}}, {"content": "49. No one in the neighbourhood will help me when I need it.", "word_count": 14, "label": "", "sentence_structure": "Subject鈥搗erb鈥搊bject", "subjective_objective": {"answer": "Objective", "rationale": "Because it is objective facts, not subjective attitudes, that anyone is willing to help."}, "sentence_sentiment": {"answer": "Negative", "rationale": "Because the description \"no one will help me\" in the entry is negative."}, "language_style": {"answer": "Calm and direct", "rationale": "The entry asks the subject of the respondent in direct language whether he or she will be assisted by his or her neighbours if he or she needs help, without too many rhetorical and emotional promotions, and with a more calm and straightforward language style."}, "time_dimension": {"answer": "Present", "rationale": "The words \"when required\" and \"no\" in the sentence would make it possible to judge that the act was occurring or was likely to occur in the future and was therefore a current state of affairs."}, "sentence_type": "Negative declarative sentence", "pos": {"nouns": {"Time": 1, "Neighbors": 2, "person": 1, "Help": 1}, "verbs": {"Yes": 2, "Help": 1}, "adverbs": {"When": 1, "Medium": 1, "Nobody": 1, "Both": 1, "not": 1}, "numerals": {"49": 1, "2": 1}, "pronouns": {"I": 1}}}, {"content": "50. My family lives in peace and harmony.", "word_count": 12, "label": "", "sentence_structure": "Subject鈥搗erb鈥揷omplement", "subjective_objective": {"answer": "Objective", "rationale": "This is an objective fact describing the family situation of the patient, based on observations and experiences. While it may affect the patient鈥檚 subjective feelings and attitudes, the main purpose of this entry is to provide factual information about the family situation and should therefore be considered objective."}, "sentence_sentiment": {"answer": "Positive", "rationale": "Because the entry refers to \"family life is easy and well-being\", it is a positive description of family harmony and happiness."}, "language_style": {"answer": "Tactful and warm", "rationale": "The language style of the entry is one of euphemism and warmth. It guides the interviewee to reflect on his family ties and well-being by describing the stability and harmony of family life, and is very friendly and emotional, and can trigger a resonance and positive response from the interviewee."}, "time_dimension": {"answer": "Past", "rationale": "The entry describes the memory and experience of the testees, the key words being \"family life is comfortable\" and \"conciliation\" -- all of which happened in the past."}, "sentence_type": "Affirmative declarative sentence", "pos": {"nouns": {"Family": 1, "Life": 1, "Easy": 1, "Get along": 1, "It's nice": 1}, "verbs": {"be": 1}, "adjectives": {"Mine": 1}, "numerals": {"50": 1}}}, {"content": "51. I moved too often to have long-term friendship with people.", "word_count": 19, "label": "", "sentence_structure": "Subject鈥揷opula鈥損redicative", "subjective_objective": {"answer": "Subjective", "rationale": "The questionnaire entries describe patients鈥?subjective feelings and experiences, and relate to their behaviour (the frequent movement) and the resulting consequences (the difficulty of maintaining long-term friendship with people)."}, "sentence_sentiment": {"answer": "Negative", "rationale": "Because the entries \"too often to move\" and \"unusual to maintain long-term friendship\" have negative emotional aspects."}, "language_style": {"answer": "Tactful and warm", "rationale": "Warm and warm."}, "time_dimension": {"answer": "Past", "rationale": "Past."}, "sentence_type": "Negative declarative sentence", "pos": {"nouns": {"I": 1, "Moving": 1, "Friendship": 1}, "verbs": {"Too often": 1, "Move": 1, "Hold on": 1, "and": 1}, "adjectives": {"Long term": 1}, "adverbs": {"So": 1}, "numerals": {"51": 1, "Frequent": 1}}}, {"content": "I get along well with each other in a relationship.", "word_count": 14, "label": "", "sentence_structure": "Subject鈥揷opula鈥損redicative", "subjective_objective": {"answer": "Subjective", "rationale": "Because \"good with each other in a relationship\" is a subjective description of the patient's situation."}, "sentence_sentiment": {"answer": "Positive", "rationale": "Because it expresses a positive emotional attitude, i.e., good to get along with each other in a relationship marriage."}, "language_style": {"answer": "Tactful and warm", "rationale": "The language style of the entry is euphemistic. It uses positive expressions to describe how you are in a relationship with each other, which is relatively soft, giving people a sense of friendship and affection. Moreover, the sentence does not contain too direct or hard words, and reduces the risk of possible resistance."}, "time_dimension": {"answer": "Present", "rationale": "\"In a relationship marriage\" means a specific period of time, and \"goodly spent\" describes the attitude or state of the person tested during this period, so the entry is a description of the current situation."}, "sentence_type": "Affirmative declarative sentence", "pos": {"nouns": {"I": 1, "Love marriage": 1}, "verbs": {"in": 1, "Get along": 1}, "adjectives": {"Good": 1}, "numerals": {"52": 1}}}, {"content": "53. I find it difficult to invite friends to work with me.", "word_count": 14, "label": "", "sentence_structure": "Subject鈥搗erb鈥搊bject", "subjective_objective": {"answer": "Subjective", "rationale": "Because finding it hard to ask friends to do things with me is the patient's subjective feeling and experience."}, "sentence_sentiment": {"answer": "Negative", "rationale": "Because the word \"hard\" in the entry expresses a negative mood and attitude."}, "language_style": {"answer": "Calm and direct", "rationale": "The language style of the entry is more calm and objective, and there is no excessive rhetorical or emotional overture, but rather a direct statement of the person ' s feelings, which is easier to understand. At the same time, the expression is relatively simple and clear and can effectively convey the message."}, "time_dimension": {"answer": "Past", "rationale": "Keywords \"discovery\" and \"exceed\" indicate that the question is asked whether the subject has ever experienced it in the past."}, "sentence_type": "Negative declarative sentence", "pos": {"nouns": {"I found out": 1, "friends": 1, "Yes": 1}, "verbs": {"Invitation": 1, "Do it": 1, "Found": 1}, "adjectives": {"Hard": 1}, "numerals": {"53": 1}}}, {"content": "54. I have had very little contact with my family.", "word_count": 8, "label": "", "sentence_structure": "Subject鈥搗erb鈥揷omplement", "subjective_objective": {"answer": "Subjective", "rationale": "Because \"I have little contact with my family\" is the patient's subjective feeling and attitude."}, "sentence_sentiment": {"answer": "Negative", "rationale": "Because the fact that \"very little contact with family\" is in the entry is an expression of a negative attitude and behaviour."}, "language_style": {"answer": "Tactful and warm", "rationale": "The entry is more adept and interactive when it is spoken in a gentle and inviting manner, rather than by direct statements or orders."}, "time_dimension": {"answer": "Past", "rationale": ""}, "sentence_type": "Negative declarative sentence", "pos": {"nouns": {"I": 1, "Family": 1}, "verbs": {"Exposure": 1, "Very few": 1}, "numerals": {"54": 1}}}, {"content": "55. Friends do not seem to be interested in me for long.", "word_count": 15, "label": "", "sentence_structure": "Subject鈥搗erb鈥搊bject", "subjective_objective": {"answer": "Subjective", "rationale": "Because the sentence expresses the patient ' s perception and feelings among friends and is a manifestation of a subjective attitude."}, "sentence_sentiment": {"answer": "Negative", "rationale": "Because the expression \"friends don't seem interested in me for long\" in the entry is negative."}, "language_style": {"answer": "Calm and direct", "rationale": "The entry expresses the subject in a more direct and objective manner, without excessive rhetoric or emotional rendering, is more plain and easier to understand and accept by the reader."}, "time_dimension": {"answer": "Present", "rationale": "In the future, the entry uses the expression \"Friends do not seem...\" , in which the term \"Looks\" indicates that it is a perception or a projection of what may occur in the future. The temporal dimension of the article is therefore judged to be the future."}, "sentence_type": "Negative declarative sentence", "pos": {"nouns": {"Friends": 1, "Time": 1, "Interest": 1}, "verbs": {"Looks like it": 1, "Yes": 1, "to": 1, "Interested": 1}, "adjectives": {"Long": 1}, "adverbs": {"not": 1, "Long time": 1}, "numerals": {"55": 1}, "pronouns": {"I": 1}}}, {"content": "There are people in the unit who understand my thoughts and beliefs.", "word_count": 14, "label": "", "sentence_structure": "Subject鈥搗erb鈥搊bject", "subjective_objective": {"answer": "Subjective", "rationale": "Because \"understanding\" and \"think and faith\" are subjective feelings and ideas of individuals, the entry is viewed from the subjective perspective of patients."}, "sentence_sentiment": {"answer": "Positive", "rationale": "Because the fact that \"someone understands my thoughts and beliefs\" in the entry is an expression of positive emotional tendencies."}, "language_style": {"answer": "Tactful and warm", "rationale": "The entry is more personal and interactive when it is spoken in a gentle and inviting manner, rather than in direct statements or orders. At the same time, the narrative is simpler and easier to understand by the interviewee."}, "time_dimension": {"answer": "Present", "rationale": "The keyword \"unit\" indicates that it occurs in the current working environment, and that the \"someone understands\" behaviour is also being performed. So, the entry describes the current feelings and experiences of the person being tested."}, "sentence_type": "Affirmative declarative sentence", "pos": {"nouns": {"Units": 1, "person": 1, "Thoughts": 1, "Faith": 1}, "verbs": {"Understood": 1, "have": 1}, "numerals": {"56": 1}}}, {"content": "I try to avoid my family as much as possible.", "word_count": 9, "label": "", "sentence_structure": "Subject鈥搗erb鈥揷omplement", "subjective_objective": {"answer": "Subjective", "rationale": "Because what I'm trying to do is subjective thinking and attitude."}, "sentence_sentiment": {"answer": "Negative", "rationale": "Because the \"screw your family\" act is negative in the entry."}, "language_style": {"answer": "Calm and direct", "rationale": "The entry is in the form of a clear statement asking the respondents whether they are avoiding family ties, with a language temperature and objectivity, and without excessive emotional rendering or rhetorical techniques, so that the language style is straightforward and calm."}, "time_dimension": {"answer": "Present", "rationale": ""}, "sentence_type": "Negative declarative sentence", "pos": {"nouns": {"Family": 1}, "verbs": {"Avoidance": 1, "Try": 1}, "adverbs": {"As far as possible": 1}, "numerals": {"57": 1}}}, {"content": "It's hard to get emotional security from a relationship.", "word_count": 18, "label": "", "sentence_structure": "Subject鈥揷opula鈥損redicative", "subjective_objective": {"answer": "Subjective", "rationale": "Because \"I have difficulty getting emotional security from a relationship\" is a patient's subjective feeling and experience. Although it is based on some objective fact (such as past experience or current circumstances), it is expressed in a way that reflects the patient's emotional state and is therefore considered subjective."}, "sentence_sentiment": {"answer": "Negative", "rationale": "Because the expression \"difficulties of emotional security\" in this article is negative."}, "language_style": {"answer": "Calm and direct", "rationale": "The entry, in a statement, clearly indicates the emotional problems of the person under investigation in a relationship marriage, without excessive rhetorical or emotional rendering, and with a more balanced and objective tone."}, "time_dimension": {"answer": "Present", "rationale": "According to the word \"hard\" in \"I'm hard\" and the general \"sweet\" in the current state, it can be seen as describing the emotional state or experience of the subject."}, "sentence_type": "Negative declarative sentence", "pos": {"nouns": {"Love marriage": 1, "Emotion": 1, "Safe": 1}, "verbs": {"Got it": 1}, "adjectives": {"It's hard": 1}, "numerals": {"58": 1}, "pronouns": {"I": 1}}}, {"content": "59. My opinion is usually taken very seriously when making decisions at home.", "word_count": 16, "label": "", "sentence_structure": "Subject鈥搗erb鈥搊bject", "subjective_objective": {"answer": "Subjective", "rationale": "My opinion is usually taken seriously in decisions made at home by patients."}, "sentence_sentiment": {"answer": "Positive", "rationale": "Because the action of \"respect my opinion\" in the entry is positive and indicates that family members respect the views and suggestions of individuals."}, "language_style": {"answer": "Tactful and warm", "rationale": "The title uses a more euphemistic approach to questioning the respondent's position and authority in the family decision-making process, using the phrase 鈥渦sually important鈥?and not too direct or hard-line, reflecting respect for and equality of the interviewee. The language style of the article is thus described as 鈥渇luent鈥?"}, "time_dimension": {"answer": "Past", "rationale": "The keyword \"at home when making decisions\" usually indicates \"a customary past behaviour, and the verb \"takes my opinion seriously\" further proves this. Thus, the time dimension of the article is the past."}, "sentence_type": "Affirmative declarative sentence", "pos": {"nouns": {"Home": 1, "Decision": 1, "Observations": 1}, "verbs": {"Do it": 1, "Value": 1}, "adverbs": {"Both": 1, "very": 1}, "numerals": {"59": 1}, "pronouns": {"I": 1}}}, {"content": "Most friends do care about my suffering.", "word_count": 13, "label": "", "sentence_structure": "Subject鈥搗erb鈥搊bject", "subjective_objective": {"answer": "Subjective", "rationale": "Because \"most friends do care about my illness\" is a subjective judgment that requires patients to answer according to their own feelings."}, "sentence_sentiment": {"answer": "Positive", "rationale": "Because the phrase \"most friends do care about my pain\" in the entry conveys positive emotional colours and attitudes."}, "language_style": {"answer": "Tactful and warm", "rationale": "The entry is more adept and interactive when it is spoken in a gentle and inviting manner, rather than by direct statements or orders."}, "time_dimension": {"answer": "Present", "rationale": "The entry \"most friends\" indicates that this is a description of the current situation, while the word \"care\" implies that it is a continuous act or attitude. So, the time dimension of this purpose is now."}, "sentence_type": "Affirmative declarative sentence", "pos": {"nouns": {"friends": 1, "It's hard": 1}, "verbs": {"Care": 1}, "adjectives": {"Most of them": 1}, "adverbs": {"Indeed": 1}}}]}

{"questionnaire_title": "Questionnaire on the Questionnaire on the Response to Nature (TCSQ)", "questionnaire_category": "Behavioral questionnaire", "item_count": 20, "emotion_shift_overall": "From positive to negative", "language_style_overall": "Tactful and warm", "items": [{"content": "I'll forget about it as soon as possible.", "word_count": 10, "label": "", "sentence_structure": "Subject鈥搗erb鈥揷omplement", "subjective_objective": {"answer": "Subjective", "rationale": "Because the ability to forget unhappy experiences as quickly as possible is the patient ' s subjective approach and ability to deal with them, it is not an objective reality or result."}, "sentence_sentiment": {"answer": "Positive", "rationale": "Because what's in the entry \"can forget\" is a positive, positive attitude."}, "language_style": {"answer": "Tactful and warm", "rationale": "The entry is more adept and interactive when it is spoken in a gentle and inviting manner, rather than by direct statements or orders."}, "time_dimension": {"answer": "Present", "rationale": "Now the entry asks the testee about his current behaviour and attitude, \"can\" means a behavior of ability or custom, and \"will forget it\" concerns the way in which past experiences are handled. So, the time dimension of the entry is now judged."}, "sentence_type": "Imperative sentence", "pos": {"nouns": {"It's not good": 1, "Forget it": 1}, "verbs": {"Will": 1, "Forget it": 1}, "adjectives": {"Come on": 1, "Speed": 1}, "adverbs": {"End of story": 1, "Quick": 2}, "classifiers": {"individual": 1}}}, {"content": "2. The easily ensnared memories and illusions of events", "word_count": 19, "label": "", "sentence_structure": "Subject鈥揷opula鈥損redicative", "subjective_objective": {"answer": "Subjective", "rationale": "Because it reflects the patient ' s own thoughts, feelings and emotions, it is a subjective description of the patient ' s experience."}, "sentence_sentiment": {"answer": "Negative", "rationale": "It's negative because the entry \"can't get rid of the memory and illusion of events.\""}, "language_style": {"answer": "Calm and direct", "rationale": "The entry asks the respondents in a concise manner whether they are prone to the recollection and illusion of events, without too many emotional accrimations or rhetorical techniques, and in a more direct manner.\""}, "time_dimension": {"answer": "Present", "rationale": "The entry describes the current behaviour or state of the testee, which is \"in the memory and illusion of events\" and indicates that this is an ongoing process and therefore is now judged."}, "sentence_type": "Affirmative declarative sentence", "pos": {"nouns": {"Questionnaire": 1, "Item": 1, "I'm a little too easy to get in": 1, "person": 1, "Events": 1, "Memories": 1, "Fantasy": 1, "Centre": 1, "Get rid of it": 1}, "verbs": {"Strap": 1, "to": 1, "No, I can't": 1, "Get rid of it": 1}, "adverbs": {"Medium": 1, "Easy": 1, "No, I can't": 1}, "numerals": {"2": 1}, "pronouns": {"鈾?And the 鈾?: 1}}}, {"content": "3. As if nothing had happened.", "word_count": 10, "label": "", "sentence_structure": "Subject鈥搗erb鈥揷omplement", "subjective_objective": {"answer": "Objective", "rationale": "Because it is the way things have been dealt with in the past, as they have not happened, and it has already happened, and therefore is an objective result."}, "sentence_sentiment": {"answer": "Negative", "rationale": "Because the \"asymmetrical never happened\" act in the entry is negative, it shows that individuals have not taken a positive attitude to face and resolve the events that occurred."}, "language_style": {"answer": "Tactful and warm", "rationale": "The entry is more euphemistic and warm, as it does not question or accuse the interviewee directly, but rather leads the other in a more inclusive and understanding way. It expresses an attitude of seeking the views and feelings of the interviewee, and also reflects respect and interest for the interviewee."}, "time_dimension": {"answer": "Past", "rationale": "According to the context, \"as\" means a subjective psychological approach, while \"not happening at all\" refers to a past event or situation. The entry therefore asks the person who was tested for a past psychological response and treatment, and the temporal dimension is a past."}, "sentence_type": "Imperative sentence", "pos": {"nouns": {"Things": 1, "Come on": 1}, "verbs": {"Consider": 1, "Not": 1}, "adverbs": {"Not at all": 1}}}, {"content": "4. Easier to be angry and more often angry", "word_count": 12, "label": "", "sentence_structure": "Subject鈥搗erb鈥搊bject", "subjective_objective": {"answer": "Subjective", "rationale": "Because temperament is a subjective emotional reaction and attitude of a person, not an objective fact or event. While it may be related to some objective circumstances (e.g., facing pressure or frustration), it is a personal decision, and therefore subjective, to choose how to deal with them and express one鈥檚 feelings."}, "sentence_sentiment": {"answer": "Negative", "rationale": "Because \"reluctant anger\" and \"frequent anger\" are negative behavioural descriptions, expressing a negative emotional tendency."}, "language_style": {"answer": "Calm and direct", "rationale": "The language style of the entry is a calm and straightforward type. It does not use excessive emotional colours or rhetorical techniques, but rather presents the facts and asks the person under investigation.\""}, "time_dimension": {"answer": "Present", "rationale": "Now the entry describes a customary behaviour of the testee, the description of \"resistence\" and \"recurring temperamentality\" all implying that it is a current and continuing behaviour. So, judging from the time dimension, the entry is now."}, "sentence_type": "Negative declarative sentence", "pos": {"nouns": {"Questionnaire": 1, "Entry": 1, "Easier to be angry": 1, "others": 1, "- Grumpy": 1}, "verbs": {"Analysis": 1, "Exercising": 1, "Let's go": 1}, "adjectives": {"Regular": 1}, "numerals": {"4": 1}}}, {"content": "5. Usually, in the best sense, try to get a little bit of it.", "word_count": 11, "label": "", "sentence_structure": "Subject鈥搗erb鈥揷omplement", "subjective_objective": {"answer": "Subjective", "rationale": "This is a way of thinking and attitude of patients and is a subjective examination."}, "sentence_sentiment": {"answer": "Positive", "rationale": "The expression \"in the best interests\" and \"in the best interests\" suggests that the entry expresses a positive attitude or quality. The answer is therefore positive."}, "language_style": {"answer": "Tactful and warm", "rationale": "The entry uses encouragement and positive language, which attempts to induce respondents to view things positively and with optimism and to communicate with a caring attitude. The language style helps create a friendly, friendly atmosphere in which the interviewer feels respected and understood. At the same time, the entry also helps to guide the interviewer to a healthier psychological model by using the verb \"thinking to the good\" and \"want to be open\" and, to some extent, positive life attitudes and values."}, "time_dimension": {"answer": "Present", "rationale": ""}, "sentence_type": "Imperative sentence", "pos": {"nouns": {"Questionnaire Entry": 1, "In terms of": 1}, "verbs": {"Yes": 2}, "adjectives": {"Okay": 1, "Open": 1}, "adverbs": {"Usually": 1, "Some": 1}}}, {"content": "Unhappy things can easily cause emotional fluctuations.", "word_count": 14, "label": "", "sentence_structure": "Subject鈥揷opula鈥損redicative", "subjective_objective": {"answer": "Objective", "rationale": "Because \"unhappy things\" and \"emotional fluctuations\" are both observable facts or outcomes, they are not subjective feelings or attitudes of patients. So this is seen from an objective perspective."}, "sentence_sentiment": {"answer": "Negative", "rationale": "Because the \"unhappy things can easily cause emotional fluctuations\" in the entry are negative."}, "language_style": {"answer": "Tactful and warm", "rationale": ""}, "time_dimension": {"answer": "Present", "rationale": "The keyword \"problems\" indicates that it is the present situation or tendency, and does not refer explicitly to past or future situations. Thus, the temporal dimension of the purpose of the article is now judged."}, "sentence_type": "Negative declarative sentence", "pos": {"nouns": {"Yes": 1, "Emotional fluctuations": 1}, "verbs": {"Cause": 1}, "adjectives": {"It's not good": 1}, "adverbs": {"very": 1, "Easy": 1}, "classifiers": {"individual": 1}}}, {"content": "I like to keep my emotions inside, but I can't forget it.", "word_count": 22, "label": "", "sentence_structure": "Subject鈥搗erb鈥搊bject", "subjective_objective": {"answer": "Subjective", "rationale": "Because \"forget it\" is the patient's subjective feeling and thought."}, "sentence_sentiment": {"answer": "Negative", "rationale": "Because behavior in the entry \"crushes the mood inside and doesn't show it\" can lead to negative emotional and psychological effects."}, "language_style": {"answer": "Tactful and warm", "rationale": "The entry is a soft, understanding conversation, rather than directly asking or accusing the other of having done so. This expression is more personal and interactive, and it allows the respondents to feel the researcher's interest in and understanding of his feelings. At the same time, the descriptions of \"insisting his heart from showing\" and \"forgetting out\" are also euphemistic expressions of some of the emotional state of the respondent, rather than pointing directly to the problem."}, "time_dimension": {"answer": "Present", "rationale": ""}, "sentence_type": "Imperative sentence", "pos": {"nouns": {"Emotion": 1, "In my heart": 1}, "verbs": {"Yeah": 1, "Press": 1, "Jean": 1, "Table": 1, "Forget it": 1, "Come out": 1}, "adverbs": {"in": 1, "Lee": 1, "not": 2}, "classifiers": {"individual": 1}, "pronouns": {"Other": 1}}}, {"content": "8. It's usually nothing compared to similar people.", "word_count": 17, "label": "", "sentence_structure": "Subject鈥揷opula鈥損redicative", "subjective_objective": {"answer": "Subjective", "rationale": "Because \"I feel nothing\" is self-evaluation and perception of patients, and it is a subjective examination."}, "sentence_sentiment": {"answer": "Negative", "rationale": "Because the attitude of \"necessary\" in the entry is negative."}, "language_style": {"answer": "Calm and direct", "rationale": "The entry is more direct and concise in language style, with no excessive emotional accoutrements or rhetorical techniques. It opens its doors to questions and asks the interviewees for comparisons, which are relatively calm and objective.\""}, "time_dimension": {"answer": "Past", "rationale": "In the entry, the word \"just\" indicates a comparison between the past and the present, implying that the testee has felt this at some point in the past and that this feeling may continue to this day. So, in terms of the dimensions of time, it is the past."}, "sentence_type": "Affirmative declarative sentence", "pos": {"nouns": {"Questionnaire Entry": 1, "person": 1, "- Yeah": 1, "Compare": 1}, "verbs": {"I think": 1, "Fine": 1, "Okay": 1}, "adjectives": {"Yeah": 1, "Category": 1, "Like": 1}, "adverbs": {"Usually": 1, "Just": 1}, "numerals": {"8": 1}, "pronouns": {"Here": 1}}}, {"content": "9. Be able to translate negative factors into positive factors, e.g., participation in events, more quickly", "word_count": 20, "label": "", "sentence_structure": "Subject鈥搗erb鈥搊bject", "subjective_objective": {"answer": "Subjective", "rationale": "This is a self-presentation of the patient ' s situation, an expression of his or her attitude or belief in a certain capacity, and a subjective examination."}, "sentence_sentiment": {"answer": "Positive", "rationale": "According to the entry, \"the ability to convert negative factors into positive ones faster\" is a positive quality."}, "language_style": {"answer": "Tactful and warm", "rationale": "The language style of the entry is relatively euphemistic and warm. It asks the respondent in an invitation manner whether he or she is able to turn negative factors into positive ones and gives examples."}, "time_dimension": {"answer": "Present", "rationale": "The act of \"can quickly turn negative into positive\" is an act or habit currently being performed by the testee, and thus the temporal dimension is now judged."}, "sentence_type": "Imperative sentence", "pos": {"nouns": {"Factors": 2, "Activities": 1}, "verbs": {"Will": 1, "Participation": 1, "Negative": 1, "_Other Organiser": 1, "For example": 1}, "adjectives": {"Compare": 1, "Positive": 1}, "adverbs": {"Faster": 1}, "numerals": {"9": 1}}}, {"content": "It's easy to cry when you're in trouble.", "word_count": 15, "label": "", "sentence_structure": "Subject鈥搗erb鈥揷omplement", "subjective_objective": {"answer": "Subjective", "rationale": "Because \"easy to think\" represents the patient's thoughts and emotional reactions, and it's subjective."}, "sentence_sentiment": {"answer": "Negative", "rationale": "Because the entry \"the trouble is easy to cry\" is negative."}, "language_style": {"answer": "Tactful and warm", "rationale": "The language style of the entry is more euphemistic, and conveys concern and understanding of the interviewee's feelings, rather than directly pointing out their problems or problems. This expression is relatively more friendly and inclusive, helping to increase the interviewee's trust and willingness to cooperate."}, "time_dimension": {"answer": "Present", "rationale": ""}, "sentence_type": "Affirmative declarative sentence", "pos": {"nouns": {"Trouble": 1, "Yes": 1, "Field": 1}, "verbs": {"Yes": 2, "Cry": 1}, "adverbs": {"very": 1, "Easy": 1, "Quietly": 1}, "numerals": {"10": 1}}}, {"content": "It's easy to get you back on your feet.", "word_count": 13, "label": "", "sentence_structure": "Subject鈥搗erb鈥揷omplement", "subjective_objective": {"answer": "Subjective", "rationale": "Because \"others can easily make you happy again\" describes patients' subjective feelings and attitudes."}, "sentence_sentiment": {"answer": "Positive", "rationale": "Because the expression \"others can easily replete you\" means positive, indicating that individuals have a high degree of dependency and expectation for emotional support and help from others."}, "language_style": {"answer": "Tactful and warm", "rationale": "The entry is more personal and interactive in its expression of a gentle and inviting tone, rather than a direct statement or order. At the same time, the expression \"others can easily replete you\" is also positive and emotional, giving the respondents a sense of respect and attention."}, "time_dimension": {"answer": "Present", "rationale": "The entry indicates that \"rejoying someone\" is a normal act, implying that the emotional state is recurrent, not at a particular point in time or in the future. Thus, the judgement is \"now\"."}, "sentence_type": "Affirmative declarative sentence", "pos": {"nouns": {"The others": 1, "Happy": 2}, "verbs": {"鈾?And I'm gonna be 鈾?: 1, "Restart": 1, "Get up": 1}, "adverbs": {"very": 1, "Easy": 1}, "numerals": {"11": 1}}}, {"content": "12. If there is conflict with a person, it is preferable to ignore the other for a long time.", "word_count": 16, "label": "", "sentence_structure": "Subject鈥搗erb鈥搊bject", "subjective_objective": {"answer": "Subjective", "rationale": "Because \"I'd rather ignore each other for a long time\" is a subjective choice and attitude of a person."}, "sentence_sentiment": {"answer": "Negative", "rationale": "Because the \"long-term disregard\" in the entry is negative."}, "language_style": {"answer": "Calm and direct", "rationale": "The language style of the entry is more direct and clear, and there is no excessive rhetorical or emotional rendering, but rather a simple presentation of the subject matter. This is more objective and rational and is more in line with the characteristics of the questionnaire."}, "time_dimension": {"answer": "Future", "rationale": "The keyword \"if\" and \"better\" both imply that it is a hypothetical or possible future. The entry therefore asks how the person tested will be treated in the future in the face of conflict, judging by the future."}, "sentence_type": "Imperative sentence", "pos": {"verbs": {"Come on": 1, "Conflict": 1, "I'd rather not": 1, "I don't care": 1}, "adjectives": {"Long term": 1}, "numerals": {"12": 1}}}, {"content": "13. Too often, the key difficulties are too difficult to be addressed.", "word_count": 16, "label": "", "sentence_structure": "Subject鈥搗erb鈥揷omplement", "subjective_objective": {"answer": "Subjective", "rationale": "Because \"uncertainty\" and \"unthinkable\" are the feelings and thoughts of the patient, they are subjective."}, "sentence_sentiment": {"answer": "Negative", "rationale": "Because the entries \"uncertainty\" and \"unthinkable\" are negative expressions."}, "language_style": {"answer": "Calm and direct", "rationale": "Because the entry is in an objective and clear manner asking the other party whether it is often hesitant in the face of major difficulties, it is not based on excessive rhetorical or emotional rendering, and it is more straightforward and straightforward."}, "time_dimension": {"answer": "Present", "rationale": "The key words \"uncertainty\" and \"unthinkable\" now indicate that the act is in its present state or custom. Moreover, the word \"often\" also indicates that this is a situation that exists in past experience, but remains the main focus of attention. The entry therefore asks about the person being tested's current behaviour or mentality."}, "sentence_type": "Negative declarative sentence", "pos": {"nouns": {"Major difficulties": 1, "I'm not sure what to do": 1, "Approach": 1}, "verbs": {"Often": 1, "I've got it": 1}, "adverbs": {"Regular": 1}, "numerals": {"13": 1}}}, {"content": "14. Be able to adapt quickly to hardship and suffering", "word_count": 11, "label": "", "sentence_structure": "Subject鈥揷opula鈥損redicative", "subjective_objective": {"answer": "Subjective", "rationale": "Because it is the feelings and perceptions of the patient himself that are able to adapt quickly to difficulties and suffering, it is a subjective perspective."}, "sentence_sentiment": {"answer": "Positive", "rationale": "Because it is a positive quality that it means that people can adapt quickly to difficulties and suffering."}, "language_style": {"answer": "Calm and direct", "rationale": "The language style of the entry is more direct, concise, and there is no excessive accommodation or emotional colour. It appears in a clear question form, asking the interviewee to answer whether he or she can adapt quickly to difficulties and suffering, which is more objective and practical."}, "time_dimension": {"answer": "Present", "rationale": "The keyword is \"fast-adapted\" and it is now judged by the ability or attitude of the testee."}, "sentence_type": "Imperative sentence", "pos": {"nouns": {"Difficulties": 1, "Pain": 1}, "verbs": {"Yes": 1, "Soon": 1, "Adaptation": 1}, "adverbs": {"very": 1}, "numerals": {"14": 1}}}, {"content": "15. Believe in difficulties and frustrations to work out.", "word_count": 12, "label": "", "sentence_structure": "Subject鈥搗erb鈥揷omplement", "subjective_objective": {"answer": "Subjective", "rationale": "Believe is subjective."}, "sentence_sentiment": {"answer": "Positive", "rationale": "Because the purpose of the article is to express the belief that difficulties and frustrations can be exercised, this attitude is positive."}, "language_style": {"answer": "Tactful and warm", "rationale": "The language style of the entry is more euphemistic and encouraging than direct statement or order. It leads the interviewee to reflect on his attitude and conviction by asking a question and conveys a positive message that he should be optimistic and confident that he can exercise in the face of difficulties and setbacks. This expression is more personal and interactive."}, "time_dimension": {"answer": "Past", "rationale": "In terms of time dimension, it has no clear past, present or future orientation, and therefore cannot be clearly classified as a past, present or future act or mentality. However, in terms of context, this entry can be considered a review of current perceptions."}, "sentence_type": "Affirmative declarative sentence", "pos": {"nouns": {"Difficulties": 1, "Frustration": 1, "person": 1, "Exercise": 1}, "verbs": {"Yes": 1, "Yeah": 1}, "numerals": {"15": 1}}}, {"content": "16. Reminiscent of the unpleasant events that have occurred in a long time.", "word_count": 17, "label": "", "sentence_structure": "Subject鈥搗erb鈥搊bject", "subjective_objective": {"answer": "Subjective", "rationale": "Because \"remembering the unpleasant things\" requires the patient's own psychological activity and emotional involvement, which is a manifestation of subjective feelings."}, "sentence_sentiment": {"answer": "Negative", "rationale": "Because the \"unhappy memories\" in the entry are negative."}, "language_style": {"answer": "Calm and direct", "rationale": "The entry directly expresses the subject of asking the other party whether he or she is constantly reminiscent of past unpleasantness, without excessive rhetoric or emotional rendering, and is more objective in tone."}, "time_dimension": {"answer": "Present", "rationale": "The entry now asks the subject about his current behaviour or state of affairs, i.e. whether they will recall in a long time the unpleasant things that he or she has experienced."}, "sentence_type": "Affirmative declarative sentence", "pos": {"nouns": {"Time": 1, "Memories": 1, "Experience": 1, "Events": 1}, "verbs": {"I met him": 1, "Backtrace": 1}, "adjectives": {"It's not good": 1}, "adverbs": {"Long": 1}, "numerals": {"16": 1, "One": 1}, "classifiers": {"individual": 1}}}, {"content": "17. Problems often blame themselves for being incompetent and resenting themselves.", "word_count": 17, "label": "", "sentence_structure": "Subject鈥搗erb鈥搊bject", "subjective_objective": {"answer": "Subjective", "rationale": "This is because patients evaluate and interpret their behaviour and is clearly subjective."}, "sentence_sentiment": {"answer": "Negative", "rationale": "Because \"criticizing myself incompetent\" and \"hate yourself\" in the entry are negative emotional expressions."}, "language_style": {"answer": "Tactful and warm", "rationale": "The entry is more gentle and caring in its language style, using terms such as \"irresponsibility\" and \"hate\" to describe negative emotions, but the overall tone is more flat, with no overly direct or sharp expression. Moreover, the word \"often\" also shows an understanding of and concern for the individual emotional state, which is more in keeping with the euphemism of the language style."}, "time_dimension": {"answer": "Present", "rationale": ""}, "sentence_type": "Negative declarative sentence", "pos": {"nouns": {"Difficulty": 1, "Blame": 1, "self": 2, "Incompetent": 1, "Hate": 1}, "verbs": {"I met him": 1, "Often": 1, "Blame": 1, "And": 1, "Hate": 1}, "adjectives": {"Difficulty": 1, "Incompetent": 1, "Hate": 1}, "adverbs": {"Often": 1}, "pronouns": {"self": 2}}}, {"content": "18. I think there's nothing wrong with the world.", "word_count": 18, "label": "", "sentence_structure": "Subject鈥搗erb鈥揷omplement", "subjective_objective": {"answer": "Subjective", "rationale": "Because \"thinks\" is the patient's subjective attitude."}, "sentence_sentiment": {"answer": "Negative", "rationale": "Because \"Nothing big\" in the entry is a negative, unimportant attitude."}, "language_style": {"answer": "Tactful and warm", "rationale": "This entry is a soft, invitation-style conversation, not a direct statement or order. This expression is more personal and interactive, making the questionnaire more participatory and attractive. It also uses some oral expressions (such as \"underground,\" \"no big deal\"), which also enhances the effectiveness of and the proximity to the respondents. Overall, the entry is more linguistically focused on communication and communication with people, a more intimate, gentle language style."}, "time_dimension": {"answer": "Present", "rationale": "Now the entry asks the testee's current attitude and opinion, and the keyword \"considers\" and \"doesn't matter\" points to the current situation."}, "sentence_type": "Affirmative declarative sentence", "pos": {"nouns": {"I'm not sure": 1}, "adjectives": {"There's no big deal in the world": 1}}}, {"content": "19. The troubles of being alone.", "word_count": 11, "label": "", "sentence_structure": "Subject鈥搗erb鈥揷omplement", "subjective_objective": {"answer": "Subjective", "rationale": "Because it's the patient's subjective attitude."}, "sentence_sentiment": {"answer": "Negative", "rationale": "Because the act of \"like one's own person in a difficult situation\" in the entry may be negative, because it may indicate that an individual does not like to seek help from others or is unwilling to share his or her emotional problems."}, "language_style": {"answer": "Calm and direct", "rationale": "The entry directly enquired about the tendency of the respondents to be troubled, not using excessive adoration or emotional colours, and to be more objective and calm."}, "time_dimension": {"answer": "Present", "rationale": ""}, "sentence_type": "Affirmative declarative sentence", "pos": {"nouns": {"It's a pain in the ass": 1, "person": 2, "Alone": 1}, "verbs": {"Yes": 2, "Hi, Hi": 1, "Alone": 1}, "numerals": {"19": 1}, "classifiers": {"individual": 1}}}, {"content": "20. Usually, it's funny to resolve an awkward situation.", "word_count": 14, "label": "", "sentence_structure": "Subject鈥搗erb鈥揷omplement", "subjective_objective": {"answer": "Subjective", "rationale": "Because the use of humor to resolve an awkward situation is an individual ' s behaviour and attitude, reflecting patients ' subjective ideas and approaches to the problem."}, "sentence_sentiment": {"answer": "Positive", "rationale": "Because the entries \"resolving the embarrassment\" and \"juspitation\" are both positive."}, "language_style": {"answer": "Tactful and warm", "rationale": "In this entry, expressions such as \"normally,\" \"jusspitablely\" and \"disturbing embarrassment\" are more mild, less lax, less direct or command-like, more acceptable and resonant. At the same time, the topic \"defusing embarrassment\" is also closer to life, and it is easier to resonate and engage the respondents."}, "time_dimension": {"answer": "Present", "rationale": "The entry asks the testees about their current behaviour habits, i.e. whether they often use humor to resolve awkward situations, and therefore it is now judged."}, "sentence_type": "Exclamatory sentence", "pos": {"nouns": {"Modalities": 1, "Unusual situation": 1}, "verbs": {"Dismantling": 1, "Usually": 1}, "adjectives": {"Humor": 1}}}]}

{"questionnaire_title": "Fear of negative evaluation (FNE)", "questionnaire_category": "Mental health", "item_count": 30, "emotion_shift_overall": "From positive to negative", "language_style_overall": "Tactful and warm", "items": [{"content": "I'm rarely afraid of being stupid in other eyes.", "word_count": 16, "label": "", "sentence_structure": "Subject鈥搗erb鈥揷omplement", "subjective_objective": {"answer": "Subjective", "rationale": "Because \"I'm rarely worried\" means it's the patient's own thoughts and attitudes."}, "sentence_sentiment": {"answer": "Positive", "rationale": "Because the phrase \"I rarely worry about being stupid in other people's eyes\" is positive, indicating that individuals have a high degree of self-confidence and self-esteem in their own appearance, abilities, etc."}, "language_style": {"answer": "Tactful and warm", "rationale": "The entry is more personal and interactive when it is spoken in a question-and-answer tone rather than in direct statements or orders. At the same time, the entry is worded in a more balanced manner, with no overly subjective or emotional bias. The language style of the questionnaire entry is therefore judged to be euphemistic."}, "time_dimension": {"answer": "Present", "rationale": "\"I have little fear\" in \"almost\" of a low frequency behaviour, while \"fear\" of a general state of affairs means that it has not only occurred in the past, but may still be ongoing. The entry therefore asks about the person's current behaviour or mental state."}, "sentence_type": "Negative declarative sentence", "pos": {"nouns": {"Questionnaire": 1, "Entry": 1, "person": 2, "In the eyes": 1, "self": 2, "Stupid": 1}, "verbs": {"I'm worried": 1, "It seems": 1, "Answer": 1}, "adjectives": {"Very few": 1}, "numerals": {"One": 2}}}, {"content": "I fear what people will think of me, although I know it doesn't matter.", "word_count": 21, "label": "", "sentence_structure": "Subject鈥搗erb鈥搊bject", "subjective_objective": {"answer": "Subjective", "rationale": "I'm afraid that people will think or feel that I'm a person, even if he knows it doesn't matter."}, "sentence_sentiment": {"answer": "Negative", "rationale": "Because the \"fear what people will think\" in the entry is a negative."}, "language_style": {"answer": "Tactful and warm", "rationale": "According to the title, \"I know it's not important,\" the author can see that he is trying to face the problem in a more positive manner. And, \"I'm afraid that I'm going to look at me,\" the part that expresses a caring attitude, which is a euphemism."}, "time_dimension": {"answer": "Present", "rationale": "The word \"I'm worried\" in it means a present mood and a mental state, and \"what people will look at me\" is also what happens now, so the sentence describes the current behaviour or mental state of the subject."}, "sentence_type": "Negative declarative sentence", "pos": {"nouns": {"person": 2, "He's": 1, "self": 1, "Problem": 1}, "verbs": {"Fear": 1, "be": 1, "Yes": 1}, "adjectives": {"What": 1, "It's important": 1}, "adverbs": {"鈾?Though 鈾?: 1}, "pronouns": {"I": 1}}}, {"content": "3. I would be very nervous to know that someone is judging me.", "word_count": 22, "label": "", "sentence_structure": "Subject鈥損redicate", "subjective_objective": {"answer": "Subjective", "rationale": "This is because of patients ' subjective feelings and reactions to others ' evaluations."}, "sentence_sentiment": {"answer": "Negative", "rationale": "Because the \"sweet\" mood in the entry is negative."}, "language_style": {"answer": "Tactful and warm", "rationale": "The language style of the entry is more euphemistic. It neither touches the sensitive nerves too directly by using hypothetical words like \"if you know\" nor makes them realize that they may feel that way, and give comfort and support. Moreover, the term \"very nervous\" expresses a emotional feeling, bringing the problem closer to the feelings and experiences inside the individual."}, "time_dimension": {"answer": "Present", "rationale": "At the same time, \"very nervous\" expresses a strong emotional state, so it can be judged that the entry is looking at the person's current emotional reactions. In conclusion, the article is now judged in terms of the temporal dimension."}, "sentence_type": "Affirmative declarative sentence", "pos": {"nouns": {"person": 1, "A review of the quality of the body": 1, "I'm nervous": 1}, "verbs": {"Yes": 3, "in": 1, "to": 1, "Do it": 1}, "adjectives": {"Tens": 1}, "adverbs": {"If": 1, "Just": 1, "very": 1}, "pronouns": {"I": 1}}}, {"content": "I don't care if I know people are creating an impression against me.", "word_count": 24, "label": "", "sentence_structure": "Subject鈥搗erb鈥搊bject", "subjective_objective": {"answer": "Subjective", "rationale": "Because \"not caring\" is a subjective attitude or feeling."}, "sentence_sentiment": {"answer": "Negative", "rationale": "Because the article contains a negative attitude that does not care about the negative perception of others."}, "language_style": {"answer": "Calm and direct", "rationale": ""}, "time_dimension": {"answer": "Past", "rationale": "In terms of time dimension, the behaviour or mentality of the purpose of this article does not occur at a specific point in time, past or future, but rather expresses a long-term, continuous psychological state. Thus, the entry describes the current psychological state."}, "sentence_type": "Negative declarative sentence", "pos": {"nouns": {"Questionnaire": 1, "Entry": 1, "person": 1, "Impression": 1}, "verbs": {"Yes": 1, "Form": 1, "I don't care": 1}, "adjectives": {"Negative": 1}, "adverbs": {"Even if": 1}, "numerals": {"4": 1}, "pronouns": {"I": 2}}}, {"content": "5. When I make a social mistake, I am very unhappy.", "word_count": 17, "label": "", "sentence_structure": "Subject鈥揷opula鈥損redicative", "subjective_objective": {"answer": "Subjective", "rationale": "Because \"very unhappy\" reflects patients' subjective feelings and emotional reactions to their social performance."}, "sentence_sentiment": {"answer": "Negative", "rationale": "Because the \"very unhappy\" mood in the entry is negative."}, "language_style": {"answer": "Tactful and warm", "rationale": "The language style of this entry is euphemism. It does not directly blame or criticize, but rather asks questions in a way that is understood and sensitive. Using the words \"mistakes\" and \"unhappy\" to express the feelings of the other in society and invite the other to share their thoughts and experiences. This expression makes the interviewee more comfortable and comfortable and willing to participate in the questionnaire."}, "time_dimension": {"answer": "Present", "rationale": "The entries \"we've made a social error\" and \"we'll be very unhappy\" describe the situation and judge it as now."}, "sentence_type": "Negative declarative sentence", "pos": {"nouns": {"Social": 1, "Error": 1}, "verbs": {"Yes": 2, "Out": 1, "Very": 1, "It's not good": 1}, "adverbs": {"When": 1}, "numerals": {"5": 1}}}, {"content": "I am not much concerned about what important people think of me.", "word_count": 15, "label": "", "sentence_structure": "Subject鈥搗erb鈥搊bject", "subjective_objective": {"answer": "Subjective", "rationale": "Because the main word is \"I\" which represents the patient's subjective attitude."}, "sentence_sentiment": {"answer": "Negative", "rationale": "Because the attitude of \"not much to worry\" is negative, it is a sign that the interviewees do not value the views of important figures."}, "language_style": {"answer": "Calm and direct", "rationale": "The entry is presented in more direct and concise language, without too much emotional or rhetorical approach, and is clearly focused and directly expresses the subject of concern about the perception of key figures."}, "time_dimension": {"answer": "Present", "rationale": "So, time dimension is now."}, "sentence_type": "Negative declarative sentence", "pos": {"nouns": {"Questionnaire Entry": 1, "Important people": 1, "Observations": 1}, "verbs": {"Analysis": 1, "I'm worried": 1, "be": 1, "have": 1}, "adjectives": {"What": 1}, "adverbs": {"not": 1}, "numerals": {"6": 1}, "pronouns": {"I": 1}}}, {"content": "7. I was often afraid that I would look funny or stupid.", "word_count": 17, "label": "", "sentence_structure": "Subject鈥搗erb鈥揷omplement", "subjective_objective": {"answer": "Subjective", "rationale": "Because \"fear\" is a subjective emotional experience that reflects the fear and fear of the patient."}, "sentence_sentiment": {"answer": "Negative", "rationale": "Because the emotional attitude in the entry, \"to fear that you will be ridiculous or stupid,\" is negative."}, "language_style": {"answer": "Tactful and warm", "rationale": "This approach is both an expression of the respect and interest of researchers in being tested and a way to reduce the pressure and discomfort of being tried to answer questions. The language style of the questionnaire entry is therefore \"precipitous\"."}, "time_dimension": {"answer": "Present", "rationale": "I was often afraid that the word \"always\" meant a regular, customary act, and that the \"fear\" used the usual current state of affairs, which meant that the act had not only happened in the past but might still be ongoing. So the entry was to ask the testee what the person was."}, "sentence_type": "Affirmative declarative sentence", "pos": {"nouns": {"self": 1, "Fear": 1, "Funny": 1, "Stupid": 1}, "verbs": {"It seems": 1, "Scared": 1, "be": 1}, "adjectives": {"Often": 1}, "adverbs": {"very": 1}, "pronouns": {"I": 1}}}, {"content": "I have little reaction to others' disagreement with me.", "word_count": 13, "label": "", "sentence_structure": "Subject鈥搗erb鈥揷omplement", "subjective_objective": {"answer": "Subjective", "rationale": "Because \"near to agree with me\" is the patient's subjective feeling and attitude."}, "sentence_sentiment": {"answer": "Negative", "rationale": "Because the description of \"unresponsiveness\" implies a lack of sensitivity to the outside world by individuals, which may lead to problems of poor communication with others, personal tension and, therefore, some negative emotional aspects."}, "language_style": {"answer": "Calm and direct", "rationale": "The language style of the entry is calm. It asks the interviewee whether he or she has little reaction to the fact that he or she does not agree with himself or herself. While this may sound a little indifferent, its direct, concise language style helps the interviewee to quickly understand the meaning of the question and to respond accurately."}, "time_dimension": {"answer": "Present", "rationale": "The entry uses the descriptive phrase \"I don't agree with others\" and expresses the current behaviour or state. It does not refer to the past, nor does it refer to the expectations of how it will change in the future, so the time dimension is now."}, "sentence_type": "Affirmative declarative sentence", "pos": {"nouns": {"person": 1, "Response": 1}, "verbs": {"to": 1, "Yes": 1, "None": 1, "have": 1}, "adjectives": {"Almost": 1}, "pronouns": {"I": 1}}}, {"content": "9. I am often afraid that others will notice my shortcomings.", "word_count": 15, "label": "", "sentence_structure": "Subject鈥搗erb鈥揷omplement", "subjective_objective": {"answer": "Subjective", "rationale": "Because \"fear\" and \"care\" experience are both personal and reflect the patient's perception and perception of his or her own weaknesses. These are subjective experiences that cannot be objectively proved or observed."}, "sentence_sentiment": {"answer": "Negative", "rationale": "Because the mood of \"fear\" and the \"noticing my shorts\" in the entry are both negative."}, "language_style": {"answer": "Tactful and warm", "rationale": "The language style of the entry is more moderate and emotional. It leads the respondents to share their feelings and ideas in a way that is asked, rather than by direct command or criticism, thereby creating a climate of goodwill and understanding. Moreover, the word \"fear\" implies compassion and care for the respondents, making the questionnaire more friendly and attractive."}, "time_dimension": {"answer": "Present", "rationale": ""}, "sentence_type": "Negative declarative sentence", "pos": {"nouns": {"Questionnaire": 1, "Entry": 1, "others": 1, "Short": 1}, "verbs": {"Scared": 1, "Attention": 1}, "adjectives": {"Regular": 1}, "numerals": {"9": 1}, "pronouns": {"I": 1}}}, {"content": "10. The disagreement of others has had little effect on me.", "word_count": 14, "label": "", "sentence_structure": "Subject鈥揷opula鈥損redicative", "subjective_objective": {"answer": "Subjective", "rationale": "Because \"others' disapproval\" and \"almost zero influence on me\" are subjective feelings and attitudes of patients."}, "sentence_sentiment": {"answer": "Neutral", "rationale": "Because the entry merely describes a fact and does not show positive or negative emotional colours."}, "language_style": {"answer": "Calm and direct", "rationale": "The language style of the entry is a calm and straightforward type. It expresses the subject matter in a concise manner, without too many emotional or speech-reading techniques, and is more objective. This expression helps to capture the real thoughts and feelings of the person under investigation, and improves the effectiveness and reliability of the questionnaire."}, "time_dimension": {"answer": "Present", "rationale": "The entry describes the current feelings and state of the subject, the keywords \"now\" and \"almost without effect\", all pointing to the current situation."}, "sentence_type": "Affirmative declarative sentence", "pos": {"nouns": {"others": 1, "I don't agree": 1, "Impact": 1}, "verbs": {"have": 1}, "adjectives": {"Almost": 1}, "numerals": {"10": 1}, "pronouns": {"I": 1}}}, {"content": "If someone were to judge me, I would easily think of the worst.", "word_count": 19, "label": "", "sentence_structure": "Subject鈥揷opula鈥損redicative", "subjective_objective": {"answer": "Subjective", "rationale": "Because \"the easy to think\" and \"the worst\" are patients' subjective feelings and thoughts."}, "sentence_sentiment": {"answer": "Negative", "rationale": "Because the \"worst opinion ever thought of\" in the entry is negative."}, "language_style": {"answer": "Tactful and warm", "rationale": "The entry is more adept and interactive when it is spoken in a gentle and inviting manner, rather than by direct statements or orders."}, "time_dimension": {"answer": "Past", "rationale": "And for the sentence \"11. If someone is judging me, I can easily think of the worst.\" There is no clear past, present or future time trail, so it can be judged that this entry does not address the dimensions of time."}, "sentence_type": "Negative declarative sentence", "pos": {"nouns": {"person": 1, "Evaluation": 2, "I": 2}, "verbs": {"Suppose": 1, "in": 1, "Yes": 1, "Present": 1}, "adjectives": {"The worst": 1}, "adverbs": {"very": 1}, "numerals": {"11": 1}}}, {"content": "12. I hardly care what I am making of others.", "word_count": 19, "label": "", "sentence_structure": "Subject鈥搗erb鈥搊bject", "subjective_objective": {"answer": "Subjective", "rationale": "Because it is a subjective description of what the patient thinks inside himself."}, "sentence_sentiment": {"answer": "Neutral", "rationale": "Because the entry does not have any emotional color."}, "language_style": {"answer": "Tactful and warm", "rationale": ""}, "time_dimension": {"answer": "Present", "rationale": "The entry is to ask the testee about the level of self-expression, the temporal dimension being judged as past and present. The keyword \"near\" indicates that the act was or is likely to be sustained, and therefore can be understood as being relevant in the past and now. At the same time, the phrase \"what is the impression of others\" indicates that it is the testee's concern about the perception of others and is also an act involving a mental state."}, "sentence_type": "Negative declarative sentence", "pos": {"nouns": {"Questionnaire": 1, "Entry": 1, "person": 1, "Impression": 1}, "verbs": {"Mind your own business": 1, "Stay": 1}, "adjectives": {"Almost": 1}, "adverbs": {"Both": 1}, "numerals": {"12": 1}, "pronouns": {"I": 1, "Don't": 1}}}, {"content": "I fear that others will disagree with me.", "word_count": 10, "label": "", "sentence_structure": "Subject鈥搗erb鈥揷omplement", "subjective_objective": {"answer": "Subjective", "rationale": "Because \"fear\" is a subjective emotional experience, and \"unconsent\" is also a matter of the views and attitudes of others, an objective fact beyond the control of the patient. The question is therefore one of subjective thinking of the patient and is a subject of subjectivity."}, "sentence_sentiment": {"answer": "Negative", "rationale": "Because \"fearing someone else won't approve of me\" is negative."}, "language_style": {"answer": "Tactful and warm", "rationale": "The language style of the entry is more euphemistic, not directly indicating whether the other side is afraid of others鈥?disapproval, but rather using a more moderate and Questing approach to guide the other side to thinking about what it feels like. This expression is more personal and interactive, and it is easier for the respondents to open their hearts and respond to them."}, "time_dimension": {"answer": "Present", "rationale": ""}, "sentence_type": "Interrogative sentence", "pos": {"nouns": {"Questionnaire Entry": 1, "person": 1}, "verbs": {"Scared": 1, "Yes": 1}, "adjectives": {"others": 1}, "numerals": {"13": 1, "Yes": 1}, "pronouns": {"I": 1}}}, {"content": "I fear that others will find my mistakes.", "word_count": 12, "label": "", "sentence_structure": "Subject鈥搗erb鈥揷omplement", "subjective_objective": {"answer": "Subjective", "rationale": "Because \"fear\" is a subjective feeling or emotion."}, "sentence_sentiment": {"answer": "Negative", "rationale": "Because fear in the entry is a negative emotional vocabulary."}, "language_style": {"answer": "Tactful and warm", "rationale": "The entry uses a more euphemistic approach to ask the respondents whether they fear criticism or accusations from others, without directly expressing them, making the language style more gentle and friendly."}, "time_dimension": {"answer": "Present", "rationale": "The keyword \"fear\" suggests that it is a present mental or emotional reaction, while the verb \"discovery\" suggests that it may be a fear or a expectation of what may happen in the future. Taken together, the entry primarily asks whether the person tested currently has any fear or fear of others finding his fault.\""}, "sentence_type": "Negative declarative sentence", "pos": {"nouns": {"Questionnaire": 1, "Entry": 1, "person": 1, "Wrong": 1}, "verbs": {"Found": 1, "Yes": 1, "Scared": 1, "be": 1}, "adjectives": {"others": 1, "I": 1}, "adverbs": {"Down there": 1, "Please": 1, "you": 1, "Analysis": 1, "Here": 1, "individual": 1}, "numerals": {"14": 1}}}, {"content": "I am not worried about what others think of me.", "word_count": 14, "label": "", "sentence_structure": "Subject鈥搗erb鈥搊bject", "subjective_objective": {"answer": "Subjective", "rationale": "Because \"not to worry about what others think of me\" is the subjective description of the patient's mental state in the face of others' views."}, "sentence_sentiment": {"answer": "Positive", "rationale": "Because the \"not to worry about what others think of me\" in the entry is positive."}, "language_style": {"answer": "Calm and direct", "rationale": "The language style of the entry is straightforward, as it expresses the subject of asking the other side whether it is disturbed by the other side鈥檚 perception of itself. It is not too much rhetorical or emotional to describe it, and it is more objective in tone."}, "time_dimension": {"answer": "Present", "rationale": ""}, "sentence_type": "Negative declarative sentence", "pos": {"verbs": {"No, it's not": 1, "I'm worried": 1}, "adjectives": {"others": 1, "For me": 1}, "adverbs": {"Yes": 1}, "numerals": {"15": 1, "I": 1}, "pronouns": {"I": 1}}}, {"content": "16. If someone is not happy, I may not care.", "word_count": 16, "label": "", "sentence_structure": "Subject鈥搗erb鈥揷omplement", "subjective_objective": {"answer": "Subjective", "rationale": "Because it is a subjective view and feeling that makes a person happy, and not a subjective attitude. The entry is therefore viewed from the patient鈥檚 subjective perspective."}, "sentence_sentiment": {"answer": "Negative", "rationale": "Because the attitude in the entry is \"may not matter,\" it's a negative attitude."}, "language_style": {"answer": "Tactful and warm", "rationale": "The entry is relatively euphemistic, expressing a more subtle, moderate attitude, and not too direct or emotional. At the same time, the sentence is accompanied by a emotional tendency to be self-comfort and inclusion, which is more likely to resonate and identify with the interviewees."}, "time_dimension": {"answer": "Present", "rationale": "The entry uses the term \"may\", which indicates that it is a speculative or predictive situation and therefore is judged as now."}, "sentence_type": "Negative declarative sentence", "pos": {"nouns": {"Questionnaire": 1, "Entry": 1, "Someone": 1, "Joy": 1}, "verbs": {"Jean": 1, "Maybe": 1, "Joy": 1}, "adjectives": {"It doesn't matter": 1, "Suppose": 1, "I'm not letting you": 1}, "numerals": {"16": 1}, "pronouns": {"I": 1}}}, {"content": "When I spoke to others, I was afraid what they would think of me.", "word_count": 18, "label": "", "sentence_structure": "Subject鈥搗erb鈥搊bject", "subjective_objective": {"answer": "Subjective", "rationale": "Because \"cooking\" is a subjective expression of emotion and feeling."}, "sentence_sentiment": {"answer": "Negative", "rationale": "Because the mental state of the entry, \"Assured what they'll think of me,\" is negative."}, "language_style": {"answer": "Tactful and warm", "rationale": "The language style of the entry is euphemism, because it uses the term \"concern\" to express emotions and does not direct orders or impose opinions on the person under investigation. At the same time, the expression of the doubt \"how I would do\" appears to be more gentle and open, making it easier for the person under investigation to relax and answer the questions honestly."}, "time_dimension": {"answer": "Present", "rationale": "The entry now describes the feelings and fears of the person being tested when talking to others, which are present. So the time dimension is now."}, "sentence_type": "Negative declarative sentence", "pos": {"nouns": {"I": 2, "they": 1}, "verbs": {"Yes": 1, "Look": 1, "Talk": 1, "I'm worried": 1}, "adjectives": {"What": 1}, "adverbs": {"When": 1, "Same": 1}, "numerals": {"17": 1}, "pronouns": {"I": 2}}}, {"content": "18. I think it is inevitable that one should make a mistake in social life, and therefore why should one be upset about it?", "word_count": 27, "label": "", "sentence_structure": "Subject鈥揷opula鈥損redicative", "subjective_objective": {"answer": "Subjective", "rationale": "Because this is the patient ' s idea and attitude, it is subjective."}, "sentence_sentiment": {"answer": "Neutral", "rationale": "Because the entry was not of a clear emotional orientation, it merely presented a point of view."}, "language_style": {"answer": "Tactful and warm", "rationale": "The language style of the entry is euphemistic. It uses a gentle and invitation-style expression like \"Why should it be so sad?\" It avoids direct accusations or criticism, but instead leads the interviewees to think about the issues and encourages them to remain positive. This is easier to trigger the echoes and cooperation of the interviewees, thereby increasing the effectiveness of the questionnaire."}, "time_dimension": {"answer": "Past", "rationale": "The words \"Feel\" and \"Why be upset about it\" in the entry indicate the views and attitudes of the subjects in the past."}, "sentence_type": "Affirmative declarative sentence", "pos": {"nouns": {"person": 1, "Social": 1, "Error": 1}, "verbs": {"I think": 1, "Out": 1, "Maybe": 1, "Avoid": 1, "Sad": 1}, "adjectives": {"It's inevitable": 1}, "adverbs": {"And so": 1}, "pronouns": {"I": 1}}}, {"content": "I am usually worried about what I am making of others.", "word_count": 21, "label": "", "sentence_structure": "Subject鈥搗erb鈥揷omplement", "subjective_objective": {"answer": "Subjective", "rationale": "Because words like \"always\" and \"fear\" all reflect patients' subjective attitudes towards their own behaviour and the perception of others."}, "sentence_sentiment": {"answer": "Negative", "rationale": "Because the \"always worry\" in the entry is negative."}, "language_style": {"answer": "Tactful and warm", "rationale": "The entry is more accessible and interactive by speaking in a question-and-answer manner rather than by direct statement or order. At the same time, the \"sweet\" language style is more appropriate for psychological questionnaires, which better guide respondents to open their minds and provide real and in-depth feedback."}, "time_dimension": {"answer": "Present", "rationale": "Now that I'm usually \"always\" in \"always\" means \"normal, customary behaviour,\" and \"in fear\" uses the usual current state of affairs, which means that this behaviour has not only happened in the past, but may still be ongoing. So, the questionnaire entry is to ask the testee about the behaviour and mentality of the subject."}, "sentence_type": "Interrogative sentence", "pos": {"nouns": {"Questionnaire Entry": 1, "others": 1, "Impression": 1}, "verbs": {"I'm worried": 1, "Stay": 1, "Here": 1, "be": 1}, "adverbs": {"Total": 1, "What the hell": 1}, "numerals": {"19": 1, "What": 1}, "pronouns": {"I": 1}}}, {"content": "I'm worried about how my boss looks at me.", "word_count": 14, "label": "", "sentence_structure": "Subject鈥揷opula鈥損redicative", "subjective_objective": {"answer": "Subjective", "rationale": "This is because of patients ' concern about their superiors ' perception and is a subjective feeling and attitude."}, "sentence_sentiment": {"answer": "Negative", "rationale": "Because the emotional attitude of \"scared\" in the entries is negative."}, "language_style": {"answer": "Tactful and warm", "rationale": "The language style of the entry is euphemism. It uses a moderate, caring and indirect language to ask if the other side is worried about his superiors. It makes people feel more comfortable and close, and it encourages interviewees to share their thoughts and feelings."}, "time_dimension": {"answer": "Past", "rationale": ""}, "sentence_type": "Interrogative sentence", "pos": {"nouns": {"Sir": 1, "Observations": 1}, "verbs": {"I'm worried": 1, "Look": 1}, "adverbs": {"very": 1}, "numerals": {"20": 1}, "pronouns": {"I": 1}}}, {"content": "I don't care when I know someone is judging me.", "word_count": 19, "label": "", "sentence_structure": "Subject鈥搗erb鈥搊bject", "subjective_objective": {"answer": "Subjective", "rationale": "Because \"not caring\" is a subjective attitude or feeling that reflects the patient's attitude towards others."}, "sentence_sentiment": {"answer": "Negative", "rationale": "Because the entry expresses an attitude of indifference to others and is negative."}, "language_style": {"answer": "Calm and direct", "rationale": "The language style of the entry is relatively calm and straightforward, with no excessive rhetorical or emotional rendering, but simply and succinctly asks the person surveyed about his or her attitude to others ' assessment."}, "time_dimension": {"answer": "Present", "rationale": ""}, "sentence_type": "Negative declarative sentence", "pos": {"nouns": {"person": 1, "Evaluation": 1, "I": 2}, "verbs": {"Yes": 1, "In the process of": 1, "I don't care": 1}, "adverbs": {"A little": 1, "Yeah": 1, "Both": 1}, "numerals": {"21": 1}}}, {"content": "I fear that people will think I am useless.", "word_count": 14, "label": "", "sentence_structure": "Subject鈥揷opula鈥損redicative", "subjective_objective": {"answer": "Subjective", "rationale": "Because \"fear\" is the patient's subjective feeling and concern about what others think."}, "sentence_sentiment": {"answer": "Negative", "rationale": "Because the descriptions of \"fear\" and \"no-use\" in the entries are negative."}, "language_style": {"answer": "Tactful and warm", "rationale": "This language style is more euphemistic and implicit in order to avoid creating confusion or conflict among the respondents."}, "time_dimension": {"answer": "Present", "rationale": "The entry describes the current state of the person's mind and feelings, and the words \"fear\" and \"yes\" are all indicative of the current state of affairs. So we can judge that this is the current fear and thinking about the person's current state of mind, \"now\"."}, "sentence_type": "Negative declarative sentence", "pos": {"nouns": {"People": 1, "Fear": 1, "A useless man": 1}, "verbs": {"I think": 1, "be": 1, "I'm worried": 1}, "adverbs": {"Yes": 1, "鈾?And the 鈾?: 1}, "numerals": {"22": 1}, "pronouns": {"I": 1}}}, {"content": "I'm not really worried about how people think about me.", "word_count": 17, "label": "", "sentence_structure": "Subject鈥搗erb鈥揷omplement", "subjective_objective": {"answer": "Subjective", "rationale": "Because it is the patient's subjective attitude and thoughts that are examined."}, "sentence_sentiment": {"answer": "Neutral", "rationale": "The entry is not a positive or negative emotional expression."}, "language_style": {"answer": "Calm and direct", "rationale": "The language style of the entry is more calm and straightforward, and there is no excessive rhetoric or emotional rendering, which clearly expresses the attitude of the person under investigation towards others鈥?views of themselves."}, "time_dimension": {"answer": "Present", "rationale": "The entry describes a personal feeling or state of affairs, indicating that the testee's concern about \"how people think of me\" is low. From a time perspective, this feeling may be either the current state or the past period. But, based on the expression \"almost no concern\" in the title, it can be assumed that it is the present, that is, the present."}, "sentence_type": "Negative declarative sentence", "pos": {"verbs": {"to": 1, "What do you think": 1, "I'm worried": 1}, "adjectives": {"Total": 1}, "adverbs": {"Almost": 1}, "numerals": {"23": 1}, "pronouns": {"He's": 1}}}, {"content": "24. Sometimes I think I care too much about what others think of me.", "word_count": 17, "label": "", "sentence_structure": "Subject鈥揷opula鈥損redicative", "subjective_objective": {"answer": "Subjective", "rationale": "Because \"care about what others think of me\" is a subjective emotion and attitude, not an objective fact or result."}, "sentence_sentiment": {"answer": "Negative", "rationale": "Because the \"too much care\" in the entry is negative."}, "language_style": {"answer": "Tactful and warm", "rationale": "The entry is more euphemistic, using the word \"self\" rather than the word \"right\" to the other, which is more respectful and understanding, and triggers reflection and resonance through the word \"question.\" At the same time, the expression \"too caring\" is relatively mild and does not have too much blame or negative tone."}, "time_dimension": {"answer": "Present", "rationale": "Now the entry uses the general current \"idems\" to indicate that it is the current state of thinking and feeling of the testee. At the same time, \"sometimes\" also indicates that the idea is not persistent, but only occasionally appears. So the questionnaire entry is asking the testee about the current behaviour or mentality."}, "sentence_type": "Affirmative declarative sentence", "pos": {"nouns": {"Time": 1, "Yes": 1, "self": 1, "Observations": 1}, "verbs": {"I care": 1, "Too much": 1, "have": 1}, "adjectives": {"Someone else's": 1, "to": 1}, "adverbs": {"Sometimes": 1, "Yes": 1}, "numerals": {"24": 1}, "pronouns": {"I": 1}}}, {"content": "I often fear that I will say or do something wrong.", "word_count": 14, "label": "", "sentence_structure": "Subject鈥搗erb鈥搊bject", "subjective_objective": {"answer": "Subjective", "rationale": "Because \"care\" is a subjective emotional experience, which is an expression of the patient's fear and apprehension that he or she may have said wrong or done wrong."}, "sentence_sentiment": {"answer": "Negative", "rationale": "Because the expression \"fears to say the wrong thing or do the wrong thing\" in the entry is negative."}, "language_style": {"answer": "Tactful and warm", "rationale": "Because it asks the other side whether it is too strong or not, in a caring and understanding tone. At the same time, this expression is more likely to give the interviewee a willingness to share his/her true feelings."}, "time_dimension": {"answer": "Present", "rationale": ""}, "sentence_type": "Negative declarative sentence", "pos": {"nouns": {"I'm worried": 1, "I will": 2, "Wrong word": 1, "You're doing something wrong": 1}, "verbs": {"Yes": 2, "Say it": 1, "Do it": 1, "Wrong": 2, "I'm worried": 1}, "adverbs": {"Often": 1}, "numerals": {"25": 1}, "pronouns": {"I": 2}}}, {"content": "26. I am not usually concerned about what others think of me.", "word_count": 14, "label": "", "sentence_structure": "Subject鈥搗erb鈥揷omplement", "subjective_objective": {"answer": "Subjective", "rationale": "Because \"the way people think about me\" is usually the subjective feeling and attitude of the individual."}, "sentence_sentiment": {"answer": "Negative", "rationale": "Because the attitude of \"not caring\" in the entries is negative."}, "language_style": {"answer": "Calm and direct", "rationale": "The entry expresses in a concise and clear language the attitude of the subject to the perception of others, the lack of over-accuracy or emotional colours used to render it, the temperature of the language and objectivity."}, "time_dimension": {"answer": "Present", "rationale": ""}, "sentence_type": "Negative declarative sentence", "pos": {"nouns": {"others": 1, "Observations": 1}, "verbs": {"to": 2, "Care": 1}, "adjectives": {"Usually": 1}, "adverbs": {"not": 1}, "numerals": {"26": 1}, "pronouns": {"I": 1}}}, {"content": "I am generally confident that others will be impressed with me.", "word_count": 14, "label": "", "sentence_structure": "Subject鈥揷opula鈥損redicative", "subjective_objective": {"answer": "Subjective", "rationale": "Because confidence is a subjective feeling and attitude."}, "sentence_sentiment": {"answer": "Positive", "rationale": "The entry is a positive emotional expression."}, "language_style": {"answer": "Tactful and warm", "rationale": "The entry is more personal and interactive when it is spoken in a gentle and inviting manner, rather than in direct statements or orders. Moreover, the tone of the article is also positive and optimistic, leaving a sense of comfort and comfort."}, "time_dimension": {"answer": "Present", "rationale": "Key words are \"general\" and \"confident\" , all describing an attitude or feeling of the testee, not past experience or future intentions. The entry therefore reflects the current situation or state of the testee, which should be classified as \"now\"."}, "sentence_type": "Affirmative declarative sentence", "pos": {"nouns": {"Questionnaire": 1, "Entry": 1, "Confidence": 1, "others": 1, "I'm impressed": 1}, "verbs": {"Yes": 1, "have": 1}, "adverbs": {"General": 1}, "numerals": {"27": 1}}}, {"content": "I am always afraid that those who are important to me will not always think of me.", "word_count": 21, "label": "", "sentence_structure": "Subject鈥搗erb鈥搊bject", "subjective_objective": {"answer": "Subjective", "rationale": "Because \"fear\" is a subjective feeling, and \"those who are important to me will always think of me\" is a subjective idea."}, "sentence_sentiment": {"answer": "Negative", "rationale": "Because the expression \"always worried\" in the entry is negative."}, "language_style": {"answer": "Tactful and warm", "rationale": "This is a more euphemistic and warm language that attracts resonance and trust. At the same time, it is more acceptable and responsive, which facilitates the smooth running of the questionnaire."}, "time_dimension": {"answer": "Past", "rationale": "The entry describes a psychological state or emotional experience of the person tested, and the keyword \"generally\" indicates that the fear is a constant and persistent emotion. Thus, the temporal dimension is judged to be the past."}, "sentence_type": "Negative declarative sentence", "pos": {"nouns": {"Questionnaire": 1, "Entry": 1, "People": 1, "I": 2}, "verbs": {"I'm worried": 1, "Always": 1, "Present": 1}, "adjectives": {"Important": 1, "Those": 1}, "adverbs": {"Total": 1}, "numerals": {"28": 1}, "pronouns": {"I": 1}}}, {"content": "I am unhappy with the way my friends see me.", "word_count": 16, "label": "", "sentence_structure": "Subject鈥揷opula鈥損redicative", "subjective_objective": {"answer": "Subjective", "rationale": "Because the perception of friends is a subjective feeling and attitude of a person, not an objective fact or result."}, "sentence_sentiment": {"answer": "Negative", "rationale": "Because the word \"unhappiness\" in the entry is negative."}, "language_style": {"answer": "Tactful and warm", "rationale": "The entry is more personal and interactive when it is presented in a gentle and inviting tone, rather than in direct statements or orders. It also implies that the interviewee may have some psychological distress and need more in-depth exploration and assistance."}, "time_dimension": {"answer": "Past", "rationale": "So we can conclude that the temporal dimension of the article is \"now\"."}, "sentence_type": "Negative declarative sentence", "pos": {"nouns": {"I": 2, "friends": 2, "Observations": 1, "I'm not happy": 1}, "verbs": {"Because": 1, "to": 1, "I'm not happy": 1}, "adverbs": {"Mine": 1}, "numerals": {"29": 1}, "pronouns": {"I": 1}}}, {"content": "30. I would be nervous to know that my superiors are judging me.", "word_count": 20, "label": "", "sentence_structure": "Subject鈥搗erb鈥搊bject", "subjective_objective": {"answer": "Subjective", "rationale": "Because \"frightful\" is the patient's subjective feeling when he knows his superiors are evaluating himself."}, "sentence_sentiment": {"answer": "Negative", "rationale": "Because the emotional attitude of \"sweet\" in the entries is negative."}, "language_style": {"answer": "Calm and direct", "rationale": "The entry directly expresses the subject of emotional reactions when asked about the fact that his superiors are evaluating themselves, without excessive rhetoric or emotional rendering, and with a more balanced and objective tone."}, "time_dimension": {"answer": "Present", "rationale": "\"will become\" means a state of impending, unfinished state, and the sentence therefore describes the current state of feeling or state."}, "sentence_type": "Affirmative declarative sentence", "pos": {"nouns": {"Sir": 1, "Evaluation": 1}, "verbs": {"Yes": 1, "鈾?And become 鈾?: 1, "I'm nervous": 1}, "adjectives": {"Nervous": 1, "Unsettled": 1}, "adverbs": {"In the process of": 1}, "pronouns": {"I": 1}}}]}

{"questionnaire_title": "Chinese version of the Family Intimacy and Adaptive Measurement Table", "questionnaire_category": "Emotion and affect", "item_count": 29, "emotion_shift_overall": "From positive to negative", "language_style_overall": "Tactful and warm", "items": [{"content": "1. In times of difficulty, family members do their utmost to support each other.", "word_count": 23, "label": "", "sentence_structure": "Subject鈥搗erb鈥搊bject", "subjective_objective": {"answer": "Subjective", "rationale": "Because this phrase expresses the patient鈥檚 expectations and trust in family members, and is a manifestation of the patient鈥檚 subjective attitude. While the behaviour of family members may objectively reflect their level of support, it is more concerned with the feelings and expectations of the patient and therefore is subjective in nature."}, "sentence_sentiment": {"answer": "Positive", "rationale": "Because the behaviour of family members in support of each other, as expressed in the entry, is positive."}, "language_style": {"answer": "Tactful and warm", "rationale": "The entry uses positive expressions to guide each other in answering questions and emphasizes the support of each other among family members, the gentler and more friendly, and the euphemism of the language."}, "time_dimension": {"answer": "Present", "rationale": "The sentence indicates a state of affairs or a state of custom whereby family members support each other in the event of difficulties."}, "sentence_type": "Affirmative declarative sentence", "pos": {"nouns": {"Time": 1, "Hard": 1, "Family members": 1, "Max": 1, "Try": 1, "To each other": 1, "Support": 1}, "verbs": {"in": 1, "have": 1, "End of story": 1, "Both": 1, "Yes": 1, "Mutual support": 1}, "adverbs": {"Max": 1}, "numerals": {"1": 2}, "classifiers": {"individual": 1}, "pronouns": {"Family members": 1}}}, {"content": "2. In our family, every member can express his or her opinion freely.", "word_count": 23, "label": "", "sentence_structure": "Subject鈥搗erb鈥搊bject", "subjective_objective": {"answer": "Subjective", "rationale": "According to the description, \"Every member can express his opinion freely\" is the patient's subjective view and assessment of the family environment. The answer is therefore \"subjective\"."}, "sentence_sentiment": {"answer": "Positive", "rationale": "For the purpose of the entry, which is to encourage an open and free atmosphere of communication, is positive."}, "language_style": {"answer": "Tactful and warm", "rationale": "This entry is an invitation to ask the respondent about the openness of his or her family members to express his or her views."}, "time_dimension": {"answer": "Present", "rationale": "\"As a matter of opinion\" describes the behaviour or state of the current family, so the temporal dimension is now judged."}, "sentence_type": "Affirmative declarative sentence", "pos": {"nouns": {"Family": 1, "Every one": 1, "Members": 1, "Observations": 1}, "verbs": {"Publication": 1, "Yeah": 1}, "adverbs": {"Whatever": 1}, "classifiers": {"Medium": 1, "individual": 1}, "pronouns": {"we": 1}}}, {"content": "3. Members of our family are more willing to discuss personal issues with their friends than with their families.", "word_count": 29, "label": "", "sentence_structure": "Subject鈥搗erb鈥搊bject", "subjective_objective": {"answer": "Subjective", "rationale": "Because \"yes\" and \"no\" are subjective assessments of the preferences of family members."}, "sentence_sentiment": {"answer": "Negative", "rationale": "Because the \"unlike to talk to family\" act in the entry is negative."}, "language_style": {"answer": "Calm and direct", "rationale": "The language style of the entry is calm and straightforward, and it clearly raises the issue of observation and research, without any excessive emotional or rhetorical approach. Such expression helps to ensure objectivity and accuracy of the study."}, "time_dimension": {"answer": "Present", "rationale": "The entry describes the current behaviour and attitude of family members, and the keywords \"more willing\" and \"not willing\" indicate that this is an assessment of the current situation and therefore the judgement is now."}, "sentence_type": "Affirmative declarative sentence", "pos": {"nouns": {"Members": 2, "Family members": 1, "friends": 1, "Personal issues": 1}, "verbs": {"Yes": 2, "Consultations": 3}, "adjectives": {"Comparison": 1, "Not very much": 1}, "adverbs": {"and": 2, "And": 1}, "numerals": {"3": 1, "2": 1}, "pronouns": {"It's from our house": 1, "they": 1}}}, {"content": "4. Every family member is involved in making significant family decisions.", "word_count": 18, "label": "", "sentence_structure": "Subject鈥搗erb鈥搊bject", "subjective_objective": {"answer": "Subjective", "rationale": "The decision-making process is subjective, since participation in major family decisions involves values and will."}, "sentence_sentiment": {"answer": "Positive", "rationale": "Because the act of \"every member of the family is involved in making significant family decisions\" in the entry is positive and reflects equality and democracy within the family."}, "language_style": {"answer": "Tactful and warm", "rationale": "This is a more euphemistic and soft language style, whereby respondents are asked to express their views on the extent of their participation in family decision-making, rather than direct orders or demands."}, "time_dimension": {"answer": "Present", "rationale": ""}, "sentence_type": "Affirmative declarative sentence", "pos": {"nouns": {"Family members": 1, "Family": 2, "Decision-making": 1}, "verbs": {"Participation": 1, "Do it": 1, "Out": 1}, "adverbs": {"Both": 1, "Significant": 1}, "numerals": {"Every one": 1}}}, {"content": "All family members gather to carry out their activities.", "word_count": 15, "label": "", "sentence_structure": "Subject鈥搗erb鈥搊bject", "subjective_objective": {"answer": "Objective", "rationale": "Because \"all family members gather to operate\" is an objective occurrence of behavior and condition, whether the patient likes it or thinks it necessary."}, "sentence_sentiment": {"answer": "Positive", "rationale": "Because the act of \"family members gather to operate\" in the entry is positive."}, "language_style": {"answer": "Tactful and warm", "rationale": "Warm and warm."}, "time_dimension": {"answer": "Present", "rationale": "The keyword \"all family members come together\" is an indication of the current family situation and emphasizes that it is an ongoing behaviour, so the temporal dimension is now judged."}, "sentence_type": "Affirmative declarative sentence", "pos": {"nouns": {"Family members": 1, "Activities": 1}, "verbs": {"Gather": 1, "Conduct": 1}, "numerals": {"All": 1}}}, {"content": "6. The older generation may express their views on the teachings of the older generation.", "word_count": 17, "label": "", "sentence_structure": "Subject鈥搗erb鈥搊bject", "subjective_objective": {"answer": "Objective", "rationale": "Because this entry is an examination of the attitude or perception of the respondent in relation to the behaviour of \"the elders can express their opinions\" and this is an objective observation. Although the respondent may have his own subjective ideas and views, the item is designed without directly asking the interviewee about his feelings or feelings, but rather whether he or she agrees with the act, and therefore the entry is objective."}, "sentence_sentiment": {"answer": "Positive", "rationale": "Because it promotes respect and trust among elders and encourages them to express their views, it is positive and emotional."}, "language_style": {"answer": "Tactful and warm", "rationale": "The entry is more moderate in its language style, using the word \"may\" as the guide, to express support and encouragement for the opinion of the younger generation, and is warm and friendly. Nor is it used in too direct or hard language to avoid stress or discomfort on the interviewee. The language style of the entry is therefore considered to be warm."}, "time_dimension": {"answer": "Present", "rationale": "The keyword \"may\" denotes a capacity or right that exists in the present. At the same time, the act of \"the teaching of elders\" is also an act that is now being performed. Thus, the temporal dimension of the article is now judged."}, "sentence_type": "Affirmative declarative sentence", "pos": {"nouns": {"Sir": 1, "Senior": 1, "Observations": 1}, "verbs": {"to": 1, "Teaching": 1, "Publication": 1}, "adverbs": {"Yeah": 1}, "pronouns": {"self": 1}}}, {"content": "At home, we all do things together.", "word_count": 10, "label": "", "sentence_structure": "Subject鈥搗erb鈥揷omplement", "subjective_objective": {"answer": "Subjective", "rationale": "Because \"Do something together\" is a common behaviour and concept of a family, reflecting the family perception and attitude of the patient and the subjective feeling of the patient."}, "sentence_sentiment": {"answer": "Positive", "rationale": "Because the purpose of the entry is to do something together."}, "language_style": {"answer": "Tactful and warm", "rationale": "The language style of the entry is more euphemistic and sweet, and a positive, cooperative tone is used to guide respondents to answer questions and encourage them to share the good times of their families."}, "time_dimension": {"answer": "Present", "rationale": "The entry describes the daily situation at home, using the general current \"yes\" as an indication of a current or frequent pattern of customary behaviour. Therefore, the temporal dimension is now judged."}, "sentence_type": "Affirmative declarative sentence", "pos": {"nouns": {"Home": 1, "Yes": 1, "Everyone": 2}, "verbs": {"Do it": 1}}}, {"content": "Family members discussed the issue together and were satisfied with its resolution.", "word_count": 21, "label": "", "sentence_structure": "Subject鈥搗erb鈥揷omplement", "subjective_objective": {"answer": "Subjective", "rationale": "It is a subjective feeling that family members are satisfied with the problem."}, "sentence_sentiment": {"answer": "Positive", "rationale": "Because the words \"discussion\" and \"satisfaction\" are positive."}, "language_style": {"answer": "Tactful and warm", "rationale": "The language style of the entry is euphemism. It asks the respondents, in a kind and caring manner, whether they often discuss the solution with their families and is satisfied with it."}, "time_dimension": {"answer": "Present", "rationale": "The entry describes the current behaviour, which is \"family members discuss the problem together\" and which results in \"satisfaction\". Thus, the temporal dimension is now."}, "sentence_type": "Affirmative declarative sentence", "pos": {"nouns": {"Family members": 1, "Problem": 2, "Resolve": 1, "Satisfied": 1}, "verbs": {"Together": 1, "Discussion": 1, "I feel": 1}, "adverbs": {"and": 1}, "numerals": {"8": 1}}}, {"content": "Family members have a closer relationship with friends than family members.", "word_count": 23, "label": "", "sentence_structure": "Subject鈥揷opula鈥損redicative", "subjective_objective": {"answer": "Subjective", "rationale": "Because this entry expresses patients ' subjective perception and evaluation of relationships between family members and friends."}, "sentence_sentiment": {"answer": "Neutral", "rationale": "Because the entry is only a comparison of the closeness of the two relationships and does not express positive or negative feelings."}, "language_style": {"answer": "Tactful and warm", "rationale": ""}, "time_dimension": {"answer": "Present", "rationale": "In this entry, \"current\" describes the current state or situation, while \"family and friends are more closely related than family members\" expresses a comparison and judgement, and it can be established that this is a matter of current state."}, "sentence_type": "Imperative sentence", "pos": {"nouns": {"Family members": 2, "friends": 1, "Relations": 2}, "verbs": {"and": 1, "More": 1, "Intimacy": 1}, "adverbs": {"- Yeah": 1, "More": 1}, "numerals": {"9": 1}}}, {"content": "10. In the family, we alternately share different household tasks.", "word_count": 15, "label": "", "sentence_structure": "Subject鈥搗erb鈥揷omplement", "subjective_objective": {"answer": "Subjective", "rationale": "This is because of patients ' subjective perception and description of their own division of labour."}, "sentence_sentiment": {"answer": "Positive", "rationale": "Because the \"turning of different household chores\" in the entry is positive."}, "language_style": {"answer": "Tactful and warm", "rationale": "The entry is more personal and interactive when it is presented in a gentle and inviting tone, rather than in direct statements or orders. At the same time, the entry does not overemphasize individual responsibility or obligations, but rather uses a relatively equal approach to the respondents ' answers, reflecting respect and understanding of the interviewee."}, "time_dimension": {"answer": "Present", "rationale": "The entry now describes a routine, customary behaviour in family life, whereby family members share different household tasks in rotation. By using the general current, it not only represents current ongoing behaviour, but may also exist in the past and in the future."}, "sentence_type": "Affirmative declarative sentence", "pos": {"nouns": {"Family": 1, "Domestic work": 2}, "verbs": {"Rotation": 1, "Share": 1}, "numerals": {"10": 1}}}, {"content": "Family members are familiar with close friends of each member.", "word_count": 18, "label": "", "sentence_structure": "Subject鈥搗erb鈥搊bject", "subjective_objective": {"answer": "Subjective", "rationale": "This is viewed from the patient ' s subjective point of view, as it is possible to familiarize family members with the views and feelings of each member ' s close friends as a personal one."}, "sentence_sentiment": {"answer": "Neutral", "rationale": "Since the entry only describes the relationship between family members, it does not involve any positive or negative emotional aspects."}, "language_style": {"answer": "Tactful and warm", "rationale": "The language style of the entry is relatively euphemistic and detailed, using softer language to guide the questions that are being answered, such as \"know each member's close friend\", which is more acclaimable and interactive. At the same time, the entire sentence expresses the researchers鈥?intentions more directly, allowing the test to better understand and answer the questions."}, "time_dimension": {"answer": "Past", "rationale": "In the entry, the act of \"knowing each member's close friend\" happened in the past, because the actions of \"know each member\" and \"each member\" had already taken place. At the same time, it was impossible to judge how long ago the action was, without specifying the time frame."}, "sentence_type": "Affirmative declarative sentence", "pos": {"nouns": {"Family members": 2, "Close friends": 1}, "verbs": {"I know you": 1, "Both": 1, "have": 1}, "numerals": {"11": 1}, "classifiers": {"individual": 1}}}, {"content": "12. When family conditions change, the normal family life patterns and family rules can easily be matched.", "word_count": 29, "label": "", "sentence_structure": "Subject鈥搗erb鈥搊bject", "subjective_objective": {"answer": "Objective", "rationale": "Family status, life patterns and family rules are objective facts, and it is objective to see if they have changed."}, "sentence_sentiment": {"answer": "Neutral", "rationale": "Because the entry is a description of a situation and does not display a positive or negative emotional colour."}, "language_style": {"answer": "Tactful and warm", "rationale": ""}, "time_dimension": {"answer": "Present", "rationale": "\"When the family's situation changes\", which indicates that it is a behaviour in a given situation, \"the normal family's life patterns and family rules can easily be changed accordingly\" suggests that it is a description of the current situation, and therefore the temporal dimension is now judged."}, "sentence_type": "Affirmative declarative sentence", "pos": {"nouns": {"Family situation": 1, "Lifestyle": 1, "Family rules": 1, "Change": 1}, "verbs": {"have": 1, "Change": 3, "Easy": 1, "It's the same": 1}, "adjectives": {"Normal": 1, "Corresponding": 1}, "numerals": {"12": 1, "One": 2, "very": 1}, "pronouns": {"Family": 2, "Here": 1}}}, {"content": "13. Family members prefer to consult with their families when making their own decisions.", "word_count": 20, "label": "", "sentence_structure": "Subject鈥搗erb鈥搊bject", "subjective_objective": {"answer": "Subjective", "rationale": "The subject is to examine the subjective orientation and attitude of patients in the face of decision-making, so it is to be seen from a subjective perspective."}, "sentence_sentiment": {"answer": "Positive", "rationale": "Because the behaviour \"likes to consult with family\" in the entry is positive, it shows that the person takes into account the participation and opinion of family members in decision-making."}, "language_style": {"answer": "Tactful and warm", "rationale": "The entry is more euphemistic and warm. It does not direct orders or require the interviewee to consult with his family in his decision-making, but rather raises questions in a suggestion or in a query that gives people a more cordial, friendly and inclusive sense of mind."}, "time_dimension": {"answer": "Present", "rationale": "The entry indicates that the testee prefers to consult with his family in the current family decision-making and therefore the test is now judged."}, "sentence_type": "Affirmative declarative sentence", "pos": {"nouns": {"Family members": 1, "Decision-making": 1, "- Let's talk": 1}, "verbs": {"You want it": 1, "Do it": 1, "Yeah": 1, "With family": 1}, "numerals": {"13": 1}, "pronouns": {"self": 1}}}, {"content": "14. When there is a conflict in the family, members make compromises among themselves.", "word_count": 20, "label": "", "sentence_structure": "Subject鈥搗erb鈥搊bject", "subjective_objective": {"answer": "Objective", "rationale": "Because \"compromise with one another\" is the act of behaviour between family members in dealing with a contradiction, and it is an objective fact. It is objective to look at such behaviour and its consequences, and it is a subjective idea to be willing to do so."}, "sentence_sentiment": {"answer": "Positive", "rationale": "Because the purpose of the entry is to make compromises with mutual humility."}, "language_style": {"answer": "Tactful and warm", "rationale": "The language style of the entry is more moderate and friendly, emphasizing harmony and mutual understanding in the family. It is more pro-people, accessible and easily resonated with readers by using polite language expressions that invite each other to think about how to deal with family conflicts and compromise through mutual humility."}, "time_dimension": {"answer": "Present", "rationale": "The entry now describes a situation or behaviour in which \"if there is a contradiction\", \"to each other's humility\" and \"to compromise\" are all expressions of the present situation, and therefore the present is judged."}, "sentence_type": "Affirmative declarative sentence", "pos": {"nouns": {"Paradoxious": 1, "Family": 1, "Members": 2, "Compromise": 1}, "verbs": {"Come on": 1, "To each other": 1, "- Happiness": 1, "Access": 1}, "numerals": {"14": 1, "STAGE": 1}, "classifiers": {"individual": 1}}}, {"content": "15. In our home, recreational activities are done by the family.", "word_count": 17, "label": "", "sentence_structure": "Subject鈥搗erb鈥搊bject", "subjective_objective": {"answer": "Subjective", "rationale": "This is because the patient ' s perception and description of his/her family is subjective."}, "sentence_sentiment": {"answer": "Positive", "rationale": "Since this entry describes the behaviour of family members involved in recreational activities, such behaviour is often seen as positive behaviour that promotes kinship and fosters teamwork."}, "language_style": {"answer": "Tactful and warm", "rationale": "The language style of the entry is more euphemistic, because it uses an invitation tone, not an order or a direct statement. Moreover, it emphasizes the interaction of families, making respondents more willing to participate and share their views and experiences. This expression is more acceptable and attracts attention and interest."}, "time_dimension": {"answer": "Past", "rationale": "The entry describes past behaviour, and the temporal dimension is a past, since \"all family members do it together\" indicates that the conduct has been completed."}, "sentence_type": "Affirmative declarative sentence", "pos": {"nouns": {"Questionnaire Entry": 1, "Activities": 1, "Family": 1}, "verbs": {"be": 1, "Go on": 1, "Do it": 1}, "adverbs": {"Both": 1}, "numerals": {"15": 1}, "classifiers": {"individual": 1}, "pronouns": {"we": 1}}}, {"content": "16. In resolving the problem, the children ' s recommendations were accepted.", "word_count": 17, "label": "", "sentence_structure": "Subject鈥搗erb鈥搊bject", "subjective_objective": {"answer": "Objective", "rationale": "It is not the patient ' s subjective attitude or experience that is the reality of the fact that the child ' s advice is accepted."}, "sentence_sentiment": {"answer": "Positive", "rationale": "Because the article is \"the child's proposal can be accepted\", it is a positive attitude and behaviour."}, "language_style": {"answer": "Tactful and warm", "rationale": "This is more a way of stimulating the interviewee鈥檚 resonance and reflection and enhancing the relevance of the questionnaire."}, "time_dimension": {"answer": "Present", "rationale": "The entry describes a current state or situation where children鈥檚 suggestions are accepted when the problem is solved. The time dimension is therefore now judged."}, "sentence_type": "Affirmative declarative sentence", "pos": {"nouns": {"Problem": 1, "Son": 1, "Recommendations": 1, "Accept": 1}, "verbs": {"Resolve": 1, "Yes": 1, "By": 1, "Accept": 1}, "numerals": {"16": 1}}}, {"content": "17. Family members are very close.", "word_count": 15, "label": "", "sentence_structure": "Subject鈥揷opula鈥損redicative", "subjective_objective": {"answer": "Objective", "rationale": "Because the closeness of family relations can be the result of objective facts through observation and investigation."}, "sentence_sentiment": {"answer": "Positive", "rationale": "Because the article is \"very close\", it is a positive assessment that indicates good relations between family members."}, "language_style": {"answer": "Tactful and warm", "rationale": "The language style of the entry is euphemistic, and it is \"very close to family members\" to generate the attention and memory of the person surveyed about family feelings, rather than directly asking or accusing."}, "time_dimension": {"answer": "Present", "rationale": "The entry describes the current situation, that is, the state of relations between family members. The term \"very close\" indicates that the situation is persistent and has no temporal limitations. So we can determine that this is a description of the current situation."}, "sentence_type": "Affirmative declarative sentence", "pos": {"nouns": {"Family members": 1, "Relations": 1, "Close": 1}, "verbs": {"be": 1, "Very": 1, "of": 1}, "adjectives": {"Very": 1}, "numerals": {"17": 1}}}, {"content": "18. Our home is a reasonable tutor.", "word_count": 10, "label": "", "sentence_structure": "Subject鈥揷opula鈥損redicative", "subjective_objective": {"answer": "Subjective", "rationale": "Because \"reasonable home teaching\" is patient opinion and evaluation."}, "sentence_sentiment": {"answer": "Neutral", "rationale": "As the entry does not describe the specific content and impact of the tutorial, there is no apparent emotional tendency."}, "language_style": {"answer": "Calm and direct", "rationale": "The entry presents the educational concept of the family of the person under investigation in an objective manner, without too much emotional or rhetorical, and with a clear and concise language, with a strong empirical character. At the same time, the question is not one of euphemism, vague language, but rather of a straightforward approach to questions, which is in keeping with the immediate and calmness of the questions."}, "time_dimension": {"answer": "Present", "rationale": "Key words, \"Our tutor\" and \"reasonable\" are words that describe the current family situation, so it can be judged now."}, "sentence_type": "Affirmative declarative sentence", "pos": {"nouns": {"Tutor": 1, "Family": 1}, "verbs": {"be": 1, "Reasonable": 1}, "adjectives": {"Reasonable": 1}, "pronouns": {"we": 1}}}, {"content": "19. In the home, each member is accustomed to operating alone.", "word_count": 13, "label": "", "sentence_structure": "Subject鈥搗erb鈥揷omplement", "subjective_objective": {"answer": "Objective", "rationale": "Since the question is about the family, the description is about the habits or behaviour of the family members, and is the result of objective observation and documentation."}, "sentence_sentiment": {"answer": "Neutral", "rationale": "Because the entry describes the customary behaviour of family members, there is no apparent emotional tendency."}, "language_style": {"answer": "Calm and direct", "rationale": "The language style of the entry is more objective and direct, rather than using too many rhetorical or emotional terms, it simply describes the customary behaviour of family members and therefore belongs to the language style of \"cool and direct\"."}, "time_dimension": {"answer": "Present", "rationale": ""}, "sentence_type": "Affirmative declarative sentence", "pos": {"nouns": {"Family": 1, "Members": 1, "Activities": 1}, "verbs": {"Custom": 1, "Alone": 1}, "numerals": {"Every": 1, "individual": 2}, "pronouns": {"Other": 1}}}, {"content": "20. Our family prefers new ways of solving the problems encountered.", "word_count": 17, "label": "", "sentence_structure": "Subject鈥搗erb鈥搊bject", "subjective_objective": {"answer": "Subjective", "rationale": "Because \"like\" is a subjective attitude or emotion, indicating the respondent鈥檚 preference for and preference for the way in which the problem is solved. And the use of new methods to solve the problem is an objective act and fact, but whether it is accepted and preferred by the interviewee is a subjective view."}, "sentence_sentiment": {"answer": "Positive", "rationale": "Because the action of \"new ways to solve problems\" in the entry is positive."}, "language_style": {"answer": "Tactful and warm", "rationale": "The entry is more personal and interactive when it is spoken in a gentle and inviting manner, rather than in direct statements or orders. At the same time, this euphemism can make the interviewees feel more comfortable and comfortable and more willing to share their thoughts and experiences."}, "time_dimension": {"answer": "Present", "rationale": "The entry now describes a current behaviour or attitude of the family that favours new methods of solving problems and therefore falls within the scope of the present."}, "sentence_type": "Affirmative declarative sentence", "pos": {"nouns": {"Problem": 1, "Methodology": 1}, "verbs": {"Yeah": 1, "Use": 1, "Resolve": 1, "I met him": 1}, "adjectives": {"New": 1}, "classifiers": {"Home": 1}}}, {"content": "21. Family members are able to do their job as determined by their families.", "word_count": 17, "label": "", "sentence_structure": "Subject鈥搗erb鈥搊bject", "subjective_objective": {"answer": "Objective", "rationale": "Because \"family members can do what they decide\" is an observed family behaviour and an objective fact."}, "sentence_sentiment": {"answer": "Positive", "rationale": "Because it is positive and positive that family members are able to do what they decide to do in accordance with the entry."}, "language_style": {"answer": "Calm and direct", "rationale": "The language style of the entry is more straightforward, and there is no excessive emotional or rhetorical approach to asking questions in a concise and objective manner."}, "time_dimension": {"answer": "Present", "rationale": "The current situation is highlighted by both \"family members\" and \"doing what the family decides\" and the entry therefore describes the current state and behaviour."}, "sentence_type": "Affirmative declarative sentence", "pos": {"nouns": {"Family members": 1, "Family": 1, "Decision": 1, "Yes": 1}, "verbs": {"Yes": 1, "Press": 1, "Do it": 1}, "adverbs": {"Both": 1}, "numerals": {"21": 1}}}, {"content": "22. In our home, each member shares family obligations.", "word_count": 15, "label": "", "sentence_structure": "Subject鈥搗erb鈥搊bject", "subjective_objective": {"answer": "Objective", "rationale": "Because \"each member shares the responsibility for the family\" is an objective situation and fact. While this situation may reflect the subjective views or attitudes of the patient or the person under investigation, it is more important to describe the situation in objective terms in this matter."}, "sentence_sentiment": {"answer": "Positive", "rationale": "Because the act of \"every member shares the family duty\" in the entry is positive."}, "language_style": {"answer": "Tactful and warm", "rationale": "The entry is more euphemistic, and leads the respondents to share their ideas and experiences by asking questions. At the same time, the question is not based on a strong command tone, but rather on an equal and respectful attitude to communicating with the respondents, in keeping with the rigour and scientific nature of the psychological questionnaire study."}, "time_dimension": {"answer": "Present", "rationale": "The entry is a question of whether the testee has shared family responsibilities in the current family environment, and therefore the temporal dimension is now judged."}, "sentence_type": "Affirmative declarative sentence", "pos": {"nouns": {"Our home": 1, "Members": 1, "Family obligations": 1}, "verbs": {"Share": 1}, "numerals": {"22": 1}}}, {"content": "23. Family members prefer to spend their leisure time together.", "word_count": 15, "label": "", "sentence_structure": "Subject鈥搗erb鈥搊bject", "subjective_objective": {"answer": "Subjective", "rationale": "Because \"like to spend time together\" is the subjective feeling and attitude of family members, not the objective fact. This involves their personal preferences, emotional state, etc., and is therefore subjective."}, "sentence_sentiment": {"answer": "Positive", "rationale": "Because the \"like to spend time together\" behavior in the entry is positive."}, "language_style": {"answer": "Tactful and warm", "rationale": "The language style of the entry is euphemismal. It asks the interviewee about the time spent in leisure time between family members in a convenient way, without strong command or critical language, but rather using a moderate expression to guide the interviewee to share his views and experiences. Such language style helps create a good atmosphere of interaction and makes the interviewee more willing to participate in the questionnaire."}, "time_dimension": {"answer": "Present", "rationale": "The words \"like\" and \"overtime\" in the entry are all the verbs of the current state, which indicate that this is an act that is currently being performed. At the same time, \"family members\" suggest that this is related to the current family relationship and can therefore be judged to be the present verb."}, "sentence_type": "Affirmative declarative sentence", "pos": {"nouns": {"Family members": 1, "Amateur Time": 1}, "verbs": {"Yeah": 1, "Pass": 1}, "numerals": {"23": 1}}}, {"content": "Despite this perception, family life patterns and family rules are difficult to change.", "word_count": 28, "label": "", "sentence_structure": "Subject鈥搗erb鈥搊bject", "subjective_objective": {"answer": "Subjective", "rationale": "The idea of a family is a subjective perception of a person, and whether the rules of life and the rules of the family can be changed is an objective result."}, "sentence_sentiment": {"answer": "Neutral", "rationale": "Because the entry does not express positive or negative emotional sentiments, it merely presents a fact."}, "language_style": {"answer": "Tactful and warm", "rationale": "The language style of the entry is more euphemistic, using expressions such as \"despite\", \"like ideas\", which are not directly critical or critical, but are dialogueable in an understanding and supportive tone. This expression is more easily accepted and responded to by respondents and contributes to the effective conduct of the questionnaire."}, "time_dimension": {"answer": "Present", "rationale": "The keywords \"someone at home has this idea\" and \"family life patterns and family rules are still difficult to change\" indicate that this is the present and therefore belongs to the present."}, "sentence_type": "Affirmative declarative sentence", "pos": {"nouns": {"Home": 1, "person": 1, "Thoughts": 1, "Family": 1, "Lifestyle": 1, "Family rules": 1}, "verbs": {"have": 1, "Change": 1}, "adjectives": {"Hard": 1}, "adverbs": {"Or is it": 1}, "numerals": {"24": 1}, "pronouns": {"Here": 1}}}, {"content": "Family members are very proactive in speaking their own thoughts to the rest of the family.", "word_count": 21, "label": "", "sentence_structure": "Subject鈥搗erb鈥揷omplement", "subjective_objective": {"answer": "Subjective", "rationale": "Because the words \"proprio motu\" and \"to speak your own mind\" are the subjective feelings and perceptions of the patient about his family."}, "sentence_sentiment": {"answer": "Positive", "rationale": "Because the \"proactive talk\" in the entry is positive."}, "language_style": {"answer": "Tactful and warm", "rationale": "The entry uses a more moderate approach to asking about communication between family members of the interviewee, without direct questioning or evaluation, but rather leads to a more inclusive and understanding tone of response, which makes it more comfortable and natural."}, "time_dimension": {"answer": "Present", "rationale": "Now, the keywords \"very proactive\" and \"both\" indicate that this is a statement of the current state of affairs, that family members are actively sharing their thoughts. And, at the same time, \"talking their own minds\" is an ongoing move that further supports this judgment. So, the entry describes the current family situation of the person being tested."}, "sentence_type": "Affirmative declarative sentence", "pos": {"nouns": {"Family members": 1, "In my heart": 1}, "verbs": {"Both": 1, "Initiatives": 1, "In": 1, "Other": 1, "person": 1, "Talk": 1}, "adverbs": {"very": 1}, "numerals": {"25": 1}, "pronouns": {"self": 1}}}, {"content": "26. At home, family members can make their own demands.", "word_count": 18, "label": "", "sentence_structure": "Subject鈥搗erb鈥搊bject", "subjective_objective": {"answer": "Objective", "rationale": "Because the fact that family members can claim their own free will is an objective fact of the family environment and is not related to the patient's subjective attitude.鈥?}, "sentence_sentiment": {"answer": "Neutral", "rationale": "Because the entry is a description of a fact and there is no apparent emotional tendency."}, "language_style": {"answer": "Calm and direct", "rationale": ""}, "time_dimension": {"answer": "Present", "rationale": "The entry describes the current pattern of rules and behaviour within the family and thus determines the present."}, "sentence_type": "Affirmative declarative sentence", "pos": {"nouns": {"Home": 1, "Family members": 1, "Request": 1}, "verbs": {"Yeah": 1, "Present": 1, "Whatever": 1}, "adverbs": {"self": 1}, "numerals": {"26": 1}}}, {"content": "27. In the family, the friends of each family member are most warmly received.", "word_count": 24, "label": "", "sentence_structure": "Subject鈥搗erb鈥搊bject", "subjective_objective": {"answer": "Objective", "rationale": "Because this is a description based on observation and experience, not on the feelings or thoughts of the patient himself. It involves a family's attitude towards friends, and it does not depend on the patient's personal or emotional state."}, "sentence_sentiment": {"answer": "Positive", "rationale": "Because it expresses a warm hospitality attitude and has a positive emotional tone."}, "language_style": {"answer": "Tactful and warm", "rationale": "The entry is a euphemism. It uses an invitation tone and a positive word of \"hot reception\" to express respect and concern for the interviewee, and to enhance the sense of interaction with the interviewee. At the same time, the entry does not directly order or compel the interviewee to answer questions, but rather takes a more moderate approach, which makes the interviewee feel comfortable and natural."}, "time_dimension": {"answer": "Past", "rationale": "The entry describes the current state and situation and does not address the concept of past or future time, and therefore the judgement is now."}, "sentence_type": "Affirmative declarative sentence", "pos": {"nouns": {"Family": 1, "Members": 1, "friends": 1, "Reception": 1}, "verbs": {"Yes": 1, "Received": 1, "Reception": 1}, "adjectives": {"Very": 1, "鈾?Hot 鈾?: 1}, "numerals": {"27": 1}}}, {"content": "When a family is in conflict, family members hide their thoughts.", "word_count": 23, "label": "", "sentence_structure": "Subject鈥搗erb鈥揷omplement", "subjective_objective": {"answer": "Subjective", "rationale": "The idea of family members is a subjective experience and a subjective perception, not an objective fact."}, "sentence_sentiment": {"answer": "Negative", "rationale": "Because the \"hidden in your mind\" act in the entry is negative."}, "language_style": {"answer": "Tactful and warm", "rationale": ""}, "time_dimension": {"answer": "Present", "rationale": "The entry \"when the family is in conflict\" means what is possible, whether current or past, and \"will hide its thoughts in its heart\" describes the behaviour in such situations and therefore judges it as now."}, "sentence_type": "Negative declarative sentence", "pos": {"nouns": {"Family": 1, "Paradoxious": 1, "Family members": 1, "Thoughts": 1}, "verbs": {"Generate": 1, "Hide in": 1, "In my heart": 1}, "numerals": {"28": 1}}}, {"content": "29. At home, we prefer to work separately and less with the family.", "word_count": 24, "label": "", "sentence_structure": "Subject鈥損redicate", "subjective_objective": {"answer": "Subjective", "rationale": "Because the \"better\" and \"not\" descriptions in the questionnaire are subjective."}, "sentence_sentiment": {"answer": "Negative", "rationale": "Because the entry \"not very willing to do it with the family\" expresses a negative sentiment, which is a preference for separation."}, "language_style": {"answer": "Calm and direct", "rationale": "The entry is more directly linguistically, is based on factual statements, is not overly rhetorical or emotional, and is more focused on the accuracy and objectivity of the message.\""}, "time_dimension": {"answer": "Present", "rationale": "Thus, this entry describes current behaviour or preference."}, "sentence_type": "Affirmative declarative sentence", "pos": {"nouns": {"Home": 1, "Yes": 2, "Family": 1}, "verbs": {"Yes": 2, "Separate": 1, "Do it": 2, "Together": 1, "Not really": 1}, "adverbs": {"More": 1, "Not really": 1}, "numerals": {"29": 1}, "pronouns": {"we": 1}}}]}

{"questionnaire_title": "PDQ-4", "questionnaire_category": "Mental health", "item_count": 104, "emotion_shift_overall": "From positive to negative", "language_style_overall": "Neutral", "items": [{"content": "I avoid working with people who might criticize myself.", "word_count": 16, "label": "", "sentence_structure": "Subject鈥搗erb鈥搊bject", "subjective_objective": {"answer": "Subjective", "rationale": "Because \"avoiding\" is the behaviour choices of the patient to avoid possible criticism, it is subjective."}, "sentence_sentiment": {"answer": "Negative", "rationale": "Because the \"avoid\" act in the entry is negative."}, "language_style": {"answer": "Calm and direct", "rationale": "The entry is more calm and direct in its language style, which directly expresses the question of whether the interviewee avoids and is likely to criticize his or her work, with no excessive emotional overtones or euphemism. Such expression is clearer and easy to understand and answer questions."}, "time_dimension": {"answer": "Present", "rationale": "In the entry, \"avoid\" means an act that is now being performed, \"working with someone who might criticize himself\" is also what is happening, so the answer is \"now\"."}, "sentence_type": "Negative declarative sentence", "pos": {"nouns": {"Questionnaire Entry": 1, "self": 1, "person": 2}, "verbs": {"Avoid": 1, "Work": 1, "Together": 1}, "adverbs": {"Maybe": 1, "and": 1}, "numerals": {"1": 2}, "pronouns": {"I": 1}}}, {"content": "2. I cannot take a decision without advice or repeated assurances from others.", "word_count": 21, "label": "", "sentence_structure": "Subject鈥損redicate", "subjective_objective": {"answer": "Subjective", "rationale": "Because \"without the advice or repeated assurances of others, I cannot make a decision\" is a self-awareness and feeling of the patient and is subjective."}, "sentence_sentiment": {"answer": "Negative", "rationale": "Because the entry \"I cannot take a decision without the advice or repeated assurances of others\" reflects a lack of trust and dependence on self-decision-making capacity and therefore has a negative emotional connotation."}, "language_style": {"answer": "Calm and direct", "rationale": "This is often more investigative and scientific."}, "time_dimension": {"answer": "Present", "rationale": ""}, "sentence_type": "Negative declarative sentence", "pos": {"nouns": {"others": 1, "- I'm not sure": 1, "Repeatedly": 1, "Promise": 1, "Decision": 1}, "verbs": {"No, I'm not": 1, "Yes": 1, "Make": 1}, "adverbs": {"Just": 1}, "pronouns": {"I": 1}}}, {"content": "I've often focused too much on detail and neglected the larger aspects.", "word_count": 16, "label": "", "sentence_structure": "Subject鈥揷opula鈥損redicative", "subjective_objective": {"answer": "Subjective", "rationale": "Because \"too much attention to detail\" and \"too much neglect\" are subjective feelings and perceptions of patients."}, "sentence_sentiment": {"answer": "Negative", "rationale": "Because the action \"too much to detail, and too much to the big\" in the entry is negative."}, "language_style": {"answer": "Tactful and warm", "rationale": "This way it is easier to accept and collaborate with respondents, thus improving the effectiveness of research."}, "time_dimension": {"answer": "Present", "rationale": "The entry indicates the current customary behaviour of the subject, which often focuses too much on detail and neglects a large aspect. While the words \"now\" are not clearly stated, by using the verbs that are generally current and descriptive ( \"I often focus too much on detail\" ), we can infer that this is a description of the current act. Thus, the temporal dimension of the article is now judged."}, "sentence_type": "Negative declarative sentence", "pos": {"nouns": {"Details": 2, "In terms of": 1}, "verbs": {"Focus": 1, "Neglect": 1}, "adjectives": {"That's too much": 1}, "numerals": {"3": 1}, "pronouns": {"I": 1}}}, {"content": "I need to be the center of attention.", "word_count": 12, "label": "", "sentence_structure": "Subject鈥搗erb鈥搊bject", "subjective_objective": {"answer": "Subjective", "rationale": "Because I represent the patient's personal views and needs here, it is the patient's personal expectations that need to be the focus of attention, so it's a subjective perspective."}, "sentence_sentiment": {"answer": "Negative", "rationale": "Because the need to be a focus of attention in the entry, which reflects over-care and neglect of others, is a self-centered manifestation."}, "language_style": {"answer": "Calm and direct", "rationale": "The entry is more straightforward, and does not use euphemisms, metaphors, etc. to express the subject. It clearly presents a point of view or a need and expects feedback or responses from the other party. It can therefore be described as a calm and straightforward style."}, "time_dimension": {"answer": "Present", "rationale": "The verb \"need\" in the entry indicates that it is a present demand or desire and does not address past or future patterns and is therefore current."}, "sentence_type": "Imperative sentence", "pos": {"nouns": {"I": 1, "Centre": 1}, "verbs": {"Yes": 1, "Be": 1}, "numerals": {"4": 1}}}, {"content": "I've achieved more than others have praised me for.", "word_count": 17, "label": "", "sentence_structure": "Subject鈥搗erb鈥揷omplement", "subjective_objective": {"answer": "Subjective", "rationale": "This is a subjective one that cannot be measured or measured objectively, but is judged by the patient himself."}, "sentence_sentiment": {"answer": "Positive", "rationale": "Because \"achieving more than others praise me\" in the entry expresses a sense of confidence and pride and is positive."}, "language_style": {"answer": "Calm and direct", "rationale": "The entry uses a more-than-prescriptive and absolute term \"more than\" and expresses a sense of confidence and strongness, and does not use too much euphemism or moderation. At the same time, it does not contain too much emotional or rhetorical acrimonymism, so it can be judged as a straightforward and calm tone."}, "time_dimension": {"answer": "Present", "rationale": "\"Now.\""}, "sentence_type": "Affirmative declarative sentence", "pos": {"nouns": {"Achievements": 1, "- I'm impressed": 1}, "verbs": {"Access": 1, "- I'm impressed": 1}, "adjectives": {"Far away": 1}, "numerals": {"5": 1, "More than": 1}, "pronouns": {"I": 1, "others": 1}}}, {"content": "I'll use excessive means to stop the people I love from leaving me.", "word_count": 18, "label": "", "sentence_structure": "Subject鈥搗erb鈥搊bject", "subjective_objective": {"answer": "Subjective", "rationale": "Because \"stop\" and \"leave\" are patients' subjective attitudes and behaviour, and whether or not to resort to excessive means is the patient鈥檚 subjective choice. So the point of view of this questionnaire is to look at the patient from the subjective perspective."}, "sentence_sentiment": {"answer": "Negative", "rationale": "Because the behavior in the entry \"stopping the people I love from leaving me\" is offensive and possibly negative."}, "language_style": {"answer": "Calm and direct", "rationale": "The language style of the entry is relatively calm and straightforward, and there is no excessive trim or emotional rendering, and the tone is a fair and objective expression of the desire to ask the other side whether it is in a violent manner to prevent the person it loves from leaving the subject."}, "time_dimension": {"answer": "Past", "rationale": "\"I will use a disproportionate means to prevent the person I love from leaving me,\" but it does not clearly indicate that the decision was taken at a point in the past or that it will happen in the future. So we cannot determine the temporal dimensions of this sentence. For the questionnaire, the time dimension is not clear. \"I will prevent the person I love from leaving me by an excessive means.\""}, "sentence_type": "Imperative sentence", "pos": {"nouns": {"Means": 1, "person": 1}, "verbs": {"Yes": 1, "Stop": 1, "Leave": 1, "Love": 1}, "adjectives": {"It's too much": 1}, "numerals": {"6": 1}, "pronouns": {"I": 1, "Sou-san": 1}}}, {"content": "I've had several legal problems (e.g. detention, prosecution or re-education through labour)", "word_count": 22, "label": "", "sentence_structure": "Subject鈥搗erb鈥搊bject", "subjective_objective": {"answer": "Subjective", "rationale": "This is an event that patients have experienced, their personal experience and a subjective examination."}, "sentence_sentiment": {"answer": "Negative", "rationale": "Because \"legal trouble\" was a negative event."}, "language_style": {"answer": "Tactful and warm", "rationale": "The language style of this entry is relatively euphemistic, not to ask the respondent directly about the legal issues, but to direct the interviewee to express his experience in a more moderate way. At the same time, the expression \"shows\" avoids being too direct or intense in its approach to questions, and to respect the privacy and feelings of the interviewee."}, "time_dimension": {"answer": "Present", "rationale": "The entry describes experience that has occurred in the past, but because \"a few times\" means a number, it can also be considered as a state of continuity. The temporal dimension of the entry can therefore be either past or present."}, "sentence_type": "Affirmative declarative sentence", "pos": {"nouns": {"Law": 1, "Trouble": 1, "Detention": 1, "Prosecution": 1, "Reeducation through labour": 1}, "verbs": {"I met him": 1, "By": 3}, "adverbs": {"A few times": 1}, "numerals": {"7": 1}}}, {"content": "I'm not interested in spending time with family or friends.", "word_count": 17, "label": "", "sentence_structure": "Subject鈥揷opula鈥損redicative", "subjective_objective": {"answer": "Subjective", "rationale": "The reason for this is that the person who is interested in spending time with a family or friends is the person who thinks and feels that he or she is."}, "sentence_sentiment": {"answer": "Negative", "rationale": "Because the entry \"very uninterested\" expresses a negative emotional attitude."}, "language_style": {"answer": "Calm and direct", "rationale": "The language style of the entry is straightforward because it is a direct statement that asks the other side whether it is not interested in spending time with family or friends, and not overdoing it with a touch of a touch of adoration and emotional rendering."}, "time_dimension": {"answer": "Present", "rationale": "The entry uses the expression \"I'm not interested in...\" which is a subjective expression, and therefore is a modern-day one. Besides, \"Sitting with family or friends\" is also an act of describing the current situation, so the time dimension of the entire sentence is now."}, "sentence_type": "Negative declarative sentence", "pos": {"nouns": {"Questionnaire Entry": 1, "Time": 1, "Family": 1, "friends": 1}, "verbs": {"to": 1, "Common": 1, "Not interested": 1}, "adjectives": {"Not interested": 1}, "adverbs": {"very": 1}, "numerals": {"8": 1}}}, {"content": "I can get special information about what's happening around here.", "word_count": 18, "label": "", "sentence_structure": "Subject鈥揷opula鈥損redicative", "subjective_objective": {"answer": "Subjective", "rationale": "Because \"access to special information\" is the subjective assessment of the patient's perception and perception."}, "sentence_sentiment": {"answer": "Positive", "rationale": "Because the behaviour in the entry \"can get special information from what happens around\" is a manifestation of positive attitude and competence, expressing the respondents' unique ability to sense and understand environmental information."}, "language_style": {"answer": "Tactful and warm", "rationale": "The entry is more adept and interactive when it is spoken in a gentle and inviting manner, rather than by direct statements or orders."}, "time_dimension": {"answer": "Present", "rationale": "The word \"can\" in \"can\" means \"power,\" and \"get it from\" indicates that it's an ongoing process, so it's judged now."}, "sentence_type": "Interrogative sentence", "pos": {"nouns": {"Things": 1, "Information": 1}, "verbs": {"Come on": 1, "Got it": 1, "Special": 1}, "adjectives": {"Special": 1}, "numerals": {"9": 1}, "pronouns": {"I": 1}}}, {"content": "I know that if I don't take care of myself, someone else will take advantage of me or try to cheat me.", "word_count": 25, "label": "", "sentence_structure": "Subject鈥搗erb鈥搊bject", "subjective_objective": {"answer": "Subjective", "rationale": "Because this is the subjective view and concern of the patient."}, "sentence_sentiment": {"answer": "Negative", "rationale": "Because \"take advantage\" and \"fake\" in the entries are negative."}, "language_style": {"answer": "Calm and direct", "rationale": "This language style helps respondents to better understand the meaning of the questions and to respond to them accurately."}, "time_dimension": {"answer": "Present", "rationale": "The entry is a question about the degree of awareness of the testee of himself and others, and there is no specific time pointer to sex, so it can be judged now."}, "sentence_type": "Affirmative declarative sentence", "pos": {"nouns": {"Grandpa": 1, "Questionnaire": 1, "Entry": 1, "self": 1, "person": 1, "Cheap": 1, "- Liar": 1}, "verbs": {"Yes": 2, "Watch out": 1, "Jim": 1, "others": 2, "Try": 1, "Answer": 1}, "adverbs": {"If": 1, "I was just": 1}, "numerals": {"10": 1}, "classifiers": {"individual": 1, "Grandpa": 1}, "pronouns": {"I": 2}}}, {"content": "I get upset sometimes.", "word_count": 7, "label": "", "sentence_structure": "Subject鈥揷opula鈥損redicative", "subjective_objective": {"answer": "Subjective", "rationale": "Because \"dry\" is a subjective way of looking at the patient's own feelings and experiences."}, "sentence_sentiment": {"answer": "Negative", "rationale": "Because the emotional state of \"disturbing\" in the entries is negative."}, "language_style": {"answer": "Tactful and warm", "rationale": "The language style of the entry is more euphemistic and gentle, rather than directly revealing the problems or negative emotions that the other side may have, but rather directing the other to share the feelings of the other side in a caring and understanding manner. This expression is more acceptable and has the potential to promote more in-depth communication and communication."}, "time_dimension": {"answer": "Present", "rationale": "According to the key words in the input sentence, \"sometimes\" and \"disturbed\", the entry describes the emotional state that the person tested may have in the past, present or future. Therefore, the temporal dimension is judged to be past, present or future. But it is not possible to determine whether this emotional state is a normal act of the past, a temporary phenomenon of the present or a future scenario, due to the lack of context information."}, "sentence_type": "Affirmative declarative sentence", "pos": {"nouns": {"Time": 1, "I'm upset": 1}, "verbs": {"have": 1}, "adverbs": {"Sometimes": 1}}}, {"content": "I only make friends with people when I'm sure they like me.", "word_count": 19, "label": "", "sentence_structure": "Subject鈥搗erb鈥搊bject", "subjective_objective": {"answer": "Subjective", "rationale": "Because it's the subjective feeling of an individual who likes it or not."}, "sentence_sentiment": {"answer": "Negative", "rationale": "Because the act of \"friending people only when they are convinced that they like me\" in the entry indicates a lack of self-confidence and the need to determine whether they are worthy of being involved with someone else's preferences, which is a negative psychological state."}, "language_style": {"answer": "Calm and direct", "rationale": "The language style of the entry is more direct and clear, and is not expressed in too many emotional or rhetorical ways, but in a concise and clear language, which is more in line with the rigour and objectivity of the questionnaire."}, "time_dimension": {"answer": "Present", "rationale": "Now, \"I'm only making friends with people when I'm sure they like me\" means the testee himself, and \"to make friends with them\" means that the act is conditional and takes place only when certain conditions are met. The entry is therefore to ask the testee about the behaviour or attitude of the person."}, "sentence_type": "Affirmative declarative sentence", "pos": {"nouns": {"Questionnaire": 1, "Entry": 1, "People": 1, "Make friends": 1}, "verbs": {"Sure": 1, "Yeah": 2, "That's right": 1, "and": 1, "friends": 1}, "adverbs": {"When": 1, "Only": 1}, "numerals": {"12": 1}, "pronouns": {"I": 1}}}, {"content": "I'd rather have others take responsibility for myself.", "word_count": 14, "label": "", "sentence_structure": "Subject鈥搗erb鈥搊bject", "subjective_objective": {"answer": "Subjective", "rationale": "This is because of the patient ' s personal thoughts and attitudes and because it is part of the subjective examination."}, "sentence_sentiment": {"answer": "Negative", "rationale": "For the purpose of the act of \"placement of responsibility for others\" is negative, indicating that an individual is unwilling to assume responsibility for himself or to evade responsibility."}, "language_style": {"answer": "Calm and direct", "rationale": "The entry is more direct and concise, is not based on excessive rhetorical or emotional rendering, and is more objective and calm. It directly expresses the subject of questioning the other party's preference in the distribution of responsibilities and leads the interviewee to think and answer by providing a specific situation (i.e., \"more willing to hold others accountable for themselves\"."}, "time_dimension": {"answer": "Present", "rationale": ""}, "sentence_type": "Negative declarative sentence", "pos": {"nouns": {"Responsibility": 1, "Undertaking": 1}, "verbs": {"Yes": 1, "Jean": 1}, "adjectives": {"Don't": 1, "From": 1, "Yes": 1, "More": 1}, "numerals": {"13": 1}, "pronouns": {"I": 1}}}, {"content": "I've spent time making things perfect.", "word_count": 14, "label": "", "sentence_structure": "Subject鈥搗erb鈥搊bject", "subjective_objective": {"answer": "Subjective", "rationale": "Because \"I've taken time\" and \"to make things perfect\" are personal feelings and decisions of patients, subjective."}, "sentence_sentiment": {"answer": "Negative", "rationale": "Because the act of \"excessive perfection\" is often considered an over-exercise, which often leads to negative emotions and effects."}, "language_style": {"answer": "Tactful and warm", "rationale": "The entry uses a soft, invitation-style tone, which is more personal and interactive, and which is conducted in a way that is not directly presented or ordered."}, "time_dimension": {"answer": "Present", "rationale": ""}, "sentence_type": "Affirmative declarative sentence", "pos": {"nouns": {"Time": 1, "Things": 1, "Perfect": 1}, "verbs": {"Cost": 1, "鈾?And I'm gonna be 鈾?: 1}, "adjectives": {"That's too much": 1}, "numerals": {"14": 1}, "pronouns": {"I": 1}}}, {"content": "I'm more sexy than most people.", "word_count": 8, "label": "", "sentence_structure": "Subject鈥揷opula鈥損redicative", "subjective_objective": {"answer": "Subjective", "rationale": "Because \"more sexy than most people\" is a self-evaluation and subjective feeling, and it is not an objective fact or result."}, "sentence_sentiment": {"answer": "Positive", "rationale": "Because the \"hot\" feature in the entry is considered positive and attractive."}, "language_style": {"answer": "Tactful and warm", "rationale": "The entry uses a more positive description in a more sexual form, and places it in a subjective and individual context, which is relatively mild and has an emotional tone that encourages and affirms. At the same time, the issue implies that the subject of the survey can be involved in the discussion through self-evaluation, and thus has some interaction."}, "time_dimension": {"answer": "Future", "rationale": "The keyword \"more sexy than most people\" expresses the testee's expectation and imagination that he might be more sexy in the future. So the time dimension of this entry is the future."}, "sentence_type": "Affirmative declarative sentence", "pos": {"nouns": {"Questionnaire": 1, "Entry": 1, "person": 1, "Sexy": 1}, "verbs": {"- Yeah": 1, "More": 1, "have": 1}, "adjectives": {"Most": 1}, "numerals": {"15": 1}, "pronouns": {"I": 1, "Guys": 1}}}, {"content": "I've often thought that I was or would be a great man.", "word_count": 19, "label": "", "sentence_structure": "Subject鈥搗erb鈥揷omplement", "subjective_objective": {"answer": "Subjective", "rationale": "Because \"often thinking\" and \"great man\" are patients' subjective attitudes."}, "sentence_sentiment": {"answer": "Positive", "rationale": "Because the entry \"wanted to be great\" expresses a positive, ambitious quality and attitude."}, "language_style": {"answer": "Tactful and warm", "rationale": ""}, "time_dimension": {"answer": "Present", "rationale": "The keyword \"always\" denotes a regular act, while the word \"want\" refers to the general current, meaning that the act has not only occurred in the past, but may still be ongoing. Thus, the entry describes the person being tested's current thoughts and perceptions, which are in the present context."}, "sentence_type": "Affirmative declarative sentence", "pos": {"nouns": {"I": 2, "person": 2}, "verbs": {"Yes": 2, "be": 1, "Be": 1}, "adjectives": {"Great": 1}, "adverbs": {"Regular": 1}}}, {"content": "I love someone or hate them, there's no middle.", "word_count": 18, "label": "", "sentence_structure": "Subject鈥揷opula鈥損redicative", "subjective_objective": {"answer": "Subjective", "rationale": "Because the sentence describes the patient ' s own emotional state and feelings, it relates to the subjective attitudes and emotional experiences of the individual."}, "sentence_sentiment": {"answer": "Negative", "rationale": "Because the expression \"love or hate someone\" in the entry is negative, indicating a duality, lack of inclusion and understanding."}, "language_style": {"answer": "Calm and direct", "rationale": "The language style of the entry is \"cool and straight.\" It expresses a point in a simple and clear language: \"I have only two emotional states -- love or hate, and there is no neutral situation.\" This expression is more straightforward, and there is no excessive emotional overturning."}, "time_dimension": {"answer": "Present", "rationale": "The words \"me,\" \"or \"or \"or \"no\" indicate the current feelings or beliefs of the speaker and suggest that they are relatively stable. Moreover, the time frame for \"now\" usually refers to what happens at the moment when the speaker is speaking or what happens recently, so it can be considered that the entry is asking about the current emotional situation of the person being tested."}, "sentence_type": "Negative declarative sentence", "pos": {"nouns": {"Situation": 1, "person": 2}, "verbs": {"be": 2, "Love": 1, "Hate": 1}, "adjectives": {"No, I'm not": 1}, "adverbs": {"not": 1, "Just": 1}, "numerals": {"17": 1, "Centre": 1}, "pronouns": {"I": 1}}}, {"content": "I was involved in a lot of fights.", "word_count": 9, "label": "", "sentence_structure": "Subject鈥搗erb鈥搊bject", "subjective_objective": {"answer": "Subjective", "rationale": "Because \"responsible involvement in fighting\" is the subjective behaviour and attitude of a person, who chose to participate himself/herself, it is viewed from the subjective perspective of the patient."}, "sentence_sentiment": {"answer": "Negative", "rationale": "Because \"reinvolved in fighting\" is a bad act that can lead to physical injury, legal liability, etc., and is therefore negative."}, "language_style": {"answer": "Calm and direct", "rationale": "The entry describes in an objective and straightforward manner the problems that the respondents may have experienced in the past, without using excessive rhetoric or emotional colours, and with a more calm and straightforward language style."}, "time_dimension": {"answer": "Present", "rationale": "The entry used to ask the testee about his past experiences and behaviour, and the temporal dimension judged the past. The keyword \"multiple\" and \"fight\" all indicate that this was a past act or experience."}, "sentence_type": "Affirmative declarative sentence", "pos": {"nouns": {"Fighting": 1, "I": 1}, "verbs": {"Intrusion": 1, "Fight": 1, "Fighting": 1}, "adverbs": {"Multiple": 1}, "numerals": {"18": 1}}}, {"content": "I'd rather do things myself than with others.", "word_count": 16, "label": "", "sentence_structure": "Subject鈥搗erb鈥揷omplement", "subjective_objective": {"answer": "Subjective", "rationale": "This is because of the patient ' s personal orientation and choice and is subjective."}, "sentence_sentiment": {"answer": "Negative", "rationale": "Because the act of \"willing to do things\" in the entry is a manifestation of individualism and self-centered tendencies, it may lead to a lack of cooperative spirit and social ties."}, "language_style": {"answer": "Calm and direct", "rationale": "The article is more direct and clear, and does not have too much accommodative or emotional expression. It simply expresses the subject of asking the other party whether it prefers to act alone or to cooperate with others, which may make the person under investigation more truthful and frank."}, "time_dimension": {"answer": "Present", "rationale": "Now, I prefer to express subjective will and sexual preference, and now it's a reminder that the act is being or is taking place, so the entry describes the current state and orientation of the testee."}, "sentence_type": "Affirmative declarative sentence", "pos": {"nouns": {"Questionnaire": 1, "Entry": 1, "Things": 1, "Others": 1}, "verbs": {"I'd rather": 1, "Do it": 2, "Together": 1}, "numerals": {"19": 1}, "pronouns": {"I": 1}}}, {"content": "I have the ability to premonition before something actually happens.", "word_count": 20, "label": "", "sentence_structure": "Subject鈥搗erb鈥揷omplement", "subjective_objective": {"answer": "Subjective", "rationale": "Because \"the ability to have a prophet's vision\" is a subjective belief or attitude."}, "sentence_sentiment": {"answer": "Positive", "rationale": "Because the term \"presuppositional ability\" in the entry is positive."}, "language_style": {"answer": "Tactful and warm", "rationale": "The entry is more proficient and interactive when it is spoken in a gentle and inviting tone, rather than in direct statements or orders. At the same time, the question is not one of strong affirmation or denial, but rather of an open and neutral approach to the question of whether the person interviewed has the ability to pre-empt, a euphemism that is more likely to arouse resonance and interest among the person interviewed, thus increasing the effectiveness and reliability of the questionnaire."}, "time_dimension": {"answer": "Future", "rationale": "The keyword \"before something actually occurs\" indicates the expectation and vision of the future, and \"presupient presupposition\" is an unprecedented and possible future scenario, so the entry is judged to be the future."}, "sentence_type": "Affirmative declarative sentence", "pos": {"nouns": {"Things": 1, "Capacity": 1}, "verbs": {"Come on": 1, "Prophet": 1, "Preception": 1}, "adverbs": {"Actual": 1}, "classifiers": {"Some": 1}, "pronouns": {"I": 1}}}, {"content": "I've often wondered if all the people I know really deserve to be trusted.", "word_count": 20, "label": "", "sentence_structure": "Subject鈥揷opula鈥損redicative", "subjective_objective": {"answer": "Subjective", "rationale": "Because \"suspicious\" is a subjective feeling or idea that shows that patients have doubts and uncertainty about whether others are worthy of trust."}, "sentence_sentiment": {"answer": "Negative", "rationale": "Because \"suspicion\" and \"untrustable\" in the entries are negative emotional."}, "language_style": {"answer": "Tactful and warm", "rationale": ""}, "time_dimension": {"answer": "Present", "rationale": "\"I'm often\" means a regular, customary act, and \"suspicious\" uses the usual current state of affairs, which means that the act has not only occurred in the past, but may still be ongoing. Thus, the entry is a description of the current situation."}, "sentence_type": "Interrogative sentence", "pos": {"nouns": {"I": 1, "People": 1, "Trust": 1}, "verbs": {"I doubt it": 1, "Yes": 1, "Really": 1, "It's worth it": 1, "Trust": 1}, "adjectives": {"True": 1, "Positive": 1}, "adverbs": {"Both": 1, "Is it possible": 1}, "numerals": {"21": 1}}}, {"content": "22. I sometimes talk about people behind my back.", "word_count": 10, "label": "", "sentence_structure": "Subject鈥搗erb鈥搊bject", "subjective_objective": {"answer": "Objective", "rationale": "Since \"people speak behind\" is the objective behaviour of the patient, whether it occurs has nothing to do with the patient's idea, and therefore is objective."}, "sentence_sentiment": {"answer": "Negative", "rationale": "Because the \"back-to-back\" act in the entry is not widely accepted by society, it is somewhat negative."}, "language_style": {"answer": "Calm and direct", "rationale": "The entry directly expresses in plain and clear language whether the other party will speak about the subject behind the back, without using too many rhetorical techniques or emotional rendering, and with a more balanced and objective tone."}, "time_dimension": {"answer": "Present", "rationale": "\"Sometimes\" means that the act is not a regular one, but not only a certain period of time in the past, but that it still exists. The move \"the talker\" is an exercise that is now under way. Thus, the time dimension of the article is now."}, "sentence_type": "Affirmative declarative sentence", "pos": {"nouns": {"Behind": 1, "Arguments": 1, "People": 1}, "verbs": {"in": 1, "be": 1, "Say it": 1}, "adverbs": {"Sometimes": 1}, "numerals": {"22": 1}}}, {"content": "I'm limiting myself to being intimate with others because I'm afraid I'm being mocked.", "word_count": 25, "label": "", "sentence_structure": "Subject鈥搗erb鈥搊bject", "subjective_objective": {"answer": "Subjective", "rationale": "I fear being mocked by the subjective idea of being a patient."}, "sentence_sentiment": {"answer": "Negative", "rationale": "Because \"restricting your intimate relationship\" and \"fearing to be mocked\" in the entries are negative behaviors and emotions."}, "language_style": {"answer": "Tactful and warm", "rationale": ""}, "time_dimension": {"answer": "Past", "rationale": "The \"fear of being mocked\" in the entry indicates an emotional and psychological state that usually reflects past experiences or events and is therefore part of the temporal dimension of the past."}, "sentence_type": "Interrogative sentence", "pos": {"nouns": {"Intimacy": 1, "Relations": 1, "self": 1, "Scoffing": 1}, "verbs": {"Limits": 1, "have": 1, "Received": 1}, "adverbs": {"Because": 1}, "numerals": {"23": 1}, "pronouns": {"I": 1}}}, {"content": "I'm afraid if I don't approve of others, I'm afraid I'll lose them.", "word_count": 21, "label": "", "sentence_structure": "Subject鈥搗erb鈥搊bject", "subjective_objective": {"answer": "Subjective", "rationale": "The purpose of the questionnaire is to examine whether the patient has one of the symptoms of social phobia - fear of being criticized or opposed by others."}, "sentence_sentiment": {"answer": "Negative", "rationale": "Because the attitude of \"fearing to lose support\" in the entry is negative."}, "language_style": {"answer": "Tactful and warm", "rationale": "This language is more moderate and thoughtful, and increases the confidence and comfort of the interviewee and facilitates more realistic and accurate answers."}, "time_dimension": {"answer": "Present", "rationale": "So the answer is \"now\"."}, "sentence_type": "Negative declarative sentence", "pos": {"nouns": {"Questionnaire": 1, "Entry": 1, "self": 1, "others": 1, "Support": 1}, "verbs": {"Scared": 1, "That's not good": 1, "Lose": 1, "Give": 1}, "adjectives": {"Down there": 1, "24": 1}, "numerals": {"Four": 1, "self": 1, "others": 1, "Support": 1}, "pronouns": {"If": 1, "Just": 1}}}, {"content": "I see work as more important than spending time or entertainment with my family or friends.", "word_count": 24, "label": "", "sentence_structure": "Subject鈥搗erb鈥搊bject", "subjective_objective": {"answer": "Subjective", "rationale": "Because \"the value of work is more important than time or entertainment with family or friends\" is the patient's values and attitude, which is subjective."}, "sentence_sentiment": {"answer": "Negative", "rationale": "Because the attitude in the entry that \"the job is seen more than time or entertainment with family or friends\" is biased towards work, may neglect family and social life and therefore have a negative emotional connotation."}, "language_style": {"answer": "Calm and direct", "rationale": "This language style is conducive to accurate and straightforward answers."}, "time_dimension": {"answer": "Present", "rationale": "The entry describes a subjective attitude or value that respondents consider work more important and that this attitude exists in the present state of affairs."}, "sentence_type": "Affirmative declarative sentence", "pos": {"nouns": {"Work": 1, "Home": 1, "Lee": 1, "friends": 1, "Time": 1, "Recreation": 1}, "verbs": {"Look": 1, "- Put it on": 1, "Common": 1, "Important": 1}, "adverbs": {"More": 1}, "numerals": {"25": 1}, "pronouns": {"I": 1}}}, {"content": "I'm easy to express my feelings.", "word_count": 9, "label": "", "sentence_structure": "Subject鈥揷opula鈥損redicative", "subjective_objective": {"answer": "Subjective", "rationale": "Because \"Easy to express my feelings\" is a patient's inner feeling and attitude, a subjective description."}, "sentence_sentiment": {"answer": "Positive", "rationale": "Because the \"Easier to express my feelings\" act is positive, and it means that individuals are willing to express their feelings."}, "language_style": {"answer": "Tactful and warm", "rationale": "The entry is more personal and interactive when it is spoken in a gentle and inviting manner, rather than in direct statements or orders. Moreover, the language style of the article is relatively simple and does not use too much rhetorical or emotional publicity to make it easy for readers to understand and answer questions."}, "time_dimension": {"answer": "Present", "rationale": "According to the entry, \"I can easily express my emotions\", the entry describes the person being tested for the current behaviour or attitude, and therefore judges it as \"now\"."}, "sentence_type": "Affirmative declarative sentence", "pos": {"verbs": {"Performance": 1, "Easy": 1, "Emotion": 1}, "adjectives": {"Easy": 1}, "numerals": {"26": 1}}}, {"content": "There are only certain special people who really appreciate me and understand me.", "word_count": 19, "label": "", "sentence_structure": "Subject鈥搗erb鈥搊bject", "subjective_objective": {"answer": "Subjective", "rationale": "Because \"appear and understand\" is a subjective feeling and perception."}, "sentence_sentiment": {"answer": "Negative", "rationale": "Because \"only some special person can really appreciate and understand me\" in the entry expresses a sense of isolation and exclusion, with negative emotional aspects."}, "language_style": {"answer": "Tactful and warm", "rationale": "The expression is more personal and interactive, in a gentle and inviting tone, rather than in direct statements or orders. At the same time, the expression \"only certain special people\" gives people a sense of special treatment, increasing the proximity between respondents and questionnaires."}, "time_dimension": {"answer": "Present", "rationale": "\"Only \"in \" means \"only\" means \"specifically\" for a particular group, and the expression \"real\" implies that this is not the capacity of all, but rather that it requires the human being to have some kind of quality. At the same time, the words \"and understand me\" and \"in\" indicate that the act is a parallel of two actions, emphasizing that understanding and appreciation are equally important. So the subject is now the perception of the testee of self-awareness."}, "sentence_type": "Affirmative declarative sentence", "pos": {"nouns": {"person": 1, "I": 1, "Understanding and appreciation": 1}, "verbs": {"have": 1, "Yes": 1}, "adverbs": {"Just": 1, "Really": 1}, "numerals": {"27": 1}}}, {"content": "I've always wondered what kind of person I am.", "word_count": 15, "label": "", "sentence_structure": "Subject鈥揷opula鈥損redicative", "subjective_objective": {"answer": "Subjective", "rationale": "Because \"whomever person is\" is a self-perceptional subjective thinking."}, "sentence_sentiment": {"answer": "Negative", "rationale": "Because the act of \"whoever you are\" in the entry is negative, showing self-doubt and confusion."}, "language_style": {"answer": "Tactful and warm", "rationale": "The entry uses a first-person-known question phrase, which is more personal and interactive in the form of a moderate and inviting tone of dialogue rather than a direct statement or order. It also encourages interviewees to reflect on their identity and to demonstrate a degree of care and support."}, "time_dimension": {"answer": "Present", "rationale": "In combination, this entry looks at the current emotional state and behavioral characteristics of the person tested. So the answer should be: \"Now.\""}, "sentence_type": "Interrogative sentence", "pos": {"nouns": {"Questionnaire": 1, "Entry": 1, "28": 1, "self": 1, "person": 1}, "verbs": {"I always do": 1, "Yes": 1, "be": 1, "Sample": 1}, "adverbs": {"What the hell": 1}, "numerals": {"28": 1}, "pronouns": {"I": 1}}}, {"content": "I didn't pay the dues on time because I didn't have a long-term, stable job.", "word_count": 23, "label": "", "sentence_structure": "Subject鈥搗erb鈥搊bject", "subjective_objective": {"answer": "Subjective", "rationale": "Because \"if there is a long-term, stable job\" is a personal judgment and interpretation."}, "sentence_sentiment": {"answer": "Negative", "rationale": "Because the \"unpaid\" act in the entry is negative."}, "language_style": {"answer": "Calm and direct", "rationale": "The language style of the entry is more straightforward, it is less over-modified or emotional, it is more objective and realistic. It clearly expresses the financial position of the person under investigation and his reasons, and it does not use euphemism or emotional expressions. It can therefore be considered as \"cooling straight.\""}, "time_dimension": {"answer": "Past", "rationale": ""}, "sentence_type": "Negative declarative sentence", "pos": {"nouns": {"Work": 1, "Money": 1}, "verbs": {"Pay": 1, "Nope": 1, "have": 1, "Hand it over": 1, "be": 1, "Here": 1}, "adjectives": {"Steady": 1}, "adverbs": {"not": 2, "On time": 1}, "numerals": {"29": 1, "One": 1}, "classifiers": {"The": 1, "individual": 1}, "pronouns": {"I": 1}}}, {"content": "The sex content can't really interest me.", "word_count": 16, "label": "", "sentence_structure": "Subject鈥揷opula鈥損redicative", "subjective_objective": {"answer": "Subjective", "rationale": "Because \"interest\" is a subjective feeling or attitude, it's seen from the patient's subjective perspective."}, "sentence_sentiment": {"answer": "Negative", "rationale": "Because the entry indicates that there is no interest in sexual content, it is a negative emotion or attitude."}, "language_style": {"answer": "Tactful and warm", "rationale": "The entry is presented in a more euphemistic way to ask each other about the extent of their interest in sexual content, rather than directly asking or using strong negative tone, which makes it easier for respondents to accept and answer questions."}, "time_dimension": {"answer": "Present", "rationale": "The keyword \"can't really interest me\" means the current feelings and attitudes, so the judgement is now."}, "sentence_type": "Interrogative sentence", "pos": {"nouns": {"Sexual": 1, "Contents": 1, "Interest": 1}, "verbs": {"Yes": 1, "Really": 1, "Cause": 1}, "adverbs": {"Really": 1}, "numerals": {"30": 1}}}, {"content": "I'm always aware of or feeling things that people don't know.", "word_count": 19, "label": "", "sentence_structure": "Subject鈥揷opula鈥損redicative", "subjective_objective": {"answer": "Subjective", "rationale": "Because it is subjective to realize or feel something that others do not know is a human experience and feeling."}, "sentence_sentiment": {"answer": "Positive", "rationale": "Because the behaviour in the entry \"aware or feel something that others do not know\" can be understood as a special cognitive ability or perceptive insight, which is usually considered a positive feature."}, "language_style": {"answer": "Tactful and warm", "rationale": "The language style of this entry is euphemism. It takes the form of questions, not direct statements or orders, and gives people a sense of invitation and questioning. At the same time, the phrase is more moderate, not too emotional or rhetorical, and it feels more intimate and natural. Such a questionnaire entry can make respondents more willing to share their feelings and views, and helps to make the results of the survey more accurate and reliable."}, "time_dimension": {"answer": "Past", "rationale": "The entry therefore relates to the current act or state."}, "sentence_type": "Affirmative declarative sentence", "pos": {"nouns": {"Questionnaire": 1, "Entry": 1, "Things": 1}, "verbs": {"Awareness": 1, "Feel": 1, "Yes": 2, "I'm aware of that": 1, "Feel it": 1}, "adjectives": {"others": 1, "Regular": 1, "I don't know": 1}, "adverbs": {"- I'm not sure": 1}, "pronouns": {"I": 1}}}, {"content": "32. People will use what I tell them or what I tell them to oppose me.", "word_count": 20, "label": "", "sentence_structure": "Subject鈥搗erb鈥搊bject", "subjective_objective": {"answer": "Subjective", "rationale": "Because the patient ' s perception and feelings about whether another person will use his or her own words or things are subjective."}, "sentence_sentiment": {"answer": "Negative", "rationale": "Because the description of \"someone will use what I tell them to oppose me\" is negative."}, "language_style": {"answer": "Calm and direct", "rationale": "The entry uses a direct, clear tone to ask if the person surveyed has ever been subjected to any use of his or her language, without any means of speech or emotional rendering."}, "time_dimension": {"answer": "Present", "rationale": "The entry asks the testee's opinion as to whether another person will use what he or she says or what he or she says, and the temporal dimension is now. Key words include \"others,\" \"will use\" and \"the words or things that they tell them,\" which all suggest that this is a matter that the testee is thinking about."}, "sentence_type": "Negative declarative sentence", "pos": {"nouns": {"person": 1, "Word": 1, "Things": 1}, "verbs": {"Yes": 1, "Utilization": 1, "Objection": 1}, "adverbs": {"EMPTY": 32}, "numerals": {"32": 1}, "pronouns": {"I": 1}}}, {"content": "There are some people I don't like.", "word_count": 8, "label": "", "sentence_structure": "Subject鈥搗erb鈥搊bject", "subjective_objective": {"answer": "Subjective", "rationale": "Because \"not like\" is a subjective feeling and attitude."}, "sentence_sentiment": {"answer": "Negative", "rationale": "Because the entry is a negative emotional tendency, which is not to like certain people."}, "language_style": {"answer": "Calm and direct", "rationale": "The entry is more calm and objective in asking the respondents in a direct manner about their attitudes towards certain persons, without using euphemism or excessive rhetorical techniques."}, "time_dimension": {"answer": "Present", "rationale": "\"Some people I don't like the word \"yes\" means \"presence\" and \"unsatisfaction\" takes the general state of the art, which means that this state may exist at some point in time. So the temporal dimension of this article is now."}, "sentence_type": "Negative declarative sentence", "pos": {"nouns": {"person": 1, "Yeah": 1}, "verbs": {"have": 1}, "adjectives": {"I don't like it": 2}, "numerals": {"33": 1}}}, {"content": "I'm more sensitive to criticism or rejection than most people.", "word_count": 14, "label": "", "sentence_structure": "Subject鈥揷opula鈥損redicative", "subjective_objective": {"answer": "Subjective", "rationale": "Because the sentence describes the patient ' s perception and perception of his or her own emotions and is subjective in nature."}, "sentence_sentiment": {"answer": "Negative", "rationale": "Because \"sensitive\" is usually associated with negative emotions and feelings, the purpose of this article is negative."}, "language_style": {"answer": "Tactful and warm", "rationale": "This entry uses a mild and invitational tone, avoiding a direct negative or offensive expression. This expression makes it easier for the respondents to accept and share their feelings and experiences, thereby increasing the effectiveness and accuracy of the questionnaire."}, "time_dimension": {"answer": "Past", "rationale": "The entry uses the comparative description of the testee's past behaviour, implying that it has already occurred. At the same time, the criterion for \"sensitive\" evaluation is based on past experience and perceptions. So the time dimension of the purpose is past."}, "sentence_type": "Negative declarative sentence", "pos": {"nouns": {"I": 1, "Most people": 1, "Criticism": 1, "Reject": 1}, "verbs": {"to": 1, "More sensitive": 1}, "adjectives": {"Sensitive": 1}, "adverbs": {"- Yeah": 1, "More": 2}, "numerals": {"34": 1}}}, {"content": "If I had to do something myself, I found it hard to start.", "word_count": 18, "label": "", "sentence_structure": "Subject鈥搗erb鈥搊bject", "subjective_objective": {"answer": "Subjective", "rationale": "Because it's the patient's subjective feelings and experiences."}, "sentence_sentiment": {"answer": "Negative", "rationale": "Because the \"recognizing hard\" behavior in the entry is negative."}, "language_style": {"answer": "Tactful and warm", "rationale": "The entry is more euphemistic, less nuanced or less nuanced, but rather more acquitting in a more accommodative and understanding way. Moreover, the entry uses the more soft expression \"receiving difficult to begin\" and avoids the use of too negative or challenging words, which are more personal and human."}, "time_dimension": {"answer": "Present", "rationale": "The term \"discovery\" indicates that it is a personal feeling or observation, and the entry is therefore a description of the current situation."}, "sentence_type": "Negative declarative sentence", "pos": {"nouns": {"Yes": 2, "person": 1}, "verbs": {"Do it": 1, "Found": 1, "Start": 1}, "adjectives": {"Hard": 1}, "adverbs": {"very": 1, "Okay": 1}, "numerals": {"35": 1}, "pronouns": {"I": 1}}}, {"content": "I have a higher moral standard than anyone else.", "word_count": 12, "label": "", "sentence_structure": "Subject鈥揷opula鈥損redicative", "subjective_objective": {"answer": "Subjective", "rationale": "Because \"a higher moral standard than anyone else\" is an individual self-evaluation and subjective attitude."}, "sentence_sentiment": {"answer": "Positive", "rationale": "Because the expression \"I have higher moral standards than anyone else\" shows that individuals have higher self-requirement and moral standards and have positive emotional tendencies."}, "language_style": {"answer": "Calm and direct", "rationale": "It asked whether respondents had raised their moral standards, without too many rhetorical or emotional features, and whether the tone was more objective and direct."}, "time_dimension": {"answer": "Present", "rationale": "The time dimension of this questionnaire entry is now, and the key word is \"I have a higher moral standard than anyone else.\" This phrase is an expression of a testee's perception and assessment of his moral values, a description of the state of affairs."}, "sentence_type": "Affirmative declarative sentence", "pos": {"nouns": {"Questionnaire Entry": 1, "person": 1, "Ethical standards": 1}, "verbs": {"have": 1, "- Yeah": 1, "Higher": 1}, "adjectives": {"Other": 1, "others": 1}, "numerals": {"36": 1}, "pronouns": {"I": 1}}}, {"content": "When I want to be noticed, I use my own appearance to attract them.", "word_count": 21, "label": "", "sentence_structure": "Subject鈥搗erb鈥搊bject", "subjective_objective": {"answer": "Subjective", "rationale": "Because \"attracting someone with an appearance\" is a subjective choice and behaviour of the patient. Although the appearance may attract attention, it is still chosen and implemented by the patient, and therefore is subjectively viewed."}, "sentence_sentiment": {"answer": "Negative", "rationale": "Because the act of \"attracting them with their own appearance\" in the entry may give the impression that it is an unhealthy self-expression."}, "language_style": {"answer": "Tactful and warm", "rationale": "The language style of the entry is euphemism. It is more intimate and interactive by a gentle and inviting tone, rather than by direct statement or order. At the same time, the entry is not too much rhetorical or emotional, and is more objective and comfortable."}, "time_dimension": {"answer": "Present", "rationale": "The entry describes the current behavior and state, both \"wanted to be noticed\" and \"into their own appearance\" are both ongoing actions. So the judgment is based on the present."}, "sentence_type": "Affirmative declarative sentence", "pos": {"nouns": {"Attention": 1, "I": 2, "Appearance": 1, "they": 1}, "verbs": {"Yes": 1, "Use": 1, "Attractive": 1}, "numerals": {"37": 1}}}, {"content": "I need to be noticed or complimented.", "word_count": 14, "label": "", "sentence_structure": "Subject鈥搗erb鈥揷omplement", "subjective_objective": {"answer": "Subjective", "rationale": "Because \"need\" and \"nomination\" are the patient's own subjective feelings and expectations."}, "sentence_sentiment": {"answer": "Negative", "rationale": "Because the attitude of \"very much needed\" in the entries is negative, and attention and praise are often given to personal needs rather than to natural emotional interactions."}, "language_style": {"answer": "Tactful and warm", "rationale": "The entry uses a more euphemistic tone to ask the other side about the level of demand for attention and praise, rather than directly speaking or ordering. At the same time, the article is more acclaimed and friendly, helping to establish a good relationship between the respondents to the questionnaire and the researchers."}, "time_dimension": {"answer": "Present", "rationale": "The entry \"I really need\" and \"Significant Me\" both indicate the immediate emotional needs, so the temporal dimension is now."}, "sentence_type": "Affirmative declarative sentence", "pos": {"nouns": {"I": 2, "others": 1}, "verbs": {"Very": 1, "Yes": 1, "Attention": 1, "Praise": 1}, "adjectives": {"38": 1}, "adverbs": {"or": 1}}}, {"content": "I tried to hurt myself or kill myself.", "word_count": 9, "label": "", "sentence_structure": "Subject鈥搗erb鈥搊bject", "subjective_objective": {"answer": "Subjective", "rationale": "Because it is the patient ' s subjective experience and will to try to harm himself or to commit suicide."}, "sentence_sentiment": {"answer": "Negative", "rationale": "Because the act of \"self-inflicted or suicide\" in the entry is negative."}, "language_style": {"answer": "Calm and direct", "rationale": "The language style of the entry is more straightforward, and it does not use any rhetorical or euphemism, but rather direct queries to direct respondents to the questions."}, "time_dimension": {"answer": "Past", "rationale": "The entry asks whether the person tested had ever tried self-inflicted injuries or suicide, which often involves the psychological state and experience of the past, and the answer should therefore be \"the past\"."}, "sentence_type": "Negative declarative sentence", "pos": {"nouns": {"39": 1, "Self-inflicted": 1, "Suicide": 2}, "verbs": {"I tried": 1}, "numerals": {"39": 1}}}, {"content": "I do a lot of things without taking consequences into account.", "word_count": 12, "label": "", "sentence_structure": "Subject鈥搗erb鈥揷omplement", "subjective_objective": {"answer": "Subjective", "rationale": "Because \"no consequences\" are the personal thoughts and attitudes of the patient and are subjective."}, "sentence_sentiment": {"answer": "Negative", "rationale": "Because \"no consequences\" usually leads to negative outcomes and effects."}, "language_style": {"answer": "Calm and direct", "rationale": "The language style of the entry is of a direct, simple type. Instead of using any rhetorical or emotional description, it is a direct statement that clearly expresses the subject of asking whether the other party does not always consider consequences when doing the matter."}, "time_dimension": {"answer": "Present", "rationale": "The entry describes the person who was tested for the present act or habit, and the \"I do many things without taking consequences\" in the \"I do many things without consequences\" suggests that it is a negative act or mentality, so it can be judged that it is the present."}, "sentence_type": "Affirmative declarative sentence", "pos": {"nouns": {"Things": 1, "Consequences": 1}, "verbs": {"Do it": 1, "Consider": 1}, "numerals": {"40": 1}, "pronouns": {"I": 1}}}, {"content": "There's hardly any activity that would interest me.", "word_count": 14, "label": "", "sentence_structure": "Subject鈥揷opula鈥損redicative", "subjective_objective": {"answer": "Subjective", "rationale": "Because \"interest\" is a personal, subjective attitude and emotional experience of the patient."}, "sentence_sentiment": {"answer": "Negative", "rationale": "Because the expression \"activities that do not interest me\" in the entry indicates a lack of interest and motivation, and is a negative emotional colour."}, "language_style": {"answer": "Calm and direct", "rationale": "The language style of the entry is relatively simple, and there is no excessive emotional rendering or rhetorical technique to express directly the subject of the problem, i.e. whether the other side is not interested in any activity. The language style therefore can be judged as being calm and straightforward."}, "time_dimension": {"answer": "Present", "rationale": ""}, "sentence_type": "Negative declarative sentence", "pos": {"nouns": {"Activities": 1}, "verbs": {"鈾?And I'm gonna be 鈾?: 1, "Interested": 1, "Almost": 1}, "adjectives": {"What": 1}, "adverbs": {"Almost": 1, "No, I'm not": 1}, "numerals": {"41": 1}}}, {"content": "It's often hard to understand what I'm saying.", "word_count": 13, "label": "", "sentence_structure": "Subject鈥搗erb鈥揷omplement", "subjective_objective": {"answer": "Subjective", "rationale": "Because it is the patient ' s own subjective feelings and experiences that the patient considers difficult for others to understand."}, "sentence_sentiment": {"answer": "Negative", "rationale": "Because the description \"ununderstandable\" is negative."}, "language_style": {"answer": "Tactful and warm", "rationale": "The entry is more adept and interactive when it is spoken in a gentle and inviting manner, rather than by direct statements or orders."}, "time_dimension": {"answer": "Past", "rationale": "The word \"normal\" in the entry denotes a regular, customary act, while the word \"hard\" uses the usual current pattern, meaning that the act has not only occurred in the past, but may still be ongoing. The time dimension of the entry should therefore be the past."}, "sentence_type": "Negative declarative sentence", "pos": {"nouns": {"person": 1, "Contents": 1}, "verbs": {"Regular": 1, "Understood": 1, "Say it": 1}, "adjectives": {"Hard": 1}, "adverbs": {"very": 1, "Regular": 1}, "numerals": {"42": 1}, "pronouns": {"I": 1}}}, {"content": "I'm keeping an eye on what people say.", "word_count": 17, "label": "", "sentence_structure": "Subject鈥搗erb鈥搊bject", "subjective_objective": {"answer": "Objective", "rationale": "Because \"suspend\" and \"sure what people really mean\" are the objective behaviour of patients in the face of others, and the perception and understanding of patients."}, "sentence_sentiment": {"answer": "Positive", "rationale": "Because the purpose of the action \"to be vigilant to ascertain what people are saying\" in the entry is positive and can enhance people's communication and understanding."}, "language_style": {"answer": "Tactful and warm", "rationale": "The language style of the entry is more euphemistic and warm, rather than using direct and command-style language to ask questions, it is expressed in a more moderate way, which is more personal and interactive. At the same time, the entry also emphasizes the importance of vigilance in order to better understand the true meaning of others, which is also a more positive and constructive attitude."}, "time_dimension": {"answer": "Present", "rationale": "The keywords \"maintain\" and \"ensure\" indicate that this is a sentence describing current behaviour or attitudes and is therefore a current state of affairs."}, "sentence_type": "Affirmative declarative sentence", "pos": {"nouns": {"Watch out": 1, "People": 1, "Yes": 1, "Really": 1, "Meaning": 1}, "verbs": {"Hold on": 1, "I know": 1}, "adjectives": {"Really": 1}, "numerals": {"43": 1}, "pronouns": {"I": 1}}}, {"content": "I never lie.", "word_count": 7, "label": "", "sentence_structure": "Subject鈥損redicate", "subjective_objective": {"answer": "Subjective", "rationale": "Because it's the objective fact that a patient experiences not lying. If it's the patient's subjective attitude that is being examined, ask the question \"Do you think you never lie?\""}, "sentence_sentiment": {"answer": "Neutral", "rationale": "Because the entry describes only an act, it does not involve emotion, attitude or evaluation and is therefore neutral."}, "language_style": {"answer": "Calm and direct", "rationale": "The language style of the entry is more direct, and it clearly asks whether the person surveyed always told the truth, without using any qualifying or emotional expression."}, "time_dimension": {"answer": "Past", "rationale": "The keyword \"Never lie\" indicates that the act has been going on for a long time and that there are no signs of change, and therefore the judgement is the past."}, "sentence_type": "Negative declarative sentence", "pos": {"nouns": {"Lies": 1}, "verbs": {"Speak": 1, "Never": 1}, "adverbs": {"Never": 1}, "numerals": {"44": 1}}}, {"content": "I'm afraid of meeting someone else because I'm uncomfortable.", "word_count": 15, "label": "", "sentence_structure": "Subject鈥搗erb鈥揷omplement", "subjective_objective": {"answer": "Subjective", "rationale": "Because fear and uncomfortable feelings are the psychological experience of the patient, and are subjective."}, "sentence_sentiment": {"answer": "Negative", "rationale": "Because the words \"fear to meet a living person\" and \"frightful\" in the entries all have some negative feelings."}, "language_style": {"answer": "Tactful and warm", "rationale": "The entry is more personal and interactive in its expression of concern, understanding and support, rather than direct statements or orders. At the same time, it is more sincere and warmer to express the feelings and distress of the other side by using the phrase \"because I feel uncomfortable\"."}, "time_dimension": {"answer": "Present", "rationale": "The entry describes the situation and feelings of the subject, in which the words \"I'm afraid\" and \"because\" indicate that this is a current emotional experience. So it's now."}, "sentence_type": "Negative declarative sentence", "pos": {"nouns": {"Livers": 1, "I": 2}, "verbs": {"Scared": 1, "I met him": 1, "I feel": 1}, "adjectives": {"I'm not comfortable": 1}, "numerals": {"45": 1}}}, {"content": "I'm so desperate to make people like me, so I'm willing to do things I don't want to do.", "word_count": 24, "label": "", "sentence_structure": "Subject鈥搗erb鈥搊bject", "subjective_objective": {"answer": "Subjective", "rationale": "Because \"I want to make people like me\" and \"I want to do things I don't want to do\" are patients' subjective thoughts or attitudes."}, "sentence_sentiment": {"answer": "Negative", "rationale": "Because the entry \"I want to make people like me\" expresses excessive desire and anxiety, and \"voluntaryly do something I don't want to do\" can have negative emotional and consequences."}, "language_style": {"answer": "Tactful and warm", "rationale": ""}, "time_dimension": {"answer": "Present", "rationale": ""}, "sentence_type": "Affirmative declarative sentence", "pos": {"nouns": {"Questionnaire": 1, "Entry": 1, "People": 1, "Things": 1}, "verbs": {"Yes": 1, "鈾?And I'm gonna be 鈾?: 1, "Do it": 3, "Yeah": 1, "Voluntary": 1, "Go on": 2}, "adjectives": {"Too much": 1, "I'd love to": 1}, "adverbs": {"And so": 1}, "numerals": {"46": 1, "One": 1, "Two": 1}, "pronouns": {"I": 2}}}, {"content": "I am saving up a lot of things I don't need, but I can't afford to throw them away.", "word_count": 22, "label": "", "sentence_structure": "Subject鈥搗erb鈥搊bject", "subjective_objective": {"answer": "Subjective", "rationale": "Because \"to build up what is not needed\" and \"to throw away\" are personal feelings and attitudes of patients, and they are subjective."}, "sentence_sentiment": {"answer": "Negative", "rationale": "Because the \"screw what you don't need\" act is negative."}, "language_style": {"answer": "Tactful and warm", "rationale": "The entry is more proficient and interactive in a mild and understanding expression of a possible problem rather than a direct accusation or order."}, "time_dimension": {"answer": "Past", "rationale": "The entry describes the current behaviour of the person being tested, i.e., possession of many unwanted items and their abandonment, which relates to the dimensions of the past of time."}, "sentence_type": "Negative declarative sentence", "pos": {"nouns": {"Something": 2, "I": 3}, "verbs": {"Storage": 1, "No, I don't": 1, "Throw it away": 1}, "adjectives": {"A lot": 1, "I'm not gonna forget it": 1}, "numerals": {"47": 1}}}, {"content": "Even though I've said a lot, people say I haven't caught the point.", "word_count": 19, "label": "", "sentence_structure": "Subject鈥損redicate", "subjective_objective": {"answer": "Subjective", "rationale": "Because \"I have said many\" and \"I have not caught the point\" are subjective views of the patient about his or her performance. Although this phrase refers to others, it does not mean that it is objective, but rather reflects the patient ' s self-evaluation and feelings. So the entry is primarily about the patient ' s subjective views."}, "sentence_sentiment": {"answer": "Negative", "rationale": "Because the \"people also say I didn't catch the point\" in the entry is negative."}, "language_style": {"answer": "Tactful and warm", "rationale": "The entry asks, in a more euphemistic way, whether the other side has problems in communicating with others, avoiding a direct tone of criticism or accusations, and showing a more understanding and careful attitude. At the same time, by asking questions, encouraging each other to think and reflect on their performance, it helps to create a positive, open and constructive atmosphere of communication."}, "time_dimension": {"answer": "Present", "rationale": ""}, "sentence_type": "Negative declarative sentence", "pos": {"nouns": {"Questionnaire": 1, "Points": 1}, "verbs": {"Yes": 1, "Hold on": 1, "Say it": 1, "No, I'm not": 1}, "adverbs": {"Many": 1, "And": 1}, "numerals": {"48": 1}, "classifiers": {"individual": 1}, "pronouns": {"I": 2, "People": 1}}}, {"content": "I expect favors from others, even if I don't usually give them.", "word_count": 21, "label": "", "sentence_structure": "Subject鈥搗erb鈥搊bject", "subjective_objective": {"answer": "Subjective", "rationale": "Because expectations and non-pity are the thoughts and attitudes of the patients, they are subjective."}, "sentence_sentiment": {"answer": "Negative", "rationale": "Because the act of \"seeing someone's favors\" in the entry is negative and it is based on the need for concessions from the other side, not on relations based on equality and mutual respect."}, "language_style": {"answer": "Tactful and warm", "rationale": "The language style of this questionnaire entry is more euphemistic. It uses a more polite expression, without direct orders or accusations. It is not too emotional, but rather a moderate expression of the desire to have others give themselves a favor. It is easier to get understanding and resonance, and it facilitates the cooperation of the subjects surveyed and the effective recovery of questionnaires."}, "time_dimension": {"answer": "Past", "rationale": "The \"I expect someone to give me mercy\" in the past: \"I expect someone to give me favors\" indicates that the testee had this psychological state in the past."}, "sentence_type": "Affirmative declarative sentence", "pos": {"nouns": {"Grandpa": 1, "Questionnaire": 1, "Entry": 1, "Grace": 2, "person": 1}, "verbs": {"Expectations": 1, "Here": 1, "Got it": 1}, "adverbs": {"Usually": 1}, "numerals": {"49": 1, "Two": 1}, "pronouns": {"I": 2, "they": 1}}}, {"content": "I'm a man of rage.", "word_count": 8, "label": "", "sentence_structure": "Subject鈥揷opula鈥損redicative", "subjective_objective": {"answer": "Subjective", "rationale": "Because the description of \"freaky\" is the patient's subjective assessment or perception of himself."}, "sentence_sentiment": {"answer": "Negative", "rationale": "Because \"freakless\" means emotional instability, it's a negative adjective."}, "language_style": {"answer": "Calm and direct", "rationale": "The entry is presented in a simple and clear positive sentence, expressing the subject of questioning whether the other party has often shown an mood of irritation, with no excessive rhetorical or emotional rendering, and with a more objective tone."}, "time_dimension": {"answer": "Present", "rationale": "The entry expresses the testee ' s self-presentation of his emotional state, and does not clearly point at sex at a time, and therefore the judgement is now."}, "sentence_type": "Affirmative declarative sentence", "pos": {"nouns": {"person": 1}, "verbs": {"be": 1, "鈾?The mood is unchanging 鈾?: 1}, "adjectives": {"Unusual": 1}, "numerals": {"50": 1}, "pronouns": {"I": 1}}}, {"content": "It's easy for me to lie. I do that all the time.", "word_count": 14, "label": "", "sentence_structure": "Subject鈥搗erb鈥揷omplement", "subjective_objective": {"answer": "Subjective", "rationale": "Because \"false is easy for me\" and \"I do it all the time\" are subjective and behavioural expressions of patients."}, "sentence_sentiment": {"answer": "Negative", "rationale": "Because lying is negative."}, "language_style": {"answer": "Calm and direct", "rationale": "The entry uses a straightforward and explicit language to describe the customary behaviour of the person under investigation, and does not use excessive rhetorical or emotional rendering, so it can be judged that the language style is straightforward and calm."}, "time_dimension": {"answer": "Present", "rationale": "The entry asks the testee's attitude and habits towards lying. In it, \"Liaring is easy for me\" indicates that the testee does not think lying is a difficult thing, and \"I do it often\" suggests that this behaviour has become the testee's customary behaviour and can therefore be judged as now."}, "sentence_type": "Affirmative declarative sentence", "pos": {"nouns": {"Lies": 1, "I": 2}, "verbs": {"Liar": 1, "Do it": 1}, "adjectives": {"Easy": 1}, "adverbs": {"very": 1, "Regular": 1}, "numerals": {"51": 1}}}, {"content": "I don't care if there's any close friends.", "word_count": 13, "label": "", "sentence_structure": "Subject鈥揷opula鈥損redicative", "subjective_objective": {"answer": "Subjective", "rationale": "Because the existence of friends is a personal feeling and perception, it is subjective."}, "sentence_sentiment": {"answer": "Neutral", "rationale": "Because the entry does not have any emotional color."}, "language_style": {"answer": "Calm and direct", "rationale": "The entry directly expresses the respondent ' s attitude to having close friends, which is more direct, concise and does not use euphemism or rhetoric, and appears to be calm and straightforward."}, "time_dimension": {"answer": "Present", "rationale": "The keyword \"not in the hands of a close friend\" expresses the current attitude and perception, so the temporal dimension is now judged."}, "sentence_type": "Negative declarative sentence", "pos": {"nouns": {"I": 1, "friends": 1, "Intimate": 1}, "verbs": {"have": 1, "None": 1, "I care": 1}, "adjectives": {"Intimate": 1}, "adverbs": {"not": 1, "It doesn't matter": 1}, "numerals": {"52": 1, "0": 1}}}, {"content": "I'm always on guard against the benefits being taken.", "word_count": 12, "label": "", "sentence_structure": "Subject鈥搗erb鈥揷omplement", "subjective_objective": {"answer": "Subjective", "rationale": "Because vigilance, precaution and so on are the subjective feelings and attitudes of the patients themselves."}, "sentence_sentiment": {"answer": "Negative", "rationale": "Because the \"prevent the benefits\" in the entry is negative."}, "language_style": {"answer": "Calm and direct", "rationale": "The entry uses a direct language style, with no excessive emotional accoutrements or rhetorical techniques. It is simple to ask whether the interviewees are often vigilant to prevent harm to their interests, and is more objective and calm."}, "time_dimension": {"answer": "Past", "rationale": "In the entry, \"I am always vigilant\" means a regular, customary act that has occurred in the past and may still be ongoing. Thus, the temporal dimension of the article is a matter of the past."}, "sentence_type": "Affirmative declarative sentence", "pos": {"nouns": {"Watch out": 1, "Benefits": 1, "Take it away": 1}, "verbs": {"Always on guard": 1, "Protection": 1, "To be taken away": 1}, "adverbs": {"Always on guard": 1}, "numerals": {"53": 1}}}, {"content": "I never forget or forgive anyone who did something wrong to me.", "word_count": 16, "label": "", "sentence_structure": "Subject鈥搗erb鈥搊bject", "subjective_objective": {"answer": "Subjective", "rationale": "Because \"forgetting\" and \"forgiving\" are patient subjective attitudes, which reflect the patient's mental state and emotional orientation. These subjective attitudes are one of the purposes of this topic."}, "sentence_sentiment": {"answer": "Negative", "rationale": "Basis of judgement: The entry expresses an attitude of intolerance towards those who have made mistakes and is therefore negative."}, "language_style": {"answer": "Tactful and warm", "rationale": "The language style of the entry is more euphemistic, using the words \"forget it\" and \"forgiving\" and not directly accusing or attacking the other. This expression is more likely to trigger resonance and collaboration among the respondents."}, "time_dimension": {"answer": "Present", "rationale": ""}, "sentence_type": "Interrogative sentence", "pos": {"nouns": {"Questionnaire Entry": 1, "person": 2}, "verbs": {"Forget it": 1, "Forgive me": 1, "Do it": 1}, "adjectives": {"Wrong": 1}, "numerals": {"54": 1}, "pronouns": {"I": 1}}}, {"content": "A nuclear war may not be a bad idea.", "word_count": 15, "label": "", "sentence_structure": "Subject鈥搗erb鈥搊bject", "subjective_objective": {"answer": "Subjective", "rationale": "Because \"not a bad idea\" is a subjective assessment, which means that the speakers think that nuclear war may not be bad."}, "sentence_sentiment": {"answer": "Negative", "rationale": "Because the concept of \"a nuclear war\" in the entry is usually considered a disaster and a destructive event, the expression of this purpose is negative."}, "language_style": {"answer": "Calm and direct", "rationale": "\"Calm down straight.\""}, "time_dimension": {"answer": "Future", "rationale": "The keyword \"may not\" and \"a very bad idea\" suggests that it is a hypothetical and subjective view and relates to situations that may occur in the future. The entry therefore points to future behaviour or mentalities."}, "sentence_type": "Negative declarative sentence", "pos": {"nouns": {"Nuclear war": 1}, "verbs": {"be": 1, "Maybe": 1, "not": 1, "It's a": 1, "Bad": 1, "Ideas": 1}, "adjectives": {"very": 2}, "numerals": {"55": 1}}}, {"content": "When I was alone, I felt helpless and unable to take care of myself.", "word_count": 20, "label": "", "sentence_structure": "Subject鈥揷opula鈥損redicative", "subjective_objective": {"answer": "Subjective", "rationale": "This is because of the subjective perception and evaluation of the patient when he is alone."}, "sentence_sentiment": {"answer": "Negative", "rationale": "Because the descriptions of \"fearless\" and \"not able to take care of yourself\" in the entries are negative."}, "language_style": {"answer": "Calm and direct", "rationale": "The language style of the entry is relatively calm and straightforward, with no excessive adoration or emotional rendering, and directly expresses the feeling of asking the other person when he or she is alone, and is more objective and peaceful."}, "time_dimension": {"answer": "Present", "rationale": ""}, "sentence_type": "Negative declarative sentence", "pos": {"nouns": {"person": 2, "Time": 1, "Helpless": 1, "self": 2}, "verbs": {"I feel": 1, "Cannot": 1, "Care": 1, "Take care of yourself": 1}, "adjectives": {"Alone": 1}, "numerals": {"56": 1}, "pronouns": {"I": 1}}}, {"content": "If someone else can't do something right, I'd rather do it myself.", "word_count": 21, "label": "", "sentence_structure": "Subject鈥搗erb鈥搊bject", "subjective_objective": {"answer": "Subjective", "rationale": "Because \"I'd rather do it myself\" is the patient's subjective attitude and will."}, "sentence_sentiment": {"answer": "Negative", "rationale": "Because the entry \"Better to do it\" shows a self-centered and negative attitude towards others' abilities, which is negative emotional."}, "language_style": {"answer": "Calm and direct", "rationale": "The language style of the entry is calm and straightforward, as it clearly expresses the attitude and the way people are treated, without too many rhetorical or emotional colours."}, "time_dimension": {"answer": "Past", "rationale": "In the entry, \"If someone else can't do something right, I would rather do it myself\" expresses a customary act or attitude. Using the current state of completion (\"if\") and the general state of affairs ( \"I would rather do it myself\"), these indicate that the act has occurred in the past and may still be going on."}, "sentence_type": "Interrogative sentence", "pos": {"nouns": {"person": 1, "Things": 2, "self": 1}, "verbs": {"Do it": 3, "Come on": 1}, "adjectives": {"Correct": 1}, "adverbs": {"If": 1, "Land": 1}, "numerals": {"57": 1}, "pronouns": {"I": 1}}}, {"content": "I have a talent for acting.", "word_count": 6, "label": "", "sentence_structure": "Subject鈥搗erb鈥搊bject", "subjective_objective": {"answer": "Subjective", "rationale": "Because \"demonstration gift\" is patient self-evaluation and perception, it is part of subjective attitudes and beliefs."}, "sentence_sentiment": {"answer": "Positive", "rationale": "Because the expression \"a talent for acting\" is positive."}, "language_style": {"answer": "Calm and direct", "rationale": "The purpose of this article is expressed in a more concise and concise manner, without too many rhetorical or emotional techniques being used to qualify it, and in a more neutral and objective manner."}, "time_dimension": {"answer": "Present", "rationale": "The entry describes the current state or quality of the testee, the keyword \"with performance gift\", which refers to the current capabilities or characteristics of the testee and therefore belongs to the present."}, "sentence_type": "Affirmative declarative sentence", "pos": {"nouns": {"Performing": 1, "Talent": 1}, "verbs": {"have": 1}, "numerals": {"58": 1}, "pronouns": {"I": 1}}}, {"content": "Some people think I take advantage of others.", "word_count": 12, "label": "", "sentence_structure": "Subject鈥搗erb鈥搊bject", "subjective_objective": {"answer": "Subjective", "rationale": "Because \"some people think\" involves the views and opinions of others, and this is considered from the patient's subjective point of view."}, "sentence_sentiment": {"answer": "Negative", "rationale": "Because the \"save\" in the entries is negative."}, "language_style": {"answer": "Tactful and warm", "rationale": "The language style of the entry is more euphemistic, using the expression \"some people think\" to avoid direct accusations against each other, and the tone is more peaceful, and attempts to communicate with respect and understanding."}, "time_dimension": {"answer": "Present", "rationale": "The words \"some people\" and \"think\" and \"take\" in \"some people think I'm taking advantage of others\" represent current behaviour or attitude, so the temporal dimension of the article is now judged."}, "sentence_type": "Negative declarative sentence", "pos": {"nouns": {"person": 1, "others": 1, "Cheap": 1}, "verbs": {"I think": 1, "Jim": 1}, "adverbs": {"Some": 1}, "numerals": {"59": 1}, "pronouns": {"I": 1}}}, {"content": "I feel that my life is monolithic and meaningless.", "word_count": 15, "label": "", "sentence_structure": "Subject鈥揷opula鈥損redicative", "subjective_objective": {"answer": "Subjective", "rationale": "Because it is a self-perception of the patient and is a subjective examination."}, "sentence_sentiment": {"answer": "Negative", "rationale": "Because the words \"life alone\" and \"no sense\" in the entries express a negative emotional attitude."}, "language_style": {"answer": "Calm and direct", "rationale": "The language style of the entry is more straightforward, and the subject of asking the other side whether it feels like it lacks pleasure and purpose in life is open to question and is more objective and cold in tone.\""}, "time_dimension": {"answer": "Present", "rationale": "The keyword \"felt,\" \"uniform\" and \"unmeaning\" all indicate that it is a description and a sense of the state of life. Therefore, the entry should be classified as a current behavior or mentality."}, "sentence_type": "Negative declarative sentence", "pos": {"nouns": {"Life": 1, "Meaning": 1}, "verbs": {"I feel": 1, "Undo": 1, "No, I'm not": 1}, "numerals": {"60": 1}, "pronouns": {"I": 1}}}, {"content": "I don't care what people say about me.", "word_count": 10, "label": "", "sentence_structure": "Subject鈥搗erb鈥揷omplement", "subjective_objective": {"answer": "Subjective", "rationale": "It is because it is not objective facts or observations that care about the assessment of others as a subjective or emotional response of a person."}, "sentence_sentiment": {"answer": "Negative", "rationale": "Because the \"no one cares what I say\" attitude in the entry is negative."}, "language_style": {"answer": "Calm and direct", "rationale": "The entry expresses the meaning of \"no one cares\" in direct and concise language, without using excessive emotional colours or rhetorical techniques and with a more objective tone."}, "time_dimension": {"answer": "Present", "rationale": "Now."}, "sentence_type": "Negative declarative sentence", "pos": {"nouns": {"others": 1, "I": 1}, "verbs": {"I don't care": 1, "Say it": 1}, "adverbs": {"not": 1}, "numerals": {"61": 1}, "pronouns": {"What": 1}}}, {"content": "62. In one-on-one situations, I have difficulty dealing with people.", "word_count": 18, "label": "", "sentence_structure": "Subject鈥搗erb鈥搊bject", "subjective_objective": {"answer": "Subjective", "rationale": "Because the entry is an inquiry into the patient ' s subjective feelings and experience, i.e. the extent of the patient ' s difficulty in dealing with others in one-on-one situations."}, "sentence_sentiment": {"answer": "Negative", "rationale": "Because the description of \"difficult\" in the entry is negative."}, "language_style": {"answer": "Tactful and warm", "rationale": "The entry is more personal and interactive in its expression of a soft, invitation-style conversation rather than a direct statement or order. At the same time, it uses a query to make respondents more willing to share their views and feelings."}, "time_dimension": {"answer": "Present", "rationale": ""}, "sentence_type": "Negative declarative sentence", "pos": {"nouns": {"Situation": 1, "One": 1, "- Yeah": 1, "person": 1, "Dealing": 1, "Difficulties": 1}, "verbs": {"in": 1, "and": 1, "have": 1}, "adverbs": {"One": 1}, "numerals": {"62": 1}, "pronouns": {"I": 1, "others": 1}}}, {"content": "People often complain that I don't feel the troubles in their hearts.", "word_count": 17, "label": "", "sentence_structure": "Subject鈥搗erb鈥搊bject", "subjective_objective": {"answer": "Subjective", "rationale": "Because the words \"appear\" and \"fear\" are the feelings and emotions of the patient, the subject's subjective attitude is being examined."}, "sentence_sentiment": {"answer": "Negative", "rationale": "Because the emotional color of \"complaining\" in the entries is negative and indicates dissatisfaction with the other side."}, "language_style": {"answer": "Tactful and warm", "rationale": "The entry is more euphemistic, using the expression \"people often complain\" to avoid too direct and critical language, and to express the attitude of the interviewee that needs to be improved. At the same time, the \"I can feel their worries\" is used to guide the other to share their feelings, making the questionnaire more personal and human."}, "time_dimension": {"answer": "Present", "rationale": ""}, "sentence_type": "Negative declarative sentence", "pos": {"nouns": {"People": 1, "- He's complaining": 1, "I": 1, "they": 1, "In my heart": 1, "Trouble": 1}, "verbs": {"Regular": 1, "- He's complaining": 2, "Feel it": 1, "Yes": 1}, "adverbs": {"Regular": 1, "No, I can't": 1}, "numerals": {"63": 1}, "pronouns": {"they": 1}}}, {"content": "When people look at me, they think I'm weird, weird or weird.", "word_count": 21, "label": "", "sentence_structure": "Subject鈥搗erb鈥搊bject", "subjective_objective": {"answer": "Subjective", "rationale": "Because \"may be considered \" , \"freak,\" \"freak\" and \"freak\" are subjective thoughts and feelings of patients, not objective facts. These evaluations are based on the views and reactions of others, not just on the actual situation of the patients themselves."}, "sentence_sentiment": {"answer": "Negative", "rationale": "Because the words \"eccentric\", \"uncool\" and \"uncharacteristic\" in the entries are all negative descriptions, suggesting that people may have unfriendly views of the interviewees."}, "language_style": {"answer": "Tactful and warm", "rationale": ""}, "time_dimension": {"answer": "Present", "rationale": "The entry \"when people look at me\" indicates an ongoing act, and therefore judges it as the present. At the same time, \"will think\" also expresses a possible view or view, and does not clearly point to past or future situations."}, "sentence_type": "Negative declarative sentence", "pos": {"nouns": {"person": 1, "Look at me": 1, "I": 1, "I think": 1, "Very": 1, "Weird": 2, "Strange": 1}, "verbs": {"Watch": 1, "Maybe": 1, "I think": 1, "Very": 1}, "adjectives": {"Weird": 3, "Strange": 1}, "adverbs": {"Maybe": 1}, "numerals": {"64": 1, "One": 2}, "pronouns": {"I": 1}}}, {"content": "I like to do dangerous things for fun.", "word_count": 11, "label": "", "sentence_structure": "Subject鈥搗erb鈥搊bject", "subjective_objective": {"answer": "Subjective", "rationale": "Because \"like\" is the patient's personal emotional attitude, and it's a subjective experience and judgment, the answer is subjective."}, "sentence_sentiment": {"answer": "Negative", "rationale": "Because the act of \"doing dangerous things for fun\" in the entry is potentially risky and negative."}, "language_style": {"answer": "Calm and direct", "rationale": "The entry is a direct expression of the subject of asking the other party whether it likes to do dangerous things, is not based on too many rhetorical or emotional expressions, and is more objective in tone. At the same time, it asks the interviewee questions in a concise and clear manner, without any ambiguity or ambiguity, and is therefore more in keeping with the straight language style of calmness."}, "time_dimension": {"answer": "Present", "rationale": "So this entry is about the person's current mentalities and behavior. So the output is:"}, "sentence_type": "Affirmative declarative sentence", "pos": {"nouns": {"Things": 1, "Enjoy": 1}, "verbs": {"Yeah": 1, "Do it": 1, "Access": 1}, "adjectives": {"Dangerous": 1}, "numerals": {"65": 1}, "pronouns": {"I": 1}}}, {"content": "I lied a lot in this questionnaire.", "word_count": 12, "label": "", "sentence_structure": "Subject鈥搗erb鈥揷omplement", "subjective_objective": {"answer": "Subjective", "rationale": "Because \"fake\" is a subjective act, determined by the person interviewed, and subjective."}, "sentence_sentiment": {"answer": "Negative", "rationale": "Because the \"fake\" act in the entry is negative."}, "language_style": {"answer": "Tactful and warm", "rationale": "The wording of the article is relatively flat and does not have a clear tone of derogatory, critical or command; 2. The term \"many\" is used to express the extent of the lie, without directly indicating the number and avoiding pressure or humiliation; 3. The phrase \"is there?\" is used at the end, with an invitation and concern, which enhances the interaction and acrimonymity with the other party."}, "time_dimension": {"answer": "Past", "rationale": "And from the \"showing a lot of lies\" in the sentence, it's an action that's already happened, so it's possible to judge the temporal dimension of the entry."}, "sentence_type": "Negative declarative sentence", "pos": {"nouns": {"Questionnaire": 2, "Lies": 1}, "verbs": {"Scram": 1}, "numerals": {"A lot": 1}, "pronouns": {"I": 1}}}, {"content": "I have difficulty controlling my anger and temper.", "word_count": 13, "label": "", "sentence_structure": "Subject鈥揷opula鈥損redicative", "subjective_objective": {"answer": "Subjective", "rationale": "Because \"unmanageable\" is the patient's self-perception and subjective feeling."}, "sentence_sentiment": {"answer": "Negative", "rationale": "Because the \"hard to control your anger and temper\" act in the entry is negative."}, "language_style": {"answer": "Calm and direct", "rationale": "The entry directly expresses the question of whether the other party has difficulty in controlling anger and temperament, is more objective and does not contain excessive rhetoric, and therefore can be judged to be calm and straightforward."}, "time_dimension": {"answer": "Present", "rationale": ""}, "sentence_type": "Negative declarative sentence", "pos": {"nouns": {"Questionnaire": 1, "Entry": 1, "Anger": 1, "- Grumpy": 1}, "verbs": {"Control": 1, "be": 1}, "adjectives": {"Hard": 1}, "adverbs": {"very": 1}, "numerals": {"67": 1}, "pronouns": {"I": 1}}}, {"content": "There are some people who envy me.", "word_count": 7, "label": "", "sentence_structure": "Subject鈥搗erb鈥揷omplement", "subjective_objective": {"answer": "Subjective", "rationale": "Because jealousy is a subjective emotion or feeling."}, "sentence_sentiment": {"answer": "Negative", "rationale": "Because the \"envious\" act in the entry is negative."}, "language_style": {"answer": "Tactful and warm", "rationale": "The entry is in a more euphemistic language, and \"some\" suggests that it may be a speculation by the other party, rather than a direct accusation, and that the tone is less comfortable and does not make the person under investigation uncomfortable."}, "time_dimension": {"answer": "Present", "rationale": ""}, "sentence_type": "Affirmative declarative sentence", "pos": {"nouns": {"person": 1, "Jealousy": 1}, "verbs": {"have": 1, "be": 1}, "adjectives": {"Some": 1}, "adverbs": {"very": 1}, "numerals": {"68": 1}, "pronouns": {"I": 1}}}, {"content": "I'm vulnerable to others.", "word_count": 9, "label": "", "sentence_structure": "Subject鈥揷opula鈥損redicative", "subjective_objective": {"answer": "Subjective", "rationale": "Because \"affected by others\" is a subjective feeling and judgement of a person, not an objective and quantifiable fact."}, "sentence_sentiment": {"answer": "Negative", "rationale": "Because the behaviour of \"vulnerable\" in the entry may lead to negative external impacts on individual decision-making and behaviour, and may have some negative effects."}, "language_style": {"answer": "Tactful and warm", "rationale": "Warm and warm."}, "time_dimension": {"answer": "Present", "rationale": "The keyword \"vulnerable to others\" indicates that the act is a customary psychological preference or attitude of the person being tested, which may still exist. Thus, the temporal dimension of the article is now judged."}, "sentence_type": "Negative declarative sentence", "pos": {"nouns": {"Impact": 1, "person": 1}, "verbs": {"Easy": 1, "Yes": 1, "Impact": 1}, "adverbs": {"Other": 1}, "numerals": {"69": 1}, "pronouns": {"I": 1}}}, {"content": "I think I'm a very timid person, but I'm considered a little timid.", "word_count": 16, "label": "", "sentence_structure": "Subject鈥搗erb鈥揷omplement", "subjective_objective": {"answer": "Subjective", "rationale": "Because \"I think I'm a little bit timid\" and \"others think I'm a little cheap\" are subjective views of patients."}, "sentence_sentiment": {"answer": "Neutral", "rationale": "Because the entry does not have a clear emotional or attitudinal orientation, it describes a difference between self-awareness and other people ' s evaluation."}, "language_style": {"answer": "Calm and direct", "rationale": "The entry directly asks respondents about the perception between self-evaluation and others' evaluation, and is more concise in language style and does not have too many rhetorical or emotional renderings.\""}, "time_dimension": {"answer": "Present", "rationale": ""}, "sentence_type": "Negative declarative sentence", "pos": {"nouns": {"self": 1, "Reveal": 1, "others": 1, "- You're a little bit of a jerk": 1}, "verbs": {"I think": 2, "very": 1, "have": 1, "be": 1}, "adjectives": {"Reveal": 1, "- You're a little bit of a jerk": 1}, "numerals": {"70": 1, "very": 1}, "classifiers": {"individual": 1}, "pronouns": {"I": 2, "But": 1}}}, {"content": "When one relationship ends, I need to establish a close relationship with another immediately.", "word_count": 27, "label": "", "sentence_structure": "Subject鈥搗erb鈥搊bject", "subjective_objective": {"answer": "Subjective", "rationale": "Because \"I need to get close to another person immediately\" is a personal need and a thought for the patient, and it's a subjective examination."}, "sentence_sentiment": {"answer": "Positive", "rationale": "Because the act in the entry is \"to establish a close relationship immediately with another person\", it expresses a positive emotional attitude."}, "language_style": {"answer": "Calm and direct", "rationale": "The entry is directly presented in an attempt to ask individuals whether they have the incentive to seek a new relationship when they face the end of a relationship, and is more objective and calm."}, "time_dimension": {"answer": "Future", "rationale": "The entry describes the response of the testee to the end of an intimate relationship, namely, finding a new intimate relationship. The keywords \"when one close relationship is over\" and \"need to establish a close relationship with another immediately\" point to what may happen in the future. The temporal dimension of the topic is therefore the future."}, "sentence_type": "Affirmative declarative sentence", "pos": {"nouns": {"Close relationship": 2, "person": 1, "Create": 1}, "verbs": {"End": 1, "Yes": 1, "Immediately": 1, "and": 1}, "adverbs": {"When": 1}, "numerals": {"71": 1}, "pronouns": {"I": 1}}}, {"content": "I'm suffering from a low self-evaluation.", "word_count": 11, "label": "", "sentence_structure": "Subject鈥揷opula鈥損redicative", "subjective_objective": {"answer": "Subjective", "rationale": "Because \"self-evaluation is too low and painful\" is the patient's subjective and emotional experience."}, "sentence_sentiment": {"answer": "Negative", "rationale": "Because the act of \"suffering from being too low for self-evaluation\" in the entry is negative."}, "language_style": {"answer": "Tactful and warm", "rationale": "The entry is more personal and interactive when it is spoken in a moderate and sensitive manner, rather than directly speaking or ordering."}, "time_dimension": {"answer": "Present", "rationale": ""}, "sentence_type": "Negative declarative sentence", "pos": {"nouns": {"Self-evaluation": 1, "Pain": 1}, "verbs": {"Because": 1, "And": 1, "Pain": 1}, "adverbs": {"72": 1}, "pronouns": {"I": 1}}}, {"content": "I don't spend time reproducing to those who insult me.", "word_count": 15, "label": "", "sentence_structure": "Subject鈥搗erb鈥搊bject", "subjective_objective": {"answer": "Subjective", "rationale": "Because the reaction and attitude of \"I\" are based on personal subjectivity and opinion, not objective facts."}, "sentence_sentiment": {"answer": "Negative", "rationale": "Because the attitude in the entries is \"no respect for the insults\" and it's a negative response."}, "language_style": {"answer": "Calm and direct", "rationale": "The language style of the entry is more direct and concise, there is no excessive rhetorical or emotional rendering, and it appears to be more objective and insensitive, and it highlights the attitude of the respondents to the attack against their own insults."}, "time_dimension": {"answer": "Present", "rationale": "\"I\" uses the first person's proxy to suggest that this is a question of the current state of affairs, that is, whether the testee will be in the present situation. And the verbs \"does not take time\" and \"remember\" exist, which is further proof of the current situation.\""}, "sentence_type": "Negative declarative sentence", "pos": {"nouns": {"Time": 1, "Insulting": 1, "person": 1}, "verbs": {"Flowers": 1, "- To you": 1}, "adverbs": {"not": 1}, "numerals": {"73": 1}, "classifiers": {"individual": 1}}}, {"content": "I'm nervous about someone else around.", "word_count": 12, "label": "", "sentence_structure": "Subject鈥揷opula鈥損redicative", "subjective_objective": {"answer": "Subjective", "rationale": "This feeling is experienced by the patient himself and is a response at the subjective level."}, "sentence_sentiment": {"answer": "Negative", "rationale": "Because the \"sweet\" sentiment in the entry is negative."}, "language_style": {"answer": "Calm and direct", "rationale": "The language style of the entry is straightforward, as it is asked whether the person surveyed is nervous in the crowd in a straightforward way, and does not use excessive glossy words or expressions."}, "time_dimension": {"answer": "Present", "rationale": "\"There are others around me who feel nervous\" expresses immediate reaction and feelings, and the description of \"there are others around\" shows that this feeling is happening. So the entry looks at the feelings and experiences of the subjects."}, "sentence_type": "Negative declarative sentence", "pos": {"nouns": {"Around": 1, "person": 1, "Nervous": 1}, "verbs": {"I feel": 1, "have": 1}, "adverbs": {"Just": 1}, "numerals": {"74": 1}}}, {"content": "75. I'm afraid of being embarrassed or embarrassed in a new occasion.", "word_count": 14, "label": "", "sentence_structure": "Subject鈥揷opula鈥損redicative", "subjective_objective": {"answer": "Subjective", "rationale": "Because fear is a subjective feeling."}, "sentence_sentiment": {"answer": "Negative", "rationale": "Because the emotional expression \"resisting of embarrassment or humiliation\" in the entry is negative."}, "language_style": {"answer": "Calm and direct", "rationale": "The entry directly expresses the subject of asking the other party whether it would be afraid of embarrassment or humiliation in a new context, without excessive rhetorical or emotional rendering, and with more sound and objective tone."}, "time_dimension": {"answer": "Future", "rationale": "The keywords \"on new occasions\" and \"on fear of embarrassment or shame\" suggest what may happen in the future, so the entry asks the testee about what might happen in the future."}, "sentence_type": "Negative declarative sentence", "pos": {"nouns": {"Event": 1, "person": 2, "Yes": 1, "I'm embarrassed": 1, "Shame": 1}, "verbs": {"Scared": 1, "in": 1, "be": 1, "have": 1}, "adverbs": {"New": 1}, "numerals": {"75": 1}, "pronouns": {"I": 2}}}, {"content": "I'm afraid of being alone.", "word_count": 9, "label": "", "sentence_structure": "Subject鈥揷opula鈥損redicative", "subjective_objective": {"answer": "Subjective", "rationale": "Because fear is the patient's subjective feeling."}, "sentence_sentiment": {"answer": "Negative", "rationale": "Because the \"fear to be alone\" act in the entry is negative."}, "language_style": {"answer": "Tactful and warm", "rationale": "The entry is more euphemistic and sweet, using a question-and-answer tone to express the researchers鈥?intentions, and does not use a negative vocabulary or overly direct expression. This expression makes it easier for respondents to accept and answer questions and creates a friendly research environment."}, "time_dimension": {"answer": "Present", "rationale": "The keyword \"fear\" and \"single alone\" means the current feelings and experiences, so the temporal dimension of the article is now judged."}, "sentence_type": "Negative declarative sentence", "pos": {"nouns": {"I": 1, "Alone": 1, "One": 1}, "verbs": {"Scared": 1, "Stay": 1}, "numerals": {"76": 1}}}, {"content": "People complain I'm stubborn like a bull.", "word_count": 12, "label": "", "sentence_structure": "Subject鈥搗erb鈥揷omplement", "subjective_objective": {"answer": "Subjective", "rationale": "Because \"complaint\" is a subjective feeling and evaluation, reflecting the perception and attitude towards others. And \"obstinate\" is also based on the observation and judgement of others and is subjective in nature. Thus, the entry is viewed from a subjective perspective."}, "sentence_sentiment": {"answer": "Negative", "rationale": "Because \"complaint\" and \"resistence\" in the entries are both negative emotional."}, "language_style": {"answer": "Tactful and warm", "rationale": "The entry uses the derogatory term \"dulging\" to describe itself, which is more powerful and direct. The expression \"people complains\" also shows dissatisfaction with themselves, rather than a moderate, inquisitive dialogue."}, "time_dimension": {"answer": "Present", "rationale": "The keywords are \"people complain\" and \"hard\" , which describe the state and behaviour of the present, so it can be judged that the entry is about the present situation."}, "sentence_type": "Negative declarative sentence", "pos": {"nouns": {"People": 1, "Problem": 1, "Questionnaire": 1, "Entry": 1, "Snail": 1, "Obstinate": 1}, "verbs": {"- He's complaining": 1, "Yeah": 1, "Same thing": 1}, "adjectives": {"Obstinate": 1}, "numerals": {"77": 1}, "pronouns": {"I": 1}}}, {"content": "I'm more serious about each other than the people around me.", "word_count": 16, "label": "", "sentence_structure": "Subject鈥搗erb鈥揷omplement", "subjective_objective": {"answer": "Subjective", "rationale": "Because \"dealing with each other\" is a personal attitude or perception, which reflects the patient's subjective attitude."}, "sentence_sentiment": {"answer": "Positive", "rationale": "Because the attitudes in the entries are positive, they indicate that they are more serious about human relationships than others."}, "language_style": {"answer": "Calm and direct", "rationale": "The entry is more directly linguistically, with no excessive rhetorical or emotional colours. It explicitly asks the respondents about their attitude to the relationship with others and quantifys it by the value \"78\", making the problem more objective and precise."}, "time_dimension": {"answer": "Present", "rationale": ""}, "sentence_type": "Affirmative declarative sentence", "pos": {"nouns": {"Relations": 1, "person": 2}, "verbs": {"Processing": 1, "- Yeah": 1, "Seriously": 1}, "adjectives": {"- A little bit": 1, "More": 1}, "numerals": {"78": 1}, "pronouns": {"I": 1, "Around": 1}}}, {"content": "I'm considered a bit of a fool.", "word_count": 13, "label": "", "sentence_structure": "Subject鈥揷opula鈥損redicative", "subjective_objective": {"answer": "Subjective", "rationale": "Because \"others think\" is a subjective expression, and \"very unthinking\" is the patient's own feeling and perception, it is a subjective perspective."}, "sentence_sentiment": {"answer": "Negative", "rationale": "Because the expression \"unbelievable\" in the entry is negative."}, "language_style": {"answer": "Tactful and warm", "rationale": "The entry uses a more moderate expression, which allows the interviewee to express his/her thoughts rather than directly evaluate or criticize. This euphemism makes it easier for the interviewee to feel comfortable and receptive and to obtain real and accurate feedback."}, "time_dimension": {"answer": "Past", "rationale": "According to the title, \"it is thought that this action has occurred in the past, and the perception and evaluation of \"others\" is based on the past's behaviour or performance. The entry therefore concerns the assessment and judgement of the past."}, "sentence_type": "Negative declarative sentence", "pos": {"nouns": {"others": 1, "person": 1, "Brain": 1}, "verbs": {"I think": 1, "I can't touch it": 1}, "adjectives": {"very": 1, "You're": 1, "Okay": 1}, "adverbs": {"very": 1}, "numerals": {"79": 1}, "pronouns": {"I": 1}}}, {"content": "When I'm under great stress, something happens to me, like delusional or temporary memory loss.", "word_count": 30, "label": "", "sentence_structure": "Subject鈥搗erb鈥搊bject", "subjective_objective": {"answer": "Subjective", "rationale": "Because it's the patient's subjective feelings and experiences."}, "sentence_sentiment": {"answer": "Negative", "rationale": "Because the act of \"a delusional or temporary memory loss\" in the entry is negative."}, "language_style": {"answer": "Calm and direct", "rationale": "The entry is a direct illustration of the specific symptoms that occur in the face of severe stress, and is not based on excessive rhetorical or emotional rendering, and is more objective in tone."}, "time_dimension": {"answer": "Present", "rationale": "In the future, the entry \"when under severe pressure\" indicates that this is a hypothetical situation, while the word \"in case\" indicates that this may happen. Thus, \"the appearance of delusions or temporary loss of memory\" is a description of a possible future stress response, and therefore the basis of judgement is the future."}, "sentence_type": "Affirmative declarative sentence", "pos": {"nouns": {"Pressure": 1, "Things": 2, "Situation": 1, "Paranoia": 1, "Memory": 1, "Loss": 1}, "verbs": {"Yes": 1, "Come on": 2, "For now": 1, "Lose": 1}, "adjectives": {"Significant": 1}, "adverbs": {"Yes": 1, "Like": 1, "Some": 1}, "numerals": {"80": 1}, "pronouns": {"I": 2}}}, {"content": "I don't care if anyone else gets hurt because I want to.", "word_count": 20, "label": "", "sentence_structure": "Subject鈥損redicate", "subjective_objective": {"answer": "Subjective", "rationale": "Because \"will\" and \"no care\" are patients' subjective attitudes or beliefs."}, "sentence_sentiment": {"answer": "Negative", "rationale": "Because the entry \"not knowing whether or not others are harmed\" expresses a negative emotional insensitivity to the interests of others."}, "language_style": {"answer": "Calm and direct", "rationale": "The entry expresses the view that, regardless of whether the other person is injured or not, he or she can be left alone if he or she so wishes. This language style is more calm and objective, and does not involve too much rhetorical or emotional overtones."}, "time_dimension": {"answer": "Present", "rationale": ""}, "sentence_type": "Affirmative declarative sentence", "pos": {"nouns": {"Questionnaire": 1, "Entry": 1, "I don't care": 1, "Hurt": 1}, "verbs": {"Yes": 1, "Received": 1}, "adverbs": {"Only": 1, "not": 1}, "numerals": {"81": 1}, "pronouns": {"I": 2, "others": 1}}}, {"content": "I'm away from other people.", "word_count": 8, "label": "", "sentence_structure": "Subject鈥搗erb鈥揷omplement", "subjective_objective": {"answer": "Subjective", "rationale": "Because \"treathering\" is a subjective choice and attitude of a person, reflecting his social orientation and his interpersonal approach."}, "sentence_sentiment": {"answer": "Negative", "rationale": "Because the act of \"showing distance\" usually means avoiding contact or being unwilling to interact with people, there is a certain negative tendency."}, "language_style": {"answer": "Tactful and warm", "rationale": "The language style of the entry is more euphemismal and warm. It uses a more gentle and friendly approach to induce respondents to answer questions, rather than direct orders or strongly suggesting how they should answer. This expression is more acceptable and understandable to the interviewees and better promotes their participation and accuracy of answers."}, "time_dimension": {"answer": "Present", "rationale": "It is not possible to determine the temporal dimension of the article according to the time term in the sentence. Therefore, it is not possible to judge whether the behaviour or mentality of the past, present or future is present. It is recommended that, when designing the questionnaire, the time frame be as clear as possible and that the corresponding time pattern be used to describe the behaviour or mentality to help researchers better understand and analyse the data."}, "sentence_type": "Affirmative declarative sentence", "pos": {"nouns": {"I": 1, "Others": 1, "Distance": 1}, "verbs": {"Hold on": 1}, "numerals": {"82": 1}}}, {"content": "I've often wondered if my wife was being faithful to me.", "word_count": 22, "label": "", "sentence_structure": "Subject鈥搗erb鈥搊bject", "subjective_objective": {"answer": "Subjective", "rationale": "Because doubt is the patient's subjective feeling and attitude."}, "sentence_sentiment": {"answer": "Negative", "rationale": "Because the doubt in the entry \"wife is not faithful to me.\""}, "language_style": {"answer": "Calm and direct", "rationale": "The language style of the entry is calm, as it is a direct expression of doubts about the partner鈥檚 infidelity, and is not used too much in rhetoric or emotional rendering."}, "time_dimension": {"answer": "Past", "rationale": "At the same time, \"Was my wife faithful to me?\" is also asking the testee's current mentality or thoughts, and therefore judging as \"now\"."}, "sentence_type": "Interrogative sentence", "pos": {"nouns": {"Grandpa": 1, "Questionnaire": 1, "Entry": 1, "83": 1, "I": 2, "Regular": 1, "Doubts": 1, "Is it possible": 1, "Wife": 1, "Husband": 1, "Girlfriend": 1, "Boyfriend": 1}, "verbs": {"I'm not loyal to you": 1}, "adverbs": {"Regular": 1}, "numerals": {"83": 1}, "classifiers": {"Grandpa": 1}, "pronouns": {"I": 2}}}, {"content": "a) Excess of expenses", "word_count": 4, "label": "", "sentence_structure": "Subject鈥搗erb鈥揷omplement", "subjective_objective": {"answer": "Subjective", "rationale": "Because it is beyond the patient's subjective judgment."}, "sentence_sentiment": {"answer": "Negative", "rationale": "Because \"overspend\" means negative economics."}, "language_style": {"answer": "Calm and direct", "rationale": "The entry uses a concise language to ask the interviewees whether there have been any over-expenditures, which have not been overly accommodative or emotional, and which are direct and objective."}, "time_dimension": {"answer": "Present", "rationale": "\"Expenses exceed\" describes the current financial position of the testee and therefore judges as now."}, "sentence_type": "Negative declarative sentence", "pos": {"nouns": {"Cost": 1, "Over": 1}, "verbs": {"Over": 1}}}, {"content": "(b) Sexual relations with people I hardly know", "word_count": 13, "label": "", "sentence_structure": "Subject鈥搗erb鈥搊bject", "subjective_objective": {"answer": "Subjective", "rationale": "Because the problem involves the feelings and attitudes of the patient, it is a subjective one that requires the patient to answer his or her own thoughts."}, "sentence_sentiment": {"answer": "Negative", "rationale": "Because the entries contain a pejorative term \"near known\" and sexual relations, which have negative emotional connotations."}, "language_style": {"answer": "Calm and direct", "rationale": "The language style of the entry is calm and straightforward. It does not use any emotional vocabulary or expression, but rather directly presents the core of the problem. It is more objective and neutral and suitable for use in such contexts as questionnaires and surveys that need to be objective and impartial."}, "time_dimension": {"answer": "Present", "rationale": "The entry asks the testee whether he has sexual relations at the current time, or whether he has a sexual behavior or mentality, and thus judges \"now\"."}, "sentence_type": "Interrogative sentence", "pos": {"nouns": {"person": 1, "Sexual relations": 1}, "verbs": {"Yes": 1, "have": 1}, "adjectives": {"Almost": 1}, "classifiers": {"One": 1}, "pronouns": {"I": 1}}}, {"content": "c) Overdrinking", "word_count": 4, "label": "", "sentence_structure": "Subject鈥搗erb鈥揷omplement", "subjective_objective": {"answer": "Subjective", "rationale": "Because excessive drinking is a subjective behaviour and attitude of patients."}, "sentence_sentiment": {"answer": "Negative", "rationale": "Because the \"overdrink\" act is negative."}, "language_style": {"answer": "Calm and direct", "rationale": "The entry directly asks the interviewees whether there is excessive drinking, the language is simple and not overly pedantic or emotional, and is a more direct way of asking questions.\""}, "time_dimension": {"answer": "Past", "rationale": "Thus, the temporal dimension is judged to be the past."}, "sentence_type": "Imperative sentence", "pos": {"nouns": {"Drink": 1, "Excess": 1}}}, {"content": "d) Use of drugs (specifically addiction-dependent drugs)", "word_count": 13, "label": "", "sentence_structure": "Subject鈥搗erb鈥搊bject", "subjective_objective": {"answer": "Subjective", "rationale": "Because \"the use of drugs\" is a question of behaviour and attitude, and is a subjective choice of the patient. While it may be related to objective facts, such as the existence of drugs, access to them, in this case, it is more concerned about whether the patient is using them, which is a subjective attitude and behaviour."}, "sentence_sentiment": {"answer": "Negative", "rationale": "Because drug use is often considered negative."}, "language_style": {"answer": "Calm and direct", "rationale": "The language style of the entry is more direct and clear, with no excessive rhetorical or emotional rendering, and is more objective in tone, with the aim of obtaining an accurate picture of whether the person surveyed is addicted to drugs."}, "time_dimension": {"answer": "Future", "rationale": "The entry contains visions and expectations for the future, with the keywords \"use of drugs\" and \"specifically refer to addiction-dependent drugs\", all pointing to situations that have not yet occurred and that may arise in the future."}, "sentence_type": "Imperative sentence", "pos": {"nouns": {"Drugs": 2, "Special finger": 1, "Dependence dependency": 1}, "verbs": {"Use": 1}}}, {"content": "(e) Drinking and drinking and driving me crazy", "word_count": 7, "label": "", "sentence_structure": "Subject鈥損redicate", "subjective_objective": {"answer": "Subjective", "rationale": "Because \"drinking, drinking and dementia\" are behavioral or psychological responses to a particular situation, they are subjective."}, "sentence_sentiment": {"answer": "Negative", "rationale": "Because \"drinking and drinking\" and \"drinking\" are both bad habits, with potentially negative consequences."}, "language_style": {"answer": "Tactful and warm", "rationale": "The entry uses a more euphemistic expression, where the terms \"drinking and drinking\" and \"drinking\" do not appear directly, but rather portray the behaviour that causes problems, which is more moderate and interactive and more acceptable to the respondents."}, "time_dimension": {"answer": "Present", "rationale": "Among them, \"drinking for fun\" and \"drinking for madness\" describe the behaviour or state of the person being tested after drinking, which suggests that this is an investigation of current or future behaviour or state. The time frame for this article should therefore include present and future, i.e. output: present/future"}, "sentence_type": "Imperative sentence", "pos": {"nouns": {"Drinking and playing": 1, "Drunk": 1}}}, {"content": "f) Routine driving or riding", "word_count": 7, "label": "", "sentence_structure": "Subject鈥損redicate", "subjective_objective": {"answer": "Subjective", "rationale": "Because reckless driving or riding is a personal manifestation of behaviour, it is the patient ' s subjective attitude."}, "sentence_sentiment": {"answer": "Negative", "rationale": "Because the act of \"brash driving or riding\" is dangerous and irresponsible and has a negative emotional connotation."}, "language_style": {"answer": "Calm and direct", "rationale": "At the same time, the wording of the entry is clear and specific, and it conveys clearly what researchers want to know."}, "time_dimension": {"answer": "Past", "rationale": "The entry uses the verbs \"open\" and \"ride\" in general past-style forms (drove/cyclid), which suggests that the act has already occurred. At the same time, \"reckless\" is also an adjective describing past behaviour, which further confirms that the temporal dimension of the entry is past."}, "sentence_type": "Imperative sentence", "pos": {"nouns": {"You're reckless": 1, "Drive": 1, "Crawling": 1}, "verbs": {"Drive recklessly": 1, "Rush-car ride": 1}}}, {"content": "(a) I'm thought to be a bullying student", "word_count": 15, "label": "", "sentence_structure": "Subject鈥搗erb鈥搊bject", "subjective_objective": {"answer": "Subjective", "rationale": "Because \"the perception\" is a subjective perception and evaluation of others, it is the views and opinions of others that are being examined."}, "sentence_sentiment": {"answer": "Negative", "rationale": "Because the \"braining\" in the entry is negative."}, "language_style": {"answer": "Tactful and warm", "rationale": "The entry uses a first person's perspective, using the expression \"the perception\" to guide the interviewee to think about his image in the hearts of others. This is a more gentle and close language that does not put the interviewee too much pressure or discomfort. At the same time, the expression \"showing the weak student\" avoids direct accusations or criticism, making the questionnaire more personal and friendly."}, "time_dimension": {"answer": "Present", "rationale": "The keyword \"the perception\" is a statement that is evaluated by others and that it is in the present or the most recent period of time."}, "sentence_type": "Imperative sentence", "pos": {"nouns": {"People": 1, "I": 2, "Students": 1}, "verbs": {"I think": 1, "be": 1, "Bullying": 1}, "adjectives": {"Strong": 1, "Weak": 1}}}, {"content": "(b) I used to start a fight with other kids.", "word_count": 15, "label": "", "sentence_structure": "Subject鈥搗erb鈥揷omplement", "subjective_objective": {"answer": "Subjective", "rationale": "Because this is a subjective description of the patient himself, describing his behaviour and thoughts."}, "sentence_sentiment": {"answer": "Negative", "rationale": "Because it's a negative act to provoke trouble and fight."}, "language_style": {"answer": "Calm and direct", "rationale": "The entry asked the interviewees in a direct and clear manner whether they had often clashed with other children in the past, without excessive ado or emotional colour, and with relatively calm and objective language style."}, "time_dimension": {"answer": "Past", "rationale": ""}, "sentence_type": "Imperative sentence", "pos": {"nouns": {"Matters": 1, "Son": 2, "Array": 1}, "verbs": {"Past": 1, "Start": 1, "Fight": 1}, "numerals": {"Regular": 1}}}, {"content": "(c) I used a murder weapon in a fight.", "word_count": 10, "label": "", "sentence_structure": "Subject鈥搗erb鈥搊bject", "subjective_objective": {"answer": "Objective", "rationale": "Because the murder weapon was used as an objective fact in the event of a fight, not as a subjective attitude or feeling of the patient."}, "sentence_sentiment": {"answer": "Negative", "rationale": "Because the use of a murder weapon is a certain dangerous and negative act."}, "language_style": {"answer": "Calm and direct", "rationale": "The entry directly asked whether the other party had ever used a murder weapon in a fight without any adornment or emotional colour, and the language style was a straightforward and calm type."}, "time_dimension": {"answer": "Past", "rationale": "The keywords \"in a fight\" and \"used\" point to past events, so the entry is to question the testee about past behaviour."}, "sentence_type": "Affirmative declarative sentence", "pos": {"nouns": {"Questionnaire": 1, "Entry": 1, "Fight": 1, "The murder weapon": 1}, "verbs": {"Use": 1}, "pronouns": {"I": 1}}}, {"content": "(d) I've robbed or killed someone.", "word_count": 10, "label": "", "sentence_structure": "Subject鈥搗erb鈥搊bject", "subjective_objective": {"answer": "Objective", "rationale": "Because robbery or assault is an objective fact that the patient has actually experienced, not the patient ' s subjective attitude or idea."}, "sentence_sentiment": {"answer": "Negative", "rationale": "Because the action in the entry \"robbery or assault\" is negative."}, "language_style": {"answer": "Tactful and warm", "rationale": "The entry uses a passive speech, which is more neutral, with no strong emotional or blameful tone. At the same time, the issue uses the term \"robbery or murder\" to understand whether the person under investigation has had a similar history of behaviour, rather than directly asking whether they are criminals. This euphemism is more euphemismal and objective, protecting the privacy and dignity of the person under investigation and making them more willing to answer the questions in a genuine manner."}, "time_dimension": {"answer": "Past", "rationale": ""}, "sentence_type": "Affirmative declarative sentence", "pos": {"nouns": {"Questionnaire": 1, "Entry": 1, "person": 1, "Robbery": 1, "Murderer": 1}, "verbs": {"Tsang": 1, "to": 1, "Robbery": 1, "Murderer": 1}, "numerals": {"One": 2}, "pronouns": {"I": 1}}}, {"content": "I've been violent to others.", "word_count": 12, "label": "", "sentence_structure": "Subject鈥搗erb鈥搊bject", "subjective_objective": {"answer": "Subjective", "rationale": "Because \"I have been subjected to brutal violence\" is a subjective feeling and attitude, it is a matter of whether the patient has ever had such an act or experience."}, "sentence_sentiment": {"answer": "Negative", "rationale": "Because the act of \"a brutal violence\" in the entry is negative."}, "language_style": {"answer": "Tactful and warm", "rationale": "The entry is more euphemistic, using a subjective perspective of \"I\" and not directly accusing or exposing others of their actions. At the same time, questions are raised in an open-ended manner, giving respondents a certain amount of space and freedom to answer. Such expression respects the feelings of the interviewee and gives them real and effective information."}, "time_dimension": {"answer": "Present", "rationale": "The keyword \"difficult violence against others\" means past experience and behaviour, but it is not clear whether it is what happened in the past or what is happening in the present. Thus, the entry is now in terms of time dimension."}, "sentence_type": "Negative declarative sentence", "pos": {"nouns": {"Violence": 1}, "verbs": {"Implementation": 1, "Here": 1, "Pass": 1}, "adjectives": {"Cruel": 1}, "pronouns": {"I": 1}}}, {"content": "(f) I'm violent against animals.", "word_count": 12, "label": "", "sentence_structure": "Subject鈥搗erb鈥搊bject", "subjective_objective": {"answer": "Subjective", "rationale": "This is a self-evaluation and attitude of the patient and is subjective."}, "sentence_sentiment": {"answer": "Negative", "rationale": "Because the act of \"violence against animals\" is negative."}, "language_style": {"answer": "Tactful and warm", "rationale": "This is a more euphemistic and interactive expression that attracts the resonance and collaboration of the interviewee."}, "time_dimension": {"answer": "Future", "rationale": "The keyword \"violence against animals in a cruel manner\" refers to acts or attitudes that may occur in the future, and the entry therefore relates to future judgements."}, "sentence_type": "Negative declarative sentence", "pos": {"nouns": {"Animals": 1, "Implementation": 1, "Violence": 1}, "verbs": {"to": 1, "Cruel": 1, "Implementation": 1}, "adjectives": {"Cruel": 1}}}, {"content": "(g) I forced him to have sex with me.", "word_count": 14, "label": "", "sentence_structure": "Subject鈥搗erb鈥搊bject", "subjective_objective": {"answer": "Objective", "rationale": "Since forcing another person to have sexual relations is an objective act, it is an objective fact that is examined."}, "sentence_sentiment": {"answer": "Negative", "rationale": "Because the act in the entry \"forced sexual intercourse with another person\" is offensive and harmful and has a negative emotional connotation."}, "language_style": {"answer": "Tactful and warm", "rationale": ""}, "time_dimension": {"answer": "Past", "rationale": "The entry is for questioning whether the person tested had had a certain act or experience, and the temporal dimension is a past.\""}, "sentence_type": "Negative declarative sentence", "pos": {"nouns": {"g": 1, "I": 2, "he": 1, "she": 1, "person": 1, "Sexual relations": 1}, "verbs": {"Forced": 1, "Come on": 1, "and": 1}}}, {"content": "鈾?I've lied many times 鈾?, "word_count": 5, "label": "", "sentence_structure": "Subject鈥搗erb鈥揷omplement", "subjective_objective": {"answer": "Subjective", "rationale": "Because \"I'm lying many times\" is a self-evaluation, involving personal values, attitudes and subjective judgment."}, "sentence_sentiment": {"answer": "Negative", "rationale": "Because it involves such negative acts of lying."}, "language_style": {"answer": "Calm and direct", "rationale": "The entry directly expresses the subject of a question about whether the other party is often lying, and does not use excessive rhetorical or emotional rendering, which is more objective in tone."}, "time_dimension": {"answer": "Present", "rationale": "The entry describes a behaviour or attitude of the person tested, and does not clearly indicate a time dimension that points to the past, present or future. Therefore, the neutral time dimension can be judged."}, "sentence_type": "Negative declarative sentence", "pos": {"nouns": {"Liar": 1}, "verbs": {"Multiple": 1, "I": 1}}}, {"content": "I'm not allowed to spend the night without my parents' permission.", "word_count": 14, "label": "", "sentence_structure": "Subject鈥搗erb鈥揷omplement", "subjective_objective": {"answer": "Subjective", "rationale": "Because \"overnight without parental permission\" is the patient's behavior and experience, and it's subjective."}, "sentence_sentiment": {"answer": "Negative", "rationale": "Because the behaviour in the entry \"sleeping out without parental permission\" is against the rules, may raise security concerns and therefore have a negative emotional connotation."}, "language_style": {"answer": "Calm and direct", "rationale": "The entry uses a more direct presentation, without excessive emotional rendering or rhetorical techniques, which is more calm and objective in tone, and directly expresses the subject of asking the other party whether he or she has spent the night outside without parental permission."}, "time_dimension": {"answer": "Past", "rationale": ""}, "sentence_type": "Negative declarative sentence", "pos": {"nouns": {"parents": 1, "Permission": 1, "Outside": 1, "Night": 1}, "verbs": {"Nope": 1, "Here": 1, "Okay": 1, "Allow": 1, "Just": 1, "in": 1, "Pass": 1, "Night": 1}, "adverbs": {"i)": 1}, "pronouns": {"I": 1}}}, {"content": "I've stolen from someone else.", "word_count": 8, "label": "", "sentence_structure": "Subject鈥搗erb鈥搊bject", "subjective_objective": {"answer": "Subjective", "rationale": "Because \"I stole from someone else\" is the patient's subjective idea and behavior, not the objective fact."}, "sentence_sentiment": {"answer": "Negative", "rationale": "Because the theft is immoral and has a negative emotional connotation."}, "language_style": {"answer": "Tactful and warm", "rationale": "The entry uses a more euphemistic language style, avoiding too direct, sharp, or irritating expressions to reduce the risk of causing psychological stress and resentment. At the same time, this moderate language is more conducive to promoting openness and honesty."}, "time_dimension": {"answer": "Past", "rationale": "The keyword is \"snatched\" , which denotes an action that has occurred and is completed, and the entry is therefore a question of past experience and behaviour of the person being questioned."}, "sentence_type": "Negative declarative sentence", "pos": {"nouns": {"Questionnaire Entry": 1, "I": 1, "others": 1, "Something": 1}, "verbs": {"Steal": 1}}}, {"content": "鈾?I've been on fire 鈾?, "word_count": 4, "label": "", "sentence_structure": "Subject鈥損redicate", "subjective_objective": {"answer": "Subjective", "rationale": "Because arson is an act that needs to be carried out, it is the subjective attitude and behaviour of the patient that is examined."}, "sentence_sentiment": {"answer": "Negative", "rationale": "Because the act of arson in the entry is negative."}, "language_style": {"answer": "Calm and direct", "rationale": "The language style of the entry is more direct, with no excessive embellishment or emotional rendering, and the expression is more objective and clear, directly asking the respondents whether they had ever had arson."}, "time_dimension": {"answer": "Past", "rationale": "\"Aft the fire\" is a past move, so the entry is a question of past behaviour or mentality."}, "sentence_type": "Affirmative declarative sentence", "pos": {"nouns": {"Questionnaire": 1, "Entry": 1, "Fire": 1}, "verbs": {"Even though": 1}}}, {"content": "(l) I've deliberately broken windows or destroyed property", "word_count": 13, "label": "", "sentence_structure": "Subject鈥搗erb鈥搊bject", "subjective_objective": {"answer": "Subjective", "rationale": "The patient ' s behaviour and attitude are described, inter alia, as deliberate, broken windows and are therefore subjective."}, "sentence_sentiment": {"answer": "Negative", "rationale": "Because the entry \"intentionally broken windows or destroyed property\" is negative."}, "language_style": {"answer": "Calm and direct", "rationale": "The entry directly asked the interviewees whether there had been any deliberate break-ups or damage to property, which was more straightforward in language style, and did not use excessive acrimonyms or emotional language to give people an objective and calm sense of calm."}, "time_dimension": {"answer": "Past", "rationale": "The keyword \"intentionally breaking windows or destroying property\" describes acts that have occurred and have occurred in the past and therefore judge as past."}, "sentence_type": "Negative declarative sentence", "pos": {"nouns": {"Window": 1, "Property": 1}, "verbs": {"On purpose": 1, "Break it up": 1, "Destruction": 1}}}, {"content": "(m) I've been out for the night not only once.", "word_count": 13, "label": "", "sentence_structure": "Subject鈥搗erb鈥揷omplement", "subjective_objective": {"answer": "Subjective", "rationale": "Because \"runaway\" and \"overnight\" are both behavioral and subjective behaviour of the patient."}, "sentence_sentiment": {"answer": "Negative", "rationale": "Because \"scrambling home\" and \"overnight\" in the entries are all negative."}, "language_style": {"answer": "Calm and direct", "rationale": "The entry is based on: 1. The entry is based on simple statements, without excessive qualifiers or emotional colours. 2. The sentence is a statement of personal experience and behaviour, not an assessment or recommendation of another person or society. 3. The entry does not display a particularly strong emotion or emotion, but is presented in a factual manner."}, "time_dimension": {"answer": "Past", "rationale": ""}, "sentence_type": "Negative declarative sentence", "pos": {"nouns": {"I": 1, "Home": 1, "Once": 1, "Run away from home": 1, "Outside": 1, "Stay the night": 1}, "verbs": {"have": 1, "More than that": 1, "Run away from home": 1, "in": 1, "External": 1, "Stay the night": 1}, "adverbs": {"Only": 1}, "numerals": {"One": 2}, "classifiers": {"individual": 1}}}, {"content": "I've been avoiding school a lot since I was 13.", "word_count": 11, "label": "", "sentence_structure": "Subject鈥搗erb鈥揷omplement", "subjective_objective": {"answer": "Subjective", "rationale": "Because the AAA is influenced and educated by parents, teachers, etc., \"starting school again and again\" is the subjective behaviour choice of the patient."}, "sentence_sentiment": {"answer": "Negative", "rationale": "Because truancy is a negative act, it suggests that the interviewee may have problems growing up."}, "language_style": {"answer": "Calm and direct", "rationale": "The entry is a direct expression of the question whether the other party has regularly escaped from school before the age of 13, without using excessive rhetoric or emotional rendering, and is more objective in tone. Moreover, the question is more straightforward and clear, and there is no ambiguity or a distorted expression."}, "time_dimension": {"answer": "Past", "rationale": ""}, "sentence_type": "Affirmative declarative sentence", "pos": {"nouns": {"I": 1, "Age": 1, "- You're not going to school": 1}, "verbs": {"Start": 1, "Run": 1}, "adverbs": {"Before": 1, "More": 1}, "numerals": {"13": 1}}}, {"content": "I broke into someone's house, building or cycling without permission.", "word_count": 17, "label": "", "sentence_structure": "Subject鈥搗erb鈥搊bject", "subjective_objective": {"answer": "Objective", "rationale": "Because \"into someone's home, building or cycling without permission\" is an act of the patient, an objective fact."}, "sentence_sentiment": {"answer": "Negative", "rationale": "Because the actions in the entries violate the rights of others, are immoral and illegal and have a negative emotional connotation."}, "language_style": {"answer": "Calm and direct", "rationale": "The language style of the entry is more straightforward, with no excessive rhetorical or emotional rendering, and less objective and incriminating, highlighting the seriousness of the violation."}, "time_dimension": {"answer": "Present", "rationale": "The entry used to ask the testee whether there had been unauthorized break-ins into another person ' s house, building or cycling in the past, and the dimension of time was judged to be past."}, "sentence_type": "Negative declarative sentence", "pos": {"nouns": {"Questionnaire": 1, "Entry": 1, "others": 1, "Home": 1, "Buildings": 1, "Car": 1}, "verbs": {"Break in": 1, "Ride": 1}, "adverbs": {"Unauthorized": 1}, "numerals": {"o": 1}, "pronouns": {"I": 1}}}]}

{"questionnaire_title": "Table of psychosocial adaptation", "questionnaire_category": "Mental health", "item_count": 21, "emotion_shift_overall": "From negative to positive", "language_style_overall": "Neutral", "items": [{"content": "1 My test scores are often lower than my real learning abilities and levels.", "word_count": 20, "label": "", "sentence_structure": "Subject鈥搗erb鈥揷omplement", "subjective_objective": {"answer": "Subjective", "rationale": "This sentence is an expression of patients ' subjective view of their test results, which is that they are more capable of learning than they are of the existing examination."}, "sentence_sentiment": {"answer": "Negative", "rationale": "Because the expression \"tests are often less than my real learning ability and level\" in the entry expresses a negative emotion and attitude."}, "language_style": {"answer": "Calm and direct", "rationale": "The entry is a calm and straightforward language style. It clearly raises the question, without using excessive rhetorical or emotional rendering, and with a more objective tone. Nor is it an attempt to resonate with the person under investigation or to provoke a strong emotional reaction, but rather to raise the question in a more neutral way."}, "time_dimension": {"answer": "Present", "rationale": ""}, "sentence_type": "Negative declarative sentence", "pos": {"nouns": {"Examinations": 1, "Learning ability": 1, "Horizontal": 1}, "verbs": {"Less than": 1, "Real": 1, "Learn": 1, "Examination": 1}, "adverbs": {"Regular": 1}, "pronouns": {"I": 2}}}, {"content": "I like to try food that I've never eaten, tasted or named.", "word_count": 21, "label": "", "sentence_structure": "Subject鈥搗erb鈥搊bject", "subjective_objective": {"answer": "Subjective", "rationale": "The taste of new things is a subjective attitude, which is a tendency for patients to explore new things."}, "sentence_sentiment": {"answer": "Positive", "rationale": "Because the \"like to try new food\" behavior in the entry is positive."}, "language_style": {"answer": "Tactful and warm", "rationale": "The entry is a soft, inviting conversation, asking if the other side likes to try the subject of new food. This expression is neither too direct, nor too direct to attract interest and participation from the other side, making the questionnaire more attractive."}, "time_dimension": {"answer": "Present", "rationale": "The words \"like\" and \"attempt\" in the entry are verbs describing current behavior, while \"never ate\" food is a thing of the past. So, the time dimension of this entry is now."}, "sentence_type": "Affirmative declarative sentence", "pos": {"nouns": {"Food": 2, "I": 1}, "verbs": {"Yeah": 1, "Try": 1, "Yes": 1}, "adjectives": {"Weird": 1}, "adverbs": {"Never": 1}, "numerals": {"2": 1}}}, {"content": "Three to a new place, I'm indigestion, skin allergic or insomnia.", "word_count": 20, "label": "", "sentence_structure": "Subject鈥搗erb鈥揷omplement", "subjective_objective": {"answer": "Subjective", "rationale": "Because indigestion, skin allergies and insomnia are subjective feelings and experiences of patients, related to the physical condition of individuals, not to objective facts."}, "sentence_sentiment": {"answer": "Negative", "rationale": "Because the symptoms in the entries are negative, such as \"indigestion\", \"skin allergy\" and \"sleep insomnia\", the emotional color of the article is negative."}, "language_style": {"answer": "Calm and direct", "rationale": "The language style of the entry is more objective and direct, with no excessive rhetorical or emotional rendering. It is a simple expression of the problems that are often encountered when trying to get to a new place, which is obvious and easy to understand."}, "time_dimension": {"answer": "Past", "rationale": "Output: Past"}, "sentence_type": "Affirmative declarative sentence", "pos": {"nouns": {"Local": 1, "Indigestion": 1, "Skin allergies": 1, "Insomnia": 1}, "verbs": {"- Yes": 1, "Go on": 1, "Regular": 1}, "adverbs": {"Present": 1, "One": 1}, "numerals": {"3": 1}}}, {"content": "I have done better in formal sports than in physical education or in regular practice.", "word_count": 23, "label": "", "sentence_structure": "Subject鈥搗erb鈥揷omplement", "subjective_objective": {"answer": "Objective", "rationale": "Because the results of the official sports events are objective and not influenced by the patient鈥檚 subjective sense of mind. Even if the patient feels that his or her performance will be better, the statement remains objective if the actual results are not as expected."}, "sentence_sentiment": {"answer": "Positive", "rationale": "The phrase \"accomplishes better than physical education or exercise in regular hours\" in the entry is positive and indicates that the person performed well in the official competition."}, "language_style": {"answer": "Tactful and warm", "rationale": "This is a euphemism that allows the person surveyed to feel comfortable and respectful and to be more friendly in language."}, "time_dimension": {"answer": "Past", "rationale": "In that entry, \"participation\" and \"achievement\" were all actions of the past, so they were judged to be the past."}, "sentence_type": "Affirmative declarative sentence", "pos": {"nouns": {"I will": 1, "Official Games": 1, "Achievements": 2, "Physical education": 1, "Practice scores on a regular basis": 1}, "verbs": {"- Yeah": 1, "Participation": 1, "Okay": 1}, "numerals": {"4": 1}, "pronouns": {"I": 2}}}, {"content": "The text of the lesson is well-readed, but the back of the classroom is wrong.", "word_count": 22, "label": "", "sentence_structure": "Subject鈥搗erb鈥揷omplement", "subjective_objective": {"answer": "Subjective", "rationale": "Because \"The text of the five is well-readed, but it goes wrong when you get to class.\" It's about the patient's own perception and feelings of the situation, so it's subjective."}, "sentence_sentiment": {"answer": "Negative", "rationale": "Because the \"fault error\" in the entry is negative."}, "language_style": {"answer": "Tactful and warm", "rationale": "The entry is more euphemistic, and it does not directly blame or criticize students, but rather uses a means of asking questions about their specific situation and needs. At the same time, the entry provides a concrete proposal that addresses this problem, namely, practice more in the classroom."}, "time_dimension": {"answer": "Present", "rationale": "The keyword \"reversed\" denotes a past completed move, but \"but\" represents a turning relationship, which raises the problem of making a mistake rereading in the classroom. The entry therefore describes the situation of the person being tested, judging by the present."}, "sentence_type": "Affirmative declarative sentence", "pos": {"nouns": {"Text": 2, "Classroom": 1}, "verbs": {"Back": 3, "Out": 1, "Bad": 1}, "adjectives": {"The": 1, "I'm good": 1}, "adverbs": {"Already": 1, "Just": 1}, "numerals": {"5": 1}}}, {"content": "I am not nervous in my statement, and I am naturally speaking in such a way as to be free to go.", "word_count": 19, "label": "", "sentence_structure": "Subject鈥搗erb鈥揷omplement", "subjective_objective": {"answer": "Subjective", "rationale": "Because \"no stress\", \"no words,\" and \"showing ourselves\" are the patient's description of his subjective feelings and abilities."}, "sentence_sentiment": {"answer": "Positive", "rationale": "Because the entries are all positive, like \"no tension\", \"no language,\" \"no matter what,\" \"no action.\""}, "language_style": {"answer": "Calm and direct", "rationale": "The entry is based on direct speech, is not subject to much rhetorical or emotional overtones, and is clear and concise; - it focuses on the behaviour and ability of individuals to speak at meetings without any emotional or subjective feeling; - it is formulated in an objective, neutral manner, without any strong emotional connotations. It is therefore judged to be the language style of the purpose of the article as being straightforward."}, "time_dimension": {"answer": "Present", "rationale": "The words \"meetings\", \"not nervous\", \"words naturally\" and \"playing free\" are all terms of the present situation or capacity, so the temporal dimension of the article is now."}, "sentence_type": "Affirmative declarative sentence", "pos": {"nouns": {"Meetings": 1, "Presentations": 1, "Nervous": 1, "Language": 1, "鈾?And the end 鈾?: 1, "Play": 1, "鈾?As if I was 鈾?: 1}, "verbs": {"Open": 1, "It's not nervous": 1, "Naturally": 1, "Play": 1}, "adjectives": {"Meetings": 1, "Presentations": 1, "Nervous": 1, "Language": 1, "鈾?And the end 鈾?: 1, "鈾?As if I was 鈾?: 1}, "adverbs": {"not": 1, "鈾?And the end 鈾?: 1}, "classifiers": {"individual": 1}}}, {"content": "I am more cold in winter than others, more hot in summer and more dark in night.", "word_count": 22, "label": "", "sentence_structure": "Subject鈥搗erb鈥揷omplement", "subjective_objective": {"answer": "Subjective", "rationale": "Because \"I'm colder in winter than others, hotter in summer, darker in night\" is a description of the patient's self-perception and subjective experience, the questionnaire entry is viewed from the patient's subjective perspective."}, "sentence_sentiment": {"answer": "Negative", "rationale": "Because the entries describe negative physical reactions, such as \"fear of cold,\" \"fear of heat\" and \"fear of darkness.\" These reactions can cause inconvenience or discomfort, and are therefore negative."}, "language_style": {"answer": "Tactful and warm", "rationale": ""}, "time_dimension": {"answer": "Past", "rationale": "The entry describes the subjective feelings and experiences of the testees, and concerns the past. The words \"winter is colder than others, summer is hoter\" and \"night is darker than others\" are all depicting the past and can therefore be judged as the past."}, "sentence_type": "Affirmative declarative sentence", "pos": {"nouns": {"Winter": 1, "others": 2, "Summer": 1, "Night": 1, "Black": 1}, "verbs": {"Yes": 4}, "adjectives": {"Cold": 1, "Hot": 1, "Dark": 1}, "numerals": {"7": 1}, "pronouns": {"I": 1}}}, {"content": "In a tumultuous environment, I do what I do and I do not lose any efficiency.", "word_count": 22, "label": "", "sentence_structure": "Subject鈥搗erb鈥搊bject", "subjective_objective": {"answer": "Objective", "rationale": "Because this phrase describes the behaviour of a person in a tumultuous environment, which is a fact that can be observed and measured, not the patient鈥檚 own subjective feelings or ideas. The perspective of this questionnaire entry is therefore objective."}, "sentence_sentiment": {"answer": "Neutral", "rationale": "Because the entry describes the behaviour and efficiency of a person in a given environment, there is no obvious emotional colour."}, "language_style": {"answer": "Tactful and warm", "rationale": "The entry uses neutral, objective language such as \"business as usual\" and \"inefficiency\", and asks about the behaviour of the other in a tumultuous environment, rather than directly criticizing or commending. At the same time, the use of \"me\" makes the tone more friendly and natural."}, "time_dimension": {"answer": "Present", "rationale": ""}, "sentence_type": "Affirmative declarative sentence", "pos": {"nouns": {"Environment": 1, "Efficiency": 1}, "verbs": {"Do it": 1, "As usual": 1, "and": 1}, "adjectives": {"Loud": 1, "Chaos": 1, "Low": 1}, "adverbs": {"in": 1}, "numerals": {"8": 1}}}, {"content": "At the time of the medical examination, my pulse, blood pressure, etc. were higher than usual due to stress.", "word_count": 21, "label": "", "sentence_structure": "Subject鈥搗erb鈥揷omplement", "subjective_objective": {"answer": "Subjective", "rationale": "Because stress is the patient's subjective feeling and attitude."}, "sentence_sentiment": {"answer": "Negative", "rationale": "It's negative to increase body indicators because of \"stretching.\""}, "language_style": {"answer": "Tactful and warm", "rationale": "It uses a care and understanding approach, conveying the researcher ' s concerns about the person under investigation in the form of inquiries, without using overly direct or critical language, which makes it easier for the person under investigation to feel comfortable and acceptable."}, "time_dimension": {"answer": "Past", "rationale": "Past."}, "sentence_type": "Affirmative declarative sentence", "pos": {"nouns": {"Medical examination": 1, "Pulse": 1, "Blood pressure": 1, "Indicators": 1, "Nervous": 1, "Normal": 1}, "verbs": {"Time": 1, "be": 1, "- Yeah": 1, "High": 1}, "adjectives": {"Nervous": 1}, "adverbs": {"Because": 1}, "numerals": {"9": 1}}}, {"content": "I can stay up all night and work with energy if I need to.", "word_count": 19, "label": "", "sentence_structure": "Subject鈥搗erb鈥搊bject", "subjective_objective": {"answer": "Objective", "rationale": "Because the overnight stay is an objective act, it is not necessary to determine the patient ' s subjective will and attitude."}, "sentence_sentiment": {"answer": "Positive", "rationale": "Because the act of \"stilling through the night, doing things with energy\" in the entry is a positive expression of attitude and quality."}, "language_style": {"answer": "Tactful and warm", "rationale": "The entry expresses, in an encouraging and supportive tone, the view of whether the other party can stay up late, with a temperature of words and a positive mood, which is not too direct or too strong. Such expression makes the respondents comfortable and comfortable to answer questions, and more willing to share their real thoughts and feelings."}, "time_dimension": {"answer": "Future", "rationale": "The keyword \"if necessary\" and \"if you can stay up all night\" expresses a hypothetical situation, while \"work with energy\" indicates the behaviour or state of the future, so the entry asks about the attitude or practice of the testee in the future when faced with similar situations."}, "sentence_type": "Affirmative declarative sentence", "pos": {"nouns": {"All night long": 1, "- The energy": 1}, "verbs": {"Yeah": 1, "- Stay tight": 1, "Do it": 1}, "adjectives": {"鈾?Full of 鈾?: 1}, "adverbs": {"If you need to": 1, "Land": 1}, "numerals": {"10": 1}}}, {"content": "When someone comes to the house, my parents let me see the guests, I don't want to go.", "word_count": 19, "label": "", "sentence_structure": "Subject鈥搗erb鈥搊bject", "subjective_objective": {"answer": "Subjective", "rationale": "Because it is the patient's subjective thoughts and wishes that determine not to go."}, "sentence_sentiment": {"answer": "Negative", "rationale": "Because the position of the entry is one of negative emotional tendencies, as it is not intended to meet guests."}, "language_style": {"answer": "Tactful and warm", "rationale": "The language style of the entry is euphemistic, because it expresses the stress and discomfort that a child may feel in the home in a gentle and understanding way. It does not direct orders or criticize children, but rather seeks to understand and accommodate their feelings and offers a solution (i.e. to meet guests). This language is easier to accept and cooperate with children and helps to establish good parenting."}, "time_dimension": {"answer": "Past", "rationale": ""}, "sentence_type": "Negative declarative sentence", "pos": {"nouns": {"Home": 1, "person": 2, "parents": 1, "Guests": 1}, "verbs": {"Jean": 1, "Go on": 2, "See": 1}, "adverbs": {"not": 1}, "numerals": {"11": 1}, "pronouns": {"I": 1}}}, {"content": "I was out of town, and I quickly became accustomed to new and changed living conditions.", "word_count": 20, "label": "", "sentence_structure": "Subject鈥搗erb鈥揷omplement", "subjective_objective": {"answer": "Subjective", "rationale": "This is a reflection of patients鈥?subjective attitudes and perceptions, as it takes time and process to get used to the new environment, which can vary from person to person."}, "sentence_sentiment": {"answer": "Positive", "rationale": "Because the entry \"can quickly get used to new and changed living conditions\" expresses positive attitudes and behaviours that are adapted to the new environment."}, "language_style": {"answer": "Calm and direct", "rationale": "The entry directly asks the testee whether he or she is able to adapt quickly to the new living conditions, expressing them more directly and clearly, without excessive rhetorical or emotional colours. \""}, "time_dimension": {"answer": "Present", "rationale": ""}, "sentence_type": "Affirmative declarative sentence", "pos": {"nouns": {"Door": 1, "External": 1, "Living conditions": 1}, "verbs": {"Yes": 1, "Custom": 1, "Change": 1}, "adjectives": {"Come on": 1, "New": 1}, "numerals": {"12": 1}, "pronouns": {"I": 1}}}, {"content": "The more I get gas in 13 sports classes or sports, the more nervous I get.", "word_count": 20, "label": "", "sentence_structure": "Subject鈥搗erb鈥揷omplement", "subjective_objective": {"answer": "Subjective", "rationale": "Because fuel, stress and so on are the experiences and feelings inside the patient, it is subjective."}, "sentence_sentiment": {"answer": "Negative", "rationale": "Because the \"I'm getting more and more nervous\" in the entry is negative."}, "language_style": {"answer": "Tactful and warm", "rationale": "The entry is more gentle and gentle in its language, using the expression \"column cheers me\" instead of pointing directly to the tension of the other, and describing it in a more objective way. At the same time, the part \"I get nervous\" expresses the other side's feelings more implicitly, avoiding situations where too direct language may cause the other side to feel uncomfortable."}, "time_dimension": {"answer": "Present", "rationale": "The keyword \"bring me more\" shows that this is about the interaction of others, and the \"I'm more nervous\" reveals the subjective experience and emotional state of the subjects."}, "sentence_type": "Affirmative declarative sentence", "pos": {"nouns": {"Physical education": 1, "Games": 1, "Class": 1, "Come on": 1, "Nervous": 1}, "verbs": {"Here": 1, "The more... the more": 1}, "adverbs": {"Tsk": 2}, "pronouns": {"I": 1}}}, {"content": "I answered the questions in class and said everything I thought was all right.", "word_count": 22, "label": "", "sentence_structure": "Subject鈥搗erb鈥搊bject", "subjective_objective": {"answer": "Subjective", "rationale": "Because \"I calmly say everything I want to do\" is the patient's assessment and perception of his behaviour."}, "sentence_sentiment": {"answer": "Positive", "rationale": "Because the behavior in the entry is positive, \"coolly, say everything you want.\""}, "language_style": {"answer": "Calm and direct", "rationale": "The language style of the entry is calm. It does not have too much emotional accoutrements and rhetorical techniques, but rather expresses the subject of the question in an objective and clear way, i.e. whether the interviewee can remain calm and express his thoughts in a complete manner in the course of his or her class. This language style is simple and easy to understand and accept."}, "time_dimension": {"answer": "Present", "rationale": ""}, "sentence_type": "Affirmative declarative sentence", "pos": {"nouns": {"Classes": 1, "Problem": 1, "Answer": 1}, "verbs": {"Go, go, go": 1, "Calm down": 1, "- Put it on": 2, "Think": 1, "Say it": 1, "Come out": 1}, "adjectives": {"Everything": 1, "Complete": 1}, "adverbs": {"Land": 1}, "numerals": {"14": 1}, "pronouns": {"I": 1}}}, {"content": "I'm doing things on my own more efficiently than working with everyone.", "word_count": 17, "label": "", "sentence_structure": "Subject鈥搗erb鈥揷omplement", "subjective_objective": {"answer": "Subjective", "rationale": "Because \"efficiency\" is a subjective evaluation, depending on the individual's feelings and experience."}, "sentence_sentiment": {"answer": "Positive", "rationale": "Because the idea that one person is more efficient than others in the entry is positive."}, "language_style": {"answer": "Calm and direct", "rationale": "The language style of the entry is one of a calm and straightforward type. It asks the respondents which way to work more efficiently when they work alone and work with teams, and does not have too many emotional renderings or rhetorical techniques. This expression is more objective, direct and clear, and suitable for a questionnaire-based situation."}, "time_dimension": {"answer": "Present", "rationale": "The sentence describes the current feelings or state of the speaker, without reference to the time dimension of the past or the future. Among them, \"I do things by myself more efficiently than I do with everyone\" is a subjective assessment that says that the speaker prefers to do his job alone. So the time line of this questionnaire is now."}, "sentence_type": "Affirmative declarative sentence", "pos": {"nouns": {"Efficiency": 1, "I": 2, "person": 1, "Together": 1}, "verbs": {"Do it": 4, "- Yeah": 1, "High": 1}, "adjectives": {"Alone": 1}, "numerals": {"15": 1}}}, {"content": "In order to live together in harmony, I would rather give up my opinion and join you.", "word_count": 20, "label": "", "sentence_structure": "Subject鈥搗erb鈥搊bject", "subjective_objective": {"answer": "Subjective", "rationale": "Because \"I'd rather give up my opinion\" and \"attached\" are personal attitudes and choices, subjective."}, "sentence_sentiment": {"answer": "Positive", "rationale": "It is positive because it expresses an attitude of willingness to compromise in order to live together in harmony."}, "language_style": {"answer": "Tactful and warm", "rationale": "Warm and warm."}, "time_dimension": {"answer": "Present", "rationale": ""}, "sentence_type": "Affirmative declarative sentence", "pos": {"nouns": {"Harmony": 1, "Get along": 1, "Observations": 1, "Append": 1, "Everyone": 1}, "verbs": {"Abandon": 1, "I'd rather": 1, "Append": 1, "Get along": 1}, "adverbs": {"For": 1, "Both": 1, "I'd rather not": 1}, "numerals": {"16": 1}, "pronouns": {"I": 1}}}, {"content": "I feel uncomfortable and uncomfortable in front of all men, living beings or persons of the opposite sex.", "word_count": 19, "label": "", "sentence_structure": "Subject鈥搗erb鈥揷omplement", "subjective_objective": {"answer": "Subjective", "rationale": "Because \"fEs\" is the patient's subjective feeling and experience of his own emotions."}, "sentence_sentiment": {"answer": "Negative", "rationale": "Because the entry \"sweet, uncomfortable\" expresses a negative emotion and attitude."}, "language_style": {"answer": "Tactful and warm", "rationale": "The entry is more euphemistic and warm, without direct statements or orders. It talks in a gentle and inviting tone, and leads to thinking, using the words \"I feel\" and so forth, making each other more willing to share their real feelings and circumstances, and thus more personal and interactive."}, "time_dimension": {"answer": "Present", "rationale": "The situation of \"in front of people, living beings or persons of opposite sex\" describes the feelings and behaviour of the subject in a given context. At the same time, the words \"fear\" and \"insatibilities\" also indicate that these feelings occur in the present moment and are therefore judged to be the present."}, "sentence_type": "Affirmative declarative sentence", "pos": {"nouns": {"People": 1, "Livers": 1, "Heterosexual": 1, "Noodles": 1, "I": 1}, "verbs": {"I feel": 1, "Boom": 1, "I'm not comfortable": 1}, "adverbs": {"In front of you": 1}, "numerals": {"17": 1}}}, {"content": "I'm more efficient doing things alone than working with everyone.", "word_count": 17, "label": "", "sentence_structure": "Subject鈥搗erb鈥搊bject", "subjective_objective": {"answer": "Subjective", "rationale": "This is a statement of personal feelings and reflects the subjective views of patients in certain circumstances."}, "sentence_sentiment": {"answer": "Positive", "rationale": "Because the expression \"I'm doing it more efficiently by myself than by all of us\" is positive and suggests that individuals may be more efficient when working independently."}, "language_style": {"answer": "Calm and direct", "rationale": "The entry presents a view in a straightforward and concise manner, without excessive rhetorical or emotional colours, and therefore language styles are straightforward and dispassionate."}, "time_dimension": {"answer": "Present", "rationale": "The entry is now the present evaluation or view of the testee, which is not clearly time-directed, and is therefore judged now."}, "sentence_type": "Affirmative declarative sentence", "pos": {"nouns": {"18": 1, "person": 1, "Work": 2, "Efficiency": 1, "Together": 1}, "verbs": {"Do it": 2, "- Yeah": 1, "High": 1}, "adjectives": {"Alone": 1, "Together": 1}, "numerals": {"18": 1, "Two": 1}, "pronouns": {"I": 1}}}, {"content": "I was calm when we had an emergency.", "word_count": 16, "label": "", "sentence_structure": "Subject鈥搗erb鈥揷omplement", "subjective_objective": {"answer": "Subjective", "rationale": "Because sedated or not is a subjective feeling and attitude."}, "sentence_sentiment": {"answer": "Positive", "rationale": "Because the \"silent\" act in the entry is positive, indicating that the person has the ability to remain calm in times of crisis."}, "language_style": {"answer": "Calm and direct", "rationale": "The language style of the entry is calm and straightforward, it clearly describes the behaviour and response of individuals in emergency situations, and does not use any euphemism or rhetoric. This expression allows the subjects to understand the problem more accurately and to give more realistic and objective answers."}, "time_dimension": {"answer": "Past", "rationale": "\"What happened\" means it's happened, \"very calm\" is a description of a state or behavior of the past. So the temporal dimension of the sentence is the past."}, "sentence_type": "Affirmative declarative sentence", "pos": {"nouns": {"Emergency situations": 1, "Everyone": 1, "Panic": 1, "I": 1, "Calm down": 1}, "verbs": {"Come on": 1, "Both": 1, "very": 1, "Calm down": 1}, "adjectives": {"Panic": 1, "Calm down": 1}, "adverbs": {"Both": 1, "very": 1}, "numerals": {"19": 1}, "pronouns": {"I": 1}}}, {"content": "I'm too tired to say anything I've thought about when I'm arguing with others.", "word_count": 21, "label": "", "sentence_structure": "Subject鈥搗erb鈥揷omplement", "subjective_objective": {"answer": "Subjective", "rationale": "Because \"speaks in common\" and \"can't say anything in advance\" are subjective feelings and experiences of patients, observations and descriptions of their own behaviour. So this questionnaire entry looks at patient performance from a subjective perspective."}, "sentence_sentiment": {"answer": "Negative", "rationale": "Because the entry says, \"20 and others are talking, and I can't say it in advance,\" and it's negative."}, "language_style": {"answer": "Calm and direct", "rationale": ""}, "time_dimension": {"answer": "Present", "rationale": "\"20\" means a certain point of time, \"I'm always talking when I'm arguing\" and \"I can't say anything in advance\" describes what happens at that point of time, so the entry describes the behaviour or state of the subject at a certain point of time."}, "sentence_type": "Affirmative declarative sentence", "pos": {"nouns": {"20": 1, "others": 1, "Argument": 1, "Psst": 1, "Before": 1, "Word": 1}, "verbs": {"Regular": 1, "Yes": 1, "Say it": 1}, "adverbs": {"No exit": 1}}}, {"content": "When I met strangers, I was less defensive and easy to talk about.", "word_count": 18, "label": "", "sentence_structure": "Subject鈥搗erb鈥搊bject", "subjective_objective": {"answer": "Subjective", "rationale": "Because \"unprecious\" and \"easy to talk\" are subjective attitudes or feelings of patients."}, "sentence_sentiment": {"answer": "Positive", "rationale": "Because the words \"unprotective\" and \"climbing up\" in the entries are open and friendly."}, "language_style": {"answer": "Tactful and warm", "rationale": ""}, "time_dimension": {"answer": "Present", "rationale": ""}, "sentence_type": "Affirmative declarative sentence", "pos": {"nouns": {"Stranger": 1, "Exposure": 1, "Defense": 1, "Talk": 1}, "verbs": {"Not much": 1, "Easy": 1, "Get up": 1}, "adverbs": {"21": 1}}}]}

{"questionnaire_title": "Cornell Medical Index (CMI)", "questionnaire_category": "Behavioral questionnaire", "item_count": 218, "emotion_shift_overall": "From positive to negative", "language_style_overall": "Calm and direct", "items": [{"content": "Do you need glasses for reading the paper?", "word_count": 10, "label": "", "sentence_structure": "Subject鈥損redicate", "subjective_objective": {"answer": "Objective", "rationale": "Because the need for glasses is an objective fact of the patient ' s time and is not influenced by the subjective will of the individual."}, "sentence_sentiment": {"answer": "Neutral", "rationale": "Because the entry does not have any emotional color."}, "language_style": {"answer": "Calm and direct", "rationale": "The entry is a simple and clear question to the person under investigation about his or her vision, without too much embellishment or emotional addition. Its language style can therefore be considered as cold and neutral."}, "time_dimension": {"answer": "Present", "rationale": ""}, "sentence_type": "Interrogative sentence", "pos": {"nouns": {"Questionnaire": 1, "Entry": 1}, "verbs": {"Read it": 1, "Dai": 1}, "adverbs": {"Time": 1}, "classifiers": {"individual": 1}}}, {"content": "Do you need glasses when you look far away?", "word_count": 11, "label": "", "sentence_structure": "Subject鈥損redicate", "subjective_objective": {"answer": "Objective", "rationale": "Because \"Whether glasses are needed\" is a fact that can be determined by observation and testing without the patient ' s subjective judgement or perception. The question is therefore considered from an objective perspective."}, "sentence_sentiment": {"answer": "Neutral", "rationale": "Because the entry is merely an objective fact and does not address emotional attitudes or behavioural tendencies."}, "language_style": {"answer": "Calm and direct", "rationale": "The language style of the entry is more direct, there is no excessive rhetorical or emotional rendering, but simply asks the interviewee whether he needs glasses to look far away, and it is more objective in tone."}, "time_dimension": {"answer": "Present", "rationale": "The entry asks the testee whether it is necessary to wear glasses when looking at a distance, and the time dimension is now judged."}, "sentence_type": "Interrogative sentence", "pos": {"nouns": {"you": 1, "Far away": 1, "Glasses": 1}, "verbs": {"Look": 1, "Dai": 1, "Yes": 1}, "numerals": {"2锛?: 1}}}, {"content": "Do you often have a temporal period of immediate blackness (declining vision or seeing nothing)?", "word_count": 27, "label": "", "sentence_structure": "Subject鈥搗erb鈥搊bject", "subjective_objective": {"answer": "Objective", "rationale": "Because \"blacking\" is the actual experience and feeling of the patient, its occurrence does not depend entirely on the patient's subjective decision and is therefore an objective one."}, "sentence_sentiment": {"answer": "Negative", "rationale": "Because the description \"A momentary immediate blacking (defunct or invisible)\" in the entry is negative."}, "language_style": {"answer": "Tactful and warm", "rationale": ""}, "time_dimension": {"answer": "Present", "rationale": "So the answer should be: \"Now.\""}, "sentence_type": "Interrogative sentence", "pos": {"nouns": {"Questionnaire": 1, "Entry": 1, "phenomena": 1, "The sight is down": 1, "I can't see anything": 1}, "verbs": {"have": 1, "Dark": 1, "Down": 1, "Yeah": 1, "Regular": 1, "Come on": 1}, "adjectives": {"Time-time": 1}, "numerals": {"3": 1}, "classifiers": {"individual": 1}}}, {"content": "Have you often blinked and cried?", "word_count": 12, "label": "", "sentence_structure": "Subject鈥搗erb鈥搊bject", "subjective_objective": {"answer": "Objective", "rationale": "The basis for the judgement: frequent blinking and tearing are physical symptoms or reactions that are objective for the patient. While these reactions may be influenced by psychological factors such as the patient鈥檚 emotional and anxiety, they are themselves still objective physiological phenomena."}, "sentence_sentiment": {"answer": "Negative", "rationale": "Because frequent blinking and tearing are often a manifestation of physical discomfort and have negative emotional connotations."}, "language_style": {"answer": "Calm and direct", "rationale": "The entry directly asked the respondents whether there were frequent blinks and tears, which were more objective and direct, were not overly emotionally pedigrees or suggestions and were consistent with the calm and direct language style."}, "time_dimension": {"answer": "Present", "rationale": "The entry asks the testee about the current behaviour or symptoms, \"Is there\" a form of a now-completed state of affairs, which means that it has been or will continue to be an act for some time. Therefore, the temporal dimension is now judged."}, "sentence_type": "Interrogative sentence", "pos": {"nouns": {"Questionnaire Entry": 1, "Blink": 1, "Tears": 1}, "verbs": {"have": 1, "Frequent": 1, "Analysis": 1}, "numerals": {"4锛?: 1}, "pronouns": {"you": 1}}}, {"content": "Do your eyes often hurt?", "word_count": 10, "label": "", "sentence_structure": "Subject鈥搗erb鈥搊bject", "subjective_objective": {"answer": "Objective", "rationale": "The entry is based on an objective, not subjective, perception or attitude. The entry is therefore subject to an objective examination."}, "sentence_sentiment": {"answer": "Negative", "rationale": "Because the \"eyes often hurt\" behavior in the entries is negative."}, "language_style": {"answer": "Calm and direct", "rationale": "The entry is dialogueable in a direct question-and-answer manner, without excessive rhetorical or emotional rendering, and is more objective in tone."}, "time_dimension": {"answer": "Present", "rationale": ""}, "sentence_type": "Interrogative sentence", "pos": {"nouns": {"Eyes": 1, "Ouch": 1}, "verbs": {"Regular": 1, "be": 1, "have": 1, "Ouch": 1}, "adverbs": {"very": 1}}}, {"content": "5 'Does your eyes often look at the blurry?", "word_count": 15, "label": "", "sentence_structure": "Subject鈥搗erb鈥搊bject", "subjective_objective": {"answer": "Subjective", "rationale": "Because the vague eye is a subjective feeling and experience of a human being, not an objective fact or phenomenon."}, "sentence_sentiment": {"answer": "Negative", "rationale": "Because the description of \"eyes often blur\" in the entries is negative."}, "language_style": {"answer": "Tactful and warm", "rationale": "The basis for the determination: the entry is more personal and interactive by means of a conversation by means of inquiry rather than by direct statements or orders. At the same time, the words \"always or often\" also appear to be more moderate and avoid pressure or discomfort on the interviewees."}, "time_dimension": {"answer": "Present", "rationale": "The keyword \"Is the eye often looking at the blurry of the object\" suggests that the question is to ask the subject whether it exists at the present time (i.e. during the investigation) and therefore to judge it now."}, "sentence_type": "Interrogative sentence", "pos": {"nouns": {"Eyes": 2, "phenomena": 1, "Objects": 1}, "verbs": {"Look": 1, "Blur": 1, "Is it possible": 1}, "adverbs": {"Regular": 1}, "numerals": {"5鈥?: 1}, "pronouns": {"you": 1}}}, {"content": "6. Do your eyes often get red or inflammation?", "word_count": 13, "label": "", "sentence_structure": "Subject鈥揷opula鈥損redicative", "subjective_objective": {"answer": "Objective", "rationale": "Because the fact that the eyes are often red or inflammated is an objective fact of the patient ' s state of health."}, "sentence_sentiment": {"answer": "Negative", "rationale": "Because \"red or inflammation\" is a health problem, it is often considered a negative situation."}, "language_style": {"answer": "Calm and direct", "rationale": "The entry appears in the form of a direct, concise question, without too much embellishment or emotional addition, and is more in line with the style of objective factual description."}, "time_dimension": {"answer": "Present", "rationale": "The entry asks the current state of the subjects, i.e. whether their eyes are often marked with red or inflammatory symptoms and therefore are in the current state."}, "sentence_type": "Interrogative sentence", "pos": {"nouns": {"Eyes": 2, "Inflammation": 1}, "verbs": {"Come on": 2, "Is it possible": 1, "Red": 1, "Inflammation": 1}, "adverbs": {"Regular": 1}, "numerals": {"6锛?: 1}}}, {"content": "Do you have a hearing back?", "word_count": 8, "label": "", "sentence_structure": "Subject鈥損redicate", "subjective_objective": {"answer": "Objective", "rationale": "Because \"heart\" and \"hearing poor\" are objective conditions for the patient's body, there is no need to consider the patient's subjective feelings."}, "sentence_sentiment": {"answer": "Negative", "rationale": "Because the entry describes a physical defect, it is usually considered negative."}, "language_style": {"answer": "Calm and direct", "rationale": "The language style of the article is calm and straightforward. It directly asks the respondents whether they have hearing problems, without any qualifier or emotional colour, and is a very concise way of presenting questions, reflecting the objectivity and scientific nature of the questionnaire study."}, "time_dimension": {"answer": "Present", "rationale": "The word \"No\" in \"are you\" indicates the negative, the current state, the \"hearing back\" refers to the current situation of the person being tested, and the entry is therefore an inquiry into the person's current behaviour and state."}, "sentence_type": "Interrogative sentence", "pos": {"nouns": {"Head back": 1, "Hearing": 1, "Bad": 1}, "verbs": {"be": 1, "have": 1}, "numerals": {"7": 1}}}, {"content": "Have you ever had ear inflammation, an ear attack?", "word_count": 12, "label": "", "sentence_structure": "Subject鈥搗erb鈥搊bject", "subjective_objective": {"answer": "Objective", "rationale": "Because \"Wasn't there an ear infection, an ear attack\" is an objective fact that patients have experienced."}, "sentence_sentiment": {"answer": "Negative", "rationale": "Because \"hear inflammation, an ear puss\" is a description of a physical discomfort or disease, with a certain negative emotional color."}, "language_style": {"answer": "Calm and direct", "rationale": "The entry is based on a direct, concise approach, which is not overly qualifier or emotional, and which is more objective and cold."}, "time_dimension": {"answer": "Past", "rationale": "The entry asks if the person tested had experienced an ear inflammation or an ear attack, and the temporal dimension is a past."}, "sentence_type": "Interrogative sentence", "pos": {"nouns": {"I'm getting ear disease": 1, "Ears": 2, "Puss": 1}, "verbs": {"have": 1, "Yes": 1, "Stream": 1}, "numerals": {"8": 1}}}, {"content": "Do you hear often?", "word_count": 22, "label": "", "sentence_structure": "Subject鈥搗erb鈥搊bject", "subjective_objective": {"answer": "Objective", "rationale": "Because the sound is a hearing that the patient can sense, and it's an objective fact."}, "sentence_sentiment": {"answer": "Negative", "rationale": "Because the \"heart ringing\" is a negative sign in the entry."}, "language_style": {"answer": "Tactful and warm", "rationale": "This is a more caring expression, which is more likely to enjoy the trust and cooperation of the person surveyed."}, "time_dimension": {"answer": "Present", "rationale": "The words \"always\" in \"always\" indicate a high frequency of behavior, while the words \"hearing\" and \"self-conscious sounds\" and \"impact hearing\" point to the current state or experience. The entry is therefore an inquiry into the present behaviour of the subject, based on the time dimension."}, "sentence_type": "Interrogative sentence", "pos": {"nouns": {"# And the ears #": 1, "Sound": 1, "Hearing": 1}, "verbs": {"Is it possible": 1, "Regular": 1, "In your ear": 1, "Self-conscious": 1, "have": 1, "Impact": 1}, "adverbs": {"Various": 1}, "numerals": {"9锛?: 1}}}, {"content": "Do you often have to cough to clear your throat?", "word_count": 14, "label": "", "sentence_structure": "Subject鈥搗erb鈥搊bject", "subjective_objective": {"answer": "Subjective", "rationale": "Because words like \"often\" and \"must\" imply that it is the patient's own feelings and experiences, not the objective facts."}, "sentence_sentiment": {"answer": "Negative", "rationale": "Because the act of \"must clean up your voice\" in the entry is uncomfortable, it implies that the health may be bad and therefore has a negative emotional color."}, "language_style": {"answer": "Calm and direct", "rationale": "The entry directly asked whether the respondents were often required to cough to clean their throats, without excessive rhetoric and emotional rendering, and with a more objective tone."}, "time_dimension": {"answer": "Present", "rationale": "The word \"always\" in \"you\" means regular, customary behaviour, and \"must\" implies a situation that requires a sustained response, so the entry is about the present."}, "sentence_type": "Interrogative sentence", "pos": {"nouns": {"Questionnaire Entry": 1, "Clear your voice": 1, "Cough": 1}, "verbs": {"Often": 1, "No way": 1, "Yes": 1, "Cough": 1}, "adverbs": {"very": 1}, "numerals": {"10锛?: 1}}}, {"content": "Do you often feel like you're having a loud voice?", "word_count": 20, "label": "", "sentence_structure": "Subject鈥搗erb鈥搊bject", "subjective_objective": {"answer": "Objective", "rationale": "This is an objective perspective, as it is a biological experience of the patient and can be observed and measured."}, "sentence_sentiment": {"answer": "Negative", "rationale": "Because \"sniffy\" is a negative experience of physical discomfort."}, "language_style": {"answer": "Calm and direct", "rationale": "The entry is a direct, concise query about the perceived speech congestion of the person under investigation, and is not overly refined or emotionally expressed, and is more calm and objective."}, "time_dimension": {"answer": "Past", "rationale": "The entry asks the testees about their past experiences and feelings, and the keyword \"often\" and \"sweeting\" points to the past."}, "sentence_type": "Interrogative sentence", "pos": {"nouns": {"you": 1, "- The voice": 1, "Flow": 1, "Feel": 2, "Throat": 1, "Something": 1}, "verbs": {"- I do": 1, "have": 1}, "adverbs": {"very": 1}, "numerals": {"11": 1}}}, {"content": "Do you sneeze often?", "word_count": 9, "label": "", "sentence_structure": "Subject鈥損redicate", "subjective_objective": {"answer": "Objective", "rationale": "Since sneezes are a physical phenomenon and are not determined by the patient ' s subjective will, the entry is considered from an objective perspective."}, "sentence_sentiment": {"answer": "Neutral", "rationale": "Because the entry does not have any emotional color."}, "language_style": {"answer": "Calm and direct", "rationale": "The language style of the entry is more straightforward, with no excessive emotional accoutrements or rhetorical techniques, but rather a simple question as to whether the other party often sneezes continuously, in a concise and straightforward language."}, "time_dimension": {"answer": "Present", "rationale": ""}, "sentence_type": "Interrogative sentence", "pos": {"nouns": {"Sneeze": 1, "Continuous": 1}, "verbs": {"Fight": 1, "Regular": 1}, "adverbs": {"- You're not": 1}, "numerals": {"12": 1}, "classifiers": {"individual": 1}}}, {"content": "Do you think your nose is always blocked?", "word_count": 10, "label": "", "sentence_structure": "Subject鈥損redicate", "subjective_objective": {"answer": "Objective", "rationale": "Because it is a sense experience that the nose is always blocked, and the perception experience is a subjective one of the patients. But the question is whether the patient has such a perception experience, so the examination is an objective one, given the perception and perception of his or her condition."}, "sentence_sentiment": {"answer": "Negative", "rationale": "Because the symptoms of \"nose is always blocked\" in the entry are often uncomfortable, they can be considered as a negative emotion or experience."}, "language_style": {"answer": "Calm and direct", "rationale": "The entry is in the form of a direct, question-and-answer session, which is more direct and concise in language, asking the respondents whether they have a nose plug."}, "time_dimension": {"answer": "Past", "rationale": ""}, "sentence_type": "Interrogative sentence", "pos": {"nouns": {"Questionnaire": 1, "Entry": 1, "Nose": 1}, "verbs": {"I think": 1, "- Yeah": 1}, "numerals": {"13": 1}}}, {"content": "Do you often flut?", "word_count": 7, "label": "", "sentence_structure": "Subject鈥損redicate", "subjective_objective": {"answer": "Objective", "rationale": "Because the fact that snot is running is an objective fact of the patient ' s state of health."}, "sentence_sentiment": {"answer": "Neutral", "rationale": "Because the entry is merely a question of fact and does not contain emotional colours."}, "language_style": {"answer": "Calm and direct", "rationale": "This language style applies to, for example, questionnaires and other places, and helps to collect accurate data and information."}, "time_dimension": {"answer": "Present", "rationale": "\"Recurring noseslides\" represent the present customary behaviour or state of the person being tested, so the temporal dimension is now judged."}, "sentence_type": "Interrogative sentence", "pos": {"nouns": {"Nasal slugs": 1, "Problem": 1}, "verbs": {"Regular": 1, "Stream": 1}, "numerals": {"14": 1}}}, {"content": "Have you ever had a bad nosebleed?", "word_count": 12, "label": "", "sentence_structure": "Subject鈥搗erb鈥搊bject", "subjective_objective": {"answer": "Objective", "rationale": "Because \"sniffed\" is an observable physiological phenomenon, whether or not the patient has bled and the extent of the haemorrhage is an objective fact."}, "sentence_sentiment": {"answer": "Negative", "rationale": "Because the \"sniffed very bad\" is negative."}, "language_style": {"answer": "Calm and direct", "rationale": "The entry is based on a simple and direct language style, which provides an open question to the interviewees about nose bleeding, not over-modified or emotionalized expressions, and is more objective and practical."}, "time_dimension": {"answer": "Present", "rationale": "The keyword \"if there is a time\" and \"sniffed very well\" are descriptions of the state of the moment, so the judgment is now."}, "sentence_type": "Interrogative sentence", "pos": {"nouns": {"Nose": 1, "Hemorrhaging": 1, "That's great": 1}, "verbs": {"have": 1, "Time": 1, "be": 1}, "adjectives": {"very": 1}, "numerals": {"15": 1}}}, {"content": "Do you often get cold?", "word_count": 9, "label": "", "sentence_structure": "Subject鈥損redicate", "subjective_objective": {"answer": "Objective", "rationale": "The disease is a fact that people experience."}, "sentence_sentiment": {"answer": "Negative", "rationale": "Because the \"heavy flu\" act is negative, indicating poor health."}, "language_style": {"answer": "Calm and direct", "rationale": "The entry asks the other party in a direct and clear manner whether there is a frequent cold, and it is not clear that it involves too many emotions or rhetorical techniques."}, "time_dimension": {"answer": "Past", "rationale": "The entry \"often\" indicates that this is a customary act, usually a situation that has occurred frequently over time. The question is about the present state of affairs, so the act can be considered as past, present or future. But since it is expressed as a customary act, it is more likely to be considered as past or present."}, "sentence_type": "Interrogative sentence", "pos": {"nouns": {"Cold": 1, "I'm having a bad cold": 1}, "verbs": {"Okay": 1}, "adjectives": {"Regular": 1}}}, {"content": "Do you always have a sore throat and a swollen flat?", "word_count": 13, "label": "", "sentence_structure": "Subject鈥搗erb鈥搊bject", "subjective_objective": {"answer": "Objective", "rationale": "Because of the frequent pain in the throat, the swelling of the tonsils is an objective fact."}, "sentence_sentiment": {"answer": "Negative", "rationale": "Because \"throat pain\" and \"tangular swelling\" are symptoms of physical discomfort and are generally considered negative."}, "language_style": {"answer": "Tactful and warm", "rationale": ""}, "time_dimension": {"answer": "Present", "rationale": ""}, "sentence_type": "Interrogative sentence", "pos": {"nouns": {"My throat hurts": 1, "tonsils": 1, "Big": 1}, "verbs": {"Regular": 1, "Ouch": 1, "Swollen": 1}, "adverbs": {"Is it possible": 1}, "numerals": {"16'": 1}}}, {"content": "17. Do you often have severe bronchitis (coughing during cold, long periods of vomiting)?", "word_count": 28, "label": "", "sentence_structure": "Subject鈥搗erb鈥搊bject", "subjective_objective": {"answer": "Objective", "rationale": "Because the presence of severe chronic bronchitis is the patient ' s objective state of health."}, "sentence_sentiment": {"answer": "Negative", "rationale": "Because \"serious chronic bronchitis\" described in the entry is a disease that is usually considered to be negative and unhealthy."}, "language_style": {"answer": "Calm and direct", "rationale": "The language style of the entry is calm. It asks the interviewee whether he has serious chronic bronchitis symptoms in an objective and direct way, without using excessive emotional accommodations or euphemisms, and therefore appears to be more calm and calm. This expression allows the interviewee to understand the meaning of the problem clearly, and helps the survey to be accurate and effective."}, "time_dimension": {"answer": "Past", "rationale": ""}, "sentence_type": "Interrogative sentence", "pos": {"nouns": {"Chronic bronchitis": 1, "Cold": 1, "Cough": 1, "Boom": 1, "Time": 1}, "verbs": {"have": 1, "be": 1, "Regular": 1, "Ahem": 1, "Spit": 1, "Drag": 1, "Long time": 1}, "adverbs": {"Serious": 1}, "numerals": {"17": 1}, "classifiers": {"individual": 1}, "pronouns": {"you": 1}}}, {"content": "18. Do you always have to sleep when you catch a cold?", "word_count": 14, "label": "", "sentence_structure": "Subject鈥搗erb鈥搊bject", "subjective_objective": {"answer": "Subjective", "rationale": "The question is whether the patient must sleep in the bed when he or she has a cold. The entry is therefore subject to subjective examination."}, "sentence_sentiment": {"answer": "Negative", "rationale": "Because the \"must bed\" act is negative."}, "language_style": {"answer": "Calm and direct", "rationale": "The language style of the entry is of a calm and straightforward type, with simple questions and no excessive emotional rendering or rhetorical techniques designed to obtain factual information."}, "time_dimension": {"answer": "Past", "rationale": "The entry asks the testee whether he or she needs bed rest during a cold, and therefore is a matter of the time dimension of the past."}, "sentence_type": "Interrogative sentence", "pos": {"nouns": {"Cold": 1, "The bed": 1}, "verbs": {"I have to": 1, "Get down": 1}, "adjectives": {"Total": 1, "you": 1}, "numerals": {"18": 1, "in": 1}, "classifiers": {"Every time": 1}, "pronouns": {"you": 1}}}, {"content": "18 'You spit a lot?", "word_count": 7, "label": "", "sentence_structure": "Subject鈥搗erb鈥搊bject", "subjective_objective": {"answer": "Subjective", "rationale": "The issue is a subjective one, as it relates to personal habits and attitudes."}, "sentence_sentiment": {"answer": "Negative", "rationale": "Because it describes an uncivil, unhealthy act, with negative emotional aspects."}, "language_style": {"answer": "Calm and direct", "rationale": ""}, "time_dimension": {"answer": "Present", "rationale": "The entry asks about the person's current behaviour or habits, and the keyword \"frequentness\" indicates that the issue is related to the current situation. Moreover, \"Are you\" also indicates that this is a current-day question, highlighting concerns about the current situation. Thus, the temporal dimension of the article can be judged to be the present."}, "sentence_type": "Interrogative sentence", "pos": {"nouns": {"18": 1, "you": 1, "Regular": 1, "Boom": 1}, "verbs": {"Is it possible": 1, "Spit": 1}, "numerals": {"18": 1}, "pronouns": {"you": 1}}}, {"content": "Is it often the cold that makes you feel bad all winter?", "word_count": 15, "label": "", "sentence_structure": "Subject鈥搗erb鈥搊bject", "subjective_objective": {"answer": "Subjective", "rationale": "Because \"worship\" is the patient's own feelings and experiences, it is subjectively viewed."}, "sentence_sentiment": {"answer": "Negative", "rationale": "Because the description of \"difficult\" in the entries is negative, indicating that the cold has had a negative impact on the lives of the interviewees."}, "language_style": {"answer": "Tactful and warm", "rationale": "The entry is more euphemistic, more nuanced, and uses a question-and-answer format to guide respondents to answer questions, and uses a few more emotional terms, \"hard\" and \"winter\" to increase kinship and appeal, making respondents more willing to answer questions. Nor is the entry used in too direct or hard language to avoid stress and discomfort."}, "time_dimension": {"answer": "Present", "rationale": "The keyword \"always\" and \"hard\" indicates that it is an inquiry into the current condition of the person being tested, and therefore the temporal dimension is now judged."}, "sentence_type": "Interrogative sentence", "pos": {"nouns": {"Cold": 2, "Winter": 1}, "verbs": {"鈾?And I'm gonna be 鈾?: 1, "It's hard": 1}, "adverbs": {"Regular": 1, "Both": 1}, "numerals": {"19": 1}}}, {"content": "Do you have allergies? (Asthmamatics induced by certain allergies, such as pollen)", "word_count": 26, "label": "", "sentence_structure": "Subject鈥搗erb鈥搊bject", "subjective_objective": {"answer": "Objective", "rationale": "Because the physical condition of an allergic asthma is objective."}, "sentence_sentiment": {"answer": "Neutral", "rationale": "The entry is merely an inquiry into whether the person interviewed suffers from a disease and does not express any emotional sentiments."}, "language_style": {"answer": "Calm and direct", "rationale": "The entry is a medical issue and requires a clear and direct inquiry into whether the person interviewed has a particular disease."}, "time_dimension": {"answer": "Past", "rationale": "The entry asks if the person tested had had a sensitive asthma experience, i.e., a disease that had been experienced at a point or time in the past."}, "sentence_type": "Interrogative sentence", "pos": {"nouns": {"Allergie asthma": 1, "Allergies": 1, "pollen": 1, "Incentives": 1, "Asthma": 1}, "verbs": {"have": 1, "Result": 1, "Yes": 2, "Initiating": 1}, "adjectives": {"Allergies": 1, "Some": 1}, "numerals": {"20": 1}, "pronouns": {"you": 1}}}, {"content": "Do you have asthma?", "word_count": 24, "label": "", "sentence_structure": "Subject鈥搗erb鈥搊bject", "subjective_objective": {"answer": "Objective", "rationale": "Because asthma is a disease, it is an objective fact that it is only subjectively perceived and felt by the patient."}, "sentence_sentiment": {"answer": "Neutral", "rationale": "Because the entry does not have any emotional color."}, "language_style": {"answer": "Calm and direct", "rationale": "The entry is more objective, direct, and does not have too many rhetorical or emotional features. It is simple to ask the person surveyed whether he or she suffers from asthma and describes the main symptoms."}, "time_dimension": {"answer": "Present", "rationale": "The entry is for the current state of the person being tested and the time dimension is now judged."}, "sentence_type": "Interrogative sentence", "pos": {"nouns": {"Asthma": 1, "Repeatedly": 1, "鈾?And the attacks 鈾?: 1, "Temporary": 1, "With him": 1, "Gas": 1, "I can't breathe": 1}, "verbs": {"have": 1}, "adjectives": {"Asthma": 1, "Repeatedly": 1, "Temporary": 1}, "numerals": {"21": 1}}}, {"content": "Are you often troubled by coughing?", "word_count": 13, "label": "", "sentence_structure": "Subject鈥搗erb鈥搊bject", "subjective_objective": {"answer": "Subjective", "rationale": "Because it's the patient's subjective feelings and emotional reactions that are being examined."}, "sentence_sentiment": {"answer": "Negative", "rationale": "Because the words \"cough\" and \"fear\" in the entries are negative."}, "language_style": {"answer": "Tactful and warm", "rationale": ""}, "time_dimension": {"answer": "Past", "rationale": "The entry asks if the testee has a behaviour or mentality that is often troubled by coughing, and the temporal dimension is judged to be past."}, "sentence_type": "Interrogative sentence", "pos": {"nouns": {"Cough": 1, "Trouble": 1}, "verbs": {"Is it possible": 1, "I feel": 1, "Regular": 1, "Because": 1, "And": 1, "I think": 1, "Trouble": 2}, "adverbs": {"Regular": 1}, "numerals": {"22锛?: 1}}}, {"content": "Have you ever coughed blood?", "word_count": 7, "label": "", "sentence_structure": "Subject鈥損redicate", "subjective_objective": {"answer": "Objective", "rationale": "Because the fact that the cough blood is in fact a fact of the patient ' s health."}, "sentence_sentiment": {"answer": "Negative", "rationale": "Because coughing blood is a disease, it's negative."}, "language_style": {"answer": "Calm and direct", "rationale": "The entry is in a concise language, asking the interviewees whether they have ever had coughing symptoms, without too many rhetorical or emotional features, and with a more calm and straightforward language style."}, "time_dimension": {"answer": "Past", "rationale": "The entry is for the testee's past health, the keyword is \"had had\" and \"cough blood\", pointing to what happened in the past, and therefore judged \"had.\""}, "sentence_type": "Interrogative sentence", "pos": {"nouns": {"Cough blood": 1}, "verbs": {"Yes": 1, "have": 1}, "numerals": {"23": 1}}}, {"content": "Do you have a heavy sweat?", "word_count": 17, "label": "", "sentence_structure": "Subject鈥搗erb鈥搊bject", "subjective_objective": {"answer": "Objective", "rationale": "Because sweat theft is a symptom or a physical phenomenon, it is not subjective or attitudinal."}, "sentence_sentiment": {"answer": "Negative", "rationale": "Because sweat theft is usually a symptom of disease, it can have a negative impact on health."}, "language_style": {"answer": "Calm and direct", "rationale": "The entry asks the interviewees in a direct manner whether there is a more serious hazing, does not use excessive rhetorical techniques or emotional rendering, and is more objective and incriminating."}, "time_dimension": {"answer": "Past", "rationale": "The entry asks if the testee had had problems with sweat theft in the past and therefore judged it to be the past."}, "sentence_type": "Interrogative sentence", "pos": {"nouns": {"- The sweat": 1, "In bed": 1, "Wake up": 1}, "verbs": {"have": 1}, "adjectives": {"Heavy": 1}, "numerals": {"24锛?: 1}}}, {"content": "Have you ever suffered from chronic respiratory diseases other than tuberculosis (chronic bronchitis, bronchial expansion, pulmonary abscess)?", "word_count": 30, "label": "", "sentence_structure": "Subject鈥搗erb鈥搊bject", "subjective_objective": {"answer": "Objective", "rationale": "This is an objective fact, as it is the patient ' s past state of health."}, "sentence_sentiment": {"answer": "Neutral", "rationale": "The entry merely asked if the interviewees had suffered from some chronic respiratory disease and did not contain any emotional colour."}, "language_style": {"answer": "Calm and direct", "rationale": ""}, "time_dimension": {"answer": "Past", "rationale": "The entry is for the purpose of asking the person who was tested for a past experience of chronic respiratory disease, and therefore the temporal dimension is a past."}, "sentence_type": "Interrogative sentence", "pos": {"nouns": {"Nodules": 1, "Chronic respiratory diseases": 1, "Chronic bronchitis": 1, "Bneumatic extension": 1, "Pulmonary abscess": 1}, "verbs": {"Yes, I did": 1, "Is it possible": 1}, "numerals": {"25": 1}}}, {"content": "25' do you have a low fever (37-38 degrees)?", "word_count": 8, "label": "", "sentence_structure": "Subject鈥搗erb鈥搊bject", "subjective_objective": {"answer": "Subjective", "rationale": "Because the problem involves patients ' subjective feelings, i.e. whether they feel they have low fever."}, "sentence_sentiment": {"answer": "Neutral", "rationale": "Because the entry does not have any emotional color."}, "language_style": {"answer": "Calm and direct", "rationale": "The entry asks the interviewees in an objective and direct manner whether the person is suffering from low fever, is not used in too many emotional or rhetorical ways, and is calm and clear."}, "time_dimension": {"answer": "Present", "rationale": "The keyword \"yes\" and \"no\" indicates that this is a question of the current state of affairs, that is, of asking the person to answer whether they are currently suffering from low fever. At the same time, the \"37-38 degrees\" also indicates the extent or extent of the disease, further confirming that it is a question of the present situation."}, "sentence_type": "Interrogative sentence", "pos": {"nouns": {"Questionnaire": 1, "Entry": 1, "Low fever": 1, "Hot": 1, "Temperature": 1, "degrees": 2}, "verbs": {"have": 1, "Heat": 1}, "adjectives": {"37鈥?8": 1}, "numerals": {"25鈥?: 1, "37": 1, "38": 1, "degrees": 2}, "classifiers": {"individual": 1, "鈥?: 1}}}, {"content": "Have you ever had tuberculosis?", "word_count": 8, "label": "", "sentence_structure": "Subject鈥損redicate", "subjective_objective": {"answer": "Objective", "rationale": "The first time I was a patient, I was a patient, and I was a patient, because I had the objective fact that tuberculosis was a patient."}, "sentence_sentiment": {"answer": "Negative", "rationale": "Basis of judgement: The entry relates to the health of the individual and may give rise to a negative emotional reaction and is therefore classified as negative."}, "language_style": {"answer": "Calm and direct", "rationale": "The entry asks the interviewees whether they have ever had tuberculosis in a direct and concise manner, without any euphemism or rhetorical language, and the language style is relatively calm and straightforward."}, "time_dimension": {"answer": "Past", "rationale": ""}, "sentence_type": "Interrogative sentence", "pos": {"nouns": {"Tuberculosis": 1}, "verbs": {"Yes": 1}, "numerals": {"26": 1}}}, {"content": "Have you lived with people who have tuberculosis?", "word_count": 14, "label": "", "sentence_structure": "Subject鈥損redicate", "subjective_objective": {"answer": "Objective", "rationale": "Because it is an objective fact to live with people who have tuberculosis."}, "sentence_sentiment": {"answer": "Neutral", "rationale": "The entry does not express positive or negative feelings or attitudes."}, "language_style": {"answer": "Tactful and warm", "rationale": ""}, "time_dimension": {"answer": "Past", "rationale": "The entry asks whether the person tested had lived with a person who received tuberculosis in the past and therefore judged it \"the past\"."}, "sentence_type": "Interrogative sentence", "pos": {"nouns": {"Tuberculosis": 1, "person": 1, "Together": 1, "I've lived here": 1}, "verbs": {"Okay": 1, "in": 1, "Stay": 1}, "adverbs": {"not": 1}, "numerals": {"27": 1}, "pronouns": {"you": 1}}}, {"content": "Did the doctor say you had high blood pressure?", "word_count": 10, "label": "", "sentence_structure": "Subject鈥搗erb鈥搊bject", "subjective_objective": {"answer": "Objective", "rationale": "Because \"Does the doctor say that blood pressure is high\" is an objective fact that patients have experienced."}, "sentence_sentiment": {"answer": "Negative", "rationale": "Because the \"high blood pressure\" in the entry is unhealthy and therefore has a negative emotional color."}, "language_style": {"answer": "Calm and direct", "rationale": "The entry is more directly linguistically, without excessive rhetorical or emotional colours, and is asked about a specific fact and is more objective in tone."}, "time_dimension": {"answer": "Past", "rationale": "The keyword is \"doctor said\" and it means what happened; and \"your blood pressure is high\" is a description of the person's state of health, which happened at the time of the past. So, the entry is an inquiry into past behavior or state."}, "sentence_type": "Interrogative sentence", "pos": {"nouns": {"Doctor": 1, "High blood pressure": 1}, "verbs": {"Say it": 1, "Pass": 1}, "adjectives": {"High": 1}, "numerals": {"28": 1}, "pronouns": {"you": 1}}}, {"content": "Did the doctor say your blood pressure was low?", "word_count": 10, "label": "", "sentence_structure": "Subject鈥搗erb鈥揷omplement", "subjective_objective": {"answer": "Objective", "rationale": "The question is whether the patient was informed by the doctor that his blood pressure was low, which is the doctor ' s description of the patient ' s examination and therefore is from an objective perspective."}, "sentence_sentiment": {"answer": "Neutral", "rationale": "As this entry is factual and does not involve feelings, attitudes or behaviour."}, "language_style": {"answer": "Calm and direct", "rationale": "The entry is presented in the form of a direct question, with no excessive qualifier or emotional expression, and is more objective and quiet, with the aim of obtaining the true situation of the person interviewed."}, "time_dimension": {"answer": "Past", "rationale": "According to the key words in the sentence that was entered, \"Doctor said\" and \"Does your blood pressure low?\" it can be seen that the entry is a matter of passing time in asking if the person tested had ever been diagnosed by a doctor as low blood pressure."}, "sentence_type": "Interrogative sentence", "pos": {"nouns": {"Doctor": 1, "Blood pressure": 1}, "verbs": {"Say it": 1, "Pass": 1, "Low": 1}, "adverbs": {"very": 1}, "numerals": {"29": 1}, "classifiers": {"individual": 1}, "pronouns": {"you": 1}}}, {"content": "You have chest or heart pain?", "word_count": 10, "label": "", "sentence_structure": "Subject鈥搗erb鈥搊bject", "subjective_objective": {"answer": "Objective", "rationale": "Since chest pain or heart pain is a physical condition of the patient, it is considered from an objective perspective."}, "sentence_sentiment": {"answer": "Negative", "rationale": "Because the \"breath or heart pain\" symptoms in the entry are negative."}, "language_style": {"answer": "Calm and direct", "rationale": "The entry uses a direct question-and-answer approach, without excessive qualifiers or emotional colours, and is calm and objective, with the aim of obtaining the actual situation of the person under investigation."}, "time_dimension": {"answer": "Past", "rationale": "The entry asks if the person tested has had or is suffering from chest or heart pain, and the temporal dimension is judged to be past and present."}, "sentence_type": "Interrogative sentence", "pos": {"nouns": {"Breasts": 1, "Department": 1, "Sector": 1, "Ouch": 2}, "verbs": {"have": 1, "Sensor": 1}, "adjectives": {"Heart": 1}, "numerals": {"30": 1}}}, {"content": "Do you often feel like you're moving too fast?", "word_count": 14, "label": "", "sentence_structure": "Subject鈥搗erb鈥搊bject", "subjective_objective": {"answer": "Subjective", "rationale": "Because a feeling of excess is a subjective feeling of a person, it can be judged by subjective perception."}, "sentence_sentiment": {"answer": "Negative", "rationale": "Because \"heart-motion\" is a pathological phenomenon, usually associated with negative emotional or health problems."}, "language_style": {"answer": "Calm and direct", "rationale": "The entry is a direct question-and-answer dialogue, without adding too much accommodative language or emotional colour and with a more objective tone."}, "time_dimension": {"answer": "Present", "rationale": "The entry asks the current state of the testees, i.e. whether they often feel their heart beat too fast, and therefore the time dimension is now judged."}, "sentence_type": "Interrogative sentence", "pos": {"nouns": {"Questionnaire Entry": 1, "Heart beat": 1, "Heart beats fast": 1}, "verbs": {"I feel": 1, "Speed": 1, "Too fast": 1}, "adjectives": {"Regular": 1, "You": 1}, "numerals": {"You": 1}}}, {"content": "32. Do you often have heart attacks?", "word_count": 18, "label": "", "sentence_structure": "Subject鈥損redicate", "subjective_objective": {"answer": "Subjective", "rationale": "Because \"always\" and \"the feeling of heart beating\" are subjective feelings of patients."}, "sentence_sentiment": {"answer": "Negative", "rationale": "Because the \"heartal\" symptoms in the entries are usually considered negative experiences."}, "language_style": {"answer": "Calm and direct", "rationale": ""}, "time_dimension": {"answer": "Present", "rationale": ""}, "sentence_type": "Interrogative sentence", "pos": {"nouns": {"Questionnaire": 1, "Entry": 1, "Heartbreak": 1, "Heart beat": 1, "Feel": 1}, "verbs": {"be": 1, "Regular": 1, "have": 1}, "adjectives": {"Calm down": 1}, "numerals": {"32": 1}}}, {"content": "\"32.\" Do you often feel the pulse stops?", "word_count": 12, "label": "", "sentence_structure": "Subject鈥搗erb鈥搊bject", "subjective_objective": {"answer": "Subjective", "rationale": "The feeling of a pulse stopping is a subjective feeling or experience. Although this feeling may be due to certain physical conditions, the perception of the patient is subjective."}, "sentence_sentiment": {"answer": "Negative", "rationale": "Because \"always feeling a pulse stops\" is a symptom of physical discomfort, which is often considered negative."}, "language_style": {"answer": "Calm and direct", "rationale": "The language style of the entry is calm, as it is straightforward, because it asks in a clear and concise manner, without too many words or emotional renderings. It asks whether the interviewee often feels a pulse stoppage, without any accommodative words or tone."}, "time_dimension": {"answer": "Present", "rationale": "According to the phrase \"whether\" and \"often feel\" the question is whether the person tested acted or felt in the current state and therefore was in the present state."}, "sentence_type": "Interrogative sentence", "pos": {"nouns": {"you": 1, "Pulse": 1, "Stop": 1}, "verbs": {"Is it possible": 1, "I feel": 1}, "numerals": {"32": 1}}}, {"content": "33. Do you often feel difficulty breathing?", "word_count": 11, "label": "", "sentence_structure": "Subject鈥損redicate", "subjective_objective": {"answer": "Subjective", "rationale": "Because \"respiration difficulties\" are subjective feelings and experiences of patients, they need to be obtained through self-reporting."}, "sentence_sentiment": {"answer": "Negative", "rationale": "Because the act of \"respiration difficulty\" in the entry is negative."}, "language_style": {"answer": "Calm and direct", "rationale": "The entry asked the respondents in brief and concise language whether they had respiratory difficulties, which were more objective and direct, and did not have too many emotional or speech skills."}, "time_dimension": {"answer": "Present", "rationale": "The entry asks about the current situation and feelings of the person being tested, and \"Is it often a breathing difficulty\" indicates that this is a current experience and can therefore be judged as the present."}, "sentence_type": "Interrogative sentence", "pos": {"nouns": {"Questionnaire": 1, "Entry": 1, "Breathe": 1, "Difficulties": 1}, "verbs": {"I feel": 1, "I can't breathe": 1, "be": 1}, "adverbs": {"Regular": 1}, "numerals": {"33": 1}}}, {"content": "Are you more likely to be short-spirited than anyone else?", "word_count": 17, "label": "", "sentence_structure": "Subject鈥搗erb鈥搊bject", "subjective_objective": {"answer": "Subjective", "rationale": "Because the \"relatively short\" feeling is the patient's subjective experience and judgment."}, "sentence_sentiment": {"answer": "Negative", "rationale": "Because \"vulnerability\" in the entry is a physical discomfort, which usually makes people uncomfortable or anxious."}, "language_style": {"answer": "Calm and direct", "rationale": "The language style of the entry is calm and straightforward. It asks the other side in an objective and concise manner whether there are short-tempered situations more often than others, and does not contain too many emotional colours or rhetorical techniques."}, "time_dimension": {"answer": "Present", "rationale": "\"Easier to happen\" implies that it is a state of present or a tendency, and \"short air\" and \"fast breath\" are also the symptoms or conditions of the present. So the temporal dimension of this entry is now judged."}, "sentence_type": "Interrogative sentence", "pos": {"nouns": {"Questionnaire Entry": 1, "person": 1, "Short air": 1, "I can't breathe": 1}, "verbs": {"Come on": 1, "- Yeah": 1, "Easy": 1}, "numerals": {"34": 1}}}, {"content": "Do you sometimes feel short when you're sitting?", "word_count": 19, "label": "", "sentence_structure": "Subject鈥損redicate", "subjective_objective": {"answer": "Objective", "rationale": "Because \"fright short\" is the objective fact of the patient's experience."}, "sentence_sentiment": {"answer": "Negative", "rationale": "Because \"fatal\" is a symptom of physical discomfort, it is usually considered negative."}, "language_style": {"answer": "Calm and direct", "rationale": "This expression is useful for obtaining accurate and accurate information."}, "time_dimension": {"answer": "Present", "rationale": "The \"sitting\" and \"sometimes feeling\" indicate that this is the current experience or state of the subject, so the temporal dimension is now."}, "sentence_type": "Interrogative sentence", "pos": {"nouns": {"Situation": 1, "Time": 1, "Short air": 1}, "verbs": {"Yes": 2, "Sensor": 1, "Sit down": 1, "Even if": 1}, "adverbs": {"Sometimes": 1, "Sitting down": 1}, "numerals": {"35": 1}}}, {"content": "Do you often have severe swellings in your lower limbs?", "word_count": 13, "label": "", "sentence_structure": "Subject鈥損redicate", "subjective_objective": {"answer": "Subjective", "rationale": "According to the entry, \"36. Do you often have a severe swelling of your lower limb?\" The subject's subjective feeling, i.e., whether the patient himself has a feeling of swelling of his lower limb. Thus, the view of the questionnaire is subjective. The basis of the assessment is that the subject's subjective feeling, i.e. whether the patient has a feeling of swelling of his lower limb, is not measured and determined by objective criteria, so the view of the questionnaire is subjective."}, "sentence_sentiment": {"answer": "Negative", "rationale": "Because \"serious swelling\" is a sign of discomfort and is usually considered negative."}, "language_style": {"answer": "Calm and direct", "rationale": "The language of this project is calm and straightforward, and it asks whether the subject is often suffering from severe hypothymic hypothyroids, without any rhetorical or emotional retrofitting, and in a more objective manner."}, "time_dimension": {"answer": "Present", "rationale": "The entry now asks the testee whether there is some symptoms at present, namely, \"Is there a severe swelling of the lower limb?\" The time dimension is therefore now judged."}, "sentence_type": "Interrogative sentence", "pos": {"nouns": {"you": 1, "Serious": 1, "Lower limbs": 1, "- Swollen": 1}, "verbs": {"have": 1, "Regular": 1}, "adjectives": {"Serious": 1}, "adverbs": {"Yes": 1, "Regular": 1}, "numerals": {"36": 1}}}, {"content": "Are you upset with cold hands and feet even in the heat?", "word_count": 16, "label": "", "sentence_structure": "Subject鈥損redicate", "subjective_objective": {"answer": "Subjective", "rationale": "The entry is viewed from the patient's subjective perspective. Because \"fool\" is the patient's own feeling and experience, it's subjective, not objective."}, "sentence_sentiment": {"answer": "Negative", "rationale": "Because the entry \"fears with cold hands and feet\" is negative."}, "language_style": {"answer": "Tactful and warm", "rationale": ""}, "time_dimension": {"answer": "Present", "rationale": ""}, "sentence_type": "Interrogative sentence", "pos": {"nouns": {"Summer": 1, "person": 1, "Hands and feet": 2, "Cool": 1, "Trouble": 1}, "verbs": {"Because": 1, "Let's go": 1, "Yeah": 1, "be": 1, "And": 1, "- You're not": 1}, "adjectives": {"Hot": 1}, "adverbs": {"Even if": 1, "Because": 1}, "numerals": {"37": 1}, "classifiers": {"锛?: 1}, "pronouns": {"you": 1}}}, {"content": "Do you have a lot of leg cramps?", "word_count": 8, "label": "", "sentence_structure": "Subject鈥損redicate", "subjective_objective": {"answer": "Objective", "rationale": "Because leg cramps are an objective fact of the patient's state of health."}, "sentence_sentiment": {"answer": "Negative", "rationale": "Because the \"legic cramp\" in the entry is negative."}, "language_style": {"answer": "Calm and direct", "rationale": "The entry uses a direct, concise language style to ask the respondents whether they often have leg cramps, do not use a rhetorical or emotional rendering, and is more balanced and objective."}, "time_dimension": {"answer": "Present", "rationale": "The keyword \"often\" indicates the frequency of the act, while \"legs' cramps\" is an action or state that usually occurs and ends at a certain point in time, and therefore the entry is a description of the person's current behaviour or condition."}, "sentence_type": "Interrogative sentence", "pos": {"nouns": {"Questionnaire": 1, "Entry": 1, "Legs": 1, "Twitches": 1}, "verbs": {"be": 1, "Yes": 1, "Regular": 1, "have": 1}, "adverbs": {"very": 1, "Not really": 1}, "numerals": {"38锛?: 1}}}, {"content": "Did the doctor say you had a heart condition?", "word_count": 11, "label": "", "sentence_structure": "Subject鈥搗erb鈥搊bject", "subjective_objective": {"answer": "Objective", "rationale": "Because \"doctor says\" and \"heart disease\" are patients' objective experiences, which can be verified by fact."}, "sentence_sentiment": {"answer": "Negative", "rationale": "Because the fact that the entry \"heart is sick\" is negative."}, "language_style": {"answer": "Calm and direct", "rationale": "The entry asks the other in a straightforward manner whether the doctor informed the other party that the heart was sick, that the tone was more objective and that there was no excessive adoration or emotional expression."}, "time_dimension": {"answer": "Past", "rationale": ""}, "sentence_type": "Interrogative sentence", "pos": {"nouns": {"Doctor": 1, "Heart": 1, "Diseases": 1, "Mistake": 1}, "verbs": {"Say it": 1, "have": 1}, "numerals": {"39": 1}}}, {"content": "Are there any heart attack members of your family?", "word_count": 12, "label": "", "sentence_structure": "Subject鈥搗erb鈥搊bject", "subjective_objective": {"answer": "Objective", "rationale": "Because the fact of a heart condition is an objective fact for the family member of the patient."}, "sentence_sentiment": {"answer": "Neutral", "rationale": "As the entry is only for questioning of the family of the interviewee, it does not cover the description of emotions, attitudes, behaviour, etc., and is therefore neutral."}, "language_style": {"answer": "Calm and direct", "rationale": ""}, "time_dimension": {"answer": "Past", "rationale": ""}, "sentence_type": "Interrogative sentence", "pos": {"nouns": {"Family": 1, "Diseases": 1, "person": 1, "Heart": 1}, "verbs": {"have": 1, "be": 1}, "numerals": {"40": 1}}}, {"content": "Have you removed more than half of your teeth?", "word_count": 14, "label": "", "sentence_structure": "Subject鈥搗erb鈥搊bject", "subjective_objective": {"answer": "Objective", "rationale": "Since more than half of the teeth are taken off is an objective condition of the patient, it is not necessary to examine subjective attitudes or ideas."}, "sentence_sentiment": {"answer": "Negative", "rationale": "Because the \"over half of the teeth\" in the entry is negative."}, "language_style": {"answer": "Calm and direct", "rationale": "This is an objective and less stylish language that applies to such occasions as survey-type questionnaires or medical-type questionnaires where specific information is needed."}, "time_dimension": {"answer": "Past", "rationale": "The entry asks the testee about his past teeth state, and therefore the temporal dimension is the past."}, "sentence_type": "Interrogative sentence", "pos": {"nouns": {"Teeth": 1, "Half": 1}, "verbs": {"Drop": 1}, "numerals": {"Forty-one": 1}}}, {"content": "Are you troubled by bleeding from the tooth?", "word_count": 13, "label": "", "sentence_structure": "Subject鈥搗erb鈥搊bject", "subjective_objective": {"answer": "Subjective", "rationale": "Because \"fear\" is a subjective feeling, it's about the emotional reaction and subjective experience of the patient."}, "sentence_sentiment": {"answer": "Negative", "rationale": "Because the emotional experience of \"blooding from teeth\" in the entries is negative."}, "language_style": {"answer": "Calm and direct", "rationale": "The entry asks the other party whether it is troubled by the bleeding of the teeth in a straightforward and clear manner, and is less serenuous and objective in terms of the language or emotional expressions that are too phrasing."}, "time_dimension": {"answer": "Present", "rationale": ""}, "sentence_type": "Interrogative sentence", "pos": {"nouns": {"Toothbite": 1, "Hemorrhaging": 1, "Trouble": 1}, "verbs": {"Because": 1, "Toothbone": 1, "Trouble": 1}, "numerals": {"42": 1}}}, {"content": "Do you often have severe toothaches?", "word_count": 11, "label": "", "sentence_structure": "Subject鈥損redicate", "subjective_objective": {"answer": "Objective", "rationale": "Because \"serious toothache\" is a symptom or sign that can be measured and can be determined by examination, it is an objective observation."}, "sentence_sentiment": {"answer": "Negative", "rationale": "Because \"serious toothache\" is a pain and a distress, a negative emotional experience."}, "language_style": {"answer": "Calm and direct", "rationale": "The entry directly asks the respondents whether there are serious toothaches, which are relatively objective, concise and do not use too many euphemisms or emotional colours, and therefore can be judged as \" calm and direct \" language styles."}, "time_dimension": {"answer": "Present", "rationale": "The entry \"often\" denotes a more frequent act, while the query uses the current state of affairs, indicating that the act is occurring or has occurred. The entry therefore asks the testee about the current situation, and the temporal dimension is now judged."}, "sentence_type": "Interrogative sentence", "pos": {"nouns": {"Toothache": 1}, "verbs": {"have": 1, "Serious": 1}, "adjectives": {"Often": 1}, "numerals": {"43": 1}}}, {"content": "Does your tongue always get thick?", "word_count": 10, "label": "", "sentence_structure": "Subject鈥損redicate", "subjective_objective": {"answer": "Objective", "rationale": "Because \"the moss are often thick\" is an objective phenomenon that can be determined by observing the patient's tongue, and not by the patient's subjective feelings."}, "sentence_sentiment": {"answer": "Neutral", "rationale": "Because the entry is only a question about the physical condition of the person under investigation, it is not clearly emotional."}, "language_style": {"answer": "Calm and direct", "rationale": ""}, "time_dimension": {"answer": "Present", "rationale": ""}, "sentence_type": "Interrogative sentence", "pos": {"nouns": {"Tequila": 1, "Thread": 1}, "verbs": {"be": 1, "Often": 1, "have": 1}, "adjectives": {"Thick": 1}, "adverbs": {"very": 1}, "numerals": {"44": 1, "Regular": 1}, "classifiers": {"individual": 1}}}, {"content": "Do you always have a bad appetite?", "word_count": 14, "label": "", "sentence_structure": "Subject鈥損redicate", "subjective_objective": {"answer": "Subjective", "rationale": "Because a good appetite is a subjective feeling and experience. Although physical condition may affect appetite, appetite is subjective in itself."}, "sentence_sentiment": {"answer": "Negative", "rationale": "Because the \"bad appetite\" state of the entry is negative."}, "language_style": {"answer": "Calm and direct", "rationale": "The entry is in a simple and clear language, asking the other side whether there is often an appetite for hunger, and there is no excessive adoration or emotional rendering. Thus, the language style can be described as \"coolness.\""}, "time_dimension": {"answer": "Present", "rationale": ""}, "sentence_type": "Interrogative sentence", "pos": {"nouns": {"A appetite": 1, "Something": 1}, "verbs": {"Is it possible": 2, "Always": 1, "Yes": 1, "Eat": 1}, "adverbs": {"not": 1, "Okay": 1}, "numerals": {"45": 1}}}, {"content": "Do you eat snacks often?", "word_count": 8, "label": "", "sentence_structure": "Subject鈥損redicate", "subjective_objective": {"answer": "Subjective", "rationale": "Because the frequent consumption of snacks is a subjective act and habit of the patient, it is not an objective fact."}, "sentence_sentiment": {"answer": "Neutral", "rationale": "Because the entry does not have any emotional color."}, "language_style": {"answer": "Calm and direct", "rationale": "The entry is in a concise language and asks the respondents whether they regularly eat snacks, without adding excessive emotional or rhetorical techniques and with a more objective tone."}, "time_dimension": {"answer": "Present", "rationale": "The entry is for the present behaviour of the testee, and the keyword \"always\" refers to the current frequency and state. Therefore, the time dimension is now judged."}, "sentence_type": "Interrogative sentence", "pos": {"nouns": {"Snacks": 1, "Questionnaire": 1, "Entry": 1}, "verbs": {"Regular": 1, "Eat": 1, "be": 1}, "adverbs": {"Is it possible": 1}, "numerals": {"46锛?: 1}}}, {"content": "Do you always swallow when you eat?", "word_count": 13, "label": "", "sentence_structure": "Subject鈥搗erb鈥搊bject", "subjective_objective": {"answer": "Subjective", "rationale": "Because \"Always\" and \"Low-Swallow\" are patients' own subjective feelings or behavioural habits, the entry looks at the patient's subjective angle."}, "sentence_sentiment": {"answer": "Negative", "rationale": "Because coyotes are a bad diet, they can cause bodily harm."}, "language_style": {"answer": "Calm and direct", "rationale": ""}, "time_dimension": {"answer": "Present", "rationale": ""}, "sentence_type": "Interrogative sentence", "pos": {"nouns": {"you": 1, "Something": 1, "Time": 2}, "verbs": {"be": 1, "Eat": 1, "I'm going to swallow it": 1}, "adverbs": {"Always": 1}, "numerals": {"47锛?: 1}}}, {"content": "Do you often get sick in the stomach?", "word_count": 10, "label": "", "sentence_structure": "Subject鈥搗erb鈥搊bject", "subjective_objective": {"answer": "Subjective", "rationale": "Because stomach discomfort is a subjective feeling and experience, although it may be influenced by biological factors, individual perceptions of such feelings are subjective."}, "sentence_sentiment": {"answer": "Neutral", "rationale": "The entry simply asked the respondents whether they were often in a bad stomach and not of a human emotional nature."}, "language_style": {"answer": "Tactful and warm", "rationale": "The entry is more personal and interactive when it is dialogueed in a query manner, rather than by direct statements or orders. Moreover, the more euphemistic wording \"attendant indisposed\" is used instead of a too direct description, reducing the discomfort that may be felt by the interviewees."}, "time_dimension": {"answer": "Present", "rationale": "The keyword \"often\" also indicates that the act is continuing."}, "sentence_type": "Interrogative sentence", "pos": {"nouns": {"Questionnaire": 1, "Entry": 1, "Stomach": 1, "I'm not well": 1}, "verbs": {"be": 1, "Not often": 1}, "adverbs": {"Yes": 1}, "numerals": {"48": 1}}}, {"content": "'Do you vomit when you're sick?", "word_count": 9, "label": "", "sentence_structure": "Subject鈥搗erb鈥搊bject", "subjective_objective": {"answer": "Objective", "rationale": "The questionnaire entry therefore examines the patient鈥檚 symptoms from an objective perspective."}, "sentence_sentiment": {"answer": "Negative", "rationale": "Because vomiting is a symptom of physical discomfort, it is usually considered negative."}, "language_style": {"answer": "Calm and direct", "rationale": "Such a design would allow the testee to answer the question more honestly and accurately, avoiding misunderstanding or misleadingness arising from ambiguity in language."}, "time_dimension": {"answer": "Present", "rationale": ""}, "sentence_type": "Interrogative sentence", "pos": {"nouns": {"Gross": 1, "- Vomit": 1}, "verbs": {"Time": 1, "Is there a problem": 1}, "adverbs": {"Sometimes": 1}, "numerals": {"48鈥?: 1}}}, {"content": "Do you often feel full after dinner?", "word_count": 17, "label": "", "sentence_structure": "Subject鈥搗erb鈥搊bject", "subjective_objective": {"answer": "Subjective", "rationale": "Because \"Does it feel like a full stomach after dinner?\" is the patient's subjective feeling, and it's a subjective view."}, "sentence_sentiment": {"answer": "Neutral", "rationale": "Because the entry describes physical symptoms and does not express positive or negative feelings."}, "language_style": {"answer": "Calm and direct", "rationale": "The entry is a direct question-and-answer dialogue, with no excessive adoration or emotional colour, and is more objective in tone."}, "time_dimension": {"answer": "Present", "rationale": "The entry asks the testee about his current state of health, so the time dimension is now judged."}, "sentence_type": "Interrogative sentence", "pos": {"nouns": {"you": 1, "After dinner": 1, "Full": 1, "The abdomen are swelling": 1}, "verbs": {"have": 1, "Feel": 1}, "adjectives": {"Regular": 1}, "numerals": {"49锛?: 1}}}, {"content": "Have you often burped after dinner?", "word_count": 10, "label": "", "sentence_structure": "Subject鈥搗erb鈥搊bject", "subjective_objective": {"answer": "Subjective", "rationale": "This is because the patient ' s observation and description of his or her situation involves personal feelings and experiences and is therefore subjective."}, "sentence_sentiment": {"answer": "Negative", "rationale": "Since hiccupation after meals is usually considered an uncivility, the expression of the purpose of the article is negative."}, "language_style": {"answer": "Calm and direct", "rationale": "The entry is in a concise language and asks the respondents whether they often hiccup after eating, without adding too many rhetorical or emotional features, and with a more direct and objective language style."}, "time_dimension": {"answer": "Present", "rationale": "The entry is an inquiry into the current behaviour of the testee, and the term \"often\" indicates that it is a question of a frequent occurrence, not of past experience or future intentions. Thus, the temporal dimension is now judged."}, "sentence_type": "Interrogative sentence", "pos": {"nouns": {"After dinner": 1, "Hiccup": 1}, "verbs": {"Is it possible": 1, "Regular": 1, "Fight": 1}, "adverbs": {"Regular": 1}, "numerals": {"50": 1}}}, {"content": "- You got a hot heart?", "word_count": 9, "label": "", "sentence_structure": "Subject鈥搗erb鈥搊bject", "subjective_objective": {"answer": "Objective", "rationale": "Because heartburn and acidic acid are symptoms of the body, not subjective feelings or attitudes of the patient."}, "sentence_sentiment": {"answer": "Negative", "rationale": "Because the symptoms in the entry are negative."}, "language_style": {"answer": "Calm and direct", "rationale": "The language style of the entry is more objective, straightforward, and there is no excessive rhetorical or emotional rendering, but rather a simple and clear question of whether the interviewee has symptoms of \"heated heart acid\". It is therefore possible to judge that the language style of the entry is straightforward and calm."}, "time_dimension": {"answer": "Present", "rationale": "The entry is for the current state of the person being tested, \"Do you have a hot heart sour water\", so the temporal dimension is now."}, "sentence_type": "Interrogative sentence", "pos": {"nouns": {"50鈥?: 1, "Heart burn": 1, "Acid": 1}, "verbs": {"have": 1}, "numerals": {"50鈥?: 1}}}, {"content": "51. Do you have stomach problems often?", "word_count": 8, "label": "", "sentence_structure": "Subject鈥搗erb鈥搊bject", "subjective_objective": {"answer": "Objective", "rationale": "Because stomach disease is an objective fact that patients may experience."}, "sentence_sentiment": {"answer": "Negative", "rationale": "Because \"recurring stomach disease\" is often associated with poor health and has a certain negative emotional impact."}, "language_style": {"answer": "Calm and direct", "rationale": "Thus, it can be judged that the entry is of a calm and straightforward language style."}, "time_dimension": {"answer": "Present", "rationale": "The word \"frequently\" in \"always having stomach problems\"? indicates frequency, while the word \"frequently\" uses the usual current state of affairs, which means that the act has not only occurred in the past but may still be ongoing. The entry therefore asks about the person being tested for his current behaviour or state, which is the current state."}, "sentence_type": "Interrogative sentence", "pos": {"nouns": {"Stomach disease": 1}, "verbs": {"Yes": 1, "Is it possible": 1, "Regular": 1}, "adverbs": {"Regular": 1}, "numerals": {"51. You": 1}}}, {"content": "Are you indigestion?", "word_count": 8, "label": "", "sentence_structure": "Subject鈥損redicate", "subjective_objective": {"answer": "Objective", "rationale": "Since indigestion is a symptom of disease, it is the physical condition of the patient and is examined objectively."}, "sentence_sentiment": {"answer": "Negative", "rationale": "Because indigestion is a state of physical discomfort, which is usually considered negative."}, "language_style": {"answer": "Calm and direct", "rationale": "The entry is asked in a straightforward manner, without any rhetorical or emotional rendering, and is more objective and calm."}, "time_dimension": {"answer": "Present", "rationale": "The keyword is \"yes\", which is an inquiry into the current state."}, "sentence_type": "Interrogative sentence", "pos": {"nouns": {"Indigestion": 1}, "verbs": {"have": 1}, "numerals": {"52": 1}}}, {"content": "53. Do you often have to bend your stomach to the ground?", "word_count": 17, "label": "", "sentence_structure": "Subject鈥搗erb鈥搊bject", "subjective_objective": {"answer": "Objective", "rationale": "It is an objective situation, not the subjective feeling of the patient, because of the stomach pain."}, "sentence_sentiment": {"answer": "Negative", "rationale": "Because the description \"severe stomach pains make you often have to bend\" in the entry is negative."}, "language_style": {"answer": "Calm and direct", "rationale": "The language style of the entry is a calm and direct type. The basis of the judgement is: - The article is formulated in a very concise manner, without any reverence or emotional rendering, and in an objective and direct manner; - \" Is the severe stomach pain forcing you to bend often?\" The question is whether the person surveyed has caused the body to bend due to stomach pain, without adding any additional information, and is calm and direct."}, "time_dimension": {"answer": "Present", "rationale": ""}, "sentence_type": "Interrogative sentence", "pos": {"nouns": {"Stomach pain": 1, "Body": 1}, "verbs": {"鈾?And I'm gonna be 鈾?: 1, "Bend": 1, "I had to": 1}, "adjectives": {"Serious": 1}, "numerals": {"53": 1}}}, {"content": "54. Do you feel a constant stomach disorder?", "word_count": 12, "label": "", "sentence_structure": "Subject鈥搗erb鈥搊bject", "subjective_objective": {"answer": "Objective", "rationale": "Because the stomach is not well enough to be the patient ' s symptoms, it is an objective description."}, "sentence_sentiment": {"answer": "Negative", "rationale": "Because the description \"continuous stomach discomfort\" in the entry is negative."}, "language_style": {"answer": "Calm and direct", "rationale": "The entry directly asked the respondents whether they felt the subject of stomach discomfort, the language style was calm and objective, and there was no excessive rhetoric or emotional rendering, which met the basic requirements of the questionnaire."}, "time_dimension": {"answer": "Past", "rationale": ""}, "sentence_type": "Interrogative sentence", "pos": {"nouns": {"Stomach": 1, "I'm not feeling well": 1}, "verbs": {"I feel": 1}, "numerals": {"54": 1}}}, {"content": "Do any of your family have stomach problems?", "word_count": 12, "label": "", "sentence_structure": "Subject鈥搗erb鈥搊bject", "subjective_objective": {"answer": "Objective", "rationale": "Because \"the person with stomach disease\" is an objective fact, independent of the patient's subjective will."}, "sentence_sentiment": {"answer": "Neutral", "rationale": "Because the entry does not have any emotional color."}, "language_style": {"answer": "Calm and direct", "rationale": "This language style is objective, concise, clear and informative and helps to obtain accurate findings."}, "time_dimension": {"answer": "Present", "rationale": "The entry asks about the current family situation of the person being tested, and the time dimension is now judged."}, "sentence_type": "Interrogative sentence", "pos": {"nouns": {"Family": 1, "Stomach disease": 1, "person": 1}, "verbs": {"have": 1, "Patient": 1}, "numerals": {"55": 1}}}, {"content": "Did the doctor say you have stomach or 12-finger ulcer?", "word_count": 16, "label": "", "sentence_structure": "Subject鈥搗erb鈥搊bject", "subjective_objective": {"answer": "Objective", "rationale": "Because the existence of stomach or ulcer is a medical history of the patient, it is an objective fact."}, "sentence_sentiment": {"answer": "Negative", "rationale": "Because of the diagnosis of disease, it is usually considered negative."}, "language_style": {"answer": "Calm and direct", "rationale": "The entry asks the respondents directly whether they have stomach or 12-finger ulcer, is more straightforward in language and does not use excessive rhetoric or euphemism."}, "time_dimension": {"answer": "Past", "rationale": "The keyword \"Doctor said\" indicates that this is an inquiry into past events, while \"brain or 12-finger ulcer\" is the result of a medical diagnosis of the past. The entry is therefore about whether the person tested had stomach or 12-finger ulcer in the past, and the temporal dimension is the past."}, "sentence_type": "Interrogative sentence", "pos": {"nouns": {"Doctor": 1, "you": 1, "Stomach": 1, "Twelve finger intestines": 1, "Erosion": 1}, "verbs": {"Say it": 1, "Yes": 1}, "adverbs": {"- You're not": 1}, "numerals": {"56": 1}}}, {"content": "Do you often get stomach pains after dinner or when you're empty?", "word_count": 15, "label": "", "sentence_structure": "Subject鈥損redicate", "subjective_objective": {"answer": "Objective", "rationale": "As stomach pain is a symptom or experience of the patient, it is the result of objective observations."}, "sentence_sentiment": {"answer": "Negative", "rationale": "Because the symptoms of \"tummy pain\" are negative."}, "language_style": {"answer": "Tactful and warm", "rationale": "The language style of the entry is more euphemistic, not too direct or too cold, but rather to direct the interviewer to think and answer by asking questions. This expression is more friendly and comfortable."}, "time_dimension": {"answer": "Past", "rationale": ""}, "sentence_type": "Interrogative sentence", "pos": {"nouns": {"After dinner": 1, "Empty stomach": 1, "Stomach pain": 1}, "verbs": {"Sensor": 1, "Regular": 1, "I'm not sure": 1, "Eat": 1}, "adverbs": {"not": 2}, "numerals": {"56": 1}, "pronouns": {"you": 1}}}, {"content": "Do you often suffer from diarrhoea (diarrhea)?", "word_count": 10, "label": "", "sentence_structure": "Subject鈥搗erb鈥搊bject", "subjective_objective": {"answer": "Objective", "rationale": "Because diarrhoea is an observable biological phenomenon, it is an objective one."}, "sentence_sentiment": {"answer": "Negative", "rationale": "Because \"diarrhea\" is negative."}, "language_style": {"answer": "Calm and direct", "rationale": "The entry directly asks whether the other party is frequently exposed to diarrhoea, is not using excessive qualifier or emotional language and is more objective and direct."}, "time_dimension": {"answer": "Present", "rationale": "So the judgment is based on \"now\"."}, "sentence_type": "Interrogative sentence", "pos": {"nouns": {"you": 1, "Diarrhoea": 1, "I'm having a stomach attack": 1}, "verbs": {"be": 1, "Regular": 1, "have": 1}, "adverbs": {"Yes": 1}, "numerals": {"57锛?: 1}}}, {"content": "58. Do you have serious blood and defecation when you are diarrhoea (black or blood visible in the flesh and blood)?", "word_count": 24, "label": "", "sentence_structure": "Subject鈥搗erb鈥搊bject", "subjective_objective": {"answer": "Objective", "rationale": "Because severe blood is the symptom of the patient, it is an objective fact."}, "sentence_sentiment": {"answer": "Negative", "rationale": "Because \"serious blood\" in the entry is a description of physical discomfort and disease, with a clear negative emotional color."}, "language_style": {"answer": "Calm and direct", "rationale": "The language style of the entry is relatively calm and straightforward, with clear, specific terms and statements used to describe the problem, and with no excessive emotional or rhetorical style, aimed at providing accurate information and providing respondents with a true answer."}, "time_dimension": {"answer": "Present", "rationale": "The entry is for the testee's current or past behaviour, and the keyword \"Is there serious blood? \" indicates that it is an inquiry into current or past behaviour. The temporal dimension is therefore judged as present and past."}, "sentence_type": "Interrogative sentence", "pos": {"nouns": {"Diarrhoea": 1, "Blood and shit": 2, "Black shit": 1, "Shit": 1, "The eye": 1, "Blood": 1}, "verbs": {"Did you": 1, "Visible": 1}, "adjectives": {"Serious": 1}, "numerals": {"58": 1}}}, {"content": "Do you have any glue in your diarrhea?", "word_count": 17, "label": "", "sentence_structure": "Subject鈥搗erb鈥搊bject", "subjective_objective": {"answer": "Objective", "rationale": "This is the observation and description of the actual state of the patient ' s health, not the subjective perception or attitude of the patient."}, "sentence_sentiment": {"answer": "Negative", "rationale": "Because the description \"sluct\" has negative meaning, suggesting that there may be problems with the body."}, "language_style": {"answer": "Calm and direct", "rationale": "The entry was in a straightforward manner to ask the respondents whether the urine contained glue, was not overdoed or emotionally rendered, and the language style was more objective and calm."}, "time_dimension": {"answer": "Past", "rationale": ""}, "sentence_type": "Interrogative sentence", "pos": {"nouns": {"Diarrhoea": 1, "Shit": 1, "Slurpion": 2, "Glue matter": 1}, "verbs": {"have": 1, "Is it possible": 1, "Here": 1}, "numerals": {"58": 1}}}, {"content": "Are you upset about having intestinal parasites?", "word_count": 17, "label": "", "sentence_structure": "Subject鈥搗erb鈥搊bject", "subjective_objective": {"answer": "Objective", "rationale": "Because this is an objective fact that patients have experienced."}, "sentence_sentiment": {"answer": "Negative", "rationale": "Because the entry is a negative emotion, which is \"fearing.\""}, "language_style": {"answer": "Tactful and warm", "rationale": "The entry uses the interview tone, which leads the interviewee to think in a moderate manner, rather than directly speaking or ordering. At the same time, it avoids the use of too negative terms and tone, which makes the interviewee feel more comfortable in answering questions."}, "time_dimension": {"answer": "Past", "rationale": "The entry is a question of the testee ' s past experience and feelings and therefore is judged to be past."}, "sentence_type": "Interrogative sentence", "pos": {"nouns": {"Intestine": 1, "A parasite": 1, "Trouble": 1}, "verbs": {"have": 1, "Sensor": 1, "Present": 1}, "numerals": {"59": 1}}}, {"content": "Do you often have serious constipation?", "word_count": 13, "label": "", "sentence_structure": "Subject鈥搗erb鈥搊bject", "subjective_objective": {"answer": "Objective", "rationale": "Because \"severe constipation\" and \"dry shit\" are the physical symptoms of the patient, not the subjective feelings."}, "sentence_sentiment": {"answer": "Negative", "rationale": "Because \"serious constipation\" in the entry is a physical discomfort and negative."}, "language_style": {"answer": "Calm and direct", "rationale": ""}, "time_dimension": {"answer": "Present", "rationale": "The entry asks if the person tested currently has a serious constipation condition, and the time dimension is now judged."}, "sentence_type": "Interrogative sentence", "pos": {"nouns": {"Serious": 1, "Decency": 1, "Shit": 1, "Dry": 1}, "verbs": {"- I'm sure": 1}, "numerals": {"60": 1}}}, {"content": "Do you have hemorrhoids (alienic pain in audibles, discomfort, or blood on the face of a poop or drop of blood on the back of a poop)?", "word_count": 29, "label": "", "sentence_structure": "Subject鈥搗erb鈥搊bject", "subjective_objective": {"answer": "Objective", "rationale": "This is a medical information that doctors need to know to evaluate and treat."}, "sentence_sentiment": {"answer": "Neutral", "rationale": "Because the entry does not have any emotional color."}, "language_style": {"answer": "Calm and direct", "rationale": "The entry is simple and clear in asking questions about hemorrhoids."}, "time_dimension": {"answer": "Present", "rationale": "Now the entry asks whether the person being tested is currently physically in a state of hemorrhoids. The keywords \"are there\" , \"when in a shit\" , \"discomfort\" and \"with blood\" point to the current state or symptoms, so it can be judged to be a question of the present."}, "sentence_type": "Interrogative sentence", "pos": {"nouns": {"Hemorrhoids": 1, "Shit": 1, "Anal": 1, "Pain": 1, "I'm not well": 1, "Hemorrhaging": 2, "Blood drops": 1}, "verbs": {"have": 1, "- Yeah": 1}, "adjectives": {"Surface": 1}, "numerals": {"61": 1}, "classifiers": {"individual": 1, "Time": 1}}}, {"content": "Have you ever had yellow stings (eye, skin, urine yellow)?", "word_count": 14, "label": "", "sentence_structure": "Subject鈥搗erb鈥搊bject", "subjective_objective": {"answer": "Objective", "rationale": "Because the existence of yellow sluice is an objective fact of the patient ' s state of health, not a subjective feeling or attitude of the patient."}, "sentence_sentiment": {"answer": "Neutral", "rationale": "Because the entry is asked about objective facts, there is no apparent emotional tendency."}, "language_style": {"answer": "Calm and direct", "rationale": "The entry is dialogueable in a direct query, with no excessive rhetorical or emotional rendering, and is more objective in tone. \""}, "time_dimension": {"answer": "Past", "rationale": "The entry asks if the testee has experienced yellow sluice in the past and therefore the temporal dimension is judged to be past."}, "sentence_type": "Interrogative sentence", "pos": {"nouns": {"Wong Shui": 1, "Eye": 1, "Skin": 1, "Pee": 1}, "verbs": {"Yes, I did": 1}, "numerals": {"62锛?: 1}}}, {"content": "Have you ever had a serious liver and choreography?", "word_count": 11, "label": "", "sentence_structure": "Subject鈥損redicate", "subjective_objective": {"answer": "Objective", "rationale": "Because severe liver and choreography is an objective fact experienced by patients, it is a source of information about their past history and is not influenced by their subjective perception."}, "sentence_sentiment": {"answer": "Negative", "rationale": "The entry is based on a judgment that involves a disease and is serious, which may have a negative impact on individuals or families and therefore has a negative emotional connotation."}, "language_style": {"answer": "Calm and direct", "rationale": "This language style is usually common in medicine or other fields of specialization, and is designed to obtain accurate and objective information."}, "time_dimension": {"answer": "Present", "rationale": "The entry now asks if the person tested is currently suffering from severe liver and choreography, and thus it is judged to be now."}, "sentence_type": "Interrogative sentence", "pos": {"nouns": {"Patronal diseases": 1, "Serious": 1}, "verbs": {"Yes": 1}, "numerals": {"63": 1}}}, {"content": "Do you often have swollen pains?", "word_count": 10, "label": "", "sentence_structure": "Subject鈥損redicate", "subjective_objective": {"answer": "Subjective", "rationale": "This is an entry that is examined from the patient ' s subjective point of view, as it relates to whether the patient has a specific symptoms experience."}, "sentence_sentiment": {"answer": "Negative", "rationale": "Because the \"mass pain\" in the entry is a negative sign of discomfort."}, "language_style": {"answer": "Calm and direct", "rationale": "The entry is based on a simple and clear inquiry into the frequency of joint pain, the absence of excessive rhetorical or emotional rendering, and the more objective tone of the speech. It can therefore be concluded that the language style of the article is straightforward and calm."}, "time_dimension": {"answer": "Past", "rationale": "The keyword \"always\" means that it is a constant question, perhaps a present or a recent situation. So the answer should be \"now\"."}, "sentence_type": "Interrogative sentence", "pos": {"nouns": {"It's a joint pain": 1}, "verbs": {"have": 1, "Off": 1, "Regular": 1, "Ouch": 1}, "adverbs": {"Yes": 1, "The long run": 1}, "numerals": {"64锛?: 1, "Regular": 1}}}, {"content": "Do you often feel stiff or stiff with your muscles and joints?", "word_count": 17, "label": "", "sentence_structure": "Subject鈥搗erb鈥搊bject", "subjective_objective": {"answer": "Objective", "rationale": "Because \"muscular and joints often feel rigid or rigid\" are symptoms of the patient and are the result of objective observations."}, "sentence_sentiment": {"answer": "Negative", "rationale": "Because the \"must and joints often feel rigid or rigid\" phenomenon in the entries is negative."}, "language_style": {"answer": "Calm and direct", "rationale": "This language style is more appropriate for situations such as surveys or questionnaires where specific information is needed."}, "time_dimension": {"answer": "Present", "rationale": "The entry asks about the current status of the subject, which relates to the term \"often\" and \"fear\", and therefore the temporal dimension is now judged."}, "sentence_type": "Interrogative sentence", "pos": {"nouns": {"Muscles": 1, "joint": 1, "Entry": 1, "Fold": 1, "Stunning": 1}, "verbs": {"I feel": 1}, "adjectives": {"Regular": 1}, "adverbs": {"very": 1}, "numerals": {"65锛?: 1}}}, {"content": "Do you often experience severe pain in your arms or legs?", "word_count": 16, "label": "", "sentence_structure": "Subject鈥搗erb鈥搊bject", "subjective_objective": {"answer": "Objective", "rationale": "The pain is an objective symptom of the patient."}, "sentence_sentiment": {"answer": "Negative", "rationale": "Because it involves physical pain, it usually brings negative emotions and experiences."}, "language_style": {"answer": "Calm and direct", "rationale": "The entry is a direct and explicit question to the interviewee about severe pain in the arm or leg, a simpler and more direct language style."}, "time_dimension": {"answer": "Present", "rationale": "So the time dimension is judged now."}, "sentence_type": "Interrogative sentence", "pos": {"nouns": {"Arms": 1, "Legs": 1, "Severe pain": 1}, "verbs": {"I feel": 1, "be": 1}, "adjectives": {"Regular": 1}, "numerals": {"66锛?: 1}}}, {"content": "67. Are you incapacitated by severe rheumatism?", "word_count": 16, "label": "", "sentence_structure": "Subject鈥搗erb鈥搊bject", "subjective_objective": {"answer": "Objective", "rationale": "The existence and severity of rheumatism and the fact of incapacity are objective."}, "sentence_sentiment": {"answer": "Negative", "rationale": "Because the description \"serious rheumatism has made you incapacitated\" in the entry is negative."}, "language_style": {"answer": "Calm and direct", "rationale": "The entry is based on a simple and objective tone: the person interviewed is asked whether he/she is inoperable due to severe rheumatism, is not using excessive rhetorical or emotional techniques."}, "time_dimension": {"answer": "Past", "rationale": ""}, "sentence_type": "Interrogative sentence", "pos": {"nouns": {"Rheumatism": 1, "Activity capacity": 1}, "verbs": {"鈾?And I'm gonna be 鈾?: 1, "Loss": 1}, "adjectives": {"Serious": 1}, "numerals": {"67": 1}}}, {"content": "'Do you have a tight shoulder, neck muscle?", "word_count": 14, "label": "", "sentence_structure": "Subject鈥搗erb鈥搊bject", "subjective_objective": {"answer": "Subjective", "rationale": "Because this is the sense and subjective observation of the patient, it is not an objective fact."}, "sentence_sentiment": {"answer": "Negative", "rationale": "Because the \"sleeves, neck muscles\" in the entries are negative."}, "language_style": {"answer": "Calm and direct", "rationale": "This entry is based on a direct question, and is not based on excessive rhetorical or emotional rendering, which is more objective and balanced."}, "time_dimension": {"answer": "Present", "rationale": "The key word for the entry in the questionnaire is \"with\" and \"a nervous effect on shoulders, neck muscles\", which indicates that the testee is describing the symptoms or circumstances of the day and thus judging the behaviour of the day."}, "sentence_type": "Interrogative sentence", "pos": {"nouns": {"67": 1, "you": 1, "Shoulders": 1, "Neck": 1, "Muscles": 1, "phenomena": 1}, "verbs": {"have": 1}, "adverbs": {"Tighten up": 1}, "numerals": {"67": 1}, "pronouns": {"Here": 1}}}, {"content": "68. Are any of your family suffering from rheumatism?", "word_count": 13, "label": "", "sentence_structure": "Subject鈥搗erb鈥搊bject", "subjective_objective": {"answer": "Objective", "rationale": "Because \"Whether or not we have rheumatism\" is an objective fact, not related to the patient's subjective attitude."}, "sentence_sentiment": {"answer": "Neutral", "rationale": "Because the entry is factual and does not involve feelings or attitudes."}, "language_style": {"answer": "Calm and direct", "rationale": "The language style of the entry is straightforward, as it is investigated in a clear question form without too much qualifier or emotionally strong vocabulary.\""}, "time_dimension": {"answer": "Present", "rationale": "The entry used to ask the testee about the past, i.e. whether there were any cases of rheumatism among his family members."}, "sentence_type": "Interrogative sentence", "pos": {"nouns": {"Family": 1, "person": 1, "Rheumatism": 1}, "verbs": {"Is it possible": 1, "have": 1, "Patient": 1}, "numerals": {"68": 1}, "pronouns": {"you": 1}}}, {"content": "69. Is your feet soft and your life seriously inconvenient by pain?", "word_count": 16, "label": "", "sentence_structure": "Subject鈥搗erb鈥搊bject", "subjective_objective": {"answer": "Objective", "rationale": "Because \"soft feet, pain\" are the objective symptoms of the patient, and \"effects on life-threatening conditions\" are also the objective results that can be observed and measured. The examination of subjective feelings (such as the extent of pain or inconvenience) may require the use of more specific questions to better understand the experience of the patient."}, "sentence_sentiment": {"answer": "Negative", "rationale": "Because the description \"soft feet, pain\" in the entry is negative."}, "language_style": {"answer": "Tactful and warm", "rationale": "The entry is more euphemistic and gentle in its language style, which guides respondents to answer questions in a question-and-answer manner, without using too direct or hard-line expressions. At the same time, the term \"serious inconvenience\" is used with a milder use, without emphasizing the difficulty of life, but rather with attention to the actual experience and feelings of the interviewee."}, "time_dimension": {"answer": "Present", "rationale": ""}, "sentence_type": "Interrogative sentence", "pos": {"nouns": {"Foot": 1, "Ouch": 1, "Life": 1, "Serious": 1, "I can't": 1}, "verbs": {"Let's go": 1, "鈾?And I'm gonna be 鈾?: 1}, "adverbs": {"Extreme": 1}, "numerals": {"69": 1}, "classifiers": {"individual": 1}}}, {"content": "'Do you often feel sore legs and feet?", "word_count": 11, "label": "", "sentence_structure": "Subject鈥搗erb鈥搊bject", "subjective_objective": {"answer": "Subjective", "rationale": "Because \"are the legs and feet sour often?\" the question is about the patient's subjective feelings and experiences, which are subjective in perspective."}, "sentence_sentiment": {"answer": "Negative", "rationale": "Because the \"often feeling of leg and foot sour\" in the entries is an uncomfortable experience, it has a negative emotional color."}, "language_style": {"answer": "Calm and direct", "rationale": "The entry is in a straightforward manner asking the other side whether it is often feeling sour feet and feet, without too many rhetorical or emotional modifications, and with a more objective and concise tone."}, "time_dimension": {"answer": "Present", "rationale": "The keyword \"often feels\" means that it is a customary feeling, and the existence of such a feeling in the immediate answer to the question is the basis for judgement, so the entry asks about the current state of the subject."}, "sentence_type": "Interrogative sentence", "pos": {"nouns": {"69": 1, "you": 1, "Legs": 1, "Foot": 1, "Acid": 1}, "verbs": {"Let's go": 1, "Sensor": 1, "Present": 1}, "numerals": {"69": 1}, "classifiers": {"individual": 1}, "pronouns": {"you": 1}}}, {"content": "70. Does the back pain amount to a level that prevents you from working?", "word_count": 18, "label": "", "sentence_structure": "Subject鈥搗erb鈥搊bject", "subjective_objective": {"answer": "Objective", "rationale": "Since the degree of back pain affects work is an objective fact that does not need to be judged by the patient ' s subjective attitude or opinion."}, "sentence_sentiment": {"answer": "Negative", "rationale": "Because the result of the entry, \"I can't keep working with back pains,\" is negative."}, "language_style": {"answer": "Calm and direct", "rationale": "This is a more straightforward, concise language style that is suitable for use in surveys of such situations as those requiring rapid access to information."}, "time_dimension": {"answer": "Present", "rationale": "Now, the entry asks about the current state of health, \"Is the back pain enough to keep you from working?\" It does not concern the past or future, but rather the testee's present state and effects. So the time dimension is now."}, "sentence_type": "Interrogative sentence", "pos": {"nouns": {"My back hurts": 1, "Work": 1}, "verbs": {"Achieved": 1, "鈾?And I'm gonna be 鈾?: 1, "Ongoing": 1}, "adjectives": {"of": 2}, "numerals": {"70": 1, "No, I can't": 1}, "classifiers": {"Level": 1, "you": 1}, "pronouns": {"Is it possible": 1}}}, {"content": "71. Are you troubled by severe functional loss or malformation (aberrational) of the body?", "word_count": 26, "label": "", "sentence_structure": "Subject鈥搗erb鈥搊bject", "subjective_objective": {"answer": "Objective", "rationale": "This is an issue of physical condition and an objective fact of the patient ' s experience."}, "sentence_sentiment": {"answer": "Negative", "rationale": "Because the \"fear\" act in the entry is negative."}, "language_style": {"answer": "Calm and direct", "rationale": "The entry is in objective language and does not have too many emotional renderings or rhetorical techniques. It directly asks the interviewee whether he or she is troubled by severe physical loss of function or deformity, and expresses it clearly."}, "time_dimension": {"answer": "Present", "rationale": ""}, "sentence_type": "Interrogative sentence", "pos": {"nouns": {"Body": 1, "Functions": 1, "Loss": 1, "Malformed": 1, "Form abnormal": 1, "Trouble": 1}, "verbs": {"have": 1, "Sensor": 1, "Present": 1}, "adjectives": {"Serious": 1, "It's a freak": 1}, "numerals": {"71": 1}}}, {"content": "72. Are your skin sensitive to temperature, pain or pressure?", "word_count": 19, "label": "", "sentence_structure": "Subject鈥損redicate", "subjective_objective": {"answer": "Subjective", "rationale": "Because \"skin is sensitive to temperature, pain or pressure\" is the patient's subjective experience and feeling."}, "sentence_sentiment": {"answer": "Negative", "rationale": "Because the expression \"skin is sensitive to temperature, pain or pressure\" in the entry is a manifestation of physical discomfort and has a negative emotional colour."}, "language_style": {"answer": "Calm and direct", "rationale": "The language style of the entry is more straightforward, there is no excessive rhetorical or emotional rendering, but rather a simple language asking the other side whether the skin is very sensitive to the temperature, pain, etc., and is calm and professional."}, "time_dimension": {"answer": "Present", "rationale": "The entry asks the testee about his current state of health and feelings, and the time dimension is now judged."}, "sentence_type": "Interrogative sentence", "pos": {"nouns": {"Skin": 1, "Temperature": 1, "Pain": 1, "Pinch": 1}, "verbs": {"be": 1, "have": 1}, "adjectives": {"Tens": 1}, "numerals": {"72": 1}, "pronouns": {"you": 1}}}, {"content": "Do you often have a small undercut hemorrhage?", "word_count": 14, "label": "", "sentence_structure": "Subject鈥搗erb鈥搊bject", "subjective_objective": {"answer": "Objective", "rationale": "Because the presence of small under-cut bleeding points is a biological phenomenon of the patient, it is an objective fact."}, "sentence_sentiment": {"answer": "Negative", "rationale": "Because the symptoms of \"minus undercut haemorrhage\" in the entry are usually considered as physical abnormalities and therefore have negative emotional connotations."}, "language_style": {"answer": "Calm and direct", "rationale": "The entry is in short and clear language and directly asks the interviewee whether there is a small undercut bleeding spot, there is no excessive adoration or emotional rendering, and the expression is more objective and cold."}, "time_dimension": {"answer": "Present", "rationale": "\"Do you often have the word \"normal\" in it, which means \"normally\" and \"yes\" in its normal state of affairs, which means that this behaviour has not only happened in the past, but may still be ongoing. So, this entry is to question the current state of the person being tested, judging by the time dimension of time."}, "sentence_type": "Interrogative sentence", "pos": {"nouns": {"Little undercut bleeding": 1, "Little red dot": 1}, "verbs": {"have": 1, "Regular": 1, "Is there a problem": 1}, "adverbs": {"Regular": 1}, "numerals": {"72鈥?: 1}}}, {"content": "Do you usually have a bad way of healing your skin?", "word_count": 16, "label": "", "sentence_structure": "Subject鈥搗erb鈥揷omplement", "subjective_objective": {"answer": "Subjective", "rationale": "Because \"healable\" or \"unrehabilitated\" is a self-impression and observation of the patient, a subjective examination."}, "sentence_sentiment": {"answer": "Negative", "rationale": "Because the entry \"incisions are usually not healing\" describes an unhealthy condition and is therefore negative."}, "language_style": {"answer": "Calm and direct", "rationale": "\"Calm down straight.\""}, "time_dimension": {"answer": "Present", "rationale": "\"Skin cuts are often not healing\" is a description of the current state of affairs, which suggests that the tester is in the present situation. Thus, the temporal dimension is now judged."}, "sentence_type": "Interrogative sentence", "pos": {"nouns": {"you": 1, "Skin": 1, "Cut": 1, "Usually": 1, "It's hard": 1, "Healing": 1, "Good-looking": 1}, "adjectives": {"Long": 1, "Okay": 1}, "adverbs": {"Usually": 1, "It's hard": 1}, "numerals": {"73锛?: 1}, "pronouns": {"you": 1}}}, {"content": "Do you always get a red face?", "word_count": 8, "label": "", "sentence_structure": "Subject鈥損redicate", "subjective_objective": {"answer": "Subjective", "rationale": "Because \"fresh-received\" is a subjective feeling or experience."}, "sentence_sentiment": {"answer": "Negative", "rationale": "Because \"regrel\" in the entry is usually seen as a negative emotional reaction."}, "language_style": {"answer": "Calm and direct", "rationale": "The language style is more objective and direct, and does not attempt to induce or influence the emotions and answers of the interviewee. It is therefore clear that the language style of the questionnaire entry is straightforward and straightforward."}, "time_dimension": {"answer": "Past", "rationale": "The entry asks about the testee's past behaviour or experience, and \"always\" indicates that the act has occurred in the past and is therefore judged to be past."}, "sentence_type": "Interrogative sentence", "pos": {"nouns": {"Questionnaire": 1, "Entry": 1, "Face": 1}, "verbs": {"Regular": 1, "Red": 1}, "adjectives": {"very": 1}, "adverbs": {"Is it possible": 1, "Regular": 1}, "numerals": {"74锛?: 1}}}, {"content": "Did you sweat a lot even in cold days?", "word_count": 12, "label": "", "sentence_structure": "Subject鈥損redicate", "subjective_objective": {"answer": "Objective", "rationale": "Since the excessive sweating is an act that occurs under the objective conditions of cold days, the objective symptoms of the patient are examined."}, "sentence_sentiment": {"answer": "Negative", "rationale": "Because \"sweat-sweat\" is usually considered an abnormal physical reaction, it may suggest some health problems."}, "language_style": {"answer": "Calm and direct", "rationale": "The entry is a direct question-and-answer dialogue, without excessive rhetorical or emotional rendering and with a more objective tone."}, "time_dimension": {"answer": "Present", "rationale": "The keyword \"even if it's in cold weather\" suggests that it's a description of the current state of health, and \"Are you sweating too?\" is asking the testee what it feels like right now. So, this entry relates to the current dimension of time."}, "sentence_type": "Interrogative sentence", "pos": {"nouns": {"Oh, my God": 1, "Khan": 1, "Cold": 1}, "verbs": {"Yeah": 1, "Sweat": 1, "Mass": 1}, "adjectives": {"Even if": 1}, "adverbs": {"in": 1, "you": 1}, "numerals": {"75锛?: 1}, "classifiers": {"EMPTY": 2, "- You're not": 1}}}, {"content": "Is it a problem for you to have a serious skin itching (a itch)?", "word_count": 17, "label": "", "sentence_structure": "Subject鈥搗erb鈥搊bject", "subjective_objective": {"answer": "Subjective", "rationale": "Because \"fearing\" is the patient's subjective feeling."}, "sentence_sentiment": {"answer": "Negative", "rationale": "Because the entries \"skin itching\" and \"fearing\" are negative."}, "language_style": {"answer": "Calm and direct", "rationale": "The article is simple and clear: it does not have too many rhetorical or emotional expressions, and it is more objective in tone. At the same time, it asks the interviewee in a direct way whether he is affected by the aching skin and the extent of his distress, which is more in keeping with the calm and direct language style."}, "time_dimension": {"answer": "Past", "rationale": ""}, "sentence_type": "Interrogative sentence", "pos": {"nouns": {"Serious": 1, "Skin": 1, "Itches": 1, "It tickles": 1, "Feel": 1, "Trouble": 1}, "verbs": {"Is it possible": 1, "鈾?And I'm gonna be 鈾?: 1, "I feel": 1}, "adjectives": {"Serious": 1}, "numerals": {"76": 1}}}, {"content": "Do you often have a rash?", "word_count": 14, "label": "", "sentence_structure": "Subject鈥搗erb鈥搊bject", "subjective_objective": {"answer": "Objective", "rationale": "Because \"out of rash\" is an objective fact, it is possible to observe and measure whether the patient himself has such a situation."}, "sentence_sentiment": {"answer": "Negative", "rationale": "Because \"out of the rash\" is a physical discomfort, the entry is negative."}, "language_style": {"answer": "Calm and direct", "rationale": "The entry is in direct and clear language, is not accompanied by excessive rhetorical or emotional rendering, is objective and aims to understand whether the person under investigation has been exposed to rashes on a regular basis."}, "time_dimension": {"answer": "Present", "rationale": "The entry uses the term \"whether\" and \"often\" to ask the testee about his current behaviour or state, so the temporal dimension is now judged."}, "sentence_type": "Interrogative sentence", "pos": {"nouns": {"Herpes": 2, "Wanders": 1, "The rash": 1}, "verbs": {"be": 1, "Out": 1, "Regular": 1}, "adverbs": {"Is it possible": 1}, "numerals": {"77锛?: 1}}}, {"content": "Do you have a swelling face?", "word_count": 9, "label": "", "sentence_structure": "Subject鈥搗erb鈥搊bject", "subjective_objective": {"answer": "Objective", "rationale": "Because the swelling of the face is an objective manifestation of the patient ' s state of health, it is not related to the patient ' s subjective consciousness."}, "sentence_sentiment": {"answer": "Negative", "rationale": "Because \"face swelling\" is a negative physical condition."}, "language_style": {"answer": "Calm and direct", "rationale": "This language style is more objective, calm, with no excessive rhetorical or emotional rendering, and is more suitable for practical scenarios such as questionnaires."}, "time_dimension": {"answer": "Present", "rationale": ""}, "sentence_type": "Interrogative sentence", "pos": {"nouns": {"Face": 1, "- Swollen": 1}, "verbs": {"Time": 1, "Is there a problem": 1, "Answer": 1}, "adjectives": {"Down there": 1, "This": 1, "Questionnaire": 1, "Entry": 1}, "numerals": {"77'": 1}, "pronouns": {"you": 1}}}, {"content": "Do you often get upset by the swelling (suppress)?", "word_count": 16, "label": "", "sentence_structure": "Subject鈥搗erb鈥搊bject", "subjective_objective": {"answer": "Objective", "rationale": "Since twitch (suppressy) is an objective fact of the patient ' s experience, it is an objective question to be examined."}, "sentence_sentiment": {"answer": "Negative", "rationale": "Because the emotional reaction of \"fearing\" in the entry is negative."}, "language_style": {"answer": "Calm and direct", "rationale": ""}, "time_dimension": {"answer": "Present", "rationale": ""}, "sentence_type": "Interrogative sentence", "pos": {"nouns": {"78": 1, "you": 1, "Regular": 1, "Because": 1, "It's swollen": 1, "Pussy": 1, "And": 1, "I feel": 1, "Trouble": 1}, "verbs": {"Is it possible": 1, "Regular": 1, "Because": 1, "Fan": 1, "I feel": 1, "Trouble": 1}, "adverbs": {"Regular": 1, "Because": 1, "And": 1, "I feel": 1, "Trouble": 1}, "numerals": {"78": 1}, "pronouns": {"you": 1}}}, {"content": "Do you often feel very bad about severe headaches?", "word_count": 18, "label": "", "sentence_structure": "Subject鈥搗erb鈥搊bject", "subjective_objective": {"answer": "Subjective", "rationale": "Because \"often\" and \"sorely\" are the subjective feelings of the patient."}, "sentence_sentiment": {"answer": "Negative", "rationale": "The description of \"suffering from severe headaches\" in the entry is negative and represents an uncomfortable experience."}, "language_style": {"answer": "Calm and direct", "rationale": "The language style of the entry is calm and straightforward. It raises questions in a concise manner, without too much emotional overture or accoutrements. This direct questioning makes it easier for the respondents to understand the intentions of the question and to answer the question accurately and directly."}, "time_dimension": {"answer": "Present", "rationale": "The entry asks how and how the person is feeling, and the keywords \"frequently\" and \"suffering very hard\" point to current behavior or emotional experience."}, "sentence_type": "Interrogative sentence", "pos": {"nouns": {"A serious headache": 1, "Sensor": 1, "Tens": 1, "It's hard": 1}, "verbs": {"Is it possible": 1, "Regular": 1, "Because": 1, "I feel": 1, "And": 1, "Tens": 1, "It's hard": 1}, "adjectives": {"Serious": 1, "Tens": 1}, "adverbs": {"Regular": 1, "very": 1}, "numerals": {"79": 1}, "classifiers": {"individual": 1}}}, {"content": "80. Do you often suffer from headaches and heavy hair?", "word_count": 19, "label": "", "sentence_structure": "Subject鈥搗erb鈥搊bject", "subjective_objective": {"answer": "Objective", "rationale": "Because headaches and sunk hair are the experiences and feelings of patients, this is an objective fact and is not related to the views or feelings of patients."}, "sentence_sentiment": {"answer": "Negative", "rationale": "Because the description of \"life pain\" in the entry is negative."}, "language_style": {"answer": "Calm and direct", "rationale": "The entry directly asked whether the person tested was often suffering from headaches and fainting, and the temperature of the language and its uncompromising nature were a relatively calm and direct expression."}, "time_dimension": {"answer": "Present", "rationale": "The entry now asks if the person tested currently has a regular headache and a stubble of hair, and it is now judged."}, "sentence_type": "Interrogative sentence", "pos": {"nouns": {"you": 2, "Headache": 1, "Hair": 1, "Sink": 1, "Life": 1, "Pain": 1}, "verbs": {"Regular": 1, "Because": 1, "I feel": 1}, "adjectives": {"80": 1}, "numerals": {"80": 1}}}, {"content": "Do you have common headaches among your family?", "word_count": 10, "label": "", "sentence_structure": "Subject鈥搗erb鈥搊bject", "subjective_objective": {"answer": "Objective", "rationale": "Since it is an objective fact that the family is often suffering from headaches, the answer can be obtained through investigation or inquiry."}, "sentence_sentiment": {"answer": "Neutral", "rationale": "According to the title, the question was simply to ask the family of the person under investigation whether there was a headache, and it did not involve any emotional or value judgement, and it was therefore neutral."}, "language_style": {"answer": "Tactful and warm", "rationale": "The entry is more personal and interactive when it is spoken in a gentle, inquisitive manner, rather than in direct statements or orders. At the same time, the issue is not one of asking the interviewees personally, but focusing on their families, which makes the matter more euphemismal and less offensive."}, "time_dimension": {"answer": "Past", "rationale": ""}, "sentence_type": "Interrogative sentence", "pos": {"nouns": {"Family": 1, "Headache": 1}, "verbs": {"Common": 1}, "numerals": {"81": 1}}}, {"content": "Have you ever had a fever, a cold?", "word_count": 15, "label": "", "sentence_structure": "Subject鈥搗erb鈥搊bject", "subjective_objective": {"answer": "Objective", "rationale": "Because heat and cold are specific physical symptoms, they are not patients ' feelings or attitudes, but rather objective phenomena."}, "sentence_sentiment": {"answer": "Negative", "rationale": "Because \"heat\" and \"cool\" are symptoms of physical discomfort, usually negative."}, "language_style": {"answer": "Tactful and warm", "rationale": "The language style of the entry is more euphemistic, and the interviewer is asked to answer the questions, while avoiding the use of too direct or strong language."}, "time_dimension": {"answer": "Present", "rationale": ""}, "sentence_type": "Interrogative sentence", "pos": {"nouns": {"Line": 2, "phenomena": 1}, "verbs": {"have": 1, "Heat": 1, "Cold": 1}, "numerals": {"82锛?: 1}}}, {"content": "Do you often feel like you're feeling a little dizzy?", "word_count": 15, "label": "", "sentence_structure": "Subject鈥搗erb鈥搊bject", "subjective_objective": {"answer": "Subjective", "rationale": "Because it is the patient ' s subjective feeling that is considered to be the patient ' s own decision."}, "sentence_sentiment": {"answer": "Negative", "rationale": "Because \"headsick\" is a negative symptom of physical discomfort."}, "language_style": {"answer": "Calm and direct", "rationale": "The entry is based on a more objective language style, without excessive emotional adoration or rhetorical techniques."}, "time_dimension": {"answer": "Past", "rationale": ""}, "sentence_type": "Interrogative sentence", "pos": {"nouns": {"Dizziness": 1, "Feel": 1}, "verbs": {"have": 1, "Regular": 1}, "adjectives": {"Serious": 1}, "numerals": {"83": 1}}}, {"content": "Do you often faint?", "word_count": 7, "label": "", "sentence_structure": "Subject鈥損redicate", "subjective_objective": {"answer": "Objective", "rationale": "The reason is that the patient is often fainted because he is suffering from an objective fact."}, "sentence_sentiment": {"answer": "Negative", "rationale": "Because fainting is an abnormal physical reaction, usually associated with disease or health problems, the expression of this purpose is negative."}, "language_style": {"answer": "Calm and direct", "rationale": "The entry asks the respondents in a direct and clear manner whether they have had frequent fainting experiences, do not have too many rhetorical techniques or emotional renderings, are more objective in tone and are consistent with the linguistic style of the questionnaire."}, "time_dimension": {"answer": "Past", "rationale": ""}, "sentence_type": "Interrogative sentence", "pos": {"nouns": {"Questionnaire": 1, "Entry": 1, "I'm fainted": 1}, "verbs": {"be": 1, "have": 1, "Yes": 1}, "adverbs": {"Regular": 1}, "numerals": {"84锛?: 1}}}, {"content": "Have you fainted twice?", "word_count": 10, "label": "", "sentence_structure": "Subject鈥損redicate", "subjective_objective": {"answer": "Subjective", "rationale": "Because \"sicking twice\" is a patient's subjective experience and judgment, and the question is asked about the patient's own situation and is therefore subjective."}, "sentence_sentiment": {"answer": "Negative", "rationale": "Because fainting is usually an unhealthy symptom, the subject can be considered negative."}, "language_style": {"answer": "Calm and direct", "rationale": "The basis for the determination: the entry is a clear question in direct, concise language, without the use of excessive rhetorical or emotional rendering, and with a more balanced and objective tone. At the same time, the question is designed in a more straightforward and specific way, which allows respondents to understand and answer. I therefore think the language style of the article is straightforward."}, "time_dimension": {"answer": "Past", "rationale": "The \"sicking twice\" in the entry is about past experience and behaviour, and the temporal dimension of the purpose of the article is therefore a matter of past."}, "sentence_type": "Interrogative sentence", "pos": {"nouns": {"Questionnaire": 1, "Entry": 1, "I'm fainted": 2, "Number of times": 1}, "verbs": {"Is it possible": 1, "I'm fainted": 1, "More than": 1}, "adjectives": {"Above": 1}, "numerals": {"85": 1, "Two": 1, "Number of times": 1}}}, {"content": "Do you feel numb or shaken in some part of your body?", "word_count": 19, "label": "", "sentence_structure": "Subject鈥搗erb鈥搊bject", "subjective_objective": {"answer": "Subjective", "rationale": "Because the feeling of numbness or tremors is the physical feeling of the patient and is assessed from a subjective perspective."}, "sentence_sentiment": {"answer": "Negative", "rationale": "Because \"often numbing or shaking\" is a negative condition of physical discomfort, it can cause concern and anxiety."}, "language_style": {"answer": "Calm and direct", "rationale": "The entry is conducted in a direct question-and-answer manner without any reticulation of rhetoric or emotional colour, with an objective and incipient tone, emphasizing the seriousness and accuracy of the investigation. \""}, "time_dimension": {"answer": "Present", "rationale": "The keyword \"yes\" indicates that the question is asked about the symptoms of the present and the temporal dimension is judged to be the present."}, "sentence_type": "Interrogative sentence", "pos": {"nouns": {"Part": 1, "Feel": 1}, "verbs": {"have": 2, "Regular": 1, "I'm numb": 1, "鈾?The tremors 鈾?: 1, "Is it possible": 1}, "adjectives": {"Some": 1, "Regular": 1}, "numerals": {"86锛?: 1}}}, {"content": "Have you ever been paralysed in any part of your body (total or partial loss of sensory and motor abilities)?", "word_count": 26, "label": "", "sentence_structure": "Subject鈥損redicate", "subjective_objective": {"answer": "Objective", "rationale": "Because it is the patient ' s objective experience to be paralysed in some part of the body, it is irrelevant to subjective thinking."}, "sentence_sentiment": {"answer": "Negative", "rationale": "Because the term \"paralyzed\" is negative and emotional, indicating that the body has suffered serious injuries."}, "language_style": {"answer": "Calm and direct", "rationale": "The entry raises questions in a straightforward way, without too much rhetorical or emotional style, and with a more calm and objective language style. This expression allows respondents to answer questions more honestly, avoiding emotional influences."}, "time_dimension": {"answer": "Past", "rationale": "The entry asks whether the testee has experienced paralysis in a part of his body in the past and therefore the temporal dimension is judged to be past."}, "sentence_type": "Interrogative sentence", "pos": {"nouns": {"Part": 1, "Body": 1, "Paralytic": 1, "Feel": 1, "Sports": 1, "Capacity": 1, "Loss": 1}, "verbs": {"Once": 1, "- Have you ever been": 1}, "adverbs": {"Total": 1, "Part": 1}, "numerals": {"87锛?: 1}}}, {"content": "88. Have you been unconscious (not knowing anything) after an impact?", "word_count": 21, "label": "", "sentence_structure": "Subject鈥搗erb鈥搊bject", "subjective_objective": {"answer": "Subjective", "rationale": "Because this is the subjective feeling and experience of the patient, it is only in the patient's own sense that the \"sensorship\" is seen and therefore viewed from a subjective perspective."}, "sentence_sentiment": {"answer": "Negative", "rationale": "Because the \"discovery\" act in the entry is negative."}, "language_style": {"answer": "Calm and direct", "rationale": "The entry is more calm and straightforward in its language, and it asks the interviewee whether there is a particular phenomenon."}, "time_dimension": {"answer": "Past", "rationale": "The keyword \"Yes\" indicates that this has happened, and the phenomenon of \"insensible after impact\" occurs at some point in the past, and the entry is therefore a past."}, "sentence_type": "Interrogative sentence", "pos": {"nouns": {"phenomena": 1, "Sensibility": 2, "Hit": 1}, "verbs": {"have": 1, "Lose": 1, "Yes": 1}, "adverbs": {"What": 1, "Both": 1, "not": 2}, "numerals": {"88": 1}, "pronouns": {"you": 1}}}, {"content": "Do you feel convulsive sometimes in your head, face, shoulder?", "word_count": 25, "label": "", "sentence_structure": "Subject鈥搗erb鈥搊bject", "subjective_objective": {"answer": "Objective", "rationale": "Because the feeling of convulsion on the head, face and shoulder is the physical state of the patient and is an objective condition."}, "sentence_sentiment": {"answer": "Neutral", "rationale": "Because the entry only describes a physical condition and does not involve any emotional colour."}, "language_style": {"answer": "Calm and direct", "rationale": "The entry is in a simple and clear language, directly asking the person interviewed about his or her state of health, without any rhetorical or emotional rendering, and is more objective in tone."}, "time_dimension": {"answer": "Past", "rationale": "The entry is for the testee to ask if he had ever felt muscle pumping before, and the time dimension is a past.\""}, "sentence_type": "Interrogative sentence", "pos": {"nouns": {"Head": 1, "Noodles": 1, "Shoulder": 1, "Feel": 1, "Twitches": 1, "Muscles": 1}, "verbs": {"have": 1, "be": 1, "Sometimes": 1, "Suddenly and quickly": 1, "Scrambling": 1, "Feel": 1}, "adjectives": {"Suddenly": 1, "Quick": 1}, "adverbs": {"Sometimes": 1}, "numerals": {"89锛?: 1}}}, {"content": "Have you ever been mad?", "word_count": 16, "label": "", "sentence_structure": "Subject鈥搗erb鈥搊bject", "subjective_objective": {"answer": "Objective", "rationale": "The reason for the incident is that the patient is suffering from a mental disorder."}, "sentence_sentiment": {"answer": "Negative", "rationale": "Because the term \"sheep horn madness\" in the entry is a disease, while the use of wind is usually considered an abnormal behaviour or symptom, the emotional colour of the purpose of the article is negative."}, "language_style": {"answer": "Calm and direct", "rationale": "The entry is based on a direct inquiry into whether the person interviewed has ever had a \"screech\" (i.e. an epilepsy) or has not used euphemism or warm expression. Nor is it overly emotional or personal, neutral, objective, and consistent with the straight language style of calm."}, "time_dimension": {"answer": "Present", "rationale": "The entry asks if the person was a former madman, the keywords \"Have you ever been mad\" and \"Episcopal\" all point to what happened to the person who was tested and has now ended. So the time dimension of the article is now."}, "sentence_type": "Interrogative sentence", "pos": {"nouns": {"Crazy": 1, "Eclampsia": 1, "The horns are crazy": 1}, "verbs": {"Smoke": 1, "Yes": 1}, "adjectives": {"Is it possible": 1}, "numerals": {"90": 1}}}, {"content": "91. Are there any epilepsy in your family?", "word_count": 11, "label": "", "sentence_structure": "Subject鈥搗erb鈥搊bject", "subjective_objective": {"answer": "Objective", "rationale": "It is an objective fact that there are family historys of persons suffering from epilepsy."}, "sentence_sentiment": {"answer": "Neutral", "rationale": "The entry merely asks whether family members are suffering from epilepsy and does not express any positive or negative attitudes, feelings or tendencies."}, "language_style": {"answer": "Calm and direct", "rationale": "The language style of the entry is relatively calm and straightforward, using simple and clear methods, without too many rhetorical or emotional features, and with an objective neutral tone."}, "time_dimension": {"answer": "Present", "rationale": "The entry asks about the current status of the person being tested, and the keyword \"are there\" indicates an inquiry about the current status and therefore the judgement is now."}, "sentence_type": "Interrogative sentence", "pos": {"nouns": {"Family": 1, "Epilepsy": 1}, "verbs": {"Yes": 1, "Okay": 0}, "numerals": {"Ninety-one": 1}}}, {"content": "92. Do you have a serious habit of biting your nails?", "word_count": 13, "label": "", "sentence_structure": "Subject鈥搗erb鈥搊bject", "subjective_objective": {"answer": "Subjective", "rationale": "Because \"serious nail-biting habits\" are the personal behaviour and experience of the patient and are subjective observations."}, "sentence_sentiment": {"answer": "Negative", "rationale": "Because \"there's a serious nail-biting habit\" is negative."}, "language_style": {"answer": "Calm and direct", "rationale": "The entry is conducted in the form of a direct question, with a clear and clear tone, with no excessive emotional or rhetorical style, and is typical of a calm and direct language style."}, "time_dimension": {"answer": "Present", "rationale": "The entry asks the testee about his current behaviour (whether there is a habit of biting his fingernails) and thus judges it now."}, "sentence_type": "Interrogative sentence", "pos": {"nouns": {"Custom": 1, "Bite your nails": 1}, "verbs": {"have": 1, "Serious": 1}, "adjectives": {"Serious": 1}, "numerals": {"92锛?: 1}}}, {"content": "Are you upset by your stammering or your stutter?", "word_count": 14, "label": "", "sentence_structure": "Subject鈥搗erb鈥搊bject", "subjective_objective": {"answer": "Subjective", "rationale": "The basis for the determination: it is the patient ' s subjective feelings and experiences to examine whether he is troubled by his stasis or stutter."}, "sentence_sentiment": {"answer": "Negative", "rationale": "The \"stammering or stuttering\" in the entry is an expression of a negative emotion and attitude."}, "language_style": {"answer": "Tactful and warm", "rationale": "Warm and warm."}, "time_dimension": {"answer": "Past", "rationale": "The entry asks whether the person tested was ever troubled by a stammer or stutter, and therefore belongs to past behaviour or experience."}, "sentence_type": "Interrogative sentence", "pos": {"nouns": {"you": 2, "Talk": 1, "Slit": 1, "Snack": 1, "Trouble": 1}, "verbs": {"Is it possible": 1, "Because": 1, "Trouble": 1}, "adjectives": {"Slit": 1, "Snack": 1}, "numerals": {"93": 1}}}, {"content": "93 'Have you ever had trouble speaking because your tongue is inflexible?", "word_count": 18, "label": "", "sentence_structure": "Subject鈥搗erb鈥搊bject", "subjective_objective": {"answer": "Subjective", "rationale": "Because it involves the patient ' s personal experience and feelings, it requires the patient ' s own answers."}, "sentence_sentiment": {"answer": "Negative", "rationale": "The result of \"challenge\" is negative, suggesting that the person under investigation may have language barriers or other health problems."}, "language_style": {"answer": "Tactful and warm", "rationale": "Although the question relates directly to the physical function of the individual, it does not ask questions in a cold or direct tone. Rather, it asks the interviewee, in a caring and understanding manner, whether he has ever experienced such a situation, and expresses concern and care for the health of the interviewee."}, "time_dimension": {"answer": "Present", "rationale": "\"93\" does you have \"Is there\" to indicate a state or a presence, and \"Has there\" is a past act, so the entry asks if the testee has experienced a situation where tongue inflexibility makes it difficult to speak. So time dimension is now."}, "sentence_type": "Interrogative sentence", "pos": {"nouns": {"tongue": 1, "Problem": 1, "Talk": 1}, "verbs": {"have": 1, "Result": 1}, "adjectives": {"Flexibility": 1, "Difficulties": 1}, "numerals": {"93'": 1}}}, {"content": "Do you have sleepwalking disorder (sleeping, not remembering what you did while you were asleep)?", "word_count": 28, "label": "", "sentence_structure": "Subject鈥搗erb鈥搊bject", "subjective_objective": {"answer": "Objective", "rationale": "Because sleepwalking is a medically ill phenomenon, it is objective. Although patients may not be able to remember their behaviour or details during sleepwalking, it does not affect the fact that the phenomenon itself is considered to be an objective pathological state."}, "sentence_sentiment": {"answer": "Neutral", "rationale": "Because the entry is merely an inquiry into whether the person under investigation has ever had a certain symptoms, and does not bear any emotional or value judgement."}, "language_style": {"answer": "Calm and direct", "rationale": ""}, "time_dimension": {"answer": "Past", "rationale": "The entry is for questioning the testee's past behaviour, and the temporal dimension is a past. The keyword \"Is there\" indicates that it is a question about past experience, and \"Scramble disease\" is also a term for describing past behaviour or symptoms."}, "sentence_type": "Interrogative sentence", "pos": {"nouns": {"Sleepwalking": 1, "Sleep": 2, "Time": 1, "Things": 1}, "verbs": {"have": 1, "Let's go": 1, "Walking around": 1, "After": 1, "No, I can't": 1, "Memories": 1, "Sleeping": 1}, "numerals": {"94": 1}}}, {"content": "95. Did you wet the bed?", "word_count": 5, "label": "", "sentence_structure": "Subject鈥搗erb鈥搊bject", "subjective_objective": {"answer": "Objective", "rationale": "Since bed wetting is an observable fact, a subjective assessment by the patient is not required."}, "sentence_sentiment": {"answer": "Negative", "rationale": "Because \"wet the bed\" is negative."}, "language_style": {"answer": "Calm and direct", "rationale": "The language style of the entry is relatively cold and straightforward, and it appears to be objective and realistic to ask respondents in a concise and clear manner whether there is wet bed, and not to use excessive rhetorical or emotional rendering."}, "time_dimension": {"answer": "Present", "rationale": ""}, "sentence_type": "Interrogative sentence", "pos": {"nouns": {"you": 1, "- Bed wet": 1}, "verbs": {"Is it possible": 1, "Pee": 1, "The bed": 1}, "numerals": {"95锛?: 1}}}, {"content": "Were you wetting your bed at the 8-14 age (primary and secondary) stage?", "word_count": 14, "label": "", "sentence_structure": "Subject鈥搗erb鈥搊bject", "subjective_objective": {"answer": "Objective", "rationale": "This is a question of past experience and does not involve subjective attitudes or emotions of patients."}, "sentence_sentiment": {"answer": "Neutral", "rationale": "Because the entry does not have any emotional color."}, "language_style": {"answer": "Calm and direct", "rationale": "The entry uses a straightforward language style, asking if the person interviewed has had a bed wettling experience at the primary and secondary levels."}, "time_dimension": {"answer": "Past", "rationale": "The entry asks about the behaviour of the person tested at the age of 8-14 and therefore determines the past."}, "sentence_type": "Interrogative sentence", "pos": {"nouns": {"Questionnaire": 1, "Entry": 1, "Age": 2, "Phase": 1, "Primary": 1, "Secondary": 1, "- Bed wet": 1}, "verbs": {"Is it possible": 1, "- Bed wet": 1}, "numerals": {"96": 1, "8": 1, "14": 1}, "classifiers": {"Age": 2, "individual": 1}}}, {"content": "Have you ever had a serious problem with your genitals?", "word_count": 15, "label": "", "sentence_structure": "Subject鈥搗erb鈥搊bject", "subjective_objective": {"answer": "Objective", "rationale": "This is an objective fact of the patient ' s state of health and does not involve subjective attitudes or feelings of the patient."}, "sentence_sentiment": {"answer": "Negative", "rationale": "The entry is concerned with the health of the individual and may cause discomfort and negative emotions."}, "language_style": {"answer": "Calm and direct", "rationale": "The entry directly and explicitly enquired about the health of the person under investigation, without any qualifying or disguised words, and the language style was of the direct type of calm."}, "time_dimension": {"answer": "Present", "rationale": "Now the entry is asking the testee about his current state or experience, the keyword being \"had had\" and \"some serious problem\" and all point to the situation."}, "sentence_type": "Interrogative sentence", "pos": {"nouns": {"Questionnaire": 1, "The genitals": 1, "Mistake": 1}, "verbs": {"Yes": 1}, "adjectives": {"Serious": 1}, "numerals": {"97": 1}}}, {"content": "Do you often experience genital pain or pain (in the event of a stroke)?", "word_count": 21, "label": "", "sentence_structure": "Subject鈥搗erb鈥搊bject", "subjective_objective": {"answer": "Objective", "rationale": "The entry is therefore based on objective observations of the patient鈥檚 genital pain or suffering."}, "sentence_sentiment": {"answer": "Negative", "rationale": "Because the \"genie pain or pain\" in the entry is negative."}, "language_style": {"answer": "Calm and direct", "rationale": "The entry is directly asked in a way that is objectively cold and does not use a rhetorical technique to increase the temperature or emotional colour of the language."}, "time_dimension": {"answer": "Present", "rationale": "The entry asks about the current behaviour or experience of the subject, and the keyword \"frequently\" and \"frequently\" points to the current situation."}, "sentence_type": "Interrogative sentence", "pos": {"nouns": {"The genitals": 1, "Pain": 2, "phenomena": 1}, "verbs": {"have": 1, "Regular": 1, "Come on": 1, "It hurts": 1}, "adverbs": {"Is it possible": 1, "Regular": 1, "It hurts when you touch it": 1}, "numerals": {"98": 1, "2": 1}, "classifiers": {"individual": 1}}}, {"content": "Have you ever been treated for genitals?", "word_count": 13, "label": "", "sentence_structure": "Subject鈥搗erb鈥搊bject", "subjective_objective": {"answer": "Objective", "rationale": "Since access to genital treatment is an objective event, it can be verified, whether or not the patient has experienced it, including through medical records."}, "sentence_sentiment": {"answer": "Negative", "rationale": "Because the entry deals with the topic of genital diseases, it can cause some discomfort or negative emotions."}, "language_style": {"answer": "Calm and direct", "rationale": "This entry uses a direct and explicit language to ask the subject whether he has had a history of genital therapy, and does not use any qualifier language or expression."}, "time_dimension": {"answer": "Past", "rationale": "The entry is for questioning of the testee ' s past experience and behaviour, and relates to the specific event of undergoing genital treatment, so that it can be judged as past."}, "sentence_type": "Interrogative sentence", "pos": {"nouns": {"The genitals": 1, "Treatment": 1}, "verbs": {"Accept": 1}, "numerals": {"99": 1}}}, {"content": "Did Dr. 10O. say you had anal disemboweled?", "word_count": 17, "label": "", "sentence_structure": "Subject鈥搗erb鈥搊bject", "subjective_objective": {"answer": "Objective", "rationale": "The absence of anal depravity is an objective condition of the patient and is not influenced by subjective will."}, "sentence_sentiment": {"answer": "Negative", "rationale": "Because \"de-alcohol\" is a disease, it's not good to be diagnosed as suffering from it."}, "language_style": {"answer": "Calm and direct", "rationale": "This is a more objective and calm language style that is suitable for use in questionnaires in areas such as investigation or medicine."}, "time_dimension": {"answer": "Past", "rationale": "The entry is for the testee's past health, and the keyword \"doctor said\" and \"had exalted\" points to the behaviour or state of the past that has occurred. The temporal dimension therefore is the past."}, "sentence_type": "Interrogative sentence", "pos": {"nouns": {"Doctor": 1, "- Getting out of my anus": 1, "Rectum": 1, "Anal": 1, "Outside": 1}, "verbs": {"Say it": 1, "have": 1, "Remove": 1}, "adverbs": {"Pass": 1}, "numerals": {"10O锛?: 1, "you": 1}, "classifiers": {"- You're not": 1}}}, {"content": "Have you ever peed blood?", "word_count": 11, "label": "", "sentence_structure": "Subject鈥搗erb鈥搊bject", "subjective_objective": {"answer": "Objective", "rationale": "Because urine blood is an objective fact that patients may experience, not their subjective attitude or feelings."}, "sentence_sentiment": {"answer": "Negative", "rationale": "Because the condition of \"ure blood\" is a symptom of physical infirmity and has negative implications."}, "language_style": {"answer": "Calm and direct", "rationale": "The entry is more calm and straightforward in its language, and in a question-and-answer manner, the person surveyed is asked whether he has had symptoms of urine blood and is not using any qualifier or rhetorical techniques, which are more objective and neutral."}, "time_dimension": {"answer": "Past", "rationale": "The entry is for the purpose of asking the person who was tested for his or her past health and therefore judging as past."}, "sentence_type": "Interrogative sentence", "pos": {"nouns": {"Pee blood": 1, "Pain": 1}, "verbs": {"have": 1}, "adjectives": {"None": 1}, "adverbs": {"Sex": 1}, "numerals": {"101": 1, "It's painless": 1}}}, {"content": "Have you ever been troubled by the difficulty of urinating?", "word_count": 12, "label": "", "sentence_structure": "Subject鈥搗erb鈥搊bject", "subjective_objective": {"answer": "Objective", "rationale": "Because the urination difficulty is an objective physical condition, it is not the patient ' s subjective attitude."}, "sentence_sentiment": {"answer": "Negative", "rationale": "Because the \"pee-difficult\" disease in the entry is negative."}, "language_style": {"answer": "Calm and direct", "rationale": "This language style is suitable for use in psychological questionnaires and more realistic and objective data can be obtained."}, "time_dimension": {"answer": "Past", "rationale": ""}, "sentence_type": "Interrogative sentence", "pos": {"nouns": {"Problem": 1, "It's hard to pee": 1, "Trouble": 1}, "verbs": {"Tsang": 1, "Because": 1}, "numerals": {"102锛?: 1, "you": 1}}}, {"content": "97. Do you often suffer from menstrual pains (acheal pain during menstruation and before and after?", "word_count": 18, "label": "", "sentence_structure": "Subject鈥搗erb鈥搊bject", "subjective_objective": {"answer": "Objective", "rationale": "Because pain is a physical condition, it is an objective fact that the patient has experienced."}, "sentence_sentiment": {"answer": "Negative", "rationale": "Because the \"the pain\" act in the entry is negative."}, "language_style": {"answer": "Tactful and warm", "rationale": ""}, "time_dimension": {"answer": "Present", "rationale": "\"Do you often suffer\" means \"recurring, habitual\" behaviour, \"the pain\" means \"bare stomach pain\" during and before the menstruation, so this entry asks if the person tested has such experience either now or in the near future. So time dimension is now."}, "sentence_type": "Interrogative sentence", "pos": {"nouns": {"Questionnaire": 1, "Entry": 1, "97": 1, "you": 1, "Is it possible": 1, "Regular": 1, "It hurts": 1, "Period of menstruation": 1, "Back and forth": 1, "My stomach hurts": 1}, "verbs": {"Ache (acheal pain during menstruation and before and after gusts)": 1}, "numerals": {"97. Do you often suffer from menstrual pains (acheal pain during menstruation and before and after": 1}}}, {"content": "98. Are you often ill or weak during menstruation?", "word_count": 16, "label": "", "sentence_structure": "Subject鈥搗erb鈥搊bject", "subjective_objective": {"answer": "Subjective", "rationale": "This is a description of the patient ' s subjective feelings, i.e. whether there is a period of menstrual disorder."}, "sentence_sentiment": {"answer": "Negative", "rationale": "This entry describes the possible physical discomfort and illness of the menstruation period, which has some negative emotional colour."}, "language_style": {"answer": "Tactful and warm", "rationale": "The entry is a moderate and sensitive dialogue, expressed as a more euphemistic and friendly form of health care for women, and uses terms such as \"disease\" and \"weak\" to increase attention and concern for each other's situation."}, "time_dimension": {"answer": "Present", "rationale": "So the temporal dimension of this entry is judged now."}, "sentence_type": "Interrogative sentence", "pos": {"nouns": {"Menstruation": 1, "Diseases": 1, "Weakness": 1}, "verbs": {"Okay": 1, "Sensor": 1, "Present": 1, "Is it possible": 1, "in": 1}, "adjectives": {"Regular": 1}, "numerals": {"98": 1}}}, {"content": "Do you often have menstrual bedtime?", "word_count": 11, "label": "", "sentence_structure": "Subject鈥搗erb鈥搊bject", "subjective_objective": {"answer": "Objective", "rationale": "Because the fact that menstrual bed rest is frequent is an objective fact of the patient ' s experience."}, "sentence_sentiment": {"answer": "Negative", "rationale": "It is a negative act because of the possibility that menstruation can lead to physical discomfort and affect normal life."}, "language_style": {"answer": "Calm and direct", "rationale": "The entry directly asks the respondents whether they are sleeping regularly during their menstrual period, and the language style is more direct and objective, without any qualifying term or emotional colour, and therefore \"cool straight\" style."}, "time_dimension": {"answer": "Present", "rationale": "The keywords \"often\" and \"yes\" indicate that this is a description of the person's current behaviour or state and therefore is the time dimension of the present."}, "sentence_type": "Interrogative sentence", "pos": {"nouns": {"Menstruation": 1, "Get in bed": 1}, "verbs": {"have": 1, "Regular": 1, "Get down": 1}, "adverbs": {"The long run": 1}, "numerals": {"99": 1}}}, {"content": "Do you have vaginal bleeding, except for menstruation?", "word_count": 13, "label": "", "sentence_structure": "Subject鈥搗erb鈥搊bject", "subjective_objective": {"answer": "Objective", "rationale": "This is a question about the patient鈥檚 state of health, and it is objective to say that there is an objective physical phenomenon, namely 鈥渧aginal bleeding鈥?"}, "sentence_sentiment": {"answer": "Negative", "rationale": "Because the \"vaginal bleeding\" in the entry is a negative condition."}, "language_style": {"answer": "Calm and direct", "rationale": "The entry is based on direct, objective language style, and the subject is questioned without excessive emotional rendering or rhetorical techniques. At the same time, the question is presented in a factual and scientific manner, with the aim of obtaining accurate information without subjective bias or inappropriate guidance."}, "time_dimension": {"answer": "Past", "rationale": "The entry asks about the testee ' s past behaviour and the temporal dimension is a past."}, "sentence_type": "Interrogative sentence", "pos": {"nouns": {"99'": 1, "Menstruation": 1, "Vagina": 1, "Blood": 1}, "verbs": {"have": 1, "Divide": 1}, "adverbs": {"External": 1}, "numerals": {"99'": 1}}}, {"content": "Have you often experienced severe and persistent facial red and sweaty?", "word_count": 18, "label": "", "sentence_structure": "Subject鈥搗erb鈥搊bject", "subjective_objective": {"answer": "Objective", "rationale": "Because \"Face and sweat\" are the objective manifestations of physical symptoms, and \"are\" the subjective perceptions of the patient's state. The subject is a combination of subjective attitudes and objective phenomena."}, "sentence_sentiment": {"answer": "Negative", "rationale": "Because the entry \"severe and severe facial red and sweating\" is a symptom of physical discomfort, it has a negative emotional connotation."}, "language_style": {"answer": "Calm and direct", "rationale": "The entry starts with a direct and clear question, without any qualifier or emotional expression, and is more calm and objective. It is designed to understand whether the person under investigation has symptoms of discomfort such as red skin and sweat, and the language style is more straightforward."}, "time_dimension": {"answer": "Present", "rationale": "The entry asks if the person tested is currently suffering from severe and persistent facial red and sweaty skin, and therefore judges it as \"now\"."}, "sentence_type": "Interrogative sentence", "pos": {"nouns": {"Face": 1, "Chim Hung": 1, "Sweat": 1}, "verbs": {"have": 1, "Ongoing": 1, "Serious": 1}, "adverbs": {"Regular": 1}, "numerals": {"100锛?: 1}}}, {"content": "101. Do you often get agitated during menstruation?", "word_count": 14, "label": "", "sentence_structure": "Subject鈥搗erb鈥搊bject", "subjective_objective": {"answer": "Subjective", "rationale": "Because \"heat\" is felt by the patient himself, it's seen from a subjective point of view."}, "sentence_sentiment": {"answer": "Negative", "rationale": "Because the entry describes an uncomfortable physical phenomenon and the emotional problems that may accompany it, it has a certain negative colour."}, "language_style": {"answer": "Calm and direct", "rationale": "The entry asks in a direct and objective manner whether the other party is often restless during menstrual periods, without excessive emotional modification or speech techniques, and with a more calm and straightforward language style."}, "time_dimension": {"answer": "Present", "rationale": "The entry asks whether the testee is often anxious or anxious during the menstruation period of the current cycle, and therefore the time dimension is now judged."}, "sentence_type": "Interrogative sentence", "pos": {"nouns": {"Menstruation": 1, "- Agitation": 1}, "verbs": {"- I do": 1}, "numerals": {"101": 1}}}, {"content": "Do you often get upset about the abnormality of your belt (white visceral viscosity)?", "word_count": 19, "label": "", "sentence_structure": "Subject鈥搗erb鈥搊bject", "subjective_objective": {"answer": "Subjective", "rationale": "Because \"fear\" is a subjective feeling or emotional reaction."}, "sentence_sentiment": {"answer": "Negative", "rationale": "Because \"fear\" in the entries is a negative emotional color."}, "language_style": {"answer": "Calm and direct", "rationale": "The entry is asked in a direct, concise language, without any aesthetic or emotional rendering, and with a calm and objective tone."}, "time_dimension": {"answer": "Present", "rationale": "The temporal dimension of the purpose of the article is therefore judged to be the present."}, "sentence_type": "Interrogative sentence", "pos": {"nouns": {"Questionnaire": 1, "Entry": 1, "you": 1, "Vagina white slime": 1, "Unusual": 1, "Trouble": 1}, "verbs": {"Is it often a problem to have a self-tape abnormality": 1}, "numerals": {"102锛?: 1, "1": 1}}}, {"content": "Do you get up every night because you pee?", "word_count": 12, "label": "", "sentence_structure": "Subject鈥搗erb鈥搊bject", "subjective_objective": {"answer": "Objective", "rationale": "Because this is an issue of the patient鈥檚 living conditions, it is not necessary to examine the patient鈥檚 subjective attitude or feelings, but simply to ask the patient whether he/she gets up every night because he/she is taking a piss."}, "sentence_sentiment": {"answer": "Negative", "rationale": "Because the act of \"getting up in the piss\" in the entry is negative."}, "language_style": {"answer": "Calm and direct", "rationale": "The language style of the entry is a relatively calm and straightforward one. It is more straightforward to ask whether the respondents often get up at night because they pee, without using excessive emotional modifications or euphemism."}, "time_dimension": {"answer": "Past", "rationale": "The entry asks about the testee's past routine behaviour, and the keyword \"getting up every night because of urination\", which refers to a repeated act at a given time (every night) and therefore is judged to be the past."}, "sentence_type": "Interrogative sentence", "pos": {"nouns": {"Oh, my God": 1, "Night": 1, "Pee": 1, "Get up": 1}, "verbs": {"Is it possible": 1, "Chile": 1, "Night": 1, "Because": 1, "Pee": 1, "Get up": 1}, "adverbs": {"you": 2, "Both": 1, "Because": 1, "Chile": 1}, "numerals": {"103": 1}, "classifiers": {"individual": 1}}}, {"content": "Do you often pee in the daytime?", "word_count": 13, "label": "", "sentence_structure": "Subject鈥搗erb鈥搊bject", "subjective_objective": {"answer": "Objective", "rationale": "Because \"the frequency of urination during the day\" is a physical symptom or phenomenon of the patient, it is an objective fact, not a subjective feeling, attitude or behaviour of the patient."}, "sentence_sentiment": {"answer": "Negative", "rationale": "Because the \"high number of day-time urination\" in the entries is negative."}, "language_style": {"answer": "Calm and direct", "rationale": "The entry is based on a direct and objective language, explicitly asking the interviewee whether there are frequent daily urinations, and not too much adoration or emotional expression. Such a design can help researchers to quickly obtain accurate data."}, "time_dimension": {"answer": "Present", "rationale": ""}, "sentence_type": "Interrogative sentence", "pos": {"nouns": {"104": 1, "Oh, my God": 1, "Pee": 1, "Number": 1, "Frequent": 1}, "verbs": {"be": 1, "Regular": 1, "Daytime": 1, "Uirate": 1}, "adjectives": {"Frequent": 1}, "numerals": {"104": 1, "1": 2, "Oh, my God": 1, "Number": 1, "Frequent": 1}, "classifiers": {"Number of times": 1}, "pronouns": {"you": 1}}}, {"content": "Do you often feel burned while peeing?", "word_count": 18, "label": "", "sentence_structure": "Subject鈥搗erb鈥搊bject", "subjective_objective": {"answer": "Objective", "rationale": "Because it is a matter of the patient ' s state of health and objective fact."}, "sentence_sentiment": {"answer": "Negative", "rationale": "Because the symptoms of \"burning\" are a sign of physical discomfort and negative."}, "language_style": {"answer": "Calm and direct", "rationale": "This language style helps to ensure the validity and scientific nature of the questionnaire, since the respondents鈥?answers are not unnecessarily disturbed or misled."}, "time_dimension": {"answer": "Present", "rationale": "The entry asks the testee about his current state of health, using the now completed time pattern \"Is it often there\" to indicate that the act has occurred in the past and may continue. Therefore, the temporal dimension is now judged."}, "sentence_type": "Interrogative sentence", "pos": {"nouns": {"Questionnaire": 1, "Entry": 1, "Pee": 1, "Burning": 1, "Pain": 1, "Fire": 1}, "verbs": {"Is it possible": 2, "- I do": 1, "have": 1, "Burn": 1, "Pain": 1, "Fire": 1}, "adjectives": {"Sample": 1, "Ouch": 1}, "adverbs": {"Regular": 1}, "numerals": {"105": 1, "One": 2}, "classifiers": {"individual": 1}, "pronouns": {"you": 1}}}, {"content": "Do you sometimes have a piss out of control (not by consciousness)?", "word_count": 19, "label": "", "sentence_structure": "Subject鈥搗erb鈥搊bject", "subjective_objective": {"answer": "Objective", "rationale": "Because control of urine is determined by the biological mechanism, not by the patient ' s subjective will."}, "sentence_sentiment": {"answer": "Negative", "rationale": "Because the control of urine is a manifestation of physical discomfort, it is usually considered negative."}, "language_style": {"answer": "Calm and direct", "rationale": "The language style of the entry is calm and straightforward, and it opens up questions without too much embellishment and emotional rendering. It is simple and clear, allowing the respondents to quickly understand the questions and answer them."}, "time_dimension": {"answer": "Past", "rationale": "The entry asks if the person tested had previously had a situation of urine out of control and the time dimension was judged to be past."}, "sentence_type": "Interrogative sentence", "pos": {"nouns": {"you": 1, "Time": 1, "Pee": 2, "Out of control": 1, "Control": 1, "Peeing": 1}, "verbs": {"have": 1, "Sometimes": 1}, "adverbs": {"No, I can't": 1, "From consciousness": 1}, "numerals": {"106锛?: 1, "1": 2}}}, {"content": "Did the doctor say you had a kidney, a bladder?", "word_count": 13, "label": "", "sentence_structure": "Subject鈥搗erb鈥搊bject", "subjective_objective": {"answer": "Objective", "rationale": "It is an objective situation for the patient to be diagnosed as a kidney or bladder disease by a doctor."}, "sentence_sentiment": {"answer": "Negative", "rationale": "Because the description \"sick\" is negative."}, "language_style": {"answer": "Calm and direct", "rationale": "The language style of the entry is cold and neutral, because it is a direct query, without any emotional or rhetorical style. It asks the interviewee in an objective manner, without any personal bias or evaluation. Such an inquiry allows the interviewee to answer the question more accurately and truthfully, thereby achieving a better research purpose."}, "time_dimension": {"answer": "Past", "rationale": ""}, "sentence_type": "Interrogative sentence", "pos": {"nouns": {"Doctor": 1, "Kidney": 1, "- The bladder": 1, "Diseases": 2}, "verbs": {"Say it": 1, "have": 1, "Pass": 1}, "numerals": {"107锛?: 1, "Yours": 1}}}, {"content": "Have you often felt tired?", "word_count": 14, "label": "", "sentence_structure": "Subject鈥損redicate", "subjective_objective": {"answer": "Subjective", "rationale": "Because fatigue is the patient's subjective feeling."}, "sentence_sentiment": {"answer": "Negative", "rationale": "Because \"a moment of fatigue\" in the entry is a negative physical symptom, indicating possible problems of physical discomfort or disease."}, "language_style": {"answer": "Calm and direct", "rationale": "The language style of the entry is calm and straightforward. It asks the respondents in a concise manner whether they are often tired, without too many rhetorical or emotional modifications, and with more objective and formal tone."}, "time_dimension": {"answer": "Present", "rationale": ""}, "sentence_type": "Interrogative sentence", "pos": {"nouns": {"Tired": 1}, "verbs": {"I feel": 1, "Is it possible": 1, "Regular": 1, "Yes": 1, "One": 2, "Line": 1, "very": 1, "For a while": 1}, "adjectives": {"of": 3, "very": 1}, "numerals": {"108": 1, "1": 2}, "classifiers": {"Line": 1}, "pronouns": {"you": 1}}}, {"content": "Do you feel exhausted by your work?", "word_count": 12, "label": "", "sentence_structure": "Subject鈥揷opula鈥損redicative", "subjective_objective": {"answer": "Subjective", "rationale": "It is the subjective feeling and experience of the patient that the work is exhausting."}, "sentence_sentiment": {"answer": "Negative", "rationale": "Because \"tired\" is a negative emotional state."}, "language_style": {"answer": "Calm and direct", "rationale": "The entry is a direct query about the status of the interviewee ' s work, with a more objective and calm language style, and no excessive emotional rendering or rhetorical techniques."}, "time_dimension": {"answer": "Present", "rationale": ""}, "sentence_type": "Interrogative sentence", "pos": {"nouns": {"Work": 1, "I'm exhausted": 1}, "verbs": {"鈾?And I'm gonna be 鈾?: 1, "I feel": 1}, "numerals": {"109": 1}}}, {"content": "Do you often get up in the morning and get tired and exhausted?", "word_count": 20, "label": "", "sentence_structure": "Subject鈥搗erb鈥搊bject", "subjective_objective": {"answer": "Subjective", "rationale": "Because \"weary and exhausted when we get up in the morning\" is the patient's subjective feeling, which is viewed from the patient's perspective."}, "sentence_sentiment": {"answer": "Negative", "rationale": "Because the words \"tired\" and \"tired\" in the entries are negative descriptions."}, "language_style": {"answer": "Tactful and warm", "rationale": "It does not ask questions about the other person's health, but rather questions in a gentle and caring tone, leading the other to think and share their feelings. Moreover, the terms \"tired\" and \"tired in the morning\" are more simple and understandable."}, "time_dimension": {"answer": "Present", "rationale": ""}, "sentence_type": "Interrogative sentence", "pos": {"nouns": {"Morning": 1, "Get up": 1, "Tired": 2, "I'm exhausted": 1}, "verbs": {"I feel": 1, "That's": 1, "Is it possible": 1, "Regular": 1, "When I get up": 1}, "adverbs": {"That's": 1, "Regular": 1}, "numerals": {"110": 1}, "classifiers": {"individual": 1}, "pronouns": {"you": 1}}}, {"content": "Do you get tired of doing a little work?", "word_count": 13, "label": "", "sentence_structure": "Subject鈥搗erb鈥搊bject", "subjective_objective": {"answer": "Subjective", "rationale": "Because \"getting tired with a little work\" is a subjective feeling and experience."}, "sentence_sentiment": {"answer": "Negative", "rationale": "Because the act of \"a little work to get tired\" is negative."}, "language_style": {"answer": "Calm and direct", "rationale": "The entry is based on a simple and straightforward language that directly asks the other party whether he or she is vulnerable to fatigue in his or her work, is not using excessive acrimonyms or emotional expressions and is more objective and direct."}, "time_dimension": {"answer": "Past", "rationale": "The entry asks the testee about past experiences and feelings and thus judges as past."}, "sentence_type": "Interrogative sentence", "pos": {"nouns": {"Work": 1, "Tired": 1}, "verbs": {"I feel": 1, "Do it": 1}, "adjectives": {"A little bit": 1}, "numerals": {"111": 1}, "classifiers": {"individual": 1}}}, {"content": "112. Do you often get tired of eating?", "word_count": 12, "label": "", "sentence_structure": "Subject鈥搗erb鈥搊bject", "subjective_objective": {"answer": "Subjective", "rationale": "Because \"often\" and \"tired\" and \"failed\" are the subjective feelings and experiences of patients."}, "sentence_sentiment": {"answer": "Negative", "rationale": "Because the \"no eating because of the tired\" in the entries is negative."}, "language_style": {"answer": "Calm and direct", "rationale": "The entry is dialogueable in a direct query, is not overly rhetorical or emotional and is more objective in tone."}, "time_dimension": {"answer": "Present", "rationale": ""}, "sentence_type": "Interrogative sentence", "pos": {"nouns": {"Entry": 1, "112": 1, "you": 1, "Regular": 1, "Because": 1, "Tired": 1, "I can't eat": 1, "Rice": 1}, "verbs": {"be": 1, "have": 1, "Because": 1, "I can't eat": 1}, "adjectives": {"Tired": 1}, "adverbs": {"Regular": 1}, "numerals": {"112": 1, "1": 2}, "classifiers": {"Entry": 1, "individual": 1}, "pronouns": {"you": 1}}}, {"content": "113. Have you had a serious nervous breakdown?", "word_count": 11, "label": "", "sentence_structure": "Subject鈥搗erb鈥搊bject", "subjective_objective": {"answer": "Subjective", "rationale": "Because nervous decay is the patient ' s own feelings and experiences, it is the result of subjective observations."}, "sentence_sentiment": {"answer": "Negative", "rationale": "Because the word \"serious\" is a negative emotional expression."}, "language_style": {"answer": "Calm and direct", "rationale": "The language style of the entry is more direct, concise and not too much rhetorical or emotional rendering, with greater emphasis on transmitting information and directing respondents to responding."}, "time_dimension": {"answer": "Past", "rationale": "The entry is for the testee ' s past health and the time dimension is judged to be past."}, "sentence_type": "Interrogative sentence", "pos": {"nouns": {"Nerve failure": 1}, "verbs": {"have": 1, "Serious": 1}, "adjectives": {"Serious": 1}, "numerals": {"113": 1, "1": 2}}}, {"content": "114. Are there any of your family members suffering from nervous breakdown?", "word_count": 15, "label": "", "sentence_structure": "Subject鈥搗erb鈥搊bject", "subjective_objective": {"answer": "Objective", "rationale": "Because \"Is there a nervously weak person in your family?\" is a question of the patient's need for real information, and there is no subjective attitude or feeling. So, it's an objective question."}, "sentence_sentiment": {"answer": "Neutral", "rationale": "Because the entry is asking for an objective fact and does not contain any emotional colour."}, "language_style": {"answer": "Calm and direct", "rationale": "The language style of the entry is relatively calm and objective, and there is no excessive emotional or oral skill, and the question is directly whether the family of the person under investigation has a state of nervous weakness. \""}, "time_dimension": {"answer": "Past", "rationale": ""}, "sentence_type": "Interrogative sentence", "pos": {"nouns": {"Family": 1, "person": 1, "Nerve failure": 1}, "verbs": {"have": 1}, "numerals": {"114": 1}, "pronouns": {"you": 1, "Medium": 1}}}, {"content": "Have you been sick a lot?", "word_count": 7, "label": "", "sentence_structure": "Subject鈥損redicate", "subjective_objective": {"answer": "Objective", "rationale": "Because the fact of illness is an objective fact, the patient cannot control whether he or she is ill or not."}, "sentence_sentiment": {"answer": "Negative", "rationale": "Because the \"disease\" act in the entry is negative."}, "language_style": {"answer": "Calm and direct", "rationale": ""}, "time_dimension": {"answer": "Present", "rationale": "The entry asks if the person tested is currently ill, and the keyword \"recurring illness\" indicates that the act is occurring now or recently. The temporal dimension therefore determines the present."}, "sentence_type": "Interrogative sentence", "pos": {"nouns": {"Diseases": 1, "Sick": 1}, "verbs": {"Is it possible": 1, "Regular": 1, "Sick": 1}, "adverbs": {"Regular": 1}, "numerals": {"115": 1}}}, {"content": "Do you often sleep in bed because of illness?", "word_count": 12, "label": "", "sentence_structure": "Subject鈥搗erb鈥搊bject", "subjective_objective": {"answer": "Objective", "rationale": "The fact that bed resting because of illness is an objective fact of the patient's experience."}, "sentence_sentiment": {"answer": "Negative", "rationale": "Because \"sleeping in bed\" is a negative description."}, "language_style": {"answer": "Calm and direct", "rationale": "The entry is more neutral and objective in terms of direct and concise inquiries about the frequent need for bed-time rest due to illness, and the absence of excessive qualifier or emotional language."}, "time_dimension": {"answer": "Past", "rationale": "So the answer is \"the past\"."}, "sentence_type": "Interrogative sentence", "pos": {"nouns": {"Questionnaire": 1, "Entry": 1, "Sick": 1, "Get in bed": 1}, "verbs": {"Is it possible": 2, "Because": 1, "Regular": 1, "Get down": 1}, "adverbs": {"Regular": 1}, "numerals": {"116锛?: 1, "1": 1}, "classifiers": {"individual": 1}}}, {"content": "Are you always in poor health?", "word_count": 9, "label": "", "sentence_structure": "Subject鈥搗erb鈥搊bject", "subjective_objective": {"answer": "Subjective", "rationale": "Because \"bad health\" is a subjective assessment and perception of the patient, and it is a subjective view."}, "sentence_sentiment": {"answer": "Negative", "rationale": "Because \"bad health\" in the entry is a negative state or result."}, "language_style": {"answer": "Calm and direct", "rationale": "The entry is simple and clear in asking the other side whether it has health problems, speech temperature and calm, is not overly rhetorical or emotionally rendered, and is directly linguistic."}, "time_dimension": {"answer": "Present", "rationale": "The entry asks about the current state of the person being tested and the time dimension is now judged."}, "sentence_type": "Interrogative sentence", "pos": {"nouns": {"117": 1, "Health": 1, "Bad": 1}, "verbs": {"be": 1, "Always": 1}, "numerals": {"117": 1, "Always": 1}}}, {"content": "118. Do you think you're sick?", "word_count": 11, "label": "", "sentence_structure": "Subject鈥搗erb鈥搊bject", "subjective_objective": {"answer": "Objective", "rationale": "This is an objective assessment, since it is assessed by others as a patient."}, "sentence_sentiment": {"answer": "Negative", "rationale": "Because the description of \"infirmity\" in the entry is negative."}, "language_style": {"answer": "Calm and direct", "rationale": "The entry asks the other side whether they believe they are infirm, infirm, in a language temperature and objective, with no excessive emotional rendering or rhetorical techniques, and more in a calm and straightforward language style."}, "time_dimension": {"answer": "Present", "rationale": "The entry asks whether the person tested had a negative assessment of his or her condition at the current point of time, and therefore the temporal dimension is now judged."}, "sentence_type": "Interrogative sentence", "pos": {"nouns": {"you": 1, "I'm sick and sick": 1}, "verbs": {"I think": 1}, "numerals": {"118": 1}}}, {"content": "119. Are there any vulnerable members of your family?", "word_count": 13, "label": "", "sentence_structure": "Subject鈥搗erb鈥搊bject", "subjective_objective": {"answer": "Objective", "rationale": "The fact that the patient is a member of the family is a matter of fact."}, "sentence_sentiment": {"answer": "Neutral", "rationale": "As this entry does not address the assessment or attitude of vulnerable persons."}, "language_style": {"answer": "Calm and direct", "rationale": "The entry is more calm and straightforward in language, without too many qualifiers or emotional terms, simply asking the family member of the interviewee whether he or she is susceptible to disease."}, "time_dimension": {"answer": "Present", "rationale": "The entry asks the subjects about the health status of their family members, and the temporal dimension is therefore now judged."}, "sentence_type": "Interrogative sentence", "pos": {"nouns": {"Family": 1, "Diseases": 1}, "verbs": {"have": 1}, "adjectives": {"Vulnerability": 1}, "numerals": {"119": 1, "1": 2}, "classifiers": {"individual": 1}}}, {"content": "Have you ever been unable to work due to severe pain?", "word_count": 15, "label": "", "sentence_structure": "Subject鈥搗erb鈥搊bject", "subjective_objective": {"answer": "Objective", "rationale": "This is an objective fact that the patient has experienced, not a subjective feeling or attitude."}, "sentence_sentiment": {"answer": "Negative", "rationale": "Because the description of \"serious pain\" in the entry is negative and may involve health problems, emotional tendencies are negative."}, "language_style": {"answer": "Calm and direct", "rationale": "I therefore think that the language style of the article is straightforward and calm."}, "time_dimension": {"answer": "Past", "rationale": "The term \"had\" in the entry indicates a certain act or state of the past, and \"serious pain without work\" is a statement describing the symptoms or experiences of the past, so the entry relates to past behaviour and experiences."}, "sentence_type": "Interrogative sentence", "pos": {"nouns": {"Pain": 1, "Work": 1}, "verbs": {"Tsang": 1, "Because": 1, "Yes": 1, "not": 1, "And": 1}, "adjectives": {"Serious": 1}, "adverbs": {"Once": 1}, "numerals": {"120锛?: 1}}}, {"content": "121. Are you always too much to take for granted because you're worried about your health?", "word_count": 18, "label": "", "sentence_structure": "Subject鈥搗erb鈥搊bject", "subjective_objective": {"answer": "Subjective", "rationale": "The subject is a matter of the patient ' s health concerns, which are subjective attitudes and feelings."}, "sentence_sentiment": {"answer": "Negative", "rationale": "Because the attitude of \"cared for your health\" in the entry is negative."}, "language_style": {"answer": "Tactful and warm", "rationale": "The entry asks questions in a sensitive and understanding manner, rather than directly accusing or questioning the other party for a morbid anxiety. This expression is more conducive to creating a good atmosphere of communication, allowing respondents to share their real thoughts and feelings."}, "time_dimension": {"answer": "Past", "rationale": "The entry asks about the experience and feelings of the testee in the past, and the expression \"always and always\" also implies that this is a question of the past."}, "sentence_type": "Interrogative sentence", "pos": {"nouns": {"you": 1, "self": 1, "Health": 1, "Entry": 1, "I'm worried": 1}, "verbs": {"Always": 1, "I can't take it": 1, "Because": 1, "be": 1, "And": 1, "I'm worried about my health": 1}, "adjectives": {"of": 4, "Always": 1, "I can't take it": 1, "Because": 1}, "adverbs": {"Always": 2}, "numerals": {"121锛?: 1, "1": 3}, "classifiers": {"Entry": 1}, "pronouns": {"you": 1, "Your own": 1}}}, {"content": "122. Are you always sick and unhappy?", "word_count": 12, "label": "", "sentence_structure": "Subject鈥搗erb鈥揷omplement", "subjective_objective": {"answer": "Subjective", "rationale": "Because \"always\" and \"unhappy\" are both patients' own feelings and subjective. And the disease can be objective, but in the context, it's more about looking at the feelings and emotions of patients, and therefore subjective."}, "sentence_sentiment": {"answer": "Negative", "rationale": "Because the words \"diseased\" and \"unhappy\" in the entries are all negative."}, "language_style": {"answer": "Calm and direct", "rationale": ""}, "time_dimension": {"answer": "Present", "rationale": "Now."}, "sentence_type": "Interrogative sentence", "pos": {"nouns": {"122": 1, "Diseases": 1, "It's not good": 1}, "verbs": {"have": 1, "be": 1}, "adverbs": {"Always": 1}, "numerals": {"122": 1, "Diseases": 1, "It's not good": 1}, "classifiers": {"122": 1, "Diseases": 1, "It's not good": 1}}}, {"content": "123. Do you often feel bad for your health?", "word_count": 16, "label": "", "sentence_structure": "Subject鈥搗erb鈥搊bject", "subjective_objective": {"answer": "Subjective", "rationale": "Because feeling unhappy is a subjective emotional experience, the question is about the patient's subjective feelings and attitudes."}, "sentence_sentiment": {"answer": "Negative", "rationale": "Because the \"frightful\" attitude is negative."}, "language_style": {"answer": "Calm and direct", "rationale": "This is a more common expression in psychological questionnaires, in order to ensure the accuracy of the questionnaire and to avoid emotional influence on the person being investigated."}, "time_dimension": {"answer": "Present", "rationale": "The entry asks if the person tested is currently feeling unfortunate for his or her health problems and therefore judges it as now."}, "sentence_type": "Interrogative sentence", "pos": {"nouns": {"Health": 1, "Unfortunately": 1}, "verbs": {"I feel": 1, "Regular": 1, "Because": 1, "not": 1}, "adjectives": {"Okay": 1}, "numerals": {"123": 1}}}, {"content": "124, have you ever had a red fever?", "word_count": 7, "label": "", "sentence_structure": "Subject鈥搗erb鈥搊bject", "subjective_objective": {"answer": "Objective", "rationale": "Because of the fact that he's had a little experience with the red fever."}, "sentence_sentiment": {"answer": "Neutral", "rationale": "Because the entry is a factual question and does not contain any emotional aspects."}, "language_style": {"answer": "Calm and direct", "rationale": "The entry uses a direct, concise language to ask the respondents whether they have had a red fever, have not had too many adorations or emotional colours, and have a more calm and straightforward language style."}, "time_dimension": {"answer": "Past", "rationale": "The entry is for questioning the testee ' s past behaviour and the temporal dimension is a past."}, "sentence_type": "Interrogative sentence", "pos": {"nouns": {"Red fever": 1}, "verbs": {"Yes": 1}, "numerals": {"124": 1}}}, {"content": "125, did you get rheumatism and four limbs?", "word_count": 15, "label": "", "sentence_structure": "Subject鈥搗erb鈥搊bject", "subjective_objective": {"answer": "Objective", "rationale": "Because when I was a child I had to experience rheumatism as an objective fact of the patient's experience."}, "sentence_sentiment": {"answer": "Neutral", "rationale": "Since the entry is merely a question of fact, it does not involve any positive or negative emotions, attitudes or behaviour."}, "language_style": {"answer": "Calm and direct", "rationale": "The language style of the entry is relatively calm and straightforward, with no rhetorical or emotional colour, and the person surveyed is asked about his or her past physical condition in an objective and neutral manner, allowing him or her to answer the question more clearly and accurately."}, "time_dimension": {"answer": "Past", "rationale": "\"Adream\" refers to the period of time, \"with rheumatism, pain in limbs?\" is a question about the history and symptoms of the disease of the past. The entry is therefore a past."}, "sentence_type": "Interrogative sentence", "pos": {"nouns": {"125": 1, "Hours": 1, "When I was little": 1, "Rheumatism": 1, "Four limbs": 1, "Pain": 1}, "verbs": {"Yes": 1, "Is it possible": 1}, "adverbs": {"very": 1}, "numerals": {"125": 1, "Hours": 1}, "pronouns": {"you": 1}}}, {"content": "Have you ever had malaria?", "word_count": 7, "label": "", "sentence_structure": "Subject鈥損redicate", "subjective_objective": {"answer": "Objective", "rationale": "Because it is the patient ' s objective experience to be sick."}, "sentence_sentiment": {"answer": "Neutral", "rationale": "Because the entry merely asked if the person interviewed had ever had a disease and did not show positive or negative emotional sentiments."}, "language_style": {"answer": "Calm and direct", "rationale": "The entry is in a concise language, asking the respondents whether they had ever had malaria, without adding any rhetorical or emotional colours, and with a more calm and objective language style."}, "time_dimension": {"answer": "Past", "rationale": ""}, "sentence_type": "Interrogative sentence", "pos": {"nouns": {"Malaria": 1}, "verbs": {"Yes, I did": 1, "- You're not": 1}, "numerals": {"126": 1}}}, {"content": "127. Have you ever been treated for severe anaemia?", "word_count": 14, "label": "", "sentence_structure": "Subject鈥搗erb鈥搊bject", "subjective_objective": {"answer": "Objective", "rationale": "Because \"severe anaemia\" and \"treated\" are objective facts that patients experience."}, "sentence_sentiment": {"answer": "Negative", "rationale": "Because \"severe anaemia\" and \"treated\" have negative meanings."}, "language_style": {"answer": "Calm and direct", "rationale": "The entry asks the respondents whether they have received treatment for severe anaemia in a straightforward manner, without any aesthetic or emotional expression, and the language style is relatively calm and straightforward."}, "time_dimension": {"answer": "Past", "rationale": ""}, "sentence_type": "Interrogative sentence", "pos": {"nouns": {"Severe anaemia": 1, "Treatment": 1}, "verbs": {"Accept": 1}, "adverbs": {"Because": 1, "Serious": 1}, "numerals": {"127": 1}}}, {"content": "Have you ever been treated for sexually transmitted diseases?", "word_count": 9, "label": "", "sentence_structure": "Subject鈥搗erb鈥搊bject", "subjective_objective": {"answer": "Objective", "rationale": "Because receiving treatment for sexually transmitted diseases is an objective outcome of behaviour, not a subjective attitude or idea of the patient."}, "sentence_sentiment": {"answer": "Negative", "rationale": "Because it involves treatment for sexually transmitted diseases, it can cause discomfort and negative emotions."}, "language_style": {"answer": "Tactful and warm", "rationale": "This is a soft and warm language because it asks the other side whether he or she has been treated with sexually transmitted diseases in a more polite and respectful manner, without using language that is too direct or offensive."}, "time_dimension": {"answer": "Past", "rationale": "The entry asks if the person tested has ever been treated for an STD and the temporal dimension is judged to be past."}, "sentence_type": "Interrogative sentence", "pos": {"nouns": {"Sexually transmitted diseases": 1, "Treatment": 1}, "verbs": {"Accept": 1}, "numerals": {"128锛?: 1}}}, {"content": "129. Do you have diabetes?", "word_count": 7, "label": "", "sentence_structure": "Subject鈥損redicate", "subjective_objective": {"answer": "Objective", "rationale": "Because diabetes is an objective fact of the patient's experience."}, "sentence_sentiment": {"answer": "Negative", "rationale": "Because the entry asks if there is a disease, which is negative emotional."}, "language_style": {"answer": "Calm and direct", "rationale": "The entry is simple, is not too much adouristic or emotional, and is directly asked whether the person surveyed is diabetic, has a strong sense of specificity and clarity."}, "time_dimension": {"answer": "Present", "rationale": "So the answer is \"now\"."}, "sentence_type": "Interrogative sentence", "pos": {"nouns": {"Diabetes": 1}, "verbs": {"have": 1, "Is it possible": 1}, "numerals": {"129": 1}}}, {"content": "Did the doctor say you had thyroid edema?", "word_count": 17, "label": "", "sentence_structure": "Subject鈥搗erb鈥搊bject", "subjective_objective": {"answer": "Objective", "rationale": "Because of the objective fact that the diagnosis of thyroid edema was carried out by a doctor."}, "sentence_sentiment": {"answer": "Neutral", "rationale": "Since the entry asks whether there is a disease, it does not contain any emotion or evaluation."}, "language_style": {"answer": "Calm and direct", "rationale": "This entry is more objective and clear, and is designed to obtain accurate information."}, "time_dimension": {"answer": "Past", "rationale": ""}, "sentence_type": "Interrogative sentence", "pos": {"nouns": {"Doctor": 1, "- Goitre": 1, "Crude neck disease": 1}, "verbs": {"Say it": 1, "have": 1}, "adverbs": {"Tsang": 1}, "numerals": {"130锛?: 1}}}, {"content": "131. Have you ever received treatment for cancer or cancer?", "word_count": 13, "label": "", "sentence_structure": "Subject鈥搗erb鈥搊bject", "subjective_objective": {"answer": "Objective", "rationale": "Because it is the patient ' s objective experience and fact that he has been treated for cancer or cancer."}, "sentence_sentiment": {"answer": "Neutral", "rationale": "Because the entry does not have any emotional color."}, "language_style": {"answer": "Calm and direct", "rationale": ""}, "time_dimension": {"answer": "Past", "rationale": "The entry asks if the person tested has ever been treated for tumours or cancers and the temporal dimension is judged to be past."}, "sentence_type": "Interrogative sentence", "pos": {"nouns": {"you": 1, "Tumours": 1, "Cancer": 1, "Treatment": 1}, "verbs": {"Accept": 1, "be": 1}, "numerals": {"131锛?: 1}}}, {"content": "I32. Are you having any chronic diseases?", "word_count": 10, "label": "", "sentence_structure": "Subject鈥損redicate", "subjective_objective": {"answer": "Subjective", "rationale": "The diagnosis of chronic diseases requires a professional medical assessment, and the question is one of the patients鈥?own perception and feelings."}, "sentence_sentiment": {"answer": "Negative", "rationale": "Because chronic diseases often have a negative impact on the physical and mental health of individuals, the expression of this article has a certain negative orientation."}, "language_style": {"answer": "Calm and direct", "rationale": "The language style of the entry is calm and straightforward. It explicitly asks the respondents whether they have chronic illnesses and do not use any qualifier or emotional expression."}, "time_dimension": {"answer": "Past", "rationale": "The entry asks if the person tested has ever suffered from a chronic disease and the temporal dimension is judged to be past."}, "sentence_type": "Interrogative sentence", "pos": {"nouns": {"Chronic diseases": 1}, "verbs": {"have": 1}, "numerals": {"132": 1, "you": 1, "What": 1}}}, {"content": "- Have you ever been exposed to atomic radiation?", "word_count": 10, "label": "", "sentence_structure": "Subject鈥搗erb鈥搊bject", "subjective_objective": {"answer": "Objective", "rationale": "Because the acceptance of atomic radiation is a specific event, an objective experience and fact of the patient, not a subjective feeling or attitude of the patient."}, "sentence_sentiment": {"answer": "Neutral", "rationale": "Because the entry was not of any emotional colour, it was merely an inquiry into the fact that atomic radiation had been accepted."}, "language_style": {"answer": "Calm and direct", "rationale": "The language style of the entry is so cold, it does not have too much emotional rendering or rhetorical techniques, but rather it gives people a serious, professional feeling in a clear, concise way. This style is more suitable for use in medical or other settings that require precise information."}, "time_dimension": {"answer": "Past", "rationale": ""}, "sentence_type": "Interrogative sentence", "pos": {"nouns": {"Atomic radiation": 1, "Radiation": 1}, "verbs": {"Accept": 1}, "numerals": {"132": 1}}}, {"content": "Are you too thin?", "word_count": 9, "label": "", "sentence_structure": "Subject鈥損redicate", "subjective_objective": {"answer": "Subjective", "rationale": "Since weight loss is a subjective feeling and experience of the patient, subjective attitudes are examined as subjective."}, "sentence_sentiment": {"answer": "Negative", "rationale": "This entry implies that weight loss is a problem and can give rise to negative emotions and concerns."}, "language_style": {"answer": "Tactful and warm", "rationale": "This is a more gentle and friendly expression, which enhances the cooperation and objectivity of the person under investigation."}, "time_dimension": {"answer": "Present", "rationale": "The entry now asks the testees about their current state of health, i.e. whether they have suffered a weight loss, and therefore the test is now judged."}, "sentence_type": "Interrogative sentence", "pos": {"nouns": {"Questionnaire": 1, "Entry": 1, "you": 1, "Weight": 1, "Mitigation": 1}, "verbs": {"Pass": 1}, "numerals": {"133锛?: 1, "1": 1}, "classifiers": {"individual": 1}}}, {"content": "Have you been overweight?", "word_count": 9, "label": "", "sentence_structure": "Subject鈥損redicate", "subjective_objective": {"answer": "Subjective", "rationale": "Because \"overweight\" is a personal subjective feeling and self-evaluation, different people may have different satisfactions and standards for body size. The problem is to look at patients ' subjective feelings and perceptions."}, "sentence_sentiment": {"answer": "Negative", "rationale": "Because \"overweight\" is a negative word, expressing dissatisfaction with the physical form."}, "language_style": {"answer": "Tactful and warm", "rationale": "The entry is more proficient and interactive when it is spoken in a gentle and inviting tone, rather than in direct statements or orders. At the same time, the term \"whether or not\" leaves room for choice and does not create pressure or a sense of blame on the other."}, "time_dimension": {"answer": "Past", "rationale": "The entry asks if the testee currently has any weight gain, and therefore the time dimension is judged to be past."}, "sentence_type": "Interrogative sentence", "pos": {"nouns": {"134": 1, "you": 1, "Overweight": 1, "Weight gain": 1}, "verbs": {"be": 1}, "adjectives": {"Fat": 1, "Increase": 1}, "numerals": {"134": 1, "1": 2}, "classifiers": {"individual": 1}, "pronouns": {"you": 1}}}, {"content": "135. Did any doctor say you had a leg vein (exposed leg dyslexia)?", "word_count": 21, "label": "", "sentence_structure": "Subject鈥搗erb鈥搊bject", "subjective_objective": {"answer": "Objective", "rationale": "It is an objective fact that the leg is diagnosed by a doctor as having a vein."}, "sentence_sentiment": {"answer": "Neutral", "rationale": "Because the entry does not have any emotional color."}, "language_style": {"answer": "Calm and direct", "rationale": "The entry is based on the following criteria: it is based on direct, clear questions, with no excessive rhetoric and emotional overturning. At the same time, the questions asked are very specific and objective, and there is no ambiguity or ambiguity, and the language style is more calm and straightforward."}, "time_dimension": {"answer": "Past", "rationale": "The entry is based on the testee ' s past medical experience and medical findings, and therefore the temporal dimension is a past."}, "sentence_type": "Interrogative sentence", "pos": {"nouns": {"Doctor": 1, "Legs": 2, "Vulcan sprite": 1, "The tan is exposed": 1}, "verbs": {"Say it": 1, "have": 1}, "numerals": {"135锛?: 1}}}, {"content": "136. Have you been hospitalized and operated?", "word_count": 9, "label": "", "sentence_structure": "Subject鈥損redicate", "subjective_objective": {"answer": "Objective", "rationale": "Because hospitalization and surgery are objective facts experienced by patients, not subjective attitudes or feelings."}, "sentence_sentiment": {"answer": "Neutral", "rationale": "Because the entry does not have any emotional color."}, "language_style": {"answer": "Calm and direct", "rationale": "The entry is more accurate in terms of the information required."}, "time_dimension": {"answer": "Past", "rationale": ""}, "sentence_type": "Interrogative sentence", "pos": {"nouns": {"you": 1, "The operation": 1}, "verbs": {"Hospitalization": 1, "Do it": 1}, "numerals": {"136锛?: 1}}}, {"content": "Have you ever had any serious trauma?", "word_count": 10, "label": "", "sentence_structure": "Subject鈥損redicate", "subjective_objective": {"answer": "Objective", "rationale": "Because the existence of a serious trauma is an objective fact that the patient has experienced."}, "sentence_sentiment": {"answer": "Negative", "rationale": "Because the experience of \"serious trauma\" in the entry is negative."}, "language_style": {"answer": "Calm and direct", "rationale": "The language style of the entry is calm, as it directly asks whether the person under investigation has had a history of severe trauma, has no oral or emotional rendering, and is more objective and concise."}, "time_dimension": {"answer": "Past", "rationale": "The keyword \"had ever\" indicates that it was an inquiry into the past, so it was judged to be the past."}, "sentence_type": "Interrogative sentence", "pos": {"nouns": {"Trauma": 1, "Serious": 1}, "verbs": {"have": 1}, "adjectives": {"Pass": 1}, "numerals": {"137": 1}}}, {"content": "138. Do you have a regular accident or trauma?", "word_count": 14, "label": "", "sentence_structure": "Subject鈥損redicate", "subjective_objective": {"answer": "Objective", "rationale": "Because \"a small accident or trauma\" is an objective experience and behaviour of the patient."}, "sentence_sentiment": {"answer": "Negative", "rationale": "Because \"a small accident or trauma\" is a negative event."}, "language_style": {"answer": "Calm and direct", "rationale": "The entry is more direct in terms of the objective and concise manner in which the person tested is asked whether he or she is regularly subjected to minor accidents or injuries, without any use of a rhetorical technique or emotional colour."}, "time_dimension": {"answer": "Present", "rationale": ""}, "sentence_type": "Interrogative sentence", "pos": {"nouns": {"Accidents": 1, "Trauma": 1}, "verbs": {"Come on": 1, "Regular": 1}, "adjectives": {"Small": 1}, "numerals": {"138锛?: 1}}}, {"content": "139. Are you having difficulty sleeping or not sleeping well?", "word_count": 19, "label": "", "sentence_structure": "Subject鈥搗erb鈥搊bject", "subjective_objective": {"answer": "Subjective", "rationale": "This is because of the subjective perception that patients are experiencing certain phenomena."}, "sentence_sentiment": {"answer": "Negative", "rationale": "Because \"sleeping is difficult\" and \"sleep is not awaken\" are sleep problems, which are usually considered negative."}, "language_style": {"answer": "Calm and direct", "rationale": "The entry describes the content of the survey in an objective, concise language, without too much emotional rendering or rhetorical techniques. It asks the person under investigation whether he or she has symptoms of a lapse in sleep, is plain, clear and easy to understand. The language style of the purpose is therefore straightforward and calm."}, "time_dimension": {"answer": "Present", "rationale": "The entry asks if the person tested is currently having difficulty sleeping or sleeping awoke, and the time dimension is now judged."}, "sentence_type": "Interrogative sentence", "pos": {"nouns": {"phenomena": 1, "Sleep": 3, "Difficulties": 1, "It's deep": 1, "Wake up": 1}, "verbs": {"Did you": 2, "Deep": 1, "Wake up": 1, "Enter": 1}, "adjectives": {"very": 1, "Difficulties": 1, "Light": 1, "Easy": 1}, "numerals": {"139": 1}, "classifiers": {"individual": 1}}}, {"content": "Do you dream often?", "word_count": 6, "label": "", "sentence_structure": "Subject鈥搗erb鈥搊bject", "subjective_objective": {"answer": "Objective", "rationale": "Because the appearance and frequency of dreams are objective biological phenomena, they are not related to the patient ' s subjective attitude."}, "sentence_sentiment": {"answer": "Neutral", "rationale": "Since the entry is concerned with personal experience, it does not address positive or negative attitudes, behaviour or objectives."}, "language_style": {"answer": "Tactful and warm", "rationale": "The language style of the entry is euphemistic, and in a gentle and caring way, asks the interviewees whether they often dream, do not express their views or assessments directly, and pay more attention to the personal experiences and feelings of the interviewees."}, "time_dimension": {"answer": "Present", "rationale": "The keywords are \"often\" and \"dreaming\" and they all point to current behavior or state."}, "sentence_type": "Interrogative sentence", "pos": {"nouns": {"Questionnaire Entry": 1, "Dreams": 2, "you": 2}, "verbs": {"Regular": 1, "Do it": 1}, "adverbs": {"139": 1}, "numerals": {"1": 1}}}, {"content": "Can't you just relax regularly every day?", "word_count": 19, "label": "", "sentence_structure": "Subject鈥搗erb鈥搊bject", "subjective_objective": {"answer": "Subjective", "rationale": "Because \"relax\" and \"re Rest\" are the needs and behaviours that patients feel subjectively, these are considered subjectively."}, "sentence_sentiment": {"answer": "Negative", "rationale": "Because the entry \"can't do a regular daily relaxing\" is negative."}, "language_style": {"answer": "Calm and direct", "rationale": "The question is presented in a more concise and concise manner, with no excessive emotional or rhetorical approach, and more in line with the objective and precise requirements of the questionnaire survey."}, "time_dimension": {"answer": "Present", "rationale": "The entry asks the testee about his current behaviour or state, and the keyword \"can't\" indicates that it is a sentence that is now complete and that it begins at a certain point in time and continues until now. The time dimension of this entry is therefore now."}, "sentence_type": "Interrogative sentence", "pos": {"nouns": {"Relax": 1, "Rest": 1}, "verbs": {"Do it": 1, "There's a regular pattern": 1}, "adverbs": {"Is it possible": 1}, "numerals": {"Chile": 1, "140锛?: 1}}}, {"content": "141. Are you not easy to practice regularly every day?", "word_count": 16, "label": "", "sentence_structure": "Subject鈥損redicate", "subjective_objective": {"answer": "Subjective", "rationale": "Because \"sequenced exercise\" is a problem with behavioral habits, with patients' subjective attitudes and behavioural choices."}, "sentence_sentiment": {"answer": "Negative", "rationale": "Because the entry \"it's not easy to exercise regularly every day\" is negative."}, "language_style": {"answer": "Tactful and warm", "rationale": "The entry is more adept and interactive when it is spoken in a gentle and inviting manner, rather than by direct statements or orders."}, "time_dimension": {"answer": "Present", "rationale": "Because the title \"Are you...\" suggests that it is a question of the current situation, and \"A regular exercise every day\" describes a customary behaviour that usually takes some time to form. The entry is therefore asking if the person tested is now used to a regular exercise every day."}, "sentence_type": "Interrogative sentence", "pos": {"nouns": {"Questionnaire": 1, "Entry": 1, "Oh, my God": 1, "Exercise": 1}, "verbs": {"Do it": 1, "Easy": 1, "There's a regular pattern": 1}, "adverbs": {"not": 1, "Land": 1}, "numerals": {"141": 1}}}, {"content": "142. Do you smoke more than 20 paper cigarettes a day?", "word_count": 11, "label": "", "sentence_structure": "Subject鈥搗erb鈥搊bject", "subjective_objective": {"answer": "Objective", "rationale": "Because more than 20 paper cigarettes are an objective act and habit of the patient."}, "sentence_sentiment": {"answer": "Negative", "rationale": "Because the \"over 20 paper cigarettes per day\" act in the entry is negative."}, "language_style": {"answer": "Calm and direct", "rationale": "The language style of the entry is of a more direct and objective type, with no excessive emotional or rhetorical style. It directly asks the respondents whether they have a habit of smoking and specifies daily quantitative requirements, is concise and clear and informative."}, "time_dimension": {"answer": "Present", "rationale": "The entry asks the testees about their current behaviour, i.e. whether they smoke more than 20 cigarettes a day, and therefore the basis of the judgement is now."}, "sentence_type": "Interrogative sentence", "pos": {"nouns": {"Oh, my God": 1, "Smoke": 1}, "verbs": {"Suck": 1}, "adjectives": {"20": 1, "Expenditure": 1, "Above": 1}, "numerals": {"Chile": 1}, "classifiers": {"Paper": 1}}}, {"content": "Do you have more tea or coffee than the usual?", "word_count": 16, "label": "", "sentence_structure": "Subject鈥搗erb鈥搊bject", "subjective_objective": {"answer": "Subjective", "rationale": "This is a subjective examination because the amount of tea or coffee is the patient ' s personal habits and choices."}, "sentence_sentiment": {"answer": "Neutral", "rationale": "Because the entry does not have any emotional color."}, "language_style": {"answer": "Calm and direct", "rationale": "The entry is simple and clear, asking directly whether the interviewees drink tea or coffee more often than the general population, and the language is more objective and cold, without excessive emotional correction."}, "time_dimension": {"answer": "Present", "rationale": "The entry asks about the testee's current habits and behaviour, which is, \"Do you drink more tea or coffee than the average person?\" So it's \"now\"."}, "sentence_type": "Interrogative sentence", "pos": {"nouns": {"Questionnaire Entry": 1, "Tea": 1, "Coffee": 1, "person": 2}, "verbs": {"Have some tea": 1, "Coffee": 1, "Yes": 1, "More": 1}, "numerals": {"143": 1, "General": 1}, "classifiers": {"individual": 1}, "pronouns": {"you": 1}}}, {"content": "Have you been drinking more than twice a day?", "word_count": 13, "label": "", "sentence_structure": "Subject鈥搗erb鈥揷omplement", "subjective_objective": {"answer": "Objective", "rationale": "Because drinking white wine several times a day is an observable behaviour and fact, not just a patient鈥檚 subjective attitude or feelings. The problem is therefore viewed from an objective perspective."}, "sentence_sentiment": {"answer": "Negative", "rationale": "The basis for the judgement. The behaviour is negative because of the health hazards to the health of the person caused by the frequent drinking."}, "language_style": {"answer": "Calm and direct", "rationale": "This language style is suitable for serious, professional questionnaires to enable respondents to understand the questions and give real answers."}, "time_dimension": {"answer": "Present", "rationale": ""}, "sentence_type": "Interrogative sentence", "pos": {"nouns": {"Oh, my God": 1, "White wine": 1}, "verbs": {"Drink": 1, "Chile": 1, "Two": 2}, "adverbs": {"Go, go, go": 1, "Here": 1, "Number of times": 1}, "numerals": {"144": 1, "More than twice": 1}}}, {"content": "145. Were you sweaty or shivering when you were tested or asked?", "word_count": 22, "label": "", "sentence_structure": "Subject鈥搗erb鈥搊bject", "subjective_objective": {"answer": "Subjective", "rationale": "Because \"if the test or the question is much sweat or shivering\" is the patient's subjective feeling, not the objective fact."}, "sentence_sentiment": {"answer": "Negative", "rationale": "Because \"the test or being questioned is sweaty or shivering\" is often negative and may affect the individual's performance and confidence."}, "language_style": {"answer": "Calm and direct", "rationale": "The entry, which is in plain and clear language, directly asks the interviewees about their physical reactions to the examination or the questions they have been asked, without excessive rhetorical or emotional colours, and with a more objective and calm tone."}, "time_dimension": {"answer": "Present", "rationale": "The words \"when tested or questioned\" , \"sweaty sweat\" and \"sweet shaking\" indicate that this is a description of the current situation, so the entry is about the present behavior or state."}, "sentence_type": "Interrogative sentence", "pos": {"nouns": {"you": 2, "Examinations": 1, "Problem": 1, "Time": 1, "Sweat": 1, "Wreck": 1, "That's great": 1}, "verbs": {"Sweat": 1, "Question": 1, "Is it possible": 1, "# Wrestle #": 1}, "adjectives": {"A lot": 1, "very": 2}, "numerals": {"145": 1}, "classifiers": {"individual": 1}, "pronouns": {"Here": 1}}}, {"content": "Are you nervous and shaking when you approach your supervisor?", "word_count": 16, "label": "", "sentence_structure": "Subject鈥搗erb鈥搊bject", "subjective_objective": {"answer": "Subjective", "rationale": "As stress and shaking are psychological responses of patients, they are subjective."}, "sentence_sentiment": {"answer": "Negative", "rationale": "Because the words \"strike\" and \"shock\" in the entries are negative, suggesting that the interviewee may have a negative emotional reaction when approaching the supervisor."}, "language_style": {"answer": "Calm and direct", "rationale": "This language style is more direct and concise, and does not involve too much embellishment or emotional rendering."}, "time_dimension": {"answer": "Past", "rationale": ""}, "sentence_type": "Interrogative sentence", "pos": {"nouns": {"Supervisor's superior": 1, "Nervous": 1, "# Wresh #": 1}, "verbs": {"Close": 1, "Is it possible": 1, "Nervous and shivering": 1}, "numerals": {"146锛?: 1}}}, {"content": "147. Are you overwhelmed when your superiors watch you work?", "word_count": 18, "label": "", "sentence_structure": "Subject鈥搗erb鈥搊bject", "subjective_objective": {"answer": "Subjective", "rationale": "Because \"unsure\" is a psychological experience or experience that is viewed from the patient's subjective perspective."}, "sentence_sentiment": {"answer": "Negative", "rationale": "Because the emotional reaction of \"unsure\" in the entries is negative."}, "language_style": {"answer": "Calm and direct", "rationale": "The entry asks the respondent whether he is in a state of uncertainty when he is under superior supervision."}, "time_dimension": {"answer": "Present", "rationale": ""}, "sentence_type": "Interrogative sentence", "pos": {"nouns": {"Superior": 1, "Work": 1}, "verbs": {"Watch": 1, "I don't know what to do": 1}, "numerals": {"147": 1}, "pronouns": {"you": 2}}}, {"content": "148. When things have to be done quickly, do you have a complete confusion?", "word_count": 22, "label": "", "sentence_structure": "Subject鈥搗erb鈥搊bject", "subjective_objective": {"answer": "Subjective", "rationale": "Because this question is asked about the feelings and experiences of individuals and is subjective."}, "sentence_sentiment": {"answer": "Negative", "rationale": "Because the \"total confusion\" in the entry is negative."}, "language_style": {"answer": "Calm and direct", "rationale": "The language style of the entry is calm and straightforward. It clearly asks whether the person surveyed has a disordered mind in case of a need for prompt action, without too much ado or emotional. This expression is simple, easy to understand, and suitable for use in psychological questionnaires."}, "time_dimension": {"answer": "Present", "rationale": ""}, "sentence_type": "Interrogative sentence", "pos": {"nouns": {"148": 1, "phenomena": 1, "Things": 1}, "verbs": {"have": 1, "be": 1, "Do it": 1, "Total": 1, "Come on": 1, "Chaos": 1}, "adverbs": {"I have to": 1, "Quick": 1}, "numerals": {"148": 1, "1": 3, "Total": 1}, "classifiers": {"individual": 1}, "pronouns": {"you": 1}}}, {"content": "149. Do you have to be slow to avoid mistakes?", "word_count": 14, "label": "", "sentence_structure": "Subject鈥搗erb鈥搊bject", "subjective_objective": {"answer": "Subjective", "rationale": "Because \"must be slow\" is the patient's subjective feeling and attitude, not an objective external situation."}, "sentence_sentiment": {"answer": "Negative", "rationale": "Because the purpose of the entry \"to avoid errors\" could be negative."}, "language_style": {"answer": "Calm and direct", "rationale": "The entry uses a simple and clear language, which is not overly phrasing or emotional, and asks the respondent in a direct manner whether he/she is accustomed to slowing down his/her work to avoid errors. This language style is more objective and neutral and does not put the respondent under too much psychological pressure."}, "time_dimension": {"answer": "Present", "rationale": ""}, "sentence_type": "Interrogative sentence", "pos": {"nouns": {"Error": 1, "Yes": 1, "Do it": 1}, "verbs": {"Avoid": 1, "Yes": 1, "Slow": 1}, "adverbs": {"very": 1}, "numerals": {"149": 1}}}, {"content": "Have you often mispercepted (understand) the instructions or intentions?", "word_count": 15, "label": "", "sentence_structure": "Subject鈥搗erb鈥搊bject", "subjective_objective": {"answer": "Subjective", "rationale": "Because \"the order or intent is often perceived (understanding) wrong\" is the subjective feeling and experience of the patient and is subjective in nature."}, "sentence_sentiment": {"answer": "Negative", "rationale": "Because the \"understanding\" act in the entry is negative."}, "language_style": {"answer": "Calm and direct", "rationale": "The entry describes the survey in short, direct language, without excessive rhetorical or emotional modifications, and is more objective and calm."}, "time_dimension": {"answer": "Present", "rationale": ""}, "sentence_type": "Interrogative sentence", "pos": {"nouns": {"Command": 1, "Intention": 1}, "verbs": {"Feel it": 1, "Understood": 1, "Wrong": 1}, "adverbs": {"Regular": 1}, "numerals": {"150": 1}}}, {"content": "151. Are you afraid of people or places that are alien?", "word_count": 15, "label": "", "sentence_structure": "Subject鈥搗erb鈥搊bject", "subjective_objective": {"answer": "Subjective", "rationale": "Because this question is about the feelings and experiences of the patient and is subjective."}, "sentence_sentiment": {"answer": "Negative", "rationale": "Because the entry expresses negative behaviour and attitudes, i.e. fear of strangers or places."}, "language_style": {"answer": "Calm and direct", "rationale": "The language style of the entry is relatively simple, and the person is asked directly about the reaction to a strange environment or person, without using excessive rhetorical techniques or emotional rendering, so that the language style can be judged as \"cool and direct\"."}, "time_dimension": {"answer": "Present", "rationale": "\"Whether\" is a choice question to ask the testee about his current state or customary behaviour, \"the alien person or place scares you\" describes a feeling and experience, all of which point to the present situation. The entry is therefore about the current behaviour and mentality."}, "sentence_type": "Interrogative sentence", "pos": {"nouns": {"person": 1, "Places": 1, "Scared": 1}, "verbs": {"鈾?And I'm gonna be 鈾?: 1, "I feel": 1}, "numerals": {"151锛?: 1}}}, {"content": "Were you scared when you didn't know anyone?", "word_count": 16, "label": "", "sentence_structure": "Subject鈥搗erb鈥搊bject", "subjective_objective": {"answer": "Subjective", "rationale": "Because panic is a subjective feeling or emotional reaction, it is viewed from the patient ' s subjective perspective."}, "sentence_sentiment": {"answer": "Negative", "rationale": "Because the \"frightfulness\" in the entry is negative."}, "language_style": {"answer": "Calm and direct", "rationale": "The entry directly raises the question of whether the other party is afraid of being unaccompanied, which is clear and direct, and does not use excessive rhetorical or emotional rendering."}, "time_dimension": {"answer": "Present", "rationale": "The entry asks the testee ' s current behaviour or state, i.e. whether he feels alone and panic when there is no acquaintance, and therefore judges it now."}, "sentence_type": "Interrogative sentence", "pos": {"nouns": {"friends": 1, "person": 1, "Loneliness": 1, "Panic": 1}, "verbs": {"have": 1, "be": 1, "Yes": 1}, "numerals": {"152": 1}, "classifiers": {"individual": 1}, "pronouns": {"you": 1}}}, {"content": "153. Are you always hard to make decisions (suspensively)?", "word_count": 14, "label": "", "sentence_structure": "Subject鈥搗erb鈥搊bject", "subjective_objective": {"answer": "Subjective", "rationale": "Because \"resolve\" and \"suspensive\" are patients' subjective psychological experiences and attitudes."}, "sentence_sentiment": {"answer": "Negative", "rationale": "The basis for the judgement: the act of \"hard to resolve\" in the entry is negative."}, "language_style": {"answer": "Calm and direct", "rationale": "This type of questionnaire entry is usually designed to obtain the specific views and attitudes of the respondents on a particular issue."}, "time_dimension": {"answer": "Present", "rationale": ""}, "sentence_type": "Interrogative sentence", "pos": {"nouns": {"Resolve": 1, "Relentless": 1}, "verbs": {"Down": 1, "Total": 1, "be": 1}, "adjectives": {"Hard": 1}, "adverbs": {"Always": 1}, "numerals": {"153": 1, "you": 1, "Is it possible": 1}}}, {"content": "Have you always wanted someone to come along and give you an idea?", "word_count": 18, "label": "", "sentence_structure": "Subject鈥損redicate", "subjective_objective": {"answer": "Subjective", "rationale": "Because \"favorite\" is a person's inner feeling and thoughts, and it's a subjective view."}, "sentence_sentiment": {"answer": "Positive", "rationale": "Because the entry \"Always wants someone to come to you with an idea\" expresses an emotional, positive feeling of looking for help and advice."}, "language_style": {"answer": "Calm and direct", "rationale": "The entry is more direct and objective in its language style, with no excessive rhetoric or emotional colour. It simply raises a question and invites respondents to answer, and is more calm and direct."}, "time_dimension": {"answer": "Present", "rationale": "The entry asks the testee about his current behaviour or thinking, and the keyword \"Whether he always wants\" indicates that this is a matter of the current state of affairs."}, "sentence_type": "Interrogative sentence", "pos": {"nouns": {"person": 1, "Ideas": 1}, "verbs": {"Here": 1}, "adverbs": {"Total": 1, "in": 1}, "numerals": {"154": 1}, "classifiers": {"you": 1}}}, {"content": "Do people think you're a stupid person?", "word_count": 13, "label": "", "sentence_structure": "Subject鈥搗erb鈥搊bject", "subjective_objective": {"answer": "Objective", "rationale": "The question is whether the patient is perceived as being stupid, and the opinion is raised by others, and therefore the objective facts are examined."}, "sentence_sentiment": {"answer": "Negative", "rationale": "Because the expression \"you're considered a very stupid person\" in the entry is a derogatory and negative emotional and negative one."}, "language_style": {"answer": "Calm and direct", "rationale": "The entry is presented in the form of a direct question, without any accoutrements or emotional colours, and is of an objective and indifferent language style."}, "time_dimension": {"answer": "Present", "rationale": "The entry asks the testee what other people think or evaluate them at the moment, \"Are you a very stupid person?\" It's a judgment in the moment. So the time dimension is now."}, "sentence_type": "Interrogative sentence", "pos": {"nouns": {"others": 1, "person": 2}, "verbs": {"Recognition": 1, "be": 1, "I think": 1, "One": 1, "individual": 1, "very": 1, "Stupid": 1, "of": 3}, "adjectives": {"very": 1, "Stupid": 1}, "numerals": {"155": 1}, "pronouns": {"you": 1}}}, {"content": "156. Are you disturbed to eat anywhere but in your own house?", "word_count": 25, "label": "", "sentence_structure": "Subject鈥搗erb鈥搊bject", "subjective_objective": {"answer": "Subjective", "rationale": "Because \"frustration\" is the patient's subjective feeling."}, "sentence_sentiment": {"answer": "Negative", "rationale": "Because the \"disturbing\" feeling in the entry is negative."}, "language_style": {"answer": "Calm and direct", "rationale": "The entry is based on a direct question-and-answer dialogue, without too many rhetorical or emotional expressions and with a more objective tone. At the same time, the purpose of the article is very straightforward and there is no redundant information that will allow the respondents to understand and answer the question quickly."}, "time_dimension": {"answer": "Past", "rationale": "The entry asks the testee about his past feelings and experiences, i.e. whether he was ever so restless at dinner elsewhere than at home. The test is therefore \"the past\"."}, "sentence_type": "Interrogative sentence", "pos": {"nouns": {"156": 1, "you": 1, "Home": 1, "Local": 2, "Food": 1, "Disturbing": 1}, "verbs": {"in": 3, "Eat": 1, "I feel": 1, "Both": 1, "Yes": 1}, "adjectives": {"Any": 1, "Other": 1, "self": 1}, "adverbs": {"Outside": 1, "Both": 1}, "numerals": {"156": 1, "2": 1}, "classifiers": {"Local": 2}, "pronouns": {"you": 1}}}, {"content": "157. Are you lonely and sad at the party?", "word_count": 14, "label": "", "sentence_structure": "Subject鈥搗erb鈥搊bject", "subjective_objective": {"answer": "Subjective", "rationale": "Because feeling alone and sad is a subjective emotional experience."}, "sentence_sentiment": {"answer": "Negative", "rationale": "Because the \"sorrowing and sorrow\" emotion in the entry is negative."}, "language_style": {"answer": "Tactful and warm", "rationale": "The entry leads the interviewee to think about his feelings in a question-and-answer manner, while using the words \"lonely\" and \"sorrowly\" to resonate, with a more euphemismal style of language."}, "time_dimension": {"answer": "Present", "rationale": "The entry now asks about the feelings and experiences of the subject at this moment, the key word being \"Do you feel lonely and sad at the party?\" and points to what is happening or going through."}, "sentence_type": "Interrogative sentence", "pos": {"nouns": {"Party": 1, "Loneliness": 1, "Sad": 1}, "verbs": {"I feel": 1, "be": 1, "in": 1}, "numerals": {"157": 1}, "classifiers": {"Yeah": 1, "and": 1, "- You're not": 1}}}, {"content": "Have you often been unhappy and depressed?", "word_count": 19, "label": "", "sentence_structure": "Subject鈥損redicate", "subjective_objective": {"answer": "Subjective", "rationale": "Because the question is about the patient ' s own emotional experience, it is a subjective examination."}, "sentence_sentiment": {"answer": "Negative", "rationale": "Because the emotional description of \"discomfort and depression\" in the entry is negative."}, "language_style": {"answer": "Tactful and warm", "rationale": "The entry is more adept and interactive when it is spoken in a gentle and inviting manner, rather than by direct statements or orders."}, "time_dimension": {"answer": "Past", "rationale": "The term \"frequently\" and \"influencing\" in the entry indicates whether the person tested has had or is in existence. The temporal dimension of the article therefore determines past or present."}, "sentence_type": "Interrogative sentence", "pos": {"nouns": {"Questionnaire Entry": 1, "158": 1, "you": 1, "Regular": 1, "I feel": 1, "It's not good": 1, "Emotional depression": 1, "I'm depressed": 1}, "verbs": {"Is it possible": 1, "I feel": 1, "Regular": 1, "It's not good": 1, "and": 1, "Emotional depression": 1, "(Emotion low)": 1, "EMPTY": 1}, "adjectives": {"Regular": 1, "It's not good": 1, "Down": 1}, "numerals": {"158": 1}, "classifiers": {"Entry": 1}, "pronouns": {"you": 1}}}, {"content": "Do you cry often?", "word_count": 6, "label": "", "sentence_structure": "Subject鈥損redicate", "subjective_objective": {"answer": "Subjective", "rationale": "Because crying is an emotional expression, a subjective feeling and experience of the patient."}, "sentence_sentiment": {"answer": "Negative", "rationale": "Because the \"often crying\" act is negative."}, "language_style": {"answer": "Tactful and warm", "rationale": "This expression reduces the discomfort of the interviewee and increases his confidence in the investigation."}, "time_dimension": {"answer": "Present", "rationale": "The keyword is \"are we crying often\" and it asks about the person's current behaviour or state, so it's now."}, "sentence_type": "Interrogative sentence", "pos": {"nouns": {"you": 1, "Cry": 1}, "verbs": {"Is it possible": 1, "Regular": 1, "Cry": 1}, "adverbs": {"Regular": 1}, "numerals": {"159锛?: 1}, "pronouns": {"you": 1}}}, {"content": "Have you always felt sad and frustrated?", "word_count": 16, "label": "", "sentence_structure": "Subject鈥搗erb鈥搊bject", "subjective_objective": {"answer": "Subjective", "rationale": "Because \"distressed and frustrated\" is the patient's subjective feeling."}, "sentence_sentiment": {"answer": "Negative", "rationale": "Because the mood of \"distress and frustration\" in the entry is negative."}, "language_style": {"answer": "Calm and direct", "rationale": "Although it involves emotions, it is more direct and objective, and it does not use excessive emotional qualifiers or rhetorical techniques. Moreover, the tone of the question is more direct, and there is no attempt to induce or direct the emotional reaction of the respondents through questioning."}, "time_dimension": {"answer": "Present", "rationale": ""}, "sentence_type": "Interrogative sentence", "pos": {"nouns": {"you": 1, "It's sad": 1, "Frustration": 1, "Disappointment": 1}, "verbs": {"I feel": 1, "Always": 1}, "adjectives": {"It's sad": 1, "Frustration": 1, "Desperate": 1, "Disappointment": 1}, "adverbs": {"Always": 1}, "pronouns": {"you": 1}}}, {"content": "161. Are you completely desperate for life?", "word_count": 12, "label": "", "sentence_structure": "Subject鈥揷opula鈥損redicative", "subjective_objective": {"answer": "Subjective", "rationale": "Because despair is a subjective feeling."}, "sentence_sentiment": {"answer": "Negative", "rationale": "Because the \"showing despair\" attitude in the entry is negative."}, "language_style": {"answer": "Calm and direct", "rationale": "The entry uses direct and clear language to ask the other side whether it feels completely desperate for life, and is not based on excessive rhetorical or emotional rendering, which is more objective and balanced."}, "time_dimension": {"answer": "Present", "rationale": "The entry is an inquiry into the current emotional state of the subject, the keywords being \"whether\" and \"bereflecting total despair\", which indicate that this is a question of the present situation."}, "sentence_type": "Interrogative sentence", "pos": {"nouns": {"Life": 1, "Despair": 1}, "verbs": {"I feel": 1, "Total": 1, "Despair": 1}}}, {"content": "Do you often want to die?", "word_count": 11, "label": "", "sentence_structure": "Subject鈥搗erb鈥搊bject", "subjective_objective": {"answer": "Subjective", "rationale": "Since this question concerns the patient鈥檚 personal will and emotional feelings, it is the subjective idea of whether the patient has a suicidal tendency."}, "sentence_sentiment": {"answer": "Negative", "rationale": "Because the \"want to die\" act in the entry is negative."}, "language_style": {"answer": "Calm and direct", "rationale": "The entry directly asks whether the other side has a desire to die, and the language style is more straightforward and concise.\""}, "time_dimension": {"answer": "Present", "rationale": ""}, "sentence_type": "Interrogative sentence", "pos": {"nouns": {"Entry": 1, "person": 1, "Die": 1}, "verbs": {"Yes": 1, "It's a matter of time": 1}, "numerals": {"162": 1}}}, {"content": "Do you often worry?", "word_count": 11, "label": "", "sentence_structure": "Subject鈥搗erb鈥搊bject", "subjective_objective": {"answer": "Subjective", "rationale": "Because \"fear\" and \"browning\" are subjective emotional experiences of patients."}, "sentence_sentiment": {"answer": "Negative", "rationale": "Because \"fear\" is negative."}, "language_style": {"answer": "Calm and direct", "rationale": "The language style of the entry is relatively calm and straightforward, and it appears in a clear question form, without excessive rhetorical or emotional rendering, with a fair and objective tone, aimed at obtaining the real thoughts and feelings of the person under investigation."}, "time_dimension": {"answer": "Past", "rationale": "The entry asks about the behaviour or state of the subject in the past, the keywords being \"frequently troubled\" and \"remotely sad\", which point to the past's experience and feelings."}, "sentence_type": "Interrogative sentence", "pos": {"nouns": {"Trouble": 1, "鈾?And the blues are on the way 鈾?: 1}, "verbs": {"be": 1, "Regular": 1}, "adverbs": {"Yes": 1}, "numerals": {"163": 1}}}, {"content": "164. Are there any of your family who are not in a state of grief?", "word_count": 14, "label": "", "sentence_structure": "Subject鈥搗erb鈥搊bject", "subjective_objective": {"answer": "Objective", "rationale": "Since it is an objective fact that the family is not in a state of constant distress, it is not necessary for the patient to make a subjective judgement."}, "sentence_sentiment": {"answer": "Negative", "rationale": "Because the description \"sweet\" is negative."}, "language_style": {"answer": "Calm and direct", "rationale": "The entry is open to dialogue in a direct question-and-answer manner, with no excessive rhetorical or emotional rendering, and is more objective in tone. \" ."}, "time_dimension": {"answer": "Present", "rationale": "\"Your family\" indicates that this is an inquiry into the current situation of the interviewee, \"Is there a sad person?\" is an inquiry into whether the interviewee's family has often shown a sad mood, and the entry is therefore a survey of the current situation."}, "sentence_type": "Interrogative sentence", "pos": {"nouns": {"Family": 1, "鈾?And the blues are on the way 鈾?: 1, "person": 1}, "verbs": {"have": 1}, "adjectives": {"鈾?And the blues are on the way 鈾?: 1}, "numerals": {"164": 1}, "pronouns": {"you": 1, "he": 1}}}, {"content": "Have you been nervous and exhausted by any small encounters?", "word_count": 18, "label": "", "sentence_structure": "Subject鈥搗erb鈥搊bject", "subjective_objective": {"answer": "Subjective", "rationale": "Because stress and fatigue are emotional experiences and are subjective feelings of patients."}, "sentence_sentiment": {"answer": "Negative", "rationale": "Because the description of \"stretch and fatigue\" in the entry is negative."}, "language_style": {"answer": "Calm and direct", "rationale": "The language style of the entry is more direct and objective, there is no excessive emotional rendering or rhetorical technique, and the main facts are used to ask the respondents a specific question."}, "time_dimension": {"answer": "Present", "rationale": "The keyword \"is it\" is a question of the current state, and \"stress and fatigue\" is also a description of the person who is being tested. The entry is therefore about the person's current state."}, "sentence_type": "Interrogative sentence", "pos": {"nouns": {"Things": 1, "Nervous": 1, "Tired": 1}, "verbs": {"Problems": 1, "鈾?And I'm gonna be 鈾?: 1}, "adjectives": {"It's nothing": 1, "- A moment": 1}, "numerals": {"165": 1}}}, {"content": "Do you think you're a neurotic person?", "word_count": 22, "label": "", "sentence_structure": "Subject鈥損redicate", "subjective_objective": {"answer": "Subjective", "rationale": "Because it was the assessment of the patient that was examined and the subjective perspective."}, "sentence_sentiment": {"answer": "Negative", "rationale": "Because the description of \"neurological (strained, irritated)\" in the entry is usually considered to be negative features or behaviours."}, "language_style": {"answer": "Calm and direct", "rationale": "The language style of the entry is relatively calm and straightforward, and the main purpose of the interviewee is to ask if there is a negative perception of himself or herself, and there is no excessive emotional rendering or rhetorical approach."}, "time_dimension": {"answer": "Present", "rationale": "\"Whether\" indicates that this is an inquiry into the current state, \"considers\" and \"yes\" both to the general current, meaning that the act has not only occurred in the past, but may still be ongoing. Thus, the entry relates to the current act or state of the person being tested, which is now."}, "sentence_type": "Interrogative sentence", "pos": {"nouns": {"person": 1, "Nerves": 1, "Nervous": 1, "Unsettled": 1, "Excited": 1}, "verbs": {"I think": 1, "be": 1}, "adjectives": {"Nervous": 1, "Easy": 1}, "adverbs": {"very": 1}, "pronouns": {"you": 1}}}, {"content": "Are there any neurotics in your family?", "word_count": 13, "label": "", "sentence_structure": "Subject鈥搗erb鈥搊bject", "subjective_objective": {"answer": "Subjective", "rationale": "Because \"nervous\" is a subjective description that represents a person's character or mental state. The question is therefore to examine the patient's subjective perception and perception."}, "sentence_sentiment": {"answer": "Neutral", "rationale": "Because the entry does not have any emotional color."}, "language_style": {"answer": "Calm and direct", "rationale": "The entry is in a concise manner, asking the interviewee, without using excessive rhetorical or emotional colours, and is more objective in tone. At the same time, the question itself does not contain any encouraging or implied language, but rather directly asks the interviewee about the real situation, so that he can be judged as calm and straightforward in the language style."}, "time_dimension": {"answer": "Past", "rationale": "The entry is for questioning the family of the person who was tested, and the temporal dimension is a judgement of the past and implies a link to the person's personal experience."}, "sentence_type": "Interrogative sentence", "pos": {"nouns": {"Family": 1, "Nerves": 1, "person": 1}, "verbs": {"have": 1}, "numerals": {"167": 1}}}, {"content": "Have you ever had a nervous breakdown?", "word_count": 9, "label": "", "sentence_structure": "Subject鈥搗erb鈥搊bject", "subjective_objective": {"answer": "Subjective", "rationale": "Since mental breakdown is a subjective experience and feeling, the definition and experience of mental breakdown may vary from one person to another."}, "sentence_sentiment": {"answer": "Negative", "rationale": "Because the term \"psychological breakdown\" has a strong negative emotional connotation."}, "language_style": {"answer": "Calm and direct", "rationale": "The entry asks the question directly whether the person surveyed has experienced a mental breakdown, a more straightforward language style, and no excessive embellishment or emotional rendering."}, "time_dimension": {"answer": "Past", "rationale": "The keyword \"had\" represented a past experience or event, and thus judged it to be a past."}, "sentence_type": "Interrogative sentence", "pos": {"nouns": {"Mental breakdown": 1, "Questionnaire Entry": 1}, "verbs": {"I've had it": 1}, "adverbs": {"Tsang": 1}, "numerals": {"168锛?: 1}}}, {"content": "Have there ever been a breakdown in your family?", "word_count": 15, "label": "", "sentence_structure": "Subject鈥搗erb鈥搊bject", "subjective_objective": {"answer": "Objective", "rationale": "Because \"persons of their families who have had a mental breakdown\" are objective facts and do not depend on the patient's subjective views or feelings."}, "sentence_sentiment": {"answer": "Negative", "rationale": "The problem could have triggered negative sentiments among the respondents."}, "language_style": {"answer": "Calm and direct", "rationale": "The entry is based on a direct inquiry into the other ' s family members, using a simple and clear language, with no excessive adoration or emotional colour, and with a more objective tone."}, "time_dimension": {"answer": "Past", "rationale": "The entry is for the interview of the family history of the person who was tested, and the keyword is \"a person who was once in existence\" or \"a person who was mentally disabled\", all of which points to past events or states. It is therefore judged to be part of the survey of the past time dimension."}, "sentence_type": "Interrogative sentence", "pos": {"nouns": {"Family": 1, "Spirit": 1, "Crash": 1, "person": 2}, "verbs": {"have": 1, "Pass": 1, "Come on": 1}, "numerals": {"169": 1, "Tsang": 1}, "classifiers": {"individual": 1}, "pronouns": {"you": 1, "of": 2}}}, {"content": "Have you seen it in a mental institution?", "word_count": 20, "label": "", "sentence_structure": "Subject鈥搗erb鈥搊bject", "subjective_objective": {"answer": "Subjective", "rationale": "The question is whether the patient has been hospitalized for mental problems, which is a personal experience and feeling of the patient and is therefore subjective."}, "sentence_sentiment": {"answer": "Negative", "rationale": "Because the terms \"psychological hospital\" and \"psychological problem\" in this entry have negative meanings and may make the interviewees uncomfortable or ostracized."}, "language_style": {"answer": "Calm and direct", "rationale": "The language style of the entry is more calm and straightforward, and the person surveyed is asked in a direct and clear manner whether he or she has been treated in a psychiatric institution, without excessive rhetoric or emotional rendering."}, "time_dimension": {"answer": "Present", "rationale": ""}, "sentence_type": "Interrogative sentence", "pos": {"nouns": {"Mental hospital": 1, "Diseases": 1, "Problem": 1}, "verbs": {"Look": 1, "Because": 1, "Okay": 1, "Pass": 1, "- You're not": 1}, "numerals": {"170": 1}, "pronouns": {"you": 1}}}, {"content": "171. Have any of your family members visited a psychiatric hospital (for reasons of their mental problems)?", "word_count": 27, "label": "", "sentence_structure": "Subject鈥搗erb鈥搊bject", "subjective_objective": {"answer": "Objective", "rationale": "It is an objective fact that the examination is to examine whether the family members of the patient have had experience in psychiatric hospitals."}, "sentence_sentiment": {"answer": "Neutral", "rationale": "Because the entry is a factual situation and does not have any emotional colour."}, "language_style": {"answer": "Calm and direct", "rationale": "The style of the entry is more straightforward, and it does not use too many rhetorical or emotional colours to qualify the problem. It asks the interviewee in a concise manner whether any family has been treated in a psychiatric institution."}, "time_dimension": {"answer": "Present", "rationale": "The entry now asks if the family members of the person tested have had past mental health problems that require them to visit a psychiatric hospital, and therefore the temporal dimension is now judged."}, "sentence_type": "Interrogative sentence", "pos": {"nouns": {"Family": 1, "person": 1, "Mental hospital": 1, "Problem": 1}, "verbs": {"Is it possible": 1, "have": 1, "Present": 1, "Yes": 1, "Because": 1}, "adjectives": {"Mental": 1}, "numerals": {"171": 1, "One": 2}, "classifiers": {"individual": 1}, "pronouns": {"Other": 1}}}, {"content": "Do you often shy and sensitive?", "word_count": 12, "label": "", "sentence_structure": "Subject鈥搗erb鈥搊bject", "subjective_objective": {"answer": "Subjective", "rationale": "Because shyness and drearyness are usually subjective feelings of patients."}, "sentence_sentiment": {"answer": "Negative", "rationale": "Because the \"weakness and sensibilities\" in the entry are negative emotional states."}, "language_style": {"answer": "Tactful and warm", "rationale": "The language style of the entry is a warmer type. By using the interview style and the gentler language, respondents are more comfortable and relaxed, and are therefore more willing to respond honestly. Moreover, the subject does not directly concern the privacy or weakness of the other party, so that the answers are accurate while respecting the interviewee."}, "time_dimension": {"answer": "Present", "rationale": "The keyword \"often\" indicates that the act is repeated, and therefore the temporal dimension is now judged."}, "sentence_type": "Interrogative sentence", "pos": {"nouns": {"172": 1, "you": 1, "Shy": 1, "You're a bit of a sane": 1}, "verbs": {"Is it possible": 1, "Regular": 1, "Shy": 1, "You're a bit of a sane": 1}, "numerals": {"172": 1, "1": 1}, "pronouns": {"you": 1}}}, {"content": "Are there any shy and sensitive members of your family?", "word_count": 17, "label": "", "sentence_structure": "Subject鈥搗erb鈥搊bject", "subjective_objective": {"answer": "Subjective", "rationale": "The objective is to determine the nature of the person ' s personality."}, "sentence_sentiment": {"answer": "Neutral", "rationale": "Because the entry does not have any emotional color."}, "language_style": {"answer": "Tactful and warm", "rationale": "The entry, which is in question-based and uses \"you\" as the dominant language, is more moderate and does not imply direct orders or judgment, expressing the desire of researchers to obtain the personal information of the person being tested, with due respect and understanding."}, "time_dimension": {"answer": "Past", "rationale": ""}, "sentence_type": "Interrogative sentence", "pos": {"nouns": {"Family": 1, "Shy": 1, "You're a bit of a sane": 1, "person": 1}, "verbs": {"have": 1}, "adverbs": {"Is it possible": 1}, "numerals": {"173": 1}, "classifiers": {"EMPTY": 1}}}, {"content": "Is your feelings vulnerable?", "word_count": 12, "label": "", "sentence_structure": "Subject鈥揷opula鈥損redicative", "subjective_objective": {"answer": "Subjective", "rationale": "Because \"if\" and \"vulnerable\" are questions based on the patient's subjective feelings."}, "sentence_sentiment": {"answer": "Negative", "rationale": "Because the description of \"emotional vulnerability\" in this entry is negative."}, "language_style": {"answer": "Tactful and warm", "rationale": "Warm and warm."}, "time_dimension": {"answer": "Present", "rationale": "The entry asks the testee about the current situation, whether the keyword \"are\" and \"are your feelings vulnerable?\" both points to the emotional state and feelings of the moment. So it's now."}, "sentence_type": "Interrogative sentence", "pos": {"nouns": {"Emotion": 1, "Hurt": 1}, "verbs": {"Yes": 1, "Present": 1}, "adverbs": {"Easy": 1}, "numerals": {"174": 1}}}, {"content": "Are you always upset when you are criticized?", "word_count": 15, "label": "", "sentence_structure": "Subject鈥搗erb鈥搊bject", "subjective_objective": {"answer": "Subjective", "rationale": "Because \"disturbed\" is an emotional reaction, a subjective feeling of the patient."}, "sentence_sentiment": {"answer": "Negative", "rationale": "Because the mood for \"disturbing\" in the entries is negative."}, "language_style": {"answer": "Calm and direct", "rationale": "The language style of the entry is more calm and straightforward, and it expresses the concern about the emotional reactions of the interviewee in the face of criticism in a direct way. There is no excessive rhetorical or emotional rendering, and the tone is objective.\""}, "time_dimension": {"answer": "Present", "rationale": "Now, the entry is about the immediate mood of the testee when he receives the criticism, which is \"always distraught.\" So the basis of the judgment is \"always\" , which means that the act is regular, customary and takes place now."}, "sentence_type": "Interrogative sentence", "pos": {"nouns": {"you": 1, "Criticism": 1, "Time": 1, "I'm upset": 1}, "verbs": {"be": 1, "in": 1, "Yes": 1, "Always": 1}, "adjectives": {"Disturbing": 1}, "adverbs": {"Always": 1}, "numerals": {"175": 1}, "pronouns": {"Here": 1}}}, {"content": "Do you think you're a picky guy?", "word_count": 12, "label": "", "sentence_structure": "Subject鈥搗erb鈥搊bject", "subjective_objective": {"answer": "Subjective", "rationale": "Because \"others think\" is a subjective view or view, which reflects the image and impression of the person interviewed in the eyes of others. This is related to the personal characteristics, behaviour and circumstances of the person interviewed."}, "sentence_sentiment": {"answer": "Negative", "rationale": "Because \"love\" is a negative adjective, describing a person's shortcomings, the question is negative emotional."}, "language_style": {"answer": "Tactful and warm", "rationale": "The language style of the entry is relatively moderate and interactive, and it does not directly express the person's assessment or accusations, but rather uses the means of asking the interviewer to give the interviewee a free answer. This design helps the interviewee to be more willing to share his real thoughts and feelings and to create a more harmonious questionnaire atmosphere."}, "time_dimension": {"answer": "Present", "rationale": "The keyword is \"others think\" , which implies that it is a subjective view and that it requires the testee to think about his impressions in the eyes of others, rather than describe past or future behaviour. Thus, the time dimension of this entry is now judged."}, "sentence_type": "Interrogative sentence", "pos": {"nouns": {"person": 1, "others": 1, "Pick": 1}, "verbs": {"be": 1, "I think": 1, "Love": 1, "Picky": 1}, "adverbs": {"- You're not": 1}, "numerals": {"176锛?: 1}, "pronouns": {"you": 1}}}, {"content": "Are you often misunderstood?", "word_count": 8, "label": "", "sentence_structure": "Subject鈥損redicate", "subjective_objective": {"answer": "Subjective", "rationale": "Because it is a subjective feeling, it is linked to the patient ' s own view."}, "sentence_sentiment": {"answer": "Negative", "rationale": "Because \"misunderstanding\" is usually unpleasant and has a negative emotional connotation."}, "language_style": {"answer": "Tactful and warm", "rationale": "The entry is more euphemistic and sensitive to the emotional experience of the interviewee, using the \"Is it often misunderstood?\" approach rather than pointing directly to the misunderstanding of the interviewee. It is more moderate, more friendly, more acceptable and willing to share its true feelings."}, "time_dimension": {"answer": "Present", "rationale": "\"Are you\" is a general current-day question, indicating that it is asking about the current situation or behaviour. At the same time, the description \"often misunderstood\" is also a current one, which means that it is a continuing problem. It can therefore be concluded that the entry is asking the testee about the present situation."}, "sentence_type": "Interrogative sentence", "pos": {"nouns": {"person": 1, "Misunderstanding": 1}, "verbs": {"By": 1, "Misunderstanding": 1}, "numerals": {"177": 1}, "classifiers": {"Regular": 1}, "pronouns": {"you": 1}}}, {"content": "Do you have to be vigilant even with your friends?", "word_count": 18, "label": "", "sentence_structure": "Subject鈥損redicate", "subjective_objective": {"answer": "Subjective", "rationale": "The problem is related to the patient ' s psychological feelings and attitudes and is a subjective experience."}, "sentence_sentiment": {"answer": "Negative", "rationale": "Because the words \"suspend\" and \"suspensive\" in the entry have a certain negative emotional connotation."}, "language_style": {"answer": "Calm and direct", "rationale": "The subject, which is asked directly, makes the respondents wonder whether they need to be vigilant about friends in their lives, and does not use speech-making or emotional profiling too much, which is more objective and balanced."}, "time_dimension": {"answer": "Past", "rationale": "Even if the word \"even\" means that the act has occurred in the past; and the word \"must be guarded\" suggests that the act may need to continue in the future. Thus, the temporal dimension of this entry contains both the past and the future."}, "sentence_type": "Interrogative sentence", "pos": {"nouns": {"friends": 1, "Watch your step": 1, "Watch out": 1}, "verbs": {"I have to": 1, "Save": 1, "Relax": 1}, "adverbs": {"Yeah": 1, "Even if": 1}, "numerals": {"178": 1}}}, {"content": "Do you always do things on an impulse?", "word_count": 13, "label": "", "sentence_structure": "Subject鈥搗erb鈥搊bject", "subjective_objective": {"answer": "Subjective", "rationale": "Because \"a moment of impulse\" is a subjective idea or attitude."}, "sentence_sentiment": {"answer": "Negative", "rationale": "Because the \"doing things with a moment's impulse\" act in the entry is negative."}, "language_style": {"answer": "Calm and direct", "rationale": "The entry directly asked whether the respondents often made their decisions out of impulse, without using euphemism or vague language to express themselves clearly and directly."}, "time_dimension": {"answer": "Present", "rationale": "The emergence of the keyword \"always\" and \"a moment's impulse\" suggests that it is a description of the present behaviour or orientation of the subject, and therefore the judgement is now."}, "sentence_type": "Interrogative sentence", "pos": {"nouns": {"Things": 1, "Expedition": 1}, "verbs": {"Do it": 1, "Is it possible": 1, "Always": 1, "Yeah": 1}, "adjectives": {"One moment": 1}, "numerals": {"You": 1}}}, {"content": "Are you agitated and angry?", "word_count": 10, "label": "", "sentence_structure": "Subject鈥搗erb鈥搊bject", "subjective_objective": {"answer": "Subjective", "rationale": "Because irritation and irritation are subjective emotional experiences."}, "sentence_sentiment": {"answer": "Negative", "rationale": "Because the mood of \"freak and anger\" in the entry is negative."}, "language_style": {"answer": "Calm and direct", "rationale": "The language style of the entry is more calm and straightforward, without too many rhetorical or emotional accents, and it is more objective and concise to ask whether the person surveyed is often troubled and angry."}, "time_dimension": {"answer": "Present", "rationale": "The entry asks the testee ' s current situation, i.e. whether there are current situations of distress and anger, and therefore the temporal dimension is now judged."}, "sentence_type": "Interrogative sentence", "pos": {"nouns": {"Problem": 1, "Entry": 1, "180": 1, "Trouble": 1, "Exasperation": 1}, "verbs": {"be": 1, "have": 1, "Easy": 1, "Trouble and anger": 1}, "adjectives": {"of": 1}, "numerals": {"180": 1}}}, {"content": "181. Are you going to collapse if you don't keep your mind down?", "word_count": 15, "label": "", "sentence_structure": "Subject鈥搗erb鈥搊bject", "subjective_objective": {"answer": "Subjective", "rationale": "Because the question is about the patient's perception of his/her own inner feelings, it is about the patient's subjective attitude."}, "sentence_sentiment": {"answer": "Negative", "rationale": "Because \"mental breakdown\" in the entry is a negative consequence, indicating that lack of self-control can lead to undesirable consequences."}, "language_style": {"answer": "Calm and direct", "rationale": "The entry uses a direct, concise language style, which is clearly asked the interviewee whether they will collapse because they cannot keep their emotions under control."}, "time_dimension": {"answer": "Present", "rationale": "The entry contains visions and expectations for the future, \"If you do not keep restraint\" indicating that it is a hypothesis, and \"if you fail?\" implying the negative consequences that such a hypothesis could have. The entry therefore asks whether the testee may be in a position to have problems in the future because of his inability to keep his mental state under constant control."}, "sentence_type": "Interrogative sentence", "pos": {"nouns": {"Questionnaire Entry": 1, "Spirit": 1, "you": 1}, "verbs": {"If": 1, "Ongoing": 1, "Restraint": 1, "Just": 1, "Falling": 1}, "adverbs": {"not": 1, "Just": 1, "- You're not": 1}, "numerals": {"181锛?: 1}}}, {"content": "182. Does it make you nervous and angry at all?", "word_count": 15, "label": "", "sentence_structure": "Subject鈥搗erb鈥搊bject", "subjective_objective": {"answer": "Subjective", "rationale": "Because the words \"stretch and temper\" are the expression of patient subjective experience, they are viewed from a subjective perspective."}, "sentence_sentiment": {"answer": "Negative", "rationale": "Because \"heat and temper\" in the entry is a negative emotional reaction."}, "language_style": {"answer": "Calm and direct", "rationale": "The entry asks the other side in a direct, concise manner whether there are situations of tension and anger in the event of minor frustration or displeasure, without excessive qualifier or emotional colours, and with a more calm and straightforward language style."}, "time_dimension": {"answer": "Present", "rationale": "The entry is for the person being tested to ask about the current or recent behaviour or state, and therefore the judgement is now."}, "sentence_type": "Interrogative sentence", "pos": {"nouns": {"Problem": 1, "Entry": 1}, "verbs": {"鈾?And I'm gonna be 鈾?: 1, "Nervous": 1, "- You're losing your temper": 1, "Is it possible": 2}, "adjectives": {"Not soon": 1}, "numerals": {"182锛?: 1}}}, {"content": "183. Are you angry when you are being pushed by others?", "word_count": 12, "label": "", "sentence_structure": "Subject鈥搗erb鈥搊bject", "subjective_objective": {"answer": "Subjective", "rationale": "Because it is an emotional experience and a subjective attitude of the patient."}, "sentence_sentiment": {"answer": "Negative", "rationale": "Because the attitude of \"freaky\" in the entries is negative."}, "language_style": {"answer": "Calm and direct", "rationale": "The language style of the entry is calm and straightforward. It raises the question in a concise manner, without too many rhetorical or emotional renderings, speaking with objectivity, and asking directly whether the person under investigation is easily angry with emotional reactions when facing instructions or requests from others."}, "time_dimension": {"answer": "Present", "rationale": "In the future, the entry asks whether the person being tested will be angry in the future because of the other person ' s fingering and therefore fall within the scope of future behaviour or mentality."}, "sentence_type": "Interrogative sentence", "pos": {"nouns": {"person": 1, "Gas": 1}, "verbs": {"Easy": 1, "Angry": 1, "Support": 1}, "numerals": {"183": 1}, "pronouns": {"you": 1}}}, {"content": "- Does anyone often make you unhappy and angry?", "word_count": 12, "label": "", "sentence_structure": "Subject鈥搗erb鈥搊bject", "subjective_objective": {"answer": "Subjective", "rationale": "Because the subject is about the patient ' s emotional reactions (discomfort and anger), it is viewed from the patient ' s subjective perspective."}, "sentence_sentiment": {"answer": "Negative", "rationale": "Because the entry \"depressing and irritating\" expresses a negative sentiment."}, "language_style": {"answer": "Calm and direct", "rationale": "The entry uses simple and clear language to ask whether the interviewee is often displeased or angry because of his human being, is not over-represented in speech or emotional rendering, and is more objective. Thus, its language style can be described as \"cooling straight.\""}, "time_dimension": {"answer": "Present", "rationale": ""}, "sentence_type": "Interrogative sentence", "pos": {"nouns": {"person": 1, "Come on": 1, "Exasperation": 1}, "verbs": {"鈾?And I'm gonna be 鈾?: 1, "Not fast": 1, "Exasperation": 1}, "adverbs": {"Regular": 1, "and": 1}, "numerals": {"184锛?: 1}}}, {"content": "When you can't get what you need right away, you get angry?", "word_count": 21, "label": "", "sentence_structure": "Subject鈥搗erb鈥搊bject", "subjective_objective": {"answer": "Objective", "rationale": "The question is whether the patient is in a certain situation in which he or she is being asked whether there is an emotional reaction, and whether such a reaction occurs is an objective fact and is not influenced by the patient ' s subjective consciousness."}, "sentence_sentiment": {"answer": "Negative", "rationale": "Because it's negative in the entry."}, "language_style": {"answer": "Calm and direct", "rationale": ""}, "time_dimension": {"answer": "Present", "rationale": "The term \"sympathetic\" in the entry indicates that it is a behaviour that may occur at any time, so the temporal dimension is now judged."}, "sentence_type": "Interrogative sentence", "pos": {"nouns": {"Time": 1, "Something": 1}, "verbs": {"Let's go": 1, "Okay": 1, "Yes": 1, "Present": 1}, "adjectives": {"Now": 1}, "adverbs": {"Just": 1}}}, {"content": "Have you been angry a lot?", "word_count": 9, "label": "", "sentence_structure": "Subject鈥損redicate", "subjective_objective": {"answer": "Subjective", "rationale": "Because \"smuggling\" is an emotional reaction, a patient's subjective experience and attitude."}, "sentence_sentiment": {"answer": "Negative", "rationale": "Because the \"dry-out\" act in the entry is negative."}, "language_style": {"answer": "Calm and direct", "rationale": "The entry clearly expresses the content of the survey in a direct question, does not have too many rhetorical or emotional colours and is more neutral and objective in tone."}, "time_dimension": {"answer": "Present", "rationale": "The keyword \"often\" and the verb \"hairing\" indicate that it is a customary act and takes place in the present or the nearest period of time, and therefore is judged to be the past."}, "sentence_type": "Interrogative sentence", "pos": {"nouns": {"- Grumpy": 1, "you": 1}, "verbs": {"Big hair": 1, "be": 1, "Let's go": 1}, "adjectives": {"Regular": 1}, "numerals": {"186": 1}}}, {"content": "187. Do you often tremble and tremble?", "word_count": 10, "label": "", "sentence_structure": "Subject鈥損redicate", "subjective_objective": {"answer": "Subjective", "rationale": "Because frequent shaking and tremors may be the subjective feeling or experience of the patient, rather than the objective facts."}, "sentence_sentiment": {"answer": "Negative", "rationale": "Because the body reaction of \"shock and thaw\" is usually negative."}, "language_style": {"answer": "Calm and direct", "rationale": "This expression makes it easier for the person surveyed to understand the problem and give real answers, in line with the general requirements of the psychological questionnaire study."}, "time_dimension": {"answer": "Past", "rationale": "The entry is for questioning the testee's past customary behaviour, and the keyword is \"frequent\", which indicates that the time dimension is a past."}, "sentence_type": "Interrogative sentence", "pos": {"nouns": {"Whim": 1, "Treasure": 1}, "verbs": {"# Wresh #": 1, "Warm": 1}, "numerals": {"187": 1}}}, {"content": "188. Are you always nervous?", "word_count": 9, "label": "", "sentence_structure": "Subject鈥損redicate", "subjective_objective": {"answer": "Subjective", "rationale": "Because \"heat and anxious\" is an emotional experience, a subjective feeling of the patient."}, "sentence_sentiment": {"answer": "Negative", "rationale": "Because \"heat\" in the entry is a negative emotional attitude."}, "language_style": {"answer": "Calm and direct", "rationale": "The entry directly expresses the subject of questioning whether the other party is often in an anxiety or stressful mood, without excessive rhetoric or emotional rendering, and is more objective in tone."}, "time_dimension": {"answer": "Present", "rationale": "The entry is for the present behaviour and psychological state of the person being tested, and the keyword \"always\" indicates that the question is asking the current frequency and extent, so it can be judged to be the present."}, "sentence_type": "Interrogative sentence", "pos": {"nouns": {"Nervous": 1, "- It's urgent": 1}, "verbs": {"Regular": 1, "be": 1}, "adverbs": {"- You're not": 1}, "numerals": {"188": 1}}}, {"content": "Will you be surprised by the sudden sound?", "word_count": 22, "label": "", "sentence_structure": "Subject鈥搗erb鈥搊bject", "subjective_objective": {"answer": "Subjective", "rationale": "The question is asked about the feelings and reactions of the individual patients and is based on their subjective experience."}, "sentence_sentiment": {"answer": "Negative", "rationale": "Because \"frightful by sudden sounds\" is a negative experience and reaction."}, "language_style": {"answer": "Calm and direct", "rationale": "The entry raises questions directly, is not too much accommodative, is clear and has a more calm and straightforward language style."}, "time_dimension": {"answer": "Present", "rationale": "The entry asks the testee whether he or she will be surprised or afraid to react in a particular situation. In terms of time dimension, the question does not clearly indicate the past, present or future time point, and therefore does not determine which period of behaviour or mental description it belongs to. However, the question can be used to understand the sensitivity of the testee to sudden sounds and how to respond, which is of some value in terms of studying stress reactions, etc."}, "sentence_type": "Interrogative sentence", "pos": {"nouns": {"Sound": 1, "Jump": 1, "That's great": 1}, "verbs": {"Yes": 1, "By": 1, "What": 1, "Jump": 1, "# Wresh #": 1}, "adjectives": {"Suddenly": 1}, "numerals": {"189": 1}}}, {"content": "190. When you're called out loud, you get scared and soft?", "word_count": 24, "label": "", "sentence_structure": "Subject鈥搗erb鈥搊bject", "subjective_objective": {"answer": "Subjective", "rationale": "Because fear is the patient's subjective feeling."}, "sentence_sentiment": {"answer": "Negative", "rationale": "Because the emotional reaction described in the entry as \"shocked and soft\" is negative."}, "language_style": {"answer": "Calm and direct", "rationale": "The entry uses a direct and objective language style, which is not a touchy or emotional. It is presented in question form and is clearly and succinctly worded, and is intended to obtain the true answers of the person surveyed."}, "time_dimension": {"answer": "Past", "rationale": "The term \"weak and soft\" in this entry describes past experiences or symptoms and therefore determines past."}, "sentence_type": "Interrogative sentence", "pos": {"nouns": {"190": 1, "person": 1, "Time": 1, "Louder": 1, "Wreck": 1, "Soft": 1}, "verbs": {"Please": 1, "I'm scared": 1}, "adverbs": {"Whenever": 1, "Both": 1}, "numerals": {"190": 1}, "classifiers": {"Large": 1}}}, {"content": "Are you afraid of sudden movements at night?", "word_count": 17, "label": "", "sentence_structure": "Subject鈥搗erb鈥搊bject", "subjective_objective": {"answer": "Subjective", "rationale": "Because fear is a subjective emotional experience and reaction."}, "sentence_sentiment": {"answer": "Negative", "rationale": "Because the \"fear\" attitude is negative."}, "language_style": {"answer": "Calm and direct", "rationale": "The entry is presented in the form of a direct question, asking the interviewee whether he or she feels afraid or afraid in the face of sudden night movements."}, "time_dimension": {"answer": "Present", "rationale": "\"Whether\" means that it is a selective question, asking the testee to answer their current feelings and experiences, so it can be judged that the entry is asking the testee what it feels like right now. And \"Your sudden movement at night\" also describes the current scene or situation."}, "sentence_type": "Interrogative sentence", "pos": {"nouns": {"you": 1, "Night": 1, "Move": 1, "Fear": 1, "Scared": 1}, "verbs": {"I feel": 1, "be": 1, "Suddenly": 1, "of": 2}, "adjectives": {"Night": 1}, "numerals": {"191锛?: 1}}}, {"content": "Have you been awakening by nightmares?", "word_count": 11, "label": "", "sentence_structure": "Subject鈥搗erb鈥搊bject", "subjective_objective": {"answer": "Objective", "rationale": "Because nightmares are the objective fact of the patient's experience."}, "sentence_sentiment": {"answer": "Negative", "rationale": "Because the word \"dream\" is often considered to be negative emotional, implying unpleasant or painful experiences."}, "language_style": {"answer": "Calm and direct", "rationale": "The entry uses a straightforward language to ask whether the person surveyed is often awakened by nightmares, speaking objectively and without any emotional colour, and suitable for use in psychological questionnaires."}, "time_dimension": {"answer": "Present", "rationale": "The words \"whether\" and \"often\" indicate the item asking the testee about the present situation, not past or future behaviour. Therefore, the temporal dimension is now."}, "sentence_type": "Interrogative sentence", "pos": {"nouns": {"you": 1, "Nightmares": 1, "Wake up": 1}, "verbs": {"Is it possible": 1, "Regular": 1, "Because": 1, "And": 1, "Wake up": 1}, "adverbs": {"Is it possible": 1, "Regular": 1}, "numerals": {"192": 1}, "pronouns": {"you": 1}}}, {"content": "193. Do you often have some kind of gruesome (horrifying) thought in your head?", "word_count": 21, "label": "", "sentence_structure": "Subject鈥搗erb鈥搊bject", "subjective_objective": {"answer": "Subjective", "rationale": "Because the idea of fear is the patient's own, and it's subjective."}, "sentence_sentiment": {"answer": "Negative", "rationale": "Because the description of \"terror idea\" in the entry is negative."}, "language_style": {"answer": "Calm and direct", "rationale": "The entry describes the terrorist ideas of the person under investigation in direct and clear language, does not use excessive rhetoric or emotional rendering, and is expressed in a more calm and objective manner."}, "time_dimension": {"answer": "Present", "rationale": "Now the entry is to ask if the subject is currently suffering from a psychological phenomenon, namely, \"Do you often repeat a terrible thought in your head?\" The keyword \"does\" and \"recurringly\" indicates that it is a description of the current state. So it is now."}, "sentence_type": "Interrogative sentence", "pos": {"nouns": {"Brain": 1, "Repeatedly": 1, "Plant": 1, "Thoughts": 1}, "verbs": {"Come on": 1, "Is it possible": 1, "Horror": 1, "Horrible": 1}, "adverbs": {"Regular": 1}, "numerals": {"193锛?: 1, "Plant": 1}, "classifiers": {"Some kind of": 1}}}, {"content": "Have you often been in fear without any reason?", "word_count": 18, "label": "", "sentence_structure": "Subject鈥搗erb鈥搊bject", "subjective_objective": {"answer": "Subjective", "rationale": "Since this issue involves personal feelings and emotional reactions, it is examined from the subjective perspective of the patient."}, "sentence_sentiment": {"answer": "Negative", "rationale": "Because \"unjustifiably sudden fear\" is negative."}, "language_style": {"answer": "Calm and direct", "rationale": "The entry asks the respondents in a direct and objective manner whether they have had an unjustified sense of fear, without excessive emotional accommodations or euphemism, and with a more calm and straightforward language style."}, "time_dimension": {"answer": "Past", "rationale": "The keyword \"unjustifiably and suddenly\" expresses what has happened in the past at a moment or in a period of time."}, "sentence_type": "Interrogative sentence", "pos": {"nouns": {"Fear": 1, "Scared": 1}, "verbs": {"I feel": 1, "Suddenly": 1, "No reason": 1}, "adverbs": {"Often": 1, "No reason": 1}}}, {"content": "Do you often sweat in cold?", "word_count": 14, "label": "", "sentence_structure": "Subject鈥損redicate", "subjective_objective": {"answer": "Objective", "rationale": "Because this is a question of whether the patient is experiencing some kind of symptoms, it is an observation and description of the patient."}, "sentence_sentiment": {"answer": "Negative", "rationale": "Because \"sweet sweat\" is a physical discomfort, usually associated with negative emotions and diseases."}, "language_style": {"answer": "Calm and direct", "rationale": "The entry is in a straightforward manner to ask the respondents whether they were suddenly sweating, without using a rhetorical technique or emotional rendering, and with a sense of temperature and objectivity."}, "time_dimension": {"answer": "Present", "rationale": ""}, "sentence_type": "Interrogative sentence", "pos": {"nouns": {"Situation": 1, "Suddenly": 1, "Sweat": 1}, "verbs": {"have": 2, "The long run": 1, "Regular": 1}, "adverbs": {"Is it possible": 1, "Regular": 1, "Suddenly": 1}, "numerals": {"195锛?: 1}}}]}

{"questionnaire_title": "Holand Job Interest Test", "questionnaire_category": "Behavioral questionnaire", "item_count": 90, "emotion_shift_overall": "From negative to positive", "language_style_overall": "Tactful and warm", "items": [{"content": "1. Strong and agile bodies are important to me.", "word_count": 13, "label": "", "sentence_structure": "Subject鈥揷opula鈥損redicative", "subjective_objective": {"answer": "Subjective", "rationale": "Because \"a strong and agile body is important to me\" is a subjective view and attitude of a human being."}, "sentence_sentiment": {"answer": "Positive", "rationale": "Because \"strong and agile body\" is a positive quality."}, "language_style": {"answer": "Tactful and warm", "rationale": "The entry is more adept and interactive when it is spoken in a gentle and inviting manner, rather than by direct statements or orders."}, "time_dimension": {"answer": "Present", "rationale": ""}, "sentence_type": "Interrogative sentence", "pos": {"nouns": {"Body": 1, "Strong": 1, "Agility": 1}, "verbs": {"to": 1, "Important": 1}, "adjectives": {"Strong": 1, "Agility": 1}}}, {"content": "I must understand the truth thoroughly.", "word_count": 13, "label": "", "sentence_structure": "Subject鈥搗erb鈥揷omplement", "subjective_objective": {"answer": "Subjective", "rationale": "Because \"the truth must be understood thoroughly\" is a person's attitude or belief, not an objective reality. This reflects the patient's determination and subjective sense of wanting to fully understand a situation."}, "sentence_sentiment": {"answer": "Positive", "rationale": "Because the purpose of the act \"requiredly understanding the truth\" in the entry is positive."}, "language_style": {"answer": "Calm and direct", "rationale": "The entry uses strong tone and positive expression, does not add too much qualifier or emotional colour, emphasizes the pursuit of the truth and the commitment to it, and is more direct and calm in language style.\""}, "time_dimension": {"answer": "Present", "rationale": "The entry indicates that the person tested has a need at the moment, that is, to know the truth about the matter, and thus to judge now."}, "sentence_type": "Interrogative sentence", "pos": {"nouns": {"Things": 1, "The truth": 1}, "verbs": {"Roger that": 1, "I have to": 1, "Full": 1}, "adjectives": {"Land": 1}, "classifiers": {"of": 2}, "pronouns": {"I": 1}}}, {"content": "My mood is greatly influenced by music, colour and beauty.", "word_count": 19, "label": "", "sentence_structure": "Subject鈥揷opula鈥損redicative", "subjective_objective": {"answer": "Subjective", "rationale": "Because \"the mood is affected\" describes the patient's own subjective feelings and perceptions."}, "sentence_sentiment": {"answer": "Positive", "rationale": "Because the description of \"the mood is greatly influenced by music, colour and beauty\" in the entry expresses the individual's sensitivity and appreciation for the good, and is a positive emotional attitude."}, "language_style": {"answer": "Tactful and warm", "rationale": "The language style of the entry is more euphemistic, because it is presented in a more friendly, friendly way, without direct orders or accusations."}, "time_dimension": {"answer": "Present", "rationale": "The keyword \"mature\" suggests that it is a current emotional experience or feeling, and suggests that the impact may be immediate or persistent. Thus, the entry describes the current state and emotional experience of the person tested, and the temporal dimension is now judged."}, "sentence_type": "Affirmative declarative sentence", "pos": {"nouns": {"The mood": 1, "Music": 1, "Colour": 1, "Beautiful things": 1}, "verbs": {"Yes": 1, "Impact": 1}, "adjectives": {"Extreme": 1}, "numerals": {"3": 1}}}, {"content": "4. Relationships with others enrich and make sense of my life.", "word_count": 19, "label": "", "sentence_structure": "Subject鈥揷opula鈥損redicative", "subjective_objective": {"answer": "Subjective", "rationale": "This expression expresses the patient ' s subjective feeling that relationships have a positive impact on the patient ' s life."}, "sentence_sentiment": {"answer": "Positive", "rationale": "Because the entry \"I enriched my life and made it meaningful\" expresses positive emotions and attitudes."}, "language_style": {"answer": "Tactful and warm", "rationale": "The entry uses a positive expression and an emotional term of \"rich\" and \"meaning\" with a more moderate tone, aimed at giving respondents a sense of attention and support and enhancing their sense of participation and belonging."}, "time_dimension": {"answer": "Present", "rationale": "The entry describes the reality or experience of interacting with others, without reference to the time dimension of the past or the future. At the same time, the verb \"rich\" or \"make\" in the sentence also indicates that it is a constant state of affairs or experience. It is therefore judged to be a reference to the current questionnaire."}, "sentence_type": "Affirmative declarative sentence", "pos": {"nouns": {"Others": 1, "Relations": 1, "Life": 1, "Meaning": 1}, "verbs": {"Rich": 1, "鈾?And I'm gonna be 鈾?: 1}, "adverbs": {"of": 4}, "numerals": {"4": 1}, "pronouns": {"he": 1}}}, {"content": "I am confident that I will succeed.", "word_count": 6, "label": "", "sentence_structure": "Subject鈥搗erb鈥揷omplement", "subjective_objective": {"answer": "Subjective", "rationale": "Because self-confidence is a subjective idea and attitude."}, "sentence_sentiment": {"answer": "Positive", "rationale": "Because the \"confidence will succeed\" in the entry expresses positive attitude and confidence."}, "language_style": {"answer": "Calm and direct", "rationale": "The entry is a straightforward question of the respondents ' perception of whether they are confident that they will succeed, is not expressed in a more concise manner by excessive rhetorical or emotional rendering."}, "time_dimension": {"answer": "Future", "rationale": "The entry expresses confidence in and expectations for future success, and the keywords are \"confidence\" and \"will succeed\" , all pointing to situations that have not yet occurred and that may occur in the future. We can therefore judge the dimension of time involved in the entry as the future."}, "sentence_type": "Affirmative declarative sentence", "pos": {"verbs": {"Yes": 1, "Success": 1}, "adjectives": {"Confidence": 1}}}, {"content": "6. I must be guided in my work.", "word_count": 11, "label": "", "sentence_structure": "Subject鈥搗erb鈥揷omplement", "subjective_objective": {"answer": "Subjective", "rationale": "The question of this questionnaire is: \"I have to be clear about what I do.\" It's a patient's subjective attitude, because it involves the psychological needs and expectations of individuals. It's not objective, because it's not a general fact or observation, but it's about the individual's personality, experience and social environment."}, "sentence_sentiment": {"answer": "Neutral", "rationale": "Because the entry does not address elements of a positive or negative emotional nature, such as emotions, attitudes, behaviour, purpose or quality, but merely expresses the objective fact that \"doing things requires clear guidance\"."}, "language_style": {"answer": "Calm and direct", "rationale": "The entry asks the other side in a direct manner whether it needs clear guidance, is speaking objectively and calmly, is not overly emotional or rhetorical and is more focused on information and facts."}, "time_dimension": {"answer": "Present", "rationale": ""}, "sentence_type": "Affirmative declarative sentence", "pos": {"verbs": {"Work": 1, "have": 1, "Guidance": 1}, "adjectives": {"Clear": 1}}}, {"content": "I am good at making and repairing my own things.", "word_count": 12, "label": "", "sentence_structure": "Subject鈥搗erb鈥揷omplement", "subjective_objective": {"answer": "Subjective", "rationale": "Because \"good\" is a subjective assessment and self-awareness."}, "sentence_sentiment": {"answer": "Positive", "rationale": "Because \"good at making and repairing\" is a demonstration of self-assertion and ability."}, "language_style": {"answer": "Tactful and warm", "rationale": ""}, "time_dimension": {"answer": "Present", "rationale": "The entry is about the current abilities and skills of the testee, \"good at\" and \"self-made, repaired\" which indicate that this is an existing capability that has been learned and can be applied. The time dimension is now judged."}, "sentence_type": "Affirmative declarative sentence", "pos": {"nouns": {"Questionnaire": 1, "Entry": 1, "I": 1, "Something": 1}, "verbs": {"Good at it": 1, "Production": 1, "Repair": 1}}}, {"content": "8. I can spend a long time trying to understand the truth.", "word_count": 17, "label": "", "sentence_structure": "Subject鈥揷opula鈥損redicative", "subjective_objective": {"answer": "Subjective", "rationale": "The question is to examine the patient ' s subjective feelings and thoughts about whether he or she is willing to spend time thinking about the problem."}, "sentence_sentiment": {"answer": "Neutral", "rationale": "Because the entry describes only one form of behaviour of an individual, it does not involve positive or negative emotional aspects."}, "language_style": {"answer": "Calm and direct", "rationale": "The language style of the entry is calm, concise, and there is no excessive emotional or rhetorical style, which directly describes the thinking habits or preferences of the subject. \""}, "time_dimension": {"answer": "Present", "rationale": "The \"may\" and \"time-consuming\" actions in the entry are indicative of the present, and therefore judgmental. And \"to think about things\" is also a description of the current capabilities and state."}, "sentence_type": "Affirmative declarative sentence", "pos": {"nouns": {"Time": 1, "Reason": 1}, "verbs": {"Yeah": 1, "Flowers": 1, "I'm thinking": 1}, "adjectives": {"Long": 1}, "numerals": {"8": 1}, "pronouns": {"I": 1}}}, {"content": "I value the beautiful environment.", "word_count": 8, "label": "", "sentence_structure": "Subject鈥揷opula鈥損redicative", "subjective_objective": {"answer": "Subjective", "rationale": "Because the importance of beautiful environments is the individual value and attitude of patients, it is subjective."}, "sentence_sentiment": {"answer": "Positive", "rationale": "Because the entry \"Early and beautiful\" expresses a positive attitude and emotion."}, "language_style": {"answer": "Tactful and warm", "rationale": "The entry is more adept and interactive when it is spoken in a gentle and inviting manner, rather than by direct statements or orders."}, "time_dimension": {"answer": "Present", "rationale": "According to the verb form in the sentence and the time mark \"appreciation\", the entry asks the testee's current attitude or view, i.e. whether they value beautiful environments. Thus, its temporal dimension is now judged."}, "sentence_type": "Affirmative declarative sentence", "pos": {"nouns": {"Beautiful": 1, "Environment": 1}, "verbs": {"Value": 1}}}, {"content": "I am willing to spend time helping others to resolve their personal crises.", "word_count": 15, "label": "", "sentence_structure": "Subject鈥搗erb鈥搊bject", "subjective_objective": {"answer": "Subjective", "rationale": "Because \"yes\" is a subjective experience and attitude."}, "sentence_sentiment": {"answer": "Positive", "rationale": "Because the behavior and attitude of \"willing to spend time helping people solve personal crises\" in the entry is positive."}, "language_style": {"answer": "Tactful and warm", "rationale": "Warm and warm."}, "time_dimension": {"answer": "Present", "rationale": "The keyword \"I'm willing to spend time\" and \"I'm willing to spend time\" expresses the subjective will and choice of behaviour of the talking person, where both \"will\" and \"time spent\" are terms of the present, implying that the act is being or has been committed."}, "sentence_type": "Affirmative declarative sentence", "pos": {"nouns": {"Time": 1, "others": 1, "Crisis": 1}, "verbs": {"Yes": 1, "Flowers": 1, "Help": 1, "Resolve": 1}, "numerals": {"10": 1}, "pronouns": {"I": 1}}}, {"content": "I like competition.", "word_count": 5, "label": "", "sentence_structure": "Subject鈥損redicate", "subjective_objective": {"answer": "Subjective", "rationale": "Like a subjective emotion and attitude of a person."}, "sentence_sentiment": {"answer": "Positive", "rationale": "Because the \"like competition\" attitude in the entries is positive."}, "language_style": {"answer": "Calm and direct", "rationale": "The entry directly asks if the interviewees prefer competition, does not use euphemism or emotional rendering, and is more straightforward and concise."}, "time_dimension": {"answer": "Present", "rationale": ""}, "sentence_type": "Affirmative declarative sentence", "pos": {"verbs": {"Yeah": 1, "Competition": 1}, "numerals": {"11": 1}}}, {"content": "12. I will spend a lot of time planning before I start a plan.", "word_count": 18, "label": "", "sentence_structure": "Subject鈥搗erb鈥搊bject", "subjective_objective": {"answer": "Subjective", "rationale": "I mean, I like it. It's subjective."}, "sentence_sentiment": {"answer": "Positive", "rationale": "Because the purpose and attitude of the act \"to plan for a lot of time\" in the entry is positive."}, "language_style": {"answer": "Calm and direct", "rationale": "The language style of the entry is calm and straightforward. It describes a simple and straightforward behaviour that takes a lot of time to plan before a plan begins, without using too much qualifier or emotional expression, which is more objective and direct."}, "time_dimension": {"answer": "Present", "rationale": "The entry is an inquiry into the behaviour of the person under examination in the formulation of the plan, and the time dimension is now judged."}, "sentence_type": "Affirmative declarative sentence", "pos": {"nouns": {"Time": 1, "Planned": 1}, "verbs": {"It's a flower": 1, "Go plan": 1}, "adverbs": {"A lot": 1, "Start": 1, "Front": 1}, "numerals": {"12": 1}}}, {"content": "13. I like to do things with my hands.", "word_count": 9, "label": "", "sentence_structure": "Subject鈥搗erb鈥搊bject", "subjective_objective": {"answer": "Subjective", "rationale": "Because the preference for using hands to do things reflects the individual preferences and preferences of the patient, it is part of the subjective sense."}, "sentence_sentiment": {"answer": "Neutral", "rationale": "Because the entry merely describes a preference or habit, there is no apparent emotional tendency."}, "language_style": {"answer": "Tactful and warm", "rationale": "The language style of the entry is more euphemistic and warm, and it is easier to get their cooperation and trust by asking if the other side likes to use their hands to understand their habits and preferences."}, "time_dimension": {"answer": "Present", "rationale": "The entry now describes the personal habits and preferences of the person being tested, with no clear time frame, and therefore the judgement is now."}, "sentence_type": "Affirmative declarative sentence", "pos": {"nouns": {"Questionnaire Entry": 1, "Hands": 2, "Things": 1}, "verbs": {"Yeah": 1, "Use": 1, "Do it": 1}, "adverbs": {"Down there": 1, "you": 1, "Please": 1}, "numerals": {"13": 1}, "classifiers": {"individual": 1}, "pronouns": {"I": 1}}}, {"content": "14. I am satisfied with exploring new ideas.", "word_count": 9, "label": "", "sentence_structure": "Subject鈥揷opula鈥損redicative", "subjective_objective": {"answer": "Subjective", "rationale": "Because \"satisfaction\" is a subjective emotional experience and attitude. While exploring new ideas can bring new insights and experiences, the satisfaction of such feelings depends on the individual ' s perception and feelings, and is therefore viewed from a subjective perspective."}, "sentence_sentiment": {"answer": "Positive", "rationale": "Because the description \"exploring new ideas to my satisfaction\" shows the positive attitude and satisfaction of individuals with the new ideas."}, "language_style": {"answer": "Tactful and warm", "rationale": ""}, "time_dimension": {"answer": "Future", "rationale": ""}, "sentence_type": "Affirmative declarative sentence", "pos": {"nouns": {"Explore": 1, "Concept": 1, "Satisfied": 1}, "verbs": {"鈾?And I'm gonna be 鈾?: 1, "Explore": 1}}}, {"content": "15. I am looking for new ways to harness my creativity.", "word_count": 15, "label": "", "sentence_structure": "Subject鈥揷opula鈥損redicative", "subjective_objective": {"answer": "Subjective", "rationale": "Since the entry for the questionnaire deals with the behaviour and attitude of the patient seeking new methods, it is the patient ' s personal choice and will and is therefore subjective in nature."}, "sentence_sentiment": {"answer": "Positive", "rationale": "Because the purpose and attitude of the action \"to find new ways to use my creativity\" in the entry is positive."}, "language_style": {"answer": "Tactful and warm", "rationale": "The entry expresses respect and understanding of the subjects in an invitation format, asking them whether they are looking for new ways to develop their own creativity."}, "time_dimension": {"answer": "Past", "rationale": "The entry asks the testee whether he or she has a tendency to seek and use new methods for creativity, and therefore is judged on the basis of the behaviour or attitude of \"seeking new methods\" and the temporal dimension does not clearly point to the past, present or future, so that it can be classified as general."}, "sentence_type": "Affirmative declarative sentence", "pos": {"nouns": {"Methodology": 1, "Creativity": 1}, "verbs": {"Seek": 1, "Play": 1}, "adverbs": {"New": 1}, "numerals": {"15": 1}, "pronouns": {"I": 1}}}, {"content": "16. I think it is important to share my anxiety with others.", "word_count": 20, "label": "", "sentence_structure": "Subject鈥搗erb鈥搊bject", "subjective_objective": {"answer": "Subjective", "rationale": "Because \"think\" represents the patient's own view and attitude, and it's a subjective feeling."}, "sentence_sentiment": {"answer": "Positive", "rationale": "Because the entry expresses a positive emotional attitude that sharing anxiety can bring benefits."}, "language_style": {"answer": "Tactful and warm", "rationale": "The entry is more adept and interactive when it is spoken in a gentle and inviting manner, rather than by direct statements or orders."}, "time_dimension": {"answer": "Present", "rationale": "The words \"concept,\" \"can\" and \"very\" in the entry indicate that this is a current attitude or view, so the temporal dimension is now judged.\""}, "sentence_type": "Affirmative declarative sentence", "pos": {"nouns": {"Anxiety": 1, "person": 1, "self": 1}, "verbs": {"I think": 1, "Yes": 1, "- Put it on": 2, "Share": 1, "be": 1, "very": 1, "Important": 1}, "adverbs": {"very": 1}, "numerals": {"6": 1}, "pronouns": {"I": 1}}}, {"content": "17. It is important to me to be a key task-holder in the group.", "word_count": 18, "label": "", "sentence_structure": "Subject鈥揷opula鈥損redicative", "subjective_objective": {"answer": "Subjective", "rationale": "Because \"for me\" is a clear subjective expression of the patient's own thoughts and attitudes."}, "sentence_sentiment": {"answer": "Positive", "rationale": "Because the purpose of the entry is to be a key task-holder in the group."}, "language_style": {"answer": "Tactful and warm", "rationale": "The reason for this is that the term \"very\" is used in the wording of the article, which indicates that it is a relatively subjective feeling, and the polite expression \"please\" is used to express respect and friendship towards the other. Moreover, the expression \"becoming a key task-holder in a group\" is also more moderate and not in a strong or direct language."}, "time_dimension": {"answer": "Present", "rationale": "The entry shows the importance to the testee of being a key task holder in the current or near-term social environment. Time dimensions are now judged."}, "sentence_type": "Affirmative declarative sentence", "pos": {"nouns": {"Groups": 1, "Key mandate holders": 1}, "verbs": {"Be": 1, "It's important": 1}, "numerals": {"17": 1}, "pronouns": {"I": 1}}}, {"content": "18. I am proud to have been able to take all the details of my work into account.", "word_count": 20, "label": "", "sentence_structure": "Subject鈥揷opula鈥損redicative", "subjective_objective": {"answer": "Subjective", "rationale": "Because pride is a subjective feeling and attitude."}, "sentence_sentiment": {"answer": "Positive", "rationale": "Because the entries \"are able to take all the details of the work\" and \"be proud\" are positive emotional expressions."}, "language_style": {"answer": "Tactful and warm", "rationale": "The entry is more adept and interactive when it is spoken in a gentle and inviting manner, rather than by direct statements or orders."}, "time_dimension": {"answer": "Present", "rationale": "The entry \"showing pride\" means the emotions and feelings of the moment, and so it's judged now."}, "sentence_type": "Affirmative declarative sentence", "pos": {"nouns": {"Grandpa": 1, "Questionnaire": 1}, "verbs": {"Please": 1, "Analysis": 1, "I feel": 1, "Proud": 1}, "adjectives": {"self": 1, "Value": 1, "All": 1, "Medium": 1, "Work": 1}, "numerals": {"18": 1, "1": 2}, "classifiers": {"individual": 1, "Grandpa": 1}, "pronouns": {"self": 1}}}, {"content": "I don't care if I work to dirty my hands.", "word_count": 10, "label": "", "sentence_structure": "Subject鈥搗erb鈥揷omplement", "subjective_objective": {"answer": "Objective", "rationale": "Because it is not about the attitude and perception of a person to dirty his hands, and it is not based on any objective facts or description of events. This is therefore viewed from a subjective perspective."}, "sentence_sentiment": {"answer": "Negative", "rationale": "Because the \"not mind your work to get your hands dirty\" act in the entry is negative."}, "language_style": {"answer": "Calm and direct", "rationale": "The entry presents in a concise and direct manner whether the subject of the investigation is comfortable with the hand being dirty and not too much adour or emotional."}, "time_dimension": {"answer": "Present", "rationale": ""}, "sentence_type": "Negative declarative sentence", "pos": {"verbs": {"I care": 1, "Smuggle it": 1, "Work": 1}, "adverbs": {"not": 1}}}, {"content": "20. I consider education to be a lifelong learning process for development and brain-building.", "word_count": 21, "label": "", "sentence_structure": "Subject鈥搗erb鈥搊bject", "subjective_objective": {"answer": "Subjective", "rationale": "Because \"think\" is a subjective term, which expresses the thoughts and views of individuals. In this sentence, \"I think\" indicates the attitude and views of the speaker, and is viewed from a subjective perspective. And the latter description is an understanding and perception of education, based on the views and experiences of individuals, and can therefore be considered subjective."}, "sentence_sentiment": {"answer": "Positive", "rationale": "Because the expression in the entry is positive, education is considered to be a useful and lifelong process."}, "language_style": {"answer": "Tactful and warm", "rationale": "The language style of the entry is more euphemistic, using the words \"thinking\" , \"development\" and \"rehearsing\" to express a view of education and to see it as a \"life-long learning process\". Such expression is both a reflection of the author's point of view and a more gentle, friendly and acceptable one."}, "time_dimension": {"answer": "Present", "rationale": ""}, "sentence_type": "Affirmative declarative sentence", "pos": {"nouns": {"Education": 1, "Development": 1, "- I'm gonna work on it": 1, "Brain": 1, "Lifelong learning": 1, "Process": 1}, "verbs": {"I think": 1, "be": 1, "individual": 1, "and": 1}, "adjectives": {"individual": 1, "Development": 1, "- I'm gonna work on it": 1, "For life": 1, "Learning": 1}, "numerals": {"20": 1, "individual": 1}, "classifiers": {"individual": 1}, "pronouns": {"I": 1}}}, {"content": "I like to dress informally and try new colors and styles.", "word_count": 17, "label": "", "sentence_structure": "Subject鈥搗erb鈥搊bject", "subjective_objective": {"answer": "Subjective", "rationale": "Because the words \"like\" and \"tried\" are the subjective attitudes and behaviour of the patient, the entry in this questionnaire is viewed from the subjective perspective of the patient."}, "sentence_sentiment": {"answer": "Positive", "rationale": "Because the behavior in the entries \"likes to wear informally, try new colours and styles\" expresses a positive, open attitude."}, "language_style": {"answer": "Tactful and warm", "rationale": ""}, "time_dimension": {"answer": "Present", "rationale": "The keywords \"like\" and \"attempt\" indicate that it is a statement describing the current interest and preference of the individual, and can therefore be judged as now."}, "sentence_type": "Affirmative declarative sentence", "pos": {"nouns": {"Yeah": 1, "Wear it": 1, "Colour": 1, "Type": 1}, "verbs": {"Try": 1}, "adjectives": {"Informal": 1}, "numerals": {"21": 1}}}, {"content": "22. I often understand the need for someone to communicate with others.", "word_count": 18, "label": "", "sentence_structure": "Subject鈥搗erb鈥搊bject", "subjective_objective": {"answer": "Subjective", "rationale": "Because \"the need to feel someone wants to communicate with someone\" is a personal feeling or perception, a subjective view of the patient."}, "sentence_sentiment": {"answer": "Positive", "rationale": "Because it is a reflection of the need for interaction among people and has a positive emotional dimension."}, "language_style": {"answer": "Tactful and warm", "rationale": "The sentence \"the need for someone to communicate with others\" is based on a caring and understanding approach, and seeks to stimulate reflection and expression of the person's feelings. 2. The sentence is simple and clear, avoiding the use of complex and difficult-to-understand professional terms or cold words, which enable the person to understand and answer questions easily."}, "time_dimension": {"answer": "Present", "rationale": "Because \"often understood\" suggests that it is a regular, customary act, and because the phrase uses the usual current state of affairs, it points to the present situation."}, "sentence_type": "Affirmative declarative sentence", "pos": {"nouns": {"Questionnaire Entry": 1, "person": 1, "Communications": 1}, "verbs": {"I'm aware of it": 1, "Yes": 1, "Communications": 1}, "adverbs": {"- I'm not sure": 1}, "numerals": {"22": 1}, "pronouns": {"Someone": 1, "Others": 1}}}, {"content": "23. I like to help others improve.", "word_count": 11, "label": "", "sentence_structure": "Subject鈥搗erb鈥揷omplement", "subjective_objective": {"answer": "Subjective", "rationale": "Because \"like\" is a term that describes personal emotional attitudes, and emotions and attitudes are subjective, the entry is subjective in its perspective."}, "sentence_sentiment": {"answer": "Positive", "rationale": "Because the entry \"likes to help people improve\" expresses positive emotions and attitudes."}, "language_style": {"answer": "Tactful and warm", "rationale": "The language style of the entry is more euphemistic. It uses positive, positive words like \"like\" and \"help\" and expresses the desire and appreciation of the other person for being helpful. This expression is relatively natural and relatively friendly, and it is more likely to inspire motivation and resonance."}, "time_dimension": {"answer": "Present", "rationale": ""}, "sentence_type": "Affirmative declarative sentence", "pos": {"nouns": {"23": 1, "Yeah": 1, "Help": 1, "others": 1, "Improvements": 1}, "verbs": {"Yeah": 1, "Help": 1, "Improvements": 1}, "adverbs": {"Constantly": 1}, "numerals": {"23": 1}, "pronouns": {"I": 1}}}, {"content": "24. I am usually reluctant to take risks in making decisions.", "word_count": 11, "label": "", "sentence_structure": "Subject鈥揷opula鈥損redicative", "subjective_objective": {"answer": "Subjective", "rationale": "Because \"no risk\" is a subjective attitude and decision-making approach of a person, it is the idea and the tendency of the patient to make decisions within the patient. While this idea may be influenced by objective factors, it is a subjective experience itself."}, "sentence_sentiment": {"answer": "Negative", "rationale": "Because the \"no risk\" act in the entry is negative."}, "language_style": {"answer": "Calm and direct", "rationale": ""}, "time_dimension": {"answer": "Present", "rationale": ""}, "sentence_type": "Negative declarative sentence", "pos": {"nouns": {"Decision-making": 1, "Risk": 1}, "verbs": {"in": 1, "Do it": 1, "I don't want to": 1, "Adventure": 1}, "adverbs": {"Usually": 1}, "numerals": {"24": 1}, "pronouns": {"I": 1}}}, {"content": "25. I like to buy small parts and make finished products.", "word_count": 12, "label": "", "sentence_structure": "Subject鈥搗erb鈥搊bject", "subjective_objective": {"answer": "Subjective", "rationale": "Because it's the patient's subjective attitude."}, "sentence_sentiment": {"answer": "Neutral", "rationale": "Because the entry does not have any emotional color."}, "language_style": {"answer": "Tactful and warm", "rationale": "The language style of the entry is euphemism. Instead of using strong or direct tone to ask each other about their interests, it raises questions in a relaxed and friendly way, making each other comfortable and willing to answer. Moreover, the words used in the entry are very mild, such as \"like to buy\" and \"doing finished products,\" which have no negative or mandatory meaning, giving people a positive and pleasant feeling."}, "time_dimension": {"answer": "Past", "rationale": "The entry describes the testee ' s past behaviour and preferences, i.e. his preference for purchasing small parts and producing finished products."}, "sentence_type": "Affirmative declarative sentence", "pos": {"nouns": {"Questionnaire Entry": 1, "Small parts": 1, "The finished product": 1}, "verbs": {"Yeah": 1, "Purchase": 1, "Do it": 1}, "numerals": {"25": 1}}}, {"content": "Sometimes I read for a long time, play puzzle games, and meditate on the nature of life.", "word_count": 19, "label": "", "sentence_structure": "Subject鈥搗erb鈥搊bject", "subjective_objective": {"answer": "Subjective", "rationale": "This is a description and experience of the patients themselves, reflects their personal interests and behaviour habits and is a subjective examination."}, "sentence_sentiment": {"answer": "Neutral", "rationale": "Because the entry does not have any emotional color."}, "language_style": {"answer": "Tactful and warm", "rationale": "The entry is more personal and interactive when it is spoken in a gentle, inviting tone, rather than directly speaking or ordering. At the same time, the article is more liberal, without too serious or tense a climate, giving people a comfortable experience of communication. The language style of the questionnaire entry can therefore be described as euphemism."}, "time_dimension": {"answer": "Present", "rationale": "The entry describes the person's current behaviour or state, \"sometimes\" indicating that it is a regular, customary act, and \"long reading\", \"split puzzles\" and \"thinking the nature of life\" are all current activities. So, the time dimension of this entry is now."}, "sentence_type": "Affirmative declarative sentence", "pos": {"nouns": {"Time": 2, "I": 1, "Long": 1, "Reading": 1, "Jigsaw Game": 1, "Meditorize": 1, "Life": 1, "Nature": 1}, "verbs": {"Sometimes": 1, "Play": 1, "Meditorize": 1}, "adverbs": {"Long time": 1}, "numerals": {"26": 1}}}, {"content": "I have a strong imagination.", "word_count": 8, "label": "", "sentence_structure": "Subject鈥搗erb鈥揷omplement", "subjective_objective": {"answer": "Subjective", "rationale": "Because I have a strong imagination, a self-evaluation, which expresses the patient's own subjective feelings and attitudes."}, "sentence_sentiment": {"answer": "Positive", "rationale": "The basis of the judgment: \"a strong imagination\" is a positive ability."}, "language_style": {"answer": "Tactful and warm", "rationale": "The entry is more personal and interactive in its expression of a gentle and inviting tone, rather than a direct statement or order. Moreover, the issue uses a positive tone, encouraging respondents to share their thoughts and views, and giving people a positive, open feeling."}, "time_dimension": {"answer": "Present", "rationale": "The keyword \"severe imagination\" suggests that it is a description or evaluation of the current state."}, "sentence_type": "Affirmative declarative sentence", "pos": {"verbs": {"have": 1}, "adjectives": {"Strong": 1, "very": 1}, "numerals": {"27": 1}}}, {"content": "28. I like to help others to develop their talents and talents.", "word_count": 14, "label": "", "sentence_structure": "Subject鈥搗erb鈥搊bject", "subjective_objective": {"answer": "Subjective", "rationale": "Because it is a subjective attitude or emotion that I like to help others to develop their talents and talents."}, "sentence_sentiment": {"answer": "Positive", "rationale": "Because the behavior in the entry \"likes to help others to develop their talents and talents\" has a positive emotional expression of the willingness to help."}, "language_style": {"answer": "Tactful and warm", "rationale": "The language style of the entry is euphemistic. It expresses the theme of a gentle and encouraging way of helping others to discover and develop their talents and talents, which is more acclaimable and motivated, and easily resonates and engages with the other."}, "time_dimension": {"answer": "Present", "rationale": ""}, "sentence_type": "Affirmative declarative sentence", "pos": {"nouns": {"Questionnaire Entry": 1, "Talent": 2}, "verbs": {"Yeah": 1, "Help": 1, "Play": 1}, "numerals": {"28": 1}}}, {"content": "I like to oversee things until they are finished.", "word_count": 11, "label": "", "sentence_structure": "Subject鈥搗erb鈥揷omplement", "subjective_objective": {"answer": "Subjective", "rationale": "Because \"like\" is the patient's own attitude or emotional reaction, and it's subjective."}, "sentence_sentiment": {"answer": "Positive", "rationale": "Because the purpose of the entry is to \"like to monitor things until they're finished.\""}, "language_style": {"answer": "Calm and direct", "rationale": ""}, "time_dimension": {"answer": "Past", "rationale": "The entry asks about the preferences or habits of the testee in the past, since the preference for \"supervising things until completion\" is a description of behaviour or attitude and does not address the time dimension of the future."}, "sentence_type": "Affirmative declarative sentence", "pos": {"verbs": {"Yeah": 1, "Oversight": 1, "Things": 1, "Completed": 1}, "adverbs": {"to": 1, "Down": 1}, "numerals": {"29": 1}}}, {"content": "If I faced a new scenario, I would be well prepared in advance.", "word_count": 20, "label": "", "sentence_structure": "Subject鈥損redicate", "subjective_objective": {"answer": "Subjective", "rationale": "Because it is the patient ' s personal will and attitude and therefore subjectively, whether or not he or she is prepared sufficiently in advance."}, "sentence_sentiment": {"answer": "Positive", "rationale": "Because the \"be prepared in advance\" act in the entry is positive."}, "language_style": {"answer": "Calm and direct", "rationale": "The language style of the entry is a calm and direct one, which directly raises a question and asks the person interviewed about his or her attitude or behaviour."}, "time_dimension": {"answer": "Present", "rationale": "The keywords \"to face a new scenario\" and \"to be\" indicate that it is a description of the person being tested for current or near-term behaviour. The entry is therefore in the context of current behaviour or mentality."}, "sentence_type": "Affirmative declarative sentence", "pos": {"nouns": {"New scene": 1, "Before": 1, "Ready": 1}, "verbs": {"Yes": 2, "Do it": 1}, "adverbs": {"Full": 1}, "numerals": {"30": 1}, "pronouns": {"I": 1}}}, {"content": "31. I like to do a task on my own.", "word_count": 11, "label": "", "sentence_structure": "Subject鈥搗erb鈥揷omplement", "subjective_objective": {"answer": "Subjective", "rationale": "The desire to perform a task independently is a subjective examination of the individual feelings and attitudes of the patient."}, "sentence_sentiment": {"answer": "Positive", "rationale": "Because the entry \"likes to do a task on its own\" shows that individuals have a positive quality and attitude."}, "language_style": {"answer": "Calm and direct", "rationale": "The entry raises questions in the form of a simple statement, and is not expressed in a more direct manner using any rhetorical or emotional language."}, "time_dimension": {"answer": "Present", "rationale": ""}, "sentence_type": "Affirmative declarative sentence", "pos": {"nouns": {"Tasks": 1}, "verbs": {"Yeah": 1, "Completed": 1}, "adjectives": {"Independence": 1}, "numerals": {"31": 1}}}, {"content": "I am eager to read or think about anything that could provoke my curiosity.", "word_count": 21, "label": "", "sentence_structure": "Subject鈥搗erb鈥搊bject", "subjective_objective": {"answer": "Subjective", "rationale": "Because \"desired\" and \"quiet\" are the subjective feelings and attitudes inside the patient."}, "sentence_sentiment": {"answer": "Positive", "rationale": "Because the words \"desired\" and \"intrigued curiosity\" in the entries are expressions of positive attitudes and behaviour."}, "language_style": {"answer": "Tactful and warm", "rationale": "The language style of the entry is more euphemistic and gentle, using positive and positive words such as \" longing,\" \"reading,\" \"thinking,\" expressing a desire for and expectation of knowledge exploration. And it also uses \"anything that can trigger my curiosity,\" which is more acclaimable, natural and easily resonant and interesting to the other."}, "time_dimension": {"answer": "Present", "rationale": "The keywords \" longing,\" \"reading\" and \"thinking\" all indicate the behaviour or state of the person in progress, indicating that the person who was tested is now keen to read and think. Moreover, \"what can cause my curiosity\" also indicates that it is a state of continuity, not a past or future act. So the entry describes the situation of the person who was tested."}, "sentence_type": "Affirmative declarative sentence", "pos": {"nouns": {"I": 1, "Reading": 1, "Think": 1, "Curiosity": 1, "Something": 1}, "verbs": {"Desire": 1, "Initiating": 1}, "numerals": {"32": 1}}}, {"content": "33. I like to try the concept of innovation.", "word_count": 10, "label": "", "sentence_structure": "Subject鈥搗erb鈥搊bject", "subjective_objective": {"answer": "Subjective", "rationale": "Because like is a subjective attitude and feeling."}, "sentence_sentiment": {"answer": "Positive", "rationale": "Because the expression \"likes to try the concept of innovation\" in the entry is positive."}, "language_style": {"answer": "Tactful and warm", "rationale": "The expression in this entry starts with the words \"I,\" which use positive words, \"like,\" and emphasizes the willingness to try to use the concept of innovation. This language style is more friendly and encouraging, and makes people feel more willing to participate in the questionnaire."}, "time_dimension": {"answer": "Present", "rationale": "So, it can be judged as \"now\"."}, "sentence_type": "Affirmative declarative sentence", "pos": {"nouns": {"Concept": 1, "Yeah": 1, "Innovation": 1}, "verbs": {"Try": 1}, "numerals": {"33": 1}}}, {"content": "34. If I clash with others, I will constantly try to make a dry end.", "word_count": 20, "label": "", "sentence_structure": "Subject鈥損redicate", "subjective_objective": {"answer": "Subjective", "rationale": "This is a description of the patient ' s subjective attitude and coping methods."}, "sentence_sentiment": {"answer": "Positive", "rationale": "It is positive because it expresses attitudes and behaviour that seek to mediate and avoid conflict in the event of a conflict."}, "language_style": {"answer": "Tactful and warm", "rationale": "This is a more resonant expression and gives researchers a sense of their feelings and attitudes."}, "time_dimension": {"answer": "Future", "rationale": ""}, "sentence_type": "Affirmative declarative sentence", "pos": {"nouns": {"Grid": 1, "The Jade Empire": 1}, "verbs": {"Yes": 3, "Try": 1, "Zero": 1, "Grid": 1}, "adverbs": {"Constantly": 1}, "numerals": {"34": 1}, "pronouns": {"I": 1, "others": 1}}}, {"content": "35. Success requires high-level goals.", "word_count": 10, "label": "", "sentence_structure": "Subject鈥搗erb鈥揷omplement", "subjective_objective": {"answer": "Subjective", "rationale": "Because this is the patient ' s subjective view and attitude to success."}, "sentence_sentiment": {"answer": "Positive", "rationale": "Because the \"must set a high target\" in the entry is an attitude of positive upwards."}, "language_style": {"answer": "Calm and direct", "rationale": ""}, "time_dimension": {"answer": "Future", "rationale": ""}, "sentence_type": "Affirmative declarative sentence", "pos": {"nouns": {"Success": 1, "Objective": 1}, "verbs": {"I have to": 1, "Freeze": 1}, "adjectives": {"High": 1}, "numerals": {"35": 1}}}, {"content": "36. I prefer to be responsible for major decisions.", "word_count": 10, "label": "", "sentence_structure": "Subject鈥搗erb鈥搊bject", "subjective_objective": {"answer": "Subjective", "rationale": "Because \"like to be responsible for making major decisions\" is the patient's personal preference and attitude, and it's subjective."}, "sentence_sentiment": {"answer": "Positive", "rationale": "Because the behavior in the entry \"likes to be responsible for major decisions\" expresses a sense of responsibility and positive attitude."}, "language_style": {"answer": "Tactful and warm", "rationale": "The language style of the entry is more euphemistic, using the term \"like\" to express a positive attitude towards being responsible for decision-making, rather than using a strong or command language directly. Such expression is more acceptable and can generate resonance and interest among the other party and facilitate the effective filling of the questionnaire."}, "time_dimension": {"answer": "Present", "rationale": "The entry asks the testee about his current mentality or attitude, because \"like to be responsible for major decisions\" describes a current state or tendency and therefore the judgement is now."}, "sentence_type": "Affirmative declarative sentence", "pos": {"nouns": {"Questionnaire Entry": 1, "Decision-making": 1, "Responsibility": 1}, "verbs": {"Yeah": 1, "Responsible": 1}, "adverbs": {"Down there": 1, "36": 1}, "numerals": {"Significant": 1}}}, {"content": "I like to be frank and not to be a fan of the horns.", "word_count": 14, "label": "", "sentence_structure": "Subject鈥搗erb鈥揷omplement", "subjective_objective": {"answer": "Subjective", "rationale": "Because \"like\" and \"not like\" are the psychological tendencies and attitudes of the patient."}, "sentence_sentiment": {"answer": "Neutral", "rationale": "Because the entry merely stated a fact, it did not express a positive or negative emotional attitude."}, "language_style": {"answer": "Calm and direct", "rationale": "The language style of the entry is cold and straightforward, using the distinct terms \"into the plain\" and \"in the corner\" and expressing a direct attitude towards both modes of communication, without too much ado or euphemism."}, "time_dimension": {"answer": "Present", "rationale": "The entry is a personal preference of the testee, with no clear temporal dimensions pointing to the past, present or future, but rather a description of the current attitudes and values of the testee. So we can judge the time dimension of the questionnaire entry as \"now\"."}, "sentence_type": "Affirmative declarative sentence", "pos": {"verbs": {"Yeah": 2, "- I'm not saying anything": 1, "Turn around and rub the horns": 1}, "adjectives": {"I'm not gonna say anything": 1, "Turning around the corner": 1}, "numerals": {"37": 1}}}, {"content": "38. I must thoroughly analyse the problem before I can resolve it.", "word_count": 18, "label": "", "sentence_structure": "Subject鈥搗erb鈥揷omplement", "subjective_objective": {"answer": "Subjective", "rationale": "Because the need for a comprehensive analysis of the problem is a subjective judgement and decision-making."}, "sentence_sentiment": {"answer": "Positive", "rationale": "This entry is an expression of a careful, serious and planned approach and methodology to work."}, "language_style": {"answer": "Tactful and warm", "rationale": "The language style of the entry is euphemism. It invites the interviewee to share his/her views and experiences in a gentle and kind manner, rather than direct orders or statements."}, "time_dimension": {"answer": "Present", "rationale": "\"I have to\" means a necessity or a compulsory act, and \"before the problem is solved\" means that the act occurs before the actual problem is solved. So, the entry is about the behavioral habits that the testee usually needs to analyse thoroughly when solving the problem, and the temporal dimension is now."}, "sentence_type": "Affirmative declarative sentence", "pos": {"nouns": {"Questionnaire": 1, "Problem": 2, "Solutions": 1, "Entry": 1}, "verbs": {"Yes": 1, "Conduct": 1, "A thorough analysis": 1}, "numerals": {"38": 1}, "pronouns": {"I": 1}}}, {"content": "39. I like to reset my environment to make it different.", "word_count": 18, "label": "", "sentence_structure": "Subject鈥搗erb鈥搊bject", "subjective_objective": {"answer": "Subjective", "rationale": "Because it's the patient's subjective attitude."}, "sentence_sentiment": {"answer": "Positive", "rationale": "Because the \"like to reset my environment\" behavior in the entry is positive, and it shows the spirit of innovation and change."}, "language_style": {"answer": "Tactful and warm", "rationale": "The entry uses a positive tone and a friendly language style, which is not directly asked or ordered to answer, but rather engages in an invitation-based dialogue to express an understanding of and respect for the preferences of the interviewee."}, "time_dimension": {"answer": "Present", "rationale": "The keywords \"like to reset my environment\" and \"to make them different\" indicate that this is an entry describing the current behaviour or attitude of the testee, so the temporal dimension is now judged."}, "sentence_type": "Affirmative declarative sentence", "pos": {"nouns": {"Questionnaire": 1, "Entry": 1, "Environment": 1, "they": 1}, "verbs": {"Yeah": 1, "Set": 1, "鈾?And I'm gonna be 鈾?: 1}, "adjectives": {"Restart": 1, "Different": 1}, "numerals": {"39": 1}, "classifiers": {"individual": 1}, "pronouns": {"I": 1}}}, {"content": "40. I often use my conversations to solve my problems.", "word_count": 18, "label": "", "sentence_structure": "Subject鈥搗erb鈥搊bject", "subjective_objective": {"answer": "Subjective", "rationale": "Because \"to solve your problems by talking to others\" is the patient's subjective behaviour and attitude."}, "sentence_sentiment": {"answer": "Positive", "rationale": "Because the purpose of the entry is to solve your problems by talking to others."}, "language_style": {"answer": "Tactful and warm", "rationale": "The language style of the entry is euphemistic. It guides the interviewee to share their experiences by asking questions, and uses more gentle, friendly language, without direct command or criticism. This way allows the interviewee to be more open to questions, and it also allows them to feel respected and understood."}, "time_dimension": {"answer": "Present", "rationale": "\"I often\" suggests that it is a customary act, \"talking to others to solve my problems\" is the way to describe it, and \"solving my problems\" in the narrative suggests that it is talking about the situation or behaviour of the person being tested. So, the temporal dimension of this entry is now."}, "sentence_type": "Affirmative declarative sentence", "pos": {"nouns": {"Problem": 1, "self": 1}, "verbs": {"Borrow": 1, "Resolve": 1, "Talk": 1}, "adverbs": {"Regular": 1}, "numerals": {"40": 1}, "pronouns": {"others": 1}}}, {"content": "41. I often want to draft a plan, and others will do the details.", "word_count": 17, "label": "", "sentence_structure": "Subject鈥搗erb鈥搊bject", "subjective_objective": {"answer": "Objective", "rationale": "Because \"always want to draft a plan\", \"details by others\" is the patient's objective behaviour and state, and does not involve the patient's psychological feelings or subjective evaluation."}, "sentence_sentiment": {"answer": "Positive", "rationale": "Because the \"wanted to draft a plan\" act in the entry is positive and it is reasonable to expect others to complete the details."}, "language_style": {"answer": "Tactful and warm", "rationale": "This is a more euphemistic way of expressing the person being questioned, which leads to the question whether the person under investigation has a regular experience of developing a plan and allowing others to complete the details."}, "time_dimension": {"answer": "Present", "rationale": "The key word is \"I often think\" , which means that the word \"want\" means \"recurring, customary\" and \"want\" means \"normally\" in its current state of affairs, which means that it not only happened in the past, but may still be going on. \"Throwing a plan, and others finish details\" involves the decision-making and behaviour of the person being tested, implying that the person being tried may hand over his ideas and plans to others for execution, and that he is responsible for the general direction."}, "sentence_type": "Affirmative declarative sentence", "pos": {"nouns": {"I": 1, "Planned": 1, "person": 2, "Details": 1}, "verbs": {"I remember": 1, "Drafting": 1, "Completed": 1}, "adverbs": {"Regular": 1}, "numerals": {"41": 1}}}, {"content": "42. punctuality is very important to me.", "word_count": 10, "label": "", "sentence_structure": "Subject鈥揷opula鈥損redicative", "subjective_objective": {"answer": "Subjective", "rationale": "Because \"it's very important to me to be on time\" is an expression of personal subjective feelings and attitudes."}, "sentence_sentiment": {"answer": "Positive", "rationale": "Because the entry indicates that punctuality is of great importance to the person and is a positive attitude and value."}, "language_style": {"answer": "Calm and direct", "rationale": "The entry expresses the importance of punctuality in a straightforward and clear manner, without excessive rhetoric or emotional colours, calm and objective tone, and with greater guidance and practicality."}, "time_dimension": {"answer": "Present", "rationale": "The sentence expresses the attitude and perception of the testee as to \"just on time\", does not relate to past or future acts or events, and therefore the judgement is the present statement of attitude."}, "sentence_type": "Affirmative declarative sentence", "pos": {"verbs": {"On time": 1, "Tell me": 1, "Very": 1}, "adjectives": {"Important": 1}, "numerals": {"42": 1}}}, {"content": "43. I am relieved by the fact that I am engaged in outdoor activities.", "word_count": 12, "label": "", "sentence_structure": "Subject鈥揷opula鈥損redicative", "subjective_objective": {"answer": "Subjective", "rationale": "This is a subjective examination of patients ' feelings and perceptions of engaging in outdoor activities."}, "sentence_sentiment": {"answer": "Positive", "rationale": "Because the description of \"single-free\" expresses a positive emotional attitude."}, "language_style": {"answer": "Tactful and warm", "rationale": "The more moderate language of the entry, which uses narratives rather than orders or questions, and also uses some visualized terms such as \"the spirit of the gods\" make the question more interesting and attracts the active participation of the respondents in the answers."}, "time_dimension": {"answer": "Present", "rationale": "The entry indicates the individual's feelings and effects of engaging in outdoor activities, is now in the state of affairs and is therefore judged to be \"now\"."}, "sentence_type": "Affirmative declarative sentence", "pos": {"nouns": {"Outdoor activities": 1, "You're": 1, "I": 1, "It's a good day": 1}, "verbs": {"Active": 1}, "adjectives": {"It's a good day": 1}, "numerals": {"43": 1}}}, {"content": "I keep asking: Why?", "word_count": 8, "label": "", "sentence_structure": "Subject鈥搗erb鈥搊bject", "subjective_objective": {"answer": "Subjective", "rationale": "Because \"suspect: Why?\" is the patient's subjective thinking and behaviour, reflecting his curiosity and desire to explore things."}, "sentence_sentiment": {"answer": "Neutral", "rationale": "Because the entry is not clearly emotional, it only describes an act."}, "language_style": {"answer": "Calm and direct", "rationale": "The language style of the entry is more calm and straightforward, rather than being rendered in too many rhetorical or emotional ways, questions are asked in an objective and concise manner. This expression may make the respondents feel more comfortable and relaxed, as it does not make them feel that their answers may affect the results of the investigation or be influenced in some way."}, "time_dimension": {"answer": "Present", "rationale": "In that sentence, \"I keep\" indicates that this is repeated, while \"question\" emphasizes that this is an ongoing move. So the entry describes the person who was tested for the present behavior or mentality."}, "sentence_type": "Interrogative sentence", "pos": {"nouns": {"I": 1, "Problem": 2}, "verbs": {"Question": 1, "be": 1}, "adverbs": {"鈾?Keep going 鈾?: 1}, "numerals": {"44": 1}}}, {"content": "45. I like the way my work can express my emotions and feelings.", "word_count": 19, "label": "", "sentence_structure": "Subject鈥搗erb鈥揷omplement", "subjective_objective": {"answer": "Subjective", "rationale": "Because \"like\" is a subjective feeling and attitude, not an objective fact. This questionnaire is designed to understand patients' emotional attitudes to work, and it is therefore a subjective examination."}, "sentence_sentiment": {"answer": "Positive", "rationale": "Because the attitude in the entry \"likes my work to express my emotions and feelings\" is positive."}, "language_style": {"answer": "Tactful and warm", "rationale": "The entry is more personal and interactive when it is spoken in a gentle and inviting tone, rather than directly speaking or ordering. It also attempts to stimulate the views of the interviewees about their work and to understand their feelings and feelings by asking them whether they like it, and the language style is more friendly and warm."}, "time_dimension": {"answer": "Present", "rationale": ""}, "sentence_type": "Affirmative declarative sentence", "pos": {"nouns": {"Work": 1, "Emotion": 1, "Feel": 1}, "verbs": {"Yeah": 1, "Da-fa": 1}, "adverbs": {"self": 1}, "numerals": {"45": 1}}}, {"content": "46. I like to help others find ways to look at each other.", "word_count": 20, "label": "", "sentence_structure": "Subject鈥搗erb鈥搊bject", "subjective_objective": {"answer": "Subjective", "rationale": "Because \"like\" is patient's subjective attitude."}, "sentence_sentiment": {"answer": "Positive", "rationale": "Because the purpose of the entry is to help others find ways to look at each other."}, "language_style": {"answer": "Tactful and warm", "rationale": "The entry is used to engage in dialogue by asking each other about their preferences for helping others. The language style is more gentle, friendly, and interactive."}, "time_dimension": {"answer": "Present", "rationale": "Keywords include \"like,\" \"helping others\", \"the way to look at each other,\" which point to current behavior or ideas."}, "sentence_type": "Interrogative sentence", "pos": {"nouns": {"Approach": 1, "person": 2, "Attention": 1}, "verbs": {"Yeah": 1, "Help": 1, "Find": 1, "Mutual attention": 1}, "adverbs": {"- Yeah, yeah": 1}, "numerals": {"46": 1}, "pronouns": {"others": 1}}}, {"content": "47. It was exciting to be able to participate in major decision-making.", "word_count": 17, "label": "", "sentence_structure": "Subject鈥揷opula鈥損redicative", "subjective_objective": {"answer": "Subjective", "rationale": "Because \"excited\" is a subjective feeling and attitude, not an objective fact or behaviour. The entry is therefore viewed from the patient's subjective perspective."}, "sentence_sentiment": {"answer": "Positive", "rationale": "Because the phrase \"involved in major decision-making is exciting\" in the entry is positive emotional."}, "language_style": {"answer": "Tactful and warm", "rationale": ""}, "time_dimension": {"answer": "Present", "rationale": "The questionnaire entry asks the respondents about their feelings and perceptions of participation in major decisions, \"enabled to participate\" indicating that it is a regular, customary act or attitude, and that it may have occurred in the past, present or future. We can therefore judge that this phrase is a state or situation, that is, \"now\"."}, "sentence_type": "Exclamatory sentence", "pos": {"nouns": {"Things": 1, "Decision-making": 1}, "verbs": {"Participation": 1, "be": 1, "It's exciting": 1, "of": 2}, "adjectives": {"Significant": 1, "It's exciting": 1}, "numerals": {"47": 1}}}, {"content": "48. I have often been clean and like to be orderly.", "word_count": 13, "label": "", "sentence_structure": "Subject鈥搗erb鈥揷omplement", "subjective_objective": {"answer": "Subjective", "rationale": "Because words like \"often\" and \"like\" indicate that it's the patient's own subjective feeling and attitude."}, "sentence_sentiment": {"answer": "Positive", "rationale": "The description is positive, expressing the interviewee ' s self-management, neat attitude and habits."}, "language_style": {"answer": "Tactful and warm", "rationale": "The language style of the entry is more euphemistic, and the use of the words \"often\" and \"like\" to express the interest and respect of the investigator for the personal habits of the person under investigation."}, "time_dimension": {"answer": "Present", "rationale": ""}, "sentence_type": "Affirmative declarative sentence", "pos": {"nouns": {"Questionnaire": 1, "Entry": 1, "Cleaning": 1, "Orderly": 1}, "verbs": {"Hold on": 1, "Yeah": 1}, "adjectives": {"Regular": 1}, "numerals": {"48": 1}}}, {"content": "49. I like the surrounding environment to be simple and practical.", "word_count": 12, "label": "", "sentence_structure": "Subject鈥搗erb鈥揷omplement", "subjective_objective": {"answer": "Subjective", "rationale": "Because I like being a patient's subjective attitude."}, "sentence_sentiment": {"answer": "Neutral", "rationale": "Because the entry does not have any emotional color."}, "language_style": {"answer": "Tactful and warm", "rationale": "The language style of the entry is more euphemistic, rather than directly asking the views or feelings of the other side, but rather allowing the other side to express their preferences in an invitation-style tone, which is more personal and interactive."}, "time_dimension": {"answer": "Present", "rationale": "The entry describes the testee ' s preferences for the surrounding environment, which is now judged by the time dimension."}, "sentence_type": "Exclamatory sentence", "pos": {"nouns": {"Yeah": 1, "Peripheral": 1, "Environment": 1, "Actual": 1}, "verbs": {"Yeah": 1}, "adjectives": {"Simple": 1, "And": 1, "Actual": 1}, "numerals": {"49": 1}}}, {"content": "50. I will keep thinking about a question until I find out the answer.", "word_count": 19, "label": "", "sentence_structure": "Subject鈥搗erb鈥揷omplement", "subjective_objective": {"answer": "Subjective", "rationale": "Because the solution to this problem depends on the patient ' s willingness to seek answers, it is the individual ' s subjective attitude and behaviour."}, "sentence_sentiment": {"answer": "Positive", "rationale": "Because the behavior in the entries \"continues to think about a question until the answer is found\" is positive, showing curiosity and commitment to knowledge and understanding."}, "language_style": {"answer": "Calm and direct", "rationale": "The entry expresses direct queries about the behaviour of the interviewees in a concise and clear language, without too much rhetorical or emotional rendering, and is of a calm and direct type."}, "time_dimension": {"answer": "Present", "rationale": "The keywords \"will\" and \"continuous\" and \"until\" indicate that it is a general, customary act and is taking place. Thus, the temporal dimension of the article is now."}, "sentence_type": "Affirmative declarative sentence", "pos": {"nouns": {"Problem": 1, "Answer": 1}, "verbs": {"Yes": 1, "Thinking": 1, "Find": 1, "Out": 1, "Stop": 1}, "adverbs": {"鈾?Keep going 鈾?: 1}, "numerals": {"50": 1}}}, {"content": "The beauty of nature touches my soul deeply.", "word_count": 14, "label": "", "sentence_structure": "Subject鈥揷opula鈥損redicative", "subjective_objective": {"answer": "Subjective", "rationale": "Because the entry describes the patient鈥檚 subjective feelings and emotional reactions to the beauty of nature, rather than the objective facts or phenomena."}, "sentence_sentiment": {"answer": "Positive", "rationale": "Because the description \"the beauty of nature deeply touches my soul\" expresses appreciation and praise for it, and it is a positive emotional tendency."}, "language_style": {"answer": "Tactful and warm", "rationale": "The entry depicts the profound influence of natural beauty on the inside of the person surveyed in a soft, compassionate language, with a sense of praise and emotion. This expression gives positive, comfortable impressions and better triggers the echoes and answers of the person under investigation."}, "time_dimension": {"answer": "Present", "rationale": ""}, "sentence_type": "Affirmative declarative sentence", "pos": {"nouns": {"Nature": 1, "Beautiful": 1, "Soul": 1}, "verbs": {"Touch": 1, "be": 1}, "adjectives": {"Deep": 1}}}, {"content": "52. Close human relationships are important to me.", "word_count": 12, "label": "", "sentence_structure": "Subject鈥揷opula鈥損redicative", "subjective_objective": {"answer": "Subjective", "rationale": "Because \"intimate relationships are important to me\" is about the patient's personal feelings and attitudes, and it's subjective."}, "sentence_sentiment": {"answer": "Positive", "rationale": "Because the expression \"intimate relationships are important to me\" in the entry expresses a positive attitude and values."}, "language_style": {"answer": "Tactful and warm", "rationale": "The entry is more intimate and interactive when it is spoken in a gentle, invitation-like tone, rather than directly speaking or ordering. At the same time, the language style of the article is simpler and less mundane, less verb or emotional, and more readily understood and accepted by the interviewees."}, "time_dimension": {"answer": "Present", "rationale": "The entry is for questioning the views and attitudes of the person in question regarding his/her interpersonal relationship, without a clear time dimension past, present or future. It is therefore not possible to classify the above-mentioned \"old/present/future\" judgement."}, "sentence_type": "Affirmative declarative sentence", "pos": {"nouns": {"Human relations": 1, "Intimacy": 1}, "verbs": {"to": 1, "Important": 1}, "adjectives": {"EMPTY": 1}, "adverbs": {"EMPTY": 1}, "numerals": {"52": 1}, "classifiers": {"EMPTY": 1}, "pronouns": {"EMPTY": 1}}}, {"content": "Promotion and progress are of great importance to me.", "word_count": 10, "label": "", "sentence_structure": "Subject鈥揷opula鈥損redicative", "subjective_objective": {"answer": "Subjective", "rationale": "Because the word \"important\" reflects the patient's personal views and values and is an expression of subjective attitudes."}, "sentence_sentiment": {"answer": "Positive", "rationale": "Because this entry indicates the importance that individuals attach to promotion and advancement, it is a positive attitude or quality."}, "language_style": {"answer": "Calm and direct", "rationale": "The entry is a concise expression of the importance that the subject attaches to promotion and progress, without any qualifying words or emotional expressions, and therefore can be judged to be calm and straightforward."}, "time_dimension": {"answer": "Present", "rationale": "The entry describes the attitude and expectations of the testee in the future, the keywords being \"upgrading\" and \"progressing\" , all pointing to what may be possible in the future. So it is judged to be the future."}, "sentence_type": "Exclamatory sentence", "pos": {"verbs": {"be": 1, "to": 1}, "adjectives": {"Extreme": 1}, "numerals": {"53": 1, "I": 1}, "pronouns": {"Here": 1}}}, {"content": "54. I will feel safer when I get my daily work programme right.", "word_count": 18, "label": "", "sentence_structure": "Subject鈥揷opula鈥損redicative", "subjective_objective": {"answer": "Subjective", "rationale": "Because \"a feeling of security\" is a subjective feeling or attitude."}, "sentence_sentiment": {"answer": "Positive", "rationale": "Because the result of the entry \"will feel safe\" is positive."}, "language_style": {"answer": "Tactful and warm", "rationale": ""}, "time_dimension": {"answer": "Present", "rationale": ""}, "sentence_type": "Affirmative declarative sentence", "pos": {"nouns": {"Programme of work": 1, "Safe": 1}, "verbs": {"- Put it on": 1, "Planned": 1, "Yes": 2, "More": 1}, "adjectives": {"Daily": 1, "have": 1}, "adverbs": {"Compare": 1}, "numerals": {"54": 1}, "pronouns": {"I": 1}}}, {"content": "55. I am not afraid of overloading and know the focus of the work.", "word_count": 18, "label": "", "sentence_structure": "Subject鈥揷opula鈥損redicative", "subjective_objective": {"answer": "Subjective", "rationale": "Because \"no fear\" and \"the focus of knowing work\" are the patient's description of his attitude, which is subjective."}, "sentence_sentiment": {"answer": "Positive", "rationale": "Because \"no fear of overload\" and \"know the focus of the job\" in this entry are positive attitudes and behaviours."}, "language_style": {"answer": "Calm and direct", "rationale": "The entry is more calm and straightforward in language, with no excessive emotional or rhetorical style. It is simple to ask the interviewee whether he is not afraid of excessive workload and clearly expresses the importance of the focus of his work, which is objective and relevant."}, "time_dimension": {"answer": "Present", "rationale": "The \"no fear\" and \"know\" times are the usual times, indicating that the act or attitude is the current."}, "sentence_type": "Affirmative declarative sentence", "pos": {"nouns": {"Workload": 1, "Work": 1, "Focus": 1}, "verbs": {"No, I'm not": 1, "Yes": 1, "Too heavy": 1}, "adjectives": {"Heavy": 1, "Mass": 1}, "numerals": {"55": 1}, "pronouns": {"I": 1}}}, {"content": "I like books that make me think and give me new ideas.", "word_count": 15, "label": "", "sentence_structure": "Subject鈥搗erb鈥揷omplement", "subjective_objective": {"answer": "Subjective", "rationale": "Because it's the patient's subjective attitude."}, "sentence_sentiment": {"answer": "Positive", "rationale": "Because the words \"like,\" \"think\" and \"new ideas\" in the entries are all positive emotional."}, "language_style": {"answer": "Tactful and warm", "rationale": ""}, "time_dimension": {"answer": "Present", "rationale": "The entry expresses the testee ' s preference for the type of book and the purpose of the book, is a current act or state and can therefore be judged as the present."}, "sentence_type": "Affirmative declarative sentence", "pos": {"nouns": {"I": 1, "Books": 1, "Think": 1, "Perceptions": 1}, "verbs": {"Yeah": 1, "鈾?And I'm gonna be 鈾?: 1, "Here": 1}, "numerals": {"56": 1}}}, {"content": "57. I hope to see artistic performances, theatres and good films.", "word_count": 17, "label": "", "sentence_structure": "Subject鈥搗erb鈥搊bject", "subjective_objective": {"answer": "Subjective", "rationale": "This is because of the patient ' s personal wishes and aspirations and because it is a subjective examination."}, "sentence_sentiment": {"answer": "Positive", "rationale": "Because hope in the entry expresses a desire and a desire, a positive emotion."}, "language_style": {"answer": "Tactful and warm", "rationale": "The language style of the entry is more euphemistic, as it expresses expectations of artistic performances, theatre and good films in a question-and-answer manner, and is more cordial and friendly, while also being open and interactive."}, "time_dimension": {"answer": "Present", "rationale": "The keyword \"Hope\" represents the current desire and expectations, so the entry is a description of the present thoughts and feelings of the testees and a judgement of the present."}, "sentence_type": "Imperative sentence", "pos": {"nouns": {"Artistic performance": 1, "Theatre": 1, "Film": 2}, "verbs": {"Yeah": 1, "Hope": 1}, "adjectives": {"Okay": 1}, "numerals": {"57": 1}}}, {"content": "58. I am quite sensitive to the low mood of others.", "word_count": 14, "label": "", "sentence_structure": "Subject鈥揷opula鈥損redicative", "subjective_objective": {"answer": "Subjective", "rationale": "Because \"I'm quite sensitive to others' moods\" describes patients' subjective feelings and attitudes."}, "sentence_sentiment": {"answer": "Negative", "rationale": "Because the description of \"fun\" in the entry is negative."}, "language_style": {"answer": "Tactful and warm", "rationale": "The entry is more personal and interactive when it is spoken in a gentle and inviting manner, rather than in direct statements or orders. At the same time, the article is more softly phrased, without too much negative emotional overturning, giving people a warm feeling."}, "time_dimension": {"answer": "Present", "rationale": "The entry describes the current feelings and perceptions of the testees, \"I am sensitive\" indicating the current state or attitude, and the temporal dimension is now."}, "sentence_type": "Affirmative declarative sentence", "pos": {"nouns": {"I": 1, "Others are depressed": 1, "Sensitive": 1}, "verbs": {"to": 1, "be": 1}, "adjectives": {"Pretty much": 1}, "numerals": {"58": 1}}}, {"content": "59. I am excited to be able to influence others.", "word_count": 11, "label": "", "sentence_structure": "Subject鈥揷opula鈥損redicative", "subjective_objective": {"answer": "Subjective", "rationale": "Because \"I'm excited to influence other people\" is a subjective feeling and emotional experience that reflects the attitude and perception of patients about their behaviour and abilities."}, "sentence_sentiment": {"answer": "Positive", "rationale": "Because the expression \"I'm excited to influence others\" shows positive emotional attitudes and behavioural tendencies."}, "language_style": {"answer": "Tactful and warm", "rationale": "The entry is more adept and interactive when it is spoken in a gentle and inviting manner, rather than by direct statements or orders."}, "time_dimension": {"answer": "Present", "rationale": "The entry describes the expectations and aspirations of the future, the behaviour that \"improves\" indicates an expectation of influence, and the \"stimulating me\" expresses a positive mindset and expectation for the future. The temporal dimension of the questionnaire is therefore judged to be the future."}, "sentence_type": "Imperative sentence", "pos": {"nouns": {"person": 1, "Excited": 1}, "verbs": {"Impact": 1, "鈾?Make you feel 鈾?: 1}, "adverbs": {"Yes": 1}, "numerals": {"59": 1}}}, {"content": "When I promised one thing, I would do everything possible to monitor all the details.", "word_count": 18, "label": "", "sentence_structure": "Subject鈥損redicate", "subjective_objective": {"answer": "Subjective", "rationale": "This is because the patient evaluates or interprets his or her behaviour and attitude and is subjective in nature."}, "sentence_sentiment": {"answer": "Positive", "rationale": "Because the \"will do everything to monitor all the details\" in the entry is positive."}, "language_style": {"answer": "Tactful and warm", "rationale": "The entry is more euphemistic, in which the interviewer is guided to share his or her views and to use a positive tone. It does not directly order or evaluate the behaviour of the interviewee, but offers a possibility for the interviewee to express his or her attitude and experience freely. Thus, the language style of the article is more \"fluent.\""}, "time_dimension": {"answer": "Present", "rationale": ""}, "sentence_type": "Affirmative declarative sentence", "pos": {"nouns": {"Yes": 1, "Oversight": 1, "Details": 1}, "verbs": {"Promise": 1, "Yes": 2, "- All of it": 1, "Oversight": 1}, "adjectives": {"One": 1, "All": 1}, "numerals": {"60": 1}}}, {"content": "61. I hope that heavy bodywork will not harm anyone.", "word_count": 17, "label": "", "sentence_structure": "Subject鈥搗erb鈥搊bject", "subjective_objective": {"answer": "Subjective", "rationale": "Because hope is the patient's subjective attitude, it's the thoughts and wishes that are inside the patient."}, "sentence_sentiment": {"answer": "Positive", "rationale": "Because the entry expresses a positive desire and attitude that heavy work will not harm others, a behaviour that is sensitive to others and is safe."}, "language_style": {"answer": "Tactful and warm", "rationale": "This entry is based on a desire and caring mood, and uses the expression of a non-direct statement, which makes the respondent feel respected and understood."}, "time_dimension": {"answer": "Present", "rationale": "The entry expresses the views and expectations of the testee now, the key word being \"fear,\" the desire and expectation for the future, and \"do not hurt anyone\" is also a current psychological state. The time dimension of this article is therefore now."}, "sentence_type": "Imperative sentence", "pos": {"nouns": {"Hope": 1, "Body": 1, "Work": 1, "person": 1}, "verbs": {"Hurt": 1, "Yes": 1, "Do it": 1}, "adjectives": {"Heavy": 1, "Anyone": 1}, "numerals": {"61": 1}, "pronouns": {"I": 1}}}, {"content": "62. I hope to study all subjects of interest to me.", "word_count": 16, "label": "", "sentence_structure": "Subject鈥搗erb鈥搊bject", "subjective_objective": {"answer": "Subjective", "rationale": "Because \"hope\" indicates that it is the patient's personal wish and expectation, it is a subjective examination."}, "sentence_sentiment": {"answer": "Positive", "rationale": "Because the expression of article expression expresses a positive mindset and enthusiasm for learning, there is no negative or neutral emotional colour."}, "language_style": {"answer": "Tactful and warm", "rationale": "This expression is more respectful of the will and interests of the person under investigation and helps to create a good atmosphere of communication."}, "time_dimension": {"answer": "Future", "rationale": "The keywords are \"hope\" and \"capable\", which point to what has not yet happened and what is to be done, and \"learning all the subjects that interest me\" indicates a vision or plan for the future. The entry should therefore be classified as the future."}, "sentence_type": "Affirmative declarative sentence", "pos": {"nouns": {"Questionnaire Entry": 1, "Subject": 1}, "verbs": {"Yes": 1, "Learning": 1, "鈾?And I'm gonna be 鈾?: 1, "Interested": 1}, "adjectives": {"All": 1, "of": 2}, "adverbs": {"Hope": 1}, "numerals": {"62": 1}, "pronouns": {"I": 1}}}, {"content": "63. I hope to do something special.", "word_count": 12, "label": "", "sentence_structure": "Subject鈥搗erb鈥搊bject", "subjective_objective": {"answer": "Subjective", "rationale": "Because \"I want to do something different\" is the patient's own desire and expectations, and it's a subjective idea of the individual."}, "sentence_sentiment": {"answer": "Positive", "rationale": "Because the entry \"want to do something different\" expresses a positive, exploratory and innovative attitude, and a positive emotional orientation."}, "language_style": {"answer": "Tactful and warm", "rationale": "The language style of the entry is a milder type of question, and it expresses a positive attitude and desire that gives people a sense of affection and encouragement. Instead of using too direct or too cold language, it seeks to create an atmosphere of friendship, openness and understanding that encourages respondents to open up and share their thoughts."}, "time_dimension": {"answer": "Present", "rationale": "The entry will contain expectations and visions for the future, and the keywords are \"can\" and \"hope\" , which point to situations that have not yet occurred and that may arise in the future. At the same time, the word \"want\" in the sentence indicates that it is a vision and a vision for the future. Thus, the judgement is based on the future."}, "sentence_type": "Imperative sentence", "pos": {"nouns": {"Grandpa": 1, "Questionnaire": 1}, "verbs": {"Hope": 1, "Yes": 1, "Do it": 1, "have": 1}, "adjectives": {"Different": 1, "of": 2}, "adverbs": {"Some": 1, "The crowd": 1}, "numerals": {"63": 1}, "pronouns": {"I": 1}}}, {"content": "64. I am happy to help others with their difficulties.", "word_count": 13, "label": "", "sentence_structure": "Subject鈥搗erb鈥揷omplement", "subjective_objective": {"answer": "Subjective", "rationale": "Because reaching out to others is a subjective act and attitude."}, "sentence_sentiment": {"answer": "Positive", "rationale": "Because the entry \"Having to help others with their difficulties\" shows a positive emotional attitude and quality, offering to help others."}, "language_style": {"answer": "Tactful and warm", "rationale": ""}, "time_dimension": {"answer": "Present", "rationale": "The keyword is \"I'm having trouble with others\" and it's the attitude and the perception that is now; \"Loving to help\" is a concrete expression of that attitude. The entry therefore asks if the person who was tested is willing to help others now with the difficulties."}, "sentence_type": "Affirmative declarative sentence", "pos": {"nouns": {"others": 1, "Difficulties": 1, "Help hands": 1}, "verbs": {"Shine": 1, "Reach out": 1, "I'm happy": 1, "Here": 1}, "adverbs": {"very": 1}, "numerals": {"64": 1}, "pronouns": {"I": 1}}}, {"content": "I am willing to take a little risk to make progress.", "word_count": 11, "label": "", "sentence_structure": "Subject鈥搗erb鈥搊bject", "subjective_objective": {"answer": "Subjective", "rationale": "Because I'm willing to express the patient's subjective attitude and will."}, "sentence_sentiment": {"answer": "Positive", "rationale": "Because the purpose of the entry is to take a little risk to progress."}, "language_style": {"answer": "Tactful and warm", "rationale": ""}, "time_dimension": {"answer": "Future", "rationale": "The entry expresses the attitude and expectations of the testees for the future, the key words being \"willing\" and \"risk for progress\", all pointing to situations that have not yet occurred and that may arise in the future. The judgement is therefore based on the future of the time dimension."}, "sentence_type": "Affirmative declarative sentence", "pos": {"verbs": {"Yes": 1, "-Put it on": 1, "Progress": 1}, "adjectives": {"A little": 1}, "numerals": {"65": 1}, "pronouns": {"I": 1}}}, {"content": "When I followed the rules, I felt safe.", "word_count": 12, "label": "", "sentence_structure": "Subject鈥揷opula鈥損redicative", "subjective_objective": {"answer": "Subjective", "rationale": "Because feeling safe is the patient's subjective feeling."}, "sentence_sentiment": {"answer": "Positive", "rationale": "Because \"compliance with the rules\" and \"fear safety\" have positive meanings."}, "language_style": {"answer": "Calm and direct", "rationale": "This is usually more common in investigations or studies, as it helps to maintain objectivity and accuracy."}, "time_dimension": {"answer": "Past", "rationale": "Past."}, "sentence_type": "Affirmative declarative sentence", "pos": {"nouns": {"Protocol": 1, "I": 2}, "verbs": {"Follow": 1, "I feel": 1}, "adjectives": {"Clear": 1}, "numerals": {"66": 1}}}, {"content": "67. When I pick a car, the first thing I'm looking at is a good engine.", "word_count": 14, "label": "", "sentence_structure": "Subject鈥搗erb鈥搊bject", "subjective_objective": {"answer": "Subjective", "rationale": "This is a priority consideration in the selection of vehicles and a manifestation of subjective attitudes and values."}, "sentence_sentiment": {"answer": "Neutral", "rationale": "Because the entry does not have any emotional color."}, "language_style": {"answer": "Calm and direct", "rationale": "The language style of the entry is more straightforward, and there is no excessive rhetorical or emotional rendering, but rather an objective and concise presentation of the factors that are of greatest concern to the person under investigation when buying the car."}, "time_dimension": {"answer": "Present", "rationale": "In this entry, both the verbs \"selecting car\" and \"notice\" are used to indicate that the testee is currently doing or has completed the act. Thus, the time dimension of this questionnaire entry is now judged."}, "sentence_type": "Affirmative declarative sentence", "pos": {"nouns": {"Car": 1, "Engine": 1}, "verbs": {"Choose": 1, "Attention": 1, "be": 1}, "adjectives": {"Okay": 1}, "numerals": {"67": 1}, "pronouns": {"I": 1}}}, {"content": "I like words that stimulate my thinking.", "word_count": 11, "label": "", "sentence_structure": "Subject鈥揷opula鈥損redicative", "subjective_objective": {"answer": "Subjective", "rationale": "Because of the subjective attitude of one person, it is personal feelings and preferences that are described here."}, "sentence_sentiment": {"answer": "Positive", "rationale": "Because \"like words that stimulate my thinking\" expresses a positive emotional attitude."}, "language_style": {"answer": "Tactful and warm", "rationale": "The language style of the entry is more euphemistic, easy to use, without direct orders or criticism, and more in the search for the views and feelings of the other side, creating a climate of friendly dialogue."}, "time_dimension": {"answer": "Present", "rationale": "\"I like\" in \"like\" means \"like\" or \"like\" in the master's words for objective things, and \"may\" in \"in the words that stimulate my thinking\" takes the usual current state of affairs, which means that the speaker is looking for such words, or has already found them. So, the entry mainly describes the current thoughts and needs."}, "sentence_type": "Affirmative declarative sentence", "pos": {"verbs": {"Yeah": 1, "Exciting": 1, "Think": 1}, "adjectives": {"Yes": 1}, "numerals": {"68": 1}}}, {"content": "69. When I do creative things, I forget all old experiences.", "word_count": 19, "label": "", "sentence_structure": "Subject鈥搗erb鈥搊bject", "subjective_objective": {"answer": "Subjective", "rationale": "Because the questionnaire entries describe a psychological state and feelings, and relate to the emotional, cognitive and so on, the subjective perspective of the individual."}, "sentence_sentiment": {"answer": "Positive", "rationale": "Because \"involve in creative things\" and \"forget all old experiences\" are positive descriptions."}, "language_style": {"answer": "Tactful and warm", "rationale": ""}, "time_dimension": {"answer": "Present", "rationale": ""}, "sentence_type": "Affirmative declarative sentence", "pos": {"nouns": {"Creative": 1, "Yes": 1, "Experience": 1}, "verbs": {"Yes": 2, "Forget it": 1, "Active": 1}, "adverbs": {"When": 1, "Everything": 1, "Old": 1}, "numerals": {"69": 1}}}, {"content": "70. I am concerned that many people in society need help.", "word_count": 17, "label": "", "sentence_structure": "Subject鈥搗erb鈥搊bject", "subjective_objective": {"answer": "Subjective", "rationale": "This is because of the patient ' s personal feelings and attitudes and because it is a subjective examination."}, "sentence_sentiment": {"answer": "Positive", "rationale": "Because the entry \"concerns the need for help for many in society\" is positive, it expresses the will of the interviewees to care for others and to work hard to help others."}, "language_style": {"answer": "Tactful and warm", "rationale": "The expression \"concern\" in the sentence expresses a caring, caring attitude, without too strong a positive or negative emotional tone, and the overall language style is more moderate."}, "time_dimension": {"answer": "Present", "rationale": "The entry indicates the feelings and attitudes of the person who was tested when he spoke, and \"for\" is a vocabulary of the current state of mind, indicating that it is an emotional response that is now being carried out."}, "sentence_type": "Affirmative declarative sentence", "pos": {"nouns": {"Social": 1, "person": 1, "Help": 1, "Attention": 1}, "verbs": {"to": 1, "I feel": 1, "Yes": 1, "Help": 1, "Attention": 1}, "adverbs": {"A lot": 1}, "numerals": {"70": 1}}}, {"content": "It was interesting to persuade others to follow the plan.", "word_count": 16, "label": "", "sentence_structure": "Subject鈥揷opula鈥損redicative", "subjective_objective": {"answer": "Subjective", "rationale": "Because \"fun\" is a feeling or emotion that belongs to the patient's own subjective attitude."}, "sentence_sentiment": {"answer": "Positive", "rationale": "Because \"convincing others to follow the plan\" is a challenging and fulfilling act, and \"interesting things\" is a positive one."}, "language_style": {"answer": "Tactful and warm", "rationale": "By describing the emotional color of \"interesting,\" the entry attempts to engage the respondents and direct them to follow the plan. This language style is more moderate, friendly, and more acceptable and responsive."}, "time_dimension": {"answer": "Present", "rationale": "The sentence says \"is a matter\" and \"is a matter\" and it says that the entry describes a state or behaviour of the moment, so the answer is \"now\"."}, "sentence_type": "Affirmative declarative sentence", "pos": {"nouns": {"Convince": 1, "others": 1, "Things": 1, "Planned": 1}, "verbs": {"Ei": 1, "Okay": 1, "be": 1, "Interesting": 1, "Do it": 1}, "adjectives": {"Annex": 1}, "numerals": {"71": 1}}}, {"content": "I am good at checking the details.", "word_count": 8, "label": "", "sentence_structure": "Subject鈥揷opula鈥損redicative", "subjective_objective": {"answer": "Subjective", "rationale": "Because \"good\" is a subjective assessment or self-perception of an individual, it is a subjective perspective."}, "sentence_sentiment": {"answer": "Positive", "rationale": "Because \"good at checking details\" in the entry is a capability or skill, usually a positive quality."}, "language_style": {"answer": "Calm and direct", "rationale": "The language style of the entry is calm and straightforward. It clearly expresses the subject of asking the other party what advantages or specialties it has, without using excessive rhetorical or emotional rendering, and with a more objective tone."}, "time_dimension": {"answer": "Present", "rationale": "The entry uses the now completed-modern \"good\" expression that has been in the state or behaviour of the past at some point, and has had an impact on the present. So, the judgement is now."}, "sentence_type": "Affirmative declarative sentence", "pos": {"verbs": {"Good at it": 1, "Inspection": 1}, "adjectives": {"Details": 1}}}, {"content": "73. I usually know how to respond to emergencies.", "word_count": 13, "label": "", "sentence_structure": "Subject鈥搗erb鈥搊bject", "subjective_objective": {"answer": "Subjective", "rationale": "Because \"know how to deal with emergencies\" requires judgement based on the experience, knowledge and abilities of the individual, and is the patient's subjective attitude."}, "sentence_sentiment": {"answer": "Positive", "rationale": "Because the \"know how to deal with emergencies\" act positive in the entry."}, "language_style": {"answer": "Calm and direct", "rationale": "The language style of the entry is calm and straightforward. It asks whether the interviewee usually knows how to respond to an emergency, without too many emotional or rhetorical modifications and with a more objective tone. The design of such a question helps the researcher to better understand the emergency response capacity of the interviewee."}, "time_dimension": {"answer": "Present", "rationale": "The entry \"usually\" denotes a normal or customary act, \"know\" and \"payback\" are actions that have occurred in the past, but they describe the current state. Thus, the temporal dimension of the entry can be judged as being now."}, "sentence_type": "Affirmative declarative sentence", "pos": {"verbs": {"Yes": 1, "Payable": 1, "Emergencies": 1}, "adjectives": {"Usually": 1}, "numerals": {"73": 1}, "pronouns": {"I": 1}}}, {"content": "74. Reading the newly discovered book is exciting.", "word_count": 16, "label": "", "sentence_structure": "Subject鈥揷opula鈥損redicative", "subjective_objective": {"answer": "Subjective", "rationale": "Because the \"excited\" experience is the patient's own subjective feeling."}, "sentence_sentiment": {"answer": "Positive", "rationale": "Because the entries \"reading the newly discovered book\" and \"excited things\" have positive emotional aspects."}, "language_style": {"answer": "Tactful and warm", "rationale": "The entry is more adept and interactive when it is spoken in a gentle and inviting manner, rather than by direct statements or orders."}, "time_dimension": {"answer": "Present", "rationale": ""}, "sentence_type": "Affirmative declarative sentence", "pos": {"nouns": {"Reading": 1, "New discoveries": 1, "Books": 1, "Events": 1, "- I'm sorry": 1, "Excited": 1}, "adjectives": {"New discoveries": 1, "Excited": 1}, "numerals": {"74": 1}}}, {"content": "I like beautiful, extraordinary things.", "word_count": 11, "label": "", "sentence_structure": "Subject鈥搗erb鈥揷omplement", "subjective_objective": {"answer": "Subjective", "rationale": "Like being patient subjective."}, "sentence_sentiment": {"answer": "Positive", "rationale": "Because \"like\" and \"beautiful, extraordinary\" in the entries are positive emotions and attitudes."}, "language_style": {"answer": "Tactful and warm", "rationale": "The entry is more personal and interactive when asked in an invitation to the other side about the love of beautiful and special things, rather than direct orders or statements. Nor is it based on excessive rhetoric or emotional rendering, which is more objective and more objective."}, "time_dimension": {"answer": "Past", "rationale": ""}, "sentence_type": "Affirmative declarative sentence", "pos": {"nouns": {"Questionnaire Entry": 1, "I like it": 1, "Beautiful": 1, "Unusual": 2, "Something": 1}, "adjectives": {"Yeah": 1, "Beautiful": 1, "Unusual": 1}, "numerals": {"75": 1}}}, {"content": "76. I often care about lonely, unfriendly people.", "word_count": 12, "label": "", "sentence_structure": "Subject鈥搗erb鈥揷omplement", "subjective_objective": {"answer": "Subjective", "rationale": "Because \"care for the lonely, unfriendly\" is a subjective attitude and behaviour of a human being, not an objective fact or result."}, "sentence_sentiment": {"answer": "Neutral", "rationale": "Because the entry does not have any emotional color."}, "language_style": {"answer": "Tactful and warm", "rationale": "The entry is more euphemistic, using the expression \"often caring\" to guide the respondents to share their behaviour and feelings. Moreover, the term \"single\" and \"unfriendly person\" is used to make the subject matter of the questionnaire more specific. In general, the entry is more moderate and friendly in its presentation, making it easier for the respondents to answer questions."}, "time_dimension": {"answer": "Present", "rationale": "Now, the sentence is based on \"recurring\" and \"care\" and it is the person who is being asked about the behaviour or state of the subject, and the time dimension is now."}, "sentence_type": "Affirmative declarative sentence", "pos": {"nouns": {"Questionnaire": 1, "Entry": 1, "person": 2, "Loneliness": 1, "Unfriendly": 1}, "verbs": {"Care": 1, "be": 1, "Regular": 1}, "adverbs": {"Regular": 1}, "numerals": {"76": 1}, "pronouns": {"I": 1}}}, {"content": "I like bargaining.", "word_count": 7, "label": "", "sentence_structure": "Subject鈥搗erb鈥揷omplement", "subjective_objective": {"answer": "Subjective", "rationale": "Because it's a subjective attitude or emotional experience."}, "sentence_sentiment": {"answer": "Neutral", "rationale": "Because the entry does not have any emotional color."}, "language_style": {"answer": "Tactful and warm", "rationale": "The language style of the entry is euphemistic. It raises a question in a question-and-answer format, and does not directly express a view or an assessment of the behaviour of \"like bargaining.\" This expression is more personal and interactive, which better guides respondents to answer questions and provide real feedback."}, "time_dimension": {"answer": "Present", "rationale": "The entry uses the verb \"like\" as an expression of personal preference and does not refer to past or future time, and therefore is judged to describe current behaviour."}, "sentence_type": "Affirmative declarative sentence", "pos": {"verbs": {"Yeah": 1, "Bargaining": 1}}}, {"content": "I spend my money with caution.", "word_count": 8, "label": "", "sentence_structure": "Subject鈥搗erb鈥揷omplement", "subjective_objective": {"answer": "Subjective", "rationale": "Because \"cautionary\" is a personal attitude or feeling."}, "sentence_sentiment": {"answer": "Neutral", "rationale": "Because the entry merely describes an act, it does not show a positive or negative emotional attitude."}, "language_style": {"answer": "Tactful and warm", "rationale": "The entry is more personal and interactive when it is spoken in a gentle and inviting manner, rather than in direct statements or orders. Moreover, the use of the term \"cautious\" makes the language style more euphemismal and less of a direct or emotional tone."}, "time_dimension": {"answer": "Present", "rationale": ""}, "sentence_type": "Affirmative declarative sentence", "pos": {"nouns": {"Money": 1}, "verbs": {"Flowers": 1, "Watch your wings": 1}, "adjectives": {"Watch out": 1}, "adverbs": {"Watch your wings": 1}, "pronouns": {"I": 1}}}, {"content": "I use exercise to keep strong bodies.", "word_count": 12, "label": "", "sentence_structure": "Subject鈥搗erb鈥搊bject", "subjective_objective": {"answer": "Subjective", "rationale": "Because the phrase \"I use\" shows that it is an action taken by the patient himself, and therefore a subjective one. Although the movement itself is an objective act, it is expressed here as a subjective choice and attitude of the individual."}, "sentence_sentiment": {"answer": "Positive", "rationale": "Because the \"movement\" behavior in the entries is positive, it helps to keep healthy and strong bodies."}, "language_style": {"answer": "Tactful and warm", "rationale": "The language style of the entry is euphemistic. It is in the form of a query, not a direct statement, making it easier for the respondents to accept and answer. At the same time, it uses the active language of \"maintenance of strong bodies\" , which is more inspiring and positive."}, "time_dimension": {"answer": "Past", "rationale": "The entry asks about the testee's past habits and behavior, and \"I use sport to keep strong bodies\" describes what has happened, so it's a past."}, "sentence_type": "Affirmative declarative sentence", "pos": {"nouns": {"Body": 1, "Sports": 1}, "verbs": {"Hold on": 1, "Use": 1, "Strong": 1}, "numerals": {"79": 1}}}, {"content": "I am often curious about the mystery of nature.", "word_count": 14, "label": "", "sentence_structure": "Subject鈥搗erb鈥揷omplement", "subjective_objective": {"answer": "Subjective", "rationale": "Because \"quiet about nature's mystery\" is a personal emotion and attitude that belongs to the patient's subjective angle."}, "sentence_sentiment": {"answer": "Positive", "rationale": "Because the act of \"often curious\" expresses a positive emotional attitude to the active exploration and love of nature."}, "language_style": {"answer": "Tactful and warm", "rationale": "The entry is more acrimony and interactive, using a gentle, invitee-like approach to questions rather than direct statements or orders. At the same time, it does not use excessive rhetorical or emotional rendering, is more objective, makes the interviewee comfortable and comfortable and more willing to share his real ideas."}, "time_dimension": {"answer": "Present", "rationale": "In the entry, \"often\" means that the act is customary, and \"quiet about the mystery of nature\" is an ongoing state or feeling, so the entry describes the current situation."}, "sentence_type": "Affirmative declarative sentence", "pos": {"nouns": {"Nature": 1, "The mystery": 1, "Curiosity": 1}, "verbs": {"I feel": 1, "Regular": 1, "to": 1, "Explore": 1}, "adjectives": {"Curious": 1}, "adverbs": {"Regular": 1}, "numerals": {"80": 1}}}, {"content": "81. It is quite interesting to try something extraordinary.", "word_count": 18, "label": "", "sentence_structure": "Subject鈥揷opula鈥損redicative", "subjective_objective": {"answer": "Subjective", "rationale": "Because \"tried something extraordinary\" is a personal choice and feeling, it's seen from the patient's subjective perspective."}, "sentence_sentiment": {"answer": "Positive", "rationale": "Because the words \"tried new things\" and \"interested\" in the entries have a positive emotional color."}, "language_style": {"answer": "Tactful and warm", "rationale": "The entry attracts the participants and answers to questions by describing the taste of new things, without too strong a plea or command tone, and is expressed in a more moderate and attractive manner."}, "time_dimension": {"answer": "Present", "rationale": "The sentence emphasizes that \"a taste of something extraordinary\" is an interesting thing, does not relate to past or future concepts and therefore belongs to the present."}, "sentence_type": "Affirmative declarative sentence", "pos": {"nouns": {"Things": 2}, "verbs": {"Try": 1, "It's a piece": 1, "It's pretty funny": 1}, "adjectives": {"New": 1, "Interesting": 1}, "adverbs": {"Unusual": 1}, "numerals": {"81": 1}}}, {"content": "82. I am a good listener when others tell me about his difficulties.", "word_count": 18, "label": "", "sentence_structure": "Subject鈥揷opula鈥損redicative", "subjective_objective": {"answer": "Subjective", "rationale": "Because the entry deals with the attitudes and views of individuals, i.e. whether they are willing to be a good listener when others are trying to talk to themselves. It is a subjective judgement based on individual feelings, will and values, and is therefore viewed from a subjective perspective."}, "sentence_sentiment": {"answer": "Positive", "rationale": "Because the act of \"I'm a good listener when others like me tell him how difficult he is\" expresses a positive quality of concern and respect for others."}, "language_style": {"answer": "Tactful and warm", "rationale": "The entry raises questions in a question-and-answer manner rather than in direct statements or orders, which is more adept and interactive. At the same time, the positive term \"good listener\" is used to describe itself, which is more moderate in tone."}, "time_dimension": {"answer": "Present", "rationale": ""}, "sentence_type": "Affirmative declarative sentence", "pos": {"nouns": {"Difficulties": 1, "I": 1, "Audience": 1}, "verbs": {"Yeah": 1, "Tell me": 1, "be": 1}, "adjectives": {"individual": 1, "Okay": 1}, "adverbs": {"When": 1}, "numerals": {"82": 1}}}, {"content": "83. I will continue to work on the failure of my work.", "word_count": 11, "label": "", "sentence_structure": "Subject鈥搗erb鈥揷omplement", "subjective_objective": {"answer": "Subjective", "rationale": "Because this is a subjective view or feeling because of the attitude and conviction of patients towards failure."}, "sentence_sentiment": {"answer": "Positive", "rationale": "Because the \"really\" attitude in the entries is positive."}, "language_style": {"answer": "Tactful and warm", "rationale": "The language style of the entry is more euphemistic, it explores the psychological state of the interviewee in a positive and upward-minded manner, encourages the interviewee to learn from his failures and is confident that he will challenge himself again. Such expressions are more acceptable to the interviewee and help to create a good atmosphere of communication."}, "time_dimension": {"answer": "Future", "rationale": "The keyword \"will build on\" means future intentions and plans, and therefore the entry is a future act or mentality."}, "sentence_type": "Affirmative declarative sentence", "pos": {"verbs": {"Do it": 1, "Failed": 1, "Yes": 2, "Keep going": 1}, "numerals": {"83": 1}, "pronouns": {"I": 1}}}, {"content": "84. I need to know exactly what people are asking me for.", "word_count": 18, "label": "", "sentence_structure": "Subject鈥搗erb鈥搊bject", "subjective_objective": {"answer": "Subjective", "rationale": "And what is subjective is that \"need\" is the patient's subjective needs and ideas."}, "sentence_sentiment": {"answer": "Positive", "rationale": "Because the \"need to know with certainty\" expression of a proactive approach to understanding is a positive act."}, "language_style": {"answer": "Tactful and warm", "rationale": "The words of the entry are more euphemistic, more modest and polite, using the expression \"I need\" and, at the same time, \"know exactly what people want from me\" are more moderate, have no too strong or direct tone, and are more acceptable and responsive."}, "time_dimension": {"answer": "Present", "rationale": ""}, "sentence_type": "Interrogative sentence", "pos": {"nouns": {"I": 2, "Requirements": 1, "Request": 1}, "verbs": {"Yes": 2, "Give": 1}, "adjectives": {"Exactly": 1}, "numerals": {"84": 1}}}, {"content": "I like to unplug things and see if I can fix them.", "word_count": 16, "label": "", "sentence_structure": "Subject鈥搗erb鈥搊bject", "subjective_objective": {"answer": "Subjective", "rationale": "Because \"like\" is a subjective attitude or tendency, and removing things from the building is a specific act and an objective fact."}, "sentence_sentiment": {"answer": "Positive", "rationale": "Because the expression of the article contains an interest in things and an desire to explore, it is a positive attitude and behaviour."}, "language_style": {"answer": "Tactful and warm", "rationale": "The entry is more personal and interactive by speaking in a gentle and inviting manner, rather than by direct statement or order. At the same time, it allows respondents to be more willing to share their ideas and experiences."}, "time_dimension": {"answer": "Past", "rationale": "The entry asks the testee about his past preferences and behaviour, the keyword being \"like to tear things apart\" and it says that this has happened in the past. And the \"see\" in \"see if they can be repaired\" also means that it is an act of exploration and experimentation, which occurred in the past. So the time dimension of the entry is the past."}, "sentence_type": "Affirmative declarative sentence", "pos": {"nouns": {"Questionnaire": 1, "Entry": 1, "Yeah": 1, "Something": 2, "Repair": 1, "they": 1}, "verbs": {"Open it": 1, "Look at that": 1, "Can you": 1, "Repair": 1}, "adverbs": {"85": 1}, "numerals": {"85": 1}, "pronouns": {"I": 1}}}, {"content": "86. I like to read all the facts before taking logical decisions.", "word_count": 19, "label": "", "sentence_structure": "Subject鈥搗erb鈥搊bject", "subjective_objective": {"answer": "Objective", "rationale": "The basis for the judgement: because it is the patient鈥檚 subjective idea to read all the facts and to make logical decisions. Although it is the patient鈥檚 own idea, it is still the patient鈥檚 subjective attitude or perception. The 鈥渞eading all the facts鈥?and 鈥渓ogical decision-making鈥?are based on these subjective attitudes or specific manifestations of perceived behaviour, and they are therefore objective."}, "sentence_sentiment": {"answer": "Positive", "rationale": "Because the entry \"likes to read all the facts, then make a logical decision\" is positive."}, "language_style": {"answer": "Calm and direct", "rationale": "The language style of the entry is relatively calm and straightforward, and the term is not used in excessive rhetorical or emotional terms, but rather expresses the attitude of the person under investigation towards the decision-making process in an objective and clear manner."}, "time_dimension": {"answer": "Present", "rationale": "Both \"like\" and \"reading\" use the current state of affairs, indicating that the act is taking place or is taking place on a regular basis. \"A logical decision\" implies the completion and outcome orientation of the act. So the entry is mainly asking the testees about their current habits and attitudes, namely, whether they are inclined to study all facts before decision-making and to follow logic."}, "sentence_type": "Interrogative sentence", "pos": {"nouns": {"The facts": 1, "Logical": 1, "Decision": 1}, "verbs": {"Yeah": 1, "Study": 1, "Do it": 1}, "adverbs": {"All": 1, "Again": 1}, "numerals": {"86": 1}}}, {"content": "87. Life without beauty is incredible to me.", "word_count": 19, "label": "", "sentence_structure": "Subject鈥揷opula鈥損redicative", "subjective_objective": {"answer": "Subjective", "rationale": "Because it expresses the patient's personal feelings and views that life without beautiful things is inconceivable to him/her. It is the expression of subjective attitudes and values."}, "sentence_sentiment": {"answer": "Positive", "rationale": "Because \"life without beauty is incredible to me\" expresses the pursuit and value of beauty, and it is a positive attitude."}, "language_style": {"answer": "Tactful and warm", "rationale": "The language style of the entry is more euphemistic, because it uses the \"unbelievable\" description, which is relatively mild and subjective, emphasizing personal feelings rather than direct assertions or orders. At the same time, the whole sentence is accompanied by an encouragement and guidance, as if it were inviting interviewees to share their views on beautiful things, which are comforting and comfortable."}, "time_dimension": {"answer": "Present", "rationale": ""}, "sentence_type": "Affirmative declarative sentence", "pos": {"nouns": {"Life": 1, "Beautiful things": 1}, "verbs": {"No, I'm not": 1, "be": 1}, "adjectives": {"Unbelievable": 1}, "adverbs": {"87": 1}}}, {"content": "88. I am often told their problems.", "word_count": 12, "label": "", "sentence_structure": "Subject鈥搗erb鈥搊bject", "subjective_objective": {"answer": "Objective", "rationale": "Because \"people often tell me their problems\" is an objective fact, not related to patients' subjective attitudes or feelings."}, "sentence_sentiment": {"answer": "Neutral", "rationale": "Because the entry does not have any emotional color."}, "language_style": {"answer": "Tactful and warm", "rationale": "The entry is a conversation in a question-and-answer manner and uses the more intimate term \"show\" to express interest and concern for each other's problems, in a more gentle and euphemism."}, "time_dimension": {"answer": "Past", "rationale": "\"Tell\" is the past, \"problem\" is the situation that exists or has existed, and the entry therefore asks whether the subjects were informed of their problems in the past."}, "sentence_type": "Affirmative declarative sentence", "pos": {"nouns": {"People": 1, "Problem": 2}, "verbs": {"Tell": 1, "be": 1, "I": 1}, "adjectives": {"Regular": 1}, "numerals": {"88": 1}}}, {"content": "89. I have often been able to reach out to others through information networks.", "word_count": 16, "label": "", "sentence_structure": "Subject鈥搗erb鈥搊bject", "subjective_objective": {"answer": "Subjective", "rationale": "Because \"often\" indicates that it is the patient's subjective feeling or attitude that the patient believes that he or she is often able to communicate with others through the network."}, "sentence_sentiment": {"answer": "Positive", "rationale": "Because the behavior in the entries is positive, \"to connect with others through information networks.\""}, "language_style": {"answer": "Tactful and warm", "rationale": ""}, "time_dimension": {"answer": "Present", "rationale": ""}, "sentence_type": "Affirmative declarative sentence", "pos": {"nouns": {"Information": 1, "Network": 1, "others": 1, "Contact": 1}, "verbs": {"I can": 1, "Borrow": 1, "Access": 1}, "adverbs": {"Regular": 1}, "numerals": {"89": 1}, "pronouns": {"I": 1}}}, {"content": "90. One matter of careful completion is one of achievement.", "word_count": 19, "label": "", "sentence_structure": "Subject鈥揷opula鈥損redicative", "subjective_objective": {"answer": "Subjective", "rationale": "Because words like \"a sense of accomplishment\" and \"a careful completion of one thing\" are subjective feelings and attitudes of patients."}, "sentence_sentiment": {"answer": "Positive", "rationale": "Because the result of \"do one thing carefully\" and \"do something productive\" in the entry is positive emotional."}, "language_style": {"answer": "Tactful and warm", "rationale": "The entry is a positive expression of satisfaction in terms of what is done and what is achieved, without direct orders or demands from the other party to take certain actions. This is more acceptable, understandable and resonant."}, "time_dimension": {"answer": "Past", "rationale": "The sentence describes a perception or a sense of past experience, and the key words are \"cautionary\" and \"complete one\" and \"a sense of achievement\" that occurred at some point or time in the past."}, "sentence_type": "Affirmative declarative sentence", "pos": {"nouns": {"Things": 2, "Achievements": 1, "Sensor": 1}, "verbs": {"Completed": 1, "be": 1, "Do it": 1}, "adjectives": {"Be careful": 1, "A sense of accomplishment": 1}, "numerals": {"90": 1}}}]}

{"questionnaire_title": "Table of events in the lives of adolescents (ASLC)", "questionnaire_category": "Behavioral questionnaire", "item_count": 27, "emotion_shift_overall": "No obvious trend", "language_style_overall": "Calm and direct", "items": [{"content": "1. Misunderstanding or error", "word_count": 7, "label": "", "sentence_structure": "Subject鈥搗erb鈥揷omplement", "subjective_objective": {"answer": "Subjective", "rationale": "Because \"the wrong person or wrong person\" is a subjective feeling and experience of a person. While this situation exists objectively (i.e., someone may indeed mispercept or wrongly wrong someone), the perception, interpretation and evaluation of this situation are psychological and emotional responses of individuals and are therefore considered subjectively."}, "sentence_sentiment": {"answer": "Negative", "rationale": "Because the experience of being misunderstood or misled in the entries can cause negative emotions, such as anger, sadness, etc."}, "language_style": {"answer": "Calm and direct", "rationale": "The entry is simple, not too much phrasing and emotionally retrofitted, but rather directly asking if the other person has ever been misinformed or misled. This expression is objective and neutral, with a high level of information and relevance."}, "time_dimension": {"answer": "Present", "rationale": "The keyword \"wrong or wrong\" expresses a situation or experience that exists and is therefore judged to be the present."}, "sentence_type": "Interrogative sentence", "pos": {"nouns": {"person": 1, "Misunderstanding": 1, "Wrong": 1}, "verbs": {"By": 1, "Error": 1, "Yes": 1, "Wrong": 1, "Weird": 1}}}, {"content": "2. Relentment in the face of discrimination", "word_count": 6, "label": "", "sentence_structure": "Subject鈥搗erb鈥揷omplement", "subjective_objective": {"answer": "Subjective", "rationale": "Because discrimination and cold encounters are emotional experiences and evaluations that patients experience subjectively."}, "sentence_sentiment": {"answer": "Negative", "rationale": "Because the \"discretionalism\" is negative."}, "language_style": {"answer": "Calm and direct", "rationale": "Such an expression would allow respondents to better face and understand the problem, while avoiding excessive emotional reactions and maintaining the objectivity and accuracy of the investigation."}, "time_dimension": {"answer": "Present", "rationale": ""}, "sentence_type": "Negative declarative sentence", "pos": {"nouns": {"person": 1, "Discrimination": 1, "I'm cold": 1}, "verbs": {"Yes": 1}}}, {"content": "3. Examination failure or unsatisfactory", "word_count": 8, "label": "", "sentence_structure": "Subject鈥搗erb鈥揷omplement", "subjective_objective": {"answer": "Subjective", "rationale": "Because failure or undesiredness is the individual feeling and experience of the patient, it is a subjective examination."}, "sentence_sentiment": {"answer": "Negative", "rationale": "The frustration and frustration that often result from failure or undesired examinations is a negative emotional experience."}, "language_style": {"answer": "Tactful and warm", "rationale": ""}, "time_dimension": {"answer": "Past", "rationale": "According to the keyword \"failure or undesiredness of the examination\", it can be seen that the entry asks whether the person tested had experienced a failure or unsatisfactory situation in the past and therefore judged the past."}, "sentence_type": "Negative declarative sentence", "pos": {"nouns": {"Examinations": 1, "Failed": 1, "I don't like it": 1}}}, {"content": "4. Disputes with classmates or friends", "word_count": 10, "label": "", "sentence_structure": "Subject鈥搗erb鈥揷omplement", "subjective_objective": {"answer": "Subjective", "rationale": "This questionnaire entry is subjective in that it focuses on patients鈥?feelings and attitudes to disputes with their classmates or friends. Although these disputes may be objective, the problem is primarily one of patient鈥檚 subjective reactions and emotional experiences."}, "sentence_sentiment": {"answer": "Negative", "rationale": "Because the \"controversial\" act in the entry is negative."}, "language_style": {"answer": "Calm and direct", "rationale": "The entry directly asks if the person interviewed had had any experience of a dispute with a fellow student or friend, the language was simple and not over-modified and emotionally retroverted. The language style of the article is therefore \"cool and direct\"."}, "time_dimension": {"answer": "Past", "rationale": ""}, "sentence_type": "Interrogative sentence", "pos": {"nouns": {"Disputes": 1, "Students": 1, "Friends": 1}, "verbs": {"Come on": 1}, "numerals": {"4": 1}}}, {"content": "5. Visible changes in living habits (e.g. diet, rest, etc.)", "word_count": 13, "label": "", "sentence_structure": "Subject鈥搗erb鈥揷omplement", "subjective_objective": {"answer": "Subjective", "rationale": "Because changes in living habits are felt and experienced by the patient himself, they are considered from the patient ' s subjective perspective."}, "sentence_sentiment": {"answer": "Negative", "rationale": "Because the result of the behaviour of \"Remarkable changes in living habits (e.g. diet, rest)\" in the entry may be negative, such as the effects on health."}, "language_style": {"answer": "Tactful and warm", "rationale": "The entry allows respondents to share in a way of questioning changes in their daily habits, rather than directly making statements or orders, which are more personal and interactive."}, "time_dimension": {"answer": "Present", "rationale": "The entry describes whether the testee's current life habits have changed significantly, and thus judges it now.\""}, "sentence_type": "Affirmative declarative sentence", "pos": {"nouns": {"Life habits": 1, "Diet": 1, "Rest": 1, "Change": 1}, "adjectives": {"Obviously": 1}, "classifiers": {"individual": 1}}}, {"content": "6. Not to go to school", "word_count": 5, "label": "", "sentence_structure": "Subject鈥搗erb鈥揷omplement", "subjective_objective": {"answer": "Subjective", "rationale": "Because \"like\" is a personal feeling and attitude, and \"school\" is an objective fact or behaviour. So the question is considered from the patient's subjective point of view."}, "sentence_sentiment": {"answer": "Negative", "rationale": "Because \"not like\" in the entries is a negative word, which means that there is a negative attitude towards schools."}, "language_style": {"answer": "Calm and direct", "rationale": "The language style of the entry is more direct and concise, and there is no excessive use of rhetorical or emotional descriptions, but rather a simple and clear expression of concern about whether the person surveyed prefers to attend school."}, "time_dimension": {"answer": "Past", "rationale": ""}, "sentence_type": "Negative declarative sentence", "pos": {"nouns": {"Questionnaire": 1, "Entry": 1}, "verbs": {"Yeah": 1, "School": 1}}}, {"content": "7. Incompetence or loss of love", "word_count": 8, "label": "", "sentence_structure": "Subject鈥搗erb鈥揷omplement", "subjective_objective": {"answer": "Subjective", "rationale": "Because the success of a relationship or the absence of a false relationship is a personal feeling and experience of the patient, it is a subjective observation."}, "sentence_sentiment": {"answer": "Negative", "rationale": "Because a relationship is not smooth or broken, it usually leads to negative emotions and effects, which are negative descriptions."}, "language_style": {"answer": "Calm and direct", "rationale": "The entry is more straightforward in language style, is not used too much rhetorical or emotionally, and is relatively objective and calm. It directly asks whether the person surveyed had had any history of untimely or broken love, and expresses the purpose and subject of the questionnaire."}, "time_dimension": {"answer": "Past", "rationale": "In the entry, \"unfavourable or broken love\" describes a past experience or state, and therefore is judged to be \"the past\"."}, "sentence_type": "Interrogative sentence", "pos": {"nouns": {"Love": 2, "Good": 1, "Broken love": 1}, "verbs": {"not": 1, "Successful or": 1}, "numerals": {"7": 1}}}, {"content": "8. Long-term absence from family reunification", "word_count": 10, "label": "", "sentence_structure": "Subject鈥搗erb鈥揷omplement", "subjective_objective": {"answer": "Subjective", "rationale": "Because it's the patient's subjective feeling and the idea."}, "sentence_sentiment": {"answer": "Negative", "rationale": "Because \"long distance from family reunification\" is often unpleasant and disappointing, it has a negative emotional connotation."}, "language_style": {"answer": "Calm and direct", "rationale": "The language style of the entry is relatively calm, direct, and there is no excessive rhetoric or emotional rendering, but simply raises questions about whether the other party is often unable to join his family for long periods of time, for example, because of work. This expression is more objective and practical, helping the interviewee to better answer the questions, while avoiding unnecessary pressure or discomfort."}, "time_dimension": {"answer": "Past", "rationale": "The term \"long term\" and \"ununununited\" in the entry describe the past and therefore the temporal dimension of the article can be judged to be the past."}, "sentence_type": "Negative declarative sentence", "pos": {"nouns": {"Long term": 1, "Family": 1, "Reunification": 1}, "verbs": {"Stay away": 1, "No, I can't": 1}, "adverbs": {"Long term": 1}, "numerals": {"8": 1, "No, I can't": 1}}}, {"content": "9. High learning burden", "word_count": 5, "label": "", "sentence_structure": "Subject鈥揷opula鈥損redicative", "subjective_objective": {"answer": "Subjective", "rationale": "According to the description of the topic, \"learning is heavy\" is a subjective experience or feeling, and it can therefore be considered a subjective perspective."}, "sentence_sentiment": {"answer": "Negative", "rationale": "Because the expression \"learning with a heavy burden\" is inherently stressful and heavy, it expresses a negative emotional attitude."}, "language_style": {"answer": "Calm and direct", "rationale": "The entry is a straightforward expression of the core of the problem, is not overly emotional or rhetorical and is more objective in tone."}, "time_dimension": {"answer": "Present", "rationale": ""}, "sentence_type": "Negative declarative sentence", "pos": {"nouns": {"Learning": 1, "Burden": 1}, "verbs": {"Heavy": 1}}}, {"content": "10. Tensions with teachers", "word_count": 7, "label": "", "sentence_structure": "Subject鈥搗erb鈥揷omplement", "subjective_objective": {"answer": "Subjective", "rationale": "Because \"stretching with teachers\" is a subjective condition of the patient's self-perception, based on personal experience and emotional perceptions."}, "sentence_sentiment": {"answer": "Negative", "rationale": "Because the description of \"relationship\" in the entry is negative."}, "language_style": {"answer": "Calm and direct", "rationale": "The entry directly asks whether the interviewee has a tense relationship with the teacher, does not have too much emotional or acrimony, and is more neutral and objective in tone. \""}, "time_dimension": {"answer": "Present", "rationale": ""}, "sentence_type": "Negative declarative sentence", "pos": {"nouns": {"Teacher": 1, "Relations": 1, "Nervous": 1}, "verbs": {"have": 1, "Relations": 1, "Nervous": 1}, "numerals": {"10": 1}}}, {"content": "I am suffering from acute illness.", "word_count": 6, "label": "", "sentence_structure": "Subject鈥搗erb鈥揷omplement", "subjective_objective": {"answer": "Subjective", "rationale": "It is an objective fact that a doctor must be sure to have a serious disease. But it is the patient鈥檚 subjective judgement and perception that he thinks he has a serious disease."}, "sentence_sentiment": {"answer": "Negative", "rationale": "Because the \"Emergency Disease\" incident in the entry is negative."}, "language_style": {"answer": "Tactful and warm", "rationale": "The entry is more euphemistic, soft, and vague in its language, with respect for each other鈥檚 feelings, and is not subject to too much direct negative description or strong guidance."}, "time_dimension": {"answer": "Present", "rationale": "While the entry in the future contains the word 鈥減ersonal鈥? it asks whether it is possible in the future that the person tested will suffer from acute illness in the future and therefore is judged to be future."}, "sentence_type": "Declarative sentence", "pos": {"nouns": {"person": 1, "It's a serious illness": 1}, "verbs": {"Patient": 1}, "numerals": {"11": 1}}}, {"content": "12. Families and friends suffer from acute illness", "word_count": 6, "label": "", "sentence_structure": "Subject鈥揷opula鈥損redicative", "subjective_objective": {"answer": "Objective", "rationale": "The test is based on the fact that \"the patient and his/her family suffer from acute illness\" is an objective fact, not a subjective attitude or idea."}, "sentence_sentiment": {"answer": "Negative", "rationale": "The entry \"Emergency\" can cause anxiety, anxiety, etc."}, "language_style": {"answer": "Tactful and warm", "rationale": "Warm and warm."}, "time_dimension": {"answer": "Present", "rationale": ""}, "sentence_type": "Interrogative sentence", "pos": {"nouns": {"Friends": 1, "Diseases": 1}, "verbs": {"Patient": 1, "Heavy": 1, "Urgent": 1}, "numerals": {"12": 1}}}, {"content": "13. Deaths of relatives and friends", "word_count": 4, "label": "", "sentence_structure": "Subject鈥搗erb鈥揷omplement", "subjective_objective": {"answer": "Subjective", "rationale": "Because the death of a relative is a subjectively felt event, and everyone has different feelings and reactions to the death of a relative and friend, it is a subjective issue to be examined."}, "sentence_sentiment": {"answer": "Negative", "rationale": "Because \"death\" is a sad thing, with obvious negative emotional aspects."}, "language_style": {"answer": "Calm and direct", "rationale": "The language style of the entry is more straightforward, not using too many euphemisms or rhetorical techniques, but rather asking the respondents in a concise and clear manner whether they have experienced the death of their relatives and friends."}, "time_dimension": {"answer": "Past", "rationale": ""}, "sentence_type": "Negative declarative sentence", "pos": {"nouns": {"Friends": 1, "Death": 1}}}, {"content": "14. Stolen or lost items", "word_count": 7, "label": "", "sentence_structure": "Subject鈥搗erb鈥揷omplement", "subjective_objective": {"answer": "Objective", "rationale": "The fact that stolen or lost is an objective fact experienced by the patient does not need to be viewed from the patient ' s subjective perspective."}, "sentence_sentiment": {"answer": "Negative", "rationale": "Because the \"snatch or lost\" in the entry is negative."}, "language_style": {"answer": "Tactful and warm", "rationale": "According to the information provided, the language style of the questionnaire entry \"Stole or Lost\" may be \"Cool Direct\". The judgement is based on the fact that the entry raises questions in a direct way, without using too many emotional modifications or subtle expressions. This is not very consistent with the language style of \"Stolely Warm.\" And, given that stolen or lost items are a sensitive and serious matter, \"Cool Direct\" is more appropriate."}, "time_dimension": {"answer": "Present", "rationale": "According to the context, \"stolen or lost\" is a description of the current situation, so it's judged to be \"now\"."}, "sentence_type": "Negative declarative sentence", "pos": {"nouns": {"Stolen": 1, "Lost": 1, "Something": 1}}}, {"content": "15. Disgrace the public.", "word_count": 5, "label": "", "sentence_structure": "Subject鈥損redicate", "subjective_objective": {"answer": "Subjective", "rationale": "Because \"disguised\" is a personal feeling and experience, a subjective observation."}, "sentence_sentiment": {"answer": "Negative", "rationale": "Because the \"disgrace\" act is negative."}, "language_style": {"answer": "Tactful and warm", "rationale": "The language style of the entry is mild, leading the respondents to think and answer questions in a question-and-answer manner, without using too direct or mandatory language. Moreover, \"public humiliation\" is a more sensitive and personal privacy issue, and asking questions in this way reduces the resistance of the respondents and makes it easier to obtain real and effective feedback."}, "time_dimension": {"answer": "Present", "rationale": ""}, "sentence_type": "Imperative sentence", "pos": {"nouns": {"Face": 1, "Drop it": 1, "Public": 1}, "numerals": {"15": 1}}}, {"content": "16. Family economic difficulties", "word_count": 6, "label": "", "sentence_structure": "Subject鈥搗erb鈥揷omplement", "subjective_objective": {"answer": "Subjective", "rationale": "Family financial difficulties are subjective perceptions and judgements of patients."}, "sentence_sentiment": {"answer": "Negative", "rationale": "Because \"family economic difficulties\" are usually negative and can cause trouble to family life."}, "language_style": {"answer": "Calm and direct", "rationale": "The entry is short and simple, and directly asks about the family ' s economic situation, without excessive emotional rendering or euphemism, and is more objective and direct."}, "time_dimension": {"answer": "Past", "rationale": "According to the description of the subject, the entry asks whether the respondent had had a family financial hardship in the past and thus judged the events of the past."}, "sentence_type": "Negative declarative sentence", "pos": {"nouns": {"Family": 1, "Economy": 1, "Difficulties": 1}}}, {"content": "17. Conflict within the family", "word_count": 7, "label": "", "sentence_structure": "Subject鈥搗erb鈥揷omplement", "subjective_objective": {"answer": "Subjective", "rationale": "The contradiction within the family is \"the patient's subjective feeling or perception."}, "sentence_sentiment": {"answer": "Negative", "rationale": "Because \"relations within the family\" are usually considered negative."}, "language_style": {"answer": "Calm and direct", "rationale": "The language style of the entry is clear, there is no use of rhetoric, and the interviewee is asked whether there are contradictions in the family, and is clear and concise, consistent with the nature of the problem itself."}, "time_dimension": {"answer": "Present", "rationale": "The entry asked about the current family situation of the person tested, and the temporal dimension was therefore judged to be the present."}, "sentence_type": "Imperative sentence", "pos": {"nouns": {"Family": 1, "Internal": 1, "Paradoxious": 1}}}, {"content": "18. Expected failure (e.g., 3 best students)", "word_count": 12, "label": "", "sentence_structure": "Subject鈥搗erb鈥揷omplement", "subjective_objective": {"answer": "Subjective", "rationale": "Because \"pregnance\" and \"failure\" are patients' subjective thoughts and feelings."}, "sentence_sentiment": {"answer": "Negative", "rationale": "Because the \"failed\" act is negative."}, "language_style": {"answer": "Tactful and warm", "rationale": "The entry is more adept and interactive when it is spoken in a gentle and inviting manner, rather than by direct statements or orders."}, "time_dimension": {"answer": "Present", "rationale": "At the same time, the phrase \"failed\" also implies that the intended selection of the testee has not met its target, and it can be assumed that its emotional state may be negative, such as loss or frustration."}, "sentence_type": "Negative declarative sentence", "pos": {"nouns": {"Projected": 1, "Selection": 1, "Like": 1, "Good student": 1}, "verbs": {"Frustration": 1}, "numerals": {"18": 1, "of": 2}}}, {"content": "19. Criticism or discipline", "word_count": 6, "label": "", "sentence_structure": "Subject鈥搗erb鈥揷omplement", "subjective_objective": {"answer": "Objective", "rationale": "(b) The fact that the person concerned has been criticized or punished as an objective fact of his or her experience."}, "sentence_sentiment": {"answer": "Negative", "rationale": "Basis of judgement: The criticism or punishment is often seen as negative experiences that may give rise to negative feelings of dissatisfaction, frustration or anxiety."}, "language_style": {"answer": "Calm and direct", "rationale": "The language style of the entry is calm and straightforward. It is simple to ask whether the person interviewed has been criticized or disciplined, without any use of rhetorical or emotional acquis, and is more objective and neutral."}, "time_dimension": {"answer": "Past", "rationale": "The keyword \"resulting or punishing\" indicates that this has been the experience of the past."}, "sentence_type": "Negative declarative sentence", "pos": {"nouns": {"Criticized": 1, "Disposition": 1}, "verbs": {"Please": 1}}}, {"content": "20. Transfer or suspension", "word_count": 5, "label": "", "sentence_structure": "Subject鈥搗erb鈥揷omplement", "subjective_objective": {"answer": "Objective", "rationale": "Because it is an objective fact to transfer or to suspend from school."}, "sentence_sentiment": {"answer": "Negative", "rationale": "Because the transfer or suspension is usually due to insurmountable difficulties faced by the students, this behaviour is negative."}, "language_style": {"answer": "Calm and direct", "rationale": "The language style of the entry is calm and straightforward. It raises questions in a concise manner, without too many rhetorical or emotional renderings, and is more objective in tone."}, "time_dimension": {"answer": "Future", "rationale": "The key words \"reconstruction\" and \"re-suspension\" make it possible to judge that the entry is in the future where the testee is likely to be in a situation of transfer or suspension."}, "sentence_type": "Imperative sentence", "pos": {"nouns": {"Transferred": 1, "School is suspended": 1}, "verbs": {"or": 1}, "numerals": {"20": 1}}}, {"content": "21. Fine", "word_count": 3, "label": "", "sentence_structure": "Subject鈥搗erb鈥揷omplement", "subjective_objective": {"answer": "Subjective", "rationale": "For example, one person may think that he or she was fined for failure to comply with the rules or for wrong behaviour, while another person may think that he or she was fined for unfair circumstances. Thus, this entry is viewed from the patient鈥檚 subjective point of view."}, "sentence_sentiment": {"answer": "Negative", "rationale": "Because \"fell\" is a negative act."}, "language_style": {"answer": "Calm and direct", "rationale": "The entry directly expresses the subject of questioning whether the other party has been fined for an offence, without using excessive rhetorical or emotional rendering, and is more objective in tone."}, "time_dimension": {"answer": "Past", "rationale": ""}, "sentence_type": "Negative declarative sentence", "pos": {"nouns": {"Fine": 1}, "verbs": {"By": 1, "Fine": 1}}}, {"content": "22. Stress for advancement", "word_count": 4, "label": "", "sentence_structure": "Subject鈥搗erb鈥揷omplement", "subjective_objective": {"answer": "Subjective", "rationale": "This is a subjective feeling to examine the pressure for advancement that patients feel."}, "sentence_sentiment": {"answer": "Negative", "rationale": "Because \"stress for advancement\" is often seen as a negative stress that can have a negative impact on people's emotional and mental health."}, "language_style": {"answer": "Calm and direct", "rationale": "The entry directly asked the interviewees whether they were under pressure to advance, were clear in language, were not subject to excessive rhetoric or emotional rendering, and were expressed in an objective and calm manner."}, "time_dimension": {"answer": "Past", "rationale": "The entry describes what the testee has experienced, that is, the pressure of the transition to school. It therefore has a temporal dimension that is in the past."}, "sentence_type": "Imperative sentence", "pos": {"nouns": {"Promotion": 1, "Pressure": 1}, "numerals": {"22": 1}}}, {"content": "23. Fighting", "word_count": 4, "label": "", "sentence_structure": "Subject鈥搗erb鈥揷omplement", "subjective_objective": {"answer": "Subjective", "rationale": "Because \"fighting with people\" is the subjective feeling and attitude of the patient to have such experience and behaviour."}, "sentence_sentiment": {"answer": "Negative", "rationale": "Because \"fighting with people\" is an act of violence, which is usually considered a negative act."}, "language_style": {"answer": "Calm and direct", "rationale": "The entry asks the testee whether he has been involved in fighting in a direct and concise language, which is more air-sensitive and has no excessive adornment or emotional colour."}, "time_dimension": {"answer": "Present", "rationale": "The key word \"fight with people\" in the entry in the future suggests that it is a possible act or event, and it points to the future. Also, \"please analyze this next questionnaire entry\" suggests that this is a question about the future."}, "sentence_type": "Imperative sentence", "pos": {"nouns": {"person": 1, "Fight": 1}, "verbs": {"and": 1, "Fight": 1}, "numerals": {"23": 1}}}, {"content": "24. Scolded by parents", "word_count": 5, "label": "", "sentence_structure": "Subject鈥搗erb鈥揷omplement", "subjective_objective": {"answer": "Subjective", "rationale": "Because being verbally abused by parents is a subjective feeling and experience of a person, it is judged by subjective factors such as the individual ' s emotions, attitudes, and not by objective facts."}, "sentence_sentiment": {"answer": "Negative", "rationale": "Because the \"scolding\" in the entry is negative."}, "language_style": {"answer": "Tactful and warm", "rationale": "The language style of the entry is more euphemistic and warm, rather than being too direct or negative, but rather asking the other in a caring and understanding way whether the other person has ever been abused by his or her parents."}, "time_dimension": {"answer": "Past", "rationale": "\"Being scolded by parents\" describes a past experience and events that can be judged as past."}, "sentence_type": "Affirmative declarative sentence", "pos": {"nouns": {"parents": 1, "Scolded": 1}, "verbs": {"Shit": 1}, "numerals": {"24": 1}}}, {"content": "25. Family puts you under study pressure.", "word_count": 10, "label": "", "sentence_structure": "Subject鈥搗erb鈥揷omplement", "subjective_objective": {"answer": "Subjective", "rationale": "The family exerts learning pressure on you is a subjective judgement that reflects patients ' feelings and perceptions of learning pressure from the family."}, "sentence_sentiment": {"answer": "Negative", "rationale": "Because \"learning pressure\" is usually negative, it is emphasized here that it is imposed by the family, and this negative impact is even more emphasized."}, "language_style": {"answer": "Calm and direct", "rationale": "The entry directly expresses the research theme in a concise and straightforward manner - whether the family exerts learning pressure on the person under investigation, does not use excessive rhetorical or emotional modifications, and is more straightforward and rigid in language style."}, "time_dimension": {"answer": "Past", "rationale": "The entry asks if the tested person has put any learning pressure on them over the past period (for example, nearly six months) and therefore judged them to be past."}, "sentence_type": "Imperative sentence", "pos": {"nouns": {"Family": 1, "you": 1, "Learning": 1, "Pressure": 1}, "verbs": {"Here": 1, "Imposition": 1}, "numerals": {"25": 1}}}, {"content": "26. Accidents and accidents", "word_count": 6, "label": "", "sentence_structure": "Subject鈥揷opula鈥損redicative", "subjective_objective": {"answer": "Objective", "rationale": "Because surprises and accidents are objective events, not subjective feelings or attitudes of patients."}, "sentence_sentiment": {"answer": "Negative", "rationale": "Because \"unforeseen shock, accident\" in the entry is a negative event that can cause panic and anxiety."}, "language_style": {"answer": "Calm and direct", "rationale": "The entry uses a simple and clear language describing the theme \"Unfortunate shock, accident\", which is not accompanied by too many emotional renderings or rhetorical techniques, and is more direct and objective. Thus, the language style can be judged as being calm and straightforward."}, "time_dimension": {"answer": "Present", "rationale": "In the future, the entry contains visions and expectations for the future, with the keywords \"unforeseen shock\" and \"accident\" pointing to situations that have not yet occurred and that may arise in the future."}, "sentence_type": "Declarative sentence", "pos": {"nouns": {"Accident": 1, "Surprise": 1, "Accidents": 2}, "numerals": {"26": 1}}}, {"content": "27. Please provide information on any other incidents.", "word_count": 9, "label": "", "sentence_structure": "Subject鈥搗erb鈥搊bject", "subjective_objective": {"answer": "Objective", "rationale": "This is an objective fact-finding exercise, which allows patients to provide any information on other situations or events that may not be covered."}, "sentence_sentiment": {"answer": "Neutral", "rationale": "Since the entry asks whether there are other incidents, there is no apparent emotional, attitude, behaviour, purpose or quality orientation and therefore a neutral expression."}, "language_style": {"answer": "Tactful and warm", "rationale": "This is a more acceptable expression and is willing to answer questions."}, "time_dimension": {"answer": "Present", "rationale": "The time dimension cannot be determined because of the lack of a specific time frame."}, "sentence_type": "Interrogative sentence", "pos": {"nouns": {"Events": 1}, "verbs": {"have": 1, "Please": 1, "Annotations": 1}, "adverbs": {"Like": 1, "Other": 1}, "numerals": {"27": 1}, "pronouns": {"Other": 1}}}]}

{"questionnaire_title": "Mental health questionnaire for university students", "questionnaire_category": "Mental health", "item_count": 63, "emotion_shift_overall": "From negative to positive", "language_style_overall": "Neutral", "items": [{"content": "One hunger strike.", "word_count": 4, "label": "", "sentence_structure": "Subject鈥搗erb鈥揷omplement", "subjective_objective": {"answer": "Objective", "rationale": "Because a person ' s appetite is a physical condition, not a subjective feeling or attitude."}, "sentence_sentiment": {"answer": "Negative", "rationale": "Because \"fatal\" is usually a negative physiological state."}, "language_style": {"answer": "Calm and direct", "rationale": "The entry describes a phenomenon in a concise and clear language, without excessive emotional and rhetorical modifications and with a more objective and calm tone."}, "time_dimension": {"answer": "Present", "rationale": "The entry now describes the current symptoms or state of the testee, and \"failure\" is a specific act, implying that it is a present situation."}, "sentence_type": "Negative declarative sentence", "pos": {"nouns": {"A appetite": 1, "Pathological": 1}, "verbs": {"It's not working": 1}}}, {"content": "Two sick, stomach-suffering, stomach-ache.", "word_count": 9, "label": "", "sentence_structure": "Subject鈥搗erb鈥揷omplement", "subjective_objective": {"answer": "Objective", "rationale": "Disgusting, stomach pain and stomach pain are all facts of the patient ' s physical condition and are objective."}, "sentence_sentiment": {"answer": "Negative", "rationale": "Because the symptoms described in the entry are ill-health and have negative emotional connotations."}, "language_style": {"answer": "Calm and direct", "rationale": "The entry directly expresses the subject of asking the other party whether there are symptoms of nausea, stomach distress, stomach pain, etc., and is not based on excessive rhetoric or emotional rendering, which is more objective and sound."}, "time_dimension": {"answer": "Present", "rationale": "The entry now describes the subjective feelings and experiences that the person under examination currently experience, such as nausea, stomach pain and other symptoms of discomfort, and thus determines the present."}, "sentence_type": "Imperative sentence", "pos": {"nouns": {"Gross": 1, "-Adulge": 1, "It's hard": 1, "- Tummy": 1, "Ouch": 1}}}, {"content": "Three is easy to diarrhea or constipation.", "word_count": 8, "label": "", "sentence_structure": "Subject鈥搗erb鈥揷omplement", "subjective_objective": {"answer": "Objective", "rationale": "Since diarrhea or constipation is an objective fact of the patient ' s state of health, it is not necessary to judge it through subjective perceptions."}, "sentence_sentiment": {"answer": "Negative", "rationale": "Because \"tummy diarrhea\" and \"concrete\" are symptoms of physical discomfort and are generally considered negative."}, "language_style": {"answer": "Tactful and warm", "rationale": ""}, "time_dimension": {"answer": "Past", "rationale": ""}, "sentence_type": "Negative declarative sentence", "pos": {"nouns": {"Easy": 1, "I'm having a stomach attack": 1, "Decency": 1}, "adjectives": {"3": 1}}}, {"content": "Four, mind your heart and pulse.", "word_count": 7, "label": "", "sentence_structure": "Subject鈥搗erb鈥揷omplement", "subjective_objective": {"answer": "Subjective", "rationale": "Because care about heart attacks and pulses is the patient ' s subjective attitude and feelings."}, "sentence_sentiment": {"answer": "Negative", "rationale": "Because \"heart and pulse\" are often a manifestation of physical discomfort, attention to them is often a negative attitude or behaviour."}, "language_style": {"answer": "Tactful and warm", "rationale": ""}, "time_dimension": {"answer": "Present", "rationale": "Now the entry describes the current behaviour or feelings of the testee, the keywords being \"Concerning\" and \"heart and pulse\" which indicate that the testee is experiencing these physiological symptoms and is using them as a focus. Thus, the temporal dimension of the article is now judged."}, "sentence_type": "Exclamatory sentence", "pos": {"nouns": {"Heartbreak": 1, "Pulse": 1}, "verbs": {"Attention": 1}, "numerals": {"4": 1}}}, {"content": "5 in good health", "word_count": 8, "label": "", "sentence_structure": "Subject鈥揷opula鈥損redicative", "subjective_objective": {"answer": "Objective", "rationale": "Because good health is the true condition of the patient ' s body, not the subjective feeling or attitude."}, "sentence_sentiment": {"answer": "Neutral", "rationale": "Because the entry only describes the good or bad state of health, it does not express any positive or negative feelings."}, "language_style": {"answer": "Tactful and warm", "rationale": "The entry uses the tone of the statement, but conveys the care and expectations of the health of the interviewee by using the active words \"body\" and \"good\" to make him feel warmer and more kind. It also does not directly ask about the interviewee's health, avoiding the resistance of the interviewee. The language style of the purpose is therefore considered euphemism."}, "time_dimension": {"answer": "Present", "rationale": ""}, "sentence_type": "Affirmative declarative sentence", "pos": {"numerals": {"5": 1}}}, {"content": "Six complaints and a lot of complaints.", "word_count": 6, "label": "", "sentence_structure": "Subject鈥搗erb鈥揷omplement", "subjective_objective": {"answer": "Subjective", "rationale": "Because \"shut and displeasure\" is a personal experience and feeling, and it is a subjective attitude of the individual."}, "sentence_sentiment": {"answer": "Negative", "rationale": "Because \"shut and disgruntled\" in the entry is a negative emotional attitude."}, "language_style": {"answer": "Calm and direct", "rationale": "Moreover, the sentence is not reminiscent of any emotional tone, but simply expresses the content of the question. So I think the language style of this questionnaire entry is straightforward and calm."}, "time_dimension": {"answer": "Present", "rationale": "The keyword is \"grabbing and discontent\", which indicates that the testees are experiencing these negative sentiments and do not explicitly refer to past or future situations, and therefore judge now."}, "sentence_type": "Imperative sentence", "pos": {"nouns": {"Scoff": 1, "Dissatisfied": 1}, "verbs": {"More": 1}, "numerals": {"6": 1}}}, {"content": "7 parents over-zealous", "word_count": 6, "label": "", "sentence_structure": "Subject鈥搗erb鈥揷omplement", "subjective_objective": {"answer": "Subjective", "rationale": "Because \"high expectations\" are patients' subjective perceptions and feelings about their parents."}, "sentence_sentiment": {"answer": "Negative", "rationale": "Because the behaviour of \"high expectations\" in the entries can create stress and negative emotions, and it is negative."}, "language_style": {"answer": "Calm and direct", "rationale": ""}, "time_dimension": {"answer": "Present", "rationale": "According to the title, \"too much expectation of parents\" is one of the behaviours or mindsets of the respondents, past, present or future, and requires a time-dimensional judgement. But before making a specific time judgement, we need to understand what the questionnaire entry means. Here, \"too much expectation of parents\" means that the respondents believe that their expectations for them exceed their real abilities or reach unrealistic goals. This is a subjective feeling and perception, and therefore the entry is more biased towards the present or future. Taken together, we can conclude that the output: \"The question is whether the respondents are or will be asking whether they are feeling too much of their parents.\""}, "sentence_type": "Negative declarative sentence", "pos": {"nouns": {"parents": 1, "Expectations": 1}, "verbs": {"Too high": 1}, "numerals": {"7": 1}}}, {"content": "Is it unfortunate that you have your own past and family?", "word_count": 13, "label": "", "sentence_structure": "Subject鈥揷opula鈥損redicative", "subjective_objective": {"answer": "Subjective", "rationale": "This is because patients are subjective about their past and family."}, "sentence_sentiment": {"answer": "Negative", "rationale": "Because \"unlucky\" in the entry is a negative emotional color."}, "language_style": {"answer": "Calm and direct", "rationale": "The entry is more directly written, not in too much euphemism or emotional rendering, but in a direct interview with the interviewee whether he or she believes that he or she has suffered an unfortunate past and family."}, "time_dimension": {"answer": "Past", "rationale": "The entry asks the person who was tested about his personal experience and family background, and the temporal dimension is a past."}, "sentence_type": "Exclamatory sentence", "pos": {"nouns": {"Past": 1, "Family": 1, "Unfortunately": 1}, "verbs": {"self": 1, "be": 1}, "adverbs": {"8": 1}}}, {"content": "Nine is too worried about the future.", "word_count": 9, "label": "", "sentence_structure": "Subject鈥揷opula鈥損redicative", "subjective_objective": {"answer": "Subjective", "rationale": "Too much fear of the future is a subjective assessment, which belongs to the feelings and thoughts of the patient."}, "sentence_sentiment": {"answer": "Negative", "rationale": "Because the \"too much fear\" in the entry is negative."}, "language_style": {"answer": "Calm and direct", "rationale": ""}, "time_dimension": {"answer": "Future", "rationale": "The entry contains ideas and concerns about the future, and the keywords are \"too much to worry\" and \"the future\" and all point to situations that have not yet occurred and that may arise in the future."}, "sentence_type": "Interrogative sentence", "pos": {"nouns": {"Things": 1, "Future": 1}, "verbs": {"I'm worried": 1, "Excess": 1}, "adverbs": {"Too much": 1}, "numerals": {"9": 1}}}, {"content": "Ten doesn't want to see anyone.", "word_count": 4, "label": "", "sentence_structure": "Subject鈥搗erb鈥揷omplement", "subjective_objective": {"answer": "Subjective", "rationale": "Because \"not seeing people\" is a subjective feeling and attitude, not an objective fact or behaviour."}, "sentence_sentiment": {"answer": "Negative", "rationale": "Because the \"no-seeing\" act is negative."}, "language_style": {"answer": "Calm and direct", "rationale": "The language style of the entry is more straightforward, with no excessive use of rhetorical techniques or euphemisms, which place greater emphasis on the substance and directness of the issue. Such expressions may give a cold, but they are also more concise, helping respondents to understand the questions accurately and to give true answers."}, "time_dimension": {"answer": "Present", "rationale": "The entry is for the testee ' s current emotional state and orientation, and the temporal dimension is now judged."}, "sentence_type": "Interrogative sentence", "pos": {"verbs": {"Yes": 1, "See": 1}, "adjectives": {"10": 1}, "adverbs": {"not": 1}}}, {"content": "I feel like I'm not.", "word_count": 8, "label": "", "sentence_structure": "Subject鈥搗erb鈥揷omplement", "subjective_objective": {"answer": "Subjective", "rationale": "Because \"is it\" is a subjective judgement, but it feels like it's a subjective assessment of itself."}, "sentence_sentiment": {"answer": "Negative", "rationale": "Because \"denying yourself\" is a negative quality or attitude."}, "language_style": {"answer": "Tactful and warm", "rationale": "The language style of the entry is more euphemistic, using the more abstract expression \"no no one\" to ask the other whether they often reject their views or ideas, and it is more moderate and acceptable. In addition, the expression \"see below for your analysis of this questionnaire\" is more cordial and friendly and helps to increase the participation of the respondents."}, "time_dimension": {"answer": "Past", "rationale": "The entry contains the old time term \"11 feels \" and the negative word \"no\" and therefore can be judged as an assessment or perception of the testee's past."}, "sentence_type": "Negative declarative sentence", "pos": {"nouns": {"Questionnaire": 1, "Entry": 1, "self": 2}, "verbs": {"I think": 1, "Yes": 1}, "adverbs": {"not": 1}, "numerals": {"11": 1}}}, {"content": "Lack of enthusiasm and motivation", "word_count": 8, "label": "", "sentence_structure": "Subject鈥搗erb鈥揷omplement", "subjective_objective": {"answer": "Subjective", "rationale": "Because lack of enthusiasm and motivation is an individual ' s feeling or attitude, it is a subjective examination."}, "sentence_sentiment": {"answer": "Negative", "rationale": "Because the \"lack of enthusiasm and motivation\" in the entry describes a negative quality."}, "language_style": {"answer": "Tactful and warm", "rationale": "While the question is directly asked whether the interviewees lack enthusiasm and motivation in some respects, it uses the invitation tone to make them more comfortable and relaxed. Moreover, the question does not express any negative feelings or assessments, but rather raises questions in a gentle and respectful manner to the interviewees."}, "time_dimension": {"answer": "Present", "rationale": "Based on the time and verb pattern in the sentence, the current state or attitude of the testee is described as \"lack\" and \"motivation\", so it is judged to be the present."}, "sentence_type": "Imperative sentence", "pos": {"nouns": {"鈾?Hot": 1, "Active": 1}, "verbs": {"Lack": 1}, "numerals": {"12": 1}}}, {"content": "13 Pessimism", "word_count": 2, "label": "", "sentence_structure": "Subject鈥揷opula鈥損redicative", "subjective_objective": {"answer": "Subjective", "rationale": "Because pessimism is a subjective emotional experience and attitude. While it may be influenced by external factors, it is ultimately determined by individual perceptions and mentalities. Thus, \"pessimism\" is subjective."}, "sentence_sentiment": {"answer": "Negative", "rationale": "Because the word \"pessimism\" is itself a negative emotional term, which is a negative, pessimistic attitude to things."}, "language_style": {"answer": "Calm and direct", "rationale": "The entry directly asked whether the respondents were pessimistic, were simpler and objective, were not overdoing or emotionally rendering, and were typical of the calm and direct language style."}, "time_dimension": {"answer": "Past", "rationale": "The temporal dimension of the purpose of the article should therefore be unclear."}, "sentence_type": "Interrogative sentence", "pos": {"nouns": {"Pessimism": 1, "Questionnaire": 1}, "verbs": {"Give": 1}, "numerals": {"13": 1}}}, {"content": "14 is not focused.", "word_count": 5, "label": "", "sentence_structure": "Subject鈥搗erb鈥揷omplement", "subjective_objective": {"answer": "Subjective", "rationale": "Because the lack of concentration of thought is the subjective feeling and experience of the patient, not the objective fact."}, "sentence_sentiment": {"answer": "Negative", "rationale": "Because \"inconsistent\" is a negative behavior or state."}, "language_style": {"answer": "Calm and direct", "rationale": "The entry is in a concise language and directly asks the respondents whether there is a lack of concentration of ideas, no excessive rhetoric or emotional rendering, and the language style is straightforward and straightforward."}, "time_dimension": {"answer": "Future", "rationale": "The key word \"inconsistent\" is described as a question or a problem that may arise in the future and is therefore judged to be the future."}, "sentence_type": "Negative declarative sentence", "pos": {"nouns": {"Ideas": 1, "Focus": 1}, "verbs": {"not": 1}, "numerals": {"14": 1}}}, {"content": "Fifteen is in a state of mood.", "word_count": 6, "label": "", "sentence_structure": "Subject鈥搗erb鈥揷omplement", "subjective_objective": {"answer": "Subjective", "rationale": "Because emotional change is the patient's own subjective experience and feelings."}, "sentence_sentiment": {"answer": "Negative", "rationale": "Because the term \"excessively infatuated\" is usually considered negative."}, "language_style": {"answer": "Tactful and warm", "rationale": "The entry is more personal and interactive in a conversation in a voice of inquiry and concern, rather than in direct statements or orders. At the same time, the use of the term \"excessive mood\" avoids undue pressure or negative emotions on the person under investigation."}, "time_dimension": {"answer": "Present", "rationale": "It can be assumed from the time of the sentence that the mood swings and rises occurred over a period of time, but that they have existed or continue during that period, and thus are judged now."}, "sentence_type": "Exclamatory sentence", "pos": {"nouns": {"15": 1, "Emotion": 1, "Rise and shine": 1, "Pass": 1}, "adverbs": {"Large": 1}}}, {"content": "16 often insomnia.", "word_count": 4, "label": "", "sentence_structure": "Subject鈥搗erb鈥揷omplement", "subjective_objective": {"answer": "Subjective", "rationale": "Because insomnia is a subjective feeling and experience."}, "sentence_sentiment": {"answer": "Negative", "rationale": "Because the \"often insomnia\" act is negative."}, "language_style": {"answer": "Calm and direct", "rationale": "The language style of the entry is more straightforward, there is no rhetorical or emotional rendering, and the tone is more objective. It simply asks the interviewee whether he/she is often insomnia, without unnecessary expressions or leading questions. This style of questionnaire is more suitable for collecting objective, specific data than for seeking in-depth communication or emotional resonance."}, "time_dimension": {"answer": "Past", "rationale": "The term \"often\" indicates that it is a regular customary act, and insomnia is usually a past experience or symptom. Thus, \"often insomnia\" describes the past's behaviour or state."}, "sentence_type": "Affirmative declarative sentence", "pos": {"nouns": {"Insomnia": 1}, "verbs": {"Often": 1, "have": 1}, "numerals": {"16": 1}}}, {"content": "Seventeen headaches.", "word_count": 2, "label": "", "sentence_structure": "Subject鈥揷opula鈥損redicative", "subjective_objective": {"answer": "Subjective", "rationale": "Because headaches are a subjective symptom, the perception and description of pain may vary from one person to another. So, \"17 headaches\" are viewed from the patient's subjective perspective."}, "sentence_sentiment": {"answer": "Neutral", "rationale": "Because the entry does not have any emotional color."}, "language_style": {"answer": "Calm and direct", "rationale": "The entry is more concise, and it is based on a simple, straightforward question about the other person's symptoms of headaches, and is not used in too many emotional and speech-making techniques, so it can be judged as a \"cool straight\" language style."}, "time_dimension": {"answer": "Present", "rationale": "The number \"17\" in this entry is a serial number used to distinguish between different problems or options, not related to the temporal dimension. Headache is a symptom, and can therefore be understood as asking if the person tested currently has a headache, so it is now judged."}, "sentence_type": "Exclamatory sentence", "pos": {"nouns": {"Headache": 1}, "numerals": {"17": 1}}}, {"content": "Eighteen necks, shoulder sores.", "word_count": 6, "label": "", "sentence_structure": "Subject鈥搗erb鈥揷omplement", "subjective_objective": {"answer": "Objective", "rationale": "Because the neck, shoulder and sourness is a perceived physical condition, it is not dependent on subjective feelings or attitudes of the patient."}, "sentence_sentiment": {"answer": "Negative", "rationale": "Because \"neck, shoulder sore\" is a pain, a negative emotional experience."}, "language_style": {"answer": "Calm and direct", "rationale": "The entry is in a straightforward manner to ask the interviewees whether they often experience a sore neck and shoulder, which is relatively simple and does not contain excessive rhetorical styles and can therefore be described as \"cool straight\"."}, "time_dimension": {"answer": "Past", "rationale": "The entry describes the current state of the person being tested, and the temporal dimension is judged to be past."}, "sentence_type": "Affirmative declarative sentence", "pos": {"nouns": {"18": 1, "Neck": 1, "Shoulders": 2, "Ouch": 1}}}, {"content": "19 chests are suffocating.", "word_count": 4, "label": "", "sentence_structure": "Subject鈥搗erb鈥揷omplement", "subjective_objective": {"answer": "Subjective", "rationale": "According to the information provided, \"19 chest strangulation\" is a subjective description of the patient and is therefore a subjective examination."}, "sentence_sentiment": {"answer": "Negative", "rationale": "Because the symptoms of \" chest strangulation\" are usually associated with physical illness or discomfort, they express negative emotions."}, "language_style": {"answer": "Calm and direct", "rationale": "The entry describes in short and direct language the symptoms of the disease that the person under investigation may experience, is not overly emotional or isphraseic and is more objective in tone."}, "time_dimension": {"answer": "Past", "rationale": "The reference to \"19 chest strangulation\" in this entry is a symptom or experience, and is usually used to describe experience that has occurred."}, "sentence_type": "Exclamatory sentence", "pos": {"nouns": {"- Chestaches": 1, "- Squeeze": 1}, "verbs": {"EMPTY": 1}, "classifiers": {"19": 1}}}, {"content": "Twenty is always so strong.", "word_count": 7, "label": "", "sentence_structure": "Subject鈥搗erb鈥揷omplement", "subjective_objective": {"answer": "Subjective", "rationale": "Because \"promoting\" is a subjective word of evaluation, which represents the sense and perception of the patient's own state."}, "sentence_sentiment": {"answer": "Positive", "rationale": "Because the description \"promoting\" is usually considered positive."}, "language_style": {"answer": "Tactful and warm", "rationale": "Because the question is not whether you are always dynamic and energetic, but rather inviting you to think about your own state in a more moderate way. At the same time, this expression is more acceptable and interactive and personal."}, "time_dimension": {"answer": "Present", "rationale": ""}, "sentence_type": "Affirmative declarative sentence", "pos": {"nouns": {"20": 1, "Total": 1, "Yes": 1, "It's a big deal": 1}}}, {"content": "21 is low.", "word_count": 3, "label": "", "sentence_structure": "Subject鈥搗erb鈥揷omplement", "subjective_objective": {"answer": "Subjective", "rationale": "This statement reflects the patient ' s assessment of his or her abilities and is a subjective feeling."}, "sentence_sentiment": {"answer": "Negative", "rationale": "Because \"low gas\" is a negative quality description."}, "language_style": {"answer": "Calm and direct", "rationale": "The entry directly expresses the subject of questioning the other party for 21 low-gas situations, without excessive rhetorical or emotional rendering, and with more objective tone."}, "time_dimension": {"answer": "Present", "rationale": "Now in the entry, \"21\" means the age information of the testee, which is a static state description; \"minor gas\" is an assessment of the character characteristics of the testee, which needs to be judged by the testee's performance in completing the questionnaire, so the time dimension is now."}, "sentence_type": "Negative declarative sentence", "pos": {"nouns": {"21": 1, "Gases": 1}, "adjectives": {"Small": 1}}}, {"content": "Twenty-two worries.", "word_count": 3, "label": "", "sentence_structure": "Subject鈥搗erb鈥揷omplement", "subjective_objective": {"answer": "Subjective", "rationale": "Because love is the patient's subjective feeling and attitude."}, "sentence_sentiment": {"answer": "Negative", "rationale": "Because \"love\" is often understood as too anxiety, worry or interference with others, it is a negative emotion and attitude."}, "language_style": {"answer": "Tactful and warm", "rationale": "The expression is more personal and interactive, in a gentle and inviting tone, rather than in direct statements or orders. At the same time, the purpose of the article is more subjective and open, leaving the respondent some space to describe his or her situation, which is also a feature of a warmer language style."}, "time_dimension": {"answer": "Past", "rationale": "The entry \"22\" indicates age, \"love\" is a character or psychological state, so it can be judged to be a description of the testee's past."}, "sentence_type": "Exclamatory sentence", "pos": {"verbs": {"Mind your own business": 1}, "adjectives": {"22": 1}}}, {"content": "23 is restless.", "word_count": 4, "label": "", "sentence_structure": "Subject鈥揷opula鈥損redicative", "subjective_objective": {"answer": "Subjective", "rationale": "Because irritation is a subjective feeling or emotional experience."}, "sentence_sentiment": {"answer": "Negative", "rationale": "Because the term \"irregular\" usually brings negative emotions and expressions of negative attitudes."}, "language_style": {"answer": "Calm and direct", "rationale": "The entry directly asks the other party whether there is a state of anxiety, a clear language, a lack of excessive emotional colours or accoutrements, and a calm and direct style."}, "time_dimension": {"answer": "Present", "rationale": "\"23 Agitation\" describes the current emotional state of the testee, which is now judged by the time dimension."}, "sentence_type": "Exclamatory sentence", "pos": {"nouns": {"- Nervous": 1, "Unsettled": 1}, "adjectives": {"- Nervous": 1, "Unsettled": 1}, "numerals": {"23": 1}}}, {"content": "24 is a little angry.", "word_count": 4, "label": "", "sentence_structure": "Subject鈥揷opula鈥損redicative", "subjective_objective": {"answer": "Subjective", "rationale": "Because anger is an emotional state, it is the subjective feeling and experience of the patient."}, "sentence_sentiment": {"answer": "Negative", "rationale": "Because \"freak\" is a negative emotional state."}, "language_style": {"answer": "Calm and direct", "rationale": "The entry uses a simple and clear language and raises questions directly, without any qualifier or emotional colour, and is more calm and straightforward."}, "time_dimension": {"answer": "Present", "rationale": "The keyword \"24 is easily angry\" means the person being tested in his present state or behaviour and therefore belongs to the description of the current time dimension."}, "sentence_type": "Exclamatory sentence", "pos": {"verbs": {"Easy": 1, "Excited": 1}, "adjectives": {"24": 1}}}, {"content": "Twenty-five wants to be light.", "word_count": 3, "label": "", "sentence_structure": "Subject鈥揷opula鈥損redicative", "subjective_objective": {"answer": "Subjective", "rationale": "Suicide is an expression of subjective emotions and attitudes."}, "sentence_sentiment": {"answer": "Negative", "rationale": "Because \"light life\" is an extremely negative act."}, "language_style": {"answer": "Calm and direct", "rationale": "The language style of the entry is more direct and concise, with no excessive accommodation and emotional rendering, with greater emphasis on the nature of the transmission of information and expression, and more objective and neutral. This style helps respondents to better understand the questions and to provide more realistic and accurate answers."}, "time_dimension": {"answer": "Present", "rationale": "In the future, the term \"intention\" appears in the entry, which expresses vision and expectations for the future; at the same time, \"inherent\" refers to the negative behaviour or mentality that may emerge in the future, and therefore the entry can be judged to be a temporal dimension of the future."}, "sentence_type": "Exclamatory sentence", "pos": {"nouns": {"25": 1, "Lightness": 1}}}, {"content": "Twenty-six is not interested in anything.", "word_count": 8, "label": "", "sentence_structure": "Subject鈥揷opula鈥損redicative", "subjective_objective": {"answer": "Subjective", "rationale": "Because \"not interested in anything\" is a person's inner feeling and attitude, a subjective view."}, "sentence_sentiment": {"answer": "Negative", "rationale": "Because the description \"no interest in anything\" in the entry expresses negative emotions and attitudes."}, "language_style": {"answer": "Calm and direct", "rationale": "The entry expresses directly, in a concise and clear manner, the subject of asking the other side whether it is not interested in anything, and is not speaking in a more objective manner, using too much acrimonym or emotional colour."}, "time_dimension": {"answer": "Future", "rationale": "The entry expresses the future attitude and expectations of the testee, and the keywords are \"26\", \"nothing\" and \"no interest.\" In it, \"26\" indicates the age or intergenerational background of the testee; \"anything\" refers to everything, including but not limited to work, learning, entertainment, etc.; \"no interest\" means that the testee is not interested in these aspects. Taken together, the questionnaire is about the future attitude and expectations of the testee, namely whether they will remain uninterested in everything for the next time."}, "sentence_type": "Negative declarative sentence", "pos": {"verbs": {"to": 1, "have": 1}, "adjectives": {"Any": 1}, "adverbs": {"Both": 1}, "numerals": {"26": 1}}}, {"content": "27 memory loss", "word_count": 5, "label": "", "sentence_structure": "Subject鈥搗erb鈥揷omplement", "subjective_objective": {"answer": "Subjective", "rationale": "Because memory loss is usually a loss of memory of the patient's self-perception, and is subjective."}, "sentence_sentiment": {"answer": "Negative", "rationale": "Because memory loss is usually a negative phenomenon or symptom."}, "language_style": {"answer": "Calm and direct", "rationale": "The question is very straightforward and clear, and there is no influence on the rhetorical or emotional approach, so that one can see that the intention is to understand whether the person under investigation has a decline in memory. This language style applies to situations where objective information is needed."}, "time_dimension": {"answer": "Future", "rationale": "The entry contains an expectation or concern for the future, and \"rememberance loss\" is an unacknowledged and potentially future situation and is judged to be the future."}, "sentence_type": "Exclamatory sentence", "pos": {"nouns": {"Memory": 1, "Declining": 1}, "verbs": {"27": 1}}}, {"content": "28 lacks endurance.", "word_count": 4, "label": "", "sentence_structure": "Subject鈥揷opula鈥損redicative", "subjective_objective": {"answer": "Subjective", "rationale": "Because lack of endurance is an individual subjective feeling and assessment, not an objective fact or phenomenon."}, "sentence_sentiment": {"answer": "Negative", "rationale": "Because the expression \"insatisfactory\" is negative."}, "language_style": {"answer": "Calm and direct", "rationale": "The entry presents a direct and concise description of the researchers ' interest in learning about the patient ' s endurance, without excessive ado or emotional, and in a more calm and objective language style."}, "time_dimension": {"answer": "Present", "rationale": "The entry describes the shortcomings or problems of the testee in certain respects, and therefore the temporal dimension is now judged."}, "sentence_type": "Negative declarative sentence", "pos": {"nouns": {"Endurance": 1, "28": 1}, "verbs": {"Lack": 1}}}, {"content": "29 Lack of discretion", "word_count": 6, "label": "", "sentence_structure": "Subject鈥揷opula鈥損redicative", "subjective_objective": {"answer": "Subjective", "rationale": "Because lack of decisive capacity is self-evaluation and subjective perception of patients, it is a subjective description."}, "sentence_sentiment": {"answer": "Negative", "rationale": "Because the expression \"lack of discretion\" is negative."}, "language_style": {"answer": "Calm and direct", "rationale": ""}, "time_dimension": {"answer": "Past", "rationale": "The entry does not clearly point at a specific time, but describes a state or characteristic and therefore judges the past."}, "sentence_type": "Negative declarative sentence", "pos": {"nouns": {"Adjudication": 1}, "verbs": {"Lack": 1}, "adjectives": {"29": 1}}}, {"content": "30 is too dependent on others.", "word_count": 6, "label": "", "sentence_structure": "Subject鈥揷opula鈥損redicative", "subjective_objective": {"answer": "Subjective", "rationale": "\"Overdependence\" is the patient's subjective feeling and attitude and is therefore subjective."}, "sentence_sentiment": {"answer": "Negative", "rationale": "Because the \"too much reliance\" in the entry is negative."}, "language_style": {"answer": "Calm and direct", "rationale": "The entry directly expresses the subject matter of the question of whether the other party is over-reliant on others, does not use excessive rhetorical or emotional rendering, and is more objective in tone."}, "time_dimension": {"answer": "Present", "rationale": "In the past, the entry used the absolute number \"30\" to indicate the number or degree of the past experience or state. At the same time, \"too much reliance on others\" is a description of the past behaviour or mentality, and it also points to the past. Thus, depending on the time dimension, the questionnaire entry is about the past of the subject."}, "sentence_type": "Negative declarative sentence", "pos": {"nouns": {"30": 1, "others": 1}, "verbs": {"Too much": 1, "Dependency": 1}}}, {"content": "31 is tired of the red.", "word_count": 6, "label": "", "sentence_structure": "Subject鈥揷opula鈥損redicative", "subjective_objective": {"answer": "Subjective", "rationale": "Because \"for the pain\" expresses the patient's subjective feelings and emotional experience."}, "sentence_sentiment": {"answer": "Negative", "rationale": "Because the word \"torture\" conveys a negative emotional color."}, "language_style": {"answer": "Tactful and warm", "rationale": "Warm and warm."}, "time_dimension": {"answer": "Present", "rationale": "The entry describes the current state or feeling of the testee, i.e. the pain of the red. The keyword \"for the distress\" indicates emotional distress and distress, implying that this is a present problem. Therefore, in the light of the time dimension, the article is intended to deal with the present question of behaviour or mental state."}, "sentence_type": "Affirmative declarative sentence", "pos": {"nouns": {"Red": 1, "Trouble": 1}, "verbs": {"Yes": 1}, "adverbs": {"Yes": 1}, "numerals": {"31": 1}}}, {"content": "32 bites of shiver.", "word_count": 6, "label": "", "sentence_structure": "Subject鈥搗erb鈥揷omplement", "subjective_objective": {"answer": "Subjective", "rationale": "Because \"sound tremors\" are a personal feeling and experience, it is subjective."}, "sentence_sentiment": {"answer": "Negative", "rationale": "Because the description \"sniffing sound\" in the entry is negative."}, "language_style": {"answer": "Tactful and warm", "rationale": "Warm and warm."}, "time_dimension": {"answer": "Past", "rationale": "The entry describes the experience of the subjects, who at one point at a time have experienced stutter and tremors, and thus judge as the past."}, "sentence_type": "Affirmative declarative sentence", "pos": {"nouns": {"32": 1, "Snack": 1, "Sound": 1, "Frightened": 1}}}, {"content": "33 bodies are cold and hot.", "word_count": 6, "label": "", "sentence_structure": "Subject鈥搗erb鈥揷omplement", "subjective_objective": {"answer": "Subjective", "rationale": "Because the feeling of cold and heat is the patient's subjective experience and observation."}, "sentence_sentiment": {"answer": "Negative", "rationale": "Because the entry \"sweet body cold and hot\" describes a feeling of discomfort, which may be related to health problems and has negative emotional connotations."}, "language_style": {"answer": "Calm and direct", "rationale": "The entry directly asked about the physical condition of the person interviewed, was calm and clear, did not add too many rhetorical or emotional colours and was more in line with the language style of the questionnaire."}, "time_dimension": {"answer": "Present", "rationale": "The entry describes the current state of the person being tested, and the temporal dimension is now judged."}, "sentence_type": "Exclamatory sentence", "pos": {"nouns": {"Body": 1, "鈾?And the cold and the heat 鈾?: 1}, "adjectives": {"鈾?And the cold and the heat 鈾?: 1}, "numerals": {"33": 1}}}, {"content": "34 is for urine and sexual organs.", "word_count": 8, "label": "", "sentence_structure": "Subject鈥搗erb鈥揷omplement", "subjective_objective": {"answer": "Objective", "rationale": "Because it is a physical reaction, it is not a subjective attitude or idea of the patient."}, "sentence_sentiment": {"answer": "Neutral", "rationale": "Because the entry does not have any emotional color."}, "language_style": {"answer": "Tactful and warm", "rationale": "This is a more accessible expression, which helps to improve the effectiveness and accuracy of the questionnaire."}, "time_dimension": {"answer": "Present", "rationale": "The entry is about the health and life habits of the person being tested, and the time dimension is now judged. Key words \"note\" , \"ure\" and \"sexual organs\" point to the current behaviour and state. Therefore, the contents of the entry relate primarily to the person being tested's current behaviour and habits."}, "sentence_type": "Exclamatory sentence", "pos": {"nouns": {"34": 1, "Attention": 1, "Peeing": 1, "Sexual organs": 1}, "numerals": {"34": 1}}}, {"content": "35 is in a good mood.", "word_count": 4, "label": "", "sentence_structure": "Subject鈥搗erb鈥揷omplement", "subjective_objective": {"answer": "Subjective", "rationale": "Because \"open\" is the subjective and emotional state of the individual, it reflects the emotional experience of the individual, not the objective fact or behavioural outcome."}, "sentence_sentiment": {"answer": "Positive", "rationale": "Because \"crazy\" means a positive emotional attitude."}, "language_style": {"answer": "Tactful and warm", "rationale": "The entry uses a positive term \"open mood\" and euphemisms to ask each other about their emotional state, showing a sense of concern and concern, so that the language style is more euphemismal."}, "time_dimension": {"answer": "Present", "rationale": "The keyword \"clause\" means the state of the mood, and the temporal dimension is now."}, "sentence_type": "Affirmative declarative sentence", "pos": {"adjectives": {"The mood": 1, "- Yes, sir": 1}, "numerals": {"35": 1}}}, {"content": "36 is strangely disturbed.", "word_count": 7, "label": "", "sentence_structure": "Subject鈥搗erb鈥揷omplement", "subjective_objective": {"answer": "Subjective", "rationale": "Because this is a subjective description of the patient ' s own feelings and experiences."}, "sentence_sentiment": {"answer": "Negative", "rationale": "Because the expression \"unbelievably uneasy\" is negative."}, "language_style": {"answer": "Tactful and warm", "rationale": "Warm and warm."}, "time_dimension": {"answer": "Present", "rationale": ""}, "sentence_type": "Imperative sentence", "pos": {"nouns": {"36": 1, "I don't know": 1, "Unsettled": 1}, "adjectives": {"It's amazing": 1}, "adverbs": {"Land": 2}, "numerals": {"36": 1}, "pronouns": {"I": 1}}}, {"content": "37 people are alone when they're upset.", "word_count": 10, "label": "", "sentence_structure": "Subject鈥揷opula鈥損redicative", "subjective_objective": {"answer": "Subjective", "rationale": "Because \"uneasy\" is the feeling and experience inside the patient, and it's a subjective examination."}, "sentence_sentiment": {"answer": "Negative", "rationale": "Because the \"uneasy\" sentiment in the entry is negative."}, "language_style": {"answer": "Calm and direct", "rationale": "The entry directly expresses the subject of questioning whether the other party will be disturbed while alone, without excessive rhetoric or emotional rendering, and is more objective in tone. \""}, "time_dimension": {"answer": "Present", "rationale": "The entry is inferred from the keyword and context to ask the testee whether he was disturbed when he was alone, and therefore the temporal dimension is now judged."}, "sentence_type": "Exclamatory sentence", "pos": {"nouns": {"37": 1, "person": 1, "Alone": 1, "Time": 1, "I feel": 1, "Unsettled": 1}, "numerals": {"37": 1}}}, {"content": "Lack of self-confidence", "word_count": 5, "label": "", "sentence_structure": "Subject鈥揷opula鈥損redicative", "subjective_objective": {"answer": "Subjective", "rationale": "Because lack of self-confidence is the patient ' s own feeling and perception, it is the patient ' s subjective view."}, "sentence_sentiment": {"answer": "Negative", "rationale": "Because lack of self-confidence is a negative emotional state."}, "language_style": {"answer": "Calm and direct", "rationale": "The entry is based on a direct, concise presentation, without excessive rhetorical or emotional overtook, but rather on questions: \"38 lacks self-confidence\", which is more objective, rational and consistent with the formal and scientific nature of the questionnaire. At the same time, this calm and straightforward language style can better guide the interviewee to reflect and answer the questions, avoiding being influenced by personal emotions or subjective bias."}, "time_dimension": {"answer": "Present", "rationale": ""}, "sentence_type": "Exclamatory sentence", "pos": {"nouns": {"Confidence": 2}, "verbs": {"Lack": 1}, "adjectives": {"38": 1}}}, {"content": "39 is a very strong one.", "word_count": 6, "label": "", "sentence_structure": "Subject鈥搗erb鈥揷omplement", "subjective_objective": {"answer": "Subjective", "rationale": "Because \"daunted tail\" is a subjective description of the patient's psychological state of affairs."}, "sentence_sentiment": {"answer": "Negative", "rationale": "Because the entry \"daunted tail\" expresses negative attitudes and behaviour, and a lack of decisiveness and enforcement."}, "language_style": {"answer": "Tactful and warm", "rationale": "The entry is more ad hoc and interactive when it is being approached in a gentle and inviting manner, asking whether the other side is acting too cautiously and hesitantly, rather than directly speaking or ordering."}, "time_dimension": {"answer": "Present", "rationale": "The entry indicates the person's current behaviour or attitude, and \"daunting tail\" is a descriptive language, indicating that the person is too careful and has no discretion in dealing with the matter. The entry therefore covers the time dimension of the present."}, "sentence_type": "Negative declarative sentence", "pos": {"nouns": {"- You're afraid of the end": 1, "39": 1, "Operations": 1}, "adverbs": {"very": 1}, "numerals": {"39": 1, "individual": 1, "- You're afraid of the end": 1, "OPERATIONS": 1}, "classifiers": {"individual": 1}}}, {"content": "Forty is easy to misconstrue.", "word_count": 6, "label": "", "sentence_structure": "Subject鈥搗erb鈥揷omplement", "subjective_objective": {"answer": "Subjective", "rationale": "Because \"facilitative\" is the result of a patient's self-perception and perception, and is a subjective description."}, "sentence_sentiment": {"answer": "Neutral", "rationale": "Because the entry does not have any emotional color."}, "language_style": {"answer": "Tactful and warm", "rationale": "The language style of the entry is more euphemistic, moderate, and not too strong or direct, but rather a way to guide the other side to think and express concern and concern that the other side may be mispercepted."}, "time_dimension": {"answer": "Present", "rationale": "The entry expresses a normal feeling and experience that the testee often feels misunderstood in the current state of life. Thus, the temporal dimension is now judged."}, "sentence_type": "Negative declarative sentence", "pos": {"nouns": {"person": 1, "Misunderstanding": 1}, "verbs": {"Easy": 1, "By": 1}, "adverbs": {"very": 1}, "numerals": {"40": 1}}}, {"content": "41 doesn't trust anyone.", "word_count": 5, "label": "", "sentence_structure": "Subject鈥搗erb鈥揷omplement", "subjective_objective": {"answer": "Subjective", "rationale": "Because \"no trust\" is the patient's subjective attitude."}, "sentence_sentiment": {"answer": "Negative", "rationale": "Because the attitude of \"no one else\" is negative."}, "language_style": {"answer": "Calm and direct", "rationale": "The entry follows a concise and fast-simplistic language style, which is often a clear and direct impression of a numerical style. Moreover, the purpose of the article is very clear, without ambiguity or too much embellishment, and it is therefore judged to be straightforward in terms of calmness."}, "time_dimension": {"answer": "Past", "rationale": "The keyword \"no confidence\" suggests that this is a subjective assessment of whether the testee himself believes in others, rather than an objective factual description or a memory of past experiences. So we can conclude that the entry to the questionnaire is about the present attitude and conviction of the testee."}, "sentence_type": "Negative declarative sentence", "pos": {"nouns": {"person": 1, "Yes": 1, "others": 1}, "verbs": {"not": 1, "Letter": 1}, "numerals": {"41": 1}}}, {"content": "42 is too suspicious.", "word_count": 4, "label": "", "sentence_structure": "Subject鈥揷opula鈥損redicative", "subjective_objective": {"answer": "Subjective", "rationale": "Because too much suspicion is the patient's subjective attitude."}, "sentence_sentiment": {"answer": "Negative", "rationale": "Because \"suspect\" is usually a negative emotion, a sign of mistrust or excessive suspicion."}, "language_style": {"answer": "Calm and direct", "rationale": "The entry directly expresses the theme \"excessive suspicion\" and does not use too much language or emotional terms, which are more objective and direct."}, "time_dimension": {"answer": "Future", "rationale": "The keyword \"exaggerous\" in the entry indicates the vision and expectations for the future, that is, the scenarios of which may arise in the future. It therefore points to situations that have not yet occurred and that may arise in the future, with the temporal dimension judged to be the future."}, "sentence_type": "Exclamatory sentence", "pos": {"nouns": {"Too much": 1, "Doubt": 1}, "verbs": {"Doubt": 1}, "adverbs": {"Too much": 1}, "numerals": {"42": 1}}}, {"content": "43 hates dating.", "word_count": 4, "label": "", "sentence_structure": "Subject鈥搗erb鈥揷omplement", "subjective_objective": {"answer": "Subjective", "rationale": "Because \"aversion to dating\" is a person's inner feeling and attitude, and it's a subjective view of the patient."}, "sentence_sentiment": {"answer": "Negative", "rationale": "Because the attitude of \"aversion to dating\" is negative."}, "language_style": {"answer": "Calm and direct", "rationale": "The language style of the entry is calm and straightforward. It clearly expresses the theme of whether the person surveyed hates to interact with someone, without using too many emotional accoutrements or euphemisms. Such language style makes the issue more straightforward and understandable."}, "time_dimension": {"answer": "Present", "rationale": "The term \"disguised association\" in this entry is a description of the current state of affairs, implying that it is a current attitude or feeling and may continue."}, "sentence_type": "Negative declarative sentence", "pos": {"nouns": {"- To be with someone": 1}, "verbs": {"Disgusting": 1}, "numerals": {"43": 1}}}, {"content": "I feel humbled.", "word_count": 4, "label": "", "sentence_structure": "Subject鈥搗erb鈥揷omplement", "subjective_objective": {"answer": "Subjective", "rationale": "Because \"perception of inferiority\" is a subjective emotional experience. Although it may be due to external factors (e.g., others' assessment, life pressure, etc.), it is mainly caused by the individual鈥檚 own thinking and feelings."}, "sentence_sentiment": {"answer": "Negative", "rationale": "Because \"supra\" in the entries is a negative emotional attitude."}, "language_style": {"answer": "Calm and direct", "rationale": "The entry directly asks whether the interviewees often feel inferior, without using excessive emotional qualifiers or euphemisms, and in a more straightforward manner."}, "time_dimension": {"answer": "Past", "rationale": ""}, "sentence_type": "Exclamatory sentence", "pos": {"nouns": {"You're a low man": 1, "44": 1}, "verbs": {"I feel": 1}}}, {"content": "Forty-five is a bad idea.", "word_count": 4, "label": "", "sentence_structure": "Subject鈥搗erb鈥揷omplement", "subjective_objective": {"answer": "Subjective", "rationale": "Because \"sweet\" is a spoken word, describing a psychological state of fear, a psychological experience of the patient."}, "sentence_sentiment": {"answer": "Negative", "rationale": "Because \"sweet\" is an overly worrying, anxious mentality, often seen as negative emotions or attitudes."}, "language_style": {"answer": "Tactful and warm", "rationale": "So, an analysis of the following questionnaire entry: \"45 is a worrying thing,\" we can see that the title starts with a number, and looks like a serial number or a number, while the latter part of \"Osquiet is a saying that describes a person's concerns about something that is unnecessary. So, in terms of language style, the topic is both a calm and a warm one (which starts with a number) and a soft one (which is a sign of thought). So, in combination, the language style of the questionnaire is \"comfort and a cooler.\""}, "time_dimension": {"answer": "Future", "rationale": "The entry asks if the testee will be as anxious and anxious as \"crazy\" in the future, and therefore the temporal dimension is the future."}, "sentence_type": "Negative declarative sentence", "pos": {"nouns": {"It's a matter of time": 1}}}, {"content": "46 is tired.", "word_count": 4, "label": "", "sentence_structure": "Subject鈥揷opula鈥損redicative", "subjective_objective": {"answer": "Objective", "rationale": "Because \"body fatigue\" is an objective symptom of the patient."}, "sentence_sentiment": {"answer": "Negative", "rationale": "Because \"body fatigue\" is a negative state."}, "language_style": {"answer": "Calm and direct", "rationale": "The entry is succinct and directly asks the testee whether he/she is often feeling tired, is not using excessive rhetorical techniques or emotional rendering, and is speaking more objectively and directly."}, "time_dimension": {"answer": "Past", "rationale": "The entry describes the current state of the person being tested and the temporal dimension is judged to be past."}, "sentence_type": "Exclamatory sentence", "pos": {"nouns": {"Body": 1, "Tired": 1}, "adjectives": {"46": 1}}}, {"content": "47 sweats when he's in a hurry.", "word_count": 7, "label": "", "sentence_structure": "Subject鈥搗erb鈥揷omplement", "subjective_objective": {"answer": "Objective", "rationale": "As sweating is a physical reaction of the patient and is not controlled by the person ' s will, it is objectively examined."}, "sentence_sentiment": {"answer": "Negative", "rationale": "Because \"cool sweat\" in the entry is a negative physical reaction."}, "language_style": {"answer": "Calm and direct", "rationale": "The language style of the entry is \"cool and direct\" because it is clear to the other side whether he sweats when he or she is nervous or anxious, and is not trimmed with any rhetorical or emotional colours. This expression is simple, easy to understand, more objective and accurate."}, "time_dimension": {"answer": "Present", "rationale": ""}, "sentence_type": "Affirmative declarative sentence", "pos": {"nouns": {"47": 1, "Urgent": 1, "Khan": 1}, "verbs": {"Out": 1}, "adjectives": {"Cold": 1}, "numerals": {"Forty-seven": 1}}}, {"content": "48 stands up and gets dizzy.", "word_count": 6, "label": "", "sentence_structure": "Subject鈥揷opula鈥損redicative", "subjective_objective": {"answer": "Objective", "rationale": "The fact that the person is physically ill because of his or her rise is an objective fact."}, "sentence_sentiment": {"answer": "Negative", "rationale": "Because \"headlight\" is a negative sign of physical discomfort, not active or neutral."}, "language_style": {"answer": "Tactful and warm", "rationale": "The entry is more adept and interactive when it is spoken in a gentle and inviting manner, rather than by direct statements or orders."}, "time_dimension": {"answer": "Past", "rationale": "The entry is for the testee ' s past symptoms and the temporal dimension is a past."}, "sentence_type": "Interrogative sentence", "pos": {"nouns": {"48": 1, "Dizziness": 1}, "verbs": {"Stop": 1, "Get up": 1}}}, {"content": "49 was unconscious, and it was a blow.", "word_count": 8, "label": "", "sentence_structure": "Subject鈥搗erb鈥揷omplement", "subjective_objective": {"answer": "Objective", "rationale": "Because the wind and the loss of consciousness are objective facts that patients experience."}, "sentence_sentiment": {"answer": "Negative", "rationale": "Because \"discretion\" and \"screech\" are terms that describe disease or health problems and are usually considered negative."}, "language_style": {"answer": "Calm and direct", "rationale": ""}, "time_dimension": {"answer": "Past", "rationale": "In the entry, \"had\" is a reference to past experience or circumstances, and therefore judged \"the past\"."}, "sentence_type": "Exclamatory sentence", "pos": {"nouns": {"Awareness": 1, "Wind": 1}, "verbs": {"Lose": 1}, "numerals": {"49": 1}}}, {"content": "Fifty people are popular.", "word_count": 6, "label": "", "sentence_structure": "Subject鈥揷opula鈥損redicative", "subjective_objective": {"answer": "Subjective", "rationale": "Because \"50 people are popular\" is a phrase that describes the patient's personal feelings and subjective views. It's not about objective facts or behaviour, but about the patient's perception of his/her social abilities. So this entry is viewed from the patient's subjective perspective."}, "sentence_sentiment": {"answer": "Positive", "rationale": "Because the \"50s are popular\" in the entry is positive emotion, attitude or quality."}, "language_style": {"answer": "Tactful and warm", "rationale": "The language style of the entry is more euphemistic and friendly, and there is no overly direct or rigid expression. The term \"popular\" is used, and the term \"50 people\" is used to quantify it, making it more specific and clear, without creating a feeling of being ordered or blamed. This expression is more acceptable and willing to answer questions."}, "time_dimension": {"answer": "Future", "rationale": "The entry asks whether the person tested will have a good personal relationship in the future and suggests that it is an expected or hypothetical result, so that it can be judged to be relevant in the future."}, "sentence_type": "Affirmative declarative sentence", "pos": {"nouns": {"Humanities": 1, "Welcome": 1}, "verbs": {"Okay": 1, "Yes": 1}, "numerals": {"50": 1}}}, {"content": "51 is too much.", "word_count": 4, "label": "", "sentence_structure": "Subject鈥搗erb鈥揷omplement", "subjective_objective": {"answer": "Subjective", "rationale": "Because too rigid a description is an evaluative one, reflecting the patient ' s subjective attitude towards himself."}, "sentence_sentiment": {"answer": "Negative", "rationale": "Because the attitude of \"too much incorruptibility\" is negative, it means that individuals are overly careful, stubborn or demanding in terms of social, behavioural and other aspects."}, "language_style": {"answer": "Tactful and warm", "rationale": "This questionnaire entry is more euphemistic and moderate, and in a way that leads the interviewee to reflect on his or her own characteristics or preferences in some respects, without too much criticism or direct criticism. This expression makes the interviewee more comfortable and comfortable, and more likely to receive a true and accurate answer."}, "time_dimension": {"answer": "Past", "rationale": "It is therefore not possible to classify them as past, present or future in terms of time dimension."}, "sentence_type": "Negative declarative sentence", "pos": {"verbs": {"Too much": 1, "- What": 1}, "adjectives": {"51": 1}}}, {"content": "52 is not comfortable without a lot of confirmation.", "word_count": 14, "label": "", "sentence_structure": "Subject鈥搗erb鈥揷omplement", "subjective_objective": {"answer": "Subjective", "rationale": "The basis for the judgement: This is a patient's subjective attitude, because it describes and explains its own behaviour. While this sense of insecurity may affect their lives and decision-making, it is subjective in itself.\""}, "sentence_sentiment": {"answer": "Negative", "rationale": "Because the attitude of \"unreconfirmed\" in the entry is negative."}, "language_style": {"answer": "Calm and direct", "rationale": "The entry is presented as a clear value, \"52\", and then asks the interviewee whether he or she is always sure of everything repeatedly. The sentence is simple, not too much embellishment or emotional rendering, and the language style is more calm and straightforward."}, "time_dimension": {"answer": "Present", "rationale": "\"Unreconfirmed\" means that the way the subject is dealing with things is a present act or attitude. So it can be concluded: \"Now\"."}, "sentence_type": "Exclamatory sentence", "pos": {"nouns": {"Yes": 1, "Love": 1, "Objects": 1}, "verbs": {"Yes": 1, "Recognition": 1, "Fire": 1}, "adjectives": {"Repeat": 1, "Any": 2}, "adverbs": {"not": 3}, "numerals": {"52": 1}, "pronouns": {"Just": 1, "Here": 1}}}, {"content": "53 for the dirty.", "word_count": 5, "label": "", "sentence_structure": "Subject鈥搗erb鈥揷omplement", "subjective_objective": {"answer": "Subjective", "rationale": "Because \"care\" is the patient's personal feelings and attitudes and is subjective."}, "sentence_sentiment": {"answer": "Negative", "rationale": "Since \"dirty\" in the entry is usually considered negative or bad, the entire description can be considered negative."}, "language_style": {"answer": "Tactful and warm", "rationale": "The language style of the entry is more euphemistic, without direct orders or criticism. It leads the interviewee to reflect on his perception of and attitude towards \"dirty\" in a way that is more personal and interactive, and that gives the interviewee a sense of respect and understanding."}, "time_dimension": {"answer": "Past", "rationale": "The time indicator in the entry is \"53 pair\", which indicates a past event or situation and therefore judgement."}, "sentence_type": "Exclamatory sentence", "pos": {"nouns": {"Dirty": 2, "I care": 1}, "adjectives": {"very": 1}, "numerals": {"53": 1}}}, {"content": "54 is not free from the meaningless idea.", "word_count": 11, "label": "", "sentence_structure": "Subject鈥搗erb鈥揷omplement", "subjective_objective": {"answer": "Subjective", "rationale": "Because \"no sense\" is a subjective description."}, "sentence_sentiment": {"answer": "Negative", "rationale": "Because the expression \"can't get rid of\" in the entry expresses a negative emotional colour by expressing inability to control or avoid these ideas."}, "language_style": {"answer": "Calm and direct", "rationale": "The language style of the entry is \"cool and straight.\" It expresses the theme \"indefinable ideas\" in a simple and clear language, without too many emotional amenity or rhetorical techniques, and is more objective in tone."}, "time_dimension": {"answer": "Present", "rationale": "Now the \"indefinite\" in the entry indicates the current state or situation, and the \"innocent idea\" is the characteristic or content of the description of the state or situation, and therefore the judgement is now."}, "sentence_type": "Negative declarative sentence", "pos": {"nouns": {"Thoughts": 1, "It's pointless": 1}, "verbs": {"Get rid of it": 1, "of": 2, "Yes": 1}, "adjectives": {"It's pointless": 1}, "numerals": {"54": 1}}}, {"content": "55 thinks he smells weird.", "word_count": 8, "label": "", "sentence_structure": "Subject鈥搗erb鈥揷omplement", "subjective_objective": {"answer": "Subjective", "rationale": "Because \"I feel like I have a strange smell\" is a subjective feeling and judgment, and it's about the patient's self-awareness and sensory ability."}, "sentence_sentiment": {"answer": "Negative", "rationale": "Because the description of \"frightful smell\" in the entry is negative, it implies that the person under investigation may have physical problems or psychological distress."}, "language_style": {"answer": "Tactful and warm", "rationale": "The entry is more personal and interactive when it is presented in a gentle and inviting tone, rather than in direct statements or orders. At the same time, it avoids the use of language that is too direct or offensive, making the respondents comfortable and respectful in their responses."}, "time_dimension": {"answer": "Present", "rationale": "The keyword \"I feel strange smell\" indicates the experience and current experience that the subject is experiencing, and it is therefore possible to judge the time dimension of the article as it is now."}, "sentence_type": "Negative declarative sentence", "pos": {"nouns": {"55": 1, "self": 1, "Smells weird": 1}, "verbs": {"I think": 1}}}, {"content": "56 people say bad things behind their backs.", "word_count": 10, "label": "", "sentence_structure": "Subject鈥搗erb鈥搊bject", "subjective_objective": {"answer": "Subjective", "rationale": "Because \"others speak bad behind their backs\" is a subjective feeling and experience."}, "sentence_sentiment": {"answer": "Negative", "rationale": "Because the \"others say bad things behind their back\" in the entry is negative."}, "language_style": {"answer": "Tactful and warm", "rationale": "The entry is more euphemistic, implicit, and does not directly blame or criticize the other party鈥檚 behaviour, but rather leads the interviewee to answer the question in a more moderate way. Moreover, the more general social context is used as the subject matter, which is more likely to resonate with the interviewee and to engage."}, "time_dimension": {"answer": "Past", "rationale": "\"56 others say bad things behind their backs,\" \"56\" shows that the act took place in the testee itself and was a passive act. The experience occurred at some point in the past. So this entry concerns the testee's past experience."}, "sentence_type": "Affirmative declarative sentence", "pos": {"nouns": {"56": 1, "person": 1, "Behind": 1, "Bad word": 1}, "verbs": {"Say it": 1}}}, {"content": "57 always watch out for the people around.", "word_count": 7, "label": "", "sentence_structure": "Subject鈥搗erb鈥搊bject", "subjective_objective": {"answer": "Subjective", "rationale": "Because \"watch the people around\" is a psychological activity, it's the patient's subjective feelings and experiences."}, "sentence_sentiment": {"answer": "Neutral", "rationale": "Because the entry does not have a clear emotional or behavioural tendency, it merely describes the focus of the person under investigation on the surrounding person and is therefore neutral."}, "language_style": {"answer": "Tactful and warm", "rationale": "The entry is more personal and interactive when it is spoken in a gentle and inviting manner, rather than in direct statements or orders. At the same time, it does not use strong adjectives or words to emphasize a certain emotion, but rather asks the person under investigation in plain language, which is euphemism."}, "time_dimension": {"answer": "Present", "rationale": "The keyword \"generally\" denotes a regular, customary act, and \"care\" adopts the general current, meaning that the act has not only occurred in the past but may still be ongoing. The entry is therefore to ask the testee about the current behaviour or mentality."}, "sentence_type": "Exclamatory sentence", "pos": {"nouns": {"person": 1}, "verbs": {"Attention": 1}, "numerals": {"57": 1}, "pronouns": {"I": 1}}}, {"content": "58 cares about people's eyes.", "word_count": 6, "label": "", "sentence_structure": "Subject鈥搗erb鈥揷omplement", "subjective_objective": {"answer": "Subjective", "rationale": "Because \"care for others\" is a subjective feeling or attitude that reflects the patient's level of awareness of what others think of themselves."}, "sentence_sentiment": {"answer": "Neutral", "rationale": "Because the entry does not have any emotional color."}, "language_style": {"answer": "Tactful and warm", "rationale": "The entry uses a more euphemistic term of \"care\" rather than a direct statement of whether the other party is very concerned about the other person's eyes, thus avoiding too much blame or criticism, more humanistic and respectful. At the same time, the whole sentence is more friendly and friendly."}, "time_dimension": {"answer": "Present", "rationale": "The entry \"Care\" indicates the current level of concern and concern, and \"Look at the other person\" refers to the eyes or views of others, judging by the time dimension of time."}, "sentence_type": "Imperative sentence", "pos": {"nouns": {"I care": 1, "others": 1, "Eyes": 1}, "adverbs": {"58": 1}}}, {"content": "I think people despise themselves.", "word_count": 8, "label": "", "sentence_structure": "Subject鈥搗erb鈥揷omplement", "subjective_objective": {"answer": "Subjective", "rationale": "Because \"the feeling\" represents the patient's own subjective feelings and thoughts."}, "sentence_sentiment": {"answer": "Negative", "rationale": "Because the feeling of being despised is often considered negative and may give rise to negative feelings of inferiority, anxiety, etc."}, "language_style": {"answer": "Tactful and warm", "rationale": "The entry is presented in a moderate and understanding way, rather than directly accusing or judging the other, which makes it easier for the interviewees to share their real thoughts and feelings."}, "time_dimension": {"answer": "Present", "rationale": ""}, "sentence_type": "Imperative sentence", "pos": {"nouns": {"59": 1, "others": 1, "self": 1}, "verbs": {"I think": 1, "Disgrace": 1}, "numerals": {"59": 1}, "pronouns": {"he": 1}}}, {"content": "60 is vulnerable to destruction.", "word_count": 6, "label": "", "sentence_structure": "Subject鈥揷opula鈥損redicative", "subjective_objective": {"answer": "Subjective", "rationale": "Because \"float-prone\" is a description of personal feelings and experiences that require patient self-evaluation and reflection."}, "sentence_sentiment": {"answer": "Negative", "rationale": "Because \"float is broken\" is a negative emotional state."}, "language_style": {"answer": "Calm and direct", "rationale": "The entry directly indicates the subject of the question, namely, whether the person surveyed is vulnerable to emotional disruption, is not using excessive adoration or emotional colours, and is speaking more objectively and calmly."}, "time_dimension": {"answer": "Present", "rationale": "The keyword \"Emperability of Emotions\" indicates that the entry describes the current state or orientation of the interviewee and does not address the temporal dimensions of the past or the future."}, "sentence_type": "Imperative sentence", "pos": {"nouns": {"Emotion": 1, "Destruction": 1}, "verbs": {"Easy to be subjected to": 1, "Destruction": 1}, "adjectives": {"60": 1}, "numerals": {"60": 1}}}, {"content": "So far, do you feel like you have problems with your health?", "word_count": 18, "label": "", "sentence_structure": "Subject鈥搗erb鈥搊bject", "subjective_objective": {"answer": "Subjective", "rationale": "The problem is a subjective one, since it concerns the patient ' s feelings and perception of his or her health status."}, "sentence_sentiment": {"answer": "Negative", "rationale": "Because the word \"problem\" in the entry conveys negative emotions and attitudes."}, "language_style": {"answer": "Calm and direct", "rationale": "The entry is based on a direct inquiry into the health of the person interviewed, without the use of excessive rhetorical techniques or emotional rendering. At the same time, the tone is more objective, and the question is not posed explicitly and directly, in a euphemism or in a tacit manner, so that the language style of the purpose of the article can be judged as being calm and straightforward."}, "time_dimension": {"answer": "Past", "rationale": "In conclusion, the answer to this question is \"the past.\""}, "sentence_type": "Interrogative sentence", "pos": {"nouns": {"Self": 1, "Health": 1, "Problem": 1}, "verbs": {"I feel": 1, "have": 1}, "adverbs": {"To date": 1}, "numerals": {"None": 1}, "pronouns": {"you": 1}}}, {"content": "Ever feel like there's a problem with mental health?", "word_count": 14, "label": "", "sentence_structure": "Subject鈥損redicate", "subjective_objective": {"answer": "Subjective", "rationale": "This is a matter of the patient ' s mental state and feelings and of subjective experience and perception."}, "sentence_sentiment": {"answer": "Negative", "rationale": "Because the phrase \"sanitary problems\" in the entry is negative."}, "language_style": {"answer": "Tactful and warm", "rationale": "The entry uses a moderate, leading language, without questioning or ordering directly, and is more personal and interactive, making the person surveyed more willing to answer the question. At the same time, the term \"psychological aspects\" is more professional, but not too complex to understand, and is understood as a question of mental health."}, "time_dimension": {"answer": "Past", "rationale": "The entry asks if the person tested had experienced problems with mental health at a certain point in time and therefore relies on the past."}, "sentence_type": "Interrogative sentence", "pos": {"nouns": {"Problem": 1, "Mental health": 1, "In terms of": 1}, "verbs": {"I think": 1, "have": 1}, "adverbs": {"Once": 1}, "pronouns": {"you": 1}}}, {"content": "So far, have you ever been given mental health counselling and treatment?", "word_count": 21, "label": "", "sentence_structure": "Subject鈥搗erb鈥搊bject", "subjective_objective": {"answer": "Subjective", "rationale": "Because \"mental counselling and treatment\" is a patient's personal experience and behaviour and is subjective."}, "sentence_sentiment": {"answer": "Neutral", "rationale": ""}, "language_style": {"answer": "Calm and direct", "rationale": "The entry is asked in a straightforward, concise manner, without too much emotional qualifier or euphemism, and in a more calm and objective manner."}, "time_dimension": {"answer": "Past", "rationale": "\"To date\" indicates that the question was one of questioning the testee's behaviour before a certain point in time in the past and therefore is judged to be the past."}, "sentence_type": "Interrogative sentence", "pos": {"nouns": {"To date": 1, "Mental health": 1, "Consultants": 1, "Treatment": 1}, "verbs": {"Yes": 1}, "adverbs": {"Once": 1}, "numerals": {"One": 2}, "pronouns": {"you": 1}}}]}

{"questionnaire_title": "Makjavili Method", "questionnaire_category": "Behavioral questionnaire", "item_count": 20, "emotion_shift_overall": "From positive to negative", "language_style_overall": "Neutral", "items": [{"content": "1. No one shall be told of the true reasons for what you have done, unless you have another purpose.", "word_count": 24, "label": "", "sentence_structure": "Subject鈥搗erb鈥搊bject", "subjective_objective": {"answer": "Objective", "rationale": "Because it is objective to tell someone whether the real cause is an objective act, and whether there are other subjective ideas aimed at the patient.鈥?The purpose of the questionnaire is to judge that the entry is to examine whether people's behaviour and decision-making are driven by their subjective intent, which is not directly visible and needs to be inferred from the actual actions of people. Thus, the entry is viewed from an objective perspective."}, "sentence_sentiment": {"answer": "Negative", "rationale": "Because the expression \"never tell anyone\" implies an emotional ambivalence of mistrust, suspicion and precaution, which is negative. And the latter condition \"unless you have another purpose\" further emphasizes this emotional ambivalence."}, "language_style": {"answer": "Calm and direct", "rationale": "The reference to the questionnaire is based on a clear and straightforward language, with no excessive emotional rendering or rhetorical techniques, and is more objective in tone. At the same time, the entry conveys the message in a command language, emphasizing that no one must be told the true reasons for what you do and setting out conditions for disclosure unless there is another purpose. This language style is more common in conveying important messages and guiding behaviour, and is more relevant and practical."}, "time_dimension": {"answer": "Present", "rationale": "Now the entry expresses a recommendation or rule and uses the current state of affairs, implying that it is a current act or state. It does not address the time dimension of the past or the future."}, "sentence_type": "Interrogative sentence", "pos": {"nouns": {"person": 2, "The real reason": 1, "Purpose": 1}, "verbs": {"Tell": 1, "Do it": 1, "Never": 1, "have": 1, "Other": 1}, "adverbs": {"Never": 1}, "numerals": {"2": 1}, "pronouns": {"you": 3, "Something": 1, "You do it": 1}}}, {"content": "2. The best way to live with people is to say what they want to know.", "word_count": 19, "label": "", "sentence_structure": "Subject鈥搗erb鈥搊bject", "subjective_objective": {"answer": "Subjective", "rationale": "Because \"the best way\" and \"the best thing to hear\" are patients' perceptions and expectations of interpersonal interaction, it's a subjective attitude to examine patients."}, "sentence_sentiment": {"answer": "Neutral", "rationale": "Since the purpose of the article is expressed without any apparent emotional tendency."}, "language_style": {"answer": "Tactful and warm", "rationale": "The language style of the entry is more euphemistic and gentle, and it does not direct or instruct the other party about what to do, but rather directs the other party to think in a question-and-answer manner. At the same time, positive language, such as \"best way\", is used in the sentence, which helps to stimulate the motivation and participation of the person under investigation."}, "time_dimension": {"answer": "Present", "rationale": ""}, "sentence_type": "Interrogative sentence", "pos": {"nouns": {"Methodology": 1, "person": 1, "Yes": 1}, "verbs": {"Get along": 1, "Say it": 1, "Yes": 2, "be": 1}, "adverbs": {"I'd better": 1}, "pronouns": {"they": 1}}}, {"content": "3. Action can only be taken if there is a conviction that it is ethical.", "word_count": 15, "label": "", "sentence_structure": "Subject鈥搗erb鈥搊bject", "subjective_objective": {"answer": "Objective", "rationale": "Since ethical criteria are an objective criterion of judgement, and action is based on this objective criterion of judgement, this is considered from an objective perspective."}, "sentence_sentiment": {"answer": "Neutral", "rationale": "Because the entry does not have any emotional color."}, "language_style": {"answer": "Calm and direct", "rationale": "The wording of the article is simple and clear, and not used too much rhetorical or emotional rendering. 2. The entry is a clear statement that opens up a dialogue, rather than leading the other party to answer in a question or recommendation. 3. The terms used in the entry are more neutral, such as \"convincing\", \"moral\", \"acting\" and \"doing\" , which are not overly emotional in the context of the language. 4. The language style of the entire sentence is more calm and objective, and there is no excessive personal emotion or subjective tendency."}, "time_dimension": {"answer": "Present", "rationale": "The entry describes an attitude or belief that it is necessary to ensure that it meets ethical standards before action is taken. This attitude is present and may have an impact on future decision-making and behaviour."}, "sentence_type": "Imperative sentence", "pos": {"nouns": {"Ethics": 1, "Action": 1}, "verbs": {"Sure": 1, "Match": 1, "Adoption": 1}, "adverbs": {"Only": 1}}}, {"content": "4. Most people are basically good and kind.", "word_count": 13, "label": "", "sentence_structure": "Subject鈥揷opula鈥損redicative", "subjective_objective": {"answer": "Subjective", "rationale": "Because \"most people are basically good and good\" is an opinion or belief, not an objective fact. This view is based on individual values, beliefs, etc., and is therefore subjective in its approach."}, "sentence_sentiment": {"answer": "Positive", "rationale": "Because it expresses a positive view of people, who think that most people are good and kind."}, "language_style": {"answer": "Tactful and warm", "rationale": "This entry is more encouraging and motivated by a moderate and positive tone of dialogue, a positive perception of people and a lack of language that is too critical or negative."}, "time_dimension": {"answer": "Present", "rationale": ""}, "sentence_type": "Affirmative declarative sentence", "pos": {"nouns": {"person": 1, "Okay": 1, "Yes": 1}, "verbs": {"Basic": 1, "be": 1}, "adjectives": {"More": 1, "Large": 1}, "adverbs": {"Both": 1, "and": 1, "Basically": 1}, "numerals": {"4": 1, "1": 3}, "pronouns": {"Here": 1}}}, {"content": "5. The safest principle of occultism is to assume that all people have an evil side and that Kim will emerge if he has the opportunity to do so.", "word_count": 36, "label": "", "sentence_structure": "Subject鈥搗erb鈥搊bject", "subjective_objective": {"answer": "Subjective", "rationale": "Because it is a subjective idea or attitude."}, "sentence_sentiment": {"answer": "Negative", "rationale": "Because the idea that \"assuming that everyone has an evil side\" in the entry is negative and emotional, implying widespread mistrust and hostility."}, "language_style": {"answer": "Calm and direct", "rationale": "The entry is based on a direct statement of a view, which is not based on excessive rhetoric or emotional rendering. At the same time, the article is more serious and objective, and does not reveal a warm emotional ambience. It is therefore considered to be a calm and straightforward language."}, "time_dimension": {"answer": "Future", "rationale": "The content of the article should therefore be classified as future."}, "sentence_type": "Affirmative declarative sentence", "pos": {"nouns": {"The principle of occultity": 1, "Clear": 1, "person": 2, "Evil": 2, "Opportunities": 1, "One side": 1, "Yes": 2}, "verbs": {"be": 1, "Assumptions": 1, "have": 2, "Come out": 1}, "adjectives": {"Safest": 1, "Average": 1, "The evil one": 1}, "numerals": {"5": 1}, "pronouns": {"All": 1, "People": 1}}}, {"content": "6. Integrity is the best policy in all circumstances.", "word_count": 12, "label": "", "sentence_structure": "Subject鈥揷opula鈥損redicative", "subjective_objective": {"answer": "Subjective", "rationale": "Because honesty is a moral norm of the individual, a value and conviction of the individual, a subjective attitude or belief. While honesty has a positive effect on building trust and good interpersonal relations, its choice depends on individual value judgements and contextual factors, and is therefore a subjective issue."}, "sentence_sentiment": {"answer": "Positive", "rationale": "Because the belief in \"Enternal is best\" in the entry is positive and advocates honesty."}, "language_style": {"answer": "Tactful and warm", "rationale": "The entry is more personal and interactive in its expression of conversation in a gentle and inviting tone, rather than in direct statements or orders. At the same time, it conveys a positive value that honesty should be maintained whenever and wherever it is. This language style is more acceptable to the interviewees and is willing to answer questions."}, "time_dimension": {"answer": "Past", "rationale": "This may have occurred, and is and will be possible, and therefore it is not possible to determine precisely the temporal dimension of the article."}, "sentence_type": "Affirmative declarative sentence", "pos": {"nouns": {"Situation": 1, "Honesty": 1, "Best practice": 1}, "verbs": {"be": 1}, "adverbs": {"Any": 1}}}, {"content": "There is no reason to lie to others.", "word_count": 11, "label": "", "sentence_structure": "Subject鈥搗erb鈥揷omplement", "subjective_objective": {"answer": "Subjective", "rationale": "Because it is a personal value and a moral standard that lies to others, it is the patient ' s subjective attitude."}, "sentence_sentiment": {"answer": "Positive", "rationale": "Because it conveys a positive value, namely honesty and respect for others, is a positive attitude and quality."}, "language_style": {"answer": "Calm and direct", "rationale": "The line clearly conveys the message of non-false in a direct statement, with a strong tone and without too much embellishment or pretence. This expression is usually used in the context of emphasis on certain principles, norms or values, with the aim of giving the person under investigation a clear understanding and acceptance of these provisions or expectations."}, "time_dimension": {"answer": "Present", "rationale": "The keyword \"no reason\" or \"false\" implies that the act is being described or considered. Moreover, \"to others\" suggests that it is a situation involving others, not the individual's inner world, which further supports the judgment of the present."}, "sentence_type": "Negative declarative sentence", "pos": {"nouns": {"Rationale": 1, "others": 1, "Lies": 1}, "verbs": {"have": 1, "Scram": 1}, "adjectives": {"Any": 1}, "adverbs": {"No, I'm not": 1}}}, {"content": "8. Generally, people are not able to work hard without pressure.", "word_count": 18, "label": "", "sentence_structure": "Subject鈥損redicate", "subjective_objective": {"answer": "Subjective", "rationale": "Because this sentence expresses the author ' s subjective view that people do not work hard when there is no pressure."}, "sentence_sentiment": {"answer": "Negative", "rationale": "Because the expression \"people cannot work hard without pressure\" has a negative attitude that people are motivated to work only when they are under pressure."}, "language_style": {"answer": "Calm and direct", "rationale": "The entry expresses the views that the investigators want to understand in a concise and clear language, which is more objective, is not accompanied by too many emotional or rhetorical techniques and is a more calm and direct expression."}, "time_dimension": {"answer": "Present", "rationale": "The entry describes a general phenomenon or view, does not clearly point at time and therefore is judged to be the present."}, "sentence_type": "Negative declarative sentence", "pos": {"nouns": {"Pressure": 1, "Work": 1}, "verbs": {"Yes": 1, "Try": 1, "Work": 1}, "adjectives": {"General": 1, "No, I'm not": 1}, "numerals": {"8": 1}, "pronouns": {"People": 1}}}, {"content": "9. In sum, the status is inferior and honesty superior to the status and deception.", "word_count": 20, "label": "", "sentence_structure": "Subject鈥搗erb鈥搊bject", "subjective_objective": {"answer": "Subjective", "rationale": "Because expressions such as \"low status and honesty\" and \"high standing and deception\" are the expression of subjective views, views and values of patients."}, "sentence_sentiment": {"answer": "Positive", "rationale": "Because the entry emphasizes \"lower status and honesty\" over \"higher status and deception\", which is a positive value."}, "language_style": {"answer": "Calm and direct", "rationale": "The entry expresses the author鈥檚 view in a concise and clear language that inferiority and honesty are more important than superiority and deception. This expression is straightforward and does not have too much rhetoric or emotional overtones."}, "time_dimension": {"answer": "Present", "rationale": "The entry now expresses a human value or belief that inferiority and honesty are more important than superiority and deception. This value or belief can influence individual decision-making and behaviour and may be reflected in the interaction with others. Thus, the entry can be considered a current state or attitude."}, "sentence_type": "Affirmative declarative sentence", "pos": {"nouns": {"Anyway": 1, "Status": 2, "Honesty": 1, "Highlight": 1, "- Liar": 1}, "verbs": {"Better than": 1}, "adverbs": {"Anyway": 1}, "numerals": {"9": 1}}}, {"content": "10. When you ask for help, it is better to say that you are asking for real reasons rather than making them up for more convincing ones.", "word_count": 31, "label": "", "sentence_structure": "Subject鈥搗erb鈥搊bject", "subjective_objective": {"answer": "Subjective", "rationale": "The choice of real reasons is based on the patient ' s personal will and decision-making."}, "sentence_sentiment": {"answer": "Neutral", "rationale": "Because the entry does not have any obvious emotional colour."}, "language_style": {"answer": "Calm and direct", "rationale": "The entry expresses the view, through a clear statement, that when seeking help from others, you should express your needs in good faith, rather than fabricating more compelling reasons. This language style does not have too much emotional or rhetorical style, but rather conveys the message in an objective and direct manner."}, "time_dimension": {"answer": "Present", "rationale": "The entry will contain suggestions and expectations for the future, and the key words are \"best\" and \"good reason, not more compelling reason,\" all pointing to situations that have not yet occurred and that may arise in the future. So I think it is about the future."}, "sentence_type": "Imperative sentence", "pos": {"nouns": {"you": 1, "others": 1, "Rationale": 2, "Really": 1, "More convincing": 1}, "verbs": {"Please": 1, "Help": 1, "I'll tell you what": 1, "Fiction": 1, "That's convincing": 1}, "adjectives": {"More": 1, "Real": 1}, "adverbs": {"I'd better": 1}, "numerals": {"10": 1, "2": 1}, "classifiers": {"individual": 1}, "pronouns": {"Here": 1}}}, {"content": "11. Most of the world ' s successful people live in a clean and moral life.", "word_count": 19, "label": "", "sentence_structure": "Subject鈥搗erb鈥揷omplement", "subjective_objective": {"answer": "Subjective", "rationale": "Because the descriptions of \"most\" and \"clean\" and \"moral\" are based on the subjective views and values of researchers."}, "sentence_sentiment": {"answer": "Positive", "rationale": "Because the entry expresses the moral qualities that successful people should have, it is a positive value."}, "language_style": {"answer": "Tactful and warm", "rationale": "It conveys a positive view to the interviewees in a positive way, and it is easy to use words and accept. It also gives people a space for reflection, giving them the opportunity to agree with or contradict this view."}, "time_dimension": {"answer": "Future", "rationale": "The entry describes a scenario or expectation that \"successful people in the world\" will lead \"a clean and moral life\". It is a vision of the future, and therefore can be judged as future behaviour."}, "sentence_type": "Affirmative declarative sentence", "pos": {"nouns": {"World": 1, "person": 1, "Life": 1, "Clean": 1, "Ethics": 1}, "verbs": {"- Yes": 1, "Success": 1}, "numerals": {"Mostly": 1, "11": 1}}}, {"content": "12. Full trust in others is tantamount to self-indulging.", "word_count": 12, "label": "", "sentence_structure": "Subject鈥揷opula鈥損redicative", "subjective_objective": {"answer": "Subjective", "rationale": "Because each person ' s opinions and attitude are different on the question of whether there should be complete trust in others, and this is viewed from a subjective perspective."}, "sentence_sentiment": {"answer": "Negative", "rationale": "Because the act of \"full trust\" in the entry is considered to be the cause of \"self-suffering\" and is therefore negative."}, "language_style": {"answer": "Tactful and warm", "rationale": "The language style of the entry is more euphemistic, using a \"reverse-speech\" approach to express the view, i.e., to express the positive view in a negative form, so that the interlocutor does not feel too direct or stingy in reading. At the same time, the expression \"self-sorture\" is more implicit, avoiding the possibility of direct attack or criticism, making it easier for readers to accept and resonate."}, "time_dimension": {"answer": "Present", "rationale": "The entry expresses the view or belief that \"a complete trust is tantamount to self-indulging\". It does not have a clear time reference, so it can be judged as now."}, "sentence_type": "Imperative sentence", "pos": {"nouns": {"Questionnaire": 1, "Entry": 1, "person": 1, "It's bitter": 1}, "verbs": {"I trust you completely": 1, "Equal": 1, "Self-provement": 1}, "adverbs": {"very": 2}, "numerals": {"12": 1}, "pronouns": {"others": 1, "self": 1}}}, {"content": "13. The greatest difference between prisoners and the general population is that they are caught for being foolish.", "word_count": 23, "label": "", "sentence_structure": "Subject鈥搗erb鈥搊bject", "subjective_objective": {"answer": "Subjective", "rationale": "This is a subjective view of the prisoner and the general population, since the entry in the questionnaire highlights the fact that the incompetence of the prisoner is the reason for their arrest."}, "sentence_sentiment": {"answer": "Negative", "rationale": "The term \"inmate\" has a derogatory and discriminatory meaning, and the term \"wilder\" has a derogatory connotation, so the emotional orientation of the purpose of the article is negative."}, "language_style": {"answer": "Calm and direct", "rationale": "The entry is more straightforward in its language style, does not use euphemisms, metaphors, etc., or expresses too much emotional. It simply and succinctly expresses that the greatest difference between the prisoner and the general population is criminal behaviour, which is caught in the hands of their stupidity and is more calm and objective."}, "time_dimension": {"answer": "Present", "rationale": "The entry now describes the current situation and state, referring to specific persons or things such as \"inmate\", \"general person\", \"stupid person\" and not to past or future acts or events. The time dimension of this questionnaire is therefore now."}, "sentence_type": "Affirmative declarative sentence", "pos": {"nouns": {"Inmate": 1, "Normal": 1, "Maximum difference": 1, "Stupid": 1, "Hold on": 1}, "verbs": {"It's": 1, "By": 2}, "adjectives": {"Stupid": 1}, "numerals": {"13": 1, "One": 1}, "classifiers": {"individual": 1}}}, {"content": "14. Most people are courageous.", "word_count": 7, "label": "", "sentence_structure": "Subject鈥揷opula鈥損redicative", "subjective_objective": {"answer": "Objective", "rationale": "Because \"most people are brave\" is an objective statement of fact, not involving patients' subjective attitudes or feelings."}, "sentence_sentiment": {"answer": "Neutral", "rationale": "As the entry describes a fact or general observation, it does not contain any emotion, attitude or evaluation and is therefore neutral."}, "language_style": {"answer": "Tactful and warm", "rationale": "The entry conveys to the interviewees a positive view in a moderate and positive tone, that most people are brave and do not speak directly critical or negative. This expression is more friendly and acceptable."}, "time_dimension": {"answer": "Present", "rationale": "The entry in the past described a general phenomenon or view, with no clear time limits, and could therefore be considered as a past act or mentality."}, "sentence_type": "Affirmative declarative sentence", "pos": {"nouns": {"Most people": 1, "Brave": 1}, "verbs": {"be": 1}, "numerals": {"14": 1}}}, {"content": "It was wise to flatter the big people.", "word_count": 11, "label": "", "sentence_structure": "Subject鈥揷opula鈥損redicative", "subjective_objective": {"answer": "Subjective", "rationale": "Because this statement reflects the subjective view or belief of the interviewees that flattering the big people is a good strategy."}, "sentence_sentiment": {"answer": "Negative", "rationale": "Because the act of \"prospering the big man\" in the entry is a negative value-oriented act."}, "language_style": {"answer": "Tactful and warm", "rationale": "According to the language style, the article is expressed more directly and with a critical tone, and in a more cynical tone. Conversely, if the sentence is spoken in a gentle and inviting manner, its language style is more euphemismal."}, "time_dimension": {"answer": "Present", "rationale": ""}, "sentence_type": "Affirmative declarative sentence", "pos": {"nouns": {"Pity": 1, "Big guy": 1, "Smart": 1, "Practice": 1}, "adverbs": {"be": 1}, "numerals": {"15": 1}}}, {"content": "16. Excellence in all areas is possible.", "word_count": 13, "label": "", "sentence_structure": "Subject鈥揷opula鈥損redicative", "subjective_objective": {"answer": "Subjective", "rationale": "Because this expression expresses a patient ' s attitude or belief that it is possible to achieve excellence in all respects, it is a subjective examination."}, "sentence_sentiment": {"answer": "Positive", "rationale": "Because the expression \"good in every aspect is possible\" in this entry is positive in attitude and conviction."}, "language_style": {"answer": "Calm and direct", "rationale": "The entry presents the idea in a more straightforward way that the possibility of being superior in every respect exists. This expression is not too emotional or rhetorical, and is more objective and rational."}, "time_dimension": {"answer": "Present", "rationale": "The keyword \"possible to reach\" means that it is a judgement about the present, and the entire sentence does not address the past or future time dimension, so it can be determined that the entry is asking the testee's opinion as to whether he or she is likely to be good in all respects in the future."}, "sentence_type": "Affirmative declarative sentence", "pos": {"nouns": {"In terms of": 1, "Excellent": 1}, "verbs": {"be": 1, "Achieved": 1}, "numerals": {"16": 1}}}, {"content": "The phrase 鈥渢he birth of a fool every minute鈥?was incorrect.", "word_count": 17, "label": "", "sentence_structure": "Subject鈥揷opula鈥損redicative", "subjective_objective": {"answer": "Objective", "rationale": "This is an assessment of objective facts and does not involve the individual feelings or attitudes of the patient."}, "sentence_sentiment": {"answer": "Neutral", "rationale": "Because the entry merely presented a statement, it did not express the author ' s emotional preference."}, "language_style": {"answer": "Calm and direct", "rationale": "The language style of the entry is more objective, direct and logical, and there is no excessive emotional or rhetorical approach that gives the impression of rigour and professionalism."}, "time_dimension": {"answer": "Past", "rationale": "The entry is now, as the sentence does not contain a description of the past or future."}, "sentence_type": "Negative declarative sentence", "pos": {"nouns": {"min": 1, "Idiot": 2, "Birth": 1}, "verbs": {"have": 1, "be": 1, "to": 1}, "adjectives": {"Every": 1, "Both": 1, "It's not right": 1}, "numerals": {"17": 1}, "pronouns": {"This one": 1}}}, {"content": "Success is difficult without shortcuts.", "word_count": 9, "label": "", "sentence_structure": "Subject鈥揷opula鈥損redicative", "subjective_objective": {"answer": "Subjective", "rationale": "Because the short cut is an individual ' s decision and attitude, this is the patient ' s subjective view."}, "sentence_sentiment": {"answer": "Negative", "rationale": "Because the \"no shortcut\" act is negative."}, "language_style": {"answer": "Calm and direct", "rationale": "The language style of the entry is calm and straightforward. It clearly expresses the view that \"it is difficult to succeed without a shortcut\" and is not used any euphemism or emotional rendering, which is more balanced and objective. It is more appropriate to use it in a formal and serious context to convey clear messages and ideas."}, "time_dimension": {"answer": "Future", "rationale": "The entry expresses expectations and visions for the future, and the phrase \"It is difficult to succeed without shortcuts\" implies that efforts are needed to achieve the goal, and therefore the temporal dimension is judged to be the future."}, "sentence_type": "Affirmative declarative sentence", "pos": {"nouns": {"Questionnaire Entry": 1, "Shortcut": 1, "Success": 1}, "verbs": {"Let's go": 1, "Hard": 1, "Access": 1}, "adjectives": {"not": 1}, "adverbs": {"very": 1}}}, {"content": "19. People suffering from incurable diseases should have the right to choose between painless and unsufferable deaths.", "word_count": 20, "label": "", "sentence_structure": "Subject鈥搗erb鈥搊bject", "subjective_objective": {"answer": "Subjective", "rationale": "The perspective in the title is one of personal ethics and values and is therefore subjective."}, "sentence_sentiment": {"answer": "Negative", "rationale": "Because the idea of the \"right to choose death without pain\" in the entry relates to the value of life and the perception of life and death, which may trigger some negative emotional reactions."}, "language_style": {"answer": "Calm and direct", "rationale": "The entry expresses a view in the language of the statement, which is not refined by euphemism or emotional acrimonym, and appears to be relatively cold and straightforward."}, "time_dimension": {"answer": "Past", "rationale": ""}, "sentence_type": "Affirmative declarative sentence", "pos": {"nouns": {"19": 1, "person": 1, "Pathological": 1, "Rights": 1, "Death": 1}, "verbs": {"Patient": 1, "Reactions": 1, "have": 1, "Selection": 1, "No pain": 1}, "adjectives": {"It's not good": 1, "鈾?And the 鈾?: 1, "Pain": 1}, "numerals": {"19": 1}, "pronouns": {"Patient": 1, "person": 1, "鈾?And the 鈾?: 1, "Death": 1}}}, {"content": "20. Most people can easily forget the death of their father, but not the loss of property.", "word_count": 24, "label": "", "sentence_structure": "Subject鈥搗erb鈥揷omplement", "subjective_objective": {"answer": "Objective", "rationale": "Because most people can easily forget the death of their father, but not the loss of property, it is a factual description, looking at objective circumstances."}, "sentence_sentiment": {"answer": "Negative", "rationale": "Because the \"lost property\" act in the entry is negative."}, "language_style": {"answer": "Tactful and warm", "rationale": "The language style of the entry is more euphemistic and warm. Although it talks about topics as heavy as death and loss, it is not too straightforward or painful. It makes the questionnaire more acceptable and understandable and may encourage more people to participate in the survey."}, "time_dimension": {"answer": "Present", "rationale": "The content of this questionnaire entry relates to the prevailing behaviour and mindset of people today and can therefore be judged as now."}, "sentence_type": "Affirmative declarative sentence", "pos": {"nouns": {"person": 1, "Father": 1, "Death": 1, "Property": 1, "Losses": 1}, "verbs": {"Yes": 1, "Forget it": 2, "Easy": 1, "Remember": 1}, "adjectives": {"Most": 1, "Easy": 1, "It's not easy": 1}, "numerals": {"20": 1}}}]}

{"questionnaire_title": "A philosophy of humanity.", "questionnaire_category": "Mental health", "item_count": 102, "emotion_shift_overall": "From positive to negative", "language_style_overall": "Neutral", "items": [{"content": "2. Most students tell their teachers when they give them a misunderstanding (even more).", "word_count": 21, "label": "", "sentence_structure": "Subject鈥搗erb鈥搊bject", "subjective_objective": {"answer": "Objective", "rationale": "This is an objective fact, not involving subjective attitudes or ideas. The entry is therefore viewed from an objective perspective."}, "sentence_sentiment": {"answer": "Neutral", "rationale": "The entry merely describes a situation and does not express an assessment or emotional colour of the student ' s behaviour."}, "language_style": {"answer": "Tactful and warm", "rationale": "The language style of the entry is more euphemistic and sensitive to the students鈥?feelings, and the subject of the survey is expressed by the term \"most students\" and the more objective terms are used to describe the problem, such as \"mistake\", \"score\" and \"specify\" without directly accusing or criticizing the teacher. This soft language makes the interviewee more receptive and willing to answer the question."}, "time_dimension": {"answer": "Past", "rationale": "The word \"when\" indicates that the act has already occurred, and the words \"mistake\" and \"multiplier\" also occurred at some point or time in the past. The entry therefore asks about the past act."}, "sentence_type": "Affirmative declarative sentence", "pos": {"nouns": {"Students": 1, "Teacher": 1, "min": 1}, "verbs": {"When": 1, "Here": 1, "Tell": 1}, "adjectives": {"Most": 1, "Wrong": 1, "More": 1}, "adverbs": {"Even": 1}, "numerals": {"2": 1}}}, {"content": "8. If you give someone a job and let him do it himself, everyone can do it.", "word_count": 28, "label": "", "sentence_structure": "Subject鈥搗erb鈥搊bject", "subjective_objective": {"answer": "Objective", "rationale": "This statement describes a general phenomenon or result of behaviour, rather than a subjective perception or attitude of the individual."}, "sentence_sentiment": {"answer": "Positive", "rationale": "Because the phrase \"generally everyone can do this well\" in the entry is positive and indicates that the task is not difficult."}, "language_style": {"answer": "Calm and direct", "rationale": "The language style of the questionnaire entry is calm, straightforward, and there is no excessive emotional colour or rhetorical technique. It expresses a clear and concise view of the capacity of the interviewee and is more objective in tone."}, "time_dimension": {"answer": "Present", "rationale": "If \"if\" indicates the scenario, \"place\" and \"do\" acts occur or will occur, so it can be judged that the item is being questioned about the current attitude or experience of the person being tested."}, "sentence_type": "Affirmative declarative sentence", "pos": {"nouns": {"you": 1, "person": 1, "Work": 1, "Item": 1}, "verbs": {"Here": 1, "Set": 1, "Do it": 2, "Jean": 1}, "adjectives": {"General": 1, "Both": 1}, "adverbs": {"If": 1, "and": 1}, "classifiers": {"Here": 1}, "pronouns": {"Someone": 1}}}, {"content": "14. People often tell the truth even when they know that it is more advantageous to withdraw a lie.", "word_count": 17, "label": "", "sentence_structure": "Subject鈥搗erb鈥搊bject", "subjective_objective": {"answer": "Subjective", "rationale": "Because it is a subjective view and understanding of a person, that is, \"the people think it's better to speak the truth\", rather than an objective description or fact."}, "sentence_sentiment": {"answer": "Neutral", "rationale": "Because the entry does not have any emotional color."}, "language_style": {"answer": "Tactful and warm", "rationale": "The entry is more euphemistic, with more objective and neutral descriptions, and not too violent expressions or emotional terms. It also presents an interesting point of view and stimulates the reader鈥檚 reflection and discussion in a question-and-answer manner."}, "time_dimension": {"answer": "Present", "rationale": "\"14.\" indicates that it is describing a general phenomenon or situation, and that \"often\" is a pattern of habitual and customary behaviour, and these keywords point to the action or state of action being undertaken. The entry is therefore a description of the current situation."}, "sentence_type": "Affirmative declarative sentence", "pos": {"nouns": {"People": 1, "Lie": 1, "The truth": 1}, "verbs": {"Yes": 1, "Good": 1, "Say it": 2}, "adverbs": {"Yeah": 1, "Often": 1}, "numerals": {"14": 1}}}, {"content": "Most students are not cheating on the examination.", "word_count": 10, "label": "", "sentence_structure": "Subject鈥搗erb鈥揷omplement", "subjective_objective": {"answer": "Objective", "rationale": "Since the test was fraudulent and was an objective result of behaviour, it was not related to the students ' subjective attitude."}, "sentence_sentiment": {"answer": "Neutral", "rationale": "Because the entry does not have any emotional color."}, "language_style": {"answer": "Calm and direct", "rationale": "The entry expresses the view that most students do not cheat on the examination in a concise and clear language, without too much accommodative or emotional verb, and with a more objective and straightforward tone."}, "time_dimension": {"answer": "Present", "rationale": "The entry is a description and judgement of the behaviour of most students in the examination today, namely, \"most students do not cheat at the examination\". The time dimension should therefore be judged now."}, "sentence_type": "Affirmative declarative sentence", "pos": {"nouns": {"Students": 1, "Examinations": 1, "Cheating": 1}, "verbs": {"not": 1}, "adjectives": {"Most": 1}, "numerals": {"20": 1}}}, {"content": "26. Most people are basically honest.", "word_count": 10, "label": "", "sentence_structure": "Subject鈥揷opula鈥損redicative", "subjective_objective": {"answer": "Subjective", "rationale": "Because \"honest\" is a moral concept, an individual's attitude or perception, it is subjective."}, "sentence_sentiment": {"answer": "Neutral", "rationale": "Since the entry is a factual description, it is not emotional and is therefore neutral."}, "language_style": {"answer": "Tactful and warm", "rationale": "The entry is more personal and interactive when it is presented in a soft, invitation-like tone, rather than in direct statements or orders. At the same time, it avoids using strong assertions or negative assessments, but instead raises questions in a more neutral manner, making them more acceptable to respondents and willing to answer."}, "time_dimension": {"answer": "Present", "rationale": ""}, "sentence_type": "Affirmative declarative sentence", "pos": {"nouns": {"Most people": 1, "Honesty": 1}, "verbs": {"be": 1}, "adjectives": {"Basic": 1}, "adverbs": {"Go, go, go": 1}, "numerals": {"26": 1, "Basically": 1}}}, {"content": "62. If you trust others, they almost always trust you.", "word_count": 20, "label": "", "sentence_structure": "Subject鈥損redicate", "subjective_objective": {"answer": "Subjective", "rationale": "Because trust is a subjective feeling, and \"Does anyone trust you the same way\" is based on subjective judgment, the purpose of this questionnaire is subjective."}, "sentence_sentiment": {"answer": "Neutral", "rationale": "As the entry is a factual description, it does not involve any emotion or attitude."}, "language_style": {"answer": "Tactful and warm", "rationale": "The entry is more personal and interactive when it is spoken in a gentle and inviting manner, rather than in direct statements or orders. At the same time, it uses the conditional phrase \"if ... would...\", making the phrase more fluid, understandable and more accessible to respondents."}, "time_dimension": {"answer": "Past", "rationale": "It is a general statement describing the characteristics of a relationship of trust that can be applied in different time contexts."}, "sentence_type": "Affirmative declarative sentence", "pos": {"nouns": {"person": 2, "you": 3, "Questionnaire": 1, "Entry": 1}, "verbs": {"Yes": 2, "Trust": 2, "Give": 1}, "adverbs": {"Almost": 1, "Both": 1, "Same thing": 1}, "numerals": {"62": 1}, "pronouns": {"you": 3}}}, {"content": "86. Most people live in a clean and decent life.", "word_count": 12, "label": "", "sentence_structure": "Subject鈥搗erb鈥揷omplement", "subjective_objective": {"answer": "Objective", "rationale": "Because this is a description of the majority and an objective fact."}, "sentence_sentiment": {"answer": "Positive", "rationale": "Because the words \"clean, decent\" in the entries convey positive qualities and behaviours."}, "language_style": {"answer": "Calm and direct", "rationale": "The entry is simple and plain, and is not used too much rhetorical to express it clearly and directly, and therefore is in the \"cool and direct\" language style."}, "time_dimension": {"answer": "Present", "rationale": "The keywords \"majority\" and \"over\" indicate that this is a description of the current state, and the temporal dimension is now."}, "sentence_type": "Affirmative declarative sentence", "pos": {"nouns": {"person": 1, "Life": 1}, "verbs": {"- Yes": 1, "Clean": 1, "Normal": 1}, "adjectives": {"Most": 1}, "adverbs": {"Most": 1}, "numerals": {"86": 1}}}, {"content": "32. Everyone claimed to have an ethical standard of honesty and ethics, but few people complied with those standards once they had happened.", "word_count": 40, "label": "", "sentence_structure": "Subject鈥搗erb鈥搊bject", "subjective_objective": {"answer": "Subjective", "rationale": "Because the sentence describes an act (a statement that ethical standards are being observed but are in fact rarely observed), it is a subjective view."}, "sentence_sentiment": {"answer": "Negative", "rationale": "Because the entries describe the fact that people are often unable to observe the ethical norms they claim when they encounter events, which is a negative behaviour or attitude."}, "language_style": {"answer": "Calm and direct", "rationale": "The language style of the entry is calm. It is a direct description of a widespread phenomenon, and does not include too many rhetorical or emotional expressions to express a view. It makes it easier for readers to understand and accept their views."}, "time_dimension": {"answer": "Present", "rationale": "The words \"claim,\" \"yes\", \"when in a situation\" in that sentence indicate that it is describing the current social phenomenon and therefore judging it as now."}, "sentence_type": "Affirmative declarative sentence", "pos": {"nouns": {"Everyone": 1, "One": 1, "Honesty": 1, "Ethics": 1, "Ethical standards": 1, "Things": 1}, "verbs": {"Claim": 1, "have": 1, "An encounter": 1, "Compliance": 1, "But few people": 1, "Say it": 1}, "adjectives": {"About": 1}, "adverbs": {"鈾?Once 鈾?: 1, "But it's rare": 1}, "numerals": {"32": 1, "One": 1}, "classifiers": {"individual": 1}, "pronouns": {"self": 1}}}, {"content": "38. If you want someone else to do something right, you have to talk and watch closely.", "word_count": 24, "label": "", "sentence_structure": "Subject鈥損redicate", "subjective_objective": {"answer": "Subjective", "rationale": "Because \"must be repeatedly told and closely monitored\" is the patient's subjective view or attitude."}, "sentence_sentiment": {"answer": "Negative", "rationale": "Because the entry implies mistrust and excessive control over others and is a negative expression."}, "language_style": {"answer": "Calm and direct", "rationale": "The language style of the entry is calm and straightforward. It clearly expresses the view that guides others in their task, underlines the importance of duplication and oversight, and does not use euphemism, incitement or over-accuracy."}, "time_dimension": {"answer": "Present", "rationale": "Now the entry describes a hypothetical situation ( \"if you want someone else to do something well\"), but it does not make it clear that it has happened or will happen in the future. It is more about exploring a possible practice or strategy, and should therefore be classified as now."}, "sentence_type": "Affirmative declarative sentence", "pos": {"nouns": {"person": 1, "One thing": 1, "Answer": 1, "Oversight": 1}, "verbs": {"Hope": 1, "Do it": 1, "Repeatedly": 1, "Close": 1}, "adverbs": {"I have to": 1}, "numerals": {"38": 1}, "pronouns": {"you": 1}}}, {"content": "44. Most people would do so if they could afford to go to people ' s cinemas and certainly not be discovered.", "word_count": 27, "label": "", "sentence_structure": "Subject鈥搗erb鈥搊bject", "subjective_objective": {"answer": "Objective", "rationale": "Because it is a question of whether people are willing to go to a cinema and see movies at the risk of being caught, it is a question of fact, not of subjective attitudes or ideas. Thus, the entry is viewed from an objective perspective."}, "sentence_sentiment": {"answer": "Negative", "rationale": "Because the acts in this entry are illegal and deceptive, a negative attitude is expressed."}, "language_style": {"answer": "Tactful and warm", "rationale": "Warm and warm."}, "time_dimension": {"answer": "Past", "rationale": "The entry asks about a hypothetical situation ( \"if you can afford to go to a movie theater and you will never be found\") and whether the person interviewed would choose to do so in such a situation."}, "sentence_type": "Affirmative declarative sentence", "pos": {"nouns": {"Money": 1, "Cinema": 1, "person": 2, "Most people": 1}, "verbs": {"Flowers": 1, "In": 1, "Fuck": 1}, "adjectives": {"Sure": 1, "No, I won't": 1, "That way": 1}, "adverbs": {"If": 1, "And": 1, "Sure": 1, "No, I won't": 1}, "numerals": {"44": 1}, "classifiers": {"individual": 1}, "pronouns": {"Most people": 1}}}, {"content": "50. The honesty of most people is not genuine, but rather fear of being caught.", "word_count": 22, "label": "", "sentence_structure": "Subject鈥揷opula鈥損redicative", "subjective_objective": {"answer": "Subjective", "rationale": "This is a subjective view and explanation of the reasons for the honesty of the majority."}, "sentence_sentiment": {"answer": "Negative", "rationale": "Because the description in the entry that \"the majority is not out of heart\" is negative, implying that the lack of honesty is motivated by self-protection and avoidance of punishment, which is often perceived as negative or negative."}, "language_style": {"answer": "Tactful and warm", "rationale": ""}, "time_dimension": {"answer": "Present", "rationale": "The keyword \"majority\" , \"innocently\" and \"intimately caught\" all indicate the understanding and perception of honesty."}, "sentence_type": "Imperative sentence", "pos": {"nouns": {"Most people": 1, "Honesty": 1, "Really": 1, "they": 1, "Scared": 1, "Hold on": 1}, "verbs": {"be": 1, "鈾?Out of": 1, "Not really": 1, "Scared": 1, "By": 1, "Hold on": 1}, "adjectives": {"Really": 1}, "numerals": {"Most": 1}, "pronouns": {"they": 1}}}, {"content": "74. Most people cheat on taxes if they have the opportunity to do so.", "word_count": 16, "label": "", "sentence_structure": "Subject鈥損redicate", "subjective_objective": {"answer": "Subjective", "rationale": "Since the title deals with the will and attitude of people, namely, \"the will of the majority to cheat in tax matters\", it is a subjective question."}, "sentence_sentiment": {"answer": "Negative", "rationale": "Because the \"cheating\" act is negative."}, "language_style": {"answer": "Calm and direct", "rationale": "The language style of the entry is calm, straightforward, and there is no excessive emotional or rhetorical style, but simply a statement of a point. This expression is more consistent with the description of objective facts and the requirements of research."}, "time_dimension": {"answer": "Present", "rationale": "The sentence describes a widespread phenomenon, namely, \"the majority cheats on taxes\", and does not address the time dimension of the past or the future, and therefore judges it now."}, "sentence_type": "Affirmative declarative sentence", "pos": {"nouns": {"Opportunities": 1, "person": 1, "Taxation": 1}, "verbs": {"Yes": 1, "in": 1, "Taxes": 1, "In terms of": 1, "Cheating": 1}, "adjectives": {"Most": 1}, "adverbs": {"If": 1}, "numerals": {"74": 1}}}, {"content": "92. The modern world has committed many crimes that have never been heard before.", "word_count": 17, "label": "", "sentence_structure": "Subject鈥搗erb鈥搊bject", "subjective_objective": {"answer": "Objective", "rationale": "Because it is an objective fact whether or not a crime has been committed, modernity is subjective in nature, unlike its predecessors."}, "sentence_sentiment": {"answer": "Negative", "rationale": "Because it expresses criticism and denial of the modern, it is negative."}, "language_style": {"answer": "Calm and direct", "rationale": "The language style of the entry is relatively calm and straightforward, with no excessive emotional or rhetorical tact, but simply a brief statement of one point."}, "time_dimension": {"answer": "Present", "rationale": "The entry describes new phenomena or situations that have emerged in modern society, and the temporal dimension is now judged. \""}, "sentence_type": "Affirmative declarative sentence", "pos": {"nouns": {"Modern people": 1, "Crimes": 1}, "verbs": {"Perpetrators": 1, "Smell": 1}, "adjectives": {"Many": 1, "Former": 1, "Unsniffed": 1}, "numerals": {"92": 1}}}, {"content": "4. In today ' s complex society, most people still want to follow the principle of humanity in the Bible.", "word_count": 26, "label": "", "sentence_structure": "Subject鈥搗erb鈥搊bject", "subjective_objective": {"answer": "Subjective", "rationale": "Because the observance of the principle of humanity in the Bible is the individual belief and value of the patient, reflects their subjective attitude."}, "sentence_sentiment": {"answer": "Positive", "rationale": "Because the act and purpose of \"following the principles of humanity in the Bible\" are positive."}, "language_style": {"answer": "Tactful and warm", "rationale": "The entry uses a more euphemistic approach to express what the surveyer wants to know, namely, whether the majority of the population still adheres to the principle of humanity in the Bible in this complex society. This approach is more friendly and respects the positions and views of the respondents, which facilitates good interaction and the effectiveness of the questionnaire."}, "time_dimension": {"answer": "Present", "rationale": "The keyword \"in a complex society like this\" means a description and observation of the society today, while \"the majority still wants to follow the principle of humanity in the Bible\" shows the behaviour or attitude of the interviewee. So the entry asks about the current thinking and behaviour of the interviewee, and the time dimension is now."}, "sentence_type": "Affirmative declarative sentence", "pos": {"nouns": {"Social": 1, "person": 2, "Principles": 1, "Bible": 1}, "verbs": {"Yes": 1, "Follow": 1}, "adjectives": {"Complex": 1, "Today": 1}, "numerals": {"Most": 1, "Four": 1}, "pronouns": {"Here": 1}}}, {"content": "10. Most people would not hesitate to help people in trouble.", "word_count": 20, "label": "", "sentence_structure": "Subject鈥搗erb鈥搊bject", "subjective_objective": {"answer": "Objective", "rationale": "Because \"the majority will not hesitate to help people in trouble\" is a social phenomenon that can be observed, and can be objectively studied and validated through statistics, surveys, etc. The examination of subjective attitudes or feelings is a subjective one."}, "sentence_sentiment": {"answer": "Positive", "rationale": "Because the \"intentionally helping people in trouble\" act is positive."}, "language_style": {"answer": "Tactful and warm", "rationale": "The entry uses an invitation language style to ask respondents whether they agree with the behaviour of the majority and do not make a direct judgement or evaluation, which is more likely to resonate and respond positively to the interviewee."}, "time_dimension": {"answer": "Future", "rationale": "\"Does not hesitate to help\" is an act or mentality that looks forward to the future, so the entry can be judged to be the future. At the same time, \"persons in trouble\" is also an untimely situation that may occur in the future. The entire sentence therefore describes the possibilities of the future."}, "sentence_type": "Affirmative declarative sentence", "pos": {"nouns": {"person": 2, "Most people": 1, "Trouble": 1}, "verbs": {"Yes": 2, "Go on": 1, "Help": 1}, "adjectives": {"I'm not hesitant": 1, "I met him": 1}, "adverbs": {"Land": 2, "- On purpose": 1}, "numerals": {"10": 1}, "pronouns": {"he": 1}}}, {"content": "16. Most people are generous when given the opportunity.", "word_count": 13, "label": "", "sentence_structure": "Subject鈥損redicate", "subjective_objective": {"answer": "Subjective", "rationale": "Because \"most people are good\" is a subjective view or hypothesis that needs to be confirmed through observation and investigation."}, "sentence_sentiment": {"answer": "Positive", "rationale": "Because the \"blessing\" act in the entry is positive."}, "language_style": {"answer": "Tactful and warm", "rationale": "The language style of the entry is euphemistic. First, it is carried out in the form of a \"if there is a chance\" scenario, giving people a sense of being kind and easy to reach; second, it is also more neutral in the choice of words, and it is not too emotional or strong in tone, giving people a gentle and friendly impression."}, "time_dimension": {"answer": "Present", "rationale": ""}, "sentence_type": "Affirmative declarative sentence", "pos": {"nouns": {"Opportunities": 1, "person": 2, "Be nice and nice": 1}, "verbs": {"have": 1, "Both": 1}, "adjectives": {"More": 1, "of": 1}, "numerals": {"16": 1}, "pronouns": {"If": 1}}}, {"content": "鈥淲hatever you want others to do to yourself, you should do to others鈥? a slander that most people have taken to heart.", "word_count": 29, "label": "", "sentence_structure": "Subject鈥搗erb鈥搊bject", "subjective_objective": {"answer": "Objective", "rationale": "Because it is an objective statement describing the code of conduct followed by the majority. While this sentence may reflect the values and beliefs of the individual, it is not in itself a subjective view or feeling."}, "sentence_sentiment": {"answer": "Negative", "rationale": "Because the expression \"what should be done to others\" in the entry has a certain sense of moral norms and obligations, while \"defamation\" implies contempt or lack of respect for such a moral norm, the article is emotionally negative."}, "language_style": {"answer": "Calm and direct", "rationale": "The entry presents the point directly in a concise and clear language, without excessive rhetoric or emotional rendering, and with a more balanced and objective tone."}, "time_dimension": {"answer": "Past", "rationale": "The entry describes a norm or concept of conduct that was generally observed in the past and therefore determines the past."}, "sentence_type": "Affirmative declarative sentence", "pos": {"nouns": {"Questionnaire Entry": 1, "person": 2, "others": 2, "self": 2, "Stigma": 1}, "verbs": {"Hope": 1, "Treatment": 2, "I think so": 1, "Kei-kyu": 1, "Answer": 1}, "adverbs": {"Most": 1}, "numerals": {"22": 1, "One": 1}, "classifiers": {"individual": 1}, "pronouns": {"Here": 1}}}, {"content": "64. The difficulties of others are generally taken seriously.", "word_count": 15, "label": "", "sentence_structure": "Subject鈥搗erb鈥揷omplement", "subjective_objective": {"answer": "Subjective", "rationale": "The basis for the decision: the entry for this questionnaire is \"to care sincerely about the difficulties of others\", and the idea and attitude are part of the patient's subjective thinking and behavioral orientation."}, "sentence_sentiment": {"answer": "Positive", "rationale": "Because the act of \"cared sincerely for the difficulties of others\" is positive."}, "language_style": {"answer": "Tactful and warm", "rationale": "The phrase \"generally caring for the difficulties of others\" is more implicit, and does not directly indicate whether the other party is often concerned about the difficulties of others, but rather leads the other to answer by asking questions, which are also in line with the psychological questionnaire study."}, "time_dimension": {"answer": "Present", "rationale": ""}, "sentence_type": "Affirmative declarative sentence", "pos": {"verbs": {"Yes": 1, "I'm really concerned": 1, "Others": 1, "of": 1, "Difficulties": 1}, "adjectives": {"General": 1}, "numerals": {"64": 1}}}, {"content": "70. In the event of a nuclear war, most of those with nuclear facilities would leave their neighbours in there.", "word_count": 25, "label": "", "sentence_structure": "Subject鈥損redicate", "subjective_objective": {"answer": "Subjective", "rationale": "Because the description of what \"if there is a nuclear facility, what would there be to protect them\" in the questionnaire is a subjective assumption or assumption. Although nuclear war is an objective threat, it is impossible to determine whether people actually keep neighbours in their nuclear facility."}, "sentence_sentiment": {"answer": "Neutral", "rationale": "As the entry merely stated a fact, it did not involve any positive or negative emotional aspects."}, "language_style": {"answer": "Calm and direct", "rationale": "The entry is more calm and objective in its language, presents a fact and raises a question, without excessive rhetorical or emotional rendering. It asks the interviewee in a neutral manner about his opinion, without any predetermined value judgement."}, "time_dimension": {"answer": "Present", "rationale": "In the future, the term \"if nuclear war\" in the entry implies an extreme scenario that may occur in the future, and the act of \"staying neighbours inside\" is also a future planning and decision-making. The temporal dimension of the entry is therefore judged in the future."}, "sentence_type": "Affirmative declarative sentence", "pos": {"nouns": {"Nuclear war": 1, "Nuclear facilities": 1, "person": 2, "Neighbors": 1, "Inside": 1}, "verbs": {"Let's go": 1, "Fan": 1, "Jean": 1, "Stay": 1}, "adverbs": {"More": 1, "Both": 1, "in": 1}, "numerals": {"70": 1, "One": 2}}}, {"content": "88. Most people stop to help the car crasher.", "word_count": 15, "label": "", "sentence_structure": "Subject鈥搗erb鈥搊bject", "subjective_objective": {"answer": "Objective", "rationale": "This is a description of the behaviour prevailing, not a subjective view or feeling of the patient."}, "sentence_sentiment": {"answer": "Positive", "rationale": "Because the majority of people are willing to stop to help people with car failures is a positive social act."}, "language_style": {"answer": "Tactful and warm", "rationale": "The entry is more adept and interactive when it is spoken in a gentle and inviting manner, rather than by direct statements or orders."}, "time_dimension": {"answer": "Present", "rationale": "The entry represents a description or observation of current social phenomena and therefore the judgement is \"now\"."}, "sentence_type": "Affirmative declarative sentence", "pos": {"nouns": {"person": 1, "Car": 1, "Faulter": 1}, "verbs": {"Stop": 1, "Help": 1, "Out": 1}, "adjectives": {"Most": 1, "Yes": 1}, "numerals": {"88": 1}}}, {"content": "40. Only a few would risk sacrificing their bodies to help others.", "word_count": 23, "label": "", "sentence_structure": "Subject鈥搗erb鈥搊bject", "subjective_objective": {"answer": "Subjective", "rationale": "Because this entry involves patients' subjective perception and assessment of the \"very few\" who are willing to risk helping others. This is not just a description based on objective facts, but rather contains patients' subjective attitudes and values."}, "sentence_sentiment": {"answer": "Negative", "rationale": "Because the \"dangerous danger of sacrificing one's body to help another\" in the entry is a very dangerous act with potentially serious consequences, it is considered negative."}, "language_style": {"answer": "Calm and direct", "rationale": "The language style of the entry is calm, as it expresses a view or fact in an objective and concise manner, without excessive emotional or rhetorical overtones. Moreover, it uses a negative expression to emphasize that only a few people would do so, which gives a calm and rational impression."}, "time_dimension": {"answer": "Present", "rationale": "The entry describes a general phenomenon that some people risk to help others. This behaviour is a current situation and does not clearly point to the past or the future. Thus, the temporal dimension of the entry is now judged."}, "sentence_type": "Affirmative declarative sentence", "pos": {"nouns": {"Ken": 1, "You're gonna have to risk it": 1, "Sacrifice": 1, "My body": 1, "Danger": 1, "Others": 1}, "verbs": {"Only a few": 1, "Go on": 1, "Help": 1}, "adjectives": {"Very few": 1, "I'm willing to risk my life": 1}, "adverbs": {"Only": 1, "have": 1, "You're gonna have to risk it": 1}, "numerals": {"40": 1, "Very few": 1}, "pronouns": {"self": 1}}}, {"content": "46. The selfless in today ' s society were too poor because so many people wanted to count them.", "word_count": 28, "label": "", "sentence_structure": "Subject鈥搗erb鈥搊bject", "subjective_objective": {"answer": "Subjective", "rationale": "Because this sentence expresses the author ' s view and assessment that the selfless are pathetic in today ' s society, and that this view is based on the author ' s subjective feelings and values."}, "sentence_sentiment": {"answer": "Negative", "rationale": "Because the expression \"selfless is too poor\" in the entry is negative emotional."}, "language_style": {"answer": "Calm and direct", "rationale": "The basis for the judgement: The entry presents a point in a direct and clear way that in this society, selfless people are often misunderstood or exploited. This expression is not too much rhetorical or emotional, but rather a straightforward way of conveying the author's position and opinion."}, "time_dimension": {"answer": "Present", "rationale": ""}, "sentence_type": "Affirmative declarative sentence", "pos": {"nouns": {"Social": 1, "person": 2, "Calculate": 1}, "verbs": {"in": 1, "be": 1, "Too much": 1, "Yes": 1, "Calculate": 1}, "adjectives": {"Selfless": 1, "Poor thing": 1, "have": 1}, "adverbs": {"Today": 1, "Too much": 1}, "numerals": {"46": 1, "Well": 1}, "classifiers": {"individual": 1}, "pronouns": {"they": 1}}}, {"content": "52. There is a apparent lack of interest in each other, but in fact less.", "word_count": 19, "label": "", "sentence_structure": "Subject鈥搗erb鈥揷omplement", "subjective_objective": {"answer": "Subjective", "rationale": "Because the sentence is an expression of subjective feelings and perceptions that the evaluation of interpersonal relationships is based on individual feelings. Thus, it is a subjective view."}, "sentence_sentiment": {"answer": "Negative", "rationale": "Because the entries express a perceived feelings of mutual concern that is not actually genuine, it is a negative attitude or quality."}, "language_style": {"answer": "Tactful and warm", "rationale": "The entry uses a moderate language expression to describe the difference between the appearance of people and the actual situation, and does not accuse people too directly or harshly. Moreover, the entry does not have too many rhetorical or emotional expressions, but is presented in an objective and balanced way, which is more acceptable and understandable."}, "time_dimension": {"answer": "Present", "rationale": "The words \"the appearance of people caring for each other\" and \"the actual lack of concern\" point to current behaviour or phenomena, indicating the current situation. So the entry is a reflection of current social behaviour or attitudes, and the temporal dimension is now."}, "sentence_type": "Negative declarative sentence", "pos": {"nouns": {"People": 1, "Surface": 1, "Go, go, go": 1, "Care for each other": 1, "Actually": 1, "No, I'm not": 1, "Well": 1, "Care": 1}, "verbs": {"- Yeah, yeah": 1, "Care": 1}, "adjectives": {"On the surface": 1, "Actually": 1, "No, I'm not": 1, "Well": 1}, "adverbs": {"On the surface": 1, "Actually": 1}, "numerals": {"52": 1}}}, {"content": "58. Most people are not willing to help others from the heart.", "word_count": 19, "label": "", "sentence_structure": "Subject鈥搗erb鈥揷omplement", "subjective_objective": {"answer": "Objective", "rationale": "Because \"willing to help others\" is an objective act or result, and most people's intentions are also detectable and measurable. The entry therefore looks at the objective situation."}, "sentence_sentiment": {"answer": "Negative", "rationale": "The entry is a negative attitude that is not willing to help others."}, "language_style": {"answer": "Tactful and warm", "rationale": "The language style of the entry is more euphemistic, expressing an attitude of understanding and support through the use of words such as \"from the innermost of the mind\" and \"unwilling to give\" without losing objectivity and neutrality. It enables respondents to feel more comfortable and free to answer questions, and thus to get more realistic and accurate answers."}, "time_dimension": {"answer": "Present", "rationale": "Therefore, the entry is now."}, "sentence_type": "Exclamatory sentence", "pos": {"nouns": {"person": 2, "Deep down": 1, "Others": 1}, "verbs": {"be": 1, "May I": 1, "From": 1, "Help": 1, "- On purpose": 1, "But": 1}, "adjectives": {"Most": 1, "Deep": 1}, "adverbs": {"Deep down": 1}, "numerals": {"58": 1}, "pronouns": {"From": 1}}}, {"content": "76. Most had exaggerated their difficulties in exchange for sympathy.", "word_count": 20, "label": "", "sentence_structure": "Subject鈥搗erb鈥搊bject", "subjective_objective": {"answer": "Subjective", "rationale": "Because \"exagger your difficulties\" and \"in exchange for sympathy\" are subjective attitudes and motivations of patients."}, "sentence_sentiment": {"answer": "Negative", "rationale": "Because the entry \"exaggers your difficulties in exchange for sympathy\" is negative."}, "language_style": {"answer": "Tactful and warm", "rationale": "The language style of the entry is more objective, rather than directly accusing or criticizing others, but rather a gentle reminder to the interviewees that their behaviour is likely to have unnecessary negative effects. Such expressions are more acceptable and open to reflection."}, "time_dimension": {"answer": "Past", "rationale": "The words \"majority\", \"exaggeration\", \"difficult\" and \"in exchange for sympathy\" in the entry point point to past behaviour or state, and therefore to the past."}, "sentence_type": "Affirmative declarative sentence", "pos": {"nouns": {"Most people": 1, "self": 1, "Difficulties": 1, "Compassion": 1}, "verbs": {"Exaggeration": 1, "In exchange for": 1}, "adverbs": {"More": 1}, "numerals": {"76": 1}, "pronouns": {"others": 1}}}, {"content": "94. Most people are committed to their own interests.", "word_count": 14, "label": "", "sentence_structure": "Subject鈥搗erb鈥搊bject", "subjective_objective": {"answer": "Subjective", "rationale": "Because \"one mind is for one's own good\" is a subjective judgement that expresses the attitude and perception of the investigator towards the majority. This view is based on the subjective perception and values of the investigator, and is not necessarily an objective fact."}, "sentence_sentiment": {"answer": "Negative", "rationale": "Because the entry \"one mind only for one's own good\" expresses a selfish and self-serving attitude, which is negative."}, "language_style": {"answer": "Calm and direct", "rationale": "The language style of the entry is relatively calm and straightforward, and there is no excessive emotional or rhetorical use. It presents a widespread phenomenon in an objective way, allowing readers to understand its meaning clearly."}, "time_dimension": {"answer": "Present", "rationale": "The \"majority\" and \"one mind only for one's own good\" in this entry indicate the state or behaviour of the moment. So the time dimension of this entry is now."}, "sentence_type": "Affirmative declarative sentence", "pos": {"nouns": {"Most people": 1, "I'm not gonna let you down": 1, "self": 1, "Interest": 1}, "verbs": {"Yes": 1}, "adverbs": {"Only": 1}, "numerals": {"94": 1}}}, {"content": "33. Most people have the courage to admit mistakes.", "word_count": 10, "label": "", "sentence_structure": "Subject鈥搗erb鈥搊bject", "subjective_objective": {"answer": "Subjective", "rationale": "This is a subjective view, because the perception and attitude of the \"majority\" is subjective. While \"recognition of error\" is a universally accepted code of conduct, perceptions of when and how to recognize error may vary according to culture, personal convictions, and other factors."}, "sentence_sentiment": {"answer": "Neutral", "rationale": "Because the entry merely stated a fact and did not express any emotional colour."}, "language_style": {"answer": "Tactful and warm", "rationale": "The entry uses a moderate tone and inspiring term, \"courage,\" which leads the interviewees to think and answer questions, and avoids the possibility of direct accusations or criticism. At the same time, the language style of the article is more positive, contributing to a good investigation atmosphere."}, "time_dimension": {"answer": "Present", "rationale": "Now the entry is asking the testee whether he has the courage to admit the wrong view or experience of the \"majority\" and is a description of the current state of affairs."}, "sentence_type": "Affirmative declarative sentence", "pos": {"nouns": {"Courage": 1, "Recognition": 1, "Error": 1}, "verbs": {"have": 1, "Recognition": 1}, "adverbs": {"Most": 1}, "numerals": {"33": 1}}}, {"content": "39. Most people are able to make their own decisions without public influence.", "word_count": 18, "label": "", "sentence_structure": "Subject鈥損redicate", "subjective_objective": {"answer": "Subjective", "rationale": "Because \"the majority can make its own decisions without public influence\" is a view or opinion that belongs to the patient's subjective attitude."}, "sentence_sentiment": {"answer": "Neutral", "rationale": "Because the entry does not have any emotional color."}, "language_style": {"answer": "Calm and direct", "rationale": "The entry raises questions in an objective manner, without the use of rhetorical or emotional colours for rendering, and the language style is relatively calm and direct.\""}, "time_dimension": {"answer": "Present", "rationale": "The sentence describes the widespread ability to make decisions autonomously and free of public influence, and whether the possession of such capacity is judged and expressed at the moment."}, "sentence_type": "Affirmative declarative sentence", "pos": {"nouns": {"Most people": 1, "Impact": 1, "Decision": 1}, "verbs": {"Yes": 1, "Do it": 1}, "adjectives": {"By the public": 1, "self": 1}, "numerals": {"39": 1}}}, {"content": "45. You have made progress today not by grouping but by working together.", "word_count": 18, "label": "", "sentence_structure": "Subject鈥搗erb鈥揷omplement", "subjective_objective": {"answer": "Subjective", "rationale": "Because \"work\" is the patient's subjective opinion and attitude."}, "sentence_sentiment": {"answer": "Positive", "rationale": "Because the \"work for progress\" act in the entry is positive."}, "language_style": {"answer": "Calm and direct", "rationale": "The language style of the entry is more straightforward, there is no excessive rhetorical or emotional rendering, and it is more objective and calm. It expresses the subject of the questionnaire in a concise language, i.e., asking whether the main reason why the interviewee is making progress today is by his own efforts."}, "time_dimension": {"answer": "Present", "rationale": "The keyword \"You achieve progress today\" means the facts or state of things that have happened today, while the verb \"does not rely on grouping but on working\" shows that the testees have made progress through their own efforts in the past. So the entry asks about the behavior and mentality of the present."}, "sentence_type": "Affirmative declarative sentence", "pos": {"nouns": {"you": 1, "Today": 1, "Progress": 1, "Together": 1, "Try": 1}, "verbs": {"Access": 1, "Shit": 1, "be": 2, "No, it's not": 1}, "adverbs": {"of": 3, "It's": 1}, "numerals": {"45": 1}}}, {"content": "51. As long as they were right, they were generally indifferent to the opposition.", "word_count": 27, "label": "", "sentence_structure": "Subject鈥損redicate", "subjective_objective": {"answer": "Subjective", "rationale": "Because this entry looks at the individual ' s conviction and attitude, namely, whether or not he or she is willing to maintain his or her views, it is subjective."}, "sentence_sentiment": {"answer": "Negative", "rationale": "Because the phrase \"maintenance of one's own view despite the opposition of others\" in this entry is often considered as stubborn and a negative attitude."}, "language_style": {"answer": "Calm and direct", "rationale": "The entry expresses the phenomenon of \"the common man holding his own view in spite of the opposition\" in an objective and straightforward manner. It is not too much rhetorical or emotional to be said in a more straightforward way. Its linguistic style can therefore be described as being calm and straightforward."}, "time_dimension": {"answer": "Present", "rationale": ""}, "sentence_type": "Affirmative declarative sentence", "pos": {"nouns": {"person": 2, "self": 3, "General": 1, "others": 1, "Views": 1}, "verbs": {"I think": 1, "Hold on": 1, "Objection": 1, "I'll give up": 1, "And": 1}, "adjectives": {"Correct": 1, "Both": 1}, "adverbs": {"General": 1}, "numerals": {"51": 1}, "pronouns": {"self": 2}}}, {"content": "57. If a student does not advocate cheating, he will not follow even if he sees many cheatings.", "word_count": 29, "label": "", "sentence_structure": "Subject鈥搗erb鈥搊bject", "subjective_objective": {"answer": "Subjective", "rationale": "Because this statement relates to individual moral and behavioural choices, it is a subjective judgement."}, "sentence_sentiment": {"answer": "Positive", "rationale": "Because the entry is intended to express an act of honesty and morality, it is positive."}, "language_style": {"answer": "Tactful and warm", "rationale": "The entry is more euphemistic in its language, because it asks the respondents in an invitation tone and uses positive language to guide the answer. At the same time, it does not directly state or order what the respondents should do, but rather presents a hypothesis and asks them to judge on their own basis, which is more personal and interactive."}, "time_dimension": {"answer": "Past", "rationale": "The entry describes a hypothetical situation where \"if a student does not advocate cheating\", the latter conduct \"even if he sees many cheating, he will not follow\" is reasoned on the assumption that this premise is based, and therefore the temporal dimension is a priori."}, "sentence_type": "Affirmative declarative sentence", "pos": {"nouns": {"Students": 1, "person": 1, "Cheating": 2, "Fuck": 1}, "verbs": {"Claim": 1, "Look": 1, "I'm not following you": 1, "Yes": 1, "Follow me": 1}, "adverbs": {"Someone": 1, "Even if": 1}, "numerals": {"57": 1}, "pronouns": {"he": 2}}}, {"content": "75. People with new ideas are respected in our society.", "word_count": 18, "label": "", "sentence_structure": "Subject鈥搗erb鈥搊bject", "subjective_objective": {"answer": "Subjective", "rationale": "Because \"respect\" is a subjective attitude or feeling that depends on individual values and beliefs."}, "sentence_sentiment": {"answer": "Positive", "rationale": "Because the entry expresses respect and recognition for people with new views, it is a positive emotional attitude."}, "language_style": {"answer": "Tactful and warm", "rationale": "The language style of the entry is euphemismal. It does not directly state \"the respect of people with new views\", but rather expresses the subject in a more moderate way, i.e., the degree to which these people are respected in this society. This expression is more friendly and more resonant with the respondents."}, "time_dimension": {"answer": "Present", "rationale": "The entry now describes a general social phenomenon or perception, which does not clearly point at sex, and therefore is judged to be the present."}, "sentence_type": "Affirmative declarative sentence", "pos": {"nouns": {"person": 1, "Views": 1, "we": 1, "Social": 1}, "verbs": {"have": 1, "Yes": 1, "Respect": 1}, "adjectives": {"New": 1}, "numerals": {"75": 1}}}, {"content": "93. Most people will manifest their faith.", "word_count": 11, "label": "", "sentence_structure": "Subject鈥搗erb鈥搊bject", "subjective_objective": {"answer": "Subjective", "rationale": "Because \"expression of one's faith\" is a subjective act that requires the will and choice of the individual. Although belief is itself an objective existence, whether or not it is expressed is the individual's subjective attitude and choice."}, "sentence_sentiment": {"answer": "Neutral", "rationale": "Since the entry merely describes a fact, it does not express any feelings or attitudes."}, "language_style": {"answer": "Tactful and warm", "rationale": "The entry is based on a more euphemistic formulation, \"showing\" that the term avoids too strong or direct a tone, and expresses the general attitude of the majority. Moreover, the term \"belief\" has some emotional and value orientation, which is also consistent with the warm expression of euphemism."}, "time_dimension": {"answer": "Present", "rationale": "The key word \"majority\" in the entry in the future would indicate a future situation in which most people may express their faith in the future. Although there may already be a large number of people expressing their faith, this does not mean that all will continue to do so in the future, so the time horizon will be the future."}, "sentence_type": "Affirmative declarative sentence", "pos": {"nouns": {"Most people": 1, "Faith": 1}, "verbs": {"It's gonna show": 1}, "adverbs": {"Most": 1}, "numerals": {"93": 1}}}, {"content": "3. When the majority faces severe criticism, even the perception of the innermost person changes completely.", "word_count": 28, "label": "", "sentence_structure": "Subject鈥搗erb鈥搊bject", "subjective_objective": {"answer": "Objective", "rationale": "Because it is a common phenomenon, where people face external pressure or criticism, they can make a superficial change to deal with this situation, without necessarily representing a change in their real mind. The questionnaire entry is therefore viewed from an objective perspective."}, "sentence_sentiment": {"answer": "Negative", "rationale": "Because the entry says \"even if it doesn't change the way it looks,\" it's negative."}, "language_style": {"answer": "Calm and direct", "rationale": "This entry is a direct statement of a point, not too emotionally or verbally. It expresses the apparent reaction that most people may have in facing criticism in an objective and calm manner. This language style is easy to convey, making it easier for readers to understand the author鈥檚 point of view."}, "time_dimension": {"answer": "Past", "rationale": "The entry describes a general social phenomenon, whereby people, when facing severe criticism, adopt coping strategies to protect their faces and dignity. Such behaviour may have occurred, present and future, but we are silently referring to it as a description of the past, because there is no explicit time reference or expression in the story."}, "sentence_type": "Affirmative declarative sentence", "pos": {"nouns": {"person": 2, "Criticism": 1, "Surface": 1}, "verbs": {"Faced": 1, "Change": 3, "Yes": 1}, "adjectives": {"Hard": 1, "Total": 1}, "adverbs": {"Most": 1}, "numerals": {"3": 1}, "pronouns": {"self": 1}}}, {"content": "9. Many people are now in a position to act until they understand what others think.", "word_count": 24, "label": "", "sentence_structure": "Subject鈥搗erb鈥揷omplement", "subjective_objective": {"answer": "Objective", "rationale": "This is a description of the general phenomenon of the human being and not from the subjective perspective of the patient."}, "sentence_sentiment": {"answer": "Negative", "rationale": "Because the \"no action\" in the entry is negative."}, "language_style": {"answer": "Tactful and warm", "rationale": "The language style of the entry is euphemism, because it uses a gentle and inviting tone to present a view rather than direct command or criticism. It tries to stimulate the other side to think and encourage them to share their ideas, rather than forcing them to accept a view. Moreover, it is easier to feel comfortable and respected, and therefore more willing to participate in a dialogue."}, "time_dimension": {"answer": "Present", "rationale": "The keyword \"there are many people\" suggests that the entry is describing the current situation and the current situation, and that the act of \"doing nothing until others are seen\" is also happening or recurrent and can therefore be judged as now."}, "sentence_type": "Affirmative declarative sentence", "pos": {"nouns": {"person": 2, "Observations": 1, "Action": 1}, "verbs": {"Now there is": 1, "Roger that": 1, "Yes": 1, "Do it": 1}, "adjectives": {"Many": 1, "others": 1}, "adverbs": {"Before": 1}, "numerals": {"9": 1}, "pronouns": {"Here": 1}}}, {"content": "15. The key to success now was not how to work hard, but how to live in harmony with people.", "word_count": 28, "label": "", "sentence_structure": "Subject鈥搗erb鈥揷omplement", "subjective_objective": {"answer": "Subjective", "rationale": "Because \"the key to success is not how to work hard, but how to live in harmony with people\" is a view or belief, and it does not involve objective facts or behaviour, and it is therefore subjective."}, "sentence_sentiment": {"answer": "Neutral", "rationale": "Since the purpose of the article was not expressed in any clear emotional terms, it was simply a statement of one view."}, "language_style": {"answer": "Calm and direct", "rationale": "The entry presents a point in a concise manner, does not move the reader with excessive rhetorical or emotional rendering, and is more calm and objective."}, "time_dimension": {"answer": "Present", "rationale": "The entry uses the verb \"acquire\" of the current state of affairs, indicating that this view is presented in the context of the current situation. At the same time, the structure of \"not ... but\" also emphasizes that this view is directed at the present situation."}, "sentence_type": "Affirmative declarative sentence", "pos": {"nouns": {"Success": 1, "Chile": 2, "How's that": 3, "Work hard": 1, "We're living in harmony with all of us": 1}, "verbs": {"Access": 1, "No, it's not": 1, "It's": 1, "What": 1, "Work": 1, "Get along": 1}, "adjectives": {"Present": 1, "How's it going": 1}, "adverbs": {"No, it's not": 2, "And": 1, "How": 1}, "numerals": {"15": 1}, "classifiers": {"individual": 1}, "pronouns": {"The crowd": 1}}}, {"content": "27. A normal student cheats when he cheats on the test, even if he has his own moral standards.", "word_count": 31, "label": "", "sentence_structure": "Subject鈥搗erb鈥搊bject", "subjective_objective": {"answer": "Subjective", "rationale": "This topic is examined from the patient ' s subjective perspective, as it relates to the patient ' s behaviour and ideas."}, "sentence_sentiment": {"answer": "Negative", "rationale": "Because the description of the article contains the negative act of \"test fraud\"."}, "language_style": {"answer": "Calm and direct", "rationale": "The entry uses direct representations to specify the moral behaviour of some students and the possible outcomes of the decision-making process."}, "time_dimension": {"answer": "Past", "rationale": "The entry describes a past act, i.e. a situation of cheating in the examination, and it is caused by the fraudulent behaviour of others. The temporal dimension is therefore a past."}, "sentence_type": "Affirmative declarative sentence", "pos": {"nouns": {"Students": 1, "Your own moral standards": 1, "others": 1, "Examinations": 1, "Cheating": 2}, "verbs": {"have": 1, "Even if": 1, "in": 1, "Yes": 1}, "adjectives": {"General": 1}, "adverbs": {"Yeah": 1, "Average": 1}, "pronouns": {"he": 1}}}, {"content": "63. Opposing the public is rare.", "word_count": 11, "label": "", "sentence_structure": "Subject鈥揷opula鈥損redicative", "subjective_objective": {"answer": "Objective", "rationale": "Because \"very few people are opposed to people\" is an objective fact that can be derived from surveys or statistics. It is not the individual feelings or thoughts of patients, but rather the phenomenon that is prevalent."}, "sentence_sentiment": {"answer": "Neutral", "rationale": "Because the entry does not address emotional, attitudinal, behavioural, etc., but rather describes the frequency of a phenomenon."}, "language_style": {"answer": "Tactful and warm", "rationale": "The entry is more personal and interactive when it is spoken in a gentle and inviting manner, rather than in direct statements or orders. Moreover, the expression \"very few people are opposed to the human being\" is more objective, neutral and does not have too much emotional or subjective judgement."}, "time_dimension": {"answer": "Past", "rationale": "The entry describes the facts or phenomena of the past, in which it is rare for people who have opposed the people. Thus, the temporal dimension is judged to be the past."}, "sentence_type": "Affirmative declarative sentence", "pos": {"nouns": {"person": 2, "People": 1, "- Yes": 1}, "verbs": {"See": 1}, "adjectives": {"Tens": 1}, "adverbs": {"Less": 1}, "numerals": {"63": 1}}}, {"content": "69. Most people have to rely on others when making major decisions for themselves.", "word_count": 20, "label": "", "sentence_structure": "Subject鈥搗erb鈥搊bject", "subjective_objective": {"answer": "Objective", "rationale": "This is a situation that most people generally encounter, not a subjective attitude or feeling of the patient."}, "sentence_sentiment": {"answer": "Negative", "rationale": "Because the \"have to rely on someone else\" act in the entry is negative."}, "language_style": {"answer": "Tactful and warm", "rationale": "The entry uses a relatively mild tone, not directly accusing or criticizing the other, but directing the other to answer the question in a more inclusive and understanding way. At the same time, the question is not too emotional, much more about a prevailing phenomenon, and can therefore be judged as a soft language style."}, "time_dimension": {"answer": "Future", "rationale": "The entry describes the possibility that in the future people may face situations in which they need to rely on others to make important decisions. While this issue does not clearly point to the past or to the present, it can be judged as the future by the use of the term \"will\"."}, "sentence_type": "Affirmative declarative sentence", "pos": {"nouns": {"person": 2, "Decision-making": 1, "Dependency": 1, "others": 1}, "verbs": {"Do it": 1, "Significant": 1, "Decision": 1, "I had to": 1, "Dependency": 1}, "adjectives": {"Significant": 1}, "adverbs": {"Most": 1, "Time": 1}, "numerals": {"69": 1}, "pronouns": {"self": 1}}}, {"content": "87. The general perception of people ' s views is not the same as that of themselves.", "word_count": 27, "label": "", "sentence_structure": "Subject鈥損redicate", "subjective_objective": {"answer": "Subjective", "rationale": "Because the reference to \"very little will be said\" is the subjective feeling and attitude that patients may have in facing this situation."}, "sentence_sentiment": {"answer": "Negative", "rationale": "Because the \"very rarely expresses your opinion\" act is negative in the entry."}, "language_style": {"answer": "Tactful and warm", "rationale": "Warm and warm."}, "time_dimension": {"answer": "Present", "rationale": ""}, "sentence_type": "Negative declarative sentence", "pos": {"nouns": {"Normal": 1, "People": 1, "Views": 2, "self": 2}, "verbs": {"Found": 1, "Show": 1, "Not at the same time": 1}, "adverbs": {"Very few": 1, "General": 1}, "numerals": {"87": 1, "2": 1}, "pronouns": {"they": 1}}}, {"content": "31. When a person has made enough effort to achieve his or her life goals, it is common for him or her to be able to achieve his or her life goals.", "word_count": 21, "label": "", "sentence_structure": "Subject鈥損redicate", "subjective_objective": {"answer": "Objective", "rationale": "Because \"a person's efforts\" and \"the achievement of his or her life goals\" are objective facts that can be observed and tested, and are not influenced by the subjective consciousness of the individual."}, "sentence_sentiment": {"answer": "Positive", "rationale": "Because the expression \"if you try hard enough, you will usually achieve your life's goals\" in the entry is positive."}, "language_style": {"answer": "Tactful and warm", "rationale": "The language style of the entry is mild, encouraging and positive, and it is not expressed in too direct or hard-line a way. It leads the interviewee to reflect in a positive manner on the relationship between personal efforts and the goal of life, and increases the motivation and self-effectiveness of the interviewee."}, "time_dimension": {"answer": "Present", "rationale": "The entry now describes a widespread view that efforts can achieve life goals. This view is not clear about the point of time, and thus it is now judged."}, "sentence_type": "Affirmative declarative sentence", "pos": {"nouns": {"person": 1, "Life goals": 1}, "verbs": {"Try": 1, "Achieved": 1}, "adjectives": {"Enough": 1}, "adverbs": {"Usually": 1}, "numerals": {"31": 1}}}, {"content": "37. The general public has a clear understanding of the motives for their actions.", "word_count": 18, "label": "", "sentence_structure": "Subject鈥揷opula鈥損redicative", "subjective_objective": {"answer": "Subjective", "rationale": "This sentence relates to patients鈥?subjective cognitive and understanding abilities, and should therefore be considered subjective. Patients鈥?understanding of the motivations for their actions is based on their personal experience and beliefs, which gives them some subjectivity."}, "sentence_sentiment": {"answer": "Neutral", "rationale": "Because the entry does not have any emotional color."}, "language_style": {"answer": "Calm and direct", "rationale": "The language style of the entry is calm, as it is a direct statement of the fact that most people have a clear understanding of their behaviour, and does not use excessive rhetorical or emotional rendering. This expression is more objective, concise and understandable."}, "time_dimension": {"answer": "Present", "rationale": "The entry is a general statement of fact and does not relate to past or future behaviour or mentalities, and therefore is judged to be \"now\"."}, "sentence_type": "Affirmative declarative sentence", "pos": {"nouns": {"General": 1, "person": 2, "Motive": 1, "Behaviour": 1}, "verbs": {"have": 1, "Roger that": 1}, "adjectives": {"Clear": 1}, "numerals": {"37": 1}}}, {"content": "43. Future wars could be avoided if people worked hard enough.", "word_count": 20, "label": "", "sentence_structure": "Subject鈥揷opula鈥損redicative", "subjective_objective": {"answer": "Subjective", "rationale": "Because \"if people try hard enough\" is a hypothetical condition, a subjective view of the interviewee."}, "sentence_sentiment": {"answer": "Positive", "rationale": "Because the entry expresses positive attitudes and beliefs that human beings are capable of avoiding war through their efforts."}, "language_style": {"answer": "Tactful and warm", "rationale": "The language style of the entry is more moderate, encouraging and hopeful, and conveys positive messages and expectations in the form of a \"if you can avoid it.\" This expression is more likely to resonate with the interviewees and encourage them to do more to achieve their goals."}, "time_dimension": {"answer": "Future", "rationale": "The entry contains visions and expectations for the future, with the keyword \"wars of the future can be avoided\", all pointing to situations that have not yet occurred and that may arise in the future. The temporal dimension of the article is therefore the future."}, "sentence_type": "Affirmative declarative sentence", "pos": {"nouns": {"People": 1, "The future": 1, "War": 1, "Try": 1}, "verbs": {"Yeah": 1, "Avoid": 1, "If": 1, "Enough": 1, "If you": 1, "be": 1}, "adjectives": {"Work hard": 1}, "adverbs": {"That's enough": 1}, "numerals": {"43": 1}, "pronouns": {"they": 1}}}, {"content": "49. The general population is basically the master of its own destiny.", "word_count": 15, "label": "", "sentence_structure": "Subject鈥揷opula鈥損redicative", "subjective_objective": {"answer": "Subjective", "rationale": "Because \"the master of his destiny\" is a subjective view or belief of a human being."}, "sentence_sentiment": {"answer": "Positive", "rationale": "The expression \"owner of his own destiny\" according to the purpose of the questionnaire shows that it expresses a positive, confident and autonomous attitude."}, "language_style": {"answer": "Calm and direct", "rationale": "The basis for the determination: the entry presents in an objective and direct manner a general view 鈥?that people are usually the masters of their own destiny. There is no excessive emotional or rhetorical style, which is more plain and clear. The language style of the article can therefore be described as being calm and straightforward."}, "time_dimension": {"answer": "Present", "rationale": "\"The general population is basically the master of its own destiny\" describes the prevailing perception or attitude, not the past or future, and thus the temporal dimension is now judged."}, "sentence_type": "Affirmative declarative sentence", "pos": {"nouns": {"General": 1, "person": 1, "Destiny": 1, "Master": 1}, "verbs": {"be": 1}, "adjectives": {"Basic": 1, "Go, go, go": 1}, "numerals": {"49": 1}}}, {"content": "55. In local or national elections, the majority will select a reasonable and logical candidate.", "word_count": 28, "label": "", "sentence_structure": "Subject鈥搗erb鈥搊bject", "subjective_objective": {"answer": "Objective", "rationale": "Because the entry describes a general phenomenon, namely, that people tend to choose rational and logical candidates in elections. This is an objective view, independent of personal feelings or subjective bias."}, "sentence_sentiment": {"answer": "Neutral", "rationale": "Because the entry does not relate to positive or negative emotions, attitudes, behaviours, aims or qualities. It simply describes a phenomenon in which people prefer to choose candidates with reason and logic in elections."}, "language_style": {"answer": "Calm and direct", "rationale": "The entry is more straightforward, is less talkative or emotional, and is calm and objective. It expresses the view in a factual way that people prefer to choose a reasonable and logical candidate in local or national elections. This is more straightforward and can send a message to the reader."}, "time_dimension": {"answer": "Present", "rationale": ""}, "sentence_type": "Affirmative declarative sentence", "pos": {"nouns": {"Local": 1, "National": 1, "Elections": 2, "Most people": 1, "Candidates": 1, "Reason": 1, "Logical": 1}, "verbs": {"Selection": 1}, "adjectives": {"- That's a reasonable one": 1, "It's logical": 1}, "numerals": {"55": 1}}}, {"content": "73. Most people have a great deal of control over what happens in their own lives.", "word_count": 22, "label": "", "sentence_structure": "Subject鈥搗erb鈥搊bject", "subjective_objective": {"answer": "Objective", "rationale": "Because \"controlling what happens in your life\" is an objective description, and it does not involve the patient's specific feelings or subjective attitudes."}, "sentence_sentiment": {"answer": "Positive", "rationale": "Because it describes people ' s ability to control their own lives and is a positive attitude and behaviour."}, "language_style": {"answer": "Calm and direct", "rationale": "The language style of the entry is more direct and objective, and there is no excessive emotional or rhetorical approach, but rather a presentation of the facts."}, "time_dimension": {"answer": "Past", "rationale": "Thus, in terms of time dimension, the issue is neither past nor future, but rather one of current state and conviction, now."}, "sentence_type": "Affirmative declarative sentence", "pos": {"nouns": {"person": 2, "Yes": 1}, "verbs": {"Yes": 1, "Control": 1, "Come on": 1}, "adjectives": {"Mostly": 1, "Level": 1, "self": 1}, "adverbs": {"To a large extent": 1}, "numerals": {"73": 1, "Both": 1}}}, {"content": "79. Most people are aware of their strengths and weaknesses.", "word_count": 19, "label": "", "sentence_structure": "Subject鈥搗erb鈥揷omplement", "subjective_objective": {"answer": "Subjective", "rationale": "Because the perception of \"your strengths and weaknesses\" is the patient's personal view and perception, it is subjective."}, "sentence_sentiment": {"answer": "Positive", "rationale": "Because it expresses the ability of people to be self-aware, it is a positive attitude and quality."}, "language_style": {"answer": "Tactful and warm", "rationale": "The language style of the entry is more euphemistic, warm, easy to use, and does not directly speak or order the other to do something. This expression is more friendly, acceptable and participatory."}, "time_dimension": {"answer": "Present", "rationale": "The \"majority\" in the entry indicates the state of the moment, and the \"aware\" also indicates that it is a state or behaviour in progress, and therefore the judgement is now."}, "sentence_type": "Affirmative declarative sentence", "pos": {"nouns": {"person": 2, "Yes": 1, "- Yes, I am": 1, "Short": 1, "Strengths": 1}, "verbs": {"have": 1}, "adjectives": {"Most": 1, "- Yes, I am": 1}, "numerals": {"79": 1}, "pronouns": {"self": 1}}}, {"content": "1. The great artists, inventors and ordinary people often have their own uninformed motivations to drive the great achievements in life ...", "word_count": 42, "label": "", "sentence_structure": "Subject鈥搗erb鈥搊bject", "subjective_objective": {"answer": "Subjective", "rationale": "Because the sentence expresses personal views or beliefs and involves an assessment and speculation of \"the great artist, inventor and ordinary person\", it can be classified as subjective."}, "sentence_sentiment": {"answer": "Positive", "rationale": "Because the great achievements of great artists, inventors and ordinary people mentioned in the entry are positive and are driven by \"ununderstood power\", it is also a positive attitude."}, "language_style": {"answer": "Tactful and warm", "rationale": "The entry uses a soft language style, which expresses an attitude of encouragement and inspiration rather than a direct statement or order. It presents an interesting point of view and invites others to reflect and discuss it, which is more personal and interactive."}, "time_dimension": {"answer": "Present", "rationale": ""}, "sentence_type": "Affirmative declarative sentence", "pos": {"nouns": {"Artist": 1, "Inventor": 1, "Normal": 1, "Life": 1, "Achievements": 2, "Power": 1}, "verbs": {"be": 1, "By": 1, "Driven by": 1, "I don't know": 1}, "adjectives": {"Great": 1, "Large": 1}, "numerals": {"1": 2}, "pronouns": {"they": 1}}}, {"content": "7. Our achievements in life are largely determined by forces beyond our control.", "word_count": 30, "label": "", "sentence_structure": "Subject鈥揷opula鈥損redicative", "subjective_objective": {"answer": "Objective", "rationale": "Because \"achievements in life\" and \"powers beyond control\" are objective facts, this statement examines the impact of these objective phenomena on individual subjective perceptions."}, "sentence_sentiment": {"answer": "Negative", "rationale": "Because the expression in the entry \"to a large extent determined by forces beyond our control\" conveys a negative attitude and view."}, "language_style": {"answer": "Tactful and warm", "rationale": "The language style of the entry is more euphemistic, presenting a point in a relatively objective and neutral way. At the same time, the use of the vague term \"to a large extent\" avoids too much absolute or strong tone. Such expression can inspire the reader to think and resonate, and is also more acceptable."}, "time_dimension": {"answer": "Present", "rationale": ""}, "sentence_type": "Negative declarative sentence", "pos": {"nouns": {"Life": 1, "Achievements": 2, "Power": 2}, "verbs": {"be": 1, "Decision": 1, "Control": 1}, "adjectives": {"A lot": 1}, "adverbs": {"in": 1, "we": 1, "By": 1, "Sou-san": 1, "No, I can't": 1, "Both": 1, "of": 4}, "numerals": {"7": 1}, "pronouns": {"we": 2}}}, {"content": "19. Attempts to recognize oneself are often futile.", "word_count": 18, "label": "", "sentence_structure": "Subject鈥揷opula鈥損redicative", "subjective_objective": {"answer": "Subjective", "rationale": "Because \"in vain\" is a subjective assessment, which means that the author believes that people's attempts are usually ineffective."}, "sentence_sentiment": {"answer": "Negative", "rationale": "Because the expression 鈥渙ften in vain鈥?in the article's purpose conveys a negative attitude."}, "language_style": {"answer": "Calm and direct", "rationale": "The basis for the judgement: The entry expresses its views on self-awareness attempts in a clear and straightforward way, without too much rhetoric or emotional rendering. The phrase \"in vain\" is used to emphasize the ineffectiveness of the attempt, which is more objective and calm in tone, and does not have overly subjective emotional aspects."}, "time_dimension": {"answer": "Past", "rationale": "The entry uses the past \"often\" to indicate that such attempts have occurred in the past. The \"in vain\" suggests that these attempts have not produced the desired results. The time dimension of the article is therefore the past."}, "sentence_type": "Negative declarative sentence", "pos": {"nouns": {"People": 1, "Self": 2, "Try": 1, "It's useless": 1}, "verbs": {"I want to meet you": 1, "be": 1}, "adjectives": {"Often": 1}, "numerals": {"19": 1}}}, {"content": "25. Man hardly has the slightest chance of changing his or her destiny.", "word_count": 15, "label": "", "sentence_structure": "Subject鈥揷opula鈥損redicative", "subjective_objective": {"answer": "Subjective", "rationale": "Because \"man's fate can be changed\" is a subjective view and conviction, and different people have different perspectives, and it is therefore a subjective perspective."}, "sentence_sentiment": {"answer": "Negative", "rationale": "Because the expression \"can't change your destiny\" has a negative emotional and attitude."}, "language_style": {"answer": "Calm and direct", "rationale": "The entry directly expresses doubts about the ability of individuals to change their own destiny, does not use excessive rhetoric or emotional rendering, and is more objective in tone.\""}, "time_dimension": {"answer": "Present", "rationale": "In conclusion, the temporal dimension of the article may be present or future."}, "sentence_type": "Negative declarative sentence", "pos": {"nouns": {"person": 1, "self": 1, "Destiny": 1}, "verbs": {"Yes": 1, "Change": 1}, "adjectives": {"Almost nothing": 1}, "numerals": {"25": 1}}}, {"content": "61. The majority is hardly able to change the reality they face.", "word_count": 17, "label": "", "sentence_structure": "Subject鈥搗erb鈥搊bject", "subjective_objective": {"answer": "Objective", "rationale": "Because \"the majority can hardly change the reality they face\" is a fact that can be observed and proven and is not determined by the patient's personal feelings or attitudes."}, "sentence_sentiment": {"answer": "Negative", "rationale": "Because the phrase \"near changing the reality they face\" in the entry is negative."}, "language_style": {"answer": "Calm and direct", "rationale": "The language style of the entry is a calm and direct type. It clearly presents a view, without using excessive rhetorical or emotional rendering. This type of language style is usually more objective, concise and suitable for delivering factual information or expressing serious views."}, "time_dimension": {"answer": "Past", "rationale": "The entry uses the term \"near changing\" to express an objective statement of the facts that occurred in the past and therefore belongs to the past."}, "sentence_type": "Negative declarative sentence", "pos": {"nouns": {"person": 1, "Reality": 1}, "verbs": {"Change": 1, "Faced": 1}, "adjectives": {"Most": 1, "Almost": 1}, "numerals": {"61": 1}, "pronouns": {"they": 1}}}, {"content": "67. Most people have unrealistic overestimations of their abilities.", "word_count": 19, "label": "", "sentence_structure": "Subject鈥搗erb鈥搊bject", "subjective_objective": {"answer": "Subjective", "rationale": "Because this evaluation is the patient ' s own perception and judgement of his or her abilities, it is subjective."}, "sentence_sentiment": {"answer": "Negative", "rationale": "Because the unrealistic overestimation of the ability of most people is a negative attitude and behaviour."}, "language_style": {"answer": "Tactful and warm", "rationale": "The language style of the entry is a euphemism, because it does not directly criticize or accuse the interviewee of a competency assessment, but rather uses a more objective and neutral language expression. It also provides an interesting question of reflection, allowing the interviewee to reflect on whether his or her capacity assessment is overestimated, and thus to direct them to a deeper self-thinking exercise."}, "time_dimension": {"answer": "Present", "rationale": "\"Most\" means that the current situation, \"there are unrealistic overestimations of their abilities\" are their behaviour or mentality, and the entry therefore describes the present situation."}, "sentence_type": "Negative declarative sentence", "pos": {"nouns": {"Capacity": 1, "Most people": 1, "Overestimate": 1}, "verbs": {"have": 1, "I think": 1, "Give": 1}, "adjectives": {"Real": 1, "not": 1}, "numerals": {"67": 1}}}, {"content": "85. The majority voted for a political candidate not on the basis of its position, but on the basis of minor subsections such as appearance or name.", "word_count": 40, "label": "", "sentence_structure": "Subject鈥搗erb鈥搊bject", "subjective_objective": {"answer": "Subjective", "rationale": "Because this view is the subjective interpretation and perception of the majority of voting behaviour by observers (questionnaire researchers). It involves the observers鈥?personal judgement and bias, and is therefore subjective."}, "sentence_sentiment": {"answer": "Negative", "rationale": "Because the act of \"majority voting for a political candidate, not on the basis of its position, but on the basis of minor subsections\" in the entry is negative."}, "language_style": {"answer": "Calm and direct", "rationale": "The entry expresses the views in a direct statement, without using rhetorical or emotional publicity. It directly points to the main reasons why the majority voted for political candidates, and refers to them as being often unrelated to the true position of the candidates, such as their appearance or name, and less important subsections. This language style is more objective and calm, allowing readers to understand the author鈥檚 intentions clearly."}, "time_dimension": {"answer": "Present", "rationale": ""}, "sentence_type": "Affirmative declarative sentence", "pos": {"nouns": {"Questionnaire": 1, "Entry": 1, "person": 1, "Political candidates": 1, "Position": 1, "Subsection": 1, "Appearance": 1, "Name": 1}, "verbs": {"Vote": 1, "Support": 1, "Based on": 2, "Not really": 1, "be": 1, "Decision": 1, "Ignore": 1, "Some": 1, "Yeah": 1, "Like what": 1, "锛?: 1}, "adjectives": {"Most": 1, "Important": 1, "Appearance": 1}, "adverbs": {"Not really": 1, "Based on": 1, "Some": 1, "Yeah": 1, "锛?: 1}, "numerals": {"85": 1}, "classifiers": {"individual": 1}, "pronouns": {"Other": 1, "it": 1, "EMPTY": 2, "Here": 1, "锛?: 1}}}, {"content": "36. I find my first impression of others often wrong.", "word_count": 19, "label": "", "sentence_structure": "Subject鈥搗erb鈥揷omplement", "subjective_objective": {"answer": "Subjective", "rationale": "Because \"first impressions are often wrong\" are subjective feelings and observations, not objective facts. This is the patient's assessment of his behaviour and views, and therefore subjective."}, "sentence_sentiment": {"answer": "Negative", "rationale": "Because the expression \"wrong\" in the entry is negative."}, "language_style": {"answer": "Calm and direct", "rationale": "The entry is not based on a direct statement, but on a more balanced and objective tone, using too many rhetorical or emotional expressions. At the same time, the entry does not use too many euphemisms, but rather opens its eyes to the mountains and expresses its views and observations, and is more calm and straightforward."}, "time_dimension": {"answer": "Present", "rationale": "The entry describes the current discovery or thinking of the person being tested, does not address past or future behaviour or mentality and therefore the judgement is now."}, "sentence_type": "Affirmative declarative sentence", "pos": {"nouns": {"self": 1, "others": 1, "First impression": 1, "Error": 1}, "verbs": {"Found": 1, "to": 1, "be": 1}, "adverbs": {"Often": 1}, "numerals": {"36": 1}}}, {"content": "42. Some people are too complicated to judge them.", "word_count": 16, "label": "", "sentence_structure": "Subject鈥揷opula鈥損redicative", "subjective_objective": {"answer": "Subjective", "rationale": "Because the complexity of some people is perceived as subjective in the light of the patient ' s experience and observations."}, "sentence_sentiment": {"answer": "Negative", "rationale": "The expression \"cannot judge it\" in the article's purpose expression is an expression of negative perception of some."}, "language_style": {"answer": "Tactful and warm", "rationale": "The language style of the entry tends to be euphemistic rather than direct statements or orders. The use of \"some\" instead of \"some\" to replace a specific individual avoids excessive blame or evaluation of the individual, while expressing their confusion and weakness by \"not able to judge them\", makes the entire sentence more personal and interactive and more resonant with the other."}, "time_dimension": {"answer": "Present", "rationale": ""}, "sentence_type": "Negative declarative sentence", "pos": {"nouns": {"person": 1, "Judgement": 1}, "verbs": {"Too much": 1, "Do it": 1}, "adjectives": {"Complex": 1, "Cannot": 1}, "adverbs": {"Yes": 2, "to": 1, "Other": 1}, "numerals": {"42": 1}, "pronouns": {"I": 1}}}, {"content": "48. I believe that it is never possible to truly understand what others feel.", "word_count": 18, "label": "", "sentence_structure": "Subject鈥搗erb鈥搊bject", "subjective_objective": {"answer": "Subjective", "rationale": "Because the title is about \"I think,\" it's about the patient's personal perception and feelings, and it's subjective."}, "sentence_sentiment": {"answer": "Negative", "rationale": "Because the entry \"never really know what people feel\" expresses a pessimism, negative emotions and attitudes."}, "language_style": {"answer": "Tactful and warm", "rationale": "Warm and warm."}, "time_dimension": {"answer": "Present", "rationale": "The keyword \"considers\" expresses a subjective view or view, \"it will never really be possible to truly understand the feelings of others\" is a current view or attitude based on personal conviction, and the temporal dimension of the article is therefore judged as it is."}, "sentence_type": "Negative declarative sentence", "pos": {"nouns": {"Questionnaire": 1, "Entry": 1, "Feel": 1}, "verbs": {"I think": 1, "Maybe": 1, "Really": 1, "Roger that": 1}, "adjectives": {"Forever": 1, "not": 1, "Maybe": 1, "Really": 1}, "adverbs": {"Yeah": 1, "From": 1}, "numerals": {"48": 1}, "classifiers": {"individual": 1}, "pronouns": {"I": 1}}}, {"content": "54. It is not possible to describe a person accurately in just a few words.", "word_count": 17, "label": "", "sentence_structure": "Subject鈥搗erb鈥揷omplement", "subjective_objective": {"answer": "Subjective", "rationale": "Because it is possible to describe accurately a person ' s subjective judgement and perception as a person in a few words."}, "sentence_sentiment": {"answer": "Neutral", "rationale": "Because the entry is not marked by any emotional tendency, it merely presents a fact."}, "language_style": {"answer": "Calm and direct", "rationale": "The language style of the entry is more calm and objective, it is not too much rhetorical or emotional to be rendered, and it is more straightforward. It expresses a point in a simple and clear language that cannot be based on a few words to describe a person accurately. This language style is suitable for views or information that need to be clearly expressed."}, "time_dimension": {"answer": "Present", "rationale": "The keyword \"unable\", \"only a few words\" and \"accurate description\" indicate that the person being tested would take more time and effort to describe, which is a description of the current state of affairs, and therefore the act to which the entry relates is present."}, "sentence_type": "Negative declarative sentence", "pos": {"nouns": {"Entry": 1, "person": 1}, "verbs": {"Come on": 1, "Description": 1, "Maybe": 1, "Only": 1, "Use": 1, "Correct": 1}, "adjectives": {"A few words": 1}, "adverbs": {"Land": 1}, "numerals": {"54": 1}}}, {"content": "60. It is impossible to divide everyone into good or bad people.", "word_count": 16, "label": "", "sentence_structure": "Subject鈥損redicate", "subjective_objective": {"answer": "Objective", "rationale": "Because \"the division of every person into good or bad\" is a point of view or judgment, not an objective fact that patients experience. So, it is an objective view."}, "sentence_sentiment": {"answer": "Neutral", "rationale": "Because the entry does not express a positive or negative emotional tendency, it is merely a statement of the facts."}, "language_style": {"answer": "Tactful and warm", "rationale": "The language style of the entry is more euphemistic, and it does not pass judgement or comment directly, but rather presents a point of view in a balanced and open manner, inviting the interviewee to reflect on his/her views. This expression neither leads to resistance from the interviewee nor makes the interviewee more willing to participate in the discussion."}, "time_dimension": {"answer": "Present", "rationale": ""}, "sentence_type": "Negative declarative sentence", "pos": {"nouns": {"Questionnaire Entry": 1, "person": 1}, "verbs": {"min": 1, "Done": 1}, "adjectives": {"Okay": 1, "Bad": 1}, "numerals": {"60": 1}, "pronouns": {"Every": 1, "One": 2}}}, {"content": "78. People are too complex to be fully understood.", "word_count": 14, "label": "", "sentence_structure": "Subject鈥揷opula鈥損redicative", "subjective_objective": {"answer": "Subjective", "rationale": "Because \"people are too complex to fully understand\" is the subjective view and opinion of people."}, "sentence_sentiment": {"answer": "Negative", "rationale": "Because the entry expresses a pessimism and a negative sentiment about human beings, the complexity of which cannot be fully understood and grasped. This view can lead to negative perceptions and behaviour in human relations, social interaction and so on, and discourages the building of positive interpersonal relations and social resilience."}, "language_style": {"answer": "Calm and direct", "rationale": "The language style of the entry is calm and straightforward. It does not use any rhetorical or emotional expression, but rather describes human complexity in an objective and concise language that shows that we have difficulty fully understanding others. It makes it easier to accept and think about this view, rather than being influenced by emotions or personal positions."}, "time_dimension": {"answer": "Past", "rationale": "The entry describes a widespread phenomenon or view and does not address the current behaviour or mentality of the person being tested, so the temporal dimension is judged to be the past."}, "sentence_type": "Negative declarative sentence", "pos": {"nouns": {"person": 2, "Complex": 1, "Roger that": 1}, "verbs": {"Too much": 1, "Maybe": 1, "Take it": 1}, "adjectives": {"Complex": 1}, "adverbs": {"Too much": 1}, "numerals": {"78": 1}, "pronouns": {"Here": 1}}}, {"content": "90. People are too complex to understand what their motivations are.", "word_count": 17, "label": "", "sentence_structure": "Subject鈥揷opula鈥損redicative", "subjective_objective": {"answer": "Subjective", "rationale": "Because \"hard to understand\" is a subjective assessment and feeling that involves the cognitive and emotional elements of the individual. And \"man is too complex\" is an objective fact. So it's a subjective question."}, "sentence_sentiment": {"answer": "Negative", "rationale": "Because \"people are too complicated to understand\" gives people a negative and helpless feeling."}, "language_style": {"answer": "Calm and direct", "rationale": "The entry is based on objective facts and presents a view, without excessive rhetoric or emotional colours, and is more calm and direct in language style."}, "time_dimension": {"answer": "Present", "rationale": "The entry is an expression of the views and opinions of the speaker and is not clearly time-directed and therefore judged to be the present-day situation."}, "sentence_type": "Negative declarative sentence", "pos": {"nouns": {"person": 2, "Motives for behaviour": 1}, "verbs": {"It's too complicated": 1, "Roger that": 1}, "adjectives": {"Hard": 1}, "adverbs": {"What": 1}, "numerals": {"90": 1}}}, {"content": "I find my first impression of others often correct.", "word_count": 19, "label": "", "sentence_structure": "Subject鈥搗erb鈥揷omplement", "subjective_objective": {"answer": "Subjective", "rationale": "Because the statement reflects the patient ' s personal views and beliefs that they consider their first impressions to be usually accurate. It is a subjective observation or observation."}, "sentence_sentiment": {"answer": "Positive", "rationale": "Because the purpose of the article is affirmative, it expresses confidence and accurate observation."}, "language_style": {"answer": "Calm and direct", "rationale": "The language style of the entry is more straightforward, not using too many rhetorical techniques or emotional renderings, but rather expressing the subject of the investigation in a simple and clear manner. At the same time, the question is not in too complex or difficult to understand, so that the person surveyed can understand and answer quickly. Thus, the language style of the purpose is more \"cool and direct.\""}, "time_dimension": {"answer": "Present", "rationale": "The sentence describes a testee's current self-awareness and perception, which is \"I find myself...\", and therefore judgment is now."}, "sentence_type": "Affirmative declarative sentence", "pos": {"nouns": {"self": 1, "First impression": 1, "others": 1}, "verbs": {"Found": 1, "be": 1, "Often": 1, "Right": 1}, "adjectives": {"of": 3}, "adverbs": {"to": 1, "Both": 1}, "pronouns": {"I": 1}}}, {"content": "A term can be used to describe a person precisely, such as 鈥渋nternal鈥? 鈥渕oral鈥?or 鈥渓ove鈥?", "word_count": 24, "label": "", "sentence_structure": "Subject鈥搗erb鈥揷omplement", "subjective_objective": {"answer": "Subjective", "rationale": "Because the character character of a person is described as subjective by the patient."}, "sentence_sentiment": {"answer": "Neutral", "rationale": "Because the entry does not have any emotional color."}, "language_style": {"answer": "Calm and direct", "rationale": "The language style of the entry is a calm and straightforward one. The entry expresses what needs to be filled out in a concise and clear way, without too much rhetorical or emotional recitation, and is more objective in tone. It also gives a specific range of answers (one word), making them clearer and more specific."}, "time_dimension": {"answer": "Present", "rationale": "The entry allows the person to describe a person ' s character in a word, which is usually a description of the present, objective circumstances and therefore the present."}, "sentence_type": "Affirmative declarative sentence", "pos": {"nouns": {"Questionnaire Entry": 1, "Someone": 1, "Word": 2, "Inner": 1, "Ethics": 1, "Communication": 1}, "verbs": {"Yeah": 1, "Description": 1, "Use": 1}, "adjectives": {"Correct": 1}, "numerals": {"One": 2}, "classifiers": {"Like": 1}}}, {"content": "It is not difficult to find what really matters to a person.", "word_count": 15, "label": "", "sentence_structure": "Subject鈥搗erb鈥搊bject", "subjective_objective": {"answer": "Subjective", "rationale": "Because \"what really matters to a person is not hard to find\" is an opinion or opinion, a subjective attitude of the patient."}, "sentence_sentiment": {"answer": "Positive", "rationale": "Because the phrase \"recovering what really matters to a person\" in the entry is positive."}, "language_style": {"answer": "Tactful and warm", "rationale": "The language style of the entry is more euphemistic and sweet. It uses a relatively easy and friendly tone, without direct orders or asking the other to answer questions, but rather to guide the interviewee to think and share his/her views in an invitation and understanding. This expression makes it easier to make the interviewee comfortable and welcome and helps to create a good questionnaire atmosphere."}, "time_dimension": {"answer": "Present", "rationale": "In conclusion, I will classify this questionnaire entry as \"the future\", which means that the testees have some expectation and exploration of how to discover what really matters to them."}, "sentence_type": "Affirmative declarative sentence", "pos": {"nouns": {"person": 1, "Important": 1}, "verbs": {"Found": 1, "It's not hard": 1}, "adjectives": {"Really": 1}, "adverbs": {"Really": 1, "not": 1}, "numerals": {"18": 1}, "classifiers": {"What": 1, "To someone": 1}, "pronouns": {"Here": 1}}}, {"content": "24. I believe that a brief conversation with someone would give a considerable understanding of its nature.", "word_count": 26, "label": "", "sentence_structure": "Subject鈥搗erb鈥搊bject", "subjective_objective": {"answer": "Subjective", "rationale": "Because \"think\" expresses the patient's subjective attitude."}, "sentence_sentiment": {"answer": "Positive", "rationale": "The article's formulation, which includes \"a considerable understanding of its nature\", expresses a positive and optimistic attitude and expectation."}, "language_style": {"answer": "Tactful and warm", "rationale": "The language style of the entry is more euphemistic and somewhat subjective, using the expression \"conceptive\" and \"to some extent\" to make the issue more open and non-judgmental, and to reflect an attitude of respect for individual differences."}, "time_dimension": {"answer": "Present", "rationale": "The entry describes the current or future view of the testee, and does not address past or future situations. In it, \"concept\" and \"capable\" use the current state of affairs, indicating that this is the testee's view of the current situation. So the temporal dimension is now."}, "sentence_type": "Affirmative declarative sentence", "pos": {"nouns": {"Questionnaire": 1, "Entry": 1, "Talk": 1, "Nature": 1}, "verbs": {"I think": 1, "Yes": 1, "have": 1, "Roger that": 1}, "adjectives": {"Someone": 1, "Short": 1, "Pretty much": 1}, "adverbs": {"After": 1}, "numerals": {"24": 1, "Level": 1}, "classifiers": {"individual": 1}, "pronouns": {"Other": 1}}}, {"content": "30. If someone among us were allowed to ask three questions about him (and assume that his answer was honest), I would have a considerable knowledge of him.", "word_count": 40, "label": "", "sentence_structure": "Subject鈥搗erb鈥搊bject", "subjective_objective": {"answer": "Subjective", "rationale": "Because a considerable degree of knowledge about a person needs to be based on observation and understanding of that person, this is the patient ' s subjective judgement and expectation."}, "sentence_sentiment": {"answer": "Neutral", "rationale": "Because the entry does not have any emotional color."}, "language_style": {"answer": "Tactful and warm", "rationale": "The language style of the entry is more euphemistic and inviting. It does not direct the respondent to answer questions, but rather requests in a friendly and understanding manner. Moreover, it assumes that the interviewee鈥檚 response is honest, which also demonstrates respect and trust for the interviewee."}, "time_dimension": {"answer": "Present", "rationale": ""}, "sentence_type": "Affirmative declarative sentence", "pos": {"nouns": {"Questionnaire Entry": 1, "I": 1, "he": 2, "Problem": 3, "An honest answer": 1}, "verbs": {"Allow": 1, "Someone else": 1, "have": 1, "Answer": 1, "Yes, I can": 1, "Level of knowledge": 1}, "adjectives": {"About him": 2}, "adverbs": {"If": 1, "And assume that": 1, "Pretty much": 1}, "classifiers": {"individual": 1}}}, {"content": "72. When I first met someone, I tried to find a basic feature that would allow me to understand him.", "word_count": 26, "label": "", "sentence_structure": "Subject鈥揷opula鈥損redicative", "subjective_objective": {"answer": "Subjective", "rationale": "According to the description in the sentence, \"I'm trying to find a basic feature to understand this person,\" which shows that it is a personal, subjective act and idea, and therefore the entry to the questionnaire is subjective."}, "sentence_sentiment": {"answer": "Neutral", "rationale": "Because the entry does not have any emotional color."}, "language_style": {"answer": "Tactful and warm", "rationale": "The entry is more gentle in its language style, using the words \"trace to find\" and \"know\" and expresses an attempt and exploration, more polite and respectful of the other. It also reflects a caring and caring attitude by asking about the behaviour of someone who first meets the other."}, "time_dimension": {"answer": "Present", "rationale": "The entry describes the behaviour and way of thinking of the person who was tested when first met someone, and is a description of the current situation, and thus a judgement of the present."}, "sentence_type": "Affirmative declarative sentence", "pos": {"nouns": {"Initial": 1, "Someone": 1, "Basic characteristics": 1, "This man": 1}, "verbs": {"Yes": 1, "Trying to find": 1, "Roger that": 1}, "adverbs": {"When": 1, "Initial": 1}, "numerals": {"72": 1}, "pronouns": {"I": 1}}}, {"content": "96. By providing me with some facts about someone, I can see if I like him.", "word_count": 25, "label": "", "sentence_structure": "Subject鈥搗erb鈥搊bject", "subjective_objective": {"answer": "Subjective", "rationale": "Because it is the patient ' s own feelings and judgment that is being examined and is subjective."}, "sentence_sentiment": {"answer": "Positive", "rationale": "Because the purpose of the action in the entry is positive, i.e. to know someone in order to decide whether he likes him or not."}, "language_style": {"answer": "Tactful and warm", "rationale": "The language style of the entry is more euphemistic and gentle, because it does not directly ask or order the person interviewed to provide some information. Rather, it uses a more open-minded tone, which attempts to channel the person to share his thoughts and feelings. This expression is more likely to resonate and engage the person interviewed, and to be more friendly and interactive."}, "time_dimension": {"answer": "Present", "rationale": "The \"Providing me\" and \"I know\" in that sentence occurred at the moment when the person who spoke thought and answered the question, so it's the act now."}, "sentence_type": "Affirmative declarative sentence", "pos": {"nouns": {"Questionnaire": 1, "The facts": 1, "person": 1}, "verbs": {"Provision": 1, "Yes": 2}, "adverbs": {"Just": 1}, "numerals": {"96": 1}, "classifiers": {"Some": 1}, "pronouns": {"I": 1, "he": 1}}}, {"content": "11. The response of one person to events has changed as a result of the change in his or her situation.", "word_count": 19, "label": "", "sentence_structure": "Subject鈥揷opula鈥損redicative", "subjective_objective": {"answer": "Subjective", "rationale": "Because \"a person's response to events changes because of his or her situation\" is the patient's perception and interpretation of his or her behaviour, feelings, etc., or that of others, and is a subjective examination."}, "sentence_sentiment": {"answer": "Neutral", "rationale": "Since the entry does not address positive or negative attitudes, behaviour, purpose or quality, it merely describes a phenomenon."}, "language_style": {"answer": "Calm and direct", "rationale": "The language style of the entry is more calm and objective, and there is no excessive emotional rendering or rhetorical technique, which directly presents a fact in a concise and clear language."}, "time_dimension": {"answer": "Past", "rationale": "The entry describes the evolution of the individual ' s response in different contexts, which is a review and summary of the past."}, "sentence_type": "Affirmative declarative sentence", "pos": {"nouns": {"person": 1, "Things": 1, "Response": 1, "Situation": 1, "Change": 2}, "verbs": {"to": 1, "Because": 1, "Change": 1}, "numerals": {"One": 3}, "pronouns": {"Other": 1}}}, {"content": "17. Different people react differently to the same situation.", "word_count": 16, "label": "", "sentence_structure": "Subject鈥揷opula鈥損redicative", "subjective_objective": {"answer": "Objective", "rationale": "This is an objective phenomenon, since it describes the different reactions of people in the same circumstances, without taking into account the subjective views of individuals."}, "sentence_sentiment": {"answer": "Neutral", "rationale": "Because the entry does not have any emotional color."}, "language_style": {"answer": "Calm and direct", "rationale": "The entry describes a general phenomenon in objective and concise language, without excessive emotional or subjective judgement, and is more calm and straightforward in tone.\""}, "time_dimension": {"answer": "Present", "rationale": "The entry describes a phenomenon or observation, which is not clearly time-directed, and therefore is judged as \"now\"."}, "sentence_type": "Imperative sentence", "pos": {"nouns": {"Situation": 1, "person": 2, "Response": 1}, "verbs": {"to": 1, "have": 1, "Same": 1, "Different": 1, "Response": 1}, "adjectives": {"Different": 1, "Same": 1}, "numerals": {"17": 1}, "classifiers": {"individual": 1}, "pronouns": {"Here": 1}}}, {"content": "23. Each individual has a different character.", "word_count": 12, "label": "", "sentence_structure": "Subject鈥揷opula鈥損redicative", "subjective_objective": {"answer": "Subjective", "rationale": "Because character is described as a subjective view of personal identity."}, "sentence_sentiment": {"answer": "Neutral", "rationale": "Because the entry merely stated a fact, and did not have any emotional colour."}, "language_style": {"answer": "Tactful and warm", "rationale": "The entry is more personal and interactive when it is spoken in a gentle and inviting manner, rather than in direct statements or orders. At the same time, by using the expression \"everyone is different from the other\" it emphasizes the uniqueness and diversity of the individual and can also trigger reflection and reflection on the character characteristics of the individual."}, "time_dimension": {"answer": "Present", "rationale": "The entry represents a general statement of fact that each individual ' s personality is unique and does not involve past or future behaviour or mentality, and should therefore be judged as the present."}, "sentence_type": "Affirmative declarative sentence", "pos": {"nouns": {"person": 1, "Character": 1}, "verbs": {"Average": 1, "Different": 1}, "numerals": {"23": 1}, "pronouns": {"Every one": 1}}}, {"content": "29. Basic interests vary widely.", "word_count": 11, "label": "", "sentence_structure": "Subject鈥揷opula鈥損redicative", "subjective_objective": {"answer": "Subjective", "rationale": "Because \"basic interests are very different\" is a subjective view and view."}, "sentence_sentiment": {"answer": "Neutral", "rationale": "Because the entry is merely a statement of fact and does not express a positive or negative emotional attitude."}, "language_style": {"answer": "Calm and direct", "rationale": "The language style of the entry is more direct, without any syllable or emotional rendering, simply stating a fact, without expressing any attitude or position, and therefore can be judged as \"cooling straight\"."}, "time_dimension": {"answer": "Past", "rationale": "The entry does not have a clear time reference point, but it describes the diversity of people ' s basic interests, which is a relatively stable fact and can be judged as a description of the past."}, "sentence_type": "Affirmative declarative sentence", "pos": {"nouns": {"People": 1, "Basic interests": 1, "Different": 1}, "adjectives": {"Large": 1}}}, {"content": "The 鈥渕otives鈥?of behaviour vary considerably from one person to another.", "word_count": 13, "label": "", "sentence_structure": "Subject鈥搗erb鈥揷omplement", "subjective_objective": {"answer": "Objective", "rationale": "Because \"motives of conduct\" refers to the driving force behind people's behaviour, it is an objective fact. While the motivations of each individual may differ, this difference can be derived from observation and research and is therefore in the objective category."}, "sentence_sentiment": {"answer": "Neutral", "rationale": "Because the entry does not have any emotional color."}, "language_style": {"answer": "Calm and direct", "rationale": "It is therefore clear that the language style is calm and straightforward."}, "time_dimension": {"answer": "Present", "rationale": "The entry describes the different characteristics of people in their current state, and the terms \"people's \"motives\" and \"many differences\" all indicate that this is the present description."}, "sentence_type": "Affirmative declarative sentence", "pos": {"nouns": {"People": 1, "Motives for behaviour": 2}, "verbs": {"Difference": 1, "A lot": 1}, "numerals": {"65": 1}}}, {"content": "83. The basic human personality is often changed by religious belief, psychological treatment, etc.", "word_count": 24, "label": "", "sentence_structure": "Subject鈥搗erb鈥搊bject", "subjective_objective": {"answer": "Objective", "rationale": "Whether or not a person鈥檚 fundamental personality changes due to religious belief, psychological treatment, etc. is a fact that can be observed and studied. While these factors may affect a person鈥檚 character or behaviour, the impact is objective and not subjective."}, "sentence_sentiment": {"answer": "Neutral", "rationale": "Because the entry does not express a positive or negative emotional attitude, it is merely a statement of the facts."}, "language_style": {"answer": "Tactful and warm", "rationale": "The entry raises questions in a gentle and exploratory way, asking if the interviewees believe that the fundamental personality of the human person will change for a number of reasons (such as religious belief or psychological therapy). This expression is a form of affection and guidance that makes people feel comfortable and willing to share their views."}, "time_dimension": {"answer": "Present", "rationale": "In the future, the entry indicates an event that is expected to occur, i.e., that the fundamental personality of the human person may change for reasons of religious belief, psychological treatment, etc. The event has not yet occurred, but it is likely to be realized in the future, so we can judge it as a future act or mentality."}, "sentence_type": "Affirmative declarative sentence", "pos": {"nouns": {"person": 2, "Personality": 1, "Religious beliefs": 1, "Reason": 1, "Psychotherapy": 1}, "verbs": {"Change": 1, "have": 1}, "adjectives": {"Basic": 1}, "numerals": {"83": 1}}}, {"content": "89. People will not be able to move forward from one occasion to another.", "word_count": 22, "label": "", "sentence_structure": "Subject鈥損redicate", "subjective_objective": {"answer": "Subjective", "rationale": "Because \"unpredictable\" is a subjective assessment or feeling that reflects expectations and perceptions of a situation, not objective facts."}, "sentence_sentiment": {"answer": "Negative", "rationale": "Because the entry describes people ' s actions as unpredictable, it is a negative situation."}, "language_style": {"answer": "Calm and direct", "rationale": "The entry is more directly linguistically, without too much qualifier or emotional expression. It is a succinct statement of the fact that human behaviour is unpredictable and is more objective and calm in tone."}, "time_dimension": {"answer": "Past", "rationale": "The entry is about the unpredictable nature of people ' s actions and does not address the time dimension of past, present or future, and therefore does not allow for time-based judgement."}, "sentence_type": "Negative declarative sentence", "pos": {"nouns": {"People": 1, "Event": 2, "Action": 1}, "verbs": {"Will": 1, "Projections": 1, "Cannot": 1}, "adjectives": {"89": 1, "Another one": 1}, "numerals": {"89": 1}, "classifiers": {"Plant": 2}}}, {"content": "35. The basic interest is very similar.", "word_count": 11, "label": "", "sentence_structure": "Subject鈥揷opula鈥損redicative", "subjective_objective": {"answer": "Objective", "rationale": "Because \"people's interest\" is the fact that you can observe, measure, not the subjective feelings or attitudes of the patient."}, "sentence_sentiment": {"answer": "Neutral", "rationale": "Because the entry describes a fact and does not involve positive or negative emotional colours."}, "language_style": {"answer": "Tactful and warm", "rationale": "The language style of this entry is more euphemistic and inclusive, and it does not directly negate or confirm the similarity of interest, but rather raises questions in a manner that is accessible and open to exploration. This expression is more likely to trigger resonance and reflection among the interviewees and to facilitate the collection of real and effective data."}, "time_dimension": {"answer": "Present", "rationale": "In conclusion, according to the sentence entered, \"35. People's basic interests are very similar.\" The content is classified as \"now\"."}, "sentence_type": "Affirmative declarative sentence", "pos": {"nouns": {"People": 2, "Basic interests": 1, "Similar": 1}, "verbs": {"have": 1, "Extreme": 1}, "adjectives": {"Similar": 1}, "adverbs": {"Extreme": 1}, "numerals": {"35": 1}, "pronouns": {"they": 1}}}, {"content": "41. People are essentially similar in character.", "word_count": 9, "label": "", "sentence_structure": "Subject鈥揷opula鈥損redicative", "subjective_objective": {"answer": "Objective", "rationale": "Since character is inherent to the individual and cannot be changed, the basic similarities of character are an objective fact."}, "sentence_sentiment": {"answer": "Neutral", "rationale": "Because the entry does not have any emotional color."}, "language_style": {"answer": "Tactful and warm", "rationale": "The language style of this entry is more euphemistic, with the term \"people\" being used instead of a specific group of people and with the emphasis on the concept of \"basic similarity\", which is expressed more broadly and in a more encouraging manner."}, "time_dimension": {"answer": "Past", "rationale": "The entry describes a general belief or perception, which does not relate to a particular period or event. It can therefore be judged to be a description of the past."}, "sentence_type": "Affirmative declarative sentence", "pos": {"nouns": {"People": 1, "Character": 1}, "verbs": {"Basic": 1, "Similar": 1}, "numerals": {"41": 1}}}, {"content": "47. It is only by understanding how two or three people react to a particular occasion that it can be assumed that the majority will respond in the same way.", "word_count": 37, "label": "", "sentence_structure": "Subject鈥搗erb鈥搊bject", "subjective_objective": {"answer": "Subjective", "rationale": "This is because the patient's assumptions or assumptions about the behaviour or way of thinking of the person are subjective."}, "sentence_sentiment": {"answer": "Neutral", "rationale": "The purpose of this questionnaire is not to express a clear positive or negative emotional expression, but rather to present a fact and view, without expressing any emotional tendencies or values. I therefore consider the entry to be neutral."}, "language_style": {"answer": "Calm and direct", "rationale": "The language style of the entry is more calm and objective, and there is no excessive emotional or rhetorical approach. It presents a point of view in a factual manner and requires the respondent to judge its identity, and therefore is a direct expression of calm."}, "time_dimension": {"answer": "Present", "rationale": ""}, "sentence_type": "Affirmative declarative sentence", "pos": {"nouns": {"person": 2, "Event": 1, "Response": 2}, "verbs": {"Roger that": 1, "And guessed": 1, "鈾?Will take 鈾?: 1}, "adverbs": {"Just": 2, "Two, three": 1, "Most": 1}, "numerals": {"Forty-seven": 1, "Two": 1, "III": 1}}}, {"content": "53. Most people react the same way in different contexts.", "word_count": 19, "label": "", "sentence_structure": "Subject鈥搗erb鈥搊bject", "subjective_objective": {"answer": "Objective", "rationale": "This is because it is describing a general phenomenon, not the subjective attitude or experience of the patient."}, "sentence_sentiment": {"answer": "Neutral", "rationale": "Because the entry merely describes a phenomenon and does not involve any emotional colour or value judgement."}, "language_style": {"answer": "Calm and direct", "rationale": "The entry is more straightforward, concise, less talkative or emotional, more objective. It expresses in a simple way the subject of asking if the other side will react to things in different contexts."}, "time_dimension": {"answer": "Present", "rationale": "The entry describes a normalized response by people in different contexts, does not address the time dimension of past or future and can therefore be judged as now."}, "sentence_type": "Affirmative declarative sentence", "pos": {"nouns": {"Most people": 1, "Event": 1, "Things": 1, "Response": 1}, "verbs": {"Adoption": 1}, "adjectives": {"Different": 1, "Same thing": 1}, "numerals": {"53": 1}}}, {"content": "59. When a person is a child, he or she is equally a person of age.", "word_count": 22, "label": "", "sentence_structure": "Subject鈥搗erb鈥搊bject", "subjective_objective": {"answer": "Subjective", "rationale": "Because \"into love\" is a subjective assessment that reflects a sense of affection and intimacy towards a person. This sense of affection and intimacy may be based on a variety of factors, such as individual qualities, behaviour patterns, and these are subjective criteria of judgment. So, \"A person who is popular as a child is equally popular as an adult.\" This is based on subjective assumptions that do not reflect reality entirely objectively."}, "sentence_sentiment": {"answer": "Neutral", "rationale": "Because the entry is a mere description of a phenomenon and does not express any positive or negative emotional colours."}, "language_style": {"answer": "Tactful and warm", "rationale": "The entry is more proficient and interactive when it is spoken in a gentle and inviting manner, rather than in direct statements or orders. At the same time, the language style of the article is more objective and neutral, with no excessive rhetorical or emotional overtones, and is more linguistically sound."}, "time_dimension": {"answer": "Past", "rationale": "The entry describes a general phenomenon whereby a person who is attracted to a child is usually treated the same way as a person who has reached the age of majority."}, "sentence_type": "Affirmative declarative sentence", "pos": {"nouns": {"Alone": 1, "When I was little": 1, "After the adult": 1, "Same thing": 1, "Loved": 2}, "verbs": {"If": 1, "be": 1, "Adult": 1, "Move": 1}, "adverbs": {"Yeah": 1, "Same thing": 1}, "numerals": {"59": 1}}}, {"content": "If I could understand the response of someone to one situation, I would know how he would react to other situations.", "word_count": 32, "label": "", "sentence_structure": "Subject鈥搗erb鈥搊bject", "subjective_objective": {"answer": "Objective", "rationale": "This is seen from an objective perspective. The sentence describes a general phenomenon or pattern, not the patient ' s subjective attitude or feelings."}, "sentence_sentiment": {"answer": "Neutral", "rationale": "Because the entry does not express any positive or negative tendencies to be emotional, attitudinal, behavioural, purpose or quality."}, "language_style": {"answer": "Calm and direct", "rationale": "The entry is more concise, is less emotional or rhetorical, and is more objective and calm. It asks the other side's opinion of \"if I can understand someone's reaction to one situation, I can know how he will react to another's situation,\" and is straightforward, without any cornering or ambiguity, and therefore can be judged as a calm and straightforward style of language."}, "time_dimension": {"answer": "Present", "rationale": "The entry discusses a hypothetical situation - understanding the reaction of a person to a particular situation, and infers what they would react to other situations. This is a description of the current way of thinking and understanding, and does not address future or past events or situations. Thus, the temporal dimension is judged as \"now\"."}, "sentence_type": "Affirmative declarative sentence", "pos": {"nouns": {"Situation": 3, "Response": 2, "person": 1}, "verbs": {"Yes": 3, "Roger that": 1, "to": 1, "How's that": 1, "Will": 1, "Response": 1}, "adverbs": {"If": 1, "Yes": 1}, "numerals": {"77": 1}, "pronouns": {"I": 1, "he": 1}}}, {"content": "95. If you seriously consider it, you find that people ' s feelings are very similar.", "word_count": 22, "label": "", "sentence_structure": "Subject鈥搗erb鈥搊bject", "subjective_objective": {"answer": "Objective", "rationale": "Because \"the composition of people's feelings\" is a fact or a state of existence, it's an objective examination."}, "sentence_sentiment": {"answer": "Neutral", "rationale": "Because the entry describes a fact, it does not express any positive or negative emotional colours."}, "language_style": {"answer": "Tactful and warm", "rationale": "The entry is more personal and interactive when it is presented in a gentle and inviting tone, rather than in direct statements or orders. At the same time, the phrase \"if you seriously consider\" is used to guide each other to further thinking and to involve respondents more actively in the questionnaire."}, "time_dimension": {"answer": "Past", "rationale": "In terms of context, this sentence may be a general description or observation of people鈥檚 feelings, not a specific time point. I am therefore unable to attribute it accurately to a certain time dimension, past, present or future, and suggest that the context information be further clarified for a more accurate analysis."}, "sentence_type": "Affirmative declarative sentence", "pos": {"nouns": {"People": 1, "Emotion": 1, "Composition": 1, "Extreme": 1, "Similar": 1}, "verbs": {"Yes": 2, "Found": 1, "Consider": 1, "Seriously": 1}, "adjectives": {"Seriously": 1, "Extreme": 1}, "adverbs": {"If": 1, "Just": 1}, "numerals": {"95": 1}, "pronouns": {"you": 1}}}, {"content": "1. Most people would have done so if they had access to people ' s cinemas without paying for them and would certainly not have been discovered.", "word_count": 31, "label": "", "sentence_structure": "Subject鈥搗erb鈥搊bject", "subjective_objective": {"answer": "Subjective", "rationale": "Because the subject is to ask people about their will and ideas, it is a subjective examination."}, "sentence_sentiment": {"answer": "Neutral", "rationale": "As this entry does not express positive or negative attitudes, behaviour, purpose or quality."}, "language_style": {"answer": "Calm and direct", "rationale": "The language style of the entry is more calm and straightforward, and dialogue is conducted in a factual manner, with no excessive emotional rendering and rhetorical techniques. This is a simple and clear expression that is more consistent with the seriousness and objectivity of the questionnaire."}, "time_dimension": {"answer": "Present", "rationale": "The keyword \"if\" and \"will\" now contained in the entry indicates that it is a hypothetical picture and description of behaviour, but does not specify whether such conduct has occurred or will occur in the past. Thus, it is now judged by the time dimension."}, "sentence_type": "Affirmative declarative sentence", "pos": {"nouns": {"Questionnaire Entry": 1, "person": 1, "Cinema": 1, "Most people": 1}, "verbs": {"Yes": 2, "Do it": 1, "In": 1, "Found": 1, "That way": 1}, "adjectives": {"No money": 1, "I'm sure it won't": 1}, "numerals": {"Most": 1, "1": 4}, "classifiers": {"individual": 1}, "pronouns": {"If": 1, "Yes": 1, "Well": 1, "That way": 1}}}, {"content": "2. Most people have the courage to recognize mistakes.", "word_count": 9, "label": "", "sentence_structure": "Subject鈥揷opula鈥損redicative", "subjective_objective": {"answer": "Objective", "rationale": "Because the majority ' s views and behaviour are objective facts, they are not subjective attitudes or feelings of the patient."}, "sentence_sentiment": {"answer": "Positive", "rationale": "Because the \"courage to recognize\" act is positive."}, "language_style": {"answer": "Tactful and warm", "rationale": "The language style of the entry is relatively warm. It uses a mild, invitational tone to engage in dialogue, rather than directly speaking or ordering. Moreover, the expression \"majority\" and \"fragmentation courage\" in the sentence give people a sense of affection, understanding and support, without too much negative emotional expression."}, "time_dimension": {"answer": "Present", "rationale": "The keyword \"courage to recognize wrong\" denotes current behaviour or attitude, does not relate to past or future information, and it is therefore possible to judge that the entry is a description of the present situation."}, "sentence_type": "Affirmative declarative sentence", "pos": {"nouns": {"person": 1, "Courage": 1}, "verbs": {"have": 1, "Wrong": 1, "Recognition": 1}, "adverbs": {"More": 1, "- The Grand Dus": 1}, "numerals": {"Most": 1}}}, {"content": "3. The general population is self-righteous.", "word_count": 12, "label": "", "sentence_structure": "Subject鈥搗erb鈥揷omplement", "subjective_objective": {"answer": "Subjective", "rationale": "Because it relates to the ideas and views of individuals, it is a reflection of their subjective attitude."}, "sentence_sentiment": {"answer": "Negative", "rationale": "Because it expresses a negative feeling that there is a general tendency to be arrogant."}, "language_style": {"answer": "Calm and direct", "rationale": "The entry is straightforward, does not have too much emotional rendering or rhetorical techniques and is a more objective and dispassionate formulation."}, "time_dimension": {"answer": "Present", "rationale": "In the entry, the word \"general\" denotes a normal or general situation, while the word \"all\" emphasizes the universality of the situation. The description \"self-righteous\" is subjective in terms of attitude, perception, and so its temporal dimension can be judged as now."}, "sentence_type": "Affirmative declarative sentence", "pos": {"verbs": {"be": 1, "Yes": 1, "I thought": 1, "You think you're great": 1}, "adjectives": {"General": 1}, "pronouns": {"Here": 1}}}, {"content": "4. Even in today ' s complex society, the majority still wishes to follow the principle of humanity in the Bible.", "word_count": 28, "label": "", "sentence_structure": "Subject鈥搗erb鈥搊bject", "subjective_objective": {"answer": "Objective", "rationale": "Because the entry describes a widespread phenomenon, in which most people wish to follow the principle of humanity in the Bible. This does not involve subjective attitudes or feelings of individuals, and is therefore viewed from an objective perspective."}, "sentence_sentiment": {"answer": "Neutral", "rationale": "Because the entry simply stated a fact and did not express any emotional tendencies."}, "language_style": {"answer": "Tactful and warm", "rationale": "The entry is more personal and interactive when it is spoken in a gentle and inviting tone, rather than in direct statements or orders. At the same time, the use of the phrase \"even if\" makes the sentence open and exploratory, and leads the interviewees to share their views and experiences."}, "time_dimension": {"answer": "Present", "rationale": "The entry appears as \"Today\" and \"Ambit\" and represents a tendency to think in the current social environment, and the temporal dimension is now."}, "sentence_type": "Affirmative declarative sentence", "pos": {"nouns": {"Social": 1, "person": 1, "Principles": 1, "Bible": 1}, "verbs": {"Follow": 1, "Yes": 1, "in": 1, "This one": 1, "Lee": 1, "Complex": 1, "Still": 1, "Compliance": 1}, "adjectives": {"Complex": 1}, "numerals": {"Most": 1}, "pronouns": {"they": 1}}}, {"content": "5. Most people stop to help people whose cars are out of order.", "word_count": 17, "label": "", "sentence_structure": "Subject鈥搗erb鈥搊bject", "subjective_objective": {"answer": "Objective", "rationale": "This is a description of a widespread social phenomenon, which does not involve subjective attitudes or experiences of individuals."}, "sentence_sentiment": {"answer": "Positive", "rationale": "Because the behavior in the entry \"stop and help people with car problems\" is positive."}, "language_style": {"answer": "Tactful and warm", "rationale": "The entry is a soft, inviting conversation, expressing concern and concern about the fact that most people are willing to stop to help people who need help with car problems. This expression is more personal and interactive."}, "time_dimension": {"answer": "Present", "rationale": "The keywords \"majority\", \"stop\" and \"help\" indicate that this is a description of current behaviour, implying that most people choose to stop and provide assistance when they encounter people who need help."}, "sentence_type": "Affirmative declarative sentence", "pos": {"nouns": {"person": 2, "Car": 1}, "verbs": {"Out": 1, "Stop": 1, "Help": 1}, "adjectives": {"More": 1}, "adverbs": {"Yes": 1, "Just": 1}, "numerals": {"5": 1}, "pronouns": {"Most people": 1}}}, {"content": "6. Even if there is a set of ethical standards, ordinary students also cheat when everyone else cheats on the exam.", "word_count": 33, "label": "", "sentence_structure": "Subject鈥搗erb鈥搊bject", "subjective_objective": {"answer": "Objective", "rationale": "Because the title describes an objective fact that in a given situation, ordinary students may also behave in a manner inconsistent with ethical standards. The moral choice of the student is examined, but whether or not it has occurred is objective and not influenced by subjective attitudes."}, "sentence_sentiment": {"answer": "Negative", "rationale": "Because the \"he can cheat\" in the entry is negative."}, "language_style": {"answer": "Tactful and warm", "rationale": "The language style of the entry is more euphemistic and implicit, rather than direct orders or statements, and the representation of the investigation is increased by introducing the \"general student\" group, thereby reducing the resistance of the person under investigation. Moreover, the expression \"even if there is a set of ethical standards, when others cheat in the examination, he also cheats\" is more guiding and can trigger the person under investigation to reflect and express his true ideas."}, "time_dimension": {"answer": "Present", "rationale": "The entry describes a hypothetical situation in which a student may behave in a certain way. Although this may not occur in all settings, it can still be considered a realistic possibility and should therefore be classified as an entry in the present state of affairs."}, "sentence_type": "Affirmative declarative sentence", "pos": {"nouns": {"Students": 1, "One": 1, "Ethical standards": 1, "person": 2, "Examinations": 1, "he": 1}, "verbs": {"have": 1, "be": 1, "Yes": 2, "Cheating": 2}, "adjectives": {"Normal": 1, "Average": 1}, "adverbs": {"Even if": 1, "When": 1, "Yeah": 1}, "numerals": {"One": 2, "Two": 1}, "classifiers": {"Set": 1, "individual": 1}, "pronouns": {"Other": 1, "he": 1}}}, {"content": "7. Most would not hesitate to help those in need.", "word_count": 19, "label": "", "sentence_structure": "Subject鈥搗erb鈥搊bject", "subjective_objective": {"answer": "Subjective", "rationale": "Because the subject is \"the attitude of the majority,\" it's subjective."}, "sentence_sentiment": {"answer": "Positive", "rationale": "Because \"without hesitation to help\" expresses a positive emotional attitude, namely, a willingness to take the initiative of caring for and helping others."}, "language_style": {"answer": "Calm and direct", "rationale": "The entry is of a calm and straightforward language, with no excessive rhetoric or emotional rendering, and is more objective."}, "time_dimension": {"answer": "Present", "rationale": "The entry now describes a widespread phenomenon or perception that most people will come to the assistance of those in difficulty, without a clear time limit, and therefore judge it as now."}, "sentence_type": "Affirmative declarative sentence", "pos": {"nouns": {"person": 2, "Help": 1, "People in difficulty": 1}, "verbs": {"Yes": 1, "Go on": 1, "Help": 1}, "adjectives": {"Most": 1, "I'm not hesitant": 1}, "numerals": {"7": 1}}}, {"content": "8. If it brings benefits, most people lie.", "word_count": 15, "label": "", "sentence_structure": "Subject鈥搗erb鈥搊bject", "subjective_objective": {"answer": "Subjective", "rationale": "Because the subject is an individual ' s view or prediction, it is subjective judgement influenced by factors such as the individual ' s values, beliefs, etc."}, "sentence_sentiment": {"answer": "Negative", "rationale": "Because the \"fake\" in the entry is negative."}, "language_style": {"answer": "Calm and direct", "rationale": "The language style of the entry is a calm and straightforward type. It expresses the subject of the survey in a concise and clear statement, without any qualifying term or emotional description, with an objective and neutral tone, which meets the rigorousness and professionalism of the research."}, "time_dimension": {"answer": "Present", "rationale": "According to the phrase \"if\" and \"the majority will lie\", it can be seen as a description of the current situation, without involving past or future factors. The answer is \"now\"."}, "sentence_type": "Affirmative declarative sentence", "pos": {"nouns": {"Liar": 1, "Benefits": 1}, "verbs": {"Bring it": 1, "Will you": 1, "Lies": 1}, "numerals": {"Most": 1}}}, {"content": "9. The selfless in today ' s society were too poor for so many people to be so careful with.", "word_count": 24, "label": "", "sentence_structure": "Subject鈥搗erb鈥搊bject", "subjective_objective": {"answer": "Subjective", "rationale": "Because the words \"too poor\" and \"calculating him\" express the patient's personal and emotional attitude."}, "sentence_sentiment": {"answer": "Negative", "rationale": "Because the expression \"the selfless is so pathetic\" and \"the calculation of his\" has a negative emotional connotation."}, "language_style": {"answer": "Calm and direct", "rationale": "The language style of the entry is a calm and straightforward one. It expresses criticism of selfish behaviour in today's society and sympathy for selfless people, with no excessive emotional or rhetorical style, and is more objective in tone."}, "time_dimension": {"answer": "Present", "rationale": "The entry describes the phenomena and attitudes of modern society and does not have clear time indicators and therefore belongs to the present."}, "sentence_type": "Affirmative declarative sentence", "pos": {"nouns": {"Social": 1, "person": 2, "Poor thing": 1, "Count it out": 1}, "verbs": {"in": 1, "be": 1, "Too much": 1, "have": 1}, "adjectives": {"Selfless": 1}, "adverbs": {"Too much": 1}, "numerals": {"9": 1}, "pronouns": {"he": 1}}}, {"content": "鈥淲hatever you want others to do to yourself, first of all, to others鈥? the motto of the majority.", "word_count": 29, "label": "", "sentence_structure": "Subject鈥搗erb鈥搊bject", "subjective_objective": {"answer": "Subjective", "rationale": "Because it is a subjective attitude or view. Although it is a long-standing adage and is followed in many cases, it nevertheless expresses a subjective value and attitude that people believe should treat themselves and others in the same way. The questionnaire is therefore viewed from the patient鈥檚 subjective perspective."}, "sentence_sentiment": {"answer": "Positive", "rationale": "Because the phrase \"that you want others to treat yourself, first and foremost to do so\" in the entry conveys a positive interpersonal attitude."}, "language_style": {"answer": "Tactful and warm", "rationale": "The language style of the entry is more euphemistic and educational, emphasizing the principles and values that people should follow when they are treated with. It is expressed not by direct command or criticism, but by a moderate way that leads people to think about the effects of their actions on others. Such a expression is more acceptable and resonant."}, "time_dimension": {"answer": "Present", "rationale": "The entry is an expression of a prevailing view or principle, which does not relate to past periods or future expectations, and therefore can be judged as the present. At the same time, \"majority adherence\" indicates that this is a widely accepted and practiced principle, and is the present."}, "sentence_type": "Affirmative declarative sentence", "pos": {"nouns": {"Questionnaire": 1, "Entry": 1, "person": 2, "self": 1, "others": 2, "The motto": 1}, "verbs": {"Hope": 1, "Reactions": 1, "Guard": 1, "Treatment": 2}, "adjectives": {"Most": 1, "grams": 1}, "numerals": {"10": 1, "2": 1}, "classifiers": {"individual": 1}, "pronouns": {"Here": 1}}}, {"content": "11. Everyone claimed to have a set of ethical standards, but few people followed them when they came into existence.", "word_count": 36, "label": "", "sentence_structure": "Subject鈥損redicate", "subjective_objective": {"answer": "Subjective", "rationale": "Because people's perception and behaviour of morality are their subjective ideas and behaviour. While they may have corresponding ethical standards, in practice they may not always follow them, which is determined by the subjective will and circumstances of the individual."}, "sentence_sentiment": {"answer": "Negative", "rationale": "Because the entry describes a phenomenon in which people often fail to observe the ethical standards they claim when they encounter things, it has a certain negative emotional connotation."}, "language_style": {"answer": "Calm and direct", "rationale": "The language style of the entry is relatively calm and straightforward, and by describing a widespread social phenomenon, it triggers a reflection on whether ethical standards can be truly respected. There is no excessive emotional or rhetorical approach that presents the problem in an objective and rational way."}, "time_dimension": {"answer": "Present", "rationale": "The entry refers to the behaviour of people in reality, that is, \"in the event of an incident\", and uses the general current, which means that this is a frequent occurrence and therefore judged to be the present. Moreover, \"everyone claims to have a set of ethical standards\" implies that it is a general phenomenon, not a particular case in the past or in the future."}, "sentence_type": "Exclamatory sentence", "pos": {"nouns": {"Everyone": 1, "Claim": 1, "One": 1, "Ethics": 1, "Ethical standards": 2, "An encounter": 1, "Time": 1, "person": 1, "Follow": 1, "These": 1, "Standard": 1}, "verbs": {"have": 1, "Claim": 1, "Follow": 1}, "adverbs": {"Very few": 1, "鈾?Once 鈾?: 1}, "numerals": {"11": 1, "2": 1, "1": 7}, "classifiers": {"One": 1, "person": 1}, "pronouns": {"self": 1, "These": 1}}}, {"content": "12. Most people will manifest their faith.", "word_count": 11, "label": "", "sentence_structure": "Subject鈥搗erb鈥搊bject", "subjective_objective": {"answer": "Subjective", "rationale": "Because both \"expression\" and \"belief\" are considered subjectively by the patient."}, "sentence_sentiment": {"answer": "Neutral", "rationale": "Since the entry does not cover biased descriptions of emotions, attitudes or behaviour, it merely presents a fact."}, "language_style": {"answer": "Calm and direct", "rationale": "The entry raises the question in a direct manner, without using any rhetorical or emotional rendering, and is more balanced and objective. Thus, its language style is straightforward and calm."}, "time_dimension": {"answer": "Past", "rationale": "The entry describes the past customary behaviour of the majority of people, i.e., their more proactive in expressing their beliefs."}, "sentence_type": "Affirmative declarative sentence", "pos": {"nouns": {"Most people": 1, "Faith": 1}, "verbs": {"Yes": 1, "Show": 1}, "numerals": {"12": 1}}}, {"content": "People seem to care for each other, but they don't.", "word_count": 17, "label": "", "sentence_structure": "Subject鈥搗erb鈥揷omplement", "subjective_objective": {"answer": "Subjective", "rationale": "Because \"the appearance of mutual interest, but not in fact\" is a subjective assessment and judgment."}, "sentence_sentiment": {"answer": "Negative", "rationale": "Because the entry \"Perceptive concern is not really the same\" is negative."}, "language_style": {"answer": "Tactful and warm", "rationale": "The language style of the entry is more euphemistic, because it expresses a perception of the apparent relationship of people in an implicit and inviting manner, without directly blaming or criticizing each other. Such expression is more acceptable and willing to reflect further on the issue."}, "time_dimension": {"answer": "Present", "rationale": ""}, "sentence_type": "Negative declarative sentence", "pos": {"nouns": {"People": 1, "On the surface": 1, "Care": 1, "Actually": 1}, "verbs": {"Care for each other": 1, "It's not true": 1}, "adjectives": {"Care": 1, "Surface": 1, "Actually": 1}, "adverbs": {"On the surface": 1, "Actually": 1}, "numerals": {"13": 1}, "pronouns": {"they": 1}}}, {"content": "Even when it is known that it is good to lie, people tend to tell the truth.", "word_count": 17, "label": "", "sentence_structure": "Subject鈥損redicate", "subjective_objective": {"answer": "Objective", "rationale": "The purpose of the questionnaire is to look at people鈥檚 preferences from an objective perspective. While the section 鈥淜nowing the benefits of lying鈥?contains a certain degree of subjective judgement, the whole sentence is centred on 鈥減eople tend to tell the truth鈥? which suggests that researchers are exploring a general phenomenon or trend, not based on subjective experience of one person."}, "sentence_sentiment": {"answer": "Neutral", "rationale": "Because the entry merely describes a phenomenon, it does not express any positive or negative emotional colours."}, "language_style": {"answer": "Calm and direct", "rationale": "The entry is more calm and straightforward in its language, without excessive rhetorical or emotional rendering. It presents a point and asks the other side to answer, and is speaking in a fair and objective manner.\""}, "time_dimension": {"answer": "Past", "rationale": "The entry describes the behaviour of people in certain circumstances, i.e., they tend to tell the truth even when they know that it is good to lie. It is not what will happen in the future, it is not what is happening now, but what has happened in the past. The temporal dimension of the entry is therefore the past."}, "sentence_type": "Affirmative declarative sentence", "pos": {"nouns": {"Lies": 1, "Benefits": 1, "person": 2, "Things": 1}, "verbs": {"Yes": 1, "Liar": 1, "Often": 1, "Speak": 1, "have": 1}, "adverbs": {"Yeah": 1, "The truth": 1}, "numerals": {"14": 1}, "pronouns": {"People": 1}}}, {"content": "15. Most people are psychologically unwilling to help others.", "word_count": 14, "label": "", "sentence_structure": "Subject鈥搗erb鈥揷omplement", "subjective_objective": {"answer": "Subjective", "rationale": "Because the willingness to help others is the subjective attitude and will of one person, not an objective reality or fact."}, "sentence_sentiment": {"answer": "Negative", "rationale": "Because the entry \"not willing to help others\" expresses negative attitudes and emotional sentiments."}, "language_style": {"answer": "Tactful and warm", "rationale": "The words \"most people\" and \"psychological\" in the sentence are also used in a more objective and realistic way, consistent with the linguistic style of the questionnaire."}, "time_dimension": {"answer": "Present", "rationale": "The entry asks the testee about his attitude to helping others, and the keywords are \"majority\" and \"no\" , all pointing to the present situation. Thus, the temporal dimension of the article is now judged."}, "sentence_type": "Affirmative declarative sentence", "pos": {"nouns": {"person": 2, "Psychological": 1, "Help": 1}, "verbs": {"From": 1, "Yes": 1, "But": 1, "Help": 1}, "adjectives": {"Most": 1, "No, I don't": 1}, "adverbs": {"Most": 1, "From": 1, "Psychological": 1, "But": 1}, "numerals": {"15": 1}}}, {"content": "16. Most people cheat on income tax if they had the opportunity.", "word_count": 16, "label": "", "sentence_structure": "Subject鈥搗erb鈥搊bject", "subjective_objective": {"answer": "Subjective", "rationale": "Because \"most people\" are a subjective view or a hypothesis, not an objective fact."}, "sentence_sentiment": {"answer": "Negative", "rationale": "Because the \"false\" in the entry is negative."}, "language_style": {"answer": "Calm and direct", "rationale": "The entry is presented in a straightforward, concise question, without any rhetorical or emotional recitation, and with relatively calm and objective language styles."}, "time_dimension": {"answer": "Present", "rationale": "\"If given the opportunity\" means that this is a hypothetical situation, while \"will\" means that the testee, in answering this question, has to consider whether they will take such an act. Thus, the entry relates to the prediction of present and future behaviour, the temporal dimension being judged as now."}, "sentence_type": "Affirmative declarative sentence", "pos": {"nouns": {"Opportunities": 1, "Income tax": 1, "Most people": 1, "Cheating": 1}, "verbs": {"Yes": 1, "in": 1}, "adverbs": {"If": 1}, "numerals": {"16": 1}}}, {"content": "17. If they think they are right, they can hold their views against the opposition of others.", "word_count": 28, "label": "", "sentence_structure": "Subject鈥損redicate", "subjective_objective": {"answer": "Subjective", "rationale": "Because \"thinking you're right\" and \"maintenance of your opinion despite the opposition\" are subjective ideas and behaviours of patients."}, "sentence_sentiment": {"answer": "Positive", "rationale": "Because the purpose of the action of \"maintenance\" in the entry is positive."}, "language_style": {"answer": "Calm and direct", "rationale": "The entry raises a question in an objective way, and does not use excessive rhetorical or emotional rendering. This language style is more straightforward, concise and easy to understand."}, "time_dimension": {"answer": "Present", "rationale": "The entry describes the reaction of a person in general to his or her conduct in a given situation, that is, \"as long as he or she thinks he or she is right,\" and \"as long as he or she is correct, he or she insists on his or her views despite the opposition of others.\" The description is a description of a general, recurrent situation, not a specific event, past or future. Thus, the basis of judgement is now."}, "sentence_type": "Affirmative declarative sentence", "pos": {"nouns": {"person": 1, "self": 2, "Views": 1}, "verbs": {"Yes": 1, "Hold on": 1, "I think": 1, "Objection": 1}, "adjectives": {"General": 1, "Correct": 1}, "adverbs": {"Just": 1, "Both": 1}, "numerals": {"17": 1}, "pronouns": {"self": 1}}}, {"content": "18. Most people would be happy to do so if they had the opportunity.", "word_count": 13, "label": "", "sentence_structure": "Subject鈥損redicate", "subjective_objective": {"answer": "Subjective", "rationale": "Because \"benevolentness\" is the expression of individual subjective will and values, and it is the patient's own view."}, "sentence_sentiment": {"answer": "Positive", "rationale": "Because \"bent and do good\" is a positive quality."}, "language_style": {"answer": "Tactful and warm", "rationale": "The entry is more adept and interactive when it is spoken in a gentle and inviting manner, rather than by direct statements or orders."}, "time_dimension": {"answer": "Present", "rationale": "The entry asks the respondents about the attitude and preferences of \"if there is a chance\" towards \"behaviour and kindness\", and therefore the temporal dimension is now judged."}, "sentence_type": "Affirmative declarative sentence", "pos": {"nouns": {"Opportunities": 1, "Most people": 1, "Be nice and nice": 1}, "verbs": {"have": 1, "Here": 1}, "adverbs": {"If": 1}, "numerals": {"8": 1}}}, {"content": "19. The honesty shown by the majority was not justified, but rather feared capture.", "word_count": 29, "label": "", "sentence_structure": "Subject鈥搗erb鈥搊bject", "subjective_objective": {"answer": "Subjective", "rationale": "Because this sentence expresses the author's view and assessment of the \"majority\" and is a subjective view."}, "sentence_sentiment": {"answer": "Negative", "rationale": "Because the description \"the honesty of the majority is not justified\" in the entry is negative."}, "language_style": {"answer": "Calm and direct", "rationale": "The entry is presented in a direct manner, without any use of rhetoric or emotional colours and with a more calm and objective tone."}, "time_dimension": {"answer": "Past", "rationale": "The entry describes a widespread act or phenomenon (i.e. \"the honesty shown by the majority is not justified\"), but does not address any specific point or period of time. It is therefore more appropriate to judge the past in terms of time dimension. At the same time, the sentence expresses an opinion or attitude to this phenomenon, which does not clearly point to what is going on in the future or now."}, "sentence_type": "Affirmative declarative sentence", "pos": {"nouns": {"Most people": 1, "Honesty": 1, "Rationale": 1, "they": 1, "Grab him": 1}, "verbs": {"Performance": 1, "Not": 1, "鈾?Out of": 1, "Yes": 1, "By": 2, "Hold on": 1}, "adverbs": {"Only": 1, "But": 1}, "pronouns": {"Sou-san": 1}}}, {"content": "20. Ordinary people would be sincere in their concern for the difficulties of others.", "word_count": 15, "label": "", "sentence_structure": "Subject鈥搗erb鈥搊bject", "subjective_objective": {"answer": "Objective", "rationale": "Because \"care for others in good faith\" is a common social habit and value of humanity, a universal phenomenon that is not influenced by individual differences. The content of this questionnaire is therefore objective."}, "sentence_sentiment": {"answer": "Positive", "rationale": "Because the \"cutely concerned about the difficulties\" in the entry is positive."}, "language_style": {"answer": "Tactful and warm", "rationale": "The language style of this entry is more euphemism, which guides the respondents to think and answer questions in a caring, understanding and supportive manner. By using the word \"clandestine\" it expresses the importance of honesty and honesty and encourages the respondents to do the same in their responses. This euphemism and warmness of presentation is more likely to resonate with the respondents and increase the completion rate of the questionnaire."}, "time_dimension": {"answer": "Present", "rationale": "The word \"now\" appears in the entry, which means that the survey is on the social phenomenon of the present. So the answer is \"now\"."}, "sentence_type": "Affirmative declarative sentence", "pos": {"nouns": {"Normal": 1, "Care": 1, "others": 1, "Difficulties": 1}, "verbs": {"All of them": 1, "-Significant": 1}, "adverbs": {"Land": 1}, "numerals": {"20": 1}}}]}

{"questionnaire_title": "Children ' s education psychological control sources are at the highest level", "questionnaire_category": "Self-cognition", "item_count": 47, "emotion_shift_overall": "From negative to positive", "language_style_overall": "Neutral", "items": [{"content": "1. What I did did did not work for my children.", "word_count": 13, "label": "", "sentence_structure": "Subject鈥搗erb鈥搊bject", "subjective_objective": {"answer": "Subjective", "rationale": "Because \"I'm doing nothing to my children\" is a patient's subjective judgment and perception, not an objective fact. This may be a subjective perception or an assessment of the outcome of his actions."}, "sentence_sentiment": {"answer": "Negative", "rationale": "Because the \"no good\" evaluation in the entry is negative."}, "language_style": {"answer": "Tactful and warm", "rationale": "The entry is more euphemistic, using a relatively vague expression such as \"no use\" to avoid direct accusations or denial of each other's behaviour and performance. At the same time, it is more moderate and respectful, giving people a sense of being heard and understood, and helps to build a good atmosphere of communication and trust."}, "time_dimension": {"answer": "Present", "rationale": "The keywords \"I did\" and \"does nothing to my children\" all indicate the current state of behaviour and the results, so it can be inferred that the questionnaire is asking the person who was tested about the current state of affairs."}, "sentence_type": "Negative declarative sentence", "pos": {"nouns": {"I": 1, "Son": 1, "Role": 1}, "verbs": {"Do it": 1, "have": 1, "be": 1}, "adverbs": {"Nope": 1}}}, {"content": "2. I cannot resolve any problems between me and the children.", "word_count": 18, "label": "", "sentence_structure": "Subject鈥搗erb鈥搊bject", "subjective_objective": {"answer": "Subjective", "rationale": "The purpose of this questionnaire is to describe the patient ' s feelings and thoughts in the event of problems with the child and is therefore a subjective examination."}, "sentence_sentiment": {"answer": "Negative", "rationale": "Because the \"no solution\" in the entry is negative."}, "language_style": {"answer": "Calm and direct", "rationale": "The language style of the entry is relatively calm, straightforward, with no excessive emotional or euphemism, and more focused on the presentation of facts and views. At the same time, the entry does not use an overly complex sentence structure, making the issue more clear, simple and understandable, in line with the questionnaire."}, "time_dimension": {"answer": "Present", "rationale": "The entry is for the purpose of asking the testee about his current behaviour or attitude, and the temporal dimension is now judged."}, "sentence_type": "Negative declarative sentence", "pos": {"nouns": {"Son": 1, "Problem": 1}, "verbs": {"Out": 1, "Resolve": 1}, "adverbs": {"鈾?Once 鈾?: 1, "I can't": 1}, "pronouns": {"I": 1}}}, {"content": "Parents should point to the children's problems, since the lack of attention to them does not disappear.", "word_count": 27, "label": "", "sentence_structure": "Subject鈥搗erb鈥搊bject", "subjective_objective": {"answer": "Objective", "rationale": "This is a description and recommendation of the behaviour of parents and is an objective view."}, "sentence_sentiment": {"answer": "Negative", "rationale": "Because the act of \"notifying the children's problems\" in the entry is based on negative attitudes (criticism and blame) and believes that ignoring the problem only makes it worse."}, "language_style": {"answer": "Tactful and warm", "rationale": ""}, "time_dimension": {"answer": "Present", "rationale": "In this entry, \"should\" and \"does not disappear without looking at the problem\" means current or future behaviour or phenomenon."}, "sentence_type": "Affirmative declarative sentence", "pos": {"nouns": {"parents": 1, "Son": 1, "Mistake": 1, "Problem": 1}, "verbs": {"It should be noted that": 1, "to": 1, "I don't see": 1, "Disappear": 1}, "adjectives": {"This": 1}, "adverbs": {"Yes": 1}, "numerals": {"III": 1}, "classifiers": {"individual": 1}, "pronouns": {"鈾?And the 鈾?: 1}}}, {"content": "If you try anything to get your kid mad, you have to stop.", "word_count": 20, "label": "", "sentence_structure": "Subject鈥搗erb鈥搊bject", "subjective_objective": {"answer": "Objective", "rationale": "This is because it describes the objective fact that, regardless of the parents ' efforts, if the child remains angry, the choice is ultimately to give up."}, "sentence_sentiment": {"answer": "Negative", "rationale": "Because the behavior in the entry \"just had to stop\" is negative."}, "language_style": {"answer": "Tactful and warm", "rationale": ""}, "time_dimension": {"answer": "Present", "rationale": "The use of the old-fashioned words \"do whatever\" and \"do what you have to do\" in the past in the entry suggests that this is a description of the experience or situation of a given period of time in the past."}, "sentence_type": "Affirmative declarative sentence", "pos": {"nouns": {"Approach": 1, "Son": 1, "- Grumpy": 1, "you": 2, "Stop": 1}, "verbs": {"Yes": 1, "End of story": 1, "Big hair": 1, "Just": 1, "Yeah": 1}, "adverbs": {"If": 1, "Or is it": 1}, "numerals": {"4": 1}}}, {"content": "My children eventually acted in their own way, so why should they do so?", "word_count": 21, "label": "", "sentence_structure": "Subject鈥搗erb鈥搊bject", "subjective_objective": {"answer": "Subjective", "rationale": "Because the entry expresses the patient ' s attitude and perception of the child ' s behaviour and is subjective."}, "sentence_sentiment": {"answer": "Negative", "rationale": "Because the expression \"Why should we care about it\" in the entry expresses a negative emotional expression of abandoning the education and guidance of children."}, "language_style": {"answer": "Calm and direct", "rationale": "The entry is more straightforward, concise and not based on too many rhetorical techniques or emotional rendering. It is more explicit and direct in its presentation of issues, and is designed to capture the views and attitudes of the other, and it appears relatively calm and objective in its tone."}, "time_dimension": {"answer": "Present", "rationale": ""}, "sentence_type": "Affirmative declarative sentence", "pos": {"nouns": {"Son": 1, "Thoughts": 1}, "verbs": {"Press": 1, "Okay": 1, "Do it": 1, "Pipe": 1}, "adjectives": {"Eventually": 1, "self": 1}, "adverbs": {"So": 1, "Why": 1}, "pronouns": {"I": 1}}}, {"content": "6. No matter how hard parents struggle, some children are not concerned.", "word_count": 20, "label": "", "sentence_structure": "Subject鈥搗erb鈥搊bject", "subjective_objective": {"answer": "Subjective", "rationale": "Because \"care\" is a subjective concept, which depends on individual perceptions and feelings, not objective facts. Although parents can observe the behaviour of their children, they cannot directly measure their inner or emotional state."}, "sentence_sentiment": {"answer": "Negative", "rationale": "Because the \"not mind\" act in the entry is negative."}, "language_style": {"answer": "Calm and direct", "rationale": "The entry uses the tone of the direct statement, without excessive emotional accoutrements or euphemisms, and is more calm and objective in its language style."}, "time_dimension": {"answer": "Present", "rationale": "\"Some children are just not concerned\" describes a status quo or behavioral behaviour that is a present phenomenon and does not clearly point to the past or the future. Thus, the temporal dimension of this entry is now."}, "sentence_type": "Imperative sentence", "pos": {"nouns": {"Questionnaire Entry": 1, "Parents": 1, "Powerful": 1, "Son": 1, "My heart": 1}, "verbs": {"Fees": 1, "Fire": 1, "in": 1}, "adjectives": {"Large": 1, "not": 1}, "adverbs": {"Just": 1, "Whatever": 1}, "numerals": {"6": 1}, "pronouns": {"they": 1}}}, {"content": "I can only predict what my children will be doing in different circumstances.", "word_count": 24, "label": "", "sentence_structure": "Subject鈥搗erb鈥搊bject", "subjective_objective": {"answer": "Subjective", "rationale": "This is because it is a subjective view, as it is an assessment and recognition of the patient ' s own abilities."}, "sentence_sentiment": {"answer": "Positive", "rationale": "Because the \"how much can be expected\" behaviour in the entries is positive, it shows that the respondents have some control and predictability over it."}, "language_style": {"answer": "Tactful and warm", "rationale": "The entry uses a soft, invitation-style tone for dialogue rather than direct statements or orders, which is more personal and interactive. At the same time, the purpose of the article is to focus more on the individual ' s feelings and circumstances, without too direct or inclement language, reflecting the respect and interest of psychological questionnaire researchers in the person under investigation."}, "time_dimension": {"answer": "Future", "rationale": ""}, "sentence_type": "Affirmative declarative sentence", "pos": {"nouns": {"Questionnaire Entry": 1, "Son": 1, "Performance": 1, "Environment": 1}, "verbs": {"I expected that": 1, "have": 1, "What kind": 1}, "adverbs": {"More": 1, "In different ways": 1}, "numerals": {"7": 1}}}, {"content": "8. It is not always wise to expect too much from my children, because many things end up as a result of good or bad luck.", "word_count": 36, "label": "", "sentence_structure": "Subject鈥揷opula鈥損redicative", "subjective_objective": {"answer": "Objective", "rationale": "Because the sentence describes a general situation or pattern, it is not specific to a subjective view or feeling of a person. While it may contain some personal emotional colours (e.g., expectations of a child), it is generally based on statements made on the basis of facts and observations and is therefore classified as objective."}, "sentence_sentiment": {"answer": "Negative", "rationale": "Because the idea that \"too much is expected of my children is not always wise\" in the entry is negative."}, "language_style": {"answer": "Calm and direct", "rationale": "The entry describes the problem of over-ambitious expectations of children in a more direct and objective language style and presents a view."}, "time_dimension": {"answer": "Present", "rationale": ""}, "sentence_type": "Affirmative declarative sentence", "pos": {"nouns": {"Son": 1, "Expectations": 1, "Smart": 1, "Things": 1, "Good luck": 1, "Bad luck": 1, "Result": 1}, "verbs": {"to": 1, "Not": 1, "Always": 1, "be": 1, "Many": 1, "In the end": 1, "But": 1, "Come on": 1}, "adjectives": {"More": 1, "Smart": 1, "Good luck or bad luck": 1}, "adverbs": {"Always": 1, "Not": 1, "Many": 1, "But": 1}, "numerals": {"8": 1}, "pronouns": {"I": 1}}}, {"content": "When my children are angry, I remain calm and calm and calm.", "word_count": 22, "label": "", "sentence_structure": "Subject鈥搗erb鈥搊bject", "subjective_objective": {"answer": "Subjective", "rationale": "Because \"I remain calm\" is what the patient thinks he can do, and it's a subjective view of the patient. And \"it always calms things down\" is an objective result, which means that the patient's behavior has had some effect."}, "sentence_sentiment": {"answer": "Positive", "rationale": "Because the entry describes the calmness and effectiveness of parents in dealing with the emotional problems of their children, it is a positive attitude."}, "language_style": {"answer": "Calm and direct", "rationale": "The entry directly expresses the subject of questioning the attitude and methods of dealing with the anger of the child, does not use excessive rhetoric or emotional rendering, and is more objective in tone. At the same time, it proposes a specific method of solving the problem (clumbing) and emphasizes the positive effect of this method (which can calm things down), which is clear in language, and which is more guiding and practical."}, "time_dimension": {"answer": "Present", "rationale": "\"When my child is angry\" is about what happens now or in the near future, and the \"I remain calm and calm\" behind it describes a current way of acting or strategy. So the whole entry focuses on the state and practice of the present, judging by \"now\"."}, "sentence_type": "Affirmative declarative sentence", "pos": {"nouns": {"Son": 1, "Anger": 1, "Yes": 1, "Gas": 1}, "verbs": {"Let's go": 1, "Jean": 1, "Quiet": 1, "Hold on": 1}, "adjectives": {"Calm down": 1}, "adverbs": {"Only": 1, "Total": 1}, "numerals": {"9": 1}, "pronouns": {"I": 2}}}, {"content": "When I plan for the future of my child, I can almost certainly help him/her achieve it.", "word_count": 26, "label": "", "sentence_structure": "Subject鈥搗erb鈥搊bject", "subjective_objective": {"answer": "Subjective", "rationale": "Because \"I can almost be sure\" in this sentence shows the confidence and conviction of the speaker, which is a subjective attitude of the individual."}, "sentence_sentiment": {"answer": "Positive", "rationale": "Because the purpose of the entry is to help him/her achieve it."}, "language_style": {"answer": "Tactful and warm", "rationale": "The basis for the judgement: the words in the entry are phrased in a \"almost certain\" expression of optimism and confidence, but also in a way that leaves room for no over-prescriptiveness. This expression gives the impression that the author is careful and thoughtful in considering the issue, while trying to create a positive atmosphere. The tone of the whole speech is more peaceful and sweet, which is consistent with the euphemism of the euphemism of the language."}, "time_dimension": {"answer": "Future", "rationale": "\"I plan for the future of my child\" and \"I can almost certainly help him/her achieve\" are all about the future, so the entry is the future."}, "sentence_type": "Affirmative declarative sentence", "pos": {"nouns": {"Son": 2, "Way forward": 1, "Planning": 1}, "verbs": {"Do it": 1, "Achieved": 1}, "adjectives": {"Sure": 1, "he": 1, "she": 1}, "adverbs": {"Almost": 1}, "numerals": {"10": 1}, "classifiers": {"individual": 1}, "pronouns": {"I": 1}}}, {"content": "No good, bad, good, good, good.", "word_count": 21, "label": "", "sentence_structure": "Subject鈥損redicate", "subjective_objective": {"answer": "Subjective", "rationale": "Because \"no good, no bad, no bad\" is the standard for researchers to evaluate children, and it's seen from a personal, subjective perspective."}, "sentence_sentiment": {"answer": "Neutral", "rationale": "The entry expresses a view or perception and does not involve positive or negative emotional attitudes or behavioural tendencies."}, "language_style": {"answer": "Tactful and warm", "rationale": "The language style of the questionnaire entry is more euphemistic. It uses an incentive expression, not direct orders or denial of the other. The sentence shows that the investigator believes that every child is unique and that parents should take responsibility for educating their children. This soft and positive expression makes it easier for the interviewee to feel respected and accepted, and therefore to participate more readily in the questionnaire."}, "time_dimension": {"answer": "Present", "rationale": "The entry shows the author's view of the concept of \"good and bad child\" that there is no absolute difference between good and bad, but rather depends on the way parents are educated and behaved. This view is the belief that the author now holds, and therefore the judgement is now."}, "sentence_type": "Negative declarative sentence", "pos": {"nouns": {"Son": 2, "Bad": 1, "Okay": 2, "parents": 2}, "verbs": {"have": 1, "Say it": 1, "Do it": 1}, "adjectives": {"It's not good": 1}, "adverbs": {"No, I'm not": 1}, "numerals": {"11": 1}}}, {"content": "12. If my child is of good character, it is because he/she is not as hard as I am.", "word_count": 21, "label": "", "sentence_structure": "Subject鈥搗erb鈥搊bject", "subjective_objective": {"answer": "Subjective", "rationale": "The reason for this is the patient's subjective view of the integrity of his child."}, "sentence_sentiment": {"answer": "Positive", "rationale": "Because \"good conduct\" and \"not bad for me\" efforts are both positive descriptions."}, "language_style": {"answer": "Calm and direct", "rationale": "The language style of the entry is more calm and straightforward, and there is no excessive rhetoric or emotional colour. It asks the interviewee in an objective manner why the child is good, rather than directing the answer through a strong emotional expression. The language style of the entry can therefore be described as \"cool and straight.\""}, "time_dimension": {"answer": "Present", "rationale": "Now the entry asks the testee's current view and conviction that they believe that their child's character is precisely because of their efforts. The keyword \"because\" indicates that it is a causal expression, implying that it is the testee's current view or experience."}, "sentence_type": "Affirmative declarative sentence", "pos": {"nouns": {"Son": 1, "Good conduct": 1, "Try": 1}, "verbs": {"Not bad": 1}, "adjectives": {"Positive": 1}, "numerals": {"XII": 1}, "pronouns": {"He/she": 1, "I": 1}}}, {"content": "Parents who cannot make their children listen to themselves have no idea how to get along with their children.", "word_count": 32, "label": "", "sentence_structure": "Subject鈥搗erb鈥搊bject", "subjective_objective": {"answer": "Objective", "rationale": "Because the sentence describes and evaluates a situation, it does not involve subjective attitudes or feelings of the patient, but is addressed from an objective perspective."}, "sentence_sentiment": {"answer": "Negative", "rationale": "Because the description of \"not having children listen\" in the entry is negative and critical of the behaviour of these parents, with negative emotional aspects."}, "language_style": {"answer": "Calm and direct", "rationale": ""}, "time_dimension": {"answer": "Past", "rationale": ""}, "sentence_type": "Affirmative declarative sentence", "pos": {"nouns": {"Son": 2, "parents": 2, "Children": 1, "Speech": 1, "Get along": 1}, "verbs": {"Jean": 1, "Listen": 1, "Yes": 1, "Same": 1, "Get along": 1}, "adjectives": {"Cannot": 1, "I don't understand": 1}, "numerals": {"13": 1}, "classifiers": {"individual": 1}, "pronouns": {"self": 2, "Theirs": 1}}}, {"content": "14. My children are at my fault if they are misbehavioured.", "word_count": 14, "label": "", "sentence_structure": "Subject鈥搗erb鈥搊bject", "subjective_objective": {"answer": "Subjective", "rationale": "Because this is a subjective view or view of the patient, who believes that the child鈥檚 mistake is entirely attributable to himself. This view is based on personal factors such as the values, beliefs and emotional attitudes of the patient, and is not a description of objective facts."}, "sentence_sentiment": {"answer": "Negative", "rationale": "Because the attitude of \"in my wrong\" in the entry is negative, which shows that parents are too responsible and disproved."}, "language_style": {"answer": "Tactful and warm", "rationale": "The entry uses a more euphemistic and polite tone to express expectations and responsibilities towards children. By turning the direct phrase \"if my child is misbehaved\" into \"me,\" it makes the phrase more cozy and educational."}, "time_dimension": {"answer": "Present", "rationale": ""}, "sentence_type": "Negative declarative sentence", "pos": {"nouns": {"Son": 1, "Misconduct": 1, "It's all about the wrong thing": 1}, "verbs": {"have": 1, "If": 1, "in": 1}, "adjectives": {"I'm the one who made the mistake": 1}, "numerals": {"14": 1}, "pronouns": {"I": 1}}}, {"content": "Those who fail to be good parents are not able to take advantage of their chances.", "word_count": 27, "label": "", "sentence_structure": "Subject鈥搗erb鈥搊bject", "subjective_objective": {"answer": "Objective", "rationale": "Because \"persons who are capable of good parents but fail\" are an objective phenomenon, and \"take the chance\" is a possible explanation or reason for this, the view of this questionnaire entry is to be seen from an objective perspective."}, "sentence_sentiment": {"answer": "Positive", "rationale": "This entry highlights the failure to seize opportunities as an incentive and motivational attitude."}, "language_style": {"answer": "Calm and direct", "rationale": "The entry expresses criticism and judgment in a direct and objective manner, without too much adoration or emotional colour being used to render it. At the same time, the language style is colder, giving people a more direct, serious sense of human affection and a lack of warm human attention."}, "time_dimension": {"answer": "Past", "rationale": ""}, "sentence_type": "Affirmative declarative sentence", "pos": {"nouns": {"Capacity": 2, "person": 1, "parents": 1, "Failed": 1, "Opportunities": 2, "Son": 1, "Hold on": 1}, "verbs": {"have": 1, "Be careful": 1, "After all": 1}, "adjectives": {"Those": 1, "Capable": 1, "Okay": 1, "Loser": 1, "of": 2}, "numerals": {"15": 1, "1": 3}, "classifiers": {"individual": 1}, "pronouns": {"they": 1}}}, {"content": "16. Children ' s misconduct is due in large part to the mistakes of their parents.", "word_count": 21, "label": "", "sentence_structure": "Subject鈥搗erb鈥搊bject", "subjective_objective": {"answer": "Subjective", "rationale": "This is a view or opinion based on subjective judgement of the relationship between the child ' s character and the conduct of the parents."}, "sentence_sentiment": {"answer": "Negative", "rationale": "The fact that there is a certain degree of responsibility and debasement in the formulation of this article has led to the stigmatization and denial of parents, which has a negative emotional connotation."}, "language_style": {"answer": "Calm and direct", "rationale": "The entry begins with a clear statement of view, without any accrimation or emotional colour, and with a more calm and objective language style."}, "time_dimension": {"answer": "Past", "rationale": "The entry \"children's misbehaviour is due mostly to the mistakes of their parents\" expresses a view or interpretation of the past and is therefore judged to be the past."}, "sentence_type": "Affirmative declarative sentence", "pos": {"nouns": {"Son": 1, "Good conduct": 1, "parents": 2, "Error": 1, "Cause": 1}, "verbs": {"be": 1, "Because": 1, "Sou-san": 1, "Cause": 1}, "adjectives": {"Unend": 1}, "numerals": {"16": 1}, "pronouns": {"they": 1}}}, {"content": "17. All those parents who had caused the children to be sore were those who had failed to employ the best means of education.", "word_count": 29, "label": "", "sentence_structure": "Subject鈥揷opula鈥損redicative", "subjective_objective": {"answer": "Subjective", "rationale": "Because \"best-of-the-art\" is a subjective judgement, depending on what is the definition and standard of \"best\" and, at the same time, \"brained parents\" is a subjective assessment that reflects the views and attitudes of observers or interviewees towards those parents."}, "sentence_sentiment": {"answer": "Negative", "rationale": "Because the word \"corroborated\" in the entry expresses a negative sentiment and implies a negative impact on children."}, "language_style": {"answer": "Calm and direct", "rationale": "The entry presents the author ' s views directly, without any accoutrements or euphemisms, and is more direct and decisive in language style."}, "time_dimension": {"answer": "Present", "rationale": "The entry describes what is happening now, and the descriptions of \"corrh\" and \"no best means of education\" are all about the current situation. The entry is therefore about the question of the current behaviour or state."}, "sentence_type": "Affirmative declarative sentence", "pos": {"nouns": {"Kids": 1, "It's a bad one": 1, "parents": 1, "Educational tools": 1}, "verbs": {"Fuck": 1, "It's": 1, "Adopt": 1}, "adjectives": {"Best": 1}, "numerals": {"17": 1}, "pronouns": {"they": 1}}}, {"content": "18. Most children do not have behavioural problems if parents are educated in a well-informed manner.", "word_count": 30, "label": "", "sentence_structure": "Subject鈥損redicate", "subjective_objective": {"answer": "Objective", "rationale": "This is a description of a hypothetical situation, in which the child鈥檚 behaviour is reduced if the parents adopt a sophisticated education. This is based on the researcher鈥檚 observations and analysis of possibilities, not on patient鈥檚 subjective thoughts or feelings."}, "sentence_sentiment": {"answer": "Neutral", "rationale": "Since the entry describes a possible situation, it does not include an evaluation of whether the methods of parental education are sufficiently sophisticated or whether there are positive or negative effects on the behaviour of the child."}, "language_style": {"answer": "Calm and direct", "rationale": "The entry is presented in a clear statement of causality, which is objective and direct, without too much emotional rendering or rhetorical, and is more calm and direct."}, "time_dimension": {"answer": "Past", "rationale": ""}, "sentence_type": "Affirmative declarative sentence", "pos": {"nouns": {"parents": 1, "Educational methods": 1, "Son": 1, "Behaviour": 1, "Problem": 1}, "verbs": {"Yes": 1, "Come on": 1, "No, I won't": 1}, "adjectives": {"Smart": 1}, "numerals": {"Most of them": 1}, "pronouns": {"they": 1}}}, {"content": "I am responsible for the behaviour of my children.", "word_count": 12, "label": "", "sentence_structure": "Subject鈥搗erb鈥搊bject", "subjective_objective": {"answer": "Subjective", "rationale": "Because \"responsible\" is the patient's subjective attitude or belief."}, "sentence_sentiment": {"answer": "Positive", "rationale": "Because the attitude in the entries is responsible."}, "language_style": {"answer": "Tactful and warm", "rationale": "The language style of the entry is more euphemistic, with a soft, invitation-style conversation, rather than a direct statement or order, which is more personal and interactive. At the same time, the issue is based on a first-named approach, making it easier for the respondents to resonate and identify and increasing the acceptability of the questionnaire."}, "time_dimension": {"answer": "Past", "rationale": ""}, "sentence_type": "Affirmative declarative sentence", "pos": {"nouns": {"Son": 1, "Behaviour": 1}, "verbs": {"Responsible": 1}, "pronouns": {"self": 1}}}, {"content": "20. Success and failure as parents are direct consequences of my own behaviour.", "word_count": 22, "label": "", "sentence_structure": "Subject鈥揷opula鈥損redicative", "subjective_objective": {"answer": "Subjective", "rationale": "It is a subjective view of the patient, since it relates to self-evaluation and to the attitude of being responsible for his or her actions."}, "sentence_sentiment": {"answer": "Positive", "rationale": "Because it expresses the attitude and belief that individuals are responsible for their actions."}, "language_style": {"answer": "Calm and direct", "rationale": "The entry uses a simple and clear language style, which directly describes the behaviour of the parent who is responsible for success or failure, and does not have too much emotional colour or accommodation, and therefore is a calm and straightforward language style."}, "time_dimension": {"answer": "Past", "rationale": "This entry asks the testee鈥檚 views about his or her success and failure as a parent, which are considered to be the direct result of his or her own behavior."}, "sentence_type": "Affirmative declarative sentence", "pos": {"nouns": {"parents": 1, "Behaviour": 1, "Result": 1}, "verbs": {"be": 1, "As": 1, "Success": 1, "Failed": 1, "Direct": 1}, "numerals": {"20": 1}, "classifiers": {"One": 1}, "pronouns": {"I": 1}}}, {"content": "21. My life is largely dominated by my children.", "word_count": 14, "label": "", "sentence_structure": "Subject鈥揷opula鈥損redicative", "subjective_objective": {"answer": "Subjective", "rationale": "Because \"life is primarily about children\" is the subjective feeling and perception of the patient, and it is subjective."}, "sentence_sentiment": {"answer": "Negative", "rationale": "Because the entry expresses a passive and helpless feeling that one ' s life is controlled and that there is some negative feeling."}, "language_style": {"answer": "Tactful and warm", "rationale": "The language style of the entry is more euphemistic, and by using the expression \"mostly my children\" it implies the extent to which the children of the person under investigation influence their lives, rather than directly."}, "time_dimension": {"answer": "Present", "rationale": ""}, "sentence_type": "Affirmative declarative sentence", "pos": {"nouns": {"Life": 1, "Son": 1}, "verbs": {"Around": 1}, "numerals": {"21": 1}, "pronouns": {"I": 1}}}, {"content": "My children have not been able to influence my life.", "word_count": 12, "label": "", "sentence_structure": "Subject鈥搗erb鈥搊bject", "subjective_objective": {"answer": "Objective", "rationale": "Because the entry describes the impact of the presence of a patient ' s child on the life of the patient, it is an objective fact and does not involve subjective attitudes or feelings of the patient."}, "sentence_sentiment": {"answer": "Neutral", "rationale": "Because the entry does not have any emotional color."}, "language_style": {"answer": "Tactful and warm", "rationale": "The language style of the entry is euphemism. It does not say \"I control my life\" directly, but it is expressed in a more relaxed, implicit way, \"My child doesn't control my life.\" It makes the person under investigation more comfortable and comfortable and easier to give true and honest answers."}, "time_dimension": {"answer": "Present", "rationale": "This sentence describes the situation, where \"my children do not control my life\" is a status quo, meaning that the current living conditions of the speakers are that their children do not disproportionately influence their lives. So, time dimensions are now judged."}, "sentence_type": "Negative declarative sentence", "pos": {"nouns": {"Son": 1, "Around": 1, "Life": 1}, "verbs": {"No, I'm not": 1, "Impact": 1}, "adverbs": {"No, I'm not": 1, "Around": 1}, "numerals": {"22": 1}, "pronouns": {"I": 1}}}, {"content": "23. My children have an impact on the number of friends I have.", "word_count": 18, "label": "", "sentence_structure": "Subject鈥搗erb鈥揷omplement", "subjective_objective": {"answer": "Subjective", "rationale": "Because the number of children affected is the patient ' s own feeling and perspective and is subjective."}, "sentence_sentiment": {"answer": "Neutral", "rationale": "Because the entry does not have any emotional color."}, "language_style": {"answer": "Calm and direct", "rationale": "The decision is based on the following: The purpose of this article is clear, concise and without any qualifying words or emotional colours. It asks whether the children of the interviewee have influenced the number of their friends' circles and is speaking with objectivity and calm."}, "time_dimension": {"answer": "Past", "rationale": "The entry asks how much the testee鈥檚 presence has affected the number of his friends鈥?circles in his past experience."}, "sentence_type": "Affirmative declarative sentence", "pos": {"nouns": {"Son": 1, "friends": 2, "Number": 1}, "verbs": {"Impact": 1, "Possession": 1}, "adverbs": {"How much": 1}, "numerals": {"23": 1}, "pronouns": {"I": 1}}}, {"content": "24. I feel that the events in my life are largely the decisions of my children.", "word_count": 20, "label": "", "sentence_structure": "Subject鈥揷opula鈥損redicative", "subjective_objective": {"answer": "Subjective", "rationale": "Because \"the feeling\" describes the patient's subjective feelings and perceptions, it means that this is the patient's personal view and not an objective fact."}, "sentence_sentiment": {"answer": "Neutral", "rationale": "Since the entry does not have a clear emotional tendency or attitude, it simply presents a fact."}, "language_style": {"answer": "Calm and direct", "rationale": "The basis for the judgment: the entry expresses a view in a direct statement that \"I feel that the events in my life are largely determined by my children.\" It does not use excessive rhetorical or emotional rendering to increase euphemism or warmth. This language style is more common in psychological questionnaire research, because it is more objective and direct and facilitates the acquisition of the real thoughts and feelings of the respondents."}, "time_dimension": {"answer": "Present", "rationale": "The keyword \"fear\" and \"the events in life are largely determined by my children\" indicate that this is a current feeling or perception and therefore is a time dimension."}, "sentence_type": "Affirmative declarative sentence", "pos": {"nouns": {"Life": 1, "Events": 1, "Son": 1}, "verbs": {"I feel": 1, "Main": 1, "Decision": 1}, "adverbs": {"Main": 1}, "numerals": {"24": 1}, "pronouns": {"I": 1}}}, {"content": "25. It is easy for me to act independently of the child ' s attempts to control me.", "word_count": 24, "label": "", "sentence_structure": "Subject鈥揷opula鈥損redicative", "subjective_objective": {"answer": "Subjective", "rationale": "Because this sentence describes the feelings and perceptions of the patient, it is subjective."}, "sentence_sentiment": {"answer": "Positive", "rationale": "Because the behavior in the entry \"to avoid the child's attempts to control me, to act independently\" is considered positive."}, "language_style": {"answer": "Calm and direct", "rationale": "The entry is more directly linguistically, with no excessive rhetorical or emotional rendering, and presents the views and feelings of the interviewees in objective and calm language, allowing the reader to understand the intended intentions of the interviewees more accurately."}, "time_dimension": {"answer": "Past", "rationale": "There is no specific point or period of time in the time dimension, and therefore the past, present or future cannot be judged."}, "sentence_type": "Interrogative sentence", "pos": {"nouns": {"I": 1, "Son": 1, "Control": 1, "Attempt": 1, "Independence": 1, "Act": 1}, "verbs": {"Avoid": 1, "be": 1, "Easy": 1}, "adjectives": {"Easy": 1}, "adverbs": {"Easy": 1}, "numerals": {"25": 1}}}, {"content": "When I make mistakes about children, I can usually correct them.", "word_count": 20, "label": "", "sentence_structure": "Subject鈥搗erb鈥搊bject", "subjective_objective": {"answer": "Subjective", "rationale": "Because \"I can usually correct\" is a patient's self-observation and self-evaluation, which is a subjective attitude and sense."}, "sentence_sentiment": {"answer": "Positive", "rationale": "Because the \"can correct\" act in the entry is positive."}, "language_style": {"answer": "Tactful and warm", "rationale": "The entry uses a more moderate and implicit expression to ask the respondents about their attitudes and behaviour in dealing with the children's errors. By using the expression \"usually able\", the possibility of direct accusations or negative evaluations of the interviewee is avoided, while leaving the interviewee with a space for self-interpretation, which is more readily acceptable to the interviewee and is willing to answer questions."}, "time_dimension": {"answer": "Present", "rationale": "The keyword \"usually\" indicates that it is a regular, customary act and can therefore be concluded for the time being."}, "sentence_type": "Affirmative declarative sentence", "pos": {"nouns": {"Son": 1, "Yes": 1}, "verbs": {"Yes": 1, "Twist": 1}, "adjectives": {"Wrong": 1}, "adverbs": {"Usually": 1}, "pronouns": {"I": 1}}}, {"content": "27. Even if your children are often angry, parents should not give up their efforts.", "word_count": 21, "label": "", "sentence_structure": "Subject鈥搗erb鈥搊bject", "subjective_objective": {"answer": "Subjective", "rationale": "Because \"not give up your efforts\" is the subjective attitude and decision of parents."}, "sentence_sentiment": {"answer": "Positive", "rationale": "The attitude in the entries is to support parents in their efforts to keep their children educated and guided."}, "language_style": {"answer": "Tactful and warm", "rationale": "The language style of the entry is euphemism, because it conveys the message in a way that is both encouraging and advising, and is more gentle and friendly. It does not direct orders or criticisms of parents, but rather reminds them not to give up easily, but to continue working. This expression is more acceptable and has positive effects."}, "time_dimension": {"answer": "Present", "rationale": "The entry will contain visions and expectations for the future, that is, parents should not give up their efforts when they are dealing with a child's temperament. The keyword \"even if\" and \"not\" point to what may happen in the future."}, "sentence_type": "Affirmative declarative sentence", "pos": {"nouns": {"Son": 1, "parents": 1, "Try": 1}, "verbs": {"- You're losing your temper": 1, "Abandon": 1, "Reactions": 1, "not": 1, "Try": 1}, "adjectives": {"Big hair": 1}, "numerals": {"27": 1}, "pronouns": {"you": 1, "they": 1}}}, {"content": "28. Being a good parent is often a matter of whether you are lucky and have a good child.", "word_count": 21, "label": "", "sentence_structure": "Subject鈥揷opula鈥損redicative", "subjective_objective": {"answer": "Subjective", "rationale": "Because the ideas of being a good parent and having a good child are based on personal values, experience and expectations, not on objective facts."}, "sentence_sentiment": {"answer": "Positive", "rationale": "Because \"be a good parent\" and \"have a good child\" are both positive descriptions."}, "language_style": {"answer": "Tactful and warm", "rationale": "By describing the importance of good parenting, the entry indirectly leads the respondents to reflect on their own parenting methods and outcomes, and to express an attitude of encouragement and expectation. At the same time, the expression \"Whether or not to be lucky\" implies that the child's growth is not entirely dependent on the efforts of the parents, but rather a complex and fluid process, which is neither objective nor emotional, and reflects a warm and gentle language style."}, "time_dimension": {"answer": "Present", "rationale": ""}, "sentence_type": "Affirmative declarative sentence", "pos": {"nouns": {"parents": 1, "Son": 1}, "verbs": {"Do it": 1, "Look": 1, "have": 1, "Lucky": 1}, "adjectives": {"Okay": 2}, "numerals": {"28": 1}, "pronouns": {"you": 1}}}, {"content": "29. I'm lucky to be a parent, and I happen to have a good kid.", "word_count": 17, "label": "", "sentence_structure": "Subject鈥搗erb鈥搊bject", "subjective_objective": {"answer": "Subjective", "rationale": "Because \"I happen to have a good child\" is patient subjective knowledge and evaluation."}, "sentence_sentiment": {"answer": "Positive", "rationale": "Because \"have a good child\" is a positive expression of the sense of happiness and satisfaction as parents."}, "language_style": {"answer": "Tactful and warm", "rationale": "The language style of the entry is euphemistic, because it expresses the praise of a parent for his children in a way that is grateful and happy. It is more gentle, friendly and emphasizes positive emotional experiences. It also uses emotional terms (such as \"fate\" and \"scene\") to express its feelings, making it easier for readers to resonate."}, "time_dimension": {"answer": "Present", "rationale": "The entry describes the current state, where the words \"good luck\" and \"yes\" indicate that this is a description of the current situation. So the temporal dimension is now."}, "sentence_type": "Affirmative declarative sentence", "pos": {"nouns": {"parents": 1, "Lucky": 1, "Son": 1}, "verbs": {"have": 1, "It happens": 1, "be": 1, "Do it": 1, "very": 1, "Lucky": 1}, "adjectives": {"Okay": 1}, "numerals": {"29": 1}, "pronouns": {"I": 1}}}, {"content": "30. I often find things to be happening, and things that should happen to my children will always happen.", "word_count": 23, "label": "", "sentence_structure": "Subject鈥搗erb鈥搊bject", "subjective_objective": {"answer": "Objective", "rationale": "Because it is a descriptive statement, it presents an objective fact without subjective explanation."}, "sentence_sentiment": {"answer": "Neutral", "rationale": "Because the entry describes a phenomenon, there is no apparent positive or negative emotional colour."}, "language_style": {"answer": "Tactful and warm", "rationale": "The language style of this entry is relatively warm. It does not directly criticize or accuse, but rather describes a phenomenon in a peaceful way and invites interviewees to share their views and experiences. Such expressions are more likely to resonate with the interviewee and to contribute to a good questionnaire-based interactive atmosphere."}, "time_dimension": {"answer": "Present", "rationale": ""}, "sentence_type": "Affirmative declarative sentence", "pos": {"nouns": {"Yes": 2, "I": 1, "Son": 1}, "verbs": {"Found": 1, "Regular": 1, "to": 1, "Yes": 2, "Come on": 2}, "adverbs": {"Often": 1, "- Come on": 1}, "numerals": {"30": 1}, "pronouns": {"I": 1, "The": 1, "of": 2}}}, {"content": "31. Destiny was too generous to me. I had no idea what to do if I had a bad boy.", "word_count": 26, "label": "", "sentence_structure": "Subject鈥搗erb鈥搊bject", "subjective_objective": {"answer": "Subjective", "rationale": "Because this is the patient ' s perception of his or her own destiny and is subjective."}, "sentence_sentiment": {"answer": "Negative", "rationale": "Because the phrase \"if I had a bad child\" implies a fear and a negative feeling for the future."}, "language_style": {"answer": "Tactful and warm", "rationale": "The entry uses a sympathetic and understanding expression of gratitude for having a good child, and suggests that without such luck, it may be confusing and helpless. This expression gives people a sense of affection and affection, which can easily resonate and be a source of affection."}, "time_dimension": {"answer": "Present", "rationale": "So the answer is \"now\"."}, "sentence_type": "Exclamatory sentence", "pos": {"nouns": {"Destiny": 1, "Son": 2}, "verbs": {"to": 1, "Too much": 1, "- Please": 1, "have": 1, "Yes": 2, "Do it": 2, "The": 1, "be": 1}, "adjectives": {"Bad": 1}, "adverbs": {"Too much": 1, "True": 1}, "numerals": {"31": 1}, "classifiers": {"individual": 1}, "pronouns": {"I": 2}}}, {"content": "32. Successful child discipline seems to be more related to the mood and emotions of the children at the time, and less to what action is taken.", "word_count": 33, "label": "", "sentence_structure": "Subject鈥搗erb鈥搊bject", "subjective_objective": {"answer": "Subjective", "rationale": "Because the \"seems more about the mood and emotions of the children at the time\" is the patient's subjective feeling and thoughts."}, "sentence_sentiment": {"answer": "Neutral", "rationale": "Because the entry is not clearly positive or negative, it is merely a statement of one view."}, "language_style": {"answer": "Calm and direct", "rationale": "The language style of the entry is calm and straightforward. It does not use euphemism or vague language to express its views, but rather it states that \"the ability to successfully discipline a child seems to be more related to the mood, emotions and actions of the child at the time.\" This expression makes the view clearer and understandable."}, "time_dimension": {"answer": "Present", "rationale": "The entry describes the view or belief that successful discipline of a child is independent of the action taken, but depends more on the emotional and emotional state of the child at the time. It therefore refers to the current perception or perception, and does not relate to past or future circumstances."}, "sentence_type": "Affirmative declarative sentence", "pos": {"nouns": {"Questionnaire Entry": 1, "Son": 1, "The mood": 1, "Emotion": 1, "Action": 1}, "verbs": {"Yes": 1, "Corrections": 1, "Success": 1, "Seems like": 1, "More with": 2, "Related": 2, "Adoption": 1}, "adverbs": {"At the time": 1}, "numerals": {"32": 1}, "classifiers": {"individual": 1}, "pronouns": {"What": 1}}}, {"content": "Neither my child nor myself would be responsible for his/her actions.", "word_count": 22, "label": "", "sentence_structure": "Subject鈥搗erb鈥搊bject", "subjective_objective": {"answer": "Subjective", "rationale": "Because this statement relates to the views and beliefs of individuals, i.e., their irresponsible attitude towards the child or their own behaviour. This is a reflection of the patient鈥檚 subjective attitude and is therefore the subject matter of the subjective examination."}, "sentence_sentiment": {"answer": "Negative", "rationale": "Because \"not responsible\" in the entry expresses a negative emotional attitude to evading and shirking responsibility."}, "language_style": {"answer": "Calm and direct", "rationale": "The entry expresses the questions of the respondents about the concept of family education responsibilities in a concise and clear language, without the use of too much acrimonym or emotional colours and with a relatively objective and calm tone."}, "time_dimension": {"answer": "Present", "rationale": ""}, "sentence_type": "Exclamatory sentence", "pos": {"nouns": {"Kids": 1, "self": 1}, "verbs": {"Yes": 1, "Responsible": 1}, "adjectives": {"Whatever": 1, "he": 2, "she": 1}, "adverbs": {"Both": 1}, "numerals": {"33": 1}, "pronouns": {"I": 1, "self": 1, "he": 2, "she": 1}}}, {"content": "34. In order to put my plans into practice, I want to make clear that they are in line with my children ' s wishes.", "word_count": 30, "label": "", "sentence_structure": "Subject鈥搗erb鈥搊bject", "subjective_objective": {"answer": "Subjective", "rationale": "The subject is subjective because it relates to the individual wishes and decision-making process of the patient and belongs to the patient ' s own thinking and judgement."}, "sentence_sentiment": {"answer": "Positive", "rationale": "This entry shows that the interviewees have a clear plan of action and are responsive and positive to the needs and wishes of the children."}, "language_style": {"answer": "Tactful and warm", "rationale": "The language style of the entry is more euphemistic. It leads the interviewee to wonder whether his plan is in keeping with the wishes of the child, not directly ordering or enforcing."}, "time_dimension": {"answer": "Present", "rationale": "The entry is to ask if the testee will take the needs and aspirations of the child into account in the implementation of the plan, and the time dimension is now. The keyword \"tweak\" means that this is an ongoing or imminent act, and therefore now. And \"Is it in keeping with my child's wishes\" is also a description of the current situation."}, "sentence_type": "Interrogative sentence", "pos": {"nouns": {"Son": 1, "Planned": 2, "Wish": 1}, "verbs": {"Yes": 2, "鈾?And I'm gonna be 鈾?: 1, "Clear": 1, "Implementation": 1, "Match": 1}, "adjectives": {"These": 1}, "numerals": {"34": 1}, "pronouns": {"I": 1}}}, {"content": "Most parents are unaware of the impact of the accident on the final performance of their children.", "word_count": 29, "label": "", "sentence_structure": "Subject鈥搗erb鈥搊bject", "subjective_objective": {"answer": "Subjective", "rationale": "Because \"most parents do not know\" is the subjective perception and perception of the subject."}, "sentence_sentiment": {"answer": "Negative", "rationale": "Because the description \"most parents are unaware of the effect of an accident on the child's performance\" in the entry is negative emotional."}, "language_style": {"answer": "Calm and direct", "rationale": "The entry presents its views in an objective manner, without excessive rhetorical or emotional rendering, and is more balanced and objective. Thus, the language style of the entry can be judged as \"cool and direct\"."}, "time_dimension": {"answer": "Present", "rationale": ""}, "sentence_type": "Negative declarative sentence", "pos": {"nouns": {"parents": 2, "Son": 1, "Performance": 1, "Impact": 1, "Accident": 1, "Eventually": 1}, "verbs": {"Recognizing": 1, "How many": 1, "Large": 1}, "adjectives": {"Most of them": 1}, "adverbs": {"More": 1, "Large": 1, "Eventually": 1}, "numerals": {"35": 1, "2": 1}, "classifiers": {"individual": 1}, "pronouns": {"they": 1}}}, {"content": "36. Genetics play a major role in determining the personality of the child.", "word_count": 16, "label": "", "sentence_structure": "Subject鈥搗erb鈥搊bject", "subjective_objective": {"answer": "Objective", "rationale": "The statement that \"genetics play a major role in determining the child's personality\" is based on a statement of scientific facts, not on subjective feelings or ideas of the patient. The content of this questionnaire is therefore described and examined from an objective perspective."}, "sentence_sentiment": {"answer": "Negative", "rationale": "Because genetics are immutable, the emphasis on their important role can create stress and anxiety for individuals."}, "language_style": {"answer": "Calm and direct", "rationale": "The entry presented a point in a concise and clear manner, with no excessive adoration or emotional colours, and was straightforward."}, "time_dimension": {"answer": "Past", "rationale": "The entry describes the effects of genetics on the personality of the child, which are already present in the past and therefore is judged to be \"the past\"."}, "sentence_type": "Interrogative sentence", "pos": {"nouns": {"Genetic": 1, "Son": 1, "Personality": 1, "Role": 1}, "verbs": {"Decision": 1, "Rise": 1}, "numerals": {"36": 1}}}, {"content": "37. Bad luck. One cannot be a good parent.", "word_count": 16, "label": "", "sentence_structure": "Subject鈥搗erb鈥揷omplement", "subjective_objective": {"answer": "Subjective", "rationale": "Because this is the patient's subjective assessment of his or her abilities."}, "sentence_sentiment": {"answer": "Negative", "rationale": "Because the idea that a person cannot be a good parent is negative."}, "language_style": {"answer": "Calm and direct", "rationale": "The entry is more directly linguistically, with no excessive rhetorical or emotional rendering, and is more resolute and resolute in tone, and can therefore be judged as being straightforward."}, "time_dimension": {"answer": "Present", "rationale": "The entry describes the view or belief that \"a person cannot be a good parent\" can only be achieved with good luck. The time dimension of the article is now due to the absence of a reference to the past or the future."}, "sentence_type": "Negative declarative sentence", "pos": {"nouns": {"Current": 1, "person": 2, "parents": 1}, "verbs": {"Do it": 1, "Maybe": 1}, "adjectives": {"Okay": 1}, "adverbs": {"not": 1, "Impossible": 1}, "numerals": {"37": 1}}}, {"content": "I always felt that there was something about children.", "word_count": 14, "label": "", "sentence_structure": "Subject鈥揷opula鈥損redicative", "subjective_objective": {"answer": "Subjective", "rationale": "Because \"the mind counts\" is a subjective feeling and perception that reflects the patient's knowledge and trust in his child. This is not an objective reality or behaviour, but a patient's subjective experience and self-evaluation."}, "sentence_sentiment": {"answer": "Positive", "rationale": "Because the words \"in the mind\" express confidence and control, and are positive emotional attitudes."}, "language_style": {"answer": "Calm and direct", "rationale": "The entry directly expresses the question of whether the other party always has confidence and control over the subject in dealing with the child, without excessive rhetorical or emotional rendering, and is more objective in tone."}, "time_dimension": {"answer": "Present", "rationale": "In the future, the entry expresses the expectations and visions of the testee for the future, and the key words are \"for the child\" and \"for the child\" and they point to what has not yet happened and what may happen in the future."}, "sentence_type": "Affirmative declarative sentence", "pos": {"nouns": {"Yes": 1, "Son": 1}, "verbs": {"I feel": 1, "I know what you're talking about": 1}, "adjectives": {"Total": 1}, "numerals": {"38": 1}, "pronouns": {"I": 1}}}, {"content": "39. Sometimes my children ' s behaviour makes me unconstrained.", "word_count": 15, "label": "", "sentence_structure": "Subject鈥搗erb鈥揷omplement", "subjective_objective": {"answer": "Subjective", "rationale": "Because \"acting makes me unconstrained\" is a subjective feeling or evaluation, depending on the interviewee鈥檚 own experience and feelings. Although the child鈥檚 behaviour is objective, how it is understood and evaluated is subjective."}, "sentence_sentiment": {"answer": "Negative", "rationale": "Because the \"unbinding\" behavior in the entry is negative."}, "language_style": {"answer": "Tactful and warm", "rationale": "By using the term \"sometimes\" the entry expresses that the problem of child behaviour is not always present, but is an occasional situation. At the same time, the term is used in a more moderate manner, without too harsh or blameful a tone, giving people a sense of affection."}, "time_dimension": {"answer": "Present", "rationale": "The entry asks whether the testee has any behaviour that makes his or her child feel uncontrollable during the current or recent period (i.e. now or in the past). Therefore, the temporal dimension is now and in the past. The answer is: Have you experienced that the child's behaviour has been unconstrained in the past or in the present for some time?"}, "sentence_type": "Negative declarative sentence", "pos": {"nouns": {"Son": 1, "Behaviour": 1, "Constraints": 1}, "verbs": {"You're": 1, "Cannot": 1}, "adverbs": {"Sometimes": 1}, "numerals": {"39": 1}, "pronouns": {"I": 1}}}, {"content": "I sometimes feel desperate about my children's behavior.", "word_count": 15, "label": "", "sentence_structure": "Subject鈥搗erb鈥揷omplement", "subjective_objective": {"answer": "Subjective", "rationale": "Because \"frightful\" is the patient's subjective feeling and emotional reaction."}, "sentence_sentiment": {"answer": "Negative", "rationale": "Because the mood of \"deep\" in the entries is negative."}, "language_style": {"answer": "Tactful and warm", "rationale": "The entry uses the term \"sometimes\" to express a relatively euphemistic and objective emotional attitude, rather than a strong emotional reaction. At the same time, the term \"depressive\" is not used too directly and intensely, but rather in a more relaxed way to convey the opinion of the surveyer about the feelings of the testee about the behaviour of the child."}, "time_dimension": {"answer": "Present", "rationale": "In the entry, \"sometimes\" indicates that this situation is not always present, but that it may recur, so it is now."}, "sentence_type": "Negative declarative sentence", "pos": {"nouns": {"Son": 1, "Behaviour": 1, "Despair": 1}, "verbs": {"to": 1, "I feel": 1}, "adjectives": {"Sometimes": 1}, "pronouns": {"I": 1}}}, {"content": "41. It is sometimes easier to let my children behave in their own way than to endure their anger.", "word_count": 30, "label": "", "sentence_structure": "Subject鈥搗erb鈥搊bject", "subjective_objective": {"answer": "Subjective", "rationale": "Because the question of the investigation is a reflection of the patient's subjective feelings and views, the question is the extent to which the patient considers it difficult to \"let the child act in his own way\" and \"to endure their anger.\""}, "sentence_sentiment": {"answer": "Negative", "rationale": "Because the \"Dirty-Dirty\" act in the entry is negative."}, "language_style": {"answer": "Tactful and warm", "rationale": "The language style of the entry is more euphemistic, using some gentle expression and invitation to communicate with the interviewee. For example, \"Let my child act in his own way,\" which avoids direct orders or blame and expresses the desire to explore and develop on his own. Moreover, \"sometimes it is easier to endure their anger\" , and the phrase \"more easy\" in which it is used also has a sense of comfort and encouragement. Thus, the language style of the article can be summarized as euphemism."}, "time_dimension": {"answer": "Present", "rationale": "The entry describes a situation and feeling in the current child-rearing process, using the current state of affairs and tone."}, "sentence_type": "Imperative sentence", "pos": {"nouns": {"Son": 2, "self": 1, "Modalities": 1, "Take it": 1, "You're so angry": 1}, "verbs": {"Jean": 1, "According to": 1, "Act": 1, "- Yeah": 1, "Easy": 1, "Much more": 1}, "adjectives": {"Large": 1}, "adverbs": {"Sometimes": 1, "More": 1}, "numerals": {"41": 1}, "pronouns": {"I": 1}}}, {"content": "42. I find that sometimes my children can let me do things I would not have wanted to do.", "word_count": 21, "label": "", "sentence_structure": "Subject鈥搗erb鈥搊bject", "subjective_objective": {"answer": "Subjective", "rationale": "Because the entry is a subjective examination of the patient ' s perception and feelings of his or her own behaviour."}, "sentence_sentiment": {"answer": "Negative", "rationale": "Because the expression \"sometimes my children can make me do something I would not want to do\" has a certain degree of coercion and passivity, and it is therefore negative."}, "language_style": {"answer": "Tactful and warm", "rationale": "The entry uses a more euphemistic and implicit language, without directly expressing strong feelings or opinions, but rather raises questions in a more peaceful manner, giving a gentle feeling."}, "time_dimension": {"answer": "Past", "rationale": "The words \"I find\" and \"Yes\" and \"Let me do\" all indicate that this is a description and a review of the past, so the temporal dimension of this article is the past."}, "sentence_type": "Affirmative declarative sentence", "pos": {"nouns": {"Questionnaire Entry": 1, "Time": 1, "Mine": 2, "Son": 1, "Yes": 3}, "verbs": {"Found": 1, "Jean": 2, "Do it": 2, "Yes": 1}, "adjectives": {"No, I don't": 1}, "numerals": {"42": 1, "Some": 1}, "classifiers": {"individual": 1}, "pronouns": {"I": 1}}}, {"content": "43. My children often do things that are far from what I ask.", "word_count": 20, "label": "", "sentence_structure": "Subject鈥搗erb鈥揷omplement", "subjective_objective": {"answer": "Subjective", "rationale": "This is because the patient views and evaluates the behaviour of his or her children and is subjective."}, "sentence_sentiment": {"answer": "Negative", "rationale": "Because the entry describes a wide gap between the child ' s behaviour and the requirements, it implies emotional expressions of dissatisfaction or disappointment with the child ' s behaviour, and is therefore negative."}, "language_style": {"answer": "Tactful and warm", "rationale": ""}, "time_dimension": {"answer": "Present", "rationale": "In the past, the entry described the feelings and evaluation of the testee ' s child behaviour, and the temporal dimension was judged to be past."}, "sentence_type": "Imperative sentence", "pos": {"nouns": {"Son": 1, "Behaviour": 1, "Request": 1}, "verbs": {"Difference": 1, "Far": 1}, "adjectives": {"Large": 1}, "adverbs": {"Often": 1}, "numerals": {"43": 1}, "classifiers": {"One": 1}, "pronouns": {"I": 1}}}, {"content": "Sometimes if I was tired, I would let the children do what I would normally not let them do.", "word_count": 27, "label": "", "sentence_structure": "Subject鈥搗erb鈥搊bject", "subjective_objective": {"answer": "Subjective", "rationale": "Because \"tired\" and \"that's what I'm not gonna let them do\" are the subjective feelings and decisions of patients when they answer."}, "sentence_sentiment": {"answer": "Negative", "rationale": "Because \"let the kids do what I shouldn't let them do\" is negative."}, "language_style": {"answer": "Tactful and warm", "rationale": "The entry expresses a possible situation by using moderate language (such as \"if I'm tired\" and \"if I don't normally let them do things\") and attempts to find each other's views or experiences. Such language style makes the questionnaire more personal and interactive."}, "time_dimension": {"answer": "Present", "rationale": "The entry describes the behaviour or decision of the testee in certain specific situations, i.e., when he/she is tired, which allows the children to do things that they are not normally allowed to do. As the act is currently being performed, the temporal dimension is now judged."}, "sentence_type": "Affirmative declarative sentence", "pos": {"nouns": {"Time": 1, "I": 2, "Kids": 1, "Yes": 2, "Normal": 1}, "verbs": {"Jean": 1, "Do it": 2}, "adjectives": {"I'm tired": 1}, "adverbs": {"Sometimes": 1}, "numerals": {"44": 1}}}, {"content": "45. Sometimes I feel that I have difficulty in controlling the way of life of my children.", "word_count": 21, "label": "", "sentence_structure": "Subject鈥揷opula鈥損redicative", "subjective_objective": {"answer": "Subjective", "rationale": "Because \"fear\" and \"uncontrollable\" are the subjective feelings and judgments of the patient. Although the way the child lives is an objective fact, the patient's response is subjective."}, "sentence_sentiment": {"answer": "Negative", "rationale": "This entry is a negative emotional attitude, as it expresses parents ' fear and sense of weakness in their children ' s way of life."}, "language_style": {"answer": "Tactful and warm", "rationale": "The entry is more personal and interactive when it is spoken in a gentle and inviting manner, rather than in direct statements or orders. At the same time, the language style of the entry is more neutral, and there is no over-emotive recitation, but it is an objective expression of concern and concern for the person under investigation."}, "time_dimension": {"answer": "Past", "rationale": "The entry refers to \"fear\" and \"hard\" and these terms represent the feelings and experiences of the subjects over time and can therefore be judged as the past."}, "sentence_type": "Affirmative declarative sentence", "pos": {"nouns": {"Time": 1, "I": 1, "Children": 1, "Lifestyle": 1, "Control": 1}, "verbs": {"I feel": 1, "Take it": 1}, "adverbs": {"Sometimes": 1}, "numerals": {"45": 1}}}, {"content": "I allow my children to do their own business.", "word_count": 11, "label": "", "sentence_structure": "Subject鈥搗erb鈥搊bject", "subjective_objective": {"answer": "Subjective", "rationale": "Because it's a person's subjective decision and attitude to leave the child to its own business.\""}, "sentence_sentiment": {"answer": "Neutral", "rationale": "Because the entry merely stated a fact, it did not express any feelings or attitudes."}, "language_style": {"answer": "Tactful and warm", "rationale": "The language style of the entry is more euphemistic and sweet, using the words \"permissive\" and \"your own business\" to express support and trust in children, without direct orders or criticism. This is more acceptable and promotes communication between the two sides."}, "time_dimension": {"answer": "Present", "rationale": "The entry expresses the attitude and behaviour of the testee towards his child, the keywords being \"permissive\" and \"proper\" and they indicate that the testee's actions and decisions are taking place or have been completed. Thus, the temporal dimension is now judged."}, "sentence_type": "Affirmative declarative sentence", "pos": {"nouns": {"I": 1, "Son": 1, "Things": 1}, "verbs": {"Allow": 1, "From": 1, "Okay": 1}, "numerals": {"46": 1}}}, {"content": "47: It's not hard to change my kids' minds.", "word_count": 19, "label": "", "sentence_structure": "Subject鈥搗erb鈥搊bject", "subjective_objective": {"answer": "Objective", "rationale": "The fact that the child is changed is not a matter for the patient to decide, so it is an objective result."}, "sentence_sentiment": {"answer": "Positive", "rationale": "Because the phrase \"to change a child's mind\" in the entry is positive, optimistic and positive."}, "language_style": {"answer": "Tactful and warm", "rationale": ""}, "time_dimension": {"answer": "Present", "rationale": "The entry shows the attitude and experience of the speaker in dealing with the behaviour of his child, in which the \"not an impossible thing\" is the subjective feeling or belief of the speaker. Thus, the temporal dimension of the entry is now judged."}, "sentence_type": "Affirmative declarative sentence", "pos": {"nouns": {"Yes": 1, "Son": 1}, "verbs": {"Jean": 1, "Do it": 1, "Change": 1}, "adjectives": {"Hard": 1, "Do it": 1}, "numerals": {"47": 1}, "pronouns": {"I": 1}}}]}

{"questionnaire_title": "Social expectations answer", "questionnaire_category": "Behavioral questionnaire", "item_count": 33, "emotion_shift_overall": "From positive to negative", "language_style_overall": "Neutral", "items": [{"content": "1. Before voting, I shall have a thorough knowledge of all the candidates.", "word_count": 18, "label": "", "sentence_structure": "Subject鈥搗erb鈥搊bject", "subjective_objective": {"answer": "Subjective", "rationale": "Because \"to know\" or \"to know\" attitudes or ideas are subjective to patients. Although understanding candidates is based on objective facts, the motivation and will behind such actions are subjective. So \"I want to know the situation of all candidates before voting\" is subjective."}, "sentence_sentiment": {"answer": "Neutral", "rationale": "Because the entry does not express any positive or negative emotional aspects. It describes only one act that a person would have done before voting, i.e., to know more about the candidates."}, "language_style": {"answer": "Calm and direct", "rationale": "The language style of the entry is calm and straightforward. It expresses in a concise and clear language the desire of voters to have a full understanding of the candidates before voting, and is objective and without excessive rhetoric."}, "time_dimension": {"answer": "Present", "rationale": "The entry describes a specific, ongoing behaviour whereby the testee intends to have a thorough knowledge of all candidates before voting, and thus the time dimension is now judged."}, "sentence_type": "Affirmative declarative sentence", "pos": {"nouns": {"Vote": 1, "Candidates": 1, "person": 1, "Situation": 1}, "verbs": {"Yes": 1, "Roger that": 1, "Full": 1}, "adverbs": {"Front": 1}, "numerals": {"One": 2}, "pronouns": {"I": 1}}}, {"content": "I've always been unshakable in giving up my own business to help people in difficulty.", "word_count": 21, "label": "", "sentence_structure": "Subject鈥搗erb鈥搊bject", "subjective_objective": {"answer": "Subjective", "rationale": "Because leaving one ' s own business to help others is a behaviour that is subjective to the patient."}, "sentence_sentiment": {"answer": "Positive", "rationale": "Because the quality of the entry, \"No hesitation to drop your own business and help people in difficulty,\" is positive."}, "language_style": {"answer": "Tactful and warm", "rationale": "The basis for the judgment: \"No hesitation in putting your own business behind you\" in the entry is a positive, enthusiastic expression, without too much calm, objective or cold language. At the same time, the act of \"helping people in distress\" conveys a warm, supportive atmosphere that is more in keeping with the euphemism of the language style."}, "time_dimension": {"answer": "Present", "rationale": "\"I'm always\" means it's a habit, and \"leave my own business\" and \"help the hard-earned\" are also descriptions of current behavior. So, the entry is now time-dimensional."}, "sentence_type": "Affirmative declarative sentence", "pos": {"nouns": {"Questionnaire": 1, "Entry": 1, "person": 1}, "verbs": {"Analysis": 1, "Total": 1, "Put it down": 1, "Do it": 1, "Help": 2, "It's hard": 1, "Fire": 1, "Location": 1}, "adjectives": {"Without hesitation": 1, "Your own": 1, "Hard to get": 1}, "numerals": {"2": 1}, "classifiers": {"individual": 1}, "pronouns": {"I": 1}}}, {"content": "3. It's hard to keep things going without others.", "word_count": 22, "label": "", "sentence_structure": "Subject鈥損redicate", "subjective_objective": {"answer": "Subjective", "rationale": "Because \"get encouraged\" and \"get things done\" are the feelings and thoughts of patients in the face of specific situations, and it is the subjective attitudes and emotional experiences of patients."}, "sentence_sentiment": {"answer": "Negative", "rationale": "Because the entry \"if I don't get encouraged, sometimes it's hard to keep things going\" is negative."}, "language_style": {"answer": "Calm and direct", "rationale": "The language style of the entry is calm and straightforward, and there is no excessive emotional rendering or rhetorical technique. It is a dialogue that takes a clear question, leads the interviewee to reflect on his situation and gives a real answer."}, "time_dimension": {"answer": "Past", "rationale": "The entry expresses an experience or feeling in which \"if encouraged by others\" indicates that this has happened and may reoccur in the future, and therefore the temporal dimension is judged to be past."}, "sentence_type": "Imperative sentence", "pos": {"nouns": {"others": 1, "Drumway": 1, "Things": 1}, "verbs": {"Got it": 1, "Do it": 1}, "adjectives": {"It's hard": 1}, "adverbs": {"Sometimes": 1}, "numerals": {"3": 1}, "pronouns": {"I": 1}}}, {"content": "I have never hated anyone in particular.", "word_count": 10, "label": "", "sentence_structure": "Subject鈥損redicate", "subjective_objective": {"answer": "Objective", "rationale": "Because \"doesn't hate someone\" is an objective fact that does not depend on a subjective individual's perception."}, "sentence_sentiment": {"answer": "Neutral", "rationale": "Because the entry is not clearly positive or negative. It simply expresses the fact that the interviewee never hated anyone in particular."}, "language_style": {"answer": "Tactful and warm", "rationale": "The language style of the questionnaire entry is euphemism. It asks the respondents, in a more subtle way, whether they have ever had a particularly annoying person or thing, without using a strong negative word to avoid offensiveness towards the person under investigation. This expression is more acceptable and more likely to get a real answer."}, "time_dimension": {"answer": "Past", "rationale": "The temporal dimension of the purpose of the article is therefore judged to be past."}, "sentence_type": "Negative declarative sentence", "pos": {"nouns": {"Questionnaire": 1, "Entry": 1}, "verbs": {"Analysis": 1, "have": 1}, "adjectives": {"Special": 1}, "adverbs": {"Never": 1}, "numerals": {"4": 1}}}, {"content": "I occasionally wonder if I have the capacity to succeed.", "word_count": 16, "label": "", "sentence_structure": "Subject鈥損redicate", "subjective_objective": {"answer": "Subjective", "rationale": "Because \"suspecting your ability to succeed\" is the patient's own idea and feeling, it's a subjective perspective."}, "sentence_sentiment": {"answer": "Negative", "rationale": "Because the attitude in the entry \"suspects that he has the ability to succeed\" is negative."}, "language_style": {"answer": "Tactful and warm", "rationale": ""}, "time_dimension": {"answer": "Present", "rationale": ""}, "sentence_type": "Interrogative sentence", "pos": {"nouns": {"Success": 1, "Capacity": 1}, "verbs": {"Doubt": 1}, "adjectives": {"Sometimes": 1}, "numerals": {"5": 1}, "pronouns": {"self": 1}}}, {"content": "6. I sometimes feel outrage at not being given what I want.", "word_count": 21, "label": "", "sentence_structure": "Subject鈥搗erb鈥揷omplement", "subjective_objective": {"answer": "Subjective", "rationale": "Because \"deep in anger\" is the emotional experience and attitude inside the patient, and it's subjective."}, "sentence_sentiment": {"answer": "Negative", "rationale": "Because the emotional reaction in the entry, \"deep in anger,\" is negative."}, "language_style": {"answer": "Calm and direct", "rationale": "The entry directly asks the interviewee about his emotional reaction when he cannot get what he wants, without too much ado or emotional expression, and is more direct and objective in tone."}, "time_dimension": {"answer": "Present", "rationale": "The entry describes the emotional reactions and feelings of the person being tested in certain circumstances, which are currently being experienced and are therefore judged as being present."}, "sentence_type": "Affirmative declarative sentence", "pos": {"nouns": {"Something": 1, "I'm angry": 1}, "verbs": {"Got it": 1, "I feel": 1}, "adjectives": {"self": 1, "What I want": 1}, "numerals": {"6": 1}, "pronouns": {"I": 1}}}, {"content": "I always look out for my clothes.", "word_count": 11, "label": "", "sentence_structure": "Subject鈥搗erb鈥揷omplement", "subjective_objective": {"answer": "Subjective", "rationale": "Because \"watch your clothes\" is the patient's subjective attitude and behaviour, not the objective fact or result."}, "sentence_sentiment": {"answer": "Positive", "rationale": "Because \"careful to look at your clothes\" is a self-care and attention expression of positive attitudes or behaviour."}, "language_style": {"answer": "Tactful and warm", "rationale": ""}, "time_dimension": {"answer": "Present", "rationale": ""}, "sentence_type": "Interrogative sentence", "pos": {"nouns": {"self": 1, "Clothes": 1}, "verbs": {"Always looking out": 1}, "adverbs": {"very": 1}}}, {"content": "8. I have always focused on the manners of the table, both at home and in restaurants.", "word_count": 21, "label": "", "sentence_structure": "Subject鈥搗erb鈥搊bject", "subjective_objective": {"answer": "Subjective", "rationale": "Because \"I'm just like the manners at the table\" is the patient's own subjective view and attitude."}, "sentence_sentiment": {"answer": "Positive", "rationale": "Because the purpose of the entry is to focus on the manners of the table."}, "language_style": {"answer": "Tactful and warm", "rationale": "The language style of the entry is more euphemistic and gentle, and the importance of table protocol is not too direct or harshly expressed by using the words \"all the same\" and \"focusing\" to make it easier for the interviewee to accept it and to create a good atmosphere of communication."}, "time_dimension": {"answer": "Present", "rationale": "The entry \"Equivalently focused on the manners of the table\" indicates the present behaviour of the testee and therefore falls within the scope of the present."}, "sentence_type": "Affirmative declarative sentence", "pos": {"nouns": {"Home": 1, "Hotel": 1, "Dinner table": 1, "Ceremony": 1}, "verbs": {"Both": 2, "Notes": 1, "Heavy": 1, "in": 2}, "adverbs": {"Same thing": 1, "Whatever": 1}, "pronouns": {"I": 1}}}, {"content": "9. I would do so if I could sneak into a cinema without paying for it and certainly not be discovered.", "word_count": 26, "label": "", "sentence_structure": "Subject鈥搗erb鈥搊bject", "subjective_objective": {"answer": "Subjective", "rationale": "This is the patient ' s subjective will and the possibility of behaviour in hypothetical circumstances, and is irrelevant to objective facts."}, "sentence_sentiment": {"answer": "Positive", "rationale": "Because the act of \"no money to sneak into a cinema\" itself has some seduction and risk-taking, and the promise of \"no one will ever find out\" has increased the appeal of the act. The emotional colour of the article is therefore more positive."}, "language_style": {"answer": "Calm and direct", "rationale": "This language style is more direct and objective than emotional or rhetorical."}, "time_dimension": {"answer": "Present", "rationale": "Time dimension is judged now."}, "sentence_type": "Affirmative declarative sentence", "pos": {"nouns": {"Film": 1, "Tickets": 0, "Money": 1, "Cinema": 2}, "verbs": {"No money": 1, "Run away": 1, "Come in": 1, "Yes": 2, "Do it": 1, "Found": 1}, "adjectives": {"Sure": 1}, "adverbs": {"If": 1, "I'm sure not": 1, "not": 1}, "numerals": {"9": 1}, "classifiers": {"Here": 1}, "pronouns": {"I": 1}}}, {"content": "10. For a few days, I gave up on certain things because I was not capable of doing so.", "word_count": 22, "label": "", "sentence_structure": "Subject鈥搗erb鈥搊bject", "subjective_objective": {"answer": "Subjective", "rationale": "Because \"thinking you're not capable\" is a subjective judgment and feeling."}, "sentence_sentiment": {"answer": "Negative", "rationale": "Because \"forgot something because you don't have enough power\" is negative."}, "language_style": {"answer": "Tactful and warm", "rationale": "The entry is asked in a mild manner, without directly rejecting or blaming the other party for its shortcomings, but rather using \"a few moments\" to reduce stress and interact and communicate by asking the other party whether or not a similar situation has occurred. Such language is easier to open up to the interviewee and to obtain real and effective questionnaire data."}, "time_dimension": {"answer": "Past", "rationale": ""}, "sentence_type": "Negative declarative sentence", "pos": {"nouns": {"Time": 2, "I": 1, "self": 1, "Capacity": 1, "Things": 1}, "verbs": {"Abandon": 1, "be": 1, "Because": 1, "Not enough": 1, "And": 1, "Give up something": 1}, "adjectives": {"Some": 1, "Few": 1}, "numerals": {"10": 1, "Few": 1}, "pronouns": {"Here": 1}}}, {"content": "I sometimes like to talk about others.", "word_count": 11, "label": "", "sentence_structure": "Subject鈥搗erb鈥揷omplement", "subjective_objective": {"answer": "Subjective", "rationale": "Because \"like to talk about people\" is patient's subjective attitude."}, "sentence_sentiment": {"answer": "Negative", "rationale": "Because the \"speaks another person's gossip\" act in the entry is negative."}, "language_style": {"answer": "Tactful and warm", "rationale": "The entry is more proficient and interactive in terms of the behaviour of the person questioned in a more moderate and caring manner, rather than directly accusing or ordering."}, "time_dimension": {"answer": "Present", "rationale": "\"Sometimes\" means occasional, intermittent behaviour, \"like to talk\" is the present habit or tendency of the testee, so the title describes the present situation."}, "sentence_type": "Affirmative declarative sentence", "pos": {"nouns": {"I": 1, "Baloney": 1}, "verbs": {"Yeah": 1, "Say it": 1}, "adverbs": {"Sometimes": 1}, "numerals": {"11": 1}}}, {"content": "12. Sometimes I want to defy those with authority, even if I know they are right.", "word_count": 26, "label": "", "sentence_structure": "Subject鈥搗erb鈥搊bject", "subjective_objective": {"answer": "Subjective", "rationale": "Because the word \"want\" indicates that it is the patient's subjective idea and emotional experience."}, "sentence_sentiment": {"answer": "Negative", "rationale": "Because the \"defiant authority\" in the entry is negative."}, "language_style": {"answer": "Tactful and warm", "rationale": "The language style of the entry is more euphemistic and understanding than direct accusations or criticism. It uses the term \"sometimes\" and \"even\" to express the investigator's understanding and acceptance of the person under investigation, without directly attacking or demeaning the views or behaviour of the person under investigation. Such a gentle and understanding can help the person surveyed to be more willing to participate in the questionnaire survey and to obtain more reliable data."}, "time_dimension": {"answer": "Past", "rationale": "The entry asks whether the person tested had had a psychological state or behavioural tendency, that is, \"sometimes\", and describes an emotional experience of inherent conflict: \"who wants to defy authority\" and \"who knows they are right\". So, in time, it is a past."}, "sentence_type": "Affirmative declarative sentence", "pos": {"nouns": {"Time": 1, "I": 1, "person": 1, "Authority": 1}, "verbs": {"Yes": 3, "Insubordination": 1, "Do it": 1}, "adverbs": {"Sometimes": 1, "Even if": 1}, "numerals": {"12": 1, "Right": 1}, "pronouns": {"they": 1}}}, {"content": "I am always a good listener, whoever I speak to.", "word_count": 17, "label": "", "sentence_structure": "Subject鈥揷opula鈥損redicative", "subjective_objective": {"answer": "Subjective", "rationale": "Because \"always\" is an expression of personal feelings and attitudes, a subjective view of patients."}, "sentence_sentiment": {"answer": "Positive", "rationale": "Because the description \"is a good listener\" in the entry is positive."}, "language_style": {"answer": "Tactful and warm", "rationale": "The entry uses a positive tone and emphasizes its merits 鈥?a more euphemistic, friendly and encouraging expression that is good at listening to others. At the same time, it is more objective and realistic without too much rhetorical and emotional."}, "time_dimension": {"answer": "Present", "rationale": "The word \"always\" indicates that such acts have been and are still being carried out, so it can be judged that this is a description of the present state."}, "sentence_type": "Affirmative declarative sentence", "pos": {"nouns": {"Talk": 1, "Listener": 1}, "verbs": {"Always": 1, "be": 1}, "adjectives": {"Okay": 1}, "adverbs": {"Whatever": 1}, "numerals": {"13": 1}, "pronouns": {"Who": 1, "I": 1}}}, {"content": "I recall cases of 鈥渟howing ill鈥?to escape certain things.", "word_count": 17, "label": "", "sentence_structure": "Subject鈥搗erb鈥揷omplement", "subjective_objective": {"answer": "Subjective", "rationale": "Because \"showing sickness\" is the subjective act of the patient to escape something."}, "sentence_sentiment": {"answer": "Negative", "rationale": "Because the act of \"loading sickness\" in the entry is negative."}, "language_style": {"answer": "Calm and direct", "rationale": "The entry was more concise in language and asked directly whether the interviewees had had experience of \"showing ill\" to avoid certain things, without too many rhetorical or emotional renderings, and with more direct and objective tone."}, "time_dimension": {"answer": "Past", "rationale": "The keyword \"Remembered Had\" means that it is a memory and description of something that happened in the past, so the entry is to ask if the person tested had been \"aggravated\" in the past to escape something."}, "sentence_type": "Affirmative declarative sentence", "pos": {"nouns": {"Situation": 1, "Diseases": 1}, "verbs": {"have": 1, "Yes": 1, "Purgatory": 1, "Run away": 1}, "adverbs": {"Pass": 1, "And": 1}, "numerals": {"14": 1}, "classifiers": {"Number of times": 1, "individual": 1}, "pronouns": {"I": 1}}}, {"content": "I used to use others.", "word_count": 10, "label": "", "sentence_structure": "Subject鈥搗erb鈥搊bject", "subjective_objective": {"answer": "Subjective", "rationale": "Because \"when there has been a use of others\" is a patient's subjective memory and self-assessment, which is an individual's subjective experience and perception."}, "sentence_sentiment": {"answer": "Negative", "rationale": "Because the \"use of others\" act in the entry is negative."}, "language_style": {"answer": "Calm and direct", "rationale": "The entry is \"cooled and direct\" in terms of direct and clear language, asking the interviewee whether he has ever used another person, without using euphemism or a more emotional expression."}, "time_dimension": {"answer": "Present", "rationale": "The entry asks if the person tested has had experience of using others, whether this has occurred in the past or in the present, without specifying the time frame, and therefore can be judged as now.\""}, "sentence_type": "Negative declarative sentence", "pos": {"verbs": {"have": 1, "Utilization": 1}, "adjectives": {"Don't": 1}, "numerals": {"15": 1}, "pronouns": {"I": 1}}}, {"content": "16. I am always willing to admit that whenever a mistake has been made.", "word_count": 13, "label": "", "sentence_structure": "Subject鈥損redicate", "subjective_objective": {"answer": "Subjective", "rationale": "Because \"fault\" and \"willing to admit\" are the attitudes and thoughts of the patients themselves, they are subjective."}, "sentence_sentiment": {"answer": "Positive", "rationale": "Because the attitude in the entries is positive, it is a sign of responsibility for their actions and of the courage to admit mistakes."}, "language_style": {"answer": "Tactful and warm", "rationale": "The entry uses a positive and self-reflective tone to guide respondents to share their attitudes and behaviours. Using the term \"willing to acknowledge\" conveys an honest and responsible attitude, which is more likely to generate resonance and trust among the interviewees."}, "time_dimension": {"answer": "Present", "rationale": ""}, "sentence_type": "Affirmative declarative sentence", "pos": {"verbs": {"Yes": 1, "Recognition": 1}, "adverbs": {"Always": 1}, "pronouns": {"I": 1}}}, {"content": "17. I always have to do what I say.", "word_count": 7, "label": "", "sentence_structure": "Subject鈥搗erb鈥揷omplement", "subjective_objective": {"answer": "Subjective", "rationale": "Because \"do what you have to do\" is a personal conviction and attitude, and it is a subjective element."}, "sentence_sentiment": {"answer": "Positive", "rationale": "Because the word \"do\" in the entry is positive."}, "language_style": {"answer": "Calm and direct", "rationale": "The entry asks the respondent whether he or she is always able to speak and act in a direct manner, without too many rhetorical or emotional renderings. This is clear, concise, convincing and direct."}, "time_dimension": {"answer": "Present", "rationale": "\"A word is always required\" is a regular, customary act, and \"A word is required\" is an ongoing act, so it is now used to do things. Nor is it clear whether it is a past or a future plan, and it is now."}, "sentence_type": "Affirmative declarative sentence", "pos": {"verbs": {"Always": 1, "Okay": 1}, "adjectives": {"Yes": 1}, "pronouns": {"I": 1}}}, {"content": "I don't feel particularly difficult to deal with a man who talks and hates.", "word_count": 22, "label": "", "sentence_structure": "Subject鈥搗erb鈥搊bject", "subjective_objective": {"answer": "Subjective", "rationale": "Because this statement reflects the attitude and feelings of patients towards dealing with people who are too talkative and annoying, it is based on personal subjective experience."}, "sentence_sentiment": {"answer": "Negative", "rationale": "Because the entry expresses a negative attitude towards people who are \"speaky and hateful\"."}, "language_style": {"answer": "Calm and direct", "rationale": "The entry describes in a more objective and calm tone the feelings of the subject of the survey with \"the mouthful and hateful person\" without too much emotional or subjective assessment. This language style is neutral and does not have any strong emotional connotations."}, "time_dimension": {"answer": "Past", "rationale": "The entry describes the sense and feeling of what happened in the past, that is, when you're with a man who is too talkative and annoying. The key word is \"not feeling particularly difficult,\" and it expresses an experience and attitude, and therefore judgement is the past."}, "sentence_type": "Affirmative declarative sentence", "pos": {"nouns": {"person": 2, "Get along": 1, "Difficulties": 1}, "verbs": {"I think": 1, "Special": 1, "and": 1}, "adjectives": {"You're full of shit": 1, "Shit": 1}, "adverbs": {"not": 1, "of": 2}, "numerals": {"18": 1}, "pronouns": {"I": 1}}}, {"content": "I sometimes want to give back my teeth, not to forgive or forget.", "word_count": 17, "label": "", "sentence_structure": "Subject鈥搗erb鈥搊bject", "subjective_objective": {"answer": "Subjective", "rationale": "Because \"want to give back\" or \"don't forgive or forget\" are the personal thoughts and attitudes of the patient, and are subjective."}, "sentence_sentiment": {"answer": "Negative", "rationale": "Because \"teeth for tooth\" is a revenge, and it's negative."}, "language_style": {"answer": "Calm and direct", "rationale": "The entry is more concise, is less talkative or emotional, and is more objective. It asks whether the person under investigation has had a revenge spirit, wants to give back his teeth, and does not forgive or forget past unpleasantness. The question is more in line with the psychological requirements of the study, and can directly access the real thoughts and feelings of the person under investigation."}, "time_dimension": {"answer": "Present", "rationale": ""}, "sentence_type": "Affirmative declarative sentence", "pos": {"nouns": {"Questionnaire Entry": 1, "Teeth": 1, "Zenium": 1, "Forgive me": 1, "Yes": 1}, "verbs": {"Yes": 2, "And": 1, "I'll give you a tooth for a tooth": 1, "not": 1, "I'm sorry": 1, "or": 1, "Forget it": 1}, "adverbs": {"Sometimes": 1}, "pronouns": {"I": 1}}}, {"content": "If I do not understand anything, I will admit it with great joy.", "word_count": 18, "label": "", "sentence_structure": "Subject鈥損redicate", "subjective_objective": {"answer": "Subjective", "rationale": "Because \"know\" and \"acknowledge\" are both described from the patient's subjective point of view. The sentence is about the patient's perception and attitude to himself, and it's subjective."}, "sentence_sentiment": {"answer": "Positive", "rationale": "Because it represents a quality of honesty and forthrightness, a positive emotional attitude."}, "language_style": {"answer": "Tactful and warm", "rationale": "The language style of the entry is more euphemistic, using a simple, friendly tone of speech to ask questions, meaning that if you do not know something, you can speak frankly. Such a phrase is more resonant and cooperative than direct orders or accusations, and helps to create a good atmosphere of communication and facilitate the smooth running of the questionnaire."}, "time_dimension": {"answer": "Present", "rationale": "\"If\" leads to scenarios, but \"will be very happy to admit\" in the main sentence indicates the attitudes and behaviour of the present. The entry describes the current behaviour or attitude of the testee, which is judged by the time dimension."}, "sentence_type": "Affirmative declarative sentence", "pos": {"verbs": {"Yes": 1, "Recognition": 1}, "adjectives": {"It was so nice": 1}, "numerals": {"20": 1}, "pronouns": {"I": 1}}}, {"content": "21. Even for those who are hard to live with, I am always polite.", "word_count": 17, "label": "", "sentence_structure": "Subject鈥損redicate", "subjective_objective": {"answer": "Subjective", "rationale": "The questionnaire entry is viewed from the patient鈥檚 subjective point of view. Because 鈥渂eneficial鈥?is a subjective attitude or behavioural choice, not just an objective fact. Although in some cases the expression of 鈥渂eneficial鈥?may be influenced by objective circumstances (for example, in an environment where professional image is required), in this particular case the patient鈥檚 perception of his attitude and behaviour choices is considered and therefore subjective."}, "sentence_sentiment": {"answer": "Positive", "rationale": "Because the behavior in the entry is \"behaved.\""}, "language_style": {"answer": "Tactful and warm", "rationale": "The entry raises questions in a manner that respects the other and avoids offending, and is expressed in a more gentle and polite manner, which makes it easier for the interviewee to feel comfortable and to answer questions. At the same time, the word \"behaved\" has a positive tone and helps to create a good atmosphere for question and answer questions."}, "time_dimension": {"answer": "Present", "rationale": ""}, "sentence_type": "Affirmative declarative sentence", "pos": {"nouns": {"person": 1, "Beam is polite": 1}, "verbs": {"to": 1, "Total": 1, "be": 1, "Jean": 1}, "adjectives": {"It's hard to get along": 1}}}, {"content": "22. Sometimes I have to insist on doing things my way.", "word_count": 16, "label": "", "sentence_structure": "Subject鈥搗erb鈥搊bject", "subjective_objective": {"answer": "Subjective", "rationale": "Because this is the patient ' s personal conviction and attitude and is a subjective idea."}, "sentence_sentiment": {"answer": "Neutral", "rationale": "Since the entry does not have a clear emotional tendency, it merely describes a habit."}, "language_style": {"answer": "Calm and direct", "rationale": "The language style of the entry is straightforward, with no excessive rhetorical or emotional rendering, and is more objective in tone.\""}, "time_dimension": {"answer": "Present", "rationale": "According to the context, \"sometimes\" means that the frequency of the act is not certain, it may be past, present or future; \"sure\", \"preservation\" and \"doing what you want\" all indicate that it is customary and emphasize what is being done. Thus, the temporal dimension of the article is now."}, "sentence_type": "Affirmative declarative sentence", "pos": {"verbs": {"Hold on": 1, "Do it": 1}, "adjectives": {"I'm sure": 1, "self": 1}, "adverbs": {"Yes": 1, "Press": 1}, "numerals": {"22": 1}, "pronouns": {"I": 1}}}, {"content": "23. Sometimes I really want to smash things.", "word_count": 8, "label": "", "sentence_structure": "Subject鈥搗erb鈥揷omplement", "subjective_objective": {"answer": "Subjective", "rationale": "Because the idea of smashing things is the patient's subjective attitude."}, "sentence_sentiment": {"answer": "Negative", "rationale": "Because the \"thumping\" act is destructive and has a negative emotional color."}, "language_style": {"answer": "Calm and direct", "rationale": "The language style of the entry is more direct and concise, without too many emotional or rhetorical techniques, and with greater emphasis on the facts that the interrogators want to know."}, "time_dimension": {"answer": "Present", "rationale": "The keyword \"I really want\" to indicate that the entry is asking the testee about his current emotional state and behavioral orientation. Besides, the word \"real\" in \"real\" implies that it is the testee's real thought or feeling. So, the time dimension of this entry is now."}, "sentence_type": "Exclamatory sentence", "pos": {"verbs": {"Yes": 1, "Smash": 1}, "adverbs": {"Sometimes": 1}}}, {"content": "I never thought I'd be asked to have it.", "word_count": 13, "label": "", "sentence_structure": "Subject鈥搗erb鈥搊bject", "subjective_objective": {"answer": "Subjective", "rationale": "Because \"thinking\" is a human inner activity, a subjective approach."}, "sentence_sentiment": {"answer": "Neutral", "rationale": "Because the entry describes an idea or belief, there is no apparent emotional tendency."}, "language_style": {"answer": "Tactful and warm", "rationale": "The language style of the entry is more euphemistic, using the expression \"never thought\" to give a gentle and inviting feeling. At the same time, there is no direct order or criticism of the other, but rather to ask the other person about his thoughts and feelings in a more caring and understanding way. So, the language style of the entry is closer to the warmer type of euphemism."}, "time_dimension": {"answer": "Past", "rationale": "The entry \"Never\" indicates a point in the past, and \"unexpected\" is also a past move or state. It is therefore a testee that can be judged whether the testee has ever thought of such an idea in the past."}, "sentence_type": "Negative declarative sentence", "pos": {"nouns": {"I": 1, "others": 1, "Yes": 1}, "verbs": {"I thought": 1, "Substitute": 1}, "adverbs": {"Never": 1}, "numerals": {"24": 1}}}, {"content": "I have no complaints about the demands that others expect me to repay.", "word_count": 17, "label": "", "sentence_structure": "Subject鈥搗erb鈥揷omplement", "subjective_objective": {"answer": "Subjective", "rationale": "Because \"no complaints\" is the patient's attitude and perception of himself, and it's subjective."}, "sentence_sentiment": {"answer": "Positive", "rationale": "Because it expresses positive attitudes and behaviour, that is, \"from no grievance\", and is a sign of willingness to give and help others."}, "language_style": {"answer": "Calm and direct", "rationale": "The language style of the entry is one of calm and straightforward. It does not use rhetorical or emotional rendering, but rather expresses an attitude to the demand for satisfaction in a simple and clear language. This language style makes the question objective, direct, easy to understand and answer by the interviewee."}, "time_dimension": {"answer": "Past", "rationale": "\"From complaint\" shows past experience and attitudes, and the entry is therefore an inquiry into the testee's past behaviour."}, "sentence_type": "Exclamatory sentence", "pos": {"verbs": {"to": 1, "Refund": 1, "Request": 1, "have": 1, "From": 1, "None": 1, "Grievances": 1}, "adverbs": {"Yes": 1, "others": 1, "I hope": 1, "I": 2, "of": 1}, "pronouns": {"Here": 1}}}, {"content": "I was never bored when others said something completely different from me.", "word_count": 24, "label": "", "sentence_structure": "Subject鈥揷opula鈥損redicative", "subjective_objective": {"answer": "Subjective", "rationale": "Because \"never getting bored\" is the patient's subjective feeling and attitude, and it's a subjective angle."}, "sentence_sentiment": {"answer": "Neutral", "rationale": "Because the entry describes only an objective phenomenon, it does not involve any positive or negative emotional colours."}, "language_style": {"answer": "Tactful and warm", "rationale": "The language style of this entry is euphemism. First, the issue is one of invitation, dialogue by asking each other how they feel. Second, the term and expression in the question are very gentle and polite, as is \"never tired\" , which is more acceptable and gives people a sense of affection. Finally, the whole subject is more relaxed and not too direct or too strong an emotional."}, "time_dimension": {"answer": "Future", "rationale": "The keyword \"never tired\" expresses a desire or a vision for the future, that is, the desire of the testees to remain open to the dissent of others in the future. The temporal dimension of the article should therefore be judged as the future."}, "sentence_type": "Affirmative declarative sentence", "pos": {"nouns": {"Observations": 1}, "verbs": {"Say it": 1, "No, I'm not": 1, "Boring": 1}, "adjectives": {"It's completely different": 1}, "adverbs": {"Never": 1}, "pronouns": {"I": 1, "they": 1}}}, {"content": "I have never been able to travel long distances without carefully checking the safety of vehicles.", "word_count": 25, "label": "", "sentence_structure": "Subject鈥搗erb鈥搊bject", "subjective_objective": {"answer": "Objective", "rationale": "Because this sentence describes a fact of conduct (whether or not the safety of the vehicle is carefully checked) without involving subjective attitudes or ideas of individuals. This is therefore an entry for objective observation or measurement."}, "sentence_sentiment": {"answer": "Positive", "rationale": "Because the behaviour in the entry \"when a long journey begins without careful examination of the safety of the vehicle\" is positive, it shows that the person attaches great importance to the safety of the vehicle."}, "language_style": {"answer": "Tactful and warm", "rationale": "Warm and warm."}, "time_dimension": {"answer": "Present", "rationale": "In the past, the entry described a past experience or behaviour of the person tested, i.e., no long journeys without careful examination of vehicle safety."}, "sentence_type": "Negative declarative sentence", "pos": {"nouns": {"Situation": 1, "Vehicles": 1, "Security": 1, "Long trip": 1}, "verbs": {"Start": 1, "Inspection": 1}, "adjectives": {"Never": 1, "Seriously": 1}, "adverbs": {"Never": 1}, "pronouns": {"I": 1}}}, {"content": "28. Sometimes I get jealous of other people ' s luck.", "word_count": 15, "label": "", "sentence_structure": "Subject鈥搗erb鈥揷omplement", "subjective_objective": {"answer": "Subjective", "rationale": "Because jealousy is a subjective emotion."}, "sentence_sentiment": {"answer": "Negative", "rationale": "Because the \"jealous\" mood in the entry is negative."}, "language_style": {"answer": "Tactful and warm", "rationale": "The language style of the entry is a softer expression, which leads the interviewee to answer questions by asking questions, without directly accusing or attacking the emotional state of the other. At the same time, it is more acceptable to use expressions like \"sometimes\" to deny people jealousy, but to emphasize their emotional state."}, "time_dimension": {"answer": "Past", "rationale": "The keyword \"sometimes\" indicates that it is a past experience or state and that it may still have an impact at present."}, "sentence_type": "Affirmative declarative sentence", "pos": {"nouns": {"Questionnaire": 1, "Entry": 1, "others": 1, "Lucky": 1}, "verbs": {"Sometimes": 1, "to": 1, "Jealousy": 1}, "adverbs": {"Pretty much": 1, "Sometimes": 1}, "numerals": {"28": 1}, "pronouns": {"I": 1}}}, {"content": "29. I have almost never felt like trying to blame anyone.", "word_count": 13, "label": "", "sentence_structure": "Subject鈥搗erb鈥揷omplement", "subjective_objective": {"answer": "Subjective", "rationale": "Because \"I feel like I want to blame someone\" is the patient's personal feeling and subjective attitude."}, "sentence_sentiment": {"answer": "Negative", "rationale": "Because the entry \"never feel like scolding anyone\" expresses a negative emotional attitude."}, "language_style": {"answer": "Tactful and warm", "rationale": "The entry is more personal and interactive when it is spoken in a gentle and inviting manner, rather than in direct statements or orders. At the same time, the article is more easy to use and peaceful and accessible, without using too strong or too cold language to give people a sense of affection and comfort."}, "time_dimension": {"answer": "Present", "rationale": "The entry therefore describes the current situation."}, "sentence_type": "Negative declarative sentence", "pos": {"nouns": {"Questionnaire": 1, "Entry": 1, "Reprisal": 1}, "verbs": {"I think": 1, "Yes": 1, "Reprisal": 1}, "adverbs": {"Almost never": 1}, "pronouns": {"I": 1}}}, {"content": "I sometimes get angry because I have to help.", "word_count": 14, "label": "", "sentence_structure": "Subject鈥搗erb鈥揷omplement", "subjective_objective": {"answer": "Subjective", "rationale": "Because \"freak\" is a subjective emotional reaction that reflects the attitude and feelings of the interviewees towards others asking for help."}, "sentence_sentiment": {"answer": "Negative", "rationale": "Because the entry \"smuggling because I'm needed to help\" is negative."}, "language_style": {"answer": "Calm and direct", "rationale": "This expression is more direct and practical and is appropriate for the needs of psychological questionnaire studies."}, "time_dimension": {"answer": "Present", "rationale": ""}, "sentence_type": "Affirmative declarative sentence", "pos": {"nouns": {"I": 1, "others": 1, "Help": 1, "Angry": 1}, "verbs": {"Sometimes": 1, "Because": 1, "Yes": 2}, "adjectives": {"30": 1, "I'm angry": 1}, "adverbs": {"Sometimes": 1}}}, {"content": "I have never felt punished for nothing.", "word_count": 18, "label": "", "sentence_structure": "Subject鈥搗erb鈥搊bject", "subjective_objective": {"answer": "Subjective", "rationale": "Because \"I feel punished for no reason\" is a subjective feeling and perception of a person."}, "sentence_sentiment": {"answer": "Negative", "rationale": "Because the \"punishment\" in the entry is negative."}, "language_style": {"answer": "Calm and direct", "rationale": "The entry, in a more calm and straightforward way, asks whether the other party has had a history of being punished for no reason, has not used excessive rhetoric or emotional rendering, and is more balanced and objective."}, "time_dimension": {"answer": "Past", "rationale": ""}, "sentence_type": "Negative declarative sentence", "pos": {"nouns": {"Questionnaire Entry": 1, "self": 1}, "verbs": {"I think": 1, "Received": 1, "Punishment": 1}, "adjectives": {"Never": 1, "For no reason": 2}, "adverbs": {"Never": 1, "Both": 1}, "numerals": {"31": 1}, "pronouns": {"I": 1}}}, {"content": "32. Sometimes I think that when people suffer, they are only given what they deserve.", "word_count": 23, "label": "", "sentence_structure": "Subject鈥搗erb鈥搊bject", "subjective_objective": {"answer": "Subjective", "rationale": "Because sometimes \"insisting\" is a subjective thought and view of the patient."}, "sentence_sentiment": {"answer": "Negative", "rationale": "Because the entry \"people who suffer from misfortunes are only entitled to\" expresses a negative attitude and belief. This view can be frustrating and helpless, while also ignoring the impact of individual efforts and efforts on the suffering of misfortune."}, "language_style": {"answer": "Calm and direct", "rationale": "The entry is based on a more objective and direct statement of a view, without excessive rhetorical or emotional rendering, and in a more balanced and calm tone. At the same time, the purpose of the article is to address the causes and consequences of human suffering, which is a more rational and in-depth reflection, and is consistent with the normal style of calm language."}, "time_dimension": {"answer": "Present", "rationale": "Sometimes it is a constant, customary idea, and the word \"want\" takes the usual current state of affairs, meaning that it has not only happened in the past, but may still be. \"When people suffer misfortune, they get what they deserve\" expresses a view or attitude, not a specific act or event of the past or the future."}, "sentence_type": "Negative declarative sentence", "pos": {"nouns": {"Questionnaire": 1, "Entry": 1, "person": 1, "Unfortunately": 1}, "verbs": {"Yes": 1, "Victims": 1, "Got it": 1}, "adjectives": {"It's what it's worth": 1}, "adverbs": {"Sometimes": 1}, "numerals": {"32": 1}, "pronouns": {"they": 1}}}, {"content": "I never said anything deliberately to hurt anyone's feelings.", "word_count": 17, "label": "", "sentence_structure": "Subject鈥損redicate", "subjective_objective": {"answer": "Subjective", "rationale": "Because \"never said anything deliberately to hurt someone's feelings\" is the patient's subjective attitude or belief. Although it may be a real objective fact (if the patient has never done so), in this sentence we are asking the patient about his thoughts and beliefs, which makes it subjective."}, "sentence_sentiment": {"answer": "Neutral", "rationale": "Because the entry describes the behaviour of a person (who has never said anything to hurt) there is no apparent emotional tendency."}, "language_style": {"answer": "Calm and direct", "rationale": "The entry directly expresses the subject of questioning whether the other party has consciously spoken words that might hurt the feelings of others, without using excessive rhetoric or emotional rendering, and is more objective in tone."}, "time_dimension": {"answer": "Present", "rationale": "Now \"I never\" means \"I never\" has never been, and \"I said\" has taken the usual past, which means that it happened in the past. So the entry asks about the person who was tested for \"I never\" for \"I never\" for \"I never\" for \"I never\" for \"I\" for \"I\" for \"I\" for \"I\" for \"I\" for \"I\" for \"I\" for \"I\" for \"I\" for \"I\" for \"I\" for \"I\" for \"I\" for \"I\" for \"I\" for \"I\" for \"I\" for \"I\" for \"I\" for \"I\" for \"I\" for \"I\" for \"I\" for \"I\" for \"I\" for \"I\" for \"I\" for \"I\" for \"I\" for \"I\" for \"I\" for \"I\" for \"I\" for \"I\" for \"I\" for \"I\" for \"I\" for \"I\" for \"I\" for \"I\" for \"I\" for \"I\" for \"I\" for \"I\" for \"I\" for \"I\" for \"I\" for \"I\""}, "sentence_type": "Negative declarative sentence", "pos": {"verbs": {"Say it": 1, "Hurt": 1, "have": 1, "Meaning": 1}, "adjectives": {"Never": 1, "Intentionally": 1, "It hurts people's feelings": 1}, "adverbs": {"Never": 1, "No, I'm not": 2, "I'm doing it": 1}, "classifiers": {"Sentence": 1}, "pronouns": {"I": 1}}}]}

{"questionnaire_title": "MMPI lie sheet", "questionnaire_category": "Behavioral questionnaire", "item_count": 56, "emotion_shift_overall": "From positive to negative", "language_style_overall": "Neutral", "items": [{"content": "1. My first impressions have often proved to be correct.", "word_count": 15, "label": "", "sentence_structure": "Subject鈥揷opula鈥損redicative", "subjective_objective": {"answer": "Subjective", "rationale": "Because \"first impression\" and \"right\" are both described in terms of patients' subjective feelings."}, "sentence_sentiment": {"answer": "Positive", "rationale": "Because the purpose of the article is to express that \"my first impressions are often proven to be correct\" in favour of the positive side, which is that it is generally considered that one's instincts and feelings are reliable."}, "language_style": {"answer": "Tactful and warm", "rationale": "The language style of the entry tends to be euphemistic and warm, conveying an uncertainty and openness through the use of the expression \"often\" that allows the interviewee to feel compelled or judged. At the same time, the term \"right\" is used to describe the first impression, not directly negates other possibilities, but rather maintains an objective and neutral attitude. This expression increases the sense of trust and comfort of the interviewee."}, "time_dimension": {"answer": "Past", "rationale": "This may be a descriptive question, asking the testee's views on his or her ability to observe and judge."}, "sentence_type": "Affirmative declarative sentence", "pos": {"nouns": {"Impression": 1, "First impression": 1}, "verbs": {"Prove it": 1, "be": 1, "By": 1, "Here": 1, "Often": 1, "Do it": 1, "Be": 1, "Correct": 1}, "adjectives": {"Mine": 1, "First": 1, "Right": 1}, "adverbs": {"Often": 1}, "numerals": {"One": 2}, "pronouns": {"I": 1}}}, {"content": "2. For me, it is difficult to change any bad habits.", "word_count": 16, "label": "", "sentence_structure": "Subject鈥揷opula鈥損redicative", "subjective_objective": {"answer": "Subjective", "rationale": "Because it expresses the patient ' s perception and feelings about changing bad habits, it is the patient ' s subjective attitude."}, "sentence_sentiment": {"answer": "Negative", "rationale": "Because \"it's hard to change any bad habits\" in the entry is a negative attitude and emotion."}, "language_style": {"answer": "Calm and direct", "rationale": "The entry expresses the subject in a more objective and direct language style, with no excessive rhetoric or emotional rendering, and is plain and unambiguous."}, "time_dimension": {"answer": "Present", "rationale": "The keyword \"to change any bad habits\" suggests that the testee is describing the current situation, and \"it is difficult\" also to highlight the current state and difficulty of the situation. Thus, the temporal dimension of the article is now judged."}, "sentence_type": "Negative declarative sentence", "pos": {"nouns": {"I": 1, "Custom": 1, "Bad habits": 1}, "verbs": {"Yes": 1, "Change": 1, "Both": 1, "It's hard": 1}, "adjectives": {"Any": 1}, "numerals": {"2": 1}}}, {"content": "I have no intention of knowing what other people think of me.", "word_count": 17, "label": "", "sentence_structure": "Subject鈥搗erb鈥搊bject", "subjective_objective": {"answer": "Subjective", "rationale": "Because it's the patient's subjective attitude and thoughts."}, "sentence_sentiment": {"answer": "Neutral", "rationale": "Because the entry does not have any emotional color."}, "language_style": {"answer": "Calm and direct", "rationale": "The entry is based on a simple and clear expression of a view or attitude, without excessive rhetorical or emotional rendering, and is more objective. At the same time, the entry is not worded in any euphemism or indirect way, but is a straightforward expression of an attitude that does not want to know what people think of themselves."}, "time_dimension": {"answer": "Present", "rationale": "The keyword is \"intentionally\" , indicating that the person's psychological state has not changed at the time of the investigation, \"to know\" is a point of time for the future, but the entire sentence is a current attitude and behaviour. The entry is therefore an inquiry into the person's current psychological state, and the temporal dimension is now judged."}, "sentence_type": "Negative declarative sentence", "pos": {"nouns": {"Questionnaire Entry": 1, "Observations": 1, "person": 1}, "verbs": {"Go on": 1, "Yes": 1, "to": 1, "have": 1, "be": 1}, "adverbs": {"What the hell": 1, "No, I didn't mean to": 1}, "numerals": {"3": 1}, "pronouns": {"I": 2, "What": 1}}}, {"content": "I'm not always faithful to myself.", "word_count": 10, "label": "", "sentence_structure": "Subject鈥搗erb鈥揷omplement", "subjective_objective": {"answer": "Subjective", "rationale": "Because \"faith\" is an attitude or value that needs to be viewed from the patient's subjective perspective."}, "sentence_sentiment": {"answer": "Negative", "rationale": "Because the \"not always faithful to yourself\" act in the entry is negative."}, "language_style": {"answer": "Tactful and warm", "rationale": "The language style of the entry is more euphemistic and implicit, avoiding direct accusations or self-aggression by expressing \"not always\" , which reduces each other's resistance and increases their interaction with questionnaires. At the same time, the \"fluent\" style of language allows respondents to feel respected and understood, and thus to share their thoughts and feelings."}, "time_dimension": {"answer": "Present", "rationale": "The key word \"not always\" means that it is a regular, customary act, while the use of the current state of \"faith\" means that the act has not only occurred in the past, but may still be ongoing. Thus, the temporal dimension of the article is now judged."}, "sentence_type": "Negative declarative sentence", "pos": {"verbs": {"and": 1, "Total": 1, "Faithful": 1}, "adjectives": {"self": 1}, "adverbs": {"not": 1, "Always": 1}, "pronouns": {"I": 1}}}, {"content": "5. I always understood why I liked something.", "word_count": 18, "label": "", "sentence_structure": "Subject鈥揷opula鈥損redicative", "subjective_objective": {"answer": "Subjective", "rationale": "Because \"like something\" is a subjective feeling and attitude of a human being, and therefore a subjective perspective."}, "sentence_sentiment": {"answer": "Positive", "rationale": "Because it represents a positive quality, that is, the ability to self-aware."}, "language_style": {"answer": "Tactful and warm", "rationale": "The entry uses a soft tone, a question-and-answer expression, gives the interviewee a more comfortable feeling of answering the questions, and avoids some direct or command-style statements. Such language is more friendly and friendly, and helps to enhance the interaction and trust of the interviewee with the questionnaire researchers."}, "time_dimension": {"answer": "Present", "rationale": "The keyword \"always\" indicates that the act is a customary, recurrent state, and thus the judgement is now."}, "sentence_type": "Affirmative declarative sentence", "pos": {"nouns": {"Questionnaire": 1, "Entry": 1, "Something": 1}, "verbs": {"Analysis": 1, "Yeah": 1}, "adjectives": {"Understood": 1}, "adverbs": {"Always": 1}, "numerals": {"5": 1}, "pronouns": {"self": 1}}}, {"content": "6. I'm emotionally distracted by a deviation in thinking.", "word_count": 15, "label": "", "sentence_structure": "Subject鈥揷opula鈥損redicative", "subjective_objective": {"answer": "Subjective", "rationale": "Because the description of \"emotional emotion\" is based on the patient's own feelings and experiences, and is subjective; and \"thinking deviations\" is an evaluation or interpretation of such experiences, and it is subjective. Thus, the entry to the questionnaire is viewed from the patient's subjective perspective."}, "sentence_sentiment": {"answer": "Negative", "rationale": "Because the \"ideal deviations when you're emotional\" in the entry are negative."}, "language_style": {"answer": "Calm and direct", "rationale": "The language style of the entry is relatively straightforward, not too much plating or rendering emotional, but rather asking questions in a concise and clear manner. This expression helps respondents to understand and answer questions more accurately."}, "time_dimension": {"answer": "Present", "rationale": "The keyword is \"in a moment of emotion,\" which means a particular moment or situation, and the entry is therefore asking the testee whether there is a deviation in the mood. The dimension of time is now."}, "sentence_type": "Affirmative declarative sentence", "pos": {"nouns": {"Time": 1, "Thinking": 1, "Offset": 1}, "verbs": {"in": 1, "Excited": 1, "Out": 1}, "adjectives": {"Emotion": 1}, "numerals": {"6": 1}}}, {"content": "Once I have made my decision, few people have changed my mind.", "word_count": 18, "label": "", "sentence_structure": "Subject鈥搗erb鈥揷omplement", "subjective_objective": {"answer": "Subjective", "rationale": "Because the entry describes the patient ' s own beliefs and attitudes to his or her own decisions, it is the patient ' s subjective view."}, "sentence_sentiment": {"answer": "Positive", "rationale": "Because the entry is a manifestation of personal determination and self-confidence and is positive."}, "language_style": {"answer": "Calm and direct", "rationale": "The language style of the entry is more direct and objective, and it does not use excessive rhetoric or emotional rendering, but rather expresses the problem in a clear and concise manner. This tone usually gives a calm and rational impression.\""}, "time_dimension": {"answer": "Present", "rationale": "The phrase \"when determined\" indicates that it is the mental state and the thinking of the person who speaks, and that \"very few people can change my mind\" is also a description of the current situation, and therefore the judgment is now."}, "sentence_type": "Affirmative declarative sentence", "pos": {"nouns": {"Resolve": 1, "others": 1, "Ideas": 1}, "verbs": {"Down": 1, "鈾?And I'm gonna be 鈾?: 1, "Change": 1}, "adverbs": {"鈾?Once 鈾?: 1, "Very few": 1}, "pronouns": {"I": 1}}}, {"content": "I am not a safe and reliable driver when speeding.", "word_count": 18, "label": "", "sentence_structure": "Subject鈥揷opula鈥損redicative", "subjective_objective": {"answer": "Subjective", "rationale": "Because the safety of speeding is a subjective judgement, not an objective fact. Different people may have different views and assessments."}, "sentence_sentiment": {"answer": "Negative", "rationale": "Because the descriptions of \"extra-speed\" and \"unsafe and reliable\" are negative."}, "language_style": {"answer": "Tactful and warm", "rationale": "The entry does not directly criticize the driving behaviour of the other party, but rather asks the interviewee in a more euphemistic way whether he thinks he is a safe and reliable driver when he is travelling at a speed. This expression is more inclusive and understandable, and better able to generate reflection and self-assessment by the interviewee."}, "time_dimension": {"answer": "Present", "rationale": ""}, "sentence_type": "Negative declarative sentence", "pos": {"nouns": {"Questionnaire Entry": 1, "Speeding": 1, "Driver": 1}, "verbs": {"be": 1, "No, it's not": 1, "Safe and reliable": 1}, "adverbs": {"No, it's not": 1}, "numerals": {"8": 1}, "pronouns": {"I": 1}}}, {"content": "9. I have firmly grasped my destiny.", "word_count": 11, "label": "", "sentence_structure": "Subject鈥搗erb鈥揷omplement", "subjective_objective": {"answer": "Subjective", "rationale": "Because \"hold your destiny firmly\" is an expression of subjective belief and attitude that reflects the patient's perception and perception of himself."}, "sentence_sentiment": {"answer": "Positive", "rationale": "Because the words \"concealed\" and \"their own destiny\" in the entry are positive, indicating that the person has a strong self-control and positive life attitude."}, "language_style": {"answer": "Tactful and warm", "rationale": "Warm and warm."}, "time_dimension": {"answer": "Present", "rationale": "The entry describes the current behaviour or mentality of the subject, and \"I hold my destiny firmly\" is a sentence describing the state of affairs, and therefore the judgment is \"now\"."}, "sentence_type": "Affirmative declarative sentence", "pos": {"nouns": {"Questionnaire Entry": 1, "I": 1, "Destiny": 1}, "verbs": {"Hold it tight": 1}, "adjectives": {"self": 1}, "adverbs": {"In the hole": 1}, "classifiers": {"individual": 1}}}, {"content": "It is difficult for me to leave the disturbing thoughts behind.", "word_count": 11, "label": "", "sentence_structure": "Subject鈥揷opula鈥損redicative", "subjective_objective": {"answer": "Subjective", "rationale": "Because the idea of \"leave\" and \"disturbing\" are the patients' own inner experience and feelings, it is a subjective examination."}, "sentence_sentiment": {"answer": "Negative", "rationale": "Because the entry \"the hard to leave the disturbing mind\" expresses a negative mood and attitude."}, "language_style": {"answer": "Tactful and warm", "rationale": "The entry expresses the concern and concern of the interviewees in a gentle and understanding way, asking them if they have difficulty in leaving out the troubles. At the same time, the expression \"hard\" is used to reduce the problem's directness and avoid putting pressure or discomfort on the interviewees."}, "time_dimension": {"answer": "Present", "rationale": "\"I can hardly leave behind\" and \"difficult\" verbs that indicate that it is a present psychological distress, not a past or future act. So, the entry is a current state of affairs."}, "sentence_type": "Negative declarative sentence", "pos": {"verbs": {"Throw it away": 1, "It's disturbing": 1, "Thoughts": 1}, "adjectives": {"It's hard": 1}}}, {"content": "I never regret my decision.", "word_count": 11, "label": "", "sentence_structure": "Subject鈥搗erb鈥揷omplement", "subjective_objective": {"answer": "Subjective", "rationale": "Since the perception and attitude to the matter is a subjective one, the content of this entry is viewed from a subjective perspective."}, "sentence_sentiment": {"answer": "Positive", "rationale": "Because the attitude in the entries is positive, \"never regret\" means that they have confidence and determination in their decisions, and that they are not negative or neutral in emotional terms."}, "language_style": {"answer": "Calm and direct", "rationale": "The entry is more directly written, not using too many qualifiers or very emotional terms, but rather asking whether the person surveyed always makes a decision without remorse, with objectivity and determination."}, "time_dimension": {"answer": "Present", "rationale": ""}, "sentence_type": "Affirmative declarative sentence", "pos": {"verbs": {"to": 1, "self": 1, "Decision": 1, "Regret": 1}, "adjectives": {"Never": 1}, "numerals": {"11": 1}}}, {"content": "I sometimes suffer from hesitation.", "word_count": 14, "label": "", "sentence_structure": "Subject鈥搗erb鈥揷omplement", "subjective_objective": {"answer": "Subjective", "rationale": "Losses due to hesitation are a subjective experience and feeling of one."}, "sentence_sentiment": {"answer": "Negative", "rationale": "Because the \"suspensive\" act in the entry is negative."}, "language_style": {"answer": "Tactful and warm", "rationale": "The entry is more personal and interactive when it is presented in a gentle and inviting tone, rather than in direct statements or orders. At the same time, it does not overemphasize or criticize the indecisiveness of the interviewees, but rather seeks to guide the respondents to reflect on whether their decision-making processes need to be improved, be somewhat instructive and constructive."}, "time_dimension": {"answer": "Present", "rationale": ""}, "sentence_type": "Negative declarative sentence", "pos": {"nouns": {"Questionnaire Entry": 1, "Losses": 1, "Relentless": 1}, "verbs": {"Victims": 1}, "adverbs": {"Sometimes": 1}, "numerals": {"12": 1}, "pronouns": {"I": 1}}}, {"content": "13. I participated because it was useful.", "word_count": 17, "label": "", "sentence_structure": "Subject鈥搗erb鈥搊bject", "subjective_objective": {"answer": "Subjective", "rationale": "Because the phrase \"why\" indicates that it is a personal cause or motive of the patient, it is subjective."}, "sentence_sentiment": {"answer": "Positive", "rationale": "Because the action in the entry \"voting\" is positive and the purpose \"it works\" is positive."}, "language_style": {"answer": "Tactful and warm", "rationale": "The language style of the entry is more euphemistic, using expressions such as \"why\" and \"because\" to guide the interviewee to think about the issue, rather than direct orders or demands. Moreover, the expression \"making a difference\" implies that the interviewee is more inspiring than ever before, as regards his acceptance of and active participation in the voting."}, "time_dimension": {"answer": "Present", "rationale": "The keywords \"voting\" and \"making a difference\" mean that it is a description of the current behaviour and therefore is the present."}, "sentence_type": "Affirmative declarative sentence", "pos": {"nouns": {"Questionnaire Entry": 1, "Vote": 1, "Role": 1}, "verbs": {"Participation": 1, "Rise": 1, "Present": 1}, "adverbs": {"鈾?And the reason 鈾?: 1}, "numerals": {"13": 1}, "pronouns": {"I": 1}}}, {"content": "14. Parents do not always punish me fairly.", "word_count": 14, "label": "", "sentence_structure": "Subject鈥揷opula鈥損redicative", "subjective_objective": {"answer": "Subjective", "rationale": "Since equity is a subjective criterion, which may appear to different people to have different judgments, it is the patient ' s subjective view."}, "sentence_sentiment": {"answer": "Negative", "rationale": "Because the expression \"punishment is not always fair\" in the entry is negative."}, "language_style": {"answer": "Calm and direct", "rationale": ""}, "time_dimension": {"answer": "Present", "rationale": "In the past, the entry described the testee ' s past experience and observations, and related to the testee ' s family environment and parent-child relationship, so that the temporal dimension was judged to be the past."}, "sentence_type": "Negative declarative sentence", "pos": {"nouns": {"parents": 2, "Penalties": 1, "Fair": 1}, "verbs": {"to": 2, "be": 1, "Not": 1}, "adjectives": {"Always": 1}, "numerals": {"14": 1}, "pronouns": {"I": 1}}}, {"content": "I am a man of complete reason.", "word_count": 10, "label": "", "sentence_structure": "Subject鈥揷opula鈥損redicative", "subjective_objective": {"answer": "Subjective", "rationale": "Because \"who is completely reasonable\" is a subjective expression of the patient's self-awareness and evaluation."}, "sentence_sentiment": {"answer": "Negative", "rationale": "Because the expression \"total reason\" is too categorical and inflexible to put pressure on the interviewees."}, "language_style": {"answer": "Calm and direct", "rationale": "The entry is relatively blunt, not based on excessive rhetorical or emotional descriptions, but rather on a concise and clear presentation of individual rationality."}, "time_dimension": {"answer": "Past", "rationale": "From a temporal perspective, the sentence does not clearly point to the nature of the time, and therefore does not make a judgement as to whether it is past, present or future. In terms of content, it describes the self-perception and conviction of the person being tested, is a statement of a subjective nature and does not relate to specific acts or events, and is therefore not appropriate to be judged by way of classification in the past, present and future."}, "sentence_type": "Affirmative declarative sentence", "pos": {"nouns": {"person": 1, "Reason": 1}, "verbs": {"be": 1}, "adjectives": {"Total": 1}, "numerals": {"15": 1}, "pronouns": {"I": 1}}}, {"content": "16. I rarely appreciate the criticisms made.", "word_count": 13, "label": "", "sentence_structure": "Subject鈥搗erb鈥搊bject", "subjective_objective": {"answer": "Subjective", "rationale": "Because gratitude is a response to others ' assessment and an expression of individual subjective feelings."}, "sentence_sentiment": {"answer": "Negative", "rationale": "Because \"very few\" in the entries imply negative, i.e. that the interviewees may have negative feelings or attitudes towards criticism from others."}, "language_style": {"answer": "Calm and direct", "rationale": "This is a more objective and neutral expression that helps to obtain more realistic and accurate answers."}, "time_dimension": {"answer": "Present", "rationale": ""}, "sentence_type": "Negative declarative sentence", "pos": {"verbs": {"Very few": 1, "to": 1, "Criticism": 1, "鈾?With my heart 鈾?: 1, "Thank you": 1}, "adverbs": {"very": 1}, "numerals": {"16": 1}}}, {"content": "17. I am very confident in my judgement.", "word_count": 11, "label": "", "sentence_structure": "Subject鈥揷opula鈥損redicative", "subjective_objective": {"answer": "Subjective", "rationale": "This is a self-evaluation and perception of patients and is a subjective examination."}, "sentence_sentiment": {"answer": "Positive", "rationale": "Because the entry \"very confident of his judgment\" expresses a positive attitude and quality."}, "language_style": {"answer": "Calm and direct", "rationale": "The language style of the entry is more calm and straightforward, without excessive rhetorical or emotional rendering, directly expressing the other party ' s confidence in its own judgement and being more objective in tone."}, "time_dimension": {"answer": "Present", "rationale": "The entry uses the now completed state of affairs \"have...\" to demonstrate that this belief existed and still exists today, and can therefore be judged as being present in behavior or mentality."}, "sentence_type": "Affirmative declarative sentence", "pos": {"verbs": {"to": 1, "of": 2, "self": 1, "Judgement": 1, "Very": 1, "Confidence": 1}, "adjectives": {"Confidence": 1}, "adverbs": {"Very": 1}, "pronouns": {"I": 1}}}, {"content": "I sometimes doubt my ability to be a lover.", "word_count": 16, "label": "", "sentence_structure": "Subject鈥搗erb鈥揷omplement", "subjective_objective": {"answer": "Subjective", "rationale": "Because suspicion is an individual idea and feeling, it is a subjective examination."}, "sentence_sentiment": {"answer": "Negative", "rationale": "Because the expression \"suspect of being a lover\" in the entry is a denial of one's abilities and has a negative emotional connotation."}, "language_style": {"answer": "Tactful and warm", "rationale": "The entry, in a more euphemistic way, asks the other side whether there is self-doubt, rather than directly accusing or denying the other. This expression is more likely to lead the other side to open up and share its thoughts."}, "time_dimension": {"answer": "Present", "rationale": "The keyword \"sometimes\" indicates that the suspicion is not always present, but is occasionally present, and therefore the judgement is now."}, "sentence_type": "Affirmative declarative sentence", "pos": {"nouns": {"Time": 3, "self": 2, "Capacity": 1}, "verbs": {"Doubt": 1, "Act as": 1}, "adverbs": {"of": 4, "have": 1, "Time": 1, "Both": 1, "not": 1, "That's enough": 1}, "numerals": {"18": 1}, "pronouns": {"I": 2}}}, {"content": "If someone happens to dislike me, it's nothing to me.", "word_count": 16, "label": "", "sentence_structure": "Subject鈥搗erb鈥揷omplement", "subjective_objective": {"answer": "Subjective", "rationale": "Because \"like or not\" is a subjective attitude or emotional reaction, not an objective reality."}, "sentence_sentiment": {"answer": "Positive", "rationale": "Because the attitude in the entries is positive."}, "language_style": {"answer": "Calm and direct", "rationale": "The entry is more calmly and straightforwardly phrased, without excessive rhetorical or emotional rendering, and expresses a view in a direct statement."}, "time_dimension": {"answer": "Past", "rationale": "The phrase \"if\" , \"scientific\" and \"like\" appears in the sentence, and all of them relate to the vision and assumptions of the past. Thus, it is possible to judge that the temporal dimension of the article is the past. At the same time, the sentence also expresses a mentality or attitude that does not care about those who do not like themselves."}, "sentence_type": "Negative declarative sentence", "pos": {"nouns": {"person": 1, "Yeah": 1, "I": 2}, "verbs": {"That's a coincidence": 1, "have": 1, "to": 1, "Nope": 1}, "adjectives": {"What": 1}, "adverbs": {"Suppose": 1}, "numerals": {"19": 1}}}, {"content": "20. I do not always understand why I do something.", "word_count": 22, "label": "", "sentence_structure": "Subject鈥揷opula鈥損redicative", "subjective_objective": {"answer": "Subjective", "rationale": "This is because patients have subjective knowledge and understanding of their motives."}, "sentence_sentiment": {"answer": "Neutral", "rationale": "Because the entry does not express a positive or negative attitude, emotion or behaviour, it merely describes a situation in which a person does not have a clear reason for doing something to himself."}, "language_style": {"answer": "Tactful and warm", "rationale": "The entry is more softly phrased, asking whether the other side is less aware of his or her motives. This expression helps create a comfortable atmosphere in which the interviewee is more willing to share his or her thoughts and feelings."}, "time_dimension": {"answer": "Present", "rationale": "The keyword \"not always very clear\" indicates that it is a state of the moment or a feeling, so the temporal dimension of this entry is now judged."}, "sentence_type": "Interrogative sentence", "pos": {"nouns": {"Questionnaire Entry": 1, "self": 1, "Things": 1}, "verbs": {"Do it": 1, "Understood": 1}, "adjectives": {"Not always": 1, "Tens": 1}, "numerals": {"20": 1}, "classifiers": {"individual": 1}, "pronouns": {"I": 2}}}, {"content": "21. If I had to, I'd have lied.", "word_count": 13, "label": "", "sentence_structure": "Subject鈥損redicate", "subjective_objective": {"answer": "Subjective", "rationale": "Because \"fake\" is the patient's subjective behavior and attitude."}, "sentence_sentiment": {"answer": "Negative", "rationale": "Because the \"false lies\" act in the entry is negative."}, "language_style": {"answer": "Calm and direct", "rationale": "The language style of the entry is relatively calm and straightforward, without too much adoration or emotional rendering, and the question of whether the other party would lie if necessary is expressed in an objective and concise manner. \""}, "time_dimension": {"answer": "Present", "rationale": ""}, "sentence_type": "Affirmative declarative sentence", "pos": {"nouns": {"Lies": 1}, "verbs": {"Say it": 1, "I had to": 1, "Sometimes": 1}, "adverbs": {"Sometimes": 1}, "numerals": {"21": 1}, "pronouns": {"I": 1}}}, {"content": "I never hide my mistakes.", "word_count": 10, "label": "", "sentence_structure": "Subject鈥搗erb鈥揷omplement", "subjective_objective": {"answer": "Subjective", "rationale": "Because \" never conceals one's own mistakes\" reflects the patient's personal attitudes and behaviour, it is the patient's own right and therefore is subjective."}, "sentence_sentiment": {"answer": "Neutral", "rationale": "Because the entry does not have any emotional color."}, "language_style": {"answer": "Calm and direct", "rationale": "The entry expresses a direct and positive tone of opinion, which is more objective and is more objective, in asking whether the other party is often open to the subject of his/her own error, without using a rhetorical or emotional projection."}, "time_dimension": {"answer": "Present", "rationale": "The entry \"I never\" denotes a customary act, and the act \"covering my own mistakes\" is also a description of the current state or tendency. The entry should therefore be judged to be a question in the light of the present situation."}, "sentence_type": "Negative declarative sentence", "pos": {"verbs": {"Cover": 1, "Error": 1}, "adverbs": {"Never": 1}, "pronouns": {"self": 1}}}, {"content": "I used to use people.", "word_count": 10, "label": "", "sentence_structure": "Subject鈥搗erb鈥搊bject", "subjective_objective": {"answer": "Subjective", "rationale": "Because this statement relates to the patient ' s own psychological activities and motivations for behaviour, it is a subjective view or experience of the individual."}, "sentence_sentiment": {"answer": "Negative", "rationale": "Because the \"use of others\" act is negative."}, "language_style": {"answer": "Calm and direct", "rationale": "The entry uses more straightforward and concise language presentation issues and does not use excessive glossing terms, so language style can be described as being straightforward."}, "time_dimension": {"answer": "Past", "rationale": "The keyword \"had\" is \"have\" and it indicates the behaviour or state that has been experienced, and the temporal dimension is a past."}, "sentence_type": "Negative declarative sentence", "pos": {"nouns": {"Time": 1, "person": 1}, "verbs": {"Yes": 1, "Utilization": 1}, "numerals": {"23": 1, "One": 1}, "pronouns": {"I": 1}}}, {"content": "I never curse anyone.", "word_count": 7, "label": "", "sentence_structure": "Subject鈥損redicate", "subjective_objective": {"answer": "Objective", "rationale": "Because it is an objective fact to curse others, not the patient ' s subjective attitude or feelings."}, "sentence_sentiment": {"answer": "Negative", "rationale": "Because the \"curse\" in the entry is negative."}, "language_style": {"answer": "Calm and direct", "rationale": "The statement of the question is simple and clear, and instead of using too many emotional or rhetorical techniques, the question is whether the other party never curses anyone. This expression is more objective and direct, and it is therefore possible to judge the language of the entry to the questionnaire as being calm and straightforward."}, "time_dimension": {"answer": "Past", "rationale": ""}, "sentence_type": "Negative declarative sentence", "pos": {"verbs": {"Curse": 1, "others": 1}, "adverbs": {"Never": 1}, "numerals": {"24": 1}}}, {"content": "25. I sometimes try to give back my teeth, not to forgive or forget.", "word_count": 17, "label": "", "sentence_structure": "Subject鈥搗erb鈥搊bject", "subjective_objective": {"answer": "Subjective", "rationale": "Because words like \"want\" and \"don't want\" express the individual emotions and attitudes of the patient."}, "sentence_sentiment": {"answer": "Negative", "rationale": "Because \"tooth for tooth\" is a retaliatory act that can lead to a diametric escalation and negative consequences, it has a negative emotional colour."}, "language_style": {"answer": "Calm and direct", "rationale": "The entry expresses a person ' s inner thoughts in a simple language, without excessive emotional modification or rhetorical techniques, which are more straightforward."}, "time_dimension": {"answer": "Present", "rationale": "The entry \"I sometimes\" indicates that it is a regular, but not necessarily a customary, act, \"I want to give back, I don't want to forgive or forget,\" and it shows the mental and behavioural orientation of the person being tested. So, the time dimension of the entry is now."}, "sentence_type": "Negative declarative sentence", "pos": {"nouns": {"Teeth": 1, "Revenge": 1, "Forgive me": 1, "Yes": 1}, "verbs": {"Yes": 1, "And": 1, "Forget it": 1, "I'll give you a tooth for a tooth": 1}, "adverbs": {"Sometimes": 1}, "numerals": {"25": 1}, "pronouns": {"I": 1}}}, {"content": "26. I have always been in compliance with the law, even if it is not possible to be discovered.", "word_count": 18, "label": "", "sentence_structure": "Subject鈥損redicate", "subjective_objective": {"answer": "Objective", "rationale": "Because compliance with the law is objective, even if not discovered. The examination of subjective attitudes is not the main purpose of the list entry and cannot therefore be considered subjective."}, "sentence_sentiment": {"answer": "Positive", "rationale": "Because the \"compliance\" in the entry is positive."}, "language_style": {"answer": "Calm and direct", "rationale": "The language style of the entry is more calm and straightforward, rather than using excessive rhetorical or emotional rendering, but expresses the subject matter and content of the question in an objective and clear manner. This style is more easily presented to the interviewee to understand and answer the question accurately."}, "time_dimension": {"answer": "Future", "rationale": "The entry indicates that the testee will choose to act in a manner consistent with the law when faced with a possible violation. This is therefore a vision and expectation for the future."}, "sentence_type": "Affirmative declarative sentence", "pos": {"nouns": {"I": 1, "Gi": 1, "FL": 1}, "verbs": {"Compliance": 1, "Found": 1}, "adjectives": {"Total": 1}, "adverbs": {"Always": 1, "Yeah": 1}, "numerals": {"26": 1}}}, {"content": "I have said bad things about friends behind my back.", "word_count": 12, "label": "", "sentence_structure": "Subject鈥搗erb鈥搊bject", "subjective_objective": {"answer": "Subjective", "rationale": "Because it is the patient's own behaviour and thoughts to say bad things about friends, it is subjective."}, "sentence_sentiment": {"answer": "Negative", "rationale": "Because the \"good friend\" thing in the entry is negative."}, "language_style": {"answer": "Calm and direct", "rationale": "The entry is more straightforward, concise, has no excessive speech or emotional rendering, and is more objective. It directly asks whether the person under investigation has ever been behind the behaviour of saying bad things about friends, and is more neutral and direct."}, "time_dimension": {"answer": "Past", "rationale": ""}, "sentence_type": "Negative declarative sentence", "pos": {"nouns": {"Questionnaire Entry": 1, "friends": 1, "Bad word": 1}, "verbs": {"In the back": 1, "be": 1}, "adverbs": {"Tsang": 1}, "numerals": {"27": 1, "I": 1}}}, {"content": "When I saw someone talking to me personally, I tried to avoid hearing.", "word_count": 21, "label": "", "sentence_structure": "Subject鈥搗erb鈥搊bject", "subjective_objective": {"answer": "Subjective", "rationale": "This is a subjective examination of the patient ' s thoughts and behaviour."}, "sentence_sentiment": {"answer": "Negative", "rationale": "Because the entry \"scramble as much as you can\" is negative."}, "language_style": {"answer": "Tactful and warm", "rationale": "The language style of the entry is more euphemistic. It does not direct or force the other to avoid private conversation, but rather expresses its behaviour in a way that respects the privacy of others and encourages the interviewee to do the same."}, "time_dimension": {"answer": "Present", "rationale": "This entry describes a current habit or behaviour that, when seen to have private conversations, avoids hearing it as much as possible. It may be because the person respects the privacy of others or wants to avoid unnecessary interference. Therefore, in terms of time dimension, the entry should be judged as \"now\"."}, "sentence_type": "Affirmative declarative sentence", "pos": {"nouns": {"Private conversation": 1, "person": 1, "Hear that": 1}, "verbs": {"Yeah": 1, "Avoid": 1, "Conduct": 1, "In case": 1}, "adverbs": {"Try": 1, "Avoid": 1}, "numerals": {"28": 1}, "pronouns": {"I": 1}}}, {"content": "I had quietly collected more money for me.", "word_count": 12, "label": "", "sentence_structure": "Subject鈥搗erb鈥搊bject", "subjective_objective": {"answer": "Subjective", "rationale": "Because \"silently take more money from me\" is the patient's subjective behavior and attitude."}, "sentence_sentiment": {"answer": "Negative", "rationale": "Because the \"silently take more money\" in the entry is negative."}, "language_style": {"answer": "Calm and direct", "rationale": "The entry is based on an objective, direct expression, without any emotional or rhetorical approach. It simply raises the question whether the interviewee has ever had a history of taking money more quietly. This language style is more appropriate for official settings such as questionnaires, and provides accurate and effective access to information."}, "time_dimension": {"answer": "Past", "rationale": ""}, "sentence_type": "Affirmative declarative sentence", "pos": {"nouns": {"Questionnaire": 1, "Entry": 1, "person": 1, "Money": 1}, "verbs": {"Take it": 1, "Keep looking": 1}, "adverbs": {"Quiet": 1}, "numerals": {"29": 1}, "pronouns": {"I": 1}}}, {"content": "30. At customs, I always declare everything.", "word_count": 14, "label": "", "sentence_structure": "Subject鈥搗erb鈥搊bject", "subjective_objective": {"answer": "Objective", "rationale": "Because \"I always declare everything in customs\" is a statement of fact, without the patient's subjective attitude or opinion."}, "sentence_sentiment": {"answer": "Positive", "rationale": "Because the \"show everything\" act in the entry is a sign of compliance and good faith, with a positive emotional tone."}, "language_style": {"answer": "Calm and direct", "rationale": "The language style of the entry is more calm and objective, without excessive rhetorical or emotional rendering, but simply a simple and straightforward statement of the fact. At the same time, the sentence structure is simple and easy to understand."}, "time_dimension": {"answer": "Past", "rationale": "The entry describes the past experience and behaviour of the person tested, judging by the time dimension of the past."}, "sentence_type": "Affirmative declarative sentence", "pos": {"nouns": {"Customs": 1, "Everything": 1}, "verbs": {"Always": 1, "I'm not sure I'm gonna tell you": 2}, "adverbs": {"Always": 1}, "numerals": {"30": 1}, "pronouns": {"I": 1}}}, {"content": "31. When I was a kid, I sometimes stole things.", "word_count": 10, "label": "", "sentence_structure": "Subject鈥搗erb鈥搊bject", "subjective_objective": {"answer": "Subjective", "rationale": "Because \"I steal sometimes when I'm a child\" is a personal experience and memory, described from a subjective perspective."}, "sentence_sentiment": {"answer": "Negative", "rationale": "Because the \"stealing\" in the entry is negative."}, "language_style": {"answer": "Calm and direct", "rationale": "The language style of the entry is more straightforward and there is no excessive rhetorical or emotional rendering aimed at conveying the message accurately and stimulating the other side ' s reflection."}, "time_dimension": {"answer": "Past", "rationale": "The keyword \"when we were kids\" means that the act took place in the testee's past, so it's judged to be \"the past\"."}, "sentence_type": "Affirmative declarative sentence", "pos": {"nouns": {"Time": 1, "Small": 1, "Something": 1}, "verbs": {"Steal": 1}, "adverbs": {"Time": 1}, "numerals": {"31": 1}, "pronouns": {"I": 1}}}, {"content": "I never throw dirty things in the streets.", "word_count": 11, "label": "", "sentence_structure": "Subject鈥損redicate", "subjective_objective": {"answer": "Objective", "rationale": "Because throwing dirty things on the street is an objective act, not a subjective attitude or emotional experience of the patient."}, "sentence_sentiment": {"answer": "Positive", "rationale": "Because the behavior in the entry \"no dirty stuff\" is positive."}, "language_style": {"answer": "Tactful and warm", "rationale": "The entry is more personal and interactive when it is spoken in an invitation-style tone, rather than in direct statements or orders. At the same time, the use of \"always\" emphasizes a positive behaviour rather than simple requirements or prohibitions."}, "time_dimension": {"answer": "Present", "rationale": "\"I never throw dirty things in the street\" and \"no\" expresses a behaviour or habit that has been maintained, that is, that the act has been, is and will be. Thus, the entry describes the current behaviour and attitude of the testee."}, "sentence_type": "Negative declarative sentence", "pos": {"verbs": {"Throw it": 1, "Dirty things": 1}, "adjectives": {"Never": 1}, "numerals": {"32": 1}}}, {"content": "I drive at speed sometimes.", "word_count": 7, "label": "", "sentence_structure": "Subject鈥損redicate", "subjective_objective": {"answer": "Subjective", "rationale": "Because this is a self-report of the patients, and relates to their ideas and behaviour choices, it is examined from a subjective point of view."}, "sentence_sentiment": {"answer": "Negative", "rationale": "Because the \"extra-speed drive\" is a traffic violation, it is a security risk and a potential risk, and it is a negative act."}, "language_style": {"answer": "Calm and direct", "rationale": "The language style of the entry is straightforward, as it clearly and succinctly describes the behaviour of the person under investigation (sometimes driving at speed)."}, "time_dimension": {"answer": "Present", "rationale": ""}, "sentence_type": "Affirmative declarative sentence", "pos": {"nouns": {"I": 1, "Time": 1, "Speed": 1, "Drive": 1}, "verbs": {"Sometimes": 1}, "adverbs": {"Speed": 1}, "numerals": {"33": 1}}}, {"content": "I never read porn.", "word_count": 9, "label": "", "sentence_structure": "Subject鈥損redicate", "subjective_objective": {"answer": "Objective", "rationale": "Because \"Whether or not to read porn\" is an objective fact, not a patient's subjective attitude or feelings."}, "sentence_sentiment": {"answer": "Neutral", "rationale": "Because the entry does not have any emotional color."}, "language_style": {"answer": "Calm and direct", "rationale": "The entry is more concise, has no more qualifier, has less temperature and degree of speech and is expressed relatively straightforwardly."}, "time_dimension": {"answer": "Present", "rationale": ""}, "sentence_type": "Negative declarative sentence", "pos": {"verbs": {"I": 1, "Never": 1, "not": 1, "Look": 1, "Pornography": 1, "Books": 1}, "adverbs": {"Never": 1, "not": 1}, "numerals": {"34": 1}, "pronouns": {"I": 1}}}, {"content": "35. I have done things that no one knows.", "word_count": 12, "label": "", "sentence_structure": "Subject鈥搗erb鈥揷omplement", "subjective_objective": {"answer": "Subjective", "rationale": "Because \"doing something nobody knows\" is a private or personal act, a subjective experience and idea of the patient."}, "sentence_sentiment": {"answer": "Neutral", "rationale": "Because the entry does not have any emotional color."}, "language_style": {"answer": "Tactful and warm", "rationale": "The entry is more personal and interactive by speaking in a gentle, invited tone, rather than by direct statement or order. At the same time, it attracts respondents to share some of their privacy or interesting stories."}, "time_dimension": {"answer": "Past", "rationale": "The entry asks the testee whether he has done something secret and has no clear time limit, and therefore cannot be accurately judged whether it is past, present or future."}, "sentence_type": "Affirmative declarative sentence", "pos": {"nouns": {"Yes": 1}, "verbs": {"Do it": 1, "Yes": 1}, "adverbs": {"Some": 1, "Nobody": 1}, "numerals": {"35": 1}, "classifiers": {"individual": 1}, "pronouns": {"I": 1}}}, {"content": "I never behaved like anything I didn't own.", "word_count": 13, "label": "", "sentence_structure": "Subject鈥搗erb鈥搊bject", "subjective_objective": {"answer": "Objective", "rationale": "Because \"have something that does not belong to you\" is an objective fact, and \"never be good\" is a patient's attitude or behavior, the main object of the questionnaire is the patient's subjective attitude."}, "sentence_sentiment": {"answer": "Neutral", "rationale": "It does not involve any positive or negative emotional aspects, but rather describes the behaviour of a person."}, "language_style": {"answer": "Calm and direct", "rationale": ""}, "time_dimension": {"answer": "Present", "rationale": "The entry asks the testee about his attitude and behaviour towards something that is not his own, and the temporal dimension is now judged."}, "sentence_type": "Negative declarative sentence", "pos": {"nouns": {"Questionnaire": 1, "Entry": 1, "person": 1, "Something": 1}, "verbs": {"belong to": 1, "not": 1, "Good boy": 1, "Take it": 1}, "adjectives": {"Not": 1, "Your own": 1}, "numerals": {"36": 1}, "pronouns": {"I": 2}}}, {"content": "I used to take sick leave at an office or school when I wasn't sick.", "word_count": 18, "label": "", "sentence_structure": "Subject鈥損redicate", "subjective_objective": {"answer": "Subjective", "rationale": "Because \"sick leave\" is the patient's subjective act, and it's the patient's idea."}, "sentence_sentiment": {"answer": "Negative", "rationale": "Because the entry \"Care leave without illness\" is a false, dishonest act with a certain negative emotional colour."}, "language_style": {"answer": "Tactful and warm", "rationale": "The language style of the entry is more euphemistic. It uses a more moderate way of asking questions, rather than a direct statement or order, which is more personal and interactive, making it easier to accept and answer questions. At the same time, the term \"had\" is used to avoid accusations or criticisms and to respect the feelings and privacy of the interviewee."}, "time_dimension": {"answer": "Past", "rationale": "The word \"I was\" indicates that this act happened in the past, and that \"in the absence of illness, sick leave\" further emphasizes this as a past act. Thus, the temporal dimension of this article is the past."}, "sentence_type": "Negative declarative sentence", "pos": {"nouns": {"Questionnaire": 1, "Entry": 1, "Sick leave": 1, "Units": 1, "School": 1}, "verbs": {"Once": 1, "- Are you there": 1, "When you're sick": 1, "Please": 1}, "adverbs": {"Nope": 1, "Please": 1}, "numerals": {"37": 1}, "pronouns": {"I": 1}}}, {"content": "I have never damaged the library's books or shop's contents without saying anything.", "word_count": 26, "label": "", "sentence_structure": "Subject鈥搗erb鈥揷omplement", "subjective_objective": {"answer": "Subjective", "rationale": "Because \"no\" is the patient's subjective attitude, the entry is viewed from the patient's subjective perspective."}, "sentence_sentiment": {"answer": "Negative", "rationale": "Because the entry \"damaged the library's books or shop's things without saying\" is negative."}, "language_style": {"answer": "Calm and direct", "rationale": "The entry is of a calm and straightforward language. It presents a direct standard of conduct (never damaged the library or shop's articles and kept silent), is not overly emotional or rhetorical and is more objective in tone."}, "time_dimension": {"answer": "Past", "rationale": "The entry asks if the testee has had unrecognized damage to public property in his past experience, and the temporal dimension is a matter of the past."}, "sentence_type": "Negative declarative sentence", "pos": {"nouns": {"Situation": 1, "Library": 1, "Books": 1, "Something": 1, "Shop": 1}, "verbs": {"Damage": 1, "Say it": 1, "Never": 1}, "adjectives": {"EMPTY": 1}, "adverbs": {"And": 2}, "numerals": {"38": 1}, "pronouns": {"I": 1}}}, {"content": "I have some very bad habits.", "word_count": 9, "label": "", "sentence_structure": "Subject鈥搗erb鈥揷omplement", "subjective_objective": {"answer": "Subjective", "rationale": "Because \"bad habits\" are self-evaluation and perception of a person, and are individual subjective feelings."}, "sentence_sentiment": {"answer": "Negative", "rationale": "Because the expression \"very bad habits\" in the entry is negative."}, "language_style": {"answer": "Tactful and warm", "rationale": "The language style of the entry is more euphemistic, by asking the other side whether they have \"very bad habits\" rather than directly blaming or criticizing their behaviour, which is more easily accepted and willing to share their true ideas. At the same time, it is more gentle and friendly, giving people a sense of care and support."}, "time_dimension": {"answer": "Present", "rationale": "The entry \"some\" and \"very bad habits\" indicate a context that describes the current behaviour or state of the person being tested, and therefore determines \"now\"."}, "sentence_type": "Negative declarative sentence", "pos": {"nouns": {"Custom": 1, "Not good": 1}, "verbs": {"have": 1, "Some": 1}, "adjectives": {"very": 1}, "numerals": {"39": 1}, "pronouns": {"I": 1}}}, {"content": "I do not speak short of others.", "word_count": 12, "label": "", "sentence_structure": "Subject鈥搗erb鈥揷omplement", "subjective_objective": {"answer": "Subjective", "rationale": "Because \"speak short\" is a subjective assessment and attitude."}, "sentence_sentiment": {"answer": "Neutral", "rationale": "Because the entry does not have any emotional color."}, "language_style": {"answer": "Calm and direct", "rationale": "The sentence is based on the following: the entry expresses the requirement of prohibiting the practice of short-term, in the form of a negative sentence, which is more direct and tough. At the same time, the phrase is simple, is not overly spelt out or emotional, and is more in keeping with the calm and direct linguistic style."}, "time_dimension": {"answer": "Present", "rationale": "The keyword is \"no,\" which means \"negative behaviour or state,\" and \"prolonged short\" is a customary move, so the entry describes the testee who is now often avoiding evaluating the things of others."}, "sentence_type": "Affirmative declarative sentence", "pos": {"verbs": {"Say it": 1, "Dow": 1, "Short": 1}, "adverbs": {"not": 1, "to": 1}, "pronouns": {"others": 1}}}, {"content": "I find myself able to help others in many ways. I agree.", "word_count": 20, "label": "", "sentence_structure": "Subject鈥搗erb鈥搊bject", "subjective_objective": {"answer": "Subjective", "rationale": "Because the words \"I find\" and \"can\" indicate that it's a subjective examination of the patient's own thoughts and feelings."}, "sentence_sentiment": {"answer": "Positive", "rationale": "Since the entry expresses an act or attitude that can help others, it is generally considered positive and positive."}, "language_style": {"answer": "Calm and direct", "rationale": "The language style of the entry is calm. It simply raises a question and asks the interviewee to answer \"yes\" or \"no\" without using too many rhetorical or emotional expressions, which are more objective. This expression is more straightforward, more concise and clear, and is conducive to the interviewee's real thoughts and feedback."}, "time_dimension": {"answer": "Present", "rationale": ""}, "sentence_type": "Interrogative sentence", "pos": {"nouns": {"self": 1, "In terms of": 1, "others": 1}, "verbs": {"Found": 1, "Yes": 1, "Help": 1, "Agreed": 1, "not": 1, "Meaning": 1}, "numerals": {"Many": 1}, "pronouns": {"I": 1}}}, {"content": "I feel that my life is better than my parents' lives at my age.", "word_count": 21, "label": "", "sentence_structure": "Subject鈥揷opula鈥損redicative", "subjective_objective": {"answer": "Subjective", "rationale": "Because \"good\" is a subjective word of evaluation, which involves the feelings and perceptions of individuals."}, "sentence_sentiment": {"answer": "Positive", "rationale": "Because the expression \"I feel better in my life than my parents' lives at my age\" is positive, indicating that the interviewee is certain and satisfied with his life."}, "language_style": {"answer": "Tactful and warm", "rationale": "The language style of the entry is more euphemistic and warm. It uses a positive, encouraging expression, rather than a direct criticism or accusation. It is easier for respondents to accept and share their views and feelings."}, "time_dimension": {"answer": "Present", "rationale": "The entry \"I feel\" uses the general current, which is the sense or attitude of the moment; and \"I'm better in life than my parents were at my age\" is an evaluation and comparison of the current life. So the time dimension of the entry is now."}, "sentence_type": "Affirmative declarative sentence", "pos": {"nouns": {"Life": 2, "parents": 1, "Age": 1}, "verbs": {"I feel": 1, "- Yeah": 1, "Okay": 1}, "adjectives": {"Mine": 1, "This": 1}, "numerals": {"2": 1}, "classifiers": {"individual": 1}, "pronouns": {"I": 1}}}, {"content": "3. Despite many changes, there is always a pattern in life.", "word_count": 20, "label": "", "sentence_structure": "Subject鈥搗erb鈥搊bject", "subjective_objective": {"answer": "Objective", "rationale": "Because \"the law of life\" is an objective phenomenon that does not depend on the patient鈥檚 subjective perception. Although patients may have different views and feelings, it does not affect their objectivity."}, "sentence_sentiment": {"answer": "Neutral", "rationale": "Because the entry does not have any emotional color."}, "language_style": {"answer": "Tactful and warm", "rationale": "The entry describes in a moderate and positive way the existence of a certain pattern of life, which is more acrimony and inspiring force."}, "time_dimension": {"answer": "Present", "rationale": "The entry describes the general phenomenon or pattern of life, and does not indicate the specific time point, which can be judged as now."}, "sentence_type": "Interrogative sentence", "pos": {"nouns": {"Change": 1, "Life": 1, "Pattern": 1}, "verbs": {"be": 1, "have": 1, "Go ahead": 1}, "adjectives": {"More": 1, "I'm sure": 1}, "adverbs": {"Always": 1}, "pronouns": {"Yeah": 1}}}, {"content": "4. A person can make friends if he/she wishes to.", "word_count": 17, "label": "", "sentence_structure": "Subject鈥損redicate", "subjective_objective": {"answer": "Subjective", "rationale": "Because \"can do\" requires patient's subjective efforts and will."}, "sentence_sentiment": {"answer": "Positive", "rationale": "Because \"can do\" in the entry is an affirmative, confident, emotional expression."}, "language_style": {"answer": "Tactful and warm", "rationale": "The language style of the entry is more euphemistic, expressing the message that you want to convey in a positive and encouraging way, rather than directly denying or accusing. This is more acceptable and understandable, and it stimulates the participation and initiative of the interviewees."}, "time_dimension": {"answer": "Future", "rationale": "The entry examines whether a person can achieve his or her goals in the future (i.e., make friends) and therefore judge as the future."}, "sentence_type": "Affirmative declarative sentence", "pos": {"nouns": {"person": 2, "friends": 1}, "verbs": {"Yes": 1, "I can do it": 1}, "adverbs": {"If": 1}, "numerals": {"4": 1}, "classifiers": {"One": 2}, "pronouns": {"he": 1, "she": 1}}}, {"content": "5. Anyone who is willing to work can raise the standard of living of the merchant.", "word_count": 17, "label": "", "sentence_structure": "Subject鈥損redicate", "subjective_objective": {"answer": "Objective", "rationale": "Because improving living standards depends on many objective factors, such as the socio-economic environment, educational resources, etc. While it is true that individual efforts can affect their own living standards, they cannot guarantee that the desired improvements will be achieved."}, "sentence_sentiment": {"answer": "Positive", "rationale": "Because this entry emphasizes the potential for raising living standards as long as there is a commitment to make efforts, it has positive incentives and motivation."}, "language_style": {"answer": "Calm and direct", "rationale": "The entry is more calm and straightforward, without too much rhetoric or emotional language. It directly articulates the idea that \"anyone who tries to do so can improve his or her standard of living\", in an objective and clear manner, without attempting to influence the reader's attitude in an emotional or persuasive manner."}, "time_dimension": {"answer": "Past", "rationale": ""}, "sentence_type": "Affirmative declarative sentence", "pos": {"nouns": {"person": 1, "Ken": 1, "Try": 1, "Standard of living": 1}, "verbs": {"Yeah": 1, "Dealer": 1}, "adverbs": {"Just": 1}, "classifiers": {"5": 1}, "pronouns": {"Anyone": 1}}}, {"content": "6. Most people do believe honesty is the best way to be executed.", "word_count": 15, "label": "", "sentence_structure": "Subject鈥搗erb鈥搊bject", "subjective_objective": {"answer": "Subjective", "rationale": "This is a subjective view and conviction of the patient and not an objective fact."}, "sentence_sentiment": {"answer": "Positive", "rationale": "Because the quality of honesty in the entry is considered to be the best way to live, it expresses a positive attitude and perspective."}, "language_style": {"answer": "Tactful and warm", "rationale": "The entry expresses in a moderate and positive way the view that honesty is very important for most people, without using hard or direct language. This expression is more resonant with the person under investigation and gives them the sense that their views are respected and shared."}, "time_dimension": {"answer": "Present", "rationale": ""}, "sentence_type": "Affirmative declarative sentence", "pos": {"nouns": {"person": 1, "Honesty": 1, "鈾?To be 鈾?: 1, "Plan": 1}, "verbs": {"Faith": 1, "be": 1, "Yes": 1, "Minori": 1}, "adjectives": {"Go, go, go": 1}, "numerals": {"More": 1}, "pronouns": {"Large": 1}}}, {"content": "7. Overall, I am satisfied with my life ' s luck.", "word_count": 16, "label": "", "sentence_structure": "Subject鈥揷opula鈥損redicative", "subjective_objective": {"answer": "Subjective", "rationale": "Because \"satisfaction\" is a subjective feeling and evaluation that reflects the patient's perception and attitude to life's luck."}, "sentence_sentiment": {"answer": "Positive", "rationale": "Because \"satisfaction with life's luck\" is a positive attitude and emotional expression."}, "language_style": {"answer": "Tactful and warm", "rationale": "The entry uses a more moderate, friendly expression to guide respondents to answer questions, without too direct or hard language. This expression makes it easier for the respondents to open themselves up and to express their views and feelings in a more realistic way."}, "time_dimension": {"answer": "Present", "rationale": ""}, "sentence_type": "Affirmative declarative sentence", "pos": {"nouns": {"Life": 1, "Lucky": 1}, "verbs": {"I feel": 1, "Satisfied": 1}, "adverbs": {"Overall": 1}, "pronouns": {"I": 1}}}, {"content": "8. If you treat people with kindness, they will do it to you.", "word_count": 14, "label": "", "sentence_structure": "Subject鈥搗erb鈥搊bject", "subjective_objective": {"answer": "Objective", "rationale": "Because \"good-faith\" and \"others will do this to you\" are objective acts or outcomes, independent of the subjective will of the individual. While the answer to this question may be based on his own subjective views, the final judgement should be based on objective facts."}, "sentence_sentiment": {"answer": "Positive", "rationale": "Because \"good-faith\" is a positive quality in the entry."}, "language_style": {"answer": "Tactful and warm", "rationale": "The language style of the entry is euphemism, because it is more polite, gentle and encourages respondents to respond positively. It does not speak directly or in a command style, but rather in a more friendly way."}, "time_dimension": {"answer": "Present", "rationale": "The keyword \"as long as\" indicates the condition, \"as long as\" means the result, all of which points to the current, immediate situation. So the entry describes the current attitude and conviction of the testee, which is judged by the time dimension."}, "sentence_type": "Affirmative declarative sentence", "pos": {"nouns": {"Makoto": 1, "person": 2, "others": 1}, "verbs": {"to": 2, "- Wait": 1, "Yes": 1}, "adverbs": {"Here": 2}, "pronouns": {"you": 1, "he": 1}}}, {"content": "It is difficult to make clear right and wrong now.", "word_count": 11, "label": "", "sentence_structure": "Subject鈥搗erb鈥搊bject", "subjective_objective": {"answer": "Subjective", "rationale": "Because it is the perception and feeling of the patient that is clear whether it is right or wrong, it is a subjective examination."}, "sentence_sentiment": {"answer": "Neutral", "rationale": "Because the entry does not have any emotional color."}, "language_style": {"answer": "Tactful and warm", "rationale": "The language style of the entry is more euphemistic, expressing the intention to question each other鈥檚 views, while avoiding too direct or strong a tone. This expression is more moderate, respects the views and feelings of the interviewees, and contributes to a good atmosphere of communication."}, "time_dimension": {"answer": "Present", "rationale": "The keyword is \"now\", which means the situation and state."}, "sentence_type": "Negative declarative sentence", "pos": {"nouns": {"Questionnaire": 1, "Entry": 1, "Present": 1, "Right and wrong": 1, "Yes, yes, yes, yes, yes, yes, yes, yes, yes": 1}, "verbs": {"Hard to say": 1, "Clear": 1}, "adjectives": {"very": 1}, "classifiers": {"individual": 1}}}, {"content": "Many of the friends of the police are simply trying to get something from you.", "word_count": 20, "label": "", "sentence_structure": "Subject鈥搗erb鈥搊bject", "subjective_objective": {"answer": "Subjective", "rationale": "Because this statement expresses the view or opinion of the interviewees that many people are only interested in establishing friendships with their friends. This involves the subjective judgement and perception of the interviewees, and therefore it is a subjective questionnaire entry."}, "sentence_sentiment": {"answer": "Negative", "rationale": "Because the statement in the entry \"Many people are friends with others only to get something\" expresses a negative attitude."}, "language_style": {"answer": "Calm and direct", "rationale": "The entry is based on a direct statement that many people are not friends with you out of genuine friendship, but for some benefit. It is not too much rhetorical or emotional to be rendered, and is more objective and consistent with the calm and direct language."}, "time_dimension": {"answer": "Present", "rationale": "Now the entry on the questionnaire is about the testee鈥檚 observation and understanding of the behaviour of others, and the keywords 鈥渕any people鈥? 鈥渇riends of your people鈥? 鈥渙nly鈥?and so on point to suggest that this is a question of investigating what is happening. So, the temporal dimension is now."}, "sentence_type": "Negative declarative sentence", "pos": {"nouns": {"Many people": 1, "Police": 1, "Something": 1}, "verbs": {"be": 1, "Got it": 1}, "adjectives": {"Many": 1}, "numerals": {"10": 1}}}, {"content": "11. If luck is bad, there can be no future.", "word_count": 14, "label": "", "sentence_structure": "Subject鈥損redicate", "subjective_objective": {"answer": "Subjective", "rationale": "Because bad luck and \"the day out\" are patients' subjective perceptions of their own situation."}, "sentence_sentiment": {"answer": "Negative", "rationale": "Because the expression \"unlucky, no day out\" in the entry has a negative emotional connotation."}, "language_style": {"answer": "Tactful and warm", "rationale": "The language style of this questionnaire entry is more euphemistic. It expresses a pessimism in a relatively mild way, not directly rejecting or attacking others, but in a euphemism. This expression is more readily acceptable and understood by the respondents, while avoiding unnecessary conflicts and contradictions."}, "time_dimension": {"answer": "Future", "rationale": "The keywords are \"unlucky\" and \"unlikely\" and point to an untimely situation and assume a possible future outcome. The temporal dimension of the article is therefore the future."}, "sentence_type": "Negative declarative sentence", "pos": {"nouns": {"Lucky": 1, "Head": 1, "Day": 1}, "verbs": {"Okay": 1, "have": 1, "Out": 1, "Maybe": 1}, "adjectives": {"not": 2, "Bad": 1}, "adverbs": {"If": 1, "Impossible": 1}, "numerals": {"11": 1}, "pronouns": {"鈾?And the 鈾?: 1}}}, {"content": "I feel strange to myself from time to time.", "word_count": 11, "label": "", "sentence_structure": "Subject鈥揷opula鈥損redicative", "subjective_objective": {"answer": "Subjective", "rationale": "Because \"fearing strangeness\" is a subjective feeling and experience of the patient and is a subjective examination."}, "sentence_sentiment": {"answer": "Negative", "rationale": "Because the entry \"unknown to itself\" expresses a negative emotional attitude, a self-perception of distress and confusion."}, "language_style": {"answer": "Tactful and warm", "rationale": "The language style of the entry is more euphemistic, not in a way that is too direct or strong, but in a way that guides respondents to share their feelings in an interview and understanding, which is more respectful and understanding of the psychological state of the person under investigation."}, "time_dimension": {"answer": "Present", "rationale": "The keyword \"at times feels\" means that the act is a regular, customary act that takes place in the life of the person being tested."}, "sentence_type": "Negative declarative sentence", "pos": {"nouns": {"I": 1, "self": 1}, "verbs": {"I feel": 1, "to": 1, "very": 1, "Strange": 1}, "adjectives": {"From time to time": 1}, "numerals": {"12": 1}}}, {"content": "13. The future seems bleak.", "word_count": 8, "label": "", "sentence_structure": "Subject鈥搗erb鈥揷omplement", "subjective_objective": {"answer": "Subjective", "rationale": "Because \"depressive\" is a subjective sense of pain or emotional experience."}, "sentence_sentiment": {"answer": "Negative", "rationale": "Because the word \"depression\" in the entry expresses a negative sentiment, it is negative."}, "language_style": {"answer": "Calm and direct", "rationale": "According to the sentence given, the question of \"the future seems bleak\" is more negative, pessimistic, without any positive vocabulary or emotional colour, and therefore it is possible to judge that its language style is a straightforward and calm type."}, "time_dimension": {"answer": "Future", "rationale": "The entry contains visions and expectations for the future, and the key words are \"the future appears to be \" and \" very bleak\" , all of which point to situations that have not yet occurred and that may arise in the future."}, "sentence_type": "Affirmative declarative sentence", "pos": {"adjectives": {"Psychic": 1}, "numerals": {"13": 1}}}, {"content": "14. I often feel that no one needs me.", "word_count": 10, "label": "", "sentence_structure": "Subject鈥搗erb鈥揷omplement", "subjective_objective": {"answer": "Subjective", "rationale": "Because \"need\" is a subjective feeling and perception that is generated by an individual's judgement of his or her own value. While this feeling may be related to objective facts (for example, if a person's social support system is less or less acceptable), it is still a subjective experience in itself."}, "sentence_sentiment": {"answer": "Negative", "rationale": "Because the phrase \"no one needs me\" in the entry is a lack of need, a negative emotional experience."}, "language_style": {"answer": "Tactful and warm", "rationale": ""}, "time_dimension": {"answer": "Present", "rationale": "\"I often feel\" means \"a high frequency behaviour, \"a feeling\" means \"a general state of affairs\" means that this behaviour not only has occurred in the past, but may still be ongoing. So, the entry is about the experience and experience of the person being tested, judging as it is now."}, "sentence_type": "Negative declarative sentence", "pos": {"nouns": {"I": 1, "person": 1}, "verbs": {"I feel": 1, "Yes": 1}, "adjectives": {"Regular": 1}, "numerals": {"14": 1}}}, {"content": "15. Life is too boring for me to do anything.", "word_count": 15, "label": "", "sentence_structure": "Subject鈥揷opula鈥損redicative", "subjective_objective": {"answer": "Subjective", "rationale": "Because \"too tired\" and \"doing nothing\" are the feelings and attitudes of the patient, and are subjective."}, "sentence_sentiment": {"answer": "Negative", "rationale": "Because the \"life is too boring\" sentiment in the entry is negative."}, "language_style": {"answer": "Calm and direct", "rationale": "The entry is simple and plain, without too much rhetoric or emotional rendering, and directly expresses the respondent ' s fatigue and negative feelings about life."}, "time_dimension": {"answer": "Present", "rationale": "The keywords \"too tired\" and \"not want to do\" point to the current feelings of the subjects."}, "sentence_type": "Exclamatory sentence", "pos": {"nouns": {"Life": 1, "person": 1}, "verbs": {"You're": 1, "I want to": 1}, "adjectives": {"Too much": 1, "Tired": 1}, "numerals": {"15": 1}, "pronouns": {"I": 1}}}, {"content": "16. To live with people, one must learn to live in masks.", "word_count": 16, "label": "", "sentence_structure": "Subject鈥搗erb鈥搊bject", "subjective_objective": {"answer": "Subjective", "rationale": "Because \"to learn to live in a mask\" is a personal feeling and attitude, a patient's subjective experience."}, "sentence_sentiment": {"answer": "Negative", "rationale": "Because life in a mask is a disguise for yourself, an unrealistic representation, not a positive attitude or behaviour."}, "language_style": {"answer": "Calm and direct", "rationale": "The entry presents its views in a short narrative, which is not overly emotional or rhetorical, and is more objective in tone. At the same time, the language style of the article is also calm and straightforward, and it is straightforward in presenting the idea of the need to wear masks in dealing with people without turning around or making it vague."}, "time_dimension": {"answer": "Present", "rationale": "The word \"to\" denotes the act that is needed and necessary, while the word \"to learn\" adopts the general current, which means that the act has not only occurred in the past, but may still be ongoing. The entry therefore asks whether the testee currently believes that living with a person requires a mask, and the time dimension is now judged."}, "sentence_type": "Affirmative declarative sentence", "pos": {"nouns": {"person": 1, "Masks": 1, "Life": 1}, "verbs": {"I have to": 1, "Learn": 1, "Dai": 1, "Get along": 1}, "adjectives": {"of": 3}, "adverbs": {"Just": 1}, "pronouns": {"you": 1}}}]}

{"questionnaire_title": "Carroll's depression table.", "questionnaire_category": "Mental health", "item_count": 52, "emotion_shift_overall": "From negative to positive", "language_style_overall": "Neutral", "items": [{"content": "I feel good about my spirit.", "word_count": 6, "label": "", "sentence_structure": "Subject鈥揷opula鈥損redicative", "subjective_objective": {"answer": "Subjective", "rationale": "Because it's a subjective experience and emotional reaction, \"I feel good\" is a subjective question."}, "sentence_sentiment": {"answer": "Positive", "rationale": "Because the \"frightful\" in the entry is a positive emotional attitude."}, "language_style": {"answer": "Tactful and warm", "rationale": "The language style of the entry is euphemism. It uses affirmative statements, expresses positive emotions, and does not directly express questions or orders about the emotional state of the respondent. Moreover, the entry contains a number of quaint words, \"fearing up the spirit,\" which are more gentle, friendly and encouraging, helping the interviewees to share their feelings more readily."}, "time_dimension": {"answer": "Present", "rationale": "The keyword \"I feel\" and \"sweet\" suggests that it is a description of the state of the moment, so the temporal dimension is now."}, "sentence_type": "Exclamatory sentence", "pos": {"verbs": {"I feel": 1, "Spirit": 1}, "adjectives": {"Okay": 1}}}, {"content": "I feel, unfortunately or often, like to cry.", "word_count": 11, "label": "", "sentence_structure": "Subject鈥揷opula鈥損redicative", "subjective_objective": {"answer": "Subjective", "rationale": "Because \"I feel\" and \"unlucky\" and \"often want to cry\" are subjective feelings and attitudes of patients."}, "sentence_sentiment": {"answer": "Negative", "rationale": "Because \"unfortunate\" and \"often feeling like crying\" in the entries are negative emotional expressions."}, "language_style": {"answer": "Tactful and warm", "rationale": "The entry is more euphemistic and warm. The words \"unfortunate\" and \"want to cry\" are used to express the emotional distress that the other party may experience, but not to directly accuse or order the other to change the emotional state, but to communicate with an understanding and support, making it easier for the other to feel the interest and acceptance."}, "time_dimension": {"answer": "Present", "rationale": ""}, "sentence_type": "Negative declarative sentence", "pos": {"nouns": {"I": 1, "Unfortunately": 1, "I think": 1, "Cry": 1}, "verbs": {"very": 1, "Often": 1, "be": 1}, "adjectives": {"Unfortunately": 1, "Yes": 1}, "adverbs": {"very": 1, "Regular": 1}, "numerals": {"2": 1}}}, {"content": "I think that I have lost hope in this situation.", "word_count": 12, "label": "", "sentence_structure": "Subject鈥搗erb鈥揷omplement", "subjective_objective": {"answer": "Subjective", "rationale": "Because \"I think this is hopeless\" is the patient's subjective judgment and feelings."}, "sentence_sentiment": {"answer": "Negative", "rationale": "Because the expression \"no hope\" in the entry is negative."}, "language_style": {"answer": "Tactful and warm", "rationale": "The entry uses the term \"situation\" instead of referring directly to the individual's feelings, which is relatively euphemistic. At the same time, \"no hope\" is not a positive or encouraging expression, but rather implies a pessimism. Such language is more concerned with the feelings and emotions of the interviewee than by pressing him directly to answer his questions."}, "time_dimension": {"answer": "Past", "rationale": "The entry contains a term that indicates the past \"in the mind\" and describes a situation that has already occurred, so it can be judged that this is a question of the past."}, "sentence_type": "Negative declarative sentence", "pos": {"nouns": {"Situation": 1, "Hope": 1}, "verbs": {"Yes": 1, "have": 1, "Here": 1}, "adjectives": {"This one": 1, "Already": 1, "Nope": 1}, "numerals": {"3": 1}, "pronouns": {"I": 1}}}, {"content": "My future can only be unfortunate.", "word_count": 10, "label": "", "sentence_structure": "Subject鈥揷opula鈥損redicative", "subjective_objective": {"answer": "Subjective", "rationale": "Because \"it's gonna be unfortunate\" is a subjective view or belief."}, "sentence_sentiment": {"answer": "Negative", "rationale": "Because it expresses a pessimism that the future of itself can only be unfortunate."}, "language_style": {"answer": "Calm and direct", "rationale": "The entry uses direct and clear language to describe the subject matter, with no excessive rhetorical or emotional rendering, and is more objective. This language style is suitable for situations like expressing negative emotions or challenging.\""}, "time_dimension": {"answer": "Future", "rationale": ""}, "sentence_type": "Negative declarative sentence", "pos": {"nouns": {"The future": 1, "Unfortunately": 1}, "verbs": {"It will be": 1}}}, {"content": "I feel like I'm a good person like everyone else.", "word_count": 13, "label": "", "sentence_structure": "Subject鈥揷opula鈥損redicative", "subjective_objective": {"answer": "Subjective", "rationale": "Because it is subjective and self-perception of the patient."}, "sentence_sentiment": {"answer": "Neutral", "rationale": "Because the entry merely expresses a view, it does not have any obvious emotional colour."}, "language_style": {"answer": "Tactful and warm", "rationale": "The language style of the entry is more euphemistic, friendly, and the expression is more personal and interactive, rather than directly evaluating the interviewees positively or negatively, but rather asking questions in a moderate manner, allowing the respondents to respond to their own reality."}, "time_dimension": {"answer": "Present", "rationale": "Keywords include \"think\" and \"me,\" which are verbs describing what is currently felt, and \"same\" which emphasize that is a testee's perception or belief in himself."}, "sentence_type": "Affirmative declarative sentence", "pos": {"nouns": {"Questionnaire Entry": 1, "person": 1, "Okay": 1}, "verbs": {"I think": 1, "be": 1, "Here": 1}, "adjectives": {"Same thing": 1}, "numerals": {"5": 1}, "pronouns": {"I": 1, "Don't": 1}}}, {"content": "6. I feel worthless and ashamed of myself.", "word_count": 16, "label": "", "sentence_structure": "Subject鈥揷opula鈥損redicative", "subjective_objective": {"answer": "Subjective", "rationale": "Because feeling worthless and ashamed of yourself is the patient's subjective emotional experience."}, "sentence_sentiment": {"answer": "Negative", "rationale": "Because \"no value\" and \"smuggling\" in the entries are negative emotions and attitudes."}, "language_style": {"answer": "Tactful and warm", "rationale": "The words \"fear\" and \"disguise yourself\" are used to make the entries more euphemistic and caring. Moreover, the question does not directly ask the interviewee about his feelings or experiences, but rather leads him to share his thoughts and feelings in a caring and supportive manner."}, "time_dimension": {"answer": "Present", "rationale": ""}, "sentence_type": "Negative declarative sentence", "pos": {"nouns": {"Value": 1, "Shame": 1}, "verbs": {"I feel": 2}, "pronouns": {"self": 1}}}, {"content": "Some things in life have upset me and upset me.", "word_count": 16, "label": "", "sentence_structure": "Subject鈥揷opula鈥損redicative", "subjective_objective": {"answer": "Objective", "rationale": "Because remorse and distress are subjective feelings or reactions of patients to their living conditions, these conditions are themselves objective."}, "sentence_sentiment": {"answer": "Negative", "rationale": "Because both the words \"repentant\" and \"disturbed\" are negative."}, "language_style": {"answer": "Calm and direct", "rationale": "The entry is more calm and straightforward, with no more qualifiers or emotional colours. It describes in an objective manner the possible remorse and distress in the lives of the interviewees, with the aim of understanding their inner feelings."}, "time_dimension": {"answer": "Present", "rationale": "The verb \"make\" is the verb that indicates the effect and result of past actions on the present, so the entry is a description of the person's current feelings and state."}, "sentence_type": "Affirmative declarative sentence", "pos": {"nouns": {"Life": 1, "Yes": 2, "Regret": 1, "I'm upset": 1}, "verbs": {"鈾?And I'm gonna be 鈾?: 4}, "adjectives": {"Some": 1}, "numerals": {"7": 1}}}, {"content": "I am being punished for the bad deeds of my past life.", "word_count": 18, "label": "", "sentence_structure": "Subject鈥揷opula鈥損redicative", "subjective_objective": {"answer": "Objective", "rationale": "Because \"punishment\" is a fact-based judgement, not a patient's subjective feeling or attitude."}, "sentence_sentiment": {"answer": "Negative", "rationale": "Because the \"punishment\" in the entry is negative."}, "language_style": {"answer": "Calm and direct", "rationale": "The entry is more direct and objective in its language style, and is not subject to excessive emotional acquitism or euphemism. It is a clear question whether the person interviewed is being punished for past wrongs. This is a more resolute and decisive tone, which is more likely to attract the attention of the person interviewed.\""}, "time_dimension": {"answer": "Past", "rationale": "\"As \"in\" means \"acting\" or \"doing\" and \"bad things in the past\" means \"acting in the past\" for a certain period of time, the entry asks about the testee's past act."}, "sentence_type": "Negative declarative sentence", "pos": {"nouns": {"Life": 1, "Bad things": 1, "Punishment": 1}, "verbs": {"Because": 1, "Fuck": 1, "Yes": 1}, "adverbs": {"Past": 1}, "numerals": {"8": 1}, "pronouns": {"I": 1}}}, {"content": "I feel that life still makes sense.", "word_count": 9, "label": "", "sentence_structure": "Subject鈥揷opula鈥損redicative", "subjective_objective": {"answer": "Subjective", "rationale": "Because \"fEs\" is the patient's subjective feeling, so it's a subjective view."}, "sentence_sentiment": {"answer": "Positive", "rationale": "Because the description of \"life still makes sense\" in the entry conveys a positive, positive emotional attitude."}, "language_style": {"answer": "Tactful and warm", "rationale": "The language style of the entry is euphemistic. It uses an incentive expression, not direct questions or orders, to make the interviewee more willing to share his thoughts and feelings. At the same time, the word \"still\" gives a positive sense of optimism, indicating that life is difficult, but that it has hope and meaning. All of this makes the language style of the questionnaire more euphemistic and warm."}, "time_dimension": {"answer": "Present", "rationale": "The entry asks the testee about the current feeling, and the keyword is \"fear,\" which shows that it is a question of the state of the moment. Moreover, \"still meaningful\" suggests that it is a statement describing subjective feelings and experiences."}, "sentence_type": "Affirmative declarative sentence", "pos": {"nouns": {"Life": 1, "Meaning": 1}, "verbs": {"I feel": 1}, "pronouns": {"I": 1}}}, {"content": "I often wish I had died.", "word_count": 11, "label": "", "sentence_structure": "Subject鈥搗erb鈥揷omplement", "subjective_objective": {"answer": "Subjective", "rationale": "Because \"I hope I'm dead\" is a subjective idea and a feeling."}, "sentence_sentiment": {"answer": "Negative", "rationale": "Because the idea of \"hope is dead\" in the entry is negative."}, "language_style": {"answer": "Calm and direct", "rationale": "The entry uses direct, concise language to ask the respondents whether they wish to die, is not overly accommodative or emotional and is expressed in a calmer and more direct manner."}, "time_dimension": {"answer": "Present", "rationale": "I often hope that the word \"normal\" indicates a regular, customary behaviour, and that \"hope\" adopts the general current, which means that this behaviour has not only happened in the past, but may still be ongoing. So this entry looks at the current state of the person being tested, both in mind and in mood."}, "sentence_type": "Negative declarative sentence", "pos": {"nouns": {"self": 1, "Die": 1}, "verbs": {"Hope": 1, "Already": 1, "Die": 1}, "adverbs": {"Often": 1}, "numerals": {"10": 1}, "pronouns": {"I": 1}}}, {"content": "I was also thinking about suicide.", "word_count": 9, "label": "", "sentence_structure": "Subject鈥搗erb鈥揷omplement", "subjective_objective": {"answer": "Subjective", "rationale": "Because it's a subjective description of what the patient feels and thinks inside himself."}, "sentence_sentiment": {"answer": "Negative", "rationale": "Because the act of suicide in the entry is negative."}, "language_style": {"answer": "Calm and direct", "rationale": "This language style better conveys the true picture of the situation when dealing with sensitive issues and helps to guide the interviewee to reflect and reflect."}, "time_dimension": {"answer": "Present", "rationale": "In the future, the entry \"suspecting\" suggests that it is a future act or idea, and therefore judgement is the future. At the same time, \"suicide\" is a future-related issue, because suicide requires certain planning and action."}, "sentence_type": "Negative declarative sentence", "pos": {"nouns": {"Think": 1, "What": 1, "Suicide": 1}, "verbs": {"Still there": 1, "Think": 1, "What": 1, "Suicide": 1}, "adverbs": {"And": 1}, "numerals": {"11": 1}}}, {"content": "12. Death is the best way to escape me.", "word_count": 11, "label": "", "sentence_structure": "Subject鈥揷opula鈥損redicative", "subjective_objective": {"answer": "Subjective", "rationale": "Because it's the patient's own point of view and opinion."}, "sentence_sentiment": {"answer": "Negative", "rationale": "Because the expression \"death is the best way to get out\" in the entry is negative."}, "language_style": {"answer": "Calm and direct", "rationale": "The entry is more blunt, has less emotional and rhetorical style, and is more objective and realistic. At the same time, the article鈥檚 purpose is more direct and clear, expressing the respondent鈥檚 views on death without any hint or euphemism."}, "time_dimension": {"answer": "Present", "rationale": "The phrase \"for me\" indicates the person who speaks, the person who says \"death is the best way out\" also implies the state of the mood or attitude. So the temporal dimension is now."}, "sentence_type": "Affirmative declarative sentence", "pos": {"nouns": {"Release": 1, "Die": 1}, "verbs": {"be": 1, "I'd better": 1}, "adverbs": {"Tell me": 1}, "numerals": {"12": 1}, "pronouns": {"I": 1}}}, {"content": "13. At night, I need longer than usual to sleep.", "word_count": 18, "label": "", "sentence_structure": "Subject鈥搗erb鈥揷omplement", "subjective_objective": {"answer": "Subjective", "rationale": "Because \"it takes longer to sleep\" is the patient's subjective experience and feelings."}, "sentence_sentiment": {"answer": "Negative", "rationale": "Because the \"sleeping\" behavior in the entry \"takes longer than usual\" is negative."}, "language_style": {"answer": "Calm and direct", "rationale": "The entry presents the problem in fact, without adding too much emotional or subjective assessment. This language style allows the person surveyed to answer the question more objectively, without emotional or other interference. Moreover, the entry is phrased in simple, easy to understand and meets the normative requirements of the questionnaire study."}, "time_dimension": {"answer": "Present", "rationale": "\"In the evening\" means a specific point or period of time, \"needs\" and \"longer\" means that it is a normal or customary act, and the entry describes the person being tested."}, "sentence_type": "Interrogative sentence", "pos": {"nouns": {"Night": 1, "Time": 1, "person": 1, "Sleep": 1}, "verbs": {"Yes": 1, "- Yeah": 1, "More": 1, "Talent": 1, "Sleep": 1}, "adjectives": {"Normal": 1}, "adverbs": {"- Yeah": 1, "More": 1, "That's right": 1}, "numerals": {"13": 1, "One": 1, "More": 1}, "classifiers": {"individual": 1}, "pronouns": {"I": 1}}}, {"content": "14. It takes me more than half an hour to sleep.", "word_count": 15, "label": "", "sentence_structure": "Subject鈥搗erb鈥揷omplement", "subjective_objective": {"answer": "Subjective", "rationale": "Because \"need\" is the patient's subjective need and feeling."}, "sentence_sentiment": {"answer": "Negative", "rationale": "Because \"it takes more than half an hour to sleep\" means there's a problem with sleep disorders, which is a negative state."}, "language_style": {"answer": "Calm and direct", "rationale": "The entry expresses the subject of questions about sleeping problems in a concise and clear language, and does not involve excessive rhetorical or emotional rendering, which is more objective and balanced."}, "time_dimension": {"answer": "Past", "rationale": "The entry asks about past sleep habits and behaviour of the person tested, and therefore the temporal dimension is a past."}, "sentence_type": "Affirmative declarative sentence", "pos": {"nouns": {"Time": 1, "Half an hour": 1}, "verbs": {"Yes": 1, "Sleep": 1}, "adjectives": {"Above": 1}, "numerals": {"Half": 1, "Small": 1, "Time": 1}, "classifiers": {"individual": 1}, "pronouns": {"I": 1}}}, {"content": "I slept in vain and in vain.", "word_count": 11, "label": "", "sentence_structure": "Subject鈥搗erb鈥揷omplement", "subjective_objective": {"answer": "Subjective", "rationale": "Because \"indefinite\" and \"indefinite\" are the subjective feelings and experiences of the patient."}, "sentence_sentiment": {"answer": "Negative", "rationale": "Because \"deprivation\" and \"real\" are positive words, and in this entry, negative forms are used to indicate that there are no such positive scenarios, so emotional is negative."}, "language_style": {"answer": "Calm and direct", "rationale": "The entry is a simple, direct interview with the other party, is not over-modified or emotionally rendered, and is more calm and straightforward in language style."}, "time_dimension": {"answer": "Past", "rationale": "The entry describes the current sleep conditions of the person tested, using the general current and negative word \"no\", indicating that this is a persistent state and therefore judgemental of the past."}, "sentence_type": "Negative declarative sentence", "pos": {"nouns": {"I": 1, "Sleep": 1, "Solve the poverty": 1, "Sleep well": 1, "Not yet": 1, "Minori": 1}, "verbs": {"have": 1}, "adjectives": {"I'm not tired": 1, "It's not true": 1}, "adverbs": {"Yeah": 1}}}, {"content": "I often wake up at night.", "word_count": 7, "label": "", "sentence_structure": "Subject鈥搗erb鈥揷omplement", "subjective_objective": {"answer": "Objective", "rationale": "Because awakening at night is an objective biological phenomenon, there is no need for subjective perceptions and attitudes on the part of the patient."}, "sentence_sentiment": {"answer": "Negative", "rationale": "Because \"often wake up\" is negative."}, "language_style": {"answer": "Calm and direct", "rationale": "The entry is more concise in language style, is not used too much rhetorical or emotionally retrofitting, and is asked directly whether the person surveyed often wakes up at night and is speaking in a more objective and cold manner."}, "time_dimension": {"answer": "Present", "rationale": "The entry is for the purpose of asking the person who was tested for his current behaviour or state, and the keyword is \"often wakes up at night\", which means that this is a recurring situation and therefore the judgement is now."}, "sentence_type": "Affirmative declarative sentence", "pos": {"nouns": {"Night": 1, "Lee": 1, "I": 1}, "verbs": {"Often": 1, "Wake up": 1}}}, {"content": "I woke up earlier than usual in the morning.", "word_count": 9, "label": "", "sentence_structure": "Subject鈥揷opula鈥損redicative", "subjective_objective": {"answer": "Objective", "rationale": "The basis for the judgement: because the morning wakes up is an objective fact of the patient ' s experience and is not influenced by the individual ' s subjective consciousness."}, "sentence_sentiment": {"answer": "Neutral", "rationale": "Since the entry merely objectively presents a fact, it does not involve any positive or negative emotional colour."}, "language_style": {"answer": "Calm and direct", "rationale": "The entry is in a concise language and asks the respondents whether the time of awakening is different from normal, the language style is calmer and more direct, and there is no excessive emotional rendering or rhetorical technique."}, "time_dimension": {"answer": "Present", "rationale": "Now."}, "sentence_type": "Affirmative declarative sentence", "pos": {"verbs": {"Wake up": 1, "Okay": 1, "- Yeah": 1, "Normal": 1}, "adjectives": {"Morning": 1}, "numerals": {"17": 1}}}, {"content": "18. I woke up too early in the morning, affecting daily life.", "word_count": 16, "label": "", "sentence_structure": "Subject鈥搗erb鈥揷omplement", "subjective_objective": {"answer": "Subjective", "rationale": "Because \"woke too early in the morning\" is the patient's own feelings and experiences."}, "sentence_sentiment": {"answer": "Negative", "rationale": "Because the result of \"impacting everyday life\" is negative."}, "language_style": {"answer": "Tactful and warm", "rationale": ""}, "time_dimension": {"answer": "Present", "rationale": "\"As early morning wakes up\" is an act that describes the current state and experience, and at the same time, \"to affect everyday life\" shows that this act has an impact on the lives of the tested and points further towards the present situation."}, "sentence_type": "Negative declarative sentence", "pos": {"nouns": {"Questionnaire": 1, "Entry": 1, "Morning": 1, "Wake up": 1, "Life": 1}, "verbs": {"Impact": 1, "Result": 1}, "adjectives": {"Too much": 1, "Morning": 1, "Day": 1}, "adverbs": {"So": 1}, "numerals": {"18": 1}}}, {"content": "I can get happiness and satisfaction from what I do.", "word_count": 17, "label": "", "sentence_structure": "Subject鈥揷opula鈥損redicative", "subjective_objective": {"answer": "Subjective", "rationale": "Because it is the individual feeling and subjective assessment of the patient that happiness and satisfaction are derived from what is done."}, "sentence_sentiment": {"answer": "Positive", "rationale": "Because the behavior in the entry is \"to get happiness and satisfaction from what you do\", it is a positive emotional attitude, expressing a sense of self-realization and achievement."}, "language_style": {"answer": "Tactful and warm", "rationale": "The language style of the entry is euphemistic, and it asks the interviewees whether they can find happiness and satisfaction in their work in a positive, encouraging and supportive manner, which makes them more friendly and comfortable."}, "time_dimension": {"answer": "Present", "rationale": "\"I can get happiness and satisfaction from what I do\" means that I have the ability to get or get a certain result or state, and these words point to the current behavior or state. The entry is therefore about asking the testee about his current feelings and attitudes and judging the dimensions now."}, "sentence_type": "Affirmative declarative sentence", "pos": {"nouns": {"I": 1, "Happy": 1, "Satisfied": 1}, "verbs": {"Yes": 1, "Okay": 1, "Do it": 1}, "adverbs": {"From": 1, "self": 1}}}, {"content": "I still like to go out and meet people.", "word_count": 10, "label": "", "sentence_structure": "Subject鈥搗erb鈥揷omplement", "subjective_objective": {"answer": "Subjective", "rationale": "Because it is the patient's subjective attitude and emotional experience of the situation that prefers to meet with someone outside."}, "sentence_sentiment": {"answer": "Positive", "rationale": "Because the tendency to act like \"remain like meeting someone outside\" is positive."}, "language_style": {"answer": "Tactful and warm", "rationale": "The title uses the word \"still\" to indicate a continuation of past habits, while expressing a positive attitude. This language style is more friendly, more cozy, more acceptable and willing to participate in the investigation."}, "time_dimension": {"answer": "Present", "rationale": "The keyword \"still likes\" to indicate continuity and persistence in past conduct, while \"out-of-person\" is an act that usually occurs in the immediate future. Thus, the time dimension of the article is now."}, "sentence_type": "Affirmative declarative sentence", "pos": {"nouns": {"I": 1, "Out": 1, "person": 1, "Meeting": 1}, "verbs": {"Still like it": 1}, "adverbs": {"Still": 1}}}, {"content": "21. I have lost many interests.", "word_count": 10, "label": "", "sentence_structure": "Subject鈥搗erb鈥揷omplement", "subjective_objective": {"answer": "Subjective", "rationale": "Because the loss of hobby is the patient ' s own perception and experience, it is part of the subjective sense."}, "sentence_sentiment": {"answer": "Negative", "rationale": "The entry is a negative expression of loss of interest."}, "language_style": {"answer": "Tactful and warm", "rationale": "The entry uses a more euphemistic formulation, using the word \"lost\" to express the fact that the interviewee lost many interests, rather than directly pointing to the loss or disappearance of the other's preferences, which is more moderate, respectful and understanding of the other's feelings. At the same time, the term \"many\" implies that this is a more general problem, not an individual or a particular situation. The language style of the article is therefore euphemism."}, "time_dimension": {"answer": "Past", "rationale": "The \"lost\" in the entry indicates the movement or state of the past at a certain point in time, so it can be judged to be a description and memory of the past."}, "sentence_type": "Negative declarative sentence", "pos": {"nouns": {"Questionnaire Entry": 1, "A hobby": 1}, "verbs": {"Loss": 1}}}, {"content": "22. I can still do what I want to do.", "word_count": 13, "label": "", "sentence_structure": "Subject鈥搗erb鈥搊bject", "subjective_objective": {"answer": "Subjective", "rationale": "Because it is the subjective attitude and behaviour of one person to insist on doing something, this is seen from the subjective perspective of the patient."}, "sentence_sentiment": {"answer": "Positive", "rationale": "Because the behavior in the entry \"suspend to what you want to do\" is positive."}, "language_style": {"answer": "Tactful and warm", "rationale": "The entry uses positive words, \"hope,\" \"dry,\" and emphasizes the ability to \"still hold.\" This expression is more encouraging and encouraging, with a certain amount of motivation and emotion.\""}, "time_dimension": {"answer": "Present", "rationale": ""}, "sentence_type": "Affirmative declarative sentence", "pos": {"nouns": {"Yes": 1, "Hope": 1}, "verbs": {"Fuck": 2, "Hold on": 1, "Yes": 1}, "adjectives": {"Still": 1}, "numerals": {"22": 1}, "pronouns": {"I": 1}}}, {"content": "My thoughts have been clear and fast.", "word_count": 10, "label": "", "sentence_structure": "Subject鈥揷opula鈥損redicative", "subjective_objective": {"answer": "Subjective", "rationale": "Because \"my mind is always clear, fast\" is a patient's self-evaluation, reflecting their subjective feelings."}, "sentence_sentiment": {"answer": "Positive", "rationale": "Because the expression \"thinking is always clear and fast\" conveys a positive quality character, which means that individuals are agile, responsive and positive emotional expressions."}, "language_style": {"answer": "Calm and direct", "rationale": "The entry directly describes the characteristics of the person surveyed in terms of reflection, without using qualifiers or highly emotional terms, and is of an objective and calm type."}, "time_dimension": {"answer": "Present", "rationale": "The keyword \"thinking is always clear and fast\" denotes the current state and state of affairs, so the basis of the judgement is now."}, "sentence_type": "Affirmative declarative sentence", "pos": {"verbs": {"have": 1, "Clear": 1, "Quick": 1}, "adjectives": {"Always": 1, "Clear": 1, "Shortcut": 1}, "numerals": {"23": 1}}}, {"content": "I was slow to speak and I was not angry.", "word_count": 9, "label": "", "sentence_structure": "Subject鈥搗erb鈥揷omplement", "subjective_objective": {"answer": "Subjective", "rationale": "Because the description of \"silent\" and \"not angry\" are subjective feelings and evaluations of patients."}, "sentence_sentiment": {"answer": "Neutral", "rationale": "Because the entry merely describes a fact and does not involve any emotional colour."}, "language_style": {"answer": "Tactful and warm", "rationale": "The language style of the entry is more euphemistic. It uses the expression \"simplistic\" instead of \"so stupid,\" avoiding harming each other's self-esteem and expressing one's feelings. It is more acceptable and willing to continue to engage in dialogue."}, "time_dimension": {"answer": "Past", "rationale": "The entry describes the situation and feelings of the testee in the past, and the \"silent\" and \"no anger\" have all happened in the past, and it is therefore judged to be the past."}, "sentence_type": "Negative declarative sentence", "pos": {"nouns": {"Talk": 1, "Slow": 1, "Angry": 1}, "verbs": {"Say it": 1, "have": 1}, "adjectives": {"Slow": 1}, "pronouns": {"I": 1}}}, {"content": "25. I have been unable to do almost anything recently.", "word_count": 12, "label": "", "sentence_structure": "Subject鈥揷opula鈥損redicative", "subjective_objective": {"answer": "Subjective", "rationale": "Because it is the patient ' s own perception and feelings of his current situation, it is subjective thinking and emotion."}, "sentence_sentiment": {"answer": "Negative", "rationale": "Because the expression \"almost nothing can be done\" in the entry suggests a negative emotional state."}, "language_style": {"answer": "Calm and direct", "rationale": "The language style of the entry is of a more direct type of description of the facts, with no excessive emotional rendering or rhetorical techniques and a fair and objective tone."}, "time_dimension": {"answer": "Past", "rationale": "The term \"recent\" in the entry refers to a period of time before the time of the inquiry, so it is judged to be the past."}, "sentence_type": "Negative declarative sentence", "pos": {"nouns": {"Yes": 1, "Questionnaire items": 1}, "verbs": {"Fuck": 1}, "adjectives": {"Almost": 1}, "adverbs": {"Most recently": 1}, "numerals": {"25": 1}}}, {"content": "I am very slow, and I need help to wash and dress.", "word_count": 16, "label": "", "sentence_structure": "Subject鈥揷opula鈥損redicative", "subjective_objective": {"answer": "Subjective", "rationale": "Because \"I'm very slow\" is a patient's assessment and feelings, a subjective examination."}, "sentence_sentiment": {"answer": "Negative", "rationale": "Because the description \"very slow, and all that needs help to wash and dress\" in the entry is negative."}, "language_style": {"answer": "Tactful and warm", "rationale": "The language style of the entry is euphemistic. It uses the first person's pronoun, \"I,\" to give people a more intimate sense of affection, and also to express the respondent's modesty towards self-evaluation. Moreover, the expression \"very slow\" does not point directly to the other party's problems, but rather expresses it in a more subtle way, avoiding the negative emotions or resistance that may be triggered."}, "time_dimension": {"answer": "Present", "rationale": ""}, "sentence_type": "Negative declarative sentence", "pos": {"nouns": {"Slow": 1, "Wash": 1, "Get dressed": 1, "Help": 1}, "verbs": {"Yes": 1, "Wash": 1, "Put it on": 1, "Help": 1}, "adjectives": {"Very": 1}, "numerals": {"26": 1}}}, {"content": "I think I look quiet.", "word_count": 10, "label": "", "sentence_structure": "Subject鈥揷opula鈥損redicative", "subjective_objective": {"answer": "Subjective", "rationale": "Because I represent the patient's subjective perspective, and describe how he thinks and feels about himself."}, "sentence_sentiment": {"answer": "Negative", "rationale": "Because \"I want to look quiet\" may indicate that the interviewee is not confident or satisfied with his appearance and has a negative emotional tendency."}, "language_style": {"answer": "Tactful and warm", "rationale": "The entry is more adept and interactive when it is spoken in a gentle and inviting manner, rather than by direct statements or orders."}, "time_dimension": {"answer": "Past", "rationale": "The main word in the entry, \"I,\" is self-evaluation by the individual, while the verb \"want\" and the \"silentness of my appearance\" in the bengue indicate a subjective view or impression. This perception or impression is not the present state, but the experience or perception of the past, and can therefore be judged by the past."}, "sentence_type": "Affirmative declarative sentence", "pos": {"nouns": {"Appearance": 1, "Quiet": 1}, "verbs": {"Yes": 1, "It seems": 1}, "pronouns": {"I": 1}}}, {"content": "I am restless and uneasy.", "word_count": 9, "label": "", "sentence_structure": "Subject鈥搗erb鈥揷omplement", "subjective_objective": {"answer": "Subjective", "rationale": "Because being restless and uneasy is the subjective feeling and experience of the patient."}, "sentence_sentiment": {"answer": "Negative", "rationale": "Because the expression \"sitting, uneasy\" in the entries is negative."}, "language_style": {"answer": "Tactful and warm", "rationale": "The entry uses a euphemismal and sensitive language style, without directly criticizing or accusing the other of having a problem of \"shaky, uneasy\" but rather asking the other side whether it feels that way in a mild tone. Such expression is more respectful of the other side's feelings and privacy and more conducive to dialogue and communication."}, "time_dimension": {"answer": "Present", "rationale": ""}, "sentence_type": "Affirmative declarative sentence", "pos": {"verbs": {"Sit tight": 1, "I'm not feeling well": 1}}}, {"content": "29. I am very upset and prone to anger.", "word_count": 11, "label": "", "sentence_structure": "Subject鈥揷opula鈥損redicative", "subjective_objective": {"answer": "Subjective", "rationale": "Because irritation and irritation are the emotional state that the patient feels and the subjective feeling of the patient."}, "sentence_sentiment": {"answer": "Negative", "rationale": "Because the expressions \"freaky\" and \"freaky\" are negative emotional attitudes."}, "language_style": {"answer": "Calm and direct", "rationale": "The entry describes possible behaviour of the person under investigation in a concise and clear language, without too many emotional qualifiers or euphemisms, and in a more direct and objective manner."}, "time_dimension": {"answer": "Present", "rationale": ""}, "sentence_type": "Affirmative declarative sentence", "pos": {"nouns": {"I'm upset": 1, "Fire": 1}, "verbs": {"It seems": 1, "Let's go": 1}, "adjectives": {"Boring": 1, "Unsettled": 1, "Easier than I thought": 1}, "adverbs": {"Easy": 1}}}, {"content": "30. For most of the time, I had to go around.", "word_count": 14, "label": "", "sentence_structure": "Subject鈥搗erb鈥揷omplement", "subjective_objective": {"answer": "Subjective", "rationale": "Because it is a behaviour, a feeling and experience of the patient, and a subjective view."}, "sentence_sentiment": {"answer": "Negative", "rationale": "Because the act of \"must be taken\" is a sign of negative emotion and attitude."}, "language_style": {"answer": "Calm and direct", "rationale": "The entry is more straightforward and simple in language, with no excessive rhetoric or emotional rendering. It is straightforward in asking whether the interviewee has a habit of walking in most of the time, and is more objective and neutral."}, "time_dimension": {"answer": "Present", "rationale": ""}, "sentence_type": "Affirmative declarative sentence", "pos": {"verbs": {"I'm gonna go around": 1, "I had to": 1}, "adjectives": {"Most of them": 1, "Time": 1}}}, {"content": "31. When reading, my attention is easily focused.", "word_count": 13, "label": "", "sentence_structure": "Subject鈥揷opula鈥損redicative", "subjective_objective": {"answer": "Subjective", "rationale": "Because \"the focus is easy\" is an individual's feeling or perception, and it's a subjective view of the patient."}, "sentence_sentiment": {"answer": "Positive", "rationale": "Because the purpose of the article is affirmative, it means that individuals have a better ability to focus their reading."}, "language_style": {"answer": "Tactful and warm", "rationale": "The entry is more personal and interactive when it is spoken in a gentle and inviting manner, rather than in direct statements or orders. Moreover, the expression \"my attention is easy to focus\" is relatively objective, neutral, not overly subjective, and corresponds to the \"sweet\" language style."}, "time_dimension": {"answer": "Present", "rationale": "The entry describes the current reading attention of the subject, does not address the time dimension of the past or the future, and therefore is judged to be \"now\"."}, "sentence_type": "Affirmative declarative sentence", "pos": {"verbs": {"Reading": 1, "Attention": 1, "Focus": 1}, "adjectives": {"Easy": 1}}}, {"content": "I feel distracted or disturbed.", "word_count": 12, "label": "", "sentence_structure": "Subject鈥搗erb鈥揷omplement", "subjective_objective": {"answer": "Subjective", "rationale": "Because \"think\" is a subjective description of the patient's own feelings and experiences."}, "sentence_sentiment": {"answer": "Negative", "rationale": "Because \"deep\" and \"sweet\" are negative emotional terms."}, "language_style": {"answer": "Calm and direct", "rationale": "It does not use excessive emotional terms or qualifiers, but rather uses a direct and objective tone of inquiry."}, "time_dimension": {"answer": "Present", "rationale": "The \"Feel\" in the entry indicates the current feelings and state of the subject, and \"Esperate or Unsettled\" is a specific emotional description, so the temporal dimension of the entry should be judged now."}, "sentence_type": "Affirmative declarative sentence", "pos": {"nouns": {"I'm not sure I can": 1, "I'm not sure": 1}, "verbs": {"I think": 1}}}, {"content": "33. In many cases, I have been inexplicably afraid.", "word_count": 15, "label": "", "sentence_structure": "Subject鈥搗erb鈥揷omplement", "subjective_objective": {"answer": "Subjective", "rationale": "Because \"fear\" is the feeling and experience of the patient's heart, and it's a subjective emotional state."}, "sentence_sentiment": {"answer": "Negative", "rationale": "Because the description of \"fear\" in the entry is negative."}, "language_style": {"answer": "Calm and direct", "rationale": "The entry is a direct expression of the subject of questioning whether the other party is often inexplicably afraid, without excessive rhetorical or emotional rendering, and with a more balanced and objective tone. Nor is it used in any euphemism or courtesy, but rather in a direct way, the question is raised, so that the language style can be judged as being calm and straightforward."}, "time_dimension": {"answer": "Present", "rationale": "The keyword \"in many cases\" means that it is a constant, customary description, and \"fear\" is an act or emotional reaction that occurred in the past, but it does not make it clear whether this feeling continues to the present. The entry can therefore be classified as now."}, "sentence_type": "Negative declarative sentence", "pos": {"nouns": {"Situation": 1, "Fear": 1}, "verbs": {"I don't know": 1, "I feel": 1, "be": 1, "have": 1}, "adjectives": {"A lot": 1}, "adverbs": {"Land": 2, "That's good": 1}, "numerals": {"33": 1}}}, {"content": "I'm in a state of panic, and I'm going crazy.", "word_count": 10, "label": "", "sentence_structure": "Subject鈥搗erb鈥揷omplement", "subjective_objective": {"answer": "Subjective", "rationale": "Because the sense of extreme panic and imminent madness is the subjective feeling inside the patient, not the objective reality of the outside world. This subjective emotional description reflects the patient鈥檚 state of mind and experience."}, "sentence_sentiment": {"answer": "Negative", "rationale": "Because the words \"extreme panic\" and \"crazy\" in the entries have negative feelings."}, "language_style": {"answer": "Calm and direct", "rationale": "The language style of the entry is relatively direct and concise, with no excessive rhetoric or emotional rendering, and is based on objectivity, fact, and better suited to the professional requirements of psychological questionnaires."}, "time_dimension": {"answer": "Present", "rationale": "The entry describes a state of emotion that may emerge in the future, the key words being \"I am in a state of extreme panic\" and \"I seem to be going crazy\", all pointing to situations that have not yet occurred and that may occur in the future. So the judgment is based on the future."}, "sentence_type": "Affirmative declarative sentence", "pos": {"verbs": {"Panic": 1, "It looks like": 1, "Crazy": 1}, "adjectives": {"Extreme": 1}, "numerals": {"34": 1}, "pronouns": {"I": 1}}}, {"content": "My digestive condition.", "word_count": 6, "label": "", "sentence_structure": "Subject鈥揷opula鈥損redicative", "subjective_objective": {"answer": "Subjective", "rationale": "Because the symptoms of indigestion are personally experienced by the patient and are subjective."}, "sentence_sentiment": {"answer": "Negative", "rationale": "Because the \"indigestion\" symptoms in the entries are negative."}, "language_style": {"answer": "Tactful and warm", "rationale": "The entry is more proficient and interactive in its expression of conversation in a conversation with a voice of enquiry and concern, rather than a direct statement or order. At the same time, the term \"indigestion\" is more professional and is not described in too popular or exaggerated language, making the questionnaire more scientific and rigorous."}, "time_dimension": {"answer": "Present", "rationale": "The entry asks about the current state of the person being tested, and the keyword is \"my indigestion\", which indicates that the entry is an investigation of the existing experience or feelings of the person being tested."}, "sentence_type": "Negative declarative sentence", "pos": {"nouns": {"Indigestion": 1}, "verbs": {"have": 1, "of": 1}, "numerals": {"35": 1}}}, {"content": "My heart beats faster than usual.", "word_count": 10, "label": "", "sentence_structure": "Subject鈥揷opula鈥損redicative", "subjective_objective": {"answer": "Subjective", "rationale": "Since heart beats are the individual biological experience and perception of the patient, they are subjective."}, "sentence_sentiment": {"answer": "Neutral", "rationale": "Because the entry describes a biological phenomenon, with no obvious emotional colour."}, "language_style": {"answer": "Tactful and warm", "rationale": "The entry uses more euphemistic language, \"sometimes\" which means that things are not frequent, while \"fast\" suggests that it may be physical discomfort, rather than deliberately exaggerating or causing panic. Such language style is more friendly and acceptable and can lead respondents to more active and truthful answers."}, "time_dimension": {"answer": "Present", "rationale": "The keywords are \"sometimes\" and \"more than usual\" and they all point to the condition of the person who was tested in reply to the questionnaire, so the entry indicates the current situation."}, "sentence_type": "Exclamatory sentence", "pos": {"nouns": {"Heart rate": 1, "Normal": 1}, "verbs": {"- Yeah": 1, "Come on": 1}, "adverbs": {"Sometimes": 1}, "numerals": {"36": 1}}}, {"content": "37. I have a heavier headache and a sense of conceit.", "word_count": 13, "label": "", "sentence_structure": "Subject鈥搗erb鈥揷omplement", "subjective_objective": {"answer": "Subjective", "rationale": "Because \"dumb and faint\" is the patient's subjective feeling."}, "sentence_sentiment": {"answer": "Negative", "rationale": "Because \"dumber and faint\" is a symptom of physical discomfort, which is usually considered negative."}, "language_style": {"answer": "Calm and direct", "rationale": "The entry \"Cool and Direct\" describes the symptoms that may arise in the person under investigation, is directly explicit and is not qualified by any rhetorical or emotional techniques, and therefore can be judged as being calm and straightforward."}, "time_dimension": {"answer": "Present", "rationale": "The entry describes the current symptoms of the testee, and the temporal dimension is now judged."}, "sentence_type": "Negative declarative sentence", "pos": {"nouns": {"I'm just a little dizzy": 1, "Fizziness": 1}, "verbs": {"have": 1}, "adjectives": {"Heavy": 1}, "numerals": {"37": 1}}}, {"content": "I've got a lot of shaking in my hands. People can see it.", "word_count": 12, "label": "", "sentence_structure": "Subject鈥揷opula鈥損redicative", "subjective_objective": {"answer": "Subjective", "rationale": "Because \"I'm shaking my hands\" and \"someone can easily detect\" are subjective feelings and perceptions of patients."}, "sentence_sentiment": {"answer": "Negative", "rationale": "Because it depicts the shaking of hands and points to the ease with which others can be perceived, it implies an emotional feeling of self-confidence or social anxiety, and therefore a negative emotional colour."}, "language_style": {"answer": "Calm and direct", "rationale": "The language style of the entry is more objective, direct, with no excessive rhetorical or emotional rendering, and it is directly asked if the person surveyed had a handshake and indicates that it is easily discovered."}, "time_dimension": {"answer": "Past", "rationale": "The keywords \"sweet\" and \"sweetly\" indicate that this has happened and has affected others, so the dimension of time is the past."}, "sentence_type": "Negative declarative sentence", "pos": {"nouns": {"I": 1, "Shake your hands": 1, "others": 1, "It's easy to detect": 1}, "verbs": {"# Shakes #": 1, "Found": 1}, "adjectives": {"That's great": 1}, "numerals": {"38": 1}}}, {"content": "39. My appetite is as good as usual.", "word_count": 11, "label": "", "sentence_structure": "Subject鈥揷opula鈥損redicative", "subjective_objective": {"answer": "Objective", "rationale": "Because \"attendance\" and \"good\" are objective facts that can be quantified and do not involve subjective factors such as the mood, attitude, etc. of the patient."}, "sentence_sentiment": {"answer": "Neutral", "rationale": "Because the entry describes the factual situation and does not contain any emotional colours."}, "language_style": {"answer": "Tactful and warm", "rationale": ""}, "time_dimension": {"answer": "Present", "rationale": ""}, "sentence_type": "Affirmative declarative sentence", "pos": {"nouns": {"-Adulge": 1, "Normal": 1}, "verbs": {"And": 1, "Same thing": 1, "Okay": 1}, "adverbs": {"Call": 1}}}, {"content": "I had to force myself to eat, even a little bit.", "word_count": 24, "label": "", "sentence_structure": "Subject鈥搗erb鈥揷omplement", "subjective_objective": {"answer": "Subjective", "rationale": "Because \"have to\" and \"have to\" are feelings and attitudes of the patient himself, it is a subjective examination."}, "sentence_sentiment": {"answer": "Negative", "rationale": "Because \"forced to eat\" is an unfree, uncomfortable experience, with a negative emotional color."}, "language_style": {"answer": "Calm and direct", "rationale": "This entry is a straightforward, concise expression of the question whether the person surveyed has \"forced feeding\" in his or her diet, is neutral and does not use excessive rhetorical or emotional terms. This expression helps to ensure accuracy and reliability of the questionnaire."}, "time_dimension": {"answer": "Present", "rationale": "In conclusion, the time dimension of the entry is now."}, "sentence_type": "Negative declarative sentence", "pos": {"nouns": {"I": 1, "self": 1, "Rice": 1, "A little bit": 1, "Something": 1}, "verbs": {"Forced": 2, "Eat": 1, "be": 1}, "adjectives": {"General": 1}, "adverbs": {"I had to": 1}, "numerals": {"40": 1}}}, {"content": "41. I am as energetic as I am normally.", "word_count": 12, "label": "", "sentence_structure": "Subject鈥揷opula鈥損redicative", "subjective_objective": {"answer": "Objective", "rationale": "Because \"energy\" is a state of physical activity that can be measured, and it doesn't need to be assessed by the patient's subjective feelings."}, "sentence_sentiment": {"answer": "Neutral", "rationale": "Because the entry does not have any emotional color."}, "language_style": {"answer": "Calm and direct", "rationale": "The basis for the determination: the purpose of this article is simple and clear, not too much adoration or emotional, but rather a direct statement of the person interviewed in a way that gives him an objective and direct sense."}, "time_dimension": {"answer": "Present", "rationale": "Now the entry is asking what the testee feels like at the moment, and the keyword \"I feel as usual\" indicates that it is a description of the current state, so the temporal dimension is now."}, "sentence_type": "Affirmative declarative sentence", "pos": {"nouns": {"I": 1, "Normal": 1, "- The energy": 1, "鈾?Full of 鈾?: 1}, "verbs": {"I feel": 1, "Same": 1}, "adverbs": {"Same thing": 1}, "numerals": {"41": 1}}}, {"content": "42. For most of the time, I was tired.", "word_count": 12, "label": "", "sentence_structure": "Subject鈥揷opula鈥損redicative", "subjective_objective": {"answer": "Subjective", "rationale": "Because \"fearing fatigue\" is a subjective experience."}, "sentence_sentiment": {"answer": "Negative", "rationale": "Because \"fearing fatigue\" is a negative emotional experience."}, "language_style": {"answer": "Calm and direct", "rationale": "The entry directly asked the respondents whether they were tired for most of the time, were not pediculated using any rhetorical or emotional colours, and were more calm and objective."}, "time_dimension": {"answer": "Present", "rationale": "The entry describes the current state of the person's sense, and the keyword \"mostly\" indicates that it is a state of continuity and therefore the judgement is now."}, "sentence_type": "Affirmative declarative sentence", "pos": {"nouns": {"Most of the time": 1, "Weary": 1}, "verbs": {"I feel": 1}, "adverbs": {"Large": 1, "Time": 1, "Department": 1, "None": 1, "Strength": 1}, "numerals": {"42": 1}}}, {"content": "43. My sexual desire is the same as before the disease.", "word_count": 9, "label": "", "sentence_structure": "Subject鈥揷opula鈥損redicative", "subjective_objective": {"answer": "Subjective", "rationale": "The information provided, \"sexuality\", is a subjective feeling and experience of the patient, and the view of this questionnaire entry is therefore subjective."}, "sentence_sentiment": {"answer": "Neutral", "rationale": "Because the entry does not have any emotional color."}, "language_style": {"answer": "Tactful and warm", "rationale": "The entry is presented in a more euphemistic and moderate manner to avoid direct questioning or touching of sensitive issues that may exist in the interviewee, while respecting the personal privacy and feelings of the interviewee."}, "time_dimension": {"answer": "Past", "rationale": "The entry indicates the status or situation of the person before the question is answered and therefore the test is judged to be past."}, "sentence_type": "Affirmative declarative sentence", "pos": {"nouns": {"Questionnaire Entry": 1, "Sexuality": 1, "Before you get sick": 1}, "verbs": {"Analysis": 1, "Same thing": 1}, "numerals": {"43": 1}}}, {"content": "I lost my sexual appetite after the disease.", "word_count": 12, "label": "", "sentence_structure": "Subject鈥揷opula鈥損redicative", "subjective_objective": {"answer": "Subjective", "rationale": "Because \"total loss of sexuality\" is a patient's own feeling and experience, and it's subjective."}, "sentence_sentiment": {"answer": "Negative", "rationale": "Because \"I lost my sexuality completely after the disease\" is negative, expressing physical discomfort and psychological distress."}, "language_style": {"answer": "Calm and direct", "rationale": "The entry directly asked the respondents whether there had been problems of sexual defunctness following the illness, which had not been accompanied by excessive rhetorical or emotional rendering, and was more balanced and objective."}, "time_dimension": {"answer": "Past", "rationale": ""}, "sentence_type": "Negative declarative sentence", "pos": {"nouns": {"After the illness": 1, "Sexuality": 1, "Total": 1, "Loss": 1}, "verbs": {"have": 1, "Loss": 1}, "adjectives": {"Total": 1}, "numerals": {"44": 1}}}, {"content": "I am too worried about the symptoms of my body.", "word_count": 9, "label": "", "sentence_structure": "Subject鈥搗erb鈥搊bject", "subjective_objective": {"answer": "Subjective", "rationale": "Because \"excessive fear\" is the patient's subjective feeling and attitude."}, "sentence_sentiment": {"answer": "Negative", "rationale": "Because the \"too much to worry\" act in the entry is negative."}, "language_style": {"answer": "Tactful and warm", "rationale": "The expression is more personal and interactive, in a gentle and inviting tone, rather than in direct statements or orders. Moreover, the use of the word \"excessive\" suggests that the problem may be related to anxiety and helps to guide respondents to reflect on their psychological state."}, "time_dimension": {"answer": "Present", "rationale": "The keyword \"excessive fear\" indicates that it is a state or a feeling, while \"physical symptoms\" concerns physical experience, so the entry focuses on the behaviour and feelings of the person being tested."}, "sentence_type": "Negative declarative sentence", "pos": {"nouns": {"Body": 1, "Symptoms": 1}, "verbs": {"That's too much": 1, "I'm worried": 1}, "adverbs": {"That's too much": 1}, "numerals": {"45": 1}, "pronouns": {"I": 1}}}, {"content": "46. I am particularly concerned about the functioning of the body.", "word_count": 10, "label": "", "sentence_structure": "Subject鈥搗erb鈥揷omplement", "subjective_objective": {"answer": "Subjective", "rationale": "Because \"special attention\" is the patient's subjective attitude and feelings."}, "sentence_sentiment": {"answer": "Neutral", "rationale": "Since the entry does not express positive or negative feelings, attitudes, behaviour, purpose or quality, it is merely a narrative description of one of the personal characteristics of the person under investigation."}, "language_style": {"answer": "Tactful and warm", "rationale": "The language style of the entry is more moderate and sensitive to the individual ' s feelings and needs, using a means of questioning rather than ordering or making direct statements, giving the impression of being friendly, caring and more acceptable and willing to cooperate with the investigation."}, "time_dimension": {"answer": "Present", "rationale": ""}, "sentence_type": "Affirmative declarative sentence", "pos": {"nouns": {"Functions": 1}, "verbs": {"Attention": 1, "Special": 1}, "adverbs": {"Special": 1}, "numerals": {"46": 1}, "pronouns": {"I": 1}}}, {"content": "My problems are caused by some serious body disease.", "word_count": 18, "label": "", "sentence_structure": "Subject鈥揷opula鈥損redicative", "subjective_objective": {"answer": "Objective", "rationale": "Because \"some kind of heavy body disease\" is the patient's objective condition, and it's not the patient's subjective feeling or idea."}, "sentence_sentiment": {"answer": "Negative", "rationale": "Because the entry refers to \"serious body disease\", this is usually considered a negative situation or experience."}, "language_style": {"answer": "Tactful and warm", "rationale": "The entry, which was dialogue in a moderate and understanding tone, showed the researchers ' concern and sympathy for the physical condition of the person under investigation, avoiding overly direct or frivolous representations and enhancing the acceptance of the person under investigation."}, "time_dimension": {"answer": "Past", "rationale": ""}, "sentence_type": "Affirmative declarative sentence", "pos": {"nouns": {"Problem": 1, "Body disease": 1, "Reason": 1}, "verbs": {"be": 1, "Cause": 1}, "adjectives": {"Serious": 1}, "numerals": {"47": 1}, "pronouns": {"I": 1}}}, {"content": "My body is broken, and it's rotting.", "word_count": 12, "label": "", "sentence_structure": "Subject鈥搗erb鈥揷omplement", "subjective_objective": {"answer": "Subjective", "rationale": "Because \"body is broken, it's rotting\" is a subjective feeling and description of the state of your body."}, "sentence_sentiment": {"answer": "Negative", "rationale": "Because the description \"body is broken, it's rotting\" in the entry is negative."}, "language_style": {"answer": "Tactful and warm", "rationale": "The language style of this entry is relatively warm. It uses a more intuitive and descriptive expression of the subject of the investigation, which does not convey a strong emotion or evaluation directly, but rather presents the facts in an objective manner. It makes the person under investigation more comfortable and respectful and more likely to be willing to share their true feelings."}, "time_dimension": {"answer": "Past", "rationale": ""}, "sentence_type": "Negative declarative sentence", "pos": {"nouns": {"Body": 1, "Inside": 1, "Rotten": 1}, "verbs": {"It's gone": 1}, "numerals": {"48": 1}}}, {"content": "49. I can recover once I have a good rest.", "word_count": 15, "label": "", "sentence_structure": "Subject鈥搗erb鈥揷omplement", "subjective_objective": {"answer": "Subjective", "rationale": "Because the possibility of a full recovery requires a medical examination and treatment, and patients鈥?subjective ideas play an important role here. While resting is a measure of help in restoring health, it is subjective to ensure that there is a full recovery."}, "sentence_sentiment": {"answer": "Positive", "rationale": "Because this entry expresses expectations and confidence in rehabilitation."}, "language_style": {"answer": "Tactful and warm", "rationale": "The entry expresses a belief or expectation, in a moderate and encouraging tone, that the person surveyed will feel that his recovery is possible. This is more acceptable and can inspire positive emotions and motivation."}, "time_dimension": {"answer": "Present", "rationale": "Now the entry describes the current state of the testee and the expected future, in which \"a good rest\" means the behaviour that is now being performed, and \"a complete recovery\" means the expectation of the future. So, in terms of time dimensions, the entry is now judged."}, "sentence_type": "Affirmative declarative sentence", "pos": {"nouns": {"I": 1, "Rehabilitation": 1}, "verbs": {"Yes": 1, "Full": 1, "Rehabilitation": 1}, "adjectives": {"Okay": 1}, "adverbs": {"A while": 1}, "numerals": {"49": 1}}}, {"content": "50. My illness is caused by the recent bad weather.", "word_count": 14, "label": "", "sentence_structure": "Subject鈥揷opula鈥損redicative", "subjective_objective": {"answer": "Subjective", "rationale": "Because \"the recent bad weather\" is an external factor, the patient believes that his or her illness is caused by such an external factor, which is the patient's subjective perception."}, "sentence_sentiment": {"answer": "Negative", "rationale": "Because the entry considers itself to be a disease caused by bad weather and has negative emotional connotations."}, "language_style": {"answer": "Calm and direct", "rationale": "The entry presents the link between disease and weather in a more objective and direct manner, with no excessive emotional rendering or euphemism."}, "time_dimension": {"answer": "Past", "rationale": ""}, "sentence_type": "Affirmative declarative sentence", "pos": {"nouns": {"Diseases": 1, "Organisation": 1}, "verbs": {"Okay": 1, "Cause": 1}, "adjectives": {"Most recently": 1, "Bad": 1}, "numerals": {"50": 1}, "pronouns": {"I": 1}}}, {"content": "51. My weight is decreasing.", "word_count": 8, "label": "", "sentence_structure": "Subject鈥搗erb鈥揷omplement", "subjective_objective": {"answer": "Subjective", "rationale": "This description concerns the weight of \"me\" and it is therefore viewed from the patient's subjective perspective."}, "sentence_sentiment": {"answer": "Positive", "rationale": "Because \"weight is diminishing\" is positive."}, "language_style": {"answer": "Tactful and warm", "rationale": "The entry uses positive tone and expresses positive changes, namely, weight loss, but does not directly ask about the other party鈥檚 physical condition or express concerns, but rather leads the other to share their own situation in a gentle way. Such language style is more friendly and interactive, which makes the interviewee more comfortable and relaxed."}, "time_dimension": {"answer": "Present", "rationale": ""}, "sentence_type": "Affirmative declarative sentence", "pos": {"nouns": {"Weight": 1, "Mitigation": 1}, "verbs": {"In the process of": 1}, "adverbs": {"Positive": 1}}}, {"content": "52. My weight can be said to have fallen considerably.", "word_count": 12, "label": "", "sentence_structure": "Subject鈥揷opula鈥損redicative", "subjective_objective": {"answer": "Objective", "rationale": "Because weight loss is an objective result that can be quantified, not the patient ' s feelings or perceptions."}, "sentence_sentiment": {"answer": "Positive", "rationale": "Because \"weight loss\" is usually considered a positive result."}, "language_style": {"answer": "Tactful and warm", "rationale": "The entry describes the change in the weight of the person interviewed in a positive way and uses the expression \"may be\" to reduce the likelihood of causing concern or criticism. The overall feeling of affection and encouragement reflects the principle that the questionnaire should be designed so as to avoid excessive direct or stabbing of the person interviewed."}, "time_dimension": {"answer": "Present", "rationale": "The entry is for the current situation of the testee and the time dimension is now judged."}, "sentence_type": "Affirmative declarative sentence", "pos": {"nouns": {"Weight": 1}, "verbs": {"Down": 1, "Yeah": 1, "Say it": 1}, "adjectives": {"Many": 1}, "numerals": {"52": 1}, "pronouns": {"I": 1}}}]}

{"questionnaire_title": "Eisenke Personality Questionnaire (EPQ) (children aged 7-15)", "questionnaire_category": "Self-cognition", "item_count": 88, "emotion_shift_overall": "From positive to negative", "language_style_overall": "Neutral", "items": [{"content": "Do you like the many things that please you around?", "word_count": 16, "label": "", "sentence_structure": "Subject鈥搗erb鈥搊bject", "subjective_objective": {"answer": "Subjective", "rationale": "Like being patient subjective."}, "sentence_sentiment": {"answer": "Positive", "rationale": "Because the entries \"like\" and \"doing what makes you happy\" both express positive emotional attitudes."}, "language_style": {"answer": "Tactful and warm", "rationale": "The language style of the entry is more euphemistic, because it uses an inquiry-style tone and \"you\" as the dominant language. This expression is more accommodative and moderate, which better guides respondents to share their real thoughts and feelings, and increases the rate of recovery and the amount of information available."}, "time_dimension": {"answer": "Present", "rationale": "The entry asks how the testee feels and how he feels, and \"likes\" and \"there are many things that please you around\" point to the current experience."}, "sentence_type": "Interrogative sentence", "pos": {"nouns": {"Questionnaire Entry": 1, "Things": 2}, "verbs": {"Yeah": 1, "have": 1}, "adjectives": {"Many": 1, "It's a pleasure": 1}, "adverbs": {"Around": 1}, "numerals": {"One": 1}, "classifiers": {"Entry": 1}, "pronouns": {"you": 1}}}, {"content": "Are you angry?", "word_count": 5, "label": "", "sentence_structure": "Subject鈥搗erb鈥揷omplement", "subjective_objective": {"answer": "Subjective", "rationale": "Because \"freak\" is an emotional experience and attitude, a subjective one."}, "sentence_sentiment": {"answer": "Negative", "rationale": "Because \"freak\" is negative, the purpose of this article is negative."}, "language_style": {"answer": "Calm and direct", "rationale": "The entry is a simple and straightforward way of asking the other side whether it is prone to anger, and the language style is more direct without qualification, which is a direct expression of calmness.\""}, "time_dimension": {"answer": "Present", "rationale": "The entry asks about the person's current state or habits, i.e. whether he/she is often angry. The time dimension therefore determines now."}, "sentence_type": "Interrogative sentence", "pos": {"verbs": {"Love": 1, "Fan": 1}, "adjectives": {"Gas": 1}}}, {"content": "Do you like to hurt people you like?", "word_count": 11, "label": "", "sentence_structure": "Subject鈥損redicate", "subjective_objective": {"answer": "Subjective", "rationale": "Like being patient subjective."}, "sentence_sentiment": {"answer": "Negative", "rationale": "Because the \"harming someone you like\" act is negative."}, "language_style": {"answer": "Calm and direct", "rationale": "The entry is of a cold and direct language style, without any euphemism, warmness, strong tone and a sensitive and offensive question."}, "time_dimension": {"answer": "Present", "rationale": "Now the entry asks the testee about his current feelings and behavioral orientation, the keywords being \"like\" and \"harm\", all pointing to the situation."}, "sentence_type": "Interrogative sentence", "pos": {"nouns": {"you": 2, "person": 2, "Hurt": 1}, "verbs": {"Yeah": 4}, "numerals": {"3": 1}}}, {"content": "Have you ever been greedy of others?", "word_count": 10, "label": "", "sentence_structure": "Subject鈥損redicate", "subjective_objective": {"answer": "Subjective", "rationale": "Because \"growing someone cheap\" is a subjective attitude or psychological state, not an objective fact or result. An examination of this subjective attitude is the main objective of the entry."}, "sentence_sentiment": {"answer": "Negative", "rationale": "Because the \"growing for others\" in the entries is negative."}, "language_style": {"answer": "Tactful and warm", "rationale": "If researchers want more honest and direct answers, they can use the \"cool and direct\" language style. This style emphasizes facts and truth, usually in the form of questions, avoiding too many emotional or rhetorical techniques. For example, \"Have you ever been greedy about others?\" If researchers want more friendly and inclusive answers, then they can use the \"false and warm\" language style. This style focuses on communication and communication with the respondents, and usually uses some inviteive words, such as \"Show your views or experience.\" For example, \"Have you ever been greedy about others' prices? We can discuss this together.\""}, "time_dimension": {"answer": "Present", "rationale": "The entry is for the purpose of questioning the testee's past behaviour, the keyword being \"a greedy advantage\" and the act has already taken place in the past and is therefore judged to be past."}, "sentence_type": "Interrogative sentence", "pos": {"nouns": {"others": 1, "Cheap": 1}, "verbs": {"A greedy picture": 1}, "adverbs": {"Pass": 1}, "pronouns": {"you": 1}}}, {"content": "5. Do you answer questions almost always quickly when you talk to others?", "word_count": 22, "label": "", "sentence_structure": "Subject鈥搗erb鈥搊bject", "subjective_objective": {"answer": "Subjective", "rationale": "Because it's the patient's subjective attitude that's being examined."}, "sentence_sentiment": {"answer": "Positive", "rationale": "Because \"answers to other people's questions\" in this entry are social interactions, and \"almost always soon\" indicates that the person is willing to engage in communication and react quickly, and that behaviour is usually seen as positive."}, "language_style": {"answer": "Calm and direct", "rationale": "The entry is a direct question-and-answer dialogue and is simple and clear, and in a more calm and objective tone.\""}, "time_dimension": {"answer": "Present", "rationale": "The entry asks the testee about his current behavioral habits, the keywords being \"when talking to someone\" and \"almost always answering the questions of others very quickly\", all pointing to the behaviour that is now being performed."}, "sentence_type": "Interrogative sentence", "pos": {"nouns": {"Talk": 1, "person": 1, "Problem": 1}, "verbs": {"Always": 1, "Answer": 1, "Talk": 1}, "adjectives": {"Come on": 1}, "adverbs": {"Almost": 1}, "numerals": {"5": 1}}}, {"content": "Is it easy to get bored?", "word_count": 9, "label": "", "sentence_structure": "Subject鈥損redicate", "subjective_objective": {"answer": "Subjective", "rationale": "Because boredom is a subjective emotional experience."}, "sentence_sentiment": {"answer": "Negative", "rationale": "Because the \"easily bored\" attitude in the entries is negative."}, "language_style": {"answer": "Tactful and warm", "rationale": "The language style of the entry is more euphemistic, leading the interviewee to reflect on his emotional state, avoiding overly direct or subjective expressions. At the same time, using the term \"easy\" reduces the negative emotional profile of the problem and allows the interviewee to answer the questions more comfortably."}, "time_dimension": {"answer": "Present", "rationale": "The entry of the questionnaire asked about the current feelings and state of the subject, and the keyword \"always bored\" pointed to the behaviour or feeling that was currently being carried out."}, "sentence_type": "Interrogative sentence", "pos": {"nouns": {"Boring": 1}, "verbs": {"I feel": 1, "Easy": 1, "Boring": 1}, "adverbs": {"very": 1, "Easy": 1}, "numerals": {"6": 1, "you": 1}, "classifiers": {"individual": 1}}}, {"content": "Do you sometimes like to make some really sad jokes?", "word_count": 18, "label": "", "sentence_structure": "Subject鈥搗erb鈥搊bject", "subjective_objective": {"answer": "Subjective", "rationale": "It's the patient's subjective attitude to like to joke."}, "sentence_sentiment": {"answer": "Negative", "rationale": "Because the \"depressing joke\" in the entry is negative."}, "language_style": {"answer": "Tactful and warm", "rationale": "The basis for the judgement: 1. \"Sometimes\" means that this is an act that is not frequent, implying that the problem is not entirely negative or accusatory. 2. \"Do you like to joke about?\" The question is also euphemistic and does not directly use negative or critical terms. 3. Throughout the sentence, there is no strong emotional or subjective assessment, and the tone is relatively neutral and peaceful. Thus, the language style of the questionnaire is considered to be euphemistic."}, "time_dimension": {"answer": "Present", "rationale": "The entry asks if the person tested is now acting or attitudinally, \"likes to make some really sad jokes.\" So the time dimension is now."}, "sentence_type": "Interrogative sentence", "pos": {"nouns": {"Questionnaire": 1, "Entry": 1, "A joke": 1}, "verbs": {"Yeah": 1, "Open": 1, "Letting": 1, "Sad": 1}, "adjectives": {"Some": 1, "Indeed": 1}, "adverbs": {"Sometimes": 1}, "numerals": {"7": 1}}}, {"content": "Do you always do what others ask you to do?", "word_count": 14, "label": "", "sentence_structure": "Subject鈥搗erb鈥搊bject", "subjective_objective": {"answer": "Subjective", "rationale": "Because \"doing what others ask you to do immediately\" is a personal attitude and behavioural choice of the patient and is subjective."}, "sentence_sentiment": {"answer": "Negative", "rationale": "Because the \"always do as others order\" in the entry is negative and lacks autonomy."}, "language_style": {"answer": "Calm and direct", "rationale": "The entry is based on a clear question expressed in a direct, concise tone, without any qualifier or emotional vocabulary. It simply asks whether the respondents are often following the instructions of others and do not attempt to influence or guide their answers. This type of questionnaire is usually considered to be more objective, effective and suitable for scientific research or other demanding situations."}, "time_dimension": {"answer": "Present", "rationale": "The keywords are \"always\" and \"right away\", which implies that the subjects are describing their behaviour and that it may be frequent. Thus, the temporal dimension of the article is now judged."}, "sentence_type": "Interrogative sentence", "pos": {"nouns": {"you": 1, "others": 1, "Yes": 1}, "verbs": {"Do it": 1, "Always": 1, "Press it now": 2}, "adverbs": {"Go on": 1}, "numerals": {"8": 1, "One": 1}}}, {"content": "Do you prefer being alone to playing with other kids?", "word_count": 21, "label": "", "sentence_structure": "Subject鈥搗erb鈥搊bject", "subjective_objective": {"answer": "Subjective", "rationale": "The question asked about the patient ' s personal preference to be more alone or to play with other children, which was a subjective assessment."}, "sentence_sentiment": {"answer": "Negative", "rationale": "Because the entry \"I prefer to be alone rather than playing with other kids\" is negative."}, "language_style": {"answer": "Calm and direct", "rationale": "This is more objective, concise and clear, making it easier for the respondents to understand and answer questions."}, "time_dimension": {"answer": "Present", "rationale": "The keyword \"I prefer to be alone\" and \"I don't want to play with other kids\" indicates the preferences or preferences of the testees, which is a description of the current state. So the temporal dimension is now."}, "sentence_type": "Interrogative sentence", "pos": {"nouns": {"you": 1, "Alone": 1, "person": 1, "Kids": 1, "Dow": 1, "Play": 1}, "verbs": {"I'd rather": 1, "I don't want to": 1, "in": 1, "Together": 1, "Play": 2}, "adverbs": {"More": 1}, "numerals": {"9": 1}}}, {"content": "Do you have a lot of ideas that take you to sleep?", "word_count": 18, "label": "", "sentence_structure": "Subject鈥搗erb鈥搊bject", "subjective_objective": {"answer": "Subjective", "rationale": "Because it's a question of whether the patient has a subjective feeling of sleeping problems."}, "sentence_sentiment": {"answer": "Negative", "rationale": "Because the \"many thoughts take your mind\" act is negative."}, "language_style": {"answer": "Calm and direct", "rationale": "The entry is asked explicitly, in the form of a direct question, whether the interviewees have many ideas that prevent them from sleeping, and is more concise and not overly pedigreed or emotional."}, "time_dimension": {"answer": "Present", "rationale": "The keywords \"many thoughts occupy your mind\" and \"not sleeping\" point to the current situation, so the temporal dimension of the issue is now."}, "sentence_type": "Interrogative sentence", "pos": {"nouns": {"Thinking": 2, "Brain": 1, "Sleep": 1}, "verbs": {"Occupation": 1, "鈾?And I'm gonna be 鈾?: 1, "Yes": 1, "Sleep": 1}, "adverbs": {"A lot": 1}, "numerals": {"10": 1}, "pronouns": {"you": 1}}}, {"content": "Have you ever violated regulations at school?", "word_count": 11, "label": "", "sentence_structure": "Subject鈥搗erb鈥搊bject", "subjective_objective": {"answer": "Subjective", "rationale": "The question of whether or not to violate regulations is a personal experience and behaviour of the patient is subjective."}, "sentence_sentiment": {"answer": "Negative", "rationale": "Because the action in the entry \"breaches the regulations\" is negative."}, "language_style": {"answer": "Calm and direct", "rationale": "The entry uses direct and clear language to ask the interviewees whether they have violated the rules in school, without excessive rhetoric or emotional rendering, and with more objective and calm tone."}, "time_dimension": {"answer": "Past", "rationale": "The entry is for questioning the behaviour of the person under examination during school, and the temporal dimension is a past."}, "sentence_type": "Interrogative sentence", "pos": {"nouns": {"you": 1, "School": 1, "Regulations": 1}, "verbs": {"Violations": 1}, "numerals": {"11": 1, "Tsang": 1}}}, {"content": "Do you like the other kids being scared of you?", "word_count": 11, "label": "", "sentence_structure": "Subject鈥搗erb鈥搊bject", "subjective_objective": {"answer": "Subjective", "rationale": "This is a subjective examination of the patient ' s perception and feelings of his image in the eyes of others."}, "sentence_sentiment": {"answer": "Negative", "rationale": "Because \"like people are afraid of you\" is negative."}, "language_style": {"answer": "Tactful and warm", "rationale": "The language style of the entry is more euphemistic, rather than asking whether the other child is afraid of him, but rather directing the respondents to think and answer questions in a light-handed manner, which reduces resistance and makes them more likely to have real and effective data."}, "time_dimension": {"answer": "Present", "rationale": "The entry is for the current situation of the subject, and the keywords are \"like\" and \"fear,\" which describe the behaviour or state that is occurring."}, "sentence_type": "Interrogative sentence", "pos": {"nouns": {"Kids": 1, "you": 1}, "verbs": {"Yeah": 1, "Yes": 1}, "numerals": {"12": 1}}}, {"content": "Are you a lively man?", "word_count": 5, "label": "", "sentence_structure": "Subject鈥搗erb鈥揷omplement", "subjective_objective": {"answer": "Subjective", "rationale": "Because \"silent\" is the subjective perception and evaluation of the character of the patient."}, "sentence_sentiment": {"answer": "Positive", "rationale": "Because \"vio\" is a positive word."}, "language_style": {"answer": "Tactful and warm", "rationale": "The language style of the entry is more euphemistic, and it uses an open question to guide the interviewee to think and share their feelings. At the same time, the question does not contain any tone of evaluation or blame, but rather questions in a friendly and caring manner."}, "time_dimension": {"answer": "Present", "rationale": "The keyword \"Are you lively?\" indicates that it's asking the testee's current state or character, so it's judged to be \"now\"."}, "sentence_type": "Interrogative sentence", "pos": {"verbs": {"very": 1, "Lively": 1}, "adjectives": {"Lively": 1}, "adverbs": {"very": 1}}}, {"content": "Is there a lot that bothers you?", "word_count": 10, "label": "", "sentence_structure": "Subject鈥損redicate", "subjective_objective": {"answer": "Subjective", "rationale": "Because \"fear\" is a subjective emotional feeling."}, "sentence_sentiment": {"answer": "Negative", "rationale": "Because the entries contain negative words of \"fear,\" they express a negative emotion."}, "language_style": {"answer": "Tactful and warm", "rationale": ""}, "time_dimension": {"answer": "Present", "rationale": "The entry asks if the person tested has many troubling matters at the moment, and the time dimension is now judged."}, "sentence_type": "Interrogative sentence", "pos": {"nouns": {"Things": 1, "Trouble": 1}, "verbs": {"鈾?And I'm gonna be 鈾?: 1, "have": 1}, "adverbs": {"Many": 1, "- You're not": 1}, "numerals": {"14": 1}}}, {"content": "Do you like killing animals in biology class?", "word_count": 13, "label": "", "sentence_structure": "Subject鈥搗erb鈥搊bject", "subjective_objective": {"answer": "Subjective", "rationale": "Because it is a manifestation of personal feelings and attitudes that like or dislikes killing animals, it is subjective."}, "sentence_sentiment": {"answer": "Negative", "rationale": "Because \"like to kill animals\" is negative."}, "language_style": {"answer": "Calm and direct", "rationale": "The entry is used in a calm and straightforward language, without excessive rhetorical or emotional rendering."}, "time_dimension": {"answer": "Past", "rationale": "The entry asks about the testee ' s past personal preferences and behaviour and therefore judge the past."}, "sentence_type": "Interrogative sentence", "pos": {"nouns": {"Biological classes": 1, "Animals": 1}, "verbs": {"Yeah": 1, "Kill": 1}, "numerals": {"15": 1}}}, {"content": "Have you ever taken anything from anyone?", "word_count": 21, "label": "", "sentence_structure": "Subject鈥搗erb鈥搊bject", "subjective_objective": {"answer": "Objective", "rationale": "Because this question is concerned with the history of the patient鈥檚 behaviour, whether there has been theft, not the patient鈥檚 subjective attitude or emotional state. This is therefore an objective examination."}, "sentence_sentiment": {"answer": "Negative", "rationale": "Because the entry \"Take something from someone else\" is negative."}, "language_style": {"answer": "Calm and direct", "rationale": "The language structure of the article is simple, it is not based on a terminological or emotional qualifier, and it is more objective and direct. It asks the interviewee whether he has taken something from someone else, which makes the questionnaire more readable and accurate."}, "time_dimension": {"answer": "Past", "rationale": ""}, "sentence_type": "Interrogative sentence", "pos": {"nouns": {"Something": 1, "A needle": 1, "Buttons": 1}, "verbs": {"Take it": 1, "Here": 1}, "numerals": {"6": 1}, "classifiers": {"individual": 2}, "pronouns": {"you": 1}}}, {"content": "Do you have many friends?", "word_count": 7, "label": "", "sentence_structure": "Subject鈥損redicate", "subjective_objective": {"answer": "Subjective", "rationale": "The issue is the subjective perception and perception of the patient."}, "sentence_sentiment": {"answer": "Neutral", "rationale": "The entry is simply an inquiry into how many friends the interviewees have and does not express any emotional tendencies."}, "language_style": {"answer": "Tactful and warm", "rationale": "The entry is more adept and interactive when it is spoken in a gentle, inquisitive manner, rather than in direct statements or orders. At the same time, the term \"many\" uses a comparative, neither too definite nor too negative, leaving space for reflection and answers."}, "time_dimension": {"answer": "Present", "rationale": "Now, the entry asks the testee about the current social situation, and the keyword \"many,\" \"friend\" suggests that this is a description of the current state, so it's now."}, "sentence_type": "Interrogative sentence", "pos": {"nouns": {"you": 1, "Many": 1, "friends": 1}, "verbs": {"have": 1}, "numerals": {"17": 1}}}, {"content": "Did you feel 鈥渨orried鈥?for no reason?", "word_count": 14, "label": "", "sentence_structure": "Subject鈥搗erb鈥搊bject", "subjective_objective": {"answer": "Subjective", "rationale": "Because the question is whether the patient has a feeling or emotional experience, it is the patient ' s subjective perspective."}, "sentence_sentiment": {"answer": "Negative", "rationale": "Because \"suffering\" in the entry is a negative expression of emotion, it can be judged as negative."}, "language_style": {"answer": "Calm and direct", "rationale": "The entry directly asked the interviewees whether they had experienced a traumatic experience without reason, and did not use too many qualifiers or emotionally strong language, so the expression was more calm and straightforward."}, "time_dimension": {"answer": "Present", "rationale": "The entry \"if it feels unprovoked\" indicates that it is a state of emotion that is currently being performed and therefore is judged as now."}, "sentence_type": "Interrogative sentence", "pos": {"nouns": {"Questionnaire": 1, "Entry": 1}, "verbs": {"I think": 1}, "adjectives": {"For no reason": 1, "It's hard": 1}}}, {"content": "Do you like to tease animals sometimes?", "word_count": 10, "label": "", "sentence_structure": "Subject鈥搗erb鈥搊bject", "subjective_objective": {"answer": "Subjective", "rationale": "The way you like to tease animals is a subjective attitude or hobby, so the answer is \"subjective.\""}, "sentence_sentiment": {"answer": "Positive", "rationale": "Because the \"like to tease animals\" behavior in the entry is positive."}, "language_style": {"answer": "Tactful and warm", "rationale": "The language style of the entry is more euphemistic, and the dialogue is conducted in a gentle and inviting tone, asking if the interviewees prefer to tease animals, which is more personal and interactive."}, "time_dimension": {"answer": "Present", "rationale": "The entry is for the purpose of asking the testee about his current behaviour or attitude and does not contain information about past or future time, and therefore is judged as \"now\"."}, "sentence_type": "Interrogative sentence", "pos": {"nouns": {"Animals": 1, "Time": 1}, "verbs": {"Yeah": 1, "- That's funny": 1}, "adverbs": {"Sometimes": 1}}}, {"content": "Did you pretend not to hear what people call you?", "word_count": 16, "label": "", "sentence_structure": "Subject鈥搗erb鈥搊bject", "subjective_objective": {"answer": "Subjective", "rationale": "Because it is a question of whether the patient has had any subjective behaviour, it is the subjective thoughts and behaviour of the patient that is examined."}, "sentence_sentiment": {"answer": "Negative", "rationale": "Because the \"fakeless\" act in the entry is negative."}, "language_style": {"answer": "Tactful and warm", "rationale": "The reasons for this are as follows: 1. The question is expressed in question, not in an order or in a direct statement, which makes the respondent more willing to answer. 2. The question is used as a proxy for \"others\", avoiding issues directly related to personal privacy and more respectful and sensitive to the person under investigation. 3. The question is more simple, day-to-day, and is more gentle, and does not put pressure or discomfort on the person under investigation."}, "time_dimension": {"answer": "Present", "rationale": "The entry now asks if the person who was tested had chosen to act as if he or she had not heard him or her, and thus judges it now."}, "sentence_type": "Interrogative sentence", "pos": {"nouns": {"person": 1, "Yes": 1}, "verbs": {"have": 1, "Please": 1, "Load": 1, "Listen": 1, "Pass": 1}, "adverbs": {"not": 1}, "numerals": {"20": 1}, "classifiers": {"individual": 1}, "pronouns": {"you": 1}}}, {"content": "Do you like to explore in an ancient haunted cave?", "word_count": 16, "label": "", "sentence_structure": "Subject鈥搗erb鈥搊bject", "subjective_objective": {"answer": "Subjective", "rationale": "It is the patient's subjective attitude to like or dislike adventures in ancient haunted caves."}, "sentence_sentiment": {"answer": "Negative", "rationale": "Because it is positive to like adventures, but it may have a security risk and a fear in an ancient haunted cave, the emotional colour of the article is negative."}, "language_style": {"answer": "Tactful and warm", "rationale": "The entry raises questions in question, not direct orders or statements, and uses the adjectives \"old\" and \"ghost\" to increase the mystery and appeal of the situation. This expression is more a source of curiosity and willingness to participate in the investigation and can therefore be considered a euphemism of language style."}, "time_dimension": {"answer": "Present", "rationale": "The entry now asks the extent to which the person tested is interested in a particular situation (i.e., exploring in an ancient haunted cave), without reference to past or future circumstances. The temporal dimension is therefore now judged."}, "sentence_type": "Interrogative sentence", "pos": {"nouns": {"Old": 1, "Ghosts": 1, "The cave": 1, "Adventure": 1}, "verbs": {"Yeah": 1, "In... in... in... in... in... in... in... in... in... in... in... in... in... in... in... in... in... in... in... in... in... in... in... in... in... in... in... in... in... in... in... in... in... in... in... in... in... in... in... in... in... in... in... in... in... in... in... in... in... in... in... in... in... in... in... in... in... in... in... in... in... in... in... in... in... in... in... in... in... in... in... in... in... in... in... in... in... in... in... in... in... in... in... in... in... in... in... in... in... in... in... in... in... in... in... in... in... in... in... in... in... in... in... in... in... in... in... in... in... in... in... in... in... in... in... in... in... in": 1}, "adjectives": {"Old": 1, "Ghosts": 1}, "numerals": {"21": 1}}}, {"content": "22. Do you always feel like life is very boring?", "word_count": 11, "label": "", "sentence_structure": "Subject鈥搗erb鈥搊bject", "subjective_objective": {"answer": "Subjective", "rationale": "Because \"very boring\" is a subjective feeling or emotional experience that reflects the subjective perception of a patient's own life."}, "sentence_sentiment": {"answer": "Negative", "rationale": "Because the \"feeling life very boring\" mood in the entry is negative."}, "language_style": {"answer": "Calm and direct", "rationale": "The entry clearly expresses the message that it wants to understand in a direct question, and does not use excessive rhetorical or emotional colours, which are more objective and balanced. Thus, its language style can be described as \"cool and straight\"."}, "time_dimension": {"answer": "Present", "rationale": "The entry asks what the testee feels now, and the time dimension is now judged."}, "sentence_type": "Interrogative sentence", "pos": {"nouns": {"Life": 1, "Tastes": 1}, "verbs": {"Feel": 1, "Regular": 1, "Tasteless": 1}, "adjectives": {"Very": 1}, "adverbs": {"Regular": 1}, "numerals": {"22": 1}}}, {"content": "You're more into fighting than most kids?", "word_count": 14, "label": "", "sentence_structure": "Subject鈥搗erb鈥搊bject", "subjective_objective": {"answer": "Objective", "rationale": "Because it is an objective fact that can be quantified and can be tested by statistical or other means, whether or not it is more likely than most children to fight."}, "sentence_sentiment": {"answer": "Negative", "rationale": "Because the behavior of \"challenge\" in the entries is offensive and conflicting, and therefore negative."}, "language_style": {"answer": "Calm and direct", "rationale": "This is a description that is appropriate for research and for obtaining accurate and accurate data."}, "time_dimension": {"answer": "Present", "rationale": "The entry asks the testee about his current state and performance, and the time dimension judge it as now. The keyword \"more than most children, fight\" indicates a comparison of a behaviour or character characteristic, and points to the current situation."}, "sentence_type": "Interrogative sentence", "pos": {"nouns": {"Questionnaire Entry": 1, "Kids": 1, "Shut up": 1, "Fight": 1}, "verbs": {"Comparison": 1, "Love": 1, "Louder": 1, "Fight": 1}, "adjectives": {"Most of them": 1}, "numerals": {"23": 1}, "pronouns": {"you": 1, "More": 1}}}, {"content": "- You always finish your homework and go to play?", "word_count": 15, "label": "", "sentence_structure": "Subject鈥損redicate", "subjective_objective": {"answer": "Objective", "rationale": "The basis for the determination is that whether or not to perform homework and play is a fact that can be observed and verified, not the patient鈥檚 subjective thoughts or feelings."}, "sentence_sentiment": {"answer": "Positive", "rationale": "Because the behaviour in the entry is positive, it expresses the importance of completing homework and encourages people to resume recreational activities after completing their studies."}, "language_style": {"answer": "Calm and direct", "rationale": "The entry is in an objective and concise manner, asking the respondents whether they will go to play after completing their homework, without using excessive rhetorical techniques or emotional rendering, and in a more straightforward manner."}, "time_dimension": {"answer": "Present", "rationale": "The entry asks the testee about the timing habits between homework and play, namely, \"Does it always finish homework before playing.\" This involves current daily living behaviour, and therefore is judged as \"now\"."}, "sentence_type": "Interrogative sentence", "pos": {"nouns": {"Homework": 1, "Play": 1}, "verbs": {"Completed": 1, "Go on": 1}, "adverbs": {"Always": 1}, "numerals": {"24": 1}}}, {"content": "- You like to do things fast?", "word_count": 14, "label": "", "sentence_structure": "Subject鈥搗erb鈥搊bject", "subjective_objective": {"answer": "Subjective", "rationale": "Like being patient subjective."}, "sentence_sentiment": {"answer": "Positive", "rationale": "Because \"likes to do something fast\" is positive."}, "language_style": {"answer": "Calm and direct", "rationale": "The language style of the entry is more direct and concise, and the preference of respondents for quick missions is not directly questioned in a concise manner using excessive rhetorical or emotional modifications."}, "time_dimension": {"answer": "Present", "rationale": "According to the phrase \"like\" and \"do something quick,\" the entry is asking the testee about his current preferences and behaviour and therefore falls within the current time dimension."}, "sentence_type": "Interrogative sentence", "pos": {"verbs": {"Yeah": 1, "Do it": 1}, "adjectives": {"Some": 1, "Actions": 1, "Come on": 1}, "numerals": {"25": 1}}}, {"content": "Are you worried something terrible might happen?", "word_count": 14, "label": "", "sentence_structure": "Subject鈥搗erb鈥搊bject", "subjective_objective": {"answer": "Subjective", "rationale": "Because it looks at the patient ' s fear and fear of what may happen in the future, it is the patient ' s inner feeling and reflection, which is subjective."}, "sentence_sentiment": {"answer": "Negative", "rationale": "Because the entries \"fear\" and \"worshipful things\" have negative emotional aspects."}, "language_style": {"answer": "Calm and direct", "rationale": "The entry is more straightforward, concise, less talkative or emotional, and more objective. It talks in a direct query, asking if the respondents are concerned about something terrible, and this expression is more targeted and accessible."}, "time_dimension": {"answer": "Present", "rationale": "The entry is for the current status or feelings of the subject, the keyword is \"you're worried\" and \"will\", which point to the current state or situation."}, "sentence_type": "Interrogative sentence", "pos": {"nouns": {"Questionnaire": 1, "Entry": 1, "Things": 1}, "verbs": {"I'm worried": 1, "Come on": 1}, "adjectives": {"Horrible": 1}, "numerals": {"26": 1}}}, {"content": "27. When you hear other kids say weird things, stop them?", "word_count": 16, "label": "", "sentence_structure": "Subject鈥搗erb鈥搊bject", "subjective_objective": {"answer": "Subjective", "rationale": "The subject is primarily the subject of subjective attitudes and behaviour of patients and is therefore of the \"subjective\" type."}, "sentence_sentiment": {"answer": "Positive", "rationale": "Because the behavior in the entry is positive, i.e. stopping the child from saying or doing bad things."}, "language_style": {"answer": "Calm and direct", "rationale": "The entry directly asked the interviewees whether they would intervene in the face of the unkind words and actions of other children, without excessive rhetoric or emotional colours, and with more direct and objective language styles."}, "time_dimension": {"answer": "Past", "rationale": "\"When you\" and \"stop them\" indicate that this is a past or present act or state of affairs that occurs in a given situation (i.e. when you hear other children swearing \" ), and it is therefore possible to judge that the entry relates to past/present behaviour."}, "sentence_type": "Interrogative sentence", "pos": {"nouns": {"Questionnaire Entry": 1, "Son": 1, "Weird": 1}, "verbs": {"Yes": 1, "Stop": 1, "Scolded": 1}, "numerals": {"27": 1}}}, {"content": "Can you make a party go smoothly?", "word_count": 13, "label": "", "sentence_structure": "Subject鈥搗erb鈥搊bject", "subjective_objective": {"answer": "Objective", "rationale": "For the success of a party would require an objective factual assessment."}, "sentence_sentiment": {"answer": "Neutral", "rationale": "Because the entry does not relate to specific emotions, attitudes or behaviours, it is merely questioning capacity."}, "language_style": {"answer": "Tactful and warm", "rationale": "The language style of the entry is more euphemistic, as it is dialogue in a question-and-answer manner, not direct command or statement. This expression is more personal and interactive, and it is better able to reach out to and obtain feedback from interviewees. It also uses positive terms (such as \"slowing down \" ), making the issue more attractive and stimulating, and making the interviewee more willing to participate."}, "time_dimension": {"answer": "Present", "rationale": "The entry now asks if the person tested has the role of coordinator and organizer at the evening, and the time dimension is now judged."}, "sentence_type": "Interrogative sentence", "pos": {"nouns": {"Party": 1}, "verbs": {"鈾?And I'm gonna be 鈾?: 1, "Open": 1}, "numerals": {"28": 1}}}, {"content": "29. Are you sad when people find out about your mistakes or shortcomings in your work?", "word_count": 24, "label": "", "sentence_structure": "Subject鈥搗erb鈥搊bject", "subjective_objective": {"answer": "Subjective", "rationale": "Because \"worship\" is a subjective emotional response. Although it may be related to objective circumstances (i.e. others discover your mistakes or shortcomings), whether or not it is a personal feeling and experience."}, "sentence_sentiment": {"answer": "Negative", "rationale": "Because the emotional reaction of \"Easier to be sad\" in the entry is negative."}, "language_style": {"answer": "Tactful and warm", "rationale": "The entry is more gentle, friendly and guided in its language style, using querying rather than direct presentation."}, "time_dimension": {"answer": "Past", "rationale": "In the entry, \"Easier to grieve\" refers to the feelings and behaviours of the past, and therefore to the past."}, "sentence_type": "Interrogative sentence", "pos": {"nouns": {"Error": 1, "Work": 1, "Disadvantages": 1}, "verbs": {"Found": 1, "Sad": 1}, "numerals": {"29": 1}}}, {"content": "Would it upset you to see a dog just died?", "word_count": 15, "label": "", "sentence_structure": "Subject鈥搗erb鈥搊bject", "subjective_objective": {"answer": "Subjective", "rationale": "Because it is an emotional reaction, it is the subjective experience and feeling of the patient."}, "sentence_sentiment": {"answer": "Negative", "rationale": "Because \"worship\" in the entry is a negative emotion."}, "language_style": {"answer": "Calm and direct", "rationale": "This entry is more concise and able to effectively capture the actual feelings and views of the interviewees."}, "time_dimension": {"answer": "Present", "rationale": "The entry asks if the person who was tested would feel sorry for seeing a dog who just died and therefore judge it as \"now\"."}, "sentence_type": "Interrogative sentence", "pos": {"nouns": {"Puppy": 1, "Sorry": 1}, "verbs": {"Yeah": 1, "I'll kill you": 1}, "adverbs": {"Yes": 1}, "numerals": {"30": 1}}}, {"content": "31. Do you always apologize to anyone when you're rude?", "word_count": 15, "label": "", "sentence_structure": "Subject鈥損redicate", "subjective_objective": {"answer": "Subjective", "rationale": "Because an apology is an individual ' s subjective attitude and choice, it is an individual ' s psychological test."}, "sentence_sentiment": {"answer": "Positive", "rationale": "Because \"apologizing\" is a positive act."}, "language_style": {"answer": "Calm and direct", "rationale": "The language style of the entry is calm and straightforward. It expresses the core of the problem in a simple and clear language, without too much embellishment or emotional expression.\""}, "time_dimension": {"answer": "Past", "rationale": "In conclusion, the content of the entries in the questionnaire is past."}, "sentence_type": "Interrogative sentence", "pos": {"nouns": {"you": 1, "Naughty": 1, "I'm sorry": 1, "Time": 1, "Apologize": 1, "others": 1}, "verbs": {"Yes": 1, "Total": 1, "In": 1}, "adjectives": {"Naughty": 1, "I'm sorry": 1}, "adverbs": {"Total": 1}, "numerals": {"31": 1}, "classifiers": {"individual": 1}, "pronouns": {"you": 1}}}, {"content": "32. Do people think you have done something they do not like and they have always wanted to get back at you?", "word_count": 26, "label": "", "sentence_structure": "Subject鈥搗erb鈥搊bject", "subjective_objective": {"answer": "Subjective", "rationale": "This is the subjective view of the patient as to whether he or she is misunderstood or retaliated."}, "sentence_sentiment": {"answer": "Negative", "rationale": "Because \"who thinks he did something wrong and wants revenge\" is negative."}, "language_style": {"answer": "Calm and direct", "rationale": "The language style of the entry is a calm and straightforward one. It raises a question directly, without using excessive rhetorical or emotional rendering, and is more objective."}, "time_dimension": {"answer": "Present", "rationale": "The entry asks about the current status or experience of the respondents, namely, \"Does it seem that you have done something you do not think you do to them, and they have always wanted to avenge you?\" So the time dimension is now."}, "sentence_type": "Interrogative sentence", "pos": {"nouns": {"person": 1, "Things": 1, "Revenge": 1}, "verbs": {"I think": 1, "Do it": 1, "Yes": 1, "Revenge": 1}, "adverbs": {"Always": 1}, "numerals": {"32": 1}, "pronouns": {"you": 1}}}, {"content": "- You think skiing's fun?", "word_count": 8, "label": "", "sentence_structure": "Subject鈥損redicate", "subjective_objective": {"answer": "Subjective", "rationale": "Because \"think\" means that it's a personal opinion or feeling of the patient, and it's a subjective view."}, "sentence_sentiment": {"answer": "Neutral", "rationale": "Because the entry does not have any emotional color."}, "language_style": {"answer": "Calm and direct", "rationale": "The issue is clear and specific, and it is more efficient to ask the interviewees directly about the views and feelings of skiing."}, "time_dimension": {"answer": "Present", "rationale": "The entry asks the testee ' s view of the current situation, so the time dimension is now judged."}, "sentence_type": "Interrogative sentence", "pos": {"nouns": {"Skiing": 1, "Okay": 1}, "verbs": {"Play": 1}, "numerals": {"33": 1}}}, {"content": "34. Do you often feel tired for no reason?", "word_count": 11, "label": "", "sentence_structure": "Subject鈥搗erb鈥搊bject", "subjective_objective": {"answer": "Subjective", "rationale": "For fatigue is a subjective feeling and experience and belongs to the patient ' s own psychological state."}, "sentence_sentiment": {"answer": "Negative", "rationale": "Because the feeling of \"tired\" in the entries is negative."}, "language_style": {"answer": "Calm and direct", "rationale": "Basis for the judgement: The purpose of the article is more objective and objective, with no excessive rhetorical or emotional overtones. It asks directly whether the interviewee is often tired and does not use euphemism or vague language, so that the language style can be described as \"cooling straight\"."}, "time_dimension": {"answer": "Past", "rationale": "The entry is a question about the testee's past, and the keywords are \"normal\" and \"tired\" , which indicate frequent, persistent conditions, usually occurring in the past or now. The temporal dimension of the article is therefore judged to be past."}, "sentence_type": "Interrogative sentence", "pos": {"nouns": {"Weary": 1}, "verbs": {"I think": 1, "Regular": 1, "For no reason": 1, "Weary": 1, "- You're not": 1}, "adverbs": {"Regular": 1, "For no reason": 1}, "numerals": {"34": 1}}}, {"content": "35. Do you like to make fun of other kids?", "word_count": 13, "label": "", "sentence_structure": "Subject鈥搗erb鈥搊bject", "subjective_objective": {"answer": "Subjective", "rationale": "Because \"like\" is a subjective attitude or emotional tendency of the patient, and \"joy other kids\" is their behaviour. So the entry is viewed from the patient's subjective point of view."}, "sentence_sentiment": {"answer": "Negative", "rationale": "Because the \"smoke other kids\" act is negative."}, "language_style": {"answer": "Calm and direct", "rationale": "The entry is more directly written, has no excessive rhetoric or emotional colour and asks each other in a concise and clear manner whether they like to make fun of other children."}, "time_dimension": {"answer": "Present", "rationale": "The entry asks about the current behaviour or attitude of the subject, the keywords being \"a lot like\" and \"cute\" and the words indicate that behaviour or attitude is taking place. Thus, the entry is now judged."}, "sentence_type": "Interrogative sentence", "pos": {"nouns": {"Kids": 2, "- You're making fun": 1}, "verbs": {"Yeah": 1}, "adjectives": {"very": 1}, "adverbs": {"And": 1}, "numerals": {"35": 1}}}, {"content": "You always keep quiet when adults talk?", "word_count": 13, "label": "", "sentence_structure": "Subject鈥損redicate", "subjective_objective": {"answer": "Subjective", "rationale": "Because it is a question of personal habits and attitudes, it is the choice of behaviour in the face of uncompassionate circumstances that is examined.\""}, "sentence_sentiment": {"answer": "Negative", "rationale": "Because the \"silent\" act in the entry is negative."}, "language_style": {"answer": "Calm and direct", "rationale": "The entry is in a simple and clear language and asks whether the interviewee has been silent in his conversations, without too much embellishment or emotional additions and with a calm and objective tone."}, "time_dimension": {"answer": "Present", "rationale": "The word \"always\" in the entry indicates that it is a customary, recurrent act, while the verb \"maintains\" adopts the general current, meaning that the act has not only occurred in the past, but may still be ongoing. Thus, the temporal dimension of the entry in the questionnaire is now judged."}, "sentence_type": "Interrogative sentence", "pos": {"nouns": {"Adult": 1, "Talk": 1}, "verbs": {"Always": 1, "Hold on": 1, "Quiet": 1}, "adverbs": {"Always": 1}, "numerals": {"36": 1}}}, {"content": "37. When you make new friends, do you usually take the initiative?", "word_count": 14, "label": "", "sentence_structure": "Subject鈥搗erb鈥搊bject", "subjective_objective": {"answer": "Subjective", "rationale": "Since the way to make new friends is usually a matter of personal choice and initiative, this is a subjective attitude to examine patients."}, "sentence_sentiment": {"answer": "Positive", "rationale": "Because the \"take the initiative\" act is positive in the entry."}, "language_style": {"answer": "Tactful and warm", "rationale": "The entry leads the interviewee to reflect on his/her behaviour, without direct orders or accusations, but with a more respectful sense of respect. At the same time, the word \"usually\" also indicates a common, relatively moderate way of acting, not a mandatory requirement. The language style of the entry can therefore be described as euphemism."}, "time_dimension": {"answer": "Past", "rationale": "\"When making new friends\" refers to acts committed at a certain point or time in the past, so the temporal dimension of the article is the past."}, "sentence_type": "Interrogative sentence", "pos": {"nouns": {"Hand it over": 1, "New friends": 1}, "verbs": {"Adoption": 1, "Initiatives": 1}, "numerals": {"37": 1}}}, {"content": "Are you angry about something?", "word_count": 10, "label": "", "sentence_structure": "Subject鈥損redicate", "subjective_objective": {"answer": "Subjective", "rationale": "The purpose of this questionnaire entry is to know whether the patient has acted with anger on certain things."}, "sentence_sentiment": {"answer": "Negative", "rationale": "Because it's negative in the entry."}, "language_style": {"answer": "Calm and direct", "rationale": "The language style of the entry is calm and straightforward. It uses an objective and concise tone, without too much emotional adoration or rhetoric, but rather simply raises a question that allows respondents to answer directly."}, "time_dimension": {"answer": "Past", "rationale": "The entry asks if the person tested had had a temperamental experience with certain events, and the time dimension is a past."}, "sentence_type": "Interrogative sentence", "pos": {"nouns": {"Things": 1, "- Grumpy": 1}, "verbs": {"Let's go": 1, "Yes": 1}, "numerals": {"38": 1}}}, {"content": "Do you fight a lot?", "word_count": 5, "label": "", "sentence_structure": "Subject鈥搗erb鈥搊bject", "subjective_objective": {"answer": "Objective", "rationale": "Because \"often fighting\" is an objective act that, while reflecting the attitude or preferences of the patient, in this situation it exists as a fact."}, "sentence_sentiment": {"answer": "Negative", "rationale": "Because \"fight\" is negative."}, "language_style": {"answer": "Calm and direct", "rationale": "The language style of the entry is relatively cold and straightforward, and it uses a direct form of questioning to ask the respondents whether they are often fighting and do not use euphemism or encouraging words, which appear to be straightforward and concise."}, "time_dimension": {"answer": "Present", "rationale": "The word \"always\" in \"Does you fight?\" denotes a regular, customary act, while the verb \"plays\" uses the usual current pattern, meaning that the act not only has occurred in the past, but may still be ongoing. The entry is therefore to question the person being tested for the current act."}, "sentence_type": "Interrogative sentence", "pos": {"nouns": {"Fight": 1}, "verbs": {"Regular": 1, "- You're not": 1}, "numerals": {"39": 1}}}, {"content": "Have you ever said bad or dirty things about anyone?", "word_count": 13, "label": "", "sentence_structure": "Subject鈥搗erb鈥搊bject", "subjective_objective": {"answer": "Subjective", "rationale": "Because it is a subjective act and idea to say bad or dirty words."}, "sentence_sentiment": {"answer": "Negative", "rationale": "Because \"bad or dirty\" is a bad thing, it hurts people's feelings and self-esteem, it's negative."}, "language_style": {"answer": "Tactful and warm", "rationale": "The language style of the entry is more euphemistic and moderate, with relatively neutral terms and without too strong acrimonyms or criticisms, and instead uses a moderate and inviting tone to engage in dialogue and encourage interviewees to answer questions honestly, which is more acclaimed and interactive."}, "time_dimension": {"answer": "Present", "rationale": "\"Yes\" means a past completed act, but in this sentence it is emphasized that the present state or customary sexual behaviour, namely, \"Do you often speak bad or dirty words.\" So the time dimension of this entry is now."}, "sentence_type": "Interrogative sentence", "pos": {"nouns": {"Questionnaire": 1, "Entry": 1, "person": 1, "Bad word": 1, "You're a dirty man": 1}, "verbs": {"Say it": 1, "Yes": 1}, "numerals": {"40": 1}}}, {"content": "Do you like to tell your friends jokes or funny stories?", "word_count": 17, "label": "", "sentence_structure": "Subject鈥搗erb鈥搊bject", "subjective_objective": {"answer": "Subjective", "rationale": "Because \"like\" is the patient's subjective attitude, it's about whether the patient is willing to tell a joke or a funny story to a friend."}, "sentence_sentiment": {"answer": "Positive", "rationale": "Because the \"like to tell jokes or funny stories to friends\" act is positive and can bring joy and happiness."}, "language_style": {"answer": "Tactful and warm", "rationale": "The language style of the entry is more euphemistic, presented in a question-and-answer format, giving a friendly and friendly feeling, and without direct orders or requests for answers."}, "time_dimension": {"answer": "Present", "rationale": ""}, "sentence_type": "Interrogative sentence", "pos": {"nouns": {"Questionnaire Entry": 1, "Yeah": 1, "friends": 1, "A joke": 1, "Funny": 1, "Story": 1}, "verbs": {"Speak": 1}, "adverbs": {"or": 1}, "numerals": {"41": 1}}}, {"content": "Did you feel dizzy for a while?", "word_count": 11, "label": "", "sentence_structure": "Subject鈥搗erb鈥揷omplement", "subjective_objective": {"answer": "Subjective", "rationale": "Because the feeling of dizzyness is the patient's subjective feeling."}, "sentence_sentiment": {"answer": "Neutral", "rationale": "Since the entry asks whether the person surveyed is often dizzy and does not have a clear positive or negative emotional tendency, the following is the most recent example:"}, "language_style": {"answer": "Calm and direct", "rationale": "The entry asks the respondents in a straightforward and concise manner whether they are often suffering from dizziness, with a more calm and objective language style, and without excessive emotional colours or rhetorical techniques."}, "time_dimension": {"answer": "Past", "rationale": "The entry is for the purpose of asking the testee whether he or she has experience or symptoms of dizziness and the temporal dimension is judged to be past."}, "sentence_type": "Interrogative sentence", "pos": {"nouns": {"Dizziness": 1, "Feel": 1}, "verbs": {"have": 1, "For a while": 1}, "adverbs": {"- You're not": 1}, "numerals": {"42": 1}}}, {"content": "43. Are you more vulnerable to punishment than most children in school?", "word_count": 16, "label": "", "sentence_structure": "Subject鈥搗erb鈥搊bject", "subjective_objective": {"answer": "Objective", "rationale": "Because \"punishment\" is a specific objective act, not a patient's subjective idea."}, "sentence_sentiment": {"answer": "Negative", "rationale": "Because the \"more vulnerable than most children\" act in the entry is negative."}, "language_style": {"answer": "Calm and direct", "rationale": "The entry uses a direct, concise language style, without any rhetorical or emotional rendering, and is a straightforward question of whether the person surveyed is more likely to be punished in school than any other fellow student."}, "time_dimension": {"answer": "Past", "rationale": "\"In school\" indicates that time occurs in school settings, \"are children more vulnerable to punishment than most children\" is a description of behaviour or outcome, and the entry is therefore primarily an inquiry into the person's past behaviour or state, so that the judgement should be made in the past."}, "sentence_type": "Interrogative sentence", "pos": {"nouns": {"School": 1, "Children": 2, "Fine": 1}, "verbs": {"- Yeah": 1, "It's easier": 1, "Penalties": 1}, "numerals": {"43": 1, "Most of them": 1}, "classifiers": {"individual": 1}}}, {"content": "Do you usually pick up paper and garbage that people throw on the floor of a classroom?", "word_count": 22, "label": "", "sentence_structure": "Subject鈥搗erb鈥搊bject", "subjective_objective": {"answer": "Objective", "rationale": "This is a description of the individual ' s behaviour and is an objective observation and record."}, "sentence_sentiment": {"answer": "Positive", "rationale": "Because the entry is a positive act, namely, \" pick up paper and garbage thrown on the floor of the classroom\"."}, "language_style": {"answer": "Tactful and warm", "rationale": "The language style of the entry is more euphemistic, because it uses the words \"pick up\" and \"slaming on the floor of the classroom\" to express a caring, willing behaviour for others. It also uses the means of questioning, rather than direct orders, to be more friendly and friendly."}, "time_dimension": {"answer": "Past", "rationale": "The entry \"usually\" indicates that the act has a certain frequency, but does not specify a specific time frame, so it can be judged to be the past. At the same time, \"cleans up\" indicates that an act has taken place, further confirming that it was the past."}, "sentence_type": "Interrogative sentence", "pos": {"nouns": {"Entry": 1, "others": 1, "Waste Paper": 1, "Garbage": 1, "Classrooms": 1, "Floor": 1}, "verbs": {"Pick it up": 1, "Throw it": 1, "Yes": 1}, "numerals": {"44": 1}}}, {"content": "Do you have much after-school hobby and entertainment?", "word_count": 12, "label": "", "sentence_structure": "Subject鈥損redicate", "subjective_objective": {"answer": "Subjective", "rationale": "Because the existence of many after-school hobbies and entertainment is a subjective criterion for assessment, different people may have different views and feelings."}, "sentence_sentiment": {"answer": "Positive", "rationale": "Because \"many after-school hobbies and entertainment\" are usually considered positive."}, "language_style": {"answer": "Tactful and warm", "rationale": "The entry is more personal and interactive when it is asked to guide the respondents to answer questions, with a speech temperature and respect for the will of the other party, and without direct orders or mandatory language."}, "time_dimension": {"answer": "Present", "rationale": "The entry is for the current situation of the subject, and the keywords \"many\" and \"afterschool hobbies and entertainment\" all indicate that this is a problem now, so the judgment is \"now\"."}, "sentence_type": "Interrogative sentence", "pos": {"nouns": {"After-school": 1, "Hobbies": 1, "Recreation": 1}, "verbs": {"have": 1, "Many": 1, "After-school hobby and entertainment": 1}, "numerals": {"45": 1}}}, {"content": "Are you feeling vulnerable?", "word_count": 8, "label": "", "sentence_structure": "Subject鈥搗erb鈥揷omplement", "subjective_objective": {"answer": "Subjective", "rationale": "Because emotional vulnerability is the patient's subjective feeling and perception."}, "sentence_sentiment": {"answer": "Negative", "rationale": "Because \"emotional vulnerability\" is usually considered a negative feature."}, "language_style": {"answer": "Tactful and warm", "rationale": "The language style of the entry is more euphemistic, frees the interviewer to answer by asking questions, without directly expressing criticism or judgment. At the same time, the phrase \"your\" is used to give people a sense of affection and respect."}, "time_dimension": {"answer": "Present", "rationale": "The questionnaire entry asks the subject about his/her current emotional state, i.e. whether he/she is easily vulnerable and therefore falls within the current category."}, "sentence_type": "Interrogative sentence", "pos": {"nouns": {"Emotion": 1, "Weakness": 1}, "verbs": {"be": 1}, "adverbs": {"very": 1}, "numerals": {"46": 1}}}, {"content": "Do you like to play games?", "word_count": 7, "label": "", "sentence_structure": "Subject鈥損redicate", "subjective_objective": {"answer": "Subjective", "rationale": "Because of the subjective attitude of people who like to tease people."}, "sentence_sentiment": {"answer": "Negative", "rationale": "Because the \"like to play\" act in the entry is negative emotional."}, "language_style": {"answer": "Tactful and warm", "rationale": "The language style of the entry is more euphemistic, with the word \"like\" used to ask questions, so that respondents do not feel too offended or blamed when they answer, and they can also be interested in sharing real ideas."}, "time_dimension": {"answer": "Present", "rationale": "The entry asks if the person tested likes to play games, and the time dimension is not clearly pointed at past, present or future. The answer is therefore \"neutral\"."}, "sentence_type": "Interrogative sentence", "pos": {"nouns": {"Questionnaire Entry": 1, "Yeah": 1, "You're making fun of me": 1}, "verbs": {"- You're kidding me": 1, "Yeah": 1}, "numerals": {"47": 1}}}, {"content": "Do you always wash your hands before dinner?", "word_count": 9, "label": "", "sentence_structure": "Subject鈥損redicate", "subjective_objective": {"answer": "Subjective", "rationale": "The question is whether the patient ' s personal habits and behaviour are the subject of his own decisions and actions before eating, and therefore is subjective."}, "sentence_sentiment": {"answer": "Positive", "rationale": "Because \"washing hands before dinner\" is a healthy and healthy act, and it's positive."}, "language_style": {"answer": "Calm and direct", "rationale": "The entry is more concise, is not used too much rhetorical or emotionally retrofitting, and is more calm and direct. It simply asks whether the person surveyed washes his hands before eating, so that the person can quickly understand and answer the questions."}, "time_dimension": {"answer": "Present", "rationale": "The expression \" always \" indicates that it is a customary, conventional act and that it is taking place, so the temporal dimension of the article is now judged."}, "sentence_type": "Interrogative sentence", "pos": {"nouns": {"Before dinner": 1, "Hands": 1}, "verbs": {"It's gotta be": 1, "Wash": 1}, "adverbs": {"in": 1}, "numerals": {"48": 1}}}, {"content": "49. In recreational activities, would you prefer to sit and watch rather than participate in it yourself?", "word_count": 20, "label": "", "sentence_structure": "Subject鈥損redicate", "subjective_objective": {"answer": "Subjective", "rationale": "Because this is the patient ' s subjective attitude, it is the patient ' s personal will to participate in recreational activities."}, "sentence_sentiment": {"answer": "Negative", "rationale": "Because the tendency in the entry \"I'd rather sit and watch than be personally involved\" is negative."}, "language_style": {"answer": "Calm and direct", "rationale": "The language style of the entry is more objective and calm, and the interviewee is directly asked about his or her preferences in recreational activities, without too much emotional or rhetorical use."}, "time_dimension": {"answer": "Present", "rationale": ""}, "sentence_type": "Interrogative sentence", "pos": {"nouns": {"Recreational activities": 1, "Activities": 1}, "verbs": {"I'd rather": 1, "Sit down": 1, "Look": 1, "not": 1, "May I": 1, "Kiss": 1, "Since participation": 2}, "adverbs": {"in": 1}, "numerals": {"49": 1}, "pronouns": {"you": 1}}}, {"content": "Do you often get tired of it?", "word_count": 8, "label": "", "sentence_structure": "Subject鈥損redicate", "subjective_objective": {"answer": "Subjective", "rationale": "Because \"tired\" is an emotional experience, a subjective feeling and perception of the patient, it is a subjective approach."}, "sentence_sentiment": {"answer": "Negative", "rationale": "Because \"weary\" is a negative emotion."}, "language_style": {"answer": "Calm and direct", "rationale": "The language style of the entry is more calm and straightforward, and it does not use any rhetorical or emotional rendering to guide the respondents' emotional response, but rather directs the respondents in a simple question-and-answer format to ask them whether they are often tired. This language style is more objective, neutral and effectively captures the actual feelings and thoughts of the respondents."}, "time_dimension": {"answer": "Present", "rationale": "Now the entry \"often\" indicates that it is a customary act or feeling, while \"sick\" describes the subjective experience of the subject. Thus, the entry is a matter of current state."}, "sentence_type": "Interrogative sentence", "pos": {"nouns": {"you": 1, "Tired": 1}, "verbs": {"I feel": 1, "Regular": 1, "- You're not": 1}, "adverbs": {"Regular": 1}, "numerals": {"50": 1}}}, {"content": "51. Do you feel funny sometimes when a group of people laughs or bullys a child?", "word_count": 24, "label": "", "sentence_structure": "Subject鈥搗erb鈥搊bject", "subjective_objective": {"answer": "Subjective", "rationale": "Because it's the patient's subjective feeling to feel fun."}, "sentence_sentiment": {"answer": "Negative", "rationale": "Because the \"smoke or bully a child\" in the entry is negative."}, "language_style": {"answer": "Calm and direct", "rationale": "This question is asked in a straightforward way, whether the person surveyed has ever been a scorn or bullying, and in a more blunt language, with no excessive adoration or emotional rendering."}, "time_dimension": {"answer": "Present", "rationale": "The entry asks about the current feelings and attitudes of the testee, which relate to the key words \"sometimes feel\" and \"do you feel funny?\" So the temporal dimension is now."}, "sentence_type": "Interrogative sentence", "pos": {"nouns": {"Questionnaire": 1, "Entry": 1, "Kids": 1}, "verbs": {"I feel": 2, "- You're making fun": 1, "Bullying": 1, "Fun": 1}, "adverbs": {"Sometimes": 1}, "numerals": {"51": 1}}}, {"content": "Do you always keep quiet in class, even when teachers are not in class?", "word_count": 21, "label": "", "sentence_structure": "Subject鈥搗erb鈥搊bject", "subjective_objective": {"answer": "Objective", "rationale": "This is an observational question of the behaviour of individuals in the classroom, and does not involve their feelings, attitudes or perceptions."}, "sentence_sentiment": {"answer": "Negative", "rationale": "Because the \"silent\" act in the entry is neutral, but it has a certain negative emotional effect when it is conditioned \"even if the teacher is not in the classroom\"."}, "language_style": {"answer": "Calm and direct", "rationale": "The entry is relatively simple and clear in language, and there is no excessive emotional or rhetorical approach. It is therefore possible to judge that the language style of the entry is straightforward and calm."}, "time_dimension": {"answer": "Present", "rationale": ""}, "sentence_type": "Interrogative sentence", "pos": {"nouns": {"Classroom": 1, "you": 1, "Quiet": 1, "Teacher": 1, "Classrooms": 1}, "verbs": {"Hold on": 1, "in": 1, "No, I'm not": 1}, "adjectives": {"I see": 1}, "adverbs": {"Regular": 1, "Even": 1}, "numerals": {"52": 1}}}, {"content": "You like to do something scary?", "word_count": 11, "label": "", "sentence_structure": "Subject鈥搗erb鈥搊bject", "subjective_objective": {"answer": "Subjective", "rationale": "Because it's the subjective feeling and the will to do something."}, "sentence_sentiment": {"answer": "Negative", "rationale": "Because \"likes to do something that scares people\" is not generally accepted in society and may harm others."}, "language_style": {"answer": "Calm and direct", "rationale": "The entry is more direct and concise in language style, without too many rhetorical or emotional descriptions. It leads the interviewee to answer with a clear question, which is more neutral and objective in tone."}, "time_dimension": {"answer": "Present", "rationale": "The entry asks about the current attitude and preferences of the testee and does not refer to past or future situations, and therefore the judgement is now."}, "sentence_type": "Interrogative sentence", "pos": {"nouns": {"you": 1, "Yes": 1}, "verbs": {"Yeah": 1, "Fuck": 1, "Frightening": 1, "person": 1}, "adverbs": {"- You're not": 1}, "numerals": {"53": 1}}}, {"content": "54. Are you so upset that you can't sit in your chair for a moment?", "word_count": 20, "label": "", "sentence_structure": "Subject鈥搗erb鈥搊bject", "subjective_objective": {"answer": "Subjective", "rationale": "Because the feelings and behaviours of unease are experienced subjectively by the patient."}, "sentence_sentiment": {"answer": "Negative", "rationale": "Because the entry \"can't sit quietly on the chair\" is negative."}, "language_style": {"answer": "Calm and direct", "rationale": "The language style of the entry is calm and straightforward. It opens up to questions about whether the interviewee was unable to sit in a chair because of his unease, and there was no excessive emotional rendering or euphemism. This expression allows the interviewee to understand the question more quickly and accurately, and the answer is more concise."}, "time_dimension": {"answer": "Present", "rationale": "The entry asks if the testee is currently disturbed and affects his sitting capacity, and the time dimension is now judged."}, "sentence_type": "Interrogative sentence", "pos": {"nouns": {"Questionnaire": 1, "Entry": 1, "person": 1}, "verbs": {"Sit down": 1, "Unsettled": 1, "Quiet": 1, "Yes": 1, "Just a minute": 1}, "adjectives": {"Silence": 1}, "adverbs": {"Sometimes": 1}, "numerals": {"54": 1}, "classifiers": {"individual": 1}, "pronouns": {"you": 1}}}, {"content": "Would you like to go to the moon alone?", "word_count": 10, "label": "", "sentence_structure": "Subject鈥搗erb鈥搊bject", "subjective_objective": {"answer": "Subjective", "rationale": "Because \"willing to go to the moon alone\" is a personal will or attitude, subjective."}, "sentence_sentiment": {"answer": "Neutral", "rationale": "Since the entry is a question of fact, the interviewee was asked whether he was willing to go to the Moon alone, without expressing any emotional tendencies or value judgement."}, "language_style": {"answer": "Tactful and warm", "rationale": "This is a more euphemistic and gentle way of giving the interviewee a question about whether he wants to go to the moon, without directly expressing his views or forcing the interviewee to accept a choice."}, "time_dimension": {"answer": "Future", "rationale": "The entry asks if the person tested would be willing to conduct a separate space trip in the future, and the time dimension will be judged in the future."}, "sentence_type": "Interrogative sentence", "pos": {"nouns": {"Questionnaire": 1, "Entry": 1, "Moon": 1}, "verbs": {"Yes": 1, "Alone": 1, "Go on": 1}, "numerals": {"55": 1}}}, {"content": "Do you always sing together when people sing?", "word_count": 15, "label": "", "sentence_structure": "Subject鈥搗erb鈥搊bject", "subjective_objective": {"answer": "Subjective", "rationale": "Because \"Do you always sing together, and you need to answer the interviewee's own will and behavior.\" This is the subject of subjective thinking."}, "sentence_sentiment": {"answer": "Positive", "rationale": "This entry represents an attitude of active participation and cooperation."}, "language_style": {"answer": "Tactful and warm", "rationale": "The language style of the entry is more euphemismal and warm. It uses a more relaxed, friendly approach to questions, without directly expressing criticism or accusations, but rather to create resonance and reflection among the respondents by describing a scene. This language is more acceptable and promotes the real answers of the respondents."}, "time_dimension": {"answer": "Present", "rationale": "According to the word \"when meeting\" and \"when singing\" always together, it can be judged that the act was a custom in the past and may still be ongoing. The entry is therefore part of a questionnaire describing the present behaviour or habits of the subject."}, "sentence_type": "Interrogative sentence", "pos": {"nouns": {"On the occasion of the meeting": 1, "others": 1, "Song": 2, "you": 1}, "verbs": {"Sing it": 3, "Together": 1}, "adverbs": {"Always together": 1}, "numerals": {"56": 1}, "pronouns": {"- You're not": 1}}}, {"content": "Do you like to join other kids?", "word_count": 11, "label": "", "sentence_structure": "Subject鈥搗erb鈥搊bject", "subjective_objective": {"answer": "Subjective", "rationale": "Because \"like\" is a term of subjective attitude, it's the patient's perception of whether or not he likes to join the other kids."}, "sentence_sentiment": {"answer": "Neutral", "rationale": "The entry does not express a positive or negative emotional profile, but is merely an objective fact of the person under investigation."}, "language_style": {"answer": "Tactful and warm", "rationale": "The entry is more personal and interactive when it is spoken in a gentle and inviting tone, rather than in direct statements or orders. At the same time, the use of words such as \"like\" and \"square\" also makes the sentence positive and emotional, and more likely to attract the interest and resonance of the other."}, "time_dimension": {"answer": "Present", "rationale": "The entry asks the testee about his or her current preference for other children and therefore the time dimension."}, "sentence_type": "Interrogative sentence", "pos": {"nouns": {"you": 1, "Yeah": 1, "Kids": 2, "Together": 1}, "verbs": {"Yeah": 1, "Together": 1}, "numerals": {"57": 1}, "pronouns": {"Something else": 1}}}, {"content": "Do you have many nightmares?", "word_count": 7, "label": "", "sentence_structure": "Subject鈥搗erb鈥搊bject", "subjective_objective": {"answer": "Subjective", "rationale": "Because dreams are usually a subjective experience of individuals. While dreams may be influenced by external factors (such as psychological stress, medication, etc.), patients鈥?feelings and experiences of nightmares are subjective."}, "sentence_sentiment": {"answer": "Negative", "rationale": "Because the \"many nightmares\" act in the entry is negative."}, "language_style": {"answer": "Calm and direct", "rationale": "The entry is based on a more direct inquiry method, without excessive qualifiers or emotional colours, and is intended to obtain actual information from the person under investigation."}, "time_dimension": {"answer": "Present", "rationale": "Now the entry asks the testees about their current behaviour, namely, whether they often have nightmares. The keyword \"do\" and \"many nightmares\" are all the words that are relevant to the present, so the answer is now."}, "sentence_type": "Interrogative sentence", "pos": {"nouns": {"Nightmares": 1}, "verbs": {"Do it": 1}, "adverbs": {"Many": 1}}}, {"content": "Are your parents very harsh on you?", "word_count": 11, "label": "", "sentence_structure": "Subject鈥搗erb鈥搊bject", "subjective_objective": {"answer": "Subjective", "rationale": "Because \"draconian\" is a subjective feeling and evaluation that reflects the patient's perception and assessment of the attitude of his or her parents towards themselves."}, "sentence_sentiment": {"answer": "Negative", "rationale": "Because the expression \"very harsh\" gives people a depressing, negative feeling, it can be judged negative."}, "language_style": {"answer": "Calm and direct", "rationale": "The entry is asked in a more direct and concise manner, without any accoutrements or guidance, and therefore can be judged as \"cool and direct\"."}, "time_dimension": {"answer": "Present", "rationale": "The keywords \"dramatic\" and \"you\" point to the situation and the feeling, so the judgment is now."}, "sentence_type": "Interrogative sentence", "pos": {"nouns": {"parents": 1, "you": 2}, "verbs": {"to": 1, "Very": 1, "Hard": 1}, "adjectives": {"Hard": 1}, "adverbs": {"Very": 1}, "numerals": {"59": 1}, "pronouns": {"you": 2}}}, {"content": "You like not telling anyone to go out there alone?", "word_count": 20, "label": "", "sentence_structure": "Subject鈥搗erb鈥搊bject", "subjective_objective": {"answer": "Subjective", "rationale": "Because \"like\" is patient's subjective attitude."}, "sentence_sentiment": {"answer": "Negative", "rationale": "Because leaving home alone can be dangerous and negative."}, "language_style": {"answer": "Calm and direct", "rationale": "The entry is based on a direct query, asking whether the person interviewed prefers to leave his home alone to travel outside, and does not use speech-making or emotional publicity to guide the person to answer questions, which is more objective and balanced. This type of questionnaire entry is usually considered to be a calm and straightforward language style."}, "time_dimension": {"answer": "Present", "rationale": "Present"}, "sentence_type": "Interrogative sentence", "pos": {"nouns": {"you": 1, "Questionnaire": 1, "Entry": 1, "person": 1, "Home": 1, "Outside": 1, "# Swimming #": 1}, "verbs": {"Yeah": 1, "Tell": 1, "Off": 1, "Go on": 2, "Alone": 1}, "adverbs": {"not": 1, "Any": 1}, "numerals": {"60": 1}, "classifiers": {"individual": 1}, "pronouns": {"Here": 1}}}, {"content": "Do you like to jump parachutes?", "word_count": 8, "label": "", "sentence_structure": "Subject鈥搗erb鈥搊bject", "subjective_objective": {"answer": "Subjective", "rationale": "Because it is the patient's personal subjective attitude or feelings that like or dislike the parachute."}, "sentence_sentiment": {"answer": "Positive", "rationale": "Because the preference for skydiving is an act of adventure and stimulation, it is often perceived as positive."}, "language_style": {"answer": "Calm and direct", "rationale": "The entry is more straightforward in language style, with no excessive adoration or emotional colour, and asks if the other side likes to jump into the parachute."}, "time_dimension": {"answer": "Present", "rationale": ""}, "sentence_type": "Interrogative sentence", "pos": {"nouns": {"Jump": 1, "Submarine": 1}, "verbs": {"Yeah": 1}}}, {"content": "If you think you've done something stupid, do you regret it for a long time?", "word_count": 18, "label": "", "sentence_structure": "Subject鈥搗erb鈥搊bject", "subjective_objective": {"answer": "Subjective", "rationale": "Because \"registering for a long time\" is a subjective emotional expression, it is the subjective perception and attitude of the patient to his past behavior."}, "sentence_sentiment": {"answer": "Negative", "rationale": "The basis for the judgment: the \"long regret\" in the entry is negative."}, "language_style": {"answer": "Calm and direct", "rationale": "The entry, in a more direct and explicit manner, asks the respondents about their reaction to making the wrong decision, without using too much acrimony or emotional colour to express it more calmly and objectively."}, "time_dimension": {"answer": "Present", "rationale": "The keyword \"do something stupid\" and \"regrets long\" indicate that the entry is asking the person who was tested what it feels and what it is."}, "sentence_type": "Interrogative sentence", "pos": {"nouns": {"you": 2, "Stupid thing": 1, "Regret": 1}, "verbs": {"I think": 1, "Fuck": 1, "Yes": 1, "If": 1, "self": 1, "Stupid thing": 1, "Regret": 1, "Long time": 1, "- You're not": 1}, "adjectives": {"Stupid": 1}, "adverbs": {"Dhu": 1}, "numerals": {"62": 1}, "pronouns": {"self": 1}}}, {"content": "Do you eat the food you put on the table often?", "word_count": 17, "label": "", "sentence_structure": "Subject鈥搗erb鈥搊bject", "subjective_objective": {"answer": "Objective", "rationale": "Because food at the table is an objective fact and whether it is the patient who eats everything is an objective act.\""}, "sentence_sentiment": {"answer": "Neutral", "rationale": "Because the entry is about behavioral habits and does not involve any emotional colour."}, "language_style": {"answer": "Calm and direct", "rationale": "The entry is in a clear and straightforward language, asking the respondents whether they often try all the food on the table during their meals, and not in euphemism, exaggeration or emotional language, and is expressed in a more calm and straightforward manner."}, "time_dimension": {"answer": "Past", "rationale": "\"Always\" means \"frequently\" high-frequency action, \"all things ate\" is a past and present act, so the temporal dimension of the article covers both the present and the past."}, "sentence_type": "Interrogative sentence", "pos": {"nouns": {"Rice": 1, "Table": 1, "Food": 2}, "verbs": {"Huh": 1, "Eat": 1}, "adjectives": {"Often": 1, "Sample": 1}, "numerals": {"63": 1}, "classifiers": {"individual": 1}}}, {"content": "64. At a party, can you take the initiative and play?", "word_count": 19, "label": "", "sentence_structure": "Subject鈥搗erb鈥搊bject", "subjective_objective": {"answer": "Subjective", "rationale": "Because \"involved and play\" is the patient's subjective idea."}, "sentence_sentiment": {"answer": "Positive", "rationale": "Because the \"can be active and play\" act is positive."}, "language_style": {"answer": "Tactful and warm", "rationale": ""}, "time_dimension": {"answer": "Present", "rationale": ""}, "sentence_type": "Interrogative sentence", "pos": {"nouns": {"Party": 1, "you": 2, "Play": 1}, "verbs": {"Yes": 2, "Participation": 1, "Anything": 1}, "adjectives": {"It's a big deal": 1}, "adverbs": {"Proactive": 1}, "numerals": {"64": 1}}}, {"content": "Do you sometimes feel that it's not worth living?", "word_count": 12, "label": "", "sentence_structure": "Subject鈥損redicate", "subjective_objective": {"answer": "Subjective", "rationale": "Because it's a subjective feeling and emotional reaction that feels that it's not worth living."}, "sentence_sentiment": {"answer": "Negative", "rationale": "Because the emotional attitude of \"I feel unworthy of living\" in the entry is negative."}, "language_style": {"answer": "Calm and direct", "rationale": "The entry, which is in direct, concise language, is not overly emotional or qualifier, and expresses the question of whether the respondents often feel that life is meaningless and is speaking in an objective and insensitive manner."}, "time_dimension": {"answer": "Present", "rationale": "\"Sometimes feel\" means that the emotional experience is a certain period of time, and the sentence is now complete, meaning that the emotional experience may still be ongoing. Thus, the temporal dimension of the article is now judged."}, "sentence_type": "Interrogative sentence", "pos": {"nouns": {"Entry": 1, "Questionnaire": 1, "you": 1, "Value": 1, "Live": 1, "Get down": 1}, "verbs": {"I think": 1, "It's not worth it": 1, "Fan": 1, "in": 1, "- You're not": 1}, "adverbs": {"Sometimes": 1}, "numerals": {"65": 1}}}, {"content": "Would you be sad about the animals that fell into the trap of the hunters?", "word_count": 15, "label": "", "sentence_structure": "Subject鈥搗erb鈥搊bject", "subjective_objective": {"answer": "Subjective", "rationale": "Because \"worship\" is a subjective emotional experience, the questionnaire entry is viewed from the patient's subjective perspective."}, "sentence_sentiment": {"answer": "Positive", "rationale": "Because the act of \"worshiping for animals trapped in hunters\" in the entries expresses compassion and concern for animals and has a positive emotional effect."}, "language_style": {"answer": "Tactful and warm", "rationale": "The entry is more personal and interactive in its expression, in a gentle and inviting tone, rather than in direct statements or orders. At the same time, the purpose of the article is to address the subject of animal protection, so that the use of moderate language styles is more likely to attract the resonance and attention of the respondents."}, "time_dimension": {"answer": "Present", "rationale": "The word \"will\" in the entry indicates the attitude or feeling of the person in the current state of affairs with respect to something, so the temporal dimension is now judged."}, "sentence_type": "Interrogative sentence", "pos": {"nouns": {"you": 1, "Hunter": 1, "Animals": 1, "A trap": 1}, "verbs": {"Yes": 1, "Fall": 1, "Sorry": 1}, "adjectives": {"The hunters are trapped": 1}, "numerals": {"66": 1}, "pronouns": {"you": 1}}}, {"content": "Have you acted with disrespect to your parents?", "word_count": 11, "label": "", "sentence_structure": "Subject鈥搗erb鈥搊bject", "subjective_objective": {"answer": "Objective", "rationale": "Because the act is an objective fact, and the other party鈥檚 feelings are the same. Even if it is subjectively believed that he did not act with disrespect, the act in fact may have hurt the parents鈥?feelings."}, "sentence_sentiment": {"answer": "Negative", "rationale": "Because the \"unrespectful parents\" act in the entry is negative."}, "language_style": {"answer": "Calm and direct", "rationale": "The entry is in plain and clear language and asks the respondents whether they have ever shown disrespect for their parents, and the language style is straightforward and free of any emotional or rhetorical modifications."}, "time_dimension": {"answer": "Past", "rationale": "The entry asks if the person tested had acted with disrespect for his parents and the temporal dimension was a past."}, "sentence_type": "Interrogative sentence", "pos": {"nouns": {"you": 1, "Unrespectful behaviour of parents": 1}, "verbs": {"have": 1}, "numerals": {"67": 1}}}, {"content": "68. Do you always decide to do a lot of things?", "word_count": 15, "label": "", "sentence_structure": "Subject鈥損redicate", "subjective_objective": {"answer": "Subjective", "rationale": "Because \"always sudden determination\" is a human experience and feeling, a subjective attitude and behaviour."}, "sentence_sentiment": {"answer": "Neutral", "rationale": "Because the entry is not clearly emotional, it is merely a question of the behaviour of the person under investigation."}, "language_style": {"answer": "Tactful and warm", "rationale": ""}, "time_dimension": {"answer": "Present", "rationale": ""}, "sentence_type": "Interrogative sentence", "pos": {"nouns": {"Decision": 1, "A lot of things": 1}, "verbs": {"Do it": 1}, "adverbs": {"Regular": 1, "Suddenly": 1}, "numerals": {"68": 1}}}, {"content": "You're not thinking well when you're doing your homework?", "word_count": 11, "label": "", "sentence_structure": "Subject鈥搗erb鈥搊bject", "subjective_objective": {"answer": "Subjective", "rationale": "The entry is based on the patient ' s state of mind at the time of his or her work and on his or her subjective feelings and behaviour."}, "sentence_sentiment": {"answer": "Negative", "rationale": "Because the \"done thinking\" act in the entry is negative."}, "language_style": {"answer": "Tactful and warm", "rationale": "The entry is more adept and interactive when it is spoken in a gentle and inviting manner, rather than by direct statements or orders."}, "time_dimension": {"answer": "Present", "rationale": "The entry asks if the person tested had acted with a lack of thought in performing the job, emphasizing the actions currently being carried out, and therefore the time dimension is now judged."}, "sentence_type": "Interrogative sentence", "pos": {"nouns": {"Operations": 1, "Ideas": 1, "I'm going to leave": 1}, "verbs": {"Do it": 1, "I'm not thinking": 1}, "numerals": {"69": 1}}}, {"content": "When other children yell at you, do you also give them back?", "word_count": 21, "label": "", "sentence_structure": "Subject鈥搗erb鈥搊bject", "subjective_objective": {"answer": "Subjective", "rationale": "Because \"and yell in return\" is the patient's subjective behavior."}, "sentence_sentiment": {"answer": "Negative", "rationale": "Because the \"scream in return\" in the entry is negative."}, "language_style": {"answer": "Calm and direct", "rationale": "The entry is more direct, clear-cut, and has no excessive rhetoric or emotional rendering. It directly asks the interviewee whether he or she will respond in the same way when he or she comes up against the screams of other children, without any euphemism or indirect expression. It is therefore a cold language style."}, "time_dimension": {"answer": "Present", "rationale": "The entry asks about the reaction of the testee to the situation (other children shouting at themselves) and the keyword \"also shout in return for them\" is a verb describing past or present behaviour. Thus, the temporal dimension is now."}, "sentence_type": "Interrogative sentence", "pos": {"nouns": {"Son": 2, "you": 1, "# Hoo-ooo #": 3}, "verbs": {"to": 1, "have": 1, "Yeah": 1, "Use": 1, "Rewards": 1}, "adverbs": {"When": 1, "Time": 1}, "numerals": {"70": 1}, "pronouns": {"they": 1}}}, {"content": "Do you like diving or diving?", "word_count": 9, "label": "", "sentence_structure": "Subject鈥搗erb鈥搊bject", "subjective_objective": {"answer": "Subjective", "rationale": "Because it's a subjective attitude or emotional experience that you like or dislike."}, "sentence_sentiment": {"answer": "Neutral", "rationale": "It is a question of personal interests and does not involve any emotional colour."}, "language_style": {"answer": "Tactful and warm", "rationale": "The language style of the entry is more euphemistic, because it does not ask the other side whether they like diving or diving, but rather raises the question in an easy and open way. This way makes the respondents feel more comfortable and comfortable, and more willing to share their real thoughts and feelings. It also leaves more freedom and choice for those who answer the questions, because they can choose whether and how to answer."}, "time_dimension": {"answer": "Present", "rationale": "The keyword \"likes\" implies that it is a question of current emotions or preferences."}, "sentence_type": "Interrogative sentence", "pos": {"nouns": {"Questionnaire": 1, "Entry": 1, "Dive": 1, "Jumping": 1}, "verbs": {"Yeah": 1}, "numerals": {"71": 1}}}, {"content": "Do you sleep by mistake because of something that bothers you?", "word_count": 17, "label": "", "sentence_structure": "Subject鈥損redicate", "subjective_objective": {"answer": "Subjective", "rationale": "Because insomnia is caused by the patient's subjective emotions.\""}, "sentence_sentiment": {"answer": "Negative", "rationale": "Because the result of insomnia in the entries is negative."}, "language_style": {"answer": "Calm and direct", "rationale": "The language style of the entry is calm and straightforward. It asks whether the person surveyed was insomnia due to the troubles of the night, without excessive adoration and emotional rendering, and with objectivity.\""}, "time_dimension": {"answer": "Past", "rationale": "The entry is for questioning the testee's past behaviour, and the temporal dimension is judged to be the past, and should therefore be labelled as the \"old\"."}, "sentence_type": "Interrogative sentence", "pos": {"nouns": {"Night": 1, "Things": 1, "Trouble": 1, "Insomnia": 1}, "verbs": {"have": 1}, "adjectives": {"Some": 1, "Pass": 1}, "numerals": {"72": 1}}}, {"content": "Do you write obscurantisms in school or library books?", "word_count": 16, "label": "", "sentence_structure": "Subject鈥搗erb鈥搊bject", "subjective_objective": {"answer": "Subjective", "rationale": "This is the subjective choice and behaviour of patients who are asked whether they will write in schools or libraries."}, "sentence_sentiment": {"answer": "Negative", "rationale": "Because \"writes in a book\" is a bad thing."}, "language_style": {"answer": "Calm and direct", "rationale": "The language style of the entry is calm, because the question is to ask the interviewees whether they are writing in a school or library in a straightforward and clear way. It does not use any euphemism or emotional language, but rather asks directly. This language style is more common in the questionnaire survey, because it allows the interviewee to understand the question more accurately and to provide a true answer."}, "time_dimension": {"answer": "Present", "rationale": ""}, "sentence_type": "Interrogative sentence", "pos": {"nouns": {"you": 1, "School": 1, "Library": 1, "Books": 1, "Fake": 2}, "verbs": {"in": 1, "of": 2, "- You're not": 1}, "adverbs": {"- You're not": 1}, "numerals": {"Do you write obscurantisms in school or library books": 1}, "pronouns": {"you": 1}}}, {"content": "Do you seem to be in a lot of trouble at home?", "word_count": 15, "label": "", "sentence_structure": "Subject鈥損redicate", "subjective_objective": {"answer": "Subjective", "rationale": "Because the question asked how the patient felt at home, it was subjective and therefore subjective."}, "sentence_sentiment": {"answer": "Negative", "rationale": "Because the description \"always troubled\" in the entry is negative."}, "language_style": {"answer": "Tactful and warm", "rationale": "Although the entry asks whether the other party is often upset at home, it does not use too direct and inclement language. Rather, it uses a more gentle and friendly tone to ask questions, making the questionnaire more acceptable and intimacy."}, "time_dimension": {"answer": "Past", "rationale": "The entry uses the words \"whether\" and \"like,\" asking if the person tested has experienced something in the past. At the same time, the word \"home\" implies that it is a memory or reflection of the past. Thus, the temporal dimension of the questionnaire is a past."}, "sentence_type": "Interrogative sentence", "pos": {"nouns": {"you": 1, "Home": 1, "Medium": 1, "Trouble": 1}, "verbs": {"I feel": 1, "be": 1, "Elephant": 1, "Always": 1}, "adjectives": {"It's a pain": 1}, "adverbs": {"Always": 1}, "numerals": {"74": 1}}}, {"content": "Do you think you're a live one?", "word_count": 9, "label": "", "sentence_structure": "Subject鈥搗erb鈥搊bject", "subjective_objective": {"answer": "Subjective", "rationale": "The question is asked about the observations and perceptions of patients and is examined from a subjective perspective."}, "sentence_sentiment": {"answer": "Positive", "rationale": "Because \"vio\" is a positive adjective."}, "language_style": {"answer": "Tactful and warm", "rationale": "This expression avoids causing discomfort or resistance to the person under investigation and increases the effectiveness and acceptability of the questionnaire."}, "time_dimension": {"answer": "Present", "rationale": "The entry asks about the current behaviour or state of the respondent, i.e. the evaluation of the interviewee by others."}, "sentence_type": "Interrogative sentence", "pos": {"nouns": {"others": 1, "you": 2, "Lively": 1}, "verbs": {"I think": 1, "Lively": 1}, "adjectives": {"very": 1}, "numerals": {"75": 1}, "pronouns": {"they": 1}}}, {"content": "Do you often feel lonely?", "word_count": 7, "label": "", "sentence_structure": "Subject鈥損redicate", "subjective_objective": {"answer": "Subjective", "rationale": "Because it's often felt that being alone is a subjective feeling."}, "sentence_sentiment": {"answer": "Negative", "rationale": "Because \"often feeling alone\" is a negative emotional experience."}, "language_style": {"answer": "Tactful and warm", "rationale": ""}, "time_dimension": {"answer": "Present", "rationale": ""}, "sentence_type": "Interrogative sentence", "pos": {"nouns": {"you": 1, "Alone": 1}, "verbs": {"I think": 1, "Regular": 1}, "adverbs": {"Regular": 1, "- You're not": 1}, "numerals": {"76": 1}}}, {"content": "Do you always take care of other people's things?", "word_count": 16, "label": "", "sentence_structure": "Subject鈥搗erb鈥搊bject", "subjective_objective": {"answer": "Subjective", "rationale": "Because the problem concerns the attitude and behaviour of patients towards the objects of others, it is a subjective perception and behaviour of the individual."}, "sentence_sentiment": {"answer": "Neutral", "rationale": "Because the entry does not have any emotional colour, it merely describes a habit."}, "language_style": {"answer": "Tactful and warm", "rationale": "The language style of the entry is euphemism. It uses a quiz, which is a gentle and inviting tone of conversation, rather than a direct statement or order, which is more personal and interactive. At the same time, the question is not too emotional or value-based, but rather an objective and neutral inquiry into the testee鈥檚 behavior, which makes you comfortable and willing to answer."}, "time_dimension": {"answer": "Present", "rationale": "The keywords \"always\" and \"special care\" both imply that this is a customary act, which occurs now. So the temporal dimension of the article should be now."}, "sentence_type": "Interrogative sentence", "pos": {"nouns": {"Something": 1, "others": 1}, "verbs": {"Always": 1, "Watch out": 1, "Love and protection": 1}, "adjectives": {"Special": 1}, "numerals": {"77": 1}, "pronouns": {"you": 1}}}, {"content": "Do you always share all your candy with others?", "word_count": 18, "label": "", "sentence_structure": "Subject鈥搗erb鈥搊bject", "subjective_objective": {"answer": "Subjective", "rationale": "The subject is examined in terms of subjective attitudes and behavioural tendencies of patients."}, "sentence_sentiment": {"answer": "Positive", "rationale": "Because sharing candy with others is a kind and generous act and a positive emotional expression."}, "language_style": {"answer": "Tactful and warm", "rationale": "The entry is more euphemistic, and asks questions that lead the respondents to reflect on their actions without direct criticism or criticism. At the same time, the words \"always\" and \"all candy\" are used to show concern and concern for the respondents. This is a way to make the respondents more receptive and willing to answer questions."}, "time_dimension": {"answer": "Present", "rationale": "The entry asks the testee about current behavior or habits, and the temporal dimension is now. The keyword \"always\", \"diversely eating\" and \"does\" all indicate that this is an ongoing act."}, "sentence_type": "Interrogative sentence", "pos": {"nouns": {"Grandpa": 1, "Candy": 2, "person": 1}, "verbs": {"Always": 1, "Will": 1, "Separate": 1, "Eat": 1}, "adverbs": {"All": 1}, "numerals": {"78": 1}, "pronouns": {"you": 1}}}, {"content": "Do you like to go out and play?", "word_count": 9, "label": "", "sentence_structure": "Subject鈥損redicate", "subjective_objective": {"answer": "Subjective", "rationale": "Because \"like\" means the attitude and preferences of the patient, it's viewed from the patient's subjective perspective."}, "sentence_sentiment": {"answer": "Positive", "rationale": "Because the act of \"like to play out\" is a positive emotion and attitude."}, "language_style": {"answer": "Tactful and warm", "rationale": ""}, "time_dimension": {"answer": "Present", "rationale": ""}, "sentence_type": "Interrogative sentence", "pos": {"nouns": {"Questionnaire Entry": 1, "Play": 1}, "verbs": {"Yeah": 1, "Out": 1}, "adjectives": {"very": 1}, "numerals": {"79": 1}}}, {"content": "Have you ever played games with false pretences?", "word_count": 12, "label": "", "sentence_structure": "Subject鈥搗erb鈥搊bject", "subjective_objective": {"answer": "Subjective", "rationale": "\"Affective play\" is the subjective behaviour and attitude of the patient, so the perspective of the questionnaire entry is seen from a subjective perspective."}, "sentence_sentiment": {"answer": "Negative", "rationale": "Because the \"fake\" in the entry is negative."}, "language_style": {"answer": "Calm and direct", "rationale": "The entry is more concise in language and asks the interviewee whether he has played a false game, without excessive ado or emotional colour, and in a calm and objective tone."}, "time_dimension": {"answer": "Present", "rationale": "The entry asks if the testee is making a false impression in the current game behaviour, and therefore the temporal dimension is now judged."}, "sentence_type": "Interrogative sentence", "pos": {"nouns": {"Game": 1, "you": 1, "You're making up a lie": 1}, "verbs": {"Yes": 1, "Play": 1}, "numerals": {"80": 1}}}, {"content": "81. Are you sometimes particularly happy for no reason, and sometimes especially sad for no reason?", "word_count": 28, "label": "", "sentence_structure": "Subject鈥搗erb鈥搊bject", "subjective_objective": {"answer": "Objective", "rationale": "Because \"sweetly happy\" and \"sweetly sad\" are the emotional states that patients experience at a given point in time, not their subjective views or attitudes. Such emotional reactions are objective facts that can be obtained by observation and measurement. So the questionnaire entry is viewed from the perspective of the patient."}, "sentence_sentiment": {"answer": "Negative", "rationale": "Because the article's formulations contain emotional fluctuations of \"excusable pleasure\" and \"excusable sorrow\" and therefore have negative emotions."}, "language_style": {"answer": "Tactful and warm", "rationale": ""}, "time_dimension": {"answer": "Present", "rationale": "The entry asks the subject about the current emotional experience, both happy and sad. Keywords such as \"sometimes,\" \"for no reason\" and \"for feeling\" all indicate that these emotions are present."}, "sentence_type": "Interrogative sentence", "pos": {"nouns": {"Time": 2, "you": 1, "For no reason": 2, "Special": 2, "I feel": 2, "Happy": 1, "Sad": 1}, "adjectives": {"For no reason": 2, "Special": 2}, "adverbs": {"Sometimes": 2}, "numerals": {"81": 1, "1": 2}, "pronouns": {"you": 1}}}, {"content": "You throw waste paper on the floor when you can't find waste paper baskets?", "word_count": 16, "label": "", "sentence_structure": "Subject鈥損redicate", "subjective_objective": {"answer": "Objective", "rationale": "The basis for the determination is that the question is one of the patient鈥檚 behaviour in a given situation, i.e., whether they will leave the paper on the ground when they do not find the waste basket. The act occurs objectively, free from the patient鈥檚 subjective attitude and is therefore objective."}, "sentence_sentiment": {"answer": "Negative", "rationale": "Because the \"screw paper on the ground\" act in the entry is uncivilized, irresponsible and negative."}, "language_style": {"answer": "Calm and direct", "rationale": "The language style of the entry is more straightforward, with no excessive rhetorical or emotional colours, but rather a simple and clear question of a specific behaviour."}, "time_dimension": {"answer": "Present", "rationale": "\"銆?}, "sentence_type": "Interrogative sentence", "pos": {"nouns": {"Waste sheet": 1, "Waste Paper": 1, "Land": 1}, "verbs": {"Find": 1, "Throw it": 1}, "adverbs": {"Time": 1}, "numerals": {"82": 1}}}, {"content": "Do you often feel happy and happy?", "word_count": 11, "label": "", "sentence_structure": "Subject鈥損redicate", "subjective_objective": {"answer": "Subjective", "rationale": "Because happiness and pleasure are subjective. While the degree of happiness and pleasure may be influenced by objective conditions, individual subjective experience is decisive."}, "sentence_sentiment": {"answer": "Positive", "rationale": "Because \"favouring happiness and happiness\" in the entry is a positive emotional description, expressing a positive emotional state."}, "language_style": {"answer": "Tactful and warm", "rationale": "The language style of the entry is euphemistic, using the \"Do you often feel happy and happy?\" to guide the respondents to answer questions, with a friendly tone and without too much subjective judgement. This expression helps to make them more open to sharing their feelings and to be positive about the questionnaire."}, "time_dimension": {"answer": "Present", "rationale": "In the entry, \"often\" means frequency, and \"favouring happiness and pleasure\" describes the subjective feeling of the testee, which can be generated at any point in time. The entry therefore looks at the subjective emotional state of the testee, and the dimension of the judgement is now."}, "sentence_type": "Interrogative sentence", "pos": {"nouns": {"Happiness": 1, "Have a nice day": 1}, "verbs": {"I feel": 1}, "adverbs": {"Regular": 1}, "numerals": {"83": 1}}}, {"content": "Do you often think about things first?", "word_count": 12, "label": "", "sentence_structure": "Subject鈥損redicate", "subjective_objective": {"answer": "Subjective", "rationale": "Because it's about the patient's personal thoughts and behaviour patterns."}, "sentence_sentiment": {"answer": "Negative", "rationale": "Because \"it's often not a thought\" is negative."}, "language_style": {"answer": "Calm and direct", "rationale": "The entry asks the respondents in a direct and uncompromising way whether they often do things without thinking."}, "time_dimension": {"answer": "Past", "rationale": "The entry is for questioning the testee ' s past habits or behaviour, and the temporal dimension is a past."}, "sentence_type": "Interrogative sentence", "pos": {"nouns": {"Things": 1}, "verbs": {"Do it": 1, "Let's see": 1, "Organisation": 1}, "adjectives": {"Often": 1}, "adverbs": {"not": 1}, "numerals": {"84": 1}}}, {"content": "Do you think you're a carefree person?", "word_count": 15, "label": "", "sentence_structure": "Subject鈥搗erb鈥搊bject", "subjective_objective": {"answer": "Subjective", "rationale": "Because \"no worries\" is a patient's self-evaluation and subjective feeling."}, "sentence_sentiment": {"answer": "Neutral", "rationale": "Because the entry asks about a person ' s own views and does not involve positive or negative behaviour, attitudes or emotional tendencies."}, "language_style": {"answer": "Tactful and warm", "rationale": "The entry uses a tone of inquiry and asks questions in a more friendly way, trying to make the interviewee comfortable and willing to share their thoughts. At the same time, \"no worries\" is a more subjective concept that requires the interviewee to respond with his own experience and feelings, so that the questionnaire can be more closely related to the actual situation and experience of the interviewee."}, "time_dimension": {"answer": "Present", "rationale": ""}, "sentence_type": "Interrogative sentence", "pos": {"nouns": {"self": 1, "person": 1, "No worries": 1}, "verbs": {"I think": 1, "be": 1}, "adjectives": {"No worries": 1}, "numerals": {"85": 1}, "pronouns": {"you": 1}}}, {"content": "Do you always need a warm friend to be with you to make you happy?", "word_count": 19, "label": "", "sentence_structure": "Subject鈥搗erb鈥搊bject", "subjective_objective": {"answer": "Subjective", "rationale": "Because \"often need\" is a subjective feeling or need that requires patient self-evaluation or judgement."}, "sentence_sentiment": {"answer": "Positive", "rationale": "Because the words \"hot friend\" and \"happy\" in the entries are positive emotional expressions, and they express a positive attitude and emotion."}, "language_style": {"answer": "Tactful and warm", "rationale": "The language style of the entry tends to be euphemistic, because it uses a friendly and invited tone to ask if the other side often needs friends to be with them to be happy. This expression is relatively more friendly and acceptable, and it also demonstrates the respect and interest of researchers in the person under investigation."}, "time_dimension": {"answer": "Past", "rationale": "So the time dimension of this entry is the past and the present. And, \"Are you happy with a friend who is interested in you?\" The question is whether the testees are often in need of the company of friends to be happy and therefore can be considered as being in a state of behavior or state."}, "sentence_type": "Interrogative sentence", "pos": {"nouns": {"friends": 1, "Happy": 1}, "verbs": {"Yes": 1, "Make you happy": 2}, "adjectives": {"Be kind": 1}, "numerals": {"86": 1}}}, {"content": "Have you ever damaged or lost someone's things?", "word_count": 15, "label": "", "sentence_structure": "Subject鈥搗erb鈥搊bject", "subjective_objective": {"answer": "Subjective", "rationale": "Because \"damaged or lost something\" is the patient's subjective behaviour and attitude."}, "sentence_sentiment": {"answer": "Negative", "rationale": "Because the behavior in the entry \"damage or lose something of another person\" is negative."}, "language_style": {"answer": "Calm and direct", "rationale": "The language style of the entry is more objective and straightforward, and there is no excessive emotional or rhetorical approach aimed at asking the other party whether it has had such experience."}, "time_dimension": {"answer": "Past", "rationale": "The entry \"had\" indicated that it was an act that had been completed and had occurred in the past."}, "sentence_type": "Interrogative sentence", "pos": {"nouns": {"Questionnaire": 1, "Entry": 1, "Something": 2}, "verbs": {"Damage": 1, "Lost": 1, "have": 1}, "numerals": {"87": 1}, "pronouns": {"you": 1, "others": 1}}}, {"content": "Do you like riding fast?", "word_count": 14, "label": "", "sentence_structure": "Subject鈥搗erb鈥搊bject", "subjective_objective": {"answer": "Subjective", "rationale": "Because \"like\" is an emotional attitude, it's subjective."}, "sentence_sentiment": {"answer": "Negative", "rationale": "Because \"quick-drive motorcycles\" are usually dangerous, which can cause negative emotional reactions."}, "language_style": {"answer": "Calm and direct", "rationale": "The entry is simple and clear to the respondents, is not too much rhetorical or emotional, and is more objective in tone. Thus, its language style is straightforward and calm."}, "time_dimension": {"answer": "Present", "rationale": ""}, "sentence_type": "Interrogative sentence", "pos": {"nouns": {"Yeah": 1, "Sit down": 1, "Soon": 1, "Motorcycle": 1}, "verbs": {"Multiply": 1, "Open": 1}, "adjectives": {"Come on": 1}, "numerals": {"88": 1}}}]}

{"questionnaire_title": "Self-confidence test.", "questionnaire_category": "Mental health", "item_count": 40, "emotion_shift_overall": "From negative to positive", "language_style_overall": "Neutral", "items": [{"content": "1. Once you have made up your mind, will you insist on doing it after all, even if no one agrees?", "word_count": 23, "label": "", "sentence_structure": "Subject鈥損redicate", "subjective_objective": {"answer": "Subjective", "rationale": "This is a subjective issue because of the attitude or conviction of the individual ' s will and perseverance."}, "sentence_sentiment": {"answer": "Positive", "rationale": "Because the purpose of the \"suspendant\" act in the entry is positive."}, "language_style": {"answer": "Calm and direct", "rationale": "This language style has direct and explicit characteristics and is a more calm and straightforward language style."}, "time_dimension": {"answer": "Present", "rationale": "\"When you have made a decision\" indicates a scenario, \"and determined\" indicates that the act has taken place or is in progress. And the latter \"will it remain in the end?\" is asking the testee's attitude in the face of this situation, namely, whether it will continue in the future. So, this entry is mainly concerned with the present and the future, and is judged on the basis of \"if you have made a determination\" and \"will it still be done?\""}, "sentence_type": "Interrogative sentence", "pos": {"nouns": {"Decision": 1, "person": 1}, "verbs": {"Down": 1, "Yes": 1, "Hold on": 1, "Do it": 1}, "adverbs": {"鈾?Once 鈾?: 1, "Still will": 1}, "numerals": {"One": 2}, "pronouns": {"you": 2}}}, {"content": "- Will you stand up until the party ends, even if you want to go to the bathroom?", "word_count": 25, "label": "", "sentence_structure": "Subject鈥損redicate", "subjective_objective": {"answer": "Subjective", "rationale": "Because this is a subjective choice of the patient in a particular situation."}, "sentence_sentiment": {"answer": "Negative", "rationale": "Because the behavior in this entry \"destroy until the party is over\" can cause physical discomfort, and it is a negative act."}, "language_style": {"answer": "Calm and direct", "rationale": "The language style of the entry is relatively calm and straightforward, and the information of the interviewee is obtained by simple and clear question-and-answer methods, and there is no excessive emotional rendering or rhetorical technique.\""}, "time_dimension": {"answer": "Present", "rationale": "The entry asks the testee about the choice of behaviour in a given situation, i.e. whether he or she will choose to stay away from the bathroom at dinner, until the party is over."}, "sentence_type": "Interrogative sentence", "pos": {"nouns": {"Dinner": 1, "Bathroom": 1, "End": 1}, "verbs": {"Participation": 1, "Yes": 2, "Hold on": 1, "Go, go, go": 1}, "adverbs": {"Even if": 1}, "classifiers": {"individual": 1}, "pronouns": {"you": 1}}}, {"content": "If you want to buy sexy underwear, would you try to mail it instead of going to the store yourself?", "word_count": 23, "label": "", "sentence_structure": "Subject鈥搗erb鈥搊bject", "subjective_objective": {"answer": "Subjective", "rationale": "Because the behaviour and choice of \"buy sexy lingerie\" and \"shopping\" is the patient's subjective idea."}, "sentence_sentiment": {"answer": "Negative", "rationale": "Because it involves the purchase and display of \"sexy lingerie\" and it can cause some negative emotional reactions. And the act of \"as much as possible mail order, not go to the store personally\" can be uncomfortable or uncomfortable and can therefore be considered negative."}, "language_style": {"answer": "Calm and direct", "rationale": "This entry is a direct question-and-answer dialogue, with no excessive rhetorical or emotional rendering, and is more balanced and objective."}, "time_dimension": {"answer": "Present", "rationale": "The entry now asks the testee about his current behavior and decision-making tendency to choose a mail order rather than a personal visit to the store when buying sexy underwear. Therefore, the time dimension is now judged."}, "sentence_type": "Interrogative sentence", "pos": {"nouns": {"Questionnaire": 1, "Entry": 1, "Undergarments": 1}, "verbs": {"Buy": 1, "Yes": 4, "Try": 1, "Mail order": 1, "No, I'm not": 1, "Go on": 1}, "adjectives": {"Sexy": 1}, "adverbs": {"If": 1, "Present": 1, "Shop": 1, "Lee": 1, "- You're not": 1, "And": 1}, "numerals": {"3": 1}, "classifiers": {"individual": 1}, "pronouns": {"you": 2}}}, {"content": "- You think you're a better person?", "word_count": 13, "label": "", "sentence_structure": "Subject鈥搗erb鈥搊bject", "subjective_objective": {"answer": "Subjective", "rationale": "Because it is a patient's assessment of his or her own perfection, it is a subjective examination."}, "sentence_sentiment": {"answer": "Negative", "rationale": "Because the expression \"the better person\" is a manifestation of narcissism, which is often seen as a negative sentiment."}, "language_style": {"answer": "Tactful and warm", "rationale": "The entry uses query-style language to understand the respondent ' s attitude and perception of self-evaluation rather than direct conclusions or criticism. At the same time, this mild approach can increase the willingness of respondents to share real ideas."}, "time_dimension": {"answer": "Present", "rationale": "The entry is an inquiry into the testee's perception and assessment of whether he or she is a 鈥渂etter person鈥?and does not address past, present or future behaviour or mentality. The temporal dimension of the article is therefore beyond judgement."}, "sentence_type": "Interrogative sentence", "pos": {"nouns": {"Questionnaire Entry": 1, "person": 2, "Perfect": 1}, "verbs": {"I think": 1, "be": 1, "Perfect": 1}, "adjectives": {"Perfect": 1}, "numerals": {"4": 1}, "classifiers": {"individual": 1}, "pronouns": {"you": 1}}}, {"content": "Five. If the shopkeeper's service is not good, will you tell their manager?", "word_count": 21, "label": "", "sentence_structure": "Subject鈥搗erb鈥搊bject", "subjective_objective": {"answer": "Subjective", "rationale": "Because \"will you tell their manager\" is an expression of subjective will, a subjective view of the patient."}, "sentence_sentiment": {"answer": "Positive", "rationale": "Because the purpose of this \"Tell the Manager\" act in the entry is to improve the service, and it's positive."}, "language_style": {"answer": "Calm and direct", "rationale": "The entry is more concise in language and asks if the interviewees will be able to report to their manager when they encounter a bad attitude of shopkeepers."}, "time_dimension": {"answer": "Present", "rationale": "The word \"will\" indicates that it is an action or decision to be taken, and \"to tell their manager\" indicates that something may happen at a certain point in the future, so the entry asks about the person's current attitude and behaviour."}, "sentence_type": "Interrogative sentence", "pos": {"nouns": {"Service attitude": 1, "Shop clerk": 1, "Manager": 1}, "verbs": {"Yes": 1, "Tell": 1, "Do it": 1}, "adjectives": {"Not good": 1}, "numerals": {"5": 1}, "pronouns": {"you": 1}}}, {"content": "- Don't you like your pictures?", "word_count": 11, "label": "", "sentence_structure": "Subject鈥損redicate", "subjective_objective": {"answer": "Subjective", "rationale": "Because \"unusually\" is a subjective attitude or view that expresses the patient's assessment and perception of his own photo."}, "sentence_sentiment": {"answer": "Negative", "rationale": "Because the \"unusually admired his photo\" act in the entry is negative."}, "language_style": {"answer": "Calm and direct", "rationale": "The entry appears in a simple question, with no excessive rhetorical or emotional rendering, and is more objective in tone. Moreover, the article uses more direct words, such as \"frequent\" or \"approval,\" and does not use vague words or expressions to guide respondents. These indicate that the language style of the article is calm and straightforward."}, "time_dimension": {"answer": "Present", "rationale": "Now, the entry \"unusual\" means less frequent behaviour, and \"approval\" behaviour should be an individual's subjective feeling, so it means the present. Besides, the word \"you\" uses the usual current pattern, which means that the question is whether the testees are often feeling this way."}, "sentence_type": "Interrogative sentence", "pos": {"nouns": {"you": 1, "self": 1, "Photos": 1}, "verbs": {"Enjoy": 1, "Regular": 1, "- You're not": 1}, "adverbs": {"not": 1}, "numerals": {"6": 1}}}, {"content": "Do you feel bad about being criticized?", "word_count": 12, "label": "", "sentence_structure": "Subject鈥搗erb鈥搊bject", "subjective_objective": {"answer": "Subjective", "rationale": "Because \"distress\" is a subjective feeling and emotional reaction of one's person, based on personal values, beliefs, experiences, etc. While criticism by others can lead to some objective consequences (such as affecting self-esteem or interpersonal relationships), the problem is primarily one of the emotional reactions of the critic, and is therefore a subjective one."}, "sentence_sentiment": {"answer": "Negative", "rationale": "Because the \"worried\" emotion in the entry is negative."}, "language_style": {"answer": "Tactful and warm", "rationale": "The language style of the entry is euphemism, as it uses a soft question-and-answer approach and a conversation with a sensitive tone. Nor is it expressed in a too direct or negative way, which makes the interviewee feel respected and understood."}, "time_dimension": {"answer": "Present", "rationale": ""}, "sentence_type": "Interrogative sentence", "pos": {"nouns": {"person": 1, "Criticism": 1, "you": 1, "Sorry": 1}, "verbs": {"Yes": 2, "I think": 1}, "numerals": {"7": 1}, "pronouns": {"others": 1}}}, {"content": "- You don't say anything to people?", "word_count": 14, "label": "", "sentence_structure": "Subject鈥搗erb鈥搊bject", "subjective_objective": {"answer": "Subjective", "rationale": "Because it takes subjective will and thought to say the truth."}, "sentence_sentiment": {"answer": "Negative", "rationale": "Because the \"very little real opinion\" act in the entry is negative."}, "language_style": {"answer": "Calm and direct", "rationale": "The language style of the entry is more calm and straightforward, the problem is clear, the language is not used too much rhetorical or emotional, and the tone is more objective and neutral.\""}, "time_dimension": {"answer": "Present", "rationale": ""}, "sentence_type": "Interrogative sentence", "pos": {"nouns": {"person": 2, "Observations": 1}, "verbs": {"Say it": 2, "Very few": 1}, "adverbs": {"very": 1}, "numerals": {"8": 1}, "pronouns": {"you": 1}}}, {"content": "- You're skeptical about the praises of others?", "word_count": 14, "label": "", "sentence_structure": "Subject鈥搗erb鈥搊bject", "subjective_objective": {"answer": "Subjective", "rationale": "Because \"suspicious attitude\" is a subjective psychological state."}, "sentence_sentiment": {"answer": "Negative", "rationale": "Because the \"suspicion\" attitude is usually negative."}, "language_style": {"answer": "Tactful and warm", "rationale": "The entry uses a more intimate and open expression that leads to sharing one鈥檚 own views by asking the other whether they have doubts about praise, which is more attractive and interactive. At the same time, it is more resonant and participatory than direct statements or orders."}, "time_dimension": {"answer": "Present", "rationale": "In the entry, \"Are you skeptical?\" is the tester's current attitude and behavior, so it's \"now\"."}, "sentence_type": "Interrogative sentence", "pos": {"nouns": {"Praise": 1, "Attitude": 1}, "verbs": {"Hold": 1, "Doubt": 1}, "adverbs": {"not": 1, "- You're not": 1}, "numerals": {"9": 1}}}, {"content": "Do you always feel worse than others?", "word_count": 12, "label": "", "sentence_structure": "Subject鈥搗erb鈥搊bject", "subjective_objective": {"answer": "Subjective", "rationale": "Since the entry examines the patient ' s own subjective feelings and perceptions, it asks whether he or she often has a self-demeanor."}, "sentence_sentiment": {"answer": "Negative", "rationale": "Because the attitude of \"I feel worse than anyone else\" in the entry is negative."}, "language_style": {"answer": "Calm and direct", "rationale": "The language style of the entry is more straightforward, with no excessive adoration or emotional colour, but rather simply raises a question and is more objective and neutral in tone."}, "time_dimension": {"answer": "Present", "rationale": "The keyword \"always\" indicates that this mentality is present and that the entry is therefore a judgement of the behaviour or mentality of the moment."}, "sentence_type": "Interrogative sentence", "pos": {"nouns": {"self": 1, "others": 1}, "verbs": {"I think": 1, "Bad": 1}, "adjectives": {"Total": 1}, "adverbs": {"Always": 1}, "numerals": {"10": 1}}}, {"content": "Are you satisfied with your appearance?", "word_count": 10, "label": "", "sentence_structure": "Subject鈥搗erb鈥搊bject", "subjective_objective": {"answer": "Subjective", "rationale": "Because satisfaction or dissatisfaction is a personal view and feeling and reflects subjective attitudes."}, "sentence_sentiment": {"answer": "Neutral", "rationale": "Because the entry is asked about the satisfaction of the individual external appearance and does not involve positive or negative emotional colours."}, "language_style": {"answer": "Tactful and warm", "rationale": "The entry is more personal and interactive when it is spoken in a gentle and inviting manner, rather than in direct statements or orders. At the same time, the article is more balanced, does not have too much emotional overtones, and prefers to allow respondents to express their satisfaction with their appearance."}, "time_dimension": {"answer": "Present", "rationale": "The entry is for the current satisfaction of the testee with his or her appearance, so the time dimension is now judged."}, "sentence_type": "Interrogative sentence", "pos": {"nouns": {"Appearance": 1, "self": 1}, "verbs": {"Satisfied": 1}, "numerals": {"11": 1}, "pronouns": {"you": 1}}}, {"content": "- Do you think you're better than anyone else?", "word_count": 13, "label": "", "sentence_structure": "Subject鈥搗erb鈥搊bject", "subjective_objective": {"answer": "Subjective", "rationale": "Because \"thinking that you are less capable than others\" is a subjective self-evaluation and perception."}, "sentence_sentiment": {"answer": "Negative", "rationale": "Because the expression \"failer\" in the entry is negative."}, "language_style": {"answer": "Calm and direct", "rationale": "The entry is more direct and objective in language style, with no excessive rhetorical or emotional rendering. It asks the respondents in a concise and clear manner about their abilities and those of others, and is calmer and more rational."}, "time_dimension": {"answer": "Past", "rationale": "The question is about the testee ' s self-awareness and evaluation of the past."}, "sentence_type": "Interrogative sentence", "pos": {"nouns": {"Capacity": 1, "person": 1}, "verbs": {"I think": 1, "- Yeah": 1, "Bad": 1, "- You're not": 1}, "adjectives": {"Your own": 1}, "adverbs": {"More than anyone else": 1}, "pronouns": {"you": 1}}}, {"content": "At the party, you're the only one who's not dressed properly.", "word_count": 23, "label": "", "sentence_structure": "Subject鈥搗erb鈥搊bject", "subjective_objective": {"answer": "Subjective", "rationale": "Because the problem is to examine how the patient is dressed, which is based on the patient ' s personal subjective experience."}, "sentence_sentiment": {"answer": "Negative", "rationale": "Because the \"frightful\" sentiment in the entry is negative."}, "language_style": {"answer": "Tactful and warm", "rationale": "The language style of the entry is euphemistic. It asks the respondents in a gentle and friendly manner about their feelings in a given setting, without direct orders or evaluating the behaviour of the other."}, "time_dimension": {"answer": "Present", "rationale": "The words \"party\" and \"will feel uncomfortable\" indicate that this act or feeling is taking place at some point in the present day, and therefore is a state of affairs. And, \"You are dressed unofficially\" means that it is an act or state of behavior that is related to the individual himself, and points further to the present."}, "sentence_type": "Interrogative sentence", "pos": {"nouns": {"Party": 1, "person": 1, "Put it on": 1, "Feel": 1}, "verbs": {"Yes": 2, "I feel": 1, "not": 2}, "adjectives": {"Formal": 1, "I'm not comfortable": 1}, "numerals": {"One": 2, "III": 1}, "classifiers": {"individual": 1}, "pronouns": {"you": 1}}}, {"content": "Are you a popular man?", "word_count": 9, "label": "", "sentence_structure": "Subject鈥損redicate", "subjective_objective": {"answer": "Subjective", "rationale": "Because \"popular\" is a subjective evaluation, depending on the individual's evaluation and perception by others."}, "sentence_sentiment": {"answer": "Neutral", "rationale": "Since the entry asks whether the individual is popular, it does not involve positive or negative behaviour or attitudes."}, "language_style": {"answer": "Tactful and warm", "rationale": "The entry is more euphemistic. It takes the form of a mild question, avoids direct orders or statements, and gives people a sense of affection and respect. Moreover, the issue does not involve the possibility of criticism or accusations, thereby reducing some of the potential negative emotional reactions."}, "time_dimension": {"answer": "Present", "rationale": "The entry asks the testee's current state and characteristics, \"Is he a popular person?\" The phrase expresses a subjective feeling, so the temporal dimension is now."}, "sentence_type": "Interrogative sentence", "pos": {"nouns": {"person": 1, "Questionnaire Entry": 1}, "verbs": {"be": 1}, "adjectives": {"individual": 1, "Welcome": 1}, "numerals": {"14": 1}, "pronouns": {"you": 1}}}, {"content": "- Do you think you're attractive?", "word_count": 10, "label": "", "sentence_structure": "Subject鈥搗erb鈥搊bject", "subjective_objective": {"answer": "Subjective", "rationale": "Because \"think\" is a subjective verb, which expresses the patient's own perception and feelings, it is viewed from the patient's subjective perspective."}, "sentence_sentiment": {"answer": "Neutral", "rationale": "The entry is concerned with the individual ' s assessment of himself and does not involve any positive or negative emotional aspects."}, "language_style": {"answer": "Calm and direct", "rationale": ""}, "time_dimension": {"answer": "Present", "rationale": "Now the entry is to ask the testee's current perception or feelings, and the temporal dimension is now. The keyword \"you think\" indicates that it is subjective and that there is no clear time limit, so it is now."}, "sentence_type": "Interrogative sentence", "pos": {"nouns": {"Charm": 1}, "verbs": {"I think": 1, "have": 1}, "adjectives": {"very": 1}, "numerals": {"15": 1}, "pronouns": {"self": 1}}}, {"content": "Do you have a sense of humor?", "word_count": 6, "label": "", "sentence_structure": "Subject鈥搗erb鈥搊bject", "subjective_objective": {"answer": "Subjective", "rationale": "Because sense of humor is a subjective attitude or character, different people may have different views and assessments of the same things."}, "sentence_sentiment": {"answer": "Positive", "rationale": "Because \"honorous\" is a positive quality."}, "language_style": {"answer": "Tactful and warm", "rationale": ""}, "time_dimension": {"answer": "Present", "rationale": ""}, "sentence_type": "Interrogative sentence", "pos": {"nouns": {"Humor": 1}, "verbs": {"have": 1}}}, {"content": "Is your job your specialty?", "word_count": 11, "label": "", "sentence_structure": "Subject鈥損redicate", "subjective_objective": {"answer": "Subjective", "rationale": "This is a self-assessment by patients, expressing their subjective views."}, "sentence_sentiment": {"answer": "Neutral", "rationale": "Because the entry does not have any emotional color."}, "language_style": {"answer": "Calm and direct", "rationale": "The language style of the entry is more straightforward, and there is no over-doping or emotional colour, and questions are asked about the factual nature of the other party ' s work as its area of expertise."}, "time_dimension": {"answer": "Present", "rationale": "The entry asks the testee about his current situation, i.e. whether he considers his work to be his specialty and therefore his current state of affairs."}, "sentence_type": "Interrogative sentence", "pos": {"nouns": {"Work": 1, "Expertise": 1}, "verbs": {"be": 1, "you": 1}, "adverbs": {"Current": 1}, "numerals": {"17": 1}, "classifiers": {"individual": 1}, "pronouns": {"- You're not": 1}}}, {"content": "- 18, you know how to fit?", "word_count": 8, "label": "", "sentence_structure": "Subject鈥損redicate", "subjective_objective": {"answer": "Subjective", "rationale": "It is a subjective ability or skill to know how to fit clothes."}, "sentence_sentiment": {"answer": "Neutral", "rationale": "Because the entry does not have any emotional color."}, "language_style": {"answer": "Tactful and warm", "rationale": "The language style of the entry is euphemism. It asks the interviewee whether he/she is good at matching clothes in a friendly and easy way, rather than directly questioning or criticizing their abilities."}, "time_dimension": {"answer": "Present", "rationale": ""}, "sentence_type": "Interrogative sentence", "pos": {"nouns": {"Match": 1, "Clothes": 1}, "verbs": {"Yes": 1}, "numerals": {"18": 1}}}, {"content": "Are you calm when you're in a crisis?", "word_count": 8, "label": "", "sentence_structure": "Subject鈥搗erb鈥搊bject", "subjective_objective": {"answer": "Subjective", "rationale": "Because \"cool\" is a subjective feeling and attitude. While it is objective to act in a critical situation, it is the individual's subjective experience and perception that is to feel calm."}, "sentence_sentiment": {"answer": "Neutral", "rationale": "Because the entry does not have any emotional color."}, "language_style": {"answer": "Calm and direct", "rationale": "The entry directly asks whether the person under investigation can remain calm in a dangerous situation, with a more straightforward language style, and without excessive rhetorical or emotional colours.\""}, "time_dimension": {"answer": "Future", "rationale": "The entry asks if the testee can remain calm at a critical time, which needs to be tested in the future when the actual situation is present, and therefore judged to be the future."}, "sentence_type": "Interrogative sentence", "pos": {"nouns": {"Emergency": 1, "Calm down": 1}, "verbs": {"have": 1}, "adverbs": {"very": 1}, "numerals": {"19": 1}}}, {"content": "Are you working with anyone else?", "word_count": 9, "label": "", "sentence_structure": "Subject鈥搗erb鈥搊bject", "subjective_objective": {"answer": "Subjective", "rationale": "Subjectivity: Because \"cooperativeness\" is a subjective feeling and evaluation. While this perception may be influenced by objective factors, it remains a subjective description."}, "sentence_sentiment": {"answer": "Positive", "rationale": "Because the \"cooperative\" attitude in the entry is positive."}, "language_style": {"answer": "Calm and direct", "rationale": "The language style of the entry is straightforward, as it asks the interviewee in a simple and straightforward manner how he or she is performing in a team effort without using any rhetorical or emotional expressions."}, "time_dimension": {"answer": "Present", "rationale": "The words \"cooperative\" and \"infinite\" in \"you work with others\"? indicate that the question is asking the testee about the current situation and is therefore a matter of current practice."}, "sentence_type": "Interrogative sentence", "pos": {"nouns": {"you": 1, "others": 1, "Cooperation": 1, "No space": 1}, "verbs": {"and": 1, "have": 1}, "adverbs": {"- You're not": 1}, "numerals": {"20": 1}, "classifiers": {"individual": 1}}}, {"content": "Do you think you're just an ordinary person?", "word_count": 12, "label": "", "sentence_structure": "Subject鈥搗erb鈥搊bject", "subjective_objective": {"answer": "Subjective", "rationale": "Because \"think\" is what patients think about themselves and evaluate them."}, "sentence_sentiment": {"answer": "Negative", "rationale": "Because the entry expresses a negative and self-demeaning emotional attitude, it is negative."}, "language_style": {"answer": "Calm and direct", "rationale": "The entry is in a concise and clear statement, asking the interviewees about their perception, without using excessive rhetoric or emotional colours, and with more objective and direct tone."}, "time_dimension": {"answer": "Past", "rationale": "The entry asks the testee about his/her self, the time of questioning is \"himself\", and does not have a clear time dimension to the future or to the present, but rather about the self-perception of the past."}, "sentence_type": "Interrogative sentence", "pos": {"nouns": {"you": 1, "self": 1, "Normal people": 1}, "verbs": {"I think": 1, "be": 1, "- You're not": 1}, "adjectives": {"individual": 1}, "numerals": {"21": 1}}}, {"content": "22, do you often wish you looked like someone else?", "word_count": 14, "label": "", "sentence_structure": "Subject鈥搗erb鈥搊bject", "subjective_objective": {"answer": "Subjective", "rationale": "Because \"favorite\" is a subjective emotion and attitude that looks like someone who's objective."}, "sentence_sentiment": {"answer": "Negative", "rationale": "Because the act of \"hope to look like someone\" in the entry may imply excessive attention and dissatisfaction with external images, and is a manifestation of negative emotions."}, "language_style": {"answer": "Calm and direct", "rationale": "This is a topic that is asked in a straightforward language, is not too much rhetorical or emotional, and is more focused on getting the real thoughts and feelings of the respondents. This style is usually more common in questionnaires, because they make it easier for respondents to understand and answer questions, thus improving the accuracy and reliability of the data."}, "time_dimension": {"answer": "Present", "rationale": "The entry is for the current behaviour and mindset of the subject, and the keyword \"often\" indicates that it is a customary act, while \"hope\" indicates that there is a expectation or desire for a certain situation. Thus, the time dimension of the entry can be judged as now."}, "sentence_type": "Interrogative sentence", "pos": {"nouns": {"self": 1, "person": 1}, "verbs": {"Hope": 1, "Long": 1}, "adverbs": {"Regular": 1}, "numerals": {"22": 1}, "pronouns": {"Somethin'": 1}}}, {"content": "- Are you always envious of what people have done?", "word_count": 11, "label": "", "sentence_structure": "Subject鈥損redicate", "subjective_objective": {"answer": "Subjective", "rationale": "Because Enviing the Achievement is a subjective feeling or attitude."}, "sentence_sentiment": {"answer": "Negative", "rationale": "Because the \"enjoyed for achievement\" act in the entry is negative."}, "language_style": {"answer": "Tactful and warm", "rationale": "The language style of the entry is more euphemistic, not directly asking the other side whether they are jealous, but in a gentle way that leads the respondents to think and express their thoughts. This expression is more acceptable and willing to share their true feelings."}, "time_dimension": {"answer": "Present", "rationale": "The keyword \"often\" indicates that this is a topic of customary sexual behaviour of the testee, and that \"envious achievement\" is a modern act, so the answer is now."}, "sentence_type": "Interrogative sentence", "pos": {"nouns": {"Achievements": 1, "Envious": 1}, "verbs": {"Regular": 1, "others": 1}, "adverbs": {"- You're not": 1}, "numerals": {"23": 1}}}, {"content": "- You give up what you like to make others feel better?", "word_count": 20, "label": "", "sentence_structure": "Subject鈥搗erb鈥搊bject", "subjective_objective": {"answer": "Subjective", "rationale": "Because \"to make others feel better\" is the patient's own perception and attitude to the situation. It involves the patient's inner world and emotional experience, and therefore falls within the scope of subjective examination."}, "sentence_sentiment": {"answer": "Negative", "rationale": "Because the \"release\" in the entry is negative."}, "language_style": {"answer": "Tactful and warm", "rationale": "The entry is asked in a gentle manner and attempts to generate reflection and self-awareness among the respondents. It does not direct orders or criticism of the behaviour of the other party, but rather encourages the other to express his thoughts and feelings through questioning, with some degree of kinship and interaction. At the same time, the language of the issue is more objective, with no excessive emotional rendering or rhetorical techniques, and more in keeping with a warmer description."}, "time_dimension": {"answer": "Past", "rationale": "From a time perspective, the question does not have a clear point of reference to the past, present or future, so the answer should be \"now\"."}, "sentence_type": "Interrogative sentence", "pos": {"nouns": {"24": 1, "Others": 1, "Sorry": 1, "Yeah": 1, "Yes": 1}, "verbs": {"Abandon": 1, "Do it": 1}, "adverbs": {"not": 1}, "numerals": {"24": 1}, "pronouns": {"you": 1}}}, {"content": "- Will you dress up to please others?", "word_count": 12, "label": "", "sentence_structure": "Subject鈥搗erb鈥搊bject", "subjective_objective": {"answer": "Subjective", "rationale": "The basis for the determination: This is a question of examining patients' subjective attitudes and asking whether they are willing to dress for the pleasure of others. Although \"dressing\" can be considered an objective act, the motivation and will of the patient is a key factor in this issue, and the issue is therefore subjective."}, "sentence_sentiment": {"answer": "Negative", "rationale": "Because the act of \"love\" in the entry is derogatory and indicates that it is a way of giving up its true thoughts and behaviour to suit others, and is therefore negative."}, "language_style": {"answer": "Tactful and warm", "rationale": "The entry is more personal and interactive when it is spoken in a gentle, inviting tone, asking whether the other party is dressed to please others rather than directly speaking or ordering. At the same time, the article is used more softly, with less emphasis or criticism, and more in a way that reflects the respect and understanding of researchers."}, "time_dimension": {"answer": "Present", "rationale": "The entry is for the testee's current behavior or habits, and the keyword \"will\" and \"dress\" point to the current state or behaviour. So it's now."}, "sentence_type": "Interrogative sentence", "pos": {"nouns": {"25": 1, "you": 1, "person": 1, "Beloved": 1, "others": 1, "Get dressed": 1}, "verbs": {"Yes": 1}, "numerals": {"25": 1}, "pronouns": {"you": 1}}}, {"content": "- You're not doing much of the things you're not willing to do?", "word_count": 15, "label": "", "sentence_structure": "Subject鈥搗erb鈥搊bject", "subjective_objective": {"answer": "Subjective", "rationale": "Because \"or not to be reluctant\" is the patient's subjective feeling and attitude, and it is a subjective examination."}, "sentence_sentiment": {"answer": "Negative", "rationale": "Because \"suspending to do a lot of things you don't want to do\" is a negative act."}, "language_style": {"answer": "Tactful and warm", "rationale": "The language style of the entry is euphemism, because it uses a question-and-answer approach, not a direct statement or an order. Nor is it using overly strong emotional vocabulary or rhetorical techniques, but rather a plain tone to guide respondents to think and share their experiences and feelings. Such expression makes it easier for respondents to feel comfortable and comfortable and to answer questions more honestly."}, "time_dimension": {"answer": "Present", "rationale": "The topic should therefore be judged now."}, "sentence_type": "Interrogative sentence", "pos": {"nouns": {"Questionnaire": 1, "Entry": 1, "Yes": 1}, "verbs": {"Rare": 1, "Do it": 1}, "adjectives": {"I don't want to": 1}, "adverbs": {"Many": 1, "- You're not": 1}, "numerals": {"26": 1}}}, {"content": "27, you let others control your life?", "word_count": 13, "label": "", "sentence_structure": "Subject鈥搗erb鈥搊bject", "subjective_objective": {"answer": "Subjective", "rationale": "Because this option concerns the patient ' s willingness to be controlled or influenced, it is a manifestation of subjective attitudes or values."}, "sentence_sentiment": {"answer": "Negative", "rationale": "Because the act of being dominated by others is negative."}, "language_style": {"answer": "Calm and direct", "rationale": ""}, "time_dimension": {"answer": "Present", "rationale": "The keywords \"already\" and \"dominantly\" suggest that this is a question of describing current behaviour and asking whether the person tested lets others control their own lives."}, "sentence_type": "Interrogative sentence", "pos": {"nouns": {"person": 1, "Life": 1}, "verbs": {"I'm not going anywhere": 1, "Rule": 1}, "numerals": {"27": 1}}}, {"content": "Do you think you have more merit than disadvantage?", "word_count": 12, "label": "", "sentence_structure": "Subject鈥搗erb鈥搊bject", "subjective_objective": {"answer": "Subjective", "rationale": "Because the entry examines the patient ' s subjective views and self-perception."}, "sentence_sentiment": {"answer": "Neutral", "rationale": "Because the entry was not emotional, it was merely an inquiry into the interviewee ' s own assessment."}, "language_style": {"answer": "Calm and direct", "rationale": "The entry directly asks the respondents about their strengths and weaknesses, the language style is more objective and direct, and there is no excessive rhetoric or emotional colour, and therefore the language style of \"cool and direct\"."}, "time_dimension": {"answer": "Present", "rationale": "The entry asks the testee about his current views or attitudes and therefore falls into the category of \"now\"."}, "sentence_type": "Interrogative sentence", "pos": {"nouns": {"Advantages": 1, "Disadvantages": 1}, "verbs": {"I think": 1, "- Yeah": 1, "More": 1}, "numerals": {"28": 1}}}, {"content": "29, do you always say sorry to people, even if it's not your fault?", "word_count": 20, "label": "", "sentence_structure": "Subject鈥搗erb鈥搊bject", "subjective_objective": {"answer": "Subjective", "rationale": "Because \"sorry\" is a subjective expression of emotion, even if it's not your fault, saying \"sorry\" is the patient's own feelings and attitude."}, "sentence_sentiment": {"answer": "Positive", "rationale": "Because the behavior in the entries \"showing people's apologies\" is usually considered positive social skills and attitudes."}, "language_style": {"answer": "Tactful and warm", "rationale": "The entry is based on a judgment that is based on asking whether the other party is often sorry, even if he is not wrong. It is a gentle, inviting, friendly and interactive expression that makes people feel comfortable and less susceptible to confrontation. At the same time, it is a euphemism that better guides the interviewee to reflect on his behaviour and answer questions honestly."}, "time_dimension": {"answer": "Present", "rationale": ""}, "sentence_type": "Interrogative sentence", "pos": {"nouns": {"Apologize": 1, "person": 1}, "verbs": {"Call": 1, "Say it": 1, "Sorry": 1, "Regular": 1, "in": 1, "Wrong": 1, "Situation": 1}, "adjectives": {"It's not your fault": 1}, "adverbs": {"Even if": 1, "Regular": 1}, "numerals": {"29": 1}, "pronouns": {"you": 1}}}, {"content": "Would it upset you if you broke someone's heart without any intention?", "word_count": 21, "label": "", "sentence_structure": "Subject鈥搗erb鈥搊bject", "subjective_objective": {"answer": "Subjective", "rationale": "The examination of the patient ' s own feelings and attitudes is subjective."}, "sentence_sentiment": {"answer": "Positive", "rationale": "Because the act of \"worship\" in the entry shows a certain sense of guilt and responsibility, which is a positive emotional attitude."}, "language_style": {"answer": "Tactful and warm", "rationale": "The entry uses a soft and gentle language style, raises questions in question and gives the other side a choice and a space to answer, and expresses them more amicably and respectfully. At the same time, the question is not spoken in too many direct languages or in a strong tone, making it easier for the interviewee to accept and give a true answer."}, "time_dimension": {"answer": "Past", "rationale": "The time dimension of this questionnaire entry is therefore present and future. That is, even if no similar situation has occurred in the past, the testee may feel sad in the future because it hurts others."}, "sentence_type": "Interrogative sentence", "pos": {"nouns": {"Situation": 1, "others": 1, "Heart": 1}, "verbs": {"Yes": 2, "Wounded": 1, "Sorry": 1, "in": 1, "Unintentionally": 1}, "adjectives": {"On purpose": 1}, "adverbs": {"If": 1}, "numerals": {"30": 1}, "pronouns": {"you": 1}}}, {"content": "31 Do you want to have more talent and talent?", "word_count": 16, "label": "", "sentence_structure": "Subject鈥搗erb鈥搊bject", "subjective_objective": {"answer": "Subjective", "rationale": "Because the desire for more talent and talent is a subjective desire of a person, reflecting his/her personal attitude and expectations."}, "sentence_sentiment": {"answer": "Positive", "rationale": "Because \"hope\" is a positive emotional color, and \"more talent and talent\" is a positive description."}, "language_style": {"answer": "Tactful and warm", "rationale": "The entry is more personal and interactive when it is spoken in a gentle, invitation-like tone, rather than in direct statements or orders. At the same time, the issue encourages respondents to reflect on themselves and share their ideas, and therefore language style is more personal and personal."}, "time_dimension": {"answer": "Future", "rationale": "The entry contains visions and expectations for the future, \"to have more of yourself\" expressing a sense of hope, while \"the future\" indicating that this expectation has not been met, and therefore judgement is the future."}, "sentence_type": "Interrogative sentence", "pos": {"nouns": {"Talent": 2, "Hope": 1}, "verbs": {"self": 2, "Existing": 1, "More": 1, "have": 1}, "adverbs": {"More": 1}, "numerals": {"31": 1}}}, {"content": "32 - Do you listen to people often?", "word_count": 11, "label": "", "sentence_structure": "Subject鈥損redicate", "subjective_objective": {"answer": "Subjective", "rationale": "Since the problem concerns patients ' subjective attitudes and behavioural tendencies, it is a subjective question to ask whether they are often heard."}, "sentence_sentiment": {"answer": "Neutral", "rationale": "Since the entry does not address specific behaviour or attitudes, it is simply a question of whether people are often heard and therefore has no obvious emotional colour."}, "language_style": {"answer": "Tactful and warm", "rationale": "The language style of the entry is more euphemistic, and the questioner does not direct or instruct the interviewee about what to do, but rather expresses his thoughts in a manner that is asked. At the same time, the polite expression \"often\" is used to make the interviewee more comfortable and respectful."}, "time_dimension": {"answer": "Present", "rationale": "The entry \"you\" means the person who was tested, while \"recurring\" means a habit of behaviour that uses the general current, so the temporal dimension of the article is now."}, "sentence_type": "Interrogative sentence", "pos": {"nouns": {"Observations": 1, "person": 1}, "verbs": {"Listen": 1, "Remove": 1, "Do it": 1}, "adverbs": {"Regular": 1}, "numerals": {"32": 1}}}, {"content": "At the party, did you wait for someone to say hello first?", "word_count": 17, "label": "", "sentence_structure": "Subject鈥搗erb鈥搊bject", "subjective_objective": {"answer": "Subjective", "rationale": "Because of this problem, patients are examined for subjective attitudes, i.e. their behaviour and reactions in social settings."}, "sentence_sentiment": {"answer": "Neutral", "rationale": "As this entry does not express any positive or negative emotional aspects, it merely asks the respondents about their behaviour during the meeting."}, "language_style": {"answer": "Tactful and warm", "rationale": ""}, "time_dimension": {"answer": "Present", "rationale": "So output is \"now\"."}, "sentence_type": "Interrogative sentence", "pos": {"nouns": {"Party": 1, "person": 1}, "verbs": {"Wait": 1, "Fight": 1, "Hello": 1}, "adverbs": {"Regular": 1}, "numerals": {"33": 1}, "pronouns": {"you": 1}}}, {"content": "34 - You look in the mirror more than three times a day?", "word_count": 11, "label": "", "sentence_structure": "Subject鈥損redicate", "subjective_objective": {"answer": "Subjective", "rationale": "Because looking in the mirror more than three times a day is a personal custom and a choice of behaviour, and is the patient ' s subjective attitude."}, "sentence_sentiment": {"answer": "Neutral", "rationale": "Because the entry does not have any emotional color."}, "language_style": {"answer": "Calm and direct", "rationale": "The language style of the entry is calm and straightforward, and the question is clearly and directly asked about the routine behaviour of the person under investigation, without too much ado or emotional expression."}, "time_dimension": {"answer": "Present", "rationale": ""}, "sentence_type": "Interrogative sentence", "pos": {"nouns": {"Mirror": 1, "Son": 1}, "verbs": {"Photo": 1, "Pass": 1, "Super": 1}, "adjectives": {"More": 1}, "adverbs": {"III": 1, "Number of times": 1}, "numerals": {"34": 1}, "classifiers": {"Oh, my God": 1}, "pronouns": {"you": 1}}}, {"content": "- You have a strong personality?", "word_count": 7, "label": "", "sentence_structure": "Subject鈥損redicate", "subjective_objective": {"answer": "Subjective", "rationale": "Because personality is a subjective character of a human being."}, "sentence_sentiment": {"answer": "Neutral", "rationale": "Because the entry does not have any emotional color."}, "language_style": {"answer": "Calm and direct", "rationale": "The language style of the entry is more calm and straightforward, and the interviewee is asked about his personality in a concise and clear question form, without adding too many emotional or rhetorical techniques, which is more objective and direct."}, "time_dimension": {"answer": "Present", "rationale": "The keyword is \"Is your personality strong?\" This is a general question of the current state of affairs, asking the testee about his current personality. So the judgment is based on the present."}, "sentence_type": "Interrogative sentence", "pos": {"nouns": {"Personality": 1, "Strong": 1}, "verbs": {"have": 1, "Here": 1}, "adjectives": {"very": 1}, "numerals": {"35": 1}}}, {"content": "- You're a good leader?", "word_count": 10, "label": "", "sentence_structure": "Subject鈥損redicate", "subjective_objective": {"answer": "Subjective", "rationale": "Because \"excellent\" is a subjective term for evaluation, different people define and define leadership differently."}, "sentence_sentiment": {"answer": "Positive", "rationale": "Great. Because the description in the entry is a recognition and praise of itself, it expresses a positive emotional attitude.\""}, "language_style": {"answer": "Tactful and warm", "rationale": "Warm and warm."}, "time_dimension": {"answer": "Future", "rationale": "The entry asks the testee about his vision and expectations of his future potential and abilities, the keywords being \"good leader\" and \"you\", all pointing to situations that have not yet occurred and that may arise in the future. The entry can therefore be judged to be future."}, "sentence_type": "Interrogative sentence", "pos": {"nouns": {"Leader": 1, "Excellent": 1, "you": 1}, "verbs": {"be": 1, "Leadership": 1}, "numerals": {"36": 1}}}, {"content": "- How's your memory?", "word_count": 7, "label": "", "sentence_structure": "Subject鈥損redicate", "subjective_objective": {"answer": "Subjective", "rationale": "Because \"good memory\" is the memory of a patient's self-assessment, it's subjective."}, "sentence_sentiment": {"answer": "Neutral", "rationale": "Because the entry does not have any emotional color."}, "language_style": {"answer": "Calm and direct", "rationale": "This language style is applied to questions of fact, description and objectivity in psychological questionnaires, and provides better access to accurate data and information."}, "time_dimension": {"answer": "Present", "rationale": "The entry is for the present situation of the person being tested, i.e. the current state of memory, and therefore for the present."}, "sentence_type": "Interrogative sentence", "pos": {"nouns": {"Memory": 1, "Okay": 1}, "verbs": {"very": 1, "- You're not": 1}, "numerals": {"37": 1}}}, {"content": "- Are you attracted to opposite sex?", "word_count": 9, "label": "", "sentence_structure": "Subject鈥搗erb鈥搊bject", "subjective_objective": {"answer": "Subjective", "rationale": "The attraction is viewed from a subjective perspective, as it is the subjective feeling and experience of the person being evaluated."}, "sentence_sentiment": {"answer": "Neutral", "rationale": "Since the entry merely asked the respondents whether they had attractive characteristics, it did not involve positive or negative attitudes or behaviour."}, "language_style": {"answer": "Calm and direct", "rationale": "The entry is asked in the form of direct, clear questions, without too many phrasing or guidance, and is used in a language that is more calm and direct. This expression may give the respondents some sense of twitch or stabbing, but in psychological studies, direct, concise language sometimes provides more accurate answers to the respondents."}, "time_dimension": {"answer": "Present", "rationale": "The entry is for the testee ' s current state and feelings and does not address past or future time dimensions, and therefore the judgement is now."}, "sentence_type": "Interrogative sentence", "pos": {"nouns": {"Heterosexual": 1, "Attractive": 1}, "verbs": {"have": 1}, "numerals": {"38": 1}}}, {"content": "- You know how to handle money?", "word_count": 6, "label": "", "sentence_structure": "Subject鈥損redicate", "subjective_objective": {"answer": "Subjective", "rationale": "Because \"know how to manage money\" is a personal concept and perception, subjective."}, "sentence_sentiment": {"answer": "Neutral", "rationale": "Because the entry asks about the individual ' s financial knowledge, it does not involve emotional or attitudinal orientation."}, "language_style": {"answer": "Calm and direct", "rationale": ""}, "time_dimension": {"answer": "Present", "rationale": "The entry asks if the testee has knowledge and skills in financial management, and the time dimension is now judged."}, "sentence_type": "Interrogative sentence", "pos": {"nouns": {"Questionnaire": 1, "Entry": 1, "Financial management": 1}, "verbs": {"Yes": 1}, "numerals": {"39": 1}}}, {"content": "Do you usually listen to others before buying clothes?", "word_count": 16, "label": "", "sentence_structure": "Subject鈥搗erb鈥搊bject", "subjective_objective": {"answer": "Subjective", "rationale": "The question is whether the patient will listen to others before buying clothes, which is the patient ' s subjective behaviour and thoughts."}, "sentence_sentiment": {"answer": "Neutral", "rationale": "Because the entry does not have any emotional color."}, "language_style": {"answer": "Tactful and warm", "rationale": ""}, "time_dimension": {"answer": "Present", "rationale": "The keyword \"before the clothes\" indicates that the act takes place with certain preconditions, while \"usually\" indicates that it is a regular act. In combination, the entry asks whether the person tested is used to listen to others before buying clothes, which is a current-day behavioural or mental description."}, "sentence_type": "Interrogative sentence", "pos": {"nouns": {"Clothes": 1, "Observations": 1}, "verbs": {"Listen": 1, "Buy": 1, "First": 1, "Remove": 1, "he": 1}, "adverbs": {"Usually": 1}, "numerals": {"40": 1, "you": 1}, "pronouns": {"Here": 1}}}]}

{"questionnaire_title": "Comprehensive mental health measurement (CPRS)", "questionnaire_category": "Mental health", "item_count": 41, "emotion_shift_overall": "From positive to negative", "language_style_overall": "Calm and direct", "items": [{"content": "1. Depression and sadness", "word_count": 4, "label": "", "sentence_structure": "Subject鈥揷opula鈥損redicative", "subjective_objective": {"answer": "Subjective", "rationale": "According to the above example, \"depressive distress\" is a state of emotional state described by the patient's subjective feeling and should therefore be classified as \"subjective\"."}, "sentence_sentiment": {"answer": "Negative", "rationale": "Because \"depressed and sad\" in the entry is a negative emotional attitude."}, "language_style": {"answer": "Calm and direct", "rationale": "The entry directly asked whether the respondents were often depressed and sad, and the language style was more objective and calm, and there was no excessive rhetoric or emotional coloring."}, "time_dimension": {"answer": "Past", "rationale": ""}, "sentence_type": "Interrogative sentence", "pos": {"nouns": {"Depression": 1, "Sad": 1}}}, {"content": "2 - High mood", "word_count": 4, "label": "", "sentence_structure": "Subject鈥搗erb鈥揷omplement", "subjective_objective": {"answer": "Subjective", "rationale": "Because \"emotional rise\" is a subjective feeling or experience that reflects the patient's subjective judgement and description of his or her emotional state, rather than an objective reality."}, "sentence_sentiment": {"answer": "Positive", "rationale": "Because \"frightful\" is a positive emotional expression."}, "language_style": {"answer": "Calm and direct", "rationale": "This is because the phrase is simple and straightforward, and it does not have any syllable or emotional repertoire, and is more objective and neutral. This expression helps the surveyer to understand more accurately the psychological condition and feelings of the interviewee, so that he can conduct in-depth research and analysis."}, "time_dimension": {"answer": "Present", "rationale": ""}, "sentence_type": "Interrogative sentence", "pos": {"verbs": {"High": 1}, "adjectives": {"Emotion": 1}}}, {"content": "Three, inner tension.", "word_count": 4, "label": "", "sentence_structure": "Subject鈥揷opula鈥損redicative", "subjective_objective": {"answer": "Subjective", "rationale": "Because \"inner tension\" is a subjective feeling or experience within a person, it cannot be directly observed or measured and therefore is subjective in its perspective."}, "sentence_sentiment": {"answer": "Negative", "rationale": "Because \"inner\" is an expression of a negative emotional state that may affect the physical, mental and quality of life of individuals."}, "language_style": {"answer": "Calm and direct", "rationale": "The entry is a straightforward way of asking the respondents whether they are nervous or not, without using excessive rhetorical techniques or emotional rendering, and relatively calm and straightforward language style."}, "time_dimension": {"answer": "Present", "rationale": "The entry indicates that the testee is experiencing or feeling emotional stress, and the temporal dimension is now judged."}, "sentence_type": "Negative declarative sentence", "pos": {"adjectives": {"Nervous": 1}}}, {"content": "4. Optimal mood", "word_count": 4, "label": "", "sentence_structure": "Subject鈥搗erb鈥揷omplement", "subjective_objective": {"answer": "Subjective", "rationale": "Because a hostile mood is the patient's subjective emotional experience and evaluation."}, "sentence_sentiment": {"answer": "Negative", "rationale": "Because the term \"hostility\" is usually negative in emotional terms, implying an unfriendly attitude or behaviour."}, "language_style": {"answer": "Calm and direct", "rationale": "The entry directly asks whether the interviewees are often hostile, are more concise and less nuanced or emotional and therefore are in a straight and calm manner."}, "time_dimension": {"answer": "Present", "rationale": "In the entry, \"hostile mood\" refers to the current subjective or emotional state of the person being tested, and therefore is judged as \"now\"."}, "sentence_type": "Imperative sentence", "pos": {"nouns": {"Hostile": 1, "The mood": 1}}}, {"content": "5. Insecurity means a subjective sense of lack of interest in the surrounding environment or interesting activities.", "word_count": 45, "label": "", "sentence_structure": "Subject鈥搗erb鈥搊bject", "subjective_objective": {"answer": "Subjective", "rationale": "Because the entry describes patients' subjective feelings and attitudes (i.e. \"inability\", \"ininterest\") and subjective emotional reactivity, it is subjective."}, "sentence_sentiment": {"answer": "Negative", "rationale": "Because the expression \"lack of interest in the surrounding environment or interesting activities\" and \"depressive ability to express sufficient emotional response\" in the entry are negative emotions and states, the judgement is negative."}, "language_style": {"answer": "Calm and direct", "rationale": "The entry is described as a direct illustration of sensory incapacity, does not use excessive rhetorical techniques or emotional rendering, and is more calm and straightforward in language style."}, "time_dimension": {"answer": "Present", "rationale": "The entry describes two possible behaviours or states: \"insensitiveness\" and \"defeating ability to express full emotional responses to environmental matters or people\" , which can be directed at the present situation or at the possibility of future. In it, terms such as \"discovery of interest\" and \"defeating\" indicate a dynamic process, and therefore can be judged to be possible in the present and in the future."}, "sentence_type": "Imperative sentence", "pos": {"nouns": {"Feel the ability to feel": 2, "Subjective": 1, "Peripheral environment": 1, "Interesting activity": 1, "Environmental matters": 1, "People": 1, "Emotional response": 1, "Capacity erosion": 1}, "verbs": {"Finger": 1, "I feel": 1, "Lack": 1, "Expression": 1, "Declining": 1}, "adjectives": {"Full": 1}, "numerals": {"5": 1}, "pronouns": {"None": 1}}}, {"content": "Negative pessimism refers to self-incrimination, self-incrimination, inferiority, remorse and self-defamation.", "word_count": 18, "label": "", "sentence_structure": "Subject鈥搗erb鈥搊bject", "subjective_objective": {"answer": "Subjective", "rationale": "Because \"self-responsible, self-incrimination, low self-esteem, remorse and self-denigration\" are subjective descriptions of the patient's mental state."}, "sentence_sentiment": {"answer": "Negative", "rationale": "Because the \"negative pessimism\" sentiment in the entry is negative."}, "language_style": {"answer": "Calm and direct", "rationale": "The entry uses short and clear language that directly describes negative pessimism, does not have too many rhetorical or emotional renderings and is more objective and incriminating."}, "time_dimension": {"answer": "Present", "rationale": "The entry describes a state of mind or emotion that the testee may have, or may have, \"negative pessimism.\" This mentality may appear now and may persist in the future. The keyword \"means\" means \"a definition or interpretation of the mentality, and \"self-responsible, self-incrimination, low self-esteem, remorse and self-denigration\" is the specific expression or characteristic of that mentality, all pointing to the state of the moment."}, "sentence_type": "Affirmative declarative sentence", "pos": {"nouns": {"Pessimism": 1, "You're gonna blame yourself": 1, "Sin": 1, "You're a low man": 1, "Regret": 1, "Self-defamation": 1}, "verbs": {"Finger": 1, "and": 1}, "adjectives": {"Negative": 1}}}, {"content": "Suicide", "word_count": 4, "label": "", "sentence_structure": "Subject鈥搗erb鈥搊bject", "subjective_objective": {"answer": "Subjective", "rationale": "Because \"suicide\" is a subjective psychological state or emotional experience of a person in a given situation."}, "sentence_sentiment": {"answer": "Negative", "rationale": "Because the suicide idea in the entry is a negative emotion and attitude."}, "language_style": {"answer": "Tactful and warm", "rationale": "The euphemism of the questionnaire entries is relatively euphemistic, using a gentle and respectful tone, which protects the privacy and emotions of the respondents, without asking them directly about sensitive topics or using too harsh language."}, "time_dimension": {"answer": "Past", "rationale": "At the same time, the term \"if or not\" indicates that it is a dual option, which is intended to understand whether the testee has such an idea."}, "sentence_type": "Interrogative sentence", "pos": {"nouns": {"Suicide": 1, "Thoughts": 1}}}, {"content": "8. Suspected diseases", "word_count": 2, "label": "", "sentence_structure": "Subject鈥搗erb鈥揷omplement", "subjective_objective": {"answer": "Subjective", "rationale": "The suspected disease is a psychological or emotional experience of the patient and is considered subjective."}, "sentence_sentiment": {"answer": "Negative", "rationale": "Because suspiciousness is a negative emotion and attitude."}, "language_style": {"answer": "Calm and direct", "rationale": "It presents a straightforward psychological symptom, with no excessive rhetorical or emotional tact, making it easier for respondents to understand and answer questions. At the same time, this calm and straightforward language style avoids unnecessary pressure or misled respondents."}, "time_dimension": {"answer": "Present", "rationale": ""}, "sentence_type": "Interrogative sentence", "pos": {"nouns": {"Suspected": 1}}}, {"content": "9 - It's a little trouble.", "word_count": 5, "label": "", "sentence_structure": "Subject鈥搗erb鈥搊bject", "subjective_objective": {"answer": "Subjective", "rationale": "Because \"fear for small things\" is a term that describes the patient's inner feelings and emotional state, and belongs to the patient's subjective thoughts and feelings."}, "sentence_sentiment": {"answer": "Negative", "rationale": "Because \"mind for small things\" in the entry is a negative behavior or attitude."}, "language_style": {"answer": "Calm and direct", "rationale": "The entry directly expresses the subject of questioning whether the other party is often troubled by minor matters, and is not using excessive rhetorical or emotional rendering, which is more objective and balanced."}, "time_dimension": {"answer": "Past", "rationale": ""}, "sentence_type": "Interrogative sentence", "pos": {"nouns": {"Things": 1, "Trouble": 1}, "verbs": {"Yes": 1}, "adjectives": {"Small": 1}, "numerals": {"Nine": 1, "Yes": 1}, "classifiers": {"individual": 1}}}, {"content": "10. The notion of coercion", "word_count": 4, "label": "", "sentence_structure": "Subject鈥搗erb鈥揷omplement", "subjective_objective": {"answer": "Subjective", "rationale": "Because \"coercive\" is a subjective experience and perception, reflecting the anxiety and the coercive thinking of the individual."}, "sentence_sentiment": {"answer": "Negative", "rationale": "Because \"coercive\" is often a negative psychological phenomenon, which means that individuals cannot escape uninvolved thinking or imagination."}, "language_style": {"answer": "Calm and direct", "rationale": "The entry describes in a calm and direct tone a common psychological phenomenon - the notion of coercion, which is not overly pedantic or emotional, so that the interviewee can view and answer the question more objectively."}, "time_dimension": {"answer": "Present", "rationale": "The keyword \"coercive perception\" denotes a problem or situation that exists today and does not have a clear time point, and therefore it is now judged."}, "sentence_type": "Interrogative sentence", "pos": {"nouns": {"The notion of coercion": 1}, "numerals": {"10": 1}}}, {"content": "Fear", "word_count": 2, "label": "", "sentence_structure": "Subject鈥揷opula鈥損redicative", "subjective_objective": {"answer": "Subjective", "rationale": "Because fear is an emotional experience, a subjective feeling for the patient."}, "sentence_sentiment": {"answer": "Negative", "rationale": "Because the word \"fear\" in the entry is negative and it means a negative emotion."}, "language_style": {"answer": "Calm and direct", "rationale": "The entry directly asks the interviewee whether he/she is afraid, and the language style is relatively simple, with no excessive rhetoric or emotional rendering, and prefers to speak in a calm and direct manner."}, "time_dimension": {"answer": "Past", "rationale": "The entry is for the purpose of asking the person being tested whether there is fear, and the temporal dimension is judged as past or present."}, "sentence_type": "Imperative sentence", "pos": {"nouns": {"Fear": 1}}}, {"content": "12. Ceremonial actions/acts", "word_count": 6, "label": "", "sentence_structure": "Subject鈥損redicate", "subjective_objective": {"answer": "Subjective", "rationale": "Because \"ceremonial actions/acts\" are often the expression of a particular custom or belief of individuals, reflecting their subjective attitudes and values. For example, \"Mediation every morning\" is a ritual exercise that demonstrates the values and beliefs of individuals and is their subjective choice."}, "sentence_sentiment": {"answer": "Neutral", "rationale": "Because the entry does not have any emotional color."}, "language_style": {"answer": "Tactful and warm", "rationale": "The language style of the entry is euphemistic and uses more moderate language to ask the other side whether they have experience and feelings about \"ceremonial actions/acts\" rather than direct orders or statements. This expression is more personal and interactive."}, "time_dimension": {"answer": "Present", "rationale": "The keyword \"ceremonial actions/acts\" indicates the actions or behaviours currently being performed, so the temporal dimension is now judged."}, "sentence_type": "This sentence is not semantic and cannot be judged. Please provide a full sentence for analysis.", "pos": {"nouns": {"Ceremony": 1, "Actions": 1, "Behaviour": 1}, "numerals": {"12": 1}}}, {"content": "13: Relentless", "word_count": 4, "label": "", "sentence_structure": "Subject鈥損redicate", "subjective_objective": {"answer": "Subjective", "rationale": "Because \"suspensive\" is the patient's own psychological state, a subjective description."}, "sentence_sentiment": {"answer": "Negative", "rationale": "Relentment is a negative attitude or behaviour."}, "language_style": {"answer": "Tactful and warm", "rationale": "The language style of the entry is more euphemistic, and the expression is more friendly and respectful by leading the interviewee to wonder whether he or she has been hesitant or not, without directly accusing or evaluating the interviewee."}, "time_dimension": {"answer": "Present", "rationale": "\"Screeching\" means that the testees are troubled and hesitant in their decision-making, a state that may have existed in the past or may have emerged only recently. The article therefore determines the time dimension of the purpose as it is."}, "sentence_type": "Exclamatory sentence", "pos": {"nouns": {"Whispering": 1, "No, I'm not": 1}}}, {"content": "14 The lack of energy refers to the difficulty and slowness with which daily activities are initiated and carried out.", "word_count": 24, "label": "", "sentence_structure": "Subject鈥搗erb鈥搊bject", "subjective_objective": {"answer": "Subjective", "rationale": "Since the description of the purpose of the questionnaire mainly concerns the feelings and experiences of the patient himself, such as \"difficulties and slowness\", it can be judged from the patient's subjective perspective."}, "sentence_sentiment": {"answer": "Negative", "rationale": "Because \"drained energy\" and \"difficult and slow\" are negative descriptions."}, "language_style": {"answer": "Tactful and warm", "rationale": "The language style of the entry is more euphemistic, using the terms \"start-up\" and \"hardness\" to express the problems that people may encounter in their daily activities, rather than using too direct or too rigid a tone, which is more gentle and friendly. Such a design makes it easier for respondents to accept and answer questions and improve the effectiveness and accuracy of questionnaires."}, "time_dimension": {"answer": "Present", "rationale": "The act of \"starting and carrying out day-to-day activities\" is a description of the current day-to-day state and therefore judgement is now."}, "sentence_type": "Interrogative sentence", "pos": {"nouns": {"- The energy": 1, "Day": 1, "Activities": 1, "Difficulties": 1, "Slow": 1}, "verbs": {"Finger": 1, "Start": 1, "Conduct": 1, "I feel": 1}, "adverbs": {"Start": 1, "and": 1}, "numerals": {"14": 1}}}, {"content": "15, Irritation", "word_count": 3, "label": "", "sentence_structure": "Subject鈥搗erb鈥揷omplement", "subjective_objective": {"answer": "Subjective", "rationale": "Because fatigue is a subjective feeling, different people have different feelings of fatigue for the same activity."}, "sentence_sentiment": {"answer": "Negative", "rationale": "Because \"retired\" is usually considered negative."}, "language_style": {"answer": "Calm and direct", "rationale": "The entry directly asks whether the interviewees are vulnerable to fatigue, are more concise and have less adoration or emotional colours, and can therefore be judged as a calm and direct expression."}, "time_dimension": {"answer": "Past", "rationale": "The entry asks the testee about past feelings or experiences, and therefore the temporal dimension is judged to be past."}, "sentence_type": "Affirmative declarative sentence", "pos": {"nouns": {"Tired": 1}, "adjectives": {"Easy": 1}, "numerals": {"15": 1}}}, {"content": "16. Focus on the difficulty of concentration", "word_count": 6, "label": "", "sentence_structure": "Subject鈥搗erb鈥揷omplement", "subjective_objective": {"answer": "Subjective", "rationale": "Because \"not focused\" is a patient's subjective feeling and experience."}, "sentence_sentiment": {"answer": "Negative", "rationale": "Because the description \"care for difficulty\" is negative."}, "language_style": {"answer": "Calm and direct", "rationale": "The language style of the entry is calm and straightforward. It asks the respondents whether they have difficulty focusing, are not too emotional or amplified, and are more objective in tone."}, "time_dimension": {"answer": "Present", "rationale": "The entry now describes the current behaviour or state of the person being tested, i.e., the difficulty of focusing. The keyword \"care to focus\" indicates that it is a description of the current act, not a prediction or expectation of the past or the future. Thus, the time dimension of the article is now."}, "sentence_type": "Declarative sentence", "pos": {"nouns": {"Focus": 1, "Difficulties": 1}, "verbs": {"Attention": 1}}}, {"content": "Memory difficulties are those that are subjectively considered more difficult to remember than ever before.", "word_count": 18, "label": "", "sentence_structure": "Subject鈥搗erb鈥搊bject", "subjective_objective": {"answer": "Subjective", "rationale": "Because \"the subjective perception is more difficult to remember than ever before\" is the personal feeling and view of the patient and is subjective."}, "sentence_sentiment": {"answer": "Neutral", "rationale": "Because the entry does not have any emotional color."}, "language_style": {"answer": "Calm and direct", "rationale": "The language style of the entry is calm and objective, and a definition or concept is presented in a simple and clear statement. It does not have too many emotional colours or rhetorical techniques to convey messages rather than to provoke specific emotional reactions."}, "time_dimension": {"answer": "Present", "rationale": "The entry describes the perception and evaluation of the person ' s memory, which is a subjective self-reported experience and can therefore be judged as the present."}, "sentence_type": "Affirmative declarative sentence", "pos": {"nouns": {"Memory difficulties": 1, "Memories": 1}, "verbs": {"Finger": 1, "I think": 1, "Conduct": 1, "Hard": 1}, "adjectives": {"Subjective": 1, "It's harder than usual": 1}, "adverbs": {"As usual": 1}, "numerals": {"17": 1}}}, {"content": "18. Decline in appetite", "word_count": 4, "label": "", "sentence_structure": "Subject鈥搗erb鈥揷omplement", "subjective_objective": {"answer": "Objective", "rationale": "Because a reduction in appetite is a biological phenomenon of the patient and is an objective fact."}, "sentence_sentiment": {"answer": "Negative", "rationale": "Because the \"eating\" act in the entry is negative."}, "language_style": {"answer": "Tactful and warm", "rationale": "The entry uses a more moderate approach to questions, is euphemistic, has no direct or strong tone, gives people a sense of affection and care. It also clearly expresses the question of whether the person under investigation has a reduced appetite, which is very sweet and caring."}, "time_dimension": {"answer": "Past", "rationale": "In this entry, \"the abated appetite\" indicates the condition or behaviour of the person tested over time, and therefore the temporal dimension is judged to be the past."}, "sentence_type": "Negative declarative sentence", "pos": {"nouns": {"A appetite": 1, "Declining": 1}, "numerals": {"18": 1}}}, {"content": "19: Sleep loss", "word_count": 4, "label": "", "sentence_structure": "Subject鈥搗erb鈥揷omplement", "subjective_objective": {"answer": "Subjective", "rationale": "Because sleep is more or less a personal feeling and experience, it is a subjective examination."}, "sentence_sentiment": {"answer": "Negative", "rationale": "Because sleep loss is usually a physical or mental health problem, it has a negative emotional effect."}, "language_style": {"answer": "Calm and direct", "rationale": "The entry directly asked the respondents whether there had been any reduction in sleep, the language was simple, the speech was not too much rhetorical or emotional and the tone was more objective."}, "time_dimension": {"answer": "Present", "rationale": "The keyword \"sleep loss\" indicates that this is an ongoing phenomenon and may be long-term."}, "sentence_type": "Negative declarative sentence", "pos": {"nouns": {"Sleep": 1, "Reduction": 1}, "numerals": {"EMPTY": 1, "19": 1}}}, {"content": "20, sleep increase", "word_count": 4, "label": "", "sentence_structure": "Subject鈥搗erb鈥揷omplement", "subjective_objective": {"answer": "Subjective", "rationale": "Because sleep gain is a subjective experience, the subjective perceptions and performance of different people may differ."}, "sentence_sentiment": {"answer": "Negative", "rationale": "Because the word \"increasing\" is often used to describe negative events or situations, it implies that sleeping may be problematic or abnormal."}, "language_style": {"answer": "Tactful and warm", "rationale": "The language style of the entry is more euphemistic. First, it uses the word \"addition,\" not \"shortfall\" or \"reduce,\" which suggests that the entry is more concerned about whether the interviewee ' s situation has improved than about the problems. Second, the title \"20, sleep gain\" itself is mild, without direct orders or threats, but rather, it raises the subject in a light-handed manner, encouraging the interviewee to respond honestly."}, "time_dimension": {"answer": "Present", "rationale": "The entry is an inquiry into the present behaviour of the testee, and the \"increasing\" of the keyword suggests that it is a process of change, implying that the issue is affecting the current state of the testee and can therefore be judged as now."}, "sentence_type": "Negative declarative sentence", "pos": {"nouns": {"Sleep": 1, "Increase": 1}, "numerals": {"20": 1}}}, {"content": "21. Declining sexual interest", "word_count": 5, "label": "", "sentence_structure": "Subject鈥搗erb鈥揷omplement", "subjective_objective": {"answer": "Subjective", "rationale": "Because the decline in sexual interest is the subjective feeling and experience of the patient."}, "sentence_sentiment": {"answer": "Negative", "rationale": "Because \"sex interest decline\" is a negative behaviour or state, it is usually associated with physical health, psychological stress, etc."}, "language_style": {"answer": "Calm and direct", "rationale": "The entry directly expresses a question about whether the other party ' s sexual interest has diminished the subject, without excessive rhetoric or emotional rendering, and with a more objective tone."}, "time_dimension": {"answer": "Present", "rationale": "The entry is for questioning the person's current behaviour or state, and the keywords are \"sexual interest\" and \"reducing\" and they all point to the current situation."}, "sentence_type": "Negative declarative sentence", "pos": {"nouns": {"Sexual interest": 1, "Declining": 1}}}, {"content": "22 Increased sexual interest", "word_count": 5, "label": "", "sentence_structure": "Subject鈥搗erb鈥揷omplement", "subjective_objective": {"answer": "Subjective", "rationale": "Because sexual interest is a subjective feeling and psychological state of the human being, not an objective fact."}, "sentence_sentiment": {"answer": "Neutral", "rationale": "Because the entry does not have any emotional color."}, "language_style": {"answer": "Tactful and warm", "rationale": "The more euphemistic and vague language of the entry, such as \"increased sexual interest\", may help respondents to feel comfortable and relaxed and to share their views. At the same time, this expression helps avoid creating feelings of discomfort or exclusion."}, "time_dimension": {"answer": "Present", "rationale": "The entry contains a key word for the person who is tested, \"Increased sexual interest\"."}, "sentence_type": "Affirmative declarative sentence", "pos": {"nouns": {"Sexual interest": 1}, "verbs": {"Increase": 1}, "numerals": {"22": 1}}}, {"content": "23, Vegetal neurosis", "word_count": 6, "label": "", "sentence_structure": "Subject鈥搗erb鈥揷omplement", "subjective_objective": {"answer": "Objective", "rationale": "Since plant neurosis is a pathological state, whether the patient has the disease can be examined and diagnosed, which is an objective examination."}, "sentence_sentiment": {"answer": "Neutral", "rationale": "Because the entry is merely a description of a disease, and it does not involve any emotional colour."}, "language_style": {"answer": "Calm and direct", "rationale": "The entry is based on a more objective, concise language style, which does not involve too much qualifier or emotional rendering, but rather directly asks the respondents whether they have experienced plant nervous disorders and appear calm and direct."}, "time_dimension": {"answer": "Future", "rationale": "The entry describes the possible occurrence of the disease of plant neurosis, which is judged by the temporal dimension to be the future."}, "sentence_type": "Affirmative declarative sentence", "pos": {"nouns": {"Plant neurosis": 1}, "numerals": {"23": 1}}}, {"content": "24. Pain", "word_count": 2, "label": "", "sentence_structure": "Subject鈥揷opula鈥損redicative", "subjective_objective": {"answer": "Subjective", "rationale": "Because pain is the patient's own subjective feeling."}, "sentence_sentiment": {"answer": "Neutral", "rationale": "As the entry merely describes whether the person under investigation has pain symptoms and is not emotional."}, "language_style": {"answer": "Calm and direct", "rationale": "The question is whether the person interviewed has pain, is not over-modified or emotionally retrofitted, and is more concise in language style."}, "time_dimension": {"answer": "Past", "rationale": "The entry asks if the person tested has experienced pain before and the time dimension is judged to be past."}, "sentence_type": "Interrogative sentence", "pos": {"nouns": {"Pain": 1}}}, {"content": "25, muscle stress.", "word_count": 4, "label": "", "sentence_structure": "Subject鈥搗erb鈥揷omplement", "subjective_objective": {"answer": "Objective", "rationale": "As muscle stress is a physical phenomenon, it is not the patient ' s subjective feeling or attitude."}, "sentence_sentiment": {"answer": "Negative", "rationale": "The basis for the judgment: \"must stress\" is a negative reaction of the body and is therefore expressed as negative."}, "language_style": {"answer": "Calm and direct", "rationale": "The entry directly asked the respondents whether there were frequent muscle stress, a clear language style, and a relatively calm and straightforward display of not much rhetorical or emotional overtones."}, "time_dimension": {"answer": "Present", "rationale": "The entry asks if the person tested is currently suffering from muscle stress, and the time dimension is now judged."}, "sentence_type": "Interrogative sentence", "pos": {"nouns": {"Muscles": 1, "Nervous": 1}, "numerals": {"25": 1}}}, {"content": "26: Loss of feeling", "word_count": 6, "label": "", "sentence_structure": "Subject鈥搗erb鈥揷omplement", "subjective_objective": {"answer": "Subjective", "rationale": "Because \"fear\" and \"feel\" are personal and subjective experiences of patients, they cannot be measured by objective criteria. So the question is the patient's subjective experience."}, "sentence_sentiment": {"answer": "Negative", "rationale": "Because the entry \"feeling loss\" is a physical illness or pain, a negative emotion and experience."}, "language_style": {"answer": "Tactful and warm", "rationale": "The language style of the entry is more euphemistic and comprehensible, which does not directly ask the respondents whether they feel the loss of movement, but rather a gentle and inviting tone of speech. This expression is more respectful of the privacy and dignity of the person under investigation and better stimulates their enthusiasm for participation. At the same time, the entry uses more common language, making it easier for the person under investigation to understand its meaning and to share his or her true feelings."}, "time_dimension": {"answer": "Future", "rationale": "The entry describes the feeling that the testee may feel \"move\" in some way in the future and thus judges it as the future."}, "sentence_type": "Imperative sentence", "pos": {"nouns": {"Feel": 1, "Actions": 1, "Loss": 1}, "numerals": {"26": 1}}}, {"content": "27. Defunct reality", "word_count": 4, "label": "", "sentence_structure": "Subject鈥揷opula鈥損redicative", "subjective_objective": {"answer": "Subjective", "rationale": "Because the disintegration of reality is an expression of individual subjective feelings."}, "sentence_sentiment": {"answer": "Negative", "rationale": "Because the term \"dissolution\" is often negative, implying bad results or destructive processes."}, "language_style": {"answer": "Calm and direct", "rationale": "The entry is expressed in direct language, without excessive rhetorical or emotional rendering, and in a more objective and calm manner describes a concept of \"real disintegration\"."}, "time_dimension": {"answer": "Future", "rationale": "The entry contains the will and the idea of a solution, namely, \"to solve the problems in reality\" and the vision and expectations for the future, \"realization.\" These points to situations that have not yet occurred and that may occur in the future."}, "sentence_type": "Negative declarative sentence", "pos": {"nouns": {"Reality": 1, "Dissolution": 1}, "verbs": {"Come on": 1}, "numerals": {"Twenty-seven": 1}}}, {"content": "28. Demolition of the personality", "word_count": 4, "label": "", "sentence_structure": "Subject鈥揷opula鈥損redicative", "subjective_objective": {"answer": "Objective", "rationale": "Since depersonalization is a symptom of mental illness, it is not something that the patient can choose or change on his own, and is therefore examined from an objective perspective."}, "sentence_sentiment": {"answer": "Negative", "rationale": "Because the disintegration of personality is usually considered a serious psychological handicap."}, "language_style": {"answer": "Calm and direct", "rationale": "The language style of the questionnaire entry is relatively calm and straightforward, rather than using too many qualifiers or emotional terms, but rather using a simple and clear language to express the problem, which is usually used in professional psychological questionnaires, which convey messages clearly and avoid causing emotional fluctuations among the subjects."}, "time_dimension": {"answer": "Present", "rationale": "The entry is for the purpose of asking the person who was tested whether there was a \"dissociation\" of personality, which is now judged in terms of time dimension."}, "sentence_type": "Interrogative sentence", "pos": {"nouns": {"Demolition": 1}}}, {"content": "29: Controlled senses", "word_count": 4, "label": "", "sentence_structure": "Subject鈥搗erb鈥揷omplement", "subjective_objective": {"answer": "Subjective", "rationale": "Because control is a psychological experience or experience, it is considered from the patient ' s subjective perspective."}, "sentence_sentiment": {"answer": "Negative", "rationale": "Because \"controllable\" is usually seen as a negative emotion and experience."}, "language_style": {"answer": "Tactful and warm", "rationale": "The entry uses a more euphemistic language style, using the theme of \"controllable\" to guide respondents to answer questions, without asking questions or ordering directly. Moreover, \"controllable\" is a more sensitive and personal topic, and a more euphemism is more likely to protect the privacy and dignity of the interviewee."}, "time_dimension": {"answer": "Present", "rationale": ""}, "sentence_type": "Interrogative sentence", "pos": {"nouns": {"Control": 1, "29": 1}, "verbs": {"By": 1, "Feel": 1}, "numerals": {"29": 1}}}, {"content": "30: A mind disorder implies a sudden pause in thinking (a mind interruption) or a mind inserted into the mind (a thought inserted); or being taken out (a thought taken away), heard or broadcast.", "word_count": 46, "label": "", "sentence_structure": "Subject鈥搗erb鈥搊bject", "subjective_objective": {"answer": "Subjective", "rationale": "Because mental disorders are the subjective feelings and experiences of the patient."}, "sentence_sentiment": {"answer": "Negative", "rationale": "Because the narratives of the articles involve negative emotional experiences, such as \"suspension of mind\" and \"into mind\" that reflect the potential for distress, anxiety and anxiety."}, "language_style": {"answer": "Calm and direct", "rationale": "The entry uses professional terms to describe symptoms and to present them in objective, concise language, without excessive rhetorical or emotional rendering. Thus, its language style can be considered as calm and straightforward."}, "time_dimension": {"answer": "Present", "rationale": "The entry describes a subjective feeling and experience, namely, \" experience a sudden pause in thinking\" and \"thinks are inserted into the brain\" and does not have a clear time point. Therefore, judging by keywords and sentences, the entry should be a current expression."}, "sentence_type": "Affirmative declarative sentence", "pos": {"nouns": {"I'm having a mental disorder": 1, "Experience": 1, "Thinking": 2, "Brain": 1, "Insert": 1, "Interrupt": 1, "Ideas": 4, "Yes": 1, "Radio": 1}, "verbs": {"Finger": 1, "鈾?And experience 鈾?: 1, "Suddenly, it stopped": 1, "Insert": 1, "Checkout": 1, "- They hear you": 1, "It's on the radio": 1}, "adverbs": {"Suddenly": 1}, "numerals": {"30": 1}}}, {"content": "31. Perceptions of victimization/false thoughts", "word_count": 6, "label": "", "sentence_structure": "Subject鈥搗erb鈥搊bject", "subjective_objective": {"answer": "Subjective", "rationale": "Because the notion of victimization/false vision is usually the idea and conviction that is deep inside the patient, it is a manifestation of subjective experience."}, "sentence_sentiment": {"answer": "Negative", "rationale": "Because \"feed/false\" is often seen as a manifestation of psychological impairment, with negative emotional connotations."}, "language_style": {"answer": "Calm and direct", "rationale": "The entry directly asked the respondents whether there was a perception of victimization/falseness, and the language style was more calm and objective, without excessive rhetoric or emotional publicity."}, "time_dimension": {"answer": "Past", "rationale": "The term \"intention of the victim\" and \"insatibilities\" in the entry are questions about the subjective feelings and experiences of individuals, which are usually in the past or in the symptoms. The temporal dimension of the entry is therefore the past."}, "sentence_type": "Interrogative sentence", "pos": {"nouns": {"31": 1, "The concept of victimization": 1, "Paranoia": 1}}}, {"content": "32 Exaggeration/false", "word_count": 6, "label": "", "sentence_structure": "Subject鈥搗erb鈥搊bject", "subjective_objective": {"answer": "Subjective", "rationale": "Because exaggerating ideas and delusions are often subjective experiences or ideas that patients themselves feel."}, "sentence_sentiment": {"answer": "Negative", "rationale": "Basis: Exaggeration/false is usually a symptom of psychological impairment that can affect individuals ' thinking and behaviour and is therefore considered to be a negative emotional attitude or quality."}, "language_style": {"answer": "Tactful and warm", "rationale": ""}, "time_dimension": {"answer": "Past", "rationale": "So the answer is \"uncertain.\""}, "sentence_type": "Interrogative sentence", "pos": {"nouns": {"Exaggeration": 1, "Paranoia": 1}, "numerals": {"32": 1}}}, {"content": "33. The delusional vision implies a serious and unreasonable feeling of presumptuousness and a sudden conviction that certain minor matters have profound and strange meanings.", "word_count": 34, "label": "", "sentence_structure": "Subject鈥搗erb鈥搊bject", "subjective_objective": {"answer": "Subjective", "rationale": "Because delusions are patients ' subjective feelings and ideas, which may not seem reasonable or realistic, they are their own perceptions and interpretations, not objective facts."}, "sentence_sentiment": {"answer": "Negative", "rationale": "Because the contents of the entry describe a pathological psychological state and are negative emotional tendencies."}, "language_style": {"answer": "Calm and direct", "rationale": "The language style of the questionnaire entry is more calm and straightforward, using the terms \"fancy\" and \"presension\" to describe symptoms, without excessive rhetorical or emotional rendering. This expression makes it easier to understand the characteristics and manifestations of the disease and to analyse and compare the data of researchers."}, "time_dimension": {"answer": "Present", "rationale": ""}, "sentence_type": "Affirmative declarative sentence", "pos": {"nouns": {"I'm just thinking about what's going on": 1, "Foreground": 1, "A little bit of a thing": 1, "Deep meaning": 1}, "verbs": {"Finger": 1, "Serious": 1, "and": 1, "It doesn't make sense": 1, "I'm sure of that": 1, "Yes": 1, "Strange": 1, "Meaning": 1}, "adjectives": {"Serious": 1, "It doesn't make sense": 1, "Fine": 1, "Deep": 1, "Strange": 1}, "numerals": {"33": 1}}}, {"content": "34 - The Vertigo.", "word_count": 4, "label": "", "sentence_structure": "Subject鈥搗erb鈥搊bject", "subjective_objective": {"answer": "Subjective", "rationale": "Because \"secret\" is an adjective that describes the feelings and experiences inside the patient and is subjectively observed."}, "sentence_sentiment": {"answer": "Negative", "rationale": "Since \"smuggling\" usually refers to a high level of excitement, pleasure or excitement that can lead to impulsive behaviour or decision-making, it is not necessarily beneficial."}, "language_style": {"answer": "Tactful and warm", "rationale": "The language style of the entry is more euphemistic, creating a romantic and mysterious atmosphere by using poetic words and adjectives, making the respondents more relaxed and willing to share their feelings in answering questions. At the same time, this expression makes the questionnaire more attractive and interactive, and better resonates with the respondents."}, "time_dimension": {"answer": "Past", "rationale": ""}, "sentence_type": "Exclamatory sentence", "pos": {"nouns": {"鈾?Smuggle 鈾?: 1}}}, {"content": "35: Pathological jealousy", "word_count": 5, "label": "", "sentence_structure": "Subject鈥搗erb鈥搊bject", "subjective_objective": {"answer": "Subjective", "rationale": "Because \"pathological jealousy\" describes the patient's psychological state and emotional experience, and the patient's subjective feeling."}, "sentence_sentiment": {"answer": "Negative", "rationale": "Because \"pathological jealousy\" is a negative emotion and attitude that represents excessive control and appetite for partners or others."}, "language_style": {"answer": "Calm and direct", "rationale": "The language style of the entry is a calm and straightforward type. It clearly presents a theme 鈥?鈥減athological jealousy鈥?鈥?and is expressed in a concise language, which is less emotional or acrimonymous, and is more objective. The advantage of this style is that it can clearly convey information, facilitate the understanding of the questions by the respondents and avoid emotional expressions that may lead to misunderstandings or deviations."}, "time_dimension": {"answer": "Future", "rationale": "The keyword \"pathological jealousy\" refers to excessive anxiety and resentment over the success of others, a mentality or behaviour that may emerge in the future."}, "sentence_type": "Interrogative sentence", "pos": {"nouns": {"Pathologicality": 1, "Jealousy": 1}, "adjectives": {"Sick": 1}, "numerals": {"35": 1}}}, {"content": "Other perceptions/falsions", "word_count": 6, "label": "", "sentence_structure": "Subject鈥搗erb鈥揷omplement", "subjective_objective": {"answer": "Subjective", "rationale": "Because \"other ideas/false ideas\" are the thoughts and views of the individual patient and are subject to subjective assessment."}, "sentence_sentiment": {"answer": "Neutral", "rationale": "Because the entry merely asks whether there are other ideas or delusions, it does not involve positive or negative emotional aspects."}, "language_style": {"answer": "Tactful and warm", "rationale": "The language style of the entry is more euphemistic, not too strong to ask the other side whether there are certain ideas or delusions, but to direct the other side to answer in a caring and understanding manner. At the same time, the question is also open-ended, allowing the interviewee to express his thoughts and feelings more freely."}, "time_dimension": {"answer": "Present", "rationale": "The entry asks the testee whether there are other ideas or delusions that do not relate to past or future behaviour or mentality and therefore determines \"now\"."}, "sentence_type": "Exclamatory sentence", "pos": {"nouns": {"Other": 1, "Perceptions": 1, "Paranoia": 1}}}, {"content": "Commentatory illusions mean that voices are spoken (or restated) about themselves, or that they are called by a third person and commented or debated.", "word_count": 43, "label": "", "sentence_structure": "Subject鈥搗erb鈥搊bject", "subjective_objective": {"answer": "Objective", "rationale": "Because the entry describes a phenomenon in the patient鈥檚 subjective experience, i.e. the person heard a voice commenting on or debating his/her ideas, which is not the patient鈥檚 own initiative. This is an observed phenomenon and therefore falls within the scope of an objective examination."}, "sentence_sentiment": {"answer": "Neutral", "rationale": "Because the entry does not have any emotional color."}, "language_style": {"answer": "Calm and direct", "rationale": "It clearly describes the definition and characteristics of \"commental illusions\" and does not have too many emotional or rhetorical features, which are more objective and professional in tone."}, "time_dimension": {"answer": "Present", "rationale": "In terms of time dimension, the entry contains a description of the symptoms that are now happening, and therefore the term \"now\"."}, "sentence_type": "Affirmative declarative sentence", "pos": {"nouns": {"Commentative Spectrum": 1, "Sound": 2, "My own mind": 1, "Name": 1, "Others": 1, "Third": 1, "Comments": 1, "Debate": 1, "Questionnaire Entry": 1}, "verbs": {"I'm talking to you": 1, "Speak up": 1, "Repeat": 1, "Name": 1, "Take it": 1, "and": 1}, "numerals": {"37": 1}, "pronouns": {"self": 2}}}, {"content": "38 - Other voices", "word_count": 4, "label": "", "sentence_structure": "Subject鈥搗erb鈥揷omplement", "subjective_objective": {"answer": "Objective", "rationale": "Because \"other hearing\" is a symptom that requires a doctor's judgement and diagnosis, not a patient's subjective feeling or attitude."}, "sentence_sentiment": {"answer": "Negative", "rationale": "Because the symptom of \"phantom\" is usually a manifestation of mental illness, the purpose of this article is negative."}, "language_style": {"answer": "Calm and direct", "rationale": "The entry uses a simple and quick language, which is used to ask the person under investigation whether the symptoms of \"other voices\" appear, and the tone is not too emotional or rhetorical."}, "time_dimension": {"answer": "Future", "rationale": "The keyword \"false hearing\" usually refers to the sound of a voice or its source, which is one of the manifestations of mental illness. Moreover, the word \"other\" also indicates that the person tested may have reported some of the common types of hearing that may be asked this time for more specific or specific symptoms. Thus, this entry may involve speculation and expectations about the future of the disease."}, "sentence_type": "Interrogative sentence", "pos": {"nouns": {"I'm hearing things": 2, "Other": 1}, "numerals": {"38": 1}}}, {"content": "39, Vision", "word_count": 2, "label": "", "sentence_structure": "Subject鈥損redicate", "subjective_objective": {"answer": "Objective", "rationale": "Since vision is an objective fact experienced by patients, not a subjective imagination or feeling, it is an objective test."}, "sentence_sentiment": {"answer": "Negative", "rationale": "Because \"discovery\" is a psychiatric condition, it is often seen as a negative emotion, attitude or behaviour."}, "language_style": {"answer": "Calm and direct", "rationale": "Because the language style of the entry is relatively simple, it does not use excessive rhetorical or emotional colours, but rather it is more objective and calm to ask the interviewee whether there is a vision."}, "time_dimension": {"answer": "Past", "rationale": "The keyword is \"discovery\", which is usually considered to be one of the symptoms of mental illness or mental health problems, and the entry is therefore more likely to ask whether the person tested has experienced vision in the past."}, "sentence_type": "Interrogative sentence", "pos": {"nouns": {"Vision": 1}}}, {"content": "40, Other hallucinations", "word_count": 4, "label": "", "sentence_structure": "Subject鈥損redicate", "subjective_objective": {"answer": "Subjective", "rationale": "Enter: \"40, other hallucinations,\" output: \"subjective because illusions are the patient's own perception, so they are subjective.\""}, "sentence_sentiment": {"answer": "Negative", "rationale": "Because \"fantasia\" in the entries is usually classified as a symptom of mental illness, this expression is considered negative."}, "language_style": {"answer": "Calm and direct", "rationale": "This is a more objective and neutral expression that facilitates accurate data."}, "time_dimension": {"answer": "Future", "rationale": "The entry contains the keyword \"other hallucinations\", implying that the testee may have such problems in the future, and thus judging as future."}, "sentence_type": "Interrogative sentence", "pos": {"nouns": {"Imagination": 2, "Other": 1}, "numerals": {"40": 1}}}, {"content": "41. Depression and sadness", "word_count": 4, "label": "", "sentence_structure": "Subject鈥搗erb鈥揷omplement", "subjective_objective": {"answer": "Subjective", "rationale": "Because depression is the emotional experience that patients feel, it is a subjective feeling and emotional state."}, "sentence_sentiment": {"answer": "Negative", "rationale": "Basis for judgement: Depression usually causes depression and sadness is one of its common symptoms and can therefore be judged negative."}, "language_style": {"answer": "Tactful and warm", "rationale": "The entry is more personal and interactive when it is spoken in a gentle and inviting tone, rather than directly speaking or ordering. At the same time, \"depressive grief\" is a more sensitive subject, which in a soft and warm language style can respect the feelings of the person under investigation and reduce their resistance."}, "time_dimension": {"answer": "Present", "rationale": "The entry asks about the current emotional state of the testee, and the temporal dimension is now judged."}, "sentence_type": "Negative declarative sentence", "pos": {"nouns": {"Sad": 1}}}]}

{"questionnaire_title": "University Mental Health Survey (UPI)", "questionnaire_category": "Mental health", "item_count": 63, "emotion_shift_overall": "From positive to negative", "language_style_overall": "Neutral", "items": [{"content": "1. Desperate appetite", "word_count": 4, "label": "", "sentence_structure": "Subject鈥搗erb鈥揷omplement", "subjective_objective": {"answer": "Subjective", "rationale": "Because the patient's subjective feelings and experiences are the strong appetite."}, "sentence_sentiment": {"answer": "Negative", "rationale": "Because the description of \"failure\" is usually seen as a disease or a negative physical reaction."}, "language_style": {"answer": "Calm and direct", "rationale": "The entry is very direct in language style, with no syllable or emotional rendering. It is simply asking if the subject is hungry, without any redundant words or qualifiers, and therefore belongs to the \"cool straight\" language style."}, "time_dimension": {"answer": "Present", "rationale": "\"Adulgence is\" describes a symptom, which is usually used to measure and diagnose a person's state at the moment. The purpose of the article is therefore primarily to describe the status of the person being tested."}, "sentence_type": "Negative declarative sentence", "pos": {"nouns": {"A appetite": 1, "It's not working": 1}}}, {"content": "Disgusting, stomach pains.", "word_count": 9, "label": "", "sentence_structure": "Subject鈥損redicate", "subjective_objective": {"answer": "Objective", "rationale": "Disgusting, stomach pain and stomach pain are symptoms of the patient and are examined from an objective perspective."}, "sentence_sentiment": {"answer": "Negative", "rationale": "Because the symptoms of \"obscenity, stomach pain\" in the entry are all manifestations of physical discomfort, a problem of negative emotional or physical health."}, "language_style": {"answer": "Tactful and warm", "rationale": "The entry uses descriptive language to describe the symptoms of physical discomfort, rather than asking directly whether the person tested is uncomfortable."}, "time_dimension": {"answer": "Past", "rationale": ""}, "sentence_type": "Exclamatory sentence", "pos": {"nouns": {"Gross": 1, "-Adulge": 1, "- Tummy": 1}, "verbs": {"It's hard": 1, "Ouch": 1}}}, {"content": "3. Easy to diarrhea or constipation", "word_count": 8, "label": "", "sentence_structure": "Subject鈥損redicate", "subjective_objective": {"answer": "Subjective", "rationale": "Because the experience of diarrhea or constipation is a subjective one."}, "sentence_sentiment": {"answer": "Negative", "rationale": "Because the description \"easy diarrhea or constipation\" is a negative health condition."}, "language_style": {"answer": "Calm and direct", "rationale": "The language style of the entry is more objective and precise, and it is appropriate to conduct a scientific psychological questionnaire."}, "time_dimension": {"answer": "Past", "rationale": "The entry \"Easy to diarrhea or constipation\" describes the health status of the person tested in the past and can therefore be judged as past."}, "sentence_type": "Affirmative declarative sentence", "pos": {"nouns": {"I'm having a stomach attack": 1, "Decency": 1}, "verbs": {"Easy": 1, "Pull": 1, "Decency": 1}, "numerals": {"3": 1}}}, {"content": "4. Focus on heart attack and pulse", "word_count": 7, "label": "", "sentence_structure": "Subject鈥搗erb鈥揷omplement", "subjective_objective": {"answer": "Subjective", "rationale": "Because the entry looks at the patient ' s subjective feelings and observations, not at objective facts."}, "sentence_sentiment": {"answer": "Neutral", "rationale": "Because the entry does not have a clear emotional, attitudinal or behavioural orientation, it merely describes a phenomenon of concern."}, "language_style": {"answer": "Calm and direct", "rationale": "It explicitly asked whether the respondents were concerned about their heart and pulse, and did not use excessive rhetorical or euphemismal language. This was therefore a more direct and specific language and was suitable for obtaining information on the respondents in questionnaires."}, "time_dimension": {"answer": "Present", "rationale": "The entry asks if the person tested is currently looking at his or her heart and pulse, and therefore the time dimension is now judged."}, "sentence_type": "Imperative sentence", "pos": {"nouns": {"Heartbreak": 1, "Pulse": 1}, "verbs": {"Attention": 1}}}, {"content": "5. Good health", "word_count": 8, "label": "", "sentence_structure": "Subject鈥揷opula鈥損redicative", "subjective_objective": {"answer": "Objective", "rationale": "Since the physical condition is an objective fact, it is not the patient ' s subjective attitude or feelings."}, "sentence_sentiment": {"answer": "Positive", "rationale": "Because \"good health\" is a positive expression, it means good health, without any negative emotional appearance."}, "language_style": {"answer": "Tactful and warm", "rationale": "The entry uses a more euphemistic and caring interview about the other ' s health, without direct questioning or orders, which makes it easier for the interviewee to open his heart and answer honestly."}, "time_dimension": {"answer": "Past", "rationale": "The entry describes the current state of health of the person being tested, does not relate to future or other present aspects and therefore determines the past."}, "sentence_type": "Affirmative declarative sentence", "pos": {"nouns": {"Body": 1, "Status": 1, "Health": 1}, "verbs": {"Good": 1}, "numerals": {"5": 1}}}, {"content": "6. Dissatisfaction and dissatisfaction", "word_count": 6, "label": "", "sentence_structure": "Subject鈥搗erb鈥揷omplement", "subjective_objective": {"answer": "Subjective", "rationale": "Because gruesome and disaffected are emotional reactions of one person and are a manifestation of subjective feelings."}, "sentence_sentiment": {"answer": "Negative", "rationale": "Because the attitude of \"shut and disgruntled\" in the entry is negative."}, "language_style": {"answer": "Calm and direct", "rationale": "The entry directly asks the respondents whether they have more complaints and grievances, the temperature of the language and its objectivity, and the lack of excessive embellishment or emotional rendering."}, "time_dimension": {"answer": "Past", "rationale": "Analysis: There is no clear time indicator in the questionnaire entry, nor does it address future plans or expectations. But the description of \"grabbing and discontent\" can be understood as a state or situation, so we can classify it as \"now\". Answer: \"now.\""}, "sentence_type": "Imperative sentence", "pos": {"nouns": {"Scoff": 1, "Dissatisfied": 1}}}, {"content": "7. Over-zealous expectations of parents", "word_count": 6, "label": "", "sentence_structure": "Subject鈥搗erb鈥揷omplement", "subjective_objective": {"answer": "Subjective", "rationale": "Because \"too much parental expectation\" is a description of the patient's subjective feelings, reflecting their feelings about the stress they face in life, learning, etc. This perception and perception are not objective realities, but are dependent on the individual's emotions and attitudes. Therefore, \"too much parental expectations\" are viewed from the patient's subjective perspective."}, "sentence_sentiment": {"answer": "Negative", "rationale": "Because the expression \"high expectations\" implies that parents are over-zealous and stressful towards their children, it may have a negative impact on the children."}, "language_style": {"answer": "Tactful and warm", "rationale": "The entry uses moderate language, not harsh language or accusatory tone, but rather an understanding and caring attitude to ask if the other side feels over-explored expectations from parents."}, "time_dimension": {"answer": "Past", "rationale": "In conclusion, the time dimension of the questionnaire entry \"Personal expectations\" is past."}, "sentence_type": "Negative declarative sentence", "pos": {"nouns": {"parents": 1, "Expectations": 2, "High": 1}, "verbs": {"Too high": 1}}}, {"content": "8. The past and the family are unfortunate.", "word_count": 11, "label": "", "sentence_structure": "Subject鈥搗erb鈥揷omplement", "subjective_objective": {"answer": "Subjective", "rationale": "The family is the patient ' s subjective perception and feelings and is a subjective one."}, "sentence_sentiment": {"answer": "Negative", "rationale": "Because the expression \"the past and family misfortune\" has a negative emotional connotation."}, "language_style": {"answer": "Tactful and warm", "rationale": "The entry is more personal and interactive when it is spoken in a gentle and inviting manner, rather than in direct statements or orders. It also does not use too negative language or emotional terms, but rather uses a more neutral and objective description."}, "time_dimension": {"answer": "Past", "rationale": "At the same time, the description of \"family misfortune\" may include present or future situations. Therefore, the emotional dimension of the problem can be considered for analysis in both present and past dimensions."}, "sentence_type": "Declarative sentence", "pos": {"nouns": {"self": 1, "Past": 1, "Family": 2, "Unfortunately": 1}, "verbs": {"have": 1}, "numerals": {"8": 1}}}, {"content": "9. Too much concern for the future.", "word_count": 9, "label": "", "sentence_structure": "Subject鈥搗erb鈥揷omplement", "subjective_objective": {"answer": "Subjective", "rationale": "This is because of patients' subjective feelings and thoughts about their future concerns."}, "sentence_sentiment": {"answer": "Negative", "rationale": "Because the \"excessive fear\" in the entry is negative."}, "language_style": {"answer": "Calm and direct", "rationale": "The entry directly expresses the question whether the other party is often over-scared about the subject in the future, is not using excessive rhetorical or emotional rendering and is more objective in tone."}, "time_dimension": {"answer": "Future", "rationale": ""}, "sentence_type": "Negative declarative sentence", "pos": {"nouns": {"What happens in the future": 1}, "verbs": {"I'm worried": 1, "That's too much": 1}, "adverbs": {"Too much": 1}}}, {"content": "10. Not to see anyone.", "word_count": 4, "label": "", "sentence_structure": "Subject鈥損redicate", "subjective_objective": {"answer": "Subjective", "rationale": "Because \"not seeing people\" is a subjective idea or emotional expression."}, "sentence_sentiment": {"answer": "Negative", "rationale": "Because the \"no-see\" act in the entry is negative."}, "language_style": {"answer": "Tactful and warm", "rationale": "The entry uses a euphemism, starting with \"Whether or not\" and using the emotional language \"Don't Want to Meet People\" to trigger emotional resonance and reflection among the respondents. This way reduces the resistance of the respondents and makes it easier to get real answers."}, "time_dimension": {"answer": "Present", "rationale": "The word \"wanted\" in \"unwanted\" uses the usual current state of affairs, indicating that the idea or emotion exists at present and may be recurrent."}, "sentence_type": "Negative declarative sentence", "pos": {"nouns": {"person": 1}, "verbs": {"Yes": 1, "See": 1}, "numerals": {"10": 1}, "classifiers": {"individual": 1}}}, {"content": "I feel like I'm not myself.", "word_count": 8, "label": "", "sentence_structure": "Subject鈥揷opula鈥損redicative", "subjective_objective": {"answer": "Subjective", "rationale": "Because \"I feel like I'm not myself\" is a subjective feeling and cognitive experience, not an objective fact or behaviour. This reflects the subjective perception and understanding of the patient of his identity, personality, etc."}, "sentence_sentiment": {"answer": "Negative", "rationale": "Because the feeling in the entry that you're not yourself is negative."}, "language_style": {"answer": "Tactful and warm", "rationale": "The entry uses a moderate tone to guide the interviewee to think and reflect on himself, without directly questioning or criticizing the interviewee, which makes it easier for the interviewee to relax and share real ideas. At the same time, the expression \"thinking that he is not himself\" is also more euphemistic, avoiding direct contact and the pain or confusion that may exist deep inside him."}, "time_dimension": {"answer": "Present", "rationale": "The entry is a question of the present feelings and self-awareness of the testee, and is judged on the time dimension."}, "sentence_type": "Negative declarative sentence", "pos": {"nouns": {"self": 2}, "verbs": {"I think": 1, "be": 1, "No, it's not": 1}, "pronouns": {"self": 1}}}, {"content": "12. Lack of enthusiasm and motivation", "word_count": 8, "label": "", "sentence_structure": "Subject鈥搗erb鈥揷omplement", "subjective_objective": {"answer": "Subjective", "rationale": "Because lack of enthusiasm and motivation is the feeling and attitude of the patient inside, it is subjective."}, "sentence_sentiment": {"answer": "Negative", "rationale": "Because the description of \"lack of enthusiasm and motivation\" in the entry is negative."}, "language_style": {"answer": "Tactful and warm", "rationale": "The entry is more personal and interactive in its expression of conversation in a gentle and inviting tone, rather than in direct statements or orders. At the same time, the term \"lack\" is used to describe enthusiasm and motivation, and it is more euphemism, avoiding too direct criticism or accusations."}, "time_dimension": {"answer": "Past", "rationale": "The entry does not address the concept of the time of the future, nor does it contain any clear action or decision, but rather describes a state or feeling and therefore judges as the past."}, "sentence_type": "Negative declarative sentence", "pos": {"nouns": {"鈾?Hot": 1, "Active": 1}, "verbs": {"Lack": 1}, "numerals": {"12": 1}}}, {"content": "13. Pessimism", "word_count": 2, "label": "", "sentence_structure": "Subject鈥搗erb鈥揷omplement", "subjective_objective": {"answer": "Subjective", "rationale": "Because \"pessimism\" is an emotion or attitude that describes how patients see things and reflects their subjective perception and emotional state."}, "sentence_sentiment": {"answer": "Negative", "rationale": "Because pessimism is a negative emotional attitude."}, "language_style": {"answer": "Calm and direct", "rationale": "The question is whether the other party is often pessimistic in a simple and straightforward way, and is not speaking too well or in a moody way, and speaking more in a balanced and objective manner. The language style of this questionnaire entry is therefore straightforward and calm."}, "time_dimension": {"answer": "Present", "rationale": "The entry asks if the testee is currently pessimistic and the time dimension is now judged."}, "sentence_type": "Interrogative sentence", "pos": {"nouns": {"Pessimism": 1}}}, {"content": "14. Lack of concentration of ideas", "word_count": 5, "label": "", "sentence_structure": "Subject鈥揷opula鈥損redicative", "subjective_objective": {"answer": "Subjective", "rationale": "Because the concentration of ideas is a subjective feeling and experience, it requires that the patient describe and evaluate it."}, "sentence_sentiment": {"answer": "Negative", "rationale": "Because \"inconsistent\" is a negative behaviour that may affect individual learning and work efficiency."}, "language_style": {"answer": "Calm and direct", "rationale": ""}, "time_dimension": {"answer": "Future", "rationale": "The keyword \"influenced\" indicates that the entry asks about the question of whether the testee will have a disfocused mind in the future."}, "sentence_type": "Negative declarative sentence", "pos": {"nouns": {"Ideas": 1, "Focus": 1}, "verbs": {"not": 1, "Focus": 1}, "adverbs": {"very": 1}}}, {"content": "15. Unnerved moods", "word_count": 6, "label": "", "sentence_structure": "Subject鈥搗erb鈥揷omplement", "subjective_objective": {"answer": "Subjective", "rationale": "The degree and cause of emotional overstretch is usually the patient ' s subjective perception and judgement, rather than the objective facts."}, "sentence_sentiment": {"answer": "Negative", "rationale": "Because the situation of excessive emotional overstretch is usually negative, it may affect the life and work of a person."}, "language_style": {"answer": "Calm and direct", "rationale": "The entry directly asks whether the respondents are prone to great emotional volatility, are more concise and have no excessive adoration or emotional colours, and therefore can be judged as \" calm and direct \" language styles."}, "time_dimension": {"answer": "Past", "rationale": "The entry uses the now completed tense \"have...\" to indicate that the emotional inactivity has occurred in the past, so the temporal dimension is a past."}, "sentence_type": "Affirmative declarative sentence", "pos": {"nouns": {"Emotion": 1, "Rise and shine": 1}, "verbs": {"Pass": 1}, "adjectives": {"Large": 1}, "numerals": {"15": 1}}}, {"content": "16. Often insomnia", "word_count": 4, "label": "", "sentence_structure": "Subject鈥搗erb鈥揷omplement", "subjective_objective": {"answer": "Subjective", "rationale": "Because insomnia is a subjective experience and feeling."}, "sentence_sentiment": {"answer": "Negative", "rationale": "Because \"insomnia\" is a negative physiological condition, which means that sleep is poor."}, "language_style": {"answer": "Calm and direct", "rationale": "The entry directly expresses the question of whether the other party is frequently insomnia, is not using excessive rhetorical techniques or emotional rendering, and is more objective in tone."}, "time_dimension": {"answer": "Present", "rationale": "The entry is an inquiry into the current state and experience of the subject, with the keywords \"often\" and \"insomnia\", all pointing to the situation currently under way."}, "sentence_type": "Affirmative declarative sentence", "pos": {"nouns": {"Insomnia": 1}, "verbs": {"Often": 1, "Insomnia": 1}}}, {"content": "17. Headaches.", "word_count": 2, "label": "", "sentence_structure": "Subject鈥揷opula鈥損redicative", "subjective_objective": {"answer": "Subjective", "rationale": "Because headaches are a subjective feeling or experience."}, "sentence_sentiment": {"answer": "Negative", "rationale": "Because the description of \"headache\" is usually considered negative."}, "language_style": {"answer": "Calm and direct", "rationale": "The entry directly asked whether the respondents often suffered from headaches, without using any qualifier or emotional expression, and was relatively simple and clear."}, "time_dimension": {"answer": "Present", "rationale": "\"Having headaches\" describe the current state of the person being tested, and do not clearly point at sex at a time, and therefore judge now."}, "sentence_type": "Exclamatory sentence", "pos": {"nouns": {"Headache": 1}}}, {"content": "18. A sore neck, shoulder", "word_count": 6, "label": "", "sentence_structure": "Subject鈥搗erb鈥揷omplement", "subjective_objective": {"answer": "Objective", "rationale": "Because \"the neck, shoulder sore\" is a physical feeling or a symptom, an objective experience of the patient."}, "sentence_sentiment": {"answer": "Negative", "rationale": "Because the entry \"neck, shoulder sore\" describes a symptom of physical discomfort, which is usually considered a negative experience."}, "language_style": {"answer": "Tactful and warm", "rationale": "The language style of the entry is euphemistic. It uses a more moderate language, such as \"neck, shoulder sore\" rather than a direct description of symptoms, which is more caring and understanding and makes it easier for the person under investigation to share his or her true feelings."}, "time_dimension": {"answer": "Past", "rationale": ""}, "sentence_type": "Affirmative declarative sentence", "pos": {"nouns": {"Neck": 1, "Shoulders": 1}, "verbs": {"Sour": 1}}}, {"content": "19. Heart pains.", "word_count": 4, "label": "", "sentence_structure": "Subject鈥搗erb鈥揷omplement", "subjective_objective": {"answer": "Objective", "rationale": "The pain in the chest is an objective fact of the patient's experience."}, "sentence_sentiment": {"answer": "Negative", "rationale": "Because chest suffocation is a physical discomfort, it is often considered a negative experience."}, "language_style": {"answer": "Calm and direct", "rationale": ""}, "time_dimension": {"answer": "Past", "rationale": "The entry describes whether the person tested has had breast pain and suffocation symptoms at a certain point in time (i.e., in the past) and therefore is judged to be \"the past\"."}, "sentence_type": "Affirmative declarative sentence", "pos": {"nouns": {"- Chestaches": 1, "- Squeeze": 1}}}, {"content": "It's always a big deal.", "word_count": 7, "label": "", "sentence_structure": "Subject鈥搗erb鈥揷omplement", "subjective_objective": {"answer": "Subjective", "rationale": "Because it is always the patient who is feeling and feeling for the patient, it is the patient who is subjective."}, "sentence_sentiment": {"answer": "Positive", "rationale": "Because \"breathing\" is a positive adjective, expressing positive and dynamic attitudes and behaviour."}, "language_style": {"answer": "Tactful and warm", "rationale": "The entry collects information by describing whether the other side is always able to maintain a positive and dynamic attitude, not using direct criticism or negative language, but rather expressing the problem in a moderate manner. Such language styles are more comfortable and respected for the interviewee and more likely to get accurate and true answers."}, "time_dimension": {"answer": "Present", "rationale": "The entry now describes the current status and performance of the testee, using the adverb \"always\" for the frequency, so the temporal dimension is now judged."}, "sentence_type": "Affirmative declarative sentence", "pos": {"verbs": {"Always": 1}, "adjectives": {"It's a big deal": 1}}}, {"content": "21. Low gas", "word_count": 3, "label": "", "sentence_structure": "Subject鈥搗erb鈥揷omplement", "subjective_objective": {"answer": "Subjective", "rationale": "Air mass is a subjective feeling and evaluation."}, "sentence_sentiment": {"answer": "Negative", "rationale": "Because \"small gas\" is a negative quality."}, "language_style": {"answer": "Calm and direct", "rationale": "The entry describes the characteristics to be assessed in a concise and clear language, with no excessive emotional or rhetorical style, and a more calm and straightforward language style."}, "time_dimension": {"answer": "Past", "rationale": "The keyword is \"low gas\", which is a term describing past performance or characteristics, and the entry therefore asks about the behaviour or character characteristics of the person being tested in the past."}, "sentence_type": "Negative declarative sentence", "pos": {"verbs": {"Small gas": 1}, "adjectives": {"Small": 1}}}, {"content": "Careless.", "word_count": 3, "label": "", "sentence_structure": "Subject鈥搗erb鈥搊bject", "subjective_objective": {"answer": "Subjective", "rationale": "Because \"love\" is a description of subjective emotional experience and attitude, which reflects the feelings and perceptions inside the patient, it is subjective."}, "sentence_sentiment": {"answer": "Negative", "rationale": "Because \"love\" is often seen as a negative quality, which means that a person is too worried and anxious and can cause stress and distress to himself and others around him."}, "language_style": {"answer": "Calm and direct", "rationale": "The language style of the entry is relatively simple and clear, there are no excessive qualifiers and rhetorical techniques, which are more direct and objective in tone, and therefore can be judged as \"cool and direct\"."}, "time_dimension": {"answer": "Present", "rationale": ""}, "sentence_type": "Exclamatory sentence", "pos": {"verbs": {"Love": 1, "Mind your own business": 1}, "adjectives": {"Minding": 1}}}, {"content": "23. Routine", "word_count": 4, "label": "", "sentence_structure": "Subject鈥揷opula鈥損redicative", "subjective_objective": {"answer": "Subjective", "rationale": "The first is that the patient has a subjective or emotional experience of being restless."}, "sentence_sentiment": {"answer": "Negative", "rationale": "Because the \"freaky\" act is negative."}, "language_style": {"answer": "Tactful and warm", "rationale": ""}, "time_dimension": {"answer": "Future", "rationale": "The entry asks if the person tested will experience \"agitation\" in the future and therefore judges it as the future."}, "sentence_type": "Exclamatory sentence", "pos": {"nouns": {"- Nervous": 1, "Unsettled": 1}, "adjectives": {"Anxiety": 1}, "adverbs": {"Unsettled": 1}, "numerals": {"23": 1}}}, {"content": "24. Easier to be angry", "word_count": 4, "label": "", "sentence_structure": "Subject鈥搗erb鈥揷omplement", "subjective_objective": {"answer": "Subjective", "rationale": "Because anger is an emotional reaction, it is a matter of the patient ' s inner experience and feelings, and it is a subjective examination."}, "sentence_sentiment": {"answer": "Negative", "rationale": "Because the \"frightful\" act in the entry is negative."}, "language_style": {"answer": "Calm and direct", "rationale": "The entry directly expresses the subject of an inquiry into the other party ' s tendency to be angry, without excessive rhetoric or emotional rendering, and with a more objective tone."}, "time_dimension": {"answer": "Past", "rationale": ""}, "sentence_type": "Exclamatory sentence", "pos": {"verbs": {"Easy": 1, "Excited": 1}}}, {"content": "25. It's no fun to live.", "word_count": 5, "label": "", "sentence_structure": "Subject鈥揷opula鈥損redicative", "subjective_objective": {"answer": "Subjective", "rationale": "Because \"living useless\" is a subjective feeling and attitude, which is perceived and experienced by the patient himself, and therefore is subjective."}, "sentence_sentiment": {"answer": "Negative", "rationale": "Because \"living is pointless\" is a negative emotional expression."}, "language_style": {"answer": "Calm and direct", "rationale": "The entry is presented directly to ask the respondents whether they feel that life is lost to pleasure or meaning, and the language style is more straightforward and straightforward."}, "time_dimension": {"answer": "Future", "rationale": "\"Live without meaning\" is an emotional expression of dissatisfaction and despair about the future living situation. Thus, in terms of time dimension, the entry points to the possibility of the future."}, "sentence_type": "Negative declarative sentence", "pos": {"nouns": {"Live": 1, "Meaning": 1}, "verbs": {"Here": 1, "Nope": 1, "Interesting": 1}, "numerals": {"25": 1}}}, {"content": "Not interested in anything.", "word_count": 9, "label": "", "sentence_structure": "Subject鈥揷opula鈥損redicative", "subjective_objective": {"answer": "Subjective", "rationale": "Because \"interest\" and \"no interest\" are the feelings and attitudes of the patients themselves, the answer to this questionnaire is subjective."}, "sentence_sentiment": {"answer": "Negative", "rationale": "Because \"no interest\" in the entry is an expression of a negative emotional attitude."}, "language_style": {"answer": "Tactful and warm", "rationale": "In response to question \"26. Nothing is interesting\" my observations and analysis are as follows: 1. The question is designed in a very direct and clear form, without using too many rhetorical or vague language expressions, so that it can be judged as a \"cooling straight\" language style. 2. The question is: The question is a very personal one, requiring the respondents to respond to their feelings and attitudes inside. This type of questionnaire is usually sensitive and personal, and therefore a more gentle, friendly inquiry may make the respondents uncomfortable or unwilling to answer."}, "time_dimension": {"answer": "Present", "rationale": "The entry is for the current psychological state of the person being tested, which is \"no interest in anything\". Although the time frame is not specified, it is now judged because it is a question of the current psychological state."}, "sentence_type": "Negative declarative sentence", "pos": {"nouns": {"Interest": 1}, "verbs": {"No, I'm not": 1, "to": 1, "Both": 1}, "adverbs": {"Any": 1}, "numerals": {"26": 1}}}, {"content": "27. Memory loss", "word_count": 5, "label": "", "sentence_structure": "Subject鈥搗erb鈥揷omplement", "subjective_objective": {"answer": "Subjective", "rationale": "Because memory loss may be a subjective feeling of the patient, not an objective fact."}, "sentence_sentiment": {"answer": "Negative", "rationale": "Because the \"rememberance loss\" in the entries is negative."}, "language_style": {"answer": "Calm and direct", "rationale": "The language style of the entry is relatively calm and straightforward, with no excessive emotional or qualifier colours, and it is more objective and professional to ask the interviewees whether there has been any memory loss."}, "time_dimension": {"answer": "Past", "rationale": "The entry asks if the person tested has suffered memory loss, and does not specify whether it occurred in the past, present or future, and therefore cannot be accurately judged on the time dimension."}, "sentence_type": "Exclamatory sentence", "pos": {"nouns": {"Memory": 1, "Declining": 1}, "numerals": {"27": 1}}}, {"content": "28. Lack of endurance", "word_count": 4, "label": "", "sentence_structure": "Subject鈥搗erb鈥揷omplement", "subjective_objective": {"answer": "Subjective", "rationale": "Because lack of endurance is a subjective feeling and evaluation."}, "sentence_sentiment": {"answer": "Negative", "rationale": "Because the lack of endurance is negative."}, "language_style": {"answer": "Tactful and warm", "rationale": "The entry is more euphemistic, using the expression \"lack\" rather than direct criticism or accusation, and is more moderate and inclusive. At the same time, the entry gives an objective description, not subjective assessment. The language style of the entry can therefore be classified as \"precipitous\"."}, "time_dimension": {"answer": "Past", "rationale": "In summary, for the entry sentence, \"Please analyse the following entry for \"28. Lack of endurance\", output: \"I think it should be answered that the entry describes a psychological state and feelings that the person tested has in a given situation and does not involve specific acts or events of the past, present or future."}, "sentence_type": "Negative declarative sentence", "pos": {"nouns": {"Endurance": 1}, "verbs": {"Lack": 1}}}, {"content": "29. Lack of decisive capacity", "word_count": 6, "label": "", "sentence_structure": "Subject鈥搗erb鈥揷omplement", "subjective_objective": {"answer": "Subjective", "rationale": "Because lack of decisive capacity is a manifestation of individual psychological characteristics and experience, it is a subjective examination."}, "sentence_sentiment": {"answer": "Negative", "rationale": "Because the term \"lack of decisiveness\" is usually considered negative quality."}, "language_style": {"answer": "Tactful and warm", "rationale": "The entry is more personal and interactive when it is presented in a gentle and inviting tone, rather than in direct statements or orders. At the same time, it avoids feeling pressure or negative emotions for the respondents, but instead leads them to think and answer questions in a more understanding and supportive manner."}, "time_dimension": {"answer": "Past", "rationale": "\"Leave of judgment\" is a description of a state or situation, indicating that the testee may have had the problem at some point in the past."}, "sentence_type": "Negative declarative sentence", "pos": {"nouns": {"Adjudication": 1, "Lack": 1}, "numerals": {"29": 1}}}, {"content": "30. Overdependence", "word_count": 6, "label": "", "sentence_structure": "Subject鈥搗erb鈥揷omplement", "subjective_objective": {"answer": "Subjective", "rationale": "Because \"reliance\" is a feeling and attitude within a person, and it's a subjective approach."}, "sentence_sentiment": {"answer": "Negative", "rationale": "Because the \"too much reliance\" in the entry is negative."}, "language_style": {"answer": "Calm and direct", "rationale": "The language style of the entry is calm and straightforward, as it directly expresses the subject matter of the inquiry as to whether the other party is too dependent on others, is not based on excessive rhetorical or emotional rendering, and is more objective in tone."}, "time_dimension": {"answer": "Present", "rationale": "The keyword is \"too much reliance on others\", describing the person being tested for the act or situation. So the temporal dimension is now."}, "sentence_type": "Negative declarative sentence", "pos": {"nouns": {"Too much": 1, "Dependency": 1, "others": 1}, "verbs": {"Dependency": 1}, "adjectives": {"Too much": 1}, "numerals": {"30": 1}}}, {"content": "31. Worried about the red face", "word_count": 6, "label": "", "sentence_structure": "Subject鈥搗erb鈥揷omplement", "subjective_objective": {"answer": "Subjective", "rationale": "Because \"Happiness for the Face\" is a subjective feeling or emotional experience, it is a emotional reaction within the patient and it is a subjective perspective."}, "sentence_sentiment": {"answer": "Negative", "rationale": "Because \"suffering\" expresses negative emotional attitudes."}, "language_style": {"answer": "Calm and direct", "rationale": "The language style of the entry is calm, straightforward, and there is no excessive adoration or emotional rendering, and the subject of asking the other side whether it is a problem with the face is simple and clear."}, "time_dimension": {"answer": "Present", "rationale": ""}, "sentence_type": "Affirmative declarative sentence", "pos": {"nouns": {"Red": 1, "Trouble": 1}, "verbs": {"Yes": 1, "Trouble": 1}, "numerals": {"31": 1}}}, {"content": "32. Sniper, loud.", "word_count": 6, "label": "", "sentence_structure": "Subject鈥搗erb鈥揷omplement", "subjective_objective": {"answer": "Objective", "rationale": "Since stutter and tremor are objective manifestations of the patient, it is not necessary to consider whether the patient is aware of these symptoms or willing to acknowledge their existence."}, "sentence_sentiment": {"answer": "Negative", "rationale": "Because stutter and sound tremors are both negative expressions."}, "language_style": {"answer": "Calm and direct", "rationale": "The entry is expressed in more direct, concise language, with no excessive adoration or emotional colour, and is more focused on the purpose of transmitting factual information and investigations. At the same time, the issue is also of a certain professional nature, using medical terms \"sniffing\" and \"throwing\" and providing a more objective and accurate picture of the symptoms of the person under investigation."}, "time_dimension": {"answer": "Past", "rationale": "Past."}, "sentence_type": "Affirmative declarative sentence", "pos": {"nouns": {"Snack": 1, "Sound": 1, "Frightened": 1}, "verbs": {"have": 1}, "numerals": {"32": 1}}}, {"content": "33. Frightened body", "word_count": 6, "label": "", "sentence_structure": "Subject鈥搗erb鈥揷omplement", "subjective_objective": {"answer": "Objective", "rationale": "The fact that the patient is suffering from the cold and the heat is an objective fact."}, "sentence_sentiment": {"answer": "Negative", "rationale": "Because \"heat and cold\" is usually a manifestation of physical discomfort, which can cause health problems, it is negative."}, "language_style": {"answer": "Tactful and warm", "rationale": "The entry is described in more moderate and polite language, does not lead to questions being answered too directly or strongly, reflecting respect for and interest in the person under investigation. At the same time, this expression is also better able to bring the person under investigation closer to him, enhance his or her participation and cooperation."}, "time_dimension": {"answer": "Past", "rationale": ""}, "sentence_type": "Exclamatory sentence", "pos": {"nouns": {"Body": 1, "Cold and hot": 1}, "verbs": {"Kub": 2}, "numerals": {"33": 1}}}, {"content": "34. Pay attention to urine and sexual organs.", "word_count": 8, "label": "", "sentence_structure": "Subject鈥搗erb鈥揷omplement", "subjective_objective": {"answer": "Subjective", "rationale": "The question is asked about the extent of the attention given to the body of the person under investigation and about the subjective consciousness and feelings of the individual."}, "sentence_sentiment": {"answer": "Negative", "rationale": "This entry may cause embarrassment and discomfort and its content is more personal."}, "language_style": {"answer": "Calm and direct", "rationale": "The language style of the entry is calm and straightforward. It raises a simple question, without any euphemism or accommodative language. This direct expression makes it easier for the person surveyed to understand the meaning of the question and provides an accurate answer."}, "time_dimension": {"answer": "Present", "rationale": "The entry asks whether the person being tested is currently focusing on urine and sexual organs, and the temporal dimension is now judged."}, "sentence_type": "Imperative sentence", "pos": {"nouns": {"Attention": 1, "Peeing": 1, "Sexual organs": 1}, "numerals": {"34": 1}}}, {"content": "35. A happy mood.", "word_count": 4, "label": "", "sentence_structure": "Subject鈥搗erb鈥揷omplement", "subjective_objective": {"answer": "Subjective", "rationale": "Because \"cream\" is a subjective feeling and emotional state."}, "sentence_sentiment": {"answer": "Positive", "rationale": "Because \"crazy\" expresses positive emotional attitudes."}, "language_style": {"answer": "Tactful and warm", "rationale": "The entry, in a positive and encouraging tone, asks whether the respondents are often in a state of open mood, soft and stimulating speech, which can trigger positive emotions and resonance among the respondents, and therefore the language style is euphemism."}, "time_dimension": {"answer": "Present", "rationale": "The word \"mature\" in \"clause\" suggests that it is a term for the inner state of the individual, and \"clause\" is an adjective for the current state and feelings of the person being tested. So, the entry is asking the person who is being tested for the current state of mood. In terms of time dimensions, it belongs to the present."}, "sentence_type": "Affirmative declarative sentence", "pos": {"nouns": {"The mood": 1, "- Yes, sir": 1}, "adjectives": {"- Yes, sir": 1}, "numerals": {"35": 1}}}, {"content": "36. Unsurprisingly uneasy.", "word_count": 7, "label": "", "sentence_structure": "Subject鈥搗erb鈥揷omplement", "subjective_objective": {"answer": "Subjective", "rationale": "Because \"unusually disturbed\" is a psychological or emotional experience, it is the sense and perception of the patient himself and is therefore a subjective examination."}, "sentence_sentiment": {"answer": "Negative", "rationale": "Because the entry \"unbelievably uneasy\" expresses a negative emotion."}, "language_style": {"answer": "Calm and direct", "rationale": "The entry directly asks whether the interviewees are often in a state of inexplicable anxiety, and does not use excessive rhetorical or emotional colours, and is more objective and direct in language style.\""}, "time_dimension": {"answer": "Present", "rationale": ""}, "sentence_type": "Imperative sentence", "pos": {"nouns": {"Questionnaire": 1, "Entry": 1}, "verbs": {"Analysis": 1, "I'm just wondering why": 1}, "adjectives": {"I don't know": 1}, "adverbs": {"Land": 2}, "numerals": {"36": 1}}}, {"content": "37. Disturbing when one is alone.", "word_count": 10, "label": "", "sentence_structure": "Subject鈥揷opula鈥損redicative", "subjective_objective": {"answer": "Subjective", "rationale": "Because \"uneasy\" is a subjective emotional experience that reflects the feelings and mindset of patients when they are alone."}, "sentence_sentiment": {"answer": "Negative", "rationale": "Because \"uneasy\" is a negative emotion."}, "language_style": {"answer": "Tactful and warm", "rationale": "The entry is more personal and interactive in terms of a moderate and understanding of the concerns and understandings that may be disturbing when the person interviewed is alone, rather than direct orders or criticism."}, "time_dimension": {"answer": "Present", "rationale": "The entry is for the testee ' s feelings in certain circumstances, and the time dimension is now judged."}, "sentence_type": "Exclamatory sentence", "pos": {"nouns": {"person": 1, "Alone": 1, "Unsettled": 1}, "verbs": {"I feel": 1, "Alone": 1, "Unsettled": 1}, "numerals": {"37": 1}}}, {"content": "Lack of self-confidence", "word_count": 5, "label": "", "sentence_structure": "Subject鈥搗erb鈥揷omplement", "subjective_objective": {"answer": "Subjective", "rationale": "Because lack of self-confidence is the patient ' s subjective assessment and perception of himself."}, "sentence_sentiment": {"answer": "Negative", "rationale": "Because the description of \"lack of self-confidence\" in the entry is negative."}, "language_style": {"answer": "Tactful and warm", "rationale": "The entry uses a soft question and avoids the use of strong negative language or overly direct expressions that make the respondents feel more comfortable and acceptable. At the same time, this expression encourages the respondents to answer questions more honestly and frankly, thereby obtaining more accurate data results."}, "time_dimension": {"answer": "Past", "rationale": "The entry is for the purpose of asking the testee about his current situation and feelings, and the time dimension is a past."}, "sentence_type": "Exclamatory sentence", "pos": {"nouns": {"Self-confidence": 1, "Lack": 1}, "verbs": {"have": 1}, "numerals": {"38": 1}}}, {"content": "39. Daunting the tail.", "word_count": 6, "label": "", "sentence_structure": "Subject鈥搗erb鈥揷omplement", "subjective_objective": {"answer": "Subjective", "rationale": "Because \"daunted tail\" is a subjective feeling and attitude."}, "sentence_sentiment": {"answer": "Negative", "rationale": "Because the word \"daunted\" in the entry is derogatory and indicates a lack of decisiveness and courage in acting."}, "language_style": {"answer": "Tactful and warm", "rationale": "The entry is more adept and interactive when it is spoken in a gentle and inviting manner, rather than by direct statements or orders."}, "time_dimension": {"answer": "Present", "rationale": ""}, "sentence_type": "Negative declarative sentence", "pos": {"nouns": {"Yes": 1, "- You're afraid of the end": 1}, "verbs": {"OPERATIONS": 1}, "adjectives": {"- You're afraid of the end": 1}, "numerals": {"39": 1}}}, {"content": "40. It's easy to misconstrue.", "word_count": 6, "label": "", "sentence_structure": "Subject鈥搗erb鈥揷omplement", "subjective_objective": {"answer": "Subjective", "rationale": "Because \"facilitate\" is a personal feeling and experience, a result of subjective patient judgement."}, "sentence_sentiment": {"answer": "Negative", "rationale": "Because the \"misunderstanding\" in the entry is negative."}, "language_style": {"answer": "Tactful and warm", "rationale": "The entry is more personal and interactive when it is spoken in a gentle and inviting manner, rather than in direct statements or orders. At the same time, this euphemism is more comfortable and willing to share its real ideas, thereby improving the effectiveness and data quality of the questionnaire."}, "time_dimension": {"answer": "Present", "rationale": "In the past, the entry asked the testee whether he had been misconstrued by others and the temporal dimension judged to be past."}, "sentence_type": "Negative declarative sentence", "pos": {"nouns": {"person": 1, "Misunderstanding": 1}, "verbs": {"Easy": 1, "By": 1, "Misunderstanding": 1}, "adverbs": {"very": 1}, "numerals": {"40": 1}}}, {"content": "41. Trust no one else.", "word_count": 5, "label": "", "sentence_structure": "Subject鈥搗erb鈥揷omplement", "subjective_objective": {"answer": "Subjective", "rationale": "Because the words \"convinced\" and \"not believed\" are subjective decisions and attitudes of patients."}, "sentence_sentiment": {"answer": "Negative", "rationale": "Because the attitude of \"no one else\" is negative."}, "language_style": {"answer": "Calm and direct", "rationale": "The entry directly asks the interviewees whether they do not trust others in certain circumstances, that the language is clear and that they do not use excessive rhetoric or emotional colours to present a calm and objective expression."}, "time_dimension": {"answer": "Present", "rationale": ""}, "sentence_type": "Negative declarative sentence", "pos": {"nouns": {"Questionnaire": 1, "Entry": 1, "others": 1}, "verbs": {"Yes": 1, "have": 1}, "numerals": {"41": 1}}}, {"content": "42. Overstretch", "word_count": 4, "label": "", "sentence_structure": "Subject鈥搗erb鈥揷omplement", "subjective_objective": {"answer": "Subjective", "rationale": "The blogger says that the idea of a person who is a patient is a subjective one or an idea."}, "sentence_sentiment": {"answer": "Negative", "rationale": "Because the act of \"too much suspicion\" is negative."}, "language_style": {"answer": "Tactful and warm", "rationale": "The language style of the entry is more euphemistic, using softer words, such as \"too much suspicion,\" and not directly accusing or criticizing the other, but rather directing the respondents to think and answer questions in an understanding and support-based manner. This expression helps to create a good atmosphere of communication, allowing the respondents to share their thoughts and feelings."}, "time_dimension": {"answer": "Present", "rationale": ""}, "sentence_type": "Negative declarative sentence", "pos": {"nouns": {"Doubt": 1, "Excess": 1}, "verbs": {"have": 1}, "adjectives": {"Too much": 1}, "numerals": {"42": 1}}}, {"content": "43. Disgusting association", "word_count": 4, "label": "", "sentence_structure": "Subject鈥搗erb鈥揷omplement", "subjective_objective": {"answer": "Subjective", "rationale": "Because \"averse to being in a relationship\" is an expression of personal feelings and attitudes, which are subjective feelings and perceptions of patients."}, "sentence_sentiment": {"answer": "Negative", "rationale": "Basis for judgement: The article is negative in its formulation, which expresses a negative emotional attitude, i.e. aversion to interpersonal interaction."}, "language_style": {"answer": "Calm and direct", "rationale": "The entry asks the other side whether it is in a direct manner that it is not in a position to engage in human interaction, that it is not using excessive emotional or rhetorical techniques and that it is more objective and straightforward."}, "time_dimension": {"answer": "Past", "rationale": "The entry indicates the respondent ' s aversion to dealing, which may have been developed and which has existed in the past."}, "sentence_type": "Negative declarative sentence", "pos": {"nouns": {"Disgusting": 1, "- To be with someone": 1}, "numerals": {"43": 1}}}, {"content": "44. Feeling inferior.", "word_count": 4, "label": "", "sentence_structure": "Subject鈥揷opula鈥損redicative", "subjective_objective": {"answer": "Subjective", "rationale": "Because \"substantial\" is a subjective emotional experience and feeling that reflects patients' subjective judgement and evaluation of their abilities and values. While feelings of inferiority may be related to objective facts (e.g., lack of self-confidence or insufficient capacity in some respects), they are subjective in themselves and depend on the patient鈥檚 inner experience and thoughts."}, "sentence_sentiment": {"answer": "Negative", "rationale": "Because the entry \"Feeling low\" expresses a negative emotional attitude."}, "language_style": {"answer": "Calm and direct", "rationale": ""}, "time_dimension": {"answer": "Past", "rationale": ""}, "sentence_type": "Affirmative declarative sentence", "pos": {"nouns": {"You're a low man": 1, "Sensor": 1}, "numerals": {"44": 1}}}, {"content": "45. The world is in a state of anxiety.", "word_count": 4, "label": "", "sentence_structure": "Subject鈥損redicate", "subjective_objective": {"answer": "Subjective", "rationale": "Because \"fearing\" is a manifestation of subjective fear and anxiety."}, "sentence_sentiment": {"answer": "Negative", "rationale": "Because \"sweet\" is a negative emotion and attitude when it comes to worrying about something that is unnecessary."}, "language_style": {"answer": "Tactful and warm", "rationale": "The language style of the entry is euphemism. It does not use a direct denial or criticism, but rather a gentle, encouraging and supportive tone to guide the interviewees to share their thoughts and feelings. This expression is more acceptable and promotes more active participation in the questionnaire."}, "time_dimension": {"answer": "Future", "rationale": "The keyword \"crazy\" implies fear and fear for the future, so the entry contains visions and expectations for the future, which can be judged as the future."}, "sentence_type": "Affirmative declarative sentence", "pos": {"nouns": {"Scratch": 1, "Oh, my God": 1}}}, {"content": "46. Physical fatigue", "word_count": 4, "label": "", "sentence_structure": "Subject鈥揷opula鈥損redicative", "subjective_objective": {"answer": "Objective", "rationale": "Because physical fatigue is the physical condition experienced by the patient and is the result of objective observations."}, "sentence_sentiment": {"answer": "Negative", "rationale": "Because \"tired\" is a term for negative feelings, implying poor health."}, "language_style": {"answer": "Calm and direct", "rationale": "The entry uses simple and clear language, directly asking the interviewee whether he is physically exhausted and not expressing too much adoration or emotionally, and therefore the language style can be judged as \"cool and straight\"."}, "time_dimension": {"answer": "Present", "rationale": "\"By-doing\" means the person being tested is in his current state of health, so it's now."}, "sentence_type": "Affirmative declarative sentence", "pos": {"nouns": {"Body": 1, "Tired": 1}, "numerals": {"46": 1}}}, {"content": "47. He sweats in a hurry.", "word_count": 7, "label": "", "sentence_structure": "Subject鈥搗erb鈥揷omplement", "subjective_objective": {"answer": "Objective", "rationale": "Because \"cool sweat\" is an expression of a physical reaction, which is objective. Although it may be related to the patient鈥檚 emotions, from this point of view it is more of an examination of the patient鈥檚 physical reaction, and should therefore be considered objective."}, "sentence_sentiment": {"answer": "Negative", "rationale": "Because the act of \"cool sweat in a hurry\" in the entry is negative."}, "language_style": {"answer": "Tactful and warm", "rationale": "The question is asked in a soft and caring manner, not in too direct or strong a language, but in a way that guides the interviewee to share their emotional reactions and experiences, making the interviewee more comfortable and comfortable in answering questions. At the same time, the question concerns the mental health of the individual, which needs to be approached in a gentle way, and therefore the use of a warmer tone is more appropriate."}, "time_dimension": {"answer": "Past", "rationale": "In the entry, \"Cool sweats in a hurry\" describes a symptom or behaviour of the past and therefore determines it to be the past."}, "sentence_type": "Exclamatory sentence", "pos": {"nouns": {"I'm in a hurry": 1, "Cold sweat": 1}, "verbs": {"Out": 1}, "adverbs": {"Just": 1}, "numerals": {"47": 1}}}, {"content": "48. Upon your feet, you're dizzy.", "word_count": 6, "label": "", "sentence_structure": "Subject鈥揷opula鈥損redicative", "subjective_objective": {"answer": "Objective", "rationale": "Because it's the patient's objective condition whether or not the vertigo occurs."}, "sentence_sentiment": {"answer": "Negative", "rationale": "Because \"get up and get dizzy\" is negative and may involve physical health."}, "language_style": {"answer": "Tactful and warm", "rationale": "The entry asks the other person about his/her condition in a way that is sensitive and understandable, without directly highlighting the seriousness of the problem or the accusations, and is more gentle and thoughtful. This expression is more reflective of the respect and concern of the researcher with the interviewee."}, "time_dimension": {"answer": "Past", "rationale": ""}, "sentence_type": "Affirmative declarative sentence", "pos": {"nouns": {"Dizziness": 1}, "verbs": {"Stand up": 1}, "numerals": {"48": 1}}}, {"content": "49. I've had unconscious, cramped.", "word_count": 8, "label": "", "sentence_structure": "Subject鈥搗erb鈥揷omplement", "subjective_objective": {"answer": "Objective", "rationale": "Physical symptoms such as unconsciousness, cramps and so forth are objective facts experienced by patients and are biological responses."}, "sentence_sentiment": {"answer": "Negative", "rationale": "Because \"de-conscious, cramped\" is a symptom of physical discomfort, it is often considered a negative experience."}, "language_style": {"answer": "Calm and direct", "rationale": "The entry directly asked whether the person surveyed had experienced \"disconscious, cramped\" and was more direct and not over-emotional."}, "time_dimension": {"answer": "Past", "rationale": "The entry asks if the testee has experienced \"disconscious, cramped\" and the temporal dimension is a past."}, "sentence_type": "Exclamatory sentence", "pos": {"nouns": {"Awareness": 1, "Twitches": 1}, "verbs": {"have": 1, "Pass": 1, "Lose": 1}, "numerals": {"49": 1}}}, {"content": "50. The human race is popular.", "word_count": 6, "label": "", "sentence_structure": "Subject鈥揷opula鈥損redicative", "subjective_objective": {"answer": "Subjective", "rationale": "Because humanity and popularity often depend on the evaluation of others, which is subjective."}, "sentence_sentiment": {"answer": "Positive", "rationale": "Because the description \"honorable\" is positive and positive."}, "language_style": {"answer": "Tactful and warm", "rationale": "The language style of the entry is euphemism, because it asks respondents, in a gentle and inviting tone, whether their human well-being is well received, and this expression is more personal and interactive. At the same time, there is no direct statement or order, but rather directs the interviewer to answer the questions in a question-and-answer manner, which makes it easier to make the interviewee comfortable and free and more likely to get the real answer."}, "time_dimension": {"answer": "Present", "rationale": ""}, "sentence_type": "Affirmative declarative sentence", "pos": {"nouns": {"Humanities": 1, "Welcome": 1}, "verbs": {"It's good": 1}}}, {"content": "51. Too much restraint.", "word_count": 4, "label": "", "sentence_structure": "Subject鈥搗erb鈥揷omplement", "subjective_objective": {"answer": "Subjective", "rationale": "Too rigid is a subjective assessment or feeling, which involves a person ' s perception and perception of his or her own behaviour or emotions, rather than a description of an objective external fact. Thus, it is a subjective question."}, "sentence_sentiment": {"answer": "Negative", "rationale": "Because the quality of \"too much muddled\" in the entries is usually considered negative."}, "language_style": {"answer": "Tactful and warm", "rationale": ""}, "time_dimension": {"answer": "Present", "rationale": "In the future, the entry contains visions and expectations for the future, and the keyword is \"too rigid\" and points to situations that have not yet occurred and that may arise in the future."}, "sentence_type": "Negative declarative sentence", "pos": {"nouns": {"- What": 1}, "verbs": {"That's too much": 1}, "adverbs": {"Pass": 1}, "numerals": {"51": 1}}}, {"content": "52. Worried by the lack of certainty", "word_count": 8, "label": "", "sentence_structure": "Subject鈥搗erb鈥揷omplement", "subjective_objective": {"answer": "Subjective", "rationale": "Because pain is a subjective feeling and experience."}, "sentence_sentiment": {"answer": "Negative", "rationale": "Because the mood of \"depression\" in the entry is negative."}, "language_style": {"answer": "Tactful and warm", "rationale": ""}, "time_dimension": {"answer": "Present", "rationale": ""}, "sentence_type": "Exclamatory sentence", "pos": {"nouns": {"Blue": 1, "No, I'm not": 1, "Trouble": 1}, "numerals": {"52": 1}}}, {"content": "53. Cares about the dirty.", "word_count": 5, "label": "", "sentence_structure": "Subject鈥搗erb鈥揷omplement", "subjective_objective": {"answer": "Subjective", "rationale": "Because \"care about the dirty\" is the patient's subjective feeling and attitude."}, "sentence_sentiment": {"answer": "Negative", "rationale": "Because the \"care about dirty\" behavior in the entries is negative."}, "language_style": {"answer": "Tactful and warm", "rationale": "The language style of the entry is more euphemistic, and the way in which the interviewee is directed to answer is not too direct or harsh. At the same time, the use of the expression \"care\" is also relatively neutral, avoiding words that could provoke negative emotions."}, "time_dimension": {"answer": "Present", "rationale": "The entry indicates the current attitude or feeling of the testee, \"care\" is an ongoing act, so the temporal dimension is now judged."}, "sentence_type": "Negative declarative sentence", "pos": {"nouns": {"Dirty": 1}, "verbs": {"I care": 1}}}, {"content": "54. I can't get rid of the meaningless idea.", "word_count": 11, "label": "", "sentence_structure": "Subject鈥搗erb鈥揷omplement", "subjective_objective": {"answer": "Subjective", "rationale": "Because \"indefinable\" means that it is a subjective feeling and experience of the patient, not an objective fact or situation."}, "sentence_sentiment": {"answer": "Negative", "rationale": "Because \"not getting rid of\" is negative, and it means you can't control your own ideas."}, "language_style": {"answer": "Tactful and warm", "rationale": "The expression in which the entry is used is more sympathetic and supportive, by euphemism, by not directly accusing or criticizing the person under investigation, but by trying to understand the distress and suffering of the person under investigation."}, "time_dimension": {"answer": "Present", "rationale": "The entry asks if the testee is currently in a situation where he is not free of meaningless ideas, and the time dimension is now judged."}, "sentence_type": "Negative declarative sentence", "pos": {"nouns": {"Thoughts": 1, "Meaning": 1}, "verbs": {"Get rid of it": 1, "have": 1}, "adjectives": {"Nothing": 1}, "numerals": {"54锛?: 1}}}, {"content": "I feel like I smell weird.", "word_count": 8, "label": "", "sentence_structure": "Subject鈥揷opula鈥損redicative", "subjective_objective": {"answer": "Subjective", "rationale": "Because feeling strange smells is a subjective feeling or perception."}, "sentence_sentiment": {"answer": "Negative", "rationale": "Because the description \"there's a strange smell\" is negative."}, "language_style": {"answer": "Tactful and warm", "rationale": "This entry expresses respect and interest in the person being investigated, by using the \"you think\" type of language, and does not directly accuse or criticize the problems that the person being investigated may have. This expression is more accompanied and moderate, and contributes to greater acceptance of the questionnaire."}, "time_dimension": {"answer": "Present", "rationale": "The entry contains a description of the current situation, and the words \"like\" and \"have\" indicate that this is a current expression, so the judgment is \"now\"."}, "sentence_type": "Negative declarative sentence", "pos": {"nouns": {"self": 1, "Smells weird": 1}, "verbs": {"I think": 1}, "adjectives": {"have": 1}, "numerals": {"55": 1}}}, {"content": "People say bad things behind their backs.", "word_count": 10, "label": "", "sentence_structure": "Subject鈥搗erb鈥揷omplement", "subjective_objective": {"answer": "Objective", "rationale": "Because \"the people who say bad things behind their back\" are the objective facts that patients experience, they do not need subjective attitudes or ideas to explain, and are therefore objective."}, "sentence_sentiment": {"answer": "Negative", "rationale": "Because the \"others say bad things behind their back\" act is negative."}, "language_style": {"answer": "Calm and direct", "rationale": "The purpose of this article is to describe the problem in a more concise and clear way, without using too many rhetorical techniques or emotionally coloured language. The question itself is more straightforward and clear, asking the respondents directly whether they have experienced the fact that others are speaking bad behind their backs, without too many guidance or implication. This language is more in keeping with the characteristics of the questionnaire, and it accurately conveys the information they want to collect."}, "time_dimension": {"answer": "Present", "rationale": ""}, "sentence_type": "Negative declarative sentence", "pos": {"nouns": {"person": 1, "Bad word": 1}, "verbs": {"Say it": 1, "Behind": 1}, "adverbs": {"In your own self": 1, "others": 1}, "numerals": {"56锛?: 1}}}, {"content": "57. Always watch out for the people around.", "word_count": 7, "label": "", "sentence_structure": "Subject鈥搗erb鈥揷omplement", "subjective_objective": {"answer": "Subjective", "rationale": "Because \"watching the people around\" is an individual's behavioural choice and preference that depends on the individual's consciousness and will to implement, it reflects the patient's subjective attitude."}, "sentence_sentiment": {"answer": "Neutral", "rationale": "The entry does not relate to any apparent emotion, attitude, behaviour, purpose or quality, and therefore there is no apparent positive or negative tendency, which is neutral."}, "language_style": {"answer": "Tactful and warm", "rationale": "The entry is more gentle and friendly, does not ask questions directly or command answers. Instead, it uses an invitation language, makes the person surveyed comfortable and respectful and more willing to participate. Moreover, the entry is not used in too many specialized or technical terms, but is expressed in a simple and understandable way, so that the person surveyed can better understand and answer the questions. Thus, the language style of the questionnaire is the \"false\" type."}, "time_dimension": {"answer": "Present", "rationale": ""}, "sentence_type": "Interrogative sentence", "pos": {"nouns": {"person": 1}, "verbs": {"Attention": 1}, "adverbs": {"Total": 1}}}, {"content": "58. Care about people looking at each other.", "word_count": 6, "label": "", "sentence_structure": "Subject鈥搗erb鈥揷omplement", "subjective_objective": {"answer": "Subjective", "rationale": "Because \"care for others\" is a subjective feeling and attitude of a person, reflecting his/her sensitivity to the outside world. It is not an objective thing that exists, but is created within the individual. So, \"care for others\" is a subjective view of the problem."}, "sentence_sentiment": {"answer": "Negative", "rationale": "Because the act of \" caring for others ' sight \" reflects excessive attention to external evaluation and anxiety, which may affect the mental health of individuals."}, "language_style": {"answer": "Tactful and warm", "rationale": "The entry uses an invitation-style question language style, and by asking the other side about the level of interest in others, it presents a kind and gentle tone that makes people feel concerned and respected. At the same time, this euphemismal language style makes it easier for respondents to open their hearts and share their true feelings."}, "time_dimension": {"answer": "Present", "rationale": "Keywords are \"care\" and \"vision\" and they represent the current state of attitude or emotion, so they are judged now."}, "sentence_type": "Imperative sentence", "pos": {"nouns": {"Eyes": 1}, "verbs": {"I care": 1}}}, {"content": "59. Thinks others despise themselves.", "word_count": 8, "label": "", "sentence_structure": "Subject鈥搗erb鈥揷omplement", "subjective_objective": {"answer": "Subjective", "rationale": "Because it's the patient's own subjective feeling that feels contempt."}, "sentence_sentiment": {"answer": "Negative", "rationale": "Because the act of \"negative\" is negative, the subject of the entry is the feeling of being despised by others, and can therefore be judged negative."}, "language_style": {"answer": "Tactful and warm", "rationale": "The language style of the entry is more euphemistic and leads the interviewee to share his/her thoughts, rather than direct command or evaluation."}, "time_dimension": {"answer": "Present", "rationale": "The entry describes the current feelings of the testee, without reference to past or future behaviour or mentalities. In it, \"the feeling\" means the subjective feeling of the testee, and \"the contempt of others\" is the cause of that feeling, both of which are current and therefore judged to be the present."}, "sentence_type": "Imperative sentence", "pos": {"nouns": {"others": 1, "self": 1}, "verbs": {"I think": 1, "Disgrace": 1}, "numerals": {"59": 1}}}, {"content": "60. Emotional damage.", "word_count": 7, "label": "", "sentence_structure": "Subject鈥揷opula鈥損redicative", "subjective_objective": {"answer": "Subjective", "rationale": "Because \"facilitation is a state of self-inflicted emotion, a subjective approach."}, "sentence_sentiment": {"answer": "Negative", "rationale": "Because of the fact that emotions are vulnerable to destruction is a negative state or characteristic."}, "language_style": {"answer": "Calm and direct", "rationale": ""}, "time_dimension": {"answer": "Present", "rationale": ""}, "sentence_type": "Negative declarative sentence", "pos": {"nouns": {"Emotion": 1, "Destruction": 1}, "verbs": {"Easy": 1}, "adverbs": {"By": 1}, "numerals": {"60": 1}}}, {"content": "So far, do you feel that you have problems with your own health?", "word_count": 18, "label": "", "sentence_structure": "Subject鈥損redicate", "subjective_objective": {"answer": "Objective", "rationale": "Because the question is asking about the patient ' s state of health, it is an objective fact description."}, "sentence_sentiment": {"answer": "Negative", "rationale": "Because the description \"there's a problem\" in the entry is negative."}, "language_style": {"answer": "Calm and direct", "rationale": "The language style of the entry is calm and straightforward. It asks the interviewee whether he has experienced or is suffering from physical health problems in a concise manner. It is not too much adour and emotional, but is expressed in simple and clear language, which meets the objectivity and neutrality of the questionnaire."}, "time_dimension": {"answer": "Present", "rationale": ""}, "sentence_type": "Interrogative sentence", "pos": {"nouns": {"Problem": 1, "Self-health": 1}, "verbs": {"I feel": 1, "have": 1}, "adverbs": {"To date": 1}, "numerals": {"61锛?: 1}}}, {"content": "62. Has there ever been a mental health problem?", "word_count": 14, "label": "", "sentence_structure": "Subject鈥損redicate", "subjective_objective": {"answer": "Subjective", "rationale": "Because this issue relates to the patient ' s self-perceptions and perceptions, it is a subjective examination."}, "sentence_sentiment": {"answer": "Negative", "rationale": "Because the phrase \"sanitary problems\" in the entry is negative."}, "language_style": {"answer": "Calm and direct", "rationale": "The entry is asked in a direct and clear language, without any verbs or qualifiers, and is more objective and calm."}, "time_dimension": {"answer": "Past", "rationale": "The entry asks about the experience or feelings of the subject in the past, and the keyword is \"had felt\" and it points to what has happened, what has happened in the past."}, "sentence_type": "Interrogative sentence", "pos": {"nouns": {"Mental health": 1, "Problem": 1}, "verbs": {"Tsang": 1, "I think": 1}, "adverbs": {"Once": 1}, "numerals": {"62": 1}}}, {"content": "63. Have you received mental health counselling and treatment so far?", "word_count": 21, "label": "", "sentence_structure": "Subject鈥搗erb鈥搊bject", "subjective_objective": {"answer": "Objective", "rationale": "This is an objective experience or fact because it concerns whether the patient has received mental health counselling and treatment."}, "sentence_sentiment": {"answer": "Neutral", "rationale": "Because the entry does not have any emotional color."}, "language_style": {"answer": "Calm and direct", "rationale": "The entry asks the person surveyed whether he or she has had a history of mental health counselling and treatment in a more objective and direct way."}, "time_dimension": {"answer": "Past", "rationale": "The entry \"To date\" indicates that it is a question of the past, and that \"had received mental health counselling and treatment\" is also a question of past behaviour or state. Thus, the temporal dimension of the entry can be judged to be the past."}, "sentence_type": "Interrogative sentence", "pos": {"nouns": {"To date": 1, "Mental health": 1, "Consultants": 1, "Treatment": 1}, "verbs": {"Yes": 1}, "adverbs": {"Once": 1, "- You're not": 1}, "numerals": {"63锛?: 1}}}]}

{"questionnaire_title": "Connors parents use questionnaires.", "questionnaire_category": "Emotion and affect", "item_count": 48, "emotion_shift_overall": "No obvious trend", "language_style_overall": "Calm and direct", "items": [{"content": "1. Some small action (biting fingernails, sucking fingers to pull hair, pulling hair on clothes)", "word_count": 21, "label": "", "sentence_structure": "Subject鈥搗erb鈥揷omplement", "subjective_objective": {"answer": "Subjective", "rationale": "Because these acts are usually self-conscious actions of patients, are their habits and preferences and are therefore viewed from a subjective perspective."}, "sentence_sentiment": {"answer": "Neutral", "rationale": "Because the entry describes only an act and does not contain any positive or negative emotional colours."}, "language_style": {"answer": "Tactful and warm", "rationale": "The language style of the questionnaire entry is more euphemistic, and it guides the respondents to think and answer questions in descriptive terms, without direct orders or criticism. This expression makes it easier for the respondents to accept and participate in the investigation, while protecting their privacy and self-esteem."}, "time_dimension": {"answer": "Present", "rationale": ""}, "sentence_type": "Interrogative sentence", "pos": {"nouns": {"Small action": 1, "Bite your nails": 1, "Suck your fingers": 1, "Pull your hair": 1, "Pull your clothes": 1, "Boob": 1}, "verbs": {"Do it": 1}, "numerals": {"Some": 1, "Plant": 1}}}, {"content": "2. Insolence to adults", "word_count": 7, "label": "", "sentence_structure": "Subject鈥搗erb鈥揷omplement", "subjective_objective": {"answer": "Subjective", "rationale": "Because \"black manners\" is a test of the patient's own attitude, and it's a subjective idea of the patient."}, "sentence_sentiment": {"answer": "Negative", "rationale": "Because \"blacky\" is a negative act or attitude, expressing dissatisfaction or disrespect for the other."}, "language_style": {"answer": "Tactful and warm", "rationale": "The entry is more adept and interactive when it is spoken in a gentle and inviting manner, rather than by direct statements or orders."}, "time_dimension": {"answer": "Past", "rationale": "The entry asks the person who was tested for past behaviour or attitude, the keyword being \"black-of-the-black\" and expresses what has happened to an act or event. The temporal dimension therefore is the past."}, "sentence_type": "Interrogative sentence", "pos": {"nouns": {"Large": 1, "person": 1, "Naughty": 1, "Insolent": 1}, "verbs": {"to": 1}}}, {"content": "Problems in making friends or maintaining friendships", "word_count": 14, "label": "", "sentence_structure": "Subject鈥搗erb鈥搊bject", "subjective_objective": {"answer": "Subjective", "rationale": "Because \"friending or maintaining friendship\" is an individual act that depends on the patient's own will and attitude, it is the patient's subjective view."}, "sentence_sentiment": {"answer": "Negative", "rationale": "Problems of making friends or maintaining friendship are generally considered to be a negative situation."}, "language_style": {"answer": "Calm and direct", "rationale": "The entry expresses the subject of the survey directly in a concise language - whether there are difficulties in making friends or maintaining friendship, without excessive qualifier or emotional colours and with a more objective tone."}, "time_dimension": {"answer": "Present", "rationale": "The entry asks if the person tested has any difficulties or problems in dealing with a person, and the time dimension is now judged."}, "sentence_type": "Affirmative declarative sentence", "pos": {"nouns": {"Make friends": 1, "Friendship": 1}, "verbs": {"Existence": 1, "Problem": 1}, "numerals": {"3": 1}}}, {"content": "4. Excited, impulsive", "word_count": 6, "label": "", "sentence_structure": "Subject鈥搗erb鈥揷omplement", "subjective_objective": {"answer": "Subjective", "rationale": "Because \"excited\" and \"excited\" are the psychological state of the patient, the subjective feeling."}, "sentence_sentiment": {"answer": "Negative", "rationale": "Because the act of excavation, excavation has a negative impact."}, "language_style": {"answer": "Tactful and warm", "rationale": "The language style of the entry is more euphemistic, using the means of questioning rather than direct statements or orders, which makes it easier for the interviewees to feel comfortable and comfortable and to gain their real feedback. At the same time, the description of \"excited, impulsive\" is also more objective and neutral, without too much emotional overturning or demeaning the other, and more easily obtains the view of the interviewee."}, "time_dimension": {"answer": "Present", "rationale": "The keyword \"excited, impulsive\" describes the person being tested in the current state of behaviour or emotion, and thus judges it as now."}, "sentence_type": "Affirmative declarative sentence", "pos": {"verbs": {"Excited": 1, "Easier than you can": 1}, "adjectives": {"Easy": 2}}}, {"content": "5. The hand of the finger.", "word_count": 5, "label": "", "sentence_structure": "Subject鈥搗erb鈥揷omplement", "subjective_objective": {"answer": "Subjective", "rationale": "Because love is an act that expresses personal attitudes and habits, it is a subjective approach."}, "sentence_sentiment": {"answer": "Negative", "rationale": "Because the \"finger-to-hand\" act in the entry is negative."}, "language_style": {"answer": "Calm and direct", "rationale": "The language style of the entry is more straightforward, there is no excessive emotional or rhetorical approach, and the person surveyed is asked whether he or she is interested in pointing and pointing and doing so in a more concise and clear manner."}, "time_dimension": {"answer": "Present", "rationale": ""}, "sentence_type": "Imperative sentence", "pos": {"verbs": {"Love": 1, "Finger": 1, "Row": 1}, "adjectives": {"Hands": 1, "Foot": 1}}}, {"content": "Suck or chew ( thumb, clothing, blankets)", "word_count": 11, "label": "", "sentence_structure": "Subject鈥搗erb鈥揷omplement", "subjective_objective": {"answer": "Objective", "rationale": "Because the act of sucking or chewing is objective, the objects chosen are also objective objects such as thumbs, clothing and blankets."}, "sentence_sentiment": {"answer": "Neutral", "rationale": "Because the entry does not have any emotional color."}, "language_style": {"answer": "Calm and direct", "rationale": "This entry describes in a concise and clear language certain customary practices that may exist in the person under investigation, without excessive emotional or rhetorical style."}, "time_dimension": {"answer": "Present", "rationale": "Now the entry describes the testee's current behavior, i.e. whether there is a sucking or chewing behaviour, and does not address the time dimension of the past or the future. So the answer is \"now\"."}, "sentence_type": "Imperative sentence", "pos": {"nouns": {"Questionnaire": 1, "Entry": 1, "Suck": 1, "Chew": 1, "Thumbs": 1, "Clothes": 1, "Blanket": 1}, "verbs": {"Please": 1, "Analysis": 1, "Suck": 1, "Chew": 1}, "numerals": {"6": 1}}}, {"content": "7. Easy or frequent crying", "word_count": 7, "label": "", "sentence_structure": "Subject鈥搗erb鈥揷omplement", "subjective_objective": {"answer": "Subjective", "rationale": "Because it is the patient ' s own feelings and experiences that cry easily or often, it is a subjective expression of emotion."}, "sentence_sentiment": {"answer": "Negative", "rationale": "Because the \"easy or frequent\" act in the entry is negative."}, "language_style": {"answer": "Calm and direct", "rationale": "The entry directly asks the respondents whether they are inclined to cry or shout, is not too emotional and is more concise in language style."}, "time_dimension": {"answer": "Past", "rationale": ""}, "sentence_type": "Imperative sentence", "pos": {"verbs": {"Cry": 2}, "adjectives": {"Easy": 1, "Regular": 1}}}, {"content": "8. A lot of temper.", "word_count": 4, "label": "", "sentence_structure": "Subject鈥搗erb鈥揷omplement", "subjective_objective": {"answer": "Subjective", "rationale": "Because \"sweet\" is a subjective evaluation or description, depending on the emotional state and the opinion of the interviewee."}, "sentence_sentiment": {"answer": "Negative", "rationale": "Because \"sweet\" is negative."}, "language_style": {"answer": "Calm and direct", "rationale": "It directly asks the other in a simple and clear language about the character of a larger temperament, without using excessive rhetorical techniques or emotional rendering. This expression is direct, clear and more accurate."}, "time_dimension": {"answer": "Past", "rationale": "The key word \"sweet\" makes it clear that the entry is asking the testee about his past behavior or state, so the answer is \"the past\"."}, "sentence_type": "Imperative sentence", "pos": {"adjectives": {"very": 1, "Large": 1}}}, {"content": "Daydreams", "word_count": 3, "label": "", "sentence_structure": "Subject鈥搗erb鈥搊bject", "subjective_objective": {"answer": "Subjective", "rationale": "Daydreams are a psychological phenomenon that belongs to the patient ' s subjective experience and feelings."}, "sentence_sentiment": {"answer": "Neutral", "rationale": "Because the entry does not have any emotional color."}, "language_style": {"answer": "Tactful and warm", "rationale": "The language style of the entry is more euphemistic and easy, and in question-words, the interviewee is guided to share his ideas and experiences, without using too direct or command-style tone. Moreover, the topic of \"Dream of the Day\" is usually considered a more private and delicate subject, so that using a moderate language style can better create a friendly, open interview environment."}, "time_dimension": {"answer": "Present", "rationale": "The entry asks if the person tested is now used to daydreaming or is acting, so the temporal dimension is now judged."}, "sentence_type": "Interrogative sentence", "pos": {"nouns": {"Daydreams": 1}}}, {"content": "10. Learning difficulties", "word_count": 4, "label": "", "sentence_structure": "Subject鈥揷opula鈥損redicative", "subjective_objective": {"answer": "Subjective", "rationale": "Learning difficulties are not an objective, verifiable or observed fact. Learning difficulties may be caused by a number of reasons, including, but not limited to, inappropriate learning methods, low concentration, emotional problems, etc. Therefore, learning difficulties are assessed by taking into account the experience, perceptions and subjective reporting of patients."}, "sentence_sentiment": {"answer": "Negative", "rationale": "Because the expression \"learning difficulties\" in the entry is negative."}, "language_style": {"answer": "Calm and direct", "rationale": "The entry directly asks the interviewees whether they have difficulties in learning, are clear and clear, are not over-modified or emotional, and therefore can be judged as being calm and straightforward."}, "time_dimension": {"answer": "Present", "rationale": "\"Learning difficulties\" are the difficulties currently faced by the testees, and therefore the temporal dimension is now judged."}, "sentence_type": "Exclamatory sentence", "pos": {"nouns": {"Learning difficulties": 1}, "numerals": {"10": 1}}}, {"content": "11. Twisting.", "word_count": 4, "label": "", "sentence_structure": "Subject鈥搗erb鈥揷omplement", "subjective_objective": {"answer": "Subjective", "rationale": "This is a term describing the emotional state of the patient, and is a subjective examination."}, "sentence_sentiment": {"answer": "Negative", "rationale": "Because \"shaking\" is a negative act."}, "language_style": {"answer": "Tactful and warm", "rationale": "The entry is more adept and interactive when it is spoken in a gentle and inviting manner, rather than by direct statements or orders."}, "time_dimension": {"answer": "Present", "rationale": "The entry describes the current behaviour or state of the person being tested, using the general current state of affairs to indicate that this is an ongoing phenomenon and thus the judgement is \"now\"."}, "sentence_type": "Imperative sentence", "pos": {"verbs": {"Wreck": 1, "Ann": 1}, "adjectives": {"Unsettled": 1}}}, {"content": "Fear (new environment, strangers, strangers, school)", "word_count": 14, "label": "", "sentence_structure": "Subject鈥搗erb鈥搊bject", "subjective_objective": {"answer": "Subjective", "rationale": "Because \"fear\" is a subjective emotional experience that reflects the patient's feelings about the new environment, strangers, strangers and school."}, "sentence_sentiment": {"answer": "Negative", "rationale": "Because the emotional attitude of fear is negative."}, "language_style": {"answer": "Calm and direct", "rationale": "The entry is based on a simple and clear language style, which does not have too many rhetorical or emotional colours, but rather is more calm and objective in terms of asking the interviewees whether they are afraid of new circumstances, strangers, strangers or school attendance."}, "time_dimension": {"answer": "Future", "rationale": "The entry contains fears and fears that may arise in the future, i.e., when entering new environments, meeting strangers or going to strange places. The keyword \"the future\" suggests that this is the expectation and the assumption of the future."}, "sentence_type": "Declarative sentence", "pos": {"nouns": {"Fear": 1, "New Environment": 1, "Stranger": 1, "Strange places": 1, "School": 1}, "adverbs": {"锛堬級": 4}, "numerals": {"12": 1}}}, {"content": "Unsettled, often 鈥渨orking鈥?, "word_count": 8, "label": "", "sentence_structure": "Subject鈥搗erb鈥揷omplement", "subjective_objective": {"answer": "Subjective", "rationale": "Because the restlessness and the busyness are subjective feelings and behaviours of patients, reflecting their internal stress and anxiety."}, "sentence_sentiment": {"answer": "Negative", "rationale": "Because \"silent\" and \"frequently \" busy\" are words that describe negative emotions or behaviour."}, "language_style": {"answer": "Tactful and warm", "rationale": "The language style of the entry is more euphemistic and is more personal and interactive, using the means of questioning rather than direct statements or orders. At the same time, quotation marks are used to highlight the meaning of \"keep\" and make it more focused, leading the respondents to in-depth reflection and answers."}, "time_dimension": {"answer": "Present", "rationale": "The keywords are \"sustained\" and \"frequently busy\", which suggest that the person tested may have feelings of anxiety or stress or be overworked at work or in life."}, "sentence_type": "Affirmative declarative sentence", "pos": {"nouns": {"Sit tight": 1, "Busy": 1}, "adverbs": {"Regular": 1}, "numerals": {"13": 1}, "classifiers": {"individual": 1}}}, {"content": "14. Disruptive", "word_count": 3, "label": "", "sentence_structure": "Subject鈥搗erb鈥揷omplement", "subjective_objective": {"answer": "Subjective", "rationale": "Because destructive descriptions are usually based on personal emotions, attitudes or values, not just objective facts or behaviour."}, "sentence_sentiment": {"answer": "Negative", "rationale": "Because the term \"destructive\" is usually considered negative."}, "language_style": {"answer": "Calm and direct", "rationale": "The language style is initially considered to be straightforward, based on the entry \"14. Disruption\" that was entered. Although the subject of the entry is destructive, it is not too emotional or rhetorical. It is a simple question, which is to ask the respondents directly about what they think or experience of \"disruption,\" and it is not too much euphemism or implication. This language style gives people an objective, straightforward sense of being used in formal surveys of the questionnaire. So, based on the above analysis, it is possible to conclude that the language style of the entry is straightforward."}, "time_dimension": {"answer": "Present", "rationale": "The entry does not have a clear time indicator or time reference point, so it is not possible to determine whether it is past, present or future. But \"destructive\" is an adjective describing behaviour characteristics, indicating that the testee's behaviour is destructive, which may mean acts that may have occurred, present or future. Thus, we can say that the entry relates to the present behaviour characteristics."}, "sentence_type": "Interrogative sentence", "pos": {"nouns": {"Disruptive": 1}}}, {"content": "15. Lies or fabrications", "word_count": 7, "label": "", "sentence_structure": "Subject鈥損redicate", "subjective_objective": {"answer": "Subjective", "rationale": "The lying or fabrication of circumstances requires the patient to have the subjective will and behaviour."}, "sentence_sentiment": {"answer": "Negative", "rationale": "The fact that the lie or the fabrication of the plot is dishonest is negative emotional."}, "language_style": {"answer": "Calm and direct", "rationale": "The style of the entry is more direct, concise, and does not use too much qualifier or emotionally strong terms, but rather raises the question in an objective and neutral manner."}, "time_dimension": {"answer": "Present", "rationale": "The entry asks whether the person tested is now guilty of lying or making the plot, and the keywords \"fiction\" and \"fiction\" point to the situation."}, "sentence_type": "Negative declarative sentence", "pos": {"nouns": {"Liar": 1, "Cases": 1}, "verbs": {"Fake": 1}, "numerals": {"15": 1}}}, {"content": "16. Shame.", "word_count": 2, "label": "", "sentence_structure": "Subject鈥搗erb鈥揷omplement", "subjective_objective": {"answer": "Subjective", "rationale": "Because \"weak\" is a subjective emotional experience and feeling."}, "sentence_sentiment": {"answer": "Negative", "rationale": "Because the feeling of shame is usually negative, indicating lack of confidence or oversensitiveness."}, "language_style": {"answer": "Tactful and warm", "rationale": "The entry begins with a more neutral narrative, \"weakness\", followed by a question-and-answer period, which leads the respondents to express their thoughts and feelings, which is relatively gentle, friendly and easier to relax and share their inner world."}, "time_dimension": {"answer": "Present", "rationale": "The keyword is \"weak,\" which indicates the person's current emotional or psychological state. Thus, the time dimension of the article is now."}, "sentence_type": "Negative declarative sentence", "pos": {"nouns": {"Shy": 1}, "adjectives": {"Yes": 1}}}, {"content": "17. More trouble than children of the same age.", "word_count": 11, "label": "", "sentence_structure": "Subject鈥揷opula鈥損redicative", "subjective_objective": {"answer": "Objective", "rationale": "Because the objective facts that make it possible to measure and compare the problems are the same as the criteria for children of the same age."}, "sentence_sentiment": {"answer": "Negative", "rationale": "Because the expression \"problems more trouble than children of the same age\" in the entry is negative and indicates that the person under investigation may have problems with behaviour, attitude, etc."}, "language_style": {"answer": "Tactful and warm", "rationale": "The entry uses a more linguistic expression, which does not directly accuse or criticize the person under investigation, but rather expresses the problem by comparing it to children of the same age, which is more moderate, friendly and acceptable. At the same time, it gives the person under investigation some self-esteem and personal space and does not make him too embarrassed or embarrassed."}, "time_dimension": {"answer": "Past", "rationale": "Because words like \"old,\" \"old,\" \"old\" and \"old\" are usually associated with the past period. So, \"problems more trouble than children of the same age\" are more likely to mean that the testees experienced more problems than their peers at some point in the past. So the output is: \"old.\""}, "sentence_type": "Imperative sentence", "pos": {"nouns": {"Trouble": 1, "Same age": 1}, "verbs": {"Cause": 1, "More": 1}, "adverbs": {"- Yeah": 1}, "numerals": {"17": 1}}}, {"content": "18. Speeches are different from children of the same age (like babies, stutters, others can't easily understand)", "word_count": 20, "label": "", "sentence_structure": "Subject鈥搗erb鈥揷omplement", "subjective_objective": {"answer": "Subjective", "rationale": "Since the entry examines whether the language skills of the patient are different among children of the same age, this is a distinctive expression based on the experience and feelings of the patient and is therefore viewed from a subjective perspective."}, "sentence_sentiment": {"answer": "Negative", "rationale": "Because the behaviour of \"talking different from children of the same age\" in the entry may be misunderstood or rejected by others, it is in a negative emotional state."}, "language_style": {"answer": "Tactful and warm", "rationale": "The language style of the entry is more euphemistic, and by using descriptions such as \"others are not easy to understand\" to suggest that the person under investigation may have communication problems, avoiding a direct tone of blame or criticism. This expression is more acceptable and understandable and does not create undue tension or anxiety among the person under investigation."}, "time_dimension": {"answer": "Past", "rationale": "In the entry, \"18.\" indicates that it is a number, and \"like a baby, ate, not easily understood\" describes the characteristics or behaviour of the past. The entry therefore asks whether the person tested spoke differently from children of the same age in the past, and is judged on the basis of the past in terms of time dimension."}, "sentence_type": "Negative declarative sentence", "pos": {"nouns": {"Talk": 1, "Same age children": 1, "Baby": 1, "Snack": 1, "others": 1, "It's easy to understand": 1}, "verbs": {"and": 1, "Different": 1}, "numerals": {"18": 1}}}, {"content": "19. Mistake or imputation of guilt", "word_count": 9, "label": "", "sentence_structure": "Subject鈥搗erb鈥搊bject", "subjective_objective": {"answer": "Subjective", "rationale": "Because the act of renouncing the wrong or attributing another person is the patient ' s subjective attitude and choice of behaviour."}, "sentence_sentiment": {"answer": "Negative", "rationale": "Because the act of \"condonation or guilt\" is an exoneration from responsibility and irresponsible behaviour, it is negative."}, "language_style": {"answer": "Tactful and warm", "rationale": "The entry is more euphemistic in its language, using an invitation tone to guide respondents to answer questions, and not to speak directly or order respondents. At the same time, the entry does not have too many emotional renderings or rhetorical techniques, but rather to ask the respondents鈥?views and feelings in a balanced and objective manner."}, "time_dimension": {"answer": "Present", "rationale": "In this entry, the actions of \"refuting wrong\" and \"incrimination of others\" are the investigation of the current mindset or behaviour of the person being tested, and therefore the temporal dimension is now judged."}, "sentence_type": "Negative declarative sentence", "pos": {"nouns": {"Refusal": 1, "Error": 1, "Attribution": 1, "Others": 1}, "verbs": {"Refusal": 1, "Attribution": 1}}}, {"content": "20. Good argument.", "word_count": 3, "label": "", "sentence_structure": "Subject鈥揷opula鈥損redicative", "subjective_objective": {"answer": "Subjective", "rationale": "Pro-challenge is a subjective attitude."}, "sentence_sentiment": {"answer": "Negative", "rationale": "Because the \"good argument\" in the entry is negative."}, "language_style": {"answer": "Calm and direct", "rationale": "This is a more direct language style, with no excessive adoration or emotional colour, and more often a question about whether the person surveyed is easily at odds with others."}, "time_dimension": {"answer": "Past", "rationale": "It is suggested that, whenever possible, the questionnaire be designed using words or phrases that indicate clearly the time in order to facilitate accurate analysis and interpretation of the data."}, "sentence_type": "Imperative sentence", "pos": {"nouns": {"Argument": 1}, "adjectives": {"Okay": 1}}}, {"content": "21. Mouth and anger", "word_count": 5, "label": "", "sentence_structure": "Subject鈥搗erb鈥揷omplement", "subjective_objective": {"answer": "Subjective", "rationale": "Because mouth and anger are emotional reactions, subjective feelings and experiences of patients."}, "sentence_sentiment": {"answer": "Negative", "rationale": "Because mouths and anger both express negative emotions."}, "language_style": {"answer": "Calm and direct", "rationale": "The entry is more concise in its language style, and it is asked whether the other person is expressing feelings, both lip-sniffing and anger, and is not over-modified or insensitive."}, "time_dimension": {"answer": "Present", "rationale": "Now the entry asks if the person tested currently has feelings or behaviours of \"sniffing\" and \"exhausting\" and therefore judges it now."}, "sentence_type": "Interrogative sentence", "pos": {"nouns": {"Poop": 1, "Angry": 1}}}, {"content": "22. Theft", "word_count": 2, "label": "", "sentence_structure": "Subject鈥損redicate", "subjective_objective": {"answer": "Subjective", "rationale": "The basis for the judgement: theft is an act, which is usually a subjective choice and decision of the individual. Although theft may be caused by some external pressure or internal distress, it is still a subjective act. Thus, the answer to this questionnaire is \"subjective\"."}, "sentence_sentiment": {"answer": "Negative", "rationale": "Because the theft is illegal and negative."}, "language_style": {"answer": "Calm and direct", "rationale": "The subject is simple and clear, and the interviewee is asked whether he has ever committed theft, is more objective and neutral, does not use excessive rhetoric or emotional colours and is in keeping with a calm and straightforward language style."}, "time_dimension": {"answer": "Past", "rationale": "The entry deals with inquiries as to whether the person tested had committed theft, and therefore determines the past."}, "sentence_type": "Exclamatory sentence", "pos": {"nouns": {"Theft": 1}}}, {"content": "23. Insubordination or refusal to obey", "word_count": 8, "label": "", "sentence_structure": "Subject鈥搗erb鈥揷omplement", "subjective_objective": {"answer": "Subjective", "rationale": "Because \"insubordination\" and \"subordination\" are both psychological feelings of patients themselves, reflecting their attitudes and feelings, and are therefore subjective in their perspective."}, "sentence_sentiment": {"answer": "Negative", "rationale": "Because insubordination is usually considered to be a negative act."}, "language_style": {"answer": "Tactful and warm", "rationale": "The language style of the entry is more euphemistic, by asking the other side about \"insubordination\" or \"subordination,\" and by directing the interviewee to think and express his thoughts, and there is no way to ask questions that are too direct or hard. This way of expressing the interviewer is more comfortable and comfortable and more willing to share real thoughts and feelings."}, "time_dimension": {"answer": "Present", "rationale": "The entry does not clearly point to the time, and therefore can be understood as current behaviour or attitude. Both \"insubordination\" and \"competence\" refer to the attitude of the person being tested when faced with a request or order, which may have an impact on the mental health of the person being tested."}, "sentence_type": "Exclamatory sentence", "pos": {"nouns": {"Insubordination": 1, "Obedience": 1}, "verbs": {"Obey": 2}}}, {"content": "24. More worries than others (loneness, disease, death)", "word_count": 14, "label": "", "sentence_structure": "Subject鈥搗erb鈥搊bject", "subjective_objective": {"answer": "Subjective", "rationale": "Because fear is more subjective than others, it is not an objective reality."}, "sentence_sentiment": {"answer": "Negative", "rationale": "Because the description \"more worried\" in the entry is negative."}, "language_style": {"answer": "Calm and direct", "rationale": "In conclusion, the language style of the article is \"precipitous and warm\"."}, "time_dimension": {"answer": "Past", "rationale": "The keywords \"awesome,\" \"lsolation\", \"disease\" and \"death\" describe the psychological state that the person was likely to have in the past, so the entry asks whether the person was in the past concerned."}, "sentence_type": "Imperative sentence", "pos": {"nouns": {"Fear": 1, "person": 1, "Loneliness": 1, "Diseases": 1, "Death": 1}, "verbs": {"- Yeah": 1, "More": 1}, "numerals": {"24": 1}}}, {"content": "25. The way things started,", "word_count": 6, "label": "", "sentence_structure": "Subject鈥搗erb鈥揷omplement", "subjective_objective": {"answer": "Subjective", "rationale": "Because \"doing everything in its own right\" is the subjective idea and feeling that patients have generated in self-evaluation, it is a subjective view."}, "sentence_sentiment": {"answer": "Negative", "rationale": "The basis for the judgement: the whole of the matter is a negative act, which means that it cannot be done or done. The purpose of the questionnaire is therefore negative."}, "language_style": {"answer": "Calm and direct", "rationale": "The language style of the entry is calm and straightforward, because it is a simple and straightforward expression of the question whether the other party often does things well but is ultimately unable to finish the subject, without using excessive rhetorical techniques or emotional rendering."}, "time_dimension": {"answer": "Past", "rationale": "The keyword \"finding\" denotes past behaviour or attitude."}, "sentence_type": "Interrogative sentence", "pos": {"nouns": {"Yes": 1, "Start": 1, "End": 1}, "verbs": {"Do it": 1, "have": 1}, "adjectives": {"None": 1, "End": 1}, "numerals": {"25": 1}}}, {"content": "26. Vulnerability of feelings", "word_count": 6, "label": "", "sentence_structure": "Subject鈥揷opula鈥損redicative", "subjective_objective": {"answer": "Subjective", "rationale": "Because the vulnerability of feelings to harm is an individual ' s feeling and perception, it is a subjective examination."}, "sentence_sentiment": {"answer": "Negative", "rationale": "Because the attitude of \"emotional vulnerability\" in the entry is negative."}, "language_style": {"answer": "Calm and direct", "rationale": "The entry directly expresses the subject of questioning the other party ' s vulnerability to emotional harm, without excessive rhetoric or emotional rendering, and is more objective in tone."}, "time_dimension": {"answer": "Present", "rationale": "Now the \"emotional vulnerability\" in the entry describes a state or feature without a clear time point. So it can be judged to be a present act or state description, and the time dimension is now."}, "sentence_type": "Negative declarative sentence", "pos": {"nouns": {"Emotion": 1, "Damage": 1}, "verbs": {"Easy": 1}, "numerals": {"26": 1}}}, {"content": "27. Bullying.", "word_count": 4, "label": "", "sentence_structure": "Subject鈥搗erb鈥揷omplement", "subjective_objective": {"answer": "Subjective", "rationale": "Because bullying is a manifestation of subjective behaviour and attitudes, determined by the mental state of the person. Although it may affect objective outcomes, such as the suffering, harm, etc., it is still the result of the subjective will and actions of the bullying person."}, "sentence_sentiment": {"answer": "Negative", "rationale": "Because bullying is a negative act, harms the victim and is therefore negative."}, "language_style": {"answer": "Calm and direct", "rationale": "The entry directly expresses the subject of questioning the other party about any experience of bullying, without excessive rhetoric or emotional rendering, and with a more objective tone."}, "time_dimension": {"answer": "Present", "rationale": "Since the title did not mention a specific time frame or time frame, it simply asked the testee whether he had had a history of bullying, and therefore judged it now."}, "sentence_type": "Imperative sentence", "pos": {"nouns": {"Bullying": 1, "person": 1}, "verbs": {"Bullying": 1}, "numerals": {"27": 1}}}, {"content": "Repetitive activities cannot be stopped", "word_count": 9, "label": "", "sentence_structure": "Subject鈥損redicate", "subjective_objective": {"answer": "Subjective", "rationale": "Because \"not stop\" and \"repeated sex\" are the patient's description and feelings, so it's subjective."}, "sentence_sentiment": {"answer": "Negative", "rationale": "Because the act of \"can't stop repetitive activity\" in the entry is negative."}, "language_style": {"answer": "Tactful and warm", "rationale": ""}, "time_dimension": {"answer": "Present", "rationale": "The entry \"cannot stop\" indicates the conduct now under way, and \"repeated activity\" is the specific description of the act and can therefore be judged as now."}, "sentence_type": "Negative declarative sentence", "pos": {"nouns": {"Questionnaire": 1, "Entry": 1, "Activities": 1}, "verbs": {"Yes": 1, "Stop": 1, "Repeat": 1, "Conduct": 1}, "adjectives": {"Sex": 1}, "adverbs": {"not": 1, "Okay": 1, "Stop": 2, "Heavy": 1, "Rhett": 1}, "numerals": {"28": 1}, "classifiers": {"individual": 1}, "pronouns": {"Here": 1}}}, {"content": "29. Cruelness", "word_count": 2, "label": "", "sentence_structure": "Subject鈥揷opula鈥損redicative", "subjective_objective": {"answer": "Subjective", "rationale": "Because \"cruel\" is a value or a criterion for evaluation, it is the subjective perception and evaluation of the conduct of the person under investigation by the investigator."}, "sentence_sentiment": {"answer": "Negative", "rationale": "Because \"cruel\" is a negative term, which is a bad behavior or attitude."}, "language_style": {"answer": "Calm and direct", "rationale": "The entry does not have too many rhetorical and emotional expressions, but rather is more serious in terms of asking the respondents in a straightforward manner about the behaviour or attitude of the person under investigation, with the aim of obtaining objective and realistic answers."}, "time_dimension": {"answer": "Present", "rationale": "The entry asks if the testee is currently displaying \"cruel\" characteristics or behaviour, and therefore the temporal dimension is now judged."}, "sentence_type": "Negative declarative sentence", "pos": {"nouns": {"Cruel": 1}}}, {"content": "30. Indulgement or indefatigability (help, attachment, often encouragement, support for what you do)", "word_count": 27, "label": "", "sentence_structure": "Subject鈥搗erb鈥揷omplement", "subjective_objective": {"answer": "Subjective", "rationale": "Because the subject is about patients鈥?subjective feelings, whether they feel naive or immature, and how they behave. These are subjective assessments and observations."}, "sentence_sentiment": {"answer": "Negative", "rationale": "Because the description of \"pubreness or incompetence\" in the entry is negative."}, "language_style": {"answer": "Tactful and warm", "rationale": "The entry is more euphemistic and warm, and not too directly or forcefully. It asks the interviewee in a more gentle and friendly way whether he is naive or premature, and emphasizes that such behaviour may require help and support. Such expression makes it easier for the interviewee to feel comfortable and to get real and accurate feedback."}, "time_dimension": {"answer": "Present", "rationale": "The entry describes the current behaviour of the testee, including the customary state of \"self-serving\" and \"enthusiast\" and \"often need encouragement, support\". So the temporal dimension is now."}, "sentence_type": "Affirmative declarative sentence", "pos": {"nouns": {"- Naughty": 1, "Immortality": 1, "Yes": 1, "person": 1, "Encourage": 1, "Support": 1}, "verbs": {"Yes": 2, "Help": 1, "Entanglement": 1}, "numerals": {"30": 1}, "pronouns": {"self": 1}}}, {"content": "31. Easier to be distracted or less focused", "word_count": 11, "label": "", "sentence_structure": "Subject鈥揷opula鈥損redicative", "subjective_objective": {"answer": "Subjective", "rationale": "As distraction and concentration are the experiences and feelings of the patients themselves, it is a subjective approach."}, "sentence_sentiment": {"answer": "Negative", "rationale": "Because \"diffic\" and \"insensitive\" are negative."}, "language_style": {"answer": "Calm and direct", "rationale": "The entry directly asks whether the other party is too distracted or distracted, does not use too much rhetorical or emotional rendering, and is more objective in tone. At the same time, the entry does not express the issue in a euphemism, but is presented in a direct and clear manner. It can therefore be concluded that the language style of the article is straightforward and calm."}, "time_dimension": {"answer": "Present", "rationale": "The keyword \"easy to distract or insensitive\" describes a current state of mind and behaviour, so it can be judged that the entry is an inquiry into the present situation of the person being tested."}, "sentence_type": "Exclamatory sentence", "pos": {"nouns": {"Discretion": 1, "Attention": 1}, "verbs": {"Easy": 1, "Focus": 1}, "numerals": {"31": 1}}}, {"content": "32. Headaches", "word_count": 2, "label": "", "sentence_structure": "Subject鈥揷opula鈥損redicative", "subjective_objective": {"answer": "Objective", "rationale": "Since headaches are an observable symptom or experience, they are not subject to subjective evaluation or perception by the patient."}, "sentence_sentiment": {"answer": "Negative", "rationale": "Because headaches are usually a negative symptom, they may be a precursor to physical health problems."}, "language_style": {"answer": "Calm and direct", "rationale": "The entry directly asked the interviewees whether they had had headaches, did not use any qualifiers or emotional language, and was more objective and insensitive."}, "time_dimension": {"answer": "Present", "rationale": "The keyword is \"headache\", which is a symptom that is now going through, so it can be judged that the entry is an inquiry into the present experience and circumstances of the subject."}, "sentence_type": "Negative declarative sentence", "pos": {"nouns": {"Headache": 1}}}, {"content": "33. The mood changed rapidly.", "word_count": 8, "label": "", "sentence_structure": "Subject鈥揷opula鈥損redicative", "subjective_objective": {"answer": "Subjective", "rationale": "Since emotional change is an individual ' s feeling and experience, it is the patient ' s subjective perspective that is examined."}, "sentence_sentiment": {"answer": "Negative", "rationale": "Because the expression \"surgery changes rapidly\" means that emotions are more volatile and can have negative effects."}, "language_style": {"answer": "Calm and direct", "rationale": "The entry presents the emotional changes in the subject ' s profile in direct and concise language, without the use of excessive rhetorical techniques or emotional colours, and with a more objective and calm language style."}, "time_dimension": {"answer": "Past", "rationale": "The entry describes the emotional behaviour of the testee in the past and therefore judges it as \"the past\"."}, "sentence_type": "Affirmative declarative sentence", "pos": {"nouns": {"Emotion": 1, "Change": 1, "Quick": 1, "Extreme": 1}, "adjectives": {"Quick": 1, "Extreme": 1}, "numerals": {"33": 1}}}, {"content": "34. Dislike or non-compliance with discipline or discipline", "word_count": 12, "label": "", "sentence_structure": "Subject鈥損redicate", "subjective_objective": {"answer": "Subjective", "rationale": "Because \"like or obey\" is the patient's subjective attitude."}, "sentence_sentiment": {"answer": "Negative", "rationale": "Because the attitude in the entry \"not to like or not to obey discipline or discipline\" is negative."}, "language_style": {"answer": "Tactful and warm", "rationale": "The language style of the entry is euphemism, by asking if the other party feels unsatisfied or unobserving with discipline or constraint, rather than directly accusing or criticizing. At the same time, the more friendly name \"you\" is used to make people feel more friendly and friendly."}, "time_dimension": {"answer": "Present", "rationale": "The term \"not like or fail to comply\" in this entry describes the attitudes and behaviour of the testee, so the temporal dimension is now judged."}, "sentence_type": "Negative declarative sentence", "pos": {"nouns": {"Discipline": 1, "Constraints": 1}, "verbs": {"Yeah": 1, "Compliance": 1}, "adjectives": {"I don't like it": 1}, "numerals": {"34": 1}}}, {"content": "35. Fighting often.", "word_count": 4, "label": "", "sentence_structure": "Subject鈥搗erb鈥揷omplement", "subjective_objective": {"answer": "Subjective", "rationale": "Because \"often\" is a subjective assessment or description of the patient."}, "sentence_sentiment": {"answer": "Negative", "rationale": "Because \"fight\" is usually considered a negative act, offensive and violent."}, "language_style": {"answer": "Calm and direct", "rationale": "The entry is based on a direct question, without any qualifier or emotional term, and is more calm and straightforward.\""}, "time_dimension": {"answer": "Present", "rationale": "The entry uses the term \"regular\" to indicate the frequency of the action, indicating that it is a customary, recurrent act, and therefore the temporal dimension is now judged."}, "sentence_type": "Affirmative declarative sentence", "pos": {"nouns": {"Fight": 1}, "verbs": {"Fight": 1, "Array": 1}, "adverbs": {"Regular": 1}, "numerals": {"35": 1}}}, {"content": "36. Not very good with brothers and sisters.", "word_count": 11, "label": "", "sentence_structure": "Subject鈥搗erb鈥揷omplement", "subjective_objective": {"answer": "Subjective", "rationale": "This is a subjective assessment of the patient ' s relationship with his siblings, and does not involve specific objective facts or behaviour."}, "sentence_sentiment": {"answer": "Negative", "rationale": "Because behavior in the entry is negative."}, "language_style": {"answer": "Tactful and warm", "rationale": "The entry is more euphemistic, using the more subtle expression \"not very comfortable with each other\" rather than pointing out the problem directly. And the expression \"please analyse below\" is more polite and respectful of each other's feelings, and is generally more gentle and kind."}, "time_dimension": {"answer": "Present", "rationale": "\"36\" may be a title number. According to the description, \"not very good with brothers and sisters\" means that the testee encounters difficulties in his or her dealings with his or her siblings. The problem may have some distress and impact on their lives. Thus, in analysing this entry, it is necessary to take into account that the scene is present and may affect the quality of life of the testee."}, "sentence_type": "Negative declarative sentence", "pos": {"nouns": {"36": 1, "Brothers and sisters": 1, "Get along": 1}, "verbs": {"and": 1, "Yes": 1, "Marriage": 1, "Location": 1}, "numerals": {"36": 1}}}, {"content": "37. Easier to ventilate in efforts", "word_count": 8, "label": "", "sentence_structure": "Subject鈥搗erb鈥揷omplement", "subjective_objective": {"answer": "Subjective", "rationale": "Because \"easiness\" is a subjective feeling and attitude of a human being."}, "sentence_sentiment": {"answer": "Negative", "rationale": "Because the \"freak-off\" act in the entry is negative."}, "language_style": {"answer": "Calm and direct", "rationale": "The language style of the entry is calm and straightforward. It is straightforward in asking the interviewee whether he is easily depressed in the course of his efforts, without using any accommodative language or acquital techniques, and is sound and objective, and is consistent with the linguistic characteristics of the psychological questionnaire."}, "time_dimension": {"answer": "Present", "rationale": "The keyword \"in an effort\" indicates that the behaviour is ongoing or has recently ended, and \"fluent\" describes a mentality or feeling, which suggests that the entry is asking about the current mindset and feelings of the subject. So the time dimension of the questionnaire entry is now."}, "sentence_type": "Interrogative sentence", "pos": {"nouns": {"Try": 1, "Depression": 1}, "verbs": {"in": 1, "Easy": 1, "Medium": 1, "Depression": 1}, "adjectives": {"Try": 1, "Easy": 1}, "numerals": {"37": 1}}}, {"content": "38. Impediment of other children", "word_count": 6, "label": "", "sentence_structure": "Subject鈥搗erb鈥搊bject", "subjective_objective": {"answer": "Subjective", "rationale": "Since \"harming other children\" is a subjective judgement, it is necessary to assess whether the behaviour or attitude of the patient is actually the subject."}, "sentence_sentiment": {"answer": "Negative", "rationale": "Because the act of \"invigorating\" in the entry is negative."}, "language_style": {"answer": "Calm and direct", "rationale": "The language style of the entry is of a calm and direct type, and it clearly expresses the subject of questioning whether the other child has been harmed, without excessive rhetorical or emotional overtook."}, "time_dimension": {"answer": "Past", "rationale": "Because the result of the act may have occurred or be continuing."}, "sentence_type": "Affirmative declarative sentence", "pos": {"nouns": {"Impairment": 1, "Other": 1, "Children": 1}, "verbs": {"Children": 1}, "numerals": {"38": 1}}}, {"content": "39. Basically, an unhappy child.", "word_count": 12, "label": "", "sentence_structure": "Subject鈥揷opula鈥損redicative", "subjective_objective": {"answer": "Subjective", "rationale": "Because \"basically\" and \"an unpleasant child\" are patients' evaluations and descriptions of themselves."}, "sentence_sentiment": {"answer": "Negative", "rationale": "Because the word \"unhappy\" in the entry means that emotional attitudes are negative."}, "language_style": {"answer": "Calm and direct", "rationale": "The language style of the entry is more straightforward, it is not over-modified and emotionally retrofitted, it is calm and objective and it is more focused on the accuracy and effectiveness of the message."}, "time_dimension": {"answer": "Past", "rationale": "According to the sentence, \"basically\" and \"a child\", it can be inferred that the question is an assessment of the testee's past character, and therefore the temporal dimension of the article is the past."}, "sentence_type": "Negative declarative sentence", "pos": {"nouns": {"Kids": 1}, "verbs": {"be": 1, "Basic": 1, "Go, go, go": 1, "One": 1}, "adjectives": {"It's not good": 1}, "adverbs": {"Basically": 1}}}, {"content": "40. Eating problems (fatal appetite, frequent running)", "word_count": 15, "label": "", "sentence_structure": "Subject鈥揷opula鈥損redicative", "subjective_objective": {"answer": "Subjective", "rationale": "The patient is subjectively suffering from hunger and frequent runaways."}, "sentence_sentiment": {"answer": "Negative", "rationale": "Because the descriptions of \"fat appetite\" and \"scrambling in food\" in the entries are negative."}, "language_style": {"answer": "Calm and direct", "rationale": "The type of questionnaire entry is more focused on data accuracy and validity, and is suitable for scientific research or statistical analysis."}, "time_dimension": {"answer": "Present", "rationale": "The keyword \"there is\" means a state of existence, \"fatal, often running away from food\" describes current behaviour or symptoms, so the entry is to ask the person who was tested for his or her current behaviour and circumstances."}, "sentence_type": "Declarative sentence", "pos": {"nouns": {"Diet": 1, "Problem": 1, "A appetite": 1, "Food": 1, "Medium": 1, "Regular": 1, "Run away": 1}, "verbs": {"have": 1, "Bad": 1, "Eat": 1, "Food": 2, "Medium": 1, "Run away": 1}, "numerals": {"40": 1}}}, {"content": "41. Stomach pain", "word_count": 2, "label": "", "sentence_structure": "Subject鈥搗erb鈥揷omplement", "subjective_objective": {"answer": "Objective", "rationale": "Because stomach pain is a physical feeling and symptoms of the patient, it is seen from an objective perspective."}, "sentence_sentiment": {"answer": "Negative", "rationale": "Because \"talmic pain\" is a physical discomfort, usually associated with negative emotions and experiences."}, "language_style": {"answer": "Calm and direct", "rationale": "The entry directly asked the respondents whether they often experienced stomach pain, did not overtly qualify the expression of verbs or emotional colours, and the language style was biased towards objectivity and calmness."}, "time_dimension": {"answer": "Past", "rationale": "The entry asks about the testee ' s past health and the time dimension is judged to be past."}, "sentence_type": "Affirmative declarative sentence", "pos": {"nouns": {"Stomach pain": 1}, "numerals": {"41": 1}}}, {"content": "42. Sleep problems (no sleep, early awakening or night rise)", "word_count": 16, "label": "", "sentence_structure": "Subject鈥搗erb鈥搊bject", "subjective_objective": {"answer": "Objective", "rationale": "Because the question of whether or not to sleep is an objective fact of the patient ' s actual experience, there is no need for subjective judgement by the patient."}, "sentence_sentiment": {"answer": "Negative", "rationale": "Because sleep problems are physical and mental health problems that may affect daily life, they are considered negative."}, "language_style": {"answer": "Calm and direct", "rationale": "The entry uses a simple and quick language, asking the interviewee whether he has sleep problems, is not over-repeated or emotionally retroverted and is speaking in a more objective and cold manner."}, "time_dimension": {"answer": "Past", "rationale": "The entry asked if the person tested had past sleep problems and the time dimension judged to be past."}, "sentence_type": "Declarative sentence", "pos": {"nouns": {"Sleep problems": 1, "Sleep": 1, "Wake up": 1, "Get up at night": 1}, "verbs": {"have": 1}, "numerals": {"42": 1}}}, {"content": "43. Other pain", "word_count": 4, "label": "", "sentence_structure": "Subject鈥搗erb鈥揷omplement", "subjective_objective": {"answer": "Objective", "rationale": "Because \"other pain\" is a physical condition or a symptom, it is not the patient's own subjective feeling or attitude, and it is therefore objective."}, "sentence_sentiment": {"answer": "Neutral", "rationale": "Because the entry does not have any emotional color."}, "language_style": {"answer": "Tactful and warm", "rationale": "The language style of the entry is euphemism, it asks questions in a caring, sensitive manner and does not directly ask about specific types or levels of pain, leaving the interviewee some space for reflection and answers, which can ease the tension of the interviewee. At the same time, the term \"other\" also reflects respect for and understanding of individual differences, and increases the inclusiveness and humanization of the questionnaire."}, "time_dimension": {"answer": "Past", "rationale": "The entry is for the testee to ask if he has had \"other pain\" before, and the temporal dimension is a past."}, "sentence_type": "Interrogative sentence", "pos": {"nouns": {"43": 1, "Other": 1, "Pain": 1}, "numerals": {"43": 1}}}, {"content": "44. Disgusting or vomiting", "word_count": 5, "label": "", "sentence_structure": "Subject鈥搗erb鈥揷omplement", "subjective_objective": {"answer": "Objective", "rationale": "Because vomiting or nausea is the symptom of the patient, it is an objective observation."}, "sentence_sentiment": {"answer": "Negative", "rationale": "Because \"pull or vomit\" is a reaction to physical discomfort, it is often seen as a negative symptom."}, "language_style": {"answer": "Calm and direct", "rationale": "The basis for the judgement: - The question is presented in an objective and neutral manner, without too many emotional terms; - Symptoms are described in a simpler and less sophisticated manner, without the use of specialized terms such as complex medical terms."}, "time_dimension": {"answer": "Present", "rationale": "The entry asks if the person under examination is currently suffering from vomiting or nausea, and therefore judged as \"now\"."}, "sentence_type": "Imperative sentence", "pos": {"nouns": {"- Vomit": 1, "Gross": 1}, "verbs": {"have": 1}, "numerals": {"44": 1}}}, {"content": "45. Feels like being cheated in a family circle.", "word_count": 11, "label": "", "sentence_structure": "Subject鈥揷opula鈥損redicative", "subjective_objective": {"answer": "Subjective", "rationale": "Because it is a subjective feeling of a person to feel deceived, not an objective fact."}, "sentence_sentiment": {"answer": "Negative", "rationale": "Because the \"smuggling\" in the entry is an expression of a negative emotion and experience."}, "language_style": {"answer": "Tactful and warm", "rationale": ""}, "time_dimension": {"answer": "Present", "rationale": "The entry asks about current feelings and experiences, and the keywords are \"fear\" and \"be deceived\", all pointing to the current situation."}, "sentence_type": "Affirmative declarative sentence", "pos": {"nouns": {"Family circle": 1, "- Liar": 1}, "verbs": {"I feel": 1, "- I'm being deceived": 1}, "numerals": {"45": 1}}}, {"content": "46. Self-incriminating or bragging", "word_count": 5, "label": "", "sentence_structure": "Subject鈥損redicate", "subjective_objective": {"answer": "Subjective", "rationale": "Because self-incriminating or bragging is a subjective attitude or behaviour, it reflects patient self-evaluation and expression."}, "sentence_sentiment": {"answer": "Negative", "rationale": "Because \"self-criticism or bragging\" is a self-centred, modest act that is usually considered negative."}, "language_style": {"answer": "Calm and direct", "rationale": "The entry is a simple question, which is whether the interviewee is easy to boast or bragg. Instead of using excessive rhetorical or emotional tacts to describe, it leads the interviewee to think and answer questions by asking questions directly and objectively. This language style makes the questionnaire more concise and clear, facilitating access to accurate information."}, "time_dimension": {"answer": "Present", "rationale": "The entry asks the testee whether he is currently boasting or bragging about or indignant, and the temporal dimension is now judged."}, "sentence_type": "Affirmative declarative sentence", "pos": {"nouns": {"- You're exaggerating": 1, "Bullshit": 1}, "verbs": {"be": 1}, "numerals": {"46": 1}}}, {"content": "47. Make yourself deceived.", "word_count": 8, "label": "", "sentence_structure": "Subject鈥搗erb鈥搊bject", "subjective_objective": {"answer": "Subjective", "rationale": "Because \"fraud\" is a subjective feeling, not an objective fact or act. The problem is to know whether the patient is vulnerable to deception by others or whether he has experienced deception by others, which is viewed from the patient's subjective point of view."}, "sentence_sentiment": {"answer": "Negative", "rationale": "Because the act of being deceived is negative."}, "language_style": {"answer": "Calm and direct", "rationale": "The entry directly expresses the subject of questioning whether the other party has experienced a situation of deception, without excessive rhetoric or emotional rendering, and with a more objective tone."}, "time_dimension": {"answer": "Past", "rationale": "The entry for this questionnaire can therefore be judged as now."}, "sentence_type": "Imperative sentence", "pos": {"nouns": {"self": 1, "others": 1, "- Liar": 1}, "verbs": {"Jean": 1, "Yes": 1}, "numerals": {"47": 1}}}, {"content": "48. Pee problems (diarrhea, defecation, constipation)", "word_count": 14, "label": "", "sentence_structure": "Subject鈥搗erb鈥搊bject", "subjective_objective": {"answer": "Objective", "rationale": "Because \"the problem of poop\" is an objective symptom or condition. It is determined by the patient's physical condition and is not influenced by the patient's subjective will."}, "sentence_sentiment": {"answer": "Negative", "rationale": "Because the entry relates to physical discomfort and may be uncomfortable."}, "language_style": {"answer": "Calm and direct", "rationale": "The language style of the entry is straightforward, using clear professional terms to describe possible problems, and not using any emotional terms or expressions."}, "time_dimension": {"answer": "Present", "rationale": "The entry asks if the testee has a problem with poop at the moment, and the time dimension is now judged."}, "sentence_type": "Declarative sentence", "pos": {"nouns": {"Shit": 1, "Diarrhoea": 1, "It's not a routine": 1, "Decency": 1}, "numerals": {"48": 1}}}]}

{"questionnaire_title": "Conners Parents Questionnaire (PSQ)", "questionnaire_category": "Behavioral questionnaire", "item_count": 47, "emotion_shift_overall": "No obvious trend", "language_style_overall": "Neutral", "items": [{"content": "1. Insolence towards adults", "word_count": 7, "label": "", "sentence_structure": "Subject鈥搗erb鈥揷omplement", "subjective_objective": {"answer": "Subjective", "rationale": "Because \"black-of-the-black\" is a subjective assessment and attitude description that reflects patients' perception and evaluation of their behaviour."}, "sentence_sentiment": {"answer": "Negative", "rationale": "Because the \"black manners\" in the entry is negative."}, "language_style": {"answer": "Calm and direct", "rationale": "The entry clearly expresses concern and concern about the behaviour of adults in a direct manner, without excessive qualifying or emotional expressions and with a more objective and calm tone."}, "time_dimension": {"answer": "Past", "rationale": "The keyword \"black-of-the-box\" indicates that it is an inquiry into the testee's past behaviour or attitude."}, "sentence_type": "Imperative sentence", "pos": {"nouns": {"Sir": 1, "How rude": 1}}}, {"content": "2. Problems in making friends or maintaining friendship", "word_count": 14, "label": "", "sentence_structure": "Subject鈥搗erb鈥揷omplement", "subjective_objective": {"answer": "Subjective", "rationale": "The reference to the questionnaire is based on the patient ' s subjective attitude to the perception and perception of his or her own problems in making friends or maintaining friendship."}, "sentence_sentiment": {"answer": "Negative", "rationale": "Because the expression \"problem\" in the entry is negative."}, "language_style": {"answer": "Tactful and warm", "rationale": "The entry is more personal and interactive when it is spoken in a gentle and inviting manner, rather than in direct statements or orders. At the same time, the theme \"problems in making friends or maintaining friendship\" does not require too much emotional overtones, so it is more appropriate to choose the \"sweet\" language style."}, "time_dimension": {"answer": "Present", "rationale": "The keyword \"problems in making friends or maintaining friendship\" describes the current challenges and difficulties faced by the subjects, so the entry asks about the present situation."}, "sentence_type": "Negative declarative sentence", "pos": {"nouns": {"friends": 1, "Make friends": 1, "Staying friendly": 1, "Problem": 1}, "verbs": {"in": 1, "have": 1}, "numerals": {"2": 1}}}, {"content": "3. Excited, impulsive", "word_count": 6, "label": "", "sentence_structure": "Subject鈥搗erb鈥揷omplement", "subjective_objective": {"answer": "Subjective", "rationale": "Because \"excited, impulsive\" is the patient's own feelings and experiences, and it is a subjective observation and description."}, "sentence_sentiment": {"answer": "Negative", "rationale": "Because \"excited, impulsive\" is a negative description, suggesting that there may be some emotional control problems."}, "language_style": {"answer": "Calm and direct", "rationale": "The language style of the entry is a straightforward type of calm, without the use of euphemisms, praises or hints, but rather a straightforward question about the other ' s excavation and impulses, and the way in which they are expressed is clear and objective."}, "time_dimension": {"answer": "Present", "rationale": "\"Easier, more impulsive\" describes a state of current emotion and behaviour that does not address the time dimension of the past or the future, and therefore judges it now."}, "sentence_type": "Imperative sentence", "pos": {"verbs": {"Excited": 1, "Easier than you can": 1}, "adjectives": {"Easy": 2}}}, {"content": "4. A man who wants to point his finger.", "word_count": 5, "label": "", "sentence_structure": "Subject鈥搗erb鈥搊bject", "subjective_objective": {"answer": "Subjective", "rationale": "Since the finger-pointing is the expression of a person ' s behaviour, it reflects the behaviour and character of the patient and is viewed from a subjective perspective."}, "sentence_sentiment": {"answer": "Negative", "rationale": "As finger-pointing is often considered to be uncivil and rude, it conveys a negative emotional tendency."}, "language_style": {"answer": "Tactful and warm", "rationale": "The entry is more adept and interactive when it is spoken in a gentle and inviting manner, rather than by direct statements or orders."}, "time_dimension": {"answer": "Past", "rationale": "It is therefore not possible to determine its temporal properties and this can be considered to be a neutral descriptive issue."}, "sentence_type": "Imperative sentence", "pos": {"verbs": {"- I'm telling you, I'm telling you": 1}, "adjectives": {"Love": 1}}}, {"content": "Suck or chew ( thumb, clothing, blanket)", "word_count": 11, "label": "", "sentence_structure": "Subject鈥搗erb鈥揷omplement", "subjective_objective": {"answer": "Objective", "rationale": "Because sucking or chewing is an objective act, whether or not the patient is aware, it exists."}, "sentence_sentiment": {"answer": "Neutral", "rationale": "Because the entry describes an act without a clear emotional tendency."}, "language_style": {"answer": "Calm and direct", "rationale": "The entry describes a common behaviour in simple and clear language, which is not over-modified or emotionally rendered, and which is more objective and direct."}, "time_dimension": {"answer": "Past", "rationale": "In this entry, \"sucking\" and \"bite\" are all actions of the past, and therefore the past is judged."}, "sentence_type": "Imperative sentence", "pos": {"nouns": {"Questionnaire": 1, "Entry": 1, "Suck": 1, "Chew": 1, "Thumbs": 1, "Clothes": 1, "Blanket": 1}, "numerals": {"5": 1}}}, {"content": "6. Easy or frequent crying", "word_count": 7, "label": "", "sentence_structure": "Subject鈥搗erb鈥揷omplement", "subjective_objective": {"answer": "Subjective", "rationale": "Because \"easy or frequent crying\" is an emotional response that reflects the feelings and experiences of the patient himself and is a subjective examination."}, "sentence_sentiment": {"answer": "Negative", "rationale": "Because \"crying\" is usually considered a negative emotional expression."}, "language_style": {"answer": "Tactful and warm", "rationale": "The entry uses a more euphemistic approach to ask the interviewees whether they are easy or often emotional to express and release, rather than to describe it directly in negative terms. This approach reflects respect and understanding of the interviewees and is better able to guide them to open their minds to real answers."}, "time_dimension": {"answer": "Past", "rationale": "\"Always\" in \"easy or frequent\" cries indicates that the act is a customary phenomenon that has existed in the past and may continue. The temporal dimension of the article therefore determines the past."}, "sentence_type": "Imperative sentence", "pos": {"nouns": {"Questionnaire": 1, "Entry": 1, "Cry": 1}, "verbs": {"Easy": 1, "Regular": 1}, "numerals": {"6": 1}}}, {"content": "7. A lot of temper.", "word_count": 4, "label": "", "sentence_structure": "Subject鈥搗erb鈥揷omplement", "subjective_objective": {"answer": "Subjective", "rationale": "Because \"bout\" describes the feelings and experiences of the patient himself, and is a subjective description."}, "sentence_sentiment": {"answer": "Negative", "rationale": "Because the expression \"sweet\" in the entry is negative emotional."}, "language_style": {"answer": "Calm and direct", "rationale": "The entry uses a simple and quick language style, with no excessive emotional accommodations or euphemisms, and is directly about the subject \"symptomatic.\" Thus, the language style is cold."}, "time_dimension": {"answer": "Past", "rationale": "According to the sentence entered, \"sweet\" describes the testee's past behavior or attitude."}, "sentence_type": "Imperative sentence", "pos": {"nouns": {"- Grumpy": 1}, "adjectives": {"Large": 1}}}, {"content": "Daydreams", "word_count": 3, "label": "", "sentence_structure": "Subject鈥搗erb鈥揷omplement", "subjective_objective": {"answer": "Subjective", "rationale": "Because daydreams are usually the scenarios or experiences that individuals imagine in a state of soberness, reflecting the inner world and imagination of the patient, and are subjective in their approach."}, "sentence_sentiment": {"answer": "Neutral", "rationale": ""}, "language_style": {"answer": "Tactful and warm", "rationale": "The entry is more euphemistic and implicit, without asking the specifics of the interviewee directly or giving clear instructions."}, "time_dimension": {"answer": "Present", "rationale": "The entry now asks the testee whether there is a \"daydream\" at the moment, without a clear time limit or a past or future pointer. So it is now."}, "sentence_type": "Interrogative sentence", "pos": {"nouns": {"Daydreams": 1}}}, {"content": "9. Learning difficulties", "word_count": 4, "label": "", "sentence_structure": "Subject鈥揷opula鈥損redicative", "subjective_objective": {"answer": "Subjective", "rationale": "Because \"learning difficulties\" are subjective assessments of a patient's abilities or circumstances, not objective facts or phenomena. Everyone can have different views and feelings about learning, so the question is one of patient's subjective attitudes and experiences, not objective realities."}, "sentence_sentiment": {"answer": "Negative", "rationale": "Because \"learning difficulties\" are usually considered negative."}, "language_style": {"answer": "Calm and direct", "rationale": "The entry is clear and simple, and the interviewee is asked whether he has any difficulty in learning. There is no accoutrement or acquitt, and it is straightforward, making it easier for the interviewee to understand and answer.\""}, "time_dimension": {"answer": "Present", "rationale": "The keyword is \"learning difficulties\", which describes the current learning situation of the subjects."}, "sentence_type": "Exclamatory sentence", "pos": {"nouns": {"Learning": 1, "Difficulties": 1}}}, {"content": "10. Wiggle.", "word_count": 4, "label": "", "sentence_structure": "Subject鈥損redicate", "subjective_objective": {"answer": "Subjective", "rationale": "Because \"sharping no-motion\" describes how the patient feels and behaves, and it is the patient's subjective view."}, "sentence_sentiment": {"answer": "Neutral", "rationale": "Because the entry does not have a clear emotional tendency or value judgement, it merely describes an act."}, "language_style": {"answer": "Tactful and warm", "rationale": "The entry is more adept and interactive when it is spoken in a gentle and inviting manner, rather than by direct statements or orders."}, "time_dimension": {"answer": "Past", "rationale": ""}, "sentence_type": "Exclamatory sentence", "pos": {"nouns": {"Wreck": 1, "Let's go": 1}, "adverbs": {"not": 1}, "numerals": {"10": 1}}}, {"content": "Fear (new environment, strangers, strangers, school)", "word_count": 14, "label": "", "sentence_structure": "Subject鈥搗erb鈥搊bject", "subjective_objective": {"answer": "Subjective", "rationale": "Because fear is a subjective emotion and attitude, it is the patient鈥檚 inner feeling and reaction to new environments, strangers, strangers, or school. Although these situations exist objectively, fear is the patient鈥檚 subjective experience and evaluation, so the entry is subjective."}, "sentence_sentiment": {"answer": "Negative", "rationale": "Because the \"fear\" in the entry is negative."}, "language_style": {"answer": "Calm and direct", "rationale": "The entry uses a concise language, which directly asks the respondents whether they are afraid of a particular scene or situation, and is not overly emotionally or verbally explicit and is more objective and clear."}, "time_dimension": {"answer": "Future", "rationale": "Fear, New Environment, Stranger Place, Stranger Place and School all suggest that the testees may encounter these situations in the future, so the temporal dimension of this article is the future."}, "sentence_type": "Interrogative sentence", "pos": {"nouns": {"New Environment": 1, "Stranger": 1, "Strange places": 1, "School": 1}, "verbs": {"Fear": 1}, "numerals": {"11": 1}}}, {"content": "Unsettled, often 鈥渨orking鈥?, "word_count": 8, "label": "", "sentence_structure": "Subject鈥搗erb鈥揷omplement", "subjective_objective": {"answer": "Subjective", "rationale": "Because the feeling of \"silent\" and \"kick\" is the subjective feeling that patients themselves have."}, "sentence_sentiment": {"answer": "Negative", "rationale": "Because being restless and often busy is usually an expression of anxiety or stress and a negative emotional experience."}, "language_style": {"answer": "Tactful and warm", "rationale": "The entry uses a more euphemistic description, with the words \"suspensive\" and \"frequently \" busy\" all of which are somewhat subjective and emotional, and do not directly ask the other side whether or not such behaviour or psychological state exists. Such language style is more accommodative, gentler, and more likely to receive positive feedback and collaboration from the interviewees."}, "time_dimension": {"answer": "Present", "rationale": "The keywords \"often\" and \"frequently\" indicate that the entry is asking the subject about the current behaviour or state of the person being tested and is therefore in the current state of affairs."}, "sentence_type": "Affirmative declarative sentence", "pos": {"nouns": {"I'm not ready": 1, "Regular": 1, "Busy": 1}, "verbs": {"be": 1, "have": 1}, "adverbs": {"Regular": 1}, "numerals": {"12": 1}, "classifiers": {"individual": 1}, "pronouns": {"Here": 1}}}, {"content": "13. Disruptive", "word_count": 3, "label": "", "sentence_structure": "Subject鈥搗erb鈥揷omplement", "subjective_objective": {"answer": "Subjective", "rationale": "Because destructive is the psychological orientation or attitude that patients may have, it is the realm of subjective judgment."}, "sentence_sentiment": {"answer": "Negative", "rationale": "Because \"destructive\" is usually seen as a negative act or quality, with a negative emotional tone."}, "language_style": {"answer": "Calm and direct", "rationale": "The language style of this questionnaire entry is relatively calm, straightforward, and not too much adoration or emotional. It simply raises the question whether the person interviewed is clearly able to answer whether his attitude or behaviour is related to destructive behaviour. This expression is more suitable for scientific research or data analysis and can be used to obtain objective information effectively."}, "time_dimension": {"answer": "Past", "rationale": "The entry does not contain clear time indicators or a description of past events, and therefore is judged to be \"the past\"."}, "sentence_type": "Imperative sentence", "pos": {"nouns": {"Disruptive": 1}}}, {"content": "14. Lies or fabrications", "word_count": 7, "label": "", "sentence_structure": "Subject鈥損redicate", "subjective_objective": {"answer": "Subjective", "rationale": "Because \"false or fabricate\" is a subjective act or attitude."}, "sentence_sentiment": {"answer": "Negative", "rationale": "Because the \"false or fabricated\" act is negative."}, "language_style": {"answer": "Calm and direct", "rationale": "Cold and sharp."}, "time_dimension": {"answer": "Future", "rationale": ""}, "sentence_type": "Negative declarative sentence", "pos": {"nouns": {"Liar": 1, "Cases": 1}, "verbs": {"Fake": 1}, "numerals": {"14": 1}}}, {"content": "15. Shame.", "word_count": 2, "label": "", "sentence_structure": "Subject鈥搗erb鈥揷omplement", "subjective_objective": {"answer": "Subjective", "rationale": "Because \"weakness\" is a psychological state or emotional experience that belongs to the patient's own subjective feelings and perceptions, not to objective facts."}, "sentence_sentiment": {"answer": "Negative", "rationale": "Because the mental state of \"screech\" is usually negative."}, "language_style": {"answer": "Tactful and warm", "rationale": "The language style of this entry is euphemism, because it is a question-and-answer tone that guides the interviewee to wonder whether he is easily shy or humbled, and is more friendly and friendly. This expression helps the interviewee to answer the questions more relaxedly, while it also allows researchers to get more realistic and accurate answers."}, "time_dimension": {"answer": "Present", "rationale": "\"Frightful\" describes a state of current mental or emotional feeling and therefore judges it now."}, "sentence_type": "Negative declarative sentence", "pos": {"nouns": {"Shame": 1}}}, {"content": "16. More trouble than children of the same age", "word_count": 11, "label": "", "sentence_structure": "Subject鈥揷opula鈥損redicative", "subjective_objective": {"answer": "Subjective", "rationale": "This is because patients are subjectively evaluating their own situation."}, "sentence_sentiment": {"answer": "Negative", "rationale": "Because the entry expresses a negative situation or quality."}, "language_style": {"answer": "Tactful and warm", "rationale": ""}, "time_dimension": {"answer": "Past", "rationale": ""}, "sentence_type": "Imperative sentence", "pos": {"nouns": {"Trouble": 1, "Son": 1}, "verbs": {"Cause": 1, "- Yeah": 1}, "adjectives": {"Same age": 1, "More": 1}, "numerals": {"16": 1}}}, {"content": "17. Speeches are different from children of the same age (like talking, eating, being unheard of).", "word_count": 22, "label": "", "sentence_structure": "Subject鈥搗erb鈥揷omplement", "subjective_objective": {"answer": "Objective", "rationale": "Because speaking is not the objective expression of the patient in real life, unlike a child of the same age, and the way in which it is described is objective and not clearly subjective."}, "sentence_sentiment": {"answer": "Negative", "rationale": "Because these acts in this entry are \"like talking, eating, not easy to understand\" are perceived as negative features of society, which may affect the social interaction and self-awareness of individuals and are therefore considered negative."}, "language_style": {"answer": "Calm and direct", "rationale": "It is not too much adoration or emotional rendering, but rather asks the interviewees in an objective and direct way to get more accurate and true answers. The language style of the entry is therefore \"cool and direct.\""}, "time_dimension": {"answer": "Present", "rationale": "The entry asks the testee about language characteristics, in which \"talking\" is the current state of affairs and \"different children of the same age\" is also a description of the current situation, so it is possible to judge the time dimension of the entry as it is."}, "sentence_type": "Exclamatory sentence", "pos": {"nouns": {"Talk": 2, "Children": 1, "Same age": 1, "Snack": 1, "person": 1, "Entry": 1}, "verbs": {"Yeah": 1, "Say it": 1, "The world": 1}, "adjectives": {"Easy": 1, "It's hard": 1}, "adverbs": {"Different": 1}, "numerals": {"17": 1}, "classifiers": {"individual": 1}, "pronouns": {"he": 1}}}, {"content": "18. Defeat or atonement of others", "word_count": 9, "label": "", "sentence_structure": "Subject鈥搗erb鈥搊bject", "subjective_objective": {"answer": "Subjective", "rationale": "Because the act of condoning or blaming another person is the patient ' s psychological orientation and attitude, it is a subjective examination."}, "sentence_sentiment": {"answer": "Negative", "rationale": "Because the \"conjecture or blame\" in the entry is negative."}, "language_style": {"answer": "Tactful and warm", "rationale": "Warm and warm."}, "time_dimension": {"answer": "Present", "rationale": "The keywords \"condemnity\" and \"incrimination\" point to the person's immediate behaviour or attitude."}, "sentence_type": "Negative declarative sentence", "pos": {"nouns": {"Refusal": 1, "Error": 1, "Attribution": 1, "Others": 1}}}, {"content": "19. Good argument.", "word_count": 3, "label": "", "sentence_structure": "Subject鈥損redicate", "subjective_objective": {"answer": "Subjective", "rationale": "Because it is a personal preference or character characteristic, it reflects the patient ' s subjective attitude and emotional experience."}, "sentence_sentiment": {"answer": "Negative", "rationale": "Because the \"good argument\" in the entry is negative."}, "language_style": {"answer": "Tactful and warm", "rationale": "Since the question is not clearly one of certainty or denial, it can be understood as an open question that requires the other party to judge and answer. Moreover, the use of the word \"good\" is somewhat subjective. Thus, the language style of this questionnaire entry is neither direct nor emotional, but rather a more moderate and euphemistic way to guide the other party to think and express its views."}, "time_dimension": {"answer": "Present", "rationale": "The keyword \"good\" means more frequent, and the word \"challenge\" indicates the nature of the act."}, "sentence_type": "Imperative sentence", "pos": {"verbs": {"Okay": 1, "Argument": 1}, "adjectives": {"None": 1}, "adverbs": {"None": 1}, "numerals": {"19": 1}, "classifiers": {"individual": 1}}}, {"content": "20. Mouth and anger", "word_count": 5, "label": "", "sentence_structure": "Subject鈥損redicate", "subjective_objective": {"answer": "Subjective", "rationale": "Because mouth and anger are emotional expressions, individual subjective feelings and behaviours."}, "sentence_sentiment": {"answer": "Negative", "rationale": "Because \"sniff\" and \"freak\" in the entries are negative emotional expressions."}, "language_style": {"answer": "Tactful and warm", "rationale": "The language style of the entry is more euphemistic, expressing concern and concern for the emotional state of the person under investigation by using the verbs of the images of \"sniffing\" and \"frumpy\" and not asking directly whether the person concerned is easily angry or emotional. At the same time, this soft language style is more likely to trigger resonance and willingness to participate in the person under investigation, and helps to improve the effectiveness and reliability of the questionnaire."}, "time_dimension": {"answer": "Past", "rationale": "The entry asks about the behaviour or habits of the person tested in the past, since the verbs \"sniff\" and \"frump\" are all the same."}, "sentence_type": "Interrogative sentence", "pos": {"nouns": {"Poop": 1, "Angry": 1}}}, {"content": "21. Theft", "word_count": 2, "label": "", "sentence_structure": "Subject鈥搗erb鈥揷omplement", "subjective_objective": {"answer": "Objective", "rationale": "Since theft is an act that is objectively present, it does not require the patient ' s subjective will or attitude to achieve it."}, "sentence_sentiment": {"answer": "Negative", "rationale": "Because \"theft\" is a violation of law and morality and has a negative emotional connotation."}, "language_style": {"answer": "Calm and direct", "rationale": "The language style of the entry is more straightforward, with no excessive qualifier or emotional expression, but rather a concise description of a problem or behaviour, which can be judged as a calm and straightforward language style."}, "time_dimension": {"answer": "Present", "rationale": "The entry is to ask the testee whether he or she has committed theft or has a tendency to steal, judging by the time dimension as now and in the past. Because the testee has not committed theft in the past, it may have occurred and may still exist. The article therefore has a time frame that covers both now and the past."}, "sentence_type": "Imperative sentence", "pos": {"nouns": {"Theft": 1}}}, {"content": "Insubordination or subservience", "word_count": 8, "label": "", "sentence_structure": "Subject鈥損redicate", "subjective_objective": {"answer": "Subjective", "rationale": "Because \"insubordination\" and \"subordination\" are subjective attitudes and ideas of patients, not objective facts."}, "sentence_sentiment": {"answer": "Negative", "rationale": "Because the insubordination in the entry is negative."}, "language_style": {"answer": "Tactful and warm", "rationale": "The language style of the entry is mild, using words such as \"insubordination\" and \"subordination\", but not too direct or strong, but rather in a more euphemistic way, expressing a question about whether there are cases of insubordination or subservience in certain circumstances. This expression is more acceptable to the interviewees and more conducive to the smoothing of the dialogue."}, "time_dimension": {"answer": "Present", "rationale": "The keyword \"insubordination or subservience\" denotes the present behaviour or attitude of the testee and therefore the present judgement."}, "sentence_type": "Exclamatory sentence", "pos": {"nouns": {"Insubordination": 1, "Obedience": 1}, "verbs": {"Please": 1, "Analysis": 1}, "numerals": {"22": 1}}}, {"content": "23. More considerations than others (awesome, lonely, sick, dead)", "word_count": 14, "label": "", "sentence_structure": "Subject鈥搗erb鈥搊bject", "subjective_objective": {"answer": "Subjective", "rationale": "The patient's feelings of anxiety, loneliness, disease and death are subjective."}, "sentence_sentiment": {"answer": "Negative", "rationale": "Because the entries \"awesome,\" \"lonely,\" \"disease\" and \"death\" are all negative emotions, the whole sentence is negative."}, "language_style": {"answer": "Calm and direct", "rationale": "The subject is based on a simple, simple, emotional or rhetorical approach that makes people feel more comfortable."}, "time_dimension": {"answer": "Past", "rationale": "The entry does not specify whether it is past, present or future behaviour or mentality, and therefore it is not possible to determine the specific temporal dimension."}, "sentence_type": "Imperative sentence", "pos": {"nouns": {"Fear": 1, "Loneliness": 1, "Diseases": 1, "Death": 1}, "verbs": {"- Yeah": 1, "More": 1}, "numerals": {"23": 1}}}, {"content": "24. The whole thing is going to be a long way.", "word_count": 6, "label": "", "sentence_structure": "Subject鈥搗erb鈥揷omplement", "subjective_objective": {"answer": "Subjective", "rationale": "Because \"doing things from the beginning\" reflects the patient's own mental state and behaviour, and it is a subjective assessment."}, "sentence_sentiment": {"answer": "Negative", "rationale": "Because the \"doing things always\" act is negative."}, "language_style": {"answer": "Tactful and warm", "rationale": "The more euphemistic and neutral terminology of \"finding\" is used in the entry, rather than pointing directly to the problems or shortcomings of the other, which is more moderate and respectful and helps to establish good investigative relationships."}, "time_dimension": {"answer": "Present", "rationale": "The entry describes the current state or orientation of the testee, with no clear time indicator or sentence structure implying past or future circumstances. Thus, we can judge it as being used to ask the testee about his current behaviour or character characteristics."}, "sentence_type": "Interrogative sentence", "pos": {"nouns": {"Yes": 1, "Start": 1, "End": 1}, "verbs": {"Do it": 1, "have": 1, "None": 1}, "adverbs": {"24": 1}, "numerals": {"24": 1}}}, {"content": "25. Vulnerability of feelings", "word_count": 6, "label": "", "sentence_structure": "Subject鈥揷opula鈥損redicative", "subjective_objective": {"answer": "Subjective", "rationale": "Because \"relationship\" is an intrinsic emotional state, it is a subjective experience and feeling of the individual, and the entry is therefore viewed from a subjective perspective."}, "sentence_sentiment": {"answer": "Negative", "rationale": "Because the entry indicates that an individual ' s feelings are vulnerable and is a negative expression."}, "language_style": {"answer": "Calm and direct", "rationale": "The entry is presented in a short and direct statement, without too much qualifier language or emotional colour, and in a simpler and more concise manner."}, "time_dimension": {"answer": "Present", "rationale": ""}, "sentence_type": "Negative declarative sentence", "pos": {"nouns": {"Emotion": 1, "Damage": 1}, "verbs": {"Easy": 1}, "numerals": {"25": 1}}}, {"content": "26. Bullying.", "word_count": 4, "label": "", "sentence_structure": "Subject鈥搗erb鈥揷omplement", "subjective_objective": {"answer": "Subjective", "rationale": "Because bullying is a subjective act and attitude of one person, reflecting its perception of others and the way they behave."}, "sentence_sentiment": {"answer": "Negative", "rationale": "Because bullying is a negative act."}, "language_style": {"answer": "Calm and direct", "rationale": "The entry is more calm and straightforward in its language, with no excessive rhetorical or emotional rendering. It is succinctly a question of whether the person under investigation has ever bullied others, and is more objective and neutral in its tone, with the aim of obtaining the actual situation of the person under investigation."}, "time_dimension": {"answer": "Future", "rationale": "The entry contains visions and expectations for the future, and \"bullying\" is a potential future event and therefore judgement is the future."}, "sentence_type": "Imperative sentence", "pos": {"nouns": {"Bullying": 1, "others": 1}, "verbs": {"Bullying": 1}, "numerals": {"26": 1}}}, {"content": "27. Repetitive activities cannot be stopped", "word_count": 9, "label": "", "sentence_structure": "Subject鈥損redicate", "subjective_objective": {"answer": "Objective", "rationale": "Because \"can't stop\" and \"repeat sex\" are depicting the patient's behaviour or state, not the patient's subjective attitude or feelings."}, "sentence_sentiment": {"answer": "Negative", "rationale": "Because the act of \"can't stop repetitive activity\" is negative."}, "language_style": {"answer": "Tactful and warm", "rationale": "The entry is spoken in a question-and-answer tone and expresses a concern and understanding using the term \"cannot stop\". This expression is more gentle and friendly than direct statements or orders, and it makes it easier for the respondents to open their minds."}, "time_dimension": {"answer": "Present", "rationale": "Now the entry uses the now completed state of 鈥渃annot stop鈥? which means that the act has been and still has an effect on the present, and therefore it is judged to be the present. At the same time, the term 鈥渞epeated activity鈥?also indicates a constant act or habit that has nothing to do with the past or the future."}, "sentence_type": "Negative declarative sentence", "pos": {"nouns": {"Activities": 1, "Repeatability": 1}, "verbs": {"Yes": 1, "Stop": 1}, "adverbs": {"not": 1}, "numerals": {"27": 1}}}, {"content": "28. Cruelness", "word_count": 2, "label": "", "sentence_structure": "Subject鈥搗erb鈥揷omplement", "subjective_objective": {"answer": "Subjective", "rationale": "Because \"cruel\" is a moral term, a subjective view and judgement of the patient's behaviour or that of others."}, "sentence_sentiment": {"answer": "Negative", "rationale": "Because the word \"cruel\" is usually considered negative."}, "language_style": {"answer": "Calm and direct", "rationale": "The rationale for the determination is that the entry uses a stronger and more violent term, such as \"cruel\", to describe an act or attitude, which is usually more direct and intense, and does not involve excessive rhetorical or emotional rendering. Thus, the language style of the article is considered to be straightforward and calm."}, "time_dimension": {"answer": "Past", "rationale": ""}, "sentence_type": "Imperative sentence", "pos": {"nouns": {"Cruel": 1}}}, {"content": "29. Indulgentity or indigence (help in matters of one ' s own mind, attachment to others, often encouraged and supported)", "word_count": 27, "label": "", "sentence_structure": "Subject鈥搗erb鈥揷omplement", "subjective_objective": {"answer": "Subjective", "rationale": "Because the entry describes a psychological characteristic or behavioural habit, it is the feelings and attitudes of the patient himself that need to be examined and is therefore subjective in nature."}, "sentence_sentiment": {"answer": "Negative", "rationale": "Because the act of \"needing encouragement and support\" in the entry is a sign of dependency and may be interpreted as negative quality."}, "language_style": {"answer": "Tactful and warm", "rationale": "The language style of the entry is more euphemistic, because it questions with a question and concern, rather than directly accusing or criticizing. The term \"pure or premature\" is used to describe a behaviour characteristic and to explain its expression by supplementary information in brackets, which is more objective and gentle and is easily accepted and understood by the interviewees."}, "time_dimension": {"answer": "Present", "rationale": "Now the entry describes the current behaviour and mentality of the subject, the key words \"pure or premature\" and \"self-problems, entanglement, often requiring encouragement and support\" that indicate that this is the current situation. Thus, the temporal dimension of the article is now judged."}, "sentence_type": "Affirmative declarative sentence", "pos": {"nouns": {"Naughty": 1, "Immortality": 1, "Yes": 1, "person": 1, "self": 1, "Encourage": 1, "Support": 1}, "verbs": {"Yes": 2, "Help": 1, "Entanglement": 1}, "numerals": {"29": 1, "Regular": 1}, "classifiers": {"individual": 1}, "pronouns": {"others": 1}}}, {"content": "30. The ease of distraction or distraction becomes a matter of concern.", "word_count": 16, "label": "", "sentence_structure": "Subject鈥損redicate", "subjective_objective": {"answer": "Subjective", "rationale": "Because \"easy distraction\" and \"not concentration\" are patients' subjective feelings."}, "sentence_sentiment": {"answer": "Negative", "rationale": "Because \"easy to distract or to focus\" in the entry is a negative quality."}, "language_style": {"answer": "Calm and direct", "rationale": "The language style of the entry is more calm and straightforward, and there is no excessive emotional or rhetorical style, but rather a simple and clear expression of the message that is sought."}, "time_dimension": {"answer": "Past", "rationale": "In conclusion, the answer to this question is: \"The past/present/future\"."}, "sentence_type": "Exclamatory sentence", "pos": {"nouns": {"Discretion": 1, "Attention": 1, "Focus": 1, "Problem": 1}, "verbs": {"Easy": 1, "Be": 1}, "adverbs": {"One": 1}, "numerals": {"30": 1}}}, {"content": "31. Headaches", "word_count": 2, "label": "", "sentence_structure": "Subject鈥搗erb鈥揷omplement", "subjective_objective": {"answer": "Objective", "rationale": "As headaches are an objective fact of the patient ' s state of health, they are not subjective feelings or attitudes of the patient."}, "sentence_sentiment": {"answer": "Negative", "rationale": "Because \"headaches\" are often considered to be a physical discomfort, with negative emotional aspects."}, "language_style": {"answer": "Calm and direct", "rationale": "The entry is in a straightforward language and asks the interviewee whether he has symptoms of headaches and has no aesthetic or emotional colour, so that the language style is straightforward and calm."}, "time_dimension": {"answer": "Present", "rationale": ""}, "sentence_type": "Negative declarative sentence", "pos": {"nouns": {"Headache": 1}}}, {"content": "32. The mood changes rapidly and dramatically.", "word_count": 8, "label": "", "sentence_structure": "Subject鈥揷opula鈥損redicative", "subjective_objective": {"answer": "Subjective", "rationale": "Because emotional change is the expression of the patient ' s own mental state, it is subjective."}, "sentence_sentiment": {"answer": "Negative", "rationale": "Because rapid and dramatic emotional changes are often negative, they may indicate that a person has emotional instability."}, "language_style": {"answer": "Calm and direct", "rationale": "The language style of the entry is calm and straightforward, as it directly asks whether the person surveyed has experienced rapid and dramatic emotional changes, without any accoutrements or emotional descriptions."}, "time_dimension": {"answer": "Present", "rationale": "According to the information provided in the title, \"Factful changes in emotions\" describe the emotional condition of the person currently tested and therefore fall within the current temporal dimension."}, "sentence_type": "Interrogative sentence", "pos": {"nouns": {"Emotional change": 1, "Extreme": 1}, "verbs": {"鈾?And become 鈾?: 1}, "adjectives": {"Quick": 1}, "numerals": {"32": 1}}}, {"content": "33. Dislike or non-obligation with discipline or discipline", "word_count": 12, "label": "", "sentence_structure": "Subject鈥搗erb鈥揷omplement", "subjective_objective": {"answer": "Subjective", "rationale": "Because \"like\" and \"compliment\" are patients' subjective attitudes."}, "sentence_sentiment": {"answer": "Negative", "rationale": "Because the \"not like or disobey discipline or discipline\" in the entry is negative."}, "language_style": {"answer": "Calm and direct", "rationale": "The language style of the entry is relatively simple and not too emotional or rhetorical, the main purpose of which is to ask the interviewee whether he or she does not like or follow discipline or discipline."}, "time_dimension": {"answer": "Present", "rationale": "The entry is for questioning the attitude and behaviour of the testee towards discipline or restraint, and the temporal dimension is now judged."}, "sentence_type": "Negative declarative sentence", "pos": {"nouns": {"Discipline": 1, "Constraints": 1}, "verbs": {"Yeah": 1, "Compliance": 1}, "numerals": {"33": 1}}}, {"content": "34. Fighting often.", "word_count": 4, "label": "", "sentence_structure": "Subject鈥搗erb鈥揷omplement", "subjective_objective": {"answer": "Subjective", "rationale": "Because \"often fighting\" is an act, a subjective act and attitude of the patient, it is subjective."}, "sentence_sentiment": {"answer": "Negative", "rationale": "Because \"fights\" are offensive and conflict-like acts, they are often considered negative."}, "language_style": {"answer": "Calm and direct", "rationale": "The entry uses a simple and clear language and asks the interviewees whether they are often fighting, without any aesthetic or emotional colour, so that the language style is \"cool and straight\"."}, "time_dimension": {"answer": "Present", "rationale": "Now. The entry uses the current-style \"often fighting\" which means it's an ongoing act or state, so the temporal dimension is now."}, "sentence_type": "Exclamatory sentence", "pos": {"nouns": {"Fight": 1}, "verbs": {"Regular": 1}}}, {"content": "35. Not very good with siblings.", "word_count": 11, "label": "", "sentence_structure": "Subject鈥搗erb鈥揷omplement", "subjective_objective": {"answer": "Subjective", "rationale": "Because the situation with siblings is a personal experience and experience of the patient, it is a subjective examination."}, "sentence_sentiment": {"answer": "Negative", "rationale": "Because the inability to live well with siblings is a negative behaviour, it can lead to problems such as family tension."}, "language_style": {"answer": "Calm and direct", "rationale": "The language style of the entry is calm and straightforward, and it clearly raises a question, without using any rhetorical or emotional means of rendering."}, "time_dimension": {"answer": "Present", "rationale": ""}, "sentence_type": "Negative declarative sentence", "pos": {"nouns": {"Brothers and sisters": 1, "Get along": 1}, "verbs": {"No, I can't": 1, "Good": 1}, "numerals": {"35": 1}}}, {"content": "36. Easier to ventilate in efforts", "word_count": 8, "label": "", "sentence_structure": "Subject鈥揷opula鈥損redicative", "subjective_objective": {"answer": "Subjective", "rationale": "Because \"discharge\" is an emotional reaction, a subjective experience."}, "sentence_sentiment": {"answer": "Negative", "rationale": "Because the \"depressive\" act is negative."}, "language_style": {"answer": "Calm and direct", "rationale": "The entry directly expresses the subject of questioning whether the other party is less embittered in the course of its efforts, does not use excessive rhetorical or emotional rendering, and is more objective in tone."}, "time_dimension": {"answer": "Present", "rationale": "The keywords \"in the effort\" and \"in the ease of depression\" indicate that the subject is asking the subject about the current behaviour or state of the subject, so the temporal dimension is now."}, "sentence_type": "Interrogative sentence", "pos": {"nouns": {"Try": 1, "Depression": 1}, "verbs": {"Easy": 1}}}, {"content": "37. Impediment of other children", "word_count": 6, "label": "", "sentence_structure": "Subject鈥搗erb鈥搊bject", "subjective_objective": {"answer": "Subjective", "rationale": "The basis for the judgement: \"Damn other children\" is the patient's subjective view and evaluation, reflecting his or her own behaviour and its possible consequences. This is not an objective fact, because the real \"harm other children\" requires a third-party assessment and confirmation."}, "sentence_sentiment": {"answer": "Negative", "rationale": "Because \"harming other children\" is a negative act."}, "language_style": {"answer": "Calm and direct", "rationale": "The entry describes the subject in a direct and clear language, namely, \"impairing other children\", without too much rhetorical or emotional projection, and is more objective. This language style helps to convey clear messages and avoids ambiguity or misunderstanding."}, "time_dimension": {"answer": "Present", "rationale": "Based on the time mark in the sentence, \"impede other children\", it can be inferred that the questionnaire entry asks whether the person who was tested had acted in the past to obstruct other children. The answer is therefore \"the past\"."}, "sentence_type": "Affirmative declarative sentence", "pos": {"nouns": {"Children": 1, "Impairment": 1}, "verbs": {"Impairment": 1, "Other": 1, "Children": 1}, "numerals": {"37": 1}}}, {"content": "38. Basically, an unhappy child.", "word_count": 12, "label": "", "sentence_structure": "Subject鈥揷opula鈥損redicative", "subjective_objective": {"answer": "Subjective", "rationale": "Because \"basically\" and \"an unpleasant child\" are described from the patient's subjective point of view."}, "sentence_sentiment": {"answer": "Negative", "rationale": "Because the word \"unhappy\" is negative."}, "language_style": {"answer": "Calm and direct", "rationale": "The language style of the entry is more direct, concise and not overly accommodative or emotional, but rather directs the subject of the investigation by asking questions."}, "time_dimension": {"answer": "Present", "rationale": "The entry is for personal characteristics or past experience of the person tested, and the keyword is \"basically an unpleasant child.\" Because the time frame for this is not clear, it can be considered a description of the past. So the output should read \"the past.\""}, "sentence_type": "Negative declarative sentence", "pos": {"nouns": {"Kids": 1}, "verbs": {"Basic": 1, "be": 1}, "adjectives": {"One": 1, "It's not pleasant": 1}, "adverbs": {"Go, go, go": 1}, "numerals": {"38": 1}}}, {"content": "39. Eating problems (fatal appetite, frequent running)", "word_count": 15, "label": "", "sentence_structure": "Subject鈥損redicate", "subjective_objective": {"answer": "Objective", "rationale": "Because eating problems are an objective one, they are a problem encountered by the patient in the course of eating."}, "sentence_sentiment": {"answer": "Negative", "rationale": "Because \"fat appetite, frequent running\" in the entry is a negative description."}, "language_style": {"answer": "Tactful and warm", "rationale": "Because the entry uses more moderate language, such as \"with eating problems\" rather than \"with problems\" and by asking questions, which allow the respondents to express their reality more freely. Moreover, the use of brackets to provide additional explanations of the appetite for bad food and the frequent running of food also makes the language more euphemism and tolerance."}, "time_dimension": {"answer": "Past", "rationale": "The description of behaviour in \"39. Eating problems (depressive appetite, frequent running)\" relates to past events, such as hunger, frequent running, etc., and therefore the time dimension of the entry is judged to be past."}, "sentence_type": "Declarative sentence", "pos": {"nouns": {"Dietary problems": 1, "A appetite": 1, "Food": 1}, "verbs": {"have": 1, "Run away": 1}, "numerals": {"39": 1}}}, {"content": "40. Stomach pain", "word_count": 2, "label": "", "sentence_structure": "Subject鈥搗erb鈥揷omplement", "subjective_objective": {"answer": "Objective", "rationale": "Because \"talache\" is a symptom, and it is only through physical examination that a patient's stomach pain can be determined, so it's an objective description."}, "sentence_sentiment": {"answer": "Negative", "rationale": "Because \"bone pain\" usually means physical discomfort, it's a negative feeling or state."}, "language_style": {"answer": "Calm and direct", "rationale": "The language style of the entry is straightforward, as it is a simple and straightforward expression of the investigator ' s desire to know whether the person under investigation has stomach disorders and does not have too many emotional modifications and rhetorical techniques."}, "time_dimension": {"answer": "Present", "rationale": ""}, "sentence_type": "Negative declarative sentence", "pos": {"nouns": {"Stomach pain": 1}}}, {"content": "41. Sleep problems (no sleep, early awakening, night rise)", "word_count": 15, "label": "", "sentence_structure": "Subject鈥搗erb鈥揷omplement", "subjective_objective": {"answer": "Objective", "rationale": "Because the problem of insomnia is the physical sleep disorder faced by the patient, it is an objective fact."}, "sentence_sentiment": {"answer": "Negative", "rationale": "Because sleep problems are usually seen as a negative emotion or state."}, "language_style": {"answer": "Calm and direct", "rationale": "The entry was in a direct and concise manner, specifically asking the interviewee whether he had sleep problems and was more calm and objective in language style."}, "time_dimension": {"answer": "Past", "rationale": "The entry asks if the person tested had a past sleep problem and the time dimension is judged to be past."}, "sentence_type": "Interrogative sentence", "pos": {"nouns": {"Sleep problems": 1, "People sleep": 1, "Wake up": 1, "Get up at night": 1}, "verbs": {"have": 1}, "numerals": {"41": 1}}}, {"content": "42. Other pain", "word_count": 4, "label": "", "sentence_structure": "Subject鈥搗erb鈥揷omplement", "subjective_objective": {"answer": "Subjective", "rationale": "Because \"other pain\" is the patient's subjective feeling and description, not an objective fact that can be observed."}, "sentence_sentiment": {"answer": "Neutral", "rationale": "Basis: The entry does not express a clear emotional tendency or attitude, but merely asks whether there are other types of pain."}, "language_style": {"answer": "Tactful and warm", "rationale": "The entry starts with a question-and-answer tone, expressing concern and concern for the pain of the other, not by asking the specific pain, but by using the word \"other\" to guide the other to speak, which is more gentle and respectful of the other's feelings. At the same time, \"other\" also leaves the other side some space to describe his pain freely, without undue care or embarrassment."}, "time_dimension": {"answer": "Past", "rationale": ""}, "sentence_type": "Interrogative sentence", "pos": {"nouns": {"Questionnaire": 1, "Entry": 1, "Pain": 1}, "verbs": {"Analysis": 1}, "numerals": {"42": 1}}}, {"content": "43. Vomit or disgusting.", "word_count": 5, "label": "", "sentence_structure": "Subject鈥搗erb鈥揷omplement", "subjective_objective": {"answer": "Objective", "rationale": "Because vomiting or nausea is a physical reaction of the patient, it is an objective fact."}, "sentence_sentiment": {"answer": "Negative", "rationale": "Because the physical reaction of \"pushing or disgusting\" in the entry is usually considered negative."}, "language_style": {"answer": "Calm and direct", "rationale": ""}, "time_dimension": {"answer": "Past", "rationale": "The entry is for the purpose of asking the person who was tested for past vomiting or disgusting experience, and the temporal dimension is judged to be past."}, "sentence_type": "Imperative sentence", "pos": {"nouns": {"- Vomit": 1, "Gross": 1}}}, {"content": "44. Feels like being cheated in a family circle.", "word_count": 11, "label": "", "sentence_structure": "Subject鈥揷opula鈥損redicative", "subjective_objective": {"answer": "Subjective", "rationale": "Because feeling deceived is a subjective emotional reaction."}, "sentence_sentiment": {"answer": "Negative", "rationale": "Because the emotional expression \"fears being deceived\" in the entry is negative."}, "language_style": {"answer": "Tactful and warm", "rationale": "The entry is more adept and interactive when it is spoken in a gentle and inviting manner, rather than by direct statements or orders."}, "time_dimension": {"answer": "Present", "rationale": "The keywords \"fear\" and \"frustration\" indicate that the entry is a description of the person's current perception or feelings, and therefore is a current state of affairs. And the phrase \"in the family circle\" also implies that this is what happens in the real life of the person interviewed, and further confirms that it is the present."}, "sentence_type": "Affirmative declarative sentence", "pos": {"nouns": {"Family circle": 1, "- Liar": 1}, "verbs": {"I feel": 1}, "numerals": {"44": 1}}}, {"content": "45. Self-incriminating and bragging", "word_count": 5, "label": "", "sentence_structure": "Subject鈥損redicate", "subjective_objective": {"answer": "Subjective", "rationale": "Because self-incriminating and bragging are subjective attitudes or behaviours of patients who self-evaluate."}, "sentence_sentiment": {"answer": "Negative", "rationale": "Because \"self-critic\" and \"blowing\" are often seen as dishonest and hypocritical, with negative emotional aspects."}, "language_style": {"answer": "Tactful and warm", "rationale": "The entry is more profane and interactive when it is spoken in a gentle, invitation-like tone, rather than directly speaking or ordering. Moreover, the terms \"self-absorbing\" and \"blowing\" are often derogatory and may be used in a context that makes the interviewee uncomfortable or unwilling to answer, so euphemistically speaking can reduce the respondent ' s resistance and improve the response rate and quality of the questionnaire."}, "time_dimension": {"answer": "Present", "rationale": "The entry is for the purpose of asking the testee whether he or she is boasting or boasting, and the temporal dimension is now judged."}, "sentence_type": "Affirmative declarative sentence", "pos": {"nouns": {"- You're exaggerating": 1, "Bullshit": 1}, "numerals": {"45": 1}}}, {"content": "46. Making yourself a fool of others", "word_count": 8, "label": "", "sentence_structure": "Subject鈥搗erb鈥揷omplement", "subjective_objective": {"answer": "Subjective", "rationale": "Because \"to be bullied\" is an act of the patient's choice, a subjective attitude and behaviour."}, "sentence_sentiment": {"answer": "Negative", "rationale": "Because the \"fouled\" act in the entry is negative."}, "language_style": {"answer": "Calm and direct", "rationale": "The entry is a direct expression of the subject of questioning whether the other party has been bullied, without using excessive rhetoric or emotional rendering, and is more objective in tone. At the same time, the direct question is more investigative and targeted."}, "time_dimension": {"answer": "Past", "rationale": ""}, "sentence_type": "Imperative sentence", "pos": {"nouns": {"self": 1, "person": 1, "- That's a lie": 1}, "verbs": {"Jean": 1, "Yes": 1}, "numerals": {"46": 1}}}, {"content": "47. Pee problems (diarrhea, defecation, constipation)", "word_count": 14, "label": "", "sentence_structure": "Subject鈥搗erb鈥揷omplement", "subjective_objective": {"answer": "Objective", "rationale": "Because \"a shit problem\" is an objective symptom or condition that a patient may experience."}, "sentence_sentiment": {"answer": "Negative", "rationale": "Because \"diarrhea\", \"unruly defecation\" and \"concrete\" are all physical health problems and have negative emotional aspects."}, "language_style": {"answer": "Calm and direct", "rationale": "This is a straightforward way of obtaining accurate and accurate information for subsequent analysis and processing."}, "time_dimension": {"answer": "Present", "rationale": "The entry asks the testee whether there is a problem with poop, and the time dimension is now judged."}, "sentence_type": "Declarative sentence", "pos": {"nouns": {"Problem": 1, "Shit": 1, "Diarrhoea": 1, "Decay": 1, "No, no, no, no": 1, "Decency": 1}, "adjectives": {"have": 1}, "numerals": {"47": 1}}}]}

{"questionnaire_title": "Newfoundland Memorial University Happiness Measure", "questionnaire_category": "Emotion and affect", "item_count": 24, "emotion_shift_overall": "From negative to positive", "language_style_overall": "Neutral", "items": [{"content": "(1) Satisfied to the extreme?", "word_count": 5, "label": "", "sentence_structure": "Subject鈥搗erb鈥揷omplement", "subjective_objective": {"answer": "Subjective", "rationale": "Because satisfaction is a subjective feeling of the patient."}, "sentence_sentiment": {"answer": "Positive", "rationale": "Because \"satisfaction to the extreme\" is a positive expression."}, "language_style": {"answer": "Calm and direct", "rationale": "The entry is presented in a simple and clear question, with no over-modified or emotional expressions, with more direct tone and a calm and direct language style."}, "time_dimension": {"answer": "Present", "rationale": "The entry does not have a clear time frame or past/future implication, but rather asks the subject for his or her current subjective feelings or state, and therefore is judged to be \"now\"."}, "sentence_type": "Imperative sentence", "pos": {"nouns": {"Questionnaire": 1, "Entry": 1}, "verbs": {"Satisfied": 1, "Present": 1, "Polar": 1}}}, {"content": "Two is good?", "word_count": 4, "label": "", "sentence_structure": "Subject鈥揷opula鈥損redicative", "subjective_objective": {"answer": "Subjective", "rationale": "Emotional and negativity is a subjective feeling."}, "sentence_sentiment": {"answer": "Negative", "rationale": "Because the entry uses the negative word \"bad\" and expresses negative emotional attitudes."}, "language_style": {"answer": "Tactful and warm", "rationale": "The entry is more adept and interactive when it is spoken in a gentle and inviting manner, rather than by direct statements or orders."}, "time_dimension": {"answer": "Present", "rationale": "The entry is for the testee ' s current emotional state, and the time dimension is now judged."}, "sentence_type": "Interrogative sentence", "pos": {"verbs": {"Good": 1}, "adjectives": {"very": 1}}}, {"content": "- ...is particularly pleased with your life?", "word_count": 9, "label": "", "sentence_structure": "Subject鈥損redicate", "subjective_objective": {"answer": "Subjective", "rationale": "Because the question is asked about the patient ' s perception and evaluation of his or her life, it is the patient ' s subjective attitude."}, "sentence_sentiment": {"answer": "Positive", "rationale": "Because the entry expresses satisfaction with one ' s life and is a positive emotion or attitude."}, "language_style": {"answer": "Calm and direct", "rationale": "It was more objective because it asked the interviewees whether they were particularly satisfied with their lives, and did not use excessive rhetoric or emotional rendering."}, "time_dimension": {"answer": "Present", "rationale": "The keyword \"special satisfaction\" also indicates a state of immediate emotion or feeling. So the answer is \"now\"."}, "sentence_type": "Interrogative sentence", "pos": {"verbs": {"to": 1, "Satisfied": 1}, "adjectives": {"Special": 1}, "numerals": {"3": 1}}}, {"content": "(4) Lucky?", "word_count": 3, "label": "", "sentence_structure": "Subject鈥搗erb鈥揷omplement", "subjective_objective": {"answer": "Subjective", "rationale": "Because \"very lucky\" is a subjective judgement or a description of feelings, reflecting the patient's perception of his own luck."}, "sentence_sentiment": {"answer": "Positive", "rationale": "Because \"good luck\" is a word of positive emotion, of the many luck and opportunities in life that the interviewees have."}, "language_style": {"answer": "Tactful and warm", "rationale": "The language style of the entry tends to be euphemistic and gentle, communicating with the interviewees in an invitation format, rather than directly ordering or making statements. This expression makes it easier for the interviewees to feel comfortable and respected and to cooperate more readily with the investigation."}, "time_dimension": {"answer": "Present", "rationale": "The entry asks whether the testee feels lucky now and the time dimension is now judged."}, "sentence_type": "Interrogative sentence", "pos": {"verbs": {"Lucky": 1, "very": 1}, "adjectives": {"Lucky": 1}, "adverbs": {"very": 1}}}, {"content": "(5) Disturbing", "word_count": 2, "label": "", "sentence_structure": "Subject鈥揷opula鈥損redicative", "subjective_objective": {"answer": "Subjective", "rationale": "Because worry is an emotional experience, a subjective feeling for the patient."}, "sentence_sentiment": {"answer": "Negative", "rationale": "Because \"fear\" is a negative emotional vocabulary."}, "language_style": {"answer": "Tactful and warm", "rationale": "The entry uses the more soft term \"fear\" to raise questions, not asking directly whether the respondent had problems or difficulties, but asking in a caring and understanding manner, which is more likely to generate resonance and trust among the respondent."}, "time_dimension": {"answer": "Present", "rationale": "There is no clear time indicator for the entry, nor does it mention specific events or situations, past or future. It therefore describes the current feelings and experiences of the testee, which is relevant now, and the answer is \"now\"."}, "sentence_type": "Interrogative sentence", "pos": {"nouns": {"Trouble": 1}}}, {"content": "(6) Very lonely or alienated?", "word_count": 9, "label": "", "sentence_structure": "Subject鈥損redicate", "subjective_objective": {"answer": "Subjective", "rationale": "Because the feeling of \"single\" or \"silent\" is the subjective experience and assessment of the individual. While these feelings may be related to objective facts, they are still subjective in themselves. The problem is therefore to examine the subjective feelings and experiences of the patient, not the objective situation."}, "sentence_sentiment": {"answer": "Negative", "rationale": "Because the words \"very lonely\" and \"sent\" in the entries are negative, expressing a negative emotional attitude."}, "language_style": {"answer": "Calm and direct", "rationale": "The language style of the entry is calm and objective, and there is no excessive emotional qualification used to ask the respondents whether they feel alone or alienated in a concise and straightforward manner."}, "time_dimension": {"answer": "Past", "rationale": "The entry asks about the testee's past behavior or mental state, so it's judged to be \"the past\"."}, "sentence_type": "Interrogative sentence", "pos": {"nouns": {"Loneliness": 1, "person": 1, "Distant": 1}, "adjectives": {"Very": 1}, "numerals": {"6": 1}}}, {"content": "(7) Discretion or very unpleasant?", "word_count": 8, "label": "", "sentence_structure": "Subject鈥揷opula鈥損redicative", "subjective_objective": {"answer": "Subjective", "rationale": "The emotional state of the patient is the result of anxiety or very unpleasant feelings."}, "sentence_sentiment": {"answer": "Negative", "rationale": "Because \"good thinking\" and \"very unhappy\" are both negative emotions, the purpose of this article is negative."}, "language_style": {"answer": "Calm and direct", "rationale": "The entry is asked in a direct question, is not described in excessive terms or in emotional colours and is more objective and calm."}, "time_dimension": {"answer": "Present", "rationale": "The entry asks if the person tested is currently worried or very unhappy, and the time dimension is now judged."}, "sentence_type": "Interrogative sentence", "pos": {"nouns": {"Fear": 1, "Precautionary": 1, "It's not good": 1}, "verbs": {"Very": 1, "Have a nice day": 1}}}, {"content": "(8) Fear because it is not known what will happen?", "word_count": 15, "label": "", "sentence_structure": "Subject鈥搗erb鈥揷omplement", "subjective_objective": {"answer": "Subjective", "rationale": "Because \"fear\" is a subjective emotional experience, and \"know what's going to happen\" is the situation that patients face, and it's an objective fact."}, "sentence_sentiment": {"answer": "Negative", "rationale": "Because the mood of \"fear\" in the entry is negative."}, "language_style": {"answer": "Tactful and warm", "rationale": "The language style of the entry is euphemistic, because it uses an invitation-style tone of questioning, without directly expressing the accusations or criticisms of the interviewees. At the same time, the more relaxed term \"fear\" is used, making the problem more moderate and friendly."}, "time_dimension": {"answer": "Future", "rationale": "The keywords are \"remorning\" and \"not knowing what's going to happen\", all of which point to situations that have not yet occurred and that may occur in the future. At the same time, \"what happens?\" This is a sign of uncertainty or concern about the future. The entry therefore relates to future behaviour or mentalities."}, "sentence_type": "Interrogative sentence", "pos": {"nouns": {"I'm worried": 1, "Problem": 1, "Situation": 1}, "verbs": {"Yes": 1, "Come on": 1}, "adverbs": {"Because": 1, "Yes, I will": 1}, "numerals": {"8": 1}}}, {"content": "(9) Feels like your life has become difficult?", "word_count": 12, "label": "", "sentence_structure": "Subject鈥搗erb鈥揷omplement", "subjective_objective": {"answer": "Subjective", "rationale": "Because \"fEss\" is the patient's own feelings and perceptions, and it's seen from a subjective perspective."}, "sentence_sentiment": {"answer": "Negative", "rationale": "Because the expression \"life becomes difficult\" in the entry is negative."}, "language_style": {"answer": "Calm and direct", "rationale": "The entry explicitly asks the other side whether they feel the dilemma of life, and the language style is more calm and straightforward, and is not too much adoration or emotional, and suitable for psychological questionnaire studies."}, "time_dimension": {"answer": "Present", "rationale": "The keyword \"fear\" and \"life becomes difficult\" all indicate that it is a description and perception of the state of affairs and should therefore be judged as the present."}, "sentence_type": "Interrogative sentence", "pos": {"nouns": {"Situation": 1, "Life": 1, "Hard": 1}, "verbs": {"I feel": 1, "鈾?And become 鈾?: 1}, "numerals": {"9": 1}}}, {"content": "(10) Generally speaking, life becomes a source of satisfaction for you?", "word_count": 16, "label": "", "sentence_structure": "Subject鈥揷opula鈥損redicative", "subjective_objective": {"answer": "Subjective", "rationale": "This is a reflection of patients ' self-perception and evaluation and of subjective attitudes and feelings."}, "sentence_sentiment": {"answer": "Neutral", "rationale": "Since the entry is asking the respondents about their satisfaction with life, there is no apparent emotional tendency or attitude."}, "language_style": {"answer": "Tactful and warm", "rationale": "The language style of the entry is more euphemistic, and the interviewer is guided by a gentle way of asking, without using too direct or strong a tone."}, "time_dimension": {"answer": "Past", "rationale": "The entry asks the testee ' s view of the satisfaction with life over time, and therefore the temporal dimension is a past."}, "sentence_type": "Interrogative sentence", "pos": {"nouns": {"General": 1, "Life": 1, "Situation": 1, "Satisfied": 1}, "verbs": {"鈾?And I'm gonna be 鈾?: 1, "I feel": 1}, "adverbs": {"- I'm not sure": 1}, "numerals": {"10": 1}}}, {"content": "(11) Is this the worst period of my life?", "word_count": 12, "label": "", "sentence_structure": "Subject鈥揷opula鈥損redicative", "subjective_objective": {"answer": "Subjective", "rationale": "Because \"most painful\" is the patient's subjective perception and assessment of the current situation."}, "sentence_sentiment": {"answer": "Negative", "rationale": "Because the description of the \"most painful period\" in the entry is negative."}, "language_style": {"answer": "Tactful and warm", "rationale": "The entry uses a relatively mild way to ask the interviewees whether they have experienced the most painful periods, which are more personal and interactive. Moreover, the use of \"Is this the most painful time of your life?\" The use of this means to ask questions and avoid the possibility of causing discomfort or resistance by saying, \"Do you have the most painful time?\""}, "time_dimension": {"answer": "Present", "rationale": "The keyword \"most painful period\" refers to current feelings and experiences, so the entry of the questionnaire asks about the present feelings of the subjects."}, "sentence_type": "Interrogative sentence", "pos": {"nouns": {"Period": 1, "It's the worst": 1}, "verbs": {"be": 1}, "adjectives": {"In my life": 1}, "adverbs": {"Here": 1}, "numerals": {"11": 1}}}, {"content": "I'm as happy as I was young?", "word_count": 9, "label": "", "sentence_structure": "Subject鈥揷opula鈥損redicative", "subjective_objective": {"answer": "Subjective", "rationale": "Because \"happiness\" is a subjective feeling or emotional state."}, "sentence_sentiment": {"answer": "Positive", "rationale": "Because the word \"happy\" in the entry is a positive emotional vocabulary."}, "language_style": {"answer": "Calm and direct", "rationale": "The entry is presented in plain and clear language, without too many rhetorical or emotional colours, and is directly asked whether the subject is still in a state of youthful pleasure and is more calm and straightforward."}, "time_dimension": {"answer": "Present", "rationale": "In the entry, \"like a young person\" means that the current emotional state is compared to the situation of a certain period of time, and \"happy\" is the current emotional experience of the person being tested. The entry therefore looks at the situation of the person being tested."}, "sentence_type": "Interrogative sentence", "pos": {"verbs": {"Elephant": 1, "Happy": 1}, "adjectives": {"Young": 1}, "numerals": {"12": 1}}}, {"content": "(13) Most of what I do is boring or monotonous?", "word_count": 17, "label": "", "sentence_structure": "Subject鈥揷opula鈥損redicative", "subjective_objective": {"answer": "Subjective", "rationale": "Because this question is an inquiry into the patient ' s views and feelings about what he does, it is a subjective examination."}, "sentence_sentiment": {"answer": "Negative", "rationale": "Because the description \"intestation or monotonous\" in the entry is negative."}, "language_style": {"answer": "Calm and direct", "rationale": "The language style of the entry is relatively calm and objective, and there is no excessive emotional or rhetorical approach, and the respondents are asked directly whether they feel bored or unspoken about what they do."}, "time_dimension": {"answer": "Present", "rationale": ""}, "sentence_type": "Interrogative sentence", "pos": {"nouns": {"Things": 1, "Questionnaire": 1, "Entry": 1}, "verbs": {"Do it": 1, "You're": 1, "Boring": 1, "Undo": 1}, "adjectives": {"Most of them": 1, "It's boring": 1, "Undo": 1}, "numerals": {"13": 1}, "pronouns": {"I": 1}}}, {"content": "(14) What I did interest me as usual.", "word_count": 14, "label": "", "sentence_structure": "Subject鈥揷opula鈥損redicative", "subjective_objective": {"answer": "Subjective", "rationale": "Because \"interest\" is a subjective feeling or attitude."}, "sentence_sentiment": {"answer": "Positive", "rationale": "Because the entry expresses a positive attitude, i.e., that there is still interest in past events and that there is no negative or neutral emotional expression."}, "language_style": {"answer": "Tactful and warm", "rationale": "The entry uses more moderate language, which allows dialogue to take place in a way that invites interviewees to share their views and experiences, rather than direct statements or orders. Moreover, it does not deal too much with specific situations or details, but rather raises questions in a more open and inclusive context, making it easier for interviewees to express their feelings and ideas freely. So I think the language style of the purpose is euphemism, more personal and interactive."}, "time_dimension": {"answer": "Past", "rationale": "The keywords \"as before\" and \"as before\" indicate that the entry asks if the person tested has acted or felt the same as before, and therefore judged as past."}, "sentence_type": "Interrogative sentence", "pos": {"nouns": {"Yes": 1, "Interest": 1}, "verbs": {"Do it": 1, "鈾?And I'm gonna be 鈾?: 1, "Elephant": 1, "Interested": 1}, "adverbs": {"Elephant": 1, "It used to be": 1}, "numerals": {"14": 1}}}, {"content": "(15) I'm quite satisfied when I look back on my life.", "word_count": 16, "label": "", "sentence_structure": "Subject鈥揷opula鈥損redicative", "subjective_objective": {"answer": "Subjective", "rationale": "Because it is the patient ' s subjective perception and evaluation of his or her life."}, "sentence_sentiment": {"answer": "Positive", "rationale": "Because the entry \"satisfactoryly satisfied\" expresses a positive emotional attitude."}, "language_style": {"answer": "Tactful and warm", "rationale": ""}, "time_dimension": {"answer": "Past", "rationale": "The entry describes the testee's self-assessment of life, in which both \"recall\" and \"satisfaction\" are points or states of time relevant to the past. The entry therefore primarily concerns the testee's past experience and feelings."}, "sentence_type": "Interrogative sentence", "pos": {"nouns": {"Life": 1, "Satisfied": 1}, "verbs": {"Recalling": 1, "I feel": 1}, "adverbs": {"When": 1, "Pretty much": 1}, "numerals": {"15": 1}, "pronouns": {"I": 1}}}, {"content": "(16) Everything gets worse with age?", "word_count": 15, "label": "", "sentence_structure": "Subject鈥揷opula鈥損redicative", "subjective_objective": {"answer": "Subjective", "rationale": "Because the title refers to \"all things getting worse with age\" as a subjective feeling or view, not an objective fact. Each person will experience different things and feelings as he or she grows older, and the question will need to be examined in the patient鈥檚 personal subjective experience."}, "sentence_sentiment": {"answer": "Negative", "rationale": "Because the phrase \"everything is worse\" in the entry is negative."}, "language_style": {"answer": "Tactful and warm", "rationale": "The entry is more personal and interactive when it is spoken in a gentle and inviting manner, rather than in direct statements or orders. At the same time, the issue is not described in too strong or negative terms, but rather in a relatively objective way, where the interviewee is comfortable and comfortable with the answers."}, "time_dimension": {"answer": "Present", "rationale": "The keyword is \"as age increases\" , which means that it is a description of the present or observation, not a past or future act. And, \"everything is worse\" is a description of the current state. So, the entry describes the current state or feeling of the person being tested, and the basis for the judgement is now."}, "sentence_type": "Interrogative sentence", "pos": {"nouns": {"Age": 1, "Things": 1}, "verbs": {"Increase": 1, "Worse": 1}, "adjectives": {"Everything": 1}, "numerals": {"16": 1}}}, {"content": "(17) How lonely do you feel?", "word_count": 10, "label": "", "sentence_structure": "Subject鈥損redicate", "subjective_objective": {"answer": "Subjective", "rationale": "Because loneliness is a subjective feeling, the degree of feeling of loneliness varies from one person to another."}, "sentence_sentiment": {"answer": "Negative", "rationale": "Because it expresses negative emotions, i.e. \"single,\" and it has a negative color."}, "language_style": {"answer": "Tactful and warm", "rationale": "The entry is more gentle in its language style and is more friendly and intimate in its approach to questions, using euphemism to ask each other about the degree of feeling, and not too direct or intensely emotional. It is therefore judged to be a warm and euphemism."}, "time_dimension": {"answer": "Past", "rationale": "The question of past/present/for future questions is about the degree of loneliness that the testee is or will be feeling, and therefore falls within the category of \"now\"."}, "sentence_type": "Interrogative sentence", "pos": {"nouns": {"Loneliness": 1, "Level": 1}, "verbs": {"I feel": 1, "How's that": 1}, "numerals": {"17": 1}}}, {"content": "Something's been bothering me this year.", "word_count": 10, "label": "", "sentence_structure": "Subject鈥揷opula鈥損redicative", "subjective_objective": {"answer": "Subjective", "rationale": "Because \"making yourself troubled\" is a subjective feeling and experience, not an objective fact or event."}, "sentence_sentiment": {"answer": "Negative", "rationale": "Because the \"fearing\" act in the entry is negative."}, "language_style": {"answer": "Calm and direct", "rationale": "The basis for the determination: the entry directly expresses the subject of asking the other side whether there are any things that bother him, without using excessive rhetorical or emotional rendering, and is more balanced and objective. So I think that the language style is straightforward."}, "time_dimension": {"answer": "Present", "rationale": "The entry is for the interview of the testee ' s feelings and experience of this year, and the time dimension is now judged."}, "sentence_type": "Interrogative sentence", "pos": {"nouns": {"Things": 1, "Trouble": 1}, "verbs": {"鈾?And I'm gonna be 鈾?: 1, "Come on": 0, "It happened": 0, "Conduct": 0, "It's going on": 0, "Completed": 0, "Done": 0, "Achieved": 0, "- It's done": 0, "Change": 0, "It's changed": 0, "Impact": 0, "It's coming": 0, "It's": 0}, "adjectives": {"This year": 1}, "adverbs": {"Some": 1}, "numerals": {"18": 1}}}, {"content": "(19) Would you like to live there if you could?", "word_count": 22, "label": "", "sentence_structure": "Subject鈥搗erb鈥搊bject", "subjective_objective": {"answer": "Subjective", "rationale": "This is viewed from the patient ' s subjective point of view. The problem is to ask the patient about his or her own will and preferences, which are personal subjective thoughts and feelings."}, "sentence_sentiment": {"answer": "Neutral", "rationale": "Because the entry asks about will, it does not contain any positive or negative emotional colours."}, "language_style": {"answer": "Tactful and warm", "rationale": "The language style of the entry is more euphemistic, and it leads the interviewee to express his will, rather than direct orders or demands. At the same time, the words are more polite, like \"you can\" and \"you want\" and make the whole conversation more friendly and friendly."}, "time_dimension": {"answer": "Present", "rationale": "The entry asks the testee about the choice of place of residence in the future, and the temporal dimension is now judged."}, "sentence_type": "Interrogative sentence", "pos": {"nouns": {"Local": 1, "Grandpa": 1}, "verbs": {"Will you": 1, "Go live": 2}, "numerals": {"19": 1}, "classifiers": {"you": 1, "Well": 1}}}, {"content": "鈾?Sometimes I feel like I'm not worth living 鈾?, "word_count": 10, "label": "", "sentence_structure": "Subject鈥揷opula鈥損redicative", "subjective_objective": {"answer": "Subjective", "rationale": "Because feeling \" alive is pointless\" is a subjective emotion or experience that can only be perceived and described by the patient himself."}, "sentence_sentiment": {"answer": "Negative", "rationale": "Because the entry \"Feeling alive is pointless\" expresses a negative emotion and attitude."}, "language_style": {"answer": "Calm and direct", "rationale": "The purpose of this article is very straightforward, and it does not use excessive rhetorical and emotional colours. It simply asks whether the interviewee sometimes feels that life is meaningless, that his tone is objective, and that he does not attempt to direct or influence the interviewee鈥檚 response."}, "time_dimension": {"answer": "Present", "rationale": "The entry now asks if the person tested has some sense of the current time frame, using the term \"sometimes\" to indicate that it does not always exist, but nevertheless appears at some point. At the same time, the expression \"fearless\" is subjective, indicating that the questionnaire designer wants to understand the person's current state and emotional experience. Thus, the time dimension of the article is now judged."}, "sentence_type": "Negative declarative sentence", "pos": {"nouns": {"Time": 1, "I": 1, "Live": 1, "Meaning": 1}, "verbs": {"Sensor": 1, "Present": 1, "Nope": 1}, "adjectives": {"Sometimes": 1}, "numerals": {"20": 1}}}, {"content": "I'm as happy as I was when I was young.", "word_count": 12, "label": "", "sentence_structure": "Subject鈥揷opula鈥損redicative", "subjective_objective": {"answer": "Subjective", "rationale": "Because the emotional joy is the patient's subjective feeling."}, "sentence_sentiment": {"answer": "Positive", "rationale": "Because \"happiness\" in the entry is a positive, positive emotional attitude."}, "language_style": {"answer": "Tactful and warm", "rationale": ""}, "time_dimension": {"answer": "Present", "rationale": ""}, "sentence_type": "Interrogative sentence", "pos": {"nouns": {"I": 1, "Young": 1, "Happy": 1}, "verbs": {"Elephant": 1, "Present": 1, "Same thing": 1, "be": 1}, "adjectives": {"Happy": 1}, "numerals": {"21": 1}}}, {"content": "Most of the time I feel like life is hard.", "word_count": 14, "label": "", "sentence_structure": "Subject鈥揷opula鈥損redicative", "subjective_objective": {"answer": "Subjective", "rationale": "Because it's the subjective view of the patient that feels like life is hard."}, "sentence_sentiment": {"answer": "Negative", "rationale": "Because it expresses a negative emotional attitude that life is hard."}, "language_style": {"answer": "Calm and direct", "rationale": "This language is more objective and neutral, and suitable for use in psychological questionnaires, etc."}, "time_dimension": {"answer": "Present", "rationale": ""}, "sentence_type": "Exclamatory sentence", "pos": {"nouns": {"Time": 1, "Life": 1, "Hard": 1}, "verbs": {"I feel": 1}, "adjectives": {"Most of them": 1}, "numerals": {"22": 1}}}, {"content": "Are you happy with your current life?", "word_count": 11, "label": "", "sentence_structure": "Subject鈥損redicate", "subjective_objective": {"answer": "Subjective", "rationale": "Since satisfaction or dissatisfaction is the emotional experience and assessment of the individual, it is a subjective examination."}, "sentence_sentiment": {"answer": "Positive", "rationale": "Because the word \"satisfactory\" in the entry is usually understood as positive emotional attitudes."}, "language_style": {"answer": "Tactful and warm", "rationale": "The entry is more personal and interactive when it is spoken in a gentle and inviting manner, rather than in direct statements or orders. At the same time, the word \"satisfaction\" is more subjective, and the use of euphemism in questioning reduces each other's resistance and makes them more willing to answer questions in a genuine manner."}, "time_dimension": {"answer": "Present", "rationale": "The keyword is \"current\", which indicates the feelings and state of the person being answered, and therefore the temporal dimension is now judged."}, "sentence_type": "Interrogative sentence", "pos": {"nouns": {"Life": 1, "Satisfied": 1}, "verbs": {"be": 1, "to": 1}, "numerals": {"23": 1}}}, {"content": "My health and my peers are better than theirs.", "word_count": 23, "label": "", "sentence_structure": "Subject鈥搗erb鈥揷omplement", "subjective_objective": {"answer": "Subjective", "rationale": "Because the entry relates to the patient's self-assessment (i.e., \"I\" health) and is therefore a subjective examination."}, "sentence_sentiment": {"answer": "Positive", "rationale": "Because the entries indicate that they are in a better health than their peers, they are a positive assessment."}, "language_style": {"answer": "Tactful and warm", "rationale": ""}, "time_dimension": {"answer": "Present", "rationale": ""}, "sentence_type": "Affirmative declarative sentence", "pos": {"nouns": {"Health": 1, "Peer": 1, "they": 1}, "verbs": {"- Yeah": 1, "Same": 1, "Okay": 1}, "adjectives": {"Mine": 2, "Even": 1}, "numerals": {"24": 1}, "pronouns": {"I": 1}}}]}

{"questionnaire_title": "Questionnaire on sick behaviour", "questionnaire_category": "Behavioral questionnaire", "item_count": 62, "emotion_shift_overall": "From negative to positive", "language_style_overall": "Calm and direct", "items": [{"content": "1. Are you very troubled by your health?", "word_count": 13, "label": "", "sentence_structure": "Subject鈥搗erb鈥搊bject", "subjective_objective": {"answer": "Subjective", "rationale": "Because trouble is a subjective feeling or experience."}, "sentence_sentiment": {"answer": "Negative", "rationale": "Because the term \"torture\" is usually considered negative and expresses a negative emotional attitude."}, "language_style": {"answer": "Calm and direct", "rationale": "The language style of the entry is a calm and straightforward one, as it raises questions directly, without using too many emotional accoutrements or euphemisms."}, "time_dimension": {"answer": "Present", "rationale": "The entry asks the person ' s current state of feeling and state, i.e. the level of concern for his or her health, so the time dimension is now judged."}, "sentence_type": "Interrogative sentence", "pos": {"nouns": {"self": 1, "Health status": 1, "Trouble": 1}, "verbs": {"very": 1, "Yes": 1, "Trouble": 1}, "adverbs": {"very": 1}, "pronouns": {"you": 1}}}, {"content": "Do you think you have a serious problem with your body?", "word_count": 15, "label": "", "sentence_structure": "Subject鈥搗erb鈥搊bject", "subjective_objective": {"answer": "Subjective", "rationale": "It is the patient ' s subjective view and assessment that he or she believes that he or she has a serious problem with his or her body."}, "sentence_sentiment": {"answer": "Negative", "rationale": "As the problem involves physical illness, it can trigger negative feelings of anxiety and anxiety."}, "language_style": {"answer": "Calm and direct", "rationale": "The entry is in the form of a straightforward question and is not expressed in any qualifier or emotional language, which is more direct and objective."}, "time_dimension": {"answer": "Present", "rationale": ""}, "sentence_type": "Interrogative sentence", "pos": {"nouns": {"Body": 1, "Serious": 1, "Problem": 1}, "verbs": {"Out": 1, "I think": 1}, "adverbs": {"self": 1}, "pronouns": {"you": 1}}}, {"content": "3. Does your illness have a great impact on life?", "word_count": 11, "label": "", "sentence_structure": "Subject鈥搗erb鈥搊bject", "subjective_objective": {"answer": "Subjective", "rationale": "Because the impact is the subjective experience that patients feel."}, "sentence_sentiment": {"answer": "Negative", "rationale": "Because the entry describes the negative impact of disease on life, it is a negative expression."}, "language_style": {"answer": "Tactful and warm", "rationale": "The entry is more euphemistic and gentle, not asking the audience directly about the seriousness of the condition, but inviting the other person to share his/her feelings in a caring and understanding manner. Moreover, the question is not too medical or technical in terms that make the respondents more comfortable and comfortable."}, "time_dimension": {"answer": "Present", "rationale": "The entry asks the extent to which the testee ' s illness has affected his or her life, and the time dimension is now judged."}, "sentence_type": "Interrogative sentence", "pos": {"nouns": {"Diseases": 1, "Life": 1}, "verbs": {"Impact": 1}, "adjectives": {"Large": 1}, "numerals": {"very": 1}}}, {"content": "Is it easy for others to get along with you after a disease?", "word_count": 13, "label": "", "sentence_structure": "Subject鈥搗erb鈥搊bject", "subjective_objective": {"answer": "Subjective", "rationale": "This is a subjective feeling and perception of the patient and involves the perception and evaluation of the attitude of others."}, "sentence_sentiment": {"answer": "Neutral", "rationale": "Because the entry does not have any emotional color."}, "language_style": {"answer": "Calm and direct", "rationale": "The entry is more straightforward, is not based on excessive rhetorical or emotional rendering, and is more objective in tone. At the same time, it asks the person in question in a clear manner about his social situation after he has developed a disease, without a personal emotion or subjective judgment, and can therefore be considered as a \"cool straight\" language style."}, "time_dimension": {"answer": "Past", "rationale": "The entry is for the testee's social relationship after the disease, and the temporal dimension is a past (because it is already \"illnessed\"."}, "sentence_type": "Interrogative sentence", "pos": {"nouns": {"Diseases": 1, "person": 2, "Get along": 1}, "verbs": {"Okay": 1, "and": 1, "Easy": 1}, "adjectives": {"Don't": 1, "Easy": 1}, "numerals": {"4": 1}, "classifiers": {"individual": 1}, "pronouns": {"you": 1}}}, {"content": "5. Are there any (similar) illnesses in your family?", "word_count": 11, "label": "", "sentence_structure": "Subject鈥搗erb鈥搊bject", "subjective_objective": {"answer": "Objective", "rationale": "This question is about whether there is an objective fact of a disease in the patient鈥檚 family, and not about the patient鈥檚 subjective attitude, feelings or experience."}, "sentence_sentiment": {"answer": "Neutral", "rationale": "Because the entry is an objective fact and does not involve a description of feelings, attitudes or behaviour."}, "language_style": {"answer": "Tactful and warm", "rationale": "The language style of the entry is more euphemistic. It is in the form of a question phrase, which is not a question about whether the other side has a family history, but rather gives the other side a space for reflection that makes the person under investigation feel more comfortable and relaxed."}, "time_dimension": {"answer": "Present", "rationale": "The entry asks the family of the tested for the disease of any kind, which is judged by the time dimension."}, "sentence_type": "Interrogative sentence", "pos": {"nouns": {"Family": 1, "Diseases": 1, "Similar": 1}, "verbs": {"have": 1, "Similar": 1}, "numerals": {"5": 1}, "classifiers": {"individual": 1}, "pronouns": {"you": 1}}}, {"content": "Do you think you are more ill than others?", "word_count": 14, "label": "", "sentence_structure": "Subject鈥搗erb鈥搊bject", "subjective_objective": {"answer": "Subjective", "rationale": "Because \"think\" represents a patient's subjective opinion or belief."}, "sentence_sentiment": {"answer": "Negative", "rationale": "Because the expression \"always ill\" in the entries is negative emotional, implying a negative perception of itself."}, "language_style": {"answer": "Calm and direct", "rationale": "The entry is more straightforward, concise, and has no excessive rhetoric or emotional colours, raising questions in an objective and neutral manner, which facilitates the acquisition of the real thoughts and feelings of the interviewees."}, "time_dimension": {"answer": "Present", "rationale": "The entry asks the testee about his current attitude and views, and the time dimension is now judged."}, "sentence_type": "Interrogative sentence", "pos": {"nouns": {"Questionnaire": 1, "Entry": 1, "Diseases": 1}, "verbs": {"I think": 1, "Sick": 1}, "adjectives": {"More": 1, "Easy": 1}, "numerals": {"6": 1}, "pronouns": {"self": 1, "others": 1}}}, {"content": "Can you believe that if the doctor told you that you were not sick?", "word_count": 20, "label": "", "sentence_structure": "Subject鈥損redicate", "subjective_objective": {"answer": "Subjective", "rationale": "Because mistrust of doctors is a subjective attitude and emotional reaction of individuals."}, "sentence_sentiment": {"answer": "Neutral", "rationale": "Because the entry does not have any emotional color."}, "language_style": {"answer": "Calm and direct", "rationale": "This entry is in a straightforward language, asking whether the person interviewed can trust the doctor鈥檚 professional judgement when facing the results of a medical test."}, "time_dimension": {"answer": "Future", "rationale": "The verbs \"said,\" \"discovery\" and \"disorder\" point to future possibilities and scenarios, so the temporal dimension of the article is future."}, "sentence_type": "Interrogative sentence", "pos": {"nouns": {"Doctor": 1, "Diseases": 1, "you": 2}, "verbs": {"Tell": 1, "Found": 1, "Yes": 2}, "adverbs": {"If": 1}, "numerals": {"7": 1}, "classifiers": {"individual": 1}}}, {"content": "8. Can you easily forget yourself for a while and think about other things?", "word_count": 22, "label": "", "sentence_structure": "Subject鈥搗erb鈥搊bject", "subjective_objective": {"answer": "Subjective", "rationale": "Because the subject is asking about the feelings and experiences of individuals, it is a subjective attitude."}, "sentence_sentiment": {"answer": "Positive", "rationale": "Because the act of \"can forget yourself for a while and think about other things\" is positive and indicates that the individual has the ability to regulate emotions and attention."}, "language_style": {"answer": "Tactful and warm", "rationale": "The entry is more euphemistic, using soft words like \"easy\" and \"for the time being forget\" and softer expression. It also takes the form of a question-and-answer sentence, which leads the respondents to think and answer, and is more interactive and personal."}, "time_dimension": {"answer": "Present", "rationale": ""}, "sentence_type": "Interrogative sentence", "pos": {"nouns": {"Questionnaire": 1, "Entry": 1, "Things": 2}, "verbs": {"Do it": 1, "Forget it": 1, "Go on": 1, "Think": 1}, "adverbs": {"Easy": 1}, "numerals": {"8": 1}}}, {"content": "When you feel sick and people say you look better, does that bother you?", "word_count": 27, "label": "", "sentence_structure": "Subject鈥搗erb鈥搊bject", "subjective_objective": {"answer": "Subjective", "rationale": "Because patients ' feelings and reactions are subjective, and the problem is viewed by the patient ' s feelings and reactions to others ' perceptions of his or her condition, not by objective facts themselves."}, "sentence_sentiment": {"answer": "Negative", "rationale": "Basis of judgement: According to the entry, \"I feel sick but I am said to look better\" may make the interviewee feel that his feelings are ignored or misunderstood, and therefore generate negative emotions."}, "language_style": {"answer": "Calm and direct", "rationale": ""}, "time_dimension": {"answer": "Past", "rationale": "The entry asks whether the testee has ever been uncomfortable in his past experience because of the lack of conformity of his or her own body assessment, and therefore is judged on the basis of the past."}, "sentence_type": "Interrogative sentence", "pos": {"nouns": {"you": 2, "self": 1, "Diseases": 1, "person": 1, "Looks like it": 1, "A lot": 1, "Yes": 1}, "verbs": {"I think": 1, "Yes": 1, "Fuck": 1}, "adjectives": {"Not fast": 1}, "adverbs": {"When": 1, "But": 1}, "numerals": {"9": 1, "More": 1}, "pronouns": {"Here": 1}}}, {"content": "Do you often realize the changes in your body?", "word_count": 15, "label": "", "sentence_structure": "Subject鈥搗erb鈥搊bject", "subjective_objective": {"answer": "Subjective", "rationale": "Because \"aware\" is the patient's subjective feeling and perception, and it's a subjective view."}, "sentence_sentiment": {"answer": "Neutral", "rationale": "Because the entry is asked about an objective fact, there is no apparent emotional tendency."}, "language_style": {"answer": "Tactful and warm", "rationale": "The entry is a soft, inviting conversation, asking if the respondents are aware of their physical changes, and are more sensitive and concerned. At the same time, the term \"often\" is used to indicate that this is a matter of ongoing concern. This expression is more personal and interactive."}, "time_dimension": {"answer": "Present", "rationale": "The keyword \"often realizes\" indicates that it is a regular act, without reference to a specific time frame, past or future. Thus, the temporal dimension of the article is now."}, "sentence_type": "Interrogative sentence", "pos": {"nouns": {"Questionnaire": 1, "Entry": 1, "Body": 1, "Various": 1, "Change": 1}, "verbs": {"I'm aware of that": 1, "Regular": 1, "be": 1, "Yes": 1, "Come on": 1}, "adverbs": {"Various": 1, "Is it possible": 2, "Regular": 1}, "numerals": {"10": 1}, "classifiers": {"individual": 1, "Plant": 1}, "pronouns": {"you": 1}}}, {"content": "鈥淒oes it ever occur to you that sickness is a form of retribution for what you have done wrong before?", "word_count": 25, "label": "", "sentence_structure": "Subject鈥搗erb鈥搊bject", "subjective_objective": {"answer": "Subjective", "rationale": "Because it's the patient's personal conviction and thoughts."}, "sentence_sentiment": {"answer": "Negative", "rationale": "Because the notion in the entry that \"illness is a form of retribution for what I did wrong\" is negative."}, "language_style": {"answer": "Tactful and warm", "rationale": "The entry leads the respondents to express their views in a question-and-answer manner, rather than directly making statements or orders, which is more personal and interactive. At the same time, the use of the \"hadn't\" approach is more euphemistic and moderate, avoiding pressure or discomfort on the respondents."}, "time_dimension": {"answer": "Past", "rationale": ""}, "sentence_type": "Interrogative sentence", "pos": {"nouns": {"Diseases": 1, "Error": 1, "One": 1, "Retribution": 1}, "verbs": {"Okay": 1, "Wrong": 1, "have": 2}, "adverbs": {"be": 1, "Before": 1, "self": 1}, "numerals": {"11": 1, "Here": 1}, "pronouns": {"you": 1, "Pass": 1}}}, {"content": "Do you think you're having a nervous breakdown?", "word_count": 14, "label": "", "sentence_structure": "Subject鈥搗erb鈥搊bject", "subjective_objective": {"answer": "Subjective", "rationale": "Because \"think\" is a term of subjective attitude, which means that patients think they have a problem with their nerves, not an objective reality."}, "sentence_sentiment": {"answer": "Negative", "rationale": "Because the expression \"neurologically ill\" is a term with negative and derogatory connotations, it is negative in emotional terms and therefore negative in its judgement."}, "language_style": {"answer": "Calm and direct", "rationale": ""}, "time_dimension": {"answer": "Past", "rationale": ""}, "sentence_type": "Interrogative sentence", "pos": {"nouns": {"Questionnaire": 1, "Entry": 1, "Nerves": 1}, "verbs": {"I think": 1, "Something's wrong": 1}, "numerals": {"12": 1}, "pronouns": {"self": 1}}}, {"content": "13. When you feel sick or upset, does it make it easier for a doctor to cheer you up?", "word_count": 24, "label": "", "sentence_structure": "Subject鈥搗erb鈥搊bject", "subjective_objective": {"answer": "Subjective", "rationale": "Since the problem involves the feelings and views of the patient, it is necessary to examine the patient ' s subjective attitude."}, "sentence_sentiment": {"answer": "Positive", "rationale": "Because the purpose of the \"Doctors Easier to Revelation\" is positive."}, "language_style": {"answer": "Calm and direct", "rationale": "The entry is directly asked in a way that is clear and straightforward, does not have too many qualifiers or emotional renderings and is of a calm and direct language style."}, "time_dimension": {"answer": "Past", "rationale": "The entry is for the interview of the person who was tested in the past and the time dimension is judged to be the past."}, "sentence_type": "Interrogative sentence", "pos": {"nouns": {"you": 2, "Doctor": 1, "Diseases": 1, "self": 1, "I'm upset": 1, "Time": 1}, "verbs": {"I feel": 1, "鈾?And I'm gonna be 鈾?: 1, "Fight": 1, "Come on": 1}, "adverbs": {"Easy": 1, "- You're not": 1}, "numerals": {"13": 1}}}, {"content": "Do you think others can understand what it's like to be sick?", "word_count": 18, "label": "", "sentence_structure": "Subject鈥搗erb鈥搊bject", "subjective_objective": {"answer": "Subjective", "rationale": "Because the questionnaire entry asks patients about their perception of others ' perception of their illness, it is the patient ' s subjective view and feeling."}, "sentence_sentiment": {"answer": "Neutral", "rationale": "Because the entry merely asks the other side for its views, it does not contain any emotional colour."}, "language_style": {"answer": "Calm and direct", "rationale": "The article is more straightforward, and does not use euphemism or excessive rhetoric. It is more objective and rational to ask the interviewee whether he or she understands the illness. We can therefore consider the language style of the purpose to be straightforward and straightforward."}, "time_dimension": {"answer": "Present", "rationale": "The entry asks the testee ' s current view of whether others are aware of the disease, and the time dimension is now judged."}, "sentence_type": "Interrogative sentence", "pos": {"nouns": {"person": 1, "Sick": 1, "It's like": 1}, "verbs": {"Yes": 1, "Recognition": 1, "Present": 1}, "numerals": {"14": 1}, "classifiers": {"individual": 1}}}, {"content": "15. Would it bother you to talk to the doctor about your illness?", "word_count": 17, "label": "", "sentence_structure": "Subject鈥搗erb鈥搊bject", "subjective_objective": {"answer": "Subjective", "rationale": "Because it is a topic of patient perception, it looks at patient subjective emotional reactions."}, "sentence_sentiment": {"answer": "Negative", "rationale": "The basis for the judgement: the act of \"disturbing\" in the entry expresses negative feelings and is therefore defined as negative."}, "language_style": {"answer": "Tactful and warm", "rationale": ""}, "time_dimension": {"answer": "Present", "rationale": "\"Talking with a doctor\" means an ongoing behavior, \"will\" means a possible future, so the entry primarily examines the emotional state of the person being tested during his conversation with the doctor, and the temporal dimension is now."}, "sentence_type": "Interrogative sentence", "pos": {"nouns": {"Doctor": 1, "Diseases": 1, "self": 1}, "verbs": {"Talk about it": 1, "鈾?And I'm gonna be 鈾?: 1, "I feel": 1, "Unsettled": 1}, "numerals": {"15": 1}, "pronouns": {"you": 1}}}, {"content": "Is there any pain that bothers you?", "word_count": 9, "label": "", "sentence_structure": "Subject鈥搗erb鈥搊bject", "subjective_objective": {"answer": "Subjective", "rationale": "Because \"there's multiple pain problems\" is a subjective pain feeling and cognitive."}, "sentence_sentiment": {"answer": "Negative", "rationale": "Because the expression \"multiple pain distress\" in the entry is negative."}, "language_style": {"answer": "Tactful and warm", "rationale": "The language style of the entry is euphemistic, using a question phrase to guide respondents to reflect on their situation, and not in too direct or command-like a tone. At the same time, the words \"multiple\" and \"problem\" express a sense of concern and understanding, making the questionnaire more friendly."}, "time_dimension": {"answer": "Present", "rationale": "The entry asks whether there are many pain problems at present, and does not address information such as past or future behaviour or mentality, and therefore determines it to be now."}, "sentence_type": "Interrogative sentence", "pos": {"nouns": {"16": 1, "Multiple": 1, "Pain": 1, "Trouble": 1}, "verbs": {"have": 1, "Answer": 1}, "numerals": {"1": 1, "Multiple": 1}, "classifiers": {"individual": 1}}}, {"content": "Is your illness such a big influence on your relationship with your family and friends?", "word_count": 17, "label": "", "sentence_structure": "Subject鈥搗erb鈥搊bject", "subjective_objective": {"answer": "Subjective", "rationale": "Because the disease is a subjective state for the patient.\""}, "sentence_sentiment": {"answer": "Negative", "rationale": "Because the description of \"diseases are very influenceful\" in the entry is negative, indicating that disease has a negative impact on interpersonal relations."}, "language_style": {"answer": "Tactful and warm", "rationale": "The entry is more personal and interactive when it is spoken in a gentle and inviting manner, rather than in direct statements or orders. At the same time, the concern is whether the relationship between the interviewee and his/her relatives is affected by disease, which makes the issue more human and compassionate and helps the interviewee to be more open to sharing his/her own situation."}, "time_dimension": {"answer": "Present", "rationale": "The keyword \"very influential\" denotes an impact on the status quo, while the entire sentence adopts the general current state of affairs and can be judged to describe the current state."}, "sentence_type": "Interrogative sentence", "pos": {"nouns": {"you": 1, "Diseases": 1, "Family": 1, "friends": 1, "- To be with someone": 1}, "verbs": {"Impact": 1}, "adjectives": {"very": 1}, "numerals": {"17": 1}}}, {"content": "Are you in a hurry?", "word_count": 6, "label": "", "sentence_structure": "Subject鈥搗erb鈥搊bject", "subjective_objective": {"answer": "Subjective", "rationale": "The lack of urgency is a subjective approach, as it is the patient ' s own feelings and perceptions."}, "sentence_sentiment": {"answer": "Negative", "rationale": "Because the \"Easy to Excited\" act in the entry is negative."}, "language_style": {"answer": "Calm and direct", "rationale": "The entry raises questions in a straightforward and concise manner, without too many rhetorical or emotional colours and with a more calm and objective language style."}, "time_dimension": {"answer": "Present", "rationale": "\"Are you in a hurry?\" is a question about the present behaviour or state of the subject, and \"easy\" is a descriptive adjective indicating that the act is relatively frequent at this stage. The entry therefore relates to the current time dimension."}, "sentence_type": "Interrogative sentence", "pos": {"verbs": {"I'm in a hurry": 1}, "adjectives": {"Easy": 1}}}, {"content": "Do you know anyone with the same illness as you?", "word_count": 13, "label": "", "sentence_structure": "Subject鈥搗erb鈥搊bject", "subjective_objective": {"answer": "Objective", "rationale": "The basis for the decision: because \"know the person who has the same disease\" is the objective fact that patients experience, it looks at their social relations and their state of illness, not their subjective ideas."}, "sentence_sentiment": {"answer": "Neutral", "rationale": "Because the entry does not have any emotional color."}, "language_style": {"answer": "Calm and direct", "rationale": ""}, "time_dimension": {"answer": "Past", "rationale": "The entry asks whether the interviewees have in the past known persons with the same illnesses as them and therefore fall within the category of past behaviour or state."}, "sentence_type": "Interrogative sentence", "pos": {"nouns": {"you": 1, "Diseases": 1}, "verbs": {"Yes": 1, "Patient": 1, "Same": 1, "Sample": 1}, "adverbs": {"Call": 1}, "numerals": {"19": 1, "Same thing": 1}, "pronouns": {"People": 1}}}, {"content": "Are you more sensitive to pain than anyone else?", "word_count": 11, "label": "", "sentence_structure": "Subject鈥搗erb鈥搊bject", "subjective_objective": {"answer": "Subjective", "rationale": "Because \"better\" is a comparative description, based on subjective judgment of the patient's own feelings and experiences."}, "sentence_sentiment": {"answer": "Negative", "rationale": "Because the expression \"more sensitive to pain than others\" in the entry is negative."}, "language_style": {"answer": "Calm and direct", "rationale": "The entry uses a simple and clear language style, without excessive rhetorical or emotional acquital, which makes it easier to understand the subject matter and give precise answers."}, "time_dimension": {"answer": "Present", "rationale": "\"Are you more sensitive to pain than anyone else?\" The word \"more\" indicates a comparison between the present and the past or the past, so it is now judged."}, "sentence_type": "Interrogative sentence", "pos": {"nouns": {"Questionnaire Entry": 1, "Pain": 1, "others": 1}, "verbs": {"More": 1, "Sensitive": 1}, "adverbs": {"More": 1}, "numerals": {"20": 1}, "classifiers": {"you": 1}}}, {"content": "Are you afraid of getting sick?", "word_count": 6, "label": "", "sentence_structure": "Subject鈥搗erb鈥搊bject", "subjective_objective": {"answer": "Subjective", "rationale": "Because fear of illness is the patient's subjective attitude."}, "sentence_sentiment": {"answer": "Negative", "rationale": "Because the attitude of \"fear of disease\" in the entries is negative."}, "language_style": {"answer": "Calm and direct", "rationale": "The entry is presented in the form of a direct question, without excessive rhetorical or emotional rendering, with a more objective and cold tone, and suitable for actual research and research."}, "time_dimension": {"answer": "Present", "rationale": "The entry asks the subject what he thinks and what he feels now, the keywords being \"fear\" and \"are you\" and they all point to the current state of mind and therefore judge it as now."}, "sentence_type": "Interrogative sentence", "pos": {"nouns": {"you": 1, "Diseases": 1}, "verbs": {"Okay": 1, "Yes": 1, "Diseases": 1}, "adverbs": {"- You're not": 1}, "numerals": {"21": 1}}}, {"content": "22. Can you express your feelings to others as you wish?", "word_count": 16, "label": "", "sentence_structure": "Subject鈥搗erb鈥搊bject", "subjective_objective": {"answer": "Subjective", "rationale": "This is the subject of the subjective attitude and ability to examine the patient."}, "sentence_sentiment": {"answer": "Positive", "rationale": "Because the \"expression of your feelings\" in the entry is positive."}, "language_style": {"answer": "Tactful and warm", "rationale": "The entry is more personal and interactive when it is spoken in a gentle and inviting tone, rather than in direct statements or orders. At the same time, the ability to express its feelings is described by the word \"su Young\" to make the language more euphemismal, gentler, easy to make the person surveyed comfortable and willing to cooperate in answering the questions."}, "time_dimension": {"answer": "Present", "rationale": "The entry asks if the testee is able to express his feelings successfully at the moment, and the time dimension is now. The keyword \"can\" represent the state of the moment, and \"express your feelings to others\" means that it is an act against others."}, "sentence_type": "Interrogative sentence", "pos": {"nouns": {"Questionnaire Entry": 1, "Feel": 1}, "verbs": {"Yes": 1, "Sun-Yung-dil": 1, "Expression": 1}, "adverbs": {"self": 1}, "numerals": {"22": 1}}}, {"content": "Are you sad when you're sick?", "word_count": 12, "label": "", "sentence_structure": "Subject鈥搗erb鈥搊bject", "subjective_objective": {"answer": "Subjective", "rationale": "Because it's a subjective emotional reaction that feels sad."}, "sentence_sentiment": {"answer": "Neutral", "rationale": "The entry is merely an inquiry into whether the person is sad about the sick interviewee and does not express any emotional tendencies."}, "language_style": {"answer": "Tactful and warm", "rationale": "The language style of this entry is more euphemistic. It uses a caring and understanding attitude to ask if the respondents feel sorry for them when they are sick, without the language of asking or ordering directly. This expression is more friendly and interactive, and it can easily guide the respondents to answer questions and share their feelings."}, "time_dimension": {"answer": "Past", "rationale": "The entry asks if the person tested was cared for and sympathized with them when they were ill, and the time dimension is judged to be past."}, "sentence_type": "Interrogative sentence", "pos": {"nouns": {"you": 1, "Diseases": 1, "person": 1, "Sorry": 1}, "verbs": {"Sensor": 1, "Present": 1, "Yes": 1}, "numerals": {"23": 1}}}, {"content": "Are you more worried about your health than most people?", "word_count": 15, "label": "", "sentence_structure": "Subject鈥搗erb鈥搊bject", "subjective_objective": {"answer": "Subjective", "rationale": "Because \"fear\" is a subjective feeling or attitude, it needs to be viewed from the personal perspective of the patient."}, "sentence_sentiment": {"answer": "Negative", "rationale": "Because the phrase \"more worried\" in the entry conveys a negative emotional attitude."}, "language_style": {"answer": "Calm and direct", "rationale": "The entry uses a direct inquiry to understand the extent of the respondent ' s concern for his or her health, the language style is more objective and calm, and there is no excessive emotional or orality."}, "time_dimension": {"answer": "Present", "rationale": "The entry asks the testee鈥檚 current state and feelings, i.e. whether they are more concerned about their health than most people at the moment. Therefore, the time dimension is now judged."}, "sentence_type": "Interrogative sentence", "pos": {"nouns": {"you": 1, "Most of them": 1, "person": 1, "self": 1, "Health": 1}, "verbs": {"More worried": 1}, "adjectives": {"More": 1}, "numerals": {"24": 1}}}, {"content": "Does your illness affect your sexual relations?", "word_count": 11, "label": "", "sentence_structure": "Subject鈥搗erb鈥搊bject", "subjective_objective": {"answer": "Subjective", "rationale": "The problem is viewed as subjective and therefore subjective."}, "sentence_sentiment": {"answer": "Negative", "rationale": "Because the entry asks about the effects of disease on sexual relations, this is a more negative topic."}, "language_style": {"answer": "Calm and direct", "rationale": "This is a more accurate and clear-cut form of questioning, with no excessive rhetoric or emotional rendering, and a more objective tone."}, "time_dimension": {"answer": "Present", "rationale": "The entry now asks the testees about their current situation, i.e. whether their illness affects their sexual relations, and therefore the temporal dimension is now judged."}, "sentence_type": "Interrogative sentence", "pos": {"nouns": {"Yours": 1, "Diseases": 1, "Sexual relations": 1}, "verbs": {"Impact": 1}, "numerals": {"25": 1}}}, {"content": "Have you suffered much for your illness?", "word_count": 14, "label": "", "sentence_structure": "Subject鈥搗erb鈥搊bject", "subjective_objective": {"answer": "Subjective", "rationale": "Because the entry of the questionnaire asks about the patient's subjective feelings, which is \"how much pain has been experienced\", it is viewed from the patient's subjective perspective."}, "sentence_sentiment": {"answer": "Negative", "rationale": "Because the words \"disease\" and \"in pain\" in the entries have a distinct negative emotional color."}, "language_style": {"answer": "Calm and direct", "rationale": "The language style of the entry is straightforward, as it directly asks whether the person under investigation has experienced physical pain, has no excessive emotional or orality."}, "time_dimension": {"answer": "Past", "rationale": "The entry asks whether the person tested had suffered from a disease in the past and therefore the time dimension can be judged to be past."}, "sentence_type": "Interrogative sentence", "pos": {"nouns": {"Pain": 1, "Diseases": 1}, "verbs": {"Experience": 1}, "adjectives": {"Many": 1}, "numerals": {"26": 1, "Many": 1}}}, {"content": "27. Is there anything in your life besides illness?", "word_count": 14, "label": "", "sentence_structure": "Subject鈥搗erb鈥搊bject", "subjective_objective": {"answer": "Objective", "rationale": "Because \"disease\" and \"other problems in life\" are objective facts that patients experience."}, "sentence_sentiment": {"answer": "Neutral", "rationale": "Because the entry is asking the respondents whether they have other problems in life, it is not emotional."}, "language_style": {"answer": "Calm and direct", "rationale": "The language style of the entry is relatively calm and straightforward, with no excessive acrimonyms or emotional colours, and questions are asked to the interviewees in a concise manner, making it easier to obtain objective answers."}, "time_dimension": {"answer": "Present", "rationale": "The entry is an inquiry into the current living conditions and distress of the person being tested, and is not clear about the temporal dimensions and can be considered as the present."}, "sentence_type": "Interrogative sentence", "pos": {"nouns": {"Diseases": 1, "Life": 1, "Problem": 1}, "verbs": {"have": 1, "And": 1, "Except": 1, "- You're not": 1}, "adverbs": {"Other": 1}, "numerals": {"27": 1}}}, {"content": "Do you care that anyone knows or is unaware of your illness?", "word_count": 15, "label": "", "sentence_structure": "Subject鈥搗erb鈥搊bject", "subjective_objective": {"answer": "Subjective", "rationale": "Because \"care\" is a subjective feeling or attitude."}, "sentence_sentiment": {"answer": "Negative", "rationale": "Because the \"intentional\" attitude in the entries is negative."}, "language_style": {"answer": "Tactful and warm", "rationale": "The language style of the entry is euphemism. It uses the tone of inquiry rather than the order or direct statement. This is more acceptable and better promotes self-expression and honesty of the person under investigation."}, "time_dimension": {"answer": "Present", "rationale": "In conclusion, for the given questionnaire entries, \"28. Do you care that anyone knows or does not know your illness?\" should be classified as \"now\"."}, "sentence_type": "Interrogative sentence", "pos": {"nouns": {"person": 1, "Diseases": 1}, "verbs": {"Meaning": 1, "Yes": 1, "Break": 1, "in": 1, "Look": 1}, "adverbs": {"very": 1}, "numerals": {"28": 1}, "classifiers": {"Grandpa": 1}}}, {"content": "Are you jealous of the health of others?", "word_count": 12, "label": "", "sentence_structure": "Subject鈥搗erb鈥搊bject", "subjective_objective": {"answer": "Subjective", "rationale": "Because jealousy is a subjective emotion and attitude, it is linked to the individual values of the patient, emotional state, etc."}, "sentence_sentiment": {"answer": "Negative", "rationale": "Because the word \"envious\" in the entry is negative emotional."}, "language_style": {"answer": "Calm and direct", "rationale": "The entry is more calmly and straightforward, and it appears objective and neutral to ask directly whether the interviewee is jealous of the health of others, without excessive embellishment or emotional rendering."}, "time_dimension": {"answer": "Past", "rationale": "The entry asks about the person ' s past feelings and emotional state, which involves experience and perceptions of the past and therefore judgement as the past."}, "sentence_type": "Interrogative sentence", "pos": {"nouns": {"others": 1, "Health": 1, "Body": 1, "Jealousy": 1}, "verbs": {"I feel": 1, "you": 1, "Jealousy": 1}, "numerals": {"29": 1}, "pronouns": {"you": 1}}}, {"content": "30. Do you have any idea how ridiculous it looks to be sick, but you can't get away with it?", "word_count": 24, "label": "", "sentence_structure": "Subject鈥搗erb鈥搊bject", "subjective_objective": {"answer": "Subjective", "rationale": "The subject of this questionnaire is to ask if the patient has a particular feeling or idea, which is the patient鈥檚 own subjective experience and perception. The entry is therefore viewed from a subjective perspective."}, "sentence_sentiment": {"answer": "Negative", "rationale": "Because the description \"Image to Forget\" in the entry is negative."}, "language_style": {"answer": "Tactful and warm", "rationale": "The reasons for this are as follows: 1. The term \"question\" is mild and does not use direct negative or positive terms; 2. The expression \"ludicrous\" and \"driving to get away\" has a sense of humor and affection, giving a sense of proximity; 3. The whole question gives the impression that the person interviewed is communicating and exploring, rather than evaluating or blaming the condition of the person interviewed."}, "time_dimension": {"answer": "Present", "rationale": ""}, "sentence_type": "Interrogative sentence", "pos": {"nouns": {"Entry": 1, "Sick": 1, "Thoughts": 2}, "verbs": {"Looks like it": 1, "Try": 1, "Get rid of it": 1}, "adjectives": {"Ridiculous": 1, "What": 1}, "adverbs": {"Yeah": 1}, "numerals": {"30": 1}, "pronouns": {"you": 1}}}, {"content": "31. Are you having financial difficulties?", "word_count": 9, "label": "", "sentence_structure": "Subject鈥損redicate", "subjective_objective": {"answer": "Objective", "rationale": "Since economic difficulties are an objective situation, there is no need for subjective evaluation by the patient."}, "sentence_sentiment": {"answer": "Negative", "rationale": "Since the entry asks whether there are economic difficulties, it is a negative event or situation and can therefore be classified as negative."}, "language_style": {"answer": "Calm and direct", "rationale": "The language style of the entry is more objective, calm and direct, and there is no excessive emotional or qualifier language, and the respondents are asked in a concise and clear language whether they have any financial difficulties."}, "time_dimension": {"answer": "Present", "rationale": "The keyword \"economically difficult\" refers to the current, present or situation. Thus, the temporal dimension is now."}, "sentence_type": "Interrogative sentence", "pos": {"nouns": {"Questionnaire": 1, "Entry": 1, "Economy": 1, "Difficulties": 1}, "verbs": {"You have": 1}, "numerals": {"31": 1}, "pronouns": {"you": 1}}}, {"content": "Are you disturbed by the way other people treat your illness?", "word_count": 18, "label": "", "sentence_structure": "Subject鈥搗erb鈥搊bject", "subjective_objective": {"answer": "Subjective", "rationale": "The questionnaire is subjective because it is about asking about patients ' feelings and experiences."}, "sentence_sentiment": {"answer": "Negative", "rationale": "Because the phrase \"to upset you\" in the entry is negative."}, "language_style": {"answer": "Calm and direct", "rationale": "The language style of the entry is calm and straightforward. It directly asks whether the person surveyed is uneasy, plain and simple in the face of the way others treat their illness, and does not use excessive rhetorical or emotional modifications."}, "time_dimension": {"answer": "Past", "rationale": "The entry is to ask the testee about the feelings and attitudes of others about their past experience of disease, and therefore to judge it as \"the past\"."}, "sentence_type": "Interrogative sentence", "pos": {"nouns": {"Diseases": 1, "Modalities": 1, "Others": 1, "you": 1}, "verbs": {"Treatment": 1, "鈾?And I'm gonna be 鈾?: 1, "I feel": 1, "Unsettled": 1}, "numerals": {"32": 1}}}, {"content": "The doctor told you there was nothing to worry about. Is it hard to believe?", "word_count": 18, "label": "", "sentence_structure": "Subject鈥搗erb鈥搊bject", "subjective_objective": {"answer": "Subjective", "rationale": "Because \"hard to believe\" is a patient's subjective feeling and attitude, and \"doctors tell you nothing to worry about\" is an objective fact."}, "sentence_sentiment": {"answer": "Negative", "rationale": "Because \"hard to believe\" is negative."}, "language_style": {"answer": "Tactful and warm", "rationale": "The language style of the entry is more euphemistic, not directly ordering or denying the other's feelings, but rather using a gentle, understanding tone, asking if the interviewees would be unbelievably surprised when they hear doctors say they need not be too worried. Such expressions are more likely to resonate and trust the interviewees, and make them more willing to share their real thoughts and feelings."}, "time_dimension": {"answer": "Present", "rationale": ""}, "sentence_type": "Interrogative sentence", "pos": {"nouns": {"Doctor": 1, "you": 2, "I'm worried": 1, "Things": 1}, "verbs": {"Tell": 1, "Yes": 1, "Fear": 1}, "adjectives": {"Hard": 1, "Yeah": 1}, "adverbs": {"very": 1}, "numerals": {"33": 1}, "pronouns": {"Here": 1}}}, {"content": "Have you often feared that you might be ill?", "word_count": 14, "label": "", "sentence_structure": "Subject鈥搗erb鈥搊bject", "subjective_objective": {"answer": "Subjective", "rationale": "Because fear is a subjective sentiment and attitude."}, "sentence_sentiment": {"answer": "Negative", "rationale": "Because the act of \"a fear of a serious illness\" in the entry is negative, expressing concern and anxiety."}, "language_style": {"answer": "Calm and direct", "rationale": ""}, "time_dimension": {"answer": "Present", "rationale": ""}, "sentence_type": "Interrogative sentence", "pos": {"nouns": {"Problem": 1, "- Oh, my God": 1}, "verbs": {"I'm worried": 1, "Okay": 1}, "adverbs": {"Regular": 1}, "numerals": {"34": 1}}}, {"content": "Did you sleep well?", "word_count": 5, "label": "", "sentence_structure": "Subject鈥損redicate", "subjective_objective": {"answer": "Subjective", "rationale": "Because sleep quality is an individual's subjective feeling."}, "sentence_sentiment": {"answer": "Neutral", "rationale": "Because the entry does not have any emotional color."}, "language_style": {"answer": "Tactful and warm", "rationale": "The language style of the entry is euphemism. It asks the respondents whether they are sleeping well, rather than asking \"How are you sleeping well.\" This expression is more acceptable to the respondents and facilitates the smooth running of the investigation."}, "time_dimension": {"answer": "Present", "rationale": "The entry asks the person who was tested for his or her present sleep, without reference to past or future behaviour or mentalities."}, "sentence_type": "Interrogative sentence", "pos": {"nouns": {"you": 1, "Sleep": 1, "Okay": 1}, "verbs": {"Okay": 1}, "adverbs": {"- You're not": 1}, "numerals": {"35": 1}}}, {"content": "When you get angry, do you prefer not to be able to make it?", "word_count": 17, "label": "", "sentence_structure": "Subject鈥搗erb鈥搊bject", "subjective_objective": {"answer": "Subjective", "rationale": "Because \"preferance\" is a term that describes the mental state and attitude of the patient, and is a subjective examination."}, "sentence_sentiment": {"answer": "Negative", "rationale": "Because \"suffer from doing it\" is negative, it means depression, not release."}, "language_style": {"answer": "Calm and direct", "rationale": "The entry is based on a specific situation and directly asks the respondent about his or her tendency to face such a situation, which is relatively calm and direct, with little linguistic reticulation or emotional rendering."}, "time_dimension": {"answer": "Present", "rationale": "The entry asks about the testee's tendency to be angry at the moment, the keywords being \"when you are angry\" and \"prefer,\" which point to the present, immediate behaviour or mentality."}, "sentence_type": "Interrogative sentence", "pos": {"nouns": {"you": 2, "Fan": 1, "Gas": 1, "Time": 1, "Preferative": 1}, "verbs": {"When": 1, "Hold on": 1, "Let's go": 1, "Do it": 1, "Come out": 1, "Prefer": 1}, "adverbs": {"not": 1}, "numerals": {"36": 1}, "classifiers": {"When": 1}, "pronouns": {"you": 2}}}, {"content": "Do you often think you'll suddenly fall ill?", "word_count": 13, "label": "", "sentence_structure": "Subject鈥搗erb鈥搊bject", "subjective_objective": {"answer": "Subjective", "rationale": "Because \"often\" is a subjective adjective, which means that the patient feels and experiences himself. And \"whom he suddenly falls ill\" is both a reflection and an imagination of the patient. So the point of this questionnaire is subjective."}, "sentence_sentiment": {"answer": "Negative", "rationale": "Because the act of \"sudden sickness\" in the entry is negative."}, "language_style": {"answer": "Calm and direct", "rationale": "The entry is a direct question, asking whether the person surveyed is often worried about his or her sudden illness."}, "time_dimension": {"answer": "Present", "rationale": "The keyword \"often\" means it's a customary act, \"sudden sickness\" is a matter to be done, so the entry is about time dimension right now."}, "sentence_type": "Interrogative sentence", "pos": {"nouns": {"you": 1, "Suddenly": 1, "You're sick": 1}, "verbs": {"Yes": 1, "Present": 1, "You're sick": 1}, "adverbs": {"Regular": 1}, "numerals": {"37": 1}}}, {"content": "38. If a disease is brought to your attention (e.g. by radio, television, newspapers or from someone you know, hear), do you fear that you will suffer from it?", "word_count": 44, "label": "", "sentence_structure": "Subject鈥搗erb鈥搊bject", "subjective_objective": {"answer": "Subjective", "rationale": "The reason is that the disease is a subjective emotional response."}, "sentence_sentiment": {"answer": "Negative", "rationale": "The article is intended to be couched in negative emotional expressions such as anxiety, tension, etc."}, "language_style": {"answer": "Calm and direct", "rationale": "The entry asks if the other person, after receiving information about a disease, fears that he or she will suffer from it."}, "time_dimension": {"answer": "Past", "rationale": "The entries contain terms such as \"if\" , \"to your attention\" , \"to see\" , \"to hear\" , which point to events that have occurred in the past and therefore to the past."}, "sentence_type": "Interrogative sentence", "pos": {"nouns": {"Diseases": 1, "Attention": 1, "Radio": 1, "Television": 1, "Newspapers": 1, "person": 1, "Patient": 1}, "verbs": {"Cause": 1, "Yeah": 1, "Yes": 1, "I'm worried": 1, "Patient": 1}, "adverbs": {"If": 1, "Like": 1}, "numerals": {"38": 1}, "pronouns": {"you": 1}}}, {"content": "Do you feel that someone has not paid much attention to your illness?", "word_count": 15, "label": "", "sentence_structure": "Subject鈥搗erb鈥搊bject", "subjective_objective": {"answer": "Subjective", "rationale": "Because \"the feeling\" means the patient's perception or feeling, it's seen from the patient's subjective perspective."}, "sentence_sentiment": {"answer": "Negative", "rationale": "Because the attitude of \"sickness is not taken seriously\" in the entries is negative."}, "language_style": {"answer": "Calm and direct", "rationale": "The entry is more straightforward, has no excessive rhetorical or emotional rendering, is more objective and aims to ask the respondents accurately and succinctly about the assessment of the attitude of the medical institutions to their services."}, "time_dimension": {"answer": "Present", "rationale": "In this entry, both \"you\" and \"think\" refer to the current behavior or state, and \"someone does not take your illness seriously\" is asking the testee what he feels at the moment. Thus, the time dimension of the article is now judged."}, "sentence_type": "Interrogative sentence", "pos": {"nouns": {"you": 2, "person": 1, "Diseases": 1, "Value": 1}, "verbs": {"I think": 1, "Not enough": 1, "Value": 1}, "adverbs": {"Is it possible": 1}, "numerals": {"39": 1}}}, {"content": "Are you disturbed by your appearance or physical condition?", "word_count": 14, "label": "", "sentence_structure": "Subject鈥搗erb鈥搊bject", "subjective_objective": {"answer": "Subjective", "rationale": "The feeling and perception of the distress is the patient ' s own, and is therefore subjective."}, "sentence_sentiment": {"answer": "Negative", "rationale": "Because the attitude of \"uneasy\" in this entry is negative."}, "language_style": {"answer": "Tactful and warm", "rationale": "The language style of the entry is euphemistic, and it guides the interviewee to express his feelings in a way that is not directly ordered or criticized. At the same time, the term \"uneasy\" is used to reflect understanding and caring for the interviewee, which is more friendly and comfortable."}, "time_dimension": {"answer": "Present", "rationale": "The \"uneasy\" in the entry indicates the state of emotion, and therefore the judgement is now."}, "sentence_type": "Interrogative sentence", "pos": {"nouns": {"Face": 1, "Body": 1, "Unsettled": 1}, "verbs": {"I feel": 1, "Because": 1, "And": 1, "you": 1}, "numerals": {"40": 1}}}, {"content": "Are there many different symptoms that disturb you?", "word_count": 12, "label": "", "sentence_structure": "Subject鈥搗erb鈥搊bject", "subjective_objective": {"answer": "Subjective", "rationale": "Because \"Is there a difference in symptoms that bother you\" is the patient's own subjective feelings and experiences."}, "sentence_sentiment": {"answer": "Negative", "rationale": "Because \"Does there be many different symptoms bothering you?\" The phrase describes a negative state of affairs that indicates that the person under investigation may be experiencing physical or psychological discomfort."}, "language_style": {"answer": "Calm and direct", "rationale": "The entry is more calm and straightforward in its language, does not have too many emotional renderings or rhetorical techniques, and asks the respondents in a concise manner whether they have multiple symptoms that bother them."}, "time_dimension": {"answer": "Present", "rationale": "The entry uses the term \"with\" and \"with many different symptoms\" which are the terms of the current state, and it can therefore be judged as asking the person who was tested what is now."}, "sentence_type": "Interrogative sentence", "pos": {"nouns": {"Symptoms": 1, "you": 1}, "verbs": {"Disturbing": 1}, "adjectives": {"Many": 1, "Different": 1}, "numerals": {"41": 1}}}, {"content": "Do you often try to explain your feelings to others?", "word_count": 15, "label": "", "sentence_structure": "Subject鈥搗erb鈥搊bject", "subjective_objective": {"answer": "Subjective", "rationale": "Because trying to explain to others what they feel is a subjective act that reflects the individual ' s thoughts and attitudes."}, "sentence_sentiment": {"answer": "Positive", "rationale": "Because the act of \"trying to explain feelings to others\" is positive and shows that individuals want to communicate and communicate effectively with others."}, "language_style": {"answer": "Calm and direct", "rationale": "The language style of the entry is more direct, concise, not overly accommodative or emotional, and is an objective and rational expression.\""}, "time_dimension": {"answer": "Present", "rationale": ""}, "sentence_type": "Interrogative sentence", "pos": {"nouns": {"you": 1, "Entry": 1, "Questionnaire": 1, "Feel": 1}, "verbs": {"Regular": 1, "Try": 1, "In": 1, "Explanation": 1, "be": 1, "- You're not": 1}, "adverbs": {"very": 1}, "numerals": {"42": 1}}}, {"content": "Is there any contradiction in your family?", "word_count": 9, "label": "", "sentence_structure": "Subject鈥損redicate", "subjective_objective": {"answer": "Subjective", "rationale": "Because \"controversial\" is usually a subjective feeling or perception, different people may have different answers and interpretations."}, "sentence_sentiment": {"answer": "Negative", "rationale": "Because the word \"controversial\" in the entries is usually negative emotional."}, "language_style": {"answer": "Calm and direct", "rationale": "The language style of the entry is calm and straightforward. It asks the respondents in a concise and clear manner whether there is any contradiction in their family, and does not use any rhetorical or emotional language to guide the respondents to answer questions."}, "time_dimension": {"answer": "Present", "rationale": "The entry asks the person ' s current family situation and the time dimension is now judged."}, "sentence_type": "Interrogative sentence", "pos": {"nouns": {"you": 1, "Home": 1, "Paradoxious": 1}, "verbs": {"have": 1}, "adverbs": {"What": 1}, "numerals": {"43": 1}}}, {"content": "Do you think there are things that are psychologically relevant to you?", "word_count": 15, "label": "", "sentence_structure": "Subject鈥搗erb鈥搊bject", "subjective_objective": {"answer": "Subjective", "rationale": "Because this is the patient's subjective view of whether certain things are psychologically relevant."}, "sentence_sentiment": {"answer": "Neutral", "rationale": "The entry is not explicitly expressing a positive or negative emotional tendency, but rather is asking the views and perceptions of the person under investigation. The entry can therefore be considered neutral."}, "language_style": {"answer": "Tactful and warm", "rationale": "The entry uses a euphemism, without direct orders or criticism of the other party ' s views, but rather a moderate way of leading the interviewees to think and express their views, a language style that is more acceptable and supportive to the interviewees."}, "time_dimension": {"answer": "Present", "rationale": "The entry asks the subject's current subjective view, \"You think\" indicates that it is a personal perception and judgment, so the temporal dimension is now."}, "sentence_type": "Interrogative sentence", "pos": {"nouns": {"Yes": 1, "Annex": 1, "Psychological": 1}, "verbs": {"Recognition": 1, "Off": 1}, "adverbs": {"and": 1, "Some": 1}, "numerals": {"44": 1}}}, {"content": "How's your diet?", "word_count": 8, "label": "", "sentence_structure": "Subject鈥損redicate", "subjective_objective": {"answer": "Subjective", "rationale": "Because the questionnaire entry is asking about the patient's personal feelings and experiences, not the objective facts that are observed. For example, the patient may answer \"I eat well\" or \"I think I eat badly,\" which is based on their subjective feelings. So, the questionnaire entry is subjective in its perspective."}, "sentence_sentiment": {"answer": "Neutral", "rationale": "Because the entry does not involve any emotional colour, it merely asks about the other party ' s diet."}, "language_style": {"answer": "Tactful and warm", "rationale": "The entry is more euphemistic, relatively gentle, and is not too direct or command-style."}, "time_dimension": {"answer": "Present", "rationale": "The entry enquires about the current diet of the person tested, does not address past or future behaviour or mentality and is therefore present."}, "sentence_type": "Interrogative sentence", "pos": {"nouns": {"you": 1, "Diet": 1, "In terms of": 1, "Okay": 1}, "verbs": {"And": 1, "- You're not": 1}, "numerals": {"45": 1}}}, {"content": "Is poor health the greatest difficulty in your life?", "word_count": 15, "label": "", "sentence_structure": "Subject鈥損redicate", "subjective_objective": {"answer": "Objective", "rationale": "Because \"poor health\" is the objective condition and experience of the patient's life, whether it is considered the most difficult depends on the patient's subjective attitude."}, "sentence_sentiment": {"answer": "Negative", "rationale": "Because the problem of \"poor health\" in the entry suggests a negative situation."}, "language_style": {"answer": "Calm and direct", "rationale": "The entry is in a direct and clear tone and asks the interviewees whether they consider health problems to be the most troubling of their lives, with a clear and concise language, and without excessive rhetoric or emotional rendering."}, "time_dimension": {"answer": "Present", "rationale": "The entry is a question about the current living situation and feelings of the person being tested, and therefore the time dimension is now judged."}, "sentence_type": "Interrogative sentence", "pos": {"nouns": {"Health": 1, "Problem": 1}, "verbs": {"be": 1, "I met him": 1}, "adjectives": {"Max": 1, "Life": 1}, "numerals": {"46": 1, "Yours": 1, "Medium": 1}, "pronouns": {"you": 1}}}, {"content": "Do you find yourself vulnerable?", "word_count": 11, "label": "", "sentence_structure": "Subject鈥損redicate", "subjective_objective": {"answer": "Subjective", "rationale": "Because \"sorture\" is a subjective emotional state, which is viewed from the patient's subjective point of view."}, "sentence_sentiment": {"answer": "Negative", "rationale": "Because the emotional state of \"easy\" in the entries is negative."}, "language_style": {"answer": "Tactful and warm", "rationale": "The entry uses a gentle and inquisitive tone rather than a direct statement or order, which is more personal and interactive, and which is more resonant with the interviewee and more willing to share his real feelings. Moreover, \"Easy\" is a more neutral description, with no over-heated emotional overtones, which make the interviewee more natural in answering questions."}, "time_dimension": {"answer": "Present", "rationale": "The keyword \"discovering itself easily sad\" suggests that this is the perception and description of the emotional state of the subject at the moment, and that it is the time dimension of the present."}, "sentence_type": "Interrogative sentence", "pos": {"nouns": {"you": 1, "self": 1, "Sorry": 1}, "verbs": {"Found": 1, "Easy": 1}, "adverbs": {"very": 1}, "numerals": {"47": 1}}}, {"content": "48. Do you even wonder about the details that most people seem to have?", "word_count": 24, "label": "", "sentence_structure": "Subject鈥搗erb鈥搊bject", "subjective_objective": {"answer": "Subjective", "rationale": "Because the entry examines patients ' perceptions and feelings about certain things and is subjective."}, "sentence_sentiment": {"answer": "Negative", "rationale": "Because the \"fearing or even fussing\" act in the entry is negative."}, "language_style": {"answer": "Tactful and warm", "rationale": ""}, "time_dimension": {"answer": "Present", "rationale": ""}, "sentence_type": "Interrogative sentence", "pos": {"nouns": {"Details": 2, "person": 1, "Trouble": 1, "It's a big deal": 1}, "verbs": {"Looks like it": 1, "Important": 1, "not": 1, "you": 1, "But": 1, "Even": 1, "It's a surprise": 1}, "adjectives": {"Most of them": 1, "of": 2, "Both": 1, "Small": 1}, "adverbs": {"It's important": 1, "not": 1, "But": 1, "Even": 1, "Large": 1}, "numerals": {"48": 1}, "classifiers": {"individual": 1}, "pronouns": {"you": 1}}}, {"content": "Are you always a patient of cooperation?", "word_count": 11, "label": "", "sentence_structure": "Subject鈥損redicate", "subjective_objective": {"answer": "Subjective", "rationale": "Because \"cooperation\" is a subjective attitude or behaviour that reflects the patient's perception and behaviour of working with a doctor or other medical practitioner."}, "sentence_sentiment": {"answer": "Positive", "rationale": "Because \"a cooperating patient\" is a positive act, it means that the patient is willing to cooperate with the treatment and the doctor's work."}, "language_style": {"answer": "Tactful and warm", "rationale": "The entry uses a more euphemistic language style, avoiding direct, hard-line attitudes or language."}, "time_dimension": {"answer": "Present", "rationale": "\"Are you always a cooperating patient?\" The phrase \"you\" in it indicates that it is a question of asking the testee about his personal characteristics or behaviour, and of asking about the situation. At the same time, \"a cooperating patient\" is also an adjective term used to describe the character or behaviour of the testee, so the entry can be judged to be the current state of affairs."}, "sentence_type": "Interrogative sentence", "pos": {"nouns": {"Patients": 1, "Cooperation": 1}, "verbs": {"be": 1}, "adverbs": {"Always": 1}, "numerals": {"49": 1}}}, {"content": "Do you always suffer from serious illness symptoms?", "word_count": 13, "label": "", "sentence_structure": "Subject鈥搗erb鈥搊bject", "subjective_objective": {"answer": "Objective", "rationale": "Because the symptoms of serious illness are a physical condition that is objectively present."}, "sentence_sentiment": {"answer": "Negative", "rationale": "Because the description \"problems of severe illness\" is negative."}, "language_style": {"answer": "Calm and direct", "rationale": "The entry is based on a direct inquiry into whether the person interviewed is often seriously ill, is not using excessive rhetorical or emotional rendering, and is more objective. This expression is more able to capture the actual situation and problems of the person interviewed and allows researchers to analyse accurate data."}, "time_dimension": {"answer": "Past", "rationale": ""}, "sentence_type": "Interrogative sentence", "pos": {"nouns": {"Symptoms of serious illness": 1, "Trouble": 1}, "verbs": {"Yes": 1, "Impact": 1, "Trouble": 2}, "numerals": {"50": 1}}}, {"content": "Are you angry?", "word_count": 8, "label": "", "sentence_structure": "Subject鈥損redicate", "subjective_objective": {"answer": "Subjective", "rationale": "Because anger and anger are emotional experiences, subjective feelings and attitudes of patients."}, "sentence_sentiment": {"answer": "Negative", "rationale": "Because \"Anger, Frenzies\" in the entry is a negative emotional expression."}, "language_style": {"answer": "Tactful and warm", "rationale": "The language style of the entry is more euphemistic and warm, rather than asking whether the other side is often angry or angry, it uses a more moderate and understanding tone to guide the respondents to answer questions. This expression makes the respondents feel more comfortable and comfortable, and more likely to share their real feelings."}, "time_dimension": {"answer": "Present", "rationale": "The entry asks the testee's current behavior or state, i.e., \"Do you have a high degree of anger and anger\", and the time dimension is now."}, "sentence_type": "Interrogative sentence", "pos": {"nouns": {"you": 1, "Angry": 1, "- Get mad": 1}, "verbs": {"Easy": 1, "Angry": 1, "- Get mad": 1, "Do it": 1}, "adverbs": {"Easy": 1}, "numerals": {"51": 1}}}, {"content": "Are you in trouble with your job?", "word_count": 8, "label": "", "sentence_structure": "Subject鈥損redicate", "subjective_objective": {"answer": "Objective", "rationale": "Because the problem of having problems at work is a factual one, it can be directly observed and verified and does not belong to the patient ' s subjective attitude or feelings."}, "sentence_sentiment": {"answer": "Negative", "rationale": "Because the expression \"work troubles\" in the entry is negative, it suggests that the interviewees may encounter difficulties or challenges in their work."}, "language_style": {"answer": "Calm and direct", "rationale": "The entry is more straightforward in language style, is not over-modified or istrophized, and is asked whether the person surveyed has encountered problems or difficulties in the course of his or her work."}, "time_dimension": {"answer": "Present", "rationale": "The entry is for questioning whether the person tested has problems or problems with the current work, and the time dimension is now judged."}, "sentence_type": "Interrogative sentence", "pos": {"nouns": {"Work": 1, "Trouble": 1}, "verbs": {"have": 1}, "numerals": {"52": 1}}}, {"content": "Do you like to keep your feelings in your own heart?", "word_count": 14, "label": "", "sentence_structure": "Subject鈥搗erb鈥搊bject", "subjective_objective": {"answer": "Subjective", "rationale": "Because \"like to leave feelings in your heart\" is a subjective attitude or emotional expression."}, "sentence_sentiment": {"answer": "Negative", "rationale": "Because the \"just stay in your own mind\" act in the entry is negative, which means that you are not willing to share your feelings with others, and may affect emotional disclosure and the development of human relations."}, "language_style": {"answer": "Calm and direct", "rationale": "The entry is based on a straightforward approach to asking the respondents whether they prefer to keep their feelings deep inside, without excessive rhetorical or emotional rendering. This expression is more objective, calm and direct."}, "time_dimension": {"answer": "Past", "rationale": "So the answer is \"now\"."}, "sentence_type": "Interrogative sentence", "pos": {"nouns": {"Feel": 1, "Heart": 1}, "verbs": {"Yeah": 1, "Stay": 1, "in": 1, "self": 1}, "adjectives": {"Only": 1}, "numerals": {"53": 1}}}, {"content": "54. Do you often get depressed?", "word_count": 8, "label": "", "sentence_structure": "Subject鈥損redicate", "subjective_objective": {"answer": "Subjective", "rationale": "Because emotions are the feelings and experiences of the patient himself, they are subjective."}, "sentence_sentiment": {"answer": "Negative", "rationale": "Because \"frustration\" in the entries is a negative word, implying a negative emotional state."}, "language_style": {"answer": "Calm and direct", "rationale": "It asked whether the other side often felt depressed and did not have too many rhetorical and emotional accents, and therefore the language style was more calm and direct."}, "time_dimension": {"answer": "Present", "rationale": ""}, "sentence_type": "Interrogative sentence", "pos": {"nouns": {"Emotion": 1, "Declining": 1}, "verbs": {"you": 1, "Regular": 1, "Yes": 1}, "adverbs": {"- You're not": 1}, "numerals": {"54": 1}}}, {"content": "55. As long as you're well, all your worries will be over, won't they?", "word_count": 19, "label": "", "sentence_structure": "Subject鈥搗erb鈥搊bject", "subjective_objective": {"answer": "Subjective", "rationale": "This expression is an expression of the subjective perception and assumption of the patient between good health and the disappearance of the pain."}, "sentence_sentiment": {"answer": "Positive", "rationale": "Because the words \"well well\" and \"all the troubles are over\" in the entries are positive, expressing positive life attitudes and beliefs."}, "language_style": {"answer": "Calm and direct", "rationale": ""}, "time_dimension": {"answer": "Past", "rationale": "The entry asks the testee's views on the relationship between good health and the disappearance of the trouble, and the temporal dimension is a past.\""}, "sentence_type": "Interrogative sentence", "pos": {"nouns": {"Body": 1, "All": 1, "Trouble": 1}, "verbs": {"Past": 1, "be": 1}, "numerals": {"55": 1, "Both": 1}, "pronouns": {"you": 1}}}, {"content": "Are you more troublesome than anyone else?", "word_count": 10, "label": "", "sentence_structure": "Subject鈥搗erb鈥搊bject", "subjective_objective": {"answer": "Subjective", "rationale": "Because \"resistence\" is a subjective assessment and feeling that reflects the subjective perception and experience of the patient of his or her emotions and behaviour."}, "sentence_sentiment": {"answer": "Negative", "rationale": "Because the attitude of \"suffering\" in the entries is negative."}, "language_style": {"answer": "Calm and direct", "rationale": "The basis for the judgement: 1. The problem is expressed in a relatively concise manner, without the use of excessive rhetorical or emotional terms; 2. The question is whether the interviewee has an anger-prone situation in a straightforward manner, which is more objective and inclement."}, "time_dimension": {"answer": "Present", "rationale": "The entry is for the testee's own characteristics and the time dimension is now judged."}, "sentence_type": "Interrogative sentence", "pos": {"nouns": {"you": 1, "person": 1, "It's annoying": 1}, "verbs": {"Easy": 1, "By": 1}, "adverbs": {"- Yeah": 1}, "numerals": {"56": 1}}}, {"content": "57. (Do you think) Could your symptoms be a source of distress (in a mood of unease)?", "word_count": 21, "label": "", "sentence_structure": "Subject鈥搗erb鈥搊bject", "subjective_objective": {"answer": "Subjective", "rationale": "Because this is the patient ' s own view and feelings, it is a subjective examination."}, "sentence_sentiment": {"answer": "Positive", "rationale": "It involves attention to and self-awareness of the mental health of the individual."}, "language_style": {"answer": "Tactful and warm", "rationale": "The entry is more euphemistic, using the way of questioning rather than direct statements or orders. At the same time, the term \"possibly\" is used to express respect and understanding of the person being investigated, without putting undue pressure on them. Such a mild tone is more likely to resonate with the person being investigated and to promote their willingness to cooperate in the completion of the questionnaire."}, "time_dimension": {"answer": "Present", "rationale": "Now the entry asks the testee about his current symptoms, and it's now judged. And \"You think\" means that this is a subjective question that requires the testee to answer it himself."}, "sentence_type": "Interrogative sentence", "pos": {"nouns": {"Symptoms": 1, "Trouble": 1, "The mood": 1, "鈾?Unsweet 鈾?: 1}, "verbs": {"be": 1, "Cause": 1}, "adverbs": {"EMPTY": 2}, "numerals": {"57": 1}, "classifiers": {"(You think)": 1, "锛?: 1, "锛?: 3}, "pronouns": {"Yours": 1}}}, {"content": "Is it easy for you to let others know that you are not happy with them?", "word_count": 22, "label": "", "sentence_structure": "Subject鈥搗erb鈥搊bject", "subjective_objective": {"answer": "Subjective", "rationale": "Because this is the subjective attitude of the patient in the particular circumstances."}, "sentence_sentiment": {"answer": "Negative", "rationale": "Because the entry \"Let's let others know you're not happy with them\" is negative."}, "language_style": {"answer": "Calm and direct", "rationale": "The entry is presented in a more direct form of question, asking whether it is easy for respondents to inform others of their dissatisfaction with them, and is of a straightforward, calm type in the language style."}, "time_dimension": {"answer": "Present", "rationale": "The word \"sure\" in \"sure\" means sexual intercourse, and the \"know\" and \"dissatisfaction\" action takes place after the main phrase, whereby the person tested must first inform others of their dissatisfaction and then answer, which suggests that the entry is a question of the person being tested's current behaviour or attitude and is therefore a current one."}, "sentence_type": "Interrogative sentence", "pos": {"nouns": {"others": 1, "Dissatisfied": 1}, "verbs": {"Jean": 1, "Yes": 2, "be": 1, "Easy": 1, "Do it": 1}, "adverbs": {"you": 2}, "numerals": {"58": 1}}}, {"content": "Are you having trouble relaxing?", "word_count": 6, "label": "", "sentence_structure": "Subject鈥損redicate", "subjective_objective": {"answer": "Subjective", "rationale": "Because \"hard to relax\" is a psychological experience or experience, and it's an assessment of the patient's subjective perspective."}, "sentence_sentiment": {"answer": "Negative", "rationale": "Because \"hard to relax\" is negative."}, "language_style": {"answer": "Tactful and warm", "rationale": ""}, "time_dimension": {"answer": "Present", "rationale": "The entry asks about the current situation of the testees, namely, whether they are having difficulty relaxing. The keyword \"hard to relax\" means that it is a survey of personal feelings and experiences, without addressing the time dimension of the past or the future. So the answer is now."}, "sentence_type": "Interrogative sentence", "pos": {"nouns": {"Questionnaire": 1, "Entry": 1, "you": 1, "Relax": 1}, "verbs": {"Hard": 1}, "adverbs": {"- You're not": 1}, "numerals": {"59": 1}}}, {"content": "Have you any personal problems that are not caused by illness?", "word_count": 14, "label": "", "sentence_structure": "Subject鈥搗erb鈥搊bject", "subjective_objective": {"answer": "Subjective", "rationale": "Because \"personal distress\" is usually the patient's subjective feeling or emotional state."}, "sentence_sentiment": {"answer": "Neutral", "rationale": "Because the entry does not have any emotional color."}, "language_style": {"answer": "Tactful and warm", "rationale": "The entry uses a gentle and caring tone to ask the other about personal distress caused by non-disease, and to express it more in a more personal and caring manner. At the same time, the article is more open-ended and encourages respondents to express their feelings and thoughts more freely."}, "time_dimension": {"answer": "Present", "rationale": "The keywords are \"you have\" and \"non-disease-induced personal distress\", which point to the current situation and do not have a clear time reference. So the entry can be judged as it is."}, "sentence_type": "Interrogative sentence", "pos": {"nouns": {"Diseases": 1, "Personal": 1, "Trouble": 1}, "verbs": {"Cause": 1, "have": 1}, "adjectives": {"not": 1}, "numerals": {"60": 1}}}, {"content": "Do you often lose patience with others?", "word_count": 11, "label": "", "sentence_structure": "Subject鈥搗erb鈥搊bject", "subjective_objective": {"answer": "Subjective", "rationale": "Because \"desatisfaction\" is an emotional response, a subjective attitude and experience of patients."}, "sentence_sentiment": {"answer": "Negative", "rationale": "Because \"desatisfaction\" is negative."}, "language_style": {"answer": "Calm and direct", "rationale": ""}, "time_dimension": {"answer": "Present", "rationale": ""}, "sentence_type": "Interrogative sentence", "pos": {"nouns": {"you": 1, "person": 1, "Lose": 1, "Patience": 1}, "verbs": {"Regular": 1, "to": 1, "Lose": 1}, "adverbs": {"Regular": 1}, "numerals": {"61": 1}}}, {"content": "Is it difficult for you to express your personal feelings to others?", "word_count": 18, "label": "", "sentence_structure": "Subject鈥搗erb鈥搊bject", "subjective_objective": {"answer": "Subjective", "rationale": "Because it is the subjective feelings and attitudes of the patient that are examined."}, "sentence_sentiment": {"answer": "Negative", "rationale": "Because the \"expression of difficulties\" in the entry is negative."}, "language_style": {"answer": "Calm and direct", "rationale": "The language style of the entry is relatively straightforward and concise, and there is no over-modified word or emotional rendering, but rather questions in an objective and clear manner. This style is more easily understood and answered by the respondents to the questionnaire.\""}, "time_dimension": {"answer": "Past", "rationale": "The entry asks about the experience and feelings of the testee in the past, i.e. whether he or she has found it difficult to express his or her feelings to others in the past. The temporal dimension is therefore a matter of the past."}, "sentence_type": "Interrogative sentence", "pos": {"nouns": {"Questionnaire Entry": 1, "Personal": 1, "Feel": 1}, "verbs": {"To others": 1, "Difficulties": 1, "Say it": 1}, "adjectives": {"Yours": 1, "of": 2, "you": 1}, "numerals": {"62": 1}, "pronouns": {"you": 2}}}]}

{"questionnaire_title": "SCI-90", "questionnaire_category": "Mental health", "item_count": 90, "emotion_shift_overall": "From negative to positive", "language_style_overall": "Calm and direct", "items": [{"content": "A headache.", "word_count": 2, "label": "", "sentence_structure": "Subject鈥搗erb鈥揷omplement", "subjective_objective": {"answer": "Objective", "rationale": "The reason for the headache is the objective fact of the patient's experience."}, "sentence_sentiment": {"answer": "Negative", "rationale": "Because \"headache\" is a symptom of physical discomfort, it is usually considered negative."}, "language_style": {"answer": "Calm and direct", "rationale": "The topic was in plain language, asking the respondents directly about headaches, not too many rhetorical and emotional renderings, and was more straightforward and concise."}, "time_dimension": {"answer": "Present", "rationale": "The entry asks if the testee is currently experiencing headaches and the time dimension is now judged."}, "sentence_type": "Interrogative sentence", "pos": {"nouns": {"Headache": 1}}}, {"content": "Two is sensitive, and they're not so strong.", "word_count": 9, "label": "", "sentence_structure": "Subject鈥搗erb鈥揷omplement", "subjective_objective": {"answer": "Subjective", "rationale": "Because \"sensitization\" and \"instantness\" are the inner feelings and experiences of patients and are subjective."}, "sentence_sentiment": {"answer": "Negative", "rationale": "Because the entries \"sensitized\" and \"insolent\" both express negative emotional attitudes."}, "language_style": {"answer": "Tactful and warm", "rationale": "The entry uses a more euphemistic language style, without direct criticism or criticism, but rather expresses concern and understanding of each other鈥檚 emotions in a question-and-answer manner."}, "time_dimension": {"answer": "Present", "rationale": "The entry describes the current state or feeling of the testee, and both \"two d茅j脿 vu\" and \"insolent\" describe the state of the immediate mood, so it can be judged as now."}, "sentence_type": "Interrogative sentence", "pos": {"nouns": {"You're a bit of a sane": 1, "Heart": 1}, "verbs": {"Medium": 1, "Pass": 1, "Fall": 1, "Minori": 1}, "adjectives": {"Not real": 1}}}, {"content": "There's no need to think or say anything in the mind.", "word_count": 15, "label": "", "sentence_structure": "Subject鈥搗erb鈥揷omplement", "subjective_objective": {"answer": "Subjective", "rationale": "Because this is the patient ' s subjective experience, it is a description of his thoughts and feelings."}, "sentence_sentiment": {"answer": "Negative", "rationale": "Because the phenomenon of \"unnecessary thinking or words \" in the entries is usually negative and may affect people's thinking and emotions."}, "language_style": {"answer": "Calm and direct", "rationale": "\"Calm down straight.\""}, "time_dimension": {"answer": "Present", "rationale": "According to the words and context of \"in the mind\" and \"in the mind\", the entry describes the current state or feeling of the person being tested and therefore the temporal dimension of the present."}, "sentence_type": "Exclamatory sentence", "pos": {"nouns": {"Brain": 1, "Thoughts": 1, "Words": 1, "Rotate": 1}, "verbs": {"have": 1, "No, I don't": 1, "Rotate": 1}, "adverbs": {"Unnecessary": 1}, "numerals": {"3": 1}}}, {"content": "Four heads fainted or fainted.", "word_count": 5, "label": "", "sentence_structure": "Subject鈥搗erb鈥揷omplement", "subjective_objective": {"answer": "Objective", "rationale": "The fact that the patient is suffering from a faint or fainting experience."}, "sentence_sentiment": {"answer": "Negative", "rationale": "Because the phenomenon of \"heads faint or fainting\" is usually associated with physical discomfort, disease, etc., it is somewhat negative."}, "language_style": {"answer": "Calm and direct", "rationale": "The entry describes possible symptoms in a direct and clear language, is not over-modified or emotionally rendered, and is more practical and objective."}, "time_dimension": {"answer": "Past", "rationale": "The entry asks whether the person tested has experienced \"dim or faint\" in the past, and therefore the temporal dimension is judged to be past."}, "sentence_type": "Exclamatory sentence", "pos": {"nouns": {"Headlight": 1, "- I'm fainted": 1}, "numerals": {"4": 1}}}, {"content": "5 Declining interest in heterosexuality", "word_count": 8, "label": "", "sentence_structure": "Subject鈥搗erb鈥揷omplement", "subjective_objective": {"answer": "Subjective", "rationale": "Because the reduction of heterosexual interest is a matter of the patient ' s own feelings and judgement and falls within the realm of subjective attitudes."}, "sentence_sentiment": {"answer": "Negative", "rationale": "Because the \"reducing interest in opposite sex\" in the entry is negative."}, "language_style": {"answer": "Calm and direct", "rationale": "The entry expresses direct and explicit queries about the decline in heterosexual interest, is not overly emotional or rhetorical and is more objective and incriminating."}, "time_dimension": {"answer": "Present", "rationale": ""}, "sentence_type": "Negative declarative sentence", "pos": {"nouns": {"Heterosexual": 2, "Interest": 1, "Declining": 1}, "verbs": {"to": 1}, "numerals": {"5": 1}}}, {"content": "Six to blame.", "word_count": 7, "label": "", "sentence_structure": "Subject鈥搗erb鈥搊bject", "subjective_objective": {"answer": "Subjective", "rationale": "Because blame is simply a subjective attitude or opinion, not an objective fact or result."}, "sentence_sentiment": {"answer": "Negative", "rationale": "Because \"acquiring for full responsibility\" in the entry is a negative act, which may have negative consequences."}, "language_style": {"answer": "Calm and direct", "rationale": "The entry is a straightforward question to whether respondents often over-require or over-require others, have a clear language and do not use excessive qualifiers or emotional expressions."}, "time_dimension": {"answer": "Present", "rationale": "According to the sentence entered, \"6 Rebukes the others\" describes a customary act or attitude that is referred to in the present state of affairs and is therefore judged to be \"now\"."}, "sentence_type": "Imperative sentence", "pos": {"nouns": {"The others": 1, "Blame": 1, "Full": 1}, "verbs": {"to": 1}, "numerals": {"6": 1}}}, {"content": "Seven feels that someone else can control your mind.", "word_count": 11, "label": "", "sentence_structure": "Subject鈥揷opula鈥損redicative", "subjective_objective": {"answer": "Subjective", "rationale": "Because \"fear\" is the patient's subjective feeling and experience."}, "sentence_sentiment": {"answer": "Negative", "rationale": "Because the \"Feeling that someone can control your mind\" behavior in the entry is negative."}, "language_style": {"answer": "Tactful and warm", "rationale": "The entry is more personal and interactive in its expression of a gentle and inviting tone, rather than a direct statement or order. It also directs respondents to share their views and experiences in a first person way, making the questionnaire process more smooth and natural."}, "time_dimension": {"answer": "Present", "rationale": "The entry now asks if the person tested feels that someone else can control his or her own mind at this point in time, without addressing the time dimension of the past or the future."}, "sentence_type": "Negative declarative sentence", "pos": {"nouns": {"you": 1, "Ideas": 1}, "verbs": {"I feel": 1, "Yes": 1, "Control": 1}, "numerals": {"7": 1}, "pronouns": {"Don't": 1, "person": 1}}}, {"content": "Eight blames others for causing trouble.", "word_count": 8, "label": "", "sentence_structure": "Subject鈥搗erb鈥搊bject", "subjective_objective": {"answer": "Subjective", "rationale": "For blaming others for causing trouble is a subjective view and attitude of one, not an objective fact."}, "sentence_sentiment": {"answer": "Negative", "rationale": "Because blaming others is a negative emotional attitude."}, "language_style": {"answer": "Calm and direct", "rationale": "It asked whether the person surveyed had been overly responsible for causing trouble, had not used excessive rhetorical techniques or emotional rendering, and was more objective in tone."}, "time_dimension": {"answer": "Past", "rationale": "The three temporal dimensions given in the title were judged to mean that the act of \"criticism\" did not clearly indicate in the sentence whether it had occurred, was present or was future, and therefore the temporal dimension of the article could not be determined accurately."}, "sentence_type": "Imperative sentence", "pos": {"nouns": {"Blame": 1, "others": 1, "Trouble": 1}, "verbs": {"Manufacturing": 1}, "adverbs": {"8": 1}}}, {"content": "Nine is a big one.", "word_count": 3, "label": "", "sentence_structure": "Subject鈥搗erb鈥揷omplement", "subjective_objective": {"answer": "Subjective", "rationale": "Because \"Big Forgetful\" is a subjective feeling and experience of a human being."}, "sentence_sentiment": {"answer": "Negative", "rationale": "Because \"Big Forgetful\" in the entry means poor memory, a negative description."}, "language_style": {"answer": "Calm and direct", "rationale": "The entry directly asks whether the interviewees often forget things, do not have too many emotional or rhetorical techniques, and the language style is more direct and objective."}, "time_dimension": {"answer": "Present", "rationale": "The entry now describes the current problem or state of the testee, the keyword being \"forgettable\" and indicates that the testee has a current memory decline."}, "sentence_type": "Interrogative sentence", "pos": {"nouns": {"Forgotten": 1, "Large": 1}, "adverbs": {"very": 1}}}, {"content": "10 is worried about their clothes and their proper manners.", "word_count": 15, "label": "", "sentence_structure": "Subject鈥揷opula鈥損redicative", "subjective_objective": {"answer": "Subjective", "rationale": "Because \"fear\" is a subjective feeling and idea of a human being."}, "sentence_sentiment": {"answer": "Negative", "rationale": "Because the word \"fear\" in the entry conveys a negative sentiment that indicates that the person is worried about his or her appearance and image, and may be feeling inferior or anxious."}, "language_style": {"answer": "Tactful and warm", "rationale": "The entry uses the relatively euphemistic expression \"concern\" to express concerns about personal image, while emphasizing the importance of proper clothing and proper tact. This way avoids direct criticism or accusations, but also allows the other to learn about the problem and to stimulate reflection and improvement."}, "time_dimension": {"answer": "Present", "rationale": "The word \"fear\" in the entry indicates that it is a state of emotion, not a state of memory or of expectation of the past. Moreover, \"dressing and the proper end of the pattern\" is a term that describes the state of affairs, further confirming that the time dimension of the entry is now. So the entry is about asking the subject about the behaviour and mindset of the present."}, "sentence_type": "Imperative sentence", "pos": {"nouns": {"self": 1, "Clothing": 1, "Ceremonial": 1}, "verbs": {"I'm worried": 1, "Clean": 1, "Positive": 1}, "adverbs": {"Quite well": 1}, "numerals": {"10": 1}, "classifiers": {"individual": 1}}}, {"content": "Eleven is a little twitchy and excited.", "word_count": 7, "label": "", "sentence_structure": "Subject鈥揷opula鈥損redicative", "subjective_objective": {"answer": "Subjective", "rationale": "The mental state of the patient is the result of the anxiety and excitement that are examined from the patient ' s subjective perspective."}, "sentence_sentiment": {"answer": "Negative", "rationale": "Because \"easy\" and \"excited\" are negative emotional expressions."}, "language_style": {"answer": "Tactful and warm", "rationale": ""}, "time_dimension": {"answer": "Present", "rationale": "The entry describes the characteristics of the person being tested for the current behaviour or mentality, and therefore the temporal dimension is now judged."}, "sentence_type": "Exclamatory sentence", "pos": {"nouns": {"Trouble": 1, "Excited": 1}, "verbs": {"Easy": 1}, "numerals": {"11": 1}}}, {"content": "Twelve chest pains.", "word_count": 2, "label": "", "sentence_structure": "Subject鈥搗erb鈥揷omplement", "subjective_objective": {"answer": "Objective", "rationale": "Because chest pain is a biological phenomenon that can be measured, it is objective."}, "sentence_sentiment": {"answer": "Negative", "rationale": "Since chest pain is usually a symptom of disease, it can cause serious health problems."}, "language_style": {"answer": "Calm and direct", "rationale": "The subject of the survey directly asked the respondents whether they had chest pain, without any use of rhetorical or emotional rendering, and with a more objective and calm language style."}, "time_dimension": {"answer": "Past", "rationale": "The entry appears with a number and time phrase \"12\", indicating that this is a specific point of time, that is, the testee's experience on the twelfth day. At the same time, \" chest pain\" is a description of behaviour or state, so the time dimension of the entry is the past."}, "sentence_type": "Interrogative sentence", "pos": {"nouns": {"- Chestaches": 1}, "numerals": {"12": 1}}}, {"content": "13 Fear of an empty place or street", "word_count": 10, "label": "", "sentence_structure": "Subject鈥搗erb鈥搊bject", "subjective_objective": {"answer": "Subjective", "rationale": "Because fear is a subjective emotion and experience."}, "sentence_sentiment": {"answer": "Negative", "rationale": "Because fear is a negative emotional color."}, "language_style": {"answer": "Tactful and warm", "rationale": "The entry is more euphemistic, using the term \"fear\" but does not directly explain the extent of or possible cause for such fear, but rather engages in dialogue in a gentle and inviting tone, more personal and interactive manner."}, "time_dimension": {"answer": "Present", "rationale": "The word \"fear\" means the state of the mood, so the time dimension of this questionnaire entry is now."}, "sentence_type": "Exclamatory sentence", "pos": {"nouns": {"Places": 1, "Street": 1}, "verbs": {"Yes": 1}, "adjectives": {"Empty": 1}, "numerals": {"13": 1}}}, {"content": "14 feels less energy and slower activity.", "word_count": 13, "label": "", "sentence_structure": "Subject鈥揷opula鈥損redicative", "subjective_objective": {"answer": "Subjective", "rationale": "Because \"fear,\" \"dreak\" and \"slow\" are subjective feelings and experiences of patients."}, "sentence_sentiment": {"answer": "Negative", "rationale": "Because \"dreak\" and \"slow\" in the entries are negative terms for describing the state of health."}, "language_style": {"answer": "Calm and direct", "rationale": "The entry expresses in a concise manner the phenomenon of asking the other side whether they feel less energy and slower activity, and the language style is more calm and straightforward, without excessive rhetoric or emotional rendering."}, "time_dimension": {"answer": "Present", "rationale": "According to the title, \"14 feels less energy and slower activity\" means the subjective feeling and experience of the subject, and \"nerdness\" and \"activity slow\" are also acts or states currently under way, so it can be judged that the entry is the present behaviour or mentality of the person being questioned."}, "sentence_type": "Affirmative declarative sentence", "pos": {"nouns": {"- The energy": 1, "Activities": 1}, "verbs": {"Down": 1, "Slow down": 1}, "numerals": {"14": 1}}}, {"content": "15 wants to end their lives.", "word_count": 8, "label": "", "sentence_structure": "Subject鈥搗erb鈥搊bject", "subjective_objective": {"answer": "Subjective", "rationale": "Because \"want to end your life\" is a subjective expression of thought and emotion."}, "sentence_sentiment": {"answer": "Negative", "rationale": "Because the \"end of your life\" act in the entry is negative."}, "language_style": {"answer": "Calm and direct", "rationale": "The language style of the entry is more calm, direct, less over-modified or emotional, and more focused on the clarity and accuracy of the message. This expression may be more likely to draw the attention of the respondents to the problem, while avoiding the negative reactions of the respondents due to the excessive linguistic and emotional colours."}, "time_dimension": {"answer": "Future", "rationale": "The entry contains visions and expectations for the future, and the key words are \"want to end\" and \"life\", all of which point to situations that have not yet occurred and that may arise in the future."}, "sentence_type": "Negative declarative sentence", "pos": {"nouns": {"15": 1, "Life": 1}, "verbs": {"Yes": 1, "End": 1}}}, {"content": "Sixteen people can't hear us.", "word_count": 10, "label": "", "sentence_structure": "Subject鈥搗erb鈥揷omplement", "subjective_objective": {"answer": "Objective", "rationale": "Because \"hearing voices that nobody can hear\" is an objective fact that can be felt."}, "sentence_sentiment": {"answer": "Neutral", "rationale": "Because the entry describes a phenomenon and does not address positive or negative attitudes, behaviour, purpose or quality."}, "language_style": {"answer": "Tactful and warm", "rationale": "The language style of the entry is more euphemistic. It guides the respondents to share their experiences of hearing voices that are not heard by others, rather than direct orders or statements. This way makes the respondents feel more comfortable and free to answer questions and more open to sharing their experiences and feelings."}, "time_dimension": {"answer": "Past", "rationale": "The phrase \"hearing\" and \"the voice of the person who cannot hear\" makes it clear that this is a question of whether the person who was tested has experienced this in the past, so the answer is the past."}, "sentence_type": "Interrogative sentence", "pos": {"nouns": {"The others": 1, "Sound": 1}, "verbs": {"Yes": 1, "I can't hear you": 1}, "adverbs": {"16": 1}}}, {"content": "Seventeen shiver.", "word_count": 2, "label": "", "sentence_structure": "Subject鈥搗erb鈥揷omplement", "subjective_objective": {"answer": "Subjective", "rationale": "Because tremors are a physical reaction or a symptom, not an objective observation or fact. It is a patient鈥檚 own feeling and experience, and it is therefore a subjective examination."}, "sentence_sentiment": {"answer": "Negative", "rationale": "Because the act of \"shocking\" is negative."}, "language_style": {"answer": "Calm and direct", "rationale": "The entry is more concise in its language style, and it directly asks whether the other party has a tremors situation, does not have too many emotional renderings or rhetorical techniques, and is more objective in its factual nature."}, "time_dimension": {"answer": "Past", "rationale": "The keyword \"shock\" indicates that it is a physical reaction or expression, which may be caused by fear, stress, cold, etc., or by a physical disease."}, "sentence_type": "Imperative sentence", "pos": {"verbs": {"# Wresh #": 1}, "adjectives": {"XVII": 1}}}, {"content": "18 feels that most people can't be trusted.", "word_count": 11, "label": "", "sentence_structure": "Subject鈥揷opula鈥損redicative", "subjective_objective": {"answer": "Subjective", "rationale": "Because \"fear\" means that it's a patient's own feeling and perception, a subjective attitude."}, "sentence_sentiment": {"answer": "Negative", "rationale": "Because the \"Feeling Untrustable\" attitude in the entries is negative."}, "language_style": {"answer": "Calm and direct", "rationale": "The entry is a direct indication of the view of the respondents that most people are not trustworthy and are not expressed in euphemism, metaphors or emotional language, and therefore are less vocal and direct."}, "time_dimension": {"answer": "Past", "rationale": ""}, "sentence_type": "Imperative sentence", "pos": {"nouns": {"person": 2, "Questionnaire Entry": 1, "Most people": 1}, "verbs": {"I feel": 1, "Trustable": 1}, "adjectives": {"No way": 1}, "numerals": {"18": 1, "Both": 1}, "pronouns": {"you": 1}}}, {"content": "19 has a bad appetite.", "word_count": 4, "label": "", "sentence_structure": "Subject鈥揷opula鈥損redicative", "subjective_objective": {"answer": "Subjective", "rationale": "Because \"bad appetite\" is a subjective feeling or experience that cannot be observed or measured."}, "sentence_sentiment": {"answer": "Negative", "rationale": "Because the expression \"bad appetite\" is usually considered negative."}, "language_style": {"answer": "Calm and direct", "rationale": "The language style of this entry is to be calm and straightforward, to ask the person under investigation whether he or she has an appetite problem, without excessive ado or emotional rendering.\""}, "time_dimension": {"answer": "Present", "rationale": "The descriptive phrase \"bad appetite\" in the sentence leads to the conclusion that the entry is about the present appetite of the testee, and therefore is judged to be \"now\"."}, "sentence_type": "Imperative sentence", "pos": {"nouns": {"-Adulge": 1}, "verbs": {"Okay": 1}, "numerals": {"19": 1}}}, {"content": "Twenty is easy to cry.", "word_count": 4, "label": "", "sentence_structure": "Subject鈥搗erb鈥揷omplement", "subjective_objective": {"answer": "Subjective", "rationale": "Because crying is an individual subjective emotional response. While the cause of crying may be objective (for example, because of grief), it is a personal choice to choose whether to cry and how to express one鈥檚 feelings, and therefore subjective."}, "sentence_sentiment": {"answer": "Negative", "rationale": "Because \"easy to cry\" is usually seen as a sign of negative emotions."}, "language_style": {"answer": "Calm and direct", "rationale": "The entry uses a direct presentation to ask the interviewees whether they are easy to cry, without any rhetorical or emotional rendering, with a more concise, clear language and more calm and objective expression."}, "time_dimension": {"answer": "Present", "rationale": ""}, "sentence_type": "Negative declarative sentence", "pos": {"nouns": {"20": 1, "Crying": 1}, "adverbs": {"Easy": 1}}}, {"content": "21 is shy and uncomfortable with opposite sex.", "word_count": 13, "label": "", "sentence_structure": "Subject鈥搗erb鈥揷omplement", "subjective_objective": {"answer": "Subjective", "rationale": "Because \"smuggling\" is a subjective emotional experience."}, "sentence_sentiment": {"answer": "Negative", "rationale": "Because the \"weakness\" in the entry is a negative emotional experience."}, "language_style": {"answer": "Tactful and warm", "rationale": ""}, "time_dimension": {"answer": "Present", "rationale": "The entry indicates the immediate feeling of the person being tested in the same way as the opposite sex, so the temporal dimension is now judged."}, "sentence_type": "Affirmative declarative sentence", "pos": {"nouns": {"21": 1, "Heterosexual": 1, "Get along": 1, "Time": 1, "Shy": 1, "I'm not comfortable": 1}, "adjectives": {"Shy": 1, "I'm not comfortable": 1}, "numerals": {"21": 1}}}, {"content": "Twenty-two felt cheated, caught in the trap or someone tried to catch you.", "word_count": 15, "label": "", "sentence_structure": "Subject鈥搗erb鈥揷omplement", "subjective_objective": {"answer": "Subjective", "rationale": "Because \"fEss\" is the patient's subjective feeling and idea, and it's a subjective view."}, "sentence_sentiment": {"answer": "Negative", "rationale": "Because all the expressions like \"fearing to be deceived, being caught in the trap\" have negative feelings."}, "language_style": {"answer": "Calm and direct", "rationale": "The entry \"Cool and Direct\" asks the other side in a direct and clear manner whether there has been a history of deception, condescending or exploitation, and the language style is calm and objective, without excessive rhetorical and emotional overtones."}, "time_dimension": {"answer": "Past", "rationale": "The keyword \"fears deceived\", \"felts\" and \"someone wants to catch you\" are all descriptions of what happened in the past, so it can be judged that the entry is asking about the testee's past experience."}, "sentence_type": "Exclamatory sentence", "pos": {"nouns": {"22": 1, "A trap": 1, "person": 1}, "verbs": {"I feel": 1, "I want to catch him": 1}, "adjectives": {"Swindled": 1}, "adverbs": {"Bingo": 1}, "numerals": {"22": 1}}}, {"content": "23 suddenly, for no reason.", "word_count": 11, "label": "", "sentence_structure": "Subject鈥搗erb鈥揷omplement", "subjective_objective": {"answer": "Subjective", "rationale": "Fear is a subjective experience and feeling, not an objective fact or event."}, "sentence_sentiment": {"answer": "Negative", "rationale": "Because the \"sudden fear without cause\" in the entry is negative."}, "language_style": {"answer": "Calm and direct", "rationale": "The entry directly expresses the subject of questioning the other party about the occurrence of unprovoked fear, without excessive rhetoric or emotional rendering, and is more objective in tone."}, "time_dimension": {"answer": "Present", "rationale": "The entry describes the emotional state that the person under examination is experiencing in reply to the questionnaire, namely, \"23 suddenly and without cause\" and therefore the temporal dimension is now judged."}, "sentence_type": "Affirmative declarative sentence", "pos": {"nouns": {"23": 1, "For no reason": 1, "Suddenly": 1, "Scared": 1}, "verbs": {"I feel": 1}}}, {"content": "24 is getting angry.", "word_count": 11, "label": "", "sentence_structure": "Subject鈥搗erb鈥揷omplement", "subjective_objective": {"answer": "Subjective", "rationale": "Because \"incontrol\" is the patient's subjective feeling and experience."}, "sentence_sentiment": {"answer": "Negative", "rationale": "Because the act of \"self-involved in a temper\" is negative."}, "language_style": {"answer": "Calm and direct", "rationale": "The entry is asked in a straightforward manner, without too much rhetorical or emotional overtook, and in a more objective and objective manner, highlighting the subject matter. At the same time, it is more conducive to the respondents to answer the questions accurately and truthfully, thereby serving the purpose of the investigation."}, "time_dimension": {"answer": "Present", "rationale": ""}, "sentence_type": "Exclamatory sentence", "pos": {"nouns": {"24": 1, "self": 1, "- Grumpy": 1}, "verbs": {"Control": 1, "Let's go": 1}, "adverbs": {"No, I can't": 1, "Land": 1}}}, {"content": "Twenty-five is afraid of going out alone.", "word_count": 5, "label": "", "sentence_structure": "Subject鈥揷opula鈥損redicative", "subjective_objective": {"answer": "Subjective", "rationale": "It is a subjective approach to the question of fear, which is the feeling and experience inside the patient."}, "sentence_sentiment": {"answer": "Negative", "rationale": "Because \"fear to go out alone\" expresses a negative, fearful feeling."}, "language_style": {"answer": "Calm and direct", "rationale": "The entry directly expresses the question of whether the other party is afraid to go out alone, does not use excessive rhetorical or emotional rendering, and is more objective in tone. It also does not use any euphemism or query structure, but rather raises the question in a straightforward manner, making it more targeted and clear."}, "time_dimension": {"answer": "Present", "rationale": "The keyword \"25 Fear of going out alone\" expresses a psychological state or feeling of the person being tested, and the fact that the fear of going out alone is not clearly stated, but that feeling or experience is present and the entry describes the state of the moment."}, "sentence_type": "Exclamatory sentence", "pos": {"nouns": {"25": 1, "Alone": 1, "Out": 1}, "adverbs": {"Yes": 1}, "numerals": {"25": 1}, "classifiers": {"individual": 1}}}, {"content": "Twenty-six often blames himself.", "word_count": 6, "label": "", "sentence_structure": "Subject鈥搗erb鈥揷omplement", "subjective_objective": {"answer": "Subjective", "rationale": "Because blame is a subjective attitude or psychological activity."}, "sentence_sentiment": {"answer": "Negative", "rationale": "Because the behavior in the entry \"criticize yourself\" is negative."}, "language_style": {"answer": "Calm and direct", "rationale": "The entry uses simple and clear language to ask whether the other party often blames his or her own subject, without too many rhetorical or emotional renderings, and is more objective in tone. It is therefore possible to judge that the language style of the entry is straightforward and calm."}, "time_dimension": {"answer": "Present", "rationale": "The term \"always\" in the entry indicates a high frequency of behaviour, and \"prission\" is a descriptive term, which is the situation that the subject often happens in the current act or state. Thus, the temporal dimension is now judged."}, "sentence_type": "Negative declarative sentence", "pos": {"nouns": {"Questionnaire Entry": 1, "self": 1}, "verbs": {"Analysis": 1, "Blame": 1}, "adjectives": {"Down there": 1, "26": 1, "Regular": 1}, "adverbs": {"Regular": 1}, "numerals": {"26": 1}, "classifiers": {"Entry": 1}, "pronouns": {"self": 1}}}, {"content": "27 waist aches.", "word_count": 2, "label": "", "sentence_structure": "Subject鈥損redicate", "subjective_objective": {"answer": "Objective", "rationale": "Since a back pain is a perceived physical condition and an objective fact that can be directly observed and measured, the angle of the examination for this article is objective."}, "sentence_sentiment": {"answer": "Negative", "rationale": "Because the symptoms of \"fear pain\" are usually considered negative."}, "language_style": {"answer": "Calm and direct", "rationale": ""}, "time_dimension": {"answer": "Present", "rationale": "The entry describes the current state of the person being tested, the keyword \"future pain\" and the number \"27\" indicating that this is an inquiry into the subjective feeling or physical condition of the person being tested. Thus, the temporal dimension of the entry is now."}, "sentence_type": "Interrogative sentence", "pos": {"nouns": {"My back hurts": 1, "The waist": 1}, "verbs": {"have": 1}, "adjectives": {"27": 1}}}, {"content": "28 felt difficult to accomplish.", "word_count": 8, "label": "", "sentence_structure": "Subject鈥揷opula鈥損redicative", "subjective_objective": {"answer": "Subjective", "rationale": "Because \"fEss\" is the patient's own feelings and perceptions, and it's a subjective observation and description."}, "sentence_sentiment": {"answer": "Negative", "rationale": "Because the description of \"difficult to accomplish\" is negative, it indicates that the testees have difficulties and challenges in facing the task."}, "language_style": {"answer": "Calm and direct", "rationale": "The language style of the entry is more straightforward, not over-modified or emotional, with more sound and objective tone, and more in line with the scientific and objective requirements of the psychological questionnaire."}, "time_dimension": {"answer": "Past", "rationale": "The entry asks if the person tested has experienced difficulties or challenges at some point in the past, and the keyword is \"difficult to accomplish the task\"."}, "sentence_type": "Interrogative sentence", "pos": {"nouns": {"Tasks": 1, "Sensor": 1}, "verbs": {"Completed": 1, "I feel": 1}, "adjectives": {"Hard": 1}, "numerals": {"28": 1}}}, {"content": "I'm lonely.", "word_count": 4, "label": "", "sentence_structure": "Subject鈥揷opula鈥損redicative", "subjective_objective": {"answer": "Subjective", "rationale": "Because feeling alone is a subjective emotional experience."}, "sentence_sentiment": {"answer": "Negative", "rationale": "Because the word \"single\" in the entry is an expression of a negative emotion."}, "language_style": {"answer": "Calm and direct", "rationale": "The entry uses a simple and clear language, which is more objective and simple, and which is therefore a calm and straightforward language."}, "time_dimension": {"answer": "Present", "rationale": ""}, "sentence_type": "Interrogative sentence", "pos": {"nouns": {"29": 1, "Loneliness": 1}, "adjectives": {"I feel": 1}}}, {"content": "30 is bored.", "word_count": 4, "label": "", "sentence_structure": "Subject鈥揷opula鈥損redicative", "subjective_objective": {"answer": "Subjective", "rationale": "Because \"silent\" is an emotional experience, a subjective feeling of the patient."}, "sentence_sentiment": {"answer": "Negative", "rationale": "Because \"silent\" expresses a negative sentiment."}, "language_style": {"answer": "Calm and direct", "rationale": "The entry is a straightforward phrase that expresses the question whether the other party is often bored with the subject, is not using excessive rhetorical techniques or emotional rendering, and is more objective in tone."}, "time_dimension": {"answer": "Present", "rationale": "The entry does not have a clear time indicator, but it can be inferred from the context that it is a current feeling or state, so the time dimension is now judged."}, "sentence_type": "Negative declarative sentence", "pos": {"nouns": {"I'm bored": 1, "30": 1}}}, {"content": "31 is too worried.", "word_count": 4, "label": "", "sentence_structure": "Subject鈥搗erb鈥揷omplement", "subjective_objective": {"answer": "Subjective", "rationale": "Excessive fear is the subjective perception and assessment of individuals, reflecting their excessive concern for uncertainty or potential threats to the future that may occur."}, "sentence_sentiment": {"answer": "Negative", "rationale": "Because \"excessive worry\" is a negative sentiment, which means that this man is too anxious or too worried."}, "language_style": {"answer": "Calm and direct", "rationale": "The language style of the entry is relatively simple, there is no excessive rhetorical or emotional rendering, and it is more oriented towards facts and problems, and it is more objective in tone. Thus, it is judged to be \"cool and direct\"."}, "time_dimension": {"answer": "Past", "rationale": "The entry asks whether the person tested had been over-aspiring and worried at a certain point in time (not specified) and therefore judged to be a past state of affairs."}, "sentence_type": "Exclamatory sentence", "pos": {"nouns": {"Grandpa": 1, "Fear": 1}, "verbs": {"That's too much": 1}, "numerals": {"31": 1}}}, {"content": "32 is not interested in things.", "word_count": 7, "label": "", "sentence_structure": "Subject鈥揷opula鈥損redicative", "subjective_objective": {"answer": "Subjective", "rationale": "Because interest is the patient ' s personal perception and emotional experience, it is subjectively observed."}, "sentence_sentiment": {"answer": "Negative", "rationale": "Because \"not interested in things\" in the entry is a negative emotional attitude."}, "language_style": {"answer": "Calm and direct", "rationale": "The entry is based on a simple and clear language style, with no excessive rhetorical or emotional rendering, and is more objective in tone, and is intended to obtain direct information on the extent of interest in the subject."}, "time_dimension": {"answer": "Present", "rationale": "In the future, the entry asks the testee about the attitude or tendency towards what may happen in the future, namely, whether they lose interest in something. The keyword \"32 does not interest in something\" expresses the possibility of the future."}, "sentence_type": "Exclamatory sentence", "pos": {"verbs": {"to": 1, "Not interested": 1}, "adjectives": {"32": 1}, "numerals": {"32": 1}}}, {"content": "Thirty-three was scared.", "word_count": 4, "label": "", "sentence_structure": "Subject鈥搗erb鈥揷omplement", "subjective_objective": {"answer": "Subjective", "rationale": "Fear is a subjective emotional response, representing the fear that patients have of something in a particular situation."}, "sentence_sentiment": {"answer": "Negative", "rationale": "Because the \"fear\" in the entry is negative."}, "language_style": {"answer": "Calm and direct", "rationale": "The language style of the questionnaire entry is more direct and concise, and it does not use excessive rhetorical or emotional publicity, but rather asks the respondents whether they are afraid, with more objective and calm tone."}, "time_dimension": {"answer": "Present", "rationale": ""}, "sentence_type": "Exclamatory sentence", "pos": {"nouns": {"33": 1, "Scared": 1}}}, {"content": "34 is vulnerable.", "word_count": 8, "label": "", "sentence_structure": "Subject鈥揷opula鈥損redicative", "subjective_objective": {"answer": "Subjective", "rationale": "Because \"emotional vulnerability\" is an emotional feeling and experience of a person, which is a subjective aspect of the individual."}, "sentence_sentiment": {"answer": "Negative", "rationale": "Because \"emotional vulnerability\" in the entry is an expression of a negative emotional state or character."}, "language_style": {"answer": "Tactful and warm", "rationale": ""}, "time_dimension": {"answer": "Present", "rationale": ""}, "sentence_type": "Negative declarative sentence", "pos": {"nouns": {"Emotion": 1, "Hurt": 1}, "verbs": {"Easy": 1, "Received": 1}, "adjectives": {"34": 1}}}, {"content": "35 others can know your secret thoughts.", "word_count": 11, "label": "", "sentence_structure": "Subject鈥搗erb鈥搊bject", "subjective_objective": {"answer": "Subjective", "rationale": "Because it's the patient's inner thoughts, it's subjective."}, "sentence_sentiment": {"answer": "Negative", "rationale": "Because \"the person in the entry knows your private thoughts\" may make the interviewee uncomfortable and feel violated in privacy, and therefore negative."}, "language_style": {"answer": "Tactful and warm", "rationale": "The entry uses a more euphemistic language style, asking the interviewees whether they are willing to share their personal thoughts and not directly stating or ordering them to feel respected and cared for."}, "time_dimension": {"answer": "Present", "rationale": "The keywords \"35th border\" and \"know\" and \"private thinking\" indicate that this is a description of the current behaviour or state, and therefore the judgement is now."}, "sentence_type": "Exclamatory sentence", "pos": {"nouns": {"The others": 1, "I'm not sure": 1}, "verbs": {"Yes": 1}, "numerals": {"35": 1}}}, {"content": "I feel like someone doesn't understand you.", "word_count": 12, "label": "", "sentence_structure": "Subject鈥揷opula鈥損redicative", "subjective_objective": {"answer": "Subjective", "rationale": "Because this feeling is a subjective emotional experience of a person, it is based on the individual鈥檚 feelings and perceptions. While this may be related to the degree of understanding of his behaviour or speech, the final feeling remains one of the subjective feelings of the individual."}, "sentence_sentiment": {"answer": "Negative", "rationale": "Because the entry is a negative emotional and experience, that is, feeling misunderstood and unsympathetic, negative emotional attitude."}, "language_style": {"answer": "Tactful and warm", "rationale": "The entry is more personal and interactive when it is spoken in a gentle and inviting tone, rather than in direct statements or orders. At the same time, the sentence is not overly emotional or strongly verbal, but rather uses a more objective language to describe the potential feelings of the respondents, which makes them comfortable and respectful."}, "time_dimension": {"answer": "Present", "rationale": ""}, "sentence_type": "Negative declarative sentence", "pos": {"nouns": {"36": 1, "others": 1, "Compassion": 1}, "verbs": {"Sensor": 1, "Understood": 1, "I don't understand": 1, "I feel": 1, "Compassion": 1}, "numerals": {"36": 1}, "classifiers": {"individual": 1}, "pronouns": {"you": 2}}}, {"content": "I feel like someone doesn't understand you.", "word_count": 12, "label": "", "sentence_structure": "Subject鈥搗erb鈥揷omplement", "subjective_objective": {"answer": "Subjective", "rationale": "Because this feeling is individual and subjective."}, "sentence_sentiment": {"answer": "Negative", "rationale": "Because \"ununderstanding\" and \"unsympathetic\" in the entries are negative terms that indicate the negative emotions that the respondents may experience in their interpersonal contacts."}, "language_style": {"answer": "Tactful and warm", "rationale": "This is not a direct or hard language, but rather a soft, invited way to get respondents to answer. This way of expressing themselves can relieve the pressure of the interviewees and encourage them to express their feelings and experiences."}, "time_dimension": {"answer": "Present", "rationale": ""}, "sentence_type": "Interrogative sentence", "pos": {"nouns": {"37": 1, "others": 1, "Compassion": 1}, "verbs": {"I feel": 1, "I don't understand": 1, "Understood": 1, "No sympathy": 1, "Compassion": 1}}}, {"content": "38 must be done slowly to ensure that it is done correctly.", "word_count": 15, "label": "", "sentence_structure": "Subject鈥搗erb鈥揷omplement", "subjective_objective": {"answer": "Subjective", "rationale": "Because \"slow\" is the patient's own subjective behaviour and ideas, it's the patient's subjective attitude."}, "sentence_sentiment": {"answer": "Negative", "rationale": "Because the \"doing things very slowly\" act in the entry is negative."}, "language_style": {"answer": "Calm and direct", "rationale": "The entry clearly states that the need to proceed slowly in order to ensure the correctness of the results is not accompanied by excessive rhetorical or emotional rendering, and is more balanced and objective, so that the language style is \"cool and direct\"."}, "time_dimension": {"answer": "Past", "rationale": "The entry describes a past habit or tendency that \"doing things very slowly\" is the way that the testee used to do in the past, in order to ensure accuracy of the work. Thus, the temporal dimension is judged to be the past."}, "sentence_type": "Exclamatory sentence", "pos": {"nouns": {"Work": 2, "Slow": 1, "Promise": 1, "Correct": 1}, "verbs": {"Do it": 2, "Slow": 1, "Promise": 1, "Okay": 1}, "adjectives": {"I have to": 1, "very": 1}, "numerals": {"38": 1}}}, {"content": "Thirty-nine heart beats.", "word_count": 5, "label": "", "sentence_structure": "Subject鈥搗erb鈥揷omplement", "subjective_objective": {"answer": "Objective", "rationale": "The fact that the heart is beating so hard is an objective fact for the patient."}, "sentence_sentiment": {"answer": "Negative", "rationale": "Because \"the heart beats hard\" in the entry means physical discomfort, and it's a negative emotion."}, "language_style": {"answer": "Calm and direct", "rationale": "The language style of the questionnaire entry is straightforward, and it is simple to ask the respondents whether they have a high heart rate, with no excessive acrimony or emotional colour, and is consistent with the language style of the general psychological questionnaire."}, "time_dimension": {"answer": "Present", "rationale": "The keyword \"39 Hearts are ABX\" implies the current physiological state of the subject, i.e., feeling an acceleration or a lack of a full heartbeat. So, this entry is a question of asking the testee about the current situation, and the temporal dimension is now."}, "sentence_type": "Exclamatory sentence", "pos": {"nouns": {"39": 1, "Heart rate": 1, "That's great": 1}, "verbs": {"Okay": 1}}}, {"content": "40 sick or stomach.", "word_count": 8, "label": "", "sentence_structure": "Subject鈥揷opula鈥損redicative", "subjective_objective": {"answer": "Objective", "rationale": "Because nausea or stomach discomfort is the symptoms of the patient, which are seen from an objective perspective."}, "sentence_sentiment": {"answer": "Negative", "rationale": "Because the description \"sick or stomach discomfort\" is a symptom of physical discomfort and is usually considered negative."}, "language_style": {"answer": "Tactful and warm", "rationale": ""}, "time_dimension": {"answer": "Present", "rationale": ""}, "sentence_type": "Imperative sentence", "pos": {"nouns": {"Gross": 1, "Stomach": 1, "I'm not feeling well": 1}, "verbs": {"have": 1, "Feel": 1}, "numerals": {"40": 1}}}, {"content": "41 feels like nothing like the others.", "word_count": 7, "label": "", "sentence_structure": "Subject鈥搗erb鈥揷omplement", "subjective_objective": {"answer": "Subjective", "rationale": "Because \"fear\" is a subjective emotional feeling, it means self-evaluation and comparative mentality of patients, and it is a subjective approach."}, "sentence_sentiment": {"answer": "Negative", "rationale": "Because the expression \"near as much as anyone else\" expresses a negative emotion and self-evaluation, which is negative."}, "language_style": {"answer": "Tactful and warm", "rationale": "The entry is more adept and interactive when it is spoken in a gentle and inviting manner, rather than by direct statements or orders."}, "time_dimension": {"answer": "Present", "rationale": "The keyword is \"fear\", which indicates the current psychological state and feelings of the person being tested, and the entry is therefore judged by the question of the person's current behaviour or mental state."}, "sentence_type": "Exclamatory sentence", "pos": {"nouns": {"I feel": 1, "person": 1}, "verbs": {"Not as good as it is": 1}, "adjectives": {"41": 1}}}, {"content": "42 muscles are sore.", "word_count": 4, "label": "", "sentence_structure": "Subject鈥搗erb鈥揷omplement", "subjective_objective": {"answer": "Objective", "rationale": "Because \"42 Muscles Aches\" is the physical feeling and experience of the patient, and it's an objective fact."}, "sentence_sentiment": {"answer": "Negative", "rationale": "Because \"muscular acid\" is a manifestation of physical discomfort, it is usually considered a negative situation."}, "language_style": {"answer": "Tactful and warm", "rationale": "The entry is more euphemistic and sensitive to the other party ' s feelings, asking the interviewee whether he has a muscular acid condition, without directly accusing or ordering an answer, and this expression is more comfortable and respectful for the interviewee."}, "time_dimension": {"answer": "Present", "rationale": "The keyword is \"muscle acid\" which describes the current state or feeling of the person being tested and therefore determines the present."}, "sentence_type": "Interrogative sentence", "pos": {"nouns": {"Muscles": 1, "Sour": 1}, "numerals": {"42": 1}}}, {"content": "I feel like someone's been watching you talking about you.", "word_count": 10, "label": "", "sentence_structure": "Subject鈥搗erb鈥揷omplement", "subjective_objective": {"answer": "Subjective", "rationale": "Because \"fear\" is a subjective feeling or perception, not an objective fact. Although it may be true, it is a subjective experience of the patient himself, and it should be viewed from a subjective perspective."}, "sentence_sentiment": {"answer": "Negative", "rationale": "Because the feeling of someone in prison talking about you is negative."}, "language_style": {"answer": "Tactful and warm", "rationale": "The entry uses more moderate language to guide the interviewee to reflect on his/her feelings, such as using \"Do you think\" or \"Do you think that there are people...\" to raise problems, without directly expressing or judging the interviewee's feelings. This language style is more personal and interactive and allows better feedback and comments from the interviewee."}, "time_dimension": {"answer": "Past", "rationale": "In the entry, \"Feeling someone talking about you\" is describing a subjective feeling or experience that usually involves memories and reflections of what happened in the past for a while. So the time dimension of the entry is the past."}, "sentence_type": "Exclamatory sentence", "pos": {"nouns": {"person": 1, "Talk": 1}, "verbs": {"Sensor": 1, "Commissioner": 1, "Talk": 1}, "adverbs": {"very": 1}, "numerals": {"43": 1}, "pronouns": {"you": 2}}}, {"content": "44 is hard to sleep.", "word_count": 4, "label": "", "sentence_structure": "Subject鈥搗erb鈥揷omplement", "subjective_objective": {"answer": "Subjective", "rationale": "Because \"sleep hard\" is a subjective feeling or experience that reflects the patient's sleep difficulties and distress."}, "sentence_sentiment": {"answer": "Negative", "rationale": "Because the \"sleeping\" behavior in the entry is negative."}, "language_style": {"answer": "Calm and direct", "rationale": "The language style of the question is very direct and straightforward, without any qualifier or emotional rendering, simply asking if the interviewee is sleeping at night, which is more easily understood and answered."}, "time_dimension": {"answer": "Present", "rationale": "The keyword \"sleeping hard\" denotes what the subject is experiencing at the moment of answering the question, and thus the temporal dimension is now judged."}, "sentence_type": "Exclamatory sentence", "pos": {"nouns": {"44": 1, "Hard": 1, "Sleep": 1}, "numerals": {"44": 1}}}, {"content": "45 must be checked over and over again.", "word_count": 8, "label": "", "sentence_structure": "Subject鈥搗erb鈥揷omplement", "subjective_objective": {"answer": "Subjective", "rationale": "Because \"repeated examination\" reflects the patient's inner thoughts and attitudes and is subjective."}, "sentence_sentiment": {"answer": "Negative", "rationale": "Because the practice of \"repeat inspection\" is often seen as overly cautious, it can lead to reduced efficiency and increased pressure. It is therefore a possible negative mood or attitude."}, "language_style": {"answer": "Calm and direct", "rationale": "The entry is simple, is not too much adoration or emotional, and is directly asked whether the other party must check repeatedly when doing the work, and is more direct and objective."}, "time_dimension": {"answer": "Past", "rationale": "The entry is an inquiry into the testee's past behavior, \"Doing Business\" which indicates that it is a regular, customary act, while \"repeat inspection\" suggests that it has occurred in the past. Therefore, according to the time dimension, the entry should be a past."}, "sentence_type": "Exclamatory sentence", "pos": {"nouns": {"45": 1, "Yes": 2, "Do it": 1, "Repeatedly": 1, "Inspection": 1}, "numerals": {"45": 1}}}, {"content": "46 Hard to make a decision.", "word_count": 6, "label": "", "sentence_structure": "Subject鈥揷opula鈥損redicative", "subjective_objective": {"answer": "Subjective", "rationale": "Because \"hard to make a decision\" is a subjective feeling and experience of a person, not an objective fact or result. This is a patient's description of his psychological state of decision-making, and therefore a subjective view."}, "sentence_sentiment": {"answer": "Negative", "rationale": "Because the \"hard to make a decision\" act is negative."}, "language_style": {"answer": "Calm and direct", "rationale": "Because the language style of the entry is more objective and direct in describing the difficulties that the respondents may encounter in making decisions, there is no excessive emotional colour or rhetorical approach."}, "time_dimension": {"answer": "Present", "rationale": "The entry is a description of the person's current behaviour or state, and the time dimension is now judged."}, "sentence_type": "Exclamatory sentence", "pos": {"nouns": {"Decision": 1}, "verbs": {"Do it": 1, "Make it": 1}, "adjectives": {"Hard": 1, "46": 1}, "numerals": {"46": 1}}}, {"content": "47 afraid of taking a train, bus, subway or train.", "word_count": 13, "label": "", "sentence_structure": "Subject鈥搗erb鈥搊bject", "subjective_objective": {"answer": "Subjective", "rationale": "Because \"fear\" is a subjective feeling and emotion, it represents the patient's fear of travelling in public transport. It is a personal psychological experience of the patient, and cannot be confirmed or denied by objective facts."}, "sentence_sentiment": {"answer": "Negative", "rationale": "Because the word \"fear\" in the entry is negative."}, "language_style": {"answer": "Tactful and warm", "rationale": "The language style of the entry is more euphemistic and uses an invitation-style tone to engage in dialogue, which makes the interviewee more comfortable and comfortable. This expression is more likely to trigger resonance and increase the likelihood of the interviewee鈥檚 willingness to participate in the investigation than direct questions or orders."}, "time_dimension": {"answer": "Past", "rationale": "The behaviour contained in this entry, \"fearing to travel by train, bus, subway or train\", is a past experience and feeling, and is therefore judged to be a past."}, "sentence_type": "Imperative sentence", "pos": {"nouns": {"47": 1, "- On the tram": 1, "Bus": 1, "Subway": 1, "Train": 1}, "verbs": {"Yes": 1}, "numerals": {"47": 1}}}, {"content": "Forty-eight has trouble breathing.", "word_count": 5, "label": "", "sentence_structure": "Subject鈥揷opula鈥損redicative", "subjective_objective": {"answer": "Objective", "rationale": "Since respiratory difficulties are a physical phenomenon that can be observed by the patient, it is not necessary to rely on the patient ' s subjective feelings."}, "sentence_sentiment": {"answer": "Negative", "rationale": "The test is that respiratory difficulties are usually a symptom and may be a sign of disease, and therefore the emotional colour of the purpose is negative."}, "language_style": {"answer": "Calm and direct", "rationale": "The entry is a straightforward question of whether the person surveyed has any breathing difficulties, is more concise and quick, has no excessive emotional modification or speech skills and is of a straightforward, calm type."}, "time_dimension": {"answer": "Past", "rationale": "The entry asks whether the person tested had had a \"respiration difficulty\" at some point in the past and can therefore be judged to be past."}, "sentence_type": "Declarative sentence", "pos": {"nouns": {"Breathe": 1, "Difficulties": 1}, "verbs": {"have": 1}, "numerals": {"48": 1}}}, {"content": "49 times cold or hot.", "word_count": 8, "label": "", "sentence_structure": "Subject鈥搗erb鈥揷omplement", "subjective_objective": {"answer": "Objective", "rationale": "Because a period of cold or fever is a symptom of the patient and is objectively observed."}, "sentence_sentiment": {"answer": "Negative", "rationale": "Because the description \"cool or heat\" is an expression of physical discomfort and is usually considered negative."}, "language_style": {"answer": "Calm and direct", "rationale": "The entry describes possible symptoms in a direct, concise language, and \"span of cold or heat\" is an objective description, with no excessive rhetoric or emotional colours, consistent with the calm and direct language style."}, "time_dimension": {"answer": "Present", "rationale": "The time word \"span\" in the entry indicates the intermittent appearance of symptoms and does not have a clear time period for describing them, and therefore determines the state of the present. At the same time, the term \"cooling or fevering\" is the verb describing the state of health, which further supports the situation."}, "sentence_type": "Affirmative declarative sentence", "pos": {"nouns": {"49": 1, "For a while": 1, "Cold": 1, "Heat": 1}}}, {"content": "50 avoids something because of fear, occasions or activities.", "word_count": 18, "label": "", "sentence_structure": "Subject鈥搗erb鈥搊bject", "subjective_objective": {"answer": "Subjective", "rationale": "Because \"fear\" is a subjective emotional experience."}, "sentence_sentiment": {"answer": "Negative", "rationale": "Because the act of \"shielding out of fear\" in the entry is negative, indicating that the person under investigation may have problems of fear, anxiety, etc."}, "language_style": {"answer": "Calm and direct", "rationale": "The language style of the entry is calm and straightforward. It clearly raises the theme that in some cases people may avoid certain things, places or activities out of fear, without using excessive rhetorical or emotional accents and speaking in a balanced and objective manner."}, "time_dimension": {"answer": "Past", "rationale": "So output is \"the past\"."}, "sentence_type": "Affirmative declarative sentence", "pos": {"nouns": {"50": 1, "Scared": 1, "Some": 1, "Something": 1, "Event": 1, "Activities": 1}, "verbs": {"Avoid": 1}, "adverbs": {"And": 1, "Because": 1}, "numerals": {"50": 1}}}, {"content": "51 is empty.", "word_count": 5, "label": "", "sentence_structure": "Subject鈥揷opula鈥損redicative", "subjective_objective": {"answer": "Subjective", "rationale": "Because brain loss is a subjective description and feeling."}, "sentence_sentiment": {"answer": "Negative", "rationale": "Because the expression \"brainless\" is negative, implying negative situations such as reduced mental capacity and memory loss."}, "language_style": {"answer": "Calm and direct", "rationale": "The entry is in plain and clear language and asks whether the other party feels he/she is retarded or incompetent, has no excessive rhetorical or emotional rendering, and is more calm and objective."}, "time_dimension": {"answer": "Present", "rationale": "The \"51 brain is empty\" in \"51\" refers to the age of the person who was tested at the time of the survey, and \"brain is empty\" is a description of the subjective feeling, so it is asking about the person who was tested in the current state of affairs, which is the current state of affairs."}, "sentence_type": "Affirmative declarative sentence", "pos": {"nouns": {"Brain": 1, "Empty": 1}, "verbs": {"Change": 1}, "numerals": {"51": 1}}}, {"content": "52 bodies numb or sting.", "word_count": 7, "label": "", "sentence_structure": "Subject鈥揷opula鈥損redicative", "subjective_objective": {"answer": "Objective", "rationale": "It is an objective fact that the symptoms of the patient are manifested by a sterilisation or sting."}, "sentence_sentiment": {"answer": "Negative", "rationale": "Because body atrophy or sting is usually a feeling of discomfort, it has a negative emotional connotation."}, "language_style": {"answer": "Calm and direct", "rationale": "The entry directly asks whether the interviewees have experienced a sterilisation or sting, a more objective, direct language style, an inordinate rhetorical approach and more efficient access to information."}, "time_dimension": {"answer": "Past", "rationale": "The entry is for the purpose of asking the person who was tested whether there was, and is, a physical atrophy or anal, and therefore covers both the past and the present."}, "sentence_type": "Affirmative declarative sentence", "pos": {"nouns": {"52": 1, "Body": 1, "Hair": 1, "Stings": 1}}}, {"content": "53 throats feel like they're infarction.", "word_count": 6, "label": "", "sentence_structure": "Subject鈥揷opula鈥損redicative", "subjective_objective": {"answer": "Objective", "rationale": "Because \"throat infarction\" is a symptom or a feeling of the patient, an objective fact that can be observed and measured, not the patient's thoughts or attitudes."}, "sentence_sentiment": {"answer": "Negative", "rationale": "Because the entry \"throat infarction\" is an uncomfortable physical condition, usually associated with negative emotions, health problems or diseases."}, "language_style": {"answer": "Tactful and warm", "rationale": ""}, "time_dimension": {"answer": "Present", "rationale": ""}, "sentence_type": "Exclamatory sentence", "pos": {"nouns": {"53": 1, "Throat": 1, "Infarction": 1}, "numerals": {"53": 1}}}, {"content": "54 feels no future, no hope.", "word_count": 10, "label": "", "sentence_structure": "Subject鈥搗erb鈥揷omplement", "subjective_objective": {"answer": "Subjective", "rationale": "The sense of hopelessness is the subjective sentiment and the idea of the patient, the vision and the evaluation of the future, and the subjective perspective."}, "sentence_sentiment": {"answer": "Negative", "rationale": "Because \"no future, no hope\" means a negative attitude and feeling."}, "language_style": {"answer": "Calm and direct", "rationale": "The basis for the judgment: the language style of the entry is relatively straightforward, not with too many rhetorical or emotional expressions, but rather with a clear and concise expression of the theme \"no future and hope,\" giving a clear sense of clarity. At the same time, this melancholy and objective attitude may be taken seriously and therefore has some authority."}, "time_dimension": {"answer": "Present", "rationale": "According to the input sentence given, \"Feeling no future, no hope\" is the testee's self-perception and description of the current situation, so it can be judged that the entry is asking the testee's current state or mood, so it's \"now\"."}, "sentence_type": "Negative declarative sentence", "pos": {"nouns": {"Future": 1, "Hope": 1}, "verbs": {"I feel": 1}, "adjectives": {"No, I'm not": 2}, "numerals": {"54": 1}}}, {"content": "55 can't focus.", "word_count": 6, "label": "", "sentence_structure": "Subject鈥搗erb鈥揷omplement", "subjective_objective": {"answer": "Subjective", "rationale": "Because concentration is an individual feeling and experience, it depends on the patient ' s subjective feelings."}, "sentence_sentiment": {"answer": "Negative", "rationale": "Because \"not focusing\" means negative behavior or lack of capacity, it's a negative emotional expression."}, "language_style": {"answer": "Calm and direct", "rationale": "The entry uses objective and concise language, clearly indicating the concerns of the respondents, without any emotional or qualifier, and therefore is more calm and straightforward."}, "time_dimension": {"answer": "Present", "rationale": "The entry is about the feelings and experiences of the testee in his current state, and \"not focusing\" is about the current state of attention of the testee, so the temporal dimension is now."}, "sentence_type": "Negative declarative sentence", "pos": {"nouns": {"Attention": 1}, "verbs": {"Focus": 1}, "adjectives": {"55": 1}, "numerals": {"not": 1, "Yes": 1}}}, {"content": "56 feels a part of the body is weak.", "word_count": 13, "label": "", "sentence_structure": "Subject鈥搗erb鈥揷omplement", "subjective_objective": {"answer": "Subjective", "rationale": "Because \"fEs\" is the patient's subjective feeling."}, "sentence_sentiment": {"answer": "Negative", "rationale": "Because the physical symptoms described in this entry are usually discomfort or anxiety, and therefore have a negative emotional connotation."}, "language_style": {"answer": "Tactful and warm", "rationale": "The entry uses a more moderate language style and leads respondents to answer questions in an interview, giving the respondents a sense of respect and concern, increasing their motivation and comfort in participating in the questionnaire. At the same time, the term \"weakness\" does not too directly or strongly stimulate the negative sentiment of the interviewees, but is presented in an objective and neutral manner."}, "time_dimension": {"answer": "Past", "rationale": "The entry asks the testee about the experience or feelings at a certain point, and the temporal dimension is judged to be the past. The keyword \"56\" may indicate a specific point or frequency, but in any case this description points to the past."}, "sentence_type": "Exclamatory sentence", "pos": {"nouns": {"Part": 1, "Body": 1, "Weakness": 1}, "verbs": {"I feel": 1}, "numerals": {"56": 1}}}, {"content": "57 is nervous or nervous.", "word_count": 9, "label": "", "sentence_structure": "Subject鈥揷opula鈥損redicative", "subjective_objective": {"answer": "Subjective", "rationale": "Because feeling nervous is a subjective emotional experience."}, "sentence_sentiment": {"answer": "Negative", "rationale": "Because the attitude of \"frightful or easy\" in the entries is negative."}, "language_style": {"answer": "Tactful and warm", "rationale": "The entry is more personal and interactive when it is presented in a gentle and inviting tone rather than in direct statements or orders. At the same time, it is more accessible and understandable, and is more easily understood and accepted by the interviewees."}, "time_dimension": {"answer": "Present", "rationale": ""}, "sentence_type": "Affirmative declarative sentence", "pos": {"nouns": {"Nervous": 2, "Easy": 1}, "verbs": {"I feel": 1}, "numerals": {"57": 1}}}, {"content": "58 feels heavy hands or feet.", "word_count": 7, "label": "", "sentence_structure": "Subject鈥搗erb鈥揷omplement", "subjective_objective": {"answer": "Objective", "rationale": "Because \"fearing a hand or a leg is a physical experience of the patient, an objective description."}, "sentence_sentiment": {"answer": "Negative", "rationale": "Because the \"fear or feet\" is negative."}, "language_style": {"answer": "Tactful and warm", "rationale": "The entry is a caring and rogatory dialogue, in which the respondents feel their feelings are valued, and they speak with a gentle and friendly tone, rather than directly stating or ordering."}, "time_dimension": {"answer": "Present", "rationale": ""}, "sentence_type": "Affirmative declarative sentence", "pos": {"nouns": {"58": 1, "Hands": 1, "Foot": 1, "Heavy": 1}}}, {"content": "59 thought of death.", "word_count": 6, "label": "", "sentence_structure": "Subject鈥搗erb鈥揷omplement", "subjective_objective": {"answer": "Subjective", "rationale": "Because \"Think of Death\" is the mind and the feeling of the patient, and it's a subjective observation of the individual."}, "sentence_sentiment": {"answer": "Negative", "rationale": "Because the subject of \"death\" is usually considered negative, it can cause negative emotions."}, "language_style": {"answer": "Calm and direct", "rationale": "The entry uses a simple language and asks the interviewees whether they have ever thought about death, without excessive embellishment or emotional rendering, and in a more calm and objective manner."}, "time_dimension": {"answer": "Present", "rationale": "The keyword \"Thinking of Death\" shows that the entry asks the subject what it thinks and what it feels now, so the temporal dimension is now."}, "sentence_type": "Exclamatory sentence", "pos": {"nouns": {"Yes": 1, "Death": 1}, "verbs": {"I thought": 1, "Do it": 1}, "numerals": {"59": 1}}}, {"content": "60 eats too much.", "word_count": 4, "label": "", "sentence_structure": "Subject鈥搗erb鈥揷omplement", "subjective_objective": {"answer": "Objective", "rationale": "Because \"60 Eat Too Too Much\" is a narrative of the facts, and does not need to involve subjective feelings or attitudes of the patient. So, it is an objective questionnaire entry."}, "sentence_sentiment": {"answer": "Negative", "rationale": "The basis for the determination: \"too much to eat\" in the entry expresses negative behaviour and attitudes and suggests possible health problems."}, "language_style": {"answer": "Calm and direct", "rationale": "The entry is an open question to the audience whether they often consume too much food, speak directly and clearly, and do not have too many rhetorical or emotional renderings, reflecting a more rigorous and objective language style. \""}, "time_dimension": {"answer": "Present", "rationale": "The entry describes the problems of the testee in the current behaviour habits, and the temporal dimension is now judged."}, "sentence_type": "Negative declarative sentence", "pos": {"nouns": {"60": 1, "Eat": 1, "Too much": 1, "More": 1}, "adjectives": {"Okay": 1, "Too much": 1}, "numerals": {"60": 1}}}, {"content": "61 feels uncomfortable when people look at you or talk about you.", "word_count": 16, "label": "", "sentence_structure": "Subject鈥搗erb鈥搊bject", "subjective_objective": {"answer": "Subjective", "rationale": "Because feeling uncomfortable is a subjective feeling that is experienced and felt by the patient himself."}, "sentence_sentiment": {"answer": "Negative", "rationale": "Because the behavior in the entry is negative."}, "language_style": {"answer": "Tactful and warm", "rationale": "So, it's a language style that can be described as \"the euphemism.\""}, "time_dimension": {"answer": "Present", "rationale": ""}, "sentence_type": "Imperative sentence", "pos": {"nouns": {"others": 1, "you": 2, "Time": 1, "Talk": 1, "I feel": 1, "I'm not comfortable": 1}, "verbs": {"Look": 1, "Watch": 1, "When": 1, "Talking about it": 1}, "adverbs": {"very": 1, "not": 1, "of": 2}, "numerals": {"61": 1}}}, {"content": "62 has ideas that don't belong to you.", "word_count": 12, "label": "", "sentence_structure": "Subject鈥搗erb鈥揷omplement", "subjective_objective": {"answer": "Subjective", "rationale": "Since the question is whether the patient has certain ideas that do not belong to him, it is the patient ' s subjective feeling and perception and is therefore subjective in nature."}, "sentence_sentiment": {"answer": "Negative", "rationale": "Because the expression \"not your own thoughts\" is negative."}, "language_style": {"answer": "Tactful and warm", "rationale": "The language style of the entry is more euphemistic and friendly, using words like \"yourself\" and \"your own mind\" to guide respondents to reflect on their own feelings, rather than direct orders or statements. At the same time, the expression \"some\" makes questions more open and inclusive, and makes them comfortable and free to express themselves."}, "time_dimension": {"answer": "Past", "rationale": "The entry does not have clear time dimension information, and therefore it is not possible to judge whether its time properties are past, present or future."}, "sentence_type": "Affirmative declarative sentence", "pos": {"nouns": {"Some": 1, "Thoughts": 1}, "verbs": {"have": 1, "belong to": 1, "not": 1}, "adjectives": {"Your own": 1}, "numerals": {"62": 1}}}, {"content": "63 has an impulse to hit or hurt someone.", "word_count": 12, "label": "", "sentence_structure": "Subject鈥搗erb鈥搊bject", "subjective_objective": {"answer": "Subjective", "rationale": "Because it's the patient's subjective feeling and the idea."}, "sentence_sentiment": {"answer": "Negative", "rationale": "Because the \"want to hit or hurt\" act in the entry is negative."}, "language_style": {"answer": "Calm and direct", "rationale": ""}, "time_dimension": {"answer": "Present", "rationale": "Keywords include \"thinking\" and \"exulting\", which indicate the current psychological state and feelings of the subject, and therefore the entry describes the situation."}, "sentence_type": "Affirmative declarative sentence", "pos": {"nouns": {"63": 1, "person": 1, "Expedition": 1}, "verbs": {"Yes": 1, "Fight": 1, "Hurt": 1}, "numerals": {"have": 1}}}, {"content": "64 was too early to wake up.", "word_count": 4, "label": "", "sentence_structure": "Subject鈥搗erb鈥揷omplement", "subjective_objective": {"answer": "Subjective", "rationale": "Because \"awaken too early\" is a personal feeling or experience, it depends on the individual's biological clock and personal habits, and it is therefore viewed from a subjective perspective."}, "sentence_sentiment": {"answer": "Negative", "rationale": "Because \"saware too early\" can affect the quality of sleep and bring negative experiences."}, "language_style": {"answer": "Calm and direct", "rationale": "The entry directly asked the person surveyed about the time of his or her daily awakening, which was not overly adorational or emotional, and was an objective and concise presentation."}, "time_dimension": {"answer": "Present", "rationale": "The entry is for the present behaviour and experience of the testee, and the time dimension is now judged."}, "sentence_type": "Negative declarative sentence", "pos": {"verbs": {"You're awake too early": 1}, "adjectives": {"Too early": 1}, "adverbs": {"very": 0, "Very": 0, "Too much": 1}, "numerals": {"64": 1}}}, {"content": "65 must wash hands repeatedly. Point numbers or touch something.", "word_count": 16, "label": "", "sentence_structure": "Subject鈥搗erb鈥揷omplement", "subjective_objective": {"answer": "Objective", "rationale": "Because \"repeat hands,\" \"number\" and \"touching something\" are objective behavioral."}, "sentence_sentiment": {"answer": "Negative", "rationale": "Because the behaviour in the entry \"repeated hand wash\" is a manifestation of coercion, usually associated with negative emotions such as anxiety, stress, etc."}, "language_style": {"answer": "Tactful and warm", "rationale": "This questionnaire entry is more euphemistic and warm, not using direct orders or critical language, but rather by asking questions to guide respondents to reflect on their habits and encourage them to reflect and change."}, "time_dimension": {"answer": "Present", "rationale": "The word \"must\" in \"must wash hands repeatedly\" means \"must do,\" while \"repeat hands washes\" means that the act is continuous, so the time dimension of this questionnaire entry is now judged."}, "sentence_type": "Interrogative sentence", "pos": {"nouns": {"65": 1, "Wash your hands": 1, "Number of points": 1, "Touch": 1, "Something": 1}, "verbs": {"I have to": 1, "Repeatedly": 1, "Wash your hands": 1, "Number of points": 1, "Touch": 1}, "adverbs": {"Repeatedly": 1}, "numerals": {"65": 1}, "classifiers": {"individual": 1}}}, {"content": "66 sleeps badly.", "word_count": 6, "label": "", "sentence_structure": "Subject鈥搗erb鈥揷omplement", "subjective_objective": {"answer": "Subjective", "rationale": "Because \"sleeping in a precarious manner\" is a subjective feeling, different people may experience different experiences. While this may be related to some objective factors (such as environmental noise, mattress hardness, etc.), it depends primarily on the individual's feelings and subjective judgment."}, "sentence_sentiment": {"answer": "Negative", "rationale": "Because \"sleeping in a state of insomnia\" is a negative quality of sleep."}, "language_style": {"answer": "Tactful and warm", "rationale": ""}, "time_dimension": {"answer": "Present", "rationale": ""}, "sentence_type": "Exclamatory sentence", "pos": {"nouns": {"66": 1, "Sleep": 1, "It's not steady": 1, "Not deep": 1}, "adjectives": {"It's not steady": 1, "Not deep": 1}, "numerals": {"66": 1}}}, {"content": "67 has an impulse to break or destroy something.", "word_count": 12, "label": "", "sentence_structure": "Subject鈥搗erb鈥搊bject", "subjective_objective": {"answer": "Subjective", "rationale": "Because \"the urge to break or destroy something\" is the patient's inner feeling and thoughts, and it's a subjective angle."}, "sentence_sentiment": {"answer": "Negative", "rationale": "Because the act of \"a impulse to break or destroy something\" is negative."}, "language_style": {"answer": "Calm and direct", "rationale": "The language style of the entry is calm, straightforward, not using excessive emotional or rhetorical techniques, but rather directly asking the interviewee whether there is some sort of impulse to behave. This expression helps the interviewee to understand the problem better and reduces the impact of subjective evaluations, thereby increasing the objectivity of the questionnaire results."}, "time_dimension": {"answer": "Present", "rationale": "The keyword \"there is\" means a state of existence, \"the urge to break or destroy something\" is a description of the inner state of the person being tested, which is the question of the present situation, so the temporal dimension of the article is now."}, "sentence_type": "Imperative sentence", "pos": {"nouns": {"Expedition": 1, "Something": 1}, "verbs": {"Yes": 1, "Broken": 1, "Destruction": 1, "have": 1}, "numerals": {"67": 1}}}, {"content": "There are ideas or ideas that no one else has.", "word_count": 13, "label": "", "sentence_structure": "Subject鈥搗erb鈥揷omplement", "subjective_objective": {"answer": "Subjective", "rationale": "Because it's the patient's subjective feeling and the idea."}, "sentence_sentiment": {"answer": "Positive", "rationale": "Because the \"some other people have no ideas or ideas\" feature in the entry is positive, indicating that this person has a unique and innovative mind and personality."}, "language_style": {"answer": "Tactful and warm", "rationale": "Warm and warm."}, "time_dimension": {"answer": "Present", "rationale": ""}, "sentence_type": "Affirmative declarative sentence", "pos": {"nouns": {"Thoughts": 2, "Thinking": 1}, "verbs": {"have": 1}, "adjectives": {"others": 1, "No, I don't": 1}, "numerals": {"68": 1}}}, {"content": "69 is very sensitive to others.", "word_count": 9, "label": "", "sentence_structure": "Subject鈥揷opula鈥損redicative", "subjective_objective": {"answer": "Subjective", "rationale": "Because \"fear\" is a subjective feeling, it is necessary to judge whether there is a sensitization to others through the patient's own perception."}, "sentence_sentiment": {"answer": "Negative", "rationale": "Because the emotional state of \"sensitized\" in the entries is negative."}, "language_style": {"answer": "Tactful and warm", "rationale": "The entry is more personal and interactive in its expression of a soft, invitation-style conversation rather than a direct statement or order. At the same time, the subject matter of the questionnaire is described in more common and understandable language, which allows participants to better understand and answer questions."}, "time_dimension": {"answer": "Present", "rationale": "The keyword \"felt\" means the person's current subjective feelings and experience, and the description \"sensitized\" is also about the current state, so the entry is about the present situation."}, "sentence_type": "Interrogative sentence", "pos": {"nouns": {"69": 1, "others": 1, "Nerves": 1, "Allergies": 1}, "verbs": {"I feel": 1, "Pass": 1}, "numerals": {"69": 1, "individual": 1, "You're a bit of a sane": 1}, "pronouns": {"to": 1}}}, {"content": "70 people are uncomfortable in shops or cinemas.", "word_count": 18, "label": "", "sentence_structure": "Subject鈥搗erb鈥揷omplement", "subjective_objective": {"answer": "Objective", "rationale": "The fact that people feel uncomfortable in places such as shops, cinemas is an objective fact of personal experience, and this feeling is not related to subjective attitudes."}, "sentence_sentiment": {"answer": "Negative", "rationale": "Because the \"frightful\" emotion in the entry is negative."}, "language_style": {"answer": "Calm and direct", "rationale": "The entry directly expresses the question of whether the other party is uncomfortable with the subject of a large number of people, is not based on excessive rhetorical or emotional rendering, and is more objective in tone."}, "time_dimension": {"answer": "Present", "rationale": "The keyword \"70\" indicates a specific point of time, while \"frightfulness in a shop or cinema is described in terms of the person's current behaviour or feelings. Thus, the time dimension of the article is now."}, "sentence_type": "Negative declarative sentence", "pos": {"nouns": {"70": 1, "person": 1, "Local": 2, "Shop": 1, "Cinema": 1, "I feel": 1, "I'm not comfortable": 1}, "verbs": {"in": 1, "Wait": 1, "I feel": 1}, "adjectives": {"More": 1, "person": 1}, "numerals": {"70": 1, "2": 1}, "classifiers": {"individual": 1}}}, {"content": "71 feels like everything is difficult.", "word_count": 10, "label": "", "sentence_structure": "Subject鈥揷opula鈥損redicative", "subjective_objective": {"answer": "Subjective", "rationale": "Because it is difficult to feel is a subjective feeling and experience of an individual."}, "sentence_sentiment": {"answer": "Negative", "rationale": "The basis for the judgment: \"Esperate that everything is difficult\" in the entry is an expression of a negative emotional state, one that is filled with frustration and weakness in life."}, "language_style": {"answer": "Calm and direct", "rationale": ""}, "time_dimension": {"answer": "Past", "rationale": ""}, "sentence_type": "Exclamatory sentence", "pos": {"nouns": {"Things": 1, "Difficulties": 1}, "verbs": {"I feel": 1}, "adjectives": {"very": 1}, "numerals": {"71": 1}}}, {"content": "A little fear or panic.", "word_count": 8, "label": "", "sentence_structure": "Subject鈥搗erb鈥揷omplement", "subjective_objective": {"answer": "Objective", "rationale": "Fear or panic is an objective fact of the patient's experience."}, "sentence_sentiment": {"answer": "Negative", "rationale": "Because \"fear or panic\" is a negative sentiment."}, "language_style": {"answer": "Calm and direct", "rationale": "The entry expresses the psychological condition that the person under investigation may have, in numbers \"72\" and in the descriptive term \"fear or panic\" 鈥?without euphemism or emotional rendering. It can therefore be considered as being calm and straightforward."}, "time_dimension": {"answer": "Past", "rationale": "The entry is for the interview of the person who was tested for a period of time, and the keyword \"for a while\" indicates that the feeling is not continuous, but is sporadic, and therefore the temporal dimension is now judged."}, "sentence_type": "Exclamatory sentence", "pos": {"nouns": {"Fear": 1, "Scared": 1}, "verbs": {"For a while": 1}, "numerals": {"72": 1}}}, {"content": "73 feels uncomfortable eating in public.", "word_count": 14, "label": "", "sentence_structure": "Subject鈥揷opula鈥損redicative", "subjective_objective": {"answer": "Subjective", "rationale": "This perception is a subjective experience and feeling of the patient, unrelated to the views or behaviour of others, and therefore a subjective examination."}, "sentence_sentiment": {"answer": "Negative", "rationale": "Because the emotional expression \"uncomfortable\" in the entry is negative."}, "language_style": {"answer": "Calm and direct", "rationale": "The entry is a straightforward question of whether the respondents are uncomfortable in public, with a more objective, concise language style, and without excessive rhetoric and emotional colours."}, "time_dimension": {"answer": "Past", "rationale": ""}, "sentence_type": "Negative declarative sentence", "pos": {"nouns": {"73": 1, "I feel": 1, "Public": 1, "Something": 1, "I'm not feeling well": 1}, "verbs": {"Eat": 1}, "numerals": {"very": 1}}}, {"content": "74 often argue with people.", "word_count": 6, "label": "", "sentence_structure": "Subject鈥搗erb鈥搊bject", "subjective_objective": {"answer": "Subjective", "rationale": "Because \"often\" and \"disputing\" are described from the patient's subjective perspective, reflecting the fact that the patient may have a strong, competitive character."}, "sentence_sentiment": {"answer": "Negative", "rationale": "Because \"controversial\" is usually considered negative."}, "language_style": {"answer": "Calm and direct", "rationale": "The entry is more personal and interactive when it is spoken in a question-and-answer tone, rather than in direct statements or orders. At the same time, the term \"often\" is used to guide the respondents to think about their own behaviour frequency, without too much emotional or strong tone, and therefore the language style is euphemismal."}, "time_dimension": {"answer": "Present", "rationale": ""}, "sentence_type": "Exclamatory sentence", "pos": {"nouns": {"person": 1, "Arguments": 1}, "verbs": {"Regular": 1}, "adjectives": {"74": 1}, "numerals": {"74": 1}}}, {"content": "75 is nervous when alone.", "word_count": 10, "label": "", "sentence_structure": "Subject鈥搗erb鈥揷omplement", "subjective_objective": {"answer": "Subjective", "rationale": "Because \"nervous\" is an emotional experience, determined by the patient's own feelings and perceptions, and therefore subjective."}, "sentence_sentiment": {"answer": "Negative", "rationale": "Because \"nervous\" is a negative sentiment."}, "language_style": {"answer": "Calm and direct", "rationale": "The entry directly expresses the question of whether the person under investigation is nervous when he is alone, is simple, has no excessive rhetoric or emotional rendering and is more objective and cold."}, "time_dimension": {"answer": "Past", "rationale": "The keyword \"when alone\" indicates the past's behavior or state, and \"nerstroke\" also shows that it is a past feeling and experience. So the entry asks if the person tested was nervous when he was alone in the past and judged the time dimension as the past."}, "sentence_type": "Exclamatory sentence", "pos": {"nouns": {"person": 1, "Nerves": 1, "Nervous": 1}, "verbs": {"75": 1, "Alone": 1, "Time": 1, "Yes": 1, "Nerves": 1}, "adverbs": {"very": 1}, "numerals": {"75": 1}, "pronouns": {"I": 1}}}, {"content": "76 people didn't properly evaluate your performance.", "word_count": 16, "label": "", "sentence_structure": "Subject鈥搗erb鈥揷omplement", "subjective_objective": {"answer": "Subjective", "rationale": "Because others are given a feedback or perception of the patient, it is the patient ' s subjective view."}, "sentence_sentiment": {"answer": "Negative", "rationale": "Because the \"not properly evaluated\" act is negative."}, "language_style": {"answer": "Tactful and warm", "rationale": "The entry is more euphemistic and warm, asking if the interviewee has encountered a situation in which his performance has not been properly evaluated, and is expressed in a more moderate and inviting manner, making it more acceptable and responsive. At the same time, the entry is not overly direct or emotional and reflects the objectivity and neutrality of the questionnaire."}, "time_dimension": {"answer": "Present", "rationale": "The entry is for the current situation of the testee, and \"no one has properly evaluated your performance\" is a status statement, so it is now judged."}, "sentence_type": "Negative declarative sentence", "pos": {"nouns": {"others": 1, "you": 2, "Achievements": 1, "Evaluation": 1}, "verbs": {"Make it": 1, "No, I'm not": 1}, "adjectives": {"Appropriate": 1}, "numerals": {"76": 1}, "pronouns": {"to": 1}}}, {"content": "77, even when you're alone with someone else.", "word_count": 13, "label": "", "sentence_structure": "Subject鈥搗erb鈥揷omplement", "subjective_objective": {"answer": "Subjective", "rationale": "Because feeling alone is a subjective feeling and experience associated with the emotional and psychological state of the individual."}, "sentence_sentiment": {"answer": "Negative", "rationale": "Because the emotional description of \"fear alone\" is negative."}, "language_style": {"answer": "Tactful and warm", "rationale": "The entry uses a \"even if\" twisting structure and a sensitive language expression that asks if the other party has a sense of isolation in interpersonal relations. This expression is more accompanimental, gentler than a direct statement or order and more likely to trigger a resonance and open response from the person under investigation."}, "time_dimension": {"answer": "Present", "rationale": "The keyword \"even if with someone else\" and \"means that the act is a description of the current situation or of the customary situation, and therefore the temporal dimension is now judged."}, "sentence_type": "Negative declarative sentence", "pos": {"nouns": {"77": 1, "others": 1, "Alone": 1}, "verbs": {"Even if": 1, "and": 1, "in": 1, "Together": 2, "I feel": 1}, "adverbs": {"Yeah": 1}, "numerals": {"77": 1}, "pronouns": {"others": 1}}}, {"content": "78 feels restless and unstable.", "word_count": 10, "label": "", "sentence_structure": "Subject鈥搗erb鈥揷omplement", "subjective_objective": {"answer": "Subjective", "rationale": "Because it's the patient's subjective feelings and experiences."}, "sentence_sentiment": {"answer": "Negative", "rationale": "Because \"silent\" and \"silent\" are negative emotional descriptions."}, "language_style": {"answer": "Calm and direct", "rationale": ""}, "time_dimension": {"answer": "Present", "rationale": ""}, "sentence_type": "Affirmative declarative sentence", "pos": {"nouns": {"Sit tight": 1, "Spirit": 1}, "verbs": {"I feel": 1, "Uncertainty": 1}, "numerals": {"78": 1}}}, {"content": "79 feels like he's of no value.", "word_count": 10, "label": "", "sentence_structure": "Subject鈥搗erb鈥揷omplement", "subjective_objective": {"answer": "Subjective", "rationale": "Because it is not an objective fact or an external event that feels that it has little value."}, "sentence_sentiment": {"answer": "Negative", "rationale": "Because \"no value\" in the entry is a negative emotional attitude."}, "language_style": {"answer": "Tactful and warm", "rationale": "The entry uses a soft, invited language style, asking if the interviewee would feel that he or she is of little value, and this expression is more personal and interactive and does not easily create pressure or anxiety among the interviewee."}, "time_dimension": {"answer": "Past", "rationale": "The entry is an expression of the current feelings or state of the person being tested, and the word \"discovery\" is an expression of a subjective sense of the current situation, which is judged by the temporal dimension as the past."}, "sentence_type": "Negative declarative sentence", "pos": {"nouns": {"79": 1, "self": 1, "Value": 1}, "verbs": {"I feel": 1, "have": 1}, "numerals": {"One": 1, "100": 1}, "pronouns": {"Where": 1}}}, {"content": "80 things that I'm familiar with become strangers or not real.", "word_count": 17, "label": "", "sentence_structure": "Subject鈥揷opula鈥損redicative", "subjective_objective": {"answer": "Subjective", "rationale": "- It's subjective to be strange or not like real is a sense or cognitive experience."}, "sentence_sentiment": {"answer": "Negative", "rationale": "Because the description \"getting familiar and becoming stranger\" in the entry is negative, implying negative emotional experience."}, "language_style": {"answer": "Tactful and warm", "rationale": "The language style of the entry is more euphemistic and sensitive to each other's feelings, and it resonates with the respondents by using specific descriptions like \"the things familiar are not known or are not real,\" rather than directly questioning or challenging them. Such expressions make it easier for the respondents to share their real thoughts."}, "time_dimension": {"answer": "Present", "rationale": "At the same time, \"fear\" also shows that it is the experience and experience of the testee, and can therefore be seen as the expression of the present."}, "sentence_type": "Exclamatory sentence", "pos": {"nouns": {"Something": 2, "True": 1, "I know you": 1}, "verbs": {"鈾?Turn into 鈾?: 1, "be": 1}, "adjectives": {"Strange": 1, "It's not like that": 1}, "adverbs": {"80": 1}}}, {"content": "81 screaming or falling.", "word_count": 6, "label": "", "sentence_structure": "Subject鈥搗erb鈥揷omplement", "subjective_objective": {"answer": "Subjective", "rationale": "Because \"81 Shout or Bite\" is an expression of emotion, reflecting the patient's subjective emotional state."}, "sentence_sentiment": {"answer": "Negative", "rationale": "Because the act of \"screaming or falling something\" is usually considered as a negative, negative emotional expression."}, "language_style": {"answer": "Tactful and warm", "rationale": "The entry uses a more moderate language style, with no too strong language or direct accusations, but rather a more open and understanding way to direct the answer. This expression is more likely to create a good atmosphere of communication and encourage respondents to share their true feelings."}, "time_dimension": {"answer": "Past", "rationale": ""}, "sentence_type": "Imperative sentence", "pos": {"nouns": {"81": 1, "Shouting": 1, "Something": 1}, "verbs": {"Fall": 1}, "adverbs": {"or": 1}, "numerals": {"81": 1}}}, {"content": "82 afraid of fainting in public.", "word_count": 10, "label": "", "sentence_structure": "Subject鈥搗erb鈥揷omplement", "subjective_objective": {"answer": "Subjective", "rationale": "Because fear is the patient's subjective emotional reaction."}, "sentence_sentiment": {"answer": "Negative", "rationale": "Because the mood of \"fearing to faint in public\" in the entry is negative."}, "language_style": {"answer": "Calm and direct", "rationale": "The language style of the entry is straightforward and it raises the question in an objective and clear manner, without adding too many emotional colours or rhetorical techniques to allow a clear understanding of the meaning of the issue."}, "time_dimension": {"answer": "Present", "rationale": "The \"fear of fainting in public\" in this entry is the current state or feeling of the person being tested, and therefore the temporal dimension is now judged."}, "sentence_type": "Exclamatory sentence", "pos": {"nouns": {"82": 1, "Scared": 1, "in": 1, "Public": 1, "- I'm fainted": 1}, "verbs": {"Yes": 1, "Scared": 1}, "numerals": {"82": 1}, "classifiers": {"individual": 1}}}, {"content": "83 feels like someone wants to take advantage of you.", "word_count": 9, "label": "", "sentence_structure": "Subject鈥揷opula鈥損redicative", "subjective_objective": {"answer": "Subjective", "rationale": "Because \"fEssing\" is a subjective feeling and perception."}, "sentence_sentiment": {"answer": "Negative", "rationale": "Because the \"others want to take advantage of you\" act is negative."}, "language_style": {"answer": "Calm and direct", "rationale": "The entry directly expresses the question whether the other party often feels that others want to take advantage of themselves, does not use excessive rhetorical or emotional renderings, and is more objective. It can therefore be concluded that the language style of the purpose is \"cool and direct\"."}, "time_dimension": {"answer": "Present", "rationale": "The \"feel\" in the entry is an expression of the current feeling or experience, and the \"others want to take advantage of you\" may be an ongoing or recent event, and therefore is a time dimension of the present."}, "sentence_type": "Imperative sentence", "pos": {"nouns": {"83": 1, "person": 1, "Cheap": 1}, "verbs": {"I feel": 1, "Yes": 1, "Jim": 1}, "numerals": {"83": 1, "One": 2}, "classifiers": {"individual": 1}, "pronouns": {"you": 1}}}, {"content": "84 was upset about some sexual ideas.", "word_count": 13, "label": "", "sentence_structure": "Subject鈥搗erb鈥揷omplement", "subjective_objective": {"answer": "Subjective", "rationale": "Because it is a subjective description of what the patient thinks inside himself."}, "sentence_sentiment": {"answer": "Negative", "rationale": "The basis for the judgement: the word \"probable\" in the entry expresses a negative emotional state, i.e. a feeling of distress and distress at the idea of sex. Thus, the emotional colour of the purpose of the article is negative."}, "language_style": {"answer": "Calm and direct", "rationale": "The entry raises the question in a direct manner, without too much qualifier or emotional colour, and with a more idiosyncratic and direct language style."}, "time_dimension": {"answer": "Present", "rationale": "The keyword \"fear\" is the emotional experience the subject is experiencing, so the time dimension of the article is now."}, "sentence_type": "Affirmative declarative sentence", "pos": {"nouns": {"84": 1, "Some": 1, "Sex": 1, "Thoughts": 1, "Trouble": 1}, "verbs": {"Yes": 1, "be": 1}, "numerals": {"84": 1, "One": 2}, "classifiers": {"Some": 1, "individual": 1}}}, {"content": "You think you should be punished for your own fault.", "word_count": 17, "label": "", "sentence_structure": "Subject鈥搗erb鈥搊bject", "subjective_objective": {"answer": "Subjective", "rationale": "It is subjective because it looks at the thoughts and opinions of the patient."}, "sentence_sentiment": {"answer": "Negative", "rationale": "Because the purpose of the \"punishment for his fault\" in the entry is negative."}, "language_style": {"answer": "Calm and direct", "rationale": "The language style of the entry is straightforward and it raises a question directly, without using excessive rhetorical or emotional rendering, and with a more balanced and objective tone."}, "time_dimension": {"answer": "Present", "rationale": "The keywords are \"conceived\" and \"required\", which indicate that the testee is thinking and judging the current situation, and therefore the time dimension should be judged now."}, "sentence_type": "Exclamatory sentence", "pos": {"nouns": {"Wrong": 1, "Punishment": 1}, "verbs": {"Reactions": 1, "Yes": 1, "I think": 1}, "adjectives": {"self": 1}, "numerals": {"85": 1}, "pronouns": {"you": 1}}}, {"content": "86 feels like it's time to finish.", "word_count": 10, "label": "", "sentence_structure": "Subject鈥搗erb鈥揷omplement", "subjective_objective": {"answer": "Subjective", "rationale": "This is often a feeling or idea of the patient, and is viewed from a subjective perspective."}, "sentence_sentiment": {"answer": "Positive", "rationale": "Because \"do it quickly\" expresses a positive attitude, namely, a sense of urgency and motivation in the task."}, "language_style": {"answer": "Tactful and warm", "rationale": "The language style of the entry is euphemism, because it is a soft, invitation-style conversation, not a direct statement or order. Moreover, it uses a means of asking people to express their feelings and ideas freely, which is more friendly and open. Such expression can facilitate good communication with the subjects of the survey, thereby obtaining more accurate and effective data."}, "time_dimension": {"answer": "Present", "rationale": "So I'm judging the time dimension of this entry for now."}, "sentence_type": "Imperative sentence", "pos": {"nouns": {"86": 1, "Things": 1}, "verbs": {"I feel": 1, "Yes": 1, "Hurry up": 1, "Finish": 1}, "adverbs": {"Hurry up": 1}, "numerals": {"86": 1}, "pronouns": {"I": 1}}}, {"content": "87 feels a serious problem with his body.", "word_count": 12, "label": "", "sentence_structure": "Subject鈥搗erb鈥揷omplement", "subjective_objective": {"answer": "Subjective", "rationale": "Because \"fE\" is the patient's subjective feeling and experience, it's the patient's own feelings and perceptions."}, "sentence_sentiment": {"answer": "Negative", "rationale": "Because the expression \"severe physical problems\" in the entry is negative."}, "language_style": {"answer": "Calm and direct", "rationale": "The entry is more calm, objective, and does not have too many rhetorical or emotional expressions, which directly convey the subject of asking the other side whether it has any health problems."}, "time_dimension": {"answer": "Present", "rationale": "The time dimension of the article can be judged by the format and examples given by the title. Because the term \"fEs\" is usually used to describe events or situations that have occurred, and in this sentence, \"feez\" is a sense of the past and a memory. Thus, \"87 feels that his body is in serious condition.\" The question is whether the person tested has had a serious physical problem in the past."}, "sentence_type": "Interrogative sentence", "pos": {"nouns": {"87": 1, "Body": 1, "Problem": 1}, "verbs": {"I feel": 1, "have": 1, "Serious": 1}, "numerals": {"87": 1, "Serious": 1}, "classifiers": {"individual": 1}}}, {"content": "88 never felt close to anyone else.", "word_count": 11, "label": "", "sentence_structure": "Subject鈥揷opula鈥損redicative", "subjective_objective": {"answer": "Subjective", "rationale": "Because it is a subjective feeling of a person, it is viewed from the subjective point of view of the patient."}, "sentence_sentiment": {"answer": "Negative", "rationale": "Because the entry \"never felt close to anyone else\" expresses a negative emotion and attitude."}, "language_style": {"answer": "Calm and direct", "rationale": "The entry asks the respondent in a direct and clear manner whether he or she has ever felt close to another person, has an objective and neutral language style and has not been subjected to excessive rhetoric or emotional rendering."}, "time_dimension": {"answer": "Present", "rationale": ""}, "sentence_type": "Imperative sentence", "pos": {"nouns": {"Questionnaire Entry": 1, "person": 1, "Other": 1}, "verbs": {"I feel": 1, "Close": 1}, "numerals": {"88": 1}}}, {"content": "89 feels guilty.", "word_count": 6, "label": "", "sentence_structure": "Subject鈥揷opula鈥損redicative", "subjective_objective": {"answer": "Subjective", "rationale": "Because feeling guilty is a feeling and self-evaluation within a person, it is viewed from a subjective perspective."}, "sentence_sentiment": {"answer": "Negative", "rationale": "Because the \"guilty\" emotion in the entry is negative."}, "language_style": {"answer": "Calm and direct", "rationale": "The entry directly asks whether the interviewees often feel guilty, do not have too many rhetorical or emotional expressions, speak with a more honest and objective tone and are of a calm and direct language style."}, "time_dimension": {"answer": "Present", "rationale": ""}, "sentence_type": "Exclamatory sentence", "pos": {"nouns": {"Sin": 1, "Sensor": 1, "self": 1}, "verbs": {"have": 1}, "numerals": {"89": 1}}}, {"content": "I feel like I'm having a brain attack.", "word_count": 10, "label": "", "sentence_structure": "Subject鈥揷opula鈥損redicative", "subjective_objective": {"answer": "Subjective", "rationale": "Because feeling a brain is a subjective feeling and an idea."}, "sentence_sentiment": {"answer": "Negative", "rationale": "Because the description of \"brain-brain\" in the entry is negative and indicates a negative attitude towards one's abilities or health."}, "language_style": {"answer": "Calm and direct", "rationale": "The language style of the entry is relatively simple and does not involve too much qualifier or emotional rendering, but rather raises questions in an objective and direct manner aimed at obtaining the true feelings and perceptions of the person under investigation."}, "time_dimension": {"answer": "Present", "rationale": "The keyword \"90%\" indicates that the subjective feelings of the subject were derived during the self-assessment; the term \"perception of a brain disorder\" is a specific description of this subjective feeling, which also implies a state of immediate mental health. Thus, it can be judged that the entry belongs to the present."}, "sentence_type": "Negative declarative sentence", "pos": {"nouns": {"90": 1, "self": 1, "Brain": 1, "Mistake": 1}, "verbs": {"I feel": 1}, "numerals": {"90": 1}, "pronouns": {"self": 1}}}]}

{"questionnaire_title": "Gas test", "questionnaire_category": "Mental health", "item_count": 60, "emotion_shift_overall": "From positive to negative", "language_style_overall": "Neutral", "items": [{"content": "1. Work as secure as possible, and generally do not do things that are uncertain.", "word_count": 15, "label": "", "sentence_structure": "Subject鈥損redicate", "subjective_objective": {"answer": "Subjective", "rationale": "Because \"showing to be safe\" and \"doing nothing\" are the thoughts and attitudes of the patients."}, "sentence_sentiment": {"answer": "Positive", "rationale": "Because the entry \"to try to be safe\" and \"to do something unsure\" expresses a cautious, responsible attitude and behaviour, and it is positive."}, "language_style": {"answer": "Tactful and warm", "rationale": "The entry is more euphemistic and implicit, rather than directly expressing the subject of the other party鈥檚 preference for caution, but rather leads the interviewee to reflect on his/her habits in a question-and-answer manner. Moreover, the entry uses positive terms (such as 鈥渃onservation鈥? 鈥渦ncertainty鈥? aimed at creating a positive and positive atmosphere for dialogue."}, "time_dimension": {"answer": "Present", "rationale": "The entry now describes the style and habits of the testee, emphasizing the current behavioural orientation and thus judging \"now\"."}, "sentence_type": "Exclamatory sentence", "pos": {"nouns": {"Yes": 2, "It's safe": 1, "Hold on": 1}, "verbs": {"Do it": 3, "Seek": 1}, "adjectives": {"General": 1}, "adverbs": {"None": 1}, "pronouns": {"I": 1}}}, {"content": "2. When you encounter something of a breath, you are in a state of rage and you want to say everything you feel.", "word_count": 23, "label": "", "sentence_structure": "Subject鈥搗erb鈥搊bject", "subjective_objective": {"answer": "Subjective", "rationale": "Because \"uncool\" and \"inspiring to say everything in your heart\" are patients' subjective feelings and thoughts."}, "sentence_sentiment": {"answer": "Negative", "rationale": "Because the entries \"uncool\" and \"shy\" express a negative sentiment."}, "language_style": {"answer": "Calm and direct", "rationale": "The basis for the determination: The entry is a direct expression of the question of whether the other party has acted with intent to speak in the face of anger, without using excessive rhetoric or emotional rendering, and is more objective in tone. At the same time, the words \"irresistible\" and \"shygienic\" express emotional colours more directly, reflecting the calmness of the language style."}, "time_dimension": {"answer": "Present", "rationale": "The use of the keyword \"attached\" and \"just \" suggests that it is a sentence describing current behaviour, and the verb \"simplified to say everything in your heart\" suggests that it is a customary mental or emotional expression. Thus, the entry is mainly concerned with the present situation."}, "sentence_type": "Exclamatory sentence", "pos": {"nouns": {"Yes": 1, "In my heart": 1, "# Happy": 1}, "verbs": {"I met him": 1, "It's a big rage": 1, "Say it": 2, "Come out": 1}, "adjectives": {"- Yeah": 1, "# Happy": 1}, "adverbs": {"Just": 1}, "numerals": {"2": 1}}}, {"content": "3. One person is preferred to one person rather than many.", "word_count": 15, "label": "", "sentence_structure": "Subject鈥搗erb鈥搊bject", "subjective_objective": {"answer": "Subjective", "rationale": "Because the terms \"better\" and \"unwilling\" in the title are the expression of individual attitudes and feelings and are subjective."}, "sentence_sentiment": {"answer": "Negative", "rationale": "For the purpose of this article, \"it is preferable to have one officer, not many people together\" to indicate a negative, self-sole attitude and behaviour."}, "language_style": {"answer": "Calm and direct", "rationale": "The language style of the entry is more calm and objective, without too much emotional rendering or rhetorical, and directly expresses the view that the interviewee prefers to perform the task on his own rather than to work with others. Such language style helps to communicate information clearly and accurately, and is suitable for use in official questionnaires."}, "time_dimension": {"answer": "Past", "rationale": "The entry describes the testee ' s past experience and preferences and therefore judges the past."}, "sentence_type": "Affirmative declarative sentence", "pos": {"verbs": {"I'd rather not": 1, "Fuck": 1, "Yes": 1, "I don't want to": 1, "A lot": 1, "person": 1, "in": 1, "Together": 1}, "adjectives": {"Alone": 1, "A lot": 1}, "numerals": {"III": 1}, "pronouns": {"Here": 1}}}, {"content": "4. A new environment will soon be adapted.", "word_count": 12, "label": "", "sentence_structure": "Subject鈥損redicate", "subjective_objective": {"answer": "Subjective", "rationale": "Because \"fast-up\" is an individual's subjective feeling and experience, it's seen from a subjective perspective."}, "sentence_sentiment": {"answer": "Positive", "rationale": "Because \"familiar\" is positive."}, "language_style": {"answer": "Tactful and warm", "rationale": "The entry is an encouraging language style, not too direct or hard-line to ask about each other鈥檚 abilities, but rather to invite the interviewee to express his or her views in a gentle and friendly tone."}, "time_dimension": {"answer": "Past", "rationale": "The words \"to a new environment\", \"to adapt soon\" contained in the entry indicate that the act has already taken place and has a temporal dimension of the past. The temporal dimension of the article can therefore be judged to be the past."}, "sentence_type": "Affirmative declarative sentence", "pos": {"verbs": {"Present": 1, "Yes": 1, "Adaptation": 1}, "adjectives": {"Come on": 1, "New": 1}, "numerals": {"4": 1}}}, {"content": "5. Disappearing of serious irritation, such as screaming, voice, dangerous lenses, etc.", "word_count": 19, "label": "", "sentence_structure": "Subject鈥搗erb鈥搊bject", "subjective_objective": {"answer": "Subjective", "rationale": "Because \"aversion\" is an emotional experience, a subjective response to a severe irritation. It's about the patient's own feelings and perceptions, so it's subjective."}, "sentence_sentiment": {"answer": "Negative", "rationale": "Because the attitude of \"obsessed\" in the entries is negative."}, "language_style": {"answer": "Calm and direct", "rationale": "The language style of the entry is calm and straightforward. It uses simple vocabulary and speech structures to express aversion to severe irritation, without any qualifier or rhetorical techniques, and therefore is more straightforward.\""}, "time_dimension": {"answer": "Present", "rationale": "The entry is for the purpose of asking the testee about his or her reactions and emotional experience in the face of severe irritation, and the temporal dimension is now judged."}, "sentence_type": "Imperative sentence", "pos": {"nouns": {"Disgusting": 1, "Serious": 1, "Exciting": 1, "Screaming": 1, "Voice": 1, "Danger": 1, "Camera": 1}, "verbs": {"Like": 1, "Wait": 1}, "adjectives": {"Those": 1}, "adverbs": {"very": 1}}}, {"content": "6. When arguing, people are always pre-emptive and tend to pick on others.", "word_count": 17, "label": "", "sentence_structure": "Subject鈥搗erb鈥搊bject", "subjective_objective": {"answer": "Subjective", "rationale": "Because \"preemptive\" and \"like to pick up people\" are patients' subjective attitudes or behavioral habits."}, "sentence_sentiment": {"answer": "Negative", "rationale": "Because the entry \"pre-emptive\" and \"crisional\" are negative."}, "language_style": {"answer": "Calm and direct", "rationale": ""}, "time_dimension": {"answer": "Present", "rationale": "Now."}, "sentence_type": "Affirmative declarative sentence", "pos": {"nouns": {"Argument": 1, "person": 1, "Maker": 1, "Picky": 1}, "verbs": {"Always": 1, "First": 1, "Yeah": 1, "Pick": 1}, "adverbs": {"First": 1}, "numerals": {"6": 1}}}, {"content": "7. A quiet environment.", "word_count": 7, "label": "", "sentence_structure": "Subject鈥揷opula鈥損redicative", "subjective_objective": {"answer": "Subjective", "rationale": "Because \"like\" is a subjective feeling and attitude."}, "sentence_sentiment": {"answer": "Neutral", "rationale": "Because the entry does not have any emotional color."}, "language_style": {"answer": "Tactful and warm", "rationale": ""}, "time_dimension": {"answer": "Present", "rationale": ""}, "sentence_type": "Affirmative declarative sentence", "pos": {"nouns": {"Environment": 1}, "verbs": {"Yeah": 1, "Quiet": 1}}}, {"content": "I am good at dealing with people.", "word_count": 7, "label": "", "sentence_structure": "Subject鈥搗erb鈥揷omplement", "subjective_objective": {"answer": "Subjective", "rationale": "Because communication is an individual's subjective capacity assessment."}, "sentence_sentiment": {"answer": "Positive", "rationale": "Because the entry \"good to be with people\" is an expression of positive personal social skills."}, "language_style": {"answer": "Tactful and warm", "rationale": "The entry is more personal and interactive when it is spoken in a gentle and inviting tone, rather than in direct statements or orders. At the same time, \"good to be with people\" is also a more positive description, without negative emotions, so it is judged to be a euphemism."}, "time_dimension": {"answer": "Present", "rationale": "The term \"good to be with people\" in this entry is a means of the ability and habit of the person being tested, which may have occurred, present and future, but in this particular questionnaire entry it is more about the person being tested. Thus, the temporal dimension is now judged."}, "sentence_type": "Affirmative declarative sentence", "pos": {"nouns": {"Questionnaire Entry": 1, "- To be with someone": 1}, "verbs": {"Good at it": 1}}}, {"content": "Envious of those who are good at restraining their feelings.", "word_count": 14, "label": "", "sentence_structure": "Subject鈥揷opula鈥損redicative", "subjective_objective": {"answer": "Subjective", "rationale": "Because envy is a subjective emotional experience and expression."}, "sentence_sentiment": {"answer": "Negative", "rationale": "Because envy often carries a certain amount of negative sentiment. While \"good to restrain\" feelings can be seen as a positive quality, it is used in this matter to describe enviable objects, and the whole emotional colour is therefore negative."}, "language_style": {"answer": "Tactful and warm", "rationale": "Because the entry uses a positive term like \"envious\" and expresses in a moderate way the appreciation and preference of people who are good at restraining their feelings, without directly criticizing or blaming each other. This expression is more acceptable and is open to questions."}, "time_dimension": {"answer": "Present", "rationale": "The time dimension of the entry cannot be determined because it does not have clear time indicators or past events. But the expression of \"envious\" is usually associated with past experience and memory, because people tend to evaluate something on the basis of their own experience and observations. Thus, while the time dimension of the entry cannot be clearly judged, it can be assumed that it may be more relevant to the emotional experience of the past."}, "sentence_type": "Affirmative declarative sentence", "pos": {"nouns": {"Questionnaire": 1, "Entry": 1, "person": 1, "Emotion": 1}, "verbs": {"Envious": 1, "Restraint": 1}, "adjectives": {"Good at it": 1}, "pronouns": {"That": 1, "self": 1}}}, {"content": "Life is regular and rarely violates the system of utensils.", "word_count": 13, "label": "", "sentence_structure": "Subject鈥搗erb鈥揷omplement", "subjective_objective": {"answer": "Objective", "rationale": "Because the regularity of life and compliance with the system are objective conditions for patients, it is not subjective feelings or ideas."}, "sentence_sentiment": {"answer": "Positive", "rationale": "Because the descriptions of \"sequence\" and \"non-violation of the system\" are positive and indicate that the person under investigation is well-intentioned."}, "language_style": {"answer": "Calm and direct", "rationale": ""}, "time_dimension": {"answer": "Present", "rationale": ""}, "sentence_type": "Affirmative declarative sentence", "pos": {"nouns": {"Life": 1, "The interest system": 1}, "verbs": {"have": 1, "Violations": 1}, "adjectives": {"Very few": 1, "Normal": 1}, "adverbs": {"very": 1}, "numerals": {"10": 1}, "classifiers": {"individual": 1}}}, {"content": "In most cases, the mood is optimistic.", "word_count": 12, "label": "", "sentence_structure": "Subject鈥揷opula鈥損redicative", "subjective_objective": {"answer": "Subjective", "rationale": "Because \"in most cases, emotions are optimistic\" is a description of the emotional state of a person, and it is subjective."}, "sentence_sentiment": {"answer": "Positive", "rationale": "According to the title, \"Empress is optimistic\" is a positive emotional attitude."}, "language_style": {"answer": "Tactful and warm", "rationale": "The language style of the entry is more euphemistic, and it uses a positive form to describe the possibility of the interviewee, i.e., the mood is optimistic in most cases. It avoids outright denial or criticism of the mood of the interviewee, but communicates with the interviewee in a more inclusive and understandable way. Moreover, the use of the phrase \"in most cases\" leaves the interviewee some room for greater comfort and comfort in answering."}, "time_dimension": {"answer": "Past", "rationale": "Although the sentence does not clearly indicate a time mark, it describes the state of the mood at hand, not past experience or future scenarios."}, "sentence_type": "Exclamatory sentence", "pos": {"nouns": {"Situation": 1, "Emotion": 1, "Optimistic": 1}, "verbs": {"be": 1, "Give": 1}, "adverbs": {"Most": 1}, "numerals": {"11": 1}}}, {"content": "12. The encounter with strangers was very ludicrous.", "word_count": 10, "label": "", "sentence_structure": "Subject鈥揷opula鈥損redicative", "subjective_objective": {"answer": "Subjective", "rationale": "It is the feelings and experiences inside the patient that are very confined to strangers and are subjective in nature."}, "sentence_sentiment": {"answer": "Negative", "rationale": "Because the attitude of \"resisting\" in the entry is negative."}, "language_style": {"answer": "Calm and direct", "rationale": "The entry uses a direct language style to ask if the interviewee is not being held hostage to strangers, without too many rhetorical or emotional renderings."}, "time_dimension": {"answer": "Present", "rationale": "The keyword is \"touch,\" which means that the act is now taking place, and \"felt\" expresses a subjective feeling, so the whole sentence describes the current state of experience or emotional state of the person being tested."}, "sentence_type": "Affirmative declarative sentence", "pos": {"nouns": {"Stranger": 1, "- You're not": 1}, "verbs": {"I've got it": 1}, "numerals": {"12": 1}}}, {"content": "13. In an outrage, self-restraint is well achieved.", "word_count": 16, "label": "", "sentence_structure": "Subject鈥搗erb鈥揷omplement", "subjective_objective": {"answer": "Objective", "rationale": "Because it is an objective fact to encounter an outrage, the ability to self-restraint is also a personal characteristic or behaviour that is considered from an objective perspective."}, "sentence_sentiment": {"answer": "Positive", "rationale": "Because the act of self-restraint in the entries is positive."}, "language_style": {"answer": "Tactful and warm", "rationale": "The language style of the entry is more euphemistic. First, the description of \"frustling an outrage\" does not directly emphasize the negative nature of the event, but rather uses a more neutral presentation; second, the evaluation of \"better self-restraint\" does not impose too much on the other side to have some capacity, but rather expresses a positive expectation and advice. This language style is more intimate and warmer."}, "time_dimension": {"answer": "Present", "rationale": "The entry describes the reaction and behaviour of the person being tested in the face of an outrage, a state or behaviour that is instantaneous or short and therefore judged \"now\"."}, "sentence_type": "Affirmative declarative sentence", "pos": {"nouns": {"Yes": 1, "Self": 1, "Restraint": 1}, "verbs": {"I met him": 1, "Yes": 1, "Very well": 1}, "adjectives": {"It's so angry": 1}, "adverbs": {"very": 1}, "numerals": {"13": 1}}}, {"content": "14. There is always a lot of energy to do.", "word_count": 10, "label": "", "sentence_structure": "Subject鈥搗erb鈥揷omplement", "subjective_objective": {"answer": "Subjective", "rationale": "Because \"always\" shows patient's subjective feelings and attitudes."}, "sentence_sentiment": {"answer": "Positive", "rationale": "Because the attitude of having a lot of energy in the entries is positive."}, "language_style": {"answer": "Tactful and warm", "rationale": "The language style of the entry is euphemism. It is more personal and interactive in its kind words of invitation to ask whether the person surveyed is always dynamic and full of spirit when doing things, rather than directly speaking or ordering."}, "time_dimension": {"answer": "Present", "rationale": "At the same time, the entry does not address what may happen in the future, so the judgment on the temporal dimension is also \"now\"."}, "sentence_type": "Affirmative declarative sentence", "pos": {"verbs": {"Do it": 1, "Yes": 1, "have": 1, "Wang-seok": 1}, "adjectives": {"Always": 1}}}, {"content": "Problems are always met with uncertainty and indecisiveness.", "word_count": 14, "label": "", "sentence_structure": "Subject鈥搗erb鈥揷omplement", "subjective_objective": {"answer": "Subjective", "rationale": "Because \"uncertainty\" and \"indecisiveness\" are patients' self-awareness and attitude. This describes how they deal with problems and how they are difficult to make decisions, reflecting their individuality and mental state. These factors are part of individual subjective experience, so the entry is viewed from a subjective point of view."}, "sentence_sentiment": {"answer": "Negative", "rationale": "Because the \"sweet indecisive\" act in the entry is negative."}, "language_style": {"answer": "Calm and direct", "rationale": "The entry is more calm and straightforward, has no excessive rhetorical or emotional rendering, and is more objective. It opens up to a question that needs to be answered, so that the interviewee can clearly and clearly know what to say.\""}, "time_dimension": {"answer": "Present", "rationale": "The entry \"Always\" indicates that this is a customary, recurring situation, while \"indecisiveness\" is a term describing personal attitudes and behaviour. The questionnaire entry therefore primarily asks about the behaviour or mental characteristics of the person being tested."}, "sentence_type": "Negative declarative sentence", "pos": {"nouns": {"Problem": 1, "Indecisiveness": 1}, "verbs": {"Always": 1, "I'm not sure what to do": 1}, "numerals": {"15": 1}}}, {"content": "16. There is never a feeling of over-restraint among the population.", "word_count": 12, "label": "", "sentence_structure": "Subject鈥揷opula鈥損redicative", "subjective_objective": {"answer": "Objective", "rationale": "It is objective because it is a subjective experience to feel too restrained, and the question is whether it is too restrained in the population."}, "sentence_sentiment": {"answer": "Neutral", "rationale": "The entry is not an expression of any emotional preference."}, "language_style": {"answer": "Tactful and warm", "rationale": "The entry is more euphemistic, not using too direct or hard language, but rather asking in a gentle, respectful and understanding way. It is introduced as a topic \"in the crowd,\" and then, by asking \"never too restrained\" it seeks to understand how the interviewees feel in social settings, and to express it in a more friendly and intimate way."}, "time_dimension": {"answer": "Present", "rationale": "The keywords \"in the crowd\" and \"never felt too restrained\" describe the current state of feeling or feeling, so the answer is now."}, "sentence_type": "Affirmative declarative sentence", "pos": {"nouns": {"Population": 1, "That's too much": 1, "Constraints": 1}, "verbs": {"I think": 1, "Never": 1}, "adverbs": {"in": 1}, "numerals": {"16": 1}}}, {"content": "17. When emotions are high, when it is fun and when it is low, it feels like nothing.", "word_count": 33, "label": "", "sentence_structure": "Subject鈥搗erb鈥揷omplement", "subjective_objective": {"answer": "Subjective", "rationale": "The purpose of the questionnaire is to describe the subjective feelings and experiences of patients, and how their emotional changes affect their interests and perceptions and are therefore subjective."}, "sentence_sentiment": {"answer": "Negative", "rationale": "Because the entry describes two extreme emotional states without any apparent positive tendencies or objectives. At the same time, the expression \"when emotions are down, feel like nothing\" has a certain negative connotation. Therefore, the emotional attitude of the purpose of the questionnaire is negative."}, "language_style": {"answer": "Tactful and warm", "rationale": "The language style of the entry is more euphemistic and moderate, and is not too direct or too strong. It guides the interviewee to share his feelings in an invitation and inquiry, rather than ordering or directly presenting the facts. This expression is more humane and acceptable."}, "time_dimension": {"answer": "Present", "rationale": "The question was about the effect of the emotional changes of the interviewees on their daily activities, and the time dimension is now judged. The key words are \"in high moods\" and \"in low moods\" , which show that the impact occurs within the current time frame."}, "sentence_type": "Affirmative declarative sentence", "pos": {"nouns": {"Time": 2, "Emotion": 2, "Things": 2}, "verbs": {"I think": 2, "Fuck": 1, "Interesting": 1, "It's not funny": 1}, "adjectives": {"High": 1, "Low": 1}, "numerals": {"17": 1}, "pronouns": {"What": 2}}}, {"content": "18. When attention is focused on one thing, it is difficult to distract me from the other.", "word_count": 20, "label": "", "sentence_structure": "Subject鈥揷opula鈥損redicative", "subjective_objective": {"answer": "Subjective", "rationale": "Because \"focus\" and \"discretion\" are subjective experiences of patients."}, "sentence_sentiment": {"answer": "Neutral", "rationale": "Because the entry does not have any emotional color."}, "language_style": {"answer": "Tactful and warm", "rationale": "The entry is more personal and interactive in its expression of a gentle and inviting tone of speech, rather than a direct statement or order. At the same time, it is a clever expression of concern and concern for the ability of respondents to focus, which is respected and understood."}, "time_dimension": {"answer": "Present", "rationale": "The entry asks the testee's current behaviour or state, i.e. whether it is easy to be disturbed by something else when you focus on it. Time dimension is now."}, "sentence_type": "Affirmative declarative sentence", "pos": {"nouns": {"Attention": 1, "Something": 1, "Yes": 2, "I": 1}, "verbs": {"Focus on the": 1, "鈾?And I'm gonna be 鈾?: 1, "Discretion": 1}, "adjectives": {"very": 1, "Hard": 1}, "adverbs": {"When": 1}, "numerals": {"18": 1}}}, {"content": "19. The problem is always understood faster than others.", "word_count": 9, "label": "", "sentence_structure": "Subject鈥搗erb鈥揷omplement", "subjective_objective": {"answer": "Subjective", "rationale": "Because \"understanding the problem is always faster\" is a subjective feeling and self-evaluation of a person, and the ability to judge one's own ability based on the individual's subjective experience. It has nothing to do with objective facts, so it is a subjective issue."}, "sentence_sentiment": {"answer": "Positive", "rationale": "Because the entry is a positive feature of personal competence, \"understanding is always faster than others\" is a positive performance."}, "language_style": {"answer": "Calm and direct", "rationale": "The questionnaire is based on the following: the subject is one sentence, with no excessive rhetorical or emotional rendering, and is more objective in tone. At the same time, it is a specific ability to ask the interviewee, and the way in which the question is asked is more direct and clear. So I think the language style of the article is straightforward and calm."}, "time_dimension": {"answer": "Present", "rationale": "The entry asks the testee whether there is a situation in the current life, namely, \"understanding the problem is always faster than others\", and therefore the time dimension is now judged."}, "sentence_type": "Affirmative declarative sentence", "pos": {"nouns": {"Problem": 1, "person": 1}, "verbs": {"Understood": 1, "- Yeah": 1, "Come on": 1}, "adverbs": {"Total": 1}, "numerals": {"19": 1}, "pronouns": {"others": 1}}}, {"content": "20. In dangerous situations, there is often a sense of extreme terror.", "word_count": 15, "label": "", "sentence_structure": "Subject鈥揷opula鈥損redicative", "subjective_objective": {"answer": "Subjective", "rationale": "Because \"extreme terror\" is the patient's subjective feeling."}, "sentence_sentiment": {"answer": "Negative", "rationale": "Because the \"extremely terror\" sentiment in the entry is negative."}, "language_style": {"answer": "Calm and direct", "rationale": "The entry describes the potential of the subject in a dangerous situation in a direct, concise language, without too much rhetorical or emotional rendering. Such language allows the respondent to understand the meaning of the question more accurately and to give a true answer."}, "time_dimension": {"answer": "Present", "rationale": "The keywords \"at risk\" and \"extreme terror\" point to the circumstances that the subject may experience at the moment."}, "sentence_type": "Affirmative declarative sentence", "pos": {"nouns": {"Dangerous situation": 1, "Horror": 1}, "verbs": {"I've got it": 1, "- I'm sure": 1}, "adjectives": {"Extreme": 1}, "numerals": {"20": 1}}}, {"content": "21. There is a high level of enthusiasm for learning and working.", "word_count": 12, "label": "", "sentence_structure": "Subject鈥搗erb鈥揷omplement", "subjective_objective": {"answer": "Subjective", "rationale": "Because passion is a subjective feeling and attitude, it is determined by the individual himself, not by the external environment or by objective facts."}, "sentence_sentiment": {"answer": "Positive", "rationale": "Because the \"high passion\" in the entry is a positive emotional attitude."}, "language_style": {"answer": "Tactful and warm", "rationale": "This entry is used in a soft, inviting tone to talk and ask the respondents whether they are enthusiastic about their studies, work, and not directly speaking or ordering."}, "time_dimension": {"answer": "Present", "rationale": "The entry describes the attitude and emotional state of the person tested, and the temporal dimension is now judged."}, "sentence_type": "Affirmative declarative sentence", "pos": {"nouns": {"Learning": 1, "Work": 1, "鈾?Hot": 1}, "adjectives": {"High": 1}, "adverbs": {"very": 1}}}, {"content": "22. A long and boring job.", "word_count": 13, "label": "", "sentence_structure": "Subject鈥揷opula鈥損redicative", "subjective_objective": {"answer": "Objective", "rationale": "Because the sentence describes a capability or skill that exists objectively, independent of the individual's subjective factors. Even if a person does not feel that the job is boring and monotonous, he/she may have such a capability."}, "sentence_sentiment": {"answer": "Negative", "rationale": "Because the \"Long-term dry, monotonous work\" act is negative."}, "language_style": {"answer": "Calm and direct", "rationale": "The language style of the entry is more direct, with no excessive qualifier or emotional colour, and a greater emphasis on the expression of facts and information."}, "time_dimension": {"answer": "Present", "rationale": "From a time perspective, the issue is not clearly time-limited and can therefore be classified as \"now\"."}, "sentence_type": "Affirmative declarative sentence", "pos": {"nouns": {"Work": 1, "Boring": 1, "Undo": 1}, "verbs": {"Yes": 1, "Do it": 1}, "adjectives": {"Long time": 1, "Bored": 1, "Unique": 1}, "numerals": {"22": 1}}}, {"content": "23. What suits your interest, and it is done in full or otherwise it is not done.", "word_count": 22, "label": "", "sentence_structure": "Subject鈥搗erb鈥揷omplement", "subjective_objective": {"answer": "Subjective", "rationale": "Because \"what suits your interests,\" \"doing everything in a good way,\" \"doing nothing else\" and so forth are the subjective feelings and attitudes of patients to their own behaviour and emotions."}, "sentence_sentiment": {"answer": "Positive", "rationale": "Because the \"doing what suits your interest, doing what you want\" behavior and attitude are positive."}, "language_style": {"answer": "Tactful and warm", "rationale": "The entry is more personal and interactive when it is spoken in a gentle and inviting tone, rather than in direct statements or orders. At the same time, it uses graphic metaphors (such as \"smokes\"), making questionnaires more interesting and attractive."}, "time_dimension": {"answer": "Present", "rationale": "The entry expresses the attitude and feelings of the testee to do what he likes, which is not about time, and therefore is judged as \"now\"."}, "sentence_type": "Affirmative declarative sentence", "pos": {"nouns": {"Things": 1, "Interest": 1, "Camera": 1, "Fuck": 2}, "verbs": {"Match": 1, "Yeah": 1, "Fuck": 2, "Just": 1}, "adjectives": {"Perfect": 1, "Otherwise": 1}, "numerals": {"23": 1}, "pronouns": {"self": 1}}}, {"content": "24. A little bit can cause emotional fluctuations.", "word_count": 12, "label": "", "sentence_structure": "Subject鈥揷opula鈥損redicative", "subjective_objective": {"answer": "Subjective", "rationale": "Because \"emotional fluctuations\" are subjective perceptions, reflecting the emotional state and level of response of the patient. Although this may have been caused by objective events, the perception, understanding and reaction to such events are subjective, so the questionnaire entry looks at the subjective perception of the patient."}, "sentence_sentiment": {"answer": "Negative", "rationale": "Because the behavior of \"floating\" in the entries is negative."}, "language_style": {"answer": "Calm and direct", "rationale": "The entry describes a phenomenon in a concise way, namely, that the person interviewed is vulnerable to the influence of small things. It is not used too much rhetorical or emotionally to make it sound, and it is more objective and calm."}, "time_dimension": {"answer": "Past", "rationale": ""}, "sentence_type": "Affirmative declarative sentence", "pos": {"nouns": {"Things": 1, "Emotional fluctuations": 1}, "verbs": {"Yes": 1, "Cause": 1}, "adjectives": {"Small": 1, "Points": 1}, "numerals": {"24": 1}}}, {"content": "25. Tired of doing the kind of work that requires patience and care.", "word_count": 13, "label": "", "sentence_structure": "Subject鈥揷opula鈥損redicative", "subjective_objective": {"answer": "Subjective", "rationale": "Because \"inflicted\" is the individual feeling and attitude of the patient, the entry is viewed from a subjective perspective."}, "sentence_sentiment": {"answer": "Negative", "rationale": "Because the \"happy\" attitude in the entries is negative."}, "language_style": {"answer": "Calm and direct", "rationale": "The entry directly expresses the subject of questioning whether the other party prefers to work with patience and detail, without excessive rhetoric or emotional rendering, and with a more objective tone."}, "time_dimension": {"answer": "Present", "rationale": "The keyword \"inadmissible\" denotes a current behaviour or attitude, \"a patient, careful work\" refers to a specific type of work, which, together, shows the current feelings and perceptions of the testee with regard to such work. The entry is therefore a question of describing the current behaviour or attitude of the testee, and the temporal dimension is now judged."}, "sentence_type": "Negative declarative sentence", "pos": {"nouns": {"Work": 1, "Patience": 1, "Quite nuanced": 1}, "verbs": {"Boring": 1}, "adverbs": {"Yes": 1, "Well": 1, "Plant": 1}, "numerals": {"25": 1}}}, {"content": "26. People are not indifferent to each other.", "word_count": 8, "label": "", "sentence_structure": "Subject鈥搗erb鈥揷omplement", "subjective_objective": {"answer": "Subjective", "rationale": "Because \"no lowness\" is an expression of an individual's attitude or attitude, it reflects the values and behaviour of patients in their interpersonal relationships and is therefore subjective in nature."}, "sentence_sentiment": {"answer": "Positive", "rationale": "Because \"no shame\" expresses a positive approach to the world."}, "language_style": {"answer": "Tactful and warm", "rationale": "The entry uses the phrase \"no lowness\" to describe attitudes in interpersonal relationships, which are more euphemistic and moderate in language. This way makes the interviewee more comfortable with the problem and more likely to share his real thoughts. At the same time, it also respects the feelings and dignity of the interviewee."}, "time_dimension": {"answer": "Present", "rationale": "The keywords \"to be in touch\" and \"to be not humbled\" indicate that it is the present behaviour or attitude of the testee, so it can be judged that the entry is about the present situation."}, "sentence_type": "Affirmative declarative sentence", "pos": {"verbs": {"- To be with someone": 1, "No shame, no shame": 1}, "adjectives": {"No shame, no shame": 1}}}, {"content": "27. A keen event was a welcome event.", "word_count": 9, "label": "", "sentence_structure": "Subject鈥揷opula鈥損redicative", "subjective_objective": {"answer": "Subjective", "rationale": "Because the desire to participate in a lively event reflects the patient ' s personal preferences and attitudes and is subjective."}, "sentence_sentiment": {"answer": "Positive", "rationale": "Because the act of \"like to go to a lively event\" is positive, it means that this person is passionate about social and activities."}, "language_style": {"answer": "Tactful and warm", "rationale": "The entry is more personal and interactive in its expression of conversation in a gentle and inviting tone, rather than a direct statement or order. At the same time, the image of \"heat activity\" has been used, making the questionnaire more interesting."}, "time_dimension": {"answer": "Present", "rationale": ""}, "sentence_type": "Affirmative declarative sentence", "pos": {"verbs": {"Yeah": 1, "Participation": 1}, "adjectives": {"Hot": 1}}}, {"content": "28. A love for the emotional detail, and a writing of the person ' s inner activity.", "word_count": 19, "label": "", "sentence_structure": "Subject鈥搗erb鈥揷omplement", "subjective_objective": {"answer": "Subjective", "rationale": "Because \"love to see\" is the patient's personal preference and tendency, and it's a subjective attitude."}, "sentence_sentiment": {"answer": "Positive", "rationale": "Because the entry \"Lictation of the mind of the person\" expresses positive emotions and attitudes."}, "language_style": {"answer": "Tactful and warm", "rationale": "The entry allows respondents to share their reading preferences in an interview, without direct conclusions or criticism of the other party鈥檚 choice. In addition, a more moderate and friendly tone is used to enhance the comfort and participation of the interviewee."}, "time_dimension": {"answer": "Present", "rationale": "So, in conclusion, \"28. Loves to be sensitive, and writes about the literary work of the person's inner activity.\" The time dimension of this questionnaire item can be judged now."}, "sentence_type": "Imperative sentence", "pos": {"nouns": {"28": 1, "Love": 1, "Emotion": 1, "Fine": 1, "Writing": 1, "People": 1, "Inner activities": 1, "Literary work": 1}, "verbs": {"Look": 1, "be": 1}, "adjectives": {"It's a delicate feeling": 1, "The inner movement of people": 1}, "numerals": {"28": 1}, "classifiers": {"individual": 1}}}, {"content": "29. Working learning is long and tired.", "word_count": 13, "label": "", "sentence_structure": "Subject鈥搗erb鈥揷omplement", "subjective_objective": {"answer": "Subjective", "rationale": "Because it is a subjective emotional experience, it is a subjective perspective."}, "sentence_sentiment": {"answer": "Negative", "rationale": "Because \"weary\" in the entries is a sign of negative emotions."}, "language_style": {"answer": "Calm and direct", "rationale": "The entry is based on a direct, concise approach that expresses the question whether the other party is often tired of the subject after a long period of work or study, without too much ado or emotional, and with a more honest and objective tone. Thus, we can consider the language style to be a straightforward type of calm."}, "time_dimension": {"answer": "Present", "rationale": "The keywords \"work learning long\" and \"often tired\" indicate that the act is regular, customary and is happening to the person who speaks. So the entry describes the current behavior or state, the answer is \"now\"."}, "sentence_type": "Affirmative declarative sentence", "pos": {"nouns": {"Work": 1, "Learning time": 1, "Tired": 1}, "verbs": {"I feel": 1, "Long": 1, "Yes": 1}, "adverbs": {"Regular": 1}, "numerals": {"29": 1}}}, {"content": "30. Not to talk about a problem for long periods of time, and to be ready to do it.", "word_count": 19, "label": "", "sentence_structure": "Subject鈥搗erb鈥揷omplement", "subjective_objective": {"answer": "Subjective", "rationale": "Because \"like\" and \"not want\" are the personal feelings and attitudes of the patient, and they are subjective."}, "sentence_sentiment": {"answer": "Positive", "rationale": "Because the behaviour of the entries \"always to talk about a problem for a long time and to actually do it\" is positive, indicating that respondents prefer actual action rather than rhetoric."}, "language_style": {"answer": "Calm and direct", "rationale": "The entry is presented in a direct way, asking the interviewee whether he does not like to discuss a problem for long periods of time, but rather prefers to act."}, "time_dimension": {"answer": "Present", "rationale": "The entries \"unwilled\" and \"will\" express the testee's current attitude and behavioral orientation, so they are judged now."}, "sentence_type": "Affirmative declarative sentence", "pos": {"nouns": {"Problem": 1, "Time": 1, "Actual": 1, "Hands and feet": 1}, "verbs": {"I don't like it": 1, "Talk": 1, "Yes": 1, "Fuck": 1}, "adjectives": {"Long time": 1}, "adverbs": {"Yeah": 1}, "numerals": {"30": 1}}}, {"content": "31. The language was more obstinate than the whispering.", "word_count": 12, "label": "", "sentence_structure": "Subject鈥搗erb鈥揷omplement", "subjective_objective": {"answer": "Subjective", "rationale": "Because \"I'd rather talk than whisper\" is the patient's personal preference and attitude, and it's subjective."}, "sentence_sentiment": {"answer": "Neutral", "rationale": "Because the entry does not have any emotional color."}, "language_style": {"answer": "Calm and direct", "rationale": "The entry is simple and straightforward, directly expressing the subject of asking the other side what mode of communication is more favourable, and is not based on too many rhetorical or emotional renderings, and is more objective in tone."}, "time_dimension": {"answer": "Present", "rationale": "Key words \"better\" and \"responsible\" indicate that this is a subjective feeling that requires the person to answer according to his or her own reality. Moreover, \"speaking\" and \"stalking\" are two different ways of communication, the former being more open, direct, and the latter being more closed and private. The entry therefore looks at the social character characteristics of the person being tested."}, "sentence_type": "Affirmative declarative sentence", "pos": {"verbs": {"I'd rather": 1, "I'm talking about it": 1, "I don't want to": 1, "The whispering": 1}}}, {"content": "32. I was often said to be depressed.", "word_count": 10, "label": "", "sentence_structure": "Subject鈥揷opula鈥損redicative", "subjective_objective": {"answer": "Subjective", "rationale": "Because \"others always say\" describes the behaviour and attitude of the patient from the perspective of others, it is viewed objectively. But the idea of \"I'm bored\" is the patient's subjective feeling, so it's subjective. Taken together, the entry contains both objective facts (the other person's assessment of the patient) and subjective experience of the patient (the patient's perception)."}, "sentence_sentiment": {"answer": "Negative", "rationale": "Because the phrase \"I'm always bored\" in the entry is negative."}, "language_style": {"answer": "Calm and direct", "rationale": "The entry describes the image of the person under investigation in the eyes of others in an objective and straightforward manner, without excessive emotional or rhetorical modification and with a greater focus on the presentation and transmission of facts."}, "time_dimension": {"answer": "Present", "rationale": "The keyword \"total\" and \"said\" indicates that it is a constant, customary description, involving current or past behaviour or state, and therefore judgement is now."}, "sentence_type": "Affirmative declarative sentence", "pos": {"nouns": {"person": 1, "It's boring": 1}, "verbs": {"Say it": 1}, "adjectives": {"It's boring": 1}, "numerals": {"32": 1}, "pronouns": {"I": 1}}}, {"content": "33. Understanding problems is often slower than others.", "word_count": 10, "label": "", "sentence_structure": "Subject鈥揷opula鈥損redicative", "subjective_objective": {"answer": "Subjective", "rationale": "Because understanding is the expression of patients ' subjective feelings and cognitive abilities."}, "sentence_sentiment": {"answer": "Negative", "rationale": "Because the \"understanding problems\" in the entry is negative."}, "language_style": {"answer": "Calm and direct", "rationale": "This is an objective, concise expression that is more in line with the linguistic characteristics and requirements of the psychological questionnaire."}, "time_dimension": {"answer": "Past", "rationale": ""}, "sentence_type": "Affirmative declarative sentence", "pos": {"nouns": {"Problem": 1, "person": 1}, "verbs": {"Understood": 1, "- Yeah": 1, "Slow": 1}, "adjectives": {"Some": 1}, "numerals": {"33": 1}}}, {"content": "34. A brief break is a time of fatigue when a person can be in a state of shock and work.", "word_count": 22, "label": "", "sentence_structure": "Subject鈥搗erb鈥搊bject", "subjective_objective": {"answer": "Subjective", "rationale": "Because \"a brief break can shake the mind\" and \"a job\" is a personal experience and a subjective one."}, "sentence_sentiment": {"answer": "Positive", "rationale": "Because the entry describes a positive expression of how a short break is needed to get back to work when we're tired."}, "language_style": {"answer": "Tactful and warm", "rationale": "The entry is more friendly and interactive when it is presented in a gentle and inviting tone rather than in direct statements or orders. At the same time, the article is more acrimony, less emotional and more focused on communication and communication with the respondents."}, "time_dimension": {"answer": "Present", "rationale": "The key words for the question about the current situation of the subject are \"tired time\", \"short rest\" and \"sweetness\" -- all behaviour or experience that describe the current state."}, "sentence_type": "Affirmative declarative sentence", "pos": {"nouns": {"Tired": 1, "Time": 1, "Short": 1, "Rest": 1, "Spirit": 1, "鈾?Swinging": 1, "Input": 1, "Work": 1}, "verbs": {"Come on": 1}, "adverbs": {"Just": 1}, "numerals": {"34": 1}}}, {"content": "35. There is a thing in mind that you prefer to think about and not say it.", "word_count": 14, "label": "", "sentence_structure": "Subject鈥損redicate", "subjective_objective": {"answer": "Subjective", "rationale": "This is because the patient ' s description and feelings of his or her mental state are subjective."}, "sentence_sentiment": {"answer": "Negative", "rationale": "Because the \"not to say\" in the entry is negative."}, "language_style": {"answer": "Tactful and warm", "rationale": "The entry uses a milder expression, \"I think I'd rather have to\" display internality, not too direct or intense emotional. Moreover, \"I don't want to say it\" has a modest and cautious attitude, which is more in keeping with the euphemism of the language."}, "time_dimension": {"answer": "Present", "rationale": "The entry describes a customary state of behaviour, whereby the person being tested psychologically tends to think alone, rather than share it with others. This behaviour may have persisted in the past, present and future, and thus the temporal dimension is now judged."}, "sentence_type": "Affirmative declarative sentence", "pos": {"verbs": {"I'm telling you something": 1, "I'd rather": 1, "Think about it": 1, "I don't want to": 1, "Say it": 1}, "adverbs": {"I'd rather not": 1}, "numerals": {"35": 1}}}, {"content": "36. The achievement of a goal is an aspiration to be achieved as soon as possible, without success.", "word_count": 21, "label": "", "sentence_structure": "Subject鈥搗erb鈥揷omplement", "subjective_objective": {"answer": "Subjective", "rationale": "Because \"the idea of a goal is to achieve it as soon as possible, without a purpose, without rest\" is the idea and attitude of the patient, and is therefore subjective."}, "sentence_sentiment": {"answer": "Positive", "rationale": "Because the entry expresses a commitment to and determination to the goal, it is a positive emotional attitude."}, "language_style": {"answer": "Calm and direct", "rationale": "The entry is in a concise language that directly describes the commitment and determination of the interviewees to accomplish their mission or objectives, is not overly emotional or rhetorical, and is more directly and logically written."}, "time_dimension": {"answer": "Present", "rationale": ""}, "sentence_type": "Affirmative declarative sentence", "pos": {"nouns": {"Objective": 1, "Achieved": 1, "Purpose": 1, "Stop": 1}, "verbs": {"Confirm": 1, "Hope": 1, "Achieved": 1}, "adjectives": {"As soon as possible": 1}, "adverbs": {"Just": 1, "No, no, no, no": 1, "Swear": 1}}}, {"content": "37. Learning, and working for the same period, is often more exhausting than others.", "word_count": 18, "label": "", "sentence_structure": "Subject鈥揷opula鈥損redicative", "subjective_objective": {"answer": "Subjective", "rationale": "Because fatigue is a feeling of the patient ' s own feelings and experiences, it is a subjective examination."}, "sentence_sentiment": {"answer": "Negative", "rationale": "Because the \"more tired\" behavior and feeling is negative."}, "language_style": {"answer": "Calm and direct", "rationale": "The entry is more direct, simple, and has no excessive emotional or rhetorical style. It is simple to ask whether the other side feels more tired than others in the same study or work, and is more objective and calm."}, "time_dimension": {"answer": "Past", "rationale": "The keyword \"often more tired than others\" denotes a constant state or habit, while the phrase \"after the same period of time\" indicates that this is the temporal dimension considered in the comparison, and the entry is therefore inclined to ask if the person tested has done so in the past."}, "sentence_type": "Affirmative declarative sentence", "pos": {"nouns": {"Learning": 1, "Work": 1, "Tired": 1}, "verbs": {"Equal": 1, "- Yeah": 1, "More": 1}, "adverbs": {"Regular": 1}, "numerals": {"37": 1}}}, {"content": "38. There was a certain amount of recklessness and often no consideration of the consequences.", "word_count": 13, "label": "", "sentence_structure": "Subject鈥搗erb鈥揷omplement", "subjective_objective": {"answer": "Subjective", "rationale": "The basis for the judgement is that \"doing things recklessly, often without regard to consequences\" is the patient's subjective attitude and behaviour, reflecting the behaviour and character characteristics of the patient, not the objective facts. The questionnaire entry is therefore viewed from a subjective perspective."}, "sentence_sentiment": {"answer": "Negative", "rationale": "Because the entry \"doing something reckless, often without consideration of consequences\" is negative."}, "language_style": {"answer": "Tactful and warm", "rationale": "The entry is more euphemistic, using more objective and neutral terms such as \"doing things recklessly\" and \"often without taking consequences\" rather than directly accusing or criticizing. This expression makes the interviewee more comfortable with understanding and being willing to receive feedback."}, "time_dimension": {"answer": "Present", "rationale": "According to the subject, \"38. acts are reckless and often \"in the light of consequences\" indicate that they are a customary act, which is being described or observed and is therefore directed at the present description."}, "sentence_type": "Negative declarative sentence", "pos": {"nouns": {"Yes": 2, "Rage": 1, "Consequences": 1}, "verbs": {"Do it": 1, "Consider": 1, "Regular": 1}, "adjectives": {"Some": 1}, "adverbs": {"Often": 1}, "numerals": {"38": 1}}}, {"content": "39. Teachers or others teach new knowledge, and when they are technical, they always wish to speak more slowly and repeat it several times.", "word_count": 26, "label": "", "sentence_structure": "Subject鈥搗erb鈥搊bject", "subjective_objective": {"answer": "Subjective", "rationale": "Because \"I'd like him to say it slowly, repeat it more often\" is the patient's personal expectations and needs, and it's subjective."}, "sentence_sentiment": {"answer": "Positive", "rationale": "Because the entry expresses the desire for more patience and care in teaching knowledge and skills, whether by teachers or others, it is a positive learning attitude."}, "language_style": {"answer": "Tactful and warm", "rationale": "The language style of the entry is more euphemistic and respects the feeling of the respondents. By using the expression \"hope\" it makes the problem more positive than direct demands or orders. Moreover, the words \"slower\" and \"repeated more than once\" also reflect a caring and supportive attitude that makes the respondents more willing to share their thoughts and feelings."}, "time_dimension": {"answer": "Present", "rationale": ""}, "sentence_type": "Imperative sentence", "pos": {"nouns": {"Teacher": 1, "Others": 1, "Knowledge": 1, "Technology": 1, "he": 1, "Again": 1}, "verbs": {"Hope": 1, "Teaching": 1, "Slow down": 1, "Repeat": 1, "More": 1}, "adjectives": {"Slow down": 1, "More": 1, "How many times": 1}, "adverbs": {"Always": 1, "Some": 1, "More": 1}, "numerals": {"Thirty-nine": 1}, "classifiers": {"individual": 1}, "pronouns": {"he": 2}}}, {"content": "40. Unhappy events could be forgotten quickly.", "word_count": 13, "label": "", "sentence_structure": "Subject鈥搗erb鈥揷omplement", "subjective_objective": {"answer": "Subjective", "rationale": "For forgetting unpleasant things is a psychological process and capacity of individuals, and is a subjective level of experience and feeling."}, "sentence_sentiment": {"answer": "Positive", "rationale": "Because the \"can forget the unpleasant things soon\" act in the entry is positive."}, "language_style": {"answer": "Tactful and warm", "rationale": ""}, "time_dimension": {"answer": "Present", "rationale": ""}, "sentence_type": "Affirmative declarative sentence", "pos": {"nouns": {"Things": 1}, "verbs": {"Forget it": 1, "Yes": 1, "Come on": 1}, "adjectives": {"very": 1, "Happy": 1}, "numerals": {"40": 1}}}, {"content": "41. It takes more time to do a job or to complete a job than others.", "word_count": 18, "label": "", "sentence_structure": "Subject鈥搗erb鈥搊bject", "subjective_objective": {"answer": "Subjective", "rationale": "Because \"more time than others\" is a subjective comparison and feeling. While the task itself is an objective fact, the sense and judgement that it takes more time to do so is subjective."}, "sentence_sentiment": {"answer": "Negative", "rationale": "Because the entry describes a negative behaviour and attitude, i.e., inefficient."}, "language_style": {"answer": "Calm and direct", "rationale": "The language style of the entry is relatively calm, straightforward, with no excessive adoration or emotional colour, and is simple in language, indicating the subject of asking the other party whether it needs to spend more time in the exercise or work."}, "time_dimension": {"answer": "Present", "rationale": "In the future, the entry \"always\" indicates the situation that the person being tested may encounter at some point in the future, and the difficulty may last for some time. It can therefore be considered as a projection and vision of the future, meeting the criteria for future judgement."}, "sentence_type": "Affirmative declarative sentence", "pos": {"nouns": {"Operations": 1, "Annex": 1, "Work": 1}, "verbs": {"Do it": 1, "Completed": 1, "- Yeah": 1, "Flowers": 1}, "adjectives": {"Don't": 1, "More": 1}, "numerals": {"41": 1}, "pronouns": {"Total": 1}}}, {"content": "42. The type of sport that is highly sportsy or involved in various arts and cultural activities is preferred.", "word_count": 23, "label": "", "sentence_structure": "Subject鈥損redicate", "subjective_objective": {"answer": "Subjective", "rationale": "Because \"like\" is patient's subjective attitude."}, "sentence_sentiment": {"answer": "Positive", "rationale": "Because the entries \"likes a big sport\" and \"participating in a variety of arts and culture\" are positive descriptions."}, "language_style": {"answer": "Tactful and warm", "rationale": "The entry is an invitation-style language, which does not directly order or ask the interviewees to answer questions, but rather leads them in a gentle way to think and express their preferences. At the same time, the language style of the article is more friendly, friendly and more resonant and active."}, "time_dimension": {"answer": "Present", "rationale": "The entry asks the testee about his current habits or hobby, the keyword \"like\" and \"participate\" and they point to the current state and behaviour."}, "sentence_type": "Affirmative declarative sentence", "pos": {"nouns": {"Movement": 1, "High physical education": 1, "Various arts and cultural activities": 1}, "verbs": {"Yeah": 1, "Participation": 1}, "adverbs": {"Large": 1, "Extreme": 1, "Or": 1}, "numerals": {"42": 1}, "classifiers": {"individual": 1}}}, {"content": "43. One cannot quickly shift attention from one thing to another.", "word_count": 22, "label": "", "sentence_structure": "Subject鈥揷opula鈥損redicative", "subjective_objective": {"answer": "Subjective", "rationale": "Because this transfer of capacity is an expression of a subjective individual's sense of mind.\""}, "sentence_sentiment": {"answer": "Negative", "rationale": "Because \"can't quickly turn attention from one thing to another\" is negative."}, "language_style": {"answer": "Calm and direct", "rationale": "The entry describes the ability of the testee to shift his attention in a straightforward manner, without using euphemism or emotional colours, but rather with a simple statement."}, "time_dimension": {"answer": "Present", "rationale": "\"Now.\""}, "sentence_type": "Negative declarative sentence", "pos": {"nouns": {"Questionnaire": 1, "Entry": 1, "Attention": 1, "Yes": 2, "Transfer": 1}, "verbs": {"We can't put it fast": 1}, "adverbs": {"From": 1, "very": 1, "Come on": 1}, "numerals": {"43": 1}, "classifiers": {"individual": 1}}}, {"content": "44. Once a mandate had been accepted, it was hoped that it would be resolved expeditiously.", "word_count": 16, "label": "", "sentence_structure": "Subject鈥損redicate", "subjective_objective": {"answer": "Subjective", "rationale": "Because the idea of resolving the problem as soon as possible is patient's subjective attitude."}, "sentence_sentiment": {"answer": "Positive", "rationale": "Because the act of \"want to get it settled quickly\" shows the spirit and determination."}, "language_style": {"answer": "Calm and direct", "rationale": "The entry is in a concise language and directly asks the interviewee about his post-mission psychological state, is not based on excessive rhetorical or emotional rendering, is more objective and is therefore judged to be \"cool and direct \" language style."}, "time_dimension": {"answer": "Present", "rationale": "The entry asks the testee about the behaviour or mentality of the person following the assignment, and the time dimension is now judged."}, "sentence_type": "Affirmative declarative sentence", "pos": {"verbs": {"Accept": 1, "Hope": 1, "Resolve": 1}, "adjectives": {"Quick": 1}, "numerals": {"44": 1}}}, {"content": "45. It was considered that discipline was better than risk-taking.", "word_count": 12, "label": "", "sentence_structure": "Subject鈥損redicate", "subjective_objective": {"answer": "Subjective", "rationale": "Because it is a personal value, it is subjective."}, "sentence_sentiment": {"answer": "Negative", "rationale": "Because the \"better of the rules than risk\" approach in the entries is negative, and tends to be conservative and not aggressive."}, "language_style": {"answer": "Calm and direct", "rationale": "The language style of the entry is more straightforward, with no excessive rhetorical or emotional tact, and the tone is more neutral and objective. At the same time, the design of the question is more direct in asking the respondents about the way they behaved and risked, without vague or leading statements."}, "time_dimension": {"answer": "Past", "rationale": "The entry describes the views and attitudes of the respondent on a particular point of view, does not involve future behavioural or mental changes and can therefore be judged to be past."}, "sentence_type": "Affirmative declarative sentence", "pos": {"nouns": {"Questionnaire Entry": 1, "Keep it in order": 1, "Risk": 1}, "verbs": {"I think": 1, "Stronger": 1}, "adverbs": {"- Yeah": 1}, "numerals": {"45": 1}}}, {"content": "46. There are several things that can be looked at at at the same time.", "word_count": 10, "label": "", "sentence_structure": "Subject鈥搗erb鈥揷omplement", "subjective_objective": {"answer": "Subjective", "rationale": "Because the ability to focus on several things at the same time is an expression of the patient ' s personal subjective perception and cognitive ability, rather than objective external facts."}, "sentence_sentiment": {"answer": "Neutral", "rationale": "Because the entry describes only a capability or characteristic, it does not express any emotional colour."}, "language_style": {"answer": "Calm and direct", "rationale": "The language style of the entry is calm and straightforward, and it clearly and succinctly asks the interviewees, without too much embellishment or emotional expression, and is objective and neutral."}, "time_dimension": {"answer": "Present", "rationale": ""}, "sentence_type": "Affirmative declarative sentence", "pos": {"verbs": {"Yes": 1, "Attention": 1, "Annex": 1}, "numerals": {"How many": 1}, "classifiers": {"individual": 1}}}, {"content": "When I was bored, it was hard for others to cheer me up.", "word_count": 17, "label": "", "sentence_structure": "Subject鈥搗erb鈥揷omplement", "subjective_objective": {"answer": "Subjective", "rationale": "Because \"silent emotions\" and \"unhappy feelings\" are the subjective experiences of patients themselves."}, "sentence_sentiment": {"answer": "Negative", "rationale": "Because the \"It's hard for me to be happy\" in the entry is negative."}, "language_style": {"answer": "Calm and direct", "rationale": "This entry directly reflects the fact that the subject of the investigation is difficult to be promoted by others when he or she is in a low mood, without excessive rhetoric or emotional rendering, and with objective and direct tone."}, "time_dimension": {"answer": "Present", "rationale": ""}, "sentence_type": "Negative declarative sentence", "pos": {"nouns": {"Time": 1, "others": 1, "Boring": 1, "I": 1, "Happy": 1}, "verbs": {"鈾?And I'm gonna be 鈾?: 1, "Get up": 1}, "adjectives": {"It's hard": 1}, "numerals": {"47": 1}}}, {"content": "48. The story of the story is acrimony and agitation.", "word_count": 13, "label": "", "sentence_structure": "Subject鈥搗erb鈥揷omplement", "subjective_objective": {"answer": "Subjective", "rationale": "Because \"love\" represents patients' subjective feelings and attitudes to novels."}, "sentence_sentiment": {"answer": "Positive", "rationale": "Because the entry \"Float, Excited novel\" shows a positive emotional attitude of love."}, "language_style": {"answer": "Tactful and warm", "rationale": "The entry is more personal and interactive when it is spoken in a gentle and inviting tone, rather than in direct statements or orders. At the same time, the words \"look\" and \"excited\" convey positive emotional sentiments that make readers more resonant."}, "time_dimension": {"answer": "Past", "rationale": ""}, "sentence_type": "Affirmative declarative sentence", "pos": {"nouns": {"Novel": 1}, "verbs": {"Love it": 1, "Excited": 1, "Rise and shine": 1}, "adjectives": {"Small": 1}, "numerals": {"48": 1}}}, {"content": "49. Work is rigorous and consistent.", "word_count": 15, "label": "", "sentence_structure": "Subject鈥搗erb鈥揷omplement", "subjective_objective": {"answer": "Subjective", "rationale": "Because attitudes towards work are subjective views and attitudes of the individual."}, "sentence_sentiment": {"answer": "Positive", "rationale": "Because the attitude in the entries is positive."}, "language_style": {"answer": "Tactful and warm", "rationale": "The entry uses an encouraging and motivated language style, which allows respondents to reflect on themselves and express their expectations and affirmations of working attitudes."}, "time_dimension": {"answer": "Present", "rationale": "\"Strateful and consistent approach to work\" describes an act or attitude that does not involve the time dimension of the past or the future and therefore determines the present."}, "sentence_type": "Affirmative declarative sentence", "pos": {"nouns": {"Work": 1, "Attitude": 1}, "verbs": {"Hug": 1, "Seriously": 1, "Strict": 1, "Always": 2}, "numerals": {"49": 1}}}, {"content": "50. Relations with the surroundings are always poor.", "word_count": 13, "label": "", "sentence_structure": "Subject鈥搗erb鈥揷omplement", "subjective_objective": {"answer": "Subjective", "rationale": "Because \"always bad\" is the patient's subjective feeling and evaluation."}, "sentence_sentiment": {"answer": "Negative", "rationale": "Because the \"bad-to-get\" behavior in the entries is negative."}, "language_style": {"answer": "Calm and direct", "rationale": "The entry is a direct expression of the subject of questioning the other party about the difficulties of interpersonal relations, and is not based on excessive rhetorical or emotional rendering, and is more objective in tone."}, "time_dimension": {"answer": "Present", "rationale": "The entry now describes the current social situation of the person being tested, using the general current \"always\" to indicate that this situation is recurrent and persistent. Thus, the temporal dimension is now judged."}, "sentence_type": "Negative declarative sentence", "pos": {"nouns": {"Around": 1, "person": 2, "Relations": 1, "Get along": 1}, "verbs": {"Always": 1, "Not good": 1}, "adverbs": {"Regular": 1}, "numerals": {"50": 1}}}, {"content": "51. The knowledge that has been studied is preferred to be repeated in skilled work.", "word_count": 19, "label": "", "sentence_structure": "Subject鈥搗erb鈥搊bject", "subjective_objective": {"answer": "Subjective", "rationale": "Because \"like\" and \"do it over and over\" are the personal feelings or attitudes of the patient, it's a subjective approach."}, "sentence_sentiment": {"answer": "Positive", "rationale": "Because \"Living knowledge\" and \"Repeating skilled work\" in the entries are positive learning attitudes and behaviours."}, "language_style": {"answer": "Tactful and warm", "rationale": "The entry is more gentle and friendly in its language, using an invitation tone to encourage respondents to share their ideas and experiences, without a sense of direct command or criticism. At the same time, the sentence is simple and easy to understand."}, "time_dimension": {"answer": "Present", "rationale": "The keywords \"like\" and \"repeat\" point to the conduct or habit in progress, so it can be established that this is a problem of the present. At the same time, the question relates to attitudes to learning and work, which further confirms that it is a question of the present investigation."}, "sentence_type": "Affirmative declarative sentence", "pos": {"nouns": {"Review": 1, "Knowledge": 1, "Work": 1}, "verbs": {"Yeah": 1, "Repeat": 1, "Do it": 1, "Skilled": 1}, "adverbs": {"Yes": 1}, "numerals": {"51": 1}}}, {"content": "52. The hope is for a change in the work that is very varied and varied.", "word_count": 12, "label": "", "sentence_structure": "Subject鈥搗erb鈥搊bject", "subjective_objective": {"answer": "Subjective", "rationale": "Because hope is a subjective attitude and desire, \"changed and varied work\" is also a subjective assessment of and expectation for work."}, "sentence_sentiment": {"answer": "Positive", "rationale": "Because \"changed, diverse\" jobs are usually fresh and stimulating, the expression of the purpose of the article is positive and emotional."}, "language_style": {"answer": "Tactful and warm", "rationale": "The language style of the entry is euphemistic. It does not directly ask or order the interviewee to do a great deal of work, but rather expresses the subject in a gentle and inviting manner, which is more personal and interactive."}, "time_dimension": {"answer": "Present", "rationale": ""}, "sentence_type": "Imperative sentence", "pos": {"nouns": {"Work": 1, "Change": 1, "- That's a trick": 1}, "verbs": {"Hope": 1, "Do it": 1}, "adjectives": {"Large": 1, "More": 1}, "numerals": {"52": 1}}}, {"content": "53. I seem to remember more than anyone else the poetry that comes to me in hours.", "word_count": 18, "label": "", "sentence_structure": "Subject鈥搗erb鈥搊bject", "subjective_objective": {"answer": "Subjective", "rationale": "Because remembering is the patient's subjective feeling and memory, not the objective fact."}, "sentence_sentiment": {"answer": "Neutral", "rationale": "Because the entry describes a personal memory phenomenon, it does not contain any apparent emotional tendencies."}, "language_style": {"answer": "Tactful and warm", "rationale": ""}, "time_dimension": {"answer": "Past", "rationale": "The key words \"hours\" and \"likely remembers more clearly than others\" in the entry indicate that this is a memory and comparison of the past. So the temporal dimension is the past."}, "sentence_type": "Affirmative declarative sentence", "pos": {"nouns": {"Hours": 1, "Poetry": 1, "person": 1, "others": 1, "Memory": 1}, "verbs": {"Back": 1, "Yes": 2}, "adverbs": {"Seems like": 1, "- Yeah": 1, "Clear": 1}, "numerals": {"53": 1}, "pronouns": {"I": 1}}}, {"content": "54. I'm not the one who says \"speaks to hurt.\"", "word_count": 16, "label": "", "sentence_structure": "Subject鈥搗erb鈥揷omplement", "subjective_objective": {"answer": "Subjective", "rationale": "Because the sentence expresses the patient's subjective feelings and self-evaluation, which is \"me being said to hurt\" but the patient does not believe that he did so, which is a rebuttal and interpretation of objective facts and is therefore subjective in nature."}, "sentence_sentiment": {"answer": "Negative", "rationale": "Because \"speaking to hurt\" is negative."}, "language_style": {"answer": "Calm and direct", "rationale": "The entry presents the criticisms of the other side in an objective and direct manner, and its own response, without too much emotional overtones or euphemism. Its language style is therefore straightforward."}, "time_dimension": {"answer": "Present", "rationale": "The word \"said\" in \"I say \"injured\" is a general past, while the word \"not\" in \"but I don't think\" indicates that this is still the case, so the entry describes the situation now."}, "sentence_type": "Imperative sentence", "pos": {"nouns": {"others": 1, "I": 2, "It hurts": 1}, "verbs": {"Say it": 1, "Not really": 1}, "adverbs": {"very": 1}}}, {"content": "55. In sports activities, slow responses often lag behind.", "word_count": 14, "label": "", "sentence_structure": "Subject鈥搗erb鈥揷omplement", "subjective_objective": {"answer": "Objective", "rationale": "Because it is an objective phenomenon that describes patients in sports."}, "sentence_sentiment": {"answer": "Negative", "rationale": "Because the expression \"slow-reactioned and backward\" is negative, it implies that the subjects are not doing well enough in sports."}, "language_style": {"answer": "Calm and direct", "rationale": "The entry directly asks the other party about the speed of reaction in sports activities in a simple and clear language, without using excessive qualifiers or emotional colours and with a more objective and cold tone."}, "time_dimension": {"answer": "Past", "rationale": "The entry asks about the behaviour of the person tested in past sports events, and therefore the temporal dimension is judged to be the past."}, "sentence_type": "Affirmative declarative sentence", "pos": {"nouns": {"Sports activities": 1, "Response": 2, "Backward": 1}, "verbs": {"in": 1, "Common cause": 1, "And behind": 1}, "adverbs": {"Slow": 1}, "numerals": {"55": 1}}}, {"content": "56. Responsive, smart.", "word_count": 8, "label": "", "sentence_structure": "Subject鈥搗erb鈥揷omplement", "subjective_objective": {"answer": "Subjective", "rationale": "Because agility and intelligence are subjective feelings and evaluations."}, "sentence_sentiment": {"answer": "Positive", "rationale": "Because \"reacting fast\" and \"mutely\" are both positive terms."}, "language_style": {"answer": "Calm and direct", "rationale": "The entry is made up of a simpler, more explicit language, with no excessive emotional or rhetorical approach, with a greater emphasis on the description of objective facts, and therefore the language style is \"cool and direct\"."}, "time_dimension": {"answer": "Present", "rationale": ""}, "sentence_type": "Affirmative declarative sentence", "pos": {"adjectives": {"Agility": 1, "Smart": 1}, "adverbs": {"56": 1}}}, {"content": "57. Work that was organized and not so cumbersome was preferred.", "word_count": 13, "label": "", "sentence_structure": "Subject鈥揷opula鈥損redicative", "subjective_objective": {"answer": "Subjective", "rationale": "Because \"like\" is the patient's personal preference and attitude, and it's subjective."}, "sentence_sentiment": {"answer": "Positive", "rationale": "Because the \"likes to be organized\" attitude in the entries is positive."}, "language_style": {"answer": "Calm and direct", "rationale": "The entry is more directly linguistically, with no excessive adoration or emotional rendering, but rather expresses preference for working methods in a concise language. At the same time, the entry does not use too formal or complex terminology and sentences, so it is more calm and objective in its overall style of language."}, "time_dimension": {"answer": "Present", "rationale": ""}, "sentence_type": "Affirmative declarative sentence", "pos": {"nouns": {"Work": 1}, "verbs": {"Yeah": 1, "It's organized": 1, "It's not a problem": 1}, "adjectives": {"Structure": 1, "It's not too much trouble": 1}, "adverbs": {"very": 1}, "numerals": {"57": 1}}}, {"content": "58. Excited things often keep me awake.", "word_count": 9, "label": "", "sentence_structure": "Subject鈥揷opula鈥損redicative", "subjective_objective": {"answer": "Subjective", "rationale": "Because insomnia is a subjective experience that the patient feels."}, "sentence_sentiment": {"answer": "Negative", "rationale": "Because the result of insomnia in the entries is negative."}, "language_style": {"answer": "Tactful and warm", "rationale": ""}, "time_dimension": {"answer": "Present", "rationale": "The keywords \"excited\" and \"insomnia\" all indicate that this is a description of the situation, so it's judged to be the present."}, "sentence_type": "Affirmative declarative sentence", "pos": {"nouns": {"Yes": 1, "Sleep": 1}, "verbs": {"鈾?And I'm gonna be 鈾?: 1, "Excited": 1, "Insomnia": 1}, "adjectives": {"Regular": 1}, "numerals": {"58": 1}}}, {"content": "59. Teachers were new and often unable to understand, but it was difficult to forget when they understood.", "word_count": 22, "label": "", "sentence_structure": "Subject鈥搗erb鈥搊bject", "subjective_objective": {"answer": "Subjective", "rationale": "Because \"often don't understand\" is the subjective assessment of the patient's ability to listen, \"to understand it\" is also the subjective feeling and perception of the patient. These are the inner experiences and feelings of the individual, and are therefore viewed from a subjective perspective."}, "sentence_sentiment": {"answer": "Neutral", "rationale": "As the entry is only an objective statement of a situation, it does not contain positive or negative emotional aspects."}, "language_style": {"answer": "Tactful and warm", "rationale": "The language style of the entry is more euphemistic and uses a number of rhetorical techniques to express the respondents' understanding and feelings about the way the teacher is taught, such as \"often incomprehensible\" and \"hard to forget\" , which are both direct and polite and, to some extent, provide more space and flexibility for respondents to respond."}, "time_dimension": {"answer": "Present", "rationale": ""}, "sentence_type": "Affirmative declarative sentence", "pos": {"nouns": {"Teacher": 1, "New concepts": 1}, "verbs": {"Speak": 1, "I don't understand": 2, "Get it": 1, "Forget it": 1}, "adjectives": {"Often": 1, "Hard": 1}, "numerals": {"59": 1}, "pronouns": {"I": 1}}}, {"content": "60. If the work is boring, it will be low soon.", "word_count": 16, "label": "", "sentence_structure": "Subject鈥損redicate", "subjective_objective": {"answer": "Subjective", "rationale": "Because \"frightful\" is the subjective feeling of a patient when he's bored and bored."}, "sentence_sentiment": {"answer": "Negative", "rationale": "Because the \"fun\" act in the entry is negative."}, "language_style": {"answer": "Calm and direct", "rationale": "The language style of the entry is more straightforward, with no excessive embellishment or emotional rendering, and in a simple language, the idea that \"if work is boring, it makes people feel less.\""}, "time_dimension": {"answer": "Present", "rationale": "\"If\" means \"if\" the scenario, \"it will be \"so soon\" is a description of the person's current emotional state. The entry therefore mainly concerns the current situation and feelings."}, "sentence_type": "Affirmative declarative sentence", "pos": {"nouns": {"Work": 1, "Boring": 1, "Tasteless": 1, "Emotion": 1, "Down": 1}, "verbs": {"Suppose": 1, "Yes": 1, "Bored and boring": 1, "Soon": 1}, "adjectives": {"Boring": 1, "Tasteless": 1, "Down": 1}, "numerals": {"60": 1}}}]}

{"questionnaire_title": "Self-measured health assessment forms (SRHMS)", "questionnaire_category": "Mental health", "item_count": 48, "emotion_shift_overall": "From negative to positive", "language_style_overall": "Neutral", "items": [{"content": "1. How's your vision?", "word_count": 7, "label": "", "sentence_structure": "Subject鈥損redicate", "subjective_objective": {"answer": "Objective", "rationale": "Since vision is a quantifiable biological indicator, independent of the patient ' s subjective consciousness, it can be derived from medical examinations."}, "sentence_sentiment": {"answer": "Neutral", "rationale": "Because the entry is only for the purpose of asking the person ' s vision and does not contain any emotional colour."}, "language_style": {"answer": "Calm and direct", "rationale": "The entry is straightforward in language, is simple and clear in its eye view, does not add too much emotional colour or rhetoric, and is more calm and straightforward."}, "time_dimension": {"answer": "Present", "rationale": "The entry asks the testee about his current visual condition, and the time dimension is now judged."}, "sentence_type": "Interrogative sentence", "pos": {"nouns": {"Visual": 1}, "verbs": {"How's it going": 1}}}, {"content": "How's your hearing?", "word_count": 7, "label": "", "sentence_structure": "Subject鈥揷opula鈥損redicative", "subjective_objective": {"answer": "Subjective", "rationale": "Because hearing is what patients feel and evaluate."}, "sentence_sentiment": {"answer": "Neutral", "rationale": "Basis: The entry is merely asking about the hearing of the other party and does not express any feelings or attitudes."}, "language_style": {"answer": "Calm and direct", "rationale": "The entry is simple and clear inquiring about the hearing of the person under investigation, without using rhetorical techniques or excessive descriptions, and is more straightforward in language style."}, "time_dimension": {"answer": "Present", "rationale": ""}, "sentence_type": "Interrogative sentence", "pos": {"nouns": {"you": 1, "Hearing": 1}, "verbs": {"How's it going": 1}}}, {"content": "How's your appetite?", "word_count": 7, "label": "", "sentence_structure": "Subject鈥搗erb鈥揷omplement", "subjective_objective": {"answer": "Subjective", "rationale": "Because appetite is the subjective feeling and experience of the patient."}, "sentence_sentiment": {"answer": "Neutral", "rationale": "Because the entry is merely an inquiry into the appetite of the person interviewed and is not of any emotional colour."}, "language_style": {"answer": "Calm and direct", "rationale": "The entry is presented in a direct interview, without a euphemism or emotional language, or pre-set or directed the person ' s feelings, and therefore can be judged as being calm and straightforward."}, "time_dimension": {"answer": "Present", "rationale": "The entry asks the testee about his current appetite, and the time dimension is now judged."}, "sentence_type": "Interrogative sentence", "pos": {"nouns": {"A appetite": 1}, "verbs": {"How's it going": 1}}}, {"content": "4. Do you often have a bad stomach (e.g., stomach swelling, diarrhea, constipation)?", "word_count": 19, "label": "", "sentence_structure": "Subject鈥搗erb鈥搊bject", "subjective_objective": {"answer": "Objective", "rationale": "Because the condition of the gastrointestinal system is an objective physical fact of the patient."}, "sentence_sentiment": {"answer": "Negative", "rationale": "Because \"the stomach and gastrointestinal indiscommodities\" are often considered as physical incomprehensible and negative."}, "language_style": {"answer": "Tactful and warm", "rationale": "The language style of the entry is euphemism, because it uses polite and invited expressions to guide respondents to answer questions. It does not ask them directly about stomach discomfort, but rather uses a more moderate way to guide them to think and remember their health. It helps to create a climate of friendship, respect and support, making them more willing to share their feelings and experiences."}, "time_dimension": {"answer": "Past", "rationale": "The entry asks about the testee ' s past symptoms, and the temporal dimension is the past."}, "sentence_type": "Interrogative sentence", "pos": {"nouns": {"- The stomach": 1, "Department": 1, "I'm not well": 1, "Bourbon": 1, "I'm having a stomach attack": 1, "Decency": 1}, "verbs": {"Regular": 1, "not": 1, "Hm, I'm sorry": 1, "Like": 1, "Boo": 1, "Quasi": 1, "Secret": 1}, "adverbs": {"you": 1, "Wait": 1, "- You're not": 1}, "numerals": {"4锛?: 1}}}, {"content": "Are you tired?", "word_count": 7, "label": "", "sentence_structure": "Subject鈥損redicate", "subjective_objective": {"answer": "Subjective", "rationale": "Because fatigue is usually a subjective feeling of the individual."}, "sentence_sentiment": {"answer": "Negative", "rationale": "Because the description \"easy to get tired\" is usually considered negative."}, "language_style": {"answer": "Tactful and warm", "rationale": ""}, "time_dimension": {"answer": "Present", "rationale": ""}, "sentence_type": "Interrogative sentence", "pos": {"nouns": {"you": 1, "Tired": 1}, "verbs": {"Easy": 1, "I feel": 1}, "numerals": {"5锛?: 1}}}, {"content": "How's your sleep?", "word_count": 7, "label": "", "sentence_structure": "Subject鈥損redicate", "subjective_objective": {"answer": "Subjective", "rationale": "Because the quality of sleep is a subjective feeling of the patient, the perception of the same problem may vary among different people and therefore need to be viewed from the patient ' s subjective perspective."}, "sentence_sentiment": {"answer": "Neutral", "rationale": "Because the entry does not have any emotional color."}, "language_style": {"answer": "Calm and direct", "rationale": ""}, "time_dimension": {"answer": "Present", "rationale": "The entry asks about the current condition of the person being tested, that is, the present state of sleep. So the answer is \"now\"."}, "sentence_type": "Interrogative sentence", "pos": {"nouns": {"you": 1, "Sleep": 1}, "verbs": {"How's it going": 1}}}, {"content": "Do you have any pains of any kind?", "word_count": 13, "label": "", "sentence_structure": "Subject鈥損redicate", "subjective_objective": {"answer": "Objective", "rationale": "Because pain or not is the patient ' s state of health, it is an objective fact."}, "sentence_sentiment": {"answer": "Negative", "rationale": "Because the description \"with varying degrees of pain\" in the entry is negative."}, "language_style": {"answer": "Calm and direct", "rationale": "The language style of the entry is calm and straightforward. It is used to ask questions about the physical condition of the person interviewed."}, "time_dimension": {"answer": "Present", "rationale": "The entry asks the person under examination about his current state of health, i.e. whether he feels pain, and the temporal dimension is now judged."}, "sentence_type": "Interrogative sentence", "pos": {"nouns": {"you": 1, "Body": 1, "Level": 1, "Pain": 1}, "verbs": {"have": 1, "Feel": 1}, "adjectives": {"Different": 1, "of": 2}, "numerals": {"7": 1}}}, {"content": "8. Are you having trouble getting dressed?", "word_count": 10, "label": "", "sentence_structure": "Subject鈥搗erb鈥揷omplement", "subjective_objective": {"answer": "Subjective", "rationale": "Because wearing clothes is a subjective act of a human being."}, "sentence_sentiment": {"answer": "Neutral", "rationale": "Because the entry is merely an inquiry into whether the person under investigation has certain physical inconveniences in life and does not reflect positive or negative emotional tendencies."}, "language_style": {"answer": "Tactful and warm", "rationale": "The entry uses a more moderate and inviting approach, which does not directly indicate whether the other party is having difficulties, but rather asks the other party for his or her own views and feelings, which is more resonant and thought-provoking. At the same time, the act of \"dressing\" itself is more private and sensitive, and is more effective in protecting the privacy and dignity of the interviewee."}, "time_dimension": {"answer": "Present", "rationale": "The description of \"the difficulty of wearing clothes\" is an inquiry into the current situation of the person being tested, so the temporal dimension is now judged."}, "sentence_type": "Interrogative sentence", "pos": {"nouns": {"self": 1, "Clothes": 1, "Difficulties": 1}, "verbs": {"Put it on": 1, "have": 1}, "adverbs": {"Hard": 1}, "numerals": {"8锛?: 1}}}, {"content": "Is it difficult to comb yourself?", "word_count": 9, "label": "", "sentence_structure": "Subject鈥搗erb鈥搊bject", "subjective_objective": {"answer": "Subjective", "rationale": "Because \"the difficulty of combing\" is the patient's own subjective feeling and assessment, which is viewed from the patient's perspective."}, "sentence_sentiment": {"answer": "Neutral", "rationale": "Because the entry does not have any emotional color."}, "language_style": {"answer": "Tactful and warm", "rationale": "The language style of the entry is euphemism, which does not directly question or accuse the other side of any problems, but rather uses a sense of concern and encouragement. By using the phrase \"Do you have difficulty combing yourself?\", the questionnaire researchers try to make the interviewees comfortable and encourage them to share their true stories without feeling pressure or embarrassment. At the same time, this euphemism creates a positive, friendly atmosphere that makes the interviewee more willing to participate in the investigation and provide real feedback."}, "time_dimension": {"answer": "Present", "rationale": "The entry asks if the person tested currently has \"a difficulty in combing\" and the time dimension is now judged."}, "sentence_type": "Interrogative sentence", "pos": {"nouns": {"Difficulties": 1, "Combination": 1}, "verbs": {"have": 1, "Difficulties": 1}, "numerals": {"9": 1}}}, {"content": "10. Are you having difficulty in carrying out your daily domestic chores?", "word_count": 14, "label": "", "sentence_structure": "Subject鈥搗erb鈥搊bject", "subjective_objective": {"answer": "Subjective", "rationale": "The entry examines the patient ' s perception and feelings of being unable to take on the day-to-day household, which is his/her subjective attitude."}, "sentence_sentiment": {"answer": "Negative", "rationale": "Because the act of \"difficulties in carrying out daily domestic chores\" is negative."}, "language_style": {"answer": "Calm and direct", "rationale": "The entry is in a concise manner, asking the respondents whether they have difficulty in carrying out their daily household chores, and whether they have not had too many emotional or speech modification techniques, which are more directly objective in tone."}, "time_dimension": {"answer": "Present", "rationale": "The entry asks the testee about the current situation, i.e. whether he has experienced difficulties in carrying out his daily household chores. The keyword \"Is there a problem\" indicates that this is a question of the state of affairs, and therefore the judgement is \"now\"."}, "sentence_type": "Interrogative sentence", "pos": {"nouns": {"Day": 1, "Domestic work": 1, "Difficulties": 1}, "verbs": {"Undertaking": 1, "It's hard": 1}, "numerals": {"10": 1, "you": 1, "of": 2}, "pronouns": {"you": 1}}}, {"content": "Can you go out alone and buy general goods?", "word_count": 13, "label": "", "sentence_structure": "Subject鈥損redicate", "subjective_objective": {"answer": "Objective", "rationale": "This is seen from an objective perspective."}, "sentence_sentiment": {"answer": "Neutral", "rationale": "Because the entry merely asked the respondent whether he or she could perform a task alone, without any emotional, attitude or evaluation."}, "language_style": {"answer": "Tactful and warm", "rationale": "The entry is more personal and interactive when it is spoken in a question-and-answer manner rather than in a direct statement or order. At the same time, the word \"single\" leaves room for self-thinking rather than forcing the other to answer a specific answer."}, "time_dimension": {"answer": "Present", "rationale": ""}, "sentence_type": "Interrogative sentence", "pos": {"nouns": {"Questionnaire Entry": 1, "person": 1, "Items": 1}, "verbs": {"Yes": 1, "Go to the street": 1, "Purchase": 1}, "numerals": {"11锛?: 1, "you": 1}}}, {"content": "12. Are you having trouble eating yourself?", "word_count": 9, "label": "", "sentence_structure": "Subject鈥損redicate", "subjective_objective": {"answer": "Subjective", "rationale": "Because \"difficulties\" are patients' subjective judgment and feelings."}, "sentence_sentiment": {"answer": "Negative", "rationale": "Because the expression \"difficult\" in the entry is negative."}, "language_style": {"answer": "Calm and direct", "rationale": "The entry is based on a direct and clear question-and-answer approach, without too much qualifier or emotional colour to allow the respondent to understand and answer the question quickly and accurately."}, "time_dimension": {"answer": "Present", "rationale": "The keyword \"difficulties\" refers to the person's current state or ability, so it's now."}, "sentence_type": "Interrogative sentence", "pos": {"nouns": {"Problem": 1, "Entry": 1, "self": 1, "Rice": 1, "Difficulties": 1}, "verbs": {"have": 1, "Eat": 1}, "numerals": {"12": 1}}}, {"content": "13. How is it that you bend and bend?", "word_count": 9, "label": "", "sentence_structure": "Subject鈥搗erb鈥揷omplement", "subjective_objective": {"answer": "Objective", "rationale": "Because it is a matter of physical function, there is no need for subjective judgement by the patient."}, "sentence_sentiment": {"answer": "Negative", "rationale": "Because it describes physical inconvenience and has negative emotional colours."}, "language_style": {"answer": "Tactful and warm", "rationale": "This soft and sensitive language style makes it easier for respondents to accept and cooperate with the questionnaire."}, "time_dimension": {"answer": "Present", "rationale": ""}, "sentence_type": "Interrogative sentence", "pos": {"nouns": {"you": 1, "Bend over": 1, "Knees": 1, "Difficulties": 1}, "verbs": {"have": 1}, "numerals": {"13": 1}}}, {"content": "14. Is it difficult to go up and down the stairs?", "word_count": 15, "label": "", "sentence_structure": "Subject鈥搗erb鈥搊bject", "subjective_objective": {"answer": "Objective", "rationale": "Because the question is asking about the experience and experience of the patient in real life, the difficulty of going up and down the stairs is that they can be drawn from direct observation and practical operations and are therefore objective in perspective."}, "sentence_sentiment": {"answer": "Negative", "rationale": "Because \"the difficulty of going up and down the stairs\" is usually considered a physical problem, which has a negative meaning."}, "language_style": {"answer": "Calm and direct", "rationale": ""}, "time_dimension": {"answer": "Present", "rationale": ""}, "sentence_type": "Interrogative sentence", "pos": {"nouns": {"you": 1, "Stairs": 2, "Difficulties": 1}, "verbs": {"have": 1, "Go, go, go": 1, "Down": 1, "Climb": 1}, "adverbs": {"At least": 1}, "numerals": {"14": 1}, "classifiers": {"Layer": 1}}}, {"content": "Is it difficult for you to walk half a mile?", "word_count": 10, "label": "", "sentence_structure": "Subject鈥搗erb鈥搊bject", "subjective_objective": {"answer": "Objective", "rationale": "Because the difficulty of walking half a mile is an objective fact that the patient actually experiences, not his subjective attitude or feeling."}, "sentence_sentiment": {"answer": "Negative", "rationale": "Because the \"hard walking half a mile\" in the entry is negative."}, "language_style": {"answer": "Tactful and warm", "rationale": "This is a euphemism, which avoids the direct expression of \"hard\" or \"no problem\" but rather uses \"walk half a mile\" as an entry point to guide the interviewee to assess his/her own health."}, "time_dimension": {"answer": "Present", "rationale": "The entry asks if the person tested has a difficult condition of walking half a mile, and the time dimension is now. The keyword \"you\" and \"inconvenient\" indicate that this is a description and a query about the current situation."}, "sentence_type": "Interrogative sentence", "pos": {"nouns": {"you": 1, "Walk": 1, "Half": 1, "- Leelu": 1, "Difficulties": 1}, "verbs": {"have": 1, "Walk": 1}, "numerals": {"15": 1, "Half": 1}, "classifiers": {"- Leelu": 1}, "pronouns": {"you": 1}}}, {"content": "How is it going to be three miles on foot?", "word_count": 10, "label": "", "sentence_structure": "Subject鈥損redicate", "subjective_objective": {"answer": "Objective", "rationale": "Since the ability to walk three miles is the objective ability of the individual, it is also the objective situation to have difficulties and does not require an examination of the patient ' s subjective feelings."}, "sentence_sentiment": {"answer": "Negative", "rationale": "Because the expression \"the three miles on foot is difficult\" is a physical inconvenience and negative."}, "language_style": {"answer": "Tactful and warm", "rationale": "The entry is more adept and interactive when it is spoken in a gentle and inviting manner, rather than by direct statements or orders."}, "time_dimension": {"answer": "Past", "rationale": "The entry asks if the testee is currently having difficulty walking three miles, and the time dimension is a past."}, "sentence_type": "Interrogative sentence", "pos": {"nouns": {"you": 1, "Walk": 1, "Three Miles": 1, "Difficulties": 1}, "numerals": {"Six": 1}}}, {"content": "17. Are you having difficulty participating in activities that are more energy-intensive (such as intense physical exercise, field manual work, heavy lifting, etc.)", "word_count": 36, "label": "", "sentence_structure": "Subject鈥搗erb鈥搊bject", "subjective_objective": {"answer": "Objective", "rationale": "This is an objective question, therefore, because the question is whether the patient is experiencing difficulties in practice, rather than the patient鈥檚 subjective feelings or perceptions."}, "sentence_sentiment": {"answer": "Negative", "rationale": "The judgement is based on the fact that the purpose of the article is to describe \"difficult\" and has a negative emotional connotation."}, "language_style": {"answer": "Calm and direct", "rationale": "The entry is more direct and clear-cut in language style, and there is no excessive emotional or rhetorical style. It simply asks the interviewee whether he has any difficulty in engaging in activities that are more energy-intensive, neutral and objective and easily understandable."}, "time_dimension": {"answer": "Present", "rationale": "The entry is a question about the current situation of the testee, and the time dimension is now. The keywords \"you're in\" and \"are you having difficulties\" indicate that this is a description of the current state."}, "sentence_type": "Interrogative sentence", "pos": {"nouns": {"Activities": 2, "Physical exercise": 1, "Field manual labour": 1, "Move heavy loads": 1, "Difficulties": 1}, "verbs": {"Participation": 1, "Consumption": 1, "have": 1, "Move": 1}, "adjectives": {"Bigger": 1, "It's violent": 1}, "numerals": {"17锛?: 1}}}, {"content": "18. What do you think of your health, in general, compared to your peers?", "word_count": 27, "label": "", "sentence_structure": "Subject鈥搗erb鈥搊bject", "subjective_objective": {"answer": "Subjective", "rationale": "Because this question is about the patient's assessment of his or her health and is a subjective view."}, "sentence_sentiment": {"answer": "Neutral", "rationale": "Since the entry does not express positive or negative feelings, attitudes, behaviours, aims or qualities, it merely asks the respondent about his or her relative assessment of his or her physical condition."}, "language_style": {"answer": "Tactful and warm", "rationale": "The entry raises questions in a comparative way about whether the interviewees have confidence in their physical condition, which is not much emotional, and the language style is euphemism."}, "time_dimension": {"answer": "Present", "rationale": "The entry asks the subject of the testee's subjective assessment of his or her health status, which does not relate to past or future behaviour or mental state, and therefore the judgement is current.鈥?}, "sentence_type": "Interrogative sentence", "pos": {"nouns": {"Questionnaire": 1, "Entry": 1, "18": 1, "you": 2, "Peer": 1, "Overall": 1, "Physical health": 1}, "verbs": {"I think": 1, "How's that": 1}, "adverbs": {"Overall": 1, "More": 1}, "numerals": {"18": 1}, "pronouns": {"Your own": 1}}}, {"content": "Are you optimistic about the future?", "word_count": 7, "label": "", "sentence_structure": "Subject鈥損redicate", "subjective_objective": {"answer": "Subjective", "rationale": "Because the future has not yet happened, patients need subjective judgment to answer this question."}, "sentence_sentiment": {"answer": "Positive", "rationale": "Because the \"optimistic future\" in the entry is a positive emotional expression."}, "language_style": {"answer": "Tactful and warm", "rationale": "The entry is more adept and interactive when it is spoken in a gentle and inviting manner, rather than by direct statements or orders."}, "time_dimension": {"answer": "Future", "rationale": ""}, "sentence_type": "Interrogative sentence", "pos": {"nouns": {"The future": 1, "Optimistic": 1}, "verbs": {"to": 1, "Look": 1}}}, {"content": "Are you satisfied with the current living conditions?", "word_count": 12, "label": "", "sentence_structure": "Subject鈥搗erb鈥搊bject", "subjective_objective": {"answer": "Subjective", "rationale": "Because \"satisfaction\" is the patient's subjective assessment of the current living conditions."}, "sentence_sentiment": {"answer": "Neutral", "rationale": "Because the entry does not have any emotional color."}, "language_style": {"answer": "Calm and direct", "rationale": ""}, "time_dimension": {"answer": "Present", "rationale": ""}, "sentence_type": "Interrogative sentence", "pos": {"nouns": {"Living conditions": 1, "Satisfied": 1}, "verbs": {"to": 1, "be": 1}}}, {"content": "Do you have confidence in yourself?", "word_count": 8, "label": "", "sentence_structure": "Subject鈥搗erb鈥搊bject", "subjective_objective": {"answer": "Subjective", "rationale": "Because confidence is a feeling and attitude within a person, it is a subjective approach."}, "sentence_sentiment": {"answer": "Neutral", "rationale": "Because the entry merely asks the other party about the level of confidence in itself, it does not involve any emotional expression."}, "language_style": {"answer": "Calm and direct", "rationale": "The entry asks the question of whether the person surveyed has confidence in himself in a direct and concise manner, without excessive emotional or rhetorical modification and with an objective and neutral tone."}, "time_dimension": {"answer": "Present", "rationale": "\"Are you confident of yourself?\" The word \"yes\" in it indicates the state or condition of the present, so the entry is the current dimension of time."}, "sentence_type": "Interrogative sentence", "pos": {"nouns": {"self": 1, "Confidence": 1}, "verbs": {"have": 1}, "numerals": {"21": 1}}}, {"content": "Are you safe from your daily life?", "word_count": 16, "label": "", "sentence_structure": "Subject鈥搗erb鈥搊bject", "subjective_objective": {"answer": "Objective", "rationale": "This is an assessment of the security of the living environment and is an objective one."}, "sentence_sentiment": {"answer": "Neutral", "rationale": "Since the entry is not clearly positive or negative, it merely asks about the security of the daily living environment."}, "language_style": {"answer": "Calm and direct", "rationale": "The entry is a direct expression of the question about whether the other party feels safe in the day-to-day environment, is not based on too many rhetorical or emotional expressions, and is more objective in tone. At the same time, it does not use euphemism to guide the interviewee to answer questions, but rather to obtain information by asking directly, so it can be judged that the language style is calm and straightforward."}, "time_dimension": {"answer": "Present", "rationale": "The entry is for the testee's feelings about the daily living environment, and the time dimension is not clearly oriented to sexual orientation, so it is judged as \"now\"."}, "sentence_type": "Interrogative sentence", "pos": {"nouns": {"you": 1, "self": 1, "Day": 1, "Life": 1, "Environment": 1, "Clear": 1}, "verbs": {"I feel": 1}, "numerals": {"22锛?: 1}}}, {"content": "Do you feel happy?", "word_count": 8, "label": "", "sentence_structure": "Subject鈥損redicate", "subjective_objective": {"answer": "Subjective", "rationale": "Because happiness is an individual subjective feeling and experience, not an objective fact or data."}, "sentence_sentiment": {"answer": "Positive", "rationale": "Because the \"happiness\" in the entry is a positive emotional experience."}, "language_style": {"answer": "Tactful and warm", "rationale": ""}, "time_dimension": {"answer": "Present", "rationale": "\"Do you feel happy?\" The word \"Yes\" in \"is the person who was tested in the state of his or her current feeling, so the entry looks at the situation now."}, "sentence_type": "Interrogative sentence", "pos": {"nouns": {"Happiness": 1, "Feel": 1}, "verbs": {"have": 1}, "numerals": {"23": 1}}}, {"content": "Are you nervous?", "word_count": 8, "label": "", "sentence_structure": "Subject鈥損redicate", "subjective_objective": {"answer": "Subjective", "rationale": "Because it's the patient's subjective feeling."}, "sentence_sentiment": {"answer": "Negative", "rationale": "Because \"sense\" is usually considered a negative emotional experience."}, "language_style": {"answer": "Calm and direct", "rationale": "So my answer is \"Calm down straight.\" This language style allows an objective understanding of the mental state of the person surveyed, and helps to make accurate data analysis and interpretation of the results."}, "time_dimension": {"answer": "Present", "rationale": "The keywords \"fear\" and \"sense\" indicate that the question is about the current mental state of the subject and is therefore about the present."}, "sentence_type": "Interrogative sentence", "pos": {"nouns": {"you": 1, "Spirit": 1, "Nervous": 1}, "verbs": {"I feel": 1}, "numerals": {"24": 1, "- You're not": 1}}}, {"content": "Are you feeling bad and depressed?", "word_count": 12, "label": "", "sentence_structure": "Subject鈥損redicate", "subjective_objective": {"answer": "Subjective", "rationale": "Because it's the patient's subjective feeling."}, "sentence_sentiment": {"answer": "Negative", "rationale": "Because the entry \"depressed, depressed\" expresses a negative emotional state."}, "language_style": {"answer": "Tactful and warm", "rationale": "Although the question is asked directly whether the interviewee feels in a bad mood or is depressed, it is not expressed in strong language, but rather in a mild tone. This language style makes the questionnaire more acceptable and answers, while reducing the discomfort of the interviewee."}, "time_dimension": {"answer": "Present", "rationale": ""}, "sentence_type": "Interrogative sentence", "pos": {"nouns": {"you": 1, "The mood": 1, "Emotion": 1, "Down": 1}, "verbs": {"I feel": 1, "not": 1, "Okay": 1}, "adjectives": {"Not good": 1, "Down": 1}, "adverbs": {"- You're not": 1}, "numerals": {"25": 1}}}, {"content": "Would you be afraid without any reason?", "word_count": 12, "label": "", "sentence_structure": "Subject鈥搗erb鈥搊bject", "subjective_objective": {"answer": "Subjective", "rationale": "Because it's a fear of what is usually the patient's subjective experience and feelings."}, "sentence_sentiment": {"answer": "Negative", "rationale": "Because the \"unjustifiably afraid\" act in the entry is negative."}, "language_style": {"answer": "Calm and direct", "rationale": "The entry is dialogueable in a direct query, without excessive rhetorical or emotional rendering, and is more objective in tone."}, "time_dimension": {"answer": "Present", "rationale": "The entry asks if the person tested currently has an unjustified fear, and the time dimension is now judged."}, "sentence_type": "Interrogative sentence", "pos": {"nouns": {"you": 1, "No reason": 1, "I'm scared": 1}, "verbs": {"Yes": 1, "I feel": 1}, "adjectives": {"Scared": 1}, "adverbs": {"No reason": 1}}}, {"content": "You're not relieved that you've been repeatedly confirmed for what you've done?", "word_count": 16, "label": "", "sentence_structure": "Subject鈥搗erb鈥搊bject", "subjective_objective": {"answer": "Subjective", "rationale": "This is a question of the patient ' s self-perception, reflects the patient ' s inner experience and attitude and is therefore a subjective examination."}, "sentence_sentiment": {"answer": "Negative", "rationale": "Because the \"reconfirmed only after a long time\" act in the entry is negative, it reflects the possibility that the interviewees may have excessive concerns and doubts."}, "language_style": {"answer": "Tactful and warm", "rationale": "The entry is a more euphemistic way of asking questions, rather than directly ordering or accusing each other. By using the term \"you\" and the questionword, the question is more open and acceptable, and it is easier to resonate and cooperate with the respondents."}, "time_dimension": {"answer": "Present", "rationale": ""}, "sentence_type": "Interrogative sentence", "pos": {"nouns": {"Things": 1, "Confirm": 1, "Don't worry": 1}, "verbs": {"Do it": 1, "Repeated confirmations": 1, "Just play it": 1}, "adverbs": {"The long run": 1, "Repeatedly": 1}, "numerals": {"27": 1}}}, {"content": "Do you feel lonely when you're with someone else?", "word_count": 14, "label": "", "sentence_structure": "Subject鈥揷opula鈥損redicative", "subjective_objective": {"answer": "Subjective", "rationale": "Because feeling alone is a subjective feeling and experience."}, "sentence_sentiment": {"answer": "Negative", "rationale": "Because feeling alone is a negative emotional experience."}, "language_style": {"answer": "Tactful and warm", "rationale": "The language style of the entry is a warmer type. Asking the other person in question whether he or she feels lonely and gentler in his or her relations with others and encouraging the interviewee to respond to it is somewhat interactive."}, "time_dimension": {"answer": "Past", "rationale": "The keyword \"when with other people\" represents the past experience and circumstances, and \"fearing alone\" describes the emotional experience of the past, so the entry asks if the person tested had experienced a similar experience in the past."}, "sentence_type": "Interrogative sentence", "pos": {"nouns": {"others": 1, "Together": 1, "Time": 1, "you": 1, "I feel": 1, "Loneliness": 1}, "verbs": {"in": 1, "Yeah": 1, "I feel": 2}, "adverbs": {"When I'm with someone else": 1}, "numerals": {"28": 1}, "pronouns": {"you": 1}}}, {"content": "Are you restless and unstable?", "word_count": 12, "label": "", "sentence_structure": "Subject鈥搗erb鈥揷omplement", "subjective_objective": {"answer": "Subjective", "rationale": "Because \"silent, unstable\" is the patient's subjective feeling and emotional experience. Although these feelings may be caused by external irritation or physical factors, they are themselves subjective perceptions of the patient's state."}, "sentence_sentiment": {"answer": "Negative", "rationale": "Because the description \"silent, unstable\" in the entry is negative."}, "language_style": {"answer": "Calm and direct", "rationale": "The entry is in plain and clear language and asks whether the respondents are often uneasy, unsure, not too emotional or rhetorical, and are more calm and straightforward.\""}, "time_dimension": {"answer": "Present", "rationale": "The entry asks the testee about the current state of the feeling, and the keywords \"frightful\" and \"shaky\" point to the experience of the present. So the time dimension is now."}, "sentence_type": "Interrogative sentence", "pos": {"nouns": {"you": 1, "I feel": 1, "Sit tight": 1, "I'm not sure I can": 1}, "verbs": {"I feel": 1, "Sit tight": 1, "I'm not sure I can": 1}, "numerals": {"29锛?: 1}}}, {"content": "Are you bored or alive for nothing?", "word_count": 17, "label": "", "sentence_structure": "Subject鈥損redicate", "subjective_objective": {"answer": "Subjective", "rationale": "It is a psychological experience and subjective feeling that is meaningless or meaningless to live."}, "sentence_sentiment": {"answer": "Negative", "rationale": "Because \"frightful\" and \"living has no meaning\" in the entries are negative emotional descriptions."}, "language_style": {"answer": "Calm and direct", "rationale": "The entry directly asked whether the respondents felt bored or felt that life was meaningless, and the language style was more direct and did not contain too much adoration or emotional colour."}, "time_dimension": {"answer": "Present", "rationale": "The keyword \"fear\" and \"no sense\" point to the state and the feeling."}, "sentence_type": "Interrogative sentence", "pos": {"nouns": {"Empty": 1, "Boring": 1, "Meaning": 1}, "verbs": {"I feel": 1, "Alive": 1, "have": 1}, "adverbs": {"or": 1}, "numerals": {"30": 1}, "pronouns": {"you": 1}}}, {"content": "How's your memory?", "word_count": 8, "label": "", "sentence_structure": "Subject鈥搗erb鈥揷omplement", "subjective_objective": {"answer": "Subjective", "rationale": "Because memory is a subjective feeling or cognitive ability, the memory experience of each individual may be different. The problem is one of perception and evaluation of the patient, and therefore subjective."}, "sentence_sentiment": {"answer": "Neutral", "rationale": "Since the entry was asked about the memory of the person under investigation, it did not contain information on emotions, attitudes or behaviour."}, "language_style": {"answer": "Tactful and warm", "rationale": "The language style of the entry is more euphemistic, moderate, and not too strong or direct, but rather a simple way of asking about each other鈥檚 memory. This expression makes the respondents feel more comfortable and comfortable and more willing to participate in the questionnaire."}, "time_dimension": {"answer": "Present", "rationale": "The entry asks the testee about his current memory and the time dimension is now judged."}, "sentence_type": "Interrogative sentence", "pos": {"nouns": {"Memory": 1}, "verbs": {"How's it going": 1}, "numerals": {"31": 1}}}, {"content": "Is it easy for you to concentrate on one thing?", "word_count": 13, "label": "", "sentence_structure": "Subject鈥損redicate", "subjective_objective": {"answer": "Subjective", "rationale": "Because it is the patient's subjective feeling of being able to concentrate."}, "sentence_sentiment": {"answer": "Neutral", "rationale": "Because the entry does not have any emotional color."}, "language_style": {"answer": "Tactful and warm", "rationale": "Warm and warm."}, "time_dimension": {"answer": "Present", "rationale": "The entry asks if the person tested is able to concentrate on his or her work at the moment, without clear time limits, and therefore judges it now."}, "sentence_type": "Interrogative sentence", "pos": {"nouns": {"Yes": 1}, "verbs": {"Do it": 1, "Focus": 1, "- The energy": 1}, "adjectives": {"Easy": 1}, "adverbs": {"very": 1}, "numerals": {"32": 1}}}, {"content": "How do you think or deal with problems?", "word_count": 16, "label": "", "sentence_structure": "Subject鈥搗erb鈥揷omplement", "subjective_objective": {"answer": "Objective", "rationale": "Because the objective ability of the patient to think and deal with the problem can be assessed through observation and testing, not just by the patient ' s own subjective evaluation."}, "sentence_sentiment": {"answer": "Neutral", "rationale": "Because the entry does not have any emotional color."}, "language_style": {"answer": "Calm and direct", "rationale": "The entry is asked in a straightforward and clear manner, without excessive qualifier or emotional expressions, and therefore is of a calm and direct language style."}, "time_dimension": {"answer": "Present", "rationale": "The entry now asks the testee about his current ability to think and deal with the problem, and the time dimension is now judged."}, "sentence_type": "Interrogative sentence", "pos": {"nouns": {"Capacity": 2, "Problem": 2}, "verbs": {"Think": 1, "Processing": 1, "How's it going": 1}, "numerals": {"33": 1}}}, {"content": "34. In general, how do you feel about your own interesting health?", "word_count": 19, "label": "", "sentence_structure": "Subject鈥損redicate", "subjective_objective": {"answer": "Subjective", "rationale": "Since the question concerns patients ' subjective assessment of themselves, it is the general perception and perception of the patients that is being questioned."}, "sentence_sentiment": {"answer": "Neutral", "rationale": "Because the entry does not have any emotional color."}, "language_style": {"answer": "Calm and direct", "rationale": "The entry is based on a simple and clear query about the other ' s state of mind, the absence of excessive rhetorical or emotional rendering, the soundness of the language and the sense of calm and direct language."}, "time_dimension": {"answer": "Present", "rationale": "The entry asks the testee about his current state of mind, and the keyword is \"in general\" and \"in the opinion\" and they point to the current situation."}, "sentence_type": "Interrogative sentence", "pos": {"nouns": {"Overall": 1, "self": 2, "Fun": 1, "Health status": 1}, "verbs": {"I think": 1, "Like": 1, "What": 1}, "adjectives": {"Okay": 1, "of": 3}, "adverbs": {"From": 1, "Go, go, go": 1}, "numerals": {"34": 1}, "pronouns": {"you": 1}}}, {"content": "Can you handle the unpleasant things that happen to you in life, in learning and in work?", "word_count": 34, "label": "", "sentence_structure": "Subject鈥搗erb鈥搊bject", "subjective_objective": {"answer": "Subjective", "rationale": "Because it looks at the attitude and ability of individuals to face unpleasant events, it is subjective."}, "sentence_sentiment": {"answer": "Positive", "rationale": "Because the \"good handling\" of the entry is positive."}, "language_style": {"answer": "Tactful and warm", "rationale": "The entry uses the tone of inquiry and has a positive term for \"good\" and \"good\" that gives encouragement and support to the sentence. At the same time, the sentence does not use negative or critical terms, nor is it too direct or commandive, so it can be considered as being a soft language."}, "time_dimension": {"answer": "Present", "rationale": "The key words of the questionnaire entry are \"life,\" \"learning\" and \"work,\" which indicate that the entry relates to what is happening now. At the same time, the question of \"good handling\" also suggests that it is about current behaviour or attitudes, not past or future. So the judgement is now."}, "sentence_type": "Interrogative sentence", "pos": {"nouns": {"Life": 1, "Learning": 1, "Work": 1, "Things": 2, "self": 1, "Body": 1, "Problem": 1}, "verbs": {"Come on": 1, "Yes": 1, "Well done": 1, "Take care of it": 1}, "adjectives": {"It's not good": 1}, "adverbs": {"Land": 2}, "numerals": {"35": 1}, "pronouns": {"you": 1}}}, {"content": "36. Can you adapt more quickly to new life, learning and working environments?", "word_count": 20, "label": "", "sentence_structure": "Subject鈥揷opula鈥損redicative", "subjective_objective": {"answer": "Subjective", "rationale": "Because \"fast-up to new living, learning and working environments\" is the subjective feeling and attitude of the patient, the entry on the questionnaire is viewed from a subjective perspective."}, "sentence_sentiment": {"answer": "Positive", "rationale": "Because the aim of the behaviour \"can adapt more quickly to new life, learning and working environments\" is positive."}, "language_style": {"answer": "Calm and direct", "rationale": "The entry raises questions in a direct and concise manner, asking whether the other party can adapt quickly to the new environment, without adding too much emotional colour or rhetoric, and with a more calm and objective language style."}, "time_dimension": {"answer": "Present", "rationale": "The phrase \"can adapt faster\" is used in this entry, emphasizing current capabilities and state, and therefore judging now."}, "sentence_type": "Interrogative sentence", "pos": {"nouns": {"Questionnaire": 1, "Entry": 1, "Life": 1, "Learning": 1, "Work": 1, "Environment": 2}, "verbs": {"Yes": 1, "Adaptation": 1, "Compare": 1, "Come on": 1, "Land": 1, "Do it": 1}, "adjectives": {"New": 1}, "adverbs": {"Compare": 1}, "numerals": {"36": 1, "2": 1}, "pronouns": {"you": 1}}}, {"content": "How do you evaluate your role in work, learning and life?", "word_count": 21, "label": "", "sentence_structure": "Subject鈥搗erb鈥搊bject", "subjective_objective": {"answer": "Subjective", "rationale": "The questionnaire entry is subjective because it is an interview with the patient ' s self-evaluation and opinion, and it looks at the patient ' s individual attitude and views."}, "sentence_sentiment": {"answer": "Neutral", "rationale": "Because the entry merely asked the respondent about his or her role and did not involve positive or negative emotional aspects."}, "language_style": {"answer": "Tactful and warm", "rationale": "The entry is more proficient and interactive when it is spoken in a gentle and inviting manner, rather than in direct statements or orders. At the same time, the language style of the article is neutral, is not overly subjective and is more favourable to the true answers of the respondents."}, "time_dimension": {"answer": "Present", "rationale": "The keyword \"how to evaluate\" suggests that it is a subjective question of the state of the present, and that \"the role played in work, learning and life\" is also a question of the role or situation of the person being tested, so the entry asks about the situation."}, "sentence_type": "Interrogative sentence", "pos": {"nouns": {"self": 2, "Work": 1, "Learning": 1, "Life": 1, "Role": 1}, "verbs": {"Evaluation": 1, "Active": 1}, "numerals": {"37": 1}}}, {"content": "38. Did you live in harmony?", "word_count": 9, "label": "", "sentence_structure": "Subject鈥揷opula鈥損redicative", "subjective_objective": {"answer": "Subjective", "rationale": "Because \"harmonization\" is a subjective feeling and an evaluation that requires the patient's own judgement and expression."}, "sentence_sentiment": {"answer": "Positive", "rationale": "Because the word \"harmonious\" in the entry is a positive emotional attitude."}, "language_style": {"answer": "Tactful and warm", "rationale": "This is a euphemism that is used in a euphemism, a way of asking about the family鈥檚 life, and a way of providing the interviewer with a real and positive answer, enhancing the interactive nature of the interview and the affection."}, "time_dimension": {"answer": "Present", "rationale": "The entry asks if the present family life of the testee is harmonious and the temporal dimension is now judged."}, "sentence_type": "Interrogative sentence", "pos": {"nouns": {"you": 1, "Family": 1, "Life": 1, "Harmony": 1}, "verbs": {"you": 1, "be": 1}, "adjectives": {"Harmony": 1}, "numerals": {"38": 1}}}, {"content": "39. Are there many colleagues, classmates, neighbours, relatives or partners close to you?", "word_count": 20, "label": "", "sentence_structure": "Subject鈥搗erb鈥揷omplement", "subjective_objective": {"answer": "Subjective", "rationale": "This question is about how many close people are around the patient, which is the patient ' s subjective perception and feelings."}, "sentence_sentiment": {"answer": "Neutral", "rationale": "The entry is only for questioning about the interpersonal relationship of the person interviewed and does not involve any emotional colour."}, "language_style": {"answer": "Calm and direct", "rationale": "The entry is more calm and straightforward in its language, and asks the interviewee about his/her relationship with the people around him in a concise and straightforward manner."}, "time_dimension": {"answer": "Present", "rationale": "Thus, in terms of time dimension, the topic looks at the current state or situation."}, "sentence_type": "Interrogative sentence", "pos": {"nouns": {"Co-workers": 1, "Students": 1, "Neighbors": 1, "Relatives": 1, "Partners": 1}, "verbs": {"More": 1, "Closely connected": 1}, "adjectives": {"Close": 1}, "numerals": {"39锛?: 1}}}, {"content": "Do you have any friends who can share happiness and grief with you?", "word_count": 17, "label": "", "sentence_structure": "Subject鈥搗erb鈥搊bject", "subjective_objective": {"answer": "Subjective", "rationale": "Because \"friends who share happiness and sorrow\" are personal feelings and attitudes of patients, and they are subjective."}, "sentence_sentiment": {"answer": "Positive", "rationale": "Since the purpose of the article is to express the existence of friends who can share pleasure and sorrow, such sharing is often considered positive."}, "language_style": {"answer": "Tactful and warm", "rationale": "The entry uses a soft and warm language style, which leads the interviewee to wonder if he/she has friends who can share pleasure and grief. This expression is easier to open up to the interviewee and is more friendly and friendly."}, "time_dimension": {"answer": "Present", "rationale": "The entry asks if the person tested currently has friends who can share pleasure and grief, and the time dimension is now judged."}, "sentence_type": "Interrogative sentence", "pos": {"nouns": {"friends": 1, "Happy": 1, "Sadness": 1}, "verbs": {"have": 1, "Share": 1}, "numerals": {"40锛?: 1}}}, {"content": "Are you talking to your friends or relatives?", "word_count": 17, "label": "", "sentence_structure": "Subject鈥搗erb鈥搊bject", "subjective_objective": {"answer": "Subjective", "rationale": "It is the patient ' s subjective idea to examine whether the patient prefers to discuss the issue with others."}, "sentence_sentiment": {"answer": "Neutral", "rationale": "Since the entry asks about the circumstances in which the act occurred, there is no apparent positive or negative emotional tendency."}, "language_style": {"answer": "Calm and direct", "rationale": "The entry is based on a direct statement of whether the person interviewed often discusses the matter with friends or relatives, without any supposition or emotional colour, and is more objective in tone. At the same time, the entry does not follow a soft and warm language style, but rather raises questions in a simple and straightforward manner, allowing the person interviewed to understand and answer clearly and accurately."}, "time_dimension": {"answer": "Present", "rationale": "The word \"now\" in the sentence makes it possible to judge that the entry is asking the subject about the current behaviour, and the temporal dimension is now."}, "sentence_type": "Interrogative sentence", "pos": {"nouns": {"friends": 1, "Relatives": 1, "Problem": 1}, "verbs": {"Talk": 1}, "numerals": {"41": 1}}}, {"content": "Do you maintain regular contact with your family and friends (e.g., visits, telephone calls, correspondence, etc.)", "word_count": 25, "label": "", "sentence_structure": "Subject鈥搗erb鈥搊bject", "subjective_objective": {"answer": "Objective", "rationale": "Because \"you keep in touch with your family and friends (e.g., visits, telephone greeting, correspondence, etc.)\" is a fact that can be observed and measured, and is considered from an objective perspective."}, "sentence_sentiment": {"answer": "Positive", "rationale": "Because the \"Remain in contact with family and friends\" act is positive."}, "language_style": {"answer": "Tactful and warm", "rationale": "The language style of the entry is euphemism, because it uses the means of questioning and is more polite and polite. At the same time, it does not speak too directly or strongly, but rather gives people a friendly and cordial feeling that helps respondents to be more willing to participate in the investigation and to provide real feedback."}, "time_dimension": {"answer": "Present", "rationale": "The keyword \"often\" and \"in touch\" means that it is a subject of questioning the present behaviour or habits of the subject, so the temporal dimension is now judged."}, "sentence_type": "Interrogative sentence", "pos": {"nouns": {"Questionnaire": 1, "Entry": 1, "Family and friends": 1, "Contact": 2, "Like": 1, "- Yeah, yeah": 1, "Visiting": 1, "Telephone": 1, "Greetings": 1, "Communications": 1}, "verbs": {"Hold on": 1, "Regular": 1, "Wait": 1, "- You're not": 1, "you": 1, "and": 1, "Like": 1, "- Yeah, yeah": 1, "Visiting": 1, "Telephone": 1, "Greetings": 1, "Communications": 1}, "adverbs": {"Regular": 1, "Wait": 1}, "numerals": {"42锛?: 1}, "pronouns": {"you": 1}}}, {"content": "Do you participate regularly in social and collective events (such as party groups, trade unions, student associations, religious, friends' gatherings, sports competitions, recreational activities, etc.)", "word_count": 35, "label": "", "sentence_structure": "Subject鈥搗erb鈥搊bject", "subjective_objective": {"answer": "Subjective", "rationale": "Since participation in social and collective activities is a subjective choice and behaviour of the patient, it is a subjective attitude and behaviour of the patient."}, "sentence_sentiment": {"answer": "Neutral", "rationale": "Because the entry does not have any emotional color."}, "language_style": {"answer": "Tactful and warm", "rationale": "The language style of the entry is euphemistic. It implies that the surveyer, by using the descriptions of \"regular\" and \"participating in some social, collective activities,\" would like to know whether the interviewee is used to active social activities. At the same time, the \"party, trade union, student, religious, friends' party, sports competition, entertainment, etc.\" lists a range of activities, giving the interviewee a broader range of options to answer questions and give people a gentle, open feeling."}, "time_dimension": {"answer": "Present", "rationale": "The entry now asks the subject about the current behaviour, and the keyword \"recurring participation\" indicates that the conduct is now being performed."}, "sentence_type": "Interrogative sentence", "pos": {"nouns": {"Activities": 2, "Party": 1, "Trade unions": 1, "Students ' Association": 1, "Religion": 1, "Friends party": 1, "Sports competitions": 1, "Recreation": 1}, "verbs": {"Participation": 1, "Regular": 1, "Social": 1, "Collective": 1}, "adverbs": {"Wait": 1}, "numerals": {"43": 1}}}, {"content": "44. Are you able to rely on your family to a great extent when you need help?", "word_count": 22, "label": "", "sentence_structure": "Subject鈥搗erb鈥搊bject", "subjective_objective": {"answer": "Subjective", "rationale": "Because this question is one of asking patients about their subjective feelings and attitudes, i.e. whether they can rely on their families when they need help. It is therefore subjective."}, "sentence_sentiment": {"answer": "Positive", "rationale": "Since the entry describes the existence of a family that can be relied upon when in need of help, this dependency is positive."}, "language_style": {"answer": "Calm and direct", "rationale": "The entry is in a concise and clear language and asks whether the person surveyed is able to rely on the family when he or she needs help, without excessive ado or emotional expression."}, "time_dimension": {"answer": "Present", "rationale": "\"Time\" in time means a certain point or time, \"when you need help\" means the situation in the current situation, when the person needs help. The entry is therefore an inquiry into the situation of the person being tested, the time dimension being judged as now."}, "sentence_type": "Interrogative sentence", "pos": {"nouns": {"Time": 1, "Help": 1, "Family": 1}, "verbs": {"Yes": 1, "Dependence": 1}, "adjectives": {"A lot": 1}, "adverbs": {"in": 1}, "numerals": {"44锛?: 1}}}, {"content": "45. Are you able to rely on friends to a great extent when you need help?", "word_count": 22, "label": "", "sentence_structure": "Subject鈥搗erb鈥搊bject", "subjective_objective": {"answer": "Objective", "rationale": "Since the question was whether the person surveyed was able to rely on friends, the existence of friends in such a situation was not a critical factor."}, "sentence_sentiment": {"answer": "Positive", "rationale": "Because the attitude of \"a great deal of reliance on friends\" in the entries is positive."}, "language_style": {"answer": "Tactful and warm", "rationale": "The entry is a euphemism, a kind expression that is not too direct or hard-line when asking if the other side can rely on friends. It is more acceptable and willing to share its true thoughts and feelings."}, "time_dimension": {"answer": "Present", "rationale": "According to the sentence, \"You can rely to a great extent on friends,\" this is a description of the current state or ability of the person being tested, and therefore the judgement is now."}, "sentence_type": "Interrogative sentence", "pos": {"nouns": {"Time": 1, "you": 2, "It's big": 1, "Level": 1, "Yes": 1, "Dependence": 1, "friends": 1}, "verbs": {"Yes": 1, "Help": 1, "In": 1, "Shit": 1}, "adjectives": {"A lot": 1}, "numerals": {"45": 1}}}, {"content": "46. Do you voluntarily seek help from others when you encounter difficulties?", "word_count": 20, "label": "", "sentence_structure": "Subject鈥搗erb鈥搊bject", "subjective_objective": {"answer": "Subjective", "rationale": "The subject is a subjective one, because it is a question of the patient ' s own behaviour and attitude."}, "sentence_sentiment": {"answer": "Positive", "rationale": "Because the \"proactively seeking help\" act is positive."}, "language_style": {"answer": "Calm and direct", "rationale": "The entry is based on a clear question-and-answer period about whether the person under investigation would actively seek help from others in the event of difficulties, without excessive rhetorical or emotional rendering, and with a more objective tone. Moreover, the question is expressed relatively directly, without any ambiguity, and in a calm and straightforward language style."}, "time_dimension": {"answer": "Present", "rationale": "The entry asks the testee's current behaviour, namely, \"Will someone ask for help if they are experiencing difficulties?\" The sentence therefore reads now."}, "sentence_type": "Interrogative sentence", "pos": {"nouns": {"Difficulties": 1, "Others": 1, "Help": 1}, "verbs": {"I met him": 1, "Initiatives": 1, "Seek": 1, "Help": 1}, "adverbs": {"Land": 1}, "numerals": {"46": 1}}}, {"content": "47. What do you think of your social functions (e.g., interpersonal relations, social interaction) in general, compared to your peers?", "word_count": 34, "label": "", "sentence_structure": "Subject鈥搗erb鈥搊bject", "subjective_objective": {"answer": "Subjective", "rationale": "This is because the patient ' s assessment and opinion of his/her social aspect are subjective in nature."}, "sentence_sentiment": {"answer": "Neutral", "rationale": "Because the entry does not have any emotional color."}, "language_style": {"answer": "Tactful and warm", "rationale": "Warm and warm."}, "time_dimension": {"answer": "Present", "rationale": "The entry asks what the testee is doing, and the time dimension is now judged. The keywords \"current\" and \"comparison with peers\" point to the current state or situation."}, "sentence_type": "Interrogative sentence", "pos": {"nouns": {"Peer": 1, "Social function": 1, "Human relations": 1, "- To be with someone": 1}, "verbs": {"Compare": 1, "I think": 1, "be": 1, "How's that": 1}, "adjectives": {"Overall": 1}, "numerals": {"47": 1}, "pronouns": {"you": 2}}}, {"content": "48. How do you feel about your health, in general, compared to your peers?", "word_count": 24, "label": "", "sentence_structure": "Subject鈥搗erb鈥搊bject", "subjective_objective": {"answer": "Subjective", "rationale": "Because the issue is examined by the respondent's self-evaluation of his health status, which is a reflection of the subjective perception of the individual."}, "sentence_sentiment": {"answer": "Neutral", "rationale": "Because the entry is merely an inquiry into an individual ' s self-assessment of his or her health status, it is not emotional."}, "language_style": {"answer": "Tactful and warm", "rationale": "This expression neither puts pressure or discomfort on the person under investigation, nor gives them a greater willingness to share their real thoughts."}, "time_dimension": {"answer": "Present", "rationale": "The entry is for the current self-evaluation of the testees, i.e. to compare their health status with that of their peers. Therefore, the time dimension is now judged."}, "sentence_type": "Interrogative sentence", "pos": {"nouns": {"Questionnaire": 1, "Entry": 1, "Peer": 1, "Health status": 2}, "verbs": {"Comparison": 1, "I think": 1}, "adverbs": {"Overall": 1}, "numerals": {"48": 1}, "pronouns": {"you": 3}}}]}

{"questionnaire_title": "The student interpersonal skills table", "questionnaire_category": "Behavioral questionnaire", "item_count": 67, "emotion_shift_overall": "From positive to negative", "language_style_overall": "Tactful and warm", "items": [{"content": "I will participate in the gathering of strangers to develop new human relationships.", "word_count": 19, "label": "", "sentence_structure": "Subject鈥搗erb鈥搊bject", "subjective_objective": {"answer": "Subjective", "rationale": "Because attending a meeting of strangers requires the active involvement of the patient, it is an expression of his or her subjective will."}, "sentence_sentiment": {"answer": "Positive", "rationale": "Because the entry is \"to attend a meeting of strangers\" and is intended to \"develop a new human relationship\", which is a positive expression."}, "language_style": {"answer": "Tactful and warm", "rationale": "The language style of the entry is euphemism. It does not directly ask or order the interviewee to attend a meeting of strangers, but rather expands relationships by asking if the interviewee is willing to do so."}, "time_dimension": {"answer": "Present", "rationale": "The keyword \"will\" indicate a will or a preference, while \"participating in a gathering of strangers to develop new human relationships\" indicates the purpose and content of such behaviour, which together indicate that the entry looks at the behaviour choices and intentions of the subject in the present state."}, "sentence_type": "Interrogative sentence", "pos": {"nouns": {"Stranger": 1, "Gathering": 1, "Human relations": 1}, "verbs": {"- Yes, I'm in": 1, "Development": 1}, "adverbs": {"Here": 1}, "pronouns": {"I": 1}}}, {"content": "I can speak with my fellow students, and I want to recognize them.", "word_count": 14, "label": "", "sentence_structure": "Subject鈥搗erb鈥揷omplement", "subjective_objective": {"answer": "Subjective", "rationale": "It is a subjective feeling or will to talk to others."}, "sentence_sentiment": {"answer": "Positive", "rationale": "Because the \"can talk to my classmates\" act positive in the entry."}, "language_style": {"answer": "Tactful and warm", "rationale": "This language helps to increase the participants鈥?willingness to participate and cooperate, and is more in line with the friendly environment and atmosphere of the questionnaire."}, "time_dimension": {"answer": "Present", "rationale": "It is judged by the words \"can\" and \"want\" that this is a description of the situation, namely, whether the person who was tested is currently able to speak to the classmates he wanted to meet."}, "sentence_type": "Interrogative sentence", "pos": {"nouns": {"Students": 1, "Yes": 1, "Talk": 1}, "verbs": {"Yes": 2, "and": 1, "Recognition": 1, "Talk": 1}, "adverbs": {"Initiatives": 1}, "pronouns": {"I": 1}}}, {"content": "3. I can clearly refuse (in euphemism or direct) when a person who normally knows you can give something valuable to you that you feel unacceptable to yourself.", "word_count": 34, "label": "", "sentence_structure": "Subject鈥搗erb鈥搊bject", "subjective_objective": {"answer": "Subjective", "rationale": "Because \"clear rejection\" or \"precise or direct\" are attitudes or behaviours that patients may adopt in the face of such situations, these reflect subjective ideas and attitudes."}, "sentence_sentiment": {"answer": "Positive", "rationale": "Because the \"can expressly reject\" behavior and attitude in the entry is positive."}, "language_style": {"answer": "Calm and direct", "rationale": "This entry contains two different ways of answering, euphemism and directness. This suggests that when facing this question, the respondents can choose the appropriate answer according to their own circumstances. At the same time, the expression \" euphemism\" reflects respect for and consideration of each other's feelings, while the expression \"direct\" is more decisive."}, "time_dimension": {"answer": "Past", "rationale": "\"I can categorically reject\" indicates that this is a past experience and behaviour, so the temporal dimension of this article is the past."}, "sentence_type": "Affirmative declarative sentence", "pos": {"nouns": {"Questionnaire": 1, "Entry": 1, "I know someone": 1, "Gifts": 1, "Accept": 1, "self": 1, "Reject": 1, "Euphemism": 1, "Direct": 1}, "verbs": {"Present": 1, "I think": 1, "No, I can't": 1, "Accept": 1, "Clear": 1, "Reject": 1, "Euphemism": 1, "Direct": 1, "be": 1}, "adjectives": {"General": 1, "Valuable": 1}, "adverbs": {"When": 1}, "numerals": {"3": 1}, "classifiers": {"individual": 1}, "pronouns": {"I": 1}}}, {"content": "4. I am willing to speak about my privacy when talking to my heterosexual friends.", "word_count": 19, "label": "", "sentence_structure": "Subject鈥搗erb鈥搊bject", "subjective_objective": {"answer": "Subjective", "rationale": "Because \"will\" expresses patients' subjective attitude to talking about their privacy."}, "sentence_sentiment": {"answer": "Positive", "rationale": "Because the entry \"willing to talk about his privacy\" expresses a positive attitude and behaviour."}, "language_style": {"answer": "Tactful and warm", "rationale": "The language style of the entry is euphemistic, implicit, using first person expression and using the term \"willing\" to ask the interviewee's wishes, making the issue more personal and interactive. At the same time, the description \"when talking to a friend of a opposite sex\" is relatively mild, avoiding the sensitive topic of direct questioning of the privacy of the individual."}, "time_dimension": {"answer": "Present", "rationale": ""}, "sentence_type": "Affirmative declarative sentence", "pos": {"nouns": {"Fae friend": 1, "self": 1, "Privacy": 1}, "verbs": {"Yes": 1, "I'm talking": 1}, "adverbs": {"in": 1, "Time": 1}, "classifiers": {"4": 1}}}, {"content": "5. When I am angry with close colleagues or friends, I can still accept that his/her views are reasonable, even if I do not agree with them.", "word_count": 48, "label": "", "sentence_structure": "Subject鈥搗erb鈥搊bject", "subjective_objective": {"answer": "Subjective", "rationale": "Because the entry examines the patient ' s thoughts and attitudes in a given context, i.e. whether he or she can accept the different opinions of others, which is the patient ' s subjective feeling and reaction."}, "sentence_sentiment": {"answer": "Positive", "rationale": "Since the expression of the purpose of the article contained attitudes of inclusion, understanding and acceptance, those attitudes were positive."}, "language_style": {"answer": "Tactful and warm", "rationale": "The entry is not expressed directly by strong emotional or command-style tone, but rather by a gentle and inviting approach. The tone of the entry is more objective, more accurate and easy to use."}, "time_dimension": {"answer": "Present", "rationale": "The entry describes a behavior or mentality of the person being tested, i.e. the way the person is responding to a class or friend. In it, the words \"can still\" , \"with reasonable grounds\" and \"even if I do not agree with this view\" indicate a customary, present attitude and reaction. Thus, the temporal dimension of the article is now."}, "sentence_type": "Imperative sentence", "pos": {"nouns": {"Students": 1, "friends": 1, "Observations": 1, "he": 1, "she": 1, "Views": 2, "Relations": 1}, "verbs": {"You're": 1, "Accept": 1, "have": 1, "Reasonable": 1, "Agreed": 1}, "adjectives": {"Close": 1}, "adverbs": {"Still": 1}, "numerals": {"5": 1}, "pronouns": {"I": 1}}}, {"content": "6. I can leave out anger or resentment when there is a quarrel between close colleagues and friends.", "word_count": 32, "label": "", "sentence_structure": "Subject鈥搗erb鈥搊bject", "subjective_objective": {"answer": "Subjective", "rationale": "This is because of patients' subjective feelings and attitudes about their ability to control their emotions after a fight."}, "sentence_sentiment": {"answer": "Positive", "rationale": "Because the \"can leave anger or resentment\" in the entry is positive."}, "language_style": {"answer": "Calm and direct", "rationale": "The entry is more direct and objective in terms of language style, and in a direct and clear manner, it asks the other party whether it can control its emotions in the face of conflict."}, "time_dimension": {"answer": "Present", "rationale": "\"Now.\""}, "sentence_type": "Affirmative declarative sentence", "pos": {"nouns": {"Argument": 1, "Students": 1, "friends": 1, "Emotion": 1}, "verbs": {"Come on": 1, "Yes": 1, "Leave it": 1}, "adjectives": {"Close": 1, "Anger": 1, "Hate": 1}, "numerals": {"6": 1}, "pronouns": {"I": 1}}}, {"content": "When there is a disagreement with a good friend, I will tell him what he thinks.", "word_count": 23, "label": "", "sentence_structure": "Subject鈥搗erb鈥搊bject", "subjective_objective": {"answer": "Subjective", "rationale": "Because \"will tell yourself the truth\" is the patient's subjective attitude."}, "sentence_sentiment": {"answer": "Positive", "rationale": "Because the entry is a positive attitude, i.e., the choice to express their views and ideas honestly when faced with problems, which facilitates communication and problem solving."}, "language_style": {"answer": "Tactful and warm", "rationale": "The more euphemistic expression, \"Tell\" replaced with \"Tell him the truth,\" indicates that the surveyer tried to understand the respondent's position and attitude in dealing with interpersonal issues in a respectful and understanding manner, avoiding direct criticism or criticism. At the same time, the expression \"unsatisfactory opinion of a good friend\" implies concern and sensitivity for the interviewee, adding a warmer language."}, "time_dimension": {"answer": "Future", "rationale": "The time dimension of this questionnaire entry is therefore the future."}, "sentence_type": "Affirmative declarative sentence", "pos": {"nouns": {"friends": 1, "Observations": 1, "Thoughts": 1}, "verbs": {"Yes": 1, "Tell": 1, "- Yeah": 1, "True": 1}, "adverbs": {"From": 1, "Land": 1}, "numerals": {"7": 1}, "classifiers": {"One": 1}, "pronouns": {"he": 1}}}, {"content": "8. I am an input listener when the emotions of close classmates or friends are low.", "word_count": 26, "label": "", "sentence_structure": "Subject鈥揷opula鈥損redicative", "subjective_objective": {"answer": "Subjective", "rationale": "\"because it involves the patient's own perception and evaluation of his own behaviour.\""}, "sentence_sentiment": {"answer": "Positive", "rationale": "Because the \"input listener\" act is positive."}, "language_style": {"answer": "Tactful and warm", "rationale": "The basis for the judgement: the entry is a soft, invited conversation, not a direct statement or an order. By using the term \"input listener,\" it expresses concern and respect for each other's emotions, while also displaying an attitude of willingness to help and support. This expression is more personal and interactive."}, "time_dimension": {"answer": "Present", "rationale": "The keyword \"when a close classmate or friend is depressed\" indicates that the act has a specific situation, and \"I am an input listener\" indicates that this is the testee's behavioral tendency in the current situation. So the entry is mainly about the testee's current situation."}, "sentence_type": "Affirmative declarative sentence", "pos": {"nouns": {"Relations": 1, "Students": 1, "friends": 2, "Emotion": 1, "Listener": 1}, "verbs": {"be": 1, "Input": 1, "Listen": 1}, "adjectives": {"Close": 1, "Low": 1}, "numerals": {"8": 1}, "pronouns": {"I": 1}}}, {"content": "I was able to listen patiently and emotionally to the feelings of the close colleagues or friends about the external distress he/she had experienced.", "word_count": 40, "label": "", "sentence_structure": "Subject鈥搗erb鈥搊bject", "subjective_objective": {"answer": "Subjective", "rationale": "The question is whether the patient has some kind of mental trait in his or her own right and needs to be examined from the point of view of the patient ' s subjective nature."}, "sentence_sentiment": {"answer": "Positive", "rationale": "Because the behaviour in the entry (the patient and emotional listening to others) is positive, it helps others to ease emotional distress."}, "language_style": {"answer": "Tactful and warm", "rationale": "The entry is more euphemistic and warm, and it asks the respondent, in a caring and understanding manner, whether he or she can listen patiently and emotionally when others are having difficulties."}, "time_dimension": {"answer": "Present", "rationale": "So the output is: \"Now.\""}, "sentence_type": "Affirmative declarative sentence", "pos": {"nouns": {"Problem": 1, "Entry": 1, "Students": 1, "friends": 1, "Emotion": 1}, "verbs": {"Listen patiently and emotionally": 1}, "adjectives": {"Close": 1, "Outside": 1}, "adverbs": {"Related": 1}, "numerals": {"9": 1}, "pronouns": {"he": 1, "she": 1}}}, {"content": "I am an interesting and willing person to share when I deal with someone I don't know.", "word_count": 22, "label": "", "sentence_structure": "Subject鈥揷opula鈥損redicative", "subjective_objective": {"answer": "Subjective", "rationale": "Because the subject is examined by the patient ' s assessment and perception of his or her social performance, it is the patient ' s own subjective feelings and attitudes."}, "sentence_sentiment": {"answer": "Positive", "rationale": "Because the description of \"interested, willing to share\" in the entry is positive and indicates a positive attitude and behaviour."}, "language_style": {"answer": "Tactful and warm", "rationale": "Warm and warm."}, "time_dimension": {"answer": "Present", "rationale": "The entry describes the behaviour of the person who was tested in his current state when he/she was dealing with someone he/she does not know, so the temporal dimension is now judged."}, "sentence_type": "Affirmative declarative sentence", "pos": {"nouns": {"person": 2, "- To be with someone": 1, "Problem": 1, "Answer": 1}, "verbs": {"be": 3, "Share": 1}, "adjectives": {"Interesting": 1, "Yes": 1, "of": 4}, "adverbs": {"Time": 1}, "numerals": {"10": 1}, "pronouns": {"I": 1}}}, {"content": "I am a man who can give the proper comfort to others.", "word_count": 16, "label": "", "sentence_structure": "Subject鈥揷opula鈥損redicative", "subjective_objective": {"answer": "Subjective", "rationale": "As this statement relates to the patient ' s self-awareness and evaluation, it is a subjective view of the individual."}, "sentence_sentiment": {"answer": "Positive", "rationale": "Because the act in the entry \"giving the proper comfort\" is positive and expresses a positive emotional attitude and quality."}, "language_style": {"answer": "Tactful and warm", "rationale": "This language style makes it easier for the interviewee to feel comfortable and acceptable, and is conducive to increasing the effectiveness of the questionnaire."}, "time_dimension": {"answer": "Present", "rationale": "The entry describes a person ' s self-awareness and current state of behaviour, that is, his/her belief in his/her ability to bring appropriate comfort to others, which involves an perception of his/her abilities and attitudes and is therefore judged to be the present."}, "sentence_type": "Affirmative declarative sentence", "pos": {"nouns": {"I": 1, "person": 1, "Consolation": 1}, "verbs": {"be": 1, "Give": 1}, "adjectives": {"Appropriate": 1}}}, {"content": "12. Telling a person of opposite sex or a good friend of something he or she did hurt himself.", "word_count": 21, "label": "", "sentence_structure": "Subject鈥搗erb鈥搊bject", "subjective_objective": {"answer": "Subjective", "rationale": "Because the entry examines whether the patient is willing to express his feelings and needs to others on his own initiative, it is the patient ' s subjective attitude."}, "sentence_sentiment": {"answer": "Positive", "rationale": "Because the \"Telling the opposite sex or best friends\" act is positive. By expressing their feelings and needs, it helps to build good relationships and solve problems."}, "language_style": {"answer": "Tactful and warm", "rationale": "Although this entry involves informing others of their feelings, it does not use direct or harsh language. Rather, it uses a gentle and caring tone, conveying their concern and understanding to the interviewees. At the same time, it provides an option to choose whether to tell the opposite sex or a good friend about his injuries, a flexible approach that also makes the interviewees feel more comfortable and comfortable."}, "time_dimension": {"answer": "Present", "rationale": "The entry is for the testee's past, the keyword is \"to tell\" and \"to do something,\" all of which point to past behavior. So the temporal dimension is the past."}, "sentence_type": "Imperative sentence", "pos": {"nouns": {"Heterosexual": 1, "Good friend": 1, "Things": 1, "self": 1}, "verbs": {"Tell": 1, "Do it": 1, "Hurt": 1}, "adverbs": {"or": 1}, "numerals": {"12": 1}, "pronouns": {"he": 1, "she": 1}}}, {"content": "I'd complain or even refuse to do things that I'm embarrassed to do when I'm close to a classmate or friend.", "word_count": 34, "label": "", "sentence_structure": "Subject鈥搗erb鈥搊bject", "subjective_objective": {"answer": "Subjective", "rationale": "Because \"intimate classmates or friends,\" \"inconvenient things,\" and \"I'll complain and even refuse to do it\" are all patients' subjective attitudes and thoughts."}, "sentence_sentiment": {"answer": "Negative", "rationale": "Because the entries \"smuggling me\" and \"satisfying me or refusing to do it\" are negative, indicating aversion to the practices of fellow students or friends, the subject's emotional color is negative."}, "language_style": {"answer": "Tactful and warm", "rationale": "The entry is more adept and interactive when it is spoken in a gentle and inviting manner, rather than by direct statements or orders."}, "time_dimension": {"answer": "Present", "rationale": "The keywords \"will raise dissatisfaction\" and \"even refuse to do it\" indicate that this is a description of the current behaviour, so the temporal dimension is now judged."}, "sentence_type": "Affirmative declarative sentence", "pos": {"nouns": {"A close classmate or friend": 2, "Things": 2, "Dissatisfied": 1, "Reject": 1}, "verbs": {"Do it": 3, "Jean": 1, "Present": 1, "Go on": 2}, "adjectives": {"I'm embarrassed": 1, "Disgusting": 1}, "adverbs": {"Even": 1}, "numerals": {"13": 1}}}, {"content": "14. In my argument, I can accept the views of close colleagues or friends and truly understand their views.", "word_count": 32, "label": "", "sentence_structure": "Subject鈥搗erb鈥搊bject", "subjective_objective": {"answer": "Subjective", "rationale": "The acceptance and understanding of opinions are subjective acts and ideas of patients."}, "sentence_sentiment": {"answer": "Positive", "rationale": "Because the behaviour in the entry \"accepts the views of a close classmate or friend and truly understands his/her views\" is positive and reflects good communication and interpersonal competence."}, "language_style": {"answer": "Tactful and warm", "rationale": ""}, "time_dimension": {"answer": "Present", "rationale": ""}, "sentence_type": "Imperative sentence", "pos": {"nouns": {"Argument": 1, "Observations": 1, "Relations": 1, "Students": 1, "friends": 1, "Views": 1}, "verbs": {"It's acceptable": 1, "Understood": 1}, "numerals": {"14": 1}, "pronouns": {"I": 1, "he": 1, "she": 1}}}, {"content": "15. I am able to give genuine emotional attention when close colleagues or friends need to speak (and perhaps I am not interested in the subject).", "word_count": 40, "label": "", "sentence_structure": "Subject鈥搗erb鈥搊bject", "subjective_objective": {"answer": "Subjective", "rationale": "This is a reflection of the individual鈥檚 choice and therefore subjective."}, "sentence_sentiment": {"answer": "Positive", "rationale": "For the entries express a positive attitude of behaviour, i.e. the ability to give sincere emotional attention to students or friends when they need to speak, which is a positive act of social support."}, "language_style": {"answer": "Tactful and warm", "rationale": "The entry uses softer tone and language in describing its behaviour and attitudes, such as \"the ability to give genuine emotional attention\" rather than direct orders or demands. Such expression is more personal and interactive, and reflects an attitude of respect for the emotions of others, and can therefore be classified as a \"sweetly\" language style."}, "time_dimension": {"answer": "Past", "rationale": "The keyword \"the past\" indicates that the act occurred at a point or time in the past."}, "sentence_type": "Affirmative declarative sentence", "pos": {"nouns": {"Relations": 1, "Students": 1, "friends": 1, "Topic": 1, "Emotional attention": 1}, "verbs": {"Tell me": 1, "Give it to me": 1, "Yes": 1}, "adjectives": {"Close": 1, "Not interested": 1}, "numerals": {"15": 1, "When": 1, "I'm sincere": 1}, "classifiers": {"One": 2}, "pronouns": {"I": 1}}}, {"content": "16. I refuse (in euphemism or directness) requests from close friends of the opposite sex that go beyond our relationship.", "word_count": 29, "label": "", "sentence_structure": "Subject鈥搗erb鈥搊bject", "subjective_objective": {"answer": "Subjective", "rationale": "Because \"rejection\" of such behaviour and attitudes is a subjective decision of the patient, and is related to their values, beliefs and personal experiences. While this may also be influenced by objective factors (such as the behaviour of the other party), in this case the main objective is the patient's attitude and decision, and therefore the subjective view."}, "sentence_sentiment": {"answer": "Positive", "rationale": "Because the attitude in the entry is positive, that is, when a close friend of the opposite sex makes a request to me that goes beyond our relationship."}, "language_style": {"answer": "Calm and direct", "rationale": "The entry is more straightforward in language style, with no excessive aesthetic and emotional rendering. It clearly sets out a theme: your attitude when a close friend of the opposite sex makes a request to you that goes beyond your relationship, and asks the interviewee to choose, be more objective and calm, and not involve too many euphemisms or warmth elements."}, "time_dimension": {"answer": "Present", "rationale": "The time dimension of the entry is now as it describes the reaction of the person being tested in a particular situation (when a person of opposite sex has requested a relationship beyond their own) which is a current or possible situation."}, "sentence_type": "Affirmative declarative sentence", "pos": {"nouns": {"Heterosexual": 1, "Intimacy": 1, "friends": 1, "I": 2, "Relations": 1, "Request": 1, "Yes": 2, "Reject": 1, "(Fructual or direct)": 1}, "adjectives": {"Over": 1}, "adverbs": {"Demeanor or directly": 1}, "numerals": {"16": 1}}}, {"content": "17. I am able to resolve the conflict between the two sides when it occurs.", "word_count": 21, "label": "", "sentence_structure": "Subject鈥搗erb鈥揷omplement", "subjective_objective": {"answer": "Objective", "rationale": "Because the existence of a conflict between others and the resolution of a conflict between the parties are objective facts and consequences.鈥?}, "sentence_sentiment": {"answer": "Positive", "rationale": "Because the action in the entry \"can resolve the conflict between the two\" is positive."}, "language_style": {"answer": "Tactful and warm", "rationale": "The language style of the entry is more moderate and friendly, using a question-and-answer tone, without a direct statement or direction of what the other party should do, which is more easily accepted and coordinated by the interviewee. At the same time, the first person is used to express his or her abilities, to enhance the trust of the interviewee and to facilitate the smooth running of the questionnaire."}, "time_dimension": {"answer": "Present", "rationale": "The keyword \"can solve\" suggests that it is a capability or skill that can be enhanced and practiced in time and practice. So \"now\" is more in line with the time description of the article."}, "sentence_type": "Affirmative declarative sentence", "pos": {"nouns": {"Paradoxious": 2, "Conflict": 1, "Both sides": 1}, "verbs": {"Come on": 1, "Dismantling": 1}, "numerals": {"17": 1}, "classifiers": {"individual": 1}, "pronouns": {"I": 1}}}, {"content": "I am able to present to my good friends the shortcomings of my personality or the more vulnerable and sensitive aspects.", "word_count": 29, "label": "", "sentence_structure": "Subject鈥搗erb鈥搊bject", "subjective_objective": {"answer": "Subjective", "rationale": "Because the disadvantage of presenting one ' s personality or the more vulnerable and sensitive aspect is the patient ' s subjective thoughts and behaviour."}, "sentence_sentiment": {"answer": "Positive", "rationale": "Because the entry describes the behaviour of a person who is willing to show his or her true side to a good friend, which is usually considered a positive quality."}, "language_style": {"answer": "Tactful and warm", "rationale": "The words used in the entry are more euphemistic, and use the terms \"presentation\" and \"feasibility\" to express an open and frank attitude, encouraging the respondents to share their true feelings, and not to ask questions in too direct or critical terms. This soft language is easier for the respondents to feel comfortable and secure and to be more willing to participate in the questionnaire."}, "time_dimension": {"answer": "Present", "rationale": "\"Can\" indicates that it is a capability, i.e. an act or condition that can be achieved in the current state, and the entry therefore describes the person being tested in the present act or condition."}, "sentence_type": "Affirmative declarative sentence", "pos": {"nouns": {"Questionnaire Entry": 1, "Good friend": 1, "Character": 1, "Disadvantages": 1, "Weakness": 1, "Sensitive": 1, "One side": 1}, "verbs": {"Yes": 1, "Present": 1}, "adjectives": {"Comparison": 1, "Weakness": 1, "Sensitive": 1}, "numerals": {"18": 1}, "classifiers": {"individual": 1}, "pronouns": {"I": 1}}}, {"content": "I would like to tell your new friend some shameful things about you.", "word_count": 24, "label": "", "sentence_structure": "Subject鈥搗erb鈥搊bject", "subjective_objective": {"answer": "Subjective", "rationale": "Because the willingness to share in personal shame is a manifestation of subjective will and attitude."}, "sentence_sentiment": {"answer": "Neutral", "rationale": "Because the entry does not have any emotional color."}, "language_style": {"answer": "Tactful and warm", "rationale": "The question is whether the interviewees are willing to share private, potentially humiliating, things. This expression is relatively more intimate and respectful of the interviewee, and is unlikely to make the interviewee feel compelled or uncomfortable. At the same time, the tone of the question is neutral, not too direct or radical, giving people a feeling of friendship and warmth."}, "time_dimension": {"answer": "Present", "rationale": ""}, "sentence_type": "Affirmative declarative sentence", "pos": {"nouns": {"Questionnaire": 1, "Entry": 1, "you": 2, "New friends": 1, "Things": 1, "I feel": 1, "Shame": 1}, "verbs": {"Yes": 1, "Tell": 1, "Related": 1}, "adjectives": {"Some": 1}, "numerals": {"19": 1}, "pronouns": {"you": 2}}}, {"content": "I can cheer people up when they are in a bad mood.", "word_count": 18, "label": "", "sentence_structure": "Subject鈥搗erb鈥搊bject", "subjective_objective": {"answer": "Subjective", "rationale": "This requires a review of the patient ' s ability to do so and is self-evaluation of the patient."}, "sentence_sentiment": {"answer": "Positive", "rationale": "Because the behavior in the entry \"can cheer him up\" is positive and positive."}, "language_style": {"answer": "Tactful and warm", "rationale": "It uses a more euphemistic language style, with the expression \"can\" as the dominant word, and the expression \"happy to him\" to emphasize his abilities rather than directly asking the other to do something. This expression is more gentle and polite."}, "time_dimension": {"answer": "Present", "rationale": "\"20. I can cheer him up when others are in a bad mood.\" The word \"can\" indicates the ability or skill, the word \"let\" indicates the will and the behaviour of the person who is being tested, and the entire sentence describes the time in which the words are now in their current state, so it can be judged that the entry is asking about the behaviour and mentality of the person being tested."}, "sentence_type": "Affirmative declarative sentence", "pos": {"nouns": {"Time": 1, "person": 1, "The mood": 1, "Happy": 1}, "verbs": {"Yes": 1, "You're": 1, "Jean": 1}, "numerals": {"20": 1}, "pronouns": {"he": 1}}}, {"content": "21. When a close student or friend puts what they can to you, you refuse.", "word_count": 32, "label": "", "sentence_structure": "Subject鈥搗erb鈥搊bject", "subjective_objective": {"answer": "Subjective", "rationale": "Because refusal is a subjective attitude and behavioural choice of one."}, "sentence_sentiment": {"answer": "Negative", "rationale": "Because the behavior described in the entry is \"when you push what they can to do, you refuse\" and may be considered as a sign of unwillingness to help others, selfishness and, therefore, a certain negative emotional ambivalence."}, "language_style": {"answer": "Tactful and warm", "rationale": "The entry is more moderate, friendly and somewhat inviting in its language, using a more euphemistic expression to guide the respondents to answer questions. It does not direct or compel the respondents to respond to questions, but rather gives the respondents the freedom to choose whether they wish to answer them by asking questions, which is easier to obtain the acceptance and cooperation of the respondents, thus improving the effectiveness and reliability of the questionnaire."}, "time_dimension": {"answer": "Present", "rationale": ""}, "sentence_type": "Negative declarative sentence", "pos": {"nouns": {"Close": 1, "Students": 1, "friends": 1, "Things": 1}, "verbs": {"Push": 1, "Do it": 1, "Yes": 1, "Reject": 1}, "adjectives": {"I can do it": 1, "Intimate": 1}, "numerals": {"21": 1}, "classifiers": {"individual": 1}, "pronouns": {"they": 1}}}, {"content": "22. I can encourage and support newly recognized peers when they are in a difficult situation.", "word_count": 26, "label": "", "sentence_structure": "Subject鈥搗erb鈥搊bject", "subjective_objective": {"answer": "Subjective", "rationale": "Because the entry describes a behaviour and attitude that encourages and supports newly recognized friends in difficult circumstances, which is patients ' subjective thinking and behaviour."}, "sentence_sentiment": {"answer": "Positive", "rationale": "Because the action and purpose of encouraging and supporting it with practical action in the entry are positive."}, "language_style": {"answer": "Tactful and warm", "rationale": ""}, "time_dimension": {"answer": "Present", "rationale": ""}, "sentence_type": "Affirmative declarative sentence", "pos": {"nouns": {"Companion": 1, "The dilemma": 1, "Action": 1, "Support": 1}, "verbs": {"in": 1, "Encourage": 1, "Yeah": 1, "Actual": 1, "be": 1, "Give it to me": 1}, "adverbs": {"When": 1, "I'm new to you": 1}, "pronouns": {"I": 1, "he": 1}}}, {"content": "23. I can listen to his/her complaints in good faith when there is a contradiction between my close classmates or friends.", "word_count": 29, "label": "", "sentence_structure": "Subject鈥搗erb鈥搊bject", "subjective_objective": {"answer": "Subjective", "rationale": "Because \"to listen to his/her complaints in good faith\" is the patient's subjective attitude and behaviour, reflecting the patient's values and way of acting in the context of interpersonal relations."}, "sentence_sentiment": {"answer": "Positive", "rationale": "Because the expression of the purpose of the article includes \"acceptance of good faith\", it demonstrates concern for and respect for the feelings of others, and is a positive attitude and quality."}, "language_style": {"answer": "Tactful and warm", "rationale": "The language style of the entry is more euphemistic and sweet, using the words \"sent fellow students or friends\" and \"accompanied listening\" in a more moderate way, without any direct criticism or criticism. This expression is more easily accepted by the respondents and is willing to share their true ideas."}, "time_dimension": {"answer": "Present", "rationale": "The word \"capable\" in the entry to the questionnaire indicates that it is a capacity or custom, and \"when it is\" means that it is a reaction in a situation. Thus, the entry describes the ability and performance of the testee in the event of a conflict with a close student or friend, as is now the case."}, "sentence_type": "Interrogative sentence", "pos": {"nouns": {"Paradoxious": 1, "Students": 1, "friends": 1, "- He's complaining": 1}, "verbs": {"Yes": 1, "- Be honest": 1, "Listen": 1}, "adjectives": {"Close": 1}, "adverbs": {"When": 1}, "numerals": {"23": 1}, "pronouns": {"he": 1, "she": 1}}}, {"content": "24. I will reject his request when close colleagues or friends want to know some of your personal secrets.", "word_count": 33, "label": "", "sentence_structure": "Subject鈥搗erb鈥搊bject", "subjective_objective": {"answer": "Subjective", "rationale": "Because this entry expresses the patient ' s own attitude and choices in this situation, it examines subjective ideas."}, "sentence_sentiment": {"answer": "Neutral", "rationale": "Because the entry does not have any emotional color."}, "language_style": {"answer": "Calm and direct", "rationale": "The entry is based on a direct and clear statement of attitude and handling of private secrets, with no excessive rhetorical or emotional rendering, and a more objective tone. Moreover, the entry uses a direct expression such as \"will reject his request,\" without leaving room for the other party or suggesting that it may be possible, and the language style is calmer and more decisive."}, "time_dimension": {"answer": "Present", "rationale": "According to the general current and the overall context of the sentence, \"24. I will reject the request of close schoolmates or friends when they want to know some of your private secrets.\" It expresses the testee's current behavior or attitude. So the answer is \"now.\""}, "sentence_type": "Affirmative declarative sentence", "pos": {"nouns": {"Relations": 1, "Intimacy": 1, "Students": 1, "friends": 1, "Time": 1, "Request": 1, "Private": 1, "Secrets": 1}, "verbs": {"Yes": 2, "I'm not going to say it": 1, "of": 2}, "adverbs": {"Yes": 2, "Some of it": 1}, "classifiers": {"individual": 1}, "pronouns": {"you": 1}}}, {"content": "I know how to advance the conversation with others and truly promote mutual understanding.", "word_count": 21, "label": "", "sentence_structure": "Subject鈥搗erb鈥搊bject", "subjective_objective": {"answer": "Subjective", "rationale": "The basis for the judgement: \"I know how to advance conversation with others and really promote mutual understanding\" in the questionnaire entry is the personal view and attitude of a person, not the objective facts. So the answer is \"subjective.\""}, "sentence_sentiment": {"answer": "Positive", "rationale": "Because the expression of the purpose of the article emphasizes the ability to know how to advance conversation with others and to promote mutual understanding, these are positive qualities or attitudes."}, "language_style": {"answer": "Tactful and warm", "rationale": "The language style of the entry tends to be euphemistic, encouraging respondents to promote dialogue with others and to enhance understanding among them in this way. This expression is relatively friendly, and helps to increase the participation and effectiveness of the respondents."}, "time_dimension": {"answer": "Present", "rationale": "The questionnaire entry asked if the person tested was currently aware of how to advance the conversation with others to promote mutual understanding, and therefore the temporal dimension was now judged."}, "sentence_type": "Affirmative declarative sentence", "pos": {"nouns": {"I": 1, "Advance": 1, "others": 1, "Talk": 1, "Mutual understanding": 1}, "verbs": {"Yes": 1, "Advance": 1, "Promotion": 1, "Roger that": 1}, "numerals": {"25": 1}}}, {"content": "I can talk to someone I've just met about my privacy.", "word_count": 19, "label": "", "sentence_structure": "Subject鈥搗erb鈥搊bject", "subjective_objective": {"answer": "Subjective", "rationale": "According to the title, the entry needs to be judged from the patient鈥檚 subjective point of view or from an objective point of view. For 鈥? I can talk to someone I've just met about my private secrets,鈥?the purpose is described as involving a subjective attitude and behaviour of the patient, and should therefore be considered subjective."}, "sentence_sentiment": {"answer": "Positive", "rationale": "Because the act of \"can talk to someone who's just met about their privacy\" in the entry is an expression of a person's openness and trust, which can be considered a positive emotional color."}, "language_style": {"answer": "Tactful and warm", "rationale": "Warm and warm."}, "time_dimension": {"answer": "Present", "rationale": "The word \"capable\" denotes the state of ability or capability, which is used to describe the current situation. And \"the person who has just met\" and \"talking about his private secrets\" all indicate that this is a description of the current behaviour, so the entry is about the present abilities and behaviour of the person being tested and should be attributed to this time dimension."}, "sentence_type": "Negative declarative sentence", "pos": {"nouns": {"person": 2, "self": 1, "Private": 1}, "verbs": {"Yes": 1, "Call": 1, "- No, no, no": 1, "I'm talking": 1}, "adverbs": {"Just now": 1}, "numerals": {"4": 1}, "pronouns": {"I": 1}}}, {"content": "5. I trust new friends and can show them your fragile and sensitive side.", "word_count": 21, "label": "", "sentence_structure": "Subject鈥搗erb鈥搊bject", "subjective_objective": {"answer": "Subjective", "rationale": "Because \"trust\" and \"showing the weak, sensitive side\" are personal feelings and attitudes of patients and are subjective."}, "sentence_sentiment": {"answer": "Positive", "rationale": "Because trust and expression of the fragile, sensitive side are positive qualities and behaviours."}, "language_style": {"answer": "Tactful and warm", "rationale": "The words are more euphemistic, gentle and emotional. By using the words \"trust\" and \"expression\" they express the expectations and trust of new friends, while also revealing an attitude that is willing to share its feelings. Such a expression is more likely to resonate and comfort with the person under investigation, and to facilitate the validity and authenticity of the questionnaire."}, "time_dimension": {"answer": "Present", "rationale": "\"I trust new friends\" shows the behaviour and attitude of the testees, and \"and I can show them your fragile, sensitive side\" implies a dynamic process in which the testees are showing their emotional state. So this entry describes the current behavior and mentality."}, "sentence_type": "Affirmative declarative sentence", "pos": {"nouns": {"friends": 2, "Weakness": 1, "Sensitive": 1}, "verbs": {"Trust": 1, "Performance": 1}, "numerals": {"5": 1}, "pronouns": {"I": 1}}}, {"content": "I am an interesting and willing person to share when I interact with people.", "word_count": 18, "label": "", "sentence_structure": "Subject鈥揷opula鈥損redicative", "subjective_objective": {"answer": "Subjective", "rationale": "Because \"interesting\" and \"willing to share\" are the feelings and attitudes of the patient, and are subjective."}, "sentence_sentiment": {"answer": "Positive", "rationale": "Because both the adjectives \"interesting\" and \"will share\" in the entries convey positive emotions."}, "language_style": {"answer": "Tactful and warm", "rationale": "The language style of the entry is more euphemistic and encouraging, and it describes the social characteristics of the subjects in a positive way, that is, they are interesting and willing to share. This expression can be accepted and appreciated, helping to build good relationships and trust."}, "time_dimension": {"answer": "Present", "rationale": ""}, "sentence_type": "Affirmative declarative sentence", "pos": {"nouns": {"person": 2, "- To be with someone": 1, "Entry": 1}, "verbs": {"be": 1, "Interesting": 1, "Yes": 1, "Share": 1}, "adjectives": {"Interesting": 1, "Yes": 1}, "adverbs": {"With people": 1}, "numerals": {"6": 1}, "pronouns": {"I": 1}}}, {"content": "7. I will tell my colleagues the secrets that cause my anxiety or fear.", "word_count": 20, "label": "", "sentence_structure": "Subject鈥搗erb鈥搊bject", "subjective_objective": {"answer": "Subjective", "rationale": "Because the feeling of \"claim,\" \"anxiety\" and \"fear\" is the patient's personal experience and feelings, it's a subjective examination."}, "sentence_sentiment": {"answer": "Positive", "rationale": "Because the act of \"showing your secrets to your peers\" in the entry is positive as it helps to relieve stress and anxiety."}, "language_style": {"answer": "Tactful and warm", "rationale": "The entry is more euphemistic, is more euphemistic, and leads respondents to share their feelings and secrets in a way that is asked, and is expressed in a more friendly way."}, "time_dimension": {"answer": "Past", "rationale": "The entry describes a past habit or behaviour whereby the testee may, in certain circumstances, reveal his or her fears or fears to his or her peers. Keywords \"will\" and \"talk to my or her fellow\" indicate that this is a regular act and has occurred repeatedly. Thus, the entry can be classified as a past act."}, "sentence_type": "Affirmative declarative sentence", "pos": {"nouns": {"Companion": 1, "Secrets": 1}, "verbs": {"Yes": 1, "Tell me": 1, "Jean": 1, "Anxiety": 1, "Scared": 1}, "adjectives": {"self": 1}, "numerals": {"7": 1}}}, {"content": "8. I am able to encourage and support newly recognized peers when they are in a difficult situation.", "word_count": 26, "label": "", "sentence_structure": "Subject鈥搗erb鈥搊bject", "subjective_objective": {"answer": "Subjective", "rationale": "Because encouragement and support are subjective attitudes and behaviours of patients."}, "sentence_sentiment": {"answer": "Positive", "rationale": "Because \"encourage and support by action\" in the entry is positive."}, "language_style": {"answer": "Tactful and warm", "rationale": "This language style is more likely to inspire self-identification and positive feelings among the respondents, making the questionnaire more friendly and friendly and more accessible to the respondents鈥?collaboration and feedback."}, "time_dimension": {"answer": "Present", "rationale": "The entry is for the purpose of asking the subject about his current behaviour or attitude, and the description of the keyword \"can\" and \"in difficult circumstances\" indicates that it is a regular act, so the temporal dimension is now judged."}, "sentence_type": "Affirmative declarative sentence", "pos": {"nouns": {"Companion": 1, "The dilemma": 1, "Support": 1}, "verbs": {"When": 1, "Yes": 2, "in": 1, "Encourage": 1, "and": 1, "Here": 1, "Practical actions": 1, "Help": 1}, "adverbs": {"New": 1}, "classifiers": {"individual": 1}, "pronouns": {"I": 1, "he": 1}}}, {"content": "9. When newly recognized colleagues speak to me, I am able to give sincere emotional attention.", "word_count": 24, "label": "", "sentence_structure": "Subject鈥搗erb鈥搊bject", "subjective_objective": {"answer": "Subjective", "rationale": "This is a self-evaluation and perception of the patient and is subjective."}, "sentence_sentiment": {"answer": "Positive", "rationale": "Because the action of \"giving genuine emotional attention\" in the entry is positive."}, "language_style": {"answer": "Tactful and warm", "rationale": "The language style of the entry is euphemism. By using the expression \"can give genuine emotional attention\" it avoids direct criticism or orders, but expresses what one needs to do in a gentle and caring manner. Moreover, using words like \"claim\" and \"partner\" conveys a friendly and friendly atmosphere."}, "time_dimension": {"answer": "Present", "rationale": ""}, "sentence_type": "Affirmative declarative sentence", "pos": {"nouns": {"Companion": 1, "Emotional attention": 1}, "verbs": {"Yes": 1, "Give it to me": 1, "Tell me": 1}, "adjectives": {"I'm new to you": 1, "I'm sincere": 2}, "numerals": {"9": 1}, "pronouns": {"I": 1}}}, {"content": "I'm a friendly talker.", "word_count": 14, "label": "", "sentence_structure": "Subject鈥揷opula鈥損redicative", "subjective_objective": {"answer": "Subjective", "rationale": "Because \"friendly talk\" is an evaluative description that reflects the perception and assessment of me by others, it is subjective."}, "sentence_sentiment": {"answer": "Positive", "rationale": "Because the \"friendly talk\" in the entry is a positive description, and he's very well-rated by the other person."}, "language_style": {"answer": "Tactful and warm", "rationale": "The entry uses more euphemistic language to avoid direct evaluation or stigmatization of the interviewee, but rather to ask the other person in a gentle and inviting manner about the image of the other person in the eyes of others, which is more personal and interactive."}, "time_dimension": {"answer": "Present", "rationale": "The entry indicates the current state or character of the testee, that is, the image in other eyes. So the judgment is \"now\"."}, "sentence_type": "Affirmative declarative sentence", "pos": {"nouns": {"others": 1, "Eye": 1, "person": 2, "individual": 1}, "verbs": {"be": 1, "Look": 1, "Friendly": 1, "Talkative": 1}, "adjectives": {"Friendly": 1, "Talkative": 1}, "numerals": {"10": 1, "One": 2}, "classifiers": {"individual": 1}, "pronouns": {"I": 1}}}, {"content": "I would care to give a good first impression to those who want to be friends or lovers.", "word_count": 27, "label": "", "sentence_structure": "Subject鈥搗erb鈥搊bject", "subjective_objective": {"answer": "Subjective", "rationale": "Because \"will\" expresses the patient's personal will and attitude, it's the patient's subjective idea."}, "sentence_sentiment": {"answer": "Positive", "rationale": "Because the behavior in the entry \"cares to make a good first impression to those who want to be friends or lovers\" is positive emotional."}, "language_style": {"answer": "Tactful and warm", "rationale": "The language style of the entry is more euphemistic, and the use of the words \"intentional\" and \"persons wishing to be friends or lovers\" is used to express concern about the interpersonal relations and self-image of the interviewee, without too much use of direct orders, threats or negative evaluations. This makes the questionnaire more friendly and acceptable."}, "time_dimension": {"answer": "Present", "rationale": ""}, "sentence_type": "Affirmative declarative sentence", "pos": {"nouns": {"Questionnaire Entry": 1, "person": 2, "First impression": 1, "friends": 1, "Lovers": 1}, "verbs": {"Yes": 1, "I care": 1, "Here": 1, "Be": 1, "Stay": 1}, "numerals": {"11": 1}, "classifiers": {"individual": 1}, "pronouns": {"you": 1}}}, {"content": "I would deny that close classmates or friends do not meet reasonable requirements.", "word_count": 22, "label": "", "sentence_structure": "Subject鈥搗erb鈥搊bject", "subjective_objective": {"answer": "Subjective", "rationale": "The fact that refusal is a personal attitude and choice of the patient is subjective. And the fact that \"intimate classmates or friends do not meet the reasonable requirements\" is in part objective, so the answer is subjective/objective."}, "sentence_sentiment": {"answer": "Negative", "rationale": "Because both the words \"no\" and \"uncomplicated\" have some negative emotions."}, "language_style": {"answer": "Calm and direct", "rationale": "The entry is more straightforward, is not expressed in too many speech-teaching or emotional terms, and is more calm and objective. It is more direct to ask whether the person under investigation will refuse to accept that a close classmate or friend does not meet the requirements of reason."}, "time_dimension": {"answer": "Present", "rationale": "The entry asks about the person's current attitude and behaviour, and the keyword \"will\" indicates that it is a customary or normal act, so the temporal dimension is now judged."}, "sentence_type": "Affirmative declarative sentence", "pos": {"nouns": {"Relations": 1, "Students": 1, "friends": 1, "Request": 1}, "verbs": {"Yes": 1, "Reject": 1, "Match": 1, "Request": 1}, "adjectives": {"Intimate": 1, "It makes sense": 1}, "numerals": {"12": 1}}}, {"content": "13. Close schoolmates or friends ask you to do what you dislike.", "word_count": 24, "label": "", "sentence_structure": "Subject鈥搗erb鈥搊bject", "subjective_objective": {"answer": "Subjective", "rationale": "This is because of patients ' subjective views and attitudes towards interpersonal relations."}, "sentence_sentiment": {"answer": "Negative", "rationale": "Because the \"do what you hate\" act in the entry is negative."}, "language_style": {"answer": "Tactful and warm", "rationale": "The entry uses a more moderate tone to ask the respondents whether they are willing to do things they do not want to do for a close friend or classmate. This expression is neither too direct nor too vague, and it is easier to resonate with the interviewees and to get an accurate answer."}, "time_dimension": {"answer": "Past", "rationale": "The entry describes the subjective perception and attitude of the testee towards past behaviour and therefore determines the past."}, "sentence_type": "Negative declarative sentence", "pos": {"nouns": {"Relations": 1, "Students": 1, "friends": 1}, "verbs": {"Jean": 1, "Do it": 2, "Go on": 2}, "adjectives": {"Intimate": 1, "Disgusting": 1}, "numerals": {"13": 1, "1": 4}, "classifiers": {"individual": 1}, "pronouns": {"you": 1}}}, {"content": "14. When close friends or friends do things that embarrass you (or disgust you), you have to complain.", "word_count": 29, "label": "", "sentence_structure": "Subject鈥搗erb鈥搊bject", "subjective_objective": {"answer": "Subjective", "rationale": "Because \"intimate classmates or friends\" are the personal relationships that patients have, and the reaction to \"satisfaction\" is also the patient's individual subjective attitude and behaviour."}, "sentence_sentiment": {"answer": "Negative", "rationale": "Because the entry \"smuggling (or aversion)\" is negative."}, "language_style": {"answer": "Calm and direct", "rationale": "The entry directly expresses the question whether the subject of grievances is directly raised when a close friend does something that embarrasss or disgusts himself, and is not presented in a more balanced and objective manner by excessive rhetorical or emotional overtones."}, "time_dimension": {"answer": "Present", "rationale": ""}, "sentence_type": "Affirmative declarative sentence", "pos": {"nouns": {"Closeness": 1, "Students": 1, "friends": 1, "Things": 1, "Dissatisfied": 1}, "verbs": {"Do it": 1, "Jean": 1, "Yes": 1, "Use": 1, "Present": 1}, "adjectives": {"I'm embarrassed": 1, "Disgusting": 1}, "numerals": {"14": 1}, "pronouns": {"you": 1}}}, {"content": "15. When a person who knows you can't accept a gift of value, you can clearly refuse it.", "word_count": 33, "label": "", "sentence_structure": "Subject鈥搗erb鈥搊bject", "subjective_objective": {"answer": "Subjective", "rationale": "Because the problem relates to the attitude and behaviour of patients towards gifts given to others, it is the patient ' s subjective thinking and attitude."}, "sentence_sentiment": {"answer": "Negative", "rationale": "Because the act of \"you can expressly refuse (in euphemism or directness) when it is not acceptable\" in the entry may make others uncomfortable or offensive and therefore negative."}, "language_style": {"answer": "Calm and direct", "rationale": "The entry is more calm and straightforward in its language, is not used in excessive speech or emotional rendering, and is more objective in tone. It asks whether the interviewee can explicitly refuse to accept the gift of a valuable gift from a person of general acquaintance, but not try to channel the interviewer鈥檚 answer in a euphemism."}, "time_dimension": {"answer": "Present", "rationale": "In terms of time dimension, the question is the reaction and attitude of the situation that is taking place, so the answer is \"now\"."}, "sentence_type": "Interrogative sentence", "pos": {"nouns": {"I know someone": 1, "Gifts": 1, "Reject": 1, "Euphemism": 1, "Direct": 1}, "verbs": {"Present": 1, "Accept": 1, "Clear": 1}, "adjectives": {"Precious": 1, "General": 1}, "numerals": {"15": 1}, "classifiers": {"individual": 1}, "pronouns": {"you": 1}}}, {"content": "16. When a close classmate or friend always puts what they can to you, you refuse.", "word_count": 34, "label": "", "sentence_structure": "Subject鈥搗erb鈥搊bject", "subjective_objective": {"answer": "Subjective", "rationale": "Because \"will refuse\" is a subjective attitude and reaction of the individual."}, "sentence_sentiment": {"answer": "Negative", "rationale": "Because the entry \" always pushes what they can to you\" is negative."}, "language_style": {"answer": "Tactful and warm", "rationale": "The language style of the entry is euphemism, which does not directly describe the behaviour of the other side as dissatisfied or uncomfortable, but rather as a way of creating resonance and reflection among the respondents by describing a scene. At the same time, the question begins with the words \"a close classmate or friend\" and gives people a sense of affection and friendship."}, "time_dimension": {"answer": "Present", "rationale": "Now the entry describes the reaction of the testee in the current circumstances, the keyword being \"will reject\" and it indicates that the answer of the testee is based on the current circumstances and experience. Thus, the temporal dimension of the questionnaire entry is now judged."}, "sentence_type": "Interrogative sentence", "pos": {"nouns": {"Students": 1, "friends": 1, "Things": 2, "you": 2}, "verbs": {"Always": 1, "- Put it on": 1, "Push it": 1, "Do it": 1, "Yes": 1, "Reject": 1}, "adjectives": {"Intimate": 1, "I can do it": 1}, "numerals": {"16": 1}, "pronouns": {"they": 1}}}, {"content": "17. When close colleagues or friends want to know some of your personal secrets that you do not want to know, you will reject his request.", "word_count": 39, "label": "", "sentence_structure": "Subject鈥搗erb鈥搊bject", "subjective_objective": {"answer": "Subjective", "rationale": "Because the willingness to reveal private secrets is a subjective attitude and choice of one."}, "sentence_sentiment": {"answer": "Negative", "rationale": "Because the words \"not knowing private secrets\" and \"not asking him back\" in the entries express a feeling of unwillingness to share and be violated, which is a negative attitude."}, "language_style": {"answer": "Calm and direct", "rationale": "The language style of the entry is more straightforward, not using too much qualifier or emotional colour, but describing the situation in an objective and practical way."}, "time_dimension": {"answer": "Past", "rationale": ""}, "sentence_type": "Imperative sentence", "pos": {"nouns": {"Relations": 1, "Students": 1, "friends": 2, "Secrets": 1, "Request": 1}, "verbs": {"Yes": 3, "Question": 1, "I'm not going to say it": 1, "His": 1, "鈾?When 鈾?: 1, "of": 4}, "adverbs": {"very": 1}, "numerals": {"One": 2, "Two": 1}, "pronouns": {"you": 1, "he": 2, "Here": 1}}}, {"content": "18. When a close friend of the opposite sex makes a request to you that goes beyond your relationship, you reject it (precipitously or directly).", "word_count": 29, "label": "", "sentence_structure": "Subject鈥搗erb鈥搊bject", "subjective_objective": {"answer": "Subjective", "rationale": "The subject is subjective because it is to examine the attitude and reaction of patients to requests from close heterosexual friends to exceed their relationship."}, "sentence_sentiment": {"answer": "Negative", "rationale": "Because the precondition of \"when a close friend of the opposite sex requests you to go beyond your relationship\" in this entry carries a certain potential risk and negative impact, and the response requested may affect the relationship between the two parties and therefore be judged to be negative."}, "language_style": {"answer": "Calm and direct", "rationale": "The language style of this questionnaire entry should therefore be calm and straightforward, as it clearly raises questions and requires the respondents to provide specific information. At the same time, the question does not contain any emotional or implied terms that would make the respondents鈥?answers more objective and accurate."}, "time_dimension": {"answer": "Present", "rationale": "The content of the topic asks how the testees would respond to requests from close friends of the opposite sex that go beyond your relationship in the current circumstances, and therefore judge it now."}, "sentence_type": "Interrogative sentence", "pos": {"nouns": {"A close friend of the opposite sex": 1, "you": 1, "Request": 1, "Relations": 1}, "verbs": {"Yes": 2, "Reject": 1, "T": 1, "Out": 1, "Demeanor or directly": 1, "to": 1}, "adjectives": {"Over": 1}, "adverbs": {"Time": 1}, "numerals": {"18": 1, "1": 6}, "classifiers": {"individual": 1, "Plant": 1}, "pronouns": {"he": 1}}}, {"content": "I can cheer people when they are in a bad mood.", "word_count": 18, "label": "", "sentence_structure": "Subject鈥搗erb鈥搊bject", "subjective_objective": {"answer": "Subjective", "rationale": "Because \"I can make him happy\" is not always true, and it's a patient's self-evaluation, so the entry is viewed from the patient's subjective point of view."}, "sentence_sentiment": {"answer": "Positive", "rationale": "Because it expresses the act of helping and supporting others when they are depressed, it has a positive emotional colour."}, "language_style": {"answer": "Tactful and warm", "rationale": "The language style of the entry is more euphemistic, and it leads the respondents to answer questions in a kind of encouragement and support. By using the \"I can\" form of affirmation and self-expression, the respondents feel more confident and comfortable sharing their own situation. Moreover, the expression \"when others are in bad mood\" is more moderate, avoiding too direct or negative emotional."}, "time_dimension": {"answer": "Present", "rationale": "The keyword \"can cheer him up\" suggests that it is a proactive act and is relevant to the present situation."}, "sentence_type": "Interrogative sentence", "pos": {"nouns": {"others": 1, "The mood": 1, "Time": 1, "I": 1, "Happy": 1}, "verbs": {"You're": 1}, "adverbs": {"It's not good": 1, "Yes": 1}, "numerals": {"19": 1}, "pronouns": {"he": 1}}}, {"content": "I can always get people to get my happy feelings.", "word_count": 16, "label": "", "sentence_structure": "Subject鈥搗erb鈥揷omplement", "subjective_objective": {"answer": "Subjective", "rationale": "Because \"to make others infected with my pleasure\" is a personal sense and attitude."}, "sentence_sentiment": {"answer": "Positive", "rationale": "Because the purpose of the entry is to get people to infect my pleasant emotions."}, "language_style": {"answer": "Tactful and warm", "rationale": "The entry is more euphemistic and gentle, resonating and reflecting on the respondents by asking them whether they have the capacity to do so, without a direct statement or order. At the same time, the term \"infection\" implies an interactive and transmissible character that makes people feel more close and friendly."}, "time_dimension": {"answer": "Future", "rationale": "The entry expresses the desire or desire of the testee to be able to convey his pleasant emotions in the future. The keyword \"always\" and \"infection\" point to future behaviour and results, and therefore to the future."}, "sentence_type": "Interrogative sentence", "pos": {"nouns": {"Emotion": 1, "Have a nice day": 1}, "verbs": {"Jean": 1, "Infection": 1}, "adverbs": {"Always": 1}, "pronouns": {"I": 1}}}, {"content": "I am a man who can give others the proper comfort.", "word_count": 16, "label": "", "sentence_structure": "Subject鈥揷opula鈥損redicative", "subjective_objective": {"answer": "Subjective", "rationale": "Because \"I am a person who can give the proper comfort\" is a patient's self-evaluation and opinion, which is a personal subjective view."}, "sentence_sentiment": {"answer": "Positive", "rationale": "Because the \"can give the proper comfort\" in the entry is positive."}, "language_style": {"answer": "Tactful and warm", "rationale": "The entry uses positive tone and a mild way to ask the other side whether it agrees with its views, giving the impression that it is positive and open. At the same time, this expression is more readily accepted and understood by the interviewees, which helps to improve the completion and recovery rates of the questionnaire."}, "time_dimension": {"answer": "Present", "rationale": ""}, "sentence_type": "Affirmative declarative sentence", "pos": {"nouns": {"person": 1, "Consolation": 1}, "verbs": {"be": 1, "Give it to me": 1}, "adjectives": {"Appropriate": 1}, "pronouns": {"I": 1}}}, {"content": "22. I can help close colleagues or friends deal with their problems with family members or with their relationship with roommates (working or staying).", "word_count": 37, "label": "", "sentence_structure": "Subject鈥搗erb鈥搊bject", "subjective_objective": {"answer": "Subjective", "rationale": "Because \"I can help\" is a patient's self-evaluation, subjective."}, "sentence_sentiment": {"answer": "Positive", "rationale": "Because the purpose of the entry is to help students or friends deal with problems."}, "language_style": {"answer": "Tactful and warm", "rationale": "The entry is more personal and interactive in its expression of conversation in a gentle and inviting tone, rather than direct statements or orders. At the same time, the phrase \"I can help\" gives a positive and sweet look at people."}, "time_dimension": {"answer": "Present", "rationale": "The temporal dimension of the entry can be judged as \"now\" based on the behaviour described in the input sentence, as it describes an act currently being performed."}, "sentence_type": "Affirmative declarative sentence", "pos": {"nouns": {"Questionnaire": 1, "Entry": 1, "Students": 1, "friends": 1, "Family members": 1, "Roommate": 1, "Relationship issues": 2}, "verbs": {"It helps": 1, "Processing": 1, "and": 1}, "adjectives": {"Close": 1}, "numerals": {"22": 1}, "classifiers": {"Other": 1, "or": 1}, "pronouns": {"I": 1}}}, {"content": "23. When I am angry with close colleagues or friends, I can accept that his/her views are reasonable, even if I do not agree with them.", "word_count": 47, "label": "", "sentence_structure": "Subject鈥揷opula鈥損redicative", "subjective_objective": {"answer": "Subjective", "rationale": "Because the phrase \"I can accept this\" and \"even if I don't agree with it\" suggests that it is a patient's description of his thoughts and attitudes, and that it is a subjective examination."}, "sentence_sentiment": {"answer": "Positive", "rationale": "Because the entry expresses an attitude of willingness to listen and understand others ' views, which is a positive emotional tendency."}, "language_style": {"answer": "Tactful and warm", "rationale": "The language style of this questionnaire entry is euphemism. It uses an invitation phrase ( \"Please analyse the next questionnaire entry\") and avoids too direct or hard language when describing the situation. At the same time, the entry also conveys a positive message that while accepting others' views, it can also maintain its own position and attitude, which is more resonant."}, "time_dimension": {"answer": "Present", "rationale": "The entry describes the behaviour and attitude of the testees in the face of a particular situation, i.e., when their classmates or friends make them angry, how they will deal with the situation."}, "sentence_type": "Affirmative declarative sentence", "pos": {"nouns": {"Relations": 1, "Students": 1, "friends": 1, "Observations": 1, "he": 2, "she": 1, "Views": 2, "Reasonable": 1, "Local": 1, "Even": 1}, "verbs": {"Accept": 1, "You're": 1, "Angry": 1, "Yes": 1, "have": 1, "Agreed": 1}, "adjectives": {"Close": 1, "Reasonable": 1}, "adverbs": {"Even": 1}, "numerals": {"23": 1, "he": 2, "she": 1, "Reasonable": 1}, "classifiers": {"One": 8}, "pronouns": {"Here": 1}}}, {"content": "24. I am able to give genuine emotional attention when close colleagues or friends need to speak (the subject may not be of interest to me).", "word_count": 40, "label": "", "sentence_structure": "Subject鈥搗erb鈥搊bject", "subjective_objective": {"answer": "Subjective", "rationale": "Because \"good emotional concern\" is the patient's subjective attitude and behavior."}, "sentence_sentiment": {"answer": "Positive", "rationale": "According to the entry expression, the act of \"capable of giving genuine emotional attention\" is positive and shows concern and support for others."}, "language_style": {"answer": "Tactful and warm", "rationale": "The entry uses a more euphemistic and caring language, which does not directly require the other party to do something or provide some feedback, but rather raises questions in a way that expresses understanding and concern."}, "time_dimension": {"answer": "Present", "rationale": "And \"when...\" also means it's a situational situation, not a long-term habit. So the temporal dimension of this questionnaire entry is \"now\"."}, "sentence_type": "Affirmative declarative sentence", "pos": {"nouns": {"Students": 1, "friends": 1, "Emotional attention": 1}, "verbs": {"Yes": 1, "Give it to me": 1, "Tell me": 1, "Maybe": 1, "Interested": 1}, "adjectives": {"Close": 1, "I'm sincere": 1}, "adverbs": {"When": 1}, "numerals": {"24": 1}, "pronouns": {"I": 1}}}, {"content": "25. No one should be angry with their fellow students and friends and avoid harming each other.", "word_count": 20, "label": "", "sentence_structure": "Subject鈥損redicate", "subjective_objective": {"answer": "Subjective", "rationale": "Because \"not to be angry with your classmates and friends\" and \"not to hurt each other\" are the patient's behavioural self-proclaims and subjective attitudes."}, "sentence_sentiment": {"answer": "Positive", "rationale": "Because the entry \"not to be angry with your classmates and friends\" is positive."}, "language_style": {"answer": "Tactful and warm", "rationale": "The language style of the entry is a soft and warmer type. It uses a gentle, inviting tone, emphasizing the importance of \"not to lose temper with friends and classmates\" and avoiding harm to each other, speaking more honestly and objectively, encouraging respondents to consider the issue from the perspective of the other, thereby promoting human relations."}, "time_dimension": {"answer": "Present", "rationale": ""}, "sentence_type": "Affirmative declarative sentence", "pos": {"nouns": {"Students": 1, "friends": 1, "- Grumpy": 1, "Hurt": 1, "You too": 1}, "verbs": {"No, it's not": 1, "It's a mess": 1, "Avoid": 1}, "adverbs": {"not": 1}, "numerals": {"25": 1}}}, {"content": "26. I am seen as a trustworthy person.", "word_count": 14, "label": "", "sentence_structure": "Subject鈥揷opula鈥損redicative", "subjective_objective": {"answer": "Objective", "rationale": "Because this is what others see as an evaluation, it is an objective perspective."}, "sentence_sentiment": {"answer": "Positive", "rationale": "Because the phrase \"I am a trustworthy person\" in the entry is positive and expresses the confidence and approval of others in the person under investigation."}, "language_style": {"answer": "Tactful and warm", "rationale": "This entry uses more euphemistic and positive language to express an assessment of the capacity of the interviewee, without directly questioning or rejecting it."}, "time_dimension": {"answer": "Present", "rationale": "The keywords are \"I'm a trustworthy person\" and \"I'm a trustworthy person\" , which emphasize observation and evaluation in the current situation, so the entry describes a state or behaviour of the present."}, "sentence_type": "Affirmative declarative sentence", "pos": {"nouns": {"person": 1, "others": 1, "In the eyes": 1}, "verbs": {"be": 1, "It's worth it": 1, "Trust me": 1}, "adverbs": {"26": 1}, "numerals": {"26": 1}, "pronouns": {"I": 1}}}, {"content": "27. I am an input listener when the emotions of close colleagues or friends are low.", "word_count": 26, "label": "", "sentence_structure": "Subject鈥揷opula鈥損redicative", "subjective_objective": {"answer": "Subjective", "rationale": "Because \"I'm an input listener\" is patient subjectivity and behavior."}, "sentence_sentiment": {"answer": "Positive", "rationale": "Because the behavior of \"input listeners\" in the entries is positive."}, "language_style": {"answer": "Tactful and warm", "rationale": "The entry uses descriptive language to express the theme, emphasizing the importance of individual listening in dealing with intimate relationships."}, "time_dimension": {"answer": "Past", "rationale": "Thus, this entry covers both the past situation (i.e., having had similar experiences) and the conduct that may be present or possible in the future (i.e., when this occurs, it becomes an input listener)."}, "sentence_type": "Imperative sentence", "pos": {"nouns": {"Relations": 1, "Students": 1, "friends": 1, "Emotion": 1, "Listener": 1}, "verbs": {"be": 1, "Input": 1, "Down": 1}, "numerals": {"27": 1}, "pronouns": {"I": 1}}}, {"content": "I was able to listen patiently and emotionally to the feelings of the close colleagues or friends about the external distress he or she had experienced.", "word_count": 40, "label": "", "sentence_structure": "Subject鈥搗erb鈥搊bject", "subjective_objective": {"answer": "Subjective", "rationale": "Because \"the patient and the emotional listening\" is the patient's attitude and behaviour in dealing with interpersonal relationships, it is a subjective entry."}, "sentence_sentiment": {"answer": "Positive", "rationale": "Because the behavior of \"suffering and emotional listening\" and \"purposes to help students or friends to solve their external problems\" in this entry are positive."}, "language_style": {"answer": "Tactful and warm", "rationale": "The language style of the questionnaire entry is euphemism. It uses words such as \"suffering\" and \"emotional\" to show that the surveyer wants the person surveyed to treat others with care, understanding and support. At the same time, the entry uses the description of \"close classmates or friends\" to give people a feeling of being closer and closer, which is also in keeping with the euphemism of the language style."}, "time_dimension": {"answer": "Present", "rationale": "The keyword \"I can\" uses the general current, which is a description of the current behaviour, and \"the patient and emotional listening\" which is a constant act or habit."}, "sentence_type": "Affirmative declarative sentence", "pos": {"nouns": {"I": 1, "Patience": 1, "Emotion": 2, "Listen": 1, "Students": 1, "friends": 1}, "verbs": {"Yes": 1, "- Out": 1, "Experience": 1, "Related": 1}, "adjectives": {"Close": 1, "Outside": 1}, "adverbs": {"And": 1, "Fill": 1}, "numerals": {"28": 1}, "pronouns": {"he": 1, "She's": 1}}}, {"content": "I can work with close colleagues or friends to solve a particular problem without the usual rhetoric (e.g., it should have been you, not me).", "word_count": 46, "label": "", "sentence_structure": "Subject鈥搗erb鈥搊bject", "subjective_objective": {"answer": "Subjective", "rationale": "Because the phrase \"I\" indicates that it is an individual view or feeling. The entry looks at whether the interviewee thinks he/she is capable of working with friends and does not take the usual attitude. This attitude and opinion is subjective, and therefore subjective."}, "sentence_sentiment": {"answer": "Neutral", "rationale": "Because the entry is not emotional. It simply describes a possible situation in which people can work with close colleagues or friends to solve a particular problem, rather than the usual rhetoric. This situation is not per se positive or negative emotional."}, "language_style": {"answer": "Tactful and warm", "rationale": "The language style of the entry is euphemism. First, it uses an invitation language to guide respondents to answer questions, such as \"please analyse below,\" which is more friendly and interactive. Second, the entry does not use direct command or speech, but presents the issue in a mild way, which makes it easier for the respondents to resonate and participate. Finally, the content of the article also reflects positive interpersonal attitudes, which help create a comfortable and pleasant environment for interviews."}, "time_dimension": {"answer": "Present", "rationale": "The entry describes what the testee can do in his current state, that is, be able to work with his classmates or friends to solve the problem. The keywords \"I can\" and \"co-solving a particular problem\" and \"without the usual rhetoric\" indicate that it is a present act or attitude, not a past or future act or attitude. So, the temporal dimension is now."}, "sentence_type": "Affirmative declarative sentence", "pos": {"nouns": {"Students": 1, "friends": 1, "Problem": 1, "Say something": 1, "That should have been": 1, "you": 1, "I": 1}, "verbs": {"Yes": 1, "Co-operative resolution": 1, "Adopt": 1, "Regular": 1, "By": 1, "Do it": 1, "Not relevant": 1}, "adverbs": {"Special": 1, "Close": 1}, "numerals": {"29": 1}, "pronouns": {"I": 1, "you": 1}}}, {"content": "30. Something that can expose its own anxiety or fear to a friend of the opposite sex.", "word_count": 23, "label": "", "sentence_structure": "Subject鈥搗erb鈥搊bject", "subjective_objective": {"answer": "Subjective", "rationale": "This is a subjective attitude to examining whether the patient is willing to reveal his or her inner anxiety or fear."}, "sentence_sentiment": {"answer": "Positive", "rationale": "This entry is a positive expression of attitudes and preferences towards frank communication and helps to build deep human relations."}, "language_style": {"answer": "Tactful and warm", "rationale": "The language style of the entry tends to be euphemistic and gentle, and to spread the subject by asking the other side whether it is willing to share its own anxiety or fear in the presence of a heterosexual friend. This is more personal and interactive, reducing the resistance of the other side and making it easier to access real data."}, "time_dimension": {"answer": "Past", "rationale": "\"Can\" indicates that the act is the person who was tested in his present state or capacity, and \"to reveal his anxiety or fear to a person of opposite sex\" is an act that can happen in the past and in the future, and that the person tested is doing or is likely to do in the future, so the entry can be seen as either the present or the future."}, "sentence_type": "Affirmative declarative sentence", "pos": {"nouns": {"Problem": 1, "Entry": 1, "Fae friend": 1, "Things": 1}, "verbs": {"Yes": 1, "Show me": 1, "self": 1, "Anxiety": 1, "Scared": 1}, "numerals": {"30": 1}}}, {"content": "31. I would like to tell your new friend some shameful things about you.", "word_count": 23, "label": "", "sentence_structure": "Subject鈥搗erb鈥搊bject", "subjective_objective": {"answer": "Subjective", "rationale": "Because \"will you tell anyone\" is patient's subjective attitude."}, "sentence_sentiment": {"answer": "Negative", "rationale": "Because the \"smuggling thing\" is usually considered negative, which suggests that the entry is in a negative emotional way."}, "language_style": {"answer": "Tactful and warm", "rationale": "The entry is more euphemistic, as it does not directly ask respondents to share their shame, but rather to raise a question to get the subject. It is more polite and respects the privacy of the interviewee and is more likely to elicit a positive response from the interviewee."}, "time_dimension": {"answer": "Present", "rationale": "The entry asks about the attitude and willingness of the testee to share certain sensitive information, and the temporal dimension is now judged."}, "sentence_type": "Interrogative sentence", "pos": {"nouns": {"friends": 1, "Things": 1, "you": 2}, "verbs": {"Tell": 1, "I feel": 1, "Yes": 1}, "adjectives": {"New": 1, "Shame": 1}, "adverbs": {"Some": 1}, "numerals": {"31": 1}}}, {"content": "32. New friends who have just met can share their interests so that they can understand you.", "word_count": 28, "label": "", "sentence_structure": "Subject鈥搗erb鈥搊bject", "subjective_objective": {"answer": "Subjective", "rationale": "Because this survey project involves personal interests and preferences, and how to show these to new friends. These are based on personal perceptions and feelings and are therefore subjective."}, "sentence_sentiment": {"answer": "Positive", "rationale": "Because the \"share your own hobby\" in the entry is positive."}, "language_style": {"answer": "Tactful and warm", "rationale": "The language style of the entry is more euphemistic, with dialogue in the form of inquiries and suggestions, and the interviewee answers questions in a relaxed atmosphere, rather than direct orders or statements. At the same time, the expression \"Let you know yourself\" is used to give greater attention to the feelings of the other side, which is respected and noted."}, "time_dimension": {"answer": "Past", "rationale": "The entry therefore covers the past and present time dimension, that is, both the present situation and future trends."}, "sentence_type": "Interrogative sentence", "pos": {"nouns": {"Questionnaire Entry": 1, "New friends": 1, "A hobby": 1, "he": 1, "she": 1}, "verbs": {"Yes": 1, "Share": 1, "鈾?And I'm gonna be 鈾?: 1, "Roger that": 1}, "adverbs": {"Just now": 1}, "numerals": {"32": 1}, "pronouns": {"self": 1}}}, {"content": "33. The ability to present to good friends the shortcomings of their personality or the more vulnerable and sensitive aspect.", "word_count": 28, "label": "", "sentence_structure": "Subject鈥搗erb鈥搊bject", "subjective_objective": {"answer": "Subjective", "rationale": "Because the subject is examined by the patient ' s personal feelings and attitudes, i.e., whether the patient is willing to show his friends any shortcomings or sensitivities in his/her personality, which is a subjective issue."}, "sentence_sentiment": {"answer": "Positive", "rationale": "Because the behaviour in the entries is to show a good friend the true self, including a defect or weakness in character, it is a manifestation of trust and sincerity."}, "language_style": {"answer": "Tactful and warm", "rationale": "This entry uses a gentle tone to ask the respondents whether they are willing to show their best friends the weaknesses and vulnerabilities of their personality, express themselves more in a friendly and interactive manner, without direct orders or statements. This makes it easier for the respondents to open their minds to questions and to reflect a more respectful and caring attitude."}, "time_dimension": {"answer": "Past", "rationale": "And \"presenting to a good friend the defect of his character or the sensitive aspect of his vulnerability\" involves the person's behaviour or behaviour, and the temporal dimension is now judged. The entry therefore asks whether the person currently has the ability to show his/her personality weaknesses to a good friend and is carrying out such an act."}, "sentence_type": "Interrogative sentence", "pos": {"nouns": {"Good friend": 1, "Character": 1, "Disadvantages": 1, "One side": 1}, "verbs": {"Present": 1, "Yes": 1, "Comparison": 1, "Weakness": 1, "Sensitive": 1}, "adjectives": {"Your own": 1, "Vulnerable and sensitive": 1}, "adverbs": {"Comparison": 1}, "numerals": {"33": 1}, "classifiers": {"individual": 1}, "pronouns": {"Here": 1}}}, {"content": "34. When talking to heterosexual friends, they are willing to talk about their privacy.", "word_count": 18, "label": "", "sentence_structure": "Subject鈥搗erb鈥搊bject", "subjective_objective": {"answer": "Subjective", "rationale": "Because the description of the questionnaire addresses patients ' perceptions and attitudes about their willingness to share their privacy when talking to heterosexual friends, it is the patient ' s subjective idea."}, "sentence_sentiment": {"answer": "Positive", "rationale": "Because the \"willing to talk about privacy\" act in the entry is positive."}, "language_style": {"answer": "Tactful and warm", "rationale": "The language style of the entry is euphemistic, using the word \"will\" to express the problem, making the questioning more gentle. Moreover, the topic \"your privacy\" is relatively sensitive, so that euphemism can avoid causing discomfort or resistance among the person under investigation."}, "time_dimension": {"answer": "Present", "rationale": "According to the word \"will\" in the sentence and the description of the sentence as a whole, the entry is to ask the subject about the current behaviour and attitude."}, "sentence_type": "Imperative sentence", "pos": {"nouns": {"Questionnaire Entry": 1, "Fae friend": 1, "Talk": 1, "Privacy": 1}, "verbs": {"Yes": 1, "and": 1, "Talk": 1, "- Yeah": 1}, "adverbs": {"34": 1}, "numerals": {"34": 1}}}, {"content": "35. It is embarrassing to tell a person of opposite sex or to have a good friend what he/she is doing.", "word_count": 21, "label": "", "sentence_structure": "Subject鈥搗erb鈥搊bject", "subjective_objective": {"answer": "Subjective", "rationale": "It is a subjective examination to tell a person of opposite sex or to make a good friend of what he/she is doing that makes it embarrassing for the patient to think that the behaviour of others makes him/her uncomfortable."}, "sentence_sentiment": {"answer": "Negative", "rationale": "Because the \"smuggling\" in the entry is negative."}, "language_style": {"answer": "Tactful and warm", "rationale": "The language style of the entry is more euphemistic, because it uses an invitation-style tone and does not direct the other to tell the opposite sex or what their best friends are doing. It makes you feel more comfortable and sweet and more acceptable."}, "time_dimension": {"answer": "Past", "rationale": ""}, "sentence_type": "Imperative sentence", "pos": {"nouns": {"Heterosexual": 1, "friends": 1, "Things": 1, "he": 1, "she": 1}, "verbs": {"Tell": 1, "Do it": 1, "Jean": 1, "I'm embarrassed": 1}, "numerals": {"35": 1}, "pronouns": {"you": 1}}}, {"content": "Tell his good friend something he did hurt you.", "word_count": 17, "label": "", "sentence_structure": "Subject鈥搗erb鈥搊bject", "subjective_objective": {"answer": "Subjective", "rationale": "This questionnaire entry examines patients鈥?subjective attitudes and behaviour. It is a subjective judgement to tell a good friend whether something harms himself, because different people may react differently to the same thing. And telling a good friend that it is an objective act itself, which has happened without the influence of personal emotions."}, "sentence_sentiment": {"answer": "Negative", "rationale": "Because the behavior in the entry \"Tell a good friend something he did hurt you\" might cause displeasure or hurt each other's feelings, and it's negative."}, "language_style": {"answer": "Calm and direct", "rationale": "The entry raises the question in a direct manner, without a euphemism or subtlety, and the language style is more straightforward."}, "time_dimension": {"answer": "Present", "rationale": "The entry asks whether the person who was tested has told or will tell his good friends that he has been harmed, and does not address past or future behaviour or mentalities."}, "sentence_type": "Imperative sentence", "pos": {"nouns": {"Good friend": 1, "Things": 1, "Hurt": 1}, "verbs": {"Tell": 1, "Do it": 1}, "adverbs": {"Him": 1}, "classifiers": {"Something": 1, "you": 1}}}, {"content": "37. Telling a heterosexual friend something he/she did is provoking you.", "word_count": 18, "label": "", "sentence_structure": "Subject鈥搗erb鈥搊bject", "subjective_objective": {"answer": "Subjective", "rationale": "It is subjective to tell a heterosexual friend that he is the patient's own initiative."}, "sentence_sentiment": {"answer": "Negative", "rationale": "Because the tone of the article is negative, the word \"inflicted\" conveys a negative emotion and attitude."}, "language_style": {"answer": "Tactful and warm", "rationale": "The language style of the entry is more euphemistic and warm, as it uses a more moderate way to ask if the other party has ever expressed dissatisfaction with a different sex. This way avoids direct accusations or attacks, but rather triggers dialogue by presenting a possible situation, while also respecting the other party鈥檚 feelings."}, "time_dimension": {"answer": "Past", "rationale": "\"The entry is for the testee's past or present behavior, and the time dimension is judged by the past and now.\""}, "sentence_type": "Imperative sentence", "pos": {"nouns": {"Questionnaire Entry": 1, "Fae friend": 1, "Something": 1, "Exasperation": 1}, "verbs": {"Tell": 1, "Do it": 1, "Excited": 1}, "numerals": {"37": 1}, "pronouns": {"he": 1, "she": 1}}}, {"content": "38. When you disagree with a good friend, you tell him what you think.", "word_count": 23, "label": "", "sentence_structure": "Subject鈥搗erb鈥搊bject", "subjective_objective": {"answer": "Subjective", "rationale": "Because \"Tell him the idea\" is a subjective act and attitude. While it is a choice of conduct in a given situation, it reflects the subjective views and values of the individual."}, "sentence_sentiment": {"answer": "Positive", "rationale": "Because the \"really tell him what you think\" act is positive."}, "language_style": {"answer": "Tactful and warm", "rationale": "The language style of the entry is mild, using \"you\" as a second term to guide respondents to self-think and express themselves, rather than direct orders or guidance on how they should do it. At the same time, the sentence encourages respondents to share their views and experiences by asking questions, a kind of language that increases the completion and effectiveness of questionnaires."}, "time_dimension": {"answer": "Present", "rationale": "The entry is a current act or attitude, i.e., a true expression of one鈥檚 own thoughts when it is not in line with the views of a good friend. While this may involve past and future factors (e.g. past experience, future expectations), it is primarily concerned with current behaviour in the case of the entry itself."}, "sentence_type": "Imperative sentence", "pos": {"nouns": {"you": 1, "Good friend": 1, "Disagreement": 1, "Thoughts": 1}, "verbs": {"Tell": 1, "Yes": 1, "It's real": 1, "and": 1, "Time": 1, "Disagreement": 1}, "adverbs": {"It's real": 1}, "numerals": {"38": 1}, "pronouns": {"you": 2, "he": 1}}}, {"content": "39. I'd like to tell your good friend about your physical defects.", "word_count": 16, "label": "", "sentence_structure": "Subject鈥搗erb鈥搊bject", "subjective_objective": {"answer": "Subjective", "rationale": "Because it is a subjective choice and decision to tell a good friend about his physical defects."}, "sentence_sentiment": {"answer": "Negative", "rationale": "Since the subject of \"physical defects\" is usually linked to negative emotions and insecurity, the expression of the article has some negative emotional connotations."}, "language_style": {"answer": "Calm and direct", "rationale": "According to the question, \"I'm willing to tell your good friend about your physical defects\" can be understood as whether the other party is willing to share some of its physical problems or shortcomings. This is very direct and clear, and there is no excessive emotional or rhetorical approach. So, I think it's more calm and straightforward."}, "time_dimension": {"answer": "Present", "rationale": "The entry asks if the person tested is willing to tell his/her own best friend about his/her physical defects at this point in time, and therefore it is now judged."}, "sentence_type": "Imperative sentence", "pos": {"nouns": {"Questionnaire": 1, "Entry": 1, "Good friend": 1, "Body": 1, "Deficiencies": 1}, "verbs": {"Tell": 1, "Yes": 1}, "adverbs": {"very": 1}, "numerals": {"39": 1}, "pronouns": {"you": 1}}}, {"content": "40. When others are in conflict, I am able to resolve the conflict between the two sides.", "word_count": 21, "label": "", "sentence_structure": "Subject鈥搗erb鈥搊bject", "subjective_objective": {"answer": "Objective", "rationale": "Since this incident has already occurred, it is the objective ability of the patient, not the subjective attitude, that is being examined."}, "sentence_sentiment": {"answer": "Positive", "rationale": "Because the action of \"resolving the conflict\" in the entry is positive."}, "language_style": {"answer": "Tactful and warm", "rationale": "Warm and warm."}, "time_dimension": {"answer": "Past", "rationale": "The entry asks about the testees' past behaviour, i.e. whether they have successfully resolved the conflict between the two sides in the event of conflict. The term \"old\" is judged because of the time dimension."}, "sentence_type": "Imperative sentence", "pos": {"nouns": {"Questionnaire": 1, "Entry": 1, "Conflict": 1, "Paradoxious": 1}, "verbs": {"Come on": 1, "Dismantling": 2}, "adverbs": {"Yes": 1}, "numerals": {"40": 1, "Both sides": 1}}}, {"content": "41. I can admit that I was wrong when the differences of opinion between my close classmates or friends were about to escalate into a war of war.", "word_count": 39, "label": "", "sentence_structure": "Subject鈥搗erb鈥搊bject", "subjective_objective": {"answer": "Subjective", "rationale": "Because the entry describes a person ' s self-awareness and behavioural response in a given context, which is based on the individual ' s feelings and experience. This requires the patient ' s own knowledge and understanding, which is usually subjective. The purpose of the article is therefore to look at the perspective subjective."}, "sentence_sentiment": {"answer": "Positive", "rationale": "Because the \"can admit it was wrong\" act is positive."}, "language_style": {"answer": "Tactful and warm", "rationale": "This topic is an invitation to ask the respondents about attitudes and behaviour in interpersonal relations, rather than direct orders or accusations, and is more gentle and friendly in language, which is easily accepted by the interviewees. At the same time, it expresses a respect, understanding and inclusiveness through the use of the term \"acknowledged error\" and reflects good interpersonal relationships."}, "time_dimension": {"answer": "Present", "rationale": ""}, "sentence_type": "Interrogative sentence", "pos": {"nouns": {"Students": 1, "Relations": 1, "friends": 1, "Dissenting views": 1, "War": 1, "self": 1, "Wrong": 1}, "verbs": {"I can admit it": 1, "Upgrade to": 1}, "adverbs": {"Upcoming": 1}, "pronouns": {"I": 2}}}, {"content": "42. When there is a quarrel between close colleagues and friends, I can put aside anger or resentment.", "word_count": 32, "label": "", "sentence_structure": "Subject鈥搗erb鈥搊bject", "subjective_objective": {"answer": "Subjective", "rationale": "Because it is a subjective attitude and behaviour of a human being to set aside anger or resentment."}, "sentence_sentiment": {"answer": "Positive", "rationale": "Because the entry \"out of anger or resentment\" is positive."}, "language_style": {"answer": "Tactful and warm", "rationale": "The entry leads the interviewees to reflect on their emotional response in dealing with interpersonal conflict, is quiet and caring, does not direct orders or criticism and is therefore a warm and euphemism."}, "time_dimension": {"answer": "Present", "rationale": "The entry is for the purpose of asking the testee about his or her behavior in a particular situation (i.e. \"when there is a quarrel between close schoolmates and friends\"), and therefore the temporal dimension is now judged."}, "sentence_type": "Affirmative declarative sentence", "pos": {"nouns": {"Argument": 1, "Students": 1, "friends": 1, "Emotion": 1}, "verbs": {"Come on": 1, "Yes": 1, "Leave it": 1, "Hate": 1}, "adjectives": {"Close": 1}, "adverbs": {"When": 1, "Time": 1}, "numerals": {"42": 1}, "pronouns": {"I": 1}}}, {"content": "I can listen to his/her complaints in good faith when there is a contradiction between my/her close colleagues or friends.", "word_count": 29, "label": "", "sentence_structure": "Subject鈥搗erb鈥揷omplement", "subjective_objective": {"answer": "Subjective", "rationale": "Since the patient ' s attitude and behaviour in the event of conflict with others are examined, the patient ' s subjective thoughts and actions need to be taken into account."}, "sentence_sentiment": {"answer": "Positive", "rationale": "Because the entry \"can listen to his/her complaints in good faith\" is positive."}, "language_style": {"answer": "Tactful and warm", "rationale": "The language style of this entry is euphemism. It asks the interviewee, in an encouraging and understanding manner, whether he can listen sincerely to others in the face of interpersonal conflicts, rather than directly ordering or criticizing. This way is more likely to resonate with the interviewee, making him feel respected and valued."}, "time_dimension": {"answer": "Present", "rationale": "The entry asks whether the person tested can listen to each other in good faith in the current situation (i.e. when there is a contradiction between a student or friend close to him)."}, "sentence_type": "Affirmative declarative sentence", "pos": {"nouns": {"Questionnaire": 1, "Entry": 1, "When": 1, "and": 2, "Relations": 1, "Close": 1, "Students": 1, "or": 1, "friends": 1, "Between": 1, "have": 1, "Paradoxious": 1, "Time": 1, "I can": 1, "Truth": 1, "Land": 1, "Listen": 1, "he": 1, "she": 1, "of": 2, "- He's complaining": 1}, "verbs": {"Yes": 1, "Listen": 1}, "adjectives": {"Close": 1, "Truth": 1}, "adverbs": {"Land": 1}, "numerals": {"43": 1}, "pronouns": {"I": 1, "he": 1, "she": 1}}}, {"content": "44. In my argument, I can accept the views of close colleagues or friends and truly understand his/her views.", "word_count": 32, "label": "", "sentence_structure": "Subject鈥搗erb鈥搊bject", "subjective_objective": {"answer": "Subjective", "rationale": "This is a subjective attitude and view of patients, and concerns their ability and preference to accept others鈥?views. While this ability may be influenced by objective conditions (e.g. their social environment, interpersonal relationships, etc.), in such cases the problem is more concerned with the attitudes and views of patients."}, "sentence_sentiment": {"answer": "Positive", "rationale": "Because the entry \"accepts the views of a close classmate or friend and truly understands his/her views\" is positive."}, "language_style": {"answer": "Tactful and warm", "rationale": "The language style of the entry is more gentle and friendly, using the words \"relative\" and \"acceptable\" and \"real understanding\" to express friendly relations with the person under investigation and to give a sense of care and respect during questioning, so that the language style can be judged as euphemism and warm."}, "time_dimension": {"answer": "Present", "rationale": "The entry now asks the testee whether he/she can accept the views of a close student or friend in a quarrel and truly understands his/her views. Although this may have happened in the past, the purpose of the questionnaire is to be expressed in the current context. So the time dimension is now judged."}, "sentence_type": "Affirmative declarative sentence", "pos": {"nouns": {"Argument": 1, "Students": 1, "friends": 2, "Observations": 1, "Views": 1}, "verbs": {"Accept": 1, "Yes": 1, "I really understand": 1}, "adjectives": {"Closely connected": 1}, "numerals": {"44": 1}, "pronouns": {"he": 1, "She's": 1}}}, {"content": "45. I will avoid talking about topics that could lead to differences and to quarrels with close colleagues and friends.", "word_count": 33, "label": "", "sentence_structure": "Subject鈥搗erb鈥搊bject", "subjective_objective": {"answer": "Subjective", "rationale": "This is the subjective idea and behaviour of the patient, which is to avoid talking about specific topics."}, "sentence_sentiment": {"answer": "Negative", "rationale": "Because the behavior in the entry \"to avoid talking to close colleagues and friends about topics that could cause disagreement and cause quarrels\" is negative."}, "language_style": {"answer": "Calm and direct", "rationale": "The sentence is based on the following: The entry expresses directly, in plain and clear language, the proposal to avoid discussions in intimate relationships that could lead to controversial topics. It does not use excessive rhetorical techniques or emotional rendering, is more objective and is consistent with the calm and direct linguistic style."}, "time_dimension": {"answer": "Past", "rationale": "\"will avoid\" indicates that it is a regular, customary act which occurred at a point or time in the past. The entry therefore describes the acts that were tried in the past."}, "sentence_type": "Affirmative declarative sentence", "pos": {"nouns": {"Students": 1, "friends": 2, "Topic": 1}, "verbs": {"Yes": 1, "Avoid": 1, "Talk": 1, "Result": 1, "Argument": 1}, "adjectives": {"Close": 1, "Maybe": 1, "Difference": 1}, "adverbs": {"and": 3, "Those": 1, "Maybe": 1, "Result": 1, "Yes": 1, "Avoid": 1, "I": 1}, "numerals": {"45": 1, "2": 1}, "pronouns": {"I": 1}}}]}

{"questionnaire_title": "Toronto Statement of Impediments", "questionnaire_category": "Behavioral questionnaire", "item_count": 26, "emotion_shift_overall": "From positive to negative", "language_style_overall": "Tactful and warm", "items": [{"content": "I know why when I cry.", "word_count": 13, "label": "", "sentence_structure": "Subject鈥揷opula鈥損redicative", "subjective_objective": {"answer": "Subjective", "rationale": "Because knowing what is the reason is the patient's subjective knowledge and judgment."}, "sentence_sentiment": {"answer": "Positive", "rationale": "Because the \"know why\" attitude in the entry is positive."}, "language_style": {"answer": "Tactful and warm", "rationale": "The language style of the entry is more euphemistic, not to ask the other side whether or why it is often crying, but to guide the other side to think about its emotions and inner world in a gentle and caring way. This expression is more acceptable to the other side and is willing to share its thoughts and feelings."}, "time_dimension": {"answer": "Past", "rationale": "The \"I Cry\" in \"I Cry\" is an expression of past events, so the time dimension of the article is the past."}, "sentence_type": "Interrogative sentence", "pos": {"nouns": {"Reason": 1, "I": 1}, "verbs": {"Yes": 1, "Crying": 1}, "adverbs": {"When": 1, "Time": 1}, "pronouns": {"What": 1}}}, {"content": "2. Ideas are a waste of time.", "word_count": 9, "label": "", "sentence_structure": "Subject鈥揷opula鈥損redicative", "subjective_objective": {"answer": "Objective", "rationale": "Because \"a mere waste of time\" is a view or an evaluation, a subjective judgement. And psychological questionnaires are usually used to understand their psychological condition and characteristics by collecting objective data, such as self-reporting or behavioural reactions, that are tested. So the entry \"a mere waste of time\" is viewed from an objective perspective."}, "sentence_sentiment": {"answer": "Negative", "rationale": "Because the expression \"time-consuming\" in the entry is negative."}, "language_style": {"answer": "Calm and direct", "rationale": "The entry expresses a negative attitude to the imagination in a concise and clear tone, with no excessive adoration or emotional colours, and a more calm and straightforward language style."}, "time_dimension": {"answer": "Future", "rationale": "The entry is not clear about timing, but the keyword \"solely waste of time\" can be judged as a vision of the future or as a reminder that time can be used more effectively in the future. Therefore, the entry is judged as the future."}, "sentence_type": "Negative declarative sentence", "pos": {"nouns": {"I'm not sure": 1, "Time": 1}, "verbs": {"Pure": 1, "be": 1, "Wasted": 1, "Analysis": 1}, "adjectives": {"Down there": 1, "This": 1, "Questionnaire": 1, "Entry": 1}, "adverbs": {"2": 1}, "pronouns": {"you": 1}}}, {"content": "I hope I'm not so shy.", "word_count": 10, "label": "", "sentence_structure": "Subject鈥搗erb鈥搊bject", "subjective_objective": {"answer": "Subjective", "rationale": "Hope is patient's subjective attitude."}, "sentence_sentiment": {"answer": "Positive", "rationale": "Because the \"hope not to be shy\" attitude in the entry is positive."}, "language_style": {"answer": "Tactful and warm", "rationale": "The entry uses a positive and encouraging tone to express the researcher ' s concern and support for the person under investigation and to attempt to inspire the other party ' s motivation and self-confidence rather than directly criticizing or rejecting their character characteristics."}, "time_dimension": {"answer": "Future", "rationale": "The key word in the entry, \"Hope,\" expresses a desire and vision for the future, while \"less shyness\" is the testee's desire to change his behaviour or mentality. The entry is therefore a future issue."}, "sentence_type": "Imperative sentence", "pos": {"nouns": {"Shy": 1, "self": 1}, "verbs": {"Hope": 1, "not": 1, "Well": 1}, "adjectives": {"Shame": 1}, "adverbs": {"Well": 1}, "pronouns": {"I": 1}}}, {"content": "I'm always confused about how I feel.", "word_count": 14, "label": "", "sentence_structure": "Subject鈥揷opula鈥損redicative", "subjective_objective": {"answer": "Subjective", "rationale": "Because it is a description of the patient ' s inner feelings, it is a subjective observation and expression."}, "sentence_sentiment": {"answer": "Negative", "rationale": "Because the \"I don't know how I feel\" act is negative."}, "language_style": {"answer": "Tactful and warm", "rationale": "The language style of the entry is more euphemistic and warm, the word \"please\" is used, and the expression is more accommodative, without direct orders or questioning, and easier for the respondents to accept and answer questions."}, "time_dimension": {"answer": "Present", "rationale": "The keyword \"I'm often\" indicates that it's a customary act, and \"unsure\" and \"like what\" indicates that the act is happening, so it can be judged as being the present behavior or mentality."}, "sentence_type": "Interrogative sentence", "pos": {"nouns": {"Questionnaire": 1, "Entry": 1, "Feel": 1}, "verbs": {"Regular": 1, "I don't know": 1, "be": 1, "What kind": 1}, "adverbs": {"self": 1}, "numerals": {"4": 1}}}, {"content": "I've always dreamed of the future.", "word_count": 7, "label": "", "sentence_structure": "Subject鈥損redicate", "subjective_objective": {"answer": "Subjective", "rationale": "\"Fantasy of the future\" is the patient's imagination and expectation."}, "sentence_sentiment": {"answer": "Positive", "rationale": "Because \"often fantasies about the future\" expresses a positive attitude and behaviour that shows that the interviewees are keen on the future."}, "language_style": {"answer": "Tactful and warm", "rationale": "The problem is explained by the following reasons: 1. The use of the term \"often\" is a more moderate expression of concern and respect for the future imagination of the interviewee. 2. The problem is not a direct question or a command-style tone, but rather a question that leads the interviewee to share their thoughts and feelings in an invitation that reflects their personal and interactive nature. 3. The use of the term \"fantasy of the future\" as a poetic expression, rather than a simple, plain expression, is also consistent with the euphemism of the euphemism."}, "time_dimension": {"answer": "Future", "rationale": ""}, "sentence_type": "Affirmative declarative sentence", "pos": {"nouns": {"Future": 1}, "verbs": {"Regular": 1, "Imagination": 1}, "adverbs": {"Regular": 1}}}, {"content": "I seem to be as easy as anyone else.", "word_count": 13, "label": "", "sentence_structure": "Subject鈥揷opula鈥損redicative", "subjective_objective": {"answer": "Subjective", "rationale": "Because \"easy\" is a subjective feeling and perception that reflects patients' subjective assessment of their ability to socialize."}, "sentence_sentiment": {"answer": "Neutral", "rationale": "Because the entry does not have any emotional color."}, "language_style": {"answer": "Tactful and warm", "rationale": "The phrase \"like\" is used to express a relatively subjective feeling, without directly stating whether it is easy to make friends. At the same time, the expression \"as easy as others\" is also more moderate, giving people a sense of equality and friendship. Overall, the entry uses a softer tone and term, which makes people feel comfortable and close."}, "time_dimension": {"answer": "Present", "rationale": "The words \"like,\" \"friending\" and \"as easy as anyone else\" indicate that the question is asked about the current feelings and circumstances of the subject, and therefore is judged to be the present."}, "sentence_type": "Interrogative sentence", "pos": {"nouns": {"friends": 1, "others": 1}, "verbs": {"Hand it over": 1, "and": 1, "Easy": 1}, "adjectives": {"Seems like": 1}, "numerals": {"6": 1}, "pronouns": {"I": 1}}}, {"content": "7. Knowledge of the answer to the question is more important than knowing its causes", "word_count": 16, "label": "", "sentence_structure": "Subject鈥揷opula鈥損redicative", "subjective_objective": {"answer": "Subjective", "rationale": "Because \"know the answer to the question is more important than know the reason\" is an opinion or belief, and it is the patient's subjective attitude."}, "sentence_sentiment": {"answer": "Neutral", "rationale": "Because the entry does not have any emotional color."}, "language_style": {"answer": "Calm and direct", "rationale": "The entry is presented directly, expressing the view that the answer to the question is more important than the reason, without the use of rhetorical techniques or excessive emotional rendering, and with a more calm and objective language style."}, "time_dimension": {"answer": "Present", "rationale": "The entry asks the respondent's views on the relative importance of \"know the answer to the question and know its cause\" and does not address specific past or future acts or events and is therefore current."}, "sentence_type": "Affirmative declarative sentence", "pos": {"nouns": {"Problem": 1, "Answer": 1, "Reason": 1}, "verbs": {"Yes": 2, "- Yeah": 1, "Important": 1}, "adverbs": {"More": 1}, "numerals": {"7": 1}}}, {"content": "I can't describe my emotions in the right words.", "word_count": 15, "label": "", "sentence_structure": "Subject鈥搗erb鈥搊bject", "subjective_objective": {"answer": "Subjective", "rationale": "Since this question is examined in terms of how the patient describes his or her feelings, it is to be answered in terms of the language and angle of self-evaluation and is therefore subjective."}, "sentence_sentiment": {"answer": "Neutral", "rationale": "The entry is merely an inquiry into whether the person surveyed can properly express his or her feelings, and does not involve any positive or negative emotional tendencies."}, "language_style": {"answer": "Tactful and warm", "rationale": "The language style of the entry is euphemistic and warm, and the difficulty of expressing an emotional description by using words such as \"hard\" and \"appropriate words\" is not the expression of direct denial or blame. This expression makes it easier to comfort and understand the interviewee and is willing to continue to participate in the questionnaire."}, "time_dimension": {"answer": "Present", "rationale": "\"I have difficulty\" means the feeling or state of the moment, and \"description\" and \"self\" in \"description of my emotions\" indicate that it is a self-observation and cognitive act, so the entry is judged now."}, "sentence_type": "Negative declarative sentence", "pos": {"nouns": {"Questionnaire Entry": 1, "Word": 1, "Emotion": 1}, "verbs": {"Description": 1, "Use": 1, "self": 1}, "adjectives": {"Appropriate": 1}, "numerals": {"8": 1}, "pronouns": {"I": 1}}}, {"content": "I like people to know how I feel about things.", "word_count": 14, "label": "", "sentence_structure": "Subject鈥搗erb鈥搊bject", "subjective_objective": {"answer": "Subjective", "rationale": "Like being patient subjective."}, "sentence_sentiment": {"answer": "Neutral", "rationale": "Because the entry does not express a clear emotional tendency, it merely describes a preference."}, "language_style": {"answer": "Tactful and warm", "rationale": "The language style of the entry is more moderate, using the more positive term \"likes\" and directing the respondents to express their views in a question-and-answer manner, without direct orders or mandatory requests for answers. This expression is more comfortable and willing to participate, thus increasing the rate of completion and recovery of questionnaires."}, "time_dimension": {"answer": "Present", "rationale": "The entry describes the current feelings and attitudes of the testee, and the keyword \"likes\" indicates an emotional tendency, while the \"attitude to things\" describes the specific direction of such an emotion. These elements are happening now, so the temporal dimension is now."}, "sentence_type": "Affirmative declarative sentence", "pos": {"nouns": {"Questionnaire Entry": 1, "Yeah": 1, "others": 1, "Attitude to things": 1}, "verbs": {"Yes": 1, "Yeah": 1}, "adverbs": {"Down there": 1, "Please": 1}, "numerals": {"9锛?: 1}, "pronouns": {"I": 1}}}, {"content": "Some of the physical feelings are not even understood by doctors.", "word_count": 13, "label": "", "sentence_structure": "Subject鈥搗erb鈥搊bject", "subjective_objective": {"answer": "Subjective", "rationale": "Because \"some physical feelings are not understood by doctors\" are the subjective perceptions and understandings of the patient of his or her own body, which are his or her subjective feelings."}, "sentence_sentiment": {"answer": "Negative", "rationale": "Because the expression \"not even a doctor understands\" implies negative situations where medical problems cannot be solved."}, "language_style": {"answer": "Tactful and warm", "rationale": "The language style of the entry is euphemism, concern and understanding. It asks the respondents in a more moderate way whether they have experienced a certain experience, i.e., physical discomfort or the difficulty of having medical interpretation. This expression avoids using too direct or hard language, but instead leads the interviewer to answer questions with a more caring and supportive attitude."}, "time_dimension": {"answer": "Present", "rationale": "The keyword \"some body feelings are not understood by doctors\" indicates that the entry asks the testee whether he or she currently has some body feeling that cannot be interpreted medically, and therefore the temporal dimension is now."}, "sentence_type": "Negative declarative sentence", "pos": {"nouns": {"Body": 1, "Feel": 1, "Doctor": 1}, "verbs": {"Understood": 1}, "numerals": {"10": 1}}}, {"content": "11. It's not enough to do work. I need to know why and how.", "word_count": 20, "label": "", "sentence_structure": "Subject鈥搗erb鈥搊bject", "subjective_objective": {"answer": "Subjective", "rationale": "Because the subject uses the subjective term \"I need\" to express personal thoughts and feelings."}, "sentence_sentiment": {"answer": "Positive", "rationale": "Because the entry \"know why and how\" expresses a positive attitude to work and a desire for career development."}, "language_style": {"answer": "Tactful and warm", "rationale": "The entry uses a soft, invitation-style tone that allows respondents to feel respected and cared for."}, "time_dimension": {"answer": "Future", "rationale": "The entry refers to needs or expectations for the future, and the keywords are \"need to know\" and \"how to do it\", which point to situations that have not yet occurred and that may occur in the future. The temporal dimension of the entry is therefore judged to be the future."}, "sentence_type": "Affirmative declarative sentence", "pos": {"nouns": {"Work": 2, "Yes": 1, "Why": 1, "How's that": 1, "Okay": 1}, "verbs": {"Do it": 1, "Yes": 2, "Okay": 1}, "adverbs": {"Only": 1, "be": 1, "Not enough": 1}, "numerals": {"11锛?: 1}, "pronouns": {"I": 1}}}, {"content": "I can easily describe my feelings.", "word_count": 12, "label": "", "sentence_structure": "Subject鈥揷opula鈥損redicative", "subjective_objective": {"answer": "Subjective", "rationale": "Because it is easy to describe one ' s feelings as a subjective assessment, it is a self-assessment of the ability of individuals to express their own perception and emotions."}, "sentence_sentiment": {"answer": "Positive", "rationale": "Because \"easy description of one's feelings\" is often seen as a positive ability to express feelings, which shows that individuals can better understand and express their feelings and experiences."}, "language_style": {"answer": "Tactful and warm", "rationale": "The entry uses the first person ' s pronoun \"I\" and uses positive tone and expression (which easily describes one ' s feelings) that gives people a more friendly and friendly sense of feeling, while also increasing the motivation and participation of the subjects of the questionnaire."}, "time_dimension": {"answer": "Present", "rationale": "By the time dimension, this questionnaire entry is looking at the current situation. So the answer is \"now.\""}, "sentence_type": "Affirmative declarative sentence", "pos": {"nouns": {"self": 1, "Feel": 1}, "verbs": {"Description": 1, "Easy": 1}, "adjectives": {"Easy": 1}, "adverbs": {"very": 1}, "numerals": {"12": 1}, "pronouns": {"I": 1}}}, {"content": "I prefer to analyze the inter-problem rather than describe it.", "word_count": 15, "label": "", "sentence_structure": "Subject鈥搗erb鈥搊bject", "subjective_objective": {"answer": "Subjective", "rationale": "Because \"like\" is a subjective feeling and attitude."}, "sentence_sentiment": {"answer": "Neutral", "rationale": "As the entry does not express positive or negative feelings, attitudes, behaviour, purpose or quality, it simply presents a fact."}, "language_style": {"answer": "Calm and direct", "rationale": "The language style of the entry is a calm and straightforward type. It clearly presents the idea that \"I prefer to analyse the problem rather than describe it\" and does not use overly refined language or emotional expressions, but rather conveys the message in an objective and direct way. This style is usually used in situations where, for example, questionnaires provide clear answers."}, "time_dimension": {"answer": "Present", "rationale": "Although the title refers to \"a preference\", the phrase still describes current trends or preferences."}, "sentence_type": "Affirmative declarative sentence", "pos": {"nouns": {"Problem": 2}, "verbs": {"Yeah": 1, "Analysis": 1, "Description": 1}, "adverbs": {"Just": 1}, "numerals": {"13": 1}, "pronouns": {"I": 1}}}, {"content": "When I'm upset, I wonder if it's sad, scared, angry?", "word_count": 17, "label": "", "sentence_structure": "Subject鈥搗erb鈥搊bject", "subjective_objective": {"answer": "Subjective", "rationale": "Because this is a matter of the patient鈥檚 own emotional feelings and understanding, it is the patient鈥檚 own choice to determine his or her emotional state."}, "sentence_sentiment": {"answer": "Negative", "rationale": "Because the entries \"worried, afraid, angry\" are all negative emotional experiences."}, "language_style": {"answer": "Tactful and warm", "rationale": "The language style of the entry is euphemistic. It is expressed in a caring and understanding way, in a question-and-answer manner, that gives people a feeling of affection that does not make them too direct or indifferent. At the same time, the words \"disturbed\" and \"worried, afraid, angry\" also make the language more relevant to life and is easily accepted and resonated with the interviewee."}, "time_dimension": {"answer": "Present", "rationale": "The entry asks about the emotional reactions that the person being tested may have in the current circumstances, so the time dimension is now judged."}, "sentence_type": "Interrogative sentence", "pos": {"nouns": {"I'm upset": 1, "Sad": 1, "Scared": 1, "Anger": 1}, "verbs": {"I don't know": 1, "be": 1}}}, {"content": "I've always had fantasies.", "word_count": 5, "label": "", "sentence_structure": "Subject鈥搗erb鈥揷omplement", "subjective_objective": {"answer": "Subjective", "rationale": "Because \"often fantasies\" reflect the patient's personal thoughts and preferences, and it's a subjective view or feeling."}, "sentence_sentiment": {"answer": "Neutral", "rationale": "Because the entry does not have any emotional color."}, "language_style": {"answer": "Tactful and warm", "rationale": "The entry is more moderate in its language style, using the term \"normal\" to suggest that the respondents have some habit, not directly critical or evaluated, but rather to ask questions in a more inclusive and understanding manner. At the same time, \"fantair\" is also a relatively neutral, objective term, with no excessive emotional colours, and is more in keeping with the euphemism of the language."}, "time_dimension": {"answer": "Past", "rationale": "The term \"often good fantasy\" indicates regular, customary behaviour, and \"good fantasy\" describes it, so the entry is mainly asking if the person tested had been often so good in the past."}, "sentence_type": "Affirmative declarative sentence", "pos": {"nouns": {"Questionnaire": 1, "Entry": 1, "Fantasy": 1}, "verbs": {"Analysis": 1, "I'm glad": 1}, "adverbs": {"Regular": 1, "Okay": 1}, "numerals": {"15": 1}}}, {"content": "16. When I have nothing to do, I think too much.", "word_count": 11, "label": "", "sentence_structure": "Subject鈥揷opula鈥損redicative", "subjective_objective": {"answer": "Subjective", "rationale": "Because \"irritive\" is the patient's subjective psychological activity."}, "sentence_sentiment": {"answer": "Negative", "rationale": "Because the \"when there's nothing to do, I'm always thinking\" act is negative."}, "language_style": {"answer": "Tactful and warm", "rationale": "The language style of the entry is more moderate and uses a few less emotional terms (e.g. \"normal\"), giving people a sense of peace and affection. This expression is more acceptable to the interviewees and has the potential to promote their real and natural answers."}, "time_dimension": {"answer": "Past", "rationale": "The entry, therefore, relates to the present behaviour and mentality of the person being tested and should fall within the scope of the present."}, "sentence_type": "Interrogative sentence", "pos": {"nouns": {"Things": 1, "I'm not sure": 1}, "verbs": {"Do it": 1, "Regular": 1, "Okay": 1, "have": 1}, "adverbs": {"When there's nothing to do": 1}, "classifiers": {"16": 1}}}, {"content": "I'm confused by the feeling inside.", "word_count": 11, "label": "", "sentence_structure": "Subject鈥揷opula鈥損redicative", "subjective_objective": {"answer": "Subjective", "rationale": "Because confusion is a subjective feeling and perception."}, "sentence_sentiment": {"answer": "Negative", "rationale": "Because the \"mucking\" emotion in the entry is negative."}, "language_style": {"answer": "Tactful and warm", "rationale": "The expression is more personal and interactive, by means of a gentle and inviting tone, rather than a direct statement or order. At the same time, the tone of the article gives a sense of consolation and support, making it easier for people to open their minds."}, "time_dimension": {"answer": "Present", "rationale": "The word \"always\" in \"I'm often confused with my body\" denotes a regular, customary behaviour, while the word \"muffled\" in \"the structure\" uses the general current, which means that this behaviour has not only occurred in the past, but may still be ongoing."}, "sentence_type": "Affirmative declarative sentence", "pos": {"nouns": {"Questionnaire": 1, "Entry": 1, "Inside": 1, "Feel": 1}, "verbs": {"Analysis": 1, "Confused": 1}, "numerals": {"17": 1}}}, {"content": "I rarely dream of day.", "word_count": 7, "label": "", "sentence_structure": "Subject鈥搗erb鈥揷omplement", "subjective_objective": {"answer": "Objective", "rationale": "Because \"dreaming\" is an objective act, whether it is not controlled by subjective intent, but rather a fact that actually occurs. So, the frequency of \"dreaming\" is also objective and can be directly observed and recorded, and not influenced by the will of the individual."}, "sentence_sentiment": {"answer": "Negative", "rationale": "The basis for the judgment: \"I rarely have daydreams\" means that this person rarely relaxes and imagines, and may be too realistic and conservative. It's a more negative attitude."}, "language_style": {"answer": "Calm and direct", "rationale": "The language style of the entry is more straightforward, concise, and does not have too many qualifiers and emotional colours, describing a behaviour characteristic more objectively. At the same time, this expression is more easily understood and answered by the interviewees."}, "time_dimension": {"answer": "Present", "rationale": "Now, the key word is \"I rarely dream of day\" and it means the person being tested in his current state or behaviour, so it can be judged that the entry refers to the current situation."}, "sentence_type": "Negative declarative sentence", "pos": {"verbs": {"Do it": 1, "Very few": 1, "Daydreams": 1}, "adjectives": {"Very few": 1}, "numerals": {"18": 1}, "pronouns": {"I": 1}}}, {"content": "I care more about what happens than what happens.", "word_count": 17, "label": "", "sentence_structure": "Subject鈥搗erb鈥搊bject", "subjective_objective": {"answer": "Objective", "rationale": "Because it is an objective fact that happens and why it happens, the patient ' s concern is subjective."}, "sentence_sentiment": {"answer": "Neutral", "rationale": "The entry is not accompanied by a clear emotional tendency or attitude."}, "language_style": {"answer": "Calm and direct", "rationale": "The language style of the entry is calm and straightforward, and there is no excessive emotional rendering or rhetorical technique that gives a concise and concise presentation of the preferences of the person questioned."}, "time_dimension": {"answer": "Past", "rationale": "\"19\" means it's the 19th question, and \"I'm more concerned about what happened, and I'm less concerned about why it happened \"incident\" and \"not paying attention\" both express past experiences and behaviours, so the entry is judged to be about the interest of the testee in the past."}, "sentence_type": "Affirmative declarative sentence", "pos": {"nouns": {"Things": 1, "Come on": 2}, "verbs": {"Care": 1, "Come on": 1, "Attention": 1}, "adjectives": {"Why": 1}, "numerals": {"19": 1}, "classifiers": {"individual": 1}, "pronouns": {"I": 1}}}, {"content": "I'm having a hard time identifying myself.", "word_count": 10, "label": "", "sentence_structure": "Subject鈥揷opula鈥損redicative", "subjective_objective": {"answer": "Subjective", "rationale": "As feelings are the subjective experience and understanding of individuals, not objective facts or external matters, they are viewed from a subjective perspective."}, "sentence_sentiment": {"answer": "Neutral", "rationale": "Because the entry describes a feeling, there is no apparent emotional bias."}, "language_style": {"answer": "Tactful and warm", "rationale": "The language style of the entry is more euphemistic, moderate, and does not directly express strong emotions or commands, but rather leads the respondents to self-reflection and exploration in a way that is asked. This expression is more human and easily resonant with the respondents."}, "time_dimension": {"answer": "Past", "rationale": "Past."}, "sentence_type": "Affirmative declarative sentence", "pos": {"nouns": {"Feel": 1, "Questionnaire": 1}, "verbs": {"Identification": 1, "have": 1}, "adjectives": {"Some": 1, "Hard": 1}, "pronouns": {"I": 1}}}, {"content": "21. Emotional communication is important.", "word_count": 10, "label": "", "sentence_structure": "Subject鈥揷opula鈥損redicative", "subjective_objective": {"answer": "Subjective", "rationale": "Because emotional communication is a subjective attitude of one person to the feelings and perception of the other person ' s own emotional interaction."}, "sentence_sentiment": {"answer": "Positive", "rationale": "Because the expression \"emotional communication is important\" in the entry is positive and emphasizes the importance of emotional communication."}, "language_style": {"answer": "Tactful and warm", "rationale": "The entry is more gentle and friendly in its language, using more moderate language to emphasize the importance of emotional communication, without direct orders or accusations. Such expression is more likely to resonate with the interviewee and make him more willing to participate in the investigation."}, "time_dimension": {"answer": "Present", "rationale": "The entry expresses a view and an understanding of the current situation, and the keywords \"emotional communication\" and \"important\" indicate that it is directed at immediate observation and evaluation and can therefore be judged as now."}, "sentence_type": "Affirmative declarative sentence", "pos": {"nouns": {"Emotion": 1, "Communications": 1}, "verbs": {"be": 1, "Important": 1}, "adjectives": {"very": 1}, "numerals": {"21": 1}}}, {"content": "I find it difficult to describe feelings for others.", "word_count": 13, "label": "", "sentence_structure": "Subject鈥揷opula鈥損redicative", "subjective_objective": {"answer": "Subjective", "rationale": "Because \"it's hard to describe feelings for others\" is a subjective feeling and perception of a person, which reflects the patient's assessment of his ability to express feelings."}, "sentence_sentiment": {"answer": "Neutral", "rationale": "Because the entry does not express specific feelings, attitudes, behaviour, purpose or quality, it merely expresses a person ' s feelings and therefore has no obvious emotional colour."}, "language_style": {"answer": "Tactful and warm", "rationale": "This entry is more personal and interactive by asking the other side about the difficulty of describing the emotions of others, and by engaging in a moderate and focused dialogue, rather than by direct presentation or command."}, "time_dimension": {"answer": "Present", "rationale": ""}, "sentence_type": "Affirmative declarative sentence", "pos": {"nouns": {"Questionnaire": 1, "Entry": 1, "Emotion": 1}, "verbs": {"I think": 1, "Description": 1, "to": 1, "others": 1}, "adjectives": {"Hard": 1}, "numerals": {"22": 1}, "pronouns": {"I": 1}}}, {"content": "I was told to express my feelings more.", "word_count": 16, "label": "", "sentence_structure": "Subject鈥搗erb鈥搊bject", "subjective_objective": {"answer": "Subjective", "rationale": "The title \"Tell me to express more about my feelings\" is a recommendation or guidance based on the views and experiences of others. Thus, it is an entry that is viewed from the patient's subjective perspective, not an objective fact or result."}, "sentence_sentiment": {"answer": "Neutral", "rationale": "Since the entry had no apparent emotional tendency, it merely made a recommendation or request."}, "language_style": {"answer": "Tactful and warm", "rationale": "The language style of the entry is more euphemistic, using an invitation tone rather than a direct command or statement. Moreover, a more verbal expression has been used, making the questionnaire more friendly and acceptable."}, "time_dimension": {"answer": "Present", "rationale": ""}, "sentence_type": "Imperative sentence", "pos": {"nouns": {"person": 1, "Feel": 1, "Tell": 1, "self": 1}, "verbs": {"Yes": 1, "Expression": 1}, "adverbs": {"More": 1}, "numerals": {"23": 1}, "pronouns": {"others": 1}}}, {"content": "24. A person should seek a deeper understanding.", "word_count": 12, "label": "", "sentence_structure": "Subject鈥損redicate", "subjective_objective": {"answer": "Subjective", "rationale": "Because \"seeing deeper understanding\" is a subjective will and attitude of a person and belongs to the inside psychological activity of the individual."}, "sentence_sentiment": {"answer": "Positive", "rationale": "Because the purpose of the action in the entry is to find a deeper understanding."}, "language_style": {"answer": "Tactful and warm", "rationale": "The entry is euphemistic, sensitive and guiding, encouraging respondents to explore deeper understanding in a non-orderal way. This expression is neither too strong nor too cold, and can stimulate the respondents to think and respond."}, "time_dimension": {"answer": "Present", "rationale": "The words \"shall\" and \"seek\" indicate that this is a current and ongoing act, while the words \"a deeper understanding\" indicate that the person being tested is now seeking understanding. Thus, the temporal dimension of the article is now."}, "sentence_type": "Imperative sentence", "pos": {"nouns": {"person": 1, "Understood": 1}, "verbs": {"Seek": 1, "Reactions": 1, "Here": 1}, "adverbs": {"Deeper": 1}, "numerals": {"24": 1}}}, {"content": "I don't know what's going on inside me.", "word_count": 13, "label": "", "sentence_structure": "Subject鈥揷opula鈥損redicative", "subjective_objective": {"answer": "Subjective", "rationale": "Because I don't know what's going on inside me is a subjective feeling and understanding, a subjective observation and assessment of the state of my mind."}, "sentence_sentiment": {"answer": "Neutral", "rationale": "Because the entry does not have any emotional color."}, "language_style": {"answer": "Tactful and warm", "rationale": "The language style of the entry is more euphemistic. Using the vague expression \"I don't know\" can ease the stress and embarrassment that the other side may feel as a result of self-evaluation or emotional problems, making it more willing to participate in the investigation and share its true feelings. And the question of what happens inside me is open, allowing the interviewee to express his own inner problems or changes."}, "time_dimension": {"answer": "Present", "rationale": "The keywords \"not knowing\" and \"what happened\" indicate the current emotional state of the subject, i.e., uncertainty and confusion about the self-emotional state of the person being tested. Thus, the temporal dimension of the article is now judged."}, "sentence_type": "Imperative sentence", "pos": {"nouns": {"Inside": 1, "Come on": 1, "Things": 1}, "verbs": {"Yes": 1, "be": 1}, "adjectives": {"What": 1}, "numerals": {"25": 1}, "pronouns": {"I": 1}}}, {"content": "I don't know why I'm angry.", "word_count": 12, "label": "", "sentence_structure": "Subject鈥揷opula鈥損redicative", "subjective_objective": {"answer": "Subjective", "rationale": "The question is about the patient's own feelings and thoughts, and is a subjective one."}, "sentence_sentiment": {"answer": "Negative", "rationale": "Because \"often angry\" is negative."}, "language_style": {"answer": "Tactful and warm", "rationale": "The rationale for the judgement: the article ' s purpose is expressed in a way that is more compassionate and friendly than in a direct order or statement. At the same time, the article ' s purpose is also more calm and moderate, and not overly emotional. The language style of the questionnaire entry is therefore considered to be euphemistic."}, "time_dimension": {"answer": "Present", "rationale": "According to the expression \"I often\" and \"I don't know\" , the word \"often\" denotes a regular, customary act, and the expression \"not knowing\" means that the act is uncertain, questionable or confused. The entry therefore asks whether the person tested has ever been, is and will be, and the time dimension can be judged by the past, by the present and by the future."}, "sentence_type": "Interrogative sentence", "pos": {"nouns": {"self": 1, "Angry": 1}, "verbs": {"Yes": 1, "Regular": 1, "Angry": 1}, "adjectives": {"Why": 1}, "adverbs": {"Regular": 1, "not": 1}, "numerals": {"26": 1, "1": 4}, "pronouns": {"I": 1}}}]}

{"questionnaire_title": "Self-assessment depression table (SDS)", "questionnaire_category": "Mental health", "item_count": 20, "emotion_shift_overall": "From negative to positive", "language_style_overall": "Tactful and warm", "items": [{"content": "1) I'm depressed, depressed.", "word_count": 11, "label": "", "sentence_structure": "Subject鈥揷opula鈥損redicative", "subjective_objective": {"answer": "Subjective", "rationale": "Because emotional feelings depend in part on the subjective feelings and emotional state of the individual."}, "sentence_sentiment": {"answer": "Negative", "rationale": "Because the mood described in the entry is negative."}, "language_style": {"answer": "Tactful and warm", "rationale": "The entry asks the respondents, in a caring and supportive tone, whether they have experienced or are experiencing negative emotions, such as depression. By using moderate and friendly language, the respondents鈥?psychological burden can be reduced and they are encouraged to share their feelings more readily."}, "time_dimension": {"answer": "Present", "rationale": ""}, "sentence_type": "Affirmative declarative sentence", "pos": {"nouns": {"Emotion": 1, "Frustration": 1, "I'm depressed": 1, "Blue": 1}, "verbs": {"I feel": 1}}}, {"content": "I feel the best in the morning.", "word_count": 13, "label": "", "sentence_structure": "Subject鈥揷opula鈥損redicative", "subjective_objective": {"answer": "Subjective", "rationale": "Because \"fear\" is a subjective feeling of the patient, the entry is considered subjectively."}, "sentence_sentiment": {"answer": "Positive", "rationale": "Because the description \"Best in the Morning\" is positive, it means that the interviewee feels happier and more fully satisfied in the morning."}, "language_style": {"answer": "Tactful and warm", "rationale": "The entry asks in a mild tone whether the interviewee feels best in the morning, rather than directly ordering or making statements. This expression is more personal and interactive, making it easier for the interviewee to accept and answer questions. At the same time, using the phrase \"morning and late\" is also intended to avoid directly pointing out words such as \"deepness at night\" that might discomfort some of the interviewees, thereby making the questionnaire more inclusive and friendly."}, "time_dimension": {"answer": "Past", "rationale": "The keyword \"best mood in the morning\" refers to feelings and experiences at some point or time in the past, and the description \"morning and late\" is also a reflection of a certain state of the past, so the entry is an inquiry into the testee's past state."}, "sentence_type": "Interrogative sentence", "pos": {"nouns": {"Morning": 1, "The mood": 1, "I'd better": 1, "It's morning and night": 1}, "verbs": {"Sensor": 1, "Present": 1, "be": 1}, "adverbs": {"Most": 1, "Okay": 1, "Morning": 1}, "numerals": {"2": 1}, "pronouns": {"I": 1}}}, {"content": "I'm gonna cry or I'm gonna cry.", "word_count": 8, "label": "", "sentence_structure": "Subject鈥搗erb鈥搊bject", "subjective_objective": {"answer": "Subjective", "rationale": "Because \"cry\" is an emotional reaction, reflects the feelings and thoughts inside the patient, and is a subjective examination."}, "sentence_sentiment": {"answer": "Negative", "rationale": "Because \"to cry or want to cry\" is negative."}, "language_style": {"answer": "Calm and direct", "rationale": "The entry directly expresses the subject of asking the other party whether it is easy to cry, without using excessive rhetoric or emotional rendering, and is more objective in tone."}, "time_dimension": {"answer": "Present", "rationale": "The key words are \"I'm gonna cry\" and \"I'm gonna cry,\" which all indicate that this is the current emotional state of the testee, which is the current state of affairs.\""}, "sentence_type": "Imperative sentence", "pos": {"nouns": {"Questionnaire": 1, "Entry": 1, "Cry": 2, "I'm crying": 1, "It's a crybaby": 1}, "verbs": {"Yes": 1, "Cry": 2, "I'm crying": 1}, "adjectives": {"Easy": 1}, "numerals": {"3": 1}}}, {"content": "I'm not sleeping well at night.", "word_count": 11, "label": "", "sentence_structure": "Subject鈥揷opula鈥損redicative", "subjective_objective": {"answer": "Subjective", "rationale": "Because sleep is bad for patients' subjective feelings."}, "sentence_sentiment": {"answer": "Negative", "rationale": "Because sleep disorders are a bad condition, they are often associated with many health problems, including anxiety, depression and other mental illnesses."}, "language_style": {"answer": "Tactful and warm", "rationale": ""}, "time_dimension": {"answer": "Present", "rationale": "The entry asks the testee's current state of affairs, i.e. whether there is a \"bad night sleep\" at the moment, and therefore the test is judged \"now\"."}, "sentence_type": "Negative declarative sentence", "pos": {"nouns": {"Night": 1, "Sleep": 2, "Sleep disorder": 1}, "verbs": {"Okay": 1}, "adverbs": {"not": 1}, "numerals": {"4": 1}}}, {"content": "I eat as much as I normally do.", "word_count": 13, "label": "", "sentence_structure": "Subject鈥搗erb鈥揷omplement", "subjective_objective": {"answer": "Objective", "rationale": "Because a reduction in appetite is a symptom of the patient and an objective factual description."}, "sentence_sentiment": {"answer": "Negative", "rationale": "Because the act of \"eating\" is negative."}, "language_style": {"answer": "Calm and direct", "rationale": "The entry uses a concise statement to ask the respondents directly if there has been any reduction in appetite, a relatively calm and objective language style, and no excessive rhetorical or emotional rendering."}, "time_dimension": {"answer": "Past", "rationale": "The entry \"eating as much as usual\" indicates the behaviour and state of the past, so the temporal dimension is judged to be the past. At the same time, the atrophy is a symptom that requires the testee to recall the past and further proves that it is a matter of the past."}, "sentence_type": "Imperative sentence", "pos": {"nouns": {"Questionnaire": 1, "Entry": 1, "Rice": 1, "Normal": 1, "A appetite": 2}, "verbs": {"Eat": 1, "Elephant": 1, "Declining": 1}, "adverbs": {"Same thing": 1, "More": 1}, "numerals": {"5": 1}, "pronouns": {"I": 1}}}, {"content": "- I'm not sexually active.", "word_count": 12, "label": "", "sentence_structure": "Subject鈥揷opula鈥損redicative", "subjective_objective": {"answer": "Objective", "rationale": "Because the normal functioning of sex and the decline in sexual interest can be determined by medical examination and scientific criteria, not just by the patient鈥檚 subjective perception. The question is therefore considered from an objective perspective."}, "sentence_sentiment": {"answer": "Negative", "rationale": "Because the decline in sexual functions is negative, it may represent a physical or psychological problem."}, "language_style": {"answer": "Calm and direct", "rationale": "The entry directly asked whether the respondents had normal sexual functions or whether there had been a decline in sexual interest, the language style was more straightforward and there was no excessive rhetorical or emotional rendering."}, "time_dimension": {"answer": "Present", "rationale": "The entry is for the testee's sexual function, and the temporal dimension is now judged. The keyword \"now\" implies that it is a description of the current situation, not a past or future event."}, "sentence_type": "Affirmative declarative sentence", "pos": {"nouns": {"Sex function": 1, "Sexual interest": 1}, "verbs": {"Normal": 1, "Declining": 1}, "numerals": {"6)": 1}}}, {"content": "7) I feel weight loss.", "word_count": 11, "label": "", "sentence_structure": "Subject鈥搗erb鈥揷omplement", "subjective_objective": {"answer": "Subjective", "rationale": "Because \"I feel\" is a patient's subjective feeling or experience, which means that the patient feels it, not an objective change in the actual weight. So, this is a subjective look."}, "sentence_sentiment": {"answer": "Negative", "rationale": "Because this entry represents a loss of weight and may cause anxiety and anxiety."}, "language_style": {"answer": "Tactful and warm", "rationale": "The entry is more adept and interactive when it is spoken in a gentle and inviting manner, rather than by direct statements or orders."}, "time_dimension": {"answer": "Past", "rationale": "The keyword \"Feeling weight loss\" indicates that the entry is a question of the person's past feelings and experience, and that the temporal dimension is a past."}, "sentence_type": "Affirmative declarative sentence", "pos": {"nouns": {"Weight loss": 2, "Weight": 1}, "verbs": {"I feel": 1}}}, {"content": "I'm worried about constipation.", "word_count": 8, "label": "", "sentence_structure": "Subject鈥搗erb鈥搊bject", "subjective_objective": {"answer": "Subjective", "rationale": "Because \"fear\" is the patient's subjective feeling."}, "sentence_sentiment": {"answer": "Negative", "rationale": "Because constipation is a physical discomfort, which leads to negative emotions and experiences."}, "language_style": {"answer": "Tactful and warm", "rationale": "The entry describes, in a gentle and caring manner, the problems that may exist in the subject of the survey, avoiding the direct use of too serious or professional terminology and tone to make it more pro-people and more acceptable."}, "time_dimension": {"answer": "Present", "rationale": "Based on the words and context of the sentence, \"I'm going through\" and \"I'm troubled by it\", it can be inferred that the entry in the questionnaire is asking about the current behaviour or state of the subject, and thus judging as now."}, "sentence_type": "Affirmative declarative sentence", "pos": {"nouns": {"Decency": 2, "Trouble": 1}, "verbs": {"Yes": 1}, "numerals": {"8": 1, "Two": 1}}}, {"content": "My heart beats faster than usual.", "word_count": 10, "label": "", "sentence_structure": "Subject鈥揷opula鈥損redicative", "subjective_objective": {"answer": "Subjective", "rationale": "The entry asks about patients鈥?subjective feelings about their own body, i.e. whether the heart beat is faster or if there is a heart attack."}, "sentence_sentiment": {"answer": "Negative", "rationale": "Because an over-heart rate may be a rational response linked to health status."}, "language_style": {"answer": "Calm and direct", "rationale": "The language style of the entry is calm and straightforward. It is presented in a simple, straightforward question form, without any rhetoric or emotional rendering, and is more objective and neutral in tone, with the aim of enabling the interviewee to answer the question accurately and honestly."}, "time_dimension": {"answer": "Present", "rationale": "The entry now asks the testees about their current state of health, i.e. whether they feel a more rapid or heart-breaking condition and therefore judge them as now."}, "sentence_type": "Affirmative declarative sentence", "pos": {"nouns": {"Heart rate": 1, "Normal": 1, "Heartbreak": 1}, "verbs": {"- Yeah": 1, "Come on": 1}, "numerals": {"9": 1}}}, {"content": "I'm tired of it for no reason.", "word_count": 9, "label": "", "sentence_structure": "Subject鈥搗erb鈥揷omplement", "subjective_objective": {"answer": "Subjective", "rationale": "Because fatigue is a perception of the patient ' s own perception and experience, it is a subjective approach."}, "sentence_sentiment": {"answer": "Negative", "rationale": "Because \"tired\" and \"tired\" are negative emotional experiences."}, "language_style": {"answer": "Tactful and warm", "rationale": ""}, "time_dimension": {"answer": "Present", "rationale": ""}, "sentence_type": "Negative declarative sentence", "pos": {"nouns": {"Tired": 2}, "verbs": {"I feel": 1, "For no reason": 1, "Tired": 1}, "adjectives": {"Easy": 1}, "adverbs": {"For no reason": 1}, "numerals": {"10": 1}}}, {"content": "My mind is as clear as usual.", "word_count": 15, "label": "", "sentence_structure": "Subject鈥揷opula鈥損redicative", "subjective_objective": {"answer": "Subjective", "rationale": "Because \"my mind is as clear as usual\" is a personal feeling and subjective judgement. Although the difficulty of thinking may have an objective impact on a person's mental ability, how to evaluate one's mental abilities is usually subjective. So, the question is the patient's subjective idea."}, "sentence_sentiment": {"answer": "Negative", "rationale": "Because the entry \"thinking hard\" expresses a negative, inactive mood or state."}, "language_style": {"answer": "Calm and direct", "rationale": "The language style of the entry is calm and straightforward, and there is no excessive emotional or rhetorical style. It conveys the subject of asking each other whether they are often thinking about difficulties in a concise and clear language."}, "time_dimension": {"answer": "Present", "rationale": ""}, "sentence_type": "Affirmative declarative sentence", "pos": {"nouns": {"Brain": 1, "Entry": 1, "Difficulties": 1}, "verbs": {"Elephant": 1, "Yes": 1, "Clear": 1}, "adjectives": {"As usual": 1, "Same thing": 1}, "numerals": {"11": 1}, "classifiers": {"individual": 1}, "pronouns": {"I": 1}}}, {"content": "I'm not as hard as I normally am.", "word_count": 18, "label": "", "sentence_structure": "Subject鈥搗erb鈥揷omplement", "subjective_objective": {"answer": "Subjective", "rationale": "Because the subject is about patients鈥?subjective feelings and perceptions, whether they feel they have more difficulties in doing things than they do in normal times. This involves their own inner experience, and is therefore subjective."}, "sentence_sentiment": {"answer": "Positive", "rationale": "Because the entry is a form of competency-based self-confidence and positive attitude."}, "language_style": {"answer": "Tactful and warm", "rationale": "Warm and warm."}, "time_dimension": {"answer": "Present", "rationale": "\"I do things as normally without difficulty\" describes a normal situation, with no clear time point, and therefore the judgment is now."}, "sentence_type": "Affirmative declarative sentence", "pos": {"nouns": {"Things": 1, "Normal": 1, "Difficulties": 1, "Capacity": 1, "Declining": 1}, "verbs": {"Do it": 1, "I feel": 1}, "adverbs": {"Elephant": 1, "not": 1, "Same thing": 1}, "numerals": {"12": 1}, "pronouns": {"I": 1}}}, {"content": "I'm not comfortable. I'm not comfortable.", "word_count": 13, "label": "", "sentence_structure": "Subject鈥搗erb鈥揷omplement", "subjective_objective": {"answer": "Subjective", "rationale": "Because \"sitting\" and \"unrequired\" are the feelings and experiences of the patient, it is viewed from a subjective perspective."}, "sentence_sentiment": {"answer": "Negative", "rationale": "Because the entries \"sitting\" and \"unusual to keep calm\" are all expressions of negative emotions, the emotional color of the article is negative."}, "language_style": {"answer": "Tactful and warm", "rationale": "The language style of the questionnaire entry is euphemistic, because it talks in a gentle, invited tone. It does not direct the respondent to answer questions or threaten to do so, but rather in a question-and-answer manner, which gives the respondent a free choice. At the same time, it is softer and more accompanied, such as \"I sit down and have difficulty maintaining calm\" , which is more likely to arouse the echoes and trust of the respondents, thereby improving access to their feedback."}, "time_dimension": {"answer": "Present", "rationale": ""}, "sentence_type": "Affirmative declarative sentence", "pos": {"nouns": {"I": 1, "Sit down": 1, "Ann": 1}, "verbs": {"Hold on": 1, "Sit down": 1}, "adjectives": {"Hard": 1, "Quiet": 1, "Calm down": 1}, "adverbs": {"not": 1}, "classifiers": {"One": 2}}}, {"content": "14) I have hope for the future.", "word_count": 11, "label": "", "sentence_structure": "Subject鈥揷opula鈥損redicative", "subjective_objective": {"answer": "Subjective", "rationale": "Because \"give hope\" and \"depression\" are patients' attitudes or feelings about the future, they are subjective."}, "sentence_sentiment": {"answer": "Positive", "rationale": "Because \"getting hopeful for the future\" expresses positive emotions and attitudes."}, "language_style": {"answer": "Tactful and warm", "rationale": "The language style of the entry is euphemistic, because it does not directly ask the respondents about their specific feelings for the future, but rather uses a gentle and inviting tone to guide the respondents to think about their own hearts. This expression is more personal and interactive, allowing the respondents to share their real thoughts in a more relaxed atmosphere."}, "time_dimension": {"answer": "Future", "rationale": "The words \"for the future\" and \"for hope\" in the entry point to situations that have not yet occurred and that may occur in the future, and therefore the temporal dimension is judged to be the future."}, "sentence_type": "Affirmative declarative sentence", "pos": {"nouns": {"The future": 1, "Hope": 1, "Despair": 1}, "verbs": {"I feel": 1}, "adjectives": {"have": 1, "of": 2}}}, {"content": "I'm more angry than usual.", "word_count": 12, "label": "", "sentence_structure": "Subject鈥揷opula鈥損redicative", "subjective_objective": {"answer": "Subjective", "rationale": "Because \"fun\" is the patient's subjective feeling and attitude."}, "sentence_sentiment": {"answer": "Negative", "rationale": "Because the description \"more anger than usual\" in the entry is negative."}, "language_style": {"answer": "Calm and direct", "rationale": "The language style of the entry is relatively simple and the question is whether the person tested is in some cases prone to anger or excitement and does not use excessive accoutrements and rhetorical techniques, which appear to be objective and calm."}, "time_dimension": {"answer": "Present", "rationale": "The entry asks the testee about his current feelings and experiences, and \"more irritating than usual\" is a comparative form, which indicates a comparison with past or normal state, and therefore judges \"now\"."}, "sentence_type": "Affirmative declarative sentence", "pos": {"nouns": {"Questionnaire": 1, "Entry": 1, "Exasperation": 1, "It's a little hot": 1}, "verbs": {"- Yeah": 1, "Easy": 1, "Exasperation": 1}, "adjectives": {"Normal": 1, "More": 1}, "numerals": {"15": 1}, "pronouns": {"I": 1}}}, {"content": "I think it's easy to decide what's going on.", "word_count": 15, "label": "", "sentence_structure": "Subject鈥搗erb鈥揷omplement", "subjective_objective": {"answer": "Subjective", "rationale": "Because it's the patient's own subjective perception and feelings of things."}, "sentence_sentiment": {"answer": "Negative", "rationale": "Because the expression \"compromise difficulty\" in the entry is negative."}, "language_style": {"answer": "Calm and direct", "rationale": "This language style is more objective and clear, and effectively conveys the intentions and problems of the researchers."}, "time_dimension": {"answer": "Present", "rationale": "\"Feel\" and \"Easy\" are terms of subjective feeling, which indicate the emotional state and attitude of the subject, so the time dimension of this entry is now."}, "sentence_type": "Negative declarative sentence", "pos": {"nouns": {"Yes": 1, "Decision": 1}, "verbs": {"I think": 1, "Easy": 1, "Decision": 1, "Tired": 1}, "adjectives": {"very": 1}, "numerals": {"16": 1, "What": 1, "Both": 1}, "pronouns": {"I": 1}}}, {"content": "17) I feel like I'm useful and indispensable.", "word_count": 19, "label": "", "sentence_structure": "Subject鈥揷opula鈥損redicative", "subjective_objective": {"answer": "Subjective", "rationale": "Because \"useful\" and \"indeed\" are the subjective feelings and perceptions of the patient."}, "sentence_sentiment": {"answer": "Positive", "rationale": "Because the entry expresses the feeling of self-confidence, self-worth and positive emotional attitudes."}, "language_style": {"answer": "Tactful and warm", "rationale": "This entry uses a soft, invitation-style tone, which attempts to give the respondents the interest and support of the researchers, while also encouraging them to share their thoughts and feelings. Such expressions can more easily gain the confidence and cooperation of the respondents and help to increase the rate and efficiency of the questionnaire."}, "time_dimension": {"answer": "Present", "rationale": "Now the entry is about the current feelings of the subject, and the word \"I feel\" in \"I feel\" means that it is subjective experience and feeling, and there is no clear time point, and it is therefore judged now."}, "sentence_type": "Affirmative declarative sentence", "pos": {"nouns": {"person": 1, "It's useless": 1}, "verbs": {"I feel": 1, "be": 1, "It works": 1, "and": 1, "No way": 1, "Missing": 1}, "adjectives": {"It's useful": 1, "Essential": 1}, "numerals": {"17": 1}, "classifiers": {"individual": 1}, "pronouns": {"I": 1}}}, {"content": "My life is very meaningful.", "word_count": 13, "label": "", "sentence_structure": "Subject鈥揷opula鈥損redicative", "subjective_objective": {"answer": "Subjective", "rationale": "Because \"meaning\" and \"emptiness\" are subjective assessments of a patient's life."}, "sentence_sentiment": {"answer": "Positive", "rationale": "Because the expression \"life makes sense\" in the entry is positive."}, "language_style": {"answer": "Tactful and warm", "rationale": "This entry provides a sense of the mental state of the person concerned by asking whether the other person鈥檚 life is meaningful and whether there is a sense of emptiness, and is directed in a moderate and encouraging way to make the person under investigation comfortable and comfortable."}, "time_dimension": {"answer": "Future", "rationale": "The keywords are \"very meaningful\" and \"life void\" and they all point to the expectations and visions of the testee for the future, so the entry is the future."}, "sentence_type": "Affirmative declarative sentence", "pos": {"nouns": {"Life": 2, "Meaning": 1, "鈾?The sense of emptiness 鈾?: 1}, "adjectives": {"very": 1, "Meaningful": 1}, "numerals": {"18": 1}}}, {"content": "19) If I die, people would be better off.", "word_count": 16, "label": "", "sentence_structure": "Subject鈥搗erb鈥揷omplement", "subjective_objective": {"answer": "Subjective", "rationale": "Because \"if I die, someone else will be better\" is a subjective idea or view that reflects the patient's perception of his value and influence in the lives of others. The idea is not an objective fact, but a result of the patient's own mental state."}, "sentence_sentiment": {"answer": "Negative", "rationale": "Because the phrase \"if I die, someone else will be better\" is negative and has a downright, self-defeating emotional dimension."}, "language_style": {"answer": "Calm and direct", "rationale": "The entry is presented directly to the respondent, asking him whether he or she is better off after his or her death, without using excessive rhetoric or emotional rendering, and in a more objective and balanced manner."}, "time_dimension": {"answer": "Present", "rationale": "According to the sentence, \"If I die\" and \"if others will be better,\" it can be seen as a hypothesis of the current situation, and that this assumption may lead to a sense of worthlessness on the part of the testee. Thus, the entry is part of an inquiry into the current behavior or mentality."}, "sentence_type": "Negative declarative sentence", "pos": {"nouns": {"I": 1, "others": 1, "Die": 1, "No sense of value": 1}, "verbs": {"If": 1, "Yes": 2}, "numerals": {"19": 1}}}, {"content": "I still love what I like.", "word_count": 18, "label": "", "sentence_structure": "Subject鈥搗erb鈥揷omplement", "subjective_objective": {"answer": "Subjective", "rationale": "Because \"love\" is the patient's subjective emotional attitude, and loss of interest is the subjective change that the patient feels. The entry therefore looks primarily at the patient's subjective feelings and thoughts."}, "sentence_sentiment": {"answer": "Positive", "rationale": "Because \"whatever you love\" in the entry is a positive attitude and behavior."}, "language_style": {"answer": "Tactful and warm", "rationale": "The entry uses a more moderate, encouraging expression that gives respondents a sense of comfort and willingness to share their ideas. At the same time, it is more acceptable to the majority and more conducive to the effectiveness and reliability of questionnaires."}, "time_dimension": {"answer": "Present", "rationale": ""}, "sentence_type": "Negative declarative sentence", "pos": {"nouns": {"Questionnaire Entry": 1, "Something": 1, "Interest": 1}, "verbs": {"Love it": 1, "Loss": 1}, "adverbs": {"Still": 1, "Normal": 1}, "classifiers": {"20)": 1}, "pronouns": {"I": 1}}}]}

{"questionnaire_title": "Multi-dimensional health psychological control source table", "questionnaire_category": "Mental health", "item_count": 18, "emotion_shift_overall": "From positive to negative", "language_style_overall": "Tactful and warm", "items": [{"content": "If I'm sick, how fast I'm going to recover is my own behavior.", "word_count": 20, "label": "", "sentence_structure": "Subject鈥搗erb鈥搊bject", "subjective_objective": {"answer": "Subjective", "rationale": "Because the speed of recovery depends on the patient's behaviour, which is the patient's subjective judgment.\""}, "sentence_sentiment": {"answer": "Positive", "rationale": "Because \"by acting\" is self-determination and motivation."}, "language_style": {"answer": "Calm and direct", "rationale": "The entry describes a hypothetical situation in an objective and direct manner, and clearly raises the question, with a more neutral and cold tone, with no excessive emotional or rhetorical style. Thus, the language style of the purpose is more \"cool and direct\"."}, "time_dimension": {"answer": "Future", "rationale": "And \"My own behaviour\" suggests that it is a self-decided act, emphasizing individual responsibility and control. Thus, the entry contains visions and expectations for the future, as well as the concept of self-decided, judged by the future of the time dimension and the self-decided nature of the behaviour/concept."}, "sentence_type": "Interrogative sentence", "pos": {"nouns": {"I": 2, "Diseases": 1, "Restore": 1, "Behaviour": 1}, "verbs": {"Suppose": 1, "have": 1, "be": 1, "By": 1}, "adverbs": {"How fast": 1}}}, {"content": "My health is in my hands.", "word_count": 9, "label": "", "sentence_structure": "Subject鈥搗erb鈥揷omplement", "subjective_objective": {"answer": "Subjective", "rationale": "Because \"your health\" is an expression of subjective feelings and attitudes, not an objective fact or observed behavioural result."}, "sentence_sentiment": {"answer": "Positive", "rationale": "Because the idea of \"health is in your hands\" in the entry is positive."}, "language_style": {"answer": "Tactful and warm", "rationale": "The entry conveys a message to the interviewees in a moderate and encouraging manner, giving them attention and support, and making them more resonant and responsive. At the same time, it is more acceptable and understandable, and contributes to improving the completion and quality of questionnaires."}, "time_dimension": {"answer": "Present", "rationale": "The entry emphasizes the current perception and attitude, the key word being \"in its own hands\", and expresses a self-responsible, proactive approach to health management, which is the expression of current behaviour or mentality."}, "sentence_type": "Affirmative declarative sentence", "pos": {"nouns": {"Health": 1, "self": 1}, "verbs": {"Got it": 1}}}, {"content": "3. If I get sick, it is my responsibility.", "word_count": 11, "label": "", "sentence_structure": "Subject鈥損redicate", "subjective_objective": {"answer": "Subjective", "rationale": "This is a self-evaluation and opinion of the patient and a subjective examination."}, "sentence_sentiment": {"answer": "Negative", "rationale": "Because the purpose of the article gives a negative, self-responsible emotional colour."}, "language_style": {"answer": "Calm and direct", "rationale": "The entry directly expresses the view that the individual is responsible for the illness, does not use any rhetorical or emotional rendering, and is more calm and direct in language style."}, "time_dimension": {"answer": "Present", "rationale": "The entry \"if I get sick\" and \"responsibility is all about myself\" are hypothetical situations, implying the testee's current beliefs or attitudes. So, time dimensions judge now."}, "sentence_type": "Affirmative declarative sentence", "pos": {"nouns": {"Responsibility": 1, "self": 1}, "verbs": {"in": 1, "Diseases": 1}, "adjectives": {"All": 1}, "pronouns": {"I": 1}}}, {"content": "4. The main factors affecting my health status are all at my own disposal.", "word_count": 22, "label": "", "sentence_structure": "Subject鈥揷opula鈥損redicative", "subjective_objective": {"answer": "Subjective", "rationale": "Because the entry emphasizes the responsibility of the individual and the ability to manage himself, it is the patient ' s subjective view."}, "sentence_sentiment": {"answer": "Positive", "rationale": "Because the entry expresses the attitude of the individual to be responsible for his or her own health, which is a positive behaviour and quality."}, "language_style": {"answer": "Calm and direct", "rationale": "The reason is that the statement of purpose of this article states directly and clearly that \"the main factors affecting my health are all the things I do myself\", that it is not too much accommodative or emotional expression, but is presented in a factual manner, which is more objective and calm."}, "time_dimension": {"answer": "Past", "rationale": "In conclusion, the temporal dimension of this questionnaire entry is neutral."}, "sentence_type": "Affirmative declarative sentence", "pos": {"nouns": {"Factors": 1, "Health status": 1, "self": 2}, "verbs": {"Impact": 1, "in": 1, "Do it": 1, "Yes": 1}, "adjectives": {"Main": 1}, "numerals": {"4": 1}, "pronouns": {"I": 1}}}, {"content": "If I had been careful, I would not have been sick.", "word_count": 13, "label": "", "sentence_structure": "Subject鈥損redicate", "subjective_objective": {"answer": "Subjective", "rationale": "Subjectivity, because it is a statement of the patient ' s views and convictions, and is subjective."}, "sentence_sentiment": {"answer": "Neutral", "rationale": "Because the entry does not express positive or negative feelings, attitudes, behaviour, purpose or quality. It merely describes a relationship between a condition and the result."}, "language_style": {"answer": "Tactful and warm", "rationale": "Although the entry directly raises a point (i.e. that it is not sick to protect itself), it is presented in a more euphemistic and polite manner, for example, by the phrase \"if you are in a situation of ...,\" without being too hard or straightforward to express it. This language style is usually more likely to generate positive feedback and reflection from the respondents."}, "time_dimension": {"answer": "Past", "rationale": ""}, "sentence_type": "Negative declarative sentence", "pos": {"verbs": {"Watch out": 1, "Sick": 1}, "adjectives": {"self": 1}}}, {"content": "If I take the right action, I'll be healthy.", "word_count": 16, "label": "", "sentence_structure": "Subject鈥搗erb鈥搊bject", "subjective_objective": {"answer": "Subjective", "rationale": "Because this phrase relates to the personal perception and conviction that I will be healthy, and it is a subjective view."}, "sentence_sentiment": {"answer": "Positive", "rationale": "Because it expresses a positive behaviour and purpose - taking appropriate action to achieve health, expressing positive emotional attitudes."}, "language_style": {"answer": "Tactful and warm", "rationale": "The entry uses a hypothetical question form, while at the same time encouraging respondents to consider the relationship between their behaviour and their outcomes by using positive language (e.g. \"appropriate action\" and \"health\"). This expression gives people a feeling of encouragement and support, which is more comfortable and persuasive.\""}, "time_dimension": {"answer": "Present", "rationale": "The keywords are \"take\" and \"take\" , which both represent a hypothetical and possible future scenario, and therefore the temporal dimension is now judged."}, "sentence_type": "Affirmative declarative sentence", "pos": {"nouns": {"Action": 1, "Health": 1}, "verbs": {"Adoption": 1, "Yes": 1}, "adjectives": {"Appropriate": 1}, "numerals": {"6": 1}, "pronouns": {"I": 1}}}, {"content": "Regular access to doctors is the best way for me to avoid illness.", "word_count": 20, "label": "", "sentence_structure": "Subject鈥揷opula鈥損redicative", "subjective_objective": {"answer": "Objective", "rationale": "Because the best way to visit a doctor on a regular basis depends on facts and evidence, not just on the patient ' s subjective view."}, "sentence_sentiment": {"answer": "Positive", "rationale": "The article expresses positive attitudes and behaviour, i.e. regular visits to a doctor help to avoid illness."}, "language_style": {"answer": "Tactful and warm", "rationale": "The entry is more adept and interactive when it is presented in a moderate and recommendive tone rather than in direct orders or statements."}, "time_dimension": {"answer": "Present", "rationale": "The entry asks the subject about his current perception and practice, the keywords being \"regular access to a doctor\", \"best way\", etc., all pointing to the current behaviour or state.\""}, "sentence_type": "Affirmative declarative sentence", "pos": {"nouns": {"Doctor": 1, "Modalities": 1}, "verbs": {"Look": 1, "Periodic": 1, "be": 1, "Avoid": 1, "Fan": 1}, "adjectives": {"Best": 1}, "numerals": {"7": 1}, "pronouns": {"I": 1}}}, {"content": "8. Whenever I feel uncomfortable, I dance to professional medical staff.", "word_count": 20, "label": "", "sentence_structure": "Subject鈥搗erb鈥搊bject", "subjective_objective": {"answer": "Subjective", "rationale": "Because \"I feel uncomfortable\" and looking for professional help are subjective experiences and choices."}, "sentence_sentiment": {"answer": "Neutral", "rationale": "Since the entry does not cover a description of emotion, attitude, behaviour, purpose or quality, it simply presents a fact."}, "language_style": {"answer": "Calm and direct", "rationale": "The entry describes in simple and clear terms the behaviour of the interviewee when it feels inappropriate, without the use of excessive rhetorical or emotional rendering, and is more objective and direct."}, "time_dimension": {"answer": "Past", "rationale": "Because \"uncomfort\" is a dynamic, frequent occurrence, \"showing to a professional \"the tester's past experience has shown that this has been done."}, "sentence_type": "Affirmative declarative sentence", "pos": {"nouns": {"I'm not feeling well": 1, "Medical personnel": 1, "Date dance": 1}, "verbs": {"Yes": 1, "In": 1, "Professional": 1, "Seek": 1, "Medical services": 1}, "adverbs": {"Every time": 1}, "numerals": {"8": 1}, "pronouns": {"I": 1}}}, {"content": "9. I am ill or in good health and have a strong relationship with my family.", "word_count": 17, "label": "", "sentence_structure": "Subject鈥搗erb鈥揷omplement", "subjective_objective": {"answer": "Objective", "rationale": "Because \"sick or healthy\" is an objective state of mind, and \"very much related to my family\" is a fact that can be observed and quantified. Although patients may have different subjective feelings and ideas, the sentence itself describes an objective phenomenon."}, "sentence_sentiment": {"answer": "Neutral", "rationale": "Because the entry is not clearly emotional, it is merely a statement of the facts."}, "language_style": {"answer": "Tactful and warm", "rationale": "The language style of the entry is more euphemistic and warm, implying close ties between a person's health and his family through the use of the expression \"great connections\". At the same time, the first person's proxies, \"I\", make the issue more human and sensitive, and give attention and attention to the interviewees."}, "time_dimension": {"answer": "Present", "rationale": "Now the entry says \"I am sick or in good health\" and \"I have a big relationship with my family,\" and this situation exists when I speak, and has some influence on the person who speaks. So the time dimension of this article is now."}, "sentence_type": "Affirmative declarative sentence", "pos": {"nouns": {"I": 2, "Diseases": 1, "Fan": 1, "Health": 1, "Home": 1, "person": 1, "It's big": 1, "Relations": 1}, "verbs": {"have": 1, "be": 1}, "adjectives": {"Body": 1}, "adverbs": {"very": 1}, "numerals": {"9": 1}, "classifiers": {"individual": 1}}}, {"content": "I am assured of my health by medical personnel.", "word_count": 11, "label": "", "sentence_structure": "Subject鈥搗erb鈥揷omplement", "subjective_objective": {"answer": "Objective", "rationale": "This is a commitment and responsibility of the medical institution and is not related to the patient ' s subjective attitude."}, "sentence_sentiment": {"answer": "Positive", "rationale": "Because the act of \"Securing my health\" in the entry is an expression of the care and commitment of the medical institutions to the patient and is positive in emotional terms."}, "language_style": {"answer": "Tactful and warm", "rationale": "This is a more appropriate expression for medical-related areas."}, "time_dimension": {"answer": "Present", "rationale": "Now."}, "sentence_type": "Affirmative declarative sentence", "pos": {"nouns": {"Medical personnel": 1, "Body": 1, "Health": 1}, "verbs": {"Promise": 1, "be": 1}, "numerals": {"10": 1}, "pronouns": {"I": 1}}}, {"content": "11. If I get sick, it is usually because of the care of others.", "word_count": 18, "label": "", "sentence_structure": "Subject鈥揷opula鈥損redicative", "subjective_objective": {"answer": "Subjective", "rationale": "Because this is the patient鈥檚 subjective view and explanation."}, "sentence_sentiment": {"answer": "Positive", "rationale": "Because the result of \"the sick\" in the entry is positive, so is the reason \"the others take care of.\""}, "language_style": {"answer": "Tactful and warm", "rationale": ""}, "time_dimension": {"answer": "Past", "rationale": "\"If I get sick\" and \"if I get sick\" are usually the result of the care of others, which is a description or a hypothesis of the past, and therefore the temporal dimension is the past."}, "sentence_type": "Affirmative declarative sentence", "pos": {"nouns": {"I": 1, "I'm sick": 1, "person": 2, "Take care of yourself": 1}, "verbs": {"be": 1, "Because": 1, "Care": 1}, "adverbs": {"Usually": 1}, "numerals": {"11": 1}, "pronouns": {"If": 1}}}, {"content": "12. For my own health, I do so only as the doctor says.", "word_count": 16, "label": "", "sentence_structure": "Subject鈥搗erb鈥搊bject", "subjective_objective": {"answer": "Subjective", "rationale": "Because \"do what the doctor says\" is a subjective decision and behaviour of a person, it examines subjective factors such as the patient's self-management capacity, level of trust, etc."}, "sentence_sentiment": {"answer": "Positive", "rationale": "Because the purpose of \"do as the doctor says\" is positive, for his own health."}, "language_style": {"answer": "Tactful and warm", "rationale": "The entry uses euphemisms, \"for his own health\", which emphasize the behaviour of individuals who actively maintain their health, and is expressed in a relatively gentle and friendly manner, without direct orders or coercion. So I think its language style is euphemism."}, "time_dimension": {"answer": "Past", "rationale": "The phrase \"do as the doctor says\" in that sentence indicates that the person was subjected to a past act or decision, and therefore is judged to be past."}, "sentence_type": "Affirmative declarative sentence", "pos": {"nouns": {"Health": 1, "Word": 1}, "verbs": {"Do it": 1}, "adverbs": {"self": 1, "Only": 1, "Both": 1}, "numerals": {"12": 1}, "pronouns": {"I": 1, "Doctor": 1}}}, {"content": "13. Whatever I do, if I get sick, I get sick.", "word_count": 17, "label": "", "sentence_structure": "Subject鈥損redicate", "subjective_objective": {"answer": "Subjective", "rationale": "Because this sentence relates to the patient鈥檚 subjective belief and expectation that if he or she feels ill, he or she will be ill. This is the patient鈥檚 subjective prediction and attitude to the future, and is therefore a subjective examination."}, "sentence_sentiment": {"answer": "Negative", "rationale": "Because the \"can get sick\" result in the entry is negative."}, "language_style": {"answer": "Tactful and warm", "rationale": "The entry is more personal and interactive when it is spoken in a gentle and inviting manner, rather than in direct statements or orders. At the same time, the language style of the article is more euphemism and understanding than cold or tough."}, "time_dimension": {"answer": "Present", "rationale": "The entry indicates the subjective perception and expectations of the person being tested about his or her physical condition, does not relate to past or future circumstances and therefore the judgement is now."}, "sentence_type": "Affirmative declarative sentence", "pos": {"nouns": {"I": 2, "Diseases": 2}, "verbs": {"Do it": 1, "Come on, come on": 1, "Yes, I do": 1}, "adverbs": {"Whatever": 1}, "numerals": {"13": 1}}}, {"content": "14. Most of the events affecting my health occurred by accident.", "word_count": 20, "label": "", "sentence_structure": "Subject鈥揷opula鈥損redicative", "subjective_objective": {"answer": "Objective", "rationale": "Because the event affecting the health situation is an objective fact, and most people are subjective in their view. The question is therefore one of patient ' s subjective ideas, but the incident itself is objective."}, "sentence_sentiment": {"answer": "Neutral", "rationale": "Because the entry merely stated a fact, it did not express a positive or negative emotional colour."}, "language_style": {"answer": "Calm and direct", "rationale": "The entry is judged by the use of the objective statement that \"most events happen by chance\" to describe the circumstances affecting the health of the interviewee, without too many rhetorical or emotional accents. This tone is more plain, simple, and directly conveys the information that researchers want to know, and therefore is a calm and straightforward language style."}, "time_dimension": {"answer": "Present", "rationale": "The words \"impact\", \"most\" and \"all\" in the entry indicate that this is a context of the current situation, and that the \"accident\" event suggests that this is happening on a continuous basis, so the temporal dimension of the entry is now judged."}, "sentence_type": "Affirmative declarative sentence", "pos": {"nouns": {"Impact": 1, "I": 1, "Health": 1, "Events": 2, "Most of them": 1, "It happens by chance": 1}, "verbs": {"be": 1, "Come on": 1}, "adjectives": {"Mostly": 1}, "adverbs": {"Both": 1}, "numerals": {"14": 1, "2": 1}, "classifiers": {"individual": 1}, "pronouns": {"of": 1}}}, {"content": "15. How fast I recovered, my luck had been very helpful.", "word_count": 20, "label": "", "sentence_structure": "Subject鈥搗erb鈥揷omplement", "subjective_objective": {"answer": "Subjective", "rationale": "Because \"the speed of recovery\" and \"luck\" are the feelings and perceptions of the patient, it's subjective."}, "sentence_sentiment": {"answer": "Positive", "rationale": "Because the expression \"good luck\" conveys a positive, positive emotional color."}, "language_style": {"answer": "Tactful and warm", "rationale": "The language style of the entry is more euphemistic, using the expression \"playing a role\" to avoid either a direct affirmation or a denial of the effect of luck on the pace of recovery. At the same time, the tone of the words is relatively soft, giving people a sense of affection."}, "time_dimension": {"answer": "Past", "rationale": "The words \"how quickly to recover\" and \"how much luck has played in it\" in the entry describe the past or experience and therefore judge it as the past."}, "sentence_type": "Interrogative sentence", "pos": {"nouns": {"I": 1, "Speed": 1, "Rehabilitation": 1, "Lucky": 1, "Role": 1}, "verbs": {"have": 1, "Rise": 1}, "adjectives": {"How fast": 1, "Large": 1}, "adverbs": {"very": 1}, "numerals": {"15": 1}}}, {"content": "16. Most of my health is a lucky relationship.", "word_count": 12, "label": "", "sentence_structure": "Subject鈥揷opula鈥損redicative", "subjective_objective": {"answer": "Subjective", "rationale": "Because the health of an individual is determined in part by a variety of factors, including genetics, life habits, and so forth, it is not a matter of luck. And the patient believes that his/her health is largely due to luck, which is his/her subjective view."}, "sentence_sentiment": {"answer": "Neutral", "rationale": "Because the entry does not express a clear emotional tendency, it merely presents a fact."}, "language_style": {"answer": "Tactful and warm", "rationale": "The entry is more euphemistic, using the expression \"mostly lucky relationships\" to express a positive view of their health, but also a modest and objective attitude. Such an expression expresses both the confidence of the interviewee in himself and the avoidance of over-examining or proudness."}, "time_dimension": {"answer": "Past", "rationale": ""}, "sentence_type": "Affirmative declarative sentence", "pos": {"nouns": {"Relations": 1, "Lucky": 1}, "verbs": {"I": 1, "be": 1, "Okay": 1, "Relations": 1, "Lucky": 1}, "adjectives": {"Half of them": 1, "Body": 1}, "adverbs": {"very": 1}, "numerals": {"16": 1}, "classifiers": {"individual": 1}, "pronouns": {"I": 1}}}, {"content": "I don't care what I do, I'm sick.", "word_count": 10, "label": "", "sentence_structure": "Subject鈥揷opula鈥損redicative", "subjective_objective": {"answer": "Subjective", "rationale": "Because \"love sick\" is the patient's subjective feeling and attitude."}, "sentence_sentiment": {"answer": "Negative", "rationale": "Because the expression \"I always love to get sick whatever I do\" reflects a negative attitude or emotion."}, "language_style": {"answer": "Tactful and warm", "rationale": "The entry asks the other side whether it is often prone to illness in a more moderate way, rather than directly pointing to the seriousness of the problem or to the shortcomings of the other side."}, "time_dimension": {"answer": "Present", "rationale": "According to the sentence entered, \" always love sick\" describes the current situation of the individual, so the temporal dimension of the article is now judged."}, "sentence_type": "Negative declarative sentence", "pos": {"nouns": {"I": 1, "Diseases": 1}, "verbs": {"Do it": 1, "Love": 1, "Fan": 1}, "adjectives": {"Total": 1}, "adverbs": {"Both": 1}}}, {"content": "18. I seem to be in good health.", "word_count": 11, "label": "", "sentence_structure": "Subject鈥揷opula鈥損redicative", "subjective_objective": {"answer": "Subjective", "rationale": "Because \"good body seems to be destined to be\" reflects the beliefs and attitudes of the patient and is a subjective examination."}, "sentence_sentiment": {"answer": "Positive", "rationale": "Because the description of \"good body\" is positive."}, "language_style": {"answer": "Tactful and warm", "rationale": "The entry is more adept and interactive when it is spoken in a gentle and inviting manner, rather than by direct statements or orders."}, "time_dimension": {"answer": "Present", "rationale": "The keyword \"seemingly bound to be\" indicates that the interviewee believes that his health is destined for fate or fate, which is a description of the current behaviour or state. Thus, the temporal dimension of the article is now judged."}, "sentence_type": "Affirmative declarative sentence", "pos": {"nouns": {"Body": 1, "Destiny": 1}, "verbs": {"Okay": 1, "Seems like": 1, "be": 1, "I see": 1}, "adverbs": {"I see": 1}, "pronouns": {"I": 1}}}]}

{"questionnaire_title": "Life events schedule", "questionnaire_category": "Mental health", "item_count": 48, "emotion_shift_overall": "From positive to negative", "language_style_overall": "Calm and direct", "items": [{"content": "2. In love or betrothal", "word_count": 5, "label": "", "sentence_structure": "Subject鈥搗erb鈥揷omplement", "subjective_objective": {"answer": "Subjective", "rationale": "Because love and betrothal are the subjective will and choice of a person and are subjective."}, "sentence_sentiment": {"answer": "Neutral", "rationale": "Because the entry is merely asking if the person tested is in love or betrothal, and does not involve any emotional or evaluative vocabulary."}, "language_style": {"answer": "Tactful and warm", "rationale": "The entry is more personal and interactive in its expression, in a gentle and inviting tone, rather than in direct statements or orders. At the same time, the topic itself involves the emotional life of the individual, and the use of a soft and warm language style better protects the privacy and self-esteem of the person under investigation."}, "time_dimension": {"answer": "Present", "rationale": "The relationship or betrothal is a state of current practice, so it can be judged that the entry refers to the present situation."}, "sentence_type": "Interrogative sentence", "pos": {"nouns": {"Love": 1, "Engagement": 1}, "verbs": {"None": 1}}}, {"content": "3. Failure in love, breakdown", "word_count": 6, "label": "", "sentence_structure": "Subject鈥搗erb鈥揷omplement", "subjective_objective": {"answer": "Subjective", "rationale": "Because love failure and break-ups are subjective feelings and experiences of people, they are viewed from the perspective of patients鈥?subjective experiences. Although these experiences are objective, their impact and significance on patients are subjective."}, "sentence_sentiment": {"answer": "Negative", "rationale": "Because \"lost love, broken love\" is a negative one."}, "language_style": {"answer": "Tactful and warm", "rationale": "The entry asks, in a sympathetic and understanding manner, whether the other party has experienced a failure or breakdown of a relationship, is more moderate in terms that do not reveal the other party ' s privacy or pain directly, and is comfortable and acceptable."}, "time_dimension": {"answer": "Past", "rationale": ""}, "sentence_type": "Negative declarative sentence", "pos": {"nouns": {"Love": 2, "Failed": 1, "Break": 1}, "numerals": {"3": 1}}}, {"content": "4. Marriage", "word_count": 2, "label": "", "sentence_structure": "Subject鈥搗erb鈥揷omplement", "subjective_objective": {"answer": "Objective", "rationale": "Since marriage is an objective fact, it is not necessary to interpret or evaluate the patient ' s subjective attitude."}, "sentence_sentiment": {"answer": "Neutral", "rationale": "Since the entry did not express any positive or negative emotional aspects, it merely asked whether or not it had been married."}, "language_style": {"answer": "Calm and direct", "rationale": "The question is whether the respondents are married, the language style is more concise, and there is no excessive use of qualifiers or emotional colours, so that the language style can be judged as \"cool and direct\"."}, "time_dimension": {"answer": "Present", "rationale": "The entry asks the person who was tested about the marital status of the marriage, and the time dimension does not specify whether the past, present or future is clearly specified and therefore cannot be judged."}, "sentence_type": "Imperative sentence", "pos": {"nouns": {"Marriage": 1}}}, {"content": "5. Being pregnant (in love)", "word_count": 6, "label": "", "sentence_structure": "Subject鈥搗erb鈥揷omplement", "subjective_objective": {"answer": "Subjective", "rationale": "Because \"pregnancy\" is a subjective state of affairs that belongs to the inner feelings and experiences of the patient or his/her lover, not just to the objective facts."}, "sentence_sentiment": {"answer": "Positive", "rationale": "Because this entry represents a happy event for individuals or lovers to give birth to a new life."}, "language_style": {"answer": "Tactful and warm", "rationale": "The entry, in a concerned and sweet tone, asks the interviewee whether he or she knows about his or her pregnancy, which makes it feel more intimate and close. At the same time, the expression \"self\" is respected and noted."}, "time_dimension": {"answer": "Future", "rationale": "The entry describes what may happen in the future, i.e. the partner of the testee may become pregnant."}, "sentence_type": "Affirmative declarative sentence", "pos": {"nouns": {"self": 1, "Lover": 1, "Pregnancy": 1}}}, {"content": "6. Abortion of oneself (love)", "word_count": 6, "label": "", "sentence_structure": "Subject鈥搗erb鈥揷omplement", "subjective_objective": {"answer": "Objective", "rationale": "Because \"self-loved abortion\" is an objective experience or event, not a patient鈥檚 subjective attitude or feeling. Even if the patient may have a subjective emotional reaction, the problem is the fact itself, not the patient鈥檚 emotional experience."}, "sentence_sentiment": {"answer": "Negative", "rationale": "The reference to \"abortion\" in the entry usually carries some negative emotional and psychological stress."}, "language_style": {"answer": "Tactful and warm", "rationale": "The entry is more euphemistic and warm. It asks the respondents about their abortion, but it does not ask them directly, but rather uses a more moderate and caring approach. It makes it easier for the interviewee to feel comfortable and to get a real and accurate answer."}, "time_dimension": {"answer": "Past", "rationale": "The entry contains the past event \"A miscarriage of oneself or of someone you love\", and therefore it is judged to be the past."}, "sentence_type": "Imperative sentence", "pos": {"nouns": {"self": 1, "Lover": 1, "Abortion": 1}, "numerals": {"6": 1}}}, {"content": "7. Family additions", "word_count": 7, "label": "", "sentence_structure": "Subject鈥搗erb鈥揷omplement", "subjective_objective": {"answer": "Subjective", "rationale": "Because the addition of new members to the family is the result of a subjective judgement, depending on the individual ' s perception and attitude towards the arrival of new life."}, "sentence_sentiment": {"answer": "Neutral", "rationale": "Because the entry does not have any emotional color."}, "language_style": {"answer": "Tactful and warm", "rationale": "The entry leads the respondents to share family-related information by means of inquiries, using the active term \"new member\" to express concern and expectations about the family life of the interviewee, and to give a warm and cordial feeling."}, "time_dimension": {"answer": "Future", "rationale": "The entry contains visions and expectations for the future, with the keyword \"new family members\" pointing to situations that have not yet occurred and that may arise in the future."}, "sentence_type": "Affirmative declarative sentence", "pos": {"nouns": {"Family": 1, "New members": 1}, "verbs": {"Add": 1}, "numerals": {"7": 1}}}, {"content": "8. Disconnect with the parents of the loved one", "word_count": 7, "label": "", "sentence_structure": "Subject鈥損redicate", "subjective_objective": {"answer": "Subjective", "rationale": "Because the discord with the parents of the loved one is an individual emotional experience, it is subjective."}, "sentence_sentiment": {"answer": "Negative", "rationale": "Because the \"unreconciliation\" in the entry is negative."}, "language_style": {"answer": "Tactful and warm", "rationale": ""}, "time_dimension": {"answer": "Present", "rationale": ""}, "sentence_type": "Interrogative sentence", "pos": {"nouns": {"Lover": 1, "parents": 1}, "verbs": {"- No, no, no": 1}}}, {"content": "9. Poor couple", "word_count": 6, "label": "", "sentence_structure": "Subject鈥揷opula鈥損redicative", "subjective_objective": {"answer": "Subjective", "rationale": "The relationship between the couple is not considered from the patient ' s subjective point of view."}, "sentence_sentiment": {"answer": "Negative", "rationale": "Because the expression \"worse couple\" is negative, it means that there is a contradiction or conflict between the couple."}, "language_style": {"answer": "Calm and direct", "rationale": ""}, "time_dimension": {"answer": "Present", "rationale": "The entry describes the emotional situation between the person tested and his partner, and is a description and evaluation of the current situation, and therefore is judged to be \"now\"."}, "sentence_type": "Negative declarative sentence", "pos": {"nouns": {"Couples": 2, "Emotion": 1, "Not good": 1}}}, {"content": "10. Separation of spouses (due to disagreement)", "word_count": 7, "label": "", "sentence_structure": "Subject鈥搗erb鈥揷omplement", "subjective_objective": {"answer": "Objective", "rationale": "Since separation of spouses (because of discord) is an objective state of affairs, it can be ascertained through investigation and knowledge, not by the patient ' s subjective feelings or thoughts."}, "sentence_sentiment": {"answer": "Negative", "rationale": "Because the \"separation\" in the entry is negative."}, "language_style": {"answer": "Tactful and warm", "rationale": "The language style of the entry is more euphemistic, because it uses the more neutral term \"separation of the couple\" and raises questions in the form of inquiries, rather than direct conclusions or accusations. This is more likely to generate resonance and response from the person under investigation."}, "time_dimension": {"answer": "Past", "rationale": ""}, "sentence_type": "Declarative sentence", "pos": {"nouns": {"Couples": 1, "Separation": 1, "- No, no, no": 1}, "verbs": {"Because": 1, "be": 1}, "numerals": {"10": 1}}}, {"content": "11. Separation of spouses (work requirements)", "word_count": 10, "label": "", "sentence_structure": "Subject鈥損redicate", "subjective_objective": {"answer": "Objective", "rationale": "Since the separation of spouses (required for work) is an objective condition of the patient ' s life."}, "sentence_sentiment": {"answer": "Neutral", "rationale": "Because the entry does not express a clear positive or negative sentiment, it merely describes a factual situation."}, "language_style": {"answer": "Calm and direct", "rationale": "The entry is simple and clear, and the interviewee is asked whether the separation of the spouses due to work has not been accompanied by excessive emotional colours and rhetorical techniques."}, "time_dimension": {"answer": "Future", "rationale": "The entry describes the possible future behaviour or state of affairs, i.e., the possibility of a future separation of spouses, because of the need for work, and therefore the judgement is the future."}, "sentence_type": "Declarative sentence", "pos": {"nouns": {"Couples": 1, "Separation": 1, "Work": 1}, "verbs": {"Yes": 1}, "numerals": {"11": 1}}}, {"content": "12. Sexually unsatisfactory or single", "word_count": 9, "label": "", "sentence_structure": "Subject鈥搗erb鈥揷omplement", "subjective_objective": {"answer": "Subjective", "rationale": "Because the satisfaction of sex and the fact that one is alone are the subjective feelings and choices of the patient."}, "sentence_sentiment": {"answer": "Negative", "rationale": "Because the terms \"sexually unsatisfactory\" and \"single\" have negative emotional connotations."}, "language_style": {"answer": "Tactful and warm", "rationale": ""}, "time_dimension": {"answer": "Present", "rationale": "The entry uses the current state of affairs, indicating that it is a question of asking the testee's current state and can therefore be judged as \"now\"."}, "sentence_type": "Interrogative sentence", "pos": {"nouns": {"Sex": 1, "Alone": 1}, "verbs": {"Dissatisfied": 1}, "numerals": {"12": 1}}}, {"content": "13. Spouses have had an affair", "word_count": 7, "label": "", "sentence_structure": "Subject鈥搗erb鈥揷omplement", "subjective_objective": {"answer": "Subjective", "rationale": "Because \"an affair\" is a patient's subjective observation or guess, it's not an objective fact."}, "sentence_sentiment": {"answer": "Negative", "rationale": "Because the \"extraordinary\" behavior in the entry is negative."}, "language_style": {"answer": "Tactful and warm", "rationale": "This expression is more respectful of the feelings and dignity of the interviewee."}, "time_dimension": {"answer": "Past", "rationale": ""}, "sentence_type": "Negative declarative sentence", "pos": {"nouns": {"Spouse": 1, "An affair": 1}, "verbs": {"have": 1}, "numerals": {"XIII": 1}}}, {"content": "14. Couples are getting back together.", "word_count": 6, "label": "", "sentence_structure": "Subject鈥搗erb鈥揷omplement", "subjective_objective": {"answer": "Subjective", "rationale": "This is the patient鈥檚 subjective idea or desire. While the 鈥渞e-integration鈥?may be an objective result (if the couple does get along well), it is subjective that the patient is expressing their hopes or expectations in this matter."}, "sentence_sentiment": {"answer": "Neutral", "rationale": "Because the entry does not have any emotional color."}, "language_style": {"answer": "Tactful and warm", "rationale": "The entry uses a more euphemistic expression, by asking the interviewees whether they have experienced \"re-integration\" and trying to understand their experiences and feelings in their marriage life, and is not too direct or stimulating in language. Thus, it is judged that the language style of the purpose is euphemism."}, "time_dimension": {"answer": "Past", "rationale": ""}, "sentence_type": "Affirmative declarative sentence", "pos": {"nouns": {"Couples": 1, "Revert": 1, "Okay": 1}}}, {"content": "15. Over-indicated fertility", "word_count": 5, "label": "", "sentence_structure": "Subject鈥搗erb鈥揷omplement", "subjective_objective": {"answer": "Subjective", "rationale": "Because the excess of the target for childbirth is the subjective behaviour of the patient or his family."}, "sentence_sentiment": {"answer": "Negative", "rationale": "Because the act of \"super-indicative fertility\" is against the rules and morality and has a negative emotional connotation."}, "language_style": {"answer": "Calm and direct", "rationale": "The language style of the entry is relatively cold and straightforward, without any rhetorical or emotional rendering, but simply raises a question of investigation. Such language styles may give a serious, calm feeling that they are suitable for use when dealing with sensitive topics or when precise data are needed."}, "time_dimension": {"answer": "Past", "rationale": "However, it is not possible to determine whether past or present behaviour is being asked for lack of a specific time frame (e.g., in the past year, in the last birth, etc.)."}, "sentence_type": "Affirmative declarative sentence", "pos": {"nouns": {"Super-indicated births": 1}}}, {"content": "16. I have myself (love) sterilization.", "word_count": 9, "label": "", "sentence_structure": "Subject鈥搗erb鈥搊bject", "subjective_objective": {"answer": "Subjective", "rationale": "Because the question is about the subjective will and choice of the individual or his/her lover, it is subjective in its perspective."}, "sentence_sentiment": {"answer": "Negative", "rationale": "Because this entry expresses a behaviour that limits fertility, it may lead to some negative emotions and attitudes."}, "language_style": {"answer": "Tactful and warm", "rationale": "The language style of the entry is relatively gentle, using the word \"personal\" rather than \"directly \"you or you\" and is more subtle in terms of the subject matter of expression, not asking too directly about personal privacy, but rather by asking whether the relevant surgery has been performed."}, "time_dimension": {"answer": "Present", "rationale": "The use of the keywords \"personal\" and \"love\" indicates that the entry is about the person being tested or his partner, and that the act of \"sterilisation\" is ongoing and thus the temporal dimension can be judged to be the present."}, "sentence_type": "Imperative sentence", "pos": {"nouns": {"In person": 1, "Lover": 1, "The operation": 1}, "verbs": {"Do it": 1, "Sterilization": 1}}}, {"content": "17. Death of spouse", "word_count": 4, "label": "", "sentence_structure": "Subject鈥揷opula鈥損redicative", "subjective_objective": {"answer": "Objective", "rationale": "Because the life and death of the spouse is an objective fact, it is not influenced by the patient ' s subjective will."}, "sentence_sentiment": {"answer": "Negative", "rationale": "Because the \"spouse death\" in the entry is negative."}, "language_style": {"answer": "Calm and direct", "rationale": "The language style of the entry is calm and straightforward. It clearly asks the interviewee whether he or she has experienced the death of a spouse in his or her past life, without using any rhetorical or emotional terms, and with a more objective and neutral tone. This expression allows the interviewee to answer the question more accurately and truthfully."}, "time_dimension": {"answer": "Past", "rationale": "The entry describes events that have occurred at a given point in time, namely, \"spouse death\". Thus, in terms of time dimension, it belongs to the past."}, "sentence_type": "Affirmative declarative sentence", "pos": {"nouns": {"Spouse": 1, "Death": 1}}}, {"content": "18. Divorce", "word_count": 2, "label": "", "sentence_structure": "Subject鈥搗erb鈥揷omplement", "subjective_objective": {"answer": "Objective", "rationale": "Because divorce is an objective fact, not a subjective feeling or attitude of the patient."}, "sentence_sentiment": {"answer": "Negative", "rationale": "Because \"divorce\" is often considered a negative social event, it may be associated with negative situations such as family breakdown, emotional injury, etc."}, "language_style": {"answer": "Calm and direct", "rationale": "The entry directly asked the respondents whether they had had any experience of divorce, were clear and made plain, and were not subject to excessive rhetoric or emotional rendering, so that they were more calm and straightforward."}, "time_dimension": {"answer": "Past", "rationale": "The keyword is \"divorce,\" which is an event that has already occurred, and therefore the temporal dimension is the past."}, "sentence_type": "Imperative sentence", "pos": {"nouns": {"Divorce": 1}}}, {"content": "19. Failure of children to pursue (employment) education", "word_count": 8, "label": "", "sentence_structure": "Subject鈥搗erb鈥搊bject", "subjective_objective": {"answer": "Subjective", "rationale": "Because \"child failure in education (employment)\" is a subjective assessment and the result of the interviewee's judgement of the child's performance based on his or her own observations and understanding."}, "sentence_sentiment": {"answer": "Negative", "rationale": "Because the \"child failure\" in the entry is negative."}, "language_style": {"answer": "Calm and direct", "rationale": "The entry uses a direct question-and-answer approach to ask if the surveyed person has experienced any failure in the promotion or employment of his or her children, with a more objective and calm language style, without excessive ado and emotional colour."}, "time_dimension": {"answer": "Present", "rationale": "The entry asks about the current status or feelings of the testee, the keyword \"child failure\" and the event has already occurred and has an impact on the testee. The temporal dimension therefore is now judged."}, "sentence_type": "Negative declarative sentence", "pos": {"nouns": {"Children": 1, "Promotion": 1, "Employment": 1, "Failed": 1}, "numerals": {"19": 1}}}, {"content": "20. Child-rearing difficulties", "word_count": 6, "label": "", "sentence_structure": "Subject鈥搗erb鈥揷omplement", "subjective_objective": {"answer": "Subjective", "rationale": "Because \"child control difficulties\" are subjective perceptions and evaluations of the patient's situation, not an objective fact or behaviour."}, "sentence_sentiment": {"answer": "Negative", "rationale": "Because the \"child control difficulties\" act is negative."}, "language_style": {"answer": "Calm and direct", "rationale": "The entry directly expresses the subject of questioning whether the other party has encountered difficulties in child discipline, without using excessive rhetoric or emotional rendering, and with a more objective tone."}, "time_dimension": {"answer": "Future", "rationale": "The entry is a forecast and a vision of possible problems or challenges in the future, with the keywords \"children\" and \"recipient difficulties\" pointing to situations that have not yet occurred and that may arise in the future. The temporal dimension of the article is therefore judged to be the future."}, "sentence_type": "Interrogative sentence", "pos": {"nouns": {"Children": 1, "Corrections": 1, "Difficulties": 1}, "numerals": {"20": 1}}}, {"content": "21. Long-term absence of children", "word_count": 6, "label": "", "sentence_structure": "Subject鈥搗erb鈥揷omplement", "subjective_objective": {"answer": "Subjective", "rationale": "The question is the patient ' s feelings and perception of the child ' s prolonged absence from the home, which is subjective."}, "sentence_sentiment": {"answer": "Negative", "rationale": "Because the \"severe-day absence\" in the entry may lead to a negative feeling of loneliness, loss, etc."}, "language_style": {"answer": "Tactful and warm", "rationale": "The entry, in a gentle and understanding way, asked the question of whether the children of the respondents had been separated for a long time, avoiding the use of language that was too rigid or offensive and was more intimate and concerned."}, "time_dimension": {"answer": "Past", "rationale": ""}, "sentence_type": "Negative declarative sentence", "pos": {"nouns": {"Children": 1, "Home": 1}, "verbs": {"Leave home": 1}, "numerals": {"21": 1}}}, {"content": "22. Parental discord", "word_count": 4, "label": "", "sentence_structure": "Subject鈥損redicate", "subjective_objective": {"answer": "Subjective", "rationale": "Because \"uncorrupt\" is a subjective view, it is suggested that there is conflict or contradiction between parents."}, "sentence_sentiment": {"answer": "Negative", "rationale": "Because the phenomenon of \"parent discord\" in the entry is usually considered negative."}, "language_style": {"answer": "Tactful and warm", "rationale": "The language style of the entry is more euphemistic, moderate, not too direct or hard-line, but rather a caring and understanding approach to guide respondents to express their views and feelings. At the same time, the more common descriptive term \"parent discord\" is used, rather than the more direct use of negative terms such as \"divorce,\" which make it easier for respondents to accept and share their real ideas."}, "time_dimension": {"answer": "Past", "rationale": ""}, "sentence_type": "Negative declarative sentence", "pos": {"nouns": {"parents": 1, "- No, no, no": 1}}}, {"content": "23. Family economic difficulties", "word_count": 6, "label": "", "sentence_structure": "Subject鈥搗erb鈥揷omplement", "subjective_objective": {"answer": "Objective", "rationale": "Since the difficulty of the family economy is an objective fact, it can be assessed and judged through statistical data, financial statements, etc."}, "sentence_sentiment": {"answer": "Negative", "rationale": "Because the description of \"family economic difficulties\" is usually considered a negative situation."}, "language_style": {"answer": "Calm and direct", "rationale": "The entry is presented in a simple statement, without too much emotional rendering or rhetorical techniques, and is more objectively and directly a matter of asking the other party whether there is family financial hardship. The language style of the purpose can therefore be described as \"cooling straight\"."}, "time_dimension": {"answer": "Past", "rationale": "The entry does not have a clear time reference point, but \"family economic difficulties\" usually refer to the current or past period, so we can judge it as past."}, "sentence_type": "Affirmative declarative sentence", "pos": {"nouns": {"Family": 1, "Economy": 1, "Difficulties": 1}, "numerals": {"23": 1}}}, {"content": "24. Over $500 in debt", "word_count": 5, "label": "", "sentence_structure": "Subject鈥搗erb鈥揷omplement", "subjective_objective": {"answer": "Subjective", "rationale": "Because everyone鈥檚 subjective judgment may differ on the question of whether or not to pay the debt, for example, some may think that over $500 is a big deal, while others may think that this is a small amount. So the question is the subjective idea of looking at patients."}, "sentence_sentiment": {"answer": "Negative", "rationale": "Because the \"debt\" act is negative."}, "language_style": {"answer": "Calm and direct", "rationale": "The language style of the entry is more straightforward, there is no excessive rhetorical or emotional rendering, and the tone is more neutral and objective, expressing a query as to whether the person interviewed is in debt of more than $500."}, "time_dimension": {"answer": "Present", "rationale": "The entry asks if the person tested is now in debt of over $500, and the keywords \"in debt\" and \"over $500\" indicate that it is asking the person tested about the current status or current situation and therefore judging as now."}, "sentence_type": "Affirmative declarative sentence", "pos": {"nouns": {"Debt": 1, "Dollar": 1}, "verbs": {"Own": 1}, "numerals": {"500": 1}}}, {"content": "25. Significant improvement in the economic situation", "word_count": 8, "label": "", "sentence_structure": "Subject鈥搗erb鈥揷omplement", "subjective_objective": {"answer": "Objective", "rationale": "Because \"a significant improvement in the economic situation\" is an objective fact of the patient's experience and can be demonstrated by data."}, "sentence_sentiment": {"answer": "Positive", "rationale": "Because the expression \"a significant improvement in the economic situation\" is positive and indicates a better economic situation."}, "language_style": {"answer": "Calm and direct", "rationale": ""}, "time_dimension": {"answer": "Present", "rationale": "The entry asks if the present economic situation of the testee has improved significantly and the time dimension is now judged."}, "sentence_type": "Affirmative declarative sentence", "pos": {"nouns": {"Economic conditions": 1, "Improvement": 1}, "verbs": {"Not significant": 1, "Increase": 1, "Improvement": 1}, "adjectives": {"Not significant": 1, "Obviously": 1, "Increase": 1}, "numerals": {"25": 1}}}, {"content": "26. Severely ill and seriously injured family members", "word_count": 8, "label": "", "sentence_structure": "Subject鈥搗erb鈥揷omplement", "subjective_objective": {"answer": "Objective", "rationale": "It is an objective fact that the family member is described as having a serious illness and serious injury as a family reality."}, "sentence_sentiment": {"answer": "Negative", "rationale": "Because \"serious illness, serious injury\" is usually negative and can cause great distress and stress."}, "language_style": {"answer": "Calm and direct", "rationale": ""}, "time_dimension": {"answer": "Present", "rationale": ""}, "sentence_type": "Declarative sentence", "pos": {"nouns": {"Family members": 1, "Serious illness": 1, "He's seriously injured": 1}, "numerals": {"26": 1}}}, {"content": "27. Death of family members", "word_count": 6, "label": "", "sentence_structure": "Subject鈥搗erb鈥揷omplement", "subjective_objective": {"answer": "Objective", "rationale": "Because \"death of a family member\" is an objective fact, independent of the patient's subjective consciousness."}, "sentence_sentiment": {"answer": "Negative", "rationale": "Because the \"death of a family member\" incident in the entry is negative."}, "language_style": {"answer": "Calm and direct", "rationale": "The subject is asked directly whether the interviewee has lost family members, the language style is relatively calm and objective, and there is no excessive emotional rendering or adoration, making the issue more straightforward."}, "time_dimension": {"answer": "Past", "rationale": "The entry is a description of the time dimension of the testee ' s experience of the death of a family member and therefore of the past."}, "sentence_type": "Negative declarative sentence", "pos": {"nouns": {"Family": 1, "Members": 1, "Death": 1}, "numerals": {"27": 1}}}, {"content": "28. I am seriously ill or seriously injured", "word_count": 7, "label": "", "sentence_structure": "Subject鈥搗erb鈥揷omplement", "subjective_objective": {"answer": "Subjective", "rationale": "Subjective: Because \"he/she is seriously ill or seriously injured\" is a patient's subjective feeling and self-judgment. While there may be some objective evidence of this, such as the doctor's diagnosis, his/her state of health, etc., knowledge of the severity of the condition depends primarily on the individual's feelings and subjective assessment."}, "sentence_sentiment": {"answer": "Negative", "rationale": "Because the entry \"seriously ill or seriously injured\" is negative."}, "language_style": {"answer": "Calm and direct", "rationale": "The entry expresses concern about the health of the person under investigation in the form of a direct query, with no excessive qualifier or emotional colour, and a more concise language style."}, "time_dimension": {"answer": "Past", "rationale": "The entry asks if the person tested has experienced serious physical illness or injury in the past and therefore determines the past."}, "sentence_type": "Negative declarative sentence", "pos": {"nouns": {"In person": 1, "Serious illness": 1, "He's seriously injured": 1}}}, {"content": "29. Housing stress", "word_count": 4, "label": "", "sentence_structure": "Subject鈥揷opula鈥損redicative", "subjective_objective": {"answer": "Subjective", "rationale": "Because housing stress is a subjective experience and feeling, different people may have different views."}, "sentence_sentiment": {"answer": "Negative", "rationale": "Basis of judgement: Housing stress is usually seen as a problem or challenge and is thus expressed as negative."}, "language_style": {"answer": "Calm and direct", "rationale": "The entry is a simple and clear expression of the pressure on the subject in the housing context, which is not based on excessive rhetorical or emotional rendering, and is more objective in tone."}, "time_dimension": {"answer": "Past", "rationale": "The entry asks about the current housing situation of the person under examination, and therefore the temporal dimension is a matter of the past."}, "sentence_type": "Negative declarative sentence", "pos": {"nouns": {"Housing": 1, "Nervous": 1}, "numerals": {"29": 1}}}, {"content": "30. Joblessness, unemployment", "word_count": 4, "label": "", "sentence_structure": "Subject鈥搗erb鈥揷omplement", "subjective_objective": {"answer": "Subjective", "rationale": "Since waiting for work and being unemployed is the patient ' s own professional status, it is a subjective information for the individual."}, "sentence_sentiment": {"answer": "Negative", "rationale": "Because \"waiting to work, unemployed\" is often considered a negative social state and has a negative emotional connotation."}, "language_style": {"answer": "Calm and direct", "rationale": "The entry is more straightforward in language style, with no excessive rhetorical or emotional rendering. It simply asks whether the person under investigation is in a state of waiting or being unemployed, is more formal and objective, lacks some kind of cultural attention or warm expression."}, "time_dimension": {"answer": "Past", "rationale": "The entry describes the current employment status of the testees, the terms \"waiting to work\" and \"unemployed\" indicating that they are currently unemployed, have experienced or are in the present."}, "sentence_type": "Negative declarative sentence", "pos": {"nouns": {"Waiting for a job": 1, "No job": 1}}}, {"content": "31. Starting employment", "word_count": 4, "label": "", "sentence_structure": "Subject鈥損redicate", "subjective_objective": {"answer": "Objective", "rationale": "Since it is an objective fact to begin employment, it is not necessary to determine whether it is possible to do so through subjective judgement."}, "sentence_sentiment": {"answer": "Neutral", "rationale": "Because the entry does not have any emotional color."}, "language_style": {"answer": "Calm and direct", "rationale": "The entry explicitly enquired about the employment situation of the respondents, without any euphemism or rhetorical techniques, was straightforward and more suitable for actual data collection and statistical analysis."}, "time_dimension": {"answer": "Future", "rationale": "The entry expresses the action that will be taken, and \"start employment\" is a future plan or decision, and therefore the temporal dimension is the future."}, "sentence_type": "Imperative sentence", "pos": {"nouns": {"Employment": 1}, "verbs": {"Start": 1}}}, {"content": "32. Failure in the examination for advanced examinations", "word_count": 4, "label": "", "sentence_structure": "Subject鈥搗erb鈥揷omplement", "subjective_objective": {"answer": "Objective", "rationale": "Because failure is an objective event, it is the result of the patient ' s experience, not their subjective attitude or emotional state."}, "sentence_sentiment": {"answer": "Negative", "rationale": "Because failure in the examination is a negative event, the emotional and psychological stress on people is negative."}, "language_style": {"answer": "Calm and direct", "rationale": "This entry is very straightforward in expressing the theme of \"failure of the High Test\", without any abbreviation or euphemism, and is more cold and plain in the tone, which is a blunt and straightforward language style."}, "time_dimension": {"answer": "Present", "rationale": ""}, "sentence_type": "Negative declarative sentence", "pos": {"nouns": {"High examination": 1, "Failed": 1}, "verbs": {"Failed": 1}, "numerals": {"32": 1}}}, {"content": "33. Deduction of bonuses or fines", "word_count": 7, "label": "", "sentence_structure": "Subject鈥搗erb鈥揷omplement", "subjective_objective": {"answer": "Subjective", "rationale": "Since the withholding of a bonus or fine is an objective fact determined by the company or organization, the patient ' s response is a personal view and perception of this objective fact and is therefore subjective."}, "sentence_sentiment": {"answer": "Negative", "rationale": "Because \"deduction of a bonus or a fine\" is a punitive act, it usually leads to negative emotional reactions."}, "language_style": {"answer": "Calm and direct", "rationale": "The entry uses a more direct language style, specifically asking the interviewees whether they have ever been subjected to a company withholding a bonus or a fine, without excessive rhetorical or emotional rendering, and with a more objective tone."}, "time_dimension": {"answer": "Present", "rationale": "The keyword \"discharge of a bonus or a fine\" suggests that it is a form of punishment for the current company and therefore a state of affairs."}, "sentence_type": "Imperative sentence", "pos": {"nouns": {"Award": 1, "Fine": 1}, "verbs": {"Block": 1}, "numerals": {"33": 1}}}, {"content": "34. Outstanding individual achievements", "word_count": 7, "label": "", "sentence_structure": "Subject鈥搗erb鈥揷omplement", "subjective_objective": {"answer": "Subjective", "rationale": "Because the outstanding personal achievement is the subjective evaluation of the patient ' s own experience."}, "sentence_sentiment": {"answer": "Positive", "rationale": "Because the expression \"prominent personal achievement\" in the entry is positive, it is an expression of the excellence and success of the respondents in certain respects."}, "language_style": {"answer": "Calm and direct", "rationale": ""}, "time_dimension": {"answer": "Present", "rationale": "The key word for the entry, \"Preminent personal achievement\", implies that the testee hopes to achieve some notable achievements in the future. The temporal dimension is therefore judged to be the future."}, "sentence_type": "Affirmative declarative sentence", "pos": {"nouns": {"Personal achievements": 1, "Highlight": 1}, "adjectives": {"Highlight": 1}, "numerals": {"34": 1}}}, {"content": "Promotion, promotion", "word_count": 4, "label": "", "sentence_structure": "Subject鈥損redicate", "subjective_objective": {"answer": "Objective", "rationale": "Since promotions, promotions are the decisions of organizations or companies, they are not the subjective feelings of the patients themselves."}, "sentence_sentiment": {"answer": "Positive", "rationale": "Since promotion, promotion or promotion means promotion to a career, it is usually considered positive behaviour."}, "language_style": {"answer": "Calm and direct", "rationale": "The language style of the entry is relatively simple and not overly retrofitted, and the interviewee is asked whether he or she is concerned about promotion or promotion and is more indifferent and objective."}, "time_dimension": {"answer": "Future", "rationale": "The entry asks if the person tested expects to be promoted or promoted in the future, and the time dimension is judged to be future."}, "sentence_type": "Imperative sentence", "pos": {"nouns": {"Promotion": 1, "Upgrade": 1}}}, {"content": "36. Dissatisfaction with current service", "word_count": 8, "label": "", "sentence_structure": "Subject鈥揷opula鈥損redicative", "subjective_objective": {"answer": "Subjective", "rationale": "Because \"unsatisfied with the job at work\" is a patient's subjective attitude or feeling, not an objective fact or result."}, "sentence_sentiment": {"answer": "Negative", "rationale": "Because the attitude in the entry \"unsatisfied with the job in the job in hand\" is negative."}, "language_style": {"answer": "Calm and direct", "rationale": "The entry is more straightforward in language style, does not have too many emotional renderings or speech techniques, and is more objective and direct in language, directly asking the other side about the satisfaction of the current job."}, "time_dimension": {"answer": "Present", "rationale": "The entry represents the current emotional state of the testee, i.e. dissatisfaction or dissatisfaction with the current job, so the temporal dimension is now judged."}, "sentence_type": "Exclamatory sentence", "pos": {"nouns": {"Current position": 1, "Work": 1}, "verbs": {"Satisfied": 1}, "numerals": {"36": 1}}}, {"content": "37. High stress in work learning (e.g. poor performance)", "word_count": 13, "label": "", "sentence_structure": "Subject鈥揷opula鈥損redicative", "subjective_objective": {"answer": "Subjective", "rationale": "Since stress and self-evaluation are patients ' subjective feelings and ideas, the fact that work learning is good and bad is an objective fact."}, "sentence_sentiment": {"answer": "Negative", "rationale": "Because the words \"pressure\" and \"bad grades\" in the entries all have negative emotional connotations."}, "language_style": {"answer": "Calm and direct", "rationale": "The entry asks the interviewees in a direct manner whether they are under stress during their work or study and do not use excessive rhetoric or emotional colours, so that the language style is more calm and straightforward."}, "time_dimension": {"answer": "Present", "rationale": "The keyword \"work learning\" indicates that it is an ongoing behavior or state, and \"pressure (e.g. poor performance)\" is a description and description of the state. The entry is therefore an inquiry into the current situation of the person being tested."}, "sentence_type": "Declarative sentence", "pos": {"nouns": {"Work": 1, "Learning": 1, "Pressure": 1, "Achievements": 1}, "verbs": {"Large": 1}, "numerals": {"37": 1}}}, {"content": "38. Tensions with superiors", "word_count": 7, "label": "", "sentence_structure": "Subject鈥搗erb鈥揷omplement", "subjective_objective": {"answer": "Subjective", "rationale": "Because tension at the higher levels is a subjective feeling and experience of a person, not an objective state or fact."}, "sentence_sentiment": {"answer": "Negative", "rationale": "Because the description of \"relationship\" in the entry is negative and indicates that there is a problem with the relationship with the superior."}, "language_style": {"answer": "Calm and direct", "rationale": "The entry is asked in an objective and direct manner, without too many emotional qualifiers or rhetorical techniques, which conveys the subject of questioning whether there is tension with the superior."}, "time_dimension": {"answer": "Present", "rationale": "The entry is for the purpose of asking the testee about his current state or feelings, and the keyword \"strained relations with superiors\" indicates a problem or contradiction at present, so the temporal dimension is now judged."}, "sentence_type": "Negative declarative sentence", "pos": {"nouns": {"38": 1, "Superior": 1, "Relations": 1, "Nervous": 1}}}, {"content": "39. Disagreement with colleagues ' neighbours", "word_count": 7, "label": "", "sentence_structure": "Subject鈥搗erb鈥揷omplement", "subjective_objective": {"answer": "Subjective", "rationale": "Because \"uncorruption with a colleague's neighbour\" is the patient's description and feelings of his or her situation, and it is subjective."}, "sentence_sentiment": {"answer": "Negative", "rationale": "Because \"uncorruptment with a colleague's neighbor\" is negative."}, "language_style": {"answer": "Calm and direct", "rationale": "The entry directly asked whether the respondents had been in conflict with their colleagues ' neighbours, had not been over-spoken or emotional, and was more objective in tone."}, "time_dimension": {"answer": "Present", "rationale": "The entry asks the testees about their current situation, i.e. whether they have problems with their colleagues ' neighbours, and therefore it is now judged."}, "sentence_type": "Interrogative sentence", "pos": {"nouns": {"Co-workers": 1, "Neighbors": 1, "and": 1}, "verbs": {"- No, no, no": 1}, "numerals": {"39": 1}}}, {"content": "First time away from home.", "word_count": 9, "label": "", "sentence_structure": "Subject鈥搗erb鈥揷omplement", "subjective_objective": {"answer": "Subjective", "rationale": "Because \"leave the country\" is a subjective experience and feeling."}, "sentence_sentiment": {"answer": "Neutral", "rationale": "Because the entry simply describes a fact, there is no apparent emotional tendency."}, "language_style": {"answer": "Tactful and warm", "rationale": ""}, "time_dimension": {"answer": "Present", "rationale": "The entry is about the testee鈥檚 past, especially with regard to leaving his home and living in another country. So the time dimension is the past. The keywords 鈥渇irst time鈥?and 鈥渄istant journey鈥?point to this past event."}, "sentence_type": "Affirmative declarative sentence", "pos": {"nouns": {"First time": 1, "Go far": 1, "Home": 1, "Foreign countries": 1}, "verbs": {"Go on": 1}, "numerals": {"40": 1}}}, {"content": "41. Significant changes in living patterns (change in eating and sleeping patterns)", "word_count": 16, "label": "", "sentence_structure": "Subject鈥搗erb鈥揷omplement", "subjective_objective": {"answer": "Objective", "rationale": "The situation is that the people are living in a situation where they are living in a situation of extreme change."}, "sentence_sentiment": {"answer": "Negative", "rationale": "Because \"a major change in the patterns of life\" is usually considered negative and may affect the physical and mental health of people."}, "language_style": {"answer": "Calm and direct", "rationale": "The language style of the entry is calm and straightforward. It describes in a concise and straightforward way a change in the life of the person under investigation, namely a major change in diet and sleep habits, without any qualifying vocabulary or emotional expression, aimed at obtaining objective information about the actual situation of the person under investigation."}, "time_dimension": {"answer": "Present", "rationale": "The entry describes the current state of life, the key words being \"life patterns\" and \"significant changes\" that the testee is experiencing or has already experienced. And, at the same time, \"reforms of eating and sleeping patterns\" are also an ongoing behavior, so time dimensions are now judged."}, "sentence_type": "Declarative sentence", "pos": {"nouns": {"Life": 1, "Change": 1, "Diet": 1, "Sleep": 1, "Pattern": 2}, "verbs": {"Significant": 1, "Change": 1}, "numerals": {"41": 1}}}, {"content": "42. I retire or do not have a specific job or other pressure", "word_count": 21, "label": "", "sentence_structure": "Subject鈥搗erb鈥搊bject", "subjective_objective": {"answer": "Subjective", "rationale": "The subject is one of the patient ' s own feelings and attitudes (with or without pressure) and is a subjective one."}, "sentence_sentiment": {"answer": "Neutral", "rationale": "Because the entry does not express a clear emotion, attitude, behaviour, purpose or quality, it merely objectively describes the person ' s state."}, "language_style": {"answer": "Tactful and warm", "rationale": "The entry, which asks the other side whether they are under pressure in a more euphemistic and polite manner to retire or not be assigned to specific tasks, is more expressions of concern and respect, making the interviewees more comfortable and willing to share their experiences."}, "time_dimension": {"answer": "Future", "rationale": "The entry contains visions and expectations for the future, with the keywords \"I retire or not have a specific job\" and \"other issues\" pointing to situations that have not yet occurred and that may arise in the future."}, "sentence_type": "Interrogative sentence", "pos": {"nouns": {"In person": 1, "Retirement leave": 1, "Not scheduled": 1, "Specific work": 1, "To-chan": 1, "Other issues": 1}, "verbs": {"Arrangements": 1, "Answer": 1}, "numerals": {"42": 1}}}, {"content": "43. Friends are seriously ill or seriously injured", "word_count": 7, "label": "", "sentence_structure": "Subject鈥搗erb鈥揷omplement", "subjective_objective": {"answer": "Subjective", "rationale": "Because the condition of a close friend is a serious illness or serious injury, it is necessary to evaluate the patient's own perception and feelings."}, "sentence_sentiment": {"answer": "Negative", "rationale": "Because the situation of \"good friends seriously ill or seriously injured\" in the entry usually causes negative feelings of grief and anxiety."}, "language_style": {"answer": "Tactful and warm", "rationale": "The entry is based on the expression \"good friend\" and uses the expression \"serious illness or serious injury\" in a more moderate way, without too direct or intense emotional colour, and more in the expression of concern and concern for friends, so the language style is more euphemistic and warm."}, "time_dimension": {"answer": "Future", "rationale": "The entry contains visions and expectations for the future, with the keyword \"serious illness or serious injury of a friend\", which points to situations that have not yet occurred and that may arise in the future."}, "sentence_type": "Exclamatory sentence", "pos": {"nouns": {"Friends": 1, "Serious illness": 1, "He's seriously injured": 1}, "numerals": {"43": 1}}}, {"content": "44. Death of a friend", "word_count": 4, "label": "", "sentence_structure": "Subject鈥搗erb鈥揷omplement", "subjective_objective": {"answer": "Subjective", "rationale": "Because the death of a friend is a subjective fact of personal experience and involves personal emotional and emotional reactions."}, "sentence_sentiment": {"answer": "Negative", "rationale": "Basis: The death of a friend is a very sad thing, and the purpose of this article is therefore negative."}, "language_style": {"answer": "Calm and direct", "rationale": "The entry directly asked the interviewees whether their friends had died, whether the language was simple and not too much emotionally modified or rendered, and how they expressed themselves calmly and objectively."}, "time_dimension": {"answer": "Past", "rationale": "The event in this entry is the deceased friend, a state that occurred at some point in the past and can therefore be judged as \"the past\"."}, "sentence_type": "Interrogative sentence", "pos": {"nouns": {"Friends": 1, "Death": 1}}}, {"content": "45. Misunderstanding, misdemeanor, false accusations, talk.", "word_count": 10, "label": "", "sentence_structure": "Subject鈥搗erb鈥揷omplement", "subjective_objective": {"answer": "Objective", "rationale": "Because \"the subject is misunderstood, misled, falsely accused, falsely spoken\" is an objective fact that a patient may encounter in life, not a subjective feeling or thought."}, "sentence_sentiment": {"answer": "Negative", "rationale": "Because the entry \"understanding, misperception, false accusations, talk\" is negative."}, "language_style": {"answer": "Calm and direct", "rationale": "The entry is more directly written, is not used too much rhetorical or emotionally, and is more objective in tone. It simply asks whether the person interviewed has been misunderstood, misled, falsely accused or talked about, a way that makes it easier to obtain real feedback and information from the person interviewed."}, "time_dimension": {"answer": "Present", "rationale": "The keyword \"understanding, miscalculation, false accusations, talk\" suggests that the act is a situation that the person being tested may encounter in the present, and thus the temporal dimension is now judged."}, "sentence_type": "Negative declarative sentence", "pos": {"nouns": {"person": 1, "Misunderstanding": 1, "Wrong": 1, "False accusations": 1, "Arguments": 1}, "verbs": {"By": 1, "Misunderstanding": 1, "Wrong": 1, "False accusations": 1, "Arguments": 1}, "numerals": {"45": 1}}}, {"content": "46. Civil legal disputes between intermediaries", "word_count": 8, "label": "", "sentence_structure": "Subject鈥搗erb鈥搊bject", "subjective_objective": {"answer": "Objective", "rationale": "Civil legal disputes are considered from an objective perspective, as they are an objective social phenomenon that is not influenced by the will of the individual."}, "sentence_sentiment": {"answer": "Negative", "rationale": "Because the \"civil law dispute\" in the entry is negative."}, "language_style": {"answer": "Calm and direct", "rationale": ""}, "time_dimension": {"answer": "Present", "rationale": ""}, "sentence_type": "Interrogative sentence", "pos": {"nouns": {"Civil legal disputes": 1}, "numerals": {"46": 1}}}, {"content": "47. Detention, trial", "word_count": 5, "label": "", "sentence_structure": "Subject鈥搗erb鈥揷omplement", "subjective_objective": {"answer": "Subjective", "rationale": "Because \"detention, trial\" is usually the result of the patient's personal experience and feelings, not of objective facts or behaviour. It is therefore a subjective view of the patient's experiences and feelings."}, "sentence_sentiment": {"answer": "Negative", "rationale": "Because the \"detention, trial\" in the entry is negative."}, "language_style": {"answer": "Tactful and warm", "rationale": "The entry uses more euphemistic language, such as \"detention\" and \"trial\" rather than direct or overly strong expressions to avoid unnecessary negative sentiments for the interviewee. Also, the \"please analyse the following questionnaire\" also uses a polite and respectful language style, which demonstrates the interest and importance of the interviewee."}, "time_dimension": {"answer": "Present", "rationale": "The entry asks whether the person tested has been detained or tried, and the temporal dimension is a matter of past."}, "sentence_type": "Affirmative declarative sentence", "pos": {"nouns": {"Detention": 1, "Trial": 1}, "verbs": {"By": 1}, "numerals": {"47": 1}}}, {"content": "48. Stolen, property losses", "word_count": 6, "label": "", "sentence_structure": "Subject鈥損redicate", "subjective_objective": {"answer": "Objective", "rationale": "The theft and the loss of property are objective facts."}, "sentence_sentiment": {"answer": "Negative", "rationale": "Because the expression \"theft, the loss of property\" is clearly negative and negative."}, "language_style": {"answer": "Calm and direct", "rationale": "The entry uses a concise language to describe the subject matter, without adding excessive adoration or emotional colour, and is more objective and direct in tone."}, "time_dimension": {"answer": "Present", "rationale": "The entry now asks the testee whether there is now a problem of theft or property loss, and the time dimension is now judged."}, "sentence_type": "Negative declarative sentence", "pos": {"nouns": {"Theft": 1, "Property": 1, "Losses": 1}, "numerals": {"48": 1}}}, {"content": "49. Accidents, accidents, natural disasters", "word_count": 12, "label": "", "sentence_structure": "Subject鈥損redicate", "subjective_objective": {"answer": "Objective", "rationale": "Because the occurrence of shock, accident and natural disaster are objective facts, they are not related to the subjective perception of the patient."}, "sentence_sentiment": {"answer": "Negative", "rationale": "Because \"unforeseen shock\", \"accident\" and \"natural disaster\" are negative events that can have negative human impacts."}, "language_style": {"answer": "Tactful and warm", "rationale": "The entry uses more neutral terms to describe the difficulties that may be encountered, avoids overly negative or violent expressions, and has some sympathy and understanding. At the same time, by using question-words, it is more friendly and friendly to give the respondents a greater sense of participation and space to express themselves."}, "time_dimension": {"answer": "Past", "rationale": "The entry describes events or situations that have occurred, including accidents, accidents and natural disasters, and therefore determines the past."}, "sentence_type": "Affirmative declarative sentence", "pos": {"nouns": {"Accident": 1, "Surprise": 1, "Accidents": 1, "Disasters": 1}, "numerals": {"49": 1}}}]}

{"questionnaire_title": "The Center uses depression tables.", "questionnaire_category": "Mental health", "item_count": 20, "emotion_shift_overall": "From negative to positive", "language_style_overall": "Tactful and warm", "items": [{"content": "1. I am upset by things that do not normally bother me.", "word_count": 15, "label": "", "sentence_structure": "Subject鈥搗erb鈥揷omplement", "subjective_objective": {"answer": "Subjective", "rationale": "Because \"fearing\" is what the patient feels like, and it's subjective."}, "sentence_sentiment": {"answer": "Negative", "rationale": "Because the \"fearing\" act in the entry is negative."}, "language_style": {"answer": "Calm and direct", "rationale": "The entry expresses a negative sentiment in a direct and concise manner, without excessive embellishment or emotionalization of the description, and is more calm and objective."}, "time_dimension": {"answer": "Present", "rationale": "The sentence expresses the state of affairs that \"some things that don't usually bother me now\" . Although these things may or will not really bother me, they bother me at this particular point in time. So, this entry is a description of what's happening."}, "sentence_type": "Negative declarative sentence", "pos": {"nouns": {"Yes": 2, "I": 1}, "verbs": {"鈾?And I'm gonna be 鈾?: 1, "I'm upset": 1}, "adjectives": {"Some": 1, "Usually": 1, "No, it's not": 1, "Trouble": 1}, "adverbs": {"Often": 1}}}, {"content": "I do not want to eat; I have a bad appetite.", "word_count": 11, "label": "", "sentence_structure": "Subject鈥搗erb鈥揷omplement", "subjective_objective": {"answer": "Subjective", "rationale": "The expression in the questionnaire entry, \"I don't want to eat\" and \"I don't want to eat\" are the feelings and experiences of the patient himself, reflecting the patient's subjective emotional and cognitive state. The content of the questionnaire should therefore be classified as \"subjective.\""}, "sentence_sentiment": {"answer": "Negative", "rationale": "Because the entries \"no food\" and \"bad appetite\" both express negative emotions and attitudes."}, "language_style": {"answer": "Tactful and warm", "rationale": "The entry uses more objective terms such as \"not wanting\" and \"not eating well\" to express the feeling of discomfort of the interviewee, and the temperature of the language and the absence of any blame or coercion are more in keeping with the euphemism of the language style."}, "time_dimension": {"answer": "Present", "rationale": "Now, the \"no food\" and \"no appetite\" in the entry show the person's current feelings and state, so the time dimension is now."}, "sentence_type": "Negative declarative sentence", "pos": {"nouns": {"Something": 1, "-Adulge": 1}, "verbs": {"Yes": 1, "Eat": 1, "have": 1}, "adjectives": {"Okay": 1}, "pronouns": {"I": 2}}}, {"content": "I feel that even with the help of a lover or friend, I cannot escape this distress.", "word_count": 22, "label": "", "sentence_structure": "Subject鈥搗erb鈥揷omplement", "subjective_objective": {"answer": "Subjective", "rationale": "I think it's the patient's personal subjective feelings and perceptions."}, "sentence_sentiment": {"answer": "Negative", "rationale": "Because the description \"can't get rid of this boredom\" in the entry is negative."}, "language_style": {"answer": "Calm and direct", "rationale": "The entry directly expresses the subject of the anguish that the other party feels insulated from when faced with a situation, and is not using excessive rhetorical or emotional rendering, which is more objective and balanced."}, "time_dimension": {"answer": "Present", "rationale": "This entry is an inquiry into the current mood and feelings of the subject, which does not address the time dimension of the past or the future, and therefore is judged as \"now\"."}, "sentence_type": "Negative declarative sentence", "pos": {"nouns": {"Questionnaire": 1, "Entry": 1, "Lover": 1, "friends": 1, "Help": 1, "I'm bored": 1}, "verbs": {"I think": 1, "have": 1, "Get rid of it": 1, "Yeah": 1}, "adjectives": {"This one": 1}, "adverbs": {"Even if": 1}, "pronouns": {"I": 1}}}, {"content": "I feel as good as anyone else.", "word_count": 9, "label": "", "sentence_structure": "Subject鈥揷opula鈥損redicative", "subjective_objective": {"answer": "Subjective", "rationale": "Because \"I feel\" is a term for patient subjective feelings, the question entry is subjective."}, "sentence_sentiment": {"answer": "Neutral", "rationale": "The entry is not a clear expression of positive or negative emotional sentiments."}, "language_style": {"answer": "Tactful and warm", "rationale": "The entry uses a more subjective expression that asks the other party whether it feels psychologically in the same position or level as others, and that is more likely to elicit resonance and emotional input from the person under investigation."}, "time_dimension": {"answer": "Past", "rationale": "It is suggested that the time frame for each question should be clearly indicated when designing the questionnaire."}, "sentence_type": "Affirmative declarative sentence", "pos": {"nouns": {"I": 1, "person": 1}, "verbs": {"Feel": 1, "Same": 1, "Okay": 1}, "adverbs": {"Same thing": 1}, "numerals": {"4": 1}}}, {"content": "5. It is difficult for me to concentrate on my work.", "word_count": 9, "label": "", "sentence_structure": "Subject鈥揷opula鈥損redicative", "subjective_objective": {"answer": "Subjective", "rationale": "Because \"hard to concentrate\" is a subjective feeling and experience, not an objective fact or result. While this may affect a person ' s behaviour and decision-making, it is an internal subjective experience of the individual, not the result of an external environment or event."}, "sentence_sentiment": {"answer": "Negative", "rationale": "Because the \"difficult to concentrate\" act in the entry is negative."}, "language_style": {"answer": "Calm and direct", "rationale": "The entry is simple and clear, does not have too many rhetorical or emotional features, directly presents the fact that the interviewee is unable to concentrate when he does his thing, and is more objective and calm."}, "time_dimension": {"answer": "Present", "rationale": "Now the entry is asking the testee's current feelings and state, and \"I have a hard time concentrating\" describes the current troubles and problems, and therefore the judgment is now."}, "sentence_type": "Negative declarative sentence", "pos": {"nouns": {"- The energy": 1, "Yes": 1}, "verbs": {"Do it": 1, "Focus": 1}, "adjectives": {"Hard": 1}}}, {"content": "I feel depressed.", "word_count": 5, "label": "", "sentence_structure": "Subject鈥揷opula鈥損redicative", "subjective_objective": {"answer": "Subjective", "rationale": "Because the word \"fEE\" shows that it is the patient's subjective feeling and experience."}, "sentence_sentiment": {"answer": "Negative", "rationale": "Basis: The entry expresses negative emotions and attitudes, and \"suffering\" is a negative emotional experience."}, "language_style": {"answer": "Calm and direct", "rationale": "The subject is a direct question to the interviewees about the depression they often feel, the language style is relatively simple, not over-modified, and is a direct expression of calm."}, "time_dimension": {"answer": "Past", "rationale": "The entry asks about the person ' s current state of feeling and emotion, and therefore the temporal dimension is a past."}, "sentence_type": "Negative declarative sentence", "pos": {"verbs": {"I feel": 1, "Repression": 1}, "adjectives": {"Repression": 1}}}, {"content": "I feel tired doing everything.", "word_count": 11, "label": "", "sentence_structure": "Subject鈥揷opula鈥損redicative", "subjective_objective": {"answer": "Subjective", "rationale": "Because this is a subjective experience, different people may feel differently about the same thing. For example, one person may feel tired doing the same work, while another person may feel very comfortable doing the same thing. So, \"I feel tired doing everything,\" is a subjective description that reflects personal feelings and experiences."}, "sentence_sentiment": {"answer": "Negative", "rationale": "Because the expression \"fears to be exhausted\" is a negative expression of emotions and attitudes."}, "language_style": {"answer": "Tactful and warm", "rationale": ""}, "time_dimension": {"answer": "Present", "rationale": ""}, "sentence_type": "Negative declarative sentence", "pos": {"verbs": {"I feel": 1, "Do it": 2, "Come on": 1}, "adjectives": {"very": 1}, "pronouns": {"I": 1}}}, {"content": "I feel that there is hope for the future.", "word_count": 8, "label": "", "sentence_structure": "Subject鈥揷opula鈥損redicative", "subjective_objective": {"answer": "Subjective", "rationale": "Because the word \"think\" expresses the patient's personal feelings and perceptions of the future, it is a subjective view of the patient."}, "sentence_sentiment": {"answer": "Positive", "rationale": "Because it expresses a sense of confidence and expectation for the future, it is positive emotional."}, "language_style": {"answer": "Tactful and warm", "rationale": ""}, "time_dimension": {"answer": "Future", "rationale": "The entry expresses the testee's vision and expectations for the future, and the word \"think\" is included to indicate a subjective feeling, and the term \"future\" suggests the temporal dimension of that feeling. The time dimension of the entry is therefore the future."}, "sentence_type": "Affirmative declarative sentence", "pos": {"verbs": {"I think": 1, "The future": 1, "Hope": 1}, "adjectives": {"Hope": 1}}}, {"content": "9. I think my life is useless.", "word_count": 11, "label": "", "sentence_structure": "Subject鈥揷opula鈥損redicative", "subjective_objective": {"answer": "Subjective", "rationale": "Because it's the patient's personal opinion and feelings."}, "sentence_sentiment": {"answer": "Negative", "rationale": "Because the entry expresses a negative attitude."}, "language_style": {"answer": "Tactful and warm", "rationale": "The language style of the entry is more euphemistic, starting with \"I think\" and leading the respondents to share their views and feelings about life, rather than directly denying or critical. This expression is more respectful of the interviewee and more effective in collecting their real thoughts and feelings."}, "time_dimension": {"answer": "Present", "rationale": "The entry expresses the current personal perception or mentality of the subject, does not address the past or future time dimension, and therefore the judgement is \"now\"."}, "sentence_type": "Negative declarative sentence", "pos": {"nouns": {"Life": 1}, "verbs": {"I think": 1, "Nothing": 1}, "adverbs": {"I": 1}}}, {"content": "I am afraid.", "word_count": 5, "label": "", "sentence_structure": "Subject鈥揷opula鈥損redicative", "subjective_objective": {"answer": "Subjective", "rationale": "Because fear is a subjective emotional experience."}, "sentence_sentiment": {"answer": "Negative", "rationale": "Because \"fear\" is a negative emotional experience."}, "language_style": {"answer": "Calm and direct", "rationale": "The entry uses a direct, clear language style to ask the subject of the fear that the other party is often afraid, without adding too many rhetorical or emotional renderings and is more objective in tone."}, "time_dimension": {"answer": "Past", "rationale": "The entry asks if the person tested had had a fearful experience at some point in the past and therefore judged it to be the past."}, "sentence_type": "Affirmative declarative sentence", "pos": {"verbs": {"I feel": 1, "Fear": 1}, "numerals": {"10": 1}}}, {"content": "I slept in the least.", "word_count": 6, "label": "", "sentence_structure": "Subject鈥搗erb鈥揷omplement", "subjective_objective": {"answer": "Subjective", "rationale": "Because \"deprivation\" is a feeling or experience, it needs to be observed and assessed from the patient's subjective perspective."}, "sentence_sentiment": {"answer": "Negative", "rationale": "Because sleep is often not enough to lead to physical and psychological fatigue."}, "language_style": {"answer": "Calm and direct", "rationale": "The entry does not use any rhetorical or emotional rendering, but rather expresses the question of the person ' s sleep problems by direct presentation. Moreover, the entry does not use any euphemism, tenderness or affection, but presents the problem in a direct, objective way. I therefore consider the language style of the entry to be \"cool and direct\"."}, "time_dimension": {"answer": "Past", "rationale": "The entry is for the current situation of the subject, does not address the time dimension of the future, and the keyword \"sleeping is not over\" indicates the current behaviour or state and is therefore judged to be past."}, "sentence_type": "Negative declarative sentence", "pos": {"verbs": {"Sleep": 1, "Solve the poverty": 1}, "adjectives": {"not": 1}, "adverbs": {"Ten": 1}, "numerals": {"One": 2}}}, {"content": "I am happy.", "word_count": 4, "label": "", "sentence_structure": "Subject鈥揷opula鈥損redicative", "subjective_objective": {"answer": "Subjective", "rationale": "Because \"very happy\" is a expression of subjective feelings or attitudes that needs to be assessed from the patient's personal experience and perception."}, "sentence_sentiment": {"answer": "Positive", "rationale": "Because the entry \"very happy\" expresses a positive emotion and attitude."}, "language_style": {"answer": "Tactful and warm", "rationale": "The entry is more euphemistic, simplistic and plain-worded. It does not use strong tone or emotional descriptions."}, "time_dimension": {"answer": "Present", "rationale": "The keyword \"very happy\" means the person who was tested for his or her current state, so the temporal dimension is now."}, "sentence_type": "Affirmative declarative sentence", "pos": {"verbs": {"very": 1, "Happiness": 1}}}, {"content": "I am less than usual.", "word_count": 7, "label": "", "sentence_structure": "Subject鈥揷opula鈥損redicative", "subjective_objective": {"answer": "Subjective", "rationale": "Because \"reduced\" is a subjective feeling and observed change that reflects the patient's subjective perception of his or her speech behaviour."}, "sentence_sentiment": {"answer": "Negative", "rationale": "Because the \"soldier\" act is negative."}, "language_style": {"answer": "Tactful and warm", "rationale": "The language style of the entry is euphemism. By using the comparison of \"more than usual\" it reduces the direct assessment of the other, avoiding the possible discomfort. At the same time, the entire sentence is more accommodative and gentler by speaking in a sense of enquiry and concern."}, "time_dimension": {"answer": "Present", "rationale": ""}, "sentence_type": "Affirmative declarative sentence", "pos": {"nouns": {"I": 1, "Word": 1, "Normal": 1}, "verbs": {"- Yeah": 1, "Less": 1, "Yes": 1}, "adverbs": {"More than usual": 1}, "numerals": {"13": 1}}}, {"content": "I feel lonely.", "word_count": 5, "label": "", "sentence_structure": "Subject鈥揷opula鈥損redicative", "subjective_objective": {"answer": "Subjective", "rationale": "Because being alone is a subjective feeling."}, "sentence_sentiment": {"answer": "Negative", "rationale": "Because \"loneliness\" in the entry is a negative emotion."}, "language_style": {"answer": "Tactful and warm", "rationale": "The language style of the entry is more euphemistic and sensitive to the individual ' s feelings, and the topic is developed in a way that invites the other party to share their own feelings, without direct accusations or questions, which are more acceptable and participatory."}, "time_dimension": {"answer": "Present", "rationale": ""}, "sentence_type": "Exclamatory sentence", "pos": {"nouns": {"Loneliness": 1}, "verbs": {"I feel": 1}}}, {"content": "15. I am not friendly.", "word_count": 7, "label": "", "sentence_structure": "Subject鈥搗erb鈥揷omplement", "subjective_objective": {"answer": "Subjective", "rationale": "The entry is subjective, since it is primarily concerned with patients鈥?subjective feelings and perceptions, i.e. whether they feel that others are not friendly with themselves."}, "sentence_sentiment": {"answer": "Negative", "rationale": "Because the \"unfriendly\" in the entry is a negative attitude."}, "language_style": {"answer": "Tactful and warm", "rationale": "This expression makes the interviewee feel more comfortable and safe and more likely to share their experiences and feelings."}, "time_dimension": {"answer": "Present", "rationale": "The entry uses the current state of affairs \"yes\", which means that it is either a current state or a regular experience, and therefore concerns the present behaviour or attitude."}, "sentence_type": "Negative declarative sentence", "pos": {"nouns": {"person": 1, "Tom": 2, "Unfriendly": 1}, "verbs": {"to": 1, "I": 1, "Friendly": 1}, "numerals": {"15": 1}}}, {"content": "I live happily ever after.", "word_count": 5, "label": "", "sentence_structure": "Subject鈥揷opula鈥損redicative", "subjective_objective": {"answer": "Subjective", "rationale": "Because happiness is a subjective feeling, each person's experience is different and one cannot be directly judged by observation as to whether one person is happy."}, "sentence_sentiment": {"answer": "Positive", "rationale": "Because the expression \"happiness in life\" is full of positive emotions."}, "language_style": {"answer": "Tactful and warm", "rationale": "The entry is more personal and interactive when it is spoken in a gentle and inviting manner, rather than in direct statements or orders. At the same time, the language style of the article is more relaxed, free of excessive pressure or stress, easier to relax the interviewee and to answer the questions in a true manner."}, "time_dimension": {"answer": "Present", "rationale": "\"I'm happy\" describes a state of affairs or a feeling, so the basis of the judgment is now."}, "sentence_type": "Affirmative declarative sentence", "pos": {"verbs": {"Life": 1, "Happy": 1}}}, {"content": "I used to cry.", "word_count": 7, "label": "", "sentence_structure": "Subject鈥搗erb鈥揷omplement", "subjective_objective": {"answer": "Objective", "rationale": "Because crying is an objective fact that patients experience, not subjective attitudes or ideas."}, "sentence_sentiment": {"answer": "Negative", "rationale": "Because the \"scream and cry\" act in the entry is negative."}, "language_style": {"answer": "Tactful and warm", "rationale": "The entry is more personal and interactive when it is spoken in a conversation with a voice of inquiry and understanding, rather than by a direct statement or order. It also gives a sense of comfort and understanding by using the \"had\" of the past."}, "time_dimension": {"answer": "Past", "rationale": "The entry \"was\" means \"screech\" is a past event, and \"screech\" is a past act. So this sentence describes an event that happened in the past, \"I used to cry.\""}, "sentence_type": "Affirmative declarative sentence", "pos": {"verbs": {"I'm crying": 1}}}, {"content": "I am sad.", "word_count": 5, "label": "", "sentence_structure": "Subject鈥揷opula鈥損redicative", "subjective_objective": {"answer": "Subjective", "rationale": "Because \"sorture\" is an emotional experience, a subjective feeling inside the patient."}, "sentence_sentiment": {"answer": "Negative", "rationale": "Because \"sort\" is negative."}, "language_style": {"answer": "Calm and direct", "rationale": "Calm down straight."}, "time_dimension": {"answer": "Past", "rationale": "The entry is for the testee ' s current feelings, and the time dimension is a past."}, "sentence_type": "Negative declarative sentence", "pos": {"verbs": {"I feel": 1, "Sad": 1}, "adjectives": {"Sad": 1}}}, {"content": "I think people hate me.", "word_count": 8, "label": "", "sentence_structure": "Subject鈥揷opula鈥損redicative", "subjective_objective": {"answer": "Subjective", "rationale": "Because \"I think\" expresses the patient's personal feelings and views, and it's a subjective examination."}, "sentence_sentiment": {"answer": "Negative", "rationale": "Because the idea of \"like people hate me\" in the entry is an expression of negative emotion."}, "language_style": {"answer": "Tactful and warm", "rationale": "The entry uses a more moderate and inviting tone of dialogue rather than a direct statement or order, which is more personal and interactive. At the same time, it avoids the use of too negative or negative language, and encourages respondents to think and share their thoughts and feelings in a more positive manner."}, "time_dimension": {"answer": "Present", "rationale": "The entry used to describe a subjective feeling or belief of the person being tested, that is, \"I think people dislike me.\" This feeling may have been caused by past experiences or events, and can therefore be judged as past."}, "sentence_type": "Negative declarative sentence", "pos": {"nouns": {"Questionnaire": 1, "Entry": 1, "person": 1, "Disgusting": 1}, "verbs": {"I think": 1, "Disgusting": 1}, "adverbs": {"Down there": 1}, "numerals": {"19": 1}, "classifiers": {"individual": 1}, "pronouns": {"I": 1}}}, {"content": "I walk very slowly.", "word_count": 5, "label": "", "sentence_structure": "Subject鈥搗erb鈥揷omplement", "subjective_objective": {"answer": "Objective", "rationale": "Because the speed of walking is a characteristic of behaviour that can be observed, it is examined from an objective perspective."}, "sentence_sentiment": {"answer": "Neutral", "rationale": "Because the entry describes a fact, it does not display a positive or negative emotional colour."}, "language_style": {"answer": "Tactful and warm", "rationale": "The language style of the entry is more euphemistic, with \"I'm slow\" to describe the pace of my walking, without too direct or negative expressions, and without a self-demeaning emotional colour, relatively gentle and respectful of each other."}, "time_dimension": {"answer": "Present", "rationale": "The entry describes the current state and condition of the person being tested, which is \"I walk very slowly\", so the temporal dimension is now."}, "sentence_type": "Affirmative declarative sentence", "pos": {"verbs": {"Let's go": 1, "Road": 1}, "adjectives": {"Slow": 1}}}]}

{"questionnaire_title": "Questionnaire on the relationship between the isolation of university students and family upbringing", "questionnaire_category": "Mental health", "item_count": 66, "emotion_shift_overall": "From positive to negative", "language_style_overall": "Tactful and warm", "items": [{"content": "I feel that my parents interfere with anything I do.", "word_count": 16, "label": "", "sentence_structure": "Subject鈥揷opula鈥損redicative", "subjective_objective": {"answer": "Subjective", "rationale": "The list of questions is based on the patient ' s subjective feelings and perceptions of his or her parents ' interference."}, "sentence_sentiment": {"answer": "Negative", "rationale": "Because the phrase \"parents interfere with anything I do\" in the entry is negative."}, "language_style": {"answer": "Calm and direct", "rationale": "The entry expresses in a straightforward manner the feelings of the surveyed person regarding the interference of parents in what they do, is not over-modified or emotionally rendered and is more objective in tone."}, "time_dimension": {"answer": "Present", "rationale": ""}, "sentence_type": "Negative declarative sentence", "pos": {"nouns": {"I": 2, "parents": 1, "Things": 1}, "verbs": {"I think": 1, "Interference": 1, "Do it": 1, "One": 1}, "adverbs": {"Any": 1}, "classifiers": {"One": 2}}}, {"content": "I can feel him/her very much in his/her words and in his/her face.", "word_count": 19, "label": "", "sentence_structure": "Subject鈥搗erb鈥揷omplement", "subjective_objective": {"answer": "Subjective", "rationale": "Because \"like me\" is an emotional attitude that requires subjective observation and interpretation by the patient to reach a conclusion."}, "sentence_sentiment": {"answer": "Positive", "rationale": "Because the entry \"likes me\" expresses a positive emotional attitude."}, "language_style": {"answer": "Tactful and warm", "rationale": "The entry is more personal and interactive when it is spoken in a gentle and inviting manner, rather than in direct statements or orders. At the same time, the entry uses the term \"sense\" to give a softer sense of the word, avoids the language style of direct accusations or criticism, and is more euphemism and warmer."}, "time_dimension": {"answer": "Past", "rationale": "The entry asks whether the person tested has experienced in the past a situation in which he or she feels liked through his or her parents鈥?words and expressions. This experience may have passed, so the temporal dimension is the past.\""}, "sentence_type": "Affirmative declarative sentence", "pos": {"nouns": {"parents": 1, "Talk": 1, "Emoticons": 1}, "verbs": {"Yes": 1, "Feel": 1}, "adjectives": {"very": 1}, "adverbs": {"Yeah": 1}, "classifiers": {"individual": 1}, "pronouns": {"I": 1, "he": 1, "she": 1}}}, {"content": "Parents love me more than my brothers and sisters.", "word_count": 15, "label": "", "sentence_structure": "Subject鈥搗erb鈥揷omplement", "subjective_objective": {"answer": "Subjective", "rationale": "This is a subjective sense and comparison of the patient, not an objective fact."}, "sentence_sentiment": {"answer": "Neutral", "rationale": "Because the entry describes an objective fact, there is no apparent emotional tendency."}, "language_style": {"answer": "Tactful and warm", "rationale": "The language style of the entry is more euphemistic and gentle, rather than directly accusing or criticizing the other, but rather raising the question in a convenient way, making the interviewee comfortable and comfortable. At the same time, the expression \"more loving\" is used to avoid too direct comparisons and to enhance objectivity and impartiality."}, "time_dimension": {"answer": "Present", "rationale": ""}, "sentence_type": "Affirmative declarative sentence", "pos": {"nouns": {"Questionnaire Entry": 1, "Brothers and sisters": 1, "parents": 2, "Pity": 1}, "verbs": {"Comparison": 1, "Pity": 1}, "numerals": {"III": 1}, "pronouns": {"I": 1}}}, {"content": "I can feel the love of my parents.", "word_count": 12, "label": "", "sentence_structure": "Subject鈥搗erb鈥揷omplement", "subjective_objective": {"answer": "Subjective", "rationale": "Because \"fear\" is the patient's subjective attitude."}, "sentence_sentiment": {"answer": "Positive", "rationale": "Because the entry \"can feel my parents' love\" expresses a positive emotion and attitude."}, "language_style": {"answer": "Tactful and warm", "rationale": "The language style of the entry is euphemistic and makes the issue more open and inclusive by using subjective expressions such as \"appreciation.\" At the same time, the issue also emphasizes emotional ties and interactions in family relationships, reflecting a caring and supportive attitude."}, "time_dimension": {"answer": "Present", "rationale": "Now the entry asks the testee how he feels about whether he or she can feel the love of his or her parents. Although this goes back to the past (for example, when the testee was a child), in this case, what matters is the current state of the testee鈥檚 feeling. So, time dimensions are now judged."}, "sentence_type": "Affirmative declarative sentence", "pos": {"nouns": {"I": 1, "parents": 1}, "verbs": {"I can feel it": 1, "Yes... love": 1}, "numerals": {"4锛?: 1}}}, {"content": "5. Even if it was a small fault, my parents punished me.", "word_count": 14, "label": "", "sentence_structure": "Subject鈥損redicate", "subjective_objective": {"answer": "Objective", "rationale": "Because the descriptions of \"small fault\" and \"parents punish me\" are based on the patient's objective experience. The patients' parents' attitudes and actions about wrong behaviour are examined, not subjective ideas."}, "sentence_sentiment": {"answer": "Negative", "rationale": "Because the \"punishment\" in the entry is negative."}, "language_style": {"answer": "Calm and direct", "rationale": "The entry is more direct in language style, with no too many emotional or rhetorical techniques. It succinctly expresses the view that the parents of the interviewee are harsh in terms of punishment for their faults as they grow up. Such tone helps the surveyer to understand more accurately the experience and feelings of the interviewee."}, "time_dimension": {"answer": "Past", "rationale": "The entry is for the purpose of questioning the testee's past experience, particularly with regard to parents' reactions to their behaviour, and therefore can be judged as \"the past\"."}, "sentence_type": "Negative declarative sentence", "pos": {"nouns": {"Wrong": 1, "parents": 1, "Punishment": 1}, "verbs": {"be": 1, "Yeah": 1, "Punishment": 1}, "adjectives": {"Very small": 1}, "numerals": {"5锛?: 1}, "pronouns": {"I": 1}}}, {"content": "Parents always try to submersion me, making me a remarkable person.", "word_count": 23, "label": "", "sentence_structure": "Subject鈥搗erb鈥揷omplement", "subjective_objective": {"answer": "Subjective", "rationale": "Because \"make someone a superior person\" is the subjective wish of parents."}, "sentence_sentiment": {"answer": "Positive", "rationale": "Because the words \"silent semantics\" and \"excellents\" in the entries are positive words that express parents' expectations and encouragement of their children."}, "language_style": {"answer": "Tactful and warm", "rationale": "The language style of the entry is euphemism. It expresses the conversation about the family relationship of the interviewee in a gentle and encouraging tone, using the terms \"smuggling\" and \"smuggling\" to convey positive messages and expectations, making the questionnaire more attractive and friendly."}, "time_dimension": {"answer": "Present", "rationale": "The behaviour of \"Parents always try to subdued me\" shows that it is a phenomenon that happens now, and \"The people who make me out of the future\" expresses expectations and visions for the future. So the entry is primarily about the sense of the testee of the current family environment and the expectations for the future."}, "sentence_type": "Affirmative declarative sentence", "pos": {"nouns": {"parents": 2, "Smuggle": 1, "Impact": 1, "person": 1, "It's a great idea": 1}, "verbs": {"Try": 1, "鈾?And I'm gonna be 鈾?: 1, "Be": 1}, "adverbs": {"Total": 1}, "classifiers": {"individual": 1}, "pronouns": {"I": 1}}}, {"content": "7. I feel that my parents have given me some uniqueness in some respects.", "word_count": 18, "label": "", "sentence_structure": "Subject鈥揷opula鈥損redicative", "subjective_objective": {"answer": "Subjective", "rationale": "Because \"think\" is a subjective term, which means the patient's feelings and perception, the perspective of this questionnaire is based on the patient's subjective perspective."}, "sentence_sentiment": {"answer": "Positive", "rationale": "According to the article, \"where I am allowed to have an exclusive presence\" can be considered as a positive emotional tendency to affirm and respect individual autonomy and personality."}, "language_style": {"answer": "Tactful and warm", "rationale": ""}, "time_dimension": {"answer": "Present", "rationale": "The entry describes the current feelings and views of the testee, using the general current. In it, \"the feeling\" suggests a subjective feeling or evaluation, and \"where I am allowed to be unique\" is a specific act or situation, which points to the current state and experience. So the answer is \"now\"."}, "sentence_type": "Affirmative declarative sentence", "pos": {"nouns": {"parents": 1, "In terms of": 1, "Where I come from": 1}, "verbs": {"Allow": 1, "have": 1}, "numerals": {"7": 1}, "pronouns": {"I": 1}}}, {"content": "8. Parents can give me what other siblings cannot get.", "word_count": 19, "label": "", "sentence_structure": "Subject鈥搗erb鈥搊bject", "subjective_objective": {"answer": "Subjective", "rationale": "Because this entry relates to patients ' perception and assessment of their parents and their subjective perception of whether they are treated with special or preferential treatment in the family."}, "sentence_sentiment": {"answer": "Positive", "rationale": "Because the purpose of the entry is to get what I can't get from other siblings."}, "language_style": {"answer": "Tactful and warm", "rationale": "The entry uses euphemism, the expression of doubt and compromise, and attempts to stimulate the reflection and reflection of the respondents and encourage them to share their views and feelings. This language makes people feel more friendly, open and equal, and more able to attract and stimulate the participants."}, "time_dimension": {"answer": "Past", "rationale": "The entry is asked about the interviewee's memory and observation of his family's past, and therefore judged as 鈥渢he past鈥?"}, "sentence_type": "Affirmative declarative sentence", "pos": {"nouns": {"parents": 1, "Brothers and sisters": 1, "Something": 1}, "verbs": {"Jean": 1, "Got it": 1}, "numerals": {"8锛?: 1}}}, {"content": "9. The punishment of my parents is fair.", "word_count": 11, "label": "", "sentence_structure": "Subject鈥揷opula鈥損redicative", "subjective_objective": {"answer": "Subjective", "rationale": "Because \"equity\" is a subjective assessment, different people have different views and feelings."}, "sentence_sentiment": {"answer": "Negative", "rationale": "Because the word \"punishment\" is derogatory and implies that there may be unfair situations."}, "language_style": {"answer": "Tactful and warm", "rationale": "The entry uses a more euphemistic approach to ask if the parents consider that their punishment is fair, and this expression is more compassionate and respectful of the views of the interviewee and more compassionate than direct questions."}, "time_dimension": {"answer": "Present", "rationale": "The keywords \"punish me\" and \"fair\" point to the state or situation, and therefore to the present."}, "sentence_type": "Affirmative declarative sentence", "pos": {"nouns": {"parents": 1, "Punishment": 1}, "verbs": {"be": 1, "Fair": 1}, "adjectives": {"Fair": 1}, "numerals": {"9锛?: 1}, "pronouns": {"I": 1}}}, {"content": "I feel that my parents are tough on me.", "word_count": 10, "label": "", "sentence_structure": "Subject鈥揷opula鈥損redicative", "subjective_objective": {"answer": "Subjective", "rationale": "Since this sentence relates to the patient ' s personal feelings and views, it is an examination of subjective views."}, "sentence_sentiment": {"answer": "Negative", "rationale": "Because the word \"draconian\" in the entry brings a negative emotional feeling."}, "language_style": {"answer": "Tactful and warm", "rationale": "The entry expresses the individual鈥檚 feelings in a first person鈥檚 words, is less scolding or critical of the language style of others, is more euphemistic and respectful. It also does not use strong adjectives or adverbs to emphasize emotions, but rather expresses its views in a peaceful way. So the language style of the questionnaire is euphemism."}, "time_dimension": {"answer": "Present", "rationale": "The main phrase in the sentence, \"I\" , shows the person who speaks, and \"I feel\" suggests that it is a subjective judgement, not a description of objective facts. So the time dimension of this entry should be now."}, "sentence_type": "Affirmative declarative sentence", "pos": {"nouns": {"parents": 1, "I": 1}, "verbs": {"to": 1, "I think": 1, "very": 1, "Hard": 1}, "adverbs": {"very": 1}, "numerals": {"10": 1}}}, {"content": "Parents always decide what I should wear and what I should dress up.", "word_count": 22, "label": "", "sentence_structure": "Subject鈥搗erb鈥搊bject", "subjective_objective": {"answer": "Subjective", "rationale": "Because \"Parents always decide what I should wear and what I should dress up\" is the subjective feeling of the patient about what he's been through."}, "sentence_sentiment": {"answer": "Negative", "rationale": "Because the expression \"parents always decide what I should wear and what I should dress\" in the entry is negative and a source of dissatisfaction with parents' interference with their dress."}, "language_style": {"answer": "Tactful and warm", "rationale": "The language style of this entry is more euphemismal. First, it uses the term \"always\" with a certain subjective connotation, expressing an emotional affirmation and emphasis that the person under investigation feels more close and understanding. Second, the phrase \"what I should wear should be dressed like\" does not use direct command or blame language, but rather conveys a sense of concern and recommendation by the word \"always\". This expression is more intimate and more responsive to the person under investigation."}, "time_dimension": {"answer": "Past", "rationale": ""}, "sentence_type": "Affirmative declarative sentence", "pos": {"nouns": {"parents": 2, "Clothes": 1, "Get dressed": 1, "Look": 1}, "verbs": {"Always": 1, "Around": 1, "Put it on": 1, "You deserve it": 1, "鈾?In a dress 鈾?: 1}, "adverbs": {"What": 2}, "numerals": {"11": 1, "2": 1}, "pronouns": {"I": 1}}}, {"content": "12. Parents do not allow me to do something other children can do because they fear something will happen to me.", "word_count": 29, "label": "", "sentence_structure": "Subject鈥搗erb鈥搊bject", "subjective_objective": {"answer": "Subjective", "rationale": "Because fear of something happens to me is the patient's subjective feeling and understanding."}, "sentence_sentiment": {"answer": "Negative", "rationale": "Because the \"not allowing me to do something other than that a child can do\" in the entry is negative."}, "language_style": {"answer": "Tactful and warm", "rationale": "The language style of the entry is more euphemistic. First, it expresses concern about the family background of the interviewee in a way that is caring and understanding, rather than directly criticizing or accusing; second, it uses the term \"because\" to explain the parents' fears, rather than simply to attribute them to their behaviour. These elements make the entry more kinetic and emotional."}, "time_dimension": {"answer": "Past", "rationale": "The sentence refers to events in the past (i.e. \"Parents do not allow me to do something other than what other children can do\") and gives reasons (i.e. \"They fear something will happen to me\". So this entry describes the behaviour or attitude of the past."}, "sentence_type": "Negative declarative sentence", "pos": {"nouns": {"parents": 1, "Things": 2, "Son": 1}, "verbs": {"Allow": 1, "Do it": 1, "Scared": 1, "Something's wrong": 1}, "adjectives": {"Other": 1}, "adverbs": {"not": 1, "Yeah": 1, "Because": 1, "Yes": 1}, "numerals": {"12": 1, "Two": 1}, "pronouns": {"I": 1}}}, {"content": "When I was a child, my parents beat me or lectured me in front of others.", "word_count": 20, "label": "", "sentence_structure": "Subject鈥搗erb鈥搊bject", "subjective_objective": {"answer": "Subjective", "rationale": "Because this is the patient ' s subjective perspective of the memory and description of his past experience."}, "sentence_sentiment": {"answer": "Negative", "rationale": "Because \"strike\" and \"teached\" in the entries are negative acts, the emotional orientation of the article is negative."}, "language_style": {"answer": "Tactful and warm", "rationale": ""}, "time_dimension": {"answer": "Past", "rationale": ""}, "sentence_type": "Affirmative declarative sentence", "pos": {"nouns": {"Time": 1, "parents": 1, "others": 1, "Noodles": 1, "Fight": 1, "Training": 1}, "verbs": {"in": 1, "In front of you": 1, "Once": 1}, "adverbs": {"When I was little": 1}, "numerals": {"13": 1}, "pronouns": {"I": 1}}}, {"content": "Parents always care about what I do at night.", "word_count": 13, "label": "", "sentence_structure": "Subject鈥搗erb鈥搊bject", "subjective_objective": {"answer": "Subjective", "rationale": "This is because of the patient ' s perception and feelings about the level of care of his or her parents and because of the patient ' s subjective experience and attitude."}, "sentence_sentiment": {"answer": "Neutral", "rationale": "As this entry does not express positive or negative emotions."}, "language_style": {"answer": "Tactful and warm", "rationale": "The entry is a more intimate and interactive language, when it is dialogue in a conversation with a voice of enquiry and concern, and does not directly order or accuse the other, but rather expresses concern and understanding in a moderate manner."}, "time_dimension": {"answer": "Present", "rationale": "The entry is for the purpose of asking the person who was tested about his or her subjective feelings about the behaviour of his or her parents, and the temporal dimension is now judged."}, "sentence_type": "Declarative sentence", "pos": {"nouns": {"parents": 1, "Night": 1, "Fuck": 1}, "verbs": {"Always": 1, "Care": 1, "Fuck": 1}, "adverbs": {"very": 1}, "numerals": {"14": 1}, "pronouns": {"I": 1}}}, {"content": "When things go wrong, I can feel that my parents are trying to encourage me to make some progress.", "word_count": 29, "label": "", "sentence_structure": "Subject鈥揷opula鈥損redicative", "subjective_objective": {"answer": "Subjective", "rationale": "Because the words \"can feel\" and \"make me feel\" indicate that it's a description of what a patient feels for himself, and it's a subjective view."}, "sentence_sentiment": {"answer": "Positive", "rationale": "Because the purpose of the entry is to \"felt my parents encouraging me as much as possible\"."}, "language_style": {"answer": "Tactful and warm", "rationale": "The entry uses more positive and encouraging words, trying to give the interviewee the support and affection of his parents and encouraging them to not give up when facing difficulties. This language style is more friendly and warm.\""}, "time_dimension": {"answer": "Present", "rationale": ""}, "sentence_type": "Affirmative declarative sentence", "pos": {"nouns": {"Yes": 1, "person": 1, "Development": 1}, "verbs": {"I met him": 1, "Sensor": 1, "Encourage": 1, "Okay": 1}, "adjectives": {"It's not easy": 1}, "numerals": {"15": 1, "Some": 1}, "classifiers": {"Some": 1}, "pronouns": {"I": 1, "in": 1}}}, {"content": "Parents are always too worried about my health.", "word_count": 12, "label": "", "sentence_structure": "Subject鈥揷opula鈥損redicative", "subjective_objective": {"answer": "Subjective", "rationale": "Because \"excessive fear\" is a subjective feeling and assessment, which represents the attitude and perception of the patient towards his parents."}, "sentence_sentiment": {"answer": "Negative", "rationale": "Because the attitude of \"too much to worry\" is negative."}, "language_style": {"answer": "Tactful and warm", "rationale": "The entry asks the other in a caring way whether the parent is feeling too worried about his or her health, rather than directly speaking or ordering, which is more personal and interactive. At the same time, the term is used in a more moderate way, without using too strong emotional colours or negative evaluations, giving people a comfortable feeling."}, "time_dimension": {"answer": "Present", "rationale": "The entry describes the situation in which the person is currently tested, i.e., the parents are over-concerned about their health and thus judged \"now\"."}, "sentence_type": "Affirmative declarative sentence", "pos": {"nouns": {"parents": 1, "Health": 1}, "verbs": {"Total": 1, "I'm worried": 1, "That's too much": 1}, "adjectives": {"Always": 1, "That's too much": 1}, "numerals": {"XVI": 1}}}, {"content": "Parents often punish me more than I deserve.", "word_count": 16, "label": "", "sentence_structure": "Subject鈥搗erb鈥揷omplement", "subjective_objective": {"answer": "Subjective", "rationale": "In the context, the entry is viewed from the perspective of the patient. This is the patient's subjective feeling, because it is about the patient's perception and perception of the punishment of his parents, not the objective fact. The output is therefore \"subjective\"."}, "sentence_sentiment": {"answer": "Negative", "rationale": "Because the \"parents often punish me more than I deserve\" act in the entry is negative."}, "language_style": {"answer": "Calm and direct", "rationale": ""}, "time_dimension": {"answer": "Past", "rationale": ""}, "sentence_type": "Affirmative declarative sentence", "pos": {"nouns": {"parents": 2, "I": 2, "Punishment": 1, "Level": 1}, "verbs": {"to": 1, "Give": 1, "Super": 1, "Pass": 1}, "adjectives": {"Often": 1}, "numerals": {"17": 1}}}, {"content": "18. If I do not do what I say at home, parents get angry.", "word_count": 16, "label": "", "sentence_structure": "Subject鈥搗erb鈥搊bject", "subjective_objective": {"answer": "Subjective", "rationale": "Because words like \"freak\" and \"failed\" are the subjective feelings and attitude of the patient."}, "sentence_sentiment": {"answer": "Negative", "rationale": "Because the \"parents get pissed off\" in the entry is negative."}, "language_style": {"answer": "Tactful and warm", "rationale": "The entry is more adept and interactive when it is spoken in a gentle and inviting manner, rather than by direct statements or orders."}, "time_dimension": {"answer": "Past", "rationale": "The phrase \"if I do not do what I say at home, parents are angry\" describes the events of the past, that is, in the past, parents are angry if they do not do what they say."}, "sentence_type": "Affirmative declarative sentence", "pos": {"nouns": {"Home": 1, "Lee": 1, "I'm not listening": 1, "parents": 2, "I'm angry": 1}, "verbs": {"Yes": 1, "- Yes, sir": 1, "I'm angry": 1}, "numerals": {"18": 1}, "pronouns": {"I": 1}}}, {"content": "19. If I do something wrong, parents always give me a sense of guilt or guilt in a sad way.", "word_count": 33, "label": "", "sentence_structure": "Subject鈥搗erb鈥搊bject", "subjective_objective": {"answer": "Subjective", "rationale": "The problem is viewed from the patient鈥檚 subjective perspective."}, "sentence_sentiment": {"answer": "Negative", "rationale": "Because the \"causing guilt or guilt\" act in the entry is negative."}, "language_style": {"answer": "Calm and direct", "rationale": "The entry directly expresses the subject of asking the other parent whether he or she has over-accusation or punishment in the family, without using excessive rhetorical or emotional rendering, and is more objective in tone. At the same time, the specific term used to describe \"the sad look\", \"the guilt or guilt\" makes the question more specific and easier for the respondents to understand and answer."}, "time_dimension": {"answer": "Present", "rationale": "It describes a phenomenon that exists, that \"I\" when doing wrong, parents use sad expressions to make \"I\" feel guilty or guilty. This is happening now, so it's based on \"now\"."}, "sentence_type": "Affirmative declarative sentence", "pos": {"nouns": {"Yes": 1, "parents": 2, "Look": 1, "- A sense of guilt": 1, "Guilt": 1}, "verbs": {"Wrong": 1, "鈾?And I'm gonna be 鈾?: 1, "have": 1}, "adjectives": {"Sad": 1, "Crime": 1, "Guilt": 1}, "numerals": {"One": 3, "Two": 1}, "classifiers": {"Plant": 2, "Sample": 1}, "pronouns": {"I": 1}}}, {"content": "20. I find it difficult for parents to get close.", "word_count": 9, "label": "", "sentence_structure": "Subject鈥揷opula鈥損redicative", "subjective_objective": {"answer": "Subjective", "rationale": "Because \"think\" is a subjective term, which expresses the feelings or perceptions of the patient, it is a subjective view."}, "sentence_sentiment": {"answer": "Negative", "rationale": "Because the description \"hard to get close\" in the entry is an expression of negative emotional attitudes."}, "language_style": {"answer": "Tactful and warm", "rationale": "The entry is more adept and interactive when it is spoken in a gentle and inviting manner, rather than by direct statements or orders."}, "time_dimension": {"answer": "Present", "rationale": "The keyword \"I find it difficult for parents to get close\" means the person's current feelings and thoughts, so it can be judged that the entry is an inquiry into the present situation."}, "sentence_type": "Negative declarative sentence", "pos": {"nouns": {"parents": 1, "Close": 1}, "verbs": {"I think": 1, "Hard": 1}, "adjectives": {"Hard": 1}}}, {"content": "I was embarrassed when parents were talking about something I had said or done in front of others.", "word_count": 30, "label": "", "sentence_structure": "Subject鈥搗erb鈥搊bject", "subjective_objective": {"answer": "Subjective", "rationale": "Because \"distressed\" is the patient's subjective feeling."}, "sentence_sentiment": {"answer": "Negative", "rationale": "Because the \"I feel embarrassed\" in the entry is negative."}, "language_style": {"answer": "Tactful and warm", "rationale": ""}, "time_dimension": {"answer": "Present", "rationale": ""}, "sentence_type": "Affirmative declarative sentence", "pos": {"nouns": {"parents": 1, "person": 1, "Word": 1, "Yes": 1}, "verbs": {"in": 1, "Yes": 2, "I feel": 1, "It's embarrassing": 1}, "adverbs": {"Tsang": 1}, "numerals": {"21": 1}, "pronouns": {"I": 1}}}, {"content": "22. I think that my parents prefer me to my brothers and sisters.", "word_count": 18, "label": "", "sentence_structure": "Subject鈥揷opula鈥損redicative", "subjective_objective": {"answer": "Subjective", "rationale": "Because \"the feeling\" expresses the views and feelings of the patient, it is a subjective attitude."}, "sentence_sentiment": {"answer": "Positive", "rationale": "Because the expression \"parents prefer me\" in the entry is positive."}, "language_style": {"answer": "Calm and direct", "rationale": "The entry is presented directly and asks the other side whether they feel that their parents prefer them, without too many rhetorical or emotional renderings, and with a more calm and straightforward language style."}, "time_dimension": {"answer": "Past", "rationale": "The entry describes the experience and experience of the testee, who feels that, for some time, parents prefer themselves over siblings."}, "sentence_type": "Affirmative declarative sentence", "pos": {"nouns": {"parents": 1, "I": 2, "Brothers and sisters": 1}, "verbs": {"More like it": 1, "be": 1, "I think": 1}, "adverbs": {"More": 1}, "numerals": {"22锛?: 1}, "pronouns": {"they": 1}}}, {"content": "23. Parents are small in terms of what I need.", "word_count": 16, "label": "", "sentence_structure": "Subject鈥搗erb鈥揷omplement", "subjective_objective": {"answer": "Subjective", "rationale": "Because \"smugness\" is a patient's subjective assessment."}, "sentence_sentiment": {"answer": "Negative", "rationale": "Because the description of \"slight\" in the entry is negative in emotional terms, it shows that parents are not generous enough to meet their children's needs."}, "language_style": {"answer": "Calm and direct", "rationale": "The entry is a direct statement of the respondent鈥檚 view of parents鈥?stinginess in meeting their needs, and does not use excessive rhetorical or emotional rendering. The language style of the questionnaire is therefore straightforward and calm."}, "time_dimension": {"answer": "Present", "rationale": "The entry describes the subjective feelings and views of the testee today, without reference to the time dimension of the past or the future. The keyword \"slight\" indicates the negative assessment of the testee's attitude towards parents, which is based on the current situation and is therefore a description of the present."}, "sentence_type": "Negative declarative sentence", "pos": {"nouns": {"Something you need": 1, "parents": 1}, "verbs": {"Satisfied": 1, "be": 1, "Here": 1}, "adjectives": {"It's so small": 1}, "numerals": {"23": 1}}}, {"content": "24. Parents often care about my score.", "word_count": 12, "label": "", "sentence_structure": "Subject鈥搗erb鈥揷omplement", "subjective_objective": {"answer": "Objective", "rationale": "Because the achievement of scores is the result of the student ' s behaviour, the parents ' views and attitudes are objective."}, "sentence_sentiment": {"answer": "Positive", "rationale": "Because the purpose of the \"I care about getting points\" in the entry is positive."}, "language_style": {"answer": "Tactful and warm", "rationale": "The entry is more adept and interactive when it is spoken in a gentle and inviting manner, rather than by direct statements or orders."}, "time_dimension": {"answer": "Past", "rationale": "The words \"often\" and \"to get points\" in the entry represent past behaviour or circumstances and are therefore judged to be past."}, "sentence_type": "Affirmative declarative sentence", "pos": {"nouns": {"parents": 1, "I": 1, "Score": 1}, "verbs": {"I care": 1, "Access": 1}, "adjectives": {"Often": 1}, "numerals": {"24锛?: 1}}}, {"content": "25. If I faced a difficult task, I could feel support from my parents.", "word_count": 23, "label": "", "sentence_structure": "Subject鈥搗erb鈥搊bject", "subjective_objective": {"answer": "Subjective", "rationale": "Because \"Sensitization of support\" is a subjective feeling or experience. Although parents may act objectively (for example, by offering help or encouraging it), it is the patient's subjective experience, so the entry is primarily about the patient's own subjective feelings."}, "sentence_sentiment": {"answer": "Positive", "rationale": "Because the \"Personal Support\" act in the entry is positive."}, "language_style": {"answer": "Tactful and warm", "rationale": "The entry gives respondents a sense of comfort and relaxation by using the term \"attribution\". At the same time, the support is described in a positive manner, emphasizing the positive impact of parents, which enhances the protege and friendship of the subject."}, "time_dimension": {"answer": "Present", "rationale": "In the future, the entry describes the potential for the testee to feel parental support in the face of future difficult tasks and therefore to judge the future."}, "sentence_type": "Affirmative declarative sentence", "pos": {"nouns": {"Tasks": 1, "Support": 1, "parents": 1}, "verbs": {"Faced": 1, "Yes": 2, "I feel it": 1, "be": 1}, "adverbs": {"If": 1, "Yes": 1, "From": 1}, "numerals": {"25": 1}, "classifiers": {"Item": 1}, "pronouns": {"I": 1}}}, {"content": "I have often been used as a 鈥渟acrifice鈥?or 鈥渁 herd of harm鈥?in my home.", "word_count": 17, "label": "", "sentence_structure": "Subject鈥揷opula鈥損redicative", "subjective_objective": {"answer": "Subjective", "rationale": "Because it is a subjective view and feeling of the patient in his or her own family environment."}, "sentence_sentiment": {"answer": "Negative", "rationale": "Because the words \"responsible\" and \"bad horse\" in the entries are negative."}, "language_style": {"answer": "Tactful and warm", "rationale": "Warm and warm."}, "time_dimension": {"answer": "Past", "rationale": ""}, "sentence_type": "Negative declarative sentence", "pos": {"nouns": {"Home": 1, "A scapegoat": 1, "The bad guys": 1}, "verbs": {"Act as": 1, "When": 1}, "adverbs": {"Often": 1}, "numerals": {"26锛?: 1}, "pronouns": {"I": 1}}}, {"content": "Parents always pick on friends I like.", "word_count": 13, "label": "", "sentence_structure": "Subject鈥搗erb鈥搊bject", "subjective_objective": {"answer": "Subjective", "rationale": "Because the words \"choot\" and \"like\" are all described from the point of view of the patient and are subjective."}, "sentence_sentiment": {"answer": "Negative", "rationale": "Because the \"parents always pick out my favorite friends\" behavior is negative."}, "language_style": {"answer": "Tactful and warm", "rationale": "The entry uses an implicit and euphemistic formulation that avoids direct accusations or criticism of the language style of the other, which is more readily accepted and understood by the interviewee. At the same time, it also allows the interviewee to feel respected and understood."}, "time_dimension": {"answer": "Present", "rationale": "The key words are \"Parents are always picky\" and \"friends I like,\" which describe a state or behavior that is in progress, so judging now."}, "sentence_type": "Negative declarative sentence", "pos": {"nouns": {"parents": 1, "friends": 1}, "verbs": {"Always": 1, "Picky": 1, "Yeah": 1}, "adverbs": {"Sou-san": 1}, "numerals": {"27锛?: 1}, "pronouns": {"I": 1}}}, {"content": "Parents always think I caused their discomfort.", "word_count": 17, "label": "", "sentence_structure": "Subject鈥搗erb鈥揷omplement", "subjective_objective": {"answer": "Subjective", "rationale": "This is because the patient鈥檚 perception and feelings of his/her relationship with his/her parents are subjective."}, "sentence_sentiment": {"answer": "Negative", "rationale": "Because the article states that \"parents always think that their discomfort is caused by me\" is derogatory, implying tension and contradictions among family members and expressing negative emotional attitudes."}, "language_style": {"answer": "Tactful and warm", "rationale": "The entry uses a gentle and understanding expression, which is more caring and respectful, by asking respondents whether they believe that their behaviour is causing displeasure among parents."}, "time_dimension": {"answer": "Present", "rationale": "The use of keywords \"total\", \"think\" and \"cause\" all point to a question of the current condition of the testee, so the temporal dimension is now judged."}, "sentence_type": "Negative declarative sentence", "pos": {"nouns": {"parents": 1, "They": 1, "Not fast": 1}, "verbs": {"I thought": 1, "Cause": 1}, "numerals": {"28锛?: 1}, "pronouns": {"I": 1}}}, {"content": "29. Parents always try to encourage me to become the best.", "word_count": 15, "label": "", "sentence_structure": "Subject鈥搗erb鈥揷omplement", "subjective_objective": {"answer": "Subjective", "rationale": "Because the criterion of \"make the man\" is based on subjective judgement and expectations of parents."}, "sentence_sentiment": {"answer": "Positive", "rationale": "Because the descriptions of \"parents always try to encourage me\" and \"make me the best\" are positive."}, "language_style": {"answer": "Tactful and warm", "rationale": "The language style of the entry is more euphemismal, because it does not use strong tone or directly express criticism or accusations against the person interviewed. Rather, it describes the behaviour of parents in a more gentle and caring way and tries to convey messages of encouragement and support. This way is more friendly and friendly, helping the person interviewed to be more willing to participate and share their feelings and experiences."}, "time_dimension": {"answer": "Present", "rationale": ""}, "sentence_type": "Affirmative declarative sentence", "pos": {"nouns": {"parents": 1, "Son": 1}, "verbs": {"Try": 1, "Encourage": 1, "鈾?And I'm gonna be 鈾?: 1, "Be": 1}, "adjectives": {"Good man": 1}, "adverbs": {"Total": 1}, "numerals": {"29锛?: 1}}}, {"content": "30. Parents always told me they loved me.", "word_count": 13, "label": "", "sentence_structure": "Subject鈥揷opula鈥損redicative", "subjective_objective": {"answer": "Subjective", "rationale": "The author of the article is a member of the public media, which is a member of the public."}, "sentence_sentiment": {"answer": "Positive", "rationale": "Because the expression \"means they love me\" in the entry is positive."}, "language_style": {"answer": "Tactful and warm", "rationale": "The wording of the entry does not directly indicate \"Does parents love you\" but uses a euphemism to guide the other party to answer."}, "time_dimension": {"answer": "Present", "rationale": "The entry describes the current feelings or experiences of the testee, namely, \"Parents always say they love me\", and does not address past or future behaviors or mentalities. Thus, the temporal dimension is now judged."}, "sentence_type": "Affirmative declarative sentence", "pos": {"nouns": {"parents": 2, "I": 1}, "verbs": {"Organisation": 1, "be": 1, "Love": 1}, "numerals": {"30": 1}, "classifiers": {"Total": 1}}}, {"content": "31. Parents have confidence in me and allow me to do something alone.", "word_count": 18, "label": "", "sentence_structure": "Subject鈥揷opula鈥損redicative", "subjective_objective": {"answer": "Objective", "rationale": "The basis for the judgement: because \"parents trust me and allow me to do something alone\" is an objective act and fact, and it is an objective situation, not a subjective attitude of the patient."}, "sentence_sentiment": {"answer": "Positive", "rationale": "Because the quality of the phrase \"parents trust me and allow me to do something alone\" in the entry is positive."}, "language_style": {"answer": "Tactful and warm", "rationale": "The language style of the entry is euphemistic, because it is expressed in a way that is not direct, but rather in a way that is a matter of inquiry. It uses a gentle and inviting tone to guide the respondents to their ideas and experiences, which is more personal and interactive, helping to create a good atmosphere of communication and to collect more realistic and effective data."}, "time_dimension": {"answer": "Present", "rationale": "\"Parents trust me and allow me to do something alone\" describes the current situation or experience of the person tested, not past or future circumstances. Thus, the time dimension of the article is now."}, "sentence_type": "Affirmative declarative sentence", "pos": {"nouns": {"parents": 1, "I": 2, "Things": 1}, "verbs": {"to": 1, "very": 1, "Trust": 1, "and": 1, "Allow": 1, "Completed": 1}, "adjectives": {"Alone": 1}, "numerals": {"31": 1}, "pronouns": {"Some": 1}}}, {"content": "32. I think my parents respect my views.", "word_count": 11, "label": "", "sentence_structure": "Subject鈥揷opula鈥損redicative", "subjective_objective": {"answer": "Subjective", "rationale": "Because \"respect\" is a subjective feeling and evaluation, the entry is viewed from the patient's subjective perspective."}, "sentence_sentiment": {"answer": "Positive", "rationale": "Because \"respect my views\" in the entry is a positive emotional attitude and quality."}, "language_style": {"answer": "Tactful and warm", "rationale": "The entry uses a positive statement and expresses respect for the interviewee, making it easier for the interviewee to accept and be willing to answer questions. Moreover, the expression is more moderate and does not create pressure or discomfort for the interviewee."}, "time_dimension": {"answer": "Past", "rationale": ""}, "sentence_type": "Affirmative declarative sentence", "pos": {"nouns": {"parents": 1, "I": 1, "Views": 1}, "verbs": {"I'm not sure": 1, "Respect": 1}, "numerals": {"32": 1}}}, {"content": "33. I feel that parents are very happy with me.", "word_count": 13, "label": "", "sentence_structure": "Subject鈥揷opula鈥損redicative", "subjective_objective": {"answer": "Subjective", "rationale": "Because \"think\" means it's a subjective feeling or view."}, "sentence_sentiment": {"answer": "Positive", "rationale": "Because the attitude of \"I feel like my parents are happy with me\" is positive."}, "language_style": {"answer": "Tactful and warm", "rationale": "The language style in this entry is more euphemistic and warm, using subjective expressions of perception rather than an objective description or direct command, making the respondent feel more comfortable and close."}, "time_dimension": {"answer": "Present", "rationale": "The keyword is \"I feel,\" which means the subject's present subjective feeling or view. And the act of \"I'm willing to be with me\" has no clear time limits, so it is the present feelings and perceptions of the testee that can be judged. So the time dimension of the entry is now."}, "sentence_type": "Affirmative declarative sentence", "pos": {"nouns": {"I": 1, "parents": 2}, "verbs": {"I think": 1, "May I": 1, "in": 1, "Together": 1}, "adjectives": {"very": 1}, "numerals": {"33": 1}}}, {"content": "34. I feel that my parents are so mean and mean to me.", "word_count": 13, "label": "", "sentence_structure": "Subject鈥揷opula鈥損redicative", "subjective_objective": {"answer": "Subjective", "rationale": "Because \"think\" is a word for personal subjective feelings, the questionnaire entry is viewed from the patient's subjective perspective."}, "sentence_sentiment": {"answer": "Negative", "rationale": "Because \"smug\" and \"smuggle\" are negative adjectives that mean \"no\" of parents' behavior."}, "language_style": {"answer": "Calm and direct", "rationale": "The entry describes, in an objective and direct tone, the subjective feelings of the surveyed person towards his parents, without too much emotional rendering or rhetorical techniques, and the language style is a direct and calm type.\""}, "time_dimension": {"answer": "Present", "rationale": "The entry is subjective and describes the person being tested ' s current thoughts and feelings, and thus the judgement is now."}, "sentence_type": "Negative declarative sentence", "pos": {"nouns": {"parents": 2, "I": 1}, "verbs": {"to": 1, "I think": 1, "- You're a little bit of a jerk": 1, "Stingy": 1}, "adjectives": {"very": 2}, "numerals": {"34": 1}}}, {"content": "Parents always say to me things like that: 鈥淚f you do this, I will be very sad鈥?", "word_count": 24, "label": "", "sentence_structure": "Subject鈥搗erb鈥搊bject", "subjective_objective": {"answer": "Subjective", "rationale": "Because it's about the patients' feelings and understanding of the way their parents talk."}, "sentence_sentiment": {"answer": "Negative", "rationale": "The basis for the determination: the entry contains the negative phrase \"worried\" and implies that parents are disgruntled and blameful for the behaviour of their children."}, "language_style": {"answer": "Calm and direct", "rationale": "The entry directly expresses the language used by parents in educating their children, without excessive adoration or emotional rendering, and is more objective and direct.\""}, "time_dimension": {"answer": "Present", "rationale": ""}, "sentence_type": "Affirmative declarative sentence", "pos": {"nouns": {"parents": 1, "Word": 1, "Sad": 1, "you": 2, "I": 1}, "verbs": {"Always": 1, "Say it": 1, "Do it": 1, "It's gonna be": 1}, "adjectives": {"Similar": 1}, "adverbs": {"Here": 1}, "numerals": {"35锛?: 1, "Here": 1, "Sample": 1}, "pronouns": {"they": 1}}}, {"content": "36. Parents have to tell me what I am doing when I ask to return to my family.", "word_count": 23, "label": "", "sentence_structure": "Subject鈥搗erb鈥搊bject", "subjective_objective": {"answer": "Objective", "rationale": "This is a rule or requirement in the patient ' s family and is not the patient ' s own subjective idea."}, "sentence_sentiment": {"answer": "Negative", "rationale": "Because the entry \"Parents have to tell me what I'm doing when I'm going home\" may cause individuals to feel bound and restrain, and therefore have negative emotional aspects."}, "language_style": {"answer": "Calm and direct", "rationale": "The entry is more concise and not based on excessive rhetorical or emotional rendering."}, "time_dimension": {"answer": "Present", "rationale": "The entry asked if the testees were often confronted with this situation in their current lives, and the temporal dimension was therefore judged as \"now\"."}, "sentence_type": "Affirmative declarative sentence", "pos": {"nouns": {"parents": 1, "I": 2, "Home": 1, "Things": 1}, "verbs": {"Request": 1, "Back": 1, "Okay": 1, "In": 1, "Annotations": 1, "Do it": 1}, "adverbs": {"I have to": 1}, "numerals": {"36锛?: 1}}}, {"content": "37. I think parents are trying to make my youth more meaningful and colorful. (For example, buy me a lot of books, arrange me for summer camps or join clubs.)", "word_count": 43, "label": "", "sentence_structure": "Subject鈥搗erb鈥搊bject", "subjective_objective": {"answer": "Subjective", "rationale": "Because the subject of the questionnaire, \"I think,\" shows that it's a subjective way of describing the patients' feelings."}, "sentence_sentiment": {"answer": "Positive", "rationale": "Because this entry describes positive things that parents do for their children, i.e. make their lives more meaningful and diverse."}, "language_style": {"answer": "Tactful and warm", "rationale": "The entry uses the first person's proxy, \"I,\" which expresses gratitude for what parents do for themselves and presents the facts in a gentle and positive way, with a more intimate and sensitive language style."}, "time_dimension": {"answer": "Past", "rationale": "The entry is about the views and feelings of the testees, \"I think parents are trying to make my youth more meaningful and colorful\" describing what happened in the past and what the testees felt."}, "sentence_type": "Affirmative declarative sentence", "pos": {"nouns": {"parents": 1, "Youth": 1, "Meaning": 1, "It's a lot of color": 1, "Books": 1, "Summer camp": 1, "Club": 1}, "verbs": {"鈾?And I'm gonna be 鈾?: 1, "have": 1, "Buy": 1, "Arrangements": 1, "Participation": 1}, "adverbs": {"Try": 1}, "numerals": {"37": 1}, "pronouns": {"I": 1}}}, {"content": "Parents often say to me the same thing: 鈥淚s this the reward we get for your daily work?鈥?, "word_count": 32, "label": "", "sentence_structure": "Subject鈥搗erb鈥搊bject", "subjective_objective": {"answer": "Subjective", "rationale": "Because the words \"often\" and \"so\" in the sentence indicate that it is the patient's own perception and feelings, and it is the patient's subjective perspective."}, "sentence_sentiment": {"answer": "Negative", "rationale": "Because the expression \"get the reward?\" in the entry is derogatory and negative, and therefore negative."}, "language_style": {"answer": "Tactful and warm", "rationale": "The topic raises questions in a more moderate and caring manner, is polite in its language, and attempts to express the love of the person under investigation. At the same time, there is no language of strong criticism or criticism in the title, nor is it used in overly direct or strong terms, which is more in the euphemism of the language."}, "time_dimension": {"answer": "Present", "rationale": "The entry describes a realistic situation that includes actions that indicate that are now being done - \"expression\" and \"get.\" So the time dimension of this questionnaire entry is now judged."}, "sentence_type": "Imperative sentence", "pos": {"nouns": {"parents": 2, "Word": 1, "Day": 1, "Refund": 1, "you": 1}, "verbs": {"Tell me": 1, "Okay": 1, "All day long": 1}, "adjectives": {"we": 1, "of": 2, "Here": 2, "And get it": 1, "Similar": 1}, "adverbs": {"- You're not": 1}, "numerals": {"38": 1, "One day": 1}, "classifiers": {"All day long": 1}, "pronouns": {"we": 1, "you": 2}}}, {"content": "Parents often use the excuse of not being comfortable with me as an excuse for not meeting my demands.", "word_count": 19, "label": "", "sentence_structure": "Subject鈥搗erb鈥搊bject", "subjective_objective": {"answer": "Subjective", "rationale": "Because \"not me\" and \"not me\" are described from the point of view of the patient, that is, the child, and are looking at the patient's subjective feelings and thoughts."}, "sentence_sentiment": {"answer": "Negative", "rationale": "Because the entry \"I can't be used to justify not meeting my demands\" is negative."}, "language_style": {"answer": "Tactful and warm", "rationale": "The entry does not directly accuse or criticize the parents of the person under investigation in their treatment of their needs, but is expressed in a gentle and understanding way, making the questionnaire more friendly and interactive. The language style of the article is therefore \"precipitous\"."}, "time_dimension": {"answer": "Past", "rationale": ""}, "sentence_type": "Negative declarative sentence", "pos": {"nouns": {"parents": 1, "- An excuse": 1, "Request": 1}, "verbs": {"Always": 1, "Cozy": 1, "Satisfied": 1}, "adverbs": {"not": 1}, "numerals": {"39": 1}, "pronouns": {"I": 2}}}, {"content": "40. I am concerned that if I do not do what my parents expect, I will be left with a conscience.", "word_count": 23, "label": "", "sentence_structure": "Subject鈥揷opula鈥損redicative", "subjective_objective": {"answer": "Subjective", "rationale": "Because \"conscientious disturbed\" is the patient鈥檚 subjective feeling and experience. Although parents鈥?expectations are an objective fact, it is still up to them to decide whether or not they want to do so, so the questionnaire is based on the patient鈥檚 subjective thoughts."}, "sentence_sentiment": {"answer": "Negative", "rationale": "It is a negative sentiment because it expresses feelings of guilt if the parents do not follow their expectations."}, "language_style": {"answer": "Tactful and warm", "rationale": "The entry is more euphemistic, using subtle language to express the pressures and concerns that may arise if it does not meet the expectations of parents. It is more able to guide the interviewee to express his thoughts and feelings in a real way."}, "time_dimension": {"answer": "Past", "rationale": "The entry \"if ... for...\" indicates the result under the scenario, indicating that it is a mental state or belief based on past memory and experience. At the same time, \"that would upset my conscience\" also reflects the effects of past experiences. The questionnaire entry therefore asks about the mental state or behaviour of the person tested in the past."}, "sentence_type": "Interrogative sentence", "pos": {"nouns": {"Questionnaire Entry": 1, "parents": 2, "Expectations": 1, "Do it": 1, "Conscience": 1, "Unsettled": 1}, "verbs": {"Yes": 1, "鈾?And I'm gonna be 鈾?: 1, "I feel": 1}, "adverbs": {"No, I don't": 1, "If": 1}, "numerals": {"40锛?: 1}, "pronouns": {"I": 1}}}, {"content": "41. I feel that parents have a high demand for my academic achievement, physical activity or similar things.", "word_count": 28, "label": "", "sentence_structure": "Subject鈥揷opula鈥損redicative", "subjective_objective": {"answer": "Subjective", "rationale": "The first is the fact that the patient's attitude and feelings are subjective."}, "sentence_sentiment": {"answer": "Negative", "rationale": "A negative emotional attitude is expressed because the act of \"high demand\" may cause some pressure and dissatisfaction among the respondents."}, "language_style": {"answer": "Tactful and warm", "rationale": ""}, "time_dimension": {"answer": "Present", "rationale": "The entry asks about the current feelings and perceptions of the person being tested, and the time dimension is now."}, "sentence_type": "Affirmative declarative sentence", "pos": {"nouns": {"parents": 1, "I": 1, "Learning achievement": 1, "Sports activities": 1, "Things": 1}, "verbs": {"I think": 1, "have": 1, "Higher": 1, "Request": 1}, "adjectives": {"of": 4, "Higher": 1, "Similar": 1}, "numerals": {"41锛?: 1}}}, {"content": "42. When I feel sad, I can get comfort from my parents.", "word_count": 20, "label": "", "sentence_structure": "Subject鈥搗erb鈥揷omplement", "subjective_objective": {"answer": "Subjective", "rationale": "Because it is the feelings and perceptions of the patient that are able to get comfort from his or her parents, it is subjective."}, "sentence_sentiment": {"answer": "Positive", "rationale": "Because this entry expresses the emotional support of a relative in the event of difficulties, which is a positive and positive expression."}, "language_style": {"answer": "Tactful and warm", "rationale": "The language style of the entry is euphemism, because it uses the expression \"can be comforted\" , which is more caring and comforting. Moreover, the term \"parents\" conveys a warm feeling of affection, which is, in general, an expression that seeks to get support and help from relatives when the investigator is sad."}, "time_dimension": {"answer": "Past", "rationale": "The entry \"when I feel sad\" indicates a past experience or situation, and \"can be comforted\" is also a past behavior or state. So the entire sentence describes a past experience or situation."}, "sentence_type": "Affirmative declarative sentence", "pos": {"nouns": {"Time": 1, "Consolation": 1, "parents": 1}, "verbs": {"Got it": 1, "I feel": 1, "Yeah": 1}, "adjectives": {"It's sad": 1}, "numerals": {"42": 1}, "pronouns": {"I": 1}}}, {"content": "43. Parents have punished me for no reason.", "word_count": 11, "label": "", "sentence_structure": "Subject鈥搗erb鈥搊bject", "subjective_objective": {"answer": "Subjective", "rationale": "Because this is the patient ' s perception and perception of past events, it is subjective."}, "sentence_sentiment": {"answer": "Negative", "rationale": "Because the experience of being punished for no reason is negative."}, "language_style": {"answer": "Tactful and warm", "rationale": "The entry is more personal and interactive when it is spoken in a gentle and inviting manner, rather than in direct statements or orders. At the same time, the term \"unprovoked\" is also more objective and neutral, without an overly emotional or subjective tendency to obtain real feedback from the interviewees."}, "time_dimension": {"answer": "Past", "rationale": "The keyword \"had\" means that this happened at some point in the past."}, "sentence_type": "Negative declarative sentence", "pos": {"nouns": {"parents": 1, "For no reason": 1, "Punishment": 1}, "verbs": {"Tsang": 1, "Punishment": 1}, "adjectives": {"For no reason": 1}, "numerals": {"43": 1}}}, {"content": "44. Parents have allowed me to do something that my friends do.", "word_count": 17, "label": "", "sentence_structure": "Subject鈥搗erb鈥搊bject", "subjective_objective": {"answer": "Subjective", "rationale": "Because it is usually the subjective feeling and perception of an individual to be allowed to do something, not the objective reality."}, "sentence_sentiment": {"answer": "Positive", "rationale": "Because the \"Let me do something that my friends do\" act positively, showing parents' faith in and respect for their children."}, "language_style": {"answer": "Tactful and warm", "rationale": "The language style of the entry is more moderate, and the interviewer is asked in an invitation tone whether his or her parents are allowed to be friends. This expression is more acceptable to the interviewee and is willing to answer questions. At the same time, the term \"some\" is used to make the questions more open and neutral, without limiting or guiding the respondents' answers."}, "time_dimension": {"answer": "Present", "rationale": "The entry describes the current situation of the testees, namely, whether they can do something like their friends. In it, \"Parents allow\" indicates that it is a continuous state, so time dimension is now judged."}, "sentence_type": "Affirmative declarative sentence", "pos": {"nouns": {"parents": 1, "I": 1, "Friends": 1, "Things": 1}, "verbs": {"Allow": 1, "Do it": 1}, "numerals": {"44锛?: 1}}}, {"content": "Parents often tell me they don't like my performance at home.", "word_count": 18, "label": "", "sentence_structure": "Subject鈥搗erb鈥搊bject", "subjective_objective": {"answer": "Subjective", "rationale": "Because \"parents often say they don't like my behaviour at home\" is a subjective statement that reflects parents' subjective perception of the patient's behaviour at home."}, "sentence_sentiment": {"answer": "Negative", "rationale": "Because the description of the act \"Parents often say they don't like my behaviour at home\" is negative."}, "language_style": {"answer": "Calm and direct", "rationale": "The entry is more directly explicit, has no excessive rhetorical or emotional colours, and is sound and objective. It asks the person surveyed in a direct way and asks the other person to answer questions about his family."}, "time_dimension": {"answer": "Present", "rationale": "The entry describes the current experience and feelings of the testee, and the key words are \"Parents often say to me\" and \"they don't like\" and points to what is happening. Thus, the time dimension of the article is now."}, "sentence_type": "Negative declarative sentence", "pos": {"nouns": {"parents": 2, "Home": 1, "Performance": 1}, "verbs": {"Say it": 1, "Yeah": 1, "in": 1}, "adverbs": {"Regular": 1, "not": 1}, "numerals": {"45": 1}, "pronouns": {"I": 1}}}, {"content": "46. Whenever I eat, my parents persuade me or force me to eat more.", "word_count": 20, "label": "", "sentence_structure": "Subject鈥搗erb鈥搊bject", "subjective_objective": {"answer": "Subjective", "rationale": "Because \"prosthetic\" and \"compulsive\" are the actions of parents, not the objective facts. The subject is subjective, considering the way people feel about their experiences."}, "sentence_sentiment": {"answer": "Negative", "rationale": "Because the behaviour in the entry \"responded or forced to eat more\" has some coercive and negative character."}, "language_style": {"answer": "Calm and direct", "rationale": "The entry uses direct language to ask if the respondents have experienced situations where parents can persuade or force them to eat more."}, "time_dimension": {"answer": "Present", "rationale": ""}, "sentence_type": "Affirmative declarative sentence", "pos": {"nouns": {"Dinner time": 1, "parents": 1, "- I'm gonna get you out of here": 1, "Forced": 1, "More": 2, "Eat": 1}, "verbs": {"Eat": 1, "- I'm gonna get you out of here": 1, "Forced": 1}, "adverbs": {"Every": 1, "Both": 1, "Again": 1}, "numerals": {"46": 1, "Some": 1}}}, {"content": "47. Parents often criticize me in front of others as lazy and useless.", "word_count": 19, "label": "", "sentence_structure": "Subject鈥搗erb鈥搊bject", "subjective_objective": {"answer": "Objective", "rationale": "Because \"Parents often criticize me in front of others for being lazy and useless\" is an objective description of the facts."}, "sentence_sentiment": {"answer": "Negative", "rationale": "Because \"criticism\" and description in the entries are negative."}, "language_style": {"answer": "Tactful and warm", "rationale": "The entry is more personal and interactive when it is spoken in a gentle and inviting tone, rather than in direct statements or orders. At the same time, the terms \"often\" and \"in front of others\" imply that the problem may have a greater psychological stress and negative impact on the person under investigation, and therefore needs to be designed with due regard for its emotional aspects and with due attention and respect."}, "time_dimension": {"answer": "Past", "rationale": "The entry asks if the person who was tested has ever experienced a situation where parents criticized their incompetence and lazyness in front of others. The keyword \"often\" and \"in front of others\" indicates that this is a continuous act and therefore a judgement of the past."}, "sentence_type": "Negative declarative sentence", "pos": {"nouns": {"parents": 1, "others": 1, "Noodles": 1, "Criticism": 1, "I": 1, "Both": 1, "Lazy": 1, "And": 1, "It's useless": 1}, "verbs": {"In front of you": 1, "Criticism": 2}, "adjectives": {"Lazy": 1, "It's useless": 1}, "numerals": {"47锛?: 1}}}, {"content": "Parents often focus on what kind of friends I make.", "word_count": 14, "label": "", "sentence_structure": "Subject鈥搗erb鈥搊bject", "subjective_objective": {"answer": "Subjective", "rationale": "Because \"often concerned\" and \"what kind of friends\" are patients' subjective feelings and thoughts."}, "sentence_sentiment": {"answer": "Negative", "rationale": "Because the word \"concern\" in the entry is usually somewhat negative and in many cases may be considered an act that infringes privacy."}, "language_style": {"answer": "Tactful and warm", "rationale": "The entry uses a euphemism to ask the other parent about the extent of his or her concern about his or her friendship, is relatively gentle and does not directly challenge or order."}, "time_dimension": {"answer": "Present", "rationale": "Now that \"often\" in the entry is a regular, customary act, \"concern\" uses the general current, which means that the act has not only occurred in the past, but may still be ongoing. So the temporal dimension of the entry is now judged."}, "sentence_type": "Affirmative declarative sentence", "pos": {"nouns": {"parents": 1, "friends": 1}, "verbs": {"Always concerned": 1, "Hand it over": 1}, "adverbs": {"Often": 1}, "numerals": {"48锛?: 1}}}, {"content": "49. I am often the only one of my brothers and sisters to be blamed for what happened.", "word_count": 24, "label": "", "sentence_structure": "Subject鈥揷opula鈥損redicative", "subjective_objective": {"answer": "Subjective", "rationale": "Because \"it's often the only thing blameable among brothers and sisters\" is a subjective feeling and observation of a human being."}, "sentence_sentiment": {"answer": "Negative", "rationale": "Because the expression \"often the only thing blames the siblings\" in the entry is negative emotional."}, "language_style": {"answer": "Tactful and warm", "rationale": "The entry uses the words \"often\" and \"sole\" to express a relatively subjective feeling. At the same time, it appears in question without direct blame or criticism, making the tone more euphemism and warmer."}, "time_dimension": {"answer": "Past", "rationale": "The entry describes an experience or feeling of the person tested and uses the past (the word \"transmit\" to refer to past events) and therefore judges \"the past\"."}, "sentence_type": "Affirmative declarative sentence", "pos": {"nouns": {"Things": 1, "Blame": 1, "Brothers and sisters": 1}, "verbs": {"Let's go": 1, "Yes": 1}, "adverbs": {"Often": 1}, "numerals": {"One": 1, "Forty-nine": 1}, "pronouns": {"I": 1}}}, {"content": "50. Parents can make me fit for their natural development.", "word_count": 12, "label": "", "sentence_structure": "Subject鈥搗erb鈥搊bject", "subjective_objective": {"answer": "Subjective", "rationale": "Because it is a statement describing patients ' subjective feelings and expectations that they want their parents to support them in their own way."}, "sentence_sentiment": {"answer": "Neutral", "rationale": "It simply describes the way parents treat their children's development, and does not evaluate or express attitudes to this way."}, "language_style": {"answer": "Tactful and warm", "rationale": "The entry is more adept and interactive when it is spoken in a gentle and inviting manner, rather than by direct statements or orders."}, "time_dimension": {"answer": "Past", "rationale": "The \"may\" action has occurred in the past and affects the present or the future, and thus the past is judged."}, "sentence_type": "Affirmative declarative sentence", "pos": {"nouns": {"parents": 2, "Development": 1}, "verbs": {"Jean": 1, "Yes": 1, "Let it be": 1, "Development": 1}, "adverbs": {"Other": 1}, "numerals": {"50": 1}, "pronouns": {"I": 1}}}, {"content": "51. Parents often behaved rudely towards me.", "word_count": 10, "label": "", "sentence_structure": "Subject鈥搗erb鈥揷omplement", "subjective_objective": {"answer": "Subjective", "rationale": "This is a subjective assessment and perception of the behaviour of the patient ' s parents, rather than an objective fact or result."}, "sentence_sentiment": {"answer": "Negative", "rationale": "Because the \"coarse and rude\" act in the entry is negative."}, "language_style": {"answer": "Calm and direct", "rationale": "The entry is presented in a direct manner, making clear that the problem faced by the respondents is that \"parents are often rude to me\", without any euphemism or rhetoric, and that the language style is relatively simple."}, "time_dimension": {"answer": "Present", "rationale": "The keyword is \"often to me\", indicating that the act is still ongoing and thus judged to be the present."}, "sentence_type": "Negative declarative sentence", "pos": {"nouns": {"parents": 1, "I": 1}, "verbs": {"to": 1, "Naughty": 1}, "adjectives": {"Nasty": 1, "Insolent": 1}, "numerals": {"51锛?: 1}}}, {"content": "52. Sometimes, even for a little bit of a little bit of a thing, my parents would punish me severely.", "word_count": 25, "label": "", "sentence_structure": "Subject鈥搗erb鈥搊bject", "subjective_objective": {"answer": "Objective", "rationale": "Because this sentence describes an objective fact that parents punish certain things severely, whether large or small."}, "sentence_sentiment": {"answer": "Negative", "rationale": "Because the \"heavy punishment\" in the entry is negative."}, "language_style": {"answer": "Calm and direct", "rationale": "The language style of the entry is a calm and direct one. It opens up to a phenomenon or situation, without too many rhetorical or emotional accents, and is more objective and direct, which facilitates the understanding and answers of the survey respondents.\""}, "time_dimension": {"answer": "Present", "rationale": "The entry describes events that the subjects have experienced in the past, i.e., the severe punishment of their parents in minor matters."}, "sentence_type": "Affirmative declarative sentence", "pos": {"nouns": {"Porky, porcelain": 1, "It's nothing": 1, "parents": 1, "I": 1}, "verbs": {"Yes": 3, "Punishment": 1, "Hard": 1}, "adjectives": {"A little": 1, "Small": 1, "It's a little puffy": 1, "Hard": 1}, "adverbs": {"Even": 1}, "numerals": {"52": 1}, "classifiers": {"One": 3}, "pronouns": {"I": 1}}}, {"content": "53. Parents beat me for no reason.", "word_count": 10, "label": "", "sentence_structure": "Subject鈥搗erb鈥搊bject", "subjective_objective": {"answer": "Objective", "rationale": "Because \"parents have hit me without reason\" is a fact that has happened, and it can be proven or perjured. This does not examine the patient's subjective thoughts or feelings. So, this is an objective entry."}, "sentence_sentiment": {"answer": "Negative", "rationale": "Because the \"hit\" in the entry is negative."}, "language_style": {"answer": "Calm and direct", "rationale": "The language style of the entry is calm and straightforward. It expresses the subject matter in a simple and clear language, without too many emotional or rhetorical techniques, and is sound and objective."}, "time_dimension": {"answer": "Past", "rationale": ""}, "sentence_type": "Negative declarative sentence", "pos": {"nouns": {"parents": 1, "For no reason": 1, "Fight": 1}, "verbs": {"Tsang": 1, "Fight": 1}, "adjectives": {"For no reason": 1}, "adverbs": {"Tsang": 1}, "numerals": {"53": 1}}}, {"content": "54. Parents usually engage in my amateur activities.", "word_count": 15, "label": "", "sentence_structure": "Subject鈥搗erb鈥搊bject", "subjective_objective": {"answer": "Subjective", "rationale": "Since this entry deals with the extent to which parents are involved in their personal hobby, it is a matter of subjective feelings and experiences of patients."}, "sentence_sentiment": {"answer": "Positive", "rationale": "Because the entry says \"Parents usually engage in my hobby\" and it's a caring and supportive act, with positive emotional aspects."}, "language_style": {"answer": "Tactful and warm", "rationale": "The entry is more adept and interactive when it is spoken in a gentle and inviting manner, rather than by direct statements or orders."}, "time_dimension": {"answer": "Present", "rationale": "The entry describes the current situation of the person tested, the key word being \"parents usually participate\", which is a current situation that is very frequent and therefore a current state of affairs."}, "sentence_type": "Affirmative declarative sentence", "pos": {"nouns": {"parents": 1, "I": 1, "Amateur activities": 1}, "verbs": {"Yes": 1, "Participation": 1}, "adverbs": {"Usually": 1}, "numerals": {"54锛?: 1}}}, {"content": "55. I was often beaten by my parents.", "word_count": 8, "label": "", "sentence_structure": "Subject鈥搗erb鈥揷omplement", "subjective_objective": {"answer": "Subjective", "rationale": "Because beating by parents is a subjective fact of the patient's experience."}, "sentence_sentiment": {"answer": "Negative", "rationale": "Because the behavior in the entry is negative."}, "language_style": {"answer": "Calm and direct", "rationale": "The entry asks the question whether the person surveyed is often subjected to corporal punishment by his parents, in a clear language, and without excessive emotional colours and rhetorical techniques. The language style of the article can therefore be considered \"cool and direct\"."}, "time_dimension": {"answer": "Past", "rationale": ""}, "sentence_type": "Affirmative declarative sentence", "pos": {"nouns": {"I": 1, "parents": 1, "Fight": 1}, "verbs": {"Scrap": 1, "be": 1}, "adverbs": {"Regular": 1}, "numerals": {"55": 1}}}, {"content": "Parents often allow me to go where I like to go, and they do not worry too much.", "word_count": 25, "label": "", "sentence_structure": "Subject鈥搗erb鈥搊bject", "subjective_objective": {"answer": "Subjective", "rationale": "Because \"parents often allow me to go where I like\" and \"they don't worry too much\" are the patient's personal views or feelings. This reflects the relationship and way of communicating between the patient and his parents, and does not involve objective facts or behavioural outcomes. So this entry is viewed from a subjective perspective."}, "sentence_sentiment": {"answer": "Positive", "rationale": "Because the \"Parents often allow me to go where I like\" in the entry is positive."}, "language_style": {"answer": "Tactful and warm", "rationale": "The question is in a more moderate language, like a friendly conversation with the interviewee, rather than direct orders or inquiries. This expression makes people feel more friendly and comfortable, more receptive and willing to answer. Moreover, the tone of the question contains elements of certainty and encouragement, such as \"parents often allow me to go where I like,\" which can be felt as respected and supported."}, "time_dimension": {"answer": "Present", "rationale": "The entry describes the current situation of the testee, namely the attitude of their parents to the testee's out. The keywords \"often\" and \"will\" indicate that this is a regular act and that this behaviour is still ongoing. Thus, the temporal dimension of the purpose is now."}, "sentence_type": "Affirmative declarative sentence", "pos": {"nouns": {"parents": 2, "Local": 1, "I": 1, "they": 1, "That's too much": 1}, "verbs": {"Allow": 1, "Go on": 1, "I'm worried": 1}, "adjectives": {"Often": 1, "Yeah": 1, "No, I won't": 1, "That's too much": 1}, "adverbs": {"And": 1}, "numerals": {"56": 1, "2": 1}, "classifiers": {"individual": 1}, "pronouns": {"EMPTY": 1}}}, {"content": "57. Parents have strict restrictions on what I should do and what I should not do, and they have no concessions.", "word_count": 25, "label": "", "sentence_structure": "Subject鈥搗erb鈥搊bject", "subjective_objective": {"answer": "Subjective", "rationale": "Because the title \"Parents have strict limits on what I should do, what they should not do, and they have no concessions,\" is a subjective feeling and understanding of the patient, not an objective fact. This is a subjective assessment and perception of the family environment, family education, etc."}, "sentence_sentiment": {"answer": "Negative", "rationale": "Because the entry expresses a negative emotional attitude - parents have too strict control and restrictions on the behaviour of the child, which may put pressure and restraint on the child."}, "language_style": {"answer": "Calm and direct", "rationale": "The entry describes the strict requirements of the interviewee鈥檚 parents for decision-making, without excessive rhetorical or emotional rendering. The language style of the purpose can therefore be described as being straightforward and calm."}, "time_dimension": {"answer": "Past", "rationale": "In this entry, the words \"restricted\" and \"absolutely not to give in\" indicate that this is an act or condition that has occurred and that existed in the past and therefore is judged to be the past."}, "sentence_type": "Negative declarative sentence", "pos": {"nouns": {"parents": 1, "I": 2, "What": 2, "What should I do": 1, "Strict": 1, "Limits": 1, "No compromise": 1}, "verbs": {"Both": 1, "have": 1, "Do it": 2, "Compliment": 1}, "adjectives": {"Strict": 1}, "numerals": {"57锛?: 1}}}, {"content": "58. Parents treat me in a way that embarrasses me.", "word_count": 15, "label": "", "sentence_structure": "Subject鈥搗erb鈥揷omplement", "subjective_objective": {"answer": "Subjective", "rationale": "Because \"the way to embarrass me\" is a description of subjective feelings, an assessment and an explanation of what patients experience."}, "sentence_sentiment": {"answer": "Negative", "rationale": "Because the \"moderate way\" in the entry is negative."}, "language_style": {"answer": "Calm and direct", "rationale": "The language style of the entry is a calm and straightforward one. It directly asks the parents of the interviewee whether they have treated them in a humiliating manner, without using excessive rhetorical or emotional rendering, and in a more objective manner."}, "time_dimension": {"answer": "Present", "rationale": "Now the entry describes the experience and feelings of the testees, that is, their parents treat them in a way that embarrasses them. So the time dimension is now."}, "sentence_type": "Negative declarative sentence", "pos": {"nouns": {"parents": 1, "Modalities": 1}, "verbs": {"鈾?And I'm gonna be 鈾?: 1, "Treatment": 1}, "adjectives": {"I'm embarrassed": 1}, "numerals": {"58锛?: 1}}}, {"content": "59. I find the fear of parents that something may happen to me exaggerated and excessive.", "word_count": 21, "label": "", "sentence_structure": "Subject鈥揷opula鈥損redicative", "subjective_objective": {"answer": "Subjective", "rationale": "Because \"the feeling\" expresses patients' subjective opinion and assessment of the matter."}, "sentence_sentiment": {"answer": "Negative", "rationale": "Because the \"fear is exaggerated, excessive\" in the entry expresses a negative emotional attitude."}, "language_style": {"answer": "Tactful and warm", "rationale": "This expression helps to create a more positive atmosphere for dialogue and reduces the risk of conflict."}, "time_dimension": {"answer": "Present", "rationale": "The entry indicates the action or state that the respondent is in progress in responding to the questionnaire and is therefore in current state."}, "sentence_type": "Affirmative declarative sentence", "pos": {"nouns": {"parents": 1, "I": 2, "Yes": 1, "I'm worried": 1}, "verbs": {"to": 1, "Maybe": 1, "Out": 1, "be": 1, "Exaggeration": 1, "It's too much": 1}, "adjectives": {"Exaggeration": 1, "That's too much": 1}, "numerals": {"59": 1}, "pronouns": {"I": 2}}}, {"content": "I feel a warm, sweet and intimate feeling of love with parents.", "word_count": 22, "label": "", "sentence_structure": "Subject鈥揷opula鈥損redicative", "subjective_objective": {"answer": "Subjective", "rationale": "Because it relates to the feelings and views of the patient himself, it is a subjective expression."}, "sentence_sentiment": {"answer": "Positive", "rationale": "Because the words \"hom, sweet, and intimate\" in the entries express positive emotions."}, "language_style": {"answer": "Tactful and warm", "rationale": "The entry conveys intimate relationships with parents by describing the feeling of \"homic, considerate and intimate\", without direct statements or orders, and is more euphemistic and acceptable in language style."}, "time_dimension": {"answer": "Present", "rationale": ""}, "sentence_type": "Affirmative declarative sentence", "pos": {"nouns": {"Grandpa": 1, "Questionnaire": 1, "Entry": 1, "I think": 1, "parents": 2, "Feel": 1}, "verbs": {"Existence": 1, "have": 1, "Warm": 1, "Kind": 1, "Intimacy": 1, "and": 1, "Between": 1}, "adjectives": {"One": 1}, "adverbs": {"I think": 1, "Between": 1}, "numerals": {"60锛?: 1}, "pronouns": {"I": 1}}}, {"content": "61. Parents can tolerate my different views.", "word_count": 15, "label": "", "sentence_structure": "Subject鈥搗erb鈥搊bject", "subjective_objective": {"answer": "Subjective", "rationale": "This is because the patient's perception and feelings towards his parents are subjective."}, "sentence_sentiment": {"answer": "Positive", "rationale": "Since this entry expresses the tolerance of parents for the different views of their children, it can be interpreted as an act that supports and encourages the children to think independently and is therefore positive emotional tendencies."}, "language_style": {"answer": "Tactful and warm", "rationale": "The entry is more moderate in its language style, using the term \"forlerable\" to give people a sense of peace and tolerance, while avoiding some hard language that may cause resistance. Moreover, the title is also expressed in a first person鈥檚 way, which is more likely to trigger a sense of resonance and self-identification among the interviewees, so that the language style can be classified as euphemism and warmness."}, "time_dimension": {"answer": "Present", "rationale": "The entry asks the respondent about the extent to which parents have accepted their differences in the current circumstances and therefore the temporal dimension is now judged."}, "sentence_type": "Affirmative declarative sentence", "pos": {"nouns": {"parents": 1, "I": 1, "Observations": 1}, "verbs": {"Yes": 1, "Tolerance": 1, "have": 1, "be": 1}, "adverbs": {"Different": 1}, "numerals": {"61锛?: 1}}}, {"content": "Parents often get mad at me without knowing why.", "word_count": 21, "label": "", "sentence_structure": "Subject鈥搗erb鈥搊bject", "subjective_objective": {"answer": "Objective", "rationale": "Because \"Big Breath\" is an objective fact, and it happens without knowing why. Although these acts may have a negative impact on patients and a subjective feeling, they are objective events."}, "sentence_sentiment": {"answer": "Negative", "rationale": "Because \"dry\" is negative."}, "language_style": {"answer": "Tactful and warm", "rationale": "The entry is more personal and interactive when it is spoken in a conversation in a conversation of inquiry and concern rather than in direct statements or orders."}, "time_dimension": {"answer": "Past", "rationale": "The \"always\" in the entry is the \"severe\" of past sexual activity, \"smuggling at me without knowing why\" event in the past, and therefore the temporal dimension is the past."}, "sentence_type": "Affirmative declarative sentence", "pos": {"nouns": {"parents": 2, "Situation": 1, "- Grumpy": 1}, "verbs": {"Often": 1, "Let's go": 1, "to": 1, "I": 1, "Yes": 1, "Reason": 1}, "adjectives": {"For no reason": 1}, "adverbs": {"in": 1, "not": 1}, "numerals": {"62": 1}, "pronouns": {"they": 1}}}, {"content": "63. When I did something successful, I felt that my parents were proud of me.", "word_count": 21, "label": "", "sentence_structure": "Subject鈥搗erb鈥搊bject", "subjective_objective": {"answer": "Subjective", "rationale": "Because \"be proud\" is a subjective feeling or attitude."}, "sentence_sentiment": {"answer": "Positive", "rationale": "Because the expression \"I feel proud of my parents\" in the entry is positive, expressing a sense of satisfaction with self-identification and achievement."}, "language_style": {"answer": "Tactful and warm", "rationale": "Warm and warm."}, "time_dimension": {"answer": "Present", "rationale": "The keyword \"thinks\" uses the general current, which means that it is a constant feeling, not a past or future. \"When it succeeds, it also indicates that it is a state and behaviour. So the entry to the questionnaire asks about the emotional state of the person being tested."}, "sentence_type": "Affirmative declarative sentence", "pos": {"nouns": {"Things": 1, "Success": 1, "parents": 2, "I": 1}, "verbs": {"Access": 1, "I think": 1, "Yes": 1, "very": 1, "Proud": 1}, "adverbs": {"When": 1, "Time": 1}, "numerals": {"63": 1}}}, {"content": "Parents often love me more than my brothers and sisters.", "word_count": 16, "label": "", "sentence_structure": "Subject鈥揷opula鈥損redicative", "subjective_objective": {"answer": "Subjective", "rationale": "Because \"preference\" is the patient's subjective assessment and perception of parental behaviour."}, "sentence_sentiment": {"answer": "Negative", "rationale": "Because it expresses \"parent preference\", this is usually considered a negative situation."}, "language_style": {"answer": "Calm and direct", "rationale": "The entry is based on a direct statement that parents have a preference for themselves and their siblings. The phrase is not based on too many rhetorical or emotional expressions, but rather on a factual description, so that its language style can be considered as calm and straightforward."}, "time_dimension": {"answer": "Present", "rationale": ""}, "sentence_type": "Affirmative declarative sentence", "pos": {"nouns": {"Questionnaire": 1, "Entry": 1, "Brothers and sisters": 2, "parents": 1, "I": 1}, "verbs": {"- Yeah": 1, "Prejudice": 1}, "adverbs": {"Often": 1}, "numerals": {"64": 1}, "pronouns": {"they": 1}}}, {"content": "65. Parents sometimes blame siblings for their responsibility even if they are wrong.", "word_count": 21, "label": "", "sentence_structure": "Subject鈥搗erb鈥搊bject", "subjective_objective": {"answer": "Subjective", "rationale": "Because this is the patient ' s perception of the parents ' responsibility and their subjective perception and view of the responsibility."}, "sentence_sentiment": {"answer": "Negative", "rationale": "Because the action of \"parents blameing siblings\" in the entry is negative."}, "language_style": {"answer": "Calm and direct", "rationale": "The sentence is simple and clear, with no rhetorical or emotional repertoire. It presents a fact directly and raises a question of not using euphemism and seductive language to draw the subject. This language style is more objective and calm, and is more suitable for use in formal questionnaires."}, "time_dimension": {"answer": "Past", "rationale": "The entry describes what the testee has experienced in the past, i.e. parents attribute responsibility to the siblings of the testee."}, "sentence_type": "Affirmative declarative sentence", "pos": {"nouns": {"Time": 1, "Error": 1, "I": 1, "parents": 2, "Responsibility": 1, "Blame": 1, "Brothers and sisters": 1}, "verbs": {"be": 1, "in": 1, "- Put it on": 1, "Blame": 1, "Return": 1}, "adverbs": {"Sometimes": 1}, "numerals": {"65锛?: 1}}}, {"content": "66. Parents often hug me.", "word_count": 7, "label": "", "sentence_structure": "Subject鈥搗erb鈥揷omplement", "subjective_objective": {"answer": "Subjective", "rationale": "Because it is a subjective feeling of a person to be embraced, not an objective fact."}, "sentence_sentiment": {"answer": "Positive", "rationale": "Because the act of \"often hugging\" in the entry expresses a positive emotional attitude."}, "language_style": {"answer": "Tactful and warm", "rationale": "The entry uses the first person's term \"you\" and the descriptive term \"often\" and \"acceptance\" , which are more natural and easily resonant with the interviewee. At the same time, the question is not directly asked about the interviewee's feelings or assessment, but is indirectly obtained by asking about the parents' behaviour, which is also relatively mild."}, "time_dimension": {"answer": "Present", "rationale": ""}, "sentence_type": "Affirmative declarative sentence", "pos": {"nouns": {"parents": 1, "Hug": 1}, "verbs": {"Regular": 1}, "adverbs": {"Regular": 1}, "classifiers": {"individual": 1}, "pronouns": {"I": 1}}}]}

{"questionnaire_title": "Mental health questionnaire for university students", "questionnaire_category": "Mental health", "item_count": 68, "emotion_shift_overall": "From negative to positive", "language_style_overall": "Calm and direct", "items": [{"content": "One hunger strike.", "word_count": 4, "label": "", "sentence_structure": "Subject鈥揷opula鈥損redicative", "subjective_objective": {"answer": "Subjective", "rationale": "\"Adulgence is a subjective description, because it involves the feelings and experiences of the patient and can be influenced by a variety of factors, including personal emotions, circumstances, etc. While appetite may be caused by objective physical conditions (such as disease or drug side effects), it is still a subjective experience in itself. The answer is, therefore, subjective."}, "sentence_sentiment": {"answer": "Negative", "rationale": "Because \"fatal\" is usually considered a health problem, with negative emotional aspects."}, "language_style": {"answer": "Tactful and warm", "rationale": "The entry is more euphemistic, avoids the use of too direct language, gives a more intimate sense of and better elicits interest and responsiveness from the other side. At the same time, it is more acceptable to express it and can gain the trust and support of the person under investigation."}, "time_dimension": {"answer": "Past", "rationale": "The entry describes the person's past behaviour or physical condition, and the keyword \"disturbed appetite\" indicates problems that have arisen over time."}, "sentence_type": "Negative declarative sentence", "pos": {"nouns": {"A appetite": 1, "Pathological conditions": 1}, "verbs": {"It's not working": 1}}}, {"content": "Two sick, stomach-suffering, stomach-ache.", "word_count": 9, "label": "", "sentence_structure": "Subject鈥搗erb鈥揷omplement", "subjective_objective": {"answer": "Subjective", "rationale": "Because nausea, stomach distress and stomach pain are subjective descriptions of the patient鈥檚 feelings and symptoms, not objective entities or events. These descriptions reflect the patient鈥檚 perception and interpretation of his or her physical state and are therefore subjective in nature."}, "sentence_sentiment": {"answer": "Negative", "rationale": "Because the symptoms in the entries are negative."}, "language_style": {"answer": "Calm and direct", "rationale": "The entry directly describes the possible symptoms of the person surveyed in a concise and clear language, does not add too much emotion or evaluation colour and is more conducive to obtaining objective and accurate feedback."}, "time_dimension": {"answer": "Past", "rationale": "The entry asks if the person tested has recently felt nausea, stomach pain or stomach pain, and the temporal dimension is a past."}, "sentence_type": "Affirmative declarative sentence", "pos": {"nouns": {"Gross": 1, "-Adulge": 1, "It's hard": 1, "- Tummy": 1, "Ouch": 1}, "adjectives": {"Serious": 1}}}, {"content": "Three is easy to diarrhea or constipation.", "word_count": 8, "label": "", "sentence_structure": "Subject鈥揷opula鈥損redicative", "subjective_objective": {"answer": "Objective", "rationale": "Because \"easy diarrhea or constipation\" is a biological phenomenon that is not controlled by the individual's will and is therefore considered from an objective perspective."}, "sentence_sentiment": {"answer": "Negative", "rationale": "Because the entry \"Easy to diarrhea or constipation\" is a physical discomfort and is usually considered negative."}, "language_style": {"answer": "Tactful and warm", "rationale": "The entry is more adept and interactive when it is spoken in a gentle and inviting manner, rather than by direct statements or orders."}, "time_dimension": {"answer": "Past", "rationale": "Answer: \"Easily diarrhea or constipation\" in the entry describes the physical condition of the person being tested, which may be a long-standing problem or a recent symptom. So, \"Easily diarrhea or constipation\" relates to the symptoms of the past and the state of the present."}, "sentence_type": "Negative declarative sentence", "pos": {"nouns": {"I'm having a stomach attack": 1, "Decency": 1}, "verbs": {"Easy": 1}, "numerals": {"3": 1}}}, {"content": "Four, focus on heart attack and pulse.", "word_count": 7, "label": "", "sentence_structure": "Subject鈥搗erb鈥揷omplement", "subjective_objective": {"answer": "Subjective", "rationale": "Because \"care\" is a subjective attitude or feeling, it is the degree of subjective concern of the patient for heart attack and pulse."}, "sentence_sentiment": {"answer": "Neutral", "rationale": "Because the entry does not display a positive or negative emotional profile, it is merely asking whether the person surveyed is concerned about his or her heart attack and pulse."}, "language_style": {"answer": "Tactful and warm", "rationale": "This language style is more likely to make the interviewee feel respected and valued, and to promote motivation and authenticity in the form of a questionnaire."}, "time_dimension": {"answer": "Present", "rationale": ""}, "sentence_type": "Exclamatory sentence", "pos": {"nouns": {"Heartbreak": 1, "Pulse": 1}, "verbs": {"Attention": 1}, "numerals": {"4": 1}}}, {"content": "5 in good health", "word_count": 8, "label": "", "sentence_structure": "Subject鈥揷opula鈥損redicative", "subjective_objective": {"answer": "Objective", "rationale": "Because good health is a description of the patient ' s health, it is an objective fact."}, "sentence_sentiment": {"answer": "Neutral", "rationale": "Because the entry does not have any emotional color."}, "language_style": {"answer": "Calm and direct", "rationale": "The entry is simple and clear in asking the person under investigation about his or her health, without excessive emotional or rhetorical style, and the language style is a direct and calm type.\""}, "time_dimension": {"answer": "Present", "rationale": ""}, "sentence_type": "Affirmative declarative sentence", "pos": {"adjectives": {"Body": 1, "Health": 1}, "numerals": {"5": 1}}}, {"content": "Six complaints and a lot of complaints.", "word_count": 6, "label": "", "sentence_structure": "Subject鈥搗erb鈥揷omplement", "subjective_objective": {"answer": "Subjective", "rationale": "Because complaints and grievances are the expression of one ' s subjective feelings and attitudes, they are not objective facts or outcomes."}, "sentence_sentiment": {"answer": "Negative", "rationale": "Because \"shut and disgruntled\" expresses a negative sentiment."}, "language_style": {"answer": "Calm and direct", "rationale": "The entry uses a clear and direct language to ask respondents about the frequency of complaints and grievances in life, with little or no rhetorical or emotional projection, and is more objective in tone."}, "time_dimension": {"answer": "Present", "rationale": "The keyword \"6 gruesome and disgruntled\" indicates that it is describing the current emotional state or feelings of the person being tested and thus judging as now."}, "sentence_type": "Imperative sentence", "pos": {"nouns": {"Scoff": 1, "Dissatisfied": 1}, "adjectives": {"More": 1}, "numerals": {"6": 1}}}, {"content": "7 parents over-zealous", "word_count": 6, "label": "", "sentence_structure": "Subject鈥搗erb鈥揷omplement", "subjective_objective": {"answer": "Subjective", "rationale": "Because \"high expectations of parents\" are the patient's perception or perception of the pressures he faces, and are a subjective description."}, "sentence_sentiment": {"answer": "Negative", "rationale": "Because the expression \"too much expectation from parents\" has negative feelings and implies possible pressures and burdens."}, "language_style": {"answer": "Calm and direct", "rationale": "The entry is investigated in a direct question-and-answer manner and is not based on any rhetorical or overly emotional language, which is more objective in tone."}, "time_dimension": {"answer": "Present", "rationale": ""}, "sentence_type": "Negative declarative sentence", "pos": {"nouns": {"parents": 1, "Expectations": 1, "High": 1}, "adverbs": {"Pass": 1}}}, {"content": "Is it unfortunate that you have your own past and family?", "word_count": 13, "label": "", "sentence_structure": "Subject鈥搗erb鈥揷omplement", "subjective_objective": {"answer": "Subjective", "rationale": "This is because the patient ' s perception and evaluation of the past and the family are subjective."}, "sentence_sentiment": {"answer": "Negative", "rationale": "Because the word \"unlucky\" in the entry is negative."}, "language_style": {"answer": "Tactful and warm", "rationale": "The language style of the entry is more euphemistic, moderate, not one that uses too direct or emotional expressions, but one that is more inquisitive, which is more resonant and comfortable for the interviewee. At the same time, the term \"unfortunate\" is not a very subjective term, but rather a relatively objective description of a state, so the language style of the entire entry tends to be more euphemistic and warm."}, "time_dimension": {"answer": "Past", "rationale": "The entry is for the purpose of asking the testee about his past experience, and the temporal dimension is a past."}, "sentence_type": "Interrogative sentence", "pos": {"nouns": {"self": 1, "Past": 1, "Family": 1, "Unfortunately": 1}, "verbs": {"Is it possible": 1}, "numerals": {"8": 1}}}, {"content": "Nine is too worried about the future.", "word_count": 9, "label": "", "sentence_structure": "Subject鈥揷opula鈥損redicative", "subjective_objective": {"answer": "Subjective", "rationale": "Because \"fear\" is a subjective emotional experience and judgment that reflects the patient鈥檚 fear and anxiety about his future. This emotional experience is the individual feeling of the patient, not an objective fact or result."}, "sentence_sentiment": {"answer": "Negative", "rationale": "Because \"too worried\" is negative."}, "language_style": {"answer": "Calm and direct", "rationale": "The entry directly expresses the subject of an over-heavy concern about the future, with no excessive rhetoric or emotional rendering, and is more objective in tone."}, "time_dimension": {"answer": "Present", "rationale": "In the future, the entry contains ideas and concerns about the future, the key words being \"too much to worry\" and \"the future\" and pointing to situations that have not yet occurred and that may arise in the future."}, "sentence_type": "Interrogative sentence", "pos": {"nouns": {"Things": 1, "Future": 1}, "verbs": {"I'm worried": 1, "Too much": 1}, "adverbs": {"Too much": 1}, "numerals": {"9": 1}}}, {"content": "Ten doesn't want to see anyone.", "word_count": 4, "label": "", "sentence_structure": "Subject鈥搗erb鈥搊bject", "subjective_objective": {"answer": "Objective", "rationale": "Because \"no person\" is the patient's subjective attitude, but here it is an objective fact to see whether the patient has really seen no one."}, "sentence_sentiment": {"answer": "Negative", "rationale": "Because the article is \"no one wants to see you,\" it's a negative emotional attitude."}, "language_style": {"answer": "Calm and direct", "rationale": "The entry directly asked whether the person surveyed was unwilling to meet with others in certain circumstances, without using any rhetorical or emotional rendering, and was more objective and quiet."}, "time_dimension": {"answer": "Present", "rationale": "Now the entry asks the testee about his current attitude and feelings, and the keyword \"no person\" shows the sense of rejection of interpersonal interaction in the current emotional state. The temporal dimension is now."}, "sentence_type": "Interrogative sentence", "pos": {"nouns": {"person": 1}, "verbs": {"Yes": 1, "See": 1}, "adjectives": {"not": 1, "Ten": 1}, "numerals": {"10": 1}}}, {"content": "Eleven thinks they're not themselves.", "word_count": 8, "label": "", "sentence_structure": "Subject鈥揷opula鈥損redicative", "subjective_objective": {"answer": "Subjective", "rationale": "Because \"not being\" is a subjective feeling and perception, not an objective fact or act."}, "sentence_sentiment": {"answer": "Negative", "rationale": "Because the expression \"not yourself\" has a negative and self-negative tendency, it is often considered a negative emotional experience."}, "language_style": {"answer": "Tactful and warm", "rationale": "It is more acceptable and inclusive because it asks the respondents in a gentle and understandable way about their feelings, rather than directly accusing or judging them."}, "time_dimension": {"answer": "Present", "rationale": "The keyword \"I feel like I'm not\" means the person who's been tested for self-awareness, and so it's now."}, "sentence_type": "Negative declarative sentence", "pos": {"nouns": {"self": 2}, "verbs": {"I'm not sure": 1, "be": 1}, "numerals": {"11": 1}}}, {"content": "Lack of enthusiasm and motivation", "word_count": 8, "label": "", "sentence_structure": "Subject鈥搗erb鈥揷omplement", "subjective_objective": {"answer": "Subjective", "rationale": "Because lack of enthusiasm and motivation is the feeling and attitude of the patient, it is subjective."}, "sentence_sentiment": {"answer": "Negative", "rationale": "Because lack of enthusiasm and motivation is a negative behaviour or attitude."}, "language_style": {"answer": "Calm and direct", "rationale": "\"Calm down straight.\""}, "time_dimension": {"answer": "Past", "rationale": "The entry describes a subjective feeling or state, and the temporal dimension does not clearly determine whether it is past, present or future."}, "sentence_type": "Negative declarative sentence", "pos": {"nouns": {"鈾?Hot": 1, "Active": 1}, "verbs": {"Lack": 1}, "numerals": {"12": 1}}}, {"content": "13 Pessimism", "word_count": 2, "label": "", "sentence_structure": "Subject鈥搗erb鈥揷omplement", "subjective_objective": {"answer": "Subjective", "rationale": "Pessimism is a subjective emotional attitude."}, "sentence_sentiment": {"answer": "Negative", "rationale": "Because \"pessimism\" is a negative emotional vocabulary."}, "language_style": {"answer": "Calm and direct", "rationale": "The subject is a direct question to the respondents about pessimism, the lack of excessive rhetorical or emotional rendering, and the more plain and direct language style."}, "time_dimension": {"answer": "Future", "rationale": "The entry expresses expectations and predictions of future emotions, and \"pessimism\" is a term describing the state of future emotions, and is therefore judged as future behaviour or mentality."}, "sentence_type": "Negative declarative sentence", "pos": {"nouns": {"Pessimism": 1, "Questionnaire": 1, "Entry": 1}, "verbs": {"Analysis": 1}, "numerals": {"13": 1}}}, {"content": "14 is not focused.", "word_count": 5, "label": "", "sentence_structure": "Subject鈥搗erb鈥揷omplement", "subjective_objective": {"answer": "Subjective", "rationale": "Because the degree of concentration is a subjective feeling and experience of one person, different people may have different assessments of the degree of concentration of thought in the same context."}, "sentence_sentiment": {"answer": "Negative", "rationale": "The \"discretionary\" act is negative."}, "language_style": {"answer": "Calm and direct", "rationale": "The entry directly expresses the subject of an inquiry into the lack of focus, with no excessive rhetoric or emotional rendering and a more objective tone."}, "time_dimension": {"answer": "Past", "rationale": "The entry asks if the testee has experienced \"inconsistent\" in the past and the temporal dimension is a past."}, "sentence_type": "Negative declarative sentence", "pos": {"nouns": {"Ideas": 1, "Focus": 1}, "verbs": {"Not focused": 1}, "numerals": {"14": 1}}}, {"content": "Fifteen is in a state of mood.", "word_count": 6, "label": "", "sentence_structure": "Subject鈥揷opula鈥損redicative", "subjective_objective": {"answer": "Subjective", "rationale": "The examples provided by the title show that the basis of judgement is \"subjective\" or \"objective\" and requires a patient's perspective. This is a subjective description of \"15 emotional inordinateness.\" Because emotions are determined by the mental state and feeling of the individual, self-reporting by the patient is subjective. The answer is therefore \"subjective.\""}, "sentence_sentiment": {"answer": "Negative", "rationale": "The basis for the judgement: excessive emotional volatility is usually considered to be an unhealthy emotional state, which can lead to high emotional volatility, anger, anxiety, etc. The purpose of the article is therefore expressed in a negative emotional way."}, "language_style": {"answer": "Tactful and warm", "rationale": "The article is expressed in a more euphemistic manner, without the excessive volatility of the mood of direct accusations or criticism of the respondents, and instead uses an inquisitorial tone to guide the respondents to their answers, which is more acrimonym and interactive. At the same time, the description of \"excessive mood\" is also mild and does not use strong terms such as \"excessive\" and \"unusual\" and is consistent with the \"false\" language style."}, "time_dimension": {"answer": "Present", "rationale": "The keyword now \"unemotionally unstable\" is a term that describes the current situation, indicating the emotional fluctuations that the testees are experiencing when they fill out the questionnaire. At the same time, \"15\" may represent a quantitative question that requires the testee to choose the corresponding answer options according to his or her actual situation, suggesting that this is a survey conducted at this time."}, "sentence_type": "Exclamatory sentence", "pos": {"nouns": {"Emotion": 1, "Rise and shine": 1}, "verbs": {"Pass": 1}, "adjectives": {"Large": 1}, "numerals": {"15": 1}}}, {"content": "16 often insomnia.", "word_count": 4, "label": "", "sentence_structure": "Subject鈥搗erb鈥揷omplement", "subjective_objective": {"answer": "Subjective", "rationale": "Because insomnia is a subjective problem for all."}, "sentence_sentiment": {"answer": "Negative", "rationale": "Because \"insomnia\" is a negative behavior or state."}, "language_style": {"answer": "Calm and direct", "rationale": "The entry uses a simple and clear language to describe a widespread sleep problem, without adding too many emotions or rhetorical techniques, so that the language style is more calm and straightforward."}, "time_dimension": {"answer": "Present", "rationale": ""}, "sentence_type": "Interrogative sentence", "pos": {"nouns": {"16": 1, "Insomnia": 1}, "verbs": {"Often": 1}}}, {"content": "Seventeen headaches.", "word_count": 2, "label": "", "sentence_structure": "Subject鈥揷opula鈥損redicative", "subjective_objective": {"answer": "Subjective", "rationale": "Because headaches are subjective."}, "sentence_sentiment": {"answer": "Negative", "rationale": "Because \"headache\" is usually a symptom of physical discomfort or disease, and it's a negative emotion."}, "language_style": {"answer": "Calm and direct", "rationale": "The entry directly asks the interviewees whether they have frequent headaches, do not use rhetorical or emotional renderings and are expressed in an objective manner, so that the language style is straightforward."}, "time_dimension": {"answer": "Future", "rationale": "The entry asks about the future headache of the testee, which includes visions and expectations for the future, and the keywords \"headache\" and \"17\", which point to situations that have not yet occurred and that may arise in the future."}, "sentence_type": "Exclamatory sentence", "pos": {"nouns": {"Headache": 1, "17": 1}, "numerals": {"17": 1}}}, {"content": "Eighteen necks, shoulder sores.", "word_count": 6, "label": "", "sentence_structure": "Subject鈥搗erb鈥揷omplement", "subjective_objective": {"answer": "Objective", "rationale": "Because \"the neck, shoulder sore\" is a physical symptom, a fact that can be confirmed by observation and examination, it is an objective evaluation criterion."}, "sentence_sentiment": {"answer": "Negative", "rationale": "Because \"sore\" usually means infirmity, it's a negative word."}, "language_style": {"answer": "Tactful and warm", "rationale": "This entry uses a mild and concerned tone to ask if the other side has a sore neck or shoulder, reflecting concern and concern for the health of the person surveyed."}, "time_dimension": {"answer": "Past", "rationale": ""}, "sentence_type": "Affirmative declarative sentence", "pos": {"nouns": {"18": 1, "Neck": 1, "Shoulders": 1, "Sour": 1}}}, {"content": "19 chests are suffocating.", "word_count": 4, "label": "", "sentence_structure": "Subject鈥搗erb鈥揷omplement", "subjective_objective": {"answer": "Subjective", "rationale": "Because chest pain is a subjective feeling of the patient."}, "sentence_sentiment": {"answer": "Negative", "rationale": "Because \"heart pain\" is a symptom of disease, which usually leads to negative physical discomfort and psychological stress."}, "language_style": {"answer": "Calm and direct", "rationale": "The language style of the entry is straightforward and calm, as it explicitly asks the testee whether he has symptoms of chest pain and suffocation, without using excessive ado or emotional colour."}, "time_dimension": {"answer": "Present", "rationale": "The previous entry had been asked about the health status and experience of the person tested and was therefore the basis for judgement in the \" past \" period."}, "sentence_type": "Exclamatory sentence", "pos": {"nouns": {"- Chestaches": 1, "- Squeeze": 1}, "numerals": {"19": 1}}}, {"content": "Twenty is always so strong.", "word_count": 7, "label": "", "sentence_structure": "Subject鈥揷opula鈥損redicative", "subjective_objective": {"answer": "Subjective", "rationale": "Because always a dynamic is an attitude or emotional experience that is felt and expressed by the interviewees themselves and is therefore subjective."}, "sentence_sentiment": {"answer": "Positive", "rationale": "Because the entry \"smoothing up\" expresses a positive emotional attitude."}, "language_style": {"answer": "Tactful and warm", "rationale": "This expression is more readily accepted and accepted by the respondents and is more conducive to the smooth running of the questionnaire study."}, "time_dimension": {"answer": "Present", "rationale": "The keyword is \"20 always,\" in which \"always\" means very high frequency, indicating that it has been going on until now. So, the sentence is describing the attitudes and behaviour of the testees."}, "sentence_type": "Affirmative declarative sentence", "pos": {"verbs": {"Total": 1, "It's a big deal": 1}, "adjectives": {"20": 1, "Bob": 1}, "numerals": {"20": 1}}}, {"content": "21 is low.", "word_count": 3, "label": "", "sentence_structure": "Subject鈥搗erb鈥揷omplement", "subjective_objective": {"answer": "Objective", "rationale": "Because gas size is one of the characteristics of human personality, it is an objective fact that can be observed and measured."}, "sentence_sentiment": {"answer": "Negative", "rationale": "Because the term \"smugly low\" is usually considered negative."}, "language_style": {"answer": "Calm and direct", "rationale": "The entry describes the question to be investigated in a short, direct language - whether the person tested is easily angry or angry, is not using euphemism or excessive emotional colours, and is more in line with the language style of the questionnaire."}, "time_dimension": {"answer": "Present", "rationale": "The entry can be judged by the keyword \"21 small gas\" to be the present behaviour or characteristics of the person being tested and therefore in the present context."}, "sentence_type": "Negative declarative sentence", "pos": {"nouns": {"Gases": 1}, "adjectives": {"Small": 1}, "adverbs": {"21": 1}}}, {"content": "Twenty-two worries.", "word_count": 3, "label": "", "sentence_structure": "Subject鈥揷opula鈥損redicative", "subjective_objective": {"answer": "Subjective", "rationale": "Because \"love\" is a term for subjective feelings or attitudes, which describe the level of fear and anxiety of patients towards themselves or others, rather than the objective existence of facts or situations. Thus, it is a subjective issue."}, "sentence_sentiment": {"answer": "Negative", "rationale": "Because \"love\" is usually seen as a negative character."}, "language_style": {"answer": "Tactful and warm", "rationale": "The language style of the entry is euphemism. Instead of asking whether the other side is easily worried or anxious, it is speaking in a gentle, inviting tone. This expression is more personal and interactive, allowing the interviewee to share his real thoughts and feelings."}, "time_dimension": {"answer": "Present", "rationale": ""}, "sentence_type": "Exclamatory sentence", "pos": {"adjectives": {"It's a matter of heart": 1}, "numerals": {"22": 1}}}, {"content": "23 is restless.", "word_count": 4, "label": "", "sentence_structure": "Subject鈥搗erb鈥揷omplement", "subjective_objective": {"answer": "Subjective", "rationale": ""}, "sentence_sentiment": {"answer": "Negative", "rationale": "Because the entry \"frightful\" expresses a negative emotional attitude."}, "language_style": {"answer": "Calm and direct", "rationale": "This topic clearly asks the respondents whether they are prone to anxiety and anxiety when facing certain things, which are more straightforward in language style, with no excessive rhetoric or emotional reticence, and can therefore be judged as the \"cool straight\" type."}, "time_dimension": {"answer": "Present", "rationale": "In the future, the entry describes an emotional state that the testee may have, i.e., a state of anxiety, and asks whether this will happen in the future. The keyword \"23\" may represent an age or a particular situation, but its specific meaning requires further context information to determine."}, "sentence_type": "Exclamatory sentence", "pos": {"verbs": {"- Nervous": 1, "Unsettled": 1}, "adjectives": {"I'm restless": 1}}}, {"content": "24 is a little angry.", "word_count": 4, "label": "", "sentence_structure": "Subject鈥揷opula鈥損redicative", "subjective_objective": {"answer": "Subjective", "rationale": "Because it's a subjective emotional reaction to a person who can be angry."}, "sentence_sentiment": {"answer": "Negative", "rationale": "Because the \"frightful anger\" act in the entry is negative."}, "language_style": {"answer": "Calm and direct", "rationale": "The entry expresses the subject in direct, concise language - easily angry, not using euphemism or accommodative terminology, and is more direct and objective."}, "time_dimension": {"answer": "Present", "rationale": "The entry indicates that the testee has the characteristic and tendency of \"excitedness\" at the current point of time, and does not explicitly refer to information such as past or future behaviour or mentality. The time dimension of the article can therefore be judged to be the present."}, "sentence_type": "Exclamatory sentence", "pos": {"nouns": {"24": 1, "Anger": 1}, "verbs": {"Move": 1}, "adjectives": {"Easier than I thought": 1}}}, {"content": "Twenty-five wants to be light.", "word_count": 3, "label": "", "sentence_structure": "Subject鈥搗erb鈥搊bject", "subjective_objective": {"answer": "Subjective", "rationale": "Because \"wanting to be light\" is the patient's subjective attitude and thought."}, "sentence_sentiment": {"answer": "Negative", "rationale": "Because \"light life\" in the entry is a negative behavior and attitude."}, "language_style": {"answer": "Calm and direct", "rationale": "The entry uses more direct and concise language to ask respondents whether they are suicidal, air-cool, do not use excessive rhetorical or emotional renderings, and is more in line with the professional requirements of psychological questionnaires."}, "time_dimension": {"answer": "Present", "rationale": "The future entry describes the ideas or intentions that the person being tested may have in the future, namely, \"want to be light\", which points to a situation that has not yet occurred and therefore is judged to be the future."}, "sentence_type": "Exclamatory sentence", "pos": {"nouns": {"25": 1, "Lightness": 1}}}, {"content": "Twenty-six is not interested in anything.", "word_count": 8, "label": "", "sentence_structure": "Subject鈥揷opula鈥損redicative", "subjective_objective": {"answer": "Subjective", "rationale": "Because \"uninterested\" is the patient's subjective feeling and attitude, it reflects the emotional and cognitive state of the patient. This is the subjective aspect of the examination."}, "sentence_sentiment": {"answer": "Negative", "rationale": "Because the description \"not interested in anything\" in the entry shows a negative emotional attitude."}, "language_style": {"answer": "Calm and direct", "rationale": "The entry is a simple and clear language style, is not over-modified or emotional and directly asks the respondents about their attitude in a particular area, which makes it more calm and objective.\""}, "time_dimension": {"answer": "Future", "rationale": "The entry describes a desire or tendency of the testee that his future be \"not interested in anything.\" This expectation may affect his behaviour and decision-making in the future. Thus, the temporal dimension of the purpose is judged by the future."}, "sentence_type": "Negative declarative sentence", "pos": {"verbs": {"to": 1, "have": 1, "Nope": 1}, "adjectives": {"Any": 1, "Interest": 1}, "adverbs": {"Both": 1}, "numerals": {"26": 1}}}, {"content": "27 memory loss", "word_count": 5, "label": "", "sentence_structure": "Subject鈥搗erb鈥揷omplement", "subjective_objective": {"answer": "Subjective", "rationale": "Because memory loss is a personal feeling and subjective assessment of the patient, not an objective and measurable fact."}, "sentence_sentiment": {"answer": "Negative", "rationale": "Because memory loss in the entries is a negative description, which is an indication of a decline in capacity in some areas."}, "language_style": {"answer": "Calm and direct", "rationale": "The entry uses a simple and clear language to ask the respondents whether there is a loss of memory and there is no excessive emotional modification or rhetoric, so that the language style is more calm and straightforward."}, "time_dimension": {"answer": "Past", "rationale": "The entry asks if the testee has any memory loss and the time dimension is judged to be past."}, "sentence_type": "Imperative sentence", "pos": {"nouns": {"Memory": 1, "Declining": 1}, "adjectives": {"27": 1}}}, {"content": "28 lacks endurance.", "word_count": 4, "label": "", "sentence_structure": "Subject鈥揷opula鈥損redicative", "subjective_objective": {"answer": "Subjective", "rationale": "Because \"insatisfactory\" is the subjective feeling and evaluation of the patient, it is a self-conscious aspect, and therefore a subjective one."}, "sentence_sentiment": {"answer": "Negative", "rationale": "Because \"insatisfactory\" is a negative quality."}, "language_style": {"answer": "Calm and direct", "rationale": "The language style of this questionnaire entry is straightforward, because it does not use excessive rhetoric or emotional rendering, but expresses the theme \"The respondent may have been in some ways intolerant\" in an objective, concise way. This expression is more easily understood and answered by the interviewee and avoids subjective and emotional interference."}, "time_dimension": {"answer": "Present", "rationale": "In the entry, \"28\" may be the testee's age or physical test score, while \"insatibilities\" is an adjective phrase describing the current condition of the testee. The entry therefore asks about the testee's current situation, and the temporal dimension is now judged."}, "sentence_type": "Negative declarative sentence", "pos": {"nouns": {"Endurance": 1}, "verbs": {"Lack": 1, "Yes": 1}}}, {"content": "29 Lack of discretion", "word_count": 6, "label": "", "sentence_structure": "Subject鈥揷opula鈥損redicative", "subjective_objective": {"answer": "Subjective", "rationale": "Because \"lack of decisive capacity\" is a subjective feeling and self-evaluation of the patient. While it may affect the patient's behaviour and decision-making, it is based on subjective judgement based on personal experience, attitudes and emotions."}, "sentence_sentiment": {"answer": "Negative", "rationale": "Because the attitude of \"lack of judgment\" is negative."}, "language_style": {"answer": "Calm and direct", "rationale": "The entry directly describes the problems that the respondents may have in making their decisions, is not overly emotional or accommodative, and is more objective and direct in language style."}, "time_dimension": {"answer": "Present", "rationale": "The \"lack\" in the entry indicates that the testee may now have the problem, and \"decisive capacity\" is the term used to describe the problem, indicating that it is a question of the state or behaviour of the time. Thus, the time dimension of the entry is now."}, "sentence_type": "Negative declarative sentence", "pos": {"nouns": {"Adjudication": 1}, "verbs": {"Lack": 1}, "numerals": {"29": 1}}}, {"content": "30 is too dependent on others.", "word_count": 6, "label": "", "sentence_structure": "Subject鈥揷opula鈥損redicative", "subjective_objective": {"answer": "Subjective", "rationale": "Because \"dependence\" is a subjective feeling and attitude that reflects the interviewee's perception and assessment of himself, rather than the objective reality."}, "sentence_sentiment": {"answer": "Negative", "rationale": "Because the act of \"too much dependence on others\" is negative."}, "language_style": {"answer": "Tactful and warm", "rationale": "The language style of the entry is more euphemistic, not to answer questions with a strong negative or accusatory tone, but rather to use a more neutral and objective approach to induce respondents to answer questions, which is more acceptable and resonates. At the same time, the word \"too much\" in the sentence also indicates that this is a relative concept, leaving the respondents some room for interpretation and adding operationality to the questionnaire."}, "time_dimension": {"answer": "Future", "rationale": "The keywords are \"too much reliance on others\" and \"30,\" which point to situations that have not yet occurred and that may occur in the future. The emergence of \"30\" also suggests that this may be a long-term trend, not a temporary state. So I think the temporal dimension of this entry is the future."}, "sentence_type": "Negative declarative sentence", "pos": {"nouns": {"30": 1, "Dependency": 1, "others": 1}, "adjectives": {"Too much": 1}}}, {"content": "3l is sore for the red.", "word_count": 6, "label": "", "sentence_structure": "Subject鈥搗erb鈥揷omplement", "subjective_objective": {"answer": "Subjective", "rationale": "Because \"for the pain\" involves the feelings and emotions of the patient, and it is a subjective examination."}, "sentence_sentiment": {"answer": "Negative", "rationale": "The term \"torture\" conveys negative sentiments, indicating that the person is troubled and discomforted in the face of the red."}, "language_style": {"answer": "Tactful and warm", "rationale": "The entry uses a mild tone, does not directly express criticism or accusations against the person under investigation, but rather asks the other person whether he or she is upset by the red face. This expression is easier to obtain the confidence and support of the person under investigation."}, "time_dimension": {"answer": "Present", "rationale": "Now the entry expresses the point where the testee is troubled by the red face at this point in time, and the dimension of time is now judged."}, "sentence_type": "Affirmative declarative sentence", "pos": {"nouns": {"3l": 1, "Red": 1, "Trouble": 1}, "verbs": {"Yes": 1, "Trouble": 1}, "numerals": {"3l": 1}}}, {"content": "32 bites of shiver.", "word_count": 6, "label": "", "sentence_structure": "Subject鈥搗erb鈥揷omplement", "subjective_objective": {"answer": "Objective", "rationale": "Because \"sniffing\" and \"throwing\" are symptoms of patient behaviour when speaking, and are objective phenomena."}, "sentence_sentiment": {"answer": "Negative", "rationale": "Because the words \"sniffing\" and \"throwing\" in the entries are negative manifestations or scenarios."}, "language_style": {"answer": "Tactful and warm", "rationale": "This is a softer language and a more friendly one that makes the person surveyed more comfortable and willing to share real ideas and feelings."}, "time_dimension": {"answer": "Present", "rationale": "The entry describes the current state or characteristics of the person being tested, i.e. whether they have problems with the stutter and sound tremor. This description is usually used to collect real-time feedback or symptoms during the study, and therefore the temporal dimension is judged to be \"now\"."}, "sentence_type": "Declarative sentence", "pos": {"nouns": {"Snack": 1, "Sound": 1, "Frightened": 1}, "verbs": {"have": 1}, "numerals": {"32": 1}}}, {"content": "33 bodies are cold and hot.", "word_count": 6, "label": "", "sentence_structure": "Subject鈥搗erb鈥揷omplement", "subjective_objective": {"answer": "Subjective", "rationale": "Because the feeling of coldness and heat is the patient鈥檚 subjective experience. Although it may have biological effects, it is more examined here as an expression of the patient鈥檚 emotional and psychological state."}, "sentence_sentiment": {"answer": "Neutral", "rationale": "Because the entry is a description of a phenomenon and there is no apparent emotional tendency."}, "language_style": {"answer": "Tactful and warm", "rationale": "The entry uses a more euphemistic approach to asking questions than directly asking about the other person鈥檚 health."}, "time_dimension": {"answer": "Past", "rationale": "According to the description of \"Cool and Hot\", this entry is a reference to the past in terms of the experience and feelings of the testees, and the time dimension is the past."}, "sentence_type": "Interrogative sentence", "pos": {"nouns": {"Body": 1, "鈾?And the cold and the heat 鈾?: 1}, "adjectives": {"鈾?And the cold and the heat 鈾?: 1}, "numerals": {"33": 1}}}, {"content": "34 is for urine and sexual organs.", "word_count": 8, "label": "", "sentence_structure": "Subject鈥搗erb鈥揷omplement", "subjective_objective": {"answer": "Subjective", "rationale": "Because attention is the patient's subjective attitude."}, "sentence_sentiment": {"answer": "Neutral", "rationale": "Since the entry is merely a question of the physical condition of the person under investigation, it does not involve any assessment of emotions, attitudes or behaviour."}, "language_style": {"answer": "Calm and direct", "rationale": "The entry is relatively simple and clear in language, is not overdoing and emotional rendering, and directly expresses the theme of the need for respondents to focus on the health of urine and sexual organs, and is more in line with the linguistic needs of the research."}, "time_dimension": {"answer": "Present", "rationale": "The entry does not contain information about past or future, and it is asked whether the person being tested is focusing on his or her urine and sexual organs and therefore judging now."}, "sentence_type": "Exclamatory sentence", "pos": {"nouns": {"Attention": 1, "Peeing": 1, "Sexual organs": 1}, "numerals": {"34": 1}}}, {"content": "35 is in a good mood.", "word_count": 4, "label": "", "sentence_structure": "Subject鈥搗erb鈥揷omplement", "subjective_objective": {"answer": "Subjective", "rationale": "Because a sense of openness is a subjective feeling or attitude."}, "sentence_sentiment": {"answer": "Positive", "rationale": "Because \"crazy\" means a positive emotional attitude."}, "language_style": {"answer": "Tactful and warm", "rationale": "The words are more gentle and friendly, using the emotional term \"clause\" and expressing a desire to remain positive and optimistic rather than direct orders or demands. This expression is more personal and interactive, and more resonant and participatory."}, "time_dimension": {"answer": "Present", "rationale": "The entry describes the current mood of the testee, and the keyword \"open mood\" indicates that this mood is now going through."}, "sentence_type": "Affirmative declarative sentence", "pos": {"verbs": {"The mood": 1, "- Yes, sir": 1}, "adjectives": {"35": 1}}}, {"content": "36 is strangely disturbed.", "word_count": 7, "label": "", "sentence_structure": "Subject鈥搗erb鈥揷omplement", "subjective_objective": {"answer": "Subjective", "rationale": "Because \"unsurprisingly uneasy\" is the patient's subjective feeling."}, "sentence_sentiment": {"answer": "Negative", "rationale": "Because the expression \"unfortunately uneasy\" expresses a negative feeling, implying anxiety and anxiety."}, "language_style": {"answer": "Tactful and warm", "rationale": "The entry uses the descriptive term \"unfortunate\" to convey more mildly and implicitly the question and exploration of a certain emotion, rather than asking directly whether it is uneasy. Nor is it too strong or tense to express a feeling of affection and understanding."}, "time_dimension": {"answer": "Past", "rationale": "The entry uses the verb \"uninvitedly disturbed\" in the past, which indicates that it is a description or feeling of the past experience of the testee and is therefore judged as the past."}, "sentence_type": "Imperative sentence", "pos": {"nouns": {"36": 1, "It's amazing": 1, "Unsettled": 1}, "adjectives": {"I don't know": 1}, "adverbs": {"Land": 1}, "numerals": {"36": 1}}}, {"content": "37 people are alone when they're upset.", "word_count": 10, "label": "", "sentence_structure": "Subject鈥揷opula鈥損redicative", "subjective_objective": {"answer": "Subjective", "rationale": "Because \"fright\" is a subjective feeling or emotional experience, it is triggered by the individual's own state of mind and is therefore considered subjective."}, "sentence_sentiment": {"answer": "Negative", "rationale": "Because the mood of \"frightful\" in the entries is negative."}, "language_style": {"answer": "Calm and direct", "rationale": "The entry directly asked the interviewees whether they were disturbed when they were alone, were not over-repeated or emotionally rendered, and were more objective and balanced."}, "time_dimension": {"answer": "Present", "rationale": "The entry \"37 One One One One One Alone\" means that in the current situation the testee is alone, and the emotional experience of \"uneasy\" is happening now. So the time dimension of the entry is now."}, "sentence_type": "Exclamatory sentence", "pos": {"nouns": {"37": 1, "person": 1, "Alone": 1, "Time": 1, "I feel": 1, "Unsettled": 1}, "verbs": {"Alone": 1, "I feel": 1}, "numerals": {"37": 1}, "classifiers": {"individual": 1}, "pronouns": {"Alone": 1}}}, {"content": "Lack of self-confidence", "word_count": 5, "label": "", "sentence_structure": "Subject鈥揷opula鈥損redicative", "subjective_objective": {"answer": "Subjective", "rationale": "Because lack of self-confidence is an individual ' s subjective feeling and assessment, not an objective fact or observation."}, "sentence_sentiment": {"answer": "Negative", "rationale": "Because \"lack of self-confidence\" is a negative quality."}, "language_style": {"answer": "Calm and direct", "rationale": "The entry is a direct indicator of the \"lack of self-confidence\" in the survey, the lack of use of excessive rhetorical or emotional rendering, and the more objective and direct tone of the survey."}, "time_dimension": {"answer": "Future", "rationale": ""}, "sentence_type": "Exclamatory sentence", "pos": {"nouns": {"Self-confidence": 1, "38": 1}, "verbs": {"Lack": 1}, "numerals": {"38": 1}}}, {"content": "39 is a very strong one.", "word_count": 6, "label": "", "sentence_structure": "Subject鈥搗erb鈥揷omplement", "subjective_objective": {"answer": "Subjective", "rationale": "Because \"daunted tail\" is a subjective attitude and psychological state that reflects patients' hesitation and fear in dealing with things."}, "sentence_sentiment": {"answer": "Negative", "rationale": "Because the act of \"daunting the tail\" is negative."}, "language_style": {"answer": "Calm and direct", "rationale": "The entry directly asked whether the respondents had shown a reluctance and timidness in dealing with the matter, and had not used excessive rhetoric or emotional rendering, and had been more objective in tone."}, "time_dimension": {"answer": "Past", "rationale": "The term \"daunted tail\" in this entry is a term describing behaviour or state, which indicates hesitation in doing things and lack of decisiveness, which is usually the result of past experience or custom. Thus, the temporal dimension of the article is a judgement of the past."}, "sentence_type": "Negative declarative sentence", "pos": {"nouns": {"Operations": 1, "- You're afraid of the end": 1}, "adjectives": {"39": 1}, "numerals": {"39": 1}}}, {"content": "Forty is easy to misconstrue.", "word_count": 6, "label": "", "sentence_structure": "Subject鈥搗erb鈥揷omplement", "subjective_objective": {"answer": "Subjective", "rationale": "Because \"vulnerable\" is a subjective feeling and experience, which involves personal emotions, attitudes and perceptions. The answer to this questionnaire should therefore be subjective."}, "sentence_sentiment": {"answer": "Negative", "rationale": "Because \"misunderstanding\" is a negative emotional experience."}, "language_style": {"answer": "Tactful and warm", "rationale": "The basis for the judgement: the article's more moderate and inviting tone of speech, rather than direct statements or orders, is more acrimony and interactive. At the same time, the use of the term \"easy\" avoids an overly categorical term, displaying an objective and honest tone."}, "time_dimension": {"answer": "Present", "rationale": ""}, "sentence_type": "Negative declarative sentence", "pos": {"nouns": {"person": 1, "Misunderstanding": 1}, "verbs": {"Easy": 1, "By": 1, "Misunderstanding": 1}, "adjectives": {"Easy": 1}, "adverbs": {"Easy": 1, "Someone else": 1}, "numerals": {"40": 1, "1": 3}}}, {"content": "41 doesn't trust anyone.", "word_count": 5, "label": "", "sentence_structure": "Subject鈥搗erb鈥搊bject", "subjective_objective": {"answer": "Subjective", "rationale": "Because \"no trust\" is the patient's subjective attitude."}, "sentence_sentiment": {"answer": "Negative", "rationale": "Because the attitude of \"no one else\" in the entry is negative."}, "language_style": {"answer": "Calm and direct", "rationale": "The language style of the questionnaire entry is more direct, and there is no excessive rhetoric or emotional rendering. It explicitly asks the respondents about their attitude to \"be sure of other people\", their temperature and objectivity, and does not put pressure on the respondents or guide them to answer a particular answer."}, "time_dimension": {"answer": "Future", "rationale": "The keyword \"confidence in others\" indicates that there is a expectation or a vision of the future, so the temporal dimension of the article is the future."}, "sentence_type": "Negative declarative sentence", "pos": {"nouns": {"person": 1, "others": 1}, "verbs": {"Yes": 1}}}, {"content": "42 is too suspicious.", "word_count": 4, "label": "", "sentence_structure": "Subject鈥搗erb鈥揷omplement", "subjective_objective": {"answer": "Subjective", "rationale": "Because too much suspicion is a subjective attitude or a mindset of one."}, "sentence_sentiment": {"answer": "Negative", "rationale": "Because the \"too suspicious\" act in the entry is negative."}, "language_style": {"answer": "Calm and direct", "rationale": "The questionnaire entry directly asked the respondents whether they were over-suspect, were clear in language, were not too emotional or rhetorical, and were more objective and calm."}, "time_dimension": {"answer": "Future", "rationale": "The entry describes a mentality or behaviour that the testee may have in the future, and \"exaggerous\" is a possible future situation and therefore judgement is a future."}, "sentence_type": "Negative declarative sentence", "pos": {"nouns": {"42": 1, "Doubt": 1}, "adjectives": {"Too much": 1}, "numerals": {"42": 1}}}, {"content": "43 hates dating.", "word_count": 4, "label": "", "sentence_structure": "Subject鈥搗erb鈥揷omplement", "subjective_objective": {"answer": "Subjective", "rationale": "Because aversion to interaction is a subjective sentiment and attitude."}, "sentence_sentiment": {"answer": "Negative", "rationale": "Because \"averse to dating\" is negative."}, "language_style": {"answer": "Calm and direct", "rationale": "The entry directly expresses the subject of questioning the other party about the aversion to association, without excessive rhetoric or emotional rendering, and is more objective in tone."}, "time_dimension": {"answer": "Present", "rationale": "According to the structure and content of the sentence, the entry asks the testee about the attitude or feelings of the person involved in the interaction, without reference to the specific temporal dimensions of the past, present or future. The answer is therefore \"no precise temporal dimensions\"."}, "sentence_type": "Negative declarative sentence", "pos": {"nouns": {"- To be with someone": 1}, "verbs": {"Disgusting": 1}, "numerals": {"43": 1}}}, {"content": "I feel humbled.", "word_count": 4, "label": "", "sentence_structure": "Subject鈥揷opula鈥損redicative", "subjective_objective": {"answer": "Subjective", "rationale": "Because feeling low is a subjective emotional experience."}, "sentence_sentiment": {"answer": "Negative", "rationale": "Because \"subsistence\" is a negative emotional and psychological state."}, "language_style": {"answer": "Calm and direct", "rationale": "The language style of the entry is a calm and straightforward type. It asks whether the interviewee has ever felt inferior, has not been over-modified or emotional, and is more objective and neutral. This expression is more accurate for the interviewee to understand the problem and to reflect his feelings."}, "time_dimension": {"answer": "Present", "rationale": "Now the entry describes the current state of the person's mind or feeling, the \"fear\" is the current state of play, which suggests that it is an ongoing emotional experience. So, the entry can be judged as now."}, "sentence_type": "Exclamatory sentence", "pos": {"nouns": {"You're a low man": 1, "44": 1}}}, {"content": "Forty-five is a bad idea.", "word_count": 4, "label": "", "sentence_structure": "Subject鈥搗erb鈥搊bject", "subjective_objective": {"answer": "Subjective", "rationale": "Because \"fearing\" is a subjective feeling of fear and anxiety."}, "sentence_sentiment": {"answer": "Negative", "rationale": "Because \"thus\" is a derogatory term, which expresses excessive anxiety and anxiety, and is a negative description."}, "language_style": {"answer": "Calm and direct", "rationale": "\"Calm down straight.\""}, "time_dimension": {"answer": "Future", "rationale": "The entry contains visions and expectations for the future, and the key words are \"crazy\" and \"show you to analyze\" and all point to situations that have not yet occurred and that may arise in the future."}, "sentence_type": "Negative declarative sentence", "pos": {"nouns": {"It's a matter of time": 1}}}, {"content": "46 is tired.", "word_count": 4, "label": "", "sentence_structure": "Subject鈥揷opula鈥損redicative", "subjective_objective": {"answer": "Subjective", "rationale": "The answer to this question requires that the patient judge his/her feelings and whether he/she is tired."}, "sentence_sentiment": {"answer": "Negative", "rationale": "Because \"body fatigue\" is usually considered a negative state or feeling."}, "language_style": {"answer": "Calm and direct", "rationale": "The language style of the questionnaire entry is calm, as it directly asks the person's health, without using any rhetoric or emotional colour to qualify the question. This simple and clear question design makes it easier for the person to understand and answer, while ensuring that accurate information is available."}, "time_dimension": {"answer": "Past", "rationale": "The entry asks about the person's physical condition at a certain point in time (i.e. \"46 body fatigue\") and can therefore be judged as past."}, "sentence_type": "Exclamatory sentence", "pos": {"nouns": {"Body": 1, "Tired": 1}, "adverbs": {"46": 1}, "numerals": {"46": 1}}}, {"content": "47 sweats when he's in a hurry.", "word_count": 7, "label": "", "sentence_structure": "Subject鈥搗erb鈥揷omplement", "subjective_objective": {"answer": "Subjective", "rationale": "Because cold sweat is a physical reaction of a human being, but it is caused by the patient's subjective feeling. It is subjective."}, "sentence_sentiment": {"answer": "Negative", "rationale": "Because the \"cool sweats in a hurry\" in the entry is negative."}, "language_style": {"answer": "Calm and direct", "rationale": "This entry is more objective and direct."}, "time_dimension": {"answer": "Present", "rationale": "The keyword \"at once\" denotes a regular, customary act, while the entire sentence adopts the general current, which means that the act has not only occurred in the past but may still be ongoing. The entry thus describes a current situation."}, "sentence_type": "Affirmative declarative sentence", "pos": {"nouns": {"47": 1, "I'm in a hurry": 1, "Cold sweat": 1}, "verbs": {"Out": 1}, "adverbs": {"One": 1}, "numerals": {"47": 1}}}, {"content": "48 stands up and gets dizzy.", "word_count": 6, "label": "", "sentence_structure": "Subject鈥揷opula鈥損redicative", "subjective_objective": {"answer": "Objective", "rationale": "Because getting up is an objective fact of the patient's experience."}, "sentence_sentiment": {"answer": "Negative", "rationale": "The entry is a symptom of physical discomfort, usually associated with illness or health problems, and therefore expresses a negative emotional tendency."}, "language_style": {"answer": "Calm and direct", "rationale": "The language style of the entry is more straightforward, with no excessive phrasing or emotional colour, and a flat tone, aimed at obtaining the actual state of the person being interviewed."}, "time_dimension": {"answer": "Past", "rationale": ""}, "sentence_type": "Affirmative declarative sentence", "pos": {"nouns": {"Dizziness": 1, "Stand up": 1}, "verbs": {"48": 1, "Get up": 1}, "numerals": {"48": 1}}}, {"content": "49 was unconscious, and it was a blow.", "word_count": 8, "label": "", "sentence_structure": "Subject鈥搗erb鈥揷omplement", "subjective_objective": {"answer": "Objective", "rationale": "Because unconsciousness and twitching are the objective experiences and manifestations of patients, they are not subjective attitudes or ideas of patients."}, "sentence_sentiment": {"answer": "Negative", "rationale": "Because venom is a pathological expression, usually associated with a physical disease. Thus, the expression of this purpose has a negative emotional tendency."}, "language_style": {"answer": "Tactful and warm", "rationale": "The entry uses relatively mild tone, avoiding direct description or command expression. Moreover, the term \"exact\" and \"screech\" is not too strong a negative emotional color, making it easier for respondents to accept and answer questions. These factors make the language style of the article more euphemismal."}, "time_dimension": {"answer": "Past", "rationale": ""}, "sentence_type": "Exclamatory sentence", "pos": {"nouns": {"Awareness": 1, "Wind": 1}, "verbs": {"Lose": 1, "Yes": 1}, "numerals": {"49": 1}}}, {"content": "Fifty people are popular.", "word_count": 6, "label": "", "sentence_structure": "Subject鈥揷opula鈥損redicative", "subjective_objective": {"answer": "Subjective", "rationale": "Because \"goodness is popular\" is a subjective feeling and an assessment."}, "sentence_sentiment": {"answer": "Positive", "rationale": "Because the expression \"good people are popular\" is positive."}, "language_style": {"answer": "Tactful and warm", "rationale": "The entry is more adept and interactive when it is spoken in a gentle and inviting manner, rather than by direct statements or orders."}, "time_dimension": {"answer": "Present", "rationale": "Now the entry asks about the current state or character of the testee, the characteristic of \"Are you popular with 50 people?\" So the time dimension is now."}, "sentence_type": "Affirmative declarative sentence", "pos": {"nouns": {"Humanities": 1, "Welcome": 1}, "verbs": {"Okay": 1, "Yes": 1, "Welcome": 1}, "adverbs": {"50": 1}, "numerals": {"50": 1}, "classifiers": {"individual": 1}}}, {"content": "51 is too much.", "word_count": 4, "label": "", "sentence_structure": "Subject鈥搗erb鈥揷omplement", "subjective_objective": {"answer": "Subjective", "rationale": "Because excessive restraints are a subjective attitude and character characteristic of a person."}, "sentence_sentiment": {"answer": "Negative", "rationale": "The basis for the judgement is that the term \"slut\" is usually used to describe negative attitudes or behaviour."}, "language_style": {"answer": "Tactful and warm", "rationale": "The phrase in this entry does not directly imply criticism or accusations, but rather asks whether the person under investigation has been overly intrusive or tendentious. This expression is more moderate than a direct indication of the problem, more respectful of the person under investigation鈥檚 feelings, and more acceptable and responsive. At the same time, the entry uses the word 鈥渆xcessive鈥?to suggest that the existence of a moderate plaque is acceptable, which would help to reduce the resistance of the person under investigation and increase the effectiveness of the questionnaire."}, "time_dimension": {"answer": "Present", "rationale": "The keyword is \"too rigid\" to indicate whether the person tested was too obscurant in a rule, custom or attitude, which is a description of the current state of conduct."}, "sentence_type": "Negative declarative sentence", "pos": {"verbs": {"That's too much": 1, "- What": 1}, "adjectives": {"That's too much": 1, "- What": 1}}}, {"content": "52 is not comfortable without a lot of confirmation.", "word_count": 14, "label": "", "sentence_structure": "Subject鈥搗erb鈥揷omplement", "subjective_objective": {"answer": "Subjective", "rationale": "Because \"unreconfirmed\" is a psychological state and feeling of a person, a subjective examination."}, "sentence_sentiment": {"answer": "Negative", "rationale": "Because the act of \"unreconfirmed and uneasy\" in the entry is negative, it suggests that the person may be too cautious, anxious or lacking a sense of trust."}, "language_style": {"answer": "Calm and direct", "rationale": "The entry directly asks the other side whether there is always a need to repeatedly identify the subject, not to use excessive rhetoric or emotional rendering, and to be more objective in tone. \""}, "time_dimension": {"answer": "Present", "rationale": "In the future, the entry asks whether the testee will continue to have \"unreconfirmed\" behaviour or attitude in the future, the keyword being \"52 for anything\" and \"not reassured without repeated confirmation\", all pointing to situations that have not yet occurred and that may arise in the future."}, "sentence_type": "Exclamatory sentence", "pos": {"nouns": {"52": 1, "Things": 1}, "verbs": {"to": 1, "not": 1, "Repeated confirmations": 1, "Just": 1, "Don't worry": 1}, "adverbs": {"Any": 1}, "numerals": {"52": 1}}}, {"content": "53 for the dirty.", "word_count": 5, "label": "", "sentence_structure": "Subject鈥搗erb鈥揷omplement", "subjective_objective": {"answer": "Subjective", "rationale": "Because \"care\" is patient's subjective attitude."}, "sentence_sentiment": {"answer": "Negative", "rationale": "Basis: The \"care\" in this entry indicates a certain aversion or rejection of the dirty, and is therefore negative."}, "language_style": {"answer": "Calm and direct", "rationale": "This article is more straightforward, does not have too many rhetorical or emotional expressions, and asks whether the person surveyed is to some extent very clean. This language style is more objective and clear, making it easier for the person surveyed to understand the meaning of the problem and to express his or her views accurately."}, "time_dimension": {"answer": "Future", "rationale": "The entry contains visions and expectations for the future, and the keywords are \"care\" and \"53 for the dirty\", all pointing to situations that have not yet occurred and that may arise in the future."}, "sentence_type": "Exclamatory sentence", "pos": {"nouns": {"Dirty": 1, "Yes": 1}, "verbs": {"in": 1, "I care": 1}, "adjectives": {"very": 1}, "numerals": {"53": 1}}}, {"content": "54 is not free from the meaningless idea.", "word_count": 11, "label": "", "sentence_structure": "Subject鈥搗erb鈥揷omplement", "subjective_objective": {"answer": "Subjective", "rationale": "Because the words \"indeed\" and \"inexplicable ideas\" express the subjective feelings and thoughts of the patient."}, "sentence_sentiment": {"answer": "Negative", "rationale": "Because the entries \"can't get rid of\" and \"no sense\" express negative feelings."}, "language_style": {"answer": "Tactful and warm", "rationale": ""}, "time_dimension": {"answer": "Present", "rationale": "The entry used the old-fashioned \"inevitability\" to show that it had happened or had happened in the near future. At the same time, the \"innocent idea\" was a description of the past, and it further emphasized that it was a question of the past. So the answer was the past."}, "sentence_type": "Negative declarative sentence", "pos": {"nouns": {"Thoughts": 1, "It's pointless": 1}, "verbs": {"I can't get rid of it": 1}, "numerals": {"54": 1}}}, {"content": "55 thinks he smells weird.", "word_count": 8, "label": "", "sentence_structure": "Subject鈥搗erb鈥揷omplement", "subjective_objective": {"answer": "Subjective", "rationale": "Because it's a subjective feeling to feel the smell."}, "sentence_sentiment": {"answer": "Negative", "rationale": "Because the description \"the strange smell\" in the entry is negative and expresses a negative emotion or attitude."}, "language_style": {"answer": "Calm and direct", "rationale": "The language style of the entry is more calm and direct, and the person surveyed's perception of his or her \"fright smell\" is directly stated by the number \"55\", which is not accompanied by excessive qualifier or rhetorical techniques, which are more objective and direct in tone."}, "time_dimension": {"answer": "Present", "rationale": ""}, "sentence_type": "Negative declarative sentence", "pos": {"nouns": {"self": 1, "Smells weird": 1}, "verbs": {"I think": 1}, "adjectives": {"have": 1, "It's weird": 1}, "numerals": {"55": 1}}}, {"content": "56 people say bad things behind their backs.", "word_count": 10, "label": "", "sentence_structure": "Subject鈥搗erb鈥搊bject", "subjective_objective": {"answer": "Objective", "rationale": "Because \"the bad word behind your own is an objective fact, independent of the subjective will of the individual."}, "sentence_sentiment": {"answer": "Negative", "rationale": "Because the entry describes a negative act."}, "language_style": {"answer": "Calm and direct", "rationale": ""}, "time_dimension": {"answer": "Present", "rationale": "\"Someone speaks bad behind his back\" describes a situation or behaviour that is not clearly time-directed, and therefore is judged now."}, "sentence_type": "Negative declarative sentence", "pos": {"nouns": {"56": 1, "others": 1, "self": 1, "Behind": 1, "Bad word": 1}, "numerals": {"56": 1}}}, {"content": "57 always watch out for the people around.", "word_count": 7, "label": "", "sentence_structure": "Subject鈥搗erb鈥揷omplement", "subjective_objective": {"answer": "Subjective", "rationale": "Because \"watching the people around\" is a subjective choice and concern of a person, not an objective reality or state of affairs."}, "sentence_sentiment": {"answer": "Neutral", "rationale": "Because the entry does not have any emotional color."}, "language_style": {"answer": "Tactful and warm", "rationale": "The language style of the entry is more euphemistic, using the word \"care\" to ask the other side whether it is a habit to focus on the surroundings, rather than directly pointing out or accusing. At the same time, the issue is not too emotional, and the tone is more neutral. The language style of the entry is therefore considered to be euphemistic."}, "time_dimension": {"answer": "Present", "rationale": "Now the word \"general\" denotes a regular, customary act, \"care\" and \"persons around\" describe the act or state of conduct currently being performed, so the temporal dimension of the article is now judged."}, "sentence_type": "Exclamatory sentence", "pos": {"nouns": {"person": 1, "Around": 1}, "verbs": {"Attention": 1, "Total": 1}, "adverbs": {"Around": 1}, "numerals": {"57": 1}}}, {"content": "58 cares about people's eyes.", "word_count": 6, "label": "", "sentence_structure": "Subject鈥搗erb鈥揷omplement", "subjective_objective": {"answer": "Subjective", "rationale": "Because caring about others is the patient's subjective feelings and experiences."}, "sentence_sentiment": {"answer": "Neutral", "rationale": "Because the entry does not express positive or negative emotional expressions, it merely describes a fact."}, "language_style": {"answer": "Tactful and warm", "rationale": "The entry is more adept and interactive when it is spoken in a gentle and inviting manner, rather than by direct statements or orders."}, "time_dimension": {"answer": "Present", "rationale": ""}, "sentence_type": "Imperative sentence", "pos": {"nouns": {"58": 1, "Eyes": 1}, "verbs": {"I care": 1}}}, {"content": "I think people despise themselves.", "word_count": 8, "label": "", "sentence_structure": "Subject鈥搗erb鈥揷omplement", "subjective_objective": {"answer": "Subjective", "rationale": "Because feeling is considered a subjective experience and feeling, it is a emotional reaction within the patient, not an objective external fact."}, "sentence_sentiment": {"answer": "Negative", "rationale": "Because the emotional expression \"likes to be despised\" is negative."}, "language_style": {"answer": "Calm and direct", "rationale": "The language style of the entry is more direct, not using too many rhetorical or euphemistic expressions, but rather asking whether the interviewee has ever felt he was being despised. This is often a direct language style that makes it easier for the interviewee to understand the meaning of the question and is more likely to provide a true and accurate answer."}, "time_dimension": {"answer": "Present", "rationale": "The entry contains a subjective description of \"I feel that I am being despised,\" which is based on the senses and observations of the present, and does not relate to the temporal dimensions of the past or the future. So the judgement is based on \"now.\""}, "sentence_type": "Imperative sentence", "pos": {"nouns": {"59": 1, "self": 1}, "verbs": {"I think": 1, "Disgrace": 1}}}, {"content": "60 is vulnerable to destruction.", "word_count": 6, "label": "", "sentence_structure": "Subject鈥揷opula鈥損redicative", "subjective_objective": {"answer": "Subjective", "rationale": "Because \"funner-prone\" describes the patient's subjective feelings and perceptions of his own emotions."}, "sentence_sentiment": {"answer": "Negative", "rationale": "Because the expression \"float is vulnerable\" is a description of the negative."}, "language_style": {"answer": "Calm and direct", "rationale": "The entry directly asks whether the other is vulnerable to emotional disruption, the language style is simpler, there is no excessive adoration or emotional rendering, and it is a more calm and direct expression."}, "time_dimension": {"answer": "Past", "rationale": "The entry is about the emotional state of the person being tested, while \"freakable\" describes an emotional characteristic or tendency that usually takes some time to form. Therefore, depending on the time dimension, the entry is more oriented towards examining the person ' s past experience and emotional state."}, "sentence_type": "Interrogative sentence", "pos": {"nouns": {"Emotion": 1, "Destruction": 1}, "verbs": {"Easy to be subjected to": 1}, "adjectives": {"60": 1}}}, {"content": "61. You consider interpersonal relations important.", "word_count": 10, "label": "", "sentence_structure": "Subject鈥搗erb鈥搊bject", "subjective_objective": {"answer": "Subjective", "rationale": "Because \"think\" is the patient's own subjective attitude, reflecting his perception of and values in human relationships."}, "sentence_sentiment": {"answer": "Positive", "rationale": "Because the idea of \"personal relationships are important\" in the entry is positive."}, "language_style": {"answer": "Tactful and warm", "rationale": "The entry uses a question-and-answer expression and gives positive expression to the view of the importance of interpersonal relations, giving a more personal and moderate sense of feeling. At the same time, the sentence does not use too direct or strong a phrase, so it can be judged as a soft and warm language style."}, "time_dimension": {"answer": "Present", "rationale": "The entry is for the purpose of asking the testee about his/her views and attitudes to interpersonal relations, and the temporal dimension is now judged."}, "sentence_type": "Interrogative sentence", "pos": {"nouns": {"Human relations": 1, "Important": 1}, "verbs": {"I think": 1, "It's important": 1}, "numerals": {"61": 1}}}, {"content": "62. You think that the purpose of love is marriage.", "word_count": 13, "label": "", "sentence_structure": "Subject鈥搗erb鈥搊bject", "subjective_objective": {"answer": "Subjective", "rationale": "Because \"conception\" expresses the patient's personal views and attitudes, it is subjective to see whether a relationship is intended to be marriage-oriented."}, "sentence_sentiment": {"answer": "Neutral", "rationale": "The entry expresses a view, which is not emotional."}, "language_style": {"answer": "Calm and direct", "rationale": "The entry raises questions in a concise manner, with no excessive emotional rendering or rhetorical techniques and a more objective tone."}, "time_dimension": {"answer": "Present", "rationale": "The entry asks the testee鈥檚 view of the purpose of the relationship, which may change with time and experience, but at this particular point in time the testee has expressed an opinion about the purpose of the relationship."}, "sentence_type": "Exclamatory sentence", "pos": {"nouns": {"Love": 1, "Purpose": 1, "Marriage": 1}, "verbs": {"I think": 1, "be": 1, "For": 1}, "numerals": {"62": 1}}}, {"content": "63. You believe that individuals should be held accountable for their sexual behaviour.", "word_count": 15, "label": "", "sentence_structure": "Subject鈥搗erb鈥搊bject", "subjective_objective": {"answer": "Subjective", "rationale": "Because the entry is an examination of the views and opinions of the interviewees themselves and is subjective in nature."}, "sentence_sentiment": {"answer": "Positive", "rationale": "Basis of judgement: The purpose of the article expresses the affirmation and importance attached to individual responsibility, without any negative emotional tendencies."}, "language_style": {"answer": "Tactful and warm", "rationale": "The language style of the entry is more euphemistic and respectful of individuals, leading the interviewees to think in an interview, without directly making moral judgements or stressing responsibility, and is more acceptable and dialogueable."}, "time_dimension": {"answer": "Present", "rationale": "The questionnaire entry asks the testee whether he should be held responsible for his personal sexual behaviour, and the time dimension is now judged."}, "sentence_type": "Interrogative sentence", "pos": {"nouns": {"Questionnaire": 1, "Entry": 1, "person": 1, "Sexuality": 1, "Responsibility": 1}, "verbs": {"I think": 1, "I think so": 1, "to": 1, "Responsible": 1}, "numerals": {"63": 1}}}, {"content": "64. You consider premarital sex acceptable.", "word_count": 14, "label": "", "sentence_structure": "Subject鈥搗erb鈥搊bject", "subjective_objective": {"answer": "Subjective", "rationale": "Because \"acceptability\" is an individual view and attitude, and it is subjective."}, "sentence_sentiment": {"answer": "Neutral", "rationale": "As the entry merely raises a question, it does not express any positive or negative emotional tendencies."}, "language_style": {"answer": "Tactful and warm", "rationale": "The language style of the entry is more euphemistic. First, it guides the other side to express their views, not direct orders or questions, which is more respectful and interactive; second, it is not too strongly emotional or morally pronounced, but rather it is open to the views of the interviewees, which is more likely to trigger in-depth reflection and communication."}, "time_dimension": {"answer": "Present", "rationale": "The entry asks the testee about the current attitude and perception of premarital sex and thus the judgement is now."}, "sentence_type": "Interrogative sentence", "pos": {"nouns": {"Premarital sex": 1, "It's acceptable": 1}, "verbs": {"I think": 1}, "adverbs": {"Yeah": 1}, "numerals": {"64": 1}}}, {"content": "65. So far, do you feel that you have problems with your own health?", "word_count": 18, "label": "", "sentence_structure": "Subject鈥損redicate", "subjective_objective": {"answer": "Subjective", "rationale": "The examination is a self-perceptional health condition of the patient and is subjective."}, "sentence_sentiment": {"answer": "Negative", "rationale": "Because the description of \"the health of the person\" in the entry is negative."}, "language_style": {"answer": "Calm and direct", "rationale": "The entry directly asks the other party whether he or she is in a health condition, does not use excessive speech techniques or emotional rendering, and is more objective and direct."}, "time_dimension": {"answer": "Past", "rationale": "The entry \"to date\" represents a time-old past, unrelated to the present and the future, and therefore judged to be the past."}, "sentence_type": "Interrogative sentence", "pos": {"nouns": {"Self-health": 1, "Problem": 1}, "verbs": {"I feel": 1, "have": 1}, "adverbs": {"To date": 1}, "numerals": {"65": 1}}}, {"content": "66. Has there ever been a mental health problem?", "word_count": 14, "label": "", "sentence_structure": "Subject鈥損redicate", "subjective_objective": {"answer": "Subjective", "rationale": "Because there is a problem with mental health is a patient ' s subjective feeling and perception."}, "sentence_sentiment": {"answer": "Negative", "rationale": "Because the expression \"sweetly problematic in mental health\" is negative."}, "language_style": {"answer": "Calm and direct", "rationale": "The entry asks the respondents, in a more neutral and objective manner, whether they have had a mental health experience, have no excessive emotional or oral style, and have a relatively direct linguistic style."}, "time_dimension": {"answer": "Past", "rationale": "The entry is for the purpose of asking the person who was tested whether he or she had experienced a mental health problem at a certain point in time, and therefore the temporal dimension is judged to be past."}, "sentence_type": "Interrogative sentence", "pos": {"nouns": {"Questionnaire": 1, "Entry": 1, "Mental health": 1, "Problem": 1}, "verbs": {"I think": 1, "have": 1}, "adjectives": {"Once": 1, "In terms of": 1}, "numerals": {"66": 1}, "pronouns": {"you": 1}}}, {"content": "67. Have you received mental health counselling and treatment so far?", "word_count": 21, "label": "", "sentence_structure": "Subject鈥搗erb鈥搊bject", "subjective_objective": {"answer": "Subjective", "rationale": "Since access to mental health counselling and treatment is a personal experience and choice of the patient, it is subjective."}, "sentence_sentiment": {"answer": "Neutral", "rationale": "Because the entry does not have any emotional color."}, "language_style": {"answer": "Tactful and warm", "rationale": "The entry is a soft and warm language. It asks the interviewee whether he has had a history of mental health counselling and treatment, and expresses his concern and understanding. At the same time, the question is not being used too directly or strongly to make the interviewee comfortable and safe."}, "time_dimension": {"answer": "Past", "rationale": ""}, "sentence_type": "Interrogative sentence", "pos": {"nouns": {"To date": 1, "Mental health": 1, "Consultants": 1, "Treatment": 1}, "verbs": {"Accept": 1, "Yes": 1}, "adverbs": {"Once": 1}, "numerals": {"67": 1}}}, {"content": "68. If you have a question about health or mental health, please write below.", "word_count": 25, "label": "", "sentence_structure": "Subject鈥搗erb鈥搊bject", "subjective_objective": {"answer": "Subjective", "rationale": "This is because of the patient ' s personal will and needs, and is subjective and thought."}, "sentence_sentiment": {"answer": "Neutral", "rationale": "As the entry does not cover any description of emotion, attitude, behaviour, purpose or quality, it simply provides a possible situation in which people can fill out their own problems."}, "language_style": {"answer": "Tactful and warm", "rationale": "The entry is more euphemistic, using a polite request \"please\" to express respect for the person under investigation. At the same time, the content of the interview is more direct and not overly refined, allowing the person to understand the purpose and content of the questionnaire more clearly."}, "time_dimension": {"answer": "Future", "rationale": "The entry is a query about the potential future needs or problems of the testee, and the keywords \"if\" and \"please write below\" suggest that this is the vision and expectation for the future."}, "sentence_type": "Interrogative sentence", "pos": {"nouns": {"Problem": 2, "Health": 1, "Mental health": 1, "Down there": 1}, "verbs": {"have": 1, "Please": 1, "Write": 1, "in": 1}, "numerals": {"68": 1}, "classifiers": {"individual": 1}, "pronouns": {"you": 1}}}]}

{"questionnaire_title": "Eisenk Personality Questionnaire (Age)", "questionnaire_category": "Self-cognition", "item_count": 88, "emotion_shift_overall": "From negative to positive", "language_style_overall": "Neutral", "items": [{"content": "Do you have many different hobbyes?", "word_count": 13, "label": "", "sentence_structure": "Subject鈥搗erb鈥搊bject", "subjective_objective": {"answer": "Subjective", "rationale": "\" 1. Do you have many different hobbyes?\" Output: Subjectivity because there are many different hobbys that are personal experiences, ideas and choices of patients and are subjective in their perspective."}, "sentence_sentiment": {"answer": "Neutral", "rationale": "Because the entry does not have any emotional color."}, "language_style": {"answer": "Tactful and warm", "rationale": "The entry is more adept and interactive when it is spoken in a gentle and inviting manner, rather than by direct statements or orders."}, "time_dimension": {"answer": "Present", "rationale": ""}, "sentence_type": "Interrogative sentence", "pos": {"nouns": {"Amateurity": 1}, "verbs": {"have": 1, "be": 1}, "numerals": {"More": 1, "not": 1, "How many": 1}, "classifiers": {"individual": 1}}}, {"content": "Do you have to stop and think carefully before you do anything?", "word_count": 20, "label": "", "sentence_structure": "Subject鈥搗erb鈥搊bject", "subjective_objective": {"answer": "Subjective", "rationale": "Because this is a matter of the patient鈥檚 behaviour and way of thinking, it needs to be judged by the patient鈥檚 personal experience and feelings. For example, some people may feel that they need to stop and think carefully before doing anything, which is their subjective idea; some people may not think so, but rather that it is a natural habit or instinct.\""}, "sentence_sentiment": {"answer": "Positive", "rationale": "Because the act of \"stop and think\" in the entry is positive, it means that this person has a habit of thinking carefully."}, "language_style": {"answer": "Calm and direct", "rationale": "This is more consistent with the objectivity and rigour of the questionnaire study."}, "time_dimension": {"answer": "Present", "rationale": "The word \"whether\" is a selective question, asking the testee what he is or what he is, \"stop and think\" is not a clear time limit and therefore belongs to the present."}, "sentence_type": "Imperative sentence", "pos": {"nouns": {"Things": 1, "Before": 1, "Think": 1}, "verbs": {"Do it": 1, "Stop": 1, "Get down": 1, "Careful": 1, "Consider": 1}, "adverbs": {"Any": 1, "Both": 1}, "numerals": {"2": 1}, "pronouns": {"you": 1}}}, {"content": "Is your heart often in a state of constant volatility?", "word_count": 10, "label": "", "sentence_structure": "Subject鈥搗erb鈥搊bject", "subjective_objective": {"answer": "Subjective", "rationale": "Because the rise and fall of the heart is usually the feeling and experience of the individual and is subjective."}, "sentence_sentiment": {"answer": "Negative", "rationale": "Because \"the heart rises and falls\" is usually considered a negative emotional state."}, "language_style": {"answer": "Tactful and warm", "rationale": "The entry is more personal and interactive in its expression of conversation in a gentle and inviting tone, rather than in direct statements or orders. At the same time, the issue is based on the neutral term \"your heart\" and avoids overly emotional or personalized descriptions, making the interviewees more willing to share their true situation."}, "time_dimension": {"answer": "Past", "rationale": "The entry is for the purpose of asking the testee about the change in his/her mind over time and the time dimension is judged to be past."}, "sentence_type": "Interrogative sentence", "pos": {"nouns": {"Heartland": 1, "Rise and shine": 1}, "verbs": {"Is there a problem": 1, "Rise and shine": 1}, "adverbs": {"Regular": 1}}}, {"content": "Have you ever been to receive a reward knowing that it was the work of others?", "word_count": 22, "label": "", "sentence_structure": "Subject鈥搗erb鈥搊bject", "subjective_objective": {"answer": "Subjective", "rationale": "Because the description of \"you\" and \"debate\" involves subjective attitudes and behaviour of individuals."}, "sentence_sentiment": {"answer": "Negative", "rationale": "Because the entry \"You're going to receive reward knowing that it's the work of others\" is immoral and has a negative emotional connotation."}, "language_style": {"answer": "Calm and direct", "rationale": "The entry is a direct question to whether the interviewee has ever had a history of taking credit for others, and is more straightforward and objective in language style."}, "time_dimension": {"answer": "Past", "rationale": "The entry asks if the testee has ever had a past in which he knows that he is the credit of others, but has to accept a reward. The keyword \"had\" and \"had\" all imply that the problem happened in the past and may not have affected it now. So the time dimension is the past."}, "sentence_type": "Interrogative sentence", "pos": {"nouns": {"Questionnaire": 1, "Entry": 1, "you": 2, "Thanks": 1, "others": 1, "Awards": 1, "Yes": 1}, "verbs": {"Yes": 2, "Accept": 1}, "adverbs": {"Tsang": 1, "And": 1, "Go on": 1}, "numerals": {"4": 1}}}, {"content": "Are you a talker?", "word_count": 5, "label": "", "sentence_structure": "Subject鈥損redicate", "subjective_objective": {"answer": "Subjective", "rationale": "Since the question of talkability is one of individual subjective perceptions and self-evaluation, it is the individual character and attitude that is examined and is subjective in nature."}, "sentence_sentiment": {"answer": "Positive", "rationale": "Because \"talking\" in this entry is usually seen as a positive feature, which means that the person is good at communication, good at mouthing, etc."}, "language_style": {"answer": "Calm and direct", "rationale": "The entry is presented in a direct question, does not use excessive rhetorical or emotional rendering, is more balanced and objective and corresponds to the language style of calm and direct."}, "time_dimension": {"answer": "Present", "rationale": "The entry asks the testee about his current state or habits, and the keyword \"problem\" indicates that it is an evaluation of the characteristics that are reflected. The temporal dimension therefore is now judged."}, "sentence_type": "Interrogative sentence", "pos": {"nouns": {"you": 1, "Talkative": 1}, "verbs": {"Is it possible": 1, "Talkative": 1}, "numerals": {"5": 1}}}, {"content": "6. Would it upset you to have debts?", "word_count": 8, "label": "", "sentence_structure": "Subject鈥損redicate", "subjective_objective": {"answer": "Subjective", "rationale": "Because the feeling of debt and the feeling of unease are part of the patient ' s subjective inner experience, it is subjective."}, "sentence_sentiment": {"answer": "Negative", "rationale": "Because the expression \"debts will upset you\" in the entry is negative and indicates that indebtedness has a negative impact on people's emotions."}, "language_style": {"answer": "Tactful and warm", "rationale": "The entry follows the query approach and uses the \"Does it bother you?\" question phrase, which is more gentle and friendly, and makes it easier for respondents to accept and answer questions. Nor does it directly point to the possible negative effects of the debt problem, avoiding pressure or discomfort on the interviewee."}, "time_dimension": {"answer": "Present", "rationale": "The keyword \"making you uncomfortable\" shows that it is an emotional or emotional reaction to the state of the present. So the entry is looking at the current psychological state and feelings of the person being tested."}, "sentence_type": "Interrogative sentence", "pos": {"nouns": {"Debt": 1, "Unsettled": 1}, "verbs": {"鈾?And I'm gonna be 鈾?: 1}, "numerals": {"Six": 1}}}, {"content": "Did you ever feel 鈥渨orried鈥?for no reason?", "word_count": 13, "label": "", "sentence_structure": "Subject鈥搗erb鈥搊bject", "subjective_objective": {"answer": "Subjective", "rationale": "Because it is a subjective feeling that the patient himself describes and judges the feeling."}, "sentence_sentiment": {"answer": "Negative", "rationale": "Because the emotional experience of feeling \"worried\" in the entry is negative."}, "language_style": {"answer": "Tactful and warm", "rationale": "The entry is a conversation in a more peaceful and friendly way, and uses the euphemism of \"for no reason\" to guide the interviewee to his emotional experience, which is more likely to trigger resonance and positive responses from the interviewee."}, "time_dimension": {"answer": "Past", "rationale": "The entry is to ask if the person tested had ever felt \"wrong\" for no reason in the past, and the dimension of time is a matter of the past."}, "sentence_type": "Interrogative sentence", "pos": {"nouns": {"you": 2, "For no reason": 1, "It's hard": 1}, "verbs": {"Tsang": 1, "I think": 1}, "adjectives": {"True": 1, "of": 2}, "adverbs": {"For no reason": 1}, "numerals": {"7": 1}}}, {"content": "Have you ever been too greedy?", "word_count": 10, "label": "", "sentence_structure": "Subject鈥搗erb鈥搊bject", "subjective_objective": {"answer": "Subjective", "rationale": "Because the scheme of greed is a subjective attitude or motivation, it reflects the individual ' s inner orientation and values."}, "sentence_sentiment": {"answer": "Negative", "rationale": "Because \"over-intensity\" is a negative act."}, "language_style": {"answer": "Calm and direct", "rationale": "The entry is in the form of a direct query, without any rhetorical or emotional colour, and is straightforward in a calm tone, with the aim of obtaining the real thoughts and answers of the person under investigation."}, "time_dimension": {"answer": "Past", "rationale": "The keyword \"had been greedy\" suggests that this is a question of past behaviour, and the concept of \"excessiveness\" also points to past behaviour or state. The entry is therefore an inquiry into whether the person tested had had experience with excesses in the past."}, "sentence_type": "Interrogative sentence", "pos": {"nouns": {"Questionnaire": 1, "Entry": 1, "Out of the way": 1, "Things": 1}, "verbs": {"A greedy picture": 1, "Tsang": 1}, "adverbs": {"That's too much": 1}, "classifiers": {"individual": 1}}}, {"content": "Did you carefully close the doors and windows at night?", "word_count": 15, "label": "", "sentence_structure": "Subject鈥搗erb鈥搊bject", "subjective_objective": {"answer": "Subjective", "rationale": "The question is one of personal habits and attitudes of the patient, and the patient is required to answer it himself."}, "sentence_sentiment": {"answer": "Neutral", "rationale": "Because the entry does not have any emotional color."}, "language_style": {"answer": "Calm and direct", "rationale": "The entry expresses the subject of asking whether the other party will carefully close the door or window at night in a concise and clear language, without excessive rhetorical or emotional rendering, and with a more objective tone."}, "time_dimension": {"answer": "Present", "rationale": "The entry asks about the current behaviour of the subject, the keywords being \"Whether or not to close the door or window at night\" and \"cautiously close the door or window\" , all pointing to the state of the moment, which is happening. So the time dimension is now."}, "sentence_type": "Interrogative sentence", "pos": {"nouns": {"you": 2, "Night": 1, "Watch out": 1, "Windows and doors": 1}, "verbs": {"Is it possible": 1, "Close it": 1}, "adjectives": {"Watch your wings": 1}, "numerals": {"9": 1}}}, {"content": "Are you more active?", "word_count": 7, "label": "", "sentence_structure": "Subject鈥搗erb鈥搊bject", "subjective_objective": {"answer": "Subjective", "rationale": "Because \"active\" is a term that describes the character or attitude of the patient, it is viewed from the subjective perspective of the patient."}, "sentence_sentiment": {"answer": "Positive", "rationale": "Because \"active\" is a positive term for description."}, "language_style": {"answer": "Tactful and warm", "rationale": "This is a more friendly and intimate way of expressing themselves, helping the person to share his or her real thoughts and feelings."}, "time_dimension": {"answer": "Present", "rationale": "The question, judged by the descriptive term \"more active\" and the structure of sentences used in the title, is to ask the testee about the current level of activity and therefore about the current behaviour or state."}, "sentence_type": "Interrogative sentence", "pos": {"adjectives": {"Comparison": 1, "Active": 1}, "numerals": {"10": 1}}}, {"content": "Is it very sad to see a child or an animal tortured?", "word_count": 24, "label": "", "sentence_structure": "Subject鈥搗erb鈥搊bject", "subjective_objective": {"answer": "Subjective", "rationale": "Because feeling sad is an emotional experience and a subjective attitude of the patient."}, "sentence_sentiment": {"answer": "Positive", "rationale": "Because the \"sweet\" sentiment in the entry is positive."}, "language_style": {"answer": "Tactful and warm", "rationale": "The entry is more personal and interactive when it is spoken in a gentle, inquisitive manner, rather than in direct statements or orders. At the same time, it uses the emotional term \"very sad\" to guide respondents to share their feelings, reflecting attention and support for the feelings of the interviewees."}, "time_dimension": {"answer": "Present", "rationale": "The entry asks if the person tested would have some emotional reaction in the current circumstances, and the temporal dimension is now. The key words are \"are you\" and \"are you very sad\" , which means a subjective feeling and emotional experience, which is created in the immediate moment of seeing a child or animal being tortured, and is therefore a state of affairs."}, "sentence_type": "Interrogative sentence", "pos": {"nouns": {"you": 1, "Kids": 1, "Animals": 1, "Torture": 1}, "verbs": {"Yes": 2, "I feel": 1, "Sorry": 1}, "adjectives": {"Very": 1}, "numerals": {"11": 1}}}, {"content": "12. Do you often do things you should not do and do things you should not say and you are nervous?", "word_count": 28, "label": "", "sentence_structure": "Subject鈥搗erb鈥搊bject", "subjective_objective": {"answer": "Subjective", "rationale": "Because it is the patient ' s own assessment and perception of his or her own behaviour, it is subjective."}, "sentence_sentiment": {"answer": "Negative", "rationale": "Because the emotional expression of \"stretch\" in the entry is negative."}, "language_style": {"answer": "Tactful and warm", "rationale": "The entry is more personal and interactive when it is spoken in a gentle and inviting tone, rather than in direct statements or orders. At the same time, the entry does not use too much emotionally nuanced terms, but rather more neutral expressions that make respondents more willing to share their true feelings."}, "time_dimension": {"answer": "Present", "rationale": "So the answer is \"now\"."}, "sentence_type": "Interrogative sentence", "pos": {"nouns": {"self": 1, "Yes": 1, "Word": 1}, "verbs": {"Do it": 1, "Say it": 1, "Nervous": 1}, "adjectives": {"Often": 1, "I shouldn't": 2}, "numerals": {"12": 1}, "pronouns": {"you": 1}}}, {"content": "Do you like to jump?", "word_count": 8, "label": "", "sentence_structure": "Subject鈥損redicate", "subjective_objective": {"answer": "Subjective", "rationale": "Because \"like\" is a subjective feeling or attitude."}, "sentence_sentiment": {"answer": "Positive", "rationale": "Because \"like\" is a positive emotional attitude."}, "language_style": {"answer": "Calm and direct", "rationale": "The language style of the entry is straightforward, without any rhetorical or emotional rendering, simply asking if the interviewee likes to jump into the parachute. This is a simple and quick language style that helps the interviewee to understand and answer the questions quickly."}, "time_dimension": {"answer": "Present", "rationale": "The temporal dimension of this purpose is therefore judged now."}, "sentence_type": "Interrogative sentence", "pos": {"nouns": {"you": 1, "Yeah": 1, "Jump": 1, "Submarine": 1}, "verbs": {"Yeah": 1, "Jump": 1}, "numerals": {"13": 1}, "pronouns": {"Here": 1}}}, {"content": "14. Can you usually play at a party?", "word_count": 16, "label": "", "sentence_structure": "Subject鈥搗erb鈥搊bject", "subjective_objective": {"answer": "Subjective", "rationale": "Because it looks at patients' subjective feelings and abilities."}, "sentence_sentiment": {"answer": "Positive", "rationale": "Because the entry \"Full play\" expresses a positive, positive emotional attitude."}, "language_style": {"answer": "Tactful and warm", "rationale": "The language style of this entry is more euphemistic, because it uses an inquisitorial phrase to guide the other to answer questions rather than to speak or order directly. It makes it easier for the interviewee to feel comfortable and respected and to establish good communication. At the same time, the use of the term \"play well\" implies that the questionnaire seeks to understand the attitude and participation of the interviewee in social activities, rather than merely to focus on his behaviour itself, which is also a display of a warmer language style."}, "time_dimension": {"answer": "Present", "rationale": "The words \"normally\" and \"capable\" in the sentence would make it clear that it is an inquiry into the present behaviour or state of the subject and therefore falls within the category of \"now\"."}, "sentence_type": "Interrogative sentence", "pos": {"nouns": {"- The party": 1}, "verbs": {"Yes": 1, "Play your game": 1}, "adverbs": {"Usually": 1}}}, {"content": "Are you excited?", "word_count": 6, "label": "", "sentence_structure": "Subject鈥損redicate", "subjective_objective": {"answer": "Subjective", "rationale": "It's seen from the patient's subjective point of view, because \"facilitation\" is a subjective emotional state of a person, and it requires the patient to answer his own feelings."}, "sentence_sentiment": {"answer": "Neutral", "rationale": "Because the entry is not of any emotional colour, it is simply a question of whether a person is easily excited and does not express a positive or negative tendency."}, "language_style": {"answer": "Tactful and warm", "rationale": "Warm and warm."}, "time_dimension": {"answer": "Past", "rationale": "The entry asks whether the testees are often excited about their past experience and therefore judge them as past."}, "sentence_type": "Interrogative sentence", "pos": {"verbs": {"Excited": 1}, "adverbs": {"Easy": 1}, "numerals": {"15": 1}}}, {"content": "Have you ever put your fault to anyone?", "word_count": 14, "label": "", "sentence_structure": "Subject鈥搗erb鈥搊bject", "subjective_objective": {"answer": "Subjective", "rationale": "The reason is that it is the subjective attitude and behaviour that pushes fault on others as patients."}, "sentence_sentiment": {"answer": "Negative", "rationale": "Because the \"putting faults on someone\" in the entry is negative."}, "language_style": {"answer": "Tactful and warm", "rationale": "The entry is more euphemistic, moderate, not too direct or harsh, but rather directed to the respondent to answer the question with concern and understanding. At the same time, the entry does not give a moral assessment or blame of the conduct, but rather raises the question in a neutral manner, leaving the respondent to judge and answer. Such expression is more comfortable and relaxed for the respondent and is more willing to answer the question in a genuine manner."}, "time_dimension": {"answer": "Past", "rationale": ""}, "sentence_type": "Interrogative sentence", "pos": {"nouns": {"Wrong": 1, "others": 1}, "verbs": {"Push": 1, "Will": 1, "Wrong": 1}, "numerals": {"16": 1}, "pronouns": {"you": 1}}}, {"content": "Do you like meeting strangers?", "word_count": 9, "label": "", "sentence_structure": "Subject鈥搗erb鈥搊bject", "subjective_objective": {"answer": "Subjective", "rationale": "Because \"like\" is a subjective feeling or attitude, and it is the patient's personal emotional experience, the question is examined from a subjective perspective."}, "sentence_sentiment": {"answer": "Neutral", "rationale": "Because the entry is not of any emotional colour, it is merely an inquiry into personal preferences."}, "language_style": {"answer": "Tactful and warm", "rationale": "The language style of the entry is relatively euphemistic and respectful of the other, and the way in which the interviewee is asked to reflect on his or her views, without giving any predetermined answer or test, is more accommodative, friendly and acceptable."}, "time_dimension": {"answer": "Present", "rationale": "The entry asks the testee's attitude and tendency towards the current situation, i.e. whether he/she prefers to be with strangers at this stage, and therefore judge as \"now\"."}, "sentence_type": "Interrogative sentence", "pos": {"nouns": {"you": 1, "Stranger": 1, "Yeah": 1, "Yes": 1}, "verbs": {"See": 1, "- You're not": 1}, "numerals": {"17": 1}}}, {"content": "18. Do you believe that the insurance system is a good way to do that?", "word_count": 15, "label": "", "sentence_structure": "Subject鈥搗erb鈥搊bject", "subjective_objective": {"answer": "Subjective", "rationale": "This is a subjective observation of patients ' perception and beliefs in the insurance system."}, "sentence_sentiment": {"answer": "Positive", "rationale": "Because the view in the entry that \"convinced that the insurance system is a good way\" expresses a positive view of the insurance system."}, "language_style": {"answer": "Calm and direct", "rationale": "The language style of the entry is more straightforward, and there is no excessive amount of embellishment or emotional rendering, but rather a simple question-and-answer period to guide the interviewee to think and answer."}, "time_dimension": {"answer": "Present", "rationale": ""}, "sentence_type": "Interrogative sentence", "pos": {"nouns": {"Insurance system": 1, "Approach": 1}, "verbs": {"Yes": 1, "be": 1, "One": 1, "Okay": 1}, "adjectives": {"Okay": 1}, "numerals": {"18": 1}, "pronouns": {"you": 1}}}, {"content": "Are you a sentimental person?", "word_count": 12, "label": "", "sentence_structure": "Subject鈥揷opula鈥損redicative", "subjective_objective": {"answer": "Subjective", "rationale": "Because \"facilitation\" is a personal feeling and attitude that belongs to the patient's own subjective perspective."}, "sentence_sentiment": {"answer": "Negative", "rationale": "Because the expression of the purpose of this article contains negative feelings, namely, \"always to hurt.\""}, "language_style": {"answer": "Tactful and warm", "rationale": "The entry is a soft, invitation-style conversation, asking if the person interviewed is vulnerable to emotional harm, not direct statements or orders. This expression is more personal and interactive, making the person interviewed more willing to participate and answer questions. And, \"Are you a person?\" The question phrase also gives the person interviewed more scope to think, to think more deeply about his or her actual situation and to give real and accurate answers."}, "time_dimension": {"answer": "Present", "rationale": "\"Are you a sentimental person?\" The options \"is\" and \"no\" are both selected by the testee at the moment, so the entry is in its current state. At the same time, the description \"the vulnerable person\" is based on an assessment of the current emotional state and character, not on past or future behaviour or results."}, "sentence_type": "Interrogative sentence", "pos": {"nouns": {"you": 1, "person": 1, "Emotion": 1}, "verbs": {"be": 1, "Wounded": 1}, "adjectives": {"One": 1, "Easy": 1, "It hurts": 1}}}, {"content": "- Are all your habits good?", "word_count": 11, "label": "", "sentence_structure": "Subject鈥損redicate", "subjective_objective": {"answer": "Subjective", "rationale": "Because custom is a long-standing pattern of behaviour of individuals and a subjective judgement as to whether a custom is \"good\"."}, "sentence_sentiment": {"answer": "Neutral", "rationale": "Because the entry asked whether all the habits of the interviewees were positive and did not have any emotional colours."}, "language_style": {"answer": "Tactful and warm", "rationale": "The language style of the questionnaire entry is more moderate, using a question and answer to express concern and concern about the interviewee rather than directly drawing conclusions or ordering."}, "time_dimension": {"answer": "Present", "rationale": "The entry is for the testee ' s current state, and the time dimension is now judged."}, "sentence_type": "Interrogative sentence", "pos": {"nouns": {"Custom": 1, "All": 1}, "verbs": {"be": 1}, "adjectives": {"Okay": 1}, "numerals": {"20": 1}, "pronouns": {"you": 1}}}, {"content": "21. Do you always hate to be seen in social settings?", "word_count": 14, "label": "", "sentence_structure": "Subject鈥搗erb鈥搊bject", "subjective_objective": {"answer": "Subjective", "rationale": "Because \"showing\" is a subjective expression and attitude, it is the patient's perception and evaluation of himself."}, "sentence_sentiment": {"answer": "Negative", "rationale": "Because the \"no-go-go\" act in the entry is negative."}, "language_style": {"answer": "Calm and direct", "rationale": "The entry raises questions in a concise manner, without excessive rhetorical or emotional rendering, and with a more objective tone."}, "time_dimension": {"answer": "Present", "rationale": "The keyword \"always unwilling\" indicates a constant, customary mentality or behaviour that is ongoing. The entry therefore asks about the social behaviour and mentality of the person being tested."}, "sentence_type": "Interrogative sentence", "pos": {"nouns": {"Socially": 1, "Show your head": 1}, "verbs": {"Is it possible": 1, "Total": 1, "May I": 1, "I don't want to": 1, "Lou": 1, "Do you always have to show up in social affairs": 1}, "adverbs": {"Total": 1, "not": 1, "May I": 1}, "pronouns": {"you": 1}}}, {"content": "Do you take strange or dangerous drugs?", "word_count": 15, "label": "", "sentence_structure": "Subject鈥搗erb鈥搊bject", "subjective_objective": {"answer": "Subjective", "rationale": "This is a subjective attitude of the patient, since it is a question of whether the patient is willing or inclined to take medications that are strange or dangerous."}, "sentence_sentiment": {"answer": "Negative", "rationale": "Because the \"taking of strange or dangerous drugs\" in the entry is negative."}, "language_style": {"answer": "Calm and direct", "rationale": "The entry is presented in the form of a straightforward question, without excessive rhetorical or emotional colours, with a more calm and objective tone, and is intended to capture the real thoughts and behavioural preferences of the interviewee."}, "time_dimension": {"answer": "Past", "rationale": "The keyword \"will\" indicates that it is an inquiry into the past or present behaviour of the person being tested, while \"the taking of a drug with a strange or dangerous effect\" points to specific actions and possibilities. Thus, the entry contains both past and present information."}, "sentence_type": "Interrogative sentence", "pos": {"nouns": {"Questionnaire": 1, "Item": 1, "Strange": 1, "Danger": 1, "Role": 1, "Drugs": 1}, "verbs": {"Take it": 1}, "adverbs": {"Yes": 1}, "numerals": {"22": 1, "you": 1, "One": 1}}}, {"content": "Do you often get tired of it?", "word_count": 8, "label": "", "sentence_structure": "Subject鈥搗erb鈥搊bject", "subjective_objective": {"answer": "Subjective", "rationale": "Because \"weary\" is a subjective emotional feeling."}, "sentence_sentiment": {"answer": "Negative", "rationale": "Because the \"tired\" feeling in the entry is negative."}, "language_style": {"answer": "Tactful and warm", "rationale": ""}, "time_dimension": {"answer": "Present", "rationale": ""}, "sentence_type": "Interrogative sentence", "pos": {"nouns": {"Tired": 1, "Sensor": 1, "you": 1}, "verbs": {"have": 1}, "adverbs": {"Regular": 1, "鈾?And the 鈾?: 1}, "numerals": {"23": 1}}}, {"content": "Have you ever taken anything from anyone?", "word_count": 16, "label": "", "sentence_structure": "Subject鈥損redicate", "subjective_objective": {"answer": "Objective", "rationale": "Because it is an objective fact to ask if the patient has ever been a thief, there is no subjective attitude or feeling on the part of the patient."}, "sentence_sentiment": {"answer": "Negative", "rationale": "Because the \"take something from someone else\" act is negative."}, "language_style": {"answer": "Calm and direct", "rationale": "The entry is directly asked whether the person interviewed has ever taken another person ' s belongings, is not using excessive emotional colours or acrimony, and is more objective and calm."}, "time_dimension": {"answer": "Past", "rationale": "The entry uses the past \"had taken\" to indicate that the question was about the testee's past behaviour and therefore the judgement is the past."}, "sentence_type": "Interrogative sentence", "pos": {"nouns": {"you": 1, "others": 1, "Something": 1, "One shot, one shot": 1}, "verbs": {"Yes": 1}, "adverbs": {"Tsang": 1}, "numerals": {"24": 1}, "classifiers": {"individual": 1}}}, {"content": "25. Do you like to go out often?", "word_count": 7, "label": "", "sentence_structure": "Subject鈥損redicate", "subjective_objective": {"answer": "Subjective", "rationale": "Because \"often love to go out\" is a personal preference and a tendency to be subjective."}, "sentence_sentiment": {"answer": "Neutral", "rationale": "Because the entry does not have any emotional color."}, "language_style": {"answer": "Calm and direct", "rationale": "The entry is more directly written, is not overdoed or emotionally retrofitted, and simply asks whether the person surveyed has a habit of frequent outing. This is a simple and clear expression that facilitates obtaining accurate information."}, "time_dimension": {"answer": "Present", "rationale": "The entry uses the term \"whether or not\" to suggest that it is a question of the current state or customary behaviour of the person being tested, so the temporal dimension is now judged. Moreover, the description of \"often love to go out\" also shows that it is a constant and continuous act, which further supports this determination."}, "sentence_type": "Interrogative sentence", "pos": {"nouns": {"you": 1, "Out": 1}, "verbs": {"Is it possible": 1, "Regular": 1, "Love": 1}, "numerals": {"25": 1}}}, {"content": "Did you enjoy hurting the person you loved?", "word_count": 17, "label": "", "sentence_structure": "Subject鈥搗erb鈥搊bject", "subjective_objective": {"answer": "Subjective", "rationale": "Because it's the patient's subjective attitude or feeling."}, "sentence_sentiment": {"answer": "Negative", "rationale": "Because the \"Funner to harm the person you love\" act is negative, derogatory and emotional."}, "language_style": {"answer": "Calm and direct", "rationale": "The entry is of a direct, concise language style, without too many rhetorical or emotional renderings. It asks the testee whether he or she is happy with someone who hurts his or her own care, and expresses it objectively and in a guiding manner, suitable for psychological questionnaire surveys."}, "time_dimension": {"answer": "Past", "rationale": "The keyword \"is it\" suggests that the question is about the testee's past experience and attitudes, and therefore the temporal dimension is a matter of the past. And, \"Favorite from harm...\", it also shows that the question relates to the testee's psychological orientation and behaviour patterns, with a greater focus on the past behavior or emotional state."}, "sentence_type": "Interrogative sentence", "pos": {"nouns": {"Questionnaire": 1, "Entry": 1, "person": 1, "Fun": 1}, "verbs": {"Is it possible": 1, "Hurt": 1, "Pity": 1, "I feel": 1}, "adverbs": {"From": 1, "And": 1}, "numerals": {"26": 1}, "pronouns": {"you": 1}}}, {"content": "27. Do you often suffer from a sense of guilt?", "word_count": 12, "label": "", "sentence_structure": "Subject鈥搗erb鈥搊bject", "subjective_objective": {"answer": "Subjective", "rationale": "Since guilt is usually the experience and feeling of the individual, it is viewed from a subjective perspective."}, "sentence_sentiment": {"answer": "Negative", "rationale": "The basis for the judgement: the \"illness of guilt\" in the entry is a negative emotion and attitude, and is therefore classified as negative."}, "language_style": {"answer": "Tactful and warm", "rationale": ""}, "time_dimension": {"answer": "Present", "rationale": "The entry asks about the current feelings of the subject, and the keyword is \"Do you often suffer?\" The word \"always\" and the verb \"problem\" in it all means that it is a constant psychological state. So, in terms of time dimension, the entry is now."}, "sentence_type": "Interrogative sentence", "pos": {"nouns": {"The feeling of guilt": 1, "Trouble": 1}, "verbs": {"Regular": 1, "Yes": 1, "Trouble": 1}, "adverbs": {"Regular": 1}, "numerals": {"27": 1}}}, {"content": "Do you ever talk about anything you don't know?", "word_count": 13, "label": "", "sentence_structure": "Subject鈥搗erb鈥搊bject", "subjective_objective": {"answer": "Subjective", "rationale": "Because \"know\" or \"under\" is the patient's own perception and understanding, it is a subjective examination."}, "sentence_sentiment": {"answer": "Negative", "rationale": "Because the act was negative."}, "language_style": {"answer": "Calm and direct", "rationale": "The entry is in a straightforward manner asking the other party whether they are not in a state of mind during their conversation, without too much embellishment or emotional rendering, and in a more calm and objective language style."}, "time_dimension": {"answer": "Present", "rationale": "The entry uses the now completed state of affairs, \"You don't know what you've done\" to indicate the impact of past experience on the present, so the time dimension is now."}, "sentence_type": "Interrogative sentence", "pos": {"nouns": {"you": 1, "Discussion": 1, "Time": 1, "I don't know what to say": 1}, "verbs": {"in": 1, "Talk": 1, "Is it possible": 1, "Sometimes": 1, "You know what I mean": 1}, "adverbs": {"Medium": 1, "Both": 1, "Time": 1}, "numerals": {"28": 1}}}, {"content": "29. Do you prefer to go to books rather than to see others?", "word_count": 15, "label": "", "sentence_structure": "Subject鈥搗erb鈥搊bject", "subjective_objective": {"answer": "Subjective", "rationale": "Because it's a subjective description of what patients like."}, "sentence_sentiment": {"answer": "Negative", "rationale": "Because the behavior in the entry \"I prefer to read to see more\" is negative and indicates that I am not willing to be with someone else."}, "language_style": {"answer": "Tactful and warm", "rationale": "The language style of the entry is more euphemistic and warm, and the interviewee's attitude to reading and interacting with people is not expressed in too direct or command language.\""}, "time_dimension": {"answer": "Present", "rationale": "The entry asks the testee's current preferences and preferences, and the keywords are \"better\" and \"no\" and \"go\" , which indicate that this is a general expression of the current state of affairs. Thus, the time dimension of the questionnaire entry is now judged."}, "sentence_type": "Interrogative sentence", "pos": {"nouns": {"you": 1, "Books": 1, "person": 1}, "verbs": {"I'd rather": 1, "Look": 1, "Go on": 4, "See you": 1}, "adverbs": {"not": 2, "May I": 1}, "numerals": {"29": 1}}}, {"content": "Have you tried to hurt your enemies?", "word_count": 10, "label": "", "sentence_structure": "Subject鈥搗erb鈥搊bject", "subjective_objective": {"answer": "Subjective", "rationale": "This is a question of asking the patient about his subjective thoughts and feelings, not about the specific behaviour or objective facts observed."}, "sentence_sentiment": {"answer": "Negative", "rationale": "Because the \"to hurt the enemy\" act in the entry is negative."}, "language_style": {"answer": "Calm and direct", "rationale": "The entry is directly explicit, is not overdoing or rendering, and is a direct expression of calmness. It asks the other side whether there is a person who wants to hurt, is more objective and neutral, and is free of any subjective emotional aspects."}, "time_dimension": {"answer": "Present", "rationale": "The entry asks if the person who was tested currently has the emotion and motive to harm a person, and the temporal dimension is now judged."}, "sentence_type": "Interrogative sentence", "pos": {"nouns": {"The enemy": 1, "Hurt": 1, "you": 1}, "verbs": {"have": 1, "Yes": 1, "Wounded": 1}, "adverbs": {"More": 1, "- You're not": 1}, "numerals": {"30": 1}}}, {"content": "31. Do you think you're a very sensitive person?", "word_count": 15, "label": "", "sentence_structure": "Subject鈥搗erb鈥搊bject", "subjective_objective": {"answer": "Subjective", "rationale": "Because \"thinking yourself\" is the patient's subjective attitude and self-awareness, it's a subjective perspective."}, "sentence_sentiment": {"answer": "Negative", "rationale": "Because the description of \"sensitization\" is negative, it suggests that the person under investigation may have feelings of anxiety or anxiety."}, "language_style": {"answer": "Calm and direct", "rationale": "The entry directly asked whether the person surveyed considered himself to be a sensitive person, was more concise in language style, was not over-repeated or emotional, and was of a calm and direct language style."}, "time_dimension": {"answer": "Present", "rationale": "The entry asks the testee's current feelings and perceptions, and therefore judges it as the present. The keyword \"self\" indicates that it is self-evaluation of the individual, and \"a sensitive person\" is the testee's current perception of his condition, pointing to the present situation."}, "sentence_type": "Interrogative sentence", "pos": {"nouns": {"self": 1, "person": 1}, "verbs": {"I think": 1, "be": 1, "You're a bit of a sane": 1, "- You're not": 1}, "adjectives": {"You're a bit of a sane": 1}, "numerals": {"31": 1}, "pronouns": {"you": 1}}}, {"content": "Do you often apologize for being rude?", "word_count": 17, "label": "", "sentence_structure": "Subject鈥搗erb鈥搊bject", "subjective_objective": {"answer": "Subjective", "rationale": "Because it is the subjective attitude and behaviour of one person to apologize."}, "sentence_sentiment": {"answer": "Positive", "rationale": "Because the \"when disrespectful\" act in the entry is negative, but the act that requires an apology is positive."}, "language_style": {"answer": "Tactful and warm", "rationale": "The entry is more personal and interactive when it is spoken in a gentle, inquisitive manner rather than directly speaking or ordering."}, "time_dimension": {"answer": "Present", "rationale": "\"Wasn't it often you apologized?\" The main phrase in \"Wasn't it always you?\" is now you, so the entry is asking the subject about the behaviour and attitude of the subject."}, "sentence_type": "Interrogative sentence", "pos": {"nouns": {"person": 1, "I'm sorry": 1, "Organisation": 1, "Sorry": 1}, "verbs": {"to": 1, "have": 1, "Regular": 1, "Yes": 1, "Organisation": 1}, "adverbs": {"Is it possible": 1, "Regular": 1}, "numerals": {"32": 1}}}, {"content": "Do you have many friends?", "word_count": 7, "label": "", "sentence_structure": "Subject鈥損redicate", "subjective_objective": {"answer": "Subjective", "rationale": "Because there are many friends who feel and know that they are a person, different people will define \"many.\" So the question is the subjective perspective of the patient."}, "sentence_sentiment": {"answer": "Neutral", "rationale": "Because the entry was not of any emotional colour, it was merely asking about the number of friends of the respondents."}, "language_style": {"answer": "Tactful and warm", "rationale": "The language style of the entry is euphemism. It asks the respondents, in a gentle and friendly tone, whether they have many friends, which is more personal and interactive. At the same time, the question is not in cold or direct language, but is presented in a more softer way, making it easier for the respondents to answer."}, "time_dimension": {"answer": "Present", "rationale": "The entry is for the current social status of the testee, and the time dimension is now judged."}, "sentence_type": "Interrogative sentence", "pos": {"nouns": {"friends": 1}, "verbs": {"have": 1}, "numerals": {"More": 1, "Many": 1}}}, {"content": "Do you like to say jokes that sometimes do hurt people?", "word_count": 17, "label": "", "sentence_structure": "Subject鈥搗erb鈥搊bject", "subjective_objective": {"answer": "Subjective", "rationale": "Because \"a love to say jokes that sometimes hurt people\" is an individual's attitude or tendency, the patient's own psychological state and feelings."}, "sentence_sentiment": {"answer": "Negative", "rationale": "Because the act of \"pronouncement of jokes that sometimes do hurt\" can have a negative impact on others and is therefore negative."}, "language_style": {"answer": "Calm and direct", "rationale": "The entry asks the other side whether they like to make a joke that may hurt, and the language style is more straightforward and not too much rhetorical or emotional. \""}, "time_dimension": {"answer": "Present", "rationale": "The entry asks about the current behaviour or habit of the testee, namely, \"Do you like to say something that sometimes hurts people?\" The phrase \"does it mean that it is a selective question, and \"do something that sometimes hurts people\" indicates that it is an act or habit. Thus, the time dimension of the article is now."}, "sentence_type": "Interrogative sentence", "pos": {"nouns": {"Questionnaire Entry": 1, "person": 2, "A joke": 1}, "verbs": {"Love it": 1, "Speak": 1, "Hurt": 1}, "adjectives": {"Sometimes": 1, "Yes, I do": 1}, "numerals": {"34": 1, "Some": 1}, "pronouns": {"you": 1}}}, {"content": "Are you a worried man?", "word_count": 11, "label": "", "sentence_structure": "Subject鈥搗erb鈥搊bject", "subjective_objective": {"answer": "Subjective", "rationale": "Because \"challenges\" are emotional or character characteristics that require patient self-assessment and description, they are subjective."}, "sentence_sentiment": {"answer": "Negative", "rationale": "Because the term \"scareful\" has a negative emotional connotation."}, "language_style": {"answer": "Tactful and warm", "rationale": "The entry is more adept and interactive when it is spoken in a gentle and inviting manner, rather than by direct statements or orders."}, "time_dimension": {"answer": "Present", "rationale": "The entry asks the current state of the subjects, i.e. whether they are often worried or worried, and therefore judges it as now."}, "sentence_type": "Interrogative sentence", "pos": {"nouns": {"person": 1, "Sad": 1, "Concerns": 1}, "verbs": {"be": 1, "More": 2}, "adjectives": {"I'm worried": 1}, "numerals": {"35": 1}}}, {"content": "36. Were you doing what you were told to do when you were a child, without complaint?", "word_count": 22, "label": "", "sentence_structure": "Subject鈥搗erb鈥搊bject", "subjective_objective": {"answer": "Subjective", "rationale": "Because it's the patient's subjective action to do what he says."}, "sentence_sentiment": {"answer": "Neutral", "rationale": "Because the entry describes a person ' s behaviour in childhood, there is no apparent emotional tendency."}, "language_style": {"answer": "Calm and direct", "rationale": "The entry is more direct and objective in terms of dialogue in a direct question and is simple and clear in language, without any accoutrements and emotional rendering."}, "time_dimension": {"answer": "Past", "rationale": "The entry is for questioning the behaviour and attitudes of the person tested during childhood, and the temporal dimension is a past."}, "sentence_type": "Interrogative sentence", "pos": {"nouns": {"you": 1, "Childhood": 1, "Yes": 1, "What": 2, "Shit": 1, "Do it": 1, "Grievances": 1}, "verbs": {"Is it possible": 1, "According to": 1, "Do it": 1, "Nothing": 1, "have": 1}, "adjectives": {"Yes": 1, "What": 2, "Shit": 1, "Do it": 1, "Grievances": 1}, "adverbs": {"Is it possible": 1, "According to": 1, "Nothing": 1}, "numerals": {"36": 1}, "pronouns": {"you": 1}}}, {"content": "Do you think you're a charisma?", "word_count": 11, "label": "", "sentence_structure": "Subject鈥搗erb鈥搊bject", "subjective_objective": {"answer": "Subjective", "rationale": "Because the \"Labourism\" is a subjective self-awareness and evaluation."}, "sentence_sentiment": {"answer": "Positive", "rationale": "Because \"the charisma\" usually refers to a positive optimism, a passionate attitude to life or character."}, "language_style": {"answer": "Tactful and warm", "rationale": "The entry is judged by the use of a question sentence, which expresses concern and understanding about the personality characteristics of the person under investigation, while encouraging the person under investigation to think and express his or her views. Moreover, the term \"the Antennaism\" itself is more positive, without too many negative emotions or mandatory requirements, and gives people a sense of comfort."}, "time_dimension": {"answer": "Present", "rationale": "The keywords \"thinks\" and \"the optimist\" both indicate that this is a description of the subjective view of the present, so the entry should be judged as it is."}, "sentence_type": "Interrogative sentence", "pos": {"nouns": {"Questionnaire": 1, "Entry": 1, "Lotte pie": 1}, "verbs": {"I think": 1, "be": 1, "One": 2, "individual": 1, "have": 1}, "adjectives": {"Lotte pie": 1}, "numerals": {"37": 1}, "pronouns": {"you": 1}}}, {"content": "Are you polite and neat?", "word_count": 10, "label": "", "sentence_structure": "Subject鈥損redicate", "subjective_objective": {"answer": "Subjective", "rationale": "The examination is based on the patient ' s personal attitudes and habits and is therefore subjective."}, "sentence_sentiment": {"answer": "Positive", "rationale": "Because \"be polite and neat\" is a positive quality."}, "language_style": {"answer": "Tactful and warm", "rationale": "The entry is more personal and interactive when it is spoken in a gentle, inquisitive manner, rather than in direct statements or orders. At the same time, the question is not too much about whether the other party should be polite and neat, but rather provides an option for the interviewee to answer the question himself, which is more respectful of the interviewee鈥檚 personal will and feelings."}, "time_dimension": {"answer": "Present", "rationale": "The entry asks about the current behaviour of the subject, the keywords being \"definable\" and \"clean,\" which imply the current state or behaviour. Thus, the temporal dimension of the purpose of the article can be judged to be the present."}, "sentence_type": "Interrogative sentence", "pos": {"nouns": {"Courage": 1, "Clean": 1}, "verbs": {"- It's a good idea": 1}, "adjectives": {"very": 1}, "numerals": {"38": 1}}}, {"content": "Are you always worried about something terrible?", "word_count": 15, "label": "", "sentence_structure": "Subject鈥揷opula鈥損redicative", "subjective_objective": {"answer": "Subjective", "rationale": "Because \"fearing something terrible\" is a subjective emotion and feeling, a feeling and experience inside the patient, not an objective fact."}, "sentence_sentiment": {"answer": "Negative", "rationale": "Because the psychological state of \"fears of something terrible\" in the entry is negative."}, "language_style": {"answer": "Tactful and warm", "rationale": "The entry is more gentle, uses a question-and-answer tone, and does not directly point to the concerns or mistakes of the other side, but rather directs the other to express their thoughts in a caring and understanding manner. This is more acceptable and less vigilant."}, "time_dimension": {"answer": "Past", "rationale": ""}, "sentence_type": "Interrogative sentence", "pos": {"nouns": {"Things": 2, "Come on": 1}, "verbs": {"I'm worried": 1}, "adjectives": {"Horrible": 1}, "numerals": {"39": 1}}}, {"content": "Have you ever damaged or lost anything from anyone?", "word_count": 14, "label": "", "sentence_structure": "Subject鈥搗erb鈥搊bject", "subjective_objective": {"answer": "Subjective", "rationale": "Because damage or loss to others is a past act of the patient, it is a matter of the patient ' s memory and self-assessment and is therefore a subjective question."}, "sentence_sentiment": {"answer": "Negative", "rationale": "Since the entry asks about acts that have damaged or lost someone else, this is usually considered irresponsible and disrespectful and therefore has a negative emotional connotation."}, "language_style": {"answer": "Calm and direct", "rationale": "The entry asks the testee whether he has damaged or lost something in a direct and concise language, which is more calm and objective and does not have too much emotional or rhetorical style."}, "time_dimension": {"answer": "Past", "rationale": "The keyword \"had\" suggested that it was an event in the past."}, "sentence_type": "Interrogative sentence", "pos": {"nouns": {"Questionnaire": 1, "Entry": 1, "person": 1, "Something": 1}, "verbs": {"Damage": 1, "Lost": 1, "Pass": 1, "Tsang": 1}, "numerals": {"40": 1}}}, {"content": "Do you usually take the initiative when making new friends?", "word_count": 14, "label": "", "sentence_structure": "Subject鈥搗erb鈥搊bject", "subjective_objective": {"answer": "Subjective", "rationale": "Because \"proprio motu\" is a description of subjective feelings or attitudes, and is the person ' s own feelings and perceptions, the entry is viewed from the patient ' s subjective perspective."}, "sentence_sentiment": {"answer": "Positive", "rationale": "Because \"take the initiative\" shows a person's openness and motivation."}, "language_style": {"answer": "Tactful and warm", "rationale": ""}, "time_dimension": {"answer": "Present", "rationale": ""}, "sentence_type": "Interrogative sentence", "pos": {"nouns": {"New friends": 1, "Initiatives": 1, "General": 1}, "verbs": {"Hand it over": 1, "Adoption": 1, "be": 2}, "adverbs": {"you": 1, "Time": 1, "- You're not": 1}, "numerals": {"41": 1}}}, {"content": "42. Do you understand what people are trying to do when they complain to you?", "word_count": 20, "label": "", "sentence_structure": "Subject鈥搗erb鈥搊bject", "subjective_objective": {"answer": "Subjective", "rationale": "Because \"easy to understand the pain\" is the patient's subjective feeling and attitude."}, "sentence_sentiment": {"answer": "Positive", "rationale": "Because the \"easy to understand their reasons\" act is positive."}, "language_style": {"answer": "Tactful and warm", "rationale": "The entry is more adept and interactive when it is spoken in a gentle and inviting manner, rather than by direct statements or orders."}, "time_dimension": {"answer": "Present", "rationale": "The entry asks about the behaviour or mentality of the person being tested in the current circumstances and therefore judges it as now."}, "sentence_type": "Interrogative sentence", "pos": {"nouns": {"Questionnaire": 1, "Entry": 1, "others": 1, "- Sorry": 1}, "verbs": {"In": 1, "Complaints": 1, "Understood": 1, "Is it possible": 1, "Easy": 1, "Yes": 1}, "adjectives": {"Easy": 1}, "adverbs": {"Is it possible": 1, "Easy": 1}, "numerals": {"42": 1}, "pronouns": {"they": 1}}}, {"content": "Do you think you're nervous, like \"stringing\"?", "word_count": 17, "label": "", "sentence_structure": "Subject鈥搗erb鈥搊bject", "subjective_objective": {"answer": "Subjective", "rationale": "Because \"thinking you're nervous\" is the patient's subjective attitude."}, "sentence_sentiment": {"answer": "Negative", "rationale": "Because words like \"sweet\" and \"string\" in entries are usually associated with negative emotions such as anxiety, anxiety, etc."}, "language_style": {"answer": "Tactful and warm", "rationale": ""}, "time_dimension": {"answer": "Present", "rationale": "The entry \"Assisted that you are nervous\", \"as if you were strung\" describes the current state and the feelings of the testee, and therefore judges it as now."}, "sentence_type": "Interrogative sentence", "pos": {"nouns": {"you": 1, "self": 1, "Nervous": 1, "String": 1}, "verbs": {"I think": 1, "Pull": 1, "Same thing": 1}, "adjectives": {"very": 1}, "numerals": {"43": 1}}}, {"content": "44. Do you throw waste paper on the floor without scrap paper?", "word_count": 18, "label": "", "sentence_structure": "Subject鈥搗erb鈥搊bject", "subjective_objective": {"answer": "Objective", "rationale": "The question is examined by the behaviour of the person under investigation and is a description of objective facts."}, "sentence_sentiment": {"answer": "Negative", "rationale": "Because the behavior in the entry \"screw paper on the floor\" is negative."}, "language_style": {"answer": "Calm and direct", "rationale": "This is a more straightforward language style, presented in question form, without any aesthetic or emotional colour."}, "time_dimension": {"answer": "Past", "rationale": "The entry asks about the testee's past customary behaviour, namely, \"Will waste paper be thrown on the floor without scrap paper?\" This behaviour is usually a long-term habit, not a short-term, present-day situation. Therefore, the time dimension is a past."}, "sentence_type": "Interrogative sentence", "pos": {"nouns": {"Waste Paper": 3, "Floor": 1}, "verbs": {"Will": 1, "Throw it": 1}, "adverbs": {"Time": 1}, "numerals": {"44": 1}}}, {"content": "45. When you're with someone else, do you have little words?", "word_count": 16, "label": "", "sentence_structure": "Subject鈥搗erb鈥搊bject", "subjective_objective": {"answer": "Subjective", "rationale": "The problem, which involves the individual feelings and behaviour of the patient in a given situation, requires self-assessment and subjective judgement by the patient."}, "sentence_sentiment": {"answer": "Negative", "rationale": "Because the \"slightly spoken\" act in the entry is negative."}, "language_style": {"answer": "Calm and direct", "rationale": "The entry is based on direct questions, with no excessive rhetorical or emotional rendering, and is more objective in tone. At the same time, the question is clear, simple and easy to understand and answer by the respondent."}, "time_dimension": {"answer": "Past", "rationale": "But because the question of whether it is \"whether\" is a question of seeking the subjective feelings and attitudes of the person being tested, it is more biased towards the present or the recent description. Thus, the temporal dimension of the article is more biased towards the present."}, "sentence_type": "Interrogative sentence", "pos": {"nouns": {"you": 2, "Time": 1, "person": 1, "Words": 1}, "verbs": {"and": 1, "in": 1, "Together": 1, "Less": 1, "Say it": 1}, "adjectives": {"More": 1}, "adverbs": {"Very few": 1}, "numerals": {"45": 1}}}, {"content": "46. Do you consider the marriage regime to be outdated and should be abolished?", "word_count": 17, "label": "", "sentence_structure": "Subject鈥搗erb鈥搊bject", "subjective_objective": {"answer": "Subjective", "rationale": "Since the issue involved a perception and evaluation of the matrimonial regime, the person interviewed was required to have his or her own subjective point of view."}, "sentence_sentiment": {"answer": "Negative", "rationale": "Basis of judgement: The reference to \"revocation\" is negative and therefore negative."}, "language_style": {"answer": "Calm and direct", "rationale": "The language style of the entry is more straightforward, with no excessive emotional colour or qualifier, and is cold and direct, with the aim of enabling the interviewee to answer the questions objectively and honestly."}, "time_dimension": {"answer": "Present", "rationale": ""}, "sentence_type": "Interrogative sentence", "pos": {"nouns": {"The smoking system": 1, "Cancel": 1}, "verbs": {"I think": 1, "I think so": 1, "Cancel": 1}, "adjectives": {"Outdated": 1}, "numerals": {"46": 1}}}, {"content": "Do you feel sorry for yourself sometimes?", "word_count": 11, "label": "", "sentence_structure": "Subject鈥損redicate", "subjective_objective": {"answer": "Subjective", "rationale": "Because feeling sorry for yourself is the subjective feeling and attitude of the patient."}, "sentence_sentiment": {"answer": "Negative", "rationale": "Because the expression of feeling sorry for itself in the entry is negative."}, "language_style": {"answer": "Tactful and warm", "rationale": "The language style of the entry, which takes the form of a question and which is expressed in the relative euphemism of the phrase \"sometimes\" without direct blame or criticism of the interviewee, is more cordial and moderate."}, "time_dimension": {"answer": "Present", "rationale": "The entry asks if the person tested has experienced \"sometimes feeling sorry\" during the current period (e.g., the past) and therefore judges it as now."}, "sentence_type": "Interrogative sentence", "pos": {"nouns": {"Questionnaire": 1, "Entry": 1, "self": 1}, "verbs": {"Is it possible": 1, "Time": 1, "I feel": 1, "Poor thing": 1}, "numerals": {"47": 1}}}, {"content": "Do you sometimes boast?", "word_count": 9, "label": "", "sentence_structure": "Subject鈥搗erb鈥搊bject", "subjective_objective": {"answer": "Subjective", "rationale": "Since this issue involves self-evaluation and attitudes of individuals, it is examined from the subjective perspective of the patient."}, "sentence_sentiment": {"answer": "Negative", "rationale": "Because \"self-indulgent\" in the entries is a negative act or attitude."}, "language_style": {"answer": "Tactful and warm", "rationale": "The entry is more personal and interactive when it is spoken in a gentle and inviting manner, rather than in direct statements or orders. At the same time, the use of words such as \"some\" and \"some\" makes language styles more euphemism and subtle, avoiding possible discomfort to the interviewees."}, "time_dimension": {"answer": "Present", "rationale": "The entry uses the current state of the time ( \"yes\") to ask the testee about the current behaviour or state, and thus to judge it as now."}, "sentence_type": "Interrogative sentence", "pos": {"nouns": {"Questionnaire": 1, "Entry": 1, "you": 1, "Time": 1, "Points": 1, "- You're exaggerating": 1}, "verbs": {"have": 1, "be": 1}, "adverbs": {"Sometimes": 1}, "numerals": {"48": 1}}}, {"content": "49. Is it easy for you to keep a silent gathering alive?", "word_count": 20, "label": "", "sentence_structure": "Subject鈥搗erb鈥搊bject", "subjective_objective": {"answer": "Subjective", "rationale": "Because \"easy to keep a silent gathering active\" is the patient's personal character and behaviour, and it's subjective."}, "sentence_sentiment": {"answer": "Positive", "rationale": "Because \"make the assembly live\" in the entry is a positive act."}, "language_style": {"answer": "Tactful and warm", "rationale": "This entry uses an invitation-style tone, asking the interviewee whether he/she is able to keep a silent gathering active, with a gentle tone and a warmer and less direct or hard language. Such an expression is more acceptable and willing to answer questions."}, "time_dimension": {"answer": "Past", "rationale": "So the answer is: past/present/future"}, "sentence_type": "Interrogative sentence", "pos": {"nouns": {"Gathering": 1, "Active": 1}, "verbs": {"Fuck": 1, "鈾?And become 鈾?: 1}, "adjectives": {"Silent": 1, "Easy": 1}, "numerals": {"49": 1}, "pronouns": {"you": 1}}}, {"content": "Do you hate people who drive with care?", "word_count": 16, "label": "", "sentence_structure": "Subject鈥搗erb鈥搊bject", "subjective_objective": {"answer": "Subjective", "rationale": "Because \"obsessed\" is a personal emotional attitude, it's seen from the subjective perspective of the patient."}, "sentence_sentiment": {"answer": "Negative", "rationale": "Because the word \"obsessed\" has a negative emotional connotation, indicating a distaste or aversion to certain behaviour."}, "language_style": {"answer": "Calm and direct", "rationale": "The entry is in the form of a question-and-answer session, which explicitly asks the interviewee about the attitude of the person who is careful to drive, and is more calm and straightforward in language, without excessive rhetorical or emotional rendering."}, "time_dimension": {"answer": "Present", "rationale": "The entry asks the testee's current attitude and feelings, and the keyword \"Do you hate\" suggests that this is a question of the moment, so it's judged now."}, "sentence_type": "Interrogative sentence", "pos": {"nouns": {"person": 1, "Car": 1, "Watch your wings": 1}, "verbs": {"Shit": 1, "Drive": 1}, "adjectives": {"Watch your wings": 1}, "numerals": {"50": 1}}}, {"content": "Are you worried about your health?", "word_count": 9, "label": "", "sentence_structure": "Subject鈥搗erb鈥搊bject", "subjective_objective": {"answer": "Subjective", "rationale": "Because it is the patient's subjective feeling and attitude."}, "sentence_sentiment": {"answer": "Negative", "rationale": "Because the \"fear\" emotion in the entry is negative."}, "language_style": {"answer": "Tactful and warm", "rationale": "The entry asks in a gentle and caring way whether the interviewee鈥檚 health is causing concern, is more intimate and concerned about the other. At the same time, the phrase 鈥測our health鈥?is used to emphasize the respect and interest of the investigator in the interviewee."}, "time_dimension": {"answer": "Present", "rationale": "The entry asks about the current state and feeling of the testee, and \"for your health\" is an action or state relevant to the present, and therefore the present is judged."}, "sentence_type": "Interrogative sentence", "pos": {"nouns": {"Health": 1}, "verbs": {"Fear": 1, "Yes": 1}, "numerals": {"51": 1}}}, {"content": "Have you ever spoken bad words about anyone?", "word_count": 11, "label": "", "sentence_structure": "Subject鈥搗erb鈥搊bject", "subjective_objective": {"answer": "Subjective", "rationale": "According to the basis of the judgment provided by the title, \"Have you ever spoken bad words about anyone?\" is the subjective attitude and behaviour of the patient, so the answer should be \"subjective\"."}, "sentence_sentiment": {"answer": "Negative", "rationale": "Because the \"who said bad things\" in the entry is negative."}, "language_style": {"answer": "Calm and direct", "rationale": "The language style of the entry is calm, because it uses direct questions and does not contain too many qualifiers or emotional colours. It simply asks the interviewee whether he has ever spoken bad words about others, without any hints or references."}, "time_dimension": {"answer": "Past", "rationale": ""}, "sentence_type": "Interrogative sentence", "pos": {"nouns": {"person": 2, "Bad word": 1}, "verbs": {"Speak": 1, "Tsang": 1, "Say it": 1}, "adjectives": {"Bad": 1}, "adverbs": {"Tsang": 1}, "numerals": {"52": 1, "you": 1}, "pronouns": {"you": 1}}}, {"content": "Do you like to tell jokes and funny stories to friends?", "word_count": 17, "label": "", "sentence_structure": "Subject鈥搗erb鈥搊bject", "subjective_objective": {"answer": "Subjective", "rationale": "Because \"like\" is a description of a subjective attitude or feeling that involves an individual's feelings and preferences, not objective facts."}, "sentence_sentiment": {"answer": "Positive", "rationale": "Because the \"like jokes and funny stories\" act in the entry is positive, expressing people's desire for humor, pleasure and love."}, "language_style": {"answer": "Tactful and warm", "rationale": "The entry is more ad hoc and interactive, using a soft, invitation-style tone, dialogue by means of inquiry, not direct statements or orders."}, "time_dimension": {"answer": "Present", "rationale": "The entry asks the testee's current preferences and habits, and the keyword \"likes\" indicates that this is a question of current behaviour or attitude."}, "sentence_type": "Interrogative sentence", "pos": {"nouns": {"friends": 1, "A joke": 1, "Story": 1}, "verbs": {"Yeah": 1, "Speak": 1}, "numerals": {"53": 1}}}, {"content": "Did you ever be rude to your parents when you were a kid?", "word_count": 13, "label": "", "sentence_structure": "Subject鈥搗erb鈥搊bject", "subjective_objective": {"answer": "Subjective", "rationale": "This is because it is a subjective view to question the behaviour and attitude of the patient."}, "sentence_sentiment": {"answer": "Negative", "rationale": "Because \"black manners\" in the entry is negative."}, "language_style": {"answer": "Calm and direct", "rationale": "The entry uses a direct and explicit language style, asking if the interviewee had behaved rudely against his parents when he was a child."}, "time_dimension": {"answer": "Past", "rationale": "The keyword \"when we were kids\" indicates that the question was asked about the testee's past experience and behaviour."}, "sentence_type": "Interrogative sentence", "pos": {"nouns": {"parents": 2, "When I was little": 1, "Brutal": 1, "Insolent": 1}, "verbs": {"Tsang": 1, "to": 1, "have": 1, "- You're not": 1}, "adverbs": {"Insolent": 1}, "numerals": {"54": 1}}}, {"content": "Do you like to hang out with people?", "word_count": 11, "label": "", "sentence_structure": "Subject鈥搗erb鈥搊bject", "subjective_objective": {"answer": "Subjective", "rationale": "Because \"like\" is a manifestation of a subjective individual feeling, the answers vary from one person to another. This is a question of examining patients' subjective attitudes."}, "sentence_sentiment": {"answer": "Neutral", "rationale": "It is merely asking whether the person under investigation is willing to interact with another person."}, "language_style": {"answer": "Tactful and warm", "rationale": "The entry is more personal and interactive when it is spoken in a gentle and inviting manner, rather than in direct statements or orders. It also implies that there are many possible answers, and it encourages respondents to answer them from their own perspective."}, "time_dimension": {"answer": "Present", "rationale": "The entry asks the testee about his current attitude or preferences, and the keyword \"likes\" indicates that this is a question about the current situation."}, "sentence_type": "Interrogative sentence", "pos": {"nouns": {"Questionnaire": 1, "Entry": 1, "person": 1}, "verbs": {"Yeah": 1, "- You're a little mixed up": 1}, "numerals": {"55": 1}}}, {"content": "56. Would it upset you if you knew you were wrong about your job?", "word_count": 20, "label": "", "sentence_structure": "Subject鈥搗erb鈥搊bject", "subjective_objective": {"answer": "Subjective", "rationale": "Because \"sorture\" is an emotional reaction, a subjective feeling of the patient."}, "sentence_sentiment": {"answer": "Negative", "rationale": "Because the \"worried\" emotion in the entry is negative."}, "language_style": {"answer": "Tactful and warm", "rationale": "The entry uses a more euphemistic language style, which guides the interviewee to answer questions by asking questions."}, "time_dimension": {"answer": "Past", "rationale": ""}, "sentence_type": "Interrogative sentence", "pos": {"nouns": {"Work": 1, "Error": 1, "Sorry": 1}, "verbs": {"Yes": 1, "鈾?And I'm gonna be 鈾?: 1, "I feel": 1}, "numerals": {"56": 1}}}, {"content": "Are you insomniatic?", "word_count": 5, "label": "", "sentence_structure": "Subject鈥損redicate", "subjective_objective": {"answer": "Objective", "rationale": "Since insomnia is a condition or a symptom, it is objective to determine whether the patient suffers from insomnia by a doctor or professional."}, "sentence_sentiment": {"answer": "Negative", "rationale": "Because \"insomnia\" is a negative word in the entries, usually associated with pain and distress."}, "language_style": {"answer": "Calm and direct", "rationale": "The entry directly asked the interviewee whether he had problems with insomnia, was of a concise language style, was not using a speech technique or emotional rendering, and was speaking with a more objective and calm tone."}, "time_dimension": {"answer": "Present", "rationale": "The keyword \"insomnia\" indicates that it is a question of the testee's present state, and that the word \"insatisfaction\" adopts the general current, meaning that the act has not only occurred in the past, but may still be ongoing."}, "sentence_type": "Interrogative sentence", "pos": {"nouns": {"Insomnia": 1}, "verbs": {"Patient": 1, "- You're not": 1}, "numerals": {"57": 1}}}, {"content": "Do you wash your hands before eating?", "word_count": 9, "label": "", "sentence_structure": "Subject鈥損redicate", "subjective_objective": {"answer": "Subjective", "rationale": "Because this is the patient ' s personal habits and attitudes and is subjective."}, "sentence_sentiment": {"answer": "Positive", "rationale": "Because hand washing is a hygiene habit, it has a positive emotional color."}, "language_style": {"answer": "Calm and direct", "rationale": "The language style of the entry is more straightforward, there is no excessive rhetorical or emotional rendering, but rather a more objective tone of the conversation in a clear question form."}, "time_dimension": {"answer": "Present", "rationale": ""}, "sentence_type": "Interrogative sentence", "pos": {"nouns": {"Rice": 1, "Hands": 1}, "verbs": {"Eat": 1, "Wash": 1}, "adverbs": {"I'm sure": 1}, "numerals": {"58": 1}}}, {"content": "Do you often feel inexhaustible and infrequent for no reason?", "word_count": 16, "label": "", "sentence_structure": "Subject鈥搗erb鈥搊bject", "subjective_objective": {"answer": "Subjective", "rationale": "Because \"always feeling inexorably inexhaustible and tired\" is the patient's subjective feeling."}, "sentence_sentiment": {"answer": "Negative", "rationale": "Because the emotions, attitudes or behaviour expressed in this entry are negative, namely, \"the feeling of inactivity and fatigue\"."}, "language_style": {"answer": "Tactful and warm", "rationale": "Reason: The entry is more personal and interactive when it is spoken in a gentle and inviting manner, rather than in direct statements or orders. At the same time, is the expression \"Do you often feel inexorably insatiable and tired?\" is also more friendly, warm, and not too direct or too cold."}, "time_dimension": {"answer": "Present", "rationale": "The entry now asks about the current status of the testee, the keywords being \"normal\" and \"fear\" and they all indicate that the act is continuing. It is therefore a questionnaire entry that can be judged to describe the current situation."}, "sentence_type": "Interrogative sentence", "pos": {"nouns": {"I'm not good at it": 1, "Tired of the window": 1}, "verbs": {"Regular": 1, "I feel": 1}, "adjectives": {"For no reason": 1}, "numerals": {"59": 1}}}, {"content": "Have you ever been a cheater when you played games with someone else?", "word_count": 15, "label": "", "sentence_structure": "Subject鈥搗erb鈥搊bject", "subjective_objective": {"answer": "Objective", "rationale": "Since deception is an objective act, it has already occurred in the game, whether or not the patient admits it."}, "sentence_sentiment": {"answer": "Negative", "rationale": "Because the act of deception in the entry is negative."}, "language_style": {"answer": "Calm and direct", "rationale": "It uses simple and straightforward language to ask whether the interviewees have ever been deceptive in playing games, without too many rhetorical or emotional expressions, and with a more objective tone. This type of questionnaire is often considered to be more objective and scientific, and can help researchers to obtain more accurate data and information."}, "time_dimension": {"answer": "Past", "rationale": "The keyword \"had committed fraud\" was a past act of conduct and a negative act, and the time frame for the purpose of the article could therefore be established."}, "sentence_type": "Interrogative sentence", "pos": {"nouns": {"person": 2, "Game": 1, "Behaviour": 1}, "verbs": {"have": 1, "- Liar": 1}, "numerals": {"60": 1, "you": 1}}}, {"content": "Do you like to do something fast?", "word_count": 16, "label": "", "sentence_structure": "Subject鈥搗erb鈥搊bject", "subjective_objective": {"answer": "Subjective", "rationale": "Because \"like\" is a subjective attitude or feeling.\""}, "sentence_sentiment": {"answer": "Positive", "rationale": "Because the \"likes to do some quick work\" in the entry is positive."}, "language_style": {"answer": "Calm and direct", "rationale": "The language style of the entry is relatively simple, there is no excessive rhetorical or emotional colour, and problems are mainly presented in the form of inquiries, which appear to be more direct and objective."}, "time_dimension": {"answer": "Present", "rationale": "The word \"now\" in the sentence makes it clear that the entry is asking the testee about the current behaviour or state, and therefore the temporal dimension is now."}, "sentence_type": "Interrogative sentence", "pos": {"nouns": {"Work": 1, "Actions": 1}, "verbs": {"Yeah": 1, "Active": 1, "Quick": 1, "Likes to be engaged": 1}, "adjectives": {"Some": 1, "Quick": 1}, "numerals": {"61": 1}}}, {"content": "Is your mother a good woman?", "word_count": 13, "label": "", "sentence_structure": "Subject鈥搗erb鈥搊bject", "subjective_objective": {"answer": "Subjective", "rationale": "The subject is subjective because it is about the patient ' s perception and assessment of the mother."}, "sentence_sentiment": {"answer": "Positive", "rationale": "Because \"good\" is a positive adjective, which describes a good quality of a person."}, "language_style": {"answer": "Tactful and warm", "rationale": "\"Slow and warm.\""}, "time_dimension": {"answer": "Present", "rationale": "The entry used to be an inquiry into the subjective perception and memory of the person tested with regard to his mother, and the temporal dimension was judged to be the past."}, "sentence_type": "Interrogative sentence", "pos": {"nouns": {"Mother": 1, "Women": 1}, "verbs": {"be": 1}, "adjectives": {"Good": 1}, "adverbs": {"EMPTY": 0}, "numerals": {"62": 1}, "classifiers": {"EMPTY": 0}, "pronouns": {"you": 1, "she": 1}}}, {"content": "Do you often find life very boring?", "word_count": 13, "label": "", "sentence_structure": "Subject鈥損redicate", "subjective_objective": {"answer": "Subjective", "rationale": "Because the feeling of a life without taste is a subjective feeling, different people have different views and feelings."}, "sentence_sentiment": {"answer": "Negative", "rationale": "Because the description \"life is very boring\" has negative emotional connotations and expresses negative attitudes."}, "language_style": {"answer": "Calm and direct", "rationale": ""}, "time_dimension": {"answer": "Present", "rationale": ""}, "sentence_type": "Interrogative sentence", "pos": {"nouns": {"Questionnaire": 1, "Entry": 1, "Life": 1}, "verbs": {"I think": 1, "Regular": 1, "have": 1, "Tasteless": 1}, "adjectives": {"Very": 1}, "numerals": {"63": 1}}}, {"content": "Have you ever used someone to gain for yourself?", "word_count": 15, "label": "", "sentence_structure": "Subject鈥搗erb鈥搊bject", "subjective_objective": {"answer": "Subjective", "rationale": "This is a subjective investigation of individual conduct and ideas."}, "sentence_sentiment": {"answer": "Negative", "rationale": "Because the behavior in the entry \"using someone else to gain for himself\" is negative."}, "language_style": {"answer": "Calm and direct", "rationale": "The entry directly asks whether the interviewee has used others for personal gain, has a relatively calm and objective language style and has no excessive emotional colour."}, "time_dimension": {"answer": "Present", "rationale": "The entry asks about the testee's past behaviour, i.e. whether someone has been used to gain benefits for himself. Although the question is expressed by the word \"had\" in the past, in practice the act may still affect the testee's current attitude and values, and therefore the temporal dimension is now judged."}, "sentence_type": "Interrogative sentence", "pos": {"nouns": {"person": 1, "Benefits": 1}, "verbs": {"Used": 1, "Access": 1}, "numerals": {"64": 1}, "classifiers": {"Some": 1}}}, {"content": "65. Do you often participate in many activities that exceed your time?", "word_count": 20, "label": "", "sentence_structure": "Subject鈥搗erb鈥搊bject", "subjective_objective": {"answer": "Subjective", "rationale": "The behaviour and timing of the event is a subjective decision of the patient."}, "sentence_sentiment": {"answer": "Negative", "rationale": "Because the entries contain a description of \"often being involved in many activities beyond the time allowed\", this suggests that there may be situations where excessive participation in activities leads to time management difficulties, which is a negative behaviour or attitude."}, "language_style": {"answer": "Calm and direct", "rationale": "The basis for the determination: the entry raises the question in a straightforward manner, \"Do you often participate in many activities, beyond your time?\" without using too many rhetorical or emotional expressions, and with a more objective tone. At the same time, the question is not answered in a soft and gentle manner, but rather in a direct way."}, "time_dimension": {"answer": "Past", "rationale": "The entry asks the testee about past customary behaviour, i.e. whether they have been involved in many activities more often than time. The time dimension of the article is therefore the past.\""}, "sentence_type": "Interrogative sentence", "pos": {"nouns": {"Activities": 2, "Time": 1}, "verbs": {"Participation": 1, "Allow": 1}, "adjectives": {"Often": 1}, "adverbs": {"More than": 1}, "numerals": {"65": 1}}}, {"content": "Do you think there are a few people who are always avoiding you?", "word_count": 11, "label": "", "sentence_structure": "Subject鈥搗erb鈥搊bject", "subjective_objective": {"answer": "Subjective", "rationale": "Because \"someone's always avoiding me\" is something that the patient feels, so it's subjective."}, "sentence_sentiment": {"answer": "Negative", "rationale": "Because the \"shun you\" act is negative in the entry."}, "language_style": {"answer": "Calm and direct", "rationale": "The entry directly asks the respondents whether there are people who avoid them, without adding any aesthetic or emotional colour, and with a more calm and objective language style."}, "time_dimension": {"answer": "Present", "rationale": "The word \"yes\" contained in the entry indicates that the issue is being investigated and that the question is about the current state or behaviour, and can therefore be judged to be the present."}, "sentence_type": "Interrogative sentence", "pos": {"nouns": {"person": 2, "Hide": 1}, "verbs": {"have": 1, "Always": 1}, "adverbs": {"How many": 1}, "numerals": {"66": 1}, "pronouns": {"you": 1}}}, {"content": "Are you very upset about your face?", "word_count": 13, "label": "", "sentence_structure": "Subject鈥搗erb鈥搊bject", "subjective_objective": {"answer": "Subjective", "rationale": "Because the pain is the patient ' s own feelings and emotional experience, it is viewed from a subjective perspective."}, "sentence_sentiment": {"answer": "Negative", "rationale": "The judgment is based on the negative emotion of the \"very upset\" attitude."}, "language_style": {"answer": "Calm and direct", "rationale": "The entry \"Calm down straight\" directly asked the interviewee about his/her own appearance, without any qualification or emotional addition, and the language style was more calm and objective."}, "time_dimension": {"answer": "Present", "rationale": "The keyword \"Is it very distressing for your appearance\" suggests that it is a direct question about the emotional state of the individual, reflecting the current emotional experience and attitude of the person being tested."}, "sentence_type": "Interrogative sentence", "pos": {"nouns": {"Looks like a": 1, "Trouble": 1}, "verbs": {"be": 1, "Yes": 1}, "adverbs": {"Very": 1}, "numerals": {"67": 1}}}, {"content": "Do you feel that people spend too much time on saving and insurance for a secure future?", "word_count": 29, "label": "", "sentence_structure": "Subject鈥搗erb鈥搊bject", "subjective_objective": {"answer": "Subjective", "rationale": "Because this is the expression of subjective attitudes about the \"time spent on saving and insurance for the future\"."}, "sentence_sentiment": {"answer": "Negative", "rationale": "Because \"Save and insure for a secure future\" in this entry is a positive act, but it is described as \"too much time spent\" and therefore has a negative emotional connotation."}, "language_style": {"answer": "Calm and direct", "rationale": "The entry expresses the view of the respondent in a simple language, by means of direct questions."}, "time_dimension": {"answer": "Present", "rationale": "The entry asks the current view of the testee, using the general current state of affairs, and the temporal dimension is now judged."}, "sentence_type": "Interrogative sentence", "pos": {"nouns": {"Questionnaire": 1, "Entry": 1, "People": 2, "The future": 1, "Safeguards": 1, "Time": 2, "Savings": 1, "Insurance": 1}, "verbs": {"I think": 1, "Processing": 1, "Flowers": 1}, "adverbs": {"Too much": 1}, "numerals": {"68": 1}, "pronouns": {"you": 1}}}, {"content": "Have you ever wished for death?", "word_count": 14, "label": "", "sentence_structure": "Subject鈥搗erb鈥搊bject", "subjective_objective": {"answer": "Subjective", "rationale": "Because it's the patient's inner thoughts and feelings, it's a subjective emotional experience."}, "sentence_sentiment": {"answer": "Negative", "rationale": "Because the idea of \"better dead\" in the entry is negative."}, "language_style": {"answer": "Calm and direct", "rationale": "The entry directly raises the question of not using any rhetorical or emotional rendering, which is more objective and direct, and does not attempt to induce or influence the feelings of the interviewees."}, "time_dimension": {"answer": "Past", "rationale": "The keyword \"had\" indicated that it was an inquiry into the testee's past experience, so the temporal dimension was a past."}, "sentence_type": "Interrogative sentence", "pos": {"nouns": {"Questionnaire": 1, "Entry": 1, "Wish": 1}, "verbs": {"have": 1, "Die": 1, "Yes": 1}, "adverbs": {"Tsang": 1, "Why don't you": 1, "Okay": 1}, "numerals": {"69": 1}}}, {"content": "If you're sure you'll never find out, will you be tax evasion?", "word_count": 19, "label": "", "sentence_structure": "Subject鈥損redicate", "subjective_objective": {"answer": "Subjective", "rationale": "Because the criteria of \"never find out\" and \"will I avoid taxes\" are based on the subjective will of the individual, not on objective facts or behavioural results."}, "sentence_sentiment": {"answer": "Negative", "rationale": "Because the entry \"tax evasion\" is illegal, irresponsible and negative."}, "language_style": {"answer": "Calm and direct", "rationale": "The language style of the entry is more calm and straightforward, and the questioner clearly indicates the core of the problem and does not contain too many rhetorical techniques or emotional rendering.\""}, "time_dimension": {"answer": "Future", "rationale": ""}, "sentence_type": "Interrogative sentence", "pos": {"nouns": {"Hold on": 1, "person": 1, "Taxes": 1}, "verbs": {"Yes": 2, "Run": 1, "Found": 1}, "adjectives": {"Forever": 1}, "adverbs": {"Never": 1}, "numerals": {"70": 1}}}, {"content": "Can you make a meeting go smoothly?", "word_count": 12, "label": "", "sentence_structure": "Subject鈥搗erb鈥搊bject", "subjective_objective": {"answer": "Subjective", "rationale": "The subjective attitude and competence of the patient are examined, not as objective facts."}, "sentence_sentiment": {"answer": "Positive", "rationale": "Because the entry expresses the ability, which is \"to enable a gathering to proceed smoothly\", and it is a positive quality and ability."}, "language_style": {"answer": "Calm and direct", "rationale": "The entry directly asked the respondents whether they were able to coordinate the organization and ensure the successful organization of the assembly, and the language style was simpler and less curvy or emotional and therefore a calm and direct language style."}, "time_dimension": {"answer": "Present", "rationale": "The keyword \"may\" indicates that it is a question of ability to describe whether the person tested currently has some skill or quality. And, since \"the assembly is going well\" is also the case, the entry is a question of the person tested's current capabilities and current status."}, "sentence_type": "Interrogative sentence", "pos": {"nouns": {"Gathering": 1, "Problem": 1}, "verbs": {"Yes": 1, "鈾?And I'm gonna be 鈾?: 1, "Conduct": 1}, "numerals": {"71": 1}, "pronouns": {"you": 1}}}, {"content": "Can you stop being rude?", "word_count": 12, "label": "", "sentence_structure": "Subject鈥損redicate", "subjective_objective": {"answer": "Subjective", "rationale": "Because the words \"restraint\" and \"insolent\" are all described from the patient's subjective point of view."}, "sentence_sentiment": {"answer": "Positive", "rationale": "Because the \"restriction of self-indignity\" in the entry is positive."}, "language_style": {"answer": "Calm and direct", "rationale": "The entry is asked explicitly, in the form of a direct question, whether the person interviewed is able to restrain his or her behaviour, is more directly linguistically, and does not have too many adorations or acrimony.\""}, "time_dimension": {"answer": "Future", "rationale": "The entry asks whether the testee will avoid being rude in the face of future circumstances (i.e. \"restrictive\"), and therefore the temporal dimension is a future."}, "sentence_type": "Interrogative sentence", "pos": {"nouns": {"Questionnaire Entry": 1, "you": 2, "person": 2}, "verbs": {"Yes": 1, "Restraint": 1, "to": 1, "Insolent": 1}, "adverbs": {"not": 2}, "numerals": {"72": 1}, "pronouns": {"Here": 1}}}, {"content": "73. Have you suffered for a long time after a difficult experience?", "word_count": 27, "label": "", "sentence_structure": "Subject鈥損redicate", "subjective_objective": {"answer": "Subjective", "rationale": "This is because it involves the emotional and subjective experience of the patient."}, "sentence_sentiment": {"answer": "Negative", "rationale": "Because \"damn experience\" and \"long suffering\" are negative emotional experiences in the entries."}, "language_style": {"answer": "Calm and direct", "rationale": "The entry asks the interviewees in an objective and direct manner whether they will continue to be negatively affected after experiencing difficult experiences, with a speech temperature and without excessive emotional rendering.\""}, "time_dimension": {"answer": "Past", "rationale": "In the entry, \"As a result of a difficult experience\" means an event that has already occurred, while \"As a long time has passed, it has been asked about the impact and feelings of the testees ' past experience on them. Thus, the entry focuses primarily on the experiences and effects of the past, which can be judged as the past."}, "sentence_type": "Interrogative sentence", "pos": {"nouns": {"Experience": 1, "I'm embarrassed": 1, "Time": 1}, "verbs": {"I met him": 1, "Feel": 1, "It's hard": 1, "For a while": 1, "And": 1}, "adjectives": {"Long": 1}, "adverbs": {"very": 1}, "numerals": {"73": 1}}}, {"content": "Do you have a \"sensitization\" condition?", "word_count": 8, "label": "", "sentence_structure": "Subject鈥搗erb鈥搊bject", "subjective_objective": {"answer": "Subjective", "rationale": "Because the existence of \"sensitized\" is a subjective feeling of the patient's own judgment, not an objective reality."}, "sentence_sentiment": {"answer": "Negative", "rationale": "Because \"sensitization\" in the entry is a negative disease, the purpose of the article is negative."}, "language_style": {"answer": "Calm and direct", "rationale": "This entry uses a more direct and explicit language style, asking if the other party has a \"sensitized\" problem, and does not use excessive rhetorical or emotional rendering."}, "time_dimension": {"answer": "Past", "rationale": "The keyword \"over\" is \"infectively allergic,\" which is a reference to what happened in the past and is now cured."}, "sentence_type": "Interrogative sentence", "pos": {"nouns": {"You're a bit of a sane": 1, "you": 1}, "verbs": {"Patient": 1, "have": 1}, "adjectives": {"He's a sensitive man": 1}, "numerals": {"74": 1}}}, {"content": "Have you ever said anything deliberately to hurt someone's feelings?", "word_count": 18, "label": "", "sentence_structure": "Subject鈥搗erb鈥搊bject", "subjective_objective": {"answer": "Subjective", "rationale": "The question is whether the patient has ever had an act or motive to hurt the feelings of others, which is the patient ' s subjective attitude and behaviour."}, "sentence_sentiment": {"answer": "Negative", "rationale": "Because the \"intentional saying something to hurt someone's feelings\" act in the entry is negative."}, "language_style": {"answer": "Calm and direct", "rationale": "This entry is presented in a direct question, with no excessive emotional rendering or rhetorical techniques, and is more calm and objective. This expression provides more direct access to the real situation of the person being tested and feedback, and has higher empirical research value."}, "time_dimension": {"answer": "Past", "rationale": "The word \"had \" in the entry indicates that the question is whether the person tested had acted in the past and therefore the temporal dimension is judged to be past."}, "sentence_type": "Interrogative sentence", "pos": {"nouns": {"Grandpa": 1, "Questionnaire": 1, "Entry": 1}, "verbs": {"On purpose": 1, "Say it": 2, "Hurt": 1, "have": 1}, "adjectives": {"Some": 1, "others": 1, "of": 3, "Emotion": 1}, "adverbs": {"- You're not": 1}, "numerals": {"75": 1}, "pronouns": {"you": 1}}}, {"content": "Is your friendship with others easily broken, though not your fault?", "word_count": 21, "label": "", "sentence_structure": "Subject鈥搗erb鈥搊bject", "subjective_objective": {"answer": "Subjective", "rationale": "Because the fragile nature of friendship is the individual feelings and perceptions of the patient."}, "sentence_sentiment": {"answer": "Negative", "rationale": "Because \"Friendbreak\" in the entry is negative."}, "language_style": {"answer": "Calm and direct", "rationale": "The language style of the entry is more objective, direct, and there is no excessive emotional or rhetorical approach, and it appears to be calmer and rational to ask the respondents in a simple and clear manner about their experiences and feelings in interpersonal relationships."}, "time_dimension": {"answer": "Present", "rationale": "The entry \"is it\" is an inquiry about the current state, and no past or future time trails are mentioned. Therefore, depending on the time dimension, the entry is now."}, "sentence_type": "Interrogative sentence", "pos": {"nouns": {"Friendship": 1, "Break": 1, "Wrong": 1}, "verbs": {"Is it possible": 1, "Easy": 1, "be": 1, "Break": 1, "Wrong": 1}, "adverbs": {"Easy": 1}, "numerals": {"76": 1}, "pronouns": {"you": 1, "others": 1}}}, {"content": "Do you often feel lonely?", "word_count": 7, "label": "", "sentence_structure": "Subject鈥損redicate", "subjective_objective": {"answer": "Subjective", "rationale": "Because feeling alone is the patient's subjective feeling."}, "sentence_sentiment": {"answer": "Negative", "rationale": "Because the \"fear alone\" sentiment in the entry is negative."}, "language_style": {"answer": "Tactful and warm", "rationale": "The language style of the entry is more euphemistic, asking the other person whether he or she feels alone in a sense of concern and understanding, rather than ordering or forcing an answer. This expression is more likely to allow the interviewee to share his or her true feelings and to establish good interaction."}, "time_dimension": {"answer": "Present", "rationale": "The keyword \"often feeling alone\" is a sense of emotional experience often experienced by the testees, and the time dimension is now judged."}, "sentence_type": "Interrogative sentence", "pos": {"nouns": {"Alone": 1, "Questionnaire": 1, "Entry": 1}, "verbs": {"I feel": 1, "Regular": 1, "Alone": 1}, "adverbs": {"Regular": 1}, "numerals": {"77": 1}}}, {"content": "78. Are you vulnerable to psychological trauma when people look for your mistakes and for shortcomings in your work?", "word_count": 29, "label": "", "sentence_structure": "Subject鈥損redicate", "subjective_objective": {"answer": "Subjective", "rationale": "Because the issue is about personal feelings and emotions, the subjective perspective is being examined."}, "sentence_sentiment": {"answer": "Negative", "rationale": "Because the emotional attitude of \"mentally impaired\" in the entry is negative."}, "language_style": {"answer": "Calm and direct", "rationale": "This language style is suitable for serious research or for the design of questionnaires in the field of expertise, which can be effective in reducing misunderstandings and deviations among the subjects and improving the accuracy and effectiveness of data."}, "time_dimension": {"answer": "Present", "rationale": "The entry describes the emotional reaction of the testee in the face of criticism or error, which may affect his or her current mental state. Therefore, the temporal dimension is now judged. \""}, "sentence_type": "Interrogative sentence", "pos": {"nouns": {"Error": 1, "Work": 1, "Disadvantages": 1, "Spirit": 1, "Frustrated": 1}, "verbs": {"Search": 1, "Find": 1, "Easy": 1}, "numerals": {"78": 1}}}, {"content": "Have you been late for a date or work?", "word_count": 12, "label": "", "sentence_structure": "Subject鈥搗erb鈥搊bject", "subjective_objective": {"answer": "Subjective", "rationale": "The content of this questionnaire is to ask patients whether they have been late, which is viewed from the patient's subjective point of view. The output is therefore \"subjective\"."}, "sentence_sentiment": {"answer": "Negative", "rationale": "Because the lateness is negative and may affect interpersonal relations and efficiency."}, "language_style": {"answer": "Calm and direct", "rationale": "The entry asks the respondent whether he has had a past experience of dating or late working, is more objective and does not have too much subjective emotional connotations."}, "time_dimension": {"answer": "Present", "rationale": ""}, "sentence_type": "Interrogative sentence", "pos": {"nouns": {"Date": 1, "Work": 1, "Late": 2}, "verbs": {"Tsang": 1, "Date": 1, "Late": 1}, "adverbs": {"Tsang": 1}, "numerals": {"79": 1}, "classifiers": {"individual": 1}}}, {"content": "Do you like to live a busy life?", "word_count": 12, "label": "", "sentence_structure": "Subject鈥搗erb鈥搊bject", "subjective_objective": {"answer": "Subjective", "rationale": "Because \"like\" is a personal emotional attitude, and it is subjective."}, "sentence_sentiment": {"answer": "Neutral", "rationale": "Because the entry does not have any emotional color."}, "language_style": {"answer": "Tactful and warm", "rationale": "The language style of the entry is more euphemistic and more intuitive and interactive, by asking in an invitation-style tone whether the other party prefers to live busy, rather than directly speaking or ordering."}, "time_dimension": {"answer": "Past", "rationale": "The entry asks the testee whether he likes his busy living, and does not address the time dimension of the past or the future, so the answer is \"now\"."}, "sentence_type": "Interrogative sentence", "pos": {"nouns": {"you": 1, "Questionnaire": 1, "Entry": 1, "Day": 1}, "verbs": {"Yeah": 1, "Pass": 1, "I'm busy": 2}, "adjectives": {"Busy": 1}, "adverbs": {"- You're not": 1}, "numerals": {"80": 1}}}, {"content": "Do you want people to be afraid of you?", "word_count": 8, "label": "", "sentence_structure": "Subject鈥搗erb鈥搊bject", "subjective_objective": {"answer": "Subjective", "rationale": "Because \"will\" is the subjective will and attitude of a person, the point of view of this questionnaire is subjective."}, "sentence_sentiment": {"answer": "Negative", "rationale": "Because the description \"others are afraid of you\" in the entry is negative."}, "language_style": {"answer": "Calm and direct", "rationale": ""}, "time_dimension": {"answer": "Future", "rationale": ""}, "sentence_type": "Interrogative sentence", "pos": {"nouns": {"person": 1, "Questionnaire": 1, "Entry": 1}, "verbs": {"Yes": 2}, "numerals": {"81": 1}, "pronouns": {"you": 1}}}, {"content": "Do you feel like sometimes you're all strung up and sometimes you're lazy?", "word_count": 21, "label": "", "sentence_structure": "Subject鈥搗erb鈥搊bject", "subjective_objective": {"answer": "Subjective", "rationale": "Because the question is asking the patient about his or her feelings and experiences, it is subjective."}, "sentence_sentiment": {"answer": "Negative", "rationale": "Because the word \"slugger\" is usually considered as a manifestation of negative emotions."}, "language_style": {"answer": "Tactful and warm", "rationale": "The language style of the entry is more euphemistic and friendly, and the way in which the interviewee is asked is to guide him to reflect on his own emotional changes rather than to make direct judgements or orders."}, "time_dimension": {"answer": "Present", "rationale": ""}, "sentence_type": "Interrogative sentence", "pos": {"nouns": {"you": 1, "Powerful": 1, "Time": 2, "Lazy": 1, "Questionnaire": 1, "Entry": 1}, "verbs": {"I think": 1, "All over": 1, "be": 1, "Lazy": 1}, "adjectives": {"Sometimes": 1, "Again": 1}, "numerals": {"82": 1}}}, {"content": "Do you sometimes drag what you have to do today to tomorrow?", "word_count": 17, "label": "", "sentence_structure": "Subject鈥搗erb鈥搊bject", "subjective_objective": {"answer": "Subjective", "rationale": "Because this is a description and reflection of the patient ' s behaviour and is a subjective feeling."}, "sentence_sentiment": {"answer": "Negative", "rationale": "Because the entry \"Tow what should be done today to tomorrow\" is negative."}, "language_style": {"answer": "Calm and direct", "rationale": "The basis of the judgement is as follows: the entry is in a concise and straightforward manner, asking whether the other party often postpones what is to be done today until tomorrow. This is a direct language that is more in keeping with the seriousness and objectivity of the questionnaire. At the same time, it is not used too much rhetorical or emotionally, is more plain, relevant, and has greater persuasiveness and credibility."}, "time_dimension": {"answer": "Present", "rationale": ""}, "sentence_type": "Interrogative sentence", "pos": {"nouns": {"Questionnaire Entry": 1, "Oh, my God": 2, "Yes": 2}, "verbs": {"- Put it on": 1, "Do it": 2, "Drag": 1}, "adverbs": {"Sometimes": 1}, "numerals": {"83": 1}, "pronouns": {"you": 1}}}, {"content": "Do you think you're alive and alive?", "word_count": 11, "label": "", "sentence_structure": "Subject鈥搗erb鈥搊bject", "subjective_objective": {"answer": "Subjective", "rationale": "This is because it is the opinion of others that patients are assessed and that they are subjective."}, "sentence_sentiment": {"answer": "Neutral", "rationale": "Because the entry asks about your evaluation, and does not concern your own emotional attitudes or behaviour."}, "language_style": {"answer": "Tactful and warm", "rationale": "The language style of the entry is more euphemistic and gentle, using the term \"others think\" to guide respondents to answer, avoiding direct denial or affirmation of their character characteristics, which would allow the respondents to leave more comfortable and open answers."}, "time_dimension": {"answer": "Present", "rationale": "The entry asks whether the present external performance or behaviour of the person tested is affected by what is assessed as \"viable\" and the time dimension is now judged."}, "sentence_type": "Interrogative sentence", "pos": {"nouns": {"others": 1, "- Yes, he's very lively": 1}, "verbs": {"I think": 1, "be": 1}, "adjectives": {"- Yes, he's very lively": 1}, "numerals": {"84": 1}, "pronouns": {"you": 1}}}, {"content": "Have you been told many lies?", "word_count": 12, "label": "", "sentence_structure": "Subject鈥搗erb鈥搊bject", "subjective_objective": {"answer": "Objective", "rationale": "Because lying is the act of others, not the subjective attitude or experience of the patient, it is objective."}, "sentence_sentiment": {"answer": "Negative", "rationale": "Because the \"fake\" in the entry is negative."}, "language_style": {"answer": "Calm and direct", "rationale": "The entry raises questions in a concise and straightforward manner, with a neutral and objective tone, without adding too many emotional or rhetorical techniques, and therefore can be judged as \"cool and direct\"."}, "time_dimension": {"answer": "Present", "rationale": "Now the key word \"does anyone else lie to you\" refers to the situation now or over time, and therefore the judgment is \"now\"."}, "sentence_type": "Interrogative sentence", "pos": {"nouns": {"others": 1, "Lies": 1}, "verbs": {"to": 1, "Say it": 1, "Do it": 1}, "adverbs": {"Many": 1}, "numerals": {"85": 1}, "pronouns": {"you": 1}}}, {"content": "86. Are you easily angry at something?", "word_count": 14, "label": "", "sentence_structure": "Subject鈥搗erb鈥搊bject", "subjective_objective": {"answer": "Subjective", "rationale": "Because the fire is a patient's emotional reaction and is subjective."}, "sentence_sentiment": {"answer": "Negative", "rationale": "Because \"flammable\" is a negative expression of behavior."}, "language_style": {"answer": "Calm and direct", "rationale": "The entry is in plain and clear language and is more direct and objective in terms of the person surveyed, who is not using excessive rhetorical techniques or highly emotional terms."}, "time_dimension": {"answer": "Past", "rationale": ""}, "sentence_type": "Interrogative sentence", "pos": {"nouns": {"Things": 1, "Fire": 1}, "verbs": {"Is it possible": 1, "Easy": 2, "-Put it on": 1}, "adjectives": {"Some": 1}, "numerals": {"86": 1}}}, {"content": "87. Do you often admit it when you make a mistake?", "word_count": 17, "label": "", "sentence_structure": "Subject鈥搗erb鈥搊bject", "subjective_objective": {"answer": "Subjective", "rationale": "Because \"often willing to admit wrong\" is an individual's choice of behaviour and attitude, and it is the patient's subjective experience."}, "sentence_sentiment": {"answer": "Positive", "rationale": "Because the act of \"acknowledgement of error\" in the entry is positive, it represents a willingness to understand and correct its own responsibility and fault."}, "language_style": {"answer": "Calm and direct", "rationale": "The entry is a simple question-and-answer dialogue, which is more direct and objective in language style, with no excessive rhetorical or emotional accent. This expression allows respondents to understand the problem more clearly and helps to get a real and reliable answer."}, "time_dimension": {"answer": "Present", "rationale": "The keyword \"when you make a mistake\" indicates that it is a description of the person's current behaviour, while the verb \"acknowledges\" in general now further emphasizes that it is a general and continuous act. So this entry asks about the person's current attitudes and behaviour."}, "sentence_type": "Interrogative sentence", "pos": {"nouns": {"Error": 1, "Recognition": 1}, "verbs": {"Yes": 2, "Recognition": 1}, "adjectives": {"Regular": 1}, "adverbs": {"Often": 1}, "numerals": {"87": 1, "1": 2}}}, {"content": "Would you be upset that an animal is caught in a trap?", "word_count": 20, "label": "", "sentence_structure": "Subject鈥搗erb鈥搊bject", "subjective_objective": {"answer": "Subjective", "rationale": "It is hard to feel the patient's subjective feelings and attitudes."}, "sentence_sentiment": {"answer": "Positive", "rationale": "Because the behavior in the entry \"can be sore for an animal caught in a trap\" expresses a sense of compassion and love, and is positive."}, "language_style": {"answer": "Tactful and warm", "rationale": "The entry uses a euphemism to ask the other side whether they have compassion and do not express their feelings or positions directly. At the same time, moderate language and tone are used, which makes respondents more willing to share their real ideas, and increases the relevance and interactive nature of the questionnaire."}, "time_dimension": {"answer": "Present", "rationale": "Keywords \"will\" and \"worried\" indicate that this is a description of the current mood, and therefore the judgment is now."}, "sentence_type": "Interrogative sentence", "pos": {"nouns": {"A trap": 1, "Animals": 1, "Sorry": 1}, "verbs": {"Yes": 1, "Catch": 1, "I feel": 1}, "numerals": {"88": 1}}}]}

